# Mapping the physiological and molecular markers of stress and SSRI antidepressant treatment in S100a10 corticostriatal neurons

**DOI:** 10.1038/s41380-019-0473-6

**Published:** 2019-08-20

**Authors:** Derya Sargin, Revathy U. Chottekalapanda, Kristina E. Perit, Victoria Yao, Duong Chu, Daniel W. Sparks, Salina Kalik, Saige K. Power, Olga G. Troyanskaya, Eric F. Schmidt, Paul Greengard, Evelyn K. Lambe

**Affiliations:** 1 Department of Physiology, University of Toronto, Toronto, ON Canada; 2 Laboratory of Molecular and Cellular Neuroscience, The Rockefeller University, New York, NY 10065 USA; 3 Department of Computer Science, Princeton University, Princeton, NJ 08544 USA; 4 Lewis-Sigler Institute for Integrative Genomics, Princeton University, Princeton, NJ 08544 USA; 5 Flatiron Institute, Simons Foundation, New York, NY 10010 USA; 6 Laboratory of Molecular Biology, The Rockefeller University, New York, NY 10065 USA; 7 Department of OBGYN, University of Toronto, Toronto, ON Canada; 8 Department of Psychiatry, University of Toronto, Toronto, ON Canada

**Keywords:** Neuroscience, Molecular biology, Depression, Physiology

## Abstract

In mood disorders, psychomotor and sensory abnormalities are prevalent, disabling, and intertwined with emotional and cognitive symptoms. Corticostriatal neurons in motor and somatosensory cortex are implicated in these symptoms, yet mechanisms of their vulnerability are unknown. Here, we demonstrate that S100a10 corticostriatal neurons exhibit distinct serotonin responses and have increased excitability, compared with S100a10-negative neurons. We reveal that prolonged social isolation disrupts the specific serotonin response which gets restored by chronic antidepressant treatment. We identify cell-type-specific transcriptional signatures in S100a10 neurons that contribute to serotonin responses and strongly associate with psychomotor and somatosensory function. Our studies provide a strong framework to understand the pathogenesis and create new avenues for the treatment of mood disorders.

## Introduction

Our understanding of mood and anxiety disorders is limited due to the complexity of the circuitry involved and the diverse symptoms manifested in these diseases. Research has largely focused on the emotional and cognitive symptoms, yet these disorders are also commonly associated with psychomotor and somatosensory abnormalities [1–6], including changes in basal activity levels and sensory perception of internal and external cues. In fact, such disturbances are considered some of the core symptoms of depression [7, 8], and account for a high proportion of the disability as well as personal and societal costs arising from mood and anxiety disorders [9].

Corticostriatal neurons are pyramidal neurons in the motor, sensory, and association areas of the cortex that coordinate goal-directed behavior across emotional, cognitive, and motor domains [10, 11]. A prominent subpopulation in the motor and somatosensory cortices has been found to be critical for the efficacy of antidepressant treatment with the selective serotonin reuptake inhibitor (SSRI) fluoxetine (Flx) [12]. This subgroup of corticostriatal neurons is characterized by expression of the mood- and antidepressant-responsive protein S100a10 (p11) [12–15]. The S100a10 protein regulates multiple serotonergic receptors and effector ion channels [16–21]. The neurotransmitter serotonin (5-HT) exerts complex effects on motor control and behavior [22–26]. However, it is not known how 5-HT modulates the physiology of the S100a10 population of motor and somatosensory neurons. Since these S100a10 corticostriatal neurons participate in a network controlling motor, premotor, and sensory function, disruption of their normal modulation may trigger psychomotor and sensory abnormalities [10, 27, 28].

In the present study, we investigated the serotonergic modulation of motor and somatosensory S100a10 corticostriatal neurons across healthy, stressed, and chronic SSRI-treated behavioral states. We used transgenic mice that label the S100a10 neurons to examine their role with respect to serotonergic neurotransmission, behavior, and antidepressant physiology. We report that these neurons have a unique ‘electrophysiological signature’ to 5-HT which is disrupted by the induction of chronic social isolation stress. This condition was previously shown to yield a constellation of behavioral changes, including depressive- and anxiety-like behaviors [29–31]. We show that socially isolated mice lose the serotonergic properties of S100a10 neurons, exhibit a reduced exploratory behavior in their homecage (a familiar environment) and exhibit hyperactivity in an open field (a novel environment). Chronic treatment of socially isolated mice with Flx restored the stress-induced modulatory electrophysiological pattern and introduced additional electrophysiological changes; however, isolation-induced changes of exploratory behavior are only partially recovered by chronic Flx. Of note, we found that a subpopulation of Flx-treated animals showed anxiety-like behavior. Anxiety effects of SSRIs have been observed in patients [32–36] subjected to chronic SSRI treatment, but the mechanism is unknown. We characterized the transcriptional alterations that mediate changes in behavior and physiology across healthy, stressed, and chronic antidepressant treatment states by profiling the translatome of motor and somatosensory S100a10 neurons using translating ribosome affinity purification (TRAP) analysis. Together, these studies provide a strong molecular framework for defining the complex physiology of a selective population of sensory and motor cortical neurons that, when perturbed, leads to mood dysfunction and may identify new avenues for treatment of sensory and motor symptoms.

## Results

### S100a10-expressing pyramidal neurons show distinctive 5-HT responses and enhanced excitability

**Fig. 1:**
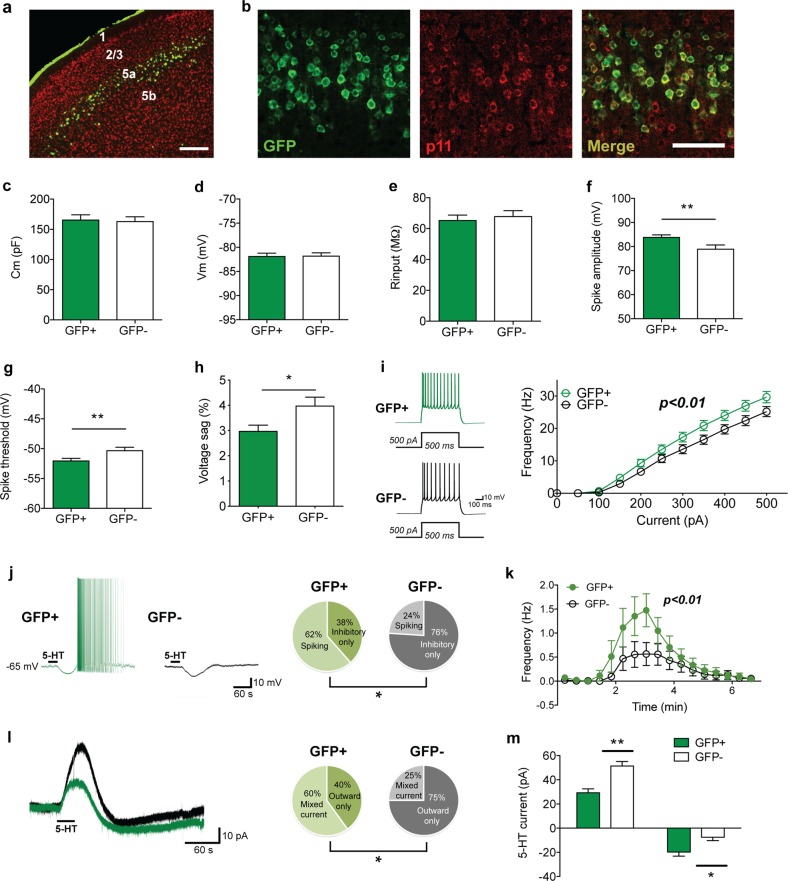
Membrane properties and 5-HT responses of S100a10 neurons compared with GFP− cells in M1 layer 5a. **a** S100a10 (GFP+) expression is restricted to layer 5a neurons in motor/somatosensory motor cortex. GFP (green) and NeuN (red) stainings are shown. Scale bar, 30 µm. **b** Double labeling with anti-GFP and anti-S100a10 antibodies in the sensorimotor cortex of S100a10 bacTRAP mice. Scale bar, 100 µm. Passive membrane properties including **c** membrane capacitance Cm (pF), **d** resting membrane potential Vm (mV), and **e** input resistance R_input_ (MΩ) were similar between layer 5a GFP + (*n* = 30) and GFP− (*n* = 24) neurons. **f** Spike amplitude (mV) of GFP+ neurons is larger compared with that of GFP− neurons (unpaired *t*-test, **p < 0.01). **g** Spike threshold (mV) of GFP+ neurons is smaller compared with that of GFP− neurons making S100a10+ neurons more excitable (unpaired *t*-test, **p < 0.01). **h** Voltage sag ratio (%) of GFP+ neurons is lower compared with that of GFP− neurons (unpaired *t*-test, *p < 0.05). **i** Representative current-clamp traces (left) from a layer 5a GFP+ and GFP− neuron in response to a 500 pA depolarizing step. Input–output curve (right) showing the frequency (Hz) of action potentials in response to a series of depolarizing current injections. GFP+ neurons (*n* = 18) fire more action potentials indicating increased intrinsic excitability compared with GFP− neurons (*n* = 15) (two-way ANOVA, *F*_(1,341)_ = 27.05). **j** Representative current-clamp traces in response to 5-HT from a GFP+ and a GFP− neuron are shown (left). Pie charts showing the distribution of 5-HT responses in current-clamp in layer 5a GFP+ and GFP− neurons (*n* = 6 mice, GFP + , *n* = 26 neurons; GFP−, *n* = 25 neurons) (right). Majority of GFP+ neurons respond to 5-HT with increased spiking. GFP− neurons are mainly inhibited (Fisher’s exact test, *p < 0.05). **k** Frequency of action *p*otentials over time in response to 5-HT in GFP+ (*n* = 26) and GFP− (*n* = 25) cells. 5-HT induced firing frequency is larger in GFP+ neurons (two-way ANOVA, *F*_(1,833)_ = 14.46). **l** Averaged voltage-clamp recordings of 5-HT currents in GFP+ (green) and GFP− (black) neurons (left). Pie charts showing the distribution of 5-HT responses in voltage-clamp in layer 5a GFP+ and GFP− neurons (right). Majority of GFP+ neurons show a mixed (inhibitory + excitatory) current response to 5-HT while GFP− neurons mostly show inhibitory responses (Fisher’s exact test, *p < 0.05). **m** The amplitude of 5-HT outward inhibitory and inward excitatory currents in layer 5a GFP+ and GFP− neurons (*n* = 6 mice, GFP+, *n* = 30 neurons; GFP−, *n* = 24 neurons). GFP+ neurons have smaller inhibitory and larger excitatory 5-HT currents compared with GFP− neurons (nonparametric, two-tailed Mann–Whitney *t*-test, *p < 0.05, **p < 0.01)

To identify S100a10-expressing neurons, we used the S100a10 bacTRAP transgenic line that expresses EGFP-tagged ribosomal protein L10a (EGFPL10a) under *S100a10* transcriptional regulatory elements [12]. EGFP fluorescence was detectable in live cortical slices and the fusion protein was restricted primarily to the soma, allowing for easy identification of labeled cells, and was costained with the p11 protein [12] (Fig. 1a, b). To decipher the unique physiological characteristics of S100a10 neurons, we performed whole-cell patch-clamp electrophysiology in cortical slices obtained from S100a10 bacTRAP mice. We focused on motor and somatosensory cortical areas which are critical for psychomotor and sensory functions and harbor an abundant expression of S100a10 in layer 5a corticostriatal neurons [12] (Fig. 1a, b).

To characterize the physiological properties of cortical layer 5a S100a10 neurons, we examined their membrane properties in comparison with the neighboring unlabeled cells. The passive membrane characteristics of layer 5a GFP+ and GFP− pyramidal neurons including the membrane capacitance (Cm), the resting membrane potential (Vm) and the input resistance (*R*_input_) did not differ (Fig. 1c–e). There were, however, differences in the active properties of these neurons. The spike amplitude of GFP+ neurons appears significantly larger and the spike threshold is significantly more hyperpolarized compared with those of unlabeled neurons (Fig. 1f, g). GFP+ neurons are weakly adapting cells (adaptation ratio; GFP+: 0.74 ± 0.02; GFP−: 0.81 ± 0.03, unpaired *t*-test, *p* = 0.074) with relatively low voltage sag rectification (Fig. 1h). These properties are consistent with the electrophysiological parameters previously reported for intratelencephalic corticostriatal neurons in adult mice [37], classifying GFP+ cells among the type 2 neurons [38–40]. Consistent with these properties, GFP+ neurons fire at a greater frequency compared with the unlabeled neurons in response to depolarizing current injections (Fig. 1i) indicating that GFP+ neurons have enhanced intrinsic excitability.

Corticostriatal S100a10 neurons have previously been suggested to be important for 5-HT signaling [17, 20, 21]. However, their electrophysiological response to 5-HT has not been characterized. Current-clamp recordings from layer 5a of motor cortex revealed that bath application of 5-HT elicited hyperpolarization followed by depolarization sufficient to trigger action potentials in the majority of GFP+ neurons (Fig. 1j). By contrast, most GFP− neurons showed only hyperpolarization in response to 5-HT (Fig. 1j). The frequency of action potentials in response to 5-HT was significantly greater in GFP+ neurons compared with GFP− neurons (Fig. 1k). As illustrated in the examples in Fig. S1, biphasic inhibitory-excitatory 5-HT responses persisted in the presence of synaptic blockers, including the AMPA/kainate receptor antagonist CNQX, the NMDA receptor antagonist AP5, and the GABA_A_ receptor antagonist picrotoxin, suggesting that they are mediated directly by 5-HT receptors on the S100a10 neurons themselves.

We next examined the distribution of 5-HT current responses in layer 5a neurons using voltage-clamp electrophysiology. In the majority of GFP+ neurons, we found mixed 5-HT current responses composed of an outward current followed by an inward current (Fig. 1l) in agreement with the current-clamp data showing that these neurons were transiently inhibited and then strongly excited by 5-HT. By contrast, the majority of unlabeled layer 5a neurons showed only the 5-HT-elicited outward current (Fig. 1l). We additionally found differences in the amplitude of 5-HT responses between S100a10 and unlabeled neurons. In GFP+ cells, the amplitude of the outward 5-HT current was significantly smaller and the inward 5-HT current was larger when compared with the amplitude of these responses in unlabeled neurons (Fig. 1m). These results further confirm greater excitatory 5-HT response in S100a10 neurons compared with neighboring cells in layer 5a.

Together these data show that layer 5a S100a10 cortical neurons display a distinctive and mainly excitatory 5-HT response that is different from the neighboring unlabeled neurons, which respond to 5-HT primarily with inhibition. S100a10 neurons are also distinctive with respect to their enhanced intrinsic excitability.

### Prolonged social isolation and chronic fluoxetine treatment affect 5-HT-elicited excitatory responses of S100a10 neurons

**Fig. 2:**
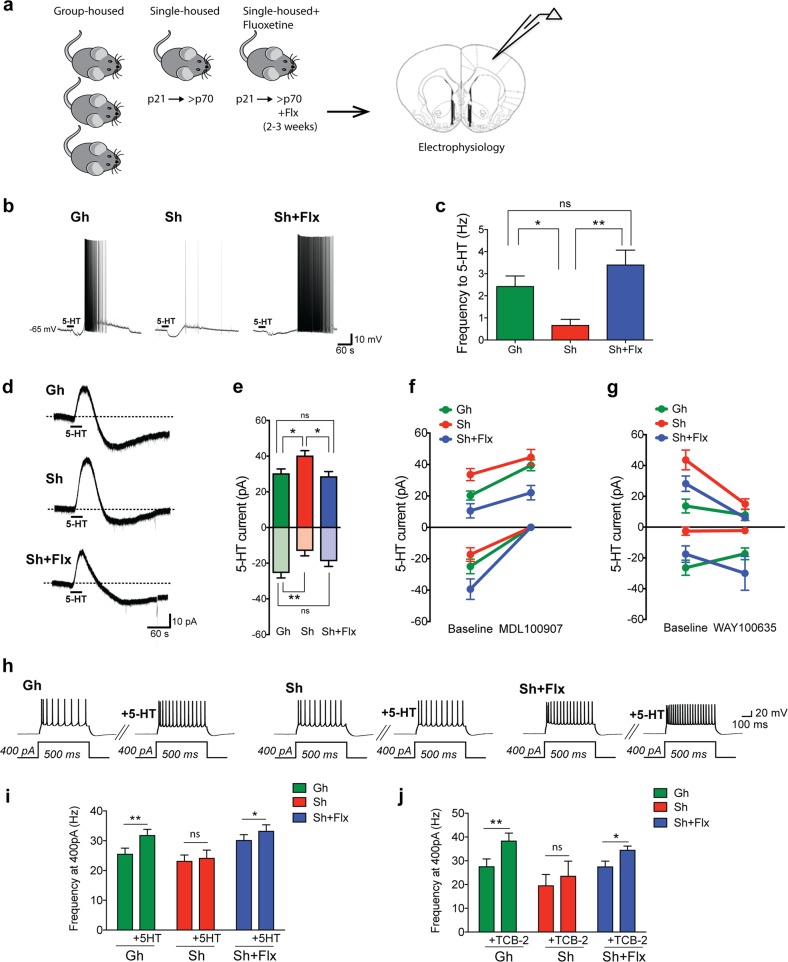
The excitatory portion of the 5-HT response in S100a10 neurons is diminished upon chronic social isolation and can be restored by chronic flx treatment. **a** Schematic representation of the experimental paradigm. **b** Representative current-clamp traces in response to 5-HT in S100a10 neurons from a group-housed (Gh), single-housed (Sh) and a single-housed mouse treated with chronic fluoxetine (Sh + Flx). **c** Quantification of spike frequency in response to 5-HT in S100a10 neurons (Gh; *n* = 6 mice, *n* = 26 neurons, Sh; *n* = 6 mice, *n* = 27 neurons, Sh + Flx; *n* = 8 mice, *n* = 35 neurons) (nonparametric, one-way Kruskal–Wallis ANOVA, *H*_(2)_ = 10.35, *p* < 0.01, Dunn’s post hoc test, *p < 0.05, **p < 0.01, ns: nonsignificant). 5-HT induced increase in the firing frequency of S100a10 neurons is reduced after social isolation and restored by chronic Flx. **d** Averaged voltage-clamp recordings of 5-HT currents in S100a10 neurons. **e** Quantification of outward inhibitory and inward excitatory currents in response to 5-HT in S100a10 neurons (Gh; *n* = 7 mice, *n* = 34 neurons, Sh; *n* = 6 mice, *n* = 38 neurons, Sh + Flx; *n* = 8 mice, *n* = 43 neurons). 5-HT inhibitory currents are increased and excitatory currents are decreased after social isolation and restored upon chronic Flx (nonparametric, one-way Kruskal–Wallis ANOVA, inhibitory currents: *H*_(2)_ = 10.01, *p* < 0.01; excitatory currents: H_(2)_ = 9.73, *p* < 0.01, Dunn’s post hoc test, ^*^p < 0.05, ns: nonsignificant). **f** The amplitude of 5-HT inhibitory currents and excitatory currents before and after application of the 5-HT_2A_R antagonist MDL100907. 5-HT inhibitory currents are increased in Gh (*n* = 5 mice, *n* = 6 neurons) and Sh + Flx (*n* = 3 mice, *n* = 4 neurons) mice after MDL100907 while the change in neurons from Sh (*n* = 3 mice, *n* = 7 neurons) mice is not significant (paired *t*-test, Gh: **p < 0.01, Sh: *p* > 0.05, Sh + Flx: **p < 0.01). MDL100907 diminished all 5-HT excitatory current responses in all of the groups (paired *t*-test, Gh: **p < 0.01, Sh: **p < 0.01, Sh + Flx: **p < 0.01). **g** The amplitude of 5-HT inhibitory currents and excitatory currents before and after application of the 5-HT_1A_R antagonist WAY100635 (Gh: *n* = 3 mice, *n* = 4 neurons; Sh: *n* = 6 mice, *n* = 7 neurons; Sh + Flx: *n* = 6 mice, *n* = 8 neurons). 5-HT inhibitory currents are decreased (paired *t*-test, Gh: *p = 0.05, Sh: **p < 0.01, Sh + Flx: **p < 0.01) while excitatory currents remain unchanged in all the groups after WAY100635 (paired *t*-test, Gh: *p* > 0.05, Sh: *p* > 0.05, Sh + Flx: *p* > 0.05). **h** Representative current-clamp traces in response to a 400 pA depolarizing step in the absence and presence of 5-HT in a S100a10 neuron from a Gh, Sh and Sh + Flx mouse. **i** 5-HT induced increase in the firing frequency of S100a10 neurons was lost after social isolation and restored with chronic Flx (Gh; *n* = 6 mice, *n* = 27 neurons, Sh; *n* = 6 mice, *n* = 24 neurons, Sh + Flx; *n* = 7 mice, *n* = 32 neurons) (paired *t*-test, Gh: **p < 0.01, Sh: *p* > 0.05, Sh + Flx: *p < 0.05). **j** TCB-2 induced increase in the firing frequency of S100a10 neurons was lost after social isolation and restored with chronic Flx (Gh; *n* = 3 mice, *n* = 5 neurons, Sh; *n* = 3 mice, *n* = 5 neurons, Sh + Flx; *n* = 3 mice, *n* = 4 neurons) (paired *t*-test, Gh: **p < 0.01, Sh: *p* > 0.05, Sh + Flx: *p < 0.05)

Chronic social isolation stress in juvenile mice has previously been shown to induce anxiety- and depressive-like phenotypes in adulthood [29–31]. We investigated the effect of chronic social isolation on the 5-HT responses of layer 5a S100a10 neurons which are critical for antidepressant responses [12]. A group of male mice was individually housed from weaning (postnatal day 21, P21) through adulthood. After a minimum of 7 weeks (>P70), a subgroup of these single-housed mice received fluoxetine (Flx; 0.167 mg/ml) in their drinking water for an additional 2–3 weeks. Group-housed littermates of the single-housed mice were used as controls. Fig. 2a illustrates the experimental design.

Current-clamp recordings in cortical slices obtained from single-housed mice showed that layer 5a S100a10 neurons responded to 5-HT with significantly fewer action potentials when compared with S100a10 neurons from control mice (Fig. 2b, c). The degree of 5-HT induced hyperpolarization was not significantly affected by single-housing (Gh: 7.9 ± 0.5 mV; Sh: 8.7 ± 0.6 mV; Sh + Flx: 7.5 ± 0.7 mV; one-way ANOVA, *F*_(2,87)_ = 0.9, *p* *=* 0.4) suggesting that near threshold, the excitatory component of the 5-HT response is specifically vulnerable to chronic social isolation. In contrast, the frequency of 5-HT induced action potentials was similar to the control levels in single-housed mice treated chronically with Flx (Fig. 2b, c). This restored excitatory effect of 5-HT in the Flx-treated mice persisted in the presence of synaptic blockers (Fig. S1, c), suggesting that chronic Flx treatment normalized the loss of direct 5-HT induced excitability caused by chronic social isolation. Voltage-clamp recordings revealed that the mixed 5-HT current responses in S100a10 neurons were also affected by chronic social isolation. The amplitude of the outward 5-HT current in S100a10 neurons of single-housed mice was significantly larger compared with those of control mice (Fig. 2d, e) and these large outward 5-HT currents persisted in the presence of synaptic blockers (Fig. S1, b). The amplitude of the inward 5-HT current was also smaller in S100a10 neurons of single-housed mice (Fig. 2d, e). In Flx-treated single-housed mice, however, the amplitudes of the outward and inward 5-HT currents in S100a10 neurons were comparable to control littermates (Fig. 2d, e). Importantly, chronic treatment with Flx was required to restore the 5-HT electrophysiological responses in S100a10 neurons of single-housed mice since a subchronic treatment (for 4 days) was not sufficient to change 5-HT responses after chronic social isolation (Fig. S2). Housing and Flx-elicited changes in the amplitude of 5-HT responses were not evident in unlabeled neurons (Fig. S3). Of note, chronic Flx treatment in group-housed mice did not alter the amplitudes of 5-HT outward and inward currents nor the intrinsic membrane properties of S100a10 neurons (Fig. S4). These findings emphasize the importance of the differential effects of antidepressant treatment under pathological versus normal/healthy conditions. Taken together, our data suggest that the S100a10 cells are uniquely responsive to chronic social isolation stress. They show a decrease in 5-HT-induced excitation that is restored with chronic treatment with Flx, while neighboring, unlabeled pyramidal neurons are unaffected by stress and Flx.

To probe whether the electrophysiological differences in S100a10 neurons are selective to the effects of 5-HT or involve additional changes, such as alterations in glutamatergic or GABAergic signaling, we examined spontaneous excitatory and inhibitory postsynaptic currents as well as intrinsic membrane properties across the treatment groups. The amplitude and inter-event interval of sEPSCs (Fig. S5a–c) and sIPSCs (Fig. S5d–f) measured from S100a10 neurons were comparable between the groups suggesting that the basal glutamate and GABA synaptic transmission were not changed after social isolation and Flx treatment. In addition, we measured current responses to bath application of muscimol, the selective GABA_A_ receptor agonist, and did not detect a difference across the groups (Fig. S5g). These data suggest a level of specificity to the stress-induced serotonergic changes detected in the S100a10 neurons. Examination of intrinsic properties across the groups revealed small but significant changes in membrane excitability (Fig. S6a, b, f) that would act to amplify the consequences of lost serotonergic excitation in social isolation and its rescue by chronic Flx.

### The role of 5-HT_1A_ and 5-HT_2A_ receptors in serotonergic responses to stress and fluoxetine in cortical S100a10 neurons

We next examined the mechanisms of the 5-HT responses in S100a10 neurons using selective blockers for the predominant 5-HT receptor subtypes in cortical pyramidal neurons. In all groups, 5-HT inward currents were completely abolished by the application of the selective 5-HT_2A_ receptor antagonist, MDL100907 (Fig. 2f). This suggests that all 5-HT inward current responses in S100a10 neurons are mediated via 5-HT_2A_ receptors. Although MDL100907 increased the 5-HT outward currents in control and Flx-treated groups, it did not have a significant effect on the 5-HT outward currents of single-housed mice (Fig. 2f). Application of the selective 5-HT_1A_ receptor antagonist WAY100635 reduced the 5-HT outward currents but did not abolish them (Fig. 2g). WAY100635 did not have an effect on the 5-HT inward currents in all groups tested (Fig. 2g). Examination of gene expression in S100a10 cells by the TRAP approach ([12]; and see below) revealed that mRNA encoding 5-HT_2A_ receptors (*Htr2a*) is greater than twofold enriched in these cells compared with the rest of cortex (Fig. S7), while 5-HT_1A_ receptors (*Htr1a*) are expressed at similar levels. Together with the pharmacological experiments, these data imply that the excitatory component of the 5-HT response in S100a10 cells is mediated by cell-type-specific enrichment of 5-HT_2A_ receptors, whereas 5-HT_1A_ receptors are only partially responsible for the inhibitory component.

To examine directly the impact of 5-HT_2A_ receptors on excitatory 5-HT responses, we compared the effects of 5-HT and the selective 5-HT_2A_ receptor agonist, TCB-2, on changes in the firing frequency of S100a10 neurons from control mice as well as from those exposed to social isolation stress with and without Flx administration. We first stimulated action potentials in layer 5a S100a10 neurons by injection of a 400 pA depolarizing current for 500 ms to establish a baseline firing frequency. Next, we measured changes in the frequency when 5-HT was applied on the slice during stimulation. S100a10 neurons from control mice responded to 5-HT with a significant average increase in the frequency of action potentials (Fig. 2h, i). S100a10 neurons from single-housed mice responded to the 400 pA stimulation; however their firing frequency was not potentiated by 5-HT application (Fig. 2h, i). Similar to our earlier findings, treatment with chronic Flx restored the 5-HT-induced frequency increase in S100a10 neurons in the single-housed mice (Fig. 2h, i). Together, these experiments showed that the 5-HT-induced excitatory effect in activated neurons is attenuated in single-housed mice but restored with chronic Flx treatment. Next we examined if stimulation of 5-HT_2A_ receptors with TCB-2 was sufficient to induce a similar change in firing frequency. We applied TCB-2 on slices along with a depolarizing current injection. TCB-2 increased the firing frequency in S100a10 neurons of control mice (Fig. 2j) while those from single-housed mice did not respond to TCB-2, indicating the absence of 5-HT_2A_ receptor mediated excitation in these neurons (Fig. 2j). Interestingly, chronic Flx treatment in single-housed mice restored the TCB-2 elicited frequency increase in S100a10 neurons (Fig. 2j). This demonstrated that selective stimulation of 5-HT_2A_ receptors increased the frequency of firing similar to 5-HT in layer 5a S100a10 neurons. This TCB-2-elicited increase in the firing frequency of S100a10 neurons was lost after chronic social isolation but could be restored by chronic Flx treatment.

We have previously shown that chronic fluoxetine treatment in the absence of stress increased *Htr4* mRNA encoding the 5-HT_4_ receptor [12]. Activation of 5-HT_4_ receptors has been reported to enhance neuronal excitability in hippocampus [41] and to modulate the excitability of cortical pyramidal neurons in prefrontal cortex [42, 43]. Therefore, we sought to determine whether 5-HT_4_ receptor function plays a role in the 5-HT mediated excitatory responses in layer 5a S100a10 neurons. Using voltage-clamp recordings, we were unable to show that the agonist BIMU 8 could mimic 5-HT induced excitation via 5-HT_4_ receptor stimulation (Fig. S8a). Furthermore, the selective 5-HT_4_ receptor antagonist GR113808 did not prevent 5-HT induced excitation observed in current-clamp recordings in S100a10 neurons (Fig. S8b, c). These experiments ruled out the contribution of 5-HT_4_ receptor function to the 5-HT mediated excitation in S100a10 neurons.

Together, these data suggest that the excitatory 5-HT responses in layer 5a S100a10 neurons are mediated by 5-HT_2A_ receptors, and that there is a specific state-dependent disruption in the 5-HT_2A_ receptor-mediated response after chronic social isolation stress which can be restored by chronic Flx.

### Behavioral heterogeneity following chronic fluoxetine treatment

5-HT depletion was previously associated with decreased exploratory locomotion without causing further sensorimotor deficits [44]. Risk assessment behaviors such as hiding, exploration and reduced activity have evolved in rodents as a defensive response to threat, can be used to investigate anxiety and depressive-like states, and respond to anti-anxiety medications [45–47]. In order to investigate the effects of alterations in 5-HT physiology on exploratory sensorimotor performance, we analyzed behavior in two different settings; a familiar homecage environment and a novel open-field environment.

**Fig. 3:**
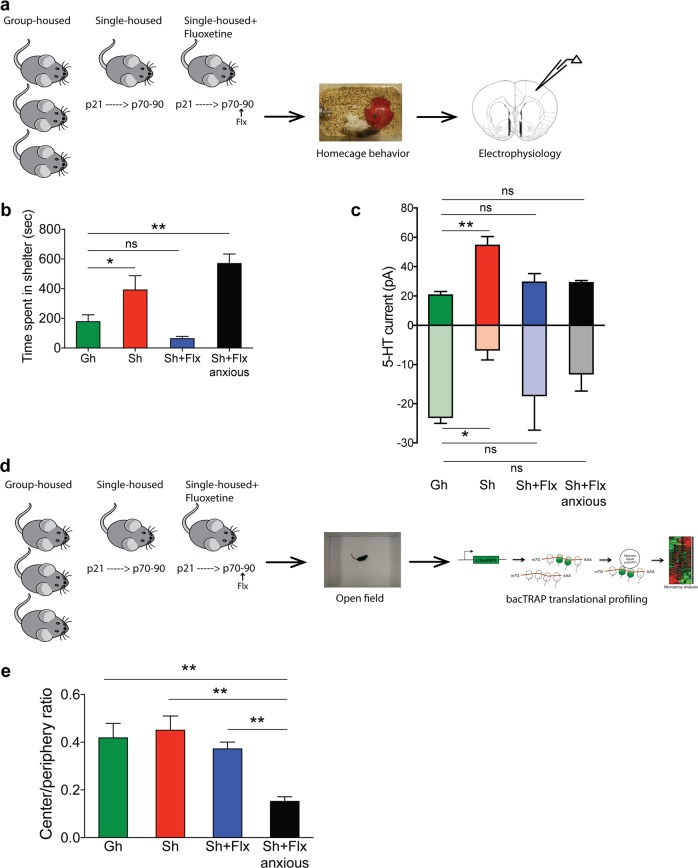
Heterogeneity emerges in the behavioral response to Flx but does not correlate with the 5-HT response. **a** Schematic representation of the experimental paradigm. Gh, Sh, and Sh + Flx mice were recorded in their homecage for 15 min before being sacrificed for electrophysiology. The time mice spent in their shelter zone was determined. **b** Sh mice (*n* = 6) spent more time in the shelter zone compared with Gh mice (*n* = 6) indicating higher anxiety. Behavior of Flx-treated Sh mice showed heterogeneity on this measure; one group (Sh + Flx; *n* = 6) with normalized anxiety and another group (Sh + Flx anxious; *n* = 7) with higher anxiety (one-way ANOVA, *F*_(3,21)_ = 14.03, *p* < 0.01, Newman–Keuls post hoc test, *p < 0.05, **p < 0.01, ns: nonsignificant). **c** The amplitude of the 5-HT currents averaged per animal is shown. 5-HT inhibitory currents are larger and excitatory currents are smaller in Sh mice (*n* = 6) compared with the currents of Gh (*n* = 6) mice. 5-HT currents of Sh + Flx (*n* = 6) and Sh + Flx anxious mice (*n* = 7) are comparable with the Gh mice. For simplicity, post hoc test results compared with controls were shown (inhibitory currents: one-way ANOVA, F_(3,21)_ = 12.19, *p* < 0.01, Newman–Keuls post hoc test; excitatory currents: nonparametric, one-way Kruskal–Wallis ANOVA, *H*_(3)_ = 8.62, *p* < 0.05, Dunn’s post hoc test, *p < 0.05, **p < 0.01, ns: nonsignificant). **d** Schematic re*p*resentation of the ex*p*erimental paradigm. Gh, Sh, and Sh + Flx mice were subjected to open field before being sacrificed for bacTRAP translational analysis. **e** Open-field analysis of the Gh (green, *n* = 11), Sh (red, *n* = 12), Sh + Flx (blue, *n* = 11) and Sh + Flx anxious mice (black, *n* = 13). Mice were habituated in the testing room in their homecage for 30 min and thigmotaxis was measured for 60 min in an open field. The Sh + Flx anxious group showed dramatically reduced center to periphery ratio compared with the other three groups groups (nonparametric, one-way Kruskal–Wallis ANOVA, *H*_(3)_ = 26.17, *p* < 0.01, Dunn’s post hoc test, **p < 0.01)

Mice use nesting/shelter for temperature regulation and protection from predators [48]. Chronic stress in mice was previously associated with increased preference for the shelter zone [49]. To investigate 5-HT responses in behaviorally-distinct conditions of chronic stress and Flx treatment, we investigated the time mice spent under the dome shelter in their homecage and performed electrophysiology after homecage analysis (Fig. 3a). We found that single-housed mice spent significantly more time under the shelter in their homecage (Fig. 3b) compared with the control mice indicating reduced exploratory behavior in a familiar environment. Interestingly, single-housed mice treated with chronic Flx could be grouped into two different behavioral profiles. Chronic Flx treatment normalized the exploratory behavior in one subpopulation of single-housed mice (Sh + Flx), while the other was resistant to such normalization (Sh + Flx anxious group; mice were classified as anxious when time spent under the shelter zone >mean ± 2 × S.E.M. of the control group) (Fig. 3b). Whole-cell electrophysiological analysis on cortical slices revealed that S100a10 neurons from single-housed mice had larger 5-HT outward currents and smaller 5-HT inward currents compared with those of control mice (Fig. 3c), similar to our previous finding. Both Flx-treated groups showed 5-HT current responses restored to control levels despite the differences in their exploratory behavior (Fig. 3c).

We next investigated the behavior of the various treatment groups in an unfamiliar environment, measuring the distance traveled and time mice spent in different zones in an open-field arena (Fig. 3d). In the open field, single-housed mice showed hyperactivity evident by an increased distance traveled over time (Fig. S9a) while Flx-treated groups had comparable levels of activity to control mice (Fig. S9b, c). Interestingly, in a subpopulation of the single-housed mice treated with Flx, we observed a decrease in the ratio of the time spent in the center relative to the time spent in the peripheral field of the arena (center to periphery ratio <0.25). This behavior was significantly different from both control and single-housed mice that did not receive Flx, indicating that chronic Flx increased anxiety levels in a subgroup of socially isolated mice (Fig. 3e).

### Molecular profiling of S100a10 neurons following chronic social isolation and Flx treatment

**Fig. 4:**
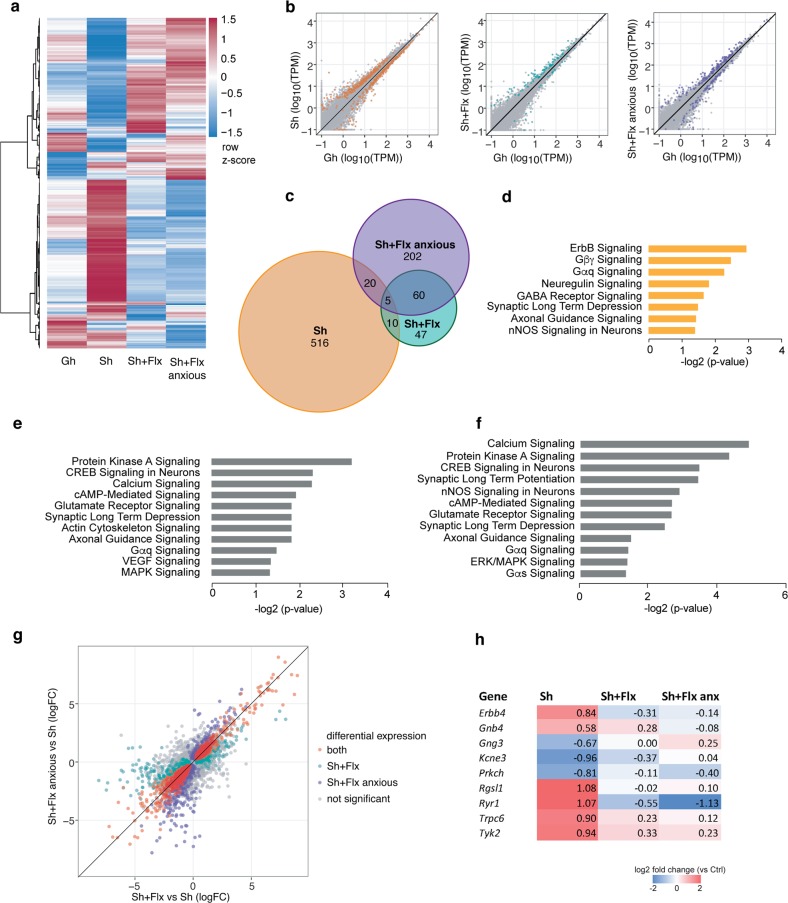
Molecular profiling of S100a10 neurons and their alteration by social isolation stress and Flx treatment. **a** Heatmap showing the hierarchical clustering of the top 10,000 variable genes (row-normalized log TPM) across all groups of Gh, Sh, Sh + Flx, and Sh + Flx anxious mice (blue, low expression; red, high expression), *n* = 4 (3 cortices pooled for each sample). **b** Scatter plots showing differentially expressed genes in the different groups (Sh versus Gh (left, orange), Sh + Flx versus Gh (center, green), Sh + Flx anxious versus Gh (right, purple). **c** Venn diagram showing the overlap between the significantly altered genes obtained by comparing the three groups (described in B and color coordinated). **d**–**f** Functional categorization of genes using ingenuity pathway analysis (IPA) for the groups Gh vs Sh, Sh vs Sh + Flx, Sh vs Sh + Flx anxious. **g** Scatter plot showing the differentially expressed genes between Sh + Flx vs Sh and Sh + Flx anxious vs Sh groups (blue, genes significantly differentially expressed in Sh + Flx vs Sh; purple, significant in Sh + Flx anxious vs Sh; orange, significant in both comparisons; gray, not significant. **h** Selected genes altered by chronic social isolation and restored by Flx

To identify molecular adaptations in the S100a10 cells that accompany the changes in physiology and behavior in response to prolonged social isolation and antidepressant treatment, we utilized the TRAP technique [50, 51]. Expression of the EGFP-tagged ribosomal protein L10a (EGFPL10a) in the S100a10 bacTRAP mice allows for the isolation of polysome-bound transcripts specifically from the S100a10-expressing cells through anti-EGFP affinity purification from whole tissue [12]. TRAP mRNA was collected from the isolated cortices of the four experimental groups: group-housed (control; Gh); single-housed (Sh); single-housed and Flx-treated mice that did not exhibit anxiety (Sh + Flx); and single-housed and Flx-treated mice that were anxious according to the results of the open-field test (Sh + Flx anxious) (see Fig. 3e). TRAP mRNA from each of the four groups was analyzed by RNA sequencing. We compared genes differentially expressed between Sh and Gh, Sh + Flx and Gh, Sh + Flx anxious and Gh mice in order to identify stress-affected genes and reveal those that are normalized by Flx treatment. The significantly altered genes for the above three comparisons are listed in Table S1a, Table S1b, and Table S1c respectively. Together, the significant changes from these comparisons are visualized as a heatmap (Fig. 4a), scatter plot (Fig. 4b) and Venn diagram (Fig. 4c).

The largest number of differentially expressed genes (551) was observed when the Sh group was compared with the Gh control (Fig. 4a, orange dots in left panel of Fig. 4b and orange circle in Fig. 4c), suggesting strong stress-induced changes in gene expression in S100a10 neurons. Functional pathway analysis revealed dysregulation of several relevant stress-regulated pathways such as ErbB, neuregulin, G protein signaling, synaptic long-term depression, and axonal guidance signaling in these cells from single-housed mice (Fig. 4d).

We found that very few of the gene changes (~6%) in the single-housed group were shared with either single-housed mice receiving Flx, regardless of the anxiety status of these Flx mice (Sh + Flx, Sh + Flx anxious) (Fig. 4c). These results suggest that Flx normalizes abnormal gene expression in S100a10 neurons of single-housed mice. The nonanxious Flx group had the smallest number of regulated genes relative to the group-housed mice (Sh + Flx, 122 genes, green dots/circle in Fig. 4b, c). In contrast, the anxious Flx group showed substantially more regulated genes (Sh + Flx anxious, 287 genes, purple dots/circle in Fig. 4b, c) but still almost half that of the untreated single-housed mice.

In order to identify the specific genes and pathways activated in response to chronic Flx, we compared gene expression between the Sh and the Sh + Flx groups. Differential expression analysis identified 3463 genes altered in the Sh + Flx group, and 4166 genes in the Sh + Flx anxious groups compared with the Sh group (Fig. S10, Table S2a, and Table S2b). Canonical pathway analysis showed that chronic Flx treatment activated several signaling pathways in both treated groups that have been previously implicated in the antidepressant response. These include protein kinase A signaling, cAMP pathway, calcium-regulated signaling, and CREB signaling pathway (Fig. 4e, f).

Next we compared the gene changes between the Sh + Flx and Sh + Flx anxious groups and identified very few differentially expressed genes as shown by the scatter plot in Fig. 4g. This indicates that the Sh + Flx and the Sh + Flx anxious animals respond to Flx treatment in a similar manner, indicating that the anxiety phenotype of the Sh + Flx anxious group is not due to a lack of molecular response to Flx (Fig. 4g, h). These findings are consistent with the physiology results where we show that the serotonergic function of the S100a10 cells in the Sh + Flx anxious animals are similar to the Sh + Flx animals. Together, these results indicate that the anxiety phenotype of Sh + Flx animals is not due to the molecular adaptations in the layer 5a S100a10 neurons but likely a result of Flx-dependent changes occurring in other cell-types or brain regions.

### Flx treatment normalizes pathways related to 5-HT_2A_ receptor signaling in S100a10 cells

**Fig. 5:**
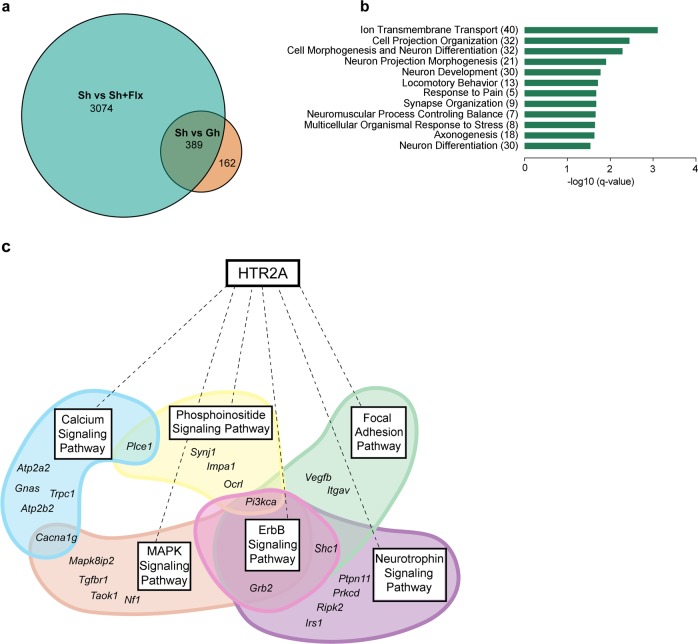
Stress-normalized genes are connected to 5-HTR_2A_ signaling and regulation. **a** Venn diagram identifying the stress-affected Flx-normalized genes (green) obtained by comparing overlapping genes between Sh versus Gh (orange) and Sh versus Sh + Flx group (cyan). **b** Functional categorization of genes using IPA. **c** Functional interaction network analysis revealing the critical pathways that mediate the connectivity between stress-normalized genes and 5-HTR_2A_ signaling

To further characterize the molecules contributing to the serotonergic phenotype, we isolated the genes that were both differentially regulated by chronic social isolation (Sh versus Gh) and normalized by Flx treatment (Sh versus Sh + Flx). We identified 389 social isolation-induced Flx-normalized genes (Fig. 5a, b and Table S3). GO analysis identified genes that control pathways involved in the regulation of neuronal differentiation, response to stress and pain, cell projection morphogenesis, regulation of ion transporter activity, and synapse organization (Fig. 5c). Our electrophysiological analysis showed that 5-HT_2A_ receptor responses were disrupted in single-housed mice (Fig. 2) suggesting that 5-HT_2A_ receptor pathways may be affected by chronic social isolation. To understand the broader implications of Flx-induced changes with respect to 5-HT signaling and regulation, we tested how the social isolation-affected Flx-normalized genes relate to 5-HT_2A_ receptor function. To do so, we utilized a cerebral cortex-specific functional gene interaction network to elucidate how genes work together through modulation of distinct interacting pathways [52]. We demonstrate that a large subset of the social isolation-affected Flx-normalized genes converge on a small number of key pathways that together regulate 5-HT_2A_ signaling (Fig. 5c and Table S4). The specific pathways in this network include Galpha_q_-coupled receptor signaling, calcium, phosphoinositide, neurotrophin, ErbB, MAPK signaling, and focal adhesion pathways (Fig. 5c and Table S4). In summary, we have identified distinct families of genes that regulate pathways relevant to 5-HT_2A_ signaling and trafficking, which are oppositely regulated by social isolation stress and Flx (Fig. S11), contributing to the serotonin response.

A striking pattern emerges from the molecular data to support the role of S100a10 neurons in the psychomotor and somatosensory aspects of a mouse endophenotype of depression. As described in Fig. S12, the gene pathways in the single-housed group contain a high frequency of associations with those implicated in motor and sensory abnormalities. Of note, these gene expression changes appear responsive to Flx, regardless of behavioral outcome. This finding gives hope that SSRIs have underappreciated potential to improve psychomotor and somatosensory abnormalities of depression [53, 54], underscoring the clinical need to understand and prevent or treat the unwanted side-effects of SSRIs.

## Discussion

Here, we have demonstrated that S100a10-expressing corticostriatal neurons in layer 5a of primary motor and somatosensory cortices have distinctive serotonergic modulation, are sensitive to perturbation by chronic social isolation stress and to restoration by antidepressant treatment. Chronic social isolation triggered a disruption in the 5-HT induced excitation of S100a10 corticostriatal neurons and promoted the inhibitory influence of 5-HT. Chronic Flx treatment during social isolation effectively restored the serotonergic excitability in these neurons. Surprisingly, the behavioral variability in the response to chronic Flx did not reflect treatment insensitivity. Instead, molecular analysis revealed a pattern of worse behavioral outcome in mice with greater transcriptional changes in the S100a10 neurons. Further investigation into the differential gene expression changes pointed to specific genes and pathways underlying these state-dependent changes in S100a10 neurons of the motor and somatosensory cortices.

Corticostriatal neurons require strong and concerted firing as a population in order to activate their downstream targets [55, 56]. The excitatory serotonergic action on the population of S100a10 corticostriatal neurons in motor and somatosensory cortices would appear to fulfill this criterion. The initial transient 5-HT inhibition of S100a10 neurons could potentially facilitate coordinated entry into “up” states of gamma activity to enhance corticostriatal population signaling across brain regions [57, 58]. Loss of excitatory serotonergic modulation through exposure to chronic social isolation stress would be expected to perturb corticostriatal signaling and the integration of sensory and emotional processing and goal-directed behavior. Of note, this predicted pattern of reduced functional corticostriatal connectivity has been seen in human imaging studies of depression [59, 60]. Interestingly, S100a10 neurons of the motor and somatosensory cortices appear to share similar serotonergic modulation to their counterparts in the prefrontal cortex [61, 62], raising the question of whether chronic social isolation stress affects the modulation of emotional and cognitive corticostriatal circuits in a manner similar to that observed in our study.

Our results point to chronic social isolation stress exerting direct changes in serotonergic modulation occurring through a downregulation of excitatory 5-HT_2A_ receptor signaling and a functional upregulation of inhibitory 5-HT receptors, including 5-HT_1A_ cortical heteroreceptors. One hypothesis is that this pattern may arise from a reduction in serotonergic stimulation related to the reduction in 5-HT neuronal excitability we have previously observed in chronic social isolation [31]. There is a considerable literature that would support a sensitization of the inhibitory 5-HT_1A_ receptor in response to reduced serotonergic stimulation [63–66], but it is less clear what this situation would predict for 5-HT_2A_ receptors [67–69]. Serotonin 5-HT_2A_ receptor dysregulation has been implicated in several neuropsychiatric diseases and modulation of the receptor function has been suggested to have numerous therapeutic implications, including improvement of depressive symptoms in treatment-resistant major depression [70]. Imaging and postmortem studies have shown some inconsistent differences in 5-HT_2A_ receptor levels in different brain regions in major depression [71–76]. Heterologous desensitization of 5-HT_2A_ receptors has also been demonstrated through tyrosine phosphorylation [77, 78], which may provide a link between the signal upregulation of 5-HT_1A_ receptors and downregulation of 5-HT_2A_ receptors through aberrant tyrosine phosphorylation [64, 78]. The last hypothesis is supported by the finding of significant upregulation of the expression of tyrosine kinase *ErbB4* in social isolation and its normalization upon Flx, as illustrated in Fig. 4h.

The effects of chronic Flx on 5-HT_2A_ receptor levels and function are also complicated. While chronic Flx has been shown to increase 5-HT_2A_ receptor levels, binding or function [79–81], nonselective 5-HT_2A_ receptor antagonists were suggested to enhance SSRI response in patients [82]. In our study, chronic Flx treatment in single-housed mice revealed a behavioral heterogeneity with a subset of mice showing greater anxiety-like behavior similar to the single-housed mice that did not receive Flx. Surprisingly, this anxious group displayed restored 5-HT_2A_ electrophysiological responses and a substantially-restored gene expression profile. These changes suggest that the anxiety effect of Flx does not reflect treatment resistance. Since cortical 5-HT_2A_ receptors play a key role in the behavioral expression of anxiety [83], this behavioral finding could be consistent with strong and prolonged serotonergic stimulation at the rescued 5-HT_2A_ receptors, since Flx would reduce local reuptake and alter autoinhibition of serotonergic neuron firing. Interestingly, Flx has previously been shown to lead to a better recovery from motor deficits when used as an add-on treatment in stroke patients [84], and to increase primary motor cortex excitability both in healthy volunteers [85] and rodents [86]. Long-term Flx treatment has been suggested to improve motor function by upregulating 5-HT_2A_ receptors [80, 87]. Supported by these studies, our data suggest that, up to a point, restoring 5-HT_2A_ receptor signaling may be specifically effective in improving psychomotor symptoms in major depression. This does not appear true for all 5-HT receptors [88].

In our previous study, which focused on gene expression profile changes in layer 5a S100a10 neurons of normal, healthy mice, we have shown that chronic fluoxetine treatment increases the mRNA expression of the 5-HT_4_ receptor [12]. In the current study, we were unable to detect electrophysiological changes in S100a10 neurons in response to activating or blocking the function of these receptors. 5-HT_4_ receptors have been previously shown to modulate neuronal excitability by affecting GABA-mediated inhibitory synaptic transmission in cortical pyramidal neurons [42]. However, we did not detect changes in the inhibitory synaptic transmission or GABA_A_-mediated currents in S100a10 neurons, ruling out the possibility of 5-HT_4_ receptor-mediated changes in GABA neurotransmission. It is important to note that Schmidt et al. examined the effects of fluoxetine in healthy, untreated mice, while the present study utilized a stress paradigm that dramatically impacted the 5-HT responses of the S100a10 cells. We did not detect a change in the mRNA levels of the 5-HT_4_ receptor in any of our experimental conditions. Our results rather indicated that 5-HT-induced excitatory effects were mediated via the 5-HT_2A_ receptor and could be completely blocked by the selective 5-HT_2A_ receptor antagonist. Yet, it is important to keep in mind that the lack of direct electrophysiological effects of 5-HT_4_ receptors in S100a10 neurons may be in part due to the trafficking of these receptors to the terminals [89].

The current lack of availability of a Cre driver line for specific genetic targeting of S100a10 neurons limits our ability to specifically manipulate the activity of layer 5a S100a10 neurons to further characterize their effects on behavior. Our current findings highlight the key role of the corticostriatal S100a10 neurons in the stress-induced effects on serotonin signaling and the restoration of these effects by chronic Flx treatment. These findings necessitate future optogenetic studies that will shed more light on the selective participation of these neurons during stress and antidepressant response. In lieu of optogenetic manipulation of S100a10 neurons, a pharmacological approach to target 5-HT_1A_ or 5-HT_2A_ receptors in vivo lacks the ability to test their cell-type-specific function and would involve manipulating a large population of neurons which also express these receptors. The development of additional tools that allow targeting the serotonergic function selectively in S100a10 neurons is required to further elucidate the direct role of 5-HT signaling in these cells in depressive-like behaviors.

Overall, our work describes the physiological and molecular markers of chronic stress and antidepressant treatment in a population of S100a10 neurons in the cerebral cortex. We have elucidated the distinctive serotonergic properties in the S100a10 neurons, their sensitivity to perturbation by chronic social isolation stress and restoration by antidepressant treatment. Strikingly, 5-HT_2A_ receptors in these neurons lose their excitatory power upon social isolation, which is restored upon chronic Flx treatment. Our cell-type-specific molecular analysis yields new insight into potential underlying mechanisms that may drive these changes through 5-HT_2A_ receptor signaling. We show that these neurons are positioned to play a key role in the psychomotor and somatosensory symptoms of depression, as well as in their response to treatment. Together, this study builds a framework to further understand the pathophysiology and treatment of depression and anxiety disorders.

## Materials and Methods

### Experimental animals

All experiments involving animals were performed in accordance with animal protocols approved by the University of Toronto and The Rockefeller University Institutional Animal Care and Use Committees. In all experiments, we used S100a10 bacTRAP transgenic mice (founder ES691) that express EGFPL10a fusion protein under the control of S100a10 regulatory elements as described previously [12]. Immediately after weaning (p21), a group of adult (>p70) male mice were individually housed for at least 7 weeks (single-housed mice; Sh). Group-housed littermates were used as controls (group-housed mice; Gh). Chronic antidepressant treatment was performed by administering 0.167 mg/ml of Fluoxetine (ANAWA) [12] in drinking water for 15–21 days. Subchronic antidepressant treatment was performed by administering the same amount of Fluoxetine for 4 days [90]. All mice were housed under a 12:12-h-light/dark cycle with *ad libitum* access to both food and water.

### Electrophysiology

Coronal cortical slices (400 μm) comprising the motor cortex and somatosensory areas were obtained using a Dosaka Linear slicer. Slicing was performed in ice cold oxygenated sucrose artificial cerebrospinal fluid (ACSF; 254 mM sucrose, 10 mM D-glucose, 24 mM NaHCO_3_, 2 mM CaCl_2_, 2 mM MgSO_4_, 3 mM KCl, 1.25 mM NaH_2_PO_4_, pH 7.4). Slices were recovered in oxygenated ACSF (128 mM NaCl, 10 mM D-glucose, 26 mM NaHCO_3_, 2 mM CaCl_2_, 2 mM MgSO_4_, 3 mM KCl, 1.25 mM NaH_2_PO_4_, pH 7.4) at 31–33 °C for at least 2 h. Recording was performed in ACSF oxygenated with 95% O_2_/5% CO_2_ and perfused at a rate of 3–4 ml/min at 31–33 °C. Internal solution for recording electrodes contained 5 mM KCl, 2 mM MgCl_2_, 4 mM K_2_-ATP, 0.4 mM Na_3_-GTP, 10 mM Na_2_-phosphocreatine, 10 mM HEPES buffer (pH 7.3). For spontaneous IPSC (sIPSC) recordings, patch electrode contained a high Cl^−^ patch solution (50 mM K-gluconate, 75 mM KCl, 2 mM MgCl_2_, 4 mM K_2_-ATP, 0.4 mM Na_3_-GTP, 10 mM Na_2_-phosphocreatine, 10 mM HEPES buffer, pH 7.3). S100a10-expressing pyramidal neurons in layer 5a were visualized with a fixed-staged microscope (Olympus BX50WI) and targeted based on the expression of EGFP. Whole-cell recordings were made in voltage-clamp or current-clamp mode with a Multiclamp 700B amplifier (molecular devices). All data were acquired at 20 kHz and low-pass filtered at 3 kHz using pClamp10.2 and Digidata1440 software.

Voltage-clamp recordings were performed at −75 mV. For illustrative purposes, voltage-clamp averaged recordings were obtained using Axograph software. 5-HT current amplitude responses were calculated by subtracting the current at the peak of the 5-HT response from the current at baseline. Current-clamp recordings were performed by injecting current into cells to maintain the cells close to a threshold of −65 mV. Peak 5-HT effect was determined by calculating the spike frequency over a 30-s period. To measure 5-HT and TCB-2 effects on spiking, 400 pA 500 ms depolarizing current injection was applied before and during drug applications. Input–output frequencies were obtained by counting the number of action potentials for each depolarizing current step of a protocol involving 50 pA 500 ms depolarizing current injections. The voltage sag ratio was quantified as the relative difference between the peak and the steady state membrane depolarizations in response to hyperpolarizing current injections [39]. Analysis of spontaneous EPSCs and IPSCs was performed from each 30 s block of 1 s sweeps using MiniAnalysis software (Synaptosoft Inc.).

### Pharmacology

5-HT responses were obtained by bath application of 10 μM 5-HT (Sigma) for 30 s. TCB-2 (1 μM, Tocris) was used as a selective agonist of 5-HT_2A_ receptors. MDL100907 (30 nM, Tocris) was applied in ACSF to block 5-HT_2A_ receptors. WAY100635 (30 nM, Sigma) was applied in bath to block 5-HT_1A_ receptors. BIMU 8 (2 μM, Tocris) was used as a selective agonist and GR113808 (200 nM, Tocris) was used as a selective antagonist of 5-HT_4_ receptors. sIPSC measurements were performed in the presence of AMPA/Kainate receptor antagonist 6-cyano-7-nitroquinoxaline-2,3-dione (CNQX; 20 μM, Tocris). Other synaptic blockers used were the NMDA receptor antagonist D-2-amino-5-phosphonovaleric acid (AP5; 50 μM, Tocris) and GABA_A_ receptor antagonist picrotoxin (100 μM, Tocris).

### Immunohistochemistry

To visualize S100a10 neurons, an adult male S100a10 bacTRAP transgenic mouse was perfused transcardially with saline followed by 4% paraformaldehyde (PFA)/0.1 M sodium phosphate buffer (PBS) under deep anesthesia. Brain was postfixed in 4% PFA overnight. Coronal sections (50 μM) were prepared by using the DSK vibratome. Free-floating sections were incubated with rabbit anti-GFP (1:500, Invitrogen), mouse anti-NeuN (1:500, Millipore) followed by goat anti-rabbit Alexa 488 (1:1000, Invitrogen), and goat anti-mouse Alexa 594 (1:1000, Invitrogen). For S100a10 staining, 40 µM sections through sensorimotor cortex were collected using a freezing microtome (Leica). Free-floating sections were incubated with chicken anti-EGFP (1:1000, Abcam) and goat anti-S100a10 (1:200, R&D Systems) followed by donkey anti-chicken Alexa 488 (1:2000, Invitrogen) and donkey anti-goat Alexa 568 (1:2000, Invitrogen). Images were obtained using either an epifluorescent microscope (Olympus BX50WI) or Zeiss LSM700.

### Behavior

#### Homecage recording

A group of S100a10 bacTRAP mice was observed in their homecage for 15 min prior to electrophysiological recordings. A transparent plexiglass sheet was used as a lid for the homecage. Video recording was performed with an upright camera. A polycarbonate round mouse igloo in the homecage provided a shelter. The time spent under the shelter zone was calculated by an observer blind to the different treatment groups.

#### Open field

Mice were habituated in the testing room in their home cages for 30 min and open-field behavior was analyzed in a square arena (50 × 50 × 22.5 cm) for 60 min. The measures were automatized using two rows of infrared photocells placed 20 and 50 mm above the floor, spaced 31 mm apart. Photocell beam interruptions were recorded on a computer using the superflex software (Accuscan Instruments). Total distance traveled was measured using the automated superflex software, from which the center–periphery ratio was calculated.

### Statistical analysis

For electrophysiological experiments, data are collected from at least three mice per group. The main findings of our study were replicated at least in three different experimental groups obtained at different times and from randomized litters. Data were analyzed using one-way ANOVA, two-way ANOVA, nonparametric, one-way Kruskal–Wallis ANOVA, Fisher’s exact test, paired two-tailed *t*-test, unpaired two-tailed *t*-test and nonparametric, two-tailed Mann–Whitney *t*-test. Newman–Keuls and Dunn’s post hoc tests were performed when appropriate. Analysis was performed using GraphPad Prism software. Data are expressed as mean ± S.E.M. Analysis was performed by experimenters blinded to the experimental groups.

### Cell-type-specific mRNA purification by translating ribosome affinity purification (TRAP)

The ribosome affinity purification and translated mRNA extractions were carried out as described [12, 91]. Three mice were pooled for each sample and four biological replicates were collected for each condition. Briefly, mice were rapidly decapitated and brains were removed. The cortical hemispheres were then dissected and the hippocampi and striata were removed from each hemisphere. Isolated cortices were rapidly dissected homogenized in extraction buffer containing 10 mM HEPES-KOH (pH 7.4), 150 mM KCl, 5 mM MgCl2, 0.5 mM DTT, 100 mg/ml cycloheximide, RNasin RNase inhibitors (Promega), and Complete-EDTA-free protease inhibitors (Roche), and cleared by centrifugation at 2000 *g*. IGEPAL CA-630 (NP-40; Sigma) and DHPC (Avanti Polar Lipids) were added to the supernatant at a final concentration of 1% for each, and the mixture was cleared by centrifugation at 20,000 *g*. Polysomes (ribosome-mRNA complexes) were immunoprecipitated with 100 mg monoclonal anti-EGFP antibodies (clones 19C8 and 19F7) bound to Protein G magnetic beads (Invitrogen), and washed with a high salt buffer containing 10 mM HEPES-KOH (pH 7.4), 350 mM KCl, 5 mM MgCl2, 1% IGEPAL CA-630, 0.5 mM DTT, 100 mg/ml cycloheximide, and RNasin RNase inhibitors (Promega). Bound mRNA was extracted, subjected to DNase digestion and purified using the RNeasy Plus Micro Kit (QIAGEN). RNA quality was analyzed on a 2100 Bioanalyzer (Agilent) using RNA Pico Chips (Agilent) to confirm RNA integrity, followed by the measurement of RNA concentrations using the Quant-iT RiboGreen RNA Assay Kit (Life Technologies). cDNAs were prepared with the Ovation RNA-Seq System V2 Kit (NuGEN), using an input of 2.5 ng RNA. 500 ng cDNA from each sample was fragmented on a Covaris S2 Focused Ultrasonicator using the operating conditions recommended by the manufacturer for a target fragment size of 250 bp. Fragment size was confirmed on a 2100 Bioanalyzer using High Sensitivity DNA Chips (Agilent). Libraries for RNA sequencing were prepared with the TruSeq RNA Sample Preparation v2 Kit (Illumina), using the manufacturer’s low-throughput protocol with the end-repair step. The concentration of the RNA-Seq libraries was determined on a 2100 Bioanalyzer using High Sensitivity DNA Chips. Subsequently, 16 samples were pooled for multiplexing with compatible adapters. After confirming the concentration of the multiplexed samples on an Agilent 2100 Bioanalyzer using High Sensitivity DNA Chips, samples were sequencing in two independent lanes on an Illumina NextSeq 500 sequencer using 75-bp paired-end sequencing.

### RNA-seq data analysis

The raw sequencing files in FASTQ format were aligned to the *Mus musculus* assembly 10 reference genome using TopHat version 2.0.11 [92] with the parameter of only one multiple hit allowed. The reference genome mm10 and genes gtf UCSC version file were downloaded from http://tophat.cbcb.umd.edu/igenomes.shtml. Cufflinks version 2.2.1 [93] was utilized to calculate the Fragments Per Kilobase of transcript per Million mapped reads for all genes in each sample. To analyze differential gene expression, edgeR version 3.8.6 was used [94]. *P* values were calculated and adjusted for multiple testing using the Benjamini–Hochberg procedure. Pathway analysis was performed using Ingenuity Pathway Analysis (Qiagen).

### 5-HT2A receptor pathway interaction analysis

In order to identify genes and corresponding pathways that may be mediating the relationship between the 5-HT_2A_ receptor and stress-affected Flx-normalized genes, we analyzed the functional interactions that connect them. After filtering the cerebral cortex functional network [95] to the top 1% of its edges, the edge weight was calculated as the reciprocal of its functional linkage score. As in [52], we calculated the betweenness centrality (BC) of every gene in between the 5-HT_2A_ receptor and stress-affected Flx-normalized genes using NetworkX [96, 97], where the betweenness centrality measure for gene g_i_ is:

$$BC\left( {g_i} \right) = \mathop {\sum }\limits_{s = 5{\mathrm{ - HT}}_{{\mathrm{2A}}},\,t \in T} \frac{{\sigma \left( {s,t|g_i} \right)}}{{\sigma \left( {s,t} \right)}},$$

where T is the set of stress-affected Flx-normalized genes, *σ*(*s*, *t*) is the number of shortest paths (weighted as described above) between s and t, and *σ*(*s*, *t*|*g*_*i*_) is the number of shortest paths between *s* and *t* that also pass through g_i_. To calculate the significance of these BC scores, we permuted the gene sets and calculated the fraction of times g_i_ had a BC score greater than or equal to its nonpermuted BC score (*n* = 100,000) and corrected for multiple hypothesis testing using the Benjamini–Hochberg correction. Genes with FDR ≤ 0.1 were considered likely potential mediators between the 5-HT_2A_ receptor and stress-affected Flx-normalized genes and were used as input into hypergeometric gene set enrichment to interpret the pathways that are overrepresented in the intermediate genes.

## Supplementary information

Legends for Supplemental Figures and Tables

Supplemental Figure S1

Supplemental Figure S2

Supplemental Figure S3

Supplemental Figure S4

Supplemental Figure S5

Supplemental Figure S6

Supplemental Figure S7

Supplemental Figure S8

Supplemental Figure S9

Supplemental Figure S10

Supplemental Figure S11

Supplemental Figure S12

Supplemental Table S1a

Supplemental Table S1b

Supplemental Table S1c

Supplemental Table S2a

Supplemental Table S2b

Supplemental Table S3

Supplemental Table S4
