# Supplementary material for: Mapping the physiological and molecular markers of stress and SSRI antidepressant treatment in S100a10 corticostriatal neurons
Source: Mol Psychiatry. 2019 Aug 20;25(5):1112–29. doi: 10.1038/s41380-019-0473-6 (PMC7031043; doi:10.1038/s41380-019-0473-6)
Supplement: Supplementary file 1 — Legends for Supplemental Figures and Tables [file 41380_2019_473_MOESM1_ESM.docx]

Supplementary Figure and Supplementary Table Legends

**Figure S1. 5-HT responses persisted in the presence of synaptic blockers.** (**a**) Representative voltage-clamp traces (left) from a Gh mouse showing 5-HT induced excitatory inward current before and after application of the AMPA/Kainate receptor antagonist CNQX, the NMDA receptor antagonist APV, and the GABA_A_ receptor antagonist picrotoxin. A representative current-clamp trace from a Gh mouse showing 5-HT elicited spiking in the presence of CNQX, APV and picrotoxin. (**b**) Representative voltage-clamp traces from a Sh mouse showing 5-HT induced inhibitory outward current before and after application of CNQX, APV and picrotoxin. (**c**) Representative voltage-clamp traces (left) from a Sh+Flx mouse showing 5-HT induced excitatory inward current before and after application of CNQX, APV and picrotoxin. A representative current-clamp trace (right) from a Sh+Flx mouse showing that 5-HT still elicited spiking in the presence of CNQX, APV and picrotoxin.

**Figure S2. Chronic Flx treatment is required to restore 5-HT responses after chronic social isolation.** Subchronic treatment with Flx for 4 days in single-housed mice was not sufficient to change the amplitude of (**a**) 5-HT inhibitory outward (Sh: n=8; Sh+subchronic Flx: n=11, nonparametric, two-tailed Mann-Whitney t-test, *p*>0.5) or (**b**) excitatory inward (Sh: n=8; Sh+subchronic Flx: n=11, nonparametric, two-tailed Mann-Whitney t-test, *p*>0.05) currents.

**Figure S3. 5-HT current responses are unchanged in GFP-negative (GFP-) neurons from Gh, Sh and Sh+Flx mice.** The amplitude of the 5-HT inhibitory currents (**a**) and excitatory currents (**b**) is similar between GFP- neurons of Gh (n=12), Sh (n=13), and Sh+Flx (n=17) mice (one-way ANOVA: inhibitory currents, F_(2,39)_ = 0.044, *p*>0.05; excitatory currents, nonparametric, one-way Kruskal-Wallis ANOVA, H_(2)_ = 1.68, *p*>0.05). The intrinsic membrane properties including the membrane capacitance Cm (pF) (**c**), resting membrane potential Vm (mV) (**d**), spike amplitude (mV) (**e**), spike threshold (mV) (**f**), and input resistance R_input_ (MΩ) (**g**) are comparable between the GFP- neurons of Gh, Sh and Sh+Flx mice (one-way ANOVA: *p*’s > 0.05).

**Figure S4. Chronic Flx treatment does not affect 5-HT current responses in GFP+ neurons from Gh mice.** The amplitude of 5-HT inhibitory outward (**a**) and excitatory inward currents (**b**) are comparable between S100a10 neurons of Gh mice (n=17) and Gh mice treated with chronic Flx (n=31) (nonparametric, two-tailed Mann-Whitney t-test: inhibitory currents, *p*>0.05; excitatory currents, *p*>0.05). The intrinsic membrane properties including the membrane capacitance Cm (pF) (**c**), resting membrane potential Vm (mV) (**d**), spike amplitude (mV) (**e**), spike threshold (mV) (**f**), and input resistance R_input_ (MΩ) (**g**) are not different between the S100a10 neurons of Gh and Gh+Flx mice (unpaired t-test; *p*’s > 0.05).

**Figure S5. No difference was observed in spontaneous excitatory or inhibitory synaptic activity or tonic GABA_A_ response in control, stress conditions and after Flx treatment.** (**a**) Representative voltage-clamp traces showing spontaneous excitatory synaptic currents in S100a10 neurons from a Gh (n=20), Sh (n=20) and Sh+Flx (n=25) mouse. Cumulative fraction plots of sEPSC amplitude (**b**) and sEPSC inter-event intervals (**c**) are shown. No significant difference was observed in the sEPSC amplitude or frequency between different groups (inset) (nonparametric, one-way Kruskal-Wallis ANOVA: sEPSC amplitude, H_(2)_ = 3.11, *p*>0.05; sEPSC frequency, H_(2)_ = 2.14, *p*>0.05). (**d**) Representative voltage-clamp traces showing spontaneous inhibitory synaptic activity in S100a10 neurons from a Gh (n=9), Sh (n=6) and Sh+Flx (n=4) mouse. Cumulative fraction plots of sIPSC amplitude (**e**) and sIPSC inter-event intervals (**f**) are shown. There were no significant differences in the sIPSC amplitude or frequency between different groups (inset) (one-way ANOVA: sIPSC amplitude, F_(2,16)_ = 0.22, *p*>0.05; sEPSC frequency, F_(2,16)_ = 0.62, *p*>0.05). (**g**) Application of the GABA_A_ receptor agonist muscimol produced an outward inhibitory current that was comparable between S100a10 neurons of Gh (n=15-17), Sh (n=11-12) and Sh+Flx (n=19-21) mice (one-way ANOVA: 1μM muscimol, F_(2,42)_ = 2.16, *p*>0.05; 3μM muscimol, F_(2,47)_ = 1.77, *p*>0.05).

**Figure S6. Effects of Flx treatment on intrinsic membrane properties and excitability in S100a10 neurons.** (**a**) Membrane capacitance Cm (pF) is similar between S100a10 neurons of Gh (n=34), Sh (n=38) and Sh+Flx (n=44) mice. (**b**) Resting membrane potential Vm (mV) is depolarized in S100a10 neurons of Sh+Flx mice compared to those of Gh and Sh mice (nonparametric, one-way Kruskal-Wallis ANOVA, H_(2)_ = 29.84, *p*<0.01, Dunn’s posthoc test, ***p*<0.01). Input resistance R_input_ (MΩ) (**c**) and spike amplitude (mV) (**d**) of S100a10 neurons are similar between all the groups. (**e**) Spike threshold (mV) of S100a10 neurons of Sh+Flx mice is smaller compared to neurons of Gh and Sh mice (one-way ANOVA, F_(2, 113)_ = 3.34, *p*<0.05, Newman-Keuls posthoc test, p>0.05; *a priori* t-test between Gh and Sh+Flx, ^#^*p* = 0.03, Sh and Sh+Flx, ^#^*p* = 0.03). (**f**) S100a10 neurons in Sh mice fire reduced number of action potentials compared to those of Gh mice. Flx treatment enhances the excitability in both groups (two-way ANOVA: Gh vs Sh, F_(1,649)_ = 9.14, ***p*<0.01; Gh vs Sh+Flx, F_(1,726)_ = 35.31, ***p*<0.01, Sh vs Sh+Flx, F_(1,693)_ = 72.28, ***p*<0.01).

**Figure S7. Serotonin 5-HT_2A_ receptors are more than two-fold enriched in S100a10 cells.** The gene expression profile of S100a10 cells obtained by the translating ribosome affinity purification (TRAP) approach is compared to a whole cortex input for several proteins, including S100a10, cell-type markers, and key 5-HT receptors. This analysis revealed that mRNA encoding 5-HT_2A_ receptors (*Htr2a*) are greater than 2-fold enriched in these cells compared to the rest of cortex, while 5-HT_1A_ receptors (*Htr1a*) are expressed at similar levels.

**Figure S8. 5-HT_4_ receptors do not contribute to the 5-HT mediated excitation in S100a10 neurons.** (**a**) Representative voltage-clamp traces from a Gh mouse in response to the bath application of the selective 5-HT_4_ receptor agonist BIMU 8 before and after application of the 5-HT_4_ receptor antagonist GR113808. (**b**) A representative current-clamp trace from a Gh mouse showing that 5-HT still elicited spiking in the presence of the 5-HT_4_ receptor antagonist GR113808. (**c**) Quantification of spike frequency in response to 5-HT in S100a10 neurons in the absence and presence of the 5-HT_4_ receptor antagonist GR113808 (n=4; paired t-test, *p*>0.05).

**Figure S9. Distance traveled in the open field.** (**a**) Sh mice (n=12) traveled greater distance in the open field over 60 minutes when compared with Gh mice (n=11) (two-way ANOVA, F_(1,252)_ = 17.18, *p* < 0.01). Distance traveled in the open field did not differ between (**b**) Gh (n=11) and Sh+Flx (n=11) mice (two-way ANOVA, F_(1,240)_ = 1.8, *p* > 0.05) and between (**c**) Sh+Flx (n=11) and Sh+Flx (n=13) anxious mice (two-way ANOVA, F_(1,264)_ = 0.08, *p* > 0.05).

**Figure S10.** Venn diagram showing the comparison between Sh versus Sh+Flx genes and Sh versus Sh+Flx anxious genes (Gene list based on Supplementary Tables S2a and S2b). Both groups show robust response to Flx treatment and no major difference between the groups was observed.

**Figure S11.** Key genes in the 5-HTR_2A_ network regulated in opposite directions by single housing (Sh) and by chronic fluoxetine (Flx).

**Figure S12.** Motor and sensory phenotype pathways altered by chronic social isolation (Sh) and restored by chronic fluoxetine (Flx).

Supplementary Table Legends:

**Tables S1a, S1b, S1c.** Gene lists representing the differentially expressed genes between the groups: Gh versus Sh, Gh versus Sh+Flx, Gh versus Sh+Flx anxious.

**Tables S2a, S2b.** Genelist representing the differentially expressed genes between the groups: Sh versus Sh+Flx, Sh versus Sh+Flx anxious.

**Table S3.** List of genes affected by single housing and normalized by Flx.

**Table S4.** List of genes and the pathways linking Flx-normalized genes to 5-HTR_2A_ signaling.
