# Supplementary figures and images for: Mapping the physiological and molecular markers of stress and SSRI antidepressant treatment in S100a10 corticostriatal neurons
Source: Mol Psychiatry. 2019 Aug 20;25(5):1112–29. doi: 10.1038/s41380-019-0473-6 (PMC7031043; doi:10.1038/s41380-019-0473-6)

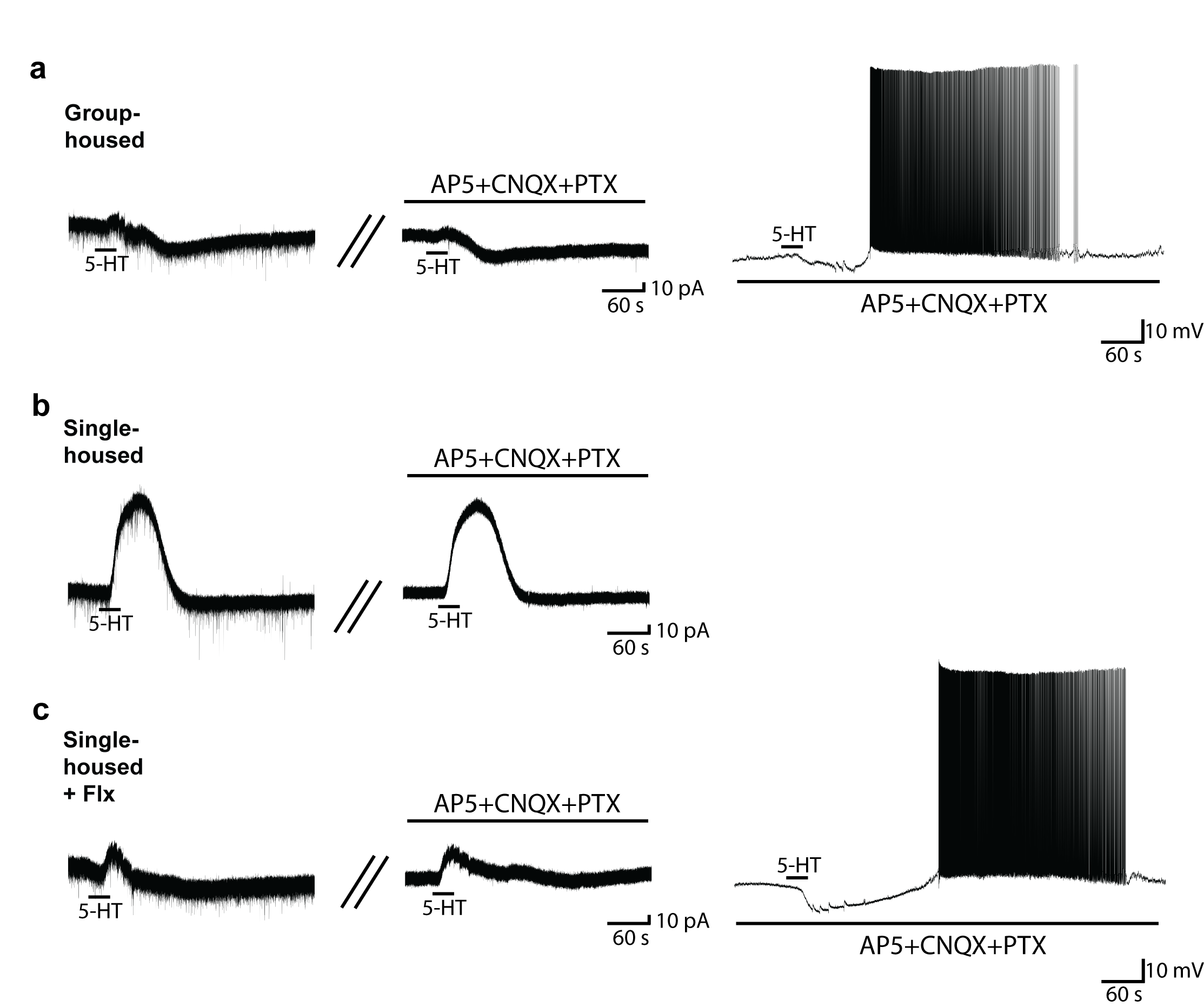

Supplement: Supplementary file 2 — Supplemental Figure S1 [file 41380_2019_473_MOESM2_ESM.tif]

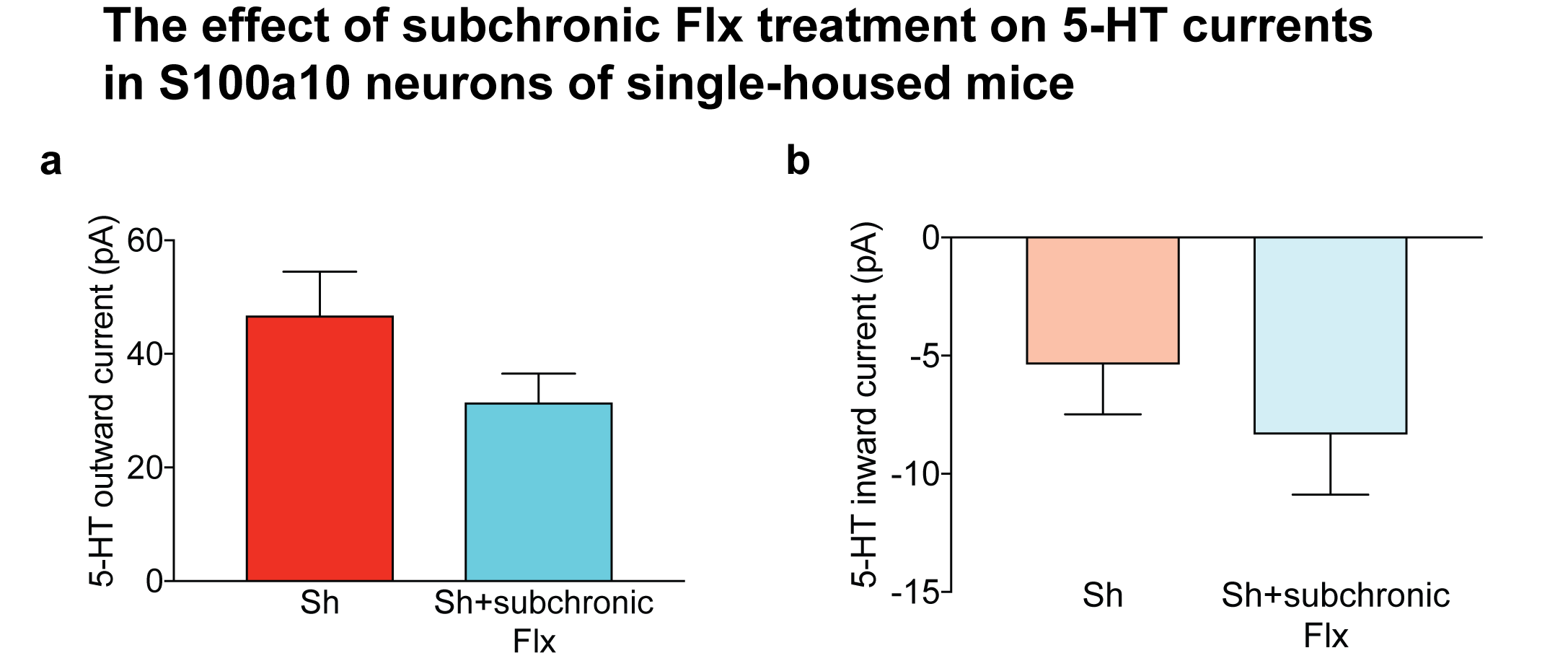

Supplement: Supplementary file 3 — Supplemental Figure S2 [file 41380_2019_473_MOESM3_ESM.tif]

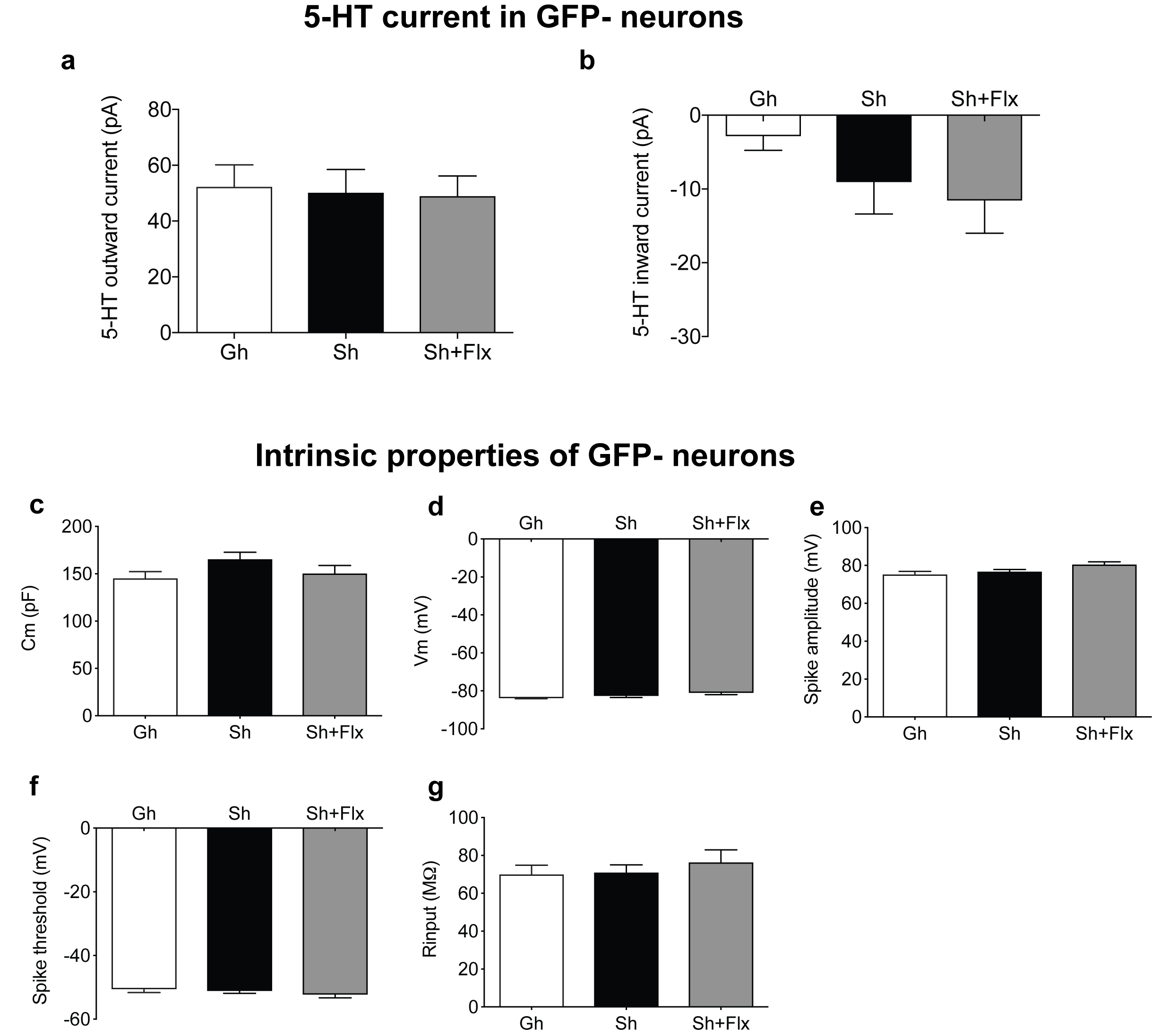

Supplement: Supplementary file 4 — Supplemental Figure S3 [file 41380_2019_473_MOESM4_ESM.tif]

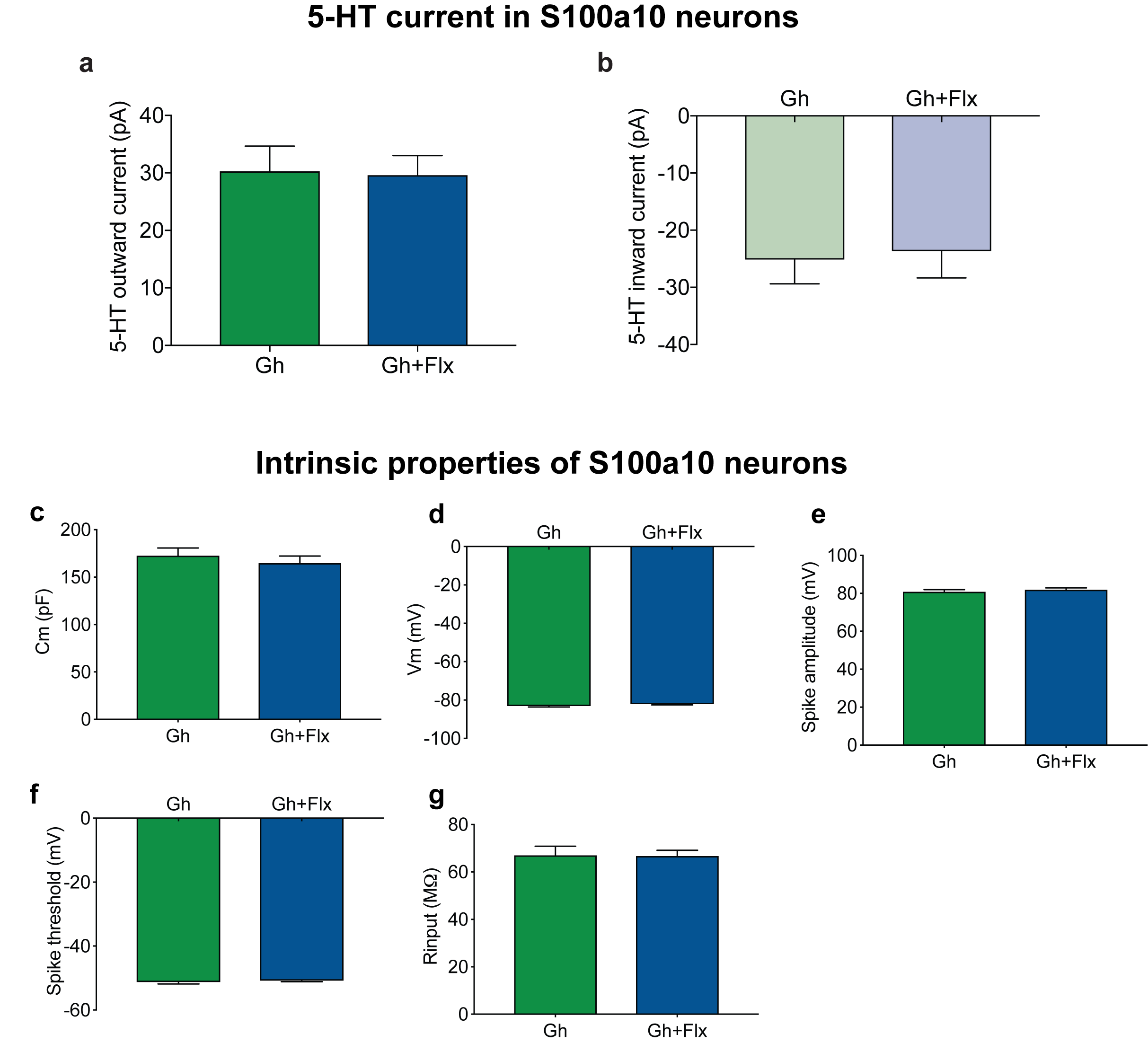

Supplement: Supplementary file 5 — Supplemental Figure S4 [file 41380_2019_473_MOESM5_ESM.tif]

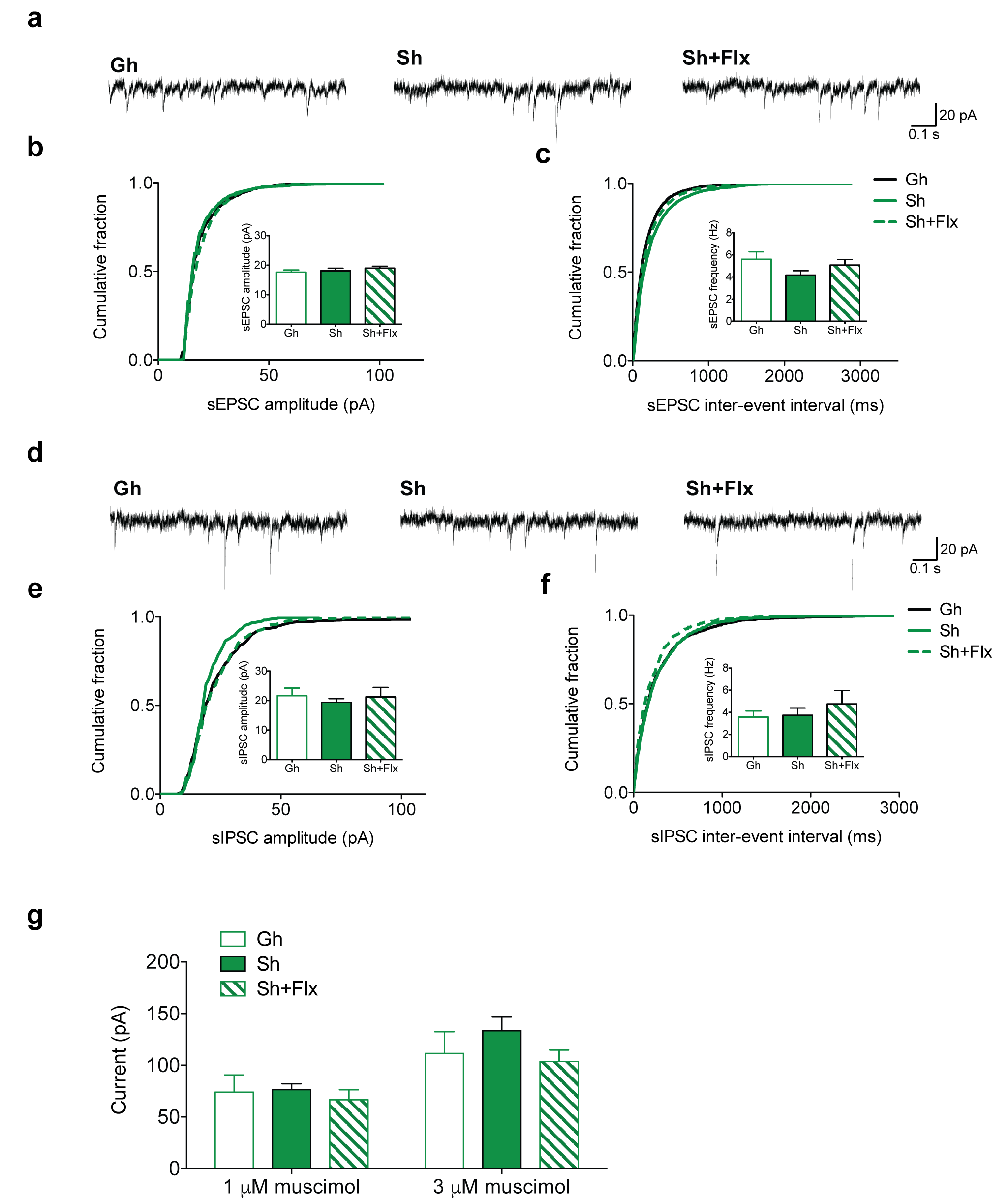

Supplement: Supplementary file 6 — Supplemental Figure S5 [file 41380_2019_473_MOESM6_ESM.tif]

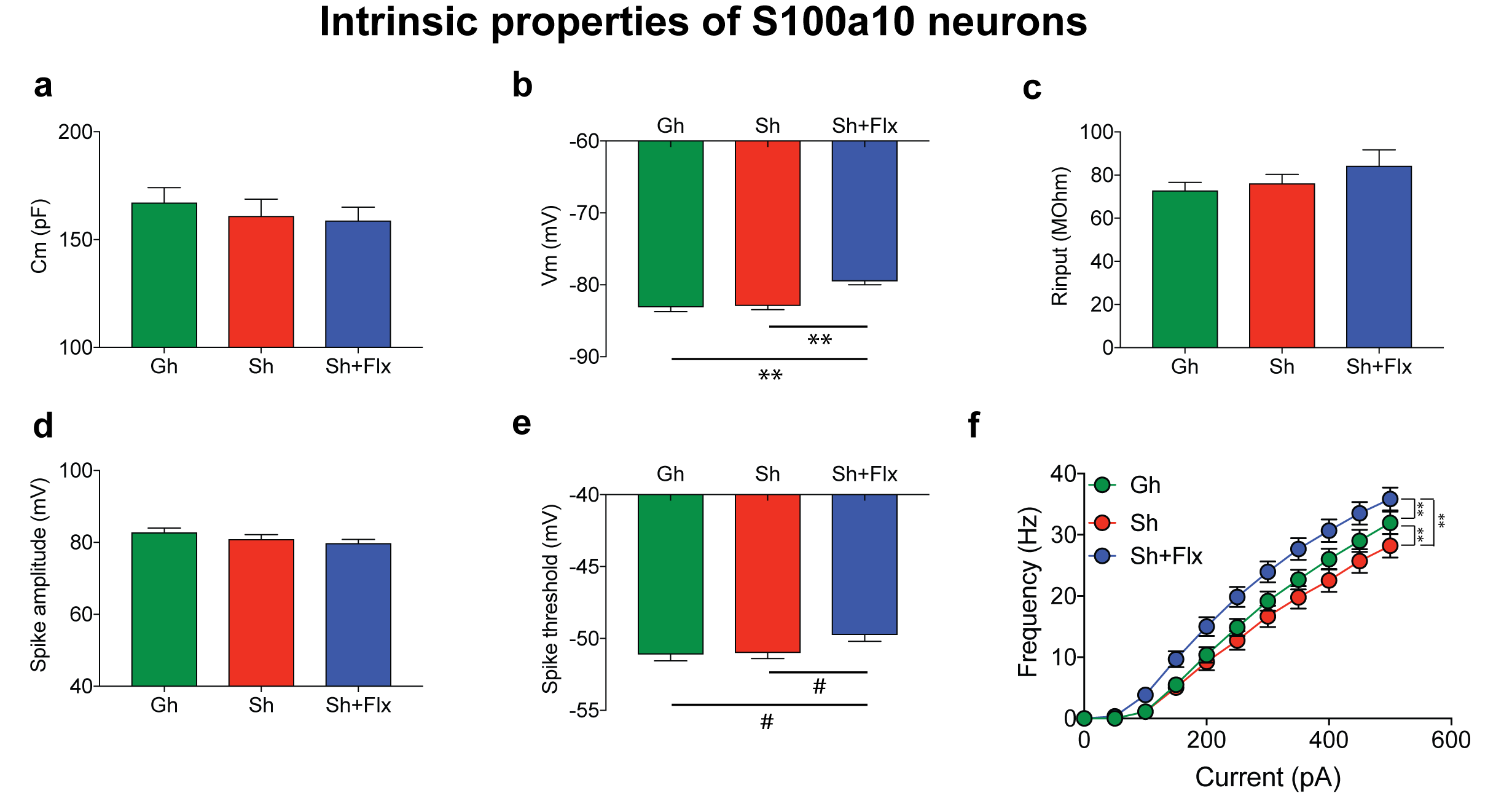

Supplement: Supplementary file 7 — Supplemental Figure S6 [file 41380_2019_473_MOESM7_ESM.tif]

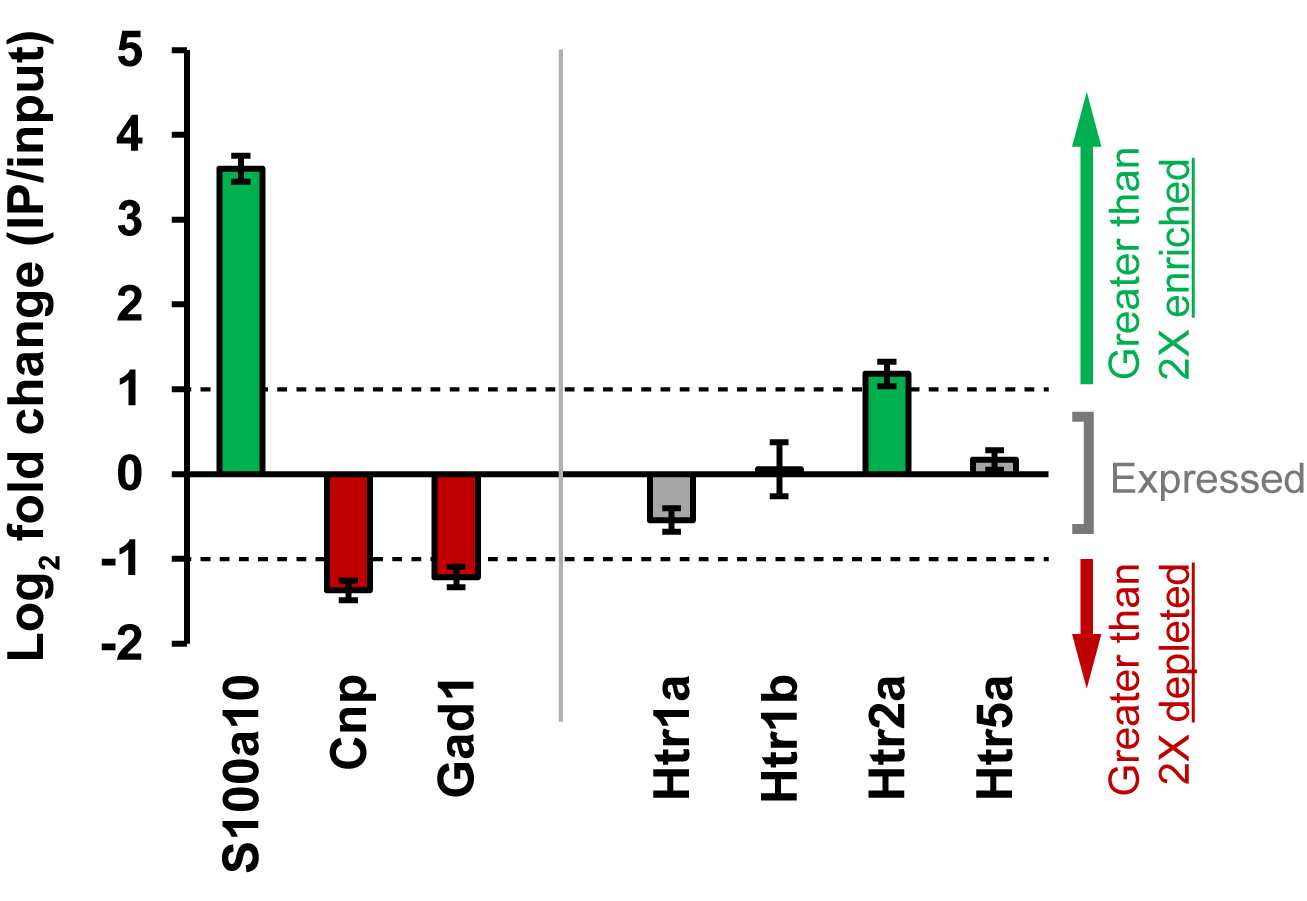

Supplement: Supplementary file 8 — Supplemental Figure S7 [file 41380_2019_473_MOESM8_ESM.tif]

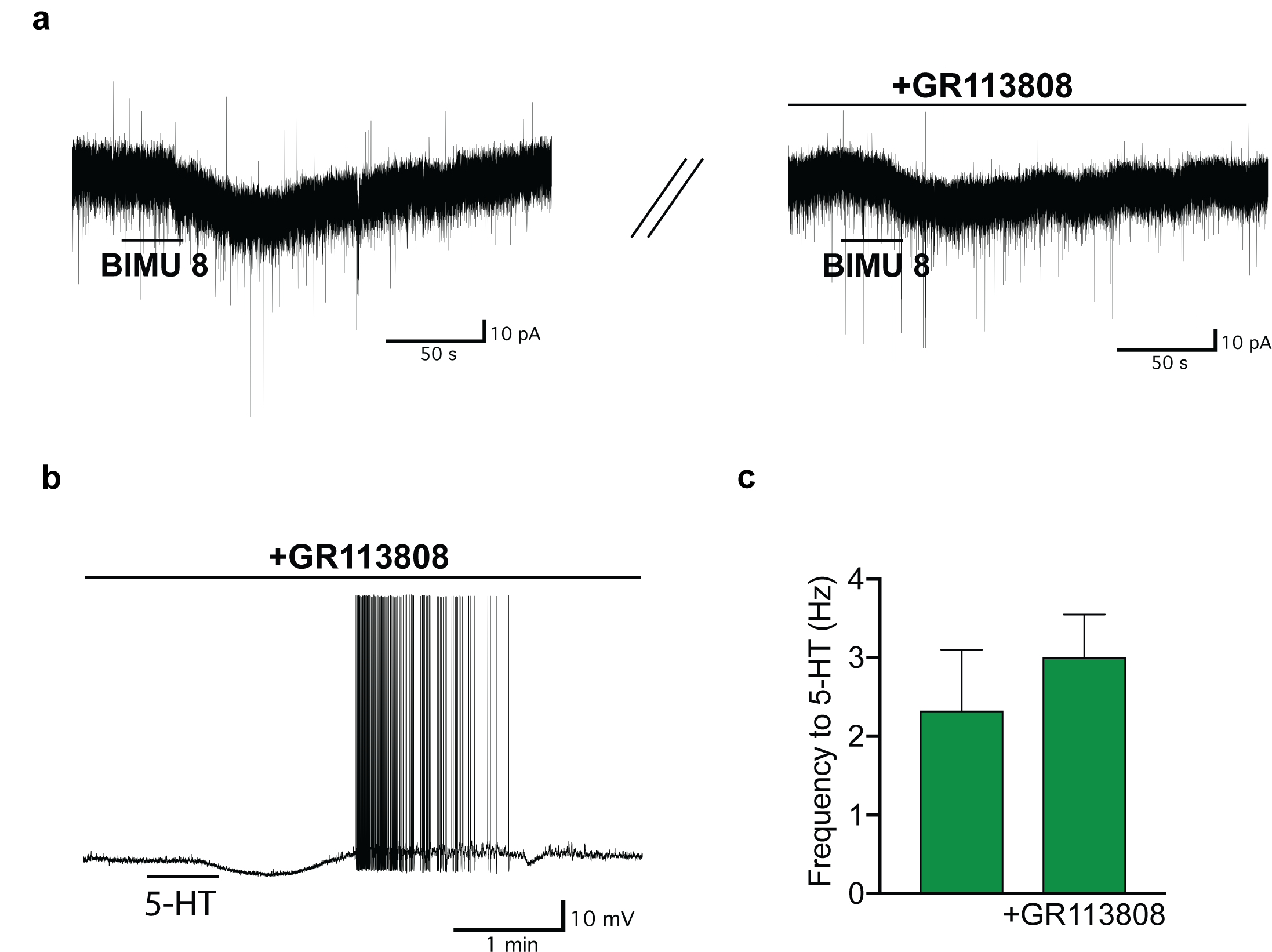

Supplement: Supplementary file 9 — Supplemental Figure S8 [file 41380_2019_473_MOESM9_ESM.tif]

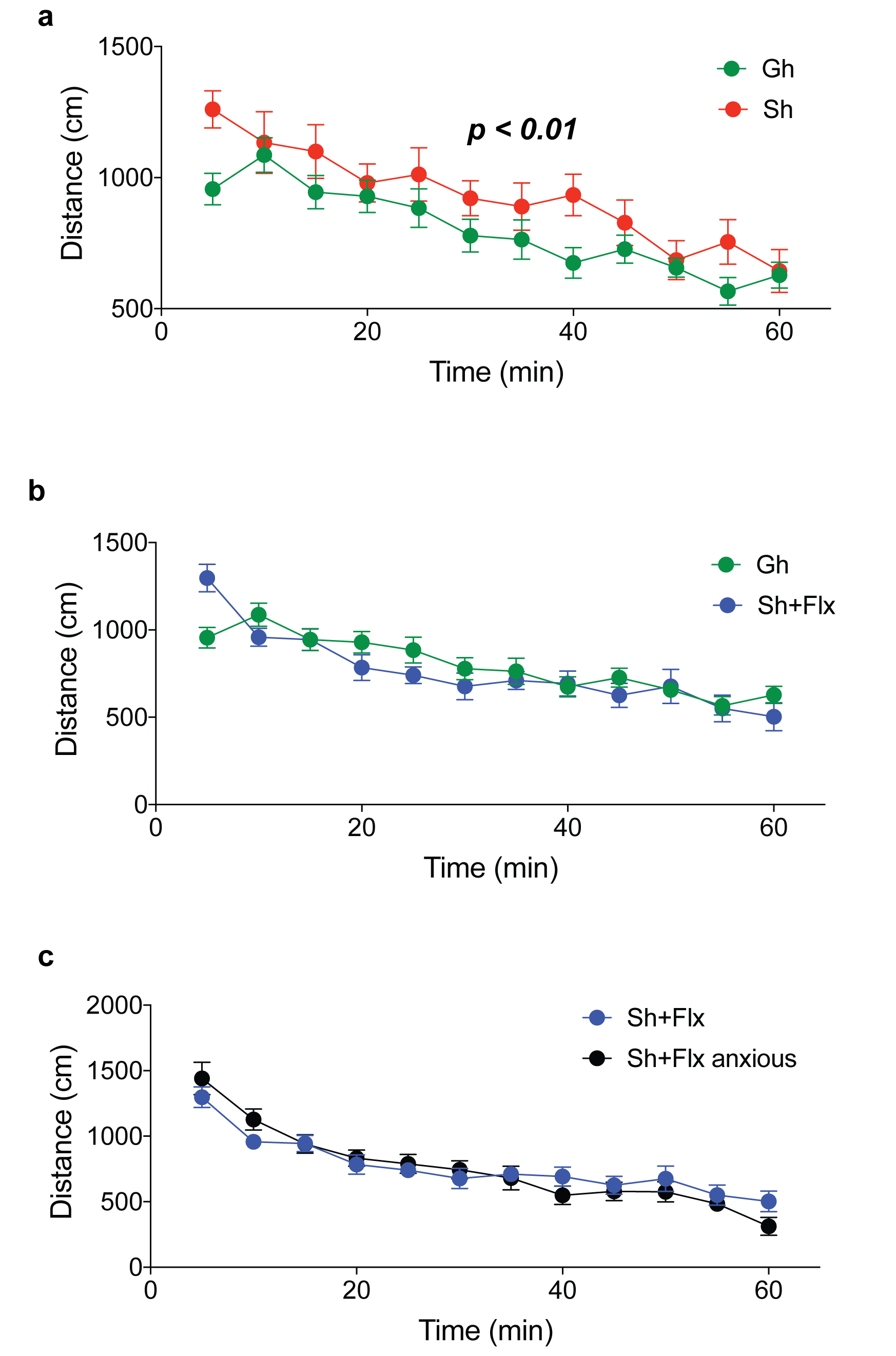

Supplement: Supplementary file 10 — Supplemental Figure S9 [file 41380_2019_473_MOESM10_ESM.tif]

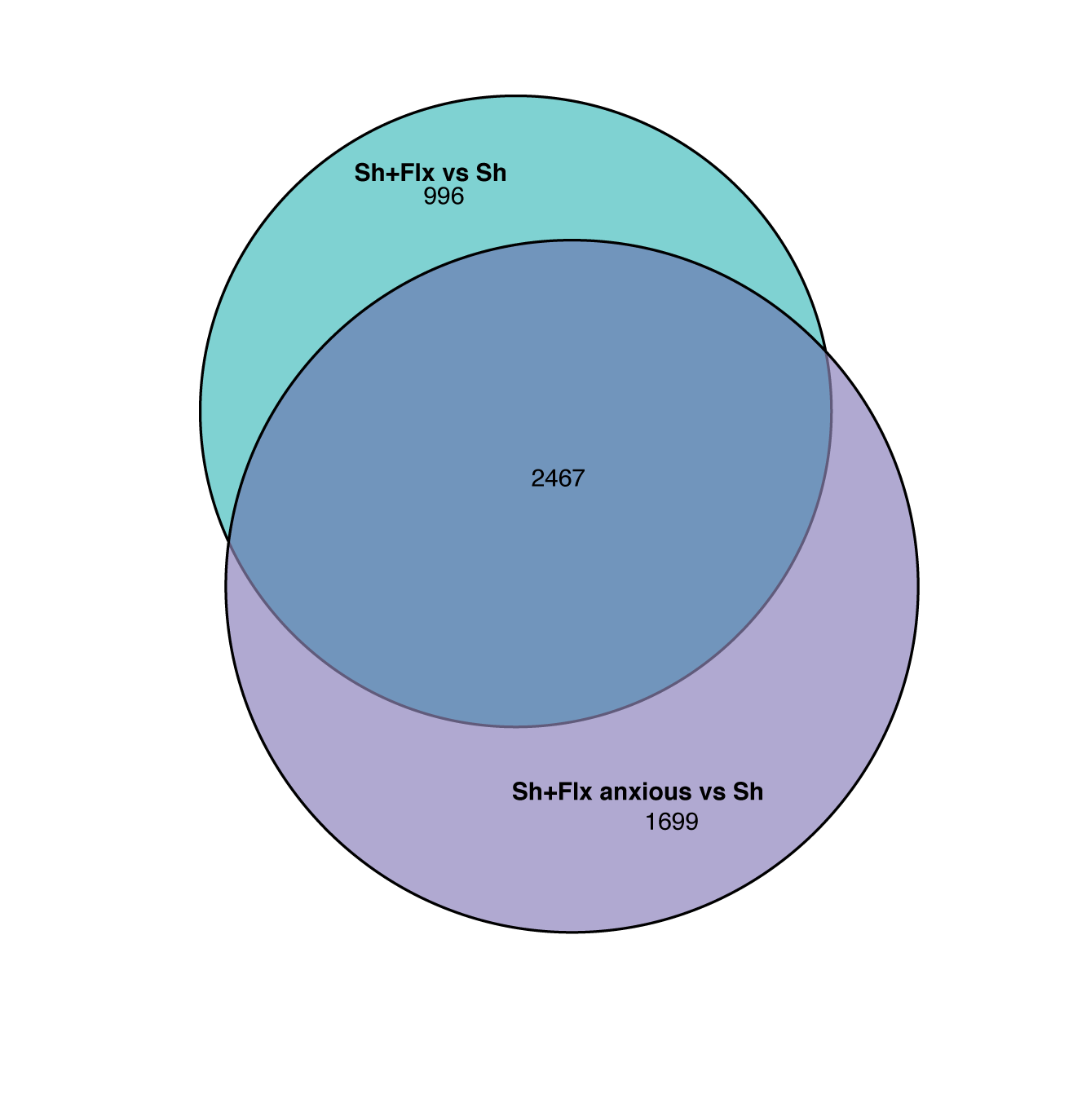

Supplement: Supplementary file 11 — Supplemental Figure S10 [file 41380_2019_473_MOESM11_ESM.tif]

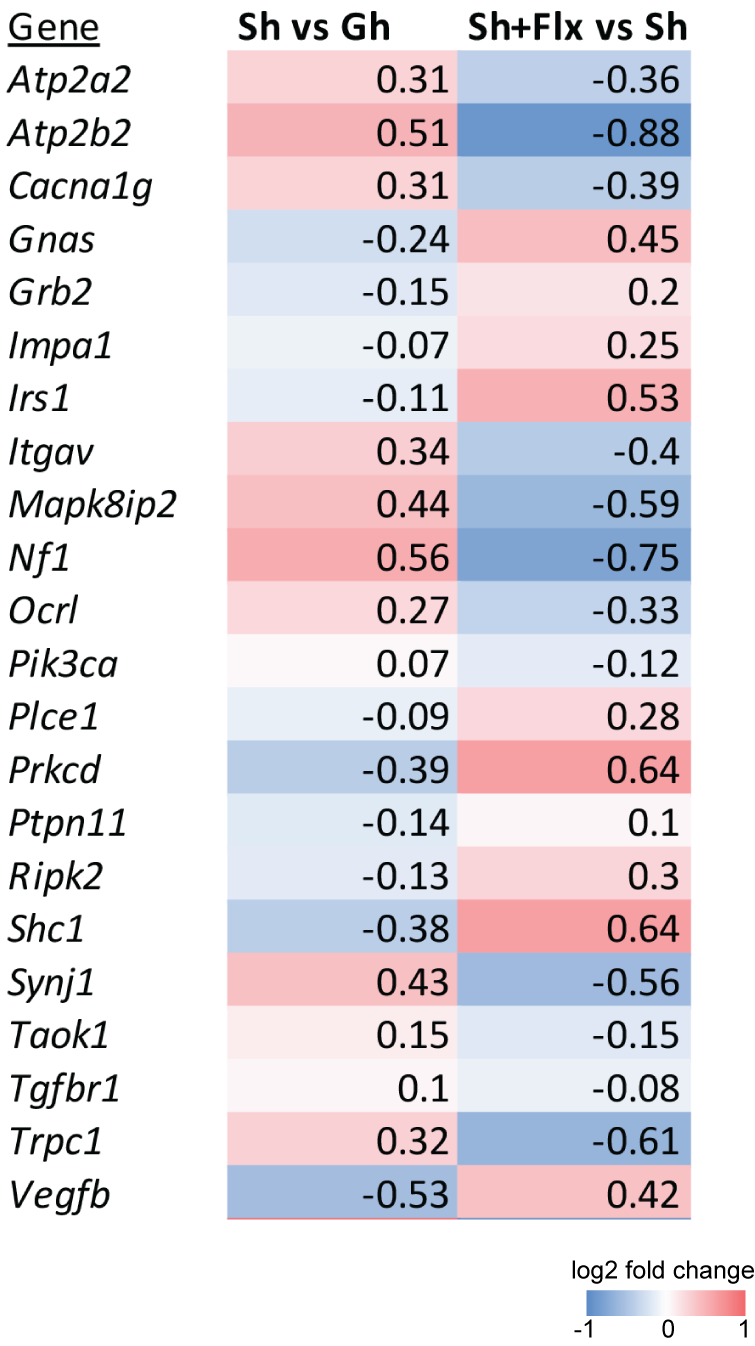

Supplement: Supplementary file 12 — Supplemental Figure S11 [file 41380_2019_473_MOESM12_ESM.tif]

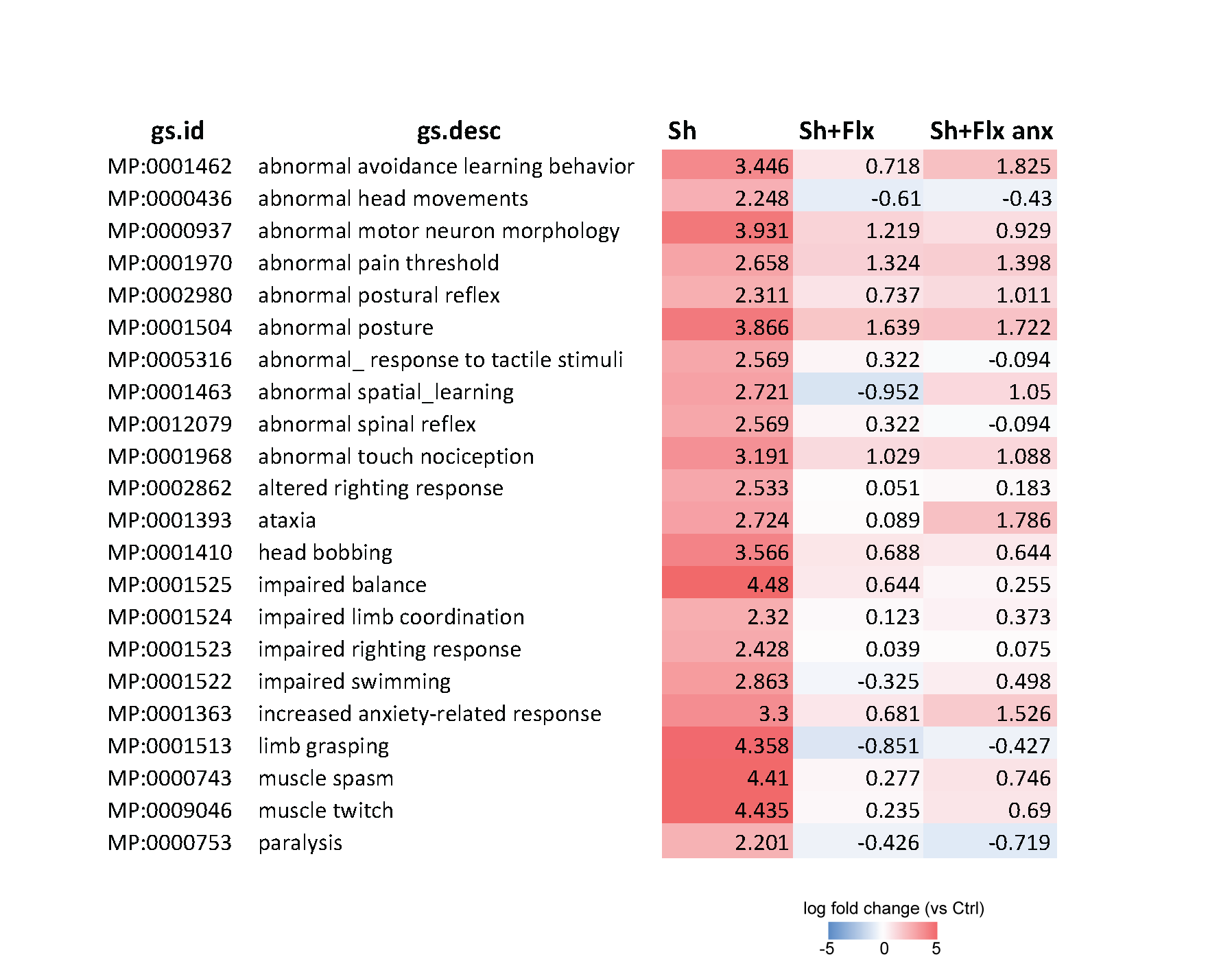

Supplement: Supplementary file 13 — Supplemental Figure S12 [file 41380_2019_473_MOESM13_ESM.tif]
