# Supplementary material for: Mapping the physiological and molecular markers of stress and SSRI antidepressant treatment in S100a10 corticostriatal neurons
Source: Mol Psychiatry. 2019 Aug 20;25(5):1112–29. doi: 10.1038/s41380-019-0473-6 (PMC7031043; doi:10.1038/s41380-019-0473-6)
Supplement: Supplementary file 14 — Supplemental Table S1a [file 41380_2019_473_MOESM14_ESM.pdf]

Suppl Table S1a: Genelist showing the differentially expressed genes (551) between the Gh and Sh groups, in the context of all the genes examined.

symbol	logFC	logCPM	F	PValue	FDR
Prl	11.4991035	1.48097504	51.5421186	1.14E-06	0.00751701
Nrbf2	-1.0194136	4.25797767	45.3361516	1.53E-06	0.00751701
Zfp873	-1.228889	3.36059903	39.803665	3.78E-06	0.01232947
Gm561	-1.1390874	3.43496853	36.7015852	6.50E-06	0.01591918
Cox5b	-0.9156881	6.31383891	33.3118226	1.22E-05	0.01806353
Lyrm7	-0.8740268	4.50608836	32.4871381	1.43E-05	0.01806353
6330403K07I	-0.6668451	7.67692203	32.0586375	1.56E-05	0.01806353
Pik3c2b	0.89824424	5.11271293	31.9132733	1.60E-05	0.01806353
Gng3	-0.7274574	8.0666176	31.0362622	1.91E-05	0.01806353
Dynlrb2	-2.8431383	0.69709176	30.2975397	2.22E-05	0.01806353
Snx2	-0.6503272	7.57439358	30.1137577	2.30E-05	0.01806353
1110059E24I	-0.6868977	5.63787582	30.0113444	2.35E-05	0.01806353
Gm14295	-0.604226	5.87168386	29.7995382	2.45E-05	0.01806353
Stmn1	-0.7219354	8.45410907	29.4248207	2.65E-05	0.01806353
Habp4	-0.5382646	7.39970962	29.1737668	2.79E-05	0.01806353
Grin1os	3.00906483	0.24889645	28.7378341	3.06E-05	0.01806353
Rasl10a	1.04020359	3.35772303	28.625639	3.13E-05	0.01806353
Nrgn	-0.6920253	7.23116509	28.3362662	3.33E-05	0.01813912
Thap11	-0.8072082	4.58550204	27.4801731	4.01E-05	0.01990873
Snca	-0.6074742	7.68402649	27.3417334	4.13E-05	0.01990873
Mtap7d3	-2.2519701	1.90900847	27.0330471	4.42E-05	0.01990873
Fam160b2	0.72433363	6.58432787	26.9752111	4.47E-05	0.01990873
Mllt11	-0.5580847	7.83720873	26.2961299	5.19E-05	0.02212352
BC048546	-0.8582245	5.10503088	25.7827513	5.83E-05	0.02302511
Coa3	-0.831825	4.39961295	25.7453302	5.88E-05	0.02302511
Oxt	-3.3594105	0.36731434	25.0257876	6.92E-05	0.02442513
Nrn1	-0.6507042	7.30682903	24.9991369	6.96E-05	0.02442513
Pitpnm2os1	4.55249192	-0.1977303	24.8702785	7.17E-05	0.02442513
0610009L18I	-1.9546802	0.8437656	24.7192075	7.43E-05	0.02442513
Skap2	-0.9389037	4.03246123	24.6521026	7.54E-05	0.02442513
1700028P14I	-2.2981771	0.53112999	24.5456314	7.73E-05	0.02442513
Med23	0.7830807	4.19630727	24.3226735	8.14E-05	0.02473279
Mrps33	-0.5963263	6.8965535	24.2218665	8.33E-05	0.02473279
Pin4	-0.8256939	4.63656401	24.049778	8.68E-05	0.02473279
Plxnd1	0.82973425	4.68512081	23.9719025	8.84E-05	0.02473279
Cytip	-1.6099717	2.74174815	23.7675808	9.27E-05	0.02488713
Tnnt2	-1.2156031	4.613484	23.7097517	9.40E-05	0.02488713
Stx1a	-0.8412501	5.57076194	23.3239782	0.00010303	0.02598569
Ppp2r2cos	3.09993696	0.74501721	23.2700794	0.00010436	0.02598569
Orc1	1.99372294	1.46819534	23.2005465	0.00010611	0.02598569
Adam10	0.67115214	5.75542415	22.6445256	0.00012134	0.02715085
Sh3bgr	-7.0963152	-0.4642722	22.4644497	0.00012677	0.02715085

Aspg	2.28588425	0.89639221	22.4035159	0.00012867	0.02715085
Efhd2	-0.6414041	6.73441745	22.368153	0.00012979	0.02715085
Ptchd4	0.97218183	4.03406526	22.3147635	0.00013149	0.02715085
AA413626	6.64232586	-0.6663741	22.3052539	0.0001318	0.02715085
4931430N09	3.14413134	1.7189984	22.1235446	0.00013781	0.02715085
Msl2	0.61983594	7.27702736	22.0748033	0.00013947	0.02715085
Sema4g	1.42739591	2.77395805	22.0496275	0.00014034	0.02715085
Bzw2	0.90802642	3.56638894	22.0175264	0.00014145	0.02715085
Erbb4	0.94033615	4.58491755	21.8361006	0.00014794	0.02715085
Medag	-0.5438921	6.21870146	21.8209005	0.0001485	0.02715085
Ndufa6	-0.7197462	5.13130613	21.7283965	0.00015195	0.02715085
Med24	0.73745712	4.25752126	21.7257101	0.00015205	0.02715085
Bmyc	-0.8028229	5.26490475	21.711949	0.00015257	0.02715085
Gm5464	2.9418034	0.45420489	21.6427222	0.00015522	0.02715085
Pnldc1	4.14453944	-0.2701485	21.4966805	0.00016098	0.02766425
Phex	1.94225174	1.95507507	21.3032435	0.00016898	0.02853745
Bloc1s3	-1.0694409	3.16661035	21.11451	0.0001772	0.02934483
Slc35b1	-0.8829638	3.70442567	21.0579958	0.00017975	0.02934483
Gpr155	0.81975592	5.73513975	20.8731246	0.00018837	0.03000085
Nfrkb	0.79285686	4.24815793	20.8414469	0.00018989	0.03000085
Cycs	-0.6836191	8.2438788	20.5182533	0.00020623	0.03159973
Adamts3	0.99609591	4.12886583	20.5137631	0.00020646	0.03159973
Katnbl1	0.95078167	4.70717542	20.4266501	0.00021113	0.03181731
Camk2n1	-0.5217018	10.5133201	20.3264573	0.00021665	0.03199712
Sfi1	1.4113135	3.39486103	20.2870879	0.00021886	0.03199712
Gosr2	-0.5239677	6.62556428	20.2119512	0.00022315	0.03214433
Pkd2l1	6.5080441	-1.3225536	20.1186067	0.00022861	0.0324532
Gabrq	1.5203003	1.88100598	19.9322771	0.00023995	0.03357639
Rilpl2	-1.2451924	3.19208681	19.6413345	0.00025891	0.03381995
Tnrc18	0.57848884	5.12993566	19.6271387	0.00025988	0.03381995
Vwa5b2	1.30111528	3.81022683	19.5558448	0.0002648	0.03381995
Col11a1	1.08809747	3.3666283	19.5185061	0.00026741	0.03381995
Emd	-0.6418869	5.92569873	19.4541757	0.00027199	0.03381995
Pcp4	-0.5701545	8.83186666	19.4281916	0.00027386	0.03381995
Gjd2	1.50978522	1.72208142	19.3843449	0.00027705	0.03381995
Strn	0.47054738	6.48418196	19.1909222	0.00029163	0.03381995
Me3	1.06181957	4.21230339	19.1697035	0.00029328	0.03381995
Erh	-0.8591701	5.18294234	19.1597423	0.00029406	0.03381995
Gldc	1.16158155	2.46772824	19.1026673	0.00029856	0.03381995
Bora	1.86274343	1.55862434	19.1017083	0.00029864	0.03381995
Mapre3	-0.5635647	7.32864478	19.0938403	0.00029926	0.03381995
2010015L04F	1.03398217	3.55873562	19.0926516	0.00029936	0.03381995
Gm20748	-1.8853942	1.58141484	19.0807539	0.00030031	0.03381995

Ryr1	1.53349134	2.63715206	19.0630367	0.00030173	0.03381995
Gm16039	-0.6305285	4.51962695	19.0556036	0.00030233	0.03381995
AB041803	1.92565748	2.42440817	19.0369947	0.00030383	0.03381995
Zmym3	0.75803641	6.02195042	18.8964492	0.00031547	0.03417929
Ralyl	-0.5145798	7.08495102	18.8868826	0.00031628	0.03417929
Tagln3	-0.6819764	6.38412678	18.853084	0.00031916	0.03417929
Filip1l	-0.5592096	5.30520595	18.8314684	0.00032102	0.03417929
Abca5	0.85666423	5.65973686	18.7458056	0.0003285	0.03427559
Rinl	-1.8728137	1.37320554	18.6770507	0.00033464	0.03427559
Dtl	-0.8103598	4.00306842	18.6572871	0.00033643	0.03427559
E330023G01	1.77671546	1.42806035	18.6380632	0.00033818	0.03427559
Pkd1	0.85771752	5.17481873	18.613914	0.00034039	0.03427559
Mrpl12	-1.0375173	3.85456759	18.5694942	0.0003445	0.03427559
Syndig1l	1.3187646	2.34801023	18.4881279	0.00035217	0.03427559
Vti1b	-0.6307287	5.76983741	18.4396658	0.00035683	0.03427559
Nxf7	-3.831241	0.83819956	18.4006355	0.00036064	0.03427559
Atf4	-0.5227742	6.67721885	18.3784636	0.00036282	0.03427559
Zfp746	-0.6745884	4.31556848	18.3775579	0.00036291	0.03427559
Cntn3	0.78362804	5.52149284	18.3442135	0.00036621	0.03427559
4933413L06F	2.71390537	-0.1724786	18.3321992	0.00036741	0.03427559
C430049B03I	-2.0448996	1.132541	18.1194906	0.00038939	0.0352489
Slc4a4	0.54285964	7.86424389	18.1147958	0.00038989	0.0352489
Ets1	-0.7369663	4.0399327	18.0746472	0.0003942	0.0352489
Calm2	-0.4451872	12.25381	18.0461512	0.0003973	0.0352489
Mdm1	1.09141927	3.14417576	18.0236582	0.00039976	0.0352489
Leng8	1.07045333	6.48428617	18.0097797	0.00040129	0.0352489
Plin4	2.27461822	0.99542139	17.993961	0.00040304	0.0352489
Cotl1	-0.9535241	3.14023198	17.9401406	0.00040905	0.03537676
Ccdc43	-0.7071684	4.71901413	17.9164947	0.00041172	0.03537676
Serinc2	2.15676827	0.7617066	17.7726832	0.0004284	0.03560378
Ndufa11	-0.8037465	3.88211188	17.6956178	0.00043764	0.03560378
6030407O03	5.76014511	-0.8430597	17.6947535	0.00043775	0.03560378
Gm9199	2.99839258	-0.4350192	17.6668145	0.00044115	0.03560378
Tppp3	-0.9353462	3.54507337	17.6275602	0.00044599	0.03560378
Fam19a3	-7.5213543	-0.4615225	18.7942972	0.00045467	0.03560378
Gabrb1	0.90320498	3.55491588	17.5547221	0.00045511	0.03560378
AI462493	-1.2803188	3.34834797	17.5242173	0.000459	0.03560378
Msl3	-0.5970872	5.48611517	17.5233659	0.00045911	0.03560378
Tnfaip8l3	-0.5229136	6.59912069	17.4722524	0.0004657	0.03560378
Il1rap	0.8480264	3.72863637	17.4538685	0.00046809	0.03560378
4930572O13	4.12683999	0.43251936	17.4399719	0.00046992	0.03560378
Cenpk	2.24009614	1.17615186	17.4342816	0.00047066	0.03560378
4833422C13I	1.72850666	2.31396796	17.3899301	0.00047654	0.03560378

Ptprz1	0.74679071	6.78132457	17.384631	0.00047724	0.03560378
Trnau1ap	-0.7640694	3.70772269	17.3617979	0.0004803	0.03560378
Atp1a4	3.38176225	-0.6699236	17.3555031	0.00048115	0.03560378
Ano4	0.83249707	3.81478993	17.2981227	0.00048895	0.03560378
Shcbp1l	2.88856624	0.19433113	17.2927651	0.00048969	0.03560378
Erlec1	0.57337181	5.82466938	17.2912864	0.00048989	0.03560378
Rspo1	-1.0837709	3.09497021	17.2854414	0.00049069	0.03560378
Rap2a	-0.4232941	7.59364331	17.2202462	0.00049976	0.03599518
Psmd6	-0.5745484	5.94318755	17.1880677	0.00050431	0.03605736
Slc27a2	1.50264219	1.809053	17.1468153	0.0005102	0.03617998
F420014N23	1.8673073	0.69867719	17.058939	0.00052301	0.03617998
Adcy5	0.46424621	6.29168631	17.0562536	0.0005234	0.03617998
Klhl3	1.24508858	2.05259147	17.0419011	0.00052553	0.03617998
Stk38l	0.52291876	5.63816732	17.0293465	0.0005274	0.03617998
Sorcs3	1.1238366	4.18264428	16.9952002	0.00053252	0.03617998
Fam184b	1.37126353	2.75541386	16.9783235	0.00053507	0.03617998
Usp29	0.73619797	5.56455857	16.9750222	0.00053557	0.03617998
Abcb6	2.24431446	1.61675515	16.9114228	0.00054531	0.03658588
Nelfe	-1.0051515	3.95887873	16.8422057	0.00055614	0.03693725
Agrn	0.83859542	5.00625141	16.8199386	0.00055968	0.03693725
Ndufs4	-0.4613245	6.61749597	16.7974435	0.00056327	0.03693725
4930579G24	1.07703948	2.52831405	16.7827303	0.00056564	0.03693725
Slc35d3	1.26839367	1.68033819	16.6907578	0.00058067	0.03709274
Mpc1	-0.5192387	6.49887657	16.6834603	0.00058188	0.03709274
Higd2a	-0.7187203	5.32361296	16.6724788	0.00058371	0.03709274
Fert2	0.55259089	4.96364727	16.657069	0.00058629	0.03709274
Tmem136	0.90002675	3.41993937	16.6355244	0.00058991	0.03709274
Chmp4b	-0.4775436	6.43768416	16.6203617	0.00059248	0.03709274
Ccpg1os	-0.9744363	2.81273087	16.599418	0.00059604	0.03709274
Ltbp1	1.11234726	4.37816882	16.5709456	0.00060092	0.03709274
Swap70	-0.6330018	4.83364218	16.5641344	0.0006021	0.03709274
Pcnx	0.64637845	7.32151792	16.4518907	0.00062182	0.03806854
Bag5	-0.5632533	6.29016191	16.4159437	0.00062829	0.03822558
Ncapd3	0.93055065	4.27461115	16.3866796	0.00063361	0.03831129
Muc6	2.01394077	1.55308457	16.3372251	0.00064271	0.03836742
Rab5a	-0.5116369	5.49876439	16.3170242	0.00064648	0.03836742
Abca1	0.62756967	5.05854337	16.3002651	0.00064961	0.03836742
Kif26b	1.31261506	1.966585	16.2971057	0.00065021	0.03836742
Mief1	-0.7918471	4.4905079	16.2621825	0.00065681	0.03848806
Stmn2	-0.430122	7.85579106	16.2407959	0.00066089	0.03848806
Dnajc30	-0.6643896	4.96880301	16.1917357	0.00067035	0.03848806
Cabin1	0.60280032	5.24959159	16.158597	0.00067682	0.03848806
Hscb	-1.1720308	4.15092299	16.1394553	0.00068059	0.03848806

Actn2	1.12910131	2.19011377	16.121629	0.00068413	0.03848806
Rtl1	1.28910401	2.9729589	16.1132592	0.00068579	0.03848806
Snx20	-1.817553	0.86909136	16.1075442	0.00068694	0.03848806
0610030E20I	0.60599212	5.46102833	16.1040643	0.00068763	0.03848806
Bambi	-1.9592331	1.23573943	16.084554	0.00069154	0.03848806
Bcas1	0.86828343	4.7132492	16.0143957	0.00070582	0.03906062
Cnrip1	-0.5356985	6.07897393	15.9624545	0.0007166	0.03909533
Mospd1	-0.6862752	6.13396769	15.9334265	0.0007227	0.03909533
Dnah7b	0.83135259	3.50336842	15.9179532	0.00072598	0.03909533
Fbxl13	6.78300724	-0.9624995	15.8704683	0.00073613	0.03909533
Sntb1	-1.3870853	1.79795292	15.8552945	0.00073941	0.03909533
Ccdc47	0.47089137	7.07371652	15.8440789	0.00074185	0.03909533
Gm16907	2.4714276	1.60754631	15.8091289	0.00074949	0.03909533
Rab36	0.64881374	3.97702559	15.8030814	0.00075082	0.03909533
Ndufa12	-0.6106283	5.67767073	15.7819201	0.0007555	0.03909533
Rheb	-0.5791723	7.10790637	15.7304507	0.00076701	0.03909533
4833420G17	0.66559179	4.88925606	15.7178521	0.00076986	0.03909533
Kcnk10	1.72808138	0.71105858	15.7032821	0.00077317	0.03909533
Ppp2ca	-0.4035231	8.45582872	15.7016373	0.00077354	0.03909533
Snrnp25	-1.3958241	2.33329262	15.6814718	0.00077815	0.03909533
Srsf1	0.45429025	7.49633913	15.6637286	0.00078223	0.03909533
Dgke	0.72440948	5.5125218	15.6626858	0.00078247	0.03909533
Batf2	-4.6049632	-0.9426477	15.6385599	0.00078806	0.03909533
Lrrtm4	0.68360317	5.30938419	15.6177872	0.0007929	0.03909533
Nwd1	0.75285848	5.14178275	15.615244	0.0007935	0.03909533
Chaf1b	-6.5349142	-1.7793604	16.6103858	0.00079493	0.03909533
Zar1l	-2.3418623	0.48899529	15.5973273	0.00079771	0.03909533
Rasl11b	-0.619737	5.61450664	15.5587708	0.00080685	0.03909533
Ftx	1.03532359	4.04392197	15.5554438	0.00080764	0.03909533
Poll	-1.7118682	0.87699302	15.5365169	0.00081218	0.03909533
Smpd4	0.72148771	5.12357101	15.5252196	0.0008149	0.03909533
Vill	1.64206103	1.24115167	15.51173	0.00081816	0.03909533
Tial1	0.54537151	5.9119415	15.4954709	0.00082211	0.03909533
Gpaa1	1.62885164	1.39963622	15.4617832	0.00083036	0.03909533
Arhgap39	0.65910113	4.98563479	15.4598384	0.00083084	0.03909533
Fbxo45	-0.492857	6.25555646	15.4542252	0.00083222	0.03909533
Lrp1b	1.07370073	5.97905963	15.4192068	0.00084092	0.03909533
Fam199x	0.65210945	4.77628233	15.4172619	0.0008414	0.03909533
Exosc1	-0.7849826	4.68225896	15.4128843	0.0008425	0.03909533
Dynll2	-0.4950608	7.03477523	15.3747704	0.00085209	0.03909533
Banf1	-0.8409777	4.45274061	15.3633021	0.00085501	0.03909533
Tceal6	-0.5460871	4.72825426	15.3356016	0.00086208	0.03909533
Pomp	-0.6424718	7.36047553	15.3179867	0.00086662	0.03909533

Meg3	1.0944981	10.8517456	15.2906061	0.00087372	0.03909533
Tmem67	1.0098932	3.81149428	15.2835983	0.00087555	0.03909533
Zmat2	-0.5001579	7.1416039	15.2450217	0.00088569	0.03909533
Myo9b	0.7065602	4.49957266	15.2438466	0.000886	0.03909533
AI467606	-6.1941442	-1.3976377	15.2399481	0.00088703	0.03909533
2610301B20	-0.5585595	5.3130088	15.2289687	0.00088994	0.03909533
Pcnxl3	0.61937627	4.69194894	15.2191581	0.00089256	0.03909533
Nat10	0.8149562	3.42472653	15.2187397	0.00089267	0.03909533
Cdca3	-4.5738736	-0.5375831	15.1969417	0.0008985	0.03909533
Utp11l	-0.8486359	4.11218496	15.1940074	0.00089929	0.03909533
Dzip3	0.68255041	6.90177053	15.1896914	0.00090046	0.03909533
Pnisr	0.78966709	7.66635585	15.1626711	0.00090777	0.03909533
Gbp10	-0.8129353	3.82201888	15.1525709	0.00091052	0.03909533
5430427O19	-3.7650849	0.2311527	15.1515716	0.00091079	0.03909533
Syt10	1.1846026	2.31250601	15.1398589	0.00091399	0.03909533
Tspan33	-1.5647241	1.50651382	15.0919176	0.00092722	0.03936348
Grwd1	-1.5456828	1.41673485	15.0880641	0.0009283	0.03936348
C030046E11	0.59028064	5.47917695	15.0618158	0.00093564	0.03938931
Ufd1l	-0.4397155	6.36786093	15.0379997	0.00094236	0.03938931
Smim11	-0.9164801	3.79796202	15.0318505	0.0009441	0.03938931
Pisd-ps1	1.18127319	5.75833039	15.0240872	0.00094631	0.03938931
Atp6v1g2	-0.481993	9.45908367	15.0043992	0.00095193	0.03938931
Gpm6b	0.65298562	8.47914724	14.9913911	0.00095566	0.03938931
Ube4a	0.5889815	5.74196263	14.9865667	0.00095705	0.03938931
Pcdhga3	0.82716633	3.43958644	14.9684107	0.0009623	0.03940335
Sumo1	-0.6631847	7.58817477	14.9576018	0.00096544	0.03940335
Usp24	0.62333729	7.4004889	14.9260633	0.00097466	0.03945959
Cfl1	-0.4844425	8.59102715	14.9253472	0.00097487	0.03945959
Chrac1	-1.0686367	2.94502002	14.8680241	0.00099189	0.03955078
Psmb1	-0.5146867	6.01550234	14.8637635	0.00099317	0.03955078
Dusp26	-0.5174174	6.07584242	14.8472319	0.00099815	0.03955078
Fancm	0.75238118	4.04748168	14.825199	0.00100482	0.03955078
Med12	0.74369465	4.99139276	14.8195507	0.00100654	0.03955078
Prrc1	-0.5880568	6.03799627	14.8153626	0.00100782	0.03955078
Nob1	-1.0110226	3.0220439	14.8151095	0.0010079	0.03955078
Ift20	-0.617053	6.78178911	14.7608639	0.0010246	0.03955078
D630045J12	0.56552095	6.85489924	14.752176	0.0010273	0.03955078
Pign	0.67112662	4.09877498	14.7519295	0.00102738	0.03955078
Gm12250	-2.3304396	0.08217832	14.7219856	0.00103676	0.03955078
Hmgb2	-0.8410876	4.01804394	14.720999	0.00103707	0.03955078
Timeless	-2.0474274	2.14805561	14.7092888	0.00104077	0.03955078
Prkg2	0.68776084	4.33622435	14.7073114	0.00104139	0.03955078
Rps4l	-1.0116477	1.95132353	14.6898437	0.00104693	0.03955078

Srfbp1	-0.7641188	3.92073567	14.6673198	0.00105413	0.03955078
Rfc2	-0.9827999	3.34450161	14.6611095	0.00105612	0.03955078
Cyb5	-0.7243092	5.88858328	14.6586209	0.00105692	0.03955078
Hspa13	0.67557069	4.88156484	14.6320053	0.00106552	0.03955078
Med31	-0.8624766	3.37068714	14.6136091	0.00107151	0.03955078
Cln8	0.70999696	3.78794767	14.6046261	0.00107444	0.03955078
Cplx1	-0.4165176	8.44920453	14.5557823	0.00109057	0.03955078
Taf10	-0.7801265	4.15935946	14.555238	0.00109075	0.03955078
Arl6	-0.5808428	5.70105526	14.5507453	0.00109225	0.03955078
Dpm1	0.69125235	5.2982306	14.5452585	0.00109408	0.03955078
Lhpp	-1.0459535	2.63005756	14.5150339	0.00110423	0.03955078
Ptprk	0.48989595	6.4903299	14.5118161	0.00110532	0.03955078
Ensa	-0.5062274	8.89617653	14.5114734	0.00110543	0.03955078
Dynlt3	-0.57858	9.11236474	14.5033438	0.00110819	0.03955078
Haus2	-0.5333011	5.4932092	14.5013601	0.00110886	0.03955078
Ift22	-0.8012777	4.09852226	14.4940148	0.00111135	0.03955078
H19	-4.9801657	-1.5509996	14.4930177	0.00111169	0.03955078
Ndufa8	-0.5708611	5.2594952	14.4896539	0.00111284	0.03955078
Anapc1	0.49908687	6.17805781	14.4748482	0.00111789	0.03955078
Lmo7	0.98084146	6.02077423	14.4695919	0.00111969	0.03955078
Svep1	1.10722134	2.75200356	14.459475	0.00112316	0.03955078
Gltpd1	-0.6930513	3.73848941	14.4448132	0.00112822	0.03955078
Pttg1	-0.542517	4.90101036	14.4380426	0.00113056	0.03955078
Sumf2	1.00201367	2.65517025	14.4168026	0.00113795	0.03957098
Fam178a	0.42435589	7.06139048	14.4131606	0.00113922	0.03957098
Slc44a3	5.76604728	-1.1805436	14.3767415	0.00115202	0.03987431
Plcg1	0.65405277	4.07223723	14.3567652	0.00115911	0.03997846
Gnb4	0.61938857	4.79186828	14.3440583	0.00116365	0.03999021
Glt8d1	0.72796097	4.23810645	14.3329792	0.00116762	0.03999021
AU022252	-1.041266	2.42007903	14.3058942	0.00117739	0.0401843
D130043K22	1.03304188	3.37003417	14.2836191	0.00118549	0.04018977
Srsf12	-0.8462553	4.02511157	14.2803081	0.0011867	0.04018977
Dyrk4	-7.3124806	-2.242214	15.1190982	0.00119155	0.04018977
Nkd2	-0.9793361	4.51143517	14.2450965	0.00119965	0.04018977
Atg5	-0.5848014	4.61398251	14.242091	0.00120076	0.04018977
L1td1	-1.0772528	2.81831236	14.2307998	0.00120495	0.04018977
Fbf1	0.69750123	4.05102096	14.2238228	0.00120755	0.04018977
Slc38a11	3.91399859	0.50440634	14.2144876	0.00121103	0.04018977
Mbp	0.77403598	7.0503577	14.1969368	0.00121761	0.04018977
B430319G15	-1.4018896	1.96980397	14.1943721	0.00121858	0.04018977
Polrmt	1.12837132	2.23954961	14.1717001	0.00122714	0.04033651
Chchd6	-0.8552462	3.43106469	14.1522953	0.00123453	0.04038384
Znhit1	-0.9053478	3.24479417	14.1212441	0.00124645	0.04038384

Tpd52l1	-0.7643094	5.00453111	14.1069372	0.00125199	0.04038384
Acn9	-0.8902242	2.90800262	14.0921922	0.00125772	0.04038384
Ep400	0.70463131	6.99752642	14.0860524	0.00126011	0.04038384
F2rl2	1.18887696	1.92100183	14.0844664	0.00126073	0.04038384
Ntrk3	0.70988231	5.67850937	14.082949	0.00126133	0.04038384
Ppp2r3c	-0.5130733	4.97086748	14.0823407	0.00126157	0.04038384
Syndig1	-0.8152001	3.49911957	14.0506867	0.00127401	0.04064948
Abcg1	0.69948973	4.94718893	14.0143278	0.00128848	0.04097759
Rpgr	0.85892527	4.34552002	13.9728454	0.00130521	0.04115339
Ccdc27	4.56228295	-1.0113339	13.9713226	0.00130583	0.04115339
Gm16675	-5.6178061	-1.3070885	13.9661276	0.00130794	0.04115339
Sema5a	0.60647971	6.42910481	13.9474068	0.00131559	0.04115339
Eif3k	-0.7857463	4.33275932	13.9406126	0.00131837	0.04115339
Nacad	0.87685047	3.25361314	13.930984	0.00132233	0.04115339
Ranbp1	-0.49313	5.21532742	13.9096656	0.00133115	0.04115339
Cstb	-0.7435962	6.70738108	13.8971291	0.00133636	0.04115339
Gpr101	1.34788224	2.41118593	13.8886795	0.00133989	0.04115339
Slc2a4rg-ps	1.70480974	1.80381614	13.8791371	0.00134389	0.04115339
Tmem26	6.16463584	-1.4309923	13.8787524	0.00134405	0.04115339
Trrap	0.84701793	6.51894758	13.8778593	0.00134442	0.04115339
Ccer1	7.1919349	-2.3162818	15.9091021	0.00136191	0.04155866
Gm16853	3.86736843	-0.9169055	13.8155692	0.00137085	0.04170162
Lamtor5	-0.7250458	5.97963691	13.7960708	0.00137924	0.04182692
Pdap1	-0.4806693	6.04391655	13.7745377	0.00138857	0.04197992
Drd1a	0.69030879	4.35712398	13.7576693	0.00139593	0.04207252
Ube2b	-0.4498698	7.64249085	13.744084	0.00140188	0.04212247
Tia1	0.77542902	5.2628024	13.6894047	0.00142615	0.04222744
Tyk2	1.1228575	2.82495666	13.6830566	0.001429	0.04222744
Gm16523	1.48217675	1.10422528	13.6825213	0.00142924	0.04222744
Cox5a	-0.5089109	6.35385492	13.6821381	0.00142941	0.04222744
Pak1	-0.4220229	9.92302229	13.6759207	0.0014322	0.04222744
Rrm2	1.93435806	1.42874558	13.6659826	0.00143668	0.04222744
Mzt1	-0.4939165	5.93690151	13.6549975	0.00144166	0.04222744
Rgs9	0.90928734	5.09525429	13.6389504	0.00144895	0.04222744
Gabarapl1	-0.4810693	8.09279506	13.6277473	0.00145407	0.04222744
Cisd1	-0.5581178	5.07555563	13.6267104	0.00145454	0.04222744
Rp9	-0.7342815	4.0520469	13.6220409	0.00145668	0.04222744
Timm8b	-0.5527251	5.55387668	13.6211114	0.00145711	0.04222744
Arnt2	0.44687126	8.03920942	13.6072512	0.00146348	0.04228705
Csrp2	-0.7007196	4.57851133	13.5934404	0.00146987	0.04234654
Slc46a1	1.33948036	1.24647663	13.5819581	0.00147519	0.04237545
1700021F05I	-0.5744344	4.52234889	13.5529635	0.00148875	0.04240547
Pou4f1	-7.4274829	-1.0522652	14.3283102	0.0014894	0.04240547

Disp2	0.62079746	7.01997622	13.5405197	0.00149461	0.04240547
Ywhah	-0.3710885	10.4947765	13.5340512	0.00149766	0.04240547
Dnah1	0.96326552	3.06537276	13.5335792	0.00149789	0.04240547
Ypel3	-0.4533969	5.84479946	13.5224327	0.00150317	0.04243238
Tmem251	-0.7666044	3.7918645	13.5007634	0.0015135	0.0425291
Cend1	-0.4455647	7.05317087	13.4858285	0.00152066	0.0425291
Gm19689	-7.782068	-1.7117989	14.2482286	0.00152396	0.0425291
Ly6e	-0.4931786	6.82283664	13.4770947	0.00152487	0.0425291
Trmt112	-0.5770377	5.06178834	13.4688523	0.00152885	0.0425291
Tbrg3	1.02142494	4.19389579	13.4610204	0.00153265	0.0425291
Lrba	0.6423214	5.84648866	13.4303998	0.00154758	0.04282224
Zyx	-0.519826	5.44226379	13.395264	0.00156492	0.04317999
Acbd3	-0.4309431	6.49257134	13.3760419	0.00157449	0.04332218
Adra1a	0.68935762	4.48599076	13.3607172	0.00158217	0.04341157
Ddn	0.49784501	6.89353307	13.3372332	0.00159403	0.04361456
Chrd	1.08744015	2.10372602	13.3191887	0.0016032	0.04365858
Avpi1	-0.7850763	3.24214639	13.3051514	0.00161037	0.04365858
Sptbn4	1.42313009	3.60591124	13.3045474	0.00161068	0.04365858
Cox14	-0.5748509	5.326743	13.2942098	0.00161599	0.04365858
Eif1b	-0.5138253	5.81897077	13.2807526	0.00162293	0.04365858
Rnf185	-0.5412304	5.79909455	13.2796331	0.00162351	0.04365858
Gm10677	1.79003415	1.60301891	13.2691478	0.00162894	0.04365858
Gabpa	-0.4810708	6.71949932	13.2646268	0.00163129	0.04365858
F630111L10I	1.36953057	2.56004193	13.2428324	0.00164266	0.04376346
Bcan	0.93910504	4.67204944	13.2377255	0.00164534	0.04376346
Cdan1	0.78410783	3.37040138	13.2314999	0.00164861	0.04376346
Mfsd3	2.53862517	0.54359466	13.1842154	0.00167369	0.04405081
Tmem198b	-1.8165454	0.79725695	13.1818499	0.00167496	0.04405081
Vps28	-0.5507113	5.80975147	13.1562634	0.00168872	0.04405081
Rgsl1	1.51416688	1.52849034	13.1548145	0.0016895	0.04405081
Lyrm1	-1.0113811	2.71571946	13.1418349	0.00169653	0.04405081
Thsd7b	1.62254801	1.27825354	13.1256705	0.00170533	0.04405081
Serac1	0.75718463	5.45659715	13.1047189	0.00171681	0.04405081
Fgf16	6.54020792	-1.5914107	13.1013392	0.00171867	0.04405081
4930449E18I	3.27970357	-0.8202355	13.0949719	0.00172219	0.04405081
Simc1	0.59016457	4.18232201	13.0937635	0.00172285	0.04405081
Hmgxb4	-0.5134958	5.2954056	13.0911644	0.00172429	0.04405081
C1ql3	0.56542364	6.79431107	13.0812561	0.00172978	0.04405081
2310047M1C	-1.0495069	2.67892349	13.0738323	0.0017339	0.04405081
Atr	0.70823714	4.55086409	13.0702448	0.0017359	0.04405081
Serpinc1	2.41315861	0.12159231	13.0650086	0.00173881	0.04405081
Pcdhb3	1.13198603	2.11823708	13.0617293	0.00174064	0.04405081
Fank1	1.84696343	1.2666404	13.0576751	0.00174291	0.04405081

Ubr4	0.82593389	7.17668192	13.049084	0.00174772	0.04405081
Bud31	-0.6134892	4.69115838	13.0264084	0.0017605	0.04405081
Bhlha15	-3.2713059	-0.1500511	13.0228258	0.00176253	0.04405081
Ppia	-0.6075114	9.86341578	13.0204955	0.00176385	0.04405081
Dtwd2	1.04388991	2.91413883	13.0197837	0.00176425	0.04405081
Kif18a	2.11905397	0.99561614	13.0140734	0.00176749	0.04405081
Rab32	-1.1280668	3.17081687	13.0081595	0.00177085	0.04405081
Snd1	0.49049292	5.02085632	12.9952738	0.00177821	0.04405081
Trank1	1.0669615	6.22042844	12.9819539	0.00178584	0.04405081
Leo1	-0.4622951	6.10134011	12.9758298	0.00178937	0.04405081
Ccdc146	-1.6767316	1.56847198	12.9724949	0.00179129	0.04405081
2310022B05I	-0.5220018	6.31292069	12.9702315	0.00179259	0.04405081
Ahi1	0.69878343	6.88549225	12.9543257	0.0018018	0.04405081
Loh12cr1	-0.9359401	2.95649361	12.9507409	0.00180388	0.04405081
Kbtbd8	0.86812733	3.16545921	12.9343493	0.00181343	0.04405081
Prdm5	0.92710292	3.03414188	12.9342731	0.00181348	0.04405081
Atad2b	0.59424961	5.16211525	12.9244685	0.00181922	0.04405081
Nicn1	-0.4287711	6.46661073	12.9192485	0.00182228	0.04405081
Gm19990	-4.699984	-0.756335	12.9177732	0.00182315	0.04405081
Exosc6	-0.7874097	3.34859601	12.9113242	0.00182695	0.04405081
Cacng5	1.21549394	2.68079669	12.9055924	0.00183033	0.04405081
Nup214	0.56567745	6.07547627	12.8662268	0.00185374	0.04418362
Chrnb1	0.93177612	2.16982591	12.8661792	0.00185377	0.04418362
Atpaf1	-0.4849782	6.0288518	12.8476627	0.00186489	0.04418362
Rpl35	-0.679864	4.96906104	12.8455336	0.00186618	0.04418362
Xpo6	0.39394101	6.87091952	12.8433219	0.00186751	0.04418362
Atp7a	0.58047117	5.55625978	12.8419182	0.00186836	0.04418362
Pts	-0.6315419	5.2535485	12.8371546	0.00187124	0.04418362
Ndufab1	-0.5137972	5.61527553	12.8303089	0.00187539	0.04418362
Med12l	0.89810895	5.30820352	12.8285733	0.00187644	0.04418362
Ttn	1.0754046	3.64854809	12.809455	0.00188809	0.04435122
Dmrta1	-1.4129333	3.04450842	12.7956819	0.00189653	0.04444287
Notch1	1.15413377	2.82737551	12.7858332	0.00190259	0.04447552
Bmpr1b	0.83180643	3.2590408	12.7786814	0.001907	0.04447552
Pdgfc	-1.0326422	2.48770178	12.7608547	0.00191805	0.04462703
Ddit3	-0.7213691	3.40717023	12.7436073	0.00192881	0.04477105
1700009P17I	-1.4577648	1.70603759	12.7159759	0.00194619	0.04506765
Plekhg5	0.62973745	4.25616956	12.6867033	0.00196479	0.04537701
Nme7	0.92667665	3.52830339	12.6750756	0.00197224	0.04537701
Mir872	-5.5657738	-1.3539564	12.6607876	0.00198143	0.04537701
Fstl4	0.76162265	3.03605396	12.6571384	0.00198378	0.04537701
Pyroxd1	0.69866121	3.35080755	12.6536806	0.00198601	0.04537701
Pcdhb22	0.98782616	3.03876864	12.6476173	0.00198994	0.04537701

Hprt	-0.4216115	7.88764741	12.6444656	0.00199198	0.04537701
Clybl	-0.6933731	4.35151724	12.633546	0.00199908	0.045433
Slc39a3	0.80073834	3.21713689	12.6263238	0.00200379	0.0454346
C1qbp	-0.4618206	5.21647744	12.605661	0.00201733	0.04563597
Bri3bp	0.5575667	5.72851111	12.580414	0.00203401	0.04585343
Celsr3	1.4370226	4.73290508	12.5769605	0.0020363	0.04585343
5830418K08I	0.78099025	5.57717409	12.5370781	0.002063	0.04634805
Snapc5	-0.6709699	6.27476818	12.5191515	0.00207512	0.04645353
BC023829	-0.8430561	3.99356688	12.5134682	0.00207898	0.04645353
Fam103a1	-0.4988577	7.87600911	12.5091555	0.00208192	0.04645353
4930419G24	1.64218668	1.826446	12.4993922	0.00208858	0.04646569
2810408M09	-0.7627271	3.2236678	12.4944648	0.00209195	0.04646569
Theg	3.18053744	-0.8968574	12.4730056	0.0021067	0.04658219
Omd	0.57476015	5.17347677	12.4729986	0.00210671	0.04658219
1110008L16F	-0.5426897	4.36276235	12.4602867	0.0021155	0.04667125
Tex35	5.58921668	-1.7940402	12.4312965	0.00213571	0.0467444
Tnc	1.36048443	1.7869043	12.4295699	0.00213692	0.0467444
Gm16702	0.78470523	4.40077098	12.4200096	0.00214363	0.0467444
Nup155	0.64544316	5.24826961	12.418883	0.00214442	0.0467444
Nckap5	0.90913793	3.2134606	12.4112754	0.00214978	0.0467444
Isoc2a	-1.1010741	1.96736771	12.4057552	0.00215368	0.0467444
Gabrg1	0.91378923	3.40402134	12.4020914	0.00215628	0.0467444
Casp4	-1.6733343	0.78416586	12.4010785	0.00215699	0.0467444
Myd88	-1.2272518	2.78060897	12.3830284	0.00216982	0.0469186
1500009C09I	-0.5709352	5.50390501	12.3738297	0.00217639	0.046957
Mlxip	0.53014965	5.16406634	12.3265328	0.00221052	0.04737501
Calcr	2.56013504	0.29845713	12.3157392	0.00221839	0.04737501
Nos1	0.96073656	3.24246223	12.3142512	0.00221948	0.04737501
Sdf2l1	-0.9158146	2.66460887	12.3122386	0.00222095	0.04737501
Pkhd1	-4.7846802	-0.6882453	12.3109916	0.00222187	0.04737501
Trim56	-0.7635863	3.52885382	12.3070079	0.00222478	0.04737501
Eri1	-0.5035348	5.71435781	12.2955204	0.00223322	0.04745155
Gfi1	-7.0324756	-1.3103246	12.9192591	0.00225226	0.0475246
2900011O08	-0.4610816	7.725197	12.2687755	0.00225301	0.0475246
Atrnl1	0.45916466	6.22704218	12.2576502	0.00226129	0.0475246
Sde2	-0.5353481	4.81445549	12.2557301	0.00226273	0.0475246
Ift27	-0.9790228	2.67768703	12.2507947	0.00226642	0.0475246
Cers3	-5.9736773	-1.6244005	12.2473277	0.00226901	0.0475246
Mctp2	1.16552709	1.62389427	12.2342222	0.00227886	0.0475246
Sqstm1	-0.9065516	8.74120663	12.2320255	0.00228051	0.0475246
A730036I17F	5.67272766	-1.0930303	12.2277852	0.00228371	0.0475246
Ppifos	4.63243929	-1.5463159	12.2195985	0.0022899	0.0475246
Yipf2	1.3968923	1.33348163	12.2150919	0.00229331	0.0475246

Ints2	0.7320012	4.08922005	12.2012439	0.00230384	0.0475246
Wdr34	0.85107865	3.15942228	12.1962622	0.00230763	0.0475246
Pomt2	0.68478454	3.59827126	12.1932184	0.00230996	0.0475246
Ndufb10	-0.5764642	5.91536809	12.1929329	0.00231018	0.0475246
Paqr8	0.49421287	5.11849638	12.1875591	0.00231429	0.0475246
Metap1	-0.4657109	6.1717032	12.172592	0.00232578	0.04766071
Pcdh15	0.87766519	4.87020321	12.1518917	0.00234178	0.04785677
Jph3	0.54806855	5.02951187	12.1475931	0.00234512	0.04785677
Slc7a1	0.52735754	5.28969118	12.133713	0.00235594	0.04790819
1810011O10	-1.2220885	4.30148064	12.1315988	0.00235759	0.04790819
Mrps15	-0.6574839	4.23879358	12.1221184	0.00236501	0.04790819
Sdk2	0.93376487	3.60874328	12.0860199	0.00239352	0.04790819
Trpc6	1.12686463	2.85249854	12.0849151	0.0023944	0.04790819
Zzef1	0.89827292	5.30413241	12.0848101	0.00239448	0.04790819
Asah2	0.56204995	5.7264065	12.0810949	0.00239744	0.04790819
Cntnap1	0.64633976	5.86954492	12.080367	0.00239802	0.04790819
Ppp1r11	-0.6185984	4.56555625	12.0774144	0.00240037	0.04790819
Coa4	-1.219492	2.07474742	12.0478438	0.00242408	0.04790819
Pcdhb9	1.10693876	2.25524528	12.0472612	0.00242455	0.04790819
Selo	1.18046343	1.26997923	12.0470247	0.00242474	0.04790819
Ncf4	-3.9453038	-0.9556413	12.045806	0.00242572	0.04790819
Nup188	0.89893743	3.73342481	12.0419429	0.00242884	0.04790819
Vegfb	-0.6016637	4.6599426	12.0377218	0.00243226	0.04790819
Txnl4a	-0.538076	6.03320287	12.0348774	0.00243456	0.04790819
Ubp1	0.53243147	6.29825145	12.0298555	0.00243863	0.04790819
4632427E13I	1.86708456	1.56758032	12.0214822	0.00244544	0.04790819
Dusp14	-0.5453669	5.93030232	12.0206851	0.00244609	0.04790819
AF357359	1.0439386	2.62008413	12.0166184	0.0024494	0.04790819
Cdk5rap1	1.75657437	1.39925974	12.0129757	0.00245237	0.04790819
Rnf11	-0.4205467	6.86253353	12.0094645	0.00245524	0.04790819
Fnbp4	0.54502716	5.76659596	11.9792923	0.00248005	0.04811128
Ddx26b	0.97537458	4.50924521	11.9773322	0.00248167	0.04811128
Crygs	-5.5090061	-2.0379571	11.9714244	0.00248657	0.04811128
2010204K13I	-0.9648243	2.63947415	11.9673622	0.00248994	0.04811128
Cyb5r2	5.50820544	-1.4312352	11.9670365	0.00249021	0.04811128
Akirin1	-0.4756774	5.78929013	11.9573613	0.00249826	0.04813012
4933431G14	2.12194343	0.30495939	11.9538733	0.00250117	0.04813012
Lphn3	0.65343731	6.76500608	11.9481818	0.00250592	0.04813012
Pate2	2.10912475	0.20605937	11.9346514	0.00251727	0.04825341
Ptprn	0.65759682	6.91147551	11.9257279	0.00252478	0.04830292
Ankrd13c	-0.4131252	6.39659034	11.8959476	0.00255004	0.04857652
Myl12b	-0.6356497	7.8337359	11.8908747	0.00255437	0.04857652
Golga7b	-0.7654607	4.36180067	11.8826841	0.00256138	0.04857652

Itga10	0.91810511	2.96327098	11.8821592	0.00256183	0.04857652
Cbr1	-0.5519721	4.80828654	11.8747281	0.00256821	0.04857652
Agbl1	-3.3501176	-0.8698047	11.8561754	0.00258421	0.04857652
Ccdc89	2.32374006	0.33416161	11.850775	0.00258889	0.04857652
Pkp2	1.14394474	2.23426187	11.8430335	0.00259561	0.04857652
D17Wsu92e	-0.3647957	7.63463128	11.8412946	0.00259712	0.04857652
Pcdh11x	0.78772485	4.67107763	11.8346626	0.0026029	0.04857652
Sp100	-0.7859312	5.07670591	11.8318174	0.00260538	0.04857652
Fnip2	0.61726014	4.74590785	11.8287464	0.00260807	0.04857652
Arhgap24	-0.8293046	3.94176612	11.8275373	0.00260912	0.04857652
Smug1	-0.5345032	4.24447993	11.8246926	0.00261161	0.04857652
Prkch	-0.9644629	2.40953756	11.822571	0.00261347	0.04857652
Ythdf2	0.52263238	5.07872959	11.7738719	0.00265654	0.04921709
Ogt	0.73339661	7.68691781	11.7720956	0.00265812	0.04921709
Prph	-4.8090234	-0.8824467	11.7666285	0.00266301	0.04921709
Alas2	1.8250548	1.07308465	11.7590974	0.00266976	0.04924888
Ttll1	-0.4365945	5.61818762	11.7487644	0.00267905	0.04930999
Mdn1	1.259959	6.51506517	11.740635	0.00268638	0.04930999
Xdh	-0.8499605	3.2821717	11.7386525	0.00268817	0.04930999
Gmps	0.47346304	6.75866356	11.7328094	0.00269346	0.04931465
Nlrp6	3.2442612	0.89625499	11.7165808	0.00270821	0.04948908
Tmem25	0.97865822	3.05454486	11.7112305	0.00271309	0.04948908
Adamts1	0.75175936	4.34942457	11.701546	0.00272195	0.04955844
Asah1	0.52286016	5.68220169	11.692706	0.00273007	0.0495704
Galnt16	0.67479879	4.41989815	11.6898148	0.00273273	0.0495704
Cyb5r4	-0.4214596	5.78849428	11.6797829	0.00274199	0.0496086
Bbs7	0.65986827	4.04676063	11.6765598	0.00274497	0.0496086
Ptpn23	0.55649402	4.86843946	11.6662852	0.00275449	0.04968908
Kirrel3	0.94002918	3.45483046	11.6557988	0.00276425	0.04970599
Gpr87	-5.5867699	-1.7229467	11.6449776	0.00277436	0.04970599
Rab12	-0.4057272	7.08669075	11.6394759	0.00277952	0.04970599
Eif4e	-0.3700553	7.4070904	11.638729	0.00278022	0.04970599
Ssh3	1.23563394	1.67855805	11.6348088	0.0027839	0.04970599
Gdap10	1.10162201	2.40450832	11.6305411	0.00278792	0.04970599
Tars2	0.81331574	2.52134656	11.6220378	0.00279594	0.04970599
Dusp28	-0.9262114	3.63802561	11.6219428	0.00279603	0.04970599
Dnajc21	-0.5317005	7.02084378	11.5928373	0.00282366	0.05002406
Smg1	0.78656094	7.69714299	11.5829161	0.00283315	0.05002406
Inf2	0.46378454	6.07676718	11.5792547	0.00283667	0.05002406
Ikbkb	-0.7009287	4.30039297	11.5691929	0.00284634	0.05002406
Slc30a1	0.46768862	5.11534121	11.5669054	0.00284854	0.05002406
Ercc4	0.70002613	3.83463509	11.5666259	0.00284881	0.05002406
Gmds	-0.8189021	3.36496846	11.5657428	0.00284967	0.05002406

Shank1	0.66994432	8.94138459	11.5456738	0.0028691	0.05018869
5730409E04I	-0.3440228	6.93327288	11.5410165	0.00287363	0.05018869
Ndufaf3	-0.6407789	4.14631992	11.5402068	0.00287441	0.05018869
Tcerg1l	0.90359986	2.69450517	11.533489	0.00288097	0.05021356
Commd10	-0.6196693	4.67204562	11.5250773	0.00288919	0.05026748
Aurkb	-4.60989	-1.0478296	11.5179363	0.00289619	0.05029998
Synpr	0.76746734	5.17764605	11.4988124	0.00291504	0.05032246
Aida	-0.4562509	6.45288865	11.4979545	0.00291589	0.05032246
Slc22a12	3.30855629	0.35222828	11.4977275	0.00291612	0.05032246
Grik2	0.79398776	5.18302285	11.4878292	0.00292593	0.05032246
Daxx	-0.5634428	3.87874798	11.4834741	0.00293026	0.05032246
Gm16596	2.20433708	0.01562541	11.4785409	0.00293517	0.05032246
Ttc33	-0.4235253	6.57140083	11.4751324	0.00293857	0.05032246
Mcee	-0.7445762	4.05548428	11.4751146	0.00293859	0.05032246
Srpx2	2.25442187	0.24432946	11.4674622	0.00294624	0.05036538
Prkd1	0.93501785	2.50520455	11.4509628	0.0029628	0.05049076
Wsb1	0.87523136	4.11688101	11.4457818	0.00296802	0.05049076
Gpr165	0.83766957	3.639751	11.4446611	0.00296915	0.05049076
Lrrc16a	0.65417273	4.29144179	11.43968	0.00297419	0.05049076
Atp11b	0.5296731	6.31297214	11.4343536	0.00297958	0.05049479
Frem2	1.18964864	1.81353757	11.4064093	0.00300805	0.05088923
Apopt1	-0.4924146	4.12066254	11.3971014	0.0030176	0.05092389
Intu	0.70719373	4.51360505	11.3925768	0.00302225	0.05092389
Exosc9	0.58339939	4.0254313	11.3806432	0.00303457	0.05092389
2700029M09	-0.4663453	5.45364033	11.3793639	0.00303589	0.05092389
Erlin2	0.46218831	5.40469007	11.3791694	0.00303609	0.05092389
Maob	0.61677928	4.16422355	11.3702934	0.00304529	0.05099089
Snrpf	-1.0742841	2.83226967	11.3506599	0.00306575	0.05121782
Gtf2f2	-0.704179	3.1438691	11.3472685	0.0030693	0.05121782
Rad23b	-0.3516206	7.99286749	11.3382619	0.00307875	0.0512234
Hnrnpa1	0.4351841	5.07049439	11.3352844	0.00308188	0.0512234
Erdr1	0.88113148	3.40101231	11.3256395	0.00309205	0.0512234
Magoh	-0.5817085	5.17826882	11.317891	0.00310024	0.0512234
Cd209c	0.94991157	2.7033176	11.3103931	0.0031082	0.0512234
Gm19466	2.62720276	-0.2042499	11.3069189	0.00311189	0.0512234
Malat1	1.24252061	12.8518958	11.3068561	0.00311196	0.0512234
Tmem86b	1.85456645	0.33394382	11.2995936	0.00311969	0.0512234
Lyg1	-4.344712	-1.5297551	11.2948724	0.00312473	0.0512234
Pddc1	-0.6905713	3.60516068	11.2940797	0.00312558	0.0512234
Rsrc1	-0.4406107	5.32700455	11.2925976	0.00312716	0.0512234
Bbip1	-0.4567466	5.93859455	11.2725626	0.00314867	0.05148954
Gcdh	-0.7650335	3.16647822	11.2565353	0.00316599	0.0516566
Arl16	-0.5312484	4.2914429	11.2530511	0.00316977	0.0516566

Vhl	-0.5042795	4.8722494	11.2485075	0.0031747	0.0516566
Btbd8	1.62892704	0.87581466	11.2335313	0.00319103	0.05175865
Oc90	6.77586523	-2.3165054	11.7869667	0.00319154	0.05175865
Ran	-0.3693259	7.71307405	11.217182	0.00320897	0.05189874
Prdx5	-0.6691021	5.90269109	11.2124826	0.00321414	0.05189874
Inhba	0.4531746	4.79140572	11.2046292	0.00322281	0.05189874
Jund	-0.5987809	5.02178906	11.2027808	0.00322486	0.05189874
Rgs13	-3.0534048	0.46691348	11.1971388	0.00323111	0.05189874
Fkbp1b	-0.73952	4.2158302	11.1913544	0.00323753	0.05189874
Pdzd2	0.59177526	6.18780981	11.1725871	0.00325846	0.05189874
Jpx	0.92268149	2.7866681	11.1688297	0.00326267	0.05189874
0610010K14I	-0.7884938	3.74429142	11.1678279	0.00326379	0.05189874
Iqck	1.31578971	2.81758821	11.16725	0.00326444	0.05189874
Slc24a4	1.23258919	2.83603818	11.1560422	0.00327703	0.05189874
Znf512b	0.48343161	4.93881231	11.1551903	0.00327799	0.05189874
Tmem200c	1.1986433	1.70233929	11.1523299	0.00328122	0.05189874
Psmb7	-0.5196945	6.85311655	11.1520949	0.00328148	0.05189874
Sox4	-0.8540472	2.90899654	11.1505299	0.00328325	0.05189874
Ahcyl2	-0.507097	8.1213262	11.1490183	0.00328495	0.05189874
Trmt61a	0.59986124	4.52357401	11.1433322	0.00329138	0.05191657
Mon2	0.53580513	6.62046016	11.1323275	0.00330386	0.05198461
Lpl	0.94088666	4.20486379	11.1267358	0.00331022	0.05198461
Zbtb24	0.57794925	5.10803084	11.1199207	0.003318	0.05198461
Tmem206	0.73390283	4.40141597	11.1116443	0.00332746	0.05198461
Telo2	1.5970518	1.49145732	11.1098751	0.00332949	0.05198461
Ufl1	0.49130867	5.79660196	11.1073772	0.00333236	0.05198461
Lrrc27	-0.9268809	3.21464008	11.1022174	0.00333828	0.05198461
Ahsa1	-0.4868109	5.77367489	11.0991607	0.0033418	0.05198461
Hist1h2bc	-0.6625746	5.43187797	11.0972232	0.00334403	0.05198461
2700060E02I	-0.4562257	5.83392768	11.093113	0.00334877	0.05198461
Snhg11	1.27022693	9.45745744	11.0875455	0.0033552	0.05200201
Syt2	0.90272761	3.39364271	11.0742098	0.00337065	0.05215905
Tac1	0.79837168	4.18556843	11.0572434	0.00339043	0.05219364
Creb3l1	-0.7924978	5.25308453	11.0550111	0.00339305	0.05219364
Spred2	-0.4286258	6.51431557	11.0483182	0.00340089	0.05219364
Mrpl9	-0.59952	4.48985531	11.0422003	0.00340808	0.05219364
Ano1	1.14220019	1.54957846	11.0420201	0.00340829	0.05219364
2310015B20I	-1.572995	1.96852753	11.0416806	0.00340869	0.05219364
Asb11	1.5926611	0.90161594	11.0372482	0.00341391	0.05219364
Psmg4	-1.4526094	1.72007138	11.0293268	0.00342326	0.05219364
Gbp6	-0.6264413	5.06947314	11.0260441	0.00342714	0.05219364
Tacc3	-1.1013404	2.22085714	11.0248176	0.0034286	0.05219364
Mvk	-0.7631465	2.941983	11.0223651	0.0034315	0.05219364

Eid1	-0.3947509	8.03895077	11.0075252	0.00344914	0.05229363
Chchd3	-0.5472516	5.11662928	11.0049917	0.00345216	0.05229363
Nop10	-0.6037043	4.74626895	11.003377	0.00345409	0.05229363
Alox8	0.78289371	3.29180948	10.9909961	0.00346891	0.05243692
Ntn3	1.65989865	1.52422669	10.9830285	0.00347848	0.05250062
Ecm2	-0.7928857	5.03263301	10.965586	0.00349954	0.05269576
Gm12359	2.34494361	0.15565247	10.963417	0.00350217	0.05269576
Mrpl44	-0.6393309	3.56917656	10.9498285	0.00351869	0.05282314
Gata3	-3.1023639	-0.4203698	10.9473516	0.00352171	0.05282314
Ttc37	0.82493062	3.70231221	10.9431712	0.00352681	0.05282314
Baiap2l1	-0.6898792	3.464308	10.9286755	0.00354458	0.05282529
Pdlim1	-0.8612662	3.85893549	10.9279759	0.00354544	0.05282529
Gm10389	0.83505384	4.2490491	10.9264703	0.00354729	0.05282529
4932438A13I	0.82805397	7.50608328	10.9183805	0.00355725	0.05282529
Kcne3	-2.9190506	-0.4643361	10.9166543	0.00355938	0.05282529
Glt25d1	0.61237216	3.99311375	10.9166235	0.00355942	0.05282529
Pdcd6	-0.5238862	5.94969641	10.9123434	0.00356471	0.05282529
Kcnt1	0.80942654	5.40537708	10.8893724	0.00359324	0.05300994
Ttk	-3.2407726	-0.5704396	10.8853454	0.00359827	0.05300994
Prr15	-2.0983522	0.78841463	10.8823343	0.00360203	0.05300994
Zbtb48	-1.4861897	1.61841727	10.8798388	0.00360515	0.05300994
Hace1	0.47910992	5.00898893	10.8773733	0.00360824	0.05300994
Zfp354c	0.48556012	5.84331025	10.8740653	0.00361239	0.05300994
Col16a1	1.06839492	1.53962728	10.8693033	0.00361837	0.05300994
Sgsm1	0.96474777	4.53170126	10.867641	0.00362046	0.05300994
Sorbs2os	1.17137098	3.15431538	10.8620526	0.0036275	0.05303371
E2f5	-0.5536121	4.32218395	10.8485121	0.00364462	0.05320453
Fnta	-0.5565003	6.63782903	10.8381768	0.00365774	0.05330383
4932418E24I	2.43191281	-0.0372914	10.8306355	0.00366735	0.05330383
A330023F24I	1.23699251	4.37251926	10.8284561	0.00367013	0.05330383
Dock3	0.87318942	7.93754843	10.8253423	0.00367411	0.05330383
Bai3	0.66118912	6.39756936	10.820467	0.00368035	0.05330383
Apobec2	-6.3449533	-1.4578593	10.8143031	0.00368826	0.05330383
Rab33a	-0.7737985	3.31455884	10.810923	0.0036926	0.05330383
Sik2	0.45053136	5.84896474	10.8025385	0.0037034	0.05330383
Rtca	-0.5042563	5.33134198	10.8010629	0.0037053	0.05330383
Rimbp2	0.78955961	5.19509569	10.7973334	0.00371012	0.05330383
C1qtnf6	-1.6267599	1.04805475	10.7964392	0.00371128	0.05330383
Nt5c3b	-0.6813832	3.23958664	10.7804923	0.00373196	0.0534928
Jak3	2.26657746	-0.2724256	10.7778845	0.00373536	0.0534928
Six4	0.9736748	3.45258233	10.7720596	0.00374295	0.05352332
Hnmt	-0.5963265	5.1539492	10.7663879	0.00375036	0.05355113
4933404O12	-0.5962754	3.94674766	10.7557906	0.00376426	0.05367125

Oasl1	4.8719065	-1.023746	10.746288	0.00377676	0.05377129
Micall2	2.48847171	0.41934992	10.7406401	0.00378422	0.05379923
Cntn5	1.3231757	2.35726734	10.7248987	0.00380508	0.05395531
Cdh11	0.37588252	6.72503246	10.723783	0.00380656	0.05395531
Cnnm2	0.75019211	3.86824534	10.71581	0.00381718	0.05395531
Ywhaz	-0.3194414	12.3202423	10.7157767	0.00381723	0.05395531
Sugp2	0.89955155	3.99885619	10.7084931	0.00382696	0.05401491
Kif20a	1.03755845	1.644675	10.683141	0.00386105	0.05418981
Gria2	0.73263706	9.08586185	10.6776814	0.00386843	0.05418981
Polr3a	0.64039406	3.97303105	10.6769324	0.00386944	0.05418981
Brip1	0.91563324	3.21142349	10.6764887	0.00387005	0.05418981
Acsl4	0.43723273	6.37633032	10.6762368	0.00387039	0.05418981
2310014L17I	2.29188059	-0.1128098	10.6694412	0.00387961	0.05418981
Dynll1	-0.4839283	8.0929737	10.6670905	0.0038828	0.05418981
Osr2	-2.6703016	-0.06452	10.6618713	0.0038899	0.05418981
Krt26	5.01101943	-2.0467071	10.6562088	0.00389763	0.05418981
2810403D21I	2.04100219	0.25113302	10.6491761	0.00390724	0.05418981
Urod	-0.4736391	5.0701814	10.6442499	0.00391399	0.05418981
Ccdc57	1.05533068	1.83468668	10.6429466	0.00391578	0.05418981
Chchd10	-0.5216048	5.60173779	10.6390447	0.00392114	0.05418981
Atg2b	0.68720119	6.04259617	10.6366292	0.00392446	0.05418981
Osgin2	-0.5603337	5.95443226	10.6358973	0.00392547	0.05418981
Atp8b2	0.69645306	4.3200366	10.6296469	0.00393408	0.05418981
Swi5	-0.6322815	5.71478352	10.6276858	0.00393679	0.05418981
Narg2	0.73118711	3.98398958	10.6261342	0.00393893	0.05418981
Ednrb	0.77632171	3.98408025	10.6159669	0.003953	0.05429642
Myom3	2.96025201	-0.2197565	10.6125355	0.00395777	0.05429642
Clasrp	0.92148414	2.54147042	10.6034736	0.00397037	0.0543932
Cntn1	0.48595501	8.31121589	10.574676	0.00401073	0.0548274
Srp19	-0.4325646	6.44334797	10.5725975	0.00401367	0.0548274
Ppapdc2	-0.3936484	6.28486106	10.5689179	0.00401886	0.0548274
Lgr5	0.99848298	2.48091856	10.5605074	0.00403076	0.05491327
Thpo	1.10526751	2.09779978	10.5444415	0.0040536	0.05514775
Fam216a	-0.4417904	5.4381747	10.5320076	0.00407138	0.05527845
Fam124a	0.58878182	4.4110444	10.5296148	0.00407481	0.05527845
Ephb1	0.69102993	3.65570156	10.5234977	0.00408359	0.05527845
A330021E22	0.65722664	4.08752451	10.517478	0.00409226	0.05527845
Pnpla1	1.48978257	0.93716736	10.5154385	0.0040952	0.05527845
Amica1	-1.4315914	1.3615346	10.507094	0.00410726	0.05527845
Tyms	0.73878939	3.66644618	10.506599	0.00410797	0.05527845
Epha6	0.64222201	4.797951	10.5063345	0.00410835	0.05527845
Bcas2	-0.3724949	5.77682026	10.492949	0.00412778	0.05535487
Ptplad2	0.79448048	3.7755559	10.4901687	0.00413183	0.05535487

Tceb2	-0.5596815	5.70765472	10.4852818	0.00413896	0.05535487
Bmper	-0.7224956	3.17057497	10.4806565	0.00414571	0.05535487
Capn15	0.9781458	1.8302036	10.4748371	0.00415423	0.05535487
Gstm6	-1.043197	2.2532875	10.471868	0.00415859	0.05535487
Tomm7	-0.5098312	4.6452699	10.4702333	0.00416099	0.05535487
Rltpr	-0.9855186	1.80579924	10.4675707	0.0041649	0.05535487
Firre	1.16215464	3.39928148	10.4616446	0.00417362	0.05535487
Pxdn	1.1697577	4.05497605	10.4581022	0.00417884	0.05535487
Clgn	1.10569561	2.17288949	10.457718	0.00417941	0.05535487
Nsun3	-0.555974	4.6415673	10.4560629	0.00418185	0.05535487
Cep19	-0.3987853	5.80327152	10.4477257	0.00419418	0.05536881
Kmt2d	0.61949977	7.22961619	10.4477057	0.00419421	0.05536881
Isy1	-0.6011275	4.96121102	10.4338808	0.00421474	0.055565
Kcnn2	0.63544938	3.8382123	10.4281433	0.00422329	0.0555862
Psma7	-0.452151	6.45837861	10.4187529	0.00423734	0.0555862
Sox18	-1.2567324	1.74663565	10.4166995	0.00424041	0.0555862
Fat1	0.51794098	6.13438627	10.4166399	0.0042405	0.0555862
1110037F02I	0.49840986	5.5306821	10.4138278	0.00424472	0.0555862
Vamp2	-0.3886052	10.2441072	10.399823	0.0042658	0.05578767
AI414108	0.87332459	4.67017966	10.3946425	0.00427363	0.05581551
Prkdc	0.72972303	5.15821367	10.3804373	0.00429517	0.05602219
Map1a	0.79711661	10.3099317	10.372587	0.00430713	0.05610345
Kcne4	-0.8852509	3.15263084	10.353735	0.004336	0.05640446
Dlg5	0.51713226	5.22165872	10.3499629	0.0043418	0.05640503
Kidins220	0.49645074	8.28762378	10.3429127	0.00435267	0.05646303
Cacna1a	0.6707943	6.4472544	10.337121	0.00436162	0.05646303
Lyst	0.71006035	6.33483125	10.3358668	0.00436356	0.05646303
Ktn1	0.40837773	7.13143191	10.3195966	0.00438882	0.05664293
Gart	-0.6234101	4.37755038	10.3194652	0.00438903	0.05664293
Hap1	0.7022899	4.20512542	10.3074729	0.00440775	0.05680976
Apol7e	-2.2407769	-0.2586059	10.2882886	0.0044379	0.05712308
Gpn1	-0.5956268	3.93186967	10.2817581	0.00444821	0.05715583
Scd1	0.44318061	6.0379921	10.2769033	0.00445589	0.05715583
Atp8a1	0.56041698	7.56623088	10.2744117	0.00445984	0.05715583
Otud6b	-0.4990205	6.33504162	10.2719293	0.00446378	0.05715583
Lrrc24	1.32692662	0.76078617	10.2665985	0.00447225	0.05718953
Bmp1	0.94460575	2.83587107	10.2614594	0.00448043	0.05721948
Adcy9	0.54172632	6.22779904	10.2555048	0.00448994	0.05724953
Zfp738	0.52914247	5.23921593	10.2488547	0.00450057	0.05724953
Hpca	-0.4334351	8.12687707	10.2472604	0.00450313	0.05724953
Cml3	-1.1987822	1.66790898	10.2453673	0.00450616	0.05724953
Ppp1r13l	-1.2599612	1.34262441	10.2325175	0.00452683	0.05743757
Yeats4	-0.5033252	5.23072457	10.210269	0.00456286	0.05777977

Actl6a	0.65517005	3.68241767	10.207471	0.00456741	0.05777977
Camsap3	0.68183303	3.48513944	10.203752	0.00457347	0.05777977
Wdr33	0.59929276	5.10123031	10.1957778	0.0045865	0.05777977
Ttll4	0.96294629	2.29826772	10.1880399	0.00459917	0.05777977
Rrp7a	-0.7373523	4.01122213	10.1813063	0.00461024	0.05777977
Cml1	-1.5107593	1.30335737	10.1801364	0.00461216	0.05777977
Herc2	0.74330554	7.52920546	10.1685103	0.00463135	0.05777977
E330020D12I	1.01332273	3.84757115	10.1640267	0.00463877	0.05777977
Map1lc3a	-0.690763	4.80328015	10.1540371	0.00465535	0.05777977
9330175M2C	1.92761583	1.18172473	10.1516561	0.00465931	0.05777977
Tmsb4x	-0.6131547	8.89562074	10.1515131	0.00465955	0.05777977
Ints6	0.50924356	4.96000102	10.1506622	0.00466097	0.05777977
Olfr1417	7.00478941	-2.0114826	10.6104228	0.0046634	0.05777977
Mrps21	-0.6332614	5.03713123	10.1475867	0.00466609	0.05777977
Tmem5	-0.5375071	4.60960499	10.1471131	0.00466688	0.05777977
Mfsd11	0.73538628	3.18360502	10.1469203	0.0046672	0.05777977
Depdc5	0.6273708	5.25652569	10.1431835	0.00467344	0.05777977
Dok5	0.70776277	3.31033104	10.1421205	0.00467522	0.05777977
Chd8	0.50662166	6.62880664	10.1408822	0.00467729	0.05777977
6330415B21I	0.86041756	3.14758581	10.1406514	0.00467767	0.05777977
Tas2r137	7.79974891	-1.9346366	11.2989755	0.00469435	0.0578043
Tenm1	0.8460284	5.44457367	10.1291873	0.00469688	0.0578043
Ubr1	0.58726408	6.15641405	10.1289032	0.00469736	0.0578043
Bcl2a1d	-1.3125873	1.18069673	10.1234714	0.0047065	0.05782325
Npas3	0.71369474	3.93110127	10.1209709	0.00471071	0.05782325
Ccdc181	-0.5164235	5.18637453	10.1136582	0.00472305	0.05787949
Cndp2	-0.5352766	4.36161415	10.1112586	0.00472711	0.05787949
Nmur2	6.7552268	-1.6835497	10.1057185	0.00473649	0.05791102
Sub1	-0.447092	8.79956942	10.1027614	0.00474151	0.05791102
Igf1r	0.43276781	6.90698274	10.0902984	0.00476271	0.0580976
Nrep	-0.5024751	7.95133817	10.0822361	0.00477649	0.05819317
Taf1	0.47587414	6.83215299	10.07314	0.00479208	0.05831064
Slc1a2	0.59886673	10.9655463	10.066711	0.00480314	0.05837267
Snrnp70	0.57520278	5.73712397	10.0599286	0.00481484	0.05844229
Arf2	-0.5495141	6.06373611	10.0542546	0.00482464	0.058444
C030013G03	-0.8029933	2.95740626	10.0529458	0.00482691	0.058444
Birc6	0.76096018	7.93359802	10.0306292	0.00486572	0.05878371
Rpl12	-0.4767342	6.78598507	10.0299157	0.00486697	0.05878371
Atp6v1d	-0.3275713	8.33107629	10.0234273	0.00487832	0.05884827
A930011O12	1.26444924	4.98274965	10.0170981	0.00488942	0.05885126
Cdc42se2	-0.3645643	6.29738969	10.0164368	0.00489059	0.05885126
1700047M11	1.13258211	1.0395494	10.0070893	0.00490704	0.05893055
Zik1	0.69785095	3.46310341	10.0058603	0.00490921	0.05893055

Gm14827	1.24265231	2.66352071	9.99732692	0.00492429	0.05900369
04-Mar	-0.4850119	4.92876459	9.99559942	0.00492735	0.05900369
Cmtm8	-2.1638812	0.26693661	9.98699074	0.00494262	0.05911436
Acsbg1	0.52581297	4.57552639	9.98304395	0.00494965	0.05912113
Gabrg2	0.48593545	6.44501646	9.97877391	0.00495726	0.05912113
Pcdhga6	0.93411189	2.36475453	9.9765086	0.0049613	0.05912113
1810026B05I	0.60042304	3.78998523	9.97034401	0.00497232	0.05914704
Itga9	0.67994639	3.28035532	9.968538	0.00497555	0.05914704
Cacna1b	0.66565502	6.84283263	9.96352995	0.00498453	0.05918194
H2-T23	0.52339765	4.24340416	9.95569157	0.00499861	0.05918934
Meig1	-1.9737965	0.54987628	9.94894528	0.00501077	0.05918934
Fam92a	-0.4264016	6.562983	9.94831388	0.00501191	0.05918934
Bag1	-0.5689537	7.80584386	9.94547534	0.00501704	0.05918934
Igf2r	0.53241052	4.7242819	9.94540475	0.00501717	0.05918934
Ywhae	-0.3832566	10.7332426	9.93945309	0.00502794	0.05918934
Psmb2	-0.5570207	5.36654514	9.93717448	0.00503207	0.05918934
Ccdc88c	0.70780303	4.2524516	9.93473136	0.0050365	0.05918934
Zfp787	-1.3894424	1.59554729	9.93306245	0.00503953	0.05918934
Rwdd1	-0.4725645	5.23543726	9.9249849	0.00505423	0.0592424
BC035044	-1.7507949	0.39691978	9.91984646	0.0050636	0.0592424
Fbn1	0.70741749	3.99856872	9.91663068	0.00506948	0.0592424
Polr2k	-0.5534292	4.07922009	9.91121751	0.00507939	0.0592424
Gm4532	4.43694249	-1.3773558	9.90731204	0.00508655	0.0592424
Polr2m	-0.4356847	8.6440255	9.89364638	0.0051117	0.0592424
Fezf1	4.64635938	-0.6692118	9.88940557	0.00511953	0.0592424
Uggt2	0.78847298	4.89630415	9.88385068	0.00512981	0.0592424
Cdk2ap1	-0.4675798	6.49375569	9.88360628	0.00513026	0.0592424
Unc80	0.78402518	8.33898979	9.88048974	0.00513604	0.0592424
0610012G03	-0.7352622	3.83728121	9.8786605	0.00513944	0.0592424
4933431E20I	-0.4107821	6.76028562	9.87742673	0.00514173	0.0592424
Rhno1	-0.7895565	2.51360383	9.87512961	0.005146	0.0592424
Dnajb6	-0.3426969	7.95725082	9.87281907	0.00515029	0.0592424
Oscar	2.32728486	0.10059127	9.8726961	0.00515052	0.0592424
Atp2b1	0.57535372	9.27705514	9.86101623	0.00517231	0.0592424
Krt1	1.51854724	1.74797374	9.85988544	0.00517442	0.0592424
Rnf141	-0.6138071	4.23117867	9.85790371	0.00517813	0.0592424
Tmx3	0.5217959	5.80840114	9.85775471	0.00517841	0.0592424
Hs3st4	-0.506159	5.45313509	9.85730765	0.00517925	0.0592424
B230118H07	-0.5192024	5.48858594	9.85670843	0.00518037	0.0592424
Tdrd6	2.7568367	-0.5008325	9.85110677	0.00519087	0.0592424
Ndfip1	-0.5317034	7.96191732	9.84910328	0.00519464	0.0592424
Leprel2	0.81698883	3.23305607	9.84801394	0.00519668	0.0592424
Calm3	-0.3458014	8.7129131	9.84613769	0.00520021	0.0592424

Unc50	-0.4956845	5.64596372	9.84346851	0.00520524	0.0592424
Chpf2	0.9883744	2.28687468	9.84234727	0.00520735	0.0592424
Ttc13	0.80229391	3.19742116	9.82597261	0.0052383	0.05943162
Bcl7b	-0.5592614	4.11000641	9.82544122	0.0052393	0.05943162
Lats2	-0.5039903	6.82253489	9.82392434	0.00524218	0.05943162
Cxcr2	0.7296662	4.02308333	9.81362474	0.00526177	0.05958472
AI504432	0.52371063	5.6203491	9.79413884	0.00529905	0.0599376
Llph	-0.5733208	7.07072829	9.79002279	0.00530696	0.05995786
Nxph1	0.56900866	4.83149152	9.77921301	0.0053278	0.06006822
Ercc6l	2.71142015	-0.7930277	9.77859749	0.00532899	0.06006822
N6amt2	-0.7282072	3.86164223	9.77223379	0.00534131	0.06013783
Pirt	-7.2165135	-0.890339	9.76816183	0.00534921	0.0601576
Ncan	0.53633595	8.37898818	9.7647704	0.00535579	0.06016261
Gapvd1	0.46654157	6.04140785	9.75938617	0.00536627	0.06021124
Tulp4	0.37209713	8.14953768	9.74697267	0.00539051	0.06041402
Atp5g1	-0.5948541	5.41654017	9.73887663	0.00540639	0.06045778
Gm4371	-6.2032044	-0.7745846	9.73868625	0.00540676	0.06045778
Aldob	2.90560749	0.34713287	9.73423881	0.0054155	0.0604865
2410089E03I	0.88452821	5.88888922	9.72520062	0.00543332	0.06058924
Sipa1l3	0.46749511	5.34766959	9.72330164	0.00543707	0.06058924
Il1rn	-4.3239808	-1.1083452	9.71922949	0.00544513	0.06061004
Paxbp1	0.66551482	5.67637254	9.71224136	0.00545898	0.06069528
Eif2s3y	0.84665505	5.08863465	9.69998093	0.00548338	0.06089746
Sez6	0.60046801	4.84745636	9.68844278	0.00550646	0.06104002
Spata2	-0.5710857	5.49015829	9.68733286	0.00550868	0.06104002
Mrps36	-0.5136184	5.16856347	9.67844384	0.00552654	0.06111264
Wnt5b	-1.4090712	1.1476272	9.6778609	0.00552771	0.06111264
Pde10a	0.66638971	7.06493354	9.66797491	0.00554766	0.06126397
Ube3a	0.48391056	6.7638615	9.66438097	0.00555492	0.06127516
Fkbp3	-0.362229	6.9856642	9.64234143	0.00559974	0.06166518
A630089N07	1.10582934	8.10768397	9.64080591	0.00560287	0.06166518
Nol12	-0.8327731	2.80881033	9.62074215	0.00564404	0.06191465
Col10a1	2.44021206	-0.5031777	9.62057598	0.00564438	0.06191465
Arhgef28	0.54848045	4.27869738	9.61397957	0.00565799	0.06191465
Pikfyve	0.53275984	6.77056476	9.61112392	0.0056639	0.06191465
Apol7b	-2.5758209	-0.4814013	9.60752608	0.00567134	0.06191465
Pcsk5	0.74742308	4.26563116	9.604554	0.0056775	0.06191465
2010107G23	-0.5928491	4.05042054	9.60378331	0.0056791	0.06191465
Eif2s2	-0.352466	7.44919119	9.60274975	0.00568125	0.06191465
Rapgefl1	0.43443471	6.31641623	9.59840903	0.00569026	0.06191465
Zfp575	0.88826486	2.81930145	9.59489205	0.00569758	0.06191465
Gm15910	0.83746313	4.50043733	9.59356301	0.00570035	0.06191465
Lrp4	0.60338054	3.95895007	9.59306378	0.00570139	0.06191465

Iqsec3	0.65218344	6.55451205	9.58966372	0.00570848	0.06192299
Zw10	1.22557973	2.49338319	9.57513726	0.00573888	0.06218387
Ppp1r3b	-0.7202032	4.93530695	9.56707909	0.00575582	0.06223715
Sh3bgrl3	-0.5277403	6.73140754	9.56530927	0.00575955	0.06223715
D030045P18	3.85291023	-0.5537525	9.56098029	0.00576868	0.06223715
Dgat1	1.40927374	1.99018369	9.55860163	0.0057737	0.06223715
Gm10471	1.22768056	1.85774165	9.55771997	0.00577556	0.06223715
C230035I16R	-2.9398268	0.05992777	9.53963672	0.00581393	0.06258172
Tmem72	-4.4608859	-1.1869953	9.53168009	0.0058309	0.0626955
Mgst3	-0.7135042	3.55588685	9.52178184	0.00585209	0.06274306
Phyh	-0.3900421	7.28550816	9.51942411	0.00585715	0.06274306
2210016L21F	-0.4565035	6.25796474	9.51889601	0.00585828	0.06274306
Iqsec2	0.51912513	5.69733138	9.51765608	0.00586094	0.06274306
Cep97	0.48343473	4.58543126	9.50599558	0.00588606	0.0629153
Lsmem1	-5.3442786	-1.4809773	9.50422574	0.00588988	0.0629153
Sox11	0.64626455	4.87642157	9.48192296	0.00593828	0.06336323
Cdh10	0.61126653	5.14094305	9.47550576	0.00595229	0.06344359
Pgk1	-0.3532457	7.98381182	9.46371625	0.00597812	0.06363119
Prdx4	0.63749903	3.41192748	9.46154993	0.00598288	0.06363119
Zfp961	-0.4221766	5.09700489	9.45475004	0.00599785	0.06368266
Dnaja3	-0.4574108	5.03685258	9.45344713	0.00600072	0.06368266
Zc3h7a	0.6317012	5.52498518	9.43403656	0.00604371	0.0640694
Slc7a14	0.49559035	7.01975861	9.42841901	0.00605621	0.06409274
Naa16	-0.645062	4.04612996	9.42716989	0.00605899	0.06409274
Tmem68	0.43407151	5.12180934	9.42028093	0.00607437	0.06418612
Pir	-0.5906665	3.46972287	9.41350467	0.00608955	0.06425107
Igdcc4	0.41692842	5.11809547	9.41167944	0.00609364	0.06425107
9830147E19I	0.95485399	1.87966411	9.39361075	0.00613433	0.06461056
Vmn2r84	2.15886872	0.21344119	9.38449508	0.00615497	0.06475835
Cdc123	-0.4280072	6.25246	9.37939243	0.00616656	0.06481067
Crcp	-0.6083681	4.59667811	9.36294859	0.00620408	0.06513504
Trim12a	-0.7955815	3.76202107	9.35729734	0.00621703	0.06519336
Leap2	3.68123403	-0.4596987	9.35432574	0.00622385	0.06519336
Fgf18	-0.9871843	2.47317753	9.34972659	0.00623442	0.06519336
Naa60	-0.3720849	6.11045487	9.34767453	0.00623915	0.06519336
BB031773	3.77537991	-1.0355317	9.34604239	0.00624291	0.06519336
Denr	-0.4384067	5.7057541	9.33928822	0.0062585	0.06527934
Mvp	-0.8601845	3.7879624	9.33352664	0.00627183	0.06527934
Rragc	-0.4112589	5.81121884	9.33098971	0.00627771	0.06527934
6330549D23I	-1.5507737	1.73944601	9.33080464	0.00627814	0.06527934
Fam96a	-0.6195175	5.17674394	9.32807968	0.00628446	0.06527934
Rrp36	-1.1153723	2.16322795	9.31644314	0.00631155	0.06549123
Fam208b	0.43070165	6.44869303	9.31056309	0.00632528	0.06549451

Brd7	-0.381341	5.86924557	9.30685793	0.00633396	0.06549451
Hgf	1.19589634	2.0473698	9.30648674	0.00633483	0.06549451
Tango6	-0.9041369	2.27746456	9.30487221	0.00633861	0.06549451
Unc13c	0.43982982	5.92405696	9.29152018	0.00637	0.06574946
Tmod4	2.60094907	-0.5114358	9.27696545	0.00640441	0.06603506
Reln	0.7966912	5.25469557	9.27123405	0.00641801	0.06610578
Dlg4	-0.4028876	7.45345862	9.26449637	0.00643405	0.06620135
Tprgl	-0.417247	6.93458413	9.26107498	0.00644221	0.06621577
Gatsl2	-0.4029614	6.17021355	9.25577149	0.00645489	0.06627648
Gm14378	2.01072965	-0.2341426	9.2529306	0.00646169	0.06627683
Rcan1	-0.48917	5.88676338	9.24661985	0.00647682	0.06636257
Cps1	4.46992595	-1.1991951	9.23814802	0.0064972	0.06643726
Eif4enif1	-0.3436432	6.6647732	9.23795017	0.00649767	0.06643726
Atp5j2	-0.5677851	5.43706898	9.21176204	0.00656113	0.06701608
Fbxo27	-0.6544087	3.83541768	9.19939974	0.00659132	0.06725434
Litaf	-0.6442832	4.59247002	9.1951142	0.00660182	0.06729141
Cetn2	-0.6436938	5.26854616	9.18554826	0.00662533	0.06739014
Tpi1	-0.476023	6.96436296	9.18040744	0.006638	0.06739014
Tmem181a	0.70558575	3.45736147	9.17861858	0.00664242	0.06739014
Slk	0.42632988	8.16449595	9.17748148	0.00664523	0.06739014
Anp32e	-0.4093473	8.05480532	9.17720755	0.00664591	0.06739014
Pnpla3	1.53925609	1.33916254	9.16613886	0.00667332	0.0674868
A330070K13	3.57734792	-0.6852585	9.16593156	0.00667384	0.0674868
Nup205	0.64964689	3.99104559	9.16501657	0.00667611	0.0674868
Rrp9	-1.3329905	1.10828275	9.15715856	0.00669566	0.06758105
Tenm2	0.73936716	7.00559318	9.15522998	0.00670046	0.06758105
Srgap2	0.3789104	6.07483362	9.15295912	0.00670613	0.06758105
Csmd1	0.86935964	6.08621205	9.14898606	0.00671605	0.06758283
Anapc11	-0.6087719	4.06063091	9.14736647	0.0067201	0.06758283
Unc45a	-0.9242285	2.75728444	9.13948375	0.00673986	0.06771196
Stag3	5.21418178	-1.6816123	9.13203046	0.0067586	0.06778142
Prr18	0.72224931	3.12852725	9.12716428	0.00677086	0.06778142
Adam9	0.42525823	4.9569587	9.12500906	0.0067763	0.06778142
Nfasc	0.50204181	7.70953215	9.12032686	0.00678813	0.06778142
Insr	0.51472801	5.99010779	9.11800239	0.00679402	0.06778142
Itga8	0.96826798	2.59275693	9.11676805	0.00679714	0.06778142
Hmgn2	-0.6437774	6.50993329	9.11320568	0.00680618	0.06778142
Sord	-0.5906532	4.3922817	9.11158188	0.0068103	0.06778142
Btbd11	0.47652341	4.43944584	9.10770846	0.00682014	0.06778142
4930570G19	0.85983434	3.53760825	9.10650227	0.00682321	0.06778142
Tgtp2	-0.8291277	3.77523968	9.10391958	0.00682979	0.06778142
Adamts4	1.16362611	1.78413118	9.10259018	0.00683317	0.06778142
Tgtp1	-0.7795546	3.77009538	9.10119603	0.00683673	0.06778142

Ccdc53	-0.6355351	3.93266763	9.09815506	0.00684449	0.06778974
Ptprf	0.45970985	4.74980605	9.08442607	0.00687965	0.0679813
Taok3	-0.4644206	5.86871677	9.08162516	0.00688684	0.0679813
Fuk	0.97899302	1.56267441	9.08033668	0.00689016	0.0679813
A230046K03	0.57470103	6.37811693	9.07977941	0.00689159	0.0679813
Pygo2	-0.5653984	3.87133083	9.07059461	0.00691527	0.06810926
Wdr3	0.76399214	3.22778779	9.06935567	0.00691847	0.06810926
Mina	-0.8192186	2.58326857	9.06467596	0.00693057	0.06815992
Nqo2	-0.5087316	5.67377076	9.05542796	0.00695456	0.06832727
Basp1	-0.4125814	9.41422249	9.05127406	0.00696537	0.06836083
Col5a2	0.70915033	2.99859565	9.04515451	0.00698132	0.06836083
Dapp1	-0.7166239	4.05572574	9.04469457	0.00698253	0.06836083
Repin1	0.5459412	4.53713337	9.04315063	0.00698656	0.06836083
Kcnk1	0.47975214	5.55611367	9.04073392	0.00699287	0.06836083
Myo10	0.4113456	5.70474364	9.03778582	0.00700059	0.06836801
Vwa8	0.5591465	5.28914095	9.02444814	0.00703561	0.06864158
Prkg1	0.38718799	5.8376894	9.00926306	0.00707572	0.06890015
Atp2c1	0.40115613	6.56727985	9.00908883	0.00707618	0.06890015
2310069B03	-7.3943025	-1.3945449	9.35896168	0.00713091	0.06929785
Zfp1	-0.4833443	4.28516805	8.98143343	0.00714989	0.06929785
Pak2	-0.399061	6.79178155	8.97954942	0.00715494	0.06929785
Pigg	0.60600255	3.45784024	8.97812202	0.00715877	0.06929785
1700008F21	1.94952685	0.94287761	8.97690756	0.00716203	0.06929785
Slc37a3	0.64842624	4.04118976	8.97625635	0.00716378	0.06929785
Fsip1	-4.8615874	-1.5380426	8.97497141	0.00716723	0.06929785
Ntmt1	-0.8491452	2.14595237	8.97259594	0.00717362	0.06929785
Higd1a	-0.4113328	6.64605834	8.95984068	0.00720803	0.06951956
Pigm	0.62945278	4.17321797	8.95754149	0.00721425	0.06951956
Med8	-0.4634849	3.81984149	8.95472195	0.00722189	0.06951956
1110004E09	-0.6150331	5.77998357	8.95359027	0.00722496	0.06951956
Gabra5	0.43397483	5.66006483	8.94256609	0.00725493	0.06971238
Hbegf	-1.1421965	1.72876483	8.9352738	0.00727483	0.06971238
Dnah10	1.42397154	1.13140033	8.9346704	0.00727648	0.06971238
Cyb561a3	0.62509932	3.48963815	8.93006283	0.00728908	0.06971238
Rspo4	-3.3220124	-1.1341276	8.92857821	0.00729315	0.06971238
Tbc1d14	-0.439502	5.17225701	8.92332686	0.00730756	0.06971238
Gm9079	2.1848234	0.57744358	8.9203913	0.00731562	0.06971238
Eif2b5	-0.5004002	4.56639285	8.91949311	0.00731809	0.06971238
Gfra4	-0.5332738	4.35583022	8.91841284	0.00732107	0.06971238
Gnb1	-0.329478	9.34935257	8.91559902	0.00732882	0.06971238
Pcna	-0.4700508	6.30910801	8.91458222	0.00733162	0.06971238
A4gnt	-4.3815846	-1.4684835	8.91127585	0.00734074	0.06971238
Ubxn6	-0.3977612	5.56864892	8.90880177	0.00734757	0.06971238

Mia3	0.49042448	6.29115697	8.90627963	0.00735455	0.06971238
Nop16	-0.9931108	2.38297078	8.9060678	0.00735513	0.06971238
Fam213b	-0.5878671	4.12437467	8.90471647	0.00735887	0.06971238
Fxyd2	-2.2130856	-0.1409644	8.89574105	0.00738376	0.06981444
Ankrd63	0.73132327	4.06143589	8.89506881	0.00738563	0.06981444
Abcc10	1.29801026	0.96648343	8.89312825	0.00739103	0.06981444
Tm2d1	0.70590434	2.9591863	8.88266951	0.00742018	0.06998495
Hax1	-0.558772	5.40342421	8.87935295	0.00742945	0.06998495
AW495222	-1.0409461	1.74746184	8.87897445	0.00743051	0.06998495
Myo7a	1.09831449	2.71220591	8.8755023	0.00744023	0.07000921
2610307P16I	-2.1628795	0.88520349	8.87059369	0.007454	0.07003356
Slmo2	-0.3819094	6.85782777	8.8668558	0.00746451	0.07003356
BC005624	-0.4823287	6.12337461	8.8665144	0.00746547	0.07003356
E130215H24I	-5.2428193	-1.8867216	8.86439858	0.00747142	0.07003356
Necap2	-0.8605284	3.41780728	8.85403664	0.00750066	0.07018963
Ap1s3	0.81144461	3.67176924	8.85166198	0.00750737	0.07018963
Acrbp	2.35286734	0.21321377	8.85016892	0.0075116	0.07018963
Ryr3	1.08622404	5.30691319	8.84686388	0.00752097	0.07018963
Vps29	-0.4400861	5.86087869	8.84271715	0.00753274	0.07018963
Inip	-0.4974754	5.15802191	8.83908905	0.00754305	0.07018963
Fam212b	-0.4200232	6.76084735	8.83870935	0.00754413	0.07018963
Tmem170	-0.7766692	3.25063668	8.83747072	0.00754766	0.07018963
Zfp618	-1.0208116	1.48784735	8.83450724	0.0075561	0.07018963
Ugt8a	0.53645412	5.3525216	8.83323623	0.00755973	0.07018963
Sin3a	0.36780642	6.53073228	8.82716277	0.00757707	0.07022341
Acsf2	-0.5528284	4.8861984	8.82694228	0.0075777	0.07022341
Fcgr3	-1.4564497	1.06475756	8.81991364	0.00759783	0.07027743
Kcnj8	2.03049375	0.66783399	8.81989719	0.00759788	0.07027743
Stk3	-0.4458908	5.66440243	8.81349441	0.00761627	0.07036572
Zfp65	0.6029781	5.05369381	8.80932071	0.00762829	0.07036572
Atp5l	-0.4883659	6.48418274	8.80908059	0.00762898	0.07036572
Cbfb	-0.5671132	6.03301844	8.80125302	0.00765157	0.07049564
Mfi2	-3.1536475	-0.3768667	8.79921746	0.00765746	0.07049564
Atf6b	-0.6897983	3.13973333	8.79384796	0.00767301	0.07051921
Hspb11	-0.7929791	3.06169139	8.79336388	0.00767442	0.07051921
Nrg3os	2.08488792	0.39788992	8.78455659	0.00770001	0.07063513
Unc79	0.79424604	5.96216454	8.78297917	0.0077046	0.07063513
Srrm4os	1.18936205	1.83106618	8.78158554	0.00770867	0.07063513
Gm14393	-0.8239969	2.67963904	8.77345735	0.0077324	0.07078639
Ppa2	-0.4270339	5.40210486	8.76766917	0.00774935	0.07084477
Pde1c	0.76930358	4.17786531	8.76370839	0.00776097	0.07084477
9430091E24I	-1.3664872	1.9031491	8.76145404	0.0077676	0.07084477
Fam84b	0.6825111	2.78382697	8.76141738	0.00776771	0.07084477

Pdzrn4	0.85825656	2.404402	8.75080721	0.00779897	0.07106373
Rab3gap1	0.53258961	4.95786349	8.74354494	0.00782045	0.07119322
Mgat1	-0.5968888	3.42055107	8.7368631	0.00784027	0.07126421
Aatf	-0.5942068	3.91732597	8.73601228	0.0078428	0.07126421
Kcnj16	0.63666151	4.19006785	8.72888704	0.007864	0.07137551
Ndufb5	-0.4442487	6.73413816	8.72512693	0.00787522	0.07137551
Psmd8	-0.4402721	5.10244073	8.72456141	0.00787691	0.07137551
Comt	-0.4018853	5.68667028	8.7201628	0.00789005	0.07138397
Alms1	0.64414097	5.05209571	8.71445394	0.00790715	0.07138397
Spag1	0.68678564	2.86066119	8.7143304	0.00790752	0.07138397
Nphp4	0.99376935	2.08215996	8.70979179	0.00792115	0.07138397
Dph5	0.6035642	4.65440441	8.70937828	0.00792239	0.07138397
Fbxo22	-0.3657131	6.86914648	8.70813198	0.00792614	0.07138397
Erlin1	0.51090988	4.56877923	8.70722865	0.00792885	0.07138397
Csmd3	0.79900733	5.37477156	8.70033443	0.00794962	0.07150522
Fry	0.76853725	8.22700154	8.69400518	0.00796874	0.07159746
Myeov2	-0.5176509	4.74117315	8.69210349	0.00797449	0.07159746
Lrp8	0.77177241	5.85580177	8.68803692	0.00798681	0.07162186
Ypel5	-0.4373274	7.68390628	8.68381423	0.00799963	0.07162186
Pcdhb20	0.79404431	3.63432425	8.6830219	0.00800204	0.07162186
Paip2b	-0.4012869	5.25895448	8.68156805	0.00800646	0.07162186
0610009B22I	-0.6927159	5.56179021	8.67911777	0.00801391	0.07162313
Cdkn1a	-0.4897108	5.39687725	8.67668062	0.00802133	0.07162412
5031434C07I	6.10276844	-1.6976484	9.01263945	0.00805441	0.07183237
Xpo5	0.53668057	4.68001948	8.66269028	0.00806409	0.07183237
1700016K19I	-1.5613587	1.06684404	8.66185302	0.00806666	0.07183237
Grin2b	0.57779319	7.05758381	8.65138062	0.00809884	0.07192497
Peli3	-2.2508595	-0.7958979	8.65078714	0.00810067	0.07192497
Rbbp5	0.3810219	5.69643705	8.64845272	0.00810786	0.07192497
Enoph1	-0.4146215	4.85653078	8.64782289	0.00810981	0.07192497
Chsy3	0.77153417	2.91210775	8.64236852	0.00812665	0.07192497
1700123M08	-1.2960182	1.25179356	8.63702746	0.00814318	0.07192497
Cops8	-0.3711809	5.82412212	8.6360394	0.00814624	0.07192497
Fstl5	0.65933298	4.1514189	8.63480923	0.00815006	0.07192497
Ept1	0.39277319	4.98612583	8.63319687	0.00815506	0.07192497
Miip	1.63199307	0.92175841	8.63313431	0.00815526	0.07192497
Yrdc	-0.6264362	3.48619489	8.63045946	0.00816356	0.07192497
Exoc4	0.38884861	5.87037846	8.62802013	0.00817115	0.07192497
Slc6a1	0.49276112	7.70563936	8.62758082	0.00817251	0.07192497
Cdca2	-3.3056124	-0.3892356	8.62519229	0.00817995	0.07192578
Hapln2	1.39198153	1.13557707	8.61444154	0.0082135	0.07205093
Plekha5	0.47393864	5.42071835	8.61280478	0.00821863	0.07205093
Esd	-0.4413055	6.08734854	8.61051254	0.00822581	0.07205093

E2f6	-0.4318813	5.50491294	8.61045349	0.00822599	0.07205093
Ccdc141	0.55512872	4.28111604	8.60886925	0.00823096	0.07205093
Maea	-0.3599603	5.48426659	8.60178561	0.00825321	0.07214689
Rsbn1l	0.41465326	5.5308702	8.60069042	0.00825665	0.07214689
Gapdhs	1.64186166	1.29571558	8.58630438	0.00830206	0.07239722
1110008P14I	-0.4509607	4.56807897	8.58620407	0.00830238	0.07239722
Llgl1	0.5019704	3.95670421	8.58365131	0.00831047	0.07239722
Idh3b	-0.3308908	7.06719223	8.58188346	0.00831607	0.07239722
Mctp1	0.45755347	5.38954545	8.57940744	0.00832393	0.07239722
Kcnc1	0.53874227	6.98292561	8.57760839	0.00832965	0.07239722
H1f0	-0.438272	6.9773006	8.5732543	0.00834349	0.0724533
Cox20	0.49280067	5.02108414	8.55977923	0.00838652	0.07262746
Tceal3	-0.450919	5.14347973	8.55966874	0.00838687	0.07262746
Polr3gl	0.68082395	2.88161822	8.55928396	0.00838811	0.07262746
Tubgcp4	0.57664956	3.73131625	8.55769119	0.00839321	0.07262746
Arpp19	-0.3185713	9.06957208	8.55353342	0.00840655	0.07265075
Cadm2	0.46985331	8.70551438	8.55053238	0.00841619	0.07265075
Ppp1r1c	-1.8422865	1.07525846	8.54992197	0.00841815	0.07265075
Nudcd2	-0.6933406	3.02746944	8.54255392	0.00844188	0.07274406
Cacna1e	0.70428311	8.22203066	8.54170104	0.00844463	0.07274406
Nhp2l1	-0.4274717	6.06380056	8.53739967	0.00845852	0.07274406
Txndc17	-0.5091188	6.32172748	8.53735519	0.00845867	0.07274406
Zfp105	-0.8703277	3.11122438	8.53040652	0.00848116	0.0728215
1700019D03I	-1.1316353	1.69475413	8.5296672	0.00848356	0.0728215
A730017L22I	0.78381079	4.89894303	8.52573776	0.00849632	0.0728215
Grhl2	1.95508746	0.49224649	8.52540175	0.00849741	0.0728215
Gm20753	3.84025922	-1.593677	8.52228722	0.00850754	0.07284455
Eif4a3	-0.4725228	5.00288545	8.51579598	0.00852869	0.0729463
Ehbp1l1	-0.4916013	4.25592144	8.51346554	0.00853629	0.0729463
Hmgcr	0.48122443	5.79284207	8.51179192	0.00854176	0.0729463
Ttyh1	0.53125747	7.60305799	8.5003086	0.00857938	0.07320377
Pi4ka	0.55874208	8.10487444	8.48950394	0.00861495	0.07333835
Cdk5r2	-0.3743138	6.69943838	8.48625954	0.00862566	0.07333835
Spef1	-1.1710715	1.53809466	8.4862202	0.00862579	0.07333835
Slc35f6	-0.9748529	1.93306923	8.48572512	0.00862743	0.07333835
Soat1	0.40075894	5.47047829	8.48275412	0.00863725	0.07333835
Tet3	0.49282925	6.03705093	8.48190095	0.00864008	0.07333835
Hist1h1a	-3.6134013	-1.2556486	8.47506982	0.00866272	0.07334732
A130077B15	0.88692832	7.65489008	8.47494646	0.00866313	0.07334732
2810408A11I	2.63603323	-0.898105	8.4731339	0.00866915	0.07334732
Tmem218	-0.7272422	3.03925079	8.47192689	0.00867316	0.07334732
Mnat1	-0.5661636	4.19867211	8.47030011	0.00867857	0.07334732
Crim1	0.52496345	6.98334758	8.46054076	0.00871111	0.07355881

Cldn1	1.60440749	3.08447347	8.45719861	0.00872228	0.07358637
Eif1a	-0.4857793	6.62350168	8.45507272	0.0087294	0.07358637
Pcdhb17	0.58757043	4.75954171	8.45036708	0.00874517	0.07363638
Olfr692	4.9408323	-1.3157961	8.44611325	0.00875945	0.07363638
Whsc1l1	0.3229294	7.71998622	8.43799409	0.00878679	0.07363638
Uqcrfs1	-0.3209133	6.5995679	8.43755729	0.00878827	0.07363638
Kmt2a	0.55042799	8.41968347	8.43583256	0.00879409	0.07363638
Lrp12	0.46341949	4.10002591	8.43455637	0.0087984	0.07363638
Tnnc1	-1.1707012	1.94600666	8.43069585	0.00881145	0.07363638
Ephb3	1.11177233	1.63705225	8.43067186	0.00881153	0.07363638
Wdr45b	-0.5055965	4.47916037	8.4302078	0.0088131	0.07363638
Gm3002	0.96561538	3.60229734	8.4278844	0.00882097	0.07363638
Nup93	0.69565232	4.53506346	8.42667479	0.00882506	0.07363638
Gcn1l1	0.65282982	4.54736474	8.42653443	0.00882554	0.07363638
Ube2s	-0.7244766	3.69044708	8.41703178	0.00885782	0.07381552
Dnajb3	-2.4468769	-0.1025041	8.40849832	0.00888691	0.07381552
Gpr162	1.25542182	2.14572844	8.40612049	0.00889504	0.07381552
Gm14327	1.09846768	2.97504976	8.40489702	0.00889922	0.07381552
Smok4a	1.37892043	1.7706538	8.4006587	0.00891373	0.07381552
Zfp260	-0.3492839	7.24177903	8.39954414	0.00891756	0.07381552
Zfp40	0.54056102	4.31869583	8.39676066	0.00892711	0.07381552
Plxna4	0.55547764	7.02214531	8.39654441	0.00892785	0.07381552
Wdfy3	0.76925875	7.86625625	8.39621982	0.00892896	0.07381552
Sox6	0.6341815	5.00814424	8.39424454	0.00893575	0.07381552
Tbc1d25	-0.7150374	3.07336258	8.39399671	0.0089366	0.07381552
Neurod6	-0.5066599	6.14249421	8.39375263	0.00893744	0.07381552
Nhp2	-0.8165506	3.21327063	8.37771837	0.00899275	0.07396422
Hcn2	1.09264977	1.3127917	8.37734265	0.00899405	0.07396422
Tmcc2	0.49352488	4.78003844	8.37609715	0.00899836	0.07396422
Ttc17	0.46796169	4.84486367	8.37536417	0.0090009	0.07396422
Rptor	0.4713696	5.34640575	8.37468526	0.00900325	0.07396422
Ahrr	4.42241634	-1.6587342	8.3735711	0.00900711	0.07396422
Ralgapa2	0.69950503	4.62057579	8.37187253	0.00901301	0.07396422
Rdh12	1.89387591	0.1413352	8.36864817	0.0090242	0.07396422
Bzrap1	1.01474467	4.8099904	8.36759503	0.00902786	0.07396422
Clcn2	0.58581789	3.90367884	8.36670619	0.00903095	0.07396422
Recql5	-0.9651086	2.43642197	8.36190452	0.00904767	0.07398987
Mrfap1	-0.514784	9.51155094	8.36146747	0.00904919	0.07398987
Nalcn	0.45664501	6.39924881	8.35391759	0.00907555	0.07414349
Kcnj10	0.51066814	5.29891954	8.34284818	0.00911435	0.07439842
Atp5g3	-0.413872	8.20651786	8.3382117	0.00913066	0.07446947
Cnbp	-0.4347523	9.17261251	8.33434327	0.00914429	0.07451701
Gng2	-0.2909489	7.99385618	8.33224189	0.0091517	0.07451701

Ovgp1	1.79278493	0.34036295	8.32985253	0.00916014	0.07452377
Sptssb	-0.7146485	4.63872135	8.32445774	0.00917922	0.07456353
Stxbp2	0.74536996	4.19154777	8.32416733	0.00918025	0.07456353
Babam1	-0.4831551	4.48843025	8.31866748	0.00919975	0.07461949
Dgki	0.67762839	4.88385758	8.31792863	0.00920238	0.07461949
Athl1	-0.7239589	3.28005213	8.31366879	0.00921752	0.07468045
Pitpnm2	0.49888458	6.50109081	8.30930444	0.00923306	0.07474455
Nme1	-0.3889674	6.28486745	8.30013174	0.00926582	0.07494783
Adamtsl1	0.67211499	3.07913631	8.2966771	0.00927819	0.07498598
Kif15	1.47380741	1.06210072	8.28444489	0.00932215	0.07527912
Galk2	-0.6075159	3.52257411	8.27239983	0.00936566	0.07556819
Net1	-0.4085595	5.59464004	8.26748852	0.00938347	0.0755974
Cacna2d1	0.53142585	7.62950505	8.26714435	0.00938472	0.0755974
St6galnac5	0.4950117	4.74676532	8.26158632	0.00940492	0.07560263
Gm5069	-0.4861659	4.42178415	8.26034172	0.00940945	0.07560263
Ttc9b	-0.8154954	3.14900253	8.259878	0.00941113	0.07560263
Cdc42	-0.335427	8.71371659	8.25642208	0.00942373	0.07560263
D930016D06	0.77931822	4.25689725	8.2554617	0.00942723	0.07560263
Gm1976	-0.5412429	4.49603841	8.25210249	0.00943949	0.07560263
Hs6st2	0.64967624	4.82029221	8.24885419	0.00945137	0.07560263
Aldh7a1	-0.4023324	4.98129847	8.24653249	0.00945987	0.07560263
Clns1a	-0.4057885	6.19127817	8.24611615	0.00946139	0.07560263
3010026O09	-0.8123356	3.29919568	8.24343887	0.00947121	0.07560263
Cadm1	0.55309822	6.13708656	8.2432058	0.00947206	0.07560263
Cdk5rap2	0.6078038	3.57249977	8.2376591	0.00949243	0.07560263
Dnajc16	0.73386628	3.07893277	8.23670831	0.00949593	0.07560263
Cybrd1	0.94104582	2.11232125	8.23586085	0.00949904	0.07560263
Hpcal1	-0.5398519	4.60151312	8.23388968	0.0095063	0.07560263
Rab30	-0.451601	4.8877884	8.23162988	0.00951463	0.07560263
Vps13d	0.70322235	6.62824952	8.22473736	0.00954007	0.07560263
Car13	-0.6756876	7.61595465	8.22426008	0.00954183	0.07560263
D030040B21	1.71562886	-0.4700405	8.22405513	0.00954259	0.07560263
Armc1	-0.3394014	7.59764091	8.22365412	0.00954407	0.07560263
Dimt1	0.52774275	5.04268064	8.22125591	0.00955295	0.07560263
Rap1gds1	-0.2853163	8.5849631	8.22065744	0.00955517	0.07560263
Nbas	0.56707029	5.07413997	8.2093407	0.00959718	0.07581494
Mtor	0.6219363	6.58603647	8.20743854	0.00960426	0.07581494
Commd6	-0.6138534	5.30398125	8.20718191	0.00960522	0.07581494
Cd6	-2.9335858	-0.0356959	8.20204715	0.00962437	0.07588819
Coa6	-0.5228107	3.73919613	8.200541	0.00962999	0.07588819
Popdc2	-1.9123736	0.81035692	8.19175278	0.00966289	0.07604475
Trub1	0.44523971	4.67288102	8.19019466	0.00966873	0.07604475
Lsm3	-0.5667303	4.10836897	8.18901798	0.00967315	0.07604475

Atrn	0.45271652	7.87994801	8.18435713	0.00969067	0.07612137
Lrp3	0.54693089	4.92686059	8.18217108	0.0096989	0.07612496
Npc1	0.4761572	4.96851897	8.17282158	0.00973418	0.07630903
Srp9	-0.4906788	6.79681905	8.1701725	0.0097442	0.07630903
Gm16712	-4.5546842	-1.9720096	8.16977071	0.00974572	0.07630903
Gm5148	-0.6380878	3.95471613	8.15927618	0.00978554	0.0764796
Cox7b	-0.4969849	7.33657115	8.15575147	0.00979896	0.0764796
Fryl	0.48974779	7.5460087	8.15567528	0.00979925	0.0764796
Zfand1	-0.6669376	4.29971391	8.15324179	0.00980853	0.0764796
Kcnj5	-3.2445495	-0.0883408	8.14932501	0.00982347	0.0764796
Ranbp2	0.54432222	7.62672975	8.14924483	0.00982378	0.0764796
4930431F12I	1.0800688	2.63778753	8.14901982	0.00982464	0.0764796
Slc16a4	1.20108898	1.73228008	8.1468328	0.009833	0.0764796
Fat3	0.81609341	8.06281523	8.1443841	0.00984237	0.0764796
Becn1	-0.5354056	5.24781277	8.14354431	0.00984558	0.0764796
Vwf	-1.0524908	2.07504973	8.14105958	0.0098551	0.07649289
Gpr88	0.49431762	6.09706618	8.1239219	0.00992105	0.07693628
Pink1	-0.2990234	7.52866259	8.12082868	0.009933	0.07693628
Dmd	0.66253237	7.88618675	8.11949328	0.00993817	0.07693628
Irs4	1.12922886	1.99826185	8.11807807	0.00994364	0.07693628
Ube2e2	-0.366756	6.58122371	8.11588687	0.00995213	0.07694118
Fam185a	-0.5641794	3.69657058	8.1137283	0.0099605	0.07694516
Acbd4	0.86364129	2.3604109	8.10493568	0.00999468	0.07714832
Zdhhc1	0.65727934	3.24992215	8.09930742	0.01001662	0.07720085
Dpysl5	0.71404352	4.75965098	8.09914746	0.01001725	0.07720085
Krt19	1.12391716	1.77903333	8.09472302	0.01003454	0.0772733
Snrpb2	-0.4708938	5.68008037	8.0879292	0.01006115	0.07741739
Shoc2	-0.3344497	8.6086196	8.07945505	0.01009446	0.07757143
Smim18	-0.9676425	2.12277284	8.07751647	0.01010209	0.07757143
Plagl2	-0.5232449	5.17042877	8.07545199	0.01011023	0.07757143
Nbea	0.7717765	8.50315609	8.07478938	0.01011285	0.07757143
Snrpb	-0.5156315	3.74360166	8.06962428	0.01013325	0.07757345
Lrrfip2	-0.3582617	5.83690377	8.06872581	0.0101368	0.07757345
Zfp831	0.78716249	5.24283615	8.06648503	0.01014567	0.07757345
3110045C21I	-3.1703318	-0.5811746	8.06477032	0.01015246	0.07757345
Sirt4	1.01603704	1.73272007	8.064013	0.01015547	0.07757345
Gad2	0.57242921	8.8081103	8.06236497	0.010162	0.07757345
Eif4ebp3	1.31442931	0.94907495	8.06071602	0.01016855	0.07757345
Fbxw8	-0.484327	3.88757682	8.05231406	0.01020196	0.07768901
Retnlg	-5.0174895	-1.3288722	8.05074776	0.0102082	0.07768901
Fam135b	0.87236015	4.6382602	8.04897819	0.01021526	0.07768901
Hcfc1r1	-0.5677826	4.47149002	8.0489391	0.01021542	0.07768901
Rab3ip	-0.4301728	5.14672072	8.04222925	0.01024223	0.07775168

Igsf21	0.75482883	3.61207176	8.04161788	0.01024468	0.07775168
Acot7	-0.4030182	6.14942926	8.04092047	0.01024747	0.07775168
Nudc	-0.4998433	5.63005536	8.03851016	0.01025713	0.07776471
Rpl4	-0.4165756	8.80304344	8.03386006	0.01027579	0.07781068
Ryr2	0.89127577	8.03232556	8.03304077	0.01027908	0.07781068
Rab3gap2	0.58022841	6.46892476	8.03044827	0.0102895	0.07782943
Ppp2cb	-0.3139998	7.09415398	8.0279972	0.01029937	0.07784394
Pdk4	0.751324	2.80876838	8.02179754	0.01032437	0.07790338
2010107E04I	-0.4844638	5.84260277	8.01961019	0.0103332	0.07790338
Exd1	-2.1245393	-0.3507734	8.01928535	0.01033452	0.07790338
Azin1	-0.3198036	7.21388816	8.01754172	0.01034157	0.07790338
Scn4b	0.60836226	4.78443793	8.01619972	0.010347	0.07790338
Nphs1	0.93680739	2.0604557	8.01236526	0.01036253	0.07796041
Tsr2	-0.4290572	4.95762152	8.00790034	0.01038065	0.07799645
Cnih3	-0.4284879	5.92678748	8.00726169	0.01038325	0.07799645
Ccdc171	0.66658624	3.20507222	8.00107882	0.01040841	0.07809094
BC002163	-0.4942662	3.58141497	8.00025321	0.01041177	0.07809094
Gstt3	-0.6361764	5.20221588	7.99824358	0.01041996	0.07809261
Pgr15l	2.63778967	0.38897474	7.9924572	0.0104436	0.07812664
Wdr54	-0.7473758	3.28234784	7.99158718	0.01044716	0.07812664
Rufy1	0.46540684	4.69976452	7.9912759	0.01044843	0.07812664
Trh	4.23765517	-1.6101563	7.98798663	0.0104619	0.07816769
Ada	-1.1273284	1.31544401	7.97924948	0.01049778	0.07818625
Pla2g4a	-0.5302285	4.59834427	7.97859502	0.01050047	0.07818625
Mrpl48	-0.4967872	4.45737419	7.97750514	0.01050495	0.07818625
Dazl	1.21327977	1.50788797	7.97661072	0.01050864	0.07818625
Igf2bp3	0.67515877	4.2010688	7.97390763	0.01051978	0.07818625
Pfn2	-0.3508517	7.71573022	7.97294502	0.01052375	0.07818625
Mir3064	2.01334649	-0.6415369	7.97115523	0.01053113	0.07818625
Myof	-0.7233282	5.02471238	7.97002819	0.01053579	0.07818625
B3gnt9	-1.118781	1.8098825	7.96992201	0.01053622	0.07818625
Skp1a	-0.4022051	8.04762672	7.96552688	0.0105544	0.07820916
Tnfaip8l1	-0.9126077	2.81437686	7.96339519	0.01056322	0.07820916
Ppef2	2.99879117	-0.8436793	7.96087961	0.01057365	0.07820916
Lrp1	0.37343147	7.3421971	7.96054087	0.01057505	0.07820916
Fut8	0.56897392	6.99074698	7.95877661	0.01058237	0.07820916
5930412G12	-1.6534937	0.82290441	7.95704257	0.01058957	0.07820916
Chd3os	-0.3044937	6.56164557	7.95568762	0.0105952	0.07820916
Ahctf1	0.46343816	6.60380263	7.9487205	0.01062421	0.07828011
Cript	-0.4627458	7.53137225	7.94735779	0.01062989	0.07828011
Bbs10	0.74938372	2.99343135	7.94690512	0.01063178	0.07828011
Zfp488	0.85703072	7.72018288	7.94428483	0.01064272	0.07828011
Arntl	0.48663904	3.98379063	7.94134003	0.01065502	0.07828011

Usp25	-0.3368753	7.47059909	7.94119726	0.01065562	0.07828011
Lmtk2	0.45213448	6.76082334	7.93997003	0.01066076	0.07828011
Abca17	3.18928127	-0.2030973	7.93673702	0.0106743	0.07828991
Ano2	2.09865554	0.14009161	7.93532543	0.01068021	0.07828991
Pgbd5	-0.3719837	7.53473833	7.93392996	0.01068607	0.07828991
Trmu	1.09975364	2.08059847	7.92872512	0.01070793	0.07832096
Arpc2	-0.390843	8.49607501	7.92771343	0.01071219	0.07832096
Dpm2	-0.4963022	4.06808179	7.92721321	0.01071429	0.07832096
AI837181	-0.5314687	4.19362542	7.92338009	0.01073044	0.07833059
Ldoc1	-3.4249175	-1.3494484	7.92310356	0.0107316	0.07833059
Atf2	-0.2925833	8.8748874	7.92013177	0.01074414	0.07836371
9030204H09I	2.89734796	-1.183812	7.90916903	0.01079053	0.07860938
Mybpc1	1.44351658	0.97446557	7.90821667	0.01079457	0.07860938
1810037I17R	-0.5383953	5.63404425	7.90454178	0.01081018	0.07860938
Nr1h2	-0.7213101	4.09397517	7.90449407	0.01081039	0.07860938
2900097C17I	-0.5009826	10.8249655	7.9027149	0.01081795	0.07860938
Lyplal1	-0.7709459	2.81065316	7.89356584	0.01085695	0.07863997
Naip6	3.86547143	-2.0934829	7.89281887	0.01086014	0.07863997
Tatdn3	0.68462607	2.52536768	7.89263968	0.01086091	0.07863997
Gnptab	0.46054904	6.15644286	7.88775035	0.01088182	0.07863997
Psme4	0.51047928	6.43544393	7.88683456	0.01088574	0.07863997
Uchl1	-0.4196089	6.11240315	7.88538452	0.01089196	0.07863997
Six3	-0.7253063	3.04223827	7.884499	0.01089575	0.07863997
Sf3b5	-0.7292618	3.86018362	7.8828973	0.01090262	0.07863997
Lcmt1	-0.4826215	4.85914656	7.88241807	0.01090468	0.07863997
Xlr	1.57311139	0.44675069	7.88182175	0.01090724	0.07863997
Zfp563	0.48759713	4.894565	7.88106918	0.01091047	0.07863997
Tmem143	-0.8829368	2.62157586	7.86616391	0.01097469	0.07903823
Vegfa	0.43602896	5.38698117	7.86450614	0.01098186	0.07903823
Catip	0.90540009	2.46427014	7.85261838	0.01103343	0.07935102
Prdm9	0.98874632	1.62547008	7.84918843	0.01104835	0.07938691
Rin1	0.67065253	3.80022868	7.84573914	0.01106339	0.07938691
Smim14	-0.5701446	7.54290449	7.84477794	0.01106758	0.07938691
Med16	0.42664758	5.29758617	7.84344947	0.01107338	0.07938691
Kcnq1ot1	1.07587611	8.05882548	7.8412593	0.01108295	0.07938691
Meiob	3.67267919	-0.9393233	7.83865577	0.01109433	0.07938691
H13	0.57788483	4.82160613	7.8384695	0.01109515	0.07938691
Pisd	0.4783799	4.45308824	7.83331325	0.01111774	0.0794519
Asic3	5.93128187	-2.0363089	8.12169623	0.01112045	0.0794519
Ctcfl	0.97300199	3.10142924	7.82686821	0.01114604	0.0795767
Bsn	0.73516998	9.30856583	7.82414081	0.01115805	0.07960438
Sdad1	0.39097513	4.79160737	7.81838807	0.01118341	0.07967222
Themis	-1.8563495	0.53572388	7.81508501	0.01119801	0.07967222

A230001M1(	1.66384797	0.24697136	7.81423786	0.01120175	0.07967222
Zcrb1	-0.3690123	6.95352091	7.81306764	0.01120693	0.07967222
Mei1	1.30265002	0.94362677	7.81131299	0.0112147	0.07967222
Eapp	-0.4295953	5.25186188	7.81093757	0.01121636	0.07967222
Zfp157	0.4649648	4.72711089	7.78840697	0.01131665	0.08032638
Nhlrc1	-0.5299412	4.10563353	7.78150386	0.01134758	0.08045977
Fam111a	-0.6021135	3.34294009	7.77998967	0.01135438	0.08045977
Cops7a	-0.4748423	6.91236937	7.77871881	0.01136009	0.08045977
Trim33	0.29697863	7.7052077	7.76956655	0.01140129	0.08069325
Rcan2	-0.2857788	7.45083841	7.767506	0.01141059	0.08070076
Flrt1	0.35764317	6.61301072	7.76337232	0.01142927	0.08074779
Ppp1r16b	0.48139631	6.6634523	7.76059335	0.01144185	0.08074779
Pcdhac2	0.58392811	4.03707919	7.76049988	0.01144227	0.08074779
Hfm1	1.32833832	1.86536128	7.75874737	0.01145021	0.08074779
Pgap3	-2.4352341	-0.2730341	7.75043009	0.01148798	0.08089059
Gramd3	-0.5708908	4.51568435	7.74913991	0.01149385	0.08089059
C230091D08	0.51984256	6.46143414	7.7483545	0.01149743	0.08089059
Atp2a3	1.30011571	0.6266662	7.74609963	0.0115077	0.08089059
Tmem132d	0.66265169	3.96671457	7.74521083	0.01151175	0.08089059
Lmbrd1	0.32673687	6.75262406	7.74254067	0.01152394	0.08090008
Phlpp1	0.4183648	6.22097017	7.74080807	0.01153185	0.08090008
Snhg8	-0.7762259	2.70202649	7.73831494	0.01154325	0.08090008
Ank3	0.72943753	9.13226804	7.73592809	0.01155417	0.08090008
Cnga4	-1.960323	0.3023142	7.73534054	0.01155686	0.08090008
Adrbk2	0.50811726	5.20441657	7.73210138	0.01157171	0.08090008
Rprd2	0.39812823	7.26401555	7.73068933	0.01157818	0.08090008
Actr1b	-0.4231811	7.25929002	7.72891584	0.01158633	0.08090008
Rbms1	-0.4093592	6.26706979	7.72791639	0.01159092	0.08090008
Nrg2	-2.5219938	-0.8635565	7.72687723	0.01159569	0.08090008
Paics	-0.4708906	8.66683901	7.72108285	0.01162237	0.08098552
1500015L24F	3.12170915	-0.0251291	7.72062493	0.01162448	0.08098552
Rian	0.71759455	6.84491912	7.71499953	0.01165044	0.08110872
Suv39h1	-0.7186566	3.57991777	7.71278156	0.0116607	0.08112246
Atp5o	-0.5240216	6.67836527	7.7100625	0.01167328	0.08115238
Tmem261	-0.5434961	3.81384098	7.70387337	0.01170199	0.08129423
C77370	0.77520505	6.42862139	7.6994057	0.01172276	0.08135516
A230056J06F	-2.9458002	-0.1013602	7.69804951	0.01172907	0.08135516
4932413F04I	1.94185058	0.1333129	7.69663131	0.01173567	0.08135516
Nudt8	-1.6935122	0.48689699	7.6921911	0.01175638	0.08143668
Mdm2	-0.298636	6.69822729	7.69054683	0.01176406	0.08143668
Trerf1	0.55174257	5.40018908	7.68804244	0.01177577	0.08145419
Chid1	0.54662591	3.41051126	7.68614201	0.01178466	0.08145419
Gadd45gip1	-0.8568806	2.62818782	7.68282645	0.01180019	0.08145419

Mrps26	-0.5087552	3.85237009	7.68273116	0.01180064	0.08145419
Enpep	1.03748752	1.97642137	7.68112514	0.01180817	0.08145419
Dcun1d5	-0.3902208	5.95831292	7.66842876	0.0118679	0.08180861
Nf1	0.53186335	8.07663234	7.66533398	0.01188251	0.08184354
Myl4	-1.115161	2.24549213	7.66381758	0.01188968	0.08184354
Nav1	0.61031816	7.3625303	7.65699838	0.01192196	0.08200816
Pcdh10	0.46602904	7.10637105	7.65368842	0.01193767	0.08205857
Srrt	0.48413509	4.88344828	7.6498489	0.01195592	0.08212638
Eml5	0.83579204	5.85855028	7.64656802	0.01197154	0.082127
Hsbp1	-0.4548998	8.97240026	7.64630764	0.01197278	0.082127
Zfp280b	0.45881531	4.89230233	7.64407298	0.01198343	0.08214255
Thnsl1	-0.6414097	3.96352648	7.64057181	0.01200014	0.08219957
Stat2	0.43058695	4.68576171	7.635231	0.01202568	0.08231094
Ccl19	-0.7352146	3.93998028	7.63264284	0.01203808	0.08231094
Pnrc1	-0.3738026	7.15483839	7.62965395	0.01205242	0.08231094
Dhx57	0.69848821	5.85111194	7.62908866	0.01205513	0.08231094
Pou2f2	0.85290507	3.6192154	7.62708912	0.01206474	0.08231094
Pebp1	-0.4624288	8.27988098	7.62605908	0.01206969	0.08231094
Ddhd2	0.35582901	6.00364545	7.62490844	0.01207522	0.08231094
Pcdhb18	0.68126187	4.15419096	7.62023183	0.01209774	0.08235542
Mettl15	-1.0763548	1.63250249	7.62006134	0.01209856	0.08235542
Haus8	-1.2744448	1.04829652	7.61180571	0.01213843	0.08256943
Zfp804b	0.89881347	2.52131327	7.60680517	0.01216265	0.0826292
Nf2	-0.3980154	6.09246586	7.60627054	0.01216524	0.0826292
Numa1	0.31752941	5.77399352	7.60477006	0.01217252	0.0826292
Mrpl36	-0.5815449	5.37409144	7.6027542	0.01218231	0.08263703
2310010J17F	-1.3737458	0.76878002	7.59942961	0.01219847	0.08263703
Ptprc	1.07061094	1.9383955	7.59928646	0.01219917	0.08263703
Rab5c	-0.3834741	6.19290957	7.59758331	0.01220746	0.08263703
Stk39	-0.4949148	7.75126051	7.59305556	0.01222953	0.08263703
5330417C22I	0.76356684	3.85966934	7.59263697	0.01223157	0.08263703
Zmat4	-0.333987	5.6454348	7.59239891	0.01223273	0.08263703
Son	0.50758613	8.07903712	7.58745463	0.01225689	0.08274315
Hspa12b	-0.7292252	3.62175018	7.582583	0.01228074	0.08284708
Smarcd3	-0.6142242	4.1281128	7.57969044	0.01229493	0.0828857
Smap1	-0.3100637	6.28610141	7.57560847	0.01231498	0.08292491
Ppdpf	-0.4700695	5.09338992	7.57506058	0.01231767	0.08292491
Snip1	-0.7150552	3.26961063	7.57266154	0.01232948	0.08294737
Zbtb39	-0.4720868	4.42513232	7.56977275	0.01234371	0.08297484
2700054A10I	0.74127825	2.52735864	7.56743573	0.01235524	0.08297484
Rhpn1	-1.136166	0.76521571	7.56651685	0.01235977	0.08297484
Nlrc4	4.33414097	-1.284615	7.56050427	0.0123895	0.08297484
Uqcrq	-0.466277	4.16383216	7.56050422	0.0123895	0.08297484

N4bp2l1	0.59280142	4.03004279	7.56043334	0.01238985	0.08297484
Tpm3	-0.3583143	7.43066738	7.5589893	0.012397	0.08297484
Celsr2	0.44364752	7.16649663	7.55658874	0.0124089	0.08297484
Ntrk2	0.52659832	8.36611345	7.55640689	0.0124098	0.08297484
Vash1	0.66573035	3.42591804	7.5508837	0.01243722	0.08310148
Zfp277	0.70290313	6.31918205	7.54641745	0.01245945	0.08317906
C030023E24l	1.22990283	2.42704436	7.54088343	0.01248705	0.08317906
Mylk4	1.27427466	1.16968029	7.54068654	0.01248804	0.08317906
Nrg3	0.38293764	6.24416023	7.53897556	0.01249658	0.08317906
Lysmd4	-0.5858045	3.30306928	7.53687208	0.0125071	0.08317906
Mrps12	-0.7337715	3.59818794	7.53656962	0.01250862	0.08317906
4931403G20	1.45634815	0.89301541	7.53639523	0.01250949	0.08317906
Usp19	0.45354502	4.7584314	7.53494071	0.01251677	0.08317906
Hdgf	-0.435627	6.54387813	7.51423948	0.0126209	0.08378576
Dnase1l2	2.61371981	-0.4876255	7.51339505	0.01262517	0.08378576
Pcdhga7	0.77690664	2.71389991	7.51156526	0.01263443	0.08379041
Bzw1	-0.4851639	8.32615048	7.50890434	0.0126479	0.08379797
Pdpr	0.62465134	3.89356158	7.50796141	0.01265268	0.08379797
4930486F22l	3.72917589	-1.2626018	7.50268837	0.01267943	0.08391152
Spag5	1.37467505	1.90163562	7.50120784	0.01268695	0.08391152
Dync2h1	0.85497423	6.15787971	7.49881541	0.01269912	0.08393533
Dennd1b	0.46838512	4.59060003	7.49546673	0.01271618	0.08399137
Magee2	0.53502318	4.78781903	7.48888082	0.01274979	0.08411917
Brox	-0.3328607	5.69031614	7.48831136	0.0127527	0.08411917
9130008F23l	-2.2783691	-0.5012529	7.48518737	0.01276868	0.08416792
Casd1	0.41865689	5.80115426	7.46883388	0.01285272	0.08463399
Nbeal2	0.81947924	1.80362844	7.46705453	0.0128619	0.08463399
Fam169b	0.96200391	1.94048087	7.46150187	0.0128906	0.08463399
Mycbp2	0.71567144	8.84042078	7.4612341	0.01289198	0.08463399
Dact1	-0.5525364	6.05623153	7.45983815	0.01289921	0.08463399
Capn11	-3.2619302	0.03771976	7.45973346	0.01289975	0.08463399
Cenpc1	0.41690806	5.59738244	7.45810894	0.01290817	0.08463399
Vgll4	-0.8582694	2.97093093	7.455061	0.01292398	0.08463399
Synm	0.47015865	5.50732263	7.45004958	0.01295001	0.08463399
Zfp236	0.50725326	5.532951	7.44829171	0.01295916	0.08463399
Plcd4	0.79467512	2.1931038	7.44566799	0.01297283	0.08463399
Strip2	0.80557686	5.1919143	7.44556676	0.01297335	0.08463399
Errfi1	-0.3473227	6.88726728	7.44475952	0.01297756	0.08463399
Gm2061	-1.2278823	1.29447359	7.44278062	0.01298788	0.08463399
Chek2	-0.9814711	2.34680415	7.44034529	0.0130006	0.08463399
Hrasls	-0.4696653	5.07368425	7.43816399	0.013012	0.08463399
Orc4	0.37081583	5.58400376	7.43753536	0.01301529	0.08463399
Dph3	-0.3794953	5.71694722	7.43574313	0.01302467	0.08463399

Pnpt1	0.70157579	3.92011407	7.43513327	0.01302786	0.08463399
Mrpl23	-0.6173255	3.13313811	7.43500548	0.01302853	0.08463399
Hrct1	-4.8992731	-2.1066727	7.43429064	0.01303227	0.08463399
Vps39	0.35902739	5.87363614	7.43382465	0.01303471	0.08463399
Tmem173	-1.0251922	1.61094865	7.43317545	0.01303811	0.08463399
Rps15a	-0.3483598	7.20599227	7.42952763	0.01305725	0.08470207
2410021H03I	2.06978528	0.56287823	7.4224968	0.01309422	0.08473212
Ero1l	0.36457283	6.37695427	7.41965437	0.01310919	0.08473212
Smim15	-0.3634915	6.1523121	7.41854711	0.01311503	0.08473212
9330179D12I	-1.454105	1.07492761	7.41747148	0.01312071	0.08473212
Nosip	-0.4806042	4.60528241	7.4168938	0.01312376	0.08473212
Pcsk2os1	0.74481434	3.00792967	7.41539471	0.01313168	0.08473212
Arl5b	0.59608663	4.18578629	7.41456723	0.01313605	0.08473212
1110002L01I	-1.2299824	1.11753927	7.414053	0.01313877	0.08473212
F8	0.58791631	3.0977305	7.41245681	0.01314721	0.08473212
Map2	0.58283359	8.3176101	7.41223421	0.01314838	0.08473212
Nlgn3	0.43901075	5.98583432	7.40944881	0.01316313	0.08477138
Cdkn3	-2.6737855	-0.6948635	7.40157727	0.0132049	0.08498451
Skida1	-0.6178662	4.1254437	7.39545703	0.01323748	0.08509343
Mrpl34	-0.81753	3.32687172	7.39513477	0.0132392	0.08509343
Vps9d1	0.53918171	3.70412847	7.39243615	0.0132536	0.0851301
Dmxl2	0.71035464	8.52492844	7.38937398	0.01326995	0.08514541
Prcp	-0.560792	3.71054934	7.38873594	0.01327336	0.08514541
Gtse1	2.6976588	-1.4834709	7.3788164	0.01332652	0.08543045
Rtf1	-0.2924339	8.31775041	7.37398779	0.01335248	0.08554089
Loxl3	1.00800052	1.77272486	7.37028847	0.01337241	0.08561257
Chac1	0.87987603	1.81672077	7.3668797	0.0133908	0.08567432
Slc39a6	0.32225269	6.58109972	7.3610676	0.01342223	0.08581933
Dynlrb1	-0.4493026	8.41788242	7.35811281	0.01343824	0.08586563
Preb	-0.5256609	5.17850217	7.35510946	0.01345453	0.08591369
Sez6l	0.40957238	6.13618277	7.34123011	0.01353011	0.08625668
Tpm1	-0.4221961	9.356263	7.34075176	0.01353272	0.08625668
Pcdhb8	1.2782771	1.09888164	7.34021043	0.01353568	0.08625668
Coq4	-0.501308	3.84372595	7.33878632	0.01354347	0.08625668
Arpc5l	-0.4449111	5.82331496	7.33567852	0.01356047	0.08630889
Eci1	-0.5366182	3.68755631	7.33264035	0.01357712	0.08635874
Ttpa	1.24000631	1.48140709	7.32272563	0.01363162	0.0866185
Gm5441	1.99849741	0.35213005	7.32199405	0.01363565	0.0866185
Snap25	-0.3007773	14.1167379	7.30521281	0.01372847	0.08715162
Cryl1	-0.6731397	3.57111571	7.30233365	0.01374447	0.08719666
Spata17	-4.2993802	-1.2522356	7.29933293	0.01376116	0.08724607
4930519F09I	0.75231944	2.53184829	7.29653088	0.01377677	0.0872712
Ythdc1	0.34857326	7.24314683	7.29141083	0.01380534	0.0872712

Rab7	-0.4006051	10.3187668	7.28874967	0.01382022	0.0872712
Catsperd	2.64675474	-0.7666412	7.28645579	0.01383306	0.0872712
Kl	-0.4441897	5.31075688	7.28274438	0.01385386	0.0872712
Sytl3	-1.1786893	0.95486318	7.2824002	0.01385579	0.0872712
Zswim5	0.44963079	4.89871036	7.28122283	0.0138624	0.0872712
Cinp	-0.5512938	4.26238492	7.27970434	0.01387092	0.0872712
Hipk4	0.66684864	3.97058021	7.27932507	0.01387305	0.0872712
Yaf2	-0.3864528	7.42070674	7.27599791	0.01389176	0.0872712
Mdga2	0.47615641	6.06321093	7.2737792	0.01390425	0.0872712
Samd1	-0.8463701	2.79565482	7.27335606	0.01390663	0.0872712
Clstn2	0.50665202	6.26457341	7.27278505	0.01390985	0.0872712
Klc4	-0.8987453	2.29237103	7.27126791	0.0139184	0.0872712
AU040972	1.48778531	1.08626308	7.27114232	0.01391911	0.0872712
Cabp7	3.16559089	-1.1968149	7.27077185	0.0139212	0.0872712
Neurl1b	0.65467062	4.89012946	7.26990419	0.01392609	0.0872712
Klhdc1	0.4742564	4.04135567	7.26949108	0.01392842	0.0872712
Atp6v1c1	-0.2793625	7.37129224	7.26843114	0.01393441	0.0872712
Atp6v1g1	-0.4755275	6.90417305	7.26596783	0.01394832	0.08730253
Ulk3	0.60244586	3.94958032	7.25999772	0.01398211	0.08745813
Cmtm7	-0.9189424	1.86623402	7.25549097	0.01400768	0.08751697
Fgd4	0.44085663	4.93470296	7.25490921	0.01401098	0.08751697
Bpgm	-0.43041	6.8275735	7.25361759	0.01401832	0.08751697
Fam160a2	0.44055238	6.65594544	7.24731543	0.01405419	0.08763276
Plxna3	0.97008896	2.16951874	7.24721569	0.01405476	0.08763276
Rpl23a	-0.4854054	7.25485524	7.23926822	0.01410014	0.0877775
Uqcrh	-0.5229394	7.05499761	7.23819646	0.01410628	0.0877775
Htr7	-0.6554436	3.40319101	7.23693795	0.01411348	0.0877775
Crem	-0.4093073	4.8377127	7.23574356	0.01412032	0.0877775
Tmem69	0.47518949	4.43179247	7.23531509	0.01412278	0.0877775
Sgpl1	0.58815554	4.29909087	7.23198705	0.01414187	0.08779283
Nhsl2	0.32324866	8.08117747	7.23058501	0.01414992	0.08779283
Zc3h15	-0.2755712	7.05509728	7.23019925	0.01415213	0.08779283
Mapkap1	-0.3245812	6.5705397	7.22308806	0.01419305	0.08799093
Usp50	4.16974459	-1.5371428	7.21910862	0.014216	0.08799896
1810058I24R	-0.6412191	4.48426675	7.21762462	0.01422457	0.08799896
Spata19	3.51409618	-2.0523574	7.2109131	0.01426341	0.08799896
Slc35d2	1.41510251	0.16421298	7.20941036	0.01427212	0.08799896
Arhgap15	-0.5567916	3.82737943	7.20849313	0.01427744	0.08799896
Mmab	-0.4471539	4.42183548	7.20552292	0.01429468	0.08799896
Zbtb6	0.37193677	5.09024405	7.20433811	0.01430157	0.08799896
Ttbk1	0.63383665	4.05638659	7.20067019	0.01432291	0.08799896
Mrpl30	-0.4480597	5.69190516	7.1993094	0.01433083	0.08799896
Tma7	-0.4706904	6.81927028	7.19847151	0.01433571	0.08799896

Mir8091	1.98499731	-0.5753025	7.19691606	0.01434478	0.08799896
Cdc40	0.33267849	6.87260442	7.19689265	0.01434492	0.08799896
Dancr	-2.1843044	-0.0093493	7.19592332	0.01435057	0.08799896
Trex1	-1.0551808	1.23374519	7.19477169	0.01435729	0.08799896
Tmtc2	0.70474206	2.97770247	7.19432041	0.01435993	0.08799896
Fmnl1	-0.5450516	5.94178596	7.19327545	0.01436603	0.08799896
Slc12a6	0.32819732	6.78989424	7.19227901	0.01437185	0.08799896
Med18	-1.2956643	1.08521293	7.19088445	0.01438001	0.08799896
Mgat5	0.50628708	4.25700485	7.19069949	0.01438109	0.08799896
Hivep3	0.68814109	6.88983087	7.19030025	0.01438342	0.08799896
Mrpl27	-0.5465716	5.32071459	7.18889711	0.01439163	0.08799896
Crct1	-4.7947166	-1.9746755	7.43534017	0.01439823	0.08799896
Sfr1	-0.4001781	6.95960474	7.18593241	0.014409	0.08799896
Cdc42se1	-0.4666689	5.51171644	7.18178856	0.01443331	0.08799896
Lmcd1	-1.1017748	2.10485348	7.17995841	0.01444406	0.08799896
Zfp830	-0.477696	5.20672535	7.179406	0.01444731	0.08799896
Mreg	0.88959008	2.40495326	7.17934685	0.01444766	0.08799896
Gm4262	0.65900077	3.20968272	7.17795575	0.01445584	0.08799896
Pkib	-0.4019952	5.513234	7.17682253	0.01446251	0.08799896
Chd6	0.46775015	7.19872025	7.17659303	0.01446386	0.08799896
Rpl7	-0.4921061	6.91340087	7.17409013	0.0144786	0.08803397
Lnp	0.46523185	6.97294153	7.17011807	0.01450203	0.08806426
Gpam	0.62721895	3.29092738	7.16986505	0.01450352	0.08806426
Alkbh7	-0.976951	1.95290312	7.16818091	0.01451347	0.08806426
Aqp4	0.56598883	5.58475126	7.16715322	0.01451954	0.08806426
D130040H23	-0.7233295	2.95254379	7.16470311	0.01453404	0.08807121
Gfod1	0.35925831	6.81594251	7.16299281	0.01454416	0.08807121
Med21	-0.6591238	5.66338597	7.1624019	0.01454766	0.08807121
Olfr113	8.51215356	-2.0314591	7.40639285	0.01455887	0.08808464
Pea15a	-0.366758	7.66623059	7.15104971	0.01461509	0.08822557
Sh2b3	-0.6445326	3.5835136	7.14698344	0.01463933	0.08822557
Agl	0.47280686	5.63069025	7.14514719	0.01465029	0.08822557
Eif4h	-0.3033053	7.04645059	7.14488056	0.01465188	0.08822557
Akr1b10	-0.4540509	3.77109963	7.14420395	0.01465592	0.08822557
Tsen15	-0.5258549	3.62941797	7.14379817	0.01465835	0.08822557
Prr11	0.6617647	3.12267496	7.14242624	0.01466655	0.08822557
Gmip	0.8914576	1.52538376	7.14159965	0.01467149	0.08822557
Tenm3	0.55519859	6.36800306	7.14145202	0.01467238	0.08822557
Ywhaq	-0.3199199	9.00817021	7.13942182	0.01468452	0.08822557
Mrpl21	-0.4869522	4.23155519	7.13530386	0.0147092	0.08822557
Dio2	0.4216752	5.92017166	7.13471847	0.01471271	0.08822557
Sphkap	0.70678216	7.2290455	7.13327192	0.0147214	0.08822557
Sytl5	0.60416976	4.73836367	7.13178138	0.01473035	0.08822557

Ybey	0.65936459	4.3752205	7.13102737	0.01473488	0.08822557
2700062C07l	-0.5807894	3.96942133	7.12997412	0.01474121	0.08822557
Akap11	0.54105812	8.45941084	7.12937733	0.0147448	0.08822557
Htr2b	1.36642732	0.19008035	7.12797469	0.01475324	0.08822557
Gm5415	1.41545902	2.10985562	7.12726778	0.01475749	0.08822557
Gas2l3	0.68016215	4.64263568	7.1264687	0.0147623	0.08822557
Grm5	0.44446013	6.71237517	7.12277473	0.01478457	0.08830111
Ttc14	0.77502286	6.58514574	7.12138219	0.01479297	0.08830111
Lrrc7	0.73211877	8.01311351	7.119634	0.01480353	0.08831031
Thap1	-0.6756796	3.74336405	7.11674384	0.014821	0.08833374
Rpl27a	-0.3970939	6.97053072	7.11600158	0.01482549	0.08833374
Ndc1	0.71047858	2.89070847	7.11333024	0.01484167	0.08837636
Dnah7a	0.70839108	2.08891377	7.11032175	0.01485991	0.08843121
Zfp518a	0.37685777	5.61763445	7.10783404	0.01487501	0.08846734
Bod1	-0.4506022	6.10494432	7.10564022	0.01488834	0.0884929
Dcaf17	0.45218099	4.46786314	7.10122846	0.01491519	0.08855213
Tmem131	0.40815942	7.23294098	7.10103229	0.01491639	0.08855213
Tceb1	-0.4119438	6.28248636	7.09721119	0.01493969	0.08860901
Bloc1s6	-0.4029691	5.60075722	7.09453729	0.01495602	0.08860901
Rpl7l1	-0.3999327	4.58990866	7.09160491	0.01497395	0.08860901
Vezt	0.44310613	4.86885438	7.09127612	0.01497597	0.08860901
Fam89b	-0.7870811	2.77397986	7.09045919	0.01498097	0.08860901
Tes	-0.5491935	3.71867944	7.08819816	0.01499482	0.08860901
Rhou	-0.4577767	6.75176283	7.08769716	0.01499789	0.08860901
Bard1	1.07560161	1.56742693	7.08654723	0.01500494	0.08860901
Prkrip1	-0.675829	3.70442885	7.0861485	0.01500738	0.08860901
Scarb1	1.04740442	2.48768877	7.08249663	0.01502981	0.08860946
Chit1	-4.5418945	-1.5464265	7.32309062	0.01503264	0.08860946
Tuba1c	-0.3077076	5.81028806	7.08171685	0.0150346	0.08860946
Atp2b2	0.48974967	9.28140881	7.07629317	0.01506798	0.08868485
Arhgap28	-0.6578015	4.00882004	7.07386286	0.01508296	0.08868485
Acsl3	0.32180077	7.2432512	7.07345371	0.01508549	0.08868485
Mylip	-0.5949613	4.11546844	7.07117769	0.01509954	0.08868485
Lhfpl3	0.56304354	3.85531894	7.06936341	0.01511075	0.08868485
Pag1	0.39400706	5.64446304	7.06832836	0.01511715	0.08868485
Hspa2	0.51714382	4.93156623	7.06638908	0.01512915	0.08868485
Murc	2.78821812	-0.1710877	7.06636007	0.01512933	0.08868485
Gpatch4	-0.3996649	4.88868949	7.0659634	0.01513179	0.08868485
Rrnad1	0.55411811	3.60625809	7.06441199	0.01514139	0.08868485
Gtf2ird1	0.50993232	3.59592891	7.06208397	0.01515583	0.08868485
Grm7	0.78319595	4.58101982	7.06101795	0.01516244	0.08868485
Dopey1	0.64099109	5.58148673	7.06059103	0.01516509	0.08868485
Ocm	-2.1848978	-0.9293338	7.05342157	0.01520966	0.08887497

Brms1	-0.7308092	2.6401315	7.05172016	0.01522026	0.08887497
Eif5a2	-0.4171326	6.5726775	7.05098899	0.01522482	0.08887497
Cryz	-0.7237768	2.82523574	7.04691248	0.01525026	0.08891868
Arfgef2	0.46640613	6.1855433	7.04560226	0.01525844	0.08891868
Kcnn3	0.67854117	4.1629489	7.04288081	0.01527546	0.08891868
Hexdc	1.0225139	1.95366144	7.04271155	0.01527652	0.08891868
Nop56	0.42602563	6.54885014	7.0425239	0.0152777	0.08891868
Rpl27	-0.4970555	6.01299865	7.03955249	0.0152963	0.08897412
Timm10b	-0.7731541	2.6479208	7.03494701	0.0153252	0.08908928
E4f1	0.76170119	2.50487204	7.03257703	0.01534009	0.08912296
Kdm5a	0.38160539	7.06546638	7.02659323	0.01537776	0.08918328
Jmjd6	-0.5592568	2.98096008	7.02579024	0.01538283	0.08918328
Zfp759	0.52701122	3.76817556	7.02339765	0.01539792	0.08918328
Kbtbd3	0.61346144	3.29849578	7.02144558	0.01541025	0.08918328
Dnajb4	-0.3181518	8.41229707	7.02007294	0.01541893	0.08918328
Glo1	-0.4463169	6.44821502	7.01958602	0.01542201	0.08918328
Tnk2	0.4449315	5.09369377	7.01910515	0.01542505	0.08918328
BC030336	0.37353671	5.66464111	7.01816398	0.01543101	0.08918328
Ccndbp1	-0.4192855	6.68427437	7.01794164	0.01543241	0.08918328
Zcchc5	-2.5341814	-0.4091356	7.01393294	0.01545781	0.0892475
Rad52	-0.5221349	3.57807302	7.01331198	0.01546175	0.0892475
Nat8l	0.52211689	6.37905119	7.01136174	0.01547413	0.08926633
LOC1005050	1.72290848	0.48424062	7.00891003	0.0154897	0.08930358
Gm15413	2.84757434	-0.5351781	7.00415874	0.01551993	0.08930495
AA987161	0.39029757	5.33446726	7.00250421	0.01553047	0.08930495
Mall	-1.865388	0.83161331	7.00218667	0.0155325	0.08930495
Cdc42ep2	-0.8847737	2.08049696	7.00068951	0.01554205	0.08930495
Fcf1	-0.4807867	4.74357637	7.00014428	0.01554553	0.08930495
Tmem104	0.57915717	3.95676381	6.9968191	0.01556676	0.08930495
Il1bos	-5.4711565	-2.0499981	7.23053079	0.01557973	0.08930495
Mab21l3	4.02317576	-1.79967	6.99445394	0.01558189	0.08930495
Dzank1	0.44702664	8.55865494	6.99282023	0.01559234	0.08930495
Zfp772	0.52817655	4.258458	6.99048884	0.01560728	0.08930495
Siah2	-0.48378	4.21540597	6.99048003	0.01560734	0.08930495
BC003965	-0.3400448	6.03354468	6.98970407	0.01561231	0.08930495
Cir1	-0.3181326	6.95479119	6.9891922	0.01561559	0.08930495
Mir32	6.63026578	-1.9347568	7.22426582	0.01561758	0.08930495
Zfp580	-0.4951104	3.72006209	6.98437461	0.01564653	0.08934686
Ccdc85c	1.0374107	0.96337472	6.98341803	0.01565268	0.08934686
Uty	0.45490722	5.05926965	6.98226127	0.01566012	0.08934686
Actg1	-0.2733967	8.97424616	6.98103066	0.01566804	0.08934686
Tdrp	-0.5149937	5.87769403	6.97943035	0.01567834	0.08934686
Pard6b	-0.6966504	3.34604039	6.97923044	0.01567963	0.08934686

Ammecr1l	-0.3228496	5.73285666	6.97455456	0.01570979	0.08943138
Meis1	1.23995339	1.67791817	6.97334436	0.01571761	0.08943138
Gon4l	0.44522473	5.89696095	6.97268708	0.01572186	0.08943138
Pgap1	0.51401748	5.42461788	6.97080781	0.01573401	0.08944643
Capn9	-5.5554058	-2.3766796	7.20366148	0.01574276	0.08944643
Cox8a	-0.4645005	7.4453694	6.96473095	0.01577337	0.08946603
Sh3gl3	-0.5379582	5.61268157	6.96180483	0.01579236	0.08946603
Mlst8	0.58262507	2.91787372	6.96171004	0.01579298	0.08946603
Eml6	0.59256849	4.31941007	6.95713618	0.01582272	0.08946603
B9d2	-0.965396	1.8269852	6.95694011	0.01582399	0.08946603
Atoh8	-0.8808574	1.84341406	6.95615389	0.01582911	0.08946603
Ptpmt1	-0.4551759	4.1804834	6.95613349	0.01582925	0.08946603
Ankfy1	0.39356277	6.00458421	6.9559254	0.0158306	0.08946603
Megf6	1.14209213	1.38357605	6.95558374	0.01583283	0.08946603
Gm14057	1.43848858	2.1774852	6.95485888	0.01583755	0.08946603
Acvr2a	0.41452841	6.16874614	6.95147573	0.01585961	0.08952735
Gm16287	1.06988509	1.40048103	6.94948417	0.01587261	0.08952735
Caml	0.50989725	3.67011036	6.9482215	0.01588086	0.08952735
2610203C20I	0.51451176	5.8366634	6.94684938	0.01588984	0.08952735
Mphosph10	-0.4548606	5.16500049	6.94387697	0.01590929	0.08952735
C130074G19	-0.4310119	6.86910222	6.94353108	0.01591155	0.08952735
Cfl2	-0.469428	7.7484807	6.94340449	0.01591238	0.08952735
Nudt16l1	-0.4320151	4.1446376	6.9409504	0.01592847	0.0895664
Gm2762	6.05578905	-1.6727129	7.16717435	0.01596726	0.08973305
Slitrk2	0.57689167	5.37782692	6.93284907	0.0159817	0.08976265
Tmco3	0.5404389	3.76678227	6.93100711	0.01599383	0.0897793
B4galnt2	0.93646969	3.0710878	6.92910322	0.01600637	0.08979828
Arf1	-0.3251005	8.31488361	6.92178426	0.01605472	0.0899171
Tnr	0.66007516	5.08287813	6.92106782	0.01605946	0.0899171
Cfp	1.42516003	1.41500791	6.92051656	0.01606311	0.0899171
Kctd12b	-0.5071837	4.98080591	6.92034034	0.01606427	0.0899171
Calml4	-1.1017651	1.39227829	6.91681724	0.01608762	0.08996981
Uso1	0.3013144	6.42363592	6.9161475	0.01609206	0.08996981
Rps2	-0.425071	6.30221728	6.91241462	0.01611684	0.0900184
Cnot7	-0.3460442	6.74951294	6.91207022	0.01611913	0.0900184
Tmem141	-0.8577284	1.88506136	6.90994652	0.01613325	0.09004593
Adh1	-1.7019732	0.18930139	6.90626269	0.01615778	0.09011485
Gm11413	3.14794738	-1.251623	6.90377799	0.01617435	0.09011485
Bcl11b	0.60553522	6.10353451	6.90348251	0.01617632	0.09011485
Mrpl41	-0.4372225	4.74946959	6.90257124	0.0161824	0.09011485
Megf8	0.58057901	5.0083475	6.89981826	0.01620079	0.09016599
Ambra1	0.46737077	5.77368778	6.89034929	0.01626422	0.09046759
Psmd7	-0.2999187	6.76951316	6.88478025	0.01630165	0.09062435

Gm1045	4.26783252	-1.1786568	6.8818219	0.01632157	0.09063536
Parp9	-0.6537624	3.39208783	6.87782632	0.01634853	0.09063536
Ephb6	0.67030124	3.56772938	6.87773957	0.01634911	0.09063536
Cep78	0.73708684	2.62711095	6.87749701	0.01635075	0.09063536
Gm20337	1.08335901	2.04686625	6.87517195	0.01636646	0.09063536
Atp9a	0.48833134	4.80433389	6.87416875	0.01637324	0.09063536
Mccc1	0.58736229	3.58728736	6.87384695	0.01637542	0.09063536
Hypk	-0.554776	6.55731281	6.87351698	0.01637765	0.09063536
Abcc1	0.66969925	3.88169339	6.87086468	0.01639561	0.0906835
Dnal1	-0.3414023	7.03713148	6.86555807	0.0164316	0.09083127
Ccl6	-1.5684453	0.33841368	6.86119037	0.01646129	0.09094407
Kctd2	-0.3990826	5.52235383	6.85632227	0.01649445	0.09107591
Samd12	-0.6926468	3.24731974	6.85476714	0.01650506	0.09108314
Mir6369	2.40166129	0.57777275	6.84955652	0.01654066	0.09122822
Hnrnpab	-0.4893948	7.5806322	6.84782695	0.0165525	0.09124213
Terf2	-0.3163754	6.26750373	6.84369934	0.01658079	0.09133504
Zcchc14	0.34802494	6.32427807	6.84256651	0.01658856	0.09133504
Htt	0.60599473	6.73840291	6.83989647	0.0166069	0.09133504
Pbld1	0.70390817	3.46365228	6.83945911	0.0166099	0.09133504
Fgf1	-0.4086743	7.40894278	6.83670592	0.01662884	0.09133504
Mrpl24	-0.7840313	2.82661338	6.83628454	0.01663174	0.09133504
Gm7173	2.00959576	-0.6906791	6.83586519	0.01663462	0.09133504
Man2a2	0.3700002	6.90128601	6.83267527	0.0166566	0.09140448
Casp8	-0.5544389	5.33351205	6.8289029	0.01668264	0.09149608
Atg3	-0.311192	6.65030074	6.82703082	0.01669557	0.09151579
Baiap3	1.29089262	1.53709293	6.81906912	0.01675071	0.0917356
Hint1	-0.4136419	5.21577441	6.8185373	0.0167544	0.0917356
Kcnq2	0.47657531	5.52989196	6.8154712	0.0167757	0.09180088
Foxl1	-1.5934092	0.5607768	6.81267027	0.01679518	0.09180475
P2ry6	1.2292337	0.78746139	6.81143833	0.01680375	0.09180475
Zfp526	-0.6584791	3.07793303	6.8098859	0.01681457	0.09180475
Rpl34	-1.0309345	0.76180846	6.80849803	0.01682424	0.09180475
Eif1	-0.443852	7.99043545	6.80822542	0.01682614	0.09180475
Aprt	-0.6464625	3.50790968	6.80466841	0.01685097	0.09180475
Trappc8	0.34824361	6.26284445	6.80412684	0.01685475	0.09180475
Atg10	-0.5920796	4.0415969	6.80295913	0.01686291	0.09180475
Apaf1	0.54665911	3.97683856	6.80229929	0.01686753	0.09180475
Madcam1	-4.1789171	-2.0561187	6.80192731	0.01687013	0.09180475
Ube2l3	-0.3667856	7.51291036	6.79993615	0.01688406	0.09182265
Ttc21b	0.5421233	4.37167452	6.79877869	0.01689217	0.09182265
Nfyb	-0.3900514	5.3894868	6.79237644	0.01693708	0.09201571
Oaz2	-0.4150354	6.79732827	6.79063993	0.01694928	0.09203097
Plag1	0.74734051	2.70955177	6.78594312	0.01698234	0.09211627

Gng4	-0.4087111	5.47719063	6.78573616	0.0169838	0.09211627
Eif1ax	-0.4987699	6.23929247	6.78325452	0.0170013	0.09213207
Lzic	-0.5426758	4.1531194	6.77949049	0.01702787	0.09213207
Cdk5r1	0.43262274	6.96452263	6.77941987	0.01702837	0.09213207
Prc1	1.2396785	1.22386664	6.77826039	0.01703657	0.09213207
1500004A13I	0.58536577	4.96989979	6.77651565	0.01704891	0.09213207
9630033F20I	-0.5113044	4.43530924	6.77543429	0.01705657	0.09213207
Zc3h8	0.66319236	2.7597881	6.77475259	0.0170614	0.09213207
Parpbp	-2.1581015	0.83810134	6.77467362	0.01706196	0.09213207
Adat2	-1.2993449	1.78734387	6.77298758	0.0170739	0.09214578
Nlrp10	2.01838788	-0.7884599	6.77024768	0.01709334	0.09214911
Cntn2	0.5626272	5.70335049	6.7686226	0.01710488	0.09214911
Asl	0.92111912	2.93065126	6.76809983	0.01710859	0.09214911
Dtd1	-0.4344242	6.04522579	6.76759925	0.01711215	0.09214911
Exoc6b	0.30525855	6.79077474	6.76471023	0.01713269	0.09216819
Tbc1d7	-0.5039306	4.04487966	6.76445482	0.01713451	0.09216819
Gpr183	1.53566086	-0.160354	6.76307152	0.01714436	0.09217055
Dst	0.73565185	9.14796673	6.75936548	0.01717078	0.09226193
Lifr	0.46825442	5.94162796	6.75733931	0.01718524	0.09228901
Ppfibp2	-0.5098552	3.58271338	6.7542645	0.01720721	0.0923124
Trpm6	-0.6710561	3.12694538	6.75409237	0.01720844	0.0923124
Grip1	0.58988907	4.08404271	6.75065725	0.01723303	0.09239369
Tlr3	0.49327886	4.87896512	6.74621522	0.01726488	0.09250363
G530011O06	0.72680049	4.65973078	6.744292	0.01727869	0.09250363
Srp54b	-0.2896555	6.34362651	6.74385006	0.01728187	0.09250363
Gm6588	3.27763508	-0.4709742	6.74063217	0.01730501	0.09257691
Mrpl16	-0.3732845	5.6404914	6.73181091	0.01736862	0.09281885
Il4ra	1.67697103	0.15441314	6.73173297	0.01736918	0.09281885
Kif4	-1.0604016	1.16208681	6.72894558	0.01738934	0.0928759
Map1lc3b	-0.4469011	7.87064155	6.72671348	0.0174055	0.09291155
Nwd2	0.87840816	5.78589024	6.72292266	0.01743298	0.0930058
Psmc3	-0.3831051	5.5806721	6.72166004	0.01744215	0.0930058
Myh10	0.5291776	8.54003851	6.71275642	0.01750693	0.09330044
Gpr89	-0.5824414	3.42303375	6.71001592	0.01752692	0.0933562
Gm19434	1.10111893	0.9469362	6.70810272	0.01754089	0.09337985
Lyg2	-7.0425319	-1.660059	6.92160565	0.01757651	0.09351863
Susd4	0.39976681	4.42295152	6.69923335	0.01760583	0.09362201
Bmx	-1.1783844	1.85250892	6.69687524	0.01762315	0.09362201
Hcls1	-0.8353635	1.43159804	6.6955842	0.01763263	0.09362201
Mapre1	-0.3785897	7.8572816	6.6950444	0.0176366	0.09362201
Ankrd13b	0.65999133	3.86267914	6.69309153	0.01765096	0.09362201
Lurap1	0.87777224	1.9634037	6.69115593	0.01766521	0.09362201
Hiatl1	0.48523085	4.0000402	6.69101737	0.01766623	0.09362201

Zc3hav1	-0.4338585	5.52843251	6.69003165	0.01767349	0.09362201
Zmynd8	0.37606481	6.82734109	6.68888294	0.01768196	0.09362201
Ccdc153	-0.9542948	1.80355475	6.68576933	0.01770493	0.09365677
Usp9x	0.48642305	9.85788988	6.6854015	0.01770765	0.09365677
Hspa1l	0.76948797	2.37598543	6.68315256	0.01772427	0.09369407
Rfx4	0.69363301	3.75945816	6.67969697	0.01774983	0.0937442
Cd320	0.71252306	2.81476072	6.67817129	0.01776113	0.0937442
Zranb2	0.31903261	8.01362559	6.6779923	0.01776246	0.0937442
Mocs2	-0.3839616	6.84766251	6.67595675	0.01777755	0.09377332
Tex12	1.98046089	-0.1499423	6.67248817	0.0178033	0.0938586
Kxd1	-0.5562532	4.23318675	6.6707689	0.01781608	0.09387544
Pmvk	-0.4920097	4.37566919	6.66860818	0.01783215	0.09388189
1700052K11l	-0.9390731	2.85164241	6.66802803	0.01783647	0.09388189
Comtd1	1.06245187	1.05456235	6.6647294	0.01786105	0.09396076
Nudcd1	0.51101411	3.65886042	6.65359157	0.01794431	0.09434813
Rcsd1	-0.6315046	4.59087504	6.65162272	0.01795908	0.0943751
Dnajc1	0.42173988	5.08351019	6.65032073	0.01796885	0.09437582
Myh3	0.71518968	2.04655776	6.64606242	0.01800085	0.09449245
Rabggtb	-0.3933383	6.33194334	6.64469511	0.01801114	0.09449245
Sgol2	-0.6554929	3.58539855	6.64261191	0.01802683	0.09449245
Olfr613	0.82458042	5.43212935	6.64223794	0.01802964	0.09449245
Aen	-0.5249342	4.24432563	6.64060071	0.01804199	0.09450657
Rnf44	-0.2662586	6.73438254	6.63838518	0.0180587	0.09452392
Dtna	0.36933532	7.13003161	6.63719134	0.01806772	0.09452392
Mre11a	0.89877817	2.75621419	6.63632701	0.01807425	0.09452392
Inpp4b	0.55970295	3.81977761	6.6313245	0.01811209	0.0946713
Tex13	1.64237331	-0.5693191	6.62819608	0.01813581	0.09474469
Ccdc90b	-0.5299777	5.8537065	6.62659047	0.01814799	0.0947578
Smco1	0.7369566	2.72570555	6.62029684	0.01819584	0.09481845
P2ry12	-0.606865	3.67863473	6.62029337	0.01819586	0.09481845
Arid2	0.3110003	6.70487632	6.62018853	0.01819666	0.09481845
Rasal2	0.43120663	7.19857233	6.61974061	0.01820007	0.09481845
Dsel	0.44739599	5.03122799	6.61869911	0.018208	0.09481845
Zfp418	0.57341503	3.63764889	6.61636322	0.01822581	0.09486075
Adam19	0.44749985	4.64126525	6.61258031	0.01825469	0.09496061
Sox2	0.66034582	3.57343566	6.61038258	0.01827149	0.09498184
Nhlrc2	0.50384674	3.76158255	6.60951028	0.01827817	0.09498184
Rac2	-0.6779352	2.78924291	6.60458481	0.0183159	0.0950453
Capzb	-0.4327507	8.59633439	6.60408146	0.01831976	0.0950453
Txndc16	0.41064792	5.40969844	6.60344853	0.01832462	0.0950453
Tmem245	0.43945701	5.51547541	6.60285285	0.01832919	0.0950453
Tmem115	-0.8339763	2.20816434	6.60130568	0.01834107	0.09505316
Rps21	-0.5458036	5.20132058	6.59925655	0.01835682	0.09505316

Dlx1as	0.54812601	3.27813681	6.59886656	0.01835982	0.09505316
Tpm4	-0.5348752	9.13505951	6.59663735	0.01837697	0.09509171
Slc23a1	1.15375714	0.69192188	6.59488838	0.01839044	0.09511117
Ctxn1	-0.2641379	7.61037203	6.58508463	0.01846616	0.09540328
Igdcc3	2.13368783	0.90123757	6.5831837	0.01848088	0.09540328
Taf2	0.37316952	5.91568039	6.58293827	0.01848278	0.09540328
Psg29	1.60941083	0.02160437	6.58146254	0.01849422	0.09540328
4930478L05	3.01575509	-1.0812398	6.58128102	0.01849563	0.09540328
Khdrbs3	-0.3353289	6.39263603	6.57987941	0.0185065	0.09540912
Lamtor3	-0.4964619	5.61417099	6.56928423	0.01858892	0.09578362
Ogfod1	0.36200389	7.29648385	6.56695509	0.01860709	0.09582686
4930470H14	0.86752948	6.76646202	6.56561011	0.01861759	0.09583057
Ndufb4	-0.6027857	5.90445898	6.56278166	0.0186397	0.09589145
Hmgb3	-0.3315023	5.81681194	6.56114925	0.01865248	0.09589145
BC048403	-0.4857767	4.04730673	6.56003483	0.01866121	0.09589145
Nsun7	1.13399165	2.25496462	6.55909344	0.01866858	0.09589145
Kalrn	-0.373852	11.7778886	6.55587182	0.01869384	0.09589302
Ccdc84	1.46944671	1.12289914	6.55329168	0.01871411	0.09589302
Csgalnact1	0.60821193	3.77298761	6.5523109	0.01872181	0.09589302
Lig1	0.45749408	4.07456712	6.55200938	0.01872418	0.09589302
Mgea5	0.39655275	8.6334026	6.55166576	0.01872689	0.09589302
Ggcx	0.57529746	3.1388994	6.54996738	0.01874025	0.09589302
Usp51	1.26290925	1.25592002	6.54945233	0.0187443	0.09589302
Dcaf12	-0.5173001	5.27104991	6.54840862	0.01875252	0.09589302
Stmnd1	-2.4754315	-0.550832	6.54656463	0.01876705	0.09589302
Ngfrap1	-0.4209479	6.52437935	6.54651048	0.01876747	0.09589302
Itgav	0.35011058	5.95813429	6.54535632	0.01877657	0.09589302
Pfkfb2	0.34733348	5.92260936	6.53956141	0.01882234	0.09607666
Rpl6	-0.4250613	7.75574068	6.53428538	0.01886412	0.09623678
Timm8a1	-0.4948898	5.39448299	6.53312003	0.01887336	0.09623678
Bnip3l	-0.3696143	8.24542799	6.53014716	0.01889696	0.09630698
Pde2a	0.46770657	6.77089871	6.52524739	0.01893592	0.09643672
Slc35d1	0.42667996	4.13099089	6.5243233	0.01894328	0.09643672
Dmxl1	0.52362026	7.30485153	6.52302609	0.01895362	0.09643672
Psma1	-0.4528038	5.5577464	6.52200055	0.0189618	0.09643672
Kntc1	2.57485518	0.00439838	6.51982716	0.01897914	0.09647482
Sgcb	0.45878854	4.81756868	6.5153652	0.01901479	0.09660593
Sft2d3	0.58680852	3.19211929	6.5086657	0.01906847	0.09675179
Mfsd4	0.60437375	7.06259956	6.5061727	0.01908848	0.09675179
Clta	-0.379665	6.84018885	6.50611093	0.01908898	0.09675179
Pebp4	2.55353466	-0.8767812	6.50605627	0.01908942	0.09675179
Tmem95	4.19756566	-2.4731922	6.50488529	0.01909883	0.09675179
Nt5dc1	0.75603132	3.02824271	6.50439599	0.01910276	0.09675179

Srrd	1.10853054	1.36859612	6.5015374	0.01912577	0.09680782
Copg2	0.51460755	5.85953571	6.50026445	0.01913602	0.09680782
Ndufaf2	-0.5894893	4.81039689	6.49933906	0.01914347	0.09680782
Baalc	-0.302876	7.07479323	6.49756732	0.01915776	0.09683007
Sav1	-0.3495092	6.59991401	6.49348204	0.01919075	0.09686036
Cyp4f13	1.04075682	1.16738394	6.49317358	0.01919324	0.09686036
Rgs11	1.49026658	1.394385	6.49315158	0.01919342	0.09686036
Agpat3	0.44505342	4.96856857	6.49016201	0.0192176	0.09693247
Kmt2b	0.50177028	4.65995809	6.4835319	0.01927136	0.09704753
Yae1d1	-0.3477438	6.50183287	6.48240251	0.01928053	0.09704753
1110004F10I	-0.4598894	6.35477478	6.48079495	0.0192936	0.09704753
Gm14440	-0.4036734	5.28812743	6.48045935	0.01929633	0.09704753
Esr1	1.26767977	1.10288743	6.47900323	0.01930817	0.09704753
Jak2	0.31200242	6.25237217	6.47859121	0.01931153	0.09704753
1700030C10I	1.24311291	1.00427737	6.47759802	0.01931961	0.09704753
Mettl6	-0.6763185	3.84319082	6.47693505	0.01932501	0.09704753
AI314180	0.34279493	7.16951713	6.47637431	0.01932958	0.09704753
5031426D15I	1.05794305	3.58407533	6.47456613	0.01934432	0.09705405
Sparcl1	0.44871347	8.69193104	6.47259873	0.01936037	0.09705405
Nme2	-0.5545657	6.80607066	6.4725703	0.01936061	0.09705405
Ubxn2a	-0.3414673	6.98286005	6.46854028	0.01939353	0.09714223
Bcap29	0.42174823	5.17023279	6.46735519	0.01940323	0.09714223
Mob3c	-0.5254674	4.65589937	6.46677853	0.01940795	0.09714223
B3gat2	0.95610207	2.95666919	6.46343046	0.01943537	0.09720718
Slc36a1os	0.88083165	2.78054286	6.46222082	0.01944529	0.09720718
Arsa	-0.7509289	2.4348493	6.46156252	0.0194507	0.09720718
Txndc5	-0.4252868	4.95852922	6.45919276	0.01947015	0.09723492
Macf1	0.63766643	9.05836431	6.45846886	0.0194761	0.09723492
Phtf2	0.40150349	5.23459006	6.45689851	0.01948901	0.0972498
1600029O15	-1.1139702	1.62135196	6.45536486	0.01950162	0.09726321
Psmd10	-0.7396748	4.10335909	6.45060005	0.01954088	0.09736793
Mras	-0.2791555	6.99979418	6.45040357	0.0195425	0.09736793
D10Bwg1379	0.6651138	7.06518929	6.44371048	0.0195978	0.09759381
Dtnbp1	-0.4568468	5.32169113	6.44114608	0.01961904	0.0976062
Crb2	-2.5558394	-0.6515591	6.44100323	0.01962022	0.0976062
Fam161a	-0.9754351	1.83373933	6.43872058	0.01963914	0.09765075
Celrr	1.91295262	0.30316582	6.43461522	0.01967323	0.09770694
Tsc1	0.50657082	5.94436256	6.43437734	0.01967521	0.09770694
Derl2	0.50178474	3.56033296	6.4337566	0.01968037	0.09770694
Cep85l	0.56283868	4.01500005	6.42783067	0.01972971	0.09775572
H2afx	-0.5406061	3.05993906	6.42724301	0.01973461	0.09775572
Adarb2	0.50946	4.9735665	6.42705628	0.01973617	0.09775572
Gpr75	0.66403498	3.63762434	6.42701098	0.01973654	0.09775572

Cep70	0.64901999	4.03986928	6.42354993	0.01976544	0.09775572
Cebpzos	-0.5761597	2.99218171	6.4232249	0.01976815	0.09775572
Ank1	0.62390567	5.05992668	6.42320402	0.01976833	0.09775572
Gm17644	0.91887677	7.68606543	6.42266758	0.01977281	0.09775572
Ltbp3	0.54550588	4.64029279	6.42180601	0.01978001	0.09775572
Hist1h1d	-2.5311428	-1.3350052	6.41480639	0.01983864	0.09792801
Tspyl1	-0.262509	7.44783897	6.41351956	0.01984944	0.09792801
Sipa1	-0.6863166	2.6000308	6.41224271	0.01986016	0.09792801
Nid2	-0.7096059	3.41703602	6.41117941	0.01986909	0.09792801
Prx	2.00666233	-0.4765211	6.41096529	0.01987089	0.09792801
Tmem181c-p	0.88730967	4.26990538	6.4104937	0.01987486	0.09792801
Kmt2c	0.42322626	8.17683856	6.40720133	0.01990256	0.09799762
Hook1	0.65884728	5.07421021	6.40527498	0.01991878	0.09799762
Vasn	0.45981385	3.78869828	6.40435904	0.01992651	0.09799762
Cyb5b	-0.2945191	7.53246763	6.40374502	0.01993168	0.09799762
Akap8l	0.77601448	3.41002979	6.40196143	0.01994673	0.09799762
Col4a2	0.46613268	4.28926856	6.4002698	0.01996102	0.09799762
Ift88	0.49303929	4.21810903	6.39997741	0.01996349	0.09799762
Mast1	0.50843783	4.88121181	6.3980576	0.01997972	0.09799762
4933421O10	0.65891784	3.5009679	6.39738728	0.01998538	0.09799762
Commd3	-0.3654614	5.0746158	6.3969563	0.01998903	0.09799762
Hgsnat	0.361939	5.17726335	6.39177093	0.02003296	0.09816385
E030024N20	-0.3498456	5.67388708	6.38901397	0.02005636	0.09819907
Slc22a15	0.63865027	2.70741686	6.38656521	0.02007717	0.09819907
4930506C21I	1.72612187	-0.2857268	6.38604953	0.02008155	0.09819907
Pigr	0.97366935	3.83540318	6.38203893	0.0201157	0.09819907
Abhd11	-0.5752384	3.15532683	6.38190061	0.02011688	0.09819907
Camk2a	0.56408684	11.2091826	6.38181361	0.02011762	0.09819907
Ccl25	1.27388493	1.70971134	6.38178604	0.02011785	0.09819907
Snord4a	1.12112096	0.58716041	6.3804143	0.02012955	0.09819907
Ppp1r8	-0.9343312	3.99769124	6.37968006	0.02013581	0.09819907
Phlda1	-0.4027737	7.72269091	6.37885886	0.02014282	0.09819907
Tmem70	-0.4397788	4.80019778	6.37703008	0.02015843	0.09819907
Hars	-0.2985063	5.89879143	6.37679383	0.02016045	0.09819907
Slc29a1	-0.5840473	2.59628195	6.37021182	0.02021676	0.09838379
Atm	0.4197863	5.99784166	6.37001395	0.02021846	0.09838379
Fam69c	1.51598907	0.11835775	6.36773834	0.02023797	0.09839325
Mesdc1	-0.4264448	4.04770127	6.3674446	0.02024049	0.09839325
Fcer2a	1.35478625	0.56786535	6.36585408	0.02025415	0.09841078
Urm1	-0.7966753	2.19931317	6.36099602	0.02029591	0.09853782
Akap8	0.48050735	5.82462808	6.36047332	0.02030041	0.09853782
Ldlrap1	-1.1895724	1.14273587	6.3584229	0.02031807	0.09853848
Grin1	0.49254428	6.12469084	6.35812193	0.02032067	0.09853848

Rpl22	-0.5550479	6.20026759	6.35352338	0.02036034	0.09866237
Arhgap20os	2.39612444	0.33173042	6.352827	0.02036636	0.09866237
3110040N11	-0.6657917	3.1539725	6.34968089	0.02039357	0.09874533
Ubxn1	-0.6522533	5.407365	6.34767276	0.02041095	0.09876276
Entpd7	0.60860692	4.12638199	6.34496759	0.0204344	0.09876276
Fhl2	-0.3668202	5.79220638	6.34471543	0.02043659	0.09876276
Cpa6	2.84324444	-1.1804844	6.34461064	0.0204375	0.09876276
Tm9sf1	0.5441864	3.98937282	6.34339305	0.02044806	0.09876509
Timm23	-0.4172704	6.17913277	6.3369138	0.02050439	0.09898833
Arhgap19	-0.6347604	3.4055371	6.33054772	0.0205599	0.09920743
Dkkl1	-0.8735241	2.23243727	6.32294042	0.02062645	0.09943392
Nt5c	-0.5966203	3.6139377	6.32196127	0.02063504	0.09943392
Scrn3	-0.4089825	4.81804462	6.32170435	0.02063729	0.09943392
4933432I03R	-2.3773677	0.10659377	6.31851039	0.02066532	0.09952004
Mccc2	0.53934886	3.4833902	6.31512169	0.02069511	0.09956762
Dus3l	0.52420088	3.75938333	6.31507377	0.02069553	0.09956762
Pdcd10	-0.3677371	6.07404911	6.31299567	0.02071383	0.0996067
Rai1	0.40270922	5.70124103	6.30757417	0.02076163	0.0997876
Ipw	1.0352059	4.05705094	6.3055122	0.02077985	0.09979908
Prex1	0.46077146	5.9249427	6.30499757	0.0207844	0.09979908
Adi1	-0.4813039	7.13727082	6.30335891	0.02079889	0.09981973
Prpf39	0.59003763	5.45336515	6.29486646	0.02087418	0.10008086
Sugt1	-0.3768527	5.59679068	6.29453704	0.0208771	0.10008086
Grm1	0.47938966	5.38932871	6.29376662	0.02088395	0.10008086
Aatk	0.69032144	5.14712158	6.29122337	0.02090657	0.10014025
Thoc2	0.43786127	7.06080005	6.28694225	0.0209447	0.10027389
Abca2	0.52411414	6.10425242	6.2817681	0.0209909	0.10040785
Tmem199	-0.41313	4.14860616	6.28151215	0.02099319	0.10040785
4933413J09F	-3.978821	-1.5657156	6.2773609	0.02103034	0.10053645
Sfrp4	0.66648956	3.4712962	6.27077127	0.02108946	0.10076991
4930547E14I	2.87574011	-0.8269126	6.26901324	0.02110526	0.10079626
Pcdhga12	0.60224125	2.95173077	6.26723141	0.0211213	0.10082367
Nagpa	-0.7868983	2.8786944	6.26509556	0.02114053	0.1008416
Slco5a1	0.86077702	2.19075446	6.26390347	0.02115128	0.1008416
Xlr4a	1.75650243	0.03908689	6.26305963	0.02115889	0.1008416
Ctxn2	-1.0787044	1.98381935	6.26224556	0.02116623	0.1008416
Ptprs	0.40713019	6.88843335	6.26036919	0.02118317	0.10086414
Mpped2	-0.3972631	6.62221731	6.25928583	0.02119296	0.10086414
1110008F13I	-0.9039857	2.33546273	6.25817033	0.02120304	0.10086414
Acot13	-0.3905627	5.31541963	6.25716244	0.02121215	0.10086414
Dsg2	0.69967009	3.13330013	6.25082119	0.0212696	0.10105656
Ppp2r3a	0.35875143	6.51542	6.25041837	0.02127325	0.10105656
Rpl36al	-0.5629139	7.29476133	6.24548767	0.02131805	0.10117729

Cstad	-0.7785878	2.55510493	6.24534733	0.02131933	0.10117729
Ptk2b	0.40665341	7.47935581	6.23873847	0.02137954	0.10131174
Coprs	-0.6090712	3.63211372	6.23763108	0.02138965	0.10131174
Zfp523	0.47273215	4.07551606	6.23677678	0.02139745	0.10131174
Supt6	0.39855618	7.40632526	6.23665224	0.02139859	0.10131174
Glrx	-0.4208723	6.11625311	6.23590118	0.02140545	0.10131174
BC018507	0.50806851	6.72558247	6.23543458	0.02140971	0.10131174
Plekhb1	0.44984094	5.81147658	6.23339769	0.02142834	0.10135092
Tmem179	0.52884329	3.41467723	6.22913362	0.02146739	0.10139541
Ces5a	-3.8826213	-1.1757707	6.22911445	0.02146757	0.10139541
9530091C08I	1.08838609	4.62543707	6.22897974	0.0214688	0.10139541
Pex10	0.6748464	2.67676415	6.22582511	0.02149775	0.10148319
Flt3	1.42650486	0.51524891	6.2246842	0.02150822	0.10148375
Rufy2	0.57592771	6.03410088	6.22084932	0.02154349	0.10152167
Vps11	0.4309372	4.10357047	6.21954289	0.02155552	0.10152167
Nrg4	1.98000461	-0.4053342	6.21858888	0.02156431	0.10152167
4933424G05	1.42700987	1.07474415	6.21729573	0.02157622	0.10152167
Ctdnep1	-0.4037008	5.82205095	6.21711541	0.02157789	0.10152167
Lipt2	-0.8493678	2.11350335	6.21705457	0.02157845	0.10152167
Snora81	2.65395586	-1.7249084	6.21060273	0.02163803	0.10174707
Ptpro	0.53176538	3.42849331	6.20961935	0.02164713	0.10174707
Tmem258	-0.697353	2.43606167	6.20282861	0.02171008	0.10196454
1700007J10F	1.52929413	0.06999481	6.20238239	0.02171422	0.10196454
Map1b	0.69893355	12.0341584	6.19401364	0.02179209	0.10220551
Etfa	-0.3677991	6.60591641	6.19366532	0.02179534	0.10220551
Rgs19	-0.4993085	4.53633584	6.19350452	0.02179684	0.10220551
Smyd3	0.32377446	6.18558953	6.19108784	0.02181939	0.10225252
Ano3	0.46409235	7.88990707	6.18744057	0.02185347	0.10225252
Chd9	0.40866735	7.98540391	6.18737888	0.02185405	0.10225252
N6amt1	-0.3344235	5.66789646	6.18724428	0.02185531	0.10225252
Alg10b	0.374683	5.55240241	6.18655636	0.02186174	0.10225252
Mrps16	-0.5879011	3.20787223	6.18572814	0.0218695	0.10225252
Gsap	0.72464853	2.23454519	6.18375298	0.02188799	0.10229018
Mettl17	0.91585905	1.69387304	6.17980482	0.02192502	0.1024142
Ccl7	-2.429517	-0.4119397	6.17869519	0.02193544	0.1024142
Mdp1	-0.4409514	4.40685737	6.17612892	0.02195956	0.10247797
Dbpht2	-0.3583113	7.6516806	6.1669523	0.02204605	0.1028326
Trio	0.50322277	7.7197757	6.16564633	0.02205839	0.10284119
Chchd2	-0.3684118	7.6487621	6.1640718	0.02207328	0.10286164
Degs2	1.06296612	1.57855019	6.16281692	0.02208516	0.10286804
Dock9	0.43691081	6.49843439	6.15818283	0.02212907	0.10302357
Bcl10	-0.6785258	4.85800539	6.15510485	0.02215828	0.10309123
Stx7	-0.3172714	7.46022408	6.15443527	0.02216465	0.10309123

Cox7c	-0.481056	7.31570746	6.15259999	0.0221821	0.10312342
Gm15800	0.7592759	8.44724005	6.15086745	0.02219858	0.10315111
Gtf3c1	0.51882119	6.48782816	6.14834936	0.02222257	0.1032136
Aox2	-4.0859042	-0.919169	6.14516686	0.02225292	0.10322925
Trib3	3.59080271	-1.1817498	6.14423582	0.02226181	0.10322925
Uap1	-0.3305164	5.8001539	6.14322425	0.02227148	0.10322925
Dhrs11	1.59135098	0.67137026	6.14304551	0.02227318	0.10322925
D130020L05I	0.66616802	2.63523436	6.14247568	0.02227863	0.10322925
Trmt61b	-0.5844423	3.3221804	6.13996382	0.02230265	0.1032917
A230073K19	1.066305	4.54489843	6.13432645	0.02235668	0.10347182
Prdm10	0.70522277	2.96049665	6.1337017	0.02236267	0.10347182
Decr1	-0.5193083	5.39052888	6.13197254	0.02237928	0.10347325
9130024F11I	0.55921951	4.23553446	6.13146971	0.02238411	0.10347325
Pcdhb5	0.67675077	1.85880226	6.12926346	0.02240532	0.10352245
Zfp952	0.37350883	4.62373598	6.12736527	0.02242359	0.10352809
Gm5820	-0.9982433	1.83755461	6.12694042	0.02242768	0.10352809
Dear1	1.31031022	1.65996176	6.12384137	0.02245754	0.10361713
Csdc2	-0.3806106	6.84565661	6.11977716	0.02249678	0.103697
Msn	-0.437815	7.6261454	6.11916594	0.02250269	0.103697
Ppcs	-0.7065262	2.41680731	6.11854541	0.02250869	0.103697
Osbpl6	0.34031932	7.06424395	6.11766518	0.0225172	0.103697
Agk	0.43516182	4.41723715	6.11274382	0.02256486	0.10385358
1700011I03R	-1.7891886	-0.0163869	6.11103811	0.02258141	0.10385358
Bmp3	0.55903511	5.23358809	6.1108735	0.02258301	0.10385358
BC033916	1.30100615	0.31825089	6.10960324	0.02259534	0.10385458
Acot2	-0.4611518	4.85835318	6.10600532	0.02263031	0.10385458
Adam22	0.42302143	7.72163475	6.10588164	0.02263151	0.10385458
P2ry13	0.89336184	2.42328425	6.10551003	0.02263513	0.10385458
Adcy1	0.49832245	8.75656607	6.10498503	0.02264024	0.10385458
Zfp653	-0.8595487	2.40126424	6.1031426	0.02265818	0.10385458
Epm2a	1.13638913	1.61414237	6.101017	0.0226789	0.10385458
Crx	2.19982075	-1.2279969	6.09944915	0.02269419	0.10385458
Cpxm2	-0.7707205	2.65931824	6.09921802	0.02269645	0.10385458
Pcdhb15	0.73344798	2.72245539	6.09896288	0.02269894	0.10385458
Ascl1	0.62639274	3.37908203	6.09821195	0.02270627	0.10385458
Tcp11l1	0.39894486	5.22320502	6.09778328	0.02271046	0.10385458
Nploc4	-0.3536215	5.5117451	6.09383527	0.02274905	0.10398254
Ubr5	0.38112601	7.85059962	6.09140092	0.02277289	0.10404294
Reep2	-0.4990873	4.64945687	6.09010173	0.02278562	0.10405258
Ubl3	-0.2829637	7.21135999	6.08720448	0.02281404	0.10409222
Cox6b1	-0.6119987	5.47414806	6.08705037	0.02281556	0.10409222
Nefh	0.67269167	5.51403652	6.08402007	0.02284533	0.10417953
Enc1	0.55517039	8.26105106	6.082308	0.02286217	0.10420782

Farp2	-0.5490867	2.80240206	6.07902178	0.02289453	0.10430679
Tmem242	-0.5898744	4.09529041	6.07443925	0.02293975	0.10446421
Rpl41	-0.5359423	8.18340747	6.06955272	0.02298808	0.10449942
Dkc1	-0.3099577	6.20082846	6.06885037	0.02299503	0.10449942
Xndc1	-0.575104	3.95280623	6.06743223	0.02300908	0.10449942
Zfyve16	0.43423967	4.90161948	6.06644051	0.02301891	0.10449942
Gzmb	2.53693668	-0.3237874	6.06567445	0.02302651	0.10449942
Atp5f1	-0.2880864	7.46224354	6.06423095	0.02304084	0.10449942
Taldo1	-0.3818867	4.07313907	6.06388481	0.02304427	0.10449942
Evi2a	0.76962483	2.7935894	6.06381919	0.02304492	0.10449942
Stat6	-0.4561976	5.05259947	6.06355129	0.02304758	0.10449942
Zrsr1	-0.2400071	6.95800016	6.0628889	0.02305416	0.10449942
Krt7	-3.9652463	-1.709268	6.0606351	0.02307656	0.10455257
Prmt5	-0.3742625	4.74281688	6.05832128	0.02309959	0.10459392
Ovol1	-1.9707685	-0.3744116	6.057572	0.02310705	0.10459392
L3hypdh	-0.8136712	2.99052929	6.05364526	0.02314619	0.10472272
Kcnb1	0.49786388	8.01709243	6.04938468	0.02318875	0.10485347
Sel1l	0.35767848	7.13736227	6.04693075	0.0232133	0.10485347
Cep85	0.52104844	3.329014	6.04647312	0.02321788	0.10485347
Gm14391	1.2248903	1.93452359	6.04606663	0.02322195	0.10485347
Pwp2	0.85154959	2.67198387	6.0454015	0.02322861	0.10485347
Slx1b	0.44563836	3.6325023	6.04258037	0.02325689	0.10493278
Nyx	2.43958232	-0.5550092	6.03781744	0.02330473	0.1051002
Tnfrsf12a	-1.8838095	-0.8392961	6.03225076	0.02336078	0.10528859
Rnf8	-0.515271	4.25082204	6.03153459	0.023368	0.10528859
Edem3	0.33045576	6.01959876	6.02987676	0.02338473	0.10530383
Mmd	0.28819101	7.33839218	6.02737394	0.02341001	0.10530383
Lnpep	0.27360654	6.88376445	6.02688023	0.023415	0.10530383
Uchl5	0.29529133	6.67267395	6.02611647	0.02342272	0.10530383
C920021L13R	0.92183616	1.96554996	6.02587753	0.02342514	0.10530383
Nup210	0.78891847	3.04798879	6.02410177	0.0234431	0.10533626
Ppfia2	0.38662357	7.76153196	6.02247622	0.02345956	0.10536189
Snrpg	-0.5071335	5.054598	6.01630584	0.02352217	0.10559463
Rbm12b2	0.52408408	4.39119605	6.01257522	0.02356011	0.1057165
Reps1	-0.3315361	6.00264591	6.01117891	0.02357433	0.10573187
Dbhos	0.82029352	3.35818867	6.00845124	0.02360213	0.10580812
Mipol1	-0.4976419	3.67320862	6.00512424	0.02363609	0.1059119
Hmgcs2	-0.5840645	4.07339553	6.00216672	0.02366633	0.10599025
B230216N24	0.80899457	2.24691743	6.00129797	0.02367522	0.10599025
Sorbs2	0.6263754	7.36528757	5.99640811	0.02372532	0.10616605
Fxyd4	2.30570339	-0.3788599	5.99395485	0.02375051	0.10623022
Dalrd3	-0.6642661	3.33754619	5.99140736	0.02377669	0.10625081
Gm5531	0.75603847	2.74342724	5.99139626	0.02377681	0.10625081

Aamdc	-0.4969896	4.22658645	5.98749243	0.023817	0.10636916
Lrrc19	1.52776234	0.34240193	5.98671485	0.02382501	0.10636916
Trappc3	-0.3945392	5.00076725	5.98537856	0.02383879	0.10638219
Jag2	1.0646961	0.88017764	5.97949257	0.02389959	0.10658722
Piwil2	2.75729592	-0.4778187	5.9788251	0.02390649	0.10658722
Cyp2j6	0.48126462	4.00812163	5.97657854	0.02392975	0.10659987
Acyp2	-0.3939574	4.62581168	5.97573679	0.02393848	0.10659987
Ngdn	-0.5570927	3.72143171	5.97539857	0.02394198	0.10659987
Gprasp1	0.6265204	8.9720936	5.97300048	0.02396685	0.1066444
Tmem254a	1.14086288	1.45185985	5.97233535	0.02397376	0.1066444
Akr7a5	-0.7351306	2.3865428	5.96947705	0.02400345	0.10672662
Esr2	-1.1921792	0.69385445	5.96845932	0.02401403	0.10672662
Ndufa5	-0.4666171	5.61427803	5.96661323	0.02403324	0.10676355
Nup133	0.37116104	4.51006149	5.96402817	0.02406017	0.10680646
Nsmaf	0.44012899	4.77577922	5.96260293	0.02407503	0.10680646
Ammecr1	0.91201291	2.35776292	5.96221122	0.02407911	0.10680646
Cggbp1	-0.3559188	7.33132985	5.96093662	0.02409241	0.10680646
Dcun1d3	-0.4031527	4.96451194	5.96039183	0.0240981	0.10680646
Tle6	-1.5449083	0.31341144	5.95835708	0.02411936	0.10680646
H2-Q4	0.94428371	1.686645	5.95671167	0.02413656	0.10680646
Car2	0.28920496	7.0909454	5.95484839	0.02415606	0.10680646
Sumo2	-0.4658553	8.73781098	5.95400206	0.02416492	0.10680646
Knop1	-0.4017349	5.63143563	5.95377869	0.02416726	0.10680646
Dstn	-0.4159286	8.25470472	5.95303493	0.02417505	0.10680646
Klf6	-0.3043576	7.42428216	5.95270424	0.02417852	0.10680646
Zfp61	0.68412692	3.04587609	5.9511397	0.02419492	0.10680646
AI464131	0.74647176	2.93921916	5.95043702	0.0242023	0.10680646
Tfrc	0.41858469	5.51718518	5.95004044	0.02420646	0.10680646
Ccna2	0.77211256	1.98448009	5.94833806	0.02422433	0.1068372
Acad10	1.13458896	0.81067931	5.94580507	0.02425095	0.10690647
Syn3	0.50183598	6.89338645	5.9436136	0.02427401	0.10692985
Kirrel2	2.76607568	-0.5200892	5.94266685	0.02428398	0.10692985
Klhl26	0.45772978	3.63165044	5.94218967	0.024289	0.10692985
Sfmbt2	0.83509295	2.5418789	5.93982772	0.0243139	0.10698313
Fkbp8	-0.4973696	5.10116843	5.93896991	0.02432295	0.10698313
Zfp429	-0.7158847	2.01858964	5.92996626	0.02441816	0.1073432
Mast4	0.34690862	6.6848335	5.92915741	0.02442673	0.1073432
Pura	-0.2512837	6.97518795	5.92409496	0.02448047	0.10746298
Slc5a5	-0.4825894	4.92902346	5.92344938	0.02448733	0.10746298
Gnb2l1	-0.3859941	5.840013	5.92276635	0.02449459	0.10746298
Stub1	-1.2625932	0.86634592	5.92222358	0.02450037	0.10746298
Dclk3	0.45083217	5.48919709	5.92078185	0.02451571	0.10746298
Actn3	-2.4836036	-0.3285975	5.91863537	0.02453857	0.10746298

Arl2	-0.5890614	3.71063299	5.91843616	0.02454069	0.10746298
Cox6c	-0.413638	7.55381088	5.91520112	0.0245752	0.10746298
05-Sep	-0.3111717	6.40078037	5.91471712	0.02458037	0.10746298
Mrpl17	-0.3334635	5.65330269	5.91382109	0.02458994	0.10746298
Rrn3	0.2936922	5.81564163	5.91364263	0.02459184	0.10746298
Sh3bp4	0.52799795	2.8722618	5.91279564	0.02460089	0.10746298
Pvrl3	0.3850479	5.40676394	5.91269183	0.024602	0.10746298
Rmi1	-0.352624	5.67301753	5.91217002	0.02460758	0.10746298
Akr1a1	-0.5033236	7.81607609	5.91090683	0.02462109	0.10747406
Zfp960	0.38058383	4.46068238	5.90615478	0.02467198	0.10763568
Lrch1	0.51702068	4.25291968	5.90456289	0.02468905	0.10763568
Ift52	-0.4341524	4.94907835	5.90386311	0.02469656	0.10763568
Rnf187	-0.3816212	6.80386205	5.90335052	0.02470207	0.10763568
4930511M06	0.83447946	3.22956521	5.90121753	0.02472498	0.10766176
AY512931	1.10958357	1.78850411	5.90049147	0.02473279	0.10766176
Spata6	-0.4693452	3.91402258	5.89773676	0.02476242	0.10766176
Ufc1	-0.4288735	5.14966962	5.89764155	0.02476345	0.10766176
Pak6	0.49582487	3.74563752	5.89690532	0.02477138	0.10766176
Cep350	0.45136317	6.68424156	5.89609198	0.02478014	0.10766176
Ccdc132	0.3556019	6.47996057	5.89485072	0.02479352	0.10766176
Herc1	0.7053225	8.10533263	5.8946222	0.02479598	0.10766176
Cxcl17	3.18458832	-1.1080661	5.89033774	0.02484223	0.10781476
Hcn1	0.50851637	7.36017206	5.88916377	0.02485492	0.10782206
Usp35	0.84138367	1.87628475	5.88487488	0.02490133	0.10797561
Icam5	0.64973233	2.88514723	5.88209337	0.02493149	0.10805853
Gm15401	1.14567421	0.45559154	5.87903906	0.02496465	0.1081544
Rnf114	-0.478159	5.39363236	5.87454442	0.02501354	0.10822512
Mtx2	-0.2840437	5.6916001	5.87408489	0.02501854	0.10822512
Kcnh3	0.48009189	3.65536925	5.87399973	0.02501947	0.10822512
1110032A03	-0.3926241	6.65671181	5.87347681	0.02502517	0.10822512
Spg11	0.42443001	5.11874808	5.86964396	0.02506697	0.10832011
Gabrb2	0.41849403	8.24512873	5.86943476	0.02506925	0.10832011
Gsk3a	-0.2655447	7.32149468	5.86193712	0.02515125	0.10850239
Gucy1a3	0.44956676	7.27725386	5.86157403	0.02515523	0.10850239
Gm21671	1.11972861	0.9986675	5.86118796	0.02515946	0.10850239
Rasgrf1	0.5289518	8.33628821	5.86090205	0.02516259	0.10850239
Gamt	-1.1770014	1.99468364	5.86051656	0.02516682	0.10850239
Shank2	0.64242428	6.98539914	5.85928435	0.02518034	0.1085129
Map2k3	-0.6365059	3.88687435	5.85761735	0.02519863	0.10851852
Chchd4	-0.4903042	4.78100268	5.85714689	0.0252038	0.10851852
Lphn1	0.40392379	8.35124724	5.85492023	0.02522827	0.10856538
Sorcs2	0.5206705	3.64157151	5.85043496	0.02527764	0.10856538
Specc1	0.3336595	6.56145607	5.84859015	0.02529797	0.10856538

Grp	-1.7177223	0.11469407	5.84853202	0.02529861	0.10856538
Phka1	0.53194139	4.255368	5.84840924	0.02529997	0.10856538
Dbi	-0.5012303	7.09650929	5.84726803	0.02531256	0.10856538
Gpr107	0.51202121	3.80767005	5.84588506	0.02532783	0.10856538
Trappc9	0.49776101	5.74787062	5.84460654	0.02534195	0.10856538
Xcr1	0.99855534	1.55130213	5.84410102	0.02534754	0.10856538
Cox7a2l	-0.4275874	7.11294571	5.84334001	0.02535595	0.10856538
Mrpl11	-0.4842739	4.64254819	5.84289575	0.02536086	0.10856538
Lpcat4	0.39241883	5.66208883	5.84246089	0.02536567	0.10856538
Sbf1	0.5803477	4.72245381	5.84233641	0.02536705	0.10856538
Mea1	-0.6138397	4.7171691	5.8418046	0.02537293	0.10856538
Dhps	-0.7964249	3.07884382	5.84083064	0.02538371	0.10856538
Tmem2	0.37823301	4.21362306	5.84008107	0.02539201	0.10856538
Bai1	0.44877401	6.13424862	5.83599719	0.02543729	0.1087115
Dnlz	-0.5241856	3.76247593	5.8317834	0.0254841	0.10886406
Pik3r6	-1.1843619	1.61009317	5.83047344	0.02549867	0.10887883
Cdt1	-2.007257	-0.1894261	5.82685098	0.02553902	0.10895791
Gdap1l1	-0.5271943	3.45144051	5.82620478	0.02554622	0.10895791
Chp1	-0.2725953	7.55719185	5.82581557	0.02555056	0.10895791
Trpm7	0.33375847	6.58706459	5.8216253	0.02559735	0.10906842
Pcdha11	1.28516569	0.37164766	5.82149986	0.02559875	0.10906842
Insig1	0.2993634	6.28332118	5.81539727	0.02566706	0.10931192
Tmem129	-0.5714704	3.06683303	5.80840396	0.02574559	0.10959873
Arsi	-1.6028877	0.61766923	5.80527845	0.02578078	0.10961517
Slc22a3	1.15577561	0.82570762	5.80514753	0.02578225	0.10961517
Dscam	0.41762599	5.64786648	5.80497786	0.02578417	0.10961517
Zcwpw1	1.34399571	0.6780819	5.80408611	0.02579422	0.10961517
Zfp59	-0.6176141	3.33000166	5.80098151	0.02582924	0.10971642
4933428C19l	-2.4119401	-0.7452506	5.79367945	0.02591183	0.10997858
Pdzd11	-0.3784875	6.13967955	5.79353983	0.02591342	0.10997858
Rps29	-0.3811841	6.7774653	5.78877559	0.02596747	0.11016025
Plec	0.44857861	6.34716249	5.78745668	0.02598245	0.11017611
Gm13446	-0.7324202	2.27103668	5.78494581	0.02601101	0.11024947
Psme1	-0.546963	7.09575098	5.78192759	0.02604538	0.11029192
Sgcg	2.4224471	-0.352772	5.78113735	0.02605439	0.11029192
Ccnt2	0.5599093	4.8957623	5.78110119	0.0260548	0.11029192
Zufsp	0.78128908	3.44116249	5.77811331	0.02608889	0.11036603
Unc13a	0.7029245	6.96026908	5.77759233	0.02609484	0.11036603
M1ap	-3.8729694	-1.5333077	5.77524622	0.02612165	0.11043006
Kif21b	0.54239725	5.75344638	5.77429528	0.02613253	0.11043006
Rnf7	-0.4281507	7.98133013	5.77136802	0.02616604	0.1104714
Cog1	0.47607007	4.11567653	5.7691615	0.02619134	0.1104714
Patl2	-5.9946444	-1.4006495	5.93586019	0.02619313	0.1104714

Erich2	-1.2976366	0.43049639	5.768885	0.02619451	0.1104714
Tgfb2	0.45192848	4.28430239	5.76780372	0.02620691	0.1104714
Ggta1	-0.997645	2.39992791	5.7668998	0.02621729	0.1104714
Myoz3	1.22541596	1.1393035	5.76655417	0.02622126	0.1104714
Cyfip1	0.33111678	6.22946683	5.7621657	0.02627171	0.11063636
Runx3	-1.1887164	0.86058091	5.75942983	0.02630322	0.11071256
Twf2	-0.5203128	3.43721528	5.75811713	0.02631835	0.11071256
Pcdhb2	1.22461593	1.80948339	5.75765235	0.02632371	0.11071256
Gm9839	3.08692654	-0.419658	5.75641186	0.02633802	0.11071394
Hp1bp3	-0.2370614	8.1320659	5.75566498	0.02634664	0.11071394
Slc27a1	0.65380073	3.86559533	5.75154447	0.02639427	0.1108665
Tbc1d30	0.43181268	6.6190001	5.74803902	0.02643486	0.1109894
Plekha2	-0.4782442	5.21607089	5.73779067	0.02655393	0.11131698
Snapc4	0.80594635	3.72206953	5.73659045	0.02656792	0.11131698
Wiz	-0.3898568	4.43720815	5.73648104	0.02656919	0.11131698
Stk33	-0.9957523	1.62331277	5.73576942	0.02657749	0.11131698
Chd5	0.51463429	6.75626534	5.7356889	0.02657843	0.11131698
Lyar	-0.3758035	4.22170673	5.73546268	0.02658106	0.11131698
Prdx2	-0.4965188	6.20444139	5.73359204	0.02660289	0.11136077
Zfp770	-0.2984511	6.22912452	5.73246167	0.02661609	0.11136842
Bend6	-0.3604331	6.64558195	5.72810499	0.02666702	0.11146721
Syt7	0.45356947	6.93971431	5.72792054	0.02666918	0.11146721
Prnp	0.35771613	8.00642486	5.72752311	0.02667383	0.11146721
2810433D01	-0.6937194	3.1447098	5.72582448	0.02669373	0.11150278
Acp5	-2.338403	-0.7192985	5.72206519	0.02673782	0.11163601
Cpt1c	0.65331134	3.36408473	5.72116254	0.02674842	0.11163601
Ubl4b	-1.707001	0.06162456	5.71976621	0.02676482	0.1116569
Slc8b1	0.80829308	1.35538975	5.71593035	0.02680995	0.11172087
Lama4	0.46852972	4.07808699	5.71374101	0.02683574	0.11172087
Gm1943	-0.5389976	3.5392695	5.71369546	0.02683628	0.11172087
Mlec	-0.3142217	6.89116658	5.7131379	0.02684285	0.11172087
Fmo1	-0.4178761	6.87527009	5.71276126	0.02684729	0.11172087
Zfp940	-0.5864543	3.31935635	5.71265133	0.02684859	0.11172087
Car11	0.36728316	5.14702599	5.70927479	0.02688845	0.1118392
Cep95	0.441847	3.92792727	5.70772289	0.02690679	0.11186799
Scn7a	-0.4297002	4.22866684	5.70598557	0.02692734	0.11190593
Pip5k1a	0.44162658	3.99435861	5.70199755	0.02697457	0.1120547
Sncg	-2.1732467	-0.3725225	5.69962155	0.02700276	0.11209861
Xpo4	0.46518833	4.12366709	5.69917759	0.02700803	0.11209861
Rnf207	1.56245313	0.9727215	5.69801701	0.02702182	0.11210832
Atp6v0a1	0.35624758	7.21774793	5.69466894	0.02706162	0.11214198
D17H6S53E	0.54061157	2.75255333	5.69462837	0.02706211	0.11214198
Ttll3	0.88185717	1.83886483	5.69429466	0.02706608	0.11214198

Ccdc91	-0.3914145	5.37046407	5.69348434	0.02707573	0.11214198
Nkx2-1	0.98566711	1.90266197	5.69138988	0.02710068	0.11216528
Zfp804a	0.50434177	5.92882127	5.69038769	0.02711262	0.11216528
Mbd3	-0.3985895	4.4107566	5.6901296	0.0271157	0.11216528
A330032B11	0.70171979	2.15244353	5.68731229	0.02714933	0.11225696
Rftn1	1.03203045	1.5953295	5.6834302	0.02719574	0.11236288
Ndufb7	-0.6597234	4.23432189	5.68325055	0.02719789	0.11236288
Eci3	-1.2516979	0.66836361	5.67907899	0.02724786	0.11248001
6330408A02	0.40688707	3.61196677	5.67896713	0.0272492	0.11248001
Lix1l	-0.6459226	6.40813005	5.67768741	0.02726456	0.11249597
Ctla2a	0.95196625	1.86945644	5.6760225	0.02728454	0.11250627
Wbp4	-0.2789839	6.16916851	5.67556639	0.02729002	0.11250627
Slc25a27	0.49842257	4.58888176	5.66818587	0.02737885	0.11277862
Hspa4l	0.4949493	7.12134736	5.6681641	0.02737911	0.11277862
Rd3	-4.4305756	-1.8484215	5.66658557	0.02739815	0.11280614
Tcea1	-0.2846778	8.06123568	5.66570118	0.02740883	0.11280614
Sae1	-0.3505823	5.57085668	5.66404248	0.02742886	0.11283027
Etv4	2.19806046	-1.3660121	5.66330868	0.02743773	0.11283027
Scap	0.4847393	4.49942401	5.65925544	0.02748676	0.11298449
Trmt44	1.34743678	0.66664518	5.65328343	0.0275592	0.11323472
Cnot6l	0.28826836	7.06788096	5.65099304	0.02758704	0.11325977
Ccdc82	0.48120738	7.54247244	5.65045133	0.02759362	0.11325977
Eogt	0.37843759	5.15256979	5.64953858	0.02760473	0.11325977
Asna1	-0.4391114	6.49108005	5.64852039	0.02761712	0.11325977
Hey1	-0.3608453	6.35228255	5.64793572	0.02762424	0.11325977
Ap5z1	0.83060658	2.61958372	5.6470796	0.02763467	0.11325977
Gemin6	-0.5299276	2.96628022	5.64585183	0.02764964	0.11327371
Efna3	-0.8715659	1.5606198	5.64477537	0.02766276	0.11328011
Rab43	-0.451896	5.4568672	5.64071743	0.02771232	0.1134356
Cops3	-0.3180078	5.48155789	5.6372594	0.02775462	0.11353786
Gpd1l	-0.2657764	7.02544809	5.63678105	0.02776048	0.11353786
Rarb	0.39235521	5.04062113	5.63396422	0.027795	0.11362067
Acap2	0.34388422	6.73780263	5.63296071	0.02780731	0.11362067
Nav2	0.56077206	7.18840989	5.63229136	0.02781552	0.11362067
Huwe1	0.52849301	9.48061089	5.63058257	0.02783651	0.11365899
Papolg	0.28172355	5.32489383	5.6280925	0.02786712	0.11373656
Apba3	0.69645342	2.53870786	5.62511089	0.02790382	0.11383892
Trim62	0.43094929	4.08115042	5.62287172	0.02793142	0.1138888
Caprin2	0.69735537	2.7154879	5.62223273	0.0279393	0.1138888
Fat4	0.33698871	5.96841046	5.6205407	0.02796018	0.11392651
Agt	1.49688243	1.29022148	5.61759597	0.02799657	0.11402733
Mib1	0.35806875	6.21587514	5.61584163	0.02801827	0.11405806
Impad1	0.2838238	7.02690267	5.615104	0.0280274	0.11405806

Cnnm3	0.38311929	4.18017385	5.6128031	0.0280559	0.11412663
Zfp60	0.31534817	6.37575966	5.61062402	0.02808292	0.11417526
Kdsr	0.40521749	4.62322859	5.6087242	0.02810651	0.11417526
E030019B13l	3.2090254	-1.3835577	5.6074666	0.02812213	0.11417526
Dnttip2	-0.2880371	6.01952394	5.60704796	0.02812733	0.11417526
Gtf2h4	-1.0092044	1.44532771	5.60562071	0.02814508	0.11417526
Mmadhc	-0.4480581	5.8456984	5.60482801	0.02815494	0.11417526
Polr2g	-0.4383234	5.38000688	5.60407148	0.02816436	0.11417526
Syne1	0.60370133	8.5747803	5.60375475	0.0281683	0.11417526
Ift122	0.48064021	3.72749146	5.60339675	0.02817276	0.11417526
Atp5k	-0.4257115	5.92428404	5.59828457	0.0282365	0.11436267
Ewsr1	0.45722393	7.69583582	5.59649156	0.0282589	0.11436267
Zfp622	-0.4373075	4.68996095	5.59628061	0.02826153	0.11436267
Nol7	-0.427251	5.77233861	5.59594684	0.0282657	0.11436267
Nsa2	-0.3320431	6.72256089	5.59217354	0.02831291	0.11448353
Hspb3	-1.3468823	0.34003333	5.5915856	0.02832027	0.11448353
Slc12a2	0.32852934	6.5705165	5.59075844	0.02833064	0.11448353
Rpl32	-0.4825858	6.79791779	5.58606784	0.02838949	0.11467403
Diap3	1.62071437	0.48127226	5.58406023	0.02841472	0.11472863
Pus7l	1.04027097	1.59734635	5.58069757	0.02845703	0.1147994
Pycr1	1.6095509	0.14120247	5.58025458	0.02846261	0.1147994
Pcdhga2	0.91931081	2.1827769	5.57917007	0.02847628	0.1147994
E2f1	-0.7326759	1.94559375	5.578495	0.02848479	0.1147994
2610028E06l	1.99122546	-0.2727317	5.57801488	0.02849084	0.1147994
Ap1ar	-0.2689945	6.76523956	5.56945682	0.028599	0.11518249
Slc13a1	3.12506776	-1.3495689	5.56863329	0.02860944	0.11518249
Aldoc	0.37220554	7.18074671	5.56607682	0.02864185	0.11524338
Vps13c	0.59216314	6.48139958	5.565585	0.02864809	0.11524338
Csrnp2	0.38299194	5.41423429	5.56399191	0.02866831	0.11524813
Nup85	0.47772983	3.70621767	5.56196724	0.02869404	0.11524813
Rmnd5b	-0.3272769	4.74085418	5.5618578	0.02869543	0.11524813
Sox7	-1.2765914	0.68090057	5.56178695	0.02869633	0.11524813
Tnik	0.50207467	7.36927851	5.55673069	0.0287607	0.11541392
5033406O09	3.15974249	-0.5531035	5.55669322	0.02876118	0.11541392
D6Wsu163e	0.41545741	4.54406392	5.55277044	0.02881123	0.11555411
Pex11b	-0.4995228	5.43222602	5.5521068	0.02881971	0.11555411
Lcp2	-0.5481306	3.02345756	5.54935273	0.02885492	0.11564241
Smox	-0.6561	2.88869692	5.54853815	0.02886534	0.11564241
Csmd2	0.77734582	4.84623264	5.5453208	0.02890655	0.11570072
Snrpe	-0.4643215	4.52125606	5.54475243	0.02891384	0.11570072
Mcm5	-1.0503147	0.90631205	5.54155836	0.02895483	0.11570072
Cdk7	0.32261575	6.25268145	5.54132989	0.02895777	0.11570072
Mirg	1.54728702	1.06503662	5.54063772	0.02896666	0.11570072

4930565N06	1.53997006	1.61358252	5.5403135	0.02897083	0.11570072
Ppp2r3d	0.67159577	3.95125935	5.54023355	0.02897185	0.11570072
Galnt7	0.51706232	3.06937041	5.54003607	0.02897439	0.11570072
Glmn	0.63943399	3.94395078	5.53906763	0.02898684	0.11570327
Scube3	1.54441774	1.4791475	5.53756495	0.02900617	0.11573327
E130309F12I	0.65126009	2.72267869	5.534789	0.02904192	0.11576564
Mc4r	2.4171041	-0.7315664	5.53469423	0.02904314	0.11576564
1700008O03	0.63805206	2.94146089	5.53418235	0.02904974	0.11576564
Spag16	-1.2446776	0.42113633	5.53254062	0.02907092	0.11578235
Zdhhc6	-0.4847528	4.61483211	5.53055325	0.02909657	0.11578235
Lsm12	-0.3218223	5.8603497	5.53030657	0.02909975	0.11578235
Smarce1	-0.3333172	6.41595894	5.53019338	0.02910122	0.11578235
Pcdhb19	0.63168558	2.92157149	5.52774764	0.02913283	0.11585167
Kdelc2	0.44786771	4.49541638	5.52665915	0.02914691	0.11585167
Spns2	0.52273978	3.5192407	5.52491375	0.0291695	0.11585167
Crtac1	0.675664	3.20349585	5.5243232	0.02917715	0.11585167
Slc8a1	0.49814205	9.18535327	5.52427525	0.02917778	0.11585167
Pcdhgb5	0.78196662	2.4519966	5.52026595	0.02922977	0.11601109
She	-1.272883	1.34917998	5.51870589	0.02925003	0.11604448
Pip4k2a	-0.2522827	7.90645861	5.51504853	0.02929759	0.11618611
Adam4	1.48483107	0.97597989	5.51401837	0.029311	0.11619225
Herc3	0.45950139	7.65233924	5.51203294	0.02933687	0.11624775
Iscu	-0.4388403	6.3829146	5.5095174	0.02936968	0.11625771
Cep164	0.51363245	3.31652975	5.50923397	0.02937338	0.11625771
Serpinb9	-0.4605283	7.10563105	5.50911068	0.02937499	0.11625771
Slc35f3	0.33749103	5.58481233	5.50726806	0.02939905	0.11630597
Hnrnpul1	-0.320561	6.59340048	5.50494067	0.02942948	0.11636867
Tnni1	2.9331547	-1.0624681	5.50402032	0.02944153	0.11636867
Hmbs	-0.5603083	3.04214054	5.50333126	0.02945055	0.11636867
Gfm2	0.73678825	3.58539059	5.50127798	0.02947744	0.11642798
Acsl6	0.3727692	6.0861071	5.49835388	0.02951579	0.11645327
Zglp1	2.77776806	-1.4615656	5.49741677	0.02952809	0.11645327
Dock1	0.35071462	5.76329246	5.49562374	0.02955165	0.11645327
Sars2	-0.8304771	1.56896706	5.49545763	0.02955383	0.11645327
Cc2d2a	0.29661941	5.78023341	5.49458402	0.02956532	0.11645327
Pitpnm3	0.37288272	6.08220485	5.49422972	0.02956998	0.11645327
Hyou1	0.47898542	5.17947452	5.49380425	0.02957558	0.11645327
Rps7	-0.3386062	6.62288406	5.4926826	0.02959033	0.11645327
Inpp1	0.65952967	3.03063513	5.49264414	0.02959084	0.11645327
Eva1b	-1.1700687	0.88173957	5.49068942	0.02961658	0.11648546
Efcab10	-1.8573219	-0.4047209	5.49021722	0.02962281	0.11648546
Tenm4	0.5201719	5.97639143	5.48698991	0.02966537	0.11655463
Acer1	1.19273338	0.2981237	5.48686271	0.02966705	0.11655463

Mmp12	-2.6357984	-1.5258281	5.48585425	0.02968037	0.11655463
Irgm2	-0.5136995	5.57099843	5.48527729	0.02968799	0.11655463
Ccdc115	-0.3654002	4.90636218	5.48212968	0.02972961	0.11667126
Igbp1	-0.435075	5.79648607	5.47942861	0.02976537	0.11670199
Parl	-0.5366503	3.34903701	5.4788761	0.0297727	0.11670199
Il17rb	1.83978294	-0.0002551	5.47883949	0.02977318	0.11670199
Hsd17b11	-0.4517931	5.02271974	5.47340439	0.02984532	0.11682385
Trim15	2.34769987	-1.2295396	5.4730856	0.02984956	0.11682385
Cnnm4	0.74449034	2.88400049	5.47006429	0.02988975	0.11682385
Palm3	0.63435761	2.25433235	5.46995696	0.02989118	0.11682385
Rictor	0.50499462	6.50234641	5.46990391	0.02989189	0.11682385
Adgb	2.9524926	-0.9356772	5.46975239	0.0298939	0.11682385
Rbm17	-0.3203792	6.6248111	5.46869786	0.02990795	0.11682385
Phf5a	-0.5467521	4.82766972	5.46798633	0.02991743	0.11682385
Klrg2	-1.9000372	-0.5935712	5.46784157	0.02991936	0.11682385
Zbtb16	0.31757126	4.67160739	5.46675756	0.02993381	0.11682385
Slc8a2	0.44022978	5.55719983	5.46625282	0.02994054	0.11682385
Cmc1	-0.5369758	3.90903451	5.46559507	0.02994932	0.11682385
Rab2a	-0.25091	9.67092269	5.46484597	0.02995931	0.11682385
Liph	3.89057195	-1.6566254	5.45730893	0.03006011	0.11715498
Dnaaf1	-3.1028146	-1.8853075	5.45670877	0.03006815	0.11715498
Nkain3	0.80502591	1.994663	5.45195523	0.03013194	0.11720774
Whsc1	0.2659416	6.66122419	5.45182025	0.03013376	0.11720774
Gm14164	2.39721977	-0.9193449	5.45153384	0.0301376	0.11720774
Dennd5b	0.51361267	6.59461121	5.45052981	0.0301511	0.11720774
A430107P09	1.59987689	-0.089564	5.45036985	0.03015325	0.11720774
Jakmip1	-0.4002419	4.70511181	5.45003354	0.03015777	0.11720774
Trpv2	-0.5362241	3.07012746	5.44946239	0.03016545	0.11720774
2610001J05F	-0.5248341	5.47852933	5.44856817	0.03017749	0.117208
Serp1	-0.5913554	7.92018947	5.44747892	0.03019215	0.11721847
Gcnt1	0.70765395	2.91051144	5.44521289	0.03022268	0.11729052
Ngfr	-0.5649978	3.55633351	5.44315911	0.03025038	0.11734245
Ifi27l2a	-0.7268771	2.32156448	5.44244494	0.03026002	0.11734245
Gnas	-0.2821374	10.8121039	5.44049983	0.03028629	0.11739785
Gpr115	0.84519527	1.61554284	5.4382523	0.03031668	0.11742778
Plxna2	0.2915713	7.727418	5.43815547	0.03031799	0.11742778
Als2	0.44061938	4.90617991	5.43494279	0.03036149	0.11750845
1300002E11I	0.37919974	5.25378338	5.43484543	0.03036281	0.11750845
St6galnac2	0.9209811	1.27839149	5.43245039	0.03039529	0.11758768
Rps13	-0.4359902	5.90492291	5.43112648	0.03041325	0.11761074
Myh8	-2.8277453	-1.5295231	5.4288162	0.03044464	0.11763701
Atp2b4	0.44355907	7.45255742	5.42796943	0.03045615	0.11763701
Ferd3l	-3.8513174	-1.5862539	5.57696163	0.03046463	0.11763701

Dsc3	-3.4596677	-1.3625269	5.42709199	0.03046809	0.11763701
Ak3	-0.3702975	8.15985105	5.42424838	0.0305068	0.11771909
Cyp2e1	0.89680702	1.04891713	5.42159137	0.03054303	0.11771909
Gm1564	1.07672793	1.12274621	5.4214044	0.03054558	0.11771909
Isca1	-0.251471	6.6628127	5.421184	0.03054858	0.11771909
Dnm3os	1.17866481	1.87406229	5.4207458	0.03055456	0.11771909
Hs6st1	0.40745647	4.62727952	5.42024116	0.03056145	0.11771909
1700063D05I	0.74633036	1.83602218	5.41744026	0.03059972	0.11777183
Rpl13	-0.4206986	7.37476852	5.41575302	0.03062279	0.11777183
Cxx1b	-0.4087239	5.87807493	5.41449517	0.03064001	0.11777183
D8Ertd82e	0.48988985	4.35499075	5.41406936	0.03064584	0.11777183
Tm7sf2	1.30345767	1.14682049	5.41393658	0.03064766	0.11777183
Drap1	-0.6024648	3.96958289	5.41387225	0.03064854	0.11777183
Ly96	-0.6077346	3.76427015	5.41246229	0.03066786	0.11777183
Ak6	-0.7020389	2.76185152	5.41162163	0.03067938	0.11777183
Rlf	0.37036504	5.84215649	5.41133185	0.03068335	0.11777183
Zfp963	-0.6317147	2.52966685	5.40790729	0.03073036	0.11786891
Fam135a	0.3789102	5.76038231	5.40738553	0.03073753	0.11786891
Slc2a3	0.37600029	5.24964401	5.4062289	0.03075342	0.11786891
Atxn7	0.34063985	5.79439811	5.40530277	0.03076616	0.11786891
Slc9a9	0.72705231	3.46094576	5.4047644	0.03077357	0.11786891
Zfr2	0.5307192	3.76353164	5.40423553	0.03078084	0.11786891
Tpo	3.07622375	-2.0661477	5.4022772	0.03080781	0.11792606
Parva	-0.4007924	7.42564118	5.39967468	0.03084369	0.11801728
Mir128-1	1.35667888	1.50571919	5.39662508	0.03088579	0.11813222
Htr5b	2.36171967	-0.3098906	5.39523903	0.03090494	0.11815935
Aldh5a1	0.3547105	6.74500979	5.39362483	0.03092727	0.11819857
Mettl10	-0.487278	5.06836517	5.3918001	0.03095253	0.11824307
4933409K07I	0.42713619	7.36388262	5.39066934	0.03096819	0.11824307
Pcdh9	0.37218867	7.3123863	5.39016921	0.03097512	0.11824307
Cox7a2	-0.4685415	6.00398457	5.38718854	0.03101647	0.11830412
Gdpd2	0.85279085	2.27646471	5.38581808	0.0310355	0.11830412
Rab11a	-0.2905518	6.56897151	5.38571884	0.03103688	0.11830412
Tiprl	-0.3052662	6.36973233	5.38521598	0.03104387	0.11830412
Hrh1	0.57743508	2.61130733	5.38466626	0.03105151	0.11830412
Rnf217	-0.3887369	4.29072797	5.38220967	0.03108567	0.11838474
Pdxdc1	-0.2709243	6.24525789	5.38067644	0.03110702	0.11838474
Tomm22	-0.4292401	5.96992969	5.38053979	0.03110892	0.11838474
Trim65	-0.5395514	4.06082884	5.37653375	0.03116478	0.11851353
Plekhg3	0.46405612	3.33748503	5.37559809	0.03117784	0.11851353
DQ267102	2.00895846	-0.3130751	5.37551054	0.03117906	0.11851353
Prkcdbp	-0.7248327	5.17983032	5.37356477	0.03120625	0.11856746
Cerkl	1.59734405	-0.1444446	5.37276274	0.03121746	0.11856746

Ak1	-0.4538153	3.98628346	5.37171532	0.03123211	0.11857713
Gas2	0.49690914	2.92876743	5.36588214	0.03131384	0.11874502
Nhs	0.50422236	3.61151607	5.36567086	0.03131681	0.11874502
Btaf1	0.43564926	6.20105171	5.3652662	0.03132249	0.11874502
Shroom3	-0.5613168	2.89460344	5.36176436	0.03137169	0.11874502
Snx6	-0.3648165	6.40291174	5.36168194	0.03137285	0.11874502
Ppp1r14c	-0.5068895	3.61494466	5.36159375	0.03137409	0.11874502
Odf3b	-1.8540493	0.00321497	5.36087697	0.03138417	0.11874502
Gm7102	0.74975056	5.46312547	5.36062599	0.0313877	0.11874502
Trmt6	-0.3779796	4.64719553	5.36052057	0.03138918	0.11874502
Zfp292	0.36117931	7.57624967	5.35992531	0.03139756	0.11874502
Prom2	3.59097129	-1.9620922	5.35726776	0.03143499	0.11874589
Setdb1	0.52013039	4.51143675	5.35658637	0.0314446	0.11874589
Eid2b	-0.3717671	4.42914406	5.35651997	0.03144553	0.11874589
Trim43a	1.50615601	-0.2022028	5.35646687	0.03144628	0.11874589
Mis18bp1	1.00505402	1.0692987	5.35243204	0.03150323	0.11891359
1700085C21I	3.21795921	-1.704339	5.34922343	0.0315486	0.11891359
Meis2	0.33460908	6.62560199	5.34890248	0.03155314	0.11891359
Klhl17	0.77134486	3.50189595	5.3486315	0.03155698	0.11891359
Msi1	-0.8893026	1.91976697	5.34826826	0.03156212	0.11891359
Cidea	-4.5771475	-1.9543343	5.49333109	0.03157139	0.11891359
Snx4	-0.2967069	6.79050908	5.34712922	0.03157825	0.11891359
Ltv1	-0.3602357	4.79175737	5.34632622	0.03158963	0.11891359
Socs2	-0.4014918	5.3342442	5.34559803	0.03159995	0.11891359
Arrdc4	-0.7262783	2.72777521	5.34371498	0.03162666	0.1189684
Baz2b	0.2639989	6.99648662	5.34130303	0.03166091	0.11905152
Eny2	-0.3045784	7.31050281	5.33498134	0.03175088	0.11934399
Atpif1	-0.3608523	7.27261637	5.33295425	0.03177979	0.11936916
R3hdm4	-0.3411564	7.68370037	5.33275259	0.03178266	0.11936916
Taf5l	-0.389212	4.1315046	5.33146642	0.03180102	0.11936916
Minpp1	0.37913274	4.91685676	5.33109587	0.03180631	0.11936916
Ubl4	-0.2572213	6.74027419	5.32900772	0.03183615	0.11943538
Gm1821	-0.4808607	4.9269643	5.32647518	0.03187239	0.11946278
Pole3	-0.6688373	2.9234239	5.32612764	0.03187736	0.11946278
Stx3	0.3516144	4.78234578	5.32594028	0.03188005	0.11946278
Coasy	0.50004078	3.65694836	5.32437492	0.03190247	0.1195011
4930447N08	-2.0659388	-0.703037	5.32309213	0.03192086	0.11952427
Cept1	0.50872228	3.88413645	5.31673704	0.03201214	0.11981215
Notch4	-0.9718647	1.02824809	5.31603775	0.03202221	0.11981215
Mpc2	-0.3468162	5.93669597	5.31397373	0.03205193	0.11987757
Atp13a3	0.26398779	7.15130974	5.31115691	0.03209254	0.11993046
Abracl	-0.4827806	4.25687212	5.31015147	0.03210705	0.11993046
Wdr16	-1.7480096	-0.2244289	5.30984463	0.03211148	0.11993046

Crybb1	-3.1194577	-1.8920045	5.30959776	0.03211504	0.11993046
AI607873	-0.8794279	1.61003838	5.30817449	0.0321356	0.11996149
Abi1	-0.252346	7.09800737	5.30583913	0.03216937	0.12003614
Ssbp1	-0.3200293	5.47566653	5.30509695	0.03218011	0.12003614
Tmem210	1.73006343	0.06352342	5.30315358	0.03220824	0.12005926
Nsun6	0.62329392	2.75050169	5.30297597	0.03221082	0.12005926
Snx9	-0.4289254	4.81473648	5.3010436	0.03223883	0.12011796
Zfyve21	-0.4965761	4.02987159	5.2996921	0.03225843	0.12013679
Rps16	-0.4771256	6.26269801	5.29900468	0.03226841	0.12013679
Atp2a2	0.33503347	9.64115715	5.29762496	0.03228845	0.12016572
Cul9	0.5770841	3.96622826	5.29565239	0.03231712	0.12022674
D17Ertd648e	1.10573416	0.72780669	5.29128204	0.03238075	0.12041257
Gpr55	1.75124472	-0.1849099	5.29053345	0.03239166	0.12041257
2610020C07I	1.20728035	0.53748742	5.2881112	0.032427	0.12047038
Hs3st5	-1.0808254	1.6804255	5.28778167	0.03243181	0.12047038
Bdnf	-0.4579668	4.26333758	5.28292263	0.03250284	0.12065012
Znfx1	0.41040504	5.36773482	5.2819863	0.03251655	0.12065012
Zfp446	0.55123516	3.31004203	5.28193261	0.03251733	0.12065012
Slc9a4	-3.6566331	-1.2674155	5.28105477	0.03253019	0.12065012
Ssna1	-0.6780491	3.00827153	5.28018346	0.03254296	0.12065012
Hes6	-0.6202887	2.20106607	5.27942392	0.0325541	0.12065012
Vdac2	-0.2662492	7.27146482	5.27705468	0.03258886	0.12070647
Brwd1	0.4126649	6.9745461	5.2759504	0.03260507	0.12070647
Itpr1	0.42531155	8.99082868	5.27586889	0.03260627	0.12070647
Tmem202	-2.1534647	0.48555398	5.27470318	0.0326234	0.12072061
Zkscan4	0.72749126	2.2997498	5.2734914	0.03264122	0.12072061
Zfp280c	0.38629157	5.37018434	5.27309383	0.03264706	0.12072061
Mterf1b	-1.2187741	0.95309795	5.2654847	0.03275921	0.1210896
Gm20063	-0.406117	3.75008213	5.26430633	0.03277662	0.12110825
Gpd2	0.26982844	6.9886585	5.26172792	0.03281474	0.12120339
Zfp366	1.95046091	-0.0905949	5.25783821	0.03287235	0.12128053
Gm14403	-0.3794457	3.69756188	5.25736796	0.03287933	0.12128053
Edc4	0.61170866	3.93410082	5.25733668	0.03287979	0.12128053
Fam19a4	-2.9858459	-1.4218659	5.25697488	0.03288516	0.12128053
Ankrd28	0.27185019	6.58204557	5.25502112	0.03291415	0.12134177
Rhbdd1	0.68188835	3.65204601	5.25267878	0.03294894	0.12142435
Zcchc2	0.33390829	6.13430018	5.24865307	0.03300885	0.12159935
Fam84a	0.34339502	4.98378711	5.24646332	0.03304148	0.12167381
Ccdc104	-0.3106917	7.65613033	5.24397556	0.0330786	0.12176473
Lrrc39	0.68408168	2.13293728	5.24147626	0.03311594	0.12183687
Ube2l6	-0.5520144	5.58533511	5.24054331	0.03312989	0.12183687
Fndc9	0.86355892	2.94928762	5.24016755	0.03313551	0.12183687
Alg6	0.56835494	3.97261279	5.23906018	0.03315208	0.12185206

Mrpl52	-0.4501635	3.85555016	5.23305248	0.03324214	0.12213699
Slc11a1	2.16641223	-0.4192487	5.2322269	0.03325454	0.12213699
Hspe1	-0.3492359	6.34454213	5.23050496	0.03328042	0.12218622
Zfp710	-0.4496757	3.6340195	5.22910987	0.0333014	0.12218982
Gins4	-0.5428624	4.78379394	5.22819282	0.0333152	0.12218982
Rnf208	-0.3331203	4.92523072	5.22667183	0.0333381	0.12218982
Gaa	0.41767901	5.861557	5.22658634	0.03333939	0.12218982
Barx2	-0.8284343	1.91715805	5.22629552	0.03334377	0.12218982
Sgce	-0.4106854	4.38630861	5.22326175	0.03338951	0.12225737
Xpot	0.26892528	7.00853197	5.22305579	0.03339262	0.12225737
Piezo1	0.96097714	1.30366693	5.22259016	0.03339965	0.12225737
S100a9	-1.906537	0.04202695	5.22045336	0.03343192	0.12228395
Serf1	-0.6147933	2.89627059	5.22039979	0.03343273	0.12228395
Spc25	-1.0501499	1.57807401	5.21901852	0.03345361	0.12228395
Ccdc88a	0.37807897	9.01307016	5.21880475	0.03345684	0.12228395
Asic1	0.42379037	4.84553209	5.21529772	0.03350993	0.12240811
Pml	-0.3496604	4.59802592	5.21490995	0.03351581	0.12240811
Phf20l1	0.40093138	5.71701924	5.21211396	0.03355821	0.12251729
Vsnl1	-0.3317871	12.0201766	5.21084497	0.03357747	0.12254195
Larp7	-0.317244	5.74968368	5.20801453	0.03362049	0.12260508
Hpse	3.39180784	-1.4409293	5.20748559	0.03362853	0.12260508
Park2	0.59485066	3.05760267	5.20643017	0.03364459	0.12260508
Clk1	0.49439228	6.46913764	5.20641391	0.03364484	0.12260508
Wash	0.44870137	3.84877299	5.20111295	0.03372563	0.1228198
Relt	-1.0172887	1.18477798	5.2009028	0.03372884	0.1228198
Hectd1	0.37816007	7.4322099	5.19960435	0.03374866	0.12284632
Isg20l2	-0.5302203	4.04315154	5.19722234	0.03378507	0.12290445
Trpv3	1.99915311	-0.520925	5.19691756	0.03378973	0.12290445
Emc1	0.33156482	4.6600872	5.19596812	0.03380425	0.12291165
Ptch1	0.34776338	4.75473834	5.19500073	0.03381906	0.12291985
Scn8a	0.58330455	8.83649402	5.19127214	0.0338762	0.12303248
Pcdhb6	1.42083981	0.85978822	5.19058792	0.03388669	0.12303248
Dctn3	-0.5154669	5.68725454	5.19052066	0.03388773	0.12303248
Naa38	-0.5886132	3.65795244	5.18944692	0.03390421	0.12304671
Tmem177	-0.5207358	3.68336884	5.18732176	0.03393685	0.12311957
Szt2	0.59443775	3.93196404	5.18519691	0.03396953	0.12316509
Fscn1	-0.4137484	5.37348676	5.18487078	0.03397455	0.12316509
Znrd1	-0.4743333	4.5581472	5.18201675	0.0340185	0.12327262
Olfm2	-0.40271	5.18180106	5.18131113	0.03402938	0.12327262
Cntnap3	0.83697204	1.93386135	5.17438313	0.03413638	0.12361452
Hells	0.98291682	1.48357957	5.17264826	0.03416324	0.12366605
Fzd3	0.31728304	7.99007841	5.17056797	0.03419547	0.12369283
Dlx6os1	0.49124526	3.48672029	5.17054083	0.03419589	0.12369283

Ermard	0.46101258	3.92559189	5.16958439	0.03421072	0.1237008
Rps6ka5	0.64424129	3.29476778	5.16763547	0.03424097	0.12372272
Slc38a6	0.72682923	3.17223248	5.16719847	0.03424775	0.12372272
Hist1h1b	-2.6456395	-1.8051552	5.1642988	0.03429282	0.12372272
Grm3	0.34267072	5.65836054	5.16413011	0.03429544	0.12372272
Kcnma1	0.44387184	7.25180016	5.1638258	0.03430017	0.12372272
Mir350	2.57597638	-2.0322071	5.16234758	0.03432318	0.12372272
Astn1	0.4439874	8.03624661	5.16198852	0.03432877	0.12372272
Hist1h2bg	-1.2681146	0.15143316	5.16102265	0.03434381	0.12372272
Taco1	-0.8299775	1.58711343	5.15975202	0.03436361	0.12372272
Gbp9	-0.5839535	3.98239406	5.15910279	0.03437374	0.12372272
Tbxas1	2.59309244	-1.1977504	5.15892258	0.03437655	0.12372272
1700028K03I	1.10160729	2.08229983	5.15823575	0.03438726	0.12372272
Zfp605	-0.3594555	4.69555872	5.15820173	0.03438779	0.12372272
Dnajc15	-0.4335298	4.25157973	5.15701214	0.03440636	0.12372272
Peg3	0.4944631	8.84130121	5.15692428	0.03440773	0.12372272
Gtf3c5	-0.5184783	3.13958845	5.15621023	0.03441888	0.12372272
Grik1	0.65393781	2.80971496	5.15515759	0.03443532	0.12373574
Cltb	-0.4076689	5.79999966	5.15436152	0.03444776	0.12373574
Twf1	-0.360372	7.41799396	5.15192374	0.0344859	0.12377997
Itgb3	0.69196335	2.3627299	5.15180031	0.03448783	0.12377997
Akap6	0.58689869	8.14468972	5.15067395	0.03450546	0.12377997
Xlr4b	-2.5345669	-0.3542584	5.15034454	0.03451062	0.12377997
AI115009	-1.2498915	0.65848687	5.14821017	0.03454408	0.1238485
Rftn2	-0.3829817	4.75878567	5.14751267	0.03455502	0.1238485
Sipa1l1	0.48167024	8.88422936	5.14448783	0.0346025	0.12397334
Zfp692	0.93889988	2.60333688	5.1435827	0.03461673	0.12397896
Ecscr	-2.1325432	-0.6788505	5.14172374	0.03464597	0.12400865
Uba1y	2.05792789	-1.1650433	5.14144567	0.03465034	0.12400865
Ankrd46	-0.2975373	6.77853635	5.13350221	0.0347756	0.12436971
Rapgef6	0.25983466	6.7995315	5.13343751	0.03477662	0.12436971
2810004N23	-0.657208	4.55543889	5.13134491	0.03480971	0.1244426
Cdca8	-1.5091323	0.45926939	5.12924908	0.03484288	0.12448693
Kctd21	-0.4685229	3.31513064	5.12895569	0.03484752	0.12448693
Palld	-0.8083879	1.61944209	5.12794745	0.0348635	0.12449858
Efr3b	0.46151891	6.81278274	5.1242065	0.03492283	0.1246387
Gm10845	0.66522264	5.57078128	5.12261766	0.03494807	0.1246387
Mien1	-0.4768453	5.3825051	5.12184909	0.03496028	0.1246387
Lipg	1.0315226	1.29673501	5.12173149	0.03496215	0.1246387
Zhx1	-0.2763683	8.00743807	5.12146729	0.03496635	0.1246387
Gstcd	0.86477958	1.73762496	5.12003444	0.03498914	0.12467456
Ccdc170	-1.168468	0.66336915	5.11547985	0.0350617	0.12481721
Tsen34	-0.4547757	4.22262638	5.11521998	0.03506584	0.12481721

Myom2	-1.9543209	0.39220815	5.11512208	0.0350674	0.12481721
Mpdz	0.30081127	6.41183289	5.11301761	0.03510099	0.12486603
Pfdn1	-0.5925209	3.51727609	5.11246888	0.03510976	0.12486603
Pcdhb4	0.88868296	2.06915542	5.11186745	0.03511936	0.12486603
Rnf34	-0.3874374	5.2729918	5.10699905	0.03519725	0.12504394
Thoc7	-0.3668017	6.34780559	5.10625242	0.03520921	0.12504394
9430037G07	0.7743953	2.05071542	5.1057476	0.0352173	0.12504394
Rpl23	-0.375168	7.17334771	5.10555039	0.03522046	0.12504394
Ddx28	-0.5780126	2.5404257	5.103299	0.03525657	0.12512679
Polr2d	-0.5550537	3.84926725	5.09904697	0.03532489	0.12532384
Arhgap36	1.50090169	0.91719473	5.09718757	0.03535481	0.12536436
Gabra2	0.48072533	4.85233987	5.09660312	0.03536422	0.12536436
Nrcam	0.39466393	7.77680341	5.09595225	0.03537471	0.12536436
Serpini1	0.34997832	7.56108133	5.09414955	0.03540376	0.12538827
Rab3a	-0.2555926	6.72958893	5.0939452	0.03540706	0.12538827
5031410I06R	0.92538369	2.61793418	5.092793	0.03542564	0.12540875
Irf4	-0.6034539	5.17414265	5.09052189	0.03546231	0.12544911
Rnf24	0.33422545	5.58235448	5.09050062	0.03546266	0.12544911
Rnf4	-0.2691644	6.47763243	5.08726812	0.03551492	0.12558864
A330076H08	0.80607774	3.78922034	5.08460762	0.035558	0.12569561
Sema3a	0.39249022	6.40552977	5.08349961	0.03557596	0.12571373
Bank1	-0.9303591	1.7660714	5.08175814	0.03560421	0.12576818
Brpf1	0.34849975	4.65622633	5.08051512	0.03562439	0.12578943
Nfs1	-0.3958421	4.3159272	5.07980578	0.03563591	0.12578943
Btbd1	-0.2644025	7.23030801	5.07782695	0.03566807	0.12582171
Psmd12	-0.2593371	6.71043969	5.0776626	0.03567075	0.12582171
Gkap1	-0.4366991	5.17658871	5.07460412	0.03572053	0.12595194
Vmn1r58	0.7672129	4.2997275	5.07133772	0.03577378	0.1259947
Etf1	-0.2787472	6.47593528	5.07051073	0.03578727	0.1259947
Oxsr1	0.29538956	5.96237437	5.07023097	0.03579184	0.1259947
Jph1	0.46935564	4.82841873	5.0702163	0.03579208	0.1259947
Dcdc2c	0.73062771	5.66716477	5.06991699	0.03579697	0.1259947
Abca13	3.19414683	-1.7270547	5.06840241	0.03582171	0.12603649
Nap1l5	-0.2568407	7.1916082	5.06661173	0.03585098	0.1260942
Cntnap4	0.66124141	3.32376177	5.06472131	0.03588191	0.12610703
Myo5a	0.60535968	9.77832343	5.06446727	0.03588607	0.12610703
Scn3a	0.62537162	5.62374621	5.0640291	0.03589325	0.12610703
Mybpc3	-4.4247015	-2.0274697	5.06234121	0.03592091	0.12615724
Mga	0.41546036	7.82664413	5.06158519	0.0359333	0.12615724
Taf1b	0.34728471	4.16744644	5.05905548	0.03597481	0.12625773
Fabp5	-0.3914263	4.98497235	5.05821852	0.03598856	0.12626073
Txn2	-0.5049759	4.68308684	5.05451304	0.03604949	0.12642921
Gabbr2	0.40106693	7.12221704	5.05312197	0.03607239	0.12646426

Dck	-0.4545635	4.99238985	5.05142055	0.03610043	0.1264927
Mrpl13	-0.3830699	4.72842033	5.0510624	0.03610633	0.1264927
Tal2	4.60441698	-2.0176317	5.04959507	0.03613053	0.12653224
Smap2	-0.2377327	7.69726981	5.04574433	0.03619414	0.12670968
Wfdc18	-2.1791301	-0.5678841	5.04378388	0.03622657	0.12673015
LOC1008622(	0.72138899	2.56939459	5.04356794	0.03623014	0.12673015
Samhd1	-0.3031876	5.59678699	5.04304496	0.0362388	0.12673015
Dctn5	-0.3621668	6.19143987	5.04220356	0.03625273	0.12673364
Clic3	-1.8946594	-0.5019365	5.04047807	0.03628133	0.12678835
Tsc22d1	-0.3395052	9.67766436	5.0382571	0.03631817	0.12687183
Lars	0.32916026	5.12564252	5.03653852	0.03634671	0.12689146
Rasgrf2	0.62908729	6.84330007	5.03567456	0.03636106	0.12689146
Krba1	0.39336326	3.86026108	5.0354869	0.03636418	0.12689146
Cers4	0.54435991	5.54302222	5.03439524	0.03638233	0.12689146
Pcdh7	0.33907533	8.15354378	5.03402096	0.03638856	0.12689146
Atp2b3	0.45307049	6.97569938	5.03318093	0.03640253	0.12689502
Tmcc1	-0.2255568	6.94096167	5.03128732	0.03643406	0.12693419
Arhgap11a	-0.6056679	3.16691669	5.03073189	0.03644332	0.12693419
Ttll5	0.34955144	5.3838044	5.02940724	0.0364654	0.12693419
Col25a1	0.41615923	5.59930305	5.02915586	0.03646959	0.12693419
Zfp781	0.39406581	6.24306577	5.02861767	0.03647856	0.12693419
Ncor2	0.34657694	6.83000484	5.02424987	0.03655151	0.12713424
Fancl	-0.5245564	2.71916364	5.02362148	0.03656201	0.12713424
Fam217b	0.38552022	4.010407	5.01924584	0.03663528	0.12734379
Fads3	0.46386085	3.57480128	5.01844882	0.03664864	0.12734505
Ntan1	-0.3975589	5.48359563	5.0166779	0.03667835	0.12740309
Mlx	-0.5482365	3.4619609	5.01464845	0.03671243	0.12742739
Col9a3	0.79292498	2.69052244	5.01378549	0.03672694	0.12742739
Anapc4	0.35053868	5.48691874	5.01291984	0.03674149	0.12742739
06-Sep	-0.2493672	6.97292922	5.01276957	0.03674402	0.12742739
Gpr64	1.38788008	1.17481498	5.01196104	0.03675762	0.12742739
Rplp2	-0.4791236	5.9945226	5.01102515	0.03677337	0.12742739
Fdxr	0.86958566	1.38721774	5.01084458	0.03677641	0.12742739
Hspb2	-1.0410868	1.09885574	5.00505384	0.03687405	0.12769726
Ubqln1	-0.2427299	7.10321703	5.00467951	0.03688037	0.12769726
3110057O12	0.48309977	3.00491508	5.00308381	0.03690733	0.12770069
Amot	0.36242256	4.95629387	5.00273908	0.03691316	0.12770069
Dot1l	0.59193466	3.89011164	5.00230674	0.03692047	0.12770069
Esco1	0.34417371	5.65472216	4.99992381	0.03696079	0.12779503
4930519G04	0.75235929	2.31728697	4.9948587	0.03704666	0.12803719
Epb4.1l4a	0.72675103	1.53030088	4.9942516	0.03705697	0.12803719
Atp13a2	0.55304368	4.17613048	4.99323759	0.0370742	0.12803898
Arhgap25	0.50934178	3.64359913	4.99220292	0.03709178	0.12803898

Agfg2	-0.4312592	3.6596711	4.99154454	0.03710297	0.12803898
Napa	-0.3252491	6.07574235	4.99114474	0.03710977	0.12803898
Actr1a	-0.2788307	6.16861461	4.98937936	0.03713981	0.1280975
Cntn4	0.44222489	5.2390942	4.98825629	0.03715894	0.12811836
Hebp2	-0.4618383	3.40226414	4.98546989	0.03720644	0.12819932
Ptma	-0.4561059	9.04456798	4.98524116	0.03721034	0.12819932
Fam193b	0.6786066	3.14884989	4.98457635	0.03722169	0.12819932
Ube2i	-0.3111484	7.37592641	4.98313543	0.03724629	0.12823896
Kif3c	-0.3254694	6.41499445	4.98150218	0.0372742	0.12828996
Rab22a	-0.3050571	5.64374601	4.9785517	0.03732467	0.12841856
9530080O11	-0.7210359	2.39329389	4.97549624	0.03737702	0.12844553
Ep300	0.31819557	8.10979494	4.97492086	0.03738689	0.12844553
Klhl30	-1.040176	0.76413979	4.97490386	0.03738718	0.12844553
Kin	0.44373445	4.30831348	4.97419592	0.03739933	0.12844553
Noxred1	2.78378979	-0.8251038	4.97411519	0.03740071	0.12844553
Mbd1	0.39076047	4.27586133	4.97350504	0.03741119	0.12844553
Jakmip3	0.65812884	4.95956322	4.96885464	0.03749111	0.12864918
Appl1	0.27333424	7.58721653	4.96811926	0.03750377	0.12864918
Dis3	0.38568518	3.97753571	4.96726204	0.03751853	0.12864918
Eif4e3	-0.3278307	5.53897092	4.96700023	0.03752304	0.12864918
A230057D06	0.70160559	3.28385481	4.96280863	0.03759531	0.12885187
Slc4a10	0.30159446	8.98850848	4.96112807	0.03762433	0.12887413
Nbeal1	0.36650761	6.22589735	4.9600837	0.03764238	0.12887413
Mfsd8	0.46637007	3.26371644	4.95867204	0.03766679	0.12887413
Aqr	0.43264892	5.22653794	4.95846986	0.03767029	0.12887413
Gm16740	2.07907596	-0.806682	4.95815529	0.03767573	0.12887413
Nlrc5	-1.5774812	0.00570201	4.95786569	0.03768075	0.12887413
Galnt5	2.37969737	-1.0142029	4.95320945	0.03776142	0.12906879
Ppm1e	0.38942557	8.03567188	4.95289908	0.03776681	0.12906879
Cox6a1	-0.337213	6.82536447	4.95170449	0.03778754	0.12906879
Ppard	-0.3356664	5.18174367	4.95149901	0.03779111	0.12906879
Nr6a1	0.60405303	3.0928936	4.95036836	0.03781075	0.12906879
Rfc4	-0.711755	1.9149827	4.95002467	0.03781672	0.12906879
Dync1h1	0.85568642	9.33827441	4.94893444	0.03783567	0.12908849
Epha5	0.39402837	6.13213742	4.94754882	0.03785977	0.12909881
Atg4c	-0.3509215	6.30646857	4.94724514	0.03786506	0.12909881
Ptpn6	-1.1201611	0.89948557	4.94523799	0.0379	0.12916547
Atg9b	1.43376874	0.83379581	4.94460805	0.03791098	0.12916547
Arhgef16	3.07380216	-0.9134374	4.94362595	0.0379281	0.12917886
Tnfrsf23	-0.9185527	1.09561265	4.94200165	0.03795643	0.12919218
Abcd4	0.78037371	1.46587845	4.94188946	0.03795839	0.12919218
Sp8	1.11141626	0.59072781	4.94005691	0.03799038	0.12925617
Eif5a	-0.5350424	7.19992216	4.93758903	0.03803352	0.129358

Gmfg	-1.4018083	1.11371591	4.93174928	0.03813581	0.12956376
Wbscr22	0.49136541	3.15028174	4.93154933	0.03813932	0.12956376
Rhox8	0.96244192	1.56502683	4.9300079	0.03816638	0.12956376
Naa15	0.25832139	6.62462264	4.92931067	0.03817862	0.12956376
Cluh	0.36734299	5.1625154	4.92928019	0.03817916	0.12956376
Ap3m2	-0.3065467	6.61854869	4.92917425	0.03818102	0.12956376
Pcsk2	0.3651888	7.2390638	4.92885632	0.0381866	0.12956376
Gm2694	1.40553464	0.04466497	4.92641845	0.03822947	0.12963395
Pglyrp3	-4.4865024	-2.0173844	5.05183559	0.03823793	0.12963395
Gm15645	0.80870911	1.94279585	4.92515396	0.03825172	0.12963395
Elmo1	-0.3031985	7.15388229	4.92449516	0.03826332	0.12963395
Cdc37	-0.3891385	5.08594465	4.92326366	0.03828501	0.12963395
Elmo3	-0.8956038	1.38740745	4.92309251	0.03828803	0.12963395
Hapln1	0.57840871	3.8075648	4.92241723	0.03829993	0.12963395
Sirpa	0.3053282	7.17435197	4.92060693	0.03833186	0.12969461
Blzf1	-0.3897016	4.90929891	4.9199002	0.03834433	0.12969461
Sertad1	-0.7130147	2.92452952	4.91703367	0.03839497	0.12969519
Terf1	0.46746609	3.52866775	4.91667748	0.03840127	0.12969519
Foxd2os	-0.6656857	2.51119551	4.9164308	0.03840563	0.12969519
Zswim8	0.38826632	5.28010502	4.91585121	0.03841588	0.12969519
Setmar	-0.772761	2.36982812	4.91559789	0.03842037	0.12969519
Sptbn1	0.48902377	10.099249	4.91539532	0.03842395	0.12969519
Tesc	-0.5639327	3.01370342	4.91425173	0.03844419	0.12971881
Rdh18-ps	1.06619097	0.35944236	4.91248306	0.03847552	0.12977982
Atp6ap1l	1.26994268	1.11229166	4.91063904	0.03850821	0.12983658
Gm5124	-0.3453449	5.01792414	4.90932707	0.03853149	0.12983658
A330049N07	1.40515418	-0.2488455	4.90929211	0.03853211	0.12983658
Gm12338	-0.4859379	4.54261356	4.90731362	0.03856725	0.12987194
Card10	1.29629713	0.38311934	4.90646546	0.03858233	0.12987194
Tspan14	0.40288386	3.50768409	4.90646236	0.03858238	0.12987194
A530058N18	0.94898781	1.14834101	4.90489071	0.03861033	0.12992138
Arrdc2	-0.9438829	1.72076775	4.90411806	0.03862408	0.12992301
Amy1	0.39026844	4.31067599	4.90256336	0.03865177	0.1299715
Ptgfrn	-0.3169034	5.24899347	4.90053351	0.03868794	0.13001406
Puf60	-0.3114674	6.5038711	4.90036384	0.03869097	0.13001406
Sgk1	-0.3021735	6.58128598	4.89920738	0.0387116	0.13003877
Serpina3g	-2.3442616	-0.4356293	4.89349745	0.03881364	0.13033686
E530011L22F	-0.5147827	2.52860136	4.89200427	0.03884038	0.13038194
Dnajc11	-0.441872	3.98636206	4.88891646	0.03889573	0.13051097
Rmnd5a	-0.2923303	7.57797669	4.88837426	0.03890546	0.13051097
Ccdc167	0.29597266	5.38308184	4.88573761	0.03895281	0.13062507
Col11a2	1.25357849	0.10976493	4.88369032	0.03898962	0.13070377
Apobec3	-0.7494387	2.31423405	4.88226149	0.03901534	0.13070692

Mnt	0.33567056	5.01566522	4.88199265	0.03902018	0.13070692
Ccdc102a	-0.9920521	1.41072203	4.87949986	0.0390651	0.13070692
Uhrf2	0.33053807	5.29468683	4.87824981	0.03908764	0.13070692
Zeb2os	0.80161291	1.51606069	4.87767889	0.03909794	0.13070692
Ckap4	-0.4421311	5.1576854	4.87722818	0.03910608	0.13070692
Epm2aip1	0.41909063	7.97617275	4.87698006	0.03911056	0.13070692
Gp5	2.63174855	-1.3607598	4.87681085	0.03911361	0.13070692
Sycp2	0.96700464	2.48123862	4.87635817	0.03912179	0.13070692
Zkscan6	0.61035168	2.9109064	4.8762355	0.039124	0.13070692
Gm7609	1.57273566	-0.6325316	4.87148266	0.03920995	0.13094939
Dhx35	0.5133921	2.62896772	4.86997252	0.0392373	0.13098048
Dscaml1	0.73185854	3.82701036	4.86949257	0.039246	0.13098048
AF357426	1.72008769	-0.8974646	4.86607573	0.03930798	0.13109914
Lif	1.97304357	-0.9504071	4.86584233	0.03931222	0.13109914
Ostc	-0.4250405	4.63120836	4.8653202	0.03932171	0.13109914
1810026J23F	-0.3276624	5.9224383	4.86384672	0.03934848	0.13112209
Gm10432	1.09674478	1.42658681	4.86282165	0.03936712	0.13112209
C4a	1.74364253	-0.5173054	4.86273211	0.03936875	0.13112209
Jagn1	0.34901223	4.86171929	4.86026052	0.03941373	0.1312273
Wdr13	0.22863469	6.73099886	4.85783973	0.03945785	0.13130758
Prr13	-0.385506	5.59039695	4.85676692	0.03947742	0.13130758
Klhl25	-0.6231536	1.87790418	4.85653044	0.03948174	0.13130758
Ccdc151	-0.9975536	0.63614155	4.85599729	0.03949147	0.13130758
Chl1	0.48165989	7.47835662	4.85352355	0.03953665	0.13140248
Dmgdh	-1.4636383	0.06703914	4.85296621	0.03954684	0.13140248
Rpl18a	-0.4488523	7.4643089	4.85167529	0.03957045	0.13143634
Ifi27	-0.5091881	4.907542	4.84975711	0.03960556	0.13150837
Id4	-0.3682035	5.33668979	4.84686577	0.03965855	0.1316397
Peli1	-0.3005534	5.95279181	4.84377812	0.03971523	0.13178318
Man1a	0.44935763	5.19943443	4.84265768	0.03973582	0.13180161
Ckap2l	-0.9732633	0.9087401	4.84201169	0.03974769	0.13180161
Ppp2r5d	-0.3763455	5.52700385	4.83858229	0.03981081	0.13193019
Naca	-0.3218603	7.84367929	4.83844142	0.03981341	0.13193019
Ahr	0.48599701	4.19486177	4.8367046	0.03984542	0.13199047
Fsd2	2.97321521	-1.7199672	4.83573508	0.0398633	0.13199047
Ankrd11	0.24763161	8.84875745	4.83526227	0.03987202	0.13199047
Tusc5	-2.3650402	0.35486227	4.83121552	0.03994678	0.13219327
Cygb	-0.4617561	4.10908569	4.8280198	0.04000593	0.1322816
Etl4	0.26416364	7.93056652	4.82794035	0.0400074	0.1322816
Gm9962	1.0700002	0.50648119	4.82726622	0.04001989	0.1322816
Arl9	-3.2823715	-1.9153914	4.82685602	0.04002749	0.1322816
AI450353	0.9600235	2.02107079	4.8258168	0.04004676	0.13229472
Slc2a13	0.33108395	6.84437649	4.82518539	0.04005847	0.13229472

1700048O20	-0.6809041	1.87947772	4.82440881	0.04007288	0.1322977
Dhodh	-0.7657787	2.11767985	4.82253042	0.04010776	0.13236824
Mif	-0.4177526	5.21874154	4.82114848	0.04013344	0.13240839
Gm16982	1.72849695	0.00829823	4.82000898	0.04015463	0.13243369
Rps5	-0.4063976	5.76664419	4.81869122	0.04017916	0.13246997
Etnk1	0.24217434	8.62575492	4.81711642	0.04020848	0.13252205
Pde7a	0.40890094	5.13023311	4.81574219	0.04023409	0.13253276
Itga7	1.82015171	-0.3260729	4.81495225	0.04024883	0.13253276
Rsf1	0.35201432	7.58645342	4.81476482	0.04025232	0.13253276
Gja6	1.86250105	-1.1012232	4.81188373	0.04030611	0.13262052
Dock7	0.35322953	6.05798925	4.81178481	0.04030795	0.13262052
Ccl27a	-0.3951223	5.16769628	4.81088011	0.04032486	0.13262052
Ppa1	-0.3294973	5.77835253	4.81043756	0.04033313	0.13262052
Gm16861	0.74205731	2.44946528	4.80890465	0.0403618	0.13267026
Tubgcp2	0.41049308	3.66953994	4.8064684	0.04040742	0.13277564
Dcx	0.3930276	5.69217413	4.80090649	0.04051178	0.13303675
Snx5	-0.2539454	7.0692407	4.79954875	0.0405373	0.13303675
A930017M01	0.60221654	2.61294087	4.79939783	0.04054014	0.13303675
Arhgdia	-0.4294917	8.02589297	4.79934077	0.04054121	0.13303675
Med19	-0.3964609	4.61208039	4.79633495	0.04059778	0.13317778
Smad1	-0.2830691	5.76445627	4.79558611	0.04061189	0.13317946
Fkbp1a	-0.3226472	9.78767574	4.79434854	0.04063522	0.13319882
Rn4.5s	1.02435615	0.52362576	4.7938303	0.04064499	0.13319882
Ubqln4	-0.3113778	6.15936586	4.79146066	0.04068971	0.13330078
Lrrfip1	0.30511774	5.98272944	4.79065953	0.04070484	0.13330577
Pgm2	0.39507447	4.06380993	4.78989712	0.04071925	0.13330838
Bub1	1.67404985	0.38533996	4.78824285	0.04075053	0.13331258
H2afz	-0.3007146	7.20723172	4.78811735	0.0407529	0.13331258
Rnaseh2a	-0.7364204	2.75816186	4.78767021	0.04076136	0.13331258
Zfp239	-0.3106633	5.66201513	4.78519172	0.04080829	0.13340076
Cdkn1b	-0.4578369	4.09793414	4.78480812	0.04081556	0.13340076
Fbxw17	-0.8054554	2.00307824	4.78361346	0.04083821	0.13343025
Bbs12	0.8473961	1.27863567	4.78234142	0.04086234	0.13343511
Gbp8	-0.9947996	1.80266192	4.78181089	0.0408724	0.13343511
Stx18	-0.5406217	3.37978799	4.78138129	0.04088056	0.13343511
Dicer1	0.33209082	6.2937579	4.77984219	0.04090979	0.13348604
Taf9	-0.3093625	5.52362682	4.77829718	0.04093916	0.13350591
Pus10	0.58032121	3.92504545	4.77808784	0.04094314	0.13350591
Abcb10	0.48874907	3.68773731	4.7768795	0.04096613	0.13350969
Ophn1	0.40589646	4.9296589	4.77659406	0.04097156	0.13350969
Rab13	-0.5747354	4.24254159	4.77565271	0.04098948	0.13352367
Lyrm2	-0.4098072	4.85568116	4.77460963	0.04100935	0.13353795
Rbm12b1	0.45480064	4.6326872	4.77399152	0.04102113	0.13353795

Lztr1	0.26319203	5.7292613	4.77315786	0.04103702	0.1335453
Aldh1a2	-0.5324262	9.18687312	4.77076932	0.04108259	0.1336492
Krt222	-0.3080981	6.63809537	4.7684124	0.04112762	0.13371519
Trappc2	-0.3954307	3.89742879	4.76806681	0.04113423	0.13371519
Tfam	-0.3699413	4.82690855	4.76756452	0.04114383	0.13371519
Srxn1	-0.2596603	6.28059447	4.76545165	0.04118426	0.13379803
Pcdhb16	0.48599059	4.67358307	4.76480517	0.04119664	0.13379803
Cacna1c	0.48531716	6.3128366	4.76367725	0.04121825	0.13382384
Ptbp2	0.3484643	6.37537676	4.75963959	0.04129571	0.13392513
Arpc5	-0.3534317	7.34556386	4.75959893	0.04129649	0.13392513
3110035E14I	-0.2549378	7.87383963	4.75865598	0.0413146	0.13392513
Adam33	1.51852518	-0.0202813	4.75849203	0.04131775	0.13392513
Hlf	-0.3098834	10.0220648	4.75808483	0.04132558	0.13392513
Scrg1	1.15670809	1.44732958	4.75777771	0.04133148	0.13392513
Psmg2	-0.5577613	3.21681785	4.75667313	0.04135272	0.13394964
Ccdc28a	-0.6513224	2.50024667	4.7541988	0.04140034	0.13405958
Dcun1d1	-0.2463975	6.8076592	4.75283291	0.04142666	0.13410046
Gad1	0.54508247	7.57420596	4.7504458	0.0414727	0.13419131
Ankrd13d	0.53987655	3.01863563	4.74995731	0.04148213	0.13419131
Polr3h	-0.4735887	2.96034667	4.74897394	0.04150111	0.13420841
Pafah1b2	-0.2300406	8.35178325	4.74733626	0.04153275	0.13426641
Cenpn	-1.2432637	1.17111086	4.74564623	0.04156544	0.13428232
Spata1	0.9720857	1.26760515	4.74536094	0.04157096	0.13428232
Fam118a	-0.3888239	4.55151073	4.7436434	0.04160421	0.13428232
09-Mar	-0.8033878	1.83379373	4.74326701	0.0416115	0.13428232
Mir3473	2.02101982	-0.4214497	4.7430671	0.04161537	0.13428232
Nptx1	0.34566822	7.55312455	4.74283179	0.04161993	0.13428232
Zfp664	-0.3157935	7.69577042	4.74073187	0.04166064	0.13436941
Nip7	-0.3234327	5.02357649	4.73839392	0.04170602	0.13446591
Snx15	-0.3393083	4.50132427	4.73777632	0.04171801	0.13446591
Lca5l	1.07752003	1.38066019	4.73654719	0.0417419	0.13446756
Lemd3	0.35008631	4.50277454	4.73633744	0.04174598	0.13446756
Gm20597	-2.6327289	-0.6734254	4.73528995	0.04176635	0.13447076
Phka2	0.62732792	4.89890177	4.73430841	0.04178545	0.13447076
Mcrs1	-0.5294002	3.23936336	4.73416932	0.04178816	0.13447076
Sbds	-0.2976086	6.7129318	4.73176572	0.04183497	0.1345772
Pcdh1	0.36327797	7.22796233	4.73064096	0.0418569	0.13460353
Capg	-0.6314494	2.58282	4.72919038	0.0418852	0.13461373
Tmem215	0.81407369	3.3389055	4.72906959	0.04188756	0.13461373
C230079O03	3.42056333	-2.0034868	4.72751812	0.04191785	0.13464209
Jag1	0.50932129	3.72956607	4.72720994	0.04192387	0.13464209
Mier2	0.9126506	1.225176	4.72633956	0.04194088	0.13465256
Fads2	0.60887627	3.20624724	4.72438605	0.04197909	0.13473106

Trim30a	0.49005856	3.79956859	4.7233361	0.04199963	0.13474844
Farsa	0.90093662	1.36654708	4.72270381	0.04201201	0.13474844
Pcdhga1	0.84482712	1.75969594	4.72009619	0.04206312	0.13483578
Shisa7	0.4309424	6.36471315	4.71901805	0.04208427	0.13483578
Elp5	-0.411782	4.81554388	4.71876534	0.04208923	0.13483578
C1galt1	0.44268115	4.66589561	4.71784663	0.04210726	0.13483578
Mettl9	-0.3506532	4.83069938	4.71602082	0.04214312	0.13483578
Sdr39u1	-0.3754041	4.98892235	4.71516216	0.04216	0.13483578
Sult1a1	-0.5622805	5.7934373	4.71334982	0.04219566	0.13483578
App	0.27500677	8.66509883	4.71277293	0.04220701	0.13483578
Tsc22d4	-0.4724201	3.17516185	4.71231465	0.04221603	0.13483578
Otof	0.84564092	2.91644019	4.71224612	0.04221738	0.13483578
Olfr1033	-0.476907	3.91660529	4.7121534	0.04221921	0.13483578
Golga7	-0.3103947	7.26421945	4.7120078	0.04222208	0.13483578
Rsad1	-0.5261342	3.20766424	4.71181182	0.04222594	0.13483578
Scn1a	0.41014319	7.07931107	4.71150608	0.04223196	0.13483578
Atp5j	-0.3388318	7.73128512	4.7073048	0.04231482	0.13505498
Lins	0.525249	3.94188219	4.70598132	0.04234096	0.13505498
Gls2	0.70906026	2.43157506	4.70529418	0.04235454	0.13505498
Tomm34	-0.3185229	5.83412048	4.70523216	0.04235577	0.13505498
Slc4a9	-3.8544549	-1.3931945	4.70435859	0.04237304	0.13506608
Klf13	0.27256836	7.23167154	4.70326248	0.04239472	0.13509124
Ptprd	0.38862463	8.31428597	4.69741219	0.04251066	0.13538248
Timmdc1	-0.4037957	4.29248554	4.69725584	0.04251376	0.13538248
Pcdhb21	0.99603821	1.24682171	4.69469324	0.04256466	0.13550053
Ndufa3	-0.4421882	4.66141307	4.69383692	0.04258169	0.13551068
Cecr6	0.54873146	4.67755729	4.69245488	0.04260918	0.13555414
Mir8115	1.84767692	-1.2524233	4.6897864	0.04266232	0.13567914
Ubtf	-0.2923151	5.83029552	4.68622973	0.04273327	0.13584943
Tnfsf12	-0.5215668	3.32585799	4.68398429	0.04277813	0.13584943
Cyp2c44	2.73895661	-0.9963417	4.68390017	0.04277982	0.13584943
Kif21a	0.43317233	8.49741799	4.68381468	0.04278152	0.13584943
Reep5	-0.2757065	8.46075093	4.6836301	0.04278521	0.13584943
Palmd	-0.3842069	5.6060739	4.68157025	0.04282642	0.13593621
Ppara	0.53238543	4.0984941	4.67968896	0.0428641	0.13601172
Polr2a	0.27989654	7.30718596	4.67648344	0.04292838	0.13611773
Ube2e1	-0.4920709	4.09330134	4.67614825	0.04293511	0.13611773
Dsn1	0.79438786	1.38617472	4.67544536	0.04294922	0.13611773
Adipor2	-0.3787115	4.69808897	4.67518234	0.0429545	0.13611773
Gstt1	-0.5932401	4.87976353	4.6741252	0.04297574	0.13611773
Isca2	-0.324148	5.90232956	4.67170136	0.04302448	0.13611773
Zfp473	2.20465488	-0.3005322	4.67157937	0.04302694	0.13611773
Igsf11	0.31677578	5.86683619	4.67155621	0.0430274	0.13611773

Bre	-0.3755052	4.14336878	4.67076827	0.04304326	0.13611773
Tacr2	1.89104384	0.30130535	4.66985248	0.0430617	0.13611773
Mfhas1	0.32175719	5.25303798	4.66976777	0.04306341	0.13611773
Myo16	0.59738217	4.34876829	4.66941871	0.04307044	0.13611773
Dsg3	-1.9371948	-0.7353267	4.66903569	0.04307816	0.13611773
Eif3a	0.33697514	9.48235141	4.66718863	0.04311539	0.13616926
Whrn	-0.5521656	3.21545349	4.66618531	0.04313564	0.13616926
E030013I19R	-2.3592721	-0.3495429	4.66615894	0.04313617	0.13616926
Cckar	-1.9976044	-0.7734753	4.66338125	0.04319227	0.13630242
Fam65b	0.40198062	5.89319166	4.66209187	0.04321834	0.13634076
Paip2	-0.2838052	7.53393563	4.6602372	0.04325586	0.13641522
Slc44a2	0.34864986	5.08209519	4.65422832	0.04337771	0.13675545
Eef1b2	-0.3858376	7.031599	4.65212203	0.04342051	0.13681045
Kat7	-0.273963	5.9074198	4.65150616	0.04343304	0.13681045
Ehd1	-0.5608951	3.5760169	4.65130847	0.04343706	0.13681045
Casp9	-0.3732539	4.67728021	4.64943852	0.04347511	0.1368863
Kcna4	0.49436123	5.66132831	4.64684424	0.04352797	0.1370087
Mrm1	-0.4459436	3.09996865	4.64443612	0.04357711	0.13708472
Nedd8	-0.5124155	5.60706888	4.64377388	0.04359063	0.13708472
Tmem8	0.56989914	2.32998663	4.64360348	0.04359411	0.13708472
Plekhn1	1.63109025	-0.0644064	4.64081133	0.04365118	0.13722014
Trappc1	-0.3847385	5.99795277	4.63812263	0.04370622	0.13734907
AI846148	0.5048535	2.95781087	4.63713509	0.04372646	0.13736859
Surf1	-0.4553281	4.01917473	4.63612677	0.04374713	0.13738947
Morc2a	0.25865492	6.17560802	4.63333338	0.04380446	0.13752542
Tmem40	-1.9604765	-0.055011	4.63219064	0.04382793	0.13755134
Ndufa4	-0.3617338	7.34644343	4.63148558	0.04384242	0.13755134
Rpl10	-0.4681977	7.8633684	4.63088174	0.04385484	0.13755134
Zfp652	-0.2957687	6.34153792	4.62808125	0.04391247	0.13768801
Tmem230	-0.292612	6.66927818	4.62683927	0.04393806	0.13772416
Scarna3a	2.01844398	-0.9411287	4.62480749	0.04397995	0.13778756
Tead3	-0.6231234	2.73021835	4.62449421	0.04398642	0.13778756
Sccpdh	-0.2494357	6.28810619	4.6230927	0.04401535	0.1377944
Cldn11	0.50585886	6.53876508	4.62176392	0.0440428	0.1377944
Psg16	-1.0769896	1.38269931	4.62164151	0.04404533	0.1377944
5430417L22R	0.25828773	6.02537351	4.62086446	0.04406139	0.1377944
Rad1	0.52020529	4.79840405	4.62052726	0.04406836	0.1377944
Aoah	1.9529447	-0.6572058	4.62030268	0.04407301	0.1377944
Abca12	2.35334721	-1.4656762	4.61953758	0.04408883	0.13779989
Slc35f5	0.44327552	4.54118939	4.61820342	0.04411645	0.13780462
Adora3	-2.5047488	-1.5513716	4.61797982	0.04412108	0.13780462
Atp9b	0.54137612	3.99564467	4.61742576	0.04413255	0.13780462
Tsfm	-0.480255	2.62494558	4.61479231	0.04418713	0.13792972

Inpp5f	0.23354576	6.79477906	4.61392999	0.04420502	0.13792972
Ubiad1	-0.721036	1.89560882	4.61263102	0.04423199	0.13792972
Camk1	-0.4320869	3.80136889	4.61214019	0.04424218	0.13792972
Ubr3	0.34320536	9.67637616	4.61209988	0.04424302	0.13792972
Dlgap1	0.39352612	8.83633306	4.60946034	0.04429789	0.13799024
Agtr2	-2.1798527	-1.1446577	4.60869245	0.04431386	0.13799024
Hexim2	-0.6135174	2.30209558	4.60854133	0.04431701	0.13799024
Atf6	0.30582778	6.94906502	4.60845617	0.04431878	0.13799024
Grm4	0.48753506	4.02745174	4.60753917	0.04433787	0.13800581
Npr2	0.37518723	4.07112397	4.60664414	0.04435651	0.13801997
Gm20767	1.03306656	1.1002341	4.60579008	0.04437431	0.1380315
Akt1s1	-0.8046185	1.7893999	4.60257054	0.04444147	0.13816926
Usp20	0.46934371	3.79233137	4.60231493	0.04444681	0.13816926
Dnajb14	-0.3568391	4.2317679	4.6009164	0.04447602	0.13817238
Nanos1	-0.2798387	5.23537647	4.59984118	0.04449849	0.13817238
BC030499	0.98894334	2.4979529	4.5989948	0.04451619	0.13817238
Rfx1	0.60098804	3.37654182	4.59838519	0.04452895	0.13817238
Dido1	0.31772177	6.59201609	4.59831197	0.04453048	0.13817238
Trpc4	0.50990499	3.39938902	4.598218	0.04453245	0.13817238
Cd226	1.16387712	0.44264566	4.59550199	0.04458933	0.13825431
1700026D08I	-1.0625859	0.68948357	4.59501575	0.04459952	0.13825431
Ptgr2	-0.381538	6.42291844	4.59406071	0.04461954	0.13825431
Stx2	0.63703321	2.63517198	4.5939961	0.0446209	0.13825431
Mrpl3	-0.2905996	5.42029151	4.59294213	0.04464301	0.13825431
Alg11	0.32975727	6.70173638	4.592917	0.04464354	0.13825431
Rita1	0.87548287	0.99608685	4.59200638	0.04466265	0.13826979
Tmem121	-4.5332014	-1.3857168	4.58870493	0.04473203	0.13837612
Rbm22	0.33995093	4.93733979	4.58705523	0.04476675	0.13837612
Casp7	-0.785942	2.71536491	4.58665664	0.04477514	0.13837612
l7Rn6	-0.3526877	6.09145303	4.58636245	0.04478133	0.13837612
Tab3	0.27225988	6.0446983	4.58629171	0.04478282	0.13837612
Pccb	-0.383108	4.70569849	4.58607173	0.04478746	0.13837612
Psma4	-0.2510495	6.88952147	4.58567162	0.04479588	0.13837612
Rnf165	0.40019226	6.31500496	4.58470304	0.0448163	0.13839553
Rbm3	-0.4114698	7.90721489	4.58365969	0.04483829	0.13841982
Tcf7	-0.415813	4.87830236	4.58255566	0.04486159	0.13842499
Foxo4	-0.7866533	1.66540675	4.58171104	0.04487941	0.13842499
Slc39a2	1.64190802	0.47883261	4.58120779	0.04489004	0.13842499
Hist2h3b	1.73723144	-1.2253731	4.58075724	0.04489956	0.13842499
Ccdc169	2.94850324	-2.2268421	4.58000083	0.04491554	0.13842499
Ndufs6	-0.46927	5.1640407	4.57956447	0.04492476	0.13842499
Gemin8	-0.4857158	3.45202186	4.57617038	0.04499657	0.13860265
R74862	-0.3697127	4.58028853	4.57393895	0.04504385	0.13870466

Hiat1	0.28326032	6.3790195	4.57270711	0.04506997	0.13874149
Cnr1	0.32663592	6.5698156	4.56930026	0.04514231	0.13892053
Fastkd3	-0.3745494	3.79209498	4.56801802	0.04516957	0.13896077
Slc24a5	0.57691855	2.28941633	4.56522268	0.04522907	0.13910011
Skp2	0.64114324	2.8285553	4.56387628	0.04525776	0.13914466
Nell1	0.40449757	4.05279299	4.56229698	0.04529144	0.13919198
Vipr1	0.82109075	2.42237839	4.56182194	0.04530157	0.13919198
Anp32a	-0.2251762	7.60656272	4.55799614	0.04538329	0.13939936
Usp34	0.38689301	8.49626078	4.55686963	0.04540739	0.13942965
Ankib1	0.24343634	6.52754493	4.55356339	0.04547819	0.13953555
5330413P13I	-0.9313666	1.58644939	4.55333605	0.04548306	0.13953555
Gprin1	-0.3935581	4.69339055	4.55321718	0.04548561	0.13953555
Fars2	-0.6216941	2.72872315	4.55259935	0.04549886	0.13953555
Tcfl5	-3.1260262	-1.4827412	4.55102392	0.04553266	0.1395955
Dnajb5	-0.3852875	5.88746748	4.54913328	0.04557326	0.13960018
Trap1	0.39045565	4.50678277	4.54911815	0.04557358	0.13960018
Gpr68	0.43698473	3.49668745	4.54896197	0.04557694	0.13960018
Ube2r2	-0.3414369	8.34444785	4.54566247	0.0456479	0.13976425
Zfp354b	0.76836423	2.41234248	4.54514479	0.04565904	0.13976425
Hivep2	0.56725748	8.90992796	4.54205588	0.04572561	0.13984956
Gm9776	0.61322354	2.31677585	4.54036254	0.04576214	0.13984956
Epb4.1l1	0.31170095	8.48495277	4.539315	0.04578476	0.13984956
Saysd1	0.59237144	2.32347982	4.539019	0.04579116	0.13984956
Pik3r4	0.35614317	5.1104775	4.53872117	0.04579759	0.13984956
Nron	1.580224	0.25872648	4.53805375	0.04581201	0.13984956
Atp5b	-0.2261563	10.5802722	4.5379302	0.04581468	0.13984956
Gpatch8	0.25286023	8.04888298	4.53792187	0.04581486	0.13984956
Smurf2	-0.2629291	7.06923446	4.53789678	0.04581541	0.13984956
St13	-0.3061879	6.32199189	4.53648991	0.04584583	0.13989882
Olfr856-ps1	0.73451211	4.31338526	4.53164277	0.04595081	0.14017552
Hsd11b1	-0.87735	1.67588969	4.53066439	0.04597204	0.14019661
2700046A07I	-0.7323511	2.66778136	4.52902684	0.04600759	0.14026135
Gm8234	0.53482732	3.69496776	4.52726151	0.04604595	0.14033462
Scaf8	0.29084543	5.88073503	4.52606676	0.04607193	0.14037012
Camsap1	0.33881899	6.94819669	4.52169933	0.04616705	0.14061619
Ralgapa1	0.48394334	7.7368995	4.51570365	0.04629799	0.14091688
Gpc6	0.43741183	7.3480851	4.51528233	0.04630721	0.14091688
Ddhd1	0.34315533	6.49345786	4.51490899	0.04631537	0.14091688
Rangap1	-0.3613127	6.60553911	4.51454631	0.04632331	0.14091688
D930015E06	0.8048224	2.15529752	4.51277451	0.04636211	0.14093333
Rbm8a	-0.4322677	5.27386236	4.51213915	0.04637604	0.14093333
Grin2a	0.41116205	6.61728008	4.51180972	0.04638326	0.14093333
Wwp2	-0.4114026	4.35375428	4.51028939	0.0464166	0.14093333

Gm12709	0.72281627	1.93978936	4.50949992	0.04643392	0.14093333
Eya2	-0.5567544	6.41533242	4.50946514	0.04643468	0.14093333
Lamtor1	-0.482253	5.52992674	4.50934696	0.04643728	0.14093333
Psmb10	-0.5911314	3.21934283	4.50855207	0.04645473	0.14093333
Edrf1	0.48204578	5.06486854	4.50839355	0.04645821	0.14093333
Zfp462	0.37056593	6.71344293	4.50661689	0.04649725	0.14098165
Srsf4	-0.5812548	2.37820875	4.50635864	0.04650292	0.14098165
4930429B21I	-0.3482942	5.16830204	4.50518296	0.04652878	0.14101639
Cacybp	-0.2790939	6.42728372	4.50379477	0.04655933	0.14106533
Akap9	0.32880035	8.44979472	4.50156979	0.04660835	0.14117017
LOC1026344	1.05082855	1.10325878	4.49991969	0.04664473	0.14120573
Inpp5b	0.33504577	4.90277006	4.49774635	0.04669271	0.14120573
Lin52	-0.334077	4.53970168	4.49763722	0.04669512	0.14120573
Esyt3	0.67080803	2.46429092	4.49757465	0.0466965	0.14120573
1700080N15	1.6725199	0.14757437	4.49757406	0.04669651	0.14120573
Slc29a4	0.87239974	1.10904848	4.49711848	0.04670658	0.14120573
Slit2	0.2951391	5.82072371	4.49567436	0.0467385	0.14124813
Pex7	-0.3663262	4.47396907	4.49513623	0.0467504	0.14124813
Cct6a	-0.2471146	8.24498773	4.49452777	0.04676386	0.14124813
Gm10791	1.4610163	0.77777269	4.49142185	0.04683264	0.14141227
Mtmr4	0.40176299	5.94243077	4.48956427	0.04687383	0.14147189
Twist2	-1.2991919	0.8075385	4.48902544	0.04688579	0.14147189
Edn1	-1.5040133	0.28649331	4.48857808	0.04689572	0.14147189
Rnf145	0.28791835	5.73026321	4.48730699	0.04692394	0.14151346
Impdh2	-0.3775984	3.73965062	4.48604867	0.0469519	0.1415542
Grhl3	2.00872333	-0.2245876	4.48521473	0.04697044	0.14156652
Glg1	0.30117524	6.66224155	4.48350916	0.04700839	0.141637
Cdo1	-0.4979285	6.85151754	4.48280818	0.04702399	0.141637
Fam21	0.33676994	6.01400352	4.48221495	0.0470372	0.141637
Mir6414	-3.3387059	-1.7663391	4.47839867	0.04712229	0.14184961
Reep6	0.34046584	4.20138878	4.47715315	0.0471501	0.14186327
Tefm	-0.5195608	3.32562014	4.47689815	0.0471558	0.14186327
Edem1	0.27965638	4.94744188	4.47624535	0.04717038	0.14186358
Polr3d	-0.5321888	3.08164623	4.47362553	0.04722897	0.14196977
Myo5b	0.56298103	4.19877001	4.47337028	0.04723468	0.14196977
Sfxn2	0.55874548	2.52378903	4.47168693	0.04727237	0.14203947
Morn4	-0.3705592	5.72266138	4.47068414	0.04729484	0.14206341
Cnot3	-0.3220198	5.42677765	4.46952227	0.04732089	0.14209808
Calm1	-0.2145118	12.308041	4.46645106	0.04738982	0.14226147
Ints1	0.44846922	4.56946716	4.4656383	0.04740809	0.14226804
Racgap1	-0.5188888	3.21053084	4.4650611	0.04742106	0.14226804
Tmsb15b2	-1.5395741	-0.3630723	4.46367577	0.04745221	0.14231792
Col6a4	2.08416429	-0.2166068	4.45965411	0.04754279	0.14250253

Fancb	1.27949527	0.97425989	4.45965085	0.04754287	0.14250253
Atp6v1e1	-0.2541373	6.40075552	4.4583693	0.04757177	0.14250843
5830415F09I	-2.1583327	-0.7140421	4.45827362	0.04757393	0.14250843
Tbrg1	-0.4455427	4.80640048	4.45217641	0.04771173	0.14276898
6530402F18I	1.03314745	1.42045997	4.4520821	0.04771387	0.14276898
Spink8	-1.5365258	-0.1941397	4.45196584	0.0477165	0.14276898
Dctn2	-0.2649051	5.87271617	4.45184609	0.04771921	0.14276898
Rsl24d1	-0.370801	5.39693246	4.45076384	0.04774372	0.14276905
Armcx5	-0.2882285	5.36847734	4.45055813	0.04774839	0.14276905
Tgfbrap1	0.29564163	5.59622484	4.44923988	0.04777827	0.14277739
Tmem109	0.40329933	3.48127732	4.44824362	0.04780086	0.14277739
Dopey2	0.43155622	5.69919646	4.4481859	0.04780217	0.14277739
Rgl2	0.63351722	2.1835214	4.4475608	0.04781635	0.14277739
Sec23b	0.39333606	4.50824447	4.44722152	0.04782406	0.14277739
Emc4	-0.2558199	6.33194098	4.4446609	0.04788222	0.14290749
Arid1b	0.40391876	6.24376872	4.44187433	0.04794561	0.14305309
Prkx	-0.4161095	4.57559272	4.43982619	0.04799226	0.14314868
Ccdc176	0.92242447	2.36353421	4.43853934	0.0480216	0.14319258
Nfat5	0.29556799	8.73989418	4.43780096	0.04803844	0.14319921
Rbm6	0.33496879	6.21619282	4.43701666	0.04805634	0.14320516
Rsph1	-0.7619571	2.13736563	4.43643253	0.04806968	0.14320516
Mir1954	5.67117765	-1.8444918	4.53871849	0.04812014	0.14322833
1500009L16F	-0.3897059	4.68452919	4.43420586	0.04812055	0.14322833
Kctd13	-0.425437	6.17116268	4.43364015	0.04813348	0.14322833
Ctnnbl1	-0.4505023	3.28848364	4.43353243	0.04813594	0.14322833
C230004F18I	0.68260573	3.88591467	4.43243658	0.04816101	0.1432594
Smc5	0.46431734	5.74348268	4.42973218	0.04822294	0.14338266
Zfp652os	-1.6046787	-0.6744618	4.42918151	0.04823556	0.14338266
Ascc3	0.49823191	5.75611155	4.42819731	0.04825813	0.14338266
Herc6	0.359866	5.29591099	4.42757651	0.04827237	0.14338266
Shc3	0.56420947	3.93974062	4.42735385	0.04827747	0.14338266
Sh2d1a	-3.1913585	-1.5924025	4.42644958	0.04829823	0.14338266
Prrc2a	0.27050482	7.7199155	4.42615846	0.04830491	0.14338266
Hrh3	-0.3728042	4.7445627	4.42499635	0.0483316	0.14341842
Wdfy4	-0.878922	1.56055721	4.42279267	0.04838226	0.14348433
Vmn2r87	1.19063353	1.17231692	4.42221915	0.04839545	0.14348433
Rundc3b	0.37713039	4.81308097	4.42079074	0.04842833	0.14348433
Ghsr	1.85833006	-0.3539409	4.4205669	0.04843349	0.14348433
Gtf2h5	-0.4178663	5.19964753	4.42051113	0.04843477	0.14348433
Lrrc42	-0.4883779	3.25022116	4.4202103	0.0484417	0.14348433
Fam131c	-2.8244262	-0.4741631	4.4195193	0.04845762	0.14348809
Dynlt1b	-0.5363357	4.20983588	4.41762258	0.04850135	0.14354668
Sdcbp2	1.62215369	-0.7021209	4.41738988	0.04850672	0.14354668

Golph3	-0.2974428	8.57650586	4.41561528	0.04854768	0.1436245
Ampd2	0.38005513	3.93877048	4.41467588	0.04856937	0.14364531
Cndp1	-3.3904811	-1.3929104	4.41334323	0.04860017	0.14369302
Neurod2	-0.2970416	5.81515841	4.41189994	0.04863356	0.14374833
E2f4	-0.386034	4.54122018	4.40735909	0.04873875	0.14396907
Rpl10a	-0.5443838	8.07832466	4.40659676	0.04875644	0.14396907
Gramd1a	0.59394746	3.06794535	4.40589182	0.0487728	0.14396907
Crbn	0.27864492	6.44617945	4.40503142	0.04879278	0.14396907
Man1b1	0.45527405	3.68547036	4.40498159	0.04879394	0.14396907
Macc1	-3.021992	-2.0982567	4.40487445	0.04879643	0.14396907
Igtp	-0.5586441	3.72896167	4.40252895	0.04885094	0.14408651
Itih3	0.8555011	1.81272516	4.40141002	0.04887697	0.14411989
Zfp113	0.4648879	4.27672379	4.40048339	0.04889854	0.14413563
Dcc	0.37430772	5.17733694	4.39947522	0.04892202	0.14413563
Wbp1	-0.5188918	3.50318168	4.39876958	0.04893846	0.14413563
Ptpn13	-0.3059282	6.26099968	4.39865336	0.04894117	0.14413563
Anxa1	-0.6501778	6.7899027	4.39767748	0.04896392	0.14414242
Ndufs3	-0.3151102	6.44442752	4.39540877	0.04901685	0.14414242
Mpg	-0.713118	1.96183919	4.39410168	0.04904738	0.14414242
Apip	0.44114451	4.17678292	4.39377636	0.04905498	0.14414242
Frem1	1.15742848	1.21713709	4.39352806	0.04906078	0.14414242
Aldh3a2	-0.251794	6.15963536	4.39345371	0.04906252	0.14414242
Lmbr1	0.32072968	5.0770825	4.39337424	0.04906438	0.14414242
Mss51	2.50120073	-0.9823114	4.39335696	0.04906478	0.14414242
Blnk	-0.5237064	3.30553498	4.39267938	0.04908062	0.14414242
Prr14l	0.33095191	7.45751808	4.39225151	0.04909063	0.14414242
1110038B12I	0.6106983	2.45169573	4.39152569	0.0491076	0.14414906
S1pr1	0.52022377	5.64766175	4.39023542	0.0491378	0.14419449
Usp33	0.30972602	6.93210108	4.38656427	0.04922384	0.14425062
Gstm7	-0.572849	4.40010318	4.38651659	0.04922496	0.14425062
Mir378b	1.70664212	0.21148148	4.38636352	0.04922855	0.14425062
Galnt14	0.73116998	2.27094978	4.38620593	0.04923225	0.14425062
Scaper	0.37463271	6.38242996	4.38576057	0.0492427	0.14425062
Ccni	-0.2754919	8.52286496	4.38565015	0.04924529	0.14425062
Flot1	-0.3096748	5.46448358	4.38491775	0.04926248	0.14425784
Tsg101	-0.3135776	5.33394706	4.38354391	0.04929475	0.14429004
Bccip	-0.3961329	5.57108292	4.38319567	0.04930294	0.14429004
Zfat	-0.6473628	2.02412204	4.38002396	0.04937754	0.14446522
Serpinb6c	-1.0730758	2.72569528	4.37930534	0.04939446	0.14447158
Map7	0.30760971	5.79155177	4.37306647	0.04954165	0.14480771
Vmn2r-ps129	1.34101624	0.85826362	4.37260288	0.04955261	0.14480771
Uchl4	1.5710384	-0.6099704	4.37255528	0.04955374	0.14480771
Zfp422	-0.3258434	6.39820939	4.37111231	0.04958786	0.14482252

Sh3tc2	2.76027383	-1.284331	4.37049252	0.04960253	0.14482252
Ikbkap	0.46090287	5.38567574	4.37046565	0.04960316	0.14482252
Grk1	0.92095886	1.35031602	4.36928825	0.04963103	0.14486072
Bptf	0.36701529	8.32255731	4.3677921	0.04966648	0.14487138
L2hgdh	-0.2495128	6.11921602	4.36705834	0.04968387	0.14487138
Emp2	0.44361098	4.33314643	4.36703142	0.04968451	0.14487138
Vps13a	0.39848231	7.32010642	4.36663788	0.04969384	0.14487138
Dnmt3a	0.32980403	6.59707257	4.36481082	0.04973719	0.14492158
Nsl1	0.62062302	4.58000397	4.36466513	0.04974065	0.14492158
Prrc2b	0.26872274	8.7073686	4.36354023	0.04976737	0.14495629
D430041D05	0.3932659	7.66574254	4.3616928	0.04981128	0.14504106
Ythdc2	0.45306448	5.51634512	4.36075629	0.04983355	0.1450417
Fer1l5	0.78154848	1.38580258	4.35931219	0.04986792	0.1450417
Slc26a11	0.77683339	2.08885462	4.35901915	0.0498749	0.1450417
Rdh1	0.8045192	2.07293096	4.3580653	0.04989762	0.1450417
Fam120b	0.30757013	6.59916245	4.35798195	0.04989961	0.1450417
Adamtsl4	0.53456431	2.8491448	4.35740957	0.04991325	0.1450417
Dmrt2	-2.2761345	-0.9402289	4.35732985	0.04991515	0.1450417
Rbfox3	-0.229359	7.81275432	4.35136695	0.05005751	0.14541224
Rif1	0.42007612	5.78944348	4.34941858	0.05010413	0.14550451
Frmd4b	-0.3345645	5.2760679	4.34674811	0.0501681	0.14564712
Fndc1	0.74655666	1.61004126	4.34507416	0.05020825	0.14570221
4932411N23	1.66138234	-0.9359805	4.34471672	0.05021683	0.14570221
Snf8	-0.4784735	4.26851453	4.34315791	0.05025426	0.14576763
Bhlhb9	0.2648356	5.47364275	4.34126586	0.05029973	0.14584457
Map2k5	-0.48068	3.60652918	4.34024151	0.05032437	0.14584457
Csrp2bp	-0.3265665	4.95271622	4.34019658	0.05032545	0.14584457
Epha7	0.38097857	6.56712493	4.33937474	0.05034523	0.14585302
Dusp22	0.31805146	4.8711312	4.33883822	0.05035815	0.14585302
St3gal4	-0.7252193	3.96093969	4.33586677	0.05042975	0.14601723
Wdr19	0.44097426	4.6043891	4.33517875	0.05044635	0.14602212
Rpl26	-1.7049862	-1.291403	4.33293072	0.05050062	0.14605701
Mff	-0.2439517	7.56692729	4.33271278	0.05050588	0.14605701
Tro	0.48007096	6.41530895	4.33185965	0.0505265	0.14605701
Ccdc101	-0.4093837	4.2368717	4.33173141	0.0505296	0.14605701
Ttc3	0.37445734	9.98771488	4.33137265	0.05053827	0.14605701
Epg5	0.57620053	5.48609808	4.33091283	0.05054939	0.14605701
Brwd3	0.31803847	6.15172761	4.33011841	0.0505686	0.14605701
Gsx1	2.97835408	-1.7004673	4.32974298	0.05057769	0.14605701
Gapdh	-0.2468402	9.52446363	4.32683779	0.05064804	0.14607666
Wdr7	0.40992942	7.54758209	4.32657507	0.05065441	0.14607666
Echdc3	-0.9217683	1.11695065	4.32650445	0.05065612	0.14607666
Fam134b	0.35227672	5.61864022	4.32601527	0.05066798	0.14607666

Tmem106b	0.27209337	7.56207036	4.32569049	0.05067586	0.14607666
Vma21	-0.2768773	6.21773459	4.32547506	0.05068108	0.14607666
Fbxl8	2.29944891	-0.7816331	4.32515353	0.05068888	0.14607666
Lrrc45	0.44725714	3.71480371	4.32210434	0.05076291	0.14624698
Inpp5k	0.32537223	4.16728098	4.32022675	0.05080856	0.14633545
Pkia	-0.2259659	8.02784986	4.31908119	0.05083643	0.14637269
Rela	-0.417895	4.25629368	4.31769795	0.05087011	0.14641843
Mrpl2	-0.5725453	3.1418661	4.31686713	0.05089036	0.14641843
Vimp	-0.3189181	6.1270351	4.31658777	0.05089716	0.14641843
Cd160	1.10191335	1.22639695	4.31535801	0.05092715	0.14646167
Dennd2c	-1.355191	-0.3429158	4.31471231	0.0509429	0.14646396
Pcdha9	1.73454006	0.22596979	4.31259458	0.0509946	0.14655085
Cyfip2	0.37626973	9.89947236	4.31224882	0.05100304	0.14655085
Dclre1b	-0.4320762	3.22638578	4.31034925	0.05104947	0.14664125
1110012L19F	-0.6805052	3.3695072	4.3062247	0.05115046	0.14670746
Scn2a1	0.47021891	8.05753643	4.30494937	0.05118173	0.14670746
Ddx5	0.26253243	9.43719994	4.30395835	0.05120604	0.14670746
Asnsd1	-0.4543026	5.29360696	4.30349741	0.05121736	0.14670746
Utp14b	0.45373777	4.91142659	4.30305719	0.05122817	0.14670746
Atad3a	0.53114944	3.12139672	4.30218429	0.0512496	0.14670746
Arl2bp	-0.3481348	5.67878258	4.30193993	0.05125561	0.14670746
Pop7	-0.5235673	2.62452614	4.30155138	0.05126515	0.14670746
Oasl2	-0.5133714	7.17688143	4.30153613	0.05126553	0.14670746
Clec4g	-2.2259782	-1.5949193	4.30135842	0.0512699	0.14670746
Zfp959	0.63933218	1.73695519	4.30128019	0.05127182	0.14670746
Ranbp3	-0.3638763	3.79785599	4.30085565	0.05128226	0.14670746
Fam192a	-0.3177257	5.25633001	4.30066705	0.05128689	0.14670746
Htr1d	1.08756264	1.15101983	4.30060293	0.05128847	0.14670746
Gtf3c3	0.33397059	5.1667821	4.30024859	0.05129718	0.14670746
Ykt6	-0.3532555	5.12626291	4.29921695	0.05132256	0.14670953
Ndufb9	-0.3880513	6.52462575	4.29900157	0.05132786	0.14670953
Fam160b1	0.35003493	5.00786603	4.29790493	0.05135485	0.14674386
Gm10785	1.03820394	0.94385693	4.29591039	0.05140399	0.14684144
Fundc2	-0.3587371	7.72147033	4.29369456	0.05145865	0.14695471
Lrrk2	0.50806679	5.84619947	4.2905615	0.05153604	0.14709381
Ap2m1	-0.2485177	7.80336144	4.29050694	0.05153739	0.14709381
Psmd9	-0.431455	4.39542341	4.28869369	0.05158224	0.14717894
Taf4a	0.31961145	4.74494453	4.28509954	0.05167128	0.14739005
Zfp951	0.53976232	2.46295741	4.28315018	0.05171964	0.14742434
Brat1	0.64516295	2.28411883	4.28297254	0.05172405	0.14742434
Zfyve19	-0.3949199	3.28719945	4.28279535	0.05172845	0.14742434
Psma3	-0.2767934	6.99917801	4.2816107	0.05175787	0.14744149
Tph1	-3.1364353	-0.7652572	4.28134104	0.05176457	0.14744149

Plxdc2	0.25307549	6.74706993	4.27871962	0.05182976	0.14754912
Capsl	-1.0514358	1.18901442	4.27861	0.05183248	0.14754912
C230029M16	-4.6136971	-1.9497572	4.2771952	0.05186771	0.14760649
Fam189a1	0.32436545	5.77277244	4.27544464	0.05191132	0.14762716
Cpm	-0.4791753	4.42576325	4.27464126	0.05193136	0.14762716
Jrkl	-0.4227032	3.30938128	4.27449056	0.05193511	0.14762716
Tomm40l	-0.3443289	3.94998471	4.27448486	0.05193526	0.14762716
Cyp51	0.37020399	5.33022634	4.27234636	0.05198863	0.147736
Kcna1	0.34392016	7.66046982	4.26910887	0.05206954	0.14792302
Nav3	0.51209806	6.74141912	4.26728439	0.05211521	0.14799653
Cpd	0.33486464	6.32169833	4.26686797	0.05212563	0.14799653
Sestd1	0.38778336	7.06403076	4.26616791	0.05214317	0.14800342
Gpr157	0.78195941	2.14962224	4.26369213	0.05220525	0.14812636
1500011K16I	-0.542524	3.942228	4.26283348	0.0522268	0.14812636
Os9	0.41532285	4.71647211	4.26234382	0.05223909	0.14812636
Myot	-2.6495863	-2.0569196	4.26184515	0.05225161	0.14812636
Klhl23	0.41270926	5.1496477	4.26142784	0.05226209	0.14812636
Abca7	0.85789309	1.81543376	4.25921882	0.05231762	0.14824085
Shc1	-0.4579907	6.42383923	4.25582214	0.05240314	0.14843697
Cand1	0.31574096	7.71060483	4.2552662	0.05241715	0.14843697
Pigt	0.34122594	5.31439876	4.25204502	0.05249841	0.14862414
Cic	0.27589914	6.64039487	4.24962341	0.0525596	0.14875438
Itgbl1	0.43297722	6.1219165	4.24844269	0.05258947	0.14879591
Mrps7	-0.3539975	5.25942909	4.24745682	0.05261442	0.14882352
Tdrd1	1.17223736	0.84020245	4.246016	0.05265091	0.14886555
Limch1	0.55097116	5.83790073	4.24567001	0.05265968	0.14886555
Ss18	-0.3064023	5.34131109	4.24487506	0.05267982	0.14887955
Slitrk1	0.28020012	7.08121807	4.24260196	0.05273749	0.14894867
Gm6583	2.44787281	-1.0788076	4.24209675	0.05275031	0.14894867
Dbf4	-0.729677	1.82206078	4.24154676	0.05276428	0.14894867
Opn4	1.7893264	-0.0898355	4.2415142	0.05276511	0.14894867
Chmp1a	-0.4255357	4.57431843	4.23986693	0.05280696	0.14898165
Dnase1	1.16220587	1.03143966	4.23931742	0.05282093	0.14898165
Def8	-0.350513	4.42867502	4.23925909	0.05282242	0.14898165
Pck2	-0.5232199	3.2418545	4.23775429	0.0528607	0.1490467
Cds1	0.3416498	5.48304788	4.23683207	0.05288418	0.14906999
Llgl2	-1.2713229	-0.2605733	4.23529329	0.05292338	0.14913757
Phykpl	0.51661915	3.26191407	4.2342577	0.05294978	0.14916905
Ms4a6d	-2.8673947	-1.2205621	4.23272853	0.05298879	0.14923603
Lama3	0.73074885	2.5107146	4.23142463	0.05302208	0.14925527
Hspb6	-0.3724463	4.31120956	4.23126743	0.05302609	0.14925527
P4ha3	0.89011349	2.42550254	4.23056921	0.05304393	0.14926258
Fopnl	-0.3765949	4.94606246	4.22784804	0.05311351	0.14940533

Hmces	0.61258022	2.01871036	4.22720441	0.05312999	0.14940533
Scai	0.33989876	7.0689649	4.22679704	0.05314042	0.14940533
Slco1a5	-2.1981128	-0.5127274	4.22409299	0.05320971	0.14953782
Wfdc17	-1.1222951	0.93154725	4.22342855	0.05322675	0.14953782
Phactr3	-0.2477531	6.15735084	4.22317156	0.05323334	0.14953782
Gga1	-0.4102404	3.54956689	4.22252344	0.05324997	0.14954166
P4ha1	0.35466019	3.95493008	4.21852549	0.0533527	0.14978719
Pde4dip	0.38459363	7.48586352	4.21283822	0.05349922	0.15013041
Ccpg1	0.28236058	6.52446713	4.21259076	0.0535056	0.15013041
2610018G03	0.84842549	1.93661506	4.2117494	0.05352732	0.15014834
Zfp101	-0.5036677	2.54349396	4.20979441	0.05357782	0.15021492
Hkdc1	-0.7722981	1.82969467	4.20964343	0.05358172	0.15021492
Tex2	-0.2319619	6.54955136	4.20832396	0.05361584	0.15026756
Dek	-0.3288585	8.40392104	4.20599772	0.05367606	0.15035611
Srp54a	-0.22196	7.11641345	4.2059174	0.05367814	0.15035611
Csnk1g1	0.2539052	6.24794993	4.20315245	0.05374981	0.15046204
Rfk	-0.5253136	9.48783152	4.20305302	0.05375239	0.15046204
Caly	-0.4753969	3.47990388	4.20268117	0.05376204	0.15046204
Dgkk	0.6543656	3.76416739	4.20170462	0.05378738	0.15048998
Nt5dc2	-1.1176172	2.65514329	4.19922831	0.05385172	0.15062695
Irf2bpl	0.30639927	5.74511218	4.19856865	0.05386887	0.15063192
Gm4841	0.74641425	2.36667917	4.19664211	0.053919	0.15064495
Vcpkmt	-0.7185725	1.7494766	4.19657682	0.0539207	0.15064495
Ppp1r9a	0.52977207	8.91038892	4.19543461	0.05395045	0.15064495
Klhdc9	-0.3969945	3.65695043	4.19509484	0.0539593	0.15064495
St8sia3	0.34815218	7.82621868	4.19491847	0.0539639	0.15064495
Laptm4b	0.28711807	5.34753559	4.19484527	0.05396581	0.15064495
Tst	-0.4841416	4.00671627	4.19328796	0.05400641	0.15071534
Slc48a1	0.35401544	4.84507852	4.18907535	0.05411642	0.15097932
Sptbn2	0.51372115	6.97958108	4.18846481	0.05413238	0.15098086
Vsig10	1.06455462	1.46708961	4.18452729	0.05423547	0.15122533
Dach1	0.7033256	2.04567433	4.18143047	0.05431671	0.15140875
Chsy1	0.30375666	5.20745645	4.17902298	0.05437996	0.15151
Prps1l3	-0.3168959	4.70122548	4.17835956	0.0543974	0.15151
Cacna2d3	0.41884194	5.15526663	4.17828229	0.05439944	0.15151
Ppp3r2	1.6994867	-0.420535	4.17705184	0.05443181	0.15151247
Dcps	-0.5713441	2.22805824	4.17700254	0.05443311	0.15151247
Colec12	0.24403932	6.68445516	4.17607512	0.05445752	0.15151247
Fam81a	-0.3107039	5.71032253	4.17557743	0.05447063	0.15151247
Rps25	-0.4942042	7.66263212	4.17499789	0.0544859	0.15151247
Zswim3	0.80734051	2.11516617	4.17442307	0.05450105	0.15151247
Heca	-0.3450194	4.53730829	4.1736273	0.05452203	0.15151247
Abcc5	0.61548538	4.93688186	4.17308524	0.05453632	0.15151247

Gm6313	0.88340107	1.20104026	4.17296345	0.05453953	0.15151247
Cenpj	-0.3905822	4.00138366	4.17237029	0.05455518	0.15151298
Inha	0.70520142	1.64997007	4.171429	0.05458003	0.15153901
Znf41-ps	0.56755454	2.72156312	4.16967356	0.05462639	0.15157056
Zbtb22	-0.4884914	3.3208382	4.16924665	0.05463768	0.15157056
Zfp882	0.34096614	4.53737201	4.1692416	0.05463781	0.15157056
Pgls	-0.8059165	1.35967136	4.16618412	0.0547187	0.15175197
Fam76a	-0.3122716	5.87616487	4.16533115	0.05474129	0.15175763
Zbtb12	-1.1967455	-0.2350096	4.16478777	0.05475568	0.15175763
Aox1	-0.5099059	3.12249429	4.16435261	0.05476722	0.15175763
Map4k2	0.36760439	4.85664931	4.16218565	0.05482469	0.15187391
Mir1931	2.45565529	-1.7815643	4.16019465	0.05487755	0.15197215
Mgat4c	0.55376236	2.88416955	4.15957779	0.05489394	0.15197215
Gpr1	-3.2290154	-1.4864563	4.15909797	0.05490669	0.15197215
Capn7	0.26497305	6.54872315	4.158321	0.05492735	0.15198638
Jmy	0.24867135	7.18953639	4.15476287	0.05502207	0.15220547
Gar1	-0.331844	4.14274439	4.15029582	0.05514124	0.15243148
Srrm2	0.36505544	10.1848417	4.15020316	0.05514372	0.15243148
Rras2	-0.4077534	4.65178085	4.14960524	0.05515969	0.15243148
Tmem55b	-0.3694077	4.99186399	4.14936863	0.05516602	0.15243148
Wdr41	-0.287662	4.83009109	4.14869282	0.05518408	0.15243839
Faim	-0.3585717	5.76111039	4.14730658	0.05522116	0.15248895
Myl6b	-0.6702744	3.78639404	4.14684475	0.05523352	0.15248895
Kdm4a	0.47899454	3.03882422	4.14609411	0.05525361	0.15249896
Prdm8	-0.4267909	5.55077021	4.14554622	0.05526828	0.15249896
Eif4a2	-0.2209468	8.98994268	4.14452612	0.05529561	0.1525314
Cmtr2	0.65792242	2.13066756	4.14174535	0.05537019	0.15269412
Rpl24	-0.3491828	6.6964983	4.13993068	0.05541892	0.15270715
Gm11127	0.51935126	3.78729841	4.13988793	0.05542007	0.15270715
Ube2q1	-0.2250534	7.10382136	4.13982775	0.05542168	0.15270715
Prrt1	-0.3539404	4.88909322	4.13701485	0.05549732	0.15283863
Arhgef37	-1.1607549	0.28806604	4.1368926	0.05550061	0.15283863
Plcd1	-0.6512185	2.01051143	4.13415984	0.05557421	0.15285534
Anapc13	-0.4708723	5.13802789	4.13355012	0.05559064	0.15285534
Aptx	-0.2945921	5.00670793	4.13342807	0.05559393	0.15285534
Zfp706	-0.2666575	7.48004342	4.13307817	0.05560337	0.15285534
Rab15	-0.244509	6.31468885	4.1330124	0.05560514	0.15285534
Lrtm1	1.15352586	3.1295136	4.13217528	0.05562773	0.15285534
Midn	-0.3390489	5.84324131	4.13178265	0.05563832	0.15285534
Sp110	-0.5660386	3.39387382	4.13145698	0.05564711	0.15285534
Chmp3	-0.3114423	6.51614272	4.13145662	0.05564712	0.15285534
Adcy3	0.6870461	2.3116347	4.12967007	0.05569537	0.15294499
Cry1	-0.3780853	4.34636312	4.12679486	0.05577312	0.15311557

Cbx1	-0.3845209	4.65570054	4.12602276	0.05579402	0.15313003
Mllt6	0.29047752	6.01059421	4.1251942	0.05581646	0.15314428
Alpk1	0.63038572	2.5294278	4.12451359	0.0558349	0.15314428
Tns1	-0.2432353	6.61314246	4.12290428	0.05587852	0.15314428
Ret	0.87775417	1.82544934	4.12236899	0.05589304	0.15314428
Kif1a	0.40062316	9.884119	4.12230903	0.05589467	0.15314428
Vps52	0.32407171	4.55164951	4.1219559	0.05590425	0.15314428
Aurkaip1	-0.416824	4.92854804	4.12143633	0.05591835	0.15314428
Deptor	-0.2474742	7.41084037	4.11999248	0.05595756	0.15314428
Arl6ip4	-0.429997	4.90499571	4.11957356	0.05596894	0.15314428
Vstm2a	0.29599941	6.5331679	4.11951447	0.05597055	0.15314428
Fbxl16	-0.3450387	7.27841856	4.11949084	0.05597119	0.15314428
Rasa2	0.29349102	5.06270366	4.11737023	0.05602885	0.15325923
Gpbp1	-0.2485904	7.99359467	4.11641502	0.05605484	0.15328753
Dennd1a	0.28762722	5.51187402	4.11474108	0.05610043	0.15336937
Gm10658	-1.6377035	-0.5900214	4.1120082	0.05617494	0.15351442
Zrsr2	-0.2616419	6.20356364	4.11164593	0.05618483	0.15351442
Fam198a	0.85832024	1.78053797	4.11055877	0.05621451	0.15355268
Itpr2	0.29150463	5.33781271	4.10782509	0.05628922	0.15367493
Nsmce4a	-0.4000866	4.58308296	4.10777319	0.05629064	0.15367493
Rara	-0.51134	5.15309412	4.10673688	0.05631899	0.15370949
2510039O18	0.60146935	3.40800812	4.10523323	0.05636016	0.15375494
Endod1	-0.3128745	4.92098218	4.10488075	0.05636981	0.15375494
Dmrta2	1.40229309	-0.2709334	4.10440921	0.05638273	0.15375494
Tle1	0.30907521	5.53266061	4.10348488	0.05640807	0.15378122
Lrrc20	-0.487501	4.21284955	4.10094927	0.05647764	0.15392803
Gna11	-0.3602816	5.92150648	4.10034978	0.0564941	0.15393007
1700012B09	-1.7008671	-0.4327784	4.09910973	0.05652817	0.15393751
Anxa4	-0.4643328	6.49079403	4.09878652	0.05653705	0.15393751
Pxn	-0.3333503	5.22610762	4.09853471	0.05654397	0.15393751
Ctdsp1	-0.3997533	6.68530634	4.09607252	0.05661171	0.15406946
Pfn1	-0.4926912	6.68741467	4.09531408	0.0566326	0.15406946
Sftpc	-3.3965034	-1.19407	4.09505879	0.05663963	0.15406946
AW046200	1.5689926	0.14785655	4.09002931	0.05677836	0.15440396
Klk14	1.24718187	-0.0143907	4.08840995	0.05682312	0.15440716
Grm2	0.37851787	3.64352703	4.08838595	0.05682378	0.15440716
Nell2	0.32391569	6.79545258	4.08787007	0.05683804	0.15440716
Kif13b	0.39519307	3.67690673	4.08770558	0.05684259	0.15440716
Tlr9	1.54681673	-0.3613039	4.08609005	0.0568873	0.154413
Plcxd3	0.38625394	5.01837522	4.08588087	0.05689309	0.154413
Zswim6	0.27233112	6.10393689	4.08568481	0.05689852	0.154413
Ccnl2	0.52966198	4.42696557	4.08534969	0.0569078	0.154413
Eci2	-0.3930644	5.4609683	4.08387593	0.05694863	0.15442119

Sgsm2	0.38027314	4.61831494	4.08384945	0.05694936	0.15442119
2310065F04I	-3.5310383	-1.6420184	4.08353394	0.05695811	0.15442119
Fam227a	0.92718708	1.77176102	4.08078664	0.05703433	0.15455675
Psmb8	-0.7691439	2.65757263	4.08016048	0.05705172	0.15455675
Hint2	-0.5691391	3.50760824	4.08002629	0.05705545	0.15455675
Ggh	0.57394355	3.23462189	4.07844953	0.05709927	0.1546099
Rabif	-0.3575833	5.84337403	4.07798025	0.05711232	0.1546099
Il1a	-2.0597817	-1.0177028	4.07761694	0.05712242	0.1546099
Fam208a	0.35267024	6.76686734	4.07669342	0.05714812	0.15463672
Wdsub1	-0.5223349	3.07045806	4.07505149	0.05719383	0.15471768
Tpt1	-0.3991774	9.33039457	4.07406908	0.0572212	0.15474899
Tmod3	-0.4120274	7.80433048	4.07327701	0.05724328	0.15476597
Ndufb8	-0.367659	4.70216124	4.07223741	0.05727228	0.15480164
C1d	-0.3408087	4.9718572	4.07105276	0.05730534	0.15484827
Gpr37l1	0.58826839	3.0098003	4.06891183	0.05736514	0.15496712
Eif4a1	-0.229697	7.37071226	4.06722866	0.05741221	0.15499479
Ubap1l	3.1403499	-1.8406109	4.06685405	0.05742269	0.15499479
Asb14	1.48543642	0.03318188	4.06684819	0.05742286	0.15499479
Lsp1	-0.5748064	3.52750994	4.06522364	0.05746834	0.15502704
Ccdc158	-1.8625644	-0.7985472	4.06456145	0.05748689	0.15502704
Gm10046	0.96427084	0.33357784	4.06440187	0.05749136	0.15502704
Erc1	0.23717724	7.67618624	4.06416094	0.05749811	0.15502704
Vstm2b	0.47476193	3.40517909	4.05992121	0.05761707	0.15530504
Gabra6	-5.4446792	-2.0502817	4.14712718	0.05767787	0.15538409
Bok	-0.4299486	4.24152508	4.05757884	0.05768292	0.15538409
Timm17a	-0.3657885	5.75091044	4.05718522	0.05769399	0.15538409
Dock5	-0.2651753	6.84778138	4.05318923	0.05780654	0.15560211
Tmsb10	-0.525533	6.42642058	4.05273014	0.05781949	0.15560211
Ttc9	-0.3498287	4.23542392	4.05156381	0.05785239	0.15560211
Ncam1	0.34672981	7.88637421	4.05119966	0.05786267	0.15560211
Rassf4	-0.5035555	3.13006963	4.05107654	0.05786615	0.15560211
Cenpq	0.81462379	2.30652887	4.05093121	0.05787025	0.15560211
Zfp58	-0.4806585	3.40158186	4.04977517	0.0579029	0.15562044
Pcsk2os2	1.46735553	1.71436286	4.04932574	0.0579156	0.15562044
Xkrx	0.7229082	2.04510831	4.04819182	0.05794765	0.15562044
Ten1	-0.5016635	3.27363178	4.04778453	0.05795917	0.15562044
Gtpbp4	-0.2273813	6.31664662	4.0475575	0.05796559	0.15562044
Gbp4	-0.5492804	2.90450644	4.04731715	0.05797239	0.15562044
Foxd2	-0.7688512	2.72874182	4.04578079	0.05801587	0.15568295
Prdm1	0.88174718	2.00380264	4.04537141	0.05802747	0.15568295
Lrrc8c	0.39882119	4.05607155	4.04368459	0.05807526	0.15574098
Greb1l	0.53525352	3.07983397	4.0434859	0.0580809	0.15574098
Prkcg	0.30526381	8.20544702	4.04131931	0.05814236	0.15582171

Gareml	0.75841965	2.21577455	4.04130313	0.05814282	0.15582171
Dgkh	0.54867066	5.56521568	4.04047166	0.05816643	0.15582971
Pes1	-0.3778705	4.47611042	4.0400775	0.05817762	0.15582971
Mafg	0.39112261	4.34596512	4.0395076	0.05819381	0.15583047
Asph	0.26302432	6.75367526	4.03877457	0.05821464	0.15584365
Sema6d	0.32173279	6.16563267	4.03821232	0.05823063	0.15584385
Piga	-0.6060857	3.12116202	4.03732098	0.05825598	0.15586911
Apmap	0.43667353	4.89839302	4.03307239	0.05837699	0.1561335
Tox2	0.47753965	2.75127914	4.03273294	0.05838668	0.1561335
Mc1r	2.25861831	-1.8869924	4.03119105	0.05843067	0.1561837
Ticam2	1.35461439	0.92254392	4.02913597	0.05848937	0.1561837
Dcaf12l1	0.295875	5.6089195	4.02894865	0.05849473	0.1561837
Mettl21c	2.28388758	-0.5973106	4.02777839	0.05852819	0.1561837
Max	-0.2980571	8.08866805	4.02776645	0.05852853	0.1561837
Arx	0.60884334	2.59868068	4.0273706	0.05853985	0.1561837
Hepacam	0.45815847	4.23256353	4.0272165	0.05854426	0.1561837
Cpsf1	0.50409116	4.0570391	4.02720082	0.05854471	0.1561837
Mpp1	-0.2532197	5.83676708	4.02705263	0.05854895	0.1561837
Gata6	-3.1125812	-1.377922	4.02395911	0.05863754	0.15635496
Gm15441	-2.672868	-1.1953439	4.02338052	0.05865413	0.15635496
Hrc	2.11597186	-0.3257219	4.02312666	0.05866141	0.15635496
Gm13298	0.4206834	5.02359113	4.02258317	0.058677	0.15635496
Vps45	0.40527669	3.79900705	4.02083572	0.05872715	0.1564402
Tmem45b	0.76244925	2.23267171	4.01973375	0.0587588	0.1564402
Rgs9bp	-2.0628896	-0.2906914	4.01931332	0.05877088	0.1564402
Pbdc1	-0.3588832	4.59042554	4.01924407	0.05877287	0.1564402
2410015M2C	-0.5911004	2.73795877	4.01795	0.05881008	0.15646058
Mir325	2.11950932	-1.4886438	4.01786668	0.05881247	0.15646058
Cnot1	0.26382197	8.09961705	4.01611786	0.0588628	0.15655193
Fundc1	-0.3169844	6.44940792	4.01432777	0.05891436	0.15657863
Uvrag	-0.2685007	5.56574296	4.01421174	0.0589177	0.15657863
Trim23	0.28976901	6.39756976	4.01410458	0.05892079	0.15657863
Mpv17	-0.3417738	5.95198813	4.01023374	0.05903248	0.15674338
Gak	0.26720214	6.70953113	4.00994993	0.05904068	0.15674338
Gm4890	-2.8127799	-1.3919237	4.0098967	0.05904221	0.15674338
Strap	-0.2102609	7.46723644	4.00973832	0.05904679	0.15674338
Ttc5	-0.3063084	4.61761479	4.00883309	0.05907295	0.15677034
Uhrf1bp1	0.46061929	3.87314258	4.00760961	0.05910833	0.15680601
Shprh	0.43289245	5.96668018	4.00644729	0.05914197	0.15680601
Zfp414	-0.4256417	4.50877681	4.0061003	0.05915201	0.15680601
Gria1	0.38742547	6.49915439	4.00601312	0.05915453	0.15680601
Ncapd2	0.5616421	2.67232371	4.00479711	0.05918975	0.15680601
Coq10a	-0.4105729	4.11376662	4.00478505	0.0591901	0.15680601

Cdk2ap2	-0.4848522	3.39186092	4.00449696	0.05919845	0.15680601
Wdr5	-0.6589355	3.23638315	4.00238896	0.05925957	0.15691734
Cep89	-0.5941645	3.02045372	4.00138029	0.05928885	0.15691734
1700018A04I	-2.7615045	-1.9461086	4.00092212	0.05930215	0.15691734
Hilpda	-0.6046583	2.63433833	4.00083908	0.05930456	0.15691734
Osbpl3	0.47219312	5.09364473	3.99915616	0.05935345	0.1570043
Rpp14	-0.320193	5.40968028	3.99831706	0.05937785	0.15701076
Epn3	-1.1907402	0.59555436	3.99796955	0.05938795	0.15701076
Ect2l	-2.1255801	-0.6733218	3.99665097	0.05942632	0.15704555
Mlkl	-1.2715303	0.73842643	3.9964153	0.05943318	0.15704555
Rasgrp4	1.36880901	-0.1143058	3.99461686	0.05948556	0.15714157
Irf3	-0.5950377	2.69589221	3.99281897	0.05953797	0.15721485
Gprasp2	0.40389288	6.17426828	3.99256446	0.0595454	0.15721485
Cxcr6	1.36614374	0.03419362	3.9902055	0.05961426	0.15732928
Selm	-0.5759578	4.47033158	3.98997957	0.05962086	0.15732928
Polr2c	-0.4279644	4.23038674	3.98787089	0.0596825	0.15741725
Vav3	0.45489051	3.29633522	3.98773974	0.05968634	0.15741725
Bhlhe40	-0.2369419	7.52034272	3.98689897	0.05971094	0.15743975
Capn6	-0.4604796	4.31627963	3.98468848	0.05977568	0.15756801
Dnaja1	-0.1987847	9.6899873	3.98315344	0.05982068	0.15762154
Cdk10	0.39041221	4.11816307	3.98289809	0.05982817	0.15762154
2310036O22	-0.4070691	4.55739908	3.98133405	0.05987406	0.15770005
Leng1	-0.5892146	4.27470386	3.97990043	0.05991617	0.15776853
Zfp874a	-0.3393645	4.88317352	3.97494034	0.06006211	0.15802384
3110002H16I	0.5841444	3.3694138	3.97468289	0.0600697	0.15802384
Ppp2r4	-0.2929465	5.70209698	3.97442535	0.06007729	0.15802384
Foxj1	-1.1993102	0.73725854	3.97441272	0.06007766	0.15802384
Slitrk6	0.98992844	0.63049086	3.97344725	0.06010612	0.15805626
Sh2d4b	1.53438495	-0.1638562	3.97136534	0.06016755	0.15817533
5430416O09	-2.7252648	-1.8621924	3.96964551	0.06021835	0.15820043
Lyl1	1.39909458	-0.0769813	3.96949399	0.06022283	0.15820043
Havcr2	-0.9442889	1.70023594	3.96940205	0.06022555	0.15820043
Tspan4	0.69838936	2.48387872	3.96606214	0.06032436	0.15839688
Slc35e3	0.33420015	4.481118	3.96537888	0.06034459	0.15839688
Mgat4b	-0.4823818	2.87867883	3.96475599	0.06036305	0.15839688
Nfkbia	-0.5517176	2.74707365	3.96468947	0.06036502	0.15839688
Ncbp2	-0.2635723	6.54868575	3.96275149	0.06042248	0.15846653
Mier1	-0.2869927	6.44738241	3.96270311	0.06042392	0.15846653
Lima1	-0.372648	7.28145281	3.96076882	0.06048134	0.15857466
Crebbp	0.24818959	8.16522246	3.95989942	0.06050716	0.15859993
Cdc7	0.73473013	2.8060365	3.95923293	0.06052697	0.15860941
Slc35b4	-0.2668579	5.64591633	3.95789104	0.06056688	0.15865197
Stx1b	-0.3010116	6.40618772	3.9575975	0.06057561	0.15865197

Wdr17	0.48997776	5.11350837	3.95677561	0.06060007	0.15865786
Nmb	1.31077489	0.12625906	3.95643356	0.06061025	0.15865786
Scyl3	0.31180359	5.298139	3.95486299	0.06065704	0.15873571
Tsen54	1.47559372	-0.256233	3.95414416	0.06067846	0.15873571
Ghdc	0.56764945	2.22972725	3.95380382	0.06068861	0.15873571
Cenpp	0.62412235	2.28834247	3.9531939	0.0607068	0.1587409
Ndufc1	-0.4122612	5.67207683	3.95168475	0.06075183	0.15881626
Mid1ip1	-0.4735594	6.08785117	3.95097947	0.06077289	0.15882653
Glra2	0.42680604	3.31837041	3.95038421	0.06079067	0.15882653
Prmt6	-0.478819	3.52929591	3.94959761	0.06081418	0.15882653
Fyco1	0.28823205	6.72397002	3.94938216	0.06082062	0.15882653
Minos1	-0.3141301	6.48496504	3.94782838	0.06086708	0.1589055
Kif14	-2.639684	-1.3150309	3.94638782	0.0609102	0.1589757
Amer1	0.3919734	4.17907539	3.94261113	0.06102341	0.15914781
Hhip	0.3752837	4.43848583	3.94200888	0.06104148	0.15914781
Ttll12	0.42493818	4.13670622	3.94145762	0.06105803	0.15914781
4833439L19F	-0.3380942	7.79754832	3.94139226	0.06105999	0.15914781
Sacm1l	0.30043689	6.00551482	3.94105453	0.06107014	0.15914781
Myo9a	0.38260021	8.3603113	3.94008282	0.06109933	0.15914781
Cd300a	0.56974091	3.03887199	3.93968185	0.06111138	0.15914781
1700006F04I	2.09354229	-0.7678995	3.93963241	0.06111286	0.15914781
Atl2	0.3175729	6.17203477	3.93931615	0.06112237	0.15914781
Cd38	0.53432476	2.81277897	3.93804642	0.06116056	0.15920492
Gm6787	2.39358728	-0.4742893	3.93713561	0.06118796	0.15923255
Kynu	-2.1929979	-0.5723934	3.93642445	0.06120937	0.15923255
Sppl2a	0.30938836	6.38309585	3.9360736	0.06121994	0.15923255
Parp2	-0.2883324	5.19107671	3.93497825	0.06125294	0.15927609
Snrnp200	0.36778891	6.89140975	3.93250987	0.06132738	0.15942733
Cobl	-0.2841779	7.98664521	3.93098303	0.06137348	0.15948413
T2	-1.2970407	-0.1876592	3.93065666	0.06138334	0.15948413
Nol9	0.47273129	3.57969393	3.9301689	0.06139807	0.15948413
Zdhhc4	-0.4429605	2.59386182	3.92929097	0.06142461	0.15951077
Col13a1	-0.8004098	2.28792226	3.92696291	0.06149504	0.15961334
Usp6nl	-0.2877023	5.91250472	3.92690818	0.0614967	0.15961334
Shb	-0.6163283	1.70974903	3.92552399	0.06153862	0.15963466
Hmox1	-0.5618367	2.83955747	3.92506597	0.0615525	0.15963466
Rad51	-1.0689275	0.79535411	3.92458895	0.06156696	0.15963466
Ppip5k1	0.43296467	5.49143345	3.92310575	0.06161194	0.15963466
Fam73a	0.29308923	6.03463599	3.92218011	0.06164003	0.15963466
Pom121	0.28807232	6.32319514	3.92213122	0.06164151	0.15963466
Smco3	-0.4867022	2.69433529	3.92196547	0.06164655	0.15963466
Jkamp	-0.375181	4.43764506	3.92185711	0.06164984	0.15963466
St6galnac3	0.48068223	3.5054826	3.92179942	0.06165159	0.15963466

Exoc2	0.3205408	5.89155414	3.91987254	0.06171012	0.15971235
Rffl	-0.4970183	3.08964374	3.91973835	0.0617142	0.15971235
Rad51d	-0.3734838	5.28271466	3.9167395	0.06180544	0.15990622
Npbwr1	-1.4457504	0.40904425	3.91463886	0.06186944	0.1600095
Slc25a42	0.28203864	4.84202156	3.91435711	0.06187803	0.1600095
Dnah8	0.95101349	1.34852445	3.91267737	0.06192927	0.16006528
Angel1	0.83345184	1.42903695	3.91166467	0.06196019	0.16006528
Kcnk3	0.57790855	1.8758333	3.91106597	0.06197847	0.16006528
Oprl1	0.41837362	3.3758433	3.91099324	0.06198069	0.16006528
Lsm14b	-0.2568178	5.55529305	3.91057327	0.06199352	0.16006528
Ccdc93	0.31901502	5.01806247	3.91043843	0.06199764	0.16006528
Cyp4f15	0.8866721	1.15049786	3.90682604	0.06210814	0.16026565
Swsap1	-0.4483609	3.36427758	3.9066764	0.06211273	0.16026565
Zfp57	0.78623626	1.89585509	3.90629726	0.06212434	0.16026565
Fam20a	0.47888932	2.57563194	3.9047424	0.06217199	0.16034634
Psg23	1.02893857	1.27596816	3.9037562	0.06220223	0.16037517
4833424O15	0.36067187	4.66209956	3.90305914	0.06222361	0.16037517
1810010H24	0.82227966	1.16088715	3.902251	0.06224842	0.16037517
Cdh7	0.40571664	3.87185716	3.90121305	0.06228029	0.16037517
Slc25a11	-0.2971334	5.65167589	3.90011354	0.06231408	0.16037517
Prr3	0.31994353	4.200943	3.89948899	0.06233328	0.16037517
Fam72a	-1.8598522	0.51817619	3.89940736	0.06233579	0.16037517
Kcnd2	0.33764974	6.04117674	3.89855257	0.06236208	0.16037517
4930578E11I	1.38699186	-0.6153435	3.89841717	0.06236625	0.16037517
Rnf157	0.33803021	7.48577637	3.89837485	0.06236755	0.16037517
Kif22	0.88783905	0.89356003	3.89768426	0.0623888	0.16037517
Tufm	-0.3750454	3.65762386	3.89637866	0.062429	0.16037517
Cspg5	0.34676751	5.25798228	3.89629711	0.06243151	0.16037517
Ascc2	0.42965359	3.30241568	3.89591012	0.06244344	0.16037517
Ebpl	-0.7142164	2.36077055	3.89546014	0.0624573	0.16037517
Cox11	0.33908033	3.91883494	3.89518662	0.06246573	0.16037517
Lonp1	0.33835511	4.27239562	3.89444515	0.06248859	0.16037517
Nrxn3	0.38932755	8.1011022	3.89428052	0.06249367	0.16037517
Nubpl	-0.5462523	2.80469616	3.89374954	0.06251005	0.16037517
Abcb8	-0.3570154	4.05750279	3.89373099	0.06251062	0.16037517
Fut9	0.39584881	7.1307687	3.89152063	0.06257885	0.16049634
Mrps18b	-0.6256217	2.96801777	3.89113966	0.06259062	0.16049634
Thy1	-0.2557613	7.45923875	3.89057321	0.06260812	0.16049921
Mtdh	0.25704942	6.76294372	3.88776687	0.06269492	0.16063422
Fam154b	-0.9029194	0.89675354	3.88771941	0.06269639	0.16063422
C130026I21R	-0.893503	1.06014838	3.88666389	0.06272907	0.16063422
Zfp35	-0.3023815	4.4899564	3.88622984	0.06274252	0.16063422
Bet1l	-0.6327206	3.14726464	3.88622132	0.06274278	0.16063422

Mum1	0.6553029	2.98551717	3.88082069	0.06291035	0.16098735
Postn	-0.5412276	2.19193298	3.88071676	0.06291358	0.16098735
Fam126a	0.3380209	4.10421944	3.87768746	0.06300781	0.16118636
Pop5	-0.3904903	4.39829903	3.87553965	0.06307471	0.16131539
Fras1	0.65148827	4.82325395	3.87443224	0.06310924	0.16136156
Copb1	0.252993	6.10022922	3.8723863	0.06317309	0.16148266
Ddx47	-0.2731195	5.91726426	3.87132481	0.06320625	0.16152526
Gpr137c	0.3647285	4.87523066	3.87062663	0.06322806	0.1615338
Ndufb11	-0.3927708	5.59865569	3.87016262	0.06324257	0.1615338
Slbp	-0.277172	5.38558167	3.86758819	0.06332312	0.16155664
Cttnbp2nl	0.26115167	6.16218878	3.86754457	0.06332448	0.16155664
Cntnap5b	0.39423221	4.15925846	3.86695309	0.06334301	0.16155664
D7Ertd715e	0.72753286	4.14583375	3.8666266	0.06335324	0.16155664
Zbed6	0.21242892	6.82936848	3.86635749	0.06336167	0.16155664
H2-T24	0.46458939	4.91991878	3.86535629	0.06339305	0.16155664
Rps28	-0.4568367	5.54608439	3.86480089	0.06341046	0.16155664
Gabarap	-0.4252566	7.19834281	3.86469897	0.06341366	0.16155664
Plscr2	-0.5594617	4.4211804	3.86356068	0.06344937	0.16155664
1810043G02	-0.461485	2.74556179	3.86311748	0.06346328	0.16155664
Tmem8b	0.35167756	5.07765794	3.86261957	0.06347891	0.16155664
Naa50	-0.226071	7.71159215	3.86240561	0.06348563	0.16155664
Rbmx2	-0.4262927	4.45926901	3.86171795	0.06350723	0.16155664
Vdac3	-0.2663742	6.33344942	3.86151898	0.06351348	0.16155664
Lrrc8a	0.24781354	6.15744372	3.86109343	0.06352685	0.16155664
Catsperg1	1.95222536	-1.0719641	3.86081346	0.06353565	0.16155664
Ccdc14	0.7106837	1.8649265	3.85987926	0.06356502	0.16155664
Wwtr1	-0.3539304	6.61329783	3.85977374	0.06356834	0.16155664
Mmp9	-2.140609	-0.7822995	3.85900932	0.06359239	0.16155664
Eif4b	-0.2071458	7.59682671	3.8584238	0.06361081	0.16155664
Cspp1	0.36831296	4.72589044	3.8582026	0.06361778	0.16155664
Bbs1	0.37274242	5.65451516	3.85811609	0.0636205	0.16155664
Cxxc5	-0.2879315	6.44888195	3.85778713	0.06363086	0.16155664
Zcchc17	-0.2891184	5.21882703	3.85550924	0.06370262	0.16169695
Tmem117	0.50566358	3.28497422	3.85447499	0.06373524	0.16173782
Ltn1	0.31519563	6.60022805	3.85310374	0.06377851	0.16180572
Kcns2	0.38969832	3.87739497	3.85252083	0.06379692	0.16181051
Adck5	0.88529761	1.01876887	3.85185826	0.06381785	0.16182169
Dpp3	-0.4510778	3.80938584	3.84790037	0.06394303	0.16209714
Klra2	1.61539367	0.277556	3.847246	0.06396375	0.16210772
Pcdha1	1.91490879	-0.9128144	3.84624174	0.06399557	0.16214389
Slc6a11	0.48931817	4.54546055	3.84575093	0.06401113	0.16214389
Ccdc108	0.93390867	1.32172893	3.84500278	0.06403485	0.16215561
Gm15133	-2.8602347	-1.759902	3.84456102	0.06404887	0.16215561

Tfe3	-0.3270507	5.30511724	3.84334875	0.06408734	0.16218297
Pcdhga5	0.45636635	3.68672093	3.84317716	0.06409279	0.16218297
Cap1	-0.2483367	7.01686267	3.84197254	0.06413104	0.16223787
Neto2	0.40193534	5.74021452	3.84069579	0.06417162	0.16229861
Nln	0.33002554	4.43820929	3.83959046	0.06420678	0.16231377
Ifnlr1	3.01331211	-0.4183534	3.83946529	0.06421076	0.16231377
Paox	1.06011925	0.96696285	3.83770127	0.06426691	0.16234735
Gcsh	-0.3610581	5.68617672	3.83709976	0.06428607	0.16234735
1700017G19	-1.2128143	0.70194423	3.83686785	0.06429346	0.16234735
Uox	-1.5923388	-0.8076337	3.83665366	0.06430029	0.16234735
Lrpprc	0.44646786	5.97469776	3.8364458	0.06430691	0.16234735
Dip2a	0.40724431	5.45376591	3.8353487	0.06434189	0.16235404
Hspbap1	0.64725647	2.22163898	3.8347446	0.06436116	0.16235404
Commd7	-0.3421542	4.6837121	3.83472222	0.06436187	0.16235404
Dpy30	-0.4513193	4.81348672	3.83302873	0.06441593	0.16235404
Echdc2	-0.6323674	2.26378607	3.83207704	0.06444632	0.16235404
Gm20751	1.9719124	-1.2658381	3.83190671	0.06445177	0.16235404
Tnfaip8	-0.4827769	5.03713846	3.8305227	0.06449601	0.16235404
Tox3	0.43432588	5.04677067	3.83024792	0.0645048	0.16235404
Tgfb1i1	-0.4416172	6.85124362	3.82962984	0.06452457	0.16235404
4930545L23F	1.54941459	0.66038367	3.82956038	0.06452679	0.16235404
Mir344c	2.53085309	-1.6073122	3.82950309	0.06452863	0.16235404
Tnks2	0.23010388	8.33239635	3.82949327	0.06452894	0.16235404
Grk4	0.57165671	4.63754829	3.82909585	0.06454166	0.16235404
Arhgap33	0.48776997	5.35412079	3.82882693	0.06455027	0.16235404
Serpinh1	-0.4913867	4.25435092	3.82857968	0.06455818	0.16235404
Scfd1	0.33516284	4.64103708	3.82780738	0.06458291	0.16237454
Etohd2	0.85851913	1.40307632	3.82488123	0.06467671	0.16254208
2610005L07F	0.34526553	6.35267353	3.82469349	0.06468273	0.16254208
Nudt18	-0.499823	3.81344971	3.82351281	0.06472063	0.16257936
Derl3	-2.0116265	-1.2662247	3.82319716	0.06473077	0.16257936
Lpar2	-0.8126147	1.631953	3.82219722	0.06476289	0.16261834
Naip2	1.14130371	0.32866889	3.82057096	0.06481517	0.1626764
Clic4	-0.4074651	8.64253682	3.82044471	0.06481923	0.1626764
Prr16	-0.3820666	4.14023859	3.81971178	0.06484281	0.1626939
Gas8	-0.5339526	3.60001775	3.81716477	0.06492482	0.16285796
Med4	-0.4270375	3.6371704	3.81546849	0.06497951	0.16292217
Mip	-1.9114703	0.37341419	3.81533908	0.06498368	0.16292217
Dnajb7	-1.6920196	-1.1210456	3.89366362	0.06504254	0.16294087
Rom1	0.78246357	1.51486163	3.8130826	0.06505652	0.16294087
Spon1	0.34161859	5.35663672	3.8129407	0.0650611	0.16294087
Senp7	0.21826349	6.97076646	3.81269813	0.06506894	0.16294087
9530052E02I	2.24535393	-0.9840896	3.81228535	0.06508228	0.16294087

Adamts13	0.50395857	4.12095028	3.81125134	0.0651157	0.16294087
A730017C20	-0.2613341	5.8893004	3.81079909	0.06513032	0.16294087
Dcbld1	0.68168875	1.98688471	3.81051076	0.06513965	0.16294087
Topaz1	-2.0079057	0.39184056	3.80998747	0.06515658	0.16294087
Gstm4	-0.6613303	1.67802119	3.80995939	0.06515749	0.16294087
Gpt2	0.29493868	4.97022842	3.80914136	0.06518396	0.1629576
Desi2	-0.2664017	6.81310931	3.80872476	0.06519745	0.1629576
Rbak	0.58754414	3.25545728	3.80792222	0.06522344	0.16298098
Qrsl1	0.49786239	2.65418748	3.80469549	0.06532807	0.16317304
Utp15	0.34863949	5.04699468	3.80452455	0.06533362	0.16317304
Trp53bp2	0.25771542	5.93993044	3.80389121	0.06535418	0.16318278
Klk11	-4.3746613	-1.816411	3.88297394	0.06537647	0.16319684
Vps13b	0.45116873	6.99017199	3.80222428	0.06540833	0.16321789
Aldh1b1	1.12587298	0.04492689	3.80191973	0.06541823	0.16321789
B230217O12	-0.6697465	1.77765777	3.80108599	0.06544534	0.16324395
G2e3	0.40516774	4.38339843	3.7988548	0.06551795	0.16335816
Wipi1	-0.4273144	4.24351571	3.79830974	0.0655357	0.16335816
Lin7b	-0.381695	3.68429884	3.79814221	0.06554116	0.16335816
Mtfr2	2.62814612	-1.0937227	3.79255238	0.06572355	0.16371749
Cav2	-0.3879497	5.18438214	3.79230767	0.06573155	0.16371749
Nfe2	-2.9325555	-1.1396766	3.79218769	0.06573547	0.16371749
4932443I19R	2.56714899	-1.4886467	3.79049905	0.06579069	0.16379367
Plxnb2	0.31254851	4.78627518	3.79022994	0.0657995	0.16379367
Rhoq	-0.3304215	6.22370793	3.78863358	0.06585176	0.16386193
Prrc2c	0.34647992	10.0574127	3.7883707	0.06586038	0.16386193
Srp68	-0.2763899	5.94571397	3.7876687	0.06588338	0.16387325
Arhgef25	-0.3111788	5.31720204	3.78672891	0.06591418	0.16387325
Zfp36	-0.5866382	4.95134142	3.78620537	0.06593135	0.16387325
Dagla	0.30863885	5.43864015	3.78619048	0.06593184	0.16387325
Tmco6	0.6991454	1.74919843	3.78520562	0.06596416	0.16389095
Gpr61	0.67434071	1.32576376	3.78495362	0.06597243	0.16389095
Aak1	0.33284374	9.72626354	3.78125836	0.06609385	0.16411384
Serhl	1.44429696	-0.4036211	3.78120334	0.06609566	0.16411384
Kcnk2	-0.2462436	7.24702257	3.78027548	0.06612619	0.16413528
2310039L15F	0.72422584	2.85952207	3.77915562	0.06616306	0.16413528
Mylk3	1.2480995	0.35676851	3.77911107	0.06616453	0.16413528
Gpr21	1.03620755	0.86234498	3.77890472	0.06617132	0.16413528
Mapk3	-0.3660701	7.23936848	3.77759653	0.06621443	0.16415167
Nudt4	-0.3616977	9.94797792	3.77716839	0.06622854	0.16415167
Snhg1	-0.4164875	4.49501652	3.77678023	0.06624134	0.16415167
Heatr5b	0.37986094	5.48811715	3.77667051	0.06624496	0.16415167
Gm14305	-0.5194334	2.61102588	3.77523923	0.06629218	0.16422714
Cttnbp2	0.41950816	6.72279741	3.77320596	0.06635934	0.16433287

Nfkbiz	0.67200423	2.15685519	3.77290057	0.06636943	0.16433287
Arf4	-0.2815503	8.85386897	3.77242369	0.06638519	0.16433287
Bmp4	-0.6184106	7.4127385	3.76956283	0.06647985	0.16450976
Smc2	0.36746594	4.51070417	3.76827767	0.06652243	0.16450976
Rprml	-0.5768925	3.00857413	3.76819586	0.06652514	0.16450976
Clrn1	2.71920488	-1.0221017	3.76811893	0.06652769	0.16450976
B3gnt3	-1.9630073	-0.0407022	3.76747354	0.06654908	0.16450976
Zfp459	0.6394622	3.00448716	3.76644127	0.06658332	0.16450976
Unc5a	0.36925259	4.9158948	3.76637144	0.06658563	0.16450976
Khsrp	-0.261278	6.76865432	3.7657663	0.06660571	0.16450976
Ict1	-0.4953873	4.30702121	3.76561129	0.06661086	0.16450976
Dlk2	0.70294142	1.71967125	3.7650166	0.0666306	0.16450976
Klhl4	0.30734462	4.81035219	3.76388913	0.06666804	0.16450976
Cpsf2	0.24675104	6.35806903	3.76380835	0.06667073	0.16450976
Nck2	-0.3368819	4.64343953	3.76347489	0.06668181	0.16450976
Arhgef3	0.35555057	5.69113974	3.7627251	0.06670673	0.16450976
Tsc2	0.38267144	5.85466584	3.76266972	0.06670857	0.16450976
Ucma	2.92988378	-0.7750345	3.75797378	0.06686491	0.16466235
Gpbp1l1	-0.3023715	6.29620693	3.75710242	0.06689396	0.16466235
D930015M05	1.68529731	0.26353852	3.75680089	0.06690402	0.16466235
Yars2	-0.3407165	4.04331491	3.75666762	0.06690846	0.16466235
Klc2	-0.3994297	5.48248672	3.75662692	0.06690982	0.16466235
Col5a1	0.42958045	3.40369357	3.75651406	0.06691359	0.16466235
Usp47	0.194875	7.1442615	3.7563903	0.06691772	0.16466235
Tbc1d9	0.30922347	5.7477432	3.75593986	0.06693275	0.16466235
Fbxl17	0.2667093	8.00944252	3.75557377	0.06694497	0.16466235
Trpm2	0.57706898	3.5551595	3.75553695	0.0669462	0.16466235
Lrfn5	0.38556375	5.23599728	3.75526259	0.06695536	0.16466235
Ash2l	0.2745367	5.00464747	3.75384282	0.06700278	0.16473761
Trnp1	-0.2740448	6.17704548	3.75328837	0.06702131	0.16474182
Gm6402	0.56278862	1.77517024	3.75159895	0.06707781	0.16480082
Nup37	-0.8500388	1.22698112	3.75142399	0.06708366	0.16480082
Ppp1ca	-0.4372257	5.51876175	3.75106172	0.06709579	0.16480082
Epha3	0.47882117	2.92841282	3.74974985	0.06713971	0.16480324
Gm9159	0.85037331	2.04989357	3.74957708	0.0671455	0.16480324
Zfp445	0.31624923	6.82886788	3.74952495	0.06714724	0.16480324
Fbxo9	-0.2300549	6.44977445	3.74605849	0.06726348	0.16496222
AI427809	-1.3243056	1.2099746	3.74493372	0.06730125	0.16496222
Hnrnpu	0.25035176	8.63388312	3.74443113	0.06731813	0.16496222
Dnajc2	-0.2711303	6.43593183	3.74442	0.0673185	0.16496222
Phf21a	0.26667645	6.60498082	3.7443722	0.06732011	0.16496222
Otop2	2.03876574	-0.9323722	3.74375927	0.06734071	0.16496222
Birc3	-0.5092287	4.01387721	3.7432249	0.06735867	0.16496222

Tmem209	0.31881141	4.22701157	3.74298721	0.06736666	0.16496222
Trpc7	-0.6335322	2.40421593	3.74278443	0.06737348	0.16496222
Scin	-2.4882748	-0.5378997	3.74257782	0.06738043	0.16496222
Rnf146	-0.2795405	5.45688731	3.7415788	0.06741404	0.16497732
Gm2518	1.67165682	-0.6249179	3.74115933	0.06742816	0.16497732
4930488L21I	1.19165309	-0.5601524	3.74013775	0.06746255	0.16497732
Gm20172	1.20004439	0.71115803	3.73989149	0.06747085	0.16497732
Kri1	0.519657	2.89361133	3.73972071	0.0674766	0.16497732
Gatad2b	-0.2064313	7.58803378	3.73884102	0.06750624	0.16497732
Stra13	-0.4894516	4.00883163	3.73845654	0.06751921	0.16497732
Eif3e	-0.257101	7.15623584	3.73839329	0.06752134	0.16497732
Dyrk2	0.55213474	3.24863198	3.73674433	0.06757696	0.16503335
Pcp2	-3.367747	-1.5685669	3.73671453	0.06757796	0.16503335
Zfp704	0.27466582	6.73180824	3.73540305	0.06762224	0.16508112
Vmn1r65	0.7673299	1.14576224	3.7351368	0.06763123	0.16508112
Gm16062	-1.3116288	-0.2612787	3.73348811	0.06768694	0.16514617
Cyp2b10	2.90024109	-1.7136659	3.73335038	0.0676916	0.16514617
Kndc1	0.47527072	6.21769581	3.73038264	0.06779203	0.16535001
Lrrc36	-1.4717993	-0.0502323	3.72979022	0.0678121	0.16535779
Fnbp1	0.22115873	6.73205691	3.72742687	0.06789223	0.16549195
Trim71	2.38607608	-0.8253599	3.72683088	0.06791245	0.16549195
Rnls	-0.8707822	1.0564206	3.72667317	0.06791781	0.16549195
Nap1l1	-0.2116768	8.68607673	3.72519029	0.06796816	0.16555676
Cpt1a	-0.3319617	5.93109532	3.72441875	0.06799438	0.16555676
Rpl15	-0.3207602	7.96298966	3.72439723	0.06799511	0.16555676
Tmem204	-0.4461601	3.94258334	3.72280143	0.06804937	0.1656477
1700034H15I	0.81236583	2.06910052	3.72221726	0.06806925	0.16565492
Nsd1	0.26974437	8.22829954	3.72046855	0.06812878	0.16573603
Dusp15	0.47941478	2.85404897	3.72024448	0.06813642	0.16573603
Rsu1	-0.3814278	6.85772679	3.71825202	0.06820434	0.16581997
Dolpp1	0.63064961	1.23214382	3.71823896	0.06820478	0.16581997
Mief2	-0.8176583	1.96346521	3.71664794	0.06825907	0.16591079
Micu2	-0.2898844	5.20628011	3.71474894	0.06832394	0.16602725
Psmd4	-0.3640054	5.3258604	3.71379024	0.06835671	0.1660657
Mrpl18	-0.3660179	5.44431311	3.71247912	0.06840157	0.1660969
Msrb1	-0.4101141	4.22744188	3.71155996	0.06843303	0.1660969
Donson	0.51021055	3.37025423	3.7114682	0.06843617	0.1660969
Eef1a1	-0.3231705	10.3156722	3.71143268	0.06843739	0.1660969
Eef1d	-0.3034722	5.27726507	3.70556348	0.0686387	0.16654423
Ppfia4	0.36663487	3.62828696	3.70460206	0.06867174	0.1665735
Pld5	-0.4585305	4.2995414	3.70385133	0.06869756	0.1665735
Spast	0.23462929	6.53538806	3.7037285	0.06870178	0.1665735
C920025E04I	-2.2957349	-1.1611135	3.70294911	0.0687286	0.16659728

Gnpda1	-0.3133425	4.59177892	3.70199388	0.06876148	0.16663574
Pdcd5	-0.3004563	6.90457778	3.70071456	0.06880554	0.16670128
Scg2	0.32769452	6.44632555	3.69924699	0.06885612	0.16678258
Kcp	1.7022899	0.03723685	3.69815645	0.06889374	0.16683245
Heatr2	-0.3289084	4.21724217	3.69674508	0.06894246	0.16690917
Rhoj	-0.4545748	5.38522687	3.69543067	0.06898787	0.16696871
Dyrk3	-0.7994358	1.85283218	3.69495349	0.06900437	0.16696871
Bin3	0.51338299	2.93786398	3.69455349	0.0690182	0.16696871
Siva1	-0.6915066	2.66080766	3.69245491	0.0690908	0.16710309
Dock10	0.47336137	5.74765679	3.6912229	0.06913347	0.16716501
Ctse	1.65416121	-0.3797391	3.68963394	0.06918854	0.16722013
Col19a1	0.57036865	3.91312099	3.68948106	0.06919385	0.16722013
Slc6a18	0.85514974	1.13879394	3.68822975	0.06923726	0.16722013
Dhtkd1	1.16140436	1.49539204	3.68813921	0.0692404	0.16722013
Hnf1a	-1.9253017	-0.9538867	3.68810399	0.06924162	0.16722013
BC031181	-0.3867193	6.05618873	3.68691318	0.06928296	0.16727873
Tead4	1.89692695	0.04047983	3.68512305	0.06934517	0.16738766
Crocc	0.82573451	2.06079015	3.68420023	0.06937726	0.16742387
H2afj	-0.5404098	3.5416245	3.68277227	0.06942696	0.16750253
2310022A10I	-0.4274301	3.59599026	3.68187809	0.06945809	0.16751365
Zfp764	-0.6890868	2.98678652	3.68135801	0.06947621	0.16751365
Kif1b	0.34231376	9.93094244	3.68116691	0.06948287	0.16751365
Lin7c	-0.2305088	7.8688952	3.67959294	0.06953774	0.16754933
Atp5c1	-0.2690899	8.18712895	3.67940479	0.06954431	0.16754933
Man2c1	0.67292839	2.65960172	3.67909457	0.06955513	0.16754933
Rps23	-0.3748356	7.24528783	3.67876649	0.06956658	0.16754933
Vit	0.88174209	2.21496262	3.67765305	0.06960544	0.16754933
Taf13	-0.2751514	6.57924145	3.67746637	0.06961196	0.16754933
Acaca	0.42887717	5.67826567	3.6773105	0.0696174	0.16754933
Abcf3	-0.3341833	5.02089209	3.67307073	0.06976566	0.16776492
Slc6a8	-0.2630266	6.34157848	3.67259215	0.06978242	0.16776492
Capza2	-0.2236571	8.06231364	3.67254869	0.06978394	0.16776492
Snora23	1.33386237	0.98583809	3.67189042	0.069807	0.16776492
Eya4	0.74457243	2.43857973	3.67186826	0.06980777	0.16776492
Top2b	0.24905839	8.14488493	3.67181186	0.06980975	0.16776492
Tsen2	0.58446421	2.55053388	3.6697131	0.06988332	0.16790055
Akr1c14	-0.4237994	3.92824185	3.66676086	0.06998697	0.16810834
Pvr	0.54874075	2.53603465	3.66576822	0.07002186	0.16815092
Ccdc134	0.7319341	0.62097452	3.66397578	0.07008492	0.16822763
Pcdhgb2	0.59474087	2.11965788	3.66301393	0.07011878	0.16822763
Gm5803	1.46357929	-0.6594237	3.66242005	0.0701397	0.16822763
Hydin	1.21750746	0.40792578	3.66213289	0.07014981	0.16822763
B3galt1	0.22792286	5.80671302	3.66199717	0.0701546	0.16822763

Arxes2	0.45854424	3.63078278	3.66186322	0.07015932	0.16822763
Pet2	1.937266	-0.6324574	3.6614457	0.07017403	0.16822763
Cenpm	-2.3602553	-1.5130958	3.65681983	0.0703373	0.16845524
Tagln2	-0.5178622	5.7784713	3.65654261	0.0703471	0.16845524
Cxcl1	-4.8483337	-1.9014253	3.65632418	0.07035482	0.16845524
Nle1	1.16037597	-0.0880185	3.6562634	0.07035697	0.16845524
Casc4	0.26582663	8.2105552	3.65615817	0.07036069	0.16845524
Rfwd2	-0.2221755	7.26114381	3.65583379	0.07037216	0.16845524
Mid2	0.29219249	5.67538724	3.65529678	0.07039115	0.16845953
Oprm1	-0.9243626	1.91344994	3.65447326	0.07042029	0.1684881
Abhd5	-0.3423577	6.67456623	3.65382885	0.0704431	0.16850152
Tcap	-0.9427723	0.86915898	3.65310052	0.07046889	0.16852206
Scgn	2.79538976	-1.7738319	3.65169986	0.07051852	0.16856977
Il13ra1	-0.4068227	4.87857504	3.65156607	0.07052326	0.16856977
Gm12429	0.78596908	0.85949264	3.65024598	0.07057008	0.1686332
AF357425	0.98698218	3.01202693	3.64984713	0.07058423	0.1686332
Tnfaip3	-0.6084088	2.85126605	3.64896401	0.07061557	0.16865771
Abhd14a	0.60418206	2.80306502	3.64858802	0.07062892	0.16865771
AK010878	-0.3826772	3.75420995	3.6470027	0.07068525	0.16871427
Gm20139	1.34541737	-0.1396949	3.64695172	0.07068706	0.16871427
Malsu1	-0.3243903	4.73281261	3.64544484	0.07074064	0.16879333
Nphp1	-0.4082884	3.83450692	3.64505135	0.07075464	0.16879333
Pcdhga9	0.54697504	2.52573333	3.64368336	0.07080334	0.1688305
Hmg20b	-0.8730121	2.17156615	3.64345844	0.07081135	0.1688305
Myo6	0.34434233	6.39830014	3.64227816	0.07085341	0.1688305
Ino80c	-0.3684084	5.69305021	3.64142558	0.0708838	0.1688305
Clec5a	-1.2844238	-0.1262084	3.64122355	0.07089101	0.1688305
Ccdc25	-0.2417981	5.86118626	3.64090851	0.07090224	0.1688305
C2cd5	0.39643467	5.79741466	3.64081276	0.07090566	0.1688305
Adcy6	0.34777909	4.09255721	3.64038012	0.07092109	0.1688305
Rgs4	-0.2610362	9.56276024	3.64026093	0.07092535	0.1688305
Nup50	0.25888189	5.45782005	3.63729435	0.07103129	0.16904162
Dnm3	0.40513001	8.50630383	3.63639883	0.07106331	0.16905072
Zfp128	-0.536378	2.6900895	3.636222	0.07106964	0.16905072
Slc25a16	-0.2823688	5.57022558	3.63327822	0.07117502	0.1692026
Gng7	0.36309789	5.43551438	3.63298173	0.07118564	0.1692026
Psmb4	-0.3492977	5.59698976	3.63288864	0.07118898	0.1692026
Syf2	-0.4319501	5.63618597	3.63250904	0.07120258	0.1692026
Dr1	-0.3025892	5.49912096	3.62838471	0.0713506	0.16951321
Ppargc1a	0.29969893	7.95652804	3.62320062	0.07153715	0.1699121
Kit	0.27479406	4.87311702	3.62275564	0.07155319	0.1699121
Ipo9	0.26884439	7.01402493	3.62101903	0.07161582	0.17001961
Fam175b	-0.2984753	4.53151692	3.61983519	0.07165856	0.17007985

Emc2	-0.3006815	6.48024617	3.61865348	0.07170124	0.17013993
Arhgap21	0.35551726	8.18769952	3.61749081	0.07174327	0.17019843
Zbtb45	0.52193878	2.41541769	3.61663002	0.0717744	0.17023106
Gatad1	-0.2466897	7.04557195	3.61452353	0.07185066	0.17031042
Dohh	-0.5018991	3.27307213	3.61443671	0.0718538	0.17031042
Rad54l2	0.22707144	5.63481962	3.61426493	0.07186002	0.17031042
Trpc1	0.3588145	4.59398598	3.61305354	0.07190393	0.17037325
Dnmt3b	1.26739486	0.32546079	3.61244874	0.07192586	0.170384
S100a10	-0.5802517	5.84680724	3.61163087	0.07195552	0.17038574
Anxa3	-0.4644786	5.81377732	3.61146939	0.07196138	0.17038574
Pspc1	0.34433202	5.2308124	3.60780569	0.07209447	0.1706596
Fuom	-0.445398	3.47949281	3.60330478	0.07225836	0.17092532
Slc25a30	0.87924201	1.14282659	3.603229	0.07226112	0.17092532
D030047H15	0.97798	0.23058066	3.60318186	0.07226284	0.17092532
Tmem223	-0.3677639	3.72335312	3.60280665	0.07227652	0.17092532
Gpr133	-0.6136359	2.64997106	3.60124262	0.07233359	0.17098678
Sh2d7	-0.9082264	1.00357488	3.60113763	0.07233742	0.17098678
Lsm2	-0.5570579	4.23845865	3.60061517	0.0723565	0.17099061
S1pr5	0.6700558	2.05751533	3.59852544	0.07243285	0.17112977
Ppp2r2d	-0.3158945	4.71076883	3.59557839	0.07254069	0.17126291
Dhx37	0.47757978	2.78793217	3.59555522	0.07254154	0.17126291
Uqcr10	-0.4354447	5.90447573	3.59555202	0.07254166	0.17126291
D7Ertd443e	0.64279969	1.54308926	3.59446594	0.07258145	0.17127747
Unc45b	-2.5068934	-0.6686464	3.59442923	0.0725828	0.17127747
Ccdc39	0.47095932	4.3535668	3.59334301	0.07262262	0.17133016
Ctbp1	-0.3644906	5.30065741	3.59229163	0.07266119	0.17136366
Trim66	0.43078217	5.4375356	3.59200226	0.07267181	0.17136366
Cdh3	-1.2647193	0.55645402	3.5905749	0.07272421	0.17143402
Speg	0.31667057	5.58177323	3.59023648	0.07273665	0.17143402
Nek7	-0.2952688	6.94421238	3.58964798	0.07275827	0.17144373
Dand5	-0.3979582	3.55729578	3.58452425	0.07294686	0.17184677
Kdelr3	0.8776897	1.07251434	3.58359682	0.07298105	0.17185705
4933428G20	0.85409176	1.69656661	3.58345435	0.07298631	0.17185705
Endou	-0.6586432	1.87124236	3.58286979	0.07300787	0.17186651
Kcnip3	-0.2036381	7.64498816	3.58201044	0.07303959	0.17189986
4930599N23	-2.4896423	-2.0599011	3.5806081	0.07309138	0.17198043
Lgals9	-0.4677478	4.40799855	3.57912741	0.07314611	0.17206787
Ptges3l	-1.0776148	1.05137096	3.57797556	0.07318872	0.17212676
Trp53i13	1.58620302	-0.2615405	3.57688299	0.07322916	0.17214902
Dgkb	0.36596754	7.92645672	3.57677039	0.07323333	0.17214902
Recql	0.36858157	3.96390845	3.57493532	0.07330132	0.17226749
Noc2l	0.39182876	4.00868273	3.57188761	0.07341439	0.17240067
Dpysl2	0.25757774	8.72760806	3.57180012	0.07341764	0.17240067

Rnpc3	0.49783256	4.82241866	3.57166654	0.0734226	0.17240067
Frg1	-0.2676737	4.81586465	3.57125957	0.07343772	0.17240067
4931414P19I	1.14429461	1.11634917	3.57052742	0.07346492	0.17240067
Prelid2	-1.9784655	-0.7033293	3.57031718	0.07347274	0.17240067
Ptk2	0.37448166	6.57805731	3.56962805	0.07349836	0.17240067
Luc7l	0.31251942	5.4931452	3.56961653	0.07349879	0.17240067
Bhlhe22	-0.3144203	5.19943466	3.568052	0.07355699	0.1724623
Fezf2	-0.2974617	5.18090564	3.56796376	0.07356027	0.1724623
Pgam1	-0.2341119	9.43813812	3.56727202	0.07358603	0.17248139
Ddb2	0.76049936	1.05610744	3.56631051	0.07362184	0.17248421
Ptges2	-0.480601	2.3907811	3.565978	0.07363423	0.17248421
Celf1	0.2685941	8.13798273	3.56547427	0.073653	0.17248421
Wdr82	-0.2178684	6.39638875	3.56469753	0.07368196	0.17248421
Dynlt1a	0.3021458	4.34694742	3.56469273	0.07368214	0.17248421
Bc1	3.64651908	-1.8486001	3.56440477	0.07369288	0.17248421
Chodl	-2.5362611	-1.4040797	3.56292914	0.07374794	0.17257185
Wdr24	0.6245493	2.33608061	3.56168839	0.07379428	0.17263903
Stx4a	-0.3320625	6.39747626	3.55947469	0.07387703	0.17279136
Proca1	-0.6232248	1.88584222	3.55885302	0.07390029	0.1728045
Rbm34	-0.2548615	5.32933223	3.55746298	0.07395232	0.17288491
Cd3g	-2.4887231	-1.3715452	3.5563385	0.07399445	0.17288919
Lmo3	-0.2869849	7.13282891	3.55622336	0.07399876	0.17288919
Slc7a6	0.67520675	2.89527166	3.55600079	0.07400711	0.17288919
Trappc6b	-0.3057621	7.79666568	3.55540962	0.07402927	0.17289056
Hoga1	-1.5979929	-0.0458098	3.5549565	0.07404626	0.17289056
Otulin	-0.5278776	3.54519405	3.554573	0.07406065	0.17289056
4930451C15I	-1.2334788	0.31137663	3.55315679	0.0741138	0.17297342
Rttn	0.63918347	2.57433777	3.55215525	0.07415141	0.17298523
Pan3	0.22598054	6.70390034	3.55208151	0.07415418	0.17298523
Dnajc4	0.60017594	2.40710364	3.55133878	0.07418209	0.17299524
Sec16a	0.26214172	6.43811088	3.5510275	0.07419379	0.17299524
Krt73	-1.961144	-1.1365842	3.54886094	0.07427529	0.17308937
Nxf1	0.24943061	5.93111729	3.54867626	0.07428224	0.17308937
Fam183b	-1.7011949	0.14856088	3.54854516	0.07428717	0.17308937
Ddx31	0.7651919	1.36652736	3.54787765	0.07431231	0.17309154
Tmem145	0.67995179	2.44946133	3.5465716	0.07436151	0.17309154
Egfl6	1.20332575	0.88646447	3.54643365	0.07436671	0.17309154
Tppp	-0.2243495	8.81381514	3.54603604	0.0743817	0.17309154
C630043F03I	-0.4171428	3.2595682	3.54559187	0.07439845	0.17309154
Gm10220	0.76320072	1.69414283	3.54546356	0.07440329	0.17309154
Uba52	-0.3916697	6.7196023	3.54523798	0.0744118	0.17309154
Grm8	0.70087638	2.66462651	3.54362859	0.07447253	0.17319169
Arpc1b	-0.6189769	6.03862312	3.54290611	0.07449982	0.17321401

Fam115a	0.34314276	6.74609982	3.54228891	0.07452313	0.17322711
Nt5m	-0.4107507	3.91316768	3.54119623	0.07456443	0.17324644
Cecr2	0.37153966	3.27743897	3.54113298	0.07456683	0.17324644
Tamm41	0.62663656	2.28639379	3.54013452	0.07460459	0.17329308
Unc13d	-1.9418471	-0.8819921	3.5394775	0.07462945	0.17329652
Sec22b	-0.2346359	7.09426359	3.53888585	0.07465185	0.17329652
Zfp341	-0.537839	3.38565362	3.53850851	0.07466613	0.17329652
Tmtc3	0.38341763	5.40573068	3.53822595	0.07467684	0.17329652
Samd14	-0.6975538	2.17700552	3.5366369	0.07473705	0.17339517
Kazald1	-0.8031571	1.58188185	3.53590157	0.07476493	0.17340488
P2rx7	0.67614963	2.03038646	3.53559294	0.07477664	0.17340488
Tead1	0.29648509	7.27776457	3.5350304	0.07479798	0.17341332
Kmo	-1.0514401	0.99366397	3.53421253	0.07482903	0.17343764
Usp49	0.41609029	3.96459954	3.53364276	0.07485066	0.17343764
Gm16617	-2.0724846	-0.99522	3.53335487	0.07486159	0.17343764
Gm9899	-0.3637589	3.86221393	3.5320398	0.07491156	0.17348863
Zfp319	0.41923114	3.1469779	3.53184361	0.07491902	0.17348863
Setx	0.37755877	7.13268707	3.5303717	0.07497501	0.17357723
Pcmt1	-0.2151297	6.42797914	3.52934703	0.07501401	0.17362649
Ptp4a3	-0.4682244	3.47659249	3.52863673	0.07504106	0.17364806
Sh3bp5	-0.2679393	6.78191508	3.52753002	0.07508322	0.1737046
Klf12	-0.2145056	7.35340552	3.52612483	0.0751368	0.17374819
Rpp25l	-0.7143605	2.05738718	3.5261055	0.07513754	0.17374819
Zmym2	0.30740321	8.1176782	3.52504398	0.07517805	0.17380083
Ncald	-0.2306118	7.7043195	3.52340878	0.07524049	0.17390415
Cacna1i	0.43047233	4.05638113	3.52179191	0.0753023	0.17400594
Cmya5	0.50922258	3.28475375	3.51967829	0.07538318	0.17408171
Setd8	-0.2795721	7.28392501	3.51944179	0.07539223	0.17408171
Grtp1	0.56835048	3.25590908	3.51911942	0.07540458	0.17408171
Armc4	1.49229359	-0.9967115	3.51907779	0.07540617	0.17408171
Tpm2	-0.5219131	6.33843715	3.51650335	0.07550486	0.17426801
Galt	0.30405209	4.43305	3.51578796	0.07553231	0.17426801
Mir28b	2.22537346	-0.4985451	3.51558124	0.07554024	0.17426801
Tmem97	-0.6611505	2.13363819	3.5129823	0.07564008	0.17444374
Fbxo43	1.15499636	-0.5212013	3.51267119	0.07565204	0.17444374
Tyrp1	4.56926959	-1.86409	3.51172333	0.07568849	0.17448672
Ddx21	0.27400306	4.80739924	3.50892204	0.07579635	0.17467853
Il10ra	1.42276775	0.14749932	3.50798784	0.07583235	0.17467853
Eid3	2.92107	-1.901409	3.50792055	0.07583495	0.17467853
Crabp2	-0.6254262	6.22136604	3.50771107	0.07584302	0.17467853
Tbca	-0.3894858	6.43366701	3.50596729	0.0759103	0.17479238
Ndufs7	-0.3574148	5.10040801	3.50510845	0.07594346	0.17480698
Spdef	-2.8203875	-1.9795163	3.50483959	0.07595385	0.17480698

E130112N10	2.21586032	-0.6904843	3.50441679	0.07597018	0.17480698
Mblac1	-0.6139828	2.67170348	3.50375142	0.07599589	0.17482508
Pcdhgc5	0.42527123	4.78780458	3.50263403	0.07603909	0.17487892
Flnb	0.24438902	6.1926105	3.50222273	0.076055	0.17487892
Wtap	0.22498255	6.35981409	3.50135694	0.07608851	0.1749149
Cystm1	-0.6583278	2.13882184	3.49964827	0.07615468	0.17496264
Ptprg	0.26798479	6.91792331	3.49894146	0.07618207	0.17496264
Ptx3	-2.050294	-1.326802	3.49816913	0.07621201	0.17496264
Trp53bp1	0.41392379	5.47033742	3.49804387	0.07621687	0.17496264
2810442I21R	3.44615168	-2.1376449	3.49795222	0.07622042	0.17496264
Kcnk6	0.80634709	1.35946233	3.49771674	0.07622956	0.17496264
Tanc2	0.40397304	8.5132507	3.49759422	0.07623431	0.17496264
Cdr2	-0.5688869	2.74416149	3.49639547	0.07628083	0.17502839
Mcmdc2	0.72744198	2.5289707	3.49562392	0.07631078	0.17505613
Rnf181	-0.3210924	6.26670878	3.49401092	0.07637346	0.17513526
Sp9	0.65313121	1.30070133	3.49381584	0.07638104	0.17513526
Ik	-0.1957118	8.56539055	3.49158723	0.07646774	0.17529303
4933412E12I	-0.6506359	1.84800618	3.48976869	0.07653858	0.17534782
Xrcc6bp1	0.79731072	1.74874407	3.48969361	0.0765415	0.17534782
Agbl3	-0.434787	3.1280662	3.48951637	0.07654841	0.17534782
Gm4814	2.33634817	-1.5837911	3.48913569	0.07656325	0.17534782
Tuba1b	-0.2159887	9.23594797	3.48757969	0.07662394	0.17540137
Zkscan16	0.4217051	5.24616112	3.48755401	0.07662494	0.17540137
Rbm42	-0.3447034	4.24635712	3.48715923	0.07664035	0.17540137
Rapgef2	0.26908991	7.49215571	3.48508574	0.07672133	0.17549399
Serpina10	3.4912012	-1.9734045	3.48474635	0.07673459	0.17549399
Alg9	0.57928075	2.99543005	3.48417142	0.07675707	0.17549399
Abca3	0.40241719	4.11377564	3.48398972	0.07676417	0.17549399
Wnt2b	0.44787619	2.90172608	3.48383048	0.0767704	0.17549399
Hist1h4d	-0.6001869	2.81423744	3.48333929	0.07678961	0.17549696
Osbpl1a	-0.2252834	8.12679639	3.48203078	0.07684082	0.17557302
Rpl36	-0.5229104	5.25284376	3.48144795	0.07686364	0.17558421
Klhl9	0.21164414	7.39308747	3.48032387	0.07690768	0.17564385
Wdr5b	-0.8580937	1.16772343	3.47857736	0.07697616	0.17575926
4930402H24I	0.34174128	5.56917673	3.47759792	0.07701459	0.17580604
Pfdn5	-0.4966685	5.7391927	3.47620997	0.0770691	0.1758867
Ppih	-0.4744379	3.16833844	3.47543749	0.07709945	0.1758867
Frmd7	1.32660338	-0.1446833	3.47524367	0.07710707	0.1758867
Slitrk5	0.36576835	5.86905806	3.47487009	0.07712175	0.1758867
Rtn2	0.60048308	2.61842012	3.47427982	0.07714496	0.17589868
B4galnt1	-0.3159567	5.39993322	3.47360806	0.07717139	0.17590927
Ttc4	-0.3080232	3.91169362	3.47289417	0.07719948	0.17590927
Lgals3bp	-0.4660358	4.89866269	3.47245128	0.07721692	0.17590927

Cebpd	-0.8548768	1.35921377	3.47183068	0.07724136	0.17590927
Arid1a	0.33280097	8.05983056	3.4712708	0.07726341	0.17590927
Slc52a2	1.15723764	0.70323119	3.47119289	0.07726648	0.17590927
Bex2	-0.3264112	6.92183574	3.47096868	0.07727532	0.17590927
Gosr1	-0.3153042	5.27713659	3.46937741	0.07733805	0.17601117
Atp6v0a4	1.11240056	0.14184209	3.46787287	0.07739742	0.17609446
Hnrnpc	-0.2157571	7.27085951	3.46710567	0.07742772	0.17609446
2410076I21R	0.95795168	0.47192172	3.46683243	0.07743851	0.17609446
Wdyhv1	-0.3555456	4.05496611	3.46662878	0.07744656	0.17609446
B230312C02	2.86953178	-1.0641934	3.46435479	0.07753646	0.17620802
Nxpe4	0.43556621	2.96070174	3.46409579	0.0775467	0.17620802
Cdr1	-0.4664897	9.91723274	3.4637384	0.07756085	0.17620802
Ints8	0.30216778	5.49096989	3.46354607	0.07756846	0.17620802
P2rx5	2.18488676	-0.9221441	3.46169365	0.07764182	0.17633377
Ikzf4	0.27272972	4.19381993	3.46033049	0.07769585	0.17636066
Ska3	0.66042204	1.45807061	3.46017844	0.07770188	0.17636066
Homer1	-0.2785344	8.40981841	3.45961228	0.07772434	0.17636066
Rpp40	0.49813425	2.58937608	3.45922934	0.07773953	0.17636066
Agpat6	-0.3302022	4.13480936	3.45900592	0.0777484	0.17636066
Msx2	-0.8179467	2.29540939	3.45857595	0.07776546	0.17636066
Slc7a5	0.3683756	4.14321219	3.45821773	0.07777968	0.17636066
Tex40	-0.857487	2.10217503	3.45654471	0.07784614	0.17637459
Aif1	-0.863908	2.15680055	3.45634044	0.07785426	0.17637459
Tbc1d4	0.51961815	3.07730751	3.45625723	0.07785757	0.17637459
Tubd1	-0.8304569	1.56401701	3.4562501	0.07785785	0.17637459
Cdk11b	-0.1990481	7.39709748	3.45516157	0.07790114	0.17643184
Ppp4r4	0.39169651	4.90833308	3.45466613	0.07792085	0.17643569
Polq	0.80004898	2.63055267	3.45313413	0.07798183	0.17651314
Invs	0.33147891	4.82609087	3.45290151	0.0779911	0.17651314
Gpr12	-0.4394317	4.05832662	3.45098112	0.07806763	0.17660237
Dok3	1.83831654	-1.3432992	3.45053399	0.07808546	0.17660237
Rgs3	-0.442365	4.10032418	3.44963084	0.07812149	0.17660237
Trim11	-0.4494732	2.75592502	3.44938268	0.0781314	0.17660237
Cdc14b	0.38640618	4.35642075	3.44809414	0.07818285	0.17660237
Pcdha10	1.35443083	-0.3966167	3.44776975	0.07819581	0.17660237
Gm19710	1.33841247	0.31012239	3.44767988	0.0781994	0.17660237
Dock6	0.36373809	3.65249162	3.44753901	0.07820502	0.17660237
Scg3	0.38525232	6.66561104	3.44743321	0.07820925	0.17660237
Rpl39	-0.4487796	7.34060567	3.44696667	0.0782279	0.17660237
Fkbp15	0.29354223	4.74414784	3.44694297	0.07822884	0.17660237
Stoml2	-0.3772462	4.28592508	3.44559183	0.07828287	0.17668282
Tsga10	0.30906179	4.98707404	3.44514985	0.07830056	0.17668282
Pomc	1.98597365	0.35685037	3.44459294	0.07832284	0.17669241

Zcchc8	0.34934052	3.8751708	3.44336971	0.07837182	0.1767622
Pot1b	0.44138498	3.44286884	3.44121724	0.0784581	0.1769072
Seh1l	-0.3298586	5.64471097	3.44069992	0.07847885	0.1769072
0610011F06I	-0.3797809	3.61598656	3.44000514	0.07850673	0.1769072
Filip1	0.43824936	3.97005883	3.43956032	0.07852458	0.1769072
Tmem181b-|	0.54230285	5.11164669	3.43836781	0.07857247	0.1769072
Acyp1	-0.3906844	5.19441005	3.43819721	0.07857933	0.1769072
Trim21	-0.4832084	3.83998389	3.43717481	0.07862042	0.1769072
Coa5	-0.2781259	7.00338621	3.43706483	0.07862484	0.1769072
Ripk1	-0.4346695	3.73532796	3.43668229	0.07864022	0.1769072
Hrg	-1.3821364	1.03122995	3.43563249	0.07868245	0.1769072
Atp6v0d1	-0.2578028	7.16095009	3.43542545	0.07869078	0.1769072
Sult4a1	-0.2297432	7.03888384	3.43539211	0.07869213	0.1769072
BC030500	-0.4126618	3.90057048	3.43534963	0.07869384	0.1769072
Wdr47	0.36873021	6.50006207	3.43451221	0.07872755	0.1769072
Chadl	-0.7327143	1.07999139	3.43415988	0.07874174	0.1769072
Slc25a43	2.60075301	-1.5902955	3.43395943	0.07874981	0.1769072
Specc1l	-0.256753	6.43890441	3.43311816	0.0787837	0.1769072
B4galt1	-0.4221484	4.19173818	3.433103	0.07878432	0.1769072
Ptges3	-0.2810049	7.90167011	3.43214222	0.07882305	0.1769072
Hspb7	1.00092608	0.47307852	3.43146226	0.07885047	0.1769072
Camkmt	-0.3161127	4.00481931	3.43034568	0.07889553	0.1769072
Zfp329	0.27041263	6.15661559	3.43027158	0.07889852	0.1769072
Ypel2	-0.2098612	6.38247528	3.42947205	0.0789308	0.1769072
Chmp6	-0.510312	2.23547176	3.42845506	0.07897189	0.1769072
Adhfe1	-0.3533658	5.11547406	3.42765805	0.0790041	0.1769072
Fam204a	-0.377512	5.54557337	3.42741184	0.07901406	0.1769072
Appl2	0.24132425	6.30559206	3.42731295	0.07901806	0.1769072
Sac3d1	-0.6420599	2.31542067	3.42731282	0.07901806	0.1769072
Mrps30	-0.4697375	3.23911452	3.4271831	0.07902331	0.1769072
L1cam	0.44763996	6.82476182	3.42715384	0.07902449	0.1769072
Kbtbd7	0.29424161	5.2378738	3.42710828	0.07902633	0.1769072
Osbpl8	0.20561817	7.70354913	3.42697894	0.07903157	0.1769072
Chad	3.22111573	-1.747927	3.42696563	0.0790321	0.1769072
Sec14l2	-0.5642058	2.46746972	3.42613939	0.07906553	0.17694159
Gm9767	-1.8066458	-1.0617499	3.42538908	0.0790959	0.17696912
Slc39a11	0.62963846	1.72164444	3.42362677	0.07916729	0.17708839
Rps24	-0.3339354	7.28769221	3.42261831	0.07920817	0.17713939
Sort1	0.26601509	7.68974787	3.42031188	0.07930176	0.17730822
Txnl1	-0.2277288	7.14529889	3.41889259	0.07935942	0.17739664
Amph	0.30273237	6.92174511	3.4177273	0.0794068	0.17742233
Stk17b	-0.3336236	5.75517255	3.4177189	0.07940714	0.17742233
Cd46	1.21271429	1.16625754	3.41604535	0.07947524	0.17752182

Lrrtm1	-0.3673359	4.64472738	3.41573395	0.07948792	0.17752182
Smarcb1	-0.3189289	4.78304177	3.41510132	0.07951368	0.17753889
Prkcd	-0.48	4.08542494	3.41430144	0.07954627	0.17757118
Dock4	0.41067324	6.18838306	3.41311973	0.07959444	0.17757902
Hspa12a	0.28046175	8.26681948	3.41271736	0.07961086	0.17757902
Tshr	2.14739825	-0.4251946	3.41266581	0.07961296	0.17757902
Cd180	-0.6368624	2.7357235	3.41243683	0.0796223	0.17757902
BC005764	0.67190584	2.18317284	3.41145368	0.07966242	0.17760437
Pcdhac1	1.30008206	-0.0962579	3.41126986	0.07966993	0.17760437
Slc26a1	1.66114242	-0.6319388	3.4106641	0.07969466	0.17761909
Arhgef2	0.45189834	5.56237743	3.40938543	0.0797469	0.17765698
Pou3f3	0.29637189	5.33868191	3.40936013	0.07974794	0.17765698
Zfp385b	-0.2886147	6.65665764	3.40793842	0.07980607	0.17771443
Cyp7b1	0.45385233	2.44135494	3.40784214	0.07981001	0.17771443
Lphn2	0.24807278	6.35521224	3.40473804	0.07993712	0.17788074
1700007F19I	-1.026241	0.05421021	3.40471661	0.079938	0.17788074
Zfand2a	-0.2563064	7.00570716	3.40468767	0.07993918	0.17788074
01-Mar	-0.3566749	5.07859103	3.40341392	0.07999141	0.17795653
Snx27	0.20870687	7.29329358	3.40268722	0.08002122	0.17796438
Mtx3	0.29418347	5.99207355	3.40196389	0.08005091	0.17796438
Gp1ba	1.54581026	0.3865583	3.40190405	0.08005337	0.17796438
Pm20d2	-0.3021123	4.21869945	3.40139896	0.08007411	0.17796438
Tex30	-0.8084618	2.38532697	3.40111475	0.08008578	0.17796438
Tmem219	-0.7454775	2.53294204	3.40053626	0.08010954	0.17797549
Nxt1	0.62044727	2.54312673	3.4001086	0.08012712	0.17797549
Zfyve28	0.51585735	3.78121154	3.39917909	0.08016533	0.17802
Map3k6	-0.5882933	1.76105039	3.39643823	0.08027813	0.17819768
Cnot10	0.35978447	4.35910618	3.39586935	0.08030157	0.17819768
Smad5	-0.2452049	6.179103	3.39559621	0.08031282	0.17819768
2310009B15I	-0.6970064	2.1245251	3.39534956	0.08032299	0.17819768
Mageb16-ps	1.72194226	-0.6955082	3.3945962	0.08035404	0.17819768
Aldh1a1	-0.4341539	7.64304832	3.39458524	0.08035449	0.17819768
Npepps	0.30117251	6.32023894	3.39322678	0.08041053	0.178277
Vps4a	-0.2721877	5.55781122	3.39283596	0.08042666	0.178277
Idua	0.43178486	3.57886831	3.39060281	0.0805189	0.17833816
Hdac9	0.21738825	6.45270884	3.39030734	0.08053112	0.17833816
Rnf14	-0.191287	8.3873547	3.39012489	0.08053866	0.17833816
6430571L13I	-1.2465413	0.33278228	3.38997283	0.08054495	0.17833816
Cacna2d2	0.40728595	4.99637062	3.3891327	0.08057969	0.17833816
Map3k7cl	1.24706387	0.50452359	3.38900708	0.08058489	0.17833816
Inpp5a	-0.3182106	4.56294931	3.38891535	0.08058868	0.17833816
Ndufa2	-0.4041731	5.45859902	3.388641	0.08060003	0.17833816
Exoc8	0.29439235	4.65866988	3.38820423	0.08061811	0.17833816

Atf3	-1.4715665	1.54733601	3.38457388	0.08076854	0.17863058
Tbc1d5	0.23098985	6.45387232	3.38224079	0.08086538	0.1788044
Lman1	0.41578373	5.02057679	3.38174796	0.08088586	0.17880931
Gpr135	-1.7295261	-0.6005813	3.38074359	0.0809276	0.17886123
Psenen	-0.5235173	5.2136911	3.37985301	0.08096464	0.17889847
Lrrc16b	1.01032133	2.98873128	3.37908837	0.08099646	0.17889847
Efhb	0.82723033	0.67924051	3.37898315	0.08100083	0.17889847
Fam228b	0.90791453	1.62913522	3.37858249	0.08101751	0.17889847
Rps3a1	-0.3313952	7.93110802	3.37733505	0.08106946	0.17897284
Myo1a	2.42903633	-1.8296905	3.3746	0.0811835	0.17918421
Snapin	-0.3549735	7.51446308	3.37250597	0.08127093	0.17933678
Oaz1	-0.3120519	5.13371728	3.37081828	0.08134148	0.17945204
Pirb	-1.2372825	-0.2529225	3.37031391	0.08136258	0.17945816
Mfsd6	0.32954875	7.207852	3.36858829	0.08143481	0.17957704
Dctpp1	-0.5683533	1.63545479	3.36672045	0.08151308	0.17970917
2810025M15	-0.5090354	2.41788734	3.36410565	0.08162279	0.17991056
Rpl17	-0.3176153	6.86589986	3.3627996	0.08167765	0.17994763
Trim9	0.37103167	8.22979145	3.36276135	0.08167926	0.17994763
Chmp2b	-0.3488485	6.34152136	3.36211734	0.08170633	0.17994763
Bbs2	0.34379594	5.17881808	3.36182846	0.08171848	0.17994763
Xpr1	0.25342281	7.89515667	3.36104159	0.08175157	0.17994763
Slc11a2	0.32590856	4.99603711	3.36104018	0.08175163	0.17994763
Slc25a20	-0.4327018	4.78265158	3.36064631	0.0817682	0.17994763
Phc2	-0.4020202	4.79911662	3.35980134	0.08180376	0.17996696
Zfhx2	0.33900202	5.15266286	3.35956456	0.08181373	0.17996696
Ube2h	-0.2115941	7.70872382	3.3590458	0.08183558	0.1799746
Fndc3b	-0.201242	5.7564573	3.35795529	0.08188152	0.18003522
Gga2	-0.362854	4.47761348	3.35729898	0.08190919	0.18005365
Actn1	0.48794344	4.61802019	3.35686268	0.08192759	0.18005365
Asgr2	3.0507376	-2.1466861	3.3564487	0.08194505	0.18005365
Eif3f	-0.2951206	5.99474716	3.3549679	0.08200754	0.18015055
Myom1	-1.2837168	0.4210617	3.3511807	0.08216762	0.18045887
Rcor1	-0.2878616	5.10840281	3.3502599	0.0822066	0.18045887
Mlc1	0.61086255	3.17657548	3.34905722	0.08225754	0.18045887
Pitpnb	-0.2284909	6.1491041	3.34890473	0.082264	0.18045887
Pgr	-0.3371709	5.93249371	3.3488972	0.08226432	0.18045887
Rps17	-0.3066228	7.26328492	3.34889351	0.08226448	0.18045887
4930453N24	-0.2739293	4.68066758	3.3483711	0.08228662	0.18045887
Pank4	0.4726101	2.77003552	3.34812705	0.08229696	0.18045887
Pcnp	-0.2659423	7.86009019	3.34773228	0.0823137	0.18045887
Smarca1	0.29372375	5.63378935	3.34682226	0.0823523	0.1805031
Gm10649	-1.7497898	-1.0484694	3.34578741	0.08239622	0.18055896
Limd2	-0.3318171	4.46135496	3.34431922	0.08245858	0.18065519

Avp	1.86390028	-1.658286	3.34237929	0.08254106	0.18074732
Akirin2	-0.2999508	6.60891221	3.3423025	0.08254432	0.18074732
Clcn4-2	0.22618887	6.19279501	3.34202842	0.08255599	0.18074732
Cuedc2	-0.3946473	4.43643921	3.34060821	0.08261644	0.1807795
Ndufv3	-0.4233645	5.13708218	3.34047947	0.08262192	0.1807795
Dennd6a	-0.2509647	6.86793009	3.34038254	0.08262605	0.1807795
6820408C15I	-1.892737	-0.5140302	3.33897735	0.08268593	0.1808701
Spop	0.2091034	7.72348367	3.33815274	0.08272109	0.18090661
Pitpna	-0.1816097	8.72835352	3.33729332	0.08275775	0.18094639
2610528A11I	-2.5523626	-1.5100274	3.33662684	0.08278619	0.18096819
Ube2k	-0.2034305	8.11082422	3.33511078	0.08285094	0.18106932
Ost4	-0.4492191	7.07732292	3.33405078	0.08289624	0.18112792
Scel	-0.4448779	3.33749651	3.33331737	0.08292761	0.18115603
Actb	-0.2825766	9.97274988	3.33287532	0.08294652	0.18115694
Drp2	0.45840852	5.99951499	3.33141937	0.08300884	0.18121425
Cebpg	-0.2346335	6.39864754	3.33139785	0.08300976	0.18121425
Ctcf	-0.2191184	6.37279785	3.33005268	0.08306738	0.18129964
Cep72	1.00698732	1.07004551	3.32652719	0.08321863	0.1815893
Rab31	-0.3135612	5.56481474	3.32504621	0.08328226	0.18168767
Slc9a8	-0.3799236	4.08268611	3.32429289	0.08331465	0.18171786
Zfp511	-0.6437915	2.57961167	3.32365743	0.08334199	0.18173701
Alms1-ps2	0.92698387	0.45228043	3.32307225	0.08336717	0.18175145
Ica1l	0.26508649	5.02285201	3.32219998	0.08340471	0.18179285
Bcl2l11	-0.4416838	5.05409116	3.3208036	0.08346487	0.18188349
Tatdn1	-0.2968437	4.60363615	3.32002556	0.0834984	0.1819161
Naip5	0.70377412	1.51567196	3.31595878	0.08367396	0.18225804
Gm2115	-0.4910192	3.08649973	3.31402809	0.08375745	0.18239935
Phf11d	-0.7725542	1.52265086	3.31313567	0.08379608	0.1824429
Mpp6	-0.3756018	8.59069649	3.31185296	0.08385163	0.18252329
Eed	-0.3207904	4.49116874	3.30991808	0.08393551	0.1826168
Cpsf3l	-0.475587	2.63719254	3.30951606	0.08395296	0.1826168
Utp20	0.42416498	4.51937359	3.30919284	0.08396698	0.1826168
Mrpl53	-0.4132462	3.73890533	3.30914255	0.08396916	0.1826168
Sirt3	-0.3605917	3.59719105	3.30337166	0.08422004	0.18310099
Zfp451	0.3410387	5.17462745	3.30316172	0.08422919	0.18310099
9430008C03I	0.73094949	1.71279956	3.30217221	0.0842723	0.18314558
Pdcd4	-0.2011169	7.48491951	3.30183276	0.08428709	0.18314558
Nudt7	-0.4137442	2.42761993	3.30096122	0.08432509	0.18318361
Ccdc58	-0.432515	3.0272777	3.30034091	0.08435215	0.18318361
Flt1	-0.3541343	4.99645471	3.30014504	0.0843607	0.18318361
Hsd3b4	1.06149497	0.76477743	3.2996575	0.08438197	0.1831892
Endov	-0.3192839	4.5500775	3.29800396	0.08445418	0.18330533
Clec11a	-0.4759603	2.21982313	3.2971502	0.08449149	0.18334525

Stx8	-0.3428017	5.33836295	3.29672663	0.08451001	0.18334525
DQ267100	1.17846339	0.15390844	3.29351247	0.08465068	0.18360977
Upp1	2.42108416	-1.5056241	3.2926436	0.08468875	0.18365168
Hapln4	0.27518437	6.04615777	3.2872037	0.08492758	0.18410677
Aqp7	1.12676946	0.05441588	3.28695443	0.08493854	0.18410677
1700125H03I	1.87828587	-0.4861726	3.28581022	0.08498888	0.18410677
Cdh22	0.79443789	1.76906801	3.28568005	0.08499461	0.18410677
Ppp2r5a	-0.2614289	5.75642456	3.28509995	0.08502015	0.18410677
Ppm1j	-2.0112684	-1.8285793	3.28492485	0.08502786	0.18410677
Mrgbp	-0.9503886	0.49676204	3.28445037	0.08504876	0.18410677
Magee1	0.36111613	6.91827672	3.2842907	0.08505579	0.18410677
Rps15	-0.4304912	6.36645525	3.28401886	0.08506777	0.18410677
Mical3	0.37842529	6.27076941	3.28239961	0.08513915	0.1842015
Gm5089	-0.4858179	4.78982062	3.28217285	0.08514915	0.1842015
Gm1123	-2.5183917	-2.3133754	3.28135956	0.08518503	0.18423843
Mppe1	-0.8376177	0.69121084	3.28071698	0.08521339	0.18425909
Slc32a1	0.40573093	4.04629258	3.27981887	0.08525305	0.18430416
Ube2j1	-0.2513988	7.45386094	3.2784772	0.08531234	0.18439164
Ctgf	-0.373627	5.68766544	3.27739102	0.08536037	0.18439668
Zbtb8a	0.65957719	2.95987828	3.27734387	0.08536246	0.18439668
Cacng3	-0.2388686	5.64058481	3.2770447	0.08537569	0.18439668
Ppp1r7	0.30642949	7.31260704	3.27672201	0.08538997	0.18439668
Rab1	-0.2326774	9.05096433	3.27437482	0.08549392	0.18458046
Lancl2	-0.2106924	8.49514295	3.27386997	0.08551629	0.18458808
Farp1	0.228085	5.39111264	3.27326607	0.08554307	0.1846052
Cux2	0.37030764	4.80386625	3.27216567	0.08559189	0.18466986
Dsg1c	-1.5845179	-0.8061699	3.27012528	0.08568249	0.18482462
Speer4b	0.87626778	0.58808674	3.26679715	0.0858305	0.18505689
9130401M01	-0.2973068	4.78490337	3.26637461	0.08584932	0.18505689
Nudt19	-0.3195512	4.87249555	3.26588073	0.08587131	0.18505689
Stc2	1.12203434	-0.1603115	3.26564485	0.08588182	0.18505689
Ehmt1	0.44593937	4.40796228	3.26558195	0.08588463	0.18505689
Tmem134	-0.4566196	2.61010811	3.26370546	0.08596828	0.1851964
Ap1s1	-0.4404382	2.8383921	3.26270309	0.086013	0.185252
Dlx1	0.4784792	3.88112312	3.26183298	0.08605184	0.18528779
Rap1b	-0.2754711	7.25010556	3.26125118	0.08607783	0.18528779
Sec11c	-0.3428888	4.53769326	3.26066111	0.08610419	0.18528779
N4bp2l2	0.20972431	6.28193823	3.26063672	0.08610528	0.18528779
Atp5s	-0.2700256	4.66930354	3.25979523	0.08614289	0.18532801
Loxl2	0.44629101	3.97633276	3.2585987	0.0861964	0.18533918
Gfpt2	0.34619735	4.19350323	3.25857312	0.08619755	0.18533918
Tdrd5	0.95852076	1.22862972	3.25840713	0.08620498	0.18533918
Aph1b	0.41009024	4.15526441	3.2579873	0.08622376	0.18533918

Nrk	-0.8205936	1.47613572	3.25710802	0.08626313	0.18534945
Ppp6c	-0.3456988	6.26254495	3.25703528	0.08626639	0.18534945
Irak2	0.33588149	3.65460377	3.25645891	0.0862922	0.18536411
Prpf18	-0.277569	4.94499553	3.25603803	0.08631106	0.18536411
Tmem252	0.99677093	-0.111035	3.25518811	0.08634916	0.18536475
Vmp1	0.27049072	5.58892093	3.2544405	0.08638268	0.18536475
1810013L24F	-0.2204731	7.41829365	3.2543767	0.08638554	0.18536475
Trim25	-0.350177	6.41306615	3.25434309	0.08638705	0.18536475
Arfgap2	-0.4218654	4.17412182	3.2534277	0.08642812	0.18541226
Sim1	1.54768703	-0.605711	3.25199423	0.08649249	0.18546244
Chfr	0.53127081	4.18743872	3.25156311	0.08651186	0.18546244
Anapc15	-0.5123178	2.43938327	3.25094239	0.08653975	0.18546244
Kank4	-0.4959707	3.92980639	3.25024831	0.08657096	0.18546244
Gpr149	1.01477601	0.73379147	3.25010078	0.08657759	0.18546244
Aspscr1	0.64488372	2.7002338	3.24987642	0.08658768	0.18546244
Tmem65	-0.2191218	8.11504766	3.24980655	0.08659082	0.18546244
Ezh1	-0.3246335	5.19034021	3.24933679	0.08661196	0.18546244
Tcp1	-0.1876495	7.08689799	3.24911542	0.08662192	0.18546244
Ube2w	-0.2150063	6.24687381	3.24836975	0.08665548	0.18549375
Dhrs13	-1.3247083	-0.3668726	3.24766494	0.08668721	0.18552114
Alkbh5	-0.2616874	6.397967	3.24677869	0.08672714	0.18554473
Rpl14-ps1	-0.543457	2.59739712	3.24657945	0.08673612	0.18554473
Ddi2	-0.5764636	2.7742584	3.24447929	0.08683083	0.18562642
Gm101	1.92311918	-1.1663423	3.24428644	0.08683953	0.18562642
Crk	-0.2018085	7.7634951	3.24427017	0.08684027	0.18562642
Fabp3	-0.3903308	3.7126582	3.24372624	0.08686482	0.18562642
Sf3b6	-0.3144351	5.00952642	3.243335	0.08688248	0.18562642
Trmt5	-0.4257294	3.47428555	3.24299117	0.08689801	0.18562642
Fastk	-0.3677253	3.68472314	3.24279301	0.08690696	0.18562642
Echdc1	-0.594353	1.77405329	3.24206025	0.08694007	0.18565665
Mcf2l	0.54437246	4.68359106	3.24146579	0.08696694	0.18567355
Asxl3	0.64706376	4.1941141	3.24054718	0.08700848	0.18572176
Gprc5c	-0.5665901	3.07802592	3.23806347	0.08712091	0.18588768
Sox10	0.39147745	4.29225309	3.23712488	0.08716344	0.18588768
Suclg1	-0.2874816	5.19103964	3.23683403	0.08717662	0.18588768
Rbm26	0.2355186	7.13250946	3.23673032	0.08718132	0.18588768
Abcc9	0.64244463	3.11914776	3.23660808	0.08718687	0.18588768
Ppap2b	0.37396019	6.43322826	3.23631682	0.08720007	0.18588768
Exo5	-0.460564	3.74028073	3.23533718	0.08724451	0.18593013
Gpsm2	0.54501201	2.5646379	3.23504102	0.08725795	0.18593013
Phf7	-1.1461589	0.91577903	3.23263536	0.08736721	0.18612245
Aldoa	-0.2100054	9.15691313	3.23163894	0.08741251	0.18617847
Exd2	0.30261975	5.36403923	3.23072558	0.08745406	0.18622647

Akr1e1	-0.2662288	5.1974539	3.22860563	0.08755059	0.1863915
Pclo	0.51838185	10.1329204	3.22740769	0.08760518	0.18646721
Upb1	1.52894929	-0.4929598	3.22690351	0.08762818	0.18646877
Baz1b	0.21466424	7.83348427	3.22655674	0.08764399	0.18646877
Tmem19	-0.3674593	3.56691785	3.22422159	0.08775059	0.18660424
Met	-0.3918657	3.05753534	3.22407715	0.08775719	0.18660424
Abcg4	0.41181622	4.08949237	3.22391029	0.08776482	0.18660424
Coro7	0.38200259	4.16065774	3.22089578	0.08790268	0.18682244
Slc25a53	0.59785202	2.57101255	3.22039933	0.08792541	0.18682244
Qrich1	-0.2075343	6.48425595	3.22037623	0.08792647	0.18682244
2510049J12F	-1.1308254	1.13682001	3.21972625	0.08795623	0.18682244
Masp1	0.61735323	1.67919418	3.21958284	0.0879628	0.18682244
Zfp369	0.31915257	5.24517541	3.21769891	0.08804916	0.18696531
Tnnt3	-2.9355545	-1.5294982	3.21648309	0.08810494	0.18704321
Zfp114	-1.0971579	0.38684237	3.21564196	0.08814356	0.18707018
Wdr73	-0.2877412	4.41027299	3.21517352	0.08816507	0.18707018
Stc1	0.58665311	2.90925401	3.21419045	0.08821024	0.18707018
Prdm11	1.78000667	-0.5723408	3.21382274	0.08822714	0.18707018
Irf2bp2	-0.1996312	8.43053574	3.21310792	0.08826001	0.18707018
Ctps2	0.22528575	5.66905144	3.21286188	0.08827133	0.18707018
B230209E15	-0.3164568	6.31479577	3.21273454	0.08827719	0.18707018
Lhfpl5	-1.9630606	-0.401819	3.21265432	0.08828088	0.18707018
Las1l	0.32375092	4.97411249	3.21221398	0.08830114	0.18707018
Robo2	0.2942357	6.52649214	3.21205137	0.08830862	0.18707018
1700047A11	2.45566808	-1.7041524	3.21122913	0.08834647	0.18710989
Atp6v1f	-0.4563772	4.75645672	3.20978255	0.08841311	0.18716195
Ddx25	0.42482841	4.1774863	3.20951339	0.08842551	0.18716195
Prep	0.2469629	4.39806506	3.20894514	0.08845171	0.18716195
Phax	-0.2549484	5.38378733	3.20864137	0.08846572	0.18716195
Pgpep1	-0.4201709	4.21898306	3.20862248	0.08846659	0.18716195
Dok2	-1.4809811	-0.7678923	3.2077629	0.08850624	0.18720541
Card14	-1.4143328	0.21782031	3.20680147	0.08855062	0.18725883
Bhmt	-2.5989422	-1.2367822	3.20585211	0.08859446	0.18731111
Atrip	-0.3804986	3.49551033	3.20465956	0.08864957	0.18738718
9330182L06F	0.3481853	5.05696034	3.20353922	0.08870138	0.18740201
Ifnar1	0.28728491	5.75464592	3.20275384	0.08873772	0.18740201
Fkbp4	-0.3828429	5.92966816	3.20271222	0.08873965	0.18740201
Ralgps1	0.34279216	6.58154748	3.20264695	0.08874267	0.18740201
D330050I16F	1.14372384	-0.4734021	3.20244013	0.08875224	0.18740201
Thoc1	0.34292091	4.76711734	3.20188717	0.08877784	0.18741566
Opa3	-0.3811766	5.05372894	3.20008557	0.08886132	0.18751922
Haghl	-0.7461151	0.89350603	3.19904279	0.08890967	0.18751922
S1pr4	1.21109384	-0.8166348	3.1989316	0.08891483	0.18751922

Ola1	-0.2018507	7.60669843	3.19874653	0.08892342	0.18751922
Cd3eap	-0.4073028	3.59037109	3.19860262	0.08893009	0.18751922
Slc4a11	-2.0178255	-0.2079535	3.19835119	0.08894176	0.18751922
Gm6938	1.87343869	-0.7268445	3.19716906	0.08899664	0.18759455
Nostrin	-1.14709	0.87042759	3.19434229	0.08912803	0.1878269
Tle4	0.29768087	6.40043195	3.19397275	0.08914522	0.1878269
Aqp9	-2.3949461	-1.2215807	3.19262123	0.08920813	0.18787937
Med25	0.42299964	2.48573837	3.19226512	0.08922472	0.18787937
9530051G07	0.92611491	0.85505439	3.19171572	0.08925031	0.18787937
Zfp932	0.41330338	4.46617735	3.19162966	0.08925432	0.18787937
Sri	-0.2665002	6.57914909	3.19137857	0.08926603	0.18787937
Chmp2a	-0.4005741	5.63235119	3.1901335	0.08932407	0.1879065
Rpl35a	-0.3851396	5.46905397	3.18971815	0.08934345	0.1879065
Mturn	-0.2226055	6.37009665	3.18942357	0.08935719	0.1879065
Cnpy4	-0.3054996	4.55218705	3.18941749	0.08935748	0.1879065
Micu3	-0.2850021	7.58963231	3.18904557	0.08937483	0.1879065
Lsm5	0.69328057	1.66590589	3.18800971	0.08942319	0.18794222
Rpp30	-0.3060767	3.992085	3.18771786	0.08943682	0.18794222
Msantd4	-0.2078522	7.36767747	3.18685563	0.0894771	0.18794222
Asic2	0.30823664	5.16739371	3.18662919	0.08948769	0.18794222
A630023P12	2.21114413	-1.2681202	3.18662768	0.08948776	0.18794222
Pfdn2	-0.3296173	5.53679415	3.18547443	0.08954168	0.18796069
Syngr1	-0.2437459	6.81639169	3.1852698	0.08955125	0.18796069
Mir376a	1.72236912	0.06383784	3.18519816	0.0895546	0.18796069
4930479D17	-0.8397519	1.0864378	3.18470269	0.08957778	0.18796069
Kcna6	0.31917872	5.75091568	3.18431561	0.0895959	0.18796069
Phldb1	0.29273235	4.12078655	3.18397842	0.08961168	0.18796069
Cops7b	-0.4742372	3.71950173	3.18317708	0.08964921	0.18799914
Ldhd	-0.7657136	1.85823546	3.18252134	0.08967993	0.18802331
Ap3m1	0.23385617	5.66474037	3.18148169	0.08972866	0.1880611
Tmtc4	0.3052568	4.07511298	3.18131756	0.08973635	0.1880611
Psmb5	-0.305427	5.10193767	3.17906679	0.08984197	0.18823509
Dhdds	0.28945255	4.89381283	3.17853007	0.08986717	0.18823509
Mtmr3	0.23965029	6.04740863	3.17832036	0.08987702	0.18823509
Tmem246	0.30906131	4.14919465	3.17769615	0.08990635	0.18825626
Col4a3	1.300077	0.00043083	3.17682402	0.08994735	0.18826536
Bcl2l1	-0.4239631	4.44907231	3.17671177	0.08995263	0.18826536
Myl6	-0.3682337	7.87742737	3.17637728	0.08996836	0.18826536
Tgds	0.47785879	2.74649204	3.1753937	0.09001463	0.18832196
Cops4	-0.2284296	6.02394846	3.17453649	0.09005498	0.18835362
Psma2	-0.2837523	6.68666278	3.17425539	0.09006822	0.18835362
Cdhr2	1.94725269	-0.9457568	3.17232563	0.09015915	0.18843132
Kctd20	-0.5324447	3.14629532	3.17213199	0.09016828	0.18843132

Abi3bp	0.39060916	4.18280323	3.17187371	0.09018046	0.18843132
Rps6	-0.3622362	8.46228235	3.17183428	0.09018232	0.18843132
1700024B18I	1.60773009	-0.3069596	3.1705115	0.09024473	0.18852151
Hic1	-0.557347	3.26674791	3.16891862	0.09031996	0.18861956
Cdc27	0.2482749	7.83018118	3.16870216	0.09033018	0.18861956
Med13l	0.36328817	6.48937418	3.16809629	0.09035882	0.1886343
Ifitm6	1.22192674	0.49823614	3.16773807	0.09037575	0.1886343
2700069I18R	0.91792528	0.80104733	3.16614323	0.0904512	0.18874955
Yipf1	0.45014002	3.00966955	3.16575625	0.09046951	0.18874955
Pbx2	-0.3259768	4.84810198	3.16522385	0.09049472	0.18876194
Zfp97	0.29957683	4.12311113	3.16442072	0.09053276	0.18880108
Tnfrsf13c	-1.1800226	0.45441152	3.16321164	0.09059007	0.18880436
Snx13	0.18085763	6.88848207	3.16319872	0.09059068	0.18880436
Clvs2	0.42440213	4.43268842	3.16282396	0.09060845	0.18880436
Hmmr	0.41855658	3.27664422	3.16262213	0.09061802	0.18880436
Trim43c	1.92146564	-1.17469	3.16235464	0.09063071	0.18880436
Esrp2	1.53518249	-0.9592268	3.16188572	0.09065296	0.18881055
Ppfibp1	0.21212665	6.07535407	3.16105032	0.09069261	0.18883903
Hsd3b3	0.99315355	0.3184857	3.16078537	0.09070519	0.18883903
Gla	0.43496013	3.05440702	3.15886273	0.09079653	0.18895637
Zfp939	0.6457567	2.6746555	3.15878706	0.09080013	0.18895637
Hcfc1	0.27626699	6.90610262	3.15806067	0.09083467	0.1889881
Tnip1	-0.3781244	4.09255579	3.1561816	0.09092409	0.18910379
Mxd1	-0.2640071	6.02346775	3.15608092	0.09092889	0.18910379
Tnfrsf14	2.74103237	-1.8318768	3.15555551	0.09095391	0.18911568
Ripk3	-1.8602996	-0.6316331	3.15463662	0.09099769	0.18916657
Wasf2	-0.2733591	6.44383348	3.15350741	0.09105153	0.18923516
Fam120aos	-0.4017222	4.61883601	3.15313428	0.09106933	0.18923516
Gm4013	-2.5782655	-1.6984571	3.15118165	0.09116254	0.18932715
Peak1	0.3440458	6.77754492	3.1508889	0.09117652	0.18932715
Stamos	2.04886872	-1.1689577	3.15053285	0.09119353	0.18932715
Pias3	0.43339527	2.71943935	3.15031334	0.09120402	0.18932715
Tyr	0.62809251	2.55561045	3.14993114	0.09122229	0.18932715
Ptpn1	-0.2559175	4.77236984	3.14955635	0.0912402	0.18932715
Chtop	-0.2404462	7.48466396	3.14937457	0.0912489	0.18932715
Scrib	0.55477123	2.10448195	3.14665265	0.09137915	0.18948849
Camk2n2	-0.2960091	4.57960333	3.14657054	0.09138309	0.18948849
Suds3	-0.3396092	5.04632402	3.14639531	0.09139148	0.18948849
Rpl11	-0.3517127	7.046423	3.14613324	0.09140403	0.18948849
Astn2	0.41972922	3.40841357	3.14566534	0.09142646	0.18949486
Nrd1	0.21773183	7.74028781	3.14429643	0.09149209	0.18959077
Ankrd35	-0.4385757	2.76425955	3.1436577	0.09152273	0.18960224
Gpr56	0.47358048	2.97073981	3.14294473	0.09155695	0.18960224

Pik3cg	0.48679222	2.45335659	3.14212355	0.09159638	0.18960224
Lsm1	-0.4145193	4.10099304	3.14164704	0.09161927	0.18960224
Etohi1	-0.3472916	4.21814731	3.14112104	0.09164454	0.18960224
Ubr2	0.34491497	6.2756488	3.1408474	0.09165769	0.18960224
Gja1	0.53051951	7.63703982	3.14059408	0.09166987	0.18960224
Prkab1	-0.4678142	3.39809455	3.14039475	0.09167945	0.18960224
2410006H16I	-0.5695794	2.57226497	3.13997894	0.09169945	0.18960224
Dram2	0.32281373	5.47488685	3.1399671	0.09170001	0.18960224
Atp5e	-0.4615458	5.23526752	3.13939289	0.09172763	0.18960224
Gpr25	-0.4400471	3.66236568	3.13934589	0.0917299	0.18960224
Mir665	1.60039237	-0.6775952	3.13828964	0.09178073	0.18962451
Rab11b	-0.2251654	9.08833055	3.13826716	0.09178181	0.18962451
Rimklb	0.36777718	3.68555585	3.13791538	0.09179875	0.18962451
Larp6	-0.342925	3.96276884	3.13697962	0.09184382	0.18964453
Cwh43	2.04359609	-1.0775898	3.13691029	0.09184716	0.18964453
Hspb8	-0.4393243	6.19497759	3.13646344	0.09186869	0.18964901
Car10	-0.2946525	8.35833458	3.1326901	0.09205076	0.18998482
Adamts15	0.5383122	2.60173174	3.13202474	0.09208291	0.19001114
Fgfr1op	-0.2835008	4.38467889	3.1296124	0.09219957	0.19020399
Lime1	0.79717474	2.74346106	3.12897133	0.0922306	0.19020399
F830016B08I	0.30929178	4.1638108	3.12888841	0.09223462	0.19020399
Pced1a	0.35568515	4.13256654	3.12683877	0.09233392	0.1903687
Klk10	1.39393685	-0.2302173	3.12592507	0.09237823	0.19041439
Rpe	-0.3007898	5.42631599	3.12516977	0.09241488	0.19041439
Rhoa	-0.4201281	8.61857226	3.12509953	0.09241829	0.19041439
Abcc8	0.55820433	2.69792828	3.12451831	0.0924465	0.19041439
Tex9	0.24390569	4.98234399	3.12436541	0.09245392	0.19041439
Il27ra	2.11648254	-1.5039429	3.12389891	0.09247658	0.19041439
Ralb	-0.3449339	4.37147083	3.12356635	0.09249273	0.19041439
Ppp1r10	-0.2256988	5.87724125	3.12305787	0.09251744	0.19041439
Zbtb7c	-0.3521556	4.09881819	3.1227779	0.09253104	0.19041439
Fam221a	-1.2789877	0.13123574	3.12198032	0.09256981	0.19045417
Pkig	-0.2582094	4.79792239	3.12146669	0.09259479	0.19046556
Ifitm3	-0.5518826	7.69025273	3.12078508	0.09262795	0.19046657
Drg1	-0.3509934	5.31877549	3.1206572	0.09263418	0.19046657
Eif4g2	-0.2563507	10.874292	3.12020233	0.09265632	0.19046734
Kcne2	-1.7489783	-0.9974753	3.11950175	0.09269043	0.19046734
Eva1a	1.00181211	1.13258846	3.11945126	0.09269289	0.19046734
Ankrd34a	-0.2701872	6.32298013	3.11653593	0.09283499	0.19071934
Cd83	0.46031872	2.18761984	3.11303821	0.09300582	0.19103022
Mpnd	-0.4724867	3.83602864	3.11167744	0.09307238	0.19110038
Pou3f3os	-0.3570232	3.71496871	3.11139186	0.09308636	0.19110038
Uaca	-0.4079899	7.66103414	3.11114368	0.09309851	0.19110038

Asun	0.26980517	4.53145219	3.10971029	0.0931687	0.1912044
Dnmt3aos	-2.5299706	-1.8159098	3.10928883	0.09318935	0.19120672
Ocrl	0.29427636	6.61188684	3.10812476	0.09324642	0.1912829
Ccdc174	-0.2914433	4.70050512	3.10773499	0.09326554	0.1912829
Acvr1	0.3366258	4.31727452	3.10683472	0.09330971	0.19133343
Cnn3	-0.3432406	6.27032767	3.10376443	0.09346054	0.19160261
Oas1a	-1.1830357	0.68271435	3.10297018	0.09349961	0.19164259
Aff4	0.19667458	8.98795918	3.10121702	0.0935859	0.19177933
Chrm2	0.68182165	1.96358718	3.09882207	0.09370394	0.19196416
B630005N14	-0.2055949	6.47885509	3.09859197	0.09371529	0.19196416
Slc24a1	1.59036463	-0.0401307	3.09740302	0.09377396	0.19200732
Npy	0.4446983	3.41690542	3.09736088	0.09377604	0.19200732
Ipo11	0.27151123	6.18942871	3.09660082	0.09381357	0.19200732
Ptch2	-2.1471372	-1.929152	3.09643706	0.09382166	0.19200732
Sash1	0.22131507	6.31869525	3.09617983	0.09383437	0.19200732
Pde6d	-0.4168935	4.93270465	3.09532547	0.09387659	0.19205359
Klhl36	-1.1528317	0.6160963	3.09280252	0.09400139	0.19226875
Mgat5b	0.32518849	3.71645702	3.09206608	0.09403786	0.1922876
Ppp1r12a	0.22035385	7.45763336	3.09148724	0.09406653	0.1922876
Ank2	0.31602002	10.1838387	3.09009121	0.09413573	0.1922876
Rnf215	0.51128689	2.67262694	3.08991782	0.09414433	0.1922876
Dhx32	0.38053792	4.10411335	3.089819	0.09414923	0.1922876
C1qb	-0.6926792	2.39464549	3.08973501	0.09415339	0.1922876
St7	0.44978516	3.42973863	3.08970214	0.09415502	0.1922876
Ttc39a	0.96706286	1.32521746	3.08856761	0.09421132	0.1922876
Tango2	-0.3404385	3.743236	3.08838478	0.09422039	0.1922876
Tnfaip1	-0.3011325	6.03251067	3.08825132	0.09422702	0.1922876
Efcab7	-0.5965004	2.55634321	3.08816715	0.0942312	0.1922876
Hspbp1	-0.5048215	2.63907772	3.08786559	0.09424617	0.1922876
Cnksr3	0.54268777	1.78761728	3.08562873	0.09435732	0.19245322
Zcchc11	0.25158966	6.83603884	3.08526289	0.09437551	0.19245322
Nadk	-0.3043576	5.45393474	3.08504631	0.09438629	0.19245322
Dcaf12l2	-1.4562006	-0.1083279	3.08250676	0.09451271	0.19265866
Dlgap2	0.36607625	6.60868622	3.08223238	0.09452638	0.19265866
Psma8	-1.1739424	0.17721155	3.08181567	0.09454715	0.1926609
Nktr	0.3171065	7.07356696	3.08000225	0.09463758	0.19280507
Ap3b2	0.39313883	5.0760068	3.07952999	0.09466115	0.19281299
Lrfn2	0.53995879	1.80541065	3.07882115	0.09469654	0.19284496
Gm4922	1.60576343	-1.3059537	3.07781355	0.09474687	0.19290391
Tbce	0.31621717	4.40846817	3.07745331	0.09476487	0.19290391
Pcm1	0.33172268	8.57560764	3.07681375	0.09479684	0.19290551
Fam117b	-0.1783693	7.00148895	3.07664964	0.09480504	0.19290551
Zfp12	0.3111119	4.74653812	3.07611317	0.09483187	0.1929147

Cuta	-0.3496974	6.28943736	3.0754676	0.09486417	0.1929147
Lrp6	0.22314434	6.82316275	3.07445177	0.09491502	0.1929147
Grsf1	-0.2056933	6.82277294	3.0740308	0.0949361	0.1929147
2700099C18I	0.7463726	1.75363503	3.07381915	0.0949467	0.1929147
Mfap3l	0.3552228	5.63323855	3.07377952	0.09494868	0.1929147
Tm2d2	-0.578263	4.24607519	3.07300128	0.09498767	0.1929147
11-Mar	-0.6950056	1.34585352	3.07294938	0.09499027	0.1929147
Wrn	-0.2270061	5.66555382	3.07286708	0.0949944	0.1929147
Mir6390	-2.0560258	0.08973674	3.0723555	0.09502004	0.1929147
Itfg2	0.37504594	3.37979046	3.07185526	0.09504513	0.1929147
Tomm5	-0.3563381	4.59282901	3.07183989	0.0950459	0.1929147
Plxna1	0.37377873	5.51998092	3.07054653	0.09511078	0.19297545
Pacrgl	-0.4994935	3.30867818	3.07045802	0.09511523	0.19297545
Ndst3	0.42143868	4.32585703	3.06932688	0.09517202	0.19302466
4933406F09I	2.98352804	-1.1412617	3.06917644	0.09517958	0.19302466
Suco	0.25719197	6.12057899	3.06879782	0.0951986	0.19302466
Ssbp3	-0.2130498	5.98619152	3.06772	0.09525277	0.19309453
Lrp10	-0.5180548	4.25497654	3.06450651	0.0954145	0.19338236
Hipk2	0.20395436	7.03675887	3.06340674	0.09546992	0.19345466
Fam19a5	-0.2451066	4.98488575	3.06070111	0.09560643	0.19369121
3110021N24	0.44414181	2.41221087	3.06005037	0.0956393	0.19371773
Uprt	-0.2802367	4.57891651	3.05924717	0.09567989	0.19374381
Rgs12	0.45300619	3.26343381	3.05878414	0.09570329	0.19374381
Ubl5	-0.3856939	5.95117824	3.05862157	0.09571151	0.19374381
Rnf219	-0.2294291	5.20945198	3.05794272	0.09574584	0.19377326
Msmo1	0.36768802	4.99067555	3.05535498	0.09587684	0.1939819
Zxdb	0.24972956	5.19819493	3.05512408	0.09588854	0.1939819
0610009O20	-0.3174934	4.90817008	3.05466282	0.09591192	0.19398912
Ydjc	0.64228973	1.79528566	3.05401728	0.09594464	0.19401525
Bnip3	-0.2424252	6.08881487	3.05284845	0.09600393	0.19405347
Srsf3	-0.2494021	7.78346233	3.05265876	0.09601356	0.19405347
Grap	-0.8787205	0.96999009	3.0521603	0.09603886	0.19405347
Tex26	-1.3515017	-0.7564321	3.05208289	0.09604279	0.19405347
Idh3a	-0.2203681	6.62865407	3.05057776	0.09611923	0.19416788
B4galnt4	0.50380075	3.61833574	3.0489573	0.09620162	0.19426824
Zbtb43	0.36569625	4.51846627	3.04882041	0.09620858	0.19426824
Mpl	-0.9851683	1.52011049	3.04837658	0.09623116	0.19427379
Spred3	0.36411655	5.07279754	3.04721348	0.09629036	0.19432451
Ephb2	0.69007977	2.12447497	3.04710351	0.09629596	0.19432451
Pctp	-0.5676007	2.59913689	3.04642551	0.0963305	0.19435416
Rgs10	-0.3243881	4.32710585	3.04537976	0.09638379	0.19440993
Ttc1	-0.2962694	6.00652084	3.04510429	0.09639783	0.19440993
Prss54	-2.1914157	-1.2922153	3.04369252	0.09646985	0.19451511

Ube3c	0.2404247	6.07983139	3.0427122	0.09651989	0.19455145
Nipbl	0.23956848	7.96640291	3.04256137	0.09652759	0.19455145
Robo3	1.29867615	2.01714321	3.04177509	0.09656775	0.19459236
Fam159b	2.3749732	-2.0502647	3.03922821	0.09669798	0.19477687
Plcb2	0.72011369	1.39988177	3.03920658	0.09669909	0.19477687
Bicc1	-0.3843118	6.88745803	3.03872169	0.09672391	0.1947868
Znrf3	0.22242033	5.9800087	3.03773402	0.09677448	0.19484859
Slitrk3	0.34860611	6.08726695	3.03630633	0.09684764	0.19489183
Vti1a	-0.2410379	6.91696689	3.0361386	0.09685624	0.19489183
Glra1	1.0377852	0.24685112	3.03600543	0.09686306	0.19489183
Junb	-0.9938837	1.53956335	3.03576213	0.09687554	0.19489183
Scaf1	-0.3016533	4.68206565	3.03467036	0.09693155	0.19496446
Dpysl4	0.49565213	2.8588484	3.03191975	0.09707282	0.19518013
Edem2	-0.6438981	1.59219095	3.03147369	0.09709576	0.19518013
Pars2	-0.6737239	1.84573629	3.03059747	0.09714082	0.19518013
Gm3414	0.45042823	3.33853117	3.03017224	0.0971627	0.19518013
Adipor1	-0.2983685	6.28407623	3.03010741	0.09716604	0.19518013
Ibtk	0.20512877	5.74726595	3.02997697	0.09717275	0.19518013
Setd1b	0.26395138	5.46467722	3.02908551	0.09721865	0.19518013
Sv2c	0.40823344	4.80415195	3.02845059	0.09725135	0.19518013
Atp13a1	0.42257978	2.84849454	3.02813796	0.09726745	0.19518013
Cstl1	1.61006945	0.25753104	3.02805404	0.09727178	0.19518013
Tmem87a	0.30641374	4.44738721	3.02797174	0.09727602	0.19518013
Taf12	-0.4670666	4.10431954	3.02793561	0.09727788	0.19518013
Gfra2	-0.2977493	7.57978057	3.02720286	0.09731565	0.19521592
Asgr1	-0.5916396	6.16241364	3.02644757	0.0973546	0.19525407
Trip11	0.19770857	7.25368334	3.02498859	0.09742989	0.19536507
Add2	0.31018019	7.61685825	3.02453374	0.09745337	0.19537216
Aimp2	-0.3952686	3.29249552	3.0230683	0.09752909	0.19544572
Vmn2r1	-1.9760488	-1.1488416	3.02305123	0.09752997	0.19544572
2210018M11	0.27964409	6.75589325	3.02198332	0.09758518	0.19549194
Ern1	0.67342182	2.21275338	3.02183325	0.09759295	0.19549194
Il2rb	-1.1430594	0.95630689	3.02035534	0.09766943	0.19556644
Gsto1	-0.4173128	4.19893539	3.02034301	0.09767007	0.19556644
Faf2	0.29964342	4.40507578	3.01889663	0.09774499	0.19563623
Mcm10	2.54060003	-1.3005088	3.01848604	0.09776627	0.19563623
Slc43a1	0.94075065	0.58700747	3.0179867	0.09779216	0.19563623
Cnbd2	0.51699771	2.38608532	3.01754016	0.09781532	0.19563623
Acr	1.49764277	-0.0750512	3.01752212	0.09781625	0.19563623
Phip	0.28793441	7.35489708	3.01731411	0.09782704	0.19563623
Mgrn1	-0.2098183	6.53038613	3.01697315	0.09784473	0.19563623
Spc24	0.60340123	2.43892939	3.01533723	0.09792965	0.19576087
4930428E07I	1.46975254	-0.2484161	3.0145052	0.09797288	0.19576087

Sumo3	-0.3486867	6.45768741	3.01446937	0.09797474	0.19576087
4930404H11l	2.52922462	-1.2410107	3.01423327	0.09798701	0.19576087
Ptrhd1	-0.3503302	3.71099187	3.01305515	0.09804826	0.1958433
Nabp1	-0.3393817	3.64228668	3.01260562	0.09807165	0.19585007
Fam174a	-0.313083	5.07082992	3.01221526	0.09809196	0.1958507
Smco4	-0.6301004	1.93044032	3.01165334	0.0981212	0.19586917
Pih1d1	-0.5435048	3.36208392	3.01016656	0.09819864	0.1959838
Gm6251	-0.8289422	0.42859106	3.00898007	0.09826048	0.19606728
Chrnb3	0.83881523	1.28994325	3.00542359	0.09844613	0.19609765
Ak7	-0.6872908	1.73166403	3.00533092	0.09845098	0.19609765
1700020L24l	1.83087462	-1.6620476	3.00504902	0.09846571	0.19609765
Nop9	-0.4208353	3.74602846	3.00504285	0.09846603	0.19609765
Sec24a	0.24833457	5.49204325	3.00501489	0.09846749	0.19609765
Tnfaip6	0.58242272	1.74255489	3.00487876	0.09847461	0.19609765
Col17a1	2.69477224	-1.226709	3.00426742	0.09850658	0.19609765
Mtch1	-0.2326654	6.41998609	3.00409906	0.09851538	0.19609765
Tbc1d24	0.31085025	6.35733208	3.00340967	0.09855144	0.19609765
Irak4	-0.4703185	3.52899172	3.00338214	0.09855288	0.19609765
C1qtnf7	-0.6667092	4.36202515	3.00336098	0.09855399	0.19609765
4921515E04l	1.61148169	-0.8939193	3.00321824	0.09856146	0.19609765
AI597479	-0.271028	5.87756521	3.00317147	0.09856391	0.19609765
Bcas3os1	1.42735066	0.45882069	3.00316735	0.09856412	0.19609765
1700017B05l	-0.4438062	2.86725022	3.00294061	0.09857599	0.19609765
Rab20	-2.5610983	-2.0306466	3.00246791	0.09860074	0.19610705
A230077H06	0.75602174	2.28415411	3.00204361	0.09862295	0.19611141
Rabggta	0.43509305	3.21503695	3.00075856	0.09869028	0.19620546
Pkm	-0.2582817	8.20468187	2.99930338	0.09876658	0.19631731
Kdm5b	0.32171679	5.90629015	2.99821103	0.0988239	0.19639139
2210013O21	-0.3427056	5.29079884	2.99658035	0.09890955	0.19651391
Pcdhb12	0.55330751	2.49227365	2.99593879	0.09894326	0.19651391
Faah	0.36845603	4.14335949	2.99589177	0.09894574	0.19651391
Mal2	-0.2091846	6.39667895	2.9949329	0.09899616	0.19657419
Prpf4	0.25412676	5.00342337	2.9944216	0.09902306	0.19658617
Il1rapl1	0.35196722	3.91836958	2.99405542	0.09904233	0.19658617
Rassf2	-0.3616344	7.49587503	2.99282571	0.09910707	0.19665234
Galnt12	0.8541765	0.82133625	2.9922579	0.09913699	0.19665234
Chd1	0.18923076	6.5805177	2.99200699	0.09915021	0.19665234
Rps4x	-0.3475783	7.89493547	2.99189763	0.09915597	0.19665234
Drd3	1.47966161	-1.0424144	2.99120603	0.09919243	0.19665723
Acsm3	1.7557974	-0.887876	2.99108917	0.09919859	0.19665723
Exph5	0.44405289	6.36395724	2.98992262	0.09926012	0.19673939
Inhbb	-0.8152947	1.70768754	2.98852843	0.09933372	0.19682478
Tmsb15b1	0.73441379	1.17472549	2.98834538	0.09934339	0.19682478

Prkd3	-0.2763693	5.66350149	2.98730118	0.09939856	0.19689426
Smarca5	0.21425838	7.57245339	2.98574307	0.09948095	0.19701762
Ehd4	0.31868912	4.01675613	2.98535338	0.09950156	0.19701862
Ces2g	0.52834106	3.49425668	2.98402333	0.09957198	0.19711819
2310035C23I	0.30559585	6.63927903	2.98233602	0.09966138	0.1972454
C630031E19I	0.81147633	1.84095033	2.98205078	0.09967651	0.1972454
Rpl30	-1.0372249	0.13246401	2.98092779	0.09973608	0.19729904
Idh1	-0.2783728	5.41998055	2.98046444	0.09976067	0.19729904
Tet2	0.37608118	6.67525259	2.9802465	0.09977223	0.19729904
Cxcl5	1.02695352	0.65863524	2.98002143	0.09978418	0.19729904
Ano5	1.14600143	0.82467018	2.97904183	0.09983621	0.19736207
Rbx1	-0.3034997	6.58727791	2.97807703	0.09988748	0.19742061
Lmo4	-0.2933886	9.51337193	2.97772626	0.09990613	0.19742061
Gdpd1	0.27005729	5.43781772	2.9747787	0.10006299	0.1976158
Bop1	-0.4647106	2.95513326	2.97448351	0.10007872	0.1976158
Xkr4	0.43375076	4.24244498	2.97411795	0.1000982	0.1976158
Dpcd	-0.5149949	3.13768392	2.97382996	0.10011354	0.1976158
Cdc14a	-0.4563417	4.04350205	2.97349592	0.10013135	0.1976158
Usmg5	-0.2956179	5.86410141	2.97345005	0.1001338	0.1976158
Zfp62	0.2710739	6.19282385	2.97321877	0.10014613	0.1976158
Snrpd1	-0.2749098	5.26247952	2.97236504	0.10019166	0.19763167
Kank1	0.36950509	3.58697731	2.97231139	0.10019452	0.19763167
Spry4	0.42014045	3.46186275	2.97120553	0.10025354	0.1976921
Cited2	-0.2591344	6.39891047	2.97034941	0.10029926	0.1976921
Rab35	-0.2595679	4.52165069	2.97034173	0.10029967	0.1976921
Batf	1.27885483	0.02424304	2.97022525	0.10030589	0.1976921
Spon2	2.22372598	-0.6560705	2.96939432	0.10035029	0.19773982
Rpl31	-0.351226	8.64150413	2.96837765	0.10040464	0.19780713
Brk1	-0.2984412	6.30129478	2.96669155	0.10049486	0.19794242
Xylt2	-0.7074001	2.16298136	2.96622219	0.10052	0.19794242
Plekhm3	0.29218527	6.33117024	2.96596193	0.10053394	0.19794242
Gm5523	-0.2644343	5.24483222	2.96410369	0.10063352	0.1980751
Pcca	-0.2629576	5.77101254	2.96395002	0.10064176	0.1980751
Atp6v1h	-0.1820051	6.29465663	2.96311431	0.10068659	0.19810656
Wdr25	-0.4827048	1.99011313	2.96289801	0.1006982	0.19810656
Tnfrsf25	2.58307015	-1.3089208	2.96156304	0.10076987	0.19820775
Hspa14	0.2783373	4.2484401	2.96034241	0.10083546	0.19826355
Nufip1	-0.3759647	4.1197829	2.9602817	0.10083872	0.19826355
Enox1	-0.3630233	3.98225846	2.95906489	0.10090415	0.19833856
Ctnnd1	0.20217389	8.01841914	2.95881921	0.10091737	0.19833856
Fam171b	0.26694618	7.11323488	2.95780574	0.10097191	0.19840595
Zfp866	0.23595799	5.11100105	2.95706934	0.10101157	0.19844406
Sdpr	-0.3940526	7.13197726	2.95647337	0.10104367	0.19845051

Igsf1	1.44800896	0.70347853	2.95625627	0.10105537	0.19845051
Ap4s1	-0.225038	5.86757563	2.95554145	0.1010939	0.19848638
Sugct	-0.6937045	1.49156978	2.95462837	0.10114314	0.19851293
Ylpm1	0.43630426	8.12727214	2.95453918	0.10114795	0.19851293
Ddx51	-0.281223	3.89237777	2.95263573	0.1012507	0.19867477
Myo1f	-0.6846125	1.27857512	2.95166832	0.10130296	0.19869946
Auts2	0.21039912	6.88281406	2.95165186	0.10130385	0.19869946
Rsph4a	-0.545579	2.93990736	2.94964479	0.10141239	0.19887253
Dip2c	0.27936214	6.83602647	2.94921383	0.10143571	0.19887845
Dirc2	-0.292797	4.94217804	2.94768443	0.10151853	0.19895667
Myh13	2.3649815	-1.4079411	2.94737801	0.10153514	0.19895667
Ssu72	-0.2943756	5.2620379	2.94735205	0.10153654	0.19895667
Bdh2	-0.6171434	3.09510841	2.9468654	0.10156292	0.19896855
Miat	0.49054798	6.21309444	2.94371229	0.10173401	0.19913209
A830009L08I	-0.7363008	0.87970727	2.94354969	0.10174284	0.19913209
A330093E20	1.33168023	0.23437656	2.94337179	0.1017525	0.19913209
Daam2	0.36015122	5.00971729	2.94332744	0.10175491	0.19913209
Rbm18	-0.258504	6.24774405	2.94327237	0.1017579	0.19913209
Zfp318	0.36173854	6.73134452	2.94258958	0.10179501	0.19913209
Praf2	-0.3533483	4.63199854	2.94255509	0.10179688	0.19913209
Shroom1	0.63214222	1.58595977	2.94233163	0.10180903	0.19913209
Fbxo28	-0.2006155	6.1643748	2.94165414	0.10184586	0.19915105
Oip5	1.44311388	0.22054863	2.94140554	0.10185939	0.19915105
Rorb	-0.2815586	8.50372588	2.93999101	0.10193636	0.19926177
Gm20199	0.78804307	2.13159792	2.93729994	0.10208299	0.19950002
Bdh1	-0.3568915	3.63196056	2.93694317	0.10210244	0.19950002
5730522E02I	0.95658636	1.39875561	2.93637675	0.10213334	0.19950002
4930593A02I	2.10846779	-1.3030035	2.93622119	0.10214183	0.19950002
Ppapdc1a	1.25384928	-0.1787357	2.93588695	0.10216007	0.19950002
Fam132b	1.36311248	-0.102842	2.93462431	0.10222902	0.19952192
Gab2	-0.2698638	6.02991746	2.93457032	0.10223197	0.19952192
Ctu1	0.63666616	1.55210896	2.93446413	0.10223777	0.19952192
Zbed5	-0.4120941	3.8677752	2.93406429	0.10225961	0.19952192
Shmt2	0.4464407	2.55247759	2.93381693	0.10227313	0.19952192
Mkl2	0.33726127	9.41262042	2.9333396	0.10229922	0.19953308
Frmpd3	0.90096864	1.33049436	2.93286369	0.10232524	0.19953405
C030018K13I	0.84923298	1.52609964	2.93252926	0.10234353	0.19953405
Auh	-0.265996	5.11797038	2.93147073	0.10240145	0.19953405
Plrg1	-0.2760637	4.51438789	2.93116978	0.10241792	0.19953405
Cd59b	-1.2241129	-0.4724651	2.93109928	0.10242178	0.19953405
Tns4	-0.8337509	1.63779668	2.93109636	0.10242194	0.19953405
Map3k19	0.54486341	2.77347339	2.93049669	0.10245478	0.19955833
Gm6710	-0.4099514	2.44380817	2.92967271	0.10249992	0.19960656

Zgpat	0.65475104	1.82987523	2.92851649	0.10256329	0.19969028
Fibp	-0.3269543	4.96051385	2.92541787	0.10273337	0.19989702
Haus6	0.3400165	3.95219475	2.92534391	0.10273743	0.19989702
S100b	-0.3617414	6.07697975	2.92528686	0.10274057	0.19989702
Nras	-0.2211391	7.27390837	2.92493493	0.10275991	0.19989702
Dennd6b	0.66906665	2.8384596	2.9247237	0.10277152	0.19989702
Eef1e1	-0.3294906	3.33863197	2.92290743	0.10287141	0.20005159
Fam53b	-0.3398544	4.28924641	2.92177511	0.10293374	0.20013307
Pcsk7	0.43129573	2.68672644	2.91911331	0.10308044	0.20037518
Pcyox1l	0.62309396	1.9727679	2.91877364	0.10309918	0.20037518
Setd2	0.24989674	7.47981448	2.91800691	0.10314149	0.20041765
Anxa2	-0.5526099	6.18191341	2.91719006	0.10318659	0.20045849
Ninj2	1.54673474	-0.9280136	2.91680418	0.1032079	0.20045849
C5ar1	1.80347255	-0.0568141	2.91651459	0.1032239	0.20045849
Pam	0.24772505	6.3274475	2.9147589	0.10332096	0.20053591
D3Bwg0562e	0.28153173	7.1409987	2.91449345	0.10333564	0.20053591
Mier3	-0.2573861	5.08618564	2.91441318	0.10334008	0.20053591
Il34	-0.3319894	3.78935797	2.91422003	0.10335077	0.20053591
Igsf10	0.41258204	3.27417852	2.9139424	0.10336613	0.20053591
Syt13	-0.2551768	6.10051852	2.91336624	0.10339802	0.20055806
Slc2a12	0.39491132	4.38770554	2.91274849	0.10343222	0.20057337
Iqub	0.70997598	1.53412581	2.91248415	0.10344686	0.20057337
Adcyap1r1	0.31015446	6.44811207	2.91193961	0.10347703	0.20059215
Dusp8	0.31316354	5.57658294	2.91154324	0.103499	0.20059503
Hist1h2bl	-0.5213128	3.11037497	2.91069226	0.10354617	0.20061055
Zic1	-0.3994195	8.07189603	2.91053034	0.10355515	0.20061055
Cdk14	-0.2317211	6.91946638	2.91029068	0.10356844	0.20061055
Rtn4	0.22376116	9.9353984	2.909789	0.10359628	0.20062479
Rpl29	-0.3344666	6.10243951	2.90848911	0.10366843	0.20066227
Gpx3	0.47883185	3.06524729	2.9074129	0.10372821	0.20066227
Cbx3	-0.255778	8.36237764	2.90679961	0.1037623	0.20066227
Sec31a	0.1769622	7.07424304	2.90668793	0.1037685	0.20066227
Cast	-0.3702492	6.45665256	2.9064146	0.1037837	0.20066227
Arhgap26	0.28208434	7.57515888	2.90640536	0.10378422	0.20066227
Slc30a5	0.29521386	3.98366903	2.9062971	0.10379024	0.20066227
Alkbh3	-0.4569184	3.75199751	2.9059714	0.10380835	0.20066227
Sema4f	0.38591958	4.77742819	2.90583994	0.10381566	0.20066227
Fam175a	0.43201267	2.6092575	2.90575317	0.10382049	0.20066227
Asb8	-0.2081907	6.2702894	2.90412445	0.10391113	0.20078003
Tbx22	-2.0846367	-1.4582454	2.90349264	0.10394632	0.20078003
Kcnrg	1.11429524	1.30895024	2.90319963	0.10396264	0.20078003
Prorsd1	-0.2965327	3.89631622	2.90318601	0.1039634	0.20078003
Ssfa2	0.23374701	5.4928508	2.90119933	0.10407416	0.20095431

Cul3	-0.1802625	8.90574059	2.90012482	0.10413412	0.2010198
Klhl41	0.71221325	1.5136362	2.89979535	0.10415252	0.2010198
Saal1	-0.5171027	2.24950395	2.89939942	0.10417463	0.2010198
Mamdc4	-1.3555275	-0.0726255	2.89912123	0.10419016	0.2010198
Jarid2	0.2400793	5.84144929	2.89812981	0.10424556	0.20108707
Psmc5	-0.239279	6.68950131	2.89657043	0.10433276	0.20118227
Pcdhb7	-0.6612953	2.24062595	2.89629368	0.10434825	0.20118227
Foxj2	-0.237119	6.55270403	2.8961457	0.10435653	0.20118227
Cxx1c	-0.2288913	5.91556429	2.89520111	0.10440941	0.20124461
1700049G17	0.3900621	3.46919692	2.89402848	0.1044751	0.2013316
Ifi47	-0.5636153	3.77676149	2.89193877	0.10459228	0.20141009
Alg8	0.33336363	3.33045314	2.89151731	0.10461593	0.20141009
Prlr	0.42322087	2.37048023	2.89131107	0.10462751	0.20141009
Dhx9	0.31268483	7.76847638	2.8908166	0.10465527	0.20141009
Glrx2	-0.1964539	6.7348839	2.89031138	0.10468364	0.20141009
1700037C18I	-1.0519131	0.04865188	2.88994214	0.10470439	0.20141009
Clec14a	0.64904748	1.55869742	2.88918209	0.1047471	0.20141009
Atp5g2	-0.4445864	4.07447845	2.8888996	0.10476298	0.20141009
Plk2	0.28386947	7.04814951	2.88878393	0.10476948	0.20141009
S100a16	-0.5880821	3.25723789	2.88834857	0.10479396	0.20141009
Wdr53	-0.4948134	3.18469403	2.88833394	0.10479478	0.20141009
Nrip3	-0.2627218	5.45211388	2.88821515	0.10480147	0.20141009
5830454E08I	-1.198286	0.68021135	2.88797859	0.10481477	0.20141009
Gna13	-0.2174276	7.53995271	2.88767799	0.10483168	0.20141009
Gm13547	2.40957191	-2.4282955	2.88746869	0.10484346	0.20141009
Stk11ip	-0.4720666	3.48361789	2.88744464	0.10484481	0.20141009
Ddx59	-0.5061746	2.5780165	2.88646633	0.10489988	0.20146657
Mtap	-0.4035922	4.5968512	2.88619157	0.10491535	0.20146657
Lrrc23	-1.0669567	0.84062034	2.88576111	0.1049396	0.20147364
Slfn8	-0.5699047	3.41249401	2.88504926	0.10497971	0.20149536
Zfp28	0.47678095	2.80136131	2.88483017	0.10499206	0.20149536
Hn1	-0.3552797	4.32365813	2.88444268	0.1050139	0.2014978
Cts8	0.88539636	0.00836896	2.88368572	0.10505659	0.20151463
Slc35e2	0.30480256	5.59113456	2.8835576	0.10506381	0.20151463
Slc2a9	1.94661029	-0.8794348	2.88307429	0.10509108	0.20152747
E130317F20I	-0.8444295	1.16468983	2.8816965	0.10516886	0.20160457
Aggf1	-0.2327294	6.57041674	2.88130863	0.10519077	0.20160457
Rev3l	0.29531734	7.29469965	2.88126854	0.10519303	0.20160457
Cpe	0.23648327	10.0402984	2.88036134	0.1052443	0.20166336
Adcy2	0.26152614	5.76768634	2.87966158	0.10528386	0.20168502
Fut11	0.36800537	3.90062084	2.87917532	0.10531136	0.20168502
Traf1	-1.1850331	0.98828572	2.87906914	0.10531737	0.20168502
Kcnd3	0.3657695	5.96922766	2.8770614	0.10543102	0.20182721

Ctnna1	-0.2890548	7.91023598	2.87702944	0.10543283	0.20182721
Lrrn1	0.32582552	6.20171724	2.87660881	0.10545666	0.20183339
B3glct	0.25336137	5.47446189	2.87498603	0.10554865	0.20196999
Magi3	0.22428572	6.70809646	2.87401431	0.10560378	0.20198595
Il31ra	-0.542215	2.40872718	2.87391239	0.10560957	0.20198595
Bche	-0.4641024	7.2717699	2.87346863	0.10563476	0.20198595
Pqlc1	-0.3168933	3.33315234	2.87338548	0.10563948	0.20198595
Lmod1	-0.425419	5.0582383	2.87192243	0.10572258	0.20210539
Gm266	0.86696262	0.6425385	2.87116534	0.10576561	0.20213438
Ago3	0.2718005	6.0504347	2.8709296	0.10577902	0.20213438
Ddt	-0.4747708	3.74680858	2.87007832	0.10582744	0.20218419
Slc16a13	0.47510963	2.78603537	2.86943539	0.10586402	0.20218419
Vldlr	0.29206682	6.06539724	2.86938299	0.105867	0.20218419
Rps10	-0.4075439	4.92802873	2.86806134	0.10594226	0.20228188
C030034L19F	2.02977856	-1.2749103	2.86775951	0.10595946	0.20228188
Ushbp1	-1.062045	0.5480966	2.86498415	0.10611773	0.20254456
Dio3os	-2.3920707	-1.726407	2.86426517	0.10615878	0.20258343
Slc22a5	0.48101527	2.58385577	2.86299102	0.10623156	0.20268284
Maoa	0.27154378	4.91687969	2.86234212	0.10626865	0.20271412
Evc	0.49285293	2.46743737	2.86183717	0.10629753	0.20272972
CK137956	-1.0261258	0.78151713	2.86115077	0.10633679	0.20274792
Psmd3	-0.2815625	4.38659442	2.86094675	0.10634847	0.20274792
Gm11837	1.22584506	-0.748374	2.85996949	0.10640441	0.20281509
Ttl	-0.20678	5.9524659	2.85942786	0.10643543	0.20282427
Arhgap32	0.34583305	9.3418549	2.85902841	0.10645831	0.20282427
Spint1	1.28871942	-0.3384106	2.85864254	0.10648042	0.20282427
Nrp1	0.30478286	4.973746	2.85843968	0.10649205	0.20282427
Atp5d	-0.431786	6.11576955	2.85672754	0.10659023	0.20293446
Pcdh12	-3.5141329	-1.9877816	2.85670829	0.10659133	0.20293446
Ankrd34c	-0.3396065	4.32065125	2.85624973	0.10661765	0.20294511
Scly	-0.4759845	3.52715264	2.85416217	0.10673754	0.2030894
Map3k15	0.87293329	0.18468205	2.85304883	0.10680155	0.2030894
Tomm20	-0.177016	7.75160618	2.85295884	0.10680672	0.2030894
Fancd2	0.6512755	1.26540415	2.85257371	0.10682888	0.2030894
Gm19705	-0.7224527	1.55104948	2.85221874	0.1068493	0.2030894
D430019H16	0.30022349	6.30631517	2.85214389	0.10685361	0.2030894
Kctd17	-0.411278	4.42842515	2.85175201	0.10687616	0.2030894
Slc43a3	1.29147238	0.2159002	2.85164624	0.10688225	0.2030894
Adra2b	1.08266158	0.18659244	2.85138456	0.10689731	0.2030894
Ing4	-0.3100299	3.88740656	2.85132426	0.10690078	0.2030894
Pon3	-0.5559371	3.32299925	2.84996501	0.10697908	0.20319873
6030443J06F	0.66255522	1.76003475	2.84792802	0.10709653	0.20338239
Apobec4	1.40069145	-0.0917944	2.84682364	0.10716027	0.20342564

Tmem47	0.29270951	7.50737928	2.84681382	0.10716084	0.20342564
Spcs1	-0.3133476	5.36136485	2.84582134	0.10721816	0.20346325
Prpf8	0.25989979	8.26146319	2.84575148	0.1072222	0.20346325
Snora28	2.59451824	-1.4223794	2.84419038	0.10731244	0.20354148
A730043L09I	2.93681625	-2.1676103	2.84337545	0.10735959	0.20354148
Efhd1	-0.3266048	4.60422175	2.8427827	0.10739389	0.20354148
Sptan1	0.48958845	9.21986903	2.84269455	0.10739899	0.20354148
Zmynd19	-0.7369927	1.55240992	2.8426757	0.10740009	0.20354148
Rtp1	2.4940659	-1.5165248	2.84262846	0.10740282	0.20354148
Cwc15	-0.2882706	6.787145	2.84205641	0.10743594	0.20354148
Nek5	-2.5203333	-1.245548	2.84201394	0.1074384	0.20354148
Far1	0.24171389	6.28201656	2.84146773	0.10747004	0.20354148
Cers6	0.34448003	5.97498481	2.84144743	0.10747122	0.20354148
Gbf1	0.26670445	5.9862477	2.84106871	0.10749316	0.20354369
Klhdc2	-0.1747968	7.86071972	2.84037941	0.10753312	0.20357999
Rps26	-0.3942789	5.54799771	2.83623306	0.10777382	0.20399626
Csnk2b	-0.266699	5.72714885	2.83575932	0.10780136	0.20400897
3830408C21I	-0.5252755	2.47583336	2.83532365	0.1078267	0.2040175
AI429214	0.53245209	3.37119213	2.83491521	0.10785046	0.20402304
Nedd4l	0.34389423	8.72859637	2.83315694	0.10795281	0.20415501
Tmem214	0.32276175	4.44956222	2.83300082	0.1079619	0.20415501
Cxcr5	0.99961005	-0.2027588	2.83147498	0.10805082	0.20428226
Alox5ap	-0.6929644	1.4941033	2.83065734	0.10809851	0.20428226
Gm15663	-0.4595071	3.1623083	2.83008817	0.10813171	0.20428226
Fxyd7	-0.5401511	2.00153272	2.82968104	0.10815548	0.20428226
Gm19461	1.63518835	-0.8248495	2.82937301	0.10817346	0.20428226
Esyt2	0.20473184	5.89773321	2.82935996	0.10817422	0.20428226
Hsd17b14	-0.8349276	0.86079391	2.82903631	0.10819312	0.20428226
Actrt3	-1.709867	-0.9276285	2.8289863	0.10819604	0.20428226
Bub1b	1.09427466	0.47634977	2.8285089	0.10822392	0.20429553
Ppp2r1a	-0.1804651	7.25493951	2.82737859	0.10828997	0.20438083
Rassf9	-0.8689439	0.90130565	2.8262661	0.10835503	0.20440916
Tomt	-0.7916589	0.54964426	2.82606779	0.10836663	0.20440916
Traf6	-0.2337238	6.18016976	2.82605143	0.10836759	0.20440916
Srpx	-1.4034248	0.14940974	2.82562892	0.10839231	0.20441643
Pggt1b	-0.3028915	4.10302833	2.82456122	0.10845481	0.20446442
Gm12505	-0.4937238	1.77653996	2.82448114	0.1084595	0.20446442
Alas1	0.24751584	4.46677621	2.82356195	0.10851335	0.20451152
Clmn	0.23485217	5.98196232	2.82334193	0.10852625	0.20451152
Spty2d1	-0.2591919	4.96492595	2.82180421	0.10861641	0.20464207
Strada	0.41091187	3.65046623	2.82086432	0.10867157	0.20470661
Zfc3h1	0.34676353	6.97548533	2.81963199	0.10874393	0.2047548
BC025920	-0.6764777	1.46221459	2.81956358	0.10874795	0.2047548

C5ar2	0.554519	1.32805436	2.81906737	0.10877711	0.2047548
Micall1	-0.2460914	5.22167441	2.81900517	0.10878076	0.2047548
Rab27a	-0.3561379	3.40232344	2.81815503	0.10883074	0.20480951
Lnx1	0.34472567	4.01709519	2.81722473	0.10888546	0.20487224
Cmpk1	-0.2313534	7.47820594	2.81687733	0.1089059	0.20487224
Skiv2l	0.32380476	5.25710978	2.81563176	0.10897923	0.20497082
Fam206a	-0.2804963	5.91732222	2.81363501	0.10909691	0.20514396
Pcdh8	0.48074445	2.58289504	2.81335923	0.10911317	0.20514396
Ppp1cb	-0.1925083	9.23777984	2.81285551	0.10914289	0.20516045
Akap12	-0.286291	8.54412102	2.81114124	0.10924408	0.20531127
Yme1l1	0.22148391	6.23404001	2.81053544	0.10927987	0.20532038
Ano10	0.4554807	2.49026703	2.81014833	0.10930275	0.20532038
Siae	0.30961719	4.5606751	2.80999499	0.10931181	0.20532038
Retnla	1.0155624	1.0485688	2.80892582	0.10937503	0.20536675
Slc39a12	0.71085654	2.63789016	2.80886828	0.10937843	0.20536675
Clk4	0.29082045	5.76616854	2.80770327	0.10944737	0.20543125
Gm17660	2.16355643	-1.4513759	2.80757902	0.10945473	0.20543125
Xrcc3	0.42376555	3.43280101	2.80642909	0.10952283	0.20551968
Rnf144a	0.29712824	4.77117646	2.80550465	0.10957761	0.2055831
Dctd	0.7412403	1.64541764	2.80443905	0.1096408	0.20566226
Hs2st1	0.23253342	6.7575588	2.80384021	0.10967633	0.20568951
Tmem132b	0.34367348	7.49325511	2.80340541	0.10970213	0.20569852
5430421F17l	0.97456333	0.8712816	2.80233204	0.10976587	0.20574923
H3f3a	-0.4051781	8.1858611	2.80185282	0.10979434	0.20574923
Edf1	-0.4409734	6.41286507	2.80159039	0.10980993	0.20574923
Ldb3	-0.6236148	2.06945824	2.80153546	0.10981319	0.20574923
Tspan1	2.18121747	-1.3200596	2.79939427	0.10994053	0.20594841
Armcx6	1.02149726	0.96305597	2.79793426	0.11002745	0.20607184
Gfpt1	0.23688328	6.60092891	2.7972168	0.1100702	0.20611079
Bcs1l	0.39402652	2.77376128	2.79667843	0.11010229	0.20611079
Slc7a10	0.99721475	0.19958365	2.79648228	0.11011398	0.20611079
Zfp85os	-0.4768923	2.59106711	2.7957844	0.1101556	0.20611079
Gorasp2	-0.2338578	7.01602837	2.79562873	0.11016488	0.20611079
Ccl2	-1.3096192	-0.4998618	2.79494301	0.1102058	0.20611079
Carf	0.27521412	4.65339293	2.79487247	0.11021001	0.20611079
Tfap2d	-1.5569776	-0.1952972	2.7946414	0.1102238	0.20611079
Psmb6	-0.3355145	4.90056129	2.79432704	0.11024257	0.20611079
Pde3a	0.87073582	1.06521131	2.79405736	0.11025867	0.20611079
Lrrtm3	0.26849863	4.97338521	2.79284518	0.11033108	0.2061761
Tor1b	0.36005233	3.76440609	2.79225274	0.11036649	0.2061761
Cxx1a	-0.2390234	6.02202972	2.79216047	0.11037201	0.2061761
Gbp3	-0.4090088	4.32332538	2.79206361	0.1103778	0.2061761
Hnrnpul2	-0.1753988	8.048024	2.79009673	0.11049547	0.20635655

Hist3h2a	-0.9323556	0.54765609	2.78875773	0.11057566	0.20645683
Rap2b	-0.2329261	5.93718849	2.78849639	0.11059132	0.20645683
Nsmce2	-0.2530486	5.28630791	2.78681722	0.110692	0.20659557
Smc6	0.18637488	7.65803915	2.78620431	0.11072878	0.20659557
Set	-0.2041266	8.09528691	2.78620211	0.11072891	0.20659557
Chia1	1.51294378	-0.1819364	2.78536738	0.11077902	0.2066497
Srcin1	0.43459578	6.08474988	2.78464313	0.11082252	0.20667449
Stat4	-0.5171609	1.82879885	2.78438309	0.11083814	0.20667449
Mylpf	1.41030087	-0.1585606	2.78409237	0.11085561	0.20667449
Fancd2os	1.2110833	0.21805888	2.7829946	0.1109216	0.20672366
Sync	-0.9664296	1.07307096	2.78251382	0.11095052	0.20672366
Fam229b	-0.4650537	2.47483404	2.78243222	0.11095543	0.20672366
Gbx2	-2.5940787	-1.7696169	2.78218865	0.11097008	0.20672366
Tgfb3	-0.485237	4.69475048	2.78187919	0.11098871	0.20672366
Chn1	-0.1872776	9.71703491	2.78154854	0.11100861	0.20672366
Rngtt	0.24100919	5.27968148	2.78036027	0.11108016	0.20681759
Mmp13	2.63070273	-1.9234618	2.77920506	0.11114977	0.20683601
Gm15787	0.86516989	0.71033111	2.77901124	0.11116146	0.20683601
Prpf38b	0.22155088	7.32605818	2.77871143	0.11117954	0.20683601
Dstyk	0.22118557	5.49522802	2.778593	0.11118668	0.20683601
Rpl38	-0.3158956	6.22195774	2.77844449	0.11119563	0.20683601
Pcyt1a	-0.2536655	5.75957855	2.77762434	0.11124511	0.20685473
Vwa5a	-0.2869515	5.49992937	2.77757761	0.11124793	0.20685473
Tmx4	0.23261639	8.3392014	2.77656734	0.11130892	0.20691965
Ip6k2	0.27942155	4.0375015	2.77629946	0.1113251	0.20691965
Ndufa1	-0.3471956	5.23904643	2.77491321	0.11140886	0.20698029
Ivns1abp	0.22195515	7.54592213	2.77481298	0.11141492	0.20698029
Cd209b	2.71196233	-1.5381916	2.77457963	0.11142902	0.20698029
Mlf1	-1.2612944	-0.3012403	2.774361	0.11144224	0.20698029
Pdss1	0.57439629	2.13099445	2.77349412	0.11149468	0.20703842
Dazap2	-0.2517839	8.82880673	2.77219798	0.11157313	0.20710858
Sec22a	-0.4361553	2.98563507	2.77205909	0.11158154	0.20710858
Bcl2a1b	-0.6438263	1.45049734	2.7718222	0.11159589	0.20710858
Anapc16	-0.4179152	5.99677279	2.7707797	0.11165906	0.20718655
1700102H20I	-2.3600013	-1.2506463	2.76989954	0.11171242	0.20724631
Celf6	-0.4269582	2.68095841	2.76882966	0.11177732	0.20732745
Pwwp2a	0.33152897	5.79995736	2.76814907	0.11181863	0.20735996
4930539J05F	0.88368417	0.32804557	2.76784351	0.11183719	0.20735996
A630033H20	1.3630791	-0.6315604	2.76635456	0.11192765	0.20739194
Tmem50b	-0.2116367	5.85195413	2.76585825	0.11195782	0.20739194
Acadsb	-0.2409527	6.82862526	2.76540002	0.11198568	0.20739194
Parvb	-0.4348967	3.54574773	2.76520927	0.11199728	0.20739194
Rnf113a2	-0.3013836	4.72744651	2.76493339	0.11201407	0.20739194

Ccbe1	0.35755772	3.84561048	2.76475715	0.11202479	0.20739194
Ap4b1	-0.587078	2.03441604	2.76444391	0.11204385	0.20739194
Elof1	-0.5293992	3.1789434	2.7643856	0.1120474	0.20739194
4930594C11I	1.41761285	1.31140351	2.76425548	0.11205532	0.20739194
Rpia	-0.4054166	2.92032853	2.76407734	0.11206616	0.20739194
C4bp-ps1	2.27570023	-1.5809795	2.76307931	0.11212693	0.20746521
Ntpcr	-0.4644597	2.61603098	2.76267877	0.11215133	0.20747117
Paip1	0.25128066	6.24463162	2.76140554	0.11222894	0.20757035
Pxdc1	-0.4902024	3.64415578	2.7611039	0.11224733	0.20757035
4930469G21	1.04874062	-0.1709537	2.76014245	0.11230599	0.20758711
Rnasek	-0.3787056	7.09958463	2.75995121	0.11231766	0.20758711
Fubp1	0.24670394	7.1259254	2.7596244	0.11233761	0.20758711
Rnf220	-0.2289997	6.94879055	2.75956616	0.11234116	0.20758711
Pomgnt1	0.41025358	2.95261862	2.75907675	0.11237104	0.20760316
Frem3	1.23101182	0.105963	2.75782302	0.11244764	0.20770549
Clcn1	1.07499289	0.05914403	2.75508104	0.11261536	0.20792242
Ptprj	0.29248605	6.83767348	2.75491832	0.11262533	0.20792242
Gcnt4	0.39105629	4.22489231	2.7548623	0.11262876	0.20792242
Pcbp2	-0.2070159	6.942454	2.75379163	0.11269434	0.20800429
Etv3	0.26406977	3.91685719	2.7529013	0.11274891	0.20805366
Fam179a	1.47616172	-0.4688586	2.75195046	0.11280722	0.20805366
Dok7	1.3897633	-0.7094101	2.75188883	0.112811	0.20805366
Nemf	0.26924207	7.13975157	2.75164671	0.11282586	0.20805366
Mms19	0.44180681	3.6826746	2.7513856	0.11284188	0.20805366
Acvr1b	0.29113131	6.06409219	2.75127728	0.11284853	0.20805366
Acp2	0.21088806	5.57459214	2.74991541	0.11293215	0.20816864
Efhc1	-0.6233519	1.78039606	2.74866556	0.11300895	0.2081888
Slc10a4	-0.4493048	2.53480028	2.74860648	0.11301259	0.2081888
Prkag2os1	1.30114453	-0.1707102	2.74817687	0.113039	0.2081888
Prss12	0.48202186	1.85980198	2.74801204	0.11304914	0.2081888
Dse	-0.5425199	3.90497036	2.74800855	0.11304935	0.2081888
Cdc42bpa	0.23661898	9.05411707	2.74702899	0.11310962	0.20826063
Ccdc61	-0.7880312	0.48748189	2.74650057	0.11314214	0.20828137
A530054K11I	0.24868412	5.33622178	2.74556865	0.11319953	0.20833957
Scrn1	0.30501357	5.45736724	2.74529647	0.1132163	0.20833957
Cs	-0.1722972	7.85166602	2.74484653	0.11324403	0.20835145
Riok1	-0.3699369	3.87879186	2.74273152	0.11337446	0.20848396
Sptssa	-0.3199607	6.40935421	2.7427014	0.11337632	0.20848396
Ino80d	0.22697688	7.35145253	2.74257259	0.11338427	0.20848396
Fam114a2	-0.3316617	5.08308205	2.74222999	0.11340542	0.20848396
Mrpl49	-0.3852763	4.77436411	2.74171933	0.11343694	0.20848396
Gpx1	-0.4780139	6.58973415	2.7416091	0.11344375	0.20848396
E2f2	-0.677324	3.00064518	2.74099338	0.11348178	0.20851473

Sprn	0.28962823	5.91404279	2.73915377	0.11359551	0.20868454
Morn5	-2.3989801	-1.4756517	2.73863256	0.11362775	0.20870464
Ndufc2	-0.3227914	5.2850835	2.73790186	0.11367297	0.20874856
Hn1l	-0.4679801	4.36652087	2.73723444	0.1137143	0.20875243
Cadps	0.35083983	8.58472785	2.73674138	0.11374484	0.20875243
Usp16	-0.2416546	6.12332536	2.73660549	0.11375326	0.20875243
Adam23	0.27771588	7.15181729	2.73649142	0.11376033	0.20875243
Agtpbp1	0.24289804	8.9554073	2.73535516	0.11383075	0.20881998
Dclre1a	-0.4127302	3.56079589	2.73520964	0.11383978	0.20881998
1700030L20F	0.7372709	1.35267767	2.73455885	0.11388014	0.20885492
Phf21b	-0.5731687	2.20298585	2.73411804	0.11390749	0.20886597
Rbmx	0.20522031	6.76613628	2.73298996	0.11397752	0.20895527
Slc22a23	-0.2271011	6.19523421	2.7323311	0.11401845	0.20899118
Sh3bgrl	-0.2800242	8.852275	2.73144508	0.11407351	0.20903187
Dna2	1.60324226	-0.6860066	2.73100523	0.11410086	0.20903187
Znhit3	-0.3776618	3.91894958	2.73020513	0.11415062	0.20903187
Clip1	0.28683039	7.59804925	2.73016869	0.11415289	0.20903187
Stxbp5	0.32830702	7.72459988	2.72967394	0.11418367	0.20903187
Maz	0.31756878	3.87511503	2.72925503	0.11420975	0.20903187
Cd109	0.60342622	3.07290153	2.72895841	0.11422821	0.20903187
Vmn2r86	0.96605458	0.46302815	2.72893514	0.11422966	0.20903187
Fgf2	-0.6059242	1.59855045	2.72888622	0.11423271	0.20903187
Rfxap	-0.4046905	4.03711884	2.72722619	0.11433613	0.20918203
Zfp623	-0.3882308	3.4205748	2.72469963	0.11449374	0.20942333
Atg12	-0.3089754	5.11943071	2.72442682	0.11451077	0.20942333
Rpl3	-0.3010157	8.2228053	2.72346275	0.11457099	0.209479
Ccdc59	-0.324795	5.04935982	2.7229366	0.11460387	0.209479
Hs3st3b1	-0.4444427	3.71015302	2.72291261	0.11460537	0.209479
Sptb	0.49450659	5.42552413	2.72043043	0.11476065	0.20972369
Pex11a	-0.7635834	1.16454539	2.71962571	0.11481104	0.20973916
Wasf3	-0.2452006	5.95092442	2.7196114	0.11481194	0.20973916
Mettl16	-0.2735492	5.66215822	2.71893156	0.11485453	0.20977784
Zbtb8os	-0.4615768	4.06613915	2.71772935	0.1149299	0.20986779
Focad	0.28759228	5.28452876	2.71746263	0.11494663	0.20986779
Epb4.1	0.22628054	4.96364631	2.7168613	0.11498436	0.20988902
Tmem130	0.32856702	5.16068055	2.71659432	0.11500111	0.20988902
Flot2	-0.244856	4.8676707	2.71600659	0.11503801	0.20991724
Itpa	-0.2743813	4.19649389	2.71533333	0.11508029	0.20995528
B4galt5	0.30398738	4.63843676	2.71455477	0.1151292	0.21000541
2610507B11I	0.18318962	8.00325485	2.71254404	0.11525565	0.2101867
Zfp169	0.31046296	4.07761419	2.71229203	0.11527151	0.2101867
Mrpl50	-0.3245957	5.7014081	2.71190427	0.11529591	0.21019208
Gnb1l	-1.1961263	0.36464636	2.71129873	0.11533404	0.21022246

Hopx	-0.5117102	3.04404463	2.7106197	0.11537681	0.21022882
Dlx5	0.47702582	2.04843014	2.71021227	0.11540249	0.21022882
Xpnpep2	1.49888031	0.25433481	2.70996194	0.11541826	0.21022882
Cox4i1	-0.4121247	6.74873163	2.70946167	0.1154498	0.21022882
Mill2	0.95007887	0.60879185	2.70901345	0.11547807	0.21022882
Ppp1r3c	-0.3202754	6.08555093	2.70895727	0.11548161	0.21022882
Mtcp1	-0.4749563	3.03143394	2.70852289	0.11550901	0.21022882
Pgp	-0.266561	3.71346144	2.70851949	0.11550922	0.21022882
Pard6g	-0.3638533	5.59374964	2.7080581	0.11553834	0.21024274
Churc1	-0.2433016	4.54674334	2.70738149	0.11558105	0.21028139
Smn1	0.36234536	4.31988517	2.70646277	0.11563907	0.21034789
Kdm7a	0.2134553	7.30540922	2.70588492	0.11567558	0.21037524
Slfn1	-1.6609988	-1.0410111	2.70476902	0.11574613	0.21044814
Ets2	0.23853203	5.6900743	2.70457121	0.11575864	0.21044814
Fam109a	0.68849095	0.94421152	2.70341279	0.11583194	0.21054233
Prdx1	-0.3045021	7.18344903	2.70178538	0.11593501	0.21069057
Atxn7l1	-0.210329	6.14296575	2.70046008	0.11601902	0.21080414
Anp32b	-0.2674262	8.28371329	2.6999642	0.11605047	0.21082219
Slc12a5	0.30389372	7.82459709	2.69740651	0.11621287	0.21103247
Gm16023	0.69094995	0.95694866	2.69730694	0.1162192	0.21103247
Mov10l1	2.43462898	-2.089242	2.69695635	0.11624148	0.21103247
Morf4l1	-0.2297737	8.47922785	2.69567217	0.11632314	0.21103247
Ucp2	-0.4860349	7.46890025	2.69562548	0.11632611	0.21103247
Samd10	-0.4471781	3.49672755	2.69558506	0.11632868	0.21103247
Ttbk2	0.2460325	7.80790211	2.69535116	0.11634356	0.21103247
Rapgef1	0.24599572	6.0110437	2.69511699	0.11635847	0.21103247
Metrnl	-0.5202394	2.14349993	2.69509089	0.11636013	0.21103247
Eif2ak4	0.48726254	3.93746764	2.69448539	0.11639867	0.21106329
Atpaf2	0.36085157	3.16296147	2.69408114	0.11642441	0.21107089
Cml5	0.99133291	0.85791997	2.69362335	0.11645357	0.21107675
Tnfrsf18	0.57546095	1.90014686	2.69278012	0.1165073	0.21107675
Ube2n	-0.1671367	7.43722179	2.69255495	0.11652165	0.21107675
Xpnpep1	-0.3089231	4.48513859	2.69253637	0.11652283	0.21107675
Madd	0.31713136	6.89785884	2.69233949	0.11653538	0.21107675
Gm765	-0.4659196	2.83983776	2.69175244	0.11657282	0.21110072
Jdp2	-0.3161219	3.35051052	2.69145612	0.11659172	0.21110072
Rhoc	-0.4653115	3.48230513	2.68991036	0.11669037	0.21122778
Baiap2	0.23872719	6.25207541	2.68968094	0.11670502	0.21122778
Wnt2	1.51747018	-1.0513622	2.68921502	0.11673479	0.21124262
Ccdc13	1.43251204	-0.3812154	2.68844935	0.11678371	0.21127515
Gje1	0.6337857	1.15646148	2.68748365	0.11684545	0.21127515
Ftsj2	-0.7019838	2.03862433	2.68699789	0.11687653	0.21127515
Mapre2	-0.1779266	9.8021625	2.68689983	0.1168828	0.21127515

Uggt1	0.38093734	5.11580547	2.68684009	0.11688662	0.21127515
Ppil1	-0.3269872	3.77002774	2.68680064	0.11688915	0.21127515
Hist1h2bb	-1.3756559	-1.2061748	2.68657243	0.11690375	0.21127515
Tmem82	0.84510189	0.41533048	2.68429364	0.11704968	0.21148608
Tlr6	2.8358987	-1.3341172	2.68348247	0.11710168	0.21148608
Npas1	1.2263993	-0.7981625	2.68342905	0.11710511	0.21148608
Tmem11	-0.3967507	3.33295823	2.6830698	0.11712815	0.21148608
Slc25a24	-0.4067145	5.32740564	2.6830657	0.11712841	0.21148608
Stk32a	0.571206	1.75903453	2.6815632	0.11722483	0.21155252
Arl4a	-0.3009786	7.29655897	2.680987	0.11726183	0.21155252
1110017D15I	1.02525071	-0.1132766	2.68085134	0.11727054	0.21155252
Ttc39b	0.28718737	7.09488054	2.68082854	0.11727201	0.21155252
Slc24a3	-0.2680187	6.88660705	2.68081003	0.11727319	0.21155252
Kcnj2	0.25847294	5.54481899	2.68047001	0.11729504	0.21155296
Cx3cl1	0.25580603	6.78151362	2.67902091	0.11738818	0.21168198
Mb21d2	-0.2810081	5.16656613	2.6783279	0.11743276	0.21172339
Pde8a	-0.2531907	4.07415265	2.67761712	0.1174785	0.21174781
Tm9sf2	0.24394611	5.92029723	2.67744556	0.11748954	0.21174781
Dtx1	-0.3073149	4.62920153	2.67655306	0.11754701	0.21177593
Cdh9	0.52912362	3.91287303	2.67653175	0.11754838	0.21177593
Gm2109	-2.7014513	-2.2724261	2.67584393	0.1175927	0.21177789
Ccdc96	-0.7432888	1.74268235	2.67584369	0.11759271	0.21177789
Gm11437	2.0253064	-1.1134879	2.67384579	0.11772154	0.21197094
Gm12070	-0.2234264	10.3711141	2.67269574	0.11779578	0.21206562
Depdc7	-0.7272808	1.14205109	2.6719756	0.11784229	0.21211037
Adam17	0.29495005	4.19544606	2.67024388	0.11795423	0.21227285
Micu1	-0.2360631	4.45383813	2.66876108	0.11805017	0.21240649
Cd163	0.81464933	1.35450107	2.66685177	0.11817385	0.21257157
Sbno1	0.30478039	8.41056419	2.66667486	0.11818532	0.21257157
Zfp715	0.29585677	4.43571062	2.66545797	0.11826424	0.21264475
Cyp20a1	0.54180496	2.77855337	2.665378	0.11826943	0.21264475
2010012O05	-0.2281442	6.00612599	2.66273091	0.11844132	0.21291473
Pdia6	0.29167583	4.75854562	2.66224661	0.1184728	0.21293003
Smim13	-0.2083976	8.64473928	2.66193126	0.11849331	0.21293003
Gm10560	2.15261649	-1.0415094	2.66134324	0.11853155	0.21295968
Taz	0.38407055	3.1171464	2.65842517	0.11872156	0.21319118
Urb2	0.34725799	4.20458844	2.65841309	0.11872234	0.21319118
Spn	-1.1300597	-0.2228099	2.65828315	0.11873081	0.21319118
Slc4a2	-0.4286632	3.65489648	2.65802777	0.11874746	0.21319118
Cad	0.60045257	1.781203	2.65645849	0.11884982	0.21330866
Sntn	-1.2419031	-0.1784704	2.65635683	0.11885645	0.21330866
Uhrf1bp1l	0.29473981	7.76480923	2.65480314	0.1189579	0.21345163
A530013C23	-1.8131314	-1.4641545	2.6540828	0.11900497	0.21348724

Slc6a7	-0.3170699	5.20504357	2.65369924	0.11903004	0.21348724
Ppwd1	0.30344303	3.72394038	2.653499	0.11904313	0.21348724
Polr3b	0.3155538	4.43934886	2.65271013	0.11909473	0.21350961
Nrm	-0.7824081	0.49915898	2.65264178	0.1190992	0.21350961
Tssc1	0.32901398	3.27212638	2.65183139	0.11915223	0.21356559
Smagp	-1.0712315	0.54925906	2.65028759	0.11925333	0.2137077
Acot12	2.24241587	-1.5535456	2.64940471	0.1193112	0.21377228
Tmc2	1.01572418	-0.2558401	2.64887104	0.11934619	0.21379587
Vmac	-0.4727687	3.71146309	2.64794925	0.11940666	0.21386509
Rnf10	-0.2441415	5.83996032	2.64641053	0.11950768	0.2140069
Lpin3	-2.5106136	-1.554597	2.64449727	0.11963344	0.2141494
Vcan	0.50004181	4.12263198	2.64449119	0.11963384	0.2141494
Rpl37a	-0.3611405	5.95519467	2.64420224	0.11965285	0.2141494
Pappa2	0.6595155	2.28047774	2.64366807	0.11968799	0.21417317
Spin4	0.53880015	2.74782339	2.64312419	0.11972379	0.21419809
Pmfbp1	1.73350024	-1.3142859	2.6427665	0.11974734	0.2142011
Cish	-0.7740251	1.17032559	2.64117733	0.11985203	0.2143299
Fsd1	-0.5729425	2.29276353	2.64019705	0.11991666	0.2143299
Ermp1	0.23903188	5.74204652	2.6400612	0.11992562	0.2143299
Gm14204	0.57956921	2.45307692	2.64004792	0.1199265	0.2143299
Kat8	0.318201	3.25460002	2.64001383	0.11992874	0.2143299
Tshb	1.98962552	-1.2726407	2.63848757	0.12002947	0.21444794
Slc5a12	1.02235541	0.11531533	2.63834959	0.12003858	0.21444794
Myef2	0.28205451	5.63887936	2.63787795	0.12006973	0.21446448
Grin2c	0.54948554	2.15088364	2.63537173	0.12023541	0.21467025
Gps2	-0.2758232	4.02309499	2.63521249	0.12024595	0.21467025
Cdv3	-0.1890553	7.74731071	2.63480861	0.12027268	0.21467025
Emx2os	0.46224173	2.96091169	2.63466253	0.12028235	0.21467025
Plxnc1	0.21149894	6.4050276	2.63447875	0.12029451	0.21467025
1700052N19	0.29679659	3.84405497	2.63409752	0.12031975	0.21467618
Pcgf3	-0.2205065	5.67129604	2.63152116	0.1204905	0.21492514
Fbxo5	0.93041845	0.50880187	2.63133009	0.12050317	0.21492514
Gse1	0.30617717	5.57081042	2.62824106	0.1207083	0.21524457
Cand2	-0.3657514	3.67553313	2.62782648	0.12073586	0.21524457
Nkiras2	1.32457267	0.14816501	2.62764104	0.12074819	0.21524457
Rhod	-0.6907203	1.72168286	2.62707063	0.12078613	0.21527303
Wdr59	0.35233526	4.08083724	2.62512346	0.12091574	0.21546483
Mtx1	-0.4574347	2.53885428	2.6243864	0.12096485	0.21551313
A930004D18	-0.5857276	2.82111606	2.6216151	0.1211497	0.2158032
Rpp25	0.33098045	3.94710413	2.61981195	0.12127015	0.21597848
Crhr1	-0.5655448	1.89336019	2.61947668	0.12129256	0.21597913
Rpgrip1l	0.33912526	5.86432918	2.61756398	0.12142051	0.21616767
Nt5c3	-0.2199973	5.14275865	2.61669808	0.12147849	0.21623159

B3galnt2	0.29940057	3.82718944	2.61577551	0.1215403	0.21623313
Plxna4os1	1.21412156	-0.4873521	2.61575449	0.12154171	0.21623313
Mrpl51	-0.2412193	5.05127658	2.61569672	0.12154558	0.21623313
Dnmt1	0.30604684	5.72564765	2.61492754	0.12159714	0.21628558
Med27	-0.4926952	3.23350047	2.6122449	0.12177718	0.21652833
Psmc2	-0.1801844	6.27084943	2.61223525	0.12177783	0.21652833
Gpr171	0.7355213	1.52802902	2.61165157	0.12181704	0.21655875
Imp4	-0.3154127	4.57065773	2.61087366	0.12186933	0.21661239
Tnrc6a	0.22980624	6.73545539	2.60901494	0.12199437	0.21677075
Vezf1	-0.2449787	7.58884059	2.60889145	0.12200269	0.21677075
Otud4	0.21760851	6.54656309	2.60787534	0.12207111	0.21680378
Stmn3	-0.2893943	5.74413792	2.60763798	0.1220871	0.21680378
Zwint	-0.1723631	6.89570744	2.60762948	0.12208768	0.21680378
Kremen2	-1.0766889	-0.7455648	2.60640604	0.12217014	0.21688506
BC051142	0.83297476	1.26833807	2.6061689	0.12218613	0.21688506
2900076A07	0.76119719	0.95672739	2.6058327	0.12220881	0.21688506
Slc12a4	0.8543186	1.46995527	2.60563686	0.12222202	0.21688506
Wnk2	0.33386556	6.04766164	2.6040255	0.12233078	0.21699605
Hcn3	0.61577701	1.66083011	2.60372877	0.12235082	0.21699605
Zfp687	-0.3603886	3.60778931	2.60372582	0.12235102	0.21699605
Gm1987	1.91457219	-1.4241787	2.60162996	0.12249269	0.21717424
Rpgrip1	-1.4305646	-0.9646415	2.60158356	0.12249583	0.21717424
Cirbp	-0.5055211	4.87862209	2.60053178	0.122567	0.21726109
Rps8	-0.3269643	7.08743797	2.59927057	0.12265241	0.21734771
Camk1g	0.42342812	4.06859878	2.59903218	0.12266856	0.21734771
Acot1	-0.2776057	3.95154939	2.59882743	0.12268243	0.21734771
Epor	0.80567967	0.39059471	2.59814942	0.12272839	0.21735836
Zfp46	-0.2083821	5.51714231	2.59808403	0.12273282	0.21735836
Myo3b	0.91417551	0.40957379	2.59703145	0.12280421	0.21743927
Cox19	-0.3537399	3.39470768	2.5967559	0.12282291	0.21743927
Actr5	1.1790189	0.08199314	2.59638038	0.12284839	0.21744509
Timm9	-0.2580656	4.92435511	2.59533531	0.12291935	0.21753138
Dlat	-0.2006167	8.21845619	2.59483868	0.12295309	0.21755178
Cct3	0.2311252	5.12571248	2.59379973	0.1230237	0.21763741
2810474O19	0.22065938	6.6693074	2.5932421	0.12306162	0.2176439
Ccnb2	1.35942663	-0.2245179	2.59309233	0.12307181	0.2176439
Rps19	-0.3790486	4.67855439	2.59186312	0.12315546	0.21774808
Fbxw10	-0.8401864	0.68857113	2.59129744	0.12319398	0.21774808
Slc5a6	0.38190781	4.25431609	2.5909904	0.12321489	0.21774808
Pde9a	0.68952003	2.03889486	2.59063626	0.12323901	0.21774808
Mettl5	-0.3535572	3.45748939	2.59059433	0.12324187	0.21774808
Gm12191	-0.5620711	1.2385392	2.58970384	0.12330256	0.21777275
Ust	-0.4335242	5.5251111	2.58946719	0.12331869	0.21777275

07-Mar	0.25832062	4.80727516	2.58941093	0.12332253	0.21777275
Prkcsh	0.37555984	3.3401779	2.58864712	0.12337463	0.21781709
Sult5a1	-1.3457639	-0.535735	2.58839082	0.12339211	0.21781709
Tcam1	-3.8460548	-1.9937716	2.62620558	0.12360958	0.21816165
Irgm1	-0.3898883	4.39098843	2.58473272	0.12364201	0.21817405
Agap1	0.244836	6.71017495	2.58401817	0.1236909	0.21817405
Pou2f3	1.03107124	1.17603717	2.58381182	0.12370502	0.21817405
9530059O14	0.89802291	2.91368661	2.58380194	0.1237057	0.21817405
Mapk11	0.38857829	3.69421599	2.58306938	0.12375584	0.21819767
Plcb4	0.27673385	7.05152563	2.58256448	0.12379042	0.21819767
2610207O16	1.02741271	0.22999278	2.58230667	0.12380808	0.21819767
Zfyve26	0.3345482	4.09534786	2.58155376	0.12385967	0.21819767
Igfbp3	-0.5079083	3.83518722	2.58120576	0.12388353	0.21819767
Mrap2	-0.9333337	1.52222856	2.58115496	0.12388701	0.21819767
Epha10	0.89539303	2.34216075	2.58107203	0.1238927	0.21819767
Syce2	0.65489553	1.79897998	2.58100493	0.1238973	0.21819767
Snrpn	-0.6420395	0.80614836	2.57965488	0.1239899	0.21823341
Ahdc1	0.27282112	4.66728877	2.57961762	0.12399246	0.21823341
Tspan9	-0.4217978	3.49513706	2.57881418	0.12404761	0.21823341
Asic4	1.00219474	-0.0935885	2.57880357	0.12404834	0.21823341
Cstf1	0.39762023	2.83750905	2.57867025	0.1240575	0.21823341
Pard3b	-0.3647199	3.32297747	2.57863274	0.12406007	0.21823341
Fndc8	-2.4612513	-2.2316128	2.57828232	0.12408414	0.21823341
Sox21	0.40275643	3.44466163	2.57794817	0.12410709	0.21823341
Grk6	0.39726517	2.89637392	2.57778792	0.1241181	0.21823341
Tjp2	-0.2382366	5.62012645	2.57731532	0.12415058	0.21825134
Rpl7a	-0.2982593	7.31122517	2.57639147	0.1242141	0.21832382
Unc93a	1.90961285	-0.8055965	2.57571745	0.12426046	0.21835914
Iqgap2	0.2910669	4.97802915	2.57545126	0.12427878	0.21835914
Paqr7	0.24432377	4.56034292	2.57455191	0.12434069	0.21838576
Phldb2	-0.3346183	7.68725526	2.57434644	0.12435484	0.21838576
2410018L13R	-0.4578916	1.73556512	2.57398924	0.12437944	0.21838576
Zfp386	-0.2297501	5.38364077	2.573936	0.12438311	0.21838576
Gm3985	1.33651298	-0.8746755	2.57289659	0.12445473	0.21847235
Irf6	-0.4529287	3.74546148	2.5725478	0.12447878	0.21847541
Iffo2	-0.2837073	4.67135398	2.57176971	0.12453244	0.21853044
Sdc3	0.24786142	5.2388551	2.57112586	0.12457686	0.21856924
Rps20	-0.294067	6.34851348	2.56991158	0.1246607	0.21867715
Il16	-0.6913173	1.13578219	2.56901982	0.12472231	0.21873008
Zfp783	0.6918558	1.36514797	2.56882852	0.12473553	0.21873008
Spata4	2.0574924	-1.0776967	2.56681363	0.12487489	0.21893526
Klc1	-0.226904	7.49123601	2.56634077	0.12490762	0.21895345
Diap1	0.24245255	4.50604362	2.56587234	0.12494005	0.21897112

Hck	-1.3312566	-0.4803346	2.56525736	0.12498265	0.21900659
AI317395	-1.6823611	-0.8851693	2.56479663	0.12501457	0.21901734
Eif2ak1	-0.2112969	6.22664605	2.56452359	0.1250335	0.21901734
Tnni2	-1.8282186	-1.3371784	2.5638674	0.12507899	0.21905785
Snurf	0.61266467	1.5189843	2.56338064	0.12511275	0.21907781
Ccl22	-2.0468029	0.31341969	2.56229787	0.12518788	0.2191591
Abcb9	-0.6365913	1.61859793	2.56206687	0.12520392	0.2191591
Sidt2	0.22307853	5.19264066	2.56164239	0.1252334	0.21917152
AI506816	1.58882855	-1.375879	2.56079143	0.12529251	0.21923451
Cfdp1	-0.3010879	6.92855852	2.56047997	0.12531415	0.21923451
Tbp	-0.1963985	5.33457464	2.55865239	0.12544124	0.21941767
Smim7	-0.3387788	6.30717233	2.5578483	0.12549721	0.21947637
Gpr18	1.33190715	-0.4416102	2.55616934	0.12561417	0.21962029
Cyp11a1	1.04089614	-0.1870064	2.55574727	0.12564359	0.21962029
Pip5kl1	1.2570845	0.28413704	2.55570167	0.12564677	0.21962029
Hoxd11	1.18799334	-0.3836331	2.55316014	0.12582411	0.21989103
Lrrc9	0.82059016	1.59318661	2.55270107	0.12585617	0.21990783
Insl6	-0.8051843	1.1793507	2.55227063	0.12588625	0.21992115
Gpr3	-0.7516091	1.08837726	2.5517056	0.12592574	0.21995091
Man1a2	0.19843088	7.88075799	2.55074478	0.12599292	0.2199757
Prps2	0.22822881	6.17915191	2.55046318	0.12601262	0.2199757
Prdx3	-0.2836586	5.17439889	2.55019377	0.12603147	0.2199757
Rps19bp1	-0.3826336	2.88831036	2.55016016	0.12603382	0.2199757
Endog	-1.3862815	-0.8303844	2.54966236	0.12606866	0.2199757
Man2a1	0.199678	6.07197675	2.54957654	0.12607467	0.2199757
Fam131a	-0.260945	5.2288787	2.54905946	0.12611087	0.21999968
Ccdc127	-0.1916944	6.5235289	2.54831953	0.1261627	0.2200509
Ptprt	0.35271264	6.91884679	2.54797051	0.12618716	0.22005437
Arl14ep	-0.2020076	6.37628164	2.54679307	0.1262697	0.22015149
Ankrd55	0.46668083	2.6483128	2.54651132	0.12628946	0.22015149
Get4	-0.3231209	3.89752722	2.54621467	0.12631027	0.22015149
3632454L22F	0.87662318	0.7864583	2.54579497	0.12633972	0.22016364
Arfgef1	0.23791804	7.73294247	2.54477014	0.12641167	0.22023906
Sra1	-0.2788011	5.14917602	2.54453805	0.12642797	0.22023906
Clstn3	0.3673227	4.49061883	2.54388182	0.12647407	0.22028019
Pithd1	-0.2914602	4.77159265	2.54355052	0.12649735	0.22028157
Vim	-0.3313934	8.07016048	2.53778813	0.12690311	0.22086466
Trappc11	0.24056717	5.46142792	2.53775027	0.12690579	0.22086466
Cog2	0.31246838	3.48085586	2.5377171	0.12690813	0.22086466
Pan2	0.40120566	3.5095207	2.53722852	0.1269426	0.22086466
Pou2af1	-0.5273206	4.8134834	2.53719561	0.12694493	0.22086466
Tax1bp3	-0.4787	4.64836874	2.53546161	0.12706738	0.22102673
Nin	0.22355297	6.83042218	2.53523758	0.12708321	0.22102673

Immp2l	-1.0027465	-0.0527315	2.53476284	0.12711677	0.22104585
Homer2	0.2078624	7.54094242	2.53438482	0.12714349	0.22105308
Rnaset2b	-0.4171111	4.08648288	2.5333133	0.12721929	0.2211074
Mx1	1.42607564	0.01692365	2.53330486	0.12721988	0.2211074
Mpzl1	-0.3681463	3.20946085	2.53266503	0.12726517	0.22114687
Pof1b	-0.9652415	0.09221184	2.53117778	0.1273705	0.22126855
Ssbp2	0.22222736	6.26453336	2.53043167	0.12742339	0.22126855
Zfhx3	-0.1910155	7.2958918	2.53037916	0.12742711	0.22126855
Stk10	-0.3288479	3.50792912	2.5298104	0.12746744	0.22126855
Bdp1	0.24751343	7.43458905	2.52971034	0.12747454	0.22126855
D630023F18	0.48936339	2.00640674	2.52964759	0.12747899	0.22126855
Prune2	-0.2062097	6.64228343	2.52944558	0.12749332	0.22126855
Idh2	-0.3801644	4.04339643	2.52750247	0.12763126	0.22141244
Hecw2	0.34721573	6.36028967	2.5273345	0.12764319	0.22141244
Fbn2	0.65038628	1.50799282	2.52732258	0.12764404	0.22141244
B3galt4	1.55788676	-0.7880389	2.52601125	0.12773724	0.22153487
1700110I01R	0.83005142	1.59061764	2.52497473	0.12781096	0.2216235
Sema3f	-0.8452475	0.80992883	2.52366572	0.12790414	0.22174582
Bcr	0.2999078	6.07003854	2.52296164	0.1279543	0.22179351
Traip	1.0865209	0.84322021	2.52142097	0.12806412	0.22194461
Bach2	0.33994551	4.7164935	2.51998217	0.12816679	0.22208324
Apol9a	1.65888006	-1.0042618	2.51871462	0.12825732	0.2222008
Col20a1	0.78311555	1.24662046	2.51784511	0.12831946	0.222236
Dcp2	-0.1997955	7.18498446	2.51779546	0.12832301	0.222236
Rbbp7	-0.206699	7.41912342	2.51745326	0.12834748	0.22223908
Gm15408	1.33203265	-0.4333354	2.51682064	0.12839273	0.22227814
Ccne2	0.5960907	1.99831562	2.5164231	0.12842117	0.22228809
Gabra4	0.27299733	5.85303945	2.51451538	0.12855777	0.22248522
Tarbp2	0.43381266	2.33320718	2.5136923	0.12861676	0.22254799
E030030I06R	0.53402648	2.81872364	2.5132353	0.12864952	0.22256537
Copz2	-0.4911067	6.78348118	2.51272783	0.12868592	0.22258574
Ube2v2	-0.199013	6.88664878	2.51243759	0.12870674	0.22258574
Myl9	-0.5152235	5.49638529	2.51062793	0.12883666	0.22277109
Uck2	-0.3428766	3.46702241	2.50967451	0.12890517	0.22283691
Vprbp	0.22260586	6.75123308	2.50946509	0.12892023	0.22283691
Slco4c1	0.53695677	2.13125651	2.50878371	0.12896922	0.22288227
Pmaip1	-0.4031462	3.4226659	2.50712877	0.12908832	0.22303701
Pmpcb	-0.2077564	5.02703198	2.50690677	0.1291043	0.22303701
Soga1	0.18603536	7.13274724	2.50645624	0.12913675	0.22305373
Bysl	-0.320787	3.83428023	2.50585851	0.12917982	0.22308877
Fbxo33	-0.2772546	4.8653221	2.50530354	0.12921982	0.22311852
Neu1	-0.3496074	3.96564936	2.50406852	0.12930889	0.22323296
Ncoa5	-0.3143024	4.6525009	2.50364651	0.12933934	0.2232376

Rbm7	-0.2950948	5.12838101	2.50322236	0.12936995	0.2232376
Psmb3	-0.2912378	4.45844632	2.50308396	0.12937995	0.2232376
Ino80	0.26721784	5.08845141	2.50274819	0.12940419	0.22323815
Tmem151b	0.40535957	4.64442946	2.5024483	0.12942585	0.22323815
Slc6a6	0.19128712	7.94764577	2.50148615	0.12949536	0.22331873
Dguok	-0.4705098	2.97350383	2.49909137	0.12966858	0.22357809
Smchd1	0.24637577	6.18192511	2.49873148	0.12969464	0.22358118
Sepn1	0.46617085	3.45527712	2.49841481	0.12971757	0.22358118
Nfkbil1	1.05914204	-0.2143319	2.49786935	0.12975708	0.22358118
Pitpnc1	0.18496954	6.72428963	2.4978059	0.12976167	0.22358118
Plch2	0.51380934	3.25218423	2.49625978	0.12987375	0.22373494
Bckdk	-0.2586637	4.30818593	2.49520127	0.12995055	0.2238236
Ifi204	1.16970358	-0.3667039	2.4949206	0.12997092	0.2238236
Sytl2	-0.2748224	5.78833679	2.49405955	0.13003344	0.2238326
Stk35	-0.2324255	5.14102998	2.49372522	0.13005773	0.2238326
Spef2	-0.8094503	0.09680215	2.49348092	0.13007548	0.2238326
BC039771	0.68078537	0.57098261	2.4933669	0.13008376	0.2238326
2410004B18	-0.235449	5.13860267	2.49323209	0.13009356	0.2238326
Capns1	-0.3164319	9.23059355	2.49296111	0.13011325	0.2238326
Evc2	0.60459727	2.24314415	2.49203684	0.13018045	0.22390889
Atg9a	0.27650983	5.13802662	2.48939802	0.13037255	0.2241735
Sorl1	0.30221102	6.15766545	2.48929474	0.13038007	0.2241735
Ubb	-0.4216199	6.41461667	2.48791587	0.13048059	0.22430366
Celf5	0.23824223	6.86974412	2.48741578	0.13051707	0.22430366
Nupr1	-0.4676447	6.67529031	2.48677801	0.1305636	0.22430366
Gm6654	-0.8048856	0.80954439	2.48666051	0.13057218	0.22430366
Spag9	0.19246799	8.37034525	2.48646201	0.13058667	0.22430366
Sf3b4	-0.2796533	4.65157292	2.48637303	0.13059317	0.22430366
Tenc1	-0.3349127	5.97022186	2.48575235	0.13063849	0.22434216
Plekhg2	-0.3835471	4.20003844	2.48471946	0.13071395	0.2243679
Rexo2	-0.2090082	6.99962684	2.4844315	0.130735	0.2243679
Zdhhc21	0.24474235	6.37299455	2.48442929	0.13073516	0.2243679
Zfp82	0.59047945	1.45211517	2.48368851	0.13078933	0.2243679
Tm6sf2	1.75669202	-1.4528194	2.48367409	0.13079038	0.2243679
Trem2	1.2187747	-0.4979594	2.4836669	0.13079091	0.2243679
Zscan18	0.31515022	3.71227459	2.48269627	0.13086192	0.22443593
Gbas	-0.2336954	6.53615095	2.48241914	0.1308822	0.22443593
Gm12504	0.92139179	0.86210628	2.48195076	0.13091649	0.22443593
Ccdc77	0.37462377	3.53799165	2.48187266	0.13092221	0.22443593
Gria3	0.28612797	8.51083982	2.48121843	0.13097013	0.22445595
Lypd1	-0.3304565	4.83227986	2.48085947	0.13099643	0.22445595
Als2cl	-0.746128	0.5495359	2.48077472	0.13100264	0.22445595
Crispld1	-0.3507589	3.89178035	2.48043133	0.13102781	0.22445981

Alg5	-0.5064924	3.24676587	2.47896381	0.13113542	0.22460096
Fancg	0.45153938	2.75922905	2.47853942	0.13116656	0.22460096
Kcnn4	-2.6604307	-1.6284853	2.47837014	0.13117899	0.22460096
Ccdc166	-0.3856901	3.31457511	2.47715808	0.13126799	0.22468738
Ruvbl2	-0.2740958	4.49916799	2.47678067	0.13129571	0.22468738
Gucd1	-0.4519068	3.58927075	2.47674577	0.13129828	0.22468738
Sars	-0.2780066	4.81691384	2.47581478	0.13136671	0.22473203
Cytl1	-1.9412611	-0.5109423	2.47528905	0.13140537	0.22473203
Braf	0.20944807	8.58365271	2.4751808	0.13141333	0.22473203
Slc45a4	0.32803984	3.61257026	2.47514252	0.13141614	0.22473203
Cox4i2	1.63003241	-1.5290112	2.47407571	0.13149464	0.22482702
A930012L18I	-0.5415412	2.57284962	2.47327724	0.13155343	0.22485689
Afap1l1	-0.3170195	5.54664804	2.4732149	0.13155802	0.22485689
Lgalsl	-0.1814021	6.23959162	2.47275754	0.13159171	0.22487524
Tada2a	0.30526517	3.75529382	2.47221732	0.13163152	0.22490403
Usp4	0.23403771	5.49972037	2.47133036	0.13169691	0.22497651
Hsd3b7	-0.3956852	2.68274283	2.47099667	0.13172152	0.22497931
Ranbp3l	-0.3183075	7.77261109	2.47005796	0.13179078	0.22498315
Ndufb6	-0.2908305	5.63196151	2.47004481	0.13179175	0.22498315
Thumpd2	0.53427245	1.67259557	2.46986664	0.1318049	0.22498315
Znrd1as	-0.3436559	3.55287635	2.46961281	0.13182364	0.22498315
Sh3pxd2b	-0.2646705	4.59955708	2.46941013	0.13183861	0.22498315
Adat1	0.5325302	1.8778403	2.46828961	0.13192138	0.22508518
Pcgf1	-0.4578538	3.18836724	2.4675636	0.13197504	0.22511855
Acadvl	-0.3146824	4.90350757	2.46740312	0.1319869	0.22511855
Pramef8	-0.3493626	4.01107825	2.4666749	0.13204076	0.2251712
Ap4e1	0.36421065	4.05886757	2.46587815	0.13209971	0.22523253
Med30	-0.4201243	3.31133533	2.46403814	0.13223598	0.22542563
Lrch2	0.35647981	4.19729802	2.46319623	0.13229839	0.22546146
Orai2	-0.3329353	3.83792651	2.46313362	0.13230303	0.22546146
Tob2	-0.2841405	5.13169507	2.46192659	0.13239257	0.2255748
Emg1	-0.4989186	2.94861394	2.46129484	0.13243946	0.22561545
Alg2	0.17231137	7.44663579	2.46027994	0.13251484	0.2257046
Zfp654	0.29516369	5.43811125	2.4598658	0.13254561	0.22571776
Ccno	1.70808858	-0.7397575	2.45941825	0.13257887	0.22573516
Acox3	0.28000717	4.07254784	2.45904188	0.13260685	0.22574357
Mtpn	-0.1972875	10.0811484	2.45857676	0.13264144	0.22575452
Uck1	-0.3142042	3.16796925	2.45820474	0.13266911	0.22575452
Stoml3	-2.4696446	-1.6978164	2.45784225	0.13269608	0.22575452
Coch	-0.2842779	7.79535415	2.45743245	0.13272658	0.22575452
Taf15	0.27082634	5.09488274	2.45740637	0.13272852	0.22575452
Osgin1	-1.1486898	0.20312214	2.45709065	0.13275203	0.2257553
Camta1	0.33832993	9.37254189	2.45675003	0.13277739	0.22575924

Fsd1l	0.29556337	6.17601251	2.45573809	0.13285277	0.2258482
Smarcal1	0.31006675	6.14881675	2.45509361	0.13290081	0.22589066
Aox3	-0.3129751	6.1406988	2.45433252	0.13295756	0.22594792
Olfr464	-2.3329376	-1.9463999	2.45372307	0.13300303	0.22595327
Fes	-1.3248167	-0.3101794	2.45367195	0.13300685	0.22595327
Slc44a5	0.58689351	1.67814149	2.45256595	0.13308941	0.22602825
Serpinb1b	1.07398198	-0.1128303	2.45246251	0.13309713	0.22602825
Capn10	-0.4328396	2.36488744	2.45086157	0.13321676	0.22619219
Nfu1	-0.2850234	4.48020055	2.44962605	0.13330917	0.22630597
Ppfia3	0.32150954	4.67632354	2.44915808	0.13334419	0.22630597
Gm6994	1.00274925	0.04688228	2.44903931	0.13335308	0.22630597
H2-M3	-0.6259393	2.40479271	2.44683621	0.13351812	0.22646441
Pet112	0.39065942	2.78670512	2.44655359	0.1335393	0.22646441
Dpp7	0.57731403	1.49041866	2.44650329	0.13354308	0.22646441
Ptrh2	0.23299534	4.96435401	2.44600891	0.13358015	0.22646441
Kif16b	-0.2081031	4.96535644	2.44540038	0.13362581	0.22646441
Svip	-0.1948799	5.08626736	2.4453979	0.13362599	0.22646441
Parp12	-0.3178762	4.35770763	2.44537059	0.13362804	0.22646441
Ppip5k2	0.2518726	5.30691516	2.44532577	0.1336314	0.22646441
Alx3	-0.5369248	3.2206397	2.44393031	0.13373617	0.22660276
Efhc2	0.71200547	2.12178194	2.44323936	0.13378808	0.22665151
Pcdh17	0.29882043	6.97977252	2.44205744	0.13387693	0.22673711
Pcdha6	0.99035343	0.30558676	2.44194471	0.13388541	0.22673711
1700123O21	1.82632167	-1.0875901	2.44157088	0.13391353	0.22673711
Rpl22l1	-0.30375	5.0394012	2.44133609	0.1339312	0.22673711
Apc	0.30157352	9.68081644	2.44084997	0.13396778	0.22675985
Tbc1d23	0.29026164	4.34883226	2.43988706	0.13404028	0.22684337
Rarres1	1.90075117	-0.6069795	2.4389389	0.13411172	0.22692505
Cap2	0.27780088	8.29989561	2.43825705	0.13416312	0.22697282
Rab1b	-0.3663545	4.50357591	2.43788766	0.13419097	0.22698074
Slc39a10	0.20928517	8.38104969	2.4364114	0.13430236	0.22712993
Col14a1	-0.7886897	0.85800448	2.43482771	0.13442198	0.22725857
Cnr2	0.6938306	0.95599685	2.4347435	0.13442835	0.22725857
Hunk	0.31250183	4.23948636	2.43424183	0.13446627	0.22725857
Itpka	0.2916967	4.18836864	2.4341762	0.13447123	0.22725857
Mrpl14	-0.5064941	3.03606529	2.43225453	0.13461662	0.22746131
Sytl1	1.29760226	0.01323853	2.43164847	0.13466251	0.22746131
Hmgcll1	0.34795715	3.35418801	2.43145069	0.13467749	0.22746131
C330006A16I	-0.2265137	5.98893754	2.43136369	0.13468408	0.22746131
Gpr35	1.16588424	0.11747477	2.43071999	0.13473285	0.22750445
Il22ra1	0.53751065	1.58253079	2.4303492	0.13476095	0.22751269
Phtf1	0.19936708	5.56154343	2.4290935	0.13485618	0.22763422
Mrpl39	-0.2449349	5.38481267	2.42786988	0.13494904	0.22770641

Cnot4	-0.2455995	7.41731286	2.42706721	0.13501001	0.22770641
Nrsn1	-0.1988212	6.50508988	2.42697682	0.13501687	0.22770641
Kat5	-0.2334917	4.68628449	2.42660652	0.13504501	0.22770641
Dnm1l	0.20764143	8.30233516	2.4265459	0.13504962	0.22770641
Krt9	0.30222243	3.95812675	2.42651391	0.13505205	0.22770641
Mdh2	-0.1792769	7.24664761	2.42638728	0.13506167	0.22770641
Rassf1	-0.4544248	3.92843638	2.42588584	0.13509979	0.22773148
0610010B08I	-0.3516254	4.54974306	2.42516064	0.13515494	0.22778525
Rptoros	1.94236175	-1.2315957	2.42382372	0.13525668	0.22786913
Snx7	-0.3831071	4.97341229	2.42375447	0.13526196	0.22786913
Umps	-0.2704138	4.08953276	2.42336539	0.13529158	0.22786913
Map4k3	0.2157065	6.59915635	2.42328428	0.13529776	0.22786913
Prom1	0.50252419	2.67292899	2.42231111	0.13537191	0.22795481
Lsm14a	-0.2122141	8.08565336	2.42145102	0.13543748	0.22801311
Fdx1	-0.3971878	5.38919564	2.42124637	0.13545308	0.22801311
Gm20187	1.28280977	0.42513865	2.42023302	0.1355304	0.2280842
Hnrnpm	0.20184214	6.77001369	2.42008247	0.13554189	0.2280842
Tor4a	0.36925129	3.20994434	2.41827347	0.13568005	0.22827748
Ech1	-0.4333686	3.948323	2.41783738	0.13571338	0.22827997
Ccdc42	1.58842779	-1.1815401	2.41748376	0.13574042	0.22827997
Ptp4a1	-0.2938034	4.59349163	2.41707633	0.13577157	0.22827997
Ehf	-0.9306243	0.60550469	2.41703478	0.13577475	0.22827997
Sh3rf2	-0.3666905	3.63581108	2.41592527	0.13585964	0.22833727
Dpf3	-1.0212024	0.0628116	2.41576774	0.1358717	0.22833727
Zbtb37	-0.4950705	2.91738233	2.41565282	0.1358805	0.22833727
Olfr78	1.33962246	-0.7827399	2.41537101	0.13590208	0.22833727
Stxbp5l	0.44724006	8.44935517	2.41409525	0.13599981	0.22846229
1110001J03F	-0.4805717	2.66001857	2.41375907	0.13602557	0.22846639
Spdl1	-1.2545532	-0.1312964	2.4126602	0.13610984	0.22856873
Ift140	0.52347223	3.26109083	2.41060739	0.13626742	0.22879413
Sh2d1b1	1.28972484	0.8331285	2.40892899	0.13639643	0.22894047
C330018D20I	-0.3055761	3.75577288	2.40886536	0.13640132	0.22894047
Mlh1	0.36804002	3.81828075	2.40793276	0.13647307	0.22902165
Hecw1	0.45034036	6.93926334	2.40646027	0.13658644	0.22917265
Tmcc3	-0.236749	6.34903671	2.40517274	0.13668567	0.22919568
Gnb2	0.34757067	3.61938385	2.40492749	0.13670458	0.22919568
Gm19757	0.43657936	4.53340146	2.40485701	0.13671002	0.22919568
Dclk2	0.33871411	4.01234502	2.40482414	0.13671255	0.22919568
Ccdc136	-0.2471596	5.29502051	2.40476436	0.13671716	0.22919568
1700071M16	1.29527238	0.65191251	2.40416162	0.13676365	0.22923439
Slc24a2	0.28745395	9.69693903	2.40358569	0.1368081	0.22923587
Tbl1x	-0.1621898	6.44132166	2.40282166	0.13686708	0.22923587
Mcf2	0.53192052	3.13970048	2.40219068	0.13691582	0.22923587

1700110K17I	-0.8570878	0.8249802	2.40202537	0.13692859	0.22923587
Gm10012	-0.2833674	3.8279026	2.40177833	0.13694768	0.22923587
Spire2	0.38651172	2.52442626	2.40167925	0.13695534	0.22923587
Rapgef5	0.19708937	6.3808839	2.40157362	0.1369635	0.22923587
Rdh13	0.2996964	3.81621794	2.40090264	0.13701537	0.22923587
D8Ertd738e	-0.5143691	3.18612086	2.40068492	0.13703221	0.22923587
9330159M07	-0.4576888	2.13112621	2.40043	0.13705193	0.22923587
Atp6v0a2	0.26502226	5.13639764	2.40003446	0.13708253	0.22923587
Eif3d	0.25938116	4.54766849	2.40002876	0.13708297	0.22923587
Rit1	-0.3083358	4.74407317	2.39991554	0.13709173	0.22923587
Ccr2	-0.5498884	2.65618563	2.39939169	0.13713227	0.22923587
Pbld2	-0.8951168	0.65689591	2.3993347	0.13713668	0.22923587
Pdp2	-0.4411632	3.55228606	2.39930493	0.13713898	0.22923587
Hsd17b10	-0.2935627	4.46645797	2.3984015	0.13720894	0.22926345
Snrpc	-0.4251023	3.22601681	2.398169	0.13722695	0.22926345
Dhrs7b	0.5555845	2.406399	2.39795719	0.13724336	0.22926345
Ercc5	0.23462258	5.02331105	2.39788303	0.1372491	0.22926345
9930104L06I	-0.4383731	3.10313452	2.39719387	0.13730251	0.22931356
B230217C12	-0.1833822	5.85137644	2.39476247	0.13749115	0.2295729
Gpr137	0.38020267	3.18893614	2.39457491	0.13750571	0.2295729
Hdac10	-0.5440966	1.601888	2.39428661	0.1375281	0.2295729
Ccdc34os	2.22501987	-1.6641624	2.39363496	0.13757873	0.22961828
Acbd6	-0.272577	4.71462232	2.39223967	0.1376872	0.22976017
Med7	-0.3163294	4.81836147	2.39139969	0.13775255	0.22980829
Etfb	-0.3748702	4.37938077	2.39126596	0.13776296	0.22980829
Gtpbp3	-0.3400388	3.02973878	2.3896528	0.13788858	0.22997868
Mau2	0.24762017	5.4300466	2.38829266	0.13799461	0.230103
Ndrg1	-0.3524132	7.57194374	2.38809397	0.1380101	0.230103
Fam43a	-0.3253491	6.20468166	2.38739846	0.13806436	0.2301543
Scx	-1.0157708	-0.0094432	2.38685013	0.13810716	0.23018018
Them4	-0.3416776	4.2544018	2.38641913	0.13814081	0.23018018
Myzap	-0.4849541	4.17181973	2.38629647	0.13815039	0.23018018
Zfp408	-0.2462751	4.68741714	2.38591598	0.13818011	0.23019054
Mroh8	1.16642052	-1.0031828	2.38542838	0.1382182	0.23021485
Fzd9	-1.4303989	-0.7379368	2.38485985	0.13826264	0.23024971
Gdf11	0.74233424	1.80607405	2.38438055	0.13830011	0.23027296
Mzt2	-0.4043618	2.97104028	2.3837806	0.13834703	0.23027452
Arl3	-0.2979506	5.64605501	2.3837674	0.13834806	0.23027452
Phlpp2	0.30392879	5.35627201	2.38339611	0.13837711	0.23028374
Cep290	0.23474116	7.10751982	2.38211244	0.1384776	0.23041009
Cd300ld	-1.3994923	0.25936695	2.38172557	0.1385079	0.23041009
Usp12	-0.1877895	5.81733712	2.38144386	0.13852997	0.23041009
Ttll7	0.2378936	7.62182125	2.38122495	0.13854712	0.23041009

1190007I07R	0.53224386	2.18462635	2.37978729	0.13865984	0.2305584
Gm20362	0.84701545	0.71752703	2.37844416	0.13876524	0.23069449
Slc19a1	0.41276968	3.08317596	2.37812306	0.13879045	0.23069725
Cacna1h	0.43947181	3.2704708	2.37779429	0.13881627	0.23070102
Cnn2	-0.4652261	6.26796327	2.37555758	0.13899209	0.2309012
Sh3rf3	0.31166355	5.61462486	2.37524694	0.13901653	0.2309012
Chrnb2	0.2887568	3.9374046	2.37520629	0.13901973	0.2309012
Gm20750	1.80454613	-1.2381917	2.37504547	0.13903238	0.2309012
Pole4	-0.2612915	5.26961281	2.37461565	0.13906621	0.2309012
Glt8d2	0.32849912	3.02758039	2.37446386	0.13907816	0.2309012
Fam129a	-0.3933283	3.7238136	2.37401581	0.13911344	0.23092063
Cox18	0.40007867	2.90177609	2.37354331	0.13915065	0.23094327
Zcchc24	-0.3436843	7.2701703	2.37215329	0.1392602	0.23105929
Hagh	-0.3040558	4.40177537	2.371748	0.13929216	0.23105929
Gpr125	0.42157109	3.35494586	2.37131638	0.1393262	0.23105929
Zfp36l1	-0.2755739	7.72852485	2.37108289	0.13934463	0.23105929
Gadd45g	-0.4634285	2.06850127	2.37089096	0.13935977	0.23105929
Prox1	-0.2685953	4.8871507	2.37086161	0.13936209	0.23105929
Sult6b1	-0.7778238	0.88888743	2.36953216	0.13946705	0.23119418
Neurl3	-0.8339819	1.02709041	2.36849899	0.13954869	0.2312615
Slc10a3	-0.5188741	2.47682987	2.36824821	0.13956851	0.2312615
Ceacam2	0.5401317	1.35594275	2.36812204	0.13957849	0.2312615
Dph7	0.30314512	3.34348573	2.36727972	0.1396451	0.23133274
Slc35a5	0.24687478	5.44790196	2.36655683	0.1397023	0.23136158
Slc30a6	-0.3505055	3.08987767	2.36617932	0.13973219	0.23136158
Tulp1	-2.4039626	-1.9917509	2.36616438	0.13973337	0.23136158
Six5	-0.5688166	3.32592734	2.36365338	0.13993233	0.23165185
Thap4	0.31378345	4.20500576	2.36301676	0.13998283	0.23166523
Car5a	1.04944046	-0.2991194	2.36295517	0.13998772	0.23166523
Samd15	0.55369844	2.41371152	2.36212558	0.14005356	0.23169915
Abtb2	-0.5343157	2.54631764	2.3618438	0.14007593	0.23169915
Nxpe3	0.42163263	4.07112781	2.36180291	0.14007918	0.23169915
1500012F01I	0.32151786	3.6609381	2.35949222	0.1402628	0.23187816
Npm1	-0.1806065	9.66262949	2.35939179	0.14027079	0.23187816
Egfr	0.31625481	5.96327822	2.35890237	0.14030972	0.23187816
Dgkq	0.50818774	3.8686968	2.35835343	0.1403534	0.23187816
Dnali1	-0.9547141	0.12482844	2.35814071	0.14037033	0.23187816
5730559C18I	1.98715549	-0.9736511	2.35789107	0.14039021	0.23187816
Cdip1	-0.1767699	6.7745802	2.35783706	0.14039451	0.23187816
Pum2	0.16493105	8.19361958	2.35781824	0.14039601	0.23187816
Psmd13	-0.3238387	3.96068932	2.35776242	0.14040045	0.23187816
Ltf	-0.9412812	0.23885672	2.35606452	0.14053572	0.23206244
Sar1b	-0.2189382	5.62421325	2.35544976	0.14058474	0.2320869

Cdadc1	0.18961176	6.00419115	2.35515543	0.14060821	0.2320869
Clint1	-0.1688097	6.88746452	2.35495951	0.14062384	0.2320869
Pou5f2	1.24949324	-0.1208998	2.35469041	0.14064531	0.2320869
AA415398	-0.4652311	2.98746953	2.35438239	0.14066989	0.23208837
Slc2a8	0.57099576	1.62197619	2.35347784	0.1407421	0.2321411
Hspd1	-0.1469636	8.27695858	2.35335716	0.14075174	0.2321411
Zfp207	0.16807802	7.90237065	2.35309162	0.14077295	0.2321411
Mmp16	0.28866484	5.36699332	2.35169464	0.14088459	0.23226748
Toe1	-0.408323	2.66854513	2.35130614	0.14091565	0.23226748
Gda	0.31016546	7.16852614	2.35124276	0.14092072	0.23226748
Vps8	0.42358645	4.47332854	2.35029455	0.14099658	0.23235342
Zmym1	0.38341297	3.92520281	2.34967714	0.14104601	0.23239576
Btbd17	0.82106014	0.43753418	2.34893939	0.14110509	0.23245401
Itga5	0.38353732	2.85314034	2.34831214	0.14115534	0.2324977
Ksr2	0.29693882	6.0655444	2.34681759	0.14127517	0.23264813
D730045A05	1.37107485	-0.3651192	2.34658073	0.14129418	0.23264813
Gm10069	0.68235701	0.87244405	2.34618136	0.14132622	0.23265242
Ifitm10	1.06253072	0.45607829	2.34553368	0.14137822	0.23265242
Dapl1	-0.3457703	5.38142757	2.34543777	0.14138592	0.23265242
Kdm8	0.71320266	1.41305886	2.34536468	0.14139178	0.23265242
Dlgap5	0.7161867	0.66188119	2.34327958	0.14155934	0.23283484
Gm20324	-1.267011	-0.5352857	2.34327097	0.14156003	0.23283484
Gm3230	-0.6788162	1.29743143	2.34309772	0.14157396	0.23283484
Nim1k	-0.3376697	4.19746749	2.34245468	0.14162569	0.23285879
Ergic1	-0.2008796	6.53841747	2.34232571	0.14163607	0.23285879
Chkb	0.52079849	1.33850918	2.34154521	0.14169889	0.2328842
Gmeb2	0.4074855	2.96301519	2.34154292	0.14169907	0.2328842
Kdm1b	-0.3031637	4.2157133	2.34077843	0.14176064	0.2329463
Sgk2	1.05604365	-0.36339	2.3401264	0.14181317	0.23296085
Serpinb6a	-0.3898287	5.68020965	2.33978839	0.14184041	0.23296085
Tm4sf20	1.67500356	-1.3584128	2.37443373	0.14184084	0.23296085
Nif3l1	0.3595354	3.38042311	2.33921335	0.14188677	0.2329874
Ppm1b	-0.1598346	8.14640098	2.3387925	0.14192072	0.2329874
Gzf1	0.22559237	5.15141052	2.33869765	0.14192837	0.2329874
Plaur	-1.6314825	-1.2438699	2.33650155	0.14210565	0.23323934
Nadk2	-0.2514048	4.75716011	2.3361156	0.14213684	0.23325144
Optc	-1.6266026	-1.6157922	2.33539685	0.14219493	0.23330769
Cmtm5	-0.5279561	2.42396211	2.33329287	0.14236516	0.23350515
Vstm5	-0.5217273	2.30334241	2.33307652	0.14238268	0.23350515
Sap30	-0.427654	3.11921207	2.33302571	0.14238679	0.23350515
Ppil2	0.2510215	4.47776369	2.33213103	0.14245926	0.23358436
1500011B03I	-0.2754804	5.03776438	2.33174376	0.14249065	0.23358436
Map3k7	0.16558491	6.60263861	2.33154657	0.14250663	0.23358436

Sdha	0.1730305	8.6038196	2.33091195	0.14255809	0.23362961
Dgkg	0.34720307	6.62842365	2.33020421	0.1426155	0.23366873
Fra10ac1	0.24449742	4.40983291	2.33002951	0.14262967	0.23366873
Diras1	-0.3019087	5.13546591	2.3284557	0.14275745	0.23380405
Nudt15	-0.5496058	1.94917808	2.32842424	0.14276	0.23380405
Stk19	-0.5798624	2.00526309	2.32700789	0.14287512	0.23395346
Gdf3	1.12376158	-0.750508	2.32607581	0.14295093	0.23400675
S100a1	-0.4677158	4.83217325	2.32596039	0.14296033	0.23400675
Nhlh1	-1.3173294	-0.2689233	2.32572679	0.14297934	0.23400675
Exosc3	-0.2981562	4.95860279	2.32528143	0.14301559	0.23402698
Ccdc12	-0.3600002	3.63339538	2.32487354	0.1430488	0.23404223
F8a	-0.2965909	3.41659898	2.32397569	0.14312194	0.23406821
Vps4b	0.21411544	5.43696052	2.32383724	0.14313322	0.23406821
A930013F10	0.78487908	1.71661462	2.32379866	0.14313636	0.23406821
Sema3e	0.60761664	4.19611162	2.32331267	0.14317597	0.2340939
Atp13a4	0.60754487	2.28564418	2.32240904	0.14324966	0.23416811
Hmcn1	0.33492392	3.75683083	2.32216988	0.14326917	0.23416811
Pcdhga10	0.26364178	3.54483059	2.32148832	0.14332479	0.23421993
Nsg1	-0.2515648	5.26403443	2.31987251	0.14345675	0.23434788
Acp1	-0.2050788	7.14125661	2.3194409	0.14349202	0.23434788
Atrx	0.24655755	9.86601145	2.31943839	0.14349223	0.23434788
Ndufs1	0.25337919	6.10677345	2.31935821	0.14349878	0.23434788
Txn1	-0.3020556	6.41714117	2.31869069	0.14355336	0.23439793
Pik3ip1	0.4175462	4.0136335	2.31764153	0.14363919	0.23449898
Agap3	-0.2013485	5.85847907	2.31702978	0.14368926	0.23454164
Mansc1	0.33679584	4.31736911	2.31566484	0.14380107	0.23468503
Casq1	1.35484208	0.15794126	2.3128281	0.14403376	0.23499123
Nr1d2	-0.1988037	7.42943897	2.31260183	0.14405234	0.23499123
2610203C22I	1.07887248	0.00997097	2.31238209	0.14407039	0.23499123
Gm5105	1.55606171	-0.6224718	2.31198512	0.144103	0.23499123
Prkag3	0.71269949	1.82360284	2.31191649	0.14410864	0.23499123
BC006965	-0.655872	1.79044994	2.31087988	0.14419383	0.23509102
Htatip2	-0.5558255	2.04140005	2.31006394	0.14426094	0.23516128
Krr1	-0.1661292	6.82468627	2.30930288	0.14432356	0.23518867
Hook2	-0.7054457	1.55443116	2.30923831	0.14432888	0.23518867
Arl5a	-0.2009637	6.9303259	2.30898452	0.14434977	0.23518867
Car3	-0.9240863	1.3697851	2.30821162	0.14441342	0.23521961
Ppap2c	-0.6184939	1.53986906	2.30817072	0.14441679	0.23521961
Osr1	-0.5134626	6.37133727	2.30704955	0.14450917	0.23533096
Mcl1	-0.2286213	8.67175862	2.3062654	0.14457383	0.23539712
Pbx3	-0.3484952	7.274062	2.30576667	0.14461498	0.2354129
Ccdc24	-1.832232	-0.9110974	2.30547117	0.14463936	0.2354129
Cln3	0.45152668	2.55672215	2.30520446	0.14466137	0.2354129

Xkr6	0.53508935	2.9998954	2.30498289	0.14467966	0.2354129
4932438H23I	1.15656779	-0.1891643	2.3042168	0.14474292	0.23547672
Pygl	0.70105538	1.09311284	2.30293141	0.14484914	0.23559919
Zfp583	0.52413137	3.39265592	2.30272371	0.14486631	0.23559919
Mast3	-0.2326094	7.30129697	2.30203894	0.14492294	0.23563626
P2ry10	-2.1014992	-1.3492971	2.30163979	0.14495596	0.23563626
Cckbr	0.28167926	4.6516168	2.30157566	0.14496127	0.23563626
N4bp1	-0.1798117	7.19119929	2.30027716	0.14506876	0.23577187
Lin37	-0.264457	4.4352741	2.29909315	0.14516686	0.23589217
Yap1	-0.2572989	5.91019961	2.29854799	0.14521206	0.23592647
Chac2	0.36001181	3.90892416	2.29759281	0.14529129	0.23601605
Fbxo44	-0.2871271	4.46392844	2.29708435	0.14533348	0.23604544
Zfp52	0.27336407	4.08566603	2.29622577	0.14540477	0.23611807
Gpalpp1	-0.2192649	5.76288545	2.29579163	0.14544083	0.23611807
Tas1r1	-1.5013308	-0.3951764	2.2956751	0.14545051	0.23611807
Atad1	0.18427157	7.21366824	2.29493837	0.14551174	0.23615408
Pxk	0.22914938	5.46625676	2.294828	0.14552091	0.23615408
Ttpal	-0.2550138	5.12525269	2.2938549	0.14560183	0.23624626
Rpl9	-0.2823393	7.1941389	2.29321539	0.14565504	0.23629346
Kbtbd2	0.18726734	5.97260635	2.29271501	0.14569669	0.23632189
Nptn	0.20529039	8.10613287	2.29216847	0.1457422	0.23635387
Dnajb2	-0.2678137	5.41496715	2.29168303	0.14578264	0.23635387
B930059L03I	-1.8717306	-1.1958104	2.29160907	0.1457888	0.23635387
Plekha1	-0.189317	6.73437233	2.29111257	0.14583018	0.23638182
Lmbr1l	0.72061957	0.32699274	2.2907028	0.14586433	0.23638487
Elk3	-0.25497	4.99464242	2.29051106	0.14588032	0.23638487
Asb6	-0.3835838	2.52934525	2.28919396	0.14599019	0.23646872
Fcgr2b	1.24210897	0.11290006	2.28892506	0.14601263	0.23646872
Ackr1	0.32954898	5.69606705	2.28866392	0.14603443	0.23646872
Nespas	-2.4143367	-1.9246916	2.2886222	0.14603791	0.23646872
Lrrtm2	0.2599381	6.66013688	2.28844426	0.14605277	0.23646872
Pno1	-0.2470954	3.98519793	2.28773415	0.14611208	0.23652565
Pycrl	-0.3733265	2.65084745	2.28714254	0.14616151	0.23656657
Hivep1	0.22034389	7.4051704	2.28622807	0.14623796	0.23660909
Mx2	0.99938744	0.06366285	2.2859808	0.14625864	0.23660909
Camk2d	0.20555297	7.31796937	2.28596158	0.14626025	0.23660909
Smpd3	0.25821229	4.76558049	2.28462658	0.14637196	0.23671333
Hfe	0.60325314	3.40174727	2.28461397	0.14637302	0.23671333
Ezh2	0.44751518	3.03078086	2.28421128	0.14640673	0.23672878
Ppm1k	0.23904722	6.66579069	2.28365819	0.14645306	0.2367646
Ppp3cb	-0.168863	8.65277141	2.28092969	0.14668185	0.23707317
Gm10033	0.38502247	4.59439636	2.28075416	0.14669659	0.23707317
4930426L09I	-0.7655921	0.84824692	2.28051647	0.14671654	0.23707317

Mag	0.41424981	2.55410134	2.27951562	0.1468006	0.237154
Med13	0.1769352	9.08293275	2.27925843	0.14682221	0.237154
Chrdl1	0.43042166	2.68327976	2.27894067	0.14684891	0.237154
Tsr3	-0.2700696	4.06406222	2.27843589	0.14689134	0.237154
Wnk4	-0.4559082	4.36271897	2.27824311	0.14690755	0.237154
Nbn	-0.2309753	4.74914227	2.2781923	0.14691182	0.237154
Cyba	-0.8310464	0.14828041	2.2776158	0.14696031	0.23719318
B830017H08	-1.0465083	-0.2125688	2.27685764	0.14702411	0.23722806
Fam32a	-0.2419554	6.0705098	2.27678338	0.14703036	0.23722806
Tnrc6c	0.21219374	6.9612226	2.27605446	0.14709173	0.23728799
Hadh	-0.3146993	4.85452117	2.27497788	0.14718243	0.23739521
Zic4	-0.3410733	6.26531762	2.27454956	0.14721853	0.23741435
Cep152	0.60584764	1.78541853	2.27389468	0.14727375	0.23746431
Vamp1	-0.1977577	6.0370303	2.27351346	0.14730591	0.23747707
Rit2	-0.1926263	6.72674347	2.2732061	0.14733184	0.23747979
Susd1	0.61511303	1.90334626	2.27252006	0.14738974	0.23753403
Zfp879	-0.5859954	2.11439961	2.27097658	0.14752012	0.23770503
Trim16	0.46628642	2.64917315	2.27010929	0.14759343	0.23778406
Slc9a3r2	-0.378108	5.10276259	2.26926502	0.14766485	0.23785999
Ube2c	0.79953309	0.65337984	2.26848248	0.14773108	0.23789724
1700109K24I	1.06159811	0.84873822	2.26816816	0.14775769	0.23789724
Zfp282	0.56651127	1.90740085	2.26813101	0.14776084	0.23789724
4933427D14I	0.45047043	4.10754685	2.2660955	0.14793332	0.23802416
Mboat7	0.22611931	5.19985747	2.26585849	0.14795342	0.23802416
Gm7854	1.19684958	0.00976066	2.26582611	0.14795617	0.23802416
Klhl22	0.2448059	5.05935465	2.26555923	0.1479788	0.23802416
Smc3	0.21413653	8.16643801	2.26539419	0.1479928	0.23802416
Usp13	0.43358346	3.87018205	2.26535396	0.14799622	0.23802416
Wdr43	-0.2392765	4.90443713	2.26493282	0.14803195	0.23802416
Galnt15	-0.8278242	1.00556472	2.26490794	0.14803406	0.23802416
Wdr35	0.29808631	4.69647187	2.26416105	0.14809747	0.23808702
Rpl5	-0.2623758	8.2506935	2.26244579	0.1482432	0.23827878
Pyurf	-0.3375929	5.11563781	2.26218467	0.1482654	0.23827878
Cryab	-0.3522821	7.16970998	2.26108434	0.14835899	0.23836469
Arg1	1.58984482	-0.9245313	2.2609841	0.14836752	0.23836469
Zfp191	-0.2786446	5.01105649	2.26047674	0.1484107	0.23836885
Wnt3	1.25024177	-0.8614481	2.26038185	0.14841878	0.23836885
Golgb1	0.19291275	7.98617775	2.25913069	0.14852534	0.23850089
Arhgap27	-0.4006499	3.24383541	2.25636131	0.14876154	0.23857568
Dpcr1	-1.6924173	-1.0147611	2.25636017	0.14876164	0.23857568
Cntnap2	0.33042311	6.26469547	2.25611842	0.14878228	0.23857568
Gm6682	-0.2031273	5.04837045	2.25605104	0.14878803	0.23857568
Dock2	-0.4192691	2.3581505	2.25602099	0.1487906	0.23857568

Ubxn11	-0.952839	1.41000936	2.25589988	0.14880094	0.23857568
Snx3	-0.2029918	6.82295888	2.2558357	0.14880642	0.23857568
Gm16897	-0.7653373	0.57425721	2.25577296	0.14881178	0.23857568
Alkbh6	-0.386938	3.12318257	2.25565526	0.14882183	0.23857568
Steap3	-0.3993804	4.09819426	2.25563619	0.14882346	0.23857568
Skap1	-1.0087216	-0.0607999	2.25544446	0.14883984	0.23857568
Scd4	0.99223485	-0.175425	2.25384371	0.14897666	0.23875592
Maats1	-1.1099583	0.57079124	2.25297186	0.14905124	0.23883638
Smr3a	-1.6339034	-0.5832651	2.25215077	0.14912152	0.23890991
Ehd3	-0.191598	7.70821092	2.25111347	0.14921037	0.23901316
Rarres2	1.25015117	0.59490737	2.25068466	0.14924712	0.23903293
Fnbp1l	0.23326786	8.5932555	2.2491684	0.14937714	0.23917709
Sap18	-0.2822777	6.70105069	2.24906563	0.14938596	0.23917709
Park7	-0.3054143	4.76409095	2.24826825	0.14945439	0.23924756
Cryba2	-1.818085	-1.2182359	2.2475074	0.14951973	0.23929435
Hint3	0.28665905	4.30640939	2.24735896	0.14953248	0.23929435
Gm5577	1.26340111	1.02627357	2.24456084	0.14977309	0.23964024
Cacna1d	0.40046366	5.10948348	2.24288981	0.149917	0.23983133
Atp10a	0.35633414	3.72381248	2.24156947	0.15003083	0.23995835
Hhipl1	-1.6345839	-1.2036781	2.24140063	0.1500454	0.23995835
Lpgat1	0.21059066	7.83784343	2.24010386	0.15015731	0.24002361
Trp53	-0.2866866	5.99533336	2.23984976	0.15017925	0.24002361
Bckdha	-0.5259955	1.13284598	2.23980145	0.15018342	0.24002361
Carkd	-0.2966914	3.50228562	2.23979219	0.15018422	0.24002361
Slco1a4	0.3349429	4.88980651	2.23922917	0.15023285	0.2400523
Fam132a	-0.5108908	1.84484177	2.23900341	0.15025236	0.2400523
Asb3	0.31069466	4.02289855	2.23854993	0.15029155	0.2400523
Trim12c	-0.3248305	5.55706008	2.23844981	0.1503002	0.2400523
Slc38a7	0.40821856	2.39339797	2.23724625	0.15040428	0.24017937
Eif4g3	0.22123748	8.94854397	2.23694363	0.15043046	0.24018203
Cisd3	-0.4230799	2.82960951	2.2357746	0.15053166	0.24030443
Fbxw9	-0.5692547	2.04056221	2.23515075	0.1505857	0.24035152
Ncor1	0.23560383	9.19656886	2.23452223	0.15064016	0.24039928
Morn1	0.90195163	0.458594	2.23275815	0.15079316	0.24060424
Nceh1	0.19021354	6.21461893	2.23136897	0.15091378	0.24074708
Rpl21	-0.2989234	7.82122317	2.23116106	0.15093184	0.24074708
Mapk8ip3	0.30342441	6.77528462	2.23028126	0.1510083	0.24075772
Eif2b2	-0.3509454	4.10672235	2.230041	0.15102918	0.24075772
Acnat1	1.01821217	0.1652493	2.23002923	0.15103021	0.24075772
Immp1l	-0.2326234	5.4472159	2.22995317	0.15103682	0.24075772
Ccnd3	-0.5654708	4.56294001	2.22908541	0.1511123	0.24083884
Mafk	0.48720863	3.20017283	2.22790728	0.15121484	0.24096307
Gm11974	-0.7654379	0.35866908	2.22710573	0.15128466	0.24098984

Cxxc1	-0.2044981	5.98700297	2.22693316	0.15129969	0.24098984
Apol7d	-2.282503	-1.7446552	2.22686705	0.15130545	0.24098984
3110082J24F	1.25069582	-0.6274529	2.22649366	0.151338	0.24100249
Mrap	-0.6360458	1.69198266	2.22483708	0.15148247	0.24119335
Bend4	-0.2895824	4.40579276	2.22416572	0.15154107	0.24122532
Pnn	0.2536408	7.701744	2.22390598	0.15156374	0.24122532
Ttyh2	0.4419453	2.6258952	2.22351908	0.15159753	0.24122532
Ppp1r2-ps3	0.9427406	-0.4670166	2.22347877	0.15160105	0.24122532
Ankrd9	-0.8127465	0.40616197	2.2227236	0.15166703	0.2412911
Pak3	0.26997854	7.31845417	2.22061277	0.15185164	0.2415409
Wipf1	-0.3435149	6.02779989	2.22021069	0.15188683	0.2415409
Scaf11	0.14530875	7.60854134	2.22008285	0.15189802	0.2415409
Gm973	0.43533849	2.46480324	2.21875955	0.15201393	0.24168047
Golga2	-0.2176602	5.49769387	2.21832221	0.15205227	0.24168047
Dok1	-1.1757873	-0.2343909	2.21823608	0.15205982	0.24168047
Ints4	0.20906756	5.51097562	2.21793307	0.15208638	0.24168348
Itga4	0.32077219	4.8409161	2.21692234	0.15217504	0.24172736
Fam188a	-0.2294931	6.11642959	2.21690667	0.15217641	0.24172736
Tmprss7	-0.8973794	0.24829116	2.21639399	0.15222141	0.24172736
Serf2	-0.3525928	6.41739383	2.2162062	0.15223789	0.24172736
Mknk1	-0.2684331	4.31864868	2.21600969	0.15225514	0.24172736
Chd2	0.20084798	6.94922404	2.21562021	0.15228935	0.24172736
Cx3cr1	-0.4017857	3.35088241	2.21539884	0.15230879	0.24172736
5031414D18I	1.44878601	-1.0537703	2.21536895	0.15231141	0.24172736
Snx29	0.48590432	2.1760274	2.21226389	0.15258446	0.2420942
Doc2a	-0.4329168	2.81656322	2.21209864	0.15259901	0.2420942
Ercc6l2	0.21986294	5.12584143	2.21189768	0.1526167	0.2420942
Ddb1	0.20771636	6.36962205	2.21075179	0.15271764	0.24213357
Tm9sf4	0.2546887	4.70297856	2.21058975	0.15273192	0.24213357
Mrps14	-0.2626865	5.65995833	2.21056602	0.15273402	0.24213357
Zyg11b	0.24549631	8.98696616	2.21049354	0.1527404	0.24213357
Clspn	1.0902853	-0.1388371	2.20927116	0.15284819	0.24222702
Adamts16	0.932898	0.94222066	2.20926416	0.15284881	0.24222702
Zdhhc12	-0.8753092	0.66138768	2.20880043	0.15288972	0.24225267
Mecom	-0.7871063	1.34295964	2.20791824	0.1529676	0.24233686
Trim26	-0.2585961	4.99294878	2.20738024	0.15301511	0.24237293
Extl2	-0.2209891	6.08279579	2.20689808	0.15305771	0.2424012
Prmt2	-0.3163746	4.78853725	2.20542737	0.15318773	0.24256588
Bfsp1	-0.9157031	0.95059007	2.20516186	0.15321122	0.24256588
Snx18	-0.2059151	6.1685314	2.20486046	0.15323788	0.2425689
4930427A07I	-1.348852	-0.0174891	2.20440433	0.15327825	0.2425936
Mtss1l	0.20097909	7.05287022	2.20401974	0.1533123	0.24260828
Plk4	-0.3192234	3.65666331	2.20279771	0.15342054	0.24274036

Sel1l3	0.38434649	4.96079136	2.20188684	0.15350129	0.24279414
Osmr	0.4084527	3.50329174	2.2016659	0.15352088	0.24279414
Pcnxl2	0.34913027	4.8164733	2.20157553	0.1535289	0.24279414
Tctn3	0.55185481	2.73399384	2.20123019	0.15355953	0.24280338
Fanci	0.45630036	2.90436969	2.20088006	0.15359059	0.24281331
Tbc1d16	0.2541157	4.73877911	2.19949618	0.15371345	0.24296832
Gna12	-0.2278153	5.26986265	2.1984379	0.15380748	0.24307773
Rps6ka4	-0.4630822	2.71149669	2.19735558	0.15390372	0.24319059
Tmem63c	0.4158517	4.14277226	2.19589686	0.15403355	0.24332566
Dpy19l3	0.26510234	5.02698243	2.19583694	0.15403889	0.24332566
Fmo5	-0.3352189	3.72328381	2.19501555	0.15411205	0.2433321
Mrpl57	-0.2474791	4.6920637	2.19500152	0.1541133	0.2433321
Ankrd32	0.30308302	4.11258224	2.1949545	0.15411749	0.2433321
Pole	0.61131332	1.08411934	2.19336879	0.15425886	0.243468
Tmprss6	2.5389368	-2.147482	2.19318732	0.15427505	0.243468
Lipt1	-0.5900203	1.47774105	2.19301529	0.1542904	0.243468
Hs1bp3	0.27440609	4.68705527	2.19260123	0.15432735	0.243468
Serping1	-0.4175176	7.04095034	2.19259573	0.15432784	0.243468
Slc25a28	-0.2741466	3.8932425	2.19192417	0.15438779	0.24351088
Hibadh	-0.2937658	5.59095261	2.19173438	0.15440474	0.24351088
Sec61b	-0.3205066	4.79413797	2.19084768	0.15448395	0.24359659
Rtn4rl2	0.67021241	0.92144409	2.19024461	0.15453785	0.24363383
Nolc1	-0.2183251	5.18570524	2.19002691	0.15455732	0.24363383
Exosc10	0.23944441	4.86434649	2.18967807	0.15458851	0.2436438
Snx10	-0.2066643	6.49559681	2.18785552	0.15475161	0.2438093
Lck	-1.0021358	0.1777984	2.18784064	0.15475295	0.2438093
Hdac5	-0.286941	5.88492629	2.18767041	0.15476819	0.2438093
Nae1	0.23926706	5.057229	2.1867563	0.15485009	0.24389908
Tmem175	0.22981177	4.99426847	2.18590787	0.15492614	0.24397921
2010315B03I	-0.2613617	4.22427496	2.18559378	0.15495431	0.24397921
Gsta4	-0.3289463	6.83520109	2.18491655	0.15501507	0.24397921
Mrpl38	-0.3392573	3.37361832	2.18484231	0.15502173	0.24397921
Bnc2	-0.3342944	7.67740496	2.1847461	0.15503036	0.24397921
Zzz3	-0.1742289	7.29131947	2.18452284	0.1550504	0.24397921
Kdm3a	0.2086004	5.9982817	2.18390581	0.1551058	0.24402717
Slc35a2	0.38761356	3.20080837	2.18286462	0.15519933	0.24413511
Allc	1.92462297	-1.1534135	2.18181486	0.1552937	0.24417299
Vps26a	-0.17307	7.2527483	2.18173677	0.15530072	0.24417299
Atp5h	-0.2848068	6.99955852	2.18157194	0.15531554	0.24417299
Tdg	0.69315547	0.31915329	2.18148771	0.15532312	0.24417299
Nfatc2ip	0.39402164	2.6767404	2.18085837	0.15537974	0.24422281
Nup43	0.47238938	2.47967835	2.18047419	0.15541432	0.24423796
Trappc2l	-0.4522582	3.37912869	2.17952645	0.15549966	0.24433288

Cyp2j12	-1.1615054	-0.5687111	2.17909185	0.15553881	0.2443552
Jam2	0.1992657	5.78114302	2.17815175	0.15562354	0.24438142
Sos1	0.18831049	7.58363665	2.1779409	0.15564255	0.24438142
Gm3893	0.33297704	5.8712045	2.17763741	0.15566992	0.24438142
Nfkbie	-0.5953752	2.13805308	2.17754099	0.15567862	0.24438142
Gsg1l	0.2284254	5.847959	2.17719579	0.15570976	0.24438142
Fgfr1op2	-0.1856776	7.74239464	2.17680046	0.15574543	0.24438142
Uqcc2	-0.3889836	3.50855036	2.1764398	0.15577798	0.24438142
Prepl	0.29573364	7.5494485	2.17621615	0.15579818	0.24438142
Pvt1	-0.4803499	2.41868138	2.17603412	0.15581461	0.24438142
Bcl7c	-0.6839965	1.35263335	2.17523804	0.15588651	0.24438142
Efcab12	0.82564137	0.82845375	2.175014	0.15590676	0.24438142
Hyi	0.59197645	1.56627172	2.1749686	0.15591086	0.24438142
Cilp	-1.1871449	-0.1917374	2.17494574	0.15591292	0.24438142
Aldh2	-0.311476	5.30454069	2.17476921	0.15592888	0.24438142
Zfp346	-0.226633	4.78813054	2.17475976	0.15592973	0.24438142
Eif3i	-0.2064923	5.80342661	2.17327622	0.15606388	0.24452294
Lsm10	-0.4150003	2.36384022	2.17320905	0.15606995	0.24452294
Slc26a6	-1.1533573	-0.1905844	2.17267195	0.15611856	0.24455997
B430212C06	2.14843042	-1.5430371	2.1714401	0.1562301	0.24467054
Nabp2	-0.2782612	5.88498159	2.17134076	0.1562391	0.24467054
Ywhab	-0.1519956	10.3409979	2.17078629	0.15628934	0.2447101
Dpysl3	0.16205116	6.30285504	2.16909211	0.15644297	0.2448607
Sec24b	0.21242509	7.02039347	2.16881227	0.15646837	0.2448607
A530046M15	0.80001267	0.47076079	2.16871734	0.15647698	0.2448607
Ttyh3	0.26853099	6.04932733	2.16862337	0.15648551	0.2448607
Zfp850	-0.4219329	2.65478176	2.16754405	0.15658352	0.24496417
Lrig1	0.33065386	3.267394	2.16734439	0.15660166	0.24496417
Gan	0.39540949	3.39614427	2.16696542	0.15663609	0.24497891
Ctage5	0.17906438	5.84547173	2.16646408	0.15668166	0.24501105
Synj1	0.34949152	9.53680777	2.165991	0.15672467	0.2450392
Lbr	0.31183649	3.4404604	2.16498993	0.15681573	0.24514245
Scand1	-1.4051946	-1.4286428	2.16299843	0.15699709	0.24530675
Pold3	-0.2389401	4.88174881	2.16286616	0.15700914	0.24530675
Gm16894	-0.6145011	1.2872279	2.16269214	0.157025	0.24530675
Gm13375	-0.5060725	2.74227121	2.16268755	0.15702542	0.24530675
Tnfrsf19	0.23642289	5.27041797	2.16219028	0.15707075	0.24530675
Ppp1r3f	-0.2613289	4.33312591	2.16210036	0.15707895	0.24530675
A930005H10	-0.4295761	3.04340925	2.16191193	0.15709614	0.24530675
Tubb4b	-0.17225	7.02528068	2.16132136	0.15715001	0.24532735
Slu7	-0.2071754	5.97942903	2.16106374	0.15717351	0.24532735
Cftr	0.73831035	1.59461546	2.16094372	0.15718446	0.24532735
Pef1	-0.2577292	4.475448	2.16034657	0.15723897	0.24537332

Magel2	1.20861803	0.39993086	2.15735094	0.15751276	0.24576141
Ccbl1	0.62010229	1.5529635	2.15672312	0.15757021	0.24576944
Nt5dc3	0.25842499	5.92130142	2.15616375	0.15762142	0.24576944
Sco1	0.3423835	3.29472571	2.15613726	0.15762385	0.24576944
Ahcy	-0.3181328	3.87366617	2.15607201	0.15762982	0.24576944
Cct4	-0.1987905	5.9909585	2.15585677	0.15764953	0.24576944
Fam129c	1.60037345	-0.3552634	2.15565031	0.15766844	0.24576944
Rps12	-0.3438621	6.21202793	2.15518239	0.15771131	0.24579715
Dpf2	-0.1907918	5.81680931	2.1533064	0.15788333	0.24597934
Lrrc10b	0.49664332	3.93809863	2.15329432	0.15788443	0.24597934
Champ1	-0.2424551	4.80465221	2.15308598	0.15790355	0.24597934
Rplp0	-0.2910234	6.63245504	2.15066035	0.15812634	0.24627517
Med14	0.24976563	7.67537787	2.15021933	0.15816688	0.24627517
Trmt13	0.54863243	1.35786916	2.14998939	0.15818803	0.24627517
Kdm3b	0.15644039	7.03143235	2.14992424	0.15819402	0.24627517
Samd3	0.72559891	1.32817497	2.14964131	0.15822004	0.24627654
Uckl1os	1.11431696	-0.4792258	2.14921338	0.15825942	0.24628335
Slc25a18	0.43709879	2.52180671	2.14904721	0.15827471	0.24628335
Zranb3	-0.4205076	2.7816883	2.1486404	0.15831215	0.24629593
Ino80dos	0.47693447	2.98122524	2.14841312	0.15833308	0.24629593
Odf2l	0.38061298	3.49166624	2.14803605	0.1583678	0.24631082
Slc15a2	0.39132745	2.85421887	2.14740735	0.15842571	0.24636178
Cd52	-0.5088417	2.35416589	2.14712874	0.15845138	0.24636259
Ogdh	0.21731245	7.67834973	2.14679489	0.15848215	0.24637132
Abca8b	0.47692541	3.33237728	2.14629509	0.15852823	0.2463934
Spen	0.25338445	6.87515701	2.14594452	0.15856056	0.2463934
Klf4	-0.3506328	6.47978248	2.14566646	0.15858621	0.2463934
Klkb1	-1.9990113	-1.2638815	2.14554855	0.15859709	0.2463934
Syngr3	0.23447684	5.08592985	2.14527717	0.15862212	0.2463934
Gap43	-0.2077484	7.23966044	2.14370718	0.15876708	0.24657946
Tex10	0.25083206	4.35355981	2.1428027	0.15885066	0.24667015
Ctsh	-0.3710264	4.97746503	2.14237647	0.15889006	0.24669223
Timm21	0.34042905	4.15049091	2.14176963	0.15894618	0.24670903
Herc4	0.27970559	4.61268462	2.14169046	0.15895351	0.24670903
Car4	-0.3832935	3.1795533	2.14135071	0.15898494	0.24670903
Ticrr	1.35506645	-0.3232316	2.14117047	0.15900162	0.24670903
Plcb1	0.30033583	8.78715465	2.14084758	0.15903151	0.24671631
Cep250	0.29893627	4.7744097	2.13986008	0.15912295	0.24681908
Mcts2	-0.2841275	3.74713524	2.13958213	0.15914869	0.24681993
4833419F23I	1.05944953	-0.1610463	2.13916939	0.15918694	0.24684017
Pdxp	-0.3388722	4.93016259	2.13735236	0.15935544	0.24706234
Rybp	-0.1998203	6.64734823	2.13669159	0.15941677	0.2470903
Ralgapb	0.23457065	7.62537635	2.13563968	0.15951446	0.2470903

Npsr1	0.69443153	1.3576542	2.1355876	0.1595193	0.2470903
Polr2e	-0.2870229	4.39097372	2.13543723	0.15953327	0.2470903
Prss16	-1.7788644	-1.0446287	2.13532362	0.15954383	0.2470903
Zcchc10	-0.3816048	3.6333882	2.13513758	0.15956112	0.2470903
Ahcyl1	-0.1852329	9.39081939	2.13483885	0.15958889	0.2470903
Spin1	-0.1444223	8.82687423	2.13442441	0.15962742	0.2470903
Ago1	0.1555106	6.22133921	2.13420795	0.15964755	0.2470903
Fignl1	1.29070517	-0.7257423	2.13402643	0.15966443	0.2470903
1600002K03I	0.99907365	0.25287878	2.13393107	0.1596733	0.2470903
Map7d2	-0.2110768	7.71414111	2.13387544	0.15967848	0.2470903
Slc10a1	1.78329068	-1.138606	2.13331917	0.15973023	0.2470903
Apobec1	0.38256627	3.35042293	2.13327629	0.15973422	0.2470903
Tmem43	0.39982323	3.88559186	2.13308684	0.15975185	0.2470903
Lsm8	-0.302942	5.66105013	2.13265055	0.15979247	0.2471141
Golga3	-0.1505489	6.79705468	2.13125233	0.15992271	0.24724497
Camk2b	0.22739605	8.06092179	2.13120014	0.15992757	0.24724497
Gpr137b	-0.3287138	2.9335845	2.13055164	0.15998803	0.24729939
Fam216b	-0.7223442	1.06230103	2.12991789	0.16004713	0.24735172
Tiam1	0.24707869	6.45558787	2.12747885	0.16027485	0.24766459
Neu3	0.34897159	3.12335179	2.12712089	0.16030831	0.24767721
Mybl1	-0.298919	4.22013849	2.12589399	0.16042304	0.24778722
1700001J11F	1.40693527	-1.5119809	2.12576359	0.16043524	0.24778722
Med10	-0.2786309	3.50898414	2.12554807	0.1604554	0.24778722
Tfap2a	-0.3514536	3.94544385	2.1249531	0.16051109	0.24780574
Tex264	-0.3067454	3.26062366	2.12487933	0.16051799	0.24780574
Exoc5	0.17639682	6.2944493	2.12291774	0.16070176	0.24804756
Tceal8	-0.331059	7.27685964	2.12237297	0.16075284	0.24804756
Wdr77	0.21628756	5.07304581	2.12213427	0.16077523	0.24804756
Plch1	0.38427565	3.10887711	2.12212687	0.16077592	0.24804756
1190005I06R	-1.4815346	-1.2426018	2.12141148	0.16084305	0.24811204
Acss3	-0.4761224	2.36379029	2.12077047	0.16090322	0.24816578
Dda1	-0.2419422	4.55962534	2.12035972	0.16094179	0.24818619
Hdac4	0.28075546	5.07433914	2.11975652	0.16099845	0.24823449
Crb1	-1.6712295	-0.9575645	2.11938427	0.16103343	0.24824935
Tipin	-0.4615771	4.23054188	2.11892583	0.16107653	0.24827671
Pcdh19	0.26206054	6.24158074	2.11780472	0.16118197	0.24840015
Hs3st1	-0.284311	4.20816129	2.11639948	0.16131426	0.24854908
Ptcra	1.07993307	-0.9409011	2.1159964	0.16135222	0.24854908
Gpr180	-0.3310314	3.9525995	2.11596973	0.16135474	0.24854908
2200002D01I	-0.7693003	0.63943445	2.11232869	0.16169821	0.24903901
Fam83h	-0.521147	1.56831649	2.11176099	0.16175185	0.24908245
Cd3e	-0.6526191	2.30550467	2.10922591	0.16199161	0.24941246
Ube2e3	-0.2337285	7.65512624	2.10803892	0.16210403	0.24949741

Zfp180	0.19293437	5.1210023	2.10780889	0.16212583	0.24949741
Ptrf	-0.4032587	6.57759735	2.10757419	0.16214807	0.24949741
Srl	-0.4631871	1.87903088	2.10748041	0.16215696	0.24949741
Pdlim5	-0.3049615	7.39530716	2.1071607	0.16218726	0.24949741
Pik3c3	0.19777471	5.50158072	2.10703038	0.16219962	0.24949741
Stk25	-0.1524385	6.73281195	2.10637814	0.16226147	0.24955337
Tcf3	-0.351987	3.66483439	2.10489738	0.16240201	0.2497
Prr5l	0.39491244	2.40754669	2.10457037	0.16243307	0.2497
Mphosph9	0.30670671	4.87189749	2.10406104	0.16248145	0.2497
Stox2	0.26114349	7.99509909	2.10397302	0.16248981	0.2497
Grhl1	0.37636067	2.97171947	2.10386109	0.16250045	0.2497
Nfe2l3	0.43197984	2.48759013	2.10376306	0.16250977	0.2497
4930412C18I	-0.791748	1.24851585	2.10313456	0.16256951	0.24970728
Rtfdc1	-0.2751846	5.58179619	2.10280421	0.16260092	0.24970728
Mrps18a	-0.4194887	3.38640429	2.10224952	0.16265368	0.24970728
Rccd1	0.55091454	1.48306808	2.10190403	0.16268656	0.24970728
Ctc1	0.25304147	4.13550488	2.10187279	0.16268953	0.24970728
Sqrdl	-0.4324913	3.40384126	2.10178945	0.16269746	0.24970728
Acsl1	-0.1887968	6.51345693	2.10143236	0.16273145	0.24970728
Cramp1l	0.19552331	6.39451023	2.10108619	0.1627644	0.24970728
Ccdc107	0.45424171	2.78089312	2.10102368	0.16277036	0.24970728
Lgr4	0.2126378	5.69464346	2.10089868	0.16278226	0.24970728
Ttc18	0.86842829	0.60106621	2.1005821	0.16281241	0.24970728
Snap23	-0.2579241	7.16619775	2.1004981	0.16282041	0.24970728
Tubgcp6	0.35676401	3.40036445	2.09878354	0.16298383	0.24991878
Slc25a17	0.26310487	5.69498941	2.0978834	0.16306971	0.24999263
Trappc4	-0.3541596	3.52625341	2.09768623	0.16308853	0.24999263
Lclat1	0.20607637	6.05359353	2.0974764	0.16310856	0.24999263
Phc1	0.18131541	6.3147553	2.09707326	0.16314705	0.2500125
Unc5d	0.28829928	6.21275361	2.0950861	0.16333693	0.25021354
Vsx2	2.55487833	-1.7368724	2.09494456	0.16335047	0.25021354
1700113A16I	0.32442341	3.91329545	2.09489854	0.16335487	0.25021354
Unc5c	0.23587053	6.71418233	2.09440197	0.16340237	0.25024717
Efna2	0.85108858	0.54814271	2.09359872	0.16347924	0.25025701
Nek1	0.25405504	6.39445689	2.0935538	0.16348354	0.25025701
Ano8	0.72338401	0.67743509	2.09353393	0.16348544	0.25025701
Gm5544	2.33946051	-0.6670952	2.09292016	0.16354421	0.25030785
Usp11	0.21754722	7.40496028	2.09190975	0.16364102	0.25041689
Mzb1	-1.8732188	-1.791709	2.0911875	0.16371026	0.25048371
Gpsm1	0.35136163	2.66469454	2.09074435	0.16375276	0.25050961
Neu4	0.4470458	2.46054325	2.08958304	0.1638642	0.25057676
Gm11201	1.61195173	-0.2656289	2.08944383	0.16387756	0.25057676
Aldh1a7	-0.8070756	0.42021628	2.08915507	0.16390529	0.25057676

Pprc1	0.32302658	3.91972088	2.08909476	0.16391108	0.25057676
Pwwp2b	-0.5155476	2.38647075	2.08892432	0.16392745	0.25057676
9130011E15I	0.30388999	3.63260374	2.08853899	0.16396446	0.25057676
Dlk1	0.41510311	3.41940784	2.088244	0.16399281	0.25057676
Gabra1	0.21637954	8.20508337	2.08815561	0.1640013	0.25057676
Carhsp1	-0.421106	5.37283522	2.08736016	0.16407777	0.25065449
Ap5s1	-0.4774637	2.35698614	2.08671456	0.16413986	0.25071025
Gm17066	0.32420232	4.80154573	2.08594054	0.16421434	0.2507849
Lap3	0.21536729	5.62660117	2.0838888	0.16441196	0.25104757
Rcn1	-0.3605414	4.76303695	2.08303131	0.16449464	0.25109373
Cep76	0.30153843	3.92024491	2.08278031	0.16451886	0.25109373
Plbd2	0.22331908	5.28181595	2.08277789	0.16451909	0.25109373
Ubfd1	-0.1759764	6.94136484	2.08119499	0.16467188	0.25128776
Aco2	0.178606	7.41179208	2.08015616	0.16477224	0.25140175
Rnf216	-0.1756104	5.97522611	2.07921366	0.16486337	0.25150161
Rbm3os	1.13727175	-0.0460533	2.07884796	0.16489874	0.2515164
Rsrp1	0.32409859	6.29280834	2.07850282	0.16493213	0.25152817
Alox12	0.65055174	1.23960461	2.07756922	0.1650225	0.25161153
Zfp281	0.22208066	6.16581119	2.07740743	0.16503817	0.25161153
H2-Ke6	-0.5863402	2.38317371	2.07625971	0.16514936	0.25174187
Hmgn1	-0.3029409	8.11437481	2.07571239	0.16520242	0.25177818
Pla2g7	0.49997553	3.67048988	2.07523939	0.16524829	0.25177818
Zfp773	0.79331263	1.1356325	2.07475405	0.16529537	0.25177818
Hmgn3	-0.2454184	6.929729	2.0747028	0.16530034	0.25177818
Vamp3	-0.2771831	7.50340041	2.07463215	0.1653072	0.25177818
Psme2	-0.3420155	5.28007112	2.07439716	0.16533	0.25177818
Gpc5	0.43834693	3.53972772	2.07415912	0.16535311	0.25177818
Fam105a	-0.3695589	3.79205502	2.07378076	0.16538984	0.25179496
Cog6	0.18715301	5.57264426	2.07301405	0.1654643	0.25186918
Ppm1a	-0.1900948	6.77648494	2.07242452	0.16552158	0.25191723
Slc25a13	0.76122675	0.32074734	2.07208988	0.16555411	0.25192759
Glrb	0.18708023	6.74614551	2.07140066	0.16562113	0.25199042
Itgb8	0.31389602	4.16989927	2.07058765	0.16570022	0.25204226
Osgep	-0.2834152	3.93937832	2.07052153	0.16570666	0.25204226
Gm17769	-0.6971431	0.14104306	2.07012047	0.16574569	0.25206249
Trpt1	-0.3418238	3.12327704	2.06883003	0.16587137	0.25211223
Plxnb1	0.29981533	4.42722131	2.06874475	0.16587968	0.25211223
Zfp385a	-0.3778169	6.61742759	2.06872844	0.16588127	0.25211223
Abat	0.21925912	6.69238941	2.06872762	0.16588135	0.25211223
Nhlh2	-1.3877696	-0.6575748	2.06751452	0.16599961	0.25225283
Atox1	-0.4070011	4.36596324	2.06720293	0.16603001	0.25225988
Tasp1	-0.2081988	5.96691952	2.06645319	0.16610317	0.25231409
Zfp398	0.25447021	4.49869841	2.0663094	0.1661172	0.25231409

Tmem263	-0.2042021	7.21113596	2.06541157	0.16620487	0.25233537
Ovol2	-0.4851811	1.7981292	2.06536162	0.16620975	0.25233537
Trim6	-1.5100898	-0.5497775	2.06535101	0.16621078	0.25233537
Col24a1	1.0129412	0.43538325	2.06511076	0.16623426	0.25233537
Far2	0.35539463	3.82220963	2.06462616	0.16628161	0.2523358
Cmtm3	-0.3594818	3.14524451	2.06444285	0.16629952	0.2523358
Ptgs2os	0.74167381	0.78329448	2.06431709	0.16631182	0.2523358
Chchd5	-0.3907567	2.63589755	2.0639397	0.16634871	0.25235269
Rad51b	1.88331434	-1.3350402	2.0634607	0.16639556	0.25238467
Atp6v1b2	-0.1853032	8.40068724	2.06244052	0.16649538	0.25249698
Lrrc8b	0.23262888	5.96179813	2.06175782	0.16656223	0.25252439
Kcnh4	0.60348704	1.83296226	2.06172931	0.16656502	0.25252439
Wbp2	-0.1832974	7.02579978	2.06013289	0.16672146	0.25263734
Mroh1	0.3640993	4.29708474	2.06004426	0.16673015	0.25263734
Aasdh	0.49506788	2.09204686	2.05984795	0.1667494	0.25263734
Srrm4	0.29039563	6.13290984	2.0597876	0.16675532	0.25263734
Fnip1	0.25464001	5.8156585	2.05965341	0.16676848	0.25263734
Gm17751	-1.7762267	-0.8407761	2.05918416	0.16681451	0.25266272
Mapkapk2	-0.3162108	4.27647673	2.05895679	0.16683682	0.25266272
Ephx4	-0.2243549	5.18556437	2.05809221	0.16692169	0.25275217
Cadps2	0.25531337	6.41826898	2.05782777	0.16694766	0.25275242
Fhdc1	0.71463059	0.87332126	2.05735661	0.16699394	0.25278233
Slc18a2	0.40324981	2.87449392	2.05710127	0.16701903	0.25278233
8430419L09I	-0.2335958	5.70735474	2.05667925	0.1670605	0.25280604
9330162012I	-1.0278104	0.74118326	2.05590089	0.16713703	0.25288278
Iqsec1	0.28734526	7.84159001	2.05553391	0.16717313	0.25289833
Abhd16a	0.22585054	4.50715658	2.05486377	0.16723907	0.25295902
Trim3	0.24043987	4.52986376	2.0542791	0.16729662	0.25300701
Kcnt2	0.34545729	5.43269242	2.05373444	0.16735026	0.25302494
Vmn2r29	0.34954347	3.38591024	2.05363412	0.16736014	0.25302494
Chic2	-0.3244244	3.94294952	2.05291493	0.16743101	0.25309301
Tusc2	-0.197616	4.92345459	2.05176943	0.16754395	0.25322466
Spock3	0.21667139	5.22420083	2.05106435	0.16761352	0.25329073
Lacc1	-0.2968149	4.5523204	2.0497821	0.16774012	0.25344294
Spns1	0.48042217	2.31003266	2.04944407	0.16777352	0.25345317
Adra2a	-0.3765608	4.09654235	2.04902389	0.16781504	0.25345317
Rcan3	-0.2149392	5.12241842	2.04892804	0.16782451	0.25345317
Cyp1b1	-0.2981399	7.03186445	2.0482363	0.1678929	0.25349581
Btbd19	0.45249098	2.62610308	2.04802321	0.16791398	0.25349581
Ccm2	-0.2822955	3.74234732	2.04785732	0.16793039	0.25349581
Tcf25	-0.1469647	8.4293522	2.04689962	0.16802515	0.25359978
Ndufv2	-0.2049312	6.21579475	2.04633951	0.16808061	0.25362134
Tmem259	0.39884675	3.37626986	2.0460061	0.16811363	0.25362134

Sardh	-0.529011	2.20150384	2.04579407	0.16813463	0.25362134
Dthd1	1.28639141	-0.4413479	2.04507223	0.16820616	0.25362134
Fas	1.15570843	0.28238961	2.04484495	0.16822869	0.25362134
Nanp	-0.3378569	4.00038521	2.04453849	0.16825907	0.25362134
Zc3hc1	-0.4317677	2.28311213	2.04447808	0.16826506	0.25362134
Gk5	-0.4031123	1.99776092	2.04445314	0.16826753	0.25362134
Il6ra	0.47625261	2.57813041	2.04440341	0.16827247	0.25362134
Hpgds	0.5160132	2.40122513	2.04322611	0.16838926	0.25374835
Hsf4	0.57822711	1.34637394	2.04277806	0.16843374	0.25374835
4921507P07I	0.86973781	0.29850225	2.04272615	0.16843889	0.25374835
Map3k3	0.3195662	3.27514667	2.04235131	0.16847611	0.25374835
Aga	-0.4520905	3.47617596	2.04224915	0.16848626	0.25374835
Ccdc80	0.60107622	2.41847797	2.04181357	0.16852953	0.25377449
Bud13	-0.3770877	2.07170836	2.04117778	0.16859271	0.25378259
Mylk	-0.2570881	6.02107429	2.0411342	0.16859704	0.25378259
H3f3b	-0.3094742	9.00360196	2.04061679	0.16864848	0.25378259
Kcnip4	-0.1978636	7.02681081	2.04055292	0.16865483	0.25378259
Slc12a9	0.65563799	1.25442533	2.04021826	0.16868811	0.25378259
Olfm1	-0.1751948	9.19770912	2.04001191	0.16870864	0.25378259
Spata32	-1.006072	-0.0808031	2.03993527	0.16871626	0.25378259
Nom1	0.23272362	4.52773846	2.03951616	0.16875796	0.25378384
Gm12657	-0.3482923	3.51445235	2.03934721	0.16877478	0.25378384
Cds2	0.22181799	7.73388419	2.03914586	0.16879482	0.25378384
Gm16386	0.48640092	2.00126101	2.0377987	0.16892898	0.25385087
Rabl2	-0.3041534	3.87155561	2.03766466	0.16894234	0.25385087
Slc35b3	0.40444653	2.18496086	2.03763879	0.16894492	0.25385087
Tcf7l1	-0.3656411	5.36479086	2.0371809	0.16899055	0.25385087
Pdzd8	0.16313988	7.10026311	2.03714208	0.16899442	0.25385087
Dnajc7	-0.1575706	6.56262307	2.03707063	0.16900154	0.25385087
Gja3	-2.942439	-1.8770498	2.03666796	0.16904169	0.25385087
2810429I04R	1.43915135	-1.2896192	2.03653331	0.16905512	0.25385087
Cited1	-0.6321196	1.57350636	2.03625241	0.16908314	0.25385087
Prelid1	-0.2787888	5.79419782	2.0359827	0.16911004	0.25385087
Cda	1.63464986	-0.9011938	2.03583806	0.16912447	0.25385087
Krtcap2	-0.3171766	3.33774937	2.03521029	0.16918713	0.25390601
Zc3h7b	0.20529343	6.02032441	2.03466405	0.16924167	0.25394895
Plb1	-0.5634571	2.38062734	2.03391329	0.16931666	0.25398705
4930480K15I	0.72811599	2.7430116	2.03389074	0.16931891	0.25398705
Angptl7	0.84097922	1.56096418	2.03308263	0.16939968	0.25401199
Gm10408	0.97082217	-0.4186977	2.03279001	0.16942894	0.25401199
Commd8	-0.306069	5.76987155	2.0327652	0.16943142	0.25401199
Anxa5	-0.374266	8.20189293	2.03268625	0.16943932	0.25401199
Lipo1	-0.3306582	4.94977586	2.03180038	0.16952794	0.25401199

Ankrd33b	0.18982246	6.30615254	2.03159451	0.16954854	0.25401199
Ppat	0.24161528	4.38681869	2.03130878	0.16957714	0.25401199
Rab11fip4	-0.2918132	5.52632492	2.03110844	0.1695972	0.25401199
Tubgcp3	0.22601819	4.42504064	2.03104892	0.16960316	0.25401199
Gm15055	1.64031075	-1.4464587	2.03065155	0.16964295	0.25401199
D15Ertd621e	-0.160961	7.09753655	2.03058654	0.16964946	0.25401199
Wdr4	0.47458471	2.39994244	2.03012956	0.16969524	0.25401199
Slc41a1	-0.3072341	7.95331276	2.03012424	0.16969577	0.25401199
C2cd3	0.29037474	5.22226321	2.03009615	0.16969859	0.25401199
Glt1d1	0.59746499	1.04808471	2.02966807	0.16974149	0.25403738
Cyp4f17	1.8715641	-1.7790445	2.02903089	0.16980536	0.25409415
Taf7l	0.74396506	1.07692648	2.02788498	0.16992031	0.25417245
Dcaf6	0.245482	6.91944124	2.02787873	0.16992094	0.25417245
Gabrg3	0.42369467	4.67302753	2.02773335	0.16993553	0.25417245
Nus1	0.16368915	6.88160066	2.02633103	0.17007634	0.25434423
Gtf2h2	-0.2121177	4.9605787	2.02554273	0.17015557	0.25442386
Abcf2	-0.2210378	5.35137765	2.0252602	0.17018397	0.25442749
Smg6	0.20331543	5.6233263	2.02483867	0.17022636	0.25445203
Scd2	0.17736686	8.33435945	2.02394964	0.1703158	0.25454688
Paqr3	0.45491601	2.38706999	2.02256443	0.17045528	0.25471648
Car7	0.55279959	1.42663876	2.02201992	0.17051015	0.2547489
5930430L01F	-0.3380773	4.70600952	2.02183294	0.17052899	0.2547489
Mest	0.34345778	6.37721065	2.02110428	0.17060246	0.25481978
Nrxn2	-0.2188008	5.40005457	2.02069881	0.17064335	0.25484161
Adsl	0.29812377	4.44688596	2.02044357	0.1706691	0.25484161
C1ra	-0.6929773	0.72250136	2.0193962	0.17077482	0.2549606
Neat1	0.24755278	5.07494143	2.01850087	0.17086525	0.254985
Frs3	0.46892517	2.42524315	2.01826314	0.17088927	0.254985
Zfp600	1.14123684	-1.1369565	2.01818145	0.17089753	0.254985
Efcab4b	1.48269247	-1.4068119	2.01812421	0.17090331	0.254985
Cpeb3	0.20468054	7.55417114	2.01794606	0.17092132	0.254985
Inpp5j	0.36798822	3.41493072	2.01703492	0.17101345	0.25504729
1700024P16I	0.94943899	0.72847317	2.01670522	0.1710468	0.25504729
Tmem154	-0.4674565	3.39839812	2.01664968	0.17105242	0.25504729
Tnxb	0.61042128	1.28082218	2.01650335	0.17106722	0.25504729
Ar	0.30533371	4.30558559	2.01606872	0.17111121	0.25504947
Gmnc	1.1270285	-0.5135682	2.01597437	0.17112076	0.25504947
Mrpl10	-0.2457949	4.71819044	2.01495531	0.17122395	0.25516444
Tfdp2	-0.1590711	6.23340646	2.01451428	0.17126863	0.25519221
Chpt1	0.27007352	5.44121006	2.0141927	0.17130122	0.25519771
Grem2	-0.2289787	5.2695346	2.01396374	0.17132443	0.25519771
Megf10	0.35771967	4.61145867	2.01322982	0.17139885	0.25526974
Dut	-0.2747195	4.83592111	2.01229576	0.17149362	0.25537205

Nfe2l1	-0.191861	7.82225763	2.01200302	0.17152333	0.25537748
Lyz2	-0.464048	3.56935062	2.01156234	0.17156808	0.2553913
Trappc5	-0.3012319	4.15310119	2.01139802	0.17158476	0.2553913
Arap3	0.68700115	1.59536031	2.01048765	0.17167725	0.25549014
Cdkn1c	-0.4127963	5.69488742	2.01011998	0.17171462	0.25550694
Ahsa2	-0.2370883	4.73143672	2.00925376	0.1718027	0.25559917
1810032O08	-1.0390232	-0.4284775	2.00796435	0.17193392	0.25575555
Smg5	0.24378536	4.39297691	2.00733506	0.17199801	0.2557565
Ap1s2	-0.2125217	6.31247399	2.00725212	0.17200645	0.2557565
Pik3cd	0.38194745	2.8153947	2.00718895	0.17201289	0.2557565
Gpr176	0.4603017	2.61658925	2.00583134	0.17215126	0.25591864
Vps37a	-0.2275882	7.7321129	2.00560641	0.1721742	0.25591864
Hip1r	0.41057469	3.19870087	2.00518898	0.17221678	0.25593524
Pim2	-0.2261133	4.10662835	2.0047391	0.17226268	0.25593524
Nox4	-0.6202253	1.22891877	2.00472861	0.17226375	0.25593524
Sh3gl2	-0.2278633	9.33445628	2.00400582	0.17233753	0.25600603
Procr	-0.8876054	1.61350465	2.00345569	0.17239371	0.25601672
Vars2	-0.4991269	2.25968597	2.00326614	0.17241308	0.25601672
Zfp457	0.89409061	0.29169321	2.00316771	0.17242314	0.25601672
Adamts10	-0.4513819	2.08207284	2.00074046	0.17267134	0.2563464
Psma5	-0.2167973	5.72397389	1.99845683	0.17290526	0.25657393
Plekha8	0.27945081	4.32360688	1.99830512	0.17292082	0.25657393
BC049352	1.63301095	-1.7776477	1.99807201	0.17294472	0.25657393
Nhlrc4	-1.3212312	-0.4151963	1.99802059	0.17294999	0.25657393
Pitrm1	0.2149959	5.2643786	1.99774369	0.17297839	0.25657393
Bloc1s5	-0.4197536	3.3249666	1.99771082	0.17298177	0.25657393
Zgrf1	0.48521327	2.5509492	1.99631356	0.17312516	0.25672058
Yy2	0.87746796	-0.0337332	1.99610531	0.17314655	0.25672058
Pask	0.78856466	0.83317781	1.99598155	0.17315926	0.25672058
Arhgap4	-1.3987078	-0.1392605	1.99485116	0.1732754	0.25681422
Cd36	1.24330219	-0.390192	1.9946573	0.17329533	0.25681422
Rnf112	0.34349164	4.83507087	1.99458407	0.17330286	0.25681422
Echs1	-0.2842114	4.38260883	1.99434639	0.17332729	0.25681422
Bbs5	0.31900516	3.73149981	1.99394648	0.17336842	0.25683631
Mme	0.27071256	4.26111758	1.9935807	0.17340605	0.25685321
Tpgs2	-0.1698013	6.19132369	1.99281082	0.17348528	0.25693171
Pou2f1	0.19487801	5.80107812	1.99049884	0.17372348	0.25724559
Pi4k2b	0.49213585	2.02012472	1.98966839	0.17380914	0.25729912
Narfl	-0.4012795	2.64718879	1.98963906	0.17381216	0.25729912
Morc4	-0.340853	2.89235091	1.98912962	0.17386474	0.25730269
Prkcb	0.22650571	11.0318329	1.98910661	0.17386711	0.25730269
Hebp1	-0.2856309	3.58687395	1.98859635	0.17391979	0.25734177
2810055G20	0.4217255	2.25090386	1.9883256	0.17394775	0.25734427

Efcab6	0.59141087	1.7914979	1.9869609	0.17408876	0.25746813
Zmat3	-0.2048912	9.37056188	1.98690801	0.17409423	0.25746813
Gm15417	-0.6194254	1.17185303	1.98651649	0.17413471	0.25746813
Ddx39b	-0.1925603	5.43056837	1.98649819	0.17413661	0.25746813
1500015A07	0.51627903	2.19845466	1.98503011	0.17428851	0.25765383
Mocos	0.63304515	1.42942326	1.98439727	0.17435404	0.25771181
Rtkn2	0.69066957	1.19225338	1.98350962	0.17444601	0.25780885
Sbspon	-0.8299903	0.73858241	1.98089016	0.17471776	0.25814025
Sprr1a	-3.2503681	-0.2489927	2.00702411	0.17473177	0.25814025
Sypl	0.25208439	6.38862586	1.9805863	0.17474931	0.25814025
Rdh10	0.26887093	3.6843256	1.98006544	0.17480342	0.25818124
Zfpm2	0.39953649	3.7385624	1.97873854	0.17494136	0.25834601
Sgtb	-0.1977608	8.05187458	1.97832915	0.17498394	0.25835772
Daam1	0.21863045	6.59201576	1.97790287	0.17502829	0.25835772
B3gnt1	-0.2323757	4.84392782	1.97790178	0.17502841	0.25835772
Ptpn7	0.39393948	3.28926987	1.9774965	0.17507059	0.25838105
Adamts8	0.90735593	-0.3330181	1.97637322	0.17518757	0.25851474
Amz1	-0.4795869	1.78497787	1.97554209	0.17527419	0.25857725
Lonrf2	0.22466015	8.07291777	1.97546015	0.17528273	0.25857725
Golim4	0.25014322	5.93506605	1.97520015	0.17530983	0.2585783
Stra6	-0.4857093	7.10475634	1.97486125	0.17534518	0.25858898
Csf2rb	-0.6345193	1.3256327	1.974266	0.17540727	0.25858898
Relb	-0.6311759	0.4167856	1.97422352	0.17541171	0.25858898
Fyb	-0.3735114	4.4935111	1.97391399	0.17544401	0.25858898
Abcc3	1.15256431	-0.6679128	1.97363204	0.17547344	0.25858898
Tbpl1	-0.1514124	6.41403785	1.97361257	0.17547547	0.25858898
Pigp	-0.2904009	4.8019085	1.97335771	0.17550208	0.25858929
Phlda3	-0.4425343	2.61215372	1.97263796	0.17557725	0.25866114
Entpd1	-0.352808	3.01205359	1.97236309	0.17560597	0.25866454
Aplp2	0.14842714	8.46249735	1.97170673	0.17567457	0.2586659
Wdr27	1.68806129	-0.7713851	1.97169469	0.17567583	0.2586659
1700025F24	-2.1588441	-2.177585	1.97159634	0.17568611	0.2586659
Amotl1	-0.2229526	6.88193783	1.97065593	0.17578446	0.2587377
Taok1	0.14982956	9.64326833	1.97038963	0.17581232	0.2587377
Enah	0.22858391	8.35869406	1.97037247	0.17581412	0.2587377
Nudt12	-0.532368	2.84423276	1.96926805	0.17592973	0.25883333
Nradd	-0.9718556	0.29732932	1.96924692	0.17593195	0.25883333
Ier3	-0.3825637	4.1511689	1.96838501	0.17602224	0.25892728
Apc2	0.35290193	4.60083919	1.96739582	0.17612594	0.25899335
Chst12	-0.4636786	1.74990168	1.96738092	0.1761275	0.25899335
Pcf11	0.18929934	6.58616928	1.96719994	0.17614648	0.25899335
Slx4ip	-0.3563322	3.03855891	1.96625424	0.17624571	0.25910035
Calcrl	-0.2843332	4.34254882	1.96429625	0.17645136	0.25936376

Tmem160	-0.7163572	1.07426555	1.96392731	0.17649014	0.25936512
Coq5	-0.1710563	6.38058398	1.96378369	0.17650524	0.25936512
E030019B06I	1.4516364	-0.5614128	1.96299768	0.17658791	0.25944768
Pdcd6ip	0.1569343	7.45387281	1.96241112	0.17664964	0.25949944
Cep57	0.2342741	4.61047186	1.96200094	0.17669282	0.25951156
Parn	0.21958377	4.67553841	1.96139267	0.17675687	0.25951156
Zdhhc17	0.22010491	6.78753137	1.96138139	0.17675806	0.25951156
Tmem41a	-0.3093038	3.74697865	1.96132637	0.17676386	0.25951156
Lrrk1	-0.3491078	5.08688029	1.96052559	0.17684823	0.25955044
Mei4	0.95509156	0.42421138	1.96026875	0.1768753	0.25955044
Mapk13	-2.0491951	-1.634588	1.96011212	0.17689182	0.25955044
Dusp23	-0.504501	2.79903088	1.96006931	0.17689633	0.25955044
Msrb2	-0.2510171	4.28512107	1.9594826	0.1769582	0.25960234
Gm15217	-1.4153101	-1.6360095	1.95901778	0.17700724	0.25963539
Sp3os	-0.4488138	3.10306267	1.95718907	0.17720033	0.25986172
Tcta	-0.3135334	4.4087972	1.95705399	0.1772146	0.25986172
Chmp5	-0.2695236	6.60861862	1.95560368	0.17736793	0.26004763
Ints12	0.22346519	4.73342716	1.95455106	0.17747932	0.260172
Agmat	-1.3278137	-0.0296026	1.95367551	0.17757204	0.26026897
D630003M21	-1.0124743	0.44311259	1.95228103	0.17771983	0.26044662
Ifltd1	-0.3278503	5.40439857	1.95193989	0.17775601	0.26046067
Pvrl2	0.68672071	1.42364104	1.9514602	0.17780689	0.26047798
Nfe2l2	-0.2336654	7.84925387	1.95132722	0.17782101	0.26047798
Sp2	-0.3529541	5.31463402	1.95073851	0.17788349	0.26053054
Gm14379	1.27732705	-0.7781729	1.94995887	0.17796628	0.26059708
Cdk15	0.7091651	0.70685136	1.94963516	0.17800067	0.26059708
Fgd6	0.24254499	6.33846098	1.94939643	0.17802603	0.26059708
Pabpc1	-0.1707856	8.19210904	1.94930888	0.17803533	0.26059708
Itga6	0.3398272	3.55203255	1.94850963	0.1781203	0.26068249
Glrx5	-0.3009653	3.73372452	1.94763999	0.1782128	0.2607789
Cdpf1	-0.4674208	3.2397897	1.94704735	0.17827587	0.26081066
Plekhg6	1.5185369	0.0553454	1.94685084	0.17829679	0.26081066
Arhgef10	-0.2121785	5.27670448	1.94668563	0.17831438	0.26081066
Iigp1	-0.3661764	4.08486279	1.94477214	0.17851826	0.26106988
Rchy1	-0.2096254	5.50211776	1.94442065	0.17855574	0.26108572
Lsm4	-0.3828375	3.33172149	1.94413106	0.17858663	0.26109191
Olfr558	-2.2024781	-1.0715801	1.9415727	0.1788598	0.26145226
Vat1l	0.30832053	5.41946063	1.94020812	0.17900571	0.26155377
Rnf166	-0.2983472	4.71944735	1.94014185	0.1790128	0.26155377
Ssh1	-0.3700718	2.40579911	1.94008703	0.17901867	0.26155377
Atp11a	0.20609418	6.90085178	1.93992458	0.17903605	0.26155377
Mob3a	0.35446479	2.63458704	1.93933806	0.17909883	0.26156726
Elac2	0.4192105	2.88312398	1.93904215	0.17913051	0.26156726

Ahnak	-0.3073117	9.0753298	1.93880858	0.17915552	0.26156726
Aldh1l1	-0.2992514	3.6319835	1.93880049	0.17915638	0.26156726
F10	1.11049156	-0.8615185	1.93859118	0.1791788	0.26156726
Rmi2	0.42331525	2.19828046	1.93782764	0.17926061	0.26164372
Mrpl1	0.25485331	4.24105416	1.93744991	0.1793011	0.26164372
Gmnn	-0.4256778	2.06183801	1.93729425	0.17931778	0.26164372
4930444F02I	1.51287235	-0.6026053	1.93710547	0.17933803	0.26164372
Nxph3	0.91929163	-0.3782904	1.93628144	0.17942641	0.26172756
Xab2	0.26361965	3.85964232	1.93607161	0.17944893	0.26172756
Med22	-0.2851967	3.78866602	1.93476136	0.1795896	0.26189373
Dyx1c1	0.51518098	2.02333161	1.93264327	0.17981729	0.26215628
Syne2	0.20008282	5.49056442	1.93258867	0.17982316	0.26215628
Cmbl	-0.4149796	6.17617277	1.93113982	0.17997912	0.2623126
Elk1	0.23599901	4.81294127	1.93109501	0.17998395	0.2623126
Arid3c	1.6705816	-1.2824569	1.93004609	0.18009696	0.26243826
Cep192	0.35128814	4.46641486	1.92958622	0.18014654	0.26244923
Rapgef4	0.21679991	7.31173449	1.92921656	0.1801864	0.26244923
Gpr34	0.41506301	2.07134329	1.92841011	0.18027341	0.26244923
Fam114a1	-0.3741598	6.31411309	1.92838837	0.18027575	0.26244923
Crip2	-0.2100182	4.55658148	1.92828396	0.18028702	0.26244923
Ccdc135	-1.0440267	-0.2724766	1.928282	0.18028723	0.26244923
Grik4	0.52658166	1.91714354	1.92823748	0.18029204	0.26244923
Casz1	-0.5924808	2.11849461	1.9275955	0.18036135	0.26249761
Gm10754	0.5305969	1.74058134	1.92743322	0.18037887	0.26249761
Uqcrb	-0.2267925	6.81664099	1.92621908	0.18051005	0.26260286
Jazf1	-0.2454903	5.2823902	1.92621235	0.18051078	0.26260286
Cyr61	-0.8013255	2.43826021	1.92601949	0.18053163	0.26260286
Ddx19b	0.24764453	5.11197622	1.92513552	0.18062722	0.26264151
Srsf6	0.16262462	5.68150112	1.925059	0.1806355	0.26264151
Trmt12	-0.3335655	3.27438155	1.92493481	0.18064894	0.26264151
Srebf1	0.31718231	2.89281282	1.92478222	0.18066545	0.26264151
Slc9a6	0.19564878	6.2789716	1.92423208	0.18072499	0.26268908
Ngrn	-0.2536895	4.21976295	1.92300272	0.18085813	0.26284304
Gm10433	-2.5319849	-1.6576177	1.92270713	0.18089016	0.26284304
9130023H24I	-0.3206695	3.76212086	1.92226839	0.18093771	0.26284304
Abi2	0.17109469	8.54721792	1.92226349	0.18093825	0.26284304
8030423F21I	0.94553881	-0.8160297	1.92147619	0.18102362	0.26287674
Fbxw5	-0.2505435	5.41476069	1.92144482	0.18102702	0.26287674
Arxes1	0.41059666	2.41449458	1.92112051	0.18106221	0.26287674
Ormdl3	-0.3479528	4.20754267	1.92105984	0.18106879	0.26287674
Wnt11	1.57217543	-1.0895182	1.91958681	0.18122871	0.2630579
Arhgap18	-0.2267607	5.25575095	1.91930353	0.18125948	0.2630579
Pzp	1.37247628	-0.8954508	1.91916868	0.18127413	0.2630579

Itgae	-1.9916028	-1.8146035	1.91861479	0.18133433	0.26310627
Polk	-0.2298523	5.18978043	1.91824259	0.1813748	0.26312601
Ivd	-0.2195915	4.16781787	1.91772357	0.18143124	0.26315791
Stk16	-0.3005998	4.51350464	1.91734538	0.18147238	0.26315791
Ablim2	0.23062466	6.07377197	1.91729946	0.18147738	0.26315791
Map3k10	0.2935036	3.26749762	1.91700936	0.18150895	0.26316472
Spg21	-0.3256083	5.13850679	1.91641099	0.18157408	0.2631777
Gm20743	1.61973217	-1.8229197	1.91606498	0.18161176	0.2631777
Mterfd2	0.20383228	5.9146448	1.91605832	0.18161249	0.2631777
Cfhr2	-0.732955	0.69676622	1.91594003	0.18162537	0.2631777
Tmf1	-0.1501882	7.01464471	1.91534377	0.18169033	0.26320927
St6gal2	0.28307392	4.83107162	1.91524677	0.1817009	0.26320927
Utrn	0.16817023	7.75997577	1.91356321	0.18188447	0.26343624
Nol11	0.25538889	4.28240854	1.91290279	0.18195655	0.26347308
Bean1	-0.2431565	3.92752223	1.91264592	0.18198459	0.26347308
Car5b	0.42056406	1.96853498	1.91259083	0.18199061	0.26347308
Phf11b	-0.9973003	0.71371584	1.91156677	0.18210246	0.26359606
Mir186	1.44706929	-0.8361679	1.91099479	0.18216498	0.26364759
Mt2	-0.3184238	5.17961798	1.91010588	0.18226218	0.26374931
C730002L08F	-0.4659857	2.30631274	1.90914404	0.18236743	0.26385148
Ebna1bp2	-0.2273721	5.39896302	1.90896841	0.18238666	0.26385148
1700001K23I	1.90480185	-1.6193535	1.90822765	0.18246778	0.26392986
Txndc11	0.27370862	3.87622766	1.90780203	0.18251441	0.26395154
Slc20a1	0.26398094	5.34830117	1.90759892	0.18253667	0.26395154
Cnot2	-0.1734954	6.21927661	1.90657823	0.18264857	0.26407437
Atxn2l	0.18377814	6.83832865	1.90607484	0.18270379	0.26410157
Slc7a15	1.49795619	-1.0209344	1.90591521	0.18272131	0.26410157
Ubox5	-0.3322257	3.32960985	1.90536723	0.18278145	0.26414952
Polr3e	0.32901921	3.83577343	1.90482945	0.18284049	0.26419587
Ceacam20	0.564872	1.81222087	1.90414817	0.18291533	0.26426502
Rnft1	0.25489613	4.55113215	1.90365527	0.1829695	0.26430429
Cacfd1	0.23194577	4.71033613	1.90288047	0.18305468	0.26433329
Myh11	0.90557011	2.52897226	1.90275818	0.18306813	0.26433329
Fgfbp3	-0.3760423	2.68347118	1.90247908	0.18309883	0.26433329
Ajuba	-0.3163758	2.82632005	1.90223151	0.18312607	0.26433329
Hmgcs1	0.22825689	7.21088302	1.90201173	0.18315025	0.26433329
Alox5	-1.4964468	-1.3258855	1.90190055	0.18316249	0.26433329
Pgbd1	0.35633489	2.33747517	1.90175534	0.18317847	0.26433329
Kctd15	0.48738578	2.12360408	1.90070599	0.18329401	0.26446107
Rilpl1	-0.2365926	5.94586796	1.89988812	0.18338413	0.2645197
Dll1	0.93067041	0.10744602	1.8998343	0.18339007	0.2645197
Ankrd29	0.30676857	4.6037215	1.89960206	0.18341567	0.2645197
Gin1	0.33797142	3.43491305	1.89891423	0.18349152	0.26458498

Nprl3	0.30933615	3.06488781	1.89856945	0.18352955	0.26458498
Cdh15	1.85084524	-1.396928	1.89845696	0.18354196	0.26458498
Rab40b	0.27031794	4.03718663	1.89820609	0.18356964	0.26458595
G730013B05	0.82000129	0.35112344	1.89795027	0.18359788	0.26458771
Nek9	-0.1602515	6.73170943	1.8970915	0.1836927	0.26468541
1810043H04I	-0.5852583	1.96213509	1.89547586	0.18387125	0.26490373
Snx14	0.23049867	5.44398163	1.89482859	0.18394285	0.2649679
3830406C13I	-0.2080743	6.13628386	1.89354875	0.18408451	0.26513298
Mink1	0.24479735	5.58472022	1.89278523	0.18416909	0.26519846
Gm4787	0.98123257	2.161135	1.89264951	0.18418413	0.26519846
Unc5b	0.41071436	2.36938693	1.89182976	0.184275	0.26529031
Mettl2	0.3346857	4.13577789	1.89136911	0.18432608	0.26532486
Cd14	-1.0115714	-0.0168069	1.89069098	0.18440132	0.26539416
Mospd2	0.22412287	5.1513818	1.88947871	0.18453592	0.26550428
Casp1	-0.5774539	1.62809504	1.88917228	0.18456996	0.26550428
Fam50a	-0.3201352	4.12554342	1.88912512	0.1845752	0.26550428
1700123O20	-0.3156337	3.87720246	1.88902559	0.18458626	0.26550428
6230400D17I	0.94066025	0.25397357	1.88732177	0.18477569	0.26573774
Cdh2	0.2537119	6.47293761	1.88665159	0.18485027	0.26577539
Hras	-0.3293489	5.0526213	1.88659889	0.18485614	0.26577539
5530401A14I	0.8641187	0.01836977	1.88594287	0.18492918	0.26584139
Ttc25	1.1896854	-0.1515874	1.88516099	0.18501629	0.26592757
Fuca1	0.30603849	5.15110516	1.88372421	0.18517648	0.26609524
Flrt2	0.16973059	7.45037819	1.88345616	0.18520638	0.26609524
Srgap3	0.25272633	9.0810211	1.88338403	0.18521443	0.26609524
Apbb1	-0.2111991	5.52168404	1.88263609	0.18529791	0.26617613
Car12	0.35695672	3.31071986	1.88210875	0.1853568	0.26620235
Sik3	0.20543239	7.39081185	1.88198591	0.18537052	0.26620235
Mcm6	-0.2580507	3.64528183	1.8811685	0.18546185	0.26629447
E030003E18I	-0.377593	2.39681955	1.88074853	0.1855088	0.26632284
Copa	0.20308489	7.7765974	1.87984968	0.18560932	0.2664099
Commd9	-0.4826804	2.15963691	1.87949436	0.18564908	0.2664099
Selk	-0.2417057	6.15916449	1.87947693	0.18565103	0.2664099
B3galt2	0.27270955	5.41767912	1.8771858	0.18590765	0.26670861
Lman2	0.21178436	5.61149008	1.87713225	0.18591365	0.26670861
Xirp2	0.58505267	2.38411212	1.8768723	0.18594279	0.26671136
Rev1	-0.2207167	4.38275609	1.87508841	0.18614294	0.26692063
Rrad	-1.4951333	-1.1122327	1.87508246	0.18614361	0.26692063
Hirip3	0.1961159	5.06218827	1.87457461	0.18620064	0.26692063
Pigk	0.26943565	5.13219269	1.87456142	0.18620213	0.26692063
Phf1	0.45602212	2.86487896	1.87435831	0.18622494	0.26692063
Plcb3	-0.3218498	4.33601191	1.87371701	0.186297	0.26698485
Vapa	-0.1793127	7.18529711	1.87295964	0.18638214	0.26702376

Cd8a	-0.6504506	0.35350132	1.87264846	0.18641714	0.26702376
Pard6a	-0.5417716	2.15301672	1.87246966	0.18643725	0.26702376
Noa1	-0.2137342	4.42358664	1.872378	0.18644756	0.26702376
Pglyrp1	0.6288132	0.74415633	1.87226343	0.18646045	0.26702376
Plekho1	-0.2660126	4.03851156	1.87109464	0.18659201	0.2671731
Ccdc114	-1.0764671	0.152478	1.87065626	0.18664139	0.26720474
Bloc1s4	-0.379396	4.26340494	1.86900361	0.18682767	0.2673799
Tymp	-0.4917929	2.53864225	1.86891422	0.18683775	0.2673799
Nisch	0.19098262	7.3684988	1.86884442	0.18684563	0.2673799
Pomgnt2	-0.4406852	2.41069811	1.86821988	0.18691609	0.26743206
Uqcr11	-0.3995867	4.69448431	1.8680025	0.18694062	0.26743206
Slfn5	0.3304323	4.46679279	1.86779556	0.18696398	0.26743206
4930447C04I	0.66412753	2.03600287	1.86718311	0.18703314	0.26748604
Tmem201	0.33317489	3.96789872	1.86680997	0.18707529	0.26748604
Srm	-0.2810176	4.05505289	1.86666899	0.18709121	0.26748604
Smndc1	-0.2453109	5.33377647	1.86649433	0.18711095	0.26748604
Cactin	-0.3605784	3.17297834	1.86485407	0.18729641	0.26767751
Pfn4	0.488389	1.95490093	1.8648264	0.18729954	0.26767751
2610027K06I	1.20118403	-0.8805224	1.86445169	0.18734194	0.26768595
Lrrc32	-0.334391	4.80677595	1.8642912	0.1873601	0.26768595
Zfp655	0.19280476	5.7401676	1.86273521	0.18753632	0.26789864
Nr4a2	0.55739884	6.88908107	1.86206197	0.18761263	0.26796294
Vwc2l	0.37140214	3.42519573	1.86185546	0.18763604	0.26796294
Calcoco2	0.74275301	-0.0155403	1.86147656	0.18767901	0.26796824
Muc15	0.66004932	2.10105816	1.86134031	0.18769447	0.26796824
Adnp2	0.25716962	4.66110951	1.85997265	0.18784969	0.26815076
Wdr95	-1.8060194	-1.3915746	1.85844887	0.18802281	0.26835879
Gabrb3	0.22247267	8.62854624	1.85516264	0.18839686	0.26882071
Popdc3	1.39201526	-0.7530781	1.85512327	0.18840134	0.26882071
Acta1	-0.6250555	0.90019356	1.85463094	0.18845746	0.26883471
Isg15	-0.4612924	1.64213574	1.85455567	0.18846604	0.26883471
Nfx1	0.16399402	6.44274509	1.8532711	0.18861257	0.26900455
Cbr3	-0.3865122	2.12842456	1.85267449	0.18868067	0.26901471
Peg13	-0.1859805	8.15164275	1.85261318	0.18868767	0.26901471
Spi1	-0.7923342	0.49073913	1.85248697	0.18870208	0.26901471
Prrg1	0.40639988	2.50080234	1.85167713	0.18879459	0.26910742
Psmb11	0.61766108	1.33147065	1.85089756	0.18888369	0.26919524
Caap1	-0.3136648	2.76923956	1.85018629	0.18896503	0.26922049
Podxl2	-0.4832334	2.17688717	1.84972448	0.18901786	0.26922049
Akt2	-0.2458757	5.97679673	1.84972196	0.18901815	0.26922049
2810001G20	-0.4058694	4.81931109	1.84946912	0.18904708	0.26922049
Serpinb8	0.41454372	3.40522974	1.84912706	0.18908624	0.26922049
Tmem248	-0.199596	5.76332469	1.84909544	0.18908986	0.26922049

Slc4a7	0.29121996	3.91386654	1.84906107	0.18909379	0.26922049
Xpo1	0.1822679	6.55374024	1.84735936	0.18928873	0.26936596
Atp2c2	-0.8405246	0.00134164	1.84722249	0.18930443	0.26936596
Cdc42bpb	0.21929902	6.53365385	1.84695985	0.18933454	0.26936596
Ccs	-0.438715	2.13602256	1.84659545	0.18937633	0.26936596
Cse1l	0.16435726	6.72638057	1.8465828	0.18937778	0.26936596
Itgam	0.36467985	3.55949503	1.84649476	0.18938788	0.26936596
Mcat	-0.4078989	2.54081818	1.84648964	0.18938846	0.26936596
Slfn3	1.38096622	-0.2510446	1.84571962	0.18947682	0.26943792
Nuak1	-0.2355526	5.19287171	1.8455688	0.18949413	0.26943792
9330188P03I	0.94944972	0.9897814	1.84530751	0.18952412	0.26943792
Samsn1	0.74837545	0.62142411	1.84503778	0.18955509	0.26943792
Bst2	-0.7976977	1.61087395	1.84479308	0.18958319	0.26943792
Themis2	-0.6597612	1.61244666	1.8441951	0.18965189	0.26943792
Nucb2	0.20982911	5.26267907	1.8440679	0.1896665	0.26943792
Sall2	0.20433301	5.73749934	1.84396031	0.18967887	0.26943792
Capn2	-0.207496	7.15307409	1.84389286	0.18968662	0.26943792
Dnajc12	-0.3459416	3.61725097	1.84326128	0.18975922	0.26947374
Marveld2	-1.0468782	0.62572504	1.84319491	0.18976686	0.26947374
Kcnb2	0.24767832	5.28418829	1.84139849	0.18997357	0.26972217
Slc4a8	0.30588405	6.84351407	1.84119603	0.18999688	0.26972217
Wisp1	-0.3992741	1.94884932	1.84084636	0.19003716	0.26974026
Abcg2	-0.2749117	5.21468383	1.84058825	0.19006689	0.26974338
9430016H08I	-0.3100939	3.49549773	1.83957059	0.19018419	0.26985612
Cgnl1	-0.2370427	6.07090284	1.839161	0.19023142	0.26985612
Klhl38	-1.0148765	0.04729006	1.83896084	0.19025451	0.26985612
Zfx	-0.2064081	6.22463205	1.83894329	0.19025653	0.26985612
Ephb4	0.51000747	3.24174514	1.83787432	0.1903799	0.26999201
Tigd5	0.80023985	0.66060187	1.83691214	0.19049102	0.27006875
Pofut1	0.29106	3.36432094	1.83690956	0.19049132	0.27006875
Clasp2	0.1778843	8.0401354	1.83648542	0.19054033	0.27006875
Ppp1r1a	-0.3014996	7.06598969	1.83631705	0.19055979	0.27006875
St7l	-0.3037249	4.22717222	1.83612882	0.19058155	0.27006875
Neb	0.63125893	2.55395156	1.83597409	0.19059944	0.27006875
Bcl2l12	0.8011521	0.23537933	1.83534478	0.19067221	0.2701105
Thnsl2	0.42981326	1.59740363	1.83524244	0.19068405	0.2701105
Spats2l	-0.1936211	5.09041211	1.83459609	0.19075884	0.27017248
Slc9a5	0.43455486	2.60183647	1.83438756	0.19078298	0.27017248
Tbx20	1.31544667	-1.4327561	1.83346361	0.19088997	0.27022909
Klrg1	-2.7555588	-2.0796857	1.83344179	0.19089249	0.27022909
Nars2	0.33701947	3.83879504	1.83305035	0.19093784	0.27022909
Atp11c	0.26879806	4.6841988	1.83295357	0.19094906	0.27022909
Fus	0.27956082	5.94252772	1.83285148	0.19096089	0.27022909

Ppp1r42	-1.1203533	-0.5472227	1.83201232	0.19105817	0.2703277
Tpd52l2	-0.2783349	6.30856718	1.83156438	0.19111012	0.27032856
Bend5	-0.3322881	3.27714719	1.83148586	0.19111923	0.27032856
Tyw1	0.54106327	2.0133922	1.83129332	0.19114157	0.27032856
Tmem45a	0.51677547	2.20741099	1.83094232	0.1911823	0.27034713
Plod3	0.43767458	2.62282767	1.83068762	0.19121186	0.27034991
Eprs	0.15568058	7.57236391	1.82995304	0.19129715	0.27043146
Fbxl12	0.37962771	2.3507409	1.82947728	0.19135242	0.27046539
Pram1	0.63020152	0.92672815	1.82865579	0.19144789	0.27046539
Itgb3bp	0.44714052	3.3353098	1.82860647	0.19145363	0.27046539
Fam13b	-0.1536333	6.9242022	1.82854708	0.19146053	0.27046539
Tstd3	-0.2206334	5.42823205	1.82833772	0.19148487	0.27046539
Ccrl2	-0.6401085	0.53143071	1.82817402	0.19150391	0.27046539
Ccdc65	-0.4539812	2.03665474	1.82805494	0.19151776	0.27046539
Cib2	-0.5880665	2.27751293	1.82763663	0.19156642	0.27046539
Nr1h4	1.49410338	-0.4247243	1.82760883	0.19156965	0.27046539
Snx19	0.22774264	4.75984491	1.82676671	0.19166766	0.27053522
Gm21119	-0.708459	0.59824028	1.82670922	0.19167436	0.27053522
1700040L02F	-0.4660989	1.83594238	1.82594056	0.19176388	0.27059973
Lrrc34	-1.501728	-1.0279857	1.82584243	0.19177531	0.27059973
Ccdc177	0.45876876	2.74825824	1.82496504	0.19187756	0.27070501
Rpl14	-0.3190344	6.55012026	1.82423714	0.19196244	0.27078577
5830416P10I	0.71883335	0.35402887	1.82264604	0.19214815	0.2710087
Arhgdib	-0.3915799	7.44678784	1.82132505	0.1923025	0.27118734
Fam149a	-0.182378	5.22175722	1.82083882	0.19235935	0.27122847
Oprd1	0.31149868	4.16299304	1.8205846	0.19238908	0.27123135
Papolb	0.95172872	0.72027379	1.81980858	0.19247987	0.2713203
Klhdc3	-0.2114577	4.44002084	1.8190778	0.19256543	0.27136524
Slc9b2	-0.3345997	4.25861711	1.8187991	0.19259806	0.27136524
Paqr5	-0.4510676	3.21834626	1.81874788	0.19260406	0.27136524
Sh3tc1	-1.4090007	-1.176216	1.81858989	0.19262257	0.27136524
Tmem189	-0.3782574	2.5608425	1.81781447	0.19271343	0.27145421
Tmem208	0.41644688	2.06204217	1.81703698	0.19280459	0.27149409
Brca2	0.27984402	3.83286062	1.8168984	0.19282084	0.27149409
Entpd5	0.23689358	4.33180174	1.81686386	0.19282489	0.27149409
Tspyl2	0.21811739	6.06718009	1.81641345	0.19287773	0.27152945
Lyve1	0.47169343	3.43353616	1.81523676	0.19301586	0.27168486
Lekr1	-0.3773738	2.74701292	1.81460564	0.19309	0.27175017
Dzip1	0.17390833	6.54829099	1.8139385	0.19316841	0.27182146
Slc16a10	-0.359449	2.28528914	1.81349235	0.19322086	0.27184547
Aes	-0.2341929	6.35405243	1.81332136	0.19324097	0.27184547
Zfp385c	0.45614168	1.90571192	1.81243258	0.19334554	0.27192547
Atp8b5	1.32372539	-0.868689	1.81236606	0.19335337	0.27192547

Fam189b	0.25491775	4.60001513	1.81211384	0.19338306	0.27192818
Clec2d	-0.5169471	2.1306809	1.81111417	0.19350078	0.27204426
Gpank1	-0.5397106	2.00662254	1.81094129	0.19352115	0.27204426
Ung	0.49614964	2.10678737	1.81065406	0.19355499	0.27205279
Wbp5	-0.2595255	6.62007551	1.80989836	0.19364408	0.27211679
Sv2a	0.23088737	5.86958726	1.80979655	0.19365608	0.27211679
Gucy1a2	0.28664422	7.95296243	1.80920878	0.19372541	0.27215035
Lamc3	0.4034498	2.59835371	1.80912299	0.19373553	0.27215035
Stk38	0.21054571	4.80460911	1.80859746	0.19379755	0.27219843
Gga3	0.20437637	5.38664136	1.8074342	0.19393491	0.27233707
Vangl1	0.37719761	3.81104559	1.80729075	0.19395186	0.27233707
B3gat3	-0.329214	3.19066001	1.80680832	0.19400887	0.27237586
Hook3	0.15833334	8.61231605	1.80658641	0.1940351	0.27237586
Pydc3	0.77098962	0.73469597	1.80570657	0.19413914	0.27244526
Fbxw15	1.25869648	-0.9967257	1.80569789	0.19414017	0.27244526
Oxsm	-0.389628	3.16295024	1.80532292	0.19418453	0.27246511
Khnyn	0.31515464	2.936284	1.80510818	0.19420994	0.27246511
Snw1	-0.1810907	6.48926014	1.80449848	0.19428212	0.27246905
Hps6	0.55007123	1.04365059	1.80447591	0.19428479	0.27246905
Rpl31-ps12	-0.261999	4.06261921	1.80437954	0.1942962	0.27246905
Adnp	0.16949736	7.86024029	1.80404178	0.1943362	0.27248613
H2-Ke2	-0.3573248	3.77446252	1.80235552	0.19453605	0.27268302
Spata24	-0.7898977	0.34077621	1.80234652	0.19453712	0.27268302
Kifc2	0.20166316	6.32616462	1.80215245	0.19456013	0.27268302
Zfp334	0.19014718	4.89297415	1.8015989	0.1946258	0.27273603
Hist1h2bk	-1.2768471	-1.1580995	1.80066274	0.19473693	0.27285272
Sod1	-0.2904379	8.00215232	1.79971155	0.19484992	0.27294885
Tbc1d15	0.20067912	5.20688553	1.79961603	0.19486127	0.27294885
Crot	0.19322593	5.34776257	1.79936746	0.19489081	0.2729512
Ap2a1	0.27718701	4.06991428	1.79912472	0.19491967	0.27295259
Zfp617	-0.1992153	5.81358953	1.79865648	0.19497535	0.27299153
Ccdc86	-0.3933449	2.91362023	1.79788949	0.19506659	0.27307913
Zbtb5	0.37255147	2.58687091	1.79736159	0.19512942	0.27307913
Josd1	-0.1817081	5.03477143	1.79729105	0.19513781	0.27307913
Fam166b	-1.1913141	-0.4522952	1.7969807	0.19517477	0.27307913
Tead2	-0.4639617	2.48379968	1.79695941	0.1951773	0.27307913
Map3k4	0.2401082	4.98220469	1.79655042	0.19522601	0.27309012
Per2	0.26596044	5.05396561	1.79580058	0.19531536	0.27309012
Dusp16	-0.2827095	4.92493437	1.7955027	0.19535086	0.27309012
Pou6f2	-0.6008093	1.93296602	1.79536532	0.19536724	0.27309012
Pdss2	-0.2745729	3.1149329	1.79507858	0.19540143	0.27309012
Npy5r	0.7156028	0.98837721	1.79494163	0.19541776	0.27309012
Rfx3	0.27744051	7.27365649	1.79462529	0.19545549	0.27309012

Sspo	1.82022332	-1.2452705	1.79449136	0.19547147	0.27309012
6430584L05F	0.36011862	3.02541422	1.79428055	0.19549662	0.27309012
Alkbh2	-0.569541	1.16747695	1.79419461	0.19550688	0.27309012
Zfp248	0.27007783	4.558211	1.79413415	0.19551409	0.27309012
Csnk1a1	-0.1338162	7.62192174	1.79408706	0.19551971	0.27309012
Wrnip1	-0.2276183	5.40301787	1.79378518	0.19555574	0.2731015
6430531B16I	1.40029218	-0.6821775	1.79289246	0.19566234	0.273184
Fau	-0.3271927	5.68970486	1.79282337	0.19567059	0.273184
Tbc1d8	0.33275097	4.20495322	1.79214157	0.19575206	0.27325879
1700071K01I	1.54019165	-0.3649096	1.79149374	0.19582951	0.27330023
Zfp160	0.24361112	4.46987999	1.79142644	0.19583755	0.27330023
Cep170	0.21404929	7.48685712	1.79063342	0.19593241	0.27339366
Sulf2	0.17133707	6.53957386	1.78945278	0.19607374	0.27354149
Fam102b	-0.1594917	5.84785971	1.78928195	0.1960942	0.27354149
Ppan	-0.521779	1.68015532	1.78801827	0.19624564	0.27370418
Ankub1	0.92747676	0.64183141	1.78784246	0.19626672	0.27370418
Wwox	-0.2483252	4.0488127	1.78729108	0.19633285	0.27375742
Zpr1	-0.2220097	4.3602774	1.7860606	0.19648052	0.27392434
E130006D01I	-1.6383266	-2.0148907	1.78542813	0.19655648	0.27399125
Strbp	0.22584105	9.34693261	1.78495672	0.19661312	0.2740312
Id3	-0.3982239	6.0191475	1.78470596	0.19664326	0.27403422
Clnk	1.2179783	-0.6436547	1.78384097	0.19674726	0.27414015
Trappc10	0.18269643	5.8965905	1.78353643	0.19678389	0.27415176
Chka	0.24788702	4.53753609	1.78330636	0.19681157	0.27415176
Adora2a	0.73613298	1.89798399	1.78303374	0.19684438	0.27415847
Tlcd2	0.45336735	1.37738879	1.78157883	0.19701957	0.27432061
Dnajb13	-1.5708653	-1.4063093	1.78139422	0.19704182	0.27432061
Rest	-0.2934968	4.90144498	1.78118582	0.19706693	0.27432061
Cyc1	-0.245787	6.32295405	1.78113701	0.19707281	0.27432061
Mapk8ip2	0.43670166	4.63969142	1.78007218	0.1972012	0.27442447
Enkur	-0.4381406	2.14024953	1.77979557	0.19723457	0.27442447
Cenpw	-0.5476862	1.57348839	1.77966027	0.19725089	0.27442447
4933400F21I	0.83658865	1.22189907	1.77958903	0.19725949	0.27442447
Pik3c2a	0.17651673	6.15002745	1.77877193	0.19735812	0.27451069
Zscan25	-1.4254664	-0.360117	1.77848045	0.19739331	0.27451069
C130026L21F	0.63490044	1.06076622	1.77817617	0.19743006	0.27451069
Nudt6	-0.316968	3.7359988	1.77814718	0.19743357	0.27451069
Plekhd1	-1.337403	-1.030758	1.77741469	0.19752207	0.27457877
Speer7-ps1	0.79667645	0.64711457	1.77727806	0.19753859	0.27457877
Sesn3	0.17909912	6.09215675	1.77650757	0.19763175	0.27466929
B230206H07	1.74421373	-1.6970281	1.77518273	0.19779207	0.27482931
Mir377	1.45378584	-0.8233461	1.77509241	0.19780301	0.27482931
Arl4d	-0.4325176	2.09657324	1.77401901	0.19793303	0.27497096

Oaz3	0.49670822	2.23339133	1.77364997	0.19797775	0.27499409
Esrrg	-0.2920502	6.04331139	1.77292735	0.19806537	0.27504388
Hist2h4	-0.8080242	0.30175153	1.77277589	0.19808374	0.27504388
Dpf1	0.41801841	2.26390189	1.77265966	0.19809784	0.27504388
Pla2g16	-0.2926594	6.73766589	1.77226589	0.19814561	0.27507123
Rbck1	-0.2617	4.11843858	1.77016597	0.19840062	0.27524438
Tsc22d3	-0.3199482	6.56716066	1.76995909	0.19842577	0.27524438
Agfg1	-0.1616811	5.82001065	1.76948027	0.19848398	0.27524438
Klhl34	0.45347368	5.45597322	1.76919686	0.19851845	0.27524438
H2-D1	-0.2737433	5.17764564	1.76893043	0.19855086	0.27524438
Kcng2	0.76160285	0.29275489	1.7688261	0.19856355	0.27524438
Rassf6	-0.8050514	0.63128141	1.76878579	0.19856846	0.27524438
Tgfbr2	-0.2864854	5.70117988	1.76870125	0.19857874	0.27524438
Ralbp1	-0.1606246	6.77749448	1.7685363	0.19859881	0.27524438
Ttll9	2.08177815	-1.3124561	1.76853127	0.19859942	0.27524438
Flad1	0.44120992	2.04431352	1.76852979	0.19859961	0.27524438
Ddx60	0.6557228	1.88541566	1.76834816	0.19862171	0.27524438
Plac9b	-0.3547437	3.93696673	1.76823371	0.19863564	0.27524438
Kdm2a	0.15929649	7.17003186	1.76731797	0.19874714	0.27535993
Zfp599	0.37798631	2.83120872	1.76697402	0.19878904	0.27537903
Hist1h4k	-0.4633424	1.94575363	1.76663253	0.19883065	0.27539773
Lyrm9	0.18987766	5.68993301	1.76627489	0.19887424	0.27541916
H2afv	-0.3341922	5.31528394	1.76561492	0.19895471	0.27549165
Wnk3	0.2477977	6.92545557	1.7646045	0.19907799	0.27562339
Gtf2b	-0.2329408	4.83713545	1.7641746	0.19913047	0.27565709
Ankrd49	-0.2977939	4.93883628	1.7631437	0.19925639	0.27579242
Gm2027	0.81696645	0.23977782	1.76245589	0.19934045	0.2758698
Parp16	-0.7765357	1.90198188	1.76132028	0.19947935	0.27602302
Pcdh20	0.37322261	3.064212	1.76066647	0.19955937	0.2760863
Lsamp	0.30712836	5.43219533	1.76048609	0.19958145	0.2760863
BC005561	0.33729308	5.15199396	1.75881774	0.19978586	0.27620674
Mrps24	-0.3351898	3.95201043	1.75877682	0.19979088	0.27620674
Serp2	-0.3127392	3.78297655	1.75871965	0.19979788	0.27620674
Rbp1	-0.4103106	7.75562961	1.75870327	0.19979989	0.27620674
Gm15698	2.01967034	-1.4406106	1.75862488	0.1998095	0.27620674
Tnfsf9	1.70703149	-2.0132422	1.75659198	0.20005896	0.27651256
Ing1	-0.2621882	4.90266404	1.7558297	0.2001526	0.27660295
Khdrbs1	0.15790091	6.94592177	1.755095	0.20024291	0.27667085
Chrna7	0.60270373	1.221174	1.75471838	0.20028922	0.27667085
Necab1	-0.1771647	7.85856048	1.75422188	0.20035029	0.27667085
03-Sep	-0.1712261	8.9122498	1.75421156	0.20035156	0.27667085
Ovca2	-0.2946778	3.35314287	1.75402049	0.20037507	0.27667085
Gdnf	1.03035411	-0.5461555	1.75345863	0.20044422	0.27667085

Dip2b	0.22499303	7.80946321	1.75327083	0.20046734	0.27667085
5830417I10R	0.2351137	6.11099523	1.75316801	0.20048	0.27667085
Akap7	-0.1936364	6.50819349	1.75313691	0.20048383	0.27667085
Xk	0.27582462	5.23281007	1.75313406	0.20048418	0.27667085
Gpr52	0.47884695	1.71756204	1.75220185	0.20059901	0.27679031
Cyb5r3	-0.3676172	7.41204742	1.75167384	0.20066409	0.27684111
5730508B09I	-0.3608203	2.79684429	1.75131877	0.20070786	0.27685979
Gm16677	-1.5071838	-1.2552854	1.75110556	0.20073416	0.27685979
Hrk	-0.3332908	4.09783662	1.7503822	0.20082339	0.27693428
A630001G21	-0.8665746	0.62575873	1.74976532	0.20089953	0.27693428
Hnf1b	1.42239527	-0.7988781	1.74974197	0.20090241	0.27693428
4931406P16I	0.19491829	5.65674613	1.74961073	0.20091861	0.27693428
Gab3	0.3521619	2.77288416	1.74942864	0.2009411	0.27693428
Gpr98	0.43562302	2.82382447	1.7492934	0.2009578	0.27693428
Stab1	0.70197692	1.19212054	1.74846832	0.20105972	0.27703577
Elfn1	0.31271663	4.83375736	1.74797561	0.20112062	0.27704754
2310068J16F	-1.2990324	-0.8088933	1.74753354	0.20117528	0.27704754
Insrr	0.9701116	0.39472325	1.74726011	0.2012091	0.27704754
Osbp2	-0.212012	5.65116238	1.74689756	0.20125395	0.27704754
Gsdma	-2.3274498	-2.0985064	1.74687085	0.20125725	0.27704754
Mitf	0.28453549	3.67275615	1.74677828	0.20126871	0.27704754
Ccny	-0.1714409	8.53740711	1.74645674	0.2013085	0.27704754
Adam28	0.75977753	0.2058532	1.74635734	0.2013208	0.27704754
Creb1	-0.131072	7.03094245	1.74625772	0.20133313	0.27704754
Phospho2	-0.2234938	5.00546569	1.74549622	0.20142742	0.27704754
Gucy2e	0.52072329	1.54458021	1.74547625	0.20142989	0.27704754
Gm608	0.17985118	8.24058658	1.74545364	0.2014327	0.27704754
Rps18	-0.3805694	6.22385773	1.7454188	0.20143701	0.27704754
Slc25a51	0.15365942	6.87552165	1.74502561	0.20148572	0.27704754
Irf5	0.68636541	0.96066234	1.7449707	0.20149252	0.27704754
Mcoln1	0.35080206	2.92515908	1.74451633	0.20154883	0.27708607
Magohb	-0.3822917	2.81648979	1.74403831	0.2016081	0.27712865
Stip1	-0.1523113	7.04168692	1.74296084	0.20174176	0.27727347
Tmem216	-0.3364357	2.85697965	1.74238606	0.2018131	0.27733261
Arhgap31	-0.2181395	6.53570096	1.74134688	0.20194217	0.27747105
Myadml2	0.84849393	0.67170072	1.74034967	0.20206613	0.27758054
5830416I19R	-1.8343582	-0.9376962	1.74024994	0.20207853	0.27758054
Mettl7a3	1.15290146	-1.7645304	1.73953447	0.20216753	0.27766385
Ccdc88b	-0.6871258	0.13683675	1.73897881	0.20223668	0.27768692
AF529169	0.58883877	1.49854143	1.73894393	0.20224103	0.27768692
Kcmf1	-0.1726858	7.50021097	1.73754693	0.20241502	0.27779177
Sbf2	0.18540222	7.08779668	1.73754397	0.20241539	0.27779177
Bambi-ps1	-1.2188598	-0.9567035	1.73720176	0.20245805	0.27779177

Fbxw11	-0.168152	7.36807432	1.7371993	0.20245835	0.27779177
Gm14325	-0.2080808	4.34187906	1.73684583	0.20250242	0.27779177
Fmn2	0.27512483	6.65774574	1.73674578	0.20251489	0.27779177
Xrcc5	-0.2681018	4.92129584	1.73671227	0.20251907	0.27779177
Tecpr1	0.36539973	3.46884039	1.73651025	0.20254427	0.27779177
Ggct	-0.2805431	4.48865281	1.73625145	0.20257655	0.27779715
Gli2	0.32462442	3.37357665	1.73593251	0.20261634	0.27781282
Mbtd1	0.2024498	5.66318019	1.73540131	0.20268263	0.27783824
Cyp4x1	0.55821686	0.99608208	1.73532944	0.2026916	0.27783824
Tssk2	2.06580933	-1.7611136	1.73476499	0.20276208	0.27787594
Map3k2	0.17396928	6.20693347	1.73465479	0.20277584	0.27787594
Scp2	-0.2974153	7.10915731	1.73309331	0.20297098	0.27798377
Rpl18	-0.3512883	5.80811809	1.73299228	0.20298362	0.27798377
Fis1	-0.2599889	3.99027107	1.73294811	0.20298914	0.27798377
4931429I11R	0.97853008	-0.0218343	1.73294486	0.20298955	0.27798377
Nudt9	-0.2601922	5.37527833	1.73280409	0.20300715	0.27798377
Fam178b	-1.4563495	-1.1721613	1.73266296	0.20302481	0.27798377
Folh1	0.51740351	1.91520935	1.73158227	0.20316004	0.2779954
Rpusd1	-0.2783128	2.98295012	1.73158103	0.2031602	0.2779954
Arpp21	0.18874749	8.75965728	1.73148446	0.20317229	0.2779954
Setd1a	0.21665161	5.5857919	1.73140124	0.20318271	0.2779954
Gltscr1	-0.2531971	4.38183036	1.73116973	0.2032117	0.2779954
Ash1l	0.18016273	9.15914459	1.73104125	0.20322779	0.2779954
Irak1	0.14460452	6.53281379	1.73092033	0.20324294	0.2779954
Katna1	-0.3605923	3.60927627	1.73078136	0.20326034	0.2779954
Apba2	-0.2391121	4.81600432	1.7303346	0.20331632	0.27803314
Scrt2	0.29363774	3.35000913	1.72961621	0.20340637	0.27811745
Olfr1372-ps1	-0.9045879	-0.054429	1.72885325	0.20350206	0.27820273
Zcchc12	0.25269079	4.29286974	1.72866607	0.20352554	0.27820273
Tmem167	-0.1848602	6.42597284	1.72820491	0.20358342	0.27821631
Rab6a	-0.1460394	10.1161715	1.72813427	0.20359228	0.27821631
Cklf	-0.6101066	0.98049407	1.72756229	0.2036641	0.27826828
Clk3	-0.2198476	4.70824977	1.72737896	0.20368713	0.27826828
4931429L15R	1.18876575	-1.1998334	1.72662579	0.20378175	0.27834257
Pld6	1.63759525	-1.921622	1.72649382	0.20379834	0.27834257
Akip1	-0.4041627	2.97505457	1.72596847	0.20386438	0.27839395
Doc2b	-0.3550773	4.21046912	1.72544346	0.20393041	0.2784453
1110020A21I	0.62222356	1.0658075	1.724597	0.20403692	0.2785519
Ccdc109b	-0.5155964	1.96654742	1.72408937	0.20410082	0.27860031
Plscr4	0.40925049	3.14324998	1.72356415	0.20416697	0.27865178
Zfp809	-0.2029965	4.84571026	1.72216719	0.20434304	0.27876046
Sclt1	0.24792435	4.66918407	1.72196615	0.2043684	0.27876046
Sprtn	0.44954584	1.57573087	1.7216445	0.20440897	0.27876046

Tmem64	-0.2539771	8.87291316	1.72162128	0.2044119	0.27876046
Ube2z	-0.2208243	6.14013848	1.72138384	0.20444186	0.27876046
Snord91a	0.3118708	3.26393971	1.72119144	0.20446614	0.27876046
Hist1h2bm	-0.5921	1.35635533	1.72085729	0.20450832	0.27876046
Pde8b	0.20214663	6.1734771	1.72063889	0.20453589	0.27876046
Egr1	-0.3259618	9.14900846	1.72055874	0.20454601	0.27876046
H1fx	-1.090264	-1.4640314	1.72050952	0.20455222	0.27876046
Smc4	0.22069264	6.28417346	1.72045072	0.20455965	0.27876046
1110058L19F	-0.2337906	4.25525354	1.71929013	0.20470627	0.27892146
Sertad2	-0.1985403	5.37338932	1.71864233	0.20478816	0.27899424
Adamts20	0.57574347	2.22272247	1.71785088	0.20488827	0.27905684
Phactr2	-0.1973672	8.26110327	1.71742765	0.20494183	0.27905684
Pawr	-0.3841112	5.34760824	1.71721071	0.20496929	0.27905684
Slfn10-ps	1.24777667	-0.7631627	1.71718995	0.20497192	0.27905684
Orc5	0.29312736	3.3294651	1.71715331	0.20497656	0.27905684
1700007G11	-0.8060413	0.29803362	1.71673313	0.20502976	0.27907354
Ltbr	0.49233958	2.37578847	1.71646216	0.20506408	0.27907354
Fam207a	-0.3477079	3.37033488	1.71638153	0.2050743	0.27907354
Cdk6	-0.5976592	2.18186163	1.71598119	0.20512502	0.27908043
Txndc9	-0.1727243	5.80377906	1.71589182	0.20513634	0.27908043
Tpp1	0.22834481	6.58033688	1.71541404	0.2051969	0.2790964
Selenbp1	-0.4499633	3.05446523	1.7153496	0.20520507	0.2790964
Fam171a2	-0.3271908	2.68908278	1.714528	0.20530926	0.27917782
AW551984	0.41259365	3.43531085	1.71442808	0.20532193	0.27917782
Kdm5d	0.3200412	4.88224008	1.71395155	0.2053824	0.27922128
G6b	1.6312578	-1.0109044	1.71288842	0.20551738	0.27936601
B230216G23	1.13644968	-0.2643548	1.71211959	0.20561506	0.27946002
Btf3l4	-0.1448632	7.05131846	1.71147601	0.20569688	0.27953243
Kcnq1	-1.628914	-1.3330321	1.71080705	0.20578196	0.27960926
L3mbtl4	0.7833468	0.00633733	1.71018516	0.2058611	0.27966271
Atp7b	1.26379491	-0.6278342	1.71004923	0.2058784	0.27966271
Mdh1	-0.1457427	8.81871868	1.70916585	0.20599089	0.27977672
Gm527	-0.4592196	2.04063156	1.70822965	0.20611019	0.27989994
Mrpl32	-0.3008247	3.95919809	1.70785519	0.20615793	0.27992597
Wdr55	-0.2274655	4.11983613	1.70739693	0.20621638	0.27996652
Arpc3	-0.2461146	4.43972326	1.70634698	0.20635037	0.2801096
Psme3	-0.1453253	6.7298075	1.7059169	0.20640528	0.28011297
Irf9	0.3067325	3.88034125	1.70587189	0.20641103	0.28011297
Lsm6	-0.1912027	5.28972953	1.70565569	0.20643864	0.28011297
Prickle2	0.23465249	7.94042736	1.70420963	0.20662345	0.28031051
Srp14	-0.226125	6.88944563	1.70405223	0.20664358	0.28031051
Mllt4	0.13434649	7.44020408	1.70384509	0.20667008	0.28031051
Cd8b1	0.8223588	-0.329234	1.70350954	0.206713	0.28032992

4732471J01F	0.4844754	1.65435426	1.70222709	0.20687717	0.28045395
Eng	-0.2669085	3.29847141	1.7020985	0.20689364	0.28045395
Fastkd5	0.26711342	3.34987176	1.70192254	0.20691618	0.28045395
Fitm2	-0.8188678	0.590713	1.70168813	0.20694621	0.28045395
Rab4b	0.26608604	3.46362841	1.70139667	0.20698356	0.28045395
Itga11	-0.3645567	2.90705307	1.70136441	0.20698769	0.28045395
R3hdm2	-0.166907	9.00287395	1.70123027	0.20700488	0.28045395
Nek4	0.24848553	4.4182294	1.70059643	0.20708614	0.28046588
Ap3s2	0.173418	6.62475984	1.70050892	0.20709737	0.28046588
Tha1	-0.8055575	0.05886685	1.70031109	0.20712274	0.28046588
Wdhd1	0.37966288	2.70306363	1.70026836	0.20712822	0.28046588
Olfr99	-1.7080945	-1.5113581	1.69894611	0.20729791	0.28063862
Vps54	0.16391304	6.89711108	1.69882792	0.20731308	0.28063862
Bcas1os2	1.06546183	-0.1968734	1.69762886	0.20746713	0.28067247
Ss18l1	0.18600321	6.11108159	1.69743388	0.20749219	0.28067247
Nmnat3	0.42312943	2.54700516	1.69734964	0.20750302	0.28067247
2900008C10I	0.94810102	0.21761927	1.69732431	0.20750628	0.28067247
Foxp4	-0.3028422	4.89409226	1.69709988	0.20753513	0.28067247
Dmap1	-0.3121606	3.50122349	1.69692726	0.20755733	0.28067247
Myc	-0.3148134	3.6606208	1.69688123	0.20756325	0.28067247
Eef2	0.1582003	8.35795737	1.6967736	0.20757709	0.28067247
Fgf7	-0.5080234	1.59761899	1.69662679	0.20759598	0.28067247
Dtx2	-0.5023031	1.76024627	1.69585791	0.20769491	0.28076748
Adamts5	0.29760667	3.69374067	1.69488109	0.20782068	0.28089787
Acss1	-0.2427688	3.75449704	1.69466341	0.20784872	0.28089787
Arap2	0.22303927	7.05856224	1.69432415	0.20789244	0.28091819
Rgmb	0.22958773	4.71335	1.69387603	0.20795019	0.28092809
Trpv4	0.95940482	-0.4879297	1.69379019	0.20796126	0.28092809
Pdzd4	-0.2101108	5.59251781	1.69359983	0.2079858	0.28092809
Mtpap	-0.2215695	4.90302838	1.69159773	0.20824414	0.28123826
Mlf2	-0.1489635	6.45549964	1.69121674	0.20829335	0.28126593
Ppil4	0.18190214	5.95392326	1.69038227	0.20840118	0.28135742
Macrod2	-0.2347686	5.09283958	1.68988918	0.20846493	0.28135742
Gdi2	-0.1373151	8.14845822	1.68970001	0.20848939	0.28135742
Tmem88b	-0.2112746	5.67365952	1.68955023	0.20850876	0.28135742
Sdhc	-0.2456926	6.84374404	1.68934317	0.20853555	0.28135742
Slc37a1	0.5449977	0.7991249	1.68907399	0.20857037	0.28135742
Gm128	-1.0787666	-0.8928464	1.6889593	0.20858521	0.28135742
Gm20752	0.61204642	0.53927889	1.68891542	0.20859089	0.28135742
Ankrd54	-0.3712136	2.01306305	1.68796868	0.20871345	0.28147997
Coro1b	-0.2324575	5.32204427	1.68776974	0.20873922	0.28147997
Napb	-0.1794902	9.75654075	1.6862369	0.20893787	0.28170907
Gm10538	1.10865044	-0.6379455	1.68536559	0.2090509	0.28182267

Ticam1	-0.2906027	4.60652184	1.684845	0.20911847	0.28187497
Nudt11	-0.2643315	4.15227388	1.68418686	0.20920393	0.28195136
Fam102a	-0.2068445	5.70098183	1.68363859	0.20927516	0.28200856
Lbp	0.58641203	3.35016585	1.68180276	0.20951388	0.28225506
Slc14a1	0.37767098	2.67003188	1.68178866	0.20951572	0.28225506
Pabpn1	0.18127287	5.54509059	1.68074318	0.20965182	0.28236213
Rpl36a	-0.2923858	6.48678995	1.68062443	0.20966729	0.28236213
Mfsd10	0.55995435	0.81582909	1.68029619	0.20971005	0.28236213
Igsf3	0.21188004	4.5003432	1.68026065	0.20971468	0.28236213
Camsap2	0.20967858	8.77325357	1.68007145	0.20973933	0.28236213
A930007I19F	1.54584551	-1.0315165	1.67909055	0.20986719	0.28249545
Elp6	-0.4688283	2.98309569	1.67883706	0.20990025	0.28250113
Nsun4	-0.2762299	2.77881446	1.67832286	0.20996733	0.28253905
Cetn3	-0.2163073	7.87997825	1.6781593	0.20998868	0.28253905
Orai1	-0.6161486	1.85472513	1.6779579	0.21001496	0.28253905
Dchs1	0.36612639	2.92556451	1.67746039	0.21007991	0.28255269
Ppp4c	-0.5005243	2.05232108	1.67740032	0.21008775	0.28255269
Rnaseh1	-0.4182704	2.79486791	1.67687369	0.21015653	0.28255269
Cdca7	-0.888962	0.33717584	1.67675873	0.21017155	0.28255269
Nfkbib	-0.2720186	3.71176623	1.67648461	0.21020736	0.28255269
Dtymk	-0.2213719	4.20043631	1.67645702	0.21021097	0.28255269
6430573F11I	-0.3482346	2.57922385	1.67633417	0.21022702	0.28255269
Zic2	-0.2866955	6.54778614	1.67607122	0.21026139	0.28256011
Pcmtd2	0.22658092	4.73890337	1.67525751	0.21036779	0.28266431
Glb1l	0.33809098	3.33240544	1.67397333	0.21053584	0.28285132
Cnpy2	-0.3075819	5.15196504	1.67294916	0.21066998	0.2829592
Etnppl	0.43797679	2.95248482	1.6729192	0.21067391	0.2829592
Stt3b	0.18002719	5.87106778	1.67214554	0.21077532	0.28305659
Ulk1	-0.2010999	6.46626402	1.67132171	0.21088337	0.28315295
Tmem55a	-0.1819296	6.17654235	1.67103973	0.21092037	0.28315295
Nr1d1	-0.183045	8.75075476	1.67093742	0.21093379	0.28315295
Ccdc155	1.03962888	-0.6128863	1.67066081	0.2109701	0.28316141
Trim14	-0.6680902	2.15536847	1.67044897	0.21099791	0.28316141
Ttc29	2.2089212	-2.3012411	1.66979056	0.21108438	0.2832161
Mpzl3	1.2611142	-0.9425043	1.66969834	0.21109649	0.2832161
Efcab14	0.19609171	7.24510589	1.66898879	0.21118973	0.2832826
Zfp266	0.14367569	6.88720034	1.66888097	0.2112039	0.2832826
Etfdh	-0.225202	5.16765696	1.66812664	0.21130309	0.28337452
Ramp3	0.97202967	-0.3875601	1.66760799	0.21137132	0.28337452
A730098P11I	-0.1751624	6.70378136	1.66753668	0.2113807	0.28337452
Kcnj11	0.26949681	3.39176252	1.66748008	0.21138815	0.28337452
Gle1	-0.1986639	4.91011353	1.6672435	0.21141928	0.28337748
Mak	0.55241927	2.07930266	1.66658019	0.21150661	0.28342418

Atl1	0.27108308	6.2395318	1.66653932	0.21151199	0.28342418
Airn	-0.4809152	1.75210693	1.66608089	0.21157238	0.28343769
Sarnp	-0.2130459	5.53756592	1.66595799	0.21158857	0.28343769
Pigz	-0.4213952	2.17195228	1.66580377	0.21160889	0.28343769
Mfn1	0.22481252	5.318936	1.66556341	0.21164056	0.28344136
3110070M22	1.25703193	-0.8977719	1.66506178	0.21170669	0.28346135
Ddx58	-0.2811577	5.01473816	1.66501114	0.21171337	0.28346135
Zfand3	-0.2050125	5.53411354	1.66470695	0.21175348	0.28347631
Nlrp4f	1.61202685	-1.9884153	1.66448424	0.21178285	0.28347689
Gm1604b	0.46503576	1.39017072	1.66413209	0.21182931	0.28348779
Blvra	-0.3760299	2.55710238	1.66398381	0.21184887	0.28348779
Milr1	1.27393237	-0.3117248	1.66351096	0.21191128	0.28350899
Lims1	-0.2392241	7.53140052	1.6630253	0.2119754	0.28350899
Ddx20	0.35196349	3.71303709	1.66282431	0.21200195	0.28350899
Fbxo42	0.18073936	5.14758796	1.66280258	0.21200482	0.28350899
Dzip1l	0.26502642	3.47305147	1.66248741	0.21204645	0.28350899
Cngb1	-0.7961418	0.65399982	1.66227075	0.21207508	0.28350899
Fbxo41	0.26357404	5.17505856	1.66221965	0.21208183	0.28350899
Adamts17	0.59977246	2.62311081	1.66190555	0.21212334	0.28350899
Tmem106c	-0.4602345	3.83407144	1.66188004	0.21212671	0.28350899
Cab39	-0.1440261	7.71350092	1.66167245	0.21215415	0.28350899
Ctso	-0.3064152	5.03282715	1.66091589	0.2122542	0.28360196
Tmed8	0.15185174	6.24397623	1.660548	0.21230287	0.28360196
Tmem30a	0.16815774	7.94358369	1.66048969	0.21231058	0.28360196
Oat	-0.2211853	8.127886	1.65999562	0.21237597	0.28365062
Wfs1	0.22807942	6.64987679	1.65947417	0.21244501	0.28368688
Stmn1-rs1	1.65050098	-1.1613984	1.65935314	0.21246104	0.28368688
Slc9a7	0.31177308	4.25515932	1.65800993	0.21263902	0.28388583
Zfp719	0.21908478	5.71662256	1.65741645	0.21271772	0.28390057
Pcnxl4	0.25649971	4.64560216	1.65738978	0.21272126	0.28390057
Tex15	0.40801315	2.37812707	1.65671422	0.21281089	0.28390057
Prkag1	-0.198004	4.21440019	1.65670397	0.21281225	0.28390057
1700008J07F	-0.3741727	3.20091997	1.65622843	0.21287537	0.28390057
Aoc2	0.58551371	1.2423825	1.65616115	0.21288431	0.28390057
Dnah2	0.50003779	1.78466754	1.65607617	0.21289559	0.28390057
Nab1	0.14724027	6.61814642	1.65598212	0.21290808	0.28390057
Arg2	0.412362	2.92564472	1.65596079	0.21291091	0.28390057
2810468N07	-0.3445712	2.59220216	1.65526602	0.2130032	0.28398497
Cyth2	-0.2618627	4.26547245	1.65411122	0.2131567	0.28415095
Phc3	0.17178784	8.00497733	1.65343784	0.21324627	0.28423168
Anapc7	-0.2867717	3.5193806	1.65238856	0.21338594	0.28437914
Atp6v0e2	-0.206061	5.29601481	1.65214898	0.21341785	0.28438297
Rbm5	0.196249	6.94168735	1.65183437	0.21345976	0.28440013

Smc1b	1.32188311	-0.7178383	1.65070597	0.21361015	0.28447983
Leprel4	0.40284464	2.95741642	1.65064289	0.21361856	0.28447983
Aim1	-0.3830658	3.96012799	1.65055342	0.2136305	0.28447983
Stpg1	-0.5798129	0.68737858	1.65051403	0.21363575	0.28447983
Ppif	-0.2719815	4.94539068	1.65015388	0.21368379	0.28450512
Tuba4a	-0.1824019	7.31249873	1.64866067	0.2138831	0.28473179
Rere	0.14010757	8.29609131	1.64812804	0.21395425	0.28478781
B930025P03	1.44770286	-1.1895272	1.64790083	0.21398461	0.28478952
Tmem74b	-1.0654891	-1.2658424	1.64688536	0.21412037	0.28493148
Rab21	-0.1682405	7.62913437	1.64635419	0.21419143	0.28495419
Jtb	-0.2261754	4.13897678	1.64623062	0.21420796	0.28495419
Grin3a	0.28024878	4.72129811	1.64610552	0.2142247	0.28495419
Lrrc3b	0.37620409	3.63449655	1.64569316	0.2142799	0.2849889
Fam198b	0.29506365	3.62236405	1.64393975	0.21451479	0.28526257
Nccrp1	-1.4199659	-1.1306751	1.64303078	0.21463668	0.28538593
Fam45a	-0.1939353	4.9588035	1.64261395	0.21469261	0.28542155
Etv1	-0.1974727	7.73736449	1.64235269	0.21472767	0.28542406
Sox5	0.22072308	5.99055607	1.64216568	0.21475278	0.28542406
Rad23a	-0.252468	4.96103067	1.64153213	0.21483785	0.28547267
Sema4b	0.31845985	2.77315186	1.64137946	0.21485836	0.28547267
1110006O24	1.14828879	-0.6314302	1.64124229	0.21487678	0.28547267
Aarsd1	-0.3007657	3.70563603	1.64095121	0.21491589	0.2854859
Slc25a37	0.19730612	5.36740566	1.64035941	0.21499543	0.28555284
Trove2	0.1736906	7.07330955	1.63934019	0.2151325	0.28569616
Iah1	-0.3699724	3.18003593	1.63808226	0.21530184	0.28580582
Slc41a2	0.2606174	4.01969231	1.63806254	0.21530449	0.28580582
1810055G02	-0.2014466	5.57403239	1.63802436	0.21530963	0.28580582
Wdr36	-0.2429503	3.83820002	1.63785986	0.21533179	0.28580582
Tbk1	0.22264792	5.39326555	1.63742687	0.21539012	0.28584451
Hnrnpa3	-0.1454745	9.05026182	1.63666866	0.21549232	0.28594139
Phpt1	-0.3061991	3.28584852	1.6362996	0.21554209	0.28596869
Tspan15	0.42720721	1.86957769	1.63593006	0.21559193	0.28599609
Radil	0.4875864	2.13082272	1.63570325	0.21562253	0.28599795
Fgf12	-0.1746167	7.94471617	1.63448724	0.21578669	0.28617693
Atg4a	0.42390919	3.5910716	1.63401748	0.21585015	0.28621656
Stard4	-0.2512973	3.99959699	1.63383351	0.21587501	0.28621656
Clic5	-0.426984	2.94378902	1.63342889	0.21592969	0.28623114
Gm13582	1.51016051	-1.3285864	1.63331227	0.21594546	0.28623114
Zfp760	-0.2051531	5.25327047	1.6331036	0.21597367	0.28623114
Cul4a	-0.1444545	7.55684241	1.63233168	0.21607807	0.28629489
Plbd1	0.73866562	0.95484593	1.63231572	0.21608023	0.28629489
Mtrf1l	-0.2553072	4.04188522	1.63121118	0.21622973	0.28645423
Ankrd24	0.36641711	2.49941167	1.63074611	0.21629272	0.28649892

Tbc1d10b	-0.1845281	4.60840359	1.63041249	0.21633792	0.28652005
Cd2	-1.2644716	-0.4858184	1.63015692	0.21637255	0.28652718
Med9	-0.2726568	4.76136036	1.62941673	0.21647289	0.2866213
Guf1	0.40507316	3.90420422	1.62917009	0.21650634	0.28662371
Rgs18	-0.9409291	-0.0944499	1.62897183	0.21653323	0.28662371
Slc35b2	0.48734472	2.09245304	1.62835011	0.21661759	0.28666753
Cacng8	0.75323767	0.70465337	1.6282965	0.21662487	0.28666753
Ptpn11	-0.1451678	7.67249814	1.62552729	0.21700113	0.28712666
D930020B18	1.70018773	-2.0613081	1.62501094	0.21707138	0.28715528
Map2k2	-0.2650336	4.35852667	1.62493738	0.21708139	0.28715528
Ybx1	-0.2019128	7.17213892	1.62380077	0.21723614	0.28732118
Ndrg2	-0.2109383	8.89316175	1.62340155	0.21729052	0.28735431
2810002D19	0.3420801	3.26031947	1.62304514	0.21733909	0.28737974
Rac1	-0.1493947	10.032103	1.62194947	0.21748849	0.28753304
Brd8	0.16680807	5.86129526	1.62156379	0.2175411	0.28753304
Mertk	0.31536209	3.7656962	1.62154922	0.21754309	0.28753304
Fkbp2	-0.2480801	4.37876841	1.62083839	0.21764011	0.28762247
Tcf7l2	-0.2604624	6.41243746	1.62041004	0.21769861	0.28766096
Tgm5	1.19428343	0.06643321	1.62001625	0.2177524	0.28769323
F2	-1.3489051	-1.044684	1.61943571	0.21783173	0.28775923
A730090N16	0.76114882	0.77945632	1.61803084	0.21802386	0.28797419
Zp3r	1.68083481	-1.4150992	1.6177332	0.21806459	0.28798916
Zfp689	-0.4885999	1.65049291	1.61726707	0.2181284	0.2880346
Nol4	-0.2018647	6.37124719	1.61669988	0.21820607	0.28809833
Nxph2	0.8214642	0.00279407	1.61629548	0.21826148	0.28813164
Bid	-0.3208946	2.81864589	1.61542532	0.21838075	0.28813164
Kdm4c	0.25831642	5.52623136	1.61532026	0.21839516	0.28813164
Thada	0.2462212	5.05442858	1.61529404	0.21839875	0.28813164
Ero1lb	0.28737264	3.76443278	1.61494492	0.21844663	0.28813164
Bcl9l	0.22801302	5.44706175	1.61491722	0.21845043	0.28813164
Neu2	-0.6206639	0.65232574	1.61488629	0.21845468	0.28813164
Cbx6	0.17538753	6.62613416	1.61460383	0.21849343	0.28813164
Acvr1c	0.27317209	4.78138108	1.61458479	0.21849604	0.28813164
Aph1a	0.37321251	2.94250574	1.6130629	0.21870499	0.28835864
Prune	0.19509792	4.58506646	1.61290223	0.21872706	0.28835864
Crtap	-0.4590009	4.08574639	1.61214307	0.21883139	0.28845737
Dapk2	-0.6321057	0.52057744	1.61177284	0.2188823	0.2884614
1700086O06	-0.9439046	-0.332981	1.61169251	0.21889335	0.2884614
Gm13102	1.2121323	-1.4191695	1.61129484	0.21894805	0.28849388
Rpl8	-0.2515427	5.66174501	1.61108509	0.2189769	0.28849388
Ptar1	0.25242853	3.79022026	1.61005397	0.21911884	0.28854122
Strip1	0.20653262	4.37981101	1.60990091	0.21913991	0.28854122
Tek	0.35478839	2.9134999	1.60989278	0.21914103	0.28854122

Jmjd1c	0.19538628	8.21493046	1.60983688	0.21914873	0.28854122
Spsb3	-0.3473332	2.64510332	1.60943309	0.21920436	0.28854122
Ubl7	-0.1573104	6.5759092	1.60942443	0.21920555	0.28854122
Nudt14	-0.6229507	1.06890462	1.60932656	0.21921903	0.28854122
Exosc2	-0.2774179	3.39289145	1.6088798	0.2192806	0.28856103
Snord47	1.31771222	-1.5171434	1.60878987	0.219293	0.28856103
Slc4a3	0.34644729	3.42105113	1.60784838	0.21942282	0.2886757
Lin28b	0.38820744	3.02215074	1.60752656	0.21946722	0.2886757
Ppp2r5c	-0.166248	7.0535404	1.60751692	0.21946856	0.2886757
E130308A19I	-0.2204696	5.17680017	1.6070174	0.21953749	0.28872761
Uhrf1	-0.851514	0.39544064	1.60652807	0.21960506	0.2887524
B3gnt7	1.67403635	-0.8629206	1.60631298	0.21963476	0.2887524
Sbk3	0.78270267	0.45675442	1.6060533	0.21967063	0.2887524
A230050P20	0.37752264	1.98851893	1.60588389	0.21969403	0.2887524
A630066F11I	-0.5240082	1.4922324	1.60581364	0.21970374	0.2887524
Sirt1	0.26177874	4.47245671	1.60499405	0.21981702	0.28886253
2210416O15	-0.96872	-0.6375409	1.60472494	0.21985423	0.28887267
Coq7	-0.3209224	4.22424086	1.60397302	0.21995825	0.28894665
Sod2	-0.1563962	8.45926204	1.60389147	0.21996953	0.28894665
Scara5	-0.4668438	1.61318864	1.60331566	0.22004923	0.28901259
Tagln	-0.4839122	3.30791348	1.60284817	0.22011397	0.28905885
Ptger3	0.42692347	3.47970624	1.60129181	0.22032966	0.28930331
Gtpbp10	-0.24055	4.49151741	1.60075891	0.22040357	0.28936158
Cdnf	0.4749059	2.31471702	1.60051972	0.22043676	0.28936636
Bmp2	-0.2985341	4.62517624	1.60018318	0.22048346	0.28938688
Hdac11	-0.1886311	5.42574727	1.59998138	0.22051147	0.28938688
Bcl2l2	-0.1451326	6.25153777	1.5993796	0.22059503	0.28945775
Dhx36	0.21118409	6.26856195	1.59898941	0.22064923	0.28949009
Hist3h2ba	-1.1541032	0.02817008	1.5985995	0.2207034	0.28951141
Fam71b	-1.4717723	-1.7554449	1.59844706	0.22072459	0.28951141
Ccdc28b	-0.2804653	3.40580795	1.59733426	0.22087932	0.28965462
Cpeb4	0.20305067	8.15948239	1.59723654	0.22089292	0.28965462
Ehmt2	0.19944097	5.08226054	1.59685494	0.22094601	0.28968547
Cox17	-0.2105758	5.38108613	1.59583664	0.22108778	0.28983255
Gm10653	0.62954552	0.53454069	1.594446	0.22128157	0.29004778
Hspa8	-0.1433831	10.7212684	1.59406434	0.2213348	0.29007872
Zfp568	-0.2867417	3.27424501	1.59374185	0.22137978	0.29009255
Ccdc142	0.84704796	-0.4755545	1.59356414	0.22140458	0.29009255
Mettl18	0.52577263	2.00591038	1.5930769	0.22147257	0.29014283
Cbr4	-0.250651	4.17034349	1.59248677	0.22155497	0.29019263
Pcyt2	-0.481377	1.66845225	1.59217325	0.22159875	0.29019263
Rpp21	-0.3591308	3.15334798	1.5921682	0.22159946	0.29019263
Arf6	-0.1948004	6.84791232	1.59134388	0.22171464	0.29024689

Cspg4	0.34042881	2.74830297	1.59125311	0.22172733	0.29024689
Irak3	-0.4494677	2.79377996	1.59123555	0.22172979	0.29024689
Bckdhb	0.40502978	2.62445648	1.59100947	0.22176139	0.29024947
Spaca6	0.63235945	2.96142993	1.59066866	0.22180905	0.29027306
Trpc5	0.29615291	4.12058133	1.59038149	0.22184922	0.29028684
Acot8	-0.5268609	1.57010583	1.58969674	0.22194503	0.29034368
Zfp423	-0.1848332	6.14028828	1.5896474	0.22195194	0.29034368
Oxld1	-0.5902694	1.12722432	1.58938731	0.22198834	0.29035253
Bcat1	0.21046462	5.79615957	1.58915752	0.22202052	0.29035584
Slc43a2	0.22443365	5.45789386	1.58872988	0.22208041	0.29039539
Aff2	0.25173905	5.87645456	1.5882174	0.2221522	0.29044528
Elfn2	0.23288927	5.01552476	1.58803431	0.22217786	0.29044528
Zmym6	0.29842877	4.80463903	1.58763545	0.22223377	0.2904796
Slc30a3	-0.265158	4.31403031	1.58656943	0.22238328	0.29054223
Omg	0.23432854	7.44931293	1.58656679	0.22238366	0.29054223
Coq2	-0.2064111	4.82113311	1.5865016	0.2223928	0.29054223
Mapk8ip1	0.18701055	5.53140948	1.58644795	0.22240033	0.29054223
Car14	0.66905388	2.80967489	1.58600921	0.22246191	0.29058392
Taf8	-0.2958359	2.78515592	1.58502055	0.22260075	0.29072651
Morc1	1.76752369	-1.4927498	1.58391164	0.22275661	0.29085385
Tecpr2	0.32699633	5.55760619	1.5839043	0.22275764	0.29085385
Zfp609	0.15237957	7.47356055	1.58270017	0.22292704	0.29103624
Wdr20	0.24527671	3.5679198	1.58207845	0.22301457	0.29108269
Ufm1	-0.1782444	6.00772638	1.58202533	0.22302205	0.29108269
Cc2d1a	0.36556305	2.91803253	1.5814249	0.22310662	0.29115428
Crtc1	0.19423056	6.31936751	1.58114215	0.22314647	0.29116748
Dnaja4	-0.2047003	4.57273453	1.58062094	0.22321993	0.29121829
Gabbr1	0.20640046	7.35813942	1.58038775	0.22325281	0.29121829
Gm19897	0.98802677	-0.0053827	1.58002824	0.22330352	0.29121829
Pfas	0.28999201	3.81241102	1.58002251	0.22330432	0.29121829
Szrd1	-0.2800896	4.64014995	1.57944196	0.22338623	0.29128632
Fam19a2	0.19234985	5.53212453	1.57895323	0.22345521	0.29133749
Parp3	-0.2963765	4.28637517	1.57862635	0.22350136	0.29135888
Pmpca	-0.1442836	6.05823535	1.57545506	0.22394976	0.29185767
Pdyn	0.30596813	3.32308681	1.5753255	0.2239681	0.29185767
Dnajb1	-0.1561461	6.61552097	1.57528824	0.22397337	0.29185767
4930481A15I	-0.6345479	1.32952791	1.57483976	0.22403689	0.29187159
Nubp2	0.30867068	3.47215544	1.57479202	0.22404365	0.29187159
Msra	0.32618826	3.48859937	1.57433314	0.22410866	0.29189687
Hist1h2bn	-0.4350303	3.01491365	1.57387379	0.22417376	0.29189687
Erv3	2.32958668	-1.2482462	1.57383123	0.22417979	0.29189687
Drd2	0.40568829	3.84286227	1.5738139	0.22418225	0.29189687
Lrrc4b	0.24128205	4.57026457	1.57319253	0.22427036	0.29197278

Ntn5	0.72043426	0.36897687	1.57266092	0.22434577	0.29202993
4933406I18R	0.89237024	-0.502122	1.57246282	0.22437388	0.29202993
Vash2	0.45298598	1.75752496	1.5715779	0.22449951	0.29215462
Akap14	1.17892307	-0.9875609	1.57133952	0.22453336	0.29215986
D830005E20	0.67553824	-0.32603	1.57045949	0.22465841	0.29228374
Ccdc121	-0.7638465	0.68327863	1.56904456	0.22485964	0.2925067
Mfsd9	0.68546543	1.13707931	1.56801448	0.22500629	0.29265859
Phxr4	0.72326895	1.70294405	1.56737626	0.22509721	0.29273798
Scpep1os	1.01588752	-1.5246449	1.56538501	0.22538118	0.29306837
Rwdd2b	-0.4640148	2.25778466	1.56516829	0.22541211	0.29306969
Ankle1	1.71434369	-1.8448087	1.5647562	0.22547094	0.29310728
Mpped1	0.18970681	6.04425152	1.56364591	0.22562956	0.29327063
Zfp142	0.22473578	5.2626512	1.56341222	0.22566297	0.29327063
Marcksl1	-0.2381458	4.29698089	1.56322845	0.22568924	0.29327063
Lsm7	-0.291976	3.70808593	1.56303436	0.22571699	0.29327063
Ogfrl1	-0.1758102	7.93342895	1.56282939	0.2257463	0.29327063
Cnksr2	0.29877152	9.57153435	1.56253888	0.22578785	0.29328571
Gstm5	-0.2576094	5.97796216	1.56188577	0.22588131	0.29331195
Pnoc	-0.8955573	-0.2246849	1.56187392	0.225883	0.29331195
Il17re	-0.6980317	0.83772249	1.56162163	0.22591912	0.29331195
Fbxl12os	0.49686703	2.29522123	1.56144051	0.22594505	0.29331195
Uhmk1	0.15923081	6.47143497	1.56135166	0.22595777	0.29331195
Copz1	-0.2395972	6.13808659	1.56019543	0.2261234	0.293433
Tgfb1	0.4073933	2.17365205	1.56010106	0.22613693	0.293433
Trim43b	0.98195412	-0.7916784	1.56007341	0.22614089	0.293433
Figf	0.60959914	1.88956967	1.55939111	0.22623872	0.29348841
Cdc37l1	-0.1476485	6.61550498	1.55921088	0.22626457	0.29348841
Sema6b	-0.3737074	2.90012105	1.55903425	0.22628991	0.29348841
Sypl2	-0.8789595	0.14248168	1.5589399	0.22630344	0.29348841
Peg10	0.2245169	6.36412456	1.55726189	0.22654434	0.29376193
Dok6	-0.4455527	2.04157033	1.55680512	0.22660998	0.29380815
Atf5	-0.3307249	3.26731139	1.55628864	0.22668422	0.29383833
Samd9l	-0.2730177	6.92484062	1.55622581	0.22669325	0.29383833
C030039L03F	0.31591078	4.05436571	1.55549159	0.22679885	0.29390524
Reep3	0.24892963	7.27510265	1.55529668	0.2268269	0.29390524
Dpy19l4	0.21946799	4.18310181	1.55506735	0.2268599	0.29390524
Scml2	-0.7665691	-0.0223448	1.55503264	0.22686489	0.29390524
Arhgef18	0.19352657	4.8838218	1.55461678	0.22692476	0.29394392
Fancc	0.41558878	1.8744647	1.55367729	0.22706006	0.29405868
C230052I12R	-0.3178262	2.85948988	1.55358481	0.22707339	0.29405868
Tlr13	0.92409447	0.9213886	1.55281139	0.22718487	0.29408408
Ptprr	0.20890738	4.20917954	1.5526349	0.22721031	0.29408408
Cox6a2	-0.4483711	1.09993149	1.55260211	0.22721504	0.29408408

Dhcr7	0.26648934	3.72930431	1.55257454	0.22721902	0.29408408
Lpcat2	0.43247522	2.55061214	1.55240739	0.22724312	0.29408408
2510002D24I	-0.3285365	3.33183668	1.55062875	0.22749984	0.2943455
Rpl37	-0.2432999	6.35124797	1.55059145	0.22750523	0.2943455
Irs3	0.49455376	2.20963821	1.54977609	0.22762303	0.29445903
Ube2g1	-0.175658	6.83465287	1.54875238	0.22777106	0.2945696
A430078G23	0.2981163	3.71615754	1.5485042	0.22780696	0.2945696
Taf6	0.20420263	4.52925267	1.54840076	0.22782193	0.2945696
Stk36	0.53141837	1.23416764	1.54835331	0.22782879	0.2945696
Clpb	0.28348218	3.74008951	1.5478955	0.22789505	0.29461638
Zmiz2	0.16639627	6.4725955	1.54766266	0.22792876	0.29462107
Rps27	-0.701044	-0.165867	1.54736278	0.22797219	0.29463832
Setd4	0.71434469	1.04697933	1.54706393	0.22801547	0.29465538
Ccdc15	-0.2706536	4.26370537	1.54609558	0.22815579	0.29478504
Rtp3	0.92852902	0.44595136	1.54595626	0.22817599	0.29478504
A230056P14I	0.25374448	4.00572703	1.54547281	0.2282461	0.29483672
9130019P16I	-0.7877677	0.2995342	1.54394692	0.22846755	0.29508387
Rbm24	-0.2798353	3.97278208	1.54232766	0.22870285	0.29534884
Tacstd2	-0.9137358	0.91133709	1.5409562	0.22890239	0.29556755
Kcnj6	0.27349396	5.88929856	1.54069933	0.22893978	0.29557688
Gcnt2	0.19156029	5.3346145	1.53994837	0.22904916	0.29567912
Lrrn3	0.16642332	5.76530635	1.53930251	0.22914328	0.29576164
Isoc2b	-0.60966	0.67709782	1.53884269	0.22921032	0.29580919
Rexo1	-0.2173037	4.84694613	1.53781893	0.22935967	0.29595733
Atp6v1a	0.18282486	9.34063743	1.53747866	0.22940934	0.29595733
Cenpo	-0.5016173	2.19298701	1.53743475	0.22941575	0.29595733
Gm16432	-0.7136389	1.11354933	1.53685283	0.22950072	0.29602796
Fez2	-0.2049793	5.48917661	1.53623873	0.22959044	0.29610469
Homez	0.19258644	4.36385714	1.53538306	0.22971552	0.29621637
Pin1	-0.29115	3.03710293	1.53523271	0.22973751	0.29621637
Pisd-ps2	0.60235832	1.82561188	1.53445512	0.22985127	0.2962782
Adprhl2	1.22855087	-0.4313696	1.53430582	0.22987312	0.2962782
Ddx6	0.12785861	8.14171641	1.53428475	0.2298762	0.2962782
C1qc	-0.6015076	2.26937786	1.53312427	0.23004613	0.29645821
Uba6	0.22146051	5.70518127	1.5326207	0.23011992	0.29651429
Rassf10	-0.3624644	2.070177	1.53176267	0.23024572	0.29663736
Parp1	-0.1435697	6.13886078	1.53140618	0.23029801	0.29664188
Sirt7	0.30900777	3.02721932	1.53112245	0.23033964	0.29664188
Gm13710	-0.6965021	0.50755034	1.5311195	0.23034008	0.29664188
Znrf2	-0.2576223	4.47525774	1.53073172	0.23039699	0.29666069
Cubn	-0.3179728	5.30480592	1.53043997	0.23043982	0.29666069
Zmynd12	1.3575894	-1.5953784	1.53040099	0.23044554	0.29666069
Snord64	0.66581201	0.11126049	1.52997536	0.23050805	0.29670216

Zbtb11	0.19844624	6.58427433	1.52927514	0.23061092	0.29679558
Galnt13	0.20047572	5.74392475	1.52853185	0.23072019	0.2968972
Prkar1b	-0.1570242	7.74528638	1.52778298	0.23083035	0.29699993
Plagl1	0.26290506	6.20316342	1.52722823	0.23091199	0.29703538
Bola2	-0.4167722	2.45401443	1.52718372	0.23091855	0.29703538
Mrs2	-0.3172701	3.96128606	1.52695535	0.23095217	0.29703962
Zc3h18	0.24086037	3.91174905	1.52673664	0.23098437	0.29704204
Tpk1	-0.2718816	4.03306475	1.52635058	0.23104123	0.29707616
Rnf39	0.43369534	1.8296328	1.52576442	0.2311276	0.29714821
Cdkn2d	-0.2401601	4.11351863	1.52540959	0.23117991	0.29717645
Mageh1	0.24456153	4.38329685	1.52460047	0.23129923	0.29729082
Tmem229a	0.20121241	6.08972277	1.52389638	0.23140313	0.29732463
Lig4	0.22676931	4.33541158	1.52381728	0.23141481	0.29732463
Clip2	-0.338272	3.95451113	1.52380519	0.23141659	0.29732463
Gm10768	2.03578222	-2.1390661	1.57643612	0.23152501	0.29742491
Gipc2	-0.4942432	1.46851857	1.52243556	0.23161889	0.29750649
Gucy2c	-1.3176324	-0.8766283	1.52166281	0.23173312	0.2976142
Git2	-0.171266	5.44319972	1.52115706	0.23180793	0.29767124
Samd4b	-0.1624036	6.83146274	1.52081556	0.23185846	0.2976971
Parp10	0.4498365	1.9135946	1.52001856	0.23197643	0.29780954
Lyrm5	0.22353332	5.17912218	1.51951933	0.23205037	0.29783888
Tubb5	-0.1641912	7.90547831	1.5194443	0.23206149	0.29783888
Diexf	0.2265656	4.40759709	1.51924845	0.23209051	0.29783888
D4Ertd617e	-1.5220129	-1.1719346	1.51853713	0.23219593	0.29792789
Nipsnap1	-0.2002073	4.73071935	1.5183701	0.2322207	0.29792789
Sdhd	-0.2625542	6.88424588	1.51682609	0.23244978	0.29817182
Fastkd2	0.4005562	2.91665665	1.51667837	0.23247171	0.29817182
Adam1a	0.70131223	1.28166211	1.51543535	0.23265637	0.2983696
0610043K17I	0.36886754	1.52594547	1.51396348	0.23287527	0.29861123
Itga3	0.3187145	2.92621062	1.51310242	0.23300345	0.29873648
Ubn2	0.19554797	7.06862462	1.51223749	0.23313229	0.29885228
Tgfbi	-0.6612487	2.29086337	1.5120866	0.23315478	0.29885228
Olr1	0.87876278	-0.3912774	1.5111764	0.23329049	0.2989871
Nxt2	-0.2171345	6.76471218	1.5099561	0.23347259	0.29916329
Yipf6	-0.148318	7.26621431	1.50984584	0.23348905	0.29916329
Padi2	0.28762201	4.03186539	1.50950051	0.23354062	0.29916641
Nuak2	-1.5065092	-0.498086	1.50942051	0.23355257	0.29916641
Osbpl2	0.16566832	5.46258645	1.50883038	0.23364073	0.29924021
Cul7	0.27734748	3.0716944	1.50780815	0.23379356	0.29935409
Ky	0.66754645	0.5283509	1.50756433	0.23383003	0.29935409
Col7a1	-1.2178335	-1.240098	1.50729036	0.23387102	0.29935409
B630019K06	-0.265627	3.26317026	1.50723244	0.23387968	0.29935409
Bves	-0.8668782	-0.6052803	1.50679548	0.23394508	0.29935409

Plp1	0.26830818	8.66461957	1.50674045	0.23395331	0.29935409
Cep135	0.29553744	4.32391521	1.5067133	0.23395738	0.29935409
Fam96b	-0.32262	2.67795125	1.50660141	0.23397413	0.29935409
Bag4	0.17723464	5.95107043	1.50604055	0.23405812	0.29940803
Agtr1a	1.79625834	-1.2617721	1.50554795	0.23413191	0.29940803
Atp6ap2	0.17028217	7.25392313	1.5053775	0.23415745	0.29940803
Dazap1	0.26729343	3.48253625	1.50532139	0.23416586	0.29940803
Elf3	0.83566012	-0.0623376	1.50529964	0.23416912	0.29940803
Ntf3	-1.8134226	-1.2165419	1.50454848	0.23428173	0.29951292
Map3k1	0.19719315	5.10802838	1.5030018	0.23451383	0.29971643
Mastl	0.55879514	1.51160158	1.50289925	0.23452922	0.29971643
Slc35f1	0.22367104	7.28824329	1.50287599	0.23453272	0.29971643
Ptcd3	0.25105904	5.21562764	1.50260926	0.23457278	0.29972852
Lin7a	-0.1691097	7.6292927	1.50233494	0.23461399	0.29973228
Clpx	0.16471167	5.3907089	1.50218231	0.23463692	0.29973228
Tctex1d2	-0.2631535	4.3958505	1.50160108	0.23472427	0.29974313
Dgcr6	-0.3110895	3.95728815	1.50155999	0.23473045	0.29974313
BC016579	2.01863823	-1.2504356	1.501515	0.23473721	0.29974313
Gemin5	0.31556229	3.93796006	1.50128922	0.23477116	0.29974414
Sv2b	0.2156394	9.37558707	1.50096698	0.23481962	0.29974414
Mns1	-0.3639618	2.41699082	1.50038014	0.2349079	0.29974414
Fam107b	-0.3217198	5.28728601	1.50033672	0.23491444	0.29974414
Rpe65	0.8936519	0.67821647	1.50014772	0.23494288	0.29974414
Pcdha12	0.61774307	1.20786734	1.5000449	0.23495836	0.29974414
Cnnm1	0.18560599	6.37053827	1.5000182	0.23496237	0.29974414
Tradd	-0.6141893	0.67360552	1.49988244	0.23498281	0.29974414
Slc41a3	0.46887958	2.46873405	1.49954211	0.23503405	0.2997682
Zbtb2	-0.1838225	4.67072501	1.49935068	0.23506288	0.2997682
Tm4sf1	-0.2627509	4.66416052	1.49907254	0.23510477	0.29977724
Epha2	1.44429434	-1.6460887	1.49889728	0.23513117	0.29977724
Alyref2	-0.3771787	1.75103512	1.49861743	0.23517334	0.29979198
Slc22a2	-0.3818085	4.58244843	1.49767354	0.23531564	0.29993434
Fbl	-0.263107	4.29211646	1.49712526	0.23539834	0.30000072
Zfp651	-0.2445036	4.59660151	1.49595009	0.23557574	0.30010445
Edn3	-0.2418424	6.20018642	1.49588512	0.23558555	0.30010445
Vps25	-0.2456398	5.12728745	1.49584624	0.23559142	0.30010445
Gpr84	1.58127396	-1.0057645	1.49552998	0.2356392	0.30010445
A330040F15	-0.985774	-0.8518091	1.49521306	0.23568708	0.30010445
Zfp345	1.01384824	-0.7265107	1.49515994	0.23569511	0.30010445
Mccc1os	0.63333774	0.88030954	1.49483037	0.23574492	0.30010445
Fcer1g	-0.3644007	3.20022187	1.49480381	0.23574894	0.30010445
Pnpla7	0.43983927	2.12670054	1.49446992	0.23579942	0.30010445
Oxnad1	-0.3271484	3.78928905	1.49428218	0.23582781	0.30010445

Mdfi	-1.4756095	-1.4504208	1.49417911	0.2358434	0.30010445
Haus3	0.22707671	4.11729922	1.49415275	0.23584738	0.30010445
Fam160a1	-0.2455628	4.32997888	1.49281838	0.23604932	0.30029807
1110015O18	0.9502822	-0.9283855	1.49274212	0.23606087	0.30029807
Ptn	-0.3541146	10.3823205	1.49186838	0.23619322	0.30039853
Mthfd1	0.28938911	3.54331546	1.49181593	0.23620117	0.30039853
Mcm2	-0.35165	2.2388127	1.49129349	0.23628036	0.30042046
Klhl15	-0.3416891	3.77533684	1.49112819	0.23630542	0.30042046
4930505A04I	-1.0223668	-0.1434764	1.49109518	0.23631042	0.30042046
Lpar5	-1.1726383	-0.7769448	1.49062196	0.23638219	0.30046891
Trip4	-0.1623485	5.85807798	1.49043945	0.23640988	0.30046891
Tmem39b	0.48056482	1.82656269	1.48882005	0.23665573	0.30074235
Sncb	-0.2401915	4.66624454	1.48814388	0.23675848	0.30083282
Ankrd39	0.45753292	1.7336555	1.48794743	0.23678834	0.30083282
Zfp11	-0.2049778	4.27418731	1.48738568	0.23687376	0.30090231
Gatad2a	-0.2068441	5.71910595	1.48679308	0.23696391	0.3009778
Acta2	-0.3313033	3.71239408	1.48558074	0.23714849	0.30107873
Glrx3	-0.1756751	5.19640005	1.48550697	0.23715972	0.30107873
Traf3	0.21021684	5.05773297	1.48531041	0.23718967	0.30107873
Kif19a	-1.0937642	-1.1562717	1.48525443	0.2371982	0.30107873
1810020O05	-0.8481274	-0.8442492	1.48514278	0.23721521	0.30107873
Lias	-0.1950322	4.59285949	1.48506017	0.2372278	0.30107873
Pth2r	-1.5599636	-1.3312473	1.48483287	0.23726244	0.30108369
Lrrc57	-0.1851145	5.83911175	1.48445578	0.23731992	0.30111762
Exosc7	0.28606226	3.35910013	1.48373269	0.2374302	0.30119722
Reep4	0.88151702	0.28493167	1.48333479	0.23749091	0.30119722
Uimc1	0.21953942	4.62464259	1.48316393	0.23751699	0.30119722
Ctsw	1.70855376	-2.2378359	1.48314624	0.23751969	0.30119722
Prkaca	-0.1829711	7.24830415	1.48283649	0.23756697	0.30119722
Zfp703	-0.2051244	4.68493457	1.48283532	0.23756715	0.30119722
Ccdc92	-0.1617378	6.70486455	1.48201846	0.2376919	0.30126758
Pa2g4	-0.1341402	6.38809118	1.48171589	0.23773813	0.30126758
Agr2	-0.7746615	-0.1481052	1.48168665	0.2377426	0.30126758
Tshz2	0.14897996	6.13773992	1.48166651	0.23774567	0.30126758
Zbtb4	-0.1295969	7.52108843	1.47988649	0.23801789	0.30157352
Zfp13	0.50759846	1.79674284	1.47853952	0.23822414	0.30170744
1700123L14I	0.98266542	0.3657633	1.47845751	0.23823671	0.30170744
Tspan32	1.42115537	-1.3717372	1.47835882	0.23825183	0.30170744
Mc5r	-0.7798457	0.66592367	1.47819176	0.23827743	0.30170744
Zfp566	-0.444326	1.84360935	1.4780208	0.23830363	0.30170744
Cldn25	0.21577207	5.89007369	1.47798973	0.2383084	0.30170744
Sdhb	-0.146802	5.60717895	1.47718119	0.23843237	0.30178952
Sh2b2	-0.8180846	0.01806364	1.47716505	0.23843485	0.30178952

Mfng	-0.8258253	-0.3038503	1.47681452	0.23848862	0.30180895
Aaed1	-0.3187787	4.32597343	1.4766404	0.23851534	0.30180895
Gpr6	1.46288279	-1.4290837	1.47636581	0.23855748	0.30180895
Prss22	-1.287651	-1.6286607	1.47607724	0.23860178	0.30180895
Clip3	-0.2047433	10.1860474	1.47606107	0.23860426	0.30180895
Rps15a-ps6	-0.3156016	2.45714199	1.475598	0.23867536	0.30185992
Fbxo7	0.26279451	3.37786508	1.47521356	0.23873442	0.30189562
Macrod1	-0.8203987	0.16466757	1.47485054	0.2387902	0.30192718
Dcdc2a	-0.2112171	7.4805659	1.47458163	0.23883153	0.30192802
Pold1	0.6071931	0.57114693	1.47444514	0.23885251	0.30192802
Gnaq	-0.1261446	8.68177688	1.47420391	0.2388896	0.30193594
Vipr2	1.02442457	0.13129878	1.47371823	0.23896429	0.30195322
Cebpa	-0.5283921	2.51636749	1.47353598	0.23899233	0.30195322
Dixdc1	0.12526521	6.91295512	1.47351373	0.23899575	0.30195322
H2-DMb2	1.52477052	-1.6585688	1.47278462	0.23910796	0.30204467
Fam181b	-0.3693871	2.12299327	1.47237567	0.23917092	0.30204467
Slc1a6	0.66151892	0.37276094	1.471996	0.2392294	0.30204467
Adam32	0.95596942	-0.4974968	1.47195707	0.2392354	0.30204467
Fance	-0.3107391	2.70443339	1.47185945	0.23925044	0.30204467
Kremen1	0.26178011	3.34457656	1.47184185	0.23925315	0.30204467
Crnde	-2.4175003	-1.7775101	1.47068304	0.23943176	0.30223121
Tyro3	0.20739827	4.93736118	1.46975542	0.23957487	0.30235987
Ngb	0.56661689	0.84514126	1.46962223	0.23959542	0.30235987
Sdccag8	-0.1668345	6.24734401	1.46919459	0.23966144	0.30237868
Lsr	-0.3450998	3.45580772	1.4690419	0.23968502	0.30237868
Fbxo11	0.13228407	8.26058227	1.46889252	0.23970808	0.30237868
Arhgef10l	0.28534371	3.02220245	1.468726	0.2397338	0.30237868
Cdca7l	0.74447821	0.60647303	1.46837372	0.23978822	0.30240838
AI662270	0.55830981	0.58541783	1.4679993	0.23984608	0.30243992
AA388235	-0.2799759	3.22421595	1.46781229	0.23987499	0.30243992
Kcnh1	0.22410872	6.07146337	1.46736394	0.23994431	0.30248839
Plk3	-0.5454405	1.70869438	1.4669942	0.24000149	0.302512
Usp15	0.17554307	6.41798032	1.46684348	0.24002481	0.302512
Trpm3	0.1695401	6.82614017	1.46572528	0.24019787	0.30269118
Pdk2	-0.1491705	7.0969543	1.46552108	0.2402295	0.30269209
Ddx39	-0.3928626	2.37267461	1.46497575	0.24031397	0.30275958
2810454H06	-1.0405758	-0.9132212	1.46407291	0.24045391	0.30289693
Slc5a3	0.1835958	5.78479516	1.46353184	0.24053783	0.30293602
Rhbdl3	0.35490495	2.78035487	1.46347404	0.2405468	0.30293602
Gpr141	-1.8144089	-0.5865768	1.46258607	0.2406846	0.3030706
Stoml1	-0.3148014	3.03885234	1.46151815	0.24085047	0.30324048
Dlst	-0.1453865	6.53853897	1.46119151	0.24090123	0.30326306
Polr2l	-0.2411787	3.98022412	1.46100438	0.24093032	0.30326306

Snap47	-0.1391417	7.28616584	1.46047683	0.24101235	0.30332733
Ankrd17	0.1701932	8.9309109	1.4598611	0.24110814	0.30338924
Mtag2	-1.1379782	-0.504034	1.45956009	0.24115498	0.30338924
Pogz	0.16091671	7.70921291	1.45939499	0.24118068	0.30338924
Gm9833	0.40166099	1.15406897	1.45926457	0.24120098	0.30338924
Trp53inp1	-0.2029322	5.78981049	1.45916547	0.24121641	0.30338924
Iqce	0.29433867	3.35711486	1.45888965	0.24125936	0.3034043
Xrn1	0.19588754	6.04494522	1.45826581	0.24135653	0.30348754
Fahd1	-0.1810935	5.2997062	1.45763672	0.24145457	0.30357186
Larp4b	-0.1198911	7.45689149	1.45725025	0.24151483	0.30357186
Ift43	-0.3989545	3.32495798	1.45715458	0.24152975	0.30357186
Gmpr2	0.30176637	3.44926098	1.45704046	0.24154755	0.30357186
Pcdhgb4	0.36975413	2.29278833	1.45525783	0.24182579	0.30386252
Slc22a18	0.78197448	1.18189298	1.45510426	0.24184978	0.30386252
Nr5a2	1.75031112	-2.0178375	1.45482817	0.24189291	0.30386252
Med17	0.19858226	3.9562245	1.4546014	0.24192835	0.30386252
Gpr19	0.30566026	3.45953435	1.45456569	0.24193393	0.30386252
Pglyrp2	2.24511239	-1.8629835	1.45420869	0.24198974	0.30389364
Spata13	0.19111365	4.58809525	1.45366706	0.24207443	0.30394497
Tmem14c	-0.3231166	3.11534299	1.45355049	0.24209266	0.30394497
Arl10	0.58547162	0.91584458	1.45300428	0.24217812	0.3039977
Manf	-0.3180128	3.8192679	1.45288537	0.24219673	0.3039977
Zfp397	0.13028405	5.88909395	1.45257581	0.24224519	0.30401956
Qsox2	0.30594005	2.93619289	1.45140755	0.24242817	0.30413821
Cln6	-0.5724748	1.96632768	1.4509674	0.24249715	0.30413821
Mcidas	1.14934834	-1.6433173	1.45094922	0.2425	0.30413821
Tmem185b	0.3970876	3.21262042	1.45079624	0.24252399	0.30413821
Mrpl42	-0.1902219	5.90486063	1.45067073	0.24254366	0.30413821
Cdk16	-0.148284	7.18779947	1.45063869	0.24254869	0.30413821
Pogk	-0.1351657	6.21945239	1.45053403	0.2425651	0.30413821
Asb7	-0.1285935	6.12224714	1.45038723	0.24258812	0.30413821
Ccng1	-0.1585238	8.15001711	1.45013525	0.24262765	0.30414883
Lhfpl4	0.15506513	6.02255288	1.44992946	0.24265993	0.30415038
Smtnl2	0.45919185	1.14257697	1.44900776	0.2428046	0.30429276
AI839979	0.75546113	0.19634029	1.44859773	0.24286899	0.30430683
Olfr539	1.18128927	-0.4667982	1.44854064	0.24287796	0.30430683
Txlnb	0.44280396	2.24854528	1.44772548	0.24300604	0.30440392
Ebf3	0.40813163	1.46044412	1.44751822	0.24303862	0.30440392
Cd247	-1.774829	-1.5631675	1.4474543	0.24304867	0.30440392
Eqtn	0.91516536	0.10408834	1.44633796	0.24322426	0.30458489
Gpm6a	0.19618783	9.42964172	1.44541368	0.24336977	0.30471269
2410002F23I	0.28781522	3.20414754	1.44529455	0.24338853	0.30471269
Rpl13a	-0.1864093	7.00463303	1.44504513	0.24342782	0.30472293

Mroh7	1.6715934	-0.4460366	1.44477225	0.24347081	0.30473781
Kdm6a	0.2114668	6.12837456	1.44425466	0.24355239	0.30480097
Bag2	-0.3876461	3.17330684	1.44388943	0.24360997	0.30480556
Gopc	0.14731996	6.07453978	1.4437379	0.24363387	0.30480556
Lrrc61	-0.1828095	5.11850472	1.44341328	0.24368507	0.30480556
Sat1	-0.1993962	5.94072351	1.44328879	0.24370471	0.30480556
Xpo7	0.12556948	7.18190454	1.44324482	0.24371165	0.30480556
Rraga	0.19993645	6.03591811	1.44265456	0.2438048	0.30485859
Gstp1	-0.2076812	4.93816257	1.44258175	0.24381629	0.30485859
Plcz1	1.26210819	-0.6400122	1.44225457	0.24386795	0.30488426
Kazn	-0.18308	6.12045193	1.44184224	0.24393307	0.30489932
Csrnp3	0.23896965	7.29463756	1.44164218	0.24396467	0.30489932
Lbh	-0.3049321	6.3473381	1.44154811	0.24397954	0.30489932
Ptpru	0.28622398	3.24593205	1.44139006	0.24400451	0.30489932
1700020I14R	-0.1859815	6.32617778	1.44118658	0.24403666	0.30490061
Smdt1	-0.2982105	5.06212921	1.44039392	0.24416198	0.30497114
Gripap1	0.20025487	4.91768759	1.44026536	0.24418232	0.30497114
Tspyl4	-0.1518538	8.38403276	1.44023877	0.24418652	0.30497114
Rbm19	-0.3761055	2.26735387	1.4394991	0.24430355	0.30507841
Gtf2f1	-0.2066325	5.9128178	1.43888665	0.24440051	0.30516058
Syn2	0.17466885	7.70541142	1.43865806	0.24443671	0.30516688
Pfkp	0.22510493	7.31963642	1.43818294	0.24451197	0.30522194
Qtrtd1	0.40228629	2.08839529	1.43600239	0.24485778	0.30552645
Thbs1	0.36797033	2.76445923	1.43537656	0.24495715	0.30552645
Fam24a	-1.8634176	-2.0965885	1.43531449	0.24496701	0.30552645
Col28a1	1.1227377	-0.372539	1.43498803	0.24501886	0.30552645
Rnf126	-0.1872388	3.99171202	1.43469944	0.24506472	0.30552645
Fam71d	-1.1975009	-1.4505967	1.43467214	0.24506906	0.30552645
Agbl2	0.47335292	1.56639521	1.43422229	0.24514055	0.30552645
Cdca4	-0.3219762	2.88013428	1.4341902	0.24514566	0.30552645
Olfml3	-0.3019021	4.9645008	1.43397991	0.24517909	0.30552645
Penk	0.22570628	7.08273198	1.43378358	0.24521031	0.30552645
Mapk14	0.14011057	6.53290444	1.43361455	0.24523719	0.30552645
Efcab2	-0.2053945	3.97879449	1.43358701	0.24524157	0.30552645
Elf4	-0.395235	3.91616552	1.43337849	0.24527474	0.30552645
Nnmt	-1.1016003	-0.2419865	1.43337358	0.24527552	0.30552645
Mgat4a	0.24286244	5.48766177	1.43334712	0.24527973	0.30552645
2410004P03I	-0.3859022	2.3157078	1.43318653	0.24530528	0.30552645
Mtg1	0.35895149	2.77387314	1.43302589	0.24533084	0.30552645
Mustn1	-0.4163763	3.85996265	1.43280979	0.24536523	0.30552645
Zfp799	0.17997924	5.66244101	1.43275053	0.24537466	0.30552645
Sf3a2	-0.2460945	3.0888591	1.43271866	0.24537974	0.30552645
Ppp1r12c	-0.1590768	5.32770825	1.43233385	0.245441	0.30556389

Capza1	-0.1720887	6.23692652	1.43177129	0.24553059	0.30563658
Hykk	-0.248129	5.34662653	1.43117013	0.24562638	0.30568267
Tnfrsf1a	-0.3951698	4.11588072	1.43114724	0.24563002	0.30568267
Spdya	-0.5749962	0.69594156	1.4301917	0.24578238	0.30583342
AW112010	0.43780299	1.65671888	1.42948875	0.24589454	0.30593412
Helb	0.27376222	3.42155083	1.42795564	0.24613939	0.3061579
Ntng2	0.44141858	1.99714029	1.4276733	0.24618451	0.3061579
Xrra1	1.31117888	-1.5196995	1.42759945	0.24619632	0.3061579
Glis1	-0.7192625	0.52950865	1.4275701	0.24620101	0.3061579
Vcpip1	0.16975489	7.35241349	1.42738446	0.24623069	0.3061579
Fbxl22	0.86698657	-0.6497519	1.42612593	0.24643201	0.30636934
Dnajc8	-0.1900832	6.22569928	1.425771	0.24648883	0.30640109
Zfp810	0.19460139	4.65836076	1.42520604	0.2465793	0.30647033
Rp2h	-0.2337999	4.92032944	1.42503247	0.24660711	0.30647033
Alkbh1	-0.2778402	4.29498988	1.42427629	0.24672829	0.30655349
Psmd2	0.17358145	5.80504036	1.42422434	0.24673661	0.30655349
Pskh1	-0.3280486	3.80027565	1.42384072	0.24679812	0.30657606
Zfp825	-0.2139569	4.52217882	1.42372063	0.24681738	0.30657606
Dhx58	-0.6941556	0.55327898	1.42344013	0.24686237	0.30659307
Gm10516	0.43985412	2.08014304	1.42289648	0.2469496	0.30663243
Polm	-0.3033282	3.19492597	1.42275065	0.24697301	0.30663243
Tmem167b	-0.2717501	6.20567732	1.42265739	0.24698798	0.30663243
Tmem170b	0.17279761	7.50671763	1.42235828	0.247036	0.30665318
Zfp87	0.18982648	5.56435072	1.42187178	0.24711413	0.30666702
Ccdc51	-0.5709033	0.54766976	1.42181487	0.24712327	0.30666702
2810049E08I	0.82357904	0.86852892	1.42170407	0.24714107	0.30666702
Tmem37	-0.9040047	0.73722853	1.42137023	0.24719471	0.30669473
Plcxd2	-0.202494	7.79209282	1.42080347	0.24728581	0.3067689
Rbm43	-0.2175344	4.53418318	1.42000133	0.24741481	0.30685534
Wdr78	0.38881481	3.27238946	1.41978808	0.24744912	0.30685534
Slc44a1	0.14742601	5.88927923	1.41978595	0.24744946	0.30685534
Dusp11	0.1614695	6.38920136	1.41877527	0.24761216	0.30699428
Nelfa	0.1903089	4.89453732	1.41870056	0.24762419	0.30699428
Igfals	-1.904133	-2.2123969	1.41835573	0.24767974	0.30702429
Trpc4ap	0.16497165	5.32545809	1.41690913	0.24791294	0.3072554
St3gal5	0.18746427	6.4731257	1.41681006	0.24792892	0.3072554
Cep41	0.41209643	2.89860501	1.41622001	0.24802412	0.30727801
Fbxo47	0.55538885	1.25856779	1.41615649	0.24803437	0.30727801
Zfp839	0.17980803	5.26943064	1.41611375	0.24804127	0.30727801
Syne4	1.45377824	-1.3494945	1.41566628	0.24811351	0.30732864
Fbxo38	0.1498658	5.22070644	1.41499838	0.24822139	0.30742339
Zbtb49	0.43314534	1.51435636	1.41358748	0.24844947	0.30766697
Zfp820	-0.5037165	1.39759634	1.41255861	0.24861597	0.30782002

Ptprb	0.39462684	5.31799751	1.4124354	0.24863592	0.30782002
Hist2h2be	-0.2416045	6.00580263	1.4120388	0.24870014	0.30785891
Wnt1	-1.0908136	-1.3555042	1.41185328	0.24873019	0.30785891
Gfap	-0.2645451	4.21164272	1.4104419	0.24895896	0.30810313
Smyd1	0.76401895	1.45294208	1.41023039	0.24899326	0.30810666
Btbd2	0.24170292	4.50350605	1.4098348	0.24905744	0.30814715
Arhgap35	0.15516675	8.93178848	1.40919204	0.24916177	0.30821129
Ankhd1	0.16939527	7.51690378	1.40912771	0.24917221	0.30821129
Cyth3	-0.2148972	6.99938131	1.40885157	0.24921706	0.30822783
S100pbp	-0.2000788	5.02257202	1.40832985	0.2493018	0.30827791
Mdga1	0.41489486	2.48382082	1.40821482	0.24932049	0.30827791
C2cd4c	-0.4176004	3.3817538	1.40770583	0.24940322	0.30834128
Arid5a	-0.4567136	1.19531805	1.40705888	0.24950841	0.30841721
Tyw3	-0.3433291	3.04954393	1.40694087	0.24952761	0.30841721
1500017E21I	-1.5316496	-1.8060662	1.40669175	0.24956813	0.30842838
Riok2	0.164183	5.09861726	1.40646786	0.24960457	0.30843449
Dap3	0.17462364	4.8301408	1.40614816	0.2496566	0.30845988
Ufsp2	-0.1904408	5.28542917	1.40518393	0.24981361	0.30861494
Gm10474	1.61564683	-1.5017368	1.40455404	0.24991625	0.30867472
Araf	0.14558245	7.24708396	1.40450022	0.24992502	0.30867472
Pick1	0.23523948	3.57902935	1.4035874	0.25007387	0.30876217
Orc2	0.170711	5.78244805	1.4035601	0.25007833	0.30876217
Hdac7	0.24409466	3.70229096	1.40348614	0.25009039	0.30876217
Prpf3	-0.2101839	4.35903595	1.40307405	0.25015763	0.30880626
Steap2	0.16988537	4.92439914	1.40208105	0.25031975	0.30896746
Rbm4b	-0.2176055	4.34630072	1.40147777	0.25041832	0.30905017
Lrrc17	-0.7410274	0.06156484	1.40021212	0.25062526	0.30925383
Ncam2	0.20259634	7.16289986	1.39962746	0.25072093	0.30925383
Elf2	-0.2177686	6.74349939	1.39958745	0.25072748	0.30925383
E330033B04I	0.49818397	2.95242948	1.39952156	0.25073826	0.30925383
Smyd4	0.31793186	2.62051006	1.39950364	0.2507412	0.30925383
C130046K22I	-0.3539458	2.95526203	1.39880761	0.25085516	0.30935544
Mtcl1	0.27647201	5.72007567	1.39756441	0.25105889	0.30956771
Wscd1	0.24441336	3.74854037	1.39720893	0.25111719	0.30960061
Ext2	0.3901031	5.05571587	1.39688073	0.25117102	0.30962802
Tns3	0.14594488	6.05708022	1.39653791	0.25122727	0.30965839
BC029214	-0.2602619	3.49030025	1.39576846	0.25135358	0.3097751
Eif6	-0.2923668	4.38628276	1.39550463	0.25139691	0.30977762
Fastkd1	0.31755706	2.74592134	1.39523423	0.25144133	0.30977762
Pcbd2	-0.3190668	4.06968922	1.39515207	0.25145483	0.30977762
Speer4e	-1.2183541	-1.3641308	1.39489211	0.25149755	0.30977762
Ap5m1	0.28643878	3.37570823	1.39479347	0.25151376	0.30977762
Rab2b	-0.157661	5.9805312	1.39459409	0.25154653	0.30977903

Canx	0.16508858	7.9635512	1.39426946	0.25159989	0.3098058
Diap2	0.17943611	7.4642199	1.3940661	0.25163334	0.30980803
Exoc1	0.23853557	5.66903012	1.3937451	0.25168613	0.30983409
Dctn6	-0.2258922	6.37633636	1.3931667	0.2517813	0.30984106
Has3	0.51202115	1.97224757	1.39315928	0.25178252	0.30984106
Rxfp1	0.55039107	2.35363465	1.39313396	0.25178668	0.30984106
Mat2b	-0.1393343	7.44759251	1.39248302	0.25189385	0.30993399
Rxrg	0.46489778	0.91500745	1.39127037	0.25209364	0.31014086
Zap70	-1.3963877	-1.6527262	1.39080565	0.25217026	0.31019616
Mrps23	-0.2187448	4.23293926	1.38966582	0.25235831	0.31037599
Tbck	0.22856228	4.68675334	1.38953556	0.25237982	0.31037599
Lgr6	1.17406612	-0.8661721	1.38926976	0.2524237	0.31039098
Cdr2l	-0.2457861	3.23007911	1.38896158	0.25247459	0.3104146
Yeats2	0.16496406	5.57972497	1.38853686	0.25254475	0.31046189
Ston1	-0.3889433	4.79793078	1.38814134	0.25261011	0.31050327
Wwp1	-0.1599397	7.5347871	1.38788991	0.25265167	0.31050327
Ebag9	-0.1370219	5.98741643	1.38775784	0.25267351	0.31050327
Fbxo21	0.19152064	5.66249624	1.3874727	0.25272066	0.31052225
Tmem60	-0.1924518	5.36769151	1.38680874	0.25283049	0.31053082
Rabep1	-0.1396915	7.91370563	1.38679294	0.2528331	0.31053082
Slc38a1	0.14884641	7.61637541	1.38655556	0.25287239	0.31053082
Kcnab1	0.17748743	5.30295476	1.38651085	0.25287979	0.31053082
Lrrc8e	0.68686387	-0.1319681	1.38618161	0.25293429	0.31053082
Slc29a3	0.21439445	5.26561223	1.38600151	0.25296411	0.31053082
Npat	0.17667571	5.99288499	1.38581396	0.25299516	0.31053082
Gins1	-0.8888462	0.15266599	1.38573975	0.25300745	0.31053082
Polr1c	0.2514292	4.05554449	1.38564617	0.25302295	0.31053082
Caprin1	0.11621348	8.54069451	1.38551518	0.25304465	0.31053082
Zfp30	-0.2381621	3.69679721	1.38507594	0.25311743	0.31057353
Ddc	0.47574379	1.34508419	1.38492243	0.25314286	0.31057353
Zc3hav1l	-0.216979	6.00486285	1.38470645	0.25317866	0.31057855
Ptchd2	0.35461629	2.78492461	1.38421368	0.25326036	0.31062015
Nmi	-0.400475	3.19911829	1.38385482	0.25331988	0.31062015
Sorbs1	0.12030497	7.52415737	1.38376554	0.2533347	0.31062015
Tacr3	0.49284171	1.90532315	1.38371797	0.25334259	0.31062015
Robo4	-0.7065672	0.64692429	1.3835459	0.25337113	0.31062015
Zfp780b	0.17777162	5.39516929	1.38333183	0.25340666	0.31062483
Ermn	0.17558996	6.68337228	1.38270779	0.25351025	0.31071293
Mex3c	-0.1646235	5.21447884	1.38236478	0.25356722	0.31074386
Myl12a	-0.3379488	6.85744348	1.38165434	0.25368525	0.31084963
Gyg	-0.1500241	5.57171995	1.38122148	0.2537572	0.3108989
Zdhhc16	-0.3244254	2.26251779	1.38014577	0.25393613	0.31107921
Emx1	-0.6595977	0.52695818	1.37909238	0.2541115	0.31125512

Actr8	0.19585681	4.68296503	1.37884832	0.25415216	0.31126599
Slc25a36	0.16701762	4.98566063	1.37836493	0.25423271	0.31132572
Prkcq	-0.3830036	2.94313215	1.37795572	0.25430092	0.31137032
Pcdha7	0.85418441	-0.6336059	1.37749864	0.25437714	0.31142472
Hnrnph2	-0.1313455	7.21234144	1.37723373	0.25442133	0.31143989
Cbx2	-0.6053605	1.04972225	1.37689041	0.25447861	0.31147109
Gcm1	1.20550409	-1.3960054	1.37642354	0.25455653	0.31152753
Snord104	-1.2226499	-1.7075835	1.37610087	0.25461041	0.31155454
Rrm1	0.19904433	4.32761159	1.37565531	0.25468482	0.31160667
Gnai1	-0.1447393	9.05039895	1.37511527	0.25477506	0.31167227
Thyn1	-0.2970377	3.4539141	1.37483305	0.25482223	0.31167227
Ccnd1	-0.2272418	6.16639767	1.37476327	0.25483389	0.31167227
Cdc34	-0.3592023	3.16329513	1.37406367	0.25495088	0.31170606
Tdrd7	0.18170082	4.68204311	1.37401243	0.25495946	0.31170606
Arhgap44	0.15194062	6.68127894	1.37378682	0.2549972	0.31170606
Gm12522	0.55359659	1.18137555	1.37352004	0.25504184	0.31170606
Ifitm1	-0.3068377	5.15849788	1.37339939	0.25506204	0.31170606
Fam168b	-0.1571285	8.42329081	1.37334522	0.2550711	0.31170606
Panx2	0.24824168	4.05650636	1.37326656	0.25508427	0.31170606
A730020E08	0.3808093	2.69495924	1.37274864	0.25517098	0.31176163
4930430F08I	-0.2428547	3.77965773	1.37261477	0.2551934	0.31176163
Nog	0.73915012	-0.0920429	1.37234367	0.25523881	0.31177822
Amer2	0.2327102	4.18477025	1.37198203	0.25529941	0.31181335
Syt11	0.18558435	8.29352381	1.37139787	0.25539732	0.31187177
Rps27a	-0.1910197	6.62252119	1.37120456	0.25542973	0.31187177
Krt18	1.5376129	-1.6566113	1.37085008	0.25548918	0.31187177
Rgs22	0.75382587	0.3420577	1.37084363	0.25549027	0.31187177
Ercc3	0.21012606	4.2118526	1.37069498	0.2555152	0.31187177
Zfp235	0.30449104	3.30221274	1.37055749	0.25553827	0.31187177
02-Mar	-0.1970761	4.75045565	1.36884243	0.25582623	0.31214685
Mettl20	-0.347892	2.58159646	1.36878109	0.25583654	0.31214685
Zfp930	0.23994414	4.10431084	1.36848911	0.25588561	0.31214685
Acot5	-0.9826156	0.37298916	1.36845628	0.25589112	0.31214685
Megf11	0.19592281	5.52647012	1.36717896	0.25610593	0.31236998
Atp8a2	0.37452066	3.82763017	1.36666587	0.25619229	0.3124057
Dmrt3	2.19681153	-1.5248204	1.36639133	0.25623851	0.3124057
Pla2g4e	0.21934652	4.55148466	1.36629883	0.25625409	0.3124057
Ssr3	-0.1910683	8.00002703	1.36624711	0.2562628	0.3124057
Tmem176a	0.37139273	4.32210892	1.36599444	0.25630535	0.3124187
Emp1	-0.4002113	3.82778592	1.36563944	0.25636515	0.31245271
Hnrnpl	0.1866312	5.22655387	1.36514401	0.25644865	0.31251559
Smad7	0.28534426	3.43137267	1.36430393	0.2565903	0.31264931
Wnt7a	0.48365016	1.63757621	1.36408042	0.25662801	0.31265472

Ubac2	0.28864	2.53218994	1.36389926	0.25665857	0.31265472
Mir181b-2	1.52794799	-1.1861624	1.36351043	0.25672419	0.31269577
Fgf9	0.2258262	4.84515337	1.36298777	0.25681244	0.31275852
Ube2g2	-0.2304121	4.67683069	1.36282709	0.25683957	0.31275852
Commd5	-0.4238213	2.05512367	1.36261363	0.25687563	0.31276355
Gjb2	-0.2702231	9.09863828	1.36229257	0.25692987	0.31279071
Abhd13	0.15768167	5.20449293	1.36159186	0.25704831	0.31285024
9430083A17	0.68951137	1.31944819	1.36154526	0.25705619	0.31285024
Zfp280d	0.22235958	6.75746108	1.36138272	0.25708367	0.31285024
Rnf152	-0.1541204	6.19103377	1.36124756	0.25710653	0.31285024
Ubald1	-0.2115901	4.48217382	1.36062544	0.25721177	0.31289605
Naif1	-0.6920042	0.03808639	1.3604534	0.25724089	0.31289605
Foxf1	-1.6885969	-1.9447467	1.36028257	0.2572698	0.31289605
Stx12	-0.1404814	7.93683339	1.36026989	0.25727194	0.31289605
Scarf2	0.65782733	1.58556916	1.35963933	0.25737871	0.31292986
Pfkfb3	0.19216661	4.54080087	1.35961822	0.25738228	0.31292986
Pparg	0.46740086	1.72385308	1.35953966	0.25739559	0.31292986
Hemk1	-0.4274298	1.69722115	1.35930928	0.25743461	0.31293164
Map7d1	-0.1852114	6.98034278	1.35899819	0.25748732	0.31293164
Aftph	-0.147402	6.94774223	1.35896529	0.25749289	0.31293164
Dner	0.25441115	5.47834739	1.35764058	0.2577175	0.31316575
Cdyl	-0.1890573	4.10914746	1.35674163	0.25787007	0.31328003
Zfp619	0.27371743	2.83341509	1.35670956	0.25787551	0.31328003
Kars	-0.1885488	5.13841216	1.35639095	0.25792961	0.3133052
Nqo1	-0.4255163	4.40171669	1.35615872	0.25796906	0.3133052
Tmem194b	0.57434915	2.10193843	1.35596655	0.2580017	0.3133052
Irf2bp1	-0.2127658	3.91958908	1.35583431	0.25802417	0.3133052
Prmt7	-0.3044407	3.30379527	1.35391088	0.25835126	0.31366349
Ilf3	0.15018912	5.74793852	1.35234684	0.25861763	0.31394797
2310009A05	-0.3880083	2.69505631	1.35161784	0.25874191	0.31405992
Lrp2bp	0.80891517	0.78292561	1.3514248	0.25877483	0.31406096
Rab37	0.67223252	0.41174114	1.35112694	0.25882564	0.31408371
Tubb3	-0.212847	4.68233189	1.35086866	0.2588697	0.31409827
Trim44	-0.1142538	9.18709954	1.35034263	0.25895949	0.31415209
Dag1	0.21007404	6.67986052	1.34966777	0.25907473	0.31415209
Opa1	0.16886894	7.78864065	1.34964503	0.25907862	0.31415209
Scnm1	-0.2580067	4.02330932	1.34961103	0.25908442	0.31415209
Fam210a	0.15248308	6.44242624	1.34957548	0.2590905	0.31415209
Ppp3cc	-0.1865722	5.04839543	1.34932475	0.25913333	0.31415209
Dbil5	1.29130415	-1.4337472	1.34929417	0.25913856	0.31415209
Samd4	0.16712401	7.26723062	1.34890524	0.25920503	0.31416207
Lpcat1	0.35730379	2.86572616	1.34881812	0.25921992	0.31416207
Fech	-0.1634257	7.02489319	1.34868307	0.25924301	0.31416207

Nkx2-2	0.59771487	0.88378594	1.34815783	0.25933282	0.31423203
AW011738	-0.4258174	2.60334174	1.34657896	0.25960306	0.31452057
Ppp3r1	-0.1274827	10.0948401	1.34619984	0.259668	0.31456035
Fut10	-0.255077	3.61978587	1.3457349	0.25974768	0.31457937
Ndufs2	-0.1879104	6.6411601	1.34573335	0.25974794	0.31457937
Slc8a3	0.23632485	3.92640813	1.34487985	0.25989428	0.3147177
Angptl3	0.87034834	0.11043517	1.34335215	0.2601565	0.31491618
Ptger1	-0.2522579	3.11132064	1.3433489	0.26015705	0.31491618
Usp39	-0.2253667	4.1659872	1.34322074	0.26017907	0.31491618
2210408F21I	-0.3742021	2.4430017	1.34317577	0.26018679	0.31491618
Krt25	1.60055673	-1.5438074	1.34257377	0.26029023	0.31500245
Opcml	0.19740098	8.17137437	1.34216673	0.2603602	0.31501347
S100a6	-0.3487444	5.94037353	1.34214579	0.2603638	0.31501347
Grik3	0.25463532	5.57650509	1.34195964	0.26039581	0.31501347
Ccdc66	0.15541129	5.59627486	1.34172988	0.26043532	0.31502236
Slc18a3	-0.8664141	0.02036782	1.34055238	0.26063794	0.31522853
9030617O03	-0.2946009	2.99011174	1.3397915	0.26076898	0.31527816
Ankrd40	-0.1289325	7.06114299	1.33943001	0.26083127	0.31527816
Ap3d1	0.21498753	6.40065025	1.33932493	0.26084938	0.31527816
Angptl4	-0.4303402	2.19976334	1.33920865	0.26086942	0.31527816
Lepre1	0.3231229	2.22410944	1.33913852	0.26088151	0.31527816
Ckmt2	1.44346871	-1.0422931	1.33909166	0.26088959	0.31527816
Gm4925	0.95435451	-0.0737922	1.3390064	0.26090429	0.31527816
Clvs1	-0.2037319	4.16527292	1.33860593	0.26097334	0.31532271
Plekhb2	0.13740333	6.30581925	1.33794853	0.26108675	0.31542082
Ece1	0.19212227	5.98034349	1.33772571	0.2611252	0.31542837
Mcm3ap	0.28607467	4.45572644	1.33665937	0.26130932	0.31558958
Foxr1	0.88762529	-1.0863952	1.33654556	0.26132898	0.31558958
Snx8	0.42610068	1.59947853	1.33639321	0.26135531	0.31558958
B130034C11I	-0.7420158	0.87810322	1.33586526	0.26144655	0.31566085
Zfp420	-0.1912266	4.40160952	1.33499584	0.2615969	0.31580345
Mttp	0.37181158	2.01336206	1.33322217	0.26190398	0.31608905
S100a11	-0.2822653	9.5078464	1.33290029	0.26195976	0.31608905
Grb2	-0.1620437	5.1641288	1.33269253	0.26199577	0.31608905
Map3k12	0.21178286	5.62385011	1.33263677	0.26200543	0.31608905
Spag4	1.30911534	-1.6186552	1.33255068	0.26202036	0.31608905
B020004J07F	0.7177801	0.1922254	1.33241133	0.26204452	0.31608905
Pde4a	0.21558406	6.32580238	1.33217272	0.26208589	0.31608905
Rfesd	-0.2204773	4.17403698	1.33201536	0.26211319	0.31608905
Dxo	0.30687662	2.09703573	1.33190676	0.26213202	0.31608905
Rps3	-0.2780896	6.97730527	1.33158539	0.26218778	0.31608905
Ebp	-0.3207782	2.6593953	1.33158152	0.26218845	0.31608905
Dcbld2	0.16821453	5.26479498	1.33120712	0.26225342	0.31609532

Traf7	-0.2150548	3.73534693	1.33117967	0.26225818	0.31609532
Mir690	1.61403873	-2.00979	1.33025857	0.26241813	0.31624918
Ccdc64	0.60331572	0.49411901	1.32961752	0.26252951	0.3163445
Dgka	-0.2013665	4.79442651	1.32920055	0.262602	0.31639292
A130010J15I	0.35452458	3.56718103	1.32892395	0.2626501	0.31640867
Slc25a15	-0.2575987	3.41197835	1.3287539	0.26267967	0.31640867
Tulp2	0.64563639	0.43315833	1.32801487	0.26280826	0.31650882
Arrdc1	0.53507325	0.91740947	1.32790464	0.26282744	0.31650882
1700105P06I	2.83129637	-1.8469964	1.32697522	0.26298929	0.31666479
Dpy19l1	0.14292291	6.7235325	1.32666056	0.26304411	0.31669188
Amn1	-0.2430075	4.58958325	1.32575002	0.26320284	0.31680776
Dnd1	1.1938795	-1.5052537	1.32571505	0.26320894	0.31680776
Anks1b	0.19849268	8.50588551	1.32540425	0.26326315	0.31680776
Rnaseh2b	-0.2556448	4.32882291	1.32525912	0.26328847	0.31680776
Acot10	1.16396593	-0.5193226	1.32518112	0.26330208	0.31680776
Csk	-0.3275646	2.98253537	1.32432602	0.26345133	0.31691462
Ppl	0.29563951	2.44147578	1.32430156	0.2634556	0.31691462
Pamr1	-0.2166437	4.03100599	1.32354904	0.26358704	0.31702976
Luzp2	0.21843862	6.55836174	1.32338302	0.26361605	0.31702976
2310067B10I	0.27275133	4.10431381	1.32289381	0.26370156	0.31705112
Sh3bp5l	-0.2344213	3.77831697	1.32286453	0.26370668	0.31705112
Kcnc3	0.28125389	5.24132334	1.32272593	0.26373091	0.31705112
Poc1a	-0.3760056	2.80503987	1.32219334	0.26382406	0.31710638
Lss	0.23285841	4.11244123	1.32207054	0.26384554	0.31710638
D11Wsu47e	-0.3084132	2.51229817	1.32176261	0.26389942	0.31710638
Zbp1	-0.5096164	1.02666579	1.32155555	0.26393566	0.31710638
Ap2s1	-0.3009636	3.45522275	1.32153793	0.26393874	0.31710638
Gm14322	-0.2130772	4.29984094	1.32099121	0.26403446	0.31718247
Ncoa2	0.16869519	8.34814056	1.32022337	0.26416896	0.31728847
Psph	-0.3402789	3.65078526	1.32005768	0.264198	0.31728847
Actr3	-0.1174351	8.04242918	1.31992716	0.26422088	0.31728847
Fbxw2	-0.1753645	5.59019633	1.31973118	0.26425523	0.31728847
Hmbox1	0.17653424	5.1907916	1.31954422	0.26428801	0.31728847
Rfc1	0.1327615	5.87408846	1.3193786	0.26431705	0.31728847
Ppp1r3g	0.61446428	0.59363059	1.318713	0.26443381	0.31738129
Cdkn2aip	-0.2117179	4.20203269	1.31856845	0.26445917	0.31738129
4930556M19	0.50701435	1.12915762	1.31776545	0.26460014	0.31745622
Snora31	1.23704222	-1.2004144	1.31761107	0.26462725	0.31745622
Cul5	0.12963156	7.02475883	1.31733823	0.26467517	0.31745622
Timp4	0.34309899	2.45770989	1.31728731	0.26468412	0.31745622
Kctd1	-0.140018	6.47743231	1.31727983	0.26468543	0.31745622
Socs4	-0.1870691	5.019161	1.31694635	0.26474403	0.31745622
Rnf103	0.14265618	6.23756175	1.31690359	0.26475154	0.31745622

Sgta	-0.2136427	4.96266654	1.31673665	0.26478088	0.31745622
Phb2	-0.1895026	5.0191574	1.31632838	0.26485266	0.31750341
2010320M18	-0.4928147	1.75692754	1.31605391	0.26490092	0.31752241
Hnrnpll	0.14398777	6.34844981	1.31583763	0.26493896	0.31752915
1700030K09I	0.50928586	1.60509031	1.31537207	0.26502087	0.31758847
Tcp10b	1.49146889	-1.9858622	1.31457298	0.26516154	0.31770145
Limd1	-0.2703576	5.16136736	1.31446801	0.26518003	0.31770145
Il2ra	0.47392932	1.922807	1.31411054	0.26524299	0.31773803
Cldn12	0.16962326	5.41596748	1.31373358	0.26530941	0.31777873
Frzb	0.28894637	3.21545134	1.31337811	0.26537207	0.31781491
Fermt3	0.69215307	0.28907828	1.31318936	0.26540534	0.31781591
Trim28	0.14800991	5.90610985	1.31296044	0.26544571	0.31782539
Tm2d3	-0.2589126	3.24777264	1.31224218	0.26557241	0.31793823
Hsp90aa1	0.16747685	10.9285529	1.31194044	0.26562566	0.31795168
Fbxo31	-0.3109967	3.94589921	1.31179174	0.26565191	0.31795168
Crip1	-0.4630499	2.42763612	1.31162684	0.26568102	0.31795168
Pcbd1	-0.3468158	1.94610971	1.31104228	0.26578425	0.31802488
Akna	-0.2446849	3.34321702	1.31072488	0.26584033	0.31802488
Tmem184b	0.17501033	4.72598387	1.31071951	0.26584128	0.31802488
Prrt4	0.92579275	-0.6122405	1.31054531	0.26587206	0.31802488
Atp1a3	0.20980613	9.48253758	1.310096	0.26595147	0.31808103
Pdk1	-0.1495333	6.19472714	1.30971395	0.26601903	0.31808695
Otx2	-0.7972459	1.57621485	1.30970071	0.26602137	0.31808695
4930444P10I	-1.4910964	-1.494696	1.30936003	0.26608163	0.31810383
Il17ra	0.17036995	4.71235539	1.30895138	0.26615393	0.31810383
Cd2ap	-0.171516	7.43091632	1.30847398	0.26623843	0.31810383
Espl1	1.18160305	-1.4992261	1.30837082	0.2662567	0.31810383
BC021785	1.24159542	-1.6363028	1.30822621	0.2662823	0.31810383
Tshz1	-0.1347941	6.00307208	1.3082039	0.26628625	0.31810383
Nap1l4	-0.1743689	5.59025974	1.30808835	0.26630672	0.31810383
Rrp8	-0.2422541	4.19096313	1.3079021	0.2663397	0.31810383
Abhd10	0.21572417	4.33952825	1.3077485	0.26636691	0.31810383
Dap	-0.275162	5.24106919	1.30767847	0.26637932	0.31810383
Klf5	-0.1852633	6.62739111	1.30753235	0.26640521	0.31810383
Apol6	0.69168895	-0.189787	1.30730772	0.26644501	0.31810383
Ache	-0.362115	2.62982925	1.30723634	0.26645766	0.31810383
Rtn1	-0.1302731	10.4495925	1.30656237	0.26657715	0.31820769
Dsp	-0.525892	1.66527481	1.30631292	0.26662139	0.31822172
Map6d1	-0.1985447	5.02829765	1.3059557	0.26668476	0.31824924
4930563F08I	1.10521148	-0.1062883	1.30571032	0.2667283	0.31824924
Wdr6	0.19124542	4.85577563	1.30556376	0.26675431	0.31824924
Aass	0.75738087	0.83297659	1.3054506	0.2667744	0.31824924
Ptpn22	-0.376085	2.35806896	1.30526023	0.26680819	0.31825079

Zmpste24	-0.2207589	5.50399362	1.304711	0.26690573	0.31831092
Ywhag	-0.1321643	10.2179561	1.30449679	0.26694378	0.31831092
Rsad2	0.57155868	1.25882069	1.30434079	0.2669715	0.31831092
Ispd	-0.2836021	3.40984449	1.30424461	0.26698859	0.31831092
Cd48	-0.813358	0.34257758	1.3035652	0.26710935	0.31841614
R3hcc1	-0.2471269	4.26773556	1.3032156	0.26717152	0.31841832
Lingo2	0.21244412	5.23399232	1.30311801	0.26718888	0.31841832
Kctd14	0.65436183	-0.0124869	1.30300657	0.2672087	0.31841832
Zfp326	0.15514354	5.58681216	1.30257209	0.267286	0.3184717
Agpat2	-0.5556033	1.41287467	1.30235293	0.26732501	0.31847943
Pramel5	1.28051798	-1.7145602	1.30190142	0.26740539	0.31853645
1700128F08I	0.94192222	-0.8445133	1.30109894	0.26754833	0.31866797
Ggps1	-0.2044236	6.34114367	1.30081666	0.26759864	0.31868914
Klf15	0.27029576	4.25353879	1.30055601	0.2676451	0.31870572
Ccdc17	-0.4826559	1.07721992	1.29971271	0.26779549	0.31884604
Palm	-0.1683379	4.89436797	1.2991342	0.26789872	0.31888349
Ilkap	-0.2055744	4.44686146	1.29902895	0.26791751	0.31888349
Erf	-0.2991158	3.33277045	1.29898924	0.2679246	0.31888349
Ncr1	-0.867705	-0.1231929	1.2983325	0.26804187	0.31895701
Rhbdf2	1.13548488	-1.7055015	1.29827861	0.2680515	0.31895701
Serinc1	0.14504417	8.80303299	1.29784119	0.26812965	0.31897597
Lgals8	-0.1677952	6.12087827	1.29782488	0.26813256	0.31897597
Ypel1	0.31093629	3.12855469	1.29675369	0.26832408	0.31916504
Pbk	-1.0184285	0.03747487	1.29566317	0.26851923	0.31935839
2810032G03	0.39500574	2.61698762	1.29531486	0.2685816	0.31936654
Cdk5rap3	-0.2814959	3.15904172	1.29526074	0.26859129	0.31936654
Ramp1	0.27728745	4.14651163	1.2949023	0.2686555	0.31940412
Cd86	0.8256448	-0.1300237	1.29313834	0.26897177	0.31974132
1700066M21	0.16264995	4.79759952	1.29255703	0.26907611	0.31977948
Gpr108	-0.3183726	3.10882504	1.29241885	0.26910092	0.31977948
Gm10778	0.25900493	3.91403951	1.29241388	0.26910181	0.31977948
Tmem120a	-0.8675073	0.1265171	1.290837	0.26938514	0.32007734
Amacr	-0.2415542	3.65200596	1.29046548	0.26945195	0.32011789
Pls3	0.11814035	7.5080234	1.29014854	0.26950896	0.32014679
Gm15880	1.62535915	-0.8696785	1.28800206	0.2698955	0.32056708
Tapt1	0.15591526	5.13774678	1.28765878	0.26995739	0.32060171
Grid1	0.30302706	2.92400738	1.28717797	0.2700441	0.32063638
Zbtb42	-0.6646797	0.59707261	1.28713391	0.27005205	0.32063638
Dhrs1	-0.1810672	5.70162098	1.286726	0.27012564	0.32068489
Cope	-0.2094352	4.71358402	1.28608064	0.27024213	0.32071226
Khdrbs2	0.3598605	1.68183347	1.28571663	0.27030787	0.32071226
Dnajc9	-0.1791535	5.91835945	1.28565588	0.27031884	0.32071226
Lipe	0.38266323	2.00487894	1.28524447	0.27039317	0.32071226

Tacr1	0.26820774	3.12697226	1.28500827	0.27043585	0.32071226
Pcdha5	0.58123473	0.8341099	1.28499598	0.27043807	0.32071226
Gde1	-0.1824708	5.24696666	1.284948	0.27044674	0.32071226
Eif3g	-0.2035276	5.42210614	1.28483992	0.27046628	0.32071226
Impa2	0.86160503	-0.1342386	1.28480381	0.2704728	0.32071226
Rps9	-0.2708125	6.45473868	1.28457213	0.27051469	0.32071226
Stk32b	-0.3351159	2.80468229	1.28449974	0.27052777	0.32071226
Colgalt2	0.39642748	1.4849493	1.28442332	0.27054159	0.32071226
Erich5	0.99875093	-0.7867221	1.28412384	0.27059575	0.32073153
Oas3	-0.8148564	0.6839544	1.28382279	0.27065021	0.32073153
Gm13826	-0.2726436	2.96121639	1.28379035	0.27065608	0.32073153
Ppie	-0.4086291	1.3935998	1.28349338	0.27070981	0.32075618
Zfp119b	0.57478128	0.7155587	1.28320121	0.27076269	0.32075618
Zc3h11a	0.12649133	6.83947605	1.28298758	0.27080137	0.32075618
Snrnp35	-0.3173532	3.60208092	1.28295172	0.27080786	0.32075618
Golga5	-0.21435	4.18905184	1.28197049	0.27098559	0.32092789
Bri3	-0.230839	4.3199298	1.2811024	0.27114296	0.32103841
Anapc5	0.13308658	6.32639075	1.28104278	0.27115378	0.32103841
Iars2	0.17594514	5.53871229	1.28091342	0.27117724	0.32103841
Stbd1	-0.2272714	4.08856523	1.2792095	0.27148653	0.32136167
A330035P11	-0.4651864	1.93256336	1.27889631	0.27154343	0.32136167
Ccnj	0.28245788	2.89291347	1.27886724	0.27154872	0.32136167
Gtdc1	0.19199725	6.26817212	1.27744722	0.27180691	0.32161034
Lhx9	1.15712837	-1.07217	1.27717886	0.27185574	0.32161034
Lancl3	0.33901393	3.95821715	1.27686516	0.27191284	0.32161034
Glipr2	-0.437695	4.05857951	1.27683537	0.27191826	0.32161034
Nova2	0.1993418	7.26096089	1.27680927	0.27192301	0.32161034
Adamts6	0.45157876	1.791435	1.27659193	0.27196258	0.32161831
Mboat2	0.30716807	4.15070648	1.27629259	0.27201709	0.32164394
Tollip	-0.1478802	6.59682381	1.27579655	0.27210745	0.32171195
Naf1	-0.2076083	4.34017599	1.27520352	0.27221553	0.32178797
Golga4	0.12667777	7.9148528	1.2750833	0.27223745	0.32178797
Bai2	0.26296126	4.57330281	1.27454484	0.27233564	0.32180199
Sepw1	-0.3466405	5.57534825	1.27448888	0.27234585	0.32180199
Kif5b	0.14377283	8.37481273	1.27447784	0.27234787	0.32180199
Mthfsd	0.42110685	1.83132044	1.27418084	0.27240205	0.32181733
Aar2	-0.298254	3.78509195	1.27404653	0.27242656	0.32181733
Hist1h4i	-0.761713	-0.2487635	1.2733597	0.27255194	0.32188993
Zfp27	0.24119062	4.00866786	1.27334983	0.27255374	0.32188993
Katnal2	1.03313615	-0.4732178	1.27246465	0.27271543	0.32204206
Prss35	0.59147868	0.77546363	1.27153277	0.2728858	0.3222044
Renbp	0.55474749	2.99842113	1.27098783	0.27298549	0.32225796
Gm5595	0.325773	2.83552425	1.27078753	0.27302215	0.32225796

Emc3	-0.2148518	5.9107534	1.27074539	0.27302986	0.32225796
Grik5	0.25259651	5.30620687	1.27055565	0.27306459	0.32226012
D1Ertd622e	-0.144618	5.78632366	1.26969852	0.27322155	0.32240651
Arih1	0.12240162	7.93081181	1.26948535	0.2732606	0.32240852
Serpinb6b	-0.3604704	5.19949551	1.26932997	0.27328907	0.32240852
Tpx2	-0.4330073	1.79765562	1.26860013	0.27342286	0.32252751
Urgcp	0.20992633	4.39058297	1.26737336	0.27364794	0.32272706
Flywch2	0.40770687	1.0455833	1.26731897	0.27365792	0.32272706
Fam219a	-0.2282497	3.871757	1.26710643	0.27369695	0.32273422
Ostf1	-0.1985055	5.90732594	1.26562776	0.27396863	0.32301569
Fam65a	0.21883767	7.33207848	1.26536104	0.27401767	0.32303464
Grb14	-0.2948516	3.48487065	1.26498155	0.27408747	0.32307804
Cacul1	-0.141898	6.85414444	1.2647641	0.27412748	0.32308632
Pou3f1	0.32614122	2.01277333	1.26449319	0.27417733	0.3231062
Itfg3	-0.3647237	1.93936477	1.26309914	0.27443405	0.32327058
Ssr2	-0.3986033	3.49506716	1.26309606	0.27443462	0.32327058
Trmt1l	0.15890323	4.88441546	1.2629722	0.27445745	0.32327058
Ube2q2	-0.1515754	6.36693787	1.26293321	0.27446463	0.32327058
9330102E08I	0.2515884	4.04629306	1.26257695	0.2745303	0.32327058
Tkt	0.22997211	4.83904856	1.26245972	0.27455191	0.32327058
Fbxo32	0.20617637	5.02528935	1.26236696	0.27456902	0.32327058
Cdc26	-0.3057014	4.58922415	1.26212877	0.27461294	0.32327058
Srrm3	-0.310901	2.95902668	1.26170955	0.27469027	0.32327058
Pth1r	0.58494368	1.07751311	1.26156608	0.27471674	0.32327058
Zfp119a	-0.4607418	1.13560126	1.26146067	0.27473619	0.32327058
Zfp871	0.15482384	9.06013318	1.26130081	0.2747657	0.32327058
Chn2	-0.1401686	5.29458178	1.2612316	0.27477847	0.32327058
Adamts2	0.29689446	3.97568395	1.26118333	0.27478738	0.32327058
Snx1	-0.1486299	5.72601252	1.26105072	0.27481186	0.32327058
Six1	-0.4069732	5.37716334	1.26061735	0.27489188	0.32332588
Cd99l2	0.20529914	5.75979085	1.2604208	0.27492818	0.32332975
5730507C01I	-0.285426	3.12580485	1.25981038	0.27504096	0.32339477
Prkca	0.20017661	8.12929589	1.25976419	0.2750495	0.32339477
Fam195a	-1.0733367	-1.2383846	1.25823941	0.2753315	0.3236836
Thsd7a	0.1655953	6.78848442	1.25807873	0.27536123	0.3236836
Oxr1	-0.1330986	8.62710565	1.2574385	0.27547977	0.32378408
Cep131	0.28758748	2.86455836	1.25686696	0.27558565	0.32384048
Soat2	0.38963745	1.76274704	1.25666437	0.27562319	0.32384048
Myt1	0.31095365	2.81373741	1.25655757	0.27564298	0.32384048
Pm20d1	-0.3751635	2.06002401	1.25646579	0.27566	0.32384048
Kcnj13	0.25101509	6.22314713	1.25593821	0.27575781	0.32391654
Gpr85	0.22273503	3.9713865	1.25537158	0.27586292	0.32400115
Gchfr	-0.8338006	0.21337602	1.25492326	0.27594612	0.32404409

Mmp14	-0.3930162	3.91261797	1.2548181	0.27596564	0.32404409
Sp4	0.13830291	5.9452757	1.25428545	0.27606454	0.32412137
2900092D14I	0.23313924	5.46346693	1.25323731	0.2762593	0.32431115
Stap2	-0.5170785	1.64210429	1.25268412	0.27636216	0.32436811
Cd82	-0.7390912	1.51331837	1.25256244	0.27638479	0.32436811
Myh2	0.9513166	0.20706863	1.25244219	0.27640716	0.32436811
Abhd2	0.15551563	6.17205916	1.2518139	0.27652407	0.32446643
Mfsd7c	0.56242699	1.40256348	1.25097745	0.27667982	0.3246103
Klhdc8b	-0.453423	2.2070895	1.25055802	0.27675796	0.32464145
Ikbkg	-0.1346757	5.88354635	1.25047918	0.27677266	0.32464145
Actr3b	0.1689583	4.85592865	1.25005966	0.27685085	0.32465673
Pde4c	0.7609651	-0.1185333	1.24975683	0.27690731	0.32465673
Zfp750	-0.5298893	2.36164251	1.24965193	0.27692687	0.32465673
Serpinb1a	0.46811836	2.21914967	1.24952172	0.27695116	0.32465673
Acad8	-0.1764443	4.24425617	1.24952042	0.2769514	0.32465673
Abca6	0.42936346	1.65908555	1.24928752	0.27699485	0.3246688
Ralgds	0.13451286	5.87226687	1.24887981	0.27707092	0.32471911
5730480H06I	0.99724288	0.11818548	1.24831599	0.27717617	0.3248036
Myrf	-0.2648614	3.72404626	1.24774826	0.2772822	0.32485351
D130017N08	0.23863815	3.83627959	1.24773279	0.27728509	0.32485351
Lrrc56	-0.7220479	0.36716053	1.24680156	0.27745913	0.32501853
Syvn1	0.22873627	4.83708926	1.24621878	0.27756812	0.32509366
Mrpl55	-0.2802622	3.61984209	1.24610376	0.27758964	0.32509366
Ctxn3	-0.2817184	7.22561565	1.24526278	0.27774703	0.32517207
Ap1g2	1.04617534	0.00113336	1.24518765	0.2777611	0.32517207
Slc9a2	-0.3974223	5.21957124	1.24517681	0.27776313	0.32517207
Hrsp12	-0.2269514	4.29304503	1.24503661	0.27778938	0.32517207
Tfap2b	-0.2247569	6.8869382	1.24438803	0.27791087	0.32524602
Cul2	0.20398001	5.27435675	1.24434489	0.27791896	0.32524602
Clstn1	0.16545097	8.08403304	1.24380217	0.27802068	0.32532619
Pls1	0.23225392	4.03669189	1.24327317	0.27811987	0.32537666
Traf4	-0.270288	2.52163121	1.24309078	0.27815408	0.32537666
2310069G16	0.52730143	1.60202341	1.2430408	0.27816346	0.32537666
Sp1	-0.134906	7.09172639	1.24205642	0.27834821	0.32555389
Cd163l1	-1.3930673	-1.1743733	1.24145191	0.27846175	0.32563342
Fam227b	0.56005471	0.68226812	1.24134044	0.27848269	0.32563342
C330024D21I	0.71163471	0.122273	1.24089164	0.27856703	0.32565519
BC037704	-0.5717427	1.27085945	1.24088753	0.2785678	0.32565519
Trappc3l	1.94652885	-2.1751454	1.23990618	0.27875234	0.32583204
Epb4.1l2	-0.1515323	7.5391637	1.23910014	0.27890404	0.32593512
Myo1h	0.86817385	-1.0196732	1.23908399	0.27890708	0.32593512
Slc2a2	0.92406861	-0.36637	1.23832817	0.27904942	0.32602614
Lrrc75a	-0.25167	3.25761938	1.23830544	0.27905371	0.32602614

Zfp536	0.14776389	4.61730141	1.2378763	0.27913457	0.32602614
Dnajc28	-0.1931316	3.87108935	1.23768152	0.27917128	0.32602614
Ecm1	0.27352835	3.89611697	1.23762962	0.27918107	0.32602614
Phactr4	-0.2451909	6.13209661	1.23761048	0.27918468	0.32602614
Rab26os	0.56978141	0.98218832	1.23681318	0.27933503	0.32616285
Mad2l1	-0.276022	4.07790964	1.23617697	0.27945509	0.32626414
Me2	-0.1783556	6.29207004	1.23566544	0.27955167	0.32629639
Ctsf	0.36337385	3.07466741	1.23539231	0.27960326	0.32629639
Dapk3	-0.4247302	1.76013072	1.23536283	0.27960882	0.32629639
Sesn2	-0.4150459	2.55196552	1.23532503	0.27961596	0.32629639
Tnip2	0.24535583	3.05890154	1.23512298	0.27965413	0.32630206
Cav1	-0.2749719	6.14573405	1.23410783	0.27984602	0.32641519
Parp11	0.28609933	3.76435497	1.23410525	0.27984651	0.32641519
Aff1	-0.16706	7.01573603	1.23396345	0.27987333	0.32641519
LOC381967	0.89189506	-0.4098663	1.23390501	0.27988438	0.32641519
Phf13	-0.2629197	3.5556069	1.23333503	0.27999222	0.32650208
Gm711	1.61765018	-1.6191454	1.23275479	0.28010205	0.32653025
Med29	-0.2025789	3.3883453	1.23274773	0.28010339	0.32653025
Ctnna3	1.03418127	-0.1813405	1.23267911	0.28011639	0.32653025
Sfswap	0.26804159	4.61788931	1.23232338	0.28018376	0.32655936
Casc1	0.85685147	-0.6692017	1.23219522	0.28020803	0.32655936
Zbtb3	1.1149576	-0.3909106	1.23189718	0.2802645	0.32658631
Stmn4	-0.2443954	6.7672225	1.23170928	0.28030011	0.32658895
Sdr9c7	1.6244519	-1.6837212	1.23108744	0.28041799	0.32662651
A230072C01	0.32061308	3.64761711	1.2310126	0.28043218	0.32662651
H60b	0.74341525	0.46473753	1.2310116	0.28043237	0.32662651
Phb	-0.2271624	3.81060631	1.23045542	0.28053787	0.32671054
Tmem192	-0.3335353	2.30239886	1.22978292	0.28066551	0.3267805
Plekhs1	0.85034828	-0.1763468	1.22961338	0.2806977	0.3267805
Scarna3b	1.25793132	-1.0973364	1.22961163	0.28069803	0.3267805
4632434I11R	-0.5216074	0.48499813	1.2292572	0.28076534	0.3267849
Alg3	-0.2615634	3.44367456	1.2291311	0.28078929	0.3267849
Ippk	0.20807316	3.8158183	1.22890061	0.28083308	0.3267849
Dnaja2	-0.1104464	7.78861547	1.22888917	0.28083525	0.3267849
Ddx3x	-0.1063126	8.72107366	1.2282173	0.28096295	0.32689465
Nyap1	0.28138931	2.96489102	1.22766726	0.28106755	0.32692608
Zswim4	0.30665123	2.37334394	1.22757121	0.28108582	0.32692608
Gm13889	-0.3759662	1.54006208	1.22752993	0.28109367	0.32692608
Ptprv	1.02995035	-0.9318184	1.22726946	0.28114323	0.32692608
Cct8l1	1.72243164	-1.1835794	1.22702485	0.28118978	0.32692608
BC004004	-0.2635646	5.27753471	1.22701727	0.28119122	0.32692608
S1pr2	-0.4957719	1.73585486	1.22684718	0.28122359	0.32692608
Pdf	-0.1888032	5.08672564	1.22594227	0.28139592	0.32708759

Eri3	-0.2067043	4.21132991	1.22538839	0.28150146	0.32717145
Arpc4	-0.2831496	5.34592983	1.22477932	0.28161758	0.32720046
Sema4a	0.28638148	3.73889398	1.22473812	0.28162544	0.32720046
Rnps1	-0.1245008	6.25131145	1.22459617	0.28165252	0.32720046
Dcdc2b	-0.2565256	3.77536492	1.22455672	0.28166004	0.32720046
Srgap1	0.28583213	5.14765201	1.22430063	0.28170889	0.32721841
Zfp560	-0.2407875	4.12025676	1.22381289	0.28180197	0.32728771
Foxk2	-0.110776	6.46145582	1.22359482	0.2818436	0.32729488
Efcab9	0.94561596	-0.5491644	1.22343053	0.28187497	0.32729488
Tchp	-0.3750199	2.32131566	1.22283372	0.28198895	0.32738842
Gnal	0.19497724	8.43372102	1.22224115	0.28210219	0.32740645
Gale	-0.8428854	-0.2678959	1.22223774	0.28210284	0.32740645
Kcnn1	0.39167222	1.94683146	1.22222773	0.28210475	0.32740645
Tmem186	0.30390941	2.68750825	1.22140392	0.28226228	0.32755047
Als2cr12	-0.8903373	-1.0692108	1.22047219	0.28244059	0.32771856
Rassf3	-0.1675729	5.51837471	1.21964997	0.28259806	0.32786244
4930563E18I	1.89427685	-1.5944678	1.21940947	0.28264415	0.32787707
Cnn1	-1.4147987	-0.3466643	1.21887088	0.28274738	0.32795799
Zfp516	0.17505338	5.28253629	1.21845107	0.28282789	0.32797377
Nat14	0.40083072	1.17082962	1.21845071	0.28282796	0.32797377
Pdk3	-0.1532707	5.03255319	1.21784136	0.28294487	0.32804764
Otud5	-0.1552769	5.85476213	1.2177696	0.28295864	0.32804764
Fcho1	0.32180656	2.7911638	1.21751862	0.28300681	0.32806466
Slc7a2	0.24357249	6.92220245	1.21702897	0.28310083	0.32813481
Ccdc160	-0.4194189	1.8488038	1.2159002	0.28331772	0.32834735
Osbpl11	0.19467287	4.16970836	1.21561104	0.28337331	0.32837293
D030056L22I	0.21759599	4.86622	1.21513326	0.28346521	0.32844057
Gm5535	1.71184372	-1.7777729	1.2147567	0.28353766	0.32848566
Pkdrej	0.73321348	0.50668192	1.21365465	0.28374985	0.32868885
Txnl4b	-0.2827728	3.25776878	1.21334106	0.28381027	0.32868885
Tsn	-0.1718411	6.2732336	1.2133232	0.28381371	0.32868885
Gm11992	-0.8824609	-0.7542537	1.2112765	0.28420847	0.32909487
Zfp184	0.30345242	2.16563795	1.21115721	0.2842315	0.32909487
Ly75	1.03535559	-0.5198203	1.21090605	0.28428	0.32911213
Ppp1r9b	0.14715841	6.66886217	1.21069158	0.28432142	0.32912118
Mesdc2	0.21358968	6.39054405	1.21012809	0.28443029	0.32919914
Mtg2	0.54791054	0.41438466	1.20999513	0.28445599	0.32919914
Xpa	-0.2116711	5.24649638	1.20960341	0.28453171	0.32924788
Cnot11	-0.1710151	4.38164142	1.20932312	0.28458591	0.3292717
Lamc1	0.16076165	6.02517539	1.20874827	0.28469711	0.32934914
Fermt1	-1.0913602	0.35393899	1.20858609	0.2847285	0.32934914
Arvcf	0.22022094	4.0585454	1.2084558	0.28475372	0.32934914
Pja2	0.1606254	8.93374676	1.20773561	0.28489315	0.3294434

Nudt3	-0.1850985	6.12764947	1.20768747	0.28490248	0.3294434
Mtmr1	0.16294599	5.14613504	1.20710226	0.28501585	0.32949276
Timm44	0.24438371	3.40871495	1.20699411	0.28503681	0.32949276
Morf4l2	-0.1622674	7.36995146	1.20694631	0.28504608	0.32949276
8430427H17I	0.13491393	6.53254101	1.20665682	0.28510219	0.32951874
Ppm1d	0.18270356	5.13616871	1.20554072	0.28531867	0.32973005
Gapt	-1.0074973	-0.1591237	1.20489247	0.28544451	0.32983656
Slc35e4	0.49755823	1.114314	1.20459838	0.28550163	0.3298414
Bcor	0.15296088	5.37892961	1.2043828	0.2855435	0.3298414
Gm8801	-0.6063001	-0.2353467	1.20435076	0.28554973	0.3298414
Tifa	-0.3018223	3.62939513	1.2039539	0.28562684	0.32989033
Spopl	0.22517426	3.63922646	1.20353321	0.28570861	0.32989033
3010001F23I	0.47095499	1.13950441	1.20348984	0.28571704	0.32989033
Sh3d21	-0.5585835	1.01929013	1.2034397	0.28572679	0.32989033
Cyp4a12b	-0.4397304	3.11657346	1.20305402	0.2858018	0.32991858
4921536K21I	0.84722131	0.51703229	1.2029675	0.28581862	0.32991858
U2surp	0.13050135	7.73191875	1.20270991	0.28586874	0.32993754
Igip	0.17237119	5.87735053	1.20175206	0.28605518	0.33007761
Vrk3	-0.3724051	2.8399183	1.20141008	0.28612179	0.33007761
Ctdsp2	-0.2034061	8.00564391	1.20123618	0.28615566	0.33007761
Galnt4	0.38448212	2.3252378	1.20115995	0.28617052	0.33007761
Tyw5	-0.2071995	4.84978132	1.20105266	0.28619142	0.33007761
Mxd4	-0.4134028	2.4850677	1.20104826	0.28619228	0.33007761
4930509J09F	0.91116397	-0.3195613	1.20085084	0.28623075	0.33008116
Dnm2	-0.1515798	5.25761461	1.20056483	0.2862865	0.33008116
Trafd1	0.25454081	5.08967615	1.20051375	0.28629646	0.33008116
Gpkow	-0.1476567	6.8147221	1.19939729	0.28651423	0.33025829
Pex19	0.15257786	6.27237414	1.19938043	0.28651752	0.33025829
Col4a3bp	0.12016338	7.25980068	1.19802167	0.28678285	0.33052523
Cdc42ep5	-0.4170814	2.49276455	1.19755453	0.28687415	0.33056342
D3Ertd254e	0.21476531	6.52483606	1.19742327	0.28689981	0.33056342
Cep104	0.17000856	4.65266436	1.19716912	0.2869495	0.33056342
Thap7	-0.328396	2.81002998	1.19716161	0.28695097	0.33056342
Cpox	-0.2124865	5.72688531	1.19690313	0.28700152	0.33058277
Gprc5a	0.85038607	0.9928613	1.19633637	0.2871124	0.33064014
H2-DMa	-0.5033836	2.02322094	1.19630351	0.28711884	0.33064014
Cdc42ep4	-0.2905811	6.00259451	1.19584702	0.28720819	0.33069465
Cenpv	-0.2432231	2.83724603	1.19571681	0.28723369	0.33069465
Twist1	-0.3320653	4.73255054	1.19491443	0.28739085	0.3308301
Ncf2	0.38234943	2.47627398	1.19466681	0.28743938	0.3308301
Snrpa1	-0.1764857	4.24823877	1.194506	0.2874709	0.3308301
Smpd5	-1.5504094	-2.011353	1.19442675	0.28748644	0.3308301
Rnf128	0.41482079	1.67447049	1.19423249	0.28752452	0.33083507

Ciita	0.41780967	1.62985973	1.19396595	0.28757679	0.33084111
Pnrc2	-0.2360819	7.27023271	1.19386124	0.28759733	0.33084111
Kcnd3os	-1.0427998	-1.0140629	1.19343006	0.28768192	0.33087788
Tmem91	-0.4716767	1.95762266	1.19335395	0.28769685	0.33087788
Pcdha2	0.69066602	0.19844634	1.19292573	0.2877809	0.33093569
Larp1b	-0.2278858	4.07434066	1.19234047	0.28789582	0.33102898
Slc30a2	0.31145098	2.67361515	1.19138131	0.2880843	0.33120682
Dnajc10	0.13601727	6.34267739	1.19114584	0.28813059	0.33120685
Abt1	-0.2549526	3.38623331	1.19103722	0.28815195	0.33120685
Lrrc41	-0.199877	4.24108537	1.19021002	0.28831468	0.33135501
Ilk	-0.2111889	6.00172694	1.18995742	0.2883644	0.33137327
Hsd3b1	1.93072951	-1.4652277	1.18957294	0.28844009	0.33140751
Hmha1	-0.4843711	1.25601341	1.1893241	0.2884891	0.33140751
Dnajb12	-0.1840369	3.90776884	1.18929063	0.28849569	0.33140751
E130307A14I	0.29124274	2.29238303	1.18901024	0.28855092	0.33143209
Leng9	0.46229461	1.11170176	1.1887754	0.2885972	0.33144637
Tgif1	-0.3050023	2.7617842	1.18842422	0.28866641	0.33148699
Wbscr27	0.16572237	4.58273635	1.18791971	0.28876588	0.33156235
E430018J23F	0.38282133	2.49952801	1.18750736	0.28884722	0.33158807
3110009E18I	-0.5864008	1.10227185	1.18746289	0.28885599	0.33158807
Xlr3b	-0.5181303	1.1616973	1.18653308	0.28903951	0.33175519
Psmd11	-0.1980842	5.94030805	1.18638219	0.28906931	0.33175519
Hspa4	0.16254896	8.78386391	1.18554149	0.2892354	0.33189401
Usp17la	0.64435573	0.44794742	1.18530939	0.28928128	0.33189401
Bet1	-0.243184	4.8666255	1.18520878	0.28930117	0.33189401
Inpp4a	0.19742168	6.53634804	1.18508222	0.28932619	0.33189401
Mxra7	-0.3332402	2.78163918	1.18483424	0.28937523	0.33189401
Eef2k	0.21076976	4.34373641	1.18474155	0.28939356	0.33189401
Gabarapl2	-0.1675411	6.7008753	1.18443027	0.28945514	0.33192577
Snai1	-0.3332523	2.27376053	1.18393564	0.28955302	0.33199914
Gpatch2	-0.1741036	4.12318121	1.18294475	0.28974924	0.33217972
Rpsa	-0.1813825	6.48231852	1.18279788	0.28977834	0.33217972
Pde6c	1.3995652	-0.9940223	1.18251264	0.28983486	0.33220564
Elovl5	0.24767801	6.21453658	1.18145076	0.29004542	0.33238298
Rmdn3	-0.1942596	3.79942027	1.18139014	0.29005744	0.33238298
Tigd2	-0.1896693	4.43055657	1.18071489	0.29019145	0.33249765
Wnt4	-0.1710845	5.58221254	1.18019904	0.29029388	0.33257611
Surf2	-0.2705524	5.00252869	1.17964599	0.29040375	0.33266307
02-Mar	-0.2625161	5.33229017	1.17882008	0.29056794	0.33281223
5930438M14	-0.9233315	-0.5977789	1.17825009	0.29068132	0.3329022
Uba1	0.1277807	7.91621844	1.17808353	0.29071446	0.3329022
B3gnt5	0.85409296	0.09588602	1.1778928	0.29075242	0.33290675
Rfx8	1.40707314	-1.9345285	1.17756727	0.29081722	0.33294202

Imp3	-0.23854	4.80297023	1.17737544	0.29085542	0.33294684
Tmem57	0.12643278	6.12425202	1.17681226	0.29096759	0.33300046
Iba57	-0.4800023	0.816488	1.17669382	0.29099119	0.33300046
Slc25a23	-0.1517109	8.69692611	1.1766283	0.29100424	0.33300046
Smim5	0.8124002	-0.0767975	1.17639687	0.29105037	0.33301433
Cep170b	0.16251484	7.77688155	1.17590336	0.29114875	0.33303066
Siglece	-0.9681406	-0.5955054	1.17589643	0.29115013	0.33303066
Zfp212	0.28843587	2.92232215	1.17576098	0.29117714	0.33303066
Ube2o	0.23598459	4.91134424	1.1756432	0.29120063	0.33303066
Fam214b	-0.2148202	4.40234151	1.1754	0.29124914	0.33304725
Wdtc1	0.17306672	5.20720695	1.17448605	0.29143155	0.33315795
BC017158	-0.2602322	3.34202633	1.17446021	0.2914367	0.33315795
Tma16	-0.2462136	3.76072172	1.17440372	0.29144798	0.33315795
Zfp81	-0.204689	4.91312818	1.17420653	0.29148736	0.33316409
Fermt2	-0.1110411	7.13789339	1.17308354	0.29171175	0.33333632
Zfp455	-0.2615012	3.18800045	1.17302545	0.29172336	0.33333632
Bcorl1	0.16698739	4.54898799	1.17294154	0.29174014	0.33333632
Barhl2	-0.9126848	-0.0727471	1.17242879	0.29184269	0.3334146
Exosc4	-0.2618709	3.40495254	1.17209001	0.29191047	0.33343245
Ddx43	1.29683902	-2.0649255	1.17182496	0.29196351	0.33343245
Trib2	-0.1810148	6.19818176	1.17175519	0.29197748	0.33343245
Ccna1	-1.010707	-1.0159597	1.17164977	0.29199858	0.33343245
Sostdc1	-0.7520789	0.73496236	1.17146402	0.29203577	0.33343245
Tmed2	0.19362858	5.71179949	1.17127943	0.29207273	0.33343245
Cgref1	0.31842706	2.1814467	1.17099046	0.2921306	0.33343245
Eef1g	-0.2029926	7.72429687	1.17085908	0.29215692	0.33343245
5430416N02	0.37306735	2.04785831	1.17074211	0.29218035	0.33343245
Api5	-0.1346268	6.94753257	1.17065049	0.29219871	0.33343245
Trmt10a	-0.2646861	2.92068667	1.17047402	0.29223407	0.33343395
Raph1	0.23330994	7.73997908	1.17030184	0.29226858	0.33343449
Cdhr1	0.29189173	2.46622717	1.16998728	0.29233164	0.33346759
Usp5	-0.2100699	4.66250852	1.16931749	0.29246596	0.33358197
Armc9	0.26028291	3.36893747	1.16889981	0.29254977	0.33363871
Pacsin2	-0.141918	6.2208514	1.16851326	0.29262737	0.33368835
Napg	-0.1310251	6.73051535	1.16764457	0.29280184	0.33384844
Dennd4b	0.28626803	3.90586378	1.16707258	0.29291679	0.33385862
1700028E10l	0.90887233	-0.7948921	1.16703578	0.29292419	0.33385862
Ccdc125	-0.3433181	2.84858073	1.16675527	0.29298059	0.33385862
Usf1	-0.3187734	2.76170238	1.16668039	0.29299565	0.33385862
Gtf2i	-0.1224931	7.28734558	1.16653413	0.29302507	0.33385862
Zer1	0.19129557	4.95266359	1.16651909	0.29302809	0.33385862
Tnfrsf11a	0.31765474	2.63779918	1.16610204	0.29311199	0.33385862
Taf5	-0.2662761	3.93774759	1.16609234	0.29311394	0.33385862

Sh2b1	-0.187738	4.06126179	1.16607458	0.29311751	0.33385862
Lhx2	-0.1720007	5.46482425	1.16586326	0.29316004	0.33386823
Slc9a1	0.19636519	5.26187894	1.16449326	0.29343594	0.33414359
Xlr4c	-1.3391011	-1.3636002	1.16404302	0.29352668	0.33420808
Tacc1	0.11399766	7.88929376	1.16382269	0.29357111	0.33421981
Kctd9	-0.1762413	4.95379289	1.16344225	0.29364783	0.3342683
Spink10	-0.9698321	-0.9899243	1.16296683	0.29374375	0.33433863
Uba2	0.13670886	5.79945367	1.16269032	0.29379955	0.3343633
Npas4	0.75710022	3.17988758	1.1621893	0.29390071	0.33440285
Zfp407	0.19028089	4.98637286	1.16218001	0.29390258	0.33440285
Whamm	0.35136088	3.09783913	1.16137057	0.2940661	0.33455004
Mzf1	0.57122351	0.2685717	1.16111349	0.29411807	0.33455104
AI197445	0.65537702	0.52961767	1.16097243	0.29414658	0.33455104
Zc3h14	-0.1363812	6.41769269	1.16085936	0.29416944	0.33455104
Lzts1	0.35049718	2.77788599	1.16054648	0.29423271	0.33458415
Itgb1bp1	-0.1658636	4.68775085	1.15950177	0.29444411	0.33478567
Erbb3	-0.9106592	-0.3009485	1.15932917	0.29447905	0.33478654
Ppil3	-0.2799489	3.79873045	1.15808444	0.29473123	0.33497183
Carns1	0.48456153	2.38018108	1.15805763	0.29473666	0.33497183
Frmd8	-0.2047146	3.95943943	1.15772358	0.29480439	0.33497183
Mprip	0.14434138	7.49135185	1.1575377	0.29484209	0.33497183
Sema7a	-0.1709769	5.34240719	1.15747183	0.29485545	0.33497183
Shpk	-0.6979208	0.61399241	1.15745516	0.29485883	0.33497183
Arntl2	0.40005029	2.04468903	1.15734382	0.29488141	0.33497183
Gm4432	0.62263889	-0.0696758	1.15704097	0.29494285	0.33500278
Xcl1	1.13314407	-0.5193357	1.15681014	0.2949897	0.33501713
Zfp317	-0.216908	4.05380451	1.15660504	0.29503133	0.33502557
Pcif1	0.18046017	3.96278739	1.15637822	0.29507737	0.33503902
Trim47	-0.51606	2.25336164	1.15606292	0.2951414	0.33505205
Dpagt1	-0.30551	3.3175558	1.15598483	0.29515726	0.33505205
Smarcd1	-0.1455813	5.84974477	1.1549604	0.29536543	0.3352495
F13a1	0.5148425	1.59449827	1.15445542	0.29546811	0.3353272
Snhg12	0.24097382	3.49717348	1.15351107	0.29566027	0.33550641
Gm6537	1.24880514	-1.9734939	1.15305725	0.29575268	0.33557239
Actr6	-0.2738642	3.63828272	1.15227678	0.29591169	0.33571392
Slc25a14	0.21434898	4.89905277	1.1516771	0.29603394	0.33581373
Pou5f1	1.449388	-2.4473957	1.15122047	0.29612708	0.33584728
Polr2f	-0.3184895	2.1390759	1.15104401	0.29616308	0.33584728
Pdpk1	0.11270708	7.65899046	1.15065064	0.29624336	0.33584728
Galnt10	-0.4313919	1.9081187	1.15058377	0.29625701	0.33584728
Lrrn4	-1.1328574	-0.7844833	1.1505836	0.29625705	0.33584728
Gemin7	-0.2843146	3.2202262	1.15052384	0.29626925	0.33584728
Ap2a2	0.12682381	6.88755799	1.14976224	0.29642477	0.33598471

Tmem256	-0.3200505	2.86839731	1.14958921	0.29646012	0.3359859
Ddx18	-0.1516161	5.05537199	1.1486029	0.29666174	0.3361755
Prickle1	0.19418491	5.67967014	1.14783007	0.29681985	0.33625457
Lrrc4	0.17247346	5.96828391	1.14776135	0.29683391	0.33625457
Tbcd	0.21789701	3.95613517	1.14775846	0.2968345	0.33625457
Sec23ip	0.15881297	5.45952368	1.14739965	0.29690795	0.33629888
H2-Q1	-0.3120457	6.0697146	1.14670748	0.29704971	0.33642054
Krt20	0.31018945	3.87469395	1.14601155	0.29719233	0.33653689
Elovl7	0.21052148	4.0967768	1.14587094	0.29722116	0.33653689
Mapkbp1	0.19313579	5.2659979	1.14545279	0.29730691	0.33659508
9630001P10I	0.60446322	0.21611996	1.14443834	0.29751508	0.33679183
Cyp26b1	0.2930783	5.38657079	1.14369869	0.29766698	0.3368866
Clu	0.26453329	7.27932893	1.14369578	0.29766758	0.3368866
Wdfy1	0.19640515	4.67529189	1.14331739	0.29774533	0.3369079
Pxmp4	-0.2204093	3.31185349	1.14314023	0.29778174	0.3369079
Hmgcl	-0.2776236	2.57252918	1.14306565	0.29779707	0.3369079
Sft2d2	-0.2238154	6.40335609	1.14293478	0.29782398	0.3369079
Irak1bp1	-0.1871944	5.9075469	1.1423264	0.29794909	0.33701051
Il7	1.34173976	-0.8826703	1.14171885	0.2980741	0.33705359
Ercc1	-0.3423049	2.21367991	1.14170598	0.29807675	0.33705359
Rasd1	0.65010313	0.3412436	1.14163965	0.29809041	0.33705359
Tpgs1	-0.3436454	3.14348779	1.14131006	0.29815826	0.33706678
Tsnax	-0.1270973	7.46016546	1.14124871	0.29817089	0.33706678
Eya1	-0.1941729	7.18135044	1.1409547	0.29823144	0.33709633
Ddx1	-0.1299583	6.99049267	1.14037254	0.29835138	0.33719299
Rpa3	-0.3047202	3.1345464	1.13961087	0.29850841	0.33730307
Scn1b	-0.1349763	5.60432202	1.13956605	0.29851765	0.33730307
Rph3a	0.17516218	8.88862341	1.13926067	0.29858064	0.33733533
Myo18a	0.16767622	6.30847376	1.13890038	0.29865498	0.3373804
Mrpl20	-0.260564	4.0552747	1.13815378	0.29880911	0.33747658
C8g	-0.7596801	0.37226714	1.1380976	0.29882071	0.33747658
Gal3st4	0.4636547	1.56458374	1.13798735	0.29884348	0.33747658
Rlim	0.12348625	7.70261019	1.13773593	0.29889542	0.33749632
Pmf1	-0.4394727	3.35773183	1.13730831	0.29898378	0.33755719
Lum	-0.2958885	5.256301	1.13674548	0.29910013	0.33764106
Daf2	0.47290513	0.64992593	1.13660394	0.2991294	0.33764106
BC030867	1.17797257	-1.5114321	1.13644886	0.29916148	0.33764106
Zfp647	-0.3879954	1.3500515	1.13596769	0.29926103	0.33767456
Ccdc126	0.34216005	2.28289532	1.1358864	0.29927785	0.33767456
Pcdhgb8	0.46365815	1.14866749	1.13580556	0.29929458	0.33767456
Arid3a	-0.3573843	2.96326975	1.13545572	0.299367	0.33771737
Strn4	0.1510542	5.44574804	1.13464921	0.29953404	0.33785398
Pvrl4	-0.4078136	1.55218341	1.13453799	0.29955708	0.33785398

Ndst4	0.33527748	3.24257886	1.13405181	0.29965785	0.33792872
Dcaf7	0.12621171	7.9744213	1.13361932	0.29974752	0.33799094
Acat1	-0.1460459	7.12008456	1.13256386	0.29996653	0.33812883
Gramd4	-0.2018996	4.03964658	1.13189697	0.30010501	0.33812883
Nmrk1	0.19329069	4.76005865	1.13180129	0.30012489	0.33812883
Tox4	-0.1369854	5.999696	1.1317641	0.30013261	0.33812883
4833411C07I	1.36351414	-1.3355056	1.13168188	0.3001497	0.33812883
Mt3	0.84231531	-1.0707301	1.13157339	0.30017224	0.33812883
Fdft1	-0.1440873	6.27779223	1.13156467	0.30017405	0.33812883
Tmem183a	-0.157058	5.63463001	1.1314546	0.30019692	0.33812883
Slc22a4	-0.2411016	3.02269968	1.13128763	0.30023163	0.33812883
Rps6kb2	0.30741839	2.55862419	1.1312369	0.30024217	0.33812883
Gm11128	0.86657616	-0.8059278	1.13120152	0.30024952	0.33812883
Mapk7	0.2733657	2.64550853	1.13000651	0.30049806	0.33836982
Zfp593	0.50532396	0.84499048	1.12912597	0.30068137	0.33853732
Slc45a3	0.84350444	0.25526345	1.12863914	0.30078279	0.33860275
Nop14	0.15417137	4.94818265	1.12846286	0.30081952	0.33860275
Dmtf1	-0.1411776	6.44770029	1.12819995	0.30087432	0.33860275
Fam78b	-0.1820966	6.81848028	1.12809245	0.30089673	0.33860275
Cdc6	-0.6220885	0.74257693	1.12796359	0.30092359	0.33860275
Plac8	1.45637412	-1.2201908	1.12785185	0.30094689	0.33860275
Nubp1	0.34094344	2.29977372	1.12739596	0.30104197	0.33867082
Ccdc63	1.52407187	-2.0957611	1.12669161	0.30118896	0.33874531
C4b	-0.3014677	2.77120039	1.12654435	0.3012197	0.33874531
Gpr50	1.62274182	-1.5457712	1.12645593	0.30123816	0.33874531
Pdcl	-0.1794217	5.15656467	1.12641593	0.30124651	0.33874531
Oser1	-0.2364013	4.12300263	1.1262364	0.301284	0.33874857
Zfp182	0.16559349	4.90802302	1.12578977	0.30137729	0.33881457
Plscr1	-0.2778065	4.17838695	1.12535917	0.30146727	0.33881754
Sertad4	-0.2830175	5.75548275	1.12520098	0.30150034	0.33881754
Lrrc58	-0.177757	10.1415831	1.12517054	0.3015067	0.33881754
Ubxn7	0.11001855	7.31127239	1.12511513	0.30151828	0.33881754
Triap1	-0.3958153	3.20084066	1.12479595	0.30158502	0.33885365
Pcp4l1	-0.2067041	6.03176788	1.1242179	0.30170593	0.33895063
Ganc	-0.2151522	4.43255697	1.12304029	0.30195246	0.33916108
Ptgis	-0.3508798	2.96369474	1.12299236	0.3019625	0.33916108
Med20	-0.1712384	5.04033689	1.12227284	0.30211328	0.33929152
Asphd2	0.26397835	2.68261618	1.12157195	0.30226024	0.33941765
Rnf115	0.15044789	5.90660914	1.12139845	0.30229664	0.33941961
Lyn	0.30548814	3.40651406	1.12095854	0.30238895	0.33948434
Cwf19l2	0.14319514	5.62558779	1.12062699	0.30245854	0.33952356
Ehd2	-0.2989183	4.92766503	1.12013709	0.30256141	0.33960012
Trub2	-0.2880163	3.0142733	1.11966985	0.30265957	0.33964608

Cbll1	-0.1752012	6.17443958	1.11961213	0.3026717	0.33964608
Cacna2d4	-0.5077959	1.19254656	1.11927298	0.30274298	0.33967389
Rcbtb2	-0.2028579	4.36717018	1.11916422	0.30276585	0.33967389
Rbm38	0.85308298	0.8031753	1.11882904	0.30283632	0.33971405
Zfp672	0.17862982	4.76502454	1.11850422	0.30290464	0.33975178
Bcmo1	1.32138555	-1.5031222	1.11810348	0.30298896	0.33979225
Apool	0.42870207	2.40628015	1.11800301	0.3030101	0.33979225
Pigf	0.39891057	1.56171039	1.11719461	0.30318031	0.3399442
Tln2	0.15048205	6.80998264	1.11664041	0.30329707	0.34003619
Tpra1	0.36642309	2.47467841	1.11632391	0.30336377	0.34007206
Noc3l	-0.1812738	4.93042067	1.11591437	0.30345012	0.34010575
Fam214a	0.17359132	5.23806352	1.11585201	0.30346327	0.34010575
Paf1	0.16169	5.95108344	1.11534808	0.30356957	0.34018596
Sdf2	-0.2287319	4.44281333	1.11491765	0.3036604	0.34024883
Nacc1	0.12396088	6.99277199	1.1144606	0.3037569	0.34027917
Wdr60	0.20565909	5.41442641	1.11425956	0.30379935	0.34027917
Acan	1.07363121	-0.8003149	1.11411197	0.30383053	0.34027917
3110043O21	-0.2659579	3.97870213	1.11405806	0.30384192	0.34027917
Lamtor2	-0.2788576	4.07058306	1.11382563	0.30389102	0.34027917
Ptp4a2	-0.1866348	9.07797715	1.11378808	0.30389896	0.34027917
Arhgap12	0.13188431	5.65828219	1.1134147	0.30397787	0.34027917
Qk	0.12895458	9.3229642	1.11340523	0.30397987	0.34027917
Pphln1	-0.1307968	7.20904503	1.11330938	0.30400013	0.34027917
Mycl	-0.3112912	2.03148345	1.11308222	0.30404816	0.34029404
Itpkb	-0.2656434	3.35426576	1.11279855	0.30410815	0.34032229
Snx17	-0.2280725	4.55007244	1.11232851	0.30420758	0.34038252
A2m	0.43344332	1.67001198	1.11215706	0.30424386	0.34038252
Tox	0.19880862	5.74876714	1.11205146	0.30426621	0.34038252
Ddx54	-0.2251228	3.98407135	1.11181261	0.30431677	0.3404002
Grid2ip	-0.551927	1.10690443	1.11100929	0.3044869	0.34051687
Wfdc15b	1.21121972	-2.0026989	1.1109695	0.30449533	0.34051687
C230024C17I	-1.5240299	-1.9188972	1.11069165	0.3045542	0.34051687
Smim22	-1.0850882	-0.4654635	1.11066372	0.30456012	0.34051687
Usp28	0.2874152	3.5545743	1.10975351	0.3047531	0.34060898
Slitrk4	0.18376913	6.29654105	1.10966813	0.30477121	0.34060898
Hsd17b7	0.15714786	5.00823565	1.10963047	0.3047792	0.34060898
Iglon5	0.24603749	3.56600338	1.10952328	0.30480194	0.34060898
Cyp4b1	-0.9967416	-1.0150473	1.10945529	0.30481637	0.34060898
F630042J09F	1.07283709	-0.9915842	1.10860022	0.30499786	0.34073221
Casp6	0.32019295	2.37330272	1.10854724	0.30500911	0.34073221
Dnaic2	-1.3215511	-1.540072	1.10840421	0.30503949	0.34073221
Tra2b	-0.1996103	6.4036067	1.10807742	0.3051089	0.34073221
Cage1	-0.5162143	1.19686726	1.1079897	0.30512754	0.34073221

Zfp862-ps	-0.3021603	2.31865458	1.10795287	0.30513536	0.34073221
Ggn	-0.8569024	-0.0259875	1.10718073	0.30529948	0.34083986
Fam46a	0.1517021	5.75775238	1.10717192	0.30530136	0.34083986
Nab2	-0.2199241	5.10920248	1.10569218	0.30561622	0.34115249
Lfng	0.74074904	-0.3731663	1.10501368	0.30576074	0.34126881
Gm10941	-1.0321639	-1.0465899	1.10487588	0.3057901	0.34126881
Cdh20	0.26746551	3.92881565	1.10439333	0.30589295	0.3413447
Prkce	0.14796439	8.83402021	1.10364895	0.30605171	0.34147459
Kdelr2	0.26405974	5.18839106	1.10352071	0.30607907	0.34147459
Cd97	-0.2920923	3.10544041	1.10246628	0.30630416	0.34163213
St8sia1	0.17608402	6.67540381	1.10236682	0.30632541	0.34163213
Mrpl28	-0.3194972	3.36146762	1.1023518	0.30632862	0.34163213
Spo11	-0.9966359	0.07250482	1.10220586	0.30635979	0.34163213
Cirh1a	0.15198057	4.83870112	1.10196312	0.30641166	0.34165107
Spats1	-0.5679687	0.64257121	1.10162177	0.30648461	0.34169352
Hnrnpk	-0.1260601	7.92294978	1.10082207	0.30665562	0.34183534
Axin1	-0.2438496	3.37018017	1.10070057	0.30668161	0.34183534
Tmem161a	0.28881933	3.19158888	1.10015117	0.30679918	0.34192748
Nr2c2ap	-0.3171374	2.05884682	1.0998012	0.30687411	0.34197207
Spryd3	0.26798085	4.21771774	1.09961329	0.30691435	0.34197801
Unc93b1	0.46009753	1.36127615	1.09933069	0.30697488	0.34200655
Exosc8	-0.1873865	3.93105156	1.09911623	0.30702082	0.34201884
Mfge8	0.31879711	3.1495473	1.09887781	0.30707191	0.34203686
Fbxl20	0.12365704	6.62663552	1.09852552	0.30714743	0.34205195
4921534H16	1.45605278	0.0222078	1.09818318	0.30722083	0.34205195
Etnk2	-0.3164708	2.60113181	1.09806156	0.30724692	0.34205195
Rbks	-0.617559	1.44680274	1.09788666	0.30728443	0.34205195
Cd79a	-0.4426752	2.13856601	1.09785067	0.30729215	0.34205195
Fam118b	-0.2178647	5.23855314	1.09769308	0.30732596	0.34205195
Brsk2	0.17753204	5.66464878	1.09753816	0.3073592	0.34205195
9930014A18	-0.5581577	0.8415086	1.09751198	0.30736482	0.34205195
Tm6sf1	-0.349043	1.87347446	1.09721224	0.30742915	0.34208468
Prdm12	0.99848499	-0.1195421	1.09638877	0.30760599	0.34224257
Zfyve9	-0.2336423	3.85483059	1.09551471	0.30779383	0.34240078
Ell	0.19014166	3.49996243	1.09540184	0.3078181	0.34240078
Eid2	-0.1503488	5.24405361	1.09490562	0.30792483	0.34243094
4930426D05	1.09423294	-0.7480271	1.09484772	0.30793728	0.34243094
Kirrel	0.27372458	4.57305455	1.09470648	0.30796767	0.34243094
Lgals12	0.78956872	-0.2240564	1.09462572	0.30798505	0.34243094
Yes1	0.17550104	4.78123407	1.09418541	0.30807982	0.34249743
Mrps6	-0.2283235	4.25592822	1.09396512	0.30812725	0.34251129
Hexb	0.17359537	4.74106074	1.0932435	0.30828268	0.34264518
Tmem50a	-0.189486	5.6736444	1.09278496	0.3083815	0.34271613

Lhcgr	-1.2451941	-1.0558066	1.09213013	0.30852271	0.34283416
Vamp5	0.20735019	5.48863501	1.09168912	0.30861785	0.34290099
Shfm1	-0.1909145	4.75682789	1.09128902	0.30870421	0.34295803
1700034I23R	0.88635234	-0.3781941	1.09085633	0.30879763	0.34299986
Serpine3	0.37829738	1.95541913	1.09079033	0.30881188	0.34299986
Ube4b	0.12527719	7.77348714	1.0903629	0.30890422	0.34304987
Wdr92	-0.1279023	5.3582105	1.09001964	0.3089784	0.34304987
Usp46	0.12262795	7.22034114	1.08975838	0.30903487	0.34304987
4921511C10I	1.5246268	-1.6615581	1.08967161	0.30905363	0.34304987
C920006O11	0.36903361	1.74888489	1.08965059	0.30905817	0.34304987
Arhgap5	0.14351727	8.64702615	1.08953764	0.3090826	0.34304987
Il18	0.16807192	4.1713392	1.08944217	0.30910324	0.34304987
Cdk12	0.13032368	7.26615932	1.08928568	0.30913709	0.34304987
Dak	-0.3656981	2.76656113	1.08739138	0.30954717	0.34346603
Ccnk	-0.1297264	6.38171229	1.08713742	0.3096022	0.34348818
Palb2	0.55972266	0.75081152	1.08692426	0.30964841	0.34350054
Galc	0.2525677	3.76757953	1.08655875	0.30972766	0.34351806
Wdr44	0.16480377	4.89162934	1.08650018	0.30974036	0.34351806
Wdr11	0.20107765	5.39380015	1.0863662	0.30976941	0.34351806
Obscn	0.56527978	0.39962601	1.08596395	0.30985668	0.34354246
Egln3	-0.2196695	7.45130752	1.08594146	0.30986156	0.34354246
Gm4349	-0.5700601	0.61440801	1.08547762	0.30996222	0.34361517
BC052688	0.3232826	2.15406068	1.0849731	0.31007177	0.34369771
Cul4b	0.12420686	7.03109806	1.08468782	0.31013373	0.3437275
Dcaf15	-0.4288828	1.33317004	1.08390055	0.31030483	0.34387822
Tuba1a	-0.113808	9.67220925	1.0837041	0.31034754	0.34388299
Begain	0.24657387	3.44830392	1.08355786	0.31037934	0.34388299
Birc2	-0.1429499	5.4885812	1.08328838	0.31043796	0.34390903
Nat2	-0.3308997	3.0417629	1.08285881	0.31053142	0.34394489
Bicd2	-0.1455192	6.4485102	1.08266203	0.31057425	0.34394489
Ncf1	-0.2696506	2.38759849	1.08265552	0.31057566	0.34394489
Gm1966	0.29894183	2.95962697	1.08157392	0.31081122	0.34412338
Abhd14b	-0.225688	5.31205164	1.08144641	0.310839	0.34412338
Pknox1	-0.1937475	3.85227558	1.08133907	0.31086239	0.34412338
Plod2	0.30881509	3.8469129	1.08127036	0.31087737	0.34412338
2310002D06I	-1.346545	-1.6989495	1.08100473	0.31093527	0.34414859
Kif3a	0.17509617	8.10931872	1.08070993	0.31099955	0.34418084
Tank	-0.1999354	6.08627914	1.0800872	0.31113539	0.34427232
Grem1	0.47452823	0.83619109	1.08000879	0.3111525	0.34427232
Gltp	-0.2202631	5.52220233	1.07946675	0.31127081	0.34433362
6330419J24R	-0.2751863	3.50590208	1.0794329	0.31127821	0.34433362
Pip4k2c	-0.1597378	6.1114067	1.07905371	0.31136101	0.34434455
Zdhhc7	-0.2790349	3.13165854	1.07900267	0.31137216	0.34434455

Sox1	0.20601252	4.72724917	1.07876724	0.31142359	0.34434455
Kcnc2	0.19409762	5.91234506	1.07850599	0.31148068	0.34434455
Epas1	-0.1339039	8.09369916	1.07850372	0.31148117	0.34434455
Clec16a	0.15445902	6.3092255	1.07842207	0.31149902	0.34434455
4931440P22l	1.00841568	-1.4315636	1.07816624	0.31155494	0.34436751
Isoc1	-0.1825202	5.90672511	1.07702402	0.31180477	0.34460477
Tarsl2	0.22316625	4.5568024	1.07650945	0.31191741	0.34462785
Tmem200b	-0.7630394	-0.2351335	1.07645173	0.31193005	0.34462785
Prpf40a	-0.146669	6.39276281	1.07644643	0.31193121	0.34462785
Mas1	0.30783109	2.57691821	1.07545914	0.3121475	0.34482792
Rin2	-0.1669184	6.55233572	1.07298121	0.31269125	0.34535528
Asap3	0.33625047	2.87495429	1.07286494	0.3127168	0.34535528
Kctd4	0.22826794	5.06433096	1.07273596	0.31274514	0.34535528
Adh7	-0.525985	1.41363985	1.07255498	0.31278491	0.34535528
Zfp677	-0.3434277	3.72824083	1.07248107	0.31280116	0.34535528
Sun1	0.18940055	4.71651478	1.07215696	0.31287241	0.34539501
Pla2g4d	1.88786129	-2.0625272	1.08137944	0.3130305	0.34549736
Noc4l	-0.2458238	3.21503517	1.07141474	0.31303567	0.34549736
Cartpt	-0.4025961	1.40408598	1.07118295	0.31308667	0.34551175
Pi16	0.96494435	-0.6008267	1.07103496	0.31311925	0.34551175
Lct	0.82895383	-0.2845821	1.07083825	0.31316255	0.3455206
Ruvbl1	-0.194882	4.59810199	1.07006059	0.31333382	0.34566785
Asf1a	-0.1358496	5.03613629	1.0699119	0.31336658	0.34566785
Stat3	-0.1234177	5.61785731	1.06913269	0.31353834	0.34581837
Kif1c	-0.2181301	5.6443033	1.06877835	0.3136165	0.34586563
Grhpr	-0.3702897	2.24002637	1.0684372	0.31369176	0.34590969
Gm16701	0.60360168	0.69909148	1.06768198	0.31385848	0.34605457
Egfem1	0.4332517	1.82446598	1.06727393	0.3139486	0.34611498
Zdhhc13	-0.2438924	4.23415247	1.06666903	0.31408227	0.34622338
Epc2	0.1142932	7.43690002	1.06631543	0.31416045	0.34627059
Mfap3	0.16288086	4.95890308	1.06582161	0.31426967	0.34634507
Rslcan18	-0.3107682	2.38677572	1.06560215	0.31431822	0.34634507
Wibg	-0.4439569	2.09290631	1.06553039	0.3143341	0.34634507
Zfp707	-0.4729729	1.37092506	1.06485609	0.31448337	0.34642567
Nt5c1a	-0.5125991	0.79955439	1.0644902	0.3145644	0.34642567
Abhd17a	-0.2281332	3.66080097	1.06446724	0.31456948	0.34642567
Kif23	0.43609282	0.84121658	1.06443385	0.31457688	0.34642567
Krt12	-0.1795045	4.21129125	1.06427361	0.31461238	0.34642567
Egfl7	-0.2936603	2.41048046	1.06424173	0.31461944	0.34642567
Alg13	0.32519692	3.17797422	1.06407443	0.31465651	0.34642754
Lmf1	0.29023225	2.93702703	1.0634367	0.31479788	0.34654423
Arl4c	-0.1851097	5.17834358	1.0628118	0.31493649	0.34662831
Ndrg4	0.13840218	10.805371	1.062718	0.3149573	0.34662831

Rsbn1	0.1344029	6.12056606	1.06248548	0.3150089	0.34662831
Adamts12	0.34076071	2.48891366	1.06230163	0.3150497	0.34662831
Pnlip	1.20614169	-1.7420603	1.06220168	0.31507189	0.34662831
Cntln	-0.1369703	6.57104022	1.06213551	0.31508658	0.34662831
Brd2	-0.1091076	7.92473855	1.0613674	0.31525718	0.34673035
Mir6336	1.08367118	-1.5248579	1.0611888	0.31529686	0.34673035
2810047C21I	-0.4090408	1.87145865	1.0611335	0.31530915	0.34673035
P4htm	0.27110437	2.39224684	1.06073271	0.31539824	0.34673035
Zfp69	0.4968294	0.82551149	1.06071371	0.31540246	0.34673035
Tap2	0.62797008	0.2777487	1.06056685	0.31543512	0.34673035
Cpne2	-0.1657581	4.41297001	1.06055942	0.31543677	0.34673035
Ptchd1	0.25066335	4.63115282	1.06041105	0.31546976	0.34673035
Plekhf1	-0.3485318	3.09606806	1.06028449	0.31549791	0.34673035
Draxin	1.36354472	-1.4922165	1.06001674	0.31555747	0.3467569
Zfml	0.15840368	7.89140434	1.0596197	0.31564582	0.34679204
Gm20594	0.42608731	0.45296374	1.05952439	0.31566703	0.34679204
Stox1	-0.3721737	1.31133111	1.05939575	0.31569566	0.34679204
Lefty2	1.26725032	-1.3405723	1.05900404	0.31578288	0.34682999
Chrm3	0.24154254	3.59287046	1.05877269	0.3158344	0.34682999
Wdr75	0.21027858	4.66345154	1.05876358	0.31583643	0.34682999
Zdhhc3	0.14033248	5.5882829	1.05819597	0.3159629	0.34692997
Il11	0.50495742	0.53524101	1.05753283	0.31611074	0.3469853
Rogdi	-0.1762104	4.61086762	1.05752739	0.31611195	0.3469853
Car15	0.4473253	2.46758281	1.05749328	0.31611956	0.3469853
Dhx8	0.13963322	5.10622165	1.05706772	0.31621449	0.34705061
Ghrl	0.70898796	-0.638868	1.05678255	0.31627812	0.34708156
Ugdh	-0.1531796	4.62494384	1.05611042	0.31642817	0.34720733
Setd6	0.15434271	4.8372087	1.05544597	0.3165766	0.34733129
H2-Ob	-1.0767361	-0.2005902	1.05423263	0.3168479	0.3475637
Gulp1	-0.2400205	5.95941799	1.0541812	0.3168594	0.3475637
Lrfn1	0.6030668	0.21389315	1.05354627	0.31700151	0.34767235
Tmc7	0.18815696	4.52991918	1.05342152	0.31702944	0.34767235
Wdr31	0.38363182	1.78852451	1.05312111	0.31709671	0.34770719
Tjp3	-0.9608398	-0.8532734	1.05249738	0.31723645	0.34782148
Gm10790	-0.779678	-0.5039736	1.0519171	0.31736653	0.34788584
Tvp23b	-0.1939089	4.76558333	1.05179726	0.3173934	0.34788584
D030028A08	-0.3777727	1.33502954	1.05167216	0.31742146	0.34788584
Atp1b3	0.22149207	8.8426417	1.05154385	0.31745024	0.34788584
Rbm4	0.68505418	-0.5383219	1.05143537	0.31747457	0.34788584
Lypla2	0.29742488	2.10910724	1.05123518	0.31751949	0.34788584
Atat1	-0.1449103	5.68342495	1.05112705	0.31754375	0.34788584
Hif1a	0.15487019	6.51471301	1.05063963	0.31765316	0.34796678
Fbxl2	0.22418494	4.66631756	1.05040493	0.31770585	0.34798559

Adh5	-0.1507425	6.35142183	1.05024202	0.31774244	0.34798675
Wif1	-0.9332281	-1.1356309	1.04976433	0.31784975	0.34806537
Aip	-0.2254928	5.1871287	1.04952767	0.31790294	0.34808469
AW209491	-0.1745793	4.72485102	1.04909111	0.31800108	0.34815323
Hyls1	-0.2920661	2.25186842	1.04883939	0.31805768	0.34817629
Rcc2	0.13196413	5.31857421	1.0485477	0.3181233	0.3482092
Scfd2	0.26098117	2.97274159	1.04815122	0.31821251	0.34822726
5031425E22I	-0.2562775	3.42439837	1.04772309	0.31830888	0.34822726
9530026P05I	0.50875212	0.5180559	1.04769817	0.31831449	0.34822726
Fbxo40	-0.9579098	-0.5305245	1.04769351	0.31831554	0.34822726
Ankef1	0.71316901	0.39636914	1.04752709	0.31835302	0.34822726
Slc17a7	-0.1437896	7.53302562	1.04752675	0.3183531	0.34822726
Rpl19	-0.193763	7.38560878	1.04716487	0.3184346	0.34824385
Gpr17	0.30036305	2.74550711	1.04703876	0.31846301	0.34824385
Scrt1	0.21777953	5.3381451	1.0469859	0.31847492	0.34824385
Btbd7	0.16863921	5.67035502	1.04679241	0.31851852	0.34825265
Gli1	-0.4566302	1.65310861	1.04589857	0.31872004	0.34843409
Npdc1	0.17659177	4.46292027	1.04552025	0.31880539	0.3484765
E430025E21I	0.13652975	6.34156917	1.04541122	0.31882999	0.3484765
Ddr2	-0.2629221	6.27923198	1.04496083	0.31893165	0.34853455
Uvssa	0.19613268	4.85422542	1.04486068	0.31895426	0.34853455
I730030J21R	-1.3542075	-1.3284042	1.04468196	0.31899461	0.34853976
Efs	-0.3346852	2.17687059	1.04429277	0.31908252	0.34859692
Glp2r	0.54522807	1.68436136	1.04379175	0.31919573	0.34868172
Zfp956	-0.4222737	2.29736313	1.04337321	0.31929035	0.3487106
Tbkbp1	0.32286752	2.13818558	1.04314115	0.31934282	0.3487106
Lag3	1.17188495	-1.3344653	1.04300879	0.31937276	0.3487106
Glis2	-0.2035859	5.21343482	1.04297969	0.31937934	0.3487106
Keap1	-0.1659867	4.39016593	1.04275007	0.31943129	0.3487106
Carm1	-0.1585672	5.24535522	1.04272026	0.31943803	0.3487106
Mpp7	0.17394433	4.60590607	1.04257291	0.31947137	0.3487106
Runx1	0.32972064	3.56880583	1.04205319	0.31958901	0.3487605
Cldn10	0.38588834	1.85072512	1.04194172	0.31961425	0.3487605
Galnt1	0.16678607	6.23301899	1.04189908	0.3196239	0.3487605
Ptafr	-1.0609556	-1.0445953	1.04148063	0.31971867	0.34878287
Emc6	-0.197603	3.83980552	1.04136957	0.31974383	0.34878287
Abcc6	-1.0011735	-0.8892767	1.04133695	0.31975122	0.34878287
Mgst1	-0.413146	3.65503979	1.04031071	0.31998385	0.34899775
Npc2	-0.296311	4.68547146	1.04003867	0.32004555	0.34902619
Dusp5	-0.6327044	-0.2021188	1.03892491	0.32029834	0.34925248
Wdr70	0.21555632	3.54999649	1.03881035	0.32032436	0.34925248
Smad2	-0.1147582	6.52510052	1.03797518	0.32051412	0.34942049
Itk	-0.8834535	-0.1746427	1.03733712	0.32065921	0.34944377

Slc26a4	0.59059678	1.05431985	1.03731364	0.32066455	0.34944377
Ifi35	-0.5047745	2.04781192	1.0372725	0.3206739	0.34944377
Xpnpep3	0.17990875	4.78115851	1.03700475	0.32073481	0.34944377
Cnih1	-0.2045522	5.24449197	1.03698518	0.32073927	0.34944377
Dnase1l1	-0.345987	2.18178417	1.03694009	0.32074953	0.34944377
Tab2	-0.1202709	8.33770501	1.03639469	0.32087366	0.34954013
Cep83os	-0.1542298	5.37662296	1.03590521	0.32098512	0.34962267
Cd200r4	-1.5389764	-2.3478976	1.03538137	0.32110447	0.34971378
Rasa4	-0.8532038	-0.4240529	1.03492081	0.32120945	0.34978922
Msantd1	1.00881442	-0.632476	1.03464787	0.32127169	0.3498181
Atp5sl	-0.2396775	3.21692517	1.03391447	0.321439	0.34992561
Agap2	0.15510472	6.9295941	1.03384256	0.32145541	0.34992561
Slirp	-0.1772257	4.7971432	1.03374537	0.32147759	0.34992561
Fam163b	-0.1500133	7.61194462	1.0334419	0.32154687	0.34994054
Srf	0.17331462	4.85046671	1.03337234	0.32156276	0.34994054
Ccr4	0.69137269	0.09339256	1.03238774	0.32178768	0.35014642
Nol6	0.16870712	5.48247362	1.03222275	0.3218254	0.35014856
Ptpn5	0.15940167	5.9042918	1.03152034	0.32198602	0.35028441
Dusp27	0.61434538	1.8053028	1.0311047	0.32208112	0.35031203
Gng11	-0.295597	5.55179355	1.03091318	0.32212495	0.35031203
Gltscr1l	0.13472881	6.39227417	1.0306965	0.32217456	0.35031203
Coro6	0.61414747	1.88178598	1.03067654	0.32217913	0.35031203
Nfib	0.11922882	8.11049038	1.03062804	0.32219023	0.35031203
1700008I05R	1.3857761	-1.3148425	1.03028646	0.32226845	0.35032511
Gpatch11	-0.1845493	5.43093597	1.03025991	0.32227453	0.35032511
Pcdhb10	0.47470953	1.33394292	1.030107	0.32230956	0.35032511
Snx24	-0.2211506	4.98465963	1.02984129	0.32237043	0.35033426
Gas2l1	-0.2440985	3.8345885	1.02975805	0.3223895	0.35033426
Gper1	-0.3710096	1.96462985	1.02954229	0.32243895	0.35034764
Ttc32	0.33476245	2.57335124	1.02939223	0.32247335	0.35034764
Ankle2	0.13806655	5.23852826	1.02899768	0.32256381	0.35040706
Klhl5	-0.1540862	5.72844719	1.02827237	0.3227302	0.35054836
4931403E22I	1.84563203	-1.295699	1.02803872	0.32278382	0.35054836
9830166K06I	-0.5638124	0.88567121	1.0279628	0.32280125	0.35054836
Sh3yl1	0.26333772	3.06946594	1.02772515	0.32285581	0.35056875
Irs1	-0.1240473	6.08048577	1.02685183	0.32305642	0.3506864
Gm3558	0.77312664	-1.2723541	1.02657084	0.323121	0.3506864
Cflar	0.12560413	6.86384183	1.02649984	0.32313733	0.3506864
Fam171a1	0.16851368	5.50197085	1.02647995	0.3231419	0.3506864
Fam172a	-0.1407134	5.60774182	1.02645923	0.32314666	0.3506864
Nrip2	0.41183448	2.07644589	1.02625501	0.32319362	0.3506864
Tanc1	0.12563016	5.909847	1.02616302	0.32321477	0.3506864
15-Sep	-0.2150941	6.70943786	1.025572	0.32335072	0.35077808

Nfkbid	0.95883206	-1.1082854	1.02548437	0.32337089	0.35077808
Fmnl3	0.29309655	3.58003564	1.02525073	0.32342466	0.35079756
2900056M2C	0.18285948	7.95961071	1.0247832	0.3235323	0.35087545
Slco3a1	0.18881539	4.96501028	1.02455557	0.32358472	0.35089346
Arfgap3	-0.2180408	4.63360754	1.02419674	0.32366739	0.35094425
Dpp10	0.17688963	6.87344246	1.02386222	0.32374448	0.35098898
Armcx2	-0.1227385	5.54431266	1.023593	0.32380653	0.35101741
Tfg	-0.1166154	6.88161521	1.02238542	0.32408511	0.35124557
Il15ra	-0.2653374	2.60517085	1.02236976	0.32408872	0.35124557
Rbm20	-0.3148466	2.41221173	1.02192331	0.3241918	0.35131841
Polr3g	-0.2212996	3.14058005	1.02061459	0.32449422	0.35160724
Gm20754	0.49368074	0.72571659	1.01999529	0.32463746	0.35172354
Psen1	0.16438863	5.05000366	1.01982959	0.3246758	0.35172618
Gm8773	-1.3192189	-1.5269507	1.01944136	0.32476566	0.35178462
Cachd1	-0.2324472	3.39907885	1.01881595	0.32491049	0.35190258
Bex4	-0.2224144	4.0910986	1.01860093	0.3249603	0.35191762
Zfp598	0.16012776	4.07853737	1.01816035	0.3250624	0.35198927
Abcg3	1.50444291	-1.7768559	1.01786726	0.32513035	0.35202393
Wdr1	0.13094593	6.28277704	1.01761119	0.32518972	0.35204931
Cluap1	0.12990576	5.50407573	1.01719742	0.32528571	0.3521143
Aplf	0.18088087	4.27157919	1.01649206	0.32544941	0.35225259
Zfp93	-0.2631133	3.83897095	1.01632066	0.32548921	0.35225674
1700094D03I	-0.4599122	1.92607072	1.0161573	0.32552715	0.35225888
Rps27l	-0.3298281	4.62792645	1.01595537	0.32557405	0.35227072
Ube2d3	-0.1323306	9.02053854	1.01559736	0.32565723	0.35227142
Ogfod2	0.24291943	3.487368	1.01552325	0.32567446	0.35227142
Picalm	-0.1044551	8.08333013	1.01548823	0.32568259	0.35227142
Trip12	0.12711176	8.84253983	1.01458398	0.32589284	0.35245991
Ppp1r3e	0.21816944	4.20352793	1.01358319	0.32612576	0.35259935
Ccdc36	1.14621194	-1.4751331	1.01358177	0.32612609	0.35259935
Pigl	0.25735461	4.09399198	1.0134893	0.32614762	0.35259935
Slain1	0.21420641	4.91292818	1.0134114	0.32616576	0.35259935
Htr3a	-0.5499484	0.66237424	1.0131033	0.32623752	0.35263802
Lgals3	0.44730386	1.77857783	1.01291776	0.32628075	0.35264583
Slc1a3	0.17113483	7.23936215	1.01193006	0.326511	0.35282294
Btc	1.54339704	-1.977228	1.01184176	0.32653159	0.35282294
Olfr920	1.17303582	-0.1309598	1.01164018	0.32657862	0.35282294
St8sia5	0.25694072	4.23302689	1.01159696	0.3265887	0.35282294
Rad54b	0.86420854	-0.1526747	1.01122378	0.32667578	0.3528781
Stac2	0.1473366	5.87709308	1.01059567	0.32682242	0.35299757
Dapk1	0.16289762	5.9237312	1.01024052	0.32690538	0.35302481
Pde6a	-0.3184356	2.04928689	1.01017913	0.32691972	0.35302481
Gp49a	0.77612356	0.04712162	1.00990246	0.32698437	0.3530557

Ifnar2	-0.2205157	5.47723388	1.00965166	0.32704299	0.35308007
Srgn	-0.2197405	5.14252766	1.00882921	0.32723532	0.35318187
2310039H08I	-0.3699352	2.04685104	1.00879492	0.32724335	0.35318187
Spry2	0.14070945	5.95328064	1.00878592	0.32724545	0.35318187
Poc5	-0.1605861	3.97921674	1.00856332	0.32729754	0.35318757
2210016F16I	-0.2027282	5.04382769	1.00845519	0.32732284	0.35318757
Asb5	-0.5249935	0.5376072	1.00721781	0.32761261	0.3534613
Rgs16	-0.2041172	3.66457542	1.00653041	0.32777374	0.35357299
Trp53cor1	0.61011073	-0.0263745	1.00638125	0.32780872	0.35357299
Henmt1	0.92651573	-0.5878007	1.00631429	0.32782442	0.35357299
Cdc42ep3	-0.1951608	3.36695661	1.00583072	0.32793787	0.3536564
Slc25a47	0.76568722	-0.6245567	1.00527604	0.32806806	0.35375786
Zfp786	0.62813143	0.13871326	1.00492593	0.32815027	0.35378877
Gm5862	0.51753675	0.79766452	1.00484636	0.32816896	0.35378877
Tnrc6b	0.14025009	8.72502466	1.00450791	0.32824847	0.35383554
1700001L19I	-0.289722	3.38356868	1.00397977	0.32837259	0.35392362
Fam126b	-0.1599951	8.29445503	1.0038528	0.32840244	0.35392362
Chpf	-0.2454168	3.44592117	1.0035024	0.32848484	0.35397348
Ctnnal1	0.17221942	4.46199785	1.00267728	0.32867898	0.35414372
Chtf18	1.02690453	-1.2665603	1.00207525	0.32882074	0.35416601
Speer8-ps1	0.67153389	0.0963112	1.00166709	0.32891689	0.35416601
Ppp1r18	-0.2958233	3.13725075	1.00163277	0.32892498	0.35416601
Tmppe	0.28313558	3.02552922	1.00163111	0.32892537	0.35416601
A830082K12I	-0.1445654	6.61205122	1.00148174	0.32896057	0.35416601
Mitd1	0.2713526	3.3516068	1.00139527	0.32898095	0.35416601
Lpar3	0.45073593	2.27219078	1.00135475	0.3289905	0.35416601
St8sia6	-0.58415	0.86685262	1.00129464	0.32900467	0.35416601
Elf1	-0.1783734	5.89231624	1.00120808	0.32902507	0.35416601
Rasa3	0.16517649	5.2610341	1.00035311	0.3292267	0.35424534
Tmem241	-0.2566053	3.00809008	1.00024943	0.32925116	0.35424534
Gpx8	-0.2908451	5.63847575	1.00020892	0.32926072	0.35424534
Adcy8	0.26713431	3.85815397	1.00009399	0.32928785	0.35424534
Usp21	-0.1423993	5.1328123	0.99998017	0.32931471	0.35424534
Tuba8	-0.2533422	2.26077848	0.99997569	0.32931577	0.35424534
Sirpb1a	1.27310097	-0.9216171	0.99953167	0.32942059	0.35431919
Apod	-0.3203924	10.3568368	0.99909575	0.32952355	0.35439102
Gtf2e2	-0.1576174	4.51126659	0.99892371	0.32956419	0.35439582
Ttc26	0.3305232	2.9379197	0.99844917	0.32967634	0.3544775
Dab1	0.18164743	6.61765701	0.99794616	0.32979527	0.35452507
Ptrh1	-1.4258328	-1.2887047	0.99785208	0.32981752	0.35452507
Hspb1	-0.3119273	5.07250514	0.99765084	0.32986513	0.35452507
Angptl1	0.73956132	0.5520539	0.99764988	0.32986536	0.35452507
1700012D01I	0.89128569	0.14562904	0.99717558	0.32997759	0.35460679

9030624G23	0.21769771	3.50587459	0.99673474	0.33008195	0.35460876
Eif3m	-0.126026	6.20702064	0.99655976	0.33012339	0.35460876
Gng13	-0.3891914	1.38099842	0.99655551	0.3301244	0.35460876
Mir5119	1.10775648	-1.4890308	0.99649801	0.33013802	0.35460876
Marf1	0.13436011	7.87549306	0.99640337	0.33016043	0.35460876
Glul	-0.1573998	10.5718556	0.99588829	0.33028247	0.3547002
Rnf113a1	0.28200671	1.95448335	0.99552361	0.33036891	0.3547002
Rgs14	-0.3260111	2.19227833	0.99542376	0.33039259	0.3547002
Mob2	-0.3295616	2.48552716	0.99519958	0.33044575	0.3547002
Zc3h13	0.1412616	8.06484843	0.99504096	0.33048337	0.3547002
Rgs7bp	-0.1488591	8.51584196	0.99501343	0.3304899	0.3547002
Maneal	-0.1726469	4.31987879	0.99497485	0.33049905	0.3547002
Zdhhc20	0.1659294	5.43041885	0.99430833	0.33065721	0.35479594
Nrros	0.54783608	1.47144779	0.99425265	0.33067042	0.35479594
2010005H15	-1.3852657	-1.5641879	0.99414104	0.33069692	0.35479594
Gm13629	0.37548486	1.52851678	0.99377957	0.33078275	0.35484916
Cebpb	-0.2752095	2.72160581	0.99355403	0.33083632	0.35486777
Ndnl2	0.23212073	3.46944245	0.99323848	0.33091129	0.35487587
Ulk4	0.54785904	1.39967079	0.99321726	0.33091633	0.35487587
Lcmt2	0.28491948	2.52533972	0.99283295	0.33100767	0.35492142
Fblim1	0.4167965	3.58566952	0.99263422	0.33105492	0.35492142
Eri2	0.20249154	4.18640994	0.99258129	0.33106751	0.35492142
Acy3	-0.3528172	1.57757437	0.99234597	0.33112347	0.35494256
Ncoa1	0.10915863	8.10415337	0.99198769	0.33120869	0.35499507
Rimbp3	0.66168074	0.85861884	0.99180943	0.33125111	0.35500169
Rnf19b	0.16441624	4.34190555	0.99160981	0.33129862	0.35501376
Igfn1	0.30538027	4.3999423	0.9912921	0.33137425	0.35501672
Nme3	-0.2321416	3.44429198	0.99116575	0.33140433	0.35501672
1700010I14R	0.56744449	-0.0902284	0.99109395	0.33142143	0.35501672
Grn	0.26827873	5.16360744	0.99098932	0.33144635	0.35501672
Dpp6	0.18969865	6.35339394	0.99046629	0.33157095	0.35511134
Abcb1b	0.31808761	1.66778348	0.99017569	0.3316402	0.35514669
Pim1	-0.5446157	0.33204306	0.98980275	0.33172911	0.35520306
D830030K20	0.64500175	0.44318328	0.98916312	0.33188168	0.35530156
Ric8b	0.19956279	5.65115964	0.98899374	0.3319221	0.35530156
Foxp1	-0.1122106	8.77787499	0.98896096	0.33192992	0.35530156
A930001C03	1.36742799	-1.796291	0.98861559	0.33201235	0.35533131
Ldb1	0.17941577	3.97055761	0.98854059	0.33203026	0.35533131
Eif2a	-0.1159226	6.74759483	0.98794763	0.33217187	0.35540682
B3gat1	0.18584977	6.02154827	0.98792328	0.33217769	0.35540682
Btg1	-0.2056395	5.37506462	0.98769579	0.33223204	0.35540682
Taf3	-0.134557	6.44110544	0.98763758	0.33224595	0.35540682
Atic	0.19548878	4.23843957	0.98722955	0.33234347	0.35541864

Spag7	0.19801993	5.28595099	0.98721157	0.33234777	0.35541864
2010300C02I	0.16694425	5.50339108	0.98713595	0.33236585	0.35541864
Tada2b	-0.1652668	4.39056583	0.9866851	0.33247367	0.35545312
Ptprm	0.22186362	5.20958612	0.98656201	0.33250311	0.35545312
Slc7a3	0.41141255	1.30527275	0.98654592	0.33250696	0.35545312
Myh1	0.68031927	0.52930343	0.98561865	0.33272889	0.35565155
Ccdc78	0.61408524	-0.2894292	0.9852266	0.33282279	0.3557131
Zfp964	-0.410555	1.2599047	0.98484235	0.33291485	0.3557384
Itgb4	0.41859565	3.19530324	0.98453436	0.33298867	0.3557384
4930455C13I	0.89682628	-0.7454366	0.98421716	0.33306472	0.3557384
Sowaha	0.1621553	7.44815938	0.9841447	0.33308209	0.3557384
Snx30	0.15936666	5.50898575	0.98366236	0.33319779	0.3557384
Mbtps1	0.15289774	5.43469457	0.98359248	0.33321456	0.3557384
Runx2	0.21841028	4.70215214	0.98353909	0.33322737	0.3557384
Kitl	-0.1865386	4.77964071	0.98351499	0.33323315	0.3557384
1700101I11R	-0.8298365	0.31317228	0.98338841	0.33326353	0.3557384
Snhg18	-0.2637459	4.28839592	0.98331477	0.3332812	0.3557384
Hnrnpf	-0.1941961	6.78931311	0.98325209	0.33329625	0.3557384
Fam19a1	-0.133587	5.87262786	0.98324447	0.33329807	0.3557384
Clec2f	-0.8599878	-0.7657779	0.98315904	0.33331858	0.3557384
Hnrnph1	0.09559766	8.53090032	0.98282745	0.33339819	0.3557846
Rpf1	-0.171902	4.10670821	0.98193866	0.33361171	0.35596755
Msh6	0.19799734	4.74445881	0.98181134	0.33364231	0.35596755
9530036O11	0.65866054	-0.2627716	0.98058354	0.33393762	0.35619018
Tmem178b	0.1564763	7.54212313	0.98051783	0.33395344	0.35619018
Rpp38	0.27481508	2.56177142	0.9803956	0.33398286	0.35619018
Glyctk	-0.4883489	1.38324795	0.9803392	0.33399643	0.35619018
Zhx2	-0.1896902	6.38843228	0.97959503	0.33417565	0.35634251
Zfp518b	-0.1240738	5.59134857	0.97928525	0.33425029	0.3563833
Olfr1393	-1.5652369	-1.883936	0.97892567	0.33433696	0.35643691
Aig1	-0.2869711	3.24048573	0.97807774	0.33454146	0.35658912
Atg14	-0.2056459	4.2719661	0.97803183	0.33455254	0.35658912
Gch1	-0.4410727	0.83721912	0.97736397	0.33471374	0.35670898
Lrrc26	1.14884322	-1.5248642	0.97701333	0.33479842	0.35670898
Slc25a12	-0.1206278	7.49870517	0.97700299	0.33480092	0.35670898
Sstr1	0.39946296	3.05387091	0.97696268	0.33481065	0.35670898
Hist1h4c	-0.7166741	-0.8188294	0.97654491	0.33491159	0.35676436
Gpx7	-0.4123107	2.44494039	0.97640955	0.3349443	0.35676436
Slc5a7	0.18606914	4.15569526	0.97629536	0.3349719	0.35676436
Ggnbp2	0.11145148	7.36108644	0.97564087	0.33513016	0.35689411
Fmo2	0.36496396	2.18109682	0.97472753	0.33535118	0.35701798
Map3k13	0.2350473	5.16798129	0.97463247	0.33537419	0.35701798
Chml	0.12537653	6.24860913	0.97462605	0.33537575	0.35701798

1700023L04I	-0.9063214	-0.0254087	0.97455781	0.33539227	0.35701798
Kcnmb2	0.53979058	0.84059901	0.97438747	0.33543352	0.3570231
Antxr1	0.17687407	6.12795934	0.97396036	0.33553698	0.35709441
2310015A10I	0.31966688	2.92595868	0.97338042	0.33567754	0.35714529
Snapc2	0.2236677	4.22325126	0.97325538	0.33570785	0.35714529
Fig4	0.16838186	4.3065797	0.97318949	0.33572383	0.35714529
Comp	1.0109328	-1.3445254	0.97309702	0.33574625	0.35714529
Cbx4	0.16829306	4.43718425	0.97301111	0.33576709	0.35714529
Zfp524	-0.6306427	-0.0109418	0.97249764	0.33589164	0.35716644
Fam193a	0.14321613	7.39228776	0.9724824	0.33589534	0.35716644
Bmf	-0.3084936	2.81806401	0.97244944	0.33590334	0.35716644
Asb4	0.68111436	0.34542722	0.97232793	0.33593283	0.35716644
Klf11	-0.1994353	4.21877842	0.97188731	0.33603979	0.35724139
Mcmbp	0.12021487	5.68264481	0.97168156	0.33608975	0.35725573
Fahd2a	-0.2779331	2.7954796	0.97106394	0.33623979	0.35728028
Wnt16	0.48839251	1.69469413	0.97088682	0.33628283	0.35728028
C1qtnf1	0.21318665	4.86034582	0.97083538	0.33629533	0.35728028
Heg1	0.1583872	6.24919506	0.97074094	0.33631829	0.35728028
Rnf2	-0.1464361	5.42801567	0.97069125	0.33633037	0.35728028
Ireb2	0.15434107	6.4449494	0.97068581	0.33633169	0.35728028
Mir1188	0.74457164	-0.5347815	0.97008514	0.33647775	0.35739668
Gpr45	-0.2639545	2.23163796	0.96965282	0.33658294	0.35746964
Arglu1	-0.1066908	6.85567336	0.96929756	0.33666941	0.35752271
Stradb	0.15434676	5.42089571	0.96897316	0.33674839	0.35753932
Ppp1r26	-0.1829638	4.18231138	0.96880114	0.33679029	0.35753932
Itgb2	0.41872781	1.44270914	0.96878361	0.33679455	0.35753932
Tex261	0.26376932	1.98762786	0.96763911	0.33707348	0.35778881
Klhl18	0.18282566	4.45705032	0.96751962	0.33710262	0.35778881
4833427F10I	0.76828249	-0.7880059	0.96661787	0.33732264	0.35798354
Alox12b	0.45735344	2.01014161	0.96635173	0.33738761	0.35801371
Ankrd16	0.37299287	2.54010471	0.96601157	0.33747068	0.35806307
Lgmn	-0.220634	4.12100921	0.96541579	0.33761625	0.35817018
Nelfb	-0.170718	5.02264515	0.96529911	0.33764477	0.35817018
1700018L02I	0.67182935	0.78479279	0.96498821	0.33772078	0.35821202
Sh3gl1	-0.2422966	3.29642853	0.96469251	0.33779309	0.35824992
Kat6b	0.11226158	7.17037169	0.96423062	0.33790608	0.35832032
Vpreb3	1.27715241	-1.2330033	0.96402068	0.33795746	0.35832032
Slc25a45	0.83240841	-0.4163997	0.96397272	0.3379692	0.35832032
Fendrr	1.01699367	-0.3723177	0.96353532	0.33807628	0.35835923
Rfx5	-0.1541832	4.85278337	0.96352393	0.33807907	0.35835923
Nsg2	-0.1260508	7.8674914	0.96276062	0.33826606	0.35851183
Scamp4	-0.231216	3.4873528	0.96263747	0.33829624	0.35851183
Fut4	-0.7317978	-0.2588624	0.96245283	0.3383415	0.35852101

Lrrc59	-0.1189111	6.71192555	0.96220982	0.33840108	0.35853388
Yipf3	-0.1540813	4.45809091	0.96183453	0.33849312	0.35853388
Ndufaf5	-0.1637891	4.79767237	0.96182462	0.33849555	0.35853388
Slc6a15	0.18658205	4.70351203	0.96180624	0.33850006	0.35853388
Syncrip	0.11149045	7.70853875	0.96160791	0.33854872	0.35854665
Myo1b	0.15454772	5.93592783	0.96121389	0.33864541	0.35861028
Sorcs1	0.229509	4.97864272	0.9606039	0.33879518	0.35869075
Unc13b	0.17033814	6.53909414	0.96060103	0.33879588	0.35869075
Gm19619	-1.1341906	-1.2366144	0.96031309	0.33886662	0.35869075
Hcfc2	-0.2054251	4.10084685	0.96020241	0.33889381	0.35869075
Dmpk	-0.2401616	3.50546501	0.95998597	0.338947	0.35869075
Twistnb	-0.1325802	5.20112615	0.95986112	0.33897768	0.35869075
Prdx6	-0.1402855	6.75152676	0.95986091	0.33897773	0.35869075
Polr2j	-0.3746348	3.17793511	0.95964414	0.33903102	0.35870839
Syna	-0.3366609	1.95238682	0.95929024	0.33911804	0.35876171
Mmachc	-0.1662093	4.6196112	0.95907926	0.33916993	0.35877786
Senp6	-0.1029251	8.47980854	0.95885816	0.33922433	0.35878215
Slc12a8	0.52318404	0.36052363	0.958765	0.33924725	0.35878215
Smpd1	0.21146477	4.97641497	0.95831276	0.33935856	0.35878232
Tom1l1	-0.1910069	3.91602837	0.95823784	0.339377	0.35878232
Poglut1	-0.1354341	5.22591774	0.95820241	0.33938572	0.35878232
Crlf3	-0.291549	3.11838493	0.95810094	0.33941071	0.35878232
Gstk1	-0.262306	3.26041485	0.95802038	0.33943055	0.35878232
Dnajc27	-0.1523257	6.17751237	0.95779059	0.33948714	0.35880342
Srpk3	0.64187099	0.31649771	0.95705981	0.33966721	0.358955
Pfdn4	-0.2456708	3.92869864	0.95686404	0.33971547	0.35896728
Calr3	-0.5783225	-0.0376434	0.95653782	0.33979591	0.35901209
Prkci	0.11375913	7.35728916	0.95639482	0.33983118	0.35901209
Bcap31	-0.2060384	5.73327821	0.9559239	0.33994737	0.35909275
Cd53	-0.2602891	2.34394497	0.95578823	0.33998085	0.35909275
Zfp109	-0.2392452	3.49465105	0.95523938	0.34011636	0.35913507
Spata21	-1.4651897	-1.3681654	0.95523735	0.34011686	0.35913507
Shisa9	0.18527802	6.09755279	0.95518046	0.34013091	0.35913507
Tbc1d8b	-0.1972161	4.33544602	0.95466177	0.34025904	0.35923164
Zfp667	-0.2305862	4.79433515	0.95427933	0.34035357	0.35929271
Rasip1	-0.2846822	2.4086378	0.9535589	0.34053172	0.3594055
Fkbpl	-0.528557	0.62039627	0.95355051	0.3405338	0.3594055
Gcfc2	-0.3416335	2.09808493	0.9529904	0.3406724	0.35943354
A330074K22	0.44184956	1.28954745	0.95295589	0.34068094	0.35943354
Zfp513	0.32599841	1.85284122	0.95286796	0.34070271	0.35943354
Zfp213	0.49567624	1.09284585	0.95284593	0.34070816	0.35943354
Zfp945	0.1501262	5.18265876	0.95270184	0.34074384	0.35943354
Gabpb1	-0.1428206	5.10050839	0.95239366	0.34082015	0.35947134

1810062G17	1.37738671	-1.2913555	0.95226078	0.34085307	0.35947134
Crkl	0.11826859	6.05458741	0.95180951	0.34096488	0.35951248
Trmt10b	-0.201154	3.70171068	0.95166015	0.3410019	0.35951248
Tet1	0.14717668	6.20291372	0.95154975	0.34102926	0.35951248
Rbbp6	0.11143183	9.12289896	0.95136071	0.34107613	0.35951248
Tnfsf15	-1.5132404	-1.6671084	0.95123431	0.34110747	0.35951248
Slc9a3r1	-0.2919781	4.2153837	0.95116298	0.34112516	0.35951248
Klrd1	0.6351654	-0.5107385	0.95106689	0.34114899	0.35951248
Gm7120	0.34497994	2.94204316	0.95018526	0.34136775	0.3596787
Scnn1a	-0.2765863	2.58433615	0.94983412	0.34145493	0.3596787
Fam129b	-0.2344688	5.3393645	0.94972963	0.34148088	0.3596787
Pnp	-0.1778283	4.58401959	0.94970921	0.34148595	0.3596787
Rab9	-0.1742033	5.70015538	0.94969163	0.34149032	0.3596787
Mt1	-0.2291983	6.01750431	0.94950249	0.3415373	0.35968951
Sephs1	-0.153516	5.67359731	0.94929081	0.34158989	0.35970622
Pcsk1	0.242796	3.26775986	0.94883065	0.34170425	0.35978797
Gm4461	0.72388815	0.55151926	0.94842593	0.34180488	0.35979715
Rfx7	0.13088282	7.87409775	0.94835254	0.34182313	0.35979715
Gm13238	0.53245448	0.09036922	0.94821157	0.3418582	0.35979715
Sec61g	-0.2249023	4.7707202	0.94819212	0.34186303	0.35979715
Smim3	-0.2739258	3.12589515	0.94800528	0.34190952	0.35979715
Bcdin3d	-0.5142577	0.61609728	0.94790945	0.34193336	0.35979715
Abcb7	0.17456841	5.77587624	0.94774323	0.34197472	0.35980202
Cbln3	0.41554342	1.1983502	0.94729987	0.34208508	0.35987948
Prickle4	1.33054438	-1.8831369	0.94692169	0.34217926	0.35991656
Tmem144	0.27431939	2.21110874	0.94686324	0.34219382	0.35991656
Zfp92	-0.3563259	2.40848391	0.94622044	0.34235399	0.36004636
Rnf182	-0.3264765	3.01037818	0.94572109	0.34247849	0.36013863
Bag3	-0.1701501	5.08106989	0.9452926	0.34258537	0.36021235
Gdf9	0.8903757	-0.9128164	0.94462408	0.34275222	0.36031593
Ubash3b	0.14982181	5.78563294	0.94460315	0.34275744	0.36031593
Vwa3a	0.42059481	2.05989752	0.94421324	0.34285481	0.36037961
Fbxw7	-0.1190125	8.24940269	0.94293915	0.34317325	0.36054006
Naprt1	-0.5575743	0.45155741	0.94291058	0.3431804	0.36054006
Lyrm4	-0.1417257	5.02712425	0.94290447	0.34318192	0.36054006
Iqcd	-1.1684247	-1.7489565	0.94290347	0.34318217	0.36054006
Gm15816	0.65813971	0.7008866	0.94286619	0.3431915	0.36054006
Pqlc3	0.22351388	3.54648936	0.94252644	0.34327649	0.36056391
Cyp4f16	-0.3974133	1.73480182	0.94235396	0.34331965	0.36056391
Heatr3	0.17240232	4.64907755	0.9421456	0.3433718	0.36056391
Cars2	0.3162288	2.19304104	0.94207209	0.3433902	0.36056391
Sec23a	0.13078703	6.49396383	0.94203994	0.34339825	0.36056391
Zcchc7	0.1654277	5.64380246	0.94179325	0.34346001	0.36058049

Bbox1	-0.5518711	0.58517396	0.94168284	0.34348766	0.36058049
C3ar1	-0.3297562	2.03882037	0.94122965	0.34360117	0.360649
Cck	-0.1202124	7.75430441	0.9410122	0.34365566	0.360649
Trim41	-0.1419652	4.34501675	0.9409814	0.34366338	0.360649
Zfp36l2	-0.1805581	6.13442197	0.94083052	0.34370119	0.36065005
Fbrsl1	0.1812291	4.62818827	0.93932541	0.34407873	0.36100753
Tigit	1.15959619	-1.5637393	0.93862139	0.34425553	0.36115435
Hexa	0.30100064	3.74109112	0.93841002	0.34430864	0.36115981
Smcr8	0.1521511	5.78459802	0.93830717	0.34433448	0.36115981
Hsbp1l1	0.35773893	1.59126542	0.93805354	0.34439823	0.36118799
Bbx	0.12436106	7.92283414	0.93767766	0.34449273	0.36121734
Fcgr1	0.77189882	-0.627529	0.9376489	0.34449996	0.36121734
Ttc7	0.30000246	3.14285201	0.93747036	0.34454486	0.36122575
Fcrlb	1.31981052	-1.4451806	0.93722707	0.34460606	0.36125125
Nme5	-0.237799	3.39963312	0.93704858	0.34465097	0.36125967
Klrb1c	1.03202375	-0.3976589	0.93652994	0.34478151	0.36135783
Mut	0.10678638	5.76690923	0.93579957	0.34496546	0.36151194
Zfp174	0.19528788	3.76830348	0.93548458	0.34504483	0.36155644
Gorab	-0.1944311	3.49205678	0.9352023	0.34511599	0.36159232
Zfp758	0.2056311	3.79792439	0.93498294	0.3451713	0.36161159
Cacna1g	0.21335781	4.47879731	0.93416979	0.34537643	0.3617878
Nfxl1	0.22785651	2.91886483	0.93388517	0.34544828	0.36182436
Fbln2	0.50185296	0.80247329	0.9336502	0.3455076	0.36184781
Zfp712	0.198729	3.87900977	0.93295806	0.34568244	0.36199221
Trip6	-0.2845306	3.17019165	0.93249905	0.34579846	0.36207032
Cd200r1	-0.6876413	-0.6579988	0.9323705	0.34583096	0.36207032
Spata5	0.20490661	4.23811925	0.93203684	0.34591534	0.36211996
Rsph3b	-0.1753405	4.40296319	0.93118592	0.34613066	0.36226315
Stx5a	0.19175395	4.58266496	0.93110839	0.34615029	0.36226315
Gm13157	-0.2261035	3.36192233	0.93105793	0.34616307	0.36226315
Reps2	-0.1135574	9.58647786	0.93061469	0.34627532	0.36234191
Tmem56	0.15101115	6.25923531	0.93044053	0.34631944	0.36234937
Tmem205	-0.3604064	1.69329535	0.92981879	0.34647701	0.36244984
Arfip2	-0.1408561	5.19645534	0.92976967	0.34648947	0.36244984
A630020A06	0.78112148	0.01746508	0.92897582	0.34669082	0.36262173
Kank2	-0.1936751	7.56540758	0.9285795	0.3467914	0.36268821
Fam120c	0.1946434	6.59807918	0.92834713	0.3468504	0.36271119
Cd164l2	1.14582848	-1.0215828	0.92769191	0.34701682	0.36272581
Pop1	0.32072526	2.24893073	0.92757693	0.34704604	0.36272581
Ranbp9	-0.1051879	7.07801354	0.9275419	0.34705494	0.36272581
Slc27a3	-1.3332749	-1.6827004	0.92742097	0.34708567	0.36272581
G630071F17	0.69778187	-0.2688964	0.92739124	0.34709323	0.36272581
Ankzf1	0.34436104	1.79473831	0.92730213	0.34711588	0.36272581

Lin9	-0.273427	3.01299053	0.92727176	0.34712359	0.36272581
Riok3	-0.1356485	6.33207179	0.92674749	0.3472569	0.3628264
Pex16	0.45552803	0.74110707	0.92650581	0.34731837	0.36285193
Peo1	0.30453929	2.5381301	0.92599324	0.3474488	0.3629466
G3bp1	-0.1451822	5.14055908	0.92585851	0.3474831	0.3629466
Cdh4	0.21120417	3.48979286	0.9256962	0.34752442	0.36295106
Shisa3	-0.308293	3.96110094	0.92521029	0.34764817	0.36300971
Ltbp4	0.16557805	4.59000141	0.92518467	0.3476547	0.36300971
9530077C05I	-0.3073421	2.2458995	0.92502654	0.34769498	0.36301308
Pmm2	-0.2237947	3.40285091	0.92481542	0.34774878	0.36302093
Ublcp1	-0.1276657	6.44773241	0.92470615	0.34777663	0.36302093
Clic1	-0.269135	4.48526169	0.92427348	0.34788693	0.36309737
Nit2	0.26724195	2.27151986	0.92393723	0.34797269	0.36314819
Ecd	-0.1800418	4.1883553	0.92323932	0.34815077	0.36329533
Syt17	-0.2944921	3.80397589	0.92268029	0.34829351	0.36338297
Nol8	0.12336227	6.46452917	0.92261985	0.34830895	0.36338297
Rsrc2	-0.111833	7.78253955	0.92144377	0.34860954	0.36365783
Cd33	-0.1970671	3.1598662	0.92116897	0.34867983	0.36369243
Baiap2l2	0.87853581	-1.0733073	0.92097315	0.34872993	0.36370595
Gm8580	-0.5779023	-0.6333046	0.92050585	0.34884952	0.36379195
Prrt2	-0.118258	7.21379978	0.9195644	0.34909065	0.36400465
Ifitm2	-0.3234415	7.3431538	0.91845962	0.34937391	0.3642557
Smim6	-1.4855035	-1.6185278	0.91824457	0.34942908	0.3642557
Pde3b	0.35512719	2.68835774	0.91792233	0.34951178	0.3642557
Polr1a	0.28920636	4.59391222	0.9178023	0.34954259	0.3642557
Hs3st2	0.20660389	4.34219434	0.91774786	0.34955657	0.3642557
Mybbp1a	0.1777066	4.39925614	0.91769495	0.34957015	0.3642557
Prkar2a	-0.1241729	6.40143471	0.91761098	0.34959171	0.3642557
Stag1	-0.1135307	7.40633933	0.91712984	0.34971529	0.3643457
Pim3	0.25505233	2.92316168	0.91677893	0.34980545	0.36440088
Adamts9	0.34560521	2.69377646	0.91633314	0.34992005	0.36444301
Mak16	-0.1565657	4.98461037	0.91623269	0.34994587	0.36444301
Neo1	0.13492876	7.54444732	0.9160366	0.3499963	0.36444301
Gbp2	-0.2998192	4.51041072	0.91600593	0.35000419	0.36444301
Tlcd1	0.38928589	2.1458999	0.91581163	0.35005417	0.36444301
Fkbp10	-0.4874721	2.21110393	0.91575348	0.35006913	0.36444301
Gm14288	-0.3660326	0.84512697	0.91504739	0.35025085	0.36455527
Ctbs	0.22797795	3.00363502	0.9150428	0.35025203	0.36455527
Rabepk	-0.2658713	2.99144991	0.91490072	0.35028862	0.36455527
Sf1	-0.1128018	6.7156002	0.91435258	0.3504298	0.36466346
Nenf	-0.1906602	4.26834623	0.91415142	0.35048164	0.36467866
Tmem30b	0.48378877	2.72185465	0.91386267	0.35055606	0.36471735
Casp3	-0.2028314	4.02964314	0.91357717	0.35062967	0.3647552

Eif2b3	0.23993638	2.84573605	0.91337183	0.35068262	0.36476532
Sertm1	-0.1614928	4.58370972	0.91325066	0.35071388	0.36476532
Lama1	0.22710928	3.50150878	0.91290503	0.35080305	0.36480416
Tle3	-0.1473509	5.22576563	0.91275691	0.35084127	0.36480416
Nxnl1	1.45888331	-1.2919197	0.91267289	0.35086296	0.36480416
Rusc2	0.17238309	6.58476708	0.91234083	0.35094867	0.36485456
Hccs	-0.1775734	4.98550885	0.91209391	0.35101244	0.36488212
Gm7694	0.31060214	1.83915516	0.91179272	0.35109023	0.36492427
Zkscan14	-0.4102451	2.42938013	0.91162877	0.35113259	0.36492957
Akr1c21	1.63967036	-1.1791494	0.91134226	0.35120663	0.3649678
Dnajc13	0.18334515	6.26013549	0.9091894	0.3517637	0.36550015
Mcts1	-0.1353047	5.13383329	0.90898586	0.35181643	0.36550015
Ist1	-0.1475245	5.83351638	0.90868945	0.35189325	0.36550015
Dedd	-0.1639943	4.20891729	0.90864067	0.35190589	0.36550015
Mypop	-0.2438148	2.50815487	0.90859964	0.35191652	0.36550015
Sf3b3	-0.1419551	5.27586135	0.9084983	0.35194279	0.36550015
Morc2b	0.51247918	1.68893426	0.90773435	0.35214092	0.36563761
Igsf9b	0.27570412	3.48985487	0.90763279	0.35216727	0.36563761
Spidr	0.29282867	2.25997588	0.90755622	0.35218714	0.36563761
Ooep	-0.8822285	-1.0041709	0.9072552	0.35226526	0.36567121
Ier5	-0.149026	6.19845767	0.90710334	0.35230469	0.36567121
Olfr287	0.75457569	0.25108927	0.90700009	0.35233149	0.36567121
Mcm8	0.20132749	3.21512786	0.90643213	0.35247901	0.36578555
Slmap	-0.1159565	8.96692926	0.90625804	0.35252424	0.36579374
Nrbp2	0.14865426	6.88218076	0.90610823	0.35256317	0.36579538
Tnfsf8	1.2145522	-0.2182772	0.90566277	0.35267897	0.3658484
Cdh13	0.23441469	4.87616683	0.90553771	0.35271149	0.3658484
Fam107a	-0.207355	7.39578337	0.9054807	0.35272631	0.3658484
Xrn2	0.12686689	5.65336846	0.90459941	0.35295561	0.36600918
Phf20	0.12757326	6.49914567	0.9045977	0.35295606	0.36600918
Grpr	0.67251492	-0.1149348	0.90420445	0.35305844	0.36607659
Rab38	1.15081708	-1.447128	0.90335546	0.35327963	0.36626717
Polr2h	-0.2484312	4.08275577	0.90308637	0.35334978	0.36630112
Lmln	0.27833588	2.3864814	0.90287597	0.35340464	0.36631923
E330009J07F	0.42594752	1.34869234	0.90271888	0.35344561	0.36632293
Dcaf11	-0.135847	4.86651126	0.90220052	0.35358085	0.36642432
Stk40	0.14422241	4.15938639	0.90202581	0.35362644	0.36643281
Myh14	0.35296851	2.29748057	0.90167721	0.35371745	0.36648834
Psmc3ip	-0.3929735	2.02747276	0.90139565	0.35379098	0.36650184
Tm7sf3	0.18290985	4.54772077	0.9012277	0.35383485	0.36650184
Derl1	0.16545265	5.09007702	0.90107857	0.35387381	0.36650184
Rars2	0.18509767	3.90870699	0.90105432	0.35388015	0.36650184
Serinc3	0.15624546	8.71528027	0.90065955	0.35398331	0.3665534

Ctu2	-0.3673722	1.72551479	0.90057745	0.35400477	0.3665534
Epcam	1.29954813	-0.4798403	0.90022799	0.35409615	0.36660926
A830082N09	-0.2409634	4.43826558	0.89972222	0.35422845	0.36668401
Luc7l3	0.12601125	8.5707348	0.8996658	0.35424321	0.36668401
Cys1	-0.219064	5.15008941	0.89941978	0.35430759	0.3667119
Ormdl2	-0.5346576	1.43733539	0.89911821	0.35438653	0.36674974
4932411E22I	0.29271721	2.56302696	0.89898337	0.35442184	0.36674974
Klrc1	0.75567924	-0.657688	0.89881592	0.35446569	0.36674974
Map10	0.45313272	1.696296	0.89870814	0.35449392	0.36674974
C530008M17	0.18675018	5.06620149	0.89854776	0.35453593	0.36675447
Nmnat1	-0.4955523	0.27411572	0.89817024	0.35463485	0.36681806
Pycard	-0.2489915	2.9570236	0.89790104	0.35470542	0.36685231
Klhdc4	0.22048636	3.05653402	0.89703208	0.35493332	0.36704927
Snord19	1.03529052	-1.6782624	0.90436424	0.35500994	0.36707841
Klf2	-0.2065185	4.01005213	0.89658753	0.35505	0.36707841
Lmx1b	1.08470892	0.19280357	0.89649639	0.35507393	0.36707841
Ndufa13	-0.2641564	5.844441	0.89583432	0.35524781	0.36721941
Peli2	-0.153777	5.70732848	0.89550124	0.35533534	0.36724457
Usp22	0.11655728	7.05458543	0.89545635	0.35534714	0.36724457
Ntf5	-1.6301745	-1.8553363	0.89510041	0.35544071	0.36730252
Gss	0.24266098	3.45881164	0.89483802	0.35550971	0.36733508
Serpina9	0.68288083	-0.6081491	0.89236514	0.35616095	0.36796916
Mcam	0.38740363	1.72639642	0.89161806	0.35635803	0.36813395
Zbtb34	0.16051552	6.30562841	0.89090608	0.356546	0.36828929
Nfil3	-0.3230594	2.54597835	0.89054809	0.35664057	0.36834813
Vamp8	-0.4302329	5.10451775	0.89016225	0.35674253	0.3684146
Rabgap1	0.11057686	7.271955	0.8897518	0.35685104	0.368415
Stx6	-0.1504768	4.9700372	0.88969691	0.35686556	0.368415
Rpain	-0.2270219	2.84744177	0.8896043	0.35689005	0.368415
Prpf19	-0.1262166	7.10692766	0.88946088	0.35692798	0.368415
Supv3l1	-0.238377	3.17870659	0.88944957	0.35693098	0.368415
Snx12	-0.1319752	7.72627146	0.88914329	0.35701201	0.36845981
1700003F12I	1.15245215	-2.0074229	0.88891303	0.35707294	0.36848387
Setdb2	0.26175541	2.9904605	0.88855553	0.35716758	0.36854271
Tyrobp	-0.3777579	1.37625052	0.88829567	0.35723639	0.36857489
Olfml1	-0.2130251	4.58663499	0.88761876	0.35741573	0.36865363
Vmn2r46	0.84092207	-1.6613097	0.8875531	0.35743313	0.36865363
Sin3b	0.174506	4.8061056	0.88747126	0.35745483	0.36865363
Cdh18	0.26290292	3.95230194	0.88743948	0.35746325	0.36865363
Rec8	-0.6206928	0.39427074	0.88721908	0.35752168	0.36867507
Zbtb44	0.11910516	7.23538293	0.88683051	0.35762474	0.36873865
Tceal5	-0.1241229	6.04172697	0.88659144	0.35768816	0.36873865
Gtf2h3	0.21221176	3.5832589	0.88656086	0.35769627	0.36873865

Mir143hg	1.0171693	-1.3937044	0.88631981	0.35776024	0.36876578
Mtbp	-0.4139337	1.38346682	0.88572617	0.35791784	0.36888942
6820431F20I	0.15560864	8.79642571	0.88525282	0.35804358	0.36895706
Abhd4	-0.1910416	5.30739511	0.88519552	0.35805881	0.36895706
Mrps2	-0.1343069	4.70560518	0.88487695	0.35814347	0.36900548
Wdr52	0.33953264	2.19941663	0.88410509	0.35834873	0.36917813
Fam131b	-0.1835149	4.95506433	0.88354256	0.35849842	0.36929351
Aamp	-0.189296	4.84802461	0.88289278	0.35867145	0.36943289
Csde1	-0.0943361	9.14077136	0.88250401	0.35877503	0.36950073
Iars	0.15795284	6.46595723	0.88199718	0.35891013	0.36958115
Klf3	-0.1710786	7.62085981	0.88192801	0.35892857	0.36958115
Celf4	0.10836802	9.32146305	0.88176947	0.35897085	0.36958583
Gpc1	0.240945	3.38315005	0.88145613	0.35905443	0.36963303
Gm5136	0.70121404	0.1255518	0.88122838	0.3591152	0.36965674
Ddx55	0.1551393	4.40733803	0.88081265	0.35922616	0.3697321
Sun2	0.18134435	6.58459989	0.8805553	0.35929487	0.3697597
Zkscan3	0.18570993	3.32695568	0.88042949	0.35932847	0.3697597
Ecsit	-0.1472671	3.81187568	0.88024855	0.3593768	0.36977059
Usp14	0.12705838	6.97105052	0.87977283	0.35950391	0.36978706
9330158H04I	1.30152256	-1.188079	0.8797524	0.35950938	0.36978706
Lst1	-0.6762032	-0.2032257	0.87964048	0.35953929	0.36978706
Mxi1	-0.1231425	6.32122359	0.87951329	0.35957329	0.36978706
Zim1	0.40227941	1.09994664	0.87948235	0.35958156	0.36978706
4930512B01I	0.50467701	0.91539927	0.8787837	0.35976842	0.36989976
Aif1l	-0.306612	2.10260785	0.87873901	0.35978037	0.36989976
Polr2b	-0.1234277	6.23667482	0.87864906	0.35980444	0.36989976
Psmc1	-0.1235294	6.37600681	0.87831366	0.35989421	0.36995321
Ints3	-0.1435904	5.30231358	0.87816844	0.35993308	0.36995435
Mcc	0.13982725	6.54266634	0.87784915	0.36001858	0.3700034
9430021M05	0.21628664	4.43883393	0.8775222	0.36010616	0.37001686
Ptk7	0.38092824	1.28696047	0.87751822	0.36010722	0.37001686
Rgs6	0.23642743	3.12285949	0.87719901	0.36019276	0.37006593
Shkbp1	-0.4785509	0.80269498	0.8761823	0.36046539	0.37028107
Ldlrad4	0.15371292	4.36462641	0.87613618	0.36047777	0.37028107
Mcph1	0.20677378	5.21063922	0.8758816	0.36054608	0.37031242
Ybx3	-0.1946001	7.39449024	0.87465483	0.36087555	0.37061194
Gpd1	-0.1769964	4.49827584	0.87449724	0.3609179	0.37061658
Gm14326	0.22421091	4.52824376	0.87405418	0.36103702	0.37069512
Arhgef9	0.13276981	9.43024303	0.87393125	0.36107007	0.37069512
Heatr6	0.16158413	4.8189271	0.87366021	0.36114298	0.37073112
Pcgf5	-0.1845041	4.11613292	0.87347247	0.36119349	0.37074412
E130102H24I	0.76823205	-1.0345136	0.87329353	0.36124165	0.37075469
C920009B18I	0.46110285	0.99448876	0.8724787	0.36146104	0.370941

Lrp11	0.1487094	5.96147788	0.87218981	0.36153887	0.37097354
Atf7	-0.2169721	4.26147139	0.87207986	0.3615685	0.37097354
Deb1	-0.1495571	4.91334652	0.87188317	0.36162151	0.37098907
Clock	-0.119646	7.60340753	0.87141641	0.36174735	0.37107931
Syt4	0.1603213	7.23979806	0.87112393	0.36182624	0.37112137
Zfp330	-0.1577054	6.1249446	0.87033274	0.36203976	0.37122524
Il1rl1	0.83716568	-0.1980598	0.87030281	0.36204784	0.37122524
Pstpip2	-0.1774067	3.88083727	0.87022418	0.36206907	0.37122524
Afg3l1	0.24388826	3.28180885	0.87018703	0.36207911	0.37122524
Gstz1	0.14176696	4.65541876	0.86996708	0.3621385	0.37124728
Hnrnph3	-0.1383044	4.49209724	0.86982635	0.36217652	0.3712474
Nudt2	-0.2356005	3.19216054	0.86963151	0.36222916	0.37126251
AW822252	0.76333907	0.07551761	0.86936015	0.36230249	0.37129882
Dvl3	-0.1222117	6.09227167	0.86888513	0.36243091	0.37139157
Usb1	-0.3613197	1.54034789	0.86836745	0.36257093	0.37149619
Rfxank	-0.2650912	2.94693999	0.86721444	0.36288309	0.37177714
Prdm16	-0.2704653	3.06366429	0.86688353	0.36297275	0.37179221
Slc39a5	0.57783224	-0.5327547	0.86679913	0.36299562	0.37179221
Vgf	0.29085024	3.63822286	0.86673993	0.36301167	0.37179221
D3Ertd751e	-0.1506749	5.72744736	0.86633684	0.36312095	0.37186525
Mapkapk3	-0.4288156	2.24862019	0.86594585	0.36322699	0.37192312
Mllt3	-0.1263737	7.72199041	0.86584854	0.36325339	0.37192312
Chic1	0.12060536	6.36591201	0.86559185	0.36332304	0.37195555
Gtpbp2	0.19167042	4.27138484	0.86498976	0.36348648	0.37197295
Armc5	-0.2284434	2.58306319	0.86481355	0.36353434	0.37197295
Atg13	-0.1100088	5.92728263	0.8647878	0.36354133	0.37197295
Raver1	-0.1861758	6.00400555	0.86474884	0.36355191	0.37197295
Ush1g	1.42184961	-1.4019684	0.86473841	0.36355475	0.37197295
Nudt17	-0.3975903	1.00296696	0.8646618	0.36357556	0.37197295
Med11	-0.3268663	2.92929786	0.86455028	0.36360585	0.37197295
Chst9	0.81961958	-0.8220098	0.8641312	0.36371973	0.37202515
Lrrc48	0.2996249	2.39259325	0.86397734	0.36376156	0.37202515
Dusp10	-0.2443422	3.731895	0.86391249	0.36377919	0.37202515
BC055324	0.46426961	0.65282369	0.86369776	0.36383758	0.37202515
Irgq	0.1270237	7.40639249	0.86342844	0.36391082	0.37202515
Bax	-0.2577522	2.61546348	0.86333731	0.36393561	0.37202515
Fah	-0.2052446	3.18799279	0.8632831	0.36395036	0.37202515
Prkab2	0.16243034	4.92458605	0.86306976	0.36400841	0.37202515
Spata2l	-0.197015	4.60710982	0.86306601	0.36400943	0.37202515
Ifitm7	-0.870663	-1.6271057	0.86296589	0.36403667	0.37202515
Crls1	-0.1755912	4.55231975	0.86260232	0.36413564	0.37207089
Ak8	-0.8021167	-0.7773796	0.86252238	0.3641574	0.37207089
Rcbtb1	0.15503037	6.01330375	0.86224087	0.36423406	0.37207645

Dll3	1.03154988	-1.1968643	0.8619929	0.36430161	0.37207645
Abce1	0.12908026	6.26132422	0.86198169	0.36430466	0.37207645
Chrna5	-0.6375344	-0.3679875	0.86190587	0.36432532	0.37207645
Nacc2	0.11354739	6.50946676	0.86180511	0.36435278	0.37207645
Bmpr2	0.13608678	9.84150137	0.8612148	0.36451368	0.37216915
Tnip3	-0.4520933	1.79841068	0.86119331	0.36451954	0.37216915
Wdr90	0.61024609	1.35133883	0.86056673	0.36469045	0.37230484
Rbpj	-0.1110178	6.51270234	0.86036152	0.36474645	0.37232321
Gdap2	-0.1302793	4.7954107	0.86021005	0.36478779	0.37232661
Prg4	0.2457566	8.48540854	0.85979497	0.36490112	0.3723438
Zbtb25	-0.2007377	3.35792443	0.8595904	0.36495699	0.3723438
Armc10	-0.2032219	4.74546226	0.85957405	0.36496146	0.3723438
Nvl	0.16524531	5.01649047	0.85941062	0.3650061	0.3723438
Ctnna2	0.13088729	6.28819644	0.85933074	0.36502792	0.3723438
Fign	-0.1935166	4.7871539	0.85931324	0.3650327	0.3723438
Ccdc106	-0.372664	1.77355217	0.85912986	0.36508282	0.37235613
Cyb561	-0.1253413	4.44562997	0.85811166	0.36536122	0.37260129
Rnf32	0.21729259	3.43782625	0.85751865	0.36552352	0.372728
C1rl	0.91429737	0.26255936	0.85730701	0.36558146	0.37274828
Gm5176	0.75955865	-0.4659472	0.85715894	0.36562201	0.37275082
Mageb16	0.6677444	1.09658276	0.85664661	0.36576236	0.3728551
L3mbtl2	-0.1796337	3.73725995	0.85607601	0.36591876	0.37297572
Zdbf2	0.2523574	5.97330262	0.85579126	0.36599685	0.3730165
Nup160	0.18325509	4.50080403	0.85460944	0.3663212	0.37330823
Trim17	0.47134023	0.58160471	0.85433753	0.36639589	0.3733455
Camk2g	0.14969945	7.23186606	0.85399713	0.36648942	0.37338913
Gtf3c4	-0.1409433	5.27152828	0.85390101	0.36651583	0.37338913
D2hgdh	0.16709985	4.1262795	0.85376558	0.36655306	0.37338913
Spata5l1	0.80690698	-0.7576528	0.852997	0.36676441	0.37356558
Sgms1	0.11959547	5.94055763	0.8521247	0.36700451	0.37364433
Tprn	0.35082894	2.34731961	0.85211501	0.36700717	0.37364433
Fgd2	0.50471122	0.37736609	0.85207162	0.36701912	0.37364433
Mrpl54	-0.2957089	2.69776809	0.85205905	0.36702259	0.37364433
Chm	0.13656734	6.37748255	0.85202318	0.36703246	0.37364433
Mfsd2b	0.90867948	-0.5072159	0.85146865	0.36718523	0.37373699
Ptpn12	0.14289216	6.02226526	0.85141582	0.36719979	0.37373699
Sil1	0.30551686	2.5779536	0.8511695	0.36726768	0.37376725
Thrsp	-0.326502	3.17040787	0.85102441	0.36730768	0.37376913
Zeb1	-0.0899519	7.53527288	0.85081665	0.36736497	0.37378859
Matk	-0.220881	3.61966561	0.85058628	0.3674285	0.3738144
1700088E04l	-1.3509749	-1.5563058	0.85004457	0.36757797	0.37388811
Pde1b	0.14583062	5.52007197	0.84999688	0.36759113	0.37388811
Tspyl5	-0.1189219	5.78190886	0.84990876	0.36761545	0.37388811

Zfp763	0.20365563	4.04657361	0.84932309	0.36777717	0.37401375
1700001K19I	0.56428169	0.15610211	0.8491268	0.3678314	0.37402778
4930404I05R	-1.159994	-1.5477678	0.84899672	0.36786734	0.37402778
Pi4kb	0.17587424	4.34799035	0.84855659	0.36798898	0.37411263
Lgi1	0.13563687	7.46443041	0.84815795	0.36809921	0.37414757
1700001L05F	-0.2529374	4.34217344	0.84815602	0.36809974	0.37414757
Dusp18	-0.1595063	5.35682692	0.84773532	0.36821612	0.37422703
Nlk	-0.1316932	8.55890243	0.84743534	0.36829914	0.37423082
Mfsd7a	1.24522513	-0.7476039	0.84739525	0.36831024	0.37423082
Odf2	0.17793602	5.56183303	0.84730771	0.36833447	0.37423082
Zc3h12d	0.80037408	-0.835249	0.84701038	0.36841679	0.37427564
Arsg	-0.2028206	3.08586952	0.846572	0.36853821	0.37431272
Slc25a19	-0.2072532	3.33440775	0.84648079	0.36856348	0.37431272
Tmem150b	-1.0447003	-0.9969211	0.84646474	0.36856793	0.37431272
Mapk4	-0.1335437	7.79315389	0.84617565	0.36864804	0.37435527
Heatr1	0.23609838	4.11923889	0.84600706	0.36869478	0.37436392
Mapkapk5	0.16432233	4.18382528	0.84534927	0.36887719	0.37446954
Sp7	-0.4678583	0.83037531	0.84510944	0.36894374	0.37446954
Ap1m1	-0.2214519	3.73255191	0.8450142	0.36897016	0.37446954
St3gal3	0.20534347	3.19153663	0.84499148	0.36897647	0.37446954
1500015O10	-0.356586	5.14687595	0.84494291	0.36898995	0.37446954
Ppfia1	0.10121875	6.96011551	0.84451399	0.36910902	0.37455158
Cmpk2	0.1374742	4.55212567	0.84370842	0.36933281	0.37471076
Edc3	-0.2435354	3.0896471	0.84366631	0.36934451	0.37471076
Apeh	-0.3391629	2.13324778	0.84353626	0.36938066	0.37471076
D930048N14	0.54302859	0.71953324	0.84306737	0.36951103	0.37479599
Bace1	0.11040663	5.79319228	0.8429589	0.3695412	0.37479599
Gpr22	0.16157703	5.80525701	0.84275622	0.36959758	0.37480627
Kcnj15	1.32475406	-0.7769083	0.84255686	0.36965305	0.37480627
Ppbp	0.99154331	-0.2778654	0.84243593	0.3696867	0.37480627
Usp7	0.10921764	7.09946502	0.84237237	0.36970439	0.37480627
Pigw	-0.3897022	1.51908733	0.84221169	0.36974912	0.37481282
Gstp2	-0.1688534	4.53982424	0.84131865	0.36999784	0.37497641
Hsf2bp	-0.3985744	2.07785124	0.84120727	0.37002887	0.37497641
Styx	0.19442566	4.25906461	0.84117738	0.37003721	0.37497641
Lmnb1	0.29712249	1.82116979	0.8410826	0.37006362	0.37497641
Scn11a	-1.7552969	-1.9906575	0.84028882	0.37028496	0.37516188
Rcn2	-0.1232643	6.30450958	0.8398802	0.37039898	0.37522739
Farsb	0.1234328	5.68963609	0.83963737	0.37046676	0.37522739
Psd	0.17219314	5.56008766	0.83952506	0.37049811	0.37522739
Ptpra	-0.1209826	6.44475606	0.83950809	0.37050285	0.37522739
Stk24	-0.1550923	7.31064576	0.83899117	0.37064721	0.37528673
Icam1	0.43846022	1.87701251	0.83897779	0.37065095	0.37528673

Cela1	-0.3488641	1.3190181	0.8388868	0.37067637	0.37528673
BC018473	1.24535926	-1.5335454	0.83827372	0.37084772	0.37540239
Frk	-0.2254203	4.02607557	0.83820382	0.37086727	0.37540239
Cd84	0.29151958	2.46459589	0.83795214	0.37093765	0.37543484
Lhx8	-0.3056732	2.6491906	0.83756001	0.37104734	0.37550706
Edaradd	1.28910847	-1.1186257	0.83731295	0.37111648	0.37553823
Bcl6	0.0930525	6.10404496	0.83684263	0.37124815	0.37563266
Plekhm1	0.13347875	5.38139561	0.83666732	0.37129724	0.37564353
Zfp120	0.2134911	4.37521183	0.8364224	0.37136585	0.37567414
Lamp5	0.13859085	5.75821944	0.83618382	0.37143269	0.37570296
Caskin1	0.17444807	5.76529907	0.83601902	0.37147888	0.37571088
Fam184a	0.19174823	4.85798108	0.83524683	0.3716954	0.37589106
Vmn2r85	0.65389941	1.10954057	0.83496493	0.37177449	0.37592381
Mmp2	-0.4472298	1.32979393	0.83485783	0.37180454	0.37592381
Suv39h2	0.24465111	3.0955712	0.83466874	0.37185762	0.37593867
Olfr239	-0.9963261	-1.2311747	0.83422116	0.37198328	0.37600325
Rhof	-0.2358495	3.497237	0.8339798	0.37205107	0.37600325
Chga	-0.1958374	4.36510179	0.83391443	0.37206943	0.37600325
Tpst1	-0.1547151	5.01559497	0.8338751	0.37208048	0.37600325
Gstm2	-0.3038514	5.29499627	0.83375786	0.37211342	0.37600325
Bace2	-0.2674044	3.59463179	0.83354649	0.37217282	0.37602448
Abra	1.2405296	-0.3836444	0.83321968	0.37226468	0.37603999
Cct2	-0.0952303	7.0039922	0.83312297	0.37229187	0.37603999
Fam195b	-0.2683814	2.5685986	0.83308216	0.37230335	0.37603999
Ostm1	0.13475861	5.44644216	0.83271075	0.37240781	0.37610672
Gprin3	0.19919494	3.67896331	0.83220617	0.37254979	0.37621132
Eif2ak2	-0.1817522	5.80721038	0.83200183	0.3726073	0.37623062
Mob1a	-0.1979165	3.39147199	0.83082037	0.37294013	0.37652787
Nup98	0.11590077	6.39272742	0.82990268	0.37319895	0.3767089
Rhbdl1	-0.3883726	0.61950411	0.82983379	0.37321839	0.3767089
Acadm	-0.191768	5.06558011	0.82977558	0.37323482	0.3767089
Cib1	-0.3406956	1.9956303	0.82949639	0.37331362	0.37674962
2310034G01	0.63279519	0.91151971	0.82912839	0.37341753	0.3767722
Ndufa9	-0.1558381	6.21260257	0.8289924	0.37345594	0.3767722
Vcp	-0.1022418	7.68924044	0.82896121	0.37346475	0.3767722
Dmkn	-0.6977296	-0.2987898	0.82884393	0.37349788	0.3767722
Ddx3y	0.1126514	6.3508727	0.82873619	0.37352832	0.3767722
Hist1h1e	-0.3312937	2.92887352	0.82805619	0.37372052	0.37689549
Acad9	0.20950941	4.13075264	0.82788019	0.37377029	0.37689549
Arhgef19	-0.3773491	1.47445845	0.82778441	0.37379738	0.37689549
Ttc38	0.18093294	3.77982838	0.82775938	0.37380446	0.37689549
Sntg2	0.49777805	0.90102064	0.82703199	0.37401028	0.3770642
Pkp4	0.10881759	8.59015621	0.82666852	0.37411319	0.37712469

Ppp1r37	-0.1666968	4.26330119	0.82654818	0.37414727	0.37712469
Zscan21	-0.190929	4.60120424	0.82633003	0.37420906	0.37714816
Kcnj3	0.17838049	5.04747371	0.82609937	0.37427442	0.37717522
Map4	-0.1016438	9.19688123	0.8257796	0.37436505	0.37719773
Syde1	-0.2478678	5.18608369	0.82547698	0.37445085	0.37719773
Senp2	-0.0963074	6.40988616	0.82539361	0.37447449	0.37719773
Col18a1	0.3276554	1.82900347	0.8253501	0.37448683	0.37719773
Tom1	-0.206379	4.10098346	0.8253414	0.37448929	0.37719773
Ecel1	-0.3163681	1.9455997	0.82475916	0.37465447	0.37732531
Cnppd1	-0.2190602	4.12454066	0.82461374	0.37469575	0.37732808
Cutc	-0.2131048	3.81524854	0.82442043	0.37475062	0.37734454
Bcat2	-0.2690863	2.1273565	0.82419543	0.3748145	0.37736744
A930011G23	0.71582848	-0.3035177	0.82395882	0.37488171	0.37736744
Zfp202	0.3337117	1.45069473	0.82375655	0.37493916	0.37736744
St8sia2	-0.275785	2.85033802	0.82371653	0.37495053	0.37736744
1700024G13	-1.3005699	-1.5267944	0.82366213	0.37496599	0.37736744
Afmid	-0.4206493	1.12223607	0.82335307	0.37505382	0.37741184
Ldha	-0.1332309	7.8797741	0.82313528	0.37511573	0.37741184
Gys1	0.35389479	1.99857252	0.82294269	0.37517049	0.37741184
Fbxo48	-0.7757509	-0.7738056	0.8227895	0.37521406	0.37741184
2310003H01	-0.3850521	0.9227629	0.82274347	0.37522715	0.37741184
Prex2	0.14694184	7.37348837	0.82269377	0.37524129	0.37741184
Nlrp1b	1.7456391	-1.6327494	0.8222088	0.37537927	0.37751186
Hdx	0.30953309	2.92416688	0.82169711	0.37552493	0.37761086
Col6a3	0.29391433	3.31318191	0.8215922	0.37555481	0.37761086
Tacc2	0.12811574	5.52330764	0.82048191	0.37587121	0.37787904
Obsl1	0.21196139	3.21369127	0.82038554	0.37589869	0.37787904
Msrb3	-0.1866407	5.39694695	0.82010887	0.3759776	0.37791959
Ttf2	0.42230685	1.79361035	0.81981023	0.3760628	0.37793395
Rabgap1l	0.13697698	8.19784169	0.81978831	0.37606906	0.37793395
Slain2	-0.1171345	6.73373401	0.81939266	0.37618199	0.37800866
C330027C09	0.34561435	1.86514799	0.81924769	0.37622338	0.37801148
Fbxo10	0.22664862	4.18832971	0.81876838	0.37636027	0.37811024
Ddah2	-0.3271961	3.18176182	0.81837725	0.37647204	0.37816069
Hdgfrp2	-0.1682855	5.05959439	0.81818349	0.37652742	0.37816069
Chrna4	0.16092211	4.50706817	0.81805295	0.37656475	0.37816069
Gm10584	-0.8434181	-1.1520838	0.81805236	0.37656492	0.37816069
Ift172	0.27034846	4.63791237	0.81764557	0.37668125	0.37821795
Cass4	-0.5672693	0.49765302	0.81758295	0.37669916	0.37821795
Clec10a	-0.9235634	-1.1430793	0.81700253	0.37686526	0.37831164
Flt4	0.66859757	1.00648881	0.81696934	0.37687475	0.37831164
Rhpn2	-0.2855102	4.0440724	0.81685202	0.37690834	0.37831164
Ccdc162	0.57659172	0.22297356	0.81647905	0.37701514	0.37836025

Sqle	0.13518858	5.75304706	0.81641313	0.37703403	0.37836025
Rab18	-0.1300323	7.34877302	0.81619376	0.37709687	0.37838455
Clpp	-0.1731521	3.49623974	0.81579525	0.37721107	0.37843592
Fam3c	-0.1096541	6.43511488	0.8156136	0.37726315	0.37843592
1700102P08I	-0.9369546	-1.0640683	0.81561075	0.37726396	0.37843592
Vsx1	-0.2784565	3.86348811	0.8154595	0.37730733	0.37844066
Tnfrsf11b	-0.3528229	3.89176417	0.81521492	0.37737747	0.37847226
Col5a3	1.09117914	-1.0560599	0.81469795	0.37752579	0.37856889
Lrriq1	0.31824013	3.09680899	0.81460972	0.37755112	0.37856889
Rprd1b	-0.1585093	5.10640068	0.81390251	0.37775418	0.37872926
Xkr8	0.36105714	2.41378249	0.81352535	0.37786254	0.37872926
Plekha7	0.20312661	2.98852326	0.81336776	0.37790783	0.37872926
Nbl1	-0.3259234	6.51471903	0.81336069	0.37790986	0.37872926
Cped1	-0.2056956	7.25689517	0.81331166	0.37792396	0.37872926
Zbtb7b	0.29236193	2.97171509	0.81324526	0.37794304	0.37872926
Bmp6	-0.2530203	6.6331763	0.81283868	0.37805994	0.37880765
2610034B18I	0.23820893	4.17684086	0.81246065	0.37816868	0.37887785
Muc2	-1.8241237	-1.8954559	0.81230722	0.37821283	0.37888333
Men1	0.20340212	4.03317558	0.81192898	0.37832169	0.37893042
Urb1	0.2669801	2.76951926	0.81187511	0.3783372	0.37893042
LOC1026341(	-1.1094198	-1.4732897	0.81149695	0.3784461	0.37900073
Msantd3	-0.2694192	2.9884941	0.81095217	0.37860305	0.37908721
Snta1	0.24475014	2.78513284	0.81077614	0.37865379	0.37908721
Lrp2	1.13943293	-1.6730941	0.81068322	0.37868057	0.37908721
Rap1gap	0.20340813	4.12132355	0.81058607	0.37870858	0.37908721
Gfod2	-0.5805956	0.02465691	0.81052581	0.37872596	0.37908721
Stat5b	-0.1721236	4.00803531	0.81020619	0.37881813	0.37913734
Cks1b	-0.3477677	3.42281938	0.81001921	0.37887207	0.37913734
Mkks	-0.1676884	5.11911302	0.8099496	0.37889215	0.37913734
Kdm5c	0.16421498	5.20889389	0.80950796	0.37901961	0.37922613
Gm17296	0.4061353	2.16490273	0.80929827	0.37908014	0.37924796
Osbp	-0.1114391	5.35473339	0.80852842	0.37930252	0.37943168
Dhrs4	0.20701582	3.26827748	0.8074312	0.37961979	0.37969449
C330007P06I	-0.1480991	7.68773185	0.8073518	0.37964276	0.37969449
Acy1	0.44339123	0.78264564	0.80714028	0.37970398	0.3797145
Tmem128	0.2142954	3.40362018	0.80692309	0.37976685	0.3797145
Stam2	-0.1281228	4.84893538	0.80672114	0.37982532	0.3797145
Sdhaf2	-0.1397335	5.20430452	0.80659254	0.37986256	0.3797145
Chdh	0.39155595	1.47474422	0.80628449	0.37995179	0.3797145
Rnf25	-0.3132778	2.08902688	0.80625262	0.37996102	0.3797145
Dhrs3	-0.2394651	4.64623871	0.80608592	0.38000932	0.3797145
Uxs1	0.16450877	3.5382047	0.80600925	0.38003154	0.3797145
Prpf6	-0.1293015	5.5425876	0.80585381	0.38007659	0.3797145

Gtsf1	0.99560063	-1.7485368	0.80575375	0.3801056	0.3797145
Mgp	-0.3045353	8.28888369	0.80567679	0.38012791	0.3797145
Rxra	-0.2271328	5.7142349	0.80567667	0.38012794	0.3797145
Pnmal2	0.13930703	6.34505682	0.80543726	0.38019736	0.37974079
Smim24	-0.3489884	2.27280505	0.8053185	0.3802318	0.37974079
Ttc9c	-0.1456943	6.09942812	0.80481567	0.38037767	0.37984775
F930015N05	-0.2073228	3.60806078	0.80454663	0.38045576	0.37985631
Myh6	0.53774919	0.26047312	0.80451892	0.3804638	0.37985631
Adam21	0.49582516	0.29743149	0.80361944	0.38072504	0.38005424
Htr4	0.42479566	0.69245636	0.80356916	0.38073965	0.38005424
Slc25a35	-0.1750056	4.99898898	0.80321728	0.38084193	0.38011566
Spock2	0.14872488	8.11714131	0.80309049	0.38087879	0.38011566
Rasgrp1	0.16591468	9.64199137	0.80294796	0.38092023	0.38011829
5730403I07R	-1.3236305	-1.970672	0.80268613	0.38099639	0.38015555
Fam173b	0.37495236	0.83055761	0.8025098	0.38104768	0.38016656
Mettl22	0.29655688	2.59116622	0.8023814	0.38108504	0.38016656
5430435G22	-0.2418386	4.44542886	0.80212421	0.38115989	0.38017519
Cyld	0.1129654	7.32138492	0.80208497	0.38117132	0.38017519
Dyrk1b	0.20191772	3.09395663	0.80165295	0.3812971	0.3802501
Plekhh1	0.19568484	4.58905436	0.8015604	0.38132405	0.3802501
Ncoa3	0.1284294	6.64765796	0.80101634	0.38148256	0.38036944
AB124611	0.79619293	-0.6492218	0.80052532	0.3816257	0.38047343
Prok2	-0.9434059	-1.3584056	0.80021074	0.38171745	0.38052617
Birc5	0.46330729	0.28308296	0.79996888	0.38178801	0.38054052
Sympk	-0.203375	4.57832281	0.79989509	0.38180954	0.38054052
Slx4	0.15489315	5.16651281	0.79964439	0.38188271	0.38057472
Gm13293	-0.4024394	0.84650605	0.79930501	0.38198179	0.38063473
4930513N10	-0.7428991	-0.4688363	0.79903975	0.38205926	0.3806732
Sec14l1	0.11854808	6.38234486	0.79884307	0.38211671	0.38068024
Dbnl	-0.1514295	5.18181118	0.79866061	0.38217002	0.38068024
Cep57l1	0.23206133	2.90026587	0.79861651	0.38218291	0.38068024
Hpgd	-0.247062	3.95251446	0.79822067	0.38229861	0.38072554
Tdp1	0.26598254	2.43650373	0.79819495	0.38230613	0.38072554
Cyb561d1	0.1820987	3.70922664	0.79756027	0.38249176	0.38087168
Rps6ka3	0.10852249	7.88393158	0.79726416	0.3825784	0.38090818
Gli3	0.12792197	5.12944884	0.79716926	0.38260618	0.38090818
Gpr65	0.52504703	0.52277596	0.79692067	0.38267895	0.38094191
Sbk1	0.17174221	4.16353769	0.79624391	0.38287718	0.3811005
Rasa1	0.10377212	6.61581885	0.79590883	0.38297538	0.38115952
Nmrk2	0.35321431	0.66187961	0.79547813	0.38310166	0.38124646
Cd93	0.35806998	3.15480574	0.79493377	0.38326135	0.3813251
Tmem165	-0.1651392	4.66271088	0.79483138	0.3832914	0.3813251
Lrrc51	-0.3794199	2.09673844	0.79479254	0.3833028	0.3813251

Aldh3a1	0.75993624	-0.7939376	0.79467808	0.38333639	0.3813251
Gpx4	-0.2743509	4.24864188	0.79442653	0.38341024	0.38133614
Lmbrd2	0.14015479	6.12720119	0.79437505	0.38342536	0.38133614
Rad50	0.15823695	6.08377612	0.79352883	0.38367396	0.38154465
Pabpc5	0.28326241	2.29010092	0.79332327	0.38373438	0.381566
Gpr126	0.19987949	3.94251813	0.79223886	0.38405338	0.38172297
Gm15881	0.62340978	0.39379139	0.79218746	0.38406851	0.38172297
Gramd1b	0.13756698	5.84688195	0.79214967	0.38407963	0.38172297
2610035D17	0.22726934	3.31067357	0.79214004	0.38408247	0.38172297
Slc33a1	0.1972777	3.78701826	0.79201188	0.3841202	0.38172297
Tmod2	-0.1039768	9.94559003	0.79190024	0.38415307	0.38172297
Timm50	-0.3439827	1.68706968	0.7918596	0.38416503	0.38172297
Cxcl12	-0.1404386	6.01762405	0.79170847	0.38420954	0.38172375
Acss2	0.27631098	4.31701525	0.79151669	0.38426603	0.38172375
Rsl1d1	-0.1158377	6.66736682	0.79145663	0.38428373	0.38172375
Odc1	-0.0956246	6.42482973	0.79119739	0.38436011	0.38172375
Ajap1	0.14104093	5.20149619	0.79114082	0.38437678	0.38172375
H2-T10	-0.3182895	1.18483638	0.79106327	0.38439964	0.38172375
Sox9	0.16324435	5.66087096	0.79024416	0.38464118	0.38188696
Jade2	0.1225308	5.26676133	0.79024148	0.38464197	0.38188696
Atxn7l3b	-0.1100914	7.34105172	0.78960153	0.38483083	0.38203575
Zfp869	0.15365916	5.11244838	0.78902971	0.38499971	0.38207951
Atp2a1	1.11618748	-0.7557197	0.78880306	0.38506667	0.38207951
Nfatc3	-0.1559892	6.91576641	0.78879364	0.38506946	0.38207951
Rer1	-0.1745987	5.71875699	0.78875783	0.38508004	0.38207951
Qars	0.1686525	3.78645225	0.78872242	0.38509051	0.38207951
Myo18b	-0.631331	-0.6149514	0.78866002	0.38510895	0.38207951
Ly6h	-0.3847496	0.87209353	0.78834988	0.38520062	0.38213176
Tmem42	0.25492703	2.41919743	0.78809642	0.38527557	0.3821674
Usp37	0.16652199	5.63517387	0.78773264	0.38538317	0.38223543
Pgam5	0.1332616	6.09809971	0.78739793	0.38548222	0.38229496
Slfn9	-0.3405381	1.62630599	0.78689568	0.38563092	0.38240371
Nat6	-0.24268	3.18126994	0.78585217	0.38594013	0.38263882
Zfp558	0.33410519	2.74543963	0.78583192	0.38594613	0.38263882
Mars	0.12386444	4.89170001	0.7850142	0.3861887	0.38284056
Lrrc40	-0.1799444	5.10632569	0.78479073	0.38625503	0.38286756
Sssca1	-0.2541597	2.92878654	0.78441303	0.38636718	0.38293998
Gps1	-0.1333363	5.13884661	0.78418342	0.38643538	0.38296339
Zfp410	0.16408236	4.00281026	0.78407025	0.386469	0.38296339
Slc26a8	0.47944522	0.88939182	0.78290332	0.38681592	0.38325683
Adcy4	-0.5896961	0.47949154	0.78281104	0.38684338	0.38325683
Mat2a	0.10366727	7.87146733	0.78252795	0.38692762	0.38330153
Scn5a	0.4743952	1.37007776	0.78236717	0.38697548	0.38331017

Txlng	0.12535012	5.65176157	0.78209847	0.38705548	0.38335065
Rap1gap2	0.1917598	6.77560268	0.78184655	0.3871305	0.38336368
Sigirr	-1.1316062	-1.8461738	0.7817635	0.38715524	0.38336368
Zfp426	-0.121895	6.10522924	0.78158334	0.38720891	0.38336368
Tmem38a	-0.1778811	5.49186598	0.78152871	0.38722519	0.38336368
Foxo1	-0.1194236	7.15529798	0.78115715	0.38733592	0.38338825
Zbtb41	0.11726639	6.45382066	0.78109379	0.38735481	0.38338825
Zfp454	-0.2466847	2.31027199	0.7810515	0.38736742	0.38338825
Gm10416	0.67875009	0.47683774	0.78028499	0.38759604	0.38353876
Fam179b	0.11407092	6.53940217	0.78025022	0.38760641	0.38353876
Pdlim3	-0.8447261	-0.6166203	0.78014784	0.38763697	0.38353876
Cdh24	0.91507858	-1.4470112	0.77987727	0.38771772	0.38356023
Psma6	-0.1188214	6.42066675	0.77973154	0.38776123	0.38356023
Rhbdd3	0.50632525	-0.0468391	0.77968163	0.38777614	0.38356023
Gm16880	1.1872058	-0.2276483	0.77946348	0.38784128	0.38358594
Dhcr24	0.17441491	5.09585216	0.7792972	0.38789095	0.38359633
Sorbs3	-0.2169195	7.07168681	0.77911253	0.38794612	0.38361216
Rab19	0.42241741	1.98296092	0.77839844	0.38815957	0.38375222
Tmem41b	-0.1329737	4.8261981	0.77837657	0.38816611	0.38375222
Faf1	0.15215425	4.73011911	0.77797242	0.388287	0.38381213
Tnpo2	-0.1100237	5.98827346	0.77779991	0.38833862	0.38381213
Gm11149	0.77842377	0.11160232	0.77778104	0.38834426	0.38381213
Klhl21	-0.1883671	4.54310308	0.77743958	0.38844647	0.3838744
Ina	-0.1443457	6.3206916	0.77695756	0.38859081	0.38397831
Nhlrc3	-0.3924069	0.51559575	0.77635313	0.38877191	0.38411852
Il7r	-0.6506202	-0.0123462	0.77621712	0.38881269	0.38412005
Gdpgp1	-0.1668013	4.8161016	0.77579382	0.38893962	0.38420465
Qpctl	0.24157282	2.65051681	0.77566998	0.38897676	0.38420465
Zscan20	0.26346316	2.05490218	0.77541819	0.3890523	0.38424052
Iqcj	-0.9893811	-1.5324239	0.77471402	0.38926368	0.38441053
Pla2g4c	-0.567927	0.09718778	0.77443147	0.38934855	0.3844449
Prrg2	-0.6277393	-0.1605994	0.7741736	0.38942602	0.3844449
Cpne7	0.2723358	2.63343277	0.7741541	0.38943189	0.3844449
Ascl2	0.99221637	-0.7872353	0.77407558	0.38945548	0.3844449
Pecam1	0.38362209	1.99038698	0.77362853	0.38958987	0.38453057
Tnks	0.14479945	5.86862362	0.77344013	0.38964652	0.38453057
Nkapl	0.51275117	0.25498264	0.77332865	0.38968005	0.38453057
4930526I15R	-0.4010027	1.65572054	0.77326466	0.3896993	0.38453057
Upf3a	-0.1810168	5.8631165	0.7726798	0.38987529	0.38466548
Crebrf	0.11360011	7.2754805	0.77239824	0.38996005	0.38471036
Nat9	-0.2786369	1.99609858	0.7721239	0.39004267	0.38475312
Uqcrc2	-0.0898651	6.7286354	0.77191074	0.39010688	0.38476641
Ak4	0.16189853	5.3752424	0.7718184	0.3901347	0.38476641

4931406H21	0.82793269	0.24026653	0.77163354	0.39019041	0.3847826
Pgm5	-0.2259074	6.55807452	0.771397	0.39026171	0.38481417
Arsj	-0.4139976	0.86203898	0.77093486	0.39040106	0.38489358
Rnpep	0.21371994	3.44437432	0.77086931	0.39042083	0.38489358
Gm17762	-0.7462529	-0.4665116	0.77052804	0.39052379	0.38494756
Trnt1	0.14835262	4.77161066	0.77042734	0.39055418	0.38494756
Tmbim1	-0.1851456	5.07240249	0.77028726	0.39059646	0.3849505
Mapk1	0.10815096	9.09375929	0.76995433	0.39069697	0.38498483
Apcdd1	-0.2774356	3.98764952	0.76989971	0.39071346	0.38498483
Hipk1	-0.0998853	8.53399763	0.76974828	0.3907592	0.38498483
Pdlim4	-0.2605582	2.4719041	0.76965123	0.39078851	0.38498483
Pcdhgc3	0.26589057	4.17040003	0.76922869	0.39091617	0.38505218
Ctr9	0.13290999	5.2111628	0.76916478	0.39093549	0.38505218
Cd74	-0.3201364	6.73494248	0.76894924	0.39100064	0.38507763
Sec24d	-0.1729317	4.40749598	0.76855966	0.39111844	0.38511071
Tjap1	0.39943733	1.35414695	0.76853691	0.39112532	0.38511071
Slc30a4	-0.1111599	6.40576099	0.7684481	0.39115218	0.38511071
Cdk18	0.20628077	4.00573225	0.76790266	0.39131722	0.38521652
Plau	0.54824769	-0.1551436	0.76783302	0.3913383	0.38521652
Hnrnpr	-0.0956267	7.8987067	0.7675139	0.39143491	0.38527291
Faim2	-0.1215292	6.72865198	0.76723885	0.39151822	0.38531618
Pabpc4l	-0.390679	2.23419676	0.76662301	0.39170482	0.3854611
Cables2	-0.1514155	5.03028034	0.76596395	0.39190468	0.38559113
B3galnt1	-0.1201414	4.8280408	0.76592764	0.39191569	0.38559113
Crlf1	0.64555819	-0.4769437	0.76575093	0.39196931	0.38560515
Gm2381	0.91691975	-1.2024585	0.76560549	0.39201344	0.38560984
Akap13	0.11197127	7.25756423	0.7648095	0.39225512	0.38580883
Pop4	-0.1964634	4.0052	0.76442474	0.39237202	0.38582154
Gm3435	0.24013661	2.79609154	0.76427114	0.3924187	0.38582154
Adam18	0.99297113	-0.7034302	0.76425455	0.39242375	0.38582154
Scube2	0.47765852	1.10588406	0.76424845	0.3924256	0.38582154
Mrps18c	-0.1908109	4.53628145	0.7640304	0.39249189	0.3858434
Zfp865	0.15185327	4.44992551	0.7639162	0.39252661	0.3858434
Rxfp2	0.62347666	-0.5855475	0.76353358	0.39264298	0.38587753
A530064D06	0.96778368	-1.7685004	0.76323238	0.39273463	0.38587753
Actn4	-0.1005934	6.28219437	0.76321253	0.39274067	0.38587753
Emc8	-0.113837	5.95088228	0.76310956	0.392772	0.38587753
Stard10	-0.1958014	3.3328825	0.76306536	0.39278546	0.38587753
Lrch3	0.15723328	4.78819514	0.76295617	0.39281869	0.38587753
Slc35f2	-0.6812959	0.09581704	0.76271339	0.39289261	0.38587753
Mettl7a1	-0.1837843	6.05892047	0.76268825	0.39290027	0.38587753
Tpd52	-0.1185337	5.82796118	0.76263697	0.39291588	0.38587753
Gucy2f	0.71415859	0.43556857	0.76222158	0.39304241	0.38596013

Pdcd2	-0.1683596	3.87021714	0.76210218	0.39307879	0.38596013
Polr3k	-0.1482501	6.06279642	0.76179648	0.39317196	0.38601292
C530005A16I	0.21243661	3.24968215	0.76142416	0.39328548	0.38608567
Ociad2	-0.105967	6.64314573	0.76083954	0.39346382	0.38621999
Tspan13	-0.1817522	8.54940171	0.76070894	0.39350368	0.38621999
Gpld1	0.1420336	4.90241886	0.76058801	0.39354059	0.38621999
Abi3	-0.2882202	1.67972369	0.76039597	0.39359921	0.38623883
Col1a2	-0.2102272	7.37951761	0.75989912	0.39375096	0.38631936
4930414L22F	-0.2152872	3.17796153	0.75985646	0.39376399	0.38631936
Lzts3	0.11618888	5.7471234	0.75973991	0.3937996	0.38631936
Tmem98	-0.2794519	3.79390521	0.75938998	0.39390655	0.38638558
Dnph1	-0.3756441	1.62737352	0.75859039	0.39415108	0.38658673
Ugcg	0.14945873	6.93736904	0.75826799	0.39424974	0.38664478
Fam150b	-1.1988735	-1.1072825	0.75800848	0.39432918	0.38665887
Zfp788	-0.1023059	6.23732821	0.75796318	0.39434305	0.38665887
Krit1	0.1482368	5.84090534	0.75773798	0.39441201	0.38668498
Trp53i11	-0.2417012	8.13256173	0.75747645	0.39449212	0.38668498
Armc3	0.79874441	-0.9834486	0.75733675	0.39453493	0.38668498
Eml2	0.21485981	2.7595467	0.75721781	0.39457137	0.38668498
Ppp1r2	-0.1121537	8.03802023	0.75713355	0.3945972	0.38668498
Gm14405	0.43551841	0.15617204	0.75709575	0.39460878	0.38668498
AI987944	-0.189677	3.80950383	0.75697429	0.39464601	0.38668498
C130030K03I	0.30285916	2.25501604	0.75684117	0.39468682	0.38668628
Abhd8	-0.1324171	4.56973272	0.75645484	0.39480529	0.38675865
2610015P09I	0.17488203	3.72388813	0.75613579	0.39490317	0.38675865
Atp6v1c2	0.77269487	-0.1751808	0.75612865	0.39490536	0.38675865
Prickle3	-0.3356347	2.54428717	0.75608544	0.39491862	0.38675865
Med6	-0.1818858	4.33643809	0.75564448	0.39505397	0.38685252
Rpap3	-0.1251574	4.49697845	0.75496239	0.39526347	0.38697769
Rnf130	0.10162639	7.06320133	0.7548635	0.39529385	0.38697769
Tdo2	0.96952839	-0.9958907	0.75483976	0.39530115	0.38697769
Fmn1	-0.1158175	6.50161144	0.75471394	0.39533982	0.38697769
Dydc2	0.84279497	-0.8900281	0.75444138	0.3954236	0.38702103
Pms2	0.20847395	3.62544284	0.75413213	0.3955187	0.38707543
Hmga2-ps1	0.5445115	0.57436353	0.7534095	0.39574104	0.38725434
Cask	0.1053949	7.6195269	0.75314262	0.39582321	0.38728444
Anxa8	-0.3236459	3.46734733	0.75305274	0.39585088	0.38728444
Pcdhgb7	-0.2509662	2.21993231	0.75280093	0.39592844	0.38732163
Klhl12	-0.140878	4.79415184	0.75231832	0.39607715	0.38742532
Utp3	0.11297543	6.12821502	0.75210406	0.39614319	0.38742532
Kat2b	-0.1220861	6.08005269	0.75203184	0.39616546	0.38742532
Adap2	-0.2412803	3.73538247	0.75194369	0.39619264	0.38742532
Dnah11	0.62813963	-0.3051368	0.75141979	0.39635423	0.3875136

Xbp1	0.12361833	6.19549155	0.75139448	0.39636204	0.3875136
Eif4ebp2	-0.148915	5.77871641	0.75008683	0.39676582	0.3878464
Safb	-0.1154402	6.30017107	0.74999358	0.39679464	0.3878464
C130036L24R	0.66741989	-0.1689288	0.74990756	0.39682122	0.3878464
Fgf10	0.39580302	2.46889666	0.74949182	0.39694975	0.38793332
Armc7	-0.2805598	2.3542711	0.7492744	0.397017	0.38795195
Cyb5d2	-0.1831612	3.33322216	0.74917409	0.39704803	0.38795195
Knstrn	-0.5042883	0.64181907	0.74865376	0.39720904	0.38802568
4932435O22	-1.7085538	-1.8514079	0.74855323	0.39724016	0.38802568
Hk1os	-0.4874293	0.80907124	0.74852517	0.39724884	0.38802568
Heph	-0.4630527	2.0407408	0.74841828	0.39728194	0.38802568
Ptov1	-0.2278545	5.14555831	0.74817461	0.3973574	0.38806068
Rpa2	0.23487205	3.4815477	0.7478699	0.39745179	0.38811417
Hhex	-0.3471401	1.7525512	0.74772606	0.39749635	0.388119
Rps6kc1	0.13385871	4.98005424	0.74753145	0.39755667	0.38813241
Zbtb10	0.11776647	5.46634052	0.74742605	0.39758934	0.38813241
Psen2	-0.3205076	1.61387442	0.74694469	0.3977386	0.38817792
Bcl2l13	0.14827262	5.02911328	0.7467316	0.3978047	0.38817792
Adc	0.32756559	1.78714451	0.74667036	0.3978237	0.38817792
Cc2d1b	0.19047367	3.62334227	0.74665843	0.3978274	0.38817792
Cbfa2t3	-0.1511653	4.7943287	0.74663682	0.3978341	0.38817792
Afg3l2	0.12994253	5.7591495	0.74606556	0.39801141	0.38831224
Lipa	0.14306734	4.63551706	0.74572247	0.39811796	0.38837751
Mepce	-0.15482	4.31707559	0.74547028	0.3981963	0.38841525
2610035F20I	-0.2041431	3.01354408	0.7446234	0.39845956	0.38860247
Txnrd2	0.39672753	1.2978111	0.74459763	0.39846757	0.38860247
Izumo4	0.31618765	1.70919881	0.7436545	0.39876106	0.38879112
Zfp91	-0.0944623	7.46042978	0.74361786	0.39877248	0.38879112
Emilin1	-0.2814367	2.02283567	0.74355144	0.39879316	0.38879112
Apex2	0.55069732	1.18741274	0.74346595	0.39881978	0.38879112
Gm4477	-0.6205122	-0.8045183	0.74322046	0.39889625	0.38882697
Soga3	0.15517428	6.51710607	0.74294602	0.39898176	0.38884704
Rimkla	0.22686601	3.67743877	0.74289957	0.39899623	0.38884704
Slco1c1	0.20339445	4.33303442	0.74268441	0.39906329	0.3888737
Gabra3	0.11920182	5.47618171	0.74236649	0.39916241	0.3889316
Fam220a	-0.1317216	4.2702704	0.7422128	0.39921035	0.38893962
Rnf5	-0.1649496	5.68227105	0.74187129	0.39931688	0.38894377
Sec61a1	-0.1667879	4.9649272	0.74179828	0.39933966	0.38894377
Mfn2	0.11101204	6.63419644	0.7417748	0.39934699	0.38894377
Pcnt	0.155141	5.11387486	0.74169006	0.39937344	0.38894377
Syk	-0.3878319	1.46868149	0.7413566	0.39947752	0.38899044
Zbtb9	0.26911769	2.9040012	0.74128212	0.39950078	0.38899044
Dppa2	1.53398317	-1.8299176	0.74096231	0.39960065	0.38904901

Rmnd1	0.29153545	1.31481141	0.74057562	0.39972147	0.38912796
Zfp777	-0.2617316	2.08717096	0.73954692	0.40004313	0.38936939
Slc30a9	-0.1127196	6.51316511	0.73952824	0.40004897	0.38936939
Kifc5b	0.9630181	-1.1644293	0.73934129	0.40010748	0.38938764
Fhad1	0.29273278	2.56366346	0.73914036	0.40017037	0.38941015
Rbpms	-0.2503341	5.27075179	0.73895278	0.40022909	0.38942861
Ikbke	0.49468773	0.5804378	0.73856276	0.40035123	0.38950877
4930507D05I	-0.8605453	-1.1882022	0.73842683	0.40039382	0.38951151
Arhgef17	0.13649641	6.61858914	0.73816703	0.40047522	0.38955201
Klhl2	0.11225831	6.90275888	0.73748301	0.40068967	0.38972191
Agtrap	-0.2746263	4.03293785	0.73731169	0.40074341	0.38973548
Phf11a	-0.6037941	-0.4520695	0.73664408	0.40095293	0.38990053
Zc3h6	0.1629464	4.89306762	0.73616973	0.4011019	0.39000668
Abca9	-0.1604511	6.25692304	0.73589143	0.40118933	0.39005298
Gm11549	0.12029737	6.88483758	0.73562203	0.401274	0.39009658
Myocd	-1.2934082	-1.5673105	0.73545102	0.40132776	0.39010138
Bicd1	0.13117946	6.59984116	0.73535295	0.4013586	0.39010138
Bmp2k	0.13492742	5.06824754	0.73519375	0.40140866	0.39011133
Itsn2	0.10822098	7.26278222	0.73386713	0.40182621	0.39041391
11-Sep	-0.0993592	7.5978951	0.73384314	0.40183376	0.39041391
Dlgap3	0.18730762	4.07743113	0.73382472	0.40183957	0.39041391
Anapc2	0.11541721	4.74039092	0.73352744	0.40193323	0.39046617
Slc16a3	0.78230653	-1.1228918	0.73312677	0.40205952	0.39055013
1700021K19I	0.12185622	5.53596705	0.73285275	0.40214592	0.39059532
Unkl	0.15748422	5.40387752	0.73209079	0.40238633	0.39079008
Gm10814	-0.6746584	0.76249945	0.73148649	0.40257715	0.39093664
Fam104a	-0.1775196	5.23095913	0.73118509	0.40267238	0.39099035
Acox1	0.10467752	6.99373077	0.73096999	0.40274036	0.3910176
Atxn3	-0.124358	5.32775843	0.73047303	0.40289748	0.39113138
1700028J19F	-0.7549773	-1.2807606	0.73030043	0.40295207	0.39114561
Mrps27	0.19527626	3.35582252	0.72996074	0.40305955	0.39121117
Prr24	0.14782635	4.53434013	0.72980231	0.40310969	0.39121938
Ncmap	1.17721542	-0.974297	0.72968167	0.40314788	0.39121938
Yipf4	0.16282084	4.77633016	0.72943801	0.40322503	0.39125548
Fbxl3	-0.0972406	7.46914678	0.7291239	0.40332451	0.39131324
Fxyd6	0.16142655	5.08938502	0.72870458	0.40345737	0.39137003
1700007L15F	0.52825585	-0.0113701	0.72866601	0.4034696	0.39137003
Tnfaip2	0.45195149	0.47359873	0.72856095	0.4035029	0.39137003
Tmem240	-0.3322221	0.89522004	0.72836954	0.40356358	0.39139013
Cep44	0.21179982	2.75179763	0.7277227	0.40376875	0.39144559
Mecr	-0.3994259	1.30272389	0.72772023	0.40376953	0.39144559
Ndufs8	-0.230162	4.82298406	0.72769824	0.40377651	0.39144559
Eif3c	0.09211427	8.03434266	0.72750787	0.40383692	0.39144559

Lsg1	0.14839314	4.02144153	0.72748293	0.40384484	0.39144559
Ubald2	-0.2547805	2.32642476	0.72734844	0.40388753	0.39144559
H2afy	-0.1324481	5.91929454	0.72730756	0.4039005	0.39144559
Ntng1	0.14381378	5.3774984	0.72699991	0.40399819	0.39150153
Sec63	0.09271196	7.07591059	0.72630237	0.40421981	0.39167754
Ppcdc	-0.2457422	2.87627421	0.72585553	0.40436187	0.39172073
Dcp1b	0.16742657	4.21256879	0.72584717	0.40436453	0.39172073
AI606473	-0.6996669	-0.0018368	0.72567808	0.40441831	0.39172073
Anxa7	-0.1107951	6.45229665	0.72565911	0.40442434	0.39172073
Tor1aip2	-0.1429631	7.06303032	0.7253825	0.40451234	0.39176723
Tifab	-0.4475383	1.68083859	0.72503118	0.40462415	0.39183677
Kcna2	0.17092538	8.75410394	0.72449595	0.40479459	0.39189374
Adora2b	0.47117021	0.44978686	0.72442888	0.40481595	0.39189374
Spata33	0.81743703	-0.1889386	0.72428155	0.40486289	0.39189374
Gm1979	0.49597105	0.01681008	0.72422693	0.40488029	0.39189374
Rnf135	-0.2239845	2.75832843	0.72421837	0.40488302	0.39189374
Lrp5	-0.3326351	3.28031446	0.72394527	0.40497005	0.39193925
Pdcd2l	-0.194279	3.02662668	0.72379152	0.40501906	0.39194796
Rnf149	0.14440396	5.59735815	0.7235988	0.40508051	0.3919687
Itpkc	0.27360687	2.14887091	0.7232014	0.40520726	0.39205262
Dpm3	-0.3199236	2.16360858	0.72265611	0.40538128	0.39218225
Myadm	-0.1000018	7.63973607	0.72197486	0.40559885	0.39232503
8430408G22	1.3364461	-1.1148148	0.72194318	0.40560897	0.39232503
Rnf123	0.1454047	4.46546386	0.72164562	0.40570406	0.39237826
Zfp263	0.18819953	4.16060069	0.72148647	0.40575493	0.39238871
2610034M16	0.59382867	0.1506247	0.72114514	0.40586407	0.39241235
Tcf4	-0.0868214	9.26007733	0.72106059	0.40589111	0.39241235
Gm1141	-1.2104688	-1.6419073	0.72089972	0.40594257	0.39241235
Polr1e	0.25043012	1.80469132	0.72086575	0.40595344	0.39241235
Rragb	0.12406611	4.62507117	0.72078375	0.40597967	0.39241235
Polr1d	-0.1507005	6.27355112	0.72046301	0.40608231	0.39247283
Lhfpl2	-0.1605801	5.18302644	0.71996826	0.40624072	0.39249535
Cradd	-0.2044626	4.87140784	0.71994824	0.40624713	0.39249535
Snx25	0.1219181	5.1798809	0.7198803	0.40626889	0.39249535
4833418N02	-0.3264597	1.55173817	0.71986601	0.40627347	0.39249535
Lemd1	1.12965309	-1.7102109	0.71965177	0.4063421	0.39249535
Tcf19	-0.3048258	2.42159714	0.7196395	0.40634603	0.39249535
Fam187b	0.78239749	0.85249679	0.71931264	0.40645077	0.39255781
Ankrd52	0.13586383	5.89362456	0.71873353	0.40663645	0.3926918
Tnfrsf9	-0.9206348	-1.4474965	0.71862993	0.40666968	0.3926918
Slc22a8	-0.2085453	8.06097155	0.71846898	0.40672132	0.39270295
Pstpip1	1.02784744	-0.5833353	0.71812402	0.40683202	0.39277111
Tbc1d10c	0.8749263	-0.871725	0.71796357	0.40688352	0.39278213

Smim19	-0.1752707	4.67526929	0.71719488	0.40713042	0.39298174
E2f7	-0.9883971	-1.079622	0.71699794	0.40719371	0.39299753
3110062M04	0.39634336	0.70944072	0.71682928	0.40724792	0.39299753
Mphosph8	0.12814609	8.41146248	0.71665991	0.40730238	0.39299753
Pear1	0.28741046	3.42299803	0.7166447	0.40730727	0.39299753
Rad9b	0.48710775	0.48771744	0.71610745	0.40748008	0.39307776
Ltk	0.3861084	1.34450816	0.71602621	0.40750622	0.39307776
Btf3	-0.1703544	6.23544685	0.71601196	0.4075108	0.39307776
Psmc4	-0.1505416	4.92455703	0.71587382	0.40755526	0.39308193
Ubap1	-0.127052	5.09841286	0.71565784	0.40762478	0.39308622
Larp1	0.10248327	7.30000833	0.71561069	0.40763996	0.39308622
Adra2c	-0.271177	2.43547941	0.71523473	0.40776103	0.39310271
Nfia	-0.1347154	9.13850662	0.71518566	0.40777683	0.39310271
Tomm6	-0.1838027	5.29163217	0.71509338	0.40780656	0.39310271
Polr3f	-0.1337988	4.75486577	0.71505914	0.40781759	0.39310271
Lrfn4	0.30531717	2.51329453	0.71470271	0.40793244	0.39317473
Sacs	0.37139669	3.7593415	0.71429565	0.40806367	0.39326251
Spice1	-0.1907385	3.42151264	0.71405002	0.40814289	0.39330016
9030025P20I	0.20565018	3.21124724	0.71301114	0.4084782	0.39357811
1700023F06I	1.05882536	-1.5503012	0.71290744	0.40851169	0.39357811
Frmd6	0.13694834	5.61707454	0.71277815	0.40855345	0.39357964
Ptms	-0.236927	6.61621914	0.71226547	0.40871913	0.39370052
Cd28	-0.5321281	0.22626198	0.71095362	0.40914352	0.39405417
Trim32	0.12429904	7.59726018	0.71088196	0.40916672	0.39405417
Hadha	-0.1250447	4.94884333	0.71062056	0.40925138	0.39409695
0610010F05I	-0.1500359	5.99731277	0.71048547	0.40929514	0.39410034
Mrpl22	-0.2102346	3.03106697	0.71017293	0.4093964	0.39415911
Wdr46	0.22173176	4.10368222	0.70995798	0.40946607	0.39415987
Gm10789	1.06197837	-1.9141048	0.70992216	0.40947768	0.39415987
Samm50	0.16444212	3.50730105	0.70963438	0.40957099	0.39420267
Gm17019	0.79530349	-1.2206057	0.70953682	0.40960263	0.39420267
Map3k8	-0.2786644	1.90323526	0.70935185	0.40966262	0.39422168
Grb10	-0.0966479	6.92938384	0.70896538	0.40978802	0.39430361
Cntnap5c	0.43519931	1.45542887	0.70861015	0.40990333	0.39437583
Rc3h1	0.09467728	7.36999408	0.70825135	0.41001986	0.39444919
Rbms2	0.16278974	5.91312169	0.70783658	0.41015462	0.39453865
Col8a1	-0.2055725	5.5011309	0.70771726	0.4101934	0.39453865
Nyap2	-0.1473939	5.85461771	0.70728659	0.41033342	0.39457214
Gm715	-0.3478457	1.23912547	0.70727067	0.4103386	0.39457214
Mex3d	0.21513638	3.9163	0.70717552	0.41036955	0.39457214
Asb1	-0.1502356	4.51615356	0.70711464	0.41038935	0.39457214
Camk4	0.13744374	9.29810961	0.70673614	0.41051249	0.39462899
Slc29a2	0.50566931	0.35312579	0.70668524	0.41052906	0.39462899

Cdyl2	-0.1533843	5.28059106	0.70645882	0.41060275	0.39465118
Cct5	-0.1011918	6.33837543	0.7063668	0.41063271	0.39465118
Syt6	0.21823855	4.7974429	0.70576288	0.4108294	0.39480147
Tmem106a	0.45696611	1.6543206	0.70522694	0.41100407	0.39493059
Impdh1	0.25077217	2.57882557	0.70507086	0.41105496	0.39494075
B330016D10	0.58006152	0.44110558	0.70466405	0.41118765	0.3949913
Zfp661	0.20137701	3.17375051	0.70466227	0.41118823	0.3949913
Nos1ap	0.21989003	3.69490422	0.70434333	0.4112923	0.39501982
Cnot6	0.13299284	6.42197688	0.70422559	0.41133074	0.39501982
Mfap1b	-0.0972307	6.89563488	0.70420059	0.4113389	0.39501982
Ing2	-0.1301979	5.0945217	0.70386483	0.41144852	0.39508635
Nlrx1	0.38622748	1.86726911	0.70345633	0.41158195	0.39513526
Hnrnpdl	-0.0894437	7.47776317	0.70345206	0.41158335	0.39513526
Efcab5	0.31185842	2.66556156	0.70333847	0.41162047	0.39513526
Mcu	0.18543185	4.64332139	0.70300616	0.41172908	0.39520079
Ccdc124	-0.2164949	4.3329808	0.70262544	0.41185357	0.39523228
Atad3aos	-0.5867172	0.04760216	0.70223869	0.41198008	0.39523228
Rab4a	0.12639186	4.98587144	0.70210428	0.41202407	0.39523228
Fam134a	-0.1229301	5.4206259	0.7020535	0.41204069	0.39523228
Acaa2	-0.1717789	4.83542078	0.70205335	0.41204074	0.39523228
Camkv	0.14180811	6.00284055	0.70202694	0.41204938	0.39523228
Nnat	-0.2597204	8.65677086	0.70189756	0.41209173	0.39523228
E2f8	-0.5531152	0.8932315	0.70177336	0.41213239	0.39523228
Rab3c	0.13407569	9.29562799	0.70164293	0.41217509	0.39523228
Rem2	0.5160891	0.43792714	0.70154875	0.41220593	0.39523228
Zfp937	0.14611776	5.13090645	0.70153638	0.41220999	0.39523228
Syt16	0.12267284	6.11761101	0.70138784	0.41225864	0.39523228
Bfsp2	0.74081857	-0.7331281	0.70130301	0.41228643	0.39523228
Nr4a3	0.26207047	4.23225603	0.70040917	0.41257939	0.39547443
Snap91	0.15311568	8.93722681	0.69919773	0.41297697	0.39581679
Mob3b	0.15070373	6.71471211	0.69840196	0.41323845	0.3960032
Maged2	-0.1394317	4.4644189	0.69835232	0.41325477	0.3960032
Hsd17b13	-1.0007856	-1.215818	0.69823682	0.41329275	0.3960032
Cherp	0.10790205	5.54780331	0.69799507	0.41337225	0.39604064
Celf2	0.12549506	9.78204798	0.69760681	0.41349998	0.3960984
Mtmr6	-0.0955518	7.40515423	0.69752272	0.41352765	0.3960984
Ropn1l	-0.8597052	-1.0281018	0.69744311	0.41355386	0.3960984
Acat2	0.1319029	5.25145575	0.69707219	0.41367597	0.39614169
Lincrna-cox2	1.08873326	-1.3891797	0.69706012	0.41367994	0.39614169
Il17rd	0.2861568	2.50258226	0.69659535	0.41383303	0.39624955
Akr1c13	-0.3537474	1.750315	0.69627386	0.41393897	0.39631226
9330159F19I	0.16526901	6.85919471	0.69587391	0.41407083	0.39637858
Tomm6os	0.66372219	-0.1003295	0.69581829	0.41408917	0.39637858

Pdzd7	0.38715075	1.33017718	0.69525466	0.41427511	0.39650758
Pdrg1	-0.1881027	5.4404089	0.69511941	0.41431975	0.39650758
Mapk10	-0.1132727	9.60904329	0.69501387	0.41435459	0.39650758
Snhg6	0.30559817	1.68394643	0.69484637	0.41440989	0.39650758
Lmo2	-0.1783859	4.39602433	0.69479657	0.41442634	0.39650758
Exoc6	0.1227614	5.37342463	0.6944115	0.41455352	0.39655856
Cd248	0.23363544	4.35918922	0.69439008	0.41456059	0.39655856
Spg20	0.12399923	5.84138243	0.69370603	0.41478667	0.39673608
Prkaa2	0.1153534	7.2938567	0.69333265	0.41491016	0.39681545
H2-DMb1	-0.5704576	0.53285769	0.69224236	0.41527107	0.39709688
Vav2	-0.2745011	1.76348009	0.69219876	0.41528551	0.39709688
Gm16576	0.42395817	0.70651927	0.69207327	0.41532708	0.39709787
Fcrl6	0.53253331	0.28566598	0.69165229	0.41546659	0.39719249
Manea	0.13467671	5.51538336	0.69119704	0.41561754	0.39729802
Sapcd2	-1.0817628	-1.1384531	0.69103644	0.41567081	0.39731018
Dnajc17	-0.3358782	2.30473884	0.69061982	0.41580905	0.39740353
1810022K09I	-0.1348883	4.99892239	0.69027039	0.41592506	0.39743763
Map4k1	0.39170074	0.43633808	0.6901718	0.4159578	0.39743763
Rcor3	-0.1207974	5.44193821	0.69014573	0.41596645	0.39743763
Pitpnm1	0.21933797	3.35286185	0.68977887	0.41608831	0.39751529
Gm15446	0.33998451	1.42491026	0.68931821	0.41624141	0.39762277
Apba1	0.13950874	6.80136776	0.68871308	0.41644265	0.39777622
Lrrc3	0.19630907	3.66983848	0.68833375	0.41656888	0.39783568
Nutm1	1.43823703	-1.5031532	0.68825697	0.41659443	0.39783568
Ica1	0.17889072	4.14762539	0.68815989	0.41662675	0.39783568
Slco2b1	0.27905338	2.70595602	0.68798046	0.41668649	0.3978383
Mir103-2	0.64149632	0.14579881	0.68774195	0.41676592	0.3978383
Adal	-0.1518062	3.96032848	0.68770642	0.41677776	0.3978383
Erap1	0.25681003	3.54311035	0.6876638	0.41679196	0.3978383
Klhl14	0.34707278	1.43658351	0.6875367	0.4168343	0.39783995
Cenpb	-0.19039	4.12701979	0.6869586	0.41702697	0.39798506
Krt77	-0.7627142	-0.5571166	0.68658811	0.41715052	0.39806419
Pkp1	-0.4415849	1.20327792	0.68627274	0.41725574	0.39809877
Sepp1	-0.1857765	8.0461809	0.68623586	0.41726804	0.39809877
1810014B01I	-0.2547756	2.62046531	0.68563397	0.41746897	0.39825168
Tceal7	-0.6874021	-0.1028315	0.68536365	0.41755926	0.39829902
Mettl21a	-0.2395748	2.89583709	0.68517209	0.41762326	0.39832128
Mbnl2	-0.1029936	9.1115468	0.68477067	0.41775743	0.39841045
Fem1b	0.09912871	7.94531779	0.68411795	0.41797572	0.39851534
Zfp444	-0.1916242	3.28469872	0.68393621	0.41803654	0.39851534
Ptger2	-0.7099711	-0.470083	0.68387032	0.41805859	0.39851534
2310061J03F	-0.324956	2.3589709	0.68386552	0.41806019	0.39851534
Gzmm	1.04092013	-1.6068137	0.68373165	0.418105	0.39851534

Stk11	-0.2050932	5.51305633	0.68366389	0.41812768	0.39851534
Hectd2	0.16650678	4.47598569	0.68346063	0.41819574	0.39851534
Pnmal1	0.19788105	3.26626482	0.68340465	0.41821448	0.39851534
G6pdx	-0.1496009	5.75914343	0.68334766	0.41823357	0.39851534
Eppk1	-0.7501074	-0.5770483	0.68305866	0.41833037	0.39856881
Scmh1	-0.1225036	6.11744616	0.68213563	0.41863978	0.39882481
Wfdc2	-0.7462684	-0.6818079	0.681798	0.41875305	0.3988753
Zmym5	0.11273011	6.59672674	0.6817349	0.41877422	0.3988753
Dennd1c	0.81908813	-0.2605778	0.68146993	0.41886315	0.39892122
Pilra	-0.6197276	-0.5315319	0.68100277	0.41902001	0.39897987
Art3	0.80648123	-0.4051055	0.68098111	0.41902729	0.39897987
Pcdha3	0.86704282	-0.4062687	0.68087243	0.41906379	0.39897987
Elmod1	0.13164079	7.65046911	0.68080138	0.41908766	0.39897987
Mterf1a	-0.4127431	1.18052134	0.68064956	0.41913867	0.39898965
Mad2l2	-0.2435004	3.11655822	0.68043718	0.41921004	0.39901882
Dnah5	0.41479996	2.34714386	0.68029988	0.4192562	0.39902397
Chaf1a	-0.3055854	1.78037866	0.67931272	0.41958825	0.39930121
C2cd2	0.15882307	4.33129603	0.67856958	0.41983849	0.39950053
Zfp507	0.1336943	4.73466432	0.67816441	0.41997503	0.39952155
Rab10	-0.1012565	8.51286959	0.67815311	0.41997883	0.39952155
Cdkn2b	-1.0809021	-1.6133933	0.67814063	0.41998304	0.39952155
Lancl1	0.11985659	6.54720907	0.67801995	0.42002372	0.39952155
Best1	-0.323942	1.46628805	0.67745897	0.42021291	0.39963976
Zfp941	0.15069575	4.53019868	0.67740949	0.4202296	0.39963976
Sh3pxd2a	-0.1585301	7.07643607	0.67706919	0.42034443	0.39967893
Txndc15	0.1479696	4.33369455	0.67704562	0.42035239	0.39967893
Dusp6	0.17761029	5.38548791	0.67673145	0.42045846	0.39971535
5031439G07	0.10769325	5.64957202	0.67669043	0.42047231	0.39971535
Fgr	0.509415	0.32425747	0.67629029	0.42060746	0.39980502
1810034E14I	-0.418516	1.11510821	0.67611909	0.42066531	0.39982121
Plaa	0.0889023	6.40093827	0.67590365	0.42073812	0.39984082
Trmt2b	-0.2158741	5.21430636	0.67570206	0.42080627	0.39984082
Tubb6	-0.2390583	3.92177905	0.67558647	0.42084535	0.39984082
Cmtm4	-0.1070703	6.3562398	0.67551502	0.42086951	0.39984082
C530044C16I	-1.1061995	-1.6134224	0.67545432	0.42089004	0.39984082
Pqbp1	-0.1294978	4.38254597	0.67491375	0.42107292	0.39997577
Kcnq5	0.15488228	6.25302514	0.67476013	0.42112492	0.39998637
Apitd1	-0.7826883	-0.4734113	0.67439718	0.4212478	0.40003027
Pradc1	-0.2349579	2.72955504	0.67438238	0.42125281	0.40003027
Elac1	-0.1252491	5.18459079	0.6738109	0.42144642	0.40017533
Il18r1	0.79756603	-0.6174719	0.67325495	0.42163489	0.40023131
Idnk	-0.2217554	3.48742722	0.67320162	0.42165298	0.40023131
Dgkz	-0.1079064	6.32633871	0.67305223	0.42170365	0.40023131

Itgb6	-1.0263493	-1.8874354	0.6729804	0.42172802	0.40023131
Ccdc159	0.58980125	0.35808225	0.67283705	0.42177665	0.40023131
Ttc30a2	-0.4981966	-0.1179123	0.67267518	0.42183158	0.40023131
Lrrn4cl	-0.1271008	4.95697795	0.67260834	0.42185427	0.40023131
Gjb6	-0.1870686	8.86000288	0.67260783	0.42185444	0.40023131
Aspm	-0.3774991	1.19774706	0.67242501	0.4219165	0.40023131
Zbtb26	0.12178082	4.37235312	0.67236584	0.42193659	0.40023131
Tnpo3	-0.1135525	6.1065511	0.67231211	0.42195483	0.40023131
Ccdc71	-0.1829027	4.2507704	0.67158851	0.42220063	0.40042568
Brsk1	-0.1690643	3.49277298	0.67122897	0.42232284	0.40047919
Psmg1	-0.2343653	3.13073301	0.67118197	0.42233882	0.40047919
Lhfp	-0.1787054	6.11296999	0.67028568	0.42264374	0.40072195
Nup88	0.12321096	5.69244822	0.6701777	0.4226805	0.40072195
Htatsf1	-0.0868718	8.39789574	0.66999691	0.42274206	0.40072195
Ninj1	-0.4130457	2.11751895	0.66982311	0.42280125	0.40072195
AW554918	-0.1192063	4.9547317	0.66969628	0.42284445	0.40072195
Cthrc1	-0.1962817	2.88728204	0.66961938	0.42287064	0.40072195
Aldh16a1	0.62680808	0.16075163	0.66958841	0.4228812	0.40072195
Atp10b	0.37519636	1.16803816	0.66937159	0.42295507	0.40072374
Fgfrl1	0.2364487	2.25860153	0.66930048	0.42297931	0.40072374
Thoc5	0.18659301	3.55371757	0.66919877	0.42301397	0.40072374
Pdgfd	0.30101712	3.02315085	0.6691027	0.42304673	0.40072374
Tmem132e	0.33559785	1.29276568	0.66869575	0.42318549	0.40079037
Trip10	-0.3540882	2.25482018	0.66865643	0.4231989	0.40079037
Tctn2	-0.2007658	2.69486415	0.66828841	0.42332446	0.40087052
Podxl	0.11779286	4.29086336	0.66814535	0.42337328	0.400878
Ppp4r2	-0.0989001	8.3018626	0.66776766	0.42350222	0.40096134
Qtrt1	0.23316181	2.71706356	0.66743917	0.42361442	0.40102879
Rnaseh2c	-0.3294014	2.73847874	0.66689507	0.42380035	0.40116605
Gba	-0.1503564	3.90078188	0.66672611	0.42385812	0.40118196
Gnpda2	-0.1368309	5.96309501	0.66652478	0.42392696	0.40120835
4430402I18R	-0.2081244	2.61787943	0.66617309	0.42404727	0.40128344
Cbx7	-0.1648357	4.92502466	0.66601522	0.4241013	0.4012958
Fgfr3	0.16677716	3.90040749	0.66545426	0.42429334	0.40140294
Gusb	0.2137531	2.80361293	0.66544506	0.42429649	0.40140294
Setd5	0.10525674	8.40831928	0.66495372	0.42446481	0.40149202
C2cd4d	-1.243296	-2.1321454	0.66493094	0.42447262	0.40149202
Rgs17	-0.1332606	8.03310869	0.66460691	0.42458369	0.40152875
Lrrc75b	0.18242334	4.44886064	0.66457846	0.42459344	0.40152875
Plin3	-0.1820274	3.68225391	0.66431671	0.42468319	0.40157486
Thbd	-0.2336287	8.3227459	0.66393822	0.42481303	0.40162924
Aebp1	-0.2428778	7.19862456	0.66391001	0.42482271	0.40162924
2810013P06I	-0.164707	4.65624148	0.66355273	0.42494534	0.40166985

Hsd11b2	1.15551589	-1.1414281	0.66354591	0.42494768	0.40166985
Synj2	0.14733455	6.45483524	0.66323453	0.42505459	0.40173215
Tmem243	0.18384436	4.06310175	0.66295582	0.42515033	0.40178386
Bphl	-0.1977344	4.07017953	0.66281405	0.42519904	0.40179113
Cxxc4	-0.1294751	6.13019003	0.66265134	0.42525495	0.4018052
Pdgfrb	0.24254166	4.4274663	0.66225749	0.42539035	0.40189436
Vps26b	-0.0872921	7.41283833	0.66199593	0.4254803	0.40194058
Hmgb1	-0.1246684	8.09298652	0.66184996	0.42553051	0.40194925
Fam180a	-0.2930244	5.14193717	0.66168102	0.42558864	0.40196539
Zfp775	-0.2835803	3.00237761	0.66135992	0.42569915	0.40202435
Tfcp2l1	-0.1926096	7.02929852	0.66126119	0.42573314	0.40202435
Bscl2	0.17373411	4.33923054	0.6610197	0.4258163	0.40206411
Rrp15	-0.2220217	2.82371295	0.66079473	0.42589378	0.40209852
Zfp2	0.14217017	4.45091169	0.66062812	0.42595119	0.40211395
Clec7a	-0.7942696	0.80824858	0.65984589	0.42622085	0.40230344
Rxfp3	0.56431126	1.2141781	0.65980761	0.42623405	0.40230344
Wbp1l	-0.1657513	5.04040967	0.65938995	0.42637814	0.40231958
Aldh9a1	-0.1228982	5.52377629	0.65935791	0.4263892	0.40231958
Lhx6	0.14880783	4.21620547	0.65923465	0.42643175	0.40231958
Smim8	-0.2318685	3.79567374	0.65917888	0.426451	0.40231958
Rims2	0.17372377	6.98291186	0.65909467	0.42648007	0.40231958
Pex1	0.16023448	4.55242646	0.65904393	0.42649758	0.40231958
Pcdhga11	0.26510651	3.13527731	0.65865395	0.42663226	0.40238416
Tspan17	-0.1581844	3.27720702	0.65830993	0.42675112	0.40238416
Cbl	0.10061701	6.56782542	0.65816686	0.42680056	0.40238416
Tmem9b	-0.1098558	5.44738389	0.65809456	0.42682555	0.40238416
Mcur1	0.18244855	5.21029881	0.6580753	0.42683221	0.40238416
Alyref	-0.2066544	2.80293546	0.65805368	0.42683969	0.40238416
Ankrd42	0.14066709	4.01704178	0.65799209	0.42686098	0.40238416
Esyt1	0.20637543	3.45697832	0.65783904	0.4269139	0.40238416
Klhl1	0.24346538	3.01700362	0.65777581	0.42693576	0.40238416
Pllp	0.199118	2.36712096	0.65737399	0.42707475	0.4024471
Papss1	-0.1181168	4.96505417	0.6573452	0.42708471	0.4024471
Arsb	0.10928979	7.01737196	0.65705365	0.4271856	0.40248186
Rb1	0.10805394	6.94098613	0.65700114	0.42720378	0.40248186
Cldn22	1.29143715	-1.6312496	0.65659327	0.427345	0.40252574
Zdhhc14	0.15807306	4.30619587	0.65657932	0.42734983	0.40252574
Tpp2	0.13812744	6.74275554	0.65651061	0.42737363	0.40252574
Brd1	-0.1028823	6.0043742	0.65585752	0.42759993	0.40270016
Fam98b	-0.1291852	6.91903186	0.65547986	0.42773088	0.40278126
Ank	0.11314134	6.15385939	0.65525734	0.42780806	0.40278126
Ribc1	0.53710351	-0.2145785	0.65525349	0.4278094	0.40278126
Fam47e	-1.0696165	-1.255421	0.65481325	0.42796217	0.40288324

Sytl4	-0.3550418	2.35993316	0.65470432	0.42799998	0.40288324
Gm12185	0.39358476	0.86068717	0.65426636	0.42815207	0.40297846
Afap1	0.15008702	7.61847143	0.65417611	0.42818342	0.40297846
1700018G05	-0.8398185	-0.9983156	0.65387954	0.42828646	0.40299921
Gpr151	1.16788283	-1.6206207	0.65360973	0.42838025	0.40299921
Vta1	-0.1361747	5.04032592	0.65356143	0.42839704	0.40299921
Wbp11	-0.0993176	6.05145539	0.65340572	0.42845118	0.40299921
1700096K18I	0.39733482	1.18970557	0.65334534	0.42847218	0.40299921
Wasf1	0.14281549	7.99226552	0.65320134	0.42852226	0.40299921
Qser1	0.12908753	6.91275708	0.65305283	0.42857392	0.40299921
Emx2	-0.2003108	2.96208256	0.6529987	0.42859275	0.40299921
Ccdc60	-0.7720745	-0.8743788	0.65265884	0.42871102	0.40299921
Utp18	-0.1346073	4.20392868	0.65261	0.42872802	0.40299921
Dph6	-0.099903	6.23839124	0.65252466	0.42875772	0.40299921
Hs6st3	0.20580239	2.87786613	0.652511	0.42876248	0.40299921
Six2	-0.2052365	5.88949627	0.652481	0.42877293	0.40299921
B3gnt8	-0.6334141	-0.1537182	0.65245638	0.4287815	0.40299921
Ghitm	-0.0897653	8.70164854	0.65233836	0.42882259	0.40299921
2310011J03F	0.19645625	2.93835107	0.65164906	0.42906272	0.40318619
Sc5d	0.13914509	4.4301661	0.65136867	0.42916045	0.40320103
Asprv1	-0.5122437	0.50430577	0.65136759	0.42916083	0.40320103
Atp10d	0.24833518	2.36718858	0.65108945	0.42925782	0.40325347
Gm2897	0.18825908	3.6036603	0.65018849	0.42957222	0.40343379
Pcbp4	0.21063457	2.70007356	0.65012144	0.42959563	0.40343379
Zfp958	0.18819324	3.44409452	0.65000545	0.42963614	0.40343379
LOC1010557(	-1.3039348	-1.8437998	0.65479323	0.42964369	0.40343379
Trim39	0.13745981	4.31409837	0.64987054	0.42968326	0.40343379
Cenpf	-0.2138846	2.51014231	0.64983155	0.42969688	0.40343379
Plekha4	0.45280401	0.34010636	0.64923084	0.42990681	0.40357814
Glra4	-0.9368606	-1.6182774	0.64911636	0.42994683	0.40357814
Tceb3	-0.1256684	6.00143541	0.64902189	0.42997986	0.40357814
Thtpa	-0.1321053	4.55589553	0.64892017	0.43001544	0.40357814
Zfp954	-0.2980258	1.98684143	0.64867255	0.43010205	0.40358578
Itgal	-0.4460096	0.64269499	0.64866133	0.43010597	0.40358578
Lix1	-0.118189	4.84179322	0.64853881	0.43014884	0.40358734
Pafah1b1	0.09451076	9.15130596	0.64815947	0.4302816	0.40367324
6030419C18I	0.28964135	2.38184951	0.64716624	0.43062953	0.40396095
Lrmp	0.75529171	-0.0754907	0.64690532	0.430721	0.40400807
Kpna4	-0.0817105	7.20475338	0.64655468	0.43084397	0.40408472
Adprm	-0.2083491	3.70174473	0.64642991	0.43088775	0.40408709
Pald1	-0.2969326	1.79750332	0.64618945	0.43097212	0.40412753
Suclg2	-0.1615503	5.58089658	0.64551185	0.43121004	0.40430001
Bend3	-0.1897424	4.24324062	0.64543048	0.43123862	0.40430001

Acss2os	-0.8870724	-1.5760205	0.64515794	0.43133438	0.40432883
Gmppb	0.40247298	0.94314568	0.64510807	0.43135191	0.40432883
Csf3r	0.35150888	1.43917202	0.6444536	0.43158201	0.40450176
Adpgk	0.29361789	2.07154623	0.64434849	0.43161899	0.40450176
Ccdc138	-0.2375733	2.60096319	0.64356646	0.43189423	0.4046888
Txnip	-0.1937823	6.72781444	0.64354669	0.43190119	0.4046888
3110001I22R	0.34116349	1.10874887	0.6428937	0.43213124	0.40486562
Akr1c12	0.92992267	-0.1706401	0.64251921	0.43226326	0.40495058
Prkacb	-0.090178	9.17765633	0.64186911	0.43249259	0.40509822
Emc7	-0.1706451	5.39308965	0.641838	0.43250357	0.40509822
Etv6	-0.1186217	5.18970227	0.6414681	0.43263415	0.40515228
Parg	0.10625551	5.62381836	0.64140691	0.43265576	0.40515228
Zbtb1	-0.1255332	4.59540125	0.64105358	0.43278055	0.40515228
Aktip	-0.1072515	6.09517602	0.64103371	0.43278757	0.40515228
Tgm1	1.04639155	-1.4017659	0.64088448	0.4328403	0.40515228
Mia	0.52675055	-0.1530321	0.64088124	0.43284144	0.40515228
Lrrc47	0.26819328	2.66498574	0.64076173	0.43288368	0.40515228
Fgd3	0.72601018	-0.2428206	0.64073766	0.43289218	0.40515228
Prdm6	-0.2323834	5.62563107	0.63966688	0.43327089	0.40546798
Ppp6r1	-0.1365012	4.81027619	0.63921686	0.4334302	0.40556019
Rusc1	0.15408823	5.8504456	0.63915465	0.43345223	0.40556019
Med1	-0.1049127	7.2607964	0.63859157	0.43365172	0.40570809
Nedd4	0.08156984	9.5001887	0.63846602	0.43369622	0.40571097
Ttc27	0.15957399	3.37302631	0.63804087	0.43384696	0.40581323
Rundc3a	-0.1365749	5.33651374	0.63763341	0.43399151	0.40587088
Ubc	-0.126329	6.71051976	0.63754521	0.43402281	0.40587088
Sf3a3	0.13009038	4.70077619	0.63742403	0.43406581	0.40587088
Gm10865	0.45122374	-0.4188497	0.63740001	0.43407434	0.40587088
Azi2	-0.1156191	6.72420063	0.63714343	0.43416543	0.40590102
Smim12	0.24299766	2.10273062	0.6370758	0.43418944	0.40590102
Pgap2	-0.1724024	4.20578138	0.63688536	0.43425708	0.40592551
Gria4	0.13621465	6.76190526	0.63621655	0.43449473	0.4061089
Gng5	-0.1642043	6.39957695	0.63584339	0.43462742	0.4061849
Srsf7	0.09510111	5.89883115	0.63564438	0.43469821	0.4061849
Rerg	0.2968125	2.91998122	0.6356381	0.43470045	0.4061849
Zkscan2	-0.1327562	4.76898938	0.63533317	0.43480895	0.40624754
Plp2	-0.2464532	4.26104445	0.63521502	0.43485101	0.40624808
Dab2ip	-0.1027586	6.24974934	0.63507094	0.4349023	0.40625725
Slc35g1	-0.2237048	4.50834341	0.63475722	0.43501401	0.40630462
Aard	0.31699636	1.58846714	0.63469556	0.43503597	0.40630462
Mpp5	-0.1010253	6.90625661	0.63394581	0.43530316	0.40650473
Arhgap42	0.1083606	5.70538165	0.63386148	0.43533323	0.40650473
Purg	-0.1367899	6.01491215	0.63373121	0.43537969	0.40650936

Insig2	-0.0940219	6.13606135	0.6335059	0.43546006	0.40654034
Mlh3	-0.1110533	5.77549953	0.63340551	0.43549587	0.40654034
Il18bp	0.254093	3.28860402	0.63311163	0.43560074	0.40659949
Tmem35	0.18829944	3.85577349	0.63276383	0.43572491	0.40666496
Nlgn2	-0.1152867	6.3200892	0.63268259	0.43575392	0.40666496
Zfp709	0.17253283	4.13899333	0.63234319	0.43587516	0.40670983
Gpr124	-0.1930027	4.91586024	0.63231554	0.43588504	0.40670983
Spr	0.24811823	2.83179434	0.63203646	0.43598477	0.40676414
Ccdc32	-0.1623912	4.90145932	0.63180018	0.43606924	0.40677821
Ppig	-0.1291924	9.2691446	0.63164788	0.4361237	0.40677821
1190002N15	0.13136718	5.34921163	0.63164581	0.43612444	0.40677821
2210039B01	0.27921348	1.77921097	0.62978507	0.43679067	0.40736082
Scoc	-0.114248	8.74565605	0.62953313	0.436881	0.40740628
Clcn3	0.10674591	6.71782553	0.62941089	0.43692483	0.40740837
Kif9	0.32051573	2.35054182	0.62918537	0.43700573	0.40741652
Tbc1d22b	-0.1670443	4.46307118	0.62915462	0.43701676	0.40741652
Col22a1	0.74767717	-0.3577219	0.62858884	0.43721983	0.40756704
4930452B06	0.19551909	3.51300428	0.6280682	0.43740682	0.40770255
Acin1	0.0924085	5.86499961	0.62793006	0.43745645	0.40771002
Zfp9	-0.112131	5.92135413	0.62723366	0.43770681	0.40790455
Pnma1	-0.3315882	1.58380208	0.62653124	0.43795957	0.40808878
Pidd1	-0.810515	-1.6530424	0.62645274	0.43798783	0.40808878
Klre1	-0.8527966	-1.0160264	0.62601447	0.43814567	0.40819702
Vip	0.21388782	2.80295463	0.62578468	0.43822847	0.4082042
Smyd2	-0.1395867	5.5721474	0.62576176	0.43823673	0.4082042
Ckap5	0.15522811	7.87263961	0.62509569	0.43847686	0.40838904
Ptpn9	-0.1135128	5.88928776	0.62417685	0.43880847	0.40860989
1110032F04	0.25554463	2.18019869	0.62411106	0.43883223	0.40860989
Kifc3	0.19047262	3.01247194	0.62409197	0.43883912	0.40860989
Nup54	-0.1584348	3.41473095	0.62375288	0.43896161	0.40868509
Fbxo17	-0.3042834	1.79926331	0.62342139	0.43908141	0.40871493
Lpin2	0.1210435	6.58671134	0.62334745	0.43910814	0.40871493
Usp36	0.14766883	4.61989909	0.62326136	0.43913926	0.40871493
L3mbtl1	0.40301564	1.58648883	0.62320244	0.43916057	0.40871493
Pyroxd2	-0.3136283	1.1896043	0.62245138	0.43943227	0.40880276
Tmem150a	-0.2246353	2.52339698	0.62238241	0.43945723	0.40880276
Mib2	0.18032779	3.61288825	0.62233334	0.43947499	0.40880276
Guk1	-0.1821498	4.85729885	0.62232668	0.4394774	0.40880276
6430503K07	0.73643307	-0.7179992	0.62225179	0.43950451	0.40880276
Pigh	0.29079572	2.03473925	0.62211774	0.43955305	0.40880276
Ccdc6	0.10240115	6.7624702	0.62198961	0.43959945	0.40880276
9330020H09	-0.6244868	-1.0428225	0.62191099	0.43962792	0.40880276
A230009B12	0.71282625	0.00823792	0.62190375	0.43963054	0.40880276

1700026L06F	0.89722189	-1.4318368	0.62160474	0.43973886	0.40884193
H2-Q5	0.5424591	-0.6068189	0.62149459	0.43977878	0.40884193
Btbd10	0.11407513	5.98224862	0.62144186	0.43979789	0.40884193
Kcnh7	0.16385418	6.28135969	0.62091666	0.43998829	0.4089467
Cpped1	0.10068408	6.46665598	0.62090067	0.43999409	0.4089467
Eps8	0.11822998	5.3883578	0.62066899	0.44007813	0.4089609
Atraid	0.13607016	4.54314961	0.62048902	0.44014342	0.4089609
Bloc1s1	-0.2596695	5.14775056	0.62048546	0.44014472	0.4089609
Creb3l4	1.03334968	-0.9489596	0.62039825	0.44017636	0.4089609
Phf19	-0.5409052	0.71528443	0.62015452	0.44026483	0.40899048
Nucks1	-0.1003891	8.85000621	0.62008046	0.44029172	0.40899048
Myo1g	0.69945585	0.13617966	0.6199115	0.44035307	0.40900869
Jmjd8	-0.1824582	3.88038761	0.61974024	0.44041527	0.40902767
Cux1	0.07649691	8.04491016	0.6192614	0.44058925	0.40908387
LOC1008616	0.5175908	-0.5326978	0.61920381	0.44061018	0.40908387
Samd5	0.17031048	3.98939051	0.61916073	0.44062584	0.40908387
Plcl1	0.13184678	5.43543837	0.61911401	0.44064282	0.40908387
Gzma	1.09581283	-0.5829634	0.61852003	0.44085883	0.40924562
Triobp	-0.193016	6.03592652	0.61823933	0.44096097	0.40930164
Zfp37	0.13519455	6.80022442	0.61791345	0.4410796	0.40936416
Acot3	-0.6150421	0.33841072	0.61775309	0.44113799	0.40936416
Gm13152	0.37604015	1.28296953	0.61770995	0.4411537	0.40936416
4732416N19	-0.9380803	-1.9310366	0.61730813	0.44130008	0.40946121
Mcfd2	-0.167225	4.96654256	0.61695447	0.44142898	0.40951393
8430431K14I	0.36908176	0.78650113	0.61692282	0.44144052	0.40951393
Fkrp	-0.1147909	5.29122087	0.6162921	0.44167056	0.40967865
B3gnt6	-1.2820736	-1.3576428	0.61618677	0.441709	0.40967865
Gm8979	-0.3299073	0.55509727	0.61609209	0.44174355	0.40967865
Mrpl33	-0.1868967	4.31556903	0.61575559	0.44186639	0.40971718
B230219D22	-0.1027736	8.00694298	0.61574911	0.44186876	0.40971718
08-Mar	-0.1007509	6.24265239	0.61495329	0.4421595	0.40994797
Gm15328	0.63062531	-0.036985	0.6146768	0.44226058	0.40996649
1600014C10I	-0.1545141	4.83188295	0.61466833	0.44226368	0.40996649
Zscan29	0.13592637	5.40459204	0.61452942	0.44231448	0.40996649
Slc1a4	0.16720076	4.33679528	0.61444079	0.4423469	0.40996649
Gm5083	-0.9201845	-0.806053	0.61404273	0.44249254	0.41002814
Porcn	0.15328419	4.36489916	0.61403016	0.44249713	0.41002814
Cwc22	0.11474629	5.52773881	0.61301679	0.44286825	0.41033047
Dhx40	0.11118909	5.37711587	0.61291054	0.44290719	0.41033047
Rock2	0.11617005	10.0961384	0.61255039	0.44303922	0.41040159
Plcxd1	0.31856419	1.75679054	0.61239765	0.44309523	0.41040159
Flcn	0.11423515	4.56344802	0.61225876	0.44314618	0.41040159
Twsg1	-0.1777916	7.87788273	0.61217188	0.44317805	0.41040159

Adra1d	-0.1458913	3.5059579	0.61194118	0.4432627	0.41040159
Taf1d	0.14709392	3.99055053	0.61192036	0.44327034	0.41040159
Fkbp7	0.22205712	3.61304404	0.61184978	0.44329625	0.41040159
Nlgn1	0.14239002	6.61629113	0.61142806	0.44345107	0.41040159
Tada3	-0.1305569	5.0122739	0.61136702	0.44347349	0.41040159
Tmtc1	0.12215907	8.45845134	0.61114938	0.44355343	0.41040159
Sucla2	-0.088248	6.74457014	0.61114747	0.44355414	0.41040159
Ramp2	-0.2152766	4.5868832	0.61112398	0.44356276	0.41040159
Fzd4	-0.2319409	2.98805303	0.61104389	0.44359219	0.41040159
Slc22a6	-0.2224181	7.15639669	0.61089956	0.44364523	0.41040159
Rgag4	0.22987239	2.68256884	0.61083241	0.44366991	0.41040159
Ptpn21	-0.1476712	4.8586225	0.61076299	0.44369542	0.41040159
Hyal2	0.31853909	1.18618416	0.61057893	0.44376308	0.41040159
Clec4a3	0.92582546	-0.2808366	0.61055396	0.44377226	0.41040159
Gba2	0.2048461	3.2358917	0.6105329	0.44378	0.41040159
Cbfa2t2	-0.0855453	6.40881928	0.6098601	0.44402749	0.41055344
E130114P18I	0.37358975	0.83679261	0.60985862	0.44402803	0.41055344
Mapk12	0.27070167	1.78775596	0.6092169	0.44426429	0.41073311
Nmt1	0.09474358	5.26669908	0.60834253	0.44458652	0.41098739
Dusp2	-0.7247652	-1.032968	0.60824295	0.44462324	0.41098739
Gm4285	-0.2897698	1.33931549	0.60702191	0.44507392	0.41135261
Fam49b	0.09953086	6.79964942	0.606945	0.44510233	0.41135261
Cpsf3	0.11701777	5.09229648	0.60679344	0.44515833	0.41136554
Ccdc30	-0.2105427	3.32439912	0.60636775	0.44531567	0.41139698
Nr4a1	-0.4060828	4.92205442	0.60631006	0.445337	0.41139698
AU023762	0.17639124	3.25929013	0.60627474	0.44535006	0.41139698
Zfp874b	-0.1442264	4.64430705	0.60624694	0.44536034	0.41139698
Olfr691	1.20553965	-2.0704792	0.60563689	0.44558601	0.41154332
Fibcd1	-0.5295133	0.43586962	0.60559156	0.44560279	0.41154332
Slc35a3	0.12066447	4.86209558	0.6051418	0.4457693	0.41165828
Nmbr	0.54365319	0.19861684	0.6049966	0.44582307	0.41166913
Pknox2	-0.1041202	7.21215062	0.60436349	0.44605767	0.4117843
Cntrl	0.16565787	4.31281987	0.60418743	0.44612295	0.4117843
Bnip2	-0.1337441	7.06219684	0.60406241	0.44616931	0.4117843
Nfix	-0.0982746	8.37197493	0.60402039	0.44618489	0.4117843
Acly	0.1218097	7.0689687	0.60400334	0.44619122	0.4117843
Tmem80	0.19452525	2.82179066	0.60384835	0.44624871	0.4117843
Rnf144b	-0.140953	4.36927858	0.60361968	0.44633355	0.4117843
Arrb1	0.10839745	7.40032448	0.60355049	0.44635922	0.4117843
Itgb1	0.15745276	6.34118709	0.60347255	0.44638815	0.4117843
Tldc1	0.32321713	1.15750519	0.60346949	0.44638929	0.4117843
Rnf170	-0.1174992	5.90689747	0.60334797	0.44643439	0.4117843
Map4k4	0.10832122	6.42734492	0.60318822	0.4464937	0.4117843

6430548M08	0.11965219	6.91741558	0.60318661	0.4464943	0.4117843
Paqr9	0.15439285	5.44706084	0.60280275	0.44663686	0.411877
Slc25a4	-0.0943079	9.98946024	0.60249514	0.44675115	0.41187899
Tmem119	-0.2514183	3.49084428	0.60248902	0.44675343	0.41187899
Tnfrsf1b	-0.4247092	1.11957083	0.60245745	0.44676516	0.41187899
4933402D24	0.54076479	0.32881142	0.6020244	0.44692615	0.4119436
Itch	0.08573485	7.05379418	0.60188351	0.44697855	0.4119436
Ptger4	0.47729425	0.6562272	0.60187737	0.44698083	0.4119436
Srbd1	0.17512721	3.49013912	0.60173558	0.44703358	0.4119436
Cd80	-0.367354	1.40087992	0.60170349	0.44704552	0.4119436
Snord42a	0.61435451	-1.1470432	0.60144261	0.44714259	0.41199429
Cep162	-0.0984541	6.06887196	0.6008593	0.44735976	0.41215562
Vars	-0.233378	2.45858868	0.60013078	0.44763123	0.41236694
Zfp358	0.17114482	3.17915035	0.59930163	0.44794052	0.41261306
Oard1	-0.1692467	4.57408272	0.59901823	0.44804632	0.41267171
E130008D07	0.30262332	2.27454662	0.59881059	0.44812386	0.41270432
Zxda	0.13249159	5.37995996	0.5985673	0.44821474	0.41274921
Crlf2	-0.3780375	0.43132701	0.59808779	0.44839394	0.41287542
Fam98a	0.10749517	5.72445129	0.59796545	0.44843968	0.41287872
Tmem53	0.41451711	1.62310313	0.59688661	0.44884336	0.41318392
Myh4	0.81378498	-0.373539	0.5968541	0.44885553	0.41318392
Gpr26	0.24834803	3.43552285	0.59629951	0.44906328	0.41331769
Olfm3	0.13686433	4.62868668	0.59624093	0.44908524	0.41331769
Rbm46	-0.4556611	1.57546975	0.59600176	0.44917488	0.41336135
Ighmbp2	0.27197661	1.95229387	0.59555157	0.44934371	0.41338211
Kctd10	-0.1526224	4.71801975	0.59550515	0.44936113	0.41338211
Ttc39d	-1.1892421	-1.637163	0.59548435	0.44936893	0.41338211
Myoc	0.2326441	2.14626982	0.5953817	0.44940744	0.41338211
Kcnip2	-0.0989648	5.69882774	0.59537901	0.44940845	0.41338211
Frmd5	-0.1589473	5.14701112	0.59521746	0.44946907	0.41339905
Tfap4	0.3048516	1.05252867	0.5949863	0.44955584	0.41344004
Cstf2	0.13687734	6.37257671	0.59466998	0.44967461	0.4134422
Hat1	-0.1242079	5.05281116	0.59460104	0.44970051	0.4134422
Nutf2	-0.1912705	3.14388551	0.59419143	0.4498544	0.4134422
Psme2b	-0.2178599	3.17254541	0.5940766	0.44989756	0.4134422
Ifngr1	0.19332315	3.18392937	0.59403323	0.44991386	0.4134422
BC030307	0.3990434	0.74594489	0.59401105	0.44992219	0.4134422
1700109H08	1.05096048	-1.4188016	0.59393112	0.44995224	0.4134422
Mpp2	-0.0985326	6.71920698	0.59389776	0.44996478	0.4134422
Wdr86	-0.5700863	1.31056023	0.59387992	0.44997149	0.4134422
LOC1012436	0.64058559	-0.5993772	0.59385658	0.44998027	0.4134422
Fmr1	-0.0916342	6.28502288	0.59359733	0.45007776	0.41349299
Phrf1	0.14548306	4.78880025	0.59319472	0.45022923	0.41359335

Gars	0.08868976	6.01948736	0.59307652	0.45027371	0.41359543
Mtus2	-0.0945876	6.24056567	0.59290269	0.45033915	0.41361675
1700003E16I	0.39422991	0.61505747	0.59274094	0.45040005	0.4136339
Klhl28	0.17825499	3.90429028	0.59250533	0.45048878	0.41367562
Calhm2	0.2800058	2.54101821	0.59239606	0.45052994	0.41367562
4930583P06I	0.96280144	-1.6649089	0.59203027	0.45066778	0.41374621
Zic3	0.34719919	1.36545791	0.59187129	0.45072771	0.41374621
2810408I11R	0.53413341	0.38143534	0.59159279	0.45083272	0.41374621
2700049A03I	0.14859243	3.7645438	0.59153015	0.45085634	0.41374621
Prcc	-0.1630562	4.29593695	0.59149528	0.45086949	0.41374621
S100a8	1.04718231	-1.1417502	0.59146831	0.45087967	0.41374621
Gtf3a	0.2218112	2.55083537	0.59140779	0.4509025	0.41374621
Trappc13	-0.0904698	6.39477302	0.59115548	0.45099769	0.4137948
Dcp1a	0.1067543	5.44539344	0.59053511	0.45123189	0.41395544
Gm9866	-0.2192445	2.57794585	0.59031818	0.45131383	0.41395544
Grina	-0.1116461	7.00624105	0.59025628	0.45133722	0.41395544
Ercc8	-0.2650311	2.35817298	0.5902441	0.45134182	0.41395544
Zfp111	0.1404829	5.16737656	0.58989037	0.4514755	0.41400733
Msantd2	0.27293369	3.04456159	0.58986006	0.45148696	0.41400733
Traf5	0.32027064	1.45352908	0.58950912	0.45161966	0.41400733
Zfp946	0.21400094	2.87394696	0.58950629	0.45162073	0.41400733
Garnl3	-0.1738275	5.19900696	0.58936019	0.45167599	0.41400733
Col6a1	0.12553365	5.05740719	0.58936013	0.45167601	0.41400733
Dync1i1	0.16564783	5.12427395	0.58931189	0.45169426	0.41400733
Icosl	-0.2964803	1.45258805	0.58890239	0.45184922	0.41411062
Hars2	0.14076991	3.90856284	0.58801273	0.45218617	0.41436573
C1ql1	0.26107228	2.81366461	0.58794408	0.45221219	0.41436573
Slamf9	-0.8243832	-0.3339572	0.58782616	0.45225689	0.41436793
Pex5l	0.12665257	6.82776309	0.58744145	0.45240276	0.41446281
Nkain2	-0.1436206	6.31843164	0.58730264	0.45245541	0.41447228
Pcbp3	-0.156076	4.77316804	0.58697643	0.45257918	0.41454689
Mir1191	0.90202897	-1.5833267	0.58676734	0.45265854	0.41458082
Coro1a	0.17142399	4.02559701	0.58615494	0.45289112	0.41472246
Lysmd3	-0.1394013	4.76163054	0.58613717	0.45289787	0.41472246
Slc22a17	0.12828289	5.86933989	0.58590954	0.45298437	0.41476289
Prss57	-1.3961385	-1.3924371	0.58551531	0.45313424	0.4148329
Coq6	0.34062004	1.27354156	0.58548564	0.45314552	0.4148329
Nup62-il4i1	-0.8529564	-1.2666709	0.58506829	0.45330428	0.41493945
Ccdc129	-0.5530787	-0.2345757	0.58437154	0.45356952	0.41514345
Sar1a	-0.1379878	7.05937469	0.58388537	0.45375474	0.41527188
Fam20b	0.11669775	4.91662611	0.58378072	0.45379463	0.41527188
4933407L21I	-0.4603986	0.14756745	0.58331235	0.45397321	0.41539386
Mon1a	-0.1871389	2.34691386	0.58312503	0.45404466	0.41539386

Atg4d	0.26233684	2.16866913	0.58309755	0.45405515	0.41539386
Neurl2	-0.6731429	-0.3121361	0.58282961	0.45415739	0.41541165
Tcte1	-0.5271873	0.71897727	0.58282431	0.45415941	0.41541165
Foxo3	-0.1078844	6.59802806	0.58267564	0.45421616	0.41542477
Pxylp1	0.1700068	3.30128163	0.58202245	0.45446563	0.41561412
Ankrd27	0.09961047	5.38224598	0.58178154	0.45455769	0.41563382
Pnma2	-0.1134254	6.40633816	0.58174403	0.45457203	0.41563382
Snora30	0.87300071	-1.4576189	0.58155066	0.45464595	0.41566261
Tmem38b	0.18238298	3.18982367	0.5814008	0.45470325	0.4156762
Asb15	0.51026399	0.58360885	0.58104553	0.45483914	0.41575309
Ssb	-0.0957165	8.09090107	0.58095901	0.45487225	0.41575309
Gem	-0.5866556	2.8642039	0.57918936	0.45555021	0.41624443
Ptplb	-0.1253185	5.00768767	0.57917511	0.45555568	0.41624443
Src	-0.1899936	3.27587709	0.57911928	0.45557709	0.41624443
Tti1	0.14430067	4.735674	0.57892868	0.45565022	0.41624443
Chrm4	0.261709	2.00723448	0.57888737	0.45566607	0.41624443
Ccnf	0.44698792	0.81005706	0.57874841	0.4557194	0.41624443
1700012D14I	0.67155994	-0.1883919	0.57871133	0.45573363	0.41624443
Acp6	-0.2611162	2.46566331	0.57866927	0.45574977	0.41624443
Gtf2a1l	-1.1834335	-1.665669	0.57831277	0.45588665	0.41633062
Tbxa2r	1.00299801	-2.105866	0.57789997	0.45604522	0.41643661
Eif2s1	-0.0927017	6.78122622	0.57684306	0.45645164	0.41676888
D330023K18	-0.3831804	1.2343076	0.57650078	0.45658339	0.41683851
9430038I01R	0.48253616	0.73306406	0.57642383	0.45661301	0.41683851
Shq1	-0.3484251	1.05411316	0.57630991	0.45665688	0.41683971
Zfp821	-0.1660359	3.53873423	0.57600657	0.45677372	0.41690751
3110039I08R	0.52593762	-0.0970578	0.57584879	0.45683451	0.41692415
Lonp2	0.09714533	5.557762	0.57554412	0.45695193	0.41698453
Il5ra	-0.8983168	-0.831976	0.57545624	0.45698582	0.41698453
Angptl6	-0.4887479	-0.1215775	0.57523405	0.45707149	0.41702386
Pkdcc	0.4079346	0.20160882	0.57509257	0.45712606	0.41703014
Ncoa7	0.10907178	6.32087926	0.57499548	0.45716352	0.41703014
Cdkl5	0.1293596	8.25420541	0.57483953	0.45722369	0.41703022
Parp6	-0.1213545	5.88867392	0.57477456	0.45724876	0.41703022
Aasdhppt	0.11016015	5.24523466	0.57420216	0.45746974	0.41713868
Mtrr	0.1480956	4.01069066	0.5739884	0.45755231	0.41713868
Cyp27a1	-0.2998452	1.73152539	0.57392835	0.45757551	0.41713868
Ankrd23	0.57442828	-0.7013477	0.57388997	0.45759034	0.41713868
Cyth1	0.18358724	3.89157414	0.57380876	0.45762172	0.41713868
Gm20125	-1.1201441	-1.3196063	0.57366369	0.45767779	0.41713868
Dact2	-0.224378	2.14960065	0.57359954	0.45770258	0.41713868
Zfand2b	0.33817843	1.87465094	0.57358458	0.45770836	0.41713868
Nipal3	0.16013574	4.4565347	0.57298382	0.45794068	0.41731158

Usp18	-0.2760218	1.69808751	0.57280231	0.45801091	0.41733675
Tmem108	0.22896246	3.46789091	0.57251188	0.45812332	0.41740035
Tlr4	-0.2040392	4.43886686	0.57233897	0.45819027	0.41742252
Tstd1	0.78454685	-1.3567026	0.57203006	0.4583099	0.41749268
Frrs1l	0.11649526	7.44286109	0.57183822	0.45838423	0.41752156
Aup1	0.23607478	3.08808871	0.57160968	0.4584728	0.41756341
Fam110b	-0.1722939	3.61497942	0.57132917	0.45858155	0.41762362
Fam53a	0.17014361	3.55264989	0.57098653	0.45871444	0.41770581
Phkg1	0.27480818	1.630539	0.57069015	0.45882944	0.41777169
Plekho2	-0.1884386	4.99004047	0.57016572	0.45903304	0.41791823
Zfp84	-0.1134775	5.15423552	0.57004716	0.45907909	0.41792131
Dctn1	0.14476476	5.19929677	0.56953057	0.45927983	0.4180652
Ncs1	0.12173244	6.14444869	0.56912665	0.45943689	0.41811264
Ncl	0.08969094	8.89214441	0.56907376	0.45945746	0.41811264
Amt	-0.3007694	1.03484184	0.56906721	0.45946	0.41811264
1700030J22F	0.19315837	3.65914995	0.56869123	0.45960629	0.41817713
Mfsd5	-0.193373	3.64305993	0.56854963	0.4596614	0.41817713
Exoc3l	-0.324041	1.39302069	0.56847874	0.459689	0.41817713
Nlrp3	-0.677134	-0.9564663	0.56844627	0.45970164	0.41817713
Mthfs	-0.2241621	2.33692126	0.56823433	0.45978416	0.41819901
Nrap	0.84470027	-1.1094914	0.56816521	0.45981108	0.41819901
Slc35c2	-0.2892808	1.83053402	0.56750461	0.46006848	0.41839427
Tmem184c	0.10757029	6.04331967	0.56657876	0.46042962	0.4186813
Egf	1.05638126	-1.2277752	0.56647638	0.46046959	0.4186813
Fxn	0.22893001	2.26426653	0.56608026	0.46062426	0.41872987
Emc10	-0.1465642	5.07381744	0.56600166	0.46065497	0.41872987
Ppp4r1	0.13582425	4.32499418	0.56595999	0.46067125	0.41872987
Npy1r	-0.1265007	5.20521963	0.56565408	0.46079078	0.41872987
5033404E19I	1.02622785	-1.5114324	0.56564852	0.46079295	0.41872987
Slc16a6	0.17786612	3.11581184	0.56558322	0.46081847	0.41872987
Rad21	-0.0864104	7.13610302	0.56557358	0.46082224	0.41872987
Zcchc16	0.21695645	2.91030763	0.56537554	0.46089966	0.41876137
Slc14a2	-0.7206941	-0.4003729	0.56524074	0.46095237	0.41877042
Ppp4r1l-ps	-0.2988846	1.82378909	0.56509711	0.46100854	0.41878261
Arfip1	-0.1472008	6.0988811	0.56464079	0.46118708	0.41889341
Orc3	0.08795577	7.85503594	0.56456677	0.46121605	0.41889341
Snx11	0.17335276	3.09313506	0.56425085	0.46133973	0.41895607
Lrwd1	0.2871657	1.80651946	0.56417209	0.46137057	0.41895607
Sec16b	0.72506955	-0.2236277	0.56342008	0.46166524	0.41918479
9530082P21I	0.11687298	5.19462914	0.56251356	0.46202087	0.41946881
Gt(ROSA)26!	0.21350823	2.76247461	0.56188779	0.46226662	0.41965303
Fxyd5	-0.2218535	6.75997088	0.56166855	0.46235277	0.41968999
Ppp2r2b	0.09576165	6.76285514	0.5614598	0.46243482	0.41968999

Shd	0.23895032	2.34245362	0.56145712	0.46243587	0.41968999
Enpp5	0.0941095	7.09987463	0.56112423	0.46256677	0.41975954
Arhgef7	-0.1049681	6.71719714	0.56101476	0.46260983	0.41975954
Clip4	0.11892875	5.24150222	0.56093537	0.46264106	0.41975954
Plk5	0.41687718	1.09162456	0.56067557	0.46274329	0.4198134
Zfp608	0.12461183	5.16319009	0.5605119	0.46280771	0.41981537
Zmynd10	-0.4339162	0.25024876	0.56045167	0.46283142	0.41981537
Nrsn2	-0.1345098	5.88585076	0.56034343	0.46287403	0.41981537
Eno1	-0.2975924	0.80270498	0.56016926	0.46294262	0.41983871
Zfp697	-0.1277591	4.89969601	0.56002878	0.46299795	0.41984004
9130221H12I	0.15976825	3.48044409	0.55994791	0.46302981	0.41984004
Mro	0.12742206	4.44287072	0.55981735	0.46308125	0.41984781
Nsmce1	-0.2950553	3.55079185	0.55953761	0.4631915	0.41985554
Hdac8	0.25051632	1.90663571	0.55945485	0.46322412	0.41985554
Sp3	-0.1021213	6.67392446	0.55928999	0.46328912	0.41985554
Atg4b	0.14881026	3.8982455	0.55921693	0.46331793	0.41985554
Myh15	0.87735277	-1.1483054	0.55914967	0.46334446	0.41985554
Xylb	0.18039914	3.45145933	0.55914335	0.46334695	0.41985554
Rplp1	-0.154794	5.20971012	0.55892573	0.46343279	0.41989448
Akt1	-0.1388768	5.08357645	0.55847762	0.46360964	0.42001586
Ptpn18	-0.9521464	-1.6842723	0.55814008	0.46374292	0.42006414
Cilp2	-0.4938141	0.61710162	0.55812546	0.4637487	0.42006414
Usp31	0.14669046	7.46671707	0.55766132	0.46393208	0.42019139
Aph1c	0.15711341	3.93626938	0.55721468	0.46410866	0.42028251
Rexo4	-0.1355311	4.59770965	0.5571898	0.46411849	0.42028251
Sft2d1	-0.1528738	3.36527521	0.55685397	0.46425134	0.42036395
Adam12	-0.1395509	5.7691662	0.55628657	0.46447594	0.42040647
Jade3	-0.2244565	4.31488406	0.55607762	0.4645587	0.42040647
Dbndd1	-0.2751254	1.76124318	0.5560457	0.46457134	0.42040647
Rwdd3	0.24009593	2.62408244	0.55595712	0.46460643	0.42040647
Nckap1	0.10879272	9.44861355	0.55592239	0.46462019	0.42040647
Gphn	0.12138715	5.91839685	0.5557694	0.46468081	0.42040647
Mettl14	0.12268333	5.82265542	0.5557678	0.46468144	0.42040647
Angpt2	-0.163984	3.99421813	0.55576628	0.46468205	0.42040647
Gal3st1	0.4393873	0.20452388	0.55575991	0.46468457	0.42040647
Ppp1r15a	-0.2999975	2.79105364	0.55527262	0.46487774	0.4204499
Ganab	0.1407425	5.05845587	0.55524476	0.46488879	0.4204499
Fbll1	-0.2514184	2.23625039	0.55523769	0.46489159	0.4204499
4921511I17R	1.38435528	-2.4337037	0.55520574	0.46490427	0.4204499
Znrf1	0.09386294	6.14635925	0.55499116	0.46498938	0.42048805
Rab9b	0.09322687	6.46327609	0.55402953	0.46537113	0.42079442
Chn1os3	0.50442012	-0.0760087	0.5535704	0.46555358	0.42092054
BC020402	-0.3550992	1.68090219	0.55331364	0.46565567	0.42097398

Kcna5	0.3561004	0.85548491	0.55291645	0.46581366	0.42107795
Nxn	-0.2197936	5.05717024	0.5527896	0.46586414	0.42108472
Psd3	0.12115978	9.82749616	0.55265931	0.46591599	0.42108982
Sat2	0.21175671	2.12702867	0.55255941	0.46595576	0.42108982
Ppm1f	0.12287497	4.58566477	0.55242401	0.46600966	0.42109013
Brdt	-0.1524131	4.23195234	0.55232815	0.46604783	0.42109013
Abtb1	-0.2572334	1.96667002	0.55221113	0.46609443	0.42109013
Sgcd	0.21933381	2.93559348	0.55207102	0.46615024	0.42109013
Pabpc1l	-1.2819445	-1.9515663	0.55201879	0.46617105	0.42109013
Tpr	0.09781383	9.14637842	0.55184086	0.46624194	0.42111533
Mis18a	0.21338723	1.95977578	0.55151004	0.4663738	0.4211956
Rprd1a	-0.1029727	6.72617376	0.55138917	0.466422	0.42120029
Gsg1	-0.7395722	-0.8084375	0.55110374	0.46653583	0.42126425
Ankrd50	0.11066812	5.18671778	0.55057281	0.4667477	0.4213934
Ankrd45	-0.1147197	6.27744958	0.55043924	0.46680103	0.4213934
Adcy7	0.33954767	1.81924374	0.55042198	0.46680792	0.4213934
Ergic2	0.12886487	4.64021062	0.55030724	0.46685374	0.42139592
Tex14	-0.7475675	-0.4598515	0.55013035	0.46692439	0.42142086
Rnh1	0.15460657	4.45654197	0.54997521	0.46698637	0.42143797
Trhr2	-0.4887448	0.03532587	0.54965016	0.46711627	0.42151636
Nono	-0.0763277	7.47503312	0.54886644	0.46742972	0.42176036
Uqcrc1	0.10061358	5.02004495	0.54839326	0.46761914	0.42187768
Vps72	-0.2788476	2.61179427	0.54832647	0.46764588	0.42187768
Flna	0.12189055	5.69101565	0.54819781	0.46769742	0.42188532
Gm17821	0.14164703	9.70641566	0.54761326	0.46793166	0.42205775
Polg2	-0.4177958	0.97007582	0.54713528	0.46812334	0.42216112
Gm10406	0.52678407	-0.4766145	0.5468807	0.46822549	0.42216112
Fchsd2	0.08931454	6.70699461	0.54686724	0.46823089	0.42216112
Csnk2a2	-0.1366739	5.19443205	0.54683984	0.46824188	0.42216112
07-Sep	-0.104412	9.11917513	0.54671951	0.46829018	0.42216112
Shc2	0.14835255	4.57055209	0.54668294	0.46830486	0.42216112
Ppp1r3fos	-0.9599899	0.05384402	0.54623934	0.46848298	0.42228283
Poln	0.71797996	-1.1123698	0.54596116	0.46859474	0.4223191
1110057K04I	0.10410248	5.54252814	0.54592456	0.46860945	0.4223191
Wipf2	-0.0926303	7.56041348	0.54543852	0.46880484	0.42245632
Kcnh2	0.24109612	2.32121856	0.54522373	0.46889123	0.4224953
Flywch1	-0.1665259	3.82844992	0.54509667	0.46894234	0.42250249
Aurka	0.43775692	-0.188556	0.54470626	0.46909945	0.42257399
Tmpo	-0.1333421	5.86284667	0.54466096	0.46911769	0.42257399
A430090L17I	0.73566061	0.76348656	0.54457794	0.46915112	0.42257399
Sparc	-0.2108228	8.82825556	0.54424638	0.46928464	0.4226554
A330076C08	-0.6256314	0.12744797	0.54385029	0.46944424	0.42276026
Cdh5	-0.1908964	5.44742373	0.54351319	0.46958014	0.4227853

Dcn	-0.216971	7.6761973	0.54346128	0.46960107	0.4227853
Dnajb11	-0.0993213	5.90712065	0.54339745	0.46962682	0.4227853
Pkn1	-0.2722533	2.85347814	0.54335313	0.46964469	0.4227853
Cd34	0.17577572	3.49869475	0.54290468	0.46982563	0.42290932
Scarf1	-0.6886702	-0.7980304	0.54273606	0.46989369	0.42293172
Gfm1	0.15681099	4.55150656	0.54257685	0.46995797	0.42295071
Helq	0.24908673	2.56920515	0.54165431	0.47033072	0.42324729
Gm3500	-0.4379375	-0.3763363	0.54133976	0.47045793	0.42328464
Lims2	0.21159846	2.67256742	0.54092566	0.47062548	0.42328464
Prrx1	-0.1538906	7.06862892	0.54091544	0.47062962	0.42328464
Enpp3	0.38674606	0.61980347	0.54090261	0.47063481	0.42328464
Ddost	0.1222411	4.27040541	0.5408311	0.47066375	0.42328464
Serinc5	0.14203892	5.0789743	0.54082452	0.47066642	0.42328464
Sdcbp	-0.0996137	9.43623157	0.54078661	0.47068176	0.42328464
Itpripl2	0.14206563	5.91294504	0.54069727	0.47071793	0.42328464
Il1r1	-0.1589487	4.30797735	0.54053874	0.47078212	0.42330351
BC029722	0.28789843	2.07928093	0.54027584	0.47088861	0.42336039
Nudt5	-0.2268967	2.90762845	0.54008569	0.47096565	0.4233908
Nrarp	-0.2407731	2.7117854	0.53978278	0.47108843	0.42342588
Nkg7	-0.7361525	0.02728111	0.53962873	0.47115089	0.42342588
Trim68	0.34029819	1.24116995	0.53948715	0.4712083	0.42342588
4930525G20	0.25191697	2.28997565	0.53937624	0.47125329	0.42342588
Slc25a46	0.09045373	7.86801122	0.53924801	0.47130531	0.42342588
Ring1	-0.1336621	4.47181518	0.53914019	0.47134906	0.42342588
Trim46	0.15962189	4.42013848	0.53911969	0.47135737	0.42342588
Emr4	0.97401495	-1.2104886	0.53907721	0.47137462	0.42342588
Fgf13	0.12004009	6.45146223	0.53903013	0.47139372	0.42342588
Mogs	0.29195825	1.72178949	0.53807497	0.47178163	0.42373546
Mok	0.21989732	2.36552259	0.53788338	0.47185951	0.42373994
Harbi1	-0.2848273	2.1471248	0.53784984	0.47187314	0.42373994
Cebpz	0.08478655	6.19603558	0.53769229	0.4719372	0.42375862
Hvcn1	0.51830279	-0.3066528	0.53702181	0.47220997	0.42396467
Rfng	0.16791539	3.51082264	0.53672159	0.47233219	0.42400885
Srek1	0.11235715	6.65390786	0.53668831	0.47234574	0.42400885
Ric8	-0.1236499	5.22884109	0.53622268	0.47253541	0.42414024
A4galt	0.2098386	2.77150386	0.53600306	0.47262492	0.42418171
Wls	0.15521251	5.22275416	0.53578775	0.4727127	0.42419426
Zc3h4	-0.1123488	5.87777576	0.53569359	0.47275109	0.42419426
Ap1b1	0.11198406	5.70390084	0.53550042	0.47282988	0.42419426
Lat2	0.47621647	-0.025745	0.53549597	0.4728317	0.42419426
Tfpi	-0.1771942	6.01795099	0.53543781	0.47285542	0.42419426
BC005537	-0.0993991	7.01994452	0.53531211	0.47290671	0.42420141
Kdm4d	-0.5910118	-0.1753256	0.53504691	0.47301493	0.42423832

Atf1	0.11106706	7.58170167	0.53492039	0.47306658	0.42423832
Tubb2b	-0.1131323	5.27879931	0.53489296	0.47307778	0.42423832
1810019D21I	0.95514498	-0.5570724	0.53452428	0.47322834	0.42433449
Abhd11os	0.39328257	0.35444913	0.53434687	0.47330083	0.42434262
3000002C10I	-0.1797854	2.14423524	0.53428749	0.47332509	0.42434262
Thsd1	-0.5073839	0.40548521	0.53407578	0.47341161	0.42434262
Pcdhga8	0.20361006	2.67989961	0.53394537	0.47346492	0.42434262
Panx1	0.2750477	2.45878713	0.53393978	0.47346721	0.42434262
Chat	0.3297144	1.53177093	0.53380124	0.47352385	0.42434262
Smyd5	0.18608958	3.00264503	0.53376016	0.47354066	0.42434262
Wdr12	0.15272065	4.64403414	0.53356674	0.47361976	0.42437468
Nasp	0.1392812	4.75297334	0.53327945	0.4737373	0.42444118
Slc25a5	-0.0820944	7.08642197	0.53277157	0.47394522	0.42446244
Col4a5	0.2267005	3.29532106	0.53265809	0.4739917	0.42446244
Klf8	-0.1516459	2.88497504	0.5326212	0.47400681	0.42446244
Snrnp40	-0.1538213	4.37384869	0.5324155	0.47409108	0.42446244
Mvd	0.22521781	1.82124357	0.53238492	0.47410361	0.42446244
Hs3st6	0.65539664	-0.5819177	0.53234965	0.47411806	0.42446244
Kcnk13	0.36407813	0.83313891	0.53227514	0.4741486	0.42446244
Dcaf5	0.09306825	7.07539042	0.53221909	0.47417157	0.42446244
Tvp23a	-0.1962359	4.20461113	0.5321674	0.47419275	0.42446244
Akr1b8	-0.2528768	2.14255392	0.53216346	0.47419437	0.42446244
1810024B03I	-0.7275856	-0.6292529	0.53178953	0.47434768	0.42456088
Orai3	-0.1720301	5.00577325	0.53154488	0.47444803	0.4246119
Wdr91	-0.1840337	3.08481468	0.53103095	0.47465896	0.42474806
Nipal4	0.31274705	0.8634244	0.53096289	0.4746869	0.42474806
Clcn7	0.18160592	3.02906878	0.53069413	0.47479728	0.42480802
Slc16a9	-0.1815244	4.83671912	0.52978305	0.47517177	0.42510425
Mfsd2a	0.33278284	1.27973298	0.52937709	0.47533879	0.42521484
Hmgxb3	0.16545022	3.94378601	0.52880915	0.47557263	0.42533408
Qsox1	0.24219515	2.23119922	0.52872813	0.475606	0.42533408
Gprc5b	0.1380995	4.83589244	0.52861157	0.47565402	0.42533408
Sf3a1	-0.131214	5.62274131	0.52858628	0.47566444	0.42533408
Dact3	0.10700123	4.11706436	0.52852619	0.4756892	0.42533408
Star	0.21170655	2.52896689	0.52807623	0.47587467	0.42543284
Ly6g6e	0.96152453	-0.7640857	0.52804751	0.47588652	0.42543284
Glt28d2	-0.2156359	3.29985925	0.52753979	0.47609595	0.42558123
Pmel	-0.7875784	-1.8486004	0.52743404	0.4761396	0.42558141
Col12a1	0.10612998	5.40904331	0.52723607	0.47622131	0.42561561
Pex6	-0.1715349	3.79924889	0.52686661	0.47637388	0.42571313
Plce1	-0.096342	4.90128955	0.52655441	0.47650287	0.42576516
Tmem86a	-0.2714592	4.25573713	0.52651528	0.47651904	0.42576516
Slc17a8	-0.2228961	2.68306358	0.52600711	0.47672913	0.42591402

Thrb	-0.1018716	7.02512197	0.52560224	0.47689663	0.42599087
2510003E04I	0.10070619	7.09266894	0.52558897	0.47690212	0.42599087
Slfn2	0.52193125	0.01257306	0.52546115	0.47695502	0.42599259
Rassf7	-0.5308889	0.14261191	0.52537418	0.47699103	0.42599259
Ripk4	-0.5618757	-0.0521389	0.52495799	0.47716337	0.42601613
Nkx3-1	-0.4535947	0.14710856	0.52491392	0.47718162	0.42601613
P4ha2	0.21807814	1.85672875	0.52486859	0.4772004	0.42601613
Mios	-0.1303399	4.46628771	0.52484426	0.47721048	0.42601613
Maml2	-0.1251969	5.76477574	0.52472427	0.47726019	0.42601613
Ankrd13a	-0.1158388	4.90859285	0.52468049	0.47727834	0.42601613
Zfp592	-0.1084688	5.65613648	0.52439206	0.47739788	0.42608401
Ago2	-0.0871031	7.24356027	0.52421692	0.4774705	0.42610999
Csnk1g2	-0.1468115	5.59108536	0.52379613	0.47764504	0.42622693
Ccdc79	0.40700094	0.91589966	0.52359089	0.47773021	0.4262641
Hmox2	-0.1568241	5.03916106	0.52344415	0.47779112	0.42627961
Dtnb	0.12922007	4.6439669	0.52301303	0.47797015	0.4264005
Atad2	0.17281662	4.80944559	0.5228469	0.47803917	0.42642324
Cd4	0.2819951	3.24034563	0.52225146	0.47828669	0.42660518
Aplnr	-0.9698856	-1.2725046	0.52208397	0.47835635	0.42662847
Rnf26	-0.1917739	3.12694688	0.52136018	0.47865758	0.42682104
Lasp1	-0.0798308	6.52538605	0.52127848	0.4786916	0.42682104
Tmem178	0.10378719	5.34075358	0.52113327	0.47875209	0.42682104
Lat	0.77961432	-1.0664694	0.52091572	0.47884272	0.42682104
Bpnt1	0.09042029	6.08443902	0.52089286	0.47885224	0.42682104
Pam16	-0.1633845	3.18915383	0.52085993	0.47886597	0.42682104
Cdc5l	-0.073889	6.18232915	0.52083277	0.47887729	0.42682104
Adar	0.13863058	6.00892083	0.52038563	0.47906369	0.42694833
Sarm1	0.21638632	3.71037135	0.52019668	0.47914249	0.42695149
Socs3	-0.2817574	1.04320367	0.5201681	0.47915441	0.42695149
Commd2	-0.1405695	4.30203494	0.51993339	0.47925234	0.42699991
Wnt5a	-0.1404705	5.5511028	0.51980653	0.47930528	0.42700265
Stxbp4	-0.104527	7.33360469	0.51958136	0.47939927	0.42700265
C1galt1c1	-0.1607537	4.60541063	0.51954054	0.47941631	0.42700265
Srek1ip1	-0.1149096	6.02457811	0.51950827	0.47942979	0.42700265
Nomo1	0.119657	5.3458177	0.51912436	0.47959014	0.42706959
Cox16	0.15296824	4.60842813	0.51890168	0.47968318	0.42706959
Sema6a	0.13058446	5.73162937	0.51883118	0.47971265	0.42706959
Pik3r2	-0.1298121	4.18510452	0.51876894	0.47973867	0.42706959
Rtn4ip1	-0.2013618	3.29227625	0.51876088	0.47974203	0.42706959
Oxtr	0.22244408	3.55185533	0.51870225	0.47976654	0.42706959
St18	-0.2088515	3.77041997	0.51834663	0.47991524	0.42716104
Zfp512	0.10374044	5.07894091	0.51824803	0.47995649	0.42716104
Fam163a	0.31822067	1.81468194	0.51801649	0.48005336	0.42720844

Pdcd7	-0.1534408	3.61848309	0.51790197	0.48010129	0.42721228
Tmem159	-0.1857929	4.62406698	0.51761591	0.48022105	0.42728003
3200001D21I	0.6007129	0.18461242	0.51742358	0.48030159	0.42731287
Spata9	0.16300335	3.51825626	0.51718926	0.48039974	0.42736139
P2ry1	0.16840561	4.02734054	0.51708472	0.48044355	0.42736154
Nifk	-0.151077	4.61760274	0.51694122	0.48050369	0.42737623
Gpr137b-ps	-0.1434701	3.63692507	0.51656023	0.48066342	0.42745588
Rbfox1	-0.1014525	9.25183504	0.51646534	0.48070321	0.42745588
Cant1	0.17756763	3.30876225	0.51637641	0.48074052	0.42745588
Cltc	0.12241003	8.89667839	0.51631138	0.4807678	0.42745588
Ncln	-0.2320093	2.44640941	0.51594688	0.48092076	0.42754902
Lrrc28	0.11946535	4.49518742	0.51585375	0.48095985	0.42754902
Map2k3os	0.61213049	-0.8604769	0.51547492	0.48111893	0.42759922
Maff	-0.2123082	3.06441849	0.51544711	0.48113062	0.42759922
Ripk2	-0.153426	4.3644406	0.51540744	0.48114728	0.42759922
Gm1673	0.37745813	0.4194867	0.51522921	0.48122216	0.42762697
Scn2b	0.11144492	6.88841078	0.51489206	0.48136387	0.42766297
Mta2	-0.1211403	4.80843552	0.5146268	0.4814754	0.42766297
Phkb	0.11845899	6.1319598	0.51450166	0.48152804	0.42766297
2700094K13I	-0.2071403	3.19657688	0.51432179	0.48160371	0.42766297
Plekhh3	0.30301338	1.54505035	0.51428027	0.48162118	0.42766297
Hps3	0.16898067	4.08700997	0.51420165	0.48165427	0.42766297
Naa20	0.12891675	4.99462686	0.51418837	0.48165986	0.42766297
1110034G24	-0.3011991	1.72551301	0.51415444	0.48167413	0.42766297
Alx4	0.18690668	5.70558227	0.51410734	0.48169396	0.42766297
Pmm1	-0.122368	4.66168759	0.51401875	0.48173125	0.42766297
St6galnac4	-0.2245899	2.46563388	0.51394307	0.48176311	0.42766297
Gm10825	0.93065422	-1.3724712	0.51383052	0.4818105	0.42766297
Nmt2	-0.0870327	6.98586461	0.51369077	0.48186935	0.42766297
Fzd5	-0.2807633	2.67247355	0.51367993	0.48187391	0.42766297
Cercam	-0.2320658	1.93764476	0.51323995	0.48205928	0.42775354
Kat6a	-0.0795507	8.11258679	0.51316664	0.48209017	0.42775354
Ccdc148	-0.1353494	4.10954772	0.51312679	0.48210697	0.42775354
Crebl2	-0.1428177	5.09833326	0.51287229	0.48221426	0.42780999
Gm609	0.92219081	-1.8422175	0.51270341	0.48228549	0.42783442
Blm	0.27479555	2.1997472	0.51238265	0.48242081	0.42791571
Glra3	0.32042686	1.20109565	0.51222315	0.48248812	0.42793667
Psip1	-0.0941038	8.79468226	0.51167491	0.48271961	0.42803716
Scyl2	0.11926877	5.8534477	0.5115096	0.48278945	0.42803716
Armcx4	0.11537759	6.06988026	0.51140974	0.48283165	0.42803716
Cpsf4	0.23450517	1.62507283	0.51140823	0.48283229	0.42803716
Rai2	-0.174434	3.16974792	0.51138496	0.48284212	0.42803716
Tjp1	0.08553778	9.48507631	0.51133411	0.48286361	0.42803716

Zfp26	0.11316633	6.27134914	0.51074917	0.48311094	0.42821765
4933427E11I	-1.0130668	-1.2907902	0.5106171	0.48316681	0.42822843
Thoc6	-0.3201344	1.21006605	0.51019864	0.48334391	0.42834663
Fam60a	0.15337101	3.5921575	0.50985903	0.48348772	0.42842112
2700097O09	-0.198411	2.8756526	0.50979361	0.48351543	0.42842112
Trim34b	-0.506344	-0.8356453	0.50919978	0.4837671	0.42860533
Lhfpl1	0.70824249	-0.7826807	0.50868117	0.48398706	0.42873858
Mir3473f	0.81873564	-1.3801563	0.50855192	0.48404191	0.42873858
Tfip11	-0.181424	3.79497696	0.50853568	0.4840488	0.42873858
Prpf38a	-0.1306931	4.10992073	0.5084055	0.48410406	0.42874875
Rbm41	-0.1326461	4.52288136	0.50820964	0.48418721	0.42878363
Mettl24	-0.4837355	-0.0698367	0.50770631	0.48440101	0.42893419
Bcas3	-0.1313278	4.43762814	0.50743093	0.48451805	0.42899905
4930503L19F	0.21178614	3.03546724	0.50720129	0.48461569	0.42904672
Rala	-0.0781746	6.48951825	0.50681198	0.4847813	0.42915454
Gpc2	-0.5768059	0.06735224	0.50612395	0.48507421	0.42937504
Gm14169	-0.2354475	1.90935333	0.50587827	0.48517887	0.42942887
Pola2	0.23342435	1.80786801	0.50569706	0.4852561	0.42945515
Cbr2	-0.3305079	1.3138646	0.50541007	0.48537844	0.42945515
Sox30	-0.7135599	-1.0289941	0.5053234	0.4854154	0.42945515
Gm16515	-0.1857559	5.5106867	0.50519498	0.48547017	0.42945515
Zfp389	-0.8474051	-1.5357067	0.5051762	0.48547818	0.42945515
Armc8	0.09412553	7.05120599	0.50515472	0.48548734	0.42945515
Crip3	-1.1883271	-1.9973046	0.50508882	0.48551545	0.42945515
Gpr27	0.68263978	-1.2313857	0.5040059	0.48597781	0.42979495
B930018H19	-0.9417574	-0.9073337	0.50398353	0.48598737	0.42979495
Haus4	0.28563148	1.58054984	0.50330712	0.48627655	0.42996941
Ube2d2a	0.10459269	9.04656971	0.50321766	0.48631482	0.42996941
Creb3	0.22381129	3.08932762	0.50321414	0.48631633	0.42996941
Vwc2	0.19032608	3.14969717	0.50205236	0.48681378	0.43035077
1700029J07F	0.22538791	2.15905679	0.50200162	0.48683552	0.43035077
Prss48	0.72767677	-0.1717178	0.50138031	0.48710194	0.43051232
5530601H04I	-0.1733398	3.42913105	0.50137041	0.48710619	0.43051232
Sumf1	0.15058563	5.03265388	0.50116498	0.48719433	0.43055137
Eml3	0.21054314	2.79223939	0.5008224	0.48734138	0.43063691
Timd4	1.22645634	-2.1091428	0.50073468	0.48737904	0.43063691
Cdhr3	0.67567723	-0.2814062	0.50052128	0.48747069	0.43067068
Gm4961	0.73141799	-1.7273926	0.50022987	0.4875959	0.43067068
Sms	-0.0844159	7.09061847	0.50021112	0.48760396	0.43067068
BC027231	0.15437938	3.97245081	0.50015664	0.48762737	0.43067068
Tcp11l2	0.10900016	4.28628694	0.50013402	0.48763709	0.43067068
Mthfd2l	-0.2788928	2.19025458	0.49959243	0.48786996	0.43081818
Gab1	-0.0920629	7.04320334	0.49954103	0.48789207	0.43081818

Cuedc1	-0.1957578	2.60927741	0.49934443	0.48797666	0.43083776
Agbl4	-0.2854928	2.42252129	0.49928505	0.48800221	0.43083776
Eif2b1	0.15243132	4.79094889	0.49894747	0.48814753	0.43089506
Ms4a4c	-0.9721666	-1.6384002	0.49892989	0.4881551	0.43089506
Nme4	0.57085986	0.20736977	0.49872444	0.48824358	0.43092211
Phf12	0.08265448	6.74279914	0.49862066	0.48828828	0.43092211
AA387883	0.44389356	0.28824158	0.49850364	0.4883387	0.43092211
Triqk	-0.1901753	3.52130228	0.49833678	0.4884106	0.43092211
B130006D01	0.79017942	0.52011361	0.49827896	0.48843552	0.43092211
Il1f9	0.25373442	2.29455078	0.49824606	0.4884497	0.43092211
Micalcl	-0.2893535	1.66296183	0.49779	0.48864634	0.43102042
Usp3	-0.1150888	4.04294891	0.49778353	0.48864913	0.43102042
Ing5	0.1383654	4.683819	0.49763192	0.48871453	0.43103929
Cct7	-0.1074711	6.67429444	0.49739109	0.48881846	0.43109213
Crtc3	-0.1271386	4.93875182	0.49711227	0.48893882	0.43115946
Runx1t1	-0.0915292	7.80809386	0.49680891	0.48906983	0.43123617
Myh9	-0.0979344	6.86419916	0.4963823	0.48925417	0.43132715
Nudt13	0.17594758	2.66711577	0.49636632	0.48926108	0.43132715
Gadd45a	-0.1584845	4.16244323	0.49603566	0.48940405	0.43141436
Tdrkh	0.1537164	4.42683345	0.49584468	0.48948666	0.43144836
Loxl4	-0.7102795	-1.1594294	0.49540432	0.48967722	0.43157749
Dhx29	0.17045307	4.80532999	0.49493035	0.48988247	0.43171954
Pwp1	0.15369745	4.19205255	0.49480971	0.48993474	0.43172677
Nefm	0.1572844	9.24354979	0.4946051	0.49002341	0.43174935
Vstm4	-0.2036355	4.68068533	0.49454715	0.49004853	0.43174935
Ipo7	0.11088628	5.83228562	0.49426021	0.49017293	0.43175427
Ythdf1	0.07295475	6.7367696	0.49424496	0.49017955	0.43175427
Vstm2l	0.39652533	-0.1881095	0.4942293	0.49018634	0.43175427
Ccrn4l	0.10821299	5.44062537	0.49410019	0.49024234	0.43175777
Gm5796	-0.5063149	-0.9775086	0.4938335	0.49035804	0.43175777
D630032N06	0.90263957	-0.9869433	0.49380399	0.49037084	0.43175777
Usp10	0.1141735	5.20254236	0.493789	0.49037735	0.43175777
Marcks	0.11627277	9.44935108	0.49361911	0.49045108	0.43175777
Mrto4	-0.1753072	3.32261323	0.49361057	0.49045479	0.43175777
Tstd2	0.18234745	2.78070103	0.49322782	0.49062098	0.43186527
Arhgap23	0.12968368	5.68416057	0.49285173	0.49078438	0.43197027
Prr12	0.11026693	5.7528392	0.49237151	0.49099315	0.43202374
Impa1	-0.0929117	5.28422292	0.49231377	0.49101827	0.43202374
Rspo3	-0.2045674	7.1031934	0.49224701	0.4910473	0.43202374
Eps15l1	0.10483972	5.75893508	0.49223908	0.49105075	0.43202374
Pcdha8	0.55897545	0.18925913	0.49209332	0.49111416	0.43202374
Dusp4	-0.3580612	1.31826327	0.4920462	0.49113466	0.43202374
Ddx23	-0.1034697	5.43142983	0.49200209	0.49115386	0.43202374

BC049715	-0.7870417	-0.4258807	0.49178991	0.4912462	0.4320271
Hus1	0.12519202	4.38056297	0.49170112	0.49128485	0.4320271
Hdgfrp3	-0.0854775	8.00669921	0.49161006	0.4913245	0.4320271
Espn	-0.4563101	-0.5472656	0.491588	0.4913341	0.4320271
Cdkal1	0.14345363	4.0708894	0.49119185	0.49150665	0.43214003
AI847159	-0.9709946	-1.8989295	0.49085857	0.49165189	0.43222893
Ankrd44	0.1340313	5.2431102	0.49064387	0.4917455	0.43225046
Slc4a1ap	-0.0988164	5.65214781	0.49059997	0.49176464	0.43225046
Casp12	-0.2251716	4.19056482	0.49033479	0.4918803	0.43231334
E330011O21	-0.4676384	-0.1778107	0.49005774	0.4920012	0.43238079
AA474331	1.06876177	-1.6410653	0.48917257	0.49238779	0.43268172
Gm5643	-0.0992658	5.48793776	0.48896696	0.49247766	0.43272187
Afap1l2	-0.1877983	2.61867956	0.48868093	0.49260273	0.43276472
Foxr2	-0.5045964	0.49524515	0.48865338	0.49261478	0.43276472
Myo1e	0.11481194	4.55199139	0.48817966	0.49282206	0.43276956
Meaf6	-0.1090134	5.01797315	0.48817718	0.49282314	0.43276956
Osbpl7	0.20385123	2.31354449	0.48802592	0.49288936	0.43276956
Best3	0.63107593	-0.0664537	0.48801315	0.49289495	0.43276956
Prpf4b	0.09335031	7.35039184	0.48788717	0.49295011	0.43276956
Erbb2	0.40405072	1.57463106	0.48772785	0.49301988	0.43276956
Kif24	0.36349698	1.04121101	0.48770484	0.49302996	0.43276956
Th	0.50175217	0.60102874	0.48768519	0.49303857	0.43276956
Rabl6	0.10479352	5.81756979	0.48758442	0.49308271	0.43276956
C330021F23I	-0.187968	2.50168453	0.48754302	0.49310086	0.43276956
Gm20939	-0.1505805	3.27695277	0.48753062	0.49310629	0.43276956
Nefl	-0.0991777	8.72747445	0.48739788	0.49316445	0.43278183
Rps6ka1	-0.2154745	2.51458449	0.48706853	0.49330882	0.43286974
Pex14	0.20174505	2.54592797	0.48691082	0.49337798	0.43289165
Pde12	0.16804789	3.23531426	0.48654398	0.4935389	0.43299406
Prim2	-0.1874252	2.69583079	0.48629692	0.49364734	0.43302069
Eif5	-0.0726699	8.75249106	0.48618342	0.49369716	0.43302069
Camta2	-0.1042396	7.54902242	0.48617268	0.49370188	0.43302069
Gm12942	0.27682828	2.89548821	0.48601614	0.49377061	0.4330231
Add1	0.09270903	7.6835705	0.48594817	0.49380047	0.4330231
Pias4	-0.2284957	1.41421414	0.48582601	0.49385413	0.4330231
Tbc1d2b	-0.1141378	4.3888856	0.48576381	0.49388145	0.4330231
Fam57b	0.24620628	2.79398044	0.48548429	0.49400428	0.43309203
Col6a2	-0.2208344	5.41390763	0.48521745	0.49412159	0.4331561
Ska2	-0.1947205	2.75900867	0.48502284	0.49420717	0.43316439
Ccbl2	0.22836088	2.49897631	0.48499485	0.49421949	0.43316439
Pdcd11	0.10913227	4.16139788	0.48461257	0.49438768	0.43325507
Zdhhc5	-0.1065869	5.9280113	0.48455865	0.49441141	0.43325507
Slc35a4	-0.1221566	5.95206393	0.48443977	0.49446374	0.43326217

Rrs1	0.09887966	4.79496528	0.48372973	0.4947765	0.43347003
Ano7	-0.7118264	-1.3843268	0.48370029	0.49478947	0.43347003
Eda2r	0.6699077	0.18129697	0.48349171	0.49488142	0.43351181
Sf3b2	-0.102733	6.8265091	0.48324633	0.49498962	0.43356782
Ptbp1	-0.157512	6.00333761	0.48284079	0.49516854	0.43363861
Zfp942	0.14395643	3.51107222	0.48282263	0.49517655	0.43363861
Mkx	0.1443937	5.3746159	0.48276214	0.49520325	0.43363861
Rab6b	-0.0847306	10.4152573	0.4825078	0.49531554	0.43369817
Clcn6	-0.1430062	3.55535968	0.48209689	0.49549703	0.43381373
Topors	0.08962526	6.03630215	0.48199618	0.49554153	0.43381373
Atg7	-0.1414523	6.32718181	0.48190827	0.49558038	0.43381373
Ccdc110	-0.5793415	0.36733429	0.48163685	0.49570036	0.43384255
Mtfp1	-0.150662	4.76527358	0.4816334	0.49570189	0.43384255
Arhgap9	-0.4988212	0.95300811	0.48115281	0.49591446	0.43393973
F5	0.26200862	2.6508564	0.48114498	0.49591793	0.43393973
Nedd9	-0.1057634	4.36676939	0.48107984	0.49594675	0.43393973
4930473A02I	0.42155435	0.53604873	0.48098184	0.49599012	0.43393973
Hps4	-0.210014	2.44925576	0.4807727	0.4960827	0.43398196
Ubtd2	-0.1094924	5.27685835	0.48061598	0.4961521	0.43400391
Heatr9	0.97189599	-1.4993693	0.4802295	0.4963233	0.43404524
Slc1a5	-0.2334336	2.10216484	0.48014909	0.49635893	0.43404524
Abcf1	-0.0873313	6.76225071	0.48007855	0.4963902	0.43404524
Nexn	0.14897356	5.68358116	0.47999023	0.49642935	0.43404524
9530027J09F	1.06481539	-0.9858513	0.47972672	0.49654618	0.43404524
LOC171588	-1.0849757	-1.1518178	0.48288855	0.49655528	0.43404524
Wars2	0.18762463	3.67473032	0.47965282	0.49657895	0.43404524
Rhot2	-0.1264638	5.09631847	0.47947349	0.4966585	0.43404524
Kbtbd4	-0.1190657	4.48817103	0.47938497	0.49669777	0.43404524
Eif3l	-0.1011004	5.5720538	0.4793443	0.49671582	0.43404524
Ninl	-0.1779134	3.10125781	0.47931215	0.49673008	0.43404524
Tctn1	-0.3434908	2.90632425	0.47930053	0.49673524	0.43404524
Med28	-0.1437911	5.31342009	0.47913238	0.49680987	0.43404524
Ddo	-0.1958775	3.49965367	0.47899883	0.49686915	0.43404524
Lrrc14b	-0.2009331	2.80334172	0.47890892	0.49690907	0.43404524
Nr1i3	-0.7009874	-1.1087511	0.4787818	0.49696552	0.43404524
Mettl25	0.24098393	2.64327942	0.4787301	0.49698848	0.43404524
Cerk	0.14851057	4.07824315	0.47871102	0.49699696	0.43404524
U2af2	-0.1631227	4.69481803	0.47845062	0.49711264	0.43407231
Adcyap1	0.17470011	2.9020933	0.47838803	0.49714045	0.43407231
Fem1a	-0.0950602	5.55688316	0.47834204	0.49716089	0.43407231
Ccl9	0.23415921	1.82352796	0.4775384	0.49751826	0.43434562
Elovl4	0.15850259	4.81666935	0.47711507	0.4977067	0.43447141
Kpna3	0.09369811	7.16999286	0.47551016	0.49842222	0.43505724

Frmpd1os	-0.7988926	-1.7054424	0.47528743	0.49852166	0.43510527
Strn3	0.08079486	8.17349927	0.47507588	0.49861614	0.43514896
Zfp747	-0.1290284	3.70442029	0.47462915	0.49881576	0.43519066
Fbrs	-0.1151438	4.54391855	0.47459	0.49883326	0.43519066
Ficd	-0.1806494	2.89199918	0.47458348	0.49883617	0.43519066
Gpr158	0.11436526	8.18522937	0.47455803	0.49884755	0.43519066
Ccdc50	-0.1012956	7.37499426	0.47447188	0.49888607	0.43519066
Eif2ak3	0.1745396	3.26880954	0.47418425	0.4990147	0.43522636
Pacrg	0.19647733	2.80278156	0.47418167	0.49901585	0.43522636
Rtn3	0.0995272	9.97579174	0.47381396	0.49918038	0.43524761
Lingo1	0.09052815	7.59023862	0.47380709	0.49918346	0.43524761
Fadd	-0.1405386	4.30094282	0.47378136	0.49919497	0.43524761
Rnf20	0.09499232	6.44450563	0.47373001	0.49921796	0.43524761
Fam222b	-0.081782	6.75289279	0.47338917	0.49937057	0.43530906
Plcl2	0.13902476	5.54410284	0.47337411	0.49937732	0.43530906
Sphk1	-0.2362392	4.58508224	0.47272204	0.49966952	0.43550463
Usp48	0.15379887	5.49429636	0.4724803	0.49977792	0.43550463
Plat	0.19449523	4.7598382	0.47246233	0.49978598	0.43550463
Rgs20	0.14724909	5.70952215	0.47231695	0.4998512	0.43550463
Ap5b1	-0.530895	-0.0106222	0.47229126	0.49986272	0.43550463
Zfp112	-0.252786	2.14004169	0.47227851	0.49986844	0.43550463
Ndn	0.12184939	6.22948305	0.47197029	0.50000676	0.4355864
Ttr	-0.2684196	5.00354118	0.47179675	0.50008467	0.43561553
Metap2	-0.0784216	7.30908151	0.47137484	0.50027416	0.43574185
Gm6225	0.69252291	-1.1707404	0.47125471	0.50032815	0.43574274
Traf3ip2	-0.1887596	2.99233899	0.47117457	0.50036416	0.43574274
Gimap5	-0.4316422	1.56612691	0.47102744	0.50043029	0.43576159
Fbxo4	-0.2171624	3.73373503	0.47087743	0.50049773	0.43578158
Lrrc1	0.16114876	4.36593242	0.47025619	0.50077721	0.43598616
1700084C01I	-0.250242	1.65505672	0.46997241	0.50090496	0.43602344
Rsph3a	-0.1164544	4.43966848	0.46993683	0.50092098	0.43602344
Maged1	0.0807963	8.26477941	0.46986448	0.50095357	0.43602344
Ndufaf1	-0.1870565	3.33368147	0.46948601	0.50112407	0.43613309
Dlx2	0.27172647	0.88539876	0.46931958	0.50119908	0.43615961
Npy2r	-0.1995559	2.84536466	0.46877523	0.50144455	0.43633447
A230070E04	-0.1514974	4.56051307	0.46810266	0.50174813	0.43644964
Mamdc2	0.47996301	0.03578776	0.46810203	0.50174841	0.43644964
Exoc3	0.0949276	6.57584827	0.46800776	0.50179099	0.43644964
Ssr4	-0.1433007	3.42232035	0.46796135	0.50181196	0.43644964
Fsbp	-0.7830912	-1.8040217	0.46790157	0.50183896	0.43644964
Gm5431	0.33917591	0.93549401	0.46781247	0.50187922	0.43644964
Trim37	-0.1081096	8.21341836	0.46779126	0.5018888	0.43644964
Taf6l	0.33681502	0.66481131	0.46708673	0.50220731	0.43668785

Supt5	0.11924046	5.64203307	0.466826	0.50232528	0.43675165
BC068281	-0.1721249	2.29279265	0.46670677	0.50237924	0.43675981
Pigyl	-0.3155891	3.02094125	0.46581395	0.50278363	0.43707258
Tpmt	-0.1712662	3.69758478	0.46540936	0.50296707	0.43719325
4930447A16	1.00085157	-1.7228355	0.46529091	0.5030208	0.43720116
Igf2bp1	-0.9796458	-1.5499557	0.46491043	0.50319345	0.43731241
Mpv17l2	-0.2369887	2.69491878	0.46481008	0.503239	0.4373132
Tmem100	-0.1315397	3.5969053	0.46444332	0.50340556	0.43741913
Tesk2	0.199699	2.09730237	0.464158	0.50353519	0.43745903
Rpusd2	-0.1217067	4.33296508	0.46411704	0.5035538	0.43745903
Rap2c	-0.0753024	6.85442395	0.46403603	0.50359062	0.43745903
Snap29	-0.0998156	6.39853214	0.46386953	0.50366632	0.43745903
Lynx1	-0.0973177	7.54082893	0.46385092	0.50367478	0.43745903
Klhl33	0.94495243	-1.944118	0.46324559	0.50395014	0.43762458
Vps35	0.08119151	6.730399	0.46323549	0.50395474	0.43762458
Il10rb	-0.1834132	3.04627877	0.46241073	0.50433034	0.43791193
Def6	-0.3642493	0.86126945	0.46229513	0.50438303	0.43791886
Supt20	0.11332832	4.95552167	0.46171641	0.50464693	0.43810915
Cgn	0.21817357	2.17936625	0.46132639	0.50482493	0.43820311
Tnfrsf21	0.13494681	6.54315861	0.46128321	0.50484464	0.43820311
Fndc3a	0.08803493	8.08988479	0.4610887	0.50493345	0.43824137
Cep63	-0.1382296	5.47835818	0.46088404	0.50502693	0.43828367
Dgcr2	-0.0959315	4.64335063	0.46039668	0.50524966	0.43843812
Abhd6	0.12753802	4.18091732	0.46024047	0.50532109	0.43844895
Epb4.1l3	0.09020389	7.79596914	0.46006752	0.50540019	0.43844895
G630090E17	1.19076738	-1.1328509	0.45989856	0.50547749	0.43844895
Pcsk4	0.22593664	1.48792254	0.45971229	0.50556273	0.43844895
Polr3c	0.13987839	3.20865398	0.45969031	0.50557279	0.43844895
Cdk2	-0.2708063	1.70180027	0.45956273	0.50563119	0.43844895
Kcnq4	-0.6623063	-1.0620554	0.45954326	0.5056401	0.43844895
Fam217a	0.67449014	-0.6141347	0.45941317	0.50569967	0.43844895
Slc46a3	-0.2787211	2.39258348	0.45940476	0.50570351	0.43844895
Gm2011	0.27379061	1.36350918	0.4592929	0.50575474	0.43844895
Neto1	0.13677006	7.50462645	0.45923462	0.50578143	0.43844895
Scd3	0.21613443	3.20431338	0.45919567	0.50579928	0.43844895
Leprotl1	-0.082745	6.11791754	0.45909649	0.50584471	0.43844953
Hdac6	0.18655049	3.12401288	0.45875966	0.50599907	0.43854452
Pak1ip1	-0.1088587	4.15962948	0.45854845	0.5060959	0.43854501
Ang	-0.3320081	2.28939294	0.45847015	0.5061318	0.43854501
Agmo	0.33564166	1.67511286	0.45846547	0.50613395	0.43854501
Sema4d	0.168185	3.84624337	0.45824339	0.50623582	0.43859448
Prelp	-0.1593071	8.14582054	0.45794004	0.50637502	0.43866117
Cacnb4	-0.0973139	9.03744874	0.45788047	0.50640236	0.43866117

Gpn2	-0.2312234	1.66041057	0.45764638	0.50650983	0.43868309
Fbxl6	0.3264643	0.94416263	0.45763027	0.50651723	0.43868309
Ccser1	0.21152896	3.31125484	0.45705283	0.50678252	0.43884828
Pnkp	0.22402268	1.65228763	0.45702009	0.50679757	0.43884828
Pygm	-0.2049325	3.78732599	0.45675308	0.50692034	0.43891579
2500004C02I	0.14351201	3.8040238	0.45593295	0.50729774	0.43920374
Ifi205	-0.5917817	-1.2619821	0.45567459	0.50741673	0.43926793
Helz2	-0.3226994	1.98281562	0.45477231	0.50783268	0.43958327
Opalin	0.30383967	1.45829791	0.45462891	0.50789884	0.43958327
Phtf1os	-0.2503483	1.14798045	0.45459255	0.50791562	0.43958327
Dbp	-0.1797753	4.08973715	0.45442413	0.50799335	0.4396117
Fam101b	0.13346233	4.20018008	0.4541839	0.50810426	0.43966884
Msi2	-0.1117704	9.31989194	0.45403352	0.50817371	0.43969009
Cct8	0.08314948	6.86970819	0.45387959	0.50824482	0.43970239
Adrb3	0.53684274	-0.894653	0.45380842	0.5082777	0.43970239
Slc10a7	-0.1351732	3.06877639	0.45352467	0.50840884	0.4397639
Klf9	-0.0927743	8.08354523	0.45346029	0.5084386	0.4397639
Dio3	-0.6051696	-0.9980248	0.45330331	0.50851119	0.43977113
Upp2	-0.1568357	3.38280111	0.45310736	0.50860181	0.43977113
Rpl28	-0.1703793	5.33779006	0.45299971	0.50865161	0.43977113
Golt1b	-0.1152536	5.46758268	0.45291824	0.50868931	0.43977113
Tusc1	0.13807014	3.18417392	0.45277604	0.50875511	0.43977113
Gm13483	0.49879671	-0.7401471	0.45270787	0.50878667	0.43977113
E030011O05	0.60845751	-0.8651926	0.45260561	0.508834	0.43977113
Pdgfrl	0.25553365	2.57959627	0.45259764	0.50883769	0.43977113
Map2k4	0.09261869	7.68792526	0.45255608	0.50885694	0.43977113
Ccr5	-0.3381493	1.19262425	0.45247189	0.50889592	0.43977113
Kif5a	0.10741409	9.49887627	0.45227728	0.50898605	0.43977887
Slc35c1	0.15595804	3.33152428	0.45225866	0.50899467	0.43977887
Trim2	0.10233658	9.04551566	0.45129006	0.50944369	0.44010509
Rqcd1	-0.1167741	4.95419579	0.4512504	0.50946209	0.44010509
Tmed5	0.12666586	5.54968377	0.45106792	0.50954677	0.44013943
Ift74	-0.0905869	5.84316581	0.45051599	0.50980304	0.44032196
Actr2	0.0723829	8.88066286	0.45030958	0.50989893	0.44036595
Aff3	-0.0948259	7.61446663	0.45003786	0.51002522	0.44043619
Fam133b	0.09269283	5.13952641	0.44990635	0.51008637	0.44044144
Adamts18	0.40640897	0.25730253	0.44983138	0.51012123	0.44044144
Brix1	-0.10607	4.72605738	0.44957924	0.51023851	0.44048226
Mtrf1	-0.1511783	3.13216346	0.4495364	0.51025844	0.44048226
Fa2h	0.17767376	4.01003315	0.44931812	0.51036002	0.44053112
Ccdc11	-0.4475295	-0.1982129	0.44920595	0.51041223	0.44053737
2010111I01R	0.1164579	4.66778239	0.44874245	0.51062808	0.44068483
Sema4c	-0.3491	0.62827725	0.4485885	0.5106998	0.44070791

Epdr1	0.11098554	5.86306948	0.44813066	0.51091323	0.44078126
Tgfa	0.08667261	5.15283698	0.44811906	0.51091864	0.44078126
Uba7	-0.3774138	0.63843414	0.44811655	0.51091981	0.44078126
Cables1	-0.1679886	3.13166928	0.44766254	0.51113161	0.4409103
5830444B04I	0.3254813	1.32362467	0.44760298	0.51115941	0.4409103
Kansl2	-0.093036	4.72657534	0.44732746	0.51128803	0.44096499
Id1	-0.2313028	3.82219196	0.44727433	0.51131284	0.44096499
Pcmtd1	0.07908254	8.28762032	0.44708591	0.51140084	0.44100205
Arhgef4	0.11923428	5.08822958	0.44695316	0.51146286	0.44101671
Ifngr2	-0.1236893	5.14388699	0.44658147	0.51163658	0.44109183
Smad3	0.09582443	6.75745112	0.44657404	0.51164005	0.44109183
1110059G10	-0.1501092	4.20899495	0.44528733	0.51224223	0.44157213
Idh3g	0.08602137	6.41635125	0.44457113	0.51257797	0.44178589
Fxr1	-0.0827465	6.88681768	0.44445853	0.51263079	0.44178589
Ocln	-0.2054885	2.73975676	0.44444173	0.51263867	0.44178589
Wdr76	0.2002658	2.1984456	0.44437363	0.51267062	0.44178589
Crym	0.12526666	3.43461405	0.44416048	0.51277065	0.44182553
Ndnf	0.10431762	5.19802815	0.44408144	0.51280775	0.44182553
Clec18a	0.51932557	0.16717546	0.44398732	0.51285194	0.44182553
Mir568	0.18045665	2.11864824	0.44352989	0.51306678	0.44197175
Gm10354	-0.9301254	-2.0395813	0.44296976	0.51333008	0.44214513
Prdm2	-0.0936165	7.02812682	0.44290969	0.51335833	0.44214513
Parp14	-0.1753058	4.15536348	0.44259029	0.51350859	0.44219931
Lrif1	0.1241321	5.127569	0.44258405	0.51351153	0.44219931
Agbl5	0.1731843	2.76543721	0.44225918	0.51366445	0.44229212
Myh7	-0.1928113	2.56956059	0.44204493	0.51376534	0.44232911
Klhdc7a	0.22445956	2.71863597	0.44194602	0.51381193	0.44232911
Fhod1	0.2236463	2.36053804	0.44188031	0.51384289	0.44232911
Thop1	-0.1996125	2.56012789	0.44137038	0.51408323	0.44249712
Klf14	0.57874531	0.34924461	0.44123749	0.51414589	0.44251217
Rad51ap1	0.61284216	-0.3231284	0.44049684	0.51449542	0.44277409
Coa7	-0.2116198	2.48376687	0.44016719	0.51465112	0.44286918
Necab2	-0.1466181	3.43692255	0.43974845	0.51484902	0.44292965
Ect2	-0.6818141	-0.849832	0.43973508	0.51485534	0.44292965
Crnkl1	0.11241025	4.56709497	0.4397315	0.51485704	0.44292965
Tmem116	-0.3853089	-0.0018565	0.43954013	0.51494753	0.44296859
2410127L17I	-0.1396416	4.94433774	0.43915457	0.51512994	0.44308659
Exoc3l4	0.5691436	-0.9109404	0.43898306	0.51521112	0.4431175
Atg2a	0.16968047	3.99208014	0.43880927	0.5152934	0.44314936
Gm8300	-0.5088704	-0.6615759	0.4386426	0.51537233	0.44317833
Pp2d1	0.32719495	0.70436595	0.43844031	0.51546816	0.44320174
Thumpd3	0.1090136	4.77480916	0.43838404	0.51549482	0.44320174
Cadm4	0.18345486	3.23482238	0.43827969	0.51554427	0.44320174

Smarcc1	0.06804276	7.39862034	0.43808268	0.51563766	0.44320174
0610007P14I	-0.1318079	4.25240262	0.43797803	0.51568728	0.44320174
Emid1	-0.3458602	0.7803174	0.43787327	0.51573695	0.44320174
Map3k11	-0.1466434	3.78564312	0.43787273	0.51573721	0.44320174
Ppp2r2a	0.07660018	7.10196112	0.43776056	0.51579041	0.44320174
Vps37c	-0.1830003	3.47069224	0.43772609	0.51580676	0.44320174
Mettl23	0.18722855	2.83220101	0.43756135	0.51588492	0.44321394
Det1	-0.2310851	1.32187188	0.43747671	0.51592508	0.44321394
Cetn4	0.17390851	4.02178654	0.43741007	0.51595671	0.44321394
Egr4	0.22063741	3.23099371	0.43710368	0.51610216	0.44330001
A930006K02	-0.3581241	0.2417027	0.43688564	0.51620572	0.44335009
Foxc2	-0.2014751	6.72668038	0.43664983	0.51631776	0.44340744
1600012H06	-0.1378813	4.59940473	0.43625232	0.51650672	0.44353083
Zfp474	-0.9028099	-1.4850592	0.43609996	0.51657919	0.44355417
Adarb1	0.10919729	6.5564066	0.43581881	0.51671294	0.44362158
Tob1	0.09266185	6.21399761	0.43574456	0.51674828	0.44362158
Fam174b	-0.1644903	6.56798464	0.43561162	0.51681155	0.44363702
Nmnat2	0.08992389	7.15343766	0.43517451	0.51701971	0.44374208
Tlr5	-0.6771679	-1.2240123	0.43493357	0.51713451	0.44374208
Tsacc	0.526419	0.05827724	0.43491988	0.51714104	0.44374208
Arl6ip5	0.11426601	3.89534564	0.43491387	0.5171439	0.44374208
Sart1	0.11580781	4.71131357	0.43487917	0.51716044	0.44374208
Ntsr1	0.27773315	1.74796493	0.43468653	0.51725227	0.44375034
Hspg2	0.12693218	3.48405132	0.43466891	0.51726067	0.44375034
Zfp3	-0.1642776	3.98973026	0.43439124	0.51739309	0.44376866
Rxrb	-0.158015	3.23806349	0.43436017	0.51740791	0.44376866
Bin1	-0.1135479	5.36717275	0.43431165	0.51743106	0.44376866
Ccdc184	-0.1396555	3.20706414	0.43424418	0.51746325	0.44376866
Tgoln1	0.10658654	6.81995248	0.43391391	0.51762086	0.44386497
BC068157	0.14845292	5.92522908	0.4337921	0.51767902	0.44387598
Camkk2	-0.1308002	6.00634976	0.433252	0.51793702	0.44402063
Sstr2	-0.1829932	2.75647597	0.43324917	0.51793837	0.44402063
Ctdspl	-0.1148003	6.4620004	0.43312003	0.5180001	0.44402833
1700110C19I	0.56968041	-0.1240952	0.43304069	0.51803803	0.44402833
Stat5a	-0.3364789	1.7590182	0.43281452	0.51814617	0.44408217
Zbbx	0.5588477	-0.6191186	0.43258124	0.51825776	0.44413895
Zfp438	0.12251947	3.94995783	0.43243898	0.51832584	0.44415843
Gpr37	0.16612724	3.7003291	0.43233502	0.51837559	0.4441622
Lrrc2	0.27155092	1.76997162	0.43187236	0.51859712	0.44427298
Ebf4	0.40815664	1.13461649	0.43177479	0.51864386	0.44427298
3110082I17R	0.20976359	2.68629787	0.43170996	0.51867493	0.44427298
Scyl1	-0.1048866	4.19231876	0.43168624	0.51868629	0.44427298
Plin2	-0.247499	3.42219361	0.43154726	0.51875289	0.44428639

Ift57	0.07885143	5.92742361	0.43146428	0.51879266	0.44428639
Mmp17	-0.1205848	5.60588234	0.43130419	0.51886941	0.44431327
Foxj3	-0.1004452	7.73374767	0.4311655	0.51893591	0.44432562
9430015G10	0.22792897	2.45774709	0.43102682	0.51900243	0.44432562
Pus7	0.1356293	3.84631136	0.43094232	0.51904297	0.44432562
Gdpd5	-0.1362418	3.75002037	0.43089582	0.51906527	0.44432562
Adrb1	0.18173401	3.6706366	0.43027911	0.51936132	0.44454019
Casc5	0.45890155	0.48233608	0.42988223	0.519552	0.44462753
Sh3bp1	-0.1615391	3.23093993	0.42987777	0.51955415	0.44462753
Grin3b	-0.77545	-1.7901405	0.42962455	0.51967587	0.44469285
Fbxo3	0.07620959	6.50548621	0.42946176	0.51975415	0.44472098
Mfap4	-0.2305646	3.76607458	0.42928056	0.51984132	0.44475672
Nanos2	1.05096327	-2.2041108	0.42857983	0.52017863	0.44498842
Rhoh	-0.4033583	-0.0962766	0.42848	0.52022672	0.44498842
Pigv	0.19014939	2.81035164	0.42843495	0.52024843	0.44498842
B3gntl1	0.1568505	2.62906946	0.42817975	0.5203714	0.44505474
Lypla1	-0.1180591	5.19566608	0.42769165	0.52060675	0.44521716
Gigyf1	0.22164037	2.40603753	0.42720607	0.52084108	0.44537867
Pdia5	0.46702418	0.10203084	0.42683488	0.52102034	0.44547965
Glce	0.09715487	6.45973608	0.42667923	0.52109554	0.44547965
Tmem88	-0.2311134	2.02488157	0.42667908	0.52109561	0.44547965
Pcsk6	0.21289611	1.62600231	0.42610338	0.52137393	0.44567869
Zfp934	-0.1534368	3.61369777	0.42585938	0.52149197	0.44574069
Fam13c	0.1175645	4.49680051	0.42485568	0.52197804	0.44611723
Efemp1	-0.1761891	7.48332412	0.42431426	0.52224059	0.44625245
Dnm1	0.10333593	8.86763299	0.42420377	0.52229419	0.44625245
Gp1bb	0.21970118	1.60154465	0.42411997	0.52233486	0.44625245
AI854517	0.13633917	3.70653765	0.4240684	0.52235989	0.44625245
Gns	0.12366214	6.50749551	0.42405984	0.52236405	0.44625245
Wfikkn2	-0.308419	2.97892266	0.42378201	0.52249892	0.44631302
Slc25a26	-0.1359902	3.09585486	0.4237261	0.52252607	0.44631302
Orc6	-0.1116785	4.67622612	0.42339978	0.52268458	0.44640948
F11r	0.19689983	2.71136474	0.42306617	0.52284673	0.44650903
Tshz3	0.09370199	6.13421341	0.42240581	0.52316795	0.44674441
Zfp286	-0.1766187	3.27813945	0.42226949	0.52323431	0.44676212
Nlrp1a	0.19937484	1.62889049	0.42182461	0.52345096	0.44690816
Dmc1	0.74706296	-1.4697402	0.42162181	0.52354978	0.44695357
Nfya	-0.0874787	5.71546218	0.42121332	0.52374894	0.44707102
Bmp7	-0.1744878	7.77650848	0.42115242	0.52377864	0.44707102
Stard7	-0.0791794	6.2672211	0.42094266	0.52388097	0.44709504
Gm20257	-0.2443145	2.45904281	0.42088339	0.52390989	0.44709504
Gm10509	0.18455669	2.66833887	0.42081406	0.52394372	0.44709504
Cars	0.153908	3.58290147	0.42070527	0.52399682	0.4471014

Hsd3b2	0.69195847	-1.284295	0.4204775	0.52410802	0.44715734
Stx16	-0.0736826	6.02270895	0.42036913	0.52416094	0.44716354
Dusp3	-0.0758852	7.09315937	0.42011738	0.52428392	0.44722951
Tmem232	0.31850169	1.34682122	0.41958182	0.52454572	0.44741386
Ccdc122	-0.2195541	2.24255344	0.41927858	0.52469406	0.44746671
Msl1	-0.069868	8.01020206	0.41926841	0.52469904	0.44746671
Ccdc103	-0.5122362	-0.0614749	0.41896497	0.52484755	0.4475544
Ubxn2b	0.09525932	6.02215154	0.41839033	0.52512902	0.44775543
Ier2	-0.2680322	1.23049555	0.41826882	0.52518857	0.44776723
Gucy1b3	0.10438493	6.61329345	0.41806936	0.52528635	0.44781162
Spsb1	-0.1963195	2.8969923	0.41780248	0.52541724	0.44788423
Dock8	-0.1803758	2.85605871	0.41762726	0.52550321	0.44791853
Chd3	0.08089696	7.99789304	0.41725836	0.52568428	0.44803388
Ppp1r14b	-0.1964382	3.52403494	0.41701903	0.52580181	0.44809507
Tram2	0.17327629	3.62401292	0.41680984	0.52590459	0.44814366
Dcun1d2	-0.1027541	5.52819333	0.41649624	0.52605872	0.44822172
Prdm4	0.12646616	4.99589618	0.41643729	0.52608771	0.44822172
Hadhb	-0.0884328	6.50866812	0.41629723	0.52615658	0.44823162
Man2b2	0.18413955	3.67084702	0.41622756	0.52619085	0.44823162
Tubg1	-0.1251076	4.38928208	0.41610521	0.52625104	0.44824391
Cfb	0.23021003	2.67106589	0.41563226	0.52648381	0.44836653
Rlbp1	0.27846026	1.98670262	0.41562671	0.52648654	0.44836653
Adrm1	-0.0985271	4.16478739	0.41540783	0.52659433	0.44841871
Brpf3	0.08575887	5.26307102	0.4153164	0.52663937	0.44841871
Ucp3	0.65809807	-0.2060946	0.41505274	0.52676929	0.44849035
Hist1h4j	-0.2906898	0.67386973	0.41462984	0.5269778	0.44859835
Galnt2	0.12422254	3.84837964	0.41459622	0.52699438	0.44859835
Qprt	-0.2714679	1.21650241	0.41444555	0.52706871	0.44859835
Bco2	-0.4727282	0.95810225	0.41442401	0.52707934	0.44859835
Mdc1	0.09714841	5.55897763	0.41423163	0.52717427	0.4486225
Prr32	-0.5031019	-0.3942978	0.41418092	0.5271993	0.4486225
Msto1	0.27096941	1.83431399	0.41402073	0.52727838	0.44865081
Zbtb8b	0.24807608	1.87284961	0.41356985	0.52750108	0.44880132
Kcng4	-0.2567632	1.67984873	0.41302125	0.52777229	0.44897523
Iqca	-0.5866396	-0.2712999	0.41289667	0.52783391	0.44897523
Nlrc3	0.682685	-0.9379213	0.41287455	0.52784485	0.44897523
2900041M22	-0.4423153	-0.0298824	0.41278565	0.52788884	0.44897523
Gpcpd1	0.09066165	6.404355	0.41266679	0.52794765	0.44897796
Ddx24	-0.0866463	5.66962142	0.41259393	0.52798372	0.44897796
Mafb	-0.0915828	5.48543859	0.41230834	0.5281251	0.44905921
Zfp7	0.20471599	2.73567721	0.41219248	0.52818248	0.44905959
E130310I04R	1.30526605	-1.5761338	0.41212231	0.52821724	0.44905959
Thumpd1	-0.1027203	5.95277989	0.4112204	0.52866436	0.4494007

Zfhx4	0.095603	8.17332463	0.41103256	0.52875757	0.44944093
Vmn2r57	0.4157292	0.70186386	0.41088723	0.5288297	0.44946324
Celsr1	0.19396188	2.35191803	0.41058658	0.52897899	0.44953873
Cog8	0.19673156	2.01771711	0.41045045	0.52904661	0.44953873
Pcsk1n	-0.110329	4.4277672	0.41043114	0.5290562	0.44953873
Gng12	-0.1166185	6.58084897	0.40998172	0.52927957	0.44964065
Sh3rf1	0.08641719	5.05297757	0.40996981	0.52928549	0.44964065
Cdipt	-0.1026155	4.67887572	0.40979883	0.52937051	0.44964065
Taf9b	0.10546422	6.91473087	0.40977203	0.52938384	0.44964065
Cr2	0.40175227	1.0796989	0.40972815	0.52940567	0.44964065
Herpud2	-0.0958409	6.21592798	0.40939984	0.52956902	0.44966805
Tpbg	0.19185584	3.35700631	0.40932192	0.5296078	0.44966805
Trf	0.13847747	5.62323471	0.40921754	0.52965976	0.44966805
Pou6f1	0.10960052	5.0576674	0.40918424	0.52967634	0.44966805
Gnat2	1.40354688	-1.6727266	0.40911059	0.52971302	0.44966805
Elmo2	0.08404206	5.92396524	0.40910675	0.52971493	0.44966805
Vps37d	-0.2933453	0.98645161	0.40901771	0.52975927	0.44966805
Spcs2	-0.1232442	5.45084433	0.4088473	0.52984415	0.44970113
Asap1	0.09427834	7.9708348	0.40871873	0.52990821	0.44971653
Tgfbr1	0.11202871	5.32494421	0.40837712	0.53007848	0.44982206
Car9	0.52151352	-0.7352023	0.40821972	0.53015697	0.44983524
Tor2a	-0.2027788	1.89822862	0.40816179	0.53018586	0.44983524
Atp6v0b	0.12256874	4.83149849	0.40792576	0.53030361	0.44989618
Tnks1bp1	-0.144552	3.62382755	0.4077046	0.53041398	0.44991657
Gm3258	-0.4559204	-0.8824314	0.40769352	0.53041951	0.44991657
Nuf2	0.6363564	-0.0548779	0.40726786	0.53063207	0.44999863
Fkbp5	-0.2097126	5.97414013	0.40710786	0.53071201	0.44999863
Tbc1d19	0.09962941	5.83751372	0.40698892	0.53077145	0.44999863
Ctsb	-0.1000458	7.9144416	0.40691528	0.53080826	0.44999863
Fbxo24	0.43692633	-0.1138298	0.40688494	0.53082342	0.44999863
Evi2a-evi2b	-0.5485262	-1.3671055	0.40687147	0.53083016	0.44999863
Lxn	-0.0883671	4.9324771	0.40685612	0.53083783	0.44999863
Lca5	0.13879694	4.18042977	0.40659726	0.53096726	0.4500694
Hic2	0.17826494	2.3305498	0.40624403	0.53114398	0.45008012
Pkp3	-0.7280649	-1.3205244	0.40622724	0.53115238	0.45008012
Ermap	0.47361372	0.46988567	0.40615134	0.53119037	0.45008012
1110065P20I	-0.4121782	0.87558483	0.40613687	0.53119761	0.45008012
Cox6b2	-0.2727089	2.88656334	0.40609061	0.53122077	0.45008012
Gimap9	-0.5426053	0.14078881	0.40602104	0.5312556	0.45008012
Lcor	0.15212105	3.98024282	0.40591834	0.53130702	0.45008475
4930578C19I	-0.8636239	-1.5017189	0.40577904	0.53137677	0.45010492
Spsb2	0.32984213	0.96639361	0.40542702	0.53155314	0.45017179
Kifc1	0.63576075	-1.4155674	0.40532057	0.53160649	0.45017179

Ccdc74a	0.18608227	2.01806722	0.4052844	0.53162462	0.45017179
Slc30a7	0.11418768	4.66352327	0.405209	0.53166242	0.45017179
Fancf	-0.2141564	3.01588235	0.40516296	0.5316855	0.45017179
Gxylt1	-0.1028459	5.66834184	0.40474893	0.53189317	0.45030869
Ccnh	0.09561348	4.97463151	0.40461152	0.53196212	0.45032815
Zmynd11	0.07216703	7.87641286	0.40371658	0.53241162	0.45066248
Dph2	0.19392602	1.76580914	0.40364205	0.53244909	0.45066248
Btbd3	0.09459602	8.37597415	0.40346641	0.5325374	0.45069829
Stard3nl	0.10982611	4.46531517	0.40334209	0.53259992	0.45071226
Atad5	0.13971594	3.88604756	0.40320353	0.53266963	0.45072061
Phf8	0.10765296	5.6399736	0.40308651	0.53272851	0.45072061
Csnk1g3	-0.0666609	7.45974778	0.40301635	0.53276381	0.45072061
Chrm5	-0.2859892	1.61380987	0.40291832	0.53281315	0.45072061
Cblb	0.0999437	6.78147852	0.40286526	0.53283986	0.45072061
G0s2	-0.3046212	1.60032905	0.40270693	0.53291958	0.45074912
Rbm33	0.11964739	5.58997343	0.40245878	0.53304456	0.4508159
Slc6a17	-0.0855338	8.25018407	0.40235837	0.53309515	0.45081976
Maf1	-0.1195144	4.57616593	0.40179124	0.53338104	0.45099027
Mllt10	0.08798551	6.2237758	0.40177576	0.53338885	0.45099027
Dsc2	0.4707033	-0.1495148	0.40146628	0.53354499	0.4510486
2700070H01I	0.53509753	-1.1984218	0.40145648	0.53354994	0.4510486
Pld1	-0.1350094	5.52894528	0.4010013	0.53377974	0.45120393
Gm15455	-0.4105811	-0.8764265	0.40089859	0.53383161	0.45120884
Psmf1	-0.1259384	4.76051962	0.40071119	0.5339263	0.45124993
Naa25	0.11395999	4.53536187	0.40052734	0.53401922	0.45126152
Dlg2	0.11011339	9.50865062	0.40050176	0.53403215	0.45126152
Ssbp4	-0.1653151	2.9201445	0.3999871	0.53429243	0.45144251
Top2a	0.20283299	2.36370115	0.39969891	0.53443828	0.45149946
Pla2g5	0.3497222	1.43780082	0.39960164	0.53448752	0.45149946
Mgme1	-0.210283	2.92595142	0.39958074	0.53449811	0.45149946
Use1	-0.1826341	4.73330947	0.39912687	0.53472801	0.45165472
Slc7a4	0.17162958	2.98030875	0.39897033	0.53480734	0.45168277
Slc45a1	0.17906995	2.61914513	0.39871827	0.53493513	0.45175175
4930539E08I	-0.1601267	3.15092154	0.39839417	0.53509953	0.45185163
Gm6525	0.98671701	-1.3451882	0.39799736	0.53530094	0.45193664
St3gal2	0.08123875	5.92266657	0.39780152	0.5354004	0.45193664
4933408N05	0.56426391	-1.0389726	0.39769042	0.53545683	0.45193664
Ubac1	-0.1291245	3.83876854	0.39768958	0.53545726	0.45193664
Ube2a	-0.1077122	6.34368022	0.39767945	0.5354624	0.45193664
Fam120a	0.07364371	8.53748752	0.39765067	0.53547702	0.45193664
4921507L20I	-0.5368497	-0.5854421	0.39723597	0.5356878	0.45196037
Oas1c	0.27286467	1.37818016	0.39714055	0.53573631	0.45196037
Kifap3	0.1139119	8.45919916	0.39699185	0.53581194	0.45196037

Cer1	-0.9199102	-1.0477751	0.39691709	0.53584997	0.45196037
Smg8	0.09673919	4.41716152	0.396869	0.53587444	0.45196037
Ncaph	0.52095844	-0.3833775	0.39683256	0.53589298	0.45196037
Cyp4a12a	-0.3897992	0.33010548	0.39682142	0.53589865	0.45196037
Gm4788	-0.3373839	0.00703406	0.39671388	0.53595337	0.45196037
Pdia3	-0.0987787	7.51848694	0.39668895	0.53596606	0.45196037
Cab39l	-0.0943257	5.7725496	0.39661496	0.53600371	0.45196037
2010109I03R	0.53805049	-1.33369	0.39653106	0.53604642	0.45196037
Ccp110	0.10054336	5.8898063	0.39643363	0.53609603	0.45196037
Dpt	-0.5592853	0.73137947	0.39641318	0.53610644	0.45196037
Ggt7	0.16766136	2.63432091	0.39631638	0.53615573	0.45196037
Hnrnpa0	-0.0711511	7.6352573	0.39623487	0.53619724	0.45196037
Vmn2r118	0.86621155	-1.2699865	0.39513165	0.53675971	0.45239554
Rgn	1.03539881	-1.5682967	0.3949355	0.53685983	0.45241706
Erich3	0.21877776	3.68636656	0.39490066	0.53687762	0.45241706
Nudt21	0.12405518	4.7723066	0.39478587	0.53693623	0.45242753
Fam46c	-0.1678166	2.4252796	0.39442903	0.53711851	0.45253101
Creld1	0.1562031	3.59084989	0.39430321	0.53718281	0.45253101
Gcc2	0.10817481	7.54554474	0.39425608	0.53720689	0.45253101
Hyal1	0.22310445	3.56170097	0.39410702	0.5372831	0.45253101
Gm15706	-0.1965148	1.90207161	0.39409345	0.53729004	0.45253101
Ndufb3	-0.1171108	5.49258129	0.3938885	0.53739484	0.45255872
Lrrd1	-0.5576364	-0.3771125	0.39384844	0.53741533	0.45255872
Lad1	0.92527934	-1.4804742	0.39372787	0.53747701	0.45257175
Spcs3	0.07763544	6.30157083	0.39325164	0.53772076	0.45267217
Zfp493	0.2356525	2.60744916	0.39321293	0.53774058	0.45267217
Extl1	0.13288165	3.57333937	0.39309353	0.53780173	0.45267217
Rgag1	-0.2891808	0.93509014	0.3930928	0.53780211	0.45267217
Gsdmc4	-0.8791883	-1.6809568	0.39303601	0.5378312	0.45267217
Tep1	-0.2072825	2.96774896	0.39287345	0.53791448	0.45267217
Krtcap3	0.48755066	-0.9806215	0.3927805	0.53796211	0.45267217
Pvalb	-0.1864373	2.39315365	0.39269052	0.53800823	0.45267217
Tcf24	0.34009185	1.26987938	0.39261719	0.53804582	0.45267217
Ablim1	-0.0732706	6.74637897	0.39259264	0.53805841	0.45267217
Nipal2	-0.1921458	2.85416739	0.39237705	0.53816895	0.45272044
Hnrnpa2b1	-0.0594234	8.6580106	0.3922291	0.53824484	0.45272044
Trim63	0.31106967	1.2032021	0.39221039	0.53825443	0.45272044
Dusp19	0.11852453	4.78410897	0.39198076	0.53837226	0.45277756
Ackr3	-0.1465203	3.93527382	0.39180664	0.53846164	0.45277756
Gpr20	-0.7039376	-1.386732	0.39173217	0.53849987	0.45277756
Abl2	0.11173763	5.655913	0.39156773	0.53858431	0.45277756
Cacng2	0.08686829	5.27518469	0.39146945	0.53863479	0.45277756
Slc4a5	-0.5083798	0.04024916	0.39145614	0.53864163	0.45277756

Tmem253	-0.722034	-1.077582	0.39137457	0.53868354	0.45277756
AA414768	0.13885262	3.09144475	0.39135784	0.53869213	0.45277756
Slc6a13	-0.179971	6.53886407	0.39115973	0.53879394	0.45282427
Usp42	0.11591449	4.91321096	0.39106834	0.53884091	0.4528249
Entpd2	0.3172803	0.55519252	0.39059889	0.53908234	0.45298892
Lgi2	-0.2083077	2.70263887	0.39022249	0.53927606	0.45311284
Nmd3	0.10411026	5.08200007	0.38990213	0.53944104	0.45318523
Eln	0.19913367	2.43103705	0.3898509	0.53946744	0.45318523
Il5	0.9982546	-1.8544092	0.38972837	0.53953057	0.45318523
Gm1715	-0.7750659	-1.7361002	0.38969593	0.53954729	0.45318523
Gpatch1	-0.0861386	5.0819513	0.38938413	0.53970801	0.4532733
Nrp2	0.09126054	5.60289257	0.38931301	0.53974468	0.4532733
2210408I21R	0.15353833	4.27033096	0.38920598	0.53979988	0.45328079
Htr2a	0.14235535	4.42755385	0.38905708	0.53987669	0.45330643
Dhfr	-0.1202079	3.80304629	0.38880362	0.54000748	0.45337739
Igsf8	0.10732263	5.06803153	0.38868393	0.54006927	0.4533904
Rbm14	-0.1802582	3.06542224	0.38838479	0.54022375	0.45348122
9030612E09I	-0.3232451	1.32594339	0.38823603	0.5403006	0.45350687
6330409D20I	0.30327012	0.73553757	0.38812323	0.54035889	0.45351693
Necab3	0.12464089	5.96483057	0.38785771	0.54049614	0.45359326
Kif5c	0.11021057	9.59216499	0.38744333	0.54071047	0.45368673
Cdk1	-0.8333245	-1.4653054	0.38743494	0.54071481	0.45368673
Cntf	0.60091564	-1.5165918	0.38737376	0.54074646	0.45368673
Ubxn10	0.38716842	0.82229865	0.38687578	0.54100428	0.45386416
Apof	-0.503435	-0.0834847	0.38666906	0.54111137	0.45388985
Gm16796	-1.2519843	-2.0218992	0.38660924	0.54114237	0.45388985
Top3b	0.15874965	3.52638291	0.38654837	0.54117391	0.45388985
Golga1	-0.0827075	5.51897548	0.38578286	0.54157093	0.45414993
Sepsecs	-0.2151825	2.25079891	0.38577166	0.54157674	0.45414993
Creld2	0.1777958	3.22286523	0.38518845	0.54187958	0.45436498
Ldlrad3	-0.1743471	5.77201379	0.38483839	0.5420615	0.45443586
Aagab	-0.0988511	4.71518943	0.3847181	0.54212404	0.45443586
Tbc1d2	-0.4623743	-0.1847319	0.38458912	0.54219112	0.45443586
Narf	-0.1071182	5.05031962	0.38430491	0.54233898	0.45443586
Arfgap1	-0.118106	4.53177529	0.38426467	0.54235992	0.45443586
Snrpd2	-0.1730801	4.07892904	0.38423742	0.5423741	0.45443586
Prox2	-0.2471414	1.61354989	0.38420271	0.54239216	0.45443586
Zc2hc1c	-0.2483414	2.21582011	0.38416761	0.54241043	0.45443586
4931408D14I	0.37970926	0.71792381	0.38414361	0.54242293	0.45443586
Mog	0.18574884	2.85453928	0.38413379	0.54242803	0.45443586
Marveld3	0.92280042	-0.9875168	0.38384042	0.54258079	0.45452496
4930524B15I	1.00400437	-1.5930791	0.3836528	0.54267852	0.45456795
Kif13a	-0.0862462	5.84859562	0.38326442	0.54288093	0.45469862

Zfhx2os	0.39191691	0.39938693	0.38295244	0.54304363	0.45479287
Zmat5	-0.2058448	2.44656038	0.3828706	0.54308633	0.45479287
6720483E21I	0.73943781	-1.7520889	0.38260681	0.54322399	0.45486927
Surf6	-0.1216221	3.73763035	0.38203318	0.54352357	0.45508122
Zfp53	-0.1501671	2.75478349	0.38182473	0.54363251	0.45513353
Npr3	0.14999133	5.29409063	0.38148543	0.54380992	0.45524315
Lama5	-0.1857088	1.76974396	0.38104621	0.54403975	0.45536475
Prkar1a	-0.0637499	9.6161682	0.38099309	0.54406756	0.45536475
H2-K2	0.58533141	-0.367524	0.38094138	0.54409463	0.45536475
Nsmf	0.09597137	5.93049297	0.38073024	0.5442052	0.45541837
E2f3	-0.1044692	5.37305131	0.38054303	0.54430327	0.45543753
Nfatc2	-0.1477294	3.35874337	0.38050903	0.54432109	0.45543753
Paqr4	0.14935407	3.91087184	0.38006142	0.54455572	0.45553019
Selt	-0.0943953	8.78450393	0.38004066	0.54456661	0.45553019
Trabd	-0.2133415	2.25259154	0.38003162	0.54457135	0.45553019
Gm7444	-0.4558787	0.29348424	0.37955664	0.54482056	0.45565897
I830012O16F	0.16425204	4.05990536	0.37951099	0.54484452	0.45565897
Kcnv1	0.14176928	6.74877182	0.37928072	0.54496543	0.45565897
Zfp287	0.14834749	4.00793578	0.37924393	0.54498475	0.45565897
Tspan8	0.21326239	4.35901052	0.37921755	0.54499861	0.45565897
Jph4	0.08051741	7.22585029	0.37911499	0.54505248	0.45565897
Fam35a	-0.2130282	2.54139424	0.37907014	0.54507604	0.45565897
Zfp273	0.14580601	3.24774517	0.37902833	0.545098	0.45565897
Abcd3	-0.0783252	6.90680663	0.37894087	0.54514396	0.45565897
Cpeb2	-0.0704014	7.36638977	0.37877717	0.54523	0.45568395
Bex1	-0.1312555	5.22081363	0.37858441	0.54533134	0.45568395
Rgs1	0.48587894	-0.295751	0.37854668	0.54535118	0.45568395
Pygo1	-0.0998194	4.86566756	0.37846514	0.54539406	0.45568395
A630075F10	0.46018613	-0.7287085	0.37844159	0.54540645	0.45568395
Ccdc55	-0.0773733	6.97211916	0.37813816	0.54556609	0.45577846
6720489N17	0.13318774	3.66694827	0.37789762	0.54569271	0.45584536
Rassf5	-0.1023712	4.01244149	0.37758648	0.54585657	0.45594335
Fignl2	0.29285252	1.1062992	0.37748054	0.54591238	0.45595109
AV039307	0.40201968	0.33744871	0.37724655	0.5460357	0.45598405
Gm20300	-0.0817302	7.24327955	0.37715459	0.54608418	0.45598405
Lrrc29	-0.6241652	-0.596089	0.37714072	0.54609149	0.45598405
BC055402	-1.0151223	-2.4646339	0.37649342	0.54643298	0.45623029
2900009J06F	-0.5967829	-0.6726362	0.3762673	0.54655236	0.45629108
Celf3	0.11292757	5.51005772	0.37601162	0.54668741	0.45636493
Acot6	-0.1729056	2.70793181	0.37585601	0.54676964	0.45639468
Ccm2l	-0.6379665	-0.5703278	0.37541507	0.54700276	0.45655036
Syne3	0.4037006	0.83033734	0.37523338	0.54709888	0.45655314
Crtc2	0.11362627	4.0438307	0.37518561	0.54712415	0.45655314

Tgm2	-0.1250567	4.09520623	0.37514449	0.54714591	0.45655314
Xylt1	0.19044253	2.83464097	0.37487656	0.54728773	0.45663257
Pou3f2	-0.1088603	4.41732911	0.37468803	0.54738756	0.45667697
Ttc19	0.09084485	6.26122451	0.37457705	0.54744635	0.45668712
1700101E01I	0.652862	-0.8806146	0.37436131	0.54756066	0.45670249
Mtm1	0.20968416	2.68765722	0.37434252	0.54757061	0.45670249
Mir6920	0.61957443	-1.5890956	0.37410906	0.54769436	0.45670249
Tert	0.49578167	-0.679246	0.37407547	0.54771217	0.45670249
2310045N01	-0.1921823	4.79237446	0.37394157	0.54778318	0.45670249
Trmt2a	-0.1399005	3.786557	0.37393371	0.54778735	0.45670249
Htra2	0.26344315	1.29477846	0.37383819	0.54783802	0.45670249
Pde4b	0.10199375	7.9733752	0.37368332	0.54792018	0.45670249
2810417H13	0.36980221	0.64624972	0.37363553	0.54794554	0.45670249
Ddx42	0.0863174	6.02745186	0.37352773	0.54800275	0.45670249
Sh3bgrl2	-0.116434	4.30173649	0.37345383	0.54804198	0.45670249
Rgs2	-0.0924604	7.30623261	0.37336541	0.54808892	0.45670249
Hdac1	-0.119143	5.29501835	0.37331191	0.54811732	0.45670249
Pde5a	-0.1327306	6.52878451	0.37331153	0.54811752	0.45670249
Zscan26	0.06536763	7.19874015	0.37311753	0.54822055	0.45670588
Got2	-0.0733055	7.01775185	0.37311303	0.54822294	0.45670588
Gmpr	-0.2059983	2.07266291	0.37304052	0.54826146	0.45670588
Cort	0.68039486	-1.6899519	0.37268276	0.54845158	0.45673618
4930405J17F	0.40108605	1.00072485	0.37266146	0.54846291	0.45673618
Igfbp4	-0.1458506	5.14860724	0.37263522	0.54847686	0.45673618
Ndst2	0.1432801	3.30347432	0.37262112	0.54848435	0.45673618
Sox17	-0.3041066	1.86351752	0.37212899	0.54874611	0.4568608
Gtf2ird2	0.18658634	2.65349216	0.37207884	0.5487728	0.4568608
Wnt10a	0.20320541	1.65062776	0.37205133	0.54878744	0.4568608
2210015D19I	0.13059925	3.178976	0.37198911	0.54882056	0.4568608
1810062O18	0.4150466	0.63893738	0.37178779	0.54892774	0.45691119
Slc25a21	0.39781651	0.19478389	0.37138284	0.54914345	0.45696745
Cdkn2aipnl	-0.1055242	4.48162468	0.37137405	0.54914813	0.45696745
Uba3	-0.0808358	6.23925471	0.37136424	0.54915336	0.45696745
Pten	-0.0711225	9.22052376	0.37131061	0.54918194	0.45696745
Arih2	-0.0957042	4.72467566	0.37113258	0.54927684	0.45697626
Msr1	-0.7634098	-1.3220509	0.37111571	0.54928583	0.45697626
Fam155a	-0.0968967	6.69012916	0.37097223	0.54936234	0.4570011
St6galnac6	-0.1299896	4.07207896	0.37076173	0.54947462	0.45703466
Uckl1	0.13396109	3.45904809	0.370594	0.54956412	0.45703466
Ldlr	0.10786446	3.69142439	0.370591	0.54956572	0.45703466
Col4a1	0.13734745	3.65295433	0.37054678	0.54958932	0.45703466
Igf2bp2	-0.2223189	1.32033371	0.3704384	0.54964717	0.45704397
Mapk1ip1	-0.0950734	4.40151255	0.37026954	0.54973733	0.4570672

Zfp316	0.14953275	3.73283924	0.37021128	0.54976844	0.4570672
4933432I09R	0.94683704	-0.7107174	0.36955576	0.55011873	0.45731962
Pip5k1b	0.18817667	2.98888508	0.36937474	0.55021554	0.45732286
Riiad1	-0.3580638	0.2172966	0.36922142	0.55029756	0.45732286
Mrpl47	0.10498904	3.55239049	0.36921614	0.55030039	0.45732286
Zfp708	0.1414516	3.49910848	0.3691866	0.55031619	0.45732286
Cyp2u1	0.27741599	0.87259289	0.36905206	0.55038819	0.45732286
Mrc1	-0.2490829	2.19715843	0.36882182	0.55051144	0.45732286
Gas7	-0.073229	9.7921324	0.3687977	0.55052436	0.45732286
Slc36a2	-0.7747675	-1.7024372	0.3687339	0.55055852	0.45732286
Cpq	0.18066769	4.27189071	0.36857616	0.55064301	0.45732286
Rnmt	-0.0773299	5.92020427	0.36857449	0.5506439	0.45732286
Dleu7	0.24766354	1.26264563	0.36839105	0.55074219	0.45732286
Nudcd3	-0.0688125	6.41496489	0.36833101	0.55077436	0.45732286
Mab21l2	0.71860357	-0.8035876	0.36822975	0.55082863	0.45732286
Gid8	-0.0713753	6.55454777	0.36822481	0.55083129	0.45732286
Nek10	-0.4090881	0.42378789	0.36817786	0.55085645	0.45732286
Slc6a20a	-0.1847981	5.60574727	0.36807263	0.55091287	0.45732286
Pik3ca	0.06623311	7.01483984	0.36806619	0.55091632	0.45732286
Dync1li1	0.10689158	5.04728039	0.36791686	0.5509964	0.45735057
2810410L24F	0.35843248	-0.0493362	0.3669671	0.55150626	0.45773498
Cdk19	0.07849646	7.00391856	0.36681165	0.55158979	0.45776482
Cd9	0.19232598	4.85260339	0.36672631	0.55163567	0.45776482
Arhgef26	0.12325374	4.33599906	0.36637124	0.55182659	0.45788447
Tspan12	0.18356927	3.01681319	0.36626317	0.55188473	0.45789392
Nhsl1	-0.1047873	7.15377938	0.36612952	0.55195665	0.4579148
Cnot8	-0.0974156	4.59883218	0.36575982	0.55215567	0.45802133
Ppap2a	-0.1404012	3.7937364	0.36571728	0.55217858	0.45802133
Sdf4	-0.0646854	7.98580178	0.36542599	0.5523355	0.45807339
Slc25a54	0.66772085	-0.925918	0.36538748	0.55235625	0.45807339
Mgst2	0.71485462	-1.4875532	0.36534039	0.55238163	0.45807339
Cnep1r1	0.08396642	5.90933978	0.36512334	0.55249864	0.45809682
Kcnk12	0.52222534	-1.2942537	0.36511446	0.55250342	0.45809682
Trmt10c	-0.1149759	4.78226766	0.3645956	0.55278332	0.4582901
Isl1	0.27665876	1.28366596	0.36433291	0.55292513	0.45833359
Nol3	0.14478542	2.90621969	0.36432508	0.55292936	0.45833359
Acaa1b	0.29141565	0.75719299	0.36402362	0.55309218	0.45838616
Sesn1	0.08088038	6.4168834	0.36398101	0.55311521	0.45838616
Acot11	0.12439993	3.82662615	0.36388582	0.55316665	0.45838616
Spa17	0.24191992	2.41346414	0.36377868	0.55322455	0.45838616
Tars	0.10241518	4.55001334	0.36373135	0.55325014	0.45838616
Sgip1	0.09946789	8.10724872	0.36368805	0.55327355	0.45838616
Kcnj12	-0.1583587	3.19352967	0.36352144	0.55336364	0.45841532

Spata3	-0.5758627	-0.8067021	0.36344985	0.55340236	0.45841532
Glis3	-0.1522622	3.09215531	0.3633196	0.55347282	0.45843492
Trim59	0.16234932	3.40018725	0.36285718	0.5537231	0.45860345
Hist1h2bj	-0.5546473	-1.5692539	0.36274792	0.55378227	0.45861368
D19Bwg1357	0.08548067	5.51629324	0.36261859	0.55385232	0.45862185
Foxm1	-0.2908495	1.41921814	0.36241716	0.55396146	0.45862185
Tchh	-0.1603132	3.01158791	0.36239703	0.55397237	0.45862185
Rpa1	0.09207156	4.58334867	0.36233322	0.55400696	0.45862185
Rs1	0.67060702	-0.2740352	0.36228272	0.55403433	0.45862185
Nek6	-0.1271959	4.83374912	0.36221128	0.55407306	0.45862185
Homer3	-0.1614807	1.81476363	0.36195376	0.5542127	0.45866547
Sla	-0.1391824	3.91966931	0.36192985	0.55422568	0.45866547
Suv420h2	0.37601659	-0.0499833	0.36184351	0.55427251	0.45866547
Gm11627	-0.2577565	1.40812479	0.36170859	0.55434572	0.45866547
Ism1	0.20819173	3.28477602	0.3616825	0.55435988	0.45866547
Fndc5	-0.0889524	5.00331875	0.36114063	0.55465409	0.45883313
Tfcp2	0.10110923	3.79045747	0.36103826	0.55470971	0.45883313
Agpat4	0.11500058	5.19971773	0.36103372	0.55471218	0.45883313
Tpcn2	0.50823085	-0.307709	0.36092759	0.55476985	0.45883313
Aspdh	-0.4411168	0.11307525	0.36087635	0.5547977	0.45883313
Erbb2ip	0.06545952	7.94454887	0.36079196	0.55484357	0.45883313
Tmem150co:	0.63985417	-0.5281174	0.36022558	0.55515163	0.45904913
Ccdc173	0.17044077	3.05128335	0.36012738	0.55520507	0.45905457
Tln1	-0.1167508	6.04945015	0.35968997	0.55544325	0.45919601
Kcnj14	0.75128006	-1.0669554	0.35957812	0.55550419	0.45919601
Folr2	-0.6631419	-0.8304092	0.35955502	0.55551678	0.45919601
Mplkip	0.09377374	4.05352396	0.35942148	0.55558955	0.45921741
Fgf5	0.19243748	2.67938052	0.35908668	0.55577208	0.45932338
Zfp51	0.15323481	4.1864609	0.35901434	0.55581154	0.45932338
Nipa2	0.10448937	4.76445489	0.3588394	0.55590698	0.45936349
Moxd1	0.16077792	2.93073035	0.35855408	0.5560627	0.45942755
Zfp300	-0.1663749	2.71413456	0.35852551	0.5560783	0.45942755
Gnat1	-0.5107963	-0.4817534	0.35806818	0.5563281	0.45959482
Sema3c	-0.1138333	3.8203494	0.35788735	0.55642692	0.45959482
Hectd3	-0.1135019	4.6912483	0.35783535	0.55645535	0.45959482
Fam58b	-0.1590168	4.09447951	0.35773973	0.55650763	0.45959482
Gsn	-0.1261812	5.4563857	0.35772559	0.55651536	0.45959482
Ctdp1	-0.1053312	3.88503483	0.35738649	0.55670084	0.45967034
Efr3a	-0.0823031	7.52803135	0.35738611	0.55670105	0.45967034
Zic5	0.24063864	1.62181319	0.35730106	0.55674759	0.45967034
Zeb2	0.08774269	8.89773701	0.35672415	0.55706347	0.45989238
Bbc3	-0.3506787	-0.1095236	0.35660073	0.5571311	0.45990945
Fam86	-0.1700396	3.38812708	0.35630445	0.5572935	0.46000474

Tinf2	-0.1703993	2.67584235	0.35612835	0.55739007	0.46004569
Tbrg4	0.11166571	3.84983377	0.35584556	0.55754521	0.46013497
E130309D14I	0.11943676	4.98591316	0.35527226	0.55786	0.46035597
Cd37	0.53739664	-0.4761001	0.35516943	0.5579165	0.46036381
Smarcd2	0.19453966	3.09274541	0.35507662	0.5579675	0.46036711
Stim1	0.11281011	4.00960311	0.35484803	0.55809315	0.4603935
Des	-0.3302298	0.62764967	0.354838	0.55809867	0.4603935
Cenpi	-0.5415607	-0.5162075	0.35476195	0.55814049	0.4603935
Rtkn	-0.150561	3.44422276	0.3545724	0.55824474	0.46044072
Fbxl15	-0.4066334	-0.4412051	0.35437	0.55835611	0.4604938
Aaas	-0.1523582	3.0916257	0.35425479	0.55841952	0.46050733
Zfp324	0.18216173	3.0189689	0.35393714	0.55859443	0.46055125
5330426P16I	-0.1943431	2.64642788	0.35383119	0.55865279	0.46055125
0610037L13I	-0.1228213	4.97435407	0.35370534	0.55872213	0.46055125
Rad54l	0.30268058	0.81036565	0.35358965	0.55878589	0.46055125
Cpb1	-0.2602512	2.22765188	0.35347376	0.55884977	0.46055125
Sall3	0.18471126	1.87691425	0.35343536	0.55887094	0.46055125
AW549877	-0.0778184	7.65307398	0.353417	0.55888106	0.46055125
Uri1	0.07513325	6.632009	0.35338381	0.55889936	0.46055125
Kti12	0.20529836	2.3447234	0.35334638	0.55892	0.46055125
Angptl2	-0.1806962	4.88653529	0.35330475	0.55894296	0.46055125
Ms4a1	-0.8179194	-1.7458976	0.35289052	0.55917148	0.4607008
Bahcc1	0.12588157	3.98732664	0.3527033	0.55927483	0.46074584
Kcnmb1	0.60448806	-1.0258641	0.35262108	0.55932023	0.46074584
Phyhd1	0.18938806	2.26409937	0.35216542	0.55957195	0.46091443
Cdkl3	0.12343689	3.66734289	0.35198406	0.5596722	0.46095825
Tmed3	0.18573144	2.97606323	0.35158346	0.55989377	0.46107991
Mir124a-2	0.45566855	-0.8553492	0.35154679	0.55991406	0.46107991
Eif3h	-0.0958331	6.19535446	0.35126907	0.56006778	0.4611391
Cdh12	0.12059554	6.34900079	0.35124683	0.56008009	0.4611391
Anln	0.08288198	4.85821024	0.35092916	0.56025603	0.4611895
Mrps28	0.14547047	2.96171414	0.35087383	0.56028668	0.4611895
Abca8a	0.19092614	2.39900376	0.35077122	0.56034354	0.4611895
Hif1an	0.12581743	4.74848081	0.35077001	0.56034421	0.4611895
Yif1a	-0.2514158	1.70687004	0.35071135	0.56037672	0.4611895
Rcl1	-0.2080889	2.11762534	0.35022527	0.56064626	0.46137257
Cpt2	-0.1314991	3.28725638	0.34978444	0.56089092	0.46152406
Ppapdc3	-0.1895795	2.36618951	0.34962151	0.5609814	0.46152406
3425401B19I	-0.2144154	3.75183628	0.34957687	0.56100619	0.46152406
Timm17b	0.26349213	2.74288703	0.34955415	0.56101881	0.46152406
Nde1	-0.1644315	3.81609371	0.3493987	0.56110518	0.46155634
Dnajc19	-0.0990681	4.74616253	0.34880873	0.56143318	0.46178738
Gnb5	-0.0893428	6.22872661	0.34862224	0.56153695	0.46183394

Uevld	-0.092549	5.20567585	0.34845473	0.56163019	0.46187185
2310033P09I	-0.1537788	3.12529055	0.34829086	0.56172142	0.4619081
Pdgfb	-0.1978315	2.23334722	0.34816662	0.56179061	0.46192622
Rdh11	-0.3110179	1.0106902	0.34795931	0.56190611	0.4619824
Tcea2	-0.171739	2.74191191	0.34761031	0.56210064	0.46207756
Mbnl3	-0.2445404	1.24336771	0.34758242	0.5621162	0.46207756
Dnmbp	-0.1293685	3.65661278	0.34712159	0.56237329	0.46220551
Fam136a	-0.1120386	3.84049672	0.34711371	0.56237768	0.46220551
BC049635	-0.183848	3.11311476	0.34704971	0.5624134	0.46220551
Zfp606	-0.1031697	4.59360883	0.34692921	0.56248068	0.46222202
Mrps34	-0.1506352	3.02931867	0.34683129	0.56253536	0.46222817
Galnt6	0.23742466	1.98311809	0.34672066	0.56259715	0.46224017
Zbtb21	0.0951063	5.05564308	0.34650378	0.56271831	0.46230094
Zfp442	0.12589114	3.50310262	0.34639736	0.56277779	0.46231103
Klhl32	0.23451887	0.85298259	0.34622952	0.56287162	0.46234933
Apoo	-0.0910389	4.83079787	0.34589677	0.56305772	0.46245491
Akt3	-0.0787182	8.0782817	0.34574753	0.56314122	0.46245491
Lrrc4c	0.09864295	8.09940003	0.34574654	0.56314178	0.46245491
Nudt22	0.20206845	1.45155025	0.34565347	0.56319387	0.46245891
Eno3	-0.168541	3.21469551	0.34540672	0.56333202	0.46247212
Ptgs1	0.13943242	3.44608602	0.34540054	0.56333548	0.46247212
Rpusd4	-0.1571144	2.7187492	0.34529475	0.56339473	0.46247212
BC026585	-0.3848985	0.725837	0.34522544	0.56343356	0.46247212
Ppp6r3	0.06811456	6.4274008	0.34518578	0.56345578	0.46247212
Pkd2	0.13543545	6.20222856	0.34511892	0.56349324	0.46247212
Rbm11	0.13321216	3.26912102	0.34502106	0.56354808	0.46247838
Chek1	-0.2855672	0.84374277	0.34484672	0.5636458	0.46251066
Haus1	-0.1733193	2.88420026	0.34478243	0.56368184	0.46251066
Prrg4	0.26908693	1.78377809	0.34458838	0.56379067	0.46252285
Cwc27	-0.0884396	5.33572549	0.34452252	0.56382761	0.46252285
Zfp521	-0.107907	5.60118123	0.34448748	0.56384727	0.46252285
Crispld2	0.26764218	2.2841983	0.34434875	0.56392512	0.46252285
Lace1	0.13364253	3.18675385	0.34433506	0.5639328	0.46252285
Btk	-0.5247799	0.14467041	0.34416425	0.56402867	0.46255181
Plgrkt	0.13617245	4.07499512	0.34405566	0.56408964	0.46255181
D630041G03	0.14126784	4.20906915	0.34401235	0.56411396	0.46255181
Fam210b	-0.1171275	5.27692419	0.34393571	0.564157	0.46255181
Zfpl1	-0.1690594	2.51937251	0.343142	0.56460312	0.46287884
Zfp955b	0.0829184	5.83765315	0.34277359	0.56481043	0.46301006
Muc1	0.41594201	-0.6133025	0.34242303	0.56500784	0.46308863
Prss23	0.13641217	3.46917784	0.34233767	0.56505593	0.46308863
3300002I08R	-0.4092697	-0.3547542	0.34230507	0.56507429	0.46308863
Amd1	0.32197716	-0.1636389	0.34226764	0.56509539	0.46308863

Rhobtb2	0.07409834	5.37579515	0.34217236	0.56514908	0.46309389
Igsf5	-1.0580606	-1.9997255	0.34195765	0.56527011	0.46315432
Sfn	0.59078471	-1.1509467	0.34175547	0.56538413	0.46320899
Gltpd2	0.86015272	-1.9420875	0.34157127	0.56548805	0.46322312
Rps11	-0.1267946	6.85286099	0.3414896	0.56553414	0.46322312
Cenpa	-0.3528929	1.06374212	0.34147346	0.56554325	0.46322312
Ncaph2	0.09489564	4.2766549	0.34124243	0.56567366	0.46327845
Cyyr1	0.50848273	0.36582894	0.34118624	0.56570539	0.46327845
4930506M07	0.07921707	5.40685335	0.34088129	0.56587765	0.46338078
Ifrd1	-0.0958043	5.97236501	0.3406919	0.56598468	0.46342968
Pcdhgb6	0.19012603	2.14496782	0.34036896	0.56616728	0.46354045
Pptc7	-0.0671821	6.66629707	0.34011908	0.56630865	0.46361744
Lepr	0.12400838	5.63711491	0.33987126	0.56644892	0.46369352
Zfp644	-0.0865294	7.40811311	0.33971244	0.56653886	0.46370317
Dnajc6	0.07765006	8.53886935	0.33968326	0.56655539	0.46370317
Clec4a2	-0.4260626	-0.2856982	0.33889306	0.56700329	0.46398404
Sp5	0.43484079	1.50954197	0.33884222	0.56703214	0.46398404
Resp18	-0.1210126	3.1582388	0.33882403	0.56704245	0.46398404
Nod1	0.19156334	2.07213642	0.33874372	0.56708802	0.46398404
Mavs	-0.1759256	4.855431	0.33832569	0.56732533	0.46413943
Gtf3c2	0.09666349	6.34947437	0.33822539	0.5673823	0.46414727
Otub1	-0.1307715	5.7233998	0.33810194	0.56745243	0.46416588
Kat2a	-0.0828685	5.89910999	0.33790496	0.56756436	0.46420654
Tmub2	-0.1175368	3.40390632	0.33782222	0.5676114	0.46420654
Unc119	-0.2372198	1.76904327	0.33773102	0.56766325	0.46420654
Prtg	0.2109645	1.53552983	0.33768099	0.5676917	0.46420654
Aldh4a1	-0.126832	4.20524868	0.33755173	0.56776521	0.46422789
Alx1	-0.3138233	2.53238733	0.33733845	0.56788655	0.46427429
Zmynd15	0.36028428	0.33496792	0.33728539	0.56791674	0.46427429
Zfp382	-0.1252464	4.01271086	0.33704063	0.56805607	0.46434943
Ddx27	-0.0938804	4.1812579	0.3368735	0.56815124	0.46438848
1700037H04	-0.1252509	4.31945239	0.33676257	0.56821443	0.46440137
Ggact	0.13901456	5.86444957	0.33629579	0.56848048	0.46458005
Smoc1	-0.1332212	4.56734441	0.33584546	0.56873738	0.46465231
Slc35f4	-0.1551222	2.69234322	0.33570947	0.56881501	0.46465231
Ltbp2	-0.7180605	-1.1453635	0.33569368	0.56882403	0.46465231
Gpr173	0.15854685	2.88554124	0.33568569	0.56882859	0.46465231
Sp6	0.59667814	-0.8815398	0.33565588	0.5688456	0.46465231
Loxl1	0.23464812	1.84589249	0.33562662	0.56886231	0.46465231
Gna15	0.86060805	-1.6031651	0.33555895	0.56890095	0.46465231
Mfsd12	0.59225319	-1.2930054	0.33535509	0.56901739	0.46470866
Rabep2	-0.1928918	2.27559331	0.33505802	0.56918716	0.4647767
Lrig2	0.08859789	5.05686369	0.33498218	0.56923052	0.4647767

Gm3383	0.33124464	-0.2915731	0.3349221	0.56926487	0.4647767
Tbl2	0.11378625	3.49257687	0.33484888	0.56930674	0.4647767
Dennd4c	-0.0857546	6.04642157	0.33479432	0.56933794	0.4647767
Wfdc1	-0.2644869	2.89032144	0.33470543	0.56938879	0.46477947
2610002M06	0.06497354	7.06876313	0.33430985	0.56961517	0.46492552
Dbx2	-0.175399	1.98242717	0.33408573	0.5697435	0.46498174
Fhl1	-0.0723044	7.70582581	0.33402378	0.56977899	0.46498174
Tmc6	0.33408157	0.25957733	0.33390719	0.56984579	0.46499405
Ms4a6c	0.33338852	0.37829737	0.33379369	0.56991082	0.46499405
Foxd1	-0.1605629	5.52951661	0.33374891	0.56993649	0.46499405
4933430I17R	-0.6970223	-1.6878358	0.33329799	0.57019505	0.46512902
Ids	-0.0807349	9.64471167	0.33329479	0.57019689	0.46512902
Nkain4	-0.2711655	1.74106904	0.33261917	0.57058474	0.46535719
4930563E22I	0.38150352	0.91985291	0.33261768	0.57058559	0.46535719
Zfp532	0.07848799	5.64739454	0.33255931	0.57061912	0.46535719
Ddit4l	-0.0838846	4.93093377	0.33192091	0.57098614	0.46561774
Npl	-0.2708014	1.59114051	0.33137875	0.5712982	0.46577659
Myo5c	-0.3520833	1.43219009	0.33124876	0.57137306	0.46577659
Tram1l1	0.10756056	4.25816909	0.33122394	0.57138736	0.46577659
Ccz1	0.09081809	4.8060253	0.33107115	0.5714754	0.46577659
Gm14634	0.40384569	-0.1042854	0.33105237	0.57148622	0.46577659
Esrra	-0.2124718	2.04728223	0.33104813	0.57148867	0.46577659
Tmem71	0.53024035	0.03806179	0.33100453	0.57151379	0.46577659
Ap3s1	-0.0904152	7.18832894	0.33022409	0.57196394	0.46610467
Slc7a6os	-0.1354726	3.9736553	0.33009573	0.57203805	0.46612628
Jak1	0.0759038	7.64637337	0.3298833	0.57216073	0.46613978
Tusc3	-0.0906544	4.91653902	0.32985224	0.57217867	0.46613978
Nrde2	0.21974656	2.0474504	0.32981987	0.57219738	0.46613978
Tmem238	-0.3965209	-0.4145217	0.32940395	0.57243776	0.46625267
Sec1	-0.3942306	-0.0709414	0.32934822	0.57246999	0.46625267
Rap1a	-0.0936191	8.59777155	0.32933307	0.57247875	0.46625267
Dnajb9	0.10909809	5.83039723	0.32916119	0.57257817	0.4662922
Lactb	-0.1115793	3.6918282	0.32908456	0.5726225	0.4662922
Cbwd1	0.10810223	4.13995644	0.32871407	0.57283694	0.46640672
Ccnt1	0.0883636	6.30201614	0.32867708	0.57285836	0.46640672
Wdr83os	-0.1168366	5.70252191	0.32832837	0.57306036	0.46650285
Adrb2	-0.2559819	2.24218647	0.32830884	0.57307168	0.46650285
Rb1cc1	0.08939438	8.50531799	0.32815712	0.57315961	0.46653566
Napsa	-0.6346885	-0.6509732	0.32772072	0.5734127	0.46668508
Mbd4	0.13719148	4.40401113	0.32766739	0.57344364	0.46668508
Poldip3	-0.1015986	5.76822409	0.32754528	0.57351451	0.46668508
Lgi3	-0.1328364	3.63126066	0.32751213	0.57353375	0.46668508
Lamp2	0.11386516	7.76757349	0.32704774	0.57380343	0.46686573

Rbm12	-0.0801929	5.25852923	0.32671828	0.57399491	0.46696976
Dmp1	0.39872147	0.28825488	0.32665872	0.57402954	0.46696976
Nr2f2	0.09111228	7.60255619	0.32658175	0.5740743	0.46696976
Erp44	-0.1098735	4.53609662	0.32621703	0.57428648	0.46710356
Pla2g6	0.15484904	2.71901809	0.32592196	0.57445826	0.46720449
Cenpl	-0.1923379	1.72929489	0.32582427	0.57451516	0.46721197
Cxcr4	0.62990522	-1.3127137	0.32566592	0.5746074	0.46724819
C030037D09I	0.29550577	1.34915979	0.32523115	0.57486083	0.46741547
Ubn1	0.07684391	5.81141917	0.32506625	0.57495701	0.46745487
Gc	0.9510412	-2.7059228	0.32659822	0.57518733	0.46760331
Ubr7	0.07963587	4.90069223	0.3242167	0.57545304	0.4677649
Smurf1	0.0900873	5.13253771	0.32409188	0.57552599	0.4677649
Arnt	-0.0800869	6.02241584	0.32406831	0.57553977	0.4677649
Nkrf	0.11145507	5.84414015	0.32397038	0.57559703	0.4677649
B4galt7	0.27691944	1.05384379	0.32392276	0.57562487	0.4677649
Gm11744	-0.4640104	-0.4086737	0.32357554	0.57582797	0.46787136
Tomm70a	-0.059018	7.12662431	0.3235284	0.57585556	0.46787136
Pdhx	0.08171014	5.1461394	0.32341037	0.57592464	0.46787136
Nfkb2	0.40764571	0.95481219	0.32337229	0.57594693	0.46787136
Gm13034	0.52152024	-1.6862613	0.32303768	0.57614288	0.46799173
Hes5	0.21319719	1.57656207	0.32289596	0.57622591	0.46802036
Zfp78	0.16563456	2.73225711	0.32252195	0.57644516	0.46815962
Fn3krp	-0.0981263	4.38382354	0.32205139	0.57672124	0.46834501
F730043M19	-0.2570559	0.97539026	0.32170636	0.57692385	0.46845198
C1qtnf2	0.30685128	1.96562314	0.3216642	0.57694861	0.46845198
Gm996	0.1169849	4.57695993	0.32147702	0.5770586	0.46847939
Rcor2	0.22843531	1.62983719	0.32142672	0.57708816	0.46847939
Dera	-0.2365336	2.55249595	0.32136259	0.57712586	0.46847939
Ddx11	-0.4100599	0.10747502	0.32116239	0.57724357	0.46852095
Fam122b	-0.0995918	4.12560287	0.32111284	0.57727271	0.46852095
St6gal1	-0.128186	6.19199686	0.32079262	0.57746111	0.46863502
Ell3	0.19081608	2.29649561	0.3206897	0.57752169	0.46864536
Ifit3	-0.1451604	5.94234365	0.32054988	0.577604	0.46867333
Tmem260	0.13080092	3.77287118	0.32017936	0.57782226	0.46879916
A330009N23	0.28444358	1.21981126	0.32005833	0.57789359	0.46879916
Mical2	-0.0817183	8.91771607	0.32004296	0.57790265	0.46879916
Psd2	-0.1160535	4.92517248	0.31987177	0.57800358	0.46881094
Retn	0.69178117	-1.5550005	0.31963809	0.5781414	0.46881094
Swt1	-0.1092052	4.57133182	0.31960945	0.5781583	0.46881094
Acot4	-0.42474	0.0646272	0.31958708	0.5781715	0.46881094
C130060C02I	0.36178463	0.02592742	0.31952671	0.57820712	0.46881094
Fgfr4	0.42536644	-0.6665554	0.31949185	0.57822769	0.46881094
Gcc1	0.09667618	4.93927588	0.31945032	0.5782522	0.46881094

1810030O07	-0.0842788	6.11730603	0.31926934	0.57835903	0.46885875
9130019O22	0.17655751	2.12545968	0.31906789	0.57847799	0.46891638
Spry1	-0.1548238	3.19488316	0.31884522	0.57860954	0.4689842
Gpr82	-0.7175337	-1.234143	0.31867076	0.57871266	0.46902897
Gsdmd	-0.207015	2.29812276	0.31849737	0.57881517	0.46907324
Ftsj3	0.09381476	5.00058319	0.31829808	0.57893304	0.46909975
Ccdc71l	-0.0938848	5.24140435	0.31828013	0.57894366	0.46909975
1700001O22	0.29446784	0.38566742	0.31790482	0.57916579	0.46922054
Ppm1l	0.11771056	4.72174536	0.31782397	0.57921367	0.46922054
Apoa1bp	0.11772004	3.85453329	0.31778551	0.57923644	0.46922054
Marveld1	-0.1599098	4.34164061	0.31769174	0.57929198	0.46922672
Acsf3	0.22983359	1.50699388	0.31713973	0.57961915	0.46945291
Usp1	-0.08718	5.3274602	0.31693603	0.57973998	0.46951195
Fam151b	0.21797943	1.36776781	0.31671124	0.57987338	0.46957095
Vps33a	-0.0806825	6.28335802	0.31665172	0.57990871	0.46957095
Nup210l	-0.6235212	-0.3714807	0.31612823	0.58021964	0.46978379
Trp63	-0.2203877	2.24132109	0.31603969	0.58027226	0.46978379
1700019G17	-0.2669248	1.04324127	0.31596706	0.58031544	0.46978379
Ccsap	0.1355423	3.95722994	0.31554593	0.58056591	0.46994724
Senp8	0.09740912	4.61752894	0.31546629	0.5806133	0.46994724
Zbtb14	0.09347193	5.14679904	0.31530125	0.58071154	0.46998128
Lta4h	0.08937307	4.27326009	0.31523443	0.58075131	0.46998128
Adk	0.09589955	4.8192347	0.3147282	0.5810529	0.47015743
Zmiz1	0.07187397	8.34258388	0.31463653	0.58110754	0.47015743
Papss2	0.10439387	5.79153678	0.3146274	0.58111298	0.47015743
Csf2ra	0.14421891	3.82945891	0.31404684	0.5814593	0.47037086
Ddx56	-0.1683303	3.08038876	0.3140242	0.58147282	0.47037086
Ccdc19	0.19377677	2.03876038	0.3139224	0.58153359	0.47038118
Mal	-0.1028975	6.0909116	0.31374029	0.58164234	0.4704303
Raf1	-0.0860551	4.73108242	0.31348413	0.58179539	0.47051523
Zfp827	0.07817768	6.30042239	0.31325882	0.58193007	0.47054766
Prpsap1	-0.0728565	4.70373033	0.31325631	0.58193157	0.47054766
Hid1	-0.0808624	6.11254569	0.3130924	0.58202958	0.47058807
Krt80	-0.1779475	3.54297797	0.31294043	0.58212049	0.47062273
Mical1	-0.164664	2.58725045	0.31259951	0.58232454	0.47073347
4931406C07l	-0.1084643	6.65583506	0.31255099	0.58235359	0.47073347
Col3a1	-0.1420386	5.0592517	0.31232191	0.58249079	0.47080553
Frmpd4	0.08821935	7.77636696	0.31184046	0.58277936	0.4709999
Cstf2t	0.06601693	6.61119104	0.31171541	0.58285437	0.47102166
Zfp14	0.15212849	3.10914981	0.31153547	0.58296233	0.47106523
Musk	-0.1475994	3.43332663	0.31137574	0.58305819	0.47106523
Nckipsd	0.11898994	4.12030892	0.31133498	0.58308266	0.47106523
Inpp5e	-0.0852191	5.0308167	0.31130502	0.58310064	0.47106523

Syt3	0.13917848	3.66352254	0.31102939	0.58326618	0.47113004
Cyp2j9	0.19410362	2.44796972	0.31101126	0.58327707	0.47113004
Nek3	0.28281684	0.95365832	0.31076726	0.58342368	0.47120821
Cr1l	-0.1054434	5.40021269	0.31053234	0.58356492	0.47120821
Gm5607	0.15507464	3.8160498	0.31029649	0.58370679	0.47120821
Zfp74	-0.1006702	5.97278665	0.31019298	0.58376907	0.47120821
Nr3c1	0.05911782	7.2766846	0.31014416	0.58379845	0.47120821
Map2k7	-0.1133584	5.36639003	0.31013251	0.58380547	0.47120821
Xrcc2	-0.1936446	2.5538066	0.31012965	0.58380719	0.47120821
A930003A15	-0.8042354	-1.9242753	0.31012718	0.58380867	0.47120821
Slc38a5	-0.5783588	-1.1945764	0.31007491	0.58384014	0.47120821
Desi1	-0.0734065	5.52702697	0.31003886	0.58386184	0.47120821
Tmem196	0.12872005	3.16291533	0.30997048	0.583903	0.47120821
Vps18	-0.126934	3.64210523	0.30958326	0.58413624	0.47129551
Sike1	-0.0666816	6.60274024	0.30956255	0.58414872	0.47129551
2810008D09	0.28824148	0.70757999	0.30950667	0.58418239	0.47129551
Plcd3	-0.1371122	3.08169908	0.30939957	0.58424694	0.47129551
Gpatch2l	-0.0805366	5.15068383	0.30927853	0.58431992	0.47129551
Gramd2	-0.4552684	-0.4528712	0.30899702	0.58448971	0.47129551
Irf8	-0.2252245	1.6282917	0.30887184	0.58456525	0.47129551
Mgmt	-0.3556544	-0.09654	0.30886604	0.58456875	0.47129551
Gdap1	0.10623119	7.46345264	0.30885506	0.58457538	0.47129551
4930529M08	-0.6065089	-0.9366219	0.30877385	0.58462439	0.47129551
Fbxl7	-0.1433216	6.20230065	0.30861004	0.58472329	0.47129551
Gm20110	0.65039404	-1.7276857	0.30854999	0.58475955	0.47129551
Tirap	-0.1391811	4.4284544	0.30852443	0.58477499	0.47129551
Cacng7	-0.0874448	5.83701289	0.30851281	0.584782	0.47129551
Kptn	0.21141278	1.85330009	0.30850996	0.58478372	0.47129551
Pfkfb1	-0.4128055	-0.0473381	0.30849353	0.58479365	0.47129551
Zfp275	0.08831519	5.65090821	0.30843481	0.58482912	0.47129551
Stt3a	0.08633902	5.16260031	0.30805151	0.58506074	0.47143944
Pex2	-0.124766	4.2015927	0.30797999	0.58510398	0.47143944
Shank3	0.11381973	4.59092802	0.30773382	0.58525287	0.47152062
Dlec1	0.67288345	-0.3980741	0.30752826	0.58537726	0.47157994
Taf1a	0.14354498	3.23536216	0.30739897	0.58545551	0.47157994
Creg2	0.08923426	7.62972907	0.30730083	0.58551494	0.47157994
6330416G13	-0.115787	3.76233321	0.307294	0.58551907	0.47157994
Ifit2	-0.10883	5.61040824	0.3069924	0.58570176	0.47166814
Sf3b1	0.06901923	8.28905656	0.30695423	0.58572489	0.47166814
Esrrb	-0.4852309	-0.3614553	0.30683428	0.58579758	0.4716879
Amdhd1	0.66234082	-1.7095124	0.30669716	0.58588071	0.47171606
Rps6kb1	0.06614488	7.12851419	0.30648195	0.58601122	0.47177753
Arhgap17	-0.1092582	4.57259103	0.30641244	0.58605339	0.47177753

Zfp395	-0.1121592	6.06510323	0.30629062	0.58612731	0.47179826
Gm14420	-0.0907855	5.40322491	0.30609607	0.58624539	0.47184406
Mrps5	-0.1569444	2.91157501	0.30603817	0.58628054	0.47184406
Col15a1	-0.2712513	1.46629283	0.30587493	0.58637967	0.47185727
Stxbp3b	-0.4020511	-0.9580444	0.3058525	0.5863933	0.47185727
Eif2s3x	0.07142043	7.27309505	0.30559117	0.58655208	0.4718881
Evl	0.11718724	4.22198237	0.30557275	0.58656327	0.4718881
Plekhm2	0.13894165	3.60509107	0.30555158	0.58657614	0.4718881
Usp45	-0.080645	6.85723407	0.30542167	0.58665511	0.47189286
8430429K09I	0.11296864	4.21523201	0.30532575	0.58671343	0.47189286
Slc18b1	0.11176849	5.1384207	0.30530414	0.58672657	0.47189286
Fam78a	0.32059398	0.64030231	0.30519601	0.58679234	0.471907
Gyk	-0.1619372	4.36426834	0.30492739	0.58695578	0.47199969
2700089E24I	-0.0775431	8.43300585	0.30457716	0.58716902	0.47213241
Usp40	0.10978351	4.56375978	0.30448007	0.58722816	0.47213879
Olfml2b	0.32633384	0.63137112	0.30440588	0.58727336	0.47213879
Tmem231	0.12453262	2.88553283	0.30429865	0.58733871	0.47213972
Jrk	0.18153817	2.19497887	0.3042458	0.58737092	0.47213972
Vipas39	-0.0763368	4.82685102	0.303453	0.58785456	0.47248971
Gm867	-0.7605482	-1.991753	0.30332722	0.58793136	0.47251267
Bst1	-0.6309944	-1.3908768	0.30321298	0.58800114	0.47252998
06-Mar	0.07412698	8.79093608	0.30293047	0.58817378	0.47262994
Capn1	-0.0928295	4.19335936	0.30280414	0.58825101	0.47265322
Clp1	0.13876278	2.44874484	0.30270791	0.58830985	0.47265416
Tagap	-0.1535232	2.53749694	0.30264441	0.58834868	0.47265416
Eef1a2	-0.0884186	5.59721423	0.30248646	0.58844531	0.47267288
Diras2	0.0681077	8.13275265	0.30244857	0.5884685	0.47267288
Uap1l1	0.23840971	4.09503862	0.30234743	0.58853039	0.47268384
Kctd18	-0.0899544	4.36837927	0.30225396	0.5885876	0.47269103
Proz	0.24310877	1.46770786	0.30175757	0.58889163	0.47286542
Chchd1	-0.1293047	3.59794453	0.30171509	0.58891767	0.47286542
Frmpd1	-0.1205425	2.76514959	0.30166304	0.58894957	0.47286542
Nfic	-0.0863085	7.69138183	0.30151887	0.58903795	0.47287319
Dcaf4	-0.1332081	3.15447989	0.30148975	0.5890558	0.47287319
AU041133	-0.1515012	2.83217733	0.30136412	0.58913285	0.47289628
Ccdc33	-0.3355467	0.10981215	0.30125944	0.58919706	0.47290907
Vdac1	-0.0615315	8.05749857	0.30105743	0.58932101	0.47296754
Gimap8	0.21201121	1.21229968	0.30098337	0.58936648	0.47296754
Alpl	-0.1915611	3.38132943	0.30083743	0.58945607	0.47300069
Zfp784	0.14214594	3.96359625	0.30051775	0.58965244	0.47308717
C1s1	0.3469295	0.94045248	0.30045302	0.58969222	0.47308717
Gm8179	0.57871268	-0.1915981	0.30042614	0.58970873	0.47308717
Limk1	-0.1506768	2.85508073	0.29994864	0.59000236	0.47328396

Mettl4	0.10125788	5.08264826	0.29966904	0.59017443	0.4733522
Chst2	0.06860828	7.83723352	0.29958787	0.5902244	0.4733522
Bcam	-0.1823996	3.57616704	0.29957489	0.59023239	0.4733522
Nkx6-1	0.54224602	-0.5665665	0.29930132	0.5904009	0.47344857
Mir6236	0.2240444	4.65422758	0.29907863	0.59053813	0.47351985
Srsf9	-0.1122946	4.93587337	0.29897832	0.59059996	0.47353067
Erich1	0.15173243	2.96128345	0.29838716	0.59096468	0.47378431
Noxo1	-0.4099182	-0.2666399	0.29816807	0.59109997	0.47379917
Slc30a10	-0.105662	5.06742658	0.29807077	0.59116007	0.47379917
Rpap1	0.13677242	2.8673749	0.29806598	0.59116303	0.47379917
Pdzd9	0.33139264	0.29976476	0.29798914	0.5912105	0.47379917
Nmral1	0.18439204	2.8431498	0.29794734	0.59123633	0.47379917
Abhd12	-0.0757529	6.33405157	0.2977723	0.59134451	0.47379917
Itgb5	0.14797326	4.05281799	0.29776828	0.591347	0.47379917
6030440G07	0.41615561	-1.2399311	0.29773079	0.59137017	0.47379917
Rasgef1b	0.08329891	5.48781559	0.29741533	0.59156527	0.47391672
Slc20a2	-0.1170943	6.03436327	0.29724666	0.59166964	0.47394717
Lpin1	0.09647947	4.6454142	0.29719752	0.59170005	0.47394717
Spag8	-0.6129295	-1.5179381	0.29695268	0.59185164	0.47402983
Lrguk	0.12506797	3.24587054	0.29648177	0.59214342	0.47422475
Igsf9	0.51347797	-0.311805	0.29637684	0.59220847	0.47423808
Cdk20	-0.2830246	1.18876467	0.29623603	0.5922958	0.47426923
Rnf13	0.08993116	7.06736367	0.29602612	0.59242603	0.47433474
Tmem18	0.10425934	4.31066498	0.29577935	0.59257921	0.4744023
Pecr	0.21901553	1.25200424	0.29573414	0.59260728	0.4744023
Pgam2	-0.2823058	0.82968011	0.29548546	0.59276175	0.47448718
Dnpep	0.13654111	2.95419845	0.29539982	0.59281496	0.474491
Ssr1	0.0738018	7.3121515	0.29528585	0.59288579	0.47449538
Smoc2	0.15136946	3.40767044	0.29521618	0.5929291	0.47449538
Crebzf	0.09683286	5.31094743	0.29508092	0.5930132	0.47449538
Eps8l2	-0.2529146	1.20284362	0.29503471	0.59304194	0.47449538
Mmgt1	-0.0881312	4.66020871	0.29498417	0.59307337	0.47449538
D5Ertd605e	-0.614032	-1.4829157	0.29492355	0.59311107	0.47449538
Dync1li2	-0.0571874	8.01244482	0.29459199	0.59331741	0.47462168
Psmd5	-0.0753791	5.36568857	0.2942709	0.59351736	0.47465615
Acmsd	0.28806997	0.69188002	0.29422234	0.59354762	0.47465615
Cops2	-0.0747198	8.04261981	0.2942117	0.59355424	0.47465615
Sash3	-0.2803759	1.90364415	0.29421157	0.59355432	0.47465615
5830432E09I	-0.5184494	-1.1119705	0.29398243	0.59369713	0.47467829
Il33	0.09412662	5.16812239	0.29395003	0.59371733	0.47467829
Trim34a	-0.1658517	3.15628048	0.2939218	0.59373492	0.47467829
Zfp94	-0.1577306	2.68639115	0.29385617	0.59377585	0.47467829
Lox	-0.1700052	3.27472047	0.29361418	0.59392678	0.4747602

Cd274	0.14099333	3.76592191	0.29310518	0.59424451	0.47497542
Ankfn1	0.22102381	2.48226336	0.29288719	0.59438069	0.47504551
Zcchc3	-0.1148373	5.00157565	0.29269006	0.59450391	0.47510522
1110007C09I	-0.1870951	2.33907643	0.2924784	0.59463626	0.47513231
Tcte2	-0.1436047	2.88138795	0.29246491	0.5946447	0.47513231
Ankk1	0.39496981	-0.2694108	0.29240315	0.59468333	0.47513231
Gas5	0.06234084	6.98860921	0.29224106	0.59478474	0.47515268
Lcat	0.19181278	2.85756413	0.29212176	0.59485941	0.47515268
Ube2cbp	0.47381733	0.2999942	0.2920955	0.59487585	0.47515268
Cryzl1	-0.087099	5.36087716	0.29205237	0.59490285	0.47515268
Ormdl1	0.16801157	3.70147365	0.29196542	0.59495729	0.47515742
Map2k1	-0.0558708	7.71419502	0.29187485	0.595014	0.47516397
Sst	0.09433535	6.90303756	0.2913784	0.59532511	0.47533947
Cenpt	0.17124888	2.40118375	0.29136927	0.59533083	0.47533947
Pigc	0.18840942	3.10018614	0.29124682	0.59540762	0.47536204
Ms4a6b	-0.4564442	0.48023595	0.29083542	0.59566576	0.47552937
Eno1b	0.06622884	6.04849842	0.2906976	0.59575229	0.47555969
Drc1	0.12738802	3.18557095	0.29052895	0.59585821	0.47557572
Zdhhc2	-0.0762262	5.21262082	0.29043079	0.59591988	0.47557572
Setd3	-0.054812	6.79583478	0.29040414	0.59593662	0.47557572
AF251705	-0.4657691	-0.5359717	0.29035648	0.59596657	0.47557572
Ghr	-0.1098311	5.29795125	0.29018669	0.5960733	0.47558805
Pabpc4	0.08924471	3.89769323	0.2901487	0.59609718	0.47558805
Chgb	0.08743286	9.20757245	0.29010019	0.59612768	0.47558805
Ccdc94	-0.1982069	1.59061397	0.28969616	0.59638184	0.47575207
Myt1l	0.09220748	8.60644459	0.28959741	0.596444	0.47575304
Gnai2	-0.1291799	7.06060397	0.28953992	0.59648019	0.47575304
Apbb3	0.18739647	2.08151078	0.28932381	0.59661628	0.47582284
Gbp11	-0.4368683	-0.1374851	0.28908506	0.59676671	0.47590406
Mthfd1l	0.13809653	3.49817884	0.28876055	0.5969713	0.4759697
Tgm3	-0.1393442	2.62821885	0.28874767	0.59697942	0.4759697
Cenph	-0.4529608	-0.5446331	0.2887233	0.59699479	0.4759697
Nub1	-0.0745909	5.30271563	0.28835583	0.59722666	0.47611581
St5	-0.1265666	4.99487358	0.28823362	0.59730382	0.47612355
Klhl11	0.09954641	4.45954021	0.28818648	0.59733359	0.47612355
Amigo3	-0.5391684	-0.5059474	0.28756676	0.59772521	0.47638041
Snx16	0.0938666	4.76565689	0.28752266	0.5977531	0.47638041
Tekt5	0.33743813	0.32940631	0.28738271	0.59784163	0.4764122
Tbc1d13	-0.1118207	4.34928635	0.28704905	0.59805279	0.47654171
Lrrcc1	0.09065632	5.61702841	0.28688234	0.59815836	0.47657957
Kansl1l	0.08140055	5.50091386	0.28682035	0.59819762	0.47657957
Mael	-0.3686974	0.65600567	0.28647278	0.59841787	0.47670638
Sos2	0.07091641	7.66871831	0.28635363	0.59849342	0.47670638

Gck	0.45140688	-0.9810828	0.2862906	0.59853339	0.47670638
Cd300lb	-0.7707592	-1.3241389	0.28625785	0.59855416	0.47670638
Senp3	-0.0890782	4.80653401	0.28618539	0.59860013	0.47670638
Ccdc113	-0.2700803	1.09575397	0.28586501	0.59880344	0.47678013
B4galt2	-0.1318217	3.39230077	0.28583862	0.59882019	0.47678013
Ccr9	-0.1431764	3.73349126	0.28580938	0.59883875	0.47678013
Zbtb20	0.06939083	6.86078302	0.28567792	0.59892223	0.47680783
Tmem62	0.14519796	3.23541605	0.28523855	0.5992014	0.47699132
Naa35	0.07073245	6.08608385	0.28493641	0.59939355	0.47710386
Kif2c	0.59630183	-1.5242468	0.28486311	0.59944018	0.47710386
Cdh6	0.11752622	2.79517801	0.28448935	0.59967809	0.47725443
1700019A02I	-0.5095027	-1.7189132	0.28440985	0.59972872	0.47725595
Tmem163	0.13434252	2.75960425	0.28420366	0.59986008	0.47729368
Kcnu1	0.2932958	0.58323831	0.28418247	0.59987358	0.47729368
Zkscan1	0.0629842	7.6698238	0.28409305	0.59993057	0.47730025
Wdr48	0.08188864	5.5655483	0.28372792	0.6001634	0.47741014
Oma1	0.10675307	3.22056231	0.28372357	0.60016617	0.47741014
Tor3a	-0.1754399	3.71345958	0.28348908	0.6003158	0.47749039
Pdpn	-0.1649257	5.22471167	0.2832416	0.6004738	0.47757728
Tmem132a	0.13697946	2.66849841	0.2830617	0.60058871	0.47762989
Etv5	0.09303894	6.16652977	0.28276527	0.60077815	0.47774176
Foxf2	-0.1517688	2.76111386	0.28257178	0.60090188	0.47780136
1700073E17I	0.21058103	2.24183211	0.28237975	0.60102473	0.47786025
Osbpl10	0.21752584	2.18542351	0.28208357	0.60121432	0.47797219
Avpr1a	-0.527378	-0.6701335	0.28148035	0.60160083	0.47824066
Chd1l	-0.1261058	3.10993716	0.28113787	0.60182052	0.47829037
Syt14	-0.2508628	1.37972022	0.2811288	0.60182634	0.47829037
Hist2h2bb	-0.5003669	-0.8157757	0.28110143	0.6018439	0.47829037
Rcc1	-0.2033422	1.73423824	0.2810784	0.60185868	0.47829037
Bfar	0.08920453	5.04974187	0.28065044	0.60213347	0.47846993
Ick	-0.0736422	6.31242919	0.28048787	0.60223793	0.47851412
Hist1h3f	0.48205871	-1.7081125	0.28017688	0.60243787	0.47859805
Syngap1	0.08910719	7.35111782	0.28017156	0.60244128	0.47859805
Clec2l	0.21771892	1.15729385	0.27989385	0.60261995	0.47864126
Gjc1	0.23106087	1.80271383	0.27986417	0.60263905	0.47864126
Serpinb9b	-0.4641805	-0.7571018	0.27985916	0.60264227	0.47864126
1700112E06I	-0.2287437	1.17197641	0.2796833	0.60275548	0.47866183
Crhr2	-0.7593073	-1.7099164	0.2796671	0.60276591	0.47866183
Id2	-0.1167591	6.09138023	0.27952545	0.60285713	0.47869547
Cops6	-0.0858145	5.82043294	0.27918119	0.60307895	0.47883278
Dmtn	-0.0920814	6.22968677	0.27898352	0.6032064	0.47886519
Scg5	-0.0766038	6.32416838	0.27896624	0.60321754	0.47886519
Tmem151a	0.10405894	4.63055063	0.27872698	0.60337189	0.47892967

Dvl2	-0.1539886	2.37289552	0.278579	0.60346739	0.47892967
AU040320	0.11584179	4.17222439	0.27850672	0.60351405	0.47892967
Slc35g2	-0.1203194	3.02421412	0.27844318	0.60355508	0.47892967
Tekt1	0.43114647	-0.1937425	0.27841515	0.60357317	0.47892967
Rbmxl1	0.08138761	5.34589188	0.27838581	0.60359212	0.47892967
Gm5141	0.15603617	3.06213134	0.27813057	0.603757	0.478976
Ranbp17	0.12339611	3.12490601	0.27810504	0.6037735	0.478976
D430036J16F	0.16486812	2.35604874	0.27806627	0.60379855	0.478976
Garem	0.09254174	4.79906231	0.2779927	0.6038461	0.478976
Eya3	0.08406777	5.04768282	0.27784906	0.60393896	0.47901087
Pfkl	-0.0991328	4.18051187	0.27773149	0.60401499	0.47903238
Gpr123	-0.0968116	6.43854164	0.27717151	0.6043774	0.479281
Zfp148	0.06052979	7.82606425	0.27693622	0.60452982	0.47933402
Bmpr1a	-0.1005754	7.88324888	0.27691721	0.60454214	0.47933402
Tbc1d22a	0.10576399	3.48816352	0.27678106	0.60463037	0.47936518
Wnt7b	0.23929535	1.64236323	0.27664072	0.60472136	0.47939851
Ncbp1	0.08199015	4.95939243	0.27649515	0.60481576	0.47941684
Fam73b	0.10739639	3.66081619	0.27645415	0.60484236	0.47941684
Tspan31	-0.1123326	5.85510856	0.27616685	0.60502879	0.47948946
Vgll3	-0.1610588	2.95816261	0.27612378	0.60505674	0.47948946
Plekhg4	0.37079963	1.36026031	0.27594243	0.6051745	0.47948946
Ogfr	-0.1866567	1.96457029	0.27587502	0.60521829	0.47948946
Gm5547	-0.8124821	-1.9888501	0.27578672	0.60527564	0.47948946
Tktl2	-0.8684439	-1.1117292	0.27577866	0.60528088	0.47948946
Gsk3b	0.05736335	9.51769032	0.27573265	0.60531078	0.47948946
9430041J12F	0.2349042	1.8680772	0.27567634	0.60534737	0.47948946
Pigq	-0.0738726	5.82510229	0.27557177	0.60541534	0.47948946
Lef1	-0.1035823	4.50686433	0.27554422	0.60543324	0.47948946
Mast2	0.06550741	6.29563596	0.27544313	0.60549896	0.47948946
Mknk2	-0.0969027	3.96354222	0.27538788	0.60553489	0.47948946
Eno2	-0.0929892	8.48693367	0.27521104	0.60564991	0.47948946
Chst1	0.08320268	5.89299389	0.2751903	0.60566341	0.47948946
Armcx3	0.05950909	7.26398778	0.27515904	0.60568375	0.47948946
Cpsf6	0.09845467	5.58266669	0.27499192	0.6057925	0.47948946
Smpdl3a	0.11914827	4.81997081	0.27492775	0.60583428	0.47948946
Antxr2	-0.2202886	3.3378226	0.27490127	0.60585151	0.47948946
Lage3	-0.1446078	3.39926527	0.27488201	0.60586405	0.47948946
Psrc1	0.20257043	1.47407062	0.27474803	0.6059513	0.47950572
Pdgfa	-0.075739	5.81482077	0.27470013	0.60598249	0.47950572
4933427I22R	0.69696317	-1.2003321	0.27461854	0.60603565	0.47950904
Grpel1	-0.1025797	4.23880688	0.27428543	0.60625274	0.47964207
Zfp846	0.12302891	3.9051803	0.27397062	0.60645806	0.47976506
Trak2	-0.0694665	6.83064815	0.27389692	0.60650616	0.47976506

Ppp1r15b	-0.0652763	5.41122845	0.27378126	0.60658164	0.47978602
Dnttip1	0.14983309	2.98877728	0.27343291	0.60680912	0.47992719
Kif20b	0.19555862	1.82200334	0.2732382	0.60693635	0.47998907
Zfp955a	0.08175473	5.05285549	0.27288433	0.60716773	0.4801143
Fchsd1	0.18228117	2.02506794	0.27280097	0.60722226	0.4801143
Txndc12	-0.1224955	3.96438473	0.27274592	0.60725828	0.4801143
Tnfrsf10b	0.12811565	3.26675785	0.27269629	0.60729076	0.4801143
Mad1l1	0.16256335	2.26136408	0.2724705	0.60743855	0.48019239
Snai2	0.18575873	3.53862021	0.27223373	0.60759362	0.48026502
Vps16	0.13905153	4.44293353	0.2721805	0.6076285	0.48026502
Srpk1	-0.0641288	6.0743028	0.27187536	0.60782851	0.48038434
1600016N20	-0.4367548	-0.9326504	0.27170884	0.60793771	0.4804046
Olfr55	-0.5988607	-1.2121881	0.2716867	0.60795223	0.4804046
Stk4	-0.0592738	5.92312332	0.27150227	0.60807324	0.48046146
Ascl4	-0.633582	-1.2625005	0.2711692	0.6082919	0.48053159
2010106C02I	0.71567249	-1.616105	0.27114625	0.60830697	0.48053159
Kansl1	-0.0542961	6.95826193	0.27114293	0.60830915	0.48053159
Pacsin3	-0.1235218	3.80814932	0.27087543	0.60848491	0.48062836
Igflr1	0.52794924	-0.7968711	0.27070318	0.60859814	0.48062836
Akr1c18	-0.2887269	1.31564521	0.27056637	0.60868811	0.48062836
C2cd2l	-0.097016	5.27762797	0.27052475	0.60871548	0.48062836
Impact	0.05996515	7.83618296	0.27049224	0.60873687	0.48062836
Cyp4f14	0.69059972	-0.5174918	0.27046758	0.60875309	0.48062836
Bsg	-0.1439579	7.36556229	0.27043407	0.60877513	0.48062836
Hist1h1c	-0.2353732	2.87540411	0.27030914	0.60885734	0.48064575
Mrgprf	0.29209202	2.03239922	0.27025148	0.60889529	0.48064575
Hist1h4a	-0.5033898	-1.4203992	0.27011386	0.60898589	0.48067853
Ankrd12	0.08400832	9.70283078	0.26973418	0.60923599	0.48083718
Gimap6	0.24639369	1.78011228	0.26958743	0.60933271	0.48085488
Spp1	0.14715521	7.62210651	0.26955121	0.60935659	0.48085488
Hist1h2ak	-0.5231989	-1.7770005	0.26928282	0.6095336	0.48095582
M6pr	-0.0926608	6.22491652	0.26906485	0.60967743	0.48100803
Asf1b	0.57122628	-1.5884799	0.26903373	0.60969797	0.48100803
Cby1	-0.1860034	3.21791179	0.26857578	0.61000043	0.48120789
Dach2	0.36941829	0.10560805	0.268364	0.61014041	0.48127955
Dcst1	-0.1967108	1.39971641	0.2682571	0.6102111	0.48128719
Amfr	-0.0780557	5.82024607	0.26820075	0.61024837	0.48128719
Anxa11	-0.0856507	3.947233	0.26766197	0.61060495	0.48152965
Mrvi1	-0.1205903	5.83222849	0.26754885	0.61067988	0.48154997
Dus1l	-0.1479631	3.06335656	0.26733662	0.61082051	0.48162209
Grip2	0.19225695	2.18847827	0.26719218	0.61091625	0.48165881
Srsf2	-0.0660011	8.34931977	0.26704209	0.61101578	0.48169851
Zfp943	0.1238355	3.03883785	0.26695385	0.61107431	0.48170589

Smarca2	0.06196595	9.78810952	0.26673856	0.61121717	0.48176245
Zfp217	0.15574597	3.61460677	0.26669749	0.61124443	0.48176245
BC100451	-0.7866688	-1.5581313	0.26646779	0.61139695	0.48184388
Sdhaf1	-0.1500919	2.31989015	0.26634867	0.61147607	0.48184742
Klhl35	-0.5251616	-0.7426236	0.26631294	0.61149981	0.48184742
Traf2	0.21669277	1.32158657	0.26595816	0.61173563	0.48199446
Gm6260	-0.241552	1.52096462	0.26573186	0.61188615	0.48206651
Hacl1	0.16631666	2.44761928	0.26567273	0.61192549	0.48206651
Trappc6a	0.30258187	1.50051565	0.26543562	0.61208332	0.48212367
Eepd1	0.13774525	2.48443429	0.26541583	0.6120965	0.48212367
Pelo	0.12961272	3.1943276	0.26532406	0.6121576	0.48213303
Klf16	0.12910039	2.45963519	0.26478281	0.6125183	0.48237338
Zfp808	-0.0899937	3.85726606	0.26471839	0.61256126	0.48237338
Emcn	0.35522613	0.59230906	0.26443856	0.61274796	0.4824332
Arl6ip6	0.09436688	4.59308749	0.26427686	0.6128559	0.4824332
Adam8	0.57811213	-0.6615818	0.26424589	0.61287658	0.4824332
Tmprss5	0.65505103	-1.6692367	0.26423958	0.6128808	0.4824332
4930487H11I	-0.5689892	-1.2748223	0.26423555	0.61288348	0.4824332
Npepl1	-0.1792946	2.06684462	0.2640046	0.61303774	0.48246737
Eif4g1	0.05753259	7.06173756	0.26399498	0.61304417	0.48246737
9930111J21F	0.16033029	2.9542156	0.26394935	0.61307465	0.48246737
Ppid	-0.0679657	6.12373406	0.26382768	0.61315597	0.4824926
Ncdn	-0.0827674	8.21970628	0.26371835	0.61322904	0.48251134
Scn10a	0.58304784	-1.6089936	0.26510024	0.61330227	0.48253019
E130012A19I	-0.1366051	2.9411	0.26317087	0.61359531	0.48264798
Arid4a	0.08107569	7.65718291	0.26315178	0.61360809	0.48264798
Rps6ka6	0.15871434	2.62959887	0.26312784	0.61362412	0.48264798
Cog4	0.08828725	4.60369643	0.26309058	0.61364907	0.48264798
Abhd17c	-0.0783168	5.2820418	0.26297761	0.61372472	0.48266873
Tcf12	-0.0801509	7.35152925	0.26286307	0.61380145	0.48269031
Gm10125	0.2414736	1.01722952	0.26272267	0.61389552	0.48269653
Slc6a14	0.72663532	-1.0287029	0.26270419	0.61390791	0.48269653
Fndc7	0.78441278	-1.3887912	0.26241451	0.61410212	0.48275791
Sult2b1	-0.2890523	0.36922458	0.26235753	0.61414034	0.48275791
Alkbh8	0.10580073	4.91475563	0.26231226	0.61417071	0.48275791
Slc2a5	-0.3658832	-0.6597049	0.26229376	0.61418311	0.48275791
Leprot	-0.1337617	5.01098668	0.26195519	0.61441035	0.48287579
Slc1a1	0.08139275	6.01790734	0.26192342	0.61443168	0.48287579
Gm15987	-0.6218384	-1.7761642	0.26177351	0.61453235	0.48291617
Polr1b	0.1990112	2.23088517	0.26168585	0.61459124	0.4829237
Kcnf1	0.10100534	5.28870068	0.26159647	0.6146513	0.48293215
Padi4	0.62687147	-2.0630303	0.2614501	0.61474969	0.48297071
Psmd1	0.05386741	7.50243881	0.26109743	0.61498687	0.4831183

Arhgef1	0.10991739	4.30203653	0.26094922	0.6150866	0.48315791
Tgm4	0.23153649	1.23746859	0.26083165	0.61516574	0.48318132
Aqp1	0.25008408	1.78608843	0.26042589	0.61543906	0.48333116
4930579G18	0.37044389	0.33076995	0.26039753	0.61545817	0.48333116
Chmp1b	-0.0938959	5.41564145	0.26028105	0.61553669	0.48333116
Clmp	-0.1018286	6.01158504	0.26025556	0.61555388	0.48333116
Gal	0.49803331	-0.4440306	0.25965656	0.61595802	0.4834221
Adam3	-0.7201956	-1.935804	0.26111471	0.6159622	0.4834221
Kpnb1	-0.0614716	8.29382347	0.25958549	0.61600601	0.4834221
Larp4	0.06281848	7.14921979	0.25954035	0.6160365	0.4834221
Ap2b1	0.06623879	7.86662395	0.25952012	0.61605015	0.4834221
Lrrc73	-0.1685657	2.43181403	0.2595032	0.61606159	0.4834221
Snord118	-0.5417583	-1.5551468	0.25949956	0.61606404	0.4834221
Fip1l1	-0.0566875	6.50639175	0.25949161	0.61606942	0.4834221
Lpxn	0.59102394	-1.4889475	0.25937895	0.61614552	0.4834221
Thbs4	-0.2752483	0.57915578	0.25930599	0.61619482	0.4834221
Vsig2	-0.1086055	3.29637463	0.25927972	0.61621258	0.4834221
Npas2	0.12738107	5.29330213	0.25896486	0.61642545	0.48354944
Spsb4	0.25246495	1.11843022	0.25889362	0.61647363	0.48354944
Sgsm3	0.1142278	3.69861639	0.2588015	0.61653595	0.48355961
Slc26a5	0.64107162	-1.149165	0.25809304	0.61701571	0.48385249
Lpcat3	0.12029727	3.53870214	0.25804067	0.61705122	0.48385249
Rhbdf1	-0.1805016	1.5426094	0.2580313	0.61705757	0.48385249
Zbed4	0.11015281	4.47113745	0.25782361	0.61719839	0.48392238
Hsf1	0.07449062	5.47374936	0.25768829	0.61729019	0.48392238
Zcchc4	0.40091957	-0.1139491	0.25753429	0.61739469	0.48392238
Fjx1	-0.0930454	4.08630098	0.25753298	0.61739558	0.48392238
Atn1	-0.0653771	7.89650302	0.2575148	0.61740792	0.48392238
Gm14446	0.11388175	3.72874383	0.25743159	0.6174644	0.48392238
Arl11	0.68207058	-0.8594678	0.25739017	0.61749252	0.48392238
Zfyve20	-0.0791428	5.76894164	0.25688049	0.61783878	0.48413348
Tmem127	0.07262643	6.90098997	0.25672535	0.61794427	0.48413348
Efnb2	-0.0735841	6.13005427	0.2567063	0.61795723	0.48413348
Cd72	0.43519359	-0.3622226	0.25670283	0.61795959	0.48413348
Tfdp1	-0.0749759	6.37155299	0.25643329	0.61814296	0.48423841
Brf2	-0.2704917	0.70218386	0.25630872	0.61822776	0.48426611
Creb3l2	-0.1134574	5.98241077	0.25595278	0.61847018	0.4843958
Gpt	0.33118148	0.46254604	0.25592042	0.61849223	0.4843958
Nckap5l	-0.2132948	1.45412957	0.25570827	0.61863682	0.48447031
Nfkb1	0.09164759	4.41632121	0.25561608	0.61869969	0.48448081
L3mbtl3	-0.0945956	4.1829686	0.25526472	0.61893939	0.48462977
Eftud1	0.12993691	3.10423985	0.25486537	0.61921209	0.48480454
Fzd2	0.14219918	4.20920458	0.25478492	0.61926706	0.48480883

Rdx	0.06166886	7.03495233	0.25469601	0.61932783	0.48481765
Slc15a4	0.15354719	2.35216293	0.25458491	0.61940377	0.48483836
Bcl9	0.06654455	6.42996014	0.25440211	0.61952878	0.48489745
Scml4	-0.1205837	4.00497164	0.25403702	0.61977861	0.48503909
Cln5	-0.1243429	6.00046903	0.25394722	0.61984009	0.48503909
Foxn3	-0.0818098	6.54156047	0.25392065	0.61985829	0.48503909
Cpne1	0.06966792	5.90281002	0.25351085	0.62013906	0.48519708
Dnah9	-0.2404911	1.50568372	0.2534814	0.62015926	0.48519708
Rab11fip2	0.0593001	6.93761623	0.25324302	0.62032272	0.48528621
Golm1	-0.0976466	3.26257839	0.25310138	0.6204199	0.48529909
Engase	0.21520481	0.75839057	0.25307459	0.62043828	0.48529909
Srprb	0.10902432	3.49806931	0.25280885	0.62062071	0.48540303
Akap17b	0.08069724	5.61061691	0.25247965	0.62084687	0.48554114
Gid4	0.06287192	5.84590795	0.25237704	0.6209174	0.48555753
Gimap3	-0.1860964	2.71471156	0.25228807	0.62097857	0.4855666
Sema5b	0.18335677	3.53938769	0.25209011	0.62111472	0.4856343
09-Sep	0.10590644	5.57392956	0.25186531	0.62126941	0.48571648
Grpel2	0.09529319	4.21739814	0.25170673	0.62137859	0.48574279
C030029H02I	-0.2594276	1.08253575	0.25162683	0.62143362	0.48574279
Ppef1	-0.4267356	-0.7102362	0.25157802	0.62146723	0.48574279
Cbln2	-0.1299797	3.41947887	0.25152287	0.62150523	0.48574279
Plekhf2	-0.1086861	4.63531593	0.25145153	0.62155438	0.48574279
H2-Eb1	0.23198059	3.08413101	0.25137971	0.62160387	0.48574279
Gbe1	0.09281628	3.96139429	0.25131249	0.6216502	0.48574279
Acsl5	-0.0696714	5.63286945	0.25116681	0.62175063	0.48578252
Ankrd10	0.10188258	3.86599986	0.25106573	0.62182034	0.48579823
Slc9b1	0.67950765	-0.7893292	0.25085386	0.6219665	0.48584243
Dcakd	-0.120888	4.5004566	0.25078345	0.6220151	0.48584243
Mtus1	0.06403	6.68441201	0.25073782	0.6220466	0.48584243
Tigd3	0.32551609	-0.2656558	0.25067015	0.62209331	0.48584243
Vamp4	-0.0547678	6.82262268	0.25062439	0.6221249	0.48584243
Pou3f4	0.22272214	0.57012501	0.25036329	0.62230525	0.48594452
Tmc4	-0.112951	3.05233319	0.24955663	0.62286316	0.48634141
Dnal4	-0.1242028	2.43868295	0.24914393	0.62314903	0.48637451
Nbr1	-0.0661494	7.89776255	0.24914148	0.62315073	0.48637451
Aqp6	-0.59406	-1.3654759	0.24903957	0.62322137	0.48637451
Casc3	0.0835215	5.49386002	0.24896285	0.62327456	0.48637451
Nup62	-0.0833171	5.49699393	0.24894332	0.6232881	0.48637451
Tmem123	-0.1185778	5.06646632	0.24886963	0.62333921	0.48637451
B130024G19	0.23217802	1.1616731	0.24885493	0.6233494	0.48637451
Caskin2	0.12742413	2.60278845	0.24884065	0.6233593	0.48637451
Arhgap27os3	-0.6726791	-1.6423363	0.24883911	0.62336037	0.48637451
Gadd45b	-0.1278343	2.14979483	0.24877897	0.62340209	0.48637451

4933411K20I	0.06721979	6.72775489	0.24867936	0.62347119	0.48638969
Ccl5	-0.2492941	1.43448762	0.24827938	0.62374887	0.48654228
Melk	-0.37638	0.17902448	0.24825453	0.62376613	0.48654228
Olfml2a	-0.1800677	3.54799309	0.24798871	0.62395084	0.48662873
Rasl11a	-0.3363805	0.00434263	0.24787887	0.62402719	0.48662873
Armc2	0.17438069	1.77471484	0.24776611	0.62410561	0.48662873
Ikzf1	-0.1098629	4.34495436	0.24767032	0.62417224	0.48662873
Pter	-0.1015146	3.4557309	0.24753259	0.62426806	0.48662873
Rint1	0.09103144	4.06276788	0.24752672	0.62427215	0.48662873
Kif26a	0.14565269	2.41192325	0.24751726	0.62427873	0.48662873
Banp	0.09365915	4.65768493	0.24742826	0.62434067	0.48662873
2410004N09	0.22367463	0.54396546	0.2473879	0.62436876	0.48662873
Zbtb38	0.05893331	6.89674946	0.24737124	0.62438037	0.48662873
Mrpl46	0.1403995	3.48417152	0.24727536	0.62444712	0.48662873
Adam15	0.09010084	4.21539987	0.24723802	0.62447313	0.48662873
Fkbp9	-0.121793	5.31413582	0.2465376	0.62496134	0.48693687
2310040G24	0.38743753	0.08428143	0.24648496	0.62499806	0.48693687
Supt3	-0.1388486	2.28573846	0.24645685	0.62501768	0.48693687
Hcrtr1	0.60743693	-1.4360629	0.24638467	0.62506805	0.48693738
Gm7361	0.38614722	-0.8434527	0.24619629	0.62519956	0.48698359
Cacng4	-0.2730282	1.11388818	0.24615729	0.62522679	0.48698359
Pgm2l1	0.1015885	10.1785393	0.24603763	0.62531036	0.48700996
Chst11	-0.0754014	5.6926055	0.24588127	0.62541961	0.48705631
Enox2	-0.1368735	4.58699672	0.24569748	0.62554808	0.48711764
Mrps11	-0.1949138	1.41683712	0.24557338	0.62563486	0.48714648
Bmp8b	0.71739055	-1.422884	0.24540504	0.62575261	0.48719944
Ebf1	-0.1169424	4.28809844	0.24520399	0.62589332	0.48727026
Mkln1os	-0.1948122	1.23498685	0.24505708	0.62599618	0.48731161
Notch2	-0.1129381	7.07450311	0.24480684	0.62617148	0.48740934
Iqgap3	0.56409968	-0.6190251	0.24445358	0.62641913	0.48756337
Gpr111	0.87137015	-2.0538352	0.2440404	0.62670908	0.48775029
Raly	0.15319809	2.78369417	0.24384878	0.62684366	0.48781627
Hibch	-0.0979004	4.11210801	0.24313062	0.62734861	0.48813113
Ece2	0.12832986	2.54732572	0.24312929	0.62734954	0.48813113
Zfp867	0.09154223	4.15322404	0.24305918	0.62739888	0.48813113
Ube2ql1	0.07592892	6.24484934	0.24299	0.62744758	0.48813113
Gm4980	-0.2370237	0.8216005	0.24274078	0.6276231	0.48822889
Optn	-0.0897078	4.13149135	0.24266503	0.62767647	0.48823013
Heatr5a	-0.0890394	4.46749891	0.24259705	0.62772437	0.48823013
Scamp2	-0.1956434	2.70673317	0.24238612	0.62787306	0.48826854
3110056K07I	-0.1283673	2.46016357	0.24229801	0.6279352	0.48826854
Gnpat	0.07972292	5.40993141	0.24228004	0.62794787	0.48826854
Mir384	0.66535117	-1.205553	0.24224422	0.62797314	0.48826854

Bap1	-0.0860042	4.87571872	0.24212497	0.62805726	0.48829518
Helz	0.09449991	6.72444191	0.24200604	0.6281412	0.48832168
Irf7	-0.3184819	0.15226717	0.24177112	0.62830705	0.48839554
Nos3	0.23507569	0.88492449	0.24171328	0.62834791	0.48839554
Ufsp1	-0.1794442	1.89725205	0.24162212	0.62841231	0.48839554
Zdhhc22	-0.1375191	2.47904651	0.24152086	0.62848386	0.48839554
Zfr	0.068374	8.62368606	0.24151853	0.6284855	0.48839554
Ackr2	-0.3153185	0.49083056	0.24143916	0.6285416	0.48840038
Prmt3	0.08913103	4.30951432	0.24136047	0.62859723	0.48840486
Eaf2	-0.1694627	1.50594615	0.24125259	0.62867351	0.48842539
Sdc2	-0.1162178	6.59430272	0.2411274	0.62876205	0.48845544
Fgd1	0.11816203	3.05365511	0.2409003	0.62892276	0.48854154
Dpys	-0.5410528	-0.9644051	0.24033644	0.62932217	0.48878214
Snx33	-0.1197454	3.96125282	0.24030373	0.62934536	0.48878214
Myct1	0.64828904	-1.6149048	0.24025177	0.6293822	0.48878214
Zfp384	0.0701043	5.17019792	0.24001813	0.6295479	0.48887207
Epc1	0.05984596	6.38698738	0.2398408	0.62967374	0.48889821
Olfm4	-0.8076093	-2.1604963	0.23983004	0.62968138	0.48889821
Sirt6	0.13576341	2.94757241	0.23935955	0.63001554	0.48910302
Evi5l	0.10713574	3.32774126	0.23925844	0.63008741	0.48910302
Rasal3	-0.429026	-0.2015139	0.23923783	0.63010205	0.48910302
Kpna6	-0.0735659	7.46646261	0.23917756	0.6301449	0.48910302
Dync2li1	-0.1104771	3.59751117	0.23868939	0.63049223	0.48925637
Fli1	-0.1199203	4.25282813	0.23867805	0.6305003	0.48925637
Pdxk	-0.0715028	7.60065812	0.23863285	0.63053248	0.48925637
Ly6c1	0.2410364	1.66203813	0.23861911	0.63054226	0.48925637
Man1c1	0.07735913	6.0539353	0.23854804	0.63059288	0.48925689
Coq3	-0.1059923	3.37844803	0.23847789	0.63064284	0.4892569
Wipf3	0.07484576	7.87146541	0.23808027	0.63092622	0.48943798
Wrap53	0.21271084	1.41100641	0.23783511	0.63110108	0.48953486
Cldn8	-0.8813909	-2.2535441	0.23744522	0.63137942	0.48969652
Dnajc5b	0.45077565	-0.5513479	0.23740315	0.63140947	0.48969652
Gcat	-0.2852525	0.83684208	0.23724825	0.63152014	0.48974357
C1ql2	-0.5998425	-1.1618038	0.23707576	0.63164343	0.48978414
Zfp553	0.1346579	3.70563064	0.23698798	0.6317062	0.48978414
Dffb	-0.2592328	1.45709339	0.23696525	0.63172245	0.48978414
Eme2	-0.1722244	1.98866727	0.23679663	0.63184306	0.48983888
Incenp	-0.1135825	3.47791435	0.23667917	0.63192711	0.48986527
Arap1	-0.1200078	3.70806444	0.23651296	0.6320461	0.48988221
Fam221b	0.38271521	-0.509092	0.23650295	0.63205326	0.48988221
P2rx3	0.22416971	1.27183356	0.23643907	0.63209901	0.48988221
Vangl2	0.15605162	1.96473114	0.23627651	0.63221545	0.48993369
Nrip1	0.07528059	6.16891455	0.23588196	0.63249827	0.49010504

Nrf1	0.09291154	3.87283049	0.23582847	0.63253663	0.49010504
Sh3d19	-0.0781936	6.73968738	0.23570104	0.63262805	0.49013711
Hmg20a	-0.094977	5.9347233	0.23544057	0.63281501	0.49016181
LOC1010560	-0.1248269	3.43306051	0.23536551	0.63286892	0.49016181
Snx21	0.18838143	1.94297433	0.23534593	0.63288298	0.49016181
Anks3	0.11893849	2.94614511	0.23534497	0.63288367	0.49016181
Mroh5	-0.3580496	-0.7387819	0.23529779	0.63291755	0.49016181
Cipc	-0.0570456	6.68247745	0.23523844	0.63296019	0.49016181
Was	-0.2205101	0.99596923	0.23508141	0.63307302	0.49017353
Ttc12	0.27185869	1.39047069	0.23507811	0.6330754	0.49017353
Isg20	-0.179493	1.67087983	0.23492155	0.63318794	0.49022192
Coro1c	-0.0838705	5.89259875	0.2345942	0.63342341	0.49033085
Cct6b	0.67473524	-1.4905165	0.2345711	0.63344003	0.49033085
D2Wsu81e	-0.132576	2.04981063	0.23451185	0.63348268	0.49033085
Scn3b	0.07632484	5.74038972	0.23430564	0.63363115	0.49033085
Lor	-0.219322	0.3638543	0.23424642	0.6336738	0.49033085
Myo19	0.14813478	2.91947592	0.23394694	0.6338896	0.49033085
Pcolce	-0.1436896	4.76115866	0.23391372	0.63391355	0.49033085
Dos	0.07869023	6.40590854	0.23391319	0.63391393	0.49033085
Ptgs2	-0.1141054	4.12798815	0.23383954	0.63396703	0.49033085
Kctd16	0.12234142	3.81506031	0.2337643	0.6340213	0.49033085
Fkbp11	0.48279805	-1.4184632	0.23376346	0.6340219	0.49033085
Nelfcd	0.09876785	3.75889393	0.2337179	0.63405476	0.49033085
Gm16938	-0.1661888	2.17302732	0.23366089	0.63409589	0.49033085
Creb5	-0.0808508	4.20964926	0.23364158	0.63410982	0.49033085
Hdhd2	-0.0685383	5.52610197	0.23354595	0.63417882	0.49033085
Tssc4	-0.1221824	2.47401417	0.23350447	0.63420875	0.49033085
Smad6	0.17439188	2.58406296	0.23348376	0.6342237	0.49033085
Man2b1	-0.1096333	4.0911535	0.23347154	0.63423252	0.49033085
Smim1	-0.2653601	1.65134295	0.23340614	0.63427973	0.49033085
Socs6	0.07666462	4.27814036	0.23324757	0.63439423	0.49037539
Epha8	0.26866745	0.67208975	0.23318753	0.63443759	0.49037539
Jam3	-0.1386108	2.89043088	0.23306858	0.63452353	0.49037539
Hfe2	0.6657746	-1.659473	0.23304911	0.63453759	0.49037539
Adamtsl3	-0.1744035	4.39157337	0.23297697	0.63458973	0.49037699
Papola	0.05871111	8.23868163	0.23286866	0.63466802	0.4903988
Inppl1	0.10690227	4.24130284	0.23250921	0.63492801	0.49054438
Fanca	0.70087597	-2.154117	0.23246972	0.63495659	0.49054438
4833417C18I	-0.5611149	-1.3667908	0.23239631	0.63500972	0.49054674
Siah1a	-0.0685443	5.65534911	0.23214722	0.63519009	0.49061876
Slc25a25	-0.0821143	5.0207656	0.23212921	0.63520313	0.49061876
Fam228a	0.27915301	1.31402514	0.23169685	0.6355165	0.49082132
Alox15	0.98908008	-1.1973716	0.23162916	0.6355656	0.49082132

Fam89a	0.8104403	-1.4200627	0.23132604	0.63578555	0.49095248
Tspan3	-0.0869376	7.0022232	0.23124178	0.63584673	0.49096101
Ptbp3	-0.0695389	7.19156415	0.23087135	0.63611583	0.49107198
Usp54	0.05227184	6.65042022	0.23086736	0.63611873	0.49107198
Mgl2	-0.2791918	0.39839275	0.23083694	0.63614084	0.49107198
Vcl	-0.0743571	6.50105909	0.23072857	0.63621962	0.49107557
Caps2	-0.4896264	-1.2710401	0.23069263	0.63624576	0.49107557
Chd7	0.11709356	4.64904099	0.23034559	0.63649823	0.49123173
Btla	0.46719621	-0.3808945	0.23023442	0.63657916	0.49125548
Adck1	-0.1088361	3.05850564	0.23015884	0.63663419	0.49125925
Cgrrf1	-0.0842328	3.86189549	0.22925934	0.63729001	0.49172657
Bmp5	-0.1421458	5.86013317	0.22895689	0.63751088	0.491741
2010002M12	-0.132852	2.29318535	0.22890759	0.6375469	0.491741
Htr5a	-0.1208613	5.37112254	0.22887766	0.63756877	0.491741
Zfp90	-0.0930588	3.80356774	0.22884718	0.63759104	0.491741
Dlg3	0.08416121	6.55474967	0.22882475	0.63760743	0.491741
Tysnd1	-0.1368795	2.27809315	0.22880299	0.63762334	0.491741
Fbln7	0.18526172	4.07381577	0.22875268	0.63766011	0.491741
Siglech	0.16477347	2.13107819	0.22856767	0.63779539	0.4918066
9430076C15I	0.61669091	-1.4963144	0.22822455	0.63804644	0.49196146
Ccdc149	0.0876809	4.44351434	0.22808606	0.63814784	0.4919654
B2m	-0.104196	9.02542822	0.22808038	0.638152	0.4919654
Nsun2	0.06569601	5.15905274	0.22780374	0.63835466	0.4920829
Tmem126a	-0.0822313	4.47075064	0.22754752	0.6385425	0.49216802
Ftl1	-0.0938545	6.19724188	0.22751277	0.63856799	0.49216802
5830403L16I	0.42637696	-0.5332773	0.22733748	0.63869658	0.49216802
Hcar1	-0.1782978	4.37220881	0.22730836	0.63871795	0.49216802
Wdr96	-0.2217669	1.22536066	0.22725694	0.63875569	0.49216802
Mta3	0.07944382	5.56696149	0.22724214	0.63876655	0.49216802
Rfwd3	0.06357641	5.39806734	0.22715187	0.63883281	0.49218036
Gm6484	0.59249506	-1.9123091	0.22706362	0.63889761	0.49218201
Morn2	0.13649632	2.89696583	0.22701211	0.63893544	0.49218201
Pik3c2g	0.66938666	-2.0787659	0.22686288	0.63904506	0.49222774
Polh	0.12709567	2.48231141	0.22674011	0.63913528	0.49225852
Zfp811	-0.1072805	3.43051123	0.22664274	0.63920686	0.49226896
Gtpbp1	0.11616421	4.45862357	0.22658495	0.63924934	0.49226896
Cemip	-0.0873248	3.50331555	0.22639887	0.63938621	0.49230976
Med15	-0.057372	6.51689822	0.22637625	0.63940285	0.49230976
Cd63	-0.1596903	4.98247981	0.22609441	0.63961028	0.49243077
Rorc	0.23349564	1.15378792	0.22583251	0.63980318	0.49254057
Ccdc87	0.236889	1.01600754	0.22571157	0.63989231	0.49257047
Ldhb	-0.0693461	8.96055698	0.22557949	0.63998967	0.49260671
Tktl1	0.51683242	-1.6232973	0.22529058	0.64020278	0.49268704

Gm6981	-0.1684054	1.51002647	0.22523599	0.64024306	0.49268704
Vkorc1l1	-0.0519707	6.22933451	0.22516383	0.64029632	0.49268704
Sgpp1	-0.059437	6.5640417	0.2250529	0.64037822	0.49268704
Cep55	0.63130007	-1.7708971	0.22504291	0.64038559	0.49268704
Trhde	-0.0981375	5.81450051	0.22502905	0.64039583	0.49268704
Sys1	-0.1216332	3.2962455	0.22495979	0.64044697	0.49268769
Itpripl1	0.27933965	1.21223054	0.22483096	0.64054214	0.49272221
St3gal1	0.07303184	5.20677659	0.22449336	0.64079169	0.49287546
1700034F02I	0.59085165	-1.1505467	0.22438219	0.64087391	0.4929
Skiv2l2	0.06273635	6.77451449	0.22430066	0.64093423	0.49290769
Fez1	0.06846931	5.52476133	0.22409011	0.64109006	0.49298882
Fggy	-0.1437413	2.85345833	0.22398967	0.64116443	0.49300038
Pds5b	0.06156982	7.92641985	0.22393388	0.64120575	0.49300038
2610020H08I	0.24331305	0.73171678	0.22380541	0.64130091	0.49303485
8030462N17	-0.061602	5.47395335	0.22340665	0.64159652	0.49320485
Cpne9	0.13042011	4.22777363	0.22335782	0.64163274	0.49320485
Rbbp4	0.06604022	5.94783195	0.22327483	0.64169432	0.49320485
Atp6v0c	0.50743479	-1.4626993	0.22323558	0.64172344	0.49320485
Srsf10	-0.0597818	6.59195119	0.22311316	0.6418143	0.49322682
Rab40c	-0.1209783	2.90796542	0.22305432	0.64185798	0.49322682
Chmp7	-0.0766349	5.62002362	0.22299358	0.64190309	0.49322682
Gpc3	0.13465104	3.71078847	0.22281033	0.6420392	0.49328357
Papln	0.49429457	-0.9964692	0.22275856	0.64207767	0.49328357
Serpina1d	-0.6585345	-1.5769784	0.22255582	0.64222835	0.49336065
Adamtsl2	-0.3736146	-0.4198794	0.22223601	0.64246623	0.49347032
Psmc6	0.06578077	5.85723152	0.22222841	0.64247188	0.49347032
9230114K14I	0.17040886	1.94223052	0.22209663	0.64256997	0.49347354
Lrfn3	0.10370185	3.13777682	0.22208742	0.64257683	0.49347354
BC024978	-0.0827242	4.79122258	0.22197414	0.64266117	0.49349962
Itsn1	0.08595972	7.01485215	0.22155694	0.64297203	0.49369962
Rpf2	-0.0741169	4.36084234	0.22148564	0.64302519	0.49370174
Rad17	-0.0932328	4.17621479	0.22118773	0.64324743	0.49382173
4930520O04	-0.5604031	-1.0839657	0.22110586	0.64330854	0.49382173
Parvg	-0.2019358	1.48542068	0.22107348	0.6433327	0.49382173
Ryk	0.10203459	4.46530085	0.22099549	0.64339093	0.49382772
Ccr10	0.58319482	-1.0131679	0.220911	0.64345403	0.49383746
Pfkfb4	0.19978406	1.19847148	0.22079748	0.64353883	0.49386384
Ms4a4b	-0.4491703	-0.5138973	0.22056593	0.64371188	0.49389148
Ing3	-0.0874291	4.2107681	0.22055102	0.64372302	0.49389148
Cpxm1	-0.141513	3.30145207	0.22052333	0.64374373	0.49389148
Tmod1	0.08080795	4.73124694	0.22047948	0.64377652	0.49389148
Gm14092	0.57843517	-1.0279516	0.22014291	0.64402831	0.49397504
Dlx6	0.21244967	0.88559198	0.22010428	0.64405723	0.49397504

Gngt2	0.24263184	0.72351475	0.22006813	0.64408429	0.49397504
Fn3k	-0.12768	3.23187119	0.2200613	0.6440894	0.49397504
Ado	0.07103976	5.37027576	0.21986008	0.64424009	0.49397504
Ppm1h	0.0596465	6.75681679	0.21972396	0.64434207	0.49397504
Tspo	-0.1709127	2.02514548	0.21969461	0.64436407	0.49397504
A530032D15	-0.2277084	0.8682512	0.21957972	0.64445017	0.49397504
Flii	0.07122297	5.38782624	0.21954374	0.64447715	0.49397504
Atmin	0.05628038	6.26839051	0.21952419	0.64449181	0.49397504
Ctbp2	0.08443989	4.21634869	0.21951484	0.64449882	0.49397504
Fam196b	0.17067674	1.52133932	0.21950939	0.6445029	0.49397504
Hsdl2	0.07994456	6.17222768	0.21941142	0.64457637	0.49397504
Acvr2b	-0.2510592	0.6247075	0.21939132	0.64459145	0.49397504
Eomes	0.30042119	0.41617846	0.21930941	0.64465289	0.49398347
9930012K11	0.23570053	1.30483916	0.21858416	0.64519754	0.49436215
Spin2c	-0.125343	2.93583366	0.21838017	0.64535093	0.49444101
Tmem51os1	-0.3549239	-0.5113356	0.21819124	0.64549309	0.49445945
Nkd1	-0.1014865	4.85827505	0.21819053	0.64549362	0.49445945
Gm10421	0.46450699	0.08783032	0.21814692	0.64552644	0.49445945
Them6	0.14294792	2.62308151	0.21786558	0.64573828	0.49452976
Trabd2b	-0.1139812	6.3225332	0.2178287	0.64576607	0.49452976
Ubap2l	0.05843047	8.54563331	0.21778274	0.64580069	0.49452976
Fam149b	-0.0685857	5.38865691	0.21775688	0.64582017	0.49452976
Atxn1l	0.06264381	6.29541844	0.21763446	0.64591243	0.49456174
Kif27	0.14567692	2.20180061	0.2173995	0.6460896	0.49463918
Shisa4	-0.1010939	4.60312327	0.21735159	0.64612573	0.49463918
Necap1	-0.0545869	7.2058062	0.21729947	0.64616506	0.49463918
Mmp15	0.20837615	1.11482654	0.21709268	0.64632112	0.4946484
Hlcs	-0.0875402	4.42716132	0.21708764	0.64632492	0.4946484
Hes7	-0.7229276	-1.7035648	0.21708277	0.6463286	0.4946484
Ngef	0.07631824	5.79286165	0.21701461	0.64638006	0.49464913
Mfsd1	0.10826442	5.17209041	0.2167576	0.64657419	0.49465624
Lpar4	0.16937543	2.27780024	0.21672433	0.64659933	0.49465624
Slc16a1	0.09048127	5.03789291	0.21670878	0.64661109	0.49465624
Kank3	-0.1857343	1.87037182	0.21669708	0.64661993	0.49465624
Ccdc62	-0.2157987	1.89830832	0.21666809	0.64664184	0.49465624
Rragd	0.05087937	7.20974552	0.21626376	0.64694761	0.4948515
Selenbp2	-0.3108609	-0.2173687	0.21608304	0.6470844	0.49491623
Tmem107	-0.1554533	1.71680392	0.21601845	0.6471333	0.49491623
Haus5	0.16851454	1.62049443	0.21559567	0.64745362	0.49512255
Ddrgk1	-0.0930799	4.10669404	0.21526897	0.64770141	0.49525082
Galk1	-0.1519661	2.74754331	0.21518287	0.64776675	0.49525082
Sag	0.35527866	-0.7599543	0.21511838	0.64781571	0.49525082
Insm1	-0.2020818	2.14257829	0.21510801	0.64782358	0.49525082

Hist1h4b	-0.4212563	-1.8082194	0.21498249	0.64791889	0.49528502
Ppt2	0.18976801	1.23233552	0.2147082	0.64812727	0.49540566
C130050O18	0.58700339	-2.0678571	0.21451953	0.64827071	0.49545715
Hdc	-0.354907	-0.2146941	0.21444782	0.64832524	0.49545715
Slc31a1	0.06779449	5.88304645	0.21442004	0.64834638	0.49545715
Slc36a4	-0.0785013	4.97063688	0.21412355	0.648572	0.4955909
Mysm1	0.06977198	6.25552941	0.21392276	0.6487249	0.49566907
Tti2	-0.0836197	4.5965	0.21373928	0.64886471	0.49573722
Tsta3	-0.1777286	2.29746459	0.21351079	0.6490389	0.49583163
Ofd1	0.10898369	4.18802212	0.21338621	0.64913393	0.49586555
Fbxo2	0.10830229	3.07285821	0.21307468	0.6493717	0.49599062
Tmem14a	-0.0807766	4.84503277	0.21301765	0.64941525	0.49599062
Gatm	-0.0577109	6.23725596	0.21297274	0.64944956	0.49599062
Anks1	-0.0891129	3.49404808	0.21280091	0.64958083	0.4960522
Vat1	-0.107425	4.62141289	0.21260883	0.64972766	0.49612565
Mtfr1l	-0.0808599	5.6179435	0.21249749	0.6498128	0.49615198
Mtmr14	-0.1179383	3.11934636	0.21236092	0.64991728	0.49617966
Sox13	-0.1461458	3.36118298	0.2122729	0.64998463	0.49617966
Mrpl35	-0.0602043	5.08137465	0.21219789	0.65004205	0.49617966
Ints10	0.09667078	4.16093091	0.21218533	0.65005166	0.49617966
Wdr26	0.05133743	8.56156612	0.21175995	0.65037751	0.49637887
Wdr37	0.0557198	7.17723915	0.21171235	0.650414	0.49637887
10-Mar	-0.3607404	-0.6737566	0.21158467	0.65051189	0.49641442
Pcgf2	-0.0948421	5.09123752	0.2114813	0.65059118	0.49641442
Rab33b	0.06029149	5.90779528	0.21145334	0.65061262	0.49641442
Adck3	0.11023184	2.71438383	0.21115937	0.65083824	0.49654788
Abcc4	0.1112493	5.12241116	0.21102491	0.6509415	0.4965662
Nxf2	-0.5439888	-1.3173708	0.21088423	0.65104958	0.4965662
Ino80e	0.15498568	2.32870918	0.2108486	0.65107696	0.4965662
Gm3086	-0.3639545	0.26584689	0.21082428	0.65109565	0.4965662
Ago4	0.1545542	3.22251351	0.21079817	0.65111571	0.4965662
C2cd4b	-0.2803162	-0.0762654	0.21051467	0.65133371	0.49669378
Gm6815	0.32303593	-0.9340087	0.21039291	0.65142739	0.49672655
Rab8a	-0.1331971	4.84206691	0.21028107	0.65151346	0.49675351
Fbxo18	-0.0593596	5.50909359	0.21020796	0.65156975	0.49675776
A830018L16Rik	-0.0736558	6.95865729	0.2099109	0.65179855	0.49688282
Mpi	-0.0835155	4.47507128	0.2098633	0.65183524	0.49688282
Arsk	-0.1248266	3.59236562	0.20961051	0.65203013	0.4969728
Pla2g2d	-0.3447126	-0.6830274	0.2095786	0.65205475	0.4969728
Spag6	0.15773688	1.99929427	0.20927335	0.6522903	0.49711365
Mab21l1	-0.2085297	1.21648583	0.20913999	0.65239327	0.49714591
Stxbp3a	-0.0637206	4.61286465	0.20908708	0.65243414	0.49714591
Cpne5	0.08772194	4.94584608	0.20875561	0.6526903	0.49730242

Stom	-0.2097111	5.20970168	0.20857965	0.65282639	0.49736742
Tgs1	0.09556442	6.52407515	0.20825793	0.65307538	0.49750888
Fam168a	-0.0457477	8.13100082	0.2082085	0.65311365	0.49750888
Wwc2	0.08137619	4.8312372	0.20800004	0.65327513	0.49756927
Wdr65	0.28474425	1.0782569	0.20797502	0.65329453	0.49756927
1110054M08	0.21406524	1.12950431	0.20766781	0.65353271	0.49759285
Mrps31	-0.0974579	4.56028874	0.20764572	0.65354984	0.49759285
Tcaim	0.09163318	4.43598128	0.20763242	0.65356016	0.49759285
4921511H03	0.59125185	-1.1054533	0.20762406	0.65356664	0.49759285
Ipmk	-0.0662902	5.56169021	0.20760752	0.65357947	0.49759285
Ddx4	0.20949534	0.98895315	0.20740536	0.65373635	0.49759789
AU022754	0.34515126	-0.2459675	0.20739539	0.65374409	0.49759789
Slit3	0.09968492	4.60546321	0.20737382	0.65376084	0.49759789
Rps14	-0.1544079	4.3577063	0.20733716	0.6537893	0.49759789
Arl8b	0.0485265	7.95545664	0.20702284	0.65403344	0.49774504
Ccdc67	0.50978441	-1.3627201	0.20678918	0.65421508	0.4978007
Rnf6	-0.0484144	6.81073045	0.20669219	0.65429051	0.4978007
Treml2	-0.5628001	-0.6834042	0.20668569	0.65429557	0.4978007
Acads	0.39816035	-0.6553317	0.20666732	0.65430986	0.4978007
Aifm3	-0.0786255	5.53542131	0.20654401	0.6544058	0.49781902
Lonrf1	-0.070746	6.32971245	0.20650575	0.65443558	0.49781902
Coro2b	0.07523187	6.52167001	0.20642359	0.65449953	0.497829
Limk2	0.07247221	5.20943889	0.20628891	0.6546044	0.49787011
Smu1	0.06794908	5.43062054	0.20604266	0.65479624	0.49794467
AF357355	0.56891829	-1.001034	0.20601062	0.65482122	0.49794467
Gpn3	0.11197307	4.49154742	0.2059661	0.65485592	0.49794467
Fam189a2	-0.2504482	0.55392825	0.20590215	0.65490577	0.49794467
Ckap2	0.31703391	0.36365987	0.20566006	0.6550946	0.49804958
Klhdc10	-0.0581801	6.89725649	0.2055645	0.65516916	0.49806761
Anpep	-0.1235703	6.47703909	0.20529057	0.65538304	0.49819154
Acad11	0.07700121	4.50550654	0.20483538	0.65573882	0.4984233
Amigo1	0.06429436	5.74003178	0.20450796	0.65599503	0.49857936
2700081O15	0.06261886	6.20960645	0.20441673	0.65606646	0.49859496
Chd4	0.05936137	7.44856066	0.20401369	0.65638227	0.49879627
Fbxo36	-0.1706374	2.9116626	0.20385561	0.65650624	0.49881344
Usp2	0.07453222	5.72951967	0.20379079	0.65655709	0.49881344
Kpna1	0.05639423	6.91615196	0.20371487	0.65661667	0.49881344
Oplah	-0.1350893	1.8057182	0.20367643	0.65664684	0.49881344
Tcea3	0.11073168	3.93148528	0.20366031	0.65665948	0.49881344
Acat3	0.25422621	0.69937389	0.20352918	0.65676242	0.49885295
Layn	-0.1837683	1.44309849	0.20318164	0.65703545	0.49902163
Rft1	0.17239988	2.59796167	0.20310802	0.65709332	0.49902351
Npnt	-0.0797931	3.77911532	0.2030489	0.6571398	0.49902351

Slco2a1	-0.1335623	4.43168881	0.20294601	0.65722071	0.49904626
Ccdc38	0.28464692	-0.1987506	0.2026332	0.65746686	0.49919447
Jup	0.07282637	5.22506196	0.20253368	0.65754523	0.49921528
Arid3b	-0.1632683	2.17230131	0.20244622	0.65761411	0.49922888
Khk	-0.2245194	2.21443771	0.20227112	0.65775208	0.49926746
Iqcb1	0.07475031	4.50208082	0.20225234	0.65776687	0.49926746
Drg2	-0.0864446	3.88772029	0.2020896	0.65789517	0.49930033
Ranbp6	0.08557026	6.39165545	0.20206811	0.65791212	0.49930033
Cd302	-0.0940557	4.11724662	0.20188862	0.65805371	0.49936909
Rgp1	0.07225269	4.49069946	0.20179113	0.65813064	0.49937963
D430020J02F	-0.3153483	0.56245737	0.20168352	0.65821559	0.49937963
Lemd2	-0.1522303	2.11383672	0.20167724	0.65822054	0.49937963
Nupl2	0.12244673	3.28139523	0.20144567	0.65840344	0.49947297
Mthfd2	0.19535723	1.46340259	0.20139238	0.65844554	0.49947297
Tmx2	0.07126019	5.98579548	0.20124724	0.65856026	0.4995213
Magi2	0.07708235	7.42040035	0.20113433	0.65864954	0.49955033
Arel1	0.06024316	6.26592541	0.2008816	0.65884948	0.49966329
Npr1	0.22402653	0.10392755	0.20013686	0.65943954	0.50006167
Chst15	0.07602568	5.98406679	0.20008075	0.65948405	0.50006167
Sall1	-0.0635796	4.82359432	0.19995707	0.65958219	0.50006167
Gpr161	-0.3021044	-0.3219541	0.19989701	0.65962986	0.50006167
Trim27	-0.0684179	4.48871746	0.19985128	0.65966616	0.50006167
Sart3	-0.10113	4.68295842	0.19978663	0.65971749	0.50006167
Zbed3	-0.0797863	5.56461188	0.19975519	0.65974246	0.50006167
Syap1	-0.1086087	5.92517928	0.19970391	0.65978318	0.50006167
Zfp503	-0.0978429	3.92476051	0.19945079	0.6599843	0.50015248
Slain1os	0.26185877	1.00212883	0.19942459	0.66000512	0.50015248
Btbd6	-0.0770227	4.22448594	0.19919923	0.66018432	0.50024958
Gnpnat1	0.0780933	4.78318396	0.19906799	0.66028873	0.50029
Sowahb	0.09936888	3.51482256	0.19899649	0.66034564	0.50029441
Cpne8	0.06251791	6.41143721	0.19833069	0.66087611	0.50065759
Emp3	-0.1486027	3.79766243	0.19823415	0.66095312	0.5006772
Chst10	0.09024438	3.87552992	0.19799923	0.6611406	0.50076774
Slc38a4	0.21214196	1.56652704	0.19795035	0.66117963	0.50076774
Atxn7l3	-0.0775986	5.96367307	0.19783508	0.66127168	0.50076774
Snhg5	-0.0882576	3.73901185	0.19781987	0.66128383	0.50076774
Cdc16	-0.0769308	4.94816157	0.19772501	0.66135962	0.50076774
Pusl1	-0.1642304	2.33169202	0.19770028	0.66137938	0.50076774
Snord71	-0.6299926	-1.3463177	0.19730446	0.66169586	0.50093662
Zfp607	0.15446728	2.48139595	0.1972934	0.66170471	0.50093662
Ccdc183	0.24919359	0.60674389	0.19721747	0.66176547	0.50094382
Uspl1	0.05994525	5.10207478	0.19715372	0.66181649	0.50094382
Tk1	0.45402096	-0.8897527	0.19699341	0.66194483	0.50100225

Dcun1d4	-0.0628673	7.403709	0.19670717	0.66217416	0.50107509
Slc16a2	0.06642629	5.39027333	0.19662386	0.66224094	0.50107509
Spag17	-0.4415016	0.24423401	0.19660293	0.66225772	0.50107509
Zranb1	0.06461198	4.76917413	0.19657618	0.66227917	0.50107509
Mbtps2	0.07786489	4.92830405	0.19655414	0.66229685	0.50107509
Entpd4	-0.0662029	6.04676775	0.19630504	0.66249667	0.50118756
Frmd4a	0.09037253	5.54003423	0.19605438	0.6626979	0.50124927
Bnip1	-0.1565156	2.16630286	0.19599423	0.66274621	0.50124927
Gm14015	-0.4814607	-0.754935	0.19598729	0.66275179	0.50124927
Patl1	-0.0620754	6.17278137	0.19592952	0.6627982	0.50124927
Large	0.07115013	6.42464331	0.19588483	0.66283411	0.50124927
Mapt	-0.0566385	6.89262027	0.19569771	0.66298451	0.50132431
Zfp428	-0.2098594	0.8567422	0.19562881	0.66303991	0.5013275
Sfpq	0.061611	7.54784806	0.19543349	0.66319703	0.50138513
Pycr2	-0.1307921	2.68835934	0.19534547	0.66326786	0.50138513
Syde2	-0.1217108	3.66367295	0.1953432	0.66326968	0.50138513
Pdia4	-0.0726377	4.46957625	0.19524888	0.66334561	0.50140383
Zfp251	-0.0936577	3.78981328	0.19512198	0.6634478	0.50141565
Ak5	0.07291192	6.20518148	0.19506259	0.66349564	0.50141565
Tagap1	0.07285161	4.77504619	0.19503878	0.66351482	0.50141565
Shf	0.20585445	1.94867076	0.19482867	0.66368415	0.50150492
Lsm11	-0.0760737	4.47219831	0.19463628	0.66383929	0.50158346
Pja1	-0.0528313	6.81192113	0.19451227	0.66393934	0.50162036
Kcnc4	-0.0959879	4.33426305	0.19436397	0.66405903	0.5016503
Ccdc22	0.21821986	0.91836404	0.19427282	0.66413263	0.5016503
Spryd4	-0.1486588	2.31508092	0.19422607	0.66417039	0.5016503
Atp8b4	-0.4189132	-0.8469891	0.19420943	0.66418382	0.5016503
Hjurp	0.13032127	2.44062211	0.1941317	0.66424662	0.50165905
Zc3h12c	-0.0489912	6.25593924	0.19390986	0.6644259	0.50175576
Rpusd3	-0.2430445	0.91871578	0.1938465	0.66447713	0.50175576
Rnf214	0.05197464	6.33644603	0.19373827	0.66456466	0.50178318
Nkap	-0.0818411	5.26887566	0.19333976	0.66488721	0.50198803
Acd	-0.0880477	3.94948651	0.19314444	0.66504544	0.50205734
Gltscr2	-0.1048671	5.54829414	0.19309991	0.66508152	0.50205734
Inadl	-0.0767285	3.94804209	0.19300233	0.66516062	0.50207836
4930500J02F	-0.4707194	-0.6155177	0.19289402	0.66524845	0.50210596
Ipo5	-0.0584772	6.79794917	0.19269063	0.66541345	0.5021918
Fktn	0.06500764	5.73022151	0.19241642	0.66563607	0.50231598
Bcar3	0.11804261	2.67844132	0.19223658	0.66578218	0.50231598
Foxq1	-0.3321431	1.12475046	0.19220502	0.66580783	0.50231598
Col27a1	0.17715049	2.12821318	0.19219744	0.66581399	0.50231598
Retsat	-0.1536779	2.56790834	0.19217233	0.6658344	0.50231598
Ppt1	-0.0593315	6.44486495	0.19210046	0.66589283	0.50232137

Arf5	-0.1225867	3.03196978	0.19178044	0.66615313	0.50242085
Rab11fip3	0.05732669	6.19412059	0.19177699	0.66615594	0.50242085
Naaladl1	0.26638529	0.48937503	0.19174917	0.66617858	0.50242085
Rem1	-0.496469	-0.5359513	0.19155077	0.66634011	0.50248567
Mms22l	0.22959162	0.73336835	0.19147962	0.66639806	0.50248567
Smek2	0.05258289	6.69850331	0.19145463	0.66641842	0.50248567
Mad2l1bp	-0.1666335	1.72148209	0.19127901	0.66656152	0.50249005
Tmem81	0.24574535	0.54016435	0.19126625	0.66657193	0.50249005
Stk32c	0.11992754	2.62635104	0.19125865	0.66657812	0.50249005
Zfp870	0.09475922	3.74149517	0.19085632	0.66690629	0.50269875
Rnf121	0.21834077	0.95704987	0.19066763	0.66706034	0.50273563
Rbp4	-0.105648	4.08437903	0.19062714	0.66709341	0.50273563
Paxip1	0.05216988	6.18251122	0.19046313	0.66722741	0.50273563
Nova1	-0.0602775	7.15070647	0.19043995	0.66724635	0.50273563
Rhebl1	0.27614848	0.05855139	0.19030145	0.66735956	0.50273563
Zfp146	-0.1040825	4.37017683	0.19020362	0.66743956	0.50273563
5330434G04	0.10603698	5.00213605	0.19019659	0.66744531	0.50273563
Rnf168	-0.058329	6.53820425	0.1901878	0.6674525	0.50273563
Wnt9a	0.11020481	3.82353206	0.19010083	0.66752364	0.50273563
Ggnbp1	0.51123913	-0.8377932	0.18996041	0.66763855	0.50273563
Arrdc3	-0.0810722	5.87080385	0.18991678	0.66767426	0.50273563
Mir684-1	-0.2150386	0.23407638	0.18989167	0.66769481	0.50273563
Iws1	-0.0544369	6.44071015	0.18988797	0.66769784	0.50273563
Bmi1	-0.0656646	6.05149084	0.18986673	0.66771524	0.50273563
Wdr61	-0.0802331	4.33102401	0.1898547	0.66772508	0.50273563
Ctsc	-0.142657	2.05099363	0.18943728	0.66806707	0.50295446
A330048O09	0.34162644	0.44010957	0.18921517	0.66824924	0.50303147
Tceanc	-0.1188895	3.2960577	0.18916403	0.66829119	0.50303147
Agtr1b	0.25843897	2.19031854	0.18911819	0.66832881	0.50303147
Itpk1	-0.0830875	3.82827909	0.18900035	0.66842554	0.50303147
Mmp24	0.14616241	2.48675089	0.18899963	0.66842613	0.50303147
Top3a	-0.1761268	0.99311995	0.18880616	0.66858501	0.50306231
Rps6kl1	-0.1486041	2.37169747	0.1888038	0.66858695	0.50306231
Mbip	0.1214263	3.81516301	0.18872425	0.6686523	0.50306231
Pik3r1	0.05655222	8.09936598	0.18869963	0.66867254	0.50306231
Taf4b	-0.1014399	2.90213099	0.18858622	0.66876575	0.5030771
G6pc3	0.09670819	3.9418678	0.18855073	0.66879493	0.5030771
Cox10	-0.0718768	4.25664074	0.1881268	0.66914369	0.5033008
Lingo4	0.5576602	-1.408254	0.18787155	0.66935391	0.50342026
Zfp938	-0.0873743	4.44416906	0.18773409	0.66946719	0.50344181
Mob4	0.04797438	7.02416311	0.1876975	0.66949736	0.50344181
Slc13a3	-0.1536371	6.93531399	0.1876436	0.66954179	0.50344181
Nsun5	0.12568318	1.70862212	0.18758739	0.66958815	0.50344181

Rmdn2	0.09330955	3.71864916	0.18734441	0.6697886	0.50349199
Ccdc163	0.3585964	0.00792254	0.18733667	0.66979499	0.50349199
Dnah17	0.41316474	-1.109053	0.18725603	0.66986155	0.50349199
Mtmr12	-0.049299	6.44446954	0.18716956	0.66993294	0.50349199
2610306M01	-0.1163983	2.61735373	0.18712134	0.66997276	0.50349199
Rel	-0.0684828	4.52771932	0.18707555	0.67001059	0.50349199
Tab1	-0.1237282	2.84188839	0.18702263	0.6700543	0.50349199
Ccng2	-0.0888934	4.33906316	0.18700835	0.6700661	0.50349199
Psmg3	0.16661527	2.00134747	0.1869147	0.67014348	0.50351151
D730005E14	-0.5749431	-1.8001265	0.18668041	0.67033718	0.50361842
Coq10b	-0.0750022	5.61281997	0.1864831	0.67050042	0.50363505
Pibf1	-0.0914574	4.56124547	0.18646099	0.67051872	0.50363505
4930452G13	-0.5679576	-1.7082756	0.18644326	0.67053339	0.50363505
Ube2v1	-0.0705699	6.89310435	0.1863974	0.67057135	0.50363505
Klhl7	0.05925887	6.31339067	0.18625899	0.67068596	0.50363505
Prpsap2	-0.0762429	4.20561193	0.18623847	0.67070295	0.50363505
Tuft1	0.26348796	0.44373696	0.18621882	0.67071922	0.50363505
Pygb	-0.0605755	6.4839507	0.18602339	0.67088114	0.50365395
Hddc2	-0.0862487	3.66227842	0.18596384	0.6709305	0.50365395
C87436	-0.1164447	3.60586379	0.18595854	0.6709349	0.50365395
Ripply3	0.20759716	1.46341984	0.18590045	0.67098306	0.50365395
Pank1	0.057183	6.29469563	0.18587822	0.67100149	0.50365395
A230103J11F	-0.1605325	1.99749863	0.18566414	0.67117906	0.50374864
Ndufa7	-0.1072891	4.6453274	0.18542994	0.67137347	0.50385594
Rasgrp2	0.10426884	2.56032541	0.18521349	0.67155326	0.50393279
Exog	-0.1108599	2.92209593	0.1851828	0.67157876	0.50393279
Tmem101	0.1815889	2.47401716	0.18494084	0.67177992	0.50396611
Plekhg1	-0.068853	5.36137989	0.1849197	0.6717975	0.50396611
Asphd1	0.1109941	2.41463671	0.18475635	0.67193341	0.50396611
2310001H17I	-0.4741683	-0.9587405	0.18470069	0.67197973	0.50396611
Pik3cb	-0.0666567	5.5200715	0.18469321	0.67198596	0.50396611
Eif2b4	-0.1083246	3.19675848	0.18468476	0.67199299	0.50396611
Sema6c	-0.2312545	0.69316727	0.18466876	0.67200631	0.50396611
Mki67	0.0880157	3.76884703	0.18463458	0.67203476	0.50396611
Mks1	0.29133512	0.20709525	0.1845319	0.67212025	0.50399164
C1qa	-0.4119123	-1.5955507	0.18446248	0.67217807	0.50399641
Smad4	0.05356627	5.86879899	0.18436161	0.67226211	0.50402084
Efna4	-0.2214627	1.84046717	0.18410665	0.67247464	0.50411361
Laptm5	-0.162218	3.30029559	0.1840897	0.67248878	0.50411361
Sik1	0.09011554	4.19170344	0.18402152	0.67254565	0.50411766
Nipa1	-0.0784515	4.82904993	0.18390332	0.67264426	0.504153
Hdac3	-0.0789207	4.7598794	0.18358328	0.67291147	0.5042766
3110052M02	0.06738756	4.84073189	0.18358249	0.67291213	0.5042766

Vnn1	-0.207649	1.71551037	0.18328553	0.67316032	0.504424
Jakmip2	0.06913461	6.83067057	0.18306691	0.67334318	0.50451654
Gm5801	-0.2723135	-0.3960546	0.18298306	0.67341335	0.50451654
Pank2	-0.0692281	5.50651976	0.18295321	0.67343833	0.50451654
Rgl3	0.25448557	0.42338172	0.18269623	0.67365353	0.50462785
Slc38a9	0.10977254	4.58404155	0.18265277	0.67368994	0.50462785
Fdxacb1	0.20125978	1.52243624	0.18236698	0.67392951	0.50469957
Ngly1	-0.067538	5.55152088	0.18230904	0.67397811	0.50469957
Ngf	0.21675625	0.84562194	0.18227743	0.67400462	0.50469957
Kdelr1	-0.0884751	4.34912127	0.18222041	0.67405246	0.50469957
Ip6k1	-0.0701273	5.79046022	0.18218996	0.67407801	0.50469957
Itga2	0.39766318	0.13157257	0.18216991	0.67409484	0.50469957
Iqcg	-0.1061209	2.95988689	0.18207651	0.67417324	0.5047015
Eif4ebp1	0.16370879	1.68990599	0.18204407	0.67420047	0.5047015
Mmp28	-0.337033	0.01821936	0.18175714	0.67444147	0.50484333
Prob1	-0.1498591	1.69995626	0.18164512	0.67453562	0.50487523
Tmbim4	0.09174234	4.59340577	0.18157596	0.67459377	0.50488017
Apex1	-0.3989764	-1.045947	0.18138901	0.67475101	0.50489931
Eif3b	0.07783054	3.89030819	0.18136425	0.67477184	0.50489931
Selplg	-0.2114366	0.91735611	0.18136171	0.67477398	0.50489931
Enpp6	0.13438244	2.77992828	0.18126753	0.67485324	0.50492005
Pnpla6	0.10502534	4.04739104	0.18113715	0.674963	0.50495887
Ttc8	0.06095583	4.70920459	0.18108344	0.67500823	0.50495887
2900005J15F	-0.1761319	2.24729623	0.18095554	0.67511596	0.5050009
Sox12	-0.1075271	2.99499347	0.18086452	0.67519266	0.5050197
Lrrc18	0.12102955	2.93618998	0.18061136	0.67540612	0.50514079
Zc4h2	0.06718453	4.71566266	0.18050134	0.67549893	0.50517163
Rufy3	0.06236183	8.36759982	0.18043234	0.67555716	0.50517661
Nfatc1	-0.082206	3.99991759	0.18020068	0.67575276	0.5052843
Lnx2	0.07107267	4.43629105	0.17993301	0.67597894	0.50532132
Hcst	0.57648975	-1.5368851	0.17987861	0.67602494	0.50532132
Mtmr9	-0.0827462	3.64425542	0.17986954	0.6760326	0.50532132
Hist1h4h	-0.4042936	-1.3963942	0.17982048	0.67607409	0.50532132
Aoc3	0.24311242	3.18181364	0.17979217	0.67609803	0.50532132
Taf11	-0.0680689	5.26365295	0.17972906	0.67615141	0.50532132
Hsph1	0.06546908	9.09662742	0.17971491	0.67616338	0.50532132
Decr2	-0.0600733	5.53858173	0.17957422	0.67628244	0.50537174
Col2a1	0.46003971	-1.1175281	0.17948049	0.67636178	0.5053805
Vasp	-0.1520264	3.48114641	0.17943848	0.67639735	0.5053805
Polg	0.09409262	3.26366115	0.17934988	0.67647239	0.50539801
Pias2	0.05373365	6.01353074	0.17910257	0.67668195	0.50541777
Megf9	-0.0646033	5.961198	0.17904503	0.67673072	0.50541777
Aldh6a1	0.06687578	5.93071237	0.17904356	0.67673197	0.50541777

Slc39a13	0.10034901	5.17208541	0.17904119	0.67673399	0.50541777
Rwdd4a	-0.0651709	5.61345418	0.17901425	0.67675682	0.50541777
Arhgef33	-0.3111995	-0.5838041	0.1787734	0.67696114	0.50550143
Poc1b	-0.0798632	4.59320195	0.17876053	0.67697206	0.50550143
Txlna	0.06407139	5.13475204	0.1786357	0.67707802	0.50554202
Fbxo25	-0.0718362	4.92372084	0.17842179	0.6772597	0.50560709
Smarcc2	-0.0474751	8.25320197	0.1783548	0.67731662	0.50560709
Ndufb2	-0.0739787	3.99487828	0.17825189	0.67740409	0.50560709
Snph	0.08247981	5.88306559	0.17822579	0.67742628	0.50560709
Evpl	-0.2153622	1.2386368	0.17817836	0.67746661	0.50560709
Cacna1f	0.44477177	-0.5969001	0.17816864	0.67747488	0.50560709
Ccdc18	0.20189247	1.71139588	0.17804499	0.67758004	0.50563345
Mrrf	-0.0761905	3.972877	0.17800573	0.67761344	0.50563345
Gyltl1b	-0.4999968	-1.9403739	0.17768442	0.67788696	0.50571445
Elmod2	-0.083532	3.45349902	0.17764757	0.67791834	0.50571445
Cdc42bpg	-0.1651702	1.33986723	0.17761034	0.67795006	0.50571445
6030458C11I	0.08716475	4.92836648	0.17758674	0.67797016	0.50571445
Slc25a39	-0.1039171	4.37736588	0.17757505	0.67798012	0.50571445
Steap4	0.48639829	0.17579235	0.17723949	0.67826618	0.50581615
Obfc1	0.11061895	2.73504634	0.17719139	0.67830722	0.50581615
Ipp	-0.0958866	3.30453463	0.17717577	0.67832055	0.50581615
Rag1	-1.0439719	-1.9203626	0.17713414	0.67835606	0.50581615
Tom1l2	0.05379242	7.57538087	0.17711236	0.67837465	0.50581615
Rab8b	-0.0564187	6.34753689	0.1767055	0.67872208	0.50601562
Mgll	-0.062648	5.42694072	0.17658378	0.6788261	0.50601562
Ilf2	-0.0750693	5.70695281	0.17657652	0.67883231	0.50601562
Pold4	-0.1580678	2.12316132	0.17655722	0.67884881	0.50601562
Pdlim2	0.1261832	3.29156799	0.17641277	0.67897234	0.50606918
Mcemp1	-0.309518	0.11998169	0.17624859	0.67911281	0.50613537
Zcchc6	-0.0439511	7.22987234	0.17595021	0.6793683	0.50625265
Ubash3a	-0.5336276	-1.2589354	0.17592546	0.6793895	0.50625265
Pafah2	0.09138068	3.40663434	0.17587337	0.67943413	0.50625265
Cog7	-0.095593	4.52669569	0.17569859	0.67958394	0.50625265
Iffo1	0.11688076	3.77255364	0.17567438	0.6796047	0.50625265
Cxcl14	0.08366049	3.20959099	0.17565313	0.67962292	0.50625265
Ccnl1	-0.1165532	4.7158139	0.1756426	0.67963195	0.50625265
Gm4944	0.07308645	4.17769705	0.17552068	0.67973653	0.50629205
Arhgdig	-0.1264767	3.12759263	0.17540885	0.67983248	0.50631801
Nynrin	0.0685577	5.67264852	0.17535959	0.67987477	0.50631801
Hsdl1	0.08803811	4.27791316	0.17526335	0.67995739	0.50634104
Slc27a4	0.09355851	3.48703901	0.17519286	0.68001792	0.50634763
AA986860	0.14435326	1.71718232	0.17502662	0.68016074	0.50641547
Tmco5	-0.2354503	0.61873959	0.17481358	0.68034388	0.50651333

Ctsa	-0.0782904	5.4161235	0.17459556	0.68053144	0.50661446
Pnp2	0.28435553	-0.4451936	0.17446001	0.68064812	0.50666282
Tm9sf3	0.05115901	7.28578233	0.1741949	0.68087648	0.50675676
Sox8	-0.1146585	3.86742114	0.17417168	0.68089649	0.50675676
Mbd5	0.06534323	7.79862583	0.17407681	0.68097827	0.50675676
Cp	-0.0740963	7.00285072	0.17407334	0.68098126	0.50675676
Hps1	-0.1757533	1.40435611	0.17376511	0.68124712	0.5069161
Zscan2	0.19253598	1.3090685	0.17361693	0.68137504	0.50697277
9930021J03F	0.06716972	8.19119709	0.17355088	0.68143207	0.50697669
Hsd17b2	0.2463277	1.4829574	0.17339831	0.68156387	0.50703624
Fyn	0.04970929	6.00425844	0.17320845	0.68172799	0.50709404
AI182371	-0.6756365	-2.1088921	0.17317156	0.68175989	0.50709404
Nsfl1c	-0.0727587	5.06797912	0.17312879	0.68179688	0.50709404
Tmem179b	0.13058978	3.23647778	0.17302011	0.68189089	0.50710678
Olig1	-0.0747908	4.07031576	0.1729893	0.68191755	0.50710678
Acbd7	-0.3840324	-1.2442013	0.17276464	0.68211202	0.5072129
Prss36	-0.2291916	0.86859551	0.17247449	0.68236341	0.50736131
Slpi	0.52587577	-1.3967674	0.17236427	0.68245897	0.50739384
Gm10336	0.09488592	4.45239221	0.17182155	0.68293003	0.50770553
S100a13	-0.1776518	3.41422825	0.17171778	0.6830202	0.50772852
Il12rb2	0.16242846	2.58358439	0.17163597	0.6830913	0.50772852
Cyb5rl	-0.4747686	-0.4919336	0.17160705	0.68311645	0.50772852
Sec13	-0.0616909	4.60747021	0.17141082	0.68328711	0.50776759
Tmem168	-0.1004521	3.25320225	0.17140144	0.68329527	0.50776759
Supt4a	-0.1129913	4.73820163	0.17136781	0.68332453	0.50776759
Fhl4	0.24869994	0.87634716	0.17123816	0.68343737	0.50779889
Dkk3	0.06458926	8.69595645	0.17120029	0.68347033	0.50779889
Rock1	0.06766329	8.00893157	0.17105298	0.68359862	0.50782463
Col4a4	-0.4761543	-0.8139241	0.17104145	0.68360867	0.50782463
Lgi4	-0.1804949	1.45022589	0.17074855	0.68386394	0.50795374
Xrcc4	0.08789523	3.43775911	0.17070739	0.68389983	0.50795374
Igf2	-0.1015995	10.4379258	0.17063778	0.68396054	0.50795374
Gm3696	0.21178007	0.04726077	0.17060414	0.68398989	0.50795374
Kcnd1	0.13751081	2.40261102	0.17041392	0.6841559	0.5080385
Pagr1a	0.08771804	3.04353143	0.17031557	0.68424177	0.50806375
Slc16a11	-0.113372	3.31827081	0.16976904	0.6847195	0.50837994
Zc2hc1a	0.05748054	7.30989653	0.16968088	0.68479664	0.50839868
Rab39b	0.06419819	6.74306171	0.16956731	0.68489606	0.50843396
Asns	0.08086017	5.25667555	0.16937401	0.68506536	0.5085211
Fbxo16	0.12762405	2.08289641	0.16926467	0.68516118	0.50855368
Timm22	-0.0839991	3.87699275	0.16909996	0.68530558	0.50862232
Clk2	-0.071752	5.15025855	0.16839779	0.68592212	0.5089372
Slc6a20b	-0.1268788	1.71811984	0.16838291	0.6859352	0.5089372

Elp3	-0.0574638	5.3579593	0.16829916	0.68600884	0.5089372
Maml1	-0.0631158	5.19911972	0.16823924	0.68606154	0.5089372
0610040B10I	0.28138578	0.65165979	0.16815936	0.68613182	0.5089372
Maml3	-0.0698305	4.82223032	0.16815673	0.68613413	0.5089372
Nop2	0.11931146	2.78938159	0.1681538	0.68613671	0.5089372
Aim2	-0.1824309	2.19248865	0.16814381	0.6861455	0.5089372
Rasgrp3	0.07779726	4.97241042	0.16807753	0.68620382	0.50894193
Ifit1	0.09947004	6.03124522	0.16788249	0.68637553	0.50903074
Mis12	-0.0665475	5.24682689	0.16778421	0.6864621	0.5090564
Vsig10l	-0.1432535	2.41814072	0.16752609	0.68668962	0.50918657
Rnf19a	-0.0510042	6.03704394	0.16733153	0.68686124	0.50927527
Hepacam2	0.28715171	0.47403687	0.1671728	0.68700134	0.5093406
Fam154a	0.60640676	-1.9889816	0.16689175	0.6872496	0.50948005
Col9a2	0.13286056	2.67963195	0.16683398	0.68730067	0.50948005
Txnrd3	-0.1012437	3.37677223	0.1667367	0.68738667	0.50948005
Cst6	-0.3642853	-0.7388505	0.16672447	0.68739749	0.50948005
Ddx46	0.0462969	7.32957545	0.16656882	0.68753516	0.50950134
Tk2	0.11765846	3.29112277	0.1664066	0.68767873	0.50950134
Ipo13	0.06728094	5.39730335	0.16634691	0.68773158	0.50950134
Pigx	-0.1186581	2.84092419	0.16626832	0.68780118	0.50950134
Dnajc14	-0.089918	4.30884159	0.16624879	0.68781848	0.50950134
Podn	0.09252082	5.0364247	0.16624277	0.68782381	0.50950134
Ptprn2	0.05556625	7.30390433	0.16614893	0.68790695	0.50950134
Tmem180	0.12135789	2.4419611	0.16611297	0.68793881	0.50950134
Prkra	-0.0806726	3.57028419	0.16610843	0.68794284	0.50950134
Ptgfr	-0.1246816	4.72271228	0.16604471	0.68799931	0.50950134
Manbal	0.10194568	3.04796041	0.16597483	0.68806126	0.50950134
Ccdc105	0.40819158	-0.7318402	0.16587488	0.68814989	0.50950134
Cyp2d22	0.09968685	3.38887593	0.16579511	0.68822066	0.50950134
A230072E10	-0.2565433	0.01454516	0.16575714	0.68825435	0.50950134
9530068E07I	-0.0925216	6.71850553	0.16575573	0.6882556	0.50950134
Tmem135	-0.0449293	6.07846201	0.16575252	0.68825845	0.50950134
Aldh3b1	0.21439323	0.86317787	0.16565365	0.68834619	0.50952779
Mark3	0.04657391	6.40504925	0.16552892	0.68845693	0.50953031
Igsf6	0.18704067	1.69934988	0.1654402	0.68853573	0.50953031
Itprip	-0.3409385	-0.6277225	0.16541523	0.68855791	0.50953031
Alk	0.23536665	0.73476148	0.16539755	0.68857362	0.50953031
Galnt3	-0.4767025	-0.752167	0.16532859	0.6886349	0.50953031
Lactb2	-0.097109	3.85021558	0.16529843	0.6886617	0.50953031
Svil	-0.0820501	4.74877453	0.16519165	0.68875662	0.50956205
D230025D16	0.0529179	5.65065119	0.16469221	0.68920106	0.50985235
1600023N17	-0.4021958	-0.9773858	0.16441807	0.68944535	0.50992605
Pigb	-0.1228056	2.54584533	0.16441623	0.68944699	0.50992605

Nap1l3	-0.0675587	5.81766145	0.16433529	0.68951916	0.50992605
Hspa1b	0.12186363	2.12639653	0.16430537	0.68954584	0.50992605
Vps53	-0.0515559	6.21974496	0.16428841	0.68956097	0.50992605
Baz1a	-0.0765208	4.11718974	0.16414907	0.68968528	0.50995856
Tinagl1	0.25835069	0.32428798	0.16412243	0.68970906	0.50995856
4933413G19	0.67734549	-1.7289615	0.163965	0.6898496	0.51002398
Ikbip	0.10970839	4.11084961	0.16387997	0.68992555	0.51004163
Gipc1	0.13235611	3.03328594	0.16366354	0.69011895	0.51012019
Ftsj1	0.0749196	3.73079047	0.1636445	0.69013597	0.51012019
Mrps35	-0.0973185	3.99390383	0.16346071	0.69030034	0.51020318
Mboat1	0.26629583	0.97998995	0.16304961	0.69066838	0.51043668
Gbp7	-0.0765446	4.86337133	0.16293324	0.69077266	0.51047214
Vwa9	0.06752626	4.48041734	0.16285914	0.69083908	0.51047214
Map6	-0.0852505	5.70222243	0.16282165	0.69087269	0.51047214
Rpn1	0.0681557	5.65877509	0.16238726	0.69126249	0.510685
Tiparp	0.05548483	4.91605335	0.1623742	0.69127422	0.510685
Bmp15	0.16354173	2.22693816	0.16226499	0.69137232	0.510685
B9d1	-0.1137022	2.37419088	0.16222575	0.69140758	0.510685
Zfp64	0.09486755	2.7789355	0.16216617	0.69146112	0.510685
Epn1	0.06588927	4.47923716	0.16215229	0.69147359	0.510685
Ankra2	0.08178022	3.44615038	0.161951	0.69165458	0.51076782
A330050F15	-0.1692596	2.27249062	0.16184565	0.69174936	0.51076782
Fxr2	-0.0845847	3.71798348	0.16183056	0.69176294	0.51076782
Dis3l	0.07107857	3.87216742	0.1617957	0.69179431	0.51076782
Zswim1	-0.0731204	3.84826167	0.16163235	0.69194136	0.5108325
Msln	0.16929239	2.22083589	0.16158255	0.69198621	0.5108325
Cdkl1	-0.1417617	2.6430747	0.16146919	0.69208833	0.51084951
Cd79b	-0.4730208	-1.9913514	0.16143251	0.69212138	0.51084951
Gm13212	-0.1307046	1.56577892	0.16134007	0.69220469	0.51084951
Mlycd	-0.0835973	3.16265208	0.16129986	0.69224094	0.51084951
Trp53inp2	-0.0424656	7.84324072	0.16126762	0.69227002	0.51084951
Gimap7	-0.3270329	0.15483927	0.16105996	0.69245733	0.51092192
Tub	0.08637062	6.11922784	0.16099069	0.69251985	0.51092192
Fntb	-0.0801278	4.03066677	0.16094312	0.69256278	0.51092192
Dab2	-0.0725533	8.40747023	0.16092763	0.69257677	0.51092192
Crhbp	0.15583199	3.05964508	0.1607417	0.69274468	0.5110073
Upf3b	0.07006379	5.6288383	0.1602957	0.69314793	0.51122554
Nadsyn1	-0.1418105	1.37774701	0.16025955	0.69318065	0.51122554
Zfp944	0.0819441	3.91730998	0.16018775	0.69324563	0.51122554
Chrna3	-0.2447416	0.04976638	0.1601131	0.69331321	0.51122554
Hcrtr2	0.21277615	0.50982421	0.16011107	0.69331505	0.51122554
Sema3b	0.18260584	2.712754	0.16006842	0.69335368	0.51122554
Nlrp5-ps	0.26769266	0.46725446	0.15994144	0.6934687	0.51125128

Plscr3	0.17867162	1.25531177	0.15989718	0.6935088	0.51125128
Tmem44	0.06797236	4.61437698	0.15985705	0.69354517	0.51125128
Atp1b2	-0.0668057	9.04784506	0.15955651	0.6938177	0.51137402
Ndel1	-0.0679692	4.91685716	0.15954995	0.69382366	0.51137402
Rab17	-0.4825325	-1.5520423	0.15944878	0.69391547	0.51137402
Synpo	-0.0508017	6.50701661	0.15944323	0.69392051	0.51137402
Wbscr16	-0.1512895	1.98241993	0.15910443	0.69422822	0.5115623
Tmed9	0.04878989	6.26273586	0.15904016	0.69428665	0.51156687
Shmt1	0.43728071	-0.4443665	0.15891728	0.69439837	0.5116107
Cxcl10	0.17867303	2.81711053	0.15875832	0.69454297	0.51164973
Fhl5	-0.9853784	-2.2783668	0.15874422	0.69455581	0.51164973
B3galt5	0.12152463	3.22309558	0.15853404	0.69474715	0.51174156
Nfam1	-0.2521144	1.15050389	0.15849253	0.69478495	0.51174156
Rnase1	-0.4511678	-0.8983568	0.15834579	0.69491866	0.51178882
Siah3	0.18763216	0.8095347	0.15830743	0.69495361	0.51178882
Tmed1	-0.2091343	1.04176317	0.15793651	0.69529193	0.51197197
Rai14	-0.0713786	6.49851665	0.15792018	0.69530684	0.51197197
Fh1	0.05853622	5.26825445	0.15777909	0.69543566	0.51200096
Kif11	-0.1395335	2.2910576	0.15776256	0.69545075	0.51200096
Elovl6	0.06475556	6.01508993	0.15760058	0.69559873	0.51205936
Srsf5	0.04230158	8.0887758	0.15747784	0.69571093	0.51205936
Aacs	-0.1024147	2.68904219	0.15743592	0.69574926	0.51205936
1200014J11F	0.053488	5.36314191	0.15742778	0.6957567	0.51205936
Sec14l5	0.39485232	-1.0991668	0.15738978	0.69579146	0.51205936
Enpp2	0.05819297	8.24836656	0.15718619	0.69597772	0.5121352
Ttll13	0.37982878	-0.9046798	0.15716286	0.69599908	0.5121352
Fxyd1	0.10045246	3.46058344	0.15704492	0.69610705	0.51217618
Lcorl	-0.0622854	5.13712707	0.15693638	0.69620647	0.51221085
Paqr6	-0.1280189	2.09604064	0.1567785	0.69635115	0.51227882
Thoc3	0.10162787	4.17805033	0.15668408	0.69643772	0.51230403
Ube2j2	-0.0950701	2.89842445	0.15656127	0.69655035	0.51231959
Cadm3	0.06573929	7.86896536	0.15654696	0.69656348	0.51231959
Chrm1	0.05938688	5.75229557	0.15617126	0.6969084	0.5125348
R3hcc1l	-0.0637532	4.57641189	0.15606811	0.69700318	0.51256602
Tmeff1	-0.0711256	5.11783559	0.15587721	0.69717869	0.51260899
Yy1	0.05403787	5.38509177	0.15587717	0.69717873	0.51260899
Arl1	-0.0959803	3.68099753	0.15579766	0.69725187	0.51260899
Mir17hg	0.58286737	-1.6099372	0.15577693	0.69727095	0.51260899
Ell2	0.05192913	6.02742888	0.15562389	0.69741179	0.5126357
Cpne3	-0.0618724	7.19702496	0.15562372	0.69741195	0.5126357
Kcng3	-0.1643236	3.24075379	0.15555248	0.69747754	0.51264544
4933412O06	-0.2408383	1.25724567	0.15540677	0.69761175	0.51270562
Ptplad1	0.04568404	6.68911998	0.15524766	0.69775839	0.51277492

Ccdc81	-0.313935	0.12320534	0.15502434	0.69796436	0.51284599
Hdac2	0.04608609	7.14680496	0.1549846	0.69800102	0.51284599
Epb4.1l4b	0.09372973	3.33579708	0.15497252	0.69801217	0.51284599
Trpa1	0.37988305	-0.9427308	0.15476283	0.69820575	0.51294974
Hk1	0.0818749	5.81242358	0.15461713	0.69834035	0.51295761
Hspa9	0.04577401	7.17277212	0.15458344	0.69837149	0.51295761
C77080	-0.0818066	5.81398474	0.1545748	0.69837947	0.51295761
Pla2g15	-0.1610911	2.06800813	0.15439464	0.69854603	0.51295761
Efemp2	0.14261123	2.75571536	0.15438974	0.69855057	0.51295761
Vapb	-0.043364	6.66791428	0.15435633	0.69858147	0.51295761
B230319C09	-0.38411	-1.0365313	0.1543518	0.69858566	0.51295761
Fzr1	-0.1190525	2.83729254	0.15429804	0.6986354	0.51295761
4921531C22I	-0.1237023	2.74290352	0.15421677	0.69871059	0.51297437
2310002F09I	0.54360547	-1.5177749	0.15401034	0.69890171	0.51307623
Zfp133-ps	-0.1941247	0.55557483	0.15394958	0.698958	0.51307909
Zfp791	0.11212156	2.83005514	0.15378935	0.69910648	0.51314963
Rab7l1	-0.1646302	4.22114935	0.1534871	0.6993868	0.5133088
Neil2	-0.3258381	0.05266386	0.15338969	0.69947723	0.5133088
Gm19395	0.44826013	-0.4158174	0.15329564	0.69956455	0.5133088
Mrps25	-0.0542426	4.61514581	0.15328345	0.69957587	0.5133088
Ski	-0.0435012	8.16273402	0.15321314	0.69964117	0.5133088
Pbxip1	-0.1076599	5.41108187	0.15316097	0.69968965	0.5133088
Wwc1	0.0684412	4.29363113	0.15316043	0.69969015	0.5133088
Txnrd1	0.06751087	4.89554205	0.15290773	0.69992506	0.51339937
Spata22	-0.4513295	-0.4635355	0.15283136	0.6999961	0.51339937
Tnfsf13b	-0.4875578	-1.2214902	0.15281956	0.70000708	0.51339937
Zfp335	0.09521137	3.92791599	0.15271407	0.70010523	0.51339937
Kcnj9	0.07153587	5.02242823	0.15268254	0.70013458	0.51339937
Xrcc6	-0.0963449	3.3703194	0.1526539	0.70016125	0.51339937
Abhd3	0.11519656	3.71128761	0.15257983	0.70023021	0.51339937
Gspt1	0.04257468	7.58456715	0.15257693	0.70023291	0.51339937
Slc12a7	-0.160728	3.82914986	0.15246021	0.70034163	0.51344066
Rnase6	0.49385877	-1.1019717	0.15219604	0.70058786	0.51358273
Il1b	-0.4464501	-1.7587399	0.15202854	0.70074412	0.51359434
Ccdc112	0.10077909	4.19145441	0.15184068	0.70091948	0.51359434
Mif4gd	0.10484453	3.18581163	0.15182781	0.70093151	0.51359434
Mfsd7b	0.10373828	2.61069559	0.15180243	0.70095521	0.51359434
Rwdd2a	0.09719039	2.99713776	0.15174117	0.70101243	0.51359434
Tmem161b	0.12281359	3.57042843	0.15166642	0.70108226	0.51359434
9330133O14	0.08788855	4.07579386	0.15165707	0.701091	0.51359434
Klhl24	0.04363977	6.75247327	0.15165583	0.70109217	0.51359434
Rbl1	0.17174468	1.59039662	0.15163377	0.70111278	0.51359434
Zc3h10	-0.1211119	3.0586151	0.15161746	0.70112802	0.51359434

Prokr2	-0.1832174	1.92358666	0.1512707	0.70145233	0.5137599
Klf7	0.05703958	6.5193186	0.15122888	0.70149147	0.5137599
Brf1	-0.0644165	4.23692636	0.15118542	0.70153215	0.5137599
E030025P04I	-0.5064814	-1.4199404	0.15115159	0.70156382	0.5137599
Trpc3	-0.0870098	3.63334456	0.15100998	0.70169645	0.51378892
Pola1	0.11192625	2.66497059	0.15099727	0.70170836	0.51378892
Dnaaf3	-0.3680582	-1.1739159	0.15066451	0.7020203	0.51395375
Map9	-0.0643411	7.90118263	0.15062904	0.70205358	0.51395375
Aldh3b2	-0.2274824	-0.2498659	0.15058927	0.70209089	0.51395375
Mex3a	-0.1075328	2.55651255	0.15048614	0.70218768	0.51398619
Clec1a	-0.1745862	1.93760054	0.15041452	0.70225491	0.513997
Srpk2	-0.0456624	7.79659098	0.15015332	0.7025003	0.51413818
Rims1	-0.0661438	6.48495215	0.15003834	0.70260838	0.51415771
Slc16a8	0.48034638	-1.1791877	0.15001327	0.70263196	0.51415771
Spp2	0.3386752	0.45542696	0.1498534	0.70278234	0.51419745
Ccnc	-0.058433	5.84716191	0.14984393	0.70279126	0.51419745
Slamf1	0.44916106	-1.076657	0.14970067	0.7029261	0.5142577
2610318N02	0.47351026	-2.0397248	0.14943401	0.7031773	0.51437032
Dpep2	-0.5909852	-1.7038124	0.14937492	0.703233	0.51437032
Stx17	-0.0573756	5.51081247	0.14937006	0.70323758	0.51437032
AI413582	0.08047714	3.26757408	0.14915023	0.70344491	0.51448356
Elavl1	-0.0446996	6.63422728	0.14900128	0.7035855	0.51454796
Zfyve27	0.06782362	4.26992673	0.14892336	0.70365906	0.51456334
Brca1	-0.1848437	1.08808677	0.14865654	0.70391117	0.51470224
Slc39a1	-0.0986025	5.19620193	0.14858689	0.70397702	0.51470224
Grcc10	-0.1402251	3.80079278	0.14855557	0.70400664	0.51470224
Tcf20	-0.0584672	8.10599558	0.1482661	0.70428056	0.51486407
Prima1	0.1305683	1.40377764	0.14788672	0.70464001	0.51508841
Ccdc157	0.08374682	3.04729122	0.14781564	0.70470742	0.51508997
Ctsl	-0.095695	6.74069323	0.14777357	0.70474732	0.51508997
4632428N05	-0.1522923	2.15941038	0.14763592	0.70487794	0.51510254
Eno4	0.15196902	1.34689556	0.14762771	0.70488572	0.51510254
Btbd16	0.33603943	-0.8711988	0.14758921	0.70492227	0.51510254
Tfec	0.59227397	-1.7338427	0.14728519	0.70521104	0.51525795
Inmt	-0.140633	3.49614324	0.14716242	0.70532776	0.51525795
Ppp2r5e	-0.053305	6.40327889	0.14712423	0.70536407	0.51525795
Zscan12	-0.0680277	4.26561548	0.14709588	0.70539103	0.51525795
2410131K14I	0.1114396	1.90497599	0.14705763	0.70542742	0.51525795
Msh3	0.07842085	4.12936455	0.14694384	0.70553568	0.51525795
Gm10190	-0.3152666	-0.2077484	0.14693725	0.70554195	0.51525795
Rtbdn	0.29206287	0.38635475	0.14689578	0.70558142	0.51525795
Dym	-0.0492414	5.63051636	0.14686747	0.70560837	0.51525795
BC037032	0.21345881	0.53871092	0.14662308	0.70584114	0.51533195

Arid4b	0.05937039	7.72302548	0.14661857	0.70584544	0.51533195
Tmem198	0.14585138	2.00437496	0.146556	0.70590507	0.51533195
Rbfox2	-0.0463879	7.5197972	0.14654018	0.70592015	0.51533195
Kdm2b	-0.0569922	4.37736772	0.1463539	0.70609777	0.5154232
Pak7	0.07247559	6.03121147	0.14617719	0.70626639	0.51546771
Ndufs5	0.35184262	-1.4944173	0.14610852	0.70633196	0.51546771
Gins3	-0.1881773	1.21726451	0.14605009	0.70638775	0.51546771
Rbm10	0.05997761	4.92040676	0.14602695	0.70640985	0.51546771
Plekhh2	-0.0593508	5.65818336	0.14596834	0.70646584	0.51546771
Cep128	-0.0918212	4.25132638	0.14594241	0.70649061	0.51546771
Mxra8	-0.0980768	5.68300485	0.14590421	0.70652712	0.51546771
D6Ertd474e	0.32471377	0.2199904	0.14581181	0.70661542	0.51549374
Sh3glb1	-0.04584	8.20721644	0.14575543	0.70666933	0.51549468
Pum1	0.04253181	6.90389033	0.14541592	0.70699417	0.51569324
Cecr5	0.20082679	0.6165469	0.14516651	0.70723309	0.51582909
Arl13b	0.06831055	4.2966268	0.14498391	0.70740815	0.51586731
Eif1ad	0.07827137	4.17200908	0.14497556	0.70741616	0.51586731
Dtx3l	-0.0559361	4.87767254	0.14494707	0.70744348	0.51586731
Zfp108	0.09371942	3.07925753	0.14485726	0.70752965	0.51589174
Abcb1a	0.09292787	5.71532328	0.14468706	0.70769302	0.51597245
Plekhj1	0.13585174	2.19298864	0.14428532	0.70807909	0.51621551
Arhgap8	-0.3425034	-0.3610325	0.14406523	0.70829085	0.51629946
Nr2e1	-0.1063621	3.69179722	0.1440374	0.70831764	0.51629946
Slc22a21	0.30776387	0.03731727	0.14393799	0.70841336	0.51629946
Zfp433	0.07069859	3.82422455	0.14389779	0.70845208	0.51629946
Heyl	-0.0731725	6.00162945	0.14378687	0.70855895	0.51629946
Phf11c	0.3609947	-1.5857981	0.14375605	0.70858865	0.51629946
Vegfc	0.11934146	2.74930106	0.14372547	0.70861812	0.51629946
Prokr1	0.41576717	-0.1820214	0.14369399	0.70864847	0.51629946
Asb13	-0.0626017	4.60256161	0.1436153	0.70872434	0.51629946
Tbx2	-0.3927345	-1.4237405	0.14357948	0.70875889	0.51629946
Fam26e	-0.1079974	4.62726743	0.14356376	0.70877404	0.51629946
Fap	0.16435544	1.52176877	0.14322948	0.70909668	0.51636277
Faxc	0.05876892	7.8893427	0.14320125	0.70912395	0.51636277
Ssx2ip	-0.043723	6.28551564	0.14312707	0.70919561	0.51636277
Efcab11	0.33630954	-0.5475158	0.14312664	0.70919603	0.51636277
Slc50a1	-0.0974892	3.05410095	0.14311809	0.70920429	0.51636277
Gpr182	-0.1348246	4.10662129	0.14293279	0.7093834	0.51636277
Prmt8	-0.0528862	7.02981199	0.14291546	0.70940016	0.51636277
Cd40	-0.3659225	-1.0778425	0.14290828	0.70940711	0.51636277
A830010M2(	0.07154877	8.6443168	0.14290089	0.70941425	0.51636277
Lzts2	-0.1067066	2.82548724	0.14286911	0.70944499	0.51636277
C030006K11I	-0.10678	2.96509353	0.14283336	0.70947957	0.51636277

Pacs1	0.04806477	6.74546738	0.14281893	0.70949353	0.51636277
Naa10	0.12234006	2.32544461	0.14270316	0.70960555	0.51640593
Ube2f	0.0660575	4.74670963	0.14259902	0.70970636	0.51644092
Ppp3ca	-0.0548099	9.63284311	0.1424641	0.70983704	0.51649021
Vwa1	-0.0798787	4.20174091	0.14234566	0.70995181	0.51649021
Por	-0.0636175	5.52808753	0.14226961	0.71002554	0.51649021
Rnpepl1	0.07582742	4.18973917	0.14220611	0.71008711	0.51649021
Sugp1	0.06685721	4.58008137	0.14217461	0.71011766	0.51649021
Gnl3l	0.04599648	8.04128811	0.14214037	0.71015087	0.51649021
Fads1	0.05551775	6.22578949	0.14208369	0.71020587	0.51649021
Slc25a40	-0.0929786	3.38294368	0.14202689	0.71026098	0.51649021
Baz2a	0.05819681	6.12712443	0.14197827	0.71030817	0.51649021
Ror2	0.21465906	0.84523662	0.1419519	0.71033377	0.51649021
Angel2	0.05305365	5.77778424	0.14187356	0.71040983	0.51649021
Slc37a4	0.11012317	1.97965061	0.14185453	0.71042832	0.51649021
Setd7	-0.0493537	8.66298225	0.14182236	0.71045956	0.51649021
Tatdn2	-0.0836745	4.03065392	0.14156706	0.71070767	0.51662921
Nxph4	0.22215173	0.63504163	0.1415171	0.71075625	0.51662921
Fosb	-0.141607	4.38460971	0.14125065	0.71101552	0.51677932
Cnga2	-0.6348805	-2.1500629	0.14185574	0.71112743	0.51682131
Bcl2	0.06682825	6.6580827	0.14108298	0.71117881	0.51682131
Srd5a1	-0.1349948	2.56381811	0.14089173	0.7113652	0.5169184
Mgat2	-0.0932919	3.28423238	0.14057134	0.71167778	0.51701871
Npc1l1	0.2862701	0.04696487	0.14056677	0.71168224	0.51701871
Rtp4	-0.1308633	3.93435672	0.14048395	0.71176311	0.51701871
Dtx3	0.06613096	5.05564026	0.14048107	0.71176592	0.51701871
AU019823	-0.0672363	5.02484557	0.14043979	0.71180624	0.51701871
Lrrn2	0.08188897	4.65525531	0.14037985	0.71186479	0.51701871
4933433G19	0.1803186	0.90088046	0.14026442	0.7119776	0.51701871
Tll1	-0.0910842	3.55557792	0.14022044	0.71202059	0.51701871
Crmp1	0.06054021	6.06433348	0.14018925	0.71205109	0.51701871
Ypel4	-0.1358198	2.45977121	0.14018406	0.71205617	0.51701871
Tfb1m	0.10805391	2.80023338	0.14010934	0.71212924	0.51701871
Kiz	0.07295756	4.925296	0.1401018	0.71213662	0.51701871
Zadh2	0.06547572	4.80412858	0.14003393	0.71220301	0.51702859
Mtf2	-0.0630328	5.55865667	0.13961974	0.7126086	0.5172259
Trim7	-0.2141811	0.29043078	0.13960316	0.71262485	0.5172259
1600020E01I	-0.2031222	1.1376363	0.13959463	0.71263321	0.5172259
Akap2	0.04624241	7.52593438	0.13950205	0.71272397	0.51725344
Glp1r	0.22904297	0.0642864	0.13937193	0.71285159	0.5173031
Il1rapl2	0.11225542	3.23313246	0.13931757	0.71290493	0.5173031
Tmem59	0.06443929	6.13848722	0.13927081	0.71295082	0.5173031
Ubtd1	-0.2197552	-0.1061916	0.13913634	0.71308283	0.51732683

Ctnnd2	0.06415492	9.36130243	0.13912991	0.71308915	0.51732683
Slc13a4	-0.0933694	8.44182862	0.13894996	0.71326593	0.51741675
Fam122a	-0.0681288	4.73155212	0.13887893	0.71333575	0.51742908
Slc16a5	0.28212416	-0.6751443	0.13878763	0.71342552	0.51743594
2610044O15	0.0681832	4.55994414	0.13876187	0.71345085	0.51743594
Inpp5d	0.10499944	2.51991615	0.1383374	0.7138687	0.51768799
Npm3	-0.2559313	-0.8634113	0.13830149	0.71390408	0.51768799
Mtfr1	-0.1036549	3.94927546	0.13821751	0.71398685	0.51769552
Kif3b	0.05634682	6.59547066	0.13812255	0.71408047	0.51769552
Ssc5d	0.11619395	2.55307744	0.13809758	0.7141051	0.51769552
Pnma3	-0.0986765	2.98502679	0.13807651	0.71412588	0.51769552
2810403A07I	0.06217571	6.56468175	0.13786973	0.71432992	0.51780512
R3hdm1	-0.0666418	9.58790115	0.137792	0.71440666	0.5178142
Tekt2	-0.3930062	-0.5607391	0.13774997	0.71444817	0.5178142
Cdk13	0.04141298	7.17706312	0.13757287	0.71462315	0.5179027
Sap130	0.04854349	6.64404836	0.13751631	0.71467906	0.5179049
Eaf1	-0.057322	5.06304878	0.13745759	0.71473711	0.51790635
Gal3st3	0.06354389	6.01131671	0.13740734	0.71478681	0.51790635
Bhmt2	0.58050727	-0.5181588	0.13715002	0.71504146	0.51803869
Hspa1a	0.07589371	3.80987944	0.13711591	0.71507523	0.51803869
Kcnab3	-0.0581047	5.49972719	0.13697596	0.71521386	0.51805814
Pcyt1b	-0.0660973	5.32802086	0.13696527	0.71522446	0.51805814
08-Sep	-0.0535717	5.95391556	0.13690852	0.71528069	0.51805814
Acap3	0.06141435	4.76959602	0.13687529	0.71531363	0.51805814
Enho	0.16451379	0.55148826	0.1367127	0.71547486	0.5181366
Pak4	-0.1265366	4.81414536	0.13658866	0.71559793	0.51818741
2810006K23I	0.06921532	4.99404715	0.1363728	0.71581226	0.5183043
Clcc1	0.06687855	4.36557929	0.13628084	0.71590362	0.51833214
Hspa5	-0.0479413	7.24254107	0.13585513	0.71632704	0.51854053
Shisa2	-0.124117	2.48056302	0.13583457	0.71634751	0.51854053
Pcdhgc4	0.21157905	0.9926195	0.13575492	0.71642682	0.51854053
Ube2m	-0.0835683	3.10543801	0.13573597	0.7164457	0.51854053
Stard6	0.11232797	2.41474258	0.13562071	0.71656052	0.51854053
Eml1	-0.0442748	5.94191498	0.13560595	0.71657523	0.51854053
Sox2ot	0.0663529	4.98179201	0.1355926	0.71658853	0.51854053
Lcn2	0.83352445	-1.2419215	0.13556609	0.71661495	0.51854053
Hist1h2bh	-0.3329463	-0.7848318	0.13550596	0.71667489	0.5185456
Isyna1	-0.0799508	6.19626698	0.13536362	0.71681685	0.51861
Rab11fip4os	0.32238776	-0.7582432	0.13521569	0.71696446	0.51865693
Usp32	0.05400087	7.80025164	0.1351925	0.71698761	0.51865693
Tbc1d32	0.08364901	4.73628723	0.13509049	0.71708946	0.51869231
Tmem191c	0.0849306	4.21084663	0.13480547	0.71737429	0.51886002
Kctd7	-0.1213401	2.79768907	0.13455624	0.71762363	0.51900204

Tmc8	0.43811927	-1.0856689	0.13437336	0.71780677	0.51909196
Gtf2e1	0.08491842	3.80428153	0.13432626	0.71785395	0.51909196
Boc	0.0715738	3.53156235	0.13421894	0.7179615	0.5191314
Otud7b	0.04331628	7.25464734	0.13378934	0.71839254	0.51940473
Leprel1	-0.1437571	2.42918779	0.1335855	0.71859733	0.51949629
AA465934	0.19147647	0.237487	0.13355773	0.71862525	0.51949629
Zfp110	-0.0718135	4.42279014	0.13348499	0.71869837	0.51951081
Cyp2r1	0.18541767	0.36074522	0.13320693	0.71897815	0.51962706
Slit1	0.10448721	2.95992299	0.1332032	0.7189819	0.51962706
Col23a1	-0.0946771	5.23591138	0.13316702	0.71901833	0.51962706
Gsto2	-0.3420513	-0.5104815	0.133067	0.71911906	0.51966151
Cbs	0.07678908	3.17066703	0.13288491	0.71930256	0.51975577
Rhot1	0.05018382	6.11619502	0.13273709	0.71945162	0.51982514
Phkg2	0.11749664	2.60356202	0.13261223	0.71957762	0.51987783
Tmed7	-0.0566025	5.69417952	0.13220555	0.71998845	0.52013627
Ccar1	-0.0478773	8.06170419	0.13187785	0.72032001	0.52023994
Zc3h12a	-0.288002	-0.5978045	0.13187681	0.72032106	0.52023994
Epha4	0.05151445	7.2171443	0.13185437	0.72034378	0.52023994
Mars2	-0.1534437	2.29555454	0.13185377	0.72034439	0.52023994
Ear2	-0.3298841	-0.4067613	0.1317826	0.72041647	0.52025364
Fcgrt	-0.1229421	3.55292364	0.13170504	0.72049504	0.52027202
Hhat	-0.3552019	-0.7515552	0.13161167	0.72058967	0.520302
Slc3a2	0.0505793	6.32439206	0.13143931	0.72076445	0.52035865
Zfand4	0.16207458	1.73462481	0.13142953	0.72077438	0.52035865
Smpdl3b	0.3330164	-0.2309417	0.13122031	0.72098672	0.52047359
Ogdhl	0.13738307	1.86515548	0.13114095	0.72106731	0.52048435
Abl1	-0.0682862	5.32165928	0.13103797	0.72117193	0.52048435
Numbl	-0.0623313	4.00453307	0.13103688	0.72117304	0.52048435
Arf3	0.05766371	10.0859814	0.13097786	0.72123302	0.52048435
Fkbp14	-0.0921106	4.22152797	0.1308755	0.72133709	0.52048435
Usp30	0.06818706	4.18557355	0.13083247	0.72138086	0.52048435
Parm1	0.06502405	4.32365914	0.13081975	0.72139379	0.52048435
05-Mar	-0.04005	6.50440038	0.13077814	0.72143612	0.52048435
Rgcc	0.0976834	2.86685262	0.13063782	0.72157891	0.52048435
Cst3	-0.0888403	7.90737531	0.13057095	0.721647	0.52048435
Tlk1	-0.0368482	7.60864649	0.13056244	0.72165566	0.52048435
Gpr62	0.19720916	0.89676421	0.13054144	0.72167705	0.52048435
Flrt3	0.05196949	5.81551562	0.13052637	0.72169239	0.52048435
Slc25a44	0.04481921	5.90625202	0.13011987	0.72210676	0.52066262
Mbd6	0.1094675	3.97290676	0.13011368	0.72211308	0.52066262
Igf1	-0.0681363	5.25462119	0.130082	0.7221454	0.52066262
Cpne6	-0.0866653	5.07405814	0.13007195	0.72215566	0.52066262
Frat1	0.14147344	1.57041128	0.13002326	0.72220534	0.52066262

Prpf40b	-0.0982046	3.81498405	0.12985746	0.72237464	0.52073856
Galntl6	-0.0636791	5.12805334	0.12981598	0.72241701	0.52073856
Rtn4r	-0.0672695	4.37400648	0.12962697	0.72261019	0.52080478
Ift80	-0.0515755	4.88958403	0.12962206	0.72261521	0.52080478
Fam222a	-0.1329488	2.20401045	0.12951567	0.72272401	0.52084487
Cd55	0.08689456	5.98307271	0.12932442	0.72291974	0.5209476
Mpv17l	-0.0548555	5.64672728	0.12903448	0.72321677	0.52112331
Atcay	-0.0631634	5.5480024	0.12897938	0.72327326	0.52112568
Pip5k1c	-0.0508858	6.37559619	0.12884071	0.7234155	0.52118982
Smtn	0.13526636	2.3287142	0.12873142	0.72352765	0.52123229
1700015F17I	-0.4633958	-0.8664059	0.12857594	0.7236873	0.52130896
Dnajc18	0.05175315	6.93329218	0.12846082	0.72380558	0.52132014
Rfc3	-0.1254784	1.77971401	0.12845201	0.72381464	0.52132014
Stau1	0.04356929	6.3092757	0.12838507	0.72388345	0.52132014
Sfrp5	-0.5959573	-2.1405377	0.12835369	0.72391571	0.52132014
Stx11	0.15350445	2.10284213	0.12822275	0.72405038	0.52137879
Ppp1r16a	0.10022465	2.41050644	0.12797404	0.72430639	0.52152481
A630072M18	0.08305455	3.50751564	0.12780472	0.72448085	0.52159691
Copg1	0.04907956	7.25418824	0.12777349	0.72451304	0.52159691
Proser2	-0.1415336	2.69453176	0.12761296	0.72467858	0.52167775
Ska1	0.59358282	-1.8990028	0.12728279	0.72501942	0.52186667
Syt15	-0.1877544	1.38226934	0.12719287	0.72511234	0.52186667
Prap1	-0.5448398	-1.6095757	0.12715865	0.72514771	0.52186667
9230112J17F	0.49062375	-1.563652	0.12708658	0.72522221	0.52186667
Kcnk9	0.12870908	2.87097366	0.12707448	0.72523473	0.52186667
Atf7ip	0.03772101	6.92989395	0.12704938	0.72526068	0.52186667
4930451G09	0.13876494	1.59307176	0.12676678	0.72555312	0.52196775
Pcx	0.11518348	2.01039584	0.12676559	0.72555435	0.52196775
C1qtnf4	-0.1181034	2.03375182	0.12675916	0.72556101	0.52196775
Xist	-0.2781764	0.81075274	0.12668283	0.72564006	0.52198628
Tspan18	-0.1321858	2.75603736	0.1264493	0.7258821	0.52211637
A830019L24I	-0.3497882	-0.6944655	0.12640551	0.72592751	0.52211637
St8sia4	0.09516012	3.27236278	0.12618602	0.72615526	0.52224183
Pi15	0.26839917	0.75635518	0.12607606	0.72626945	0.52228561
Katnb1	-0.1090622	2.94699057	0.12593353	0.72641754	0.52235375
0610040J01F	-0.2433179	0.12504341	0.12581949	0.7265361	0.52240066
Ano6	0.07274014	6.43153776	0.12573521	0.72662375	0.52242534
B4galt6	0.03836934	7.17140345	0.12558172	0.72678348	0.52250182
Bbs4	-0.0443157	5.56476616	0.1253973	0.72697554	0.52260154
Cdh23	-0.3297582	-1.1264785	0.124992	0.72739819	0.522867
3110007F17I	-0.1815585	1.17181934	0.12490412	0.72748993	0.52289457
Mrpl45	-0.0877649	3.64461514	0.12460308	0.72780449	0.52308229
Hrh2	0.12655931	2.16821105	0.12448949	0.72792329	0.52312928

Per1	0.06263797	5.52922724	0.12411616	0.72831417	0.5233718
Hey2	-0.1040947	2.26917077	0.12401641	0.72841873	0.52338784
Tprkb	0.04372928	5.39818019	0.12397043	0.72846694	0.52338784
Zfp85	-0.0897577	2.44957893	0.12394196	0.7284968	0.52338784
Adam1b	-0.2276697	0.2342261	0.12380567	0.72863978	0.52345218
2610008E11I	0.06151821	5.29799621	0.12371975	0.72872995	0.52347857
A530072M11	-0.2660353	0.62588716	0.12358705	0.72886931	0.52351488
Ctif	-0.0501168	7.02124935	0.12356983	0.7288874	0.52351488
Arl15	-0.0459847	6.06996797	0.12347357	0.72898855	0.52354309
1700003D09I	0.3689452	-0.5231101	0.12343073	0.72903357	0.52354309
Rabgef1	-0.0729765	4.26776228	0.12336259	0.72910521	0.52355615
Rarg	-0.095345	2.85799831	0.12323363	0.72924086	0.52361518
Rassf8	-0.1146596	3.3170677	0.12310025	0.72938124	0.52367758
Dnase1l3	0.44701667	-0.8624302	0.12304611	0.72943824	0.52368012
Clasp1	0.05138052	7.49695129	0.1228874	0.72960543	0.52376176
Insc	0.2003654	0.14504822	0.1228321	0.72966372	0.52376522
Vtn	-0.0993032	6.16509271	0.12277937	0.72971931	0.52376674
Ebf2	0.10873098	3.16521469	0.12272714	0.72977438	0.52376789
Gm11186	0.43055855	-1.441342	0.12265461	0.72985088	0.52378442
Rfc5	0.10576199	2.67648638	0.12257747	0.72993227	0.52379607
Tbr1	0.06505471	6.77187326	0.12250831	0.73000527	0.52379607
Ccdc150	0.55227682	-1.6023388	0.12248723	0.73002753	0.52379607
Bag6	0.05943949	5.6876848	0.12231039	0.73021431	0.5238917
C130083M11	-0.1027978	2.78221445	0.12219322	0.73033814	0.52394217
BC028528	-0.137637	1.6431558	0.1220548	0.73048453	0.52400882
Ptpn4	0.05878638	7.20235562	0.12198621	0.73055709	0.52402249
Atp1a1	0.05588967	7.63614666	0.12190848	0.73063937	0.52403455
Ifrd2	-0.1627118	1.23966364	0.12174586	0.73081157	0.52403455
Trappc12	-0.0492905	5.73215981	0.12171923	0.73083979	0.52403455
Mpst	0.20388004	0.44164435	0.12171764	0.73084148	0.52403455
Lztfl1	0.05159929	6.37156356	0.12168571	0.73087531	0.52403455
Card6	-0.0676362	4.85767612	0.12166723	0.73089489	0.52403455
Lmo1	0.28414636	-0.3943841	0.12134505	0.73123658	0.52424116
Thap6	-0.0764799	3.86927998	0.12123849	0.73134971	0.52428389
Ddx10	0.04230239	5.55496738	0.12114331	0.7314508	0.52431799
Gm166	0.16055031	0.64345671	0.12102779	0.73157356	0.52436761
Deaf1	-0.0740623	4.04081843	0.12095349	0.73165254	0.52438585
Adamtsl5	0.08349336	2.76696844	0.12061993	0.73200749	0.52460186
Pid1	0.05252812	6.78804196	0.12040303	0.73223859	0.52472909
D330050G23	0.15329645	1.95972551	0.1202035	0.73245139	0.52482125
Vps51	0.08939518	3.72065299	0.12018199	0.73247434	0.52482125
Mipep	0.07824996	4.032367	0.11994273	0.73272981	0.52496589
Aifm1	0.07598229	4.10845366	0.11976711	0.7329175	0.52506196

Tbx21	-0.4361289	-0.7649604	0.11954592	0.73315413	0.52517345
Ido1	0.1578111	1.31686761	0.1194806	0.73322405	0.52517345
Mb21d1	0.16605521	0.81745462	0.11943732	0.73327039	0.52517345
Vac14	0.06607613	4.71468339	0.11942125	0.73328759	0.52517345
Il21r	0.36846307	-0.8992308	0.11930797	0.73340895	0.5251827
Cpne4	0.05698854	6.36856692	0.11928813	0.7334302	0.5251827
Pcdhgb1	0.09979714	4.09657467	0.11925907	0.73346135	0.5251827
Ccdc175	-0.5116999	-1.7965656	0.11916949	0.73355738	0.52521307
Fam101a	0.15294803	2.83924127	0.11904787	0.73368782	0.52524209
Zswim7	0.09974794	2.12660425	0.1190317	0.73370516	0.52524209
4933434E20I	0.05190545	4.51298392	0.11893631	0.73380753	0.52527698
Acer3	0.09310227	3.65386616	0.11871426	0.734046	0.52536703
Mphosph6	-0.0849923	4.50521503	0.1186996	0.73406176	0.52536703
Wdpcp	-0.0610039	3.98123373	0.11866939	0.73409422	0.52536703
Sfrp1	0.08659583	6.64119233	0.11849124	0.73428577	0.52542788
Smg7	0.03642084	8.02274237	0.11849053	0.73428654	0.52542788
Zfp658	0.09990775	2.26400875	0.1183687	0.73441762	0.52545126
Gypc	-0.0997002	5.39498822	0.11836046	0.73442649	0.52545126
Abr	0.05663613	7.69705062	0.11820859	0.73459001	0.52550794
H2-Aa	-0.1502724	4.46773061	0.11810208	0.73470476	0.52550794
Hif3a	-0.1019261	2.85379318	0.11805249	0.73475821	0.52550794
2810428I15R	-0.1191813	2.82113477	0.11804153	0.73477002	0.52550794
Fbxo30	-0.0712735	5.38115965	0.11801916	0.73479414	0.52550794
Metap1d	-0.125761	2.28054549	0.11795815	0.73485991	0.52550794
1700019L03R	-0.2485506	-0.6529311	0.11793837	0.73488125	0.52550794
Mtf1	0.04233803	5.99906572	0.11757942	0.7352687	0.5257117
Aldh18a1	0.07779774	3.57618485	0.11757495	0.73527353	0.5257117
Bhlhe41	0.05694862	7.69616051	0.11747512	0.73538141	0.52575045
Rbl2	-0.0392871	7.24597562	0.11739573	0.73546724	0.5257651
Prrt3	0.14668542	1.74678824	0.11734752	0.73551937	0.5257651
Calb1	-0.0506228	6.05115069	0.11730311	0.73556741	0.5257651
Nprl2	-0.0954641	2.467394	0.11717224	0.73570902	0.5257651
Klhl29	0.08866595	5.07510909	0.11716747	0.73571419	0.5257651
Dmwd	-0.0608849	4.86534183	0.11715845	0.73572395	0.5257651
Crat	0.04884317	5.36198451	0.11697213	0.73592574	0.52587094
Artn	-0.4985908	-2.0795552	0.11688274	0.7360226	0.52590179
Rell1	0.08514399	5.182429	0.11667539	0.73624747	0.52599334
Aifm2	-0.121212	2.59545827	0.11666558	0.73625812	0.52599334
Esm1	0.12876216	2.24517017	0.11656871	0.73636325	0.52603008
Mbnl1	-0.0517345	9.06154523	0.11642168	0.73652293	0.52610577
Klhl6	0.22932384	0.02189517	0.11621788	0.73674443	0.52619949
Apln	-0.1193959	2.58939571	0.11620212	0.73676157	0.52619949
3632451O06	-0.0811716	4.23193063	0.11603597	0.73694233	0.52629022

Cdc25c	-0.3594162	-1.6134283	0.11582012	0.73717737	0.5264197
Gpr174	-0.4240714	-1.3478259	0.115738	0.73726687	0.52644523
Ndufa10	-0.0491439	6.26299292	0.11559168	0.7374264	0.52652076
Hdlbp	-0.0346037	8.49839793	0.11550471	0.73752129	0.52655013
Zfp931	-0.0825211	2.57628972	0.11534291	0.7376979	0.52663783
Slc25a33	-0.0880471	3.31598457	0.11524509	0.73780475	0.52667573
Cd81	0.08532279	7.79192516	0.11504479	0.7380237	0.52679363
Mospd3	-0.0979168	5.64086596	0.11493483	0.73814398	0.52683545
Gfy	0.63835678	-1.9307506	0.11489292	0.73818984	0.52683545
Lcp1	-0.071964	4.76173276	0.11474119	0.73835596	0.52691561
Ptdss1	-0.0518248	4.8909108	0.11469023	0.73841178	0.5269169
BC061194	-0.2031019	0.34113822	0.11462096	0.73848767	0.5269169
Mid1	-0.0784952	4.12698169	0.11459225	0.73851914	0.5269169
Rhobtb3	-0.0649246	4.45855426	0.1144438	0.73868191	0.52699464
Gm5617	-0.1830508	1.57345453	0.11414508	0.73900979	0.52714346
Ocel1	-0.1347187	1.7625037	0.11413293	0.73902314	0.52714346
Osbpl9	-0.0502547	6.74841289	0.11409799	0.73906152	0.52714346
Ulk2	-0.0399892	7.46675213	0.11405772	0.73910578	0.52714346
Zkscan5	0.10588168	3.89448709	0.11394363	0.73923118	0.52719452
A930015D03	0.15020856	1.37320484	0.11371079	0.73948734	0.52731555
2310061I04R	-0.0647303	4.20453282	0.11364787	0.73955661	0.52731555
Kctd8	-0.1324105	2.1487354	0.11364262	0.73956239	0.52731555
Fbxl18	-0.1310436	1.31069494	0.11344149	0.73978398	0.52739674
Spaca5	0.40017487	-1.6395404	0.11343212	0.73979431	0.52739674
Gpr132	0.3467289	-1.4548746	0.11337886	0.73985302	0.52739674
Vps37b	0.07801355	3.12214722	0.11334385	0.73989163	0.52739674
Ccnd2	-0.045558	7.42819143	0.11321662	0.74003197	0.52742023
Zak	0.04512809	6.48262938	0.11321636	0.74003227	0.52742023
Timp3	-0.0861055	9.35869266	0.11293006	0.7403484	0.52752197
Ppp1cc	-0.0384333	7.62011982	0.11292829	0.74035036	0.52752197
Kcns3	0.11284184	1.97479653	0.11291251	0.7403678	0.52752197
Proser1	-0.0561087	5.5896054	0.11289203	0.74039043	0.52752197
Akap10	-0.0523302	4.87047953	0.11246742	0.74086022	0.52779521
Fbxo34	-0.0574383	5.26287799	0.11241368	0.74091975	0.52779521
Oacyl	0.25351369	0.29856542	0.1123994	0.74093558	0.52779521
Mansc4	-0.1523271	1.99202878	0.11223775	0.74111474	0.52788444
Stim2	0.05974081	5.92475523	0.11216552	0.74119485	0.52790311
Cry2	-0.0486798	6.33826493	0.11203117	0.74134392	0.52794237
Cacnb1	0.05002998	5.12663718	0.11198128	0.7413993	0.52794237
Ablim3	-0.0396565	5.35289374	0.11196274	0.74141989	0.52794237
Acpp	-0.1409854	4.28697878	0.11192162	0.74146555	0.52794237
Gm5860	0.09716595	2.07446429	0.11171973	0.74168988	0.52803957
Opn3	-0.0843118	3.12974357	0.11170173	0.74170989	0.52803957

Stard3	-0.1004304	2.37085646	0.11161436	0.74180705	0.52807036
Rnmtl1	-0.1206156	2.06520423	0.11141995	0.7420234	0.52818599
Tomm40	0.07661904	3.08970157	0.11134509	0.74210676	0.52820694
Sec24c	0.04628722	6.32172526	0.11127565	0.74218411	0.52822362
Letmd1	0.05610685	4.8416147	0.11110752	0.74237152	0.52829524
Mrpl40	-0.0818918	3.57713701	0.11108861	0.7423926	0.52829524
4930404N11	-0.1934808	0.04994917	0.11089956	0.74260354	0.52838674
Thg1l	-0.1074122	2.94982883	0.11087669	0.74262907	0.52838674
Hhatl	-0.1921745	0.58083269	0.11074998	0.74277058	0.52844904
P2rx1	0.5416044	-2.3316039	0.11061818	0.74291786	0.52851543
Ampd3	0.05111334	5.59985315	0.11047939	0.74307306	0.5285682
Gm13315	-0.4581688	-1.3209303	0.11044079	0.74311625	0.5285682
Spata7	0.05905392	4.05031783	0.11040713	0.74315391	0.5285682
Cox7a1	-0.1192421	2.11222014	0.11035854	0.74320829	0.5285685
Serpina3n	-0.1308606	2.68992215	0.11029835	0.74327567	0.52857804
Gng10	-0.0806079	4.81533876	0.11023824	0.74334298	0.52858753
Ethe1	-0.1126924	2.6815575	0.11006866	0.74353301	0.52868427
Ctf1	-0.1267988	2.64594289	0.10985781	0.74376949	0.52879245
Rtcb	0.05884024	4.1718067	0.10983676	0.74379311	0.52879245
Sstr4	0.11707278	4.50972522	0.1096975	0.74394945	0.52886521
Surf4	0.06459761	4.57231606	0.10964662	0.74400661	0.52886746
Col1a1	-0.0833068	6.95686148	0.10956947	0.74409329	0.5288907
Tbx3	-0.0743065	3.70014722	0.10936318	0.74432524	0.52901718
Ptgds	-0.1244274	12.2504338	0.1091984	0.7445107	0.5291106
Polb	-0.0702493	4.32665146	0.10909504	0.74462711	0.52915494
Rabl3	-0.0598991	4.40385949	0.10894816	0.74479263	0.52923417
Stag2	0.04129663	6.69822651	0.10876519	0.74499901	0.52931593
E230016M11	-0.2367962	0.20227686	0.10875034	0.74501577	0.52931593
Itgax	-0.2881417	-0.4385055	0.10865513	0.74512324	0.52935269
Hbp1	0.04231132	6.72167701	0.10859433	0.74519189	0.52935269
BC018242	-0.0573568	4.75332154	0.10856092	0.74522963	0.52935269
4932416H05	0.08512068	2.63823692	0.10844439	0.74536131	0.52940783
Senp5	-0.0402497	5.58786185	0.10835551	0.74546179	0.52943318
Rnasel	-0.0570956	4.6797789	0.10831721	0.7455051	0.52943318
Zdhhc15	0.08913014	3.45046656	0.10824762	0.74558383	0.52945071
Kank4os	0.38232035	-1.3756295	0.1081542	0.74568955	0.52948739
Ttc22	-0.464019	-1.6064773	0.10797187	0.74589605	0.52956485
Rnf17	0.19383628	0.73325128	0.10789012	0.74598868	0.52956485
Snrpa	-0.0719042	4.66726131	0.10781016	0.74607934	0.52956485
Ccdc120	0.10374032	2.46031896	0.1078029	0.74608757	0.52956485
Map3k5	0.07751476	5.08153796	0.10779677	0.74609453	0.52956485
Lenep	-0.160787	1.41408675	0.10777166	0.746123	0.52956485
Dgcr14	-0.0678815	3.75036642	0.10770632	0.74619713	0.52957908

Ccr7	-0.4680766	-1.9770979	0.10734819	0.74660384	0.52979176
Ankdd1b	0.06728146	3.20222513	0.10730839	0.74664908	0.52979176
Casp2	0.06023517	3.86831317	0.10729962	0.74665905	0.52979176
Rad9a	-0.0709402	3.49725422	0.10705594	0.74693629	0.52993735
Pqlc2	0.16872257	0.36876817	0.10702418	0.74697245	0.52993735
AI118078	-0.2353932	-0.438439	0.1069737	0.74702994	0.52993976
Magt1	0.06588109	5.22966798	0.10690659	0.74710638	0.52994002
Cyp2f2	-0.155279	4.94083326	0.10686733	0.74715111	0.52994002
Kcnip1	-0.0794948	4.52694561	0.10679181	0.7472372	0.52994002
Slc6a12	0.11119767	3.69420656	0.1067644	0.74726844	0.52994002
Epha1	0.52322087	-1.3427968	0.106736	0.74730082	0.52994002
Hsf5	0.37685066	0.24239207	0.10663696	0.74741379	0.52998176
Zfp949	0.05566162	4.59449231	0.10653389	0.74753142	0.53002681
Gm6498	0.25663157	-1.0240939	0.10646057	0.74761513	0.53004779
3110079O15	0.33365044	-1.8125332	0.10627739	0.74782442	0.5301578
Eps15	0.04150391	8.62271066	0.10588305	0.74827562	0.53043929
Ssh2	0.04545465	6.53010038	0.1056254	0.74857094	0.53056167
Nid1	-0.0599933	7.06377941	0.10558396	0.74861848	0.53056167
5830418P13I	-0.2178404	0.17613779	0.10556929	0.7486353	0.53056167
Ciart	-0.0893453	3.12972536	0.10554348	0.74866493	0.53056167
Mtmr7	-0.0742075	5.04746558	0.10543687	0.74878729	0.53059755
Kctd5	-0.0876773	2.84732475	0.10540499	0.74882389	0.53059755
2810021J22F	0.0624447	4.0634388	0.10524098	0.7490123	0.53068464
Cyp19a1	0.50793331	-1.5822901	0.10572322	0.74905516	0.53068464
Slc16a7	0.0637064	4.68758201	0.10510076	0.74917351	0.53072628
Cd151	-0.1092801	5.12328317	0.10505107	0.74923068	0.53072628
Dhdh	-0.0549336	6.4983225	0.10501127	0.74927647	0.53072628
Enkd1	-0.1524674	0.55606555	0.10493233	0.74936733	0.53073078
Kif6	-0.1883117	0.20610313	0.10486224	0.74944802	0.53073078
Zfp296	0.23957015	-0.1268593	0.10480617	0.7495126	0.53073078
Ate1	0.04473323	6.22399955	0.10480409	0.74951499	0.53073078
Mirlet7bhg	0.26958451	0.81513679	0.10477046	0.74955374	0.53073078
Mkrn2	-0.0548819	5.14989952	0.10468715	0.74964975	0.5307604
Itih5	0.05856832	6.2904773	0.10453881	0.74982081	0.53082624
Amdhd2	-0.1172578	1.22239547	0.10444557	0.74992838	0.53082624
Tmem87b	0.05911354	4.94297907	0.10443539	0.74994013	0.53082624
Sdsl	-0.3600688	-0.6387176	0.1044186	0.74995952	0.53082624
Aven	0.12036361	1.31727111	0.10434353	0.75004619	0.53082882
Zfp768	-0.1063973	2.42165946	0.10426705	0.75013452	0.53082882
Fpgt	-0.0523458	4.93554178	0.1042338	0.75017294	0.53082882
Atl3	0.04424812	7.4225482	0.10422775	0.75017992	0.53082882
Nudt1	-0.2381318	0.61450683	0.10411	0.75031602	0.53084541
A430105I19F	-0.0886837	3.33664494	0.10386935	0.75059444	0.53084541

Fmod	-0.091512	7.83887884	0.10386862	0.75059529	0.53084541
Smarca4	0.05975979	7.01594341	0.10382416	0.75064676	0.53084541
Notum	-0.1037227	1.64644829	0.10380645	0.75066727	0.53084541
Poldip2	0.05342835	5.13046729	0.10380127	0.75067328	0.53084541
Gm10635	0.19913572	0.85486743	0.1037926	0.75068331	0.53084541
Fzd8	-0.141582	1.75858584	0.10378943	0.75068698	0.53084541
Arrb2	0.06862741	4.08125527	0.10378586	0.75069112	0.53084541
Sergef	-0.1200937	1.78404877	0.10365422	0.75084364	0.53089943
Zbtb7a	0.0612871	6.03367033	0.10362638	0.75087591	0.53089943
Aars	-0.0526423	5.61114746	0.1034583	0.75107083	0.53099328
Prpf31	0.05446687	4.10967263	0.10338585	0.7511549	0.53099328
Mvb12a	-0.0966172	1.88568296	0.10337176	0.75117126	0.53099328
A930009A15	0.38778637	-1.9983995	0.10293733	0.75167612	0.53127447
Tecr	0.06220569	7.85813035	0.10291428	0.75170294	0.53127447
Gemin2	-0.0988793	2.12116911	0.10286498	0.75176032	0.53127447
Zkscan7	-0.1369826	1.50064791	0.10274506	0.75189994	0.53127447
1700034J05F	0.5768406	-1.6206311	0.10263145	0.75203229	0.53127447
Slc25a34	-0.17336	0.06385238	0.10257054	0.75210329	0.53127447
Sphk2	0.06154397	4.07618968	0.1025349	0.75214484	0.53127447
Cat	-0.0529621	8.12823621	0.10250708	0.75217728	0.53127447
Prkrir	-0.0410812	5.34315048	0.10248086	0.75220787	0.53127447
Tle2	-0.1129127	1.5759767	0.10245931	0.752233	0.53127447
Cdc20	0.25919388	-0.3598917	0.10245864	0.75223379	0.53127447
Arhgap1	-0.0524599	5.65617668	0.1023878	0.75231643	0.53127447
Tubb2a	0.0593749	7.72927064	0.1023612	0.75234748	0.53127447
Mpp4	-0.2535475	-1.0414371	0.10235781	0.75235143	0.53127447
Clec2i	0.36282684	-1.1682639	0.10230775	0.75240986	0.53127447
Mtif2	0.04717746	5.36245845	0.10226164	0.7524637	0.53127447
Ccdc152	-0.1020943	1.90916393	0.10223819	0.75249109	0.53127447
Crybg3	-0.0664925	4.24807861	0.10198943	0.75278181	0.53144142
Atg101	-0.0981158	2.8013093	0.1017895	0.75301574	0.53156825
D830031N03	-0.0497251	5.26126611	0.10163452	0.75319726	0.53165808
Phactr1	-0.0434927	7.94788212	0.10126766	0.75362753	0.53192346
Lrig3	0.14423433	1.08464464	0.10077481	0.75420695	0.53229407
Pex5	-0.0612503	3.90300021	0.10071074	0.75428239	0.53230896
Tfpt	-0.0955249	2.7330613	0.10064536	0.75435939	0.5323098
Entpd3	0.10769031	1.63353035	0.10056697	0.75445176	0.5323098
Itih2	-0.0716338	5.04283293	0.10055537	0.75446543	0.5323098
Tril	0.06330287	3.67077525	0.10052524	0.75450095	0.5323098
Dok4	-0.1226388	2.3734439	0.10044334	0.75459751	0.53233958
Qrfpr	0.23316403	0.13814898	0.10018551	0.75490181	0.53240262
Nhej1	-0.2221708	1.11955873	0.10017756	0.7549112	0.53240262
Wdr62	0.1679499	1.01093731	0.1001457	0.75494884	0.53240262

E530001F21I	-0.3482338	0.14723667	0.1001333	0.75496348	0.53240262
Map3k9	0.06994762	6.22637479	0.10009166	0.75501268	0.53240262
Mlip	0.06231882	4.86704619	0.1000914	0.75501299	0.53240262
Cst7	0.47514307	-1.9428477	0.10001328	0.75510532	0.5324294
Brinp2	0.05336527	4.4590988	0.09974022	0.75542837	0.53260291
Cnst	-0.0485233	6.16085831	0.09971204	0.75546174	0.53260291
Sirt5	-0.0798878	2.29635344	0.09966747	0.75551452	0.53260291
Trip13	0.23685514	0.72257965	0.09959173	0.75560424	0.53262783
Dolk	0.09177521	2.47522989	0.09945502	0.75576629	0.53270373
Asxl2	0.04465991	6.49427482	0.09934712	0.75589428	0.5327556
Phf3	0.03751733	8.18853207	0.09921906	0.75604628	0.53280897
Apoa2	0.30903955	-1.2367279	0.09916931	0.75610536	0.53280897
Sh2d5	0.06078369	4.63060511	0.09913289	0.75614863	0.53280897
Tsr1	0.05375767	6.03519977	0.09905747	0.75623824	0.53280897
Hist1h2be	-0.0920975	2.78253402	0.09905433	0.75624197	0.53280897
Wee1	0.0469216	5.51696953	0.0990021	0.75630406	0.53281439
Apobr	-0.2257447	0.34536625	0.09894191	0.75637562	0.53281812
Gemin4	0.08542539	2.31786914	0.0988616	0.75647115	0.53281812
Zfp189	-0.0683989	3.26918649	0.09878385	0.75656367	0.53281812
Oraov1	-0.0742373	3.70779662	0.09876878	0.75658162	0.53281812
Bgn	-0.0810996	8.30310633	0.0987631	0.75658837	0.53281812
4930590J08F	-0.2952234	-0.7086782	0.09872333	0.75663572	0.53281812
Kcnh8	0.37871502	-1.0813013	0.09866344	0.75670704	0.53283004
Cd22	-0.4369248	-2.1449166	0.09838937	0.75703373	0.53292331
Csgalnact2	-0.0657129	3.38256503	0.09838356	0.75704066	0.53292331
Acbd5	0.03167615	7.15738997	0.09837877	0.75704637	0.53292331
Gm5512	0.09971698	1.38961734	0.09835777	0.75707142	0.53292331
Pik3ap1	0.10232001	3.13275307	0.09832416	0.75711154	0.53292331
Memo1	-0.0533118	4.53854241	0.09817867	0.75728522	0.53300726
Safb2	-0.0501392	5.15612092	0.09809192	0.75738886	0.5330419
Mamld1	0.04755426	6.24014348	0.09801918	0.75747579	0.53306479
Clptm1	0.04535927	6.04131309	0.0978631	0.75766244	0.53315784
Immt	0.0434585	6.62963615	0.09779815	0.75774016	0.53317423
Ginm1	-0.0780303	5.23454548	0.09773275	0.75781846	0.53319102
Pnpo	-0.0699893	3.78994723	0.09755554	0.75803073	0.53330206
Serpind1	-0.0953904	4.92559936	0.0973851	0.75823511	0.53340754
Fdps	0.0741895	3.83319565	0.09728549	0.75835464	0.53343089
Capn3	0.12576494	0.85097629	0.09722624	0.75842577	0.53343089
Klk6	-0.3113772	-1.3802806	0.09722132	0.75843168	0.53343089
Frrs1	-0.1722599	1.18737088	0.09691953	0.75879437	0.53359164
Gm13749	-0.1940307	0.63489644	0.09691557	0.75879913	0.53359164
4930538K18I	-0.2050078	0.49385641	0.09685825	0.75886809	0.53359164
4930486L24F	0.53374561	-2.2845267	0.09680984	0.75892634	0.53359164

Vav1	0.17064376	0.25682111	0.09680465	0.7589326	0.53359164
Mn1	-0.0672546	4.58576522	0.09671636	0.75903889	0.5336241
Dusp1	-0.0845041	6.28480359	0.09667581	0.75908773	0.5336241
9930111J21F	-0.1042008	3.80923068	0.09646167	0.75934581	0.53376722
Slc26a2	0.0905316	6.09247466	0.0963743	0.75945121	0.533803
Zfp639	-0.0722919	4.46191698	0.0962891	0.75955403	0.53383677
Brd9	0.03737735	5.50512023	0.0962377	0.75961608	0.53383677
Ednra	-0.0588712	5.01863621	0.09616229	0.75970716	0.53383677
2610507I01R	0.04591823	4.73203653	0.09615395	0.75971724	0.53383677
Glipr1	-0.3395552	-0.9652955	0.09599315	0.75991159	0.5338812
A830080D01	-0.0563251	4.31144439	0.09598761	0.75991829	0.5338812
Rab34	-0.0809741	4.25800237	0.09594856	0.75996552	0.5338812
Psmd14	-0.0369634	6.24716733	0.09592092	0.75999895	0.5338812
Ncapg2	-0.0963819	2.56099685	0.09587626	0.76005299	0.5338812
Tnpo1	0.03820535	6.64202774	0.09575081	0.76020483	0.53388205
Ccdc68	-0.1700328	0.39708641	0.09573851	0.76021972	0.53388205
Ttc30a1	0.15194378	1.65587813	0.09570857	0.76025598	0.53388205
Psapl1	-0.5291744	-1.7375642	0.09565703	0.76031842	0.53388205
Bcl2l15	-0.3432843	-0.0955266	0.09565018	0.76032672	0.53388205
Cxcl16	-0.1096829	3.95304305	0.09557644	0.76041608	0.53390652
4930483K19I	-0.2335794	-0.4326204	0.09540025	0.76062975	0.53401827
Oas1g	-0.2448044	-0.3560232	0.0951848	0.76089132	0.53404504
Otogl	-0.307285	-1.2053507	0.09516759	0.76091223	0.53404504
Aspa	-0.0502879	6.03393762	0.0951529	0.76093008	0.53404504
Zbtb18	-0.0419915	7.35838544	0.09507928	0.76101955	0.53404504
Ccdc116	0.18624524	0.07424863	0.0950704	0.76103035	0.53404504
Gxylt2	-0.0785818	3.90150015	0.09504764	0.76105802	0.53404504
Cnp	-0.0500915	6.61886777	0.09502345	0.76108743	0.53404504
Jun	-0.0381781	6.02552746	0.09500979	0.76110404	0.53404504
Dscr3	-0.0588374	4.05739242	0.09492814	0.76120336	0.53407647
Fzd7	-0.0920158	5.93664349	0.09483136	0.76132113	0.53407848
Patz1	-0.0512451	4.85691423	0.09473584	0.76143745	0.53407848
Col6a5	0.43881069	-1.8134956	0.09472893	0.76144586	0.53407848
Lats1	0.03751838	7.15099515	0.09469826	0.76148323	0.53407848
Tmie	0.11200905	1.66140643	0.09469263	0.76149008	0.53407848
Anapc10	0.05954391	4.5033661	0.09465711	0.76153336	0.53407848
Gstt2	0.14696483	2.13162719	0.09452418	0.76169541	0.53413457
BC064078	-0.1893724	0.67834504	0.09442337	0.76181839	0.53413457
Gins2	0.11870501	2.15849279	0.0944181	0.76182481	0.53413457
Mtmr11	0.06600015	3.419479	0.0943868	0.76186302	0.53413457
Pvrl1	-0.0512379	4.31616693	0.09436797	0.761886	0.53413457
Kdm1a	-0.0490193	5.91142414	0.09418608	0.76210813	0.53425207
4930550C14I	-0.1642019	1.73402426	0.09413377	0.76217206	0.53425865

Foxc1	-0.0578668	8.36033334	0.0940086	0.76232511	0.53432769
Amigo2	-0.1037325	2.41715299	0.09382596	0.76254862	0.53444611
Tspan5	-0.0377574	7.34070433	0.0936441	0.76277143	0.53456402
A730020M07	-0.0782471	3.94037188	0.09348085	0.76297163	0.53466608
Gpsm3	-0.1470104	2.31143864	0.09337175	0.76310553	0.53472165
Fgfr1	-0.0675511	6.04436897	0.09322003	0.7632919	0.53476631
Dock11	0.0492045	5.48991872	0.09315229	0.76337515	0.53476631
Gtf3c6	-0.0685412	5.50182977	0.0931472	0.7633814	0.53476631
Brcc3	-0.0485914	5.43400162	0.09309774	0.76344222	0.53476631
Trim36	0.07626313	3.36901537	0.09305701	0.76349231	0.53476631
Erp29	-0.0713294	4.632178	0.0929478	0.76362669	0.53476631
Gm13308	-0.3059933	-1.3918595	0.09290327	0.76368151	0.53476631
Mcm3	-0.1149101	0.97874568	0.09285102	0.76374583	0.53476631
Rbm15b	0.05624699	3.8137189	0.09284008	0.76375932	0.53476631
Dcaf10	0.05071573	5.20849913	0.09281122	0.76379486	0.53476631
1700025G04	-0.0411712	6.95265813	0.09279405	0.76381601	0.53476631
Cep83	0.04398925	5.98075362	0.09273986	0.76388278	0.53476631
Mycn	0.09666158	2.05725625	0.09267645	0.76396093	0.53476631
Dynlt1f	0.1245275	0.61524014	0.09266462	0.76397551	0.53476631
0610031J06F	-0.0866566	4.49173626	0.09265436	0.76398817	0.53476631
Lpp	-0.0445918	7.6917757	0.09259285	0.76406403	0.53478119
Iqgap1	-0.0373407	6.34663726	0.09243141	0.76426323	0.5348824
Lmna	-0.0700047	3.66476007	0.09228391	0.76444542	0.53497168
Lysmd1	-0.0735624	3.29837465	0.0922246	0.76451872	0.53498476
Atxn2	0.04737321	8.07817741	0.0921439	0.7646185	0.53501636
Trmt11	0.10535412	1.67032125	0.09205317	0.76473072	0.5350226
Ccdc137	-0.0759117	4.23153916	0.09204837	0.76473666	0.5350226
Nt5c2	-0.0406659	5.54623857	0.09196838	0.76483567	0.53502545
Cnih4	-0.0439885	5.19353871	0.09195682	0.76484998	0.53502545
Papd4	0.05787826	5.05321007	0.09168591	0.76518566	0.53520646
Sobp	-0.0349951	7.44545726	0.0916006	0.76529148	0.53520646
Nos2	-0.5671201	-1.9593026	0.09159379	0.76529992	0.53520646
Cald1	-0.0574923	10.2790295	0.09154355	0.76536227	0.53520646
Tsku	-0.1308723	2.14957255	0.09147278	0.76545012	0.53520646
Snhg4	0.07260115	3.32764924	0.09137001	0.76557776	0.53520646
Il20rb	-0.3627242	-0.6847299	0.09133965	0.76561549	0.53520646
Fam169a	-0.0679453	5.76125406	0.0913295	0.7656281	0.53520646
Zfp574	0.06222526	4.88659637	0.09132558	0.76563297	0.53520646
Ndfip2	0.04395429	6.27191141	0.09128823	0.7656794	0.53520646
Nr2f6	0.08572689	2.59815813	0.09124061	0.76573859	0.53520646
Ccdc37	-0.1664437	0.91688166	0.0912187	0.76576584	0.53520646
Tgfbr3	-0.0717071	6.77981759	0.09117593	0.76581904	0.53520646
Dennd3	-0.1189244	1.70226287	0.09104118	0.76598671	0.53528545

Serinc4	0.3627818	-1.0261286	0.09090904	0.76615128	0.53533595
Rph3al	-0.1456482	1.23331176	0.09089539	0.76616828	0.53533595
Gnai3	0.04956999	6.40500411	0.09070576	0.7664047	0.53543637
Mdfic	-0.0981591	5.61031651	0.09069242	0.76642134	0.53543637
A930024E05	-0.1837807	0.66919933	0.09063171	0.76649708	0.5354511
Rps19-ps3	-0.1393156	0.61789326	0.09053423	0.76661876	0.53549791
Ccdc3	0.04525023	5.39949026	0.09046781	0.76670172	0.53550823
Fyttd1	0.03937827	7.36104306	0.09043488	0.76674286	0.53550823
Cdh8	-0.0489975	5.4722849	0.09037359	0.76681946	0.53552354
Tmsb15l	-0.1516407	1.11771483	0.0902454	0.76697974	0.53559729
Cbx5	0.03531063	9.15622761	0.09016274	0.76708316	0.5356033
Pkmyt1	-0.2030344	-0.4137615	0.09011028	0.76714883	0.5356033
Ccl17	-0.133597	1.45136625	0.09010744	0.76715239	0.5356033
Lrrc8d	0.04730596	5.77363906	0.08996462	0.76733126	0.53569
Ppp6r2	0.05799676	4.59010303	0.08991247	0.76739661	0.53569744
4933406C10I	-0.2780978	-1.0047284	0.08973591	0.76761803	0.53581383
Slc38a3	0.07093865	4.10921612	0.08948705	0.76793052	0.53599376
Ndrg3	-0.0300062	8.73840103	0.08914973	0.76835486	0.53625172
Zan	0.50257384	-2.1385419	0.08910146	0.76841565	0.53625594
Zfp595	-0.0549731	3.35783293	0.08895966	0.76859434	0.53634243
Cdc45	-0.1598055	0.92619424	0.08853626	0.76912883	0.53667718
Cmc2	-0.0597577	3.25622659	0.0884597	0.76922563	0.53670649
Mtr	0.10864949	2.80101481	0.08822428	0.76952356	0.53686625
Cisd2	0.04093087	6.17756167	0.08819218	0.76956422	0.53686625
Srebf2	-0.0583207	5.20482012	0.08811598	0.76966077	0.53689537
Ctnnbip1	0.05197803	3.99076024	0.08796113	0.7698571	0.53693541
A430035B10	0.07716203	2.99546973	0.08794127	0.7698823	0.53693541
Sec11a	-0.0772885	4.0758586	0.08792099	0.76990803	0.53693541
Ctnnb1	0.03220597	9.22493966	0.08789509	0.76994089	0.53693541
Extl3	-0.0395414	6.75161783	0.08783955	0.77001139	0.53693541
Hmgn5	0.04777179	7.16198763	0.08781144	0.77004707	0.53693541
Ccdc34	0.04938065	6.08041037	0.08763387	0.77027266	0.53702057
Gabrr2	0.34290306	-0.870399	0.087629	0.77027885	0.53702057
Cyhr1	0.0464364	5.5707049	0.08747702	0.77047213	0.53711709
Fbxl5	0.03864087	5.6184531	0.08736681	0.7706124	0.53716307
Pias1	-0.0323338	6.84402528	0.08733904	0.77064776	0.53716307
Bms1	-0.0410661	6.0653261	0.0872366	0.77077825	0.5372158
Stxbp1	-0.0415617	10.0420214	0.08709719	0.77095598	0.53730144
Nanog	-0.1950725	-0.5025616	0.0867551	0.77139273	0.5375251
Ackr4	0.4045428	-1.4397916	0.0867212	0.77143608	0.5375251
Klhl13	-0.0367078	6.10968308	0.08671693	0.77144153	0.5375251
Vtcn1	0.20102329	-0.1211964	0.08664426	0.77153446	0.53755162
Ilvbl	0.09345205	2.36640121	0.08643118	0.77180717	0.5376714

Rasal1	-0.0993685	3.28396186	0.08639704	0.77185089	0.5376714
Smc1a	0.04759802	8.10839609	0.0863291	0.77193794	0.5376714
Has1	0.18435763	0.34808261	0.08632557	0.77194247	0.5376714
1700003M07	-0.0547116	3.86364677	0.08629563	0.77198084	0.5376714
Capn5	0.04132065	5.56500213	0.0859376	0.77244029	0.53795315
Piezo2	-0.0889766	2.92854699	0.08566757	0.77278751	0.53813115
Palm2	0.05884692	7.15217427	0.08564199	0.77282043	0.53813115
Gm2a	-0.0831524	5.00654987	0.08559167	0.77288521	0.53813115
Tubgcp5	0.07314767	4.13761734	0.08552847	0.7729666	0.53813115
Kbtbd11	0.03584449	7.70405204	0.08552539	0.77297056	0.53813115
Clca2	0.30616085	-1.5911448	0.08544804	0.77307023	0.53816229
Ier5l	0.28615457	-1.2465365	0.0852596	0.77331322	0.53829318
Gm13807	-0.3881566	-2.139352	0.08514715	0.77345837	0.53833748
Ciao1	-0.0423217	5.39363612	0.08512515	0.77348677	0.53833748
4930577N17	-0.1452007	0.10867021	0.0849769	0.77367832	0.53843254
Camk1d	-0.0346472	8.32294529	0.08480183	0.77390474	0.53855185
C2	0.1121434	2.81686402	0.08475214	0.77396905	0.53855834
Myrip	-0.0532745	6.38887838	0.08465545	0.77409425	0.5386072
Tef	0.03095386	6.79325453	0.08440247	0.77442221	0.53870639
Ccdc85b	-0.0532707	4.38375005	0.08438671	0.77444265	0.53870639
Cog5	-0.0631302	4.63452983	0.08436841	0.7744664	0.53870639
Aldh1a3	0.21763265	-0.040302	0.08436613	0.77446936	0.53870639
Csf1	-0.0646612	4.87426401	0.08429471	0.77456206	0.53870639
Apold1	0.1246657	1.93923786	0.08427093	0.77459294	0.53870639
Mbd2	-0.0464572	7.38678796	0.08424872	0.77462178	0.53870639
Tmem63a	0.06419291	4.55997756	0.08410624	0.7748069	0.53874634
Slc19a3	-0.2010332	0.32608447	0.0840402	0.77489277	0.53874634
Zfp72	-0.0858359	3.12617452	0.08402645	0.77491064	0.53874634
Foxo6	-0.0791784	2.60702137	0.08401733	0.77492251	0.53874634
Cox15	-0.0464776	5.47204878	0.08397271	0.77498054	0.53874634
Csrnp1	-0.1241014	1.76997254	0.08395067	0.77500922	0.53874634
Mtif3	-0.0644919	3.36360623	0.08386571	0.77511981	0.5387517
Dync1i2	0.04001216	7.48767004	0.08386023	0.77512694	0.5387517
Ogg1	-0.1227003	0.72943137	0.08381076	0.77519136	0.53875825
Lrrc38	0.12076187	1.44435448	0.0836427	0.77541035	0.53877507
Olig2	-0.0619329	3.43458375	0.08363566	0.77541954	0.53877507
Commd4	-0.0870958	3.50891298	0.08361569	0.77544557	0.53877507
Cmas	0.03022376	6.76787663	0.08360909	0.77545417	0.53877507
Vrk2	0.18825285	0.45857261	0.0835746	0.77549916	0.53877507
Stap1	-0.1707963	0.31756925	0.08352652	0.77556189	0.53877507
Supt16	0.05743172	6.65731618	0.08349686	0.77560059	0.53877507
Lmtk3	0.14879423	1.61875562	0.08330611	0.77584968	0.53890988
Ptpre	0.05599403	5.24251314	0.08312928	0.77608087	0.53903224

1700001D01I	0.18244273	1.20263267	0.08307123	0.77615682	0.53904678
Hcn4	0.10953556	1.30114906	0.08302446	0.77621804	0.53905107
Sertad3	0.1808183	0.88470497	0.08292689	0.7763458	0.53910158
Mllt1	0.05321522	4.59534888	0.08273055	0.77660316	0.53924206
Ncapg	-0.2613272	-0.3341187	0.0826667	0.77668692	0.539262
Klrb1f	-0.205563	-0.1107929	0.08261412	0.77675592	0.53926304
Fam13a	0.04752823	3.99578644	0.08258167	0.77679853	0.53926304
Hexim1	-0.0405951	5.41505595	0.08247647	0.77693668	0.53927756
Gimap1	0.08505559	3.12082073	0.08245666	0.77696272	0.53927756
Luzp1	0.03605337	8.22082905	0.08237715	0.77706721	0.53927756
Cmtm6	0.08980502	4.86642247	0.08232965	0.77712967	0.53927756
Wars	-0.0460602	5.11435786	0.08229313	0.7771777	0.53927756
Ggt5	0.15810094	1.43336164	0.08228472	0.77718876	0.53927756
Tmem255b	-0.3318219	-0.9751836	0.0822725	0.77720483	0.53927756
Rcn3	-0.1297088	3.12705558	0.08217576	0.77733214	0.53932769
Gm12992	-0.1097042	1.37021767	0.08188688	0.77771279	0.53955358
Vbp1	0.04476119	5.18967613	0.08179502	0.77783398	0.53959101
Ddx52	-0.0377332	4.67861184	0.08174835	0.77789558	0.53959101
Idi1	0.05114964	5.81647658	0.08172076	0.77793201	0.53959101
Slco4a1	0.13072066	1.80494302	0.08166998	0.77799907	0.53959932
Fam188b	0.10960881	1.34550415	0.08149672	0.77822804	0.53971991
Gm5065	-0.2842564	-1.8556745	0.0813949	0.77836272	0.5397751
Galnt11	0.06450733	3.29136292	0.08129673	0.77849267	0.53979061
Traf3ip3	-0.3094586	-0.8302029	0.08129474	0.77849531	0.53979061
Cd209a	-0.1190101	3.352711	0.08117919	0.77864837	0.53985853
Wasl	-0.0336775	8.11217707	0.08106581	0.77879866	0.5398724
Pmepa1	-0.039325	7.36000559	0.08105434	0.77881388	0.5398724
4833412C05I	-0.1746115	0.25183559	0.08100241	0.77888276	0.5398724
Btrc	0.04167846	6.32207989	0.08099784	0.77888883	0.5398724
Mybpc2	0.13630007	1.39858629	0.08094951	0.77895295	0.53987555
Fgf23	-0.3173205	-1.0142725	0.08091135	0.77900362	0.53987555
Tada1	0.04659655	5.26378133	0.08061694	0.77939483	0.54010846
Rras	-0.0899946	4.72190055	0.08051927	0.77952478	0.5401603
Enthd2	0.11856702	1.43722193	0.08047186	0.7795879	0.54016582
Slc36a1	-0.0470315	4.99323155	0.08038456	0.77970417	0.54020818
Yars	0.04595179	5.01004981	0.08022085	0.77992239	0.54032115
Tor1aip1	0.04426131	7.34527973	0.08009036	0.7800965	0.54033531
Mthfr	0.08578104	2.89904496	0.08008521	0.78010337	0.54033531
Tmem237	-0.0787457	3.40213193	0.0800815	0.78010832	0.54033531
Synrg	0.03414393	6.70088302	0.07997589	0.78024936	0.54039479
Fbxl4	0.0546052	3.57468846	0.07983386	0.7804392	0.5404434
Cers5	0.03958031	4.92314804	0.07980221	0.78048152	0.5404434
Mfap1a	-0.0291331	7.41490946	0.07976547	0.78053067	0.5404434

Slc26a7	-0.100091	4.94168083	0.07975832	0.78054024	0.5404434
Pon2	-0.0772659	5.932534	0.0796746	0.78065226	0.54045232
Agpat1	0.05238429	4.8021746	0.07965857	0.78067373	0.54045232
Lonrf3	0.04440725	5.72486877	0.07962503	0.78071864	0.54045232
Gpr160	-0.1870084	0.01767484	0.07957086	0.78079119	0.54046434
Gm21284	0.16244022	0.13904433	0.0794869	0.78090369	0.54050402
B4galt3	0.08695167	1.94859098	0.07937429	0.78105469	0.54057034
Copb2	0.02915596	7.23618966	0.07929003	0.78116774	0.54061039
Gm4951	-0.1100595	3.36598933	0.07922726	0.78125202	0.54063051
I830077J02R	0.1375635	0.98222716	0.07898375	0.78157925	0.5408043
10-Sep	-0.0778341	2.8240973	0.07890861	0.78168034	0.5408043
Hinfp	0.08977054	1.97206335	0.07888654	0.78171005	0.5408043
Zfp185	0.09129964	3.37363519	0.07887617	0.78172399	0.5408043
Ide	0.03061549	7.24622988	0.07874668	0.78189836	0.54086746
Ints7	0.06716407	3.58778534	0.07872637	0.78192572	0.54086746
Dars	0.03953048	5.23673454	0.07866103	0.78201379	0.54089018
Alkbh4	0.11174576	1.04714267	0.07852859	0.78219239	0.54092317
Rpn2	-0.0560149	5.31139917	0.07849009	0.78224434	0.54092317
Tlr7	-0.1110887	1.59115268	0.0784476	0.78230168	0.54092317
Krt10	-0.0866007	2.23718246	0.07844131	0.78231018	0.54092317
Pgf	-0.1828481	1.21134176	0.078421	0.7823376	0.54092317
Ttc30b	0.05597405	3.5675271	0.07833873	0.78244871	0.54096181
Casr	-0.1196961	1.89347907	0.07826437	0.78254919	0.5409931
Ipo4	0.05032075	4.14921411	0.07815758	0.7826936	0.54103087
Trps1	0.04599648	6.29006397	0.07814149	0.78271536	0.54103087
Tap1	0.11593846	1.05045136	0.07810144	0.78276954	0.54103087
Arhgef15	0.08910728	2.8955382	0.0780023	0.78290374	0.54103118
Zkscan17	-0.0646075	3.86490782	0.0779612	0.78295941	0.54103118
Chst14	-0.1133208	1.60237469	0.07789404	0.7830504	0.54103118
Stam	-0.0359098	6.06935406	0.07786496	0.7830898	0.54103118
Polr2i	-0.0824788	3.0562357	0.07785977	0.78309684	0.54103118
Tspan6	-0.0782454	3.9849307	0.07785641	0.78310139	0.54103118
Aste1	0.08591001	2.36355824	0.07775506	0.78323881	0.54108288
Ssrp1	0.048174	6.70649479	0.0776918	0.78332464	0.54108288
Kdelc1	0.07059097	2.8426093	0.0776753	0.78334703	0.54108288
Msh5	0.48789876	-1.8124745	0.07759839	0.78345145	0.54108288
Tmem255a	0.05531821	4.74803875	0.07759768	0.78345241	0.54108288
Gm15421	0.08482002	1.85128343	0.07749616	0.78359031	0.54113118
Psat1	-0.0377948	6.48892202	0.07745655	0.78364415	0.54113118
Parp8	0.05470712	4.80146209	0.07742423	0.78368808	0.54113118
Cmip	0.04110676	9.08758755	0.07727535	0.78389059	0.54120174
Zdhhc18	0.07282	3.24459797	0.07726788	0.78390076	0.54120174
Prr14	-0.0504709	5.45232595	0.07714153	0.78407281	0.54128237

Thra	0.04983121	5.82827563	0.07709711	0.78413333	0.541286
Nck1	-0.0519827	4.98985231	0.07700459	0.78425945	0.54133491
Aloxe3	-0.0975641	1.83074161	0.07695515	0.78432687	0.5413433
Ints9	0.04357735	4.3612064	0.07684116	0.78448242	0.54141251
Rilp	-0.2224024	-0.4836868	0.07672592	0.78463979	0.54148297
Dexi	-0.0502482	3.84167745	0.07666945	0.78471697	0.54148908
C030034I22R	0.10138581	1.65412909	0.07663854	0.78475921	0.54148908
03-Mar	-0.213355	0.04069443	0.07655898	0.78486801	0.54152601
Slc17a5	0.05145874	3.89423497	0.07637822	0.78511542	0.54165856
Gne	-0.0386582	5.51174018	0.07624182	0.78530232	0.54173082
Pdcl3	-0.0626879	4.225778	0.07622108	0.78533077	0.54173082
Snord99	0.4100207	-1.8805611	0.07617238	0.78539754	0.54173873
Pex26	-0.0593091	3.8248316	0.0760486	0.78556739	0.54179393
Rapgef3	0.06570129	2.66865458	0.07603345	0.7855882	0.54179393
Pex11g	0.28890259	-1.2928139	0.07591811	0.78574661	0.54186504
Ergic3	0.06116323	5.24047087	0.07575026	0.78597738	0.54198602
Eltd1	0.10421024	2.92488026	0.07568116	0.78607247	0.54199948
Nans	-0.0979085	2.40515802	0.07565566	0.78610757	0.54199948
Tbc1d1	-0.0428509	4.55994656	0.07560055	0.78618345	0.54201365
Smek1	0.04005097	5.62499511	0.0755266	0.78628532	0.54204573
Anxa6	0.04942403	5.87471101	0.07537143	0.78649925	0.54215505
Egr2	0.13607953	3.93185383	0.07531891	0.78657171	0.54216685
6430550D23I	0.21990305	-0.2776649	0.07519336	0.78674503	0.54224816
Pafah1b3	-0.1181696	2.03369673	0.07505312	0.78693883	0.54231041
Hes1	-0.0600694	3.68862672	0.07494278	0.78709144	0.54231041
Add3	-0.0354604	8.2735249	0.07491628	0.78712811	0.54231041
Emc9	-0.0767524	2.43498504	0.07491483	0.78713013	0.54231041
Arcn1	-0.027007	7.30276195	0.07490823	0.78713925	0.54231041
Tmc3	-0.1458654	0.99473456	0.0748878	0.78716754	0.54231041
Rnf125	-0.1175969	1.24006064	0.07474463	0.78736581	0.54240886
Rhob	-0.0362349	7.36944358	0.07470022	0.78742737	0.54241312
Slc22a14	0.52659005	-1.9966081	0.07462814	0.7875273	0.54243077
Pi4k2a	0.04809185	4.91082753	0.07460186	0.78756375	0.54243077
Dnaic1	0.12260631	0.56456003	0.07453979	0.78764987	0.5424504
Cntrob	0.09902624	2.14754063	0.07448072	0.78773185	0.5424504
Cdc73	-0.0377464	6.15338114	0.07446162	0.78775838	0.5424504
Asxl1	0.04761354	5.35960439	0.07425804	0.78804126	0.54255344
2300009A05I	-0.0834049	2.43271049	0.07423495	0.78807336	0.54255344
Otud1	-0.0475555	6.12233648	0.07423436	0.78807419	0.54255344
Kif18b	0.32685975	-1.7836768	0.07414177	0.788203	0.54260398
Zfp39	0.04059983	5.36008907	0.07368096	0.78884539	0.54297849
Mir425	0.31350105	-1.6929276	0.07367202	0.78885788	0.54297849
Pros1	-0.0657591	5.1652713	0.07360785	0.78894751	0.54300203

Pla2g3	-0.2224362	-0.0423008	0.07321825	0.78949262	0.54333902
C1qtnf3	0.47951502	-2.2217414	0.07310299	0.78965417	0.5433722
Pld2	-0.0960249	2.40034598	0.07307026	0.78970008	0.5433722
Pkn2	-0.0368691	6.06826145	0.07306515	0.78970725	0.5433722
Nap1l2	-0.0562619	5.39456633	0.0730104	0.78978407	0.54338431
Zfp362	-0.0429921	5.19322583	0.07297355	0.78983578	0.54338431
Slc25a38	0.06282196	2.90799022	0.07291768	0.78991423	0.54340011
Rnf31	-0.0703725	3.23321975	0.07285849	0.78999736	0.54341913
Sez6l2	0.04787187	5.43861925	0.07265618	0.7902818	0.54354819
Cdc25b	0.071847	2.97562223	0.07264611	0.79029596	0.54354819
4930467D21	0.31609773	-1.3164276	0.07260333	0.79035617	0.54355144
Trim45	0.07972094	2.49172665	0.07254846	0.79043341	0.54356639
Acer2	-0.0882697	3.38377005	0.07242651	0.7906052	0.54364636
Grid2	0.09036332	2.47251112	0.07238245	0.79066731	0.54365091
Abcd1	0.0795143	3.7141478	0.07222804	0.79088513	0.54375216
Klrk1	-0.1714715	0.53259997	0.07219937	0.7909256	0.54375216
D430042O09	0.06501482	3.42769263	0.07206435	0.79111631	0.54383333
Mov10	-0.0760637	2.92325618	0.07203719	0.79115469	0.54383333
Gnaz	-0.0408045	5.82216852	0.0719573	0.79126765	0.54387281
Mark2	-0.0408578	6.32552604	0.07187364	0.791386	0.54391598
Muc5b	-0.2884753	-1.4645111	0.07203991	0.79163643	0.54404993
Ankrd1	-0.2687707	-1.3890658	0.07159231	0.79178455	0.54408298
Pttg1ip	0.07020788	6.25434244	0.07158452	0.79179561	0.54408298
Tmco1	0.07122614	4.77673781	0.0714268	0.79201943	0.54418856
Btbd9	0.04866118	4.98296484	0.07139797	0.79206036	0.54418856
Pde7b	-0.029191	6.60018981	0.07109981	0.79248431	0.54444164
LOC1016697	0.28607891	-0.978259	0.07098556	0.792647	0.54451523
Stab2	-0.296052	-1.1978547	0.07089449	0.79277679	0.54456619
Prim1	0.08280902	2.94450573	0.07073813	0.79299983	0.54462594
Atxn7l2	0.12544889	2.34763128	0.07069592	0.79306009	0.54462594
Srr	-0.040221	6.94514693	0.07064987	0.79312586	0.54462594
H2afy2	-0.0572581	4.52595508	0.07064298	0.79313569	0.54462594
Usp43	-0.2034152	1.0019832	0.07060909	0.79318411	0.54462594
Tesk1	0.05293083	4.95553184	0.0705716	0.79323768	0.54462594
Chtf8	-0.0492078	5.11956091	0.07055688	0.79325873	0.54462594
Cbln4	0.04856015	4.04057016	0.070522	0.79330858	0.54462594
Fzd10	0.12053835	1.03771457	0.07037086	0.79352478	0.54473619
Tph2	0.36551514	-1.2085196	0.07016357	0.79382172	0.54490185
Wnt6	0.10541399	3.15703096	0.06994554	0.79413455	0.54507838
G630025P09	0.37866996	-1.6225258	0.0699022	0.7941968	0.54508291
Ttc28	-0.0413285	5.76228808	0.06983556	0.79429255	0.54509087
Fbxl14	0.04366436	5.24253951	0.06981669	0.79431968	0.54509087
Krcc1	-0.0686507	7.09047023	0.06965861	0.79454705	0.5452087

Rangrf	-0.0749787	2.89797601	0.06951345	0.79475607	0.54529712
Cabp1	0.07299229	2.88542464	0.06949181	0.79478725	0.54529712
Sowahc	-0.0455018	4.15343982	0.0692534	0.79513112	0.54547057
Apbb2	-0.0316611	7.4120643	0.06922823	0.79516746	0.54547057
Pifo	-0.26411	-0.2393641	0.06916608	0.79525722	0.54547057
Arl8a	0.0368318	6.34525013	0.06913623	0.79530035	0.54547057
Synpo2	-0.0709753	3.37274146	0.06912368	0.79531849	0.54547057
Ndufv1	-0.0507468	4.74009569	0.06908479	0.79537471	0.54547094
Actr10	0.03053277	7.66079374	0.06903838	0.7954418	0.54547876
Hyal3	0.14898322	0.5348794	0.06891657	0.79561803	0.54551925
U2af1l4	-0.0874193	2.09191891	0.068904	0.79563623	0.54551925
Kif17	-0.071063	3.1648129	0.06888211	0.79566793	0.54551925
Tbccd1	0.04548694	4.58301558	0.0688261	0.79574904	0.54553668
Ercc6	0.05702579	5.60019108	0.06874156	0.79587153	0.54556624
Ctsk	0.10301698	2.38531761	0.06871947	0.79590355	0.54556624
Chordc1	-0.0320859	6.72134123	0.06863721	0.79602284	0.54560983
Slc16a14	0.07163058	4.04172305	0.06859872	0.79607869	0.54560993
Tlr2	-0.3189061	-1.1053509	0.06854365	0.79615862	0.54562653
Sod3	-0.0686955	5.39044112	0.06847855	0.79625313	0.5456307
Snn	-0.031741	6.63171243	0.06842931	0.79632467	0.5456307
Sirt2	-0.0493923	6.66112863	0.0684244	0.79633181	0.5456307
Trhr	0.1086988	1.50417131	0.06830518	0.79650511	0.54571127
Tcof1	0.04820034	4.23575164	0.06819989	0.79665831	0.54577806
Slc35g3	-0.3378973	-1.5128325	0.06799396	0.79695831	0.5459009
Sgcz	0.12736398	1.8960049	0.0679554	0.79701453	0.5459009
Hlx	-0.3173728	-1.893623	0.06790518	0.79708779	0.5459009
Rab24	-0.0434851	5.10408853	0.06789395	0.79710417	0.5459009
Myg1	-0.0714429	3.22637071	0.06788566	0.79711627	0.5459009
5730420D15I	0.3290912	-0.8912027	0.06775103	0.79731282	0.54599733
Ddah1	0.03557364	7.1291285	0.06759679	0.79753827	0.54601801
Mmrn2	-0.225979	-0.3197743	0.06759221	0.79754497	0.54601801
Calu	0.04584479	6.14089054	0.06754829	0.79760921	0.54601801
Zscan22	-0.0416361	5.38400301	0.06754746	0.79761043	0.54601801
Sipa1l2	0.06367113	4.53337432	0.06753974	0.79762172	0.54601801
Cntn6	-0.1023876	3.21774274	0.06749096	0.79769312	0.54602415
Plekha6	0.03470943	6.85142814	0.06739004	0.79784089	0.54602415
Msx1	-0.1021213	2.36231787	0.06738976	0.7978413	0.54602415
Sfxn1	-0.033669	5.91828744	0.06715827	0.79818074	0.54602415
Cd2bp2	-0.0495313	4.98529416	0.067137	0.79821195	0.54602415
Zbtb33	0.04052749	5.58479729	0.06708957	0.7982816	0.54602415
Rnf139	-0.0378346	5.09145467	0.06706577	0.79831654	0.54602415
Rnf138rt1	0.27700617	-1.5642962	0.0670628	0.79832091	0.54602415
Letm1	0.0454421	5.94169306	0.06706107	0.79832345	0.54602415

Nol10	0.06339884	4.15900657	0.06704807	0.79834254	0.54602415
2810029C07I	-0.1274797	2.23293347	0.06704752	0.79834335	0.54602415
Dhx30	0.0667578	4.70608379	0.06704264	0.79835051	0.54602415
Gskip	0.05146527	4.39527717	0.06703934	0.79835537	0.54602415
Ccl3	-0.4933085	-2.0153871	0.06693991	0.79850149	0.54608596
B930041F14	-0.0448868	5.11013948	0.06681828	0.7986804	0.54617019
Coil	-0.0663243	3.23445408	0.0664889	0.79916576	0.54646394
Creg1	0.0596795	4.80450992	0.06639654	0.79930209	0.54650558
Six3os1	0.11908209	0.77632868	0.06637207	0.79933823	0.54650558
Ppic	-0.0800218	4.60365247	0.06632132	0.7994132	0.5465095
Otud7a	0.08754215	3.09168606	0.06629266	0.79945555	0.5465095
Xxylt1	-0.0568435	3.52555456	0.06621232	0.79957431	0.54655254
Utp14a	0.05164693	3.98157565	0.0661184	0.79971326	0.54660741
Tmem176b	-0.0718641	5.40405704	0.06608265	0.79976618	0.54660741
Fam219b	0.06201274	3.70312131	0.06601612	0.79986469	0.54663659
4921504A21I	-0.0712459	3.35380676	0.06595387	0.79995691	0.54665762
Tmub1	-0.0761636	2.31800996	0.06592003	0.80000707	0.54665762
Vcam1	0.04424587	5.84331922	0.0658728	0.8000771	0.54666733
Ddx50	-0.0321088	6.27247617	0.06577454	0.80022287	0.5466993
D10Jhu81e	0.0522264	3.58095012	0.06576602	0.80023551	0.5466993
Gm5126	0.07860224	2.50523899	0.06565649	0.80039814	0.54671001
Mrpl43	-0.0492731	5.11496143	0.06565198	0.80040484	0.54671001
Mst1	0.32122298	-1.388693	0.0656427	0.80041863	0.54671001
Tfb2m	-0.0419254	5.65396134	0.06556211	0.8005384	0.54675369
Tmbim6	0.04863757	6.63596553	0.06542172	0.80074723	0.54685819
Btn2a2	-0.3296814	-0.4733493	0.06521266	0.80105864	0.54701482
Slc25a1	-0.074283	3.82764877	0.06519279	0.80108827	0.54701482
Mef2a	0.02840974	8.26170554	0.06514493	0.80115965	0.54702543
Fgl2	-0.0620719	4.78953892	0.06497661	0.80141092	0.54715885
Mrc2	-0.0836886	4.96143287	0.06490829	0.80151299	0.54719041
Rab42	-0.2729252	-1.7234385	0.06485637	0.80159061	0.54720525
Gimap4	-0.0984856	3.0918841	0.06463579	0.80192074	0.54739247
4933416C03I	-0.4129381	-1.228785	0.06458187	0.80200152	0.54740947
Dctn4	-0.0300898	7.41932633	0.0644804	0.80215365	0.54747515
Dhx15	0.02733913	6.58366258	0.06434581	0.80235564	0.54757486
Angpt1	0.05905911	4.59662105	0.06419412	0.80258356	0.54769224
Sltm	-0.0341408	7.70883421	0.06414881	0.8026517	0.54770059
Sdc4	-0.0809173	5.26521449	0.064012	0.80285758	0.54780163
Ubap2	0.03047053	6.09196296	0.06394244	0.80296236	0.54780163
Ccdc8	-0.2274938	-0.2951193	0.06393899	0.80296756	0.54780163
Tmem222	0.08513393	4.53161946	0.06375224	0.80324914	0.54792068
Prps1	-0.0302988	5.87724468	0.06374907	0.80325392	0.54792068
Rspry1	-0.0398495	4.75303855	0.06368794	0.8033462	0.54792738

Cks2	0.135365	0.30209845	0.06366845	0.80337562	0.54792738
B930003M22	-0.1832544	-0.1766797	0.06351499	0.80360751	0.54802249
Dysf	0.18957503	0.33250589	0.06350212	0.80362697	0.54802249
AA543186	-0.2348415	-1.0680148	0.06342864	0.80373813	0.54804648
Icmt	-0.0366522	4.61061729	0.0634049	0.80377405	0.54804648
Pthlh	0.08150757	2.27733895	0.06332886	0.80388916	0.54808682
H2-K1	0.06083232	5.06587241	0.06323227	0.8040355	0.54814844
Ceacam1	-0.0762816	2.96410889	0.06311069	0.80421986	0.54817826
Efcc1	-0.0723624	2.53421232	0.06310987	0.80422111	0.54817826
Eda	0.08484306	2.48147763	0.06309271	0.80424714	0.54817826
Suz12	-0.0319555	6.88359314	0.06301122	0.80437085	0.54822443
Dnah6	0.14620437	0.7087383	0.06294609	0.80446976	0.5482537
Snrk	-0.0302321	6.38919124	0.06286065	0.80459962	0.54825875
Gjc2	-0.1545083	0.31923454	0.06285576	0.80460706	0.54825875
Spryd7	-0.0333779	5.47150062	0.06276784	0.80474078	0.54825875
Galr1	0.30864686	-1.8175192	0.06276631	0.80474311	0.54825875
Manba	-0.0888718	2.7862483	0.06275717	0.80475703	0.54825875
Itm2b	0.05271203	8.98079647	0.06263519	0.80494274	0.54834714
Pds5a	-0.0339022	6.86280437	0.06257265	0.80503802	0.54837391
Phf2	-0.0260734	6.53371212	0.06237352	0.80534178	0.54854267
Disp1	-0.0634405	3.22651825	0.06233286	0.80540387	0.54854682
Rab11fip1	0.05503859	3.66126826	0.0622239	0.80557035	0.54862206
Efna5	-0.0375841	6.08444567	0.06216013	0.80566787	0.54863335
Zfp456	0.06823651	3.08223601	0.06208273	0.80578629	0.54863335
1110038F14I	-0.0682732	3.35510242	0.06206506	0.80581333	0.54863335
Fhl3	-0.1043049	2.04694674	0.06206176	0.80581839	0.54863335
Il17d	-0.0938646	0.95456877	0.06196978	0.80595925	0.54863335
1110051M2C	0.04557408	4.1589489	0.06196286	0.80596985	0.54863335
Calr	-0.0346831	6.40600111	0.06194558	0.80599632	0.54863335
Gas1	0.06566412	5.78647272	0.06191379	0.80604504	0.54863335
Gmppa	0.04174038	3.73672678	0.06184834	0.80614539	0.54863335
Lrrc6	0.05578011	3.88873187	0.06184728	0.80614702	0.54863335
Mamstr	0.14334535	0.20664591	0.06165557	0.80644127	0.54877168
Mdm4	-0.0287307	6.15415626	0.06164186	0.80646233	0.54877168
Zhx3	-0.0349726	6.21195891	0.06145073	0.8067562	0.54893352
Tbata	-0.1834237	-0.0328198	0.06120108	0.8071408	0.54903433
Pgd	-0.0503084	5.24899362	0.06118703	0.80716247	0.54903433
4931440F15I	0.1897122	-0.2478327	0.06117267	0.80718462	0.54903433
Slmo1	0.05233744	5.01944233	0.06114297	0.80723045	0.54903433
Mettl3	0.05271064	3.85454975	0.06111539	0.807273	0.54903433
Pigu	-0.0580875	3.77721647	0.06108086	0.80732631	0.54903433
Dlx4	-0.3799486	-1.9372752	0.06097698	0.80748675	0.54903433
Trim13	0.07389293	2.67528309	0.06097123	0.80749564	0.54903433

Snapc1	0.03093802	5.51212667	0.06093418	0.8075529	0.54903433
Slc25a3	-0.0269996	7.93988783	0.06089197	0.80761816	0.54903433
AI854703	-0.0707244	3.07551616	0.06084956	0.80768376	0.54903433
Snrnp27	-0.0534437	5.43105277	0.06080195	0.80775742	0.54903433
Ppp1r1b	0.05414844	5.39614067	0.06079049	0.80777516	0.54903433
Morc3	0.05408793	4.5466549	0.06078818	0.80777873	0.54903433
Zfp668	-0.0542665	3.70242168	0.0607807	0.80779031	0.54903433
Map2k6	-0.047453	4.00294987	0.06077368	0.80780117	0.54903433
Myh7b	0.10660146	2.32790488	0.06071677	0.80788929	0.54905612
Tax1bp1	-0.027462	8.62016464	0.06066459	0.80797012	0.54907296
Epyc	-0.1745482	-0.2199656	0.06055276	0.80814348	0.54915267
Lpo	0.27381575	-0.8109951	0.06036529	0.80843448	0.54927488
Glyr1	-0.0379667	5.78046531	0.06035499	0.80845049	0.54927488
Gm19522	-0.0737464	2.40790063	0.06032857	0.80849154	0.54927488
Gm11944	0.13714952	0.61037529	0.06027618	0.80857297	0.5492921
Gm13251	-0.0794718	2.15003669	0.06020483	0.80868394	0.54930235
Ciapin1	-0.0385655	4.72236937	0.06018812	0.80870994	0.54930235
Tfr2	-0.1222463	0.97778747	0.06015833	0.8087563	0.54930235
Pole2	-0.1523006	1.03558652	0.06004601	0.80893119	0.54938304
Bsdc1	0.03772373	5.28190997	0.05986698	0.80921033	0.54950011
Cit	0.03896632	7.31494479	0.0598635	0.80921576	0.54950011
Cbln1	0.09142106	1.84551883	0.05979113	0.80932872	0.54951175
Zfp740	0.04675844	4.79995904	0.05977719	0.8093505	0.54951175
Vmn2r18	-0.3778799	-1.8672575	0.05968929	0.80948782	0.54951175
A330041J22R	0.17162552	0.08516935	0.05968116	0.80950052	0.54951175
D16Ertd472e	0.04511172	6.32211038	0.05966727	0.80952223	0.54951175
Sgsh	0.09549143	1.3039999	0.05960862	0.80961394	0.54951175
Ccdc40	-0.1675943	0.06775867	0.05958348	0.80965326	0.54951175
Rbbp8	0.05706083	4.13428988	0.0595653	0.8096817	0.54951175
Dusp7	-0.0289743	6.05121561	0.05948566	0.80980634	0.54955827
Zfp354a	0.04781817	3.36857815	0.05942844	0.80989596	0.54958101
Glcci1	-0.0286077	6.88902066	0.05937665	0.8099771	0.549598
Tlk2	-0.0309514	7.11573256	0.05927167	0.81014171	0.54967161
Matn2	-0.0685463	3.70694898	0.05911967	0.81038031	0.54979542
Slc26a10	0.11628197	0.66412926	0.05906618	0.81046436	0.54980282
Tmem221	0.1986663	-0.4644933	0.05903883	0.81050735	0.54980282
Rps15a-ps4	-0.0918593	1.32191386	0.05899968	0.8105689	0.54980282
Gm10409	0.05978629	4.00369907	0.05896292	0.81062671	0.54980282
Mir1b	0.37544325	-1.1700818	0.05893423	0.81067186	0.54980282
Kpna2	-0.031191	5.1767939	0.0588475	0.81080837	0.54984625
Gtf2a2	-0.0401675	4.82817	0.05876964	0.81093101	0.54984625
Tnfaip8l2	0.20866016	-0.6473809	0.05875335	0.81095669	0.54984625
Slc25a10	0.11108213	1.47873183	0.05871848	0.81101166	0.54984625

Unk	0.05539974	3.96633081	0.05871536	0.81101657	0.54984625
04-Sep	0.03421195	5.93010736	0.05862235	0.81116328	0.54989083
1700086L19I	0.06135145	3.09670287	0.05856285	0.81125719	0.54989083
Ptpla	0.06747893	2.93398082	0.05853915	0.81129461	0.54989083
Sap30bp	0.05098428	3.65865418	0.05850342	0.81135103	0.54989083
Csnk1d	-0.0269175	6.50616897	0.05849584	0.81136301	0.54989083
Trim8	0.03658544	6.14101651	0.05842704	0.81147173	0.54991091
Rbfa	-0.0714815	2.63680944	0.05838669	0.81153552	0.54991091
Ptcd1	-0.0513365	3.44479309	0.05837054	0.81156107	0.54991091
Pgm1	0.04464721	3.70359012	0.05822308	0.81179443	0.55003099
Hddc3	-0.0604698	2.87659112	0.05815288	0.81190562	0.55006828
Cep112	-0.0515511	3.64725574	0.05809452	0.81199813	0.55009291
Smarca5-ps	-0.0778097	1.23453082	0.05805572	0.81205966	0.55009654
Dpp8	-0.0233187	8.3975238	0.05797778	0.81218333	0.55014227
Zfand5	0.0260944	8.01763302	0.05772042	0.81259228	0.55036038
Slc31a2	-0.0671261	5.11246442	0.05757338	0.81282636	0.55036038
Stau2	-0.0309428	7.12925765	0.05755941	0.81284862	0.55036038
Snhg3	0.08644688	3.02494622	0.05755574	0.81285447	0.55036038
Rsl1	0.07252597	2.89096568	0.0575486	0.81286585	0.55036038
Bcl6b	-0.1375699	0.05208606	0.05750347	0.81293778	0.55036038
Klf10	-0.0330932	5.6640606	0.05749676	0.81294847	0.55036038
Ikzf3	0.07324764	2.779429	0.05749278	0.81295481	0.55036038
Rnft2	-0.0509935	4.54258712	0.05743952	0.81303975	0.55037984
Ppox	-0.1250955	1.015352	0.05736676	0.81315584	0.55042039
Atp6ap1	0.03889685	7.63568664	0.05724643	0.81334802	0.55051244
Fzd6	0.07015865	3.99486454	0.05717556	0.8134613	0.55055107
2310030G06	-0.087643	2.80094313	0.05712382	0.81354404	0.55056902
Syt12	0.04374238	4.19268637	0.05695026	0.81382191	0.55068892
Rnf38	0.02650978	7.97757377	0.05690276	0.81389803	0.55068892
Eefsec	-0.0713864	2.69284834	0.05689703	0.81390723	0.55068892
Gm8615	-0.0576048	2.82423481	0.05686376	0.81396057	0.55068892
Cd300lg	-0.2096995	-1.1975361	0.05683773	0.81400231	0.55068892
Nppc	0.18486239	-0.4988418	0.05677084	0.81410964	0.5507235
Atg16l2	-0.118927	1.46873458	0.05666263	0.8142834	0.55080301
Phyhip	-0.0392253	6.76786975	0.0565906	0.81439916	0.55084327
9330117O12	-0.0963568	1.48878023	0.05654382	0.81447437	0.55085611
E130309D02I	-0.0465879	4.011427	0.05648494	0.81456911	0.55088214
Fut2	-0.1483986	0.57040956	0.05629305	0.81487819	0.55097336
Poli	-0.0456137	3.37439859	0.05620907	0.81501363	0.55097336
Sdc1	0.09606633	2.17858721	0.05615572	0.81509973	0.55097336
Klhl8	-0.0461058	4.31491677	0.05615194	0.81510584	0.55097336
Pcdhb13	0.07176535	3.50224641	0.05612054	0.81515653	0.55097336
Inhbe	0.42362094	-1.9838091	0.05611266	0.81516926	0.55097336

Rmst	0.16702802	0.98297744	0.0561119	0.81517048	0.55097336
Negr1	-0.0324497	8.24486592	0.05605325	0.81526522	0.55097336
Kcns1	0.08255286	1.60185337	0.05603545	0.81529399	0.55097336
1700124L16F	-0.2724596	-1.713115	0.05599586	0.81535798	0.55097336
1700013F07I	0.1290292	-0.0736069	0.05598968	0.81536798	0.55097336
Sppl3	0.03101366	5.25228808	0.05596521	0.81540755	0.55097336
Mmgt2	-0.0599656	3.31545724	0.0559481	0.81543522	0.55097336
Fam173a	-0.0621707	3.09933044	0.05590661	0.81550234	0.5509807
Wrb	-0.0310017	5.3922587	0.05581602	0.81564899	0.55104178
Eva1c	-0.0897947	2.79785056	0.05563533	0.81594185	0.55120162
Ppp1r12b	0.06174665	5.67617441	0.05546269	0.81622214	0.55132333
Notch3	0.09661306	2.36530039	0.05545502	0.8162346	0.55132333
Nop58	0.0351781	6.14567815	0.05524921	0.81656938	0.55151143
Mdk	-0.0714544	3.66848016	0.0550932	0.81682359	0.55158436
Oprk1	0.07644381	3.81311892	0.05506106	0.81687601	0.55158436
Rapsn	-0.3572627	-2.5007466	0.05500787	0.81696279	0.55158436
Tmem126b	-0.0488868	4.18323739	0.0549958	0.81698249	0.55158436
Tceal1	0.03879748	5.42049778	0.05499405	0.81698535	0.55158436
Emr1	0.15058083	0.09863425	0.05497574	0.81701523	0.55158436
Mcm4	-0.0445093	4.37157769	0.05485366	0.81721465	0.55168097
Fem1c	0.03551793	5.33679925	0.05478222	0.81733145	0.55171063
Eps8l1	-0.0570632	2.75832042	0.05466925	0.81751631	0.55171063
P4hb	0.05411012	5.58337131	0.05466268	0.81752708	0.55171063
Ch25h	0.29853891	-1.5622507	0.05462353	0.81759119	0.55171063
Pcdh18	-0.0536197	3.19480884	0.05450904	0.81777883	0.55171063
Dmrtc1a	0.10776877	0.89570495	0.05448416	0.81781965	0.55171063
Gm5	-0.2429693	-0.5194814	0.05448076	0.81782522	0.55171063
Ccdc130	-0.0855022	2.27195659	0.05446973	0.81784331	0.55171063
Rep15	-0.317889	-1.3864379	0.05444726	0.81788019	0.55171063
Lypd6b	0.05206818	3.94970159	0.05439532	0.81796544	0.55171063
Clic6	0.0769543	2.60919011	0.05438854	0.81797656	0.55171063
Wdr83	0.06168876	2.6962975	0.05438545	0.81798163	0.55171063
Sla2	-0.1483633	-0.1584206	0.05436485	0.81801546	0.55171063
4933405O20	-0.3463482	-0.526677	0.05433642	0.81806215	0.55171063
Spata18	0.12667857	0.3596755	0.05431129	0.81810344	0.55171063
Nphs2	-0.0627536	3.92360048	0.054152	0.81836535	0.55184926
Sidt1	0.04448113	5.12946762	0.05410147	0.81844852	0.55186736
Fam43b	-0.0929526	2.4659987	0.05404408	0.81854305	0.55187364
Slc17a6	0.03924724	4.81099216	0.05400021	0.81861532	0.55187364
Spaca1	0.18439239	0.59477305	0.05399321	0.81862686	0.55187364
Vopp1	-0.0307802	5.84434405	0.05383695	0.8188846	0.5520094
Piwil4	0.24106275	-0.897219	0.05364762	0.8191974	0.55212434
Gpatch3	0.17387105	-0.187732	0.05364293	0.81920516	0.55212434

Efcab4a	0.11147523	0.98823308	0.05363142	0.8192242	0.55212434
BB557941	0.31692946	-0.6396822	0.05357678	0.8193146	0.55214727
Ikzf5	-0.0335023	5.07149542	0.05351007	0.81942503	0.5521837
Ace	-0.0762495	5.32388205	0.05342072	0.81957306	0.55224546
Sap30l	0.05026135	3.93973451	0.05336861	0.81965945	0.55226569
Neurl1a	-0.0526292	4.46459299	0.05332275	0.81973551	0.55227895
Lin54	-0.0381522	4.48532347	0.05321777	0.81990977	0.55235836
Cabyr	0.05832024	4.12377264	0.05315867	0.82000796	0.55238652
Tmeff2	-0.0315401	6.92265816	0.05310302	0.82010045	0.55241083
Cryga	0.31081476	-1.8631762	0.05265055	0.82085446	0.55287427
Dnajc24	-0.0439333	3.81975444	0.05262248	0.82090136	0.55287427
Zfp790	-0.0446052	4.40422388	0.05255001	0.82102247	0.55288271
4933416I08R	0.22558333	-1.0641654	0.05254744	0.82102677	0.55288271
Fam57a	-0.0940399	1.66580717	0.0525097	0.82108988	0.5528872
Spock1	0.03370838	8.18431266	0.05244831	0.82119259	0.55291835
Rbms3	0.03767739	7.34695524	0.05232335	0.82140185	0.55302124
Ptprh	0.2028269	-0.9851058	0.05222424	0.82156801	0.55309509
Tulp3	-0.0518921	5.2594078	0.05216619	0.8216654	0.55310704
Sell	0.29952565	-1.0459632	0.05209538	0.8217843	0.55310704
Znhit6	0.03361475	5.17501458	0.05208649	0.82179924	0.55310704
Mfap2	-0.1372494	-0.0144527	0.05207911	0.82181163	0.55310704
Nkain1	-0.036089	4.40543864	0.051923	0.82207407	0.55322544
Papd5	-0.0359614	6.11240003	0.05190729	0.82210051	0.55322544
Kras	-0.0340092	8.1423861	0.05172433	0.82240867	0.55338915
Lrrc49	0.04786198	4.99706323	0.05166183	0.82251407	0.55338915
4930579K19I	0.34188404	-1.3326872	0.05163384	0.82256129	0.55338915
Ap4m1	-0.0987885	1.14471779	0.05162882	0.82256977	0.55338915
Ppargc1b	-0.0674114	3.29665832	0.05153137	0.8227343	0.55346183
BC022687	-0.0768449	1.99778136	0.05139302	0.82296818	0.55357177
Pard3	-0.032225	5.12882172	0.05134479	0.82304978	0.55357177
Pdzrn3	0.02885332	7.30350977	0.05124469	0.82321927	0.55357177
Glod4	-0.0299733	5.51592075	0.05122091	0.82325958	0.55357177
Grasp	0.04066746	4.23156831	0.05120612	0.82328464	0.55357177
Hsd17b4	-0.0388135	5.79095532	0.05118468	0.82332098	0.55357177
Pdha1	-0.0234638	7.41076837	0.05111617	0.82343715	0.55357177
Gm10548	-0.0879878	1.74627685	0.0510725	0.82351126	0.55357177
Wdr74	-0.0643186	2.90109075	0.05107192	0.82351224	0.55357177
Nkx6-2	0.13955667	0.35293073	0.0510073	0.82362195	0.55357177
H6pd	0.05420917	3.62020881	0.05096497	0.82369385	0.55357177
Mgat3	-0.0310161	6.48403666	0.05095294	0.8237143	0.55357177
Chst13	0.27778868	-0.7207541	0.05094111	0.82373441	0.55357177
Perp	-0.0805658	6.65280061	0.05089529	0.8238123	0.55357177
Fst	-0.140086	0.61537621	0.05088982	0.8238216	0.55357177

2900026A02	-0.0357991	5.8920017	0.0508827	0.8238337	0.55357177
Romo1	-0.0695232	4.01738062	0.05086814	0.82385846	0.55357177
Ascc1	0.06579821	3.49080484	0.05073946	0.82407746	0.55364825
Srsf11	-0.0301214	6.73223638	0.05073484	0.82408533	0.55364825
Mpp3	0.04721029	3.41134994	0.05061888	0.82428296	0.55374305
Gm5113	0.05614871	4.18435604	0.05053977	0.82441791	0.5537655
Fasn	0.04423383	6.43402626	0.05050831	0.82447161	0.5537655
Nsf	0.03285406	9.99472431	0.05049989	0.82448598	0.5537655
Zfp629	-0.044576	4.52124725	0.05030813	0.8248137	0.55394762
Gkn3	-0.3154328	-1.1762182	0.05015504	0.8250758	0.5540843
Nfatc4	0.08957545	3.20919778	0.05008258	0.82520002	0.5540843
Kcnk5	0.09595332	2.33406875	0.05008066	0.82520331	0.5540843
Aars2	0.08318458	1.65531529	0.05003238	0.82528611	0.5540843
Srp72	-0.0230804	7.21966283	0.05002427	0.82530003	0.5540843
Pinx1	0.05253822	3.43679909	0.04987868	0.82555004	0.55421416
Ints5	-0.0629034	3.12011438	0.04972933	0.82580689	0.55434696
Kctd3	-0.0406559	5.13243541	0.04969787	0.82586104	0.55434696
Bcar1	0.03642107	4.23651133	0.04954614	0.8261225	0.55448447
2610524H06	-0.0595917	2.15892431	0.0494029	0.82636971	0.55461238
Ipo8	0.03553715	5.43226085	0.04930309	0.82654219	0.55468082
Cd1d1	0.27675739	-1.2586938	0.04927837	0.82658494	0.55468082
Sfxn3	-0.0319391	7.52997578	0.04921862	0.8266883	0.55468538
Nts	-0.1166864	1.01467383	0.04917351	0.82676637	0.55468538
Tcirg1	-0.078475	1.52990941	0.04911132	0.82687409	0.55468538
2410016O06	-0.0555652	3.82287711	0.04901877	0.82703451	0.55468538
Gm3604	0.05395007	2.73309904	0.04900706	0.82705483	0.55468538
Tmem220	-0.0675122	2.86846777	0.04899333	0.82707864	0.55468538
2010109A12	0.27213821	-2.023385	0.04896507	0.82712767	0.55468538
Fosl2	0.06987377	6.03954939	0.04896312	0.82713105	0.55468538
Mr1	-0.0539093	4.41173809	0.04895549	0.8271443	0.55468538
4632415L05	0.03254068	5.48944619	0.04894759	0.827158	0.55468538
Sned1	-0.0607095	4.23200509	0.04884117	0.82734282	0.55477134
Catsper2	0.07407996	1.9578967	0.04874375	0.82751218	0.55484692
5730405O15	0.17569218	-0.293786	0.0486861	0.82761248	0.55484833
6330403A02	0.03648012	7.47245341	0.04865005	0.82767524	0.55484833
Nsdhl	0.037095	4.56977349	0.04864489	0.82768422	0.55484833
Cdk17	0.03298202	7.87426799	0.04827042	0.82833758	0.55521158
Samd8	0.0296097	6.05743869	0.04821326	0.82843755	0.55521158
Scpep1	0.04586701	5.11264015	0.04818037	0.8284951	0.55521158
Rhobtb1	0.06578988	2.61378833	0.04811821	0.82860392	0.55521158
Ttc23	-0.0628478	2.80822105	0.04810079	0.82863443	0.55521158
BC052040	0.04127925	4.25305052	0.04807382	0.82868168	0.55521158
Tlr1	-0.2785238	-0.9829122	0.04802736	0.82876311	0.55521158

Rrp12	-0.0462958	3.92891724	0.0480234	0.82877004	0.55521158
Spint2	-0.0643978	2.87373047	0.04798567	0.82883621	0.55521158
Cdc23	-0.0338474	5.00918478	0.04796983	0.82886398	0.55521158
Trim30d	0.04914912	3.62302109	0.04794575	0.82890622	0.55521158
Aimp1	-0.0472426	4.50395021	0.04791013	0.82896874	0.55521158
Sgms2	0.08406011	3.45145195	0.04790042	0.82898579	0.55521158
Tdrd9	0.3162986	-1.9587224	0.04785018	0.829074	0.55521158
Cdh1	-0.0689951	6.50236452	0.04780815	0.82914784	0.55521158
Sgpp2	-0.0416028	3.73622858	0.0477931	0.82917429	0.55521158
Cldn2	0.14110615	1.2896757	0.04778435	0.82918967	0.55521158
Rnf138	-0.0445792	3.49491587	0.04769521	0.82934642	0.55526371
Syt1	0.03151926	9.90610824	0.04767561	0.8293809	0.55526371
Gm6086	-0.2237601	-1.5354807	0.04758889	0.82953359	0.55532798
Ccdc23	0.07469729	3.16987757	0.04729208	0.83005724	0.55561349
Socs1	0.2828592	-0.8725271	0.04726028	0.83011347	0.55561349
Ifi30	0.09025564	2.01187958	0.04725079	0.83013024	0.55561349
Ppib	0.05866839	2.66338172	0.04717126	0.8302709	0.55563697
Fgf11	0.03131071	6.14600193	0.04716682	0.83027876	0.55563697
Fam213a	-0.0477956	7.66914769	0.04707202	0.83044662	0.55570617
Uba5	0.02734595	5.87438216	0.04702565	0.8305288	0.55570617
Bhlha9	-0.2759845	-1.2344932	0.04701235	0.83055237	0.55570617
Tmem120b	-0.1693881	0.44603953	0.04688694	0.83077485	0.55579716
Syp	-0.0450302	8.81758959	0.04687173	0.83080185	0.55579716
Sbsn	-0.0790353	1.79275705	0.0467971	0.8309344	0.55584788
Hsd17b12	0.05463069	3.64403364	0.04670882	0.83109136	0.55591491
Dusp9	0.11909427	0.92120224	0.04662689	0.83123715	0.55597446
Gabpb2	0.02933984	6.14422032	0.04657565	0.83132842	0.55599754
Gm5086	-0.2109838	-0.7664952	0.04643136	0.83158567	0.55608076
Gen1	-0.1202376	0.53316377	0.04642408	0.83159865	0.55608076
Zfyve1	0.03489809	4.19700661	0.04641035	0.83162315	0.55608076
Rbm27	-0.0236309	7.46391826	0.04636787	0.83169899	0.55609351
Pigo	-0.0796324	1.91965557	0.04629796	0.83182388	0.55613905
Cmklr1	0.09032555	1.08346113	0.04616619	0.83205953	0.55620322
Coro2a	0.04972356	5.32555707	0.04612924	0.83212568	0.55620322
Gpr150	0.0915107	0.66432843	0.04612835	0.83212727	0.55620322
Fth1	-0.0253338	9.06580264	0.04611734	0.83214699	0.55620322
BC021891	0.08894964	1.57565952	0.04596174	0.83242585	0.55631643
Hltf	-0.0371115	4.91250401	0.04590328	0.83253077	0.55631643
Dhrs7	0.05876911	3.8290547	0.04586226	0.83260441	0.55631643
Ly86	0.08111367	2.08681599	0.04580991	0.83269846	0.55631643
Phf14	0.02507492	6.44424764	0.04580052	0.83271535	0.55631643
Oxa1l	-0.036982	5.17887572	0.0457815	0.83274953	0.55631643
Cpeb1	0.04019582	5.01021217	0.04578066	0.83275103	0.55631643

Nt5e	0.05772559	4.27306299	0.04576971	0.83277072	0.55631643
Mutyh	-0.125155	-0.0979963	0.04571976	0.83286054	0.55633778
Tmem164	-0.0328645	4.58664058	0.045687	0.83291948	0.55633778
Smo	-0.0627936	6.95353589	0.04565723	0.83297306	0.55633778
Clec9a	0.19560705	-0.0266248	0.04560352	0.83306977	0.55634714
Tapbp	0.06783158	4.62292862	0.04558479	0.83310352	0.55634714
Ankrd37	0.08517421	1.64700432	0.04555485	0.83315747	0.55634714
Rbm15	-0.0357867	3.93178982	0.04548854	0.83327702	0.55637426
Alad	-0.0562096	2.53834977	0.04544333	0.83335859	0.55637426
Hsf2	0.03346748	5.63223317	0.04543784	0.83336849	0.55637426
Tmem132c	0.05936353	2.32488406	0.04540217	0.83343287	0.55637933
Shh	0.08712561	2.02679904	0.04529388	0.83362851	0.556472
Slc13a5	0.06496561	2.26735821	0.04514939	0.83388992	0.55660857
Slc16a12	-0.0584173	4.08482467	0.04504503	0.834079	0.55666289
Ndst1	0.02794445	5.73500255	0.04499296	0.83417343	0.55666289
Ttf1	0.03096555	4.43710407	0.04497422	0.83420743	0.55666289
Lrtm2	0.04073361	5.89053661	0.04492628	0.83429443	0.55666289
Zfp748	0.03736089	4.81917853	0.04491045	0.83432318	0.55666289
Cdk4	0.05082487	4.37868643	0.04490612	0.83433103	0.55666289
Gm12060	-0.2021732	-1.0311945	0.04488516	0.8343691	0.55666289
Itm2a	0.05678325	6.66213787	0.04452793	0.83501929	0.55702958
Otud3	-0.07493	1.74259567	0.04451233	0.83504774	0.55702958
Trpm4	-0.0860379	2.0419583	0.04448953	0.83508934	0.55702958
Plek	0.03451662	4.63025579	0.04445225	0.83515738	0.55703704
Zfp637	-0.0501537	3.80381429	0.04424263	0.83554052	0.55725464
Gpr146	0.04494965	4.1900935	0.04418468	0.83564661	0.5572646
Arhgef11	-0.0273864	6.85563668	0.04417232	0.83566924	0.5572646
Ncoa4	0.02367938	7.81367737	0.0440118	0.83596352	0.55740717
Tmem9	0.04625022	4.96445114	0.04399365	0.83599684	0.55740717
Igfbp2	-0.0612966	4.96392241	0.04387952	0.83620645	0.55750898
Hbs1l	-0.0274446	5.48903497	0.04377578	0.83639724	0.55759823
Exo1	0.17464966	-0.6416641	0.04364381	0.83664029	0.5577223
2810442N19	0.15377338	0.21262058	0.0435461	0.83682048	0.55776754
Ctdspl2	-0.0269055	6.57527525	0.0435152	0.83687752	0.55776754
Tcn2	0.06337698	4.22294844	0.04351441	0.83687898	0.55776754
Zdhhc8	-0.0433835	4.78650998	0.04347632	0.8369493	0.55777646
Nup107	-0.0452374	3.78393651	0.04340121	0.83708809	0.557831
Cdsn	-0.1002491	0.95293506	0.04329924	0.83727668	0.55791872
Phf10	-0.0300173	5.32366642	0.04315432	0.83754514	0.55805543
Slc7a11	-0.0426288	9.83007909	0.043122	0.83760508	0.55805543
Tube1	0.1265459	0.36225688	0.04307704	0.83768848	0.55805543
Slc2a1	-0.0468984	4.64273172	0.04306559	0.83770973	0.55805543
Txk	-0.2277595	-1.7280901	0.04299411	0.83784247	0.5581059

Acaa1a	0.0410238	3.87713101	0.04292239	0.83797574	0.55815672
Rnf41	-0.028822	5.35339329	0.0427863	0.83822899	0.55824923
Lmnb2	-0.0513128	3.01885766	0.04276801	0.83826304	0.55824923
Opn1mw	-0.2061518	-1.6765362	0.04275591	0.83828559	0.55824923
Adss	0.02185221	7.09678092	0.04270356	0.83838315	0.55827624
Agpat9	0.08281316	1.27578134	0.0426631	0.83845861	0.55828853
5930403L14R	-0.0561987	4.76609012	0.04257487	0.83862324	0.55828896
Vdr	0.10298706	0.75906916	0.04256779	0.83863646	0.55828896
Tdrd3	0.028226	5.51620388	0.04249573	0.83877108	0.55828896
Med26	-0.0519102	2.57431246	0.04248597	0.83878933	0.55828896
Clec12a	-0.2050905	-0.500665	0.04244778	0.83886074	0.55828896
Elmsan1	0.02782792	5.92131118	0.04244614	0.83886381	0.55828896
Ppm1g	0.03215375	4.91335133	0.04241084	0.83892982	0.55828896
Cd24a	0.04361502	5.29033458	0.04237318	0.8390003	0.55828896
Flnc	0.07610185	2.89884862	0.04233984	0.83906273	0.55828896
Pcid2	0.05237392	4.26634617	0.04233912	0.83906408	0.55828896
Dfna5	0.04458746	4.32154807	0.0423273	0.8390862	0.55828896
Mst1r	-0.1521417	-0.7294972	0.04225666	0.83921856	0.5583391
1700120C14R	-0.1719971	-1.1689078	0.04219713	0.8393302	0.55835075
Nagk	0.05162236	3.23250208	0.04218653	0.83935007	0.55835075
Primpol	-0.0786945	2.5564363	0.04213355	0.8394495	0.55836077
Fam98c	0.16226358	-0.7052618	0.04210917	0.83949529	0.55836077
Hist1h2bf	-0.1674861	-0.8437802	0.04208741	0.83953615	0.55836077
Thbs2	0.05437864	5.06590599	0.04204618	0.83961362	0.5583705
0610038B21R	0.23794668	-1.8223318	0.04201896	0.83966479	0.5583705
Dbndd2	0.02595126	5.98223656	0.04183765	0.84000605	0.55855952
Arhgap20	0.02676927	8.42146037	0.04173356	0.84020231	0.5586521
Kcng1	-0.1554959	0.10079227	0.04156237	0.84052563	0.55875616
Traf3ip1	0.02867141	4.88522269	0.04150796	0.84062856	0.55875616
Ppp5c	-0.0333775	4.63015193	0.04149379	0.84065537	0.55875616
Cdkl2	-0.0281891	6.06379876	0.04148255	0.84067663	0.55875616
Ildr2	0.03210643	9.87099209	0.04146212	0.84071531	0.55875616
Vrk1	-0.0410759	3.86179314	0.04140864	0.8408166	0.55875616
Cd200	0.03359173	6.86633435	0.0413962	0.84084016	0.55875616
Fan1	0.06916199	1.65864258	0.04135222	0.84092351	0.55875616
Mobp	-0.0405648	5.81453183	0.04129938	0.84102372	0.55875616
Herpud1	0.03690108	4.41628755	0.04129392	0.84103409	0.55875616
P2rx4	-0.0526328	3.21501847	0.04127355	0.84107273	0.55875616
Pstk	-0.0483763	4.18438685	0.04123481	0.84114627	0.55875616
Sdccag3	-0.03361	4.74850971	0.04122916	0.84115699	0.55875616
Sh3glb2	0.02810741	5.23675854	0.04122893	0.84115742	0.55875616
Gm4759	-0.2220793	-0.9557459	0.04111438	0.84137509	0.55886285
P2ry14	0.07945175	1.47512137	0.04101091	0.84157196	0.5589508

Tubg2	-0.0399376	4.31544614	0.04098483	0.84162162	0.5589508
F3	-0.0457363	5.60082471	0.04087682	0.84182748	0.55904962
Katnal1	0.02836147	6.85502897	0.04084273	0.84189253	0.55905491
Syt9	-0.0476933	3.34875711	0.04077919	0.8420138	0.55909754
Tmem169	0.04443765	2.9231717	0.0406886	0.84218691	0.55917458
5730455P16I	0.02402291	7.14297718	0.04057586	0.8424026	0.55920718
Arid5b	0.028308	6.23806089	0.04057075	0.84241239	0.55920718
Ppil6	0.08764048	2.35318332	0.04054685	0.84245815	0.55920718
Pnkd	-0.0270049	6.53540609	0.04053096	0.84248858	0.55920718
Fen1	0.05372351	2.73471219	0.04045788	0.84262865	0.55920718
Dlc1	0.02899344	6.60840981	0.04045436	0.84263541	0.55920718
Cdca5	0.24211715	-1.4969449	0.04045424	0.84263564	0.55920718
Rubie	-0.2437299	-1.4883069	0.04034798	0.84283955	0.55923917
Nudt10	-0.0399788	3.41269048	0.04034517	0.84284494	0.55923917
Foxn2	-0.0368928	4.77039956	0.04033987	0.84285512	0.55923917
Lig3	0.03599234	4.99211735	0.04020867	0.84310729	0.5593686
Dcaf8	-0.0241554	5.70611161	0.04010807	0.84330095	0.55945919
Sulf1	0.046207	5.8986324	0.04002626	0.84345862	0.55948998
9630028B13I	0.09095794	2.68441156	0.04002471	0.8434616	0.55948998
Nr1h3	-0.07751	2.61585111	0.03997342	0.84356053	0.55951772
Clec4a1	0.18248412	-0.821454	0.0399305	0.84364337	0.55953477
Oaf	-0.0839814	1.012557	0.03980459	0.84388667	0.5596257
Hk2	-0.054452	2.80291057	0.03977391	0.84394601	0.5596257
Bak1	-0.0519069	3.90940784	0.03977088	0.84395186	0.5596257
Cd59a	-0.0484245	4.58348747	0.03973264	0.84402588	0.55963689
Itga2b	-0.1290869	-0.091508	0.0396098	0.84426385	0.55974654
Casp8ap2	0.02247224	6.4656115	0.0395883	0.84430553	0.55974654
Cyb5r1	0.05171465	2.67456624	0.03955688	0.84436649	0.55974907
Etaa1	-0.0401014	4.87202741	0.03949013	0.84449604	0.55979707
Ap1g1	0.0324257	7.3243594	0.03942563	0.84462134	0.55980596
Frat2	0.07339398	3.01946885	0.03942439	0.84462376	0.55980596
Ephx1	-0.0573664	4.19076574	0.03939036	0.84468991	0.55981192
Gpr83	0.06426595	2.73427723	0.03926122	0.84494123	0.55993839
Ugp2	-0.0233426	6.43308734	0.03921768	0.84502605	0.55993839
Ccar2	-0.0444573	4.23538832	0.0391867	0.84508646	0.55993839
Sirpb1b	0.28170789	-1.704659	0.03916805	0.84512281	0.55993839
Gucy2g	0.07918491	1.35849486	0.03912709	0.84520271	0.55993839
Josd2	0.06635255	2.23009201	0.03911633	0.84522371	0.55993839
Tmem33	0.02339056	6.24306401	0.03907631	0.84530183	0.55995227
Nptx2	0.04531818	4.03919698	0.03898789	0.84547457	0.55998568
Ppp1r13b	0.03067229	5.6077684	0.03896871	0.84551207	0.55998568
Crh	-0.1344061	0.14931193	0.03894662	0.84555527	0.55998568
Chuk	0.03885906	4.87758158	0.0389335	0.84558093	0.55998568

Rdh14	-0.0359631	5.61458882	0.03881205	0.84581871	0.56003761
Avl9	-0.0304725	6.00225198	0.03880598	0.8458306	0.56003761
Dennd5a	-0.0205061	7.52315641	0.03878548	0.84587079	0.56003761
Arhgap10	0.0385166	4.4409268	0.03874114	0.84595772	0.56003761
Fhod3	0.04135999	5.14905749	0.03873642	0.84596699	0.56003761
2010107G12	-0.2986481	-1.5028494	0.03871837	0.84600239	0.56003761
Bub3	-0.0256365	5.8548539	0.03866242	0.8461122	0.56007245
Atp5a1	-0.0196983	9.40853935	0.03862436	0.84618695	0.56008408
Arl5c	0.18257426	-0.6989785	0.03856671	0.84630023	0.56012121
Nkx2-2os	0.26184065	-0.6508866	0.0384466	0.84653655	0.56023976
Gramd1c	0.09166325	1.68330996	0.03830093	0.84682368	0.56039192
Col6a6	0.23880137	-1.4085711	0.03822909	0.8469655	0.56041312
Nr2f1	-0.031482	6.76098752	0.03822674	0.84697014	0.56041312
Ptpn2	0.04811089	4.93947843	0.0381253	0.84717061	0.56045893
Tbc1d10a	-0.0876009	1.66524401	0.03811503	0.84719093	0.56045893
Mettl8	-0.0490772	3.77776576	0.03809181	0.84723686	0.56045893
Kif7	-0.092633	1.49510603	0.03805816	0.84730347	0.56045893
Hnrnpd	0.02119274	8.29361653	0.03804705	0.84732546	0.56045893
1700007K13I	-0.1258139	0.10950649	0.0380063	0.84740615	0.560459
Myo1c	0.04368519	5.19518825	0.03796255	0.84749285	0.560459
Pcdhb11	0.11155042	0.96598835	0.03793562	0.84754624	0.560459
Fam162a	-0.0433421	5.00443547	0.03793149	0.84755443	0.560459
Qdpr	-0.0252305	5.53356808	0.03785728	0.84770166	0.56046548
Cntfr	-0.0577993	4.14223416	0.03785636	0.84770348	0.56046548
Alcam	0.04101738	9.49713943	0.03784004	0.84773588	0.56046548
Upf1	0.03645529	4.27540549	0.037806	0.84780349	0.56047235
Klhl10	0.20173626	-0.8904987	0.03774962	0.84791551	0.56050858
Rnf150	0.03332992	7.33779721	0.03765032	0.84811306	0.56060133
Drosha	-0.0244678	6.89887873	0.03761346	0.84818645	0.56060501
Prrx2	-0.0946057	3.43939736	0.03759005	0.84823309	0.56060501
Ndufa4l2	0.13185209	-0.0866325	0.03750846	0.84839574	0.56067468
Fiz1	0.04223289	3.76023	0.03743563	0.84854107	0.56068093
Gm11346	0.21511472	-0.647839	0.03737507	0.84866204	0.56068093
Reck	-0.040231	5.35084657	0.03730553	0.84880106	0.56068093
Dennd2d	-0.1640148	-0.2058797	0.03724889	0.84891439	0.56068093
Eral1	-0.0429948	3.54601251	0.03723218	0.84894784	0.56068093
Xrcc1	-0.0473183	2.99943505	0.03715261	0.84910726	0.56068093
Hr	0.06786107	3.0286007	0.03712797	0.84915666	0.56068093
Sec22c	-0.0332889	4.63441973	0.03711876	0.84917511	0.56068093
5031425F14I	0.3891433	-2.2204453	0.03711118	0.84919032	0.56068093
Ptges	-0.0512469	4.28233446	0.03710257	0.8492076	0.56068093
Arc	0.07487407	5.37353445	0.03706934	0.84927426	0.56068093
Shroom2	-0.0206678	6.79998881	0.0370513	0.84931048	0.56068093

Gm6277	0.0643394	2.28629519	0.03701941	0.84937449	0.56068093
Htr2c	-0.0391492	4.57506655	0.03694077	0.8495325	0.56068093
Snx22	0.12293569	-0.2486315	0.03688898	0.84963667	0.56068093
Uchl3	-0.0393418	3.58882508	0.03688345	0.8496478	0.56068093
Lrpap1	-0.0373467	5.78732057	0.03688189	0.84965094	0.56068093
B430010I23F	-0.188878	-0.1740072	0.03685028	0.84971454	0.56068093
Timm13	-0.0670359	2.54144414	0.03679577	0.84982432	0.56068093
Fgfbp1	0.06822011	4.61375719	0.03675415	0.84990819	0.56068093
Rhbdd2	0.03618377	4.33556615	0.03674912	0.84991832	0.56068093
Hsp90b1	0.02789448	9.12486298	0.03674155	0.8499336	0.56068093
Fos	-0.1284783	3.49210614	0.03673116	0.84995455	0.56068093
Sycp3	0.07294576	2.03633713	0.0367057	0.85000588	0.56068093
Rab5b	0.02528361	7.01530066	0.03670397	0.85000938	0.56068093
Zfp688	-0.0916427	1.2095014	0.03669833	0.85002075	0.56068093
Per3	0.02434345	5.90032191	0.03666883	0.85008027	0.56068093
Gdi1	0.02545104	8.88966639	0.03666594	0.8500861	0.56068093
LOC1026324	-0.4506882	-1.9097647	0.03663787	0.85014277	0.56068093
Lamb3	0.24350654	-1.016154	0.03663341	0.85015179	0.56068093
Rims3	-0.1027685	0.8032777	0.03661943	0.85018001	0.56068093
Fam117a	-0.0805619	3.77824039	0.03659128	0.85023687	0.56068093
Fgfr2	-0.0375362	6.83047451	0.03654233	0.85033581	0.56070843
Msx1os	-0.250361	-1.7882486	0.03649091	0.85043979	0.56073925
Ift81	0.02294331	5.57400414	0.03644768	0.85052729	0.56075919
Gm15412	0.39284022	-1.650277	0.0364172	0.85058902	0.56076215
1810041L15F	0.04155792	5.52631739	0.03634287	0.85073965	0.56076328
Smad9	-0.0301213	5.40048518	0.03634264	0.85074012	0.56076328
C7	0.10083826	0.35357518	0.03633161	0.85076247	0.56076328
Tslp	0.24023825	-1.0755982	0.03624356	0.85094115	0.56084331
Gfer	0.04925542	3.00280689	0.03615969	0.85111153	0.56088035
BC065397	-0.1020167	0.92368029	0.03615952	0.85111188	0.56088035
Gnao1	0.02619079	10.1861888	0.03606557	0.85130299	0.56093228
Tut1	0.06255072	2.2972698	0.03606448	0.85130521	0.56093228
Lypd6	-0.034408	4.9247082	0.03585845	0.85172523	0.56108963
Tmem150c	0.04416743	4.08315618	0.0358499	0.8517427	0.56108963
BC037034	0.03817984	3.84047215	0.03584977	0.85174297	0.56108963
Vps33b	0.03330171	5.18058997	0.035835	0.85177314	0.56108963
Zfp219	-0.0598787	1.95613144	0.03578647	0.85187227	0.5611172
Stat1	-0.0301819	5.50301959	0.03574129	0.85196465	0.56114032
2700038G22	-0.112716	-0.1344479	0.03571068	0.85202727	0.56114383
Tmem203	0.04741578	2.72407557	0.03565358	0.85214415	0.56114954
Ly6c2	-0.1719817	-0.5892945	0.03562786	0.85219683	0.56114954
E130311K13I	-0.0462577	3.48396889	0.03559804	0.85225792	0.56114954
Gpc4	0.03576285	4.23406779	0.03558499	0.85228467	0.56114954

Ido2	0.06680365	2.00854124	0.0355666	0.85232237	0.56114954
Klri2	-0.1275259	0.40890264	0.03550367	0.85245147	0.56117309
Ccdc85a	-0.0306316	5.81768813	0.03547195	0.85251658	0.56117309
Dffa	0.02741204	5.08935317	0.03546541	0.85253002	0.56117309
AI661453	-0.0757654	1.73037844	0.03537838	0.85270882	0.56119894
S100a4	-0.0880999	3.08503307	0.03533145	0.85280536	0.56119894
Pla2r1	-0.1159076	-0.1361902	0.03533102	0.85280624	0.56119894
Rnf169	-0.0209379	6.75839916	0.03531709	0.8528349	0.56119894
Cybb	-0.1207784	0.85518632	0.03529277	0.85288496	0.56119894
Slc39a14	-0.0427457	3.35867201	0.03527913	0.85291304	0.56119894
Gm8787	-0.178815	-1.3720546	0.03523555	0.85300281	0.5612203
Gldn	0.10771875	0.93540306	0.03519669	0.85308288	0.56122464
Acacb	0.09241722	1.28171749	0.03516468	0.85314889	0.56122464
Kcnab2	0.03428518	5.95193359	0.03514897	0.85318129	0.56122464
Ncoa6	-0.0263865	8.53285239	0.03507994	0.85332379	0.56125728
Cacnb3	-0.0240382	6.89215654	0.03505865	0.85336775	0.56125728
Gm6623	-0.0543371	1.5751644	0.03504168	0.8534028	0.56125728
Susd2	0.03643461	5.00079841	0.03500874	0.8534709	0.56126438
Ppp1r36	-0.1665868	-0.2099402	0.03492595	0.85364214	0.56131909
Grin2d	-0.0614935	1.80761077	0.03491312	0.85366869	0.56131909
Snapc3	0.03017133	4.89748963	0.03485436	0.8537904	0.56132802
Gm19557	0.1745795	-0.643446	0.03485123	0.85379689	0.56132802
Clca1	-0.1833312	-0.4968022	0.03481286	0.85387641	0.56134262
Osbpl5	-0.0364487	3.92473795	0.03474691	0.85401322	0.56139489
Scara3	-0.058479	6.56283487	0.03462652	0.85426328	0.56152158
Suox	-0.0424388	4.24986719	0.03451828	0.85448851	0.56163194
Zfp383	-0.0531608	2.57221034	0.03444304	0.85464528	0.56166907
Rab26	-0.0509327	3.91471612	0.03442999	0.85467248	0.56166907
Il17rc	-0.0722573	1.90977284	0.03440863	0.85471702	0.56166907
Trit1	0.04821103	3.53345895	0.03435857	0.85482148	0.56167222
Serpine1	0.13190677	-0.2823831	0.03435138	0.85483649	0.56167222
Trak1	-0.0221017	7.52589125	0.03429198	0.85496056	0.56168141
Tug1	-0.0198427	8.05181296	0.03427035	0.85500577	0.56168141
Nov	-0.0581921	7.77909032	0.03426234	0.85502251	0.56168141
Rex2	0.18637857	-1.6257951	0.03421917	0.85511278	0.56169187
Sec14l3	0.18576129	-0.5228344	0.03419989	0.85515311	0.56169187
Gm10536	0.18182891	-1.3984401	0.03415938	0.85523791	0.5617099
Ctla2b	0.19363941	-0.6607534	0.03411216	0.8553368	0.56173719
Rims4	0.21055583	-1.1305353	0.0339545	0.85566754	0.56190259
Ciz1	-0.0272255	5.70076852	0.0339026	0.8557766	0.56190259
Sgk3	0.0285118	5.20054935	0.03388061	0.85582282	0.56190259
Oas1b	0.1298196	0.04731173	0.03387171	0.85584154	0.56190259
Naa30	0.0255838	5.57942222	0.03384452	0.85589872	0.56190259

Fam167a	-0.0634853	2.37068262	0.03380251	0.85598713	0.56190259
Dusp12	0.05972567	2.30696083	0.03376984	0.85605592	0.56190259
Foxl2	0.21417151	-0.4672663	0.0337343	0.85613079	0.56190259
Gigyf2	0.02759137	6.95812281	0.03370707	0.85618819	0.56190259
Zfp935	0.03681627	4.49689751	0.03370009	0.8562029	0.56190259
Jade1	0.02507674	6.43364774	0.03363354	0.8563433	0.56190259
Ak2	-0.0409757	4.85332912	0.03363222	0.85634609	0.56190259
Entpd6	0.04465385	3.09505747	0.03361241	0.8563879	0.56190259
Gm11696	-0.0741429	1.18376986	0.03361059	0.85639175	0.56190259
Ckb	-0.0286182	7.92797394	0.03355775	0.85650334	0.56193817
Oas2	-0.1157015	0.13255888	0.03348912	0.85664844	0.56199448
Plxdc1	-0.0397933	2.91556988	0.0334629	0.85670392	0.56199448
Fam63b	0.02213174	7.76590666	0.03330555	0.85703731	0.56210687
4933407K13I	-0.0856201	1.84513764	0.03330319	0.85704232	0.56210687
Myb	-0.0890451	0.61825725	0.03330079	0.85704741	0.56210687
Lyz1	-0.1209326	1.04164161	0.0331191	0.85743342	0.56231993
Fbxl21	0.03780237	3.14489174	0.0330727	0.85753216	0.56231993
Prss53	0.23927845	-1.4968797	0.03305867	0.85756203	0.56231993
Amer3	0.04213155	3.42676157	0.03303014	0.85762281	0.56231993
Ctns	0.04849131	2.65639179	0.03301302	0.85765929	0.56231993
Pex12	-0.0322793	4.06742378	0.03296181	0.85776845	0.56232066
Rnf180	0.05693301	2.8396008	0.03295864	0.85777522	0.56232066
Pla2g12a	0.04171381	3.72783429	0.03291366	0.8578712	0.56234594
Urah	-0.1178939	0.09704359	0.03271213	0.85830202	0.5625907
Tbx18	-0.0370069	6.20995825	0.03260417	0.85853338	0.5627047
Aco1	0.03100096	4.27600672	0.03257117	0.85860418	0.56271345
Gm13031	-0.1883668	-0.4832155	0.03247732	0.85880573	0.56274386
Rrbp1	0.02887223	6.08677972	0.03246992	0.85882163	0.56274386
Mchr1	-0.0596317	2.58860554	0.03246931	0.85882293	0.56274386
Rnd3	0.03524697	5.26196549	0.03232557	0.85913225	0.56282087
2810459M11	0.07029868	1.84804127	0.0323191	0.85914619	0.56282087
Grb7	-0.0880504	0.847069	0.03231887	0.85914669	0.56282087
Galns	0.09064602	0.66042022	0.03228261	0.85922484	0.56282087
Lamc2	0.04297749	2.97337274	0.03228046	0.85922946	0.56282087
Alg1	0.1415119	0.0254511	0.03225461	0.85928521	0.56282087
Lrrc55	0.02872657	4.49946179	0.03214557	0.8595206	0.56293741
Hipk3	0.01832855	7.79073933	0.03211519	0.85958625	0.56294045
Kiss1r	-0.1037612	0.15055536	0.03209024	0.85964019	0.56294045
Mapk1ip1l	-0.0277715	8.02239861	0.03178712	0.86029731	0.56331344
Upk1b	-0.1055559	1.27671909	0.03177448	0.86032478	0.56331344
Cntd1	0.04712404	2.33775579	0.03174217	0.86039502	0.56331358
Nusap1	-0.0678729	2.16744972	0.03172149	0.86044002	0.56331358
Wapal	-0.0185263	7.56975924	0.03167102	0.86054984	0.5633373

Ehbp1	-0.0258041	6.77901696	0.031652	0.86059127	0.5633373
Rab23	-0.0252261	5.37717903	0.03159194	0.86072213	0.56335009
Tsnaxip1	0.2935653	-1.2806416	0.03159025	0.86072583	0.56335009
Got1	0.02461149	8.34719757	0.03153427	0.86084792	0.56339235
Taf7	0.03591873	3.82423093	0.03145093	0.8610299	0.56347381
Skil	-0.0206315	7.34534605	0.03137297	0.86120036	0.56353457
Cd47	-0.0282947	7.49962198	0.03133271	0.86128849	0.56353457
D10Wsu102e	-0.0254181	5.43301154	0.03129539	0.86137021	0.56353457
Bahd1	0.04349916	4.09485007	0.03129526	0.8613705	0.56353457
Sstr3	0.0338206	3.54620134	0.03127705	0.86141041	0.56353457
Rhog	-0.0452272	3.23796966	0.03123044	0.86151258	0.56356377
Plod1	-0.047466	3.01885443	0.03113534	0.8617213	0.56357111
Tal1	0.12918174	0.26914581	0.03111685	0.86176192	0.56357111
Zfp551	0.06994839	2.48571282	0.03109661	0.86180639	0.56357111
Mndal	-0.0494289	2.99456607	0.03109535	0.86180916	0.56357111
Mum1l1	0.09756165	1.70224745	0.03109431	0.86181147	0.56357111
Il6st	0.02847027	7.38933824	0.030889	0.8622635	0.5638202
Tapbpl	-0.0340941	3.28314003	0.03086906	0.8623075	0.5638202
2900052N01	-0.0558325	2.81930191	0.03079607	0.86246862	0.56388791
Dtd2	-0.0449104	4.09246295	0.03063428	0.8628265	0.56406779
Elavl2	0.02573967	6.93896308	0.03059059	0.86292331	0.56406779
Pced1b	-0.0408711	3.1020314	0.03058662	0.86293211	0.56406779
AW146154	-0.0476871	3.00440561	0.03056769	0.86297408	0.56406779
Glud1	0.01781422	8.41518438	0.03049133	0.86314351	0.56406927
Dlgap4	-0.0217875	7.19902997	0.03044046	0.86325652	0.56406927
Tbc1d12	0.03603419	3.91318309	0.03043603	0.86326637	0.56406927
Edil3	0.03199659	6.55862889	0.03040917	0.86332607	0.56406927
Fbln1	0.05164858	4.89785489	0.03039055	0.86336748	0.56406927
Tmed10	-0.0228437	6.82479277	0.03038828	0.86337253	0.56406927
Mrps9	-0.0386758	3.40868777	0.03038517	0.86337944	0.56406927
Mrps17	0.03633123	4.15799816	0.03029372	0.86358303	0.56412257
Furin	0.05162786	2.94407601	0.0302625	0.8636526	0.56412257
Gas2l2	0.19162836	-0.998051	0.03025724	0.86366433	0.56412257
Dnajc25	0.05687415	2.95692566	0.03021446	0.86375972	0.56412257
1810021B22I	-0.1102306	0.54550243	0.03020125	0.86378918	0.56412257
Rab3il1	-0.0417856	4.12135723	0.03019346	0.86380657	0.56412257
Avil	0.21390037	-1.4523535	0.03013427	0.86393873	0.56413661
Sfxn5	-0.033192	4.82337831	0.03013224	0.86394326	0.56413661
Ppm1m	0.04146888	3.80344664	0.03002242	0.86418884	0.5642271
Cdk5	0.03323013	4.20561656	0.03001875	0.86419704	0.5642271
Greb1	-0.1018518	0.39296859	0.02989813	0.86446732	0.56436595
Nebl	-0.0250375	6.61374962	0.02985849	0.86455624	0.56438638
Nup35	-0.0390588	3.28593471	0.02978838	0.86471371	0.56445156

Pld3	-0.0322098	7.04304457	0.02971727	0.8648736	0.56451831
Pltp	-0.05182	4.01039227	0.02960342	0.86513003	0.56461576
Frmd3	0.05817396	2.67448322	0.02956911	0.86520741	0.56461576
Purb	0.02029701	9.71124071	0.02956459	0.86521761	0.56461576
Pkhd1l1	0.15936218	-0.6430587	0.02951827	0.86532214	0.56461576
Tiam2	0.03386511	4.92606776	0.02951166	0.86533706	0.56461576
Slc39a9	0.02675421	4.89213791	0.02945794	0.86545843	0.56461576
Frs2	0.01913597	7.4298957	0.02944524	0.86548713	0.56461576
Mir344b	0.22090322	-2.0874716	0.02942023	0.86554369	0.56461576
Smarcad1	0.03262167	5.2613754	0.02939768	0.8655947	0.56461576
Lamb2	0.05346435	4.32199266	0.02939168	0.86560826	0.56461576
Top1	-0.0236979	8.05108416	0.0293389	0.86572776	0.56461576
Tmem125	0.09124486	0.65984188	0.02930369	0.86580753	0.56461576
Mecp2	-0.0218567	8.93917506	0.02927346	0.86587606	0.56461576
Sntg1	0.03076031	5.58941701	0.02927245	0.86587834	0.56461576
Inca1	-0.0687175	0.79195085	0.02922639	0.86598283	0.56461576
Cttn	0.02412963	5.83338286	0.02918913	0.86606742	0.56461576
Olfr316	0.1931173	-0.6026175	0.02917501	0.8660995	0.56461576
Slc35a1	0.03742889	5.25560159	0.02915791	0.86613834	0.56461576
Synj2bp	0.01958639	7.03416657	0.02914262	0.86617307	0.56461576
Dclre1c	0.04349349	5.24539351	0.02914145	0.86617573	0.56461576
AK129341	0.04212167	5.21366025	0.02892279	0.86667367	0.56489252
Tsc22d2	0.02035764	6.84533582	0.0289044	0.86671564	0.56489252
Gnptg	-0.0289863	4.63941865	0.02884316	0.86685549	0.56490808
Pianp	0.02385429	6.45296759	0.0288117	0.8669274	0.56490808
Secisbp2l	0.02060095	7.76977179	0.02879458	0.86696655	0.56490808
Ube2t	-0.1805956	-1.0713827	0.02878863	0.86698015	0.56490808
Tbc1d17	-0.055702	2.57286383	0.02875372	0.86706002	0.56490808
Pde1a	0.01685023	9.4446545	0.02869437	0.86719592	0.56490808
Abhd17b	-0.0283403	5.76779969	0.02866734	0.86725786	0.56490808
Sybu	0.03036157	5.28269704	0.02865858	0.86727794	0.56490808
Wnt9b	0.24594486	-1.9055539	0.02862991	0.86734367	0.56490808
Trp53rk	-0.0864359	2.02455285	0.02862351	0.86735835	0.56490808
Dll4	-0.1917394	-1.5188964	0.02860935	0.86739084	0.56490808
Htr1b	0.05958305	1.29237253	0.02856154	0.8675006	0.56490808
Zfp597	0.02416452	4.89162682	0.02854653	0.86753507	0.56490808
Mus81	-0.0493022	2.15460591	0.02854137	0.86754691	0.56490808
Htr6	-0.1866734	-1.4043342	0.02847082	0.86770909	0.56493509
Cdc42ep1	-0.0811967	2.57236804	0.02845805	0.86773848	0.56493509
Zfp579	0.0525077	1.47293401	0.02841771	0.86783131	0.56493509
Il2rg	-0.1332205	0.43366703	0.02840354	0.86786394	0.56493509
Nat8	-0.1226079	0.23578524	0.02839798	0.86787676	0.56493509
Pyhin1	-0.1007477	1.688976	0.0283376	0.8680159	0.56498811

Kcnmb4	-0.0303834	3.94159509	0.02800039	0.86879583	0.565409
Atp1b1	-0.0183564	10.1417314	0.02795092	0.86891066	0.565409
A730056A06	0.06990918	1.59917993	0.02788635	0.86906069	0.565409
Sec62	0.02157126	7.55522414	0.02787514	0.86908676	0.565409
Meox1	0.15296657	-1.373733	0.02787389	0.86908967	0.565409
Htra4	-0.0516221	3.17792456	0.02786598	0.86910805	0.565409
Ace2	0.24882333	-0.716021	0.02784997	0.86914531	0.565409
2900079G21	0.06020891	1.16559202	0.02782205	0.86921027	0.565409
Speer4a	-0.0897986	0.36362606	0.02780752	0.86924409	0.565409
Rtn4rl1	0.02172913	6.19990827	0.0277763	0.8693168	0.565409
Cwf19l1	-0.0429815	3.90492963	0.02777238	0.86932594	0.565409
Rbm45	0.03522324	3.49260112	0.02775983	0.86935518	0.565409
Tmem39a	0.02769462	4.96808589	0.02772504	0.86943628	0.56542419
Pank3	-0.021154	7.493322	0.02766205	0.86958326	0.56542662
Sema3d	0.04880193	6.49635754	0.02765874	0.86959098	0.56542662
Rac3	-0.1505494	-0.9383691	0.02764923	0.86961319	0.56542662
Igfbp5	-0.0372584	8.64917915	0.02760248	0.8697224	0.5654601
9030624J02F	0.02062254	5.22420034	0.0275255	0.86990244	0.56553962
Gmcl1	0.02772621	4.31263932	0.02744711	0.87008606	0.56562145
Trib1	0.02579134	5.66745826	0.0273993	0.87019817	0.56565679
Mocs1	0.04672203	4.31887356	0.0273166	0.87039235	0.56570923
Mfap5	0.0952125	2.15899869	0.02727072	0.87050021	0.56570923
Zfp322a	0.02284565	6.03840484	0.02721732	0.87062584	0.56570923
Slc39a8	0.06104568	2.78541867	0.02721097	0.8706408	0.56570923
Pipox	0.08256492	0.57711512	0.02717926	0.87071549	0.56570923
A630007B06	0.02316673	7.27132585	0.02716783	0.8707424	0.56570923
Anxa9	-0.1236233	-0.5916334	0.02716747	0.87074326	0.56570923
Lefty1	0.06795741	1.93851202	0.02715117	0.87078167	0.56570923
Calcoco1	-0.019259	7.47821036	0.02714398	0.87079862	0.56570923
Lmf2	0.04494109	2.46143487	0.02711219	0.87087359	0.56572042
Ociad1	-0.0190857	7.1434542	0.02706239	0.87099112	0.56575879
Mrps10	0.03951069	2.80224304	0.02703823	0.87104817	0.56575879
Perm1	0.13897901	-0.4116374	0.02695773	0.87123847	0.56584487
Alg14	-0.0434939	4.58603862	0.02688122	0.87141961	0.56588888
Agps	0.01956499	7.19752054	0.02687273	0.87143971	0.56588888
C130021I20R	0.09586196	1.23443208	0.02679791	0.87161714	0.56588888
Atp13a5	0.03184328	4.94737896	0.02679542	0.87162305	0.56588888
Nxpe2	0.08443516	1.13167238	0.02679248	0.87163002	0.56588888
Appbp2	0.02577728	5.28876882	0.02678286	0.87165286	0.56588888
LOC1005047(	-0.1336618	-0.317366	0.02667301	0.8719139	0.56602083
Mex3b	-0.0378121	3.37780557	0.02661557	0.87205059	0.56607205
Zfp641	-0.0543077	3.02665455	0.02656293	0.87217601	0.56611524
Efnb3	0.03426971	4.91469848	0.02653585	0.87224058	0.56611524

Timp1	-0.0939463	1.52982041	0.02647919	0.8723758	0.56611524
Ubqln2	-0.0239278	7.64015162	0.02636645	0.87264528	0.56611524
Rab3d	-0.0339276	3.19566862	0.02636464	0.87264961	0.56611524
Dleu2	-0.053621	2.42882056	0.02633951	0.87270975	0.56611524
Prkcz	-0.0217858	6.3385358	0.02633503	0.87272049	0.56611524
LOC1005034	-0.1378073	-1.3524867	0.02631475	0.87276906	0.56611524
Amotl2	0.04816631	3.80934103	0.02631009	0.87278022	0.56611524
Fam83b	-0.133181	-0.6156451	0.02630744	0.87278655	0.56611524
Fibin	0.04536614	4.43120011	0.02630546	0.87279131	0.56611524
Atp1a2	0.03636412	11.2666227	0.02629739	0.87281065	0.56611524
Mlxipl	0.07654692	0.95267483	0.0262573	0.87290673	0.56614007
Xlr3c	-0.1616904	-1.4935788	0.02622232	0.87299065	0.56615701
Zfp771	-0.0908537	0.69994245	0.02613908	0.87319056	0.56621333
Ptpn14	-0.0379386	6.35989005	0.02613802	0.8731931	0.56621333
Dus4l	-0.0488987	2.58436004	0.02607727	0.87333922	0.56627059
Shc4	0.04373873	2.54008987	0.02605145	0.87340137	0.5662734
Sspn	0.04635506	4.45629155	0.02601729	0.87348365	0.56628591
Mapk9	0.01954225	8.50240878	0.02599545	0.87353628	0.56628591
Gm16845	0.11983099	0.69117642	0.02593307	0.87368674	0.56634596
Mmaa	0.04189205	3.75448196	0.02590192	0.87376193	0.56635723
1110019D14	0.04307247	2.68830163	0.02586411	0.87385326	0.56637895
Tac2	-0.0732355	1.01105244	0.02568506	0.87428674	0.56656281
Rprm	-0.0349184	3.97565688	0.02566835	0.87432728	0.56656281
Bin2	0.04748512	3.02808699	0.02564586	0.87438185	0.56656281
D17Wsu104	0.05943187	2.07184343	0.02564388	0.87438665	0.56656281
Gbgt1	-0.0970432	0.52653388	0.02562762	0.87442614	0.56656281
Gpr116	-0.0595651	4.84174366	0.02558954	0.87451863	0.56658526
Cyb5d1	-0.0632008	1.39283836	0.02550626	0.87472116	0.56667899
N28178	0.02946501	7.04077347	0.02539821	0.87498444	0.56678798
Il11ra1	-0.0264968	4.34286303	0.0253779	0.87503399	0.56678798
Klra9	-0.1891697	-0.9669091	0.02535626	0.87508682	0.56678798
Ubxn8	-0.0352411	4.33700244	0.02533206	0.87514591	0.56678798
Git1	-0.0229488	6.87270412	0.02531863	0.87517871	0.56678798
Klhdc8a	-0.0341841	3.8166038	0.02529199	0.87524382	0.56679268
Dld	-0.0191022	7.47831163	0.02523585	0.87538115	0.56684413
Mrpl15	-0.0438898	4.9439857	0.02517738	0.87552434	0.56689547
Rnase4	-0.0421004	5.15514776	0.02515622	0.87557617	0.56689547
Sncaip	-0.0381334	5.65640111	0.02511613	0.8756745	0.56692166
Lrrc14	-0.0417799	2.72830869	0.02508259	0.8757568	0.56693346
Zfp691	-0.0581414	2.53282717	0.02506155	0.87580848	0.56693346
Gspt2	0.03032906	4.58635054	0.02502783	0.87589132	0.56694947
Zdhhc24	0.02953918	5.97825576	0.02500438	0.87594898	0.56694947
Rnd2	0.06744969	1.72906481	0.0249299	0.87613226	0.56703063

2610305D13I	-0.0939739	0.60259501	0.02478366	0.87649294	0.56719919
Prkd2	-0.0481405	2.88515912	0.02477736	0.87650851	0.56719919
Tnfrsf4	0.24203774	-2.2440682	0.02474814	0.87658071	0.56720844
Taf1c	0.0723101	1.20429894	0.02467531	0.87676088	0.56728342
Scamp5	-0.0264079	6.44709612	0.0246545	0.87681242	0.56728342
Gm6548	-0.0446573	3.51256874	0.02459326	0.87696419	0.56729581
Ift46	0.03668341	4.57608285	0.02454476	0.87708454	0.56729581
Abhd15	0.15813133	-1.3237041	0.0245415	0.87709265	0.56729581
Mkrn1	0.02005282	6.7341484	0.02453803	0.87710125	0.56729581
Eea1	-0.0204949	7.47743477	0.02453002	0.87712114	0.56729581
Dclk1	0.02763816	10.5375499	0.02450081	0.87719371	0.56730529
Calb2	0.05925341	1.07864787	0.02442638	0.87737878	0.56738752
Fam203a	-0.0524578	2.59822023	0.02428962	0.87771962	0.56751205
Pde11a	0.10171449	-0.3706956	0.02428937	0.87772025	0.56751205
Pydc4	0.18449852	-1.4334036	0.02427938	0.87774517	0.56751205
Aplp1	0.02304538	8.34531989	0.02424604	0.87782845	0.56752844
Hs3st3a1	0.04327842	4.59110995	0.02418238	0.87798757	0.56759385
Napepld	-0.0289463	6.43308313	0.02411875	0.87814684	0.56762964
Fpgs	-0.0804762	0.54372643	0.02403958	0.87834534	0.56762964
Kcna3	0.06550378	1.38186323	0.024028	0.87837438	0.56762964
Gm14005	-0.0955293	0.45134043	0.02401877	0.87839755	0.56762964
Arl6ip1	-0.0172392	7.00630343	0.02399819	0.87844922	0.56762964
Xlr3a	-0.0854899	0.44850432	0.02399642	0.87845368	0.56762964
Rnf43	-0.0468862	3.23811628	0.02397801	0.8784999	0.56762964
4831440E17I	0.06458892	1.79053662	0.02397538	0.87850653	0.56762964
Scube1	0.02241766	5.95682339	0.02394089	0.87859321	0.56764469
Pln	0.09798843	0.61041831	0.023897	0.87870363	0.56764469
Sh2d2a	-0.0691307	1.19444342	0.02385565	0.87880774	0.56764469
Mpeg1	0.05410799	2.86253962	0.02381967	0.87889839	0.56764469
Asap2	0.02565791	6.25243567	0.0238174	0.87890412	0.56764469
Rab14	-0.0171734	7.96281547	0.02381497	0.87891026	0.56764469
Pex13	0.02085139	5.35870662	0.02380168	0.87894376	0.56764469
Ctsz	0.03950103	3.99981656	0.02378199	0.87899343	0.56764469
Znhit2	-0.0384741	3.10195257	0.02375078	0.8790722	0.56765814
Rabac1	-0.0540047	3.07237434	0.0237078	0.87918075	0.56769081
Epb4.1l5	0.02611421	4.6767128	0.02357681	0.87951226	0.56782517
Fam186b	0.17934439	-0.8546847	0.02355635	0.8795641	0.56782517
Pemt	0.1434429	-0.981126	0.02354124	0.87960243	0.56782517
Stambp	0.02529895	4.3459198	0.02349263	0.87972576	0.56782517
Chst3	-0.1210391	0.66964206	0.02344783	0.87983957	0.56782517
Calr4	0.18438707	-1.2051204	0.02343058	0.8798834	0.56782517
Ankrd34b	0.03596665	4.5032415	0.023426	0.87989505	0.56782517
Pepd	-0.031484	3.62132166	0.02339611	0.87997109	0.56782517

Mblac2	0.02843264	6.02592494	0.0233762	0.88002174	0.56782517
2210404O09	-0.0489168	2.13870663	0.02333554	0.88012528	0.56782517
Rgma	0.03778499	4.29821178	0.02333444	0.88012808	0.56782517
Ercc2	-0.0609127	1.24485289	0.02332492	0.88015235	0.56782517
Hmgb1-rs17	0.06746336	0.38820929	0.02332368	0.8801555	0.56782517
Pdzk1ip1	-0.079876	1.63263449	0.02327885	0.8802698	0.56782517
Ctps	-0.0323445	3.98879188	0.0232706	0.88029085	0.56782517
Cited4	-0.0653017	0.97690506	0.02322239	0.8804139	0.56782517
Pgrmc2	-0.0219585	5.49575319	0.02321615	0.88042984	0.56782517
Dbt	0.02587511	5.64794495	0.0232152	0.88043227	0.56782517
Plcg2	-0.0455524	1.88534038	0.02313684	0.88063261	0.56782599
Rars	0.02248566	5.45583611	0.02312854	0.88065384	0.56782599
Dus2	0.04460866	2.42156585	0.02311453	0.8806897	0.56782599
Wbscr17	-0.0327921	3.82824717	0.02310095	0.88072448	0.56782599
Foxk1	0.01992373	7.16862433	0.02309529	0.88073897	0.56782599
Tpcn1	-0.0399249	5.97215689	0.02307874	0.88078136	0.56782599
Slc34a2	-0.0856479	0.83213554	0.02301885	0.88093491	0.56785858
Nr2c2	-0.015419	7.33174205	0.0230138	0.88094786	0.56785858
Svopl	0.12757685	-0.2633265	0.02294895	0.88111438	0.56787892
Cdk8	-0.0188147	5.70955149	0.02293394	0.88115295	0.56787892
Gjb1	0.12783482	-0.6787146	0.02293347	0.88115417	0.56787892
Mef2c	0.02437051	10.0758779	0.02291124	0.88121131	0.56787892
Ifih1	0.02438538	5.32452961	0.02283387	0.88141045	0.56796989
Gm3219	0.05976164	0.84330458	0.02280857	0.88147566	0.56797454
Zfp143	0.03711072	3.39032296	0.02273048	0.88167711	0.56806155
4930523C07I	0.03815626	3.74449244	0.02271129	0.88172668	0.56806155
Chp2	0.06521527	0.84874003	0.0226511	0.88188226	0.56812442
Pcdhb14	0.040982	3.02501865	0.02230013	0.88279374	0.56867421
Prkaa1	-0.0193757	5.69360873	0.02224146	0.88294683	0.56873543
Tbc1d9b	0.01862741	6.59204366	0.02218747	0.88308789	0.56878831
Ccdc9	0.03443789	2.73401385	0.02215904	0.88316225	0.56878831
Cyth4	0.05278061	1.85872667	0.02214341	0.88320314	0.56878831
Acadl	0.02908141	5.29580048	0.02211826	0.88326898	0.56879332
Wtip	0.053395	2.38718565	0.02198914	0.88360756	0.56897395
Kcnq3	-0.0220879	5.53464675	0.02196068	0.88368235	0.5689847
Ppp1r21	-0.0188418	5.54478482	0.02190941	0.88381716	0.56900165
Fat2	-0.0524864	2.77609445	0.0218876	0.88387456	0.56900165
Zfp106	0.01500554	9.16699433	0.02188208	0.8838891	0.56900165
Rpap2	-0.0219412	4.70556522	0.02184013	0.88399958	0.56900165
Nthl1	-0.0934255	0.3650133	0.02183098	0.88402371	0.56900165
Wisp2	-0.1801243	-1.02055	0.02181828	0.8840572	0.56900165
Ppp1r3d	0.06969408	1.13591682	0.02175936	0.88421266	0.56905418
Idi2	-0.1029011	-0.0722269	0.02172151	0.88431264	0.56905418

Cdkl4	0.0295013	4.17292672	0.02170761	0.8843494	0.56905418
Arhgef12	-0.0148481	9.52684002	0.02168427	0.88441111	0.56905418
Yif1b	-0.0533586	1.7760898	0.02167739	0.88442929	0.56905418
Prkag2	-0.0214323	6.12234664	0.02161096	0.88460518	0.56912997
Esam	0.07959381	1.35258999	0.02155033	0.88476595	0.56919601
Jmjd7	0.11501378	-0.6699958	0.02148981	0.88492667	0.56925813
Blvrb	0.06155069	1.2397928	0.02144055	0.88505764	0.56925813
Gm15545	0.12738288	-0.7285073	0.02142772	0.88509177	0.56925813
Mir9-2	0.17483719	-0.9702704	0.02140538	0.88515125	0.56925813
Fcrls	-0.069363	1.54344306	0.02139296	0.88518433	0.56925813
Gm3716	0.12408331	-0.725553	0.02136529	0.88525803	0.56925813
Asrgl1	-0.0266906	5.83523342	0.02135584	0.88528322	0.56925813
Ndufaf6	-0.0513709	1.59740847	0.02133926	0.88532743	0.56925813
Cd244	-0.1760705	-0.6517929	0.02125185	0.8855608	0.5693399
Crabp1	0.25624533	-1.4468871	0.02124809	0.88557084	0.5693399
Gdf10	0.06708303	2.35877311	0.02117885	0.88575607	0.56940361
Cyp3a13	0.13166958	-1.1165901	0.0211557	0.88581806	0.56940361
Rsph9	0.0673282	2.03446966	0.02114589	0.88584433	0.56940361
Hgs	0.03108191	4.12396268	0.02110876	0.88594386	0.56942434
Blcap	0.03014868	5.14326123	0.0210905	0.88599285	0.56942434
Ddx49	0.07086215	0.76089997	0.02100443	0.88622401	0.56953554
Ddx17	-0.0253454	8.05832796	0.02098168	0.88628519	0.56953749
Mettl11b	-0.2457835	-2.3411546	0.02092682	0.88643285	0.56958268
Tmem63b	-0.025127	5.59252783	0.02090964	0.88647914	0.56958268
Zfp41	-0.0418572	1.96882306	0.02089079	0.88652996	0.56958268
Cnpy3	-0.0361328	3.44903708	0.02086085	0.88661069	0.56959719
Btg2	-0.0450982	3.76373562	0.02082756	0.88670056	0.56960412
Hmga2	-0.0275048	3.850615	0.02081377	0.88673779	0.56960412
Tnfsf10	-0.0378313	3.01706064	0.02064476	0.88719526	0.56984703
Satb1	-0.024814	7.71689747	0.02058902	0.88734656	0.56984703
Dph1	-0.1468709	-1.3555422	0.02054384	0.88746934	0.56984703
Matr3	0.02162581	8.16150937	0.02052279	0.88752658	0.56984703
Uqcc1	0.03129167	3.87512325	0.02050828	0.88756607	0.56984703
4930515G01	0.1490875	-0.7787779	0.02049667	0.88759767	0.56984703
Eif5b	0.02183092	9.35457205	0.02047251	0.88766345	0.56984703
Ccdc69	0.04327325	2.06926137	0.02045705	0.88770559	0.56984703
Me1	0.01828197	5.34578068	0.02039517	0.88787435	0.56984703
Ralgps2	-0.017832	6.33811271	0.0203946	0.88787588	0.56984703
Cd164	-0.0334346	7.83696566	0.0203806	0.8879141	0.56984703
Letm2	-0.0339824	3.25028569	0.02037905	0.88791836	0.56984703
Klrb1b	-0.1365971	0.72419676	0.02037542	0.88792826	0.56984703
Cyp2t4	0.11546507	-0.8172949	0.02037065	0.8879413	0.56984703
Uros	-0.0313839	3.53309665	0.02034181	0.88802007	0.56984703

Wdr45	-0.0272479	4.46017267	0.02033206	0.88804673	0.56984703
Nme6	0.04966903	1.77490388	0.02022973	0.8883268	0.5699894
Rdh5	-0.1561706	-1.2298068	0.02013939	0.88857467	0.57003282
Lgals1	0.05059561	4.62330975	0.02013719	0.88858072	0.57003282
Dram1	-0.0775758	0.62220117	0.02010465	0.88867016	0.57003282
Hsp90ab1	0.01695498	10.3381856	0.02010278	0.88867529	0.57003282
Nnt	0.02002475	5.08269873	0.02009909	0.88868544	0.57003282
Pdgfra	0.02542374	5.49716989	0.02000555	0.88894296	0.57013288
6430562O15	-0.2050014	-1.7198949	0.02000015	0.88895784	0.57013288
C230037L18F	-0.0681581	1.60175458	0.01993519	0.88913707	0.57021049
Susd5	0.04889107	2.31888443	0.0198796	0.88929068	0.57025108
Slc2a6	0.07223629	1.04842691	0.01986291	0.88933682	0.57025108
Sntb2	-0.0210283	5.67243718	0.01984911	0.88937501	0.57025108
Elavl4	0.01980528	6.90619641	0.01978872	0.88954226	0.57032099
Tmem200a	-0.0404075	3.68035073	0.01971225	0.88975443	0.57038154
Csnk2a1	-0.0157058	6.99510857	0.01970171	0.8897837	0.57038154
Gm5795	0.19422282	-2.0561598	0.01969174	0.88981139	0.57038154
Ube3b	-0.0213591	6.09056482	0.01965794	0.88990537	0.57040445
Cenpe	-0.0416592	2.89436466	0.01960938	0.89004048	0.57041703
G3bp2	0.01952582	9.47815298	0.01960903	0.89004146	0.57041703
4932441J04F	0.11999074	-0.8893644	0.01948875	0.89037691	0.57059468
Rrp1b	-0.027724	3.45718733	0.01946441	0.89044492	0.57060093
Ankmy2	0.01586284	6.26991117	0.01943367	0.8905309	0.5706187
Vps36	0.02873985	3.86772111	0.0193442	0.89078145	0.57071088
Gm16973	0.03588311	2.88073708	0.0193407	0.89079128	0.57071088
Setbp1	-0.0169941	6.75198855	0.01926007	0.89101763	0.57078671
Csad	-0.0246432	4.22252035	0.01920869	0.89116211	0.57078671
Shcbp1	-0.1238259	-0.5415224	0.01919696	0.89119514	0.57078671
Zcchc18	-0.0192292	7.20061704	0.0191965	0.89119643	0.57078671
Dennd2a	-0.0261552	4.23267622	0.01918638	0.89122492	0.57078671
Cplx3	0.0548821	1.32700046	0.01917418	0.89125928	0.57078671
Fbxo46	0.04063695	1.66258558	0.0191443	0.89134348	0.57080332
Fuca2	0.02143761	4.54226025	0.01910646	0.89145018	0.57080429
Elp2	0.024116	5.93591872	0.01910243	0.89146154	0.57080429
Wsb2	0.01469286	7.7269333	0.01906053	0.89157985	0.57080911
Glb1	-0.0405054	3.05622481	0.01901339	0.89171313	0.57080911
Tmed4	0.030099	5.53251403	0.01896833	0.89184066	0.57080911
Cpb2	0.19767722	-1.7620521	0.01896793	0.89184179	0.57080911
Gm15760	-0.048175	2.13793902	0.01896086	0.89186183	0.57080911
B4galt4	0.02947753	4.72188672	0.0189575	0.89187136	0.57080911
Pld4	0.085927	-0.3204414	0.01894425	0.89190888	0.57080911
Gorasp1	0.03404814	3.03344982	0.01893495	0.89193525	0.57080911
Foxp2	-0.0238696	7.30765241	0.01887675	0.89210035	0.57087747

Bclaf1	0.01426153	9.51920161	0.01879173	0.89234201	0.57098042
Egr3	-0.0221315	7.83055763	0.01877916	0.89237781	0.57098042
9430020K01I	0.01514778	8.95856324	0.0187329	0.89250957	0.57102742
Nars	-0.015392	7.85975066	0.01869421	0.89261992	0.57103941
Tmem234	0.02527991	4.73707194	0.01868545	0.89264489	0.57103941
Epn2	-0.0143811	6.4669456	0.01864559	0.89275873	0.57107373
Ldoc1l	-0.0220111	4.91591851	0.01862584	0.89281515	0.57107373
B230208H11	0.13404708	-0.0817268	0.01855392	0.89302096	0.57116807
Scamp1	-0.0157574	7.87224213	0.018505	0.89316118	0.57118037
Elovl1	0.04939618	3.3782113	0.01849041	0.89320304	0.57118037
Gnl3	0.02504029	4.71939905	0.0184862	0.89321512	0.57118037
Trpv6	0.05015505	1.64659565	0.01840039	0.89346165	0.57129805
Htra3	-0.0490639	4.06121175	0.01835648	0.89358802	0.57129805
Rrp1	0.01975681	7.75798968	0.01835544	0.89359103	0.57129805
Arhgef40	-0.026233	3.63309355	0.01834106	0.89363244	0.57129805
Ybx2	0.09534236	-0.0781036	0.01830439	0.89373813	0.57131798
Dennd4a	-0.018045	7.418427	0.01828833	0.89378447	0.57131798
Igj	0.06512355	1.01319306	0.01826958	0.8938386	0.57131798
Adap1	0.02544681	4.20432379	0.01822675	0.89396229	0.57135977
Taok2	-0.022165	5.310984	0.01819805	0.89404531	0.57136744
Nup153	-0.0188754	6.16442377	0.01815537	0.89416882	0.57136744
1700007P06I	0.09677057	-0.667829	0.01814063	0.89421153	0.57136744
Jsrp1	-0.1196854	-0.75672	0.01811276	0.89429229	0.57136744
Wdr8	0.04370217	2.26818252	0.01806837	0.89442109	0.57136744
Malt1	-0.0309176	3.60673066	0.0180555	0.89445848	0.57136744
Rab39	-0.0723838	0.57454384	0.01805139	0.89447042	0.57136744
Gna14	0.08494396	1.3106898	0.01803675	0.89451295	0.57136744
Gpr139	0.10542567	-0.5549748	0.01800886	0.89459402	0.57136744
Trdn	0.0816261	0.16367472	0.01800773	0.8945973	0.57136744
Ntsr2	0.03826078	3.21728665	0.018001	0.89461688	0.57136744
Tspan2	-0.0218412	6.3946554	0.01797004	0.89470698	0.57136744
Asb2	0.10491205	0.02670531	0.01796124	0.8947326	0.57136744
9230110C19I	-0.032817	3.02451979	0.01791659	0.89486269	0.57141326
Rasl10b	-0.0327034	3.49792742	0.01780979	0.89517457	0.57157516
Gatc	-0.0224111	7.36425707	0.01778958	0.89523371	0.57157565
Jmjd4	0.02251693	4.63418793	0.01761494	0.89574603	0.57184152
Eftud2	0.02279454	5.01527971	0.01760785	0.89576687	0.57184152
Clcn5	0.05177533	2.3199123	0.01754624	0.89594829	0.57192006
Gjc3	0.02687851	5.44559443	0.01749002	0.8961141	0.57196328
Prrg3	-0.0153147	6.63317947	0.01748141	0.89613953	0.57196328
Gpr4	0.03525231	5.56002445	0.01746063	0.89620089	0.57196328
Trim67	0.05596177	1.22140215	0.01743049	0.89628995	0.57196328
Ifi203	0.04790992	3.12728401	0.01741737	0.89632877	0.57196328

Shroom4	0.03755099	3.13879811	0.01740466	0.89636635	0.57196328
Gata2	0.09013829	0.77249264	0.01731107	0.89664369	0.57210298
Slc23a2	-0.0168985	7.23608384	0.01718065	0.89703142	0.5722589
Nufip2	-0.0159615	7.74209213	0.01715881	0.89709649	0.5722589
Zc3h3	-0.0404391	2.62834499	0.01715049	0.8971213	0.5722589
Serpine2	-0.0196863	5.98452131	0.01715034	0.89712175	0.5722589
Pkd1l3	0.06492672	0.8831859	0.01712044	0.89721092	0.5722675
Zmat1	0.02178958	5.17673557	0.01709197	0.89729593	0.5722675
Mapk6	-0.0145456	7.5119382	0.01707021	0.89736092	0.5722675
Irs2	0.03262119	4.35157986	0.01706753	0.89736893	0.5722675
Fgd5	-0.0733905	1.84175982	0.01702404	0.89749898	0.57231318
Nampt	0.01693359	7.31073047	0.01698555	0.89761425	0.57234943
Ubxn4	-0.0140545	7.28314811	0.01686249	0.89798361	0.5725205
Tbcb	-0.0281226	4.55125127	0.01685723	0.89799944	0.5725205
Col4a6	-0.040163	2.4222693	0.01678367	0.89822092	0.57262443
Ptcd2	-0.0242932	4.69005806	0.01674262	0.89834475	0.57266611
Fam110a	-0.0628338	1.23693417	0.01670724	0.89845157	0.57269693
Rc3h2	0.01634071	7.92859234	0.01668566	0.89851679	0.57270123
Rtel1	-0.041551	2.53186171	0.0166218	0.89871007	0.57278716
Rora	0.01656606	8.37690505	0.01659071	0.89880427	0.57280992
Cd44	-0.0300111	3.13111967	0.01657076	0.89886479	0.57281122
4930528A17	0.18188218	-1.4834842	0.0165412	0.89895453	0.57282112
Rgs5	0.03045246	4.11792046	0.01652712	0.89899727	0.57282112
4933408B17	0.21725481	-1.1572245	0.0164637	0.89919015	0.57290569
Zkscan8	0.01934966	6.10581544	0.01644504	0.89924697	0.57290569
Aspn	0.12382419	-0.6780731	0.01642543	0.8993067	0.57290648
N4bp2	-0.0313558	3.4697598	0.01638979	0.89941538	0.57292034
Apol9b	-0.1031982	-0.6994516	0.01635449	0.89952313	0.57292034
Mpdu1	-0.0369748	2.850041	0.01635396	0.89952477	0.57292034
Pgs1	0.0230595	4.41509529	0.01633549	0.8995812	0.57292034
Secisbp2	0.02625014	3.83289147	0.0163225	0.8996209	0.57292034
Cdc25a	-0.0323969	2.73292837	0.01627323	0.89977165	0.57294584
Wnk1	0.01374335	10.008663	0.01627118	0.89977793	0.57294584
Fbxo6	-0.0343693	2.55592837	0.01623736	0.89988155	0.57297458
Zfp948	0.02975928	3.61939955	0.01619313	0.90001724	0.5730099
Mlph	0.04544004	2.44175312	0.01618114	0.90005403	0.5730099
Bloc1s2	0.03069815	3.29573558	0.01615144	0.90014529	0.57303076
Pf4	-0.115216	-1.4000512	0.01611373	0.90026127	0.57306734
Fam69a	-0.0203347	4.66369222	0.01607178	0.90039046	0.57307959
Pot1a	-0.0222019	5.1867932	0.01606936	0.9003979	0.57307959
As3mt	0.03196097	3.54657067	0.01603335	0.90050895	0.57307959
Rab27b	0.02059978	5.41007318	0.01603154	0.90051453	0.57307959
Cd68	-0.0447895	2.3580621	0.0159876	0.90065019	0.57312869

Rab11fip5	0.01939485	5.95716067	0.01594937	0.9007684	0.57316667
Kdm6b	-0.0241826	5.61535267	0.01590606	0.90090246	0.57321475
Pilrb2	-0.1864211	-0.5024789	0.01588163	0.90097818	0.57322569
Pdzk1	0.03392109	2.85859714	0.01584986	0.9010767	0.57325114
Shisa5	0.0307561	5.06994187	0.01578859	0.90126704	0.57333499
Slc37a2	0.10745427	-0.1409012	0.01570208	0.90153641	0.57346911
Trp73	-0.1118021	-0.2048053	0.01563158	0.9017565	0.57357186
Mnda	0.08737853	-0.6338785	0.01556163	0.90197538	0.57366669
Bcl7a	0.02294764	4.93507583	0.01552802	0.9020807	0.57366669
Dhx33	0.02764955	4.90226004	0.01550521	0.90215227	0.57366669
Gmfb	0.01514296	8.2222009	0.01550133	0.90216446	0.57366669
Kcnj4	-0.0298663	3.36991447	0.01549051	0.90219842	0.57366669
Wscd2	-0.0290798	3.52687442	0.01544331	0.90234673	0.57372375
Stil	-0.0667911	0.88091538	0.01539465	0.90249988	0.57378388
Lars2	-0.02061	13.4159353	0.01534752	0.90264841	0.57383391
9330151L19R	0.02705444	4.78112007	0.01533253	0.90269574	0.57383391
Numb	-0.0205741	5.65734998	0.01527928	0.90286392	0.57390358
Btd	0.03683681	3.78710427	0.01521404	0.90307042	0.57397536
Stard13	-0.0175696	5.39133096	0.01520659	0.90309404	0.57397536
Ebi3	-0.1529348	-1.0286193	0.01517135	0.90320578	0.57400914
Rbbp9	0.02204274	6.4796997	0.01512317	0.9033588	0.57406914
4930528D03R	0.08281359	-0.1350901	0.01505319	0.90358149	0.57409694
Mir22hg	-0.0253555	3.66681709	0.01505164	0.90358642	0.57409694
Dcxr	0.08465371	0.43505574	0.01504555	0.90360583	0.57409694
Pde4d	0.02094412	7.08529817	0.01502612	0.90366777	0.57409694
4732491K20R	-0.0443801	2.09539837	0.0150114	0.9037147	0.57409694
Sh3bp2	0.05191241	1.47945493	0.01498121	0.90381106	0.57409694
Chrna2	0.0948429	-0.6586616	0.01498066	0.90381281	0.57409694
Svop	0.02382269	5.76057809	0.01492471	0.90399167	0.57414083
Galnt18	-0.0372741	2.29504213	0.01491798	0.9040132	0.57414083
Zwilch	0.05606983	1.4763998	0.01490406	0.90405776	0.57414083
Igf2os	-0.0428903	3.56174372	0.01482562	0.90430925	0.57426332
Grk5	-0.0192545	5.33355787	0.01477108	0.90448452	0.57431029
Hpcal4	0.01857068	9.96877456	0.01476612	0.90450048	0.57431029
Eif4e2	0.01848757	5.28784106	0.01469857	0.90471806	0.57441121
2610037D02R	0.07667354	0.16203569	0.01461483	0.90498847	0.57454565
Tmco4	0.05120969	2.26886504	0.01457087	0.90513074	0.57457178
Mmp19	0.04371319	2.61996574	0.01456587	0.90514695	0.57457178
Serbp1	0.01264002	9.37863843	0.01454628	0.90521042	0.57457484
Sec61a2	0.0275233	4.92170337	0.01452364	0.90528383	0.5745842
Slc47a1	-0.0421786	5.38233735	0.01447552	0.90544009	0.57464614
Tdp2	0.02751862	3.61974665	0.01441868	0.90562496	0.57471537
Lamtor4	-0.056197	1.98318415	0.01440593	0.90566651	0.57471537

Prmt10	0.03874576	3.33900613	0.01435884	0.90582004	0.57476083
Cstf3	0.0228272	5.29501296	0.01434797	0.9058555	0.57476083
Slc25a32	-0.0245648	3.88956618	0.01426709	0.90611989	0.57489135
Rnf167	0.02871531	4.18757965	0.01422391	0.90626137	0.57491426
Mettl1	0.10807658	-0.7518971	0.01421895	0.90627765	0.57491426
Laptm4a	0.02449274	8.0527121	0.01418636	0.90638456	0.57491426
Sfrp2	-0.0649749	0.51677032	0.01417571	0.90641954	0.57491426
Lair1	0.03955456	3.29310948	0.0141666	0.90644947	0.57491426
2310057M21	-0.0210189	4.09603702	0.01411248	0.90662746	0.57496773
Ndp	0.08171264	1.01292183	0.01409683	0.906679	0.57496773
Supt7l	0.01902839	5.59171236	0.01408745	0.90670987	0.57496773
Gdf5	-0.1230377	-1.6391325	0.01399285	0.90702211	0.5750795
Drd5	-0.0743975	0.25089244	0.01398127	0.9070604	0.5750795
Suv420h1	0.01324203	6.72862008	0.01398071	0.90706226	0.5750795
Diablo	-0.0286965	4.26106158	0.01380293	0.90765217	0.57535788
Zpbp	-0.0920466	0.39865645	0.01379787	0.90766902	0.57535788
Rnf111	-0.0123098	7.07452193	0.01378683	0.90770579	0.57535788
Mybl2	0.10256746	-1.316841	0.01377768	0.90773629	0.57535788
Aox4	-0.0719596	0.66151393	0.01369643	0.90800741	0.57549249
Psap	-0.0184868	8.49462902	0.01366882	0.90809975	0.57551377
Ankrd6	0.02301562	5.51365967	0.01360788	0.90830384	0.57560588
Cep120	0.01685578	6.33522438	0.01354902	0.90850145	0.57568597
Mycbp	0.02254377	5.63413358	0.01353524	0.90854778	0.57568597
Pgm3	-0.0227888	4.00884134	0.01349234	0.90869213	0.57571385
Cabp4	0.14220515	-0.8660315	0.01348723	0.90870933	0.57571385
Psmb9	-0.0451403	3.32181791	0.01342657	0.90891392	0.57571713
Cox8b	-0.1879273	-1.4825481	0.0134092	0.90897256	0.57571713
Gm960	-0.0957678	-0.5551087	0.01337675	0.90908226	0.57571713
Rdh9	-0.0737117	0.40008975	0.01336823	0.90911108	0.57571713
Bola3	-0.0286418	3.33328221	0.01336691	0.90911554	0.57571713
Cth	-0.0631174	0.94286867	0.01336217	0.90913159	0.57571713
Mrpl37	0.02098201	4.02283892	0.01333274	0.90923125	0.57571713
Oxct1	-0.0139229	8.33302897	0.01332253	0.90926586	0.57571713
Sdr42e1	-0.0267964	2.65229571	0.01331856	0.9092793	0.57571713
Ctss	-0.0328024	3.74244017	0.01331179	0.90930225	0.57571713
Icam4	0.08937921	-0.0270766	0.01326245	0.90946972	0.57578595
Yod1	-0.0208262	4.82945894	0.01322394	0.90960065	0.57583162
Gm7008	0.07657738	-0.366684	0.01315305	0.90984216	0.57594729
Nipsnap3b	-0.0238959	3.68325496	0.01308556	0.91007269	0.57600126
Ranbp10	-0.0183942	4.71513143	0.0130789	0.91009546	0.57600126
Sppl2b	0.04626021	1.50331952	0.01307646	0.91010383	0.57600126
Stac	-0.0875014	0.1183344	0.01305036	0.91019319	0.5760206
Gpr63	-0.0290375	3.16893815	0.01301497	0.91031449	0.5760284

Pnpla2	0.02894384	4.47056317	0.01299658	0.91037756	0.5760284
Spats2	-0.0244188	3.28971686	0.0129946	0.91038436	0.5760284
Ntn1	-0.0302395	3.73349064	0.01297818	0.91044075	0.5760284
Akap5	0.01473181	8.01809372	0.01293744	0.91058078	0.57607979
Elp4	-0.0180271	4.2881464	0.012908	0.91068211	0.57610669
P2rx6	0.05856487	1.20179979	0.01283034	0.91095001	0.57622155
Wdr89	0.04180342	1.09939631	0.01278287	0.91111415	0.57622155
Efnb1	-0.0402365	3.42047178	0.01274043	0.91126119	0.57622155
Uxt	-0.0327693	3.52008913	0.01273494	0.91128023	0.57622155
D630013N20	0.16236041	-0.8906889	0.01272559	0.91131265	0.57622155
Map1s	0.02575693	3.04419634	0.01272532	0.91131358	0.57622155
Cmss1	0.05324528	2.41005536	0.01269934	0.91140376	0.57622155
Nek2	-0.0643512	1.56168566	0.01268381	0.9114577	0.57622155
Itga1	-0.0202152	4.19163823	0.01268354	0.91145863	0.57622155
Adm	0.03426224	2.24747852	0.01266851	0.91151087	0.57622155
A330102I10F	-0.0597285	1.29368866	0.01265267	0.91156598	0.57622155
Ddit4	0.03421239	2.67190027	0.01265163	0.91156959	0.57622155
Kif2a	0.01413863	7.65851713	0.01263445	0.91162939	0.57622216
Cul1	-0.0119618	7.35300591	0.01259168	0.91177844	0.57627035
Foxred1	0.02907262	3.05111748	0.01256119	0.91188486	0.57627035
Adcy10	-0.062538	0.20138722	0.01255242	0.91191548	0.57627035
Pacs2	0.01355793	6.07020537	0.01251849	0.91203408	0.57627035
Mmd2	0.02347167	4.00550522	0.01248561	0.91214918	0.57627035
Nckap1l	-0.0274708	3.03439757	0.01248207	0.91216159	0.57627035
Cnih2	0.0214796	3.54137883	0.01247148	0.9121987	0.57627035
Syn1	0.02535133	10.2351077	0.01244794	0.91228123	0.57627035
Nphp3	0.03333003	2.49082718	0.01242032	0.91237818	0.57627035
Vkorc1	-0.0332507	5.37488519	0.01238568	0.91249994	0.57627035
Rfx2	-0.0324693	2.14871108	0.01238415	0.91250529	0.57627035
Nes	0.01949566	4.26178602	0.01236442	0.91257471	0.57627035
Fbxo8	-0.016699	4.84429171	0.01236312	0.91257929	0.57627035
Rnd1	-0.0430187	1.29585045	0.01235607	0.91260412	0.57627035
Brinp1	0.01710583	6.39757904	0.01234915	0.91262849	0.57627035
Insl5	0.11952052	-1.3287275	0.01237609	0.91272959	0.57627035
Elmod3	-0.0344601	3.29131426	0.01230298	0.91279126	0.57627035
AW549542	0.06835332	-0.2372378	0.01229841	0.9128074	0.57627035
Metrn	-0.0784107	-0.6406921	0.01229387	0.91282342	0.57627035
Gm15319	0.06180876	0.17664311	0.01224601	0.91299254	0.57633997
Casq2	-0.0772626	-0.2411714	0.01219012	0.9131905	0.57642779
Mettl13	-0.0300276	2.10148209	0.01214497	0.9133507	0.5764632
Efna1	0.07176621	0.04723327	0.01214115	0.9133643	0.5764632
Pdcd1lg2	-0.0680443	0.07482019	0.01209466	0.91352962	0.57653039
Fbln5	-0.0441358	4.21843074	0.01205315	0.9136775	0.57658658

Naga	-0.0426976	3.0896614	0.01196689	0.91398564	0.57673682
Zbtb17	0.03237338	2.67988355	0.01195356	0.91403335	0.57673682
Il18rap	0.09846375	-0.4302662	0.01186458	0.91435259	0.57689465
Arv1	-0.0529637	0.87145989	0.01182869	0.91448168	0.57689465
Qpct	-0.0456529	1.21858287	0.01182292	0.91450245	0.57689465
Haus7	0.03923427	2.08117045	0.01181831	0.91451906	0.57689465
Cmah	0.01850127	6.36639796	0.01178268	0.9146475	0.57690231
Chil1	-0.0733309	0.532544	0.01178226	0.914649	0.57690231
Klhl20	0.01884476	5.71641113	0.01174677	0.91477711	0.57694597
Tmem17	0.03738502	1.7627744	0.01168929	0.91498502	0.57703994
Clptm1l	-0.0218203	4.51045933	0.01157465	0.91540125	0.57721786
Neurl4	0.02128117	4.54166249	0.01156925	0.9154209	0.57721786
Morn3	-0.1757603	-1.2840511	0.01156293	0.91544391	0.57721786
Bivm	0.01484937	4.9555411	0.01144552	0.91587258	0.57745097
Sufu	-0.019018	4.10405418	0.01141606	0.91598048	0.57748183
Tonsl	0.06938841	0.46502871	0.01138481	0.91609511	0.57751693
Nr3c2	0.01620742	5.95236095	0.01135052	0.91622105	0.57755915
Agpat5	-0.0144687	5.497355	0.01131673	0.91634534	0.57760033
Naip1	0.14659803	-1.843122	0.01128606	0.91645834	0.57763439
Hps5	-0.0241255	3.60626407	0.0112488	0.9165958	0.5776667
Fbxw4	0.02877548	2.72014757	0.01121804	0.91670949	0.5776667
Pih1d2	-0.0656254	0.08336365	0.01121232	0.91673064	0.5776667
Alg12	-0.038346	2.79775609	0.01119263	0.91680349	0.5776667
Gstm3	0.03759389	1.53275135	0.01119236	0.91680448	0.5776667
Thap2	-0.0181018	5.05056081	0.01116282	0.91691393	0.57767415
Ap3b1	0.01381727	6.59633929	0.01115638	0.9169378	0.57767415
Gclc	0.01402549	6.26031202	0.01114144	0.91699322	0.57767415
Erg	0.04924742	1.59530933	0.01112441	0.91705643	0.57767682
Naalad2	-0.04266	1.33162238	0.01108811	0.91719135	0.57772276
Gm10767	0.03283803	1.92261135	0.01107307	0.91724732	0.57772276
Cfh	-0.0263858	7.13021819	0.01102141	0.91743981	0.57776896
Mkrn3	0.08918594	-0.2692378	0.01101827	0.91745155	0.57776896
Rps6ka2	0.01873008	5.22080832	0.01100113	0.91751554	0.57776896
Rhov	0.0714684	1.2476218	0.01096937	0.91763423	0.57776896
Cobll1	-0.0192634	4.89653014	0.01096923	0.91763476	0.57776896
Neil1	-0.0690608	-0.0590332	0.01095858	0.91767458	0.57776896
Upf2	-0.0122761	6.89054025	0.01092431	0.91780293	0.57777616
Rspo2	-0.0314247	4.39083096	0.01092403	0.91780398	0.57777616
C130060K24I	-0.0928996	0.00503325	0.01088255	0.91795957	0.5778142
Htr1a	0.02273453	3.55770126	0.01087648	0.91798238	0.5778142
Ly6a	0.05516006	2.3561402	0.01085716	0.91805496	0.57782275
Adck4	0.05198681	1.29627474	0.0108178	0.91820309	0.5778631
Tmem138	0.037933	1.17105868	0.010786	0.91832292	0.5778631

Gm13139	0.03519354	2.32441717	0.01078328	0.9183332	0.5778631
Ccnyl1	-0.0165472	5.32079068	0.01075266	0.91844878	0.5778631
Dcaf13	-0.0152859	4.45411103	0.01074964	0.91846019	0.5778631
BC051226	-0.1048959	-1.6089049	0.01072655	0.91854751	0.5778631
Arhgap22	-0.0781677	0.14062593	0.01071684	0.91858425	0.5778631
Senp1	0.01765864	5.22528024	0.01069161	0.91867977	0.5778631
Zfp54	-0.052173	1.59679142	0.01068525	0.91870387	0.5778631
Dnase2a	-0.067845	-0.3412987	0.01068389	0.91870901	0.5778631
Itpr3	0.03308446	2.12556191	0.01064768	0.91884639	0.57788932
1110046J04F	-0.0442391	1.55816368	0.01064181	0.91886868	0.57788932
Zfp947	-0.0310621	2.25516998	0.01062326	0.91893917	0.57789655
Arhgap30	0.03493125	2.85648946	0.01056859	0.91914721	0.57795634
Gpr156	-0.0556016	1.13406632	0.01056727	0.91915225	0.57795634
Dhx34	0.03537692	1.42848274	0.01053465	0.91927667	0.57798446
Fam53c	-0.014482	5.54866896	0.01051029	0.91936973	0.57798446
Gmeb1	0.01371609	5.29855396	0.01050917	0.919374	0.57798446
Emilin2	-0.110896	-1.0549577	0.01046764	0.91953287	0.57800865
Cd27	0.11964414	-1.5497778	0.01044055	0.9196367	0.57800865
Xaf1	0.02406824	3.44495522	0.01041098	0.91975017	0.57800865
Dad1	-0.0283465	5.04388937	0.01040139	0.91978702	0.57800865
Dis3l2	-0.0217508	3.42555374	0.01040098	0.91978856	0.57800865
Naa40	0.02403941	2.94332388	0.01038805	0.91983825	0.57800865
Pomt1	-0.0445231	1.47726047	0.01038729	0.91984119	0.57800865
Rell2	0.03742923	1.68782175	0.01037602	0.91988455	0.57800865
Thbs3	0.04638915	1.10642805	0.01023277	0.92043749	0.57831899
Foxred2	-0.0244903	2.99096199	0.01021171	0.92051911	0.57833318
Bcl2a1a	0.05125039	0.16812951	0.0101696	0.92068259	0.5783592
Pms1	-0.0262581	2.75300023	0.01016343	0.92070656	0.5783592
Topbp1	-0.0180427	5.30103597	0.01015543	0.92073765	0.5783592
Gm5088	0.0325414	1.55621154	0.01008947	0.92099457	0.57848348
Atp6v0e	-0.0275896	4.75238444	0.01005361	0.92113459	0.57853434
Ndc80	0.09094076	-0.3951648	0.01002348	0.92125245	0.57857126
D5Ertd579e	0.01373809	7.30516997	0.00999967	0.92134573	0.5785724
Tspyl3	-0.0144872	4.6678116	0.0099894	0.92138596	0.5785724
Prr5	0.04214982	1.40299752	0.0099778	0.92143147	0.5785724
Robo1	0.01621724	7.25073871	0.00994599	0.92155639	0.57861375
Atg16l1	-0.0235368	3.88649107	0.00990639	0.92171217	0.57867447
Gca	0.01707081	6.18271984	0.00987864	0.9218215	0.57870602
Hira	-0.0163097	4.13802974	0.00983521	0.92199294	0.57875434
Wdfy2	0.03923952	1.70188876	0.00981763	0.92206244	0.57875434
Msc	-0.1195023	-0.979222	0.00981428	0.92207572	0.57875434
Irf1	0.03182399	2.9745514	0.00976086	0.92228734	0.57875717
Tubb4a	0.01657574	10.3898448	0.00975568	0.92230792	0.57875717

Tspan7	0.01425671	8.1016807	0.00974742	0.92234068	0.57875717
Onecut2	-0.0240414	3.59482213	0.00974356	0.92235601	0.57875717
Gm10638	-0.0770582	-0.6071244	0.00973291	0.92239832	0.57875717
Siah1b	0.03797445	1.8626382	0.00972251	0.92243965	0.57875717
N4bp3	-0.0451676	1.3835988	0.00970888	0.92249383	0.57875717
Ptgr1	0.037006	2.92611121	0.00965744	0.92269873	0.57884865
Trim24	0.01380706	5.55712033	0.00963602	0.92278422	0.57886521
Axin2	0.01979521	4.98891397	0.00958299	0.92299629	0.57896116
Ei24	0.01652071	6.08275607	0.00956531	0.92306713	0.57896852
Ccnjl	0.05403203	1.08242256	0.00947362	0.92343549	0.57913962
Pfkm	-0.0171496	5.87960746	0.00946799	0.92345816	0.57913962
Egln1	-0.0122671	6.86090147	0.00942183	0.92364437	0.57919059
Tmem194	0.03587422	2.91223127	0.00941444	0.92367423	0.57919059
Dlg1	-0.0125047	7.4792636	0.0094039	0.92371683	0.57919059
Rrm2b	-0.0122919	6.74187979	0.00935999	0.92389456	0.57926495
Wac	-0.0136359	8.63974807	0.00933882	0.92398038	0.57928168
Kmt2e	0.01258441	9.52673944	0.00931067	0.92409465	0.57928993
C78339	0.02609641	2.77947753	0.0092989	0.92414252	0.57928993
Cntnap5a	0.02366878	4.3575796	0.0092919	0.92417096	0.57928993
Pkd2l2	0.02015725	3.72470118	0.00926765	0.92426967	0.57931333
Neurod4	-0.064987	0.44780469	0.00925368	0.92432657	0.57931333
Ccnb1	0.0968922	-1.0194549	0.00922407	0.92444735	0.57935196
4930581F22I	-0.0557197	0.41831874	0.00919945	0.92454793	0.57935952
Zfp68	-0.0163108	6.18202329	0.00917491	0.9246483	0.57935952
Ddr1	0.01796292	3.43016774	0.00916056	0.92470708	0.57935952
Dnajc5	-0.0101295	7.92614819	0.00915973	0.92471046	0.57935952
Tie1	0.07675207	0.39482434	0.00914883	0.92475514	0.57935952
Unc119b	0.02390441	4.03047117	0.00911285	0.92490278	0.57939767
Sp140	-0.0154026	4.89215056	0.00910516	0.92493434	0.57939767
Gm7904	-0.2286688	-1.4868777	0.00906477	0.9251005	0.5794647
Tbc1d22bos	-0.122893	-1.0139922	0.00903535	0.92522177	0.5795036
Esf1	-0.0110948	7.18536144	0.00900395	0.92535139	0.57951347
Pofut2	0.02546667	3.73010993	0.00900287	0.92535586	0.57951347
Pla1a	0.05951786	0.99689346	0.00898161	0.92544378	0.57952296
Slc2a4	-0.0609634	0.01525547	0.0089706	0.92548934	0.57952296
Cops5	0.01046934	6.0242677	0.00894323	0.92560273	0.5795309
Prosc	0.01299959	5.53997079	0.00893898	0.92562035	0.5795309
Armcx1	-0.0127166	5.69029124	0.00890871	0.92574602	0.57957254
Dgkd	-0.0182518	5.44293754	0.00885871	0.92595406	0.57963639
Scaf4	-0.0113431	6.40873382	0.00885575	0.92596636	0.57963639
Mcm9	0.0352089	2.0178278	0.00877963	0.92628426	0.57977631
Adprh	0.0141796	4.24486342	0.0087739	0.92630825	0.57977631
Arhgap29	-0.0191097	9.98480511	0.00871697	0.92654697	0.57987004

Grap2	0.0402603	1.1940081	0.00870099	0.92661413	0.57987004
Lman2l	-0.0212139	3.23296751	0.00867019	0.92674375	0.57987004
Mrpl19	0.01390326	5.02303377	0.00866797	0.92675309	0.57987004
Gm13498	-0.0577482	-0.4467189	0.00866775	0.92675401	0.57987004
Gtpbp8	0.01631278	3.77131635	0.00864708	0.92684115	0.57988753
Brms1l	0.01287995	5.94753582	0.00861931	0.92695833	0.57991326
Gm2382	-0.0266515	2.975679	0.00860929	0.92700068	0.57991326
Nkiras1	0.01104567	6.11160246	0.00856744	0.92717781	0.57998703
Slc25a29	0.04637184	0.83597894	0.00846991	0.92759227	0.58019142
Pbx4	-0.0509735	0.13138919	0.00846269	0.92762302	0.58019142
Tmem59l	0.02289268	4.17803681	0.00844021	0.92771895	0.58021437
Zfp467	0.02264101	4.00171468	0.00841281	0.92783599	0.58022528
Rad18	0.02308542	4.2203029	0.0084084	0.92785486	0.58022528
Bcl3	-0.0425391	0.8728901	0.00837257	0.92800829	0.58028418
Gm6568	0.0403551	0.79574085	0.00834889	0.92810987	0.58031065
Cyp39a1	-0.0263651	3.46184472	0.00832987	0.92819159	0.5803247
Ldb2	-0.0116846	6.02685681	0.00827546	0.92842581	0.58041315
Blmh	-0.0141728	5.01121896	0.00826547	0.92846887	0.58041315
Rab28	0.0128156	5.36756956	0.00825576	0.92851081	0.58041315
Itm2c	-0.0201124	7.70303371	0.00819873	0.92875744	0.58053027
Tlr8	-0.1158287	-1.0531026	0.00810712	0.92915545	0.58071964
Dnaaf2	0.02607082	3.59941112	0.00810172	0.92917897	0.58071964
Otub2	0.01933399	3.60956246	0.00807395	0.92930012	0.5807497
Igfbp7	-0.0223457	5.10106368	0.00805039	0.92940306	0.5807497
Mtmr2	0.01279379	6.65554123	0.0080353	0.92946905	0.5807497
Ankrd26	0.01369513	5.96875239	0.0080182	0.92954392	0.5807497
Dpyd	0.02218174	3.2659172	0.00801714	0.92954859	0.5807497
Thsd4	0.02595516	6.29216156	0.00800933	0.92958281	0.5807497
Ppme1	0.01459431	5.17759303	0.00796686	0.92976924	0.58078153
Gadl1	0.06583531	-0.5549758	0.00795799	0.92980823	0.58078153
Galm	-0.0328044	2.35655813	0.00795722	0.92981163	0.58078153
Tcerg1	-0.011861	6.61335519	0.00790843	0.93002653	0.58083322
Cyp46a1	0.01557838	4.74106673	0.00790463	0.93004329	0.58083322
Ythdf3	-0.0103586	7.18928412	0.00787709	0.93016493	0.58083322
Armc6	-0.0286298	1.69641095	0.00787115	0.93019121	0.58083322
Tc2n	-0.0439326	0.86585266	0.00786923	0.93019967	0.58083322
Mon1b	-0.0108346	5.95988164	0.00785152	0.93027807	0.58083322
Gpi1	0.01120202	7.06928865	0.00784443	0.93030946	0.58083322
Cacnb2	0.01226422	6.33615313	0.00782643	0.93038924	0.58083707
D630024D03	0.1238822	-1.7869437	0.00781113	0.93045713	0.58083707
Fhit	-0.0539286	1.2402851	0.00780293	0.93049352	0.58083707
A430033K04	0.01621556	5.56796504	0.00774852	0.93073564	0.58095119
Wbscr25	0.08541974	-0.9108051	0.00772934	0.93082117	0.58096755

Gfra1	-0.0196149	3.28474915	0.00768735	0.93100886	0.58104768
Rbpms2	0.03209484	2.82537584	0.00766976	0.9310876	0.5810598
Ndor1	-0.022963	3.23268518	0.0076556	0.93115109	0.5810624
Zfa-ps	0.03500581	1.11562698	0.00762791	0.93127536	0.58108878
Dedd2	-0.0371825	1.48902123	0.00761976	0.93131202	0.58108878
Ier3ip1	-0.0150057	5.53606747	0.00756283	0.93156838	0.58114097
Tbcc	-0.018225	3.28109105	0.00756002	0.93158107	0.58114097
Toporsos	0.0319112	1.98353634	0.00755497	0.93160387	0.58114097
Tbl3	0.02963387	2.07913153	0.00754853	0.93163297	0.58114097
Mmp23	0.0741677	-0.4723101	0.00752928	0.93171997	0.58115823
Fam134c	0.02689107	2.95746964	0.00750892	0.93181209	0.58117868
Sptlc2	-0.0120987	5.09740509	0.00740674	0.93227644	0.58139933
Oscp1	0.02013068	3.51831819	0.00739784	0.93231702	0.58139933
4930432K21I	-0.0509385	-0.3257549	0.00738064	0.93239556	0.58139933
Il12a	0.03078298	1.92491101	0.00737895	0.93240328	0.58139933
Bbs9	-0.012098	5.07388828	0.00736376	0.93247268	0.58140559
Cchcr1	-0.0433365	0.99538075	0.0073158	0.9326924	0.5814898
Pcgf6	0.02334683	3.32218114	0.00730838	0.93272646	0.5814898
Gm9958	0.03163978	1.63163496	0.00724261	0.93302905	0.58164143
Pip4k2b	-0.0116436	7.02044146	0.00722877	0.93309293	0.58164423
Stxbp6	0.01326702	6.56505081	0.00715617	0.93342888	0.58181662
Slc6a9	-0.019069	4.07559452	0.00713377	0.93353292	0.58182977
Papd7	0.01543934	4.69030791	0.00712605	0.93356878	0.58182977
Ppp2r2c	-0.0124161	9.02177401	0.00708224	0.93377281	0.58188639
Emb	-0.0238017	6.18088226	0.00707361	0.93381306	0.58188639
Gm17801	-0.0867592	-0.6337183	0.007065	0.93385327	0.58188639
5031434O11	-0.074587	0.19315469	0.00705559	0.93389724	0.58188639
Raver2	0.01893946	3.14227939	0.00702099	0.9340591	0.58195022
Slc38a10	-0.0179269	3.43456733	0.00696884	0.93430388	0.5820657
Tmem236	0.11898977	-1.7102304	0.00694132	0.93443339	0.58207405
Nr2c1	-0.0157222	4.14095446	0.00694074	0.93443612	0.58207405
Fndc4	-0.0185976	3.68251726	0.0069237	0.93451644	0.58208706
Alpk2	0.11230726	-1.4775081	0.00688395	0.93470424	0.58216702
Stambpl1	0.01641117	3.56400113	0.00683921	0.93491625	0.58226205
Slc39a7	-0.0177463	4.19422238	0.00682424	0.93498735	0.58226931
Axl	0.02356728	5.27439036	0.00677229	0.93523469	0.5823833
Fam161b	-0.0190066	3.87855137	0.00675167	0.93533313	0.5823833
Islr	-0.0274812	6.83435633	0.0067484	0.93534876	0.5823833
Gclm	-0.0143559	5.32642741	0.00669183	0.93561967	0.58250081
Gm14436	-0.0137479	5.06734564	0.00668418	0.93565643	0.58250081
9630013A20I	0.03090989	1.48920394	0.00659275	0.93609696	0.58271794
Flt3l	0.03323322	1.70226686	0.00658064	0.93615554	0.58271794
Mynn	0.01139834	5.5697216	0.00655583	0.93627576	0.58271794

Dtwd1	0.03329797	2.03221828	0.00655031	0.93630251	0.58271794
Slc25a22	0.01247026	4.94675892	0.00655028	0.93630265	0.58271794
Ears2	-0.0463258	0.22658193	0.00650712	0.93651239	0.58281145
Mtfmt	-0.0215028	3.0333309	0.00638551	0.93710709	0.58309393
4931428F04I	0.03479737	1.13749693	0.00638305	0.93711918	0.58309393
Strc	0.35466172	-1.2857172	0.15895613	0.93725226	0.58309393
Gm7457	-0.0811059	-0.9471554	0.00635451	0.9372596	0.58309393
Terf2ip	0.010909	6.07454767	0.00635131	0.93727536	0.58309393
Rnf40	0.01883939	3.98065516	0.00634155	0.93732348	0.58309393
Plekha3	0.01180207	5.32988785	0.00630629	0.93749759	0.58316521
Nrxn1	0.01357924	9.77366721	0.00628059	0.93762477	0.58317006
0610040F04I	-0.0446726	0.96442766	0.00627538	0.9376506	0.58317006
C130071C03I	0.02263117	2.7042152	0.00626721	0.93769109	0.58317006
Zfp458	0.02064833	4.61657535	0.00625665	0.93774353	0.58317006
Mta1	0.01244332	4.54349994	0.0061846	0.93810222	0.5833331
Dbn1	0.01537611	4.44211037	0.0061597	0.93822671	0.5833331
Lingo3	-0.0222432	3.01449084	0.0061532	0.93825925	0.5833331
Ncstn	-0.0151225	5.1058978	0.00615005	0.93827498	0.5833331
Shisa6	0.01760373	5.30408921	0.00614437	0.93830346	0.5833331
Sh2d3c	0.01267981	4.98566397	0.00608925	0.93858022	0.58346813
Rbm25	-0.0114835	8.74047397	0.00607161	0.93866904	0.58348631
Myo1d	0.01351644	4.42317551	0.00603898	0.93883371	0.58355164
Phyhipl	0.01063417	7.4588456	0.00601023	0.93897919	0.58360503
Pet100	-0.0246713	3.54535669	0.00597976	0.93913373	0.58362788
Ttc34	-0.0579394	-0.1334546	0.00597949	0.93913512	0.58362788
Mesp2	-0.0481563	1.96370812	0.0059634	0.9392169	0.58364168
Ppp2r5b	0.01679924	3.29609995	0.00591979	0.93943907	0.5837427
Rreb1	0.01513861	5.25007658	0.00588131	0.93963584	0.58376461
Dgcr8	0.01276379	4.13811439	0.00588121	0.93963632	0.58376461
Abcb4	0.04483369	0.37409322	0.00587417	0.93967238	0.58376461
Tmem74	-0.0302397	1.24584302	0.00586631	0.93971271	0.58376461
Smim20	-0.0195406	3.66801231	0.00580097	0.9400487	0.58392788
Pcyox1	0.01347509	5.62445195	0.00578358	0.94013843	0.58392788
Ikzf2	-0.0147296	4.23824318	0.00576688	0.94022478	0.58392788
Ckmt1	-0.0142277	5.28012258	0.00576661	0.94022617	0.58392788
Tmem110	0.01676068	2.80599648	0.0057494	0.94031524	0.58392788
2410007B07I	-0.1089128	-1.9733839	0.00572941	0.9404189	0.58392788
1700034G24	0.06774762	-0.0836493	0.00572066	0.94046429	0.58392788
Ptdss2	-0.0172996	3.24280538	0.00569326	0.94060677	0.58392788
Scamp3	0.01831939	3.63923408	0.00569277	0.94060929	0.58392788
E030018B13I	0.15042847	-1.4538829	0.0056877	0.94063572	0.58392788
Nucb1	-0.0189187	4.56765034	0.00567153	0.94072001	0.58392788
Tmem229b	-0.0193262	3.13060807	0.00567081	0.94072376	0.58392788

Dnajc3	-0.0143934	6.76679914	0.00566539	0.94075201	0.58392788
Cenpu	-0.0406513	-0.0263099	0.00565426	0.94081011	0.58392788
Snrpd3	-0.0230932	2.63607777	0.00561547	0.9410131	0.58399917
Mef2d	0.00975684	7.08971438	0.00560861	0.94104909	0.58399917
Gsr	0.00987732	5.88993082	0.00559818	0.94110383	0.58399917
Zfp276	-0.0215923	2.6787564	0.00556212	0.94129345	0.58404189
Brd3	-0.0098219	6.20578828	0.00554703	0.94137296	0.58404189
Bach1	0.01212085	5.05667399	0.00554265	0.9413961	0.58404189
Pgrmc1	-0.0149759	7.58892138	0.00553384	0.94144257	0.58404189
Zfp612	-0.0132702	7.4305425	0.0055285	0.9414708	0.58404189
Tfeb	0.03372848	1.47424651	0.00550071	0.94161778	0.58407386
Cpa2	-0.0501399	-0.4612285	0.00549622	0.94164159	0.58407386
Luc7l2	0.0106365	7.39714737	0.00546286	0.94181865	0.58414669
Nrbp1	-0.0105154	5.94088008	0.00542719	0.94200853	0.58422747
Adam5	0.06046626	0.8451942	0.00541123	0.94209369	0.58423388
A830052D11	0.0415104	0.26860189	0.00540291	0.94213815	0.58423388
Irf2	-0.0127058	5.24195731	0.00536474	0.9423425	0.58430217
1600002H07	-0.018791	3.04316158	0.00536007	0.94236757	0.58430217
Dpp9	0.0121982	4.59523009	0.00531608	0.94260408	0.58439508
Cpsf7	0.01217711	5.71114193	0.00531002	0.94263674	0.58439508
Prss8	0.18003157	-2.1549414	0.00527038	0.94285087	0.58449084
Ipcef1	-0.0115723	7.00043625	0.00524513	0.94298767	0.58453865
Sdk1	0.01834778	4.14071295	0.00520235	0.94322025	0.58462598
Tmx1	0.01435455	4.8957351	0.00519727	0.94324792	0.58462598
Bod1l	0.00991952	8.59520974	0.00517339	0.94337819	0.58466973
Lrriq3	0.04589467	0.57315114	0.00513933	0.94356459	0.58467353
Kctd11	0.02495837	3.32432119	0.00512481	0.94364419	0.58467353
Zfp367	-0.0161307	3.52014188	0.00510956	0.94372795	0.58467353
Cwc25	0.0120028	4.51326967	0.00510501	0.94375298	0.58467353
Tra2a	0.01039894	6.08364828	0.00508923	0.94383983	0.58467353
Gls	0.01216449	9.46596455	0.00508523	0.94386186	0.58467353
Zfp229	-0.0162237	3.67899273	0.00507722	0.94390599	0.58467353
Rdm1	-0.029879	2.51132514	0.00507678	0.94390847	0.58467353
Adora1	-0.0104662	7.10715741	0.00507441	0.94392154	0.58467353
Pus1	-0.0239226	1.66141706	0.00503222	0.94415474	0.58478101
Pacsin1	-0.0106591	6.57730162	0.00501319	0.94426025	0.58480937
Il13ra2	0.03510585	1.98597913	0.00498883	0.94439559	0.5848405
Tmem158	-0.0131834	4.10897851	0.00498266	0.94442992	0.5848405
Ccdc117	0.01391214	4.88265275	0.0048785	0.94501282	0.58516447
Col26a1	0.02611819	1.50329749	0.00484445	0.94520471	0.5852251
Zfp472	-0.0240347	1.75869423	0.00482452	0.94531735	0.5852251
Fam76b	0.01540591	4.65486334	0.00481388	0.94537759	0.5852251
Cldn5	0.06621568	-0.2046789	0.00481128	0.9453923	0.5852251

Pmp22	0.01665849	8.57001292	0.00480825	0.94540947	0.5852251
Atxn10	-0.0074081	7.66352388	0.00477927	0.94557394	0.58528993
Abcd2	-0.0148084	4.77158187	0.00472596	0.94587788	0.5853504
Scarb2	0.01800217	4.27595107	0.00472069	0.945908	0.5853504
Wdr81	-0.0191679	3.02157766	0.00471838	0.94592119	0.5853504
Erich6	-0.0389319	1.27272317	0.00471415	0.94594539	0.5853504
Usp27x	0.0110525	4.93859851	0.00470146	0.94601808	0.5853504
Tmem66	-0.0104855	5.98328088	0.00469935	0.94603018	0.5853504
Socs5	-0.0092698	6.54267267	0.00465604	0.94627904	0.58546739
Srrm1	-0.0067329	7.56337242	0.00463606	0.94639424	0.58550169
Rmdn1	0.01630297	3.73525051	0.00458182	0.94670826	0.58564161
Rgs8	-0.0123926	6.68073307	0.00457636	0.94673999	0.58564161
Pik3r3	-0.007655	6.59840432	0.00456075	0.94683075	0.58566077
Sfxn4	-0.0199419	3.64508379	0.00453261	0.94699477	0.58572524
Trmt1	0.02048555	2.8026084	0.00452214	0.9470559	0.58572606
Elavl3	0.01017466	7.19794494	0.0045068	0.94714565	0.58573494
Tnfsf13	0.06660894	-1.1613654	0.00449925	0.94718985	0.58573494
Fto	-0.0080547	7.00755629	0.00448487	0.94727422	0.58575013
Pigs	-0.0123432	4.61576132	0.00447356	0.94734059	0.5857542
Dkk2	-0.0835284	-0.8060264	0.00445553	0.94744669	0.58578282
Sephs2	-0.0129433	4.04139257	0.0044348	0.94756887	0.58582139
Pax6	0.02178962	2.24170717	0.0043869	0.94785237	0.58594467
Kcnh6	-0.0399497	-0.1846566	0.00438091	0.94788792	0.58594467
Gpr179	-0.0621616	-0.4016833	0.004367	0.94797057	0.58595879
Gnl2	0.00876021	5.97776156	0.00435627	0.94803445	0.58596129
Tmem51	-0.0246779	2.21709795	0.0043299	0.94819177	0.58602156
Acot9	-0.0130417	3.80085597	0.00430821	0.94832149	0.58606475
Rbm39	-0.007212	7.64977397	0.00428035	0.94848859	0.58613104
Tnfrsf22	0.02307691	1.74743848	0.00421998	0.94885258	0.58631898
C330013E15I	-0.0357115	0.41328429	0.00419323	0.94901471	0.58638217
Rad51ap2	0.0349358	1.2965391	0.00408384	0.9496832	0.58659861
Vil1	-0.0486931	-0.0528895	0.00407421	0.94974244	0.58659861
Tec	-0.0153268	5.07150878	0.00406318	0.94981044	0.58659861
Tas1r3	-0.0421643	-0.1782649	0.00405048	0.94988883	0.58659861
Srd5a3	-0.0225663	2.96750846	0.00403999	0.94995366	0.58659861
Chmp4c	-0.0819847	-1.6063499	0.00403933	0.94995777	0.58659861
Actg2	-0.0609242	-0.8348818	0.00403792	0.94996646	0.58659861
Zfp800	-0.0115805	5.95938566	0.00403006	0.95001516	0.58659861
Ppp2r1b	-0.008609	5.52140425	0.00402453	0.95004937	0.58659861
Rundc1	0.01068756	5.45873072	0.00402286	0.95005972	0.58659861
Rgl1	0.00663434	7.58694642	0.00402221	0.95006374	0.58659861
Serpinb10	-0.0498526	-0.6672712	0.00401901	0.95008362	0.58659861
Wipi2	-0.0121003	5.00363007	0.00400644	0.95016162	0.58660979

Gstm1	-0.0143217	7.64972735	0.00396038	0.95044852	0.58674993
Brinp3	-0.0108986	4.53933013	0.00393058	0.95063504	0.58678784
Zdhhc23	-0.0331734	1.06906349	0.00392014	0.95070055	0.58678784
Tspan11	0.01965045	3.38958987	0.00391949	0.95070465	0.58678784
Neurod1	-0.0164484	3.51486705	0.00391235	0.95074953	0.58678784
Gm13242	-0.054135	-0.9866561	0.00388985	0.95089111	0.58681756
Pcdhga4	0.02171032	1.68857709	0.00388567	0.95091752	0.58681756
Stard8	0.00762156	5.63681122	0.00386195	0.95106736	0.58687306
BC017643	-0.0201419	2.48039427	0.00383461	0.95124061	0.58694299
Cmtr1	-0.0127924	5.59587889	0.00379851	0.95147035	0.58704097
Amz2	0.01078935	4.75288145	0.00378341	0.95156677	0.58704097
Naglu	-0.0293354	1.20985482	0.00378147	0.95157919	0.58704097
Fv1	0.03523668	0.54461146	0.00375513	0.9517479	0.58710807
Pelp1	-0.0145822	3.28073169	0.00373213	0.95189572	0.58715491
Map4k5	0.00735496	5.95929711	0.00372013	0.95197301	0.58715491
Glb1l2	0.05322171	-0.4162008	0.00371294	0.95201941	0.58715491
Il1rl2	0.06448337	-1.0983278	0.00370609	0.95206359	0.58715491
Lpar1	0.01537768	6.85129751	0.00369639	0.95212631	0.58715662
Adrbk1	-0.010018	5.09070677	0.00366377	0.95233775	0.58725004
Scn9a	-0.023558	2.68024145	0.00362189	0.95261057	0.58728018
Pcolce2	0.0315971	1.14660412	0.00361057	0.95268462	0.58728018
Fam212a	-0.0577303	-0.7309088	0.003601	0.95274725	0.58728018
4930429F24I	-0.0335812	0.47399518	0.00357704	0.9529045	0.58728018
Ppapdc1b	-0.0105766	4.27415139	0.0035667	0.95297256	0.58728018
2610100L16I	-0.0202108	2.15137763	0.00356038	0.95301421	0.58728018
4930405A21I	-0.0311714	1.03742886	0.0035595	0.95302001	0.58728018
Tgif2	-0.0213376	2.2958135	0.00355786	0.95303082	0.58728018
Klhl42	-0.0071629	6.43862941	0.00355762	0.95303236	0.58728018
Lrrc71	0.06769169	-1.4883196	0.00355738	0.953034	0.58728018
Lamp1	-0.0130162	7.56325449	0.00355554	0.95304613	0.58728018
Chrnb4	-0.032009	0.14843537	0.00352004	0.95328079	0.58738783
Gsta3	-0.0339939	0.88015063	0.00348535	0.95351132	0.58749291
Zfp953	0.01637663	2.98051226	0.00345208	0.95373347	0.58759283
Fzd1	-0.0130479	6.13564224	0.00344014	0.95381341	0.58760109
Pdhb	-0.0061602	6.93228658	0.00343218	0.95386685	0.58760109
Tbl1xr1	-0.0063087	7.63651991	0.0034138	0.95399036	0.58764022
Daglb	0.01391728	3.13088302	0.00339138	0.95414156	0.5876668
Dgat2	-0.0125427	3.75689684	0.00338961	0.9541535	0.5876668
Spred1	0.00734204	8.20895939	0.00337773	0.9542338	0.5876793
Ttll11	0.01299898	4.20607825	0.00334082	0.95448422	0.58779657
Cysltr1	0.03894342	-0.4593609	0.00326375	0.95501173	0.58806381
Gm684	0.0131901	3.50100373	0.0032599	0.95503825	0.58806381
Dbr1	-0.0112607	3.96110004	0.00323782	0.95519055	0.58811254

Ogfod3	0.02208482	2.08932277	0.00323104	0.95523747	0.58811254
Rasl12	-0.0417593	-0.0654377	0.00320638	0.95540841	0.58818082
Gtf2h1	0.0104958	5.09621696	0.00311799	0.95602663	0.58849541
Zfand6	0.01044211	5.61016138	0.00310771	0.9560991	0.58849541
Syngr2	-0.0245608	1.68918734	0.00310764	0.95609965	0.58849541
Acad12	0.04172226	-0.9552592	0.00309326	0.95620121	0.58850406
Snupn	0.01404361	3.39514449	0.00308864	0.95623386	0.58850406
Sh3kbp1	-0.0077563	6.38425475	0.00306872	0.95637507	0.58851003
Dvl1	0.01020605	4.07621373	0.00306093	0.95643043	0.58851003
Cyb561d2	0.02432828	1.76477958	0.00304978	0.95650977	0.58851003
Slc28a3	0.0323324	1.09393552	0.00304765	0.95652494	0.58851003
2700046G09	-0.0240504	1.75503727	0.00303048	0.95664749	0.58851003
Ddx19a	0.00968134	5.4407088	0.00302839	0.9566624	0.58851003
LOC106740	0.01152443	4.1747146	0.00302168	0.95671036	0.58851003
Cd7	0.1534845	-1.7326019	0.01676164	0.95672929	0.58851003
Usp38	0.00927562	5.03510708	0.0030017	0.95685363	0.58851003
Sfmbt1	0.00712652	5.97509888	0.00299697	0.95688757	0.58851003
Sigmar1	-0.0109058	3.01872859	0.00299462	0.95690445	0.58851003
Smim4	0.01677188	2.15559357	0.0029675	0.95709982	0.58859323
Msh2	-0.0098022	4.64569386	0.00292789	0.95738681	0.58873276
Sass6	0.01141816	3.77705377	0.00290048	0.95758656	0.58879832
Arhgef6	0.00789857	6.45736664	0.00289677	0.95761365	0.58879832
Zbtb40	0.02165862	2.50864861	0.00288055	0.9577324	0.58880854
Trim35	0.0066133	6.9522191	0.00287808	0.95775049	0.58880854
Fstl1	0.01661781	7.22418471	0.00285233	0.95793969	0.58886364
Lpar6	0.01662291	2.23537527	0.00284953	0.95796035	0.58886364
Gm15713	-0.0509982	-0.6500398	0.00283658	0.95805589	0.58886699
Ntrk1	0.04867166	-1.4088131	0.0028261	0.95813338	0.58886699
Itgb7	0.04999767	-1.4779286	0.00282234	0.95816118	0.58886699
Ror1	-0.0304358	0.88849533	0.00281042	0.95824955	0.58886699
Cysltr2	-0.0572172	-0.9832844	0.00280763	0.95827026	0.58886699
Elovl2	-0.0171556	2.66654295	0.00280006	0.9583265	0.58886699
Aldh1l2	-0.021753	2.81231289	0.00278491	0.95843931	0.58889937
Rce1	-0.0346508	0.41775303	0.00277321	0.95852658	0.58891605
Msl3l2	0.01172324	4.12858652	0.00274845	0.95871196	0.588993
Gng8	0.03926663	-0.4881482	0.00273525	0.95881113	0.58901618
Dyrk1a	0.00587075	7.22479528	0.00271834	0.95893852	0.58901618
Doc2g	-0.0514922	-0.3671259	0.00269643	0.95910418	0.58901618
Eml4	0.00712191	6.15080929	0.0026886	0.95916355	0.58901618
Tunar	-0.0156301	3.21410415	0.00268857	0.9591638	0.58901618
Arpc1a	0.00784635	6.16045961	0.00267976	0.95923065	0.58901618
Serpinf1	-0.0150747	5.60135265	0.00267795	0.95924448	0.58901618
Yipf5	-0.0065838	5.41711625	0.00267201	0.95928966	0.58901618

Chrna1	-0.019475	2.07646178	0.00266849	0.95931644	0.58901618
Elk4	-0.0062659	6.40798049	0.00266395	0.95935101	0.58901618
Impg1	-0.0328343	0.27161682	0.0026203	0.95968512	0.58911985
Mocs3	-0.0350049	-0.9356207	0.00261929	0.95969285	0.58911985
Aqp11	0.01607024	1.77804299	0.00261315	0.95974012	0.58911985
Rn45s	0.0118883	14.5621267	0.00261051	0.95976044	0.58911985
4930539N22	0.04034174	0.71910845	0.00259269	0.9598979	0.58912177
Tardbp	-0.0059073	7.45746874	0.00258783	0.95993544	0.58912177
Pappa	-0.0237845	2.78235808	0.00258673	0.95994399	0.58912177
Actl6b	0.0147359	3.2826385	0.002573	0.96005027	0.58914028
Vps41	-0.0074304	7.02432777	0.00256731	0.96009445	0.58914028
Usp53	0.00680616	6.92540021	0.00254906	0.96023643	0.5891606
Tram1	0.00994626	5.97538072	0.00254759	0.96024786	0.5891606
Syce1	0.07462743	-2.0044352	0.00252864	0.96039587	0.58921451
Akap1	0.01178511	3.33678222	0.00250475	0.96058323	0.58929255
Kcnh5	0.01044342	5.78019818	0.00249699	0.96064431	0.58929311
Lax1	0.05183391	-0.2643545	0.00247701	0.96080192	0.58935289
Ripply2	-0.0342966	-0.3361722	0.00243201	0.96115929	0.58950929
Syt5	0.00846621	5.40819515	0.00242976	0.96117726	0.58950929
Slc2a10	0.02366928	0.93217711	0.00241704	0.96127896	0.58953475
Egflam	0.01282682	3.52709281	0.00238441	0.96154094	0.5896585
Ptpdc1	-0.0066541	5.41754893	0.00236046	0.96173446	0.58973959
Rab3b	0.0067262	5.12020472	0.00235316	0.96179359	0.58973959
Tmem147	0.01098822	3.44846783	0.00234122	0.96189055	0.58976213
4930509E16I	-0.0517792	-1.2491604	0.00232824	0.96199629	0.58976449
1700003M02	0.03030282	0.2590926	0.00232597	0.96201482	0.58976449
Cplx2	0.007811	9.63292106	0.00230452	0.96219019	0.58983508
Mto1	0.01039939	3.51939617	0.00229361	0.96227979	0.5898531
Pus3	0.01186535	3.36515777	0.00228221	0.96237356	0.58987366
Ankrd66	0.07558883	-2.1740184	0.0022548	0.96259999	0.58990517
Enpp4	0.00950367	4.28764148	0.00225351	0.96261072	0.58990517
Ric3	0.00679321	6.1222974	0.00225125	0.96262944	0.58990517
Zcchc9	0.00661355	5.82683185	0.00223906	0.96273066	0.58990517
Mrgpre	-0.0129586	3.56459171	0.00223827	0.96273725	0.58990517
Dars2	-0.0135459	3.49596305	0.00223237	0.9627863	0.58990517
Cpz	-0.0592292	-1.0697219	0.00221634	0.96292006	0.5899335
Brd4	-0.0059756	8.73212684	0.0022124	0.962953	0.5899335
Kctd6	-0.0077866	5.85232124	0.00219969	0.96305947	0.58996184
Satb2	0.00813348	7.21935707	0.00217598	0.96325901	0.59004717
Kcnk4	0.02415105	0.31919566	0.00216516	0.96335035	0.59006622
Meis3	0.01190118	3.60716697	0.00215191	0.96346261	0.59009808
Itfg1	0.00575966	7.75586571	0.00211739	0.96375665	0.59018836
Top1mt	-0.0113459	3.13439273	0.00210946	0.96382449	0.59018836

Slc40a1	0.01446875	2.11802595	0.0021033	0.96387737	0.59018836
Foxp3	0.03845343	-0.7855002	0.00210268	0.96388262	0.59018836
Ttc39c	0.01417443	2.23883921	0.00209628	0.96393765	0.59018836
Fubp3	-0.00678	5.88427179	0.0020856	0.96402952	0.59018836
F2r	-0.0120144	2.66735324	0.00208534	0.96403178	0.59018836
Spire1	-0.0077517	7.44840204	0.00207354	0.96413362	0.59021382
Vwa5b1	-0.025954	0.4933153	0.00206458	0.9642111	0.59022437
Cbx8	0.0292303	0.15420196	0.00205166	0.96432323	0.59022711
Exosc5	-0.0199653	0.96836672	0.00205018	0.96433609	0.59022711
Srpr	-0.009895	6.37658832	0.00201463	0.96464639	0.59030349
Kdm4b	0.01163733	2.7623584	0.00201224	0.96466734	0.59030349
Csf1r	-0.0100952	3.45706931	0.00201082	0.96467981	0.59030349
Misp	0.05042514	-1.123436	0.0020083	0.96470194	0.59030349
Fam20c	-0.0151247	2.99503013	0.00198355	0.96491997	0.59039173
Ephx2	-0.0083242	3.85688729	0.00197827	0.96496668	0.59039173
Ptpn3	0.00752175	6.04739918	0.00196902	0.96504863	0.59040498
Ext1	-0.0055001	5.68457115	0.00195354	0.96518613	0.59041958
Htra1	0.00957489	3.91348664	0.00195277	0.96519304	0.59041958
Zfp868	-0.0083981	4.89066314	0.00192631	0.96542951	0.59042552
Nit1	0.01028366	2.91999726	0.00192628	0.96542976	0.59042552
Rasgef1a	0.0067965	6.81493714	0.00192416	0.96544876	0.59042552
Atp6v0c-ps2	0.02656512	-0.3216039	0.00192149	0.96547278	0.59042552
Nat1	-0.0187695	2.09121257	0.00191252	0.96555337	0.59042552
Rbm47	-0.0090717	4.57140377	0.00191129	0.96556441	0.59042552
Cog3	-0.0065521	5.52673204	0.00187544	0.96588875	0.59056943
Camkk1	-0.0090647	4.88035327	0.00187196	0.96592034	0.59056943
Grrp1	-0.0405227	-1.7128194	0.00186142	0.96601638	0.59059129
Nek11	0.02347313	0.02576182	0.00183151	0.96629034	0.59072191
Dao	0.07764985	-1.5808403	0.00181919	0.9664038	0.59072681
Zfp711	-0.0100902	4.20917401	0.00181275	0.96646332	0.59072681
Lamb1	-0.0074262	4.17024606	0.00181102	0.96647928	0.59072681
Lurap1l	0.01088963	2.60578454	0.00180151	0.96656737	0.59074379
Dpp4	-0.0134516	5.50219731	0.00179186	0.96665699	0.59076171
Zfp646	0.00841787	3.74038985	0.0017841	0.96672924	0.590769
Arfrp1	0.00929888	3.99464723	0.0017716	0.96684587	0.59078269
Gabrd	0.01521383	2.84095757	0.00176237	0.96693229	0.59078269
Hist1h2ai	0.03607583	-1.5684036	0.00176234	0.96693258	0.59078269
AI593442	0.00582189	8.70047291	0.00175424	0.96700861	0.5907923
Ston2	-0.0097757	4.6411271	0.0017426	0.96711821	0.5908224
Timm10	0.01177999	3.84783903	0.00173499	0.96719004	0.59082944
Prkar2b	-0.0057113	5.63127322	0.00168314	0.96768371	0.5910395
Mkln1	-0.0066918	6.15447147	0.00167187	0.96779208	0.5910395
Stard5	0.01291819	3.55374453	0.00166987	0.9678113	0.5910395

Cdk9	0.00720032	4.38479661	0.00166857	0.96782378	0.5910395
Arhgef5	0.00981585	5.78450739	0.00165578	0.96794732	0.5910395
Pex3	-0.006407	5.12396252	0.00165456	0.9679591	0.5910395
Sharpin	0.00856394	3.8065174	0.00165174	0.96798645	0.5910395
Gm15708	0.04011665	-1.0342753	0.00164448	0.96805685	0.5910395
Pbx1	0.00470122	9.2799897	0.00163683	0.96813116	0.5910395
Cdkn2c	0.01548519	2.73098207	0.00163312	0.96816725	0.5910395
Klhl40	-0.0278994	0.1157573	0.0016176	0.96831876	0.5910395
Osgepl1	0.00773665	4.77018591	0.00161201	0.96837351	0.5910395
Bad	-0.0174381	2.66095758	0.00160942	0.96839891	0.5910395
Fuz	-0.0227942	0.47719722	0.00160401	0.96845213	0.5910395
Zmym4	0.00554273	7.67947104	0.00159463	0.96854443	0.5910395
Gja4	0.02693817	-0.5079181	0.00158242	0.96866504	0.5910395
Hc	0.08354801	-1.9135058	0.0015696	0.96879216	0.5910395
Lysmd2	0.00635108	4.50476051	0.00156662	0.96882175	0.5910395
Mtss1	-0.0066664	6.68507177	0.00155887	0.96889895	0.5910395
Mapk8	-0.0060288	7.5751734	0.00155675	0.96892011	0.5910395
Parp4	-0.0057564	5.70580261	0.00155375	0.96894998	0.5910395
Fn1	-0.0103933	8.00392047	0.00155166	0.96897083	0.5910395
Pnck	0.00692941	3.71850949	0.00155004	0.96898706	0.5910395
Exoc7	0.00661855	4.63607834	0.00154307	0.96905684	0.5910395
Slc19a2	-0.0093565	3.68181895	0.00153486	0.96913923	0.5910395
Kctd12	-0.0044428	7.42126274	0.00153457	0.96914219	0.5910395
4933439C10I	0.0149144	1.82049563	0.00153249	0.96916306	0.5910395
Phf6	-0.0050654	5.27930574	0.00152605	0.96922786	0.59104222
Spata45	-0.045218	-0.5529667	0.0014812	0.96968323	0.59126625
Slc7a7	-0.0216444	1.12585442	0.001478	0.96971596	0.59126625
Snx32	0.00715057	4.27697155	0.00146202	0.96988006	0.5913295
1810044D09	0.03529324	-1.5291417	0.00144845	0.97002004	0.59137803
Tor1a	-0.0098547	2.96947977	0.00143106	0.97020049	0.59142696
Magi1	-0.0066089	6.35945009	0.00142908	0.97022106	0.59142696
Ogn	0.00917164	8.11652656	0.00141081	0.97041196	0.59150652
Dtx4	-0.0057304	5.31482988	0.00140034	0.9705219	0.59153673
Ehhadh	0.01606534	1.10449656	0.00139158	0.97061416	0.59155615
Clec3b	-0.0193107	0.90492489	0.00138129	0.97072306	0.59156017
Adam11	-0.0102304	3.57860668	0.00137954	0.97074155	0.59156017
Fam49a	-0.0048649	8.23706648	0.00137057	0.97083674	0.59158138
Pcbp1	-0.0054763	5.75618372	0.0013502	0.97105417	0.59163479
Ezr	0.00497972	6.23592406	0.00134105	0.97115237	0.59163479
Mtch2	-0.0044397	5.59819807	0.00133717	0.97119416	0.59163479
Lypd2	-0.0222634	1.82578607	0.00133707	0.97119524	0.59163479
Csnk1e	-0.0055202	6.14590417	0.00133286	0.97124058	0.59163479
Zfp628	-0.0110404	2.2903361	0.00132858	0.97128679	0.59163479

Smpd2	0.01141416	2.31042198	0.00129493	0.97165259	0.59177171
Zfp131	0.00599055	5.28940919	0.00129271	0.97167683	0.59177171
Nrg1	0.00972305	3.36162218	0.00129125	0.97169281	0.59177171
Brap	0.00564812	5.40008628	0.00127174	0.97190742	0.59186561
Tpst2	-0.0099333	2.1402579	0.00125368	0.97210752	0.59195066
Cyp2s1	0.01226856	3.8419522	0.00123436	0.97232313	0.59204515
Zc3h12b	0.0053677	5.05571314	0.00119914	0.97272077	0.59225046
Xpc	0.00677423	3.9902574	0.00118064	0.97293193	0.5923422
Mark4	-0.0071435	3.63180136	0.00116384	0.97312502	0.59242294
Col8a2	0.01482694	2.09764553	0.0011448	0.97334577	0.59242384
Fam219aos	0.01029364	3.09071208	0.00113945	0.97340808	0.59242384
Sgol1	-0.0166545	0.8431864	0.00113807	0.97342418	0.59242384
Pold2	-0.0139169	2.57042042	0.00112562	0.97356988	0.59242384
Mterfd3	-0.0085692	2.97547655	0.00112446	0.97358344	0.59242384
Rasgef1c	0.00815235	3.5194525	0.00111491	0.9736959	0.59242384
Mcm7	-0.008896	2.98666999	0.00110874	0.97376876	0.59242384
Apbb1ip	-0.0081675	3.19513467	0.00110413	0.97382325	0.59242384
Cxcl9	0.03308562	-0.5124598	0.0010988	0.97388647	0.59242384
Nudt16	0.00634065	4.68686686	0.00109711	0.97390661	0.59242384
Mri1	-0.0134585	1.14525126	0.00109218	0.97396529	0.59242384
Fam109b	-0.0202883	1.55754912	0.00109162	0.97397198	0.59242384
Gm13051	0.03224198	-1.3911024	0.00108791	0.97401621	0.59242384
Golph3l	-0.0059082	5.99608675	0.00108652	0.97403282	0.59242384
Ttc7b	0.00591935	8.19378473	0.00108644	0.97403369	0.59242384
Ccdc97	0.00518273	5.32492824	0.00101813	0.97486295	0.59285759
Opn1sw	0.02900694	0.40682071	0.00101773	0.9748679	0.59285759
Galnt9	-0.0058951	4.68290137	0.00100124	0.97507227	0.5928594
Tdgf1	0.04161764	-0.1595847	0.00098817	0.97523551	0.5928594
Pdik1l	-0.0059624	3.96548955	0.00098732	0.9752461	0.5928594
Ccl28	0.0132003	1.68767328	0.00098371	0.97529137	0.5928594
Cyp4v3	-0.0081337	4.68104747	0.0009808	0.97532803	0.5928594
Erc2	0.00560274	8.10052414	0.00098023	0.97533511	0.5928594
Pnpla8	-0.0039914	6.88366748	0.00097467	0.97540522	0.5928594
Slc52a3	-0.0129595	1.79376373	0.00097119	0.97544916	0.5928594
Pik3r5	0.00737495	2.80084635	0.00096346	0.97554692	0.5928594
Ccser2	0.00337476	8.27618302	0.000951	0.97570561	0.5928594
Epsti1	0.01640058	1.15801116	0.00094979	0.97572105	0.5928594
Foxg1	0.00508154	6.13935789	0.00094973	0.97572178	0.5928594
Gm16532	-0.021003	1.04913494	0.00094639	0.97576447	0.5928594
2610316D01	-0.0111009	2.20637587	0.00094419	0.97579263	0.5928594
Mdh1b	-0.0337483	-1.1226519	0.00093979	0.97584913	0.5928594
Fabp4	0.02094177	-0.6828978	0.0009389	0.97586057	0.5928594
Zxdc	0.00451367	5.4891973	0.00093391	0.97592479	0.5928594

Tmem79	-0.0356218	-0.2191697	0.00093088	0.97596387	0.5928594
Dhx16	0.00851113	2.95949162	0.00092618	0.97602457	0.5928594
Pomk	-0.00518	4.76491231	0.0009218	0.97608137	0.5928594
Slc7a8	0.00549963	5.5378534	0.00091168	0.97621291	0.59286091
2510009E07I	0.00410186	6.8544813	0.00091064	0.97622653	0.59286091
Tceanc2	-0.0045364	4.63240654	0.00090368	0.9763175	0.59286091
Mrps22	0.00438883	4.4030088	0.00090303	0.97632595	0.59286091
Fam3a	0.00610364	3.56031125	0.0008489	0.97704628	0.59320444
Gm11517	0.02277671	-1.0909567	0.00084727	0.97706832	0.59320444
Zfp933	-0.0052642	4.53456843	0.00084478	0.97710211	0.59320444
Ndufaf4	0.0042901	5.76558867	0.00084243	0.97713391	0.59320444
Arr3	-0.0325843	-1.161148	0.00083786	0.97719594	0.59320533
D330045A20	0.01603982	-0.0948508	0.0008328	0.97726492	0.59321044
Atp6v0d2	-0.0323385	0.3918407	0.00082603	0.97735745	0.59322984
Gpr153	0.00788858	3.00274264	0.00081835	0.97746298	0.59323508
Mrpl4	-0.0065756	4.75516618	0.00081659	0.9774872	0.59323508
Ppp1r35	0.01065516	1.49108591	0.0008102	0.97757552	0.59325192
Psd4	-0.0184492	0.02147733	0.00079869	0.97773526	0.59328636
Naaa	-0.0069417	3.92316073	0.00079739	0.97775341	0.59328636
6720468P15I	0.0364975	-0.4914859	0.00076189	0.97825413	0.59352028
Maf	0.00496948	7.20796103	0.00076147	0.97826009	0.59352028
E230016K23I	0.02675472	-0.708949	0.00075187	0.97839747	0.59356686
Nek8	0.01670432	1.27127554	0.0007421	0.9785383	0.59361553
Ctsd	-0.0068698	7.20349076	0.00073428	0.97865165	0.59364753
Ccdc73	0.00718772	3.33422071	0.00072383	0.97880403	0.5937032
Synb	-0.0205265	-0.6087097	0.00071547	0.97892686	0.59373083
Efcab1	-0.0062939	3.56238764	0.00070803	0.97903665	0.59373083
Gbp5	-0.0124947	2.13971357	0.00070722	0.97904869	0.59373083
BC053749	-0.0099546	2.84818733	0.00070429	0.97909204	0.59373083
Tmem29	-0.0033244	4.81473657	0.00069845	0.97917883	0.59374671
Atp8b1	0.00756353	2.4639012	0.00068837	0.9793296	0.59379144
Csrp1	0.00633534	7.56769806	0.00068543	0.97937384	0.59379144
Rin3	-0.0067944	4.36278488	0.00065612	0.9798196	0.59398785
Fbxl19	0.00597282	3.63288	0.00065601	0.97982125	0.59398785
Impg2	0.03760976	-0.3888372	0.00065221	0.97987971	0.59398785
Coq9	0.00485605	4.37311304	0.00064294	0.98002328	0.59403811
Reep1	-0.0040612	6.96590109	0.00063228	0.98018946	0.59407274
4933424G06	0.0183811	0.47933401	0.0006315	0.9802017	0.59407274
Adssl1	0.0073022	2.68975469	0.00061728	0.98042589	0.59412719
BC031361	-0.0069127	2.8200458	0.00061218	0.98050686	0.59412719
B3gnt2	-0.0043516	5.73961934	0.00060973	0.98054593	0.59412719
Mpzl2	-0.0053159	7.15656014	0.00060783	0.98057624	0.59412719
Map3k14	-0.0108922	1.77007305	0.00060667	0.98059482	0.59412719

Rasd2	0.00425907	4.98101848	0.00059197	0.98083119	0.59422234
Evi5	-0.0028005	7.79948001	0.00058938	0.98087318	0.59422234
Wdr18	0.00396583	3.64050136	0.00057936	0.98103644	0.59428449
LOC1000389	0.04125728	-1.5510624	0.00057419	0.98112133	0.59429916
Commd1	0.00625686	4.15830275	0.00056418	0.98128651	0.59431777
Cxcl11	0.10453438	-2.3691744	0.00056413	0.98128726	0.59431777
Lrsam1	-0.0060384	3.61904986	0.00056131	0.98133408	0.59431777
Prmt1	0.00751224	2.8682413	0.00055756	0.98139668	0.59431894
1700027H10	0.0146373	0.39054886	0.00055003	0.98152256	0.59432548
Fmnl2	0.00323646	8.09144302	0.00054966	0.98152883	0.59432548
Zfp365	0.00431213	9.10482669	0.00054451	0.98161551	0.59434123
Cd276	0.00743865	2.49201803	0.00053617	0.98175691	0.5943901
Gm5468	-0.0118436	1.88273939	0.00051783	0.98207151	0.59453131
Pbrm1	0.00248809	7.48916556	0.00051288	0.98215744	0.59453131
U2af1	-0.0043935	4.10283801	0.00051203	0.98217224	0.59453131
Fabp7	0.00449102	4.52045423	0.00050468	0.98230053	0.59457223
Fam63a	0.00621602	4.35498773	0.00050119	0.98236195	0.59457266
Prdm15	0.00562849	3.40894954	0.00048731	0.98260784	0.59457697
Slc38a2	-0.00583	9.37180083	0.00048646	0.98262303	0.59457697
Chst8	0.01153946	0.65193605	0.0004855	0.98264009	0.59457697
Pdlim7	0.00391252	4.32830393	0.00048063	0.98272742	0.59457697
5430405H02	-0.008599	1.41100039	0.00047979	0.98274247	0.59457697
Rae1	-0.004033	3.83274446	0.00047838	0.98276794	0.59457697
Islr2	-0.0059709	2.79239509	0.00047693	0.98279396	0.59457697
Fam71e1	0.01128394	0.25455267	0.00046259	0.98305453	0.59469788
Utp6	-0.0029522	6.67201553	0.00045253	0.9832399	0.59473811
Mkl1	-0.0027402	5.55924494	0.00045166	0.983256	0.59473811
Cdon	-0.0051114	5.1561861	0.00044456	0.98338812	0.59473811
Caln1	-0.0037271	4.57744271	0.00044001	0.98347331	0.59473811
Cers2	-0.0045423	6.07253664	0.00043502	0.9835673	0.59473811
Mmp11	-0.012163	1.13783293	0.00043404	0.98358577	0.59473811
Enpp1	-0.0055587	5.3449305	0.00043315	0.98360258	0.59473811
Iqcc	-0.00675	2.65551747	0.00043293	0.98360677	0.59473811
Fam115c	-0.009762	1.3926034	0.00041872	0.98387808	0.59482961
Ndufaf7	0.00308667	4.55722598	0.00041864	0.98387954	0.59482961
Nedd1	-0.0060764	2.72720241	0.00041326	0.98398339	0.59485567
Trdmt1	-0.0066097	2.84535639	0.00040581	0.98412835	0.59488795
Gm10440	-0.062754	-1.531503	0.00040429	0.98415823	0.59488795
Phf23	0.00393085	4.64131352	0.00039111	0.98441854	0.59500858
Pdp1	0.00320183	7.11894952	0.00038486	0.98454355	0.59501839
Slamf7	-0.0104998	1.12128087	0.00038422	0.98455627	0.59501839
Egln2	0.00472903	4.09706298	0.00037657	0.98471094	0.59507515
02-Sep	-0.0027061	7.89735262	0.00036831	0.98487954	0.59514032

B3galt6	0.00691135	2.33602443	0.0003607	0.98503646	0.59519842
Spg7	-0.0046607	3.55351131	0.00034777	0.98530698	0.59531161
Ksr1	0.00372086	3.86342717	0.0003452	0.98536136	0.59531161
Csmd2os	0.01550711	-0.3659152	0.0003431	0.9854061	0.59531161
Mettl7a2	-0.0057433	1.73110516	0.00032865	0.98571661	0.59546247
Gnl1	0.00229487	5.89680834	0.00032136	0.98587582	0.59552192
LOC1005036	-0.0053964	3.67730937	0.00031225	0.98607744	0.59560698
Fgf14	0.00233761	6.28372255	0.00030733	0.98618762	0.59563671
Eif2d	0.00397264	3.93549472	0.00030464	0.98624828	0.59563671
Iqch	0.01402822	-0.5441629	0.00029773	0.98640513	0.59567003
Aebp2	0.00198622	6.84929857	0.00029685	0.98642507	0.59567003
Gpnmb	-0.0112241	1.08924575	0.00028219	0.98676463	0.59574746
Il15	0.00607746	1.90573341	0.00027816	0.98685936	0.59574746
Ddx41	-0.0036084	3.8381311	0.00027787	0.98686633	0.59574746
Snhg10	-0.0080329	0.7763282	0.000276	0.98691061	0.59574746
01-Sep	0.00613385	2.38936797	0.00027549	0.98692264	0.59574746
Slc35e1	0.00301338	4.92752732	0.00027234	0.98699764	0.59574746
4921524J17F	0.00536258	4.04418156	0.00027211	0.98700302	0.59574746
3110039M2C	0.00719507	2.13060271	0.00027057	0.98703984	0.59574746
E230029C05I	-0.0094891	0.54626243	0.00026492	0.98717589	0.59578883
Apoe	0.00530992	6.97856543	0.00026269	0.98723004	0.59578883
Mul1	-0.00357	3.14208276	0.00025143	0.98750678	0.59585669
Htr1f	0.006477	1.8051509	0.0002481	0.98758961	0.59585669
S1pr3	-0.0035214	3.33109425	0.00024573	0.9876491	0.59585669
Kcne1l	0.02068091	-1.2949601	0.00024296	0.98771883	0.59585669
Tbc1d31	-0.0033204	3.31663818	0.00023854	0.98783114	0.59585669
Degs1	0.00293666	6.56259852	0.00023842	0.98783404	0.59585669
Bcl11a	0.00222805	6.56535469	0.0002383	0.98783722	0.59585669
Card11	0.01168811	-1.0961286	0.00023717	0.98786608	0.59585669
A730046J19F	-0.0096175	0.59754944	0.00023606	0.98789457	0.59585669
Cxadr	0.00342188	6.56603292	0.00023387	0.98795079	0.59585669
1700029I15R	0.01206726	-0.841606	0.00022543	0.9881703	0.59593677
Mark1	0.00197325	5.97795323	0.0002241	0.98820524	0.59593677
BC003331	-0.0018223	6.91312747	0.00022114	0.98828338	0.5959472
Ptgdr	-0.0035207	5.28157991	0.00020926	0.98860227	0.5961028
Accs	-0.0042216	2.8710364	0.00020403	0.98874556	0.5961525
Cdh19	0.00653087	1.52153738	0.00019378	0.98903203	0.59623175
Sbno2	0.00454206	1.71964168	0.00019357	0.98903787	0.59623175
Skint3	0.00678147	1.16596641	0.00018932	0.98915888	0.59623175
Gm6297	-0.0084046	1.00891899	0.00018875	0.98917528	0.59623175
Mtmr10	0.00227643	4.27423478	0.00018762	0.9892078	0.59623175
5430402O13	-0.0102867	-0.5565172	0.00018279	0.98934756	0.59623175
Rgs7	-0.0020083	7.1722786	0.00018239	0.98935905	0.59623175

H2-Ab1	0.00538118	4.25397424	0.00018223	0.98936394	0.59623175
Aim1l	-0.0098651	-0.2877581	0.00017522	0.98957036	0.59626962
Fam69b	0.00382526	3.04810245	0.00017391	0.98960936	0.59626962
Adck2	0.00366298	2.23540847	0.00017225	0.98965919	0.59626962
Tbc1d20	-0.0030008	4.21044894	0.00016983	0.98973196	0.59626962
Gas6	-0.0023098	4.3991346	0.00016559	0.98986096	0.59626962
Rad51c	-0.0055143	1.97713349	0.00016519	0.98987341	0.59626962
Snhg7	-0.0048546	1.28793666	0.00016372	0.98991856	0.59626962
Cep68	-0.0023181	5.0034212	0.00016226	0.98996363	0.59626962
Rnf122	-0.0071126	0.02224621	0.0001619	0.98997465	0.59626962
A330069E16	0.00544734	0.70240005	0.00015627	0.99015051	0.59629284
St3gal6	0.00254316	4.08416693	0.00015526	0.99018237	0.59629284
Lect1	0.01159064	-0.5496984	0.00015483	0.99019581	0.59629284
Arhgap6	0.00221199	4.73769687	0.00015203	0.99028506	0.59630992
Mvb12b	0.00159523	6.44063794	0.00014735	0.99043581	0.59632214
Kdr	0.00293779	2.63592826	0.00014698	0.99044764	0.59632214
Lilrb4	0.00729199	0.43424165	0.00014574	0.99048799	0.59632214
Thrap3	-0.0014322	7.46786872	0.00013999	0.99067775	0.59639973
Capn12	-0.0093281	-0.7080199	0.00013492	0.99084784	0.59644357
Zdhhc9	-0.0017241	6.30578159	0.0001342	0.99087236	0.59644357
Bend7	-0.0049678	0.64875533	0.00012459	0.99120526	0.5965916
A430005L14l	-0.0030851	2.80963571	0.00012306	0.99125936	0.5965916
Gtf2a1	-0.0012843	6.99718016	0.00012189	0.991301	0.5965916
Mterfd1	-0.0018565	5.07451548	0.00011262	0.99163836	0.59675037
Acvrl1	0.00515269	0.83253302	0.00011132	0.99168664	0.59675037
Mycbpap	0.004842	0.72901751	0.00010359	0.99198079	0.5968907
Abca4	0.0038508	3.09333688	0.00010126	0.9920712	0.59690843
Zbtb46	0.00438971	1.31475956	9.43E-05	0.99234889	0.59702321
Timp2	0.00159921	8.97279797	9.34E-05	0.99238385	0.59702321
Car8	-0.0027601	2.79708353	8.74E-05	0.99263561	0.59703457
Tbx15	0.00217547	6.7955854	8.72E-05	0.99264261	0.59703457
Ube2d1	0.00153056	6.92626022	8.56E-05	0.99271125	0.59703457
Sptlc1	0.0016732	3.62416493	8.45E-05	0.99275904	0.59703457
Rbm48	0.00173124	3.43229954	8.33E-05	0.99280994	0.59703457
Nupl1	-0.0012262	6.08892636	8.31E-05	0.99281946	0.59703457
9430018G01	0.001487	-1.5023933	8.28E-05	0.9928294	0.59703457
Plvap	0.00440122	0.6205832	7.40E-05	0.99322284	0.59714069
Amn	-0.005223	-0.2705762	7.23E-05	0.99330057	0.59714069
Phgdh	0.00273885	2.53464317	7.16E-05	0.99333516	0.59714069
Xiap	0.00123805	7.12351358	7.09E-05	0.99336488	0.59714069
Zbtbd6	-0.0079255	0.54639363	6.81E-05	0.9934957	0.59714069
Schip1	0.00162506	3.86375504	6.73E-05	0.99353688	0.59714069
Hist1h2bp	0.00682244	-0.7282023	6.72E-05	0.99354127	0.59714069

Bola1	-0.0028998	1.69514703	6.68E-05	0.99356083	0.59714069
Usf2	-0.0012175	4.98736857	6.60E-05	0.99359651	0.59714069
Tbcel	0.00138204	5.2480881	6.57E-05	0.99361549	0.59714069
2610002J02F	0.00272084	3.24413391	6.27E-05	0.99376195	0.59716145
Ino80b	-0.0045224	-0.0544744	6.25E-05	0.99377196	0.59716145
6330418K02I	-0.00349	1.11067126	6.00E-05	0.99389497	0.59719873
Trim52	0.00632594	-1.6565991	5.24E-05	0.99429584	0.59740296
Fam26f	0.0042893	1.22710449	4.98E-05	0.99443936	0.59742713
Usp8	-0.0007544	7.66784425	4.95E-05	0.99445806	0.59742713
Gm1653	0.00678642	0.50863292	4.74E-05	0.99457262	0.59745931
Gm12216	-0.0055916	-0.754129	4.58E-05	0.99466801	0.59747997
Rbm28	0.00106403	4.98405403	3.91E-05	0.99507102	0.59763235
Thap3	-0.0025025	2.29989503	3.80E-05	0.99514279	0.59763235
Dhx38	0.0010753	4.56252447	3.78E-05	0.99515429	0.59763235
Utp23	-0.0011268	4.38435016	3.72E-05	0.99519559	0.59763235
Prodh	0.00190915	2.14169972	3.62E-05	0.99525688	0.59763235
Plk1	0.01064505	-0.9043404	3.58E-05	0.99528775	0.59763235
Fcho2	0.0008514	6.99913835	3.26E-05	0.99550111	0.59772382
Mob1b	-0.0007805	5.1918482	3.06E-05	0.99564151	0.59776856
Nfyc	-0.0010375	4.60424449	2.98E-05	0.99569767	0.59776856
Pxmp2	0.00143281	2.49324457	2.77E-05	0.99584963	0.59781348
Ccne1	0.00176285	1.77384286	2.71E-05	0.99589455	0.59781348
4933426M11	-0.0009242	5.71371529	2.62E-05	0.99596464	0.59781891
Anks6	0.00144079	2.0881777	2.47E-05	0.99608434	0.59785413
Zfp449	0.00099829	5.20703437	2.12E-05	0.99637284	0.59799064
Mnd1	-0.003123	0.36796527	1.97E-05	0.99650366	0.59801186
Socs7	0.0006661	6.96844492	1.87E-05	0.99658982	0.59801186
Snrnp48	0.00068687	4.90671398	1.87E-05	0.99659135	0.59801186
Plxnb3	-0.0016015	2.04041531	1.47E-05	0.99697464	0.59820521
Zfpm1	0.00098212	1.76314135	1.08E-05	0.99740808	0.59838733
Adra1b	0.00070541	3.92520741	1.06E-05	0.99743328	0.59838733
Kansl3	0.00045166	5.88337963	9.94E-06	0.99751558	0.59838733
Igfbp6	-0.0009485	4.92640641	9.89E-06	0.99752251	0.59838733
Pcsk9	-0.0010195	-0.0783968	8.03E-06	0.99776778	0.59849781
Hdhd3	-0.0014847	0.2830346	5.24E-06	0.99819634	0.5986933
Ttc16	-0.0013465	-1.5557674	4.79E-06	0.99827619	0.5986933
Gtl3	0.00030315	4.59835817	4.78E-06	0.99827705	0.5986933
Smg9	0.00060792	2.90670721	4.33E-06	0.99836095	0.59869957
Med9os	0.00362416	-1.163347	3.97E-06	0.99843043	0.59869957
Ntn4	0.00066888	1.3451484	3.77E-06	0.99847086	0.59869957
Atxn1	0.00029073	8.6929335	3.25E-06	0.99857958	0.59872811
Hip1	-0.0003285	4.55084302	2.81E-06	0.99867879	0.59873037
Ntm	0.00020433	7.47633413	2.55E-06	0.99874106	0.59873037

4930592I03R	-0.0047765	-1.8628536	2.41E-06	0.99878043	0.59873037
Ifi44	-0.000399	3.33645629	1.78E-06	0.99894975	0.59873037
Csf2rb2	-0.0015555	-0.1943771	1.76E-06	0.9989533	0.59873037
Mypn	0.00029744	1.79093751	1.65E-06	0.99898903	0.59873037
Veph1	0.00127064	-0.1458469	1.35E-06	0.99908379	0.59873037
Lama2	-0.0002108	5.21919044	1.22E-06	0.99913026	0.59873037
Tnnt1	0.00053392	1.24684882	1.21E-06	0.99913348	0.59873037
Gatsl3	-0.0002603	1.40357407	9.44E-07	0.99923453	0.5987543
Rab10os	0.00011984	3.09165259	2.78E-07	0.9995846	0.59884664
Apol8	5.84E-05	-0.2681025	2.61E-07	0.99959713	0.59884664
Tmem28	0.00016612	1.70191866	1.58E-07	0.99968635	0.59884664
2900055J20R	-0.0001061	3.12873871	1.11E-07	0.99973733	0.59884664
Fam196a	5.25E-05	4.27266502	7.41E-08	0.99978549	0.59884664
Chchd7	-1.32E-05	3.63145787	1.25E-08	0.99991177	0.59884664
Otx1	4.40E-05	2.08237612	8.37E-09	0.9999279	0.59884664
2310007B03I	0.14350914	-1.8865621	7.10E-10	0.99997911	0.59884664
Nkx1-2	-0.0269067	-1.9523449	4.22E-11	0.99999491	0.59884664
Fetub	-0.0464614	-0.5004889	-4.34E-11	1	0.59884664
