# Supplementary material for: Mapping the physiological and molecular markers of stress and SSRI antidepressant treatment in S100a10 corticostriatal neurons
Source: Mol Psychiatry. 2019 Aug 20;25(5):1112–29. doi: 10.1038/s41380-019-0473-6 (PMC7031043; doi:10.1038/s41380-019-0473-6)
Supplement: Supplementary file 15 — Supplemental Table S1b [file 41380_2019_473_MOESM15_ESM.pdf]

Suppl Table S1b: Genelist representing the differentially expressed genes (122) between the Gh and Sh+Flx groups, in the context of all the genes examined.

symbol	logFC	logCPM	F	PValue	FDR
Cstb	1.72064962	6.70738108	82.7277023	1.58E-08	0.000157
Ecm1	2.118859	3.89611697	75.9453304	3.15E-08	0.000157
Emd	1.2566404	5.92569873	74.9352785	3.50E-08	0.000157
Maff	2.33643958	3.06441849	66.6745523	8.77E-08	0.00028447
Fam46a	1.16750945	5.75775238	65.0874048	1.06E-07	0.00028447
Nr4a3	2.47242532	4.23225603	61.0484432	1.73E-07	0.00038753
Nptx2	1.71350777	4.03919698	58.6857187	2.33E-07	0.00044803
Inhba	0.95929566	4.79140572	50.4976004	7.09E-07	0.0011931
Rgs2	1.05455624	7.30623261	48.6530016	9.28E-07	0.00138785
Rasl10a	1.33345193	3.35772303	47.5060116	1.10E-06	0.00148204
Eprs	0.76540188	7.57236391	44.0082976	1.89E-06	0.00214379
Sdpr	1.48777378	7.13197726	43.9396636	1.91E-06	0.00214379
Tmcc3	1.01404609	6.34903671	43.4196387	2.08E-06	0.00215024
Wdr92	0.76784185	5.3582105	39.5411957	3.95E-06	0.00379586
Aqp1	2.88289824	1.78608843	34.5744702	9.61E-06	0.00861837
Rbp4	1.34470542	4.08437903	33.0573962	1.28E-05	0.01078019
Slc25a17	1.01404826	5.69498941	32.1120907	1.54E-05	0.01152496
Adamts1	1.19353483	4.34942457	31.9973637	1.58E-05	0.01152496
Penk	1.0705366	7.08273198	31.8350172	1.63E-05	0.01152496
Hpcal4	0.86096998	9.96877456	31.2436784	1.83E-05	0.01188365
Gprc5a	4.14083396	0.9928613	31.1779014	1.85E-05	0.01188365
Ucma	7.62099195	-0.7750345	30.5379674	2.11E-05	0.01217405
Cdkn1a	0.88907159	5.39687725	30.5134535	2.12E-05	0.01217405
Mpp7	0.94048827	4.60590607	30.396914	2.17E-05	0.01217405
Hrg	3.26769028	1.03122995	30.1873815	2.27E-05	0.0121974
Fabp7	1.0885234	4.52045423	29.9798749	2.36E-05	0.01223757
Dnajb7	-8.1951264	-1.1210456	32.5699841	2.62E-05	0.0122698
Rarb	0.87849423	5.04062113	29.4582884	2.63E-05	0.0122698
Mvk	1.20678597	2.941983	29.4002746	2.67E-05	0.0122698
Htra4	1.62101258	3.17792456	29.2489578	2.75E-05	0.0122698
Aox3	1.06292682	6.1406988	29.1181819	2.83E-05	0.0122698
Gcnt1	1.56237775	2.91051144	28.6350221	3.13E-05	0.01312601
Ptgs2	1.28436206	4.12798815	28.4884214	3.23E-05	0.01312601
Slc2a12	1.21457351	4.38770554	28.3599243	3.32E-05	0.01312601
Cldn1	2.93921603	3.08447347	28.1009021	3.50E-05	0.01325685
Nlrp6	4.86745624	0.89625499	28.0453681	3.55E-05	0.01325685
Ccdc69	1.54897203	2.06926137	27.3635998	4.11E-05	0.01453688
Hapln4	0.78385869	6.04615777	27.3577479	4.11E-05	0.01453688
Ext2	1.75652229	5.05571587	27.2475623	4.21E-05	0.01453688
Rdh10	0.98041028	3.6843256	27.1262392	4.33E-05	0.01455345
Cab39l	0.74928187	5.7725496	26.3570699	5.12E-05	0.01656632
Rgs13	3.32040224	0.46691348	26.3018136	5.19E-05	0.01656632

Coq10b	0.84529659	5.61281997	26.1775438	5.33E-05	0.01656632
1700110K17I	-3.4280678	0.8249802	26.1032708	5.42E-05	0.01656632
D130040H23	-1.3498281	2.95254379	26.007833	5.54E-05	0.01656632
Ankrd55	1.50326761	2.6483128	25.7317164	5.89E-05	0.01695484
Camk2n1	-0.5871452	10.5133201	25.7111841	5.92E-05	0.01695484
Col5a2	1.17180837	2.99859565	25.2008427	6.65E-05	0.01844922
Fbxl16	-0.8342353	7.27841856	25.0712627	6.85E-05	0.01844922
Arl15	0.65292374	6.06996797	25.0670557	6.86E-05	0.01844922
Insig2	0.58302528	6.13606135	24.4979276	7.82E-05	0.0206224
Wfs1	0.88149799	6.64987679	24.3683393	8.05E-05	0.02084414
Ppl	1.24617696	2.44147578	24.1361762	8.50E-05	0.02140349
Casr	2.07093234	1.89347907	24.0933013	8.59E-05	0.02140349
Rassf3	-0.7451593	5.51837471	23.9580354	8.87E-05	0.02169205
Spata32	-4.4175913	-0.0808031	23.8549818	9.08E-05	0.02182768
Heyl	0.90322127	6.00162945	23.5016952	9.88E-05	0.02331463
Ythdf2	0.73195059	5.07872959	23.1716195	0.00010685	0.02392592
Txndc11	0.9482214	3.87622766	23.0913488	1.09E-04	0.02392592
Cd300lg	-6.6379413	-1.1975361	23.0888991	0.00010899	0.02392592
Stra6	1.53643398	7.10475634	23.0519093	0.00010997	0.02392592
E530011L22F	-1.1210461	2.52860136	23.0416047	0.00011024	0.02392592
Capn6	1.06870172	4.31627963	22.7863694	0.00011724	0.02461328
Tiparp	0.65411739	4.91605335	22.7514577	0.00011823	0.02461328
Sfrp4	1.22406801	3.4712962	22.7284009	0.00011889	0.02461328
Vamp5	0.90994498	5.48863501	22.5881715	0.00012301	0.02502905
Cdk11b	0.50827068	7.39709748	22.5346178	0.00012462	0.02502905
Stip1	0.54363318	7.04168692	22.2750071	0.00013278	0.02620142
Bmp3	1.0387458	5.23358809	22.2055327	0.00013506	0.02620142
Ampd3	0.71238176	5.59985315	22.1647256	0.00013642	0.02620142
Ppp1r1c	-2.9381194	1.07525846	22.1076127	0.00013835	0.02620142
S100a6	1.32313908	5.94037353	22.0538104	0.00014019	0.02620142
C1ql3	0.72053647	6.79431107	21.9216583	0.00014484	0.02669928
Stard13	0.66127184	5.39133096	21.8454097	0.0001476	0.02684017
Fetub	7.28266813	-0.5004889	26.4722446	0.0001519	0.02725356
Fosb	1.75377347	4.38460971	21.5843196	0.0001575	0.02788592
Habp4	-0.4603751	7.39970962	21.4378509	0.00016337	0.02854963
Gm11549	0.65335519	6.88483758	21.3802803	0.00016574	0.02859301
Dgkz	-0.5917797	6.32633871	21.2753575	0.00017016	0.02898452
Rspo1	-1.1091964	3.09497021	21.1372597	0.00017619	0.0296351
Tubb6	1.27272681	3.92177905	21.0201913	0.00018147	0.03014792
Zfp873	-0.8716841	3.36059903	20.9150162	0.00018638	0.03058462
Isyna1	0.94559615	6.19626698	20.8017064	0.00019182	0.03109885
Kcnip2	-0.584494	5.69882774	20.7234765	0.00019568	0.03134721
Sccpdh	0.52443719	6.28810619	20.6142556	0.00020122	0.03185472

Topaz1	3.77062059	0.39184056	20.5206894	0.0002061	0.03224783
Insl5	-7.230125	-1.3287275	21.8560167	0.00022036	0.03408296
Crem	0.68209786	4.8377127	20.1954374	0.0002241	0.03426825
Bend6	-0.6443441	6.64558195	20.0358188	0.00023357	0.03531444
Caml	0.85620761	3.67011036	19.8201592	0.00024707	0.03694017
Zfp46	-0.5738354	5.51714231	19.7135552	0.00025406	0.03756761
Aldoa	0.5108571	9.15691313	19.6133646	0.00026082	0.03814908
Slc34a2	2.38069804	0.83213554	19.5576864	0.00026467	0.03829544
Dnaja1	0.44047556	9.6899873	19.5151227	0.00026765	0.03831503
Ltbp1	1.14602707	4.37816882	19.2788186	0.0002849	0.04035503
Cdk5r2	-0.5777326	6.69943838	19.1424471	0.00029541	0.04140797
Dpm1	0.78874647	5.2982306	19.0440248	0.00030326	0.04207033
F5	-1.6837638	2.6508564	18.9828497	0.00030826	0.04232709
Klhl13	0.54073452	6.10968308	18.9285195	0.00031277	0.04251303
Oasl1	6.3359785	-1.023746	18.7913146	0.0003245	0.04360501
Errfi1	0.55525814	6.88726728	18.7594983	0.00032729	0.04360501
Nr4a2	1.84877139	6.88908107	18.4914318	0.00035186	0.04581023
Ypel2	-0.4875075	6.38247528	18.4806395	0.00035289	0.04581023
Slc35d3	1.32460304	1.68033819	18.4684928	0.00035405	0.04581023
Medag	0.49535659	6.21870146	18.3727362	0.00036338	0.04605522
Vimp	0.6545288	6.1270351	18.3111433	0.00036953	0.04605522
Tpm4	0.82414603	9.13505951	18.2912432	0.00037154	0.04605522
Arhgap29	0.8571784	9.98480511	18.2641072	0.0003743	0.04605522
Pde1a	0.42520874	9.4446545	18.2358531	0.00037719	0.04605522
Gm16617	-5.6711723	-0.99522	18.2116835	0.00037969	0.04605522
Mx2	2.86771027	0.06366285	18.2096147	0.00037991	0.04605522
Pex12	0.74947171	4.06742378	18.0874463	0.00039282	0.04703114
Tspan2	-0.7038601	6.3946554	18.0677988	0.00039495	0.04703114
A230057D06	-1.3307582	3.28385481	18.0136935	0.00040086	0.04731625
Dhrs1	0.65704399	5.70162098	17.9486144	0.0004081	0.04775186
Vcl	0.65439075	6.50105909	17.7942375	0.00042585	0.04908565
Akap12	0.70528802	8.54412102	17.7862827	0.00042679	0.04908565
Ctxn1	-0.4332208	7.61037203	17.7149063	0.00043531	0.04939102
Krt19	1.61334139	1.77903333	17.6888852	0.00043846	0.04939102
Krt12	-0.7317594	4.21129125	17.6650391	0.00044137	0.04939102
Tmc7	-0.7715844	4.52991918	17.624355	0.00044638	0.04939102
Brsk1	-0.8964368	3.49277298	17.6129839	0.0004478	0.04939102
Pelo	1.04213639	3.1943276	17.5188971	0.00045968	0.05028935
Caprin1	0.41225434	8.54069451	17.4072405	0.00047423	0.05146325
Map6d1	-0.6908342	5.02829765	17.3231145	0.00048554	0.05172053
Stk38l	0.52554038	5.63816732	17.3204889	0.00048589	0.05172053
Osbpl1a	-0.5022199	8.12679639	17.2847067	0.0004908	0.05172053
Cck	0.51469933	7.75430441	17.276121	0.00049198	0.05172053

05-Sep	-0.5303962	6.40078037	17.1960926	0.00050317	0.05248691
Fam101a	1.74085613	2.83924127	17.1591377	0.00050843	0.05262784
9330159M07	-1.2278901	2.13112621	17.108073	0.0005158	0.05290311
D630023F18	1.25645707	2.00640674	17.0833124	0.00051942	0.05290311
Serpinf1	1.12521201	5.60135265	17.059767	0.00052289	0.05290311
Ppfia4	-0.7872779	3.62828696	16.8446403	0.00055576	0.05580928
Wisp1	1.17046402	1.94884932	16.707406	0.00057792	0.05729142
Car3	2.36342193	1.3697851	16.7006542	0.00057903	0.05729142
Ezr	0.55484728	6.23592406	16.6646531	0.00058502	0.05746121
Homer2	-0.510725	7.54094242	16.5499515	0.00060455	0.05850355
Col24a1	-3.5599726	0.43538325	16.5332518	0.00060745	0.05850355
Theg	3.64758529	-0.8968574	16.5262717	0.00060867	0.05850355
Grasp	0.72121625	4.23156831	16.4818573	0.00061649	0.05883444
Klhl18	-0.7805131	4.45705032	16.4420992	0.00062358	0.05909195
Nrp2	0.58776866	5.60289257	16.3738343	0.00063596	0.05974349
Phex	1.64431578	1.95507507	16.3555066	0.00063933	0.05974349
Akr1b10	0.67432895	3.77109963	16.327084	0.0006446	0.05982024
Bche	-1.0747281	7.2717699	16.2744311	0.00065448	0.06015006
AI593442	-0.5402004	8.70047291	16.2504615	0.00065904	0.06015006
Lin28b	-1.198543	3.02215074	16.2104808	0.00066671	0.06015006
Flrt1	0.53018421	6.61301072	16.2072559	0.00066734	0.06015006
Nnat	1.12996661	8.65677086	16.1909299	0.0006705	0.06015006
Sytl2	-0.6885887	5.78833679	16.1542815	0.00067767	0.06039045
Fam71b	-6.6505553	-1.7554449	16.02797	0.00070303	0.06223825
5530601H04	-0.9785253	3.42913105	15.9643713	0.0007162	0.0629893
Ftx	-1.0213591	4.04392197	15.9400431	0.0007213	0.06302658
Dthd1	-4.7110059	-0.4413479	15.8452727	0.00074159	0.06394249
Pnldc1	3.50302411	-0.2701485	15.8371727	0.00074335	0.06394249
Idi1	0.68378451	5.81647658	15.8248508	0.00074604	0.06394249
Hs3st4	-0.6398718	5.45313509	15.731844	0.0007667	0.06484669
Gadd45a	0.82819645	4.16244323	15.7196812	0.00076945	0.06484669
Ddx51	-0.6476393	3.89237777	15.6995625	0.00077402	0.06484669
Dnajc21	-0.6002356	7.02084378	15.6914538	0.00077587	0.06484669
Ralgds	0.47408486	5.87226687	15.5890348	0.00079966	0.06642286
Adam19	0.68317292	4.64126525	15.5407827	0.00081115	0.06696381
Phactr3	-0.4728236	6.15735084	15.4176696	0.0008413	0.06902923
Kifc2	-0.5715971	6.32616462	15.2779494	0.00087703	0.07129892
P2ry1	0.91661192	4.02734054	15.2682808	0.00087956	0.07129892
Mut	0.42960644	5.76690923	15.2377413	0.00088761	0.07152109
Scn10a	-6.8770239	-1.6089936	16.1590586	0.00089663	0.07181724
Lipg	1.78957853	1.29673501	15.155522	0.00090971	0.0719172
Wtap	0.46728475	6.35981409	15.1534102	0.00091029	0.0719172
Msl2	0.50792171	7.27702736	15.140154	0.00091391	0.0719172

Acot13	0.6068301	5.31541963	15.0862971	0.00092879	0.07265562
Il17ra	-0.5785572	4.71235539	15.0673323	0.00093409	0.07265562
Tfrc	-0.664741	5.51718518	14.9825414	0.00095821	0.07394556
Sncaip	0.87687295	5.65640111	14.9706028	0.00096167	0.07394556
Osbp2	-0.6056791	5.65116238	14.9178427	0.00097708	0.07420339
Grin2c	-1.3479725	2.15088364	14.8805693	0.00098814	0.07420339
Ubr3	0.61925275	9.67637616	14.8749261	0.00098983	0.07420339
F8	-0.83889	3.0977305	14.8399915	0.00100034	0.07420339
1110019D14	1.01847814	2.68830163	14.8336178	0.00100227	0.07420339
Slc16a13	1.07713545	2.78603537	14.8307012	0.00100315	0.07420339
A530046M15	-2.471625	0.47076079	14.8078573	0.00101011	0.07420339
Cd34	-0.921362	3.49869475	14.7905928	0.00101541	0.07420339
Aspg	1.83749638	0.89639221	14.7882302	0.00101614	0.07420339
Mcm5	-1.7319159	0.90631205	14.7751822	0.00102016	0.07420339
F2rl2	1.19532706	1.92100183	14.7100319	0.00104053	0.07527801
Fosl2	1.36943365	6.03954939	14.6651541	0.00105482	0.07590389
Lamc3	1.12271912	2.59835371	14.6389684	0.00106326	0.0761042
Dynlrb2	-1.8760897	0.69709176	14.5692357	0.0010861	0.07732774
Scn1a	-0.6901516	7.07931107	14.5477254	0.00109326	0.07742747
Mzt1	-0.5139051	5.93690151	14.5265632	0.00110035	0.07752159
Rapgef5	0.4821193	6.3808839	14.4883574	0.00111328	0.07802397
Atp11b	-0.5775386	6.31297214	14.4636313	0.00112173	0.07820934
Arhgef12	0.38342652	9.52684002	14.4359563	0.00113128	0.07846861
1700007G11	-2.462713	0.29803362	14.4174905	0.00113771	0.07850938
Cd8a	-1.8496903	0.35350132	14.3996497	0.00114395	0.07853744
Mdh2	0.43530674	7.24664761	14.3460373	0.00116294	0.07879048
Mal2	-0.4572291	6.39667895	14.3453698	0.00116318	0.07879048
Ddit4l	-0.551176	4.93093377	14.3397173	0.0011652	0.07879048
Cebpb	1.04445495	2.72160581	14.3216746	0.00117168	0.07883272
Glt28d2	1.12601957	3.29985925	14.2579506	0.0011949	0.07967477
Mbd1	0.65540807	4.27586133	14.2046313	0.00121472	0.07967477
Xrcc2	-1.2653525	2.5538066	14.1899854	0.00122023	0.07967477
Pou2f3	2.51547991	1.17603717	14.1882301	0.00122089	0.07967477
Atp8b4	-4.8526714	-0.8469891	14.1864255	0.00122157	0.07967477
Ppfia1	0.41437241	6.96011551	14.1731402	0.0012266	0.07967477
Sulf1	0.85384186	5.8986324	14.1621818	0.00123076	0.07967477
4833422C13	1.53827421	2.31396796	14.1459298	0.00123696	0.07967477
6030407O03	5.09929243	-0.8430597	14.1385789	0.00123978	0.07967477
Pgr15l	3.52088349	0.38897474	14.1291339	0.00124341	0.07967477
Tmem100	0.71406421	3.5969053	14.0266586	0.00128355	0.08185739
Nova1	-0.5101584	7.15070647	14.0011687	0.00129376	0.08202225
Tfcp2l1	0.80831351	7.02929852	13.9757223	0.00130404	0.08202225
Gpr87	-5.4674516	-1.7229467	13.9747739	0.00130443	0.08202225

Catip	1.22579841	2.46427014	13.9107381	0.0013307	0.08328548
Acot9	0.71229535	3.80085597	13.8686243	0.00134831	0.08399645
Por	0.60686801	5.52808753	13.8144906	0.00137131	0.08503594
Foxf2	1.05009749	2.76111386	13.7720365	0.00138966	0.08577827
Eya1	0.64200287	7.18135044	13.7070385	0.00141827	0.08690605
Rhobtb2	-0.4686224	5.37579515	13.689674	0.00142603	0.08690605
AA415398	-1.1346245	2.98746953	13.6715112	0.00143419	0.08690605
Dnah6	-2.4851693	0.7087383	13.6678314	0.00143585	0.08690605
Mansc4	1.61410336	1.99202878	13.6581659	0.00144022	0.08690605
Trib1	0.58409262	5.66745826	13.6301356	0.00145298	0.08724538
Kctd10	0.70176542	4.71801975	13.6080717	0.00146311	0.08724538
A2m	1.44979714	1.67001198	13.6015194	0.00146613	0.08724538
Bach1	0.58936374	5.05667399	13.5893159	1.47E-03	0.08724538
Dnah9	-1.8935381	1.50568372	13.5749014	0.00147848	0.08725836
Cd24a	-0.7756428	5.29033458	13.5231245	0.00150284	0.08769719
Xbp1	0.51179765	6.19549155	13.5217786	0.00150348	0.08769719
Pex19	0.51062821	6.27237414	13.5175939	0.00150547	0.08769719
Lrrc55	-0.6008781	4.49946179	13.4905355	0.0015184	0.08806922
Spp1	0.95400296	7.62210651	13.4696472	0.00152847	0.08827262
Abi1	0.40081193	7.09800737	13.4426182	0.0015416	0.08865078
Rprm	0.78998913	3.97565688	13.4011977	0.00156198	0.08944015
Ccp110	-0.5735897	5.8898063	13.3788025	0.00157311	0.08969632
Rgcc	0.98383891	2.86685262	13.363555	0.00158075	0.08975132
Tagln2	0.91949126	5.7784713	13.2730538	0.00162692	0.09198448
Eno3	1.01943498	3.21469551	13.2472703	0.00164034	0.09235546
Ppp4r2	0.44046959	8.3018626	13.205138	0.00166254	0.09276954
Piwil4	3.72012262	-0.897219	13.1845543	0.00167351	0.09276954
Gm2115	0.9378078	3.08649973	13.1821566	0.00167479	0.09276954
Upp2	-0.8950115	3.38280111	13.1812621	0.00167527	0.09276954
Zfp710	-0.7108558	3.6340195	13.1482176	0.00169307	0.09315661
Pkd2l2	-0.7496923	3.72470118	13.1170757	0.00171003	0.09315661
4930572O13	3.49814438	0.43251936	13.1145308	0.00171143	0.09315661
Zkscan2	-0.6023315	4.76898938	13.1143092	0.00171155	0.09315661
Iqub	-1.5433082	1.53412581	13.1046044	0.00171688	0.09315661
Csnk1a1	0.36011103	7.62192174	13.0136147	0.00176775	0.09544422
Uchl5	0.4332178	6.67267395	13.004001	0.00177322	0.09544422
Pkig	-0.5261175	4.79792239	12.9645484	0.00179588	0.09627842
4933424G05	-2.2135283	1.07474415	12.9235899	0.00181973	0.09653328
Syn2	0.52348697	7.70541142	12.921333	0.00182106	0.09653328
Mkx	0.75135955	5.3746159	12.9194724	0.00182215	0.09653328
Gm21119	-1.8859118	0.59824028	12.9053364	0.00183048	0.09659416
1500015O10	1.3043867	5.14687595	12.8905004	0.00183926	0.09667863
Vwa1	0.72995019	4.20174091	12.8304445	0.00187531	0.09778411

Rltpr	-1.1137113	1.80579924	12.8281471	0.0018767	0.09778411
Nucb2	0.55051451	5.26267907	12.7944884	0.00189726	0.09778411
Tmem200c	1.22552272	1.70233929	12.7843346	0.00190351	0.09778411
Capg	1.00696612	2.58282	12.7842599	0.00190356	0.09778411
1110008P14I	-0.5466773	4.56807897	12.7837104	0.0019039	0.09778411
Lgals3	1.57402146	1.77857783	12.7672465	0.00191408	0.09793349
Npnt	-0.6429099	3.77911532	12.6801291	0.001969	0.10011944
Zic4	0.75055817	6.26531762	12.6704865	0.00197518	0.10011944
Sod3	0.88522529	5.39044112	12.6643535	0.00197913	0.10011944
Cd7	4.50089144	-1.7326019	12.627934	0.00200273	0.10093419
Apip	0.7532695	4.17678292	12.5889592	0.00202834	0.10141281
Tacr2	3.23513099	0.30130535	12.5809823	0.00203363	0.10141281
Pgk1	0.40655532	7.98381182	12.5770027	0.00203627	0.10141281
Thbd	0.93976202	8.3227459	12.5678321	0.00204238	0.10141281
Adamts12	1.15020403	2.48891366	12.5290005	0.00206845	0.10232993
Per3	-0.4499882	5.90032191	12.5147782	0.00207809	0.10243037
Cacng7	-0.5816536	5.83701289	12.4867985	0.00209721	0.10262394
Dcx	-0.6090184	5.69217413	12.4812816	0.002101	0.10262394
Sh2d4b	2.75143323	-0.1638562	12.4746033	0.0021056	0.10262394
Gdpd2	-1.4064964	2.27646471	12.4645794	0.00211253	0.10262394
Pcsk2os1	-0.970041	3.00792967	12.4518603	0.00212135	0.10268193
Picalm	0.36525535	8.08333013	12.4133994	0.00214829	0.10347123
Dlst	0.4227282	6.53853897	12.4066695	0.00215304	0.10347123
Irs1	0.42908626	6.08048577	12.3720969	0.00217763	0.10359318
Fra10ac1	0.5593869	4.40983291	12.3710501	0.00217838	0.10359318
Fmo1	0.5939963	6.87527009	12.3706465	0.00217867	0.10359318
Col3a1	0.82507568	5.0592517	12.3258378	0.00221103	0.10442081
Pcdh18	-0.8098097	3.19480884	12.3250578	0.0022116	0.10442081
Sin3b	0.65578188	4.8061056	12.2898111	0.00223743	0.10456351
Sgpp2	-0.6690769	3.73622858	12.2879611	0.0022388	0.10456351
Aff4	0.38772716	8.98795918	12.2862143	0.00224009	0.10456351
LOC1008622	1.1136196	2.56939459	12.2784964	0.00224579	0.10456351
Cyp1b1	0.68018254	7.03186445	12.2655086	0.00225544	0.10456351
Scml2	-2.2329726	-0.0223448	12.2367772	0.00227693	0.10456351
1700019D03I	-1.393616	1.69475413	12.2222919	0.00228786	0.10456351
Rbm24	-0.7795693	3.97278208	12.2222765	0.00228787	0.10456351
Fmn1	-0.4564432	6.50161144	12.2138865	0.00229422	0.10456351
Rab3a	-0.3953138	6.72958893	12.2020245	0.00230324	0.10456351
Slc25a25	0.58915281	5.0207656	12.201864	0.00230336	0.10456351
Ifrd1	0.5837052	5.97236501	12.194988	0.00230861	0.10456351
0610030E20I	0.51240022	5.46102833	12.1858015	0.00231564	0.10456351
Wipf3	0.54895992	7.87146541	12.1531085	0.00234084	0.10534809
Lancl2	0.40564961	8.49514295	12.1319321	0.00235733	0.10557648

Gm20752	-1.6954194	0.53927889	12.1264635	0.00236161	0.10557648
Sprr1a	5.70845681	-0.2489927	12.6968393	0.002409	0.10704606
Ranbp17	-0.8257895	3.12490601	12.0648801	0.00241039	0.10704606
Apol9a	3.79473385	-1.0042618	12.0545086	0.00241871	0.10706243
Micalcl	-1.4402098	1.66296183	12.0007662	0.00246237	0.10863728
Insig1	0.42896639	6.28332118	11.9842198	0.00247598	0.10884421
Nmt1	0.41820347	5.26669908	11.9671637	0.0024901	0.10884421
Slc7a1	0.52185543	5.28969118	11.9656958	0.00249132	0.10884421
Pou5f2	-3.1342515	-0.1208998	11.9441187	0.00250932	0.10927595
Fert2	0.46493738	4.96364727	11.9321235	0.0025194	0.10936058
Adam3	-5.7376455	-1.935804	12.5136215	0.00254736	0.11021904
Comtd1	1.40828864	1.05456235	11.8771483	0.00256613	0.11040402
Ung	1.26170565	2.10678737	11.8749121	0.00256805	0.11040402
Hspa1b	1.0156273	2.12639653	11.8478979	0.00259138	0.11104176
Zfand6	0.62167171	5.61016138	11.8386932	0.00259939	0.11104176
Mybpc2	1.64126733	1.39858629	11.8108818	0.00262374	0.11172734
Mmp17	0.60826081	5.60588234	11.784234	0.00264731	0.11237541
Gem	2.7229659	2.8642039	11.7679692	0.00266181	0.11263568
Tmem194b	-1.8834184	2.10193843	11.7443794	0.002683	0.11317642
Haus3	0.61517347	4.11729922	11.6942695	0.00272863	0.11446049
C3ar1	-1.1951761	2.03882037	11.6835973	0.00273846	0.11446049
Ube2a	0.58686863	6.34368022	11.6830613	0.00273896	0.11446049
Myrf	-0.846219	3.72404626	11.6733557	0.00274793	0.11448004
Ppp1r42	-3.0969149	-0.5472227	11.6414452	0.00277767	0.11536189
Fam189a2	-2.0299826	0.55392825	11.5678509	0.00284763	0.11790354
Tppp3	-0.7288126	3.54507337	11.5407161	0.00287392	0.11862689
Srpx2	2.23721776	0.24432946	11.5231249	0.0028911	0.11891288
Shroom2	0.3635595	6.79998881	11.5138146	0.00290024	0.11891288
Ift57	0.40610868	5.92742361	11.5054263	0.00290851	0.11891288
Ciart	-0.9224992	3.12972536	11.4911451	0.00292264	0.11891288
Cyp2f2	1.56796567	4.94083326	11.4771166	0.00293659	0.11891288
Notum	-1.1065432	1.64644829	11.4725727	0.00294113	0.11891288
Bdnf	0.67315348	4.26333758	11.4709903	0.00294271	0.11891288
Melk	-3.0704451	0.17902448	11.4391307	0.00297474	0.11984758
Mmp12	-4.6138047	-1.5258281	11.3775507	0.00303777	0.12151872
Kdm8	1.55685609	1.41305886	11.3746375	0.00304079	0.12151872
Spef2	-1.7781385	0.09680215	11.3722001	0.00304331	0.12151872
Ccdc148	-0.6378362	4.10954772	11.3084839	0.00311023	0.12338382
Klf14	-3.4080383	0.34924461	11.3027498	0.00311633	0.12338382
Dcp2	-0.4232707	7.18498446	11.2917726	0.00312804	0.12338382
Rbm47	0.66523129	4.57140377	11.2901529	0.00312978	0.12338382
Fndc9	1.25299983	2.94928762	11.2774131	0.00314344	0.12338382
5031434C07I	6.83645133	-1.6976484	11.8308283	0.00314783	0.12338382

4930473A02	-2.0815517	0.53604873	11.2674231	0.00315421	0.12338382
Ccl27a	-0.5726157	5.16769628	11.2448047	0.00317873	0.12398267
Atp13a4	-1.2965556	2.28564418	11.2253501	0.00319999	0.12445123
Zfp963	-0.9149131	2.52966685	11.2008727	0.00322697	0.12497188
Lig1	0.59531306	4.07456712	11.1947079	0.0032338	0.12497188
Stx11	1.40141845	2.10284213	11.1872873	0.00324205	0.12497188
Amer3	-0.7854843	3.42676157	11.1796792	0.00325053	0.12497188
Cemip	-0.6332765	3.50331555	11.1280276	0.00330875	0.12684798
Nptx1	0.54010193	7.55312455	11.1044411	0.00333573	0.1275187
Clca1	-4.0556527	-0.4968022	11.088173	0.00335447	0.12787203
Mtpn	0.41881378	10.0811484	11.0536611	0.00339463	0.1290372
Khdrbs1	0.39245632	6.94592177	11.0431922	0.00340691	0.12913941
Cytip	-1.0501869	2.74174815	10.9760968	0.00348684	0.13144931
Krt222	-0.4654706	6.63809537	10.9715304	0.00349235	0.13144931
Zfp64	0.76907693	2.7789355	10.9456419	0.0035238	0.13144931
Ankmy2	0.3754193	6.26991117	10.941957	0.0035283	0.13144931
Pygb	0.43771798	6.4839507	10.9365689	0.00353489	0.13144931
Asic3	7.16184968	-2.0363089	11.4540578	0.00354654	0.13144931
Zfp804b	-1.0741908	2.52131327	10.9239458	0.00355039	0.13144931
Tcap	-1.6765582	0.86915898	10.9227419	0.00355188	0.13144931
Lins	0.81231156	3.94188219	10.9195837	0.00355577	0.13144931
Ube2d2a	0.47420646	9.04656971	10.8943879	0.00358699	0.13224014
Wnt10a	1.06830167	1.65062776	10.8753258	0.00361081	0.13275465
Pappa	1.45938128	2.78235808	10.8546023	0.00363691	0.13334981
Dcps	-0.9136954	2.22805824	10.8235762	0.00367637	0.13443048
2610035D17	0.80571782	3.31067357	10.8021459	0.00370391	0.13503952
Miat	-0.9210365	6.21309444	10.7950326	0.0037131	0.13503952
Ovol2	-1.1124835	1.7981292	10.7833561	0.00372824	0.13505822
2900026A02	-0.5171624	5.8920017	10.7791686	0.00373369	0.13505822
Crhr1	-1.0821822	1.89336019	10.7574119	0.00376213	0.13572219
Paqr6	-1.1097056	2.09604064	10.7297449	0.00379864	0.13667311
Tmem44	-0.5558922	4.61437698	10.6982587	0.00384068	0.13781706
Pik3r5	-0.7800416	2.80084635	10.6622318	0.00388941	0.1388427
Cdh3	-2.1903124	0.55645402	10.6618754	0.0038899	0.1388427
Adora3	-4.7683596	-1.5513716	10.6390105	0.00392119	0.13924397
A730020M07	0.79476039	3.94037188	10.6320939	0.00393071	0.13924397
Panx2	-0.7301537	4.05650636	10.624397	0.00394133	0.13924397
Ankrd44	-0.6245516	5.2431102	10.6235287	0.00394253	0.13924397
Col19a1	-0.9415095	3.91312099	10.6148056	0.00395461	0.1393051
Slco2a1	0.94760166	4.43168881	10.571026	0.00401588	0.14109397
Ap2a2	0.37641083	6.88755799	10.5471488	0.00404974	0.14191304
1700020I14R	-0.5042608	6.32617778	10.5085803	0.00410511	0.14253819
Naa40	-0.7686551	2.94332388	10.5039327	0.00411183	0.14253819

Mob3a	0.83682756	2.63458704	10.4987952	0.00411928	0.14253819
Dhodh	-1.087418	2.11767985	10.4939357	0.00412635	0.14253819
Srxn1	0.38345492	6.28059447	10.4892965	0.0041331	0.14253819
Aspa	0.51653135	6.03393762	10.4819343	0.00414385	0.14253819
Impg2	-4.0064586	-0.3888372	10.4811258	0.00414503	0.14253819
Cenpf	-0.865522	2.51014231	10.4761404	0.00415232	0.14253819
D030045P18	4.24112327	-0.5537525	10.4588168	0.00417778	0.14304731
Akr1c14	-0.7134771	3.92824185	10.4476818	0.00419424	0.14314759
Havcr2	-1.5146094	1.70023594	10.4424571	0.00420199	0.14314759
Ifitm1	0.79315133	5.15849788	10.4312874	0.0042186	0.1433507
Slc39a6	0.3825569	6.58109972	10.4185311	0.00423767	0.14363582
Mybpc1	1.65840156	0.97446557	10.4089959	0.00425198	0.1437016
Tec	0.74046564	5.07150878	10.403028	0.00426097	0.1437016
Efcab10	-2.770628	-0.4047209	10.3850671	0.00428814	0.14425638
Ifnlr1	4.72722564	-0.4183534	10.3701928	0.00431079	0.14431243
Ints12	0.51181799	4.73342716	10.3670796	0.00431554	0.14431243
Armcx6	1.98828195	0.96305597	10.3628738	0.00432198	0.14431243
Osmr	0.87347811	3.50329174	10.3116819	0.00440117	0.14626264
Rnasel	-0.5572786	4.6797789	10.3110722	0.00440212	0.14626264
1500009C09I	-0.4978474	5.50390501	10.3002588	0.00441906	0.14646375
Arf4	0.45419806	8.85386897	10.2597727	0.00448312	0.1474252
Large	0.49695362	6.42464331	10.2536191	0.00449295	0.1474252
Hdac11	-0.4663202	5.42574727	10.2533307	0.00449341	0.1474252
Rpap3	0.45693346	4.49697845	10.2476207	0.00450255	0.1474252
Dgat1	1.43436034	1.99018369	10.247435	0.00450285	0.1474252
Narf	-0.5513849	5.05031962	10.2160754	0.00455342	0.14805978
Gm9199	2.20270155	-0.4350192	10.2131783	0.00455813	0.14805978
Ascl2	3.76734892	-0.7872353	10.2088243	0.00456521	0.14805978
Snx20	-1.4048507	0.86909136	10.2045093	0.00457224	0.14805978
Nkx1-2	7.41535092	-1.9523449	11.3801238	0.00458348	0.14805978
Sufu	0.56260022	4.10405418	10.1947068	0.00458825	0.14805978
Gm2762	7.54261206	-1.6727129	10.6316874	0.00463078	0.14879323
Slc22a2	0.91478585	4.58244843	10.1638392	0.00463908	0.14879323
Mrps6	0.69179086	4.25592822	10.160779	0.00464415	0.14879323
Pik3c2a	-0.4148094	6.15002745	10.1462562	0.00466831	0.14921201
Itgbl1	0.6520983	6.1219165	10.1218127	0.00470929	0.1493081
Top1mt	0.78281524	3.13439273	10.1207607	0.00471106	0.1493081
Prpf19	0.40756925	7.10692766	10.1176644	0.00471628	0.1493081
C030013G03	-0.7892462	2.95740626	10.1121487	0.0047256	0.1493081
Gck	-2.9340806	-0.9810828	10.1083502	0.00473203	0.1493081
Ace2	4.55809155	-0.716021	10.1004836	0.00474537	0.1493081
Jag1	0.71053832	3.72956607	10.0848018	0.0047721	0.1493081
Nat8	-2.3626495	0.23578524	10.0769018	0.00478563	0.1493081

Zfp52	0.56852677	4.08566603	10.0746663	0.00478946	0.1493081
Plk5	-1.801392	1.09162456	10.0746359	0.00478952	0.1493081
Meiob	4.09006825	-0.9393233	10.0701059	0.0047973	0.1493081
Tas1r3	-2.2729712	-0.1782649	10.0659418	0.00480447	0.1493081
Atg7	0.62765364	6.32718181	10.0584702	0.00481735	0.14936371
Hspa13	0.56362818	4.88156484	10.0428857	0.00484436	0.14966907
E030003E18I	0.84908783	2.39681955	10.0399622	0.00484945	0.14966907
Gria4	-0.5174758	6.76190526	10.0315001	0.0048642	0.14978085
Vkorc1l1	-0.3464052	6.22933451	10.0229574	0.00487915	0.14989799
Gm16596	2.02517507	0.01562541	10.0050942	0.00491056	0.1504644
Mettl24	-2.363338	-0.0698367	9.99978155	0.00491994	0.1504644
Chgb	0.51412485	9.20757245	9.98607797	0.00494425	0.15086476
Unc5c	0.51015418	6.71418233	9.9612271	0.00498866	0.15187555
Ido2	-1.14464	2.00854124	9.94350532	0.0050206	0.15233822
Cdr1	-0.7653466	9.91723274	9.94024767	0.0050265	0.15233822
Serbp1	0.33019127	9.37863843	9.91180965	0.0050783	0.15324882
Smyd3	0.4217463	6.18558953	9.91010141	0.00508143	0.15324882
Grik4	-1.2189911	1.91714354	9.90504521	0.00509071	0.15324882
Bend4	-0.6105791	4.40579276	9.89618132	0.00510702	0.15339669
Cd84	-1.0030837	2.46459589	9.87455156	0.00514707	0.15402069
Ptger3	1.02103184	3.47970624	9.86728076	0.00516061	0.15402069
Folh1	-1.2716862	1.91520935	9.86117147	0.00517202	0.15402069
Cnga2	-5.8464451	-2.1500629	10.2981382	0.00517358	0.15402069
Ankrd13d	-0.8313382	3.01863563	9.85108429	0.00519092	0.1541744
Tbccd1	0.54120567	4.58301558	9.84005784	0.00521166	0.1541744
Thbs1	-0.9836683	2.76445923	9.83928631	0.00521312	0.1541744
Klra2	-2.7697683	0.277556	9.82199882	0.00524584	0.15462825
Ltbp2	-5.0719997	-1.1453635	9.81904773	0.00525145	0.15462825
Asl	1.0901965	2.93065126	9.80122437	0.00528546	0.15528999
Map3k15	-1.6784201	0.18468205	9.78397726	0.00531861	0.15549257
Fam132a	-1.0680538	1.84484177	9.7768357	0.0053324	0.15549257
Prkg2	0.55715195	4.33622435	9.77472191	0.00533649	0.15549257
Eif5a2	-0.4650341	6.5726775	9.7688169	0.00534794	0.15549257
Apln	-1.1062496	2.58939571	9.76314651	0.00535895	0.15549257
Dusp28	-0.8500346	3.63802561	9.75502375	0.00537477	0.15549257
Zcchc9	0.42344237	5.82683185	9.74240258	0.00539947	0.15549257
Mafk	0.97633074	3.20017283	9.74195138	0.00540035	0.15549257
Acvr2b	-1.7349712	0.6247075	9.73777469	0.00540855	0.15549257
Xrcc3	0.78530198	3.43280101	9.73330272	0.00541735	0.15549257
Pak6	-0.6357327	3.74563752	9.7155513	0.00545241	0.15549257
Pvt1	-1.0744391	2.41868138	9.70549278	0.0054724	0.15549257
Pcolce2	-1.8714431	1.14660412	9.70129023	0.00548077	0.15549257
Mc4r	3.05772295	-0.7315664	9.69683511	0.00548966	0.15549257

Gmpr2	0.76320173	3.44926098	9.695245	0.00549284	0.15549257
Pdzd4	-0.5014715	5.59251781	9.6873061	0.00550874	0.15549257
Nefl	-0.4312395	8.72747445	9.68718027	0.00550899	0.15549257
Cyb5r2	4.8451527	-1.4312352	9.68650332	0.00551035	0.15549257
1700110C19I	-2.8669546	-0.1240952	9.68262899	0.00551812	0.15549257
Shisa6	-0.7077068	5.30408921	9.67997389	0.00552346	0.15549257
Clic5	-1.0035265	2.94378902	9.67264516	0.00553822	0.15558267
Bmp2	0.71175394	4.62517624	9.65309982	0.00557781	0.15636832
Gpsm1	-0.7597626	2.66469454	9.64616157	0.00559194	0.15643848
Gm14092	-4.4383906	-1.0279516	9.61022547	0.00566576	0.15772361
Tjp2	0.45913497	5.62012645	9.60727368	0.00567187	0.15772361
Ddx18	0.43528915	5.05537199	9.60670723	0.00567304	0.15772361
Adamts15	0.92319117	2.60173174	9.58738471	0.00571324	0.15831557
Fanci	-0.9347233	2.90436969	9.58226255	0.00572394	0.15831557
Gm7457	-4.0281719	-0.9471554	9.57954831	0.00572963	0.15831557
Glra4	-4.5996991	-1.6182774	9.57169979	0.0057461	0.15844529
Gm527	-1.1009315	2.04063156	9.55808388	0.00577479	0.15854063
Ocm	-2.6270888	-0.9293338	9.55658419	0.00577796	0.15854063
Tagap	-0.845654	2.53749694	9.54812589	0.00579588	0.15854063
Cox7a1	1.05654942	2.11222014	9.54392952	0.00580479	0.15854063
Plekhh1	-0.6892588	4.58905436	9.54220391	0.00580846	0.15854063
Kitl	-0.5586245	4.77964071	9.53258028	0.00582898	0.15877568
Lin7b	-0.6002458	3.68429884	9.52710818	0.00584067	0.15877568
Rnf144b	-0.5590534	4.36927858	9.51801563	0.00586017	0.15898451
Ism1	1.05284739	3.28477602	9.4786273	0.00594547	0.16074134
Nol8	0.38549794	6.46452917	9.4770939	0.00594882	0.16074134
Zfp618	-1.0401155	1.48784735	9.46459236	0.0059762	0.16115752
Ptcd1	0.62559858	3.44479309	9.44958604	0.00600925	0.16172465
Arl4c	-0.5522192	5.17834358	9.43666289	0.00603787	0.16217067
Hspa5	0.39877638	7.24254107	9.43065496	0.00605123	0.16220571
Capn1	0.51666937	4.19335936	9.42162252	0.00607138	0.16242219
Tmem215	1.1102089	3.3389055	9.41110875	0.00609492	0.16272853
9130221H12I	0.64829928	3.48044409	9.38027857	0.00616455	0.16424194
Arl2bp	-0.523573	5.67878258	9.34988433	0.00623406	0.16424194
Zc3h12d	2.63713214	-0.835249	9.3480588	0.00623826	0.16424194
Ptges	0.80298103	4.28233446	9.34563992	0.00624384	0.16424194
Gfap	0.67812226	4.21164272	9.34501739	0.00624527	0.16424194
Tti2	-0.5531094	4.5965	9.34363924	0.00624845	0.16424194
Gm20199	-1.4450074	2.13159792	9.34081757	0.00625496	0.16424194
Tax1bp1	0.3408736	8.62016464	9.33131038	0.00627697	0.16424194
Ebf3	-1.0577682	1.46044412	9.32569977	0.00628999	0.16424194
2310007B03I	6.95928676	-1.8865621	10.3181027	0.00631153	0.16424194
Ier5	0.45795996	6.19845767	9.30636539	0.00633511	0.16424194

Zbtb12	-1.8068327	-0.2350096	9.30499323	0.00633833	0.16424194
D330045A20	-2.0920951	-0.0948508	9.29571748	0.00636011	0.16424194
Map3k3	0.68583388	3.27514667	9.28483949	0.00638576	0.16424194
Arhgef16	4.26266533	-0.9134374	9.27781339	0.0064024	0.16424194
Klf10	0.42473639	5.6640606	9.27746381	0.00640323	0.16424194
A730036I17F	4.87585209	-1.0930303	9.27613201	0.00640638	0.16424194
Upp1	4.10514475	-1.5056241	9.27384666	0.00641181	0.16424194
Clec11a	-0.7939277	2.21982313	9.26739492	0.00642715	0.16424194
AI661453	1.17156303	1.73037844	9.26544072	0.0064318	0.16424194
Tcea3	0.71508004	3.93148528	9.26480154	0.00643332	0.16424194
Fam192a	0.46031375	5.25633001	9.25714995	0.00645159	0.16424194
Clmn	-0.4179805	5.98196232	9.2537442	0.00645974	0.16424194
Mib1	0.457289	6.21587514	9.25003258	0.00646863	0.16424194
Sdhb	0.36525648	5.60717895	9.24957754	0.00646972	0.16424194
11-Sep	0.35218092	7.5978951	9.23495258	0.0065049	0.16424194
Usp29	-0.5488479	5.56455857	9.23177265	0.00651258	0.16424194
Adam23	-0.4910361	7.15181729	9.23029329	0.00651616	0.16424194
Arl9	-5.4328179	-1.9153914	9.22113922	0.00653833	0.16424194
Ly6g6e	3.71946554	-0.7640857	9.21434896	0.00655483	0.16424194
Eno1b	0.37131286	6.04849842	9.21408398	0.00655547	0.16424194
Flt1	-0.5935263	4.99645471	9.21324876	0.00655751	0.16424194
2310003H01	-1.3566889	0.9227629	9.21144924	0.00656189	0.16424194
Tbc1d30	-0.5475367	6.6190001	9.20951865	0.00656659	0.16424194
Adamts10	-0.9333954	2.08207284	9.2014169	0.00658638	0.16443123
Car2	0.35858849	7.0909454	9.18148655	0.00663534	0.16534678
Rorb	-0.4727187	8.50372588	9.15887271	0.00669139	0.16643514
Ldb2	0.38657792	6.02685681	9.12737436	0.00677033	0.168088
Gjc3	-0.5892605	5.44559443	9.11446233	0.00680299	0.16855213
Slc25a5	0.33871332	7.08642197	9.11009517	0.00681408	0.16855213
Sh3bp4	0.65035219	2.8722618	9.10064165	0.00683814	0.16883709
Aptx	-0.4349785	5.00670793	9.09016426	0.00686493	0.16918796
Sned1	0.79875513	4.23200509	9.07076152	0.00691484	0.17004251
Fndc7	4.50240151	-1.3887912	9.0643977	0.00693129	0.17004251
Aoah	2.70464081	-0.6572058	9.06199828	0.00693751	0.17004251
Ntn4	-1.1269193	1.3451484	9.03821574	0.00699946	0.17124909
Pak7	-0.5708197	6.03121147	9.02377759	0.00703738	0.17186416
Serinc2	1.51238294	0.7617066	9.01162791	0.00706946	0.17205122
Pcdha11	1.60498493	0.37164766	9.00810338	0.00707879	0.17205122
Casp9	-0.5043167	4.67728021	8.99762007	0.00710664	0.17205122
Pdia3	0.47054248	7.51848694	8.99760566	0.00710668	0.17205122
Nuak1	-0.5097546	5.19287171	8.98669702	0.00713579	0.17205122
Actg1	0.3099735	8.97424616	8.98331059	0.00714486	0.17205122
Tcf12	0.46493015	7.35152925	8.97878622	0.00715699	0.17205122

Ttc4	-0.49303	3.91169362	8.97768571	0.00715994	0.17205122
Irs4	-1.2166522	1.99826185	8.97762376	0.00716011	0.17205122
Thbs3	-1.4351195	1.10642805	8.96927359	0.00718257	0.17210345
Cirh1a	0.43049386	4.83870112	8.96558732	0.00719251	0.17210345
Asb5	-1.6061383	0.5376072	8.96044943	0.00720639	0.17210345
Htr7	-0.7234315	3.40319101	8.95784177	0.00721344	0.17210345
Gpr1	-4.6638702	-1.4864563	8.95024452	0.00723404	0.17228946
Mzb1	-4.874315	-1.791709	8.92566481	0.00730114	0.17328212
Kcnj15	4.18308285	-0.7769083	8.92554222	0.00730148	0.17328212
Pdha1	0.30972569	7.41076837	8.92076694	0.00731459	0.17328778
Fbf1	0.5484361	4.05102096	8.9125613	0.00733719	0.17328985
Anp32a	0.31442016	7.60656272	8.91026937	0.00734352	0.17328985
Polr2k	-0.5134892	4.07922009	8.90215856	0.00736596	0.17328985
Csnk1e	-0.438143	6.14590417	8.89614788	0.00738263	0.17328985
Sart1	0.48770867	4.71131357	8.89161659	0.00739523	0.17328985
Vgf	0.89610007	3.63822286	8.88934398	0.00740156	0.17328985
Slc44a3	4.47640657	-1.1805436	8.88215241	0.00742163	0.17328985
Mdh1	0.33207135	8.81871868	8.87977498	0.00742827	0.17328985
Ptbp2	0.45404644	6.37537676	8.87529048	0.00744083	0.17328985
Map3k9	-0.6803697	6.22637479	8.87435204	0.00744346	0.17328985
Chac2	0.6895027	3.90892416	8.86897719	0.00745854	0.17334115
Npbwr1	-2.4043603	0.40904425	8.85700736	0.00749226	0.17382456
Gm11517	-3.5916907	-1.0909567	8.84185106	0.0075352	0.17451204
Vtcn1	1.99290414	-0.1211964	8.83741059	0.00754783	0.17451204
Col4a2	0.54637138	4.28926856	8.80862156	0.0076303	0.17594532
Igsf6	-1.4959168	1.69934988	8.80665457	0.00763597	0.17594532
B230217C12	-0.3508339	5.85137644	8.79302162	0.00767541	0.17655168
Mir1954	8.58495648	-1.8444918	9.13540312	0.0077124	0.17672952
Fbxo8	0.44222763	4.84429171	8.78016502	0.00771281	0.17672952
Cap1	0.37446114	7.01686267	8.76855187	0.00774676	0.17672952
Sbspon	-1.6505504	0.73858241	8.76777604	0.00774904	0.17672952
Grhl3	2.82578554	-0.2245876	8.7670581	0.00775114	0.17672952
Prps2	0.41187232	6.17915191	8.75921937	0.00777417	0.17672952
Emc1	-0.4302098	4.6600872	8.75707105	0.0077805	0.17672952
Gcfc2	-1.1263216	2.09808493	8.75445368	0.00778821	0.17672952
Swap70	-0.4550171	4.83364218	8.7448041	0.00781672	0.17707786
Nfatc1	0.55428021	3.99991759	8.73769083	0.00783781	0.17725724
Mall	1.68858929	0.83161331	8.72793487	0.00786684	0.17761526
Srsf1	0.33818643	7.49633913	8.72299071	0.0078816	0.17765038
2610528A11	-4.1802708	-1.5100274	8.70653612	0.00793094	0.1784635
Chst11	0.44625248	5.6926055	8.69619267	0.00796212	0.17866204
Adcy2	-0.4531765	5.76768634	8.68695757	0.00799009	0.17866204
Zgrf1	-0.9799198	2.5509492	8.67839179	0.00801612	0.17866204

Ecm2	-0.6812411	5.03263301	8.67796911	0.00801741	0.17866204
P2rx5	3.58765252	-0.9221441	8.6747537	0.00802721	0.17866204
Xrcc4	0.62093428	3.43775911	8.66988837	0.00804206	0.17866204
Ercc1	0.92837586	2.21367991	8.66813397	0.00804742	0.17866204
Krt26	4.45923753	-2.0467071	8.6676751	0.00804883	0.17866204
Sqstm1	0.75402328	8.74120663	8.65920037	0.0080748	0.17866204
Mir181b-2	-4.0619645	-1.1861624	8.65793753	0.00807867	0.17866204
Myo3b	1.66869231	0.40957379	8.65355842	0.00809214	0.17866204
Mst1	-3.8868997	-1.388693	8.65130126	0.00809908	0.17866204
Adam1b	-1.9552254	0.2342261	8.62887728	0.00816848	0.17989796
A630075F10	-2.3590181	-0.7287085	8.61219404	0.00822054	0.18072772
Ndrg2	0.47892789	8.89316175	8.60821271	0.00823302	0.18072772
Rcan1	0.46895168	5.88676338	8.59469347	0.00827555	0.18075854
Hnf1b	-4.1356293	-0.7988781	8.59348498	0.00827936	0.18075854
Mturn	-0.3650589	6.37009665	8.5916164	0.00828526	0.18075854
Myh8	-3.6695047	-1.5295231	8.59070115	0.00828815	0.18075854
Gm4841	1.02682556	2.36667917	8.58053884	0.00832034	0.18111309
Gm21284	-1.694259	0.13904433	8.57707864	0.00833133	0.18111309
Msx1os	-4.2329748	-1.7882486	8.56806248	0.00836004	0.18144417
2410018L13F	-0.8313807	1.73556512	8.55898645	0.00838906	0.18153136
Haghl	-1.2902733	0.89350603	8.55223184	0.00841073	0.18153136
Hrasls	-0.5007149	5.07368425	8.55160325	0.00841275	0.18153136
Wls	0.61199348	5.22275416	8.54996188	0.00841802	0.18153136
Fam174b	0.6809433	6.56798464	8.5330841	0.00847249	0.18241357
Tspan17	-0.5674797	3.27720702	8.50916703	0.00855034	0.18291065
Rtl1	-0.9248667	2.9729589	8.50767343	0.00855523	0.18291065
N6amt2	-0.7054658	3.86164223	8.50357723	0.00856866	0.18291065
Prune2	-0.3767879	6.64228343	8.49896203	0.00858381	0.18291065
Gpr68	0.59261229	3.49668745	8.49712388	0.00858985	0.18291065
Nlrp10	2.2772553	-0.7884599	8.49602836	0.00859345	0.18291065
Myo1f	-1.1669724	1.27857512	8.49346059	0.00860191	0.18291065
C2	1.07646825	2.81686402	8.49272851	0.00860432	0.18291065
Ehd3	0.37187751	7.70821092	8.48407068	0.0086329	0.18291948
Phf21b	-1.0150008	2.20298585	8.48040894	0.00864502	0.18291948
Rhou	0.47103552	6.75176283	8.47970003	0.00864737	0.18291948
Ccdc142	-2.0739619	-0.4755545	8.47373089	0.00866717	0.18291948
Sec13	0.43015958	4.60747021	8.47206604	0.0086727	0.18291948
Zfp114	-1.8032004	0.38684237	8.46790744	0.00868654	0.18292458
Pdzd9	-1.8331006	0.29976476	8.45603746	0.00872617	0.18346418
Dusp9	1.56532899	0.92120224	8.45156785	0.00874114	0.18346418
Ddx20	0.76542561	3.71303709	8.44174273	0.00877416	0.18346418
Fmo2	1.07384139	2.18109682	8.43662774	0.0087914	0.18346418
Fbxo36	1.04352269	2.9116626	8.43506795	0.00879667	0.18346418

LOC171588	-7.273801	-1.1518178	8.75728719	0.00882222	0.18346418
Gm12070	0.38470495	10.3711141	8.42681286	0.0088246	0.18346418
Add3	0.375325	8.2735249	8.42621949	0.00882661	0.18346418
Ngf	1.40566566	0.84562194	8.42173839	0.00884181	0.18346418
Cyp26b1	-0.7592207	5.38657079	8.41976964	0.0088485	0.18346418
Rspo4	-2.9752387	-1.1341276	8.41160212	0.00887632	0.18375771
Chrnb4	-1.6279611	0.14843537	8.39427646	0.00893564	0.18394623
Mecom	-1.4876307	1.34295964	8.39362276	0.00893789	0.18394623
Itm2a	0.69821202	6.66213787	8.39318208	0.0089394	0.18394623
Ifngr1	0.72222515	3.18392937	8.39242803	0.00894199	0.18394623
Nomo1	0.47922884	5.3458177	8.38900639	0.00895377	0.18394623
Emr4	-5.0279237	-1.2104886	8.37885714	0.00898881	0.18397213
Mfsd11	0.64426255	3.18360502	8.37805261	0.00899159	0.18397213
Ccnf	1.64572243	0.81005706	8.37676513	0.00899605	0.18397213
Hkdc1	-1.0650103	1.82969467	8.37019683	0.00901882	0.18415803
Gm8787	-3.1760488	-1.3720546	8.36452678	0.00903854	0.18428091
Alox5ap	-1.2193998	1.4941033	8.35786573	0.00906176	0.18447482
Idh3g	0.36462229	6.41635125	8.33905917	0.00912767	0.18464876
Cmtm4	-0.380813	6.3562398	8.33847049	0.00912975	0.18464876
Rbp1	0.83647781	7.75562961	8.33708607	0.00913462	0.18464876
Olfr558	-5.4420278	-1.0715801	8.33556622	0.00913997	0.18464876
Klf8	-0.5973954	2.88497504	8.33190812	0.00915288	0.18464876
Tmco6	1.05657875	1.74919843	8.32859795	0.00916457	0.18464876
Rars	0.42530707	5.45583611	8.32809356	0.00916635	0.18464876
Rnh1	0.57543959	4.45654197	8.31735569	0.00920441	0.1851382
Lag3	3.38735534	-1.3344653	8.29871295	0.0092709	0.18575908
Snapc2	0.62698658	4.22325126	8.29340461	0.00928993	0.18575908
Sdcbp2	2.20492674	-0.7021209	8.29081585	0.00929923	0.18575908
Rsad2	1.44184077	1.25882069	8.28748538	0.0093112	0.18575908
Cnn1	3.18901221	-0.3466643	8.28197663	0.00933105	0.18575908
Erlec1	0.40192358	5.82466938	8.27851837	0.00934353	0.18575908
2610035F20I	0.67271858	3.01354408	8.27755154	0.00934702	0.18575908
Ptprm	-0.6584669	5.20958612	8.27736814	0.00934769	0.18575908
Eif5	0.2998029	8.75249106	8.274096	0.00935952	0.18575908
Egln3	0.56156523	7.45130752	8.26583486	0.00938947	0.18584418
Tdrd1	1.60899933	0.84020245	8.26529578	0.00939143	0.18584418
Dtl	-0.5302888	4.00306842	8.24104644	0.00947999	0.18709229
Cdk19	-0.3721275	7.00391856	8.24041384	0.00948231	0.18709229
Vim	0.56410027	8.07016048	8.23342589	0.00950801	0.18710892
Fh1	0.42044303	5.26825445	8.22825915	0.00952706	0.18710892
D4Ertd617e	-3.9154356	-1.1719346	8.22107701	0.00955361	0.18710892
Vangl1	0.80072495	3.81104559	8.21905381	0.00956111	0.18710892
Med26	0.71497207	2.57431246	8.21872337	0.00956233	0.18710892

Sulf2	0.36594167	6.53957386	8.21757746	0.00956658	0.18710892
Rpp25	0.59236002	3.94710413	8.21203962	0.00958714	0.18723894
Gm10409	-0.7058586	4.00369907	8.19021541	0.00966866	0.18855725
Gm10220	-1.1337614	1.69414283	8.18004949	0.00970689	0.18891282
Nkain4	-1.3097436	1.74106904	8.17790774	0.00971497	0.18891282
Bloc1s3	-0.677257	3.16661035	8.16931455	0.00974745	0.18895822
Rab9b	0.34461485	6.46327609	8.16721279	0.00975541	0.18895822
Shc2	-0.5636283	4.57055209	8.16615278	0.00975943	0.18895822
St3gal1	0.43792634	5.20677659	8.14783041	0.00982918	0.1896314
Rbbp9	0.51743967	6.4796997	8.14751266	0.0098304	0.1896314
Kif18a	1.64647232	0.99561614	8.1401867	0.00985845	0.1896314
Brpf3	-0.379248	5.26307102	8.13438943	0.00988071	0.1896314
Ttk	-2.7597287	-0.5704396	8.1325074	0.00988795	0.1896314
4931440P22I	-3.540313	-1.4315636	8.12767112	0.00990658	0.1896314
Cadps2	-0.4949658	6.41826898	8.12495082	0.00991707	0.1896314
Crtac1	0.81685676	3.20349585	8.12391251	0.00992108	0.1896314
Larp1b	0.60367251	4.07434066	8.12085031	0.00993292	0.1896314
Frmpd1	-0.6253598	2.76514959	8.12028046	0.00993512	0.1896314
Aars	0.44694732	5.61114746	8.10019877	0.01001314	0.1908499
Myo1b	-0.4479967	5.93592783	8.0912984	0.01004794	0.19104562
Kctd6	0.47573782	5.85232124	8.09031197	0.01005181	0.19104562
Gapdh	0.33742317	9.52446363	8.08319034	0.01007976	0.1913067
Lrrc75a	-0.6382694	3.25761938	8.07768508	0.01010143	0.19144792
Dusp3	-0.3327842	7.09315937	8.07067847	0.01012908	0.19154572
Atp6v1b2	0.35338707	8.40068724	8.06688353	0.01014409	0.19154572
Slc30a4	-0.3493246	6.40576099	8.06557087	0.01014929	0.19154572
9330102E08I	-0.6341563	4.04629306	8.05987605	0.01017188	0.19170315
Zfp641	-0.9725105	3.02665455	8.04780671	0.01021994	0.1922987
Pgbd1	0.72651548	2.33747517	8.03909318	0.01025479	0.1922987
Fgfr3	-0.6122135	3.90040749	8.03688642	0.01026364	0.1922987
Sdhaf2	0.43393556	5.20430452	8.03293031	0.01027952	0.1922987
4933431E20I	-0.3615353	6.76028562	8.0319709	0.01028338	0.1922987
Camk2n2	-0.4547794	4.57960333	8.02245092	0.01032173	0.1922987
Krt1	1.34672772	1.74797374	8.02157528	0.01032526	0.1922987
Fuca2	0.43547069	4.54226025	8.02014091	0.01033106	0.1922987
Zmynd19	-1.2073522	1.55240992	8.01988391	0.0103321	0.1922987
Dhrs4	0.64404941	3.26827748	8.01049177	0.01037013	0.19272271
Ncdn	0.44077013	8.21970628	8.00402169	0.01039642	0.19272271
Idh3b	0.31358907	7.06719223	7.99940725	0.01041522	0.19272271
C5ar1	2.96169863	-0.0568141	7.99778785	0.01042182	0.19272271
Aspm	-1.377111	1.19774706	7.99664495	0.01042649	0.19272271
Gm11744	-2.3202018	-0.4086737	7.98890909	0.01045812	0.19304228
Rps4l	-0.7286353	1.95132353	7.98522101	0.01047324	0.19305655

DQ267100	-1.949947	0.15390844	7.97216319	0.01052697	0.19328544
Zbtb34	-0.4567811	6.30562841	7.97211858	0.01052716	0.19328544
Nqo2	0.47307013	5.67377076	7.97173174	0.01052875	0.19328544
Dach2	2.02538236	0.10560805	7.95881184	0.01058223	0.19400245
Ndnf	-0.4412466	5.19802815	7.95254987	0.01060825	0.19421499
Wfdc17	-1.5216832	0.93154725	7.94482263	0.01064047	0.19454011
Pld5	-0.7000368	4.2995414	7.92071232	0.01074169	0.19600934
4932411E22I	-0.870367	2.56302696	7.91327693	0.01077312	0.19600934
Scd3	0.85860126	3.20431338	7.91164239	0.01078005	0.19600934
Zc3h7b	-0.4089847	6.02032441	7.91157299	0.01078034	0.19600934
Fgf7	1.06182799	1.59761899	7.90655593	0.01080162	0.19600934
Rbm34	0.37693922	5.32933223	7.90312781	0.01081619	0.19600934
Rad52	-0.5629592	3.57807302	7.89908841	0.01083339	0.19600934
Taf5l	-0.4711146	4.1315046	7.89815691	0.01083736	0.19600934
Trib3	4.0512533	-1.1817498	7.89425865	0.01085399	0.19604662
Dstn	0.47002337	8.25470472	7.88763181	0.01088233	0.19629496
Rel	0.44092681	4.52771932	7.87095255	0.01095402	0.19732356
Usp47	0.2817606	7.1442615	7.86562399	0.01097703	0.19747376
Gm20110	-3.8244455	-1.7276857	7.84358511	0.01107279	0.19882334
Mgat1	-0.560155	3.42055107	7.83826955	0.01109602	0.19882334
Cpt2	0.59865128	3.28725638	7.83230655	0.01112215	0.19882334
Exph5	-0.6874816	6.36395724	7.82685636	0.0111461	0.19882334
Mpzl2	0.58164427	7.15656014	7.82585189	0.01115052	0.19882334
Dyrk4	-3.9181425	-2.242214	8.10079459	0.01120681	0.19882334
Srp72	0.28805746	7.21966283	7.81244025	0.01120971	0.19882334
Rpusd1	-0.5890129	2.98295012	7.81155249	0.01121364	0.19882334
Cblb	0.42932526	6.78147852	7.81130359	0.01121474	0.19882334
C130030K03I	-0.9650361	2.25501604	7.80565265	0.0112398	0.19882334
Hspa1a	0.57103404	3.80987944	7.80492499	0.01124303	0.19882334
Gpr37	-0.6956111	3.7003291	7.79706337	0.011278	0.19882334
Myh1	1.93381283	0.52930343	7.79537917	0.01128551	0.19882334
Csmd2os	-2.5196533	-0.3659152	7.79484613	0.01128789	0.19882334
Bpnt1	0.34861622	6.08443902	7.7929652	0.01129628	0.19882334
Slc25a42	0.39558791	4.84202156	7.79110867	0.01130457	0.19882334
AI317395	-3.3325359	-0.8851693	7.78963974	0.01131114	0.19882334
Ccndbp1	0.44551249	6.68427437	7.78810466	0.01131801	0.19882334
Cog4	0.47643264	4.60369643	7.77542345	0.0113749	0.19936737
A930004D18	-1.1218391	2.82111606	7.77460069	0.01137861	0.19936737
Rassf6	-1.7155022	0.63128141	7.77041902	0.01139745	0.19943777
Kcnj10	-0.4939782	5.29891954	7.76295837	0.01143114	0.19954873
Tcte2	0.73425049	2.88138795	7.76244962	0.01143345	0.19954873
Gfra1	-0.6039297	3.28474915	7.75456116	0.01146921	0.19991356
Naaa	-0.677033	3.92316073	7.73244897	0.01157011	0.20134654

Ryr1	-0.9784729	2.63715206	7.73000112	0.01158134	0.20134654
A330102I10F	-1.4454369	1.29368866	7.72651453	0.01159736	0.20136487
Pdlim4	0.80153638	2.4719041	7.7123316	0.01166278	0.20206332
Adamts16	-1.8369069	0.94222066	7.7070336	0.01168732	0.20206332
Pcp4	-0.3582013	8.83186666	7.70600288	0.0116921	0.20206332
1700096K18I	1.34582971	1.18970557	7.70337865	0.01170428	0.20206332
Mycn	-0.8806673	2.05725625	7.70157404	0.01171267	0.20206332
Rufy1	0.45432226	4.69976452	7.68924435	0.01177015	0.20255671
Cpm	0.61656271	4.42576325	7.6878301	0.01177676	0.20255671
Kcnk10	1.2255041	0.71105858	7.68349907	0.01179704	0.20255671
Baalc	-0.3283711	7.07479323	7.68255098	0.01180148	0.20255671
Sumf2	0.7435468	2.65517025	7.67293178	0.01184668	0.2030734
09-Sep	0.55902145	5.57392956	7.66882867	0.01186601	0.20314608
Atp6ap2	0.37470623	7.25392313	7.66033721	0.01190614	0.2035741
Anxa5	0.64941588	8.20189293	7.63731068	0.01201573	0.20483169
Slc16a10	-0.7360944	2.28528914	7.63685753	0.0120179	0.20483169
Cdhr3	-3.09095	-0.2814062	7.63312613	0.01203576	0.20483169
Dxo	0.72342233	2.09703573	7.62977772	0.01205182	0.20483169
LOC1010560	0.68567084	3.43306051	7.62802865	0.01206022	0.20483169
Eif2b1	0.56294993	4.79094889	7.62578085	0.01207102	0.20483169
Camkk2	-0.5285247	6.00634976	7.62058106	0.01209606	0.20499792
Dnajc28	0.47339562	3.87108935	7.61184381	0.01213824	0.20527172
Pik3ap1	0.85140732	3.13275307	7.61091892	0.01214272	0.20527172
Gm19689	-4.5399074	-1.7117989	7.87065366	0.0122097	0.20614506
1700112E06I	1.16022053	1.17197641	7.58752628	0.01225654	0.20667648
Dnali1	-1.7083657	0.12482844	7.57650917	0.01231055	0.20732752
Igfbp6	0.79916154	4.92640641	7.56186112	0.01238278	0.20769735
Mterfd2	0.40355211	5.9146448	7.56162568	0.01238395	0.20769735
Ociad2	0.33325385	6.64314573	7.56131618	0.01238548	0.20769735
Ppif	0.55606883	4.94539068	7.55758	0.01240398	0.20769735
Nup37	-1.2711379	1.22698112	7.55642965	0.01240969	0.20769735
Psmd13	0.55684827	3.96068932	7.54590084	0.01246202	0.20831421
Atg10	-0.6223453	4.0415969	7.53662063	0.01250836	0.20882935
Hdac3	-0.5045317	4.7598794	7.53113984	0.01253582	0.20902839
Sik1	0.59116611	4.19170344	7.52582301	0.01256252	0.20921433
3110007F17I	-1.3778916	1.17181934	7.51355435	0.01262437	0.20979484
Mmp19	0.98854759	2.61996574	7.5127262	0.01262855	0.20979484
BC061194	-1.7693767	0.34113822	7.50759083	0.01265455	0.20988679
Slc39a3	0.61124814	3.21713689	7.50547495	0.01266528	0.20988679
Fxyd7	-0.8471727	2.00153272	7.49082047	0.01273988	0.21086329
Vwf	-0.9956715	2.07504973	7.48180456	0.01278602	0.21134422
Kcnq3	-0.4039714	5.53464675	7.4790115	0.01280035	0.21134422
A730043L09I	4.73332687	-2.1676103	7.47319008	0.01283028	0.21157873

Zfp712	-0.5486737	3.87900977	7.46390786	0.01287816	0.21202556
Zfp212	0.71881651	2.92232215	7.46050317	0.01289577	0.21202556
Itih2	0.59270092	5.04283293	7.45689002	0.01291449	0.21202556
Col9a2	0.85697546	2.67963195	7.45575078	0.0129204	0.21202556
Enpp6	-0.8434154	2.77992828	7.44127312	0.01299575	0.21211883
Abhd8	-0.413418	4.56973272	7.44099068	0.01299723	0.21211883
Zmynd15	-1.9346755	0.33496792	7.43576079	0.01302457	0.21211883
Tcfl5	-4.2853493	-1.4827412	7.43395216	0.01303404	0.21211883
A830082N09	-0.6696761	4.43826558	7.43011804	0.01305415	0.21211883
Amph	0.44252945	6.92174511	7.42912919	0.01305934	0.21211883
Gm684	0.6121315	3.50100373	7.42536482	0.01307912	0.21211883
Ptpla	-0.7761694	2.93398082	7.42370857	0.01308784	0.21211883
Oxt	-1.6292397	0.36731434	7.42234097	0.01309504	0.21211883
2900092D14	-0.5596413	5.46346693	7.42091295	0.01310256	0.21211883
Irf2bpl	0.38626129	5.74511218	7.41595603	0.01312871	0.21211883
Stat3	0.32349959	5.61785731	7.4157191	0.01312996	0.21211883
Arl4d	-0.8844149	2.09657324	7.41371693	0.01314054	0.21211883
Osgin2	0.46237428	5.95443226	7.41034403	0.01315839	0.21211883
Myl6b	-0.8532711	3.78639404	7.40845582	0.01316839	0.21211883
Grid2ip	-1.5663536	1.10690443	7.40493944	0.01318704	0.21211883
Afg3l2	0.4079443	5.7591495	7.40361744	0.01319406	0.21211883
Nol3	0.64490135	2.90621969	7.38136595	0.01331283	0.21377293
Zc3h15	0.27736565	7.05509728	7.3761029	0.0133411	0.21397151
Fam160a1	0.54005243	4.32997888	7.35958366	0.01343026	0.2151451
Vamp2	-0.325952	10.2441072	7.3504745	0.01347972	0.21568052
Slco1c1	-0.6409912	4.33303442	7.34063582	0.01353336	0.21628162
Igf2	-0.6307715	10.4379258	7.33730277	0.01355158	0.21631598
Tle3	0.41227365	5.22576563	7.32209896	0.01363507	0.21716551
Haus7	0.97429155	2.08117045	7.32173416	0.01363708	0.21716551
Ppef1	-2.4167273	-0.7102362	7.30198858	0.01374639	0.21841867
4930578C19	-3.8349553	-1.5017189	7.29667509	0.01377597	0.21841867
AB124611	2.40420267	-0.6492218	7.29664531	0.01377613	0.21841867
Gbp4	-0.7453061	2.90450644	7.29582612	0.0137807	0.21841867
Mapk14	0.31524866	6.53290444	7.28647851	0.01383293	0.21880208
Rora	-0.3374275	8.37690505	7.28155708	0.01386052	0.21880208
Tgfb2	0.49131115	4.28430239	7.280663	0.01386554	0.21880208
AI467606	-3.0441847	-1.3976377	7.27818525	0.01387946	0.21880208
Col12a1	0.39223295	5.40904331	7.2742527	0.01390158	0.21880208
2410007B07	3.38734903	-1.9733839	7.27123019	0.01391861	0.21880208
Map3k7	0.29203049	6.60263861	7.27121279	0.01391871	0.21880208
Rpp40	0.71650818	2.58937608	7.25631261	0.01400301	0.21987042
Fam229b	-0.7231098	2.47483404	7.25282645	0.01402282	0.21992478
Gm15881	-2.0640591	0.39379139	7.2463079	0.01405993	0.22011155

Serpina3n	1.02694602	2.68992215	7.24438605	0.0140709	0.22011155
Msln	1.0751445	2.22083589	7.24212719	0.0140838	0.22011155
Itpkc	0.84785285	2.14887091	7.23302425	0.01413591	0.22066978
Ndufb6	0.47342976	5.63196151	7.22217766	0.0141983	0.22138676
Plekhm2	-0.7010288	3.60509107	7.21643768	0.01423143	0.22153029
Nudt10	0.52699316	3.41269048	7.21351152	0.01424836	0.22153029
Dnase1l2	-2.8171973	-0.4876255	7.21203821	0.01425689	0.22153029
Col1a1	0.63536947	6.95686148	7.2015191	0.01431796	0.2220718
Kcnj9	-0.4901468	5.02242823	7.20035402	0.01432475	0.2220718
Lrrc56	-1.7040861	0.36716053	7.1964804	0.01434732	0.22216584
Vmn2r86	-1.5520297	0.46302815	7.19206765	0.01437309	0.22230901
Hsd17b2	1.60454718	1.4829574	7.18833049	0.01439495	0.22239153
Trim41	-0.3903652	4.34501675	7.18153113	0.01443482	0.2225472
Hpse	3.82169224	-1.4409293	7.18097248	0.0144381	0.2225472
Ik	0.2801788	8.56539055	7.16445454	0.01453551	0.22342844
Fbxl21	-0.5558273	3.14489174	7.16100038	0.01455597	0.22342844
2810408A11I	2.46428482	-0.898105	7.16063847	0.01455811	0.22342844
Xab2	0.50370506	3.85964232	7.15942695	0.0145653	0.22342844
Cyp4a12b	-1.0722156	3.11657346	7.15723721	0.0145783	0.22342844
Invs	0.46362285	4.82609087	7.15088484	0.01461607	0.22375259
Lmcd1	1.01799264	2.10485348	7.14461703	0.01465346	0.22396195
Cage1	-1.3005369	1.19686726	7.14301365	0.01466304	0.22396195
Nek10	-2.0494888	0.42378789	7.13140379	0.01473262	0.22455756
Zfp940	-0.6236828	3.31935635	7.13093909	0.01473541	0.22455756
AI427809	-1.7181124	1.2099746	7.12159615	0.01479168	0.22516013
Ano1	-0.916461	1.54957846	7.11195173	0.01485002	0.22579278
Ptpmt1	-0.4582835	4.1804834	7.10184511	0.01491144	0.22635763
Tagap1	-0.4506425	4.77504619	7.1003058	0.01492082	0.22635763
Aldh1a2	0.59592758	9.18687312	7.09676871	0.01494239	0.22636471
Pgm1	0.48686241	3.70359012	7.09471639	0.01495493	0.22636471
Ccdc89	1.78883959	0.33416161	7.0825228	0.01502964	0.22724007
Stat4	-0.8578235	1.82879885	7.07687206	0.01506441	0.2275101
Ift74	0.35757425	5.84316581	7.06126858	0.01516089	0.2285799
Fignl1	2.35293463	-0.7257423	7.05479059	0.01520114	0.2285799
Cycs	0.3982156	8.2438788	7.05468706	0.01520178	0.2285799
Zfp207	0.29077321	7.90237065	7.05446039	0.0152032	0.2285799
Zfp655	0.37445704	5.7401676	7.04796092	0.01524371	0.22865501
Tbx18	0.5305516	6.20995825	7.04519895	0.01526096	0.22865501
Als2cl	-1.2679158	0.5495359	7.04412216	0.0152677	0.22865501
Frzb	-0.6923063	3.21545134	7.04052178	0.01529023	0.22865501
Amz1	-0.907464	1.78497787	7.03976289	0.01529499	0.22865501
E4f1	0.75521854	2.50487204	7.03734508	0.01531015	0.22865501
Gm10471	-1.0276968	1.85774165	7.03418565	0.01532998	0.22869741

Ccdc25	0.33431821	5.86118626	7.02320277	0.01539915	0.22937006
Rasl10b	-0.6512524	3.49792742	7.02121899	0.01541169	0.22937006
Fgd2	1.43722734	0.37736609	7.01892287	0.01542621	0.22937006
Dock10	-0.6318373	5.74765679	7.0002798	0.01554466	0.23087625
Tbrg3	-0.7508806	4.19389579	6.99641315	0.01556936	0.23092785
Synpr	0.59213218	5.17764605	6.99436459	0.01558246	0.23092785
Rtp1	3.85121373	-1.5165248	6.98901078	0.01561676	0.23100866
2900097C17I	-0.481486	10.8249655	6.98815527	0.01562225	0.23100866
Slc25a35	0.49744257	4.99898898	6.98405045	0.01564861	0.23114451
Mtbp	-1.1803535	1.38346682	6.97321879	0.01571842	0.23173866
Ccrn4l	0.40435182	5.44062537	6.97134452	0.01573054	0.23173866
Sox2	0.67451405	3.57343566	6.96980439	0.0157405	0.23173866
Prg4	0.68247418	8.48540854	6.96018089	0.01580291	0.23240328
Pdcd6ip	0.29071427	7.45387281	6.9548671	0.0158375	0.23265255
Ggact	0.59258059	5.86444957	6.95226798	0.01585444	0.23265255
Phlda1	0.41670566	7.72269091	6.94628861	0.0158935	0.2329717
Spata2	-0.4651618	5.49015829	6.93734025	0.01595216	0.23322913
Blmh	0.4078886	5.01121896	6.93631036	0.01595893	0.23322913
Fhl3	1.08460745	2.04694674	6.93543833	0.01596466	0.23322913
Hrh1	-0.6602962	2.61130733	6.93153549	0.01599035	0.23322913
Gm20743	3.04538453	-1.8229197	6.92663924	0.01602263	0.23322913
Psmb5	0.43313832	5.10193767	6.92621425	0.01602544	0.23322913
Fbxl8	2.97027895	-0.7816331	6.92516103	0.01603239	0.23322913
Rrp1	0.37732168	7.75798968	6.9211712	0.01605877	0.23331004
Zc3hav1	0.43208283	5.52843251	6.91907834	0.01607263	0.23331004
Arhgap15	-0.5406693	3.82737943	6.90865941	0.01614182	0.23351708
Poc1b	0.49175413	4.59320195	6.90657391	0.01615571	0.23351708
Slc30a9	-0.3402105	6.51316511	6.90479132	0.01616759	0.23351708
Gpr141	-3.8907038	-0.5865768	6.90374724	0.01617455	0.23351708
Rassf10	0.75179669	2.070177	6.90146035	0.01618982	0.23351708
Utp20	-0.5966602	4.51937359	6.90128084	0.01619102	0.23351708
Tmem167	-0.3534527	6.42597284	6.89730286	0.01621761	0.23358345
Gucy2e	1.0322825	1.54458021	6.89540209	0.01623034	0.23358345
Trp53inp2	0.27741439	7.84324072	6.88860136	0.01627595	0.23398973
Plcxd2	-0.4265528	7.79209282	6.87610249	0.01636017	0.2346149
Nsun5	-0.7632506	1.70862212	6.86745067	0.01641875	0.2346149
Slc44a1	-0.3241412	5.88927923	6.8658382	0.0164297	0.2346149
Pls1	-0.5373333	4.03669189	6.86331764	0.01644682	0.2346149
Ferd3l	3.03518247	-1.5862539	7.08998125	0.01645433	0.2346149
Jagn1	0.41271424	4.86171929	6.86187682	0.01645662	0.2346149
Lima1	0.49168977	7.28145281	6.86031251	0.01646726	0.2346149
Srsf12	-0.5400914	4.02511157	6.85705886	0.01648943	0.2346149
Igf1r	-0.3432959	6.90698274	6.85651132	0.01649316	0.2346149

Cdh13	0.64427841	4.87616683	6.85510408	0.01650276	0.2346149
Epha4	-0.3649426	7.2171443	6.85386349	0.01651123	0.2346149
Fam228a	-1.57702	1.31402514	6.84768934	0.01655344	0.23470579
Rab4b	0.53328864	3.46362841	6.84424319	0.01657706	0.23470579
Capn15	0.78447568	1.8302036	6.84368249	0.0165809	0.23470579
Fkbp7	0.71599452	3.61304404	6.84247293	0.0165892	0.23470579
Tomm20	-0.2738697	7.75160618	6.8401965	0.01660483	0.23470579
D19Bwg1357	0.3761953	5.51629324	6.82523282	0.01670801	0.23528862
Adk	0.43923619	4.8192347	6.82193563	0.01673084	0.23528862
Zdhhc8	-0.5454175	4.78650998	6.82057545	0.01674026	0.23528862
5430427O19	-2.1898353	0.2311527	6.82022508	0.01674269	0.23528862
Ntng1	-0.4296493	5.3774984	6.81913832	0.01675023	0.23528862
St3gal4	-0.8877372	3.96093969	6.81827303	0.01675624	0.23528862
Hic2	-0.7281116	2.3305498	6.8149166	0.01677955	0.23528862
Gpr64	1.61372975	1.17481498	6.81261516	0.01679556	0.23528862
Npy1r	-0.4376436	5.20521963	6.81036816	0.01681121	0.23528862
Zfp316	-0.6692574	3.73283924	6.80897417	0.01682092	0.23528862
Olfr78	-2.6107683	-0.7827399	6.80273174	0.0168645	0.23565326
C130071C03I	-0.7493066	2.7042152	6.79819611	0.01689625	0.23585192
Gramd4	-0.4907185	4.03964658	6.79498571	0.01691876	0.23592139
9530026P05I	1.26520814	0.5180559	6.77872985	0.01703325	0.23691378
Mme	-0.5188087	4.26111758	6.76989186	0.01709586	0.23691378
Adamts5	0.60667189	3.69374067	6.76937774	0.01709951	0.23691378
Aqp6	-3.8408876	-1.3654759	6.76759027	0.01711221	0.23691378
Tmem191c	-0.6026575	4.21084663	6.76509673	0.01712994	0.23691378
Zhx2	0.48220594	6.38843228	6.76292596	0.0171454	0.23691378
St8sia5	0.644603	4.23302689	6.76023214	0.01716459	0.23691378
Vps36	0.53951879	3.86772111	6.75904628	0.01717305	0.23691378
2700069I18R	1.32344495	0.80104733	6.7559842	0.01719492	0.23691378
Mfsd3	1.75550426	0.54359466	6.75508887	0.01720132	0.23691378
Spry1	0.68008701	3.19488316	6.75387834	0.01720997	0.23691378
Me2	0.41390449	6.29207004	6.7524055	0.01722051	0.23691378
Mogs	-1.0569026	1.72178949	6.75205667	0.01722301	0.23691378
5730405O15	-2.1760497	-0.293786	6.74812971	0.01725114	0.23691378
Sh3gl3	-0.5219069	5.61268157	6.74730471	0.01725706	0.23691378
Pde1c	0.66367518	4.17786531	6.7427102	0.01729006	0.23691378
Phyhipl	0.35971593	7.4588456	6.74117486	0.0173011	0.23691378
4930479D17I	-1.2988742	1.0864378	6.74037802	0.01730683	0.23691378
Asgr1	0.77978257	6.16241364	6.73250113	0.01736363	0.23744973
Nab1	0.29362899	6.61814642	6.72699834	0.01740344	0.23775243
Olfr692	4.33751115	-1.3157961	6.71999793	0.01745422	0.23800319
Fam214a	-0.426925	5.23806352	6.71959253	0.01745717	0.23800319
Cmpk2	0.38524096	4.55212567	6.71372305	0.01749988	0.23817061

H2-Q5	-1.8491901	-0.6068189	6.71155761	0.01751567	0.23817061
Scara3	0.77548897	6.56283487	6.70955415	0.01753029	0.23817061
Omd	-0.4141683	5.17347677	6.70819166	0.01754024	0.23817061
0610040J01F	-1.8736999	0.12504341	6.70048561	0.01759665	0.23852544
Acat2	0.41258784	5.25145575	6.69814901	0.01761379	0.23852544
Slc38a11	2.57217537	0.50440634	6.69628558	0.01762748	0.23852544
Plk1	-2.9829582	-0.9043404	6.69406192	0.01764382	0.23852544
Morc2a	-0.3103148	6.17560802	6.69254213	0.01765501	0.23852544
Pin4	-0.4318342	4.63656401	6.68887673	0.01768201	0.23865063
Uba3	0.34161753	6.23925471	6.68594577	0.01770363	0.23868037
Barhl2	-2.5761703	-0.0727471	6.68377229	0.01771968	0.23868037
Aldh2	0.54504314	5.30454069	6.67370172	0.01779429	0.23929489
Bmper	-0.5693468	3.17057497	6.67281477	0.01780087	0.23929489
Jdp2	-0.4936261	3.35051052	6.66924226	0.01782743	0.23941276
Macc1	-3.3195903	-2.0982567	6.66149234	0.0178852	0.23994911
Bmx	-1.2046357	1.85250892	6.65820543	0.01790977	0.24003936
Gnl3	0.47155484	4.71939905	6.65426931	0.01793924	0.24019505
Bank1	-1.136936	1.7660714	6.64768282	0.01798867	0.24029328
Pi16	2.39259774	-0.6008267	6.64724179	0.01799198	0.24029328
Exosc4	-0.60698	3.40495254	6.64534328	0.01800626	0.24029328
Strc	-2.6018916	-1.2857172	6.6388486	0.0180552	0.24029328
Mrps25	0.35411696	4.61514581	6.63502026	0.01808412	0.24029328
Nos1	0.68658566	3.24246223	6.63433339	0.01808932	0.24029328
Dbn1	-0.4850045	4.44211037	6.63283864	0.01810063	0.24029328
Hist1h2be	0.74274712	2.78253402	6.63195574	0.01810731	0.24029328
Fli1	0.60808581	4.25282813	6.63065016	0.0181172	0.24029328
Zfp788	-0.3020681	6.23732821	6.62960214	0.01812514	0.24029328
Clic4	0.50638532	8.64253682	6.62023455	0.01819631	0.24099932
Cyp3a13	-2.5419948	-1.1165901	6.61494857	0.01823661	0.24107111
Gm14827	-1.0083574	2.66352071	6.61302598	0.01825129	0.24107111
Gmip	0.87332719	1.52538376	6.61247786	0.01825548	0.24107111
4931430N09	1.63095826	1.7189984	6.60281196	0.01832951	0.24181141
Cnot11	0.39595702	4.38164142	6.5949275	0.01839014	0.24237373
Zfp438	0.47344104	3.94995783	6.58855882	0.01843929	0.24243866
2610034B18I	0.65258481	4.17684086	6.58808077	0.01844298	0.24243866
1110032F04I	0.81682581	2.18019869	6.58728679	0.01844912	0.24243866
Nyap2	-0.4383615	5.85461771	6.58427772	0.0184724	0.24250782
Unc93a	3.12813441	-0.8055965	6.56489898	0.01862315	0.24424855
Uso1	0.29269848	6.42363592	6.56032349	0.01865894	0.24447972
Grn	0.66416329	5.16360744	6.54583358	0.01877281	0.24573236
Pdk2	0.31423889	7.0969543	6.53358662	0.01886966	0.24614978
Klhdc9	-0.4907274	3.65695043	6.53346758	0.0188706	0.24614978
Arhgap6	0.46521054	4.73769687	6.533381	0.01887129	0.24614978

Catsper2	-0.8508135	1.9578967	6.53255149	0.01887787	0.24614978
Tmem261	-0.5067787	3.81384098	6.52054165	0.01897343	0.24700434
Pex10	0.68317448	2.67676415	6.51926811	0.0189836	0.24700434
Fgd1	0.6088208	3.05365511	6.51535705	0.01901486	0.24700434
Glod4	0.33640564	5.51592075	6.51354993	0.01902932	0.24700434
Lcorl	-0.3859882	5.13712707	6.51140688	0.01904648	0.24700434
Chst2	-0.3202153	7.83723352	6.508844	0.01906704	0.24700434
4930444F02I	-3.2547897	-0.6026053	6.50767705	0.0190764	0.24700434
Tbc1d14	-0.3701909	5.17225701	6.5030571	0.01911353	0.24700434
Aldoc	0.40205246	7.18074671	6.50119121	0.01912855	0.24700434
Ajuba	-0.5823817	2.82632005	6.50077606	0.0191319	0.24700434
Zfp839	0.39099324	5.26943064	6.49910974	0.01914532	0.24700434
Mfap1a	0.26245987	7.41490946	6.48994388	0.01921937	0.24704522
Adra2c	-0.8135843	2.43547941	6.48726578	0.01924107	0.24704522
Pkd2l1	3.6380491	-1.3225536	6.48454769	0.01926311	0.24704522
Zfand3	0.40217181	5.53411354	6.48408056	0.0192669	0.24704522
Bag3	0.43685169	5.08106989	6.48309673	0.01927489	0.24704522
Vmn2r1	-2.8275059	-1.1488416	6.47355004	0.01935261	0.24704522
Rassf2	-0.4871902	7.49587503	6.47166153	0.01936803	0.24704522
Kctd4	0.54388255	5.06433096	6.47127269	0.0193712	0.24704522
Ccdc88a	0.4041716	9.01307016	6.47061402	0.01937658	0.24704522
Gm4349	-1.3664917	0.61440801	6.46666056	0.01940891	0.24704522
Ltbp4	-0.4364802	4.59000141	6.46279261	0.0194406	0.24704522
0610040F04I	-1.4449725	0.96442766	6.46213555	0.01944599	0.24704522
Bcap29	0.41968786	5.17023279	6.4617715	0.01944898	0.24704522
Prx	2.05005844	-0.4765211	6.46152144	0.01945103	0.24704522
Trim16	0.77462084	2.64917315	6.46079244	0.01945702	0.24704522
D130017N08	-0.5433615	3.83627959	6.45941944	0.01946829	0.24704522
Mob1b	0.35711865	5.1918482	6.45382784	0.01951428	0.24704522
Snapc3	0.40833549	4.89748963	6.45314488	0.0195199	0.24704522
Nxph1	0.46018313	4.83149152	6.45248534	0.01952534	0.24704522
E030019B06	-2.9561543	-0.5614128	6.45079018	0.01953931	0.24704522
Vdr	1.24858769	0.75906916	6.45051523	0.01954158	0.24704522
Cd28	1.58642584	0.22626198	6.447128	0.01956954	0.24704522
Psap	0.40136559	8.49462902	6.44698208	0.01957075	0.24704522
Epdr1	0.41953715	5.86306948	6.43414257	0.01967716	0.24780583
Il31ra	0.78174153	2.40872718	6.4335862	0.01968178	0.24780583
Gm5523	0.37995005	5.24483222	6.43304902	0.01968625	0.24780583
Olig1	-0.4557159	4.07031576	6.4264691	0.01974106	0.24796184
Slc7a4	0.69388528	2.98030875	6.42491852	0.01975401	0.24796184
Gpatch4	-0.3790371	4.88868949	6.4239249	0.0197623	0.24796184
Trim2	0.37600303	9.04551566	6.42272207	0.01977235	0.24796184
Gm13749	-1.6560911	0.63489644	6.41846924	0.01980794	0.24815584

Apc2	-0.6125519	4.60083919	6.41630971	0.01982603	0.24815584
Clu	0.61093751	7.27932893	6.41426894	0.01984315	0.24815584
Brox	0.30566044	5.69031614	6.40861103	0.01989069	0.24851945
Nupl1	0.33663652	6.08892636	6.39852597	0.01997575	0.24918085
Coro7	-0.5390915	4.16065774	6.39794545	0.01998066	0.24918085
Kcnh4	-1.0864923	1.83296226	6.39251535	0.02002665	0.24945166
Gcc1	0.43088795	4.93927588	6.39046139	0.02004407	0.24945166
Ednrb	0.59807594	3.98408025	6.38882163	0.02005799	0.24945166
Hnrnpa2b1	0.23965121	8.6580106	6.38172809	0.02011835	0.24997122
Hsdl2	0.41562718	6.17222768	6.36966273	0.02022147	0.25102076
Rab30	-0.4063296	4.8877884	6.36229303	0.02028475	0.25157423
Impdh2	0.44283264	3.73965062	6.35519285	0.02034593	0.25210062
Pdlim1	0.61980155	3.85893549	6.35117642	0.02038063	0.25229825
Itpk1	0.475828	3.82827909	6.34720954	0.02041497	0.25249103
Spin2c	0.69384901	2.93583366	6.34171723	0.02046261	0.25254954
Clip2	-0.6617613	3.95451113	6.34051916	0.02047302	0.25254954
Msl1	-0.271527	8.01020206	6.33813296	0.02049377	0.25254954
Rnf44	-0.2595511	6.73438254	6.33801874	0.02049477	0.25254954
Cstad	-0.7667306	2.55510493	6.33365924	0.02053274	0.25258098
Zgpat	0.9375652	1.82987523	6.33341654	0.02053486	0.25258098
Tmem71	2.33656323	0.03806179	6.31968954	0.02065497	0.25351323
Il6st	0.40671732	7.38933824	6.31897119	0.02066128	0.25351323
Rffl	-0.6641104	3.08964374	6.31654703	0.02068258	0.25351323
2700038G22	-1.5750243	-0.1344479	6.31193865	0.02072314	0.25351323
Gpi1	0.30455473	7.06928865	6.31164043	0.02072576	0.25351323
Rgs9bp	-2.7313901	-0.2906914	6.31071488	0.02073392	0.25351323
Mxra7	-0.7490033	2.78163918	6.30973863	0.02074253	0.25351323
Zyx	0.3483775	5.44226379	6.30755582	0.02076179	0.2535184
Scg3	0.51686105	6.66561104	6.29729477	0.02085262	0.25439657
Fhl1	0.31024197	7.70582581	6.29426638	0.02087951	0.25439741
Napsa	-2.7276824	-0.6509732	6.29303007	0.0208905	0.25439741
Slc30a7	-0.4645522	4.66352327	6.2885927	0.02092999	0.25440997
Relb	1.09886166	0.4167856	6.28826994	0.02093287	0.25440997
Fzd9	-2.3715409	-0.7379368	6.28654482	0.02094825	0.25440997
Pld6	-3.4006417	-1.921622	6.28298121	0.02098006	0.25456654
Hsd17b10	0.4634177	4.46645797	6.27504654	0.02105108	0.2551982
Cybb	-1.7037662	0.85518632	6.27232294	0.02107552	0.25526452
Trem2	1.90915883	-0.4979594	6.26798208	0.02111454	0.25550715
Mir32	6.22617593	-1.9347568	6.4498731	0.02121447	0.25631536
Rbpms2	0.87137948	2.82537584	6.2563586	0.02121943	0.25631536
Sppl2b	-1.0278459	1.50331952	6.25300931	0.02124976	0.25645152
Ywhab	0.25795699	10.3409979	6.25005716	0.02127653	0.25654457
Tmem260	-0.5584149	3.77287118	6.24583827	0.02131486	0.25662538

Rsl24d1	-0.4473513	5.39693246	6.2451221	0.02132137	0.25662538
Il7r	-1.9388404	-0.0123462	6.24131585	0.02135603	0.2567272
Slc19a2	-0.5924145	3.68181895	6.23868958	0.02137999	0.2567272
Slc35e1	-0.4578611	4.92752732	6.23791334	0.02138707	0.2567272
Gpr116	-0.8886599	4.84174366	6.2277945	0.02147967	0.25711758
Ubiad1	-0.8038913	1.89560882	6.22646934	0.02149183	0.25711758
Mfap1b	0.28837619	6.89563488	6.22136473	0.02153875	0.25711758
Atp13a5	0.48301987	4.94737896	6.219185	0.02155881	0.25711758
Ssh2	-0.3445963	6.53010038	6.2157026	0.02159092	0.25711758
Kcnh3	-0.4931151	3.65536925	6.21540151	0.0215937	0.25711758
Slc4a9	-5.3098371	-1.3931945	6.21524091	0.02159518	0.25711758
Ttl	-0.303961	5.9524659	6.21459739	0.02160112	0.25711758
Urm1	-0.7490384	2.19931317	6.21385685	0.02160796	0.25711758
Tapbpl	-0.4815766	3.28314003	6.21329195	0.02161317	0.25711758
4930539N22	-2.1231109	0.71910845	6.2114957	0.02162978	0.25711758
Ifi204	1.76275337	-0.3667039	6.20460021	0.02169363	0.25724701
Hmgxb4	-0.3620014	5.2954056	6.20447576	0.02169479	0.25724701
Ppp1r9b	-0.3346767	6.66886217	6.20412803	0.02169801	0.25724701
Prlr	-0.6230506	2.37048023	6.19929074	0.02174295	0.25755283
G630090E17	-4.9777489	-1.1328509	6.19486214	0.02178418	0.25760248
4930447C04I	-1.1907413	2.03600287	6.19220871	0.02180893	0.25760248
Rims2	-0.5019329	6.98291186	6.19022971	0.0218274	0.25760248
Thap4	0.52448778	4.20500576	6.1891151	0.02183782	0.25760248
Pdzrn3	0.31098711	7.30350977	6.18689871	0.02185854	0.25760248
Arhgap18	0.39967232	5.25575095	6.18555417	0.02187112	0.25760248
Mllt4	0.25573556	7.44020408	6.18411823	0.02188457	0.25760248
Ccdc134	-0.9600587	0.62097452	6.17951556	0.02192774	0.25760248
Pdgfc	-0.7038009	2.48770178	6.17904692	0.02193214	0.25760248
Apobec1	0.64750312	3.35042293	6.17836147	0.02193858	0.25760248
Hace1	-0.3598267	5.00898893	6.15966527	0.02211501	0.25919767
Zswim5	-0.4008516	4.89871036	6.15602136	0.02214958	0.25919767
Snrnp70	-0.4467682	5.73712397	6.15471676	0.02216197	0.25919767
Pcdhga1	0.92568405	1.75969594	6.15395807	0.02216918	0.25919767
Adrbk2	-0.4636057	5.20441657	6.15379406	0.02217074	0.25919767
Khsrp	-0.333191	6.76865432	6.15090915	0.02219819	0.25929323
4930404H11I	3.71728846	-1.2410107	6.14527341	0.02225191	0.25963506
Ptgfrn	-0.3618179	5.24899347	6.14210634	0.02228216	0.25963506
Nrk	1.07807767	1.47613572	6.14177455	0.02228533	0.25963506
Plekhg4	1.54185663	1.36026031	6.13524579	0.02234786	0.26013825
R3hdm1	-0.4254948	9.58790115	6.12812087	0.02241631	0.2607096
Cyp4a12a	-1.533601	0.33010548	6.12159788	0.02247919	0.26121516
Cc2d1a	-0.7246074	2.91803253	6.11883596	0.02250588	0.26129959
Mtfmt	-0.6640181	3.0333309	6.11680057	0.02252557	0.26130272

Fbxl13	4.18536584	-0.9624995	6.11024027	0.02258915	0.26137516
Ggh	0.70672272	3.23462189	6.10906065	0.02260061	0.26137516
Yes1	0.41526228	4.78123407	6.1088133	0.02260301	0.26137516
Zfp59	-0.5901717	3.33000166	6.10770219	0.02261381	0.26137516
Ifitm6	1.68831029	0.49823614	6.1052466	0.02263769	0.26137516
Acss1	-0.4672604	3.75449704	6.10244494	0.02266498	0.26137516
Tle6	-1.5335207	0.31341144	6.10215735	0.02266778	0.26137516
Capzb	0.39962821	8.59633439	6.09657838	0.02272223	0.26177867
Tmem163	0.62342602	2.75960425	6.08621277	0.02282378	0.26252989
Parp6	-0.3920065	5.88867392	6.08594079	0.02282645	0.26252989
9330182L06F	-0.4553977	5.05696034	6.08162505	0.02286889	0.26264669
Serpinb8	0.7245868	3.40522974	6.07475055	0.02293667	0.26264669
A830009L08I	-1.0829991	0.87970727	6.07472129	0.02293696	0.26264669
Zfp619	-0.5803871	2.83341509	6.07459794	0.02293818	0.26264669
Rbm11	0.56787505	3.26912102	6.07286488	0.02295531	0.26264669
1700008I05R	3.39866077	-1.3148425	6.07184835	0.02296536	0.26264669
4933439C10I	-0.9362188	1.82049563	6.07105184	0.02297324	0.26264669
Gramd3	0.49355324	4.51568435	6.06477348	0.02303545	0.26305031
Samd4b	0.3189021	6.83146274	6.0623445	0.02305957	0.26305031
Rcan2	-0.2521235	7.45083841	6.06157828	0.02306719	0.26305031
Psmd12	0.28227513	6.71043969	6.05637835	0.02311894	0.26341723
Ahrr	3.57044919	-1.6587342	6.04236765	0.02325903	0.26421506
Acot4	-1.8852364	0.0646272	6.04158306	0.0232669	0.26421506
Ciita	-0.9491885	1.62985973	6.04051025	0.02327767	0.26421506
Dnah11	-1.8413192	-0.3051368	6.03875696	0.02329529	0.26421506
AF357425	-1.3130391	3.01202693	6.03690359	0.02331392	0.26421506
Anks1	-0.4648546	3.49404808	6.03572718	0.02332576	0.26421506
Taf15	0.41664342	5.09488274	6.035663	0.02332641	0.26421506
Rin2	0.39576948	6.55233572	6.03232968	0.02335998	0.26437287
Zpr1	0.4000505	4.3602774	6.02762249	0.0234075	0.26454125
Filip1l	-0.3138987	5.30520595	6.02608024	0.02342309	0.26454125
2900052N01	-0.7767372	2.81930191	6.02267224	0.02345758	0.26454125
Ttc9	-0.4230631	4.23542392	6.02029632	0.02348166	0.26454125
Ppifos	3.18026084	-1.5463159	6.01687	0.02351644	0.26454125
Ncln	-0.8198934	2.44640941	6.016533	0.02351986	0.26454125
Uqcrfs1	0.26973147	6.5995679	6.01559089	0.02352943	0.26454125
LOC106740	-0.5318284	4.1747146	6.01383584	0.02354728	0.26454125
Psmd4	0.44116425	5.3258604	6.01339188	0.0235518	0.26454125
Mre11a	0.84918956	2.75621419	6.00959823	0.02359043	0.26475423
Slc26a8	-1.4252343	0.88939182	5.99795101	0.0237095	0.26586879
Fam179a	-2.5795523	-0.4688586	5.9930249	0.02376006	0.26621394
Krt9	0.48543389	3.95812675	5.99076012	0.02378335	0.26623357
Zfp286	-0.6937704	3.27813945	5.98900784	0.02380139	0.26623357

Zbtb3	2.42674973	-0.3909106	5.98680807	0.02382405	0.26626573
5031425F14I	3.93182437	-2.2204453	5.98141454	0.02387972	0.26666641
0610009L18F	-0.9111888	0.8437656	5.97667757	0.02392873	0.26674177
Trim35	0.29109351	6.9522191	5.97538536	0.02394212	0.26674177
Bclaf1	0.25441591	9.51920161	5.97501741	0.02394593	0.26674177
Grip2	-0.9084853	2.18847827	5.96992212	0.02399882	0.26678171
Fibcd1	-1.6852872	0.43586962	5.96857389	0.02401284	0.26678171
Dhrs11	1.54586777	0.67137026	5.96768791	0.02402206	0.26678171
Ecsit	0.37947767	3.81187568	5.96703762	0.02402882	0.26678171
Mpzl1	-0.5626857	3.20946085	5.9646624	0.02405356	0.26679812
Hps1	-1.0569279	1.40435611	5.96308935	0.02406995	0.26679812
B3gntl1	-0.5865775	2.62906946	5.96109131	0.0240908	0.26680939
Atp6v0d2	2.98888733	0.3918407	5.95637723	0.02414006	0.2671351
Tnip3	1.18635141	1.79841068	5.94551972	0.02425395	0.26797151
Timp2	0.39504546	8.97279797	5.94351513	0.02427504	0.26797151
Tanc2	-0.5005794	8.5132507	5.94348275	0.02427539	0.26797151
Abcb6	1.27955998	1.61675515	5.94153105	0.02429594	0.2679786
Zw10	0.92467861	2.49338319	5.93737844	0.02433975	0.26803088
Atp1b3	0.51429605	8.8426417	5.93666654	0.02434727	0.26803088
Tex35	3.81583644	-1.7940402	5.9331466	0.02438448	0.26803088
Vamp1	-0.319133	6.0370303	5.9327245	0.02438895	0.26803088
Kcnj16	-0.5251471	4.19006785	5.93165423	0.02440028	0.26803088
Ccl6	-1.507763	0.33841368	5.92880824	0.02443043	0.26814326
Dyrk1b	-0.548004	3.09395663	5.92262272	0.02449612	0.26843082
Tpx2	-0.9264937	1.79765562	5.9225844	0.02449653	0.26843082
Adgb	3.06054628	-0.9356772	5.92036946	0.0245201	0.26847049
Sbds	0.32829377	6.7129318	5.91734412	0.02455234	0.26860489
Hyou1	0.48476786	5.17947452	5.91143003	0.02461549	0.26907705
Ostf1	0.41216291	5.90732594	5.90468869	0.0246877	0.2694486
Serpinb10	-1.9754982	-0.6672712	5.90451855	0.02468953	0.2694486
Zmiz1	0.31035186	8.34258388	5.90201907	0.02471637	0.2695229
Esr1	1.19929959	1.10288743	5.8991517	0.02474719	0.26964055
Il1rapl1	-0.4800305	3.91836958	5.89154504	0.02482919	0.27031503
Cxcl14	-0.4827315	3.20959099	5.8840531	0.02491024	0.27097822
Adam9	0.33978059	4.9569587	5.87681465	0.02498883	0.27161361
Gss	0.60290262	3.45881164	5.87049109	0.02505772	0.27194168
9430076C15I	3.1815632	-1.4963144	5.86880986	0.02507607	0.27194168
4930509J09F	-2.2744191	-0.3195613	5.86291268	0.02514056	0.27194168
D5Ertd579e	-0.3323754	7.30516997	5.86231214	0.02514714	0.27194168
Lekr1	-0.6739325	2.74701292	5.85947463	0.02517825	0.27194168
Ccdc160	0.8876086	1.8488038	5.85851495	0.02518878	0.27194168
Mex3b	-0.5707801	3.37780557	5.85686656	0.02520688	0.27194168
3110001I22R	1.0164428	1.10874887	5.85545181	0.02522242	0.27194168

T2	-1.5844651	-0.1876592	5.85500417	0.02522734	0.27194168
Slc24a4	0.8803559	2.83603818	5.8536122	0.02524265	0.27194168
Pabpc4	0.39732896	3.89769323	5.85143672	0.0252666	0.27194168
Bcor	0.33506557	5.37892961	5.84971919	0.02528552	0.27194168
Ubc	0.36313691	6.71051976	5.84899801	0.02529347	0.27194168
Efemp1	0.60787482	7.48332412	5.84822998	0.02530194	0.27194168
Tbx22	-3.2169725	-1.4582454	5.84560028	0.02533097	0.27203637
Dzip1l	-0.4965685	3.47305147	5.83188262	0.025483	0.27322344
Dusp26	-0.3320911	6.07584242	5.83145816	0.02548772	0.27322344
BB031773	2.90265035	-1.0355317	5.82927993	0.02551196	0.27322344
Bbs9	0.3379239	5.07388828	5.82831308	0.02552273	0.27322344
Fzd1	0.51986376	6.13564224	5.82306829	0.02558122	0.273632
Dlgap1	-0.4246402	8.83633306	5.81655685	0.02565406	0.27419316
Brca1	-1.1901994	1.08808677	5.80885918	0.02574047	0.27489836
Rps15a-ps6	-0.6241638	2.45714199	5.8054857	0.02577844	0.27508557
Mpl	-1.3616108	1.52011049	5.80357363	0.02579999	0.27509737
Spast	0.29084984	6.53538806	5.80120371	0.02582673	0.27516444
Gars	0.27636475	6.01948736	5.79899754	0.02585165	0.27521204
Rxfp3	1.64494246	1.2141781	5.79209338	0.02592981	0.2757893
Hpcal1	0.44071848	4.60151312	5.78946874	0.02595959	0.2757893
Pnp	0.43086753	4.58401959	5.78759062	0.02598093	0.2757893
4930512B01	-1.3729738	0.91539927	5.78698118	0.02598786	0.2757893
Adam1a	-1.4987894	1.28166211	5.78206071	0.02604386	0.27595861
Tmem86b	1.28658738	0.33394382	5.78197623	0.02604483	0.27595861
Etv3	0.37969347	3.91685719	5.77792389	0.02609106	0.27623093
Hnrnph3	0.35314719	4.49209724	5.77263277	0.02615156	0.2766538
Il4ra	1.55631565	0.15441314	5.76757297	0.02620956	0.27704961
Asprv1	-1.5383299	0.50430577	5.76428327	0.02624735	0.27713615
Dlat	0.29877	8.21845619	5.75986463	0.02629821	0.27713615
Fyttd1	0.30703573	7.36104306	5.75984816	0.0262984	0.27713615
Gadd45b	0.604447	2.14979483	5.75871404	0.02631147	0.27713615
Eml6	-0.5535495	4.31941007	5.75615787	0.02634095	0.27713615
Egfl6	1.51508439	0.88646447	5.75427609	0.02636269	0.27713615
Smad3	0.34280858	6.75745112	5.7537474	0.02636879	0.27713615
Evl	0.49383677	4.22198237	5.75256094	0.02638251	0.27713615
Pbx3	0.50190685	7.274062	5.74931985	0.02642002	0.27731367
Lrrc8b	-0.3982557	5.96179813	5.74718379	0.02644477	0.27735714
Tacc1	0.25309982	7.88929376	5.74336608	0.02648908	0.27754564
Zfp217	0.7024246	3.61460677	5.74036309	0.02652399	0.27754564
Cnppd1	-0.5572316	4.12454066	5.74030852	0.02652462	0.27754564
Fancb	-1.6306884	0.97425989	5.73477881	0.02658904	0.27800352
3110045C21	-2.655603	-0.5811746	5.73295688	0.0266103	0.27800983
Tchp	-0.852328	2.32131566	5.72916351	0.02665464	0.27825697

Strip2	0.64632432	5.1919143	5.72140862	0.02674553	0.27849151
Fam216b	-1.1875207	1.06230103	5.72068317	0.02675405	0.27849151
Ctsa	0.44583288	5.4161235	5.71730986	0.02679371	0.27849151
1810058I24R	0.54699968	4.48426675	5.71544856	0.02681562	0.27849151
Cyp2j6	0.4670139	4.00812163	5.71523234	0.02681817	0.27849151
Tmem214	0.45923475	4.44956222	5.71402838	0.02683235	0.27849151
Ppp2r1a	0.25578882	7.25493951	5.71292853	0.02684532	0.27849151
Gpr137	0.58198292	3.18893614	5.71157724	0.02686126	0.27849151
Ccdc153	-0.8640295	1.80355475	5.71038122	0.02687538	0.27849151
Esr2	-1.122685	0.69385445	5.70861047	0.0268963	0.27849151
Gatad2b	0.25471494	7.58803378	5.70678683	0.02691786	0.27849151
Kctd1	-0.2919249	6.47743231	5.70589315	0.02692843	0.27849151
Pip5k1c	-0.3406947	6.37559619	5.7043962	0.02694615	0.27849151
Fam196b	-0.9170236	1.52133932	5.69884116	0.02701203	0.2786088
Fzd4	-0.750021	2.98805303	5.69724273	0.02703102	0.2786088
Cml3	-0.8792489	1.66790898	5.69598462	0.02704597	0.2786088
Bcl7b	-0.4209618	4.11000641	5.69427333	0.02706633	0.2786088
Atp5c1	0.32813339	8.18712895	5.69316154	0.02707957	0.2786088
Gins3	1.13817422	1.21726451	5.69298023	0.02708173	0.2786088
Dock2	-0.6625395	2.3581505	5.6880858	0.02714009	0.27880166
1700094D03I	-1.0616442	1.92607072	5.68649643	0.02715907	0.27880166
Trim11	-0.5692322	2.75592502	5.68610666	0.02716373	0.27880166
Camk2d	0.31764787	7.31796937	5.68446552	0.02718335	0.27880166
Slc38a4	1.10389091	1.56652704	5.67195632	0.02733343	0.28009935
Id4	-0.3972052	5.33668979	5.67045465	0.02735151	0.28009935
L1td1	-0.6340964	2.81831236	5.66640534	0.02740033	0.2803859
Nudt3	-0.3882994	6.12764947	5.66207537	0.02745264	0.28056432
Nyap1	-0.624105	2.96489102	5.66017315	0.02747565	0.28056432
Arl6ip5	0.41569723	3.89534564	5.6589124	0.02749092	0.28056432
Hspa8	0.26417315	10.7212684	5.65806696	0.02750116	0.28056432
Cd37	2.11356967	-0.4761001	5.65175722	0.02757774	0.28060428
Acta2	-0.6673353	3.71239408	5.65117083	0.02758487	0.28060428
Isg20	0.85108225	1.67087983	5.65114003	0.02758525	0.28060428
Ogfrl1	-0.3189249	7.93342895	5.65015299	0.02759725	0.28060428
Gltpd1	-0.4240093	3.73848941	5.64915945	0.02760934	0.28060428
Ddah1	0.33302571	7.1291285	5.63515318	0.02778042	0.28190041
Bcl3	1.08183768	0.8728901	5.63359968	0.02779947	0.28190041
A930012L18I	-0.7755588	2.57284962	5.63212501	0.02781757	0.28190041
Rnf181	0.39267561	6.26670878	5.63187222	0.02782067	0.28190041
Stbd1	0.47055451	4.08856523	5.62879919	0.02785843	0.28207059
Hecw2	-0.5054221	6.36028967	5.62401116	0.02791737	0.28245487
Abhd17c	-0.3713779	5.2820418	5.61937017	0.02797464	0.28282163
Nptn	0.32109742	8.10613287	5.61460557	0.02803357	0.2830469

Mtap7d3	-0.9592197	1.90900847	5.61416791	0.02803899	0.2830469
Qprt	1.00670567	1.21650241	5.60683209	0.02813002	0.28375293
Coil	0.5986189	3.23445408	5.60374421	0.02816843	0.2839276
Mcm3ap	-0.5706494	4.45572644	5.60104085	0.02820211	0.28404298
Gm1123	-3.6905497	-2.3133754	5.59866649	0.02823173	0.28404298
Slc35b4	-0.317129	5.64591633	5.59770493	0.02824374	0.28404298
Sash1	0.30104561	6.31869525	5.59605802	0.02826431	0.28404298
Rrs1	0.33391387	4.79496528	5.58989822	0.02834142	0.28460531
Wdr81	-0.6429734	3.02157766	5.58700085	0.02837777	0.28475783
Dgka	0.40593952	4.79442651	5.58152092	0.02844666	0.28523646
Acsl1	-0.3072796	6.51345693	5.57193636	0.02856762	0.28589325
Sst	0.42196489	6.90303756	5.57158624	0.02857205	0.28589325
Crip2	0.35313773	4.55658148	5.57128136	0.02857591	0.28589325
F730043M19	-1.0818308	0.97539026	5.5631983	0.0286784	0.28651852
Ankrd1	-2.3015275	-1.3890658	5.56299431	0.02868099	0.28651852
Strn4	-0.333909	5.44574804	5.55929416	0.02872805	0.28676756
Slain1os	-1.3752159	1.00212883	5.55613204	0.02876833	0.28676756
Nkain1	-0.3721088	4.40543864	5.55438661	0.0287906	0.28676756
A630001G21	-1.4800449	0.62575873	5.55434247	0.02879116	0.28676756
6230400D17	-1.669505	0.25397357	5.55101394	0.02883367	0.28682377
Ttr	-0.8930403	5.00354118	5.55045412	0.02884083	0.28682377
Nid1	0.41489103	7.06377941	5.54710065	0.02888375	0.28682377
Psmc1	0.31195601	6.37600681	5.54563427	0.02890254	0.28682377
Trappc3	0.36938746	5.00076725	5.54556837	0.02890338	0.28682377
Hnrnpll	0.29456395	6.34844981	5.54020689	0.0289722	0.2872948
Zfp28	-0.6637776	2.80136131	5.53644736	0.02902056	0.28737398
Kctd21	0.47769303	3.31513064	5.5338779	0.02905367	0.28737398
Alas1	0.34444847	4.46677621	5.5335934	0.02905734	0.28737398
Grem2	0.3773601	5.2695346	5.53295219	0.02906561	0.28737398
Sptlc2	-0.3299134	5.09740509	5.53094871	0.02909146	0.28741845
Dcp1a	0.32535102	5.44539344	5.52783279	0.02913173	0.28760509
Cep83os	-0.3551096	5.37662296	5.52380987	0.02918381	0.28783933
Mast3	-0.3474333	7.30129697	5.52269665	0.02919823	0.28783933
Prdm1	1.02437477	2.00380264	5.51720049	0.02926959	0.28833158
Ankrd34c	-0.4716595	4.32065125	5.50805498	0.02938877	0.28929381
Zfp281	0.34894302	6.16581119	5.50584726	0.02941763	0.28931937
Ccdc171	0.54899589	3.20507222	5.50265149	0.02945945	0.28931937
Cx3cr1	-0.6475001	3.35088241	5.50087109	0.02948277	0.28931937
B230217O12	-0.800058	1.77765777	5.50079074	0.02948383	0.28931937
Vpreb3	3.01621746	-1.2330033	5.49964339	0.02949887	0.28931937
Gzmb	2.39475943	-0.3237874	5.49413504	0.02957122	0.28981774
P2ry14	-0.9341879	1.47512137	5.48870172	0.02964279	0.29019889
1700028J19	-2.1525071	-1.2807606	5.48754047	0.02965811	0.29019889

Eif3m	0.29234704	6.20702064	5.48627491	0.02967481	0.29019889
Urb1	-0.6928734	2.76951926	5.48429155	0.02970102	0.29024421
Pafah1b3	0.95226999	2.03369673	5.48151519	0.02973774	0.2903922
Slc30a1	0.31994572	5.11534121	5.47781489	0.02978677	0.29066001
Scrg1	1.25924574	1.44732958	5.47487431	0.02982579	0.29082992
Mgat4b	-0.5944301	2.87867883	5.47150643	0.02987056	0.2908653
Gm15910	-0.6070583	4.50043733	5.47126375	0.02987379	0.2908653
Baiap2	0.33313649	6.25207541	5.46972513	0.02989427	0.2908653
Capn5	0.33184532	5.56500213	5.46433282	0.02996616	0.29135419
Tcp11l2	0.35781901	4.28628694	5.46203076	0.02999692	0.29144262
Prmt8	-0.3195944	7.02981199	5.45901781	0.03003722	0.29162366
Ect2l	-2.5150223	-0.6733218	5.44862531	0.03017672	0.29235109
Tpst1	-0.3889977	5.01559497	5.44849528	0.03017847	0.29235109
Gls2	0.7681794	2.43157506	5.44491912	0.03022664	0.29235109
Dock5	0.30389485	6.84778138	5.4440219	0.03023874	0.29235109
Ccdc129	1.55567009	-0.2345757	5.44324469	0.03024923	0.29235109
Ankrd10	-0.4619096	3.86599986	5.44305229	0.03025182	0.29235109
Gpr55	1.73287588	-0.1849099	5.44213317	0.03026423	0.29235109
Bard1	0.89861085	1.56742693	5.43652469	0.03034006	0.29287338
Appl2	-0.31147	6.30559206	5.43056825	0.03042083	0.2933282
Asap2	-0.3832183	6.25243567	5.42912747	0.03044041	0.2933282
Ap5s1	0.74290598	2.35698614	5.42823251	0.03045257	0.2933282
Frmd8	0.4382622	3.95943943	5.42201166	0.03053729	0.2938484
0610043K17I	-0.7008456	1.52594547	5.41881245	0.03058096	0.2938484
Kazn	-0.345078	6.12045193	5.41763747	0.03059702	0.2938484
Map2k5	-0.5282891	3.60652918	5.41383867	0.030649	0.2938484
BC048546	-0.379031	5.10503088	5.41130983	0.03068366	0.2938484
Lin7a	-0.3084275	7.6292927	5.41122737	0.03068479	0.2938484
Cenpj	-0.4415798	4.00138366	5.41119924	0.03068517	0.2938484
Epm2a	1.05675038	1.61414237	5.41116317	0.03068567	0.2938484
Tcf24	1.21058427	1.26987938	5.40989131	0.03070312	0.2938484
Iqsec1	-0.4480322	7.84159001	5.40589268	0.03075805	0.2941649
Gaa	0.41799803	5.861557	5.40263197	0.03080292	0.29438485
Trim17	1.16874534	0.58160471	5.39808215	0.03086566	0.29446411
Ap4m1	-1.0707008	1.14471779	5.39778239	0.0308698	0.29446411
Syt5	0.38397918	5.40819515	5.39727098	0.03087686	0.29446411
Rab15	-0.2743052	6.31468885	5.39387012	0.03092387	0.29470357
Thumpd3	0.37966998	4.77480916	5.39196918	0.03095019	0.29470612
Pgr	-0.4030371	5.93249371	5.38915589	0.03098917	0.29470612
Prom1	-0.774829	2.67292899	5.38910749	0.03098985	0.29470612
Snx1	0.30581524	5.72601252	5.38077121	0.0311057	0.29543238
Srp68	0.3277266	5.94571397	5.3804536	0.03111012	0.29543238
Fgf23	-2.9645644	-1.0142725	5.37683507	0.03116057	0.29570278

Calm2	-0.2424768	12.25381	5.37418722	0.03119755	0.29584503
Glt8d2	0.48899417	3.02758039	5.36714097	0.03129619	0.29653392
Col6a3	0.72459899	3.31318191	5.36438354	0.03133488	0.29653392
Jam3	-0.6626217	2.89043088	5.36428224	0.0313363	0.29653392
Pogz	-0.2993035	7.70921291	5.36102574	0.03138208	0.29675837
Klhl3	0.68900441	2.05259147	5.35774841	0.03142822	0.29698598
2810468N07	-0.6157457	2.59220216	5.35594513	0.03145364	0.29701762
Mpeg1	-0.7836934	2.86253962	5.35329642	0.03149102	0.29716207
Pitpna	0.22980102	8.72835352	5.34989414	0.03153911	0.29722527
Ncald	0.28175888	7.7043195	5.34917983	0.03154922	0.29722527
Pink1	-0.2422312	7.52866259	5.34772681	0.03156979	0.29722527
Clcn3	-0.3095785	6.71782553	5.34397495	0.03162297	0.29722527
Nfkb2	1.56925006	0.95481219	5.34266724	0.03164153	0.29722527
Zdhhc2	-0.326028	5.21262082	5.34221872	0.0316479	0.29722527
Abca8a	-0.720402	2.39900376	5.34190672	0.03165233	0.29722527
Meg3	-0.6082676	10.8517456	5.33949065	0.03168667	0.29734024
Plagl2	-0.4264862	5.17042877	5.33482336	0.03175313	0.29764092
Col6a4	2.16455587	-0.2166068	5.33413417	0.03176295	0.29764092
Sdf4	-0.2470346	7.98580178	5.33053117	0.03181438	0.29791537
Hey2	-0.6767046	2.26917077	5.32840168	0.03184482	0.2979846
Snx13	-0.2345837	6.88848207	5.32655663	0.03187122	0.2979846
Pappa2	0.8999303	2.28047774	5.3253703	0.03188821	0.2979846
Med8	-0.3534221	3.81984149	5.32168397	0.03194106	0.29827136
Cml5	1.33297141	0.85791997	5.31866007	0.03198449	0.29846978
E330011O21	-1.6556251	-0.1778107	5.31424871	0.03204797	0.29860143
Rarres1	2.86812082	-0.6069795	5.31319599	0.03206313	0.29860143
Cdk18	0.54506706	4.00573225	5.31232822	0.03207564	0.29860143
Pcdhb15	-0.7136906	2.72245539	5.31151585	0.03208736	0.29860143
Fbrsl1	-0.42588	4.62818827	5.30570215	0.03217135	0.29877976
Mamld1	0.34516707	6.24014348	5.30520002	0.03217861	0.29877976
Cxx1c	0.30687423	5.91556429	5.30441474	0.03218998	0.29877976
Luzp2	0.41871915	6.55836174	5.3025728	0.03221666	0.29877976
Snta1	0.6194216	2.78513284	5.29867407	0.03227321	0.29877976
Kcnip3	0.24729887	7.64498816	5.2981708	0.03228052	0.29877976
Fmnl2	0.31308874	8.09144302	5.29810541	0.03228147	0.29877976
Tnfrsf1b	-1.3272374	1.11957083	5.29705578	0.03229672	0.29877976
Dusp27	1.36248514	1.8053028	5.29608022	0.0323109	0.29877976
Wdr45b	-0.3915096	4.47916037	5.29348866	0.0323486	0.29877976
Iqcj	-2.6168597	-1.5324239	5.29222194	0.03236705	0.29877976
Cd74	-0.7913702	6.73494248	5.29181579	0.03237297	0.29877976
Flna	0.37372854	5.69101565	5.28552735	0.03246474	0.29927996
H2-Aa	-0.9251848	4.46773061	5.28342268	0.03249552	0.29927996
Ezh1	-0.4102009	5.19034021	5.28217262	0.03251382	0.29927996

Sox7	-1.2286119	0.68090057	5.28108801	0.03252971	0.29927996
Leprel1	-0.9925352	2.42918779	5.28049684	0.03253837	0.29927996
Lrrc4	-0.3616516	5.96828391	5.26591803	0.03275281	0.30099865
Nrgn	-0.2964904	7.23116509	5.26465502	0.03277147	0.30099865
Pcdh20	-0.6423209	3.064212	5.26324322	0.03279233	0.30099865
Pnmal1	0.53780106	3.26626482	5.25699786	0.03288481	0.30164177
Pgd	0.44581435	5.24899362	5.24782873	0.03302113	0.30268578
Oscar	1.77802152	0.10059127	5.23919785	0.03315002	0.30366043
Cgnl1	0.38824684	6.07090284	5.2346029	0.03321887	0.30405929
Pmpca	-0.2623159	6.05823535	5.2332782	0.03323876	0.30405929
Dmrtc1a	1.03549957	0.89570495	5.22778015	0.03332141	0.30426653
Cpped1	0.29108742	6.46665598	5.2274519	0.03332635	0.30426653
Fam188a	-0.3508584	6.11642959	5.22725985	0.03332924	0.30426653
Dkk3	0.36164026	8.69595645	5.22573585	0.0333522	0.3042697
Tgif1	0.63338827	2.7617842	5.22404212	0.03337774	0.30427339
Slc6a17	0.30021285	8.25018407	5.2227105	0.03339783	0.30427339
Kcnh7	-0.4474535	6.28135969	5.2188065	0.03345682	0.30442257
Caap1	-0.5226848	2.76923956	5.2174367	0.03347754	0.30442257
Bves	-1.666799	-0.6052803	5.21713718	0.03348207	0.30442257
Ddost	0.37736154	4.27040541	5.21291365	0.03354608	0.30479244
Erich1	0.60344348	2.96128345	5.21146516	0.03356806	0.30479244
Thsd1	-1.6584844	0.40548521	5.20837773	0.03361496	0.30501254
Zfp772	0.47397431	4.258458	5.20624244	0.03364745	0.30510157
Emc9	0.63312506	2.43498504	5.19597324	0.03380418	0.30604886
Frk	0.54521981	4.02607557	5.19179669	0.03386815	0.30604886
Zfyve27	-0.3992308	4.26992673	5.1916805	0.03386993	0.30604886
Rbm20	-0.7062498	2.41221173	5.189286	0.03390668	0.30604886
Stat1	0.36449921	5.50301959	5.1891167	0.03390928	0.30604886
Snca	-0.2671835	7.68402649	5.18718291	0.03393899	0.30604886
Sema3f	-1.2601253	0.80992883	5.18643012	0.03395056	0.30604886
Rragb	0.3301807	4.62507117	5.18599399	0.03395727	0.30604886
AI854517	-0.4661636	3.70653765	5.1850187	0.03397227	0.30604886
Clpx	0.30462766	5.3907089	5.18383955	0.03399042	0.30604886
Phykpl	0.56813282	3.26191407	5.18200137	0.03401874	0.30604886
Pkd1l3	-1.1307929	0.8831859	5.17999737	0.03404964	0.30604886
Rab3il1	0.52150291	4.12135723	5.17807927	0.03407925	0.30604886
Uba5	0.28789987	5.87438216	5.17722175	0.03409249	0.30604886
Armc8	0.30247364	7.05120599	5.17718399	0.03409308	0.30604886
Enpp2	-0.3336915	8.24836656	5.16632713	0.03426129	0.30694214
Cmtr1	-0.4958644	5.59587889	5.1608394	0.03434667	0.30694214
Gatsl2	-0.2996485	6.17021355	5.15944113	0.03436846	0.30694214
Pogk	-0.2544718	6.21945239	5.15866952	0.03438049	0.30694214
Flnc	0.79385254	2.89884862	5.1580758	0.03438976	0.30694214

Tekt5	-1.4484919	0.32940631	5.1578466	0.03439333	0.30694214
1700003M02	-1.4713219	0.2590926	5.15688663	0.03440832	0.30694214
Tmem132c	-0.6476625	2.32488406	5.15534479	0.0344324	0.30694214
Cenpk	1.20858061	1.17615186	5.15475945	0.03444154	0.30694214
Ltbp3	0.47574075	4.64029279	5.15430891	0.03444859	0.30694214
Ppp1r1b	0.48173447	5.39614067	5.15139261	0.03449421	0.30694214
Ube2j1	0.3120093	7.45386094	5.15044757	0.03450901	0.30694214
Vsx2	3.9615378	-1.7368724	5.14721398	0.0345597	0.30694214
Hrh2	-0.8213786	2.16821105	5.14571672	0.0345832	0.30694214
2610507I01R	-0.3347911	4.73203653	5.14424287	0.03460635	0.30694214
Lrrfip1	0.31208234	5.98272944	5.14407683	0.03460896	0.30694214
Sat2	0.63937799	2.12702867	5.14367045	0.03461535	0.30694214
Skap2	-0.420898	4.03246123	5.14330883	0.03462104	0.30694214
Exosc2	-0.493098	3.39289145	5.14299424	0.03462598	0.30694214
Cacnb4	-0.3158039	9.03744874	5.13943609	0.03468198	0.30723615
Ythdc1	0.2844581	7.24314683	5.13561954	0.03474216	0.30723697
Dnajb5	0.39067066	5.88746748	5.13476557	0.03475564	0.30723697
Tmx3	0.35921457	5.80840114	5.13426982	0.03476347	0.30723697
Mirg	-1.5462421	1.06503662	5.13364126	0.0347734	0.30723697
Pkhd1	-2.6270504	-0.6882453	5.13089942	0.03481675	0.30723806
Krt20	-0.6574045	3.87469395	5.13074544	0.03481919	0.30723806
Galnt6	-0.9609091	1.98311809	5.12706442	0.03487749	0.30731114
Sfr1	0.3349881	6.95960474	5.12682369	0.03488131	0.30731114
Postn	-0.6252847	2.19193298	5.12441372	0.03491954	0.30731114
Ipo5	0.29200856	6.79794917	5.12304647	0.03494126	0.30731114
Ccl19	-0.5769172	3.93998028	5.12302107	0.03494166	0.30731114
Ndst4	0.67935182	3.24257886	5.1200625	0.0349887	0.30749875
Car12	0.59265031	3.31071986	5.11769698	0.03502636	0.30749875
Tmem116	-1.3223601	-0.0018565	5.1173713	0.03503155	0.30749875
Mtag2	-2.3716511	-0.504034	5.11436402	0.0350795	0.30755814
Atp1a3	0.41582497	9.48253758	5.11408039	0.03508402	0.30755814
Piga	-0.6778674	3.12116202	5.1122158	0.0351138	0.30761874
Jak2	0.27639686	6.25237217	5.11025011	0.03514522	0.30769366
Coq4	-0.4125112	3.84372595	5.1076823	0.03518631	0.30785311
Cenpi	-2.1088052	-0.5162075	5.10558389	0.03521993	0.30794703
Ppp4r1l-ps	-0.9156576	1.82378909	5.10378956	0.0352487	0.30799851
Plekhb2	0.26360669	6.30581925	5.09940595	0.03531912	0.30839587
Smad1	0.28976159	5.76445627	5.09810686	0.03534001	0.30839587
Frmd3	-0.7364855	2.67448322	5.09546986	0.03538248	0.30842059
Stx1a	-0.3690075	5.57076194	5.09508452	0.03538869	0.30842059
Pcgf3	0.30485169	5.67129604	5.09188169	0.03544035	0.30867093
Arhgap25	0.52778084	3.64359913	5.08835475	0.03549734	0.30881261
Jpx	-0.6530697	2.7866681	5.08802774	0.03550263	0.30881261

Adcy8	-0.6203413	3.85815397	5.08600129	0.03553543	0.30881261
Ica1	-0.4758858	4.14762539	5.08519942	0.03554842	0.30881261
Cstl1	1.96737261	0.25753104	5.08187225	0.03560236	0.308957
Filip1	0.51960222	3.97005883	5.08134262	0.03561096	0.308957
Gpr63	-0.615447	3.16893815	5.0771866	0.03567849	0.30930297
Jup	0.35498451	5.22506196	5.07439804	0.03572388	0.30930297
Tmem104	0.48463203	3.95676381	5.07389517	0.03573208	0.30930297
Arl5b	0.49925182	4.18578629	5.07323854	0.03574278	0.30930297
Scg2	0.3831124	6.44632555	5.06854143	0.03581943	0.30957199
Slc35f5	0.4579268	4.54118939	5.06582455	0.03586386	0.30957199
LOC1005036	0.70481186	3.67730937	5.06528291	0.03587272	0.30957199
Mon1b	0.2741805	5.95988164	5.064766	0.03588118	0.30957199
5430402O13	-1.9705322	-0.5565172	5.06314239	0.03590778	0.30957199
Ptgs2os	1.15684238	0.78329448	5.06283892	0.03591275	0.30957199
Pde12	0.52989093	3.23531426	5.05990079	0.03596093	0.30957199
Fap	-0.9896003	1.52176877	5.05914348	0.03597337	0.30957199
Dok7	-1.9499342	-0.7094101	5.0586838	0.03598092	0.30957199
Ddx3x	0.21564644	8.72107366	5.05672588	0.03601309	0.30959343
Mapk6	-0.2503081	7.5119382	5.05573252	0.03602942	0.30959343
Zfp704	-0.3145923	6.73180824	5.04862353	0.03614657	0.31020575
Arl4a	0.3865435	7.29655897	5.04861032	0.03614679	0.31020575
Syap1	0.53481939	5.92517928	5.04569394	0.03619497	0.31024928
Carm1	0.33991461	5.24535522	5.04542394	0.03619944	0.31024928
Uck1	0.44228375	3.16796925	5.043015	0.0362393	0.31024928
Kcnt2	-0.505242	5.43269242	5.04272581	0.03624408	0.31024928
Slc22a3	1.06648246	0.82570762	5.0386056	0.03631239	0.31027635
Chd3os	-0.2415762	6.56164557	5.03857037	0.03631297	0.31027635
Rit2	0.28558947	6.72674347	5.0383625	0.03631642	0.31027635
Ikzf2	0.41097562	4.23824318	5.03600395	0.03635559	0.3104139
Ranbp1	-0.2944382	5.21532742	5.03337362	0.03639933	0.31059028
Erf	-0.5658655	3.33277045	5.02751274	0.036497	0.31115977
Prmt1	0.67970877	2.8682413	5.02389256	0.03655748	0.31115977
Rrnad1	0.45604349	3.60625809	5.02385678	0.03655808	0.31115977
Nr2f2	0.34229917	7.60255619	5.02382787	0.03655856	0.31115977
Yap1	0.36845485	5.91019961	5.01843685	0.03664884	0.31168883
Gas5	-0.2578017	6.98860921	5.0173514	0.03666705	0.31168883
A930011G23	1.65442894	-0.3035177	5.01404348	0.0367226	0.31196397
Rnd1	-0.90606	1.29585045	5.00970096	0.03679567	0.3123875
Cept1	0.48891928	3.88413645	5.00718734	0.03683804	0.31252188
Sox18	-0.9069208	1.74663565	5.00600746	0.03685795	0.31252188
Jtb	-0.3986596	4.13897678	5.00130103	0.03693748	0.31282463
C2cd2	0.42760515	4.33129603	5.00039374	0.03695284	0.31282463
Tktl1	-2.7953388	-1.6232973	4.99976998	0.0369634	0.31282463

Clrn1	2.94491044	-1.0221017	4.99542043	0.03703713	0.31320343
Blnk	0.55866705	3.30553498	4.99412796	0.03705907	0.31320343
Ephb2	-0.9285224	2.12447497	4.99207919	0.03709388	0.31320343
Tmem109	0.42349762	3.48127732	4.99164551	0.03710126	0.31320343
Pcyt1a	0.34041977	5.75957855	4.98888727	0.03714819	0.31340304
Gsdma	-4.3112885	-2.0985064	4.98184841	0.03726828	0.31396238
Nrbf2	-0.3301393	4.25797767	4.9802221	0.03729608	0.31396238
Synb	-1.9083921	-0.6087097	4.97960579	0.03730663	0.31396238
Vcpkmt	-0.7651556	1.7494766	4.97953617	0.03730782	0.31396238
Epcam	-3.0638875	-0.4798403	4.97694229	0.03735224	0.3141397
Cpa6	2.60327581	-1.1804844	4.97324334	0.03741568	0.31425654
Haus1	0.62687836	2.88420026	4.97214364	0.03743456	0.31425654
Ide	0.24404965	7.24622988	4.97103963	0.03745353	0.31425654
Cpne4	-0.3588314	6.36856692	4.97069	0.03745954	0.31425654
Folr2	2.22762107	-0.8304092	4.96819889	0.0375024	0.31442002
Nol10	-0.5439571	4.15900657	4.96555478	0.03754794	0.31460585
Klhdc1	-0.3980463	4.04135567	4.96371483	0.03757967	0.31467578
Fam102a	-0.3598335	5.70098183	4.95322083	0.03776123	0.3159994
Pvrl4	-0.8513569	1.55218341	4.94702177	0.03786894	0.31670385
Sp8	1.10086955	0.59072781	4.94217856	0.03795334	0.31704849
Mtf2	-0.3632254	5.55865667	4.94195318	0.03795728	0.31704849
Orai2	-0.4712989	3.83792651	4.93499714	0.03807888	0.31786693
Uchl1	0.31579856	6.11240315	4.92448991	0.03826341	0.31920928
Cdc42se2	-0.25464	6.29738969	4.92305827	0.03828863	0.31922178
Cops4	0.28258999	6.02394846	4.91874718	0.03836469	0.31965787
Tnfrsf9	-2.5202182	-1.4474965	4.91200914	0.03848392	0.32005325
Gpr180	-0.5000158	3.9525995	4.91178824	0.03848784	0.32005325
Spink10	-2.0157834	-0.9899243	4.90975363	0.03852392	0.32005325
Zranb2	0.26837349	8.01362559	4.90883445	0.03854024	0.32005325
Oprk1	-0.7449493	3.81311892	4.90843929	0.03854726	0.32005325
Cebpz	0.25531293	6.19603558	4.90544982	0.03860039	0.32005325
Cct7	0.32239913	6.67429444	4.90511218	0.03860639	0.32005325
Syne1	-0.5235529	8.5747803	4.90453647	0.03861664	0.32005325
Sra1	0.39041726	5.14917602	4.90399863	0.03862621	0.32005325
Lrp2	-3.2141697	-1.6730941	4.9014812	0.03867105	0.32022761
Rgl1	0.23123401	7.58694642	4.89587395	0.03877114	0.32085896
Nif3l1	0.51481526	3.38042311	4.89227368	0.03883555	0.32100063
Adap2	-0.6179069	3.73538247	4.89225079	0.03883596	0.32100063
Drg2	0.41888755	3.88772029	4.88413897	0.03898155	0.321947
Nat1	0.94027081	2.09121257	4.88244585	0.03901202	0.321947
Hmgb2	-0.4724125	4.01804394	4.88160601	0.03902714	0.321947
Glra1	1.3030424	0.24685112	4.88055031	0.03904616	0.321947
Stamos	2.70417485	-1.1689577	4.87807998	0.0390907	0.3221169

Klk10	1.72118534	-0.2302173	4.87656875	0.03911798	0.32214441
Gjb2	0.46298929	9.09863828	4.87484689	0.03914909	0.32220339
Dcaf17	-0.3627461	4.46786314	4.87093568	0.03921985	0.32258849
Tomm70a	0.22843586	7.12662431	4.86648352	0.03930058	0.32305501
Grtp1	0.6561469	3.25590908	4.86412694	0.03934339	0.32306388
Ak5	0.35587435	6.20518148	4.86300138	0.03936385	0.32306388
Trim37	-0.33543	8.21341836	4.86246074	0.03937368	0.32306388
Rpusd2	-0.3922974	4.33296508	4.85541857	0.03950203	0.32320202
Slc25a38	0.50775926	2.90799022	4.85541684	0.03950206	0.32320202
Snord64	-1.2107109	0.11126049	4.85518337	0.03950633	0.32320202
Hydin	-1.3954064	0.40792578	4.85417787	0.03952469	0.32320202
Rnf19b	0.36097346	4.34190555	4.85365188	0.0395343	0.32320202
Camk2a	-0.4703732	11.2091826	4.85233993	0.03955829	0.32320202
Ap3m2	-0.290471	6.61854869	4.85232021	0.03955865	0.32320202
Slc15a2	-0.5936583	2.85421887	4.84382578	0.03971435	0.3237533
Clcn6	-0.4524345	3.55535968	4.84169883	0.03975345	0.3237533
Sec1	-1.5898578	-0.0709414	4.8406556	0.03977264	0.3237533
Las1l	-0.3941826	4.97411249	4.83959278	0.0397922	0.3237533
Ubl7	0.27155936	6.5759092	4.83957957	0.03979244	0.3237533
Mdm4	-0.2540816	6.15415626	4.83935311	0.03979661	0.3237533
Fzd5	-0.8873437	2.67247355	4.83885023	0.03980588	0.3237533
Nespas	-3.3791557	-1.9246916	4.83815936	0.0398186	0.3237533
Stk19	-0.7974336	2.00526309	4.8260338	0.04004274	0.32511933
Fbxo24	-1.5478198	-0.1138298	4.82529013	0.04005653	0.32511933
1700030C10I	1.04977789	1.00427737	4.82272332	0.04010418	0.32511933
Pla2g4e	-0.4002887	4.55148466	4.82265402	0.04010546	0.32511933
Ifit1	0.52257125	6.03124522	4.82254893	0.04010742	0.32511933
Cchcr1	-1.0933346	0.99538075	4.81905631	0.04017236	0.32542354
Nek6	0.45172107	4.83374912	4.81721045	0.04020673	0.32542354
Hltf	-0.3649648	4.91250401	4.81663273	0.0402175	0.32542354
Smarcd1	-0.3003064	5.84974477	4.80971628	0.04034662	0.32602019
Slc9a4	-3.0903091	-1.2674155	4.8089584	0.0403608	0.32602019
Casp1	-0.9290138	1.62809504	4.80879175	0.04036392	0.32602019
Myzap	0.66398714	4.17181973	4.80585667	0.04041888	0.32618947
Dnajc16	0.54808236	3.07893277	4.80508452	0.04043336	0.32618947
Abca13	3.10078112	-1.7270547	4.80355092	0.04046212	0.32622595
Pik3cd	-0.5818886	2.8153947	4.80212622	0.04048886	0.3262461
Ncoa4	0.24705199	7.81367737	4.80083402	0.04051314	0.32624633
Zfp445	-0.3512738	6.82886788	4.79751031	0.04057565	0.3265543
Nup188	0.57010194	3.73342481	4.79089395	0.04070041	0.3269243
Parp10	0.77407962	1.9135946	4.79074283	0.04070327	0.3269243
Ccdc97	0.35327426	5.32492824	4.79041971	0.04070937	0.3269243
Zfp472	-0.7601638	1.75869423	4.78992066	0.0407188	0.3269243

Psmc6	0.30373194	5.85723152	4.78290318	0.04085168	0.32779554
Foxred2	-0.5298199	2.99096199	4.77997974	0.04090718	0.32802025
Gdpd5	0.45182468	3.75002037	4.77758595	0.04095269	0.32802025
Mlf1	-1.6408341	-0.3012403	4.7775793	0.04095281	0.32802025
Shq1	-1.0077228	1.05411316	4.7761088	0.0409808	0.32804913
Tmem181b-ı	-0.6178919	5.11164669	4.77146236	0.04106937	0.32842605
Ppp1r16a	-0.612146	2.41050644	4.77096255	0.04107891	0.32842605
08-Mar	-0.2751241	6.24265239	4.76836127	0.0411286	0.32842605
Samd12	-0.5793585	3.24731974	4.76781947	0.04113896	0.32842605
Cox11	0.37262111	3.91883494	4.76548324	0.04118366	0.32842605
Smarce1	0.30693927	6.41595894	4.76493787	0.0411941	0.32842605
Cenpc1	0.33110233	5.59738244	4.76469593	0.04119873	0.32842605
Anxa7	0.28125308	6.45229665	4.76125166	0.04126476	0.32874591
Tead1	0.32829207	7.27776457	4.76005566	0.04128772	0.32874591
Zswim6	0.28886448	6.10393689	4.75745825	0.04133762	0.32894862
Atp5g3	0.31697864	8.20651786	4.75522451	0.04138059	0.32895632
C030029H02	-1.2367418	1.08253575	4.75486677	0.04138748	0.32895632
Rpe65	-1.7773943	0.67821647	4.75283152	0.04142669	0.32901761
Tubb4b	0.25479837	7.02528068	4.75192857	0.04144409	0.32901761
Bhlha15	-1.8106419	-0.1500511	4.74624767	0.0415538	0.32969406
Prex2	-0.3354734	7.37348837	4.74420749	0.04159328	0.3298096
Palm2	-0.4170187	7.15217427	4.7422791	0.04163064	0.3298096
H1f0	0.32354712	6.9773006	4.74112169	0.04165308	0.3298096
Speg	-0.3482124	5.58177323	4.73993441	0.04167611	0.3298096
Cdh19	-1.0350732	1.52153738	4.7391718	0.04169091	0.3298096
Atp5a1	0.21815618	9.40853935	4.73753954	0.04172262	0.32986646
AW146154	0.58651538	3.00440561	4.73599827	0.04175257	0.32990949
Fabp3	-0.4487853	3.7126582	4.73236163	0.04182336	0.33023916
Gtf2e2	0.33978836	4.51126659	4.72967136	0.04187582	0.33023916
Amn1	-0.4444608	4.58958325	4.7272562	0.04192297	0.33023916
Evc	0.61639114	2.46743737	4.72655451	0.04193668	0.33023916
Cxxc5	0.31311886	6.44888195	4.72647234	0.04193829	0.33023916
Prr16	-0.4221155	4.14023859	4.72594886	0.04194852	0.33023916
Ntrk3	0.41476739	5.67850937	4.72295848	0.04200703	0.33023916
Pkib	-0.3243403	5.513234	4.72276607	0.0420108	0.33023916
Shank1	-0.4165152	8.94138459	4.72254264	0.04201517	0.33023916
Aph1c	-0.4581482	3.93626938	4.71767221	0.04211068	0.33070417
Ppp1cc	0.24811844	7.62011982	4.71702044	0.04212348	0.33070417
Pitpnm2os1	1.89530956	-0.1977303	4.71495171	0.04216414	0.33083035
Otof	-0.8895533	2.91644019	4.71254198	0.04221156	0.33100938
Sergef	-0.8052231	1.78404877	4.70967231	0.0422681	0.33125975
AU022252	-0.5809085	2.42007903	4.7051347	0.04235769	0.3315851
Atp2b2	-0.3870059	9.28140881	4.70436163	0.04237298	0.3315851

AW554918	-0.3202176	4.9547317	4.70300138	0.04239989	0.3315851
Sertad2	-0.3260309	5.37338932	4.70258207	0.04240818	0.3315851
Sat1	0.36600907	5.94072351	4.70031555	0.04245308	0.33174333
Rbl2	0.24722054	7.24597562	4.69762463	0.04250644	0.33196758
Syndig1	-0.4598826	3.49911957	4.69284407	0.04260144	0.33218839
Mapk12	0.71995449	1.78775596	4.69226444	0.04261297	0.33218839
Pgpep1	0.47731189	4.21898306	4.69169401	0.04262433	0.33218839
Pkhd1l1	-2.5560919	-0.6430587	4.69123526	0.04263346	0.33218839
Rab19	1.06343687	1.98296092	4.68883687	0.04268125	0.33231868
Maged1	0.25519157	8.26477941	4.6879182	0.04269957	0.33231868
Mog	-0.6762564	2.85453928	4.68008335	0.0428562	0.33333852
Sox12	-0.5359065	2.99499347	4.67722425	0.04291351	0.33333852
Acat1	0.2926117	7.12008456	4.67671159	0.0429238	0.33333852
Klrc1	1.72442327	-0.657688	4.67540091	0.04295011	0.33333852
Atp8b2	-0.4509594	4.3200366	4.6739683	0.04297889	0.33333852
Gpr98	0.6933608	2.82382447	4.67395083	0.04297924	0.33333852
Pdzd11	0.33785306	6.13967955	4.67066813	0.04304528	0.33353938
Tdp2	0.47577275	3.61974665	4.67019927	0.04305472	0.33353938
Larp7	0.29834699	5.74968368	4.66597847	0.04313981	0.3340063
Smtnl2	-0.8101984	1.14257697	4.66343862	0.04319111	0.33421116
Lama4	0.42071329	4.07808699	4.66037828	0.04325301	0.33432353
Smyd5	0.54406087	3.00264503	4.65998751	0.04326092	0.33432353
4831440E17I	-0.9411815	1.79053662	4.65862853	0.04328845	0.33432353
Prodh	-0.6867515	2.14169972	4.65781125	0.04330501	0.33432353
Fat4	-0.2946382	5.96841046	4.64703092	0.04352417	0.33582281
Elk3	-0.3540602	4.99464242	4.63981703	0.04367153	0.33658696
Pcdhb14	-0.5721439	3.02501865	4.63973373	0.04367323	0.33658696
Ddx55	-0.3549783	4.40733803	4.63274494	0.04381654	0.33716078
Lgals3bp	0.53342486	4.89866269	4.63182744	0.0438354	0.33716078
Lzts3	-0.2865882	5.7471234	4.63104797	0.04385142	0.33716078
Galm	0.77565243	2.35655813	4.63035274	0.04386572	0.33716078
Cyb5r1	0.55268194	2.67456624	4.63000042	0.04387297	0.33716078
Gm8979	-0.9041558	0.55509727	4.62813385	0.04391139	0.33722716
Fpgs	-1.138128	0.54372643	4.62518015	0.04397227	0.33722716
Zmynd12	2.28282284	-1.5953784	4.62498558	0.04397628	0.33722716
Zfp365	-0.38297	9.10482669	4.62395268	0.04399759	0.33722716
Zic1	0.45203362	8.07189603	4.62350154	0.04400691	0.33722716
Disp2	0.34970688	7.01997622	4.62030565	0.04407295	0.33754098
Mlxipl	-0.9997264	0.95267483	4.618458	0.04411118	0.3376357
Irak1	0.23577441	6.53281379	4.61728354	0.0441355	0.3376357
Rrn3	0.25860484	5.81564163	4.61433607	0.0441966	0.3378374
Cluap1	0.27564713	5.50407573	4.61204624	0.04424414	0.3378374
Zfp592	-0.3196701	5.65613648	4.61111468	0.04426349	0.3378374

Crebrf	-0.2764419	7.2754805	4.6103679	0.04427901	0.3378374
Prkag1	0.32708862	4.21440019	4.60996499	0.04428739	0.3378374
Zfp689	-0.8041256	1.65049291	4.60803665	0.04432751	0.33789598
Smoc1	0.48133179	4.56734441	4.60718277	0.04434529	0.33789598
Trabd2b	0.50010012	6.3225332	4.60552657	0.0443798	0.33796754
Nkx6-1	2.12566247	-0.5665665	4.59836718	0.04452932	0.33891442
Ptprd	-0.3722439	8.31428597	4.59389974	0.04462292	0.33939396
Tmem88b	-0.3417707	5.67365952	4.59295327	0.04464278	0.33939396
Zfp709	-0.4749835	4.13899333	4.58649117	0.04477862	0.34012041
Elovl7	-0.4311418	4.0967768	4.58600405	0.04478888	0.34012041
Hfe	0.83244482	3.40174727	4.58266128	0.04485936	0.3403749
Slc16a6	0.50127724	3.11581184	4.58201564	0.04487298	0.3403749
Acsl6	-0.3457461	6.0861071	4.5792345	0.04493174	0.3404418
Mctp2	0.7064813	1.62389427	4.5775311	0.04496776	0.3404418
Frmpd1os	-3.2815317	-1.7054424	4.57625195	0.04499484	0.3404418
Ctsz	0.5175676	3.99981656	4.57594704	0.0450013	0.3404418
Mir6390	-2.3790494	0.08973674	4.57561619	0.0450083	0.3404418
Ptpn7	-0.5773933	3.28926987	4.57441368	0.04503378	0.34044316
Sugp1	0.37674989	4.58008137	4.57228734	0.04507888	0.34059271
Hspg2	0.40760563	3.48405132	4.56749461	0.04518071	0.3406301
Pnma1	0.83882005	1.58380208	4.56660541	0.04519963	0.3406301
Smurf1	0.34199182	5.13253771	4.56651142	0.04520163	0.3406301
Endov	-0.3795746	4.5500775	4.56598808	0.04521277	0.3406301
Qrsl1	0.55356656	2.65418748	4.56575111	0.04521781	0.3406301
Dpy19l1	-0.2652711	6.7235325	4.56491108	0.04523571	0.3406301
Astn2	-0.5076759	3.40841357	4.56123387	0.04531412	0.34094176
Irak4	0.56236411	3.52899172	4.56059465	0.04532777	0.34094176
Sdk1	0.52635681	4.14071295	4.55431104	0.04546217	0.34152462
Tpm1	0.32080363	9.356263	4.55336002	0.04548255	0.34152462
Rbm10	0.33300504	4.92040676	4.55210477	0.04550947	0.34152462
Enoph1	0.29744723	4.85653078	4.55131287	0.04552646	0.34152462
Cxxc1	0.29619944	5.98700297	4.55006233	0.0455533	0.34152462
Mta1	-0.3366823	4.54349994	4.54986501	0.04555754	0.34152462
Hmox1	0.59166657	2.83955747	4.54756669	0.04560693	0.3416892
Islr	0.6852207	6.83435633	4.54531655	0.04565535	0.3416892
Uck2	0.45728361	3.46702241	4.54530136	0.04565567	0.3416892
Tmc4	-0.4781543	3.05233319	4.54258523	0.04571419	0.3419137
Dera	0.83203431	2.55249595	4.53782972	0.04581685	0.3419137
Snapc1	0.26614346	5.51212667	4.53640801	0.0458476	0.3419137
Tsr2	-0.3248696	4.95762152	4.53594378	0.04585764	0.3419137
Unc13c	-0.3019503	5.92405696	4.53432278	0.04589273	0.3419137
4931406P16I	-0.3063605	5.65674613	4.53286808	0.04592425	0.3419137
B230216N24	0.69171215	2.24691743	4.5324985	0.04593226	0.3419137

Rev1	-0.3411819	4.38275609	4.53243403	0.04593366	0.3419137
St3gal2	0.27325973	5.92266657	4.53224894	0.04593767	0.3419137
Slc5a7	-0.4117964	4.15569526	4.53204787	0.04594203	0.3419137
Jakmip1	-0.3468118	4.70511181	4.53026374	0.04598073	0.3419137
Abcd2	-0.4402393	4.77158187	4.52981016	0.04599058	0.3419137
Lct	-1.7110529	-0.2845821	4.52664931	0.04605926	0.34192423
Sncb	-0.4047294	4.66624454	4.52664805	0.04605929	0.34192423
Cast	0.44136256	6.45665256	4.52413538	0.04611396	0.34192423
Gm1976	-0.3883133	4.49603841	4.52340958	0.04612977	0.34192423
Zfp287	-0.5091714	4.00793578	4.52055632	0.04619198	0.34192423
Neu2	-1.0347705	0.65232574	4.51934398	0.04621843	0.34192423
Mfsd4	-0.4785323	7.06259956	4.51896481	0.04622671	0.34192423
St3gal3	0.47070471	3.19153663	4.51890879	0.04622794	0.34192423
Zfp202	-0.7956565	1.45069473	4.51758261	0.04625691	0.34192423
Vstm2a	0.30709575	6.5331679	4.51718956	0.0462655	0.34192423
Agt	1.30493546	1.29022148	4.51643621	0.04628197	0.34192423
Chrm2	0.7838831	1.96358718	4.51575273	0.04629691	0.34192423
Sema6c	-1.2447917	0.69316727	4.51417576	0.04633142	0.34199139
Manbal	0.52605028	3.04796041	4.50877611	0.04644981	0.34267728
Vps4b	0.29893554	5.43696052	4.50357844	0.04656409	0.34333216
Myh3	-0.5862847	2.04655776	4.49751375	0.04669785	0.34390276
Dpysl2	-0.2848869	8.72760806	4.49695225	0.04671025	0.34390276
Ppid	0.2789047	6.12373406	4.4965948	0.04671815	0.34390276
Zfp672	0.3562689	4.76502454	4.49464478	0.04676127	0.34403198
Ppp1r16b	-0.3657909	6.6634523	4.49300565	0.04679755	0.34411077
Ucp3	2.00662194	-0.2060946	4.49103032	0.04684132	0.34424447
Jmjd4	-0.3582495	4.63418793	4.48658515	0.04693998	0.34478123
Vac14	0.40317818	4.71468339	4.48109699	0.04706211	0.34538817
Oat	0.35147782	8.127886	4.47898977	0.0471091	0.34538817
Gtf2e1	0.48649673	3.80428153	4.47838085	0.04712269	0.34538817
Yipf1	0.52992093	3.00966955	4.47728144	0.04714724	0.34538817
AI450353	-0.9336337	2.02107079	4.4770707	0.04715194	0.34538817
9030025P20I	-0.5127483	3.21124724	4.4756744	0.04718314	0.34538817
Clec1a	0.96102877	1.93760054	4.47481841	0.04720228	0.34538817
Nek11	1.15104609	0.02576182	4.47345015	0.04723289	0.34542431
Bcas1	-0.4611785	4.7132492	4.47042309	0.04730069	0.34543024
Spg21	0.46919543	5.13850679	4.47035454	0.04730223	0.34543024
Lman2l	0.47565895	3.23296751	4.46864139	0.04734065	0.34543024
Yipf2	0.83533624	1.33348163	4.46809019	0.04735302	0.34543024
Chaf1a	-0.8203849	1.78037866	4.46768759	0.04736205	0.34543024
Pafah2	0.45575282	3.40663434	4.46529724	0.04741575	0.34563454
Dok6	-0.7459955	2.04157033	4.46239352	0.04748107	0.34576623
Gc	-3.9298881	-2.7059228	4.56731742	0.04749722	0.34576623

Prrx1	0.42269602	7.06862892	4.45956999	0.04754469	0.34576623
Nlrc4	3.34379489	-1.284615	4.45902313	0.04755702	0.34576623
Pacsin2	0.27654824	6.2208514	4.45878938	0.04756229	0.34576623
Gm16982	-1.707334	0.00829823	4.4553376	0.04764023	0.3461458
Gcn1l1	0.47316424	4.54736474	4.45307122	0.04769148	0.34620329
9330175M2(	-1.3001198	1.18172473	4.45271253	0.0476996	0.34620329
Tmem173	-0.7861783	1.61094865	4.45092718	0.04774002	0.34630692
Hgf	0.8131422	2.0473698	4.44980966	0.04776535	0.34630692
Stac2	0.3088592	5.87709308	4.44674749	0.04783482	0.34662008
Csf2rb	-0.9612627	1.3256327	4.44525879	0.04786863	0.34662008
Rpa1	0.32025034	4.58334867	4.4445026	0.04788582	0.34662008
Cntn2	-0.4509568	5.70335049	4.44273631	0.04792599	0.34672435
Cdc14a	-0.5370699	4.04350205	4.44108121	0.04796367	0.34680269
Pcdhac2	0.44245503	4.03707919	4.43999724	0.04798836	0.34680269
Uspl1	0.28281562	5.10207478	4.43837521	0.04802534	0.34688363
Med29	0.37885019	3.3883453	4.435791	0.04808433	0.34703108
2310067B10	0.47743184	4.10431381	4.43453965	0.04811292	0.34703108
Zwint	0.22409033	6.89570744	4.43409307	0.04812312	0.34703108
Abcf1	0.26467914	6.76225071	4.42751093	0.04827387	0.34758669
Syngr1	-0.2858262	6.81639169	4.42677645	0.04829072	0.34758669
Tac1	0.50433965	4.18556843	4.42580352	0.04831306	0.34758669
Cml1	-0.9011674	1.30335737	4.42525091	0.04832575	0.34758669
Rasgrp3	-0.4094876	4.97241042	4.42473098	0.0483377	0.34758669
Pik3cb	-0.3255594	5.5200715	4.42362396	0.04836314	0.34758669
P2ry12	-0.5007865	3.67863473	4.42258795	0.04838697	0.34758669
Hnrnpr	0.22934535	7.8987067	4.41796931	0.04849335	0.34758669
Sall1	0.29618544	4.82359432	4.41648146	0.04852768	0.34758669
Fam71d	-2.170386	-1.4505967	4.41605947	0.04853742	0.34758669
Adh5	0.30794725	6.35142183	4.41533723	0.0485541	0.34758669
Cdk5rap1	1.04195613	1.39925974	4.41519356	0.04855742	0.34758669
Pfkfb1	-1.5767765	-0.0473381	4.41383258	0.04858886	0.34758669
A230072E10	-1.3589904	0.01454516	4.41340637	0.04859871	0.34758669
Slbp	0.2941378	5.38558167	4.41158968	0.04864074	0.34758669
Eef1e1	-0.401676	3.33863197	4.41104056	0.04865345	0.34758669
Foxm1	0.99102898	1.41921814	4.41055724	0.04866464	0.34758669
Myo1h	1.61845723	-1.0196732	4.41053613	0.04866512	0.34758669
Cxx1b	0.37319329	5.87807493	4.40833333	0.04871616	0.34768919
Drp2	-0.48323	5.99951499	4.40768685	0.04873115	0.34768919
Igfbp4	0.47794744	5.14860724	4.40560001	0.04877958	0.34785025
Odc1	0.22482006	6.42482973	4.40071325	0.04889319	0.34816611
Trmt61b	0.49279779	3.3221804	4.40038489	0.04890083	0.34816611
Eif2b2	0.49483366	4.10672235	4.40031091	0.04890255	0.34816611
Klf4	0.49267208	6.47978248	4.39856496	0.04894323	0.34816611

Entpd3	-0.711366	1.63353035	4.39813541	0.04895324	0.34816611
Clec9a	-2.2146086	-0.0266248	4.39121762	0.04911481	0.34913072
Zfp446	0.49735068	3.31004203	4.38846897	0.04917918	0.34940369
Gm6225	2.01500983	-1.1707404	4.38633627	0.04922919	0.34941971
Snhg11	-0.7556877	9.45745744	4.38543072	0.04925044	0.34941971
Wnt2	1.96013676	-1.0513622	4.3850518	0.04925933	0.34941971
Slfn5	-0.4986576	4.46679279	4.38144587	0.04934408	0.34967981
Zfp658	-0.6082077	2.26400875	4.38128032	0.04934797	0.34967981
Flad1	0.68342202	2.04431352	4.37822714	0.04941987	0.35000493
Sfrp1	-0.5065444	6.64119233	4.37710843	0.04944624	0.35000749
Smarcb1	0.35730003	4.78304177	4.375276	0.04948947	0.35012932
Gdnf	1.60104589	-0.5461555	4.36712276	0.04968235	0.35122547
Kcnh5	-0.4222009	5.78019818	4.36473922	0.04973889	0.35122547
Ap5m1	-0.5100856	3.37570823	4.36368809	0.04976386	0.35122547
Fmnl1	-0.4088677	5.94178596	4.3634842	0.0497687	0.35122547
Elovl2	0.67255103	2.66654295	4.36322266	0.04977491	0.35122547
Dynlt1a	0.33223988	4.34694742	4.36051729	0.04983924	0.35149506
Pilrb2	-2.9050496	-0.5024789	4.35650609	0.04993479	0.35162235
Sp7	1.03676185	0.83037531	4.35621138	0.04994182	0.35162235
Kcnmb1	-2.497728	-1.0258641	4.3550908	0.04996855	0.35162235
Gm12060	-2.3367219	-1.0311945	4.3527596	0.05002422	0.35162235
Flt3l	-0.8423049	1.70226686	4.35274455	0.05002458	0.35162235
Cpsf7	-0.3481852	5.71114193	4.35272856	0.05002496	0.35162235
Ube2d3	0.26733476	9.02053854	4.35209094	0.0500402	0.35162235
Olfr99	-2.8167699	-1.5113581	4.34971463	0.05009704	0.3516257
4931406C07I	0.39093574	6.65583506	4.34879651	0.05011902	0.3516257
Nucb1	0.50237089	4.56765034	4.34879454	0.05011907	0.3516257
Mir3473f	-2.5553146	-1.3801563	4.34549847	0.05019807	0.35188673
01-Mar	-0.3904812	5.07859103	4.34465029	0.05021843	0.35188673
Zbtb39	-0.3541147	4.42513232	4.34397121	0.05023473	0.35188673
Jph4	-0.2806096	7.22585029	4.34213245	0.0502789	0.35201291
Zbtb8b	-0.8181889	1.87284961	4.33814125	0.05037493	0.35238843
Utp3	0.26439312	6.12821502	4.33530163	0.05044338	0.35238843
Chga	0.42913733	4.36510179	4.33378945	0.05047988	0.35238843
Fbln1	0.58168535	4.89785489	4.33330895	0.05049148	0.35238843
4930453N24	-0.3097904	4.68066758	4.33325174	0.05049286	0.35238843
Rsrc2	0.24221556	7.78253955	4.33249925	0.05051104	0.35238843
Prl	4.58616849	1.48097504	4.39421395	0.05053536	0.35238843
Cttnbp2	-0.4405458	6.72279741	4.33121682	0.05054204	0.35238843
Hbp1	0.26660749	6.72167701	4.32565758	0.05067666	0.35289973
Klf11	-0.4050532	4.21877842	4.32522346	0.05068719	0.35289973
Cpne2	0.3315486	4.41297001	4.32494053	0.05069405	0.35289973
Gm4814	-2.8812365	-1.5837911	4.32375737	0.05072276	0.35291705

Dnaja4	0.33480625	4.57273453	4.32034449	0.0508057	0.35331139
A330050F15	0.84411574	2.27249062	4.31700068	0.0508871	0.35366079
Sppl3	-0.2717704	5.25228808	4.31612257	0.0509085	0.35366079
Dnajc13	-0.385403	6.26013549	4.31337066	0.05097564	0.35371338
3300002I08R	-1.5015227	-0.3547542	4.31159481	0.05101902	0.35371338
Celrr	1.52530803	0.30316582	4.31156618	0.05101972	0.35371338
Gtpbp8	0.3608381	3.77131635	4.31025525	0.05105177	0.35371338
Bivm	0.28838535	4.9555411	4.31014275	0.05105452	0.35371338
Tph1	-2.9382767	-0.7652572	4.30925785	0.05107617	0.35371338
Atxn7l3	-0.3592006	5.96367307	4.30828131	0.05110008	0.35371338
Htr3a	-1.1532813	0.66237424	4.30556766	0.05116657	0.35399152
Ldhb	0.29179947	8.96055698	4.30372168	0.05121185	0.35412277
Endod1	-0.3184397	4.92098218	4.29988797	0.05130605	0.3543528
P4ha3	0.87122135	2.42550254	4.29927098	0.05132123	0.3543528
Atp10d	-0.612122	2.36718858	4.29915347	0.05132412	0.3543528
Tmem2	0.32268971	4.21362306	4.2929878	0.05147609	0.35502522
Hnrnpk	0.24672108	7.92294978	4.29108772	0.05152303	0.35502522
2900011O08	-0.2685489	7.725197	4.29104915	0.05152398	0.35502522
Nrp1	0.36595959	4.973746	4.29092522	0.05152705	0.35502522
Stk10	-0.4242973	3.50792912	4.28832947	0.05159125	0.35528569
Fut4	1.39840922	-0.2588624	4.28389561	0.05170114	0.35569058
2510039O18	0.61305108	3.40800812	4.28300166	0.05172333	0.35569058
4930593A02	2.48932029	-1.3030035	4.2827592	0.05172935	0.35569058
5930412G12	-1.1899047	0.82290441	4.27959862	0.05180789	0.35578749
Slc40a1	-0.6362315	2.11802595	4.27902387	0.05182219	0.35578749
Gpr135	-1.952871	-0.6005813	4.27900073	0.05182276	0.35578749
Fndc1	-0.7661086	1.61004126	4.27792256	0.05184959	0.35579019
Sparc	0.55701967	8.82825556	4.27637456	0.05188815	0.35587326
Dsp	-0.9005076	1.66527481	4.27034425	0.05203865	0.35672365
Magee1	-0.3865736	6.91827672	4.26918385	0.05206767	0.35673519
Kcnma1	-0.3964406	7.25180016	4.2681573	0.05209335	0.35673519
Nlk	-0.2814226	8.55890243	4.26652162	0.05213431	0.35683407
Abhd17b	0.32993561	5.76779969	4.25764677	0.05235718	0.3580129
Fgfbp3	-0.5855028	2.68347118	4.25703673	0.05237254	0.3580129
Galnt7	-0.4550154	3.06937041	4.25648811	0.05238636	0.3580129
Mrps26	-0.3904326	3.85237009	4.25264244	0.05248333	0.35849356
Ubxn4	0.22282427	7.28314811	4.2483965	0.05259064	0.35874424
Ptprz1	-0.3635206	6.78132457	4.24807729	0.05259872	0.35874424
Tpd52l2	0.41210494	6.30856718	4.24802613	0.05260001	0.35874424
Eif4g2	0.29331766	10.874292	4.24480899	0.0526815	0.359118
Batf	1.4968347	0.02424304	4.24301079	0.05272711	0.35924694
Lgals12	-1.6055933	-0.2240564	4.23865417	0.0528378	0.35946033
F2	-2.3455665	-1.044684	4.23851541	0.05284133	0.35946033

Ndufab1	0.29280822	5.61527553	4.23845519	0.05284287	0.35946033
Ocln	-0.6258886	2.73975676	4.23757425	0.05286528	0.35946033
9430018G01	-2.9980462	-1.5023933	4.23511276	0.05292798	0.35959463
Ccdc138	-0.6323553	2.60096319	4.2334988	0.05296913	0.35959463
Cpxm2	-0.6391771	2.65931824	4.23342179	0.0529711	0.35959463
Fam19a2	0.30624872	5.53212453	4.23260555	0.05299193	0.35959463
Cdc6	1.10516842	0.74257693	4.22926052	0.05307738	0.35998021
Midn	0.3377344	5.84324131	4.22828818	0.05310225	0.35998021
Apobec2	-2.742383	-1.4578593	4.22615602	0.05315683	0.35999736
Gm6787	2.41674002	-0.4742893	4.22609924	0.05315829	0.35999736
Skil	0.2393058	7.34534605	4.22384544	0.05321606	0.36020729
Obfc1	0.55141422	2.73504634	4.2202636	0.05330801	0.36064829
Tmem168	-0.48701	3.25320225	4.21894042	0.05334202	0.36069707
B230206H07	-2.7641722	-1.6970281	4.21751136	0.05337879	0.36076438
Pcsk1n	-0.3453282	4.4277672	4.21506305	0.05344184	0.36085751
Parp2	0.29597163	5.19107671	4.21458842	0.05345408	0.36085751
Tmem231	0.4579621	2.88553283	4.21385374	0.05347302	0.36085751
4833427F10I	1.5663803	-0.7880059	4.21205487	0.05351943	0.36090869
Ifi27	0.44387598	4.907542	4.21148117	0.05353425	0.36090869
Gm4980	-0.9705275	0.8216005	4.2097365	0.05357932	0.36103169
Zranb3	-0.5906647	2.7816883	4.20674685	0.05365666	0.3610896
Ly6e	-0.274742	6.82283664	4.20571153	0.05368347	0.3610896
Fam43b	0.77992965	2.4659987	4.20502538	0.05370125	0.3610896
Tmem175	0.31658248	4.99426847	4.20432124	0.0537195	0.3610896
Clk2	-0.3577819	5.15025855	4.20422149	0.05372208	0.3610896
Hecw1	-0.556813	6.93926334	4.20037214	0.05382199	0.3615805
Dner	0.4284996	5.47834739	4.19540622	0.05395119	0.36223136
Kpna6	-0.2938868	7.46646261	4.19458038	0.05397271	0.36223136
4930447N08	-1.8828844	-0.703037	4.19257714	0.05402495	0.36240123
Cort	-2.4945978	-1.6899519	4.19018851	0.05408732	0.36247155
Kcnk5	0.82559195	2.33406875	4.19011244	0.05408931	0.36247155
Aars2	-0.7624321	1.65531529	4.18803653	0.05414358	0.36249931
Rassf4	-0.5067262	3.13006963	4.1878933	0.05414733	0.36249931
Ptplb	-0.3313316	5.00768767	4.18649681	0.05418388	0.36256361
Zfp366	1.74418874	-0.0905949	4.18290359	0.05427805	0.36301322
Cd248	0.55957063	4.35918922	4.18124642	0.05432154	0.36310248
Hopx	-0.5984498	3.04404463	4.18033948	0.05434536	0.36310248
Mylpf	1.68177119	-0.1585606	4.17476242	0.0544921	0.36390223
Tesk2	0.59276085	2.09730237	4.17182652	0.05456953	0.36423855
Naa30	0.28287184	5.57942222	4.16827569	0.05466335	0.36462933
1810041L15F	-0.4279082	5.52631739	4.16717562	0.05469245	0.36462933
Plekhn1	1.52436739	-0.0644064	4.16653641	0.05470937	0.36462933
Fer1l5	0.76479436	1.38580258	4.16252362	0.05481572	0.36502585

Lrrn3	-0.2733791	5.76530635	4.16022675	0.0548767	0.36502585
Surf1	-0.4410471	4.01917473	4.15933135	0.05490049	0.36502585
Mettl21a	0.60222734	2.89583709	4.15927087	0.0549021	0.36502585
Zdhhc13	-0.4663191	4.23415247	4.15856142	0.05492096	0.36502585
Nbas	-0.4003542	5.07413997	4.15687633	0.05496578	0.36502585
Stap1	-1.3283643	0.31756925	4.1536666	0.05505129	0.36502585
Ptafr	-2.2291966	-1.0445953	4.15251128	0.0550821	0.36502585
Kcnab3	-0.3115975	5.49972719	4.15246733	0.05508327	0.36502585
Dock4	-0.4473341	6.18838306	4.15237581	0.05508571	0.36502585
Dcn	0.53689046	7.6761973	4.15116981	0.0551179	0.36502585
Gabrd	-0.7571493	2.84095757	4.1497212	0.0551566	0.36502585
F830016B08I	0.35512318	4.1638108	4.14914394	0.05517202	0.36502585
2310009B15I	-0.7360404	2.1245251	4.14899561	0.05517599	0.36502585
Cd22	-3.3355583	-2.1449166	4.14842282	0.0551913	0.36502585
5031410I06R	-0.8349479	2.61793418	4.1479894	0.05520289	0.36502585
Hexim1	0.28664023	5.41505595	4.14394183	0.05531127	0.36556286
Stk40	0.30640138	4.15938639	4.14060597	0.05540078	0.3657071
Prdx6	0.29036616	6.75152676	4.13993287	0.05541886	0.3657071
Gareml	0.75996109	2.21577455	4.13956785	0.05542867	0.3657071
Tmem170b	-0.2827019	7.50671763	4.13907894	0.05544181	0.3657071
Sfxn5	-0.3990009	4.82337831	4.13686585	0.05550133	0.36591344
Cckar	-2.0257649	-0.7734753	4.13459105	0.05556259	0.36591344
Taf1b	0.31209752	4.16744644	4.13312681	0.05560206	0.36591344
Hspd1	0.19459077	8.27695858	4.13255406	0.05561751	0.36591344
Vps11	0.33987104	4.10357047	4.13146599	0.05564687	0.36591344
Nat8l	-0.3858791	6.37905119	4.13136431	0.05564961	0.36591344
Trnt1	0.33784632	4.77161066	4.13085231	0.05566343	0.36591344
Gspt1	0.2211501	7.58456715	4.12725028	0.0557608	0.36637447
Myo6	-0.3584712	6.39830014	4.12606784	0.0557928	0.36640583
Tbc1d22a	0.40502346	3.48816352	4.12245538	0.0558907	0.36686974
Hip1	-0.4081079	4.55084302	4.12050707	0.05594358	0.36698939
Mars	0.28226719	4.89170001	4.11977485	0.05596347	0.36698939
Cela1	0.74514674	1.3190181	4.118453	0.0559994	0.36704611
Dpm3	0.72218556	2.16360858	4.11548226	0.05608024	0.36739096
5430421F17I	1.1763287	0.8712816	4.11451389	0.05610662	0.36739096
Sh2d3c	0.32822746	4.98566397	4.10815425	0.05628022	0.36834846
Tspan11	-0.6400235	3.38958987	4.10641174	0.05632789	0.36848125
Swsap1	0.45556086	3.36427758	4.10371798	0.05640168	0.36864262
Zc3h6	-0.3674826	4.89306762	4.10351118	0.05640735	0.36864262
Cul5	0.22829198	7.02475883	4.10052726	0.05648922	0.36891381
Unc50	-0.3060092	5.64596372	4.09733648	0.05657693	0.36891381
Ifi44	0.60607949	3.33645629	4.09644062	0.05660158	0.36891381
Kcne3	-1.640486	-0.4643361	4.09441542	0.05665735	0.36891381

Ccdc81	-1.5737848	0.12320534	4.09391123	0.05667125	0.36891381
Ogg1	0.83453281	0.72943137	4.09329293	0.05668829	0.36891381
Prosc	0.28018531	5.53997079	4.092554	0.05670867	0.36891381
Prkag2	0.28632038	6.12234664	4.09182234	0.05672886	0.36891381
Tbxas1	2.32734422	-1.1977504	4.09155755	0.05673617	0.36891381
Clnk	1.87827428	-0.6436547	4.09132279	0.05674265	0.36891381
Apol7b	-1.549863	-0.4814013	4.08945426	0.05679425	0.36891381
Plgrkt	0.45696894	4.07499512	4.08919906	0.0568013	0.36891381
Cand2	0.44222274	3.67553313	4.08905626	0.05680525	0.36891381
Smarcad1	-0.3723811	5.2613754	4.0871095	0.05685908	0.36892857
Tle1	-0.2976678	5.53266061	4.08699123	0.05686236	0.36892857
Kcnn4	-3.4753505	-1.6284853	4.08429011	0.05693715	0.3691183
U2surp	-0.2396746	7.73191875	4.08375901	0.05695187	0.3691183
Stambpl1	-0.4034841	3.56400113	4.08285311	0.05697699	0.3691183
D630041G03	-0.4576957	4.20906915	4.0815836	0.05701221	0.3691183
Ptgdr	0.4828672	5.28157991	4.08098765	0.05702875	0.3691183
Elmsan1	0.27120401	5.92131118	4.077925	0.05711385	0.36949139
U2af1	-0.3888675	4.10283801	4.0767449	0.05714668	0.36952612
Cygb	-0.4203765	4.10908569	4.07529272	0.05718711	0.36960993
Heatr5b	-0.3926501	5.48811715	4.07364971	0.05723289	0.36972824
Htr1f	-0.8204038	1.8051509	4.06884164	0.05736711	0.37023299
2310034G01	1.3814406	0.91151971	4.06790013	0.05739343	0.37023299
Gtsf1	-2.3948809	-1.7485368	4.06789524	0.05739357	0.37023299
Chrna2	-1.6496422	-0.6586616	4.06682181	0.0574236	0.37024921
2010002M12	0.55126666	2.29318535	4.06486828	0.05747829	0.37042438
Gm17821	-0.3674561	9.70641566	4.0637115	0.05751071	0.37042878
Wnt7b	0.88143441	1.64236323	4.06287968	0.05753403	0.37042878
Tm2d3	-0.4504694	3.24777264	4.06013513	0.05761106	0.37074735
Kctd17	-0.4717078	4.42842515	4.05884299	0.05764737	0.37080366
Cdkl4	-0.3965984	4.17292672	4.05760411	0.05768221	0.37085046
C030018K13I	-1.0646742	1.52609964	4.05517812	0.05775049	0.37101006
Myom3	1.79197455	-0.2197565	4.05476341	0.05776217	0.37101006
2610306M01	0.52417455	2.61735373	4.04495613	0.05803923	0.37261176
Exosc7	0.47451328	3.35910013	4.03971197	0.058188	0.37325579
Brdt	-0.4100665	4.23195234	4.03917578	0.05820324	0.37325579
Fndc3b	0.21945969	5.7564573	4.03822238	0.05823034	0.37325579
Fancd2os	1.45775424	0.21805888	4.03751368	0.0582505	0.37325579
Klkb1	-2.7825639	-1.2638815	4.03614442	0.05828946	0.37332769
Spcs3	-0.2483837	6.30157083	4.0350725	0.05831999	0.3733455
Ttc9b	-0.5403449	3.14900253	4.02959125	0.05847636	0.37411123
Mettl2	-0.4819936	4.13577789	4.02893189	0.05849521	0.37411123
Chsy1	0.29763702	5.20745645	4.02792195	0.05852408	0.37411809
Sirt5	-0.5136063	2.29635344	4.02600903	0.05857882	0.3742902

Yeats2	0.27984041	5.57972497	4.02395173	0.05863776	0.37431676
Prss23	0.46380741	3.46917784	4.02392182	0.05863861	0.37431676
Wnt9a	-0.4890156	3.82353206	4.01811261	0.0588054	0.37520344
Pou3f3os	-0.4015593	3.71496871	4.01610079	0.05886329	0.37532038
Slc27a2	0.71483549	1.809053	4.01553736	0.05887951	0.37532038
Rab11fip4	-0.417505	5.52632492	4.01351353	0.05893783	0.37551424
Gm5607	-0.550349	3.8160498	4.01002039	0.05903864	0.37580531
2410076I21R	-1.0506313	0.47192172	4.00999522	0.05903937	0.37580531
Tas1r1	-1.7335471	-0.3951764	4.00720423	0.05912006	0.37583011
Arglu1	-0.2166364	6.85567336	4.00717674	0.05912085	0.37583011
Nono	0.20586893	7.47503312	4.00618813	0.05914947	0.37583011
Zfp455	-0.4702738	3.18800045	4.00368962	0.05922185	0.37583011
Vill	0.81252177	1.24115167	4.00255854	0.05925465	0.37583011
5031434O11	-1.8328717	0.19315469	4.00253421	0.05925536	0.37583011
Rab7	0.29571465	10.3187668	4.00215178	0.05926646	0.37583011
Pgbd5	0.25810739	7.53473833	4.00179875	0.0592767	0.37583011
Zbtb16	-0.2710598	4.67160739	4.00114131	0.05929578	0.37583011
Hnrnpc	0.23120393	7.27085951	4.00021927	0.05932256	0.37583011
Pigm	-0.4261665	4.17321797	3.99818136	0.05938179	0.37584079
Eif3g	0.34794587	5.42210614	3.99528564	0.05946607	0.37584079
Ankrd45	-0.2939307	6.27744958	3.99392431	0.05950574	0.37584079
Tubg1	0.37687692	4.38928208	3.99387487	0.05950718	0.37584079
Tmtc2	0.51866732	2.97770247	3.9930703	0.05953064	0.37584079
Pes1	0.3719477	4.47611042	3.99269225	0.05954167	0.37584079
Lars2	-0.3268442	13.4159353	3.99244214	0.05954897	0.37584079
Emp3	0.6260228	3.79766243	3.99243944	0.05954904	0.37584079
Fank1	0.98892617	1.2666404	3.99149406	0.05957663	0.37584079
Zbtb26	0.29483873	4.37235312	3.99057218	0.05960355	0.37584079
Enpp3	1.07043679	0.61980347	3.98764497	0.05968911	0.37611528
Wdr52	-0.7228329	2.19941663	3.98703796	0.05970687	0.37611528
Ssbp3	0.24044863	5.98619152	3.98621599	0.05973093	0.37611528
Rslcan18	-0.6296727	2.38677572	3.9840906	0.0597932	0.37633125
Kat2a	0.28374888	5.89910999	3.98263049	0.05983602	0.37642467
Retnlg	-2.808426	-1.3288722	3.98108073	0.0598815	0.37653478
Ddx46	0.22605301	7.32957545	3.97852388	0.05995663	0.37669261
Gm765	-0.5534984	2.83983776	3.97812049	0.05996849	0.37669261
Parp3	0.44491983	4.28637517	3.97736963	0.05999058	0.37669261
Enpp4	-0.3782703	4.28764148	3.97416676	0.06008491	0.37705773
N4bp1	-0.2359342	7.19119929	3.97280582	0.06012504	0.37705773
Fam196a	-0.4019676	4.27266502	3.97180819	0.06015448	0.37705773
Tmem192	-0.5979325	2.30239886	3.97159358	0.06016081	0.37705773
Boc	-0.3889388	3.53156235	3.97026867	0.06019994	0.3771273
Tppp	-0.237647	8.81381514	3.9685978	0.06024932	0.37726104

Cspp1	-0.368895	4.72589044	3.96733313	0.06028673	0.3773197
Slc29a4	0.80652334	1.10904848	3.96595464	0.06032754	0.37735333
Lrrc32	0.47943939	4.80677595	3.96525741	0.06034819	0.37735333
Ccdc155	1.52602955	-0.6128863	3.96416398	0.06038059	0.37736169
Nr4a1	1.03671053	4.92205442	3.95921918	0.06052738	0.37736169
Nudt6	0.46573025	3.7359988	3.95893652	0.06053579	0.37736169
Ddx39b	0.26036883	5.43056837	3.95831567	0.06055425	0.37736169
Pard3	0.28080275	5.12882172	3.9569634	0.06059448	0.37736169
Gmps	0.2733717	6.75866356	3.95612253	0.06061952	0.37736169
Garnl3	-0.4359993	5.19900696	3.95593992	0.06062495	0.37736169
Alx4	0.48836582	5.70558227	3.95543042	0.06064013	0.37736169
Vmn2r85	-1.3550959	1.10954057	3.95499499	0.0606531	0.37736169
Rnf40	-0.4597397	3.98065516	3.95469575	0.06066202	0.37736169
Gdpd1	0.30999179	5.43781772	3.95451928	0.06066728	0.37736169
Noxred1	2.39983471	-0.8251038	3.95388965	0.06068605	0.37736169
BC022687	0.64722848	1.99778136	3.9525272	0.06072669	0.37743997
Vps51	0.49292229	3.72065299	3.95121968	0.06076572	0.37750819
Smyd1	1.24878983	1.45294208	3.94791852	0.06086439	0.37783048
Cdsn	0.91174037	0.95293506	3.94760549	0.06087375	0.37783048
Angptl2	-0.5881054	4.88653529	3.94324244	0.06100447	0.37834119
Ybx1	0.30409473	7.17213892	3.94229808	0.0610328	0.37834119
Mfap3	-0.3129529	4.95890308	3.94204546	0.06104038	0.37834119
Lmtk2	-0.3117936	6.76082334	3.93941353	0.06111944	0.37835394
Slc25a12	0.24621867	7.49870517	3.93935673	0.06112115	0.37835394
Ssc5d	0.60110009	2.55307744	3.93864794	0.06114246	0.37835394
Zfp317	-0.3758483	4.05380451	3.93823411	0.06115491	0.37835394
Cramp1l	-0.2591968	6.39451023	3.93289724	0.06131569	0.37846507
Fam216a	-0.2674454	5.4381747	3.93065685	0.06138333	0.37846507
Pop4	0.44285108	4.0052	3.92951181	0.06141793	0.37846507
Hjurp	-0.5794226	2.44062211	3.9290997	0.06143039	0.37846507
Ikzf4	-0.2899387	4.19381993	3.92852165	0.06144787	0.37846507
Ptpre	-0.4029228	5.24251314	3.92809505	0.06146078	0.37846507
Gnb4	0.32273927	4.79186828	3.92783077	0.06146878	0.37846507
Ccdc107	0.61364028	2.78089312	3.92768976	0.06147304	0.37846507
4933412O06	-1.1872659	1.25724567	3.92686977	0.06149786	0.37846507
Gxylt1	-0.3174213	5.66834184	3.92561904	0.06153574	0.37846507
Xylb	-0.4788204	3.45145933	3.92459394	0.06156681	0.37846507
Mbnl2	0.24614057	9.1115468	3.92399428	0.06158499	0.37846507
Kif5a	-0.3175498	9.49887627	3.92258166	0.06162784	0.37846507
Zfp182	-0.3093101	4.90802302	3.92250651	0.06163012	0.37846507
Erbb3	-1.7553928	-0.3009485	3.9216746	0.06165538	0.37846507
Znhit2	0.4904468	3.10195257	3.92161539	0.06165718	0.37846507
4933404O12	-0.3555653	3.94674766	3.92111547	0.06167236	0.37846507

Arhgef25	-0.3149341	5.31720204	3.92078741	0.06168232	0.37846507
Xrcc6bp1	0.83771789	1.74874407	3.9199669	0.06170726	0.37846507
Sepn1	0.58176464	3.45527712	3.91899792	0.06173671	0.37847325
Cd80	-0.904915	1.40087992	3.91626715	0.06181982	0.37881016
Fbxo45	-0.2466291	6.25555646	3.91210722	0.06194667	0.37941467
Myoc	-0.5915227	2.14626982	3.90970662	0.06202001	0.3795521
Jun	0.24406465	6.02552746	3.90952632	0.06202552	0.3795521
E130309D02I	0.37870236	4.011427	3.90767951	0.06208202	0.37972512
Rnf113a2	0.34972379	4.72744651	3.90497637	0.06216481	0.37994271
Gimap3	0.68495529	2.71471156	3.90467469	0.06217406	0.37994271
Emilin1	0.6279941	2.02283567	3.90207731	0.06225375	0.380257
Acss2	0.59328352	4.31701525	3.89905526	0.06234662	0.38065146
Gspt2	0.36683897	4.58635054	3.89743612	0.06239644	0.38078287
Wdr31	0.71148692	1.78852451	3.89253486	0.06254753	0.38127144
Snrpa	0.41131228	4.66726131	3.89243324	0.06255067	0.38127144
Kcnh8	-2.930281	-1.0813013	3.89070786	0.06260396	0.38127144
Uggt2	-0.4727881	4.89630415	3.8887226	0.06266534	0.38127144
Zmat4	-0.2376268	5.6454348	3.88797784	0.06268839	0.38127144
Phf11d	-0.8439119	1.52265086	3.88652618	0.06273334	0.38127144
Nipal2	-0.6219128	2.85416739	3.8864267	0.06273642	0.38127144
Cabyr	-0.4878956	4.12377264	3.88642083	0.0627366	0.38127144
Riok2	0.27144318	5.09861726	3.88573382	0.06275788	0.38127144
Pcdhb16	-0.4352235	4.67358307	3.88567082	0.06275984	0.38127144
Kdm4b	-0.4860702	2.7623584	3.88310724	0.06283934	0.38132384
1700019G17	0.88898244	1.04324127	3.88234024	0.06286315	0.38132384
Trim67	-0.7971303	1.22140215	3.88107024	0.0629026	0.38132384
Atp5b	0.20892147	10.5802722	3.88091331	0.06290747	0.38132384
Hdlbp	0.20048778	8.49839793	3.88082715	0.06291015	0.38132384
C7	-1.0523131	0.35357518	3.87649192	0.06304504	0.38163101
Cstf1	0.48300575	2.83750905	3.87647339	0.06304562	0.38163101
Pla2g4d	-4.3935881	-2.0625272	3.95801543	0.06307379	0.38163101
Ablim1	-0.2298354	6.74637897	3.87555384	0.06307427	0.38163101
Tmem150b	-2.4026112	-0.9969211	3.87432287	0.06311265	0.38168752
4930506C21I	1.36218882	-0.2857268	3.8734354	0.06314034	0.38168752
Sp9	0.68130437	1.30070133	3.86978236	0.06325446	0.38218588
Atp5g1	0.35698847	5.41654017	3.86814207	0.06330578	0.38218588
Ctgf	0.38162099	5.68766544	3.8680715	0.06330799	0.38218588
Nfyb	-0.2920623	5.3894868	3.86564084	0.06338413	0.38247393
Tmem219	-0.7772972	2.53294204	3.86252966	0.06348173	0.38273055
C4b	-0.5485342	2.77120039	3.86246569	0.06348374	0.38273055
Apba1	-0.3373937	6.80136776	3.86156668	0.06351198	0.38273055
Nr1h3	0.71472319	2.61585111	3.85403063	0.06374926	0.3839224
Bfsp2	1.72131386	-0.7331281	3.85307548	0.06377941	0.3839224

Cenpu	-1.0591157	-0.0263099	3.85181287	0.06381928	0.3839224
Mrps28	0.47657605	2.96171414	3.85112182	0.06384112	0.3839224
Bag2	-0.6028351	3.17330684	3.85076438	0.06385242	0.3839224
Cat	0.30776408	8.12823621	3.8469909	0.06397183	0.38413819
Slc38a6	-0.6078845	3.17223248	3.84692562	0.0639739	0.38413819
Dnajc11	0.3706829	3.98636206	3.84548394	0.0640196	0.38413819
Spata24	-1.0719055	0.34077621	3.84459875	0.06404767	0.38413819
Gria3	-0.3405328	8.51083982	3.84376239	0.06407421	0.38413819
Ppm1h	0.24649832	6.75681679	3.84374261	0.06407484	0.38413819
Lemd3	-0.3264888	4.50277454	3.84332365	0.06408814	0.38413819
Has3	-0.8432481	1.97224757	3.83963437	0.06420538	0.3846696
Psma6	0.26448234	6.42066675	3.8378406	0.06426248	0.38484033
Asf1b	2.13766663	-1.5884799	3.83542214	0.06433955	0.38513048
Cyp51	0.34246291	5.33022634	3.83356415	0.06439883	0.38516765
Tmem176a	0.61261018	4.32210892	3.83296492	0.06441796	0.38516765
Tdp1	0.57292173	2.43650373	3.83152011	0.06446412	0.38516765
Snx25	-0.280723	5.1798809	3.83019578	0.06450647	0.38516765
Tshr	2.26534062	-0.4251946	3.82839863	0.06456398	0.38516765
Bcas1os2	-1.5864767	-0.1968734	3.8269496	0.06461039	0.38516765
Peg13	-0.2618979	8.15164275	3.8268258	0.06461436	0.38516765
5730409E04I	-0.197415	6.93327288	3.82619109	0.0646347	0.38516765
Ccl2	1.45923291	-0.4998618	3.82462615	0.06468489	0.38516765
Nol4	-0.2983888	6.37124719	3.82390083	0.06470817	0.38516765
Slc4a11	1.80418907	-0.2079535	3.82369256	0.06471486	0.38516765
E330033B04I	-0.826768	2.95242948	3.82324877	0.06472911	0.38516765
Sytl1	1.57943219	0.01323853	3.82299293	0.06473732	0.38516765
4933402D24I	-1.4784992	0.32881142	3.82266499	0.06474786	0.38516765
Taco1	-0.7013878	1.58711343	3.82103627	0.0648002	0.38516765
Cited2	-0.2830602	6.39891047	3.82046455	0.06481859	0.38516765
Cox20	0.32337427	5.02108414	3.8200365	0.06483236	0.38516765
8430431K14I	-0.9092787	0.78650113	3.81766009	0.06490886	0.38527856
Trnp1	0.27528019	6.17704548	3.81710302	0.06492681	0.38527856
Rad23b	0.2036147	7.99286749	3.81620878	0.06495564	0.38527856
Jrk	0.62980602	2.19497887	3.81590122	0.06496555	0.38527856
2810442N19	-1.4218212	0.21262058	3.81376971	0.06503433	0.38538719
Cuedc2	0.40430743	4.43643921	3.81273242	0.06506783	0.38538719
Dopey2	-0.3910031	5.69919646	3.81204653	0.06508999	0.38538719
Jam2	0.26257077	5.78114302	3.81178551	0.06509843	0.38538719
Myom1	-1.344039	0.4210617	3.81064699	0.06513524	0.38543556
Znhit6	0.28510119	5.17501458	3.80903133	0.06518753	0.38545031
Hsp90b1	0.28410211	9.12486298	3.80879967	0.06519503	0.38545031
Gm15055	2.24047118	-1.4464587	3.80418256	0.06534472	0.38603336
Nfatc4	0.77747426	3.20919778	3.80398859	0.06535102	0.38603336

Gulp1	0.4246067	5.95941799	3.80280882	0.06538934	0.38609021
Apopt1	-0.2812679	4.12066254	3.80102917	0.06544719	0.3862623
Tigd2	-0.3372753	4.43055657	3.79814117	0.06554119	0.3863689
Serpinh1	0.47172375	4.25435092	3.79720024	0.06557185	0.3863689
Ccdc65	-0.6519672	2.03665474	3.79709886	0.06557516	0.3863689
Spryd7	0.25864539	5.47150062	3.79694723	0.0655801	0.3863689
Akap5	0.25156252	8.01809372	3.79261905	0.06572137	0.3870091
Akr1b8	0.64739369	2.14255392	3.79185683	0.06574628	0.3870091
0610009O20	-0.3462504	4.90817008	3.7899136	0.06580985	0.38721391
Zfp566	-0.6979642	1.84360935	3.78845389	0.06585765	0.38732578
Ppp2ca	0.1976585	8.45582872	3.7860257	0.06593725	0.38754783
Casp6	0.57142073	2.37330272	3.78509189	0.06596789	0.38754783
Unc45b	-2.2914489	-0.6686464	3.78408112	0.06600107	0.38754783
Pycrl	0.4705156	2.65084745	3.78274635	0.06604493	0.38754783
Gm19395	-2.2861114	-0.4158174	3.78211477	0.06606569	0.38754783
Actb	0.28897974	9.97274988	3.78095058	0.06610397	0.38754783
Cmah	-0.3156169	6.36639796	3.78047427	0.06611965	0.38754783
Nsl1	-0.5559096	4.58000397	3.7802355	0.06612751	0.38754783
Zfp866	-0.2662485	5.11100105	3.77941218	0.06615461	0.38754783
Sav1	0.26580155	6.59991401	3.77772309	0.06621026	0.38770503
Smr3a	-2.2506704	-0.5832651	3.77237354	0.06638685	0.38842761
Ephx2	0.35937505	3.85688729	3.77223637	0.06639139	0.38842761
Eya2	0.44280592	6.41533242	3.76669079	0.06657504	0.38933281
Bhlhb9	0.24565557	5.47364275	3.76500045	0.06663113	0.38949159
Snd1	0.26245168	5.02085632	3.76078307	0.06677133	0.38969066
Klf15	-0.4603786	4.25353879	3.76066557	0.06677524	0.38969066
Slc29a3	-0.344213	5.26561223	3.75994206	0.06679933	0.38969066
1700003F12I	-2.8301884	-2.0074229	3.75892486	0.06683321	0.38969066
Arv1	-0.991352	0.87145989	3.75602411	0.06692994	0.38969066
Rfx1	-0.5251815	3.37654182	3.75598036	0.0669314	0.38969066
Ttc1	0.32208592	6.00652084	3.75505883	0.06696216	0.38969066
Ppil6	-0.8696228	2.35318332	3.7541089	0.06699389	0.38969066
Kcne2	-1.9228525	-0.9974753	3.75407318	0.06699508	0.38969066
Zdhhc17	-0.3118678	6.78753137	3.75346661	0.06701535	0.38969066
Pcbp1	0.28717713	5.75618372	3.75297111	0.06703192	0.38969066
Ccne1	-0.6588247	1.77384286	3.75285301	0.06703587	0.38969066
Cnnm4	0.61273025	2.88400049	3.75267954	0.06704167	0.38969066
Mn1	-0.4038892	4.58576522	3.75098179	0.06709846	0.3898524
Cbx2	-0.9569354	1.04972225	3.7490866	0.06716193	0.39004151
Sowahb	-0.4443624	3.51482256	3.74827944	0.06718898	0.39004151
Elmo2	0.253135	5.92396524	3.74720244	0.0672251	0.39008288
Nrxn1	-0.3136236	9.77366721	3.74575173	0.06727378	0.39013798
Zscan12	0.34196891	4.26561548	3.74519185	0.06729258	0.39013798

Cacna1h	-0.5263117	3.2704708	3.74264331	0.06737823	0.3904663
Rom1	0.7636721	1.51486163	3.73794681	0.06753639	0.39121441
A330035P11	0.75994264	1.93256336	3.73580113	0.06760879	0.39126185
Chit1	2.21890187	-1.5464265	3.81223296	0.0676375	0.39126185
Tmem121	2.58884873	-1.3857168	3.73422229	0.06766213	0.39126185
Tox2	0.45525456	2.75127914	3.73407318	0.06766717	0.39126185
Card14	-1.4212221	0.21782031	3.7333988	0.06768996	0.39126185
Agpat6	-0.3404493	4.13480936	3.72927788	0.06782946	0.39189984
Bambi-ps1	1.66041835	-0.9567035	3.72769925	0.06788299	0.39194085
Vav2	0.62289768	1.76348009	3.72735068	0.06789482	0.39194085
Erbb4	-0.3807999	4.58491755	3.72412025	0.06800452	0.39240583
Slc25a22	0.29451589	4.94675892	3.7199606	0.06814609	0.39269276
Med15	0.23188484	6.51689822	3.71968621	0.06815544	0.39269276
Nfatc2ip	0.51349664	2.6767404	3.71885831	0.06818366	0.39269276
Klrk1	1.21526722	0.53259997	3.71856017	0.06819383	0.39269276
Cobll1	-0.3565552	4.89653014	3.71837441	0.06820016	0.39269276
Fdft1	0.26002872	6.27779223	3.71060493	0.06846574	0.39381722
Nop9	0.46678925	3.74602846	3.70926518	0.06851165	0.39381722
Maged2	0.3157433	4.4644189	3.70859613	0.0685346	0.39381722
Gm4371	2.25800198	-0.7745846	3.70818222	0.06854879	0.39381722
Gja1	0.5527489	7.63703982	3.70755959	0.06857016	0.39381722
Pdcd10	0.26992004	6.07404911	3.70698509	0.06858988	0.39381722
A330032B11	-0.5719385	2.15244353	3.7066811	0.06860032	0.39381722
Chek2	-0.6440629	2.34680415	3.7054188	0.06864367	0.39382654
Dzip1	-0.2447471	6.54829099	3.70492997	0.06866047	0.39382654
Cog2	0.37499354	3.48085586	3.69947697	0.06884819	0.39473502
Tll1	0.46586225	3.55557792	3.69735009	0.06892157	0.39498744
Chm	-0.2770404	6.37748255	3.69513582	0.06899806	0.39525746
Ssb	0.23560572	8.09090107	3.69247997	0.06908994	0.39531898
Csf2ra	-0.5013281	3.82945891	3.69178268	0.06911408	0.39531898
Nron	-1.4595534	0.25872648	3.69131224	0.06913038	0.39531898
Pcdhb20	-0.5424988	3.63432425	3.68934236	0.06919866	0.39531898
1700123O20	0.43985142	3.87720246	3.68927713	0.06920092	0.39531898
Il2ra	0.80446629	1.922807	3.68837407	0.06923225	0.39531898
Nfkbid	1.87660955	-1.1082854	3.68790377	0.06924857	0.39531898
Bmp15	0.76367766	2.22693816	3.68785306	0.06925033	0.39531898
Mettl9	-0.307833	4.83069938	3.68635351	0.06930241	0.39531898
Slk	0.26073987	8.16449595	3.68557108	0.0693296	0.39531898
Ell3	0.67120226	2.29649561	3.68550309	0.06933196	0.39531898
Cd3g	-2.7097589	-1.3715452	3.68367214	0.06939564	0.39551446
Phf23	0.36652865	4.64131352	3.68138305	0.06947534	0.39567166
Cx3cl1	0.29919648	6.78151362	3.68119115	0.06948203	0.39567166
Lsg1	0.33173872	4.02144153	3.67766425	0.06960505	0.39591368

Tktl2	-2.7988322	-1.1117292	3.67714598	0.06962315	0.39591368
Epha8	-1.0900225	0.67208975	3.67685673	0.06963325	0.39591368
Hdac4	0.36800235	5.07433914	3.67660024	0.06964221	0.39591368
Hbs1l	-0.250798	5.48903497	3.6753211	0.06968692	0.39600055
Csmd3	-0.503226	5.37477156	3.67315944	0.06976255	0.39605912
Txnl1	0.23564237	7.14529889	3.67306068	0.06976601	0.39605912
Slc36a4	-0.3363585	4.97063688	3.67161933	0.06981649	0.39605912
Dnpep	0.47209174	2.95419845	3.67133796	0.06982635	0.39605912
Ccdc71	0.41075454	4.2507704	3.67079401	0.06984542	0.39605912
Xpo6	0.21002661	6.87091952	3.66778521	0.06995099	0.39605912
Xrcc6	0.46758604	3.3703194	3.66752415	0.06996016	0.39605912
Ywhaz	0.18668397	12.3202423	3.66590362	0.0700171	0.39605912
Lipe	0.63248541	2.00487894	3.6657363	0.07002299	0.39605912
Cyb561a3	-0.4000232	3.48963815	3.66530102	0.07003829	0.39605912
Ndufaf1	0.51733253	3.33368147	3.66511335	0.07004489	0.39605912
Lpar3	-0.8541369	2.27219078	3.6638736	0.07008851	0.39605912
Mum1	0.64575289	2.98551717	3.66352627	0.07010074	0.39605912
Hadhb	0.25710271	6.50866812	3.662724	0.07012899	0.39605912
Ammecr1	0.7023031	2.35776292	3.66244765	0.07013872	0.39605912
Egflam	0.48088681	3.52709281	3.65841609	0.07028091	0.39669554
Chchd7	-0.4206909	3.63145787	3.65667728	0.07034234	0.39687579
Cdca3	-1.6772989	-0.5375831	3.65348456	0.07045529	0.39734646
Cul3	0.20219812	8.90574059	3.65259591	0.07048676	0.39735744
Ddit3	-0.3786138	3.40717023	3.6449736	0.07075741	0.39871613
Egr1	-0.44871	9.14900846	3.64326952	0.07081808	0.39889097
Hipk2	-0.2301623	7.03675887	3.64076697	0.07090729	0.39922634
Fbxl20	0.22462523	6.62663552	3.6327818	0.07119281	0.40051175
Crlf3	-0.5453149	3.11838493	3.63271723	0.07119512	0.40051175
Lztr1	0.22877871	5.7292613	3.63122801	0.07124852	0.40064465
Raver1	0.3646543	6.00400555	3.62737462	0.0713869	0.40125512
Zbtb18	-0.2549643	7.35838544	3.62439084	0.07149427	0.40169081
Myh7	0.55304862	2.56956059	3.62063736	0.0716296	0.40204654
Mpv17	0.32067034	5.95198813	3.6191348	0.07168385	0.40204654
Asf1a	0.24791889	5.03613629	3.61852425	0.07170591	0.40204654
Chrna4	-0.3410763	4.50706817	3.61814354	0.07171967	0.40204654
Hfm1	-0.9213153	1.86536128	3.61792012	0.07172775	0.40204654
Trpc3	-0.4240923	3.63334456	3.61766838	0.07173685	0.40204654
Aldob	1.64065803	0.34713287	3.6129563	0.07190745	0.4028349
Pla2g16	0.39651661	6.73766589	3.61002116	0.07201396	0.40314679
Rbm12	0.26721348	5.25852923	3.60956886	0.07203039	0.40314679
Anxa1	0.52646432	6.7899027	3.60834612	0.07207482	0.40314679
1700016K19I	-1.0205682	1.06684404	3.60733951	0.07211142	0.40314679
Chrna3	-1.1534763	0.04976638	3.60722052	0.07211575	0.40314679

Mgrn1	0.22756893	6.53038613	3.60647482	0.07214288	0.40314679
Babam1	-0.314968	4.48843025	3.60234485	0.07229336	0.40315748
Cit	0.30625481	7.31494479	3.59952001	0.0723965	0.40315748
Mmp13	3.04847551	-1.9234618	3.59680726	0.0724957	0.40315748
Cyfip2	-0.3294043	9.89947236	3.59674348	0.07249804	0.40315748
Phax	0.26801829	5.38378733	3.5962997	0.07251428	0.40315748
Fam115c	0.88887248	1.3926034	3.59571242	0.07253579	0.40315748
Mical3	-0.4023695	6.27076941	3.59547273	0.07254456	0.40315748
Fdps	0.43926098	3.83319565	3.59530418	0.07255074	0.40315748
Rd3	-3.7231782	-1.8484215	3.59487032	0.07256663	0.40315748
Arx	-0.6001607	2.59868068	3.59454807	0.07257844	0.40315748
Trim23	0.27218869	6.39756976	3.59417264	0.0725922	0.40315748
Usp50	2.73750692	-1.5371428	3.59395017	0.07260036	0.40315748
Snx9	0.34659468	4.81473648	3.59362704	0.0726122	0.40315748
H2-Ob	-2.185795	-0.2005902	3.59347636	0.07261773	0.40315748
Tgm2	0.36820114	4.09520623	3.59298628	0.0726357	0.40315748
Ppp1r8	-0.6452765	3.99769124	3.59279345	0.07264277	0.40315748
Thoc5	0.41297147	3.55371757	3.59248408	0.07265412	0.40315748
Tor1aip2	0.30940812	7.06303032	3.59110717	0.07270466	0.40325954
Dok5	0.41773557	3.31033104	3.59035058	0.07273245	0.40325954
1700001K23I	2.62596912	-1.6193535	3.58827519	0.07280874	0.40338397
Cysltr2	-2.2229312	-0.9832844	3.58810918	0.07281485	0.40338397
Utp15	-0.3442102	5.04699468	3.58503622	0.07292799	0.40384448
Gemin7	-0.4951803	3.2202262	3.58327094	0.07299307	0.40396168
Ncf2	-0.6655105	2.47627398	3.58207781	0.0730371	0.40396168
Tcf3	0.45871194	3.66483439	3.58187887	0.07304445	0.40396168
Gucy2f	-1.5890112	0.43556857	3.58118404	0.0730701	0.40396168
Ccbe1	0.41667658	3.84561048	3.57999138	0.07311417	0.40396168
Tnfrsf25	3.37714541	-1.3089208	3.57958275	0.07312927	0.40396168
Itgam	-0.5055338	3.55949503	3.57754867	0.07320452	0.40421138
Zfp944	0.3807522	3.91730998	3.57504233	0.07329735	0.40438416
Map3k10	-0.3998872	3.26749762	3.57441071	0.07332077	0.40438416
1110057K04I	0.26527096	5.54252814	3.57427059	0.07332596	0.40438416
Snap91	0.33162127	8.93722681	3.57261432	0.07338741	0.40455725
Aldh1l1	0.38632944	3.6319835	3.56974447	0.07349403	0.40497908
Lage3	0.50457974	3.39926527	3.56659576	0.07361121	0.40545877
Cdh6	-0.4146455	2.79517801	3.56299001	0.07374567	0.40603317
Gm3086	-1.5628699	0.26584689	3.56143939	0.07380358	0.40618582
Chp2	-0.829905	0.84874003	3.55819083	0.07392507	0.40647158
Hnrnpa1	0.24275654	5.07049439	3.55763872	0.07394574	0.40647158
A230073K19	-0.7739507	4.54489843	3.5576286	0.07394612	0.40647158
Flii	0.28275896	5.38782624	3.55525328	0.07403513	0.40677009
Scaf1	-0.3340549	4.68206565	3.55456661	0.07406089	0.40677009

Frmpd4	0.28652671	7.77636696	3.55163759	0.07417086	0.40691093
Cct6a	0.2159613	8.24498773	3.55149566	0.07417619	0.40691093
B4galnt2	-0.6459595	3.0710878	3.55134107	0.074182	0.40691093
Lyrm5	0.33653164	5.17912218	3.54943701	0.07425361	0.40691093
Pip5k1b	-0.5711859	2.98888508	3.54830001	0.0742964	0.40691093
Cped1	0.3992234	7.25689517	3.54826583	0.07429769	0.40691093
Cenpq	0.76461287	2.30652887	3.5478645	0.0743128	0.40691093
Aen	0.37384129	4.24432563	3.54744925	0.07432844	0.40691093
Usp53	0.24782521	6.92540021	3.54647776	0.07436505	0.40694578
Micu3	-0.2844979	7.58963231	3.54446235	0.07444106	0.40717494
Rnf169	-0.2096016	6.75839916	3.54370882	0.0744695	0.40717494
Atad2	-0.4358214	4.80944559	3.54151613	0.07455234	0.40717494
Kat2b	0.26085204	6.08005269	3.54126234	0.07456193	0.40717494
Serpinb6c	-0.8731081	2.72569528	3.54014251	0.07460429	0.40717494
Kdm5b	-0.3482627	5.90629015	3.53980776	0.07461695	0.40717494
Xcl1	1.98623251	-0.5193357	3.53951077	0.07462819	0.40717494
Tusc2	-0.2581375	4.92345459	3.53896105	0.074649	0.40717494
Mrps23	0.35351422	4.23293926	3.53811685	0.07468097	0.40718426
Nphp1	-0.3917909	3.83450692	3.5349244	0.074802	0.40745335
Gm10538	-1.71048	-0.6379455	3.5345476	0.07481631	0.40745335
Tcea2	-0.5201107	2.74191191	3.53250005	0.07489407	0.40745335
Ptges2	-0.4713331	2.3907811	3.53243511	0.07489654	0.40745335
Pkp1	0.96694125	1.20327792	3.53141371	0.07493537	0.40745335
Tmem87a	-0.3311142	4.44738721	3.53005992	0.07498687	0.40745335
Bicc1	0.37834628	6.88745803	3.52958036	0.07500512	0.40745335
Il1f9	-0.6532323	2.29455078	3.52917845	0.07502043	0.40745335
4930469G21	-1.2996716	-0.1709537	3.52892327	0.07503014	0.40745335
Best1	-0.73444	1.46628805	3.52884514	0.07503312	0.40745335
Gchfr	-1.4762077	0.21337602	3.52614942	0.07513586	0.4075955
Tspan1	-2.9961805	-1.3200596	3.52513997	0.07517438	0.4075955
Sez6	0.36489378	4.84745636	3.52477279	0.0751884	0.4075955
Pcdhgb4	-0.5736337	2.29278833	3.52446251	0.07520024	0.4075955
Wdr62	0.98947355	1.01093731	3.52418743	0.07521075	0.4075955
Tnfsf9	-2.7942203	-2.0132422	3.52226076	0.07528437	0.40783023
Txlng	0.26078273	5.65176157	3.52127434	0.07532209	0.40783645
5430417L22F	-0.2242929	6.02537351	3.52064599	0.07534613	0.40783645
Sox10	0.40707233	4.29225309	3.51723518	0.07547679	0.40794567
Lonrf3	0.29680872	5.72486877	3.51700065	0.07548579	0.40794567
Nt5e	0.49653658	4.27306299	3.51696286	0.07548724	0.40794567
Capn2	0.28594221	7.15307409	3.51642377	0.07550791	0.40794567
Ntm	-0.241632	7.47633413	3.51494783	0.07556456	0.40794567
Sp140	0.30288918	4.89215056	3.51406095	0.07559862	0.40794567
Gpc3	0.5221493	3.71078847	3.5139192	0.07560407	0.40794567

Pcdhgb7	-0.5705566	2.21993231	3.51159496	0.07569343	0.40794567
Cttnbp2nl	0.24338761	6.16218878	3.51130596	0.07570455	0.40794567
Prdm5	0.48273866	3.03414188	3.51064769	0.07572988	0.40794567
Crygs	-2.6241165	-2.0379571	3.51037979	0.0757402	0.40794567
Atg16l2	-0.9297308	1.46873458	3.51031477	0.0757427	0.40794567
Scrt1	-0.3918093	5.3381451	3.5096236	0.07576932	0.40794567
Prkaa1	0.24260779	5.69360873	3.50906754	0.07579074	0.40794567
Kif2c	-2.1940742	-1.5242468	3.50681821	0.07587746	0.40824917
Kctd7	0.61642638	2.79768907	3.50405923	0.075984	0.40831852
Ascl1	-0.4761721	3.37908203	3.50333805	0.07601187	0.40831852
Fzd6	0.52660512	3.99486454	3.50240667	0.07604789	0.40831852
Rtfdc1	0.33861308	5.58179619	3.50195223	0.07606547	0.40831852
E130012A19I	-0.4826258	2.9411	3.50142189	0.07608599	0.40831852
Cd38	0.49542676	2.81277897	3.50105737	0.0761001	0.40831852
Hspa4	0.27111213	8.78386391	3.50098871	0.07610276	0.40831852
Pnrc1	0.24584505	7.15483839	3.49893655	0.07618226	0.40853589
Tyk2	0.56098118	2.82495666	3.49799173	0.07621889	0.40853589
Adamts20	-0.8429746	2.22272247	3.49759299	0.07623436	0.40853589
Fam131b	-0.3825756	4.95506433	3.49643548	0.07627927	0.40861387
9430021M05	-0.415122	4.43883393	3.49378209	0.07638235	0.40877641
Uxs1	-0.3413083	3.5382047	3.49332937	0.07639996	0.40877641
Rmdn3	-0.3369617	3.79942027	3.49330899	0.07640075	0.40877641
Fam81a	-0.2802021	5.71032253	3.49092189	0.07649365	0.40900381
Fads2	0.51655395	3.20624724	3.49065507	0.07650404	0.40900381
Car13	0.4128958	7.61595465	3.48809127	0.07660398	0.40923377
Rhoa	0.42193781	8.61857226	3.48799133	0.07660788	0.40923377
Top1	0.25006588	8.05108416	3.4860838	0.07668234	0.40945686
Loh12cr1	-0.4880294	2.95649361	3.48299745	0.07680299	0.40945686
Pamr1	-0.3494461	4.03100599	3.48224642	0.07683238	0.40945686
Gmds	-0.4247604	3.36496846	3.47979173	0.07692854	0.40945686
Arhgdig	-0.5757038	3.12759263	3.47838315	0.07698378	0.40945686
Cnr1	0.27853012	6.5698156	3.47814402	0.07699316	0.40945686
Rbm5	-0.2785896	6.94168735	3.47800851	0.07699848	0.40945686
Arntl2	-0.6861716	2.04468903	3.47753416	0.0770171	0.40945686
Creb3l2	0.39860401	5.98241077	3.47705259	0.077036	0.40945686
9330188P03I	1.27473179	0.9897814	3.47672437	0.07704889	0.40945686
Ttc33	-0.233544	6.57140083	3.47654318	0.07705601	0.40945686
Ddx11	-1.3484814	0.10747502	3.47633083	0.07706435	0.40945686
Dcun1d1	0.20989805	6.8076592	3.4759678	0.07707861	0.40945686
Bhlhe22	-0.3080019	5.19943466	3.47423421	0.07714676	0.40945686
Edn1	-1.2647609	0.28649331	3.47114821	0.07726824	0.40945686
Gm10336	-0.4081921	4.45239221	3.47082437	0.077281	0.40945686
Zfp964	-0.7747403	1.2599047	3.47035608	0.07729946	0.40945686

Prima1	0.62076761	1.40377764	3.470083	0.07731023	0.40945686
4930452G13	-2.6343705	-1.7082756	3.46981472	0.07732081	0.40945686
Tmem38b	-0.4456364	3.18982367	3.46900213	0.07735286	0.40945686
9130008F23I	-1.4340369	-0.5012529	3.46883665	0.07735939	0.40945686
Mbd2	0.28837599	7.38678796	3.4673102	0.07741964	0.40945686
Hnrnpd	0.20221604	8.29361653	3.46696699	0.0774332	0.40945686
Cyp2j9	-0.6749339	2.44796972	3.46693768	0.07743435	0.40945686
Asb7	-0.198227	6.12224714	3.46551943	0.0774904	0.40945686
Fbn1	0.41494175	3.99856872	3.46497995	0.07751173	0.40945686
Erh	-0.3736858	5.18294234	3.46454529	0.07752892	0.40945686
Arhgef6	0.26348808	6.45736664	3.46437481	0.07753566	0.40945686
4930427A07I	-1.7164266	-0.0174891	3.46322968	0.07758098	0.40945686
Man2b1	0.39726298	4.0911535	3.46250625	0.07760963	0.40945686
Prpf4	0.27126582	5.00342337	3.4611965	0.07766152	0.40945686
Oprd1	0.43467876	4.16299304	3.46102914	0.07766815	0.40945686
6820431F20I	-0.291563	8.79642571	3.46064542	0.07768336	0.40945686
Tbl3	0.60294158	2.07913153	3.4603588	0.07769473	0.40945686
Cntn1	0.27079813	8.31121589	3.4595526	0.07772671	0.40945686
Spaca5	-2.6162292	-1.6395404	3.45881354	0.07775603	0.40945686
Rrbp1	0.2893761	6.08677972	3.45832315	0.0777755	0.40945686
Irak1bp1	-0.3210736	5.9075469	3.45743383	0.07781082	0.40948259
Lats2	-0.3015584	6.82253489	3.45610596	0.07786358	0.40960009
Nt5c3	0.25115698	5.14275865	3.45362687	0.07796221	0.40974733
Pvrl3	-0.2895451	5.40676394	3.45331116	0.07797478	0.40974733
Robo4	-1.0814682	0.64692429	3.45310673	0.07798292	0.40974733
Card10	1.08738855	0.38311934	3.44980864	0.0781144	0.41027794
9330133O14	0.40098361	4.07579386	3.44721807	0.07821785	0.41040529
Slc25a43	2.53635947	-1.5902955	3.44708769	0.07822306	0.41040529
Etnk2	0.53960643	2.60113181	3.4469105	0.07823014	0.41040529
St3gal6	-0.3800207	4.08416693	3.44514506	0.07830075	0.4106156
Tspan4	-0.6739238	2.48387872	3.44393008	0.07834938	0.41071058
Icam1	0.90500257	1.87701251	3.44122183	0.07845791	0.41111935
Tgfb1	0.59158877	2.17365205	3.43596252	0.07866917	0.41179431
Slc30a6	-0.4138976	3.08987767	3.43576993	0.07867692	0.41179431
Bcl6	0.18798036	6.10404496	3.43573002	0.07867853	0.41179431
Ralyl	-0.2116739	7.08495102	3.43481959	0.07871517	0.41182592
Ube2s	-0.440395	3.69044708	3.42787891	0.07899517	0.41304287
Akap8	-0.3459356	5.82462808	3.42718742	0.07902313	0.41304287
Hnmt	-0.3324709	5.1539492	3.42664236	0.07904518	0.41304287
Amotl2	-0.5612185	3.80934103	3.42587634	0.07907618	0.41304287
Mospd2	0.29471571	5.1513818	3.42500708	0.07911137	0.41304287
Snord118	-2.0446515	-1.5551468	3.42280192	0.07920072	0.41304287
Sertad1	0.56144537	2.92452952	3.42229744	0.07922118	0.41304287

Dbpht2	-0.2544924	7.6516806	3.42196904	0.0792345	0.41304287
Fgf1	0.28451895	7.40894278	3.42165773	0.07924713	0.41304287
Eef1a2	0.28519354	5.59721423	3.4210727	0.07927088	0.41304287
Ubxn6	0.23990106	5.56864892	3.42071437	0.07928542	0.41304287
Eif2b4	0.46246553	3.19675848	3.41965807	0.07932832	0.41310641
Bin3	0.48646223	2.93786398	3.41394018	0.07956099	0.41404109
B3galt5	-0.5841991	3.22309558	3.41296803	0.07960063	0.41404109
Kcnd1	-0.6472889	2.40261102	3.41226689	0.07962923	0.41404109
A4galt	0.51905783	2.77150386	3.41222654	0.07963088	0.41404109
Hps6	0.75315659	1.04365059	3.40950634	0.07974196	0.41421801
Praf2	0.36798836	4.63199854	3.40922928	0.07975329	0.41421801
Cd83	0.47700021	2.18761984	3.40818365	0.07979604	0.41421801
Fblim1	0.71751657	3.58566952	3.4076948	0.07981604	0.41421801
Gm9079	1.28292304	0.57744358	3.40637917	0.07986989	0.41421801
Fbxw7	-0.2259934	8.24940269	3.40615628	0.07987901	0.41421801
Hoga1	-1.5567705	-0.0458098	3.40612284	0.07988038	0.41421801
Mcmdc2	-0.7124351	2.5289707	3.40163585	0.08006438	0.41499638
Tcf20	-0.2789812	8.10599558	3.40095918	0.08009217	0.41499638
Dmrt2	-2.1292682	-0.9402289	3.398163	0.08020713	0.41543206
Srp54b	0.20464375	6.34362651	3.39546606	0.08031818	0.41584722
Ttll4	-0.5453516	2.29826772	3.39427311	0.08036737	0.41586117
Prdm15	-0.4787948	3.40894954	3.39390163	0.08038269	0.41586117
Sil1	0.58450144	2.5779536	3.39169077	0.08047395	0.4161733
Lmo2	0.39370598	4.39602433	3.39001772	0.08054309	0.41631479
Gsg1	-2.0224012	-0.8084375	3.38953175	0.08056319	0.41631479
Gla	0.4462129	3.05440702	3.38758651	0.08064368	0.4165708
Sh3gl2	0.28031655	9.33445628	3.38409468	0.08078842	0.4171583
Sidt1	-0.3486669	5.12946762	3.38149683	0.08089629	0.4175551
Vps45	0.3541463	3.79900705	3.3799429	0.0809609	0.41756732
Klrb1b	-1.7224218	0.72419676	3.37938435	0.08098414	0.41756732
Gnas	0.22237534	10.8121039	3.37920129	0.08099176	0.41756732
Snrpa1	0.2934815	4.24823877	3.37688846	0.08108807	0.41790376
Ift46	0.40568567	4.57608285	3.37481556	0.0811745	0.41805603
Hspb3	-1.016662	0.34003333	3.37451564	0.08118702	0.41805603
Calb1	0.27049591	6.05115069	3.37373125	0.08121976	0.41805603
Tjp1	0.21984067	9.48507631	3.37320139	0.08124188	0.41805603
Emilin2	2.1196071	-1.0549577	3.36876251	0.08142751	0.41862188
Gpr115	0.66218423	1.61554284	3.36843271	0.08144133	0.41862188
Igfn1	-0.5514691	4.3999423	3.36745461	0.0814823	0.41862188
Lrrc34	-2.0385006	-1.0279857	3.36708289	0.08149788	0.41862188
Ndufa10	0.25341727	6.26299292	3.36685595	0.0815074	0.41862188
BB557941	2.5547296	-0.6396822	3.36571909	0.08155507	0.41870693
Trappc9	-0.3713871	5.74787062	3.36417999	0.08161967	0.41881633

Dap	0.41814265	5.24106919	3.36372847	0.08163863	0.41881633
Mlph	0.64272633	2.44175312	3.36221389	0.08170227	0.41898309
Jak3	1.25549895	-0.2724256	3.36050266	0.08177424	0.41911317
03-Sep	-0.2425764	8.9122498	3.36013	0.08178993	0.41911317
Arrdc4	-0.5875244	2.72777521	3.35861919	0.08185355	0.41925135
Pcdh8	0.51690822	2.58289504	3.35708235	0.08191832	0.41925135
1810055G02	0.28638821	5.57403239	3.35639731	0.08194722	0.41925135
Pcdhb6	-1.1893034	0.85978822	3.35629213	0.08195165	0.41925135
Gm10649	-1.6920976	-1.0484694	3.35579386	0.08197268	0.41925135
Mapre3	0.23589633	7.32864478	3.352833	0.08209774	0.41973145
Pkm	0.25690269	8.20468187	3.35092811	0.08217831	0.41996198
Tmcc2	-0.3116254	4.78003844	3.35012221	0.08221243	0.41996198
Ptprf	0.27747061	4.74980605	3.34751071	0.0823231	0.41996198
Selenbp1	-0.5981301	3.05446523	3.34654781	0.08236395	0.41996198
Ifitm2	0.57862548	7.3431538	3.34609415	0.0823832	0.41996198
Plxnb3	-0.7411615	2.04041531	3.34546398	0.08240995	0.41996198
Efcab2	0.31024696	3.97879449	3.34493819	0.08243228	0.41996198
Col16a1	0.5860977	1.53962728	3.34237148	0.08254139	0.41996198
Pik3cg	0.49470236	2.45335659	3.34198298	0.08255792	0.41996198
Fbxl14	-0.3015826	5.24253951	3.34105586	0.08259738	0.41996198
Rbfox1	-0.2495302	9.25183504	3.33997643	0.08264335	0.41996198
Top3a	-0.7397091	0.99311995	3.33963103	0.08265807	0.41996198
Sdr42e1	0.41999289	2.65229571	3.33911466	0.08268007	0.41996198
Slc12a6	-0.2234426	6.78989424	3.33900476	0.08268476	0.41996198
Aox4	-1.1291017	0.66151393	3.33846556	0.08270775	0.41996198
Csrp2	0.33041552	4.57851133	3.33810307	0.0827232	0.41996198
Slc3a2	0.25390913	6.32439206	3.33799431	0.08272784	0.41996198
Rpa3	-0.5023706	3.1345464	3.33768515	0.08274103	0.41996198
Ctps	-0.3858212	3.98879188	3.33727705	0.08275844	0.41996198
Figf	0.88743611	1.88956967	3.33702492	0.0827692	0.41996198
Rab10os	-0.4400282	3.09165259	3.33634498	0.08279823	0.41996198
Hilpda	0.52239506	2.63433833	3.33435647	0.08288318	0.4201131
Rtn4rl1	0.23707578	6.19990827	3.33418607	0.08289046	0.4201131
Slc38a5	-1.9452187	-1.1945764	3.32819888	0.08314687	0.42052294
Zcchc17	-0.2666929	5.21882703	3.32815008	0.08314897	0.42052294
Zfat	-0.5497422	2.02412204	3.32733851	0.0831838	0.42052294
B3gnt8	-1.4371844	-0.1537182	3.32453997	0.08330403	0.42052294
Pdia6	0.33426035	4.75854562	3.32421336	0.08331807	0.42052294
Grp	-1.2688856	0.11469407	3.32342122	0.08335215	0.42052294
Slc14a2	-1.9349352	-0.4003729	3.32324565	0.0833597	0.42052294
Gm5820	-0.716756	1.83755461	3.32295361	0.08337227	0.42052294
Glyctk	-0.8936318	1.38324795	3.32124315	0.08344593	0.42052294
Ap1s1	-0.4306492	2.8383921	3.32065284	0.08347136	0.42052294

Coq6	0.78771338	1.27354156	3.32046445	0.08347948	0.42052294
Sclt1	-0.3332575	4.66918407	3.3203969	0.0834824	0.42052294
Nfil3	0.60628052	2.54597835	3.3199822	0.08350027	0.42052294
Kcnc3	-0.4516003	5.24132334	3.31940435	0.08352519	0.42052294
Icmt	-0.2640009	4.61061729	3.31940349	0.08352523	0.42052294
Malat1	-0.616354	12.8518958	3.31926528	0.08353119	0.42052294
Eif3c	0.19658246	8.03434266	3.31893209	0.08354556	0.42052294
Plcb4	-0.3026801	7.05152563	3.31857956	0.08356078	0.42052294
Tm7sf3	-0.3487311	4.54772077	3.31847951	0.08356509	0.42052294
Lrrc29	-1.7502551	-0.596089	3.31677929	0.0836385	0.42071809
Lamp5	-0.2753246	5.75821944	3.31613359	0.0836664	0.42071809
Ccdc57	0.58360803	1.83468668	3.31471562	0.08372771	0.42073439
Clcn2	-0.3669618	3.90367884	3.31373367	0.08377019	0.42073439
Ddx24	-0.2396762	5.66962142	3.31323781	0.08379166	0.42073439
Btc	-3.0159229	-1.977228	3.3128238	0.08380958	0.42073439
Cpeb2	-0.2080176	7.36638977	3.31175615	0.08385583	0.42073439
Cbfa2t3	-0.3153269	4.7943287	3.31111032	0.08388382	0.42073439
Mael	-1.3146632	0.65600567	3.31100203	0.08388851	0.42073439
Tnni1	2.23549628	-1.0624681	3.30676535	0.0840724	0.42149958
Hs3st5	-0.7796452	1.6804255	3.30593999	0.08410828	0.42152241
Paqr7	-0.2757355	4.56034292	3.30337683	0.08421982	0.42179922
Cdh9	0.57072043	3.91287303	3.30323012	0.08422621	0.42179922
Minos1	0.28319601	6.48496504	3.30101213	0.08432287	0.42199553
Tst	-0.4180581	4.00671627	3.30089163	0.08432813	0.42199553
Stk32a	-0.6353802	1.75903453	3.29846482	0.08443405	0.42219466
Elovl5	0.39646179	6.21453658	3.29786681	0.08446017	0.42219466
Rapgef3	-0.4315441	2.66865458	3.29782393	0.08446204	0.42219466
Hist1h2bc	0.34479851	5.43187797	3.29565375	0.08455693	0.42251201
Nup155	-0.3310003	5.24826961	3.29484257	0.08459243	0.42253249
Prrg3	-0.212303	6.63317947	3.29301232	0.08467259	0.42277595
Myo18a	-0.2739791	6.30847376	3.28965963	0.08481966	0.42317236
N4bp3	0.82047576	1.3835988	3.28941884	0.08483023	0.42317236
Apoo	0.27284351	4.83079787	3.28905244	0.08484633	0.42317236
Timp1	1.01972872	1.52982041	3.28585355	0.08498698	0.4237168
Mtmr11	0.38318161	3.419479	3.28416977	0.08506112	0.42392938
Faim2	-0.2487227	6.72865198	3.28281695	0.08512074	0.42397795
Crcp	-0.3539623	4.59667811	3.28181588	0.08516489	0.42397795
Cd209c	-0.5029888	2.7033176	3.28180474	0.08516539	0.42397795
Jund	-0.312065	5.02178906	3.2805702	0.08521987	0.42408707
Ltv1	0.27888213	4.79175737	3.27875442	0.08530008	0.42408707
Slc38a1	-0.2314465	7.61637541	3.27842481	0.08531465	0.42408707
Neat1	-0.3146996	5.07494143	3.27789721	0.08533798	0.42408707
Arhgap36	1.22261794	0.91719473	3.27774117	0.08534488	0.42408707

Skiv2l2	0.23869019	6.77451449	3.27590627	0.08542608	0.42433384
Vps18	-0.4122769	3.64210523	3.27419778	0.08550176	0.42435081
Ergic2	-0.3143374	4.64021062	3.27394617	0.08551292	0.42435081
Ccdc170	-0.9725576	0.66336915	3.27369384	0.0855241	0.42435081
Clta	0.27053965	6.84018885	3.27113185	0.08563778	0.42475821
Dmrta1	-0.7308535	3.04450842	3.26717826	0.08581354	0.42526755
Ptpn13	0.26046629	6.26099968	3.26671912	0.08583398	0.42526755
Adat1	-0.6530624	1.8778403	3.26668996	0.08583528	0.42526755
Plxna1	-0.4042412	5.51998092	3.26536257	0.0858944	0.42528086
Cxx1a	0.2490609	6.02202972	3.26518279	0.08590241	0.42528086
Slc25a46	0.21365199	7.86801122	3.26443749	0.08593563	0.42528086
Clgn	0.61533185	2.17288949	3.26354864	0.08597527	0.42528086
Csf3r	-0.8079186	1.43917202	3.26247233	0.0860233	0.42528086
Gpr101	0.64846711	2.41118593	3.26169728	0.0860579	0.42528086
3632454L22R	0.98527515	0.7864583	3.2614251	0.08607006	0.42528086
4930528D03R	-1.364209	-0.1350901	3.2608089	0.08609758	0.42528086
Tial1	-0.2433449	5.9119415	3.26025348	0.08612241	0.42528086
Spata21	2.17806815	-1.3681654	3.2589759	0.08617953	0.42540533
Gadd45g	0.52859955	2.06850127	3.25774176	0.08623476	0.42540533
Fam78b	-0.2909975	6.81848028	3.25756984	0.08624245	0.42540533
Exosc9	0.30995766	4.0254313	3.25379909	0.08641146	0.42608278
Steap2	0.25759236	4.92439914	3.25307441	0.08644398	0.42608702
St8sia4	-0.4792063	3.27236278	3.25049044	0.08656007	0.426503
A930015D03	0.78650555	1.37320484	3.24978356	0.08659186	0.42650346
Cmpk1	0.25165794	7.47820594	3.24740798	0.08669879	0.4267175
Thbs4	-1.0140087	0.57915578	3.24598465	0.08676293	0.4267175
Cabp7	2.05654862	-1.1968149	3.2459389	0.08676499	0.4267175
Dbndd2	-0.2278914	5.98223656	3.24548202	0.08678559	0.4267175
Thsd7a	-0.253345	6.78848442	3.24508359	0.08680356	0.4267175
Mrpl4	0.39733127	4.75516618	3.24459556	0.08682558	0.4267175
Vgll3	-0.5386423	2.95816261	3.24260123	0.08691562	0.42677557
Mycl	-0.5271476	2.03148345	3.24236905	0.08692611	0.42677557
Il10ra	1.35191006	0.14749932	3.24066222	0.08700327	0.42677557
Ipo13	-0.3049371	5.39730335	3.2406428	0.08700415	0.42677557
Sh2b2	-1.1894968	0.01806364	3.23950767	0.08705551	0.42677557
Hmces	0.53154042	2.01871036	3.23906781	0.08707542	0.42677557
Ccdc78	1.10760004	-0.2894292	3.2390146	0.08707783	0.42677557
Acad11	0.30439939	4.50550654	3.23868807	0.08709262	0.42677557
Suv39h1	-0.4687139	3.57991777	3.23719138	0.08716042	0.42677557
Arfgef2	0.30975184	6.1855433	3.2371558	0.08716204	0.42677557
Igf2bp2	0.64021877	1.32033371	3.23642735	0.08719506	0.42677557
Fezf1	2.62538508	-0.6692118	3.23564165	0.0872307	0.42677557
Psip1	0.23034799	8.79468226	3.23522284	0.0872497	0.42677557

Aff1	0.26916648	7.01573603	3.23377121	0.0873156	0.42694273
Ccz1	0.27764328	4.8060253	3.23236519	0.08737949	0.42699496
4930455C13I	-1.7015912	-0.7454366	3.23102688	0.08744035	0.42699496
Tlr5	-1.9749163	-1.2240123	3.22969978	0.08750075	0.42699496
Pms1	-0.4627049	2.75300023	3.22967464	0.0875019	0.42699496
Cd180	-0.6129279	2.7357235	3.22891698	0.0875364	0.42699496
Tnfaip8l3	-0.2210466	6.59912069	3.22833657	0.08756284	0.42699496
4930506M07	0.24238644	5.40685335	3.22777644	0.08758837	0.42699496
Mei4	1.14677216	0.42421138	3.22756889	0.08759783	0.42699496
Gm3230	-0.7529681	1.29743143	3.22655566	0.08764404	0.42699496
Mnt	-0.2722897	5.01566522	3.22583433	0.08767696	0.42699496
Ttc39d	-2.9265046	-1.637163	3.22553271	0.08769072	0.42699496
Kif20b	-0.7144168	1.82200334	3.22486335	0.08772128	0.42699496
Trim7	-1.0909839	0.29043078	3.22447976	0.0877388	0.42699496
Pigz	-0.5609658	2.17195228	3.2234815	0.08778441	0.42706248
Prps1l3	-0.2765461	4.70122548	3.22227912	0.08783938	0.42717548
Cdh15	-2.8140042	-1.396928	3.2211621	0.08789049	0.42723694
Osbpl2	0.23843609	5.46258645	3.22061525	0.08791552	0.42723694
Wdr76	-0.521408	2.1984456	3.21821501	0.08802549	0.42755557
Scgn	2.48901286	-1.7738319	3.21719656	0.0880722	0.42755557
Isg15	0.59923777	1.64213574	3.21710486	0.08807641	0.42755557
Speer4a	-0.9483768	0.36362606	3.21600586	0.08812685	0.427642
Fam173b	0.73696879	0.83055761	3.2139316	0.08822214	0.427642
Syt10	0.54268949	2.31250601	3.21370071	0.08823275	0.427642
Zfp362	0.28274949	5.19322583	3.21298367	0.08826573	0.427642
6430571L13F	-1.1875672	0.33278228	3.21278094	0.08827505	0.427642
Kdelr1	0.36674493	4.34912127	3.2122057	0.08830152	0.427642
Lrig2	-0.2714257	5.05686369	3.21146651	0.08833554	0.427642
Grin1os	0.98407687	0.24889645	3.21076197	0.08836799	0.427642
Mark4	-0.3721279	3.63180136	3.21000714	0.08840276	0.427642
Meox1	-1.7658508	-1.373733	3.20980629	0.08841201	0.427642
Fgd4	0.29243358	4.93470296	3.20783431	0.08850295	0.42782787
Gnb2	0.38520519	3.61938385	3.20750514	0.08851814	0.42782787
Gpr34	0.52931518	2.07134329	3.20690533	0.08854582	0.42782787
Dclk3	0.32732387	5.48919709	3.20617292	0.08857964	0.42783765
Zswim4	0.48509943	2.37334394	3.20425307	0.08866836	0.42804705
Kcnab2	-0.3158132	5.95193359	3.20385843	0.08868662	0.42804705
Gm7008	1.19572506	-0.366684	3.20185701	0.08877924	0.42834047
Trpv6	0.62365595	1.64659565	3.20050901	0.08884169	0.42848813
Rab3ip	-0.2686465	5.14672072	3.19846478	0.08893649	0.42862332
Hid1	0.25109961	6.11254569	3.19746766	0.08898277	0.42862332
AI462493	-0.5030135	3.34834797	3.19736986	0.08898732	0.42862332
Cabp1	-0.4819186	2.88542464	3.1971585	0.08899713	0.42862332

Sema3a	0.30904926	6.40552977	3.19482584	0.08910554	0.42898783
Araf	0.21934632	7.24708396	3.19390788	0.08914824	0.42898783
Cyp2u1	0.78541041	0.87259289	3.19347351	0.08916846	0.42898783
Leng9	0.757435	1.11170176	3.18890265	0.0893815	0.42985911
Pnpla1	-0.8166933	0.93716736	3.18449789	0.08958737	0.43069524
Cdo1	0.39953807	6.85151754	3.18260127	0.08967618	0.43094035
F11r	-0.5271759	2.71136474	3.18204175	0.0897024	0.43094035
Tmem245	0.29679774	5.51547541	3.18106165	0.08974835	0.43100723
Cbln3	-0.7622374	1.1983502	3.17964364	0.08981488	0.43117286
Inf2	0.24054982	6.07676718	3.17692211	0.08994274	0.43140508
Rnpep	0.43429091	3.44437432	3.17672871	0.08995183	0.43140508
Paip2	0.23359876	7.53393563	3.17606294	0.08998314	0.43140508
Lpar2	-0.7629249	1.631953	3.17588539	0.0899915	0.43140508
4930592I03R	-3.1216109	-1.8628536	3.23335798	0.09002948	0.43141165
Rbm42	0.32534597	4.24635712	3.17449393	0.09005699	0.43141165
Slc12a5	-0.3176743	7.82459709	3.17322359	0.09011683	0.43154467
Hdx	-0.586522	2.92416688	3.17032087	0.09025373	0.43204653
Gpnmb	1.18830063	1.08924575	3.1674009	0.0903917	0.43255309
Mgp	0.54712134	8.28888369	3.16669543	0.09042507	0.43255895
Tmem80	-0.4448629	2.82179066	3.16565791	0.09047417	0.43262762
C1qtnf3	3.15176912	-2.2217414	3.16503376	0.09050372	0.43262762
E030019B13I	2.29711677	-1.3835577	3.16238897	0.09062908	0.43285012
Gm13139	0.59623474	2.32441717	3.16167076	0.09066316	0.43285012
Stard3	-0.5109046	2.37085646	3.16138439	0.09067675	0.43285012
Gm12250	-0.9850361	0.08217832	3.1610968	0.0906904	0.43285012
Bms1	-0.2444198	6.0653261	3.16030338	0.09072808	0.43285012
Spata13	0.28028498	4.58809525	3.15987966	0.0907482	0.43285012
Pm20d2	-0.2892097	4.21869945	3.15930648	0.09077544	0.43285012
Camk4	-0.2852739	9.29810961	3.1561905	0.09092367	0.43325465
Ptges3l	-1.0222076	1.05137096	3.15564055	0.09094986	0.43325465
Cct2	0.18492427	7.0039922	3.15513309	0.09097404	0.43325465
Tyr	-0.6158621	2.55561045	3.15481773	0.09098906	0.43325465
Tas2r137	4.25738477	-1.9346366	3.29511967	0.09110171	0.43363759
Tmem74	-0.7031735	1.24584302	3.14913806	0.09126021	0.43412377
Tmem56	-0.2754756	6.25923531	3.14896723	0.09126838	0.43412377
Clvs1	0.30085134	4.16527292	3.14401116	0.09150577	0.43509916
Zfp354a	-0.3458152	3.36857815	3.14245502	0.09158046	0.43530047
Akr1e1	0.26316556	5.1974539	3.14120381	0.09164056	0.43543235
Fam47e	2.16029088	-1.255421	3.14035848	0.09168119	0.43547165
Smad6	0.62043897	2.58406296	3.13604501	0.09188886	0.43630402
Dnaja2	0.17620471	7.78861547	3.1351806	0.09193054	0.43634421
Col23a1	0.4466733	5.23591138	3.13436001	0.09197013	0.43634421
Vps25	0.35283728	5.12728745	3.1319212	0.09208791	0.43634421

She	-0.8926741	1.34917998	3.13054534	0.09215443	0.43634421
Gm10125	-0.8245593	1.01722952	3.13008625	0.09217664	0.43634421
Pcdhb4	0.69123876	2.06915542	3.129481	0.09220593	0.43634421
Thap1	0.45375289	3.74336405	3.12890002	0.09223406	0.43634421
Sec22c	-0.3044489	4.63441973	3.12842901	0.09225687	0.43634421
1700019A02I	-1.7380786	-1.7189132	3.12813937	0.0922709	0.43634421
Pim3	0.44957903	2.92316168	3.12635963	0.09235716	0.43634421
9430037G07	0.61069269	2.05071542	3.12515303	0.09241569	0.43634421
Gm11837	1.28578564	-0.748374	3.12503302	0.09242152	0.43634421
Apol8	-1.0986358	-0.2681025	3.12478908	0.09243336	0.43634421
Bcl9	-0.240504	6.42996014	3.12440412	0.09245204	0.43634421
Gpr155	0.30543341	5.73513975	3.1242124	0.09246135	0.43634421
Tasp1	-0.2545517	5.96691952	3.12411826	0.09246593	0.43634421
Psmd2	0.2546511	5.80504036	3.12356379	0.09249286	0.43634421
Lnx2	0.29402145	4.43629105	3.12302196	0.09251918	0.43634421
Gm20300	-0.2294282	7.24327955	3.12117616	0.09260893	0.43634421
Whrn	-0.4449322	3.21545349	3.12090893	0.09262193	0.43634421
Scly	0.47886927	3.52715264	3.12071941	0.09263115	0.43634421
Mybbp1a	-0.3231151	4.39925614	3.12048141	0.09264273	0.43634421
Cda	1.98255274	-0.9011938	3.11935292	0.09269768	0.43634421
Slc25a3	0.19313585	7.93988783	3.11934841	0.09269789	0.43634421
Tcf7	0.32695674	4.87830236	3.11856519	0.09273605	0.43634421
Tcp10b	2.31155438	-1.9858622	3.11821864	0.09275294	0.43634421
Morf4l2	0.25734553	7.36995146	3.11760417	0.09278289	0.43634421
Ythdf3	0.20008916	7.18928412	3.11714517	0.09280527	0.43634421
Ctc1	-0.3052751	4.13550488	3.11437863	0.09294031	0.43651124
Serpinb1b	1.20388605	-0.1128303	3.11370426	0.09297326	0.43651124
Slitrk4	-0.3047485	6.29654105	3.11353007	0.09298178	0.43651124
Zeb1	-0.1718867	7.53527288	3.11250682	0.09303181	0.43651124
Cops6	0.27273168	5.82043294	3.11226046	0.09304386	0.43651124
Rbm48	-0.3329923	3.43229954	3.11173002	0.09306981	0.43651124
Nr2f6	0.49741589	2.59815813	3.11080474	0.0931151	0.43651124
Nanos1	-0.228259	5.23537647	3.1100918	0.09315001	0.43651124
Eif2s1	0.21700563	6.78122622	3.10988446	0.09316017	0.43651124
Acot1	0.29955265	3.95154939	3.10978194	0.09316519	0.43651124
Slc1a4	0.36579955	4.33679528	3.10716842	0.09329333	0.43695949
Otud6b	-0.2808882	6.33504162	3.10416205	0.09344099	0.437375
Micu1	-0.2529948	4.45383813	3.10403875	0.09344705	0.437375
Notch3	-0.7399066	2.36530039	3.10334415	0.09348121	0.43738273
Pcsk1	-0.4259629	3.26775986	3.10235157	0.09353004	0.43739474
Npepl1	0.61921919	2.06684462	3.10197085	0.09354878	0.43739474
Zbtb8a	0.64379253	2.95987828	3.10119175	0.09358715	0.43742211
9330158H04I	2.35541441	-1.188079	3.09984472	0.09365352	0.43758033

Traf3ip3	-1.7909128	-0.8302029	3.09830325	0.09372953	0.43776947
Vsnl1	0.24862006	12.0201766	3.09770486	0.09375906	0.43776947
Rsrp1	-0.3864392	6.29280834	3.09458147	0.09391337	0.43805981
Sv2b	0.29941511	9.37558707	3.094031	0.0939406	0.43805981
Mageb16	-1.3083161	1.09658276	3.09351548	0.0939661	0.43805981
Wapal	0.18298837	7.56975924	3.09340682	0.09397148	0.43805981
Gpt2	0.26440428	4.97022842	3.09315349	0.09398402	0.43805981
Neurl2	1.41029204	-0.3121361	3.09121438	0.09408005	0.43835559
Tbc1d4	0.48167288	3.07730751	3.08932677	0.09417364	0.43853025
Krt10	0.54216575	2.23718246	3.08885313	0.09419714	0.43853025
Mal	-0.3223151	6.0909116	3.08792945	0.094243	0.43853025
Hp1bp3	0.17336158	8.1320659	3.08783087	0.09424789	0.43853025
Dok3	1.7618651	-1.3432992	3.08634415	0.09432175	0.43872221
Csnk2a2	0.31353251	5.19443205	3.08297678	0.0944893	0.43933679
Glis1	1.02200554	0.52950865	3.0823769	0.09451918	0.43933679
Col4a1	0.3747151	3.65295433	3.08143022	0.09456636	0.43940431
C1ra	-0.8207843	0.72250136	3.08063812	0.09460586	0.43943611
Trpc7	-0.586014	2.40421593	3.07703858	0.0947856	0.43985435
Pex5	-0.3334679	3.90300021	3.07694989	0.09479003	0.43985435
Rangrf	-0.4923495	2.89797601	3.07687122	0.09479397	0.43985435
Siah1a	-0.2501802	5.65534911	3.07564489	0.0948553	0.43996948
Chdh	-0.773507	1.47474422	3.07393123	0.09494108	0.43996948
Tuba1c	0.20136012	5.81028806	3.0736009	0.09495763	0.43996948
Prkaca	-0.2506182	7.24830415	3.07273079	0.09500123	0.43996948
Wasf2	0.268536	6.44383348	3.07256322	0.09500963	0.43996948
H2-Ab1	-0.6741407	4.25397424	3.07245699	0.09501495	0.43996948
Hectd3	-0.3314101	4.6912483	3.07135538	0.0950702	0.44007385
Tm6sf2	-2.0491986	-1.4528194	3.07025259	0.09512554	0.4401786
Spr	0.53703974	2.83179434	3.06664642	0.09530677	0.4408656
Rps6ka1	-0.5203213	2.51458449	3.06527247	0.09537592	0.44092265
1700008O03	-0.4709301	2.94146089	3.06509948	0.09538463	0.44092265
Gm16386	-0.5697759	2.00126101	3.06244797	0.09551827	0.44128168
Mobp	-0.3563615	5.81453183	3.06174151	0.09555392	0.44128168
Lamc1	0.254961	6.02517539	3.06039699	0.09562179	0.44128168
Slc25a33	0.43475272	3.31598457	3.05978433	0.09565274	0.44128168
Armcx2	0.21098028	5.54431266	3.05830976	0.09572728	0.44128168
Emid1	-0.9069331	0.7803174	3.05722546	0.09578213	0.44128168
Serpine2	0.2614685	5.98452131	3.05675427	0.09580598	0.44128168
Clpp	0.32801931	3.49623974	3.05503629	0.09589299	0.44128168
Txk	1.83209648	-1.7280901	3.05503372	0.09589312	0.44128168
Chad	3.01714851	-1.747927	3.05472553	0.09590874	0.44128168
Tmem241	-0.4513081	3.00809008	3.05463641	0.09591326	0.44128168
C330018D20	-0.3407281	3.75577288	3.05442631	0.09592391	0.44128168

Smtn	0.62364312	2.3287142	3.05425997	0.09593234	0.44128168
Vamp4	0.19065687	6.82262268	3.0527105	0.09601093	0.44128168
3830408C21I	-0.541156	2.47583336	3.05202164	0.0960459	0.44128168
Raly	0.51966447	2.78369417	3.05192316	0.0960509	0.44128168
Tmem159	0.42795796	4.62406698	3.05168527	0.09606298	0.44128168
Neurl4	-0.3439051	4.54166249	3.05086909	0.09610443	0.44128168
Wdr12	0.34835357	4.64403414	3.0506928	0.09611339	0.44128168
Gm14378	-1.2535779	-0.2341426	3.05059857	0.09611818	0.44128168
Frrs1l	-0.2599438	7.44286109	3.04993798	0.09615175	0.44128526
Desi1	-0.2293359	5.52702697	3.04894403	0.09620229	0.44136669
Gm867	-2.2529474	-1.991753	3.04743797	0.09627893	0.44156776
Edil3	0.31515746	6.55862889	3.04637132	0.09633326	0.44166638
Mis12	0.28085516	5.24682689	3.04473791	0.09641652	0.44189754
Atxn3	-0.263419	5.32775843	3.04344453	0.0964825	0.44194067
9930104L06F	-0.505123	3.10313452	3.04326607	0.09649161	0.44194067
Ak7	-0.6836534	1.73166403	3.04221775	0.09654514	0.44201446
Pcca	0.26819757	5.77101254	3.04166421	0.09657342	0.44201446
Maf	0.29907555	7.20796103	3.03986114	0.0966656	0.44228592
Cbl	0.21357161	6.56782542	3.03720191	0.09680174	0.44269914
Pcolce	0.48606345	4.76115866	3.03463518	0.09693335	0.44269914
Twsg1	0.36347659	7.87788273	3.03297086	0.09701881	0.44269914
Dhx37	-0.4420312	2.78793217	3.03200455	0.09706846	0.44269914
Tnfrsf13c	-1.140345	0.45441152	3.03186243	0.09707577	0.44269914
Spata33	1.67419865	-0.1889386	3.03146472	0.09709622	0.44269914
2010015L04F	0.4078435	3.55873562	3.03021724	0.09716039	0.44269914
Rmnd5b	0.23917858	4.74085418	3.02968211	0.09718793	0.44269914
Mtx2	0.20264882	5.6916001	3.02954018	0.09719524	0.44269914
Lamb1	0.30131691	4.17024606	3.02892196	0.09722707	0.44269914
Agmat	1.58017173	-0.0296026	3.0282478	0.0972618	0.44269914
2810403D21I	1.06432283	0.25113302	3.02786546	0.0972815	0.44269914
Syt2	0.48224988	3.39364271	3.02670567	0.09734129	0.44269914
Chrnb1	0.44742674	2.16982591	3.02650092	0.09735185	0.44269914
Prorsd1	0.29933791	3.89631622	3.0263002	0.0973622	0.44269914
B430319G15	-0.6162118	1.96980397	3.02597221	0.09737912	0.44269914
Gm10432	0.85234235	1.42658681	3.02548824	0.0974041	0.44269914
Bbox1	-0.9731551	0.58517396	3.02451642	0.09745427	0.44269914
Csrp1	0.39486537	7.56769806	3.02198948	0.09758487	0.44269914
Lrriq1	-0.5811994	3.09680899	3.02160774	0.09760461	0.44269914
Hyal2	-0.7112914	1.18618416	3.01934159	0.09772194	0.44269914
1700001K19I	-1.0759685	0.15610211	3.01924467	0.09772696	0.44269914
Ptpn6	-0.8483706	0.89948557	3.01803901	0.09778945	0.44269914
Tceb1	-0.2567297	6.28248636	3.01691064	0.09784797	0.44269914
Trpm7	-0.2435972	6.58706459	3.01566696	0.09791253	0.44269914

Ankrd11	-0.1952291	8.84875745	3.01556538	0.09791781	0.44269914
Cmtm8	-1.089878	0.26693661	3.01543729	0.09792446	0.44269914
Pwp2	0.58011021	2.67198387	3.01511801	0.09794104	0.44269914
Mapkapk2	0.37166608	4.27647673	3.01473017	0.09796119	0.44269914
Dgcr8	-0.2878368	4.13811439	3.01454509	0.09797081	0.44269914
Pacsin3	0.39372501	3.80814932	3.01346226	0.09802709	0.44269914
1110046J04R	-0.7522822	1.55816368	3.01322957	0.09803919	0.44269914
Fbxl12	-0.4870248	2.3507409	3.01290356	0.09805615	0.44269914
Aspscr1	0.59570186	2.7002338	3.01137629	0.09813563	0.44269914
Mrpl16	0.2532048	5.6404914	3.01114066	0.0981479	0.44269914
Ylpm1	-0.4164224	8.12727214	3.01078424	0.09816646	0.44269914
Mrps22	0.25147851	4.4030088	3.01058012	0.09817709	0.44269914
Epha6	-0.333894	4.797951	3.00991402	0.0982118	0.44269914
Usp33	-0.2491843	6.93210108	3.00898977	0.09825998	0.44269914
Slc23a2	-0.2235084	7.23608384	3.00777077	0.09832356	0.44269914
Ifi35	0.80254163	2.04781192	3.00689536	0.09836926	0.44269914
Usp42	-0.3130464	4.91321096	3.00643325	0.09839339	0.44269914
Smim11	-0.4173219	3.79796202	3.006284	0.09840118	0.44269914
Hspa1l	0.51185918	2.37598543	3.00619377	0.09840589	0.44269914
Mphosph8	0.24915463	8.41146248	3.00607229	0.09841224	0.44269914
Agrn	-0.361908	5.00625141	3.00565504	0.09843404	0.44269914
Cd209a	-0.6945363	3.352711	3.00542956	0.09844582	0.44269914
Rgp1	0.27704512	4.49069946	3.00512444	0.09846177	0.44269914
08-Sep	-0.2502762	5.95391556	3.00479947	0.09847876	0.44269914
Slc25a23	-0.2380465	8.69692611	3.00471514	0.09848316	0.44269914
Card6	0.32947979	4.85767612	3.00334893	0.09855462	0.44269914
2410006H16I	-0.5454267	2.57226497	3.00199058	0.09862573	0.44269914
Dnmt3aos	-2.3798033	-1.8159098	3.00157472	0.09864751	0.44269914
Cd52	-0.5880156	2.35416589	3.00113518	0.09867054	0.44269914
Necab2	0.37889435	3.43692255	3.00026925	0.09871593	0.44269914
Ctsb	0.26955778	7.9144416	2.99971248	0.09874512	0.44269914
Atxn10	0.18533021	7.66352388	2.99927978	0.09876782	0.44269914
Fn3krp	-0.3093235	4.38382354	2.99908549	0.09877801	0.44269914
Spag17	-1.8727603	0.24423401	2.99794338	0.09883795	0.44269914
Pstk	-0.3906451	4.18438685	2.99769135	0.09885119	0.44269914
Rab33a	-0.3996643	3.31455884	2.99751178	0.09886062	0.44269914
Ciao1	-0.2502395	5.39363612	2.99741731	0.09886558	0.44269914
Rftn1	0.76404994	1.5953295	2.99663385	0.09890673	0.44269914
Tmem95	2.82795061	-2.4731922	2.99570973	0.09895531	0.44269914
Grhl1	-0.4492002	2.97171947	2.99522873	0.0989806	0.44269914
Tmem254a	0.80694462	1.45185985	2.99358605	0.09906704	0.44269914
Smarcc1	0.17770818	7.39862034	2.99335645	0.09907912	0.44269914
Macrod2	0.31019452	5.09283958	2.99314636	0.09909019	0.44269914

Slc12a2	-0.2317646	6.5705165	2.9921613	0.09914208	0.44269914
9330159F19I	-0.3254146	6.85919471	2.99201214	0.09914994	0.44269914
C130046K22I	-0.5125564	2.95526203	2.99140789	0.09918178	0.44269914
Thnsl1	-0.3782509	3.96352648	2.99117385	0.09919412	0.44269914
Zswim3	0.67365464	2.11516617	2.99057968	0.09922546	0.44269914
Tcp11l1	0.2772503	5.22320502	2.98926235	0.09929497	0.44269914
Car4	-0.4479929	3.1795533	2.98924513	0.09929588	0.44269914
2900009J06F	-1.7276794	-0.6726362	2.98889486	0.09931437	0.44269914
1700029J07F	-0.5480115	2.15905679	2.98834709	0.09934329	0.44269914
9530036O11	-1.1304684	-0.2627716	2.9876322	0.09938106	0.44269914
Plod1	0.45867038	3.01885443	2.98667458	0.09943168	0.44269914
Fhad1	0.58965066	2.56366346	2.98643584	0.0994443	0.44269914
Spns1	-0.5811996	2.31003266	2.98642854	0.09944469	0.44269914
Usp31	-0.3296974	7.46671707	2.98601279	0.09946668	0.44269914
Ap1s3	-0.4618407	3.67176924	2.98529194	0.09950482	0.44269914
Kdm6a	-0.2892537	6.12837456	2.98478154	0.09953183	0.44269914
Cst6	1.46887463	-0.7388505	2.98398843	0.09957382	0.44269914
Ormdl1	0.53044753	3.70147365	2.98377318	0.09958522	0.44269914
Meig1	-1.045212	0.54987628	2.98288726	0.09963216	0.44276154
Atp13a1	-0.4058511	2.84849454	2.98206489	0.09967576	0.44280903
Pcdhb8	0.80280184	1.09888164	2.98132827	0.09971483	0.4428364
Mtcp1	-0.4850237	3.03143394	2.9794011	0.09981713	0.44288278
Baiap2l1	0.35154376	3.464308	2.9793952	0.09981744	0.44288278
Stx8	0.31521211	5.33836295	2.97927149	0.09982401	0.44288278
Cep170b	-0.2525712	7.77688155	2.97754129	0.09991596	0.44314464
Gata6	-2.6295288	-1.377922	2.97570434	0.1000137	0.44341169
Shisa3	-0.5521585	3.96110094	2.97517134	0.10004208	0.44341169
Pde6d	0.3891628	4.93270465	2.97125591	0.10025085	0.44407865
Cyp7b1	-0.4228512	2.44135494	2.97111144	0.10025856	0.44407865
Nell2	0.28128948	6.79545258	2.97003415	0.1003161	0.44418729
Csrnp2	0.27663141	5.41423429	2.96822592	0.10041276	0.44437225
Ccdc80	0.70466741	2.41847797	2.96771474	0.1004401	0.44437225
Tpbg	0.5111587	3.35700631	2.96735036	0.1004596	0.44437225
Rhpn2	-0.5211556	4.0440724	2.96576101	0.1005447	0.44437225
Nmrk2	-0.6862183	0.66187961	2.96569809	0.10054807	0.44437225
Tmem199	-0.280883	4.14860616	2.96554987	0.10055601	0.44437225
Xlr4a	1.19196748	0.03908689	2.96223855	0.1007336	0.4450109
Plin4	0.91694171	0.99542139	2.9615781	0.10076906	0.44502147
E130006D01I	-2.2279654	-2.0148907	2.95970759	0.10086959	0.44523358
Anln	-0.2384652	4.85821024	2.95945314	0.10088327	0.44523358
Tmem179b	0.53743552	3.23647778	2.95868942	0.10092435	0.44526886
Cdk14	-0.2321304	6.91946638	2.95635133	0.10105025	0.44567818
2010109A12I	-2.2438273	-2.023385	2.95553489	0.10109425	0.44572617

Mc5r	-1.0948579	0.66592367	2.95381673	0.10118693	0.44598867
Tecpr2	-0.4174161	5.55760619	2.95304517	0.10122858	0.44602616
2310036O22	-0.3455012	4.55739908	2.95222074	0.10127311	0.44607212
Gm5862	-0.8924477	0.79766452	2.95162478	0.10130532	0.44607212
Rlf	-0.2685505	5.84215649	2.95058261	0.10136166	0.44617421
Galk2	0.35519093	3.52257411	2.94663524	0.1015754	0.44689534
Alg5	-0.5715523	3.24676587	2.94633075	0.10159191	0.44689534
Pcdhgb5	0.56348606	2.4519966	2.94557273	0.10163302	0.44693009
Acn9	-0.3982826	2.90800262	2.94388856	0.10172443	0.44718595
Zc2hc1c	-0.676999	2.21582011	2.94287418	0.10177954	0.44728207
Ppp1r14c	-0.3733352	3.61494466	2.94208592	0.10182239	0.44730003
Snai1	0.52240371	2.27376053	2.94157613	0.10185011	0.44730003
Ston2	-0.3896575	4.6411271	2.93984365	0.10194438	0.44748874
Mrpl38	0.3871835	3.37361832	2.93956439	0.10195959	0.44748874
Cog7	-0.3637815	4.52669569	2.93586816	0.1021611	0.44791863
Tmem25	0.48265413	3.05454486	2.93495791	0.1022108	0.44791863
Trpc1	-0.3239846	4.59398598	2.93492837	0.10221241	0.44791863
Clstn2	0.30977966	6.26457341	2.93463887	0.10222822	0.44791863
Gm5148	-0.3569636	3.95471613	2.93417094	0.10225379	0.44791863
Trmu	0.65325745	2.08059847	2.93396456	0.10226506	0.44791863
Qser1	0.25892993	6.91275708	2.93349833	0.10229055	0.44791863
Icosl	0.64169794	1.45258805	2.93114581	0.10241924	0.44833626
Asrgl1	0.30645594	5.83523342	2.92957172	0.10250545	0.44856774
11-Mar	-0.6676709	1.34585352	2.92829448	0.10257547	0.44872821
Dnah8	0.82199873	1.34852445	2.9258235	0.10271109	0.44917547
Atp6v0c	1.76123912	-1.4626993	2.92279256	0.10287773	0.44965611
Gtf3c4	-0.2592783	5.27152828	2.92260905	0.10288783	0.44965611
Slc17a6	-0.2927128	4.81099216	2.91648747	0.1032254	0.45081293
Gstt2	0.76529587	2.13162719	2.91605094	0.10324952	0.45081293
Fdxr	0.65690168	1.38721774	2.91598746	0.10325303	0.45081293
Them6	-0.5053001	2.62308151	2.91514918	0.10329937	0.45086897
Wipi1	0.36629489	4.24351571	2.91445028	0.10333803	0.45089144
Ccdc87	0.81143987	1.01600754	2.9136595	0.10338179	0.45093615
Tceal5	0.2240179	6.04172697	2.91261305	0.10343972	0.45104267
Gtpbp3	-0.3587658	3.02973878	2.9075915	0.10371829	0.45209906
Hapln2	-0.8074266	1.13557707	2.90703547	0.10374919	0.45209906
2310002D06I	1.95918373	-1.6989495	2.90559924	0.10382905	0.4523006
Kcnu1	0.91447943	0.58323831	2.90458113	0.10388571	0.45240095
Nipa2	0.29556231	4.76445489	2.90323821	0.10396049	0.45258017
Gp49a	1.36774558	0.04712162	2.90211266	0.10402322	0.45262575
G630071F17	-1.1722599	-0.2688964	2.90184344	0.10403824	0.45262575
Camk2g	-0.2693478	7.23186606	2.90036207	0.10412088	0.45269525
Ccdc151	-0.753093	0.63614155	2.90035106	0.10412149	0.45269525

Nop14	0.24596941	4.94818265	2.8989	0.10420252	0.45288848
Lhfpl3	-0.3598197	3.85531894	2.89835006	0.10423325	0.45288848
Tmem88	-0.5925356	2.02488157	2.89710795	0.1043027	0.45304393
Tm9sf1	0.36526096	3.98937282	2.89548411	0.10439356	0.4532923
Lyrm1	-0.4772492	2.71571946	2.89327906	0.1045171	0.45368233
Snx4	0.21784292	6.79050908	2.89136591	0.10462443	0.45400175
Ttpal	-0.2796615	5.12525269	2.89010442	0.10469527	0.45416269
Slc45a3	1.34141989	0.25526345	2.88933619	0.10473844	0.45420353
Got1	0.233689	8.34719757	2.88753755	0.10483959	0.4544036
Ehd2	0.43594895	4.92766503	2.88710794	0.10486376	0.4544036
Tmem74b	-1.3985888	-1.2658424	2.88671504	0.10488588	0.4544036
Pon3	0.53081406	3.32299925	2.88249551	0.10512375	0.45528755
Rnmt	-0.2154981	5.92020427	2.8788536	0.10532956	0.45559824
Ebf2	0.52264397	3.16521469	2.87882263	0.10533132	0.45559824
Per2	-0.3295153	5.05396561	2.87865586	0.10534075	0.45559824
Slc25a44	0.21007193	5.90625202	2.87825464	0.10536346	0.45559824
Adck2	-0.4715572	2.23540847	2.87789734	0.10538368	0.45559824
Neb	-0.7730874	2.55395156	2.87763335	0.10539863	0.45559824
Usp17la	-0.9850537	0.44794742	2.87659765	0.10545729	0.45570543
Mip	-1.608138	0.37341419	2.87483294	0.10555734	0.45587147
Slc25a18	0.50225995	2.52180671	2.87469754	0.10556502	0.45587147
Kpnb1	0.19647739	8.29382347	2.87412763	0.10559735	0.45587147
Tsc22d1	0.25299893	9.67766436	2.87257953	0.10568525	0.45604823
AI464131	0.52245874	2.93921916	2.87221286	0.10570608	0.45604823
Helq	-0.5534215	2.56920515	2.86752284	0.10597294	0.45705304
BC035044	-0.9163273	0.39691978	2.86648102	0.10603233	0.45707985
Lgalsl	-0.1946816	6.23959162	2.86622213	0.1060471	0.45707985
BC049635	-0.5451272	3.11311476	2.86389836	0.10617972	0.45741517
Lin37	0.2918349	4.4352741	2.86340851	0.10620771	0.45741517
Gm609	-2.27658	-1.8422175	2.86302721	0.1062295	0.45741517
Armc5	0.40935277	2.58306319	2.86199718	0.10628838	0.45741517
Smarca5	0.20305539	7.57245339	2.8618839	0.10629486	0.45741517
Gdf9	1.50689421	-0.9128164	2.86063691	0.1063662	0.45757585
Maoa	0.26296428	4.91687969	2.85813572	0.10650947	0.45767723
Lace1	-0.3849435	3.18675385	2.85787926	0.10652417	0.45767723
Catsperd	1.62363193	-0.7666412	2.85779363	0.10652908	0.45767723
F3	0.38039086	5.60082471	2.8571304	0.10656712	0.45767723
Adam15	-0.3053802	4.21539987	2.8569234	0.10657899	0.45767723
Itga2	-1.6077446	0.13157257	2.85611656	0.10662529	0.45767723
Nt5dc1	0.49724122	3.02824271	2.85551993	0.10665954	0.45767723
Pcna	0.26035018	6.30910801	2.85547956	0.10666186	0.45767723
Rnd3	-0.3293405	5.26196549	2.8543013	0.10672955	0.45779411
Gm21671	-0.7912226	0.9986675	2.85382115	0.10675714	0.45779411

Cd163l1	-1.9962043	-1.1743733	2.85288	0.10681126	0.45788025
AW112010	0.60891773	1.65671888	2.85217256	0.10685196	0.45788243
Nup153	-0.2310155	6.16442377	2.85168841	0.10687982	0.45788243
Rad54l2	-0.2012254	5.63481962	2.84885805	0.10704289	0.45814034
Timm10b	-0.4962264	2.6479208	2.84885761	0.10704291	0.45814034
H2afy	0.26028024	5.91929454	2.84828853	0.10707573	0.45814034
Pdzd8	-0.1907505	7.10026311	2.84787378	0.10709966	0.45814034
Grin3b	1.84343315	-1.7901405	2.84769017	0.10711026	0.45814034
Klhl6	-1.1397955	0.02189517	2.84628231	0.10719153	0.45821488
Efhc1	0.62483362	1.78039606	2.84620867	0.10719579	0.45821488
Sepw1	-0.4863293	5.57534825	2.84554381	0.1072342	0.45823351
4932435O22	2.84181816	-1.8514079	2.84451268	0.1072938	0.45834266
Mrps5	-0.4842693	2.91157501	2.84023445	0.10754152	0.45925508
Polr2e	0.3345816	4.39097372	2.83898876	0.10761378	0.45935738
Rnf4	-0.2003433	6.47763243	2.83758555	0.10769524	0.45935738
Pgam1	0.20697647	9.43813812	2.8371822	0.10771867	0.45935738
Lrrc4c	-0.2693878	8.09940003	2.83624174	0.10777332	0.45935738
Fgfr4	-1.3193091	-0.6665554	2.83554037	0.1078141	0.45935738
Lgr6	1.61935791	-0.8661721	2.8353615	0.1078245	0.45935738
Exd1	-1.1877017	-0.3507734	2.83408546	0.10789874	0.45935738
Nup160	-0.3325453	4.50080403	2.83143673	0.10805305	0.45935738
2610018G03	0.67829685	1.93661506	2.83114696	0.10806995	0.45935738
Stk11ip	-0.4382823	3.48361789	2.83057332	0.10810341	0.45935738
Adcy1	-0.3250812	8.75656607	2.8294785	0.1081673	0.45935738
07-Sep	-0.2280351	9.11917513	2.82916725	0.10818547	0.45935738
Ipw	-0.7216782	4.05705094	2.82781582	0.10826442	0.45935738
Rab1	0.21503502	9.05096433	2.82766367	0.10827331	0.45935738
Slc30a2	-0.5075545	2.67361515	2.82639677	0.10834738	0.45935738
Wbscr25	-1.8990869	-0.9108051	2.82555109	0.10839686	0.45935738
Myh2	1.30003226	0.20706863	2.825139	0.10842098	0.45935738
Ushbp1	-1.0412994	0.5480966	2.82513698	0.1084211	0.45935738
Morn5	-2.8717644	-1.4756517	2.82508917	0.1084239	0.45935738
Fam89a	-2.6633875	-1.4200627	2.8248628	0.10843715	0.45935738
Sugt1	0.25229652	5.59679068	2.82469686	0.10844687	0.45935738
9630013A20I	-0.6705854	1.48920394	2.82450775	0.10845795	0.45935738
Scpep1	0.35032075	5.11264015	2.82412108	0.10848059	0.45935738
Pcp2	-2.3991007	-1.5685669	2.82401855	0.1084866	0.45935738
Amy1	-0.2955381	4.31067599	2.82350374	0.10851676	0.45935738
Aldh1a1	0.3830141	7.64304832	2.82342053	0.10852164	0.45935738
Tfg	0.19333984	6.88161521	2.82279106	0.10855854	0.45935738
Plcxd3	0.30821966	5.01837522	2.82274299	0.10856135	0.45935738
Casq1	-1.6425791	0.15794126	2.82266026	0.10856621	0.45935738
Usp10	0.27182594	5.20254236	2.82195984	0.10860728	0.45935738

Thra	-0.3081387	5.82827563	2.82116526	0.1086539	0.45935738
4833411C07I	2.04771214	-1.3355056	2.82078178	0.10867641	0.45935738
Parm1	0.30079585	4.32365914	2.82051633	0.108692	0.45935738
Dgcr2	-0.2361841	4.64335063	2.81954965	0.10874877	0.45937413
Ccdc91	0.26528707	5.37046407	2.81922227	0.10876801	0.45937413
Usp48	-0.3709419	5.49429636	2.81790077	0.10884569	0.45937413
Cd320	0.45902249	2.81476072	2.81785475	0.1088484	0.45937413
Themis2	-0.7729467	1.61244666	2.81611248	0.10895092	0.45937413
Prkd1	-0.4632624	2.50520455	2.81595527	0.10896018	0.45937413
A930011O12	-0.6337284	4.98274965	2.81586463	0.10896552	0.45937413
Hsd11b1	0.64463068	1.67588969	2.81577101	0.10897103	0.45937413
Ehhadh	0.67567026	1.10449656	2.81425731	0.10906022	0.45937413
Gm11437	-2.0867655	-1.1134879	2.81336894	0.1091126	0.45937413
Ecel1	0.57108422	1.9455997	2.81284544	0.10914348	0.45937413
Palm	-0.2465177	4.89436797	2.81265591	0.10915466	0.45937413
Adipor2	-0.2910245	4.69808897	2.81238096	0.10917089	0.45937413
Pak1ip1	0.26665994	4.15962948	2.81233006	0.10917389	0.45937413
Inhbb	-0.8070754	1.70768754	2.81136588	0.10923082	0.45940586
Dapk1	-0.27152	5.9237312	2.8110459	0.10924972	0.45940586
Abca8b	-0.5245818	3.33237728	2.80861802	0.10939324	0.45986568
Stmn1-rs1	-2.2493019	-1.1613984	2.80702454	0.10948756	0.46011844
Vav3	0.37762529	3.29633522	2.80562133	0.10957069	0.46016881
Apol7e	-1.1117267	-0.2586059	2.80539022	0.10958439	0.46016881
Gna15	2.43468602	-1.6031651	2.80509097	0.10960214	0.46016881
Mvd	0.50841698	1.82124357	2.80403497	0.10966477	0.46018725
Dusp6	0.35095829	5.38548791	2.80296303	0.10972839	0.46018725
Irf2bp2	-0.1863094	8.43053574	2.80289498	0.10973243	0.46018725
Gabrb1	0.35744324	3.55491588	2.80271162	0.10974332	0.46018725
4833417C18I	-1.9747285	-1.3667908	2.801774	0.10979902	0.46025969
Spata5l1	1.54794165	-0.7576528	2.8012694	0.10982901	0.46025969
Senp7	-0.1870096	6.97076646	2.80053824	0.10987248	0.46027475
Plekha7	-0.3751041	2.98852326	2.79984251	0.10991386	0.46027475
Stx12	0.20113252	7.93683339	2.79948358	0.10993521	0.46027475
Rpl35	-0.3021778	4.96906104	2.79858557	0.10998867	0.46035531
Akip1	0.5000674	2.97505457	2.79725747	0.11006778	0.46043979
Grb7	0.8197949	0.847069	2.79690468	0.1100888	0.46043979
Grk5	0.25684962	5.33355787	2.79652385	0.1101115	0.46043979
9130024F11I	0.36512557	4.23553446	2.79542902	0.1101768	0.460477
Avpi1	-0.3491877	3.24214639	2.79522718	0.11018884	0.460477
Ubtd1	0.95721048	-0.1061916	2.79378907	0.11027469	0.46049248
Surf4	0.31640468	4.57231606	2.7937714	0.11027575	0.46049248
Slc35g2	-0.3816193	3.02421412	2.79344552	0.11029521	0.46049248
Btbd16	-1.467577	-0.8711988	2.79282864	0.11033207	0.46050348

Msmo1	0.34774686	4.99067555	2.79105997	0.11043782	0.46080196
Slc6a20b	-0.5107838	1.71811984	2.78823573	0.11060694	0.46101345
Pde2a	0.3147853	6.77089871	2.78821111	0.11060842	0.46101345
Dscr3	0.31565232	4.05739242	2.78802716	0.11061945	0.46101345
Alg6	0.39176174	3.97261279	2.78736365	0.11065923	0.46101345
Tnrc6a	-0.2332936	6.73545539	2.78735395	0.11065981	0.46101345
Dbnl	0.28059068	5.18181118	2.78634529	0.11072032	0.46106173
Acy3	0.59005991	1.57757437	2.78601854	0.11073993	0.46106173
Megf8	0.35903042	5.0083475	2.78241874	0.11095624	0.46144373
Gm10785	-0.8957693	0.94385693	2.7816725	0.11100114	0.46144373
Abcg4	-0.3737419	4.08949237	2.78121877	0.11102846	0.46144373
Ndfip2	-0.2397499	6.27191141	2.78100185	0.11104152	0.46144373
Tcerg1l	0.43908316	2.69450517	2.78077867	0.11105496	0.46144373
Tbck	-0.3143137	4.68675334	2.78068892	0.11106036	0.46144373
Tmem66	0.25417895	5.98328088	2.78030287	0.11108362	0.46144373
Trappc12	0.23468507	5.73215981	2.77993112	0.11110601	0.46144373
Myl4	0.6525524	2.24549213	2.77882815	0.1111725	0.46149783
Nebl	0.23245095	6.61374962	2.77677466	0.1112964	0.46149783
Trim71	2.06929055	-0.8253599	2.77649633	0.11131321	0.46149783
Rmi2	-0.4943362	2.19828046	2.77645559	0.11131567	0.46149783
Calr3	-0.9813642	-0.0376434	2.77632157	0.11132376	0.46149783
Cst3	0.38740815	7.90737531	2.77590948	0.11134865	0.46149783
Spidr	0.51221943	2.25997588	2.77564053	0.1113649	0.46149783
Spata19	-2.5025556	-2.0523574	2.77516882	0.11139341	0.46149783
Onecut2	-0.3908469	3.59482213	2.77362685	0.11148665	0.46174196
Sorcs3	0.44147694	4.18264428	2.77054464	0.11167331	0.46211081
Dgkg	0.37357983	6.62842365	2.77009737	0.11170042	0.46211081
Apba2	-0.2940405	4.81600432	2.76991833	0.11171128	0.46211081
C130036L24R	1.33279128	-0.1689288	2.76968178	0.11172563	0.46211081
Arsa	-0.4873509	2.4348493	2.76864583	0.11178848	0.46211081
Nrbp2	-0.2522775	6.88218076	2.76843213	0.11180145	0.46211081
Serpina1d	1.91117381	-1.5769784	2.76819087	0.1118161	0.46211081
Tbc1d16	0.28359392	4.73877911	2.76691439	0.11189362	0.46217648
Lgr4	0.23712803	5.69464346	2.76679827	0.11190068	0.46217648
1810019D21R	2.05547877	-0.5570724	2.76571038	0.11196681	0.46220322
Rai14	0.28686674	6.49851665	2.76522706	0.1119962	0.46220322
Ptpn1	-0.2381167	4.77236984	2.76495793	0.11201257	0.46220322
Cd2bp2	0.30700665	4.98529416	2.7639672	0.11207287	0.46220322
Tagln	-0.6251507	3.30791348	2.76386817	0.11207889	0.46220322
4933428C19R	-1.7380685	-0.7452506	2.76025041	0.1122994	0.46297066
Tmem134	-0.4153684	2.61010811	2.7577691	0.11245093	0.46345339
Neurl1b	-0.3979009	4.89012946	2.75683254	0.11250819	0.4635474
Snx22	-1.1220869	-0.2486315	2.75581993	0.11257014	0.46366066

Zbed6	0.1789054	6.82936848	2.75365185	0.1127029	0.46406547
Coasy	0.35660309	3.65694836	2.75283321	0.11275308	0.46413006
Mtr	-0.5723878	2.80101481	2.75021529	0.11291373	0.4646492
Plp2	0.48906729	4.26104445	2.74756645	0.11307655	0.464817
Bahd1	0.40504219	4.09485007	2.74753101	0.11307873	0.464817
Wdr53	0.46612712	3.18469403	2.7474284	0.11308504	0.464817
Arrdc3	-0.2989117	5.87080385	2.7473043	0.11309268	0.464817
Paqr5	-0.5329719	3.21834626	2.74629444	0.11315484	0.46483325
Naa50	0.19047452	7.71159215	2.7461177	0.11316572	0.46483325
Pacs1	0.21051406	6.74546738	2.74548725	0.11320455	0.46485085
Pcdhga3	-0.3705964	3.43958644	2.74442742	0.11326986	0.46497714
H2-T23	-0.2738295	4.24340416	2.74324616	0.11334271	0.46513429
Med24	0.26002254	4.25752126	2.73956314	0.11357019	0.46563938
9430041J12F	-0.7494616	1.8680772	2.7389878	0.11360577	0.46563938
Rasgef1b	-0.2529291	5.48781559	2.73872934	0.11362176	0.46563938
Ccnl2	-0.4290128	4.42696557	2.73855591	0.11363249	0.46563938
Aldh1l2	-0.668615	2.81231289	2.73833352	0.11364626	0.46563938
Cers5	-0.2311311	4.92314804	2.73763188	0.11368969	0.46563938
Impg1	-1.0817843	0.27161682	2.73713193	0.11372065	0.46563938
Atp6v0d1	0.22069227	7.16095009	2.73610903	0.11378402	0.46563938
Mapk9	-0.1983173	8.50240878	2.73607943	0.11378586	0.46563938
Nrip2	0.65740625	2.07644589	2.73566051	0.11381182	0.46563938
Fbxw2	-0.2559002	5.59019633	2.73404349	0.11391212	0.46567027
Kctd12	0.18589488	7.42126274	2.73399723	0.11391499	0.46567027
Cdc25a	0.40960352	2.73292837	2.73253332	0.11400589	0.46567027
Zfp169	-0.3174841	4.07761419	2.73226438	0.11402259	0.46567027
4933411K20I	0.21693196	6.72775489	2.73211668	0.11403177	0.46567027
Unc80	-0.3796647	8.33898979	2.7320081	0.11403852	0.46567027
A430078G23	-0.3797809	3.71615754	2.73059573	0.11412632	0.46567027
E130008D07I	-0.6541125	2.27454662	2.73044432	0.11413574	0.46567027
Pla2r1	0.99673331	-0.1361902	2.7302895	0.11414537	0.46567027
Kptn	-0.6130889	1.85330009	2.72996702	0.11416543	0.46567027
Mylip	-0.3623862	4.11546844	2.72709857	0.11434408	0.46604086
Tmem204	-0.3807239	3.94258334	2.72685837	0.11435905	0.46604086
Ogfod3	0.62627888	2.08932277	2.72645307	0.11438433	0.46604086
Gm6484	-2.1680329	-1.9123091	2.72628479	0.11439482	0.46604086
Ubox5	0.37723151	3.32960985	2.72523783	0.11446014	0.46611454
Sdcbp	0.2203506	9.43623157	2.72437654	0.11451391	0.46611454
Nol11	0.29693019	4.28240854	2.7243299	0.11451683	0.46611454
Gm5176	-1.3061953	-0.4659472	2.72284083	0.11460986	0.46623566
Plekha3	0.2422509	5.32988785	2.72249079	0.11463174	0.46623566
Ube2h	0.19023108	7.70872382	2.72178191	0.11467608	0.46623566
Slc35f6	-0.5524536	1.93306923	2.72054304	0.1147536	0.46623566

Ankrd29	0.34772486	4.6037215	2.72051609	0.11475529	0.46623566
Smu1	0.24747715	5.43062054	2.72019767	0.11477522	0.46623566
Preb	-0.3296843	5.17850217	2.71997574	0.11478912	0.46623566
Nme7	0.40833752	3.52830339	2.7193132	0.11483062	0.46626348
Slc30a10	-0.3159423	5.06742658	2.71776183	0.11492786	0.46651757
Tmem144	0.47561093	2.21110874	2.71516137	0.11509109	0.46670934
Whsc1	0.18722966	6.66122419	2.71421498	0.11515056	0.46670934
Dlg5	-0.2673893	5.22165872	2.71413019	0.11515589	0.46670934
Hyal3	0.86038279	0.5348794	2.71315836	0.115217	0.46670934
Kdm4d	-1.270355	-0.1753256	2.71306218	0.11522305	0.46670934
Dhrs7	0.43990137	3.8290547	2.71236491	0.11526692	0.46670934
Met	0.35200165	3.05753534	2.71208352	0.11528463	0.46670934
Txnl4a	-0.2506531	6.03320287	2.71149998	0.11532137	0.46670934
Abca1	0.25452326	5.05854337	2.71109391	0.11534694	0.46670934
Mccc2	0.35281468	3.4833902	2.71096721	0.11535492	0.46670934
Adam12	0.30255936	5.7691662	2.71094015	0.11535662	0.46670934
Neurod1	-0.4053582	3.51486705	2.70993828	0.11541975	0.46682439
Ak2	0.35268683	4.85332912	2.70794725	0.11554533	0.46713928
Mterfd3	0.41125383	2.97547655	2.70760336	0.11556704	0.46713928
Gp5	1.9914388	-1.3607598	2.70189468	0.11592808	0.46839993
Slc29a1	0.37090358	2.59628195	2.70157192	0.11594853	0.46839993
Tyw3	-0.4895186	3.04954393	2.69772345	0.11619273	0.46924555
Spred1	0.20044692	8.20895939	2.69682773	0.11624965	0.46927832
Rcbtb1	-0.2573885	6.01330375	2.69599949	0.11630232	0.46927832
Ccdc3	0.24305723	5.39949026	2.69563083	0.11632577	0.46927832
Gm20125	-3.2642916	-1.3196063	2.69540179	0.11634034	0.46927832
Cdkn2aipnl	0.27825812	4.48162468	2.69307947	0.11648822	0.46953572
Eif4ebp3	0.76232506	0.94907495	2.69298339	0.11649434	0.46953572
Cct3	0.23433879	5.12571248	2.69275599	0.11650883	0.46953572
Ddn	-0.217746	6.89353307	2.6917211	0.11657482	0.46964294
Akap11	-0.3180063	8.45941084	2.69124412	0.11660524	0.46964294
Tet2	-0.3408503	6.67525259	2.68831115	0.11679254	0.4700773
6430548M08	0.24970834	6.91741558	2.68825	0.11679645	0.4700773
Angptl7	0.95276844	1.56096418	2.68791472	0.11681789	0.4700773
Apool	0.65635111	2.40628015	2.68701642	0.11687534	0.47016788
Dlgap3	-0.3543401	4.07743113	2.68535447	0.11698172	0.4702847
Keap1	-0.2600413	4.39016593	2.68508773	0.11699881	0.4702847
Sun1	-0.2980067	4.71651478	2.6846609	0.11702615	0.4702847
Scml4	0.3762546	4.00497164	2.68389283	0.11707537	0.4702847
Ajap1	-0.2640365	5.20149619	2.68383434	0.11707912	0.4702847
1810034E14I	0.8377788	1.11510821	2.68120621	0.11724775	0.47077381
Golga7b	-0.3396129	4.36180067	2.68084637	0.11727086	0.47077381
Rell2	-0.6044726	1.68782175	2.68015431	0.11731532	0.47081184

Pcdhb13	-0.5020364	3.50224641	2.67910518	0.11738276	0.47094204
Lcor	-0.3865267	3.98024282	2.67851406	0.11742078	0.47095416
Tsen54	1.2266005	-0.256233	2.67732062	0.11749758	0.47112178
Wsb1	-0.4204524	4.11688101	2.67565929	0.1176046	0.4712907
Sh3yl1	0.40646566	3.06946594	2.67557917	0.11760976	0.4712907
Fam19a5	-0.2277418	4.98488575	2.67495725	0.11764985	0.471311
2700046G09	-0.693534	1.75503727	2.67299784	0.11777627	0.47142384
2310015A10I	-0.5229669	2.92595868	2.67274724	0.11779245	0.47142384
Prdm6	0.42339044	5.62563107	2.6720721	0.11783605	0.47142384
Sema4f	-0.3854437	4.77742819	2.67189848	0.11784727	0.47142384
Slc37a3	0.34446338	4.04118976	2.67119951	0.11789244	0.47142384
Zfp948	-0.3996235	3.61939955	2.6710959	0.11789914	0.47142384
AI846148	0.37476709	2.95781087	2.67048288	0.11793877	0.47142384
Foxr2	-1.180805	0.49524515	2.67018106	0.11795829	0.47142384
Flrt3	-0.2275572	5.81551562	2.66893117	0.11803916	0.47147969
Susd5	-0.5522633	2.31888443	2.66860796	0.11806009	0.47147969
Scn9a	-0.600995	2.68024145	2.66810213	0.11809284	0.47147969
Atg3	0.19385308	6.65030074	2.66779996	0.11811241	0.47147969
Syt15	-0.8299874	1.38226934	2.66528036	0.11827576	0.47199173
Calcoco2	-0.9100091	-0.0155403	2.66306426	0.11841966	0.4721806
Nacad	0.38117621	3.25361314	2.66299644	0.11842407	0.4721806
Bbs7	0.30776279	4.04676063	2.66234287	0.11846654	0.4721806
Tor1b	-0.3457168	3.76440609	2.66180223	0.1185017	0.4721806
Asph	-0.2151013	6.75367526	2.66152067	0.11852001	0.4721806
Aatf	-0.3178139	3.91732597	2.66131127	0.11853363	0.4721806
2310014L17I	1.13052298	-0.1128098	2.66064682	0.11857686	0.47221303
2310001H17I	1.9125395	-0.9587405	2.65912847	0.11867573	0.47246691
Tril	-0.3244298	3.67077525	2.65814681	0.1187397	0.47255636
L3mbtl1	-0.7918765	1.58648883	2.65739934	0.11878844	0.47255636
Hsf2	0.25229748	5.63223317	2.65716764	0.11880355	0.47255636
Aktip	-0.2193989	6.09517602	2.65320472	0.11906238	0.47335864
Copb2	0.16833025	7.23618966	2.65300251	0.1190756	0.47335864
Slc2a3	0.26919869	5.24964401	2.65212076	0.11913329	0.47337808
Arhgap1	0.25845929	5.65617668	2.65071521	0.11922532	0.47337808
Pdp2	-0.4487028	3.55228606	2.65021968	0.11925778	0.47337808
Arap3	0.74328269	1.59536031	2.65019768	0.11925922	0.47337808
Gpm6b	0.2686785	8.47914724	2.64984456	0.11928236	0.47337808
Ppt2	0.64311009	1.23233552	2.64945096	0.11930816	0.47337808
Spred2	-0.2089536	6.51431557	2.64895811	0.11934048	0.47337808
Ccdc11	-1.1067737	-0.1982129	2.64833385	0.11938142	0.47337808
Cxxc4	-0.2581012	6.13019003	2.64805416	0.11939978	0.47337808
E130311K13I	0.39390988	3.48396889	2.64745966	0.11943879	0.47337808
Ica1l	-0.2351095	5.02285201	2.64651665	0.11950071	0.47337808

Zfp459	-0.5197544	3.00448716	2.64590674	0.11954078	0.47337808
Phyh	0.2045383	7.28550816	2.64566829	0.11955645	0.47337808
Rdh11	0.82931069	1.0106902	2.64541654	0.119573	0.47337808
C1qtnf1	0.33952783	4.86034582	2.64448675	0.11963413	0.47348081
Abra	-1.9276809	-0.3836444	2.64271893	0.11975047	0.47380188
Usp43	-1.155906	1.0019832	2.64170834	0.11981703	0.47381458
Ncmap	-1.949207	-0.974297	2.64160111	0.1198241	0.47381458
Gm16432	-0.9438743	1.11354933	2.64041746	0.11990212	0.47398382
Alas2	0.86241895	1.07308465	2.63938746	0.11997007	0.47399021
Pprc1	-0.3698966	3.91972088	2.63932506	0.11997419	0.47399021
Dynll2	-0.2036878	7.03477523	2.63812444	0.12005345	0.47403105
Ccdc136	-0.2524203	5.29502051	2.63810127	0.12005498	0.47403105
Cisd3	-0.4416113	2.82960951	2.63755608	0.12009099	0.47403416
Gm6938	1.67455033	-0.7268445	2.63655123	0.1201574	0.47406688
2610001J05F	0.35422668	5.47852933	2.63631552	0.12017299	0.47406688
Pspc1	0.2896417	5.2308124	2.63558575	0.12022125	0.47406688
Rmi1	-0.2399396	5.67301753	2.63529933	0.1202402	0.47406688
Gdap1	-0.3004003	7.46345264	2.6342035	0.12031274	0.4741759
AA987161	0.23405886	5.33446726	2.63381698	0.12033833	0.4741759
Stox2	-0.2785496	7.99509909	2.63283372	0.12040347	0.4742937
Olfr1033	0.339909	3.91660529	2.63181259	0.12047117	0.47442148
Uxt	0.4567712	3.52008913	2.62998175	0.12059266	0.47474042
Ldha	0.22892665	7.8797741	2.62952907	0.12062272	0.47474042
Selplg	-0.7966223	0.91735611	2.62779352	0.12073805	0.4750554
Fndc5	-0.2386889	5.00331875	2.62708344	0.12078528	0.47510229
Ybx2	-1.204477	-0.0781036	2.62624081	0.12084135	0.47518393
Rmdn1	-0.3965758	3.73525051	2.62526411	0.12090638	0.47528553
9130011E15I	0.33980917	3.63260374	2.62436558	0.12096624	0.47528553
Batf2	-1.5393104	-0.9426477	2.62405244	0.12098711	0.47528553
Gusb	-0.4230129	2.80361293	2.62242523	0.12109562	0.47528553
Msrb3	0.32046213	5.39694695	2.62200951	0.12112337	0.47528553
Fam109b	0.93242747	1.55754912	2.62158585	0.12115165	0.47528553
Tuba8	-0.4062883	2.26077848	2.62148434	0.12115843	0.47528553
A330048O09	-1.2086815	0.44010957	2.62125021	0.12117406	0.47528553
Dhrs13	-1.169499	-0.3668726	2.62108533	0.12118507	0.47528553
Ccno	1.80542901	-0.7397575	2.61962372	0.12128273	0.47550611
Uqcrb	0.25771161	6.81664099	2.61885307	0.12133426	0.47550611
Zfp628	0.47472927	2.2903361	2.61865774	0.12134732	0.47550611
Mrps17	0.34019432	4.15799816	2.61770055	0.12141137	0.47552832
Samd8	-0.218443	6.05743869	2.61749884	0.12142487	0.47552832
Map2k3	-0.4087894	3.88687435	2.61619561	0.12151215	0.47552832
Slc39a9	-0.2559547	4.89213791	2.61578263	0.12153982	0.47552832
Myh13	2.14121016	-1.4079411	2.61277353	0.12174168	0.47552832

Mill2	0.9146456	0.60879185	2.61251355	0.12175914	0.47552832
1700007L15F	-1.0542135	-0.0113701	2.61223954	0.12177754	0.47552832
Smim3	0.4483765	3.12589515	2.61218338	0.12178131	0.47552832
Gxylt2	-0.4009725	3.90150015	2.61157126	0.12182244	0.47552832
Ahcyl1	0.20448129	9.39081939	2.61129831	0.12184078	0.47552832
1700017B05I	-0.4093569	2.86725022	2.6110096	0.12186019	0.47552832
Ccdc114	-1.1865079	0.152478	2.61050578	0.12189407	0.47552832
Arpc5l	-0.2547351	5.82331496	2.60952902	0.12195977	0.47552832
Sf3b1	0.20133419	8.28905656	2.60950038	0.1219617	0.47552832
1110020A21I	0.75604541	1.0658075	2.60948776	0.12196255	0.47552832
Dnajc7	0.17771144	6.56262307	2.60944576	0.12196538	0.47552832
Lyrm9	-0.227066	5.68993301	2.60899914	0.12199544	0.47552832
Nfkbie	0.66067172	2.13805308	2.60823712	0.12204674	0.47552832
Zfp84	-0.2376892	5.15423552	2.60792529	0.12206775	0.47552832
Spats1	-0.8285911	0.64257121	2.60753952	0.12209374	0.47552832
Man2a2	-0.2299936	6.90128601	2.60737061	0.12210512	0.47552832
Rad1	-0.3742452	4.79840405	2.6069949	0.12213044	0.47552832
6330409D20I	-0.7856141	0.73553757	2.60334658	0.12237664	0.47592638
Nxn	0.42888894	5.05717024	2.60333599	0.12237736	0.47592638
Prkar1b	0.20233321	7.74528638	2.6031398	0.12239062	0.47592638
Prom2	2.47502228	-1.9620922	2.6022673	0.12244959	0.47592638
Ndufa1	0.32551041	5.23904643	2.60112741	0.12252669	0.47592638
Ttc18	-1.0475129	0.60106621	2.60076158	0.12255145	0.47592638
Pls3	0.16752549	7.5080234	2.60060935	0.12256175	0.47592638
Prkrir	-0.2064623	5.34315048	2.6005732	0.1225642	0.47592638
Zbtb25	-0.3470312	3.35792443	2.60053415	0.12256684	0.47592638
1810032O08	-1.1934423	-0.4284775	2.60024588	0.12258636	0.47592638
Cpt1a	-0.2709707	5.93109532	2.59903446	0.12266841	0.47610756
2810442I21R	2.92485156	-2.1376449	2.59771286	0.12275799	0.47631788
Gsx1	2.32916417	-1.7004673	2.59614013	0.1228647	0.4765945
Lingo2	-0.2849556	5.23399232	2.59472411	0.12296087	0.4768301
Prdm16	-0.4583251	3.06366429	2.59330472	0.12305737	0.4770668
Usf2	-0.2399248	4.98736857	2.59089018	0.12322171	0.47724873
Nipsnap1	0.25920907	4.73071935	2.58981905	0.12329471	0.47724873
Stc1	-0.5474003	2.90925401	2.58978557	0.12329699	0.47724873
Crkl	0.19195079	6.05458741	2.58931891	0.12332881	0.47724873
Pdgfd	0.59008892	3.02315085	2.58867241	0.1233729	0.47724873
Tars	0.27183674	4.55001334	2.58856679	0.12338011	0.47724873
Ccna1	-1.6499757	-1.0159597	2.5884995	0.1233847	0.47724873
Zap70	-1.7797036	-1.6527262	2.58845069	0.12338803	0.47724873
Pdxdc1	-0.1871762	6.24525789	2.58631134	0.1235341	0.47764247
Samd9l	0.33856673	6.92484062	2.58592034	0.12356082	0.47764247
Ppp1r12c	-0.2127605	5.32770825	2.58411305	0.12368441	0.47798292

Pgm3	-0.3004753	4.00884134	2.58344694	0.12372999	0.47802181
Nbr1	0.2125098	7.89776255	2.57760343	0.12413078	0.47924114
1700123O21	1.7455527	-1.0875901	2.57758086	0.12413233	0.47924114
Cacng5	0.54555601	2.68079669	2.57725462	0.12415475	0.47924114
Rab6b	-0.1958097	10.4152573	2.57611504	0.12423311	0.47924114
Bcl2l11	0.37474684	5.05409116	2.57586898	0.12425004	0.47924114
Cacna1a	-0.3182034	6.4472544	2.57566442	0.12426411	0.47924114
Katnal2	1.43323935	-0.4732178	2.57521698	0.1242949	0.47924114
Med23	-0.2538598	4.19630727	2.57434217	0.12435513	0.4792685
Epb4.1l2	0.21796725	7.5391637	2.5737854	0.12439348	0.4792685
Slc41a3	0.60949778	2.46873405	2.57265484	0.1244714	0.4792685
Ifitm10	1.08644039	0.45607829	2.57185942	0.12452625	0.4792685
Psmb8	-0.5972368	2.65757263	2.57136285	0.12456051	0.4792685
Chn2	-0.1991932	5.29458178	2.57084952	0.12459594	0.4792685
Necab1	0.20766025	7.85856048	2.57077024	0.12460141	0.4792685
Pi4k2b	0.56343343	2.02012472	2.57065213	0.12460956	0.4792685
Thtpa	0.26065054	4.55589553	2.57046399	0.12462255	0.4792685
Gm2897	-0.3727297	3.6036603	2.56885194	0.12473391	0.47955971
Med13l	-0.3234688	6.48937418	2.5670108	0.12486124	0.4797914
2510049J12F	-1.0189148	1.13682001	2.56694948	0.12486548	0.4797914
Tlr3	0.30158126	4.87896512	2.56519958	0.12498665	0.48011989
Faxc	-0.2468624	7.8893427	2.56436231	0.12504468	0.4802057
Nck1	-0.2957596	4.98985231	2.56142571	0.12524844	0.48026033
Hexim2	0.44716373	2.30209558	2.5610034	0.12527778	0.48026033
Prpf4b	0.20713142	7.35039184	2.56097524	0.12527974	0.48026033
Mab21l3	2.55961984	-1.79967	2.56074396	0.12529581	0.48026033
Slc8a2	-0.3037814	5.55719983	2.5599473	0.12535118	0.48026033
Wrb	-0.2093136	5.3922587	2.55990467	0.12535414	0.48026033
Aig1	0.44367975	3.24048573	2.55967149	0.12537036	0.48026033
Exoc6	0.23462307	5.37342463	2.55948374	0.12538341	0.48026033
Tmprss6	2.59056319	-2.147482	2.55898869	0.12541785	0.48026033
Nanog	-1.0599757	-0.5025616	2.55883271	0.1254287	0.48026033
Gng4	-0.2441276	5.47719063	2.55850507	0.1254515	0.48026033
BC030500	-0.343677	3.90057048	2.55672593	0.12557538	0.4804544
Ppara	0.38756089	4.0984941	2.55609623	0.12561926	0.4804544
Dnm2	-0.2199958	5.25761461	2.55585076	0.12563637	0.4804544
Avl9	-0.2350131	6.00225198	2.55572687	0.12564501	0.4804544
Epas1	-0.201962	8.09369916	2.55486448	0.12570515	0.48054781
Suclg2	0.30381093	5.58089658	2.55432605	0.12574272	0.4805549
Hlx	-1.9956246	-1.893623	2.55310421	0.12582801	0.48074435
Shank3	-0.3215904	4.59092802	2.54915964	0.12610386	0.48155927
Spice1	-0.3576229	3.42151264	2.54903078	0.12611288	0.48155927
Gm13157	0.36818883	3.36192233	2.54733974	0.12623137	0.48173842

Pcmtd2	-0.2702996	4.73890337	2.54663723	0.12628063	0.48173842
Cyp2b10	2.30711664	-1.7136659	2.54648282	0.12629146	0.48173842
U2af2	-0.3600325	4.69481803	2.54518402	0.1263826	0.48173842
Col17a1	2.20888693	-1.226709	2.54488123	0.12640386	0.48173842
2410004P03I	-0.5286216	2.3157078	2.54484613	0.12640633	0.48173842
Kcnk4	-0.8276826	0.31919566	2.54478817	0.1264104	0.48173842
Qpctl	0.42491938	2.65051681	2.54190392	0.12661314	0.48228
Mapkapk5	0.28563481	4.18382528	2.54119216	0.12666323	0.48228
5031426D15I	-0.6771802	3.58407533	2.54073	0.12669577	0.48228
Ppp2r3c	-0.2158112	4.97086748	2.54042051	0.12671756	0.48228
Ddx6	0.16438455	8.14171641	2.53936962	0.1267916	0.48228
Actn4	-0.180752	6.28219437	2.53870526	0.12683843	0.48228
Surf6	0.31075655	3.73763035	2.53837384	0.1268618	0.48228
Osbpl6	-0.2187848	7.06424395	2.53763408	0.12691398	0.48228
Pacs2	-0.192663	6.07020537	2.53753324	0.1269211	0.48228
Dhx32	0.3362103	4.10411335	2.53726248	0.12694021	0.48228
Gyk	-0.4522741	4.36426834	2.53716964	0.12694676	0.48228
Myo1d	-0.2732164	4.42317551	2.53584483	0.1270403	0.48249917
Golgb1	-0.2011912	7.98617775	2.53424099	0.12715366	0.48265715
Cpe	0.2217398	10.0402984	2.53324389	0.1272242	0.48265715
Prr14	0.28244357	5.45232595	2.53231583	0.12728989	0.48265715
Tbc1d15	0.23708031	5.20688553	2.53108228	0.12737727	0.48265715
Reep1	0.25929902	6.96590109	2.53102316	0.12738146	0.48265715
Tsc22d2	0.18468373	6.84533582	2.53086139	0.12739293	0.48265715
5031439G07	0.207536	5.64957202	2.52977163	0.12747019	0.48265715
Dpys	-1.5825974	-0.9644051	2.52916428	0.12751328	0.48265715
9230114K14I	0.57022554	1.94223052	2.52901691	0.12752373	0.48265715
Sema4d	-0.4019307	3.84624337	2.52900529	0.12752456	0.48265715
Ttbk2	-0.2328305	7.80790211	2.52877153	0.12754115	0.48265715
Smarca2	-0.1904621	9.78810952	2.52871315	0.12754529	0.48265715
Tox3	0.36488718	5.04677067	2.52753616	0.12762886	0.48265715
Prmt3	0.28960122	4.30951432	2.52727396	0.12764749	0.48265715
Ak8	-1.3585465	-0.7773796	2.52659936	0.12769543	0.48265715
Dhrs7b	0.54505384	2.406399	2.52652481	0.12770073	0.48265715
Olfm4	2.25735785	-2.1604963	2.52533973	0.12778499	0.48265715
Kpna2	0.2030818	5.1767939	2.52452927	0.12784266	0.48265715
Necap1	0.18473602	7.2058062	2.52437648	0.12785354	0.48265715
Fam53b	0.30050692	4.28924641	2.52405325	0.12787655	0.48265715
Gm5535	2.61369121	-1.7777729	2.52391328	0.12788651	0.48265715
Tspan9	-0.3960059	3.49513706	2.52238224	0.12799558	0.48265715
Zfp882	0.26437447	4.53737201	2.5223817	0.12799562	0.48265715
Fgf5	0.49022842	2.67938052	2.52230684	0.12800096	0.48265715
Katnbl1	0.32070688	4.70717542	2.52213669	0.12801309	0.48265715

Shmt2	0.42073225	2.55247759	2.51943577	0.1282058	0.48265715
Gtf3c5	-0.3560524	3.13958845	2.51832543	0.12828513	0.48265715
A230103J11F	0.5770068	1.99749863	2.51824289	0.12829103	0.48265715
Hspa12b	-0.4218347	3.62175018	2.51819619	0.12829436	0.48265715
Slc25a16	-0.233897	5.57022558	2.51813803	0.12829852	0.48265715
Ntsr2	-0.4630476	3.21728665	2.5163905	0.1284235	0.48265715
Sh2d7	-0.7456137	1.00357488	2.51632511	0.12842818	0.48265715
Naip6	2.23693611	-2.0934829	2.51585	0.12846219	0.48265715
Mlip	-0.2978553	4.86704619	2.51578421	0.1284669	0.48265715
Med16	-0.2383937	5.29758617	2.51556256	0.12848277	0.48265715
Tlr2	1.84844488	-1.1053509	2.5153676	0.12849672	0.48265715
Serinc5	-0.3160774	5.0789743	2.51507102	0.12851797	0.48265715
Tspan13	0.324641	8.54940171	2.51466848	0.1285468	0.48265715
Coro1c	0.25558109	5.89259875	2.51461581	0.12855057	0.48265715
Sorbs1	0.16195729	7.52415737	2.51425147	0.12857668	0.48265715
Tsnaxip1	-2.8073918	-1.2806416	2.51419379	0.12858081	0.48265715
Slc13a5	-0.5044859	2.26735821	2.51408827	0.12858838	0.48265715
2310022B05I	0.22720748	6.31292069	2.5125234	0.12870059	0.48293404
4933413J09F	-1.9853654	-1.5657156	2.51205885	0.12873392	0.48293404
Ccdc27	1.88363392	-1.0113339	2.51112218	0.12880116	0.48301775
Depdc7	-0.7045947	1.14205109	2.51035936	0.12885596	0.48301775
St8sia6	-0.8974328	0.86685262	2.50996628	0.1288842	0.48301775
Fxr2	-0.3308022	3.71798348	2.50925261	0.1289355	0.48301775
Ror2	0.88156355	0.84523662	2.50883382	0.12896562	0.48301775
Dffa	0.22905899	5.08935317	2.508528	0.12898762	0.48301775
Tob1	0.22180353	6.21399761	2.50825158	0.1290075	0.48301775
Ywhag	0.18347452	10.2179561	2.50760076	0.12905434	0.4830587
Lrtm2	-0.3080311	5.89053661	2.50647425	0.12913545	0.48311502
Gnpda1	0.25592153	4.59177892	2.50639466	0.12914119	0.48311502
Gm16523	-0.6408441	1.10422528	2.50580136	0.12918394	0.48314062
Cops5	0.17452738	6.0242677	2.50472534	0.12926151	0.48318729
Sema3d	0.44071902	6.49635754	2.5034227	0.12935549	0.48318729
Anxa8	0.55845172	3.46734733	2.50303417	0.12938354	0.48318729
Cherp	0.20348751	5.54780331	2.50184157	0.12946968	0.48318729
Wdr16	-1.2072813	-0.2244289	2.50146586	0.12949683	0.48318729
Mrc1	-0.6384523	2.19715843	2.50090627	0.12953728	0.48318729
Snx10	0.21090117	6.49559681	2.5005633	0.12956208	0.48318729
Mus81	0.46757469	2.15460591	2.50019704	0.12958857	0.48318729
Zfp92	-0.6138504	2.40848391	2.49916435	0.1296633	0.48318729
Vwa5b1	-0.9014385	0.4933153	2.49888623	0.12968343	0.48318729
Zfp747	0.29326731	3.70442029	2.49877886	0.12969121	0.48318729
Gm16023	-0.6949642	0.95694866	2.49846557	0.12971389	0.48318729
Rbm33	-0.2915064	5.58997343	2.49830825	0.12972529	0.48318729

Sgol1	-0.7737245	0.8431864	2.49822101	0.1297316	0.48318729
Rs1	-2.2377598	-0.2740352	2.49710367	0.12981256	0.48318729
Myt1	-0.4635077	2.81373741	2.49667486	0.12984365	0.48318729
Rpp30	0.26755753	3.992085	2.49666328	0.12984449	0.48318729
Cog1	0.306937	4.11567653	2.49638346	0.12986478	0.48318729
Foxc1	0.28615575	8.36033334	2.49579372	0.12990756	0.48318729
Synm	0.27060096	5.50732263	2.49569702	0.12991457	0.48318729
Ttc9c	0.25610866	6.09942812	2.49500352	0.1299649	0.48324091
Asap3	0.50360277	2.87495429	2.49370693	0.13005906	0.48345741
Xpo4	-0.2969572	4.12366709	2.49241195	0.13015318	0.48367249
Poldip3	0.2764259	5.76822409	2.49192204	0.1301888	0.48367249
Dlk2	-0.5638993	1.71967125	2.48893268	0.13040646	0.48423906
Stard4	0.2936418	3.99959699	2.48883908	0.13041328	0.48423906
Snai2	0.55330748	3.53862021	2.48702435	0.13054563	0.48459676
Cilp	-1.247007	-0.1917374	2.48464292	0.13071955	0.48489143
Ret	0.64281568	1.82544934	2.48460925	0.13072201	0.48489143
Rc3h2	0.18968113	7.92859234	2.48440997	0.13073657	0.48489143
Tgds	0.42117437	2.74649204	2.48396449	0.13076914	0.48489143
Zfp949	-0.2674542	4.59449231	2.48257938	0.13087048	0.48513349
Mir3473	1.53838976	-0.4214497	2.48183297	0.13092512	0.48520238
Gm20594	-0.652963	0.45296374	2.48057644	0.13101717	0.48531187
Ints4	0.2207967	5.51097562	2.48044516	0.13102679	0.48531187
Cables1	-0.388346	3.13166928	2.47842049	0.13117529	0.4857282
Ctdnep1	0.24278071	5.82205095	2.475796	0.13136809	0.48630827
Tnfrsf10b	-0.3709525	3.26675785	2.47405856	0.1314959	0.48644809
Adora1	-0.2272286	7.10715741	2.47368439	0.13152345	0.48644809
Setd2	-0.2223408	7.47981448	2.47351192	0.13153615	0.48644809
Zcchc5	-1.4939122	-0.4091356	2.47331764	0.13155046	0.48644809
Atp5f1	0.18327417	7.46224354	2.47050631	0.1317577	0.48708056
Il11	0.76497237	0.53524101	2.46950457	0.13183163	0.48722005
Noc4l	0.36633337	3.21503517	2.46855883	0.13190148	0.48732521
Smim13	-0.2006199	8.64473928	2.46790051	0.13195013	0.48732521
Psmg1	0.42367114	3.13073301	2.466626	0.13204437	0.48732521
Brca2	-0.3193366	3.83286062	2.46564179	0.13211721	0.48732521
Josd2	-0.5249715	2.23009201	2.46460202	0.1321942	0.48732521
Eml2	-0.3796835	2.7595467	2.46433434	0.13221403	0.48732521
Cyc1	0.27776807	6.32295405	2.46414038	0.13222841	0.48732521
Gje1	-0.6063322	1.15646148	2.46406859	0.13223373	0.48732521
Cep89	-0.4377145	3.02045372	2.46386281	0.13224898	0.48732521
Arnt2	0.18864074	8.03920942	2.46382621	0.13225169	0.48732521
Plxna2	-0.1959477	7.727418	2.4637349	0.13225846	0.48732521
Podn	0.3456273	5.0364247	2.46153728	0.13242146	0.48743833
Nlrc5	-1.0714623	0.00570201	2.46147146	0.13242635	0.48743833

Htr1d	0.83324355	1.15101983	2.4607298	0.13248142	0.48743833
Palld	0.5431768	1.61944209	2.46070353	0.13248337	0.48743833
Zfp804a	-0.3286217	5.92882127	2.46035805	0.13250903	0.48743833
Cd59a	0.36782256	4.58348747	2.46016324	0.13252351	0.48743833
Irs2	0.38329056	4.35157986	2.45902348	0.13260822	0.48743833
Dph7	0.30613279	3.34348573	2.45895121	0.13261359	0.48743833
Bin2	0.46011642	3.02808699	2.45892999	0.13261517	0.48743833
AI597479	0.24013342	5.87756521	2.45761437	0.13271304	0.48749887
Tgfb1i1	0.3435311	6.85124362	2.45760145	0.132714	0.48749887
Trip10	0.65270403	2.25482018	2.45724782	0.13274033	0.48749887
Lipt1	-0.6389185	1.47774105	2.45455179	0.13294121	0.48788018
Th	1.08677759	0.60102874	2.45442941	0.13295034	0.48788018
4930478L05F	1.78637638	-1.0812398	2.45423603	0.13296476	0.48788018
Amer2	0.29428026	4.18477025	2.45390873	0.13298918	0.48788018
Pccb	-0.276549	4.70569849	2.45342247	0.13302546	0.48788028
Herc1	-0.4296848	8.10533263	2.45213913	0.13312129	0.48808345
Wdr37	-0.1873775	7.17723915	2.45170925	0.1331534	0.48808345
Grm2	-0.2917168	3.64352703	2.44993462	0.13328608	0.48843675
Ets2	0.22590716	5.6900743	2.44928247	0.13333488	0.48843729
Spdya	-0.7486051	0.69594156	2.44896255	0.13335883	0.48843729
Gpr126	0.34833138	3.94251813	2.4479894	0.1334317	0.48845417
Rbms3	0.25078027	7.34695524	2.44761872	0.13345947	0.48845417
Zfp408	-0.2478747	4.68741714	2.44683086	0.13351852	0.48845417
Cd300ld	-1.4867895	0.25936695	2.44648039	0.13354479	0.48845417
Wdr73	-0.2490998	4.41027299	2.44647854	0.13354493	0.48845417
Yipf3	0.24334358	4.45809091	2.44292176	0.13381195	0.48925954
Ggt7	-0.4254824	2.63432091	2.44257733	0.13383784	0.48925954
Acot3	-1.2294095	0.33841072	2.44205387	0.1338772	0.48927051
Eif5b	0.22786677	9.35457205	2.44097208	0.13395859	0.48932172
Tspan12	0.46819762	3.01681319	2.44055461	0.13399001	0.48932172
Fyn	0.18603901	6.00425844	2.44041791	0.13400031	0.48932172
Uevld	-0.2522379	5.20567585	2.43883377	0.13411964	0.48962461
Morc2b	-0.8591578	1.68893426	2.43579754	0.13434871	0.49021293
Rsu1	0.29545987	6.85772679	2.43568255	0.1343574	0.49021293
Lyrm7	-0.2345868	4.50608836	2.43524987	0.13439008	0.49021293
6330549D23I	-0.7036378	1.73944601	2.43395234	0.13448816	0.49038705
Mir124a-2	-1.2481587	-0.8553492	2.43365424	0.1345107	0.49038705
Sntb1	0.50784442	1.79795292	2.43307027	0.13455488	0.49041523
Stard8	-0.1907541	5.63681122	2.43133792	0.13468603	0.49070589
Vps13b	-0.346019	6.99017199	2.43105373	0.13470756	0.49070589
Lmnb2	0.38122084	3.01885766	2.4284797	0.13490275	0.49128393
Hdac5	-0.3060275	5.88492629	2.4273149	0.13499119	0.491473
Ntn1	0.40814808	3.73349064	2.42515771	0.13515516	0.49193689

Fitm2	-1.0192781	0.590713	2.42291107	0.13532619	0.49225604
Exosc6	-0.3343574	3.34859601	2.42224958	0.1353766	0.49225604
Adamts6	-0.5937232	1.791435	2.42213503	0.13538533	0.49225604
Slitrk6	-0.7926296	0.63049086	2.42160964	0.13542538	0.49225604
Mfn1	-0.2704261	5.318936	2.42158509	0.13542725	0.49225604
Eno1	0.60203518	0.80270498	2.42096095	0.13547485	0.49225604
Pqbp1	0.24298984	4.38254597	2.42007629	0.13554236	0.49225604
Mitd1	0.41729116	3.3516068	2.4195243	0.1355845	0.49225604
Ctxn3	0.36237456	7.22561565	2.41921916	0.1356078	0.49225604
Mppe1	-0.7063441	0.69121084	2.41916972	0.13561158	0.49225604
Senp8	0.26133383	4.61752894	2.41872891	0.13564525	0.49225604
Il17re	-0.855333	0.83772249	2.41580459	0.13586888	0.49268334
Gm101	1.62702582	-1.1663423	2.41541529	0.13589869	0.49268334
Otop2	1.6275055	-0.9323722	2.41497376	0.1359325	0.49268334
Commd7	-0.2663892	4.6837121	2.41464964	0.13595733	0.49268334
Gtpbp4	0.17482052	6.31664662	2.41451141	0.13596792	0.49268334
Spsb2	0.76309323	0.96639361	2.41397897	0.13600872	0.49268334
D10Jhu81e	0.31402893	3.58095012	2.41370008	0.13603009	0.49268334
Aldh3b2	0.88513422	-0.2498659	2.41336344	0.1360559	0.49268334
Impact	0.17914809	7.83618296	2.41142928	0.1362043	0.49288526
Nhej1	1.08867841	1.11955873	2.41124382	0.13621854	0.49288526
Vezf1	0.23276283	7.58884059	2.41120467	0.13622155	0.49288526
Polr2f	0.45285297	2.1390759	2.40746938	0.13650873	0.49370122
Cts8	-0.8085366	0.00836896	2.40731734	0.13652044	0.49370122
C630043F03I	-0.334103	3.2595682	2.40546322	0.13666328	0.49408498
Pcbd2	0.39664262	4.06968922	2.40344033	0.13681932	0.49448264
Pisd-ps1	-0.4352601	5.75833039	2.40308483	0.13684676	0.49448264
2310069B03I	-3.2277717	-1.3945449	2.43833568	0.13692128	0.49461909
C630031E19I	-0.7277625	1.84095033	2.39968547	0.13710953	0.4951662
Golph3l	0.26886679	5.99608675	2.39826511	0.1372195	0.49543039
Nmnat2	-0.2104512	7.15343766	2.39723001	0.13729971	0.49548451
Fam174a	-0.2762966	5.07082992	2.39705616	0.13731319	0.49548451
Fut2	-0.9533445	0.57040956	2.39664645	0.13734496	0.49548451
Paox	0.8445424	0.96696285	2.39494766	0.13747677	0.4957611
Edaradd	2.15298278	-1.1186257	2.39430981	0.1375263	0.4957611
Fam181b	-0.490555	2.12299327	2.39302452	0.13762617	0.4957611
Nudt21	0.30016896	4.7723066	2.39279996	0.13764363	0.4957611
Poc1a	0.45790973	2.80503987	2.39252829	0.13766475	0.4957611
Bean1	-0.2701081	3.92752223	2.39140651	0.13775202	0.4957611
Dnajc9	0.24229485	5.91835945	2.39107803	0.13777759	0.4957611
Aatk	-0.4030381	5.14712158	2.39071229	0.13780606	0.4957611
Mfsd6	0.2767464	7.207852	2.39058894	0.13781566	0.4957611
Mir186	-1.6461382	-0.8361679	2.39054425	0.13781914	0.4957611

Xirp2	-0.6525386	2.38411212	2.38887483	0.13794921	0.4957611
Pcdhgc5	0.34474735	4.78780458	2.38882559	0.13795305	0.4957611
Exd2	-0.263808	5.36403923	2.3886809	0.13796433	0.4957611
Trhde	-0.3130191	5.81450051	2.38867741	0.1379646	0.4957611
Zfp457	-0.9486484	0.29169321	2.38849853	0.13797855	0.4957611
Syncrip	0.17552645	7.70853875	2.38808118	0.1380111	0.4957611
Nusap1	-0.5860288	2.16744972	2.38721867	0.1380784	0.49583332
Alpl	0.51110154	3.38132943	2.38687908	0.1381049	0.49583332
Tmem43	0.41822495	3.88559186	2.38521406	0.13823495	0.49616784
Vmn2r18	-2.5259041	-1.8672575	2.38304126	0.13840488	0.49640635
Shisa7	-0.3024041	6.36471315	2.3829745	0.13841011	0.49640635
Dcakd	0.35325005	4.5004566	2.38281315	0.13842274	0.49640635
Wdr34	0.37205182	3.15942228	2.38232893	0.13846065	0.49640635
2310011J03R	0.36876145	2.93835107	2.3820071	0.13848585	0.49640635
Emc8	0.2003399	5.95088228	2.38152659	0.13852349	0.49640903
Carkd	-0.2967176	3.50228562	2.38055131	0.13859992	0.49649229
Pcdhb19	-0.4368362	2.92157149	2.37956698	0.13867712	0.49649229
Pphln1	0.18741248	7.20904503	2.37922411	0.13870402	0.49649229
Smim5	1.15863394	-0.0767975	2.3790014	0.1387215	0.49649229
Mrpl54	0.46869826	2.69776809	2.37887772	0.13873121	0.49649229
Sgpl1	0.32580679	4.29909087	2.37644258	0.13892249	0.49704468
C130021I20R	0.86419234	1.23443208	2.37346733	0.13915664	0.49775006
Mmd2	0.30966409	4.00550522	2.37220549	0.13925608	0.49792473
Kcna2	-0.2953698	8.75410394	2.37153663	0.13930883	0.49792473
Gm12185	-0.7459711	0.86068717	2.37046319	0.13939353	0.49792473
Vps52	0.24414032	4.55164951	2.36949123	0.13947028	0.49792473
Slc39a10	0.20590298	8.38104969	2.36790077	0.13959598	0.49792473
Pou3f3	-0.2369287	5.33868191	2.3677095	0.13961111	0.49792473
Rundc1	0.26415877	5.45873072	2.36756989	0.13962215	0.49792473
Mypn	-0.7040059	1.79093751	2.36671273	0.13968996	0.49792473
Elavl4	0.20898312	6.90619641	2.36605856	0.13974175	0.49792473
Zfp420	-0.2438403	4.40160952	2.36596874	0.13974886	0.49792473
Fuca1	0.33577496	5.15110516	2.3646637	0.13985224	0.49792473
Eml3	0.44463146	2.79223939	2.36449934	0.13986526	0.49792473
Pdlim2	0.44047027	3.29156799	2.3644856	0.13986635	0.49792473
Entpd1	-0.3815943	3.01205359	2.36446749	0.13986779	0.49792473
Socs1	-1.9570607	-0.8725271	2.3642267	0.13988687	0.49792473
4930529M08	-1.8744144	-0.9366219	2.36419583	0.13988932	0.49792473
Lgals1	0.51418543	4.62330975	2.36417777	0.13989075	0.49792473
Rev3l	-0.2532837	7.29469965	2.36402477	0.13990288	0.49792473
9430020K01I	-0.1699971	8.95856324	2.3639536	0.13990853	0.49792473
Rnf170	-0.2272943	5.90689747	2.36330644	0.13995985	0.49797568
Nudt16	0.28221899	4.68686686	2.36125227	0.14012291	0.49815631

AF357359	-0.4586078	2.62008413	2.36107884	0.14013668	0.49815631
Gas2	0.32652186	2.92876743	2.35964609	0.14025056	0.49815631
Acrbp	1.17287701	0.21321377	2.35942319	0.14026829	0.49815631
Sall3	-0.4765305	1.87691425	2.35940085	0.14027007	0.49815631
Pnpo	0.33612295	3.78994723	2.35926974	0.14028049	0.49815631
Unc13a	-0.4159705	6.96026908	2.35925782	0.14028144	0.49815631
Vps37b	0.35162641	3.12214722	2.35856091	0.14033689	0.49815631
Ckmt1	0.26924006	5.28012258	2.35847408	0.1403438	0.49815631
Slc9b1	-2.0137436	-0.7893292	2.35651245	0.14050002	0.49853634
Chrdl1	-0.4376583	2.68327976	2.35553048	0.1405783	0.49853634
Mkrn2	0.255484	5.14989952	2.35541569	0.14058745	0.49853634
Ppap2b	0.31600235	6.43322826	2.35527022	0.14059906	0.49853634
Elovl6	-0.2402121	6.01508993	2.35454542	0.14065688	0.49860999
Fam84b	0.35085768	2.78382697	2.35356055	0.1407355	0.49875728
Il1rn	-1.6401705	-1.1083452	2.35250725	0.14081963	0.49889325
1700084C01l	-0.5631582	1.65505672	2.35106231	0.14093516	0.49889325
Gigyf2	-0.22439	6.95812281	2.35045679	0.1409836	0.49889325
Klhl34	-0.4814879	5.45597322	2.34999515	0.14102055	0.49889325
Jph3	-0.2383201	5.02951187	2.34967332	0.14104631	0.49889325
Zfp458	-0.3763797	4.61657535	2.34944816	0.14106434	0.49889325
Gar1	-0.2475609	4.14274439	2.34879542	0.14111662	0.49889325
2210404O09	-0.4874385	2.13870663	2.34857286	0.14113445	0.49889325
Atp10b	-0.7079173	1.16803816	2.34811685	0.14117099	0.49889325
Bckdhb	-0.4975141	2.62445648	2.34806071	0.14117549	0.49889325
Cpne8	-0.2122961	6.41143721	2.3477746	0.14119843	0.49889325
Trhr2	-0.9786643	0.03532587	2.34738906	0.14122934	0.49889325
Dkkl1	-0.510952	2.23243727	2.34663386	0.14128991	0.49889325
Mphosph10	-0.2603077	5.16500049	2.3465965	0.14129291	0.49889325
Smim20	0.38040735	3.66801231	2.34519956	0.14140504	0.49910921
Gm5643	0.21613547	5.48793776	2.34491054	0.14142826	0.49910921
Has1	-0.9304878	0.34808261	2.3443056	0.14147686	0.49911243
Parp4	0.21513555	5.70580261	2.34397593	0.14150335	0.49911243
Hlcs	0.28606839	4.42716132	2.34349101	0.14154234	0.4991191
Ttc17	-0.2601509	4.84486367	2.34217026	0.14164858	0.49936287
Gmeb1	-0.2042103	5.29855396	2.34147261	0.14170473	0.49943
Pole4	0.25027705	5.26961281	2.34024724	0.14180343	0.49959722
C1s1	-0.9379423	0.94045248	2.33996184	0.14182643	0.49959722
Ddx49	0.70693441	0.76089997	2.338443	0.14194891	0.49977568
Glrx	0.24943977	6.11625311	2.33796988	0.14198709	0.49977568
Flnb	0.19717929	6.1926105	2.33795219	0.14198852	0.49977568
A230056P14	-0.2983402	4.00572703	2.33655893	0.14210102	0.50000537
Gm20324	-1.2433096	-0.5352857	2.33622384	0.14212809	0.50000537
Bzw1	0.26035405	8.32615048	2.3346891	0.14225217	0.5001884

Ndufa9	0.25272901	6.21260257	2.33398524	0.14230911	0.5001884
4931414P19I	0.90604659	1.11634917	2.3337622	0.14232716	0.5001884
Igsf9b	0.4266792	3.48985487	2.33374198	0.1423288	0.5001884
Pvrl2	0.75119244	1.42364104	2.33308136	0.14238228	0.50024571
Gm5088	0.48642408	1.55621154	2.33216404	0.14245659	0.50028891
Kdr	-0.3763971	2.63592826	2.33201168	0.14246894	0.50028891
Zbtb7c	0.30020769	4.09881819	2.33121132	0.14253381	0.50038614
Ibtk	0.17929607	5.74726595	2.32938577	0.14268192	0.50077545
A330076H08	-0.5106018	3.78922034	2.32850213	0.14275368	0.50089665
Lrp6	-0.1939853	6.82316275	2.32663594	0.14290537	0.50117759
Fgl2	0.36299977	4.78953892	2.32658794	0.14290927	0.50117759
Mtmr12	-0.1732665	6.44446954	2.32594888	0.14296126	0.50117759
Prr12	-0.2430974	5.7528392	2.32504681	0.14303469	0.50117759
Mrps34	0.37524244	3.02931867	2.32464865	0.14306711	0.50117759
Itprip	-1.2802265	-0.6277225	2.32426821	0.1430981	0.50117759
Tmem42	-0.450528	2.41919743	2.32365941	0.14314771	0.50117759
Chpf2	0.47541416	2.28687468	2.32365794	0.14314783	0.50117759
Ddx43	-2.2603863	-2.0649255	2.32339886	0.14316895	0.50117759
Nadk2	0.24594606	4.75716011	2.32249103	0.14324297	0.50130632
Gad2	0.29872173	8.8081103	2.32120646	0.1433478	0.50154273
Perm1	-1.3069972	-0.4116374	2.32014956	0.14343411	0.50171427
Ebna1bp2	0.23949044	5.39896302	2.31694119	0.14369652	0.50217311
A130010J15I	0.47549329	3.56718103	2.3167109	0.14371537	0.50217311
A730056A06	0.61734459	1.59917993	2.31594093	0.14377844	0.50217311
Amdhd1	1.7086355	-1.7095124	2.31591713	0.14378039	0.50217311
Tpk1	0.33262156	4.03306475	2.31565506	0.14380187	0.50217311
Ppp4r4	-0.3074644	4.90833308	2.31531607	0.14382965	0.50217311
Dleu7	0.62480923	1.26264563	2.31521769	0.14383771	0.50217311
Ppp1r13b	-0.2367258	5.6077684	2.31445994	0.14389985	0.50217311
Zbtb42	0.85888725	0.59707261	2.31444397	0.14390116	0.50217311
5330413P13I	-0.623005	1.58644939	2.31385724	0.14394929	0.50221084
Npy5r	0.83075286	0.98837721	2.31183259	0.14411553	0.50266049
Gas2l2	-1.6089038	-0.998051	2.3111707	0.14416993	0.50271991
Mbp	-0.3000826	7.0503577	2.30888142	0.14435826	0.50312346
Kcnj12	0.38334232	3.19352967	2.30863393	0.14437864	0.50312346
Ube2r2	0.24216052	8.34444785	2.30794513	0.14443537	0.50312346
Morn3	-1.7310968	-1.2840511	2.30690043	0.14452147	0.50312346
Otx1	-0.4719759	2.08237612	2.306708	0.14453733	0.50312346
Cyp4f17	2.01798944	-1.7790445	2.30655301	0.14455011	0.50312346
Bai1	-0.2774998	6.13424862	2.30613257	0.14458479	0.50312346
Senp2	-0.1605559	6.40988616	2.30584978	0.14460812	0.50312346
Plce1	0.20028351	4.90128955	2.30567962	0.14462216	0.50312346
Aoc3	-0.8518263	3.18181364	2.30460765	0.14471064	0.50317898

Itch	0.16757966	7.05379418	2.30458027	0.1447129	0.50317898
Fibin	-0.404791	4.43120011	2.30407786	0.1447544	0.50318446
Mustn1	0.48859119	3.85996265	2.30365578	0.14478927	0.50318446
Pon2	0.39467457	5.932534	2.30292663	0.14484953	0.50322007
Gm13315	1.9294006	-1.3209303	2.30262695	0.14487431	0.50322007
Wdr41	-0.2127733	4.83009109	2.30015958	0.1450785	0.50379928
Tbc1d9b	0.18910692	6.59204366	2.29820375	0.1452406	0.50423208
Vps26a	0.17478644	7.2527483	2.29668958	0.14536625	0.50452203
Map3k1	0.24281504	5.10802838	2.29616278	0.14541	0.50452203
1700003D09	-1.6245558	-0.5231101	2.29563653	0.14545371	0.50452203
St7l	-0.3176585	4.22717222	2.29513379	0.14549549	0.50452203
Wdr45	0.27772211	4.46017267	2.29494015	0.14551159	0.50452203
Atxn7	-0.2214625	5.79439811	2.29351826	0.14562984	0.50455834
Prok2	-1.5678968	-1.3584056	2.29318531	0.14565755	0.50455834
Birc2	0.20442339	5.4885812	2.29233872	0.14572803	0.50455834
Gp1ba	1.30744473	0.3865583	2.29145355	0.14580176	0.50455834
Mrrf	0.26966541	3.972877	2.29005248	0.14591856	0.50455834
Msh2	0.27048896	4.64569386	2.28953229	0.14596196	0.50455834
Trmt61a	0.26439922	4.52357401	2.28899608	0.1460067	0.50455834
Vamp8	0.64673185	5.10451775	2.28859823	0.14603991	0.50455834
Tslp	2.20837958	-1.0755982	2.2880138	0.14608872	0.50455834
Lcmt1	0.2596012	4.85914656	2.28754058	0.14612825	0.50455834
Ostm1	0.22238802	5.44644216	2.28742967	0.14613752	0.50455834
Katnb1	0.44351323	2.94699057	2.28722626	0.14615451	0.50455834
Smco3	-0.3680223	2.69433529	2.28689775	0.14618197	0.50455834
Kctd20	0.41328341	3.14629532	2.28680582	0.14618965	0.50455834
Impa2	-1.2118451	-0.1342386	2.28587211	0.14626773	0.50455834
Fam101b	0.29739999	4.20018008	2.28551437	0.14629766	0.50455834
Lgi3	-0.3636937	3.63126066	2.28550164	0.14629872	0.50455834
Gm16039	-0.2154718	4.51962695	2.28546221	0.14630202	0.50455834
Cul9	-0.3913374	3.96622826	2.28525058	0.14631973	0.50455834
Tnnt1	-0.6450461	1.24684882	2.28501538	0.14633941	0.50455834
Lrrk2	-0.3546467	5.84619947	2.28498018	0.14634236	0.50455834
Atf5	0.37804475	3.26731139	2.28444152	0.14638745	0.50455834
Slc39a2	-1.2006	0.47883261	2.28429725	0.14639953	0.50455834
Hist1h4d	-0.4598175	2.81423744	2.28352733	0.14646402	0.50455834
A330023F24	-0.5719337	4.37251926	2.28332748	0.14648077	0.50455834
Hunk	-0.3014514	4.23948636	2.28313428	0.14649696	0.50455834
Wnt4	0.23665583	5.58221254	2.2807308	0.14669855	0.50511472
Pcbp3	0.29568813	4.77316804	2.2795744	0.14679566	0.50511472
Purb	-0.1733417	9.71124071	2.27926615	0.14682156	0.50511472
Rad50	-0.2612157	6.08377612	2.27918288	0.14682855	0.50511472
Kif19a	-1.3583846	-1.1562717	2.27814289	0.14691598	0.50511472

Adcy10	-0.8348903	0.20138722	2.27742628	0.14697626	0.50511472
Cep57	0.25088392	4.61047186	2.27726491	0.14698983	0.50511472
06-Sep	0.16628199	6.97292922	2.27640693	0.14706205	0.50511472
Mtx1	-0.4136468	2.53885428	2.27587234	0.14710707	0.50511472
Cox8b	-2.0587585	-1.4825481	2.27538476	0.14714814	0.50511472
Kif23	-0.6399613	0.84121658	2.27506561	0.14717503	0.50511472
Pdhb	0.15823048	6.93228658	2.27441085	0.14723023	0.50511472
Kif21b	-0.3312811	5.75344638	2.27429033	0.14724039	0.50511472
Dram1	-0.8372267	0.62220117	2.2735943	0.14729909	0.50511472
Zfp354c	0.21712484	5.84331025	2.27358943	0.1472995	0.50511472
Sap18	0.27174055	6.70105069	2.27349065	0.14730783	0.50511472
Kcnq1	-1.8214036	-1.3330321	2.27318684	0.14733347	0.50511472
Lpp	0.2129173	7.6917757	2.27317847	0.14733417	0.50511472
Ippk	0.28130438	3.8158183	2.2721284	0.14742281	0.50528987
Cdk8	0.18699842	5.70955149	2.27138736	0.14748541	0.50537568
B3gnt5	-1.3095845	0.09588602	2.27078035	0.1475367	0.50542275
Ppp2r3a	-0.2075029	6.51542	2.26785327	0.14778436	0.50567588
Nars	0.16598861	7.85975066	2.2675226	0.14781237	0.50567588
Dag1	0.26463243	6.67986052	2.26729563	0.14783159	0.50567588
Ankhd1	-0.2053658	7.51690378	2.26724348	0.14783601	0.50567588
Cdca8	-1.007582	0.45926939	2.26719529	0.1478401	0.50567588
Plk4	-0.3224543	3.65666331	2.26643759	0.14790431	0.50567588
Sh3bgr	-1.3235853	-0.4642722	2.2663686	0.14791016	0.50567588
Slc31a1	0.2119027	5.88304645	2.26635609	0.14791122	0.50567588
Nyx	1.4887509	-0.5550092	2.26553985	0.14798045	0.50570459
Zfp593	0.6975276	0.84499048	2.26537081	0.14799479	0.50570459
Il10rb	-0.3941926	3.04627877	2.26442274	0.14807525	0.50585108
Inpp5a	0.25760748	4.56294931	2.26329402	0.14817111	0.50605008
Tie1	-1.2197291	0.39482434	2.26245266	0.14824261	0.50606879
Eif2s3y	0.37920282	5.08863465	2.26234457	0.1482518	0.50606879
Mbnl3	-0.61969	1.24336771	2.26038899	0.14841817	0.50626021
Cmklr1	0.62613897	1.08346113	2.25995199	0.14845538	0.50626021
Mplkip	0.23317656	4.05352396	2.25994759	0.14845576	0.50626021
Wdr59	-0.3260652	4.08083724	2.25926289	0.14851408	0.50626021
Sema4c	0.73259173	0.62827725	2.25872107	0.14856025	0.50626021
Cs	0.15599557	7.85166602	2.25848396	0.14858046	0.50626021
B230209E15	-0.2471315	6.31479577	2.2583965	0.14858792	0.50626021
Tuba4a	0.20874007	7.31249873	2.25777573	0.14864085	0.50626021
Tmem253	-1.6943696	-1.077582	2.25770971	0.14864648	0.50626021
1500011K16I	-0.3955266	3.942228	2.25619187	0.14877601	0.50655626
Itgb3bp	0.46800758	3.3353098	2.25580907	0.14880869	0.50655626
BC048403	-0.2754055	4.04730673	2.2541818	0.14894775	0.50689799
Col14a1	-0.7504624	0.85800448	2.25338149	0.14901619	0.50689799

Cdc16	0.25048025	4.94816157	2.25331253	0.14902209	0.50689799
Gdi2	0.15841792	8.14845822	2.25251938	0.14908997	0.5070007
Tmprss5	-2.1515864	-1.6692367	2.25202788	0.14913204	0.50701566
Nfyc	0.27010322	4.60424449	2.25134374	0.14919064	0.50708676
Gmfb	0.18232972	8.2222009	2.24998365	0.14930721	0.50722822
AA465934	0.79335239	0.237487	2.24977077	0.14932547	0.50722822
Ap3s1	0.23122066	7.18832894	2.2488163	0.14940735	0.50722822
Arap2	-0.2466989	7.05856224	2.24752649	0.14951809	0.50722822
Elfn2	-0.2685338	5.01552476	2.24718506	0.14954742	0.50722822
Krt73	-1.4805801	-1.1365842	2.24675541	0.14958434	0.50722822
Zfp345	1.24855273	-0.7265107	2.24639152	0.14961562	0.50722822
Mesdc1	-0.2506659	4.04770127	2.24636052	0.14961828	0.50722822
Ecd	0.27131713	4.1883553	2.24630479	0.14962308	0.50722822
Birc3	0.3905463	4.01387721	2.24587042	0.14966042	0.50722822
Tes	-0.3049039	3.71867944	2.24581628	0.14966508	0.50722822
Dab2ip	-0.1896658	6.24974934	2.24523437	0.14971513	0.50722822
Det1	-0.5335725	1.32187188	2.24515121	0.14972229	0.50722822
Pdcd2l	0.33726973	3.02662668	2.2437883	0.1498396	0.5074979
Rab31	-0.2521833	5.56481474	2.24284872	0.14992055	0.50760074
Pdyn	0.36174017	3.32308681	2.24135361	0.15004945	0.50760074
Nfix	-0.1889734	8.37197493	2.24118354	0.15006413	0.50760074
Rimbp2	0.35111783	5.19509569	2.24084338	0.15009348	0.50760074
Rusc2	0.26464737	6.58476708	2.24081962	0.15009553	0.50760074
Cd300a	-0.4210281	3.03887199	2.23992377	0.15017286	0.50760074
Abcc4	0.36533256	5.12241116	2.23962294	0.15019884	0.50760074
Dppa2	2.70522343	-1.8299176	2.23941422	0.15021687	0.50760074
Slc16a8	1.95804739	-1.1791877	2.23909605	0.15024436	0.50760074
Cbln4	0.27240758	4.04057016	2.23906324	0.15024719	0.50760074
Dpep2	-2.1828673	-1.7038124	2.23840055	0.15030446	0.50766677
Pou3f2	-0.2578576	4.41732911	2.23754948	0.15037805	0.50778787
Eva1a	0.83823031	1.13258846	2.23655128	0.15046442	0.50795205
Lif	-1.3652236	-0.9504071	2.23573333	0.15053524	0.50806365
Rarg	0.39704194	2.85799831	2.23219979	0.15084163	0.50870321
Tm4sf20	1.69214733	-1.3584128	2.26326724	0.15092291	0.50870321
Nt5c1a	0.71719062	0.79955439	2.23113318	0.15093426	0.50870321
Ada	-0.5869427	1.31544401	2.23087022	0.15095711	0.50870321
Satb2	-0.2531817	7.21935707	2.23082537	0.15096101	0.50870321
Rem1	-1.6956358	-0.5359513	2.23058611	0.1509818	0.50870321
Olfr1417	3.14832054	-2.0114826	2.26214024	0.15101856	0.50870321
Cdk7	-0.2039114	6.25268145	2.23006425	0.15102716	0.50870321
Creg2	0.23032047	7.62972907	2.22881029	0.15113624	0.5089432
Inpp5d	-0.4195507	2.51991615	2.22831227	0.15117958	0.5089618
Tjp3	1.35321962	-0.8532734	2.22785404	0.15121948	0.50896878

D730005E14	-2.2575677	-1.8001265	2.22705666	0.15128893	0.50907521
Gm5547	-2.2225934	-1.9888501	2.22642577	0.15134391	0.5091329
Dnajc15	-0.27316	4.25157973	2.22587138	0.15139225	0.50916821
Adam17	-0.2655166	4.19544606	2.22530809	0.15144137	0.50920616
Panx1	0.54041568	2.45878713	2.22358662	0.15159163	0.50951048
Mcf2l	-0.4273117	4.68359106	2.2231414	0.15163053	0.50951048
Cdca7l	0.90279933	0.60647303	2.22297032	0.15164547	0.50951048
Slc25a4	0.18064805	9.98946024	2.22104793	0.15181356	0.5099479
Dnah7b	-0.3092141	3.50336842	2.22016777	0.15189059	0.5099787
C430049B03	-0.6715073	1.132541	2.22007712	0.15189852	0.5099787
Pclo	-0.4132176	10.1329204	2.21959435	0.1519408	0.50999339
Zfp738	0.23759531	5.23921593	2.21835038	0.1520498	0.51023197
Cwf19l2	0.20031764	5.62558779	2.21634591	0.15222563	0.51054266
Peg10	0.25254362	6.36412456	2.21619266	0.15223908	0.51054266
Ccdc157	-0.3166012	3.04729122	2.21554807	0.15229568	0.51054266
Fam78a	0.85063648	0.64030231	2.21554805	0.15229568	0.51054266
Rcbtb2	-0.2847393	4.36717018	2.21505123	0.15233933	0.51054266
Klhl40	0.99280072	0.1157573	2.21432672	0.152403	0.51054266
Abcc6	-1.4486061	-0.8892767	2.21418744	0.15241524	0.51054266
St7	0.37205648	3.42973863	2.21383864	0.15244591	0.51054266
Gadl1	1.06773786	-0.5549758	2.21337633	0.15248657	0.51055176
Erg	-0.6770711	1.59530933	2.21140312	0.15266026	0.51100616
Arhgef4	-0.2634066	5.08822958	2.2108764	0.15270666	0.51103437
Prickle1	-0.270142	5.67967014	2.2094372	0.15283354	0.51118324
Abhd16a	0.232756	4.50715658	2.20887574	0.15288308	0.51118324
Rnf111	0.15549381	7.07452193	2.20834745	0.1529297	0.51118324
St6galnac4	-0.4599156	2.46563388	2.20779079	0.15297885	0.51118324
Celf4	0.17127173	9.32146305	2.20769795	0.15298705	0.51118324
Kiz	0.28393916	4.925296	2.20646828	0.15309569	0.51118324
Dpp3	0.32223292	3.80938584	2.20513657	0.15321345	0.51118324
Pf4	-1.4136998	-1.4000512	2.20476363	0.15324645	0.51118324
Gm15706	-0.4636268	1.90207161	2.20450133	0.15326967	0.51118324
Grk1	-0.6406686	1.35031602	2.20418378	0.15329778	0.51118324
1700015F17	-1.7693715	-0.8664059	2.20401467	0.15331275	0.51118324
S100a8	1.92749108	-1.1417502	2.20342555	0.15336492	0.51118324
Clp1	0.36966868	2.44874484	2.20333231	0.15337318	0.51118324
Il5ra	-1.5535539	-0.831976	2.20318809	0.15338596	0.51118324
Ccdc127	-0.1726552	6.5235289	2.20292796	0.153409	0.51118324
Pcbd1	0.44435454	1.94610971	2.20261703	0.15343656	0.51118324
Irf6	-0.4057119	3.74546148	2.20228307	0.15346616	0.51118324
Dnal1	-0.1929656	7.03713148	2.20165011	0.15352228	0.51118324
Zak	-0.1922261	6.48262938	2.20146075	0.15353908	0.51118324
9530080O11	0.44551055	2.39329389	2.20114228	0.15356733	0.51118324

Smc2	0.27896664	4.51070417	2.20101922	0.15357825	0.51118324
Rbbp7	0.19312162	7.41912342	2.20052783	0.15362185	0.51118324
Crlf2	-0.7210796	0.43132701	2.19984112	0.15368282	0.51118324
Map2k1	0.15309278	7.71419502	2.19926392	0.15373408	0.51118324
Gab3	0.39131582	2.77288416	2.19902983	0.15375488	0.51118324
Atad5	-0.3087022	3.88604756	2.19865224	0.15378843	0.51118324
Adamts4	0.56639664	1.78413118	2.198605	0.15379263	0.51118324
Actr8	0.24917321	4.68296503	2.19835536	0.15381482	0.51118324
Dmc1	-1.7799182	-1.4697402	2.1969997	0.15393538	0.51145054
Spop	-0.1695181	7.72348367	2.19659647	0.15397127	0.51145054
Apbb1	0.22124649	5.52168404	2.19576148	0.15404561	0.51157118
Hmmr	-0.3429211	3.27664422	2.19485422	0.15412643	0.51163652
Gm17751	-1.7105962	-0.8407761	2.19468702	0.15414133	0.51163652
Scube3	-0.946348	1.4791475	2.192604	0.1543271	0.51212684
Gprin3	-0.3360076	3.67896331	2.1916116	0.1544157	0.51214256
Unc5a	0.28085354	4.9158948	2.19112366	0.15445929	0.51214256
Lrrcc1	-0.2425432	5.61702841	2.19070361	0.15449683	0.51214256
Slc6a1	0.24769157	7.70563936	2.19056594	0.15450913	0.51214256
Hdac1	0.28903327	5.29501835	2.19040548	0.15452347	0.51214256
Zfp346	-0.2261519	4.78813054	2.18998322	0.15456122	0.51214256
Cd79b	-1.8393118	-1.9913514	2.1895691	0.15459826	0.51214256
Nrde2	0.55723292	2.0474504	2.1887045	0.15467561	0.5122727
Slc43a1	0.80748811	0.58700747	2.18822284	0.15471873	0.51228941
Tph2	1.81348678	-1.2085196	2.18613905	0.15490541	0.51272347
Bin1	-0.2402335	5.36717275	2.18550356	0.1549624	0.51272347
Fgf11	0.21236772	6.14600193	2.18525686	0.15498453	0.51272347
Pkdrej	0.95825687	0.50668192	2.18481344	0.15502432	0.51272347
Cbwd1	0.28185681	4.13995644	2.18437889	0.15506332	0.51272347
Pdcd7	-0.3033081	3.61848309	2.18389107	0.15510712	0.51272347
Tgif2	0.50558066	2.2958135	2.18369814	0.15512445	0.51272347
Twist1	0.41199945	4.73255054	2.18336196	0.15515464	0.51272347
Dusp16	-0.321405	4.92493437	2.18262015	0.1552213	0.5128178
Nkd2	-0.3812009	4.51143517	2.18161789	0.15531141	0.51298957
Hyal1	0.49091323	3.56170097	2.18038715	0.15542215	0.51304288
Lims1	0.26431982	7.53140052	2.18021153	0.15543796	0.51304288
Gdf10	0.66147754	2.35877311	2.1801674	0.15544194	0.51304288
Trmt10c	0.27093778	4.78226766	2.17957764	0.15549505	0.51304288
Tpi1	0.22294577	6.96436296	2.17932076	0.15551818	0.51304288
Vegfa	-0.2292457	5.38698117	2.17785199	0.15565057	0.51335376
Ctla2b	1.54817758	-0.6607534	2.17714112	0.15571469	0.51338382
Fam120a	0.17217083	8.53748752	2.17516446	0.15589316	0.51338382
Hsf2bp	-0.6001786	2.07785124	2.17511769	0.15589739	0.51338382
Kpna4	0.14957128	7.20475338	2.17444822	0.15595789	0.51338382

Gm5531	-0.4437569	2.74342724	2.17413089	0.15598658	0.51338382
Ube2f	0.25744173	4.74670963	2.17369556	0.15602595	0.51338382
Prrt4	-1.2352431	-0.6122405	2.17365115	0.15602996	0.51338382
Tapt1	-0.201913	5.13774678	2.17338405	0.15605412	0.51338382
Gpr19	-0.3701511	3.45953435	2.17271897	0.1561143	0.51338382
Bud13	-0.385836	2.07170836	2.17193389	0.15618538	0.51338382
Vegfc	-0.4586801	2.74930106	2.17186211	0.15619188	0.51338382
Tcf4	0.15063496	9.26007733	2.17184465	0.15619346	0.51338382
Nhsl1	0.24491745	7.15377938	2.17167708	0.15620863	0.51338382
Ccdc64	-0.7760994	0.49411901	2.17136567	0.15623684	0.51338382
Scrib	-0.4606744	2.10448195	2.16996232	0.15636404	0.51338382
Rap1gap2	0.31611936	6.77560268	2.16939862	0.15641517	0.51338382
Tpra1	0.50025658	2.47467841	2.16912082	0.15644037	0.51338382
Sqle	0.22514521	5.75304706	2.1687194	0.1564768	0.51338382
Nt5m	0.3187118	3.91316768	2.16824435	0.15651992	0.51338382
Tinagl1	-1.0307529	0.32428798	2.16775404	0.15656444	0.51338382
Znf41-ps	0.40645134	2.72156312	2.16741716	0.15659505	0.51338382
Shh	0.57360497	2.02679904	2.16737621	0.15659876	0.51338382
Cdc5l	0.15017014	6.18232915	2.1672921	0.15660641	0.51338382
Nell1	0.27633694	4.05279299	2.16722859	0.15661218	0.51338382
Sema3e	0.56939359	4.19611162	2.1670835	0.15662536	0.51338382
Spg20	0.21316409	5.84138243	2.16663418	0.15666619	0.51338382
Trim3	0.24528031	4.52986376	2.16625815	0.15670038	0.51338382
4933433G19	-0.7070105	0.90088046	2.16595507	0.15672794	0.51338382
Pigc	0.49351473	3.10018614	2.16425016	0.15688307	0.51373381
Abhd5	0.25140454	6.67456623	2.1639419	0.15691114	0.51373381
Nostrin	-0.8974053	0.87042759	2.16191011	0.1570963	0.51416596
Zbtb20	0.18985529	6.86078302	2.16085042	0.15719298	0.51416596
Ccdc146	-0.6702707	1.56847198	2.16066764	0.15720966	0.51416596
Cd33	-0.2997021	3.1598662	2.15956094	0.15731072	0.51416596
Tubd1	-0.6182636	1.56401701	2.15949642	0.15731661	0.51416596
Pirb	-0.9922858	-0.2529225	2.1582656	0.1574291	0.51416596
Zfp697	-0.2556586	4.89969601	2.15800119	0.15745328	0.51416596
Car7	0.56596642	1.42663876	2.15790912	0.1574617	0.51416596
Adssl1	0.42538178	2.68975469	2.15749731	0.15749937	0.51416596
AI847159	1.93061744	-1.8989295	2.1574635	0.15750246	0.51416596
Lgi4	-0.6256546	1.45022589	2.15715411	0.15753077	0.51416596
S100pbp	-0.2467108	5.02257202	2.15649034	0.15759152	0.51416596
Fbxl19	-0.3342235	3.63288	2.15635	0.15760437	0.51416596
6330415B21	-0.3981511	3.14758581	2.15574089	0.15766015	0.51416596
R3hdm2	-0.1859414	9.00287395	2.15560627	0.15767248	0.51416596
Bend7	0.6291524	0.64875533	2.155233	0.15770668	0.51416596
Scx	-0.9594341	-0.0094432	2.1552102	0.15770876	0.51416596

Abcc5	-0.4350062	4.93688186	2.1544586	0.15777765	0.51416596
Ttc22	-2.2667073	-1.6064773	2.15429548	0.15779261	0.51416596
Atp5g2	-0.3615055	4.07447845	2.15375146	0.1578425	0.51416596
Gpr133	-0.4771847	2.64997106	2.15371827	0.15784554	0.51416596
1300002E11I	0.24421831	5.25378338	2.1523304	0.15797291	0.51434029
Rasip1	-0.428557	2.4086378	2.15220744	0.1579842	0.51434029
Rasal1	-0.4938639	3.28396186	2.15160002	0.15803999	0.51434029
4930599N23	-1.7698215	-2.0599011	2.15146977	0.15805195	0.51434029
Metap1d	-0.5420871	2.28054549	2.15012461	0.15817559	0.51449002
Vhl	-0.2181075	4.8722494	2.1492775	0.15825352	0.51449002
Rab22a	0.19950525	5.64374601	2.14926297	0.15825485	0.51449002
Crhbp	0.55429085	3.05964508	2.14901878	0.15827732	0.51449002
Tmem240	-0.5680449	0.89522004	2.14860384	0.15831552	0.51449002
Aco2	0.18099946	7.41179208	2.14847514	0.15832737	0.51449002
9030624J02F	0.18117766	5.22420034	2.14783436	0.15838637	0.51455751
Ptprc	0.56052611	1.9383955	2.14703195	0.1584603	0.51459639
Vps28	0.21839606	5.80975147	2.14687438	0.15847483	0.51459639
8430419L09F	0.22665648	5.70735474	2.14535151	0.15861527	0.51492816
Gpr171	-0.7153988	1.52802902	2.14469285	0.15867605	0.51500126
Nek3	-0.7392816	0.95365832	2.14287246	0.15884421	0.51531613
Slc1a5	-0.4948132	2.10216484	2.14255406	0.15887364	0.51531613
Urgcp	0.27175223	4.39058297	2.142095	0.15891609	0.51531613
Gtf2h4	-0.5969722	1.44532771	2.14168833	0.15895371	0.51531613
Gm6297	-0.8894157	1.00891899	2.14157117	0.15896454	0.51531613
Tfap2a	0.34599785	3.94544385	2.14068829	0.15904625	0.51545682
Plscr1	0.3654047	4.17838695	2.14027357	0.15908465	0.51545712
BC030307	0.76634831	0.74594489	2.139501	0.15915621	0.51546867
Eif3b	0.26314932	3.89030819	2.13940799	0.15916483	0.51546867
Fuk	0.47245782	1.56267441	2.1372919	0.15936105	0.51597997
Rwdd3	-0.4634689	2.62408244	2.13513156	0.15956168	0.51650528
Sf3a3	0.23664469	4.70077619	2.13449947	0.15962044	0.51657123
Uaca	0.30948835	7.66103414	2.13390379	0.15967584	0.51662626
Vasn	-0.264132	3.78869828	2.13307673	0.15975279	0.516751
Aip	0.30465213	5.1871287	2.131026	0.15994381	0.51724453
Lrrtm2	-0.2461686	6.66013688	2.12995759	0.16004343	0.51734997
Syt12	-0.2705794	4.19268637	2.1296723	0.16007005	0.51734997
Gm16796	2.64764467	-2.0218992	2.12914381	0.16011936	0.51734997
Rad51b	1.80575154	-1.3350402	2.12902773	0.1601302	0.51734997
Nol12	-0.3864107	2.80881033	2.12781011	0.1602439	0.51754133
C230037L18F	-0.7191117	1.60175458	2.12724501	0.16029671	0.51754133
Fkbp15	-0.2297738	4.74414784	2.12684164	0.16033441	0.51754133
Elf4	0.46719317	3.91616552	2.12671391	0.16034635	0.51754133
Plscr4	0.43984837	3.14324998	2.12633557	0.16038173	0.51754133

Eid1	-0.1728034	8.03895077	2.12367588	0.16063071	0.51820212
Uvrag	-0.1943479	5.56574296	2.12310069	0.16068461	0.51820212
Miip	0.77903335	0.92175841	2.12273154	0.16071922	0.51820212
Elovl4	0.31704708	4.81666935	2.12223064	0.16076619	0.51820212
Reep6	0.23267391	4.20138878	2.12209349	0.16077906	0.51820212
Fpgt	-0.2350238	4.93554178	2.12159807	0.16082554	0.51822781
Itsn1	-0.2607506	7.01485215	2.1207815	0.16090218	0.51826425
2210408I21R	-0.3414399	4.27033096	2.12065698	0.16091387	0.51826425
Nans	0.48791205	2.40515802	2.1195988	0.16101327	0.51834675
Pank4	0.36031033	2.77003552	2.11945609	0.16102668	0.51834675
Slc7a10	-0.949681	0.19958365	2.11891279	0.16107775	0.51834675
Dnd1	1.49996591	-1.5052537	2.11845561	0.16112074	0.51834675
Zmynd10	-0.8863326	0.25024876	2.11833492	0.16113209	0.51834675
Grsf1	0.17274013	6.82277294	2.11751516	0.16120922	0.51839672
Cd3e	-0.6412739	2.30550467	2.11735091	0.16122468	0.51839672
Cenpa	-0.8387241	1.06374212	2.11614991	0.16133776	0.51842627
Fam135b	0.44223386	4.6382602	2.11591292	0.16136009	0.51842627
Cul4b	0.17136084	7.03109806	2.11552323	0.16139681	0.51842627
Cyp2t4	-1.1902685	-0.8172949	2.11548905	0.16140003	0.51842627
Med20	0.23901403	5.04033689	2.11520822	0.1614265	0.51842627
Unc13b	-0.2475262	6.53909414	2.11454198	0.16148932	0.51850426
Mtus1	0.18051537	6.68441201	2.11345258	0.1615921	0.51851172
Dock8	-0.4022571	2.85605871	2.11326368	0.16160993	0.51851172
Pja1	0.17369685	6.81192113	2.11325273	0.16161096	0.51851172
Mettl15	-0.5489912	1.63250249	2.1122943	0.16170146	0.51851172
Myh15	-1.97236	-1.1483054	2.11182245	0.16174604	0.51851172
E130317F20I	-0.6935405	1.16468983	2.11117785	0.16180696	0.51851172
Nsmf	-0.2184547	5.93049297	2.11077579	0.16184498	0.51851172
Bbs5	0.32460472	3.73149981	2.11054656	0.16186665	0.51851172
Zfp62	0.21947744	6.19282385	2.11025444	0.16189428	0.51851172
Murc	1.4332596	-0.1710877	2.10998637	0.16191964	0.51851172
Kif1c	-0.2949915	5.6443033	2.10966758	0.16194981	0.51851172
Arhgef2	-0.3357987	5.56237743	2.10962294	0.16195404	0.51851172
Tmem91	0.58202883	1.95762266	2.10815829	0.16209272	0.51862483
Ifitm3	0.42583074	7.69025273	2.10814183	0.16209428	0.51862483
Mirlet7bhg	-1.2943332	0.81513679	2.10802881	0.16210499	0.51862483
Dld	0.17397304	7.47831163	2.10753741	0.16215155	0.5186505
Rpa2	0.37668275	3.4815477	2.10605791	0.16229185	0.51892425
Zfp874a	-0.2387391	4.88317352	2.1058217	0.16231427	0.51892425
Fam159b	1.98239828	-2.0502647	2.10515392	0.16237765	0.51896387
Rad23a	0.28263582	4.96103067	2.1048786	0.16240379	0.51896387
Tnnc1	-0.5586494	1.94600666	2.10379273	0.16250695	0.51908598
Mynn	-0.1978848	5.5697216	2.10346493	0.1625381	0.51908598

Cobl	0.19923458	7.98664521	2.10264154	0.16261639	0.51908598
Col5a3	1.69650337	-1.0560599	2.10216837	0.1626614	0.51908598
Magoh	-0.2447139	5.17826882	2.10206927	0.16267083	0.51908598
Cenpe	-0.4304347	2.89436466	2.10161132	0.16271441	0.51908598
Vnn1	-0.6847636	1.71551037	2.10152166	0.16272295	0.51908598
Ggct	0.29536484	4.48865281	2.10123106	0.16275061	0.51908598
Snap29	0.20898517	6.39853214	2.10023255	0.16284571	0.51916866
Actr3b	-0.2179495	4.85592865	2.09997807	0.16286996	0.51916866
Olfr113	4.2488852	-2.0314591	2.12854164	0.16290432	0.51916866
Snhg4	0.34528195	3.32764924	2.09919632	0.16294447	0.51916866
Rfesd	-0.2731597	4.17403698	2.09884297	0.16297817	0.51916866
Pus7l	0.63232197	1.59734635	2.09852992	0.16300802	0.51916866
Tmc3	-0.7709056	0.99473456	2.09759753	0.16309699	0.51925199
Inmt	0.49065059	3.49614324	2.09744698	0.16311137	0.51925199
Aurkb	1.2978675	-1.0478296	2.09566574	0.16328152	0.51967071
Clmp	0.27642128	6.01158504	2.09511718	0.16333396	0.51971471
Mcf2	-0.5095945	3.13970048	2.09404014	0.16343699	0.5199196
Abcc3	1.18136904	-0.6679128	2.09327582	0.16351015	0.52002713
Tmed10	-0.1865812	6.82479277	2.09281088	0.16355468	0.52002713
Sema6d	-0.2464289	6.16563267	2.0920675	0.1636259	0.52002713
Wbp11	0.17696395	6.05145539	2.09194492	0.16363764	0.52002713
Gm5431	-0.7133132	0.93549401	2.09106855	0.16372166	0.52002713
Dus2	-0.4127301	2.42156585	2.09077404	0.16374991	0.52002713
3632451O06	0.33430827	4.23193063	2.09001564	0.16382267	0.52002713
Sirt7	-0.3712637	3.02721932	2.08980949	0.16384246	0.52002713
Litaf	0.29419788	4.59247002	2.08980632	0.16384276	0.52002713
Cep78	0.40315981	2.62711095	2.08965539	0.16385725	0.52002713
Ftsj3	0.23821878	5.00058319	2.08826312	0.16399097	0.52032879
Anapc1	-0.188691	6.17805781	2.0866012	0.16415076	0.52071302
Olfr613	-0.4447585	5.43212935	2.08571171	0.16423636	0.52086177
Mink1	-0.2498384	5.58472022	2.08516353	0.16428914	0.52090639
Timeless	-0.7083756	2.14805561	2.08314709	0.16448348	0.52139971
Efemp2	0.50529467	2.75571536	2.08244181	0.16455151	0.52149253
Myg1	0.38011676	3.22637071	2.08093674	0.16469682	0.52169289
Tmem232	0.70187015	1.34682122	2.08084185	0.16470599	0.52169289
Atp5d	0.34580935	6.11576955	2.08058249	0.16473105	0.52169289
Epb4.1l5	0.2440004	4.6767128	2.07894473	0.16488938	0.52207146
Cdca4	-0.3936547	2.88013428	2.07850822	0.16493161	0.52208233
Sox9	0.26189974	5.66087096	2.07792262	0.16498829	0.52213891
Cdk9	-0.2524887	4.38479661	2.07649487	0.16512657	0.52225276
Cwc25	0.24371039	4.51326967	2.07603545	0.1651711	0.52225276
Tmem145	-0.5434688	2.44946133	2.07538578	0.16523409	0.52225276
Dennd1c	-1.3799565	-0.2605778	2.07476202	0.1652946	0.52225276

Ankle1	-2.3622219	-1.8448087	2.07471333	0.16529932	0.52225276
4930444P10I	-1.7085124	-1.494696	2.07469306	0.16530129	0.52225276
Arhgap8	-1.3995676	-0.3610325	2.0743814	0.16533153	0.52225276
St8sia1	-0.2409842	6.67540381	2.07434825	0.16533475	0.52225276
Cnrip1	0.19096696	6.07897393	2.0737079	0.16539691	0.52230538
Nhlh2	-1.40781	-0.6575748	2.07294016	0.16547148	0.52230538
Sptb	-0.4196945	5.42552413	2.07288582	0.16547676	0.52230538
Scmh1	0.21335139	6.11744616	2.07205935	0.16555708	0.52230538
A630020A06	-1.0909065	0.01746508	2.07201656	0.16556124	0.52230538
Ugp2	0.16919173	6.43308734	2.07177937	0.1655843	0.52230538
Mettl1	1.30455607	-0.7518971	2.07040512	0.16571799	0.52253112
Zfp746	-0.2233468	4.31556848	2.07002765	0.16575473	0.52253112
Ptger4	-0.892827	0.6562272	2.06984656	0.16577236	0.52253112
Grid1	-0.3835124	2.92400738	2.06727139	0.16602333	0.52297011
Chchd10	0.22360068	5.60173779	2.06707386	0.1660426	0.52297011
Oacyl	-1.2258862	0.29856542	2.06702761	0.16604711	0.52297011
Atp6v1a	0.21156829	9.34063743	2.0668229	0.16606708	0.52297011
Eif2b3	-0.357713	2.84573605	2.06595022	0.16615227	0.52311593
Ninl	-0.3715189	3.10125781	2.06553456	0.16619286	0.52312133
Nubp1	0.45509653	2.29977372	2.06387467	0.16635507	0.52319721
AK129341	-0.3312433	5.21366025	2.06378713	0.16636363	0.52319721
Pygo2	-0.265975	3.87133083	2.0636778	0.16637433	0.52319721
Scarf1	-1.3451629	-0.7980304	2.06269612	0.16647037	0.52319721
Lrrn4	-1.671905	-0.7844833	2.0626823	0.16647172	0.52319721
4930404I05R	-1.9397467	-1.5477678	2.06165927	0.16657188	0.52319721
Tsen15	-0.2816318	3.62941797	2.06144876	0.1665925	0.52319721
Gimap1	-0.4142512	3.12082073	2.06140891	0.1665964	0.52319721
Dnajb11	0.19285073	5.90712065	2.06096361	0.16664003	0.52319721
Impa1	0.18899256	5.28422292	2.06074293	0.16666166	0.52319721
Micall2	1.10498815	0.41934992	2.06042567	0.16669275	0.52319721
Tenm2	-0.3277479	7.00559318	2.06015154	0.16671963	0.52319721
Dcaf15	-0.5906352	1.33317004	2.06012309	0.16672242	0.52319721
Fam213a	0.30499665	7.66914769	2.05815912	0.16691512	0.52355436
Ccdc163	-1.178751	0.00792254	2.05782835	0.1669476	0.52355436
Pmaip1	0.34552249	3.4226659	2.05777386	0.16695295	0.52355436
Ccdc93	0.22658142	5.01806247	2.05583582	0.16714343	0.52402956
Hsd17b4	0.24512161	5.79095532	2.05478287	0.16724703	0.5240636
Slc29a2	0.82405601	0.35312579	2.05460826	0.16726421	0.5240636
Usp54	-0.1557041	6.65042022	2.05453809	0.16727112	0.5240636
Rhobtb3	-0.2764452	4.45855426	2.0540764	0.16731658	0.524084
D6Ertd474e	1.18200478	0.2199904	2.05218151	0.16750331	0.52446484
Gabra4	-0.2365642	5.85303945	2.05167955	0.16755282	0.52446484
4930451G09	-0.5766473	1.59307176	2.05134221	0.1675861	0.52446484

1810044D09I	-1.4253554	-1.5291417	2.05126145	0.16759407	0.52446484
Wdr70	-0.3054123	3.54999649	2.05036923	0.16768214	0.52448275
Spc24	-0.4773343	2.43892939	2.05020601	0.16769825	0.52448275
Mettl5	-0.3114821	3.45748939	2.04977087	0.16774123	0.52448275
9830166K06I	-0.7475421	0.88567121	2.04944245	0.16777368	0.52448275
Gm10406	-1.0086318	-0.4766145	2.04850927	0.16786591	0.52448275
Dbx2	0.4129509	1.98242717	2.04812024	0.16790438	0.52448275
Acot2	0.25995204	4.85835318	2.04777338	0.16793869	0.52448275
Gstm6	-0.4520827	2.2532875	2.04741324	0.16797432	0.52448275
N28178	-0.2703046	7.04077347	2.0469036	0.16802476	0.52448275
Gabrq	-0.5044019	1.88100598	2.04629882	0.16808464	0.52448275
Dact3	-0.2096404	4.11706436	2.04609895	0.16810443	0.52448275
Cmtm5	-0.5021138	2.42396211	2.04546124	0.16816761	0.52448275
Lamtor5	-0.2620222	5.97963691	2.04540501	0.16817318	0.52448275
Triobp	0.31997137	6.03592652	2.04510243	0.16820316	0.52448275
Slc22a21	-1.1730105	0.03731727	2.04498201	0.1682151	0.52448275
Chst1	-0.2175	5.89299389	2.04447592	0.16826528	0.52448275
Ifnar2	0.29992468	5.47723388	2.04438277	0.16827451	0.52448275
Zfp770	-0.1764193	6.22912452	2.04409293	0.16830326	0.52448275
Vmn2r-ps12!	-0.8844774	0.85826362	2.04334395	0.16837757	0.52448275
Cartpt	-0.5546458	1.40408598	2.04332624	0.16837932	0.52448275
Yif1b	0.5109349	1.7760898	2.04209212	0.16850185	0.52474295
Ppp1r12b	-0.3560224	5.67617441	2.04130891	0.16857967	0.52486382
Snip1	0.35734515	3.26961063	2.0406737	0.16864282	0.52493897
Sfmbt1	-0.1825394	5.97509888	2.04014922	0.16869498	0.52497989
Mms22l	-0.7536113	0.73336835	2.03877717	0.16883152	0.52516754
Dis3l	-0.2509846	3.87216742	2.03857761	0.16885139	0.52516754
Acot7	0.19619272	6.14942926	2.03830389	0.16887865	0.52516754
Pold4	0.51937298	2.12316132	2.03764381	0.16894441	0.52516754
Rbm41	-0.2641005	4.52288136	2.0372957	0.16897911	0.52516754
Supt7l	0.22498263	5.59171236	2.03719203	0.16898944	0.52516754
Gpr137c	-0.2601371	4.87523066	2.0364784	0.1690606	0.52526735
Zfp787	-0.6271553	1.59554729	2.03503506	0.16920462	0.52549655
Fndc8	-1.9001746	-2.2316128	2.03495646	0.16921247	0.52549655
Ptpn2	-0.3329419	4.93947843	2.03411317	0.16929669	0.52563679
Dync2h1	-0.409011	6.15787971	2.03141051	0.16956696	0.52623822
H60b	0.9911903	0.46473753	2.03097186	0.16961087	0.52623822
Ddx56	0.40894023	3.08038876	2.03096689	0.16961137	0.52623822
Rasgef1c	-0.3504743	3.5194525	2.03061285	0.16964683	0.52623822
Glis2	0.27314256	5.21343482	2.02957309	0.16975101	0.52644002
Pgap1	-0.2648546	5.42461788	2.02751807	0.16995714	0.52651345
Sgta	0.25288727	4.96266654	2.02673531	0.17003573	0.52651345
Plk2	0.23298161	7.04814951	2.02671698	0.17003757	0.52651345

Fam131c	1.46711436	-0.4741631	2.026217	0.1700878	0.52651345
Sco1	0.32946748	3.29472571	2.02615922	0.17009361	0.52651345
Slc22a23	-0.1948344	6.19523421	2.02558471	0.17015135	0.52651345
Cntnap5c	-0.7736714	1.45542887	2.02537314	0.17017261	0.52651345
Tmem72	-1.7349377	-1.1869953	2.02349759	0.1703613	0.52651345
Hsph1	0.21641969	9.09662742	2.02233539	0.17047836	0.52651345
Pot1b	-0.3309325	3.44286884	2.02180414	0.17053189	0.52651345
Stx4a	0.23822811	6.39747626	2.02153893	0.17055863	0.52651345
Mycbpap	-0.6696258	0.72901751	2.02149536	0.17056302	0.52651345
D17Wsu92e	-0.1503283	7.63463128	2.0212705	0.17058569	0.52651345
Pde4c	-0.9534792	-0.1185333	2.02119486	0.17059332	0.52651345
Tbc1d31	-0.3017513	3.31663818	2.01984668	0.17072934	0.52651345
Mef2a	0.15798577	8.26170554	2.01935093	0.17077939	0.52651345
Zc3h4	-0.2171651	5.87777576	2.01835427	0.17088006	0.52651345
Pou2af1	0.45249864	4.8134834	2.01818124	0.17089755	0.52651345
Idh1	0.22686729	5.41998055	2.01786636	0.17092938	0.52651345
Lmod1	-0.3339822	5.0582383	2.01779174	0.17093692	0.52651345
Rnf39	0.49663976	1.8296328	2.01772295	0.17094387	0.52651345
Rangap1	0.22122733	6.60553911	2.01765502	0.17095074	0.52651345
Fxyd2	-0.9646988	-0.1409644	2.01722362	0.17099436	0.52651345
Chtop	0.18852759	7.48466396	2.01717511	0.17099927	0.52651345
Mettl11b	2.09722455	-2.3411546	2.01687559	0.17102956	0.52651345
Ttll9	1.85605074	-1.3124561	2.01624285	0.17109358	0.52651345
Mok	-0.4217951	2.36552259	2.01609342	0.17110871	0.52651345
BC049715	-1.6948235	-0.4258807	2.01606392	0.17111169	0.52651345
Ppp1r15a	0.55278878	2.79105364	2.01575157	0.17114331	0.52651345
Mrpl45	0.33926219	3.64461514	2.01462981	0.17125693	0.52651345
Ifitm7	-1.3211702	-1.6271057	2.01460106	0.17125984	0.52651345
Gimap5	-0.8650636	1.56612691	2.01455927	0.17126407	0.52651345
Tmem151a	-0.2787985	4.63055063	2.01446847	0.17127327	0.52651345
Ifi27l2a	0.43965316	2.32156448	2.01424574	0.17129585	0.52651345
Gm11186	1.65788304	-1.441342	2.01419098	0.1713014	0.52651345
Mettl18	0.59711317	2.00591038	2.01405955	0.17131472	0.52651345
Iglon5	-0.332856	3.56600338	2.01403556	0.17131715	0.52651345
Rasgrf1	-0.3047568	8.33628821	2.0138471	0.17133626	0.52651345
1700001O22	-0.7309368	0.38566742	2.01316671	0.17140525	0.52651345
4930511M06	-0.4770596	3.22956521	2.01311518	0.17141048	0.52651345
Alox12	0.64217298	1.23960461	2.01292689	0.17142958	0.52651345
Psd	-0.2631393	5.56008766	2.01277205	0.17144529	0.52651345
Ing5	0.27445862	4.683819	2.01265495	0.17145717	0.52651345
Dnajb2	0.25045229	5.41496715	2.0122555	0.1714977	0.52651554
Trank1	-0.4070713	6.22042844	2.01187722	0.1715361	0.52651554
Camsap3	0.29372771	3.48513944	2.01104696	0.17162042	0.526585

Kcnd3os	-1.4073795	-1.0140629	2.01088376	0.171637	0.526585
Rrm2	0.71994909	1.42874558	2.01007241	0.17171946	0.5266427
2010106C02I	-2.1401452	-1.616105	2.00946009	0.17178172	0.5266427
Vat1l	0.3179176	5.41946063	2.00915497	0.17181275	0.5266427
Hddc3	0.34980778	2.87659112	2.00890137	0.17183855	0.5266427
Taf6	0.2266558	4.52925267	2.0087742	0.17185149	0.5266427
Dcaf10	-0.2261601	5.20849913	2.00715019	0.17201684	0.52702938
Taf8	0.32814384	2.78515592	2.00673123	0.17205953	0.52704017
Nr1d2	-0.1800016	7.42943897	2.00587881	0.17214642	0.52718632
Klhl22	-0.2287585	5.05935465	2.00372248	0.17236647	0.5277401
Ldlr	0.24828767	3.69142439	2.00251472	0.17248987	0.52778498
Gpr82	-1.8777073	-1.234143	2.00238306	0.17250332	0.52778498
Caprin2	-0.4061998	2.7154879	2.00228315	0.17251354	0.52778498
Chuk	-0.2640826	4.87758158	2.00204377	0.17253801	0.52778498
Gstm1	0.30428553	7.64972735	2.00056122	0.17268969	0.52812889
Vps35	0.16817863	6.730399	2.00013273	0.17273356	0.52814302
Arr3	-1.6682908	-1.161148	1.99966357	0.1727816	0.52816992
Siva1	-0.4782791	2.66080766	1.9979397	0.17295829	0.52844866
Prkd3	0.22173506	5.66350149	1.99776126	0.17297659	0.52844866
Rbmx2	-0.2926391	4.45926901	1.99729591	0.17302433	0.52844866
Ovca2	-0.3106226	3.35314287	1.99724185	0.17302988	0.52844866
Polr2g	0.25294745	5.38000688	1.99581191	0.17317668	0.528777
Exosc10	0.22738985	4.86434649	1.99478842	0.17328185	0.5289119
Ccdc135	-0.991844	-0.2724766	1.99461697	0.17329947	0.5289119
Kcmf1	0.18435368	7.50021097	1.99239491	0.1735281	0.52928571
Ndrg1	0.29678811	7.57194374	1.99221588	0.17354654	0.52928571
Strbp	-0.2297491	9.34693261	1.99194792	0.17357414	0.52928571
Slc46a3	-0.5571873	2.39258348	1.9918979	0.17357929	0.52928571
Celsr1	-0.4333463	2.35191803	1.99053464	0.17371979	0.52946031
Vwc2	0.37151732	3.14969717	1.99032133	0.17374178	0.52946031
Zfp932	0.3233073	4.46617735	1.9901972	0.17375459	0.52946031
Igsf11	0.20618087	5.86683619	1.98859963	0.17391945	0.5298427
Chrna1	0.50253873	2.07646178	1.9876444	0.17401812	0.53002329
Arpp21	-0.1999562	8.75965728	1.98691999	0.17409299	0.53013135
Tek	-0.3924533	2.9134999	1.98590195	0.17419828	0.5302085
Pdhx	0.20145094	5.1461394	1.98583313	0.1742054	0.5302085
Ddi2	0.41548196	2.7742584	1.98552276	0.17423752	0.5302085
Ttc14	-0.3838767	6.58514574	1.98515157	0.17427594	0.5302085
Plekhg5	-0.2495657	4.25616956	1.9847056	0.17432211	0.53022909
Hist1h2bn	0.46191067	3.01491365	1.98306329	0.17449228	0.53062674
Dnajc4	0.45111875	2.40710364	1.98245541	0.17455531	0.53067912
Tmem165	-0.2545169	4.66271088	1.98213668	0.17458838	0.53067912
Ndufv2	0.19933271	6.21579475	1.98066451	0.17474119	0.53102366

Nudt8	-0.7905323	0.48689699	1.98006055	0.17480393	0.53109438
Phf7	-0.8821253	0.91577903	1.97902958	0.17491109	0.5313
Cdca2	-1.4810714	-0.3892356	1.97852261	0.17496381	0.53130935
Sgcg	-1.4429115	-0.352772	1.97824074	0.17499314	0.53130935
Tyrp1	3.29375079	-1.86409	1.97640747	0.175184	0.53155295
Ear2	-1.2506552	-0.4067613	1.97585028	0.17524206	0.53155295
Gm14393	-0.3797391	2.67963904	1.9751656	0.17531344	0.53155295
4933431G14	0.83129051	0.30495939	1.97501515	0.17532913	0.53155295
Serinc4	-1.7599886	-1.0261286	1.97487989	0.17534323	0.53155295
Kcnip1	0.32639402	4.52694561	1.9738386	0.17545188	0.53155295
Ttc37	0.34143578	3.70231221	1.97376852	0.17545919	0.53155295
Etf1	0.17306938	6.47593528	1.97347829	0.17548949	0.53155295
Ikbke	0.77645763	0.5804378	1.97342248	0.17549532	0.53155295
AA414768	0.30831528	3.09144475	1.97330983	0.17550708	0.53155295
4930550C14I	0.73288844	1.73402426	1.9732693	0.17551131	0.53155295
Icam5	0.3708254	2.88514723	1.9720651	0.17563711	0.53155295
Gak	0.18338703	6.70953113	1.9718691	0.1756576	0.53155295
Npy2r	-0.3816374	2.84536466	1.97138444	0.17570826	0.53155295
Pcsk9	-0.8269172	-0.0783968	1.97137419	0.17570934	0.53155295
Kctd13	-0.2834267	6.17116268	1.97107491	0.17574063	0.53155295
Gm20753	1.80052677	-1.593677	1.97103408	0.1757449	0.53155295
Nudt17	-0.6167424	1.00296696	1.97031822	0.1758198	0.53165996
Pigw	-0.6213243	1.51908733	1.96983709	0.17587015	0.53169275
Slc22a6	-0.3565625	7.15639669	1.96903831	0.1759538	0.53182614
Anapc13	0.3051575	5.13802789	1.96828676	0.17603254	0.53194465
Parp1	0.16113064	6.13886078	1.96737908	0.17612769	0.53211271
Ube2c	-0.7429215	0.65337984	1.9667564	0.17619301	0.53219055
Smap2	0.14806732	7.69726981	1.96572683	0.17630107	0.5323311
Clec18a	1.03123936	0.16717546	1.96500494	0.17637689	0.5323311
Srbd1	0.31613984	3.49013912	1.96421661	0.17645973	0.5323311
Gpr107	0.28804844	3.80767005	1.96421023	0.1764604	0.5323311
Agpat4	0.26307096	5.19971773	1.96419165	0.17646235	0.5323311
Ctsd	0.34022784	7.20349076	1.96372739	0.17651116	0.5323311
Npy	0.35677023	3.41690542	1.96364191	0.17652015	0.5323311
Pdcd1lg2	-0.8740405	0.07482019	1.96307535	0.17657974	0.5323311
Dusp4	0.67302995	1.31826327	1.96290237	0.17659794	0.5323311
Cdc27	0.1883635	7.83018118	1.96227519	0.17666394	0.5323311
Prr18	0.32468421	3.12852725	1.96217302	0.1766747	0.5323311
Kirrel3	0.3794189	3.45483046	1.96098498	0.17679982	0.53246998
Actr1b	-0.2064576	7.25929002	1.96098393	0.17679993	0.53246998
Arhgdia	0.2619685	8.02589297	1.96037473	0.17686413	0.53254414
Atp2a3	0.64586182	0.6266662	1.95963137	0.17694251	0.53266095
Tbc1d10a	0.62566246	1.66524401	1.95854593	0.17705704	0.5328865

Mapkapk3	0.62353574	2.24862019	1.95788739	0.17712656	0.53291572
Cep41	0.47729063	2.89860501	1.95753698	0.17716357	0.53291572
Pctp	0.43663052	2.59913689	1.95732888	0.17718556	0.53291572
Zfp146	0.32569904	4.37017683	1.95560171	0.17736814	0.53323221
Sox13	-0.4273021	3.36118298	1.95558377	0.17737004	0.53323221
Pts	-0.2497465	5.2535485	1.95417509	0.17751913	0.53348209
Ptpn23	0.23060741	4.86843946	1.95346814	0.177594	0.53348209
Zdhhc22	0.38995656	2.47904651	1.95286242	0.17765819	0.53348209
Ercc6	-0.2952686	5.60019108	1.95237543	0.17770982	0.53348209
Sbk1	-0.2677013	4.16353769	1.95180482	0.17777033	0.53348209
Gm13807	1.77596325	-2.139352	1.95160629	0.17779139	0.53348209
Ttc5	-0.2122507	4.61761479	1.95112751	0.1778422	0.53348209
Chrna5	-0.9552508	-0.3679875	1.94984593	0.17797827	0.53348209
Sar1a	0.24517252	7.05937469	1.94963589	0.17800059	0.53348209
Zfp488	-0.4067978	7.72018288	1.94937673	0.17802812	0.53348209
Ptgr1	0.50135689	2.92611121	1.94906447	0.17806131	0.53348209
Musk	0.35659219	3.43332663	1.94794825	0.17818	0.53348209
1110001J03F	0.42969067	2.66001857	1.94783565	0.17819198	0.53348209
Pex16	0.6540619	0.74110707	1.94770064	0.17820634	0.53348209
Gm16740	1.29923195	-0.806682	1.94752513	0.17822502	0.53348209
Magi3	0.18124337	6.70809646	1.94705662	0.17827488	0.53348209
Ccdc77	0.32944918	3.53799165	1.94669753	0.17831311	0.53348209
Srp54a	0.15059442	7.11641345	1.94664201	0.17831902	0.53348209
Piezo2	-0.4122739	2.92854699	1.94614948	0.17837147	0.53348209
Cpne9	-0.3712731	4.22777363	1.94580332	0.17840835	0.53348209
Tspan32	1.72333511	-1.3717372	1.9455376	0.17843666	0.53348209
Srrd	0.58760961	1.36859612	1.94540304	0.178451	0.53348209
Lrba	-0.2350834	5.84648866	1.94529506	0.17846251	0.53348209
Dlx6os1	-0.306961	3.48672029	1.9451529	0.17847766	0.53348209
Ppm1m	0.32812487	3.80344664	1.94510396	0.17848288	0.53348209
Pcsk5	0.3320435	4.26563116	1.94509404	0.17848394	0.53348209
Avp	1.38536269	-1.658286	1.94338136	0.17866662	0.53375412
Tmem63a	-0.3089333	4.55997756	1.94318418	0.17868767	0.53375412
Daam2	-0.28908	5.00971729	1.94247001	0.17876393	0.53375412
Psen1	0.22598849	5.05000366	1.94211058	0.17880232	0.53375412
Cetn3	0.22831537	7.87997825	1.94178602	0.178837	0.53375412
Mir384	-2.019315	-1.205553	1.94162391	0.17885433	0.53375412
Rbm3os	1.04306636	-0.0460533	1.9414856	0.17886911	0.53375412
Camk1	-0.2768721	3.80136889	1.94126887	0.17889228	0.53375412
Rbms1	0.20281765	6.26706979	1.9401215	0.17901498	0.53383772
Rgs18	0.99480588	-0.0944499	1.93994682	0.17903367	0.53383772
Gm4922	-1.286093	-1.3059537	1.93989412	0.17903931	0.53383772
Dennd2c	0.85456766	-0.3429158	1.93884143	0.179152	0.53405539

Fam155a	-0.2087315	6.69012916	1.93794697	0.17924782	0.53414203
Mettl13	-0.37707	2.10148209	1.93782909	0.17926045	0.53414203
Arfgap2	-0.322198	4.17412182	1.93677287	0.17937369	0.53429824
Lamtor4	-0.6182699	1.98318415	1.93659951	0.17939229	0.53429824
Atl3	0.18781698	7.4225482	1.9356156	0.17949787	0.53437824
Hsp90aa1	0.19410062	10.9285529	1.93560904	0.17949857	0.53437824
Zfp563	-0.2409208	4.894565	1.93362944	0.17971123	0.53476678
Iffo1	-0.3864385	3.77255364	1.93347987	0.17972731	0.53476678
Ccr9	-0.3636668	3.73349126	1.93328459	0.17974831	0.53476678
Aebp1	0.3761817	7.19862456	1.93197637	0.17988905	0.5350672
Anapc15	0.38661625	2.43938327	1.93083459	0.180012	0.53523872
Kif15	-0.7332065	1.06210072	1.93029893	0.18006971	0.53523872
Traf2	-0.5766153	1.32158657	1.93006798	0.1800946	0.53523872
Serf1	-0.3813902	2.89627059	1.92995663	0.18010661	0.53523872
Kif17	0.35388369	3.1648129	1.92959498	0.1801456	0.53523872
Bcl2l15	-1.7249229	-0.0955266	1.92874968	0.18023677	0.53539139
Prrt3	0.57168469	1.74678824	1.92794529	0.18032358	0.53545819
Col7a1	-1.3514412	-1.240098	1.92780393	0.18033884	0.53545819
Atp13a3	0.15875159	7.15130974	1.92695369	0.18043067	0.53561266
Efna1	-0.9249105	0.04723327	1.92622311	0.18050961	0.53564503
Dhrs3	0.35391391	4.64623871	1.92611607	0.18052119	0.53564503
Neurod6	-0.2281154	6.14249421	1.92526616	0.18061309	0.53566226
Gpank1	0.50487325	2.00662254	1.92463831	0.18068102	0.53566226
Trpc4ap	0.19139106	5.32545809	1.92372977	0.18077937	0.53566226
Nsf	0.19927387	9.99472431	1.92315261	0.18084189	0.53566226
Six3	-0.3616031	3.04223827	1.92300047	0.18085837	0.53566226
Hnrnpab	0.25048084	7.5806322	1.92175897	0.18099295	0.53566226
Slc6a20a	-0.3954843	5.60574727	1.92161455	0.18100861	0.53566226
Bdh1	0.28520704	3.63196056	1.9203733	0.1811433	0.53566226
Snhg8	-0.3788009	2.70202649	1.92009292	0.18117374	0.53566226
A230056J06F	-1.3045109	-0.1013602	1.91975987	0.18120991	0.53566226
Cenpo	-0.5249242	2.19298701	1.91965912	0.18122085	0.53566226
Prmt7	-0.3725819	3.30379527	1.91954313	0.18123345	0.53566226
Upb1	1.15755914	-0.4929598	1.91941287	0.1812476	0.53566226
Mir17hg	-2.4630086	-1.6099372	1.91938758	0.18125035	0.53566226
Rragd	-0.1514813	7.20974552	1.91931351	0.1812584	0.53566226
Dusp15	-0.3415863	2.85404897	1.918442	0.18135312	0.53566226
Rasd1	0.84420128	0.3412436	1.91844031	0.1813533	0.53566226
Elf3	-0.9607734	-0.0623376	1.91825362	0.1813736	0.53566226
Snx3	0.18738447	6.82295888	1.91718355	0.18148999	0.53566226
Tsfm	-0.3051508	2.62494558	1.91710993	0.181498	0.53566226
Arf2	-0.2329543	6.06373611	1.91709011	0.18150016	0.53566226
Atg4d	-0.4758597	2.16866913	1.91696504	0.18151377	0.53566226

1110007C09I	0.4589942	2.33907643	1.9169283	0.18151777	0.53566226
Dclre1c	-0.3480062	5.24539351	1.91676318	0.18153574	0.53566226
Appbp2	-0.2106452	5.28876882	1.91675442	0.1815367	0.53566226
Mss51	1.5932002	-0.9823114	1.91652211	0.18156199	0.53566226
Trim65	-0.3116882	4.06082884	1.91581113	0.18163941	0.53576543
Cmya5	-0.3754362	3.28475375	1.91546988	0.18167659	0.53576543
Farsb	0.18549617	5.68963609	1.91487849	0.18174104	0.53578025
Pdk4	0.36501628	2.80876838	1.91469309	0.18176125	0.53578025
Tmem161a	-0.4013409	3.19158888	1.91374217	0.18186495	0.53578067
Bcl6b	-0.7763499	0.05208606	1.91326024	0.18191754	0.53578067
Col4a6	0.42222275	2.4222693	1.91275497	0.18197269	0.53578067
2210015D19I	0.29364713	3.178976	1.91164281	0.18209416	0.53578067
Stmn1	-0.1844064	8.45410907	1.91163032	0.18209552	0.53578067
Mdk	0.41457888	3.66848016	1.91156528	0.18210263	0.53578067
Gba	-0.2531445	3.90078188	1.91155594	0.18210365	0.53578067
Fam83b	-1.1376549	-0.6156451	1.91102409	0.18216178	0.53578067
Rmnd1	0.46090119	1.31481141	1.91091861	0.18217331	0.53578067
Psmd14	0.16444914	6.24716733	1.91065625	0.18220199	0.53578067
Cr2	-0.8723995	1.0796989	1.91054466	0.18221419	0.53578067
Stxbp3a	-0.1917311	4.61286465	1.91016866	0.18225532	0.53578067
Iffo2	-0.2401084	4.67135398	1.90961129	0.1823163	0.53578067
Hgs	0.28794833	4.12396268	1.90947843	0.18233084	0.53578067
H6pd	-0.3242925	3.62020881	1.90835813	0.18245349	0.53578067
Grik1	-0.3975055	2.80971496	1.90827605	0.18246248	0.53578067
Nefm	-0.2909514	9.24354979	1.90826335	0.18246387	0.53578067
Slc18a2	-0.3867475	2.87449392	1.90810074	0.18248169	0.53578067
Wdr27	-1.8296271	-0.7713851	1.90777023	0.1825179	0.53578067
Tma7	-0.2422349	6.81927028	1.90714279	0.18258667	0.53586565
Rnf144a	0.24124656	4.77117646	1.90600628	0.18271131	0.53589091
Cyb5d1	0.53310955	1.39283836	1.90581624	0.18273217	0.53589091
Afmid	0.62229456	1.12223607	1.90581056	0.18273279	0.53589091
Zcchc24	0.28736208	7.2701703	1.90558699	0.18275733	0.53589091
Plxdc2	0.16842389	6.74706993	1.90524929	0.1827944	0.53589091
LOC1016697	-1.3890522	-0.978259	1.90466392	0.18285867	0.53591609
Gm5126	0.41272249	2.50523899	1.90378305	0.18295545	0.53591609
Gm16702	-0.3004066	4.40077098	1.90343369	0.18299385	0.53591609
H3f3b	0.2879898	9.00360196	1.90338049	0.1829997	0.53591609
Gm3383	-0.7911886	-0.2915731	1.90335849	0.18300212	0.53591609
Ctu2	0.51839003	1.72551479	1.90133226	0.18322505	0.53645218
Bnc2	0.30051975	7.67740496	1.899001	0.18348195	0.53697248
St6galnac2	0.53899292	1.27839149	1.89899538	0.18348257	0.53697248
Wfdc2	1.23633141	-0.6818079	1.89792702	0.18360045	0.53720063
Zfp35	-0.2093401	4.4899564	1.89614968	0.18379676	0.53765813

Rpn2	0.27323945	5.31139917	1.89558353	0.18385935	0.53766965
Fancl	-0.3171118	2.71916364	1.89539123	0.18388061	0.53766965
Card11	-1.2217147	-1.0961286	1.89213851	0.18424077	0.53808554
Adcyap1	0.34683043	2.9020933	1.89179966	0.18427833	0.53808554
Rbm28	0.23229623	4.98405403	1.89088773	0.18437949	0.53808554
Map3k2	0.18049441	6.20693347	1.89056823	0.18441494	0.53808554
Ccl22	-1.533325	0.31341969	1.89055602	0.1844163	0.53808554
Dennd6b	-0.5306213	2.8384596	1.8905327	0.18441889	0.53808554
Pbld1	-0.3598922	3.46365228	1.89004591	0.18447293	0.53808554
Gm14436	0.22206775	5.06734564	1.8898647	0.18449305	0.53808554
Fubp3	-0.204057	5.88427179	1.88980282	0.18449992	0.53808554
Sesn1	0.18286361	6.4168834	1.88971609	0.18450955	0.53808554
Gmeb2	0.3628527	2.96301519	1.88946007	0.18453799	0.53808554
Gm16897	-0.6910827	0.57425721	1.88934605	0.18455065	0.53808554
LOC1005034!	-1.2188456	-1.3524867	1.88912441	0.18457527	0.53808554
Nup205	-0.2875195	3.99104559	1.88905784	0.18458267	0.53808554
Ldlrad3	-0.3505905	5.77201379	1.88776445	0.18472645	0.53837175
Kcnc2	-0.2446843	5.91234506	1.88745503	0.18476087	0.53837175
Hras	-0.3101035	5.0526213	1.88617414	0.18490343	0.53846198
Aasdhppt	-0.1944128	5.24523466	1.8857361	0.18495221	0.53846198
Stxbp5l	-0.3739334	8.44935517	1.88570562	0.18495561	0.53846198
Snrnp40	0.2864512	4.37384869	1.88564074	0.18496284	0.53846198
Neu3	-0.3383388	3.12335179	1.88537974	0.18499191	0.53846198
Lypd1	-0.2858703	4.83227986	1.88489907	0.18504548	0.53850141
Kmt2a	-0.2509405	8.41968347	1.88401812	0.1851437	0.53867074
Coro1b	0.23325985	5.32204427	1.88310329	0.18524576	0.53876421
Osbpl3	0.30787024	5.09364473	1.88301248	0.1852559	0.53876421
Irf9	0.32185421	3.88034125	1.88237998	0.18532651	0.5388531
Rasl11b	-0.2056824	5.61450664	1.88083446	0.18549919	0.53923867
Rbm4b	-0.2511469	4.34630072	1.87997367	0.18559545	0.53937707
Ctcfl	-0.4556502	3.10142924	1.87962041	0.18563497	0.53937707
Diap3	0.9538103	0.48127226	1.87933381	0.18566705	0.53937707
Mkrn1	0.17227021	6.7341484	1.87809315	0.18580597	0.53940032
Syt7	-0.274195	6.93971431	1.87761456	0.18585959	0.53940032
Sema5a	0.21209869	6.42910481	1.87756233	0.18586544	0.53940032
Magel2	-1.1305205	0.39993086	1.87740712	0.18588284	0.53940032
Hrc	1.40244138	-0.3257219	1.8767658	0.18595473	0.53940032
Sec11c	-0.2440674	4.53769326	1.87642262	0.18599322	0.53940032
Olfr464	-1.935776	-1.9463999	1.87619585	0.18601866	0.53940032
4833419F23I	0.97588013	-0.1610463	1.87558335	0.18608739	0.53940032
Cdk12	0.1654346	7.26615932	1.87550581	0.18609609	0.53940032
Acadvl	0.25316204	4.90350757	1.87494833	0.18615867	0.53940032
Map1a	-0.3128539	10.3099317	1.87491027	0.18616295	0.53940032

Mybpc3	-2.1411911	-2.0274697	1.87463901	0.18619341	0.53940032
Otud4	-0.1779094	6.54656309	1.87461452	0.18619616	0.53940032
C1d	-0.2299163	4.9718572	1.87335641	0.18633753	0.53969367
Exoc2	-0.2200061	5.89155414	1.8728676	0.18639249	0.53973669
Slc26a11	-0.5166719	2.08885462	1.87214022	0.18647432	0.53985745
Gca	-0.2341182	6.18271984	1.87165311	0.18652914	0.53990001
Jrkl	-0.2770773	3.30938128	1.87100235	0.18660241	0.53992423
Gng12	0.23532728	6.58084897	1.870586	0.1866493	0.53992423
Shc1	0.28589391	6.42383923	1.87050988	0.18665788	0.53992423
Ypel4	0.47575429	2.45977121	1.86928247	0.18679622	0.54000143
Cidea	1.90826004	-1.9543343	1.89315593	0.18679773	0.54000143
Slc11a1	1.27773295	-0.4192487	1.86920501	0.18680496	0.54000143
Ash2l	0.19272669	5.00464747	1.86643408	0.18711776	0.54062004
Cadm4	0.37504298	3.23482238	1.866262	0.1871372	0.54062004
Cpeb1	-0.2527437	5.01021217	1.86590433	0.18717763	0.54062004
Gm1979	-0.8193287	0.01681008	1.86588637	0.18717966	0.54062004
Rap1gds1	0.13572883	8.5849631	1.8653998	0.18723468	0.5406629
Blvra	0.38710303	2.55710238	1.86452509	0.18733363	0.54083258
Rpia	-0.3319949	2.92032853	1.86394855	0.18739889	0.54090493
2700054A10I	0.36418192	2.52735864	1.86268446	0.18754207	0.54110365
Stam2	0.19334985	4.84893538	1.86263071	0.18754816	0.54110365
Gm7173	-1.0490602	-0.6906791	1.86216883	0.18760051	0.54113867
Kif2a	0.16596605	7.65851713	1.8601403	0.18783065	0.5412291
Gprasp1	-0.3238183	8.9720936	1.86013521	0.18783123	0.5412291
Pttg1	-0.1924589	4.90101036	1.86001804	0.18784453	0.5412291
Scn2a1	-0.2860801	8.05753643	1.85989285	0.18785875	0.5412291
Mcts1	0.1923119	5.13383329	1.85970268	0.18788035	0.5412291
4930525G20	0.45860669	2.28997565	1.85956475	0.18789601	0.5412291
Zfp85	0.34388365	2.44957893	1.85941158	0.18791341	0.5412291
Ccdc15	-0.2949199	4.26370537	1.85895646	0.18796512	0.54123097
Rsl1	0.39672664	2.89096568	1.85869792	0.1879945	0.54123097
Krr1	0.148491	6.82468627	1.85831033	0.18803856	0.54124202
Dnah5	-0.6607928	2.34714386	1.85659846	0.18823332	0.54157258
Pomc	-1.4354877	0.35685037	1.85643652	0.18825175	0.54157258
Mettl8	-0.3467631	3.77776576	1.85623983	0.18827415	0.54157258
Igflr1	-1.4435526	-0.7968711	1.85536572	0.18837371	0.5416331
Tra2b	0.25396039	6.4036067	1.85534839	0.18837569	0.5416331
Rps15a	-0.1727379	7.20599227	1.85414532	0.18851283	0.54181441
Cep120	-0.1937275	6.33522438	1.85408885	0.18851928	0.54181441
Serpinb9b	-1.2624748	-0.7571018	1.85338729	0.18859931	0.54192869
Znhit1	-0.3251559	3.24479417	1.85221297	0.18873338	0.54210155
Cnot2	0.17018094	6.21927661	1.8521546	0.18874004	0.54210155
Rab32	-0.4102385	3.17081687	1.84873442	0.18913119	0.54310909

Ntf3	-1.8284593	-1.2165419	1.84834613	0.18917566	0.54312089
Actl6a	0.28094073	3.68241767	1.84758266	0.18926314	0.54315824
Wnt11	1.61943265	-1.0895182	1.84752804	0.1892694	0.54315824
Lipa	0.22356919	4.63551706	1.84690533	0.18934079	0.54318984
Lysmd2	-0.2187829	4.50476051	1.84672781	0.18936115	0.54318984
Twistnb	-0.183116	5.20112615	1.84617117	0.189425	0.54324359
Zfp292	-0.2036816	7.57624967	1.8458607	0.18946062	0.54324359
Eif1ax	0.24969719	6.23929247	1.84393518	0.18968176	0.54333401
Ttc23	0.38624417	2.80822105	1.84345058	0.18973746	0.54333401
Drd1a	0.25153922	4.35712398	1.84326047	0.18975932	0.54333401
Focad	-0.2363748	5.28452876	1.84262023	0.18983295	0.54333401
Ngrn	-0.246559	4.21976295	1.84221725	0.18987932	0.54333401
Acaa1a	-0.2600863	3.87713101	1.84221318	0.18987979	0.54333401
Odf3b	-1.0667899	0.00321497	1.84116114	0.1900009	0.54333401
Mrps30	-0.3426309	3.23911452	1.84105341	0.19001331	0.54333401
Kras	-0.1942483	8.1423861	1.84066064	0.19005855	0.54333401
Arid2	0.15876582	6.70487632	1.84015348	0.19011699	0.54333401
Tamm41	0.46265677	2.28639379	1.84013271	0.19011938	0.54333401
Myo1g	1.15858447	0.13617966	1.8401325	0.19011941	0.54333401
Vwa5b2	-0.3976233	3.81022683	1.84006977	0.19012664	0.54333401
Cyp39a1	0.37309918	3.46184472	1.8397599	0.19016236	0.54333401
Abcd4	-0.4758805	1.46587845	1.83944371	0.19019882	0.54333401
Cdc23	0.20871461	5.00918478	1.83911856	0.19023631	0.54333401
Tor1a	0.33847643	2.96947977	1.83764425	0.19040646	0.54333401
Bsg	0.3572687	7.36556229	1.83652195	0.19053611	0.54333401
Stk3	-0.2037306	5.66440243	1.83644006	0.19054557	0.54333401
Sema3c	-0.2633471	3.8203494	1.83582172	0.19061706	0.54333401
5430416N02	0.45236986	2.04785831	1.83489937	0.19072374	0.54333401
Nat9	0.42153319	1.99609858	1.8346657	0.19075078	0.54333401
Psmg4	-0.5700555	1.72007138	1.83424649	0.19079931	0.54333401
Gm6682	0.18157646	5.04837045	1.83392955	0.190836	0.54333401
Isg20l2	-0.3103568	4.04315154	1.83388016	0.19084172	0.54333401
Fam50a	-0.3024914	4.12554342	1.83327308	0.19091204	0.54333401
Mir28b	1.54229446	-0.4985451	1.83300422	0.19094319	0.54333401
Srrm1	-0.1337562	7.56337242	1.83263645	0.19098581	0.54333401
Kcp	1.21126986	0.03723685	1.83258497	0.19099178	0.54333401
Nrip1	0.20929308	6.16891455	1.832221	0.19103397	0.54333401
Celf3	-0.2397065	5.51005772	1.83187685	0.19107388	0.54333401
Hsd17b12	0.33902296	3.64403364	1.83170594	0.1910937	0.54333401
Cebpd	0.59841578	1.35921377	1.83167145	0.1910977	0.54333401
Vps13c	-0.3200102	6.48139958	1.83131719	0.1911388	0.54333401
Spon1	0.23469007	5.35663672	1.83085499	0.19119243	0.54333401
Bag6	0.21581921	5.6876848	1.83043126	0.19124162	0.54333401

Golim4	0.23501244	5.93506605	1.83032674	0.19125376	0.54333401
Lin9	-0.3636039	3.01299053	1.8300166	0.19128977	0.54333401
Rb1	0.18017145	6.94098613	1.82993749	0.19129896	0.54333401
Lrrc73	-0.4306112	2.43181403	1.82906757	0.19140003	0.54333401
Abhd6	0.25519616	4.18091732	1.82883874	0.19142662	0.54333401
4930404N11	0.77743731	0.04994917	1.82865191	0.19144834	0.54333401
Vps33a	-0.1905097	6.28335802	1.82848598	0.19146763	0.54333401
Tac2	0.60921558	1.01105244	1.82831245	0.19148781	0.54333401
Lingo3	0.3696215	3.01449084	1.82804134	0.19151934	0.54333401
Tnfsf10	-0.3644587	3.01706064	1.82717819	0.19161977	0.54333401
Dynlt1b	0.32505087	4.20983588	1.82674308	0.19167042	0.54333401
C330024D21I	-0.9314002	0.122273	1.82670658	0.19167466	0.54333401
Metap2	0.15601879	7.30908151	1.8265678	0.19169082	0.54333401
Neurod4	-0.8889012	0.44780469	1.82605745	0.19175026	0.54333401
Calr4	1.61875314	-1.2051204	1.8259567	0.191762	0.54333401
4930577N17	0.66014983	0.10867021	1.82546465	0.19181933	0.54333401
Larp4	0.16091853	7.14921979	1.82519137	0.19185118	0.54333401
Dsg1c	-1.116994	-0.8061699	1.82482341	0.19189407	0.54333401
Hagh	0.25769605	4.40177537	1.82477902	0.19189925	0.54333401
Galnt4	0.4653047	2.3252378	1.82477122	0.19190016	0.54333401
Jkamp	-0.2475268	4.43764506	1.82465177	0.19191409	0.54333401
Tbcb	0.28762703	4.55125127	1.82461279	0.19191863	0.54333401
Gm15408	-1.1799922	-0.4333354	1.82408883	0.19197974	0.54333401
Fam204a	-0.2767426	5.54557337	1.82389379	0.1920025	0.54333401
5033406O09	1.86241656	-0.5531035	1.82368844	0.19202646	0.54333401
Rab35	0.20129082	4.52165069	1.82296478	0.19211093	0.54333401
D3Ertd254e	-0.2548517	6.52483606	1.822677	0.19214453	0.54333401
Zfp865	0.23472412	4.44992551	1.82262598	0.19215049	0.54333401
Tmem181c-p	-0.4543865	4.26990538	1.82142969	0.19229026	0.54333401
Tal2	2.41106241	-2.0176317	1.82116373	0.19232136	0.54333401
Ilf3	0.17347305	5.74793852	1.82028909	0.19242365	0.54333401
Gm1604b	0.47548031	1.39017072	1.82025337	0.19242783	0.54333401
Zfp385a	0.33544547	6.61742759	1.8197225	0.19248995	0.54333401
Gm10754	-0.5210564	1.74058134	1.81953454	0.19251195	0.54333401
Aspdh	-0.9763784	0.11307525	1.81931013	0.19253822	0.54333401
Mylk3	0.86209161	0.35676851	1.81908865	0.19256415	0.54333401
Arhgef3	-0.2467556	5.69113974	1.81867894	0.19261214	0.54333401
Rere	0.14704253	8.29609131	1.81840247	0.19264453	0.54333401
Scamp2	0.53527281	2.70673317	1.81823622	0.19266401	0.54333401
Pbx1	0.15667936	9.2799897	1.81806848	0.19268366	0.54333401
Ppil2	0.2158347	4.47776369	1.81791737	0.19270137	0.54333401
5830416P10I	-0.708924	0.35402887	1.81743782	0.19275759	0.54333401
2610037D02I	-0.8540909	0.16203569	1.81713233	0.19279341	0.54333401

Rilp	1.04105357	-0.4836868	1.81704646	0.19280348	0.54333401
Ap3d1	-0.2418152	6.40065025	1.81677779	0.19283499	0.54333401
B230312C02	2.08352342	-1.0641934	1.81670303	0.19284376	0.54333401
Gm13547	-1.9236902	-2.4282955	1.81640523	0.1928787	0.54333401
Cdc20	1.06322174	-0.3598917	1.81636108	0.19288388	0.54333401
Obscn	0.72538798	0.39962601	1.81558442	0.19297504	0.5433708
Rfxap	-0.3238275	4.03711884	1.81556175	0.1929777	0.5433708
Npas2	-0.3288548	5.29330213	1.813658	0.19320139	0.54380941
Dbr1	-0.2664749	3.96110004	1.81354816	0.1932143	0.54380941
Usp3	0.21761379	4.04294891	1.81287631	0.19329333	0.54391806
Fmnl3	0.39083404	3.58003564	1.81227842	0.19336368	0.54395398
Gm4944	0.23356557	4.17769705	1.81208088	0.19338694	0.54395398
Coro6	-0.7810988	1.88178598	1.81170838	0.19343079	0.54396363
Frrs1	-0.8027723	1.18737088	1.81080293	0.19353745	0.54414986
Man1a	0.26770235	5.19943443	1.80967156	0.19367082	0.54431436
Paics	0.22603252	8.66683901	1.80962038	0.19367686	0.54431436
Pros1	0.31168239	5.1652713	1.80829796	0.19383291	0.54447651
Tcte1	0.84408294	0.71897727	1.80808979	0.19385748	0.54447651
1110038B12	-0.387014	2.45169573	1.80779633	0.19389214	0.54447651
Dab2	0.23359633	8.40747023	1.8075375	0.19392271	0.54447651
Dpyd	0.32659901	3.2659172	1.80723165	0.19395884	0.54447651
Bmp1	-0.395699	2.83587107	1.80601096	0.19410314	0.54447651
Fbxo11	0.1464836	8.26058227	1.80545455	0.19416896	0.54447651
Zdhhc4	-0.2954282	2.59386182	1.80509295	0.19421175	0.54447651
Gm3500	-0.7897843	-0.3763363	1.80471265	0.19425676	0.54447651
Copb1	0.1695649	6.10022922	1.80453135	0.19427823	0.54447651
Pde3b	-0.4874263	2.68835774	1.80447712	0.19428465	0.54447651
Galnt10	-0.5425461	1.9081187	1.80438849	0.19429514	0.54447651
1700007F19	-0.7255495	0.05421021	1.80407412	0.19433237	0.54447651
Slc2a1	-0.2978582	4.64273172	1.80400993	0.19433997	0.54447651
Tom1l2	0.17164357	7.57538087	1.80373424	0.19437263	0.54447651
Mfsd2b	-1.3043578	-0.5072159	1.8036555	0.19438196	0.54447651
Acbd5	0.13541762	7.15738997	1.80236894	0.19453446	0.54470704
Pcdhga6	0.39489699	2.36475453	1.80217708	0.19455721	0.54470704
Rpl23a	-0.2360243	7.25485524	1.80171795	0.19461168	0.54470704
Rps23	-0.2484221	7.24528783	1.80075781	0.19472564	0.54470704
Ptov1	0.33681013	5.14555831	1.80014475	0.19479845	0.54470704
Zfp605	-0.2108602	4.69555872	1.80014021	0.19479899	0.54470704
Nras	-0.1727829	7.27390837	1.79997456	0.19481867	0.54470704
Cep290	0.19724347	7.10751982	1.79979329	0.19484021	0.54470704
Nrsn1	-0.1706339	6.50508988	1.79940641	0.19488618	0.54470704
Heg1	-0.2148043	6.24919506	1.79831932	0.19501545	0.54470704
Gpr165	-0.3423442	3.639751	1.79809093	0.19504262	0.54470704

Tex14	-1.4270099	-0.4598515	1.79799326	0.19505424	0.54470704
Lonrf1	0.20347325	6.32971245	1.7978194	0.19507493	0.54470704
Stk25	-0.1405054	6.73281195	1.79774063	0.1950843	0.54470704
Gtf2b	0.23028406	4.83713545	1.79737665	0.19512762	0.54470704
Slc16a9	0.3253775	4.83671912	1.79728815	0.19513816	0.54470704
Fbxl5	-0.1748688	5.6184531	1.79716845	0.19515241	0.54470704
Atp6v0a2	0.2281872	5.13639764	1.79558043	0.1953416	0.54510539
Rps14	-0.4269806	4.3577063	1.7950837	0.19540082	0.54510539
Klra9	1.5981221	-0.9669091	1.79495088	0.19541666	0.54510539
Gm15663	-0.3413333	3.1623083	1.79415679	0.19551139	0.54525662
Myo1c	0.28919756	5.19518825	1.79321717	0.19562356	0.54545639
Pyroxd1	0.26104347	3.35080755	1.79177369	0.19579603	0.54559237
Gapvd1	-0.1989685	6.04140785	1.79159915	0.1958169	0.54559237
Gng11	0.35073295	5.55179355	1.79126052	0.19585739	0.54559237
Lims2	-0.3796461	2.67256742	1.79061065	0.19593514	0.54559237
Mfap3l	0.26506584	5.63323855	1.79033139	0.19596855	0.54559237
Rrp9	-0.5655741	1.10828275	1.78956837	0.1960599	0.54559237
Gm15446	-0.548444	1.42491026	1.78956715	0.19606005	0.54559237
Prpf6	0.19265082	5.5425876	1.78946528	0.19607225	0.54559237
Dcun1d3	-0.2184037	4.96451194	1.78932514	0.19608903	0.54559237
Gm10845	-0.3808275	5.57078128	1.78931614	0.19609011	0.54559237
Lrp8	0.32425359	5.85580177	1.78908056	0.19611833	0.54559237
Peg3	-0.2765853	8.84130121	1.78856189	0.19618047	0.54565245
Hnf1a	-1.1561138	-0.9538867	1.78748164	0.19630999	0.54578515
2310040G24	1.0759033	0.08428143	1.78737978	0.19632221	0.54578515
Pptc7	0.1523422	6.66629707	1.78714921	0.19634986	0.54578515
Fam171b	-0.2024748	7.11323488	1.78571213	0.19652237	0.54606944
Gm19757	-0.3662037	4.53340146	1.78521755	0.19658178	0.54606944
Elf1	0.231814	5.89231624	1.78508547	0.19659765	0.54606944
Gm9776	0.37878238	2.31677585	1.78450974	0.19666685	0.54606944
Nrg2	-1.1915892	-0.8635565	1.78438406	0.19668195	0.54606944
Gm10658	-1.0272029	-0.5900214	1.78427036	0.19669562	0.54606944
Myt1l	0.21964186	8.60644459	1.78380561	0.19675151	0.54611193
Ppp1r7	-0.2168887	7.31260704	1.78247611	0.1969115	0.54642019
Ehbp1l1	-0.2224772	4.25592144	1.78220801	0.19694379	0.54642019
Pla2g4c	0.811646	0.09718778	1.78028554	0.19717547	0.5468656
Shisa5	0.3087218	5.06994187	1.7802015	0.1971856	0.5468656
Eef2k	-0.2617939	4.34373641	1.77968347	0.19724809	0.54692619
Dad1	0.35386427	5.04388937	1.77851687	0.19738892	0.54698483
Sidt2	0.18505482	5.19264066	1.7784191	0.19740072	0.54698483
Arel1	-0.1832382	6.26592541	1.77823843	0.19742254	0.54698483
Abcc9	-0.4666384	3.11914776	1.77770691	0.19748676	0.54698483
Herc2	-0.2928861	7.52920546	1.77760395	0.1974992	0.54698483

Ado	-0.2014648	5.37027576	1.77743366	0.19751978	0.54698483
Tmub2	-0.2677917	3.40390632	1.77715235	0.19755379	0.54698483
Lypd2	-0.7977947	1.82578607	1.77607057	0.19768462	0.54723447
Kif16b	0.17597252	4.96535644	1.7755098	0.19775248	0.54730974
Bbs10	0.35227476	2.99343135	1.77477149	0.19784187	0.54733806
Scarb2	0.35218941	4.27595107	1.77437458	0.19788995	0.54733806
Prss22	1.29046135	-1.6286607	1.77350176	0.19799572	0.54733806
Yaf2	-0.1867709	7.42070674	1.77305408	0.19805	0.54733806
Abce1	0.17921338	6.26132422	1.7729848	0.1980584	0.54733806
Tk1	-1.4219423	-0.8897527	1.772967	0.19806056	0.54733806
Ldb1	0.23869218	3.97055761	1.77275863	0.19808583	0.54733806
Cpsf3	0.19888818	5.09229648	1.77245611	0.19812253	0.54733806
Gm10584	-1.2157516	-1.1520838	1.77231395	0.19813978	0.54733806
Rbbp5	-0.1721369	5.69643705	1.77206934	0.19816946	0.54733806
Rundc3a	-0.2205793	5.33651374	1.77167515	0.19821731	0.54735786
Slco4a1	0.59605074	1.80494302	1.77099172	0.1983003	0.54738125
Sh3bgrl	-0.2148596	8.852275	1.77093541	0.19830714	0.54738125
Trerf1	-0.2694188	5.40018908	1.76930557	0.19850523	0.54750026
Slc37a2	-1.0576417	-0.1409012	1.76928527	0.1985077	0.54750026
Mrap	0.53679522	1.69198266	1.76923076	0.19851433	0.54750026
Mettl14	0.22028758	5.82265542	1.76917393	0.19852124	0.54750026
Hivep1	-0.189926	7.4051704	1.7689072	0.19855369	0.54750026
E430025E21I	0.17233487	6.34156917	1.7681749	0.1986428	0.54760219
BC068281	-0.3323056	2.29279265	1.76767659	0.19870346	0.54760219
Vsig10l	-0.4577057	2.41814072	1.76760043	0.19871274	0.54760219
Cabin1	0.19808248	5.24959159	1.76711542	0.19877181	0.54765283
Tgs1	-0.2619806	6.52407515	1.7662488	0.19887742	0.54783163
Dmp1	0.93062216	0.28825488	1.76550493	0.19896812	0.54796931
Sypl2	-0.9041465	0.14248168	1.76505802	0.19902264	0.5480073
Crat	0.18706212	5.36198451	1.76401968	0.19914938	0.54813618
Actn1	0.34631004	4.61802019	1.7638697	0.1991677	0.54813618
Ccnh	0.19874601	4.97463151	1.76367357	0.19919165	0.54813618
Ddx25	0.30593299	4.1774863	1.76302681	0.19927067	0.54820779
Api5	0.16475973	6.94753257	1.76274349	0.19930529	0.54820779
Ttll3	-0.4788225	1.83886483	1.76239531	0.19934786	0.54820779
Megf10	-0.3210665	4.61145867	1.76198924	0.19939751	0.54820779
Lrrc2	-0.5259034	1.76997162	1.76179417	0.19942137	0.54820779
Tmem194	-0.5033165	2.91223127	1.76032713	0.19960092	0.54847964
Wdr11	-0.257126	5.39380015	1.75980117	0.19966534	0.54847964
Tvp23b	-0.2483698	4.76558333	1.75944845	0.19970855	0.54847964
Lime1	-0.6066465	2.74346106	1.75935442	0.19972008	0.54847964
Psme1	0.27400145	7.09575098	1.75834721	0.19984355	0.54847964
4930481A15I	0.63915124	1.32952791	1.75797621	0.19988906	0.54847964

Ptpn5	0.20409479	5.9042918	1.7577929	0.19991155	0.54847964
Cd200	0.21063672	6.86633435	1.75744657	0.19995405	0.54847964
Ccdc103	-1.057313	-0.0614749	1.75731919	0.19996968	0.54847964
Pde7b	0.1447626	6.60018981	1.75698915	0.2000102	0.54847964
Dcaf13	0.19398293	4.45411103	1.75698307	0.20001094	0.54847964
Axin2	-0.2637414	4.98891397	1.75605437	0.200125	0.54847964
Sertm1	0.22239978	4.58370972	1.7559296	0.20014033	0.54847964
N6amt1	-0.1769405	5.66789646	1.75562638	0.20017759	0.54847964
Atp5o	0.23568226	6.67836527	1.755541	0.20018808	0.54847964
Cacnb2	-0.1787508	6.33615313	1.75540154	0.20020522	0.54847964
Fam122b	0.23432527	4.12560287	1.7553368	0.20021318	0.54847964
Zfp191	-0.2410614	5.01105649	1.75435002	0.20033453	0.54870036
Rnpc3	-0.3304797	4.82241866	1.75296578	0.20050491	0.54897194
Cntnap3	-0.5159247	1.93386135	1.75260446	0.20054941	0.54897194
Mrps9	0.28291262	3.40868777	1.75255034	0.20055608	0.54897194
Fat2	0.45692793	2.77609445	1.75152517	0.20068241	0.54903054
Mrfap1	0.23218094	9.51155094	1.7513808	0.20070021	0.54903054
Icam4	-1.0105704	-0.0270766	1.75116105	0.20072731	0.54903054
Pea15a	0.17819393	7.66623059	1.75064606	0.20079083	0.54903054
Snf8	0.28620793	4.26851453	1.75064594	0.20079085	0.54903054
Abhd4	0.25830869	5.30739511	1.7503911	0.20082229	0.54903054
Chst3	-1.0933926	0.66964206	1.74924164	0.20096419	0.54910373
Slc22a18	0.83759272	1.18189298	1.74901198	0.20099255	0.54910373
Fbxo6	0.35144676	2.55592837	1.7490012	0.20099388	0.54910373
Wbscr17	-0.2968616	3.82824717	1.74819207	0.20109386	0.54910373
Psg16	-0.5897939	1.38269931	1.74723227	0.20121254	0.54910373
Eps8l1	0.32160238	2.75832042	1.74670903	0.20127727	0.54910373
Actrt3	-1.2304334	-0.9276285	1.74653746	0.20129851	0.54910373
Comt	0.17668138	5.68667028	1.74590705	0.20137654	0.54910373
Sucla2	-0.1486062	6.74457014	1.74574305	0.20139685	0.54910373
Kirrel2	1.47920313	-0.5200892	1.74571111	0.20140081	0.54910373
Prkdc	-0.2842877	5.15821367	1.74556843	0.20141848	0.54910373
Stoml3	-1.8564962	-1.6978164	1.74542441	0.20143632	0.54910373
Aoc2	-0.5900765	1.2423825	1.74529531	0.20145231	0.54910373
Rpl30	-0.7711502	0.13246401	1.74526793	0.2014557	0.54910373
Eln	-0.4216742	2.43103705	1.74517187	0.2014676	0.54910373
Lamb2	0.3929995	4.32199266	1.74359248	0.20166339	0.54910373
Car15	-0.6005448	2.46758281	1.74280815	0.2017607	0.54910373
Wbscr27	0.19967277	4.58273635	1.74271773	0.20177193	0.54910373
Cryz	-0.3534134	2.82523574	1.74239043	0.20181256	0.54910373
Psma7	0.18221608	6.45837861	1.74211794	0.20184639	0.54910373
Mb21d2	-0.2300286	5.16656613	1.74207713	0.20185146	0.54910373
Gdap1l1	0.27025459	3.45144051	1.74202223	0.20185828	0.54910373

Eif3f	0.21119276	5.99474716	1.74192043	0.20187092	0.54910373
Phlpp2	-0.2474367	5.35627201	1.74166528	0.20190261	0.54910373
C130083M11	-0.4069069	2.78221445	1.74162519	0.20190759	0.54910373
Spryd4	0.4338029	2.31508092	1.74120799	0.20195943	0.54910373
Ccdc63	1.8974812	-2.0957611	1.74037818	0.20206258	0.54910373
Gpc5	0.40708677	3.53972772	1.74035019	0.20206606	0.54910373
Champ1	-0.2163799	4.80465221	1.74019696	0.20208512	0.54910373
Cldn25	0.23078579	5.89007369	1.73980709	0.20213361	0.54910373
Glt8d1	-0.2536356	4.23810645	1.73958592	0.20216113	0.54910373
Gng13	-0.5068695	1.38099842	1.73934668	0.2021909	0.54910373
2410089E03I	-0.3557605	5.88888922	1.73930831	0.20219567	0.54910373
Crtc3	-0.2351269	4.93875182	1.73878377	0.20226097	0.54917021
Rims1	-0.2206139	6.48495215	1.73790943	0.20236986	0.54924995
Mterf1b	-0.6572374	0.95309795	1.73789249	0.20237197	0.54924995
Zfp235	-0.3385264	3.30221274	1.73584467	0.2026273	0.54973827
Ifih1	0.20680843	5.32452961	1.73560585	0.2026571	0.54973827
Pglyrp3	1.8756493	-2.0173844	1.7563737	0.2027063	0.54973827
Zfp612	-0.2234602	7.4305425	1.73513957	0.2027153	0.54973827
Agpat3	0.22923277	4.96856857	1.73462521	0.20277954	0.54976536
Lpar4	-0.4748861	2.27780024	1.73440531	0.202807	0.54976536
Sgol2	-0.332956	3.58539855	1.733497	0.20292051	0.54989014
Retnla	0.76804602	1.0485688	1.7331187	0.20296781	0.54989014
Col20a1	0.62843504	1.24662046	1.73242691	0.20305434	0.54989014
Nfkbib	0.2734492	3.71176623	1.73242474	0.20305461	0.54989014
Wdr13	-0.1362751	6.73099886	1.73230043	0.20307016	0.54989014
Krt18	1.59982411	-1.6566113	1.73207617	0.20309822	0.54989014
Crnkl1	-0.2223808	4.56709497	1.73107952	0.203223	0.54992353
Smad5	-0.1716164	6.179103	1.73059118	0.20328417	0.54992353
Tekt1	1.06080209	-0.1937425	1.73057464	0.20328624	0.54992353
Bag5	0.1794613	6.29016191	1.73054566	0.20328987	0.54992353
Ppp1r1a	0.27667765	7.06598969	1.73006909	0.20334959	0.54992353
Itm2c	0.28142232	7.70303371	1.7300199	0.20335576	0.54992353
Htra2	0.56102936	1.29477846	1.72951658	0.20341886	0.54994443
Chrm1	-0.1964798	5.75229557	1.72930632	0.20344523	0.54994443
Crct1	1.63014006	-1.9746755	1.74990921	0.20349968	0.54998114
Slc35f2	-1.0267063	0.09581704	1.72849818	0.20354661	0.54998733
5730455P16I	0.15501712	7.14297718	1.72820258	0.20358371	0.54998733
Tnnt2	0.30449541	4.613484	1.72710211	0.2037219	0.55022764
Prkar1a	0.13567371	9.6161682	1.72684307	0.20375445	0.55022764
2810001G20	-0.3569349	4.81931109	1.72610782	0.20384686	0.55036675
Hist1h2bp	0.92683165	-0.7282023	1.72508256	0.20397581	0.55056403
Gcnt4	-0.3059578	4.22489231	1.724725	0.2040208	0.55056403
Myl6	0.25866142	7.87742737	1.72455125	0.20404267	0.55056403

Ntf5	-2.2995454	-1.8553363	1.7233893	0.204189	0.5508484
Pmepa1	0.18082785	7.36000559	1.72227234	0.20432979	0.55111771
Dcaf12l2	-1.0066181	-0.1083279	1.72151006	0.20442593	0.55126655
Zmym1	-0.3140548	3.92520281	1.72029157	0.20457975	0.55157079
Ak4	0.23341451	5.3752424	1.71967704	0.20465737	0.55166585
Ppa1	0.19524242	5.77835253	1.71936347	0.204697	0.55166585
Tada2b	-0.216653	4.39056583	1.71838313	0.20482094	0.55188936
Prph	-1.7411782	-0.8824467	1.71798047	0.20487188	0.55191608
5330417C22I	0.34644669	3.85966934	1.71713802	0.2049785	0.55201249
Dock3	-0.3246453	7.93754843	1.71572173	0.2051579	0.55201249
Smarca1	0.20825488	5.63378935	1.71540567	0.20519796	0.55201249
Nipa1	-0.2395227	4.82904993	1.71513758	0.20523195	0.55201249
Tnks1bp1	-0.2846486	3.62382755	1.71504197	0.20524407	0.55201249
Podxl2	-0.453993	2.17688717	1.71491499	0.20526017	0.55201249
Nop16	-0.4086674	2.38297078	1.71485978	0.20526717	0.55201249
Fxn	-0.3949545	2.26426653	1.71395717	0.20538169	0.55201249
Trh	1.88875123	-1.6101563	1.71316752	0.20548193	0.55201249
Tsacc	1.02029467	0.05827724	1.71304292	0.20549776	0.55201249
Stk32b	-0.3899476	2.80468229	1.71281428	0.2055268	0.55201249
Rhobtb1	-0.4042376	2.61378833	1.71276898	0.20553255	0.55201249
Gcnt2	0.20577373	5.3346145	1.71260707	0.20555312	0.55201249
Acvr1c	0.27699651	4.78138108	1.71254315	0.20556124	0.55201249
Lrrc47	0.41934924	2.66498574	1.71245648	0.20557225	0.55201249
Dll3	1.44120922	-1.1968643	1.71234748	0.2055861	0.55201249
Pih1d1	-0.3884496	3.36208392	1.71185673	0.20564847	0.55201249
Mdfic	0.38527405	5.61031651	1.71149466	0.20569451	0.55201249
Lurap1	0.45207487	1.9634037	1.71127699	0.20572219	0.55201249
5430435G22	0.33802182	4.44542886	1.71079889	0.205783	0.55201249
Fbxo7	-0.2811679	3.37786508	1.71055262	0.20581433	0.55201249
Sp6	1.30998908	-0.8815398	1.71049498	0.20582167	0.55201249
4930487H11I	-1.5809954	-1.2748223	1.71019121	0.20586033	0.55201249
Banf1	-0.2631572	4.45274061	1.70965232	0.20592894	0.55201249
Thyn1	0.31560435	3.4539141	1.70911419	0.20599747	0.55201249
Srf	0.22425959	4.85046671	1.70902482	0.20600886	0.55201249
Serping1	0.33587862	7.04095034	1.70897448	0.20601527	0.55201249
Mybl1	-0.2657061	4.22013849	1.7081434	0.20612119	0.55218634
Mrps2	0.18506412	4.70560518	1.70755342	0.20619642	0.55227555
BC005561	-0.3149299	5.15199396	1.70711492	0.20625236	0.55227555
Rgma	-0.3146887	4.29821178	1.70640823	0.20634255	0.55227555
Angel1	-0.5533614	1.42903695	1.70635551	0.20634928	0.55227555
Gm6583	-1.5541302	-1.0788076	1.70616268	0.2063739	0.55227555
Zfp790	0.25059587	4.40422388	1.70595242	0.20640074	0.55227555
04-Mar	-0.1985137	4.92876459	1.70388751	0.20666465	0.55271655

Clec4a2	-0.898258	-0.2856982	1.70372019	0.20668605	0.55271655
Snx32	0.23262801	4.27697155	1.70338306	0.20672919	0.55271655
Xlr	0.72457349	0.44675069	1.70260651	0.20682858	0.55271655
Dusp8	-0.2378572	5.57658294	1.70260186	0.20682918	0.55271655
Brms1	-0.3407485	2.6401315	1.70220833	0.20687957	0.55271655
Blvrb	0.5308749	1.2397928	1.70209303	0.20689434	0.55271655
Ints10	-0.2691735	4.16093091	1.70206549	0.20689786	0.55271655
Prss16	-1.3560011	-1.0446287	1.70165345	0.20695065	0.55271655
Sema4a	0.32189982	3.73889398	1.70145325	0.2069763	0.55271655
Ace	-0.4029035	5.32388205	1.70010606	0.20714904	0.55300893
Ccdc85a	-0.2037456	5.81768813	1.69975771	0.20719373	0.55300893
Slc25a45	1.08994215	-0.4163997	1.69963807	0.20720908	0.55300893
Ralgps1	-0.2382974	6.58154748	1.69812893	0.20740287	0.55333588
Wnt5a	-0.2446358	5.5511028	1.69804359	0.20741383	0.55333588
Htr5b	1.34081715	-0.3098906	1.69730643	0.20750858	0.55347891
Terf2ip	0.17625173	6.07454767	1.69657749	0.20760232	0.55361921
Phf20	0.17393019	6.49914567	1.69355656	0.20799138	0.55452965
Taf9b	0.20927357	6.91473087	1.69305524	0.20805603	0.55452965
Edem2	-0.4737758	1.59219095	1.69269704	0.20810224	0.55452965
Ubap2l	0.15902043	8.54563331	1.69262224	0.2081119	0.55452965
Plaa	0.14027485	6.40093827	1.69232871	0.20814977	0.55452965
Asb11	0.61988099	0.90161594	1.69178892	0.20821946	0.55460549
Fam105a	-0.330822	3.79205502	1.69103065	0.20831739	0.55475654
Pus3	0.32075881	3.36515777	1.68971809	0.20848705	0.55509451
Rae1	0.23805875	3.83274446	1.68941073	0.20852681	0.55509451
Rlbp1	0.54360933	1.98670262	1.68859051	0.20863294	0.55526719
Cdca7	-0.9111864	0.33717584	1.6866173	0.20888855	0.55583755
Cetn4	0.34247319	4.02178654	1.685548	0.20902724	0.55597301
Igsf3	-0.2112433	4.5003432	1.68499058	0.20909957	0.55597301
Tmem106b	0.169575	7.56207036	1.68460253	0.20914995	0.55597301
Nme6	0.45889048	1.77490388	1.68450256	0.20916293	0.55597301
Rgs6	-0.327064	3.12285949	1.68438014	0.20917883	0.55597301
Xpa	0.24374541	5.24649638	1.68404793	0.20922198	0.55597301
Sars	-0.2172229	4.81691384	1.68388066	0.20924371	0.55597301
Pcdh1	0.22235616	7.22796233	1.68367835	0.20926999	0.55597301
BC030499	-0.5799187	2.4979529	1.68311258	0.20934353	0.55602174
Josd1	0.17445805	5.03477143	1.68221547	0.20946019	0.55602174
Gpr156	0.67961888	1.13406632	1.68185255	0.2095074	0.55602174
Tmem129	-0.3028024	3.06683303	1.68177661	0.20951729	0.55602174
Ano3	0.23222987	7.88990707	1.68165464	0.20953316	0.55602174
Megf9	-0.1972575	5.961198	1.68163083	0.20953626	0.55602174
Fam220a	-0.1970453	4.2702704	1.68124099	0.209587	0.55604674
Snrpd3	0.40547974	2.63607777	1.67952414	0.20981066	0.55623941

Slc35a1	-0.2904344	5.25560159	1.67943722	0.20982199	0.55623941
Vps39	0.16969978	5.87363614	1.67937571	0.20983001	0.55623941
Nf2	-0.1794952	6.09246586	1.67904024	0.20987375	0.55623941
Hvcn1	0.95331382	-0.3066528	1.67895452	0.20988493	0.55623941
Nlrp1a	-0.3957816	1.62889049	1.678496	0.20994474	0.55623941
Narg2	0.28114305	3.98398958	1.67843527	0.20995267	0.55623941
Mthfd1l	-0.3319106	3.49817884	1.67814671	0.20999032	0.55623941
Sdhc	0.22961126	6.84374404	1.67726021	0.21010605	0.55642316
Slc12a8	-0.6975091	0.36052363	1.67698197	0.21014239	0.55642316
Sh3d19	0.20119502	6.73968738	1.67577532	0.21030007	0.55673114
Opa3	0.2595486	5.05372894	1.67504815	0.21039517	0.55683
Ccr5	-0.6510707	1.19262425	1.67472701	0.21043719	0.55683
Ces5a	1.3734791	-1.1757707	1.67414251	0.21051369	0.55683
Klhl14	-0.5168826	1.43658351	1.6740261	0.21052893	0.55683
Rgag1	0.5690494	0.93509014	1.67390852	0.21054432	0.55683
Tm7sf2	0.73462365	1.14682049	1.67354243	0.21059226	0.55684735
Alpk2	1.6818769	-1.4775081	1.67305129	0.2106566	0.55690804
3110070M22	1.18298224	-0.8977719	1.67247739	0.21073181	0.55699162
Fancc	0.42216933	1.8744647	1.67217844	0.210771	0.55699162
Zfp87	0.20187945	5.56435072	1.6710848	0.21091445	0.5571788
BC065397	-0.6983445	0.92368029	1.67084548	0.21094586	0.5571788
Ptpn3	0.20788653	6.04739918	1.67069165	0.21096605	0.5571788
Capza1	0.18352078	6.23692652	1.66913868	0.21117003	0.55750101
Cacna1i	-0.2994425	4.05638113	1.66913193	0.21117091	0.55750101
9530052E02I	1.44606879	-0.9840896	1.66875885	0.21121995	0.5575211
B3galnt2	-0.2380957	3.82718944	1.66713327	0.21143379	0.55794905
Itm2b	0.26749936	8.98079647	1.66649145	0.2115183	0.55794905
Mrps27	0.29256489	3.35582252	1.66633437	0.21153899	0.55794905
Dlx2	-0.5141661	0.88539876	1.6662664	0.21154794	0.55794905
Zfp521	0.23015069	5.60118123	1.66525291	0.21168149	0.55802508
Arhgap35	-0.1681943	8.93178848	1.66517309	0.21169201	0.55802508
Ogfod2	0.31088905	3.487368	1.66503705	0.21170995	0.55802508
Zbed3	0.22557174	5.56461188	1.6647891	0.21174264	0.55802508
Slc35a3	-0.2053364	4.86209558	1.66443085	0.21178989	0.55804031
Ktn1	-0.1587168	7.13143191	1.66339534	0.21192654	0.55812396
Zfp780b	0.18736706	5.39516929	1.66334892	0.21193267	0.55812396
Dcdc2a	-0.2164478	7.4805659	1.66302988	0.2119748	0.55812396
Ap3m1	0.16838937	5.66474037	1.66268147	0.21202081	0.55812396
Klhl17	-0.4323182	3.50189595	1.66261931	0.21202903	0.55812396
I730030J21R	-1.5530153	-1.3284042	1.66217569	0.21208764	0.55816906
Dock7	-0.2014385	6.05798925	1.66107445	0.21223322	0.55819035
Siah2	0.23356403	4.21540597	1.6606452	0.21229001	0.55819035
Mir22hg	-0.2718155	3.66681709	1.66042531	0.2123191	0.55819035

Tmem150c	-0.2999091	4.08315618	1.66022549	0.21234554	0.55819035
Proser1	-0.2166135	5.5896054	1.66014912	0.21235565	0.55819035
Far2	-0.3211765	3.82220963	1.65982925	0.21239799	0.55819035
Itgb8	-0.278955	4.16989927	1.65967676	0.21241818	0.55819035
Vdac2	0.14885583	7.27146482	1.65948545	0.21244351	0.55819035
Dvl1	-0.2362625	4.07621373	1.6591344	0.21249001	0.55819035
Lap3	0.19155084	5.62660117	1.65897937	0.21251054	0.55819035
Il13ra2	0.63082414	1.98597913	1.65796872	0.21264449	0.55838375
Hist1h2bl	0.36716265	3.11037497	1.65758039	0.21269598	0.55838375
Ythdf1	0.13333103	6.7367696	1.65747763	0.21270961	0.55838375
Cradd	0.30177475	4.87140784	1.65717192	0.21275016	0.55838375
Ccnt1	-0.1977571	6.30201614	1.65538751	0.21298706	0.55884435
D830005E20	-0.6966936	-0.32603	1.65451908	0.21310247	0.55884435
Pdap1	-0.1655917	6.04391655	1.65425596	0.21313745	0.55884435
Asb13	-0.2118186	4.60256161	1.65420913	0.21314368	0.55884435
Pisd	0.21782361	4.45308824	1.65399949	0.21317156	0.55884435
1700020L24F	1.30428411	-1.6620476	1.65397485	0.21317483	0.55884435
Zfp133-ps	-0.6109254	0.55557483	1.65337495	0.21325464	0.55894467
Asah1	0.19568624	5.68220169	1.65296202	0.21330959	0.55897983
Ankrd13a	0.20428151	4.90859285	1.65219366	0.2134119	0.55901275
Tox	0.24970558	5.74876714	1.65212316	0.21342129	0.55901275
Fam161b	-0.2816119	3.87855137	1.65193174	0.21344679	0.55901275
Pthlh	0.41125746	2.27733895	1.65121797	0.2135419	0.55915302
Aldh7a1	0.17820632	4.98129847	1.65071584	0.21360884	0.55916601
Cct4	0.17131385	5.9909585	1.65048424	0.21363972	0.55916601
Dnm1l	0.16579686	8.30233516	1.65024584	0.21367152	0.55916601
Zfp296	-0.9448128	-0.1268593	1.64953915	0.21376581	0.55922606
Ksr2	-0.2489175	6.0655444	1.64945094	0.21377758	0.55922606
Xkr4	-0.3219036	4.24244498	1.64861571	0.2138891	0.55927629
Mto1	0.27300373	3.51939617	1.64823787	0.21393958	0.55927629
Cd3eap	-0.2854768	3.59037109	1.64812117	0.21395517	0.55927629
Mecr	0.57903937	1.30272389	1.6480623	0.21396303	0.55927629
Ptbp3	0.17972089	7.19156415	1.64730755	0.21406391	0.55943131
Farsa	0.5290704	1.36654708	1.64661275	0.21415683	0.55943593
8430427H17I	-0.1572888	6.53254101	1.64645606	0.2141778	0.55943593
Gm1715	-1.5544563	-1.7361002	1.6456939	0.2142798	0.55943593
Smpd1	0.26643923	4.97641497	1.64554801	0.21429933	0.55943593
Ankrd34b	0.28734241	4.5032415	1.64552288	0.21430269	0.55943593
Cdh8	-0.2077888	5.4722849	1.64543	0.21431513	0.55943593
Ctsc	0.41269922	2.05099363	1.64409319	0.21449422	0.55979482
Tmem248	-0.1851211	5.76332469	1.64344085	0.21458168	0.55991449
Fbxo22	0.15609217	6.86914648	1.64302143	0.21463794	0.5599527
Kdm6b	-0.230596	5.61535267	1.64252295	0.21470482	0.56001595

2610002M06	0.14373839	7.06876313	1.64200706	0.21477407	0.56001595
Acvr1	0.23309457	4.31727452	1.64179258	0.21480287	0.56001595
Rmnd5a	-0.1635312	7.57797669	1.6416006	0.21482865	0.56001595
Hcst	-1.6805591	-1.5368851	1.64124215	0.2148768	0.56002482
Stx6	0.20277994	4.9700372	1.64095569	0.21491529	0.56002482
Eif4ebp2	-0.21821	5.77871641	1.63969785	0.21508439	0.56019968
Sema7a	-0.1967879	5.34240719	1.6396003	0.21509751	0.56019968
Pofut2	-0.3456218	3.73010993	1.63952762	0.21510729	0.56019968
Calhm2	-0.4574054	2.54101821	1.6391275	0.21516112	0.56023146
Zfp579	0.39261066	1.47293401	1.6382631	0.21527748	0.56037337
Csgalnact2	-0.2712575	3.38256503	1.63781069	0.21533841	0.56037337
Ptbp1	0.27487022	6.00333761	1.63779479	0.21534056	0.56037337
Syndig1l	0.38666286	2.34801023	1.63518969	0.21569184	0.56117899
Sh3pxd2b	-0.2134486	4.59955708	1.63467159	0.21576179	0.56125246
Smco1	-0.3574001	2.72570555	1.63391876	0.21586349	0.56128088
Slpi	1.53976187	-1.3967674	1.6336787	0.21589593	0.56128088
BC052040	0.23213086	4.25305052	1.63366282	0.21589807	0.56128088
Dedd	0.21505827	4.20891729	1.63235652	0.21607471	0.56128088
Cstf2t	0.15056276	6.61119104	1.63220219	0.21609559	0.56128088
Txnrd2	0.58678675	1.2978111	1.6319912	0.21612414	0.56128088
Vrk1	-0.2600109	3.86179314	1.63188871	0.21613801	0.56128088
Tmem198	0.47755791	2.00437496	1.63168815	0.21616516	0.56128088
Pgap3	-1.0180418	-0.2730341	1.63165292	0.21616993	0.56128088
Cope	0.22510343	4.71358402	1.63150588	0.21618983	0.56128088
2810410L24F	0.74220228	-0.0493362	1.63098596	0.21626023	0.56131043
Nubp2	0.31047517	3.47215544	1.63066798	0.2163033	0.56131043
Txndc17	0.21851062	6.32172748	1.63049784	0.21632635	0.56131043
Esm1	-0.4718987	2.24517017	1.63005895	0.21638583	0.56135651
Unc45a	0.35225174	2.75728444	1.62970964	0.21643318	0.56137112
Mief1	-0.2411161	4.4905079	1.62852917	0.21659329	0.56154424
Eif2d	0.28734411	3.93549472	1.62841753	0.21660844	0.56154424
4933432I03R	-1.1200521	0.10659377	1.62829467	0.21662512	0.56154424
Dedd2	0.52422771	1.48902123	1.62731738	0.21675781	0.56168885
Lilrb4	-0.7981844	0.43424165	1.62715179	0.2167803	0.56168885
Pdrg1	0.2728145	5.4404089	1.62686213	0.21681966	0.56168885
Klhl11	-0.2360386	4.45954021	1.62665453	0.21684787	0.56168885
Gltscr1	-0.2333089	4.38183036	1.62603408	0.21693221	0.56175371
Sstr4	0.42064241	4.50972522	1.62561719	0.2169889	0.56175371
Rrp12	0.2622134	3.92891724	1.62554922	0.21699815	0.56175371
Ubtf	0.17057632	5.83029552	1.62437494	0.21715795	0.56205767
Hif3a	-0.3818723	2.85379318	1.62407215	0.21719918	0.56205767
Isl1	-0.6043019	1.28366596	1.62347575	0.21728041	0.56205767
Apoa1bp	0.26626394	3.85453329	1.62345939	0.21728264	0.56205767

Kbtbd8	-0.3053429	3.16545921	1.62240456	0.21742642	0.56227593
Prkab2	0.22784588	4.92458605	1.62177819	0.21751185	0.56227593
Dll4	-1.4443218	-1.5188964	1.62164396	0.21753016	0.56227593
Fam198b	0.2852077	3.62236405	1.62139623	0.21756397	0.56227593
Gnb1	-0.1392363	9.34935257	1.62130849	0.21757594	0.56227593
Fam13c	-0.2337336	4.49680051	1.62099591	0.21761861	0.56227821
Fras1	-0.395264	4.82325395	1.61965938	0.21780116	0.56264184
Hspb8	-0.298912	6.19497759	1.61877737	0.21792174	0.56270037
Sez6l	-0.1940075	6.13618277	1.61800703	0.21802711	0.56270037
Wipf2	-0.1571037	7.56041348	1.61796761	0.21803251	0.56270037
Alkbh1	0.30026545	4.29498988	1.61779433	0.21805622	0.56270037
A330009N23	0.60248246	1.21981126	1.61767165	0.21807301	0.56270037
Fcrl6	-0.7628364	0.28566598	1.61739029	0.21811153	0.56270037
Dhx16	-0.3560927	2.95949162	1.61735372	0.21811653	0.56270037
Mark3	0.14507834	6.40504925	1.61624855	0.21826791	0.56292877
Serpina10	2.4459986	-1.9734045	1.61609657	0.21828873	0.56292877
Socs3	0.48421613	1.04320367	1.61548318	0.21837282	0.5630377
Ythdc2	-0.2677721	5.51634512	1.61506664	0.21842994	0.56307709
Zdhhc15	-0.3375813	3.45046656	1.61390897	0.2185888	0.56322577
Msi1	-0.472396	1.91976697	1.61375842	0.21860946	0.56322577
Plcb1	-0.2475877	8.78715465	1.61373134	0.21861318	0.56322577
Epg5	-0.3254966	5.48609808	1.61321747	0.21868375	0.56329973
Pstpip1	1.4241552	-0.5833353	1.61180748	0.21887754	0.56359595
Csnk1d	0.14088676	6.50616897	1.61177127	0.21888252	0.56359595
Lhfpl5	-1.1549085	-0.401819	1.61127447	0.21895085	0.56366404
Grap2	-0.5437445	1.1940081	1.61027798	0.21908799	0.56390921
Pcdhga9	0.36116324	2.52573333	1.60990522	0.21913932	0.56393346
3110039M2(	-0.5370574	2.13060271	1.60874494	0.21929919	0.56400596
Unc79	-0.3293427	5.96216454	1.60809583	0.21938869	0.56400596
Sult4a1	-0.1568038	7.03888384	1.607685	0.21944536	0.56400596
Il6ra	0.41099111	2.57813041	1.60758229	0.21945954	0.56400596
Fam120aos	-0.2771178	4.61883601	1.60699233	0.21954096	0.56400596
Map3k5	-0.292461	5.08153796	1.60693553	0.2195488	0.56400596
Ctnna1	0.21093021	7.91023598	1.60669403	0.21958214	0.56400596
Traf3ip1	-0.1783819	4.88522269	1.60586862	0.21969614	0.56400596
Ddx23	0.18589044	5.43142983	1.60572392	0.21971614	0.56400596
Pram1	-0.5938334	0.92672815	1.60550749	0.21974605	0.56400596
Acap2	0.17579239	6.73780263	1.60515643	0.21979457	0.56400596
Timp3	0.30252419	9.35869266	1.60514278	0.21979646	0.56400596
Ackr1	0.26385806	5.69606705	1.60510671	0.21980145	0.56400596
Txndc16	0.19884235	5.40969844	1.60505358	0.21980879	0.56400596
Chrnb2	-0.2294612	3.9374046	1.60501848	0.21981364	0.56400596
Rnf25	-0.4253381	2.08902688	1.60474512	0.21985144	0.56400596

Wiz	-0.2047642	4.43720815	1.60398663	0.21995637	0.56400596
Lgals9	0.29381729	4.40799855	1.6038392	0.21997677	0.56400596
Cdc42ep5	-0.4585587	2.49276455	1.60360291	0.22000947	0.56400596
Rdh5	-1.4988429	-1.2298068	1.60344952	0.2200307	0.56400596
Tulp1	-1.835059	-1.9917509	1.60303572	0.220088	0.56400596
Sprn	0.21341554	5.91404279	1.60302416	0.2200896	0.56400596
Gm15319	-0.6911465	0.17664311	1.60265646	0.22014052	0.56402905
Uqcrc2	0.12911328	6.7286354	1.60165806	0.22027888	0.56427609
Zfp7	0.39703474	2.73567721	1.60070281	0.22041135	0.56441506
Cep85	0.26536158	3.329014	1.60066201	0.22041701	0.56441506
Zkscan3	-0.2488187	3.32695568	1.60010927	0.22049372	0.56450405
Prr5l	0.34023614	2.40754669	1.59966319	0.22055565	0.56455518
Cd274	-0.327927	3.76592191	1.59922592	0.22061637	0.56460322
Serhl	0.97028904	-0.4036211	1.59781128	0.22081298	0.56470309
Ccl5	-0.6030311	1.43448762	1.59740784	0.22086909	0.56470309
Gale	-0.9664691	-0.2678959	1.59739227	0.22087125	0.56470309
Rell1	0.3065157	5.182429	1.59736474	0.22087508	0.56470309
Lig3	0.22180972	4.99211735	1.59723332	0.22089337	0.56470309
Zfp846	-0.2874975	3.9051803	1.59713397	0.22090719	0.56470309
Cnih3	-0.1872154	5.92678748	1.59649384	0.22099627	0.56482351
Zfp646	0.245901	3.74038985	1.59502712	0.22120056	0.56505646
Zfp526	-0.3116964	3.07793303	1.59472009	0.22124336	0.56505646
Galnt13	-0.1959468	5.74392475	1.59460669	0.22125917	0.56505646
Peli3	-0.8955585	-0.7958979	1.59405009	0.22133678	0.56505646
Htr1a	-0.274028	3.55770126	1.59362897	0.22139553	0.56505646
Pla2g12a	-0.2952024	3.72783429	1.59360254	0.22139922	0.56505646
Rac1	0.14801903	10.032103	1.59332959	0.2214373	0.56505646
Galns	0.62159048	0.66042022	1.59304652	0.22147681	0.56505646
Tbcc	0.2680574	3.28109105	1.59235054	0.22157399	0.56505646
Bbs12	-0.4905488	1.27863567	1.59227863	0.22158403	0.56505646
Ccdc68	-0.7261059	0.39708641	1.59212035	0.22160614	0.56505646
Ap1m1	-0.2912033	3.73255191	1.5917818	0.22165344	0.56505646
Mfng	-0.8702831	-0.3038503	1.59177757	0.22165403	0.56505646
Vps13a	-0.2313988	7.32010642	1.59162535	0.2216753	0.56505646
Ghsr	-1.1449209	-0.3539409	1.59064601	0.22181222	0.56507097
4833420G17	0.20981417	4.88925606	1.59064491	0.22181237	0.56507097
AA543186	1.17204727	-1.0680148	1.59060865	0.22181744	0.56507097
Zfp759	-0.250243	3.76817556	1.59020102	0.22187446	0.56507097
Rplp2	-0.2539278	5.9945226	1.59008312	0.22189096	0.56507097
Vps9d1	0.24826507	3.70412847	1.5893719	0.2219905	0.56513278
Gsto1	0.28913278	4.19893539	1.58930956	0.22199923	0.56513278
Lrrc27	-0.3428008	3.21464008	1.58813019	0.22216443	0.56515124
Lgi2	-0.4311643	2.70263887	1.58750277	0.22225237	0.56515124

Pacsin1	-0.1826836	6.57730162	1.58724724	0.2222882	0.56515124
Mlf2	-0.1439053	6.45549964	1.5870457	0.22231647	0.56515124
Nog	-0.8364438	-0.0920429	1.58696206	0.2223282	0.56515124
Cyb5b	0.14630818	7.53246763	1.58645805	0.22239891	0.56515124
Ddr2	-0.2989725	6.27923198	1.58634341	0.222415	0.56515124
Scn5a	0.66362323	1.37007776	1.58623541	0.22243016	0.56515124
Efcab1	0.28913908	3.56238764	1.5861959	0.2224357	0.56515124
Hmgb3	0.16212248	5.81681194	1.58599414	0.22246402	0.56515124
Cyyr1	-1.1037966	0.36582894	1.58596248	0.22246847	0.56515124
Gabrb3	-0.1993233	8.62854624	1.58489717	0.22261808	0.56540743
Rab20	-1.7721779	-2.0306466	1.58464617	0.22265335	0.56540743
Btbd11	0.19781831	4.43944584	1.58311178	0.22286911	0.56574725
Ccdc12	0.28178737	3.63339538	1.58309655	0.22287126	0.56574725
Zfpl1	0.34689248	2.51937251	1.58242852	0.22296528	0.56577778
Fgf16	2.14689326	-1.5914107	1.58241362	0.22296737	0.56577778
Mgat5b	-0.2318287	3.71645702	1.58174236	0.2230619	0.56583882
Lrrc3b	0.35469259	3.63449655	1.58164562	0.22307553	0.56583882
Psmd3	0.20410724	4.38659442	1.58015692	0.22328536	0.56626433
Acer3	-0.3267364	3.65386616	1.57902927	0.22344448	0.56630433
Pus1	0.41844412	1.66141706	1.57894671	0.22345613	0.56630433
Dlg1	-0.1594765	7.4792636	1.57802299	0.22358659	0.56630433
Mavs	0.35064541	4.855431	1.57778853	0.22361971	0.56630433
Rgs9	-0.3072554	5.09525429	1.57753306	0.22365582	0.56630433
Ttc28	0.19195848	5.76228808	1.57746919	0.22366484	0.56630433
Cp	0.21578304	7.00285072	1.57745107	0.22366741	0.56630433
Ctsw	-2.0422632	-2.2378359	1.57739495	0.22367534	0.56630433
Pvalb	0.37009567	2.39315365	1.57734537	0.22368235	0.56630433
Tlk1	-0.1279382	7.60864649	1.57690442	0.22374469	0.56630433
Grm8	0.44883525	2.66462651	1.57668195	0.22377615	0.56630433
Lrrc49	-0.2515692	4.99706323	1.57606595	0.22386329	0.56630433
AA986860	0.42685839	1.71718232	1.57582289	0.22389769	0.56630433
Klhl10	1.26437325	-0.8904987	1.575698	0.22391537	0.56630433
Spryd3	-0.3063714	4.21771774	1.57482956	0.22403833	0.56630433
Accs	-0.3556379	2.8710364	1.57467569	0.22406013	0.56630433
Cdhr1	0.33568258	2.46622717	1.57457717	0.22407408	0.56630433
Klc1	0.17232348	7.49123601	1.57447487	0.22408858	0.56630433
Lum	0.31916194	5.256301	1.57431227	0.22411162	0.56630433
D930015E06	-0.4555228	2.15529752	1.57409202	0.22414283	0.56630433
Mir425	1.41875326	-1.6929276	1.5733614	0.22424641	0.56645967
Tnip1	0.26272783	4.09255579	1.57267382	0.22434394	0.56659968
Cdr2l	0.25776457	3.23007911	1.57223355	0.22440642	0.56665112
Nrip3	-0.1871531	5.45211388	1.57000034	0.22472368	0.56717876
Lrig1	0.27885532	3.267394	1.56966849	0.22477088	0.56717876

Brinp3	-0.2163343	4.53933013	1.56960637	0.22477971	0.56717876
Bbs1	0.23699818	5.65451516	1.56934805	0.22481646	0.56717876
Bmyc	0.21035201	5.26490475	1.56879065	0.22489578	0.56717876
Tmem198b	-0.623082	0.79725695	1.5679334	0.22501784	0.56717876
Foxo1	0.16561069	7.15529798	1.56790094	0.22502246	0.56717876
Timmdc1	-0.2231523	4.29248554	1.5678845	0.2250248	0.56717876
Slc8a3	-0.2549085	3.92640813	1.5678493	0.22502981	0.56717876
Slc10a4	-0.3343562	2.53480028	1.56768376	0.22505339	0.56717876
Gucy2c	1.32210821	-0.8766283	1.56750388	0.22507902	0.56717876
Vmn1r58	-0.4033269	4.2997275	1.56690169	0.22516484	0.56718492
Epha5	0.22029203	6.13213742	1.56635392	0.22524294	0.56718492
Usp14	0.16350572	6.97105052	1.56625687	0.22525679	0.56718492
Ido1	0.58321725	1.31686761	1.56577445	0.2253256	0.56718492
Smndc1	0.22874541	5.33377647	1.56566953	0.22534057	0.56718492
Nmrk1	-0.2229748	4.76005865	1.56555817	0.22535646	0.56718492
Cttn	0.17621084	5.83338286	1.56519389	0.22540846	0.56718492
Col26a1	-0.4771988	1.50329749	1.56494994	0.22544328	0.56718492
Fam189b	0.22930333	4.60001513	1.56473943	0.22547334	0.56718492
Cox6b2	-0.5164109	2.88656334	1.56436626	0.22552664	0.56718492
Emx2	0.3055421	2.96208256	1.56423689	0.22554512	0.56718492
Cdan1	-0.2691568	3.37040138	1.56376334	0.22561278	0.56724906
1190002N15	-0.1981944	5.34921163	1.56277385	0.22575424	0.5674987
Ankrd16	-0.4749178	2.54010471	1.56228673	0.22582393	0.56756785
Fmo5	0.26970261	3.72328381	1.56194568	0.22587273	0.5675845
Uvssa	-0.2267096	4.85422542	1.56096825	0.22601268	0.56775724
Tmem128	0.29043368	3.40362018	1.5608762	0.22602586	0.56775724
Gal3st1	-0.7489302	0.20452388	1.56054693	0.22607303	0.56776975
Osbpl9	0.18152097	6.74841289	1.56007497	0.22614067	0.56783363
Mrgbp	-0.6346735	0.49676204	1.55952245	0.22621988	0.56790546
Naa10	0.39402492	2.32544461	1.55921272	0.2262643	0.56790546
Fut8	-0.2380971	6.99074698	1.55878513	0.22632565	0.56790546
Lrrc10b	0.39712493	3.93809863	1.55818105	0.22641235	0.56790546
Khnyn	0.28858483	2.936284	1.55793903	0.22644709	0.56790546
Gm3696	-0.6504489	0.04726077	1.55790455	0.22645204	0.56790546
Serpina9	0.91339992	-0.6081491	1.5578164	0.2264647	0.56790546
3000002C10I	0.30174093	2.14423524	1.55723755	0.22654784	0.56795878
Cpne3	0.19188137	7.19702496	1.5570807	0.22657038	0.56795878
Aurkaip1	0.24355214	4.92854804	1.55612339	0.22670798	0.56819787
Gpr111	-1.9664706	-2.0538352	1.55450481	0.22694088	0.56867565
Dok1	-1.0293351	-0.2343909	1.55415296	0.22699154	0.56869672
Htt	-0.2653835	6.73840291	1.55367504	0.22706039	0.56876331
Ccdc177	0.39989379	2.74825824	1.55250171	0.22722952	0.56894023
Sh3tc2	1.65989411	-1.284331	1.55194918	0.22730922	0.56894023

Tmem117	-0.3245121	3.28497422	1.55145872	0.22738	0.56894023
Etfa	0.18548348	6.60591641	1.55129406	0.22740377	0.56894023
Tex12	0.96091823	-0.1499423	1.55126121	0.22740851	0.56894023
Utp6	0.1718305	6.67201553	1.55117438	0.22742105	0.56894023
Wnt7a	-0.5102527	1.63757621	1.55035668	0.22753914	0.56894023
Tstd1	-1.3245298	-1.3567026	1.55028867	0.22754897	0.56894023
Atp6ap1	0.2021736	7.63568664	1.55017699	0.2275651	0.56894023
Sharpin	0.25364312	3.8065174	1.55016489	0.22756685	0.56894023
Fam19a4	-1.4463675	-1.4218659	1.54996242	0.22759611	0.56894023
Pcx	-0.3984836	2.01039584	1.5488607	0.22775539	0.56903879
Lrrc19	-0.7981744	0.34240193	1.54809995	0.22786546	0.56903879
Arhgap19	0.29832062	3.4055371	1.54803767	0.22787447	0.56903879
Clcc1	-0.224463	4.36557929	1.54797065	0.22788418	0.56903879
Nup210l	-1.4496694	-0.3714807	1.54769017	0.22792478	0.56903879
Ano4	0.24077522	3.81478993	1.54752456	0.22794876	0.56903879
Ascc2	0.26976981	3.30241568	1.54747147	0.22795645	0.56903879
Cntln	-0.1589956	6.57104022	1.54715376	0.22800246	0.56903879
Gabarap	0.24880871	7.19834281	1.54705939	0.22801613	0.56903879
Samd4	-0.1728662	7.26723062	1.54592455	0.22818059	0.56934364
Ddx50	-0.1570379	6.27247617	1.54559657	0.22822815	0.56935069
Hs1bp3	-0.2281307	4.68705527	1.54525932	0.22827706	0.56935069
9830147E19l	-0.3858693	1.87966411	1.54495418	0.22832133	0.56935069
Pnmal2	0.18757869	6.34505682	1.54473831	0.22835266	0.56935069
Col11a2	-0.7191667	0.10976493	1.54412836	0.2284412	0.56941927
Thap3	-0.494453	2.29989503	1.54388636	0.22847634	0.56941927
Sec22a	-0.3212298	2.98563507	1.54350207	0.22853216	0.56941927
Pirt	1.49269701	-0.890339	1.54338321	0.22854943	0.56941927
Lancl3	0.35264336	3.95821715	1.54299527	0.2286058	0.56945428
Skiv2l	0.24541225	5.25710978	1.54235511	0.22869886	0.56947942
Glra2	0.26485823	3.31837041	1.54219646	0.22872193	0.56947942
Psma3	0.16600287	6.99917801	1.54135337	0.22884458	0.56947942
Tmem29	0.15528231	4.81473657	1.54128515	0.22885451	0.56947942
Mpdz	0.16443087	6.41183289	1.54075073	0.2289323	0.56947942
Aga	0.38160061	3.47617596	1.54040267	0.22898298	0.56947942
Gpr108	-0.3487271	3.10882504	1.54033557	0.22899276	0.56947942
Nek5	-1.7189572	-1.245548	1.54020707	0.22901147	0.56947942
Slc7a14	0.19442822	7.01975861	1.54011977	0.22902419	0.56947942
Pdpr	0.28450257	3.89356158	1.53964046	0.22909402	0.56947942
Psmb9	-0.4690059	3.32181791	1.53948323	0.22911694	0.56947942
Chmp1b	0.22402938	5.41564145	1.53943657	0.22912374	0.56947942
Trim28	0.15968985	5.90610985	1.53913136	0.22916823	0.56948481
Vta1	0.20649379	5.04032592	1.53882198	0.22921334	0.56949174
Zfp428	-0.5906795	0.8567422	1.53819689	0.22930452	0.56958321

Ergic3	0.26039294	5.24047087	1.53798926	0.22933481	0.56958321
Dnah17	-1.1791997	-1.109053	1.53675647	0.22951479	0.56967162
Gtf3a	0.34940037	2.55083537	1.53646692	0.22955709	0.56967162
Ano5	0.80928806	0.82467018	1.5362921	0.22958264	0.56967162
Ldb3	-0.4807456	2.06945824	1.53592393	0.22963644	0.56967162
G530011O06	0.35991491	4.65973078	1.53541235	0.22971124	0.56967162
Abhd14b	-0.2578264	5.31205164	1.53533912	0.22972195	0.56967162
Rb1cc1	-0.1835488	8.50531799	1.53520409	0.22974169	0.56967162
Tead3	-0.3543202	2.73021835	1.53463147	0.22982546	0.56967162
S100a11	0.27731171	9.5078464	1.53455101	0.22983724	0.56967162
Snap23	0.2136954	7.16619775	1.53445324	0.22985154	0.56967162
Ubqln1	0.13391321	7.10321703	1.53403912	0.22991216	0.56967162
1600014C10I	-0.2339202	4.83188295	1.5336904	0.22996321	0.56967162
Fnbp1l	0.18561931	8.5932555	1.53341816	0.23000308	0.56967162
Gltpd2	1.75412564	-1.9420875	1.53334938	0.23001316	0.56967162
Cxcl9	-1.2592052	-0.5124598	1.53291646	0.23007658	0.56967162
Mmgt2	0.31213582	3.31545724	1.53253091	0.23013308	0.56967162
Abi3	-0.4077456	1.67972369	1.53147925	0.23028729	0.56967162
Ptch1	-0.1880556	4.75473834	1.53136689	0.23030378	0.56967162
Gtf2a1l	2.00691748	-1.665669	1.53132118	0.23031048	0.56967162
Acy1	0.61151152	0.78264564	1.53110702	0.23034191	0.56967162
Trim34a	0.38049343	3.15628048	1.53106832	0.23034759	0.56967162
Zfp821	0.26389484	3.53873423	1.5308432	0.23038063	0.56967162
Pradc1	-0.3692518	2.72955504	1.530747	0.23039475	0.56967162
Hemk1	-0.4445118	1.69722115	1.53048855	0.23043269	0.56967162
Chac1	0.39678382	1.81672077	1.53048845	0.2304327	0.56967162
Ncapd2	-0.3505508	2.67232371	1.53022683	0.23047112	0.56967162
Camta2	-0.181524	7.54902242	1.52957495	0.23056687	0.56978416
Oas1g	0.99826484	-0.3560232	1.52934041	0.23060133	0.56978416
B4galt3	-0.3777073	1.94859098	1.5284438	0.23073314	0.57000517
Prune	0.19080048	4.58506646	1.52807596	0.23078724	0.57003418
Mtmr7	-0.2751219	5.04746558	1.52760534	0.23085649	0.57010057
Zdbf2	-0.3125145	5.97330262	1.52577096	0.23112664	0.57066298
Arsj	0.58209161	0.86203898	1.52544845	0.23117418	0.57067564
Timm21	0.27764192	4.15049091	1.52501775	0.23123768	0.57072771
Smug1	-0.1896762	4.24447993	1.52365034	0.23143945	0.57112095
Cxcl17	-1.6575382	-1.1080661	1.52265804	0.23158601	0.57132397
Emp1	0.40017692	3.82778592	1.52251845	0.23160664	0.57132397
Scfd2	-0.314574	2.97274159	1.52158903	0.23174404	0.57155812
Foxj1	-0.7063037	0.73725854	1.52090463	0.23184528	0.57156343
Bmi1	-0.186385	6.05149084	1.52082341	0.2318573	0.57156343
Caskin2	0.31185667	2.60278845	1.52071313	0.23187362	0.57156343
Pfn1	0.27746007	6.68741467	1.51928891	0.23208451	0.57165765

Spata6	0.23528978	3.91402258	1.51887415	0.23214597	0.57165765
Nudt4	-0.2215425	9.94797792	1.51870067	0.23217169	0.57165765
Thumpd1	0.19649761	5.95277989	1.51860241	0.23218625	0.57165765
Ak3	0.18853546	8.15985105	1.51845964	0.23220742	0.57165765
Gde1	0.196987	5.24696666	1.51830014	0.23223107	0.57165765
Tmtc1	0.19053306	8.45845134	1.51809429	0.2322616	0.57165765
Dnajc12	0.29901494	3.61725097	1.51788137	0.23229318	0.57165765
Atxn7l3b	-0.1522278	7.34105172	1.5178746	0.23229418	0.57165765
Scn8a	-0.2976245	8.83649402	1.51739599	0.23236519	0.57167898
Hsf1	0.17852611	5.47374936	1.51724352	0.23238782	0.57167898
Ror1	0.69243416	0.88849533	1.51682375	0.23245013	0.57172774
Ly6a	-0.6586738	2.3561402	1.51501009	0.23271959	0.57191908
Sowahc	0.21110782	4.15343982	1.51481502	0.23274859	0.57191908
Ubr1	-0.2212928	6.15641405	1.51466755	0.23277052	0.57191908
2610034M16	0.8238424	0.1506247	1.51429697	0.23282565	0.57191908
Mrpl1	-0.2220206	4.24105416	1.51397799	0.23287311	0.57191908
Mkl2	0.23449123	9.41262042	1.51383739	0.23289403	0.57191908
Olfm2	-0.2172069	5.18180106	1.51352128	0.23294108	0.57191908
Hck	-0.9996271	-0.4803346	1.51344425	0.23295255	0.57191908
Ctif	0.17073857	7.02124935	1.51334751	0.23296695	0.57191908
Xrn1	-0.1915168	6.04494522	1.51333648	0.2329686	0.57191908
Pabpc4l	-0.5543904	2.23419676	1.51315619	0.23299544	0.57191908
Mir6236	-0.4839824	4.65422758	1.51243783	0.23310244	0.57207738
Dmwd	-0.2127857	4.86534183	1.51161887	0.23322451	0.57220556
Cntnap5b	-0.2455915	4.15925846	1.51129568	0.2332727	0.57220556
9430091E24I	-0.5250728	1.9031491	1.51123167	0.23328224	0.57220556
Ndst1	-0.1615934	5.73500255	1.51032563	0.23341742	0.5724328
Arhgap30	-0.4035144	2.85648946	1.50992962	0.23347654	0.57247344
Slc2a8	-0.458047	1.62197619	1.50955192	0.23353294	0.57250741
Atp1a4	0.96508993	-0.6699236	1.50911389	0.23359837	0.5725635
Prss12	-0.3559246	1.85980198	1.50792844	0.23377557	0.57289347
Dbndd1	-0.4355219	1.76124318	1.50757152	0.23382895	0.57291996
Palm3	-0.3316714	2.25433235	1.50654941	0.23398192	0.57306104
A330093E20	-0.9060131	0.23437656	1.50624708	0.23402718	0.57306104
Prmt5	-0.1792886	4.74281688	1.50595824	0.23407044	0.57306104
Tmem40	0.91288222	-0.055011	1.50540744	0.23415297	0.57306104
Xdh	-0.2980834	3.2821717	1.50527206	0.23417326	0.57306104
Fmn2	-0.25002	6.65774574	1.50507382	0.23420297	0.57306104
Sbno2	0.39211431	1.71964168	1.50469711	0.23425945	0.57306104
Mipep	0.27153847	4.032367	1.50466048	0.23426494	0.57306104
Scamp4	0.27964946	3.4873528	1.50462787	0.23426983	0.57306104
Atxn2l	0.16451089	6.83832865	1.504344	0.2343124	0.57306104
Fastkd1	-0.3294118	2.74592134	1.502619	0.23457131	0.57359002

4933416I08R	-1.3450954	-1.0641654	1.50165453	0.23471624	0.57365415
Pemt	1.12271418	-0.981126	1.50156711	0.23472938	0.57365415
Abhd11os	0.64311235	0.35444913	1.50149042	0.23474091	0.57365415
Ranbp6	-0.2219739	6.39165545	1.50130979	0.23476806	0.57365415
Fam72a	1.03659036	0.51817619	1.50073693	0.23485422	0.57366365
Reps1	-0.1649691	6.00264591	1.50061151	0.23487309	0.57366365
Prss36	-0.6568658	0.86859551	1.50043145	0.23490018	0.57366365
Tfb2m	0.20067678	5.65396134	1.49998616	0.2349672	0.57366365
1700018G05	-1.265667	-0.9983156	1.49986715	0.23498511	0.57366365
Sorl1	0.22936092	6.15766545	1.49854746	0.23518389	0.57404477
Slco1a4	-0.2734791	4.88980651	1.49805051	0.23525879	0.57411906
AF357355	-1.6271056	-1.001034	1.49773906	0.23530576	0.57411906
Epsti1	0.60641166	1.15801116	1.49749663	0.23534232	0.57411906
Wdfy2	-0.4738506	1.70188876	1.49627014	0.23552741	0.57446644
Smim4	0.36845978	2.15559357	1.49598709	0.23557015	0.57446657
Aldh9a1	0.18453322	5.52377629	1.49536178	0.23566461	0.57450331
Gab1	0.15910731	7.04320334	1.49516622	0.23569416	0.57450331
1700012D01	1.00605636	0.14562904	1.49503958	0.2357133	0.57450331
Tex30	-0.5149857	2.38532697	1.49418999	0.23584175	0.57470208
Begain	-0.2832592	3.44830392	1.49393534	0.23588027	0.57470208
Pdk3	0.17067045	5.03255319	1.49324881	0.23598416	0.57479802
Zdhhc21	-0.1821415	6.37299455	1.49311058	0.23600508	0.57479802
Pdgfa	-0.1742285	5.81482077	1.49202494	0.2361695	0.57504371
Shroom4	-0.3477101	3.13879811	1.49160574	0.23623302	0.57504371
Plekhg6	1.33252711	0.0553454	1.49109324	0.23631072	0.57504371
Qrich1	-0.1408292	6.48425595	1.49090032	0.23633997	0.57504371
Psapl1	-2.0534656	-1.7375642	1.49082826	0.2363509	0.57504371
Zfp334	0.17220298	4.89297415	1.49075269	0.23636236	0.57504371
Mgst3	-0.2756428	3.55588685	1.48871607	0.23667152	0.57551624
Mllt10	-0.1652955	6.2237758	1.4886725	0.23667814	0.57551624
Nhlrc4	-1.169122	-0.4151963	1.48862807	0.23668489	0.57551624
Spats2	0.25802302	3.28971686	1.48774036	0.23681982	0.57570005
Wnk3	-0.2216195	6.92545557	1.48756786	0.23684605	0.57570005
Dnm3os	-0.5976505	1.87406229	1.48713925	0.23691124	0.57575352
Tfap4	0.47293026	1.05252867	1.48677705	0.23696635	0.57575352
Mcts2	0.23804338	3.74713524	1.48608416	0.23707182	0.57575352
Cep70	0.30045616	4.03986928	1.48607664	0.23707297	0.57575352
Pcdha3	1.15173828	-0.4062687	1.48588083	0.23710278	0.57575352
Adarb1	-0.201711	6.5564066	1.48573644	0.23712477	0.57575352
Mphosph9	-0.244798	4.87189749	1.48497028	0.2372415	0.5758721
Lrrc16a	0.2272742	4.29144179	1.48485357	0.23725928	0.5758721
Nudt1	-0.8898712	0.61450683	1.4844125	0.23732652	0.5758721
Ddhd1	0.1916732	6.49345786	1.48393538	0.23739928	0.5758721

Ncbp1	-0.1889414	4.95939243	1.48377354	0.23742397	0.5758721
Cachd1	-0.2780727	3.39907885	1.48343729	0.23747527	0.5758721
Plekho2	0.28137608	4.99004047	1.48332849	0.23749187	0.5758721
Plekha5	-0.1984476	5.42071835	1.48317055	0.23751598	0.5758721
Gm13446	-0.3611206	2.27103668	1.4821497	0.23767185	0.57614622
Glt1d1	0.5124044	1.04808471	1.48159712	0.23775628	0.57624707
Sec24b	0.1698577	7.02039347	1.4809937	0.23784852	0.57625885
Polr3gl	0.28067767	2.88161822	1.48098528	0.23784981	0.57625885
Zdhhc20	-0.1952951	5.43041885	1.48072499	0.23788961	0.57625885
Prtg	-0.4423277	1.53552983	1.48025984	0.23796076	0.57631259
Mtm1	-0.4166243	2.68765722	1.47929707	0.23810812	0.57631259
Arhgef5	0.28409896	5.78450739	1.47908454	0.23814066	0.57631259
Rnf14	0.12623398	8.3873547	1.47907708	0.2381418	0.57631259
Fbxo16	-0.3715995	2.08289641	1.47886265	0.23817464	0.57631259
Fancd2	0.4643964	1.26540415	1.47867347	0.23820362	0.57631259
Sptan1	-0.3403792	9.21986903	1.47862144	0.23821159	0.57631259
Apol9b	-0.9974793	-0.6994516	1.47744175	0.23839241	0.5765533
Calcrl	-0.2476089	4.34254882	1.47723112	0.23842471	0.5765533
Mir1191	-1.6162277	-1.5833267	1.47709307	0.23844589	0.5765533
Dact1	-0.2399879	6.05623153	1.47665036	0.23851381	0.5765533
Srsf11	-0.1621448	6.73223638	1.47657535	0.23852532	0.5765533
Tet1	-0.1781458	6.20291372	1.47623018	0.2385783	0.576562
Slc17a5	0.22575015	3.89423497	1.47599361	0.23861462	0.576562
Pifo	-1.1581387	-0.2393641	1.47527087	0.23872561	0.57660561
Nsun4	-0.2564488	2.77881446	1.47506708	0.23875692	0.57660561
Gin1	0.29854105	3.43491305	1.47503918	0.23876121	0.57660561
Ppp1cb	0.13939466	9.23777984	1.47422489	0.23888637	0.57673322
Vti1b	-0.176957	5.76983741	1.47413774	0.23889977	0.57673322
P2rx4	-0.3225935	3.21501847	1.47369407	0.23896801	0.57674018
A230050P20	-0.345172	1.98851893	1.47356168	0.23898837	0.57674018
Elk4	-0.1446087	6.40798049	1.47284656	0.23909842	0.57690229
Rnf122	0.71900725	0.02224621	1.47247167	0.23915614	0.5769381
Ube2w	-0.1440447	6.24687381	1.47137107	0.23932569	0.57714636
Anp32e	0.16203109	8.05480532	1.47135446	0.23932825	0.57714636
Arhgef1	0.26532412	4.30203653	1.47076034	0.23941984	0.57716334
Ankrd13c	0.14438551	6.39659034	1.47044872	0.2394679	0.57716334
A630089N07	-0.4095461	8.10768397	1.47025144	0.23949833	0.57716334
Myrip	-0.2142823	6.38887838	1.47018084	0.23950922	0.57716334
Gm7120	0.41414688	2.94204316	1.46991819	0.23954975	0.57716334
Scarna3b	-1.389221	-1.0973364	1.46917243	0.23966486	0.5772195
Dact2	0.35238619	2.14960065	1.46914581	0.23966897	0.5772195
Polk	-0.1999629	5.18978043	1.46835698	0.23979081	0.5772195
Polb	-0.2490039	4.32665146	1.46803908	0.23983994	0.5772195

Rfx3	0.2406718	7.27365649	1.46797658	0.2398496	0.5772195
Adrb3	-0.9903404	-0.894653	1.46765905	0.23989868	0.5772195
C920021L13F	0.45295944	1.96554996	1.46765288	0.23989963	0.5772195
Snap47	0.13898353	7.28616584	1.46730555	0.23995334	0.5772195
Ntn3	0.60454566	1.52422669	1.46726816	0.23995912	0.5772195
Ccdc82	-0.2346918	7.54247244	1.46598124	0.24015824	0.57753901
Rcor3	-0.1771478	5.44193821	1.46585504	0.24017778	0.57753901
Gabarapl1	0.15344532	8.09279506	1.46499205	0.24031145	0.57775717
Fcgrt	0.40551348	3.55292364	1.46431874	0.2404158	0.57782257
Poglut1	-0.1665901	5.22591774	1.46416903	0.24043901	0.57782257
Nod1	-0.3961139	2.07213642	1.46398549	0.24046747	0.57782257
BC049352	1.36791442	-1.7776477	1.46324822	0.24058183	0.5779016
Rtf1	-0.1300605	8.31775041	1.46321301	0.2405873	0.5779016
Il1rap	-0.2441368	3.72863637	1.46286993	0.24064054	0.5779016
Kat5	-0.1818808	4.68628449	1.46239099	0.24071489	0.5779016
D630013N20	-1.9860173	-0.8906889	1.46196465	0.2407811	0.5779016
Top2a	-0.4047768	2.36370115	1.46182035	0.24080352	0.5779016
Zfp36l1	0.2072167	7.72852485	1.46131991	0.24088128	0.5779016
Fam26e	0.33182731	4.62726743	1.46112257	0.24091195	0.5779016
Engase	-0.5109295	0.75839057	1.46086674	0.24095172	0.5779016
Gm14379	1.09586394	-0.7781729	1.46043217	0.2410193	0.5779016
Dlec1	-1.3532533	-0.3980741	1.4603231	0.24103626	0.5779016
Snx16	0.20250733	4.76565689	1.460252	0.24104732	0.5779016
Ago3	0.18373577	6.0504347	1.45931865	0.24119256	0.5779016
Itgb4	0.4982738	3.19530324	1.45887064	0.24126232	0.5779016
Slc7a6os	0.28140858	3.9736553	1.45790986	0.241412	0.5779016
Ncam1	0.20499998	7.88637421	1.45773611	0.24143908	0.5779016
4930524B15I	1.81472501	-1.5930791	1.45722595	0.24151862	0.5779016
Uqcr10	0.26224786	5.90447573	1.45706255	0.2415441	0.5779016
1700007J10F	0.72975665	0.06999481	1.45692463	0.24156562	0.5779016
Ndufa5	0.22019575	5.61427803	1.4568817	0.24157231	0.5779016
Gm10635	-0.7290019	0.85486743	1.45658541	0.24161854	0.5779016
Aaed1	0.29662138	4.32597343	1.45658457	0.24161867	0.5779016
Zfp110	0.23433804	4.42279014	1.45648594	0.24163406	0.5779016
Ngb	0.5469126	0.84514126	1.45608554	0.24169655	0.5779016
Apitd1	-1.1408817	-0.4734113	1.45601768	0.24170714	0.5779016
Pfdn2	-0.2269336	5.53679415	1.45597956	0.24171309	0.5779016
Mccc1os	0.60848144	0.88030954	1.45569259	0.24175789	0.5779016
Madd	0.22259684	6.89785884	1.45562123	0.24176904	0.5779016
4930547E14I	1.41955905	-0.8269126	1.45554242	0.24178134	0.5779016
Efnb1	0.40372432	3.42047178	1.45535105	0.24181123	0.5779016
Zmym6	-0.2729622	4.80463903	1.45521999	0.2418317	0.5779016
Zfp91	-0.1318533	7.46042978	1.45468185	0.24191578	0.57795475

Plp1	-0.2536581	8.66461957	1.45447996	0.24194733	0.57795475
Smg8	-0.1843885	4.41716152	1.4542531	0.24198279	0.57795475
Hnrnph2	0.13472155	7.21234144	1.45307417	0.24216719	0.57801165
Sycp2	-0.5039557	2.48123862	1.45279013	0.24221164	0.57801165
1600002K03I	0.78000369	0.25287878	1.45224057	0.24229768	0.57801165
Dnlz	-0.2465669	3.76247593	1.45201632	0.2423328	0.57801165
Doc2a	0.33433121	2.81656322	1.45173275	0.24237721	0.57801165
Vti1a	-0.1565743	6.91696689	1.4517265	0.24237819	0.57801165
Ppapdc2	-0.1451918	6.28486106	1.45137105	0.24243389	0.57801165
Ccdc112	0.2900884	4.19145441	1.45132064	0.24244179	0.57801165
Lyst	-0.2563555	6.33483125	1.45110196	0.24247606	0.57801165
Skp1a	-0.1694234	8.04762672	1.45092999	0.24250302	0.57801165
Ehmt2	0.18624677	5.08226054	1.45081396	0.24252121	0.57801165
S1pr5	0.43328042	2.05751533	1.45080845	0.24252207	0.57801165
Syt16	-0.1733973	6.11761101	1.44972656	0.24269177	0.57831366
Commd6	0.24580835	5.30398125	1.4478062	0.24299335	0.57889279
Rnf112	-0.2854285	4.83507087	1.44763132	0.24302084	0.57889279
Cd209b	1.90952432	-1.5381916	1.44714088	0.24309795	0.57895478
Tmem55b	0.20754882	4.99186399	1.44655792	0.24318965	0.57895478
Nrn1	-0.1554916	7.30682903	1.44636391	0.24322018	0.57895478
Tubb5	0.15341978	7.90547831	1.44587855	0.24329657	0.57895478
Skap1	0.81755739	-0.0607999	1.44579423	0.24330985	0.57895478
Gm20172	-0.7822726	0.71115803	1.44574442	0.24331769	0.57895478
Phxr4	-0.70048	1.70294405	1.44555169	0.24334804	0.57895478
Fam154a	-2.4064251	-1.9889816	1.44459791	0.24349829	0.57910726
Scn2b	-0.1857747	6.88841078	1.44416819	0.24356602	0.57910726
Dpp6	0.23204637	6.35339394	1.44412541	0.24357277	0.57910726
Ankub1	-0.809367	0.64183141	1.44405244	0.24358427	0.57910726
Zfp456	-0.3250188	3.08223601	1.44345311	0.24367879	0.57922963
Olfm3	-0.216543	4.62868668	1.44316654	0.243724	0.57923478
Tmem119	0.37417438	3.49084428	1.44284826	0.24377423	0.57925184
E130112N10	-1.5740913	-0.6904843	1.44200889	0.24390675	0.57931982
Mgea5	0.18076419	8.6334026	1.4419305	0.24391913	0.57931982
Trmt6	-0.1964735	4.64719553	1.44161571	0.24396885	0.57931982
Zdhhc12	-0.6990725	0.66138768	1.44157653	0.24397504	0.57931982
Fcgr3	-0.5638181	1.06475756	1.44047045	0.24414988	0.57963269
Hist1h4k	0.40810339	1.94575363	1.44005859	0.24421502	0.57968507
Fam193b	-0.3569509	3.14884989	1.43940127	0.24431904	0.579728
Mcm6	0.22302095	3.64528183	1.43939977	0.24431927	0.579728
Hps3	-0.2747301	4.08700997	1.43892325	0.24439471	0.57979008
Nr5a2	1.64056283	-2.0178375	1.43869025	0.24443161	0.57979008
Hspa14	0.19276939	4.2484401	1.43822346	0.24450555	0.57986326
Pold2	0.46604783	2.57042042	1.43651448	0.24477651	0.57989804

Mrps31	0.25317106	4.56028874	1.43618348	0.24482904	0.57989804
Pwwp2a	-0.225759	5.79995736	1.43480926	0.24504727	0.57989804
Clk3	-0.1891914	4.70824977	1.43472808	0.24506017	0.57989804
Sipa1l1	-0.2402007	8.88422936	1.43454462	0.24508932	0.57989804
Ifit3	0.29469681	5.94234365	1.43418743	0.2451461	0.57989804
Rnf139	0.17384137	5.09145467	1.43411682	0.24515732	0.57989804
Uba2	0.15131132	5.79945367	1.43401434	0.24517361	0.57989804
Eva1c	0.43880974	2.79785056	1.43388753	0.24519378	0.57989804
Krt7	-1.9213291	-1.709268	1.43387634	0.24519556	0.57989804
Tub	-0.2520549	6.11922784	1.43365376	0.24523095	0.57989804
2310022A10	-0.2563881	3.59599026	1.43358133	0.24524247	0.57989804
Gm13375	-0.4006184	2.74227121	1.43344182	0.24526467	0.57989804
H2-K1	0.28347232	5.06587241	1.43341575	0.24526881	0.57989804
Celf1	0.16504448	8.13798273	1.43341524	0.24526889	0.57989804
Fbxl15	-0.8017938	-0.4412051	1.43315444	0.24531039	0.57989804
Car11	-0.1833963	5.14702599	1.43298877	0.24533675	0.57989804
E030030I06R	-0.3864732	2.81872364	1.43297418	0.24533907	0.57989804
A130077B15	-0.3473508	7.65489008	1.43187638	0.24551385	0.57989804
Iqgap1	0.14857326	6.34663726	1.43183159	0.24552098	0.57989804
Mir344b	-1.7398751	-2.0874716	1.43127807	0.24560918	0.57989804
Fbn2	-0.500899	1.50799282	1.4312014	0.24562139	0.57989804
Scn7a	-0.2113429	4.22866684	1.43112297	0.24563389	0.57989804
Lmbrd2	-0.1812282	6.12720119	1.43056128	0.24572344	0.57989804
Fam71e1	-0.6204711	0.25455267	1.43038595	0.2457514	0.57989804
Zdhhc14	0.23086713	4.30619587	1.43028641	0.24576727	0.57989804
Mmp16	-0.2168127	5.36699332	1.43007241	0.24580141	0.57989804
Slc20a1	-0.2223535	5.34830117	1.43003469	0.24580743	0.57989804
Scube2	0.64916047	1.10588406	1.42986717	0.24583415	0.57989804
Pin1	-0.2782198	3.03710293	1.4296187	0.2458738	0.57989804
Dcdc2b	0.27361585	3.77536492	1.42896696	0.24597784	0.57989804
Rab39b	-0.1859722	6.74306171	1.42831956	0.24608124	0.57989804
Gpd1l	0.13333552	7.02544809	1.42823141	0.24609532	0.57989804
Cdc14b	-0.2421839	4.35642075	1.42803549	0.24612663	0.57989804
Smim12	0.36336053	2.10273062	1.42790557	0.24614739	0.57989804
Pebp4	1.16959129	-0.8767812	1.42787406	0.24615242	0.57989804
AK010878	-0.2395868	3.75420995	1.42769608	0.24618087	0.57989804
Bhmt	1.68690069	-1.2367822	1.42750421	0.24621154	0.57989804
Prdx4	0.24239238	3.41192748	1.42728491	0.2462466	0.57989804
Olfml1	-0.2603873	4.58663499	1.42710937	0.24627467	0.57989804
Ipo9	-0.1683158	7.01402493	1.42618312	0.24642286	0.57989804
Crispld2	0.52012494	2.2841983	1.42554346	0.24652526	0.57989804
Nrg3	0.15949646	6.24416023	1.4251517	0.24658801	0.57989804
Sybu	-0.2096487	5.28269704	1.42507496	0.2466003	0.57989804

Wif1	1.04055315	-1.1356309	1.42499894	0.24661248	0.57989804
Ripply3	-0.5486765	1.46341984	1.42469294	0.24666151	0.57989804
Klrg1	1.77548158	-2.0796857	1.42454026	0.24668598	0.57989804
P2ry13	0.4291958	2.42328425	1.42447253	0.24669683	0.57989804
Ganab	0.222035	5.05845587	1.42438543	0.24671079	0.57989804
Agbl1	-1.0132684	-0.8698047	1.42430219	0.24672413	0.57989804
Kctd12b	0.23328136	4.98080591	1.42359436	0.24683763	0.57989804
1110004E09I	0.23956065	5.77998357	1.42326157	0.24689102	0.57989804
Snhg12	0.27165731	3.49717348	1.42299164	0.24693433	0.57989804
Lepre1	-0.3314099	2.22410944	1.42257964	0.24700046	0.57989804
Rab36	0.19344652	3.97702559	1.42256327	0.24700309	0.57989804
Gpt	0.76316414	0.46254604	1.42245645	0.24702024	0.57989804
Dpy19l3	-0.2087766	5.02698243	1.42211736	0.24707468	0.57989804
Usp12	-0.1445217	5.81733712	1.42199636	0.24709412	0.57989804
Frat1	0.46056126	1.57041128	1.42193952	0.24710325	0.57989804
Usp6nl	-0.165234	5.91250472	1.42159618	0.2471584	0.57989804
Cdc37	0.20111267	5.08594465	1.42100306	0.24725372	0.57989804
Gng5	0.2351851	6.39957695	1.42099585	0.24725488	0.57989804
Tusc5	-1.1725165	0.35486227	1.42094039	0.24726379	0.57989804
Cacng2	-0.1687628	5.27518469	1.42083871	0.24728014	0.57989804
Fhl2	0.16903328	5.79220638	1.42058219	0.24732138	0.57989804
1700052K11I	-0.4048622	2.85164241	1.4197367	0.24745739	0.57998179
Zfyve26	0.24050388	4.09534786	1.41891345	0.24758991	0.57998179
Gabbr1	-0.1916312	7.35813942	1.41884412	0.24760107	0.57998179
C1rl	1.07247195	0.26255936	1.41881954	0.24760503	0.57998179
Dennd3	-0.4671632	1.70226287	1.41862198	0.24763685	0.57998179
Fbl	0.24721442	4.29211646	1.41857015	0.2476452	0.57998179
Gfm1	-0.2509511	4.55150656	1.41834202	0.24768195	0.57998179
H2-Eb1	-0.5210104	3.08413101	1.41819243	0.24770605	0.57998179
Slc5a5	-0.2287146	4.92902346	1.41795063	0.24774501	0.57998179
Zfp53	-0.2866453	2.75478349	1.41698783	0.24790024	0.58005546
Fzd8	0.53351678	1.75858584	1.41692992	0.24790958	0.58005546
Fezf2	-0.1819128	5.18090564	1.41661187	0.24796089	0.58005546
Mier3	-0.1784551	5.08618564	1.41647551	0.24798289	0.58005546
Spin4	0.39452364	2.74782339	1.41631114	0.24800942	0.58005546
Iqck	0.47279364	2.81758821	1.41600268	0.2480592	0.58005546
Osbpl8	0.13121135	7.70354913	1.41588484	0.24807823	0.58005546
Arhgef10l	-0.2838272	3.02220245	1.41482011	0.24825019	0.58032376
Pomt1	-0.5328692	1.47726047	1.41438828	0.24831998	0.58032376
Nolc1	0.17036246	5.18570524	1.41422649	0.24834614	0.58032376
Pycr2	-0.3447039	2.68835934	1.41400209	0.24838242	0.58032376
Eif4h	-0.1344308	7.04645059	1.41362141	0.24844398	0.58032376
4930452B06I	0.2928046	3.51300428	1.41357349	0.24845173	0.58032376

Gnb2l1	-0.1817888	5.840013	1.41219842	0.24867429	0.58041119
Rbm14	0.32943675	3.06542224	1.41090014	0.24888465	0.58041119
Hs3st6	0.99764436	-0.5819177	1.41045691	0.24895652	0.58041119
Cdon	-0.2745162	5.1561861	1.41040951	0.24896421	0.58041119
Slc7a6	0.43486496	2.89527166	1.41007325	0.24901875	0.58041119
Hapln1	0.29506958	3.8075648	1.40988394	0.24904947	0.58041119
Gm19434	-0.5074856	0.9469362	1.40958661	0.24909772	0.58041119
2310015B20I	0.5046867	1.96852753	1.40936638	0.24913347	0.58041119
Daam1	-0.1778598	6.59201576	1.40930463	0.24914349	0.58041119
Plscr2	-0.3107681	4.4211804	1.40910033	0.24917666	0.58041119
Rdh14	0.21562013	5.61458882	1.40906691	0.24918209	0.58041119
Erdr1	-0.3259413	3.40101231	1.40899287	0.24919411	0.58041119
Zfp507	-0.1917106	4.73466432	1.40894046	0.24920262	0.58041119
Parn	0.18743458	4.67553841	1.40832668	0.24930232	0.58041119
Des	0.64685171	0.62764967	1.40829471	0.24930751	0.58041119
Gm10389	-0.2898717	4.2490491	1.40805557	0.24934637	0.58041119
Itpka	-0.2206655	4.18836864	1.40780751	0.24938669	0.58041119
Prdx1	0.20843705	7.18344903	1.40712804	0.24949716	0.58041119
4932443I19R	1.52039399	-1.4886467	1.40691115	0.24953244	0.58041119
Galr1	1.51345537	-1.8175192	1.4068601	0.24954075	0.58041119
Ces2g	-0.3625148	3.49425668	1.40683211	0.2495453	0.58041119
Cdk6	-0.532825	2.18186163	1.40666144	0.24957307	0.58041119
Spata45	-1.7333894	-0.5529667	1.40666115	0.24957311	0.58041119
Saal1	-0.3554528	2.24950395	1.40653249	0.24959405	0.58041119
Dyrk2	0.33577715	3.24863198	1.40638477	0.24961809	0.58041119
H2afz	-0.1597276	7.20723172	1.40600753	0.24967949	0.58041119
Zc3h7a	0.24723296	5.52498518	1.40571675	0.24972683	0.58041119
Usp18	0.42757857	1.69808751	1.405656	0.24973672	0.58041119
LOC1010557(	-1.9838954	-1.8437998	1.41990643	0.24987536	0.58041119
Nrap	1.35664713	-1.1094914	1.40479748	0.24987658	0.58041119
Nktr	-0.2072544	7.07356696	1.40470673	0.24989137	0.58041119
Zcchc12	-0.2269914	4.29286974	1.40457691	0.24991252	0.58041119
Eci2	0.22001435	5.4609683	1.40457673	0.24991255	0.58041119
Cnih4	0.17095765	5.19353871	1.404212	0.24997201	0.5804491
Tcam1	2.21485962	-1.9937716	1.41851785	0.25009801	0.58060064
L3mbtl3	0.22167738	4.1829686	1.40328278	0.25012357	0.58060064
Mr1	0.27586299	4.41173809	1.40211571	0.25031409	0.58094268
Tmem189	-0.328773	2.5608425	1.40130811	0.25044604	0.58112372
Ccdc47	0.13967847	7.07371652	1.40110974	0.25047847	0.58112372
Prdm2	-0.1616175	7.02812682	1.40010233	0.25064322	0.58140571
Bambi	-0.5256567	1.23573943	1.39951263	0.25073972	0.58152932
Gm14295	-0.1297147	5.87168386	1.39915984	0.25079748	0.58156304
4930581F22I	-0.7103297	0.41831874	1.39820757	0.25095347	0.5818093

Ctsf	-0.379748	3.07466741	1.39798371	0.25099016	0.5818093
Calb2	-0.4436384	1.07864787	1.39767895	0.25104012	0.58182488
Map3k13	-0.2719989	5.16798129	1.39713875	0.2511287	0.58192996
Bmf	0.36213965	2.81806401	1.396509	0.25123202	0.58200457
Riiad1	0.68156392	0.2172966	1.39641528	0.2512474	0.58200457
Adcy4	-0.7576224	0.47949154	1.39599358	0.25131662	0.58206472
Gpr3	0.52260453	1.08837726	1.39476751	0.25151802	0.58215118
Inca1	-0.4704319	0.79195085	1.39463188	0.25154031	0.58215118
Mkrn3	-1.2155881	-0.2692378	1.39448302	0.25156479	0.58215118
Mettl3	0.24813775	3.85454975	1.39427876	0.25159837	0.58215118
Cnih2	-0.22479	3.54137883	1.39424855	0.25160333	0.58215118
Hspb6	0.21777415	4.31120956	1.39398636	0.25164645	0.58215118
Dgkb	0.22138756	7.92645672	1.39357954	0.25171337	0.58215118
Rnf11	-0.143403	6.86253353	1.39356522	0.25171572	0.58215118
Prpf31	0.19863111	4.10967263	1.39329472	0.25176023	0.58215118
Syngap1	-0.1912398	7.35111782	1.39313464	0.25178657	0.58215118
2810408M09	-0.2516965	3.2236678	1.39267055	0.25186297	0.58222777
Drc1	-0.2728751	3.18557095	1.39238858	0.2519094	0.58223507
Fah	-0.258003	3.18799279	1.39186656	0.25199539	0.5823338
BC029722	0.4465037	2.07928093	1.39106502	0.25212749	0.58252311
2310009A05	-0.3718537	2.69505631	1.39082434	0.25216717	0.58252311
Ptrf	0.29611871	6.57759735	1.39053282	0.25221525	0.58252311
Cenpw	-0.4650742	1.57348839	1.38978129	0.25233925	0.58252311
Chst9	1.02605724	-0.8220098	1.38958011	0.25237246	0.58252311
Arf6	0.17904138	6.84791232	1.38945815	0.25239259	0.58252311
Unkl	0.20988911	5.40387752	1.38939393	0.2524032	0.58252311
Mtch2	0.14243315	5.59819807	1.38927019	0.25242363	0.58252311
Rnf113a1	0.32997477	1.95448335	1.38678432	0.25283453	0.58327179
Nuf2	-1.144308	-0.0548779	1.38678304	0.25283474	0.58327179
Slco3a1	0.2185454	4.96501028	1.38605984	0.25295445	0.5833235
Grik3	-0.2600024	5.57650509	1.38574346	0.25300684	0.5833235
Tmem17	-0.4237106	1.7627744	1.3856628	0.2530202	0.5833235
Zbtb37	-0.3535287	2.91738233	1.38519532	0.25309764	0.5833235
Sec24c	0.15808464	6.32172526	1.38509614	0.25311408	0.5833235
Rimklb	-0.2346813	3.68555585	1.38489709	0.25314707	0.5833235
Tsta3	-0.434532	2.29746459	1.3848154	0.2531606	0.5833235
Zfp874b	0.20929371	4.64430705	1.38377988	0.25333232	0.58356609
Timm22	-0.2416695	3.87699275	1.38333729	0.25340575	0.58356609
Tspan33	-0.4178709	1.50651382	1.38317674	0.2534324	0.58356609
3110002H16	0.33601309	3.3694138	1.38313481	0.25343936	0.58356609
Fbxw5	-0.2135439	5.41476069	1.38183304	0.25365555	0.58384232
Mapkbp1	0.20239015	5.2659979	1.38122583	0.25375648	0.58384232
Cds2	-0.1829573	7.73388419	1.38114206	0.25377041	0.58384232

Efr3a	-0.155239	7.52803135	1.38108773	0.25377944	0.58384232
Trim66	-0.2675175	5.4375356	1.38073108	0.25383875	0.58384232
Bcan	-0.327193	4.67204944	1.38072357	0.25384	0.58384232
Papss2	0.21361216	5.79153678	1.38058508	0.25386304	0.58384232
A630066F11	0.47422417	1.4922324	1.38008421	0.25394638	0.58393418
Atp2b4	0.22085554	7.45255742	1.37942669	0.25405583	0.58401222
Dpp8	-0.1136857	8.3975238	1.3793589	0.25406712	0.58401222
LOC1005047	0.92702686	-0.317366	1.37887928	0.254147	0.58409607
Cdk20	-0.6110336	1.18876467	1.37849262	0.25421143	0.58410188
Tram2	0.30725216	3.62401292	1.3783431	0.25423634	0.58410188
Csmd2	-0.3917484	4.84623264	1.37796785	0.2542989	0.58413261
Arsk	-0.3247521	3.59236562	1.37763738	0.254354	0.58413261
Six3os1	-0.5468927	0.77632868	1.37733018	0.25440524	0.58413261
Kcnb1	-0.2280733	8.01709243	1.37722156	0.25442336	0.58413261
Pde8a	-0.1801808	4.07415265	1.37676668	0.25449926	0.58420719
App	0.14820206	8.66509883	1.37642864	0.25455568	0.58423705
Romo1	0.34738987	4.01738062	1.37594016	0.25463724	0.58424813
Gpr137b-ps	-0.2326033	3.63692507	1.37587969	0.25464734	0.58424813
Il16	0.49515396	1.13578219	1.37492464	0.25480692	0.58451458
Tmem221	0.96688849	-0.4644933	1.3745544	0.25486881	0.58452423
Mpp2	-0.1524586	6.71920698	1.37397519	0.25496569	0.58452423
Nudt14	0.53991271	1.06890462	1.37395551	0.25496898	0.58452423
Smad9	0.18200084	5.40048518	1.37386049	0.25498488	0.58452423
Akap6	-0.2854089	8.14468972	1.37303867	0.25512242	0.58473991
Whsc1l1	0.12965485	7.71998622	1.37232511	0.25524192	0.58491419
5830417I10R	-0.2079086	6.11099523	1.37201966	0.2552931	0.58493185
Coq9	0.21727803	4.37311304	1.37114064	0.25544045	0.58510661
Gpr56	-0.3194952	2.97073981	1.37104592	0.25545634	0.58510661
Spaca6	-0.6306682	2.96142993	1.37061245	0.25552905	0.58517354
Arhgap27	-0.3054177	3.24383541	1.37009193	0.25561639	0.58527397
Sbk3	-0.7171728	0.45675442	1.36969584	0.25568289	0.58532662
Slc25a53	-0.3891002	2.57101255	1.36929619	0.25575	0.58533155
Vmn2r87	-0.6576276	1.17231692	1.36916498	0.25577204	0.58533155
Abt1	0.26594567	3.38623331	1.36845317	0.25589165	0.5855057
Sepp1	0.24163966	8.0461809	1.36811131	0.25594911	0.58553763
Trpc4	0.26603671	3.39938902	1.36692834	0.25614811	0.58589326
Fam73a	-0.1721497	6.03463599	1.36651115	0.25621834	0.58595429
Myh7b	0.49075425	2.32790488	1.36601643	0.25630165	0.58599393
Wdr4	-0.3905913	2.39994244	1.36589106	0.25632276	0.58599393
Dse	-0.3651289	3.90497036	1.36477493	0.25651087	0.58632435
Papss1	0.1688464	4.96505417	1.36401949	0.25663829	0.58643153
Gabra1	-0.1691603	8.20508337	1.36398018	0.25664492	0.58643153
Gpam	0.27213829	3.29092738	1.36322243	0.25677281	0.58651083

Kmt2c	-0.1870239	8.17683856	1.36312739	0.25678886	0.58651083
Dnajb1	0.14277741	6.61552097	1.36299994	0.25681038	0.58651083
Dcaf5	-0.1512884	7.07539042	1.36271519	0.25685847	0.58652111
Rnf208	-0.168532	4.92523072	1.3610913	0.25713296	0.58689793
D130043K22	0.30944564	3.37003417	1.36052152	0.25722936	0.58689793
Pik3r2	0.20848812	4.18510452	1.36047488	0.25723725	0.58689793
Tmem68	0.16419134	5.12180934	1.36042754	0.25724526	0.58689793
Fam160b1	0.19533079	5.00786603	1.36024609	0.25727597	0.58689793
Daf2	0.5123596	0.64992593	1.36003453	0.25731179	0.58689793
Mylk4	-0.5195929	1.16968029	1.35993405	0.2573288	0.58689793
Cdc42bpa	-0.1627121	9.05411707	1.35923041	0.25744797	0.58702737
Pacrg	0.34626828	2.80278156	1.35901992	0.25748364	0.58702737
Siah3	-0.5581278	0.8095347	1.358676	0.25754192	0.58702737
Eci3	-0.6016756	0.66836361	1.35856904	0.25756005	0.58702737
A330076C08	-1.0026923	0.12744797	1.35716306	0.25779853	0.58739242
Pcdhb9	0.38366813	2.25524528	1.35710647	0.25780813	0.58739242
Gpd1	0.2105081	4.49827584	1.3568529	0.25785118	0.58739242
Zfp955a	-0.1746908	5.05285549	1.35598569	0.25799845	0.58753183
Trex1	-0.4335344	1.23374519	1.35597835	0.2579997	0.58753183
Arhgdib	0.30588488	7.44678784	1.35531041	0.25811321	0.58769087
Gm10069	-0.5185432	0.87244405	1.35476195	0.25820646	0.58780374
Egfl7	-0.3214242	2.41048046	1.35448371	0.25825379	0.58781203
Phf14	-0.1359718	6.44424764	1.35417602	0.25830614	0.58783175
Phtf1	-0.1483043	5.56154343	1.35341397	0.25843585	0.58792777
Zbbx	-0.9717488	-0.6191186	1.35326016	0.25846204	0.58792777
Tollip	0.14913636	6.59682381	1.35315817	0.2584794	0.58792777
Gja4	-0.846978	-0.5079181	1.35275641	0.25854784	0.58798404
Anxa4	0.24506537	6.49079403	1.35146966	0.25876718	0.58827118
Cars2	0.37475646	2.19304104	1.35120092	0.25881301	0.58827118
01-Sep	0.40363687	2.38936797	1.35118257	0.25881615	0.58827118
Wsb2	-0.1235866	7.7269333	1.3505904	0.25891719	0.58827118
Sipa1l2	-0.2776504	4.53337432	1.35058718	0.25891774	0.58827118
Mapt	-0.1432993	6.89262027	1.35047784	0.2589364	0.58827118
Chmp2a	0.24564233	5.63235119	1.34996316	0.25902428	0.58837149
Ptprj	-0.1958685	6.83767348	1.3487268	0.25923553	0.58875196
Tmem97	-0.400854	2.13363819	1.34798875	0.25936174	0.58886975
Wrn	-0.1497601	5.66555382	1.34765426	0.25941897	0.58886975
Galnt16	-0.2401295	4.41989815	1.34741887	0.25945926	0.58886975
Prkcq	-0.3834832	2.94313215	1.34720338	0.25949614	0.58886975
Mir128-1	-0.6522664	1.50571919	1.34661467	0.25959694	0.58886975
Haus8	-0.5145075	1.04829652	1.34656285	0.25960582	0.58886975
Cacna1d	-0.3026391	5.10948348	1.34642289	0.25962979	0.58886975
Uap1l1	0.47202355	4.09503862	1.34637795	0.25963749	0.58886975

Ms4a6d	-1.4017028	-1.2205621	1.34560837	0.25976936	0.5889525
Nfib	0.13418564	8.11049038	1.34543984	0.25979826	0.5889525
Tspo	0.41333476	2.02514548	1.34512984	0.25985141	0.5889525
Fam35a	-0.3818715	2.54139424	1.34501387	0.2598713	0.5889525
Olfr920	-1.4280826	-0.1309598	1.34488843	0.25989281	0.5889525
L1cam	0.27639023	6.82476182	1.34462701	0.25993766	0.58895494
Fabp4	-1.3861457	-0.6828978	1.34199444	0.26038982	0.58965939
Hmha1	-0.5008878	1.25601341	1.34196371	0.2603951	0.58965939
Rhoj	0.25238921	5.38522687	1.34178901	0.26042515	0.58965939
Matk	-0.2615566	3.61966561	1.3417094	0.26043884	0.58965939
Btla	-1.1907968	-0.3808945	1.34149523	0.26047568	0.58965939
Zfp300	-0.3236537	2.71413456	1.34121086	0.2605246	0.58965939
Cryba2	1.19134685	-1.2182359	1.34094139	0.26057098	0.58965939
Slc19a3	-0.808001	0.32608447	1.34075937	0.26060231	0.58965939
Chtf18	-1.270261	-1.2665603	1.34037566	0.26066837	0.58965939
Slc39a13	0.26837259	5.17208541	1.3402688	0.26068677	0.58965939
Mterfd1	0.19846924	5.07451548	1.33953199	0.2608137	0.5898374
Rgs22	0.73315382	0.3420577	1.33930314	0.26085314	0.5898374
Psmg3	-0.4364916	2.00134747	1.3388983	0.26092292	0.58989608
Gpatch3	0.85873899	-0.187732	1.33791117	0.26109319	0.59018187
Mfge8	0.34927366	3.1495473	1.33712297	0.26122925	0.59024586
Trp53bp1	-0.2435203	5.47033742	1.33680588	0.26128401	0.59024586
Slamf1	-1.3589598	-1.076657	1.33645982	0.2613438	0.59024586
Lsmem1	-1.302906	-1.4809773	1.33620958	0.26138704	0.59024586
Slc25a21	-0.7577309	0.19478389	1.33579105	0.26145938	0.59024586
C330021F23I	-0.2999369	2.50168453	1.33525505	0.26155206	0.59024586
Cdkn2aip	0.21121358	4.20203269	1.33513225	0.2615733	0.59024586
1110012L19F	0.36848593	3.3695072	1.33485393	0.26162145	0.59024586
Acox1	0.14094268	6.99373077	1.33459697	0.26166592	0.59024586
Lox	0.33548475	3.27472047	1.33456867	0.26167081	0.59024586
Adc	-0.4259172	1.78714451	1.33456505	0.26167144	0.59024586
Glb1l	0.30236059	3.33240544	1.33456304	0.26167179	0.59024586
Ptprb	-0.3828866	5.31799751	1.33388768	0.2617887	0.59024586
Aven	0.42726949	1.31727111	1.3338352	0.26179779	0.59024586
Setdb2	0.32977195	2.9904605	1.33364421	0.26183087	0.59024586
Gm2061	-0.4983514	1.29447359	1.33358447	0.26184122	0.59024586
Tmeff1	-0.2080261	5.11783559	1.33326978	0.26189573	0.59024586
Rem2	0.69066857	0.43792714	1.33290553	0.26195885	0.59024586
Phf10	0.16114556	5.32366642	1.33275283	0.26198532	0.59024586
Ptplad1	0.13354735	6.68911998	1.33267515	0.26199878	0.59024586
Glis3	-0.2874649	3.09215531	1.33161894	0.26218195	0.59055966
Pcdhga12	-0.2759981	2.95173077	1.3312425	0.26224728	0.59060794
Ano6	0.22672058	6.43153776	1.33098536	0.26229191	0.59060961

Bfsp1	-0.7347439	0.95059007	1.33047526	0.26238049	0.59068232
Cyb5r3	0.2954058	7.41204742	1.33029386	0.262412	0.59068232
Ugt8a	-0.2093931	5.3525216	1.32893923	0.26264744	0.59110054
Misp	1.33646434	-1.123436	1.32871956	0.26268565	0.59110054
Hectd2	-0.2209619	4.47598569	1.32781277	0.26284344	0.59129357
Rexo2	0.15371744	6.99962684	1.32772158	0.26285931	0.59129357
Srgap2	0.14385956	6.07483362	1.32653578	0.26306586	0.59165928
Yrdc	-0.2441487	3.48619489	1.32616004	0.26313135	0.59170768
Sec14l3	-1.1198842	-0.5228344	1.32590269	0.26317622	0.5917097
Plag1	-0.3390468	2.70955177	1.32530249	0.2632809	0.59171923
Sfswap	-0.2681361	4.61788931	1.32508246	0.26331929	0.59171923
Farp2	-0.2524424	2.80240206	1.32485361	0.26335923	0.59171923
Csnk2b	0.17780419	5.72714885	1.32473	0.2633808	0.59171923
Ccdc109b	0.43751516	1.96654742	1.32446314	0.26342739	0.59171923
Htr2a	0.25132864	4.42755385	1.32427707	0.26345988	0.59171923
Tox4	0.14780393	5.999696	1.32403755	0.26350171	0.59171923
Tex2	-0.1296757	6.54955136	1.32367015	0.26356588	0.59171923
Entpd4	-0.1707544	6.04676775	1.32350889	0.26359406	0.59171923
Gm19461	-1.1169889	-0.8248495	1.32328686	0.26363286	0.59171923
Ino80dos	-0.3680463	2.98122524	1.32298704	0.26368526	0.59171923
Sstr1	0.45461089	3.05387091	1.32271623	0.26373261	0.59171923
Adrb2	-0.4942989	2.24218647	1.32242822	0.26378298	0.59171923
Sf3b6	0.19902905	5.00952642	1.32232394	0.26380121	0.59171923
4930583P06I	1.34803913	-1.6649089	1.32210187	0.26384006	0.59171923
Gdpgp1	-0.2209056	4.8161016	1.32097143	0.26403792	0.59177355
Pcdhb22	-0.3212871	3.03876864	1.3207291	0.26408036	0.59177355
Kremen2	-0.7642798	-0.7455648	1.32031993	0.26415205	0.59177355
Lrrd1	-0.9517953	-0.3771125	1.32028732	0.26415776	0.59177355
Fbxl6	-0.5424374	0.94416263	1.32024837	0.26416459	0.59177355
Purg	-0.1866694	6.01491215	1.32017481	0.26417748	0.59177355
Gstp1	0.19146133	4.93816257	1.32009163	0.26419205	0.59177355
Agtr1a	1.62070561	-1.2617721	1.31984491	0.2642353	0.59177355
Vps33b	0.19683593	5.18058997	1.31948642	0.26429814	0.59177355
Ech1	0.3124448	3.948323	1.3193547	0.26432124	0.59177355
Xk	-0.2315822	5.23281007	1.31820583	0.26452282	0.59177355
Timm10	0.31402265	3.84783903	1.31818634	0.26452624	0.59177355
Rab10	0.14069944	8.51286959	1.31760793	0.2646278	0.59177355
Pcp4l1	0.21296689	6.03176788	1.31733657	0.26467546	0.59177355
Col13a1	-0.443725	2.28792226	1.31728596	0.26468436	0.59177355
Ip6k2	0.19124657	4.0375015	1.31720324	0.26469889	0.59177355
Pianp	-0.1657985	6.45296759	1.31634611	0.26484954	0.59177355
Hspa9	0.13334856	7.17277212	1.31595422	0.26491845	0.59177355
6030419C18I	0.39577821	2.38184951	1.31592798	0.26492307	0.59177355

Tenm4	-0.2474785	5.97639143	1.31568801	0.26496528	0.59177355
Laptm4b	0.1601246	5.34753559	1.3153573	0.26502347	0.59177355
Ttc19	-0.1669431	6.26122451	1.31514062	0.26506161	0.59177355
Cops3	0.15239742	5.48155789	1.31512885	0.26506368	0.59177355
Cwh43	1.30268106	-1.0775898	1.31496152	0.26509313	0.59177355
Prkag3	-0.537729	1.82360284	1.31479401	0.26512262	0.59177355
Triap1	-0.4036195	3.20084066	1.31472884	0.2651341	0.59177355
Mzt2	0.29490514	2.97104028	1.3145406	0.26516724	0.59177355
Kcnk13	0.563675	0.83313891	1.31453724	0.26516784	0.59177355
Zfp607	-0.3957358	2.48139595	1.31410432	0.26524409	0.59177355
Hipk1	-0.1302467	8.53399763	1.31395298	0.26527075	0.59177355
Ramp2	0.29207619	4.5868832	1.31388762	0.26528227	0.59177355
Mcfd2	0.24229773	4.96654256	1.31360693	0.26533173	0.59177355
Emc4	0.13834441	6.33194098	1.31290008	0.26545635	0.59177355
Mcrs1	-0.2670837	3.23936336	1.31277651	0.26547815	0.59177355
Kcnf1	0.22354674	5.28870068	1.31267417	0.2654962	0.59177355
AU022754	-0.8867321	-0.2459675	1.31255547	0.26551713	0.59177355
Tmem130	0.22829679	5.16068055	1.31229175	0.26556366	0.59177355
Trim30a	0.26448763	3.79956859	1.31210324	0.26559693	0.59177355
Kdm3b	-0.1220832	7.03143235	1.31204767	0.26560674	0.59177355
Pan2	-0.2837324	3.5095207	1.3119534	0.26562337	0.59177355
Ppp6r2	-0.2150931	4.59010303	1.31167502	0.26567251	0.59178505
Stoml2	0.22205898	4.28592508	1.31090106	0.2658092	0.59186196
Steap4	-1.4564211	0.17579235	1.31076197	0.26583377	0.59186196
Dot1l	0.3050373	3.89011164	1.3107324	0.265839	0.59186196
Mcam	-0.4832124	1.72639642	1.31028907	0.26591735	0.59193678
Ddo	0.30792702	3.49965367	1.3100445	0.26596058	0.59193678
Uba6	0.19526054	5.70518127	1.30942841	0.26606953	0.59200332
Ebf1	-0.2719603	4.28809844	1.30930138	0.266092	0.59200332
Mob3b	0.19731179	6.71471211	1.30912922	0.26612246	0.59200332
2310068J16F	1.05559699	-0.8088933	1.307948	0.26633157	0.59229598
Rabep2	0.37877555	2.27559331	1.30778685	0.26636012	0.59229598
Fahd1	0.17228658	5.2997062	1.30764035	0.26638607	0.59229598
Gpr25	-0.2672962	3.66236568	1.30735253	0.26643707	0.59231151
Wibg	0.47021891	2.09290631	1.30704881	0.2664909	0.59233332
Trappc6b	-0.1913172	7.79666568	1.30611086	0.26665723	0.59252836
Spire1	-0.1860979	7.44840204	1.30605738	0.26666672	0.59252836
Aftph	-0.1441663	6.94774223	1.30498297	0.26685742	0.59276066
Asb15	0.77932163	0.58360885	1.30481282	0.26688764	0.59276066
Bcar1	0.18503624	4.23651133	1.3045977	0.26692585	0.59276066
Pde4dip	0.20460057	7.48586352	1.30407713	0.26701835	0.59276066
Shroom1	0.41661102	1.58595977	1.30399349	0.26703321	0.59276066
Ap3b1	0.14877076	6.59633929	1.30386316	0.26705638	0.59276066

4931403E22I	-1.9568814	-1.295699	1.30373243	0.26707962	0.59276066
Calml4	-0.4583855	1.39227829	1.30327379	0.26716117	0.59284388
Mapk3	0.20175018	7.23936848	1.30217913	0.26735595	0.59317828
Aimp1	0.236775	4.50395021	1.30095499	0.26757398	0.59338702
Kpna1	0.14248193	6.91615196	1.30094572	0.26757563	0.59338702
Herc6	-0.1865603	5.29591099	1.30089393	0.26758486	0.59338702
D10Wsu102e	0.15788145	5.43301154	1.30066078	0.26762642	0.59338702
Ntan1	0.19954977	5.48359563	1.30034421	0.26768286	0.59339164
Me3	0.28222242	4.21230339	1.30013849	0.26771954	0.59339164
Swi5	-0.2119257	5.71478352	1.2999072	0.26776079	0.59339164
Efna2	0.66660507	0.54814271	1.2990332	0.26791675	0.59363949
Chat	0.50953558	1.53177093	1.29865103	0.26798499	0.59369292
Inip	0.19205727	5.15802191	1.29787652	0.26812334	0.59390165
2310039L15I	-0.4099434	2.85952207	1.2972613	0.2682333	0.5940063
Hsd3b4	-0.6704781	0.76477743	1.29708755	0.26826437	0.5940063
Mlst8	0.24961607	2.91787372	1.29687146	0.26830301	0.5940063
Grina	0.16310059	7.00624105	1.29661235	0.26834936	0.59401117
Hnrnpa0	0.1284975	7.6352573	1.29625368	0.26841353	0.5940555
Serpinb1a	0.47055032	2.21914967	1.29596337	0.26846549	0.59407278
Golph3	0.15723902	8.57650586	1.29524489	0.26859413	0.59407892
Kat8	0.22097744	3.25460002	1.29499987	0.26863802	0.59407892
Rab28	0.15881435	5.36756956	1.29440132	0.26874527	0.59407892
Rab5c	0.15356882	6.19290957	1.29440072	0.26874538	0.59407892
4932441J04I	0.98465358	-0.8893644	1.29420246	0.26878092	0.59407892
Soat1	0.15599574	5.47047829	1.29410948	0.26879759	0.59407892
Coch	0.20927622	7.79535415	1.29404086	0.26880989	0.59407892
Sh3bp5	0.16130528	6.78191508	1.2939764	0.26882145	0.59407892
Aldh1b1	0.64590086	0.04492689	1.29263934	0.26906133	0.59447102
Leng8	-0.2761932	6.48428617	1.29244894	0.26909552	0.59447102
Adcy6	0.20556212	4.09255721	1.29224903	0.26913141	0.59447102
Myef2	-0.1915839	5.63887936	1.29176858	0.26921771	0.59447886
Sys1	-0.2788531	3.2962455	1.29173738	0.26922331	0.59447886
Fam117a	-0.4491731	3.77824039	1.29125981	0.26930913	0.5945036
Csf1	-0.2425469	4.87426401	1.29109611	0.26933856	0.5945036
Fut10	-0.2488313	3.61978587	1.29093754	0.26936706	0.5945036
Ppp2r2b	0.14055247	6.76285514	1.29051218	0.26944355	0.59452094
Zfp606	-0.1936711	4.59360883	1.29005933	0.26952501	0.59452094
Dpf3	-0.7294324	0.0628116	1.29004025	0.26952845	0.59452094
Sptbn1	-0.2452568	10.099249	1.28975782	0.26957927	0.59452094
F930015N05	-0.2604041	3.60806078	1.28946944	0.26963118	0.59452094
Prr32	-0.9021789	-0.3942978	1.28940511	0.26964276	0.59452094
Podxl	-0.1628288	4.29086336	1.28905568	0.26970567	0.59452094
Alg1	-0.9266946	0.0254511	1.28888565	0.2697363	0.59452094

Irf4	-0.2855102	5.17414265	1.28868438	0.26977255	0.59452094
Neurl1a	-0.2458978	4.46459299	1.2875736	0.26997275	0.59469274
Itga4	-0.2321745	4.8409161	1.2873443	0.2700141	0.59469274
Ppp2r2c	-0.1652727	9.02177401	1.2871214	0.2700543	0.59469274
Zfp81	-0.2146012	4.91312818	1.28672938	0.27012503	0.59469274
Mob3c	0.22830616	4.65589937	1.28667329	0.27013515	0.59469274
Fam60a	-0.2418522	3.5921575	1.28661774	0.27014518	0.59469274
Trpc6	0.35549899	2.85249854	1.2863902	0.27018625	0.59469274
Kndc1	-0.2754108	6.21769581	1.28629152	0.27020406	0.59469274
Pithd1	-0.2033348	4.77159265	1.28592568	0.27027011	0.59474084
Elavl3	0.17651365	7.19794494	1.28512349	0.27041503	0.59483864
Mon2	0.17772775	6.62046016	1.28496999	0.27044277	0.59483864
Arhgap4	-1.0279952	-0.1392605	1.28494561	0.27044717	0.59483864
Gm14164	1.10519072	-0.9193449	1.28413223	0.27059423	0.59506483
Gm16576	-0.5942796	0.70651927	1.28370214	0.27067204	0.59513866
Ppp1r3f	-0.1996963	4.33312591	1.28308723	0.27078333	0.59528609
Slc2a2	-0.9580044	-0.36637	1.28262678	0.2708667	0.59537211
Lphn2	-0.1518226	6.35521224	1.28191778	0.27099515	0.59546085
Mesdc2	0.21492574	6.39054405	1.28177709	0.27102064	0.59546085
Mapk8ip3	-0.2253929	6.77528462	1.28167126	0.27103983	0.59546085
Gzf1	0.16623878	5.15141052	1.28116073	0.27113239	0.59556696
Gpr21	0.58582102	0.86234498	1.28001759	0.27133979	0.59592527
Dnajc10	-0.1399933	6.34267739	1.27884958	0.27155192	0.59629384
Txndc9	0.14840652	5.80377906	1.27772601	0.27175619	0.59654652
Mapre2	0.12262167	9.8021625	1.27748989	0.27179915	0.59654652
Tbc1d9	-0.1764387	5.7477432	1.27748525	0.27179999	0.59654652
Lrrc57	0.17062194	5.83911175	1.27700236	0.27188786	0.59656823
Cog3	0.16770796	5.52673204	1.27640579	0.27199647	0.59656823
Arl2	-0.2741704	3.71063299	1.27638339	0.27200055	0.59656823
Tnfaip8	0.26432358	5.03713846	1.27632412	0.27201135	0.59656823
Zfp61	0.31341558	3.04587609	1.27621319	0.27203155	0.59656823
Reep2	-0.2128053	4.64945687	1.27596363	0.27207701	0.5965707
Armcx4	-0.1848961	6.06988026	1.27485337	0.27227937	0.59691715
Rtbdn	-0.8479667	0.38635475	1.27428182	0.27238363	0.59704843
Gcat	-0.6312053	0.83684208	1.27367738	0.27249393	0.59719294
Abhd13	0.15159002	5.20449293	1.27284083	0.2726467	0.59740108
Cnn3	0.21183347	6.27032767	1.27244841	0.2727184	0.59740108
Nup54	0.22461564	3.41473095	1.27210467	0.27278123	0.59740108
Patl2	1.95948265	-1.4006495	1.2850235	0.27278357	0.59740108
Acmsd	-0.5844892	0.69188002	1.271724	0.27285083	0.59740108
Grwd1	-0.428967	1.41673485	1.27169965	0.27285528	0.59740108
Twist2	-0.6371586	0.8075385	1.27123478	0.27294031	0.5974324
Efcab9	0.92757255	-0.5491644	1.27113598	0.27295838	0.5974324

Derl3	-1.1660529	-1.2662247	1.27060536	0.27305549	0.59754775
Kcnk1	0.17896752	5.55611367	1.27008076	0.27315154	0.59766074
H2afj	-0.3003679	3.5416245	1.26946378	0.27326455	0.59779575
Gm8580	-0.6670361	-0.6333046	1.26918116	0.27331635	0.59779575
Smim15	0.15087907	6.1523121	1.26892716	0.2733629	0.59779575
Atp10a	0.26665233	3.72381248	1.26877423	0.27339094	0.59779575
Ccdc58	-0.2652336	3.0272777	1.26688003	0.27373852	0.59845012
Psme3	0.12485711	6.7298075	1.26637513	0.27383127	0.59845012
Marf1	-0.147546	7.87549306	1.26631574	0.27384218	0.59845012
Anxa2	0.31898414	6.18191341	1.26601167	0.27389806	0.59845012
Rtn1	-0.1281422	10.4495925	1.26593269	0.27391257	0.59845012
Tmem178b	-0.1829389	7.54212313	1.26550065	0.273992	0.59852648
Cebpa	-0.4668037	2.51636749	1.26446751	0.27418206	0.59878626
Zfp786	0.69363499	0.13871326	1.26437046	0.27419992	0.59878626
5830444B04I	-0.5838652	1.32362467	1.2640574	0.27425755	0.59881493
Gpr61	0.38582054	1.32576376	1.2635484	0.27435129	0.59884835
Cep250	0.22217029	4.7744097	1.26349098	0.27436186	0.59884835
Zfp93	-0.2844141	3.83897095	1.26315783	0.27442324	0.59888518
Arid3b	-0.3958912	2.17230131	1.26261037	0.27452414	0.5989566
Pard6g	0.24265685	5.59374964	1.26249727	0.27454499	0.5989566
Zfp629	-0.2214088	4.52124725	1.26181097	0.27467156	0.59912562
Zmym3	-0.1976297	6.02195042	1.26141454	0.27474471	0.59912562
P4hb	0.2506848	5.58337131	1.26135315	0.27475604	0.59912562
Trp53	0.21061783	5.99533336	1.26065904	0.27488418	0.59930793
Tiprl	0.14419219	6.36973233	1.26031975	0.27494685	0.59934745
Lrrc26	1.25119096	-1.5248642	1.25994616	0.27501587	0.59940081
Nt5dc2	-0.5893255	2.65514329	1.25874601	0.27523776	0.59961716
Dnaaf1	-1.2303182	-1.8853075	1.25864567	0.27525632	0.59961716
Scn11a	1.66410939	-1.9906575	1.2583789	0.27530568	0.59961716
Crb1	-1.153149	-0.9575645	1.25821709	0.27533563	0.59961716
D630024D03	-1.6203973	-1.7869437	1.2582046	0.27533794	0.59961716
AW551984	-0.3472099	3.43531085	1.25764708	0.27544115	0.59974486
Dctpp1	0.33642397	1.63545479	1.25718927	0.27552593	0.59983242
Dcbld2	-0.1629699	5.26479498	1.2564502	0.27566289	0.60003349
Paxbp1	-0.2326015	5.67637254	1.25620728	0.27570792	0.60003446
Slc9a8	-0.2363332	4.08268611	1.2555714	0.27582585	0.60019404
Gm3435	-0.2996722	2.79609154	1.25528779	0.27587847	0.60021148
Ilvbl	-0.3434414	2.36640121	1.25491384	0.27594787	0.60026542
Sult5a1	0.87735153	-0.535735	1.25450616	0.27602355	0.60033301
Slc7a11	0.22045663	9.83007909	1.25390316	0.27613555	0.60047954
Gm10046	0.5313553	0.33357784	1.25366253	0.27618026	0.60047973
Necap2	-0.3054755	3.41780728	1.25319621	0.27626694	0.60057114
4930519G04	-0.3684082	2.31728697	1.25275027	0.27634985	0.60065436

Dusp10	-0.2804385	3.731895	1.2522995	0.27643371	0.60068255
5430416O09	-1.3914585	-1.8621924	1.2522006	0.27645211	0.60068255
Ube2v2	0.13820723	6.88664878	1.25194716	0.27649927	0.60068803
Dtwd1	0.44952574	2.03221828	1.25082089	0.27670899	0.60089921
Lbh	-0.2683457	6.3473381	1.25079295	0.27671419	0.60089921
Kcnj4	-0.2643374	3.36991447	1.25066851	0.27673738	0.60089921
Pfkfb2	0.14729831	5.92260936	1.25046609	0.27677509	0.60089921
Slc43a3	0.88115967	0.2159002	1.24977617	0.27690371	0.60106194
Map7	-0.1639913	5.79155177	1.24958484	0.27693939	0.60106194
Lpxn	1.23442234	-1.4889475	1.24899024	0.27705031	0.60120573
Cd53	-0.2947597	2.34394497	1.24829672	0.27717977	0.60138966
Irgm1	0.27857684	4.39098843	1.24763814	0.27730277	0.60155955
Usp20	0.24301845	3.79233137	1.24718184	0.27738804	0.60162919
Mrps18c	0.23955697	4.53628145	1.24681539	0.27745654	0.60162919
Rnls	-0.4829153	1.0564206	1.24674875	0.277469	0.60162919
Rnf115	0.15404361	5.90660914	1.24646932	0.27752126	0.60164554
Zfp748	-0.1869528	4.81917853	1.24517384	0.27776368	0.60195558
Stxbp6	-0.1664473	6.56505081	1.24503716	0.27778928	0.60195558
Gm3002	-0.3661774	3.60229734	1.24498806	0.27779847	0.60195558
Usp25	0.13296445	7.47059909	1.24470122	0.2778522	0.60197506
Bloc1s1	0.33001908	5.14775056	1.24430403	0.27792661	0.60198538
Tmem143	-0.3332633	2.62157586	1.24419826	0.27794643	0.60198538
Atp5j	0.17330679	7.73128512	1.24351674	0.27807419	0.60216517
Pde11a	-0.7679395	-0.3706956	1.24318535	0.27813634	0.60220285
Slit3	0.23630376	4.60546321	1.24241224	0.27828141	0.60236684
Kcnj2	-0.1708376	5.54481899	1.24209565	0.27834085	0.60236684
Rps6ka3	0.13055218	7.88393158	1.24166771	0.27842121	0.60236684
Cox14	-0.172274	5.326743	1.24156577	0.27844036	0.60236684
Mrpl39	0.17387338	5.38481267	1.24114853	0.27851875	0.60236684
Arg1	1.18220232	-0.9245313	1.24111959	0.27852419	0.60236684
Klre1	-1.292119	-1.0160264	1.24096633	0.27855299	0.60236684
Rcc2	0.14297983	5.31857421	1.24079408	0.27858537	0.60236684
Lypla1	0.19997679	5.19566608	1.24056757	0.27862795	0.60236684
Gmppb	0.57360467	0.94314568	1.24039856	0.27865973	0.60236684
Tinf2	0.31535874	2.67584235	1.23977595	0.27877684	0.6025232
Rbm46	0.62807132	1.57546975	1.23949252	0.27883018	0.6025417
Otub2	0.23558342	3.60956246	1.23851533	0.27901417	0.60284247
Taf4a	-0.1712511	4.74494453	1.23767632	0.27917226	0.60308723
Akirin2	0.18443875	6.60891221	1.23686027	0.27932615	0.60309532
Siglece	-1.0213589	-0.5955054	1.2367335	0.27935007	0.60309532
Zkscan7	-0.4801306	1.50064791	1.23663386	0.27936887	0.60309532
Arfip1	0.20707345	6.0988811	1.23623435	0.27944426	0.60309532
Igsf9	0.94949337	-0.311805	1.23622236	0.27944652	0.60309532

1700024G13	-2.0315501	-1.5267944	1.23616585	0.27945719	0.60309532
Gnpda2	0.18460298	5.96309501	1.23599341	0.27948974	0.60309532
E2f6	-0.1603849	5.50491294	1.23452337	0.27976746	0.60313328
Ncr1	-0.7687415	-0.1231929	1.23435762	0.27979879	0.60313328
Ap4b1	0.36105442	2.03441604	1.23418788	0.27983089	0.60313328
Gm2518	0.92178742	-0.6249179	1.234177	0.27983294	0.60313328
Psmd10	-0.2979095	4.10335909	1.23386874	0.27989124	0.60313328
Ank3	-0.2703173	9.13226804	1.23382381	0.27989974	0.60313328
Mthfr	-0.3332571	2.89904496	1.23341679	0.27997675	0.60313328
Mcc	0.16556633	6.54266634	1.23341204	0.27997765	0.60313328
Exosc3	0.21438792	4.95860279	1.23310687	0.2800354	0.60313328
Uqcr11	0.30126108	4.69448431	1.23297886	0.28005963	0.60313328
Zfp354b	0.38055667	2.41234248	1.23293398	0.28006813	0.60313328
Bola2	-0.3576202	2.45401443	1.23283762	0.28008637	0.60313328
Mocos	0.48149026	1.42942326	1.23281841	0.28009001	0.60313328
Plekhg1	0.17714617	5.36137989	1.2321951	0.28020805	0.60324215
Lat2	0.71277404	-0.025745	1.23207806	0.28023023	0.60324215
Hyls1	0.3140923	2.25186842	1.23164202	0.28031285	0.6033235
Sptbn2	-0.2599547	6.97958108	1.23086442	0.28046029	0.60340197
1810022K09I	0.17721819	4.99892239	1.23075037	0.28048192	0.60340197
Chp1	0.12483805	7.55719185	1.23058565	0.28051317	0.60340197
Prkcsh	0.26113542	3.3401779	1.23050387	0.28052868	0.60340197
Vwa5a	0.18656013	5.49992937	1.23012321	0.28060091	0.6034294
A730090N16	-0.6542789	0.77945632	1.22996408	0.28063112	0.6034294
Zfp410	-0.2046638	4.00281026	1.22919381	0.28077738	0.60357399
Ndst2	-0.2590367	3.30347432	1.22866916	0.28087706	0.60357399
Bbx	-0.140268	7.92283414	1.2283889	0.28093033	0.60357399
Ndufaf7	-0.1663753	4.55722598	1.22814856	0.28097602	0.60357399
Diras1	-0.2248114	5.13546591	1.22814066	0.28097752	0.60357399
Ccnj	-0.2761381	2.89291347	1.22798967	0.28100623	0.60357399
Rarres2	0.90296846	0.59490737	1.22776039	0.28104983	0.60357399
Pde5a	0.22420506	6.52878451	1.2275543	0.28108903	0.60357399
Psmb11	-0.4935522	1.33147065	1.22748588	0.28110205	0.60357399
Cnksr2	-0.2473802	9.57153435	1.22717618	0.28116098	0.6036042
Chmp3	0.16673353	6.51614272	1.22681929	0.2812289	0.60365372
Doc2b	-0.285148	4.21046912	1.22625078	0.28133715	0.60365502
Snx8	0.4025904	1.59947853	1.22618082	0.28135047	0.60365502
2810454H06I	-1.0116575	-0.9132212	1.22610934	0.28136409	0.60365502
Fbxl22	0.84984114	-0.6497519	1.22583965	0.28141547	0.603669
Hook1	0.27140591	5.07421021	1.22524116	0.28152953	0.60381741
Tuba1b	0.12640222	9.23594797	1.2249649	0.2815822	0.60383413
1700001D01I	-0.6767074	1.20263267	1.22445602	0.28167925	0.60386874
Lactb	-0.2127221	3.6918282	1.22419796	0.28172848	0.60386874

Nxf2	-1.2824772	-1.3173708	1.22415252	0.28173715	0.60386874
Jmjd1c	-0.1641815	8.21493046	1.22393931	0.28177784	0.60386874
Rec8	-0.6920529	0.39427074	1.22367882	0.28182756	0.60387912
Hspbp1	-0.3091511	2.63907772	1.2230283	0.28195178	0.6040491
3110039I08R	-0.7654837	-0.0970578	1.22227698	0.28209534	0.60426046
Timm23	0.17448949	6.17913277	1.22184936	0.28217709	0.60432956
Csrnp3	-0.2077398	7.29463756	1.22163845	0.28221742	0.60432956
Efna3	-0.3912875	1.5606198	1.22111055	0.28231841	0.60435197
Zbtb11	-0.1709388	6.58427433	1.22110789	0.28231892	0.60435197
Kcns3	0.36737439	1.97479653	1.22087949	0.28236262	0.60435197
2310061I04R	-0.2097632	4.20453282	1.22052011	0.28243141	0.60440307
Fam217a	1.16901808	-0.6141347	1.2199024	0.2825497	0.60454876
Gfod1	-0.1478879	6.81594251	1.21946729	0.28263307	0.60454876
Sec24d	0.22309366	4.40749598	1.21891472	0.28273898	0.60454876
Esrrg	-0.2273897	6.04331139	1.2185579	0.2828074	0.60454876
Tmem223	-0.2112225	3.72335312	1.21819554	0.28287691	0.60454876
Zfp867	-0.200252	4.15322404	1.21801444	0.28291165	0.60454876
Nqo1	0.37483202	4.40171669	1.2179724	0.28291972	0.60454876
Tnfrsf11b	-0.4281277	3.89176417	1.21793143	0.28292758	0.60454876
Zfp503	0.23201021	3.92476051	1.21788424	0.28293664	0.60454876
Rnf215	0.31779423	2.67262694	1.21782106	0.28294876	0.60454876
Tubgcp2	0.20514115	3.66953994	1.21670821	0.28316244	0.60490394
Klrd1	-0.7289411	-0.5107385	1.21648719	0.2832049	0.60490394
Rab40b	-0.2196183	4.03718663	1.21617262	0.28326535	0.60493703
Arfgap1	0.20326353	4.53177529	1.21479237	0.2835308	0.60532021
Fam203a	0.35927649	2.59822023	1.21433713	0.28361842	0.60532021
Rplp1	-0.2168645	5.20971012	1.21427236	0.28363089	0.60532021
Fktn	-0.1609551	5.73022151	1.21413496	0.28365735	0.60532021
Fbln2	-0.5686535	0.80247329	1.21385051	0.28371213	0.60532021
Eid2b	0.17454164	4.42914406	1.21371636	0.28373797	0.60532021
Acly	0.16938048	7.0689687	1.21356329	0.28376745	0.60532021
Nfe2l1	-0.147819	7.82225763	1.2132596	0.28382597	0.60532021
Pou6f1	-0.1867811	5.0576674	1.21313618	0.28384975	0.60532021
Ryr2	-0.3276703	8.03232556	1.2125156	0.28396939	0.60532021
Apol6	-0.6757821	-0.189787	1.21245076	0.2839819	0.60532021
Ebp	0.30082253	2.6593953	1.21239649	0.28399236	0.60532021
Scai	-0.1742994	7.0689649	1.21220357	0.28402957	0.60532021
C130074G19	-0.1720404	6.86910222	1.21139772	0.28418507	0.6054532
Vldlr	-0.1897405	6.06539724	1.21138844	0.28418686	0.6054532
Camkv	-0.1811176	6.00284055	1.21118075	0.28422696	0.6054532
Nlrp4f	1.42656968	-1.9884153	1.21041797	0.28437428	0.60567114
Etnk1	0.12124134	8.62575492	1.21007223	0.28444109	0.60571756
Gtse1	1.05529989	-1.4834709	1.20982666	0.28448855	0.60572279

Rhno1	-0.2705093	2.51360383	1.20912346	0.28462453	0.60577922
Ciz1	0.16092262	5.70076852	1.20898048	0.28465219	0.60577922
Nkain2	-0.2032406	6.31843164	1.20833959	0.28477621	0.60577922
AW495222	-0.3710698	1.74746184	1.20813283	0.28481623	0.60577922
Gltp	0.22990334	5.52220233	1.20786141	0.28486879	0.60577922
Sc5d	0.1882845	4.4301661	1.20760336	0.28491877	0.60577922
Zfp768	0.34321501	2.42165946	1.20747232	0.28494415	0.60577922
Syce1	-1.5463812	-2.0044352	1.2074462	0.28494921	0.60577922
Rin3	0.2769295	4.36278488	1.20723878	0.2849894	0.60577922
Col4a3	0.77087065	0.00043083	1.20709614	0.28501704	0.60577922
Pcsk7	-0.2673923	2.68672644	1.20694159	0.28504699	0.60577922
Kctd3	-0.1942883	5.13243541	1.20676596	0.28508103	0.60577922
Tbx21	1.27789567	-0.7649604	1.2066666	0.28510029	0.60577922
Frmd5	-0.2216808	5.14701112	1.20624577	0.28518189	0.60585694
Pdcd2	0.20985142	3.87021714	1.20595782	0.28523774	0.60587994
Htatsf1	0.11644104	8.39789574	1.2053491	0.28535586	0.60592954
Chic2	-0.2479096	3.94294952	1.20512221	0.28539991	0.60592954
Setd8	0.16171845	7.28392501	1.20474906	0.28547236	0.60592954
Dlgap5	-0.5013139	0.66188119	1.20457672	0.28550583	0.60592954
Rxrg	-0.4325764	0.91500745	1.20453052	0.28551481	0.60592954
Dcdc2c	-0.3444522	5.66716477	1.2044012	0.28553993	0.60592954
Smpd3	-0.1865715	4.76558049	1.20396575	0.28562454	0.60592954
Agap1	-0.1690903	6.71017495	1.20358334	0.28569887	0.60592954
Stmnd1	-0.9850327	-0.550832	1.20355788	0.28570382	0.60592954
Zbp1	0.47365758	1.02666579	1.20351891	0.28571139	0.60592954
Got2	0.13134656	7.01775185	1.20304463	0.28580362	0.60602963
Vmn2r84	-0.8143694	0.21344119	1.20222017	0.28596404	0.60614529
Zfp189	0.23304295	3.26918649	1.20220119	0.28596774	0.60614529
Wnt16	0.52638512	1.69469413	1.2019349	0.28601958	0.60614529
Slc6a9	-0.2424008	4.07559452	1.20176206	0.28605323	0.60614529
Dap3	0.16065073	4.8301408	1.20149442	0.28610536	0.60614529
Gm7904	2.37948842	-1.4868777	1.20137592	0.28612844	0.60614529
Eps8l2	-0.5073593	1.20284362	1.20073998	0.28625236	0.60631235
4930519F09I	-0.2962229	2.53184829	1.19976037	0.28644338	0.60654335
S1pr1	0.27261122	5.64766175	1.19962794	0.28646922	0.60654335
Notch1	-0.3541901	2.82737551	1.19933147	0.28652707	0.60654335
4932438A13I	-0.2569604	7.50608328	1.19909751	0.28657274	0.60654335
Hif1a	-0.1574897	6.51471301	1.1990255	0.28658679	0.60654335
Ctns	-0.2875921	2.65639179	1.19873544	0.28664343	0.60656781
Dalrd3	-0.2809816	3.33754619	1.19812075	0.28676349	0.60661886
Clec5a	-0.7015233	-0.1262084	1.19753525	0.28687792	0.60661886
Cdh10	0.21858873	5.14094305	1.1974627	0.2868921	0.60661886
Angpt2	0.23587185	3.99421813	1.1973252	0.28691898	0.60661886

Ulk4	-0.6209835	1.39967079	1.19712623	0.28695789	0.60661886
Crot	0.15932726	5.34776257	1.1970294	0.28697682	0.60661886
Gsdmc4	-1.5309642	-1.6809568	1.19699723	0.28698311	0.60661886
Dok2	0.86229973	-0.7678923	1.19609954	0.28715876	0.60677749
Ggnbp1	-1.2026228	-0.8377932	1.19595748	0.28718656	0.60677749
Adck1	-0.2398302	3.05850564	1.19580081	0.28721724	0.60677749
Zan	-1.7313907	-2.1385419	1.19569206	0.28723853	0.60677749
Dpt	0.86392254	0.73137947	1.1942582	0.28751948	0.60727566
Mapk1ip1l	0.1649031	8.02239861	1.19329612	0.2877082	0.60757888
Klhl33	1.45781727	-1.944118	1.19284039	0.28779765	0.60767167
Rpusd3	-0.5750866	0.91871578	1.19206739	0.28794947	0.60767167
Rad54b	0.89297342	-0.1526747	1.19203762	0.28795531	0.60767167
Pcf11	0.14352725	6.58616928	1.19194673	0.28797317	0.60767167
Prdx3	0.18886849	5.17439889	1.19192249	0.28797794	0.60767167
Golga7	-0.1550501	7.26421945	1.19108154	0.28814324	0.60780365
Snora28	1.49532171	-1.4223794	1.19105136	0.28814917	0.60780365
Ammecr1l	-0.1327721	5.73285666	1.19091502	0.28817598	0.60780365
Letmd1	-0.1830611	4.8416147	1.18980663	0.28839408	0.60789944
Ccl9	-0.3645275	1.82352796	1.18972681	0.2884098	0.60789944
03-Mar	-0.8295294	0.04069443	1.18950042	0.28845437	0.60789944
Nt5dc3	-0.1916916	5.92130142	1.1893871	0.28847669	0.60789944
Rps28	-0.2366971	5.54608439	1.18936366	0.28848131	0.60789944
Mcm3	-0.4078044	0.97874568	1.18879257	0.28859381	0.60789944
Sigirr	-1.5601006	-1.8461738	1.1887346	0.28860524	0.60789944
Celsr3	-0.4141397	4.73290508	1.18862823	0.2886262	0.60789944
Cdh2	0.20746559	6.47293761	1.18861918	0.28862798	0.60789944
Gpr17	0.31740623	2.74550711	1.18833403	0.28868419	0.60792266
Dtwd2	0.31142436	2.91413883	1.1876375	0.28882154	0.60811674
Oscp1	0.26232371	3.51831819	1.186204	0.2891045	0.60856086
Eml5	-0.3034943	5.85855028	1.18589386	0.28916577	0.60856086
Sox2ot	-0.199205	4.98179201	1.18588182	0.28916815	0.60856086
Olfr856-ps1	-0.3582384	4.31338526	1.18486434	0.28936928	0.60884303
4933430I17R	1.28631811	-1.6878358	1.18433795	0.2894734	0.60884303
Pigs	0.1977489	4.61576132	1.18399682	0.28954091	0.60884303
Srrm4	-0.2123538	6.13290984	1.18395493	0.2895492	0.60884303
Arl5a	-0.1390745	6.9303259	1.18378999	0.28958185	0.60884303
Phtf2	0.1712057	5.23459006	1.18371647	0.2895964	0.60884303
2810047C21I	0.42573397	1.87145865	1.18360259	0.28961895	0.60884303
Pop1	0.3662269	2.24893073	1.18232117	0.28987281	0.60928152
Ccdc8	0.92434302	-0.2951193	1.18148204	0.29003921	0.60953607
Layn	0.43648559	1.44309849	1.1802984	0.29027415	0.60985878
Gpc6	0.21696957	7.3480851	1.18025175	0.29028341	0.60985878
Slc16a3	0.98668556	-1.1228918	1.179914	0.2903505	0.60990451

Gorasp2	0.14768375	7.01602837	1.17903054	0.29052609	0.61015569
Slc2a9	1.34287906	-0.8794348	1.17885614	0.29056077	0.61015569
Nelfb	0.1868621	5.02264515	1.17820267	0.29069075	0.61028199
Grm5	-0.173573	6.71237517	1.17808136	0.29071489	0.61028199
Csde1	0.10890865	9.14077136	1.17698481	0.29093322	0.61028199
Ubxn7	0.11232113	7.31127239	1.17646481	0.29103683	0.61028199
Imp4	-0.203042	4.57065773	1.17643937	0.2910419	0.61028199
Lmna	0.24649194	3.66476007	1.1762976	0.29107015	0.61028199
Far1	0.14872548	6.28201656	1.17629478	0.29107071	0.61028199
Rnps1	0.12150563	6.25131145	1.17609914	0.29110971	0.61028199
Asxl1	0.18306567	5.35960439	1.17592153	0.29114513	0.61028199
A730017C20	-0.1421181	5.8893004	1.17576944	0.29117545	0.61028199
Smpd4	0.19560553	5.12357101	1.17561395	0.29120646	0.61028199
Epha10	-0.5884138	2.34216075	1.17554895	0.29121943	0.61028199
Coa5	-0.1601648	7.00338621	1.17547009	0.29123516	0.61028199
Prr15	-0.586192	0.78841463	1.17536638	0.29125585	0.61028199
Atl1	-0.2160745	6.2395318	1.17511775	0.29130546	0.6102909
Ube3b	0.16474604	6.09056482	1.17427254	0.29147418	0.61054919
Spry4	0.26301733	3.46186275	1.17388149	0.29155228	0.61054919
Izumo4	-0.4020104	1.70919881	1.1736378	0.29160097	0.61054919
Ntng2	0.38788465	1.99714029	1.17359144	0.29161024	0.61054919
Pcdhgc4	0.59324009	0.9926195	1.17322895	0.29168268	0.61060587
Gm16675	0.95900344	-1.3070885	1.17299322	0.29172981	0.61060953
Etnppl	0.37178248	2.95248482	1.17246956	0.29183453	0.61067062
Rsad1	-0.2461918	3.20766424	1.17239345	0.29184976	0.61067062
Mtmr10	-0.1790594	4.27423478	1.17197493	0.2919335	0.61068954
Ppp1r10	0.13753622	5.87724125	1.17189463	0.29194957	0.61068954
Gm6537	-1.2719806	-1.9734939	1.17022599	0.29228378	0.61127872
L3hypdh	-0.3530342	2.99052929	1.16994884	0.29233934	0.61127872
Foxp2	0.18089494	7.30765241	1.16975667	0.29237788	0.61127872
Ankrd33b	0.14336536	6.30615254	1.16958182	0.29241294	0.61127872
Gm14325	-0.1666388	4.34187906	1.16877601	0.29257462	0.6115217
Arf1	0.13190762	8.31488361	1.16778257	0.29277411	0.61176706
Zfp160	0.18564984	4.46987999	1.167724	0.29278588	0.61176706
Atg4c	-0.1612744	6.30646857	1.16739859	0.29285126	0.61176706
Dnajb13	-1.1838774	-1.4063093	1.16726078	0.29287896	0.61176706
Plch1	-0.2849145	3.10887711	1.16677918	0.29297578	0.61176706
Apold1	0.46461722	1.93923786	1.16657867	0.29301611	0.61176706
Gm15441	1.13943234	-1.1953439	1.16626591	0.29307902	0.61176706
Hspb2	-0.4725506	1.09885574	1.16615931	0.29310047	0.61176706
Bcs1l	0.25242602	2.77376128	1.16604797	0.29312287	0.61176706
Fam117b	-0.1095879	7.00148895	1.16592984	0.29314664	0.61176706
Cep128	-0.2570061	4.25132638	1.16547435	0.29323833	0.61186351

Tsn	-0.1670916	6.2732336	1.1648026	0.29337361	0.61196951
Col5a1	-0.2381416	3.40369357	1.16420603	0.29349383	0.61196951
Lhfpl1	1.00324901	-0.7826807	1.16356474	0.29362313	0.61196951
Eid3	1.58889718	-1.901409	1.16338635	0.29365911	0.61196951
Ilk	0.19634125	6.00172694	1.16335806	0.29366481	0.61196951
Mertk	-0.2564128	3.7656962	1.16299941	0.29373717	0.61196951
Anapc2	0.14448727	4.74039092	1.16267724	0.29380219	0.61196951
Slc32a1	0.24301936	4.04629258	1.16266385	0.2938049	0.61196951
Smc6	-0.1197565	7.65803915	1.16253159	0.2938316	0.61196951
A330021E22	-0.2237466	4.08752451	1.16242742	0.29385262	0.61196951
Ears2	-0.6211066	0.22658193	1.16235795	0.29386665	0.61196951
Zp3r	-1.7365765	-1.4150992	1.16216141	0.29390634	0.61196951
Reps2	0.12304432	9.58647786	1.16212081	0.29391454	0.61196951
Adpgk	0.39493672	2.07154623	1.16206493	0.29392582	0.61196951
Wasf1	-0.1873516	7.99226552	1.16137371	0.29406547	0.61212254
Trp73	-0.8566715	-0.2048053	1.16125083	0.29409031	0.61212254
Tmed5	0.19260211	5.54968377	1.16078999	0.29418347	0.61222176
Insc	0.60383989	0.14504822	1.16055994	0.29422999	0.61222389
Cald1	0.19683106	10.2790295	1.16005586	0.29433196	0.61234139
Tmcc1	-0.1079672	6.94096167	1.15894506	0.29455684	0.6126327
Tsg101	0.16030371	5.33394706	1.15882266	0.29458163	0.6126327
Gjd2	-0.370405	1.72208142	1.15868969	0.29460857	0.6126327
Cdadc1	0.13396205	6.00419115	1.15820082	0.29470764	0.61274402
07-Mar	0.17119159	4.80727516	1.15783421	0.29478196	0.61280386
Crlf1	0.76224396	-0.4769437	1.15755351	0.29483888	0.61282751
Ercc8	-0.3696961	2.35817298	1.15702137	0.29494683	0.61290833
Gdap10	-0.3568779	2.40450832	1.15680373	0.294991	0.61290833
Nop58	-0.153236	6.14567815	1.15666141	0.29501988	0.61290833
Gatm	-0.1345626	6.23725596	1.156464	0.29505996	0.61290833
Cyth2	0.20904615	4.26547245	1.15546999	0.29526185	0.61311902
Fam104a	0.2137312	5.23095913	1.15531353	0.29529365	0.61311902
Ept1	-0.1432633	4.98612583	1.15529174	0.29529807	0.61311902
Aldh5a1	-0.1665926	6.74500979	1.1547544	0.29540731	0.6132213
Edem1	0.1413151	4.94744188	1.15449964	0.29545912	0.6132213
Cbfb	-0.1968451	6.03301844	1.15437708	0.29548405	0.6132213
BC052688	-0.3305795	2.15406068	1.153591	0.295644	0.61335181
Gnai3	0.17375464	6.40500411	1.15356117	0.29565008	0.61335181
Polr2a	0.13681924	7.30718596	1.15339611	0.29568368	0.61335181
Rassf9	-0.5652171	0.90130565	1.15301358	0.29576158	0.61336941
Puf60	0.1448611	6.5038711	1.15264118	0.29583743	0.61336941
Pign	0.18421977	4.09877498	1.15191944	0.29598453	0.61336941
Gjb6	0.23451542	8.86000288	1.15191647	0.29598514	0.61336941
Mrpl37	-0.1943415	4.02283892	1.15176247	0.29601654	0.61336941

Il17rc	-0.397042	1.90977284	1.15167321	0.29603474	0.61336941
Prps1	-0.1283211	5.87724468	1.15125396	0.29612025	0.61336941
Polm	0.2671557	3.19492597	1.15123447	0.29612423	0.61336941
Abtb1	-0.3661844	1.96667002	1.15107546	0.29615667	0.61336941
Rrp15	0.28451474	2.82371295	1.15091122	0.29619018	0.61336941
Fbxl17	-0.1436465	8.00944252	1.15089461	0.29619357	0.61336941
Galntl6	-0.1805421	5.12805334	1.15053737	0.29626648	0.61337867
Gm15328	-0.8632336	-0.036985	1.15036686	0.29630129	0.61337867
6430573F11I	-0.2852358	2.57922385	1.15003374	0.29636932	0.61337867
1700003E16I	0.53239824	0.61505747	1.14970916	0.29643562	0.61337867
Henmt1	-0.9969585	-0.5878007	1.14970092	0.2964373	0.61337867
Mcm8	-0.2253242	3.21512786	1.14949525	0.29647932	0.61337867
Creb5	-0.1782842	4.20964926	1.14931028	0.29651712	0.61337867
Lpgat1	-0.1466152	7.83784343	1.14876923	0.29662773	0.61351316
Mgl2	-0.6276825	0.39839275	1.14838131	0.29670706	0.61355637
Clstn1	0.15860046	8.08403304	1.14822122	0.29673981	0.61355637
Pax6	-0.3464199	2.24170717	1.14746017	0.29689556	0.61377009
Xrra1	1.09885703	-1.5196995	1.14700805	0.29698814	0.61377009
Cyb5rl	-1.104684	-0.4919336	1.14677021	0.29703686	0.61377009
Galnt12	0.52840239	0.82133625	1.1466238	0.29706685	0.61377009
Kcnj3	-0.2194161	5.04747371	1.14660242	0.29707123	0.61377009
C330013E15I	0.56938843	0.41328429	1.14620896	0.29715186	0.61379227
E430018J23F	0.36124393	2.49952801	1.14610487	0.2971732	0.61379227
Slc25a20	0.23880692	4.78265158	1.14534204	0.29732962	0.61402111
Slc35a2	-0.2801269	3.20080837	1.14484662	0.29743127	0.61404323
9530077C05I	0.34103576	2.2458995	1.14476727	0.29744756	0.61404323
Fam212a	0.96127141	-0.7309088	1.1446227	0.29747723	0.61404323
Oard1	0.225217	4.57408272	1.14434941	0.29753334	0.61404512
Cckbr	0.19712507	4.6516168	1.14417371	0.29756941	0.61404512
Nrbp1	0.14902427	5.94088008	1.14354158	0.29769926	0.61413547
Mzf1	-0.5749615	0.2685717	1.1435162	0.29770448	0.61413547
Plekha8	-0.2107354	4.32360688	1.14301366	0.29780776	0.61424335
1700024P16I	0.71934428	0.72847317	1.14281763	0.29784807	0.61424335
Mga	-0.1893337	7.82664413	1.14194642	0.29802727	0.61444922
Zhx3	0.14730396	6.21195891	1.14183617	0.29804996	0.61444922
Extl1	-0.2250651	3.57333937	1.14159098	0.29810043	0.61444922
Gpr158	-0.1707639	8.18522937	1.14144469	0.29813054	0.61444922
Arhgap33	-0.2681556	5.35412079	1.14095005	0.2982324	0.61449583
Atxn7l1	-0.1362092	6.14296575	1.14081486	0.29826025	0.61449583
Snhg10	-0.5084318	0.7763282	1.14046887	0.29833153	0.61449583
1700071M16	-1.0813985	0.65191251	1.14044805	0.29833582	0.61449583
Fmr1	-0.1257474	6.28502288	1.139736	0.2984826	0.61470407
Rab27a	-0.2200865	3.40232344	1.13929842	0.29857285	0.61474399

Stmn3	-0.1816608	5.74413792	1.13919905	0.29859336	0.61474399
Svop	0.20167657	5.76057809	1.13860775	0.29871538	0.61479183
Commd3	-0.1547618	5.0746158	1.13842579	0.29875294	0.61479183
Lphn1	-0.1748075	8.35124724	1.13842233	0.29875366	0.61479183
Slc35b3	-0.3013104	2.18496086	1.13812337	0.29881539	0.61480685
Slc35c2	0.38737782	1.83053402	1.13794451	0.29885233	0.61480685
Bscl2	0.22275581	4.33923054	1.13728518	0.29898856	0.61487579
Zbtb9	-0.3310037	2.9040012	1.13688273	0.29907175	0.61487579
Trpm4	-0.426937	2.0419583	1.13632116	0.29918789	0.61487579
1700028E10I	-0.9241489	-0.7948921	1.13616196	0.29922083	0.61487579
E230016K23I	1.06568735	-0.708949	1.13590314	0.29927439	0.61487579
Zfp276	-0.3096787	2.6787564	1.13587984	0.29927921	0.61487579
4930470H14I	-0.3409505	6.76646202	1.13584014	0.29928742	0.61487579
AI429214	0.33013306	3.37119213	1.13581562	0.2992925	0.61487579
Rock1	0.16479696	8.00893157	1.13544868	0.29936845	0.61487579
Smim6	-1.5227777	-1.6185278	1.13535793	0.29938724	0.61487579
Pcdhb7	-0.4066972	2.24062595	1.13529174	0.29940095	0.61487579
9230110C19I	0.25925517	3.02451979	1.1351313	0.29943417	0.61487579
Aloxe3	-0.3800667	1.83074161	1.13434397	0.29959729	0.61499142
Aimp2	0.23744347	3.29249552	1.13326997	0.29981999	0.61499142
Gm15133	1.3082818	-1.759902	1.13304051	0.2998676	0.61499142
Usp8	0.11402991	7.66784425	1.13256763	0.29996574	0.61499142
Lrrc4b	-0.2025901	4.57026457	1.13229545	0.30002225	0.61499142
Acap3	-0.1762028	4.76959602	1.13224711	0.30003229	0.61499142
Btaf1	-0.1937454	6.20105171	1.13202273	0.30007889	0.61499142
Gapdhs	0.58144859	1.29571558	1.1320008	0.30008345	0.61499142
BC030867	-1.1794358	-1.5114321	1.13197487	0.30008883	0.61499142
Ccs	0.3444419	2.13602256	1.13165927	0.3001544	0.61499142
Serac1	0.21200679	5.45659715	1.13156839	0.30017328	0.61499142
Dnaic2	-1.3079284	-1.540072	1.13144028	0.3001999	0.61499142
Slc31a2	0.28658871	5.11246442	1.13139445	0.30020942	0.61499142
Tmco1	0.28093682	4.77673781	1.13136189	0.30021619	0.61499142
Inpp5e	0.15451539	5.0308167	1.13130661	0.30022768	0.61499142
B630019K06	-0.2277054	3.26317026	1.13105818	0.30027932	0.61499142
Asb1	-0.1891002	4.51615356	1.13105397	0.3002802	0.61499142
Mark1	0.13912818	5.97795323	1.13050772	0.30039379	0.61499142
D930020B18	1.37994242	-2.0613081	1.13043706	0.30040848	0.61499142
Trmt1	-0.308685	2.8026084	1.1302866	0.30043978	0.61499142
E230016M11	-0.7327181	0.20227686	1.13023633	0.30045024	0.61499142
Dnmt3b	0.69663553	0.32546079	1.12901828	0.30070381	0.61529479
R3hdm4	-0.1514805	7.68370037	1.12883839	0.30074128	0.61529479
Pof1b	-0.6329357	0.09221184	1.12866271	0.30077788	0.61529479
F630111L10I	0.39622048	2.56004193	1.12864602	0.30078136	0.61529479

Cdk1	-1.6642349	-1.4653054	1.12824328	0.30086529	0.61537293
Coprs	-0.254131	3.63211372	1.12759498	0.30100046	0.61555585
Zmat3	-0.1514042	9.37056188	1.12682504	0.3011611	0.61570019
Usp32	-0.1509007	7.80025164	1.12653633	0.30122137	0.61570019
Naga	0.40689452	3.0896614	1.12641538	0.30124663	0.61570019
Wdr17	-0.2608097	5.11350837	1.12637975	0.30125406	0.61570019
Bcar3	0.28162516	2.67844132	1.12606649	0.30131949	0.61574038
Ralgapb	-0.1648797	7.62537635	1.1256449	0.30140756	0.6158159
Ctf1	0.38079837	2.64594289	1.12535737	0.30146765	0.6158159
Hmgcr	0.1707199	5.79284207	1.12505861	0.3015301	0.6158159
Emg1	-0.3180642	2.94861394	1.12501365	0.3015395	0.6158159
Psme2b	-0.2975497	3.17254541	1.1244543	0.30165647	0.61596131
Arid5b	-0.1432463	6.23806089	1.12419864	0.30170996	0.61597705
Fry	-0.2573611	8.22700154	1.12381769	0.30178969	0.61604635
Zdhhc18	0.27510981	3.24459797	1.12247785	0.30207031	0.61624392
Zfp26	-0.1582292	6.27134914	1.12235335	0.3020964	0.61624392
Rpsa	-0.1696846	6.48231852	1.12232952	0.30210139	0.61624392
Rpe	0.18193668	5.42631599	1.12216556	0.30213576	0.61624392
Fhl5	2.2353566	-2.2783668	1.12172004	0.30222918	0.61624392
Hspb1	0.29618239	5.07250514	1.12167651	0.30223831	0.61624392
Mob4	0.11706069	7.02416311	1.12153051	0.30226893	0.61624392
Gm15417	0.45015338	1.17185303	1.12119643	0.30233902	0.61624392
Luc7l	-0.1786669	5.4931452	1.12119612	0.30233909	0.61624392
Tmem201	-0.2493917	3.96789872	1.12117067	0.30234443	0.61624392
Telo2	0.49783317	1.49145732	1.12065221	0.30245325	0.61637235
Fam84a	-0.1627122	4.98378711	1.11993958	0.3026029	0.61653002
Creb3l1	0.23357968	5.25308453	1.1197827	0.30263586	0.61653002
Fam135a	0.16694227	5.76038231	1.11962944	0.30266807	0.61653002
Ncoa5	-0.2088201	4.6525009	1.11932528	0.30273199	0.61653302
Notch2	0.22909802	7.07450311	1.11907793	0.30278399	0.61653302
Nrsn2	-0.1894129	5.88585076	1.11876412	0.30284998	0.61653302
Kctd5	0.28448969	2.84732475	1.11865075	0.30287382	0.61653302
4921511I17R	1.81733387	-2.4337037	1.1184694	0.30291197	0.61653302
2010111I01R	-0.1828139	4.66778239	1.11831506	0.30294444	0.61653302
Plxna4os1	-0.7981601	-0.4873521	1.11806123	0.30299785	0.61654847
Dmtf1	0.13714623	6.44770029	1.11768922	0.30307616	0.61661456
Man1c1	0.1627359	6.0539353	1.11743253	0.3031302	0.61663129
Erc1	0.12406867	7.67618624	1.1167146	0.30328143	0.61676734
AF529169	0.47390706	1.49854143	1.11644289	0.30333869	0.61676734
Fgd5	-0.5990051	1.84175982	1.11630051	0.3033687	0.61676734
Dclre1b	-0.2170128	3.22638578	1.11602149	0.30342753	0.61676734
Ecscr	-0.905316	-0.6788505	1.11583536	0.30346678	0.61676734
Tbc1d5	0.12939038	6.45387232	1.11581016	0.30347209	0.61676734

Alx1	0.52133336	2.53238733	1.11536252	0.30356652	0.61680865
Gon4l	-0.1777531	5.89696095	1.1152137	0.30359792	0.61680865
Rps27	-0.592732	-0.165867	1.11499652	0.30364376	0.61680865
Mrpl42	0.16464312	5.90486063	1.11484484	0.30367577	0.61680865
Tmem110	-0.2317968	2.80599648	1.11431841	0.30378693	0.6169413
Ppp1r9a	-0.2583718	8.91038892	1.11404152	0.30384541	0.61696696
Pcif1	0.17593361	3.96278739	1.11295528	0.304075	0.61722409
Pik3c2g	-1.5728692	-2.0787659	1.11240266	0.30419189	0.61722409
Tdgf1	-1.2160684	-0.1595847	1.11237338	0.30419809	0.61722409
Gm16287	-0.4358196	1.40048103	1.11228808	0.30421614	0.61722409
Snapin	0.19851911	7.51446308	1.11197868	0.30428162	0.61722409
Kcnip4	-0.1403606	7.02681081	1.11186346	0.304306	0.61722409
Atad3aos	-0.7208352	0.04760216	1.11183391	0.30431226	0.61722409
Traf5	0.45311426	1.45352908	1.11155107	0.30437214	0.61722409
Helb	0.23801703	3.42155083	1.111491	0.30438486	0.61722409
Gpx7	0.41055734	2.44494039	1.11066875	0.30455905	0.61747635
Epc1	-0.1283565	6.38698738	1.11035917	0.30462467	0.61747635
Gpr137b	0.22938604	2.9335845	1.11018216	0.3046622	0.61747635
Slc9a3r2	-0.2497788	5.10276259	1.11003777	0.30469282	0.61747635
Nfya	0.14143308	5.71546218	1.10932522	0.30484396	0.61751071
Cpne5	0.19577535	4.94584608	1.10925975	0.30485786	0.61751071
Lysmd1	0.24530807	3.29837465	1.10911336	0.30488893	0.61751071
Scnn1a	-0.3059009	2.58433615	1.10899192	0.3049147	0.61751071
F2r	-0.2817668	2.66735324	1.10887644	0.30493922	0.61751071
Gm8615	0.25219497	2.82423481	1.10826612	0.30506882	0.6176802
Vamp3	0.19120078	7.50340041	1.10715376	0.30530522	0.61801297
5930438M14	-0.8145441	-0.5977789	1.10706062	0.30532503	0.61801297
Bop1	0.27637157	2.95513326	1.10652154	0.30543969	0.61815208
Trabd	-0.3497067	2.25259154	1.1059944	0.30555188	0.61817928
Kcnq5	-0.1986332	6.25302514	1.10588085	0.30557605	0.61817928
Myadm	0.12355822	7.63973607	1.10578719	0.30559599	0.61817928
Mir3064	-0.7436616	-0.6415369	1.1054817	0.30566104	0.61817928
Grin2a	-0.1975201	6.61728008	1.1053794	0.30568283	0.61817928
Cisd2	-0.1418968	6.17756167	1.10479568	0.30580719	0.61833785
Adra1d	0.19319787	3.5059579	1.10403458	0.30596945	0.61857298
Smoc2	0.2903074	3.40767044	1.10290299	0.30621091	0.61861256
Ldhd	0.41767419	1.85823546	1.10282761	0.306227	0.61861256
Dst	-0.2783505	9.14796673	1.10271812	0.30625038	0.61861256
Tom1l1	-0.2032101	3.91602837	1.10271256	0.30625157	0.61861256
Rab5a	-0.1319323	5.49876439	1.10267788	0.30625897	0.61861256
Ndufa12	-0.1581469	5.67767073	1.10252076	0.30629253	0.61861256
Arpc1a	-0.1564527	6.16045961	1.10243507	0.30631083	0.61861256
4930428E07I	0.88348837	-0.2484161	1.10214548	0.30637269	0.61864465

Reep3	0.20133887	7.27510265	1.10171889	0.30646385	0.61866497
AU040972	-0.5900664	1.08626308	1.10153839	0.30650243	0.61866497
Gm3414	0.26766138	3.33853117	1.10126718	0.30656042	0.61866497
Hcn3	0.39058049	1.66083011	1.10067613	0.30668684	0.61866497
Dph5	-0.2071209	4.65440441	1.10063301	0.30669606	0.61866497
Mpped2	0.16551616	6.62221731	1.09978057	0.30687852	0.61866497
Pcbp4	-0.2776374	2.70007356	1.09959328	0.30691863	0.61866497
Eif2ak3	-0.2631452	3.26880954	1.09957025	0.30692356	0.61866497
Rnf138	-0.2108546	3.49491587	1.09891791	0.30706332	0.61866497
Med18	-0.4556058	1.08521293	1.09890785	0.30706548	0.61866497
Peo1	0.32853501	2.5381301	1.09882065	0.30708416	0.61866497
Exoc7	0.17696827	4.63607834	1.0987507	0.30709916	0.61866497
Gfra2	0.1696171	7.57978057	1.09866119	0.30711834	0.61866497
Rn4.5s	-0.492414	0.52362576	1.09857022	0.30713785	0.61866497
Zfp358	0.22992475	3.17915035	1.09848891	0.30715528	0.61866497
Rrm2b	0.13280555	6.74187979	1.09846028	0.30716142	0.61866497
AI197445	0.62813918	0.52961767	1.0983977	0.30717483	0.61866497
Nanos2	1.55475988	-2.2041108	1.09772764	0.30731855	0.61866497
Nradd	0.67896127	0.29732932	1.09754942	0.30735679	0.61866497
Carns1	0.4395212	2.38018108	1.09742005	0.30738455	0.61866497
Shfm1	0.18936175	4.75682789	1.09738698	0.30739165	0.61866497
Slc5a12	-0.6503962	0.11531533	1.09737498	0.30739422	0.61866497
Lyg1	1.01054128	-1.5297551	1.09680984	0.30751555	0.61881659
Slit1	0.30658503	2.95992299	1.09611464	0.30766488	0.6189701
Dnajc5b	0.957943	-0.5513479	1.09602648	0.30768383	0.6189701
Trpc5	-0.2404135	4.12058133	1.09549437	0.30779821	0.61910764
Kctd11	0.34532757	3.32432119	1.09491815	0.30792213	0.61917614
Gtf2h1	0.19154975	5.09621696	1.09467609	0.30797421	0.61917614
Alg8	0.20323906	3.33045314	1.09445038	0.30802278	0.61917614
Dph2	0.31635994	1.76580914	1.09391019	0.30813907	0.61917614
1110034G24	-0.4338698	1.72551301	1.09378882	0.30816521	0.61917614
Gltscr1l	-0.1384823	6.39227417	1.09368638	0.30818727	0.61917614
Srsf6	-0.12223	5.68150112	1.0931991	0.30829225	0.61917614
Sp110	0.27997835	3.39387382	1.09295398	0.30834507	0.61917614
Glul	0.16486919	10.5718556	1.09284294	0.30836901	0.61917614
H2-DMa	-0.4616586	2.02322094	1.09246425	0.30845065	0.61917614
Smurf2	-0.1296307	7.06923446	1.0921455	0.30851939	0.61917614
Htr4	-0.4912282	0.69245636	1.09201781	0.30854693	0.61917614
Slc35b1	-0.1943781	3.70442567	1.09144985	0.30866949	0.61917614
Rab26os	-0.5385123	0.98218832	1.09144476	0.30867059	0.61917614
Efnb3	0.21851958	4.91469848	1.09132544	0.30869634	0.61917614
Timm17a	0.18024524	5.75091044	1.09046248	0.3088827	0.61917614
Nhsl2	0.12535988	8.08117747	1.09034949	0.30890712	0.61917614

Man2a1	0.13005693	6.07197675	1.09011759	0.30895723	0.61917614
Sycp3	-0.3820755	2.03633713	1.0896992	0.30904766	0.61917614
Cltb	0.18606185	5.79999966	1.08957572	0.30907436	0.61917614
Psmc2	0.11587905	6.27084943	1.08935848	0.30912134	0.61917614
Amot	0.16585222	4.95629387	1.08887942	0.30922497	0.61917614
Pdxk	0.1458633	7.60065812	1.08870062	0.30926366	0.61917614
Ppp2r5c	-0.1355813	7.0535404	1.08869736	0.30926437	0.61917614
Ccdc34	-0.1699733	6.08041037	1.08851099	0.3093047	0.61917614
Cpeb3	0.14870647	7.55417114	1.08847304	0.30931292	0.61917614
Alg14	0.26870161	4.58603862	1.08832249	0.30934551	0.61917614
Dlx6	-0.4645689	0.88559198	1.08825037	0.30936112	0.61917614
Brms1l	0.14443249	5.94753582	1.08817436	0.30937758	0.61917614
Dnal4	0.25539427	2.43868295	1.08813183	0.30938679	0.61917614
Med10	0.19647375	3.50898414	1.08801638	0.30941179	0.61917614
Pole2	-0.651528	1.03558652	1.08753838	0.30951532	0.61917614
1700034F02I	-1.3826205	-1.1505467	1.08681134	0.30967289	0.61917614
Nxt1	0.3564564	2.54312673	1.08680348	0.30967459	0.61917614
Parp9	-0.2512231	3.39208783	1.08638756	0.30976478	0.61917614
Fam219aos	0.30610266	3.09071208	1.08637954	0.30976652	0.61917614
1700021K19I	0.14744653	5.53596705	1.08628105	0.30978788	0.61917614
Akap10	-0.1620376	4.87047953	1.08617527	0.30981083	0.61917614
Tgfb3	-0.2980849	4.69475048	1.08605488	0.30983695	0.61917614
Smad4	0.12956957	5.86879899	1.0858832	0.3098742	0.61917614
Itga3	-0.2688166	2.92621062	1.08565282	0.30992419	0.61917614
Ifngr2	-0.1894493	5.14388699	1.08556797	0.30994261	0.61917614
Aldh16a1	0.84406701	0.16075163	1.08551002	0.30995519	0.61917614
Tfeb	0.4597947	1.47424651	1.08544214	0.30996992	0.61917614
Il17rb	0.79107451	-0.0002551	1.08532603	0.30999513	0.61917614
Nmur2	2.10137596	-1.6835497	1.08505251	0.31005452	0.61917614
Klhl41	-0.4289684	1.5136362	1.08505144	0.31005476	0.61917614
Cecr2	-0.205122	3.27743897	1.08476549	0.31011686	0.61917614
B3gnt7	1.22855224	-0.8629206	1.08463289	0.31014567	0.61917614
Ift88	-0.2019098	4.21810903	1.08461597	0.31014934	0.61917614
Anxa11	-0.1708006	3.947233	1.08440037	0.31019619	0.61917614
Pdgfra	0.18553227	5.49716989	1.08426789	0.31022498	0.61917614
Uhrf1bp1l	0.1820925	7.76480923	1.08369943	0.31034856	0.61933093
Psat1	0.14387559	6.48892202	1.08341383	0.31041067	0.61936302
Tmem200a	0.28840744	3.68035073	1.08305057	0.31048969	0.61937707
B3galt2	-0.2039176	5.41767912	1.08288462	0.3105258	0.61937707
Akirin1	-0.1381508	5.78929013	1.0825717	0.31059391	0.61937707
Exosc8	-0.1836737	3.93105156	1.08253535	0.31060182	0.61937707
Exoc1	0.20783268	5.66903012	1.08186781	0.31074719	0.61954924
3110079O15	-1.1252118	-1.8125332	1.08171605	0.31078025	0.61954924

D930048N14	0.61771862	0.71953324	1.08108868	0.31091697	0.61972999
Rab3d	0.21467671	3.19566862	1.08060467	0.3110225	0.61984853
Uhrf1	-0.7265521	0.39544064	1.07920279	0.31132845	0.6201981
Ctnnbip1	0.18128792	3.99076024	1.07918431	0.31133249	0.6201981
Airn	0.35751078	1.75210693	1.0788163	0.31141287	0.6201981
Coq10a	-0.2029244	4.11376662	1.07878641	0.3114194	0.6201981
Aldh3a1	0.83949389	-0.7939376	1.0786526	0.31144864	0.6201981
Myo1a	-1.5341327	-1.8296905	1.07853449	0.31147445	0.6201981
Prim1	0.32332458	2.94450573	1.07820727	0.31154597	0.62021232
Cxcl1	1.60468554	-1.9014253	1.07808009	0.31157377	0.62021232
Jmjd6	0.2148791	2.98096008	1.07764549	0.3116688	0.62024737
Polr3g	-0.2253497	3.14058005	1.07751602	0.31169712	0.62024737
Glt25d1	-0.1918776	3.99311375	1.07736728	0.31172966	0.62024737
Fkbp2	-0.1946297	4.37876841	1.07631369	0.31196028	0.62055209
Commd5	0.36331285	2.05512367	1.07623118	0.31197835	0.62055209
Ccdc162	-0.6597358	0.22297356	1.07559461	0.31211781	0.62055209
Cdk4	0.24275565	4.37868643	1.07558535	0.31211984	0.62055209
Med12	-0.198585	4.99139276	1.07532152	0.31217766	0.62055209
Dsg3	-0.8621398	-0.7353267	1.07517281	0.31221026	0.62055209
Mrpl15	0.26508789	4.9439857	1.07512212	0.31222138	0.62055209
Capn12	-0.8252298	-0.7080199	1.07498365	0.31225174	0.62055209
Cpsf3l	-0.2657716	2.63719254	1.07443787	0.31237144	0.620655
Kcna6	0.18469808	5.75091568	1.07432703	0.31239576	0.620655
Mfsd12	1.04670595	-1.2930054	1.07366912	0.31254017	0.62071674
C1qb	0.38961995	2.39464549	1.07357542	0.31256074	0.62071674
04-Sep	-0.143084	5.93010736	1.07322655	0.31263735	0.62071674
Grpr	0.72790657	-0.1149348	1.07314577	0.3126551	0.62071674
Sh2d1a	-1.3006348	-1.5924025	1.07313492	0.31265748	0.62071674
Btn2a2	-1.2855023	-0.4733493	1.07241748	0.31281514	0.62093812
Fam227a	-0.4824639	1.77176102	1.07150567	0.31301566	0.62098831
Bcl2l2	-0.1184258	6.25153777	1.07146802	0.31302394	0.62098831
Isy1	0.18581919	4.96121102	1.071391	0.31304089	0.62098831
Eed	0.18076533	4.49116874	1.07137412	0.3130446	0.62098831
Ccdc167	0.1398796	5.38308184	1.07125341	0.31307116	0.62098831
Yipf5	0.13083764	5.41711625	1.07102553	0.31312132	0.62099626
Tgm5	-0.9427818	0.06643321	1.06910341	0.3135448	0.62166473
Antxr2	0.40598839	3.3378226	1.06894367	0.31358003	0.62166473
Ralgapa1	-0.2238574	7.7368995	1.06879417	0.31361301	0.62166473
Mtus2	-0.1242965	6.24056567	1.06865742	0.31364317	0.62166473
BC023829	-0.2373982	3.99356688	1.0673303	0.31393615	0.62192436
Mknk2	0.1849895	3.96354222	1.06660654	0.31409609	0.62192436
Tshz3	-0.1438294	6.13421341	1.06625886	0.31417296	0.62192436
Ccdc6	0.13410362	6.7624702	1.06623117	0.31417908	0.62192436

A930001C03	1.55138539	-1.796291	1.06612826	0.31420184	0.62192436
Fam160b2	0.14294202	6.58432787	1.065968	0.31423728	0.62192436
Spire2	-0.2571314	2.52442626	1.06534325	0.31437552	0.62192436
Leo1	0.13148757	6.10134011	1.06524003	0.31439837	0.62192436
Tmem200b	0.73508278	-0.2351335	1.06523484	0.31439951	0.62192436
Actr10	-0.1195951	7.66079374	1.06520139	0.31440692	0.62192436
Gabrg3	-0.2952035	4.67302753	1.06517629	0.31441247	0.62192436
Kif11	-0.3599166	2.2910576	1.06517164	0.3144135	0.62192436
Rpp25l	-0.3884745	2.05738718	1.06475699	0.31450531	0.62192436
Polr1c	0.23092416	4.05554449	1.06466124	0.31452652	0.62192436
Rock2	0.14571659	10.0961384	1.06462913	0.31453363	0.62192436
Itpr1	-0.183966	8.99082868	1.06453576	0.31455431	0.62192436
A730098P11	-0.1379087	6.70378136	1.06451064	0.31455987	0.62192436
Zbtb22	-0.2383408	3.3208382	1.06348472	0.31478723	0.62228245
Hhat	-1.0153726	-0.7515552	1.06316069	0.31485909	0.62229143
BC003331	0.12476511	6.91312747	1.06294892	0.31490606	0.62229143
Myo19	-0.3140126	2.91947592	1.06282122	0.3149344	0.62229143
Atp11a	0.15235771	6.90085178	1.06263032	0.31497675	0.62229143
Slc37a1	-0.4322855	0.7991249	1.06212419	0.3150891	0.62242199
Klhl4	0.16270105	4.81035219	1.06161409	0.31520238	0.62254884
Zfp58	0.24641004	3.40158186	1.06117209	0.31530058	0.62254884
Copz2	0.2813033	6.78348118	1.0607952	0.31538435	0.62254884
Mex3a	0.28633306	2.55651255	1.06051675	0.31544626	0.62254884
Cln3	0.30368484	2.55672215	1.06035563	0.31548209	0.62254884
Metrn	0.71848039	-0.6406921	1.06029519	0.31549553	0.62254884
Tcf7l1	0.24337928	5.36479086	1.05992068	0.31557884	0.62254884
H1fx	-0.8289515	-1.4640314	1.0596551	0.31563794	0.62254884
Ncf1	-0.2631517	2.38759849	1.05950853	0.31567056	0.62254884
Ndufs5	0.92001407	-1.4944173	1.05946588	0.31568005	0.62254884
AU041133	-0.2813211	2.83217733	1.05943758	0.31568635	0.62254884
Nosip	0.17383326	4.60528241	1.05933816	0.31570848	0.62254884
Map3k7cl	-0.7013044	0.50452359	1.05896854	0.31579078	0.62260362
Scamp1	0.11903009	7.87224213	1.05846707	0.31590249	0.62260362
Recql	-0.1940534	3.96390845	1.05832921	0.31593321	0.62260362
Erich6	-0.5948167	1.27272317	1.05828857	0.31594226	0.62260362
1700102H20	-1.3039904	-1.2506463	1.05817486	0.3159676	0.62260362
Tmco4	0.40619203	2.26886504	1.05779948	0.31605128	0.62263651
Zfp473	1.0125651	-0.3005322	1.05737737	0.31614541	0.62263651
Rab38	1.21316407	-1.447128	1.05727989	0.31616715	0.62263651
Fam111a	0.21774002	3.34294009	1.05726991	0.31616938	0.62263651
Nol9	0.24482311	3.57969393	1.05679716	0.31627486	0.62270306
Tmed2	-0.1809676	5.71179949	1.05670367	0.31629572	0.62270306
Mrpl36	-0.2125297	5.37409144	1.05550006	0.31656451	0.62309701

Asun	-0.1566178	4.53145219	1.05539301	0.31658844	0.62309701
Wdr36	-0.193851	3.83820002	1.05488057	0.31670298	0.6232097
Eif6	0.24461645	4.38628276	1.05460497	0.31676461	0.6232097
Ccng2	-0.2091687	4.33906316	1.05449546	0.3167891	0.6232097
Orc4	0.1387396	5.58400376	1.05364051	0.31698041	0.6232097
2810013P06I	0.19916037	4.65624148	1.05343397	0.31702665	0.6232097
Zfp280c	-0.1652454	5.37018434	1.05330392	0.31705577	0.6232097
Gemin5	0.25653584	3.93796006	1.05321497	0.31707569	0.6232097
H13	0.20411174	4.82160613	1.0528606	0.31715506	0.6232097
Dnajb14	-0.1693572	4.2317679	1.05284824	0.31715783	0.6232097
5730559C18I	1.35618018	-0.9736511	1.05279303	0.3171702	0.6232097
Exoc4	-0.1353674	5.87037846	1.05234201	0.31727127	0.6232097
Dnaja3	-0.151291	5.03685258	1.05215229	0.3173138	0.6232097
Igfbp3	-0.3095902	3.83518722	1.05210126	0.31732524	0.6232097
C130060C02I	-0.6996138	0.02592742	1.05150333	0.31745933	0.6232097
Rrad	1.01193546	-1.1122327	1.05133644	0.31749677	0.6232097
Tnfrsf4	1.87137386	-2.2440682	1.05123475	0.31751958	0.6232097
Myo5a	-0.2553539	9.77832343	1.0511782	0.31753227	0.6232097
Pam	0.1450468	6.3274475	1.0511426	0.31754026	0.6232097
Gm11944	-0.5982194	0.61037529	1.0509361	0.31758661	0.6232097
Snx15	0.15791748	4.50132427	1.05091432	0.31759149	0.6232097
Pole3	-0.2806215	2.9234239	1.05079499	0.31761828	0.6232097
Atg9b	0.64441114	0.83379581	1.05053369	0.31767694	0.62323393
Ccdc34os	-1.4334569	-1.6641624	1.04999967	0.31779688	0.62337834
Slc22a5	-0.304782	2.58385577	1.04941242	0.31792884	0.6235463
Ino80	-0.1769383	5.08845141	1.04659175	0.31856375	0.62457411
Acan	-1.0397792	-0.8003149	1.04658043	0.3185663	0.62457411
Mid1ip1	0.23159435	6.08785117	1.04641445	0.31860371	0.62457411
Cbln2	-0.2588788	3.41947887	1.04588797	0.31872243	0.62457411
Gtf2a1	0.11683255	6.99718016	1.04495026	0.31893403	0.62457411
Tmem255a	0.20218076	4.74803875	1.04493759	0.31893689	0.62457411
B230219D22	0.13128064	8.00694298	1.04484716	0.31895731	0.62457411
Cbx7	0.20519253	4.92502466	1.04473902	0.31898173	0.62457411
D10Bwg137[illegible]	-0.2517705	7.06518929	1.04470093	0.31899033	0.62457411
Ranbp9	0.11125106	7.07801354	1.04408761	0.31912887	0.62457411
Rbms2	0.18878583	5.91312169	1.04363026	0.31923223	0.62457411
Ccdc84	0.577382	1.12289914	1.04355025	0.31925032	0.62457411
Dhcr24	0.20059547	5.09585216	1.04342925	0.31927768	0.62457411
Sostdc1	0.68822206	0.73496236	1.04342809	0.31927794	0.62457411
Zc3h3	0.31084785	2.62834499	1.04333808	0.31929829	0.62457411
Mib2	0.23614912	3.61288825	1.04320528	0.31932832	0.62457411
Col1a2	0.22836043	7.37951761	1.04316132	0.31933826	0.62457411
Zfp131	-0.1696956	5.28940919	1.04293366	0.31938975	0.62457411

Sgk1	0.13839372	6.58128598	1.0428451	0.31940979	0.62457411
Vwc2l	0.26597891	3.42519573	1.04281931	0.31941562	0.62457411
Diras2	-0.1239325	8.13275265	1.04276633	0.31942761	0.62457411
Unc5b	0.30221374	2.36938693	1.04230486	0.31953204	0.62468753
Epha2	1.26465009	-1.6460887	1.04203258	0.31959368	0.62471727
Gm17644	-0.3532753	7.68606543	1.0416227	0.31968649	0.62477374
Ubap1	-0.1526344	5.09841286	1.04149498	0.31971542	0.62477374
Ube2k	0.11356988	8.11082422	1.04103785	0.319819	0.62488539
Prkce	0.14558457	8.83402021	1.03985914	0.32008628	0.62525704
Acnat1	-0.6638518	0.1652493	1.03953015	0.32016093	0.62525704
Prrg4	-0.4484927	1.78377809	1.03938422	0.32019406	0.62525704
Sdccag8	-0.1398369	6.24734401	1.03937976	0.32019507	0.62525704
Rad9a	-0.2200745	3.49725422	1.03899114	0.3202833	0.62529522
Mab21l2	-1.1267887	-0.8035876	1.03880381	0.32032585	0.62529522
Klc4	-0.3119169	2.29237103	1.03865489	0.32035967	0.62529522
Adap1	0.18376675	4.20432379	1.03847518	0.3204005	0.62529522
A730017L22I	-0.2616669	4.89894303	1.03808839	0.32048839	0.62537605
Arid3c	-1.3919268	-1.2824569	1.03786753	0.32053859	0.62538333
Cab39	0.11344699	7.71350092	1.03719089	0.32069247	0.62540106
Emc3	0.19074022	5.9107534	1.03711943	0.32070872	0.62540106
Ranbp2	-0.1861779	7.62672975	1.037102	0.32071269	0.62540106
Wdr6	-0.1668334	4.85577563	1.03682532	0.32077564	0.62540106
4930565N06	0.62567651	1.61358252	1.03650937	0.32084755	0.62540106
Fbxl18	-0.3828757	1.31069494	1.03644986	0.3208611	0.62540106
Kif14	-1.2813101	-1.3150309	1.03638	0.32087701	0.62540106
Auh	0.15483027	5.11797038	1.03619338	0.32091949	0.62540106
Myl9	-0.3095628	5.49638529	1.03553326	0.32106986	0.62560348
Klhl30	-0.4630083	0.76413979	1.03456384	0.32129085	0.62575293
Maats1	-0.7512939	0.57079124	1.03437667	0.32133354	0.62575293
Slc39a11	0.35468664	1.72164444	1.03428332	0.32135484	0.62575293
Elac2	0.29841123	2.88312398	1.03418775	0.32137664	0.62575293
Pabpc5	-0.3204069	2.29010092	1.0341771	0.32137907	0.62575293
Klrg2	-0.7756161	-0.5935712	1.03380287	0.32146447	0.6258049
Utp14a	0.20319845	3.98157565	1.03365259	0.32149877	0.6258049
Mas1	0.29992078	2.57691821	1.03342508	0.32155071	0.62581547
Zfp14	-0.2680005	3.10914981	1.03318514	0.3216055	0.62583159
Arhgef28	-0.1821898	4.27869738	1.0322456	0.32182018	0.62612755
Eri2	-0.2072032	4.18640994	1.03191923	0.32189479	0.62612755
Prr3	0.16348966	4.200943	1.03190877	0.32189718	0.62612755
Slc30a3	-0.200541	4.31403031	1.03139899	0.32201378	0.62614409
Ptn	0.28364582	10.3823205	1.03138843	0.3220162	0.62614409
Snx19	-0.1754	4.75984491	1.03079271	0.32215253	0.62614409
2210408F21I	-0.3315735	2.4430017	1.03076553	0.32215875	0.62614409

Bcas2	-0.1159855	5.77682026	1.03064602	0.32218611	0.62614409
Clstn3	0.22571773	4.49061883	1.03064102	0.32218726	0.62614409
Arhgap24	-0.2352227	3.94176612	1.03007313	0.32231731	0.62614409
Rpl36al	0.21872456	7.29476133	1.02984544	0.32236948	0.62614409
1700010I14R	0.57354294	-0.0902284	1.02942881	0.32246496	0.62614409
Rrm1	-0.1710826	4.32761159	1.02942486	0.32246587	0.62614409
Pam16	0.22594889	3.18915383	1.02908709	0.32254331	0.62614409
AA387883	-0.6369577	0.28824158	1.02882624	0.32260313	0.62614409
Lrpap1	0.19625196	5.78732057	1.02847617	0.32268343	0.62614409
Hn1l	0.27588051	4.36652087	1.02833086	0.32271678	0.62614409
Dynll1	0.15024142	8.0929737	1.02806169	0.32277855	0.62614409
Tm4sf1	-0.2104133	4.66416052	1.02782856	0.32283207	0.62614409
Thoc1	-0.1853853	4.76711734	1.02768744	0.32286447	0.62614409
Mad2l1bp	0.38538973	1.72148209	1.02763255	0.32287708	0.62614409
Fgd6	0.16827699	6.33846098	1.02755806	0.32289418	0.62614409
Tpm2	0.2565073	6.33843715	1.0275129	0.32290455	0.62614409
Ephx4	-0.157354	5.18556437	1.0271534	0.32298713	0.62614409
Rgs14	-0.3264861	2.19227833	1.0271242	0.32299384	0.62614409
Tctn3	-0.3720342	2.73399384	1.02699526	0.32302346	0.62614409
Rnase6	-1.2216269	-1.1019717	1.02693587	0.32303711	0.62614409
Adam5	0.79817447	0.8451942	1.02658464	0.32311783	0.62614409
Xpnpep3	0.1773612	4.78115851	1.02635934	0.32316963	0.62614409
Srd5a1	-0.3561347	2.56381811	1.02619439	0.32320756	0.62614409
Rab3gap1	-0.1815397	4.95786349	1.0260807	0.3232337	0.62614409
2610020H08	-0.5188923	0.73171678	1.02577743	0.32330346	0.62614409
Ccdc125	0.31231122	2.84858073	1.02565561	0.32333149	0.62614409
Cth	-0.5499786	0.94286867	1.02522348	0.32343093	0.62614409
02-Mar	-0.1715269	4.75045565	1.02510875	0.32345734	0.62614409
Atg14	-0.2169832	4.2719661	1.02501642	0.3234786	0.62614409
Lrrc8d	-0.1568	5.77363906	1.02497662	0.32348776	0.62614409
Cib2	-0.4148333	2.27751293	1.02449981	0.32359757	0.62626654
Olfr287	0.79225824	0.25108927	1.02412654	0.32368356	0.62630737
Ctso	0.22951622	5.03282715	1.02394726	0.32372488	0.62630737
Pi4k2a	0.17123214	4.91082753	1.02380229	0.32375829	0.62630737
Gm1045	1.61462905	-1.1786568	1.0229966	0.32394408	0.6264169
Prkcd	0.24535375	4.08542494	1.02268538	0.32401588	0.6264169
Cnot8	0.16169238	4.59883218	1.02262687	0.32402938	0.6264169
Il13ra1	0.20739275	4.87857504	1.02242947	0.32407494	0.6264169
Rnase1	-1.2072486	-0.8983568	1.02237687	0.32408708	0.6264169
Tmem256	-0.2860657	2.86839731	1.02218732	0.32413084	0.6264169
Dsc2	-0.8087432	-0.1495148	1.02147982	0.32429424	0.6264169
Rftn2	0.16886085	4.75878567	1.0214452	0.32430223	0.6264169
Ccrl2	-0.4719201	0.53143071	1.02137975	0.32431736	0.6264169

Mycbp	0.18985593	5.63413358	1.0213693	0.32431977	0.6264169
Ctsk	0.38208522	2.38531761	1.02119235	0.32436066	0.6264169
Ppapdc1a	0.76514603	-0.1787357	1.020969	0.32441228	0.6264169
Ccdc32	0.20272071	4.90145932	1.0209229	0.32442294	0.6264169
Pcsk6	-0.3275454	1.62600231	1.02062104	0.32449273	0.6264169
Brip1	-0.2836652	3.21142349	1.02039162	0.32454578	0.6264169
Pdik1l	-0.1903103	3.96548955	1.02033126	0.32455974	0.6264169
Gnpat	-0.1678781	5.40993141	1.01991608	0.32465579	0.62649397
Pdcl3	0.22313261	4.225778	1.01975619	0.32469279	0.62649397
Hcrtr1	-1.2873823	-1.4360629	1.01895198	0.32487898	0.62676335
Eif4a1	0.11482905	7.37071226	1.01831556	0.32502643	0.62695792
Naa35	0.13339555	6.08608385	1.01793644	0.32511431	0.6270144
Gm5124	-0.1618266	5.01792414	1.01778726	0.32514889	0.6270144
Pdgfrb	-0.2846361	4.4274663	1.01747727	0.32522078	0.62706317
Bfar	0.17212647	5.04974187	1.01594691	0.32557602	0.62724972
0610010K14I	0.23114628	3.74429142	1.01568412	0.32563707	0.62724972
Ap2b1	0.12796265	7.86662395	1.01547365	0.32568598	0.62724972
Alox5	-1.0321579	-1.3258855	1.01545901	0.32568939	0.62724972
Gm14057	0.52608373	2.1774852	1.01540119	0.32570282	0.62724972
Hn1	0.20603619	4.32365813	1.01540022	0.32570305	0.62724972
2810417H13I	-0.5980402	0.64624972	1.01539972	0.32570317	0.62724972
Gan	-0.2724732	3.39614427	1.0152106	0.32574713	0.62724972
Asb8	0.12241017	6.2702894	1.01504007	0.32578677	0.62724972
Gjb1	-0.8585178	-0.6787146	1.0149402	0.32581	0.62724972
Setx	-0.1940182	7.13268707	1.01470242	0.32586529	0.62724972
Slc22a12	1.02323136	0.35222828	1.01465251	0.3258769	0.62724972
Col6a1	-0.1639095	5.05740719	1.0144452	0.32592513	0.62725282
Tex13	-0.6390566	-0.5693191	1.01423001	0.3259752	0.62725946
Glcci1	-0.1180483	6.88902066	1.01374129	0.32608895	0.62738863
Il33	-0.1747823	5.16812239	1.01340966	0.32616617	0.62744749
Il15	0.36748129	1.90573341	1.01076295	0.32678336	0.62854493
Ripk3	-1.0576381	-0.6316331	1.01037744	0.32687339	0.62854707
Hmbs	0.23229646	3.04214054	1.01035819	0.32687789	0.62854707
Gm6588	1.15385883	-0.4709742	1.00958702	0.3270581	0.62880374
6330403A02I	0.16452944	7.47245341	1.00916121	0.32715766	0.62883949
Efcab4a	0.48200848	0.98823308	1.00892999	0.32721175	0.62883949
Zfp763	-0.2131304	4.04657361	1.00890798	0.3272169	0.62883949
Syngr2	0.42770378	1.68918734	1.00829698	0.32735987	0.62902443
Psmg2	-0.2492832	3.21681785	1.00768781	0.3275025	0.6291336
Erp29	0.22474808	4.632178	1.00744634	0.32755907	0.6291336
Incenp	0.22339515	3.47791435	1.00723578	0.3276084	0.6291336
Birc5	-0.5196604	0.28308296	1.00716844	0.32762418	0.6291336
Pmvk	-0.1810085	4.37566919	1.00669093	0.3277361	0.6291336

Mrpl17	0.13643934	5.65330269	1.00630507	0.32782659	0.6291336
BC055324	0.49336454	0.65282369	1.00626062	0.32783701	0.6291336
Phf20l1	-0.1675514	5.71701924	1.00602394	0.32789253	0.6291336
Stard10	0.21935054	3.3328825	1.00572051	0.32796373	0.6291336
Ercc5	0.15126894	5.02331105	1.00572026	0.32796379	0.6291336
Nagpa	-0.3250201	2.8786944	1.00555937	0.32800155	0.6291336
Nnt	0.14087576	5.08269873	1.00550853	0.32801348	0.6291336
Med25	0.23475892	2.48573837	1.00521223	0.32808304	0.6291336
Cdc42bpg	-0.3910836	1.33986723	1.00511599	0.32810564	0.6291336
Aldh4a1	0.21480054	4.20524868	1.00494208	0.32814648	0.6291336
Ccdc85b	-0.1821086	4.38375005	1.00461779	0.32822265	0.6291336
Tubb2b	0.1463912	5.27879931	1.00461536	0.32822322	0.6291336
Klhdc10	-0.1283918	6.89725649	1.00446625	0.32825826	0.6291336
Snrnp200	-0.185423	6.89140975	1.00403096	0.32836056	0.62920437
Mcur1	0.21696147	5.21029881	1.00387747	0.32839664	0.62920437
Hspb7	0.54393099	0.47307852	1.00353585	0.32847697	0.62920437
Zc3hav1l	-0.1794176	6.00486285	1.00351354	0.32848222	0.62920437
Hmga2	0.18864206	3.850615	1.00293434	0.32861848	0.6292896
Cd44	-0.2364055	3.13111967	1.00292686	0.32862024	0.6292896
Asxl2	-0.1372339	6.49427482	1.00170763	0.32890734	0.62974976
Dak	0.33528517	2.76656113	1.00105462	0.32906125	0.62995481
Foxd2os	-0.2940734	2.51119551	1.00061628	0.32916462	0.6300369
Loxl1	0.39163255	1.84589249	1.00047573	0.32919777	0.6300369
Golt1b	-0.1676376	5.46758268	1.00013301	0.32927864	0.63010205
1110006O24	0.87456322	-0.6314302	0.99974692	0.32936977	0.63010848
Cpb2	-1.3818016	-1.7620521	0.99972199	0.32937565	0.63010848
Wdfy1	0.18779381	4.67529189	0.99951105	0.32942546	0.63011418
Chst8	0.52514403	0.65193605	0.99924488	0.32948832	0.63014485
Ank1	0.22843754	5.05992668	0.99903722	0.32953737	0.6301491
Nfe2l2	0.16165464	7.84925387	0.9988343	0.32958532	0.63015123
Aldh3b1	0.50881735	0.86317787	0.99812324	0.3297534	0.63038302
Diap1	-0.1530858	4.50604362	0.99758175	0.32988147	0.63053829
Cstf2	0.17284736	6.37257671	0.99685939	0.33005244	0.63077547
Cldn8	-1.565009	-2.2535441	0.99646061	0.33014688	0.63079314
Drd3	0.86668632	-1.0424144	0.99642445	0.33015544	0.63079314
Coq5	0.12132783	6.38058398	0.99582977	0.33029634	0.63085779
Ccr10	1.20389166	-1.0131679	0.99579859	0.33030373	0.63085779
Atm	-0.1605963	5.99784166	0.99554101	0.33036479	0.63085779
Naip5	-0.3838555	1.51567196	0.99509649	0.3304702	0.63085779
Vstm5	-0.33044	2.30334241	0.99506952	0.33047659	0.63085779
Dancr	-0.7240398	-0.0093493	0.99504725	0.33048188	0.63085779
Grm3	-0.1482477	5.65836054	0.99467912	0.33056921	0.63085779
Pigr	-0.359811	3.83540318	0.99460937	0.33058576	0.63085779

Six5	-0.3444531	3.32592734	0.99411309	0.33070356	0.63085779
Neto2	-0.1958495	5.74021452	0.99390429	0.33075313	0.63085779
Mad2l2	0.29077846	3.11655822	0.99387027	0.33076121	0.63085779
Hint3	-0.1876333	4.30640939	0.99374496	0.33079097	0.63085779
Acvrl1	-0.4818556	0.83253302	0.99371224	0.33079874	0.63085779
Acad10	0.46156577	0.81067931	0.99294118	0.33098195	0.63104129
Kif6	-0.5696664	0.20610313	0.99288346	0.33099567	0.63104129
Kif13a	-0.1380493	5.84859562	0.99259669	0.33106385	0.63104129
Rhbdl1	-0.4187092	0.61950411	0.99251805	0.33108254	0.63104129
Seh1l	-0.1733915	5.64471097	0.99212708	0.33117553	0.63112913
1810062O18	0.62654211	0.63893738	0.9918597	0.33123915	0.63116098
Lama3	-0.3447871	2.5107146	0.99127053	0.33137938	0.63133879
Olig2	-0.2197729	3.43458375	0.99072635	0.33150899	0.63136178
Git2	0.13737264	5.44319972	0.99059442	0.33154042	0.63136178
Hivep2	-0.2440051	8.90992796	0.99048622	0.3315662	0.63136178
Marcks	0.15880204	9.44935108	0.99034544	0.33159975	0.63136178
Asb2	0.72445062	0.02670531	0.99023509	0.33162604	0.63136178
Bcmo1	1.19397604	-1.5031222	0.9895346	0.33179306	0.63152215
D8Ertd82e	-0.2102084	4.35499075	0.98948813	0.33180414	0.63152215
Tra2a	0.13972126	6.08364828	0.9890376	0.33191163	0.63163738
Vps54	-0.1230822	6.89711108	0.98860475	0.33201494	0.63174465
Ccdc55	0.12189379	6.97211916	0.98773872	0.33222178	0.63200718
Hinfp	0.32635036	1.97206335	0.98753632	0.33227015	0.63200718
Stag1	0.11762288	7.40633933	0.98727022	0.33233375	0.63200718
Marveld1	0.25832238	4.34164061	0.98724081	0.33234078	0.63200718
Pcdhga10	-0.1711814	3.54483059	0.98582559	0.33267934	0.63251128
Igdcc3	0.79556722	0.90123757	0.98562128	0.33272826	0.63251128
Rapgefl1	-0.1388242	6.31641623	0.98554349	0.33274689	0.63251128
Pter	0.20099541	3.4557309	0.98534727	0.33279388	0.63251128
Ehf	-0.5958383	0.60550469	0.98515087	0.33284093	0.63251136
Stxbp2	-0.2502995	4.19154777	0.98459412	0.33297435	0.63262909
Gm973	0.28464077	2.46480324	0.98440316	0.33302012	0.63262909
Aldh18a1	0.22408848	3.57618485	0.98430389	0.33304392	0.63262909
Tpt1	0.18946521	9.33039457	0.98408321	0.33309684	0.6326403
Arhgap11a	-0.2617685	3.16691669	0.98377177	0.33317154	0.63268124
Sf3a2	-0.2014377	3.0888591	0.98360134	0.33321243	0.63268124
Tbc1d10c	0.94489026	-0.871725	0.98194443	0.33361032	0.6330289
Pcdha8	-0.8217834	0.18925913	0.98128988	0.33376769	0.6330289
Fcho1	-0.2966007	2.7911638	0.98120625	0.3337878	0.6330289
Iqgap3	1.07413766	-0.6190251	0.9812013	0.33378899	0.6330289
Mob1a	0.21198408	3.39147199	0.98053542	0.3339492	0.6330289
Fam126b	0.15008018	8.29445503	0.9805018	0.3339573	0.6330289
Clip1	-0.1655186	7.59804925	0.98040691	0.33398014	0.6330289

Sema6b	-0.2897476	2.90012105	0.98036708	0.33398972	0.6330289
Zfp213	0.48108753	1.09284585	0.98023593	0.3340213	0.6330289
Tmem115	-0.3031046	2.20816434	0.98021031	0.33402747	0.6330289
Esam	-0.5179717	1.35258999	0.98017748	0.33403537	0.6330289
Clcn4-2	-0.122219	6.19279501	0.98008685	0.33405719	0.6330289
AI506816	0.99209049	-1.375879	0.97986182	0.33411138	0.6330289
N4bp2l2	0.11461452	6.28193823	0.97977829	0.3341315	0.6330289
Msl3l2	0.21902319	4.12858652	0.97939823	0.33422306	0.6330289
Fbxo33	-0.1719733	4.8653221	0.9790074	0.33431726	0.6330289
Nup50	0.13376655	5.45782005	0.9789855	0.33432253	0.6330289
B3gnt2	0.1700899	5.73961934	0.97893411	0.33433492	0.6330289
Ebi3	1.16254242	-1.0286193	0.97856747	0.33442333	0.6330289
Urod	-0.142183	5.0701814	0.97837217	0.33447043	0.6330289
Ankk1	-0.6937139	-0.2694108	0.97803488	0.3345518	0.6330289
Zcrb1	-0.1301102	6.95352091	0.97798592	0.33456361	0.6330289
Laptm5	-0.3580096	3.30029559	0.97765498	0.33464348	0.6330289
Actn2	-0.2769	2.19011377	0.97759781	0.33465729	0.6330289
Txnip	0.2282559	6.72781444	0.97757088	0.33466379	0.6330289
6530402F18I	0.48040719	1.42045997	0.9770293	0.33479456	0.6330289
Atp8b1	0.27621366	2.4639012	0.9767814	0.33485445	0.6330289
Afg3l1	0.25540814	3.28180885	0.97663153	0.33489066	0.6330289
A230046K03	-0.1844233	6.37811693	0.97662152	0.33489308	0.6330289
Popdc2	-0.633117	0.81035692	0.97651484	0.33491886	0.6330289
Necab3	-0.1995402	5.96483057	0.976261	0.33498021	0.6330289
Tomm22	0.17387825	5.96992969	0.97622351	0.33498927	0.6330289
Dmap1	0.23746978	3.50122349	0.97619016	0.33499733	0.6330289
Fibp	0.18589177	4.96051385	0.97560557	0.3351387	0.6330289
Rims3	-0.5051731	0.8032777	0.97557993	0.3351449	0.6330289
Hes1	-0.208754	3.68862672	0.97531599	0.33520875	0.6330289
Lsm5	0.38919748	1.66590589	0.97493975	0.3352998	0.6330289
Med12l	-0.2447251	5.30820352	0.97490001	0.33530942	0.6330289
Vmn2r57	0.60174844	0.70186386	0.97467422	0.33536408	0.6330289
Krtcap3	-0.7631572	-0.9806215	0.97446365	0.33541507	0.6330289
4933428G20	-0.4762684	1.69656661	0.97444572	0.33541941	0.6330289
Lrrc7	-0.2531274	8.01311351	0.97443534	0.33542193	0.6330289
Ube2cbp	-0.8340046	0.2999942	0.97431265	0.33545164	0.6330289
9530027J09F	-1.2869644	-0.9858513	0.97419936	0.33547908	0.6330289
Acaa1b	0.47970519	0.75719299	0.97406149	0.33551248	0.6330289
Ralgps2	-0.1228875	6.33811271	0.97186059	0.33604628	0.63381115
Cd200r1	-0.6921703	-0.6579988	0.97157926	0.33611459	0.63381115
Ufd1l	0.11104433	6.36786093	0.97144446	0.33614734	0.63381115
Upf3a	0.1944266	5.8631165	0.97126389	0.3361912	0.63381115
Ets1	0.16710847	4.0399327	0.97120649	0.33620515	0.63381115

Dexi	0.17736396	3.84167745	0.97098251	0.33625957	0.63381115
Acaca	-0.2176977	5.67826567	0.97085959	0.33628945	0.63381115
Calcr	0.71400804	0.29845713	0.97080017	0.33630389	0.63381115
Kcng2	-0.5783822	0.29275489	0.97057041	0.33635974	0.63382764
Rab27b	0.15944341	5.41007318	0.97009655	0.33647498	0.63387224
Plcg2	-0.2921023	1.88534038	0.9699026	0.33652216	0.63387224
9530051G07	0.50042493	0.85505439	0.96980951	0.33654481	0.63387224
Lrrfip2	-0.1235045	5.83690377	0.96905066	0.33672952	0.63387224
Eml4	-0.1342448	6.15080929	0.96865777	0.33682521	0.63387224
Ppil4	0.13516856	5.95392326	0.96856067	0.33684886	0.63387224
Ndufs1	0.16013647	6.10677345	0.96781105	0.33703155	0.63387224
Dicer1	-0.147307	6.2937579	0.96772264	0.33705311	0.63387224
Cep104	0.15175806	4.65266436	0.96743828	0.33712246	0.63387224
Rhpn1	-0.3905144	0.76521571	0.96730457	0.33715507	0.63387224
Klhl26	-0.1839221	3.63165044	0.96728231	0.3371605	0.63387224
Sdhaf1	0.2801117	2.31989015	0.9672603	0.33716587	0.63387224
Nhs	-0.2201019	3.61151607	0.96674139	0.33729249	0.63387224
Sugp2	-0.2733283	3.99885619	0.96654934	0.33733937	0.63387224
4921511H03l	-1.2009467	-1.1054533	0.96650106	0.33735115	0.63387224
4933427I22R	-1.2631153	-1.2003321	0.96623694	0.33741564	0.63387224
Oplah	-0.2912366	1.8057182	0.96594222	0.33748762	0.63387224
Zfp945	0.1504136	5.18265876	0.96589663	0.33749876	0.63387224
Adam28	-0.5716063	0.2058532	0.96587982	0.33750287	0.63387224
Hnrnpf	0.18390535	6.78931311	0.96565599	0.33755755	0.63387224
1700047A11l	-1.4640786	-1.7041524	0.96560446	0.33757014	0.63387224
Ppapdc3	-0.3181659	2.36618951	0.96558176	0.33757569	0.63387224
Gm10440	1.66333823	-1.531503	0.9655498	0.3375835	0.63387224
2810025M15	-0.2736295	2.41788734	0.9655477	0.33758401	0.63387224
Mfsd7c	0.49293517	1.40256348	0.96530269	0.33764389	0.63387224
Ctnnb1	0.106718	9.22493966	0.9650217	0.33771259	0.63387224
Trap1	0.17938597	4.50678277	0.96489249	0.33774418	0.63387224
Itgb2	0.41228477	1.44270914	0.96486051	0.337752	0.63387224
Mbip	-0.2596242	3.81516301	0.96471175	0.33778838	0.63387224
Gm14440	-0.1535838	5.28812743	0.96427678	0.33789479	0.63387224
Stub1	-0.4647373	0.86634592	0.9642387	0.33790411	0.63387224
Itih3	-0.4211234	1.81272516	0.96411025	0.33793554	0.63387224
Jmjd8	-0.2230313	3.88038761	0.96410059	0.3379379	0.63387224
Spata2l	-0.2059104	4.60710982	0.96347005	0.33809227	0.63398604
Ccdc40	0.64518855	0.06775867	0.96303987	0.33819763	0.63398604
Serinc1	0.1248904	8.80303299	0.96285254	0.33824353	0.63398604
3110043O21	-0.2349705	3.97870213	0.9628209	0.33825129	0.63398604
Sike1	0.11727378	6.60274024	0.96272601	0.33827454	0.63398604
Ranbp3l	0.18596346	7.77261109	0.96269859	0.33828126	0.63398604

Glyr1	0.15067861	5.78046531	0.96233386	0.33837067	0.63400478
Gtf2h2	0.14500176	4.9605787	0.96227339	0.33838549	0.63400478
Rrp8	0.20228805	4.19096313	0.96194031	0.33846717	0.63403589
Zbtb33	0.14949181	5.58479729	0.96182142	0.33849634	0.63403589
Rpl22	-0.2012595	6.20026759	0.96095261	0.33870955	0.63413273
BC027231	0.21548802	3.97245081	0.96054277	0.33881019	0.63413273
Hdac8	-0.3270273	1.90663571	0.96034337	0.33885918	0.63413273
Slc24a1	0.85771616	-0.0401307	0.96032631	0.33886337	0.63413273
Pbx2	-0.1741915	4.84810198	0.95998986	0.33894604	0.63413273
Nploc4	0.13914118	5.5117451	0.95983702	0.3389836	0.63413273
Otud3	-0.3382656	1.74259567	0.95964852	0.33902994	0.63413273
Gucy1b3	-0.1560482	6.61329345	0.95960588	0.33904042	0.63413273
Agtr1b	-0.5928254	2.19031854	0.95950605	0.33906497	0.63413273
Siah1b	-0.3714892	1.8626382	0.95945262	0.33907811	0.63413273
Dbhos	-0.3133959	3.35818867	0.95926881	0.33912331	0.63413273
Ghitm	0.10878459	8.70164854	0.95922731	0.33913352	0.63413273
Dlg4	-0.1234035	7.45345862	0.95911694	0.33916066	0.63413273
BC037032	-0.5499783	0.53871092	0.95879087	0.33924088	0.6341362
Dars	0.13728191	5.23673454	0.95852861	0.33930543	0.6341362
Kcna5	-0.4618106	0.85548491	0.95848282	0.33931669	0.6341362
Rgag4	-0.2896738	2.68256884	0.95821575	0.33938244	0.6341362
Tssk2	1.69642085	-1.7611136	0.95798866	0.33943836	0.6341362
Myot	-1.1592989	-2.0569196	0.95796057	0.33944527	0.6341362
Map4	-0.1094318	9.19688123	0.95757659	0.33953986	0.63419699
Tmem55a	-0.1357178	6.17654235	0.95707514	0.33966343	0.63419699
Src	-0.2448112	3.27587709	0.95698257	0.33968625	0.63419699
Tgtp2	-0.2631578	3.77523968	0.9568499	0.33971896	0.63419699
Ntpcr	0.26774147	2.61603098	0.95669435	0.33975731	0.63419699
Foxo6	0.26306843	2.60702137	0.95668104	0.33976059	0.63419699
Ccdc149	-0.1789921	4.44351434	0.9560802	0.3399088	0.63438563
1700013F07I	-0.5359507	-0.0736069	0.95543151	0.34006891	0.63451904
Dnajc24	-0.1880908	3.81975444	0.95513033	0.34014329	0.63451904
Itga6	-0.2370809	3.55203255	0.9549218	0.3401948	0.63451904
Bet1	0.208024	4.8666255	0.95465009	0.34026193	0.63451904
Timm9	-0.1580327	4.92435511	0.95394845	0.34043537	0.63451904
Rcn1	0.24119478	4.76303695	0.9539111	0.34044461	0.63451904
Tgm3	-0.2509544	2.62821885	0.95371457	0.34049321	0.63451904
Gm7361	-0.7395896	-0.8434527	0.95359818	0.340522	0.63451904
Usp39	0.18172593	4.1659872	0.95345405	0.34055766	0.63451904
Kdm4c	-0.1972877	5.52623136	0.95335226	0.34058285	0.63451904
Ndc80	-0.8561309	-0.3951648	0.95297347	0.34067659	0.63451904
Sh2b3	0.2207046	3.5835136	0.95280389	0.34071857	0.63451904
Ptpn21	-0.1820301	4.8586225	0.95266949	0.34075185	0.63451904

Bco2	-0.6747179	0.95810225	0.95254086	0.3407837	0.63451904
Scn1b	-0.1228377	5.60432202	0.95250905	0.34079158	0.63451904
1600029O15	0.40671842	1.62135196	0.9521919	0.34087013	0.63451904
D2Wsu81e	0.26331391	2.04981063	0.95189283	0.34094423	0.63451904
Gm5089	0.24810281	4.78982062	0.95172853	0.34098495	0.63451904
Asphd1	-0.2495154	2.41463671	0.95138039	0.34107125	0.63451904
Ckmt2	-1.1728488	-1.0422931	0.95101059	0.34116295	0.63451904
Agap3	-0.1276819	5.85847907	0.9509474	0.34117863	0.63451904
Abcb8	-0.1744186	4.05750279	0.95063995	0.3412549	0.63451904
Gtdc1	-0.1622982	6.26817212	0.950471	0.34129683	0.63451904
B930025P03	1.10385744	-1.1895272	0.95036448	0.34132326	0.63451904
Prkacb	-0.10961	9.17765633	0.95034853	0.34132722	0.63451904
Bzrap1	-0.3132658	4.8099904	0.95020565	0.34136269	0.63451904
Tnpo1	0.11993943	6.64202774	0.95017622	0.34136999	0.63451904
Ube2g1	0.13696398	6.83465287	0.9501222	0.3413834	0.63451904
Six4	0.27696118	3.45258233	0.95011892	0.34138422	0.63451904
Ing2	-0.1460982	5.0945217	0.94979597	0.3414644	0.63451904
Zfp180	0.12895697	5.1210023	0.94945641	0.34154875	0.63451904
Zik1	0.2058997	3.46310341	0.94932215	0.3415821	0.63451904
Adora2b	-0.5736283	0.44978686	0.94917732	0.34161809	0.63451904
Scin	1.03935028	-0.5378997	0.94913168	0.34162943	0.63451904
Fgf9	-0.1814153	4.84515337	0.949049	0.34164998	0.63451904
Rab21	0.12762479	7.62913437	0.94893697	0.34167782	0.63451904
Grk4	-0.2696536	4.63754829	0.94873987	0.34172682	0.63452246
Gpr12	-0.2219502	4.05832662	0.94829122	0.34183838	0.63455484
1700101E01I	-1.1900656	-0.8806146	0.9481866	0.34186441	0.63455484
Lsm4	-0.2562453	3.33172149	0.94810089	0.34188573	0.63455484
Dopey1	-0.2252397	5.58148673	0.94768406	0.34198945	0.6346598
Pp2d1	-0.4627501	0.70436595	0.94737538	0.34206628	0.63471487
Klhdc8a	-0.2076596	3.8166038	0.94683864	0.34219995	0.63483486
P4ha2	-0.2918435	1.85672875	0.94673684	0.34222531	0.63483486
Arhgap9	-0.6882764	0.95300811	0.94652617	0.3422778	0.63484471
Def8	-0.1641479	4.42867502	0.94598747	0.34241206	0.63500622
Gm608	-0.1263666	8.24058658	0.94573111	0.34247599	0.63503725
Nccrp1	-1.1389924	-1.1306751	0.94513272	0.34262526	0.6352265
Tmem154	-0.3099472	3.39839812	0.9441723	0.34286504	0.63550261
Intu	-0.204467	4.51360505	0.94411805	0.34287859	0.63550261
Stk39	0.16853912	7.75126051	0.94396882	0.34291587	0.63550261
Alox15	-1.6872202	-1.1973716	0.94371216	0.34298	0.63553393
Ipo8	-0.1554377	5.43226085	0.94326599	0.34309152	0.63565305
Immp1l	0.14865621	5.4472159	0.94257669	0.34326392	0.63588491
Vps13d	-0.2267224	6.62824952	0.94236675	0.34331645	0.63589468
Ccdc28a	0.27479061	2.50024667	0.94176411	0.34346731	0.63596151

Lin52	-0.1546292	4.53970168	0.94166533	0.34349204	0.63596151
Rfc1	0.11181602	5.87408846	0.94165626	0.34349431	0.63596151
Slc4a8	-0.2100262	6.84351407	0.94129121	0.34358575	0.63603202
Unc119b	0.2357532	4.03047117	0.94112685	0.34362693	0.63603202
Stx5a	0.18446531	4.58266496	0.94039112	0.34381135	0.63628584
E330023G01	-0.4078484	1.42806035	0.94009474	0.34388568	0.63631861
Id2	-0.2025413	6.09138023	0.93980956	0.34395723	0.63631861
Srcin1	-0.2478713	6.08474988	0.93975499	0.34397092	0.63631861
Fam195a	0.86853787	-1.2383846	0.93913788	0.34412582	0.63640383
Bcl2a1d	-0.3875697	1.18069673	0.93886447	0.34419447	0.63640383
Aspn	-0.9694996	-0.6780731	0.938857	0.34419635	0.63640383
Dagla	-0.1500415	5.43864015	0.93879798	0.34421117	0.63640383
Il18rap	0.85632782	-0.4302662	0.93844321	0.3443003	0.63640383
Tldc1	-0.393857	1.15750519	0.9383325	0.34432812	0.63640383
Rprd1a	-0.1309579	6.72617376	0.93824548	0.34434999	0.63640383
Lrrc36	-0.706859	-0.0502323	0.93806503	0.34439534	0.63640383
Tbc1d13	0.19841037	4.34928635	0.93748453	0.3445413	0.63658613
Snord99	1.2591061	-1.8805611	0.93712288	0.34463228	0.6366668
Zfp558	0.3719272	2.74543963	0.93564697	0.34500391	0.63726587
Pdlim7	0.17090754	4.32830393	0.93456709	0.34527618	0.6376107
Mef2d	0.12622441	7.08971438	0.93424147	0.34535834	0.6376107
Ppwd1	-0.1797205	3.72394038	0.93407933	0.34539926	0.6376107
Mlycd	-0.2004213	3.16265208	0.93384092	0.34545945	0.6376107
Pard3b	0.21567437	3.32297747	0.93372839	0.34548786	0.6376107
Prkx	0.18836211	4.57559272	0.93370373	0.34549409	0.6376107
Zbtb6	-0.133511	5.09024405	0.93359206	0.34552228	0.6376107
Ogt	-0.1997945	7.68691781	0.93316259	0.34563076	0.63772343
Asb6	-0.2421893	2.52934525	0.93257963	0.34577809	0.63778373
Lrp12	0.15301362	4.10002591	0.93252065	0.345793	0.63778373
Wdr5	-0.3059424	3.23638315	0.93243873	0.34581371	0.63778373
Rnf8	-0.1980486	4.25082204	0.9322832	0.34585303	0.63778373
D930016D06	-0.2728242	4.25689725	0.93159987	0.34602589	0.6378187
Lhcgr	-1.1676433	-1.0558066	0.93148479	0.34605501	0.6378187
Supt20	-0.1587235	4.95552167	0.93146354	0.34606039	0.6378187
Zfp956	0.38396025	2.29736313	0.93141234	0.34607335	0.6378187
Rpl37a	-0.2026036	5.95519467	0.93084858	0.34621608	0.6378187
Zkscan8	-0.1404726	6.10581544	0.93052371	0.34629837	0.6378187
Plekha1	-0.1209268	6.73437233	0.93040094	0.34632947	0.6378187
Loxl2	-0.230592	3.97633276	0.93020938	0.34637801	0.6378187
Adrbk1	0.15799573	5.09070677	0.93020425	0.34637931	0.6378187
M1ap	-1.4672583	-1.5333077	0.93019273	0.34638223	0.6378187
Otx2	-0.6985363	1.57621485	0.93013985	0.34639563	0.6378187
Ing1	0.18266532	4.90266404	0.92983268	0.34647349	0.6378187

Bex4	-0.2108496	4.0910986	0.92976267	0.34649124	0.6378187
Atf6b	-0.2143678	3.13973333	0.9295878	0.34653558	0.6378187
Slc48a1	0.1614209	4.84507852	0.92874128	0.34675034	0.6381051
Chchd6	-0.2143614	3.43106469	0.92860067	0.34678603	0.6381051
Lamtor3	0.18192275	5.61417099	0.9280256	0.34693205	0.63812674
Neo1	0.13557825	7.54444732	0.92791401	0.34696039	0.63812674
Snord71	-1.5333344	-1.3463177	0.92785737	0.34697478	0.63812674
Lancl1	0.14145832	6.54720907	0.92780739	0.34698748	0.63812674
2610028E06I	0.77774559	-0.2727317	0.92737082	0.34709842	0.63824353
Clcn7	0.23646417	3.02906878	0.92707917	0.34717256	0.63829264
4933416C03I	-1.4725349	-1.228785	0.92677172	0.34725073	0.63834915
Pold3	-0.1551064	4.88174881	0.92627713	0.34737655	0.63843608
Xpnpep2	0.89223287	0.25433481	0.92621284	0.34739291	0.63843608
Snhg7	-0.3565727	1.28793666	0.92562856	0.34754164	0.63861373
Srprb	0.21419166	3.49806931	0.92543835	0.34759008	0.63861373
4930480K15I	-0.4863388	2.7430116	0.92527058	0.34763281	0.63861373
Pcdh15	0.23848089	4.87020321	0.92488462	0.34773115	0.63861373
Sf3b4	0.16446925	4.65157292	0.92474931	0.34776563	0.63861373
Oaz1	-0.1597507	5.13371728	0.92452953	0.34782165	0.63861373
Lhx8	-0.3183545	2.6491906	0.92443521	0.34784569	0.63861373
Pxk	0.14034913	5.46625676	0.92434285	0.34786924	0.63861373
Spsb3	0.25870146	2.64510332	0.92392298	0.34797632	0.63865536
Rps6	0.18783149	8.46228235	0.92388173	0.34798684	0.63865536
Pknox2	-0.1330864	7.21215062	0.92357551	0.34806497	0.63871163
6330416G13	-0.1987473	3.76233321	0.92336216	0.34811942	0.63872444
Ermap	-0.7163732	0.46988567	0.92172144	0.34853854	0.63937522
Gm20750	1.18879199	-1.2381917	0.9215157	0.34859114	0.63937522
Gm2694	0.59844903	0.04466497	0.92131633	0.34864213	0.63937522
1700017G19	-0.5504053	0.70194423	0.92109982	0.34869752	0.63937522
Pcyt1b	0.17105049	5.32802086	0.92104447	0.34871168	0.63937522
H2afx	-0.2009169	3.05993906	0.92040297	0.34887586	0.63958911
Amer1	-0.185424	4.17907539	0.91983748	0.34902068	0.63976744
Hist3h2a	-0.5126788	0.54765609	0.91960927	0.34907915	0.63978746
Mrpl10	-0.1629024	4.71819044	0.91882062	0.34928131	0.64003613
Sertad3	-0.6339379	0.88470497	0.91861293	0.34933458	0.64003613
Dsg2	0.27157113	3.13330013	0.91852208	0.34935788	0.64003613
Rabep1	-0.1106146	7.91370563	0.91789207	0.34951955	0.64003613
Clvs2	0.22711529	4.43268842	0.9178581	0.34952827	0.64003613
Atraid	0.16486248	4.54314961	0.91785766	0.34952838	0.64003613
C530005A16I	0.23069853	3.24968215	0.91778212	0.34954778	0.64003613
Sec31a	0.09922894	7.07424304	0.9169852	0.34975245	0.64023104
Amdhd2	0.34021269	1.22239547	0.91697649	0.34975469	0.64023104
Itga7	-0.8182658	-0.3260729	0.91664085	0.34984094	0.64023104

Higd1a	-0.1284181	6.64605834	0.91662686	0.34984454	0.64023104
Osbpl10	0.38834539	2.18542351	0.91481721	0.35031012	0.64095467
Hadh	0.19163911	4.85452117	0.91471976	0.35033522	0.64095467
Mad2l1	-0.2425846	4.07790964	0.91419897	0.35046938	0.64102913
Dpysl3	-0.105013	6.30285504	0.91419196	0.35047119	0.64102913
Ppp1r13l	-0.3608527	1.34262441	0.91370996	0.35059543	0.6411124
Mansc1	0.20677969	4.31736911	0.91364567	0.35061201	0.6411124
Gpc1	0.23514837	3.38315005	0.91322086	0.35072156	0.64121607
Foxd2	-0.3457412	2.72874182	0.91305615	0.35076405	0.64121607
Stag3	1.65458576	-1.6816123	0.91281495	0.35082629	0.64121607
4933412E12I	-0.3229778	1.84800618	0.91268702	0.35085931	0.64121607
Gbp8	0.41339893	1.80266192	0.91236299	0.35094295	0.64128184
Vps72	-0.3437053	2.61179427	0.91189202	0.35106458	0.64128688
Sox1	0.18938297	4.72724917	0.91170566	0.35111273	0.64128688
Fam179b	-0.1224379	6.53940217	0.91139342	0.35119341	0.64128688
Ctdsp1	0.17974468	6.68530634	0.91130242	0.35121693	0.64128688
Rbl1	0.40600992	1.59039662	0.91127015	0.35122527	0.64128688
Pnkd	-0.1237379	6.53540609	0.91113141	0.35126114	0.64128688
Dscam	-0.1614078	5.64786648	0.91106112	0.35127931	0.64128688
Nr1i3	-0.9996109	-1.1087511	0.91065119	0.35138531	0.64139338
Fig4	-0.1625058	4.3065797	0.90993891	0.35156962	0.6415199
Repin1	0.17196127	4.53713337	0.90963686	0.35164781	0.6415199
Btbd6	-0.1612357	4.22448594	0.90951576	0.35167917	0.6415199
Ophn1	-0.1786561	4.9296589	0.90934242	0.35172406	0.6415199
Usp36	-0.1779215	4.61989909	0.90888344	0.35184297	0.6415199
Rgs4	-0.1272134	9.56276024	0.90865764	0.35190149	0.6415199
Kars	0.14900358	5.13841216	0.908504	0.35194132	0.6415199
4933406F09I	-1.4582901	-1.1412617	0.9083947	0.35196965	0.6415199
Pola1	-0.2632113	2.66497059	0.90828038	0.35199929	0.6415199
Camkk1	0.19947768	4.88035327	0.90821288	0.3520168	0.6415199
Thrap3	0.1143495	7.46786872	0.90818872	0.35202306	0.6415199
Mroh8	0.70827578	-1.0031828	0.90799118	0.35207429	0.6415199
Baz1a	-0.173693	4.11718974	0.90797333	0.35207892	0.6415199
Ppp1r3b	-0.2195761	4.93530695	0.90780701	0.35212207	0.6415199
Gpr162	0.39661569	2.14572844	0.90756661	0.35218444	0.64154668
Cxcl12	-0.1484604	6.01762405	0.90722185	0.35227392	0.64162282
Lactb2	0.21578783	3.85021558	0.90665407	0.35242135	0.64180448
Camsap2	-0.1466439	8.77325357	0.90550839	0.35271911	0.6422479
Mief2	-0.4096455	1.96346521	0.90535004	0.3527603	0.6422479
Gemin6	0.20784303	2.96628022	0.90448804	0.3529846	0.64249954
Gstm2	0.29816272	5.29499627	0.90445192	0.35299401	0.64249954
Tgfbi	-0.526212	2.29086337	0.90366415	0.35319919	0.64270266
Ankfn1	-0.3724519	2.48226336	0.9036567	0.35320113	0.64270266

Gbf1	-0.1455366	5.9862477	0.90309332	0.35334797	0.64288293
Gbx2	-1.4111328	-1.7696169	0.90239745	0.35352946	0.64312618
Prickle3	-0.3408873	2.54428717	0.90142636	0.35378296	0.6433598
Pmp22	0.21867771	8.57001292	0.90117558	0.35384847	0.6433598
Lpcat1	-0.2914686	2.86572616	0.90103052	0.35388636	0.6433598
Sfxn1	-0.1236255	5.91828744	0.90093299	0.35391185	0.6433598
Sgpp1	-0.1186059	6.5640417	0.90087887	0.35392599	0.6433598
Eif4e	-0.1026613	7.4070904	0.90077471	0.35395322	0.6433598
Slc7a5	0.18676475	4.14321219	0.90062418	0.35399256	0.6433598
LOC1000389	1.25305493	-1.5510624	0.90035498	0.35406294	0.64340081
Zfp959	0.28935966	1.73695519	0.89945221	0.3542991	0.64371978
Fez1	-0.1339158	5.52476133	0.89931833	0.35433415	0.64371978
Snx11	-0.2198021	3.09313506	0.8988783	0.35444936	0.64381646
Txndc5	0.15641301	4.95852922	0.89868112	0.354501	0.64381646
Ssfa2	-0.127276	5.4928508	0.89856699	0.3545309	0.64381646
Myo9a	-0.1745662	8.3603113	0.89741433	0.35483304	0.6442782
Nanp	-0.2196151	4.00038521	0.89627345	0.35513246	0.6447212
Cnbp	0.141081	9.17261251	0.89601175	0.3552012	0.6447212
Cpne6	-0.220688	5.07405814	0.89567797	0.35528889	0.6447212
Klhl1	-0.2840615	3.01700362	0.89567701	0.35528915	0.6447212
C130026L21	-0.4470213	1.06076622	0.8955726	0.35531658	0.6447212
Ddx41	0.19889002	3.8381311	0.89532355	0.35538204	0.64475304
Lrrc14b	-0.2627957	2.80334172	0.89496092	0.35547739	0.64476517
Fbxw9	-0.3545103	2.04056221	0.89453763	0.35558872	0.64476517
Arhgef19	0.39286111	1.47445845	0.89428972	0.35565396	0.64476517
Slc35a4	0.15775444	5.95206393	0.89413579	0.35569447	0.64476517
Ppp2cb	0.10435828	7.09415398	0.89410228	0.35570329	0.64476517
Khk	-0.4455474	2.21443771	0.89382734	0.35577567	0.64476517
Itfg1	0.11819656	7.75586571	0.8936757	0.3558156	0.64476517
Lca5l	-0.4829874	1.38066019	0.89351475	0.35585798	0.64476517
Klhl42	0.1141691	6.43862941	0.8932526	0.35592704	0.64476517
Snx18	0.13385886	6.1685314	0.89316341	0.35595053	0.64476517
Orc1	0.38371191	1.46819534	0.89288263	0.35602452	0.64476517
Gm8234	-0.2369707	3.69496776	0.89243394	0.35614281	0.64476517
Slc17a8	0.29114174	2.68306358	0.89242314	0.35614565	0.64476517
Gstp2	0.16742965	4.53982424	0.89232912	0.35617045	0.64476517
Nedd1	0.27909517	2.72720241	0.89223567	0.35619509	0.64476517
Igfbp5	0.20530976	8.64917915	0.89203883	0.35624701	0.64476517
Ptger2	-0.7561385	-0.470083	0.89199782	0.35625783	0.64476517
Soga3	-0.1657487	6.51710607	0.89199336	0.35625901	0.64476517
Cd164l2	1.11293283	-1.0215828	0.89184131	0.35629912	0.64476517
E030018B13	1.39498523	-1.4538829	0.89042109	0.35667412	0.64507359
Uchl4	0.73515835	-0.6099704	0.89028323	0.35671056	0.64507359

Slc25a37	-0.1449209	5.36740566	0.89020921	0.35673012	0.64507359
Zfp369	0.17044179	5.24517541	0.88971541	0.35686067	0.64507359
2310061J03F	0.352004	2.3589709	0.88940323	0.35694323	0.64507359
Adra2b	0.59564971	0.18659244	0.88927968	0.35697592	0.64507359
Rabl2	-0.1993973	3.87155561	0.88919344	0.35699874	0.64507359
Gm7444	-0.6488432	0.29348424	0.88901768	0.35704525	0.64507359
Zfp119b	0.46936989	0.7155587	0.88900356	0.35704898	0.64507359
Tmem186	-0.2610754	2.68750825	0.88881463	0.35709899	0.64507359
Slc35f4	-0.2496458	2.69234322	0.88881059	0.35710006	0.64507359
Frem2	-0.330966	1.81353757	0.88880268	0.35710215	0.64507359
Cgref1	0.27191708	2.1814467	0.88871199	0.35712616	0.64507359
Tada3	0.15549298	5.0122739	0.88800298	0.35731392	0.64507359
Nsfl1c	0.15756558	5.06797912	0.88780558	0.35736622	0.64507359
Rela	0.1916093	4.25629368	0.88776311	0.35737748	0.64507359
Snap25	0.10416704	14.1167379	0.88764396	0.35740905	0.64507359
Xpc	-0.1848637	3.9902574	0.88761561	0.35741657	0.64507359
Hexb	-0.1577435	4.74106074	0.88749166	0.35744942	0.64507359
Hic1	0.27950082	3.26674791	0.88733863	0.35748999	0.64507359
Capns1	0.17620752	9.23059355	0.88704479	0.3575679	0.64507359
9430008C03I	0.38161599	1.71279956	0.88691781	0.35760158	0.64507359
Nfkbia	0.26473314	2.74707365	0.88683606	0.35762326	0.64507359
Zfp94	-0.271006	2.68639115	0.88675438	0.35764493	0.64507359
C1galt1	0.19058808	4.66589561	0.8865282	0.35770494	0.64507359
Lhx2	0.14935049	5.46482425	0.88620314	0.3577912	0.64507359
Acyp2	0.14998554	4.62581168	0.8855762	0.35795767	0.64507359
Cep152	-0.3909726	1.78541853	0.88516935	0.35806576	0.64507359
Rgs7	-0.1340937	7.1722786	0.88493735	0.35812742	0.64507359
Acad8	0.14707623	4.24425617	0.88474001	0.35817988	0.64507359
Nicn1	0.111528	6.46661073	0.8846611	0.35820085	0.64507359
Tdrd9	-1.2349198	-1.9587224	0.88453149	0.35823532	0.64507359
Kcna4	0.20450378	5.66132831	0.88449486	0.35824506	0.64507359
Rilpl1	0.15670803	5.94586796	0.88448296	0.35824822	0.64507359
Carhsp1	-0.2595805	5.37283522	0.88447104	0.35825139	0.64507359
Dennd4b	-0.2386447	3.90586378	0.88441936	0.35826513	0.64507359
Ndufs2	0.14504228	6.6411601	0.88434987	0.35828362	0.64507359
Gbgt1	0.55593918	0.52653388	0.88410068	0.3583499	0.64507359
Lepr	0.19163168	5.63711491	0.8839099	0.35840066	0.64507359
Cdk17	-0.1363692	7.87426799	0.88390978	0.35840069	0.64507359
Afap1l1	-0.1829873	5.54664804	0.88378074	0.35843503	0.64507359
Trmt10a	-0.2278545	2.92068667	0.88350622	0.35850809	0.64510708
Brsk2	-0.1586441	5.66464878	0.88335061	0.35854952	0.64510708
Lsm6	0.13679751	5.28972953	0.88227151	0.35883699	0.64522747
Rpf2	0.14676445	4.36084234	0.88222081	0.35885051	0.64522747

Plrg1	0.14987922	4.51438789	0.88186431	0.35894556	0.64522747
Ift172	-0.2586255	4.63791237	0.88184753	0.35895003	0.64522747
Cpne7	0.29213403	2.63343277	0.88180812	0.35896054	0.64522747
Gm5069	0.15667428	4.42178415	0.88165974	0.35900011	0.64522747
A730046J19F	0.621421	0.59754944	0.88151518	0.35903868	0.64522747
Tank	0.17212005	6.08627914	0.88150367	0.35904175	0.64522747
Rnf157	-0.1594596	7.48577637	0.88134978	0.3590828	0.64522747
Cndp2	-0.1563206	4.36161415	0.88110484	0.35914817	0.64522747
Tonsl	-0.575462	0.46502871	0.88090329	0.35920196	0.64522747
Xkr6	-0.3134806	2.9998954	0.88029203	0.35936519	0.64522747
C230004F18I	-0.2891557	3.88591467	0.88026348	0.35937281	0.64522747
Liph	1.61590092	-1.6566254	0.88018966	0.35939254	0.64522747
1700008F21I	0.5828666	0.94287761	0.88011698	0.35941195	0.64522747
Anxa6	0.16351992	5.87471101	0.88011403	0.35941274	0.64522747
Hykk	0.18953806	5.34662653	0.87966335	0.35953318	0.64522747
Fto	-0.1113568	7.00755629	0.87956245	0.35956015	0.64522747
Zfp112	-0.348682	2.14004169	0.8793775	0.35960959	0.64522747
Pgrmc1	0.18627406	7.58892138	0.87918679	0.35966059	0.64522747
Trak2	-0.1275738	6.83064815	0.87911121	0.35968081	0.64522747
Sbf2	-0.129799	7.08779668	0.87902914	0.35970276	0.64522747
Gm15413	0.96640406	-0.5351781	0.87875671	0.35977564	0.64522747
Rab42	-0.9433578	-1.7234385	0.87847544	0.3598509	0.64522747
Fam20b	-0.1440784	4.91662611	0.87804763	0.35996543	0.64522747
Decr2	-0.133712	5.53858173	0.87788747	0.36000832	0.64522747
Timd4	1.61559138	-2.1091428	0.87757298	0.36009255	0.64522747
Lmx1b	0.98692196	0.19280357	0.87729197	0.36016785	0.64522747
Zfp879	-0.3551435	2.11439961	0.8772818	0.36017057	0.64522747
Gbp9	-0.2320199	3.98239406	0.87706194	0.3602295	0.64522747
4931428F04I	0.40354872	1.13749693	0.87706102	0.36022975	0.64522747
1110038F14I	0.25653689	3.35510242	0.87704092	0.36023513	0.64522747
Vma21	-0.1241329	6.21773459	0.87703042	0.36023795	0.64522747
Man2c1	-0.3246987	2.65960172	0.87695606	0.36025788	0.64522747
Hdhd2	0.1320618	5.52610197	0.87681882	0.36029467	0.64522747
Meis1	0.43355725	1.67791817	0.87648056	0.36038538	0.64525588
BC021891	-0.3832419	1.57565952	0.87614156	0.36047632	0.64525588
Ankle2	-0.1270502	5.23852826	0.87588063	0.36054634	0.64525588
BC002163	0.16069776	3.58141497	0.87574954	0.36058153	0.64525588
Igf2os	-0.3167189	3.56174372	0.87574406	0.360583	0.64525588
Wdfy4	-0.383052	1.56055721	0.87562837	0.36061405	0.64525588
Tubgcp4	0.18215432	3.73131625	0.87550862	0.3606462	0.64525588
Noxo1	-0.6938726	-0.2666399	0.87432924	0.36096306	0.64549182
Kpna3	0.12253668	7.16999286	0.87374844	0.36111924	0.64549182
A630033H20	0.77354112	-0.6315604	0.87357737	0.36116527	0.64549182

Abat	-0.1385009	6.69238941	0.87356795	0.3611678	0.64549182
Tmem184c	0.130848	6.04331967	0.87344332	0.36120134	0.64549182
Taz	-0.2195783	3.1171464	0.87303268	0.36131186	0.64549182
Vps37d	-0.414494	0.98645161	0.87294954	0.36133424	0.64549182
Mfsd9	0.49950764	1.13707931	0.87287301	0.36135485	0.64549182
Treml2	-1.0056385	-0.6834042	0.87280507	0.36137314	0.64549182
Gk5	0.25880612	1.99776092	0.87275275	0.36138723	0.64549182
Myh6	-0.5540699	0.26047312	0.87261283	0.36142491	0.64549182
Fyco1	0.12990633	6.72397002	0.87255621	0.36144016	0.64549182
Cdkn3	-0.7746002	-0.6948635	0.87227136	0.3615169	0.64549182
Ftsj1	-0.171982	3.73079047	0.872182	0.36154097	0.64549182
Mb21d1	-0.4666339	0.81745462	0.87206425	0.36157271	0.64549182
Rnf220	-0.1240446	6.94879055	0.87164468	0.3616858	0.64549182
Ttc7b	-0.1579406	8.19378473	0.87146526	0.36173418	0.64549182
Snapc5	-0.1715753	6.27476818	0.87132211	0.36177278	0.64549182
Ackr2	0.57339467	0.49083056	0.87113307	0.36182377	0.64549182
Slc25a27	-0.190306	4.58888176	0.87106473	0.36184221	0.64549182
Tmem151b	-0.246993	4.64442946	0.87091813	0.36188176	0.64549182
Tdg	0.4320311	0.31915329	0.8708025	0.36191296	0.64549182
Inpp5f	-0.1012214	6.79477906	0.87029194	0.36205078	0.64549182
Mak	-0.3955474	2.07930266	0.87024019	0.36206475	0.64549182
Gps1	0.13666509	5.13884661	0.87019109	0.36207801	0.64549182
Zdhhc3	-0.1268653	5.5882829	0.8701859	0.36207941	0.64549182
Ptpn4	-0.1504334	7.20235562	0.87009899	0.36210288	0.64549182
Ssh1	-0.2439522	2.40579911	0.86994082	0.3621456	0.64549182
Minpp1	0.15293919	4.91685676	0.86985349	0.36216919	0.64549182
Trp53i13	0.75206535	-0.2615405	0.86898958	0.36240267	0.64582241
S1pr3	-0.2147102	3.33109425	0.86852024	0.3625296	0.64592537
Zfp37	0.15561558	6.80022442	0.86842099	0.36255645	0.64592537
BC005764	-0.3254352	2.18317284	0.86775696	0.36273616	0.64605235
Ctse	0.78235188	-0.3797391	0.86763503	0.36276918	0.64605235
Ano10	0.25076027	2.49026703	0.86762552	0.36277175	0.64605235
B4galnt4	0.2604633	3.61833574	0.86724634	0.36287445	0.64608841
Hprt	0.110034	7.88764741	0.86680939	0.36299284	0.64608841
Ocel1	0.36398428	1.7625037	0.86643082	0.36309546	0.64608841
Lrrc6	-0.209155	3.88873187	0.86615559	0.3631701	0.64608841
Grap	-0.4397602	0.96999009	0.8659027	0.3632387	0.64608841
2700070H01	-0.7695511	-1.1984218	0.86586446	0.36324907	0.64608841
Acsl5	-0.1256229	5.63286945	0.86559229	0.36332292	0.64608841
Etv4	0.83920181	-1.3660121	0.86540869	0.36337275	0.64608841
Bid	-0.2330092	2.81864589	0.86516385	0.36343921	0.64608841
Endou	-0.3180953	1.87124236	0.86502975	0.36347563	0.64608841
Cic	0.1274818	6.64039487	0.86500918	0.36348121	0.64608841

Mrpl55	0.22136556	3.61984209	0.86498626	0.36348743	0.64608841
Rps29	-0.147885	6.7774653	0.86470609	0.36356353	0.64608841
Ccdc13	0.80825299	-0.3812154	0.86441998	0.36364126	0.64608841
Cers3	-1.1083093	-1.6244005	0.86441876	0.36364159	0.64608841
Gm16853	0.97637126	-0.9169055	0.86433046	0.36366558	0.64608841
Lamtor2	0.23169633	4.07058306	0.86408165	0.3637332	0.64608841
Gps2	-0.1564501	4.02309499	0.86406986	0.36373641	0.64608841
Nup43	0.28694429	2.47967835	0.86385343	0.36379525	0.64608841
Plcd3	-0.2207427	3.08169908	0.86378317	0.36381435	0.64608841
Csad	0.16339687	4.22252035	0.86364142	0.3638529	0.64608841
Slc9a7	-0.2324105	4.25515932	0.86362038	0.36385862	0.64608841
Pgrmc2	0.13330577	5.49575319	0.86348177	0.36389632	0.64608841
Fgd3	-0.9676544	-0.2428206	0.86297173	0.36403508	0.64624952
Spag5	-0.4799097	1.90163562	0.86235625	0.36420264	0.64635008
Rapsn	1.33969811	-2.5007466	0.86220384	0.36424415	0.64635008
Mpv17l	-0.1386528	5.64672728	0.86194625	0.36431432	0.64635008
Glb1l2	-0.8615054	-0.4162008	0.86193783	0.36431661	0.64635008
Prob1	-0.3232735	1.69995626	0.86186926	0.3643353	0.64635008
Efna5	-0.1369788	6.08444567	0.86137378	0.36447034	0.64635008
Rab13	0.23519474	4.24254159	0.86129217	0.36449259	0.64635008
Ipmk	-0.1348354	5.56169021	0.86125188	0.36450357	0.64635008
Eme2	-0.3211255	1.98866727	0.86117685	0.36452403	0.64635008
H2-DMb1	-0.5988399	0.53285769	0.8608615	0.36461003	0.64638514
Slc25a26	0.19154415	3.09585486	0.86073347	0.36464496	0.64638514
Samd14	-0.3386709	2.17700552	0.86057604	0.36468791	0.64638514
Kcnv1	-0.2044424	6.74877182	0.86036111	0.36474656	0.64640395
Derl1	-0.1594295	5.09007702	0.86002402	0.36483857	0.64648187
Mras	-0.1016612	6.99979418	0.85965021	0.36494065	0.64657761
Ube2d1	-0.1513962	6.92626022	0.85901183	0.36511507	0.64669992
Ascc1	0.26102488	3.49080484	0.85882669	0.36516568	0.64669992
Rbks	-0.5098145	1.44680274	0.8587439	0.36518831	0.64669992
Ldlrap1	-0.4323137	1.14273587	0.85869411	0.36520193	0.64669992
Nme4	0.76622211	0.20736977	0.85803356	0.36538259	0.64685373
Ccdc28b	0.19964844	3.40580795	0.85802504	0.36538492	0.64685373
4930486L24I	1.4053821	-2.2845267	0.85784708	0.36543362	0.64685484
Gm10941	-0.8771861	-1.0465899	0.85730203	0.36558282	0.64703383
Otud7a	-0.3064965	3.09168606	0.8568324	0.36571145	0.64709155
Dennd6a	-0.1243341	6.86793009	0.85663678	0.36576505	0.64709155
Ran	0.10171723	7.71307405	0.85623009	0.36587652	0.64709155
Prss35	0.48192469	0.77546363	0.85619625	0.36588579	0.64709155
Mgat5	-0.1807664	4.25700485	0.85602858	0.36593177	0.64709155
Pde4b	-0.1547817	7.9733752	0.85594696	0.36595415	0.64709155
Mpi	-0.1681903	4.47507128	0.855239	0.36614837	0.64709155

Slc25a39	0.21578164	4.37736588	0.85518975	0.36616188	0.64709155
Tob2	0.1604555	5.13169507	0.85509098	0.36618899	0.64709155
Sh3bgrl2	0.1767799	4.30173649	0.85508982	0.36618931	0.64709155
Gprasp2	-0.1858786	6.17426828	0.85483401	0.36625954	0.64709155
Zfp433	-0.1714896	3.82422455	0.85455626	0.36633581	0.64709155
Rad51ap1	0.85968244	-0.3231284	0.85407598	0.36646775	0.64709155
Cyp4f15	-0.4171933	1.15049786	0.85380523	0.36654216	0.64709155
Lrif1	0.16333485	5.127569	0.85368121	0.36657625	0.64709155
Gbe1	0.16841143	3.96139429	0.85367187	0.36657882	0.64709155
10-Mar	-0.7194397	-0.6737566	0.85343595	0.36664368	0.64709155
Itgb3	0.27762297	2.3627299	0.85343422	0.36664416	0.64709155
Plvap	-0.4802192	0.6205832	0.85325547	0.36669332	0.64709155
Ppp1r3g	-0.4818126	0.59363059	0.85292934	0.36678303	0.64709155
Pag1	-0.1366408	5.64446304	0.85286708	0.36680016	0.64709155
Clec2f	0.75346641	-0.7657779	0.85286086	0.36680187	0.64709155
Zfp277	-0.2258014	6.31918205	0.85276227	0.366829	0.64709155
Arl8a	-0.12626	6.34525013	0.85265786	0.36685773	0.64709155
Fam132b	-0.7416375	-0.102842	0.85261917	0.36686838	0.64709155
Gm1673	0.47117606	0.4194867	0.85254363	0.36688917	0.64709155
Tango2	-0.1765276	3.743236	0.85245407	0.36691382	0.64709155
Rnf149	-0.154174	5.59735815	0.85218684	0.36698739	0.64713649
Acadsb	-0.1354538	6.82862526	0.85191437	0.36706243	0.647184
Trim56	0.19586029	3.52885382	0.85171785	0.36711657	0.64719465
Calcoco1	0.10769099	7.47821036	0.85134087	0.36722044	0.64728637
Ppil1	-0.1880807	3.77002774	0.85117342	0.3672666	0.64728637
Cyp27a1	-0.3577741	1.73152539	0.85068721	0.36740066	0.64728637
Fbln5	-0.3343838	4.21843074	0.85055479	0.36743719	0.64728637
Tapbp	0.28521996	4.62292862	0.85051432	0.36744835	0.64728637
Mir1188	0.66283074	-0.5347815	0.85047853	0.36745822	0.64728637
Ttll7	-0.1380736	7.62182125	0.85030785	0.36750531	0.64728637
Inpp5b	0.1487171	4.90277006	0.84997931	0.36759598	0.64736132
Cend1	0.10985774	7.05317087	0.84937031	0.36776413	0.64744045
Slc26a4	0.54264268	1.05431985	0.84934942	0.3677699	0.64744045
Bloc1s2	0.2238715	3.29573558	0.84929385	0.36778525	0.64744045
Cd86	0.66810061	-0.1300237	0.84898607	0.36787028	0.64750542
Hps5	0.20788794	3.60626407	0.84799207	0.36814509	0.64783435
Fendrr	-1.0304654	-0.3723177	0.8479104	0.36816768	0.64783435
Zfp1	-0.1467647	4.28516805	0.84769202	0.3682281	0.64783435
Dgkk	-0.2947371	3.76416739	0.84761387	0.36824973	0.64783435
Timm8a1	-0.1737373	5.39448299	0.84683995	0.36846399	0.64812654
Kif18b	1.07760256	-1.7836768	0.84662298	0.36852409	0.64812738
Smc5	-0.1932785	5.74348268	0.8464905	0.36856079	0.64812738
Prr13	0.15633153	5.59039695	0.84587624	0.36873104	0.64820828

Mrps16	0.2131616	3.20787223	0.84579953	0.36875231	0.64820828
Slc17a7	0.12340869	7.53302562	0.84553905	0.36882455	0.64820828
Zfp12	-0.1622145	4.74653812	0.84529818	0.36889137	0.64820828
Tmem125	-0.4818686	0.65984188	0.84504992	0.36896025	0.64820828
Tcn2	0.26908277	4.22294844	0.84489306	0.36900379	0.64820828
Hes6	0.24163806	2.20106607	0.84480004	0.36902961	0.64820828
Fgfr2	0.17338132	6.83047451	0.84439387	0.36914238	0.64820828
Atg4a	0.30262402	3.5910716	0.8441134	0.36922028	0.64820828
Fam154b	-0.4082154	0.89675354	0.84396671	0.36926103	0.64820828
Sms	-0.1092825	7.09061847	0.8436843	0.36933951	0.64820828
Galnt2	-0.1766562	3.84837964	0.84362249	0.36935669	0.64820828
Mpc1	-0.1144157	6.49887657	0.84359365	0.3693647	0.64820828
Ahnak	0.18908591	9.0753298	0.84334506	0.36943381	0.64820828
Gpx8	0.25053827	5.63847575	0.84288814	0.36956088	0.64820828
Slc6a18	-0.4065831	1.13879394	0.84281645	0.36958082	0.64820828
Igf1	-0.1711338	5.25462119	0.84230102	0.36972425	0.64820828
Ube2ql1	0.13782833	6.24484934	0.84222437	0.36974559	0.64820828
1700071K01I	-1.1453599	-0.3649096	0.84205587	0.3697925	0.64820828
Vps53	0.11380286	6.21974496	0.84187998	0.36984147	0.64820828
1810010H24I	0.37599958	1.16088715	0.84180609	0.36986205	0.64820828
Ms4a6b	0.76566267	0.48023595	0.84179919	0.36986397	0.64820828
Dda1	-0.1511977	4.55962534	0.84177821	0.36986981	0.64820828
Stim2	-0.1590694	5.92475523	0.84152506	0.36994033	0.64820828
Mef2c	-0.1432873	10.0758779	0.84137929	0.36998094	0.64820828
Igf2r	-0.1539354	4.7242819	0.84137183	0.36998302	0.64820828
Gm1966	-0.2714277	2.95962697	0.84124045	0.37001963	0.64820828
AW549542	-0.5726154	-0.2372378	0.84107436	0.37006592	0.64820828
Zfp395	0.17745626	6.06510323	0.84085255	0.37012775	0.64820828
Gm4532	1.24304883	-1.3773558	0.84052461	0.37021919	0.64820828
Rnf41	-0.1271573	5.35339329	0.84046564	0.37023564	0.64820828
2200002D01I	-0.4616962	0.63943445	0.84039005	0.37025672	0.64820828
Clca2	-0.9633863	-1.5911448	0.84031216	0.37027845	0.64820828
Pim2	0.14457109	4.10662835	0.84029586	0.370283	0.64820828
Nhlrc1	-0.1720393	4.10563353	0.84024655	0.37029675	0.64820828
Bace2	0.26000293	3.59463179	0.84008809	0.37034096	0.64820828
Taldo1	0.14023905	4.07313907	0.8394859	0.37050905	0.64841813
Cux1	-0.0889862	8.04491016	0.8392979	0.37056154	0.64842567
2210016L21I	0.12990322	6.25796474	0.83866049	0.37073961	0.64865292
Ccdc22	-0.4583989	0.91836404	0.83845992	0.37079567	0.64866665
Ncstn	0.17223237	5.1058978	0.83755872	0.3710477	0.64890141
Ltf	-0.5617324	0.23885672	0.83750454	0.37106286	0.64890141
Zadh2	0.15474867	4.80412858	0.83746285	0.37107453	0.64890141
Slc24a3	-0.1431547	6.88660705	0.83709703	0.37117692	0.64899612

Hibch	-0.1806974	4.11210801	0.83689704	0.37123291	0.64900969
Zfp551	-0.368155	2.48571282	0.83617407	0.37143543	0.64927938
Gsr	-0.1203181	5.88993082	0.83509768	0.37173724	0.64965758
1110008F13I	-0.3121316	2.33546273	0.83505813	0.37174834	0.64965758
Aste1	-0.2822225	2.36355824	0.83482848	0.37181278	0.64968582
Grb10	-0.104649	6.92938384	0.83448597	0.37190892	0.64976944
Iqgap2	-0.1664665	4.97802915	0.83384312	0.37208947	0.64987654
Pnlip	-1.0646456	-1.7420603	0.83366707	0.37213893	0.64987654
Inpp4a	-0.1605824	6.53634804	0.83364247	0.37214585	0.64987654
Lrguk	0.207526	3.24587054	0.83357998	0.37216341	0.64987654
Mvb12b	-0.1197141	6.44063794	0.8326969	0.3724117	0.6502038
Cox16	-0.1843113	4.60842813	0.83243954	0.37248411	0.6502038
Stt3a	0.141736	5.16260031	0.83229267	0.37252544	0.6502038
Taf11	0.14721111	5.26365295	0.83222639	0.37254409	0.6502038
Hsbp1	0.14580845	8.97240026	0.83066544	0.37298381	0.65081751
Zc4h2	0.14314567	4.71566266	0.83063387	0.37299271	0.65081751
Zbtb46	0.39429931	1.31475956	0.83046322	0.37304083	0.65081751
Nbl1	0.31050684	6.51471903	0.83015049	0.37312903	0.65088701
Actg2	0.86575602	-0.8348818	0.82918169	0.37340248	0.65115715
Tex261	0.24162712	1.98762786	0.82914577	0.37341262	0.65115715
Cdt1	0.62775501	-0.1894261	0.82908754	0.37342907	0.65115715
Alkbh2	-0.3824203	1.16747695	0.82887499	0.3734891	0.65117746
Abr	-0.1428096	7.69705062	0.82830294	0.37365076	0.65133778
Arid1a	-0.1559192	8.05983056	0.82805241	0.37372159	0.65133778
Gm2382	0.25606816	2.975679	0.82788795	0.37376809	0.65133778
Abcd3	0.11406268	6.90680663	0.82743214	0.37389703	0.65133778
Snx12	0.12676428	7.72627146	0.82739535	0.37390744	0.65133778
Tnfaip3	0.27834654	2.85126605	0.82729375	0.37393619	0.65133778
Micu2	0.13772235	5.20628011	0.82725451	0.3739473	0.65133778
Zmym4	-0.1215382	7.67947104	0.82710642	0.37398921	0.65133778
Eogt	-0.1445027	5.15256979	0.82685815	0.3740595	0.65133778
D630003M21	-0.6716493	0.44311259	0.82683836	0.3740651	0.65133778
Arl5c	0.81510306	-0.6989785	0.82652467	0.37415393	0.65134587
Ccdc117	0.18032051	4.88265275	0.82632266	0.37421115	0.65134587
Wars	0.14099094	5.11435786	0.82626723	0.37422686	0.65134587
Gimap8	-0.3495396	1.21229968	0.82606624	0.37428381	0.65134587
Pcnxl3	-0.1438597	4.69194894	0.82596759	0.37431176	0.65134587
Depdc5	-0.1778925	5.25652569	0.82573312	0.37437822	0.65137728
Bloc1s5	-0.2707837	3.3249666	0.82548149	0.37444957	0.65141718
D7Ertd443e	-0.3094057	1.54308926	0.82519898	0.37452969	0.65147234
Ercc6l	0.76636236	-0.7930277	0.82441494	0.37475218	0.6517751
Dll1	0.60130764	0.10744602	0.82382138	0.37492075	0.6518668
Tmem246	0.15617644	4.14919465	0.82372685	0.3749476	0.6518668

Zfp444	-0.2081885	3.28469872	0.82371759	0.37495023	0.6518668
Dph6	0.11176884	6.23839124	0.82305422	0.37513878	0.65191109
Pik3r6	-0.4334379	1.61009317	0.82280113	0.37521075	0.65191109
Ms4a4b	0.83657299	-0.5138973	0.82278304	0.37521589	0.65191109
Gramd2	-0.7460273	-0.4528712	0.8226312	0.37525908	0.65191109
Adarb2	-0.1812075	4.9735665	0.82249343	0.37529828	0.65191109
Sdsl	-0.9440594	-0.6387176	0.82196675	0.37544817	0.65191109
Slc36a2	-1.2357866	-1.7024372	0.82193992	0.3754558	0.65191109
Klhl32	0.35314021	0.85298259	0.82183571	0.37548547	0.65191109
Nppc	-0.7645992	-0.4988418	0.82130911	0.37563544	0.65191109
Dlg2	-0.1517278	9.50865062	0.8212683	0.37564707	0.65191109
Fst	-0.5298486	0.61537621	0.82114179	0.37568312	0.65191109
Pom121	-0.1297022	6.32319514	0.82108278	0.37569993	0.65191109
St8sia3	-0.1476415	7.82621868	0.82107704	0.37570156	0.65191109
Trove2	0.11752528	7.07330955	0.82104555	0.37571054	0.65191109
Gpr4	-0.2336717	5.56002445	0.82080182	0.37578	0.65191109
Igj	-0.4198805	1.01319306	0.82079875	0.37578088	0.65191109
Elp5	0.16791873	4.81554388	0.82073412	0.3757993	0.65191109
Cnot6l	-0.109094	7.06788096	0.82002045	0.37600282	0.65218007
Kdelr2	0.2195021	5.18839106	0.81961593	0.37611825	0.65223465
Gart	-0.1732208	4.37755038	0.81939901	0.37618017	0.65223465
R3hcc1l	0.14489864	4.57641189	0.81935993	0.37619133	0.65223465
Pvr	-0.2515559	2.53603465	0.8192021	0.3762364	0.65223465
Syt14	-0.4141126	1.37972022	0.81906117	0.37627664	0.65223465
Irgq	0.12707053	7.40639249	0.81827977	0.3764999	0.65253759
Tbc1d22b	-0.181425	4.46307118	0.81767148	0.37667384	0.6525802
Rspo2	0.25725106	4.39083096	0.81763627	0.37668391	0.6525802
Plac9b	-0.2446746	3.93696673	0.81758824	0.37669765	0.6525802
Amica1	-0.38726	1.3615346	0.81745593	0.3767355	0.6525802
Hadha	0.13288239	4.94884333	0.81734596	0.37676697	0.6525802
Tceal8	0.19104287	7.27685964	0.8171705	0.37681718	0.65258317
Fcgr1	0.7202396	-0.627529	0.81658995	0.37698338	0.652787
Grik2	-0.2082133	5.18302285	0.8161252	0.37711652	0.65285569
Sgcz	-0.4151521	1.8960049	0.81611273	0.37712009	0.65285569
Slco4c1	-0.308761	2.13125651	0.81571404	0.37723435	0.6529695
Snora81	0.95067412	-1.7249084	0.81525503	0.37736597	0.6531133
Ppp2r2cos	0.55372472	0.74501721	0.81495574	0.37745182	0.65315594
Rsph1	-0.3198248	2.13736563	0.81483076	0.37748768	0.65315594
Tubgcp3	-0.1424888	4.42504064	0.81413839	0.37768643	0.6533262
Luc7l2	-0.1245597	7.39714737	0.81401015	0.37772326	0.6533262
Ust	0.23742321	5.5251111	0.81386424	0.37776517	0.6533262
Papln	-0.9450234	-0.9964692	0.8136754	0.37781942	0.6533262
Gm12505	-0.2604336	1.77653996	0.81332899	0.37791897	0.6533262

Gsta4	0.18826127	6.83520109	0.81327742	0.3779338	0.6533262
BC018507	-0.1756926	6.72558247	0.8128797	0.37804815	0.6533262
Zfp105	-0.2657551	3.11122438	0.81221599	0.37823908	0.6533262
Bmp7	0.22204718	7.77650848	0.8120715	0.37828067	0.6533262
Apoe	-0.2807025	6.97856543	0.81203418	0.37829141	0.6533262
Rttn	-0.2996398	2.57433777	0.8119463	0.37831671	0.6533262
Mageh1	0.1725306	4.38329685	0.81188736	0.37833368	0.6533262
Gna11	0.15292695	5.92150648	0.81178934	0.3783619	0.6533262
Tssc1	0.18085917	3.27212638	0.81177467	0.37836612	0.6533262
Tmx2	0.13839142	5.98579548	0.81166512	0.37839766	0.6533262
Arhgef9	-0.1268844	9.43024303	0.81166091	0.37839888	0.6533262
Spock3	-0.1306374	5.22420083	0.81161721	0.37841146	0.6533262
Fbxo38	-0.1131695	5.22070644	0.81109939	0.37856063	0.65349989
Itsn2	0.10960428	7.26278222	0.81061494	0.37870026	0.65365708
Col25a1	0.1613154	5.59930305	0.81020237	0.37881923	0.65368793
Bace1	-0.1079461	5.79319228	0.8100375	0.37886679	0.65368793
Ttc12	0.49641566	1.39047069	0.8099621	0.37888854	0.65368793
AF357426	-0.7086821	-0.8974646	0.80987926	0.37891245	0.65368793
Lmtk3	-0.4552544	1.61875562	0.80908318	0.37914225	0.65392602
Ntmt1	-0.2482255	2.14595237	0.80906449	0.37914765	0.65392602
Stat5a	-0.4292629	1.7590182	0.80872597	0.37924544	0.65393788
Phip	-0.1420169	7.35489708	0.80870422	0.37925172	0.65393788
Mat2b	-0.1060009	7.44759251	0.80818646	0.37940136	0.65411208
Tmf1	0.09733996	7.01464471	0.80790929	0.3794815	0.65411856
Appl1	0.10832632	7.58721653	0.80783722	0.37950234	0.65411856
Mbtps2	0.15406561	4.92830405	0.80729167	0.37966016	0.65430281
Rdh12	0.57263021	0.1413352	0.80695784	0.37975679	0.65430281
Upf2	-0.1048998	6.89054025	0.80685191	0.37978745	0.65430281
Neil1	-0.5896027	-0.0590332	0.80663844	0.37984927	0.65430281
Cercam	0.28949725	1.93764476	0.80662777	0.37985236	0.65430281
Trp53inp1	-0.145401	5.78981049	0.80619008	0.37997914	0.65435985
Ppfibp2	0.17291845	3.58271338	0.80614359	0.37999261	0.65435985
Thumpd2	0.30759473	1.67259557	0.80584949	0.38007784	0.65435985
Plxna4	0.1702776	7.02214531	0.80584211	0.38007998	0.65435985
Rab33b	0.11726625	5.90779528	0.80551302	0.38017539	0.65436913
Ywhaq	0.10729999	9.00817021	0.80535631	0.38022083	0.65436913
Tnfsf13	-0.9182441	-1.1613654	0.805312	0.38023368	0.65436913
Drd2	0.28110408	3.84286227	0.80494158	0.38034114	0.65436913
Fam189a1	-0.1398307	5.77277244	0.80485701	0.38036568	0.65436913
Bcl11a	-0.1291767	6.56535469	0.8047913	0.38038475	0.65436913
Cse1l	0.10529303	6.72638057	0.80464991	0.38042578	0.65436913
Rnaseh2b	0.19536831	4.32882291	0.80433808	0.3805163	0.65444118
Rab3c	0.13850998	9.29562799	0.80408815	0.38058888	0.65446029

Sac3d1	-0.3042536	2.31542067	0.8039321	0.3806342	0.65446029
Pxylp1	-0.1994162	3.30128163	0.80379744	0.38067332	0.65446029
Upf3b	-0.1480251	5.6288383	0.80341898	0.3807833	0.65448611
Ogfr	0.31412285	1.96457029	0.8033416	0.38080579	0.65448611
Setmar	0.30060178	2.36982812	0.80324368	0.38083425	0.65448611
C230079O03	1.3493601	-2.0034868	0.80240354	0.3810786	0.65470796
C030006K11I	-0.2505709	2.96509353	0.80174266	0.38127097	0.65470796
Adm	-0.2794945	2.24747852	0.80148659	0.38134555	0.65470796
Olfm1	-0.10888	9.19770912	0.80136985	0.38137956	0.65470796
Ppp1r14b	0.25927671	3.52403494	0.8013531	0.38138444	0.65470796
Snx14	-0.1442574	5.44398163	0.80120749	0.38142686	0.65470796
Gpsm3	-0.4114103	2.31143864	0.80120301	0.38142817	0.65470796
Il34	-0.1701908	3.78935797	0.80072343	0.38156794	0.65470796
Ppp2r2a	0.10326656	7.10196112	0.8007031	0.38157387	0.65470796
Eif1a	0.14586502	6.62350168	0.80058207	0.38160915	0.65470796
Orc5	-0.1989742	3.3294651	0.80048921	0.38163623	0.65470796
Tdrkh	0.18844318	4.42683345	0.80047058	0.38164167	0.65470796
Chmp2b	0.16620664	6.34152136	0.80034975	0.38167691	0.65470796
Eno2	0.1510229	8.48693367	0.80034215	0.38167912	0.65470796
Tmem26	1.44649794	-1.4309923	0.80029403	0.38169316	0.65470796
Pcdhb21	0.40627503	1.24682171	0.79998403	0.38178359	0.65477962
Adnp	0.10975722	7.86024029	0.79938858	0.38195739	0.65485174
Muc2	-1.7486035	-1.8954559	0.79915996	0.38202415	0.65485174
Zfp871	-0.1176323	9.06013318	0.79904419	0.38205796	0.65485174
Rnf32	-0.2196529	3.43782625	0.79887479	0.38210744	0.65485174
Gm8179	-0.9761456	-0.1915981	0.79869194	0.38216087	0.65485174
AI314180	0.11533376	7.16951713	0.79867817	0.38216489	0.65485174
Speer4b	-0.4206817	0.58808674	0.79867335	0.3821663	0.65485174
Rph3a	-0.1423369	8.88862341	0.79843886	0.38223483	0.65488577
Haus5	0.32557568	1.62049443	0.79823081	0.38229565	0.65488929
Trappc4	0.21092394	3.52625341	0.79800778	0.38236086	0.65488929
Nalcn	-0.1345715	6.39924881	0.79777593	0.38242866	0.65488929
Iqcc	0.28378951	2.65551747	0.79776606	0.38243155	0.65488929
1810020O05	-0.6162317	-0.8442492	0.79729338	0.38256985	0.65493968
Ndc1	0.24220906	2.89070847	0.7972431	0.38258457	0.65493968
Lztfl1	0.12754721	6.37156356	0.79715639	0.38260995	0.65493968
Rp9	-0.1729561	4.0520469	0.7969536	0.38266931	0.65493968
BC005537	-0.1206882	7.01994452	0.79683398	0.38270434	0.65493968
Il27ra	1.04308844	-1.5039429	0.79646435	0.38281259	0.6550375
Agk	-0.155931	4.41723715	0.79620836	0.38288759	0.6550375
Lysmd3	0.15859525	4.76163054	0.79614032	0.38290753	0.6550375
Lhfp	0.19058976	6.11296999	0.79542658	0.38311678	0.65531214
Shprh	-0.1872666	5.96668018	0.7948509	0.38328567	0.6554431

Mndal	-0.2503716	2.99456607	0.79481506	0.38329619	0.6554431
Eral1	-0.1980687	3.54601251	0.79447113	0.38339715	0.6554431
Arhgap21	-0.1627562	8.18769952	0.79435417	0.38343149	0.6554431
Ankrd46	0.11305724	6.77853635	0.79421406	0.38347264	0.6554431
Mcmbp	0.10834623	5.68264481	0.79416995	0.38348559	0.6554431
Carf	-0.1455366	4.65339293	0.79385102	0.38357928	0.65548641
Stx16	-0.100932	6.02270895	0.79373656	0.38361291	0.65548641
Atp1a1	-0.1421166	7.63614666	0.79344597	0.38369831	0.65548641
Hrk	-0.2194864	4.09783662	0.79302285	0.38382272	0.65548641
Cdh7	-0.1853399	3.87185716	0.79301892	0.38382387	0.65548641
AI115009	-0.4717079	0.65848687	0.79239863	0.38400635	0.65548641
Mmadhc	0.16327901	5.8456984	0.79234697	0.38402156	0.65548641
Uqcrh	0.1626786	7.05499761	0.79216343	0.38407558	0.65548641
Rragc	-0.1173723	5.81121884	0.79211392	0.38409016	0.65548641
Gm9899	0.16977945	3.86221393	0.79204469	0.38411054	0.65548641
Mir377	-0.9903049	-0.8233461	0.79202498	0.38411634	0.65548641
Itgb5	0.22897054	4.05281799	0.79199793	0.3841243	0.65548641
2610002J02F	0.28735379	3.24413391	0.79163473	0.38423126	0.65548641
B130034C11	-0.576943	0.87810322	0.79162327	0.38423464	0.65548641
Pla1a	0.52173851	0.99689346	0.79159958	0.38424161	0.65548641
Fam208a	-0.1487849	6.76686734	0.79056213	0.38454739	0.65592468
Mtrf1l	0.17586531	4.04188522	0.79038257	0.38460035	0.65592468
Zbed4	-0.1915163	4.47113745	0.79023201	0.38464476	0.65592468
Galt	0.1432425	4.43305	0.78984296	0.38475956	0.65603732
Brinp1	-0.1288603	6.39757904	0.78931602	0.38491514	0.65604864
Nxf1	-0.1174298	5.93111729	0.78909504	0.38498041	0.65604864
Opcml	-0.14662	8.17137437	0.78896128	0.38501992	0.65604864
Hook2	-0.3828191	1.55443116	0.7889602	0.38502024	0.65604864
5031425E22I	0.21859815	3.42439837	0.78849526	0.38515764	0.65604864
D17H6S53E	-0.2004494	2.75255333	0.78837227	0.385194	0.65604864
Wdr89	0.3248094	1.09939631	0.78832105	0.38520915	0.65604864
Pglyrp2	1.77402945	-1.8629835	0.78828266	0.3852205	0.65604864
Plcxd1	0.35649348	1.75679054	0.78825896	0.3852275	0.65604864
Ermard	-0.1791942	3.92559189	0.78815479	0.38525831	0.65604864
Rnf38	-0.0985653	7.97757377	0.7879676	0.38531367	0.65604864
Gstm3	0.3090548	1.53275135	0.78783485	0.38535294	0.65604864
Tmem229a	-0.1374313	6.08972277	0.78767575	0.38540001	0.65604864
Trim43c	0.91396203	-1.17469	0.78709822	0.38557094	0.65614679
Arntl	-0.1523387	3.98379063	0.78676023	0.38567103	0.65614679
Tpo	1.13707098	-2.0661477	0.78652225	0.38574153	0.65614679
Mpp5	0.1122277	6.90625661	0.78641463	0.38577342	0.65614679
Tstd2	0.22753558	2.78070103	0.78631397	0.38580324	0.65614679
Ddx19a	0.15323933	5.4407088	0.78612274	0.38585992	0.65614679

Camta1	-0.1802102	9.37254189	0.78611299	0.38586281	0.65614679
Lyve1	0.29881542	3.43353616	0.78587013	0.3859348	0.65614679
Clic1	0.22905954	4.48526169	0.78580607	0.3859538	0.65614679
Fam89b	0.25126013	2.77397986	0.7856679	0.38599477	0.65614679
Socs4	-0.1438569	5.019161	0.78560058	0.38601473	0.65614679
Sh3glb1	0.10392395	8.20721644	0.78550595	0.3860428	0.65614679
Bc1	-1.7131651	-1.8486001	0.78507726	0.38616999	0.65628007
Kcng4	0.35059181	1.67984873	0.78468033	0.38628781	0.6563974
Efnb2	-0.1281732	6.13005427	0.78386119	0.38653112	0.65663029
Wdr83	0.22571607	2.6962975	0.78360248	0.38660801	0.65663029
Lmln	-0.2504105	2.3864814	0.78356904	0.38661795	0.65663029
Kif4	-0.3515318	1.16208681	0.78356199	0.38662005	0.65663029
AB041803	0.37431502	2.42440817	0.78318129	0.38673324	0.65667072
Coa4	0.29831217	2.07474742	0.78313179	0.38674796	0.65667072
Prrg1	-0.2597131	2.50080234	0.78298961	0.38679026	0.65667072
Gdi1	0.11321216	8.88966639	0.78276507	0.38685706	0.65670128
Immp2l	0.54211693	-0.0527315	0.78161088	0.38720071	0.65710322
Zfp574	0.17493029	4.88659637	0.78155074	0.38721863	0.65710322
Irf3	0.25477887	2.69589221	0.78147787	0.38724034	0.65710322
Hpca	-0.1152032	8.12687707	0.78111368	0.38734888	0.65720454
Blm	-0.3335869	2.1997472	0.78075917	0.38745458	0.65722902
Serp1	0.2114319	7.92018947	0.78058263	0.38750724	0.65722902
Rab8a	0.2415367	4.84206691	0.78049655	0.38753292	0.65722902
Mex3d	-0.2233845	3.9163	0.78041019	0.38755868	0.65722902
Ankfy1	-0.1295935	6.00458421	0.77977169	0.38774925	0.65735093
Bcl2a1a	0.44042947	0.16812951	0.77963908	0.38778884	0.65735093
4930545L23R	0.67801997	0.66038367	0.7795833	0.3878055	0.65735093
Lpo	-1.0631841	-0.8109951	0.77919736	0.38792078	0.65735093
Brd7	-0.1096492	5.86924557	0.77915703	0.38793283	0.65735093
Bri3	-0.1775494	4.3199298	0.77908471	0.38795444	0.65735093
Ttpa	-0.3962605	1.48140709	0.77902419	0.38797252	0.65735093
1700030L20R	-0.3806168	1.35267767	0.77875586	0.38805272	0.65740403
Ngef	-0.1402383	5.79286165	0.77858017	0.38810524	0.65741024
Mpst	0.51123807	0.44164435	0.7782364	0.38820804	0.65746255
Ryk	-0.1875663	4.46530085	0.77805899	0.3882611	0.65746255
Gtf2i	-0.0999298	7.28734558	0.77797027	0.38828764	0.65746255
Gpc2	-0.7342409	0.06735224	0.77747413	0.38843612	0.65746255
Hist1h2bm	0.37538977	1.35635533	0.77746996	0.38843737	0.65746255
Angpt1	0.19876059	4.59662105	0.77722513	0.38851067	0.65746255
Nbea	-0.2251786	8.50315609	0.7770116	0.38857462	0.65746255
Arl3	0.16093494	5.64605501	0.77683489	0.38862755	0.65746255
Fis1	-0.1650172	3.99027107	0.77680697	0.38863592	0.65746255
Cep170	0.13669372	7.48685712	0.77680426	0.38863673	0.65746255

Fam169a	-0.188621	5.76125406	0.77661612	0.3886931	0.65746255
Fjx1	0.15728039	4.08630098	0.77634441	0.38877453	0.65746255
Hcn1	0.17554322	7.36017206	0.77609314	0.38884986	0.65746255
Il12a	0.30999384	1.92491101	0.77605783	0.38886044	0.65746255
Sox21	0.21807396	3.44466163	0.77602927	0.38886901	0.65746255
Nup88	0.12994921	5.69244822	0.7755835	0.38900271	0.65760597
2610027K06l	0.79425502	-0.8805224	0.77536278	0.38906893	0.65763531
Ndufa8	0.13018937	5.2594952	0.77502583	0.38917006	0.65772363
Mir6369	0.73388481	0.57777275	0.77435176	0.38937249	0.65775034
Rfc4	-0.2749351	1.9149827	0.7742796	0.38939417	0.65775034
Nfs1	-0.1528816	4.3159272	0.7741717	0.3894266	0.65775034
Sgsh	0.33739226	1.3039999	0.7740758	0.38945542	0.65775034
Mrpl21	0.16113029	4.23155519	0.77406876	0.38945753	0.65775034
Zfhx2	-0.1622732	5.15266286	0.773994	0.38948	0.65775034
Efcab6	0.3679209	1.7914979	0.7736864	0.38957247	0.65775034
Gpatch11	0.15937163	5.43093597	0.77360597	0.38959665	0.65775034
Prr11	-0.2139724	3.12267496	0.77344263	0.38964577	0.65775034
D7Ertd715e	-0.3095317	4.14583375	0.77313981	0.38973686	0.65775034
Tbc1d24	0.15263478	6.35733208	0.7731295	0.38973996	0.65775034
2810428I15R	-0.2908442	2.82113477	0.77302157	0.38977243	0.65775034
Cers4	0.2105286	5.54302222	0.7725235	0.38992234	0.65777857
Atp6v1g1	0.15642791	6.90417305	0.77251361	0.38992531	0.65777857
Zc3h14	0.11086421	6.41769269	0.77241031	0.38995642	0.65777857
Hdgfrp3	0.10492564	8.00669921	0.77231643	0.38998469	0.65777857
Klf9	0.11953237	8.08354523	0.77145005	0.39024572	0.65813635
Pla2g6	0.23576	2.71901809	0.77037833	0.39056897	0.65845178
Ltn1	-0.1396945	6.60022805	0.77035696	0.39057542	0.65845178
Mkl1	0.11282618	5.55924494	0.77034327	0.39057955	0.65845178
Gatsl3	0.39810705	1.40357407	0.76998458	0.39068784	0.65849407
A230001M1(	0.515631	0.24697136	0.76993597	0.39070251	0.65849407
Pced1b	-0.2033023	3.1020314	0.76919243	0.39092713	0.65879014
Fbxo9	-0.1056165	6.44977445	0.76867288	0.3910842	0.65889285
Soat2	-0.298904	1.76274704	0.76866689	0.39108601	0.65889285
Gna13	0.10935018	7.53995271	0.76762259	0.39140201	0.65908838
Vrk3	-0.282572	2.8399183	0.767583	0.39141399	0.65908838
Svopl	0.68653768	-0.2633265	0.76753033	0.39142994	0.65908838
Cpsf4	-0.2824305	1.62507283	0.76735357	0.39148347	0.65908838
Kcnd2	-0.1426369	6.04117674	0.76703021	0.39158142	0.65908838
Tbl1xr1	-0.0944134	7.63651991	0.7670157	0.39158582	0.65908838
Lyl1	0.61545815	-0.0769813	0.76691977	0.39161489	0.65908838
Mrvi1	0.19132041	5.83222849	0.76691094	0.39161756	0.65908838
Cyth4	-0.3080146	1.85872667	0.76682737	0.39164289	0.65908838
Ddhd2	0.11250625	6.00364545	0.76646699	0.39175212	0.65918261

Ier2	-0.3575075	1.23049555	0.76627501	0.39181033	0.65918261
Ubald2	0.25446555	2.32642476	0.76608617	0.3918676	0.65918261
Pxmp4	0.17812026	3.31185349	0.76599641	0.39189483	0.65918261
Fut9	-0.1662709	7.1307687	0.76566118	0.39199654	0.65920179
Nudt19	-0.1528315	4.87249555	0.76536159	0.39208747	0.65920179
Rpl39	-0.1947013	7.34060567	0.76534934	0.39209119	0.65920179
Fdxacb1	-0.4173593	1.52243624	0.76525073	0.39212113	0.65920179
Hs3st2	0.18120442	4.34219434	0.76501271	0.3921934	0.65920179
Msx1	0.32979455	2.36231787	0.76484931	0.39224303	0.65920179
Rnft1	0.15867567	4.55113215	0.76461665	0.39231371	0.65920179
1700027H10I	-0.5585146	0.39054886	0.76432133	0.39240345	0.65920179
Fam133b	0.12202809	5.13952641	0.76408346	0.39247576	0.65920179
Pstpip2	0.16384921	3.88083727	0.76407203	0.39247923	0.65920179
Ttc30a1	-0.4227739	1.65587813	0.76404768	0.39248663	0.65920179
Sv2c	-0.2075566	4.80415195	0.76394443	0.39251802	0.65920179
Malsu1	0.14744723	4.73281261	0.76386204	0.39254308	0.65920179
Dync1h1	-0.3211667	9.33827441	0.76349158	0.39265576	0.65926396
1700001J11F	-0.8596308	-1.5119809	0.76341819	0.39267809	0.65926396
Apoa2	-0.8467059	-1.2367279	0.76294773	0.39282126	0.65942207
Fkbpl	0.45496467	0.62039627	0.76269631	0.39289781	0.65944197
Oxsm	-0.2496336	3.16295024	0.76258688	0.39293113	0.65944197
Grrp1	-0.8285754	-1.7128194	0.76231483	0.393014	0.659495
Gm20187	0.70224818	0.42513865	0.76215748	0.39306194	0.659495
Agmo	0.4253379	1.67511286	0.76200054	0.39310976	0.659495
Mapre1	0.1262879	7.8572816	0.76153781	0.39325082	0.6596494
Sumf1	0.17906707	5.03265388	0.76134748	0.39330886	0.65966453
Aff2	0.17019298	5.87645456	0.76086542	0.39345592	0.65982289
Tmem126a	-0.1493923	4.47075064	0.76071657	0.39350135	0.65982289
Rprd1b	0.15188915	5.10640068	0.76023181	0.39364934	0.65993533
Tmem14a	-0.1516068	4.84503277	0.76017565	0.39366649	0.65993533
Ormdl2	-0.4590378	1.43733539	0.75975117	0.39379616	0.66007048
Zfp827	0.12107401	6.30042239	0.75929194	0.39393652	0.66014955
Arid1b	-0.1632277	6.24376872	0.75925754	0.39394703	0.66014955
Cplx1	-0.0948698	8.44920453	0.75911534	0.39399051	0.66014955
Mc1r	0.98430216	-1.8869924	0.7588633	0.39406759	0.66019649
Tdo2	0.98250519	-0.9958907	0.75863118	0.3941386	0.66022389
Gopc	0.10646759	6.07453978	0.75845943	0.39419115	0.66022389
Psmc3ip	-0.3497459	2.02747276	0.75832878	0.39423113	0.66022389
Tmem64	0.1607867	8.87291316	0.75802124	0.39432528	0.66029937
Adrm1	0.13191398	4.16478739	0.75752883	0.39447608	0.66039091
Sema4b	0.21897546	2.77315186	0.75752225	0.39447809	0.66039091
Nudcd3	-0.0984945	6.41496489	0.75727972	0.3945524	0.66043314
Wdr61	0.15793232	4.33102401	0.75698511	0.39464269	0.66050212

Arhgap32	-0.1706313	9.3418549	0.75654404	0.39477793	0.6606463
Catsperg1	0.84984707	-1.0719641	0.75618038	0.39488949	0.66067395
Pir	0.16398778	3.46972287	0.75617006	0.39489266	0.66067395
Omg	0.16498908	7.44931293	0.75596289	0.39495623	0.66069817
Zc3h12b	0.13403435	5.05571314	0.75569033	0.39503989	0.66071357
Osbpl5	0.16871812	3.92473795	0.755613	0.39506364	0.66071357
Ccnl1	-0.2278611	4.7158139	0.75523973	0.39517826	0.66074657
Vps26b	0.09309115	7.41283833	0.75489465	0.39528428	0.66074657
Rabgap1	-0.1016001	7.271955	0.75482922	0.39530439	0.66074657
Vmn2r118	-1.2039351	-1.2699865	0.75469659	0.39534515	0.66074657
Crip1	-0.3278006	2.42763612	0.75442484	0.39542869	0.66074657
Tex15	-0.2877743	2.37812707	0.75432679	0.39545884	0.66074657
BC018473	-1.2320032	-1.5335454	0.7541423	0.39551557	0.66074657
Vapa	0.11325117	7.18529711	0.75402964	0.39555022	0.66074657
A530058N18	-0.3689843	1.14834101	0.7539212	0.39558358	0.66074657
Rabgef1	-0.1821288	4.26776228	0.75386306	0.39560146	0.66074657
Gm3604	-0.2084526	2.73309904	0.75333386	0.39576433	0.66074657
Usp27x	-0.1346339	4.93859851	0.75325799	0.39578769	0.66074657
B4galt6	-0.0938357	7.17140345	0.75323145	0.39579586	0.66074657
Stt3b	0.12044634	5.87106778	0.75302583	0.39585917	0.66074657
H2-DMb2	1.15378146	-1.6585688	0.75293158	0.3958882	0.66074657
Mprip	-0.1161502	7.49135185	0.75265609	0.39597306	0.66074657
Tmod1	0.15139835	4.73124694	0.75242144	0.39604536	0.66074657
Ankrd28	0.1013678	6.58204557	0.75227346	0.39609097	0.66074657
Aamp	-0.1685015	4.84802461	0.75210808	0.39614195	0.66074657
Ddit4	0.26006768	2.67190027	0.75208125	0.39615022	0.66074657
Dhdh	0.1443863	6.4983225	0.7520769	0.39615157	0.66074657
Paqr8	0.12256792	5.11849638	0.75188215	0.39621161	0.66074657
Cd72	0.74293703	-0.3622226	0.75187848	0.39621274	0.66074657
Pcdhga4	-0.3057857	1.68857709	0.75149304	0.39633163	0.66086293
Ccdc94	-0.3132141	1.59061397	0.75112441	0.39644538	0.66093735
Pdlim5	0.16877804	7.39530716	0.75103008	0.3964745	0.66093735
Zfp266	-0.0961491	6.88720034	0.75062255	0.39660032	0.66096282
Fam102b	0.10288592	5.84785971	0.750509	0.39663539	0.66096282
Zer1	0.15022118	4.95266359	0.75050338	0.39663713	0.66096282
B230208H11	-0.8540049	-0.0817268	0.75015823	0.39674376	0.66105864
Suox	0.18757001	4.24986719	0.74924315	0.39702666	0.66119495
Taf12	0.22297577	4.10431954	0.74907028	0.39708014	0.66119495
AI987944	0.18771296	3.80950383	0.7488924	0.39713518	0.66119495
Cd244	1.00216499	-0.6517929	0.74886338	0.39714416	0.66119495
Alg13	-0.2725241	3.17797422	0.74883948	0.39715156	0.66119495
Prkca	-0.1472476	8.12929589	0.74880764	0.39716141	0.66119495
Hmg20a	0.16378579	5.9347233	0.74877677	0.39717096	0.66119495

Degs2	-0.3842997	1.57855019	0.74813924	0.39736835	0.66119495
4930539J05F	-0.451564	0.32804557	0.7481272	0.39737208	0.66119495
Kcnh2	-0.2805443	2.32121856	0.74807944	0.39738687	0.66119495
Hdac9	-0.103082	6.45270884	0.74783267	0.39746332	0.66119495
Pank3	-0.1071397	7.493322	0.74776041	0.39748571	0.66119495
Ripk2	0.18407474	4.3644406	0.7476453	0.39752138	0.66119495
Kbtbd2	0.10658791	5.97260635	0.74687602	0.39775989	0.66119495
Zfp60	-0.1135011	6.37575966	0.74677264	0.39779196	0.66119495
Rreb1	-0.1704029	5.25007658	0.74674946	0.39779916	0.66119495
Rabac1	0.2879901	3.07237434	0.74671541	0.39780972	0.66119495
Ncf4	-0.727696	-0.9556413	0.74670036	0.39781439	0.66119495
5330426P16I	0.27892785	2.64642788	0.74663516	0.39783462	0.66119495
Ppapdc1b	0.15155179	4.27415139	0.7465896	0.39784876	0.66119495
Dph3	-0.1189127	5.71694722	0.74653278	0.39786638	0.66119495
Gimap7	-0.6627788	0.15483927	0.74628831	0.39794226	0.66119495
Ppp3r1	0.09489675	10.0948401	0.74624499	0.3979557	0.66119495
Mtif2	-0.1238718	5.36245845	0.74577894	0.39810042	0.66134746
Btbd19	-0.2724079	2.62610308	0.74563287	0.39814579	0.66134746
Pias4	-0.2806044	1.41421414	0.74539701	0.39821907	0.66138754
Clec7a	-0.7651058	0.80824858	0.74515032	0.39829573	0.66143323
Cpz	-1.0425975	-1.0697219	0.74457604	0.39847429	0.6615334
1700034G24	0.72058092	-0.0836493	0.74455892	0.39847961	0.6615334
Tmpo	0.15352376	5.86284667	0.744482	0.39850354	0.6615334
Brwd1	-0.1529816	6.9745461	0.74393726	0.39867304	0.66173314
Ap1ar	-0.0976372	6.76523956	0.74357277	0.39878652	0.6617405
Pdss2	0.17406674	3.1149329	0.743371	0.39884935	0.6617405
Oser1	0.18889748	4.12300263	0.74315958	0.39891521	0.6617405
Pced1a	0.1721637	4.13256654	0.74304091	0.39895219	0.6617405
Tmem8	0.22724221	2.32998663	0.74303679	0.39895347	0.6617405
Csf1r	-0.1919349	3.45706931	0.74297562	0.39897253	0.6617405
A230009B12	-0.8196302	0.00823792	0.74241317	0.39914786	0.66181515
Zfp961	0.11713047	5.09700489	0.74226063	0.39919543	0.66181515
Tor3a	-0.2764345	3.71345958	0.74225519	0.39919712	0.66181515
Gm15545	-0.7633846	-0.7285073	0.74220021	0.39921427	0.66181515
4930505A04I	-0.6537949	-0.1434764	0.74169193	0.39937285	0.66199649
Stk35	-0.1259716	5.14102998	0.74136941	0.39947352	0.66208181
Rit1	0.17351456	4.74407317	0.74086179	0.39963205	0.66220099
Zfp595	-0.1572848	3.35783293	0.7406703	0.39969188	0.66220099
Ubxn11	-0.4954268	1.41000936	0.74061252	0.39970994	0.66220099
Txlna	0.12984979	5.13475204	0.74050902	0.39974228	0.66220099
Lyrm2	-0.1540643	4.85568116	0.73971196	0.3999915	0.66234964
Inpp5k	0.13364943	4.16728098	0.73963131	0.40001673	0.66234964
Pi15	-0.6368309	0.75635518	0.7394835	0.40006297	0.66234964

Vps29	-0.1263116	5.86087869	0.739374	0.40009724	0.66234964
Dos	-0.1421052	6.40590854	0.73933915	0.40010814	0.66234964
2310065F04I	-1.6660031	-1.6420184	0.73912434	0.40017538	0.66234964
Car14	0.44138164	2.80967489	0.73898854	0.40021789	0.66234964
Zfp952	0.12906595	4.62373598	0.73896333	0.40022579	0.66234964
Mgat2	-0.2104729	3.28423238	0.73875642	0.40029058	0.6623754
2510002D24I	-0.2312151	3.33183668	0.73845883	0.40038379	0.66244817
Spty2d1	0.13000731	4.96492595	0.73818717	0.40046891	0.66246345
Decr1	0.17568752	5.39052888	0.73798436	0.40053247	0.66246345
Chek1	-0.4131118	0.84374277	0.73772047	0.4006152	0.66246345
Slc25a13	0.44638103	0.32074734	0.73756518	0.4006639	0.66246345
Nacc1	0.10064311	6.99277199	0.73746898	0.40069407	0.66246345
Lurap1l	0.2180644	2.60578454	0.73733262	0.40073685	0.66246345
Mrto4	0.21623758	3.32261323	0.73724866	0.40076319	0.66246345
Kcnab1	0.12850978	5.30295476	0.73678362	0.40090912	0.66246345
Commd9	0.28220489	2.15963691	0.73672513	0.40092748	0.66246345
Foxl1	-0.4864318	0.5607768	0.73666974	0.40094487	0.66246345
Dvl2	-0.252902	2.37289552	0.73663093	0.40095706	0.66246345
Prdx2	0.16858401	6.20444139	0.73640239	0.40102882	0.66246345
Clns1a	0.11713622	6.19127817	0.73638903	0.40103302	0.66246345
Hoxd11	-0.641483	-0.3836331	0.73600179	0.40115466	0.6625495
Morn2	0.24956963	2.89696583	0.7357257	0.40124142	0.6625495
Akap1	-0.2006181	3.33678222	0.73553501	0.40130136	0.6625495
Mag	-0.2388845	2.55410134	0.73550138	0.40131193	0.6625495
Pfkfb3	0.14040474	4.54080087	0.73533115	0.40136545	0.6625495
Il20rb	-1.0679457	-0.6847299	0.73528319	0.40138053	0.6625495
Gpx3	0.24094327	3.06524729	0.73490291	0.40150014	0.66255099
Adamts9	0.30985843	2.69377646	0.7346229	0.40158825	0.66255099
Pak1	-0.0975552	9.92302229	0.73412159	0.40174607	0.66255099
Acin1	-0.0996395	5.86499961	0.73404509	0.40177016	0.66255099
Mical1	0.2535717	2.58725045	0.73347588	0.40194948	0.66255099
Prss53	1.05695598	-1.4968797	0.73332551	0.40199687	0.66255099
A330049N07	-0.5507526	-0.2488455	0.733296	0.40200617	0.66255099
Lta4h	-0.1376968	4.27326009	0.73327607	0.40201245	0.66255099
Pcdh11x	-0.1916569	4.67107763	0.73314852	0.40205266	0.66255099
Rtp4	0.30293076	3.93435672	0.73313159	0.402058	0.66255099
Fubp1	0.12366451	7.1259254	0.73285594	0.40214492	0.66255099
Muc6	0.41300094	1.55308457	0.73250792	0.40225469	0.66255099
Rxra	0.20392503	5.7142349	0.73237104	0.40229788	0.66255099
Trappc3l	-1.3111661	-2.1751454	0.73219641	0.40235299	0.66255099
Syne3	0.56181828	0.83033734	0.73194217	0.40243325	0.66255099
Ndufb11	0.16486399	5.59865569	0.73184285	0.40246461	0.66255099
Cnga4	0.53563936	0.3023142	0.73171137	0.40250612	0.66255099

Dmd	0.19038185	7.88618675	0.73161603	0.40253624	0.66255099
Slc6a8	0.11387932	6.34157848	0.7315551	0.40255548	0.66255099
Stk33	-0.3622528	1.62331277	0.73123957	0.40265516	0.66255099
Mtmr1	0.12510079	5.14613504	0.73111864	0.40269338	0.66255099
Acvr2a	0.12921047	6.16874614	0.73109551	0.40270069	0.66255099
Chrac1	-0.2289676	2.94502002	0.73099512	0.40273241	0.66255099
Smpd5	1.12425011	-2.011353	0.73091592	0.40275745	0.66255099
Adamtsl1	0.19784716	3.07913631	0.73073993	0.40281309	0.66255099
Stard6	-0.2559477	2.41474258	0.73073184	0.40281564	0.66255099
Adora2a	0.46285756	1.89798399	0.73050273	0.40288809	0.66255099
1110037F02I	-0.1314271	5.5306821	0.73015647	0.40299762	0.66255099
Plau	0.51681171	-0.1551436	0.73011516	0.40301069	0.66255099
Sptlc1	-0.1545967	3.62416493	0.72996791	0.40305728	0.66255099
Sntn	-0.5922238	-0.1784704	0.72986502	0.40308984	0.66255099
Aplnr	0.95855325	-1.2725046	0.72967747	0.40314921	0.66255099
Ifltd1	-0.1965929	5.40439857	0.72966917	0.40315184	0.66255099
Ndufs8	0.21395571	4.82298406	0.72966686	0.40315257	0.66255099
Bmpr1b	-0.1951329	3.2590408	0.72910797	0.40332955	0.66255099
Celf5	0.13079117	6.86974412	0.72880708	0.40342489	0.66255099
AA474331	-1.4394954	-1.6410653	0.72873673	0.40344718	0.66255099
Spag9	-0.1030478	8.37034525	0.72863851	0.40347831	0.66255099
Htra1	0.18016438	3.91348664	0.72852599	0.40351398	0.66255099
Rnf6	0.09075253	6.81073045	0.72852346	0.40351478	0.66255099
Cox7b	0.14455093	7.33657115	0.72823884	0.40360502	0.66255099
Tsc1	-0.1654147	5.94436256	0.72817204	0.40362621	0.66255099
Gm1821	0.16981352	4.9269643	0.72791767	0.40370689	0.66255099
Gm14005	-0.5241748	0.45134043	0.72789241	0.4037149	0.66255099
Lrrc17	-0.5224964	0.06156484	0.72788171	0.40371829	0.66255099
Neto1	-0.1656832	7.50462645	0.72778653	0.40374849	0.66255099
Ptgfr	-0.2538755	4.72271228	0.72767046	0.40378532	0.66255099
Fbxo40	0.72571879	-0.5305245	0.72765704	0.40378958	0.66255099
Mblac2	-0.1535258	6.02592494	0.72764293	0.40379406	0.66255099
Cmbl	0.22699651	6.17617277	0.72746845	0.40384943	0.66256052
Chn1os3	-0.584332	-0.0760087	0.72679908	0.40406198	0.66256052
Best3	0.7538564	-0.0664537	0.72651326	0.40415279	0.66256052
Nadk	-0.1510032	5.45393474	0.72650959	0.40415395	0.66256052
Slc4a1ap	0.11967249	5.65214781	0.72646438	0.40416832	0.66256052
Sla	-0.2043306	3.91966931	0.72633971	0.40420794	0.66256052
Prss57	1.30866661	-1.3924371	0.72613426	0.40427325	0.66256052
Mro	0.14357411	4.44287072	0.72606235	0.40429611	0.66256052
Cdh20	-0.2139311	3.92881565	0.72605428	0.40429867	0.66256052
Ap1b1	0.12866222	5.70390084	0.72598522	0.40432063	0.66256052
Sin3a	0.1051929	6.53073228	0.72591963	0.40434148	0.66256052

Mtap	0.19612814	4.5968512	0.72543569	0.40449542	0.66264092
Dsel	0.14426445	5.03122799	0.72530015	0.40453855	0.66264092
Abhd14a	0.26061287	2.80306502	0.72525765	0.40455207	0.66264092
Ggcx	0.19004352	3.1388994	0.72514625	0.40458753	0.66264092
Ezh2	-0.243168	3.03078086	0.72486605	0.40467672	0.66267029
Ccdc173	0.22709755	3.05128335	0.72477694	0.4047051	0.66267029
Fam178a	0.09484763	7.06139048	0.72462591	0.4047532	0.66267029
Maml1	0.13038441	5.19911972	0.72395438	0.40496715	0.66285175
Zfhx2os	-0.5348149	0.39938693	0.72375211	0.40503163	0.66285175
Macrod1	-0.5511937	0.16466757	0.72362953	0.40507071	0.66285175
9330151L19F	-0.1820692	4.78112007	0.72359735	0.40508097	0.66285175
Dlx4	-1.2575528	-1.9372752	0.72344447	0.40512973	0.66285175
Sh3pxd2a	0.15852341	7.07643607	0.72335086	0.40515958	0.66285175
Gm15401	-0.4051097	0.45559154	0.72314647	0.40522479	0.66287783
D15Ertd621e	-0.0957541	7.09753655	0.72256571	0.40541014	0.66309707
Ppp1r37	0.1542392	4.26330119	0.7224178	0.40545737	0.66309707
Avil	-1.0774999	-1.4523535	0.72193939	0.40561018	0.66326492
2700029M09	-0.1166133	5.45364033	0.72178792	0.40565858	0.66326492
Elmod2	-0.1669385	3.45349902	0.72145432	0.40576521	0.66335865
Gpcpd1	-0.1186663	6.404355	0.72104811	0.4058951	0.6634087
Baiap3	-0.4282981	1.53709293	0.7210101	0.40590726	0.6634087
Clybl	-0.1575598	4.35151724	0.72077801	0.40598151	0.6634087
Scd1	0.11691005	6.0379921	0.7205465	0.40605559	0.6634087
Bcat2	0.24822467	2.1273565	0.72043397	0.40609161	0.6634087
Espl1	0.80879515	-1.4992261	0.72034508	0.40612006	0.6634087
Cdk5rap2	0.17838587	3.57249977	0.72027991	0.40614093	0.6634087
Bphl	-0.204322	4.07017953	0.71918306	0.40649231	0.66374753
Cdnf	0.31930654	2.31471702	0.71909014	0.4065221	0.66374753
Golga4	0.09503814	7.9148528	0.7189995	0.40655116	0.66374753
Unc13d	0.72381802	-0.8819921	0.71893703	0.40657119	0.66374753
2900041M22	0.55461375	-0.0298824	0.71859428	0.40668112	0.66374753
Ubn2	-0.1282399	7.06862462	0.71823011	0.40679797	0.66374753
Wash	0.16957066	3.84877299	0.71813758	0.40682767	0.66374753
Limk1	0.22606266	2.85508073	0.71806962	0.40684948	0.66374753
Naip1	-1.286683	-1.843122	0.71801346	0.40686751	0.66374753
4921524J17F	-0.255668	4.04418156	0.7179712	0.40688107	0.66374753
Ddx4	-0.3934405	0.98895315	0.71794046	0.40689094	0.66374753
Kazald1	-0.3585082	1.58188185	0.71773114	0.40695815	0.66376869
Ccdc102a	0.36182882	1.41072203	0.71755514	0.40701468	0.66376869
Adck5	0.37997989	1.01876887	0.71743925	0.4070519	0.66376869
Snrpf	-0.262964	2.83226967	0.71687423	0.40723347	0.66388879
Tnfaip1	-0.1400138	6.03251067	0.71681224	0.4072534	0.66388879
Emc2	0.13068935	6.48024617	0.71670647	0.40728741	0.66388879

Zfp609	-0.1006418	7.47356055	0.7164181	0.40738014	0.66388879
Wbscr16	-0.3153753	1.98241993	0.7162844	0.40742315	0.66388879
Brd8	-0.1105834	5.86129526	0.7162574	0.40743183	0.66388879
Fbxl3	0.09619486	7.46914678	0.7160338	0.40750377	0.66388879
Ptchd1	-0.1983663	4.63115282	0.71596199	0.40752688	0.66388879
St13	0.12104703	6.32199189	0.71569022	0.40761435	0.66388879
Dab1	-0.1542381	6.61765701	0.71567605	0.40761892	0.66388879
Dtd1	-0.1341999	6.04522579	0.71545319	0.40769067	0.6639253
Rnf145	0.11472187	5.73026321	0.71520696	0.40776997	0.66392629
Cdc42ep1	-0.3755991	2.57236804	0.7151449	0.40778996	0.66392629
Tenm3	-0.170854	6.36800306	0.71497174	0.40784575	0.66393679
Zbtb1	0.13169215	4.59540125	0.71458233	0.40797124	0.66406075
Hccs	0.15622003	4.98550885	0.71416732	0.40810506	0.66419822
Galnt15	0.44266381	1.00556472	0.71386869	0.40820139	0.66426188
Rasa4	-0.6849142	-0.4240529	0.71374	0.40824291	0.66426188
Acadm	0.17103109	5.06558011	0.71297074	0.40849124	0.66455114
Zfp651	-0.1622518	4.59660151	0.71288341	0.40851945	0.66455114
Kri1	0.2333606	2.89361133	0.71263791	0.40859876	0.66459982
Firre	-0.2918528	3.39928148	0.7124185	0.40866966	0.6646348
Tbl1x	0.0879736	6.44132166	0.71179923	0.40886988	0.66479886
Rab23	0.11914671	5.37717903	0.71172379	0.40889428	0.66479886
Mocs3	0.5634648	-0.9356207	0.71164816	0.40891875	0.66479886
Ccdc51	0.39320136	0.54766976	0.71128236	0.40903711	0.66491095
Cadps	-0.170376	8.58472785	0.71104591	0.40911364	0.66495503
Spag8	0.90765703	-1.5179381	0.71034886	0.40933939	0.66513408
Txnl4b	0.20863184	3.25776878	0.71034102	0.40934193	0.66513408
Mmp9	0.79593398	-0.7822995	0.71024795	0.40937209	0.66513408
Txn1	0.15768519	6.41714117	0.70966479	0.40956113	0.66536088
Ggnbp2	0.09482781	7.36108644	0.70923525	0.40970045	0.66545745
Tefm	-0.2030884	3.32562014	0.70917661	0.40971947	0.66545745
Akap14	-0.7341434	-0.9875609	0.70810497	0.41006741	0.66590519
Rusc1	-0.1607212	5.8504456	0.70794251	0.41012019	0.66590519
Snx17	0.17940569	4.55007244	0.70787047	0.4101436	0.66590519
Napb	0.1131549	9.75654075	0.70720356	0.41036043	0.66609009
Slc35e2	-0.1451128	5.59113456	0.70712871	0.41038477	0.66609009
Tstd3	-0.1363388	5.42823205	0.70688757	0.41046322	0.66609009
Ireb2	-0.1274886	6.4449494	0.70658953	0.41056021	0.66609009
Otud5	0.11489718	5.85476213	0.70651113	0.41058573	0.66609009
Ccdc53	-0.1706979	3.93266763	0.70643422	0.41061076	0.66609009
Gbas	0.12089707	6.53615095	0.70642705	0.41061309	0.66609009
Shc3	0.22493153	3.93974062	0.70623593	0.41067532	0.66609009
Dynlt1f	-0.3417514	0.61524014	0.705969	0.41076225	0.66609009
Psme4	0.14674462	6.43544393	0.70595893	0.41076553	0.66609009

Zxda	-0.1370724	5.37995996	0.70584702	0.41080199	0.66609009
Usp30	0.15449262	4.18557355	0.70564218	0.41086873	0.66611804
Nrxn2	-0.1233215	5.40005457	0.70542789	0.41093857	0.666151
AA413626	-1.4711006	-0.6663741	0.70523882	0.4110002	0.66617066
6330408A02I	0.14254817	3.61196677	0.70483244	0.41113272	0.66621607
Aldh1a7	0.4437889	0.42021628	0.70478318	0.41114879	0.66621607
Commd1	0.21133446	4.15830275	0.70469748	0.41117674	0.66621607
Cd200r4	-1.2112884	-2.3478976	0.7044462	0.41125873	0.66626869
2310010J17F	-0.4003463	0.76878002	0.70392824	0.41142781	0.66632256
Rgs20	0.17883478	5.70952215	0.70391251	0.41143295	0.66632256
Suz12	-0.1065824	6.88359314	0.70375839	0.41148328	0.66632256
Rtkn2	-0.4176479	1.19225338	0.70373766	0.41149005	0.66632256
C1qtnf6	-0.3833707	1.04805475	0.70333502	0.41162159	0.66645536
Ccdc73	-0.2217478	3.33422071	0.70286737	0.41177445	0.66650208
Vangl2	-0.2648267	1.96473114	0.70264204	0.41184814	0.66650208
Ehmt1	0.19980871	4.40796228	0.70258536	0.41186668	0.66650208
Srpx	-0.6891276	0.14940974	0.70249093	0.41189756	0.66650208
Ei24	-0.1353022	6.08275607	0.70233836	0.41194747	0.66650208
Dhx34	-0.2845337	1.42848274	0.70233785	0.41194764	0.66650208
Fiz1	0.1750286	3.76023	0.7019679	0.4120687	0.66656714
Serpinb6a	0.20165107	5.68020965	0.70174029	0.41214322	0.66656714
Timm8b	-0.1217326	5.55387668	0.70157871	0.41219612	0.66656714
Usp38	-0.1413034	5.03510708	0.7015067	0.41221971	0.66656714
Cep350	-0.1499812	6.68424156	0.70142461	0.41224659	0.66656714
Iars	-0.1348652	6.46595723	0.70124531	0.41230533	0.66656714
Rhoc	0.22610291	3.48230513	0.70088328	0.41242396	0.66656714
Lrrc75b	-0.1804149	4.44886064	0.7007606	0.41246417	0.66656714
Sap30	0.2166547	3.11921207	0.70073014	0.41247415	0.66656714
Tbx15	0.18350876	6.7955854	0.70070253	0.4124832	0.66656714
Scaf4	0.10048915	6.40873382	0.69984644	0.412764	0.6669408
Mtmr14	-0.2119927	3.11934636	0.69947189	0.41288694	0.66705936
Zmynd8	0.11741413	6.82734109	0.69910501	0.41300742	0.66715489
Plxnc1	-0.1064002	6.4050276	0.69898752	0.41304602	0.66715489
Prex1	0.14930936	5.9249427	0.69883901	0.41309481	0.66715489
Ncl	0.09679355	8.89214441	0.69865787	0.41315433	0.66717094
Zc2hc1a	0.11445105	7.30989653	0.69847446	0.41321462	0.66718823
Mboat1	-0.5212548	0.97998995	0.69804561	0.41335562	0.66733583
9930111J21F	-0.2595537	2.9542156	0.69772734	0.41346032	0.66734637
Ece2	0.21087348	2.54732572	0.6976199	0.41349567	0.66734637
Nnmt	0.75307381	-0.2419865	0.69748849	0.41353892	0.66734637
Ube2l6	0.19714627	5.58533511	0.69715838	0.41364759	0.66734637
Mrs2	-0.2077623	3.96128606	0.69673379	0.41378742	0.66734637
Fem1b	0.0999494	7.94531779	0.6966395	0.41381848	0.66734637

Pla2g4a	0.15142561	4.59834427	0.69645668	0.41387872	0.66734637
Uchl3	0.16444773	3.58882508	0.6964367	0.4138853	0.66734637
Nabp2	0.15445018	5.88498159	0.69634504	0.41391551	0.66734637
Cspg4	-0.2255123	2.74830297	0.69629534	0.41393189	0.66734637
Qsox1	0.26846217	2.23119922	0.69605324	0.4140117	0.66734637
Cdh24	-0.8486184	-1.4470112	0.69589211	0.41406483	0.66734637
Pcyox1l	-0.2910885	1.9727679	0.69571422	0.41412349	0.66734637
Pfn4	0.29157864	1.95490093	0.69556167	0.41417381	0.66734637
Snx29	0.26437487	2.1760274	0.69535715	0.41424129	0.66734637
Dusp19	0.15913781	4.78410897	0.69529109	0.41426309	0.66734637
Nf1	-0.1537069	8.07663234	0.69524255	0.41427911	0.66734637
Mtmr3	-0.1118781	6.04740863	0.69517015	0.414303	0.66734637
Tfam	0.13762744	4.82690855	0.69516584	0.41430443	0.66734637
Ccm2	-0.162268	3.74234732	0.6943436	0.41457595	0.66770379
Galnt1	0.13546206	6.23301899	0.69312168	0.41497996	0.6682745
Bicd2	-0.1161287	6.4485102	0.69290078	0.41505306	0.66831223
Gpatch2	-0.1321697	4.12318121	0.69235766	0.41523288	0.66848009
Erap1	-0.2598611	3.54311035	0.6921285	0.41530878	0.66848009
Mast4	0.11831581	6.6848335	0.6920993	0.41531846	0.66848009
Klf6	-0.1032787	7.42428216	0.69190072	0.41538426	0.66848009
Shisa2	-0.2761969	2.48056302	0.69162553	0.41547546	0.66848009
Pid1	0.11911873	6.78804196	0.69156447	0.41549571	0.66848009
Slitrk1	0.11376492	7.08121807	0.6914356	0.41553843	0.66848009
Foxc2	0.22557347	6.72668038	0.69138645	0.41555473	0.66848009
Pik3ip1	0.2247937	4.0136335	0.69123507	0.41560493	0.66848093
Epb4.1l4a	0.26669927	1.53030088	0.6910288	0.41567335	0.66851107
4833418N02	-0.3285078	1.55173817	0.69018857	0.41595222	0.6688227
Tmem242	-0.185521	4.09529041	0.69014552	0.41596652	0.6688227
Suv420h1	-0.0928271	6.72862008	0.68946713	0.41619191	0.66905887
Sass6	-0.1697711	3.77705377	0.68940413	0.41621285	0.66905887
C030046E11I	0.12564513	5.47917695	0.68925253	0.41626324	0.66905996
Ncaph2	0.13387702	4.2766549	0.68879999	0.41641374	0.6691762
Ufsp1	0.30415041	1.89725205	0.68860838	0.41647749	0.6691762
Clasrp	-0.2312029	2.54147042	0.68850105	0.4165132	0.6691762
Gnao1	0.11203569	10.1861888	0.6884371	0.41653448	0.6691762
Lsp1	-0.2214738	3.52750994	0.68819309	0.4166157	0.66922678
Fcer1g	-0.2402734	3.20022187	0.68800121	0.41667958	0.66924951
Svil	0.16124019	4.74877453	0.6877471	0.41676421	0.66929142
Acad9	0.18453331	4.13075264	0.6875717	0.41682264	0.66929142
Slc35g3	-1.0376313	-1.5128325	0.68735294	0.41689553	0.66929142
Cnot10	-0.1610593	4.35910618	0.68732563	0.41690463	0.66929142
E330020D12I	-0.2538109	3.84757115	0.68703415	0.41700178	0.66930913
Fam13a	0.13609678	3.99578644	0.6869776	0.41702064	0.66930913

Nxf7	-0.5828674	0.83819956	0.68673028	0.4171031	0.66930913
Brpf1	0.12722434	4.65622633	0.68669576	0.41711462	0.66930913
Rad9b	-0.4919339	0.48771744	0.68653901	0.4171669	0.66931114
Abracl	0.16818813	4.25687212	0.68600159	0.41734623	0.66931114
Gpr22	0.1396188	5.80525701	0.68599319	0.41734903	0.66931114
Cct5	0.09922073	6.33837543	0.68594706	0.41736443	0.66931114
Unc5d	-0.1588846	6.21275361	0.68594665	0.41736457	0.66931114
A4gnt	0.93471083	-1.4684835	0.68564416	0.41746557	0.66939333
Tnfsf15	1.36725711	-1.6671084	0.68500293	0.41767979	0.66952951
Crls1	0.15639256	4.55231975	0.68498445	0.41768597	0.66952951
Ick	-0.1145234	6.31242919	0.68452494	0.41783959	0.66952951
Utp23	-0.1531836	4.38435016	0.6843313	0.41790435	0.66952951
Rnf167	0.20494507	4.18757965	0.68431535	0.41790968	0.66952951
Hist1h2ak	-0.8144477	-1.7770005	0.68410184	0.41798111	0.66952951
Calr	0.11491123	6.40600111	0.68400378	0.41801393	0.66952951
Casc5	-0.5809065	0.48233608	0.68387988	0.41805539	0.66952951
Hint1	0.12957533	5.21577441	0.68383149	0.41807158	0.66952951
Ccbl1	0.34736822	1.5529635	0.68376956	0.41809231	0.66952951
Gm10653	-0.4089378	0.53454069	0.68374481	0.4181006	0.66952951
Wdr3	0.2062154	3.22778779	0.68345541	0.41819749	0.66952951
Cdyl	-0.1334294	4.10914746	0.68325818	0.41826354	0.66952951
Smpd2	-0.2604396	2.31042198	0.68323077	0.41827272	0.66952951
Fkbp1b	-0.1743587	4.2158302	0.68296039	0.4183633	0.66952951
Chrna7	0.36131178	1.221174	0.68289556	0.41838502	0.66952951
Gabrb2	-0.1358982	8.24512873	0.68286177	0.41839634	0.66952951
Sh3d21	-0.4101951	1.01929013	0.68231763	0.41857875	0.66974175
Eif2ak1	-0.1084432	6.22664605	0.68103875	0.41900793	0.67034225
Ttc16	-1.1028393	-1.5557674	0.68090252	0.41905368	0.67034225
Chmp7	0.13057037	5.62002362	0.68055655	0.41916993	0.6704485
Ccnd3	0.29833676	4.56294001	0.68035587	0.41923738	0.67047669
Tfcp2	0.13768182	3.79045747	0.68004924	0.41934047	0.67056186
Ino80d	0.1133744	7.35145253	0.67950468	0.41952365	0.67071414
Hrh3	-0.1471277	4.7445627	0.67946982	0.41953538	0.67071414
Zbtb21	0.13250482	5.05564308	0.67917669	0.41963404	0.67079217
Tmem45b	-0.3003454	2.23267171	0.67887347	0.41973614	0.67087568
Ccdc85c	0.32040537	0.96337472	0.67864186	0.41981415	0.67092067
Lrrc28	-0.1367821	4.49518742	0.67840384	0.41989434	0.67096914
Zfp947	0.24806276	2.25516998	0.67806828	0.42000743	0.67107017
Cdkl3	-0.1701699	3.66734289	0.67769817	0.42013223	0.67118986
B3glct	-0.1226006	5.47446189	0.67741383	0.42022814	0.67124513
Ybey	-0.1967974	4.3752205	0.6772276	0.42029097	0.67124513
Srd5a3	-0.2889586	2.96750846	0.67713254	0.42032305	0.67124513
Ndufaf5	0.13615223	4.79767237	0.67700427	0.42036635	0.67124513

Lrrc39	-0.2459374	2.13293728	0.6767043	0.42046762	0.67128896
Il1rapl2	-0.242945	3.23313246	0.67662745	0.42049357	0.67128896
Limk2	-0.1300163	5.20943889	0.67645272	0.42055259	0.67130353
Tmem82	-0.4264363	0.41533048	0.67601787	0.42069951	0.67145483
Rabl3	0.15182349	4.40385949	0.67575929	0.42078692	0.67145483
F8a	-0.1581136	3.41659898	0.67558882	0.42084455	0.67145483
Mdc1	0.12227067	5.55897763	0.67553453	0.42086291	0.67145483
Mmachc	0.13795742	4.6196112	0.67543413	0.42089687	0.67145483
Foxl2	0.87097725	-0.4672663	0.67491451	0.42107266	0.67158783
Sdhd	0.17130932	6.88424588	0.67459532	0.42118071	0.67158783
Nuak2	0.91050712	-0.498086	0.6744826	0.42121887	0.67158783
Map3k6	0.2536129	1.76105039	0.67417209	0.42132404	0.67158783
Sec23a	-0.1102025	6.49396383	0.67407744	0.4213561	0.67158783
2300009A05I	-0.2564609	2.43271049	0.67407335	0.42135749	0.67158783
Fkbp4	0.16577238	5.92966816	0.67392743	0.42140693	0.67158783
Fut11	0.17185256	3.90062084	0.67387294	0.42142539	0.67158783
Ahctf1	-0.1330925	6.60380263	0.67364122	0.42150393	0.67158783
Gm4461	-0.6488405	0.55151926	0.67360328	0.42151679	0.67158783
B3galnt1	-0.1120483	4.8280408	0.67356656	0.42152924	0.67158783
Rbbp6	0.0930561	9.12289896	0.67242632	0.42191605	0.67203379
Pxmp2	-0.2233516	2.49324457	0.67226667	0.42197026	0.67203379
Glb1	0.2408399	3.05622481	0.67206444	0.42203893	0.67203379
Naprt1	-0.4579377	0.45155741	0.67202547	0.42205217	0.67203379
Mlh3	-0.1136386	5.77549953	0.67159828	0.42219731	0.67203379
C030039L03I	-0.2059997	4.05436571	0.67135944	0.42227849	0.67203379
Paip2b	-0.1107512	5.25895448	0.67123657	0.42232026	0.67203379
Tm9sf4	0.13966733	4.70297856	0.67123075	0.42232223	0.67203379
Snrpb	0.14631102	3.74360166	0.67101105	0.42239694	0.67203379
Hnrnpa3	-0.0930988	9.05026182	0.6709395	0.42242128	0.67203379
4930405J17I	-0.5489344	1.00072485	0.6708742	0.42244349	0.67203379
Phf6	0.10566937	5.27930574	0.67069891	0.42250312	0.67203379
Sgsm1	0.22986792	4.53170126	0.67067533	0.42251114	0.67203379
Cd68	0.28070196	2.3580621	0.67064987	0.4225198	0.67203379
Slc36a1os	-0.2806071	2.78054286	0.6703097	0.42263557	0.67203379
Nol7	-0.1442843	5.77233861	0.67020043	0.42267276	0.67203379
Usmg5	0.13918064	5.86410141	0.67003448	0.42272926	0.67203379
Ccdc61	-0.381338	0.48748189	0.66996619	0.42275252	0.67203379
Stau2	-0.1021995	7.12925765	0.66994998	0.42275804	0.67203379
Kirrel	0.2081227	4.57305455	0.66969152	0.42284607	0.67208001
1700102P08I	-0.7849545	-1.0640683	0.66947653	0.42291932	0.67208001
Rtn4	0.10448091	9.9353984	0.66942476	0.42293695	0.67208001
Rian	-0.1963811	6.84491912	0.66875298	0.42316597	0.67236454
Syvn1	0.16645379	4.83708926	0.66859167	0.42322099	0.67236926

Itga9	0.1742814	3.28035532	0.66825763	0.42333496	0.67236926
Taf3	-0.1056216	6.44110544	0.66824162	0.42334043	0.67236926
Egr3	0.13122419	7.83055763	0.66791329	0.4234525	0.67236926
Pbk	0.67946914	0.03747487	0.66782676	0.42348204	0.67236926
4632428N05	-0.3313557	2.15941038	0.66775789	0.42350556	0.67236926
Zfx	-0.1243752	6.22463205	0.66771938	0.42351871	0.67236926
Tshz1	-0.096008	6.00307208	0.6671049	0.42372863	0.67262317
Dctd	0.37491276	1.64541764	0.66661303	0.42389679	0.67273368
Dimt1	0.14281805	5.04268064	0.66642839	0.42395993	0.67273368
Sntg1	-0.1381288	5.58941701	0.66635952	0.42398349	0.67273368
Car10	-0.1275232	8.35833458	0.66608898	0.42407605	0.67273368
Gm5136	-0.6466696	0.1255518	0.66606692	0.4240836	0.67273368
Polr1d	0.1405018	6.27355112	0.66596355	0.42411898	0.67273368
5830403L16F	0.70231642	-0.5332773	0.66587816	0.4241482	0.67273368
Insrr	0.5962933	0.39472325	0.66544164	0.42429766	0.67284632
Rfx7	-0.1044747	7.87409775	0.66536343	0.42432445	0.67284632
Cbs	0.17039724	3.17066703	0.66508402	0.42442016	0.67284632
Ncam2	0.13323949	7.16289986	0.66504405	0.42443386	0.67284632
Lypd6b	-0.178316	3.94970159	0.66466733	0.42456297	0.67284632
6430562O15	-1.2500928	-1.7198949	0.66461359	0.4245814	0.67284632
AF251705	0.64915152	-0.5359717	0.66454704	0.42460421	0.67284632
Cxcl11	1.46340886	-2.3691744	0.66433684	0.42467629	0.67284632
Arnt	0.10982152	6.02241584	0.6643192	0.42468234	0.67284632
Clec4a1	-0.8275831	-0.821454	0.66401933	0.4247852	0.67284632
Abhd11	-0.1820705	3.15532683	0.66376719	0.42487172	0.67284632
Crocc	-0.3652674	2.06079015	0.66364547	0.4249135	0.67284632
Slc35b2	0.29677138	2.09245304	0.66342663	0.42498863	0.67284632
Tnfrsf19	0.13440343	5.27041797	0.66341933	0.42499113	0.67284632
Dennd1b	0.13891772	4.59060003	0.66316847	0.42507728	0.67284632
Mrpl2	0.22308613	3.1418661	0.66315533	0.42508179	0.67284632
Cenpm	-1.0326422	-1.5130958	0.66309713	0.42510178	0.67284632
Zfp608	0.13663171	5.16319009	0.66295753	0.42514974	0.67284632
Kank3	-0.3224871	1.87037182	0.66290069	0.42516927	0.67284632
Asb4	-0.5726756	0.34542722	0.66211476	0.42543943	0.67306385
C130050O18	0.96850342	-2.0678571	0.66208687	0.42544902	0.67306385
Ankrd34a	-0.1214942	6.32298013	0.66193149	0.42550246	0.67306385
1700028K03I	-0.3749222	2.08229983	0.66191888	0.4255068	0.67306385
Cul1	0.08644935	7.35300591	0.66088028	0.42586431	0.67350837
Pcnp	-0.1173109	7.86009019	0.66053521	0.4259832	0.67350837
Anapc4	-0.1266213	5.48691874	0.66047959	0.42600237	0.67350837
Efcab5	-0.2968881	2.66556156	0.66042508	0.42602115	0.67350837
Zfp418	-0.1758774	3.63764889	0.66037599	0.42603807	0.67350837
Tmem87b	0.14620034	4.94297907	0.66009066	0.42613643	0.67358473

4931406H21I	0.74956514	0.24026653	0.65957366	0.42631475	0.67378744
Trappc10	-0.1108209	5.8965905	0.6593297	0.42639894	0.67381601
Lsm2	0.22200525	4.23845865	0.65923109	0.42643298	0.67381601
Gfod2	-0.5050208	0.02465691	0.65895154	0.42652948	0.67388937
Gm1943	0.18336121	3.5392695	0.65834062	0.42674051	0.67409118
Ddx17	-0.1361378	8.05832796	0.65829173	0.42675741	0.67409118
Homer1	-0.1149098	8.40981841	0.65802409	0.42684991	0.67415817
0610012G03	0.18816859	3.83728121	0.65773648	0.42694936	0.67421763
Tap2	0.4941431	0.2777487	0.65735286	0.42708206	0.67421763
Ttc39a	0.42976361	1.32521746	0.65719997	0.42713496	0.67421763
Trim33	0.08622433	7.7052077	0.65708853	0.42717353	0.67421763
Rtn3	0.11638052	9.97579174	0.65685173	0.4272555	0.67421763
Ddx21	0.11632571	4.80739924	0.65678583	0.42727832	0.67421763
Rlim	0.09371543	7.70261019	0.65667119	0.42731801	0.67421763
Arxes1	0.23791364	2.41449458	0.65649998	0.42737731	0.67421763
Surf2	-0.1914135	5.00252869	0.65614517	0.42750023	0.67421763
Ints7	0.19135317	3.58778534	0.6560839	0.42752146	0.67421763
Slc8b1	-0.27278	1.35538975	0.65607429	0.42752479	0.67421763
Kctd2	-0.1273481	5.52235383	0.65604864	0.42753369	0.67421763
Vbp1	0.12493367	5.18967613	0.655877	0.42759318	0.67421763
Srrm2	0.13860854	10.1848417	0.65568257	0.42766058	0.67421763
2810408I11R	-0.580966	0.38143534	0.65552464	0.42771535	0.67421763
Adcyap1r1	0.14965356	6.44811207	0.65521658	0.4278222	0.67421763
Zfp39	-0.1228872	5.36008907	0.65518111	0.42783451	0.67421763
Coro2b	-0.1339872	6.52167001	0.65506087	0.42787623	0.67421763
Tardbp	0.09388598	7.45746874	0.65504159	0.42788292	0.67421763
Ftl1	0.15334889	6.19724188	0.6550222	0.42788965	0.67421763
Eaf2	0.2768278	1.50594615	0.65439308	0.42810805	0.67448279
Ss18l1	0.115072	6.11108159	0.65404527	0.42822888	0.67459416
Rybp	-0.1059024	6.64734823	0.65355789	0.42839827	0.67478201
Tmem237	-0.2143836	3.40213193	0.65331881	0.4284814	0.67483396
Grin1	0.15349757	6.12469084	0.65283466	0.42864983	0.67502022
Cdkl2	0.11321562	6.06379876	0.65221558	0.42886535	0.67525663
Masp1	-0.2792735	1.67919418	0.65203762	0.42892733	0.67525663
Fkrp	-0.1170011	5.29122087	0.65197111	0.4289505	0.67525663
Fgf14	-0.1049903	6.28372255	0.6517908	0.42901332	0.67527653
Alkbh6	-0.1990355	3.12318257	0.65162559	0.42907089	0.67528816
Gpn1	-0.1477117	3.93186967	0.65147778	0.42912242	0.67529027
Ubl5	-0.1665155	5.95117824	0.65108868	0.42925809	0.67538848
Fam162a	0.17531	5.00443547	0.65101092	0.42928521	0.67538848
Trim46	0.16637215	4.42013848	0.65076173	0.42937214	0.67544627
Hacl1	0.25399645	2.44761928	0.65044828	0.42948152	0.67553938
Wdr48	-0.1183128	5.5655483	0.64971592	0.42973727	0.67581801

Mttp	-0.258727	2.01336206	0.64963572	0.4297653	0.67581801
Klf2	-0.1795281	4.01005213	0.64950969	0.42980934	0.67581801
Cacybp	-0.1035974	6.42728372	0.64931354	0.42987789	0.67584683
Timm17b	0.34938851	2.74288703	0.64897231	0.4299972	0.67587145
4930507D05I	0.78034702	-1.1882022	0.64885729	0.43003742	0.67587145
Tmppe	-0.2390743	3.02552922	0.64883783	0.43004423	0.67587145
Ccr7	1.06278689	-1.9770979	0.64858304	0.43013336	0.67593258
6030443J06F	-0.3097712	1.76003475	0.64816014	0.43028137	0.67608621
Gamt	-0.3578028	1.99468364	0.64758925	0.4304813	0.67628628
Lclat1	0.11331792	6.05359353	0.6474726	0.43052216	0.67628628
Negr1	-0.1065881	8.24486592	0.64732823	0.43057275	0.67628628
Gnai1	0.09676564	9.05039895	0.6467517	0.43077487	0.67628628
Chordc1	0.09827531	6.72134123	0.6466359	0.43081548	0.67628628
Mta2	0.13576594	4.80843552	0.64662876	0.43081799	0.67628628
Slc9a6	0.11345632	6.2789716	0.64659159	0.43083103	0.67628628
Zfp3	-0.1975967	3.98973026	0.64644927	0.43088095	0.67628628
Cipc	0.09428118	6.68247745	0.64643464	0.43088609	0.67628628
Kif1a	-0.1542336	9.884119	0.64636284	0.43091128	0.67628628
Sel1l3	-0.2073447	4.96079136	0.64558132	0.43118564	0.67663795
Btg2	0.24942811	3.76373562	0.64493109	0.43141411	0.67684435
Bcl2	-0.1385832	6.6580827	0.64479683	0.43146131	0.67684435
Trappc11	-0.120886	5.46142792	0.64472325	0.43148718	0.67684435
Lrrk1	0.18562658	5.08688029	0.64463458	0.43151836	0.67684435
Hspb11	-0.2161677	3.06169139	0.64447438	0.4315747	0.67685382
BC039771	-0.3438322	0.57098261	0.6440168	0.4317357	0.6769467
Ube2q2	-0.1075988	6.36693787	0.64401227	0.43173729	0.6769467
Psma4	0.09366918	6.88952147	0.64387716	0.43178484	0.6769467
Rps6kc1	0.12149116	4.98005424	0.64371915	0.43184047	0.67695504
Dock9	-0.1378775	6.49843439	0.64345608	0.4319331	0.67698347
Pex11b	-0.1645795	5.43222602	0.64322351	0.43201503	0.67698347
Atp8b5	-0.7927261	-0.868689	0.64319841	0.43202387	0.67698347
C77080	-0.1641188	5.81398474	0.64299211	0.43209656	0.67698347
Foxd1	0.20628691	5.52951661	0.64295353	0.43211016	0.67698347
Ndufa13	0.20558237	5.844441	0.64242473	0.43229658	0.67719669
Stx1b	-0.1184848	6.40618772	0.64201807	0.43244003	0.67724322
Vprbp	-0.1093731	6.75123308	0.64187506	0.43249049	0.67724322
Atp5k	0.1396636	5.92428404	0.64177505	0.43252579	0.67724322
Cacnb3	-0.1025231	6.89215654	0.64176994	0.43252759	0.67724322
B3gat1	-0.1451695	6.02154827	0.64119238	0.43273152	0.67731582
Pcdh7	-0.1153262	8.15354378	0.6410844	0.43276967	0.67731582
Syt1	0.11538064	9.90610824	0.64099384	0.43280166	0.67731582
Slmo2	0.10184259	6.85782777	0.64080691	0.43286771	0.67731582
Kalrn	0.11666795	11.7778886	0.64076807	0.43288144	0.67731582

4930431F12I	-0.296736	2.63778753	0.64058312	0.43294681	0.67731582
Lhfpl4	-0.1028137	6.02255288	0.6403228	0.43303885	0.67731582
Xrn2	0.10641198	5.65336846	0.64026222	0.43306027	0.67731582
Tbc1d19	0.12363223	5.83751372	0.64022908	0.43307199	0.67731582
Vcan	-0.2355294	4.12263198	0.6400507	0.43313508	0.67731582
Slc4a2	-0.2061116	3.65489648	0.63973599	0.43324643	0.67731582
Cdc123	-0.1074765	6.25246	0.63962165	0.43328689	0.67731582
Rps12	-0.1761367	6.21202793	0.63961403	0.43328959	0.67731582
Hist1h1d	-0.7016773	-1.3350052	0.63950035	0.43332983	0.67731582
Fryl	-0.132746	7.5460087	0.63937421	0.43337449	0.67731582
Rwdd2b	0.29060896	2.25778466	0.63868353	0.43361913	0.67731582
Gtf2ird1	0.15253741	3.59592891	0.63860969	0.4336453	0.67731582
Osbp	-0.098564	5.35473339	0.63849094	0.43368739	0.67731582
Cln6	-0.3713996	1.96632768	0.63842724	0.43370997	0.67731582
Elp4	0.12579216	4.2881464	0.63839232	0.43372234	0.67731582
Tcerg1	-0.103503	6.61335519	0.63823791	0.43377709	0.67731582
Usb1	0.30300362	1.54034789	0.63800874	0.43385836	0.67731582
Pramel5	-0.945479	-1.7145602	0.63798181	0.43386791	0.67731582
Hsp90ab1	0.0952924	10.3381856	0.63792059	0.43388962	0.67731582
Lix1l	0.20313917	6.40813005	0.63771855	0.4339613	0.67731582
9930014A18I	0.41479624	0.8415086	0.63768302	0.43397391	0.67731582
Ptgis	-0.2572431	2.96369474	0.63767884	0.43397539	0.67731582
Zfp516	0.1249455	5.28253629	0.6376347	0.43399105	0.67731582
Cldn12	-0.1174715	5.41596748	0.63751463	0.43403366	0.67731582
Cep162	-0.0987658	6.06887196	0.63694583	0.4342356	0.67750138
BC031361	-0.2190886	2.8200458	0.63685292	0.4342686	0.67750138
Sparcl1	0.14046837	8.69193104	0.63675436	0.43430361	0.67750138
Lyn	0.21891632	3.40651406	0.63633304	0.43445332	0.67753072
Hebp1	0.15951539	3.58687395	0.63630055	0.43446487	0.67753072
Gm10190	-0.6881094	-0.2077484	0.63627637	0.43447347	0.67753072
Sertad4	-0.1986104	5.75548275	0.63590747	0.43460463	0.67764279
Pcdhac1	-0.5947244	-0.0962579	0.63578899	0.43464677	0.67764279
Uckl1os	0.57692384	-0.4792258	0.63560069	0.43471376	0.67764279
Pde9a	-0.3341404	2.03889486	0.63550789	0.43474678	0.67764279
Rtn2	0.26493282	2.61842012	0.63535822	0.43480004	0.67764417
Sgtb	-0.1082306	8.05187458	0.6348878	0.43496751	0.67764417
Fam175a	0.20042862	2.6092575	0.63477879	0.43500633	0.67764417
Marcksl1	-0.1491586	4.29698089	0.6346289	0.43505972	0.67764417
Pfdn4	-0.1953117	3.92869864	0.63459494	0.43507182	0.67764417
Ddb2	-0.3199674	1.05610744	0.63452312	0.4350974	0.67764417
Gm15787	0.3970185	0.71033111	0.63451535	0.43510017	0.67764417
Tlcd2	-0.2730898	1.37738879	0.6342661	0.43518899	0.67770406
1700086L19I	-0.1944571	3.09670287	0.633756	0.43537085	0.67776125

Rprml	-0.2256202	3.00857413	0.63372341	0.43538247	0.67776125
Ahr	0.1675097	4.19486177	0.63368646	0.43539565	0.67776125
Tmed7	-0.1235496	5.69417952	0.6334814	0.43546879	0.67776125
Pdcd11	0.12394296	4.16139788	0.6333655	0.43551015	0.67776125
Ddah2	0.27568364	3.18176182	0.6333157	0.43552792	0.67776125
Ppat	0.13803708	4.38681869	0.6330633	0.435618	0.67776648
Rpl27a	-0.117604	6.97053072	0.63299248	0.43564328	0.67776648
Lingo1	0.10245498	7.59023862	0.63288295	0.43568238	0.67776648
Sirpb1a	0.92226822	-0.9216171	0.63241103	0.43585093	0.6779503
Cdh4	0.17888954	3.48979286	0.63206516	0.43597452	0.67806416
Tmco5	-0.4525536	0.61873959	0.63185086	0.43605112	0.67810493
Cgn	-0.2600683	2.17936625	0.63139529	0.43621404	0.6782411
Rraga	0.12343982	6.03591811	0.63132416	0.43623949	0.6782411
Prdm12	-0.7593524	-0.1195421	0.63098525	0.43636077	0.67830593
Spp2	-0.6531027	0.45542696	0.6309259	0.43638201	0.67830593
Yars	0.12731095	5.01004981	0.63050411	0.43653303	0.6784623
B3gnt6	1.07384439	-1.3576428	0.63019405	0.43664409	0.67855655
Ankib1	-0.0904211	6.52754493	0.63003447	0.43670128	0.67856705
Rsbn1	-0.1032732	6.12056606	0.62965772	0.43683632	0.67859001
Vegfb	-0.1348139	4.6599426	0.62955464	0.43687328	0.67859001
Cfl2	0.13789054	7.7484807	0.62920802	0.4369976	0.67859001
Pdgfb	-0.2640271	2.23334722	0.62885266	0.43712512	0.67859001
Abcc8	-0.240408	2.69792828	0.62878822	0.43714825	0.67859001
Olfr1393	1.0318826	-1.883936	0.62873574	0.43716709	0.67859001
Ulk2	-0.0926234	7.46675213	0.62851546	0.43724617	0.67859001
Ccdc116	-0.4799704	0.07424863	0.62842814	0.43727753	0.67859001
Ptcd3	-0.1590925	5.21562764	0.62840128	0.43728717	0.67859001
Zfp57	-0.3324044	1.89585509	0.62830632	0.43732128	0.67859001
Caln1	-0.1439092	4.57744271	0.6282652	0.43733605	0.67859001
S100a16	-0.2570574	3.25723789	0.62823354	0.43734742	0.67859001
E130309F12I	-0.2183546	2.72267869	0.62816614	0.43737163	0.67859001
Hist1h2bg	0.41639665	0.15143316	0.62776293	0.43751652	0.67873654
Myh9	0.10995707	6.86419916	0.62723438	0.43770656	0.67884844
Bmp4	-0.2275363	7.4127385	0.62719019	0.43772245	0.67884844
B4galt1	0.1783981	4.19173818	0.62714142	0.43773999	0.67884844
Olfml3	0.19100999	4.9645008	0.62679083	0.43786613	0.67896581
LOC1026341(	-0.9072942	-1.4732897	0.62652639	0.43796132	0.67903516
Fam134a	0.11287977	5.4206259	0.62615552	0.43809487	0.67906689
Mgll	-0.1174336	5.42694072	0.62605661	0.4381305	0.67906689
Epb4.1l3	0.10274228	7.79596914	0.62560929	0.43829168	0.67906689
Tceal6	-0.1088661	4.72825426	0.6256064	0.43829272	0.67906689
Extl3	-0.1053359	6.75161783	0.62550592	0.43832894	0.67906689
Acp2	-0.10028	5.57459214	0.6254594	0.43834571	0.67906689

Ubqln2	0.11096216	7.64015162	0.62543134	0.43835583	0.67906689
Ucp2	0.21406852	7.46890025	0.62469515	0.43862137	0.67906689
Sh2b1	0.13535161	4.06126179	0.62441094	0.43872395	0.67906689
D330050I16F	0.49846503	-0.4734021	0.6242372	0.43878668	0.67906689
Idi2	-0.5725773	-0.0722269	0.6241321	0.43882463	0.67906689
Traf1	-0.5156763	0.98828572	0.62406168	0.43885006	0.67906689
Ano8	0.39176321	0.67743509	0.62401899	0.43886548	0.67906689
Ppp1r3fos	-0.9594411	0.05384402	0.62368305	0.43898684	0.67906689
Mina	-0.2089185	2.58326857	0.62352445	0.43904416	0.67906689
Rnaset2b	0.19408964	4.08648288	0.62351131	0.43904891	0.67906689
Bcl2a1b	0.2998818	1.45049734	0.62349652	0.43905425	0.67906689
Stard7	-0.096188	6.2672211	0.62333359	0.43911315	0.67906689
Scamp5	-0.1361851	6.44709612	0.62322804	0.43915131	0.67906689
Dido1	-0.1143776	6.59201609	0.623092	0.4392005	0.67906689
Polr2h	0.20451743	4.08275577	0.62304925	0.43921596	0.67906689
Myoz3	-0.3943281	1.1393035	0.62295507	0.43925002	0.67906689
Snx7	-0.1903987	4.97341229	0.62280952	0.43930267	0.67906689
Dctn2	0.09847843	5.87271617	0.62275746	0.43932151	0.67906689
Tacstd2	-0.5374109	0.91133709	0.62263487	0.43936586	0.67906689
Slc26a7	0.25698256	4.94168083	0.62263147	0.43936709	0.67906689
Wnk1	-0.0849821	10.008663	0.62237138	0.43946122	0.67906689
Angptl4	0.27950062	2.19976334	0.62219344	0.43952564	0.67906689
Rcl1	-0.2735039	2.11762534	0.6221784	0.43953108	0.67906689
Cyp2d22	-0.1919152	3.38887593	0.62214041	0.43954484	0.67906689
Nsmce2	0.11848543	5.28630791	0.62203473	0.43958311	0.67906689
Atp6v1c2	0.69682771	-0.1751808	0.62199733	0.43959665	0.67906689
Chsy3	-0.2042229	2.91210775	0.6215941	0.43974272	0.67921456
Bnip3	0.10884609	6.08881487	0.62135402	0.43982972	0.67927097
Spon2	0.97097027	-0.6560705	0.62112402	0.4399131	0.67930371
Plekhf1	-0.2570884	3.09606806	0.62094393	0.4399784	0.67930371
Sla2	0.48966802	-0.1584206	0.62072168	0.44005901	0.67930371
Ubb	0.1991056	6.41461667	0.62068765	0.44007136	0.67930371
Chka	0.14517072	4.53753609	0.62059951	0.44010333	0.67930371
Fam221b	0.58819354	-0.509092	0.62041334	0.44017089	0.67933006
Sri	0.11282593	6.57914909	0.62010126	0.44028416	0.67939164
Ulk3	-0.1670066	3.94958032	0.6199854	0.44032623	0.67939164
Flt3	0.44190375	0.51524891	0.61975699	0.44040918	0.67939164
Map3k8	-0.2627154	1.90323526	0.61957102	0.44047674	0.67939164
Cad	0.27751237	1.781203	0.61936613	0.44055118	0.67939164
Kcnk12	-0.6760716	-1.2942537	0.61909628	0.44064927	0.67939164
Spock1	0.1150124	8.18431266	0.61908565	0.44065313	0.67939164
Gm10768	1.27228732	-2.1390661	0.63293761	0.44066327	0.67939164
Lfng	0.54953437	-0.3731663	0.61885405	0.44073734	0.67939164

Alkbh8	0.16487948	4.91475563	0.61869936	0.4407936	0.67939164
Ino80b	0.49173469	-0.0544744	0.61862314	0.44082132	0.67939164
Scoc	0.11056281	8.74565605	0.61854736	0.44084889	0.67939164
Zim1	-0.3486587	1.09994664	0.61840011	0.44090246	0.67939164
Gtf2h5	0.15264081	5.19964753	0.61812888	0.44100117	0.67939164
Ndufv1	-0.1450708	4.74009569	0.61812312	0.44100327	0.67939164
Klhl28	-0.1828627	3.90429028	0.61808098	0.44101861	0.67939164
Parp11	-0.1964073	3.76435497	0.61788798	0.44108887	0.6794221
Kcnn2	-0.1494862	3.8382123	0.61757355	0.44120339	0.67948588
Pcdhb3	0.2441646	2.11823708	0.61739206	0.4412695	0.67948588
Cwf19l1	-0.2042591	3.90492963	0.61734082	0.44128817	0.67948588
Pcdh9	-0.1253365	7.3123863	0.61721984	0.44133226	0.67948588
Cntnap5a	-0.1842325	4.3575796	0.61633621	0.44165447	0.67965352
Cyp4b1	-0.7669375	-1.0150473	0.61619191	0.44170712	0.67965352
Leprot	0.19924295	5.01098668	0.61618573	0.44170938	0.67965352
Pcyt2	-0.2872782	1.66845225	0.61603	0.44176621	0.67965352
Adamts18	-0.4742566	0.25730253	0.61598275	0.44178346	0.67965352
Fads1	0.11525652	6.22578949	0.61595649	0.44179305	0.67965352
Tyrobp	-0.3120942	1.37625052	0.61595197	0.4417947	0.67965352
Lrfn4	0.28147655	2.51329453	0.61554172	0.4419445	0.67980625
Dnajc5	0.08296306	7.92614819	0.61538439	0.44200197	0.67981694
Zfp78	0.22675606	2.73225711	0.61514245	0.44209037	0.67987519
Rnf17	0.43994434	0.73325128	0.61476359	0.44222885	0.67991314
Mt1	0.16844033	6.01750431	0.61467163	0.44226247	0.67991314
Dsn1	0.28885266	1.38617472	0.61460675	0.4422862	0.67991314
Nrros	0.41327634	1.47144779	0.61444778	0.44234434	0.67991314
Gm16677	-0.8180572	-1.2552854	0.61438396	0.44236768	0.67991314
4933405O20	-1.1558708	-0.526677	0.61379165	0.44258443	0.6801686
Ttf1	0.11361213	4.43710407	0.61343901	0.44271356	0.6802435
Mrpl46	-0.2114856	3.48417152	0.61338247	0.44273427	0.6802435
Trmt44	-0.4851726	0.66664518	0.61278318	0.44295387	0.68049754
Gm6548	0.21688121	3.51256874	0.61265529	0.44300076	0.68049754
1810013L24F	0.09406708	7.41829365	0.61238438	0.4431001	0.68057245
Zfhx3	-0.0937564	7.2958918	0.6118695	0.44328901	0.6807849
4930523C07I	0.18951283	3.74449244	0.61155936	0.44340286	0.6807858
Lrfn3	0.17205733	3.13777682	0.6114248	0.44345227	0.6807858
Itfg3	-0.2505649	1.93936477	0.61137855	0.44346926	0.6807858
Gbp2	0.22810713	4.51041072	0.61131672	0.44349197	0.6807858
Dtnbp1	-0.1369131	5.32169113	0.61029134	0.44386884	0.68112144
Ifnar1	-0.1288205	5.75464592	0.61027071	0.44387642	0.68112144
Gm4925	0.65386819	-0.0737922	0.60999881	0.44397644	0.68112144
Dusp2	0.72388198	-1.032968	0.60997304	0.44398593	0.68112144
Pllp	-0.1905846	2.36712096	0.60994666	0.44399563	0.68112144

Vash1	-0.1876009	3.42591804	0.60989588	0.44401432	0.68112144
Triqk	0.20326548	3.52130228	0.60966162	0.44410054	0.68113819
Npr1	-0.3911228	0.10392755	0.60954336	0.44414407	0.68113819
Cys1	-0.1714904	5.15008941	0.60932378	0.44422493	0.68113819
Fbxw15	-0.7244316	-0.9967257	0.60917951	0.44427806	0.68113819
Fau	0.17810914	5.68970486	0.60917877	0.44427833	0.68113819
Usp11	-0.116943	7.40496028	0.60830764	0.44459938	0.68142815
Tprn	-0.2934038	2.34731961	0.60826387	0.44461553	0.68142815
Madcam1	-0.989831	-2.0561187	0.60825342	0.44461938	0.68142815
Klhl2	0.09997892	6.90275888	0.6081161	0.44467003	0.68142816
Luzp1	-0.0937031	8.22082905	0.60795849	0.44472817	0.68143965
Edn3	-0.1513258	6.20018642	0.60778776	0.44479117	0.68145858
Sec24a	0.11071033	5.49204325	0.60763307	0.44484825	0.68146846
Gm12359	-0.5363722	0.15565247	0.60713489	0.44503219	0.68167263
2010109I03R	-0.6676801	-1.33369	0.60694833	0.4451011	0.68167485
Tns1	0.0930089	6.61314246	0.60681317	0.44515104	0.68167485
Rnpepl1	0.15534704	4.18973917	0.6067196	0.44518562	0.68167485
Zfand1	-0.1738266	4.29971391	0.60639834	0.44530436	0.68174214
Cxcr2	-0.1756977	4.02308333	0.60632659	0.44533089	0.68174214
Hist3h2ba	0.63780574	0.02817008	0.60609088	0.44541805	0.68179801
Ftsj2	0.3116302	2.03862433	0.60558851	0.44560392	0.68196128
Pitpnm3	-0.1217844	6.08220485	0.60552865	0.44562607	0.68196128
Il1rl2	-0.8473034	-1.0983278	0.60479715	0.44589696	0.68229823
Ccl3	1.24153214	-2.0153871	0.60443964	0.44602945	0.68234645
Samd5	-0.1669102	3.98939051	0.60437638	0.44605289	0.68234645
Trit1	-0.2063222	3.53345895	0.60430166	0.4460806	0.68234645
Mtmr9	-0.1503652	3.64425542	0.60386709	0.44624175	0.6824058
Neu4	0.23070813	2.46054325	0.60384501	0.44624994	0.6824058
Arcn1	0.07652463	7.30276195	0.60342556	0.44640559	0.6824058
Dohh	-0.1999814	3.27307213	0.60336063	0.44642969	0.6824058
Fam43a	-0.1582377	6.20468166	0.60331576	0.44644635	0.6824058
Arl1	-0.1805484	3.68099753	0.60322799	0.44647893	0.6824058
5930403L14F	-0.207461	4.76609012	0.60304771	0.44654588	0.6824058
Igsf5	-1.2603726	-1.9997255	0.6028574	0.44661656	0.6824058
Vkorc1	0.2092686	5.37488519	0.60283759	0.44662391	0.6824058
Gfm2	0.23910483	3.58539059	0.60283057	0.44662652	0.6824058
Proz	0.32559027	1.46770786	0.60220581	0.4468587	0.68245761
H2-T10	-0.271961	1.18483638	0.60209992	0.44689807	0.68245761
Tmco3	0.1619257	3.76678227	0.60193178	0.4469606	0.68245761
Mmp2	0.3626384	1.32979393	0.60192192	0.44696427	0.68245761
Ckap2	0.53969124	0.36365987	0.60183133	0.44699796	0.68245761
Fip1l1	0.08607808	6.50639175	0.6016949	0.44704871	0.68245761
Zbtb14	0.13054048	5.14679904	0.60143391	0.44714583	0.68245761

Tspyl5	0.09934182	5.78190886	0.60133766	0.44718165	0.68245761
Klf12	-0.0884115	7.35340552	0.60097264	0.44731755	0.68245761
Hcls1	0.24257209	1.43159804	0.60086804	0.4473565	0.68245761
4933427E11I	0.99134428	-1.2907902	0.60086089	0.44735917	0.68245761
Dgkd	-0.146114	5.44293754	0.60085324	0.44736202	0.68245761
Enox1	-0.1647947	3.98225846	0.60078558	0.44738722	0.68245761
N4bp2	-0.1891309	3.4697598	0.600701	0.44741873	0.68245761
Cables2	-0.1323648	5.03028034	0.60064515	0.44743953	0.68245761
Utrn	0.0940883	7.75997577	0.60039111	0.44753419	0.68245761
Pex5l	-0.1265062	6.82776309	0.60038507	0.44753644	0.68245761
Olr1	0.53540257	-0.3912774	0.60028611	0.44757333	0.68245761
Ttbk1	-0.1801039	4.05638659	0.59951689	0.44786019	0.68279189
Ripk4	0.5818952	-0.0521389	0.59930921	0.4479377	0.68279189
1700012B09I	-0.6200429	-0.4327784	0.59929023	0.44794478	0.68279189
Ppbp	-0.7770637	-0.2778654	0.59858594	0.44820778	0.68297878
Dip2c	-0.1207426	6.83602647	0.59853772	0.44822579	0.68297878
Nufip2	0.09125346	7.74209213	0.59845948	0.44825502	0.68297878
Tmem160	-0.3745221	1.07426555	0.59841829	0.44827041	0.68297878
Mfsd7a	1.01565444	-0.7476039	0.59821687	0.44834569	0.68301613
C530044C16I	0.95151951	-1.6134224	0.59786131	0.44847862	0.68314131
Ndufa11	0.14812964	3.88211188	0.59757992	0.44858387	0.68318095
Mfsd1	0.17708271	5.17209041	0.59748879	0.44861796	0.68318095
Ddx26b	-0.2121809	4.50924521	0.59730787	0.44868566	0.68318095
Tunar	0.22631416	3.21410415	0.59724892	0.44870772	0.68318095
Zfp661	-0.1928587	3.17375051	0.59696911	0.44881246	0.68326311
Khdrbs2	-0.2372552	1.68183347	0.59677179	0.44888636	0.68329829
Sbno1	-0.1382173	8.41056419	0.59663426	0.44893786	0.6832994
5031414D18I	0.70808462	-1.0537703	0.59630694	0.4490605	0.68332801
Fam180a	-0.2561013	5.14193717	0.59614053	0.44912287	0.68332801
Srp19	-0.1022878	6.44334797	0.59595054	0.44919409	0.68332801
Mier2	0.32720884	1.225176	0.59590674	0.44921051	0.68332801
Ptp4a1	0.14485878	4.59349163	0.59587837	0.44922115	0.68332801
Nedd9	0.11663612	4.36676939	0.59563496	0.44931243	0.68332801
A430090L17I	0.73789338	0.76348656	0.59545487	0.44937999	0.68332801
Inha	-0.26464	1.64997007	0.59545144	0.44938127	0.68332801
Elp3	-0.107622	5.3579593	0.59536504	0.44941369	0.68332801
Pnpla7	-0.29068	2.12670054	0.59498797	0.44955521	0.68337007
Dzip3	0.12801452	6.90177053	0.59498179	0.44955753	0.68337007
Slc45a1	-0.2183891	2.61914513	0.59488543	0.4495937	0.68337007
Supt6	-0.1221904	7.40632526	0.59456594	0.44971369	0.68347524
1810026J23F	0.11241506	5.9224383	0.59370531	0.45003715	0.68388959
Ms4a1	-1.0467521	-1.7458976	0.59326898	0.45020128	0.68402687
Amacr	0.16316073	3.65200596	0.59315008	0.45024603	0.68402687

Tmem18	-0.1515292	4.31066498	0.59298233	0.45030916	0.68402687
Rpl35a	-0.1575412	5.46905397	0.59289499	0.45034204	0.68402687
Slitrk2	-0.1692636	5.37782692	0.59278979	0.45038165	0.68402687
Cacng3	-0.10106	5.64058481	0.59246092	0.45050551	0.68409821
Ccnyl1	0.12194936	5.32079068	0.5923951	0.4505303	0.68409821
Eps8	0.11606577	5.3883578	0.59222023	0.45059619	0.68412106
Med6	0.15552899	4.33643809	0.59206169	0.45065593	0.68413458
Tm6sf1	-0.2466222	1.87347446	0.59186443	0.45073029	0.68417027
Slfn9	0.28147279	1.62630599	0.59172214	0.45078394	0.68417453
Zfp764	-0.2614531	2.98678652	0.59150445	0.45086603	0.68422196
0610031J06F	0.20997644	4.49173626	0.59114775	0.45100061	0.68430399
Atp6v0b	0.14322709	4.83149849	0.5910525	0.45103655	0.68430399
Ppme1	-0.1211986	5.17759303	0.59095686	0.45107265	0.68430399
Ttc34	-0.559094	-0.1334546	0.59081144	0.45112755	0.68431012
Mfsd8	-0.1617126	3.26371644	0.5903739	0.45129278	0.68448215
F13a1	0.35194468	1.59449827	0.59000251	0.45143312	0.68448215
Erbb2ip	0.08362159	7.94454887	0.58980159	0.45150906	0.68448215
Rab43	0.13912833	5.4568672	0.58971091	0.45154335	0.68448215
Gpaa1	0.31548326	1.39963622	0.58959492	0.45158721	0.68448215
Zfp319	0.17024833	3.1469779	0.58957106	0.45159623	0.68448215
Acsbg1	0.13293892	4.57552639	0.58938322	0.45166727	0.68448215
Rxfp1	-0.3730768	2.35363465	0.5893334	0.45168612	0.68448215
Bcam	0.24504508	3.57616704	0.58891871	0.45184304	0.68448215
Ubqln4	0.10766678	6.15936586	0.58885597	0.45186679	0.68448215
6430584L05F	0.19710539	3.02541422	0.58878796	0.45189254	0.68448215
Sec63	-0.0833556	7.07591059	0.58874338	0.45190941	0.68448215
Rhoq	0.12683066	6.22370793	0.58863719	0.45194962	0.68448215
Kcnk3	0.21766088	1.8758333	0.58856625	0.45197648	0.68448215
Gm20748	-0.3084719	1.58141484	0.58849369	0.45200396	0.68448215
Fbxl4	-0.1470073	3.57468846	0.5877396	0.4522897	0.68480464
Naaladl1	-0.4796384	0.48937503	0.58760183	0.45234193	0.68480464
Gpr161	-0.5134668	-0.3219541	0.58744374	0.45240189	0.68480464
Utp18	-0.1267928	4.20392868	0.58714676	0.45251455	0.68480464
Lrrc61	0.11577866	5.11850472	0.58703603	0.45255656	0.68480464
Snrpb2	0.12372637	5.68008037	0.58701627	0.45256406	0.68480464
Plcz1	0.77641814	-0.6400122	0.58689284	0.4526109	0.68480464
Cox6c	0.12608467	7.55381088	0.58684296	0.45262984	0.68480464
Zfp652os	-0.5499304	-0.6744618	0.58661184	0.45271758	0.68480464
Adam33	0.54077005	-0.0202813	0.58659011	0.45272583	0.68480464
Dlx1	0.20139944	3.88112312	0.58623456	0.45286087	0.68493191
Cd2ap	0.11523011	7.43091632	0.58581859	0.45301894	0.68499965
Larp4b	0.07588496	7.45689149	0.58575979	0.45304129	0.68499965
Mex3c	0.10638849	5.21447884	0.58545404	0.45315754	0.68499965

Ttyh1	-0.1362139	7.60305799	0.58544549	0.45316079	0.68499965
2310069G16	0.35671442	1.60202341	0.58540536	0.45317605	0.68499965
Wwp1	-0.102105	7.5347871	0.58519349	0.45325664	0.68499965
Hcn2	-0.2884589	1.3127917	0.58507726	0.45330086	0.68499965
Slc12a7	-0.3134275	3.82914986	0.58504563	0.4533129	0.68499965
Fam186b	0.9774304	-0.8546847	0.58486704	0.45338086	0.68502543
Rnf182	-0.2629491	3.01037818	0.58462053	0.4534747	0.68506206
Mitf	0.16284028	3.67275615	0.58428881	0.45360103	0.68506206
Lmbr1	-0.1165934	5.0770825	0.58421328	0.4536298	0.68506206
Ltbr	0.27582795	2.37578847	0.58356939	0.45387519	0.68506206
Npr3	-0.1786208	5.29409063	0.58327043	0.4539892	0.68506206
D17Wsu104e	0.27896215	2.07184343	0.58321189	0.45401153	0.68506206
Knop1	-0.1222649	5.63143563	0.58310802	0.45405115	0.68506206
Oprm1	-0.3400457	1.91344994	0.58304155	0.45407652	0.68506206
Serpine1	-0.5650703	-0.2823831	0.58292575	0.4541207	0.68506206
Cmc2	0.15193955	3.25622659	0.58289706	0.45413165	0.68506206
10-Sep	0.21344177	2.8240973	0.58288414	0.45413658	0.68506206
Polr3e	0.18435704	3.83577343	0.58282927	0.45415752	0.68506206
Arc	-0.2815487	5.37353445	0.5827381	0.45419232	0.68506206
Lynx1	-0.1068676	7.54082893	0.58273643	0.45419296	0.68506206
Slc7a3	0.31506795	1.30527275	0.58272719	0.45419649	0.68506206
H2-K2	0.70337104	-0.367524	0.58253771	0.45426882	0.68506206
Kank1	-0.1685893	3.58697731	0.58240925	0.45431787	0.68506206
Hsd3b7	0.18810904	2.68274283	0.58227687	0.45436843	0.68506206
A330074K22	-0.3606071	1.28954745	0.58226648	0.4543724	0.68506206
Ppil3	0.19627156	3.79873045	0.58189657	0.45451373	0.68518277
Gm15713	0.74031047	-0.6500398	0.5817904	0.4545543	0.68518277
Ap3b2	-0.1657768	5.0760068	0.58154299	0.45464888	0.68522971
Slc6a6	0.09212846	7.94764577	0.58131272	0.45473694	0.68522971
Thada	-0.1443981	5.05442858	0.58130939	0.45473821	0.68522971
Cog8	0.22632021	2.01771711	0.58109484	0.45482028	0.68527578
Cyfip1	-0.1035669	6.22946683	0.58096323	0.45487063	0.68527578
Rpl38	-0.1371139	6.22195774	0.58074742	0.45495322	0.68528529
A630023P12	-0.9959375	-1.2681202	0.58068059	0.4549788	0.68528529
Rbm12b1	0.15802066	4.6326872	0.58035764	0.45510245	0.68539481
Gpr123	-0.1353102	6.43854164	0.58010473	0.45519932	0.68542067
Thsd7b	0.32380866	1.27825354	0.5800332	0.45522672	0.68542067
Psma8	-0.4919127	0.17721155	0.57954768	0.4554128	0.68542067
Rnf121	0.36133157	0.95704987	0.57953361	0.4554182	0.68542067
Ctbp1	-0.1385296	5.30065741	0.57949374	0.45543349	0.68542067
Gen1	-0.4321628	0.53316377	0.57942889	0.45545835	0.68542067
Pum1	-0.0852308	6.90389033	0.5793824	0.45547618	0.68542067
Ddx3y	0.09391859	6.3508727	0.578951	0.45564166	0.68556066

B4galt2	-0.1753429	3.39230077	0.57882769	0.45568897	0.68556066
Prmt2	0.16070696	4.78853725	0.57867702	0.4557468	0.68556066
Apobec3	-0.2574451	2.31423405	0.57860879	0.45577299	0.68556066
Gm13212	0.2441312	1.56577892	0.57841886	0.45584591	0.68559371
Ptprh	0.656813	-0.9851058	0.5782609	0.45590657	0.68560637
Aqp4	0.15995449	5.58475126	0.57811174	0.45596386	0.68560637
Pdia4	-0.1241299	4.46957625	0.57799898	0.45600718	0.68560637
Sde2	0.11472093	4.81445549	0.57775604	0.45610054	0.68564085
Mir872	-1.009107	-1.3539564	0.57767412	0.45613202	0.68564085
Casp3	0.16182252	4.02964314	0.57730534	0.45627381	0.6857407
Adam18	0.79613441	-0.7034302	0.57713811	0.45633813	0.6857407
Kif26a	0.21586367	2.41192325	0.57691717	0.45642312	0.6857407
4930515G01	0.78044822	-0.7787779	0.57665889	0.45652252	0.6857407
Fcgr2b	0.59767125	0.11290006	0.57665885	0.45652253	0.6857407
Dock11	0.12228096	5.48991872	0.57655973	0.45656069	0.6857407
Brk1	0.12570961	6.30129478	0.57650344	0.45658236	0.6857407
BC016579	-1.1535072	-1.2504356	0.57633704	0.45664643	0.6857407
Dbf4	-0.2572943	1.82206078	0.57630835	0.45665748	0.6857407
Mrps33	0.09267943	6.8965535	0.57612753	0.45672712	0.6857407
Alox8	0.17740474	3.29180948	0.57590803	0.45681168	0.6857407
4933406C10I	-0.6876497	-1.0047284	0.57583156	0.45684115	0.6857407
Klk14	-0.4503624	-0.0143907	0.57562638	0.45692023	0.6857407
Abca6	-0.2873384	1.65908555	0.57557077	0.45694166	0.6857407
3110035E14I	0.08842596	7.87383963	0.5755158	0.45696286	0.6857407
Tspan15	-0.2520342	1.86957769	0.57491161	0.45719588	0.68601388
Ooep	0.63932494	-1.0041709	0.57445999	0.45737018	0.6861989
Zfp120	-0.1726569	4.37521183	0.57429769	0.45743285	0.68621641
A430005L14I	0.20967157	2.80963571	0.57403289	0.45753512	0.68626016
Tmem141	-0.2415372	1.88506136	0.57395812	0.45756401	0.68626016
1700023L04I	-0.6916687	-0.0254087	0.57364485	0.45768507	0.68635163
Ctage5	-0.091919	5.84547173	0.57353636	0.45772701	0.68635163
Ska2	-0.2143451	2.75900867	0.57293621	0.4579591	0.68662314
Lmnb1	0.24231605	1.82116979	0.57266751	0.45806308	0.68670252
Sesn2	0.26631136	2.55196552	0.5717765	0.45840815	0.68703907
Gm5141	-0.2124013	3.06213134	0.57171817	0.45843075	0.68703907
Frs3	0.24433018	2.42524315	0.57138936	0.45855821	0.68703907
Ttf2	0.33621547	1.79361035	0.57137898	0.45856223	0.68703907
Efcab7	-0.2473251	2.55634321	0.571294	0.45859518	0.68703907
Tlr7	-0.2901325	1.59115268	0.57122697	0.45862117	0.68703907
Gtf2a2	0.12339149	4.82817	0.57100892	0.45870575	0.68703907
Mbnl1	0.11116231	9.06154523	0.57090314	0.45874679	0.68703907
Csrp2bp	-0.1165849	4.95271622	0.5708062	0.4587844	0.68703907
Snurf	-0.2794668	1.5189843	0.57077079	0.45879814	0.68703907

St3gal5	-0.1123657	6.4731257	0.570458	0.45891955	0.6871444
Sult6b1	-0.3710612	0.88888743	0.57011486	0.4590528	0.68722273
Nsun6	-0.2023286	2.75050169	0.56999102	0.4591009	0.68722273
Gpr124	-0.1758488	4.91586024	0.5699288	0.45912507	0.68722273
Pus10	0.19206987	3.92504545	0.56957531	0.45926244	0.68723124
Dnajc25	-0.2436023	2.95692566	0.56954753	0.45927324	0.68723124
Sdc1	-0.298903	2.17858721	0.56950409	0.45929012	0.68723124
Mms19	0.19289437	3.6826746	0.56937022	0.45934217	0.68723124
Mvp	-0.199467	3.7879624	0.5692572	0.45938612	0.68723124
Eya4	0.29155938	2.43857973	0.5687759	0.45957334	0.68725811
Rhebl1	-0.5071987	0.05855139	0.56862934	0.45963038	0.68725811
Slc52a2	0.45571925	0.70323119	0.56861261	0.45963689	0.68725811
Nphs1	-0.2467075	2.0604557	0.56857527	0.45965142	0.68725811
Arhgef17	-0.1194083	6.61858914	0.56823987	0.459782	0.68725811
Dock1	0.11259065	5.76329246	0.56821089	0.45979329	0.68725811
Exo5	-0.1839952	3.74028073	0.56820786	0.45979447	0.68725811
Celf6	-0.1928253	2.68095841	0.56812034	0.45982855	0.68725811
Brap	0.11884216	5.40008628	0.56799692	0.45987663	0.68725811
Plxnb1	-0.1608391	4.42722131	0.56785111	0.45993343	0.68725811
Enkur	-0.2462625	2.14024953	0.56776783	0.45996589	0.68725811
Tmem185b	-0.2460232	3.21262042	0.56735229	0.46012786	0.6874238
Gm2a	0.19956286	5.00654987	0.56688849	0.46030875	0.68761771
Fbxo48	-0.6434031	-0.7738056	0.56655972	0.46043705	0.68766963
Smarca4	0.13432	7.01594341	0.56639771	0.4605003	0.68766963
Poll	-0.3062941	0.87699302	0.56624777	0.46055884	0.68766963
Ncapg2	-0.2271987	2.56099685	0.56620013	0.46057745	0.68766963
Was	-0.3539611	0.99596923	0.56614277	0.46059985	0.68766963
Pick1	0.14809964	3.57902935	0.56601403	0.46065013	0.68766963
Zfyve16	0.12853029	4.90161948	0.5657645	0.46074763	0.68773887
Zfp575	0.21208068	2.81930145	0.56507886	0.46101568	0.68800219
Fahd2a	-0.2091565	2.7954796	0.56505173	0.46102629	0.68800219
Zfp553	0.20263768	3.70563064	0.56483614	0.46111063	0.68805175
Cd79a	-0.3016776	2.13856601	0.56430742	0.46131758	0.68828422
Inpp1	-0.219296	3.03063513	0.56368689	0.46156066	0.68853868
Mdn1	-0.2567696	6.51506517	0.56351043	0.46162982	0.68853868
Tusc3	-0.1146195	4.91653902	0.56328358	0.46171876	0.68853868
C230029M16	1.04938857	-1.9497572	0.56318627	0.46175692	0.68853868
Ubr7	0.10439549	4.90069223	0.5630044	0.46182826	0.68853868
Gas6	-0.133596	4.3991346	0.56297556	0.46183957	0.68853868
Capsl	-0.3718416	1.18901442	0.56288024	0.46187697	0.68853868
Arl16	-0.1171328	4.2914429	0.5626383	0.46197191	0.68853868
Tsc22d3	-0.1755961	6.56716066	0.56250202	0.4620254	0.68853868
Cdk5r1	-0.1226062	6.96452263	0.56248458	0.46203224	0.68853868

Efcc1	-0.2136914	2.53421232	0.56243684	0.46205098	0.68853868
Nlrp5-ps	0.50237469	0.46725446	0.56164879	0.46236053	0.68892367
Ctu1	0.27598579	1.55210896	0.56148639	0.46242436	0.6889425
Tmem132b	-0.1530562	7.49325511	0.56111996	0.46256845	0.68907666
Lbp	0.33053352	3.35016585	0.56099696	0.46261683	0.68907666
Heatr1	-0.1830546	4.11923889	0.56066272	0.46274834	0.68919626
Pum2	-0.0802661	8.19361958	0.55998573	0.46301491	0.68933937
Tmem30b	0.37057149	2.72185465	0.55997105	0.46302069	0.68933937
Vars	-0.2229302	2.45858868	0.55982214	0.46307936	0.68933937
Slc27a1	0.19797093	3.86559533	0.55970815	0.46312428	0.68933937
Vcp	0.08371429	7.68924044	0.55968062	0.46313513	0.68933937
Ccdc101	0.14648337	4.2368717	0.55961288	0.46316183	0.68933937
Zbtb41	-0.0979691	6.45382066	0.55950836	0.46320303	0.68933937
Wasf3	-0.1118195	5.95092442	0.55914088	0.46334793	0.68947875
Cep72	-0.4293673	1.07004551	0.55892887	0.46343156	0.68952695
Zswim8	-0.1255733	5.28010502	0.55876646	0.46349564	0.68954605
Actr1a	0.09154687	6.16861461	0.55845237	0.46361961	0.68961415
Fam160a2	-0.1182479	6.65594544	0.55839082	0.46364391	0.68961415
2310033P09I	0.19101364	3.12529055	0.55806004	0.46377453	0.68966815
Gpm6a	0.11911644	9.42964172	0.55788609	0.46384326	0.68966815
Banp	-0.1432553	4.65768493	0.55778132	0.46388465	0.68966815
Gm15645	-0.2630084	1.94279585	0.55777987	0.46388523	0.68966815
Fam129c	0.73442864	-0.3552634	0.55752014	0.46398788	0.68969146
Serpinc1	-0.4957584	0.12159231	0.55748086	0.46400341	0.68969146
Fcrls	0.34292466	1.54344306	0.55699914	0.46419391	0.68985973
Slco1a5	0.72555685	-0.5127274	0.55693533	0.46421915	0.68985973
Macf1	-0.1807368	9.05836431	0.55670544	0.46431012	0.68988095
Leprel2	0.19267235	3.23305607	0.55647034	0.46440318	0.68988095
Fam120c	-0.1438985	6.59807918	0.55645503	0.46440924	0.68988095
Psmb4	0.13453364	5.59698976	0.55638112	0.4644385	0.68988095
Car9	0.60962632	-0.7352023	0.55618087	0.4645178	0.68992258
Rnf24	0.10744835	5.58235448	0.55550286	0.46478646	0.69024541
Pml	0.11294284	4.59802592	0.55532792	0.46485581	0.6902588
Cox8a	0.12179256	7.4453694	0.55511402	0.46494064	0.6902588
Pld1	0.15535284	5.52894528	0.5547937	0.46506773	0.6902588
Map3k14	-0.3271161	1.77007305	0.55478802	0.46506998	0.6902588
Lgals8	0.10924469	6.12087827	0.55476226	0.4650802	0.6902588
Hirip3	0.10613204	5.06218827	0.5545273	0.46517346	0.6902588
Zic3	-0.3311884	1.36545791	0.55447728	0.46519332	0.6902588
Trp53i11	0.18839662	8.13256173	0.55431119	0.46525927	0.6902588
Tex264	0.15437952	3.26062366	0.55421039	0.46529929	0.6902588
Pcm1	-0.1346209	8.57560764	0.55407307	0.46535384	0.6902588
Cd81	0.17811192	7.79192516	0.5537937	0.46546484	0.6902588

Fbxw17	-0.2617535	2.00307824	0.55375456	0.46548039	0.6902588
Ash1l	-0.0979533	9.15914459	0.55358738	0.46554684	0.6902588
Gpr88	-0.1283658	6.09706618	0.55355381	0.46556018	0.6902588
Cbx1	0.13790215	4.65570054	0.55350389	0.46558003	0.6902588
Pkn2	0.10033145	6.06826145	0.55339453	0.46562351	0.6902588
Irf5	0.38065127	0.96066234	0.55328388	0.4656675	0.6902588
Myc	-0.1766034	3.6606208	0.55294958	0.46580048	0.69026375
Trim30d	0.16487365	3.62302109	0.5529107	0.46581595	0.69026375
Pcgf2	-0.1464231	5.09123752	0.5527309	0.4658875	0.69026375
Prrx2	0.33320586	3.43939736	0.55262463	0.4659298	0.69026375
Pno1	0.11995646	3.98519793	0.55248299	0.46598618	0.69026375
Frat2	-0.2615882	3.01946885	0.55248221	0.46598649	0.69026375
Gm17066	-0.1581963	4.80154573	0.55234322	0.46604183	0.69026375
Trim14	-0.3796903	2.15536847	0.55224432	0.46608122	0.69026375
Gm5577	-0.5838753	1.02627357	0.55203061	0.46616634	0.69031384
Myo5c	-0.4273444	1.43219009	0.55160444	0.46633617	0.69032748
Rsf1	-0.1148796	7.58645342	0.55149917	0.46637814	0.69032748
Mir9-2	-0.8295162	-0.9702704	0.55144701	0.46639893	0.69032748
Pwwp2b	-0.258808	2.38647075	0.55138247	0.46642467	0.69032748
Arhgap31	0.11980574	6.53570096	0.55136393	0.46643206	0.69032748
Hmgn5	0.11437584	7.16198763	0.55096503	0.46659117	0.69044193
Ndufa6	-0.1132336	5.13130613	0.5509128	0.46661201	0.69044193
Opa1	-0.1069435	7.78864065	0.55076125	0.46667248	0.69045549
Nop56	-0.1171399	6.54885014	0.55025933	0.46687287	0.69061074
4933426M11	-0.1341877	5.71371529	0.55024132	0.46688006	0.69061074
Lrp4	-0.1436863	3.95895007	0.54974068	0.46708009	0.69078743
Znrf1	-0.0930774	6.14635925	0.54968541	0.46710218	0.69078743
Pygl	-0.3443444	1.09311284	0.54895111	0.46739584	0.69090629
Sytl5	0.16403379	4.73836367	0.54870934	0.46749259	0.69090629
Mtmr2	-0.1055041	6.65554123	0.54861669	0.46752968	0.69090629
Gm5464	0.4468176	0.45420489	0.54853272	0.4675633	0.69090629
Rufy3	0.10847979	8.36759982	0.54849296	0.46757921	0.69090629
Nes	-0.1288803	4.26178602	0.54804358	0.4677592	0.69090629
Pzp	-0.7353718	-0.8954508	0.54790454	0.46781491	0.69090629
Rtp3	-0.5803415	0.44595136	0.54781674	0.4678501	0.69090629
Acad12	-0.6035817	-0.9552592	0.5477823	0.4678639	0.69090629
Ccdc130	-0.2730822	2.27195659	0.54776842	0.46786946	0.69090629
Nsun7	0.30679952	2.25496462	0.54756375	0.4679515	0.69090629
Padi2	-0.1752914	4.03186539	0.54750777	0.46797395	0.69090629
Snrnp25	0.2378156	2.33329262	0.54747507	0.46798706	0.69090629
Gzma	0.89489402	-0.5829634	0.54727967	0.46806542	0.69090629
Trub2	-0.1983108	3.0142733	0.54719571	0.4680991	0.69090629
Ppard	0.1109217	5.18174367	0.54704815	0.46815829	0.69090629

4921511C10I	-1.168021	-1.6615581	0.54704383	0.46816003	0.69090629
Cntf	-0.715679	-1.5165918	0.54685883	0.46823426	0.69090629
Hk1os	-0.4012403	0.80907124	0.54677785	0.46826676	0.69090629
Nxpe3	-0.1996892	4.07112781	0.54677618	0.46826743	0.69090629
Fam122a	0.13452753	4.73155212	0.54667877	0.46830653	0.69090629
Ppp1r26	0.13293262	4.18231138	0.54655453	0.46835641	0.69090629
2900005J15F	0.30092161	2.24729623	0.54653693	0.46836347	0.69090629
Aprt	0.17839046	3.50790968	0.54618223	0.46850593	0.69104067
Diap2	0.10647951	7.4642199	0.54576556	0.46867335	0.69121186
Col6a6	0.90293196	-1.4085711	0.5454647	0.46879431	0.69124168
D8Ertd738e	0.23467335	3.18612086	0.54545973	0.46879631	0.69124168
Gm5083	-0.846777	-0.806053	0.54526747	0.46887363	0.69127994
Tmem35	0.16586055	3.85577349	0.54501341	0.46897584	0.69131705
Ypel3	-0.0903125	5.84479946	0.54494951	0.46900155	0.69131705
Lck	-0.4721983	0.1777984	0.54436858	0.46923542	0.69158602
Zbtb43	0.1535677	4.51846627	0.54368919	0.46950918	0.69191372
Mea1	-0.174991	4.7171691	0.54351435	0.46957967	0.69194183
Naif1	0.41799201	0.03808639	0.54305433	0.46976524	0.69213946
2010107G23	-0.1387789	4.05042054	0.54262436	0.46993879	0.69231936
Wfdc15b	-0.8945294	-2.0026989	0.54236894	0.47004193	0.69233302
Camkmt	-0.1211458	4.00481931	0.54233262	0.4700566	0.69233302
Rpgrip1	-0.6367073	-0.9646415	0.54196839	0.47020376	0.69233302
Ptges3	0.10977923	7.90167011	0.54176626	0.47028546	0.69233302
Hcfc2	-0.1530757	4.10084685	0.5414151	0.47042745	0.69233302
Cd46	-0.4877953	1.16625754	0.54136988	0.47044574	0.69233302
Unk	0.1725839	3.96633081	0.54126748	0.47048716	0.69233302
Kctd15	-0.264408	2.12360408	0.54123957	0.47049846	0.69233302
Tmem30a	0.09427817	7.94358369	0.54117706	0.47052375	0.69233302
Pex11a	-0.3339687	1.16454539	0.5411521	0.47053384	0.69233302
Mknk1	0.13138694	4.31864868	0.5410787	0.47056354	0.69233302
Cyp2r1	0.3651324	0.36074522	0.54097734	0.47060456	0.69233302
Cds1	-0.1223934	5.48304788	0.54075617	0.47069409	0.69233302
Syt6	0.18326173	4.7974429	0.54060109	0.47075688	0.69233302
Ddx52	0.09639446	4.67861184	0.54053319	0.47078437	0.69233302
Gripap1	0.12177354	4.91768759	0.5404835	0.4708045	0.69233302
Alk	-0.4212442	0.73476148	0.54032594	0.47086831	0.69233302
Dnaaf3	0.65442406	-1.1739159	0.54015432	0.47093784	0.69233302
Ppa2	-0.1041841	5.40210486	0.54010629	0.47095731	0.69233302
Nek2	-0.4065548	1.56168566	0.54005753	0.47097707	0.69233302
Rbbp8	0.16819388	4.13428988	0.53974044	0.47110559	0.69240159
Gm1987	0.83041184	-1.4241787	0.53956906	0.47117508	0.69240159
Lrrc71	-0.866355	-1.4883196	0.53956168	0.47117808	0.69240159
Gm16532	-0.5179313	1.04913494	0.53915871	0.47134154	0.69249804

Adamts13	0.18661	4.12095028	0.53900425	0.47140422	0.69249804
Tfb1m	0.20751985	2.80023338	0.5389285	0.47143497	0.69249804
Tns3	-0.090405	6.05708022	0.53878162	0.47149459	0.69249804
Zfyve9	0.15894469	3.85483059	0.53876579	0.47150102	0.69249804
Senp6	0.07711712	8.47980854	0.53862136	0.47155967	0.69250859
Msantd2	-0.2520789	3.04456159	0.53814321	0.4717539	0.69264649
Mlx	0.17405314	3.4619609	0.53784435	0.47187538	0.69264649
Casc1	-0.5704853	-0.6692017	0.53769871	0.47193459	0.69264649
Serpinb9	-0.140379	7.10563105	0.53769745	0.4719351	0.69264649
Pebp1	0.11749482	8.27988098	0.53760141	0.47197416	0.69264649
Bmp8b	0.92244671	-1.422884	0.53751213	0.47201047	0.69264649
Zic5	0.28262126	1.62181319	0.53750371	0.47201389	0.69264649
Gbp3	0.1803388	4.32332538	0.53735921	0.47207267	0.69265676
Phf1	-0.2568015	2.86487896	0.53714675	0.47215912	0.69265676
Zfp36l2	0.13161837	6.13442197	0.53710697	0.47217531	0.69265676
Sgce	-0.130276	4.38630861	0.53690803	0.47225628	0.69267051
Med14	-0.1241951	7.67537787	0.53683102	0.47228763	0.69267051
Gm12504	-0.4184447	0.86210628	0.53632537	0.47249357	0.69285799
Lbr	-0.1537337	3.4404604	0.53624635	0.47252577	0.69285799
4930594C11I	-0.6589658	1.31140351	0.53613797	0.47256993	0.69285799
Zfp385b	-0.1078214	6.65665764	0.53590352	0.4726655	0.69287388
Crbn	0.09293575	6.44617945	0.53585874	0.47268375	0.69287388
Ccar1	0.09208781	8.06170419	0.53562018	0.47278104	0.69293123
Fxyd4	0.69188503	-0.3788599	0.53535894	0.4728876	0.69293123
Gapt	-0.6581943	-0.1591237	0.53533298	0.47289819	0.69293123
Churc1	-0.1073493	4.54674334	0.53525783	0.47292886	0.69293123
Mbtd1	-0.1099063	5.66318019	0.53506087	0.47300924	0.69294932
Ticam1	-0.1585263	4.60652184	0.53497524	0.47304419	0.69294932
Lrrc45	-0.1507259	3.71480371	0.53462161	0.47318859	0.69296323
Trappc2l	-0.2096559	3.37912869	0.53453154	0.47322538	0.69296323
Pyhin1	-0.4182452	1.688976	0.53442586	0.47326855	0.69296323
St18	-0.2099681	3.77041997	0.5343399	0.47330367	0.69296323
Thrsp	0.24641302	3.17040787	0.53417935	0.47336928	0.69296323
Idh3a	0.09040536	6.62865407	0.53390982	0.47347946	0.69296323
St6galnac5	0.12532033	4.74676532	0.53365582	0.47358333	0.69296323
Qsox2	-0.1844479	2.93619289	0.53358107	0.4736139	0.69296323
Gtf2h3	-0.1635622	3.5832589	0.53354034	0.47363056	0.69296323
Gm10536	-0.6939689	-1.3984401	0.53345338	0.47366614	0.69296323
Rbm15	-0.1216723	3.93178982	0.53337557	0.47369798	0.69296323
1810043H04I	-0.3078063	1.96213509	0.53335846	0.47370498	0.69296323
Fam86	0.2041066	3.38812708	0.53331403	0.47372316	0.69296323
Hfe2	-1.0067356	-1.659473	0.53305087	0.47383086	0.6929706
Avpr1a	-0.709545	-0.6701335	0.53288619	0.47389829	0.6929706

Sema4g	0.23098804	2.77395805	0.53280963	0.47392963	0.6929706
Rps10	-0.1679659	4.92802873	0.53269477	0.47397667	0.6929706
Arhgap39	0.12131284	4.98563479	0.53267278	0.47398568	0.6929706
Zfpm2	-0.2067138	3.7385624	0.53204206	0.47424414	0.69327314
St5	-0.164609	4.99487358	0.53165027	0.4744048	0.69331596
Mkln1os	-0.2844873	1.23498685	0.53159824	0.47442614	0.69331596
Asns	0.13787384	5.25667555	0.53154403	0.47444838	0.69331596
Adal	-0.1320047	3.96032848	0.5310681	0.47464371	0.69331596
Rnf217	0.12311302	4.29072797	0.53102835	0.47466003	0.69331596
Irs3	0.27488499	2.20963821	0.53102585	0.47466105	0.69331596
Evpl	0.36146975	1.2386368	0.53088507	0.47471886	0.69331596
Kif27	-0.2301708	2.20180061	0.53072166	0.47478597	0.69331596
Abhd10	-0.1397707	4.33952825	0.53069467	0.47479705	0.69331596
Gm11346	-0.7570614	-0.647839	0.53058526	0.474842	0.69331596
Smg9	0.20433438	2.90670721	0.5305805	0.47484396	0.69331596
Mafb	0.10324688	5.48543859	0.53034769	0.47493962	0.69331596
Setd5	-0.0906934	8.40831928	0.5303389	0.47494323	0.69331596
Cebpg	-0.0932468	6.39864754	0.53001439	0.47507663	0.69338497
Chmp6	-0.1960092	2.23547176	0.5299732	0.47509357	0.69338497
Npc1	-0.1262135	4.96851897	0.52936516	0.4753437	0.69363751
Higd2a	-0.1241421	5.32361296	0.52930201	0.47536969	0.69363751
Gramd1b	-0.113662	5.84688195	0.52912413	0.47544292	0.69366913
A930005H10	0.21349139	3.04340925	0.52885232	0.47555485	0.69375722
Dmrt3	1.38167228	-1.5248204	0.52860233	0.47565783	0.69377495
Pkia	0.07891148	8.02784986	0.5285725	0.47567012	0.69377495
Chd6	-0.123178	7.19872025	0.52832371	0.47577265	0.69380727
Gm2109	-1.267761	-2.2724261	0.52826773	0.47579572	0.69380727
Lmo3	-0.1058914	7.13282891	0.52814346	0.47584695	0.69380727
Mcm4	-0.1372664	4.37157769	0.52794421	0.47592912	0.69385188
Clic6	-0.2328086	2.60919011	0.52753644	0.47609734	0.69395202
Rpl7a	0.12781414	7.31122517	0.52752769	0.47610095	0.69395202
Ncs1	0.11509781	6.14444869	0.52672944	0.47643055	0.693964
Rcn3	0.30722057	3.12705558	0.52672876	0.47643083	0.693964
Hist2h4	-0.4404628	0.30175153	0.52652048	0.47651689	0.693964
Ctdp1	0.12551524	3.88503483	0.5258637	0.47678845	0.693964
Il1rl1	-0.7043358	-0.1980598	0.52578122	0.47682257	0.693964
Smim19	0.14493049	4.67526929	0.52575336	0.4768341	0.693964
Tsnax	-0.0860714	7.46016546	0.52562568	0.47688693	0.693964
Mfhas1	-0.1075063	5.25303798	0.5256215	0.47688866	0.693964
Sdc2	0.16171994	6.59430272	0.5255272	0.47692769	0.693964
Nap1l2	-0.1429678	5.39456633	0.52549285	0.4769419	0.693964
Stk38	0.11283083	4.80460911	0.5254791	0.4769476	0.693964
Nfrkb	0.12511639	4.24815793	0.52541273	0.47697507	0.693964

6030458C11I	-0.1488516	4.92836648	0.5254001	0.47698029	0.693964
Zfp384	0.10314808	5.17019792	0.52534338	0.47700377	0.693964
Sik3	-0.1097179	7.39081185	0.52509264	0.4771076	0.693964
Bhmt2	-0.9807298	-0.5181588	0.52507238	0.47711599	0.693964
Rps24	-0.1264864	7.28769221	0.5248255	0.47721825	0.693964
Dctn1	-0.1361755	5.19929677	0.52474559	0.47725136	0.693964
4930432K21I	0.42185673	-0.3257549	0.52439458	0.47739684	0.693964
Il1r1	-0.1561302	4.30797735	0.52430777	0.47743283	0.693964
Ppp1r35	0.26700103	1.49108591	0.52421474	0.4774714	0.693964
Atp9b	-0.1803936	3.99564467	0.52421277	0.47747222	0.693964
1600002H07I	-0.1858619	3.04316158	0.5242062	0.47747494	0.693964
Map2k7	-0.142448	5.36639003	0.52416719	0.47749112	0.693964
Myo10	-0.0989043	5.70474364	0.52407276	0.47753028	0.693964
Asb3	0.14313042	4.02289855	0.52400197	0.47755965	0.693964
Cntrob	-0.2672115	2.14754063	0.52399416	0.47756288	0.693964
Mypop	-0.1817467	2.50815487	0.52387223	0.47761346	0.693964
Pde6c	-0.9747012	-0.9940223	0.52366639	0.47769888	0.693964
Sdk2	0.19524502	3.60874328	0.52356528	0.47774084	0.693964
Fastkd3	-0.125309	3.79209498	0.52353504	0.47775339	0.693964
P2rx1	-1.1170649	-2.3316039	0.52352042	0.47775946	0.693964
Enpp1	0.17997837	5.3449305	0.52338894	0.47781404	0.69396838
Shcbp1l	0.49076816	0.19433113	0.52307646	0.4779438	0.69405103
Rras	0.21370392	4.72190055	0.52300351	0.47797411	0.69405103
Prkcz	-0.0966545	6.3385358	0.52280777	0.47805543	0.69406353
F420014N23	-0.3235221	0.69867719	0.5227345	0.47808588	0.69406353
Mthfd2	0.3095847	1.46340259	0.52219297	0.47831101	0.6942884
Zglp1	-0.9089884	-1.4615656	0.52210427	0.47834791	0.6942884
1110004F10I	-0.124121	6.35477478	0.52190517	0.47843073	0.6942884
Adck3	-0.1725092	2.71438383	0.52186572	0.47844715	0.6942884
Hexdc	0.26955785	1.95366144	0.5215203	0.47859091	0.69430336
Cd247	-0.9802313	-1.5631675	0.52150466	0.47859743	0.69430336
Cfp	0.38474195	1.41500791	0.52146906	0.47861225	0.69430336
Ist1	0.10573557	5.83351638	0.52116395	0.47873931	0.69439397
Fam65b	-0.1334623	5.89319166	0.52106613	0.47878006	0.69439397
Toe1	0.1884913	2.66854513	0.5209474	0.47882952	0.69439397
02-Mar	0.16569943	5.33229017	0.52072374	0.47892272	0.69445429
Trim12a	-0.1854202	3.76202107	0.520526	0.47900516	0.69446485
Ebf4	-0.443634	1.13461649	0.52045867	0.47903323	0.69446485
Klrb1f	-0.5226204	-0.1107929	0.51989017	0.47927037	0.6947338
Pbx4	-0.394429	0.13138919	0.51963186	0.47937819	0.69475399
Zfp777	-0.2164005	2.08717096	0.51960151	0.47939086	0.69475399
Gm5796	-0.5161831	-0.9775086	0.51943638	0.47945981	0.69475399
Mycbp2	-0.1788332	8.84042078	0.51936212	0.47949082	0.69475399

Nsun2	-0.0987677	5.15905274	0.51874664	0.47974799	0.69493893
Ikbkap	-0.1570929	5.38567574	0.51868647	0.47977314	0.69493893
1700018A04I	-0.8697412	-1.9461086	0.51842058	0.47988432	0.69493893
Skint3	-0.3486065	1.16596641	0.51836176	0.47990892	0.69493893
Oraov1	-0.1692901	3.70779662	0.51829808	0.47993555	0.69493893
2010300C02I	-0.1192527	5.50339108	0.51821425	0.47997062	0.69493893
Lman2	0.10877218	5.61149008	0.51813245	0.48000485	0.69493893
Pcgf1	0.20292315	3.18836724	0.51806847	0.48003161	0.69493893
A930024E05	0.43558092	0.66919933	0.51767806	0.48019502	0.69497281
Tbx20	0.6983824	-1.4327561	0.51765908	0.48020297	0.69497281
Tap1	-0.2931917	1.05045136	0.5176424	0.48020995	0.69497281
Tc2n	-0.3521468	0.86585266	0.51727659	0.48036316	0.69511977
Zfp583	0.23785799	3.39265592	0.51710048	0.48043695	0.69515179
Pomt2	0.13972527	3.59827126	0.51680767	0.48055967	0.6952546
Senp3	-0.1189849	4.80653401	0.51664947	0.480626	0.6952595
Snph	-0.140973	5.88306559	0.516224	0.48080446	0.6952595
Grip1	0.1637175	4.08404271	0.51610975	0.4808524	0.6952595
Slc25a19	-0.1609844	3.33440775	0.51607023	0.48086898	0.6952595
Kat7	0.09068988	5.9074198	0.51606637	0.48087061	0.6952595
Rgs19	0.14182352	4.53633584	0.51601821	0.48089082	0.6952595
Specc1	0.10014698	6.56145607	0.51591194	0.48093543	0.6952595
Eno4	-0.2803128	1.34689556	0.51571414	0.48101847	0.6952595
Cnot1	-0.092247	8.09961705	0.51569128	0.48102807	0.6952595
Btbd17	-0.4004485	0.43753418	0.51550773	0.48110515	0.69529206
Ypel1	-0.2017832	3.12855469	0.5153916	0.48115393	0.69529206
Cops2	0.09707082	8.04261981	0.51469721	0.48144579	0.69562079
Lsm8	-0.1419489	5.66105013	0.51455039	0.48150754	0.69562079
Slc26a1	-0.6310857	-0.6319388	0.51443933	0.48155426	0.69562079
Yy2	-0.4517148	-0.0337332	0.51435866	0.4815882	0.69562079
Gramd1a	-0.205469	3.06794535	0.51399913	0.48173951	0.69573589
Tmbim6	0.13180803	6.63596553	0.51369524	0.48186746	0.69573589
Cd300lb	0.99736268	-1.3241389	0.51357664	0.48191742	0.69573589
Zfp941	-0.1314202	4.53019868	0.51355907	0.48192482	0.69573589
Ublcp1	0.09540841	6.44773241	0.51344027	0.48197486	0.69573589
Mthfd1	-0.1760621	3.54331546	0.51343258	0.48197811	0.69573589
Gm15816	0.45270844	0.7008866	0.51319261	0.48207923	0.69580722
Npepps	-0.1144774	6.32023894	0.51294852	0.48218212	0.69588109
Simc1	-0.1162566	4.18232201	0.51264447	0.48231035	0.69598149
Psmd11	0.12462778	5.94030805	0.5125383	0.48235513	0.69598149
Tyms	-0.1551136	3.66644618	0.51222381	0.48248784	0.69608993
Irgm2	0.1564883	5.57099843	0.51211506	0.48253375	0.69608993
Ipo11	0.1063393	6.18942871	0.51180774	0.48266351	0.69609119
Arsi	0.44173992	0.61766923	0.5116566	0.48272735	0.69609119

Tmbim1	-0.1447148	5.07240249	0.51160756	0.48274806	0.69609119
Btd	-0.2129211	3.78710427	0.51153708	0.48277784	0.69609119
Fam92a	0.09559295	6.562983	0.51150057	0.48279327	0.69609119
Zfp426	0.09605695	6.10522924	0.51091422	0.48304113	0.69633333
Tram1l1	-0.1327542	4.25816909	0.51068803	0.4831368	0.69633333
Nemf	-0.1114857	7.13975157	0.51063323	0.48315998	0.69633333
Pitpnm2	0.12118389	6.50109081	0.51052081	0.48320755	0.69633333
Espn	-0.456529	-0.5472656	0.51044369	0.48324019	0.69633333
Mpg	-0.2316	1.96183919	0.51036924	0.4832717	0.69633333
Arpc1b	0.2158301	6.03862312	0.51004442	0.48340921	0.69645294
Mettl17	-0.2680597	1.69387304	0.50992867	0.48345823	0.69645294
Tubb2a	0.12585527	7.72927064	0.50977987	0.48352125	0.69646918
Tnfsf13b	-0.8747118	-1.2214902	0.50938946	0.48368668	0.6966329
Ccar2	-0.1583647	4.23538832	0.50915219	0.48378727	0.69667689
Gm10548	-0.2690293	1.74627685	0.50899678	0.48385318	0.69667689
Gemin2	-0.2187799	2.12116911	0.50895111	0.48387255	0.69667689
Nus1	0.08190728	6.88160066	0.50848563	0.48407005	0.69688668
Ptrh2	0.10569869	4.96435401	0.50822276	0.48418164	0.69693909
Gria1	0.1356863	6.49915439	0.50796739	0.48429009	0.69693909
Sorbs3	0.16384991	7.07168681	0.50793034	0.48430583	0.69693909
Desi2	-0.0970341	6.81310931	0.50791199	0.48431362	0.69693909
Serinc3	0.11451178	8.71528027	0.50760693	0.48444324	0.69695452
Pycr1	0.48812161	0.14120247	0.50743621	0.48451581	0.69695452
Mbd6	-0.2063743	3.97290676	0.50742581	0.48452023	0.69695452
1500015L24F	-0.789903	-0.0251291	0.50725606	0.4845924	0.69695452
Mrpl57	-0.118787	4.6920637	0.50718249	0.48462369	0.69695452
Gm19466	-0.5506759	-0.2042499	0.50707331	0.48467012	0.69695452
Tnfaip6	-0.2463477	1.74255489	0.50695253	0.4847215	0.69695452
Myl12a	0.19055078	6.85744348	0.50691211	0.4847387	0.69695452
Neil2	-0.6129206	0.05266386	0.50659362	0.48487422	0.69704888
Zfand5	0.07722462	8.01763302	0.50651444	0.48490793	0.69704888
Rab9	0.12093153	5.70015538	0.50632954	0.48498665	0.69708758
Rnf146	-0.1022298	5.45688731	0.50611935	0.48507617	0.69714178
Npr2	-0.122422	4.07112397	0.50575119	0.48523302	0.6972631
Cep192	-0.1734551	4.46641486	0.50554145	0.48532242	0.6972631
E2f3	-0.1213372	5.37305131	0.50523119	0.48545473	0.6972631
Klf3	0.12451697	7.62085981	0.50522118	0.48545899	0.6972631
Plcd4	-0.210501	2.1931038	0.50515407	0.48548762	0.6972631
Ncapg	-0.615215	-0.3341187	0.50512325	0.48550077	0.6972631
Sorcs1	-0.1591516	4.97864272	0.50501637	0.48554636	0.6972631
Hipk3	0.0725918	7.79073933	0.50475946	0.48565599	0.6972631
Cd55	0.16538829	5.98307271	0.50466909	0.48569457	0.6972631
Gabpa	0.09181306	6.71949932	0.50459161	0.48572764	0.6972631

Rpain	-0.1690472	2.84744177	0.50458475	0.48573057	0.6972631
Tnfsf12	-0.16825	3.32585799	0.50376463	0.48608092	0.69757841
Dnajb4	0.08411322	8.41229707	0.50365742	0.48612675	0.69757841
Anapc7	-0.1548809	3.5193806	0.50354999	0.48617268	0.69757841
Btg1	0.14653375	5.37506462	0.50343722	0.48622091	0.69757841
4921531C22I	0.21784426	2.74290352	0.50343063	0.48622373	0.69757841
Alkbh7	-0.2411988	1.95290312	0.50332479	0.48626899	0.69757841
Gpd2	0.08323354	6.9886585	0.50304372	0.48638924	0.69757841
Lmf1	0.19772471	2.93702703	0.50295003	0.48642934	0.69757841
Cnnm2	0.16056424	3.86824534	0.50286572	0.48646542	0.69757841
Cpsf2	0.0899189	6.35806903	0.50281627	0.48648659	0.69757841
Ccnb2	0.57262258	-0.2245179	0.50266506	0.48655132	0.69757841
Phf13	0.16501245	3.5556069	0.50259516	0.48658125	0.69757841
Hs6st2	0.15940915	4.82029221	0.5022738	0.4867189	0.69757841
Mis18bp1	-0.3048738	1.0692987	0.50223557	0.48673527	0.69757841
Leprotl1	-0.0862533	6.11791754	0.50199537	0.48683821	0.69757841
Commd4	0.19807318	3.50891298	0.50187652	0.48688915	0.69757841
Lpcat3	-0.1746663	3.53870214	0.50171234	0.48695953	0.69757841
Ticrr	-0.6685309	-0.3232316	0.5017111	0.48696007	0.69757841
4632427E13I	0.36463474	1.56758032	0.50147671	0.48706059	0.69757841
Lect1	0.49380236	-0.5496984	0.50138102	0.48710163	0.69757841
M6pr	0.12525756	6.22491652	0.50118565	0.48718546	0.69757841
Parp12	-0.1405915	4.35770763	0.50116414	0.48719469	0.69757841
Sltm	-0.0909206	7.70883421	0.50114734	0.4872019	0.69757841
Gm12338	-0.1479848	4.54261356	0.50106067	0.4872391	0.69757841
Vav1	0.39653291	0.25682111	0.50104405	0.48724623	0.69757841
Mrpl14	-0.217858	3.03606529	0.50077787	0.4873605	0.69762678
Tmed9	0.08632394	6.26273586	0.50068514	0.48740032	0.69762678
Golga2	0.1028002	5.49769387	0.50060312	0.48743554	0.69762678
Tctn1	-0.3410704	2.90632425	0.50044645	0.48750284	0.69764053
Gnpnat1	0.12350406	4.78318396	0.50033938	0.48754884	0.69764053
Atf3	0.56842768	1.54733601	0.4998579	0.4877558	0.69774086
Hbegf	0.2622886	1.72876483	0.49979688	0.48778203	0.69774086
Fzd7	-0.2045122	5.93664349	0.49966981	0.48783668	0.69774086
Mrpl27	0.13785412	5.32071459	0.49966236	0.48783988	0.69774086
Akap9	-0.1060243	8.44979472	0.4993784	0.48796204	0.69774086
Ctnna3	0.64598624	-0.1813405	0.4991821	0.48804652	0.69774086
Magee2	-0.1381165	4.78781903	0.49907903	0.48809089	0.69774086
Zfp939	-0.2499087	2.6746555	0.49898705	0.48813049	0.69774086
Eef1b2	-0.1250913	7.031599	0.49884465	0.48819181	0.69774086
Fgfbp1	0.24475147	4.61375719	0.49847115	0.48835269	0.69774086
Gm14326	-0.159503	4.52824376	0.49846666	0.48835463	0.69774086
Ebag9	-0.0817813	5.98741643	0.49844566	0.48836368	0.69774086

Oc90	1.12641698	-2.3165054	0.50143153	0.48851869	0.69774086
Il2rb	-0.4142709	0.95630689	0.49805238	0.48853319	0.69774086
Oxa1l	0.12667198	5.17887572	0.49801897	0.4885476	0.69774086
Cox5a	0.09420832	6.35385492	0.4979748	0.48856665	0.69774086
Traf3ip2	-0.1836929	2.99233899	0.49777423	0.48865314	0.69774086
Snhg3	0.2498353	3.02494622	0.49714832	0.48892325	0.69774086
Bdh2	-0.2451812	3.09510841	0.49701603	0.48898037	0.69774086
Arxes2	0.16793372	3.63078278	0.49678964	0.48907816	0.69774086
Klf13	-0.088654	7.23167154	0.49675194	0.48909444	0.69774086
Pex13	-0.0949565	5.35870662	0.49669563	0.48911877	0.69774086
Chrnb3	-0.3505686	1.28994325	0.49668998	0.48912121	0.69774086
Cdh18	0.19172519	3.95230194	0.49662725	0.48914831	0.69774086
Nlgn1	0.12270893	6.61629113	0.49662026	0.48915134	0.69774086
Morn4	-0.1173711	5.72266138	0.49661247	0.4891547	0.69774086
Plaur	0.69981842	-1.2438699	0.49657653	0.48917023	0.69774086
Socs7	-0.1065443	6.96844492	0.49647289	0.48921502	0.69774086
Ttc3	-0.1226561	9.98771488	0.49641478	0.48924014	0.69774086
Prickle4	0.9428661	-1.8831369	0.4963998	0.48924661	0.69774086
Gas8	-0.1855574	3.60001775	0.49633489	0.48927467	0.69774086
Sorbs2os	-0.249602	3.15431538	0.49615025	0.4893545	0.69774086
Atpaf2	0.15392876	3.16296147	0.49614926	0.48935492	0.69774086
Atp7a	-0.1117105	5.55625978	0.49608679	0.48938194	0.69774086
Hmcn1	-0.1535072	3.75683083	0.49586083	0.48947967	0.69777088
5730403I07R	-1.1776364	-1.970672	0.49579835	0.4895067	0.69777088
Zbtb10	0.09541403	5.46634052	0.49563078	0.48957921	0.69780032
Sh3bgrl3	-0.1137821	6.73140754	0.49530572	0.48971991	0.6979024
Itgax	-0.6074434	-0.4385055	0.49503319	0.48983793	0.6979024
Syk	-0.3329332	1.46868149	0.49500005	0.48985228	0.6979024
Whamm	-0.2286968	3.09783913	0.4948775	0.48990537	0.6979024
Tigit	0.86395812	-1.5637393	0.49476962	0.48995211	0.6979024
Speer4e	-0.6448661	-1.3641308	0.49456115	0.49004246	0.6979024
Sorcs2	-0.1527422	3.64157151	0.49447501	0.4900798	0.6979024
Znfx1	-0.1235586	5.36773482	0.4942923	0.49015902	0.6979024
Col15a1	-0.3335265	1.46629283	0.49427112	0.4901682	0.6979024
Ahi1	-0.1273452	6.88549225	0.49426819	0.49016948	0.6979024
Lrrc24	0.28760455	0.76078617	0.49354728	0.49048226	0.69812262
Ciapin1	-0.1100698	4.72236937	0.49346418	0.49051834	0.69812262
Gm19705	0.29153235	1.55104948	0.49338485	0.49055279	0.69812262
Gm7102	-0.2197059	5.46312547	0.49331915	0.49058132	0.69812262
Lrrc40	0.13322112	5.10632569	0.49320985	0.49062879	0.69812262
Med9os	-0.7409579	-1.163347	0.49309403	0.4906791	0.69812262
Tmem161b	-0.2168662	3.57042843	0.49307513	0.49068731	0.69812262
Msi2	0.11292314	9.31989194	0.49279852	0.4908075	0.6982198

2810474O19	0.09607833	6.6693074	0.49267343	0.49086188	0.69822334
Uty	-0.1170186	5.05926965	0.49245726	0.49095586	0.6982342
Pggt1b	0.12500356	4.10302833	0.49241719	0.49097329	0.6982342
Nup35	-0.157867	3.28593471	0.49203303	0.4911404	0.69839804
Pate2	0.40324883	0.20605937	0.49167231	0.4912974	0.69844318
Naalad2	0.26754488	1.33162238	0.49162797	0.4913167	0.69844318
Eif2ak2	0.13495038	5.80721038	0.4915637	0.49134468	0.69844318
Ssr1	0.09499269	7.3121515	0.49148315	0.49137976	0.69844318
Ssbp4	-0.1765069	2.9201445	0.49038163	0.49185987	0.69891317
Gper1	0.25407334	1.96462985	0.490338	0.4918789	0.69891317
Msto1	0.30029434	1.83431399	0.49029159	0.49189915	0.69891317
Ybx3	0.13963641	7.39449024	0.490248	0.49191817	0.69891317
Tmem120b	0.54478509	0.44603953	0.490053	0.49200326	0.69896027
Traf7	0.12891784	3.73534693	0.48979178	0.4921173	0.69904847
Cdc42ep2	0.2181254	2.08049696	0.48953119	0.4922311	0.69911721
Rps6ka5	-0.1969054	3.29476778	0.48944306	0.4922696	0.69911721
Mllt11	-0.0758062	7.83720873	0.48926598	0.49234696	0.69912485
Pbrm1	0.07656369	7.48916556	0.48915845	0.49239396	0.69912485
Atg2a	-0.182278	3.99208014	0.48907405	0.49243085	0.69912485
Gm16938	-0.2338982	2.17302732	0.48873988	0.49257695	0.69914856
Fxyd6	0.12668118	5.08938502	0.48867919	0.49260349	0.69914856
D830030K20	-0.4362301	0.44318328	0.4886567	0.49261333	0.69914856
Aplp1	0.1050703	8.34531989	0.48855272	0.49265882	0.69914856
Fndc3a	0.08827814	8.08988479	0.48823466	0.49279799	0.69914856
9630033F20I	-0.1355218	4.43530924	0.48822485	0.49280228	0.69914856
Gpr157	0.26015126	2.14962224	0.48811566	0.49285007	0.69914856
Cyp2s1	0.23508515	3.8419522	0.48808568	0.4928632	0.69914856
Fam198a	-0.2922042	1.78053797	0.48784672	0.49296782	0.6991869
Eci1	-0.1364865	3.68755631	0.48778662	0.49299415	0.6991869
Vipr1	0.25989086	2.42237839	0.48738211	0.49317136	0.69928044
Apc	-0.1280774	9.68081644	0.48728994	0.49321176	0.69928044
Cirbp	-0.2057455	4.87862209	0.48728026	0.493216	0.69928044
Cldn10	-0.2667984	1.85072512	0.48706989	0.49330822	0.6993071
Abcf3	-0.1228641	5.02089209	0.4869379	0.4933661	0.6993071
Acvr1b	-0.1259318	6.06409219	0.48688178	0.49339071	0.6993071
Rn45s	-0.1545603	14.5621267	0.48603788	0.49376107	0.69972215
Mov10l1	-0.9520791	-2.089242	0.48597759	0.49378755	0.69972215
Tmem147	0.15777335	3.44846783	0.48581195	0.4938603	0.69975156
Sh3gl1	-0.1696505	3.29642853	0.48549898	0.49399783	0.6997999
Lrtm1	-0.3934192	3.1295136	0.48549761	0.49399843	0.6997999
Erv3	1.43431888	-1.2482462	0.48510137	0.49417264	0.69997299
Mrm1	-0.1420692	3.09996865	0.48472203	0.49433951	0.7001213
AI413582	-0.1456061	3.26757408	0.48462684	0.4943814	0.7001213

Ube2e3	0.10716882	7.65512624	0.48438701	0.49448697	0.7001697
Tmem176b	0.18378232	5.40405704	0.48431281	0.49451964	0.7001697
Il7	0.86838422	-0.8826703	0.48408241	0.49462111	0.7001752
Rbm38	0.53425672	0.8031753	0.48406769	0.49462759	0.7001752
Hmbox1	-0.1067291	5.1907916	0.4838742	0.49471284	0.70022221
Haus6	0.13747028	3.95219475	0.48368199	0.49479754	0.70026482
Alpk1	0.220007	2.5294278	0.48356973	0.49484702	0.70026482
Mocs2	0.10465862	6.84766251	0.48332767	0.49495375	0.7003344
Cd8b1	-0.4350766	-0.329234	0.4831724	0.49502223	0.7003344
Son	-0.1236584	8.07903712	0.48310418	0.49505232	0.7003344
Mpnd	0.17256275	3.83602864	0.48270405	0.49522889	0.70051054
Pds5b	-0.0881705	7.92641985	0.48236846	0.49537707	0.70064649
Olfml2a	0.2429415	3.54799309	0.48217167	0.49546399	0.7006826
Slmo1	-0.1412329	5.01944233	0.4820749	0.49550675	0.7006826
Ndufc2	0.12882121	5.2850835	0.48182958	0.49561516	0.70072562
Lmbrd1	0.08142617	6.75262406	0.4816995	0.49567267	0.70072562
2210018M11	0.10636582	6.75589325	0.48165262	0.49569339	0.70072562
Fam63a	0.17692279	4.35498773	0.48148547	0.4957673	0.70075649
Phka2	-0.1847985	4.89890177	0.48126438	0.4958651	0.7008211
Fam107b	0.16564755	5.28728601	0.48114497	0.49591793	0.70082217
Nkiras2	0.58138957	0.14816501	0.48070637	0.49611207	0.70097125
Golga3	0.07131163	6.79705468	0.48067127	0.49612761	0.70097125
Rps27a	-0.109792	6.62252119	0.48043026	0.49623436	0.70104846
Dmrta2	0.48273867	-0.2709334	0.48018003	0.49634522	0.70106127
Efhd2	0.08966949	6.73441745	0.48017461	0.49634762	0.70106127
Cyth1	-0.1683205	3.89157414	0.4799589	0.49644323	0.70112272
Slc39a12	-0.2926543	2.63789016	0.47964909	0.4965806	0.70116294
2010107E04I	0.11372128	5.84260277	0.4795726	0.49661453	0.70116294
Ubash3a	0.83932227	-1.2589354	0.47954217	0.49662803	0.70116294
Efr3b	-0.1386514	6.81278274	0.47923625	0.49676376	0.701281
Gstz1	0.10674784	4.65541876	0.47892895	0.49690017	0.70131128
Mocs1	0.18575422	4.31887356	0.47888409	0.49692009	0.70131128
Evc2	-0.2791569	2.24314415	0.47870586	0.49699925	0.70131128
Snora30	-0.8512878	-1.4576189	0.47858616	0.49705242	0.70131128
Terf1	-0.1415227	3.52866775	0.47857169	0.49705885	0.70131128
Tef	-0.0735609	6.79325453	0.47839425	0.49713769	0.70131128
Spdef	-0.9141652	-1.9795163	0.47821093	0.49721916	0.70131128
Tspan14	-0.1252032	3.50768409	0.47804188	0.49729432	0.70131128
Olfr316	-0.8083649	-0.6026175	0.47803101	0.49729915	0.70131128
Tceal7	-0.5719601	-0.1028315	0.47792906	0.49734449	0.70131128
Fzd2	0.18697248	4.20920458	0.47763778	0.49747405	0.70131128
Col6a2	-0.2155489	5.41390763	0.47754062	0.49751728	0.70131128
Ptpn22	0.21655015	2.35806896	0.47752107	0.49752598	0.70131128

Trim43b	-0.5417254	-0.7916784	0.47749631	0.497537	0.70131128
Cnep1r1	-0.0934928	5.90933978	0.47737614	0.49759048	0.70131128
Ccdc66	0.09011503	5.59627486	0.47728305	0.49763191	0.70131128
Proser2	-0.2648367	2.69453176	0.47709783	0.49771438	0.70131128
Pip4k2a	-0.0740967	7.90645861	0.47693238	0.49778806	0.70131128
Ccne2	-0.2466007	1.99831562	0.4766995	0.4978918	0.70131128
Xlr4c	0.78798267	-1.3636002	0.47656512	0.49795168	0.70131128
Slc25a11	0.10238654	5.65167589	0.4765592	0.49795432	0.70131128
Fam184b	0.2310405	2.75541386	0.47650985	0.49797631	0.70131128
Cdk15	0.34606164	0.70685136	0.47649279	0.49798392	0.70131128
Pdlim3	-0.6380506	-0.6166203	0.47631915	0.49806132	0.70132659
Clec10a	-0.6766699	-1.1430793	0.47623458	0.49809903	0.70132659
Ccdc120	-0.2133757	2.46031896	0.4758694	0.4982619	0.70136647
Adam11	0.18062521	3.57860668	0.47578006	0.49830176	0.70136647
5033404E19I	0.9750624	-1.5114324	0.47575023	0.49831507	0.70136647
Gata2	-0.4695109	0.77249264	0.47563482	0.49836657	0.70136647
Rep15	0.87871733	-1.3864379	0.47558689	0.49838796	0.70136647
Tmie	0.24124585	1.66140643	0.4752199	0.49855181	0.70148152
Micall1	-0.1020097	5.22167441	0.47487534	0.49870573	0.70148152
C230052I12R	-0.1737843	2.85948988	0.47485322	0.49871562	0.70148152
BC003965	-0.0893385	6.03354468	0.47482113	0.49872995	0.70148152
Hspa12a	-0.1015923	8.26681948	0.47479178	0.49874307	0.70148152
Nmbr	0.481406	0.19861684	0.47445361	0.49889423	0.70148152
Chrm3	-0.1670336	3.59287046	0.47437353	0.49893004	0.70148152
Prmt10	0.21966234	3.33900613	0.47428543	0.49896944	0.70148152
Gpr125	0.18810648	3.35494586	0.47425544	0.49898286	0.70148152
Tbxa2r	0.92671969	-2.105866	0.47423718	0.49899102	0.70148152
C1ql2	-0.8311784	-1.1618038	0.47398644	0.4991032	0.70154316
Tnni2	-0.7189834	-1.3371784	0.47390612	0.49913914	0.70154316
Trps1	-0.1104413	6.29006397	0.47372036	0.49922228	0.70158674
Sp5	0.51343411	1.50954197	0.47331621	0.49940325	0.70176778
Msantd4	0.07990449	7.36767747	0.47308508	0.4995068	0.70183999
Bmp5	0.18340923	5.86013317	0.47293871	0.49957239	0.70184462
Fars2	-0.1926854	2.72872315	0.47274583	0.49965885	0.70184462
Spsb4	-0.3477642	1.11843022	0.47272863	0.49966656	0.70184462
Setdb1	-0.1511137	4.51143675	0.47246934	0.49978283	0.70193467
Rpl8	0.13051409	5.66174501	0.4722349	0.49988801	0.70194237
Kdm2a	-0.082693	7.17003186	0.47222455	0.49989265	0.70194237
Ece1	0.11319115	5.98034349	0.47188942	0.50004306	0.70197145
Rpl19	0.12428253	7.38560878	0.47180547	0.50008075	0.70197145
Fstl1	0.2058175	7.22418471	0.47170729	0.50012484	0.70197145
C030034L19F	0.79904882	-1.2749103	0.47166795	0.5001425	0.70197145
Gm6568	-0.2981001	0.79574085	0.4715974	0.50017419	0.70197145

Itfg2	0.14833129	3.37979046	0.47125663	0.50032728	0.7020233
Bicd1	-0.1018529	6.59984116	0.47118838	0.50035795	0.7020233
Tgm4	0.30584469	1.23746859	0.47107148	0.50041049	0.7020233
Haus4	0.27198047	1.58054984	0.47105074	0.50041982	0.7020233
Zfp708	0.15837823	3.49910848	0.47088487	0.50049439	0.70205086
AI606473	0.54865289	-0.0018368	0.47076797	0.50054696	0.70205086
Eif1ad	-0.140025	4.17200908	0.4705161	0.50066025	0.70205086
Rps19bp1	0.16266169	2.88831036	0.47043747	0.50069563	0.70205086
Pwp1	-0.1547513	4.19205255	0.4704146	0.50070592	0.70205086
Tnpo2	-0.0841962	5.98827346	0.4703111	0.5007525	0.70205086
Socs5	-0.093143	6.54267267	0.47006869	0.50086161	0.70213068
Naip2	-0.3950689	0.32866889	0.46969803	0.50102854	0.70215361
Gfer	-0.1763534	3.00280689	0.4695041	0.50111591	0.70215361
Cdc45	0.3567336	0.92619424	0.4694184	0.50115454	0.70215361
Osbpl11	0.12007193	4.16970836	0.46935738	0.50118204	0.70215361
Itgb6	0.77059333	-1.8874354	0.4693287	0.50119497	0.70215361
Zfp36	-0.192576	4.95134142	0.46919979	0.50125308	0.70215361
Shisa9	0.12419245	6.09755279	0.46916313	0.5012696	0.70215361
Fkbp9	0.16011777	5.31413582	0.46910589	0.50129541	0.70215361
Rsbn1l	-0.0965219	5.5308702	0.46873125	0.50146439	0.70227298
Plch2	-0.2169135	3.25218423	0.46868554	0.50148501	0.70227298
Sf3b2	0.09747261	6.8265091	0.46838222	0.5016219	0.70239159
Ptpra	-0.0901266	6.44475606	0.46825453	0.50167955	0.70239922
Pyroxd2	-0.2701256	1.1896043	0.46770446	0.50192802	0.70264345
Kdm1a	-0.1102625	5.91142414	0.46759282	0.50197848	0.70264345
Dydc2	0.65899159	-0.8900281	0.46750345	0.50201887	0.70264345
Rad51	0.35105822	0.79535411	0.46723494	0.50214028	0.70264345
Tmem183a	0.09707291	5.63463001	0.46722464	0.50214494	0.70264345
Ifit2	0.13550443	5.61040824	0.4670674	0.50221606	0.70264345
Wnt1	0.61385291	-1.3555042	0.46692823	0.50227902	0.70264345
I830012O16F	0.18135471	4.05990536	0.46678687	0.50234298	0.70264345
Prox1	-0.1190187	4.8871507	0.46666959	0.50239606	0.70264345
Zufsp	-0.2205679	3.44116249	0.46663679	0.50241091	0.70264345
Tango6	-0.1964476	2.27746456	0.46647886	0.50248241	0.70264345
Trpa1	0.65863388	-0.9427308	0.46616154	0.50262612	0.70264345
Dcc	-0.121299	5.17733694	0.46615261	0.50263017	0.70264345
Pcdha12	-0.3436643	1.20786734	0.46612666	0.50264193	0.70264345
Mcidas	0.64022091	-1.6433173	0.46601199	0.50269388	0.70264345
Ccdc24	-0.7233996	-0.9110974	0.46591673	0.50273705	0.70264345
Ptpn14	0.15422092	6.35989005	0.46590652	0.50274168	0.70264345
Gipc2	-0.2687923	1.46851857	0.46575879	0.50280863	0.70265275
Spint2	0.1987412	2.87373047	0.46566144	0.50285276	0.70265275
Asxl3	-0.2259656	4.1941141	0.46525846	0.50303552	0.7028056

Stpg1	0.29996767	0.68737858	0.46518994	0.50306661	0.7028056
Hsd3b2	-0.7318065	-1.284295	0.46459983	0.50333447	0.70310681
Zfp263	-0.1453043	4.16060069	0.46421434	0.50350959	0.70325577
Msr1	-0.8076186	-1.3220509	0.46403959	0.50358901	0.70325577
Creb3l4	0.89159557	-0.9489596	0.46402005	0.50359789	0.70325577
Dcbld1	-0.2347501	1.98688471	0.46351939	0.50382556	0.70340682
Cdr2	-0.201805	2.74416149	0.46343955	0.50386188	0.70340682
Orai3	-0.1530674	5.00577325	0.46340112	0.50387936	0.70340682
Nom1	0.11059416	4.52773846	0.46332248	0.50391514	0.70340682
Tmem69	0.11949392	4.43179247	0.46320037	0.50397072	0.70341142
Fn1	0.16389774	8.00392047	0.46291923	0.50409871	0.70348159
Gm12709	-0.2291272	1.93978936	0.46285613	0.50412744	0.70348159
Tmem70	-0.1158372	4.80019778	0.4627306	0.50418461	0.70348159
Alkbh3	0.17999936	3.75199751	0.46263073	0.50423011	0.70348159
Myo1e	0.11128424	4.55199139	0.46245654	0.50430947	0.70351937
Timm50	-0.2633444	1.68706968	0.46219093	0.50443053	0.70358834
Ablim3	0.08016857	5.35289374	0.46211866	0.50446348	0.70358834
Maz	0.12970827	3.87511503	0.46178589	0.50461524	0.70365962
Fam24a	-0.9782046	-2.0965885	0.46160928	0.50469582	0.70365962
Nacc2	-0.0806934	6.50946676	0.46154358	0.5047258	0.70365962
Cdk5rap3	-0.1660781	3.15904172	0.46148202	0.50475389	0.70365962
Myo5b	0.18551491	4.19877001	0.46141105	0.50478628	0.70365962
Tmem206	-0.1463676	4.40141597	0.4613189	0.50482834	0.70365962
Slc10a1	0.76779486	-1.138606	0.46102569	0.50496223	0.70377334
Nfatc3	0.11860402	6.91576641	0.46049858	0.50520308	0.7040361
Six2	0.15470685	5.88949627	0.46031821	0.50528554	0.7040781
Mcm2	-0.1924808	2.2388127	0.46017606	0.50535054	0.70408921
Fam168b	-0.0888615	8.42329081	0.46007195	0.50539816	0.70408921
1500004A13	-0.1503081	4.96989979	0.45932453	0.50574026	0.7043973
Wdpcp	0.1189611	3.98123373	0.45928184	0.5057598	0.7043973
Acpp	-0.2770249	4.28697878	0.45924571	0.50577635	0.7043973
Nckap5	-0.1813097	3.2134606	0.45912598	0.5058312	0.70440078
Atxn1	-0.103351	8.6929335	0.45890181	0.50593391	0.70444668
Limd1	0.1520794	5.16136736	0.45882557	0.50596886	0.70444668
Atpaf1	-0.0910852	6.0288518	0.45832186	0.50619982	0.70462931
Cd1d1	0.85349124	-1.2586938	0.45831109	0.50620476	0.70462931
Ttyh2	0.20186656	2.6258952	0.45804565	0.50632655	0.70470898
Pyurf	0.1424089	5.11563781	0.45795807	0.50636674	0.70470898
Pja2	-0.0977443	8.93374676	0.45766356	0.50650195	0.70482426
Ache	-0.2112226	2.62982925	0.45738985	0.50662766	0.70489357
Psmd5	0.09354266	5.36568857	0.45727362	0.50668106	0.70489357
Gtpbp10	0.12707113	4.49151741	0.45721301	0.50670891	0.70489357
Pnpla2	0.16175145	4.47056317	0.45672877	0.50693152	0.70513035

Lgi1	0.09702939	7.46443041	0.4565778	0.50700096	0.70515404
Zbtb17	-0.1964518	2.67988355	0.45644119	0.5070638	0.70516856
Gja6	-0.5496342	-1.1012232	0.45597542	0.50727818	0.70534575
Tatdn3	-0.1644024	2.52536768	0.45593662	0.50729605	0.70534575
Fancf	0.21708588	3.01588235	0.45562753	0.50743841	0.70542592
Mfi2	0.57360431	-0.3768667	0.45558381	0.50745855	0.70542592
Atp7b	0.68375415	-0.6278342	0.45527824	0.50759937	0.70554879
Ankrd35	0.16389092	2.76425955	0.45497505	0.50773917	0.70567021
Gm10516	0.24671404	2.08014304	0.45481373	0.50781357	0.70570073
Gm17296	-0.3040143	2.16490273	0.45463199	0.50789742	0.70574437
Gpr20	0.68931095	-1.386732	0.4544327	0.5079894	0.70579929
Mul1	-0.148351	3.14208276	0.45416823	0.5081115	0.70589605
Kcnk2	-0.0846625	7.24702257	0.45389734	0.50823662	0.70599699
Grm1	-0.1239912	5.38932871	0.45362124	0.5083642	0.70610132
09-Mar	0.23270676	1.83379373	0.45342248	0.50845608	0.70613394
Hax1	0.12115835	5.40342421	0.45332028	0.50850334	0.70613394
Sdad1	0.09367509	4.79160737	0.45299699	0.50865287	0.70613394
Slc39a7	-0.1399014	4.19422238	0.45294945	0.50867487	0.70613394
Tmem181a	0.15642729	3.45736147	0.45270728	0.50878694	0.70613394
Rbm7	0.12202399	5.12838101	0.45268927	0.50879528	0.70613394
Slc11a2	-0.1210357	4.99603711	0.45257954	0.50884607	0.70613394
Rfc2	-0.1598031	3.34450161	0.45249188	0.50888666	0.70613394
Gm15987	-0.7684765	-1.7761642	0.45245494	0.50890376	0.70613394
Rdx	0.08145475	7.03495233	0.45241274	0.50892331	0.70613394
Wbp1l	0.13669514	5.04040967	0.45232289	0.50896492	0.70613394
Entpd2	0.33851195	0.55519252	0.45165249	0.5092756	0.70649213
Sgk2	0.45786617	-0.36339	0.45146936	0.50936052	0.70652523
C2cd4d	-1.0159241	-2.1321454	0.45124667	0.50946382	0.70652523
Zfp951	0.17398932	2.46295741	0.45116654	0.509501	0.70652523
Rbm8a	-0.1284069	5.27386236	0.45107705	0.50954253	0.70652523
Hmgb1-rs17	-0.2943422	0.38820929	0.45103514	0.50956198	0.70652523
Ryr3	-0.2347038	5.30691319	0.45036939	0.50987114	0.70672068
Lrch2	0.14855256	4.19729802	0.45024986	0.50992669	0.70672068
Gli2	0.16043245	3.37357665	0.45019029	0.50995437	0.70672068
BC020402	-0.3113642	1.68090219	0.44996627	0.51005851	0.70672068
Sphkap	-0.1626076	7.2290455	0.44988549	0.51009607	0.70672068
Nhlrc3	-0.2953958	0.51559575	0.44987632	0.51010033	0.70672068
Abhd2	0.09430564	6.17205916	0.44981193	0.51013028	0.70672068
Cadm2	-0.1030158	8.70551438	0.44954631	0.51025383	0.70672068
Nfat5	0.0906187	8.73989418	0.44944436	0.51030127	0.70672068
Cnih1	-0.1332712	5.24449197	0.4494385	0.510304	0.70672068
Hnrnpdl	0.07134052	7.47776317	0.44940515	0.51031952	0.70672068
Mgat3	0.0917843	6.48403666	0.44930536	0.51036596	0.70672068

Rbm25	-0.0935255	8.74047397	0.44918541	0.51042179	0.70672068
1700128F08I	-0.5574271	-0.8445133	0.44907369	0.5104738	0.70672068
Fsip1	-0.735668	-1.5380426	0.44898123	0.51051686	0.70672068
Aplf	0.11778452	4.27157919	0.448891	0.51055888	0.70672068
Maob	0.12016321	4.16422355	0.44881177	0.51059578	0.70672068
Kif26b	-0.2267681	1.966585	0.44863574	0.51067779	0.7067615
Phc1	0.08363119	6.3147553	0.44837725	0.51079826	0.70684044
Arvcf	0.13337323	4.0585454	0.44828795	0.51083989	0.70684044
Chd9	-0.1045779	7.98540391	0.44798466	0.51098132	0.70696344
Col22a1	0.59520439	-0.3577219	0.44755576	0.51118145	0.70706995
Ambra1	-0.1155726	5.77368778	0.44748593	0.51121404	0.70706995
Runx1t1	-0.0823665	7.80809386	0.4471819	0.51135601	0.70706995
Tmem28	0.26937555	1.70191866	0.44716577	0.51136354	0.70706995
Uqcc1	-0.1405496	3.87512325	0.44699819	0.51144182	0.70706995
BC024978	-0.1166834	4.79122258	0.44681792	0.51152605	0.70706995
Trappc6a	0.37813106	1.50051565	0.44679616	0.51153622	0.70706995
Polr2b	-0.0854915	6.23667482	0.44665828	0.51160067	0.70706995
Slc46a1	0.23967761	1.24647663	0.44660734	0.51162448	0.70706995
Arfrp1	-0.1421175	3.99464723	0.44657714	0.5116386	0.70706995
Lamp1	0.14150381	7.56325449	0.44644862	0.51169869	0.70706995
Olfr539	0.68798291	-0.4667982	0.44626115	0.51178637	0.70706995
Sgsm3	-0.1491383	3.69861639	0.44622007	0.51180558	0.70706995
Efs	0.21403683	2.17687059	0.4461959	0.51181689	0.70706995
2510003E04I	0.0918205	7.09266894	0.44613263	0.51184649	0.70706995
Lrrc1	0.15012969	4.36593242	0.4459786	0.51191856	0.70709692
Slc26a5	-0.8547633	-1.149165	0.44550642	0.51213961	0.70732268
Kifc5b	-0.7536711	-1.1644293	0.44536217	0.51220718	0.70732268
Hibadh	0.12938751	5.59095261	0.44529275	0.51223969	0.70732268
Bcl11b	0.15275822	6.10353451	0.44509839	0.51233077	0.70735479
A530032D15	0.31077819	0.8682512	0.44490565	0.51242111	0.70735479
Akap13	0.08355972	7.25756423	0.44490317	0.51242227	0.70735479
Ell2	0.08481453	6.02742888	0.44465654	0.51253791	0.70735479
Noc3l	-0.1156305	4.93042067	0.44461748	0.51255623	0.70735479
Nip7	0.09810956	5.02357649	0.44457032	0.51257835	0.70735479
Mrps24	0.1572717	3.95201043	0.44419244	0.51275565	0.70746153
Gm10509	0.18745966	2.66833887	0.44418136	0.51276085	0.70746153
Ptchd2	-0.1963128	2.78492461	0.44403698	0.51282862	0.7074825
Nkap	-0.1171723	5.26887566	0.44382567	0.51292784	0.70754684
Kif5c	-0.1129968	9.59216499	0.44360929	0.51302948	0.70757113
Slc16a1	0.12445026	5.03789291	0.44352435	0.51306938	0.70757113
Dmgdh	0.39689611	0.06703914	0.44344427	0.51310701	0.70757113
Zmynd11	0.0743056	7.87641286	0.44334049	0.51315578	0.70757113
D430020J02F	0.46285062	0.56245737	0.44318956	0.51322673	0.70759645

Rassf7	-0.4602162	0.14261191	0.44305189	0.51329146	0.70761319
Bcat1	0.10957999	5.79615957	0.44253717	0.51353359	0.7077424
Ctbp2	0.11976982	4.21634869	0.4425286	0.51353762	0.7077424
Gdf3	-0.487775	-0.750508	0.44251723	0.51354297	0.7077424
Olfml2b	0.39140261	0.63137112	0.44236255	0.51361578	0.70777026
Tprkb	-0.0823125	5.39818019	0.44209185	0.51374324	0.70787341
Smc3	0.09060276	8.16643801	0.44180698	0.51387744	0.70798582
Mxd1	-0.098546	6.02346775	0.44142164	0.51405906	0.70803622
B3gat2	0.24094465	2.95666919	0.44140275	0.51406796	0.70803622
Nudt12	-0.2388871	2.84423276	0.44139447	0.51407187	0.70803622
A330070K13	0.79176535	-0.6852585	0.44126311	0.51413381	0.70804513
Arl11	0.83504587	-0.8594678	0.44114966	0.51418732	0.70804513
Krt77	-0.6154469	-0.5571166	0.44092412	0.51429372	0.70804513
Calu	0.11692057	6.14089054	0.44074129	0.51438001	0.70804513
Nrm	-0.3087258	0.49915898	0.44070598	0.51439668	0.70804513
Ccdc50	0.09485308	7.37499426	0.44051384	0.51448739	0.70804513
Nsmaf	-0.1152162	4.77577922	0.44047665	0.51450495	0.70804513
Kctd16	-0.1611562	3.81506031	0.44040149	0.51454044	0.70804513
Rasgrf2	-0.1768197	6.84330007	0.44031121	0.51458309	0.70804513
Stk32c	0.18552797	2.62635104	0.43988722	0.51478342	0.70804513
Gmcl1	0.11053157	4.31263932	0.43987719	0.51478816	0.70804513
Atg16l1	-0.1513613	3.88649107	0.43970754	0.51486837	0.70804513
Smc1a	-0.1032995	8.10839609	0.43967482	0.51488384	0.70804513
Pla2g5	0.36197964	1.43780082	0.43956033	0.51493798	0.70804513
Ubl3	-0.0758042	7.21135999	0.43953766	0.5149487	0.70804513
Rbm43	0.11977182	4.53418318	0.43950126	0.51496592	0.70804513
4932413F04I	0.44714519	0.1333129	0.43946691	0.51498216	0.70804513
Ugcg	-0.1126232	6.93736904	0.43937537	0.51502547	0.70804513
Ubl4	0.07361718	6.74027419	0.43924736	0.51508603	0.70805605
Zfp831	0.1757247	5.24283615	0.43911712	0.51514766	0.70806844
Ipcef1	-0.1012285	7.00043625	0.43883417	0.51528161	0.70816529
Elmod1	-0.104501	7.65046911	0.43855225	0.51541513	0.70816529
Cox10	-0.1085091	4.25664074	0.43847378	0.5154523	0.70816529
Cnp	-0.1081694	6.61886777	0.43843053	0.5154728	0.70816529
Pias2	-0.0838336	6.01353074	0.43841266	0.51548126	0.70816529
Rbm3	-0.119616	7.90721489	0.43795414	0.5156986	0.70822169
Uhrf1bp1	-0.150523	3.87314258	0.43794906	0.51570101	0.70822169
Esrrb	0.53650352	-0.3614553	0.43792892	0.51571056	0.70822169
Tmem123	0.1502652	5.06646632	0.43767524	0.51583088	0.70822169
Tmem164	0.10099351	4.58664058	0.4375031	0.51591256	0.70822169
Lrrc18	-0.1810843	2.93618998	0.43728065	0.51601814	0.70822169
Rab39	-0.3527279	0.57454384	0.43695283	0.5161738	0.70822169
Fez2	0.10375658	5.48917661	0.43695051	0.51617491	0.70822169

Rabif	0.11352246	5.84337403	0.43668078	0.51630305	0.70822169
Pde8b	-0.0995704	6.1734771	0.43663372	0.51632542	0.70822169
Mdm1	-0.1745886	3.14417576	0.43636724	0.51645208	0.70822169
Tfe3	0.1055877	5.30511724	0.43624347	0.51651093	0.70822169
Itpripl2	0.1213895	5.91294504	0.43623187	0.51651645	0.70822169
Phtf1os	-0.2435336	1.14798045	0.4361969	0.51653308	0.70822169
Gm11128	0.5286244	-0.8059278	0.43619357	0.51653466	0.70822169
1700025G04	-0.0888493	6.95265813	0.43614044	0.51655993	0.70822169
Dapp1	0.15172043	4.05572574	0.43609011	0.51658387	0.70822169
Syt13	-0.0980395	6.10051852	0.436062	0.51659724	0.70822169
05-Mar	0.07295901	6.50440038	0.43602061	0.51661693	0.70822169
Kti12	0.2260271	2.3447234	0.43574594	0.51674762	0.70822169
Slc12a9	0.29420087	1.25442533	0.43569719	0.51677082	0.70822169
Helz	-0.1218812	6.72444191	0.43564926	0.51679364	0.70822169
Mterf1a	-0.3302362	1.18052134	0.43563228	0.51680172	0.70822169
Chaf1b	-0.7029243	-1.7793604	0.43831827	0.51685716	0.70822169
Vopp1	0.08720211	5.84434405	0.43550694	0.51686139	0.70822169
Cacng8	0.38384671	0.70465337	0.43535197	0.51693518	0.70822169
Alcam	0.13569332	9.49713943	0.43533482	0.51694335	0.70822169
Csmd1	-0.1737991	6.08621205	0.43500095	0.5171024	0.70823382
Opn3	0.16462261	3.12974357	0.43485609	0.51717144	0.70823382
Slc47a1	0.22656264	5.38233735	0.43482684	0.51718538	0.70823382
Wfdc18	0.53356438	-0.5678841	0.4348162	0.51719045	0.70823382
AI118078	-0.4895013	-0.438439	0.43476393	0.51721537	0.70823382
Irf7	-0.4179327	0.15226717	0.43446826	0.51735635	0.70829046
Pex3	-0.1033673	5.12396252	0.4344564	0.51736201	0.70829046
Tmem222	0.21175925	4.53161946	0.43385256	0.51765015	0.70861267
Dnah2	-0.2550456	1.78466754	0.43374256	0.51770268	0.70861267
2210016F16I	0.12970443	5.04382769	0.43360625	0.51776777	0.70862968
Cks2	0.3544178	0.30209845	0.43328046	0.51792342	0.70876465
Arsg	-0.1434815	3.08586952	0.4331334	0.51799371	0.70876465
Mios	-0.1172958	4.46628771	0.43295581	0.51807861	0.70876465
Mnda	0.46422918	-0.6338785	0.43291507	0.51809809	0.70876465
Map4k4	0.08946163	6.42734492	0.43283527	0.51813625	0.70876465
Tmx4	0.09166798	8.3392014	0.43266117	0.51821952	0.70876465
Ano2	0.50266795	0.14009161	0.43262864	0.51823509	0.70876465
Rwdd4a	0.09763589	5.61345418	0.43213417	0.51847174	0.7090125
Myh14	-0.2408145	2.29748057	0.43202986	0.51852169	0.7090125
Slc6a14	0.95303555	-1.0287029	0.43167855	0.51868998	0.70907215
Dhx58	-0.3698684	0.55327898	0.43165309	0.51870217	0.70907215
Robo1	0.10650105	7.25073871	0.43147956	0.51878534	0.70907215
Ston1	-0.1920151	4.79793078	0.4313065	0.5188683	0.70907215
Zfhx4	0.09447983	8.17332463	0.43124261	0.51889894	0.70907215

Copz1	0.12005478	6.13808659	0.43116266	0.51893728	0.70907215
Tmem209	-0.1077292	4.22701157	0.43115919	0.51893894	0.70907215
Wwtr1	0.11339573	6.61329783	0.43105926	0.51898687	0.70907215
Cpne1	0.09058524	5.90281002	0.43038928	0.51930841	0.70943943
Bloc1s6	-0.0983462	5.60075722	0.43010863	0.51944321	0.70946699
Elmo3	0.25471962	1.38740745	0.43005135	0.51947074	0.70946699
Iscu	0.11586214	6.3829146	0.42996973	0.51950995	0.70946699
Nat2	0.20547617	3.0417629	0.42981689	0.51958341	0.70946699
Zbtb40	0.25546791	2.50864861	0.42948898	0.51974107	0.70946699
Vmac	-0.1763992	3.71146309	0.42939877	0.51978445	0.70946699
Dnajc8	-0.1024944	6.22569928	0.42938931	0.519789	0.70946699
Xpnpep1	0.12224102	4.48513859	0.42933705	0.51981414	0.70946699
Ttc38	0.12944267	3.77982838	0.42927509	0.51984395	0.70946699
5730507C01l	0.16420435	3.12580485	0.42911472	0.51992111	0.70946699
Tubb4a	-0.1068381	10.3898448	0.42905948	0.5199477	0.70946699
Rpl36a	-0.1398554	6.48678995	0.42901871	0.51996732	0.70946699
Zfp622	-0.1226611	4.68996095	0.42878879	0.520078	0.70946699
Gpr27	0.61903208	-1.2313857	0.42872689	0.52010781	0.70946699
Psme2	0.15195285	5.28007112	0.42860678	0.52016565	0.70946699
Sel1l	0.09368579	7.13736227	0.42859326	0.52017216	0.70946699
Ethe1	0.21078734	2.6815575	0.42826067	0.5203324	0.70961361
Lat	-0.7282723	-1.0664694	0.42782257	0.5205436	0.70982971
Rbfa	0.18818457	2.63680944	0.42745956	0.52071873	0.70999656
Gid8	0.07673919	6.55454777	0.42721827	0.52083519	0.71008341
Zscan21	0.13588991	4.60120424	0.42698138	0.52094958	0.71016648
Ckap4	-0.1292316	5.1576854	0.4267726	0.52105043	0.71016648
Pcnxl4	-0.129826	4.64560216	0.42676428	0.52105445	0.71016648
Psd3	-0.1018495	9.82749616	0.4262149	0.52131999	0.71030276
Enc1	0.13723619	8.26105106	0.42613998	0.52135623	0.71030276
Apba3	0.19411837	2.53870786	0.42603638	0.52140634	0.71030276
Kntc1	-0.6326289	0.00439838	0.42574499	0.52154733	0.71030276
Snord104	-0.658693	-1.7075835	0.4255789	0.52162772	0.71030276
Cryga	-0.8603059	-1.8631762	0.42557169	0.52163121	0.71030276
Mfsd5	0.15959686	3.64305993	0.42548003	0.52167559	0.71030276
Kmt2d	-0.1207886	7.22961619	0.42546133	0.52168464	0.71030276
Atat1	0.09320657	5.68342495	0.42539059	0.5217189	0.71030276
Drap1	0.16580494	3.96958289	0.42525862	0.52178281	0.71030276
Scd2	-0.0836453	8.33435945	0.42525185	0.52178609	0.71030276
Cenpv	-0.1437877	2.83724603	0.42523815	0.52179272	0.71030276
Chchd5	0.17320283	2.63589755	0.42504057	0.52188844	0.71030276
Blzf1	-0.1164313	4.90929891	0.42503026	0.52189344	0.71030276
Parg	-0.082927	5.62381836	0.42488825	0.52196226	0.71032457
Hs3st1	0.12589786	4.20816129	0.42449471	0.52215306	0.71048248

Nudt7	-0.1460752	2.42761993	0.42439441	0.52220171	0.71048248
Thap11	-0.0981737	4.58550204	0.4243223	0.52223669	0.71048248
1700012D14I	0.55853788	-0.1883919	0.42403332	0.52237692	0.71051376
Tbc1d1	0.09938696	4.55994656	0.42388078	0.52245096	0.71051376
Usp4	0.09657712	5.49972037	0.42380578	0.52248738	0.71051376
Wdr26	0.07256814	8.56156612	0.42338042	0.52269399	0.71051376
Ufsp2	0.10441045	5.28542917	0.42327946	0.52274305	0.71051376
Pdgfrl	0.23411347	2.57959627	0.42259471	0.52307603	0.71051376
B3galt6	-0.2279876	2.33602443	0.42257944	0.52308345	0.71051376
Tyw5	-0.1200825	4.84978132	0.42249262	0.5231257	0.71051376
Atad3a	-0.1669826	3.12139672	0.42245918	0.52314197	0.71051376
1700088E04I	0.83753988	-1.5563058	0.42223574	0.52325073	0.71051376
Itgb7	-0.6390422	-1.4779286	0.42195538	0.52338726	0.71051376
Pagr1a	-0.1368201	3.04353143	0.4219392	0.52339514	0.71051376
Commd2	0.12801752	4.30203494	0.42191091	0.52340892	0.71051376
Armc2	-0.2349824	1.77471484	0.42185823	0.52343459	0.71051376
Rhbdd2	0.11852183	4.33556615	0.42178093	0.52347225	0.71051376
Syt4	-0.1077304	7.23979806	0.42173154	0.52349631	0.71051376
Rpl14-ps1	-0.1831888	2.59739712	0.42168654	0.52351824	0.71051376
2810029C07I	-0.311752	2.23293347	0.42148005	0.52361888	0.71051376
Pik3c3	0.08814156	5.50158072	0.42143998	0.52363841	0.71051376
Inpp5j	-0.1619436	3.41493072	0.42141813	0.52364907	0.71051376
Gna14	-0.3968516	1.3106898	0.42141355	0.5236513	0.71051376
E130102H24I	-0.5584509	-1.0345136	0.4213532	0.52368072	0.71051376
Alkbh4	0.25491536	1.04714267	0.42127834	0.52371723	0.71051376
LOC1026324:	-1.4146358	-1.9097647	0.42119948	0.52375568	0.71051376
Prap1	1.04350429	-1.6095757	0.42119827	0.52375627	0.71051376
Lacc1	0.12908279	4.5523204	0.42112382	0.52379258	0.71051376
Srgn	0.13625085	5.14252766	0.42109567	0.52380632	0.71051376
Mrpl43	0.11927469	5.11496143	0.42080329	0.52394897	0.71051376
Xylt2	-0.2672111	2.16298136	0.42075414	0.52397296	0.71051376
H2-T24	-0.1463086	4.91991878	0.42070189	0.52399847	0.71051376
Dnaaf2	0.18559972	3.59941112	0.42058231	0.52405684	0.71051376
Zmat2	-0.0801531	7.1416039	0.42048894	0.52410243	0.71051376
Il18r1	0.62476744	-0.6174719	0.42046572	0.52411377	0.71051376
Soga1	-0.0754	7.13274724	0.42041804	0.52413705	0.71051376
Vars2	0.21783803	2.25968597	0.42031667	0.52418656	0.71051376
Primpol	0.24227609	2.5564363	0.42026735	0.52421066	0.71051376
Rest	0.13903875	4.90144498	0.42017646	0.52425506	0.71051376
Lefty2	0.78428114	-1.3405723	0.42015378	0.52426614	0.71051376
Satb1	-0.1084551	7.71689747	0.419595	0.52453928	0.7107965
Kcnq1ot1	-0.238025	8.05882548	0.41943225	0.52461888	0.7107965
Dlk1	0.1913497	3.41940784	0.4193639	0.52465231	0.7107965

Foxred1	0.16145495	3.05111748	0.41902824	0.52481658	0.7107965
Cubn	-0.1510748	5.30480592	0.41894501	0.52485732	0.7107965
Agpat1	-0.1182699	4.8021746	0.4189352	0.52486213	0.7107965
Map2k2	-0.1295346	4.35852667	0.41866271	0.52499557	0.7107965
Iars2	0.09625697	5.53871229	0.41865239	0.52500062	0.7107965
Mccc1	-0.142831	3.58728736	0.4186486	0.52500248	0.7107965
Mrpl12	-0.148086	3.85456759	0.41855455	0.52504855	0.7107965
Cystm1	-0.2161864	2.13882184	0.41853164	0.52505978	0.7107965
Gtf2f1	0.10908683	5.9128178	0.41840711	0.52512079	0.7107965
Cgrrf1	0.11269868	3.86189549	0.41832414	0.52516146	0.7107965
Smim18	-0.2003685	2.12277284	0.41813228	0.5252555	0.71083907
Bptf	-0.1097362	8.32255731	0.41804446	0.52529856	0.71083907
Tecr	0.12137551	7.85813035	0.41787652	0.52538092	0.71087904
Sntb2	-0.0955817	5.67243718	0.41725331	0.52568676	0.71119386
Tecpr1	-0.1854744	3.46884039	0.41718704	0.5257193	0.71119386
Chd1	0.07046594	6.5805177	0.41698694	0.52581757	0.7112553
Dbp	-0.1644248	4.08973715	0.41638555	0.52611315	0.71148752
Dnmt1	-0.1183533	5.72564765	0.41625406	0.52617782	0.71148752
Gpr89	-0.1470855	3.42303375	0.41619521	0.52620676	0.71148752
Glud1	-0.0657802	8.41518438	0.4161558	0.52622615	0.71148752
Alyref	0.16493556	2.80293546	0.41606976	0.52626848	0.71148752
Frmpd3	0.33819497	1.33049436	0.41597171	0.52631672	0.71148752
Klk11	-1.2763455	-1.816411	0.41835193	0.52644354	0.71148752
Ppap2a	0.14282565	3.7937364	0.41557208	0.52651344	0.71148752
Ndrg4	-0.0871441	10.805371	0.41536139	0.52661721	0.71148752
Spats2l	-0.0927386	5.09041211	0.41529858	0.52664815	0.71148752
Specc1l	-0.0864627	6.43890441	0.41525759	0.52666835	0.71148752
Srgap1	0.15888663	5.14765201	0.41525484	0.5266697	0.71148752
Rtkn	-0.1620827	3.44422276	0.41515902	0.52671691	0.71148752
Ccm2l	0.66352509	-0.5703278	0.41513351	0.52672948	0.71148752
Slc5a3	0.0961295	5.78479516	0.41481269	0.52688763	0.71162338
Alx3	0.21090058	3.2206397	0.41471493	0.52693583	0.71162338
Chmp1a	0.12887706	4.57431843	0.41443952	0.52707168	0.71173542
Me1	-0.0821016	5.34578068	0.41414061	0.5272192	0.71186318
Stra13	0.15951791	4.00883163	0.41385335	0.52736104	0.71192453
Wdr95	-0.9938428	-1.3915746	0.41371606	0.52742885	0.71192453
Cep131	0.15862706	2.86455836	0.41362413	0.52747427	0.71192453
Ttc21b	-0.1344074	4.37167452	0.41359502	0.52748865	0.71192453
Bcas3os1	-0.6054379	0.45882069	0.41344208	0.52756423	0.71192453
Setd3	0.06524237	6.79583478	0.41340596	0.52758208	0.71192453
Cyhr1	0.09708453	5.5707049	0.41328095	0.52764387	0.71193653
Ggta1	-0.2485246	2.39992791	0.41279017	0.5278866	0.71218991
Sdr9c7	0.92652491	-1.6837212	0.41268728	0.52793751	0.71218991

Acp1	0.08642148	7.14125661	0.41248759	0.52803636	0.71222962
A930013F10	-0.3395145	1.71661462	0.41241395	0.52807281	0.71222962
Tmed4	-0.1365202	5.53251403	0.41170992	0.5284216	0.71260602
Fen1	-0.1705778	2.73471219	0.41163689	0.5284578	0.71260602
Cmtr2	-0.2055815	2.13066756	0.41096935	0.52878894	0.71292523
Frem1	0.36485412	1.21713709	0.41094609	0.52880049	0.71292523
Tatdn1	0.10350962	4.60363615	0.41077652	0.52888467	0.71296729
Zfp11	0.10686184	4.27418731	0.41036397	0.52908957	0.71308102
Tgfa	0.08254062	5.15283698	0.41013328	0.52920422	0.71308102
Nfe2	-0.8417032	-1.1396766	0.41011109	0.52921525	0.71308102
Adam22	-0.107038	7.72163475	0.410083	0.52922921	0.71308102
Ndrg3	-0.0643236	8.73840103	0.410061	0.52924015	0.71308102
Bend5	0.15241269	3.27714719	0.4099668	0.52928699	0.71308102
Fkbp11	0.62515875	-1.4184632	0.4097695	0.5293851	0.71309593
1700008J07F	0.18373563	3.20091997	0.40963866	0.52945018	0.71309593
E130309D14	-0.1260794	4.98591316	0.40962489	0.52945703	0.71309593
Rc3h1	0.0719137	7.36999408	0.40946374	0.52953722	0.71313255
Thbs2	0.16243465	5.06590599	0.40914151	0.52969762	0.71327718
Lst1	0.44115104	-0.2032257	0.40891043	0.5298127	0.71336076
Agap2	0.09382039	6.9295941	0.40824595	0.53014389	0.71367674
Celsr2	0.09943992	7.16649663	0.40822677	0.53015345	0.71367674
Spcs1	-0.115707	5.36136485	0.40799373	0.5302697	0.71375899
Trhr	0.26018732	1.50417131	0.40779174	0.53037049	0.71375899
Iqce	0.15532096	3.35711486	0.40778535	0.53037368	0.71375899
Scfd1	0.10656004	4.64103708	0.40750834	0.53051197	0.71387246
Pnrc2	0.1297733	7.27023271	0.40740395	0.5305641	0.71387246
Rdh1	-0.2379656	2.07293096	0.40705107	0.53074039	0.71403826
1500011B03	-0.1185642	5.03776438	0.40672404	0.53090387	0.71418679
Zfp933	-0.1113367	4.53456843	0.40624133	0.53114534	0.7144402
Cnpy2	0.14282308	5.15196504	0.40605195	0.53124012	0.71449628
Zfp101	-0.1511483	2.54349396	0.40583136	0.53135057	0.71452231
9430015G10	-0.2176783	2.45774709	0.40554999	0.53149152	0.71452231
Fignl2	-0.2930351	1.1062992	0.40551424	0.53150943	0.71452231
Pip5kl1	-0.4944935	0.28413704	0.4054438	0.53154473	0.71452231
Tgoln1	0.10282523	6.81995248	0.40534463	0.53159443	0.71452231
Tmem63b	-0.1123083	5.59252783	0.40533308	0.53160022	0.71452231
Apobec4	-0.5407014	-0.0917944	0.40527134	0.53163117	0.71452231
Gfy	1.02843882	-1.9307506	0.40483932	0.53184782	0.71467437
Calm3	0.07000018	8.7129131	0.40483391	0.53185053	0.71467437
Tesk1	0.12044556	4.95553184	0.40468628	0.53192461	0.71470253
Zfp715	0.11454284	4.43571062	0.40452493	0.53200558	0.71473997
Gsdmd	-0.2330457	2.29812276	0.40426971	0.53213372	0.71484075
Il12rb2	-0.2467294	2.58358439	0.4039204	0.53230918	0.71500508

Frmd4a	-0.1253083	5.54003423	0.4033583	0.53259177	0.71521343
Gm5415	-0.3227778	2.10985562	0.4033135	0.5326143	0.71521343
Ap1s2	0.09266404	6.31247399	0.40319905	0.53267188	0.71521343
Huwe1	-0.1341727	9.48061089	0.40303885	0.53275249	0.71521343
Dlg3	-0.1103953	6.55474967	0.40297854	0.53278284	0.71521343
Atrip	-0.1331178	3.49551033	0.4028905	0.53282716	0.71521343
Gldc	0.17174447	2.46772824	0.40286241	0.5328413	0.71521343
Bmpr1a	-0.1163085	7.88324888	0.4026623	0.53294205	0.71521343
Polr1b	-0.2394115	2.23088517	0.40258079	0.5329831	0.71521343
Slc24a2	-0.1155796	9.69693903	0.40255558	0.5329958	0.71521343
Cdca5	0.75717088	-1.4969449	0.40243714	0.53305546	0.71522216
Eif2s2	0.07194396	7.44919119	0.40223838	0.53315561	0.71528521
Cisd1	-0.0932775	5.07555563	0.40212705	0.53321173	0.71528918
Spa17	-0.2555703	2.41346414	0.40195095	0.5333005	0.71532546
Cep97	0.09878152	4.58543126	0.40186254	0.53334508	0.71532546
Adrb1	-0.1733832	3.6706366	0.40170613	0.53342397	0.71535996
Tmtc4	0.10784606	4.07511298	0.40157477	0.53349024	0.71537754
Slc13a4	-0.1441336	8.44182862	0.40133397	0.53361177	0.71539047
Mta3	-0.1051986	5.56696149	0.40131638	0.53362064	0.71539047
Psrc1	0.24095928	1.47407062	0.40121542	0.53367161	0.71539047
Ric8	0.1066162	5.22884109	0.40110405	0.53372785	0.71539047
Ier3	-0.1612873	4.1511689	0.4009707	0.53379519	0.71539047
Rac2	-0.1659544	2.78924291	0.40083592	0.53386327	0.71539047
Kmo	0.33348879	0.99366397	0.40081858	0.53387203	0.71539047
Dusp22	-0.096251	4.8711312	0.40066773	0.53394826	0.71542137
BC051142	-0.3288195	1.26833807	0.40029215	0.53413812	0.71558643
Cxcl10	0.26863113	2.81711053	0.40021369	0.53417781	0.71558643
Lrriq3	0.40612616	0.57315114	0.39990035	0.53433633	0.71572753
Cplx3	0.24886471	1.32700046	0.39950451	0.53453671	0.71589177
Spata22	0.73378344	-0.4635355	0.39943001	0.53457444	0.71589177
Pet2	-0.628668	-0.6324574	0.39928411	0.53464834	0.71589177
Zfp398	-0.1114722	4.49869841	0.39898039	0.53480224	0.71589177
Sec14l5	0.64234549	-1.0991668	0.39889779	0.53484411	0.71589177
Leprel4	-0.1883915	2.95741642	0.39889049	0.53484781	0.71589177
6720483E21I	-0.7260649	-1.7520889	0.39886316	0.53486167	0.71589177
Mllt3	-0.0840939	7.72199041	0.39878482	0.53490139	0.71589177
Kbtbd7	0.09967873	5.2378738	0.39871311	0.53493775	0.71589177
Krcc1	0.14920348	7.09047023	0.39847661	0.53505771	0.7159811
Ch25h	0.71243197	-1.5622507	0.39826245	0.53516638	0.71598997
Ndufb7	-0.1628728	4.23432189	0.39825381	0.53517076	0.71598997
D430036J16I	-0.1903129	2.35604874	0.39782297	0.5353895	0.71620079
Pcdhga5	0.1449964	3.68672093	0.39755838	0.53552392	0.71620079
Slc22a15	-0.164064	2.70741686	0.39732315	0.53564348	0.71620079

Ost4	0.14426667	7.07732292	0.39730095	0.53565476	0.71620079
Nmnat3	0.20568946	2.54700516	0.39720097	0.53570559	0.71620079
Phrf1	-0.1184207	4.78880025	0.39711347	0.53575009	0.71620079
Tmod4	0.51684385	-0.5114358	0.39687832	0.5358697	0.71620079
Fam46c	0.166185	2.4252796	0.39684004	0.53588917	0.71620079
Acp5	0.53963223	-0.7192985	0.39653196	0.53604597	0.71620079
9430016H08I	-0.1368781	3.49549773	0.39646596	0.53607956	0.71620079
Smek2	-0.0755122	6.69850331	0.39643225	0.53609673	0.71620079
Eps15l1	-0.0953234	5.75893508	0.39617877	0.53622582	0.71620079
Mpp6	0.12347839	8.59069649	0.39579298	0.53642241	0.71620079
Aph1b	0.1413976	4.15526441	0.39570824	0.5364656	0.71620079
Ppp1r2-ps3	0.39419052	-0.4670166	0.39567261	0.53648377	0.71620079
Nmral1	0.20732577	2.8431498	0.39565003	0.53649528	0.71620079
Abcb10	0.13915942	3.68773731	0.39564929	0.53649566	0.71620079
Bub1	-0.4502672	0.38533996	0.3956313	0.53650483	0.71620079
Slc23a1	-0.282713	0.69192188	0.39552303	0.53656004	0.71620079
LOC381967	0.48722215	-0.4098663	0.39533911	0.53665386	0.71620079
Cspg5	-0.1125314	5.25798228	0.39530432	0.5366716	0.71620079
Klhl5	-0.0949548	5.72844719	0.39527884	0.5366846	0.71620079
Hspa4l	0.1204975	7.12134736	0.39517987	0.5367351	0.71620079
Sgcd	-0.1777733	2.93559348	0.39501344	0.53682004	0.71620079
Get4	0.12718152	3.89752722	0.39500263	0.53682556	0.71620079
Map6	0.12514678	5.70222243	0.39491197	0.53687184	0.71620079
Ska3	-0.2279418	1.45807061	0.39468692	0.53698676	0.71620079
Phb2	-0.1027956	5.0191574	0.39457121	0.53704587	0.71620079
LOC1008616	-0.4125285	-0.5326978	0.39438995	0.53713848	0.71620079
Poln	0.58369577	-1.1123698	0.39425075	0.53720962	0.71620079
Tufm	-0.1180494	3.65762386	0.39396371	0.53735638	0.71620079
Stx2	0.18215297	2.63517198	0.39378591	0.53744732	0.71620079
Tmub1	0.18209229	2.31800996	0.39364938	0.53751717	0.71620079
1700105P06I	1.45286946	-1.8469964	0.39358057	0.53755238	0.71620079
Ppp2r5a	0.09003866	5.75642456	0.3935541	0.53756593	0.71620079
Mtss1l	0.08464099	7.05287022	0.39332577	0.53768281	0.71620079
Mfap5	-0.3532865	2.15899869	0.39317652	0.53775923	0.71620079
Fam69b	0.16684728	3.04810245	0.39313442	0.53778079	0.71620079
Nkd1	0.1317482	4.85827505	0.39303031	0.53783412	0.71620079
Tmem108	-0.1863501	3.46789091	0.39294751	0.53787653	0.71620079
Scrn1	0.11733702	5.45736724	0.39293832	0.53788125	0.71620079
Tyw1	-0.2628108	2.0133922	0.39291545	0.53789296	0.71620079
Nckap1	-0.0885721	9.44861355	0.39290647	0.53789756	0.71620079
Spopl	-0.1278756	3.63922646	0.39286444	0.5379191	0.71620079
Blcap	-0.1268836	5.14326123	0.39285571	0.53792357	0.71620079
Psmb7	0.092392	6.85311655	0.39282727	0.53793814	0.71620079

Rpusd4	-0.1671593	2.7187492	0.39278996	0.53795726	0.71620079
Vps16	-0.1658099	4.44293353	0.39272244	0.53799187	0.71620079
Trpm3	0.0864838	6.82614017	0.39272139	0.53799241	0.71620079
Tuba1a	-0.0684605	9.67220925	0.39264908	0.53802947	0.71620079
Slfn3	0.64648558	-0.2510446	0.39234363	0.53818609	0.71620079
Mtfr1l	0.10898558	5.6179435	0.39221646	0.53825132	0.71620079
Nbn	0.09493806	4.74914227	0.39221147	0.53825388	0.71620079
Hist1h4j	-0.2758835	0.67386973	0.39213927	0.53829092	0.71620079
Stac	0.46047685	0.1183344	0.39195336	0.53838632	0.71620079
Kcnh6	0.36924443	-0.1846566	0.39193673	0.53839486	0.71620079
Ntn5	0.34926962	0.36897687	0.39191539	0.53840581	0.71620079
Ccdc37	-0.3423368	0.91688166	0.39188815	0.53841979	0.71620079
Gm5803	0.47004278	-0.6594237	0.39179316	0.53846856	0.71620079
Gne	0.08732733	5.51174018	0.39142589	0.53865717	0.71630356
1700025F24I	-0.8819078	-2.177585	0.39135354	0.53869434	0.71630356
Map3k4	0.11107464	4.98220469	0.39133178	0.53870552	0.71630356
Uprt	-0.0991912	4.57891651	0.39117536	0.5387859	0.71633966
Sfmbt2	-0.2185488	2.5418789	0.3910284	0.53886145	0.71636833
Plekhb1	-0.1137674	5.81147658	0.3908321	0.53896238	0.71636833
Efcab11	0.53659698	-0.5475158	0.39082278	0.53896717	0.71636833
Slc4a7	-0.1276671	3.91386654	0.39057298	0.53909567	0.71637915
Pdzk1	-0.1754826	2.85859714	0.39042553	0.53917155	0.71637915
Smchd1	-0.0940157	6.18192511	0.39035357	0.53920858	0.71637915
Zfp800	0.10787697	5.95938566	0.39022998	0.5392722	0.71637915
Pabpc1l	0.96178505	-1.9515663	0.39017324	0.53930142	0.71637915
Ddx10	0.07561107	5.55496738	0.39014122	0.53931791	0.71637915
Rnf168	0.08334388	6.53820425	0.39008281	0.53934798	0.71637915
Sstr2	0.17287134	2.75647597	0.38987103	0.53945707	0.71645332
Cox6a2	0.2190188	1.09993149	0.38968121	0.53955487	0.71647005
Ndufa2	0.13019756	5.45859902	0.38963992	0.53957615	0.71647005
Birc6	-0.1416742	7.93359802	0.38934423	0.53972858	0.71649569
Slc6a13	-0.1637911	6.53886407	0.38933259	0.53973458	0.71649569
Tdrd6	0.55517849	-0.5008325	0.38927011	0.5397668	0.71649569
Klhl24	-0.0697851	6.75247327	0.38906271	0.53987379	0.71649569
Cbx8	-0.3944659	0.15420196	0.38904373	0.53988358	0.71649569
Phgdh	0.19521549	2.53464317	0.38894468	0.53993469	0.71649569
Slc25a14	0.12059782	4.89905277	0.38878818	0.54001545	0.71649569
St6galnac3	-0.151359	3.5054826	0.38877661	0.54002143	0.71649569
Foxr1	-0.469338	-1.0863952	0.38844682	0.54019171	0.71660872
Skp2	-0.1901132	2.8285553	0.38832135	0.54025652	0.71660872
Zfp318	-0.1251175	6.73134452	0.38830226	0.54026638	0.71660872
Lpar6	0.18309527	2.23537527	0.38819176	0.54032348	0.71661381
Prrc2c	-0.1065646	10.0574127	0.3880885	0.54037684	0.71661396

Cdc40	0.07642187	6.87260442	0.38783216	0.54050935	0.71671905
Tmem39a	-0.1031098	4.96808589	0.38760369	0.54062751	0.71677236
Flt4	-0.443783	1.00648881	0.38734557	0.54076105	0.71677236
Meis2	0.0901812	6.62560199	0.3871735	0.54085012	0.71677236
Htr2b	0.31717476	0.19008035	0.38710038	0.54088797	0.71677236
Lyz2	-0.1882404	3.56935062	0.3870727	0.5409023	0.71677236
Opn1sw	0.5554013	0.40682071	0.38699262	0.54094377	0.71677236
Rfx5	-0.0973761	4.85278337	0.38698044	0.54095008	0.71677236
1110059E24I	-0.0770722	5.63787582	0.38683783	0.54102394	0.71677236
Baz2a	-0.0971315	6.12712443	0.386607	0.54114353	0.71677236
Mlc1	0.20625569	3.17657548	0.3865752	0.54116001	0.71677236
Endog	-0.5156161	-0.8303844	0.38645728	0.54122113	0.71677236
Tmem19	-0.1272914	3.56691785	0.38644493	0.54122753	0.71677236
Cdc42bpb	0.0980324	6.53365385	0.38641697	0.54124202	0.71677236
Trmt5	-0.1403075	3.47428555	0.38628014	0.54131296	0.71679576
Pcdhb10	-0.2996602	1.33394292	0.38604305	0.54143592	0.7168288
Fam161a	-0.2236701	1.83373933	0.38602661	0.54144445	0.7168288
Rint1	-0.1163768	4.06276788	0.38569461	0.54161672	0.71685492
Cacna2d4	-0.3100445	1.19254656	0.38566629	0.54163143	0.71685492
Gm6277	0.20200275	2.28629519	0.38449826	0.54223838	0.71685492
Pxdn	0.21107784	4.05497605	0.38420939	0.54238869	0.71685492
Dcaf12l1	0.09090502	5.6089195	0.38418109	0.54240342	0.71685492
1700073E17I	-0.2473864	2.24183211	0.38417403	0.54240709	0.71685492
Cox18	0.15937861	2.90177609	0.38412063	0.54243488	0.71685492
Gm10789	0.76903509	-1.9141048	0.38405385	0.54246965	0.71685492
Letm1	0.10881083	5.94169306	0.38401425	0.54249027	0.71685492
B3gnt9	0.23105712	1.8098825	0.38399495	0.54250032	0.71685492
2310047M1C	0.17823824	2.67892349	0.38385828	0.54257149	0.71685492
Anks6	0.17219677	2.0881777	0.38380947	0.54259691	0.71685492
Mdp1	-0.1073405	4.40685737	0.38375636	0.54262457	0.71685492
Naf1	0.11315136	4.34017599	0.38370967	0.54264889	0.71685492
Zfp600	0.4989122	-1.1369565	0.38368436	0.54266208	0.71685492
Klf5	-0.0976041	6.62739111	0.38355132	0.54273139	0.71685492
Lrrc9	-0.303066	1.59318661	0.38344062	0.54278908	0.71685492
Adamts17	0.28584839	2.62311081	0.38334487	0.54283899	0.71685492
Tor2a	0.1949209	1.89822862	0.3830908	0.54297147	0.71685492
Samd1	-0.1845234	2.79565482	0.38307917	0.54297753	0.71685492
Sestd1	-0.1114048	7.06403076	0.38292076	0.54306016	0.71685492
Gm6654	0.30063685	0.80954439	0.38273656	0.54315627	0.71685492
4930538K18I	-0.3958532	0.49385641	0.38269668	0.54317708	0.71685492
Ube2n	0.06287528	7.43722179	0.3826329	0.54321037	0.71685492
Ak6	0.18212378	2.76185152	0.38253291	0.54326256	0.71685492
Rbck1	-0.1173485	4.11843858	0.38252189	0.54326832	0.71685492

Ube2g2	0.11568973	4.67683069	0.38221317	0.54342953	0.71685492
Cep57l1	0.15935258	2.90026587	0.38193767	0.54357348	0.71685492
Gpr151	0.87282136	-1.6206207	0.38192924	0.54357789	0.71685492
Dclk2	0.13626601	4.01234502	0.3819048	0.54359066	0.71685492
Ggps1	0.107598	6.34114367	0.38181302	0.54363863	0.71685492
Ankrd50	-0.0930587	5.18671778	0.38180878	0.54364085	0.71685492
Kcnmb2	-0.3389246	0.84059901	0.38178268	0.54365449	0.71685492
Memo1	0.10009289	4.53854241	0.38177123	0.54366048	0.71685492
Mtdh	0.08128301	6.76294372	0.3814791	0.54381324	0.71685492
Coq2	-0.1023202	4.82113311	0.3813382	0.54388694	0.71685492
Dmxl2	-0.1510123	8.52492844	0.38131911	0.54389693	0.71685492
Cand1	0.0944203	7.71060483	0.38131908	0.54389695	0.71685492
Wbscr22	0.13504074	3.15028174	0.38115312	0.54398379	0.71685492
Pdcd4	0.06817664	7.48491951	0.38110697	0.54400795	0.71685492
Ikzf3	0.18673735	2.779429	0.38089486	0.54411899	0.71685492
BC053749	0.22368723	2.84818733	0.38060908	0.54426867	0.71685492
Rps19-ps3	0.2696146	0.61789326	0.38060172	0.54427253	0.71685492
1190007I07R	-0.2132991	2.18462635	0.38052829	0.54431099	0.71685492
Park7	0.11949929	4.76409095	0.38052662	0.54431187	0.71685492
Kank2	-0.1163602	7.56540758	0.38041773	0.54436893	0.71685492
Yipf4	0.11422352	4.77633016	0.38040538	0.5443754	0.71685492
Npas4	0.45739135	3.17988758	0.38036579	0.54439615	0.71685492
Ccnd2	-0.0811232	7.42819143	0.3802766	0.5444429	0.71685492
Arl14ep	0.07642802	6.37628164	0.38020775	0.544479	0.71685492
Zdhhc9	0.09150144	6.30578159	0.38015877	0.54450468	0.71685492
Zfp474	-0.7655843	-1.4850592	0.38011951	0.54452526	0.71685492
Ngfr	-0.1430306	3.55633351	0.3801057	0.54453251	0.71685492
Rasa3	-0.0990539	5.2610341	0.38001866	0.54457815	0.71685492
Psmb1	0.08250998	6.01550234	0.38001217	0.54458155	0.71685492
Pqlc3	-0.1410431	3.54648936	0.37998697	0.54459477	0.71685492
2700049A03I	0.11321128	3.7645438	0.37998541	0.54459559	0.71685492
Echdc1	0.19872316	1.77405329	0.37994079	0.54461899	0.71685492
Tmem150a	0.17492724	2.52339698	0.37991026	0.54463501	0.71685492
Ndufa4	0.10244054	7.34644343	0.37965843	0.54476714	0.71685492
Kank4os	0.67928729	-1.3756295	0.37957731	0.54480971	0.71685492
Fn3k	0.16686749	3.23187119	0.3795678	0.5448147	0.71685492
Rspry1	0.0968553	4.75303855	0.3795536	0.54482216	0.71685492
Fermt1	-0.5765569	0.35393899	0.37941129	0.54489687	0.71685492
Fam96a	-0.1171097	5.17674394	0.37935408	0.54492691	0.71685492
Ercc4	-0.1259955	3.83463509	0.37918393	0.54501626	0.71690238
Mir6920	-0.6385925	-1.5890956	0.3788251	0.5452048	0.71704162
Btbd2	0.11956652	4.50350605	0.37877966	0.54522869	0.71704162
Zdhhc6	-0.1199569	4.61483211	0.3784999	0.54537578	0.71716497

Cmss1	0.28432881	2.41005536	0.37827207	0.54549563	0.71725247
Irf1	0.19884231	2.9745514	0.37788418	0.54569978	0.71745081
Lmbr1l	-0.2917422	0.32699274	0.377583	0.5458584	0.71758924
Fbxo31	-0.1552197	3.94589921	0.37727397	0.54602125	0.7177332
D16Ertd472e	0.11070733	6.32211038	0.37716134	0.54608062	0.71774113
Prokr1	0.67482309	-0.1820214	0.37698043	0.54617602	0.7177964
Arhgef11	0.07876228	6.85563668	0.37664909	0.54635081	0.71786381
Kdelc2	0.11382182	4.49541638	0.37660508	0.54637404	0.71786381
Snx21	-0.2352856	1.94297433	0.37640978	0.54647713	0.71786381
Gm2027	0.3759213	0.23977782	0.37638476	0.54649034	0.71786381
Epha1	-0.8761385	-1.3427968	0.37635195	0.54650766	0.71786381
Tm9sf2	0.09117933	5.92029723	0.37627671	0.54654739	0.71786381
Sprtn	-0.2072069	1.57573087	0.37608297	0.54664972	0.71792813
Tmem220	-0.1775963	2.86846777	0.37588073	0.54675657	0.71799839
Ptk2b	-0.0987829	7.47935581	0.37560283	0.54690347	0.71803025
Xpo7	-0.0639604	7.18190454	0.3753962	0.54701274	0.71803025
Serp2	0.1457607	3.78297655	0.37530692	0.54705997	0.71803025
Gpn2	-0.2052919	1.66041057	0.37518334	0.54712535	0.71803025
Grm4	0.13536406	4.02745174	0.3751265	0.54715543	0.71803025
Wwp2	-0.1173547	4.35375428	0.37507455	0.54718293	0.71803025
Ptpn18	0.71669528	-1.6842723	0.37501722	0.54721327	0.71803025
Dpy19l4	-0.1068138	4.18310181	0.37501547	0.54721419	0.71803025
1700001L19R	-0.1649237	3.38356868	0.37492691	0.54726108	0.71803025
Flrt2	0.07599498	7.45037819	0.3748057	0.54732525	0.71804444
Gm13308	-0.5930348	-1.3918595	0.37452935	0.54747162	0.71816645
Mtpap	-0.1044171	4.90302838	0.37406176	0.54771944	0.71821686
Ndn	0.10924696	6.22948305	0.37400762	0.54774815	0.71821686
Osbpl7	0.17630958	2.31354449	0.37372973	0.54789555	0.71821686
Sfrp2	0.31985492	0.51677032	0.37369828	0.54791224	0.71821686
Zdhhc24	-0.1134984	5.97825576	0.37357734	0.54797642	0.71821686
Klhl21	0.12660687	4.54310308	0.37349622	0.54801947	0.71821686
Psmd6	0.08389541	5.94318755	0.37349484	0.5480202	0.71821686
Cog5	-0.1339616	4.63452983	0.37345026	0.54804387	0.71821686
Ate1	-0.0843212	6.22399955	0.37334605	0.54809919	0.71821686
Snord47	0.62647253	-1.5171434	0.37327919	0.5481347	0.71821686
Zcchc7	0.10039212	5.64380246	0.3732648	0.54814234	0.71821686
Tsr3	0.10816402	4.06406222	0.37324935	0.54815054	0.71821686
Csk	-0.1669907	2.98253537	0.37293069	0.54831981	0.71836871
Mt3	-0.4895031	-1.0707301	0.37264408	0.54847215	0.71843745
Selenbp2	-0.403607	-0.2173687	0.37261426	0.548488	0.71843745
Amigo1	0.0850077	5.74003178	0.37247892	0.54855996	0.71843745
Baz2b	-0.0692741	6.99648662	0.37223204	0.54869128	0.71843745
Mysm1	-0.0888085	6.25552941	0.3722059	0.54870519	0.71843745

2310045N01	0.18309598	4.79237446	0.37218144	0.54871821	0.71843745
Actr3	-0.0623028	8.04242918	0.37212334	0.54874912	0.71843745
4931403G20	0.32111478	0.89301541	0.37159158	0.54903224	0.71843745
9130023H24	-0.1363065	3.76212086	0.37158057	0.5490381	0.71843745
Parvg	0.26424188	1.48542068	0.37151476	0.54907316	0.71843745
Gm12216	-0.5452081	-0.754129	0.37140137	0.54913358	0.71843745
Xlr3a	-0.3276504	0.44850432	0.37135417	0.54915873	0.71843745
Samd15	-0.2169887	2.41371152	0.3712847	0.54919575	0.71843745
Prdm8	0.1200068	5.55077021	0.37127056	0.54920329	0.71843745
Shisa4	0.12594902	4.60312327	0.3712498	0.54921435	0.71843745
Fam173a	0.1526025	3.09933044	0.3711003	0.54929405	0.71843745
Rheb	0.088273	7.10790637	0.37107794	0.54930597	0.71843745
S1pr2	-0.258872	1.73585486	0.37102668	0.54933331	0.71843745
Smim7	-0.1215445	6.30717233	0.37091088	0.54939506	0.71844839
1500009L16	-0.1092645	4.68452919	0.37059208	0.54956514	0.71849887
Ubtd2	-0.0935628	5.27685835	0.37036968	0.54968386	0.71849887
Trp63	-0.2441608	2.24132109	0.37036574	0.54968596	0.71849887
Zc3h11a	-0.0678504	6.83947605	0.37025496	0.54974512	0.71849887
Nasp	0.11537006	4.75297334	0.37024402	0.54975095	0.71849887
Sdccag3	-0.1025789	4.74850971	0.37008721	0.54983471	0.71849887
Rab11fip1	0.13276184	3.66126826	0.37005435	0.54985226	0.71849887
1700028P14	-0.2579041	0.53112999	0.37003831	0.54986083	0.71849887
Sag	-0.4659331	-0.7599543	0.36984311	0.54996513	0.71850969
Pa2g4	0.06677376	6.38809118	0.36982295	0.5499759	0.71850969
Nxph3	-0.4012562	-0.3782904	0.3696559	0.55006519	0.71855658
Mir6414	-0.776006	-1.7663391	0.36952205	0.55013676	0.71855869
9330179D12	-0.3087431	1.07492761	0.3694362	0.55018267	0.71855869
Jag2	0.26257141	0.88017764	0.36933281	0.55023797	0.71855869
Taf10	0.12056653	4.15935946	0.36912151	0.55035103	0.71855869
Mrpl53	-0.1305194	3.73890533	0.36907042	0.55037836	0.71855869
Phpt1	0.14634522	3.28584852	0.36905391	0.5503872	0.71855869
Fstl5	0.13725216	4.1514189	0.36889092	0.55047445	0.71856173
Rita1	0.247807	0.99608685	0.36856309	0.55065001	0.71856173
Atp2c2	0.36330663	0.00134164	0.36855217	0.55065586	0.71856173
Emc7	0.12286729	5.39308965	0.36855107	0.55065645	0.71856173
Lrrc41	0.10887419	4.24108537	0.36851099	0.55067792	0.71856173
A230077H06	0.26154111	2.28415411	0.36845126	0.55070993	0.71856173
Arhgap12	-0.0752655	5.65828219	0.36824509	0.55082042	0.71863621
Ankrd37	-0.2485993	1.64700432	0.3674168	0.55126473	0.71914617
Ghdc	0.17353207	2.22972725	0.36731625	0.55131872	0.71914688
Maea	-0.0737845	5.48426659	0.3671606	0.55140231	0.7191862
2010204K13	-0.1638114	2.63947415	0.36687934	0.55155342	0.71927304
Lrp5	-0.2263641	3.28031446	0.36668474	0.55165801	0.71927304

Pcdha6	-0.3675906	0.30558676	0.36666597	0.5516681	0.71927304
Pou3f1	0.17089775	2.01277333	0.36663881	0.5516827	0.71927304
Irf2	-0.1039501	5.24195731	0.36638813	0.55181751	0.71928751
Tmem39b	-0.2423572	1.82656269	0.36633981	0.5518435	0.71928751
Maml2	0.10226929	5.76477574	0.36632	0.55185416	0.71928751
Npm3	0.40547967	-0.8634113	0.36612295	0.55196018	0.71931668
Rpl3	0.10536617	8.2228053	0.36607971	0.55198345	0.71931668
Hyi	0.23747822	1.56627172	0.36557708	0.5522541	0.71934382
Srsf3	0.0838733	7.78346233	0.3654507	0.55232218	0.71934382
Tmsb15b2	0.40775231	-0.3630723	0.36539524	0.55235207	0.71934382
Mtf1	-0.0743992	5.99906572	0.36536491	0.55236842	0.71934382
Polr2m	0.08339314	8.6440255	0.36534118	0.55238121	0.71934382
Ccdc122	-0.2023673	2.24255344	0.36525865	0.55242569	0.71934382
Anapc5	0.07086881	6.32639075	0.36514032	0.55248948	0.71934382
Hcn4	0.22645787	1.30114906	0.36505891	0.55253338	0.71934382
Aifm1	0.13064402	4.10845366	0.36505763	0.55253407	0.71934382
Mrpl13	-0.101767	4.72842033	0.36504875	0.55253886	0.71934382
Tomm6	0.12416372	5.29163217	0.36472241	0.55271489	0.71944071
Bsn	-0.1498049	9.30856583	0.36465395	0.55275183	0.71944071
Cox4i2	0.61261167	-1.5290112	0.364612	0.55277447	0.71944071
Tomm40l	-0.099454	3.94998471	0.3644622	0.55285532	0.71944071
Dusp23	0.20247576	2.79903088	0.36441538	0.5528806	0.71944071
Iqch	-0.4641569	-0.5441629	0.36423227	0.55297948	0.71945666
Esrra	-0.2220395	2.04728223	0.36419466	0.55299979	0.71945666
Rex2	0.57665802	-1.6257951	0.36401018	0.55309945	0.71951675
Kif5b	0.0746238	8.37481273	0.36373115	0.55325025	0.71964317
Hcrtr2	-0.3142019	0.50982421	0.36353866	0.55335433	0.71964317
4933407L21I	-0.3704079	0.14756745	0.36341888	0.55341911	0.71964317
Wdr24	0.19527903	2.33608061	0.36337896	0.5534407	0.71964317
Ckap2l	-0.2592524	0.9087401	0.36333583	0.55346403	0.71964317
Dguok	0.17577776	2.97350383	0.36304457	0.55362165	0.71974402
Fam166b	0.49954646	-0.4522952	0.36298229	0.55365536	0.71974402
Anxa3	0.1374246	5.81377732	0.36289605	0.55370205	0.71974402
Pepd	0.11834556	3.62132166	0.36267687	0.55382075	0.71976774
Ndor1	-0.1622912	3.23268518	0.36266482	0.55382728	0.71976774
Nudt5	-0.1781863	2.90762845	0.36247962	0.55392762	0.71982862
Ccdc79	0.34006701	0.91589966	0.36210072	0.554133	0.71998787
Qtrt1	-0.1667591	2.71706356	0.36200298	0.55418601	0.71998787
Bcr	-0.1135524	6.07003854	0.36179135	0.55430081	0.71998787
Clint1	0.06591856	6.88746452	0.36170915	0.55434541	0.71998787
Alg10b	0.09027271	5.55240241	0.36169498	0.5543531	0.71998787
Hipk4	-0.1508563	3.97058021	0.36164433	0.55438059	0.71998787
Trpm2	-0.1770444	3.5551595	0.36139072	0.55451826	0.71998787

Akt2	0.10347246	5.97679673	0.36130832	0.55456301	0.71998787
Gpn3	0.13997985	4.49154742	0.36125195	0.55459363	0.71998787
Chpt1	0.11141161	5.44121006	0.36116775	0.55463936	0.71998787
Wrnip1	-0.0992614	5.40301787	0.36106314	0.55469619	0.71998787
Slc41a2	0.12512316	4.01969231	0.36099642	0.55473245	0.71998787
Chd3	0.07524849	7.99789304	0.36097197	0.55474574	0.71998787
Ruvbl1	-0.1124778	4.59810199	0.36055157	0.55497428	0.72006217
Fads3	-0.1249645	3.57480128	0.3605083	0.55499782	0.72006217
Eri3	0.11133569	4.21132991	0.36042161	0.55504497	0.72006217
Fbxo30	-0.1180839	5.38115965	0.36039	0.55506217	0.72006217
Hes7	-0.8467996	-1.7035648	0.36037461	0.55507054	0.72006217
Gas2l1	-0.147118	3.8345885	0.36020972	0.55516026	0.72010914
Tspan18	0.21005135	2.75603736	0.3598936	0.55533235	0.72022392
Nav3	-0.1370682	6.74141912	0.35979034	0.55538858	0.72022392
Exoc3	-0.0816615	6.57584827	0.35970169	0.55543687	0.72022392
Cdc25b	0.15470039	2.97562223	0.359608	0.55548791	0.72022392
Kat6b	0.06842446	7.17037169	0.35935585	0.55562533	0.72022392
Ceacam20	-0.2315728	1.81222087	0.35926079	0.55567715	0.72022392
4930402H24I	-0.1076278	5.56917673	0.35911135	0.55575863	0.72022392
Abcb9	-0.2329585	1.61859793	0.3590163	0.55581047	0.72022392
Aqp9	-0.6857064	-1.2215807	0.35894033	0.55585191	0.72022392
Entpd6	-0.1489868	3.09505747	0.35891811	0.55586403	0.72022392
Tceb3	0.09079742	6.00143541	0.35890255	0.55587252	0.72022392
Ramp3	0.44691252	-0.3875601	0.35886864	0.55589103	0.72022392
Myo16	0.1633599	4.34876829	0.35876724	0.55594635	0.72022626
Pitrm1	0.09066829	5.2643786	0.3585007	0.55609185	0.72034539
Tmem136	0.13402479	3.41993937	0.35780416	0.5564724	0.72076897
Gm10677	0.27884121	1.60301891	0.35762949	0.55656792	0.7208233
Cbx4	0.09779068	4.43718425	0.35737255	0.55670847	0.72093594
Aaas	0.14605128	3.0916257	0.35710676	0.55685393	0.72105493
Uqcc2	-0.1491336	3.50855036	0.35688665	0.55697446	0.72114161
Tlr9	0.46330206	-0.3613039	0.35670494	0.557074	0.72120109
Uba1	0.07016692	7.91621844	0.35646825	0.5572037	0.72128075
Zfp781	-0.0992564	6.24306577	0.35614019	0.55738357	0.72128075
Xcr1	-0.2428592	1.55130213	0.35613718	0.55738522	0.72128075
Trim45	0.17483883	2.49172665	0.35601812	0.55745053	0.72128075
Ppp1r12a	0.07241489	7.45763336	0.35590587	0.55751212	0.72128075
Rsrc1	-0.0775798	5.32700455	0.3558934	0.55751896	0.72128075
2410002F23I	-0.1419285	3.20414754	0.3558238	0.55755716	0.72128075
Gm6402	0.17243084	1.77517024	0.35581069	0.55756435	0.72128075
Bhlhe41	0.09599803	7.69616051	0.35557682	0.55769273	0.72136744
Apaf1	0.12637531	3.97683856	0.35540166	0.55778892	0.72136744
Rgs12	-0.1656273	3.26343381	0.35514757	0.55792851	0.72136744

Phf12	0.06955761	6.74279914	0.35512667	0.55793999	0.72136744
Cep44	0.14689126	2.75179763	0.3550454	0.55798466	0.72136744
Gm12942	-0.2285718	2.89548821	0.35497309	0.5580244	0.72136744
Zfand2b	-0.2665465	1.87465094	0.35483373	0.55810102	0.72136744
Tusc1	0.1211798	3.18417392	0.35481446	0.55811161	0.72136744
1700018L02F	0.39408816	0.78479279	0.35477916	0.55813102	0.72136744
Tmem178	0.08515105	5.34075358	0.35463615	0.55820968	0.72136744
Furin	-0.1833549	2.94407601	0.35458884	0.5582357	0.72136744
Rpl29	-0.1100126	6.10243951	0.35451803	0.55827465	0.72136744
Slc25a32	0.12148867	3.88956618	0.35392664	0.55860021	0.72165747
Gm166	0.26991304	0.64345671	0.35382541	0.55865598	0.72165747
Lcp1	-0.122922	4.76173276	0.35381809	0.55866001	0.72165747
Pmm2	0.13634335	3.40285091	0.35363125	0.55876296	0.72172118
Cyba	-0.3106568	0.14828041	0.3532261	0.55898633	0.72192918
Gda	-0.1145291	7.16852614	0.35314459	0.55903129	0.72192918
Slc22a4	0.13274709	3.02269968	0.35290937	0.55916108	0.72193262
Hc	-0.8790536	-1.9135058	0.35287031	0.55918263	0.72193262
Ago1	0.06302323	6.22133921	0.35275241	0.55924771	0.72193262
Zbtb24	0.09931192	5.10803084	0.35273071	0.5592597	0.72193262
4930429B21I	0.09671331	5.16830204	0.35260376	0.55932979	0.72193262
Alg2	0.06508717	7.44663579	0.3524822	0.55939692	0.72193262
Eif4a2	-0.0643892	8.98994268	0.35245941	0.55940951	0.72193262
Cd4	0.23026323	3.24034563	0.35225839	0.55952057	0.7220067
Naa15	0.06874822	6.62462264	0.351943	0.5596949	0.72216241
Fam212b	0.08127678	6.76084735	0.35151379	0.55993232	0.72231352
Pvrl1	0.09497786	4.31616693	0.35149901	0.5599405	0.72231352
Taf1	-0.0873065	6.83215299	0.35138029	0.56000621	0.72231352
Fam13b	-0.0671021	6.9242022	0.35111443	0.56015341	0.72231352
Mamdc4	-0.4596208	-0.0726255	0.35110723	0.56015739	0.72231352
Zfp799	-0.0847896	5.66244101	0.35100984	0.56021134	0.72231352
Cav1	-0.1361563	6.14573405	0.35100238	0.56021546	0.72231352
Zfr2	0.13929931	3.76353164	0.35093842	0.56025089	0.72231352
Esf1	-0.0691578	7.18536144	0.3508207	0.56031612	0.72231352
Gm6994	-0.3889013	0.04688228	0.35072184	0.56037091	0.72231352
Tert	0.47038271	-0.679246	0.35066489	0.56040247	0.72231352
Jakmip3	-0.1630369	4.95956322	0.3505298	0.56047736	0.72234085
Fan1	0.20285065	1.65864258	0.35039703	0.56055098	0.72236655
Mir1931	-0.7574101	-1.7815643	0.3501754	0.56067392	0.72245579
Prcc	0.12058594	4.29593695	0.35001088	0.56076522	0.72250424
Txlnb	-0.2136909	2.24854528	0.34983653	0.560862	0.7225494
Zc3h10	0.18311268	3.0586151	0.3497338	0.56091903	0.7225494
Gsk3a	0.06466248	7.32149468	0.34957722	0.561006	0.7225494
Plekhj1	0.20286063	2.19298864	0.34954839	0.56102201	0.7225494

Tia1	0.12134605	5.2628024	0.34935139	0.56113147	0.7225494
Tcf7l2	0.11491503	6.41243746	0.34926335	0.56118039	0.7225494
Tal1	-0.4182808	0.26914581	0.34917116	0.56123164	0.7225494
Azin1	0.06652734	7.21388816	0.34915092	0.56124289	0.7225494
Suv420h2	0.35468685	-0.0499833	0.34905653	0.56129537	0.7225494
Echs1	-0.1126454	4.38260883	0.34898126	0.56133722	0.7225494
Galnt14	0.20132131	2.27094978	0.34872081	0.5614821	0.7226241
Wwc2	0.10469019	4.8312372	0.34868386	0.56150266	0.7226241
Pex1	-0.1146644	4.55242646	0.34845873	0.56162796	0.72271623
Atf4	0.0714244	6.67721885	0.34823119	0.56175465	0.72281014
H2afv	-0.1395601	5.31528394	0.3475732	0.56212134	0.7232128
Fgf2	0.2050903	1.59855045	0.34711811	0.56237523	0.72338501
Ssr3	0.09480145	8.00002703	0.34699292	0.56244511	0.72338501
Tro	-0.128225	6.41530895	0.34682485	0.56253895	0.72338501
S100b	-0.1182659	6.07697975	0.34674453	0.56258381	0.72338501
Tmem258	-0.1613039	2.43606167	0.34665099	0.56263606	0.72338501
Usp40	-0.1137894	4.56375978	0.34658929	0.56267053	0.72338501
Gm15800	-0.1671387	8.44724005	0.34658164	0.56267481	0.72338501
Mecp2	-0.0767762	8.93917506	0.34652375	0.56270715	0.72338501
Tnc	0.22440978	1.7869043	0.34646675	0.56273901	0.72338501
Tma16	-0.1330379	3.76072172	0.34607596	0.56295748	0.72347363
Tulp3	0.12818073	5.2594078	0.34606261	0.56296495	0.72347363
Ogdhl	-0.215998	1.86515548	0.34591781	0.56304595	0.72347363
Ccng1	0.07536245	8.15001711	0.34584675	0.5630857	0.72347363
Snora31	0.62740921	-1.2004144	0.34575962	0.56313446	0.72347363
Dbi	0.11667278	7.09650929	0.34573108	0.56315043	0.72347363
Rps7	-0.0843454	6.62288406	0.34552275	0.56326705	0.72347363
Oaf	0.24363086	1.012557	0.34528859	0.56339818	0.72347363
Slc16a5	0.43922298	-0.6751443	0.34528122	0.56340231	0.72347363
Chil1	0.38165227	0.532544	0.34523407	0.56342872	0.72347363
Cct8l1	0.89787569	-1.1835794	0.34517736	0.56346049	0.72347363
Scd4	0.38024457	-0.175425	0.34508908	0.56350996	0.72347363
Git1	0.0807707	6.87270412	0.34492788	0.5636003	0.72347363
Ptprr	0.09783465	4.20917954	0.34475225	0.56369877	0.72347363
A430105I19F	0.16251619	3.33664494	0.34474959	0.56370026	0.72347363
Bai3	-0.1148969	6.39756936	0.34473482	0.56370854	0.72347363
Car5a	-0.3962086	-0.2991194	0.34465563	0.56375295	0.72347363
Erlin2	0.08005116	5.40469007	0.34461505	0.56377571	0.72347363
Npc1l1	-0.46341	0.04696487	0.34385289	0.56420351	0.72395357
Fam129a	0.14727718	3.7238136	0.34319031	0.56457595	0.72423962
Ilkap	0.10506722	4.44686146	0.34304312	0.56465875	0.72423962
Gimap9	0.48952268	0.14078881	0.34296659	0.56470181	0.72423962
Trnau1ap	-0.1053432	3.70772269	0.3429471	0.56471278	0.72423962

Morc1	-0.7897103	-1.4927498	0.34293949	0.56471706	0.72423962
Gm3558	-0.4411568	-1.2723541	0.34282035	0.56478411	0.72423962
Senp5	0.07189496	5.58786185	0.34267083	0.56486828	0.72423962
Zfp330	0.09634748	6.1249446	0.34236942	0.56503804	0.72423962
Vwa3a	-0.2561016	2.05989752	0.342339	0.56505518	0.72423962
Taf6l	0.28381868	0.66481131	0.34229645	0.56507915	0.72423962
Afap1	0.10497369	7.61847143	0.34224656	0.56510726	0.72423962
Thoc6	0.25801673	1.21006605	0.34207784	0.56520236	0.72423962
Fbxo18	-0.0753984	5.50909359	0.34206602	0.56520902	0.72423962
Robo2	-0.0935553	6.52649214	0.3418275	0.5653435	0.72423962
Cers2	0.12092502	6.07253664	0.34173265	0.56539701	0.72423962
4930426L09F	-0.2764732	0.84824692	0.34169077	0.56542063	0.72423962
Dhx8	0.07902916	5.10622165	0.34167661	0.56542862	0.72423962
Syna	-0.1982219	1.95238682	0.34164137	0.5654485	0.72423962
Sart3	0.12529853	4.68295842	0.34164038	0.56544906	0.72423962
Lor	-0.260573	0.3638543	0.34145674	0.56555269	0.72427706
Aqp7	-0.3534926	0.05441588	0.34139784	0.56558593	0.72427706
Mon1a	0.14096585	2.34691386	0.34118522	0.56570597	0.72436184
Dip2b	-0.0968288	7.80946321	0.34089675	0.56586892	0.7244028
Nkx6-2	0.34789178	0.35293073	0.34076861	0.56594132	0.7244028
Dph1	-0.6421408	-1.3555422	0.34071038	0.56597423	0.7244028
Zfyve19	-0.1102474	3.28719945	0.34062117	0.56602466	0.7244028
B930003M22	0.42095091	-0.1766797	0.34060066	0.56603626	0.7244028
Ddrgk1	0.11110797	4.10669404	0.34055697	0.56606096	0.7244028
4930590J08F	-0.5367104	-0.7086782	0.34036639	0.56616873	0.72440442
Lrsam1	-0.1505112	3.61904986	0.34036434	0.56616989	0.72440442
Slc39a8	-0.2123682	2.78541867	0.34007322	0.5663346	0.72454627
Chn1	-0.0654592	9.71703491	0.33982268	0.56647643	0.72465882
Chd1l	0.1375354	3.10993716	0.33967879	0.56655792	0.72469417
Tigd3	0.37818376	-0.2656558	0.339524	0.5666456	0.72470845
Fcrlb	0.84088389	-1.4451806	0.33931359	0.56676484	0.72470845
Ptp4a3	-0.1446289	3.47659249	0.3392474	0.56680236	0.72470845
Acadl	0.11219735	5.29580048	0.339202	0.56682809	0.72470845
Dmkn	0.43656311	-0.2987898	0.3391744	0.56684374	0.72470845
Gm10560	0.71943422	-1.0415094	0.33902655	0.56692758	0.72470845
Fam222b	0.06899442	6.75289279	0.33887904	0.56701125	0.72470845
Styx	-0.1198103	4.25906461	0.33883264	0.56703757	0.72470845
Smpdl3b	0.52419898	-0.2309417	0.33880406	0.56705379	0.72470845
Klc2	-0.1092397	5.48248672	0.33836286	0.56730422	0.72482931
Etfb	0.13387248	4.37938077	0.33824838	0.56736924	0.72482931
Ddt	0.15123127	3.74680858	0.33823648	0.567376	0.72482931
Emc6	0.1118623	3.83980552	0.33809855	0.56745435	0.72482931
Rad51c	0.24656175	1.97713349	0.33808459	0.56746229	0.72482931

Ubxn2b	0.08828445	6.02215154	0.33806829	0.56747155	0.72482931
Mrpl3	-0.0770799	5.42029151	0.33760541	0.56773468	0.72494676
1700019L03F	-0.4164119	-0.6529311	0.33759843	0.56773865	0.72494676
Ext1	-0.072025	5.68457115	0.33756218	0.56775927	0.72494676
Tle2	-0.1978032	1.5759767	0.33752749	0.567779	0.72494676
Procr	0.32813171	1.61350465	0.33719412	0.56796869	0.72505893
9330117O12	0.23480223	1.48878023	0.33718371	0.56797461	0.72505893
Grm7	-0.1605005	4.58101982	0.33666499	0.56827003	0.72535759
Vipr2	0.50010329	0.13129878	0.33651284	0.56835674	0.72535759
Slc15a4	-0.1707091	2.35216293	0.33648016	0.56837537	0.72535759
Herc4	0.10972194	4.61268462	0.33624655	0.56850856	0.72535759
Gfi1	0.68479357	-1.3103246	0.33812395	0.56858527	0.72535759
Pgs1	0.10295059	4.41509529	0.33600994	0.56864352	0.72535759
Egfr	-0.1146298	5.96327822	0.33587113	0.56872274	0.72535759
Lpar1	0.1381514	6.85129751	0.33586914	0.56872387	0.72535759
Acot8	-0.2400937	1.57010583	0.33576231	0.56878484	0.72535759
Gm20754	-0.2800009	0.72571659	0.33571897	0.56880958	0.72535759
Rab11fip4os	-0.5380331	-0.7582432	0.33569201	0.56882498	0.72535759
Armc3	0.51526664	-0.9834486	0.3356158	0.56886849	0.72535759
Strn3	0.0678454	8.17349927	0.33547903	0.5689466	0.72535759
Crmp1	-0.0897248	6.06433348	0.3354499	0.56896323	0.72535759
Mab21l1	-0.2589416	1.21648583	0.33535024	0.56902016	0.72536145
Cyld	0.07386455	7.32138492	0.33473495	0.5693719	0.72568046
Mgst2	-0.6714361	-1.4875532	0.33447541	0.5695204	0.72568046
Hes5	-0.213482	1.57656207	0.33426494	0.56964088	0.72568046
Gtf3c6	0.12952452	5.50182977	0.33415762	0.56970233	0.72568046
Commd8	-0.122444	5.76987155	0.33403215	0.56977419	0.72568046
Akr1c21	0.97274131	-1.1791494	0.33401955	0.56978141	0.72568046
Fam19a1	-0.0756947	5.87262786	0.33396669	0.56981169	0.72568046
Tex10	0.09846166	4.35355981	0.33385349	0.56987656	0.72568046
Grik5	0.12432483	5.30620687	0.33378159	0.56991776	0.72568046
Kdm3a	0.07947037	5.9982817	0.33376793	0.56992559	0.72568046
Pdzd2	-0.0983176	6.18780981	0.3337352	0.56994434	0.72568046
C2cd4c	0.19422104	3.3817538	0.33372672	0.56994921	0.72568046
Ascl4	-0.7260084	-1.2625005	0.33368785	0.56997149	0.72568046
Nxpe4	0.13455804	2.96070174	0.33358161	0.57003239	0.72568934
Trip6	0.16777307	3.17019165	0.33338989	0.57014234	0.72573802
Zc3h13	0.07907566	8.06484843	0.33332685	0.57017849	0.72573802
Nsmce4a	0.11385802	4.58308296	0.33304644	0.57033939	0.7257804
Skida1	0.12390213	4.1254437	0.3329791	0.57037804	0.7257804
Abl2	-0.1010525	5.655913	0.33292023	0.57041184	0.7257804
Hsd11b2	0.81258345	-1.1414281	0.33282778	0.57046492	0.7257804
Aim1l	-0.462394	-0.2877581	0.33279896	0.57048147	0.7257804

Hmga2-ps1	-0.351612	0.57436353	0.33268355	0.57054775	0.7257961
Pelp1	-0.1329607	3.28073169	0.33235116	0.57073874	0.72597042
Sft2d1	-0.1172742	3.36527521	0.33195041	0.57096917	0.72608855
Taf7	0.11246722	3.82423093	0.33181662	0.57104614	0.72608855
Pcgf6	0.1515736	3.32218114	0.33176546	0.57107558	0.72608855
Dolpp1	-0.1873762	1.23214382	0.3317526	0.57108298	0.72608855
Greb1l	-0.1526758	3.07983397	0.33172059	0.5711014	0.72608855
Mir5119	0.67730832	-1.4890308	0.33162369	0.57115717	0.72609085
Col4a4	-0.561769	-0.8139241	0.33147326	0.57124377	0.72613235
Polr2l	0.11012775	3.98022412	0.33123678	0.57137997	0.72616446
Cfdp1	-0.1025908	6.92855852	0.33108487	0.57146749	0.72616446
Vat1	0.12982455	4.62141289	0.33102935	0.57149949	0.72616446
Gal3st3	-0.0984638	6.01131671	0.33101657	0.57150685	0.72616446
Lrrc20	0.13742336	4.21284955	0.3309515	0.57154435	0.72616446
Cep63	0.11335532	5.47835818	0.33086725	0.57159292	0.72616446
Znrf2	-0.1158602	4.47525774	0.33069419	0.57169271	0.72616446
Cdv3	0.06792448	7.74731071	0.33068024	0.57170075	0.72616446
Apex2	-0.3518702	1.18741274	0.33043278	0.5718435	0.72623302
Smarcc2	0.06448312	8.25320197	0.33039957	0.57186267	0.72623302
Ednra	0.10899284	5.01863621	0.32990457	0.57214844	0.72651021
AW209491	0.09721824	4.72485102	0.32983451	0.57218892	0.72651021
Cltc	-0.0938167	8.89667839	0.32936791	0.5724586	0.72655346
Vtn	-0.1526497	6.16509271	0.32926715	0.57251688	0.72655346
Cars	0.13279378	3.58290147	0.32915085	0.57258415	0.72655346
Mmp28	0.43387466	0.01821936	0.32914688	0.57258644	0.72655346
Zfp423	-0.0836465	6.14028828	0.32899002	0.5726772	0.72655346
Snora23	0.37044305	0.98583809	0.32894207	0.57270495	0.72655346
Sec61a1	-0.1075943	4.9649272	0.32893799	0.57270731	0.72655346
Rasal3	0.51128388	-0.2015139	0.32891145	0.57272268	0.72655346
Iqcb1	-0.0948661	4.50208082	0.3288958	0.57273174	0.72655346
Dtx3l	0.08379184	4.87767254	0.32881284	0.57277976	0.72655346
Zfp598	0.0903345	4.07853737	0.3286759	0.57285904	0.72655346
Zfp691	-0.2112304	2.53282717	0.32861833	0.57289238	0.72655346
Adra1b	0.1223543	3.92520741	0.32856219	0.5729249	0.72655346
Hdac2	0.06761027	7.14680496	0.32839312	0.57302284	0.72660919
Gm15760	-0.2103631	2.13793902	0.32825831	0.57310096	0.72662315
Ube2e2	-0.0732661	6.58122371	0.32818777	0.57314184	0.72662315
Trafd1	0.13045374	5.08967615	0.32808147	0.57320347	0.72663281
Ralgapa2	-0.1367747	4.62057579	0.32798508	0.57325936	0.72663521
Arrb2	0.12168823	4.08125527	0.32775823	0.57339094	0.72673354
Srl	-0.1862993	1.87903088	0.32724717	0.57368758	0.72674866
Rorc	0.27259402	1.15378792	0.32723122	0.57369685	0.72674866
Dym	0.07316494	5.63051636	0.32722734	0.5736991	0.72674866

Sytl4	0.23362716	2.35993316	0.32713791	0.57375105	0.72674866
Asgr2	0.88751909	-2.1466861	0.32701316	0.57382352	0.72674866
Pfas	-0.134118	3.81241102	0.3269254	0.57387452	0.72674866
Gm20337	-0.227302	2.04686625	0.32673653	0.5739843	0.72674866
Fam118a	0.10290566	4.55151073	0.32653611	0.57410084	0.72674866
4930449E18I	0.49402378	-0.8202355	0.32651363	0.57411391	0.72674866
Otud7b	0.0675203	7.25464734	0.32645509	0.57414797	0.72674866
Hist1h2bf	0.45559174	-0.8437802	0.32644261	0.57415522	0.72674866
Slc16a7	-0.1069934	4.68758201	0.32626515	0.57425848	0.72674866
Etv6	0.08332564	5.18970227	0.32620641	0.57429266	0.72674866
Rpgrip1l	-0.1145801	5.86432918	0.32612904	0.57433769	0.72674866
BC051226	0.58543123	-1.6089049	0.32596476	0.57443334	0.72674866
Zfp775	-0.1887861	3.00237761	0.32590302	0.57446929	0.72674866
Zfp784	0.14268997	3.96359625	0.32584849	0.57450105	0.72674866
Med19	-0.1040977	4.61208039	0.32581154	0.57452257	0.72674866
Sgsm2	-0.1071658	4.61831494	0.32578725	0.57453672	0.72674866
Chchd3	0.09273825	5.11662928	0.32570005	0.57458751	0.72674866
Rcor1	0.08898901	5.10840281	0.32558308	0.57465567	0.72674866
Tmem50b	-0.0722796	5.85195413	0.32556227	0.5746678	0.72674866
Dzank1	-0.0942195	8.55865494	0.32554701	0.57467669	0.72674866
Atic	-0.1092694	4.23843957	0.3254744	0.57471901	0.72674866
Ruvbl2	-0.0973833	4.49916799	0.32541598	0.57475306	0.72674866
Ppfibp1	0.06779905	6.07535407	0.32490545	0.57505083	0.72695977
Sult2b1	-0.3171294	0.36922458	0.32486474	0.57507459	0.72695977
Cnot3	0.08638629	5.42677765	0.32485187	0.5750821	0.72695977
Rara	0.13256146	5.15309412	0.32475608	0.57513801	0.72696216
Il17d	-0.2123883	0.95456877	0.32457443	0.57524407	0.72696497
Egf	0.82292209	-1.2277752	0.32456721	0.57524829	0.72696497
Tbx3	0.12737834	3.70014722	0.32439882	0.57534663	0.72702098
Sympk	-0.1271859	4.57832281	0.32430583	0.57540096	0.72702136
Nr2c1	-0.1068448	4.14095446	0.32360578	0.57581028	0.72742283
Smc4	0.09568343	6.28417346	0.32357751	0.57582682	0.72742283
Fbxw4	-0.1525669	2.72014757	0.3232411	0.57602374	0.72748237
Crim1	0.09932178	6.98334758	0.32323666	0.57602634	0.72748237
Ctsh	0.13395525	4.97746503	0.32318633	0.57605581	0.72748237
Kcnb2	-0.0994773	5.28418829	0.32301206	0.57615789	0.72748237
Vsx1	0.17166477	3.86348811	0.32299591	0.57616735	0.72748237
Adcy5	0.06366941	6.29168631	0.32283028	0.5762644	0.72748237
Ankrd32	-0.1103819	4.11258224	0.32262663	0.57638378	0.72748237
Smok4a	-0.2553091	1.7706538	0.32261435	0.57639098	0.72748237
Rhot1	-0.0779036	6.11619502	0.32260298	0.57639764	0.72748237
Zmym5	0.0749083	6.59672674	0.32257409	0.57641458	0.72748237
Als2cr12	-0.4461775	-1.0692108	0.3222751	0.57658995	0.72763546

Slc4a4	-0.0721728	7.86424389	0.32212636	0.57667724	0.72767345
Map4k3	-0.076837	6.59915635	0.32173761	0.57690549	0.72767345
Timm13	0.18876129	2.54144414	0.32169102	0.57693286	0.72767345
BC033916	0.28964194	0.31825089	0.32161831	0.57697557	0.72767345
Oxtr	-0.1762395	3.55185533	0.32156886	0.57700463	0.72767345
2810049E08I	-0.3787918	0.86852892	0.32155616	0.57701209	0.72767345
Wdsub1	-0.1404667	3.07045806	0.32151418	0.57703676	0.72767345
Derl2	0.11132148	3.56033296	0.32148711	0.57705267	0.72767345
Gsta3	0.31207955	0.88015063	0.3210752	0.57729485	0.72791063
Rcsd1	-0.1338604	4.59087504	0.32089443	0.5774012	0.7279353
Tbc1d32	-0.1262819	4.73628723	0.32072659	0.57749997	0.7279353
Ckb	0.08507819	7.92797394	0.32057704	0.57758801	0.7279353
Anp32b	0.09155104	8.28371329	0.32056375	0.57759584	0.7279353
Sumo2	0.10486318	8.73781098	0.32049442	0.57763666	0.7279353
Rngtt	0.08147614	5.27968148	0.32032643	0.57773561	0.7279353
Igsf21	-0.149878	3.61207176	0.3203069	0.57774711	0.7279353
Camsap1	0.08964253	6.94819669	0.32030679	0.57774718	0.7279353
Xndc1	-0.1306775	3.95280623	0.32021037	0.57780399	0.72793871
Emx2os	0.15841403	2.96091169	0.31994934	0.57795784	0.72801622
Ube2b	-0.0684878	7.64249085	0.31992242	0.57797371	0.72801622
Fcf1	0.09953804	4.74357637	0.31973341	0.57808517	0.72806679
Apbb3	-0.1923618	2.08151078	0.31967086	0.57812207	0.72806679
Fbxo46	-0.1648952	1.66258558	0.31951952	0.57821136	0.72811109
4930509E16I	0.66084755	-1.2491604	0.31928202	0.57835154	0.72818133
Inadl	0.09778244	3.94804209	0.31924167	0.57837537	0.72818133
Mbd4	-0.1289834	4.40401113	0.31910757	0.57845455	0.7282127
Syn3	0.11041488	6.89338645	0.31894556	0.57855026	0.7282127
2700099C18I	-0.2380657	1.75363503	0.31886799	0.57859608	0.7282127
Pak2	0.07471633	6.79178155	0.31878174	0.57864706	0.7282127
Dnm1	0.08690887	8.86763299	0.31865341	0.57872291	0.7282127
Gns	0.10625074	6.50749551	0.3186499	0.57872499	0.7282127
Gm2381	-0.575118	-1.2024585	0.31836502	0.57889344	0.72834102
Ccdc106	-0.2113722	1.77355217	0.31829441	0.57893521	0.72834102
Plekhg3	0.11240618	3.33748503	0.31818665	0.57899898	0.72835314
Lats1	0.06860449	7.15099515	0.31778011	0.57923964	0.72857614
Snw1	0.07537937	6.48926014	0.31767016	0.57930477	0.72857614
Mir325	-0.5927694	-1.4886438	0.31751476	0.57939683	0.72857614
Ncaph	-0.4587317	-0.3833775	0.31734025	0.57950026	0.72857614
2810429I04R	-0.5468386	-1.2896192	0.31724995	0.57955379	0.72857614
Aff3	-0.0757359	7.61446663	0.31723569	0.57956225	0.72857614
Mrps7	-0.0916626	5.25942909	0.3172343	0.57956308	0.72857614
Zcchc8	-0.1053849	3.8751708	0.31709484	0.57964578	0.72857614
Prox2	-0.2214647	1.61354989	0.31698566	0.57971053	0.72857614

Uox	-0.4380456	-0.8076337	0.31690265	0.57975978	0.72857614
D030028A08	-0.2042828	1.33502954	0.31682039	0.5798086	0.72857614
Sirt2	0.10106504	6.66112863	0.3167911	0.57982598	0.72857614
Gt(ROSA)26	-0.1498398	2.76247461	0.31593019	0.58033736	0.72911643
Slc25a54	0.61253038	-0.925918	0.31586605	0.58037549	0.72911643
Glrb	0.07386398	6.74614551	0.31579372	0.58041851	0.72911643
Rnf125	-0.2440546	1.24006064	0.31568303	0.58048434	0.72913107
1810011O10	0.17304561	4.30148064	0.31547279	0.58060943	0.72914954
Atf7	0.12956952	4.26147139	0.31545431	0.58062043	0.72914954
Gatc	0.09127156	7.36425707	0.31538512	0.58066161	0.72914954
Cbr1	-0.0887087	4.80828654	0.31443104	0.58123007	0.7297463
Trmt2a	0.12042787	3.786557	0.31440549	0.58124531	0.7297463
Pou2f1	0.07748921	5.80107812	0.31428027	0.58132	0.72977199
Gm5105	-0.5605668	-0.6224718	0.3140738	0.58144321	0.72980059
Slc1a1	0.0890148	6.01790734	0.31405942	0.58145179	0.72980059
Myo7a	0.20083014	2.71220591	0.31396948	0.58150549	0.72980059
1500015A07	0.20478503	2.19845466	0.31377015	0.58162451	0.72982444
Rpl34	-0.2125295	0.76180846	0.31371615	0.58165676	0.72982444
Hectd1	-0.0909277	7.4322099	0.31365559	0.58169294	0.72982444
Caps2	-0.5609246	-1.2710401	0.31357441	0.58174144	0.72982444
5830418P13	0.35554068	0.17613779	0.31319004	0.58197119	0.72994409
Rab14	0.06222197	7.96281547	0.31317626	0.58197943	0.72994409
Iqca	-0.5098248	-0.2712999	0.31314263	0.58199954	0.72994409
Pcdhb11	-0.341056	0.96598835	0.31270459	0.58226163	0.73020473
Arsb	0.07615617	7.01737196	0.312504	0.58238172	0.73023708
Gm9839	0.67682947	-0.419658	0.31239197	0.58244882	0.73023708
Kdm5c	0.10729403	5.20889389	0.31238963	0.58245022	0.73023708
Srpk3	0.33768506	0.31649771	0.3122096	0.58255808	0.73030427
Dennd5b	-0.1143831	6.59461121	0.31199376	0.58268745	0.73039839
Armc9	-0.1367269	3.36893747	0.31171943	0.58285196	0.73040976
Nvl	-0.0948841	5.01649047	0.31163418	0.58290309	0.73040976
Tmem81	0.31062218	0.54016435	0.31156244	0.58294614	0.73040976
Dbt	0.09270782	5.64794495	0.31154317	0.5829577	0.73040976
Slc6a15	0.10223673	4.70351203	0.31138056	0.5830553	0.73040976
Mcl1	-0.0809065	8.67175862	0.31135514	0.58307056	0.73040976
Chst10	-0.112303	3.87552992	0.3112591	0.58312822	0.73040976
Dleu2	-0.1774267	2.42882056	0.31116759	0.58318317	0.73040976
Srebf2	0.10544646	5.20482012	0.31097713	0.58329757	0.73040976
Morn1	0.33455695	0.458594	0.31090307	0.58334207	0.73040976
Lrp1b	-0.1444191	5.97905963	0.31090079	0.58334344	0.73040976
Tbc1d10b	0.07986828	4.60840359	0.3108934	0.58334788	0.73040976
Xpo1	0.07447401	6.55374024	0.31012123	0.58381226	0.73073376
Supt16	-0.1060434	6.65731618	0.31008895	0.58383168	0.73073376

Dtx4	0.08491717	5.31482988	0.3100626	0.58384755	0.73073376
B020004J07F	-0.34207	0.1922254	0.31000227	0.58388387	0.73073376
Tmem9b	-0.0749037	5.44738389	0.3099512	0.58391461	0.73073376
Braf	-0.0713174	8.58365271	0.30970225	0.58406455	0.73073376
Akt1s1	-0.2082346	1.7893999	0.30951095	0.58417981	0.73073376
Ccdc33	-0.3519508	0.10981215	0.30942937	0.58422898	0.73073376
Rabggtb	0.08207278	6.33194334	0.30942832	0.58422962	0.73073376
Trappc2	0.09896969	3.89742879	0.3093258	0.58429142	0.73073376
Marveld2	0.40695622	0.62572504	0.30931584	0.58429743	0.73073376
Magi1	-0.0945035	6.35945009	0.30929528	0.58430982	0.73073376
Ptpdc1	-0.076983	5.41754893	0.30925085	0.58433661	0.73073376
Nkrf	-0.1041573	5.84414015	0.30915411	0.58439495	0.73073376
Pxdc1	0.15572776	3.64415578	0.30911059	0.5844212	0.73073376
Coq7	0.14003331	4.22424086	0.30864151	0.58470428	0.73087924
Ckap5	-0.102671	7.87263961	0.30861875	0.58471803	0.73087924
Supt4a	0.14812478	4.73820163	0.3085044	0.58478708	0.73087924
Adamtsl5	0.13220748	2.76696844	0.30841539	0.58484084	0.73087924
Lcn2	-1.1427535	-1.2419215	0.30794029	0.58512799	0.73087924
Asic4	0.3372598	-0.0935885	0.30793555	0.58513086	0.73087924
1810021B22I	-0.3317561	0.54550243	0.30791528	0.58514311	0.73087924
Peak1	-0.1083511	6.77754492	0.30789973	0.58515252	0.73087924
Adam10	0.07727414	5.75542415	0.30788379	0.58516216	0.73087924
Zscan20	0.16151768	2.05490218	0.30780857	0.58520766	0.73087924
Astn1	-0.1044391	8.03624661	0.30778035	0.58522472	0.73087924
Rspo3	-0.1495492	7.1031934	0.30768797	0.58528061	0.73087924
Clic3	0.44195392	-0.5019365	0.30768436	0.58528279	0.73087924
Aldh6a1	-0.0859752	5.93071237	0.30763528	0.58531249	0.73087924
5730522E02I	-0.3179315	1.39875561	0.30756952	0.58535228	0.73087924
Cd82	0.33641995	1.51331837	0.30731954	0.5855036	0.73100036
Npdc1	0.09514841	4.46292027	0.30693698	0.58573534	0.73117277
A230072C01I	-0.1591331	3.64761711	0.30691217	0.58575038	0.73117277
Zfp407	0.09956785	4.98637286	0.30648591	0.58600882	0.73142753
Pold1	-0.2753183	0.57114693	0.30636183	0.58608409	0.73145363
Nr3c1	0.05865019	7.2766846	0.3060939	0.5862467	0.73155735
2810021J22F	0.10731055	4.0634388	0.30604578	0.58627592	0.73155735
Sox6	0.1193052	5.00814424	0.3056962	0.58648825	0.73174047
Slc22a14	-1.1857215	-1.9966081	0.30562513	0.58653144	0.73174047
Ift140	0.18708774	3.26109083	0.30534741	0.58670026	0.73186821
Pgap2	-0.1168562	4.20578138	0.30522601	0.58677409	0.73186821
Ermn	0.08360344	6.68337228	0.30507255	0.58686745	0.73186821
Zfp157	-0.0916804	4.72711089	0.30506274	0.58687341	0.73186821
Pip5k1a	0.10054884	3.99435861	0.30490246	0.58697095	0.73186821
Ripk1	0.12541841	3.73532796	0.30486887	0.5869914	0.73186821

Pms2	0.13008522	3.62544284	0.30476102	0.58705706	0.73186821
Aox2	-0.7181648	-0.919169	0.30464441	0.58712806	0.73186821
Gm13238	-0.299058	0.09036922	0.30462135	0.5871421	0.73186821
Arhgap44	0.07144004	6.68127894	0.30456289	0.58717771	0.73186821
Myct1	0.73401674	-1.6149048	0.30436857	0.5872961	0.73191281
Ipp	0.12504287	3.30453463	0.3043256	0.58732228	0.73191281
Pofut1	-0.1176066	3.36432094	0.30408818	0.58746701	0.73202538
Pnkp	0.18123238	1.65228763	0.30387078	0.58759959	0.73212279
Colec12	-0.0653996	6.68445516	0.30372211	0.5876903	0.73216801
Rars2	-0.1064271	3.90870699	0.30356087	0.5877887	0.73222282
Snrpd2	0.14737358	4.07892904	0.30300602	0.5881276	0.73256129
Pecr	0.21735673	1.25200424	0.30291565	0.58818284	0.73256129
Mapk11	0.12814899	3.69421599	0.30284875	0.58822373	0.73256129
Bckdk	0.0892187	4.30818593	0.30260671	0.58837175	0.73267782
Ints1	-0.1130251	4.56946716	0.3024222	0.58848463	0.73270665
Them4	-0.1254014	4.2544018	0.3023156	0.58854987	0.73270665
Mdfi	-0.5877696	-1.4504208	0.30230191	0.58855825	0.73270665
Lamp2	0.10678482	7.76757349	0.30212092	0.58866905	0.73270686
Esd	0.08116226	6.08734854	0.30200885	0.58873769	0.73270686
Slc35f3	-0.0783182	5.58481233	0.301943	0.58877802	0.73270686
4930578E11I	0.38148946	-0.6153435	0.30188661	0.58881257	0.73270686
Gpr150	-0.2349487	0.66432843	0.30185705	0.58883067	0.73270686
Cmtm7	-0.180671	1.86623402	0.30176621	0.58888634	0.73270837
Bhlha9	-0.7240741	-1.2344932	0.30159722	0.58898991	0.73271865
Sec16b	0.5074946	-0.2236277	0.3014812	0.58906105	0.73271865
5730480H06I	0.4686454	0.11818548	0.301435	0.58908938	0.73271865
Malt1	-0.1313083	3.60673066	0.30139745	0.58911241	0.73271865
9530059O14	-0.3117697	2.91368661	0.30119432	0.58923701	0.73277379
Rab34	0.13599403	4.25800237	0.30114764	0.58926566	0.73277379
Grin3a	0.11455451	4.72129811	0.3008551	0.58944523	0.73292936
Piwil2	0.599711	-0.4778187	0.30033342	0.58976573	0.73318642
Susd1	0.21995183	1.90334626	0.30021847	0.58983639	0.73318642
Cacfd1	0.09151395	4.71033613	0.30021335	0.58983954	0.73318642
Thnsl2	-0.1732221	1.59740363	0.30016398	0.58986991	0.73318642
Def6	0.27715645	0.86126945	0.29986915	0.59005127	0.73322538
Rps26	-0.1190736	5.54799771	0.29985164	0.59006204	0.73322538
Glipr2	0.20386499	4.05857951	0.29984471	0.59006631	0.73322538
Zfp143	0.13429977	3.39032296	0.29975876	0.59011921	0.73322538
9130019O22	-0.1699577	2.12545968	0.29941255	0.59033238	0.73322769
Trf	0.11787993	5.62323471	0.29940115	0.59033939	0.73322769
Gnb5	-0.0837565	6.22872661	0.2993702	0.59035846	0.73322769
Tspan7	-0.0790111	8.1016807	0.29936596	0.59036107	0.73322769
Ostc	-0.1018199	4.63120836	0.29931004	0.59039552	0.73322769

Ccdc14	0.19851069	1.8649265	0.29921399	0.5904547	0.73322769
Rsph4a	0.1640579	2.93990736	0.29895764	0.59061272	0.73322769
Sar1b	-0.0778127	5.62421325	0.29887512	0.5906636	0.73322769
Zscan18	-0.1120522	3.71227459	0.2987761	0.59072467	0.73322769
Eapp	-0.0827435	5.25186188	0.29873814	0.59074808	0.73322769
Pipox	-0.2717456	0.57711512	0.29870495	0.59076856	0.73322769
E2f8	0.32858536	0.8932315	0.29869461	0.59077494	0.73322769
4930483K19I	0.40259631	-0.4326204	0.298498	0.59089626	0.73331062
Sapcd2	0.70221573	-1.1384531	0.298194	0.59108395	0.73347591
Zfp811	0.12031975	3.43051123	0.29786831	0.59128517	0.73359519
Cacna2d1	0.0974181	7.62950505	0.29786194	0.59128911	0.73359519
D2hgdh	0.0979939	4.1262795	0.29726961	0.59165543	0.73371875
Zfp512	0.07762247	5.07894091	0.2972202	0.59168602	0.73371875
Spred3	-0.1109531	5.07279754	0.29720213	0.5916972	0.73371875
Dgke	0.10209989	5.5125218	0.29717966	0.59171111	0.73371875
Elovl1	0.18659706	3.3782113	0.29709387	0.59176421	0.73371875
Armc7	-0.1731609	2.3542711	0.29697427	0.59183827	0.73371875
Nipal4	-0.2332829	0.8634244	0.29685776	0.59191043	0.73371875
Fkbp1a	0.07732295	9.78767574	0.29681814	0.59193497	0.73371875
Loxl3	-0.204058	1.77272486	0.29677017	0.59196469	0.73371875
Pigx	-0.154101	2.84092419	0.29673469	0.59198667	0.73371875
Pibf1	-0.1144111	4.56124547	0.29673176	0.59198849	0.73371875
Crebzf	-0.0935588	5.31094743	0.29645311	0.59216119	0.73380989
Pmfbp1	0.55014426	-1.3142859	0.29638923	0.5922008	0.73380989
3110056K07I	0.13932279	2.46016357	0.29634919	0.59222562	0.73380989
Rps6kb2	0.15127969	2.55862419	0.2954662	0.59277371	0.73442139
Cuta	0.10567704	6.28943736	0.29529629	0.5928793	0.73447832
Pgm2	0.09726547	4.06380993	0.29520888	0.59293364	0.73447832
Slc2a4	-0.352035	0.01525547	0.29510642	0.59299734	0.73447832
Prmt6	0.12723826	3.52929591	0.29504105	0.59303799	0.73447832
Rundc3b	0.0964603	4.81308097	0.29474542	0.5932219	0.7345688
Hmgn3	-0.0904708	6.929729	0.29464435	0.59328481	0.7345688
Ttc39c	0.16428545	2.23883921	0.29451101	0.59336782	0.7345688
Col18a1	0.19301453	1.82900347	0.29441065	0.59343032	0.7345688
Nos3	-0.2612045	0.88492449	0.29418929	0.59356821	0.7345688
Uckl1	0.11986631	3.45904809	0.29391571	0.59373872	0.7345688
Vps8	-0.1485251	4.47332854	0.29388107	0.59376032	0.7345688
Fbxo32	0.09562868	5.02528935	0.29375598	0.59383832	0.7345688
Ctnnd2	-0.0890278	9.36130243	0.2937468	0.59384405	0.7345688
Fam208b	-0.0737078	6.44869303	0.29373413	0.59385195	0.7345688
Stab2	0.58973744	-1.1978547	0.29370684	0.59386897	0.7345688
Med31	0.11490135	3.37068714	0.29364004	0.59391065	0.7345688
Zfp69	-0.2595029	0.82551149	0.29352872	0.5939801	0.7345688

Rpl13a	0.08048138	7.00463303	0.29340009	0.59406037	0.7345688
Dpm2	0.09424394	4.06808179	0.29312535	0.59423191	0.7345688
Patz1	-0.0910047	4.85691423	0.29307951	0.59426054	0.7345688
Rbak	-0.1719617	3.25545728	0.2929804	0.59432246	0.7345688
Aar2	-0.1355022	3.78509195	0.29296681	0.59433095	0.7345688
Rtn4r	-0.0982225	4.37400648	0.29281584	0.59442529	0.7345688
Hap1	0.12081894	4.20512542	0.29259627	0.59456255	0.7345688
Lamb3	0.70686039	-1.016154	0.29255399	0.59458899	0.7345688
Themis	0.33087826	0.53572388	0.29237148	0.59470314	0.7345688
Pqlc1	-0.0999262	3.33315234	0.29216707	0.59483105	0.7345688
4933413G19	-0.7491761	-1.7289615	0.2920892	0.59487979	0.7345688
Usp5	-0.0996556	4.66250852	0.29195744	0.59496229	0.7345688
Ankrd39	-0.2004569	1.7336555	0.29193478	0.59497648	0.7345688
Pcnt	-0.094901	5.11387486	0.2919268	0.59498147	0.7345688
Nr2e1	-0.148697	3.69179722	0.29181163	0.59505361	0.7345688
Noa1	-0.0835595	4.42358664	0.29179093	0.59506657	0.7345688
Traf4	-0.1295419	2.52163121	0.29176461	0.59508306	0.7345688
3110052M02	-0.0838291	4.84073189	0.29175455	0.59508936	0.7345688
Jazf1	-0.0929364	5.2823902	0.2916686	0.59514321	0.7345688
Acss2os	-0.5963475	-1.5760205	0.29165502	0.59515172	0.7345688
Patl1	0.07548616	6.17278137	0.29161966	0.59517388	0.7345688
Atp5s	0.08009074	4.66930354	0.29161008	0.59517988	0.7345688
Zscan29	-0.0911296	5.40459204	0.2915352	0.59522681	0.7345688
Zbtb48	-0.2308966	1.61841727	0.29147793	0.59526271	0.7345688
E2f1	-0.1637137	1.94559375	0.2914163	0.59530134	0.7345688
Ubac2	0.13247322	2.53218994	0.29134848	0.59534387	0.7345688
Tmem14c	0.14225125	3.11534299	0.29133734	0.59535085	0.7345688
Fam49b	-0.0688087	6.79964942	0.29130217	0.59537291	0.7345688
Ten1	-0.1281011	3.27363178	0.29116574	0.59545847	0.7345688
Crym	0.10054952	3.43461405	0.29105245	0.59552954	0.7345688
Nenf	0.10432406	4.26834623	0.29088869	0.59563232	0.7345688
Rps21	-0.1068314	5.20132058	0.29088693	0.59563342	0.7345688
Ccdc43	-0.0915012	4.71901413	0.29082187	0.59567426	0.7345688
Evi5l	-0.1168777	3.32774126	0.29073096	0.59573134	0.7345688
Rpl21	-0.1028061	7.82122317	0.2906266	0.59579687	0.7345688
Nr1h2	-0.1297299	4.09397517	0.29049322	0.59588065	0.7345688
Ppox	0.2679802	1.015352	0.29044084	0.59591356	0.7345688
Tbcel	0.09082701	5.2480881	0.29037934	0.59595221	0.7345688
G6b	0.70348701	-1.0109044	0.29036079	0.59596387	0.7345688
Nim1k	0.11824869	4.19746749	0.29029649	0.59600427	0.7345688
Zcchc6	0.05632732	7.22987234	0.29014484	0.59609961	0.73461279
Coa6	-0.0968853	3.73919613	0.29006605	0.59614914	0.73461279
Lrrc8e	-0.3040046	-0.1319681	0.2899513	0.59622131	0.73463444

Jph1	0.10943557	4.82841873	0.28959708	0.59644421	0.73466849
Zcchc2	-0.0780805	6.13430018	0.28949927	0.59650578	0.73466849
Srgap3	-0.095362	9.0810211	0.28947711	0.59651973	0.73466849
Atg5	-0.0822472	4.61398251	0.28933684	0.59660808	0.73466849
Optn	-0.0971597	4.13149135	0.28932421	0.59661603	0.73466849
Slc9a2	-0.1781281	5.21957124	0.2892545	0.59665994	0.73466849
Zkscan1	0.06151287	7.6698238	0.28924587	0.59666538	0.73466849
Gm14391	-0.2611979	1.93452359	0.28917685	0.59670887	0.73466849
Mks1	0.36040573	0.20709525	0.28899188	0.59682544	0.73466849
Cdh1	0.16145049	6.50236452	0.28892011	0.59687068	0.73466849
Scaper	-0.0937695	6.38242996	0.28887761	0.59689748	0.73466849
Agbl2	-0.2102063	1.56639521	0.288856	0.59691111	0.73466849
Polr1a	-0.1496655	4.59391222	0.28864878	0.59704179	0.73466849
Aacs	-0.1433473	2.68904219	0.28838809	0.5972063	0.73466849
Mxd4	-0.1903782	2.4850677	0.28820996	0.59731876	0.73466849
Lrrn4cl	-0.0825701	4.95697795	0.28818561	0.59733413	0.73466849
Nudt22	0.18523755	1.45155025	0.28813655	0.59736512	0.73466849
Tlr4	0.13822059	4.43886686	0.28805245	0.59741824	0.73466849
Zfp771	0.29164006	0.69994245	0.28803035	0.5974322	0.73466849
Exoc3l4	0.48469095	-0.9109404	0.287949	0.5974836	0.73466849
Hmgcll1	0.1184413	3.35418801	0.28790621	0.59751063	0.73466849
Csnk2a1	0.05947934	6.99510857	0.2878801	0.59752713	0.73466849
Klhl20	0.0895113	5.71641113	0.2878291	0.59755936	0.73466849
Hs2st1	-0.0716504	6.7575588	0.28776151	0.59760208	0.73466849
Fscn1	-0.0929811	5.37348676	0.28774288	0.59761386	0.73466849
Diablo	-0.1266401	4.26106158	0.28749821	0.59776856	0.73470916
Gm14405	-0.2668817	0.15617204	0.28748481	0.59777704	0.73470916
Gm10012	-0.0970427	3.8279026	0.28743152	0.59781074	0.73470916
Tmem41a	0.11525352	3.74697865	0.28718624	0.59796595	0.73483279
Etl4	-0.064251	7.93056652	0.28667169	0.59829181	0.73508621
9930021J03F	-0.0814155	8.19119709	0.28663779	0.59831329	0.73508621
4930556M19	0.23342809	1.12915762	0.28660188	0.59833604	0.73508621
Smad2	0.06008683	6.52510052	0.28629547	0.59853031	0.73522468
Usp51	-0.2641824	1.25592002	0.28625175	0.59855803	0.73522468
Wdr7	-0.1021485	7.54758209	0.28596425	0.59874044	0.73534184
Tmprss7	0.31232172	0.24829116	0.28592917	0.5987627	0.73534184
Jade3	0.14962466	4.31488406	0.28568676	0.59891661	0.73536957
Epb4.1l4b	0.13048569	3.33579708	0.28566918	0.59892778	0.73536957
Slc22a8	0.11863662	8.06097155	0.285624	0.59895647	0.73536957
Eomes	0.34067579	0.41617846	0.28554937	0.59900388	0.73536957
Ift122	0.10768095	3.72749146	0.28524888	0.59919484	0.73543923
Slc25a1	0.14957561	3.82764877	0.2851271	0.59927227	0.73543923
2900079G21	-0.1922436	1.16559202	0.28512701	0.59927232	0.73543923

Ccr2	0.18920342	2.65618563	0.28492759	0.59939916	0.73543923
Ttc7	0.15997934	3.14285201	0.2848594	0.59944254	0.73543923
Xxylt1	-0.1168678	3.52555456	0.28484955	0.59944881	0.73543923
C1qa	-0.541293	-1.5955507	0.28484494	0.59945174	0.73543923
Msantd1	0.49759118	-0.632476	0.28470036	0.59954375	0.73543923
Pde4d	0.08843868	7.08529817	0.2846866	0.59955251	0.73543923
Wdr5b	0.23818589	1.16772343	0.28451381	0.59966252	0.73547839
Prss8	-0.8833781	-2.1549414	0.28445521	0.59969983	0.73547839
Hsf4	-0.2126398	1.34637394	0.28429629	0.59980106	0.73547839
Shf	-0.2456047	1.94867076	0.28424523	0.59983359	0.73547839
Cyp4x1	-0.2350887	0.99608208	0.28420737	0.59985772	0.73547839
Casd1	0.08339964	5.80115426	0.28401836	0.59997818	0.73555907
Tmem107	0.17489117	1.71680392	0.28387042	0.60007251	0.73559646
Tmem138	-0.1979178	1.17105868	0.28377175	0.60013544	0.73559646
Nupl2	0.14758671	3.28139523	0.28371337	0.60017268	0.73559646
Klhl12	-0.0839999	4.79415184	0.28361757	0.6002338	0.73560437
Ubxn10	0.32269825	0.82229865	0.28328729	0.60044462	0.7356353
Ppp2r5b	0.11496375	3.29609995	0.28328661	0.60044505	0.7356353
1700034J05F	0.83460501	-1.6206311	0.28317128	0.60051871	0.7356353
Mpv17l2	-0.1798569	2.69491878	0.28310981	0.60055797	0.7356353
Galc	-0.1281134	3.76757953	0.28310856	0.60055877	0.7356353
Vcam1	-0.0913771	5.84331922	0.28300615	0.6006242	0.7356353
Usp45	-0.0740878	6.85723407	0.28297874	0.60064172	0.7356353
Bok	-0.1077487	4.24152508	0.28258068	0.60089619	0.73584414
Pomk	-0.0902373	4.76491231	0.28254096	0.6009216	0.73584414
Mageb16-ps	0.47399611	-0.6955082	0.28240598	0.60100795	0.73586188
Spag4	0.64425698	-1.6186552	0.28234736	0.60104546	0.73586188
Nifk	0.11172585	4.61760274	0.28223697	0.60111611	0.73588142
Top3b	0.12781782	3.52638291	0.28209441	0.60120738	0.7359262
Bub1b	0.36168334	0.47634977	0.28186322	0.60135544	0.73604049
Itk	-0.447681	-0.1746427	0.28147639	0.60160337	0.736254
Myo9b	0.09311603	4.49957266	0.28142035	0.60163931	0.736254
Ywhae	0.06339255	10.7332426	0.28112915	0.60182611	0.73636689
Gas7	0.06365291	9.7921324	0.28104893	0.60187759	0.73636689
Gtf3c1	-0.1074247	6.48782816	0.28096649	0.60193051	0.73636689
Taf1c	-0.2459488	1.20429894	0.28086166	0.60199781	0.73636689
Spsb1	-0.1580909	2.8969923	0.28078741	0.60204549	0.73636689
Alkbh5	-0.0752011	6.397967	0.280765	0.60205989	0.73636689
Nmd3	-0.0879459	5.08200007	0.28051025	0.60222355	0.73645112
Lzts1	0.1703773	2.77788599	0.28048742	0.60223822	0.73645112
Rpl31-ps12	-0.1018958	4.06261921	0.28030218	0.60235729	0.73648594
Zfp282	0.19470573	1.90740085	0.28020372	0.6024206	0.73648594
Crtc2	0.09756418	4.0438307	0.28013804	0.60246285	0.73648594

Rpp21	-0.1479938	3.15334798	0.28010263	0.60248562	0.73648594
Fnip1	-0.0897505	5.8156585	0.27995318	0.60258177	0.73649845
Wdr75	0.10567296	4.66345154	0.27984712	0.60265002	0.73649845
Ykt6	0.09034162	5.12626291	0.27975786	0.60270748	0.73649845
Gm5617	0.27420096	1.57345453	0.2797465	0.60271479	0.73649845
Bcl10	0.13415834	4.85800539	0.27947193	0.6028916	0.73655252
G6pc3	0.1157818	3.9418678	0.27934347	0.60297436	0.73655252
Zfp40	-0.0967342	4.31869583	0.27931244	0.60299436	0.73655252
Ergic1	-0.0690379	6.53841747	0.27926721	0.60302351	0.73655252
Smco4	-0.1878144	1.93044032	0.27925293	0.60303271	0.73655252
Krtcap2	-0.1143959	3.33774937	0.27873459	0.60336697	0.7368939
Tns4	-0.2507387	1.63779668	0.27862322	0.60343885	0.7369148
Pip4k2b	-0.0731356	7.02044146	0.27818883	0.60371936	0.73702657
Rai2	0.12743935	3.16974792	0.27815197	0.60374318	0.73702657
Ifrd2	-0.2362135	1.23966364	0.27812241	0.60376227	0.73702657
Cyp11a1	-0.3376396	-0.1870064	0.2780428	0.60381372	0.73702657
Plbd1	0.30053457	0.95484593	0.27799587	0.60384405	0.73702657
Prkch	-0.1437392	2.40953756	0.27797274	0.603859	0.73702657
S100a4	0.22576068	3.08503307	0.27736735	0.60425061	0.737223
Cer1	-0.7003886	-1.0477751	0.27727076	0.60431314	0.737223
Ccbl2	-0.1761176	2.49897631	0.27724324	0.60433095	0.737223
Ankrd42	0.09061788	4.01704178	0.27719968	0.60435916	0.737223
Zfp335	0.12657661	3.92791599	0.27716326	0.60438275	0.737223
Il11ra1	-0.0871835	4.34286303	0.27715852	0.60438582	0.737223
Nfkbiz	0.18071228	2.15685519	0.27703499	0.60446583	0.737223
Ceacam1	-0.1600399	2.96410889	0.27703384	0.60446657	0.737223
Arrdc1	0.24262504	0.91740947	0.27696215	0.60451302	0.737223
Rbfox2	0.06377993	7.5197972	0.27687323	0.60457064	0.73722645
Nup98	-0.0666952	6.39272742	0.27670855	0.60467738	0.7372898
Eif3d	0.08753254	4.54766849	0.276283	0.6049534	0.73755952
Zfp462	-0.0895143	6.71344293	0.27597436	0.60515377	0.73758279
Lrrc3	0.12279968	3.66983848	0.27590234	0.60520054	0.73758279
Zfyve28	-0.1427788	3.78121154	0.27587821	0.60521621	0.73758279
Srsf9	0.10549292	4.93587337	0.27584587	0.60523722	0.73758279
Ier3ip1	-0.0876037	5.53606747	0.27583151	0.60524655	0.73758279
Smg7	-0.055542	8.02274237	0.27569106	0.60533781	0.73758843
Mettl20	0.15050953	2.58159646	0.27560541	0.60539347	0.73758843
Arhgef40	0.10075353	3.63309355	0.27557133	0.60541562	0.73758843
Riok1	-0.1117555	3.87879186	0.27542161	0.60551296	0.73764024
Ppih	0.12571164	3.16833844	0.27531289	0.60558367	0.73765959
Sptssb	0.12479426	4.63872135	0.27514242	0.60569456	0.73770554
Spg11	-0.0897732	5.11874808	0.27501843	0.60577525	0.73770554
Med4	-0.1117212	3.6371704	0.27500214	0.60578585	0.73770554

Ddb1	0.07122274	6.36962205	0.27470707	0.60597797	0.73778347
1700085C21I	0.69243394	-1.704339	0.27464707	0.60601706	0.73778347
Prpf39	0.11683899	5.45336515	0.2746249	0.6060315	0.73778347
Kifc1	0.51488153	-1.4155674	0.27448199	0.60612462	0.73778347
Fem1a	-0.0718678	5.55688316	0.27446218	0.60613753	0.73778347
Pnpla3	-0.2759006	1.33916254	0.27411863	0.60636151	0.73778347
Cyp19a1	0.66822663	-1.5822901	0.27560559	0.60641135	0.73778347
Kcnrg	-0.3297703	1.30895024	0.27399998	0.60643891	0.73778347
Ctxn2	-0.2218014	1.98381935	0.27398511	0.60644861	0.73778347
Taf1a	-0.1336839	3.23536216	0.27389791	0.60650551	0.73778347
Gm9866	-0.1455007	2.57794585	0.27381643	0.60655869	0.73778347
Oaz2	0.08113463	6.79732827	0.27357183	0.60671838	0.73778347
Tsku	0.21673517	2.14957255	0.27354186	0.60673795	0.73778347
Pomp	-0.0815055	7.36047553	0.27352657	0.60674794	0.73778347
Cd59b	0.34073981	-0.4724651	0.27351908	0.60675283	0.73778347
Ndst3	-0.1224958	4.32585703	0.27337204	0.60684889	0.73778347
Gm6086	-0.5196281	-1.5354807	0.27336306	0.60685476	0.73778347
Aim1	0.14585312	3.96012799	0.27327413	0.60691287	0.73778347
Usp1	0.08063598	5.3274602	0.27320796	0.60695611	0.73778347
Mrpl22	0.12783346	3.03106697	0.27310019	0.60702656	0.73778347
Mrpl41	-0.0859969	4.74946959	0.27304091	0.60706532	0.73778347
Cdc42se1	-0.0883389	5.51171644	0.27302042	0.60707872	0.73778347
Dio3	-0.4631259	-0.9980248	0.27295042	0.6071245	0.73778347
Lmo7	0.13024005	6.02077423	0.27287865	0.60717144	0.73778347
Rpl9	-0.0931704	7.1941389	0.27266202	0.60731318	0.73778347
Tirap	0.12875224	4.4284544	0.27255962	0.60738021	0.73778347
Spag16	-0.2674651	0.42113633	0.27247345	0.60743663	0.73778347
Nkg7	0.50501109	0.02728111	0.27234563	0.60752033	0.73778347
Clspn	-0.378718	-0.1388371	0.27226622	0.60757234	0.73778347
Sgip1	-0.0836153	8.10724872	0.27222395	0.60760003	0.73778347
Ccdc181	0.08418667	5.18637453	0.27214555	0.6076514	0.73778347
Ptpn9	0.07550313	5.88928776	0.27203981	0.6077207	0.73778347
Wdtc1	-0.0819007	5.20720695	0.27198396	0.6077573	0.73778347
Zfp111	0.09789661	5.16737656	0.27197859	0.60776083	0.73778347
Gm7609	0.3707693	-0.6325316	0.27188891	0.60781962	0.73778347
Dennd4a	-0.0669881	7.418427	0.27187078	0.60783151	0.73778347
Itih5	-0.092871	6.2904773	0.27179914	0.60787849	0.73778347
Gm10033	-0.1290014	4.59439636	0.27161462	0.60799952	0.73780048
1110002L01F	0.21812745	1.11753927	0.27161059	0.60800216	0.73780048
Tmem47	-0.0881666	7.50737928	0.27135222	0.60817173	0.73786702
Akr7a5	0.15203778	2.3865428	0.27129526	0.60820912	0.73786702
Fgr	0.31745405	0.32425747	0.27127057	0.60822533	0.73786702
Gucy2g	-0.2069205	1.35849486	0.27119291	0.60827633	0.73786702

Myb	0.24833073	0.61825725	0.27096962	0.60842301	0.7378911
Zfp493	0.19853126	2.60744916	0.27096961	0.60842302	0.7378911
Parp14	-0.1316232	4.15536348	0.27091229	0.60846069	0.7378911
Ube2l3	0.07164604	7.51291036	0.2707784	0.60854869	0.73793131
Rxfp2	-0.3638298	-0.5855475	0.27057427	0.60868291	0.73802757
Slx1b	-0.0938887	3.6325023	0.27044771	0.60876616	0.73806201
Tchh	-0.1348874	3.01158791	0.27031423	0.60885399	0.73810199
Rgs10	0.09340477	4.32710585	0.26999496	0.60906419	0.73818474
Oas1a	-0.3413459	0.68271435	0.26997239	0.60907905	0.73818474
Ctdspl2	-0.0659696	6.57527525	0.26990277	0.6091249	0.73818474
Smim24	0.19415615	2.27280505	0.26981423	0.60918324	0.73818474
Psmf1	-0.0991952	4.76051962	0.26969876	0.60925933	0.73818474
Ppm1d	0.08591756	5.13616871	0.26963958	0.60929833	0.73818474
Bckdha	-0.178952	1.13284598	0.26962755	0.60930626	0.73818474
A930017M01	-0.1430274	2.61294087	0.26944901	0.60942398	0.73826089
Sfn	-0.5430453	-1.1509467	0.26928764	0.60953042	0.73832336
E130307A14	0.13685073	2.29238303	0.26888441	0.60979656	0.73841355
Ube2o	0.10713294	4.91134424	0.26888075	0.60979897	0.73841355
4930412C18I	-0.2710833	1.24851585	0.26887966	0.60979969	0.73841355
Gid4	-0.0646273	5.84590795	0.26884229	0.60982437	0.73841355
Hsd17b11	0.0989216	5.02271974	0.26861602	0.60997384	0.7385122
Mx1	0.46913229	0.01692365	0.26855282	0.61001561	0.7385122
Col4a3bp	0.05680412	7.25980068	0.26844467	0.61008708	0.73853229
Hs3st3b1	0.14086896	3.71015302	0.26823804	0.61022371	0.73854114
Ddr1	-0.0965151	3.43016774	0.26812843	0.61029621	0.73854114
Psma5	-0.0772259	5.72397389	0.2680426	0.61035299	0.73854114
Matr3	-0.0767194	8.16150937	0.26802995	0.61036136	0.73854114
Nxph4	0.29406904	0.63504163	0.26801868	0.61036882	0.73854114
Rab11fip2	-0.0616903	6.93761623	0.26785915	0.6104744	0.73854645
Ldoc1	-0.613646	-1.3494484	0.2678462	0.61048297	0.73854645
Efhb	-0.2298002	0.67924051	0.26749732	0.61071402	0.73874285
Trim13	0.15572369	2.67528309	0.26738064	0.61079133	0.73874285
Laptm4a	-0.1015037	8.0527121	0.26732882	0.61082568	0.73874285
Dnajc2	0.07221501	6.43593183	0.26726092	0.61087068	0.73874285
Akr1c12	0.57062543	-0.1706401	0.26713606	0.61095346	0.73874285
Smarcd3	-0.109501	4.1281128	0.26710402	0.61097471	0.73874285
Igfbp7	-0.125242	5.10106368	0.26672038	0.61122924	0.73893134
Hira	-0.0844179	4.13802974	0.26670352	0.61124043	0.73893134
B3galt4	-0.4935843	-0.7880389	0.26648614	0.61138476	0.73903943
Gcm1	-0.5022797	-1.3960054	0.26629733	0.61151018	0.73912464
Rgs5	-0.1201652	4.11792046	0.26620094	0.61157423	0.73913566
Ankrd26	0.07626819	5.96875239	0.26594117	0.61174693	0.73918031
Tnxb	0.21771369	1.28082218	0.26589684	0.61177641	0.73918031

Asnsd1	0.10788097	5.29360696	0.26588407	0.6117849	0.73918031
Ilf2	0.08859186	5.70695281	0.2658149	0.61183091	0.73918031
Zfp229	0.11196841	3.67899273	0.26559829	0.61197504	0.73928807
Btbd8	0.24673557	0.87581466	0.2653854	0.61211676	0.73931305
Homez	0.07968206	4.36385714	0.26535991	0.61213373	0.73931305
Slc52a3	0.20729146	1.79376373	0.26523369	0.61221779	0.73931305
Nat6	0.14115568	3.18126994	0.26522967	0.61222047	0.73931305
1200014J11F	-0.0690343	5.36314191	0.26515469	0.61227042	0.73931305
Cyb561d2	-0.2127675	1.76477958	0.26465179	0.61260569	0.73961441
Net1	-0.0746846	5.59464004	0.26432642	0.61282281	0.73961441
Scap	0.10393581	4.49942401	0.26426873	0.61286133	0.73961441
A930006K02	-0.2876115	0.2417027	0.26422305	0.61289183	0.73961441
4930405A21	-0.2719906	1.03742886	0.26416941	0.61292765	0.73961441
Znrd1	0.10446194	4.5581472	0.26410707	0.61296929	0.73961441
9130019P16	0.30817838	0.2995342	0.26403857	0.61301504	0.73961441
Pcnxl2	-0.1169596	4.8164733	0.26394266	0.61307912	0.73961441
Sgcb	-0.0955332	4.81756868	0.26393584	0.61308368	0.73961441
Cntn4	-0.0974361	5.2390942	0.2638824	0.61311939	0.73961441
Pak4	-0.1683109	4.81414536	0.26374758	0.61320951	0.73961441
Ninj1	0.24476925	2.11751895	0.26371325	0.61323246	0.73961441
Palb2	-0.27402	0.75081152	0.26371015	0.61323453	0.73961441
Nr6a1	-0.1391955	3.0928936	0.2636014	0.61330725	0.73963582
Cntnap1	-0.0936378	5.86954492	0.26343519	0.61341842	0.73964119
Pdss1	0.17122812	2.13099445	0.26343039	0.61342163	0.73964119
Mrps18b	0.1573332	2.96801777	0.26324571	0.61354522	0.73970777
Celf2	0.07387017	9.78204798	0.2631836	0.61358679	0.73970777
Pik3ca	-0.0558986	7.01483984	0.26305224	0.61367474	0.73974752
Sssca1	0.14147374	2.92878654	0.26265852	0.61393852	0.73993847
Kctd8	-0.1943671	2.1487354	0.26263273	0.61395581	0.73993847
Zfp451	-0.0943746	5.17462745	0.26244751	0.61407999	0.73993847
Fxyd1	-0.126304	3.46058344	0.26244728	0.61408014	0.73993847
Cerkl	-0.349057	-0.1444446	0.26240561	0.61410809	0.73993847
Nup210	0.16592514	3.04798879	0.26223029	0.6142257	0.74001392
1700086O06	0.37325512	-0.332981	0.2620173	0.61436865	0.74002303
Ndufa4l2	0.33727807	-0.0866325	0.26201371	0.61437106	0.74002303
Ivns1abp	-0.0679563	7.54592213	0.2619463	0.61441631	0.74002303
Uqcrc1	0.06913741	5.02004495	0.26153186	0.61469472	0.74002303
Ska1	0.87966558	-1.8990028	0.26123035	0.61489745	0.74002303
Timp4	-0.157087	2.45770989	0.26120079	0.61491733	0.74002303
2900055J20F	-0.1476908	3.12873871	0.26104933	0.61501923	0.74002303
Akr1c18	-0.2825067	1.31564521	0.26102398	0.61503629	0.74002303
Stc2	-0.3199442	-0.1603115	0.26093865	0.61509371	0.74002303
Hist2h3b	0.40589489	-1.2253731	0.26091721	0.61510815	0.74002303

Elavl1	0.05900511	6.63422728	0.26090764	0.61511459	0.74002303
Rtn4ip1	-0.1439283	3.29227625	0.26090054	0.61511937	0.74002303
Rmst	-0.3302153	0.98297744	0.26088368	0.61513072	0.74002303
Glp1r	0.30482272	0.0642864	0.26083337	0.61516459	0.74002303
Pcdh10	-0.0854302	7.10637105	0.26076591	0.61521001	0.74002303
Rnf123	0.08360557	4.46546386	0.2606232	0.61530612	0.74002303
A930009A15	-0.6144048	-1.9983995	0.26057339	0.61533968	0.74002303
Ero1lb	-0.1188224	3.76443278	0.26052604	0.61537157	0.74002303
Mcemp1	0.36304355	0.11998169	0.26026679	0.6155463	0.74002303
Xkr8	0.19987899	2.41378249	0.26017556	0.61560782	0.74002303
Tatdn2	-0.116151	4.03065392	0.2601746	0.61560846	0.74002303
B930041F14	-0.0879607	5.11013948	0.2601148	0.6156488	0.74002303
Pou2f2	-0.14927	3.6192154	0.26010951	0.61565236	0.74002303
Zfp106	-0.0517433	9.16699433	0.26008669	0.61566775	0.74002303
Med27	0.14700742	3.23350047	0.25993244	0.61577181	0.74002303
Pcsk2	0.08091172	7.2390638	0.25990148	0.6157927	0.74002303
Wipf1	0.10891488	6.02779989	0.25987517	0.61581045	0.74002303
Trappc13	-0.0598121	6.39477302	0.25981201	0.61585308	0.74002303
Srpk1	-0.0624295	6.0743028	0.25967587	0.61594498	0.74002303
Gstt3	0.1102317	5.20221588	0.2596636	0.61595327	0.74002303
Gm20362	-0.2701744	0.71752703	0.25959644	0.61599861	0.74002303
Fam131a	-0.0795664	5.2288787	0.25950111	0.616063	0.74002303
Vmn2r29	0.11960752	3.38591024	0.25950015	0.61606365	0.74002303
Chst13	-0.6054975	-0.7207541	0.25944178	0.61610307	0.74002303
Runx2	0.10742383	4.70215214	0.25908502	0.61634419	0.74013612
Cd47	-0.0800066	7.49962198	0.25899	0.61640844	0.74013612
Pgam2	-0.2634266	0.82968011	0.25895854	0.61642972	0.74013612
Slc9a1	0.08959484	5.26187894	0.25895447	0.61643247	0.74013612
Spns2	-0.1128468	3.5192407	0.25889567	0.61647224	0.74013612
1700001L05	-0.1354455	4.34217344	0.25877954	0.61655081	0.74016441
Ptprv	0.4843345	-0.9318184	0.25847213	0.6167589	0.74028934
Pcdha9	0.44669737	0.22596979	0.25840133	0.61680684	0.74028934
Esyt3	0.16090168	2.46429092	0.25838203	0.61681992	0.74028934
Sf3a1	0.0888062	5.62274131	0.25807389	0.61702869	0.74047386
Nln	0.08499484	4.43820929	0.25785671	0.61717595	0.74058453
Gm10814	-0.3787443	0.76249945	0.25775671	0.61724377	0.74059064
Dyrk3	0.19932681	1.85283218	0.25764899	0.61731685	0.74059064
Trim34b	0.3489381	-0.8356453	0.2574267	0.61746772	0.74059064
Snx30	-0.0823425	5.50898575	0.25732417	0.61753733	0.74059064
Gnptg	-0.0871459	4.63941865	0.25730321	0.61755157	0.74059064
Fat3	-0.1333589	8.06281523	0.2572957	0.61755667	0.74059064
Zbtb7a	-0.0967004	6.03367033	0.25722062	0.61760766	0.74059064
Meaf6	-0.0800748	5.01797315	0.2572005	0.61762133	0.74059064

G3bp2	0.06991245	9.47815298	0.25691717	0.61781385	0.74075363
Pbdc1	0.09156303	4.59042554	0.25675998	0.61792072	0.74075363
Ero1l	-0.0672505	6.37695427	0.25675751	0.6179224	0.74075363
Rhox8	-0.2122093	1.56502683	0.25640944	0.6181592	0.74097148
Tmem38a	-0.0964095	5.49186598	0.25613517	0.61834593	0.74110464
Ankrd54	-0.1425053	2.01306305	0.25608451	0.61838043	0.74110464
Gm20939	0.10535627	3.27695277	0.25587386	0.61852395	0.74121062
Ccdc150	-0.5869948	-1.6023388	0.25568025	0.61865593	0.7412974
Apex1	-0.4507587	-1.045947	0.25553334	0.61875611	0.7412974
Tmem9	-0.1064446	4.96445114	0.25542268	0.6188316	0.7412974
Lrrtm3	-0.080231	4.97338521	0.25538232	0.61885914	0.7412974
Tsc2	0.09819988	5.85466584	0.25536375	0.61887181	0.7412974
Mrpl32	0.11100117	3.95919809	0.25519723	0.61898546	0.74132258
Ankrd66	0.98036988	-2.1740184	0.25517152	0.61900301	0.74132258
Smg1	-0.1101036	7.69714299	0.25499567	0.61912309	0.74135333
Mroh5	0.36481688	-0.7387819	0.25497256	0.61913887	0.74135333
Zfp937	-0.0859493	5.13090645	0.25474083	0.61929719	0.74136938
D430019H16	-0.088269	6.30631517	0.25470837	0.61931938	0.74136938
Zrsr1	0.04903799	6.95800016	0.25464257	0.61936436	0.74136938
Tmem158	0.0956122	4.10897851	0.25454471	0.61943125	0.74136938
Tnip2	-0.1120457	3.05890154	0.25436278	0.61955568	0.74136938
Tenm1	0.12946269	5.44457367	0.25433108	0.61957737	0.74136938
F630042J09F	-0.5177325	-0.9915842	0.25431569	0.61958789	0.74136938
Sord	-0.0962736	4.3922817	0.25427151	0.61961812	0.74136938
Msrb2	0.0891793	4.28512107	0.25422766	0.61964813	0.74136938
Prkra	0.09847286	3.57028419	0.25390204	0.61987103	0.74151915
Paip1	0.07511347	6.24463162	0.2538838	0.61988352	0.74151915
Cript	-0.0818423	7.53137225	0.25367555	0.62002618	0.74162388
Lrp3	0.09916024	4.92686059	0.25345218	0.62017929	0.74174107
Scn3b	-0.0800198	5.74038972	0.2532382	0.62032603	0.74180481
Ppp1r36	0.42424633	-0.2099402	0.25316413	0.62037685	0.74180481
Ago2	-0.060549	7.24356027	0.25313255	0.62039852	0.74180481
Gm5595	0.14126078	2.83552425	0.25305303	0.62045308	0.74180481
Gm12992	-0.1932367	1.37021767	0.25243775	0.62087566	0.74216614
Nipsnap3b	0.10303363	3.68325496	0.25243167	0.62087985	0.74216614
B830017H08	-0.3169794	-0.2125688	0.25237214	0.62092077	0.74216614
Prpf38a	-0.0930163	4.10992073	0.2522084	0.62103336	0.74221911
Zar1l	-0.2715599	0.48899529	0.25214727	0.6210754	0.74221911
Ppp2r3d	-0.1357273	3.95125935	0.25182652	0.62129612	0.7423644
Nup93	-0.1192209	4.53506346	0.25158507	0.62146238	0.7423644
Mboat2	0.13274968	4.15070648	0.25156673	0.62147501	0.7423644
Hmgn2	-0.1039129	6.50993329	0.25153984	0.62149353	0.7423644
Tnfrsf11a	-0.1484925	2.63779918	0.25149364	0.62152536	0.7423644

Rnf180	-0.1591832	2.8396008	0.25147861	0.62153572	0.7423644
Trak1	-0.0598034	7.52589125	0.25133817	0.6216325	0.7423644
Gm14305	-0.1262528	2.61102588	0.25126023	0.62168622	0.7423644
Mmp14	-0.1643128	3.91261797	0.25110339	0.62179436	0.7423644
Kidins220	-0.075826	8.28762378	0.25101985	0.62185198	0.7423644
Cdkl5	-0.0857539	8.25420541	0.25082062	0.62198945	0.7423644
Zfp820	0.21086418	1.39759634	0.25080391	0.62200098	0.7423644
Akt1	-0.0879934	5.08357645	0.25074485	0.62204174	0.7423644
Adcy7	0.2256943	1.81924374	0.25068478	0.62208321	0.7423644
Nhlrc2	0.09809149	3.76158255	0.25062382	0.6221253	0.7423644
Mapk1	0.06090556	9.09375929	0.25062045	0.62212763	0.7423644
Gm13629	0.17947665	1.52851678	0.25061	0.62213484	0.7423644
Arf5	-0.1342832	3.03196978	0.25043983	0.62225237	0.74242235
Proca1	0.16150842	1.88584222	0.2503265	0.62233067	0.74242235
Hdgf	0.07705887	6.54387813	0.25030008	0.62234892	0.74242235
Doc2g	-0.4974151	-0.3671259	0.250155	0.6224492	0.7424534
Pdf	0.08737257	5.08672564	0.25010278	0.6224853	0.7424534
Wdr86	0.37564825	1.31056023	0.24986246	0.6226515	0.74251335
Cadm3	0.08087465	7.86896536	0.24979876	0.62269557	0.74251335
Cactin	-0.1251765	3.17297834	0.24975146	0.6227283	0.74251335
Atp6v1h	0.05259601	6.29465663	0.24957477	0.6228506	0.74251335
Ndp	0.31357129	1.01292183	0.24957417	0.62285101	0.74251335
Gad1	0.11493758	7.57420596	0.24941336	0.62296237	0.74251335
Fermt2	0.0511071	7.13789339	0.24935616	0.62300199	0.74251335
Hspa2	-0.0967775	4.93156623	0.24931189	0.62303265	0.74251335
Cacna1e	-0.1147894	8.22203066	0.24930673	0.62303622	0.74251335
Myom2	-0.4050109	0.39220815	0.2492065	0.62310567	0.74251335
Tgtp1	-0.1298397	3.77009538	0.24911582	0.62316851	0.74251335
Hr	0.17092578	3.0286007	0.24888002	0.623332	0.74251335
1110058L19I	0.09097994	4.25525354	0.24885725	0.62334779	0.74251335
Robo3	-0.3556512	2.01714321	0.24884894	0.62335355	0.74251335
Pydc4	0.57352562	-1.4334036	0.24883495	0.62336326	0.74251335
C2cd4b	0.30492442	-0.0762654	0.24830327	0.62373228	0.74265142
Cpq	0.1362399	4.27189071	0.24822432	0.62378711	0.74265142
Ankrd6	-0.0950582	5.51365967	0.24815618	0.62383445	0.74265142
Rqcd1	-0.0868574	4.95419579	0.24801474	0.62393274	0.74265142
Kcnj8	0.3322725	0.66783399	0.24800294	0.62394095	0.74265142
Fam63b	-0.0596665	7.76590666	0.24798295	0.62395484	0.74265142
Rps16	-0.0978804	6.26269801	0.24788879	0.6240203	0.74265142
Hmgcs1	0.07965665	7.21088302	0.24787743	0.62402819	0.74265142
Nmb	0.32547097	0.12625906	0.24783737	0.62405605	0.74265142
Zfp41	0.14246271	1.96882306	0.24780294	0.62407999	0.74265142
Chmp5	0.09511672	6.60861862	0.24779393	0.62408626	0.74265142

Mboat7	0.07449793	5.19985747	0.24764207	0.62419189	0.74271144
Ssbp1	-0.068688	5.47566653	0.24749772	0.62429233	0.74273148
Fgfrl1	-0.143483	2.25860153	0.2474337	0.62433688	0.74273148
Trim15	-0.4879682	-1.2295396	0.24728497	0.62444043	0.74273148
Copg2	0.09539362	5.85953571	0.24726501	0.62445433	0.74273148
Plod3	-0.159693	2.62282767	0.24722138	0.62448471	0.74273148
Ormdl3	0.12032467	4.20754267	0.24702907	0.62461868	0.74276865
Ccdc152	-0.1569267	1.90916393	0.24701805	0.62462636	0.74276865
Caly	-0.1119631	3.47990388	0.24684831	0.62474466	0.74283506
Gm10791	-0.3320595	0.77777269	0.24677953	0.62479261	0.74283506
Ppm1g	0.07727928	4.91335133	0.24668276	0.62486008	0.74284964
Nme2	-0.1019114	6.80607066	0.24634227	0.62509764	0.74306641
Fopnl	0.08960591	4.94606246	0.24611928	0.62525334	0.74308242
Iah1	0.13746959	3.18003593	0.24610466	0.62526355	0.74308242
Tfdp2	-0.0553952	6.23340646	0.24593927	0.62537908	0.74308242
Fchsd2	0.05885941	6.70699461	0.24587852	0.62542153	0.74308242
Tpst2	0.13770013	2.1402579	0.24582264	0.62546059	0.74308242
Iqsec3	-0.097092	6.55451205	0.24581668	0.62546475	0.74308242
Pitpnb	-0.0616767	6.1491041	0.2457696	0.62549766	0.74308242
Fam227b	0.24664847	0.68226812	0.2456031	0.62561407	0.7431551
Dnm3	-0.0993864	8.50630383	0.2454052	0.62575251	0.7432408
4932418E24I	-0.3514765	-0.0372914	0.24526972	0.62584731	0.7432408
Basp1	-0.0663735	9.41422249	0.24522077	0.62588158	0.7432408
Bach2	0.10301667	4.7164935	0.24517654	0.62591253	0.7432408
Hnrnpul1	-0.0661445	6.59340048	0.24510534	0.62596239	0.7432408
Lxn	0.06813664	4.9324771	0.24494675	0.62607345	0.74330709
2700081O15	-0.0682732	6.20960645	0.24476032	0.62620408	0.74339659
Gm14015	-0.5250981	-0.754935	0.2446215	0.62630139	0.74339896
Dusp1	-0.1348812	6.28480359	0.24459985	0.62631657	0.74339896
Zcchc18	0.06841053	7.20061704	0.24436053	0.6264844	0.74353259
Gm128	-0.3873388	-0.8928464	0.24419102	0.62660335	0.74360817
Tspyl2	0.07743787	6.06718009	0.24409187	0.62667295	0.74362518
Zfp414	-0.1006056	4.50877681	0.24397218	0.62675698	0.74364332
Dennd1a	0.07078381	5.51187402	0.24380438	0.62687485	0.74364332
Jarid2	-0.0682607	5.84144929	0.2437698	0.62689915	0.74364332
Pnn	-0.0807384	7.701744	0.24375536	0.62690929	0.74364332
Shd	-0.157373	2.34245362	0.24347193	0.62710851	0.74381407
Slc9a9	-0.1529665	3.46094576	0.24337764	0.62717482	0.74382716
Il18bp	-0.1530636	3.28860402	0.24285291	0.62754412	0.74411215
Ppm1l	-0.1050507	4.72174536	0.24279323	0.62758616	0.74411215
Cenpl	-0.1662292	1.72929489	0.24278297	0.62759338	0.74411215
Gm17660	-0.6738645	-1.4513759	0.24249282	0.62779784	0.74411215
Caskin1	0.09149133	5.76529907	0.24232039	0.62791941	0.74411215

Scrt2	0.11221636	3.35000913	0.24231501	0.62792321	0.74411215
Ikbkb	-0.0999964	4.30039297	0.24228222	0.62794633	0.74411215
Dusp5	-0.296541	-0.2021188	0.24224768	0.6279707	0.74411215
Ccdc169	0.66662959	-2.2268421	0.24215976	0.62803272	0.74411215
Mthfd2l	-0.1827845	2.19025458	0.24214334	0.62804431	0.74411215
Kcne4	-0.1269586	3.15263084	0.24211974	0.62806096	0.74411215
Arhgef7	0.0682886	6.71719714	0.2420946	0.6280787	0.74411215
Helz2	0.23263793	1.98281562	0.24196172	0.62817248	0.74415773
Rmdn2	-0.1034655	3.71864916	0.24185483	0.62824794	0.74418162
Ydjc	0.18534122	1.79528566	0.2417552	0.6283183	0.74419945
Mamdc2	0.3628871	0.03578776	0.24163484	0.62840332	0.74423464
Pde6a	0.15309121	2.04928689	0.2414622	0.62852532	0.74424762
Gabra3	0.06772782	5.47618171	0.24139437	0.62857326	0.74424762
4930539E08I	-0.1239349	3.15092154	0.24137198	0.62858909	0.74424762
Cenph	-0.4043799	-0.5446331	0.24130632	0.62863551	0.74424762
Kcng3	-0.1842526	3.24075379	0.2411627	0.62873708	0.74425183
Dcaf8	-0.0590306	5.70611161	0.24114488	0.62874969	0.74425183
Trim39	-0.0832855	4.31409837	0.24095488	0.62888412	0.74428213
Rad54l	-0.2449317	0.81036565	0.24095236	0.62888591	0.74428213
0610010B08I	-0.1088586	4.54974306	0.2407756	0.62901104	0.74436475
Hs3st3a1	0.13123604	4.59110995	0.24062104	0.6291205	0.74441789
Ddx28	-0.122894	2.5404257	0.24055599	0.62916658	0.74441789
Cct8	0.06037426	6.86970819	0.24035576	0.62930847	0.74452031
Aim2	0.20955864	2.19248865	0.24011482	0.62947932	0.74462525
Star	-0.1456731	2.52896689	0.24001192	0.62955231	0.74462525
Usp24	-0.0793619	7.4004889	0.23991261	0.62962277	0.74462525
Gkap1	0.09356302	5.17658871	0.23983743	0.62967613	0.74462525
Atpif1	0.07580297	7.27261637	0.23979441	0.62970667	0.74462525
Nt5c	0.11654161	3.6139377	0.23974147	0.62974425	0.74462525
Ctdsp2	-0.0878982	8.00564391	0.23968474	0.62978453	0.74462525
Ptgr2	0.08355056	6.42291844	0.23946113	0.62994336	0.74474331
Ctss	0.13452168	3.74244017	0.23938835	0.62999507	0.74474331
Denr	-0.0710096	5.7057541	0.23927185	0.63007787	0.74477576
Atp6v0e2	0.0760504	5.29601481	0.23905166	0.63023444	0.7448954
Anapc10	0.09383198	4.5033661	0.2389461	0.63030953	0.74491776
Tysnd1	-0.1366773	2.27809315	0.23886944	0.63036408	0.74491776
Flywch1	-0.10565	3.82844992	0.23853422	0.63060272	0.74513433
Pigg	0.0982013	3.45784024	0.23832353	0.63075281	0.74524625
Fam222a	-0.1814058	2.20401045	0.23812303	0.63089573	0.74534966
Cnpy3	0.12079534	3.44903708	0.23797005	0.63100482	0.7454131
Usf1	0.13941321	2.76170238	0.2378424	0.63109588	0.74545523
Gpr176	0.15497389	2.61658925	0.23743597	0.63138603	0.74573249
Ss18	-0.0734587	5.34131109	0.23721178	0.6315462	0.74585621

1700009P17I	-0.1871853	1.70603759	0.23697618	0.63171463	0.74594321
Rps6kl1	0.15579673	2.37169747	0.23695367	0.63173074	0.74594321
Wnt9b	0.68826824	-1.9055539	0.23687593	0.63178634	0.7459434
Pomgnt1	-0.1196976	2.95261862	0.23670681	0.63190733	0.7460075
Cggbp1	-0.07055	7.33132985	0.23658851	0.63199201	0.7460075
Rnf34	0.08197395	5.2729918	0.23648928	0.63206305	0.7460075
Ptchd4	-0.0989479	4.03406526	0.23628055	0.63221255	0.7460075
Npas3	0.10793248	3.93110127	0.23626824	0.63222137	0.7460075
Sdf2l1	-0.1236422	2.66460887	0.23625964	0.63222754	0.7460075
Pfdn1	-0.1203382	3.51727609	0.23625801	0.6322287	0.7460075
4930486F22I	-0.671508	-1.2626018	0.23597651	0.63243047	0.74617172
Snrnp48	0.07881538	4.90671398	0.23590914	0.63247878	0.74617172
Cldn11	0.109316	6.53876508	0.23579408	0.6325613	0.74620007
Lpl	0.13188217	4.20486379	0.23572102	0.63261372	0.74620007
Casp8	0.10029161	5.33351205	0.23552856	0.63275184	0.74629758
Trim43a	-0.3136131	-0.2022028	0.23538156	0.63285739	0.74630086
Fchsd1	-0.1649687	2.02506794	0.23511201	0.63305103	0.74630086
Esco1	0.07219542	5.65472216	0.23504151	0.6331017	0.74630086
Vasp	0.16313117	3.48114641	0.23503698	0.63310495	0.74630086
Eif2a	-0.0564111	6.74759483	0.23493642	0.63317725	0.74630086
Tmem255b	-0.5502095	-0.9751836	0.23489722	0.63320544	0.74630086
Ift27	0.12652354	2.67768703	0.23489145	0.63320958	0.74630086
Ulk1	-0.0751741	6.46626402	0.23478228	0.63328809	0.74630086
Msl3	-0.0684009	5.48611517	0.23477402	0.63329403	0.74630086
Eppk1	-0.4516979	-0.5770483	0.23472098	0.63333219	0.74630086
Agtr2	-0.5219501	-1.1446577	0.23455886	0.63344884	0.74630086
Ndel1	0.07854932	4.91685716	0.23444473	0.633531	0.74630086
Slx4	0.08339234	5.16651281	0.23434525	0.63360262	0.74630086
Pitpnc1	-0.056558	6.72428963	0.23433772	0.63360805	0.74630086
Atf6	-0.0694542	6.94906502	0.23433263	0.63361171	0.74630086
Zfp397	0.05216798	5.88909395	0.23425275	0.63366924	0.74630086
Lrrc58	0.07884197	10.1415831	0.23421358	0.63369746	0.74630086
Nrarp	-0.1524563	2.7117854	0.23411764	0.63376658	0.74631694
Lipo1	0.10755568	4.94977586	0.23402186	0.6338356	0.74633291
Ints8	0.07819634	5.49096989	0.2337837	0.6340073	0.74646977
Ppp6r1	-0.0815726	4.81027619	0.23369664	0.6340701	0.74647839
Xpr1	-0.0640572	7.89515667	0.2335594	0.63416912	0.74649391
Rnf130	0.05641608	7.06320133	0.2333947	0.63428799	0.74649391
Cbr2	-0.2206664	1.3138646	0.23336573	0.6343089	0.74649391
Gabra2	-0.0995835	4.85233987	0.23332177	0.63434064	0.74649391
Syt3	0.11837504	3.66352254	0.23323124	0.63440602	0.74649391
Plekha2	0.09185808	5.21607089	0.23317052	0.63444988	0.74649391
Ptch2	-0.5433945	-1.929152	0.23310971	0.63449381	0.74649391

AU023762	-0.1093657	3.25929013	0.23306366	0.63452709	0.74649391
Cyp46a1	-0.081751	4.74106673	0.23289111	0.63465179	0.74649477
Arid4a	0.07331054	7.65718291	0.23286253	0.63467245	0.74649477
Phkb	-0.0796795	6.1319598	0.23277257	0.6347375	0.74649477
Tln1	-0.0887051	6.04945015	0.23275567	0.63474972	0.74649477
Islr2	-0.130182	2.79239509	0.23248051	0.63494878	0.74657546
Deaf1	-0.0977911	4.04081843	0.23240235	0.63500535	0.74657546
Taf9	-0.0680363	5.52362682	0.2323424	0.63504874	0.74657546
Tmem135	0.05298633	6.07846201	0.2322438	0.63512014	0.74657546
Tenc1	-0.0941703	5.97022186	0.23202788	0.63527654	0.74657546
Mir8115	-0.4034053	-1.2524233	0.23170615	0.63550975	0.74657546
Ndufb9	0.08497202	6.52462575	0.23163778	0.63555934	0.74657546
Cdyl2	0.0871079	5.28059106	0.23155906	0.63561644	0.74657546
Mlec	-0.0624258	6.89116658	0.23145837	0.6356895	0.74657546
Zfp518a	-0.0678344	5.61763445	0.2314414	0.63570182	0.74657546
Cd164	0.10815715	7.83696566	0.23133294	0.63578054	0.74657546
Nrd1	0.05733813	7.74028781	0.23132409	0.63578696	0.74657546
Clec3b	-0.2414111	0.90492489	0.23129416	0.6358087	0.74657546
Rbm22	0.07870218	4.93733979	0.23128121	0.6358181	0.74657546
2010320M18	0.20238508	1.75692754	0.23124802	0.63584219	0.74657546
Mcoln1	0.12184299	2.92515908	0.23121694	0.63586476	0.74657546
Atp8a2	0.15007965	3.82763017	0.23109459	0.63595362	0.74657546
Nedd4l	0.09350113	8.72859637	0.23103591	0.63599625	0.74657546
Mast2	0.05974427	6.29563596	0.23099938	0.63602278	0.74657546
4930503L19R	0.13335595	3.03546724	0.23097867	0.63603783	0.74657546
Usp19	-0.0774593	4.7584314	0.23088428	0.63610643	0.74657546
Rasgrp4	-0.3198008	-0.1143058	0.23080601	0.63616332	0.74657546
Ccdc96	-0.209843	1.74268235	0.23077493	0.63618591	0.74657546
Mrpl52	-0.0927977	3.85555016	0.23075735	0.63619869	0.74657546
C1ql1	0.15997881	2.81366461	0.23064858	0.63627779	0.74657546
Pde10a	0.10159434	7.06493354	0.23057203	0.63633347	0.74657546
Lonrf2	0.0744666	8.07291777	0.23050737	0.6363805	0.74657546
Fsd1	-0.1591312	2.29276353	0.2304566	0.63641744	0.74657546
Mif	0.08794515	5.21874154	0.23033852	0.63650337	0.74657546
Fign	-0.1037106	4.7871539	0.23029025	0.63653851	0.74657546
Enthd2	0.19713358	1.43722193	0.23022726	0.63658437	0.74657546
LOC1005050	0.31552262	0.48424062	0.2301972	0.63660626	0.74657546
Scyl3	0.07139251	5.298139	0.23009068	0.63668383	0.74657546
Cep164	-0.1061883	3.31652975	0.23006203	0.6367047	0.74657546
Tead2	-0.1576995	2.48379968	0.22990105	0.63682198	0.74660934
Frs2	-0.0533434	7.4298957	0.2297893	0.63690342	0.74660934
Dnajc18	0.06841419	6.93329218	0.22964816	0.63700633	0.74660934
Acbd7	-0.4364012	-1.2442013	0.22964266	0.63701034	0.74660934

BC018242	0.08213815	4.75332154	0.22964174	0.63701101	0.74660934
Reep4	-0.3483418	0.28493167	0.22940431	0.6371842	0.74668472
Crnde	0.79395743	-1.7775101	0.22940143	0.6371863	0.74668472
Polh	0.12573216	2.48231141	0.2293238	0.63724296	0.74668608
Pdia5	0.33908848	0.10203084	0.22907904	0.63742166	0.74672559
Pus7	0.09766757	3.84631136	0.22896594	0.63750427	0.74672559
Casp7	-0.1675518	2.71536491	0.22888602	0.63756266	0.74672559
Nupr1	-0.1326202	6.67529031	0.22884414	0.63759326	0.74672559
Prpf40b	-0.1247293	3.81498405	0.2288278	0.63760521	0.74672559
4833439L19R	-0.0774623	7.79754832	0.22865947	0.63772826	0.74672559
Dtna	0.06775514	7.13003161	0.22865374	0.63773245	0.74672559
Gpatch2l	-0.0689516	5.15068383	0.22856705	0.63779584	0.74672559
Hdhd3	0.29908958	0.2830346	0.22849859	0.63784591	0.74672559
Clptm1l	-0.0941798	4.51045933	0.22844855	0.63788252	0.74672559
D17Ertd648e	-0.2249759	0.72780669	0.2284423	0.63788709	0.74672559
Eef2	0.05717583	8.35795737	0.22806649	0.63816217	0.74684237
Rap1a	0.07755261	8.59777155	0.22806058	0.6381665	0.74684237
Txn2	-0.098749	4.68308684	0.22798716	0.63822027	0.74684237
Mrps21	0.09422354	5.03713123	0.22787796	0.63830027	0.74684237
2700046A07I	-0.1578196	2.66778136	0.22762857	0.63848307	0.74684237
Papd5	-0.0728988	6.11240003	0.2276096	0.63849697	0.74684237
Srsf5	0.05081014	8.0887758	0.22755996	0.63853337	0.74684237
Pafah1b1	0.05559812	9.15130596	0.22754108	0.63854722	0.74684237
Nfkb1	-0.0870225	4.41632121	0.22745784	0.63860828	0.74684237
Hist1h2bk	0.41875188	-1.1580995	0.22737018	0.63867259	0.74684237
Fance	0.12061309	2.70443339	0.226997	0.63894654	0.74684237
Amn	-0.3239845	-0.2705762	0.22692165	0.63900189	0.74684237
Plekha6	0.06346868	6.85142814	0.22691441	0.6390072	0.74684237
Slc18b1	0.09429479	5.1384207	0.22690269	0.63901581	0.74684237
Txndc15	0.08808428	4.33369455	0.2268799	0.63903256	0.74684237
4732491K20I	-0.170383	2.09539837	0.22686544	0.63904318	0.74684237
Hddc2	0.09376263	3.66227842	0.22659647	0.63924088	0.74684237
Mpp3	0.09894458	3.41134994	0.22656801	0.6392618	0.74684237
Ddx1	0.05780001	6.99049267	0.22646658	0.6393364	0.74684237
Spef1	-0.1737479	1.53809466	0.22641793	0.63937218	0.74684237
Olfr55	-0.5921975	-1.2121881	0.22639906	0.63938607	0.74684237
Prrc2a	0.06188097	7.7199155	0.22638573	0.63939587	0.74684237
Azi2	-0.0654881	6.72420063	0.22633266	0.63943492	0.74684237
Prokr2	0.23706355	1.92358666	0.22627067	0.63948053	0.74684237
Pmm1	0.0804893	4.66168759	0.22624323	0.63950073	0.74684237
Hist1h3f	0.42142098	-1.7081125	0.22618682	0.63954225	0.74684237
Nfasc	-0.0783509	7.70953215	0.22610949	0.63959918	0.74684237
Utp14b	-0.10467	4.91142659	0.22590257	0.63975157	0.74684237

Fes	-0.386726	-0.3101794	0.22582349	0.63980983	0.74684237
Sdc3	-0.0747112	5.2388551	0.22578493	0.63983824	0.74684237
9630001P10I	-0.2759763	0.21611996	0.2255973	0.63997654	0.74684237
Chst14	-0.1911802	1.60237469	0.22557931	0.6399898	0.74684237
Eef1d	0.07408429	5.27726507	0.22552331	0.6400311	0.74684237
Oprl1	-0.1047516	3.3758433	0.22547619	0.64006585	0.74684237
Plscr3	-0.2089808	1.25531177	0.22544365	0.64008985	0.74684237
Cyb5d2	0.09827375	3.33322216	0.22543638	0.64009521	0.74684237
0610010F05I	-0.080604	5.99731277	0.22537084	0.64014356	0.74684237
Crabp1	0.83803328	-1.4468871	0.22495966	0.64044707	0.74684237
Atg9a	-0.0826766	5.13802662	0.22490484	0.64048757	0.74684237
Sars2	-0.1635451	1.56896706	0.22487437	0.64051007	0.74684237
Synrg	0.05715116	6.70088302	0.22483165	0.64054163	0.74684237
Tarbp2	-0.1293368	2.33320718	0.22478238	0.64057803	0.74684237
Gm6260	-0.2111608	1.52096462	0.22477137	0.64058618	0.74684237
Ptx3	-0.498154	-1.326802	0.22476028	0.64059437	0.74684237
Dfna5	0.09723282	4.32154807	0.22474545	0.64060533	0.74684237
Ndufs4	0.05303705	6.61749597	0.22472613	0.6406196	0.74684237
Ccdc124	0.11822332	4.3329808	0.22468907	0.64064699	0.74684237
Nup107	-0.1018964	3.78393651	0.22468377	0.64065091	0.74684237
Vsig2	-0.1003131	3.29637463	0.22448902	0.64079489	0.74688274
Tmem203	-0.1177582	2.72407557	0.22448678	0.64079655	0.74688274
Susd4	0.07272022	4.42295152	0.2241699	0.641031	0.74709129
E330009J07F	0.20743524	1.34869234	0.22379182	0.64131099	0.74734314
Prkcdbp	0.13046931	5.17983032	0.22352625	0.64150783	0.74734314
Drosha	0.05746472	6.89887873	0.22347634	0.64154484	0.74734314
Yod1	-0.0809175	4.82945894	0.22337009	0.64162365	0.74734314
Rnf219	0.06157222	5.20945198	0.22336813	0.6416251	0.74734314
Slc13a1	0.62305291	-1.3495689	0.22331328	0.64166579	0.74734314
Acd	0.09419204	3.94948651	0.22329473	0.64167956	0.74734314
Plek	0.07644129	4.63025579	0.22324701	0.64171496	0.74734314
2500004C02I	-0.0999328	3.8040238	0.22320392	0.64174694	0.74734314
Mest	0.1085628	6.37721065	0.22285186	0.64200834	0.74749053
Ptpru	0.10788164	3.24593205	0.22277829	0.64206301	0.74749053
Gmfg	-0.2781185	1.11371591	0.22274417	0.64208836	0.74749053
Renbp	0.21349751	2.99842113	0.22269043	0.6421283	0.74749053
Gli3	0.0672489	5.12944884	0.22246723	0.64229423	0.74749053
Tnk2	-0.0779947	5.09369377	0.22246143	0.64229854	0.74749053
Ly75	0.43439249	-0.5198203	0.22245381	0.64230421	0.74749053
Ints9	0.0736097	4.3612064	0.2224354	0.6423179	0.74749053
Chrm5	-0.2032651	1.61380987	0.22220332	0.64249056	0.74761915
Etohi1	-0.0907955	4.21814731	0.2221352	0.64254126	0.74761915
Stat6	0.08353621	5.05259947	0.22190068	0.64271588	0.74761915

Dusp7	-0.0557755	6.05121561	0.22189847	0.64271752	0.74761915
AU019823	0.08338007	5.02484557	0.22180565	0.64278667	0.74761915
Muc15	-0.2245342	2.10105816	0.22175229	0.64282643	0.74761915
Zkscan6	0.13188355	2.9109064	0.2216699	0.64288782	0.74761915
Rhoh	0.28481878	-0.0962766	0.22163369	0.64291481	0.74761915
Pgp	0.07533868	3.71346144	0.22161539	0.64292845	0.74761915
Smarca5-ps	-0.1503292	1.23453082	0.22149004	0.6430219	0.74763737
D630032N06	0.64522645	-0.9869433	0.22138358	0.6431013	0.74763737
Med17	-0.0771091	3.9562245	0.22129036	0.64317085	0.74763737
Rgs16	0.09802813	3.66457542	0.22125039	0.64320067	0.74763737
Cdhr2	-0.4702336	-0.9457568	0.22122191	0.64322192	0.74763737
AI607873	-0.1826236	1.61003838	0.22064536	0.6436525	0.7480714
Frmd4b	0.07497111	5.2760679	0.22056608	0.64371177	0.7480714
Cstf3	-0.0910043	5.29501296	0.2204177	0.64382271	0.7480714
1700006F04I	0.4830175	-0.7678995	0.22038232	0.64384918	0.7480714
1700109K24I	-0.3520563	0.84873822	0.2202277	0.64396485	0.7480714
1110015O18	-0.3937569	-0.9283855	0.22015414	0.64401991	0.7480714
Pias3	-0.1140635	2.71943935	0.22012496	0.64404175	0.7480714
Gorab	0.09193538	3.49205678	0.21989883	0.64421106	0.7480714
Glrx3	0.06725035	5.19640005	0.21984698	0.6442499	0.7480714
Sipa1	0.12382919	2.6000308	0.21982759	0.64426443	0.7480714
Deb1	-0.0744358	4.91334652	0.21974912	0.64432322	0.7480714
Ndnl2	0.1037943	3.46944245	0.21962761	0.64441428	0.7480714
Mthfsd	0.17369664	1.83132044	0.21956686	0.64445982	0.7480714
Naglu	-0.2216327	1.20985482	0.21950121	0.64450904	0.7480714
Cpeb4	-0.0717475	8.15948239	0.21938688	0.64459478	0.7480714
Lipt2	0.15441762	2.11350335	0.21932418	0.64464181	0.7480714
Ogn	-0.1103241	8.11652656	0.2192634	0.64468741	0.7480714
Gm10825	-0.5770619	-1.3724712	0.21925049	0.6446971	0.7480714
Gpatch1	-0.064283	5.0819513	0.2192401	0.6447049	0.7480714
Hnrnpul2	0.04909377	8.048024	0.21919874	0.64473593	0.7480714
Sp2	-0.1080694	5.31463402	0.2191179	0.6447966	0.7480714
Mmd	-0.0548296	7.33839218	0.2190889	0.64481838	0.7480714
Arpc2	0.06420128	8.49607501	0.2188888	0.64496863	0.74816631
Mir684-1	-0.2282991	0.23407638	0.21883188	0.64501139	0.74816631
Rnase4	-0.1201939	5.15514776	0.21842948	0.64531385	0.74836009
Oxnad1	0.12526579	3.78928905	0.21841004	0.64532847	0.74836009
Dok4	0.1852609	2.3734439	0.21837293	0.64535639	0.74836009
Phf11b	0.31100387	0.71371584	0.2183129	0.64540154	0.74836009
Nts	-0.2468992	1.01467383	0.21821739	0.64547341	0.74836009
Dhfr	-0.0859921	3.80304629	0.21816594	0.64551213	0.74836009
Ccdc19	0.16147334	2.03876038	0.2179276	0.64569157	0.74850363
Zfp677	0.15634327	3.72824083	0.21768038	0.64587782	0.74859188

Qars	0.08974748	3.78645225	0.21761222	0.6459292	0.74859188
Junb	0.24092698	1.53956335	0.21751778	0.64600039	0.74859188
Fam169b	-0.1660877	1.94048087	0.21747954	0.64602923	0.74859188
Mapk8	0.06745582	7.5751734	0.21744166	0.6460578	0.74859188
Nfu1	-0.0849204	4.48020055	0.21723646	0.6462126	0.74859188
Gng10	-0.1092626	4.81533876	0.21721533	0.64622854	0.74859188
Ak1	0.09031716	3.98628346	0.21719918	0.64624073	0.74859188
Kmt2e	-0.058558	9.52673944	0.21709705	0.64631782	0.74859188
Nub1	0.06437358	5.30271563	0.21708885	0.64632401	0.74859188
Wbp2	0.05923884	7.02579978	0.2170147	0.64637999	0.74859228
Abtb2	0.15251904	2.54631764	0.21663869	0.64666406	0.74876057
Zfp429	0.13342534	2.01858964	0.21662027	0.64667798	0.74876057
Vapb	0.05125796	6.66791428	0.21660142	0.64669223	0.74876057
P2ry10	-0.5967658	-1.3492971	0.21647222	0.64678992	0.74880925
Matn2	-0.1288979	3.70694898	0.21633932	0.64689044	0.74886119
Gdap2	0.06487351	4.7954107	0.2160834	0.64708412	0.74902097
Gm13051	0.48146415	-1.3911024	0.21595014	0.64718503	0.74907334
Dpp9	0.07612007	4.59523009	0.21574709	0.64733885	0.74909611
Polg	-0.1009307	3.26366115	0.21571158	0.64736576	0.74909611
Taf5	-0.1137102	3.93774759	0.21570374	0.64737171	0.74909611
Dync1li1	0.08127951	5.04728039	0.21553024	0.64750323	0.74912721
Rnf103	-0.0575948	6.23756175	0.21552141	0.64750993	0.74912721
Agpat2	0.22639725	1.41287467	0.21536518	0.64762842	0.7491837
Dnajb9	0.08340459	5.83039723	0.21524869	0.6477168	0.7491837
Bgn	-0.1129465	8.30310633	0.21523686	0.64772578	0.7491837
Spata3	0.43369788	-0.8067021	0.21509258	0.64783529	0.74924558
Slc6a12	0.15101054	3.69420656	0.21501969	0.64789064	0.74924558
Tug1	0.0496072	8.05181296	0.21471514	0.648122	0.74944872
Sh3rf3	-0.0882005	5.61462486	0.21442495	0.64834264	0.74951476
Tbata	0.32774641	-0.0328198	0.21438543	0.6483727	0.74951476
Prc1	0.22178929	1.22386664	0.2143554	0.64839555	0.74951476
Fam129b	0.10792305	5.3393645	0.21434705	0.64840191	0.74951476
Gm4285	-0.1695662	1.33931549	0.21426325	0.64846567	0.74952408
Shoc2	0.05377583	8.6086196	0.21397721	0.64868343	0.74964893
Snx6	0.07032369	6.40291174	0.21397501	0.6486851	0.74964893
Acss3	0.14993173	2.36379029	0.21383385	0.64879264	0.74970464
Adamts8	-0.3100969	-0.3330181	0.21376547	0.64884474	0.74970464
Dnah10	0.22195678	1.13140033	0.21360134	0.64896985	0.74974257
Vmn2r46	-0.4144481	-1.6613097	0.21355521	0.64900503	0.74974257
Lsamp	0.10881838	5.43219533	0.21350316	0.64904472	0.74974257
Phc2	-0.096039	4.79911662	0.213357	0.64915621	0.74980699
Prpf18	0.0691094	4.94499553	0.21310532	0.64934831	0.74987515
Anapc11	-0.09123	4.06063091	0.21304297	0.64939591	0.74987515

Acsm3	-0.4535131	-0.887876	0.21287628	0.64952324	0.74987515
Slco5a1	-0.1593632	2.19075446	0.21273932	0.6496279	0.74987515
Papd4	0.08503562	5.05321007	0.21271219	0.64964864	0.74987515
1700048O20	-0.1390304	1.87947772	0.21271013	0.64965021	0.74987515
Psen2	0.16824689	1.61387442	0.21270126	0.64965699	0.74987515
Gm3985	0.37117424	-0.8746755	0.21269598	0.64966103	0.74987515
Klhl15	-0.1282963	3.77533684	0.21259299	0.64973977	0.74990171
Rpl41	-0.0942227	8.18340747	0.21229689	0.64996627	0.75000374
Mast1	-0.0911045	4.88121181	0.21227709	0.64998143	0.75000374
Rbm39	0.0507328	7.64977397	0.21225886	0.64999538	0.75000374
Lmo4	-0.0761601	9.51337193	0.21218304	0.65005342	0.75000639
1700023F06I	0.55705893	-1.5503012	0.21205145	0.65015418	0.75005834
Prkab1	0.11553182	3.39809455	0.21179724	0.65034893	0.75021869
Ap4e1	-0.1058129	4.05886757	0.21167808	0.65044027	0.75025366
Trim9	0.08941478	8.22979145	0.21161224	0.65049075	0.75025366
Fam73b	0.09624087	3.66081619	0.2114896	0.65058481	0.75029783
Itpa	0.07566483	4.19649389	0.21137373	0.6506737	0.75033604
Trio	-0.0902953	7.7197757	0.21129643	0.65073302	0.75034015
Tlcd1	-0.1906452	2.1458999	0.21112199	0.65086694	0.750349
1700125H03I	-0.4901825	-0.4861726	0.21108999	0.65089152	0.750349
Fam109a	-0.1962679	0.94421152	0.21106855	0.65090798	0.750349
Magt1	-0.0903672	5.22966798	0.21092703	0.65101669	0.75041003
Rpl12	-0.0696911	6.78598507	0.21076107	0.65114424	0.75046072
B3gat3	-0.109848	3.19066001	0.21072468	0.65117221	0.75046072
Sphk2	-0.0864439	4.07618968	0.21063283	0.65124283	0.75047783
Psg23	-0.232826	1.27596816	0.21054108	0.65131339	0.75049488
Gm5065	-0.4456199	-1.8556745	0.21024665	0.65153996	0.75049846
Sema3b	-0.2065077	2.712754	0.21016924	0.65159956	0.75049846
Rnf10	-0.0679552	5.83996032	0.21013227	0.65162802	0.75049846
Akna	0.09810025	3.34321702	0.21010471	0.65164925	0.75049846
Poldip2	-0.0745058	5.13046729	0.21010351	0.65165018	0.75049846
Lama5	-0.136131	1.76974396	0.21010226	0.65165113	0.75049846
Znf512b	0.06599682	4.93881231	0.2098855	0.65181813	0.75057352
Gm266	-0.2292575	0.6425385	0.20985477	0.65184182	0.75057352
Ercc6l2	-0.0679884	5.12584143	0.20975927	0.65191543	0.75057352
Erich3	-0.1491967	3.68636656	0.20972814	0.65193942	0.75057352
Cdk2ap2	0.10599443	3.39186092	0.20935538	0.65222698	0.75082241
Porcn	-0.0884767	4.36489916	0.20930327	0.6522672	0.75082241
Rnf166	0.09391083	4.71944735	0.2091121	0.65241481	0.75092809
Stard3nl	-0.0795954	4.46531517	0.20874326	0.65269985	0.75119191
Ropn1l	-0.4568555	-1.0281018	0.20857868	0.65282714	0.75121383
Prdx5	0.0862753	5.90269109	0.20856443	0.65283816	0.75121383
Hist1h4c	-0.319102	-0.8188294	0.20844024	0.65293425	0.75121383

Mdga2	-0.0767693	6.06321093	0.20841984	0.65295004	0.75121383
Snrpc	-0.1225827	3.22601681	0.20825972	0.65307399	0.75121383
Eif1b	0.065134	5.81897077	0.2082404	0.65308895	0.75121383
Synpo2	-0.1183496	3.37274146	0.20821363	0.65310968	0.75121383
Slc44a5	0.16603942	1.67814149	0.20784446	0.65339572	0.75146927
Rnf126	-0.0706562	3.99171202	0.20770976	0.65350017	0.75146927
Pigp	0.09260494	4.8019085	0.20758327	0.65359828	0.75146927
Cacna2d2	0.0990616	4.99637062	0.20756137	0.65361528	0.75146927
Lenep	0.20674178	1.41408675	0.20755197	0.65362257	0.75146927
Daglb	-0.1041144	3.13088302	0.20749493	0.65366683	0.75146927
Pygm	-0.1428052	3.78732599	0.20740815	0.65373418	0.7514825
Smagp	-0.2836378	0.54925906	0.20711336	0.65396311	0.75155523
Nbeal2	0.1370003	1.80362844	0.20705892	0.6540054	0.75155523
Add2	-0.0821399	7.61685825	0.20705475	0.65400865	0.75155523
BC004004	0.10093488	5.27753471	0.20703903	0.65402086	0.75155523
Lasp1	-0.0501391	6.52538605	0.20688186	0.65414302	0.75163142
2310035C23I	0.07702921	6.63927903	0.20665276	0.65432119	0.75177138
Kcnt1	-0.1060619	5.40537708	0.20658159	0.65437656	0.75177138
Glmn	-0.123371	3.94395078	0.20612426	0.65473266	0.75211627
2810032G03	0.15952726	2.61698762	0.20583144	0.65496091	0.75222876
Rgs17	0.07053643	8.03310869	0.20580709	0.6549799	0.75222876
Mtss1	0.07417194	6.68507177	0.20577201	0.65500726	0.75222876
Tars2	0.10747768	2.52134656	0.20571185	0.65505419	0.75222876
Mospd3	0.11941457	5.64086596	0.20541624	0.65528489	0.75242947
Kif7	-0.2105776	1.49510603	0.20521707	0.65544046	0.75254055
Hepacam2	-0.3171104	0.47403687	0.20500933	0.6556028	0.75254055
H2-D1	0.08864548	5.17764564	0.20495899	0.65564216	0.75254055
Snrpe	0.08592944	4.52125606	0.20492122	0.65567169	0.75254055
Mgst1	-0.1629267	3.65503979	0.20488953	0.65569647	0.75254055
E030025P04I	-0.579034	-1.4199404	0.20486305	0.65571718	0.75254055
Rpl13	-0.0780935	7.37476852	0.2047754	0.65578574	0.75255505
H2-Q1	-0.1197824	6.0697146	0.20447807	0.65601843	0.75256711
Enho	-0.20077	0.55148826	0.20436204	0.6561093	0.75256711
Bcl7c	-0.1952976	1.35263335	0.20434394	0.65612347	0.75256711
Sftpc	-0.5866169	-1.19407	0.20415682	0.65627008	0.75256711
Mrpl48	0.07842894	4.45737419	0.20414256	0.65628125	0.75256711
N4bp2l1	0.09340246	4.03004279	0.20412965	0.65629137	0.75256711
Rpl28	0.1078065	5.33779006	0.20411151	0.65630559	0.75256711
Atp2b3	-0.0915684	6.97569938	0.20400422	0.6563897	0.75256711
Ephx1	-0.1243233	4.19076574	0.2039544	0.65642876	0.75256711
Pcdha5	0.24381718	0.8341099	0.20394398	0.65643694	0.75256711
Plekhh3	-0.1870476	1.54505035	0.20375188	0.65658763	0.75256711
Ndufv3	0.09997838	5.13708218	0.20374448	0.65659343	0.75256711

Prpf8	-0.0693142	8.26146319	0.20371116	0.65661958	0.75256711
Auts2	0.05582328	6.88281406	0.20369763	0.6566302	0.75256711
Cluh	0.07258731	5.1625154	0.20368065	0.65664352	0.75256711
Gm13293	-0.20116	0.84650605	0.20362006	0.65669108	0.75256711
Fzd3	-0.0604992	7.99007841	0.20342856	0.65684144	0.75257196
Papolb	-0.3345899	0.72027379	0.20330956	0.65693492	0.75257196
Tspan5	0.05557819	7.34070433	0.20328202	0.65695656	0.75257196
Trpv3	0.39550726	-0.520925	0.2032744	0.65696255	0.75257196
Rchy1	0.06800914	5.50211776	0.20320861	0.65701425	0.75257196
Ppp1r3e	0.09700073	4.20352793	0.20318413	0.65703348	0.75257196
Vstm4	-0.1195167	4.68068533	0.20311632	0.65708679	0.75257196
Wdr65	0.26058651	1.0782569	0.20293805	0.65722697	0.75263348
Deptor	0.05436667	7.41084037	0.20287492	0.65727663	0.75263348
Ddx19b	0.07716614	5.11197622	0.20282128	0.65731884	0.75263348
Ccdc60	-0.4294709	-0.8743788	0.20270343	0.65741158	0.75263348
Kdm5a	-0.0638572	7.06546638	0.20267397	0.65743477	0.75263348
Rccd1	-0.1733375	1.48306808	0.2024857	0.65758301	0.75263348
Rpl4	-0.0646832	8.80304344	0.20235364	0.65768704	0.75263348
Drg1	-0.0846615	5.31877549	0.20233654	0.65770052	0.75263348
Adprm	-0.1157564	3.70174473	0.20227948	0.65774549	0.75263348
Cnn2	0.12380057	6.26796327	0.20223604	0.65777973	0.75263348
Plcg1	-0.0771673	4.07223723	0.20219812	0.65780961	0.75263348
Itga2b	-0.2800004	-0.091508	0.20213281	0.6578611	0.75263348
Nos2	-0.6809798	-1.9593026	0.20212455	0.65786762	0.75263348
1110065P20I	-0.2764508	0.87558483	0.20185436	0.65808074	0.75276628
Chd5	0.09149829	6.75626534	0.20183555	0.65809559	0.75276628
Mrc2	0.13897943	4.96143287	0.20168801	0.65821204	0.7528355
Smo	-0.1216823	6.95353589	0.2015516	0.65831976	0.75289471
S100a9	-0.3467198	0.04202695	0.20130944	0.65851109	0.75304952
Glrx5	-0.0919355	3.73372452	0.20106712	0.65870269	0.75306401
Fam98b	0.06993526	6.91903186	0.20106483	0.6587045	0.75306401
Sap130	0.0585266	6.64404836	0.200936	0.65880642	0.75306401
Wipi2	-0.082536	5.00363007	0.20090935	0.65882752	0.75306401
Ids	-0.0602691	9.64471167	0.20089529	0.65883864	0.75306401
Gm7854	-0.3581725	0.00976066	0.20086889	0.65885954	0.75306401
Hist2h2be	0.08711733	6.00580263	0.20048216	0.65916579	0.75335006
Lrrc59	0.05267451	6.71192555	0.20030623	0.65930523	0.75340719
Gjc1	-0.2027255	1.80271383	0.20023044	0.65936532	0.75340719
4930563F08I	0.40447307	-0.1062883	0.20019977	0.65938965	0.75340719
Iqcd	-0.4829113	-1.7489565	0.20013661	0.65943974	0.75340719
Fanca	0.69074319	-2.154117	0.19997248	0.65956996	0.75346431
Mcph1	0.09847054	5.21063922	0.19990464	0.6596238	0.75346431
Tigd5	0.25596984	0.66060187	0.19986192	0.65965771	0.75346431

Dnttip1	-0.1264818	2.98877728	0.19967634	0.65980508	0.75356867
Magi2	-0.0743679	7.42040035	0.19937791	0.66004223	0.75359595
Map2k3os	0.40533749	-0.8604769	0.19922744	0.66016188	0.75359595
BC021785	-0.4718936	-1.6363028	0.19920777	0.66017753	0.75359595
Cybrd1	-0.1505768	2.11232125	0.1991781	0.66020113	0.75359595
Nutm1	0.63760056	-1.5031532	0.1990894	0.6602717	0.75359595
Gm4951	0.16741327	3.36598933	0.19907253	0.66028512	0.75359595
Rpl32	-0.0879542	6.79791779	0.19905601	0.66029827	0.75359595
6720489N17	0.09423051	3.66694827	0.19905214	0.66030135	0.75359595
Cntn3	-0.0771416	5.52149284	0.19901237	0.660333	0.75359595
Hnrnpu	0.05642522	8.63388312	0.19890526	0.66041826	0.75362934
Apod	0.13197045	10.3568368	0.1988022	0.66050033	0.75365908
Nxt2	-0.0758705	6.76471218	0.19832648	0.66087947	0.75381647
Rgs11	-0.2526656	1.394385	0.19824156	0.66094721	0.75381647
Nynrin	-0.0734368	5.67264852	0.19816725	0.6610065	0.75381647
Plat	0.11868119	4.7598382	0.19815711	0.66101459	0.75381647
Zfp935	-0.084965	4.49689751	0.19811766	0.66104607	0.75381647
Slc6a7	0.08088283	5.20504357	0.19810117	0.66105923	0.75381647
Pcsk2os2	0.31353784	1.71436286	0.19807873	0.66107714	0.75381647
Cntn6	-0.1672991	3.21774274	0.19806711	0.66108642	0.75381647
Nucks1	0.05664255	8.85000621	0.19796859	0.66116506	0.75384226
Pfn2	-0.0532947	7.71573022	0.19783507	0.66127169	0.75389996
Srek1	-0.0645846	6.65390786	0.19770084	0.66137893	0.75393588
Adcy9	0.07834327	6.22779904	0.19765538	0.66141526	0.75393588
Scara5	0.16556568	1.61318864	0.19750388	0.66153636	0.75401005
Pcdha2	-0.2796822	0.19844634	0.19729625	0.66170243	0.75404673
Gch1	0.19046903	0.83721912	0.19721002	0.66177143	0.75404673
Hsd17b14	0.2148077	0.86079391	0.19718446	0.66179189	0.75404673
Map4k2	-0.079637	4.85664931	0.19714661	0.66182218	0.75404673
Rnf26	0.11530038	3.12694688	0.19711346	0.66184872	0.75404673
2810403A07I	-0.0709123	6.56468175	0.19687792	0.66203733	0.75419776
Htr5a	-0.1083086	5.37112254	0.19672906	0.66215661	0.75420165
Gm16515	-0.105075	5.5106867	0.19664724	0.6622222	0.75420165
Gm17019	-0.4100779	-1.2206057	0.19654179	0.66230675	0.75420165
Wdr55	-0.0763349	4.11983613	0.19653585	0.66231151	0.75420165
1110017D15I	-0.2791981	-0.1132766	0.19652403	0.66232099	0.75420165
Tmem11	0.1060213	3.33295823	0.19633646	0.66247146	0.75424298
Dbil5	0.50395178	-1.4337472	0.19620352	0.66257815	0.75424298
Grb2	0.06177966	5.1641288	0.19617429	0.66260162	0.75424298
Gm4432	-0.255712	-0.0696758	0.19617338	0.66260234	0.75424298
Stk11	0.10541888	5.51305633	0.19612587	0.66264049	0.75424298
Rab7l1	0.17516285	4.22114935	0.19605975	0.66269359	0.75424298
2810433D01I	-0.1249231	3.1447098	0.19572857	0.66295969	0.75448203

Slc4a3	-0.119654	3.42105113	0.19542685	0.66320236	0.75465764
Cbr4	0.08454927	4.17034349	0.19539728	0.66322616	0.75465764
Syf2	-0.094568	5.63618597	0.19506693	0.66349214	0.7547021
Snupn	0.10670571	3.39514449	0.19506509	0.66349363	0.7547021
2510009E07I	0.05836788	6.8544813	0.19498615	0.66355723	0.7547021
Cacna2d3	-0.0876477	5.15526663	0.1949545	0.66358273	0.7547021
Rogdi	-0.0751071	4.61086762	0.19494391	0.66359127	0.7547021
Lnp	-0.0760026	6.97294153	0.1949309	0.66360175	0.7547021
Ankdd1b	0.08987718	3.20222513	0.19485049	0.66366655	0.75471201
Znrd1as	-0.0956889	3.55287635	0.19457907	0.66388544	0.75472287
Bccip	-0.079325	5.57108292	0.19455257	0.66390682	0.75472287
Lrch3	-0.0770048	4.78819514	0.19454127	0.66391594	0.75472287
Thap6	-0.0966577	3.86927998	0.19448674	0.66395994	0.75472287
Lnpep	0.0490096	6.88376445	0.19445635	0.66398447	0.75472287
Usp7	0.05230219	7.09946502	0.19424743	0.66415313	0.75472287
Gmppa	0.07330754	3.73672678	0.19422242	0.66417334	0.75472287
1700066M21	0.06274963	4.79759952	0.19420789	0.66418507	0.75472287
Ccdc38	-0.2750698	-0.1987506	0.19418961	0.66419984	0.75472287
Abca12	0.47411916	-1.4656762	0.19414363	0.66423697	0.75472287
Tax1bp3	0.12627534	4.64836874	0.19395039	0.66439314	0.75472349
Ubxn2a	0.05875022	6.98286005	0.19392181	0.66441624	0.75472349
Zfp960	0.06859159	4.46068238	0.1938811	0.66444915	0.75472349
9430083A17I	0.26318224	1.31944819	0.193829	0.66449128	0.75472349
Zfp560	-0.0966022	4.12025676	0.19370902	0.66458832	0.75472349
Mapk8ip1	-0.0635481	5.53140948	0.19370222	0.66459382	0.75472349
E130308A19I	0.0733732	5.17680017	0.19365735	0.66463012	0.75472349
Snhg1	-0.0917934	4.49501652	0.19332065	0.66490268	0.75486973
Hgsnat	0.06272887	5.17726335	0.19330755	0.66491329	0.75486973
Bod1	0.07065543	6.10494432	0.19329037	0.6649272	0.75486973
Zfp955b	0.05970809	5.83765315	0.19304427	0.66512663	0.75500006
Tmem196	-0.1009028	3.16291533	0.19301023	0.66515422	0.75500006
Fbxo2	0.10102704	3.07285821	0.19284789	0.66528586	0.75508579
Ptprs	0.06926133	6.88843335	0.1925481	0.66552914	0.7552934
Faim	-0.0737457	5.76111039	0.19248418	0.66558104	0.7552934
Ccdc184	0.0921966	3.20706414	0.1924075	0.66564332	0.75530038
Mgat4c	-0.119138	2.88416955	0.19219987	0.66581201	0.75530421
Zcchc4	-0.3395213	-0.1139491	0.19216964	0.66583659	0.75530421
Nrg1	-0.1135851	3.36162218	0.19213619	0.66586377	0.75530421
Alms1-ps2	-0.2200709	0.45228043	0.19203124	0.66594911	0.75530421
Wdr60	-0.0817442	5.41442641	0.19202385	0.66595511	0.75530421
Inpp4b	0.09960242	3.81977761	0.1919374	0.66602542	0.75530421
Slc20a2	-0.0914451	6.03436327	0.19190394	0.66605265	0.75530421
Rasl11a	-0.3085948	0.00434263	0.19185097	0.66609574	0.75530421

Fam163b	-0.0613344	7.61194462	0.19175064	0.66617738	0.75533314
Mctp1	0.06940413	5.38954545	0.19088616	0.66688194	0.75601388
Rilpl2	-0.1150447	3.19208681	0.19087612	0.66689013	0.75601388
Fhit	-0.250254	1.2402851	0.19070513	0.66702972	0.75603743
Fam19a3	0.38219522	-0.4615225	0.19170855	0.66703982	0.75603743
Trim27	0.06624248	4.48871746	0.1906217	0.66709785	0.75603743
Lnx1	-0.0889372	4.01709519	0.19048594	0.66720876	0.75603743
Ar	-0.0935804	4.30558559	0.19048423	0.66721016	0.75603743
Nfatc2	-0.1034439	3.35874337	0.19034827	0.66732129	0.75603743
Tspan8	0.1447696	4.35901052	0.190312	0.66735094	0.75603743
Gpr37l1	-0.1275435	3.0098003	0.19003009	0.66758152	0.75603743
Syt17	-0.1322438	3.80397589	0.19001254	0.66759588	0.75603743
Mettl6	0.11653931	3.84319082	0.19000997	0.66759799	0.75603743
Rab37	-0.2698509	0.41174114	0.18992669	0.66766615	0.75603743
Nudt9	0.08273474	5.37527833	0.18990482	0.66768406	0.75603743
Calm1	-0.0441891	12.308041	0.1898556	0.66772435	0.75603743
Gpx1	0.11573866	6.58973415	0.18977724	0.66778852	0.75603743
Aplp2	-0.0460078	8.46249735	0.18968432	0.66786462	0.75603743
Greb1	0.23836647	0.39296859	0.18962447	0.66791366	0.75603743
Tbx2	0.43265281	-1.4237405	0.18962093	0.66791656	0.75603743
Apcdd1	0.12929366	3.98764952	0.18957289	0.66795592	0.75603743
Abca9	0.0823295	6.25692304	0.18948786	0.66802562	0.75603743
8030423F21I	0.29347401	-0.8160297	0.18946106	0.66804758	0.75603743
Brat1	0.13451979	2.28411883	0.18937136	0.66812113	0.75603743
Tctex1d2	-0.0902414	4.3958505	0.18933984	0.66814697	0.75603743
Plekhm1	0.06317909	5.38139561	0.18921539	0.66824906	0.75608937
Grb14	0.1055345	3.48487065	0.18899862	0.66842696	0.75614048
Acot12	-0.5672798	-1.5535456	0.18898721	0.66843633	0.75614048
Lcat	-0.1567377	2.85756413	0.18888555	0.6685198	0.75614048
Pcdhga11	0.13508933	3.13527731	0.18885994	0.66854084	0.75614048
4430402I18R	0.10949825	2.61787943	0.18872466	0.66865197	0.75614048
Ppm1j	-0.4470029	-1.8285793	0.18872426	0.6686523	0.75614048
Tet3	-0.0727916	6.03705093	0.18868133	0.66868757	0.75614048
Tmem5	0.06993348	4.60960499	0.18790041	0.66933014	0.75658713
Dpysl4	-0.124179	2.8588484	0.18787544	0.66935071	0.75658713
Kcnj11	-0.0899624	3.39176252	0.18785683	0.66936604	0.75658713
Sephs1	0.06791356	5.67359731	0.18782898	0.66938899	0.75658713
Tiam1	-0.0708797	6.45558787	0.18780347	0.66941001	0.75658713
Zfp750	-0.1964069	2.36164251	0.18773649	0.66946521	0.75658713
Dek	0.06768119	8.40392104	0.18762668	0.66955574	0.75658713
BC030336	0.06083501	5.66464111	0.18761892	0.66956214	0.75658713
Epm2aip1	-0.0795046	7.97617275	0.1875569	0.66961328	0.75658713
Rap1gap	-0.0947293	4.12132355	0.18751866	0.66964482	0.75658713

Akt3	0.05717584	8.0782817	0.18732912	0.66980121	0.75663959
Lpin2	0.06636569	6.58671134	0.18732022	0.66980856	0.75663959
Cd36	0.38045852	-0.390192	0.18724557	0.66987018	0.75663959
Sgms2	-0.1678511	3.45145195	0.18713763	0.66995931	0.75663959
Mettl21c	-0.5054004	-0.5973106	0.18712178	0.6699724	0.75663959
Zfp536	0.05713827	4.61730141	0.18702766	0.67005014	0.75666388
Bloc1s4	0.11561217	4.26340494	0.186785	0.67025069	0.75681606
Crispld1	-0.0932985	3.89178035	0.18672853	0.67029738	0.75681606
Nit2	0.12120164	2.27151986	0.18639558	0.67057285	0.75689752
Fbxo25	-0.0738196	4.92372084	0.18639455	0.67057371	0.75689752
Vrk2	0.27768396	0.45857261	0.18632266	0.67063323	0.75689752
Rwdd1	-0.0641257	5.23543726	0.1862298	0.67071013	0.75689752
Setd4	0.25038942	1.04697933	0.18616785	0.67076144	0.75689752
Gprin1	0.07699385	4.69339055	0.18610862	0.67081052	0.75689752
Rpl26	-0.3324334	-1.291403	0.18604307	0.67086483	0.75689752
Lsm12	-0.0586182	5.8603497	0.18600653	0.67089512	0.75689752
Rgmb	-0.0745305	4.71335	0.18599419	0.67090535	0.75689752
Klhl36	-0.2592829	0.6160963	0.18584353	0.67103025	0.75689752
Med28	-0.0856735	5.31342009	0.18579116	0.67107368	0.75689752
Fbxo44	-0.0792475	4.46392844	0.18575585	0.67110298	0.75689752
Kif9	-0.1693878	2.35054182	0.18573825	0.67111757	0.75689752
Dnttip2	-0.052128	6.01952394	0.18549689	0.67131787	0.75689752
Gm9962	0.21054746	0.50648119	0.18547351	0.67133729	0.75689752
Tmem263	0.06022351	7.21113596	0.18546651	0.6713431	0.75689752
Fam126a	0.07346511	4.10421944	0.18546492	0.67134442	0.75689752
Cir1	0.05155751	6.95479119	0.18540019	0.67139817	0.75689752
Thpo	-0.1451334	2.09779978	0.18535193	0.67143825	0.75689752
Top2b	-0.0550672	8.14488493	0.18524856	0.67152412	0.75693091
Zrsr2	-0.055336	6.20356364	0.18504825	0.67169061	0.75700046
Tln2	0.06032262	6.80998264	0.18503895	0.67169834	0.75700046
Zfp109	0.10360239	3.49465105	0.1849451	0.67177638	0.75700635
Eif4g3	0.06195127	8.94854397	0.18483375	0.671869	0.75700635
Qk	0.05191907	9.3229642	0.18482975	0.67187233	0.75700635
6030440G07	-0.3254926	-1.2399311	0.18454158	0.6721122	0.75705544
Tbc1d8b	-0.0835826	4.33544602	0.18453129	0.67212077	0.75705544
Cilp2	0.28557792	0.61710162	0.18449222	0.6721533	0.75705544
AW046200	-0.3378628	0.14785655	0.1843733	0.67225237	0.75705544
Smyd2	0.0749234	5.5721474	0.1842805	0.67232971	0.75705544
Lars	0.06272284	5.12564252	0.18427938	0.67233064	0.75705544
Dnmt3a	-0.0679599	6.59707257	0.18421216	0.67238667	0.75705544
Ddx59	0.12386588	2.5780165	0.18394709	0.67260774	0.75705544
Kdm1b	-0.0842504	4.2157133	0.18394111	0.67261273	0.75705544
Nsa2	0.05885905	6.72256089	0.18387704	0.67266619	0.75705544

Faf2	-0.0762701	4.40507578	0.18383467	0.67270155	0.75705544
Gm10767	-0.132137	1.92261135	0.18380532	0.67272606	0.75705544
Bbs4	0.05340378	5.56476616	0.18374874	0.67277329	0.75705544
Spata18	-0.2323678	0.3596755	0.18374674	0.67277496	0.75705544
Gucy1a2	-0.086109	7.95296243	0.18371728	0.67279956	0.75705544
Nek1	0.07152985	6.39445689	0.18369751	0.67281607	0.75705544
Arhgef10	-0.0647376	5.27670448	0.18361205	0.67288744	0.75707244
Loxl4	-0.4258377	-1.1594294	0.18345193	0.67302122	0.75715119
Raver2	0.09592991	3.14227939	0.18335206	0.67310469	0.75715119
Epor	-0.2149585	0.39059471	0.18332629	0.67312624	0.75715119
Ralb	0.08162037	4.37147083	0.18314475	0.67327805	0.7571515
Prkg1	-0.0550241	5.8376894	0.18313753	0.67328409	0.7571515
Cdc7	0.16446008	2.8060365	0.1830834	0.67332938	0.7571515
Kifc3	-0.1025423	3.01247194	0.18305687	0.67335158	0.7571515
Svep1	0.11963631	2.75200356	0.18285464	0.67352085	0.75725943
Orc6	-0.0733291	4.67622612	0.18271708	0.67363607	0.75725943
Ncan	0.07359544	8.37898818	0.18267929	0.67366772	0.75725943
Fbxo34	-0.072062	5.26287799	0.18261821	0.6737189	0.75725943
Smpdl3a	0.09487728	4.81997081	0.18260623	0.67372894	0.75725943
Kctd14	0.24397228	-0.0124869	0.18250357	0.67381499	0.75729289
Edf1	0.10563576	6.41286507	0.18225169	0.67402622	0.75746702
Rfx8	-0.591075	-1.9345285	0.18217824	0.67408785	0.75747302
Rab4a	0.06400738	4.98587144	0.18197843	0.67425558	0.75751746
Ttll12	-0.0932989	4.13670622	0.18194701	0.67428197	0.75751746
Rbfox3	0.04677852	7.81275432	0.18192998	0.67429627	0.75751746
Gins2	0.15930456	2.15849279	0.18184544	0.67436728	0.75753398
Slc4a10	0.05669677	8.98850848	0.18177313	0.67442803	0.75753899
Aph1a	-0.1258528	2.94250574	0.18168925	0.67449853	0.75755368
Dcst1	-0.1598297	1.39971641	0.18158116	0.67458939	0.75755368
Ssrp1	0.0700322	6.70649479	0.18155665	0.67461001	0.75755368
Trpv2	-0.0967144	3.07012746	0.18139842	0.67474309	0.75763991
Fam172a	0.05887848	5.60774182	0.18108457	0.67500728	0.75778187
Sult1a1	0.10469973	5.7934373	0.18106526	0.67502354	0.75778187
3110082J24F	-0.3596304	-0.6274529	0.18098754	0.675089	0.75778187
Fastkd2	0.14147357	2.91665665	0.18098069	0.67509478	0.75778187
2610100L16F	-0.1377859	2.15137763	0.18060549	0.67541107	0.75789225
Tspyl1	-0.0439123	7.44783897	0.18058671	0.67542691	0.75789225
Mrap2	0.23637799	1.52222856	0.18052359	0.67548016	0.75789225
Brd4	0.05195858	8.73212684	0.18051836	0.67548458	0.75789225
Stradb	-0.0639363	5.42089571	0.18042435	0.67556391	0.75789225
Gja3	0.65461412	-1.8770498	0.18038511	0.67559703	0.75789225
Txnrd3	0.10396917	3.37677223	0.18037952	0.67560175	0.75789225
Atp1a2	-0.0927772	11.2666227	0.18025417	0.67570758	0.75789225

Cdh12	-0.0840734	6.34900079	0.18022979	0.67572817	0.75789225
Rps27l	0.12732085	4.62792645	0.18015792	0.67578888	0.75789225
Thrb	0.05903939	7.02512197	0.1799514	0.67596339	0.75789225
Ppargc1b	-0.1204076	3.29665832	0.17993216	0.67597966	0.75789225
Cysltr1	-0.293006	-0.4593609	0.17990472	0.67600286	0.75789225
Ssbp2	-0.0581772	6.26453336	0.17978802	0.67610155	0.75789225
Tmem79	-0.4681857	-0.2191697	0.17976392	0.67612193	0.75789225
Mllt1	-0.0753722	4.59534888	0.17975779	0.67612711	0.75789225
Herpud2	0.06345559	6.21592798	0.17969757	0.67617806	0.75789225
Armc6	-0.1350127	1.69641095	0.17966346	0.67620691	0.75789225
Nxpe2	-0.2168603	1.13167238	0.17954265	0.67630916	0.75793796
Gbp6	-0.0802979	5.06947314	0.17948218	0.67636035	0.75793796
Tfap2d	-0.3870449	-0.1952972	0.17926511	0.6765442	0.75808085
Itga11	-0.1166715	2.90705307	0.1791199	0.67666726	0.75815561
Smim22	-0.4145465	-0.4654635	0.17896867	0.67679548	0.75823613
Sort1	-0.0613343	7.68974787	0.17868552	0.67703572	0.75835368
Uri1	0.05326121	6.632009	0.17867937	0.67704095	0.75835368
Zfyve21	-0.0896126	4.02987159	0.17864578	0.67706947	0.75835368
Ap3s2	-0.0552559	6.62475984	0.17856413	0.6771388	0.75836821
Creld2	0.11954938	3.22286523	0.17848653	0.6772047	0.7583789
Pkd1	-0.0836136	5.17481873	0.1780592	0.67756795	0.75864687
3010026O09	-0.1112077	3.29919568	0.17803881	0.6775853	0.75864687
Mgmt	-0.2684994	-0.09654	0.1780061	0.67761312	0.75864687
A630072M18	0.09584687	3.50751564	0.17786197	0.67773578	0.7587187
Wnk2	0.08483771	6.04766164	0.17779822	0.67779005	0.7587187
Rnf114	-0.0803959	5.39363236	0.17763346	0.67793037	0.7587487
Irak3	-0.1435867	2.79377996	0.17753342	0.67801559	0.7587487
Acot6	0.11701545	2.70793181	0.17748519	0.6780567	0.7587487
Frmd7	0.28700829	-0.1446833	0.17740902	0.67812162	0.7587487
Manf	-0.1108995	3.8192679	0.17739873	0.6781304	0.7587487
Dcp1b	-0.0789057	4.21256879	0.17736967	0.67815517	0.7587487
1700011I03R	-0.2773265	-0.0163869	0.17723051	0.67827385	0.75881839
Rcn2	0.0563897	6.30450958	0.17697827	0.6784891	0.75894691
Bex2	-0.0711782	6.92183574	0.17695243	0.67851116	0.75894691
Jakmip2	-0.0653916	6.83067057	0.17686223	0.67858819	0.75894691
Arhgef15	-0.1385548	2.8955382	0.17677417	0.67866341	0.75894691
3110062M04	-0.1980954	0.70944072	0.17673713	0.67869505	0.75894691
Rpl37	-0.0776786	6.35124797	0.17649517	0.67890186	0.75894691
Arhgap27os3	-0.4917901	-1.6423363	0.17638252	0.67899822	0.75894691
Tlr13	-0.2956514	0.9213886	0.17629445	0.67907356	0.75894691
Tmem126b	0.08696757	4.18323739	0.17625995	0.67910309	0.75894691
Trpt1	0.09734841	3.12327704	0.17613386	0.67921101	0.75894691
Rab26	0.11848136	3.91471612	0.17610522	0.67923554	0.75894691

Lama1	-0.0992156	3.50150878	0.17604104	0.6792905	0.75894691
Grk6	0.10377936	2.89637392	0.17602395	0.67930513	0.75894691
Dand5	0.08624517	3.55729578	0.175806	0.67949186	0.75894691
4933407K13I	-0.1943612	1.84513764	0.1758042	0.67949341	0.75894691
Tmem57	-0.0487449	6.12425202	0.17577201	0.679521	0.75894691
Cux2	0.0856347	4.80386625	0.17571446	0.67957034	0.75894691
Mrps18a	-0.1153297	3.38640429	0.17564677	0.67962837	0.75894691
Sec61a2	-0.09232	4.92170337	0.1756197	0.67965158	0.75894691
Eea1	0.05268862	7.47743477	0.17553077	0.67972787	0.75894691
Gucd1	0.11431322	3.58927075	0.17551109	0.67974475	0.75894691
Nprl3	-0.0933178	3.06488781	0.1754836	0.67976833	0.75894691
C1qtnf7	-0.1501166	4.36202515	0.17541714	0.67982537	0.75894691
Nrf1	0.07949248	3.87283049	0.17537423	0.6798622	0.75894691
Gm20597	-0.4809425	-0.6734254	0.17536781	0.67986771	0.75894691
Phb	0.0824471	3.81060631	0.17536345	0.67987145	0.75894691
Gorasp1	0.09975402	3.03344982	0.17531098	0.6799165	0.75894691
Slitrk3	-0.082922	6.08726695	0.17524142	0.67997621	0.75894691
Gcsh	0.07327119	5.68617672	0.17517214	0.68003571	0.75894691
Atp6v0a4	-0.2514144	0.14184209	0.17511972	0.68008075	0.75894691
Nudc	-0.0705008	5.63005536	0.17503076	0.68015718	0.75896926
Cinp	-0.0838541	4.26238492	0.17496184	0.68021641	0.75897242
Chrd	-0.1234822	2.10372602	0.17480942	0.68034745	0.7590187
Rer1	-0.0773957	5.71875699	0.17474224	0.68040523	0.7590187
Cnnm3	-0.0693555	4.18017385	0.17456478	0.68055793	0.7590187
Pcdhga8	0.11740053	2.67989961	0.17455699	0.68056463	0.7590187
Ppia	0.06701411	9.86341578	0.17454672	0.68057347	0.7590187
Cdkn2b	0.50533452	-1.6133933	0.17452017	0.68059633	0.7590187
Klhl23	0.08112063	5.1496477	0.17435374	0.68073963	0.75911373
Hs6st3	-0.1049941	2.87786613	0.1742902	0.68079436	0.75911373
Cbx5	-0.0486555	9.15622761	0.17417109	0.680897	0.75916526
Tbc1d22bos	-0.4948441	-1.0139922	0.17408844	0.68096824	0.75918179
Trmt10b	-0.08658	3.70171068	0.17395335	0.68108472	0.75924874
Pi4ka	0.07630245	8.10487444	0.17383471	0.68118706	0.75924905
Sfxn3	-0.0575511	7.52997578	0.17382222	0.68119784	0.75924905
Nin	-0.0575957	6.83042218	0.17362289	0.68136989	0.7593334
Nprl2	-0.1183882	2.467394	0.1735747	0.68141151	0.7593334
Nudt16l1	0.06671386	4.1446376	0.17350567	0.68147112	0.7593334
Tmem106c	-0.1440381	3.83407144	0.17345139	0.68151802	0.7593334
Prdm4	0.0813482	4.99589618	0.17340781	0.68155567	0.7593334
Epn3	-0.2413497	0.59555436	0.1731653	0.6817653	0.75940017
D330023K18	0.20276238	1.2343076	0.17313738	0.68178945	0.75940017
Abhd3	-0.1251121	3.71128761	0.17308702	0.68183301	0.75940017
Afap1l2	-0.1064383	2.61867956	0.17307739	0.68184134	0.75940017

Tab1	-0.1174122	2.84188839	0.17289756	0.68199695	0.75946636
Slc35a5	-0.0665709	5.44790196	0.17283162	0.68205403	0.75946636
Fam163a	-0.1845595	1.81468194	0.17281307	0.68207009	0.75946636
C130026I21R	0.18290662	1.06014838	0.17272501	0.68214634	0.75948559
Alox12b	0.19455452	2.01014161	0.17266279	0.68220024	0.75948559
Mis18a	-0.1214728	1.95977578	0.17245951	0.68237639	0.75954883
Arhgef26	0.08534447	4.33599906	0.17226931	0.68254133	0.75954883
Rala	0.04540258	6.48951825	0.17219126	0.68260904	0.75954883
Rps17	-0.0671289	7.26328492	0.17213372	0.68265897	0.75954883
Lrrc38	0.17188035	1.44435448	0.1721263	0.68266541	0.75954883
Tbce	-0.0724818	4.40846817	0.17212457	0.68266691	0.75954883
Zmym2	0.06473626	8.1176782	0.17209189	0.68269527	0.75954883
Ppp3cc	0.06415046	5.04839543	0.17197125	0.68280001	0.75954883
Col9a3	-0.1516353	2.69052244	0.17197016	0.68280095	0.75954883
Bai2	-0.0936387	4.57330281	0.1719465	0.6828215	0.75954883
Clasp2	-0.0534148	8.0401354	0.17184528	0.68290941	0.75958383
Zfp389	0.48301381	-1.5357067	0.17172152	0.68301695	0.7595929
Morf4l1	0.05722338	8.47922785	0.17164658	0.68308208	0.7595929
Ncbp2	0.05377726	6.54868575	0.17163019	0.68309633	0.7595929
Bcl2l1	-0.0933909	4.44907231	0.17140227	0.68329454	0.7595929
Bcl2l13	0.070897	5.02911328	0.17119205	0.68347751	0.7595929
Ict1	-0.100269	4.30702121	0.17119048	0.68347887	0.7595929
Prkcg	0.06380852	8.20544702	0.17111334	0.68354604	0.7595929
Sigmar1	-0.0817178	3.01872859	0.17105768	0.68359452	0.7595929
Lonp1	0.07044882	4.27239562	0.17104438	0.68360611	0.7595929
Elavl2	0.05900524	6.93896308	0.1709765	0.68366525	0.7595929
Nmt2	-0.0501132	6.98586461	0.17097579	0.68366587	0.7595929
Thg1l	-0.1322804	2.94982883	0.17092682	0.68370854	0.7595929
Lin7c	0.04950713	7.8688952	0.17089364	0.68373745	0.7595929
Adam8	0.42210184	-0.6615818	0.17085608	0.68377019	0.7595929
Clcn5	-0.1610456	2.3199123	0.17081166	0.68380891	0.7595929
Foxk1	-0.0527877	7.16862433	0.17077974	0.68383674	0.7595929
2700094K13I	-0.1151559	3.19657688	0.17073335	0.68387719	0.7595929
Zfp719	-0.0673842	5.71662256	0.17059905	0.68399433	0.75966031
Phf2	-0.0430032	6.53371212	0.17042338	0.68414764	0.75966058
Tmem132a	0.10488643	2.66849841	0.17041358	0.68415619	0.75966058
Xpo5	-0.0744031	4.68001948	0.17027673	0.68427569	0.75966058
Rprd2	-0.0578017	7.26401555	0.17024086	0.68430702	0.75966058
P4htm	-0.1080651	2.39224684	0.17016868	0.68437007	0.75966058
Chmp4c	0.52947358	-1.6063499	0.17016447	0.68437376	0.75966058
Zcchc10	-0.1080774	3.6333882	0.17014616	0.68438976	0.75966058
Pcnx	0.06306342	7.32151792	0.16985906	0.68464075	0.7598765
Scnm1	0.08955253	4.02330932	0.16974546	0.68474013	0.75992412

3110082I17R	0.1297827	2.68629787	0.16959529	0.68487156	0.7600073
Wwox	0.07461337	4.0488127	0.16939364	0.68504816	0.76004781
Rsl1d1	0.05345939	6.66736682	0.16938287	0.6850576	0.76004781
Ppp2r4	-0.0583914	5.70209698	0.16936014	0.68507751	0.76004781
Cwc15	0.06862008	6.787145	0.16917044	0.68524378	0.76008683
Etaa1	0.0813726	4.87202741	0.16907613	0.68532648	0.76008683
Shmt1	-0.4228444	-0.4443665	0.16907429	0.6853281	0.76008683
Ctsl	0.09801662	6.74069323	0.16895928	0.68542898	0.76008683
Nckap1l	-0.1003031	3.03439757	0.16893452	0.68545071	0.76008683
Coro1a	0.08755128	4.02559701	0.16888495	0.68549421	0.76008683
Kcnd3	0.08907668	5.96922766	0.16886914	0.68550809	0.76008683
Tmem62	0.11042595	3.23541605	0.16871703	0.68564163	0.76012801
Syngr3	0.06798346	5.08592985	0.16869332	0.68566244	0.76012801
Pnpt1	-0.1026028	3.92011407	0.16854798	0.68579011	0.76012801
Gm19897	0.33754826	-0.0053827	0.16850809	0.68582517	0.76012801
A530054K11	0.05984687	5.33622178	0.16850526	0.68582766	0.76012801
Zfp687	-0.0923847	3.60778931	0.16826328	0.68604039	0.76030116
Wdr43	-0.0647112	4.90443713	0.16811244	0.6861731	0.76035979
Tubgcp6	-0.0972576	3.40036445	0.16806079	0.68621856	0.76035979
1700113A16	-0.0864805	3.91329545	0.16799544	0.68627608	0.76035979
Pdxp	-0.0903247	4.93016259	0.16783075	0.68642111	0.76035979
Chd8	-0.064511	6.62880664	0.16771208	0.68652566	0.76035979
Fkbp5	-0.1203249	5.97414013	0.16764245	0.68658703	0.76035979
Cacng4	-0.220062	1.11388818	0.16762822	0.68659957	0.76035979
1700123M08	0.16870661	1.25179356	0.16736064	0.68683556	0.76035979
Ptp4a2	0.07027515	9.07797715	0.1673501	0.68684485	0.76035979
Pfkm	0.06874101	5.87960746	0.16720476	0.68697313	0.76035979
Atxn7l2	0.18799732	2.34763128	0.1672024	0.68697521	0.76035979
Col2a1	-0.4709077	-1.1175281	0.16716988	0.68700392	0.76035979
Rbm18	0.06124162	6.24774405	0.16699813	0.68715561	0.76035979
Arhgap23	-0.0722372	5.68416057	0.16695049	0.6871977	0.76035979
Shkbp1	-0.1948377	0.80269498	0.16692785	0.6872177	0.76035979
Chrm4	-0.135362	2.00723448	0.16689647	0.68724543	0.76035979
Pak3	-0.0736706	7.31845417	0.16687758	0.68726212	0.76035979
Pnp2	0.27136163	-0.4451936	0.16685762	0.68727977	0.76035979
E2f2	-0.1559452	3.00064518	0.16685122	0.68728543	0.76035979
Elmo1	0.0542958	7.15388229	0.16662702	0.68748368	0.76035979
Nubpl	-0.1111196	2.80469616	0.16657541	0.68752933	0.76035979
Slc9b2	0.09975205	4.25861711	0.166551	0.68755093	0.76035979
Gtf2f2	-0.0834441	3.1438691	0.16654955	0.68755221	0.76035979
Kif24	0.21301547	1.04121101	0.1663492	0.68772955	0.76035979
C87436	0.10937579	3.60586379	0.16620578	0.68785658	0.76035979
Gphn	0.0667795	5.91839685	0.16617781	0.68788136	0.76035979

Ndufb10	0.06340556	5.91536809	0.16610197	0.68794856	0.76035979
Gm4013	-0.4881838	-1.6984571	0.16604888	0.68799561	0.76035979
Nt5c3b	-0.08245	3.23958664	0.16587945	0.68814584	0.76035979
Slc6a11	-0.1016534	4.54546055	0.16574891	0.68826165	0.76035979
Mettl25	-0.1402152	2.64327942	0.16572425	0.68828353	0.76035979
Zfp617	0.06033669	5.81358953	0.16567765	0.68832489	0.76035979
Timm44	0.08984457	3.40871495	0.16561899	0.68837696	0.76035979
Lss	0.08271851	4.11244123	0.16556691	0.6884232	0.76035979
Impad1	0.04868938	7.02690267	0.16556485	0.68842502	0.76035979
Slc16a12	0.10545638	4.08482467	0.16552227	0.68846284	0.76035979
C2cd5	0.08198803	5.79741466	0.1654998	0.68848279	0.76035979
Rps19	-0.0902709	4.67855439	0.16549337	0.6884885	0.76035979
Slc16a4	0.17336443	1.73228008	0.16533144	0.68863236	0.76035979
Gm16712	-0.4560402	-1.9720096	0.16516585	0.68877956	0.76035979
Glra3	-0.1798687	1.20109565	0.16513569	0.68880638	0.76035979
Ehd4	0.07451848	4.01675613	0.16513094	0.6888106	0.76035979
Plekhs1	0.30809179	-0.1763468	0.16507848	0.68885726	0.76035979
B9d2	-0.1430399	1.8269852	0.16504256	0.68888921	0.76035979
Ints2	0.08325491	4.08922005	0.16500957	0.68891856	0.76035979
Fam213b	0.07613992	4.12437467	0.16493417	0.68898565	0.76035979
Fam171a2	-0.1018342	2.68908278	0.16487861	0.68903509	0.76035979
Fam83h	0.14291502	1.56831649	0.16483281	0.68907586	0.76035979
Nr3c2	0.06046957	5.95236095	0.16481633	0.68909053	0.76035979
Mt2	0.0911336	5.17961798	0.16471435	0.68918134	0.76035979
Zc3hc1	-0.1206876	2.28311213	0.16465834	0.68923123	0.76035979
Zfp82	0.15034183	1.45211517	0.16461672	0.68926831	0.76035979
Cbx3	0.05936196	8.36237764	0.16439937	0.68946202	0.76035979
Tpp2	0.06521328	6.74275554	0.16439093	0.68946955	0.76035979
Mrpl18	-0.0779962	5.44431311	0.1643048	0.68954635	0.76035979
Dnajc19	-0.0685064	4.74616253	0.16428248	0.68956626	0.76035979
Cerk	0.08648334	4.07824315	0.16426537	0.68958152	0.76035979
Pkd2	-0.0886331	6.20222856	0.16420029	0.68963959	0.76035979
Gadd45gip1	-0.1180524	2.62818782	0.1641862	0.68965215	0.76035979
Fam57b	0.13946715	2.79398044	0.16404881	0.68977478	0.76035979
Gpr84	0.48412308	-1.0057645	0.16390143	0.68990638	0.76035979
Ypel5	0.05864731	7.68390628	0.16384873	0.68995345	0.76035979
Echdc3	0.16583368	1.11695065	0.16379845	0.68999837	0.76035979
A330040F15	-0.3165541	-0.8518091	0.16371217	0.69007548	0.76035979
Map2k4	-0.0539812	7.68792526	0.16370085	0.6900856	0.76035979
Park2	0.10420463	3.05760267	0.16369957	0.69008674	0.76035979
Zfp830	-0.0703172	5.20672535	0.16363453	0.69014488	0.76035979
Fastk	0.08156175	3.68472314	0.16360427	0.69017194	0.76035979
Copa	-0.0589009	7.7765974	0.16354166	0.69022793	0.76035979

Anpep	0.10136402	6.47703909	0.16353466	0.69023419	0.76035979
Zfp532	0.05485336	5.64739454	0.16342227	0.69033473	0.76035979
Dnajc3	0.07651048	6.76679914	0.16327193	0.69046927	0.76035979
Osr2	-0.3154106	-0.06452	0.16326375	0.6904766	0.76035979
Draxin	0.533972	-1.4922165	0.16324558	0.69049286	0.76035979
Bysl	0.08096595	3.83428023	0.16311357	0.69061108	0.76035979
Sec22b	-0.0501697	7.09426359	0.16308603	0.69063575	0.76035979
Iigp1	0.10334831	4.08486279	0.16301367	0.69070058	0.76035979
Gpr45	0.10852494	2.23163796	0.1630128	0.69070136	0.76035979
Klf16	0.10053212	2.45963519	0.16292554	0.69077956	0.76035979
Cryab	0.08914026	7.16970998	0.16290689	0.69079627	0.76035979
Rab1b	0.08970964	4.50357591	0.16288421	0.6908166	0.76035979
Wnt6	-0.1519166	3.15703096	0.16277263	0.69091665	0.76035979
Foxj3	-0.0584224	7.73374767	0.16270959	0.69097319	0.76035979
Ttc13	-0.1020912	3.19742116	0.16266672	0.69101165	0.76035979
Thoc2	-0.067215	7.06080005	0.1626667	0.69101166	0.76035979
Slc12a4	0.21049848	1.46995527	0.16260579	0.69106632	0.76035979
Sema5b	-0.1411045	3.53938769	0.16260072	0.69107087	0.76035979
Map1b	-0.1044345	12.0341584	0.1625486	0.69111764	0.76035979
Plac8	0.59829262	-1.2201908	0.16247186	0.69118653	0.76035979
Col6a5	0.57490609	-1.8134956	0.16245219	0.69120419	0.76035979
Klhl7	0.05628249	6.31339067	0.16241751	0.69123533	0.76035979
Mmaa	0.10372859	3.75448196	0.16217447	0.69145366	0.76045212
Rfwd2	-0.0472578	7.26114381	0.16216632	0.69146098	0.76045212
Slc35c1	-0.0928593	3.33152428	0.16213538	0.69148879	0.76045212
Gpr182	-0.1316762	4.10662129	0.16200502	0.691606	0.76047909
Use1	-0.1099176	4.73330947	0.16198239	0.69162635	0.76047909
Crebbp	0.04930363	8.16522246	0.16190023	0.69170025	0.7604982
Tnfrsf1a	0.12262853	4.11588072	0.16177919	0.69180917	0.76055581
Lcmt2	0.11306249	2.52533972	0.16170762	0.69187359	0.7605645
Gbp11	-0.3124258	-0.1374851	0.16163825	0.69193605	0.76057102
Eif4ebp1	0.14843314	1.68990599	0.16146989	0.6920877	0.76067558
4931429I11R	0.29288872	-0.0218343	0.16109798	0.69242303	0.7607879
Rgs3	-0.0952326	4.10032418	0.16107724	0.69244173	0.7607879
Lingo4	-0.5293095	-1.408254	0.1610691	0.69244908	0.7607879
Zfp810	0.06512139	4.65836076	0.16098999	0.69252048	0.7607879
Klhl25	-0.1113853	1.87790418	0.16085545	0.69264194	0.7607879
Hist1h4b	-0.3537237	-1.8082194	0.16076042	0.69272777	0.7607879
Ptprn	0.07664038	6.91147551	0.1606865	0.69279456	0.7607879
Ap4s1	-0.0536194	5.86757563	0.16057128	0.69289869	0.7607879
Rdh13	0.07740378	3.81621794	0.16053555	0.69293099	0.7607879
Shcbp1	-0.3452548	-0.5415224	0.16050191	0.6929614	0.7607879
Rrp1b	-0.0790797	3.45718733	0.16045033	0.69300805	0.7607879

Slc26a6	-0.3016251	-0.1905844	0.16041428	0.69304065	0.7607879
Il15ra	-0.1032348	2.60517085	0.16039408	0.69305892	0.7607879
Gm8300	0.29001085	-0.6615759	0.16037533	0.69307588	0.7607879
8430429K09l	0.08129448	4.21523201	0.16027776	0.69316416	0.7607879
Lrmp	0.35820316	-0.0754907	0.16017903	0.69325353	0.7607879
Rasl12	0.2846468	-0.0654377	0.16015969	0.69327103	0.7607879
Oas3	-0.2933954	0.6839544	0.16013625	0.69329225	0.7607879
Rnf214	-0.0471681	6.33644603	0.16012339	0.69330389	0.7607879
Mapk4	-0.056329	7.79315389	0.1601049	0.69332064	0.7607879
Rasal2	-0.0653056	7.19857233	0.15990419	0.69350245	0.76092535
Sox8	0.10512984	3.86742114	0.15968771	0.69369869	0.7610083
Hs6st1	0.07032256	4.62727952	0.15953136	0.69384053	0.7610083
Fbxo43	-0.2458818	-0.5212013	0.15944564	0.69391832	0.7610083
Plk3	-0.1669422	1.70869438	0.15932148	0.69403104	0.7610083
E230029C05l	-0.2334978	0.54626243	0.15927062	0.69407724	0.7610083
Pde1b	0.06159771	5.52007197	0.15921361	0.69412902	0.7610083
Heatr5a	-0.0719108	4.46749891	0.15918149	0.6941582	0.7610083
Srsf10	0.04989054	6.59195119	0.15912355	0.69421085	0.7610083
Mtcl1	-0.0872991	5.72007567	0.15911697	0.69421683	0.7610083
Pot1a	-0.0659583	5.1867932	0.15910368	0.69422891	0.7610083
Eefsec	-0.1158792	2.69284834	0.15908872	0.6942425	0.7610083
1700026L06F	0.44048799	-1.4318368	0.1590687	0.6942607	0.7610083
Tpgs2	0.0480421	6.19132369	0.15901089	0.69431325	0.7610083
Gm17762	-0.315257	-0.4665116	0.15888366	0.69442894	0.76104631
Ankrd13b	-0.0994743	3.86267914	0.15884838	0.69446104	0.76104631
Nphp3	0.11331461	2.49082718	0.15850536	0.69477327	0.76129041
Brf1	0.06540369	4.23692636	0.15847938	0.69479693	0.76129041
Samhd1	0.05343384	5.59678699	0.158377	0.69489021	0.76133062
Pde3a	0.2071389	1.06521131	0.15822448	0.69502924	0.76138019
Cyr61	-0.2308955	2.43826021	0.15820322	0.69504862	0.76138019
Mipol1	-0.0791619	3.67320862	0.15813592	0.69511	0.76138544
Disp1	-0.1000595	3.22651825	0.15781746	0.69540062	0.76151308
Wars2	-0.1081827	3.67473032	0.15781102	0.6954065	0.76151308
Sod2	-0.0490419	8.45926204	0.1577001	0.69550781	0.76151308
AI504432	-0.0656304	5.6203491	0.1576674	0.69553768	0.76151308
Clk4	-0.0659482	5.76616854	0.15764296	0.69556001	0.76151308
2410127L17F	0.08149577	4.94433774	0.15763632	0.69556607	0.76151308
Fam118b	0.08053324	5.23855314	0.15734667	0.69583088	0.76157238
Pfkp	0.07419694	7.31963642	0.15729649	0.69587679	0.76157238
Srpr	0.08245737	6.37658832	0.1572796	0.69589224	0.76157238
Rhof	0.10124711	3.497237	0.15726724	0.69590355	0.76157238
Fam175b	-0.0625108	4.53151692	0.15722862	0.69593889	0.76157238
Cdkl1	-0.1333415	2.6430747	0.15720576	0.69595981	0.76157238

Pbld2	0.22028663	0.65689591	0.15700446	0.69614411	0.7617121
Tsc22d4	0.08462774	3.17516185	0.15689127	0.6962478	0.76173049
Slc22a17	-0.0655401	5.86933989	0.15674197	0.69638463	0.76173049
Jsrp1	-0.3394245	-0.75672	0.15673957	0.69638683	0.76173049
Dpf1	0.12107336	2.26390189	0.15671881	0.69640587	0.76173049
Magohb	-0.1149645	2.81648979	0.15666834	0.69645214	0.76173049
Fbxl7	0.09833617	6.20230065	0.15661555	0.69650056	0.76173049
Garem	0.06911426	4.79906231	0.15636293	0.69673237	0.76189708
Lpar5	-0.3895469	-0.7769448	0.15632617	0.69676612	0.76189708
Atp6v1e1	0.0472786	6.40075552	0.15580588	0.69724431	0.76220928
Actn3	-0.3545715	-0.3285975	0.15569584	0.69734557	0.76220928
BC025920	-0.1546563	1.46221459	0.1556767	0.69736318	0.76220928
Neu1	-0.0853297	3.96564936	0.15565666	0.69738162	0.76220928
Smap1	0.04422982	6.28610141	0.15564579	0.69739163	0.76220928
Zmat1	0.06246442	5.17673557	0.15561527	0.69741972	0.76220928
Atp5h	0.07293408	6.99955852	0.15558442	0.69744813	0.76220928
Msh3	0.07936203	4.12936455	0.15532907	0.69768335	0.76240442
E530001F21I	-0.4643461	0.14723667	0.15513713	0.69786031	0.76241441
Tmem132d	0.09437916	3.96671457	0.1551302	0.69786669	0.76241441
Ing4	-0.0703551	3.88740656	0.15511675	0.69787911	0.76241441
Gpr26	0.12458475	3.43552285	0.15500513	0.69798208	0.76241441
Lhx6	0.07168551	4.21620547	0.15500486	0.69798233	0.76241441
Acyp1	0.07868887	5.19441005	0.15479012	0.69818055	0.76241441
1700080N15	0.32366639	0.14757437	0.15474041	0.69822646	0.76241441
Syne2	-0.0564543	5.49056442	0.15472155	0.69824388	0.76241441
A330069E16	0.16856029	0.70240005	0.15469533	0.69826811	0.76241441
Zic2	-0.0827747	6.54778614	0.15467491	0.69828697	0.76241441
Hnrnpl	0.06051335	5.22655387	0.154593	0.69836265	0.76241441
Fus	-0.0772658	5.94252772	0.1545776	0.69837689	0.76241441
Cd63	-0.1236248	4.98247981	0.15443842	0.69850555	0.76241441
Ccdc126	-0.1255167	2.28289532	0.15435428	0.69858336	0.76241441
Stxbp3b	0.28404286	-0.9580444	0.15423483	0.69869388	0.76241441
Armcx5	-0.0530746	5.36847734	0.1541793	0.69874527	0.76241441
Zbtb8os	0.10703331	4.06613915	0.15413202	0.69878904	0.76241441
Sarnp	0.06608102	5.53756592	0.1541025	0.69881637	0.76241441
Zfp623	-0.0881679	3.4205748	0.1540856	0.69883202	0.76241441
Glipr1	0.41748387	-0.9652955	0.15406032	0.69885543	0.76241441
Fuz	0.22178986	0.47719722	0.15403127	0.69888232	0.76241441
Tada2a	0.07576163	3.75529382	0.15386898	0.69903267	0.76247476
Ctla2a	-0.1528037	1.86945644	0.15384924	0.69905097	0.76247476
Srp9	-0.0654172	6.79681905	0.15363642	0.69924827	0.76258909
Gm16973	-0.1002485	2.88073708	0.15359595	0.69928582	0.76258909
Dpagt1	-0.1066204	3.3175558	0.15354507	0.69933302	0.76258909

Gprc5b	-0.073932	4.83589244	0.15332456	0.69953769	0.76258909
4930579G18	0.28719452	0.33076995	0.15331274	0.69954867	0.76258909
E030024N20	-0.0541233	5.67388708	0.15327349	0.69958512	0.76258909
Cbx6	0.05396291	6.62613416	0.15320351	0.69965013	0.76258909
DQ267102	-0.3589157	-0.3130751	0.15319991	0.69965346	0.76258909
Gatad2a	0.06576906	5.71910595	0.15318661	0.69966583	0.76258909
Smg6	0.05498908	5.6233263	0.15292859	0.69990566	0.7627887
Bsdc1	0.06047299	5.28190997	0.15273735	0.70008357	0.76285699
Slc30a5	-0.067344	3.98366903	0.15271458	0.70010477	0.76285699
Topors	0.05028685	6.03630215	0.15267845	0.70013839	0.76285699
Igip	-0.0588941	5.87735053	0.15257033	0.70023906	0.7628584
Arfgef1	0.05597019	7.73294247	0.15248185	0.70032147	0.7628584
Gm14322	0.06951081	4.29984094	0.15247201	0.70033063	0.7628584
4933424G06	-0.2713451	0.47933401	0.15243357	0.70036645	0.7628584
L2hgdh	0.04633078	6.11921602	0.15230067	0.7004903	0.76293155
Zfp870	0.0834363	3.74149517	0.15212369	0.70065534	0.76303565
Serpina3g	-0.3710963	-0.4356293	0.15207658	0.70069929	0.76303565
Athl1	0.09582576	3.28005213	0.15192419	0.70084151	0.76312876
Slc9a5	-0.1187684	2.60183647	0.15177802	0.700978	0.7631691
Paqr4	-0.0936331	3.91087184	0.1515586	0.70118304	0.7631691
1110051M2C	0.07191387	4.1589489	0.15154214	0.70119843	0.7631691
4833424O15	-0.0686776	4.66209956	0.15145012	0.70128447	0.7631691
Fbxw11	-0.0492868	7.36807432	0.15144338	0.70129078	0.7631691
Fancm	0.07776277	4.04748168	0.1514392	0.70129468	0.7631691
Ncoa3	0.05736443	6.64765796	0.15139911	0.70133219	0.7631691
Bmp2k	0.06103984	5.06824754	0.15139902	0.70133227	0.7631691
Dcxr	-0.2524721	0.43505574	0.15132633	0.70140028	0.76318138
Baz1b	-0.045974	7.83348427	0.15124225	0.70147896	0.76320528
Sft2d2	0.07872426	6.40335609	0.15098527	0.7017196	0.7633011
Srsf2	0.04954934	8.34931977	0.15098515	0.70171971	0.7633011
Id1	-0.1284955	3.82219196	0.15089348	0.70180561	0.7633011
Ccdc23	0.12980473	3.16987757	0.15086316	0.70183403	0.7633011
Suds3	0.07010364	5.04632402	0.15076948	0.70192185	0.7633011
Flywch2	-0.1397546	1.0455833	0.15073739	0.70195195	0.7633011
Cxcr4	0.42260252	-1.3127137	0.15061124	0.70207027	0.7633011
Runx3	0.18358067	0.86058091	0.15055163	0.70212621	0.7633011
Leng1	0.11239976	4.27470386	0.15054728	0.7021303	0.7633011
St6gal1	0.08315182	6.19199686	0.15051875	0.70215707	0.7633011
Dynlt3	0.05816772	9.11236474	0.15043901	0.70223193	0.7633011
Kin	0.0750803	4.30831348	0.15042218	0.70224772	0.7633011
Mmrn2	0.33748515	-0.3197743	0.15016613	0.70248825	0.76348603
Eid2	-0.056821	5.24405361	0.15012029	0.70253134	0.76348603
Sumo3	-0.073645	6.45768741	0.14997047	0.70267221	0.76357746

Selk	0.06821681	6.15916449	0.14980273	0.70283003	0.76368729
Tle4	-0.0615271	6.40043195	0.14965902	0.70296532	0.76371821
Ndufs7	0.06989857	5.10040801	0.14965193	0.702972	0.76371821
Pank1	-0.0523577	6.29469563	0.14953899	0.70307838	0.76377212
Ppp1r18	0.10882501	3.13725075	0.14942251	0.70318814	0.76382969
Hps4	-0.1165361	2.44925576	0.14925488	0.70334619	0.7639261
Sos1	0.04738418	7.58363665	0.14911991	0.70347352	0.7639261
Cct6b	0.54477042	-1.4905165	0.14909143	0.7035004	0.7639261
Ppcdc	-0.1076776	2.87627421	0.14908763	0.70350398	0.7639261
Ppt1	-0.0521118	6.44486495	0.148963	0.70362163	0.76399221
Taok3	-0.0581721	5.86871677	0.14877046	0.7038035	0.76412803
Cnpy4	-0.065221	4.55218705	0.14850059	0.70405865	0.76428518
Tceal3	-0.0574521	5.14347973	0.14840244	0.7041515	0.76428518
Galnt9	0.06975006	4.68290137	0.1483928	0.70416063	0.76428518
Gm15421	-0.1165291	1.85128343	0.14833716	0.70421328	0.76428518
Med22	0.07776533	3.78866602	0.14826394	0.7042826	0.76428518
Slfn1	-0.3553691	-1.0410111	0.14811042	0.70442799	0.76428518
Zkscan5	-0.1134018	3.89448709	0.1478661	0.70465957	0.76428518
Zxdc	0.05654427	5.4891973	0.14777947	0.70474173	0.76428518
Gm5512	0.12061754	1.38961734	0.14773207	0.70478669	0.76428518
Pex6	-0.0929612	3.79924889	0.14767749	0.70483849	0.76428518
Trim26	-0.0662536	4.99294878	0.14767421	0.7048416	0.76428518
Ccdc108	-0.1770585	1.32172893	0.14766894	0.7048466	0.76428518
Pgls	-0.1505666	1.35967136	0.14765447	0.70486032	0.76428518
Fxr1	0.04762969	6.88681768	0.147598	0.70491392	0.76428518
6430550D23I	-0.3043057	-0.2776649	0.14757974	0.70493126	0.76428518
Snn	-0.0465096	6.63171243	0.14757629	0.70493454	0.76428518
Hepacam	-0.0887604	4.23256353	0.14752851	0.7049799	0.76428518
Dcun1d4	0.05198775	7.403709	0.14749193	0.70501464	0.76428518
Gm19990	-0.357702	-0.756335	0.14747848	0.70502741	0.76428518
Casz1	0.1570806	2.11849461	0.14737751	0.70512332	0.76432758
Tpcn1	0.09707685	5.97215689	0.14712858	0.70535994	0.76446771
Plcb2	-0.1592668	1.39988177	0.14707382	0.70541201	0.76446771
Shroom3	0.09099434	2.89460344	0.14706224	0.70542303	0.76446771
Pcid2	0.09667372	4.26634617	0.14668029	0.70578663	0.76478681
1600020E01I	-0.2039628	1.1376363	0.14663356	0.70583115	0.76478681
Mesp2	0.22308625	1.96370812	0.14657271	0.70588914	0.76478806
Tfip11	0.09672528	3.79497696	0.14645072	0.70600544	0.76485249
Cops7a	0.06261261	6.91236937	0.14631111	0.7061386	0.76493516
Zdhhc7	0.1032349	3.13165854	0.14610279	0.70633743	0.7649689
H3f3a	0.08737451	8.1858611	0.14607619	0.70636283	0.7649689
Map2	-0.0760116	8.3176101	0.14604548	0.70639216	0.7649689
Pth2r	-0.4527327	-1.3312473	0.14604026	0.70639714	0.7649689

Csnk1g1	-0.0464537	6.24794993	0.14591262	0.70651908	0.7650312
Setd1a	0.06356614	5.5857919	0.14578554	0.70664054	0.7650312
Gm4477	-0.2617772	-0.8045183	0.14571152	0.70671132	0.7650312
Strip1	0.06176629	4.37981101	0.14569349	0.70672857	0.7650312
Ctnnbl1	0.07982701	3.28848364	0.14568265	0.70673893	0.7650312
Gm1653	-0.385395	0.50863292	0.14555931	0.70685693	0.76504196
Prss48	-0.3896647	-0.1717178	0.1455534	0.70686258	0.76504196
Mum1l1	0.2095282	1.70224745	0.14541968	0.70699057	0.76511895
Artn	0.49225846	-2.0795552	0.14532288	0.70708327	0.76515772
Stap2	-0.1708286	1.64210429	0.14523691	0.70716562	0.76518531
Foxn3	-0.0613396	6.54156047	0.14510163	0.70729528	0.76522006
Grpel2	-0.072472	4.21739814	0.14508473	0.70731148	0.76522006
Pip4k2c	0.05639314	6.1114067	0.14492457	0.70746507	0.76526913
Heatr3	0.06721824	4.64907755	0.14491884	0.70747057	0.76526913
Abcg3	-0.5660367	-1.7768559	0.14477765	0.70760605	0.76535416
3110057O12	-0.0817662	3.00491508	0.14464938	0.70772921	0.76542584
Pacrgl	-0.1011515	3.30867818	0.14427536	0.70808866	0.76575305
Ralbp1	0.04581634	6.77749448	0.14407799	0.70827857	0.76589687
Lax1	0.3780144	-0.2643545	0.14383146	0.70851598	0.76609203
4930447A16	0.51858946	-1.7228355	0.14376079	0.70858408	0.76610411
Tbr1	-0.0678751	6.77187326	0.14361926	0.70872052	0.76619006
Ino80e	-0.12245	2.32870918	0.14348784	0.70884728	0.76626554
Eef1a1	0.06158243	10.3156722	0.14325498	0.70907206	0.76641618
Adam32	-0.3055517	-0.4974968	0.14318091	0.7091436	0.76641618
Gm14288	0.14191783	0.84512697	0.14303111	0.70928835	0.76641618
Trappc1	-0.0637359	5.99795277	0.14302027	0.70929883	0.76641618
Mob2	-0.1192671	2.48552716	0.1430119	0.70930691	0.76641618
Cotl1	0.08206265	3.14023198	0.1429897	0.70932837	0.76641618
Gm11974	-0.1887161	0.35866908	0.14289937	0.70941572	0.76644902
Zfp185	-0.1212055	3.37363519	0.14266996	0.70963768	0.76662727
Fancg	0.10727393	2.75922905	0.14257916	0.7097256	0.7666607
Pop7	0.09345771	2.62452614	0.14242539	0.70987454	0.76676004
Phkg1	-0.1390371	1.630539	0.14199703	0.71028996	0.76701171
Shank2	-0.0966846	6.98539914	0.14193294	0.71035218	0.76701171
Arf3	-0.0576394	10.0859814	0.14185293	0.71042987	0.76701171
Ksr1	-0.0749268	3.86342717	0.14182855	0.71045355	0.76701171
Zfp664	-0.0537305	7.69577042	0.14181259	0.71046905	0.76701171
Sp100	0.08159701	5.07670591	0.1417941	0.71048701	0.76701171
Ablim2	0.06213022	6.07377197	0.14167716	0.71060063	0.76701171
Gng7	0.07136752	5.43551438	0.1416394	0.71063734	0.76701171
Fam49a	-0.0482932	8.23706648	0.1416162	0.71065989	0.76701171
Wdr33	-0.0681039	5.10123031	0.14159804	0.71067754	0.76701171
Yipf6	0.04532569	7.26621431	0.14153063	0.71074308	0.76702093

Pear1	0.12035573	3.42299803	0.14131203	0.71095576	0.76718891
Tmem184b	-0.0571865	4.72598387	0.14115087	0.71111267	0.76729671
Nsmce1	0.13923332	3.55079185	0.14089024	0.71136665	0.76743181
Slc10a3	-0.124995	2.47682987	0.14087918	0.71137743	0.76743181
Gle1	-0.0572683	4.91011353	0.14065975	0.71159149	0.76743181
Znrf3	0.04843772	5.9800087	0.14063246	0.71161811	0.76743181
Nle1	0.22500046	-0.0880185	0.14062904	0.71162145	0.76743181
A430035B10	-0.0944832	2.99546973	0.14061442	0.71163573	0.76743181
Fgf10	-0.1826073	2.46889666	0.14061301	0.71163711	0.76743181
Gm13152	-0.1764966	1.28296953	0.14050936	0.7117383	0.76745835
2010005H15	0.46380237	-1.5641879	0.14047098	0.71177578	0.76745835
Fsd1l	-0.0676258	6.17601251	0.14034433	0.7118995	0.76753025
Gm6498	-0.2842675	-1.0240939	0.14019329	0.71204714	0.7676108
Kansl1l	0.05680213	5.50091386	0.14015119	0.71208831	0.7676108
Zfp184	-0.1024949	2.16563795	0.13981626	0.71241607	0.76781974
Tubgcp5	-0.0916031	4.13761734	0.13976027	0.71247091	0.76781974
Cenpb	0.08524964	4.12701979	0.13975172	0.71247928	0.76781974
Csf2rb2	-0.2910755	-0.1943771	0.13971998	0.71251037	0.76781974
Tram1	-0.0707731	5.97538072	0.13942994	0.71279468	0.76784214
Rpl17	-0.0614653	6.86589986	0.1393971	0.7128269	0.76784214
Mrps14	0.06288037	5.65995833	0.13932795	0.71289474	0.76784214
Cbfa2t2	-0.0407454	6.40881928	0.13918506	0.71303499	0.76784214
Itgav	-0.050903	5.95813429	0.13918273	0.71303728	0.76784214
Cxcr5	0.22030293	-0.2027588	0.13917798	0.71304194	0.76784214
Gstcd	0.14684766	1.73762496	0.13904567	0.71317189	0.76784214
Angel2	0.05140789	5.77778424	0.1390112	0.71320575	0.76784214
Ppp4c	-0.1363588	2.05232108	0.13899479	0.71322188	0.76784214
Bnip3l	0.0534887	8.24542799	0.13893648	0.71327918	0.76784214
Taok2	0.05940093	5.310984	0.13891327	0.71330199	0.76784214
Aqr	0.07200979	5.22653794	0.13881037	0.71340316	0.76784214
Cnnm1	-0.0553715	6.37053827	0.13865466	0.71355632	0.76784214
Ddx58	0.07908243	5.01473816	0.1386035	0.71360666	0.76784214
Rps3a1	-0.0648765	7.93110802	0.13859594	0.71361411	0.76784214
Trmt1l	-0.0523603	4.88441546	0.13851778	0.71369104	0.76784214
Mxra8	0.09171553	5.68300485	0.13847232	0.7137358	0.76784214
Snhg6	0.13706369	1.68394643	0.1383393	0.71386683	0.76784214
Aagab	0.05892128	4.71518943	0.13828401	0.71392131	0.76784214
Zcchc3	-0.0766036	5.00157565	0.13822602	0.71397846	0.76784214
Hmgxb3	-0.0815434	3.94378601	0.13822269	0.71398175	0.76784214
Adam21	0.20397739	0.29743149	0.13805185	0.71415021	0.76784214
Orc2	0.05306882	5.78244805	0.13799809	0.71420324	0.76784214
Trip11	0.04144414	7.25368334	0.1377714	0.71442701	0.76784214
D6Wsu163e	0.06496279	4.54406392	0.13771903	0.71447873	0.76784214

Eda	0.1223809	2.48147763	0.13769465	0.71450281	0.76784214
D330050G23	0.15960314	1.95972551	0.13760872	0.71458772	0.76784214
Zranb1	-0.0537109	4.76917413	0.13755332	0.71464248	0.76784214
Zfp568	-0.08335	3.27424501	0.13751239	0.71468294	0.76784214
Rnf207	0.25044105	0.9727215	0.13746849	0.71472634	0.76784214
Kctd18	-0.0602578	4.36837927	0.13746807	0.71472676	0.76784214
Lpin1	0.06732907	4.6454142	0.13744624	0.71474835	0.76784214
Ndufaf2	-0.0839561	4.81039689	0.13740811	0.71478605	0.76784214
Psmd9	0.07339138	4.39542341	0.13738978	0.71480418	0.76784214
Atp5j2	0.06774837	5.43706898	0.13735389	0.71483968	0.76784214
Usp37	0.06633488	5.63517387	0.13725094	0.71494155	0.76784214
Bri3bp	-0.0595454	5.72851111	0.13721749	0.71497466	0.76784214
Plekhh2	-0.0573968	5.65818336	0.1372028	0.71498921	0.76784214
Cdh22	-0.1628327	1.76906801	0.13711439	0.71507673	0.76784214
Rad51d	-0.0678926	5.28271466	0.13700085	0.7151892	0.76784214
Tmem41b	-0.0554043	4.8261981	0.13695202	0.71523758	0.76784214
Mtrr	-0.0718653	4.01069066	0.13693357	0.71525587	0.76784214
Sos2	-0.0478847	7.66871831	0.13691344	0.71527581	0.76784214
Sash3	-0.1817537	1.90364415	0.13689762	0.7152915	0.76784214
Msantd3	0.10756006	2.9884941	0.13686309	0.71532573	0.76784214
Cecr5	0.18710375	0.6165469	0.13680639	0.71538194	0.76784214
Gm11992	0.28042532	-0.7542537	0.13666186	0.71552529	0.76784214
Fos	-0.2406812	3.49210614	0.13666035	0.71552679	0.76784214
Atg2b	-0.076671	6.04259617	0.13663799	0.71554897	0.76784214
Man2b2	0.10073053	3.67084702	0.13663552	0.71555143	0.76784214
Pigb	-0.1125778	2.54584533	0.13657752	0.71560898	0.76784214
Ccdc39	-0.0869869	4.3535668	0.13656338	0.71562301	0.76784214
Gtpbp2	-0.0739768	4.27138484	0.13654024	0.71564598	0.76784214
Rictor	-0.0769799	6.50234641	0.13653182	0.71565434	0.76784214
Sugct	-0.14188	1.49156978	0.13645929	0.71572636	0.76784214
Prss54	-0.4851865	-1.2922153	0.13645902	0.71572662	0.76784214
Ighmbp2	-0.1306346	1.95229387	0.13631796	0.71586674	0.7678818
Slfn10-ps	0.32119853	-0.7631627	0.1363069	0.71587772	0.7678818
Tmem236	-0.4912156	-1.7102304	0.13605675	0.71612641	0.76792506
Dck	-0.0739388	4.99238985	0.13605507	0.71612808	0.76792506
Papolg	0.04363128	5.32489383	0.136044	0.71613909	0.76792506
Esyt2	0.04473091	5.89773321	0.13603673	0.71614632	0.76792506
Usp22	-0.0452932	7.05458543	0.1359271	0.7162554	0.76794389
Cplx2	-0.0595666	9.63292106	0.13578334	0.71639852	0.76794389
Mrpl50	0.06971933	5.7014081	0.13578009	0.71640176	0.76794389
Psd4	0.23988628	0.02147733	0.13571924	0.71646235	0.76794389
B230319C09	-0.3613783	-1.0365313	0.13571805	0.71646354	0.76794389
Casp12	0.11160824	4.19056482	0.13567513	0.7165063	0.76794389

Prdm9	0.13370876	1.62547008	0.13557452	0.71660655	0.76795909
Zc3h8	0.09303034	2.7597881	0.13554636	0.71663462	0.76795909
Zfp9	0.05186368	5.92135413	0.13541475	0.71676584	0.7679832
Abcd1	0.10545191	3.7141478	0.13533674	0.71684367	0.7679832
Aebp2	-0.0423457	6.84929857	0.13533102	0.71684936	0.7679832
Csnk1g2	-0.0724197	5.59108536	0.13518518	0.71699492	0.7679832
Ccny	-0.0465361	8.53740711	0.1351528	0.71702725	0.7679832
Sec14l2	0.10991925	2.46746972	0.13515126	0.71702878	0.7679832
Clptm1	-0.053169	6.04131309	0.13510972	0.71707026	0.7679832
Dazap2	0.05456705	8.82880673	0.13506623	0.7171137	0.7679832
Dync1i1	0.07833416	5.12427395	0.13490515	0.71727464	0.76809443
Vwa8	0.06600185	5.28914095	0.1346874	0.71749239	0.76824566
Map7d1	0.05611304	6.98034278	0.13464976	0.71753005	0.76824566
Ntsr1	0.15478561	1.74796493	0.13446677	0.71771321	0.76834374
Otud1	0.06481785	6.12233648	0.13438001	0.7178001	0.76834374
Map9	-0.0581081	7.90118263	0.13436685	0.71781329	0.76834374
Ttc32	-0.1162688	2.57335124	0.13432377	0.71785645	0.76834374
Hist1h1b	-0.3788612	-1.8051552	0.13424006	0.71794033	0.76834374
Prkar2a	-0.0479155	6.40143471	0.13416579	0.71801479	0.76834374
Bst2	-0.2179	1.61087395	0.13411047	0.71807027	0.76834374
Tcaim	0.07116759	4.43598128	0.13410231	0.71807845	0.76834374
Snord19	-0.407159	-1.6782624	0.1347102	0.71815023	0.76835945
C1qtnf4	-0.1167432	2.03375182	0.13378587	0.71839602	0.76854015
Mmp11	0.22347573	1.13783293	0.13371223	0.71846998	0.76854015
Epha7	-0.0636582	6.56712493	0.13369185	0.71849046	0.76854015
Oxct1	-0.0440453	8.33302897	0.13355654	0.71862644	0.76854948
Dusp11	0.04727212	6.38920136	0.13355375	0.71862925	0.76854948
Cacna1b	-0.0720133	6.84283263	0.13351269	0.71867053	0.76854948
Sh3bp2	0.15217914	1.47945493	0.13322583	0.71895912	0.76856968
Gpr35	0.27061502	0.11747477	0.13318167	0.71900357	0.76856968
2310030G06	0.12852908	2.80094313	0.13315523	0.7190302	0.76856968
Ptger1	0.07847899	3.11132064	0.13315171	0.71903375	0.76856968
Gimap6	0.17714234	1.78011228	0.13312215	0.71906352	0.76856968
Dtx2	-0.1337234	1.76024627	0.13310819	0.71907758	0.76856968
Mri1	-0.1417809	1.14525126	0.13308683	0.71909908	0.76856968
Trim32	0.0521887	7.59726018	0.13303992	0.71914634	0.76856968
Cnot6	0.05665645	6.42197688	0.13295057	0.71923638	0.76860486
Tmem179	-0.0765685	3.41467723	0.13281054	0.71937755	0.76865652
Tmem170	-0.0935976	3.25063668	0.1327668	0.71942166	0.76865652
1810062G17	0.49610728	-1.2913555	0.132685	0.71950418	0.76865652
Ift80	-0.0519268	4.88958403	0.13265226	0.71953722	0.76865652
Nphp4	-0.1208639	2.08215996	0.13261529	0.71957453	0.76865652
Ctnna2	-0.0496364	6.28819644	0.13256286	0.71962745	0.76865652

Rnaseh2c	-0.1398007	2.73847874	0.13239677	0.71979518	0.76870921
Gbp5	0.17008955	2.13971357	0.13237216	0.71982005	0.76870921
Ddx5	0.0458034	9.43719994	0.13232365	0.71986907	0.76870921
Lpcat4	0.0609606	5.66208883	0.13228781	0.71990528	0.76870921
Etfdh	0.06254246	5.16765696	0.13215907	0.72003544	0.76877861
Ino80c	0.06844876	5.69305021	0.13210565	0.72008947	0.76877861
Ang	-0.1756147	2.28939294	0.13205404	0.72014168	0.76877861
Secisbp2	0.07396432	3.83289147	0.13194185	0.72025522	0.7688055
Acot5	-0.3399427	0.37298916	0.13191625	0.72028113	0.7688055
Lrfn5	0.06913242	5.23599728	0.1317846	0.72041444	0.7688868
Aggf1	-0.0481557	6.57041674	0.13165482	0.72054594	0.76889406
Slc2a13	-0.0522777	6.84437649	0.13160269	0.72059878	0.76889406
Ank2	0.06201533	10.1838387	0.13155854	0.72064353	0.76889406
Exoc5	-0.0437809	6.2944493	0.13147264	0.72073064	0.76889406
Clip3	0.05809876	10.1860474	0.13146972	0.72073361	0.76889406
Kcnk9	-0.1235532	2.87097366	0.13127445	0.72093175	0.76889406
Pcdhb17	-0.0734166	4.75954171	0.13120328	0.72100401	0.76889406
Rpn1	0.05963856	5.65877509	0.13112826	0.7210802	0.76889406
Slc45a4	-0.0751779	3.61257026	0.13108828	0.72112081	0.76889406
Hsd3b1	-0.8451694	-1.4652277	0.13101707	0.72119318	0.76889406
Klhl8	-0.0700747	4.31491677	0.13089484	0.72131742	0.76889406
Msrb1	-0.0731414	4.22744188	0.13083553	0.72137774	0.76889406
Wdr82	-0.0415584	6.39638875	0.13083101	0.72138233	0.76889406
Zfp54	0.18833472	1.59679142	0.13077923	0.72143501	0.76889406
Hmgb1	0.05399386	8.09298652	0.13068698	0.72152888	0.76889406
Myo18b	0.24855397	-0.6149514	0.13061477	0.72160238	0.76889406
Nfe2l3	0.10639855	2.48759013	0.1305497	0.72166864	0.76889406
Plxdc1	-0.0780894	2.91556988	0.13053915	0.72167938	0.76889406
D130020L05I	-0.0963144	2.63523436	0.13050033	0.72171892	0.76889406
Prelid2	-0.3428635	-0.7033293	0.13047704	0.72174264	0.76889406
Chd4	-0.0473255	7.44856066	0.13045302	0.72176711	0.76889406
2610005L07I	-0.0625014	6.35267353	0.13038012	0.72184139	0.76889406
D3Ertd751e	0.05682774	5.72744736	0.13029911	0.72192396	0.76889406
Wbp1	-0.0878663	3.50318168	0.13014941	0.72207663	0.76889406
Znhit3	0.07849536	3.91894958	0.13014063	0.72208558	0.76889406
Mov10	-0.0994391	2.92325618	0.13012912	0.72209732	0.76889406
Arfgap3	0.07604183	4.63360754	0.13007334	0.72215424	0.76889406
Gpr132	0.36875111	-1.4548746	0.13002837	0.72220013	0.76889406
Ptrhd1	-0.0733593	3.71099187	0.13001695	0.72221179	0.76889406
Fhl4	-0.210228	0.87634716	0.12998572	0.72224367	0.76889406
Srrm3	0.09341755	2.95902668	0.1299835	0.72224594	0.76889406
Arpc4	-0.0869901	5.34592983	0.12997979	0.72224972	0.76889406
Stau1	0.04372038	6.3092757	0.12988974	0.72234167	0.76893111

Xlr3c	0.31312442	-1.4935788	0.12958301	0.72265514	0.76909529
Zfp367	0.08213851	3.52014188	0.1295486	0.72269033	0.76909529
Atp6v1d	0.03714747	8.33107629	0.12953034	0.72270901	0.76909529
Btf3	0.06891596	6.23544685	0.12943501	0.72280654	0.76909529
Chpf	0.08313594	3.44592117	0.12942516	0.72281662	0.76909529
Zfp65	0.06947363	5.05369381	0.12937644	0.72286649	0.76909529
1700123L14F	-0.2887676	0.3657633	0.12930771	0.72293684	0.76909529
Iqcg	-0.0868515	2.95988689	0.12918733	0.72306013	0.76909529
Ndufb8	-0.0621636	4.70216124	0.1291506	0.72309777	0.76909529
Cebpzos	-0.0798337	2.99218171	0.12910058	0.72314902	0.76909529
Ufm1	0.04959834	6.00772638	0.12907784	0.72317232	0.76909529
D1Ertd622e	-0.0455856	5.78632366	0.12903793	0.72321323	0.76909529
Cabp4	0.43988622	-0.8660315	0.12901288	0.72323892	0.76909529
Dennd2d	-0.3011848	-0.2058797	0.12881234	0.7234446	0.7692006
0610009B22I	-0.0792278	5.56179021	0.12880308	0.7234541	0.7692006
Fbxl12os	-0.1372681	2.29522123	0.12872385	0.72353542	0.7692006
Lrrc51	-0.1452266	2.09673844	0.12869348	0.7235666	0.7692006
Wdr8	-0.1157006	2.26818252	0.12839549	0.72387273	0.76933046
E130310I04R	-0.608772	-1.5761338	0.12832108	0.72394924	0.76933046
Spc25	-0.155853	1.57807401	0.12831526	0.72395522	0.76933046
Ep300	0.04899181	8.10979494	0.12824054	0.72403208	0.76933046
Nkx2-2os	0.44300057	-0.6508866	0.12820043	0.72407335	0.76933046
Trmt12	-0.0847649	3.27438155	0.12818765	0.72408649	0.76933046
Fam96b	-0.0916163	2.67795125	0.12818525	0.72408896	0.76933046
Tmem202	-0.3117003	0.48555398	0.12812373	0.72415227	0.76933698
Cdpf1	0.11281713	3.2397897	0.12806535	0.72421237	0.76934009
Ppp3cb	-0.0399441	8.65277141	0.12792365	0.72435829	0.76943436
Ankrd52	0.05859045	5.89362456	0.12776498	0.72452181	0.7695089
Nckap5l	-0.1492601	1.45412957	0.12774458	0.72454284	0.7695089
Cbll1	-0.0556234	6.17443958	0.12737035	0.72492898	0.76970519
Fnip2	-0.0621116	4.74590785	0.12731185	0.7249894	0.76970519
D930015M05	-0.3313131	0.26353852	0.12724	0.72506363	0.76970519
Dhx30	0.09175369	4.70608379	0.12723392	0.72506991	0.76970519
Med9	-0.0718946	4.76136036	0.12714708	0.72515967	0.76970519
Apbb1ip	0.0866121	3.19513467	0.12700439	0.72530722	0.76970519
Zscan2	0.16235538	1.3090685	0.12696166	0.72535142	0.76970519
Arhgap5	0.04680257	8.64702615	0.12688885	0.72542676	0.76970519
Olfr239	-0.4414997	-1.2311747	0.12688366	0.72543212	0.76970519
Adsl	-0.0744046	4.44688596	0.12685571	0.72546106	0.76970519
Morc4	-0.0849633	2.89235091	0.12675755	0.72556268	0.76970519
B4galt7	-0.1716077	1.05384379	0.12672263	0.72559884	0.76970519
Btbd1	-0.0420989	7.23030801	0.12664067	0.72568374	0.76970519
Ccdc132	0.05150034	6.47996057	0.12661859	0.72570662	0.76970519

Cul4a	0.04015569	7.55684241	0.12656545	0.72576169	0.76970519
Pgm2l1	0.06886819	10.1785393	0.12645345	0.7258778	0.76970519
Ly86	-0.132538	2.08681599	0.12633361	0.72600209	0.76970519
1700052N19	-0.0650042	3.84405497	0.12619487	0.72614608	0.76970519
Cdip1	0.04052607	6.7745802	0.12619174	0.72614932	0.76970519
Mical2	0.04990397	8.91771607	0.12618965	0.7261515	0.76970519
Tmem53	0.18044217	1.62310313	0.12618496	0.72615637	0.76970519
Arl6ip1	-0.0394471	7.00630343	0.12610713	0.72623717	0.76970519
Stk36	0.15203547	1.23416764	0.12606199	0.72628406	0.76970519
Ppdpf	0.0592938	5.09338992	0.12606162	0.72628444	0.76970519
Hlf	0.05019154	10.0220648	0.1260486	0.72629797	0.76970519
Sacs	-0.1480952	3.7593415	0.12603056	0.72631671	0.76970519
Rps5	-0.0632165	5.76664419	0.12602966	0.72631764	0.76970519
Coro2a	-0.0768238	5.32555707	0.12601129	0.72633674	0.76970519
Arap1	-0.0840147	3.70806444	0.12591148	0.72644045	0.76970519
Pet100	0.10828002	3.54535669	0.12590839	0.72644367	0.76970519
Paf1	0.05250362	5.95108344	0.12564647	0.72671609	0.7698114
Hcfc1r1	-0.0676888	4.47149002	0.12551763	0.72685021	0.7698114
Ccdc92	0.04684743	6.70486455	0.12550725	0.72686101	0.7698114
Sae1	0.0509762	5.57085668	0.12544731	0.72692344	0.7698114
Fermt3	0.2117127	0.28907828	0.12539384	0.72697914	0.7698114
Rnf141	-0.0679073	4.23117867	0.12538363	0.72698978	0.7698114
Wdyhv1	-0.0667675	4.05496611	0.12534911	0.72702575	0.7698114
Ctnnal1	-0.0603428	4.46199785	0.12534024	0.72703499	0.7698114
Synj1	-0.0800526	9.53680777	0.12531742	0.72705878	0.7698114
Cdc73	0.04832683	6.15338114	0.12516935	0.72721315	0.76981776
Gnaz	0.05475575	5.82216852	0.12515062	0.72723268	0.76981776
Amigo2	0.11794485	2.41715299	0.12503831	0.72734985	0.76981776
9330020H09	-0.2819973	-1.0428225	0.12491751	0.72747596	0.76981776
Gins1	0.25790708	0.15266599	0.12484957	0.7275469	0.76981776
Eda2r	-0.3285729	0.18129697	0.12484636	0.72755026	0.76981776
Ikbip	0.09455588	4.11084961	0.12479869	0.72760005	0.76981776
Cyp2e1	0.13444271	1.04891713	0.12475754	0.72764304	0.76981776
Ipo4	-0.0620836	4.14921411	0.12467722	0.72772698	0.76981776
Creld1	-0.0872725	3.59084989	0.12466287	0.72774198	0.76981776
Hist1h2ai	0.29507045	-1.5684036	0.12460602	0.72780142	0.76981776
Pla2g7	0.12475507	3.67048988	0.12459953	0.7278082	0.76981776
Twf1	0.05442201	7.41799396	0.1245671	0.72784211	0.76981776
Napa	-0.049582	6.07574235	0.12449194	0.72792072	0.76981776
Rpl14	-0.0784365	6.55012026	0.12445786	0.72795638	0.76981776
Mmp23	-0.3257649	-0.4723101	0.12443517	0.72798012	0.76981776
Mxi1	0.0460157	6.32122359	0.12426901	0.72815406	0.76982329
C330007P06l	0.05709608	7.68773185	0.12421627	0.72820929	0.76982329

Scn4b	-0.0738208	4.78443793	0.12419492	0.72823165	0.76982329
B430010I23F	0.38552133	-0.1740072	0.12410984	0.7283208	0.76982329
Tmem37	-0.2528712	0.73722853	0.12402519	0.72840952	0.76982329
Lrig3	-0.1566435	1.08464464	0.1238694	0.7285729	0.76982329
Sh3kbp1	0.04850585	6.38425475	0.12385634	0.72858661	0.76982329
Rad18	0.08693137	4.2203029	0.12382164	0.72862301	0.76982329
Cby1	-0.1222642	3.21791179	0.12375417	0.72869382	0.76982329
Tlr6	-0.5046676	-1.3341172	0.12374431	0.72870417	0.76982329
Polq	-0.1446953	2.63055267	0.12374352	0.728705	0.76982329
Plod2	-0.0995133	3.8469129	0.1236799	0.72877179	0.76982329
Nrcam	0.05984088	7.77680341	0.12366858	0.72878368	0.76982329
Gclc	-0.0465876	6.26031202	0.12365655	0.72879631	0.76982329
C77370	-0.0913207	6.42862139	0.12357261	0.72888448	0.76982329
Dtx1	0.06313321	4.62920153	0.12352646	0.72893297	0.76982329
Ankrd24	-0.1000766	2.49941167	0.12349284	0.72896829	0.76982329
BC028528	0.13406748	1.6431558	0.12343991	0.72902393	0.76982329
Sirt4	0.13163018	1.73272007	0.12339387	0.72907233	0.76982329
Cdc42ep4	-0.0897725	6.00259451	0.12328357	0.72918832	0.76988536
Map1lc3a	-0.07165	4.80328015	0.12305211	0.72943193	0.76996139
Rad21	-0.0402299	7.13610302	0.12299523	0.72949183	0.76996139
Prrg2	-0.251607	-0.1605994	0.12297864	0.7295093	0.76996139
C920009B18I	0.17084805	0.99448876	0.12294477	0.72954499	0.76996139
Tpd52l1	0.07147706	5.00453111	0.1229434	0.72954643	0.76996139
Xrcc1	0.085078	2.99943505	0.12270554	0.72979716	0.77011056
Rbm4	0.23036814	-0.5383219	0.12270073	0.72980223	0.77011056
Ubr4	-0.0760944	7.17668192	0.12253663	0.72997538	0.77023287
Aass	0.22508164	0.83297659	0.12231898	0.73020523	0.77041498
Dapl1	-0.076718	5.38142757	0.12223694	0.73029193	0.77042557
Spag7	0.06548778	5.28595099	0.12218891	0.73034269	0.77042557
Dnaic1	-0.1599246	0.56456003	0.12214699	0.73038703	0.77042557
Cr1l	0.06350515	5.40021269	0.12197241	0.7305717	0.7704999
Slc1a3	-0.0592802	7.23936215	0.12197212	0.73057201	0.7704999
Nwd2	0.11442039	5.78589024	0.12180173	0.7307524	0.77062975
Rab17	0.40954928	-1.5520423	0.12173442	0.7308237	0.77064454
Qdpr	0.04504025	5.53356808	0.12167778	0.73088371	0.77064744
Camk1g	-0.0935033	4.06859878	0.1216183	0.73094676	0.77065352
Bbs2	-0.0645983	5.17881808	0.1214813	0.73109202	0.77074629
Wnk4	-0.0999858	4.36271897	0.12135977	0.73122096	0.77082184
Tgfbr3	0.07861532	6.77981759	0.12126885	0.73131747	0.77086318
Vps37c	0.09963523	3.47069224	0.12116255	0.73143036	0.77088528
Arl6ip6	-0.0636817	4.59308749	0.12114123	0.73145301	0.77088528
Cdc26	0.09333904	4.58922415	0.12105513	0.73154449	0.77089859
Trim44	-0.0341688	9.18709954	0.1209512	0.73165498	0.77089859

P2rx6	-0.179447	1.20179979	0.12082487	0.73178935	0.77089859
Sorbs2	-0.0834633	7.36528757	0.12080852	0.73180674	0.77089859
Tmem167b	-0.0727874	6.20567732	0.12065461	0.73197056	0.77089859
AI854703	-0.1030167	3.07551616	0.12057954	0.73205051	0.77089859
4930520O04	-0.3745161	-1.0839657	0.12057502	0.73205532	0.77089859
Acat3	-0.1891306	0.69937389	0.12057067	0.73205996	0.77089859
B130024G19	-0.1602475	1.1616731	0.12054511	0.73208718	0.77089859
Zfp341	-0.0969933	3.38565362	0.1205119	0.73212256	0.77089859
Abca5	-0.0660547	5.65973686	0.12049588	0.73213964	0.77089859
Mrpl49	0.07827256	4.77436411	0.12048324	0.73215311	0.77089859
Camk2b	-0.0520754	8.06092179	0.12026247	0.73238848	0.77097767
Ccni	-0.0459489	8.52286496	0.12014583	0.73251294	0.77097767
Tex40	-0.1626427	2.10217503	0.12006697	0.73259712	0.77097767
Lamc2	0.08252614	2.97337274	0.12006069	0.73260383	0.77097767
Arid4b	-0.0508628	7.72302548	0.12001915	0.73264818	0.77097767
Hist1h2bh	0.29668959	-0.7848318	0.11991156	0.73276311	0.77097767
Gabrr2	-0.4937187	-0.870399	0.11990223	0.73277308	0.77097767
Prkrip1	-0.0820252	3.70442885	0.1198999	0.73277557	0.77097767
Gm960	0.29580077	-0.5551087	0.11986796	0.7328097	0.77097767
1110059G10	0.07414968	4.20899495	0.11985292	0.73282577	0.77097767
C030034I22R	-0.1257038	1.65412909	0.11982234	0.73285845	0.77097767
Dennd2a	-0.0667017	4.23267622	0.11967985	0.73301082	0.77100206
Ms4a6c	-0.203103	0.37829737	0.11964541	0.73304766	0.77100206
Chd2	-0.0466788	6.94922404	0.11960831	0.73308736	0.77100206
Rfc5	-0.1017282	2.67648638	0.1195837	0.73311369	0.77100206
Vash2	-0.1207254	1.75752496	0.11953284	0.73316812	0.77100206
Emb	0.09548948	6.18088226	0.11944019	0.73326732	0.77103875
Clock	-0.042959	7.60340753	0.11936618	0.73334658	0.77103875
Susd2	0.06689866	5.00079841	0.11926751	0.7334523	0.77103875
Gm11149	0.31935052	0.11160232	0.11917063	0.73355616	0.77103875
Sbsn	0.12638459	1.79275705	0.1191009	0.73363093	0.77103875
Taf4b	-0.0790718	2.90213099	0.11898602	0.73375418	0.77103875
Setd6	-0.0515919	4.8372087	0.11897737	0.73376346	0.77103875
Rasgrp2	0.08269543	2.56032541	0.11896832	0.73377317	0.77103875
Kl	-0.0558346	5.31075688	0.11896742	0.73377415	0.77103875
Tmem229b	0.08710013	3.13060807	0.11891924	0.73382586	0.77103875
Dync1li2	-0.0363028	8.01244482	0.11886749	0.73388142	0.77103875
BC017158	0.08026408	3.34202633	0.11880312	0.73395054	0.77103875
Ptpro	-0.0731072	3.42849331	0.11877028	0.73398581	0.77103875
Slc19a1	0.09123212	3.08317596	0.1187174	0.73404263	0.77103875
Polrmt	0.10219578	2.23954961	0.11862063	0.73414664	0.77103875
Pigu	0.0812648	3.77721647	0.11856665	0.73420467	0.77103875
Popdc3	-0.3517544	-0.7530781	0.11855493	0.73421727	0.77103875

Brd9	-0.0413195	5.50512023	0.11850301	0.73427311	0.77103875
Rasd2	-0.0600797	4.98101848	0.11842442	0.73435766	0.77103875
Fam151b	0.13163009	1.36776781	0.11842422	0.73435788	0.77103875
Casc4	0.04686434	8.2105552	0.11815829	0.7346442	0.77103875
Ptma	-0.0675517	9.04456798	0.11815691	0.73464568	0.77103875
Cd226	0.18362325	0.44264566	0.11815629	0.73464635	0.77103875
Hhex	0.13705243	1.7525512	0.11815268	0.73465024	0.77103875
Slc2a4rg-ps	-0.1552082	1.80381614	0.11809809	0.73470906	0.77103875
Fam195b	0.09940101	2.5685986	0.11809301	0.73471453	0.77103875
E030011O05	0.29206131	-0.8651926	0.11795065	0.73486801	0.77103875
Scyl1	0.05441663	4.19231876	0.11795017	0.73486852	0.77103875
Ccdc104	0.04570123	7.65613033	0.11793731	0.7348824	0.77103875
2610301B20	-0.0485899	5.3130088	0.11785832	0.7349676	0.77103875
Taf1d	-0.0639518	3.99055053	0.11784748	0.7349793	0.77103875
Col10a1	-0.2835664	-0.5031777	0.11752805	0.7353242	0.77134045
Rpl36	-0.0895705	5.25284376	0.11718186	0.73569861	0.77146558
Pfkl	0.06177965	4.18051187	0.1171577	0.73572477	0.77146558
Zswim1	-0.0617994	3.84826167	0.11711728	0.73576852	0.77146558
Usp34	-0.0589728	8.49626078	0.11709105	0.73579692	0.77146558
Pigv	0.09918905	2.81035164	0.11701994	0.73587394	0.77146558
Cdk13	-0.0376222	7.17706312	0.11700404	0.73589117	0.77146558
Otulin	0.08944638	3.54519405	0.11700375	0.73589148	0.77146558
Myh10	-0.0659436	8.54003851	0.11698472	0.73591209	0.77146558
BC037034	-0.0677213	3.84047215	0.116919	0.73598331	0.77146558
Abcb1a	0.08171366	5.71532328	0.11688809	0.7360168	0.77146558
Sfrp5	-0.5432886	-2.1405377	0.11656954	0.73636236	0.7716512
Rnf187	0.05089385	6.80386205	0.11645249	0.73648946	0.7716512
Slc25a29	-0.1678838	0.83597894	0.1164464	0.73649607	0.7716512
Fam185a	-0.0663086	3.69657058	0.11638661	0.73656103	0.7716512
4933400F21	-0.2156827	1.22189907	0.1163396	0.73661211	0.7716512
Pole	0.14005613	1.08411934	0.11631094	0.73664326	0.7716512
5430405H02	0.13446701	1.41100039	0.1162844	0.7366721	0.7716512
1700120C14	0.2914236	-1.1689078	0.11625481	0.73670428	0.7716512
Fbxl2	-0.0725067	4.66631756	0.11624955	0.73671	0.7716512
Nme1	0.04556136	6.28486745	0.11610464	0.7368676	0.77166587
Ccr4	-0.2200513	0.09339256	0.11608342	0.73689069	0.77166587
Ufc1	-0.0575297	5.14966962	0.11604804	0.73692919	0.77166587
Atp2a1	0.39914354	-0.7557197	0.11602176	0.73695779	0.77166587
Fasn	0.0648484	6.43402626	0.11597313	0.73701073	0.77166587
Zfp275	0.05398535	5.65090821	0.11588115	0.7371109	0.7717107
Scel	0.08298566	3.33749651	0.11580056	0.73719869	0.77174256
Wdr90	-0.2106261	1.35133883	0.11556466	0.73745587	0.77195174
Kcna1	0.05461265	7.66046982	0.11535979	0.73767947	0.77211155

Aqp11	-0.1059095	1.77804299	0.11528914	0.73775663	0.77211155
Vstm2b	0.0803594	3.40517909	0.11526712	0.73778068	0.77211155
2610524H06I	-0.0897765	2.15892431	0.11518148	0.73787425	0.77214432
Gpkow	0.04554629	6.8147221	0.11513346	0.73792675	0.77214432
Pcdh17	0.06289572	6.97977252	0.11500042	0.73807223	0.7721594
Urah	0.21860249	0.09704359	0.1149605	0.7381159	0.7721594
Llgl1	-0.0578724	3.95670421	0.11495003	0.73812735	0.7721594
Asna1	0.05902319	6.49108005	0.11490096	0.73818104	0.7721594
Mrpl47	0.05808541	3.55239049	0.11485757	0.73822854	0.7721594
Atrn	0.05271432	7.87994801	0.11469277	0.738409	0.7721594
Lypd6	-0.0625287	4.9247082	0.11466328	0.7384413	0.7721594
Crh	-0.2308912	0.14931193	0.11459567	0.7385154	0.7721594
Plin2	0.11927721	3.42219361	0.11459404	0.73851718	0.7721594
Ap1g1	-0.0527605	7.3243594	0.11450555	0.73861419	0.7721594
2610203C22I	-0.2360567	0.00997097	0.11450438	0.73861548	0.7721594
Rhbdd3	0.19309052	-0.0468391	0.11445259	0.73867227	0.7721594
Ngdn	-0.0752628	3.72143171	0.11443283	0.73869395	0.7721594
Gyltl1b	0.38919125	-1.9403739	0.11438672	0.73874452	0.7721594
Gm715	-0.1347026	1.23912547	0.11426411	0.73887909	0.77224007
Zfand4	-0.1475293	1.73462481	0.11417172	0.73898054	0.77228612
Nox4	-0.1439612	1.22891877	0.11388253	0.73929837	0.77255828
Exosc1	-0.0672595	4.68225896	0.11367456	0.73952722	0.77263856
Rfng	-0.0767475	3.51082264	0.11363148	0.73957467	0.77263856
Npas1	0.24706375	-0.7981625	0.11362524	0.73958153	0.77263856
Clec16a	0.05008818	6.3092255	0.11360405	0.73960487	0.77263856
Ssr4	0.06877505	3.42232035	0.11353039	0.73968601	0.77266333
Gm20767	-0.1568131	1.1002341	0.11346589	0.73975708	0.7726776
Tor4a	0.0794993	3.20994434	0.11335824	0.73987576	0.77274157
Pfdn5	0.08462137	5.7391927	0.11329106	0.73994984	0.77275897
Tmsb15l	0.15980968	1.11771483	0.11311618	0.74014284	0.77278717
Scaf11	-0.0327534	7.60854134	0.1130656	0.74019868	0.77278717
Erich5	0.29718843	-0.7867221	0.11305492	0.74021048	0.77278717
Arl8b	0.03582447	7.95545664	0.11303232	0.74023544	0.77278717
Cep135	0.08031345	4.32391521	0.11296465	0.74031018	0.77278717
Snrpd1	-0.0530978	5.26247952	0.11295227	0.74032386	0.77278717
Pskh1	-0.0875261	3.80027565	0.11290251	0.74037886	0.77278717
Spaca1	-0.2428458	0.59477305	0.11281595	0.74047454	0.7728271
Atr	0.06563542	4.55086409	0.11268079	0.74062403	0.77292317
Osgepl1	0.06488176	4.77018591	0.11258959	0.74072496	0.77296855
Dynlrb1	0.05378977	8.41788242	0.11235535	0.74098439	0.77317932
Mbd3	0.05528371	4.4107566	0.11211514	0.74125074	0.77334299
Gp1bb	0.1094076	1.60154465	0.11207484	0.74129545	0.77334299
Atp5l	-0.0550725	6.48418274	0.11205844	0.74131365	0.77334299

Cyth3	0.05740333	6.99938131	0.11196969	0.74141217	0.77338581
Bud31	0.05567633	4.69115838	0.11181977	0.7415787	0.77345987
Papd7	-0.0614252	4.69030791	0.1117461	0.74166057	0.77345987
Rbpms	0.0916183	5.27075179	0.111708	0.74170292	0.77345987
Gm6710	0.07875501	2.44380817	0.11169885	0.74171309	0.77345987
Cpsf6	-0.0602092	5.58266669	0.11159661	0.7418268	0.7735185
Spink8	-0.2263349	-0.1941397	0.111501	0.74193318	0.77356948
Hist1h2bb	0.26267107	-1.2061748	0.1111455	0.74232917	0.77374049
Mir378b	-0.2790412	0.21148148	0.11109364	0.742387	0.77374049
Lman1	0.07506468	5.02057679	0.1110075	0.74248308	0.77374049
Rac3	-0.2892882	-0.9383691	0.11097283	0.74252177	0.77374049
Lysmd4	0.06923683	3.30306928	0.11086367	0.74264361	0.77374049
Gls	-0.054712	9.46596455	0.11082103	0.74269122	0.77374049
Wdr35	0.0651458	4.69647187	0.11081611	0.74269671	0.77374049
Wdfy3	-0.0821502	7.86625625	0.11063824	0.74289544	0.77374049
Zfp667	-0.0758211	4.79433515	0.11062117	0.74291452	0.77374049
Pik3r4	0.05541682	5.1104775	0.11058452	0.74295549	0.77374049
Nrep	0.05236037	7.95133817	0.11053363	0.74301239	0.77374049
Ly6h	-0.1422692	0.87209353	0.11051283	0.74303566	0.77374049
Tmem51os1	-0.2487647	-0.5113356	0.11049145	0.74305958	0.77374049
Plbd2	0.0512323	5.28181595	0.11046555	0.74308855	0.77374049
Ankra2	0.06722297	3.44615038	0.11042864	0.74312984	0.77374049
Etv5	0.05674354	6.16652977	0.11038748	0.7431759	0.77374049
Rnf31	-0.0834057	3.23321975	0.11038609	0.74317746	0.77374049
Stmn4	-0.0680326	6.7672225	0.11036493	0.74320114	0.77374049
Irf2bp1	-0.0600589	3.91958908	0.11018088	0.74340724	0.77374049
Itgal	-0.1800424	0.64269499	0.1101352	0.74345842	0.77374049
Msh6	-0.0646521	4.74445881	0.11008886	0.74351036	0.77374049
Resp18	0.068158	3.1582388	0.11006098	0.74354161	0.77374049
Cftr	-0.170038	1.59461546	0.11001739	0.74359048	0.77374049
Dnajc27	0.0506319	6.17751237	0.10995282	0.7436629	0.77374049
Hk1	0.06679109	5.81242358	0.10992681	0.74369207	0.77374049
Chtf8	-0.0583754	5.11956091	0.10987711	0.74374783	0.77374049
Peli1	0.04499331	5.95279181	0.10969621	0.7439509	0.77374049
Pttg1ip	0.08306951	6.25434244	0.10958376	0.74407723	0.77374049
Alms1	-0.0693131	5.05209571	0.10955333	0.74411143	0.77374049
Cd27	0.3897111	-1.5497778	0.10949677	0.74417501	0.77374049
B430212C06	0.46241524	-1.5430371	0.10948183	0.7441918	0.77374049
Csnk1g3	-0.0346919	7.45974778	0.1094643	0.74421152	0.77374049
Slc44a2	0.05325487	5.08209519	0.10942807	0.74425225	0.77374049
Mrpl20	0.07934937	4.0552747	0.10934943	0.74434071	0.77374049
Alg3	0.0751416	3.44367456	0.10931497	0.74437948	0.77374049
Ptpn12	-0.051151	6.02226526	0.10929559	0.7444013	0.77374049

Gnat2	0.63520611	-1.6727266	0.10925046	0.74445209	0.77374049
1700030J22F	0.08431161	3.65914995	0.10919961	0.74450933	0.77374049
Bend3	-0.0760365	4.24324062	0.10916959	0.74454314	0.77374049
Hcfc1	-0.0508637	6.90610262	0.10916025	0.74455366	0.77374049
Mien1	-0.0650239	5.3825051	0.10911695	0.74460242	0.77374049
Rpl10	0.06807125	7.8633684	0.10908677	0.74463642	0.77374049
Ift22	-0.0691595	4.09852226	0.1090675	0.74465814	0.77374049
Oxr1	-0.0380915	8.62710565	0.1089906	0.74474479	0.77374049
Otub1	0.07034174	5.7233998	0.10893887	0.7448031	0.77374049
C130060K24I	0.2753931	0.00503325	0.10893643	0.74480586	0.77374049
Crybb1	-0.3781138	-1.8920045	0.10889308	0.74485474	0.77374049
Fam76a	0.04879497	5.87616487	0.10883488	0.74492038	0.77374049
Pigl	-0.0818747	4.09399198	0.10880325	0.74495606	0.77374049
Stx3	-0.0500827	4.78234578	0.10875183	0.74501409	0.77374049
Lrwd1	0.12589897	1.80651946	0.10873517	0.74503288	0.77374049
Scarf2	0.18039651	1.58556916	0.10866161	0.74511592	0.77374049
Fam168a	0.03302442	8.13100082	0.10863612	0.74514471	0.77374049
Ubl4b	0.2303023	0.06162456	0.10856751	0.74522219	0.77376124
Orc3	0.03763751	7.85503594	0.10848077	0.74532019	0.77380329
Slc5a6	-0.0757271	4.25431609	0.10841642	0.74539293	0.7738191
Glce	0.04887102	6.45973608	0.10831048	0.74551272	0.77388376
Mcu	0.07038714	4.64332139	0.10801778	0.74584403	0.77405536
Wdr54	0.08290296	3.28234784	0.10800033	0.74586379	0.77405536
Itga5	0.08119821	2.85314034	0.10794322	0.7459285	0.77405536
Dolk	-0.0950861	2.47522989	0.10790427	0.74597265	0.77405536
Myeov2	0.05675527	4.74117315	0.10782099	0.74606706	0.77405536
Vdac3	0.04286639	6.33344942	0.10770249	0.74620147	0.77405536
Megf6	0.14135796	1.38357605	0.1076504	0.74626058	0.77405536
A830019L24I	-0.3238674	-0.6944655	0.10759319	0.74632552	0.77405536
Narfl	-0.0871954	2.64718879	0.10758892	0.74633037	0.77405536
Tulp2	-0.1818626	0.43315833	0.10749821	0.74643338	0.77405536
Wscd1	-0.0670479	3.74854037	0.1074776	0.74645679	0.77405536
Wisp2	0.39934667	-1.02055	0.10745208	0.74648578	0.77405536
Chia1	0.2952953	-0.1819364	0.10737914	0.74656866	0.77405536
Eif2ak4	0.09807866	3.93746764	0.10733963	0.74661357	0.77405536
Gltscr2	0.07368636	5.54829414	0.10732435	0.74663094	0.77405536
Ssu72	0.05458514	5.2620379	0.10731249	0.74664442	0.77405536
Tmem251	0.06653807	3.7918645	0.10730237	0.74665593	0.77405536
2210013O21	-0.0634408	5.29079884	0.10710313	0.74688258	0.77413519
Angptl1	-0.2466318	0.5520539	0.10706671	0.74692403	0.77413519
Arl13b	0.05706094	4.2966268	0.10700921	0.7469895	0.77413519
Slc25a47	-0.2461472	-0.6245567	0.10698438	0.74701777	0.77413519
Pcdhb12	0.10453693	2.49227365	0.10688462	0.74713142	0.77413519

Hivep3	-0.0783216	6.88983087	0.10674821	0.7472869	0.77413519
Wdhd1	-0.0942357	2.70306363	0.10667359	0.747372	0.77413519
Rab2a	-0.035024	9.67092269	0.10666681	0.74737973	0.77413519
Dnajb3	-0.2471167	-0.1025041	0.1066501	0.74739879	0.77413519
Parp8	-0.0639219	4.80146209	0.10664032	0.74740996	0.77413519
Slc25a24	0.07444699	5.32740564	0.10663187	0.7474196	0.77413519
Dcaf6	-0.0538634	6.91944124	0.10654096	0.74752334	0.77413519
Prpf3	0.05633359	4.35903595	0.10637423	0.74771374	0.77413519
Map3k12	-0.0583052	5.62385011	0.10633769	0.7477555	0.77413519
Gm20751	0.31431689	-1.2658381	0.10633163	0.74776241	0.77413519
Ramp1	-0.0760684	4.14651163	0.10631207	0.74778477	0.77413519
Arid5a	-0.1245122	1.19531805	0.10623601	0.74787172	0.77413519
Kcnn1	-0.1187923	1.94683146	0.10619581	0.74791768	0.77413519
Med13	-0.0371361	9.08293275	0.10616957	0.74794769	0.77413519
Rhob	-0.0431362	7.36944358	0.10604287	0.74809264	0.77413519
Rab18	-0.045382	7.34877302	0.10591316	0.74824114	0.77413519
Map10	0.15172754	1.696296	0.10587477	0.74828512	0.77413519
Suclg1	-0.0499845	5.19103964	0.1058234	0.74834396	0.77413519
Fundc1	-0.0500786	6.44940792	0.10576651	0.74840916	0.77413519
Cxcl5	-0.1817922	0.65863524	0.10573957	0.74844004	0.77413519
Zscan22	-0.0508694	5.38400301	0.10566456	0.74852603	0.77413519
Scpep1os	-0.2622772	-1.5246449	0.10563503	0.7485599	0.77413519
Txnrd1	0.0575988	4.89554205	0.10562959	0.74856614	0.77413519
D430041D05	0.05836837	7.66574254	0.10561972	0.74857745	0.77413519
Mpped1	-0.0490783	6.04425152	0.10558849	0.74861328	0.77413519
Rab24	0.05528858	5.10408853	0.10552917	0.74868134	0.77413519
Hrct1	-0.3764085	-2.1066727	0.10551384	0.74869894	0.77413519
Zfp280d	-0.0606411	6.75746108	0.10547779	0.74874031	0.77413519
Itpripl1	0.18063297	1.21223054	0.10539867	0.74883115	0.77413519
Racgap1	0.07900618	3.21053084	0.10538388	0.74884813	0.77413519
Dpy30	0.07032129	4.81348672	0.10536947	0.74886469	0.77413519
Eaf1	0.0501756	5.06304878	0.10529778	0.74894704	0.77413519
Oaz3	-0.1200659	2.23339133	0.10526008	0.74899035	0.77413519
BC037704	0.16007059	1.27085945	0.10525793	0.74899283	0.77413519
Cnot7	0.0425236	6.74951294	0.10521477	0.74904243	0.77413519
Cmc1	0.07408788	3.90903451	0.10517196	0.74909164	0.77413519
Hip1r	0.09473818	3.19870087	0.10497069	0.74932317	0.774201
H2afy2	-0.0689171	4.52595508	0.10484472	0.7494682	0.774201
Snrpg	0.06495741	5.054598	0.10480851	0.74950991	0.774201
Mir143hg	0.33278157	-1.3937044	0.10467712	0.74966131	0.774201
Mcm9	-0.1197602	2.0178278	0.10465676	0.74968478	0.774201
Fam214b	-0.0617892	4.40234151	0.10462142	0.74972552	0.774201
Acr	-0.2536663	-0.0750512	0.10455778	0.74979892	0.774201

Pxn	0.05182041	5.22610762	0.10451837	0.74984438	0.774201
Rbm17	0.04467248	6.6248111	0.10450548	0.74985925	0.774201
Zfp518b	-0.0403289	5.59134857	0.10440502	0.74997519	0.774201
Ticam2	-0.2023179	0.92254392	0.10436616	0.75002006	0.774201
Gmnc	-0.2693248	-0.5135682	0.10428744	0.75011097	0.774201
Gabpb2	0.04373174	6.14422032	0.10423711	0.75016912	0.774201
Supv3l1	0.07771765	3.17870659	0.10421214	0.75019797	0.774201
Eif3k	0.06476499	4.33275932	0.10419889	0.75021327	0.774201
Dis3	0.05573479	3.97753571	0.10414825	0.7502718	0.774201
Capza2	0.03756735	8.06231364	0.10412779	0.75029546	0.774201
Aurka	-0.189664	-0.188556	0.10412764	0.75029563	0.774201
Epb4.1l1	0.04645247	8.48495277	0.10404449	0.75039178	0.774201
Tmem131	-0.0486737	7.23294098	0.10402297	0.75041667	0.774201
Sim1	-0.2737746	-0.605711	0.10401355	0.75042757	0.774201
Sepsecs	-0.1112565	2.25079891	0.10395444	0.75049596	0.774201
Ptgs1	0.07720952	3.44608602	0.10387531	0.75058754	0.774201
Abcf2	0.04970736	5.35137765	0.10382933	0.75064078	0.774201
Ccdc110	0.26390859	0.36733429	0.10381992	0.75065168	0.774201
Amotl1	0.04934912	6.88193783	0.10379814	0.7506769	0.774201
Gpr139	-0.2508984	-0.5549748	0.10377063	0.75070876	0.774201
Dazl	0.1350967	1.50788797	0.10358628	0.75092239	0.77431971
Ngly1	0.05066505	5.55152088	0.10355123	0.75096304	0.77431971
Gm8773	0.38694351	-1.5269507	0.10344386	0.75108759	0.77431971
Sik2	-0.0438562	5.84896474	0.10341476	0.75112135	0.77431971
Ganc	0.06245494	4.43255697	0.10339631	0.75114276	0.77431971
Lrrn2	-0.0699969	4.65525531	0.1033736	0.75116912	0.77431971
Trip4	-0.04137	5.85807798	0.10329195	0.75126392	0.77435811
Nipbl	-0.0419557	7.96640291	0.10323415	0.75133106	0.77436799
Psmd8	-0.0466887	5.10244073	0.10314668	0.75143268	0.77439039
Gabrg1	-0.0845482	3.40402134	0.10311639	0.75146788	0.77439039
Cd302	0.06532254	4.11724662	0.10304276	0.75155349	0.77441931
Atxn1l	0.04283238	6.29541844	0.10292578	0.75168956	0.7744377
Zdhhc5	0.0488169	5.9280113	0.10287927	0.75174369	0.7744377
Rdh18-ps	0.15636063	0.35944236	0.10287205	0.75175209	0.7744377
Il22ra1	0.11375992	1.58253079	0.10277362	0.75186667	0.7744377
Ccdc159	-0.226054	0.35808225	0.10272588	0.75192227	0.7744377
Pla2g3	0.25635848	-0.0423008	0.10259954	0.75206948	0.7744377
Zfp273	-0.0746947	3.24774517	0.10249974	0.75218585	0.7744377
Gm19619	0.34292892	-1.2366144	0.10249012	0.75219706	0.7744377
2700097O09	-0.0892874	2.8756526	0.10245362	0.75223964	0.7744377
Nav1	-0.0649898	7.3625303	0.10241999	0.75227887	0.7744377
B9d1	-0.0899068	2.37419088	0.10233856	0.7523739	0.7744377
Nsg2	0.04108266	7.8674914	0.10233228	0.75238123	0.7744377

Rnf165	-0.0571428	6.31500496	0.1021819	0.75255683	0.7744377
Abca4	0.11755927	3.09333688	0.10217608	0.75256363	0.7744377
Sez6l2	-0.0546189	5.43861925	0.1021404	0.75260532	0.7744377
Nmnat1	0.16218382	0.27411572	0.10213528	0.7526113	0.7744377
Dnah1	0.08023319	3.06537276	0.10205731	0.75270244	0.7744377
Uhrf2	0.04761972	5.29468683	0.1020228	0.75274278	0.7744377
Heatr9	0.41086309	-1.4993693	0.1019967	0.7527733	0.7744377
Mak16	0.05196348	4.98461037	0.10198232	0.75279012	0.7744377
S100a13	0.12675583	3.41422825	0.10190339	0.75288245	0.7744377
Fam210b	0.06147604	5.27692419	0.10188711	0.7529015	0.7744377
Ptplad2	0.07566295	3.7755559	0.10186346	0.75292917	0.7744377
Rgn	0.47195106	-1.5682967	0.10170615	0.75311334	0.7744377
Runx1	0.10083602	3.56880583	0.10169649	0.75312465	0.7744377
Thap2	0.05412367	5.05056081	0.10167753	0.75314686	0.7744377
Zfp128	0.08956063	2.6900895	0.10166358	0.7531632	0.7744377
Sync	-0.1739325	1.07307096	0.10153003	0.75331972	0.7744377
Pkp3	0.3459397	-1.3205244	0.10151233	0.75334048	0.7744377
Fam58b	0.08058527	4.09447951	0.10141139	0.75345885	0.7744377
AI182371	-0.5460868	-2.1088921	0.10138265	0.75349257	0.7744377
Prep	0.04365122	4.39806506	0.1012938	0.75359685	0.7744377
Faah	0.06709798	4.14335949	0.10128665	0.75360525	0.7744377
Vps4a	0.04493326	5.55781122	0.10122917	0.75367272	0.7744377
Dhx9	-0.0560726	7.76847638	0.10114961	0.75376618	0.7744377
1810014B01	0.09487166	2.62046531	0.10114386	0.75377293	0.7744377
Sbf1	0.07529757	4.72245381	0.10112995	0.75378928	0.7744377
Eng	0.06407802	3.29847141	0.10110771	0.75381541	0.7744377
Fam134b	0.05288801	5.61864022	0.10110732	0.75381587	0.7744377
Wdr46	0.08087298	4.10368222	0.10092703	0.75402783	0.77448311
Rfwd3	-0.0422603	5.39806734	0.10091072	0.75404701	0.77448311
Nlgn2	-0.0448821	6.3200892	0.10089198	0.75406905	0.77448311
Leap2	0.37522573	-0.4596987	0.10087393	0.75409029	0.77448311
Isca2	0.04636883	5.90232956	0.10067005	0.75433031	0.77450697
Dnase1l3	0.37335246	-0.8624302	0.10047788	0.75455679	0.77450697
Kctd9	0.05131147	4.95379289	0.10044184	0.75459929	0.77450697
Tomm34	0.04616172	5.83412048	0.10042118	0.75462365	0.77450697
Ky	0.172207	0.5283509	0.10038777	0.75466307	0.77450697
Cadm1	-0.0602736	6.13708656	0.10037542	0.75467764	0.77450697
Lzts2	-0.0878506	2.82548724	0.10033106	0.75472998	0.77450697
Fam53a	0.06985723	3.55264989	0.10032984	0.75473142	0.77450697
Naa20	0.05721204	4.99462686	0.10030506	0.75476066	0.77450697
Gsn	0.06414391	5.4563857	0.10026258	0.75481081	0.77450697
Ccdc59	-0.0607012	5.04935982	0.10021465	0.7548674	0.77450697
Rin1	-0.0774504	3.80022868	0.10017279	0.75491683	0.77450697

Zfp707	0.14365125	1.37092506	0.10016127	0.75493044	0.77450697
Tbc1d23	0.05673941	4.34883226	0.10012931	0.7549682	0.77450697
Btbd7	0.05106747	5.67035502	0.10012196	0.75497688	0.77450697
Tgfbr2	-0.0657818	5.70117988	0.09974778	0.75541942	0.77477606
Ankef1	-0.2213228	0.39636914	0.09970947	0.75546478	0.77477606
Gm9767	-0.2696275	-1.0617499	0.09960422	0.75558945	0.77477606
Gm4890	-0.3590264	-1.3919237	0.09958339	0.75561413	0.77477606
Nfic	0.04904319	7.69138183	0.09956338	0.75563783	0.77477606
2810006K23I	0.05899326	4.99404715	0.0995513	0.75565215	0.77477606
Ssh3	0.11264824	1.67855805	0.09948131	0.75573512	0.77477606
Nefh	-0.0803803	5.51403652	0.09947006	0.75574846	0.77477606
Zfp930	0.06420038	4.10431084	0.09938074	0.7558544	0.77477606
Tmem132e	-0.1292368	1.29276568	0.09906607	0.75622802	0.77477606
Nfx1	-0.0378299	6.44274509	0.09900322	0.75630272	0.77477606
6720468P15I	0.53648242	-0.4914859	0.09899607	0.75631122	0.77477606
Pik3r1	-0.039757	8.09936598	0.09896007	0.75635402	0.77477606
Pramef8	0.06947169	4.01107825	0.09894609	0.75637065	0.77477606
Pnck	-0.0549507	3.71850949	0.09894188	0.75637566	0.77477606
Mtor	-0.0650279	6.58603647	0.09891259	0.7564105	0.77477606
Exo1	0.25992643	-0.6416641	0.09889658	0.75642953	0.77477606
BC064078	-0.1912838	0.67834504	0.09887237	0.75645833	0.77477606
3830406C13I	-0.0466975	6.13628386	0.09884758	0.75648783	0.77477606
Ghrl	0.21307446	-0.638868	0.09881122	0.7565311	0.77477606
Zfp85os	-0.0905269	2.59106711	0.09875564	0.75659725	0.77477606
Pknox1	0.05729976	3.85227558	0.09872927	0.75662865	0.77477606
Siglech	-0.102493	2.13107819	0.09868058	0.75668663	0.77477606
Ldlrad4	-0.0513556	4.36462641	0.09867726	0.75669059	0.77477606
A830080D01	0.05649241	4.31144439	0.09855076	0.75684129	0.77477606
Slc9a3r1	0.08657258	4.2153837	0.09847719	0.756929	0.77477606
Cops8	-0.0393841	5.82412212	0.09846605	0.75694228	0.77477606
Adamtsl2	-0.2510133	-0.4198794	0.09842372	0.75699275	0.77477606
Tmem50a	0.05567353	5.6736444	0.09834334	0.75708865	0.77477606
Tcof1	-0.0571669	4.23575164	0.09831121	0.75712698	0.77477606
Ninj2	-0.2772115	-0.9280136	0.09829259	0.75714921	0.77477606
Pnpla8	-0.0384953	6.88366748	0.09827748	0.75716724	0.77477606
Ggt5	0.16778425	1.43336164	0.09825629	0.75719254	0.77477606
Tubg2	0.06266225	4.31544614	0.09825271	0.75719681	0.77477606
Cdk5	0.05743305	4.20561656	0.09819136	0.75727006	0.77479209
4921534H16I	0.43640943	0.0222078	0.09801424	0.75748169	0.7749497
Slc38a9	-0.0758735	4.58404155	0.09788981	0.75763048	0.77499671
Per1	0.05418688	5.52922724	0.09787949	0.75764283	0.77499671
Prepl	0.05849969	7.5494485	0.09777542	0.75776736	0.77501365
Cetn2	-0.0635796	5.26854616	0.0977694	0.75777458	0.77501365

Pkp4	-0.036415	8.59015621	0.09753973	0.75804968	0.7751861
Klhl38	-0.2341887	0.04729006	0.09749354	0.75810505	0.7751861
Gramd1c	-0.1506716	1.68330996	0.09746523	0.75813899	0.7751861
Zfp72	0.08857219	3.12617452	0.09737863	0.75824287	0.7751861
Speer7-ps1	-0.1879184	0.64711457	0.09733107	0.75829993	0.7751861
Cyp4v3	0.07813318	4.68104747	0.09728002	0.75836121	0.7751861
Lix1	-0.045485	4.84179322	0.09722456	0.75842778	0.7751861
Bet1l	-0.0971156	3.14726464	0.09722385	0.75842864	0.7751861
Slc4a5	-0.271405	0.04024916	0.09719635	0.75846166	0.7751861
Stxbp4	-0.043696	7.33360469	0.0971107	0.75856454	0.77519637
Ccdc166	0.07499389	3.31457511	0.09709208	0.75858692	0.77519637
Nr2c2	0.0316149	7.33174205	0.09701354	0.75868131	0.77523395
Entpd7	0.07253489	4.12638199	0.09691155	0.75880397	0.77524583
2810459M11	-0.1244026	1.84804127	0.09683391	0.75889738	0.77524583
Usp49	0.06584556	3.96459954	0.09682396	0.75890935	0.77524583
Ube4b	0.03727866	7.77348714	0.09679919	0.75893917	0.77524583
2610008E11I	0.05381963	5.29799621	0.09676444	0.758981	0.77524583
Rpp38	-0.0857784	2.56177142	0.0966476	0.7591217	0.77527094
Oas1b	0.21546953	0.04731173	0.09664165	0.75912888	0.77527094
Tmbim4	-0.0660788	4.59340577	0.09660053	0.75917842	0.77527094
Il21r	0.32869205	-0.8992308	0.09653143	0.7592617	0.77529714
Bbc3	0.17681626	-0.1095236	0.09624874	0.75960275	0.77558655
Yme1l1	0.0408594	6.23404001	0.09611202	0.7597679	0.77561224
Gpr50	0.41349347	-1.5457712	0.09610091	0.75978133	0.77561224
Traf3	-0.05243	5.05773297	0.09608477	0.75980084	0.77561224
02-Sep	0.04319136	7.89735262	0.09591726	0.76000338	0.77573698
Rptor	0.05051032	5.34640575	0.09588837	0.76003833	0.77573698
Gm12191	-0.1058396	1.2385392	0.09583115	0.76010757	0.77574881
Dgat2	0.06507763	3.75689684	0.09572163	0.76024017	0.7758253
Igfbp2	-0.0843407	4.96392241	0.09564549	0.7603324	0.77586058
Relt	0.13380326	1.18477798	0.09551504	0.76049051	0.77596308
Zfp639	-0.0690845	4.46191698	0.09545275	0.76056606	0.77598132
Numb	0.05102494	5.65734998	0.09539539	0.76063565	0.77599348
Itpr3	-0.0986714	2.12556191	0.09523578	0.7608294	0.77613231
D3Bwg0562e	0.05015784	7.1409987	0.09514378	0.76094117	0.77618748
Ewsr1	-0.0594157	7.69583582	0.09507879	0.76102014	0.7762092
Smim1	-0.1590399	1.65134295	0.0949797	0.76114064	0.77627326
Zfp644	-0.0433904	7.40811311	0.09489691	0.76124136	0.77631714
Naa16	-0.0624283	4.04612996	0.09457436	0.76163422	0.77660527
Ocrl	-0.0498322	6.61188684	0.09457019	0.76163931	0.77660527
Cxcl16	0.1082979	3.95304305	0.09446223	0.76177098	0.77665695
Abcg2	-0.0625698	5.21468383	0.09443399	0.76180543	0.77665695
Gm14169	-0.0991804	1.90935333	0.09425831	0.76201989	0.77675737

Lrrc48	-0.0970924	2.39259325	0.09424851	0.76203186	0.77675737
Zfp809	-0.0471618	4.84571026	0.09420942	0.76207961	0.77675737
Ubfd1	-0.036503	6.94136484	0.09412728	0.76217998	0.77675737
Synj2	0.05467861	6.45483524	0.09410752	0.76220414	0.77675737
Megf11	-0.0487399	5.52647012	0.09406979	0.76225028	0.77675737
Rnf43	-0.0900804	3.23811628	0.0938621	0.76250438	0.77695308
Pabpn1	0.04262333	5.54509059	0.09381846	0.76255781	0.77695308
Fam69a	0.04870499	4.66369222	0.0934692	0.76298593	0.77727889
Tceal1	0.04969589	5.42049778	0.09344594	0.76301446	0.77727889
A530072M11	-0.215949	0.62588716	0.0934057	0.76306386	0.77727889
Ppfia3	0.06010237	4.67632354	0.09331979	0.76316934	0.77727889
Cyp4f13	0.12325874	1.16738394	0.09329888	0.76319501	0.77727889
Fcho2	0.04343375	6.99913835	0.09327516	0.76322416	0.77727889
Fam98c	-0.2376448	-0.7052618	0.09313307	0.76339878	0.77739606
Rerg	0.11485577	2.91998122	0.09307474	0.76347051	0.77739606
Foxo4	-0.106964	1.66540675	0.0929894	0.7635755	0.77739606
Tbk1	-0.0524669	5.39326555	0.09297444	0.7635939	0.77739606
Pdpn	-0.0893387	5.22471167	0.09294667	0.76362808	0.77739606
Tcp1	0.03163432	7.08689799	0.09281181	0.76379413	0.77750629
Rab11fip5	0.04671464	5.95716067	0.09269686	0.76393577	0.77754308
Slc50a1	0.07705692	3.05410095	0.09264475	0.76400002	0.77754308
Larp6	0.05819436	3.96276884	0.09259244	0.76406452	0.77754308
4930419G24	0.13877087	1.826446	0.09257271	0.76408886	0.77754308
Ptprt	-0.0632061	6.91884679	0.09254812	0.76411919	0.77754308
AI837181	-0.0544837	4.19362542	0.09235759	0.76435439	0.77772361
Flot1	0.04314337	5.46448358	0.09230256	0.76442237	0.77773396
4921515E04I	-0.2884433	-0.8939193	0.09224837	0.76448934	0.77774329
Brd3	0.04105619	6.20578828	0.09218384	0.7645691	0.77776564
Hpgd	-0.0796088	3.95251446	0.09200244	0.76479351	0.77792873
Ptprn2	0.041257	7.30390433	0.09195905	0.76484722	0.77792873
Oip5	-0.2543281	0.22054863	0.09191412	0.76490286	0.77792873
Ccl28	0.12330112	1.68767328	0.09183934	0.76499548	0.77794392
Cited4	-0.1265082	0.97690506	0.09172557	0.76513648	0.77794392
Tomt	-0.1393395	0.54964426	0.09167921	0.76519397	0.77794392
Rcan3	-0.0451039	5.12241842	0.09144674	0.76548245	0.77794392
Tdrp	-0.0560289	5.87769403	0.09140143	0.76553874	0.77794392
Spata4	0.38969633	-1.0776967	0.09130384	0.76566	0.77794392
Sncg	0.26200366	-0.3725225	0.09129803	0.76566721	0.77794392
Zfp174	0.06066613	3.76830348	0.09129752	0.76566785	0.77794392
C1qtnf2	0.15882831	1.96562314	0.09128665	0.76568136	0.77794392
Tspyl3	0.04354504	4.6678116	0.0912473	0.76573028	0.77794392
Bbip1	-0.0408054	5.93859455	0.09124464	0.76573359	0.77794392
Tom1	0.0673125	4.10098346	0.09120376	0.76578442	0.77794392

Mrpl51	0.04505195	5.05127658	0.09118138	0.76581226	0.77794392
Apeh	-0.1076966	2.13324778	0.09117649	0.76581833	0.77794392
Naa38	-0.0753398	3.65795244	0.09116139	0.76583712	0.77794392
Lig4	-0.0550949	4.33541158	0.09115683	0.7658428	0.77794392
Itga10	0.07948177	2.96327098	0.09072442	0.76638141	0.77829632
Cklf	-0.1371341	0.98049407	0.09065402	0.76646924	0.77829632
Rfxank	-0.0846787	2.94693999	0.09063112	0.76649781	0.77829632
Plekha4	0.16647753	0.34010636	0.09061268	0.76652083	0.77829632
Serpini1	0.04496005	7.56108133	0.0906096	0.76652467	0.77829632
Zfp386	-0.0428202	5.38364077	0.09051786	0.76663921	0.77829632
Prim2	0.07987132	2.69583079	0.09049391	0.76666911	0.77829632
2700089E24I	0.04166563	8.43300585	0.09047028	0.76669863	0.77829632
Ptrh1	-0.4051956	-1.2887047	0.09025836	0.76696353	0.77829632
Zfp236	0.05483404	5.532951	0.09025515	0.76696754	0.77829632
Jade1	0.03908594	6.43364774	0.09023899	0.76698776	0.77829632
Tdrd3	0.04077706	5.51620388	0.09020324	0.76703248	0.77829632
Gm13483	-0.2180825	-0.7401471	0.0901287	0.76712577	0.77829632
Dmpk	-0.0719683	3.50546501	0.09012392	0.76713175	0.77829632
Ppp4r1	0.05387646	4.32499418	0.09011207	0.76714659	0.77829632
Fam3c	-0.0363545	6.43511488	0.09010396	0.76715674	0.77829632
Ipo7	0.04631398	5.83228562	0.09007682	0.76719072	0.77829632
Pla2g2d	0.22166782	-0.6830274	0.0900448	0.76723081	0.77829632
Pigf	-0.1137071	1.56171039	0.08996033	0.76733664	0.77830117
Amt	-0.1179042	1.03484184	0.08991188	0.76739735	0.77830117
Sdf2	0.06179873	4.44281333	0.08987767	0.76744024	0.77830117
Stard5	-0.0919302	3.55374453	0.08982522	0.767506	0.77830117
Mrpl34	0.08680643	3.32687172	0.08978384	0.7675579	0.77830117
Pex7	-0.0525719	4.47396907	0.08976413	0.76758263	0.77830117
Barx2	-0.1051417	1.91715805	0.08963591	0.76774355	0.77840568
Agtpbp1	-0.0424733	8.9554073	0.08958701	0.76780495	0.77840929
Atp1b2	0.04956781	9.04784506	0.08952654	0.76788091	0.77842765
Cdh11	0.03422869	6.72503246	0.08938055	0.7680644	0.7785327
Asap1	0.04277386	7.9708348	0.08930744	0.76815636	0.7785327
Ifi30	0.12606159	2.01187958	0.08930605	0.76815811	0.7785327
Zyg11b	-0.0476026	8.98696616	0.08923595	0.76824632	0.77856346
Ahcy	0.06228588	3.87366617	0.08914319	0.7683631	0.77862317
Gprc5c	0.09018506	3.07802592	0.08908611	0.76843499	0.77863739
Fa2h	-0.0800439	4.01003315	0.088978	0.76857122	0.77871679
Ccdc42	-0.3007695	-1.1815401	0.08890132	0.76866792	0.77872693
Sirt6	0.08292235	2.94757241	0.08885359	0.76872811	0.77872693
Arid3a	-0.1001752	2.96326975	0.08883241	0.76875484	0.77872693
Tmod3	0.05820705	7.80433048	0.08867078	0.76895887	0.77887497
Bdp1	0.04413994	7.43458905	0.08850994	0.7691621	0.77902218

2010315B03I	-0.0520528	4.22427496	0.0883001	0.76942756	0.77923239
Orai1	0.13353849	1.85472513	0.08813845	0.76963228	0.77934724
Zfpm1	-0.0902946	1.76314135	0.08811912	0.76965679	0.77934724
Metrnl	0.0912811	2.14349993	0.08793737	0.76988725	0.77950033
Naca	0.04407124	7.84367929	0.08790853	0.76992384	0.77950033
Dpcr1	0.3095795	-1.0147611	0.08777998	0.77008702	0.77959964
Rasa1	0.03435929	6.61581885	0.08771687	0.77016719	0.77959964
Arfip2	0.04296468	5.19645534	0.08757569	0.77034663	0.77959964
Pask	-0.1743094	0.83317781	0.08752122	0.7704159	0.77959964
Mdh1b	-0.3177992	-1.1226519	0.08749484	0.77044945	0.77959964
E030013I19R	0.27263907	-0.3495429	0.08749482	0.77044949	0.77959964
Rpl15	-0.0473426	7.96298966	0.08745769	0.77049672	0.77959964
Gm9159	-0.1242644	2.04989357	0.08738892	0.77058425	0.77959964
Mmgt1	0.04767615	4.66020871	0.08736307	0.77061716	0.77959964
Igfals	-0.5261088	-2.2123969	0.08731949	0.77067266	0.77959964
Tmsb4x	-0.0564459	8.89562074	0.0871955	0.77083064	0.77959964
Wdr25	-0.0812212	1.99011313	0.08712571	0.77091961	0.77959964
Fbrs	-0.048853	4.54391855	0.08710737	0.770943	0.77959964
Rad51ap2	-0.1561778	1.2965391	0.08708798	0.77096773	0.77959964
Prkag2os1	-0.2296721	-0.1707102	0.08707168	0.77098852	0.77959964
Nov	0.09091833	7.77909032	0.08705347	0.77101175	0.77959964
2610203C20I	-0.0555215	5.8366634	0.0870362	0.77103378	0.77959964
Zkscan4	0.0939803	2.2997498	0.08701191	0.77106477	0.77959964
Dkk2	0.37330273	-0.8060264	0.08690055	0.77120693	0.77968479
Polr2j	0.10790075	3.17793511	0.08675435	0.7713937	0.77975703
Slc2a10	0.13909327	0.93217711	0.08671339	0.77144605	0.77975703
4933434E20I	-0.0442649	4.51298392	0.08670856	0.77145223	0.77975703
Ints5	-0.08196	3.12011438	0.08652197	0.77169092	0.77993971
Il17rd	-0.0974676	2.50258226	0.08637423	0.77188012	0.78005874
Eny2	0.03866332	7.31050281	0.08632675	0.77194096	0.78005874
Gzmm	0.37872127	-1.6068137	0.08629426	0.7719826	0.78005874
Lphn3	0.05610323	6.76500608	0.08613179	0.77219097	0.78007991
Pnoc	-0.2082243	-0.2246849	0.08611455	0.7722131	0.78007991
Lad1	0.50687617	-1.4804742	0.08611454	0.77221311	0.78007991
Ift43	0.09271097	3.32495798	0.08609714	0.77223543	0.78007991
Tpp1	0.05028698	6.58033688	0.08592002	0.77246288	0.78025109
Tmem98	-0.0915803	3.79390521	0.08577512	0.77264915	0.78035358
Nt5c2	-0.0391348	5.54623857	0.08575088	0.77268032	0.78035358
Ppp2r5e	0.04002194	6.40327889	0.08569296	0.77275483	0.78037025
Pigyl	0.12923744	3.02094125	0.08562095	0.77284752	0.78040216
Qpct	-0.1219522	1.21858287	0.08553997	0.77295179	0.78040216
Tnrc6c	-0.0407821	6.9612226	0.08550604	0.7729955	0.78040216
Igsf10	0.07044923	3.27417852	0.08548826	0.7730184	0.78040216

Pm20d1	-0.0964752	2.06002401	0.08534508	0.77320295	0.78042629
Mapk8ip2	-0.091967	4.63969142	0.08528895	0.77327536	0.78042629
Kif1b	-0.050433	9.93094244	0.08525636	0.7733174	0.78042629
Ctnnd1	0.03426317	8.01841914	0.08525282	0.77332197	0.78042629
Rce1	-0.1899932	0.41775303	0.08524482	0.77333229	0.78042629
Usp21	0.04129347	5.1328123	0.08511342	0.77350192	0.78053894
Kif20a	0.09477482	1.644675	0.08494687	0.77371713	0.7806699
Cox4i1	-0.0691465	6.74873163	0.08491331	0.77376053	0.7806699
Cacna1g	-0.0615276	4.47879731	0.08482889	0.77386973	0.7806699
Spata7	0.05141339	4.05031783	0.08479162	0.77391795	0.7806699
Crhr2	-0.3899617	-1.7099164	0.0847661	0.77395098	0.7806699
Slfn2	0.20765361	0.01257306	0.08471804	0.7740132	0.7806699
Rgs8	-0.0518184	6.68073307	0.08468589	0.77405483	0.7806699
Reep5	-0.0366121	8.46075093	0.08465424	0.77409582	0.7806699
D230025D16	0.03779089	5.65065119	0.0846038	0.77416117	0.78067729
2810002D19I	-0.0766687	3.26031947	0.08432493	0.77452283	0.78094954
Xaf1	-0.0681488	3.44495522	0.08430614	0.77454722	0.78094954
Eif5a	0.06577372	7.19992216	0.0841551	0.77474339	0.78106813
Phkg2	-0.0862838	2.60356202	0.08412622	0.77478093	0.78106813
Uba52	-0.0566672	6.7196023	0.08399861	0.77494685	0.78113329
Cd97	-0.0797774	3.10544041	0.08397499	0.77497758	0.78113329
Sgk3	0.04449909	5.20054935	0.08394261	0.77501971	0.78113329
Rexo1	-0.0491467	4.84694613	0.08380471	0.77519924	0.78120309
Mrgprf	0.15951946	2.03239922	0.08380023	0.77520508	0.78120309
Dcaf7	0.03421606	7.9744213	0.08361684	0.77544407	0.78129129
Ccl25	0.14311068	1.70971134	0.08356464	0.77551216	0.78129129
Exoc3l	-0.119829	1.39302069	0.08355533	0.77552429	0.78129129
Srrt	0.05062088	4.88344828	0.08352075	0.77556941	0.78129129
Prnp	0.04180409	8.00642486	0.08351042	0.7755829	0.78129129
Sh3tc1	0.28398838	-1.176216	0.08344046	0.77567421	0.78132478
Ugdh	0.04273812	4.62494384	0.08337869	0.77575487	0.78134754
Csdc2	-0.0441394	6.84565661	0.08328272	0.77588025	0.78135663
Tmem101	-0.1192316	2.47401716	0.08324053	0.77593539	0.78135663
Cish	0.13776647	1.17032559	0.08322107	0.77596083	0.78135663
Nlrx1	0.12973105	1.86726911	0.08319405	0.77599616	0.78135663
Zfp30	0.05758414	3.69679721	0.08307585	0.77615078	0.78140409
Zkscan17	0.06432925	3.86490782	0.08304449	0.77619182	0.78140409
Tmem45a	0.10686993	2.20741099	0.08302487	0.7762175	0.78140409
Nampt	-0.0369331	7.31073047	0.08292563	0.77634746	0.78145686
C530008M17	-0.0562982	5.06620149	0.08289615	0.77638607	0.78145686
Grhpr	0.09840117	2.24002637	0.08284428	0.77645404	0.78146682
Tacc3	0.09030033	2.22085714	0.08270905	0.77663136	0.78147829
Als2	-0.0544215	4.90617991	0.08270436	0.77663751	0.78147829

Ccdc176	-0.1179642	2.36353421	0.08270272	0.77663966	0.78147829
Dcun1d5	0.04020892	5.95831292	0.08254947	0.7768408	0.78153676
Fbxo42	-0.0400912	5.14758796	0.08254444	0.77684741	0.78153676
Dync2li1	0.06416892	3.59751117	0.08252559	0.77687216	0.78153676
Ptprk	-0.0367996	6.4903299	0.08243218	0.77699488	0.78153676
Ttc26	0.09385404	2.9379197	0.08242429	0.77700524	0.78153676
Agps	-0.0341085	7.19752054	0.08239309	0.77704625	0.78153676
Tex9	-0.0394393	4.98234399	0.08228709	0.77718565	0.78156459
Cpd	-0.0461959	6.32169833	0.08220969	0.77728748	0.78156459
Sf3b5	-0.0732396	3.86018362	0.08215505	0.77735941	0.78156459
Chml	0.03537776	6.24860913	0.08207365	0.77746661	0.78156459
Rps15	-0.0638966	6.36645525	0.08206891	0.77747285	0.78156459
Ube3a	-0.0422599	6.7638615	0.0819808	0.77758896	0.78156459
Traf6	-0.0395661	6.18016976	0.0819678	0.77760608	0.78156459
Wdr96	0.13150087	1.22536066	0.08193963	0.77764322	0.78156459
Cdk10	0.05584757	4.11816307	0.08188821	0.77771103	0.78156459
Tnfrsf23	0.11210445	1.09561265	0.0818594	0.77774903	0.78156459
Ttll13	-0.2837102	-0.9046798	0.08179125	0.77783895	0.78156459
Snx5	-0.033056	7.0692407	0.08178996	0.77784066	0.78156459
Fgfr1op	-0.0454866	4.38467889	0.08174356	0.77790191	0.78156459
Dirc2	-0.0483979	4.94217804	0.08163665	0.77804309	0.78156459
Angptl3	-0.2239384	0.11043517	0.08162199	0.77806246	0.78156459
Add1	0.03739079	7.6835705	0.08160919	0.77807937	0.78156459
Thsd4	0.0795288	6.29216156	0.08156916	0.77813228	0.78156459
Zfp90	0.05498826	3.80356774	0.08138148	0.77838048	0.78156459
Naa25	-0.0518447	4.53536187	0.08134394	0.77843017	0.78156459
Zc3h18	0.0552309	3.91174905	0.08131297	0.77847117	0.78156459
Rab12	0.03370135	7.08669075	0.08129058	0.77850081	0.78156459
Atp2a2	-0.0413438	9.64115715	0.08128725	0.77850522	0.78156459
Klhdc2	-0.029527	7.86071972	0.08128017	0.7785146	0.78156459
Trpv4	0.20640121	-0.4879297	0.08126125	0.77853966	0.78156459
Cmtm3	0.0694749	3.14524451	0.08124382	0.77856274	0.78156459
Hsdl1	-0.0581064	4.27791316	0.08121044	0.77860695	0.78156459
Pycard	-0.0748471	2.9570236	0.08114024	0.77869999	0.78156459
9230112J17F	0.3525317	-1.563652	0.08114007	0.77870021	0.78156459
Atp8a1	0.04738829	7.56623088	0.08087536	0.77905139	0.78172892
Pih1d2	0.17699504	0.08336365	0.08086955	0.77905911	0.78172892
Lonp2	0.03626435	5.557762	0.08086612	0.77906366	0.78172892
Actr5	0.20800731	0.08199314	0.08083484	0.7791052	0.78172892
Ints3	-0.0416648	5.30231358	0.0807322	0.77924157	0.78172892
Foxp3	-0.2406281	-0.7855002	0.08071309	0.77926698	0.78172892
Hist1h1e	0.09712784	2.92887352	0.0807008	0.77928331	0.78172892
Cxadr	0.06236531	6.56603292	0.08066668	0.77932868	0.78172892

Tgfbr1	0.0492165	5.32494421	0.08056874	0.77945895	0.78180131
Igsf1	0.23965142	0.70347853	0.08050478	0.77954408	0.78182842
Zbtb2	0.04228051	4.67072501	0.08029693	0.77982095	0.78195883
Gli1	0.12536333	1.65310861	0.08029203	0.77982748	0.78195883
Gas2l3	-0.0703939	4.64263568	0.0802763	0.77984844	0.78195883
Efhd1	-0.0560923	4.60422175	0.08013796	0.78003297	0.78200584
Cenpt	-0.0907984	2.40118375	0.08011342	0.78006572	0.78200584
Uap1	-0.0374748	5.8001539	0.08011047	0.78006966	0.78200584
Pcdha10	0.20279361	-0.3966167	0.0799954	0.7802233	0.78201826
Gm5086	-0.2812443	-0.7664952	0.07997987	0.78024404	0.78201826
Taf7l	-0.1504342	1.07692648	0.07995144	0.78028203	0.78201826
Gm5468	-0.1465108	1.88273939	0.07992712	0.78031452	0.78201826
B3gnt3	-0.2439682	-0.0407022	0.07986675	0.78039522	0.78204089
Wdr1	-0.0355148	6.28277704	0.07965964	0.7806723	0.78222132
Npl	0.13296892	1.59114051	0.07964527	0.78069153	0.78222132
Rap2c	-0.0311146	6.85442395	0.07955736	0.78080928	0.78228105
Epn2	0.02961621	6.4669456	0.07950047	0.78088551	0.78229918
Tmem180	-0.0864692	2.4419611	0.07932388	0.78112233	0.78247817
Hat1	0.04405629	5.05281116	0.07925215	0.78121859	0.78250205
Atg101	0.0826496	2.8013093	0.0792064	0.78128002	0.78250205
Mki67	-0.057336	3.76884703	0.07915197	0.78135314	0.78250205
Aifm3	0.04773693	5.53542131	0.07913289	0.78137877	0.78250205
BC055402	0.33798508	-2.4646339	0.07907907	0.78145109	0.78251624
Dchs1	0.07933068	2.92556451	0.07899573	0.78156314	0.7825702
Ptpn11	-0.0319004	7.67249814	0.0787765	0.7818582	0.7826997
4833412C05I	-0.1701899	0.25183559	0.07873539	0.78191357	0.7826997
Ola1	0.03159772	7.60669843	0.07873465	0.78191457	0.7826997
A430033K04	-0.0522439	5.56796504	0.07872681	0.78192513	0.7826997
Kansl2	0.03872308	4.72657534	0.0786585	0.7820172	0.78272138
Tmem218	0.0695262	3.03925079	0.07862443	0.78206312	0.78272138
Mrps35	-0.0701888	3.99390383	0.07851034	0.78221701	0.78281718
Hhip	-0.0526401	4.43848583	0.07845617	0.78229013	0.78283213
Ankzf1	0.10018086	1.79473831	0.07836771	0.78240956	0.7828548
C230024C17I	-0.366718	-1.9188972	0.07830227	0.78249797	0.7828548
Ahdc1	0.04629355	4.66728877	0.07825627	0.78256015	0.7828548
Gskip	0.05609868	4.39527717	0.0782548	0.78256214	0.7828548
Atp2b1	-0.0489399	9.27705514	0.07822407	0.78260367	0.7828548
Enah	-0.0435662	8.35869406	0.07810162	0.7827693	0.78294301
Rassf1	0.08056208	3.92843638	0.07807286	0.78280822	0.78294301
Evi5	-0.0320367	7.79948001	0.07789635	0.78304726	0.78294945
Fth1	0.03290178	9.06580264	0.07786743	0.78308646	0.78294945
Yif1a	0.11258315	1.70687004	0.07784729	0.78311375	0.78294945
2810004N23	-0.0752837	4.55543889	0.07783623	0.78312875	0.78294945

Ctcf	0.03333573	6.37279785	0.07781349	0.78315957	0.78294945
Synj2bp	-0.0319555	7.03416657	0.07780714	0.78316819	0.78294945
Rps3	-0.0629666	6.97730527	0.07776491	0.78322546	0.78294945
Htr2c	-0.0580605	4.57506655	0.0777246	0.78328014	0.78294945
Nipal3	0.05826647	4.4565347	0.07764931	0.78338232	0.78299342
Ppfia2	-0.0424454	7.76153196	0.07748024	0.78361195	0.7831385
Cd93	-0.1108862	3.15480574	0.07738765	0.78373783	0.7831385
Ppm1e	0.04731521	8.03567188	0.0773818	0.78374577	0.7831385
Mapk10	-0.0377873	9.60904329	0.07737115	0.78376026	0.7831385
Gm14420	-0.0446262	5.40322491	0.07728688	0.7838749	0.7831949
Osgin1	-0.1937943	0.20312214	0.07713534	0.78408125	0.7833429
Mfsd2a	0.12322697	1.27973298	0.07708418	0.78415095	0.78335438
Tmem106a	-0.1521708	1.6543206	0.07689946	0.78440285	0.78349493
Chchd2	0.04134468	7.6487621	0.07686743	0.78444657	0.78349493
Slc43a2	-0.0492117	5.45789386	0.07680409	0.78453303	0.78349493
Itpkb	0.06864638	3.35426576	0.0767599	0.78459339	0.78349493
2410004B18I	-0.0410525	5.13860267	0.07670174	0.78467284	0.78349493
Cog6	-0.0358888	5.57264426	0.07668755	0.78469223	0.78349493
Dars2	0.07873799	3.49596305	0.07668243	0.78469923	0.78349493
Ppp6r3	-0.0320065	6.4274008	0.07660441	0.78480588	0.78354328
Oxld1	-0.1236617	1.12722432	0.07656039	0.78486609	0.78354526
Umps	0.04750154	4.08953276	0.07648957	0.78496297	0.78358385
Mroh1	0.06921143	4.29708474	0.07636572	0.78513254	0.78369498
Mkln1	-0.0433093	6.15447147	0.07609708	0.78550085	0.78394022
Ahsa2	0.045687	4.73143672	0.07606317	0.78554739	0.78394022
Kdelr3	-0.1275122	1.07251434	0.07605908	0.78555301	0.78394022
Gm3893	-0.0623582	5.8712045	0.07579789	0.78591187	0.78424018
Gsg1l	-0.0432341	5.847959	0.07575041	0.78597718	0.7842472
Htr1b	-0.096252	1.29237253	0.07566523	0.7860944	0.784306
Agpat9	0.10909161	1.27578134	0.07555159	0.78625089	0.78437719
Ppm1f	-0.045062	4.58566477	0.07552877	0.78628233	0.78437719
Xkrx	0.09410198	2.04510831	0.07525529	0.78665952	0.78459938
Snord42a	0.21495031	-1.1470432	0.07522054	0.7867075	0.78459938
Spo11	-0.2524619	0.07250482	0.07521757	0.78671161	0.78459938
Psg29	-0.1738168	0.02160437	0.07514474	0.78681221	0.78459938
Rnf152	-0.0360831	6.19103377	0.07514344	0.786814	0.78459938
8430408G22	0.39192481	-1.1148148	0.07507155	0.78691336	0.78459938
Mbd5	-0.0411478	7.79862583	0.07504015	0.78695677	0.78459938
Dnajc30	-0.0438472	4.96880301	0.0750139	0.78699306	0.78459938
Tmx1	0.05442668	4.8957351	0.0749507	0.78708049	0.78459938
Arhgap17	0.05339521	4.57259103	0.07494518	0.78708813	0.78459938
Arhgap28	0.06548828	4.00882004	0.07481138	0.78727336	0.78472589
1810037I17R	0.05076455	5.63404425	0.07473199	0.78738333	0.78475611

Vstm2l	0.1514008	-0.1881095	0.07470531	0.78742032	0.78475611
Ccdc88c	-0.0607221	4.2524516	0.07452483	0.78767063	0.78494745
Tjap1	0.12306097	1.35414695	0.0743859	0.78786354	0.78508155
Nsd1	-0.0371527	8.22829954	0.07429135	0.78799493	0.78508323
Mat2a	0.03189528	7.87146733	0.07425915	0.78803971	0.78508323
Gpr149	-0.1530683	0.73379147	0.07425876	0.78804025	0.78508323
Ncor1	0.04082467	9.19656886	0.07413491	0.78821254	0.78512628
Elp2	0.04679688	5.93591872	0.07411928	0.78823431	0.78512628
Dtymk	0.04607979	4.20043631	0.0739602	0.78845586	0.78512628
Gm20257	-0.1018621	2.45904281	0.07393065	0.78849704	0.78512628
Cited1	-0.1183744	1.57350636	0.07389914	0.78854097	0.78512628
Foxf1	-0.3383864	-1.9447467	0.0738844	0.78856151	0.78512628
Pdcd6	-0.0438339	5.94969641	0.07387528	0.78857424	0.78512628
Trim59	0.07164112	3.40018725	0.07382377	0.78864608	0.78512628
Sh3glb2	-0.0369583	5.23675854	0.07376462	0.7887286	0.78512628
4632415L05R	0.0398126	5.48944619	0.07374647	0.78875394	0.78512628
Syne4	0.3233638	-1.3494945	0.07373305	0.78877266	0.78512628
Dysf	0.19913656	0.33250589	0.07368474	0.78884012	0.78512628
Hist1h2bj	0.24137108	-1.5692539	0.07366659	0.78886546	0.78512628
Trip12	-0.0331717	8.84253983	0.07362894	0.78891805	0.78512628
Ephb4	0.10053332	3.24174514	0.07359986	0.78895867	0.78512628
Strn	-0.0290291	6.48418196	0.07346092	0.7891529	0.78518714
Psmd1	-0.0285528	7.50243881	0.07345529	0.78916077	0.78518714
Ppm1k	-0.0424666	6.66579069	0.07343091	0.78919488	0.78518714
Mlkl	0.16342734	0.73842643	0.07321275	0.78950032	0.78529089
Iws1	0.03284637	6.44071015	0.07319974	0.78951855	0.78529089
Amigo3	0.26806627	-0.5059474	0.07313438	0.78961016	0.78529089
Abcg1	-0.0508768	4.94718893	0.07309384	0.78966702	0.78529089
Nxnl1	0.42703538	-1.2919197	0.07307617	0.78969179	0.78529089
Slc18a3	-0.1988184	0.02036782	0.07301686	0.789775	0.78529089
Samd10	-0.0706375	3.49672755	0.07301619	0.78977595	0.78529089
Txndc12	0.06286667	3.96438473	0.07301409	0.78977889	0.78529089
Aifm2	0.09702206	2.59545827	0.07298167	0.78982439	0.78529089
Isca1	0.02904662	6.6628127	0.07286686	0.7899856	0.78531623
Tmem8b	0.04780351	5.07765794	0.07278225	0.7901045	0.78531623
Atp6v1g2	-0.0331449	9.45908367	0.07276151	0.79013365	0.78531623
Pikfyve	0.04427112	6.77056476	0.07273251	0.79017442	0.78531623
Pde7a	0.04877201	5.13023311	0.07270937	0.79020697	0.78531623
Zfp647	-0.0961615	1.3500515	0.07270404	0.79021446	0.78531623
Pld3	0.05004634	7.04304457	0.07267281	0.79025839	0.78531623
Rsph9	-0.1247029	2.03446966	0.07250285	0.79049764	0.7854479
Gm14204	-0.0949555	2.45307692	0.07246969	0.79054436	0.7854479
Axin1	0.0617051	3.37018017	0.07245433	0.790566	0.7854479

4930451C15I	0.17136727	0.31137663	0.07228893	0.79079921	0.78562159
Tbrg1	0.05337941	4.80640048	0.07221393	0.79090505	0.78566874
Ptcra	-0.1999809	-0.9409011	0.07216508	0.79097401	0.78567924
P4ha1	0.0465286	3.95493008	0.07207648	0.79109917	0.78568946
Idua	0.06226566	3.57886831	0.0720568	0.79112697	0.78568946
Fyb	-0.0668424	4.4935111	0.0720061	0.79119864	0.78568946
Setbp1	0.03169249	6.75198855	0.07197584	0.79124142	0.78568946
Adprhl2	0.26099563	-0.4313696	0.07192914	0.79130747	0.78568946
Usp13	-0.0783009	3.87018205	0.07187126	0.79138938	0.78568946
Grin2d	0.08679574	1.80761077	0.07186868	0.79139302	0.78568946
Lrrc16b	-0.1498285	2.98873128	0.07175562	0.79155309	0.78573385
1810024B03I	-0.268474	-0.6292529	0.07175462	0.79155452	0.78573385
Ep400	-0.0483342	6.99752642	0.07151648	0.79189213	0.78580371
Mdga1	0.09492965	2.48382082	0.07149167	0.79192733	0.78580371
Mfsd7b	0.06974741	2.61069559	0.07145785	0.79197533	0.78580371
Cep95	-0.0490619	3.92792727	0.07144659	0.79199132	0.78580371
Imp3	-0.058563	4.80297023	0.07141889	0.79203065	0.78580371
Yeats4	0.04162725	5.23072457	0.07138799	0.79207454	0.78580371
Sox4	-0.0669944	2.90899654	0.07136025	0.79211395	0.78580371
Yae1d1	0.03635423	6.50183287	0.071316	0.79217681	0.78580371
Cdkn1c	-0.0693094	5.69488742	0.07130872	0.79218717	0.78580371
Nkx2-1	0.10795048	1.90266197	0.07129346	0.79220885	0.78580371
Fam107a	0.0577241	7.39578337	0.07112803	0.79244414	0.78597915
Isoc2a	-0.0795596	1.96736771	0.07104612	0.79256075	0.78603687
Krt80	-0.0810043	3.54297797	0.07098583	0.79264661	0.7860641
C5ar2	0.08690271	1.32805436	0.07092264	0.79273666	0.78607214
Crip3	-0.4945607	-1.9973046	0.07087739	0.79280117	0.78607214
Faf1	-0.0457309	4.73011911	0.0708195	0.79288373	0.78607214
4933413L06I	-0.1631428	-0.1724786	0.07080782	0.79290039	0.78607214
Tcirg1	-0.0932055	1.52990941	0.07077529	0.7929468	0.78607214
Plin3	0.05797244	3.68225391	0.07073035	0.79301094	0.78607781
Dmxl1	-0.0516796	7.30485153	0.07061722	0.79317249	0.78618004
Limch1	-0.0685865	5.83790073	0.07054051	0.79328212	0.78620799
Tfpt	-0.0763379	2.7330613	0.07049927	0.79334108	0.78620799
Plcd1	0.0810405	2.01051143	0.07047487	0.79337598	0.78620799
Slc25a10	0.11825195	1.47873183	0.07035226	0.79355141	0.78629493
Slc16a2	-0.0391381	5.39027333	0.07033189	0.79358057	0.78629493
Gnl1	-0.0345914	5.89680834	0.07023927	0.79371323	0.78633131
Arpc5	0.04251091	7.34556386	0.07019186	0.79378117	0.78633131
Dhdds	-0.0437584	4.89381283	0.07018389	0.7937926	0.78633131
Adcy3	0.08853	2.3116347	0.07007079	0.79395479	0.78643408
Rplp0	0.04949558	6.63245504	0.0699809	0.79408379	0.7864691
Erlin1	-0.0453994	4.56877923	0.06980173	0.79434118	0.7864691

Dusp18	-0.0442129	5.35682692	0.06977122	0.79438505	0.7864691
Tmem205	-0.1002437	1.69329535	0.06969354	0.79449677	0.7864691
Gm6313	-0.1139628	1.20104026	0.06968875	0.79450367	0.7864691
Ispd	-0.06685	3.40984449	0.06964515	0.79456641	0.7864691
Tmed3	-0.0792022	2.97606323	0.06964512	0.79456646	0.7864691
Rnaseh1	0.08415753	2.79486791	0.06961839	0.79460493	0.7864691
Gm5860	-0.0752531	2.07446429	0.06960239	0.79462797	0.7864691
Lsm7	-0.0603605	3.70808593	0.06958666	0.79465062	0.7864691
Srr	0.03826778	6.94514693	0.06951706	0.79475087	0.7864691
Ppp6c	-0.048083	6.26254495	0.06946841	0.79482098	0.7864691
Iba57	-0.1140655	0.816488	0.06946781	0.79482184	0.7864691
9530091C08I	-0.1106511	4.62543707	0.06945736	0.7948369	0.7864691
Pmel	-0.3033834	-1.8486004	0.06940899	0.79490664	0.7864691
Arhgef18	-0.0407346	4.8838218	0.06939606	0.79492528	0.7864691
Pim1	0.13891516	0.33204306	0.06934644	0.79499686	0.78648209
Thy1	-0.0333786	7.45923875	0.06914505	0.79528762	0.78667144
Pdp1	0.04210535	7.11894952	0.06913289	0.79530518	0.78667144
Atp1b1	0.02884119	10.1417314	0.06904293	0.79543523	0.78671425
Pars2	-0.0997756	1.84573629	0.06899806	0.79550011	0.78671425
Pomgnt2	0.08309699	2.41069811	0.0689455	0.79557617	0.78671425
Tnfaip2	0.13685596	0.47359873	0.06888225	0.79566773	0.78671425
4932416H05I	0.06772589	2.63823692	0.06885185	0.79571174	0.78671425
Strap	0.02748917	7.46723644	0.06881712	0.79576205	0.78671425
Gpr146	-0.0543649	4.1900935	0.06880403	0.79578101	0.78671425
Ccnjl	-0.1433591	1.08242256	0.06877976	0.79581617	0.78671425
Jak1	0.03454848	7.64637337	0.06861615	0.79605339	0.78682037
Ncoa2	-0.0374398	8.34814056	0.06853239	0.79617496	0.78682037
Prkd2	0.07962471	2.88515912	0.0685259	0.79618438	0.78682037
Gpr179	0.29485006	-0.4016833	0.06850824	0.79621002	0.78682037
Usp2	0.04319978	5.72951967	0.06849492	0.79622937	0.78682037
Clec12a	0.25710263	-0.500665	0.06846395	0.79627435	0.78682037
Trim36	-0.0632706	3.36901537	0.06826968	0.79655675	0.78686435
Dnajc1	-0.0418748	5.08351019	0.06826102	0.79656935	0.78686435
Polr3h	0.0556527	2.96034667	0.06824139	0.79659791	0.78686435
Nhlh1	-0.2156018	-0.2689233	0.06818249	0.79668364	0.78686435
Baiap2l2	0.23629266	-1.0733073	0.06816714	0.79670599	0.78686435
Rsph3a	0.04511751	4.43966848	0.06815298	0.79672661	0.78686435
Pcyox1	-0.0452721	5.62445195	0.06815189	0.79672819	0.78686435
Casc3	-0.0449469	5.49386002	0.06806819	0.79685011	0.786927
Cox5b	-0.0399463	6.31383891	0.06802484	0.79691329	0.78693164
Mid1	0.06192131	4.12698169	0.06779653	0.79724637	0.78717928
Atp2c1	-0.0333263	6.56727985	0.06777278	0.79728106	0.78717928
Cd99l2	-0.0461246	5.75979085	0.06754899	0.79760819	0.78744449

BC029214	-0.0563224	3.49030025	0.06743033	0.79778188	0.7874851
Tbkbp1	-0.0812855	2.13818558	0.06735021	0.79789925	0.7874851
Stambp	0.04253203	4.3459198	0.06730195	0.79796999	0.7874851
Usp16	0.03749901	6.12332536	0.06727352	0.79801167	0.7874851
Plxnd1	0.04383777	4.68512081	0.06726673	0.79802162	0.7874851
Napg	-0.0310903	6.73051535	0.06724615	0.7980518	0.7874851
Spata5	0.05247493	4.23811925	0.06723394	0.79806972	0.7874851
Foxp4	0.05844489	4.89409226	0.06720136	0.7981175	0.7874851
Btbd10	-0.0388876	5.98224862	0.06702162	0.7983814	0.78765013
Dnase1	0.14139152	1.03143966	0.06700771	0.79840183	0.78765013
Slc33a1	-0.0562895	3.78701826	0.06693451	0.79850943	0.78769853
Ube2m	0.05721592	3.10543801	0.0667961	0.79871304	0.78784163
Pou3f4	-0.1136689	0.57012501	0.06670669	0.79884469	0.78785222
C1qbp	0.03321354	5.21647744	0.06668114	0.79888233	0.78785222
Eftud1	-0.0659529	3.10423985	0.06666954	0.79889942	0.78785222
Aco1	-0.0440552	4.27600672	0.06659863	0.79900393	0.78789754
Prpf38b	0.03267533	7.32605818	0.06651739	0.79912373	0.78795471
Grid2	-0.0870159	2.47251112	0.0663904	0.79931115	0.78795471
Rbx1	0.04336962	6.58727791	0.06637162	0.79933889	0.78795471
Egr4	0.07964062	3.23099371	0.06634872	0.79937271	0.78795471
Rps4x	-0.0495307	7.89493547	0.0663465	0.79937599	0.78795471
Tnks	-0.0408476	5.86862362	0.06632129	0.79941324	0.78795471
Rft1	-0.0990424	2.59796167	0.06625934	0.79950479	0.78798722
Tmem65	-0.0312269	8.11504766	0.06608052	0.79976933	0.78808731
Wdr91	0.06756913	3.08481468	0.06602089	0.79985763	0.78808731
Sesn3	-0.0346857	6.09215675	0.06600243	0.79988498	0.78808731
Galnt18	0.07616808	2.29504213	0.06600194	0.7998857	0.78808731
Gstm5	-0.0501961	5.97796216	0.06595828	0.79995038	0.78808731
Psmc4	0.04494309	4.92455703	0.06595332	0.79995774	0.78808731
Ahcyl2	-0.0380561	8.1213262	0.06590742	0.80002576	0.78809663
Irak2	0.04753737	3.65460377	0.0658162	0.80016105	0.7881722
Gm6251	-0.1199799	0.42859106	0.06564705	0.80041216	0.78830371
Dpp7	-0.0942583	1.49041866	0.06563211	0.80043436	0.78830371
4930429F24I	-0.1465907	0.47399518	0.06560792	0.80047031	0.78830371
Rinl	0.10371875	1.37320554	0.06547654	0.80066565	0.78837131
Cks1b	0.09515956	3.42281938	0.06546552	0.80068205	0.78837131
Agtrap	0.07913818	4.03293785	0.06544357	0.80071471	0.78837131
Rabgap1l	0.03655087	8.19784169	0.06527484	0.80096597	0.78839648
4732471J01F	0.09436149	1.65435426	0.06526872	0.80097508	0.78839648
Eif4e3	-0.0368823	5.53897092	0.06525903	0.80098953	0.78839648
Cthrc1	-0.0603728	2.88728204	0.06524158	0.80101554	0.78839648
Akap8l	-0.0781229	3.41002979	0.06520771	0.80106602	0.78839648
Crb2	-0.212199	-0.6515591	0.06519041	0.80109182	0.78839648

Nap1l5	0.02899392	7.1916082	0.06508164	0.80125409	0.78849672
Ccdc175	-0.2955416	-1.7965656	0.06502905	0.8013326	0.78849672
Gm4262	0.06248533	3.20968272	0.06500436	0.80136946	0.78849672
Ptar1	-0.0512238	3.79022026	0.06495204	0.80144762	0.78851126
Exog	-0.0657946	2.92209593	0.06491603	0.80150143	0.78851126
Snx27	-0.02878	7.29329358	0.06481997	0.80164504	0.78851676
Ski	-0.0282549	8.16273402	0.06474596	0.80175577	0.78851676
Usp9x	-0.0459494	9.85788988	0.06469395	0.80183364	0.78851676
1700110I01R	0.12431108	1.59061764	0.06469168	0.80183704	0.78851676
Ndufb4	0.05574697	5.90445898	0.0646649	0.80187713	0.78851676
Ubash3b	-0.0390969	5.78563294	0.06463905	0.80191586	0.78851676
E130114P18I	-0.1203633	0.83679261	0.06461044	0.80195871	0.78851676
Rhog	-0.0643713	3.23796966	0.06456651	0.80202455	0.78851676
Spn	-0.1701655	-0.2228099	0.06455993	0.80203441	0.78851676
Zfp740	0.04733475	4.79995904	0.06446899	0.80217077	0.78851677
Atp5e	0.06186295	5.23526752	0.0644603	0.8021838	0.78851677
Golga5	-0.047667	4.18905184	0.06443325	0.80222439	0.78851677
Rps6kb1	-0.0297754	7.12851419	0.06438921	0.80229049	0.78851677
Msra	-0.0654337	3.48859937	0.06436461	0.80232741	0.78851677
Phlda3	0.07563544	2.61215372	0.06428467	0.80244748	0.78857718
Mcm10	0.332504	-1.3005088	0.06415625	0.8026405	0.78870927
Socs6	0.03989405	4.27814036	0.06402256	0.80284168	0.78880797
Ccdc67	-0.2792132	-1.3627201	0.0639936	0.80288529	0.78880797
Usp15	0.03660203	6.41798032	0.06397267	0.80291681	0.78880797
Pi4kb	0.04664811	4.34799035	0.06389189	0.80303853	0.78881207
Mnat1	0.04831922	4.19867211	0.06385912	0.80308793	0.78881207
Pcmt1	0.02882736	6.42797914	0.06385321	0.80309684	0.78881207
Gnl2	0.03335444	5.97776156	0.06377882	0.80320903	0.78886177
Apobr	0.1751564	0.34536625	0.06372682	0.80328749	0.78886177
Sh2d1b1	0.21074048	0.8331285	0.06359426	0.8034877	0.78886177
Lemd1	0.35390559	-1.7102109	0.06357357	0.80351895	0.78886177
Gpr85	-0.049691	3.9713865	0.06352379	0.8035942	0.78886177
Mtch1	0.03329489	6.41998609	0.06351675	0.80360486	0.78886177
Lrrtm1	0.04903019	4.64472738	0.06348517	0.8036526	0.78886177
4921507P07I	-0.1561856	0.29850225	0.06343571	0.80372742	0.78886177
4632434I11R	-0.1179374	0.48499813	0.06343403	0.80372996	0.78886177
Phlpp1	-0.0377131	6.22097017	0.06343158	0.80373368	0.78886177
Cmtm6	-0.0750008	4.86642247	0.06337988	0.80381192	0.78888102
Zfp219	-0.078796	1.95613144	0.06331741	0.8039065	0.78890032
Brd2	-0.0265863	7.92473855	0.06320316	0.80407962	0.78890032
Mir103-2	0.18565624	0.14579881	0.06315693	0.80414972	0.78890032
Gnl3l	-0.0306059	8.04128811	0.06311276	0.80421672	0.78890032
Cndp1	-0.402766	-1.3929104	0.06306084	0.80429552	0.78890032

Eepd1	0.06663975	2.48443429	0.06304022	0.80432681	0.78890032
Eps15	0.03087422	8.62271066	0.06303764	0.80433073	0.78890032
Kremen1	0.05369604	3.34457656	0.06301166	0.80437017	0.78890032
Selo	-0.0848246	1.26997923	0.06300397	0.80438185	0.78890032
2410004N09	0.11275482	0.54396546	0.06298026	0.80441786	0.78890032
Lrrc42	-0.0586217	3.25022116	0.06284333	0.80462596	0.78897437
Exosc5	0.10616078	0.96836672	0.06283627	0.8046367	0.78897437
1700040L02I	0.08485001	1.83594238	0.06281486	0.80466926	0.78897437
Ncoa7	-0.0353378	6.32087926	0.0627733	0.80473247	0.78897886
Atrx	0.0385589	9.86601145	0.06264895	0.80492178	0.78903621
Tnrc18	0.03251798	5.12993566	0.06259407	0.80500539	0.78903621
Rtca	-0.0380294	5.33134198	0.06255027	0.80507214	0.78903621
Capn11	-0.2915139	0.03771976	0.06250809	0.80513645	0.78903621
Gm996	-0.0499483	4.57695993	0.06249362	0.80515852	0.78903621
Rps2	0.03861252	6.30221728	0.062454	0.80521895	0.78903621
Gm561	-0.045307	3.43496853	0.06240506	0.80529364	0.78903621
Nutf2	0.05911242	3.14388551	0.06238936	0.80531761	0.78903621
Slc7a2	-0.0543814	6.92220245	0.0623769	0.80533663	0.78903621
Rtcb	-0.0441395	4.1718067	0.06230636	0.80544435	0.78903621
Nelfe	-0.058906	3.95887873	0.06230255	0.80545017	0.78903621
Etv1	-0.0369475	7.73736449	0.06221432	0.805585	0.78903621
4930579G24	-0.0653173	2.52831405	0.06219755	0.80561065	0.78903621
Myl12b	-0.0435849	7.8337359	0.06219674	0.80561188	0.78903621
Parvb	-0.0659163	3.54574773	0.06213042	0.80571332	0.78907813
Hhatl	0.1378518	0.58083269	0.06207289	0.80580134	0.78910691
Cntnap2	0.05245513	6.26469547	0.06192583	0.80602659	0.78925668
Sp1	0.02966783	7.09172639	0.06185841	0.80612995	0.78925668
Otogl	0.23989525	-1.2053507	0.06185821	0.80613025	0.78925668
Cd6	-0.2123577	-0.0356959	0.06180704	0.80620874	0.7892761
Eif3i	-0.0350669	5.80342661	0.06176268	0.80627681	0.78928532
Pln	-0.1573016	0.61041831	0.06169061	0.80638744	0.7893362
Itga8	-0.0778629	2.59275693	0.06158148	0.80655512	0.7894429
4921507L20I	-0.2094361	-0.5854421	0.06146765	0.80673017	0.78949605
Pcdhgb8	0.10609045	1.14866749	0.06144049	0.80677197	0.78949605
S1pr4	-0.1669495	-0.8166348	0.06143174	0.80678543	0.78949605
4921536K21I	-0.1839165	0.51703229	0.06131821	0.80696026	0.78960971
D030040B21	-0.1465368	-0.4700405	0.06125899	0.80705153	0.78964159
Slc26a2	-0.0694223	6.09247466	0.06119604	0.80714858	0.78967914
Syn1	-0.0534495	10.2351077	0.06113571	0.80724165	0.78971278
Parp16	0.13052724	1.90198188	0.06101767	0.80742389	0.78971626
1190005I06R	0.2276694	-1.2426018	0.06099244	0.80746287	0.78971626
Nop2	-0.0735225	2.78938159	0.06096492	0.80750538	0.78971626
Cbr3	-0.0689934	2.12842456	0.06090182	0.80760294	0.78971626

Nob1	-0.059329	3.0220439	0.06084149	0.80769624	0.78971626
Abcc10	-0.1071489	0.96648343	0.06083678	0.80770352	0.78971626
Abhd17a	-0.0521971	3.66080097	0.06080014	0.80776021	0.78971626
2310039H08I	0.09031796	2.04685104	0.06067639	0.80795184	0.78971626
Slc25a28	0.0436079	3.8932425	0.06067163	0.80795921	0.78971626
Mkks	-0.0457077	5.11911302	0.06066941	0.80796266	0.78971626
Kmt2b	0.04845851	4.65995809	0.06065432	0.80798604	0.78971626
Mtmr4	0.0454034	5.94243077	0.06064979	0.80799306	0.78971626
Pecam1	0.10689181	1.99038698	0.06063912	0.80800959	0.78971626
Dio3os	-0.3969648	-1.726407	0.06060219	0.80806684	0.78971626
Mfsd10	-0.105677	0.81582909	0.06053496	0.80817109	0.78972815
Gm9833	0.0811691	1.15406897	0.06051866	0.80819639	0.78972815
Nedd8	-0.056318	5.60706888	0.0603232	0.80849988	0.78990527
Myh4	-0.2808909	-0.373539	0.06027372	0.8085768	0.78990527
Snhg18	0.06162334	4.28839592	0.06024602	0.80861987	0.78990527
Rtel1	-0.079701	2.53186171	0.06021208	0.80867266	0.78990527
Pdcl	-0.0401661	5.15656467	0.06014366	0.80877913	0.78990527
B630005N14	0.02899361	6.47885509	0.06014224	0.80878134	0.78990527
Ank	0.03448275	6.15385939	0.06010758	0.8088353	0.78990527
Sptssa	-0.0462673	6.40935421	0.06002205	0.80896852	0.78990527
Rpl10a	0.05759523	8.07832466	0.0600188	0.8089736	0.78990527
Ube2q1	-0.0270138	7.10382136	0.06000824	0.80899005	0.78990527
Nkapl	-0.1508235	0.25498264	0.05996974	0.80905005	0.78990527
Tcf19	-0.0836003	2.42159714	0.05990756	0.80914702	0.78990527
Cox15	0.03912396	5.47204878	0.05987632	0.80919575	0.78990527
4932438H23I	-0.1842675	-0.1891643	0.05982142	0.80928144	0.78990527
Cyp2c44	0.28987767	-0.9963417	0.05977174	0.809359	0.78990527
Fam188b	-0.0934207	1.34550415	0.05974343	0.80940322	0.78990527
Gria2	-0.0506743	9.08586185	0.05972114	0.80943804	0.78990527
Zfp260	-0.0293685	7.24177903	0.05967598	0.80950862	0.78990527
Slc41a1	-0.0504891	7.95331276	0.05959211	0.80963975	0.78990527
Paxip1	0.02906944	6.18251122	0.05949614	0.80978994	0.78990527
l7Rn6	0.03976456	6.09145303	0.0594423	0.80987425	0.78990527
C030023E24I	-0.1057569	2.42704436	0.05934538	0.81002613	0.78990527
Prpsap2	-0.0427408	4.20561193	0.05932085	0.81006458	0.78990527
Gpalpp1	0.03557799	5.76288545	0.05931791	0.81006918	0.78990527
Aes	0.04006906	6.35405243	0.05929425	0.8101063	0.78990527
Nek9	-0.0282561	6.73170943	0.05929388	0.81010688	0.78990527
Noc2l	-0.0500395	4.00868273	0.05928388	0.81012255	0.78990527
Fsbp	-0.2664526	-1.8040217	0.05928091	0.81012721	0.78990527
Wwc1	-0.0416702	4.29363113	0.0592092	0.81023973	0.78990527
Hk2	0.06546809	2.80291057	0.05914417	0.81034183	0.78990527
Smarcal1	-0.0463098	6.14881675	0.05914262	0.81034428	0.78990527

Stim1	0.04638989	4.00960311	0.05909674	0.81041634	0.78990527
St6gal2	-0.0470287	4.83107162	0.05908499	0.81043481	0.78990527
Cdc34	-0.0693467	3.16329513	0.05907636	0.81044836	0.78990527
Ogdh	-0.0359498	7.67834973	0.05901897	0.81053857	0.78990527
1500012F01I	-0.0503236	3.6609381	0.05893504	0.81067058	0.78990527
Gm3219	0.09521555	0.84330458	0.05890262	0.8107216	0.78990527
Cyp20a1	-0.0799996	2.77855337	0.05889419	0.81073486	0.78990527
Lyrm4	0.03499851	5.02712425	0.05887484	0.81076533	0.78990527
Anxa9	0.17783365	-0.5916334	0.05887216	0.81076955	0.78990527
Stk24	-0.0396995	7.31064576	0.05886272	0.8107844	0.78990527
Ankrd40	-0.0268956	7.06114299	0.05859486	0.81120665	0.7900839
Gstm4	-0.0799794	1.67802119	0.05858939	0.81121528	0.7900839
Tradd	0.11574574	0.67360552	0.05858263	0.81122595	0.7900839
Rbm6	0.0375379	6.21619282	0.05856583	0.81125247	0.7900839
Ccl17	0.10552969	1.45136625	0.05851453	0.81133348	0.7900839
Pgam5	0.03611311	6.09809971	0.05849235	0.81136853	0.7900839
Atad1	0.02938487	7.21366824	0.05848364	0.81138229	0.7900839
Slc26a10	-0.1122842	0.66412926	0.05844872	0.81143747	0.7900839
1810026B05I	-0.0459227	3.78998523	0.05839348	0.81152479	0.79011175
Hsf5	-0.2717648	0.24239207	0.05824434	0.81176076	0.79020747
Kif3c	-0.0338222	6.41499445	0.05818407	0.81185621	0.79020747
Rpl31	-0.0465442	8.64150413	0.05815469	0.81190276	0.79020747
Fem1c	0.03649767	5.33679925	0.05813726	0.81193038	0.79020747
Fam149a	-0.0324296	5.22175722	0.05813643	0.8119317	0.79020747
Tctn2	-0.0588389	2.69486415	0.05810878	0.81197552	0.79020747
Gm13710	-0.1301075	0.50755034	0.05807179	0.81203417	0.79020747
Numbl	0.0415691	4.00453307	0.05799046	0.8121632	0.79020882
Rfx4	0.06643646	3.75945816	0.05796618	0.81220173	0.79020882
2900008C10I	0.1726782	0.21761927	0.05795988	0.81221173	0.79020882
Muc5b	0.27675189	-1.4645111	0.05814488	0.81235197	0.79028812
2410015M20	0.06752091	2.73795877	0.0578108	0.81244855	0.79032494
Dnajc17	-0.088672	2.30473884	0.05767173	0.81266975	0.79048298
Map4k5	0.02884017	5.95929711	0.0575791	0.81281726	0.79048364
Fam98a	0.03320549	5.72445129	0.05756345	0.81284218	0.79048364
Cep19	-0.0293733	5.80327152	0.05750928	0.81292851	0.79048364
Ssna1	0.06741797	3.00827153	0.05748419	0.81296851	0.79048364
Fam199x	-0.0378982	4.77628233	0.05747917	0.81297652	0.79048364
Rbm15b	0.04386909	3.8137189	0.05745008	0.8130229	0.79048364
2610316D01I	-0.0861762	2.20637587	0.0573922	0.81311524	0.7905163
Zfp783	-0.1058667	1.36514797	0.05723758	0.81336215	0.79064443
Cwc27	0.03507195	5.33572549	0.0572039	0.81341599	0.79064443
Gm13102	0.22513743	-1.4191695	0.05719932	0.8134233	0.79064443
Rnf128	-0.0901732	1.67447049	0.0571277	0.81353784	0.79069864

Vezt	-0.0387348	4.86885438	0.05703536	0.8136856	0.79072825
Adamts3	0.05295032	4.12886583	0.05703523	0.81368582	0.79072825
A330041J22F	-0.1656132	0.08516935	0.05695833	0.81380898	0.79077293
A930003A15	-0.3233948	-1.9242753	0.05693314	0.81384934	0.79077293
G6pdx	0.04115	5.75914343	0.05684641	0.81398839	0.79085093
Ssx2ip	-0.0274626	6.28551564	0.05679065	0.81407785	0.79088075
Nme3	0.05345219	3.44429198	0.056617	0.81435673	0.79090425
Fmod	0.06282002	7.83887884	0.05659314	0.81439507	0.79090425
Qrfpr	-0.1906625	0.13814898	0.05652132	0.81451058	0.79090425
Sspn	-0.0652336	4.45629155	0.05648705	0.81456572	0.79090425
Sec23ip	-0.0351139	5.45952368	0.05645773	0.81461291	0.79090425
Fam207a	-0.0604968	3.37033488	0.05642609	0.81466384	0.79090425
Ccdc113	0.11779704	1.09575397	0.056424	0.8146672	0.79090425
Rap2a	-0.0241318	7.59364331	0.05639079	0.81472069	0.79090425
Ccser2	-0.0259528	8.27618302	0.0563343	0.8148117	0.79090425
Rap1b	0.03595894	7.25010556	0.05632993	0.81481875	0.79090425
Ube2t	0.23478139	-1.0713827	0.05631224	0.81484725	0.79090425
Nek4	0.04503619	4.4182294	0.05630409	0.81486039	0.79090425
Sox17	-0.1109601	1.86351752	0.05626266	0.81492719	0.79090425
Gm6981	0.08237708	1.51002647	0.05622996	0.81497994	0.79090425
4930500J02F	-0.2384964	-0.6155177	0.05622764	0.81498368	0.79090425
Klk6	-0.2457845	-1.3802806	0.05614757	0.81511289	0.79096745
Aamdc	-0.0455966	4.22658645	0.05611445	0.81516636	0.79096745
Slfn8	0.07681629	3.41249401	0.05604189	0.81528359	0.79100776
Selt	0.03525529	8.78450393	0.055991	0.81536585	0.79100776
Klhl29	-0.0571502	5.07510909	0.05597961	0.81538425	0.79100776
Dlgap2	-0.0475706	6.60868622	0.05590648	0.81550255	0.79106549
Acp6	-0.0825371	2.46566331	0.05586057	0.81557684	0.79108053
Colgalt2	0.08194969	1.4849493	0.05564577	0.81592491	0.79129909
Sdha	-0.0267182	8.6038196	0.05561704	0.81597153	0.79129909
Plcb3	-0.0528343	4.33601191	0.05558977	0.81601578	0.79129909
Gm13826	-0.0547222	2.96121639	0.05538086	0.81635516	0.79129909
Ppp3r2	-0.1879004	-0.420535	0.05537707	0.81636133	0.79129909
Krba1	0.04112035	3.86026108	0.05537244	0.81636885	0.79129909
Krit1	-0.0397606	5.84090534	0.05534317	0.81641646	0.79129909
Fam219a	0.04774671	3.871757	0.05533157	0.81643533	0.79129909
Smyd4	-0.0619032	2.62051006	0.05528238	0.81651538	0.79129909
Kcns2	-0.0471123	3.87739497	0.05527853	0.81652165	0.79129909
Copg1	0.03113528	7.25418824	0.05524559	0.81657527	0.79129909
BC006965	-0.0949408	1.79044994	0.05517675	0.81668741	0.79129909
Fv1	0.13039296	0.54461146	0.05516168	0.81671196	0.79129909
Prickle2	-0.0399484	7.94042736	0.05509262	0.81682454	0.79129909
Zfp513	0.07719138	1.85284122	0.05504345	0.81690474	0.79129909

Psmc5	0.03159279	6.68950131	0.05504173	0.81690755	0.79129909
Srp14	-0.0411304	6.88944563	0.05503447	0.81691939	0.79129909
A230070E04	-0.0492134	4.56051307	0.05502477	0.81693522	0.79129909
Ccdc74a	-0.0680273	2.01806722	0.05501698	0.81694793	0.79129909
Sec11a	0.06034122	4.0758586	0.05499839	0.81697827	0.79129909
Radil	0.08879408	2.13082272	0.05492088	0.81710482	0.79134651
Slc25a34	-0.1319265	0.06385238	0.05484569	0.81722767	0.79134651
Thoc3	0.05651315	4.17805033	0.05483966	0.81723752	0.79134651
Lefty1	-0.0959819	1.93851202	0.05482441	0.81726246	0.79134651
Col11a1	-0.0569642	3.3666283	0.05477404	0.81734482	0.79136931
Mrps12	-0.0606763	3.59818794	0.05473796	0.81740386	0.79136953
Zcchc11	0.03215181	6.83603884	0.05457883	0.81766443	0.79156485
Fam110b	-0.0508849	3.61497942	0.05451386	0.81777093	0.79158272
Pddc1	-0.0465504	3.60516068	0.05449581	0.81780054	0.79158272
Eif4a3	0.03746022	5.00288545	0.05445555	0.81786658	0.7915897
Nhp2l1	-0.0330097	6.06380056	0.05435536	0.81803104	0.79167315
Gm19710	-0.1824068	0.31012239	0.05430618	0.81811184	0.79167315
Btbd3	0.0331143	8.37597415	0.05429555	0.8181293	0.79167315
Mrpl35	-0.0302704	5.08137465	0.05417273	0.81833124	0.79175019
Vwa9	-0.0386703	4.48041734	0.05416368	0.81834615	0.79175019
Kcnq2	-0.0422514	5.52989196	0.05407681	0.81848913	0.79175019
Dpcd	0.06790537	3.13768392	0.05402321	0.81857743	0.79175019
C1qc	-0.1098643	2.26937786	0.05396206	0.81867822	0.79175019
Dstyk	-0.0306939	5.49522802	0.05395955	0.81868234	0.79175019
Mars2	0.09234848	2.29555454	0.05395683	0.81868684	0.79175019
Gm17769	-0.1093663	0.14104306	0.05391931	0.8187487	0.79175019
Dctn6	-0.0426944	6.37633636	0.05389449	0.81878965	0.79175019
Trim47	-0.1047546	2.25336164	0.05388985	0.8187973	0.79175019
Sphk1	-0.0750197	4.58508224	0.05372565	0.81906842	0.79185006
Wdr74	0.06303244	2.90109075	0.05367129	0.81915827	0.79185006
Fnbp1	-0.0264708	6.73205691	0.05361791	0.81924655	0.79185006
Cd40	0.21044833	-1.0778425	0.05361445	0.81925226	0.79185006
Ddx42	0.03254459	6.02745186	0.05353499	0.81938377	0.79185006
Nup62	0.03717015	5.49699393	0.05349736	0.81944608	0.79185006
Opn1mw	-0.2263992	-1.6765362	0.05344972	0.819525	0.79185006
Grin2b	-0.0437839	7.05758381	0.05344889	0.81952638	0.79185006
Ccdc174	-0.0379133	4.70050512	0.0534199	0.81957442	0.79185006
Fnta	-0.0390501	6.63782903	0.05339326	0.81961858	0.79185006
Nelfa	-0.0357957	4.89453732	0.05339001	0.81962396	0.79185006
Tk2	-0.0696898	3.29112277	0.05336147	0.81967128	0.79185006
Pcdhb18	-0.0549206	4.15419096	0.05328125	0.81980438	0.79185006
9930111J21F	0.07537252	3.80923068	0.05324189	0.81986973	0.79185006
Maml3	0.0391229	4.82223032	0.05323956	0.81987358	0.79185006

Ptgds	-0.0796818	12.2504338	0.05319352	0.81995005	0.79185006
Mcm7	-0.0626449	2.98666999	0.05317416	0.81998222	0.79185006
Ubap2	0.02768136	6.09196296	0.05312008	0.82007209	0.79185006
Cass4	0.1403867	0.49765302	0.05311686	0.82007745	0.79185006
Gm11201	0.24044566	-0.2656289	0.05298524	0.82029641	0.79185006
Elac1	0.03480908	5.18459079	0.05292101	0.82040335	0.79185006
Plb1	0.09002921	2.38062734	0.05289539	0.82044604	0.79185006
E2f7	0.25950324	-1.079622	0.05286968	0.82048888	0.79185006
4921504A21I	0.05986187	3.35380676	0.0528185	0.82057419	0.79185006
Fkbp14	0.05655958	4.22152797	0.0527976	0.82060903	0.79185006
Polr2i	-0.0657268	3.0562357	0.05279695	0.82061013	0.79185006
Trim6	-0.2173367	-0.5497775	0.05275267	0.82068399	0.79185006
Tnik	-0.0454611	7.36927851	0.05274369	0.82069898	0.79185006
Lpcat2	0.07910631	2.55061214	0.05271384	0.8207488	0.79185006
Nat10	0.04759543	3.42472653	0.05270973	0.82075565	0.79185006
Cacna1f	0.22514135	-0.5969001	0.05265859	0.82084103	0.79185006
Dyx1c1	0.08464053	2.02333161	0.0526453	0.82086323	0.79185006
Ppm1a	-0.0300422	6.77648494	0.05258762	0.82095961	0.79185006
Efcab14	-0.0342212	7.24510589	0.0525339	0.82104941	0.79185006
Akr1a1	0.04466047	7.81607609	0.05253032	0.82105539	0.79185006
Rgl3	-0.1352998	0.42338172	0.05252398	0.82106599	0.79185006
Tbpl1	-0.024602	6.41403785	0.05251687	0.82107788	0.79185006
C230035I16R	-0.192497	0.05992777	0.05243939	0.82120751	0.79191832
Gtpbp1	-0.0528146	4.45862357	0.05228711	0.82146258	0.79198498
Eif1	0.03808919	7.99043545	0.05221581	0.82158215	0.79198498
Rps25	-0.0518183	7.66263212	0.05221526	0.82158307	0.79198498
Ankrd23	0.17148697	-0.7013477	0.05218773	0.82162926	0.79198498
Guk1	0.04956286	4.85729885	0.05218208	0.82163874	0.79198498
Car5b	-0.0689273	1.96853498	0.05207058	0.82182595	0.79198498
Napepld	-0.0408976	6.43308313	0.05206564	0.82183425	0.79198498
Sirpa	-0.032083	7.17435197	0.05205334	0.82185492	0.79198498
Kif3a	-0.0375351	8.10931872	0.05203272	0.82188957	0.79198498
Gnptab	0.03604551	6.15644286	0.05200122	0.82194252	0.79198498
Rapgef1	0.03268166	6.0110437	0.05192176	0.82207616	0.79198498
Wrap53	-0.0966804	1.41100641	0.05190901	0.82209761	0.79198498
Ccnd1	0.04177869	6.16639767	0.05185481	0.82218884	0.79198498
Vit	0.10372795	2.21496262	0.05183796	0.82221722	0.79198498
Cuedc1	-0.0619816	2.60927741	0.05179523	0.82228919	0.79198498
Snapc4	0.0722793	3.72206953	0.05167358	0.82249426	0.79198498
Yy1	0.03038103	5.38509177	0.05165646	0.82252314	0.79198498
Zmiz2	-0.0294782	6.4725955	0.05164933	0.82253516	0.79198498
Mia3	0.03605449	6.29115697	0.05163016	0.82256751	0.79198498
Pnma2	0.03358966	6.40633816	0.05162365	0.82257849	0.79198498

Reck	0.04708843	5.35084657	0.0515192	0.82275485	0.79198498
Cpb1	-0.0953741	2.22765188	0.05151138	0.82276808	0.79198498
Hspbap1	-0.0732335	2.22163898	0.05150867	0.82277264	0.79198498
Slc35e4	-0.1009709	1.114314	0.05147235	0.82283403	0.79198498
Adra1a	0.04189152	4.48599076	0.05146694	0.82284317	0.79198498
Ing3	-0.0419112	4.2107681	0.05145463	0.82286399	0.79198498
Crtap	0.07799744	4.08574639	0.05141514	0.82293076	0.79198498
Dus1l	-0.0621296	3.06335656	0.05141491	0.82293115	0.79198498
Erbb2	0.12473008	1.57463106	0.05135064	0.82303987	0.79198498
Fhdc1	0.11256133	0.87332126	0.0513492	0.82304232	0.79198498
Lyz1	0.14126411	1.04164161	0.05125194	0.82320699	0.79208679
Cdkn1b	0.04553915	4.09793414	0.05115401	0.82337299	0.79218987
Rai1	-0.0365675	5.70124103	0.05104136	0.82356412	0.7922646
Tgfbrap1	0.03056844	5.59622484	0.05100615	0.8236239	0.7922646
Rnmtl1	0.07819779	2.06520423	0.05100416	0.82362729	0.7922646
Ptprg	-0.0313368	6.91792331	0.05095564	0.82370972	0.79228726
Wasl	0.02666293	8.11217707	0.05090399	0.82379751	0.7923104
B930018H19	-0.3313962	-0.9073337	0.05087221	0.82385154	0.7923104
BC068157	-0.0497347	5.92522908	0.05080326	0.82396885	0.79236659
Trim24	0.03149665	5.55712033	0.05072697	0.82409875	0.79237924
Wfikkn2	-0.0986918	2.97892266	0.05072266	0.82410609	0.79237924
Ncor2	0.03412136	6.83000484	0.0506918	0.82415867	0.79237924
2610044O15	0.04057992	4.55994414	0.05054459	0.82440969	0.79256396
Mir1b	0.36374211	-1.1700818	0.05045378	0.82456473	0.79265638
Riok3	-0.0301953	6.33207179	0.05037726	0.82469549	0.79268523
Zfp703	0.03745037	4.68493457	0.05036727	0.82471255	0.79268523
Sox30	-0.2233166	-1.0289941	0.0503197	0.82479391	0.7927068
Slc16a11	0.05860157	3.31827081	0.05025143	0.82491072	0.79276245
Dazap1	0.04789007	3.48253625	0.05004157	0.82527035	0.7930047
Gabra6	0.28194394	-2.0502817	0.05027008	0.82528066	0.7930047
4930528A17	0.29437717	-1.4834842	0.04997145	0.82539069	0.79304388
Atl2	-0.0349263	6.17203477	0.04973999	0.82578854	0.79304388
Creb3	-0.0697962	3.08932762	0.04972076	0.82582164	0.79304388
Atf7ip	0.02353647	6.92989395	0.04965753	0.82593051	0.79304388
A830010M2(	-0.0401754	8.6443168	0.04965042	0.82594275	0.79304388
Hint2	0.05864299	3.50760824	0.04964613	0.82595015	0.79304388
Sp4	-0.0274246	5.9452757	0.04962485	0.82598681	0.79304388
Fbxo5	0.12776592	0.50880187	0.04961968	0.82599573	0.79304388
Gm7694	0.07187413	1.83915516	0.04958851	0.82604943	0.79304388
Alg11	-0.0329997	6.70173638	0.04957867	0.82606641	0.79304388
Tmem208	-0.0681197	2.06204217	0.04956292	0.82609356	0.79304388
Eltd1	-0.0843708	2.92488026	0.0495397	0.8261336	0.79304388
3110040N11	0.05740153	3.1539725	0.04953632	0.82613942	0.79304388

Cers6	0.04555022	5.97498481	0.04946954	0.82625465	0.79304388
Ndufa7	-0.0520047	4.6453274	0.04946544	0.82626172	0.79304388
Cdc42ep3	-0.0427849	3.36695661	0.04944649	0.82629444	0.79304388
Nlrc3	-0.2310299	-0.9379213	0.04938619	0.82639856	0.79304388
Parva	0.03719026	7.42564118	0.04937213	0.82642286	0.79304388
Rpl18	0.05543206	5.80811809	0.04936152	0.82644119	0.79304388
Trappc8	0.02878682	6.26284445	0.04931976	0.82651336	0.79305658
Fam45a	-0.0333551	4.9588035	0.04927848	0.82658474	0.79306457
Arhgap22	0.16626802	0.14062593	0.04924678	0.82663956	0.79306457
Pnisr	0.04199682	7.66635585	0.04909253	0.82690665	0.7931772
Med21	-0.051011	5.66338597	0.04892541	0.82719651	0.7931772
Hiatl1	-0.0406719	4.0000402	0.04892537	0.82719657	0.7931772
Agbl4	0.08432947	2.42252129	0.04889826	0.82724364	0.7931772
Eif4enif1	0.02487831	6.6647732	0.04888386	0.82726865	0.7931772
Ackr4	-0.2652442	-1.4397916	0.04888282	0.82727046	0.7931772
Smad7	0.05524561	3.43137267	0.04878958	0.82743248	0.7931772
Ranbp3	0.03828097	3.79785599	0.04878042	0.8274484	0.7931772
C1galt1c1	-0.0489256	4.60541063	0.048771	0.82746478	0.7931772
Spag6	-0.0745525	1.99929427	0.04871283	0.82756596	0.7931772
Abca3	0.04762576	4.11377564	0.04871015	0.82757063	0.7931772
Atn1	-0.0283973	7.89650302	0.04869744	0.82759274	0.7931772
Xpot	0.02592547	7.00853197	0.0486661	0.82764729	0.7931772
Acsl3	-0.0265979	7.2432512	0.04864526	0.82768358	0.7931772
Frmd6	-0.0351032	5.61707454	0.04862793	0.82771376	0.7931772
Pitpnm1	0.05659858	3.35286185	0.04861446	0.82773722	0.7931772
Ppargc1a	-0.033035	7.95652804	0.04853743	0.82787145	0.7931772
Atp6ap1l	0.12593138	1.11229166	0.04849683	0.82794224	0.7931772
Gpr160	0.14727559	0.01767484	0.04849631	0.82794314	0.7931772
Dnase1l1	0.07379236	2.18178417	0.04842303	0.828071	0.7931772
Ube2v1	0.03418592	6.89310435	0.04834446	0.8282082	0.7931772
Plec	0.0394385	6.34716249	0.04828823	0.82830646	0.7931772
Fadd	-0.0442712	4.30094282	0.04825302	0.82836801	0.7931772
Spag1	0.05081497	2.86066119	0.04823202	0.82840474	0.7931772
Hsd3b3	0.11868521	0.3184857	0.04821514	0.82843427	0.7931772
Sec14l1	-0.0297141	6.38234486	0.04819182	0.82847508	0.7931772
Iqsec2	0.0361253	5.69733138	0.04819112	0.82847629	0.7931772
Chst12	-0.0710097	1.74990168	0.04816495	0.8285221	0.7931772
BC017643	-0.072839	2.48039427	0.04815023	0.82854786	0.7931772
Ndfip1	-0.0352032	7.96191732	0.04813144	0.82858075	0.7931772
Gm4961	0.22202731	-1.7273926	0.04812945	0.82858424	0.7931772
Clpb	0.05011012	3.74008951	0.04809145	0.82865079	0.79318447
Zfp97	0.03641831	4.12311113	0.04804115	0.82873894	0.79320202
Mrgpre	0.05883849	3.56459171	0.04797063	0.82886258	0.79320202

Tbc1d17	0.07104553	2.57286383	0.04789224	0.82900014	0.79320202
Cnksr3	-0.0662295	1.78761728	0.04788561	0.82901177	0.79320202
Sell	-0.2868468	-1.0459632	0.04786562	0.82904688	0.79320202
Bzw2	0.04208571	3.56638894	0.04785609	0.82906363	0.79320202
Map1lc3b	-0.035714	7.87064155	0.04782112	0.82912505	0.79320202
E2f4	-0.0392282	4.54122018	0.04776687	0.8292204	0.79320202
Mgat4a	-0.0423733	5.48766177	0.04774314	0.82926211	0.79320202
Bcorl1	-0.033581	4.54898799	0.04768849	0.82935823	0.79320202
Idnk	-0.0566724	3.48742722	0.04768374	0.82936659	0.79320202
Ripply2	-0.1557252	-0.3361722	0.04766168	0.82940542	0.79320202
Tceanc	-0.0572021	3.2960577	0.04751712	0.82966004	0.79320202
Smg5	0.03713722	4.39297691	0.04745775	0.82976474	0.79320202
Zfp808	-0.0376274	3.85726606	0.04745459	0.82977032	0.79320202
Notch4	0.08878328	1.02824809	0.04742702	0.82981896	0.79320202
Mylk	-0.0374434	6.02107429	0.04741424	0.82984151	0.79320202
Dvl3	0.02869218	6.09227167	0.04738767	0.8298884	0.79320202
Olfr691	-0.2816305	-2.0704792	0.04735301	0.82994961	0.79320202
Pik3c2b	0.03416832	5.11271293	0.04730282	0.83003828	0.79320202
Dhx15	-0.0233877	6.58366258	0.04728384	0.83007181	0.79320202
Mettl7a2	-0.0677092	1.73110516	0.04724782	0.8301355	0.79320202
Rptoros	0.23450241	-1.2315957	0.04724187	0.830146	0.79320202
Gemin8	0.04872202	3.45202186	0.04722367	0.83017819	0.79320202
Cntd1	-0.057087	2.33775579	0.04713558	0.83033406	0.79320202
Rps20	-0.0391355	6.34851348	0.04713377	0.83033726	0.79320202
Il1a	0.20391318	-1.0177028	0.04708299	0.8304272	0.79320202
Gimap4	-0.0795819	3.0918841	0.04707907	0.83043413	0.79320202
Syp	0.04384239	8.81758959	0.04706669	0.83045606	0.79320202
Dpp10	-0.0376112	6.87344246	0.0470339	0.83051417	0.79320202
Swt1	-0.0416442	4.57133182	0.04698402	0.8306026	0.79320202
Acot11	-0.0444589	3.82662615	0.04698272	0.8306049	0.79320202
Prdm10	0.06049938	2.96049665	0.04697095	0.83062577	0.79320202
Ect2	-0.2162065	-0.849832	0.04691805	0.83071962	0.79320202
Bod1l	-0.0286287	8.59520974	0.04683676	0.83086395	0.79320202
Haus2	0.02998756	5.4932092	0.04680647	0.83091776	0.79320202
Atox1	0.05631508	4.36596324	0.04679367	0.83094051	0.79320202
Sdr39u1	0.03578833	4.98892235	0.04670463	0.83109882	0.79320202
Capn3	0.08636747	0.85097629	0.04667757	0.83114696	0.79320202
Tmem59l	-0.0537672	4.17803681	0.04663897	0.83121565	0.79320202
Dus3l	-0.0448058	3.75938333	0.04649118	0.83147896	0.79320202
D430042O09	-0.052004	3.42769263	0.04647007	0.8315166	0.79320202
Smim14	0.04148074	7.54290449	0.04643201	0.83158452	0.79320202
C4bp-ps1	0.32181383	-1.5809795	0.04639644	0.83164798	0.79320202
06-Mar	0.02894847	8.79093608	0.04630911	0.83180395	0.79320202

Cdh23	-0.1905806	-1.1264785	0.04629833	0.83182321	0.79320202
Tfr2	-0.1136032	0.97778747	0.04626704	0.83187914	0.79320202
A530064D06	0.23255257	-1.7685004	0.04626012	0.83189151	0.79320202
Saysd1	-0.05931	2.32347982	0.04625943	0.83189275	0.79320202
Nae1	-0.0349494	5.057229	0.04622989	0.83194556	0.79320202
Zfp142	-0.0384106	5.2626512	0.04615338	0.83208246	0.79320202
Galnt11	-0.0483553	3.29136292	0.0461294	0.83212539	0.79320202
Flcn	0.03116266	4.56344802	0.04609628	0.8321847	0.79320202
Tomm40	0.04880008	3.08970157	0.04608091	0.83221222	0.79320202
Harbi1	0.08294406	2.1471248	0.04606544	0.83223995	0.79320202
Nos1ap	0.05593332	3.69490422	0.04605109	0.83226566	0.79320202
Fbxo27	-0.0440898	3.83541768	0.04604274	0.83228062	0.79320202
A930007I19F	-0.2503571	-1.0315165	0.046037	0.8322909	0.79320202
Ppp1r21	-0.0272116	5.54478482	0.04603151	0.83230075	0.79320202
Maf1	-0.039307	4.57616593	0.04597149	0.83240837	0.79320202
Zfp599	-0.0607438	2.83120872	0.04597038	0.83241036	0.79320202
Cox7a2l	0.03592228	7.11294571	0.04596494	0.83242013	0.79320202
Igbp1	-0.039131	5.79648607	0.04596136	0.83242654	0.79320202
Isoc1	-0.0363843	5.90672511	0.04595292	0.83244169	0.79320202
Hdac7	0.04212879	3.70229096	0.04588415	0.83256512	0.79326346
Zfp934	0.0487027	3.61369777	0.04576933	0.83277141	0.79340383
Fggy	-0.0642093	2.85345833	0.04560561	0.83306602	0.79362832
Tceb2	-0.0353826	5.70765472	0.04555721	0.83315322	0.79365521
Cfl1	0.02662528	8.59102715	0.04552266	0.83321549	0.79365633
Plekho1	0.04009225	4.03851156	0.04549113	0.83327236	0.79365633
Zbed5	-0.0485712	3.8677752	0.04543229	0.83337852	0.79370126
Nup214	-0.0329667	6.07547627	0.04534143	0.83354258	0.79375533
Npc2	-0.0583423	4.68547146	0.0453031	0.83361185	0.79375533
Pnpla6	0.0500976	4.04739104	0.04528534	0.83364395	0.79375533
Dffb	0.11025828	1.45709339	0.04527025	0.83367124	0.79375533
Stom	-0.0909272	5.20970168	0.04519234	0.83381216	0.79378999
Pou6f2	-0.0874152	1.93296602	0.04516067	0.83386949	0.79378999
Eya3	0.03521413	5.04768282	0.04513069	0.83392379	0.79378999
Klhl9	-0.0240504	7.39308747	0.04508105	0.83401372	0.79378999
Rpl11	-0.040072	7.046423	0.04506674	0.83403965	0.79378999
Cav2	-0.0397985	5.18438214	0.04503371	0.83409953	0.79378999
Gstt1	0.0531313	4.87976353	0.04502211	0.83412057	0.79378999
Tbcd	-0.0417385	3.95613517	0.04495488	0.83424252	0.7938499
Tmeff2	-0.0286699	6.92265816	0.04489239	0.83435597	0.79388736
Toporsos	-0.0763791	1.98353634	0.04486822	0.83439988	0.79388736
Ankrd49	0.04530519	4.93883628	0.04483467	0.83446082	0.79388921
Slc2a5	0.14886933	-0.6597049	0.04479846	0.83452664	0.7938957
Dnajb12	0.0353377	3.90776884	0.0447237	0.83466264	0.79396895

Krt25	-0.3183431	-1.5438074	0.04460044	0.83488709	0.79411114
Tmsb15b1	0.0897742	1.17472549	0.04452096	0.835032	0.79411114
Ackr3	-0.0479852	3.93527382	0.04448681	0.83509431	0.79411114
Dr1	0.03254111	5.49912096	0.04438475	0.83528065	0.79411114
Fxyd5	0.05719644	6.75997088	0.04435677	0.83533178	0.79411114
Agbl3	-0.0483105	3.1280662	0.04434625	0.83535101	0.79411114
Anks1b	-0.0353791	8.50588551	0.04428696	0.83545942	0.79411114
Ccdc158	0.18092801	-0.7985472	0.04426751	0.835495	0.79411114
Gm20063	0.0367238	3.75008213	0.04426173	0.83550558	0.79411114
Zfp706	0.02750869	7.48004342	0.04423079	0.83556219	0.79411114
Abi2	-0.0253942	8.54721792	0.04416406	0.83568438	0.79411114
Zfp580	0.03872216	3.72006209	0.04411247	0.8357789	0.79411114
Kcnh1	0.03783809	6.07146337	0.04409862	0.83580429	0.79411114
Stoml1	0.05592347	3.03885234	0.0440928	0.83581496	0.79411114
Pld2	0.07122151	2.40034598	0.04406161	0.83587215	0.79411114
Fbxo3	-0.0243668	6.50548621	0.04401882	0.83595064	0.79411114
Lias	-0.0332708	4.59285949	0.04393515	0.83610424	0.79411114
Idh2	-0.0478049	4.04339643	0.04391433	0.83614248	0.79411114
Mia	0.13002766	-0.1530321	0.04389903	0.83617059	0.79411114
1600012H06	-0.0446446	4.59940473	0.04388164	0.83620256	0.79411114
Klri2	-0.1465754	0.40890264	0.04387321	0.83621805	0.79411114
Gm12429	0.0850688	0.85949264	0.04380994	0.8363344	0.79411114
Slc16a14	0.05695192	4.04172305	0.04376964	0.83640854	0.79411114
AW549877	-0.0268047	7.65307398	0.04376529	0.83641655	0.79411114
Slc1a6	-0.112984	0.37276094	0.04374753	0.83644923	0.79411114
Zfp449	-0.0441491	5.20703437	0.04373739	0.8364679	0.79411114
Dusp14	0.0304571	5.93030232	0.04369853	0.83653946	0.79411114
Edc3	-0.053236	3.0896471	0.04364489	0.83663829	0.79411114
Aif1	0.09680877	2.15680055	0.04356399	0.83678747	0.79411114
Fam65a	-0.0396645	7.33207848	0.04349124	0.83692175	0.79411114
Rims4	0.22808747	-1.1305353	0.04347936	0.83694369	0.79411114
Mtx3	0.03242825	5.99207355	0.04346019	0.83697909	0.79411114
Pura	0.02145547	6.97518795	0.04345645	0.836986	0.79411114
Canx	0.02910737	7.9635512	0.04344603	0.83700525	0.79411114
Cdc37l1	-0.0247736	6.61550498	0.04338678	0.83711476	0.79411114
Cpa2	0.14136909	-0.4612285	0.04337873	0.83712964	0.79411114
Cul2	0.03694865	5.27435675	0.04332715	0.83722505	0.79411114
Mospd1	-0.0353972	6.13396769	0.04331933	0.83723951	0.79411114
Adamtsl4	0.05290876	2.8491448	0.04329009	0.83729362	0.79411114
Mid2	0.03195815	5.67538724	0.04328801	0.83729748	0.79411114
Fabp5	0.035838	4.98497235	0.04324734	0.83737277	0.79411114
Tnfrsf12a	-0.1453368	-0.8392961	0.04324361	0.83737968	0.79411114
Gm15412	-0.3872811	-1.650277	0.04320795	0.83744574	0.79411114

Dync1i2	0.02729252	7.48767004	0.04319124	0.83747671	0.79411114
Eif3h	0.03213916	6.19535446	0.04317333	0.83750989	0.79411114
Supt5	0.03494088	5.64203307	0.0431406	0.83757057	0.79411114
Vipas39	-0.0286187	4.82685102	0.04313241	0.83758578	0.79411114
Ccl7	0.18323544	-0.4119397	0.04297115	0.83788512	0.79429147
Brwd3	-0.0303375	6.15172761	0.04296635	0.83789403	0.79429147
Elof1	0.06316493	3.1789434	0.04292207	0.83797635	0.79431354
Cngb1	0.12363173	0.65399982	0.04287428	0.83806522	0.79432104
Gm10408	-0.1361154	-0.4186977	0.04281435	0.83817676	0.79432104
Ndufs3	0.02980785	6.44442752	0.04280154	0.8382006	0.79432104
Tubb3	-0.0361857	4.68233189	0.04279092	0.83822038	0.79432104
Zfp385c	-0.0682364	1.90571192	0.04266881	0.83844795	0.79445948
Ccdc183	0.11489273	0.60674389	0.04264236	0.83849728	0.79445948
Cnst	-0.0316302	6.16085831	0.04261754	0.83854359	0.79445948
Slc27a3	-0.2593226	-1.6827004	0.04251213	0.83874044	0.79452027
C030037D09I	-0.1024932	1.34915979	0.04249772	0.83876738	0.79452027
Smek1	-0.0286391	5.62499511	0.04247813	0.838804	0.79452027
Rab8b	0.02738434	6.34753689	0.04244875	0.83885892	0.79452027
Cflar	0.02476509	6.86384183	0.04237452	0.8389978	0.79452027
Eif3a	0.03082184	9.48235141	0.04236929	0.83900758	0.79452027
Hcar1	0.07343022	4.37220881	0.04236209	0.83902106	0.79452027
Urb2	0.04263277	4.20458844	0.04230369	0.83913043	0.79452081
Morc3	0.04496996	4.5466549	0.04229864	0.83913989	0.79452081
Rps9	0.04642355	6.45473868	0.04226722	0.83919877	0.79452081
Tnks2	-0.0237355	8.33239635	0.04221073	0.83930469	0.79456519
Brinp2	-0.0345477	4.4590988	0.04205714	0.83959302	0.79474236
Akap17b	-0.0313352	5.61061691	0.04204813	0.83960996	0.79474236
Coa3	0.03291598	4.39961295	0.04200144	0.83969773	0.79476954
Tbc1d25	-0.0475178	3.07336258	0.04192149	0.83984814	0.79485599
Gm16701	-0.1196154	0.69909148	0.04186504	0.83995444	0.79490069
Arl6	0.03138772	5.70105526	0.04175601	0.84015995	0.79495179
B3gnt1	0.0338173	4.84392782	0.04174586	0.8401791	0.79495179
Bola1	0.07318254	1.69514703	0.04174238	0.84018567	0.79495179
Rab3gap2	-0.0414635	6.46892476	0.04158022	0.84049189	0.79518561
Sema6a	-0.0358675	5.73162937	0.04154124	0.8405656	0.79519944
Gabbr2	0.03554586	7.12221704	0.04142298	0.84078943	0.79535527
Kcnq4	0.20079238	-1.0620554	0.04137023	0.84088937	0.79535811
Mrpl11	0.03845452	4.64254819	0.04133162	0.84096257	0.79535811
Mtrf1	-0.0452826	3.13216346	0.04132783	0.84096975	0.79535811
Cacna1c	-0.0413526	6.3128366	0.04127081	0.84107792	0.79540451
Retn	0.24939343	-1.5550005	0.04115216	0.84130326	0.79549299
Dhtkd1	0.11912769	1.49539204	0.04114571	0.84131552	0.79549299
Hist1h1c	0.08529336	2.87540411	0.04110457	0.84139375	0.79549299

Gm5441	0.15185701	0.35213005	0.04109125	0.84141907	0.79549299
Cxcr6	0.13730351	0.03419362	0.04106352	0.84147183	0.79549299
Dclre1a	-0.0512591	3.56079589	0.04102969	0.84153621	0.79549299
B4galt5	-0.0377255	4.63843676	0.04098391	0.84162337	0.79549299
Lrrn1	0.03876363	6.20171724	0.04096114	0.84166676	0.79549299
Mmp24	0.06735729	2.48675089	0.04094184	0.84170353	0.79549299
Slc38a10	-0.0430719	3.43456733	0.04088364	0.84181448	0.79554197
Rubie	-0.2641975	-1.4883069	0.04079943	0.84197517	0.7955705
Tpcn2	-0.1795089	-0.307709	0.04074448	0.84208011	0.7955705
Snord4a	0.08893802	0.58716041	0.04074142	0.84208595	0.7955705
Clec14a	-0.0766824	1.55869742	0.04072839	0.84211084	0.7955705
Gosr2	0.02333954	6.62556428	0.04068229	0.84219896	0.7955705
Sh2d2a	0.08676963	1.19444342	0.04066271	0.8422364	0.7955705
Tomm5	-0.0394101	4.59282901	0.04065115	0.84225853	0.7955705
Cep68	-0.0372022	5.0034212	0.04058358	0.84238781	0.79558588
Os9	-0.0403216	4.71647211	0.04058084	0.84239306	0.79558588
Gm11696	0.08055926	1.18376986	0.04050971	0.84252931	0.79562413
Tbc1d7	0.03914752	4.04487966	0.04047002	0.84260538	0.79562413
Ptdss2	-0.0449858	3.24280538	0.04046712	0.84261093	0.79562413
Ap5b1	-0.1480969	-0.0106222	0.04039173	0.84275556	0.79570486
Nab2	-0.0409935	5.10920248	0.04034597	0.8428434	0.79570642
9030612E09I	-0.1026515	1.32594339	0.04030632	0.84291956	0.79570642
Xist	-0.1482519	0.81075274	0.04029849	0.84293461	0.79570642
Mutyh	0.1166714	-0.0979963	0.04022718	0.84307169	0.79577999
Coq3	-0.0423357	3.37844803	0.04016481	0.84319169	0.79583667
Gal	0.18862529	-0.4440306	0.04013451	0.84325002	0.79583667
Bnip1	0.06943669	2.16630286	0.04008481	0.84334575	0.7958712
Katnal1	0.0276498	6.85502897	0.04001382	0.84348261	0.79592541
Gpr18	-0.1682471	-0.4416102	0.03997166	0.84356394	0.79592541
Pard6b	0.0517971	3.34604039	0.03996168	0.84358319	0.79592541
Cyp4f16	0.07859154	1.73480182	0.03991859	0.84366637	0.79592541
0610007P14I	0.03950173	4.25240262	0.03988969	0.84372218	0.79592541
Samd3	0.09706983	1.32817497	0.03977409	0.84394567	0.79592541
Gm15455	0.12643385	-0.8764265	0.03973793	0.84401563	0.79592541
Nme5	-0.0470515	3.39963312	0.03970086	0.84408739	0.79592541
Fam136a	0.03777311	3.84049672	0.03968035	0.84412712	0.79592541
Tnfrsf14	-0.2757242	-1.8318768	0.03967876	0.84413021	0.79592541
Cntrl	-0.0420807	4.31281987	0.03965852	0.84416941	0.79592541
Htatip2	0.06981473	2.04140005	0.03965761	0.84417119	0.79592541
Dnajb6	-0.0216765	7.95725082	0.03965712	0.84417213	0.79592541
Qtrtd1	-0.064813	2.08839529	0.03945087	0.8445723	0.7961995
Ephb1	-0.0420751	3.65570156	0.03943974	0.84459392	0.7961995
1700024B18I	-0.1823109	-0.3069596	0.03941585	0.84464035	0.7961995

Emcn	-0.1369969	0.59230906	0.03936988	0.84472974	0.796228
Pkdcc	-0.108508	0.20160882	0.03920832	0.8450443	0.79645898
Ndufb5	0.02892882	6.73413816	0.0391641	0.84513052	0.79645898
Rxrb	-0.0459525	3.23806349	0.03915289	0.84515237	0.79645898
Apmap	0.04259644	4.89839302	0.03910194	0.84525179	0.79646663
Ddx27	0.03173468	4.1812579	0.03908807	0.84527886	0.79646663
Kcna3	-0.082978	1.38186323	0.03902165	0.84540859	0.7965196
Slc25a51	-0.0226892	6.87552165	0.03898549	0.84547926	0.7965196
Siae	0.03718151	4.5606751	0.03896841	0.84551265	0.7965196
Bcl9l	0.03399786	5.44706175	0.03888814	0.8456697	0.79655592
Dao	0.30552757	-1.5808403	0.03886148	0.84572189	0.79655592
Il5	-0.273375	-1.8544092	0.03885796	0.8457288	0.79655592
Tomm7	0.03150575	4.6452699	0.03878156	0.84587846	0.79664112
Tmem216	0.04893754	2.85697965	0.03868722	0.84606353	0.79675374
Tep1	-0.0647558	2.96774896	0.03865209	0.84613248	0.79675374
Epyc	-0.1432386	-0.2199656	0.03860196	0.84623096	0.79675374
Tmod2	0.02296536	9.94559003	0.03859996	0.84623488	0.79675374
Tnfaip8l1	-0.0598513	2.81437686	0.03853652	0.8463596	0.79680228
Snrnp35	0.05185215	3.60208092	0.03851351	0.84640486	0.79680228
Slc1a2	-0.036589	10.9655463	0.038441	0.84654758	0.79682854
Sox5	-0.0327824	5.99055607	0.03843494	0.84655951	0.79682854
Kcnj14	0.23649638	-1.0669554	0.03833631	0.84675389	0.79682854
Zfp524	-0.1220751	-0.0109418	0.03827776	0.84686941	0.79682854
Fam167a	0.06725962	2.37068262	0.03826454	0.8468955	0.79682854
Atad2b	0.03105022	5.16211525	0.03821655	0.84699027	0.79682854
Prpsap1	0.02528018	4.70373033	0.03821201	0.84699922	0.79682854
1700029I15R	-0.1509047	-0.841606	0.03821072	0.84700178	0.79682854
Snrk	-0.0234747	6.38919124	0.03821007	0.84700306	0.79682854
Nisch	0.02680612	7.3684988	0.0381718	0.84707869	0.79682854
Brix1	0.03068768	4.72605738	0.03816904	0.84708413	0.79682854
2310057M21	0.03425199	4.09603702	0.03805693	0.84730591	0.79693195
Sspo	-0.2695017	-1.2452705	0.03805359	0.84731251	0.79693195
Snrnp27	-0.040158	5.43105277	0.03787372	0.84766903	0.79721155
Tbca	-0.0380022	6.43366701	0.03774295	0.84792879	0.79735287
Ddc	-0.0776489	1.34508419	0.03773164	0.84795127	0.79735287
Cln5	0.04534748	6.00046903	0.03762725	0.84815899	0.79735287
Nudt18	0.04653749	3.81344971	0.03755675	0.84829944	0.79735287
H2-Ke2	0.04976094	3.77446252	0.0375334	0.848346	0.79735287
Wdr19	-0.0397416	4.6043891	0.03751108	0.84839049	0.79735287
Zfp637	0.04533067	3.80381429	0.03750876	0.84839514	0.79735287
Cnr2	-0.0854925	0.95599685	0.03750432	0.84840399	0.79735287
Art3	-0.1791066	-0.4051055	0.03748021	0.84845208	0.79735287
Dtd2	-0.0488461	4.09246295	0.03745224	0.84850792	0.79735287

Ube2i	-0.0265318	7.37592641	0.03743493	0.84854246	0.79735287
Atp6v0a1	0.02826637	7.21774793	0.03740892	0.8485944	0.79735287
Sipa1l3	-0.0296403	5.34766959	0.03740096	0.84861031	0.79735287
Ubxn1	-0.0465045	5.407365	0.03738166	0.84864887	0.79735287
C8g	-0.1342101	0.37226714	0.03730039	0.84881133	0.79744984
Gga1	0.0380198	3.54956689	0.03725807	0.84889601	0.79747371
Marveld3	-0.2503646	-0.9875168	0.03718854	0.84903525	0.79750339
Plxnb2	-0.0308624	4.78627518	0.03715748	0.8490975	0.79750339
Itgb1bp1	0.02948539	4.68775085	0.03714911	0.84911427	0.79750339
Akr1c13	-0.0813899	1.750315	0.03708577	0.8492413	0.79750339
Rpl23	-0.0315788	7.17334771	0.03708553	0.84924178	0.79750339
Ap2s1	-0.0479366	3.45522275	0.03706489	0.84928319	0.79750339
Ttc39b	0.03242844	7.09488054	0.03695091	0.84951213	0.7976084
Abi3bp	-0.040433	4.18280323	0.03691288	0.84958859	0.7976084
Eif4e2	0.02907012	5.28784106	0.03690207	0.84961035	0.7976084
Pgm5	0.04556126	6.55807452	0.03689114	0.84963233	0.7976084
F10	-0.1507125	-0.8615185	0.03686178	0.8496914	0.7976084
Gpbp1l1	0.02984917	6.29620693	0.03677808	0.84985996	0.79767641
Cry1	0.03502967	4.34636312	0.03676694	0.8498824	0.79767641
Fbxo21	0.03192704	5.66249624	0.03673012	0.84995665	0.79769045
Fam178b	0.18903922	-1.1721613	0.03667224	0.85007339	0.79769882
Ngfrap1	0.03038982	6.52437935	0.03666334	0.85009136	0.79769882
Rrp7a	0.04424432	4.01122213	0.03661943	0.85018001	0.79769882
Prkci	0.02212093	7.35728916	0.03657916	0.85026135	0.79769882
Cask	-0.0226075	7.6195269	0.03656874	0.85028241	0.79769882
C330006A16I	-0.0275272	5.98893754	0.03648957	0.85044252	0.79769882
Psmc3	-0.0281322	5.5806721	0.03645044	0.8505217	0.79769882
Bmp6	0.04846697	6.6331763	0.03644356	0.85053564	0.79769882
Tlr1	0.22888459	-0.9829122	0.03637411	0.85067631	0.79769882
Elmod3	-0.0578308	3.29131426	0.0363534	0.85071829	0.79769882
Ptdss1	0.02899114	4.8909108	0.0363529	0.8507193	0.79769882
6430531B16I	-0.1967961	-0.6821775	0.0362692	0.85088908	0.79769882
Chchd4	-0.0365675	4.78100268	0.0362214	0.85098614	0.79769882
Phf19	-0.1189818	0.71528443	0.03618846	0.85105307	0.79769882
Meis3	-0.0494387	3.60716697	0.03618707	0.85105589	0.79769882
Fat1	0.0295466	6.13438627	0.03616187	0.8511071	0.79769882
Ndufaf3	0.03521108	4.14631992	0.03612309	0.85118595	0.79769882
Arhgap20os	0.16562369	0.33173042	0.03608804	0.85125726	0.79769882
Secisbp2l	0.02273571	7.76977179	0.03604943	0.85133585	0.79769882
Rnf13	0.03070313	7.06736367	0.03604805	0.85133867	0.79769882
Pex14	0.05493027	2.54592797	0.0360281	0.8513793	0.79769882
Slirp	0.03217241	4.7971432	0.03600605	0.8514242	0.79769882
Zbtb49	0.06875138	1.51435636	0.03594845	0.85154161	0.79769882

0610037L13I	0.0373596	4.97435407	0.03594034	0.85155814	0.79769882
Igf2bp3	0.04512789	4.2010688	0.035924	0.85159148	0.79769882
Zfp931	-0.045163	2.57628972	0.03590963	0.85162079	0.79769882
1700037C18I	0.11050299	0.04865188	0.03590799	0.85162413	0.79769882
Lgr5	-0.057621	2.48091856	0.03590736	0.85162542	0.79769882
C920006O11	0.06794896	1.74888489	0.03587255	0.85169646	0.79770983
Ppap2c	-0.0742715	1.53986906	0.03581188	0.85182035	0.79777035
Lemd2	-0.0641558	2.11383672	0.03577554	0.85189463	0.79778438
Ociad1	0.02186925	7.1434542	0.03566127	0.8521284	0.79784587
Uimc1	0.0336361	4.62464259	0.03563293	0.85218644	0.79784587
Lca5	0.03943742	4.18042977	0.03560584	0.85224193	0.79784587
Gcdh	0.04105485	3.16647822	0.03558174	0.85229134	0.79784587
Mtg1	-0.057368	2.77387314	0.03556814	0.85231922	0.79784587
Cep112	-0.0399948	3.64725574	0.03552389	0.85240997	0.79784587
Polr3c	-0.0385148	3.20865398	0.03550958	0.85243933	0.79784587
Rbm19	-0.0591536	2.26735387	0.03547976	0.85250055	0.79784587
Spry2	0.02631442	5.95328064	0.03544792	0.85256592	0.79784587
Slamf9	-0.2019837	-0.3339572	0.03539732	0.8526699	0.79784587
Cfhr2	-0.0970425	0.69676622	0.03537089	0.85272424	0.79784587
Arhgap10	0.03650468	4.4409268	0.03536424	0.85273791	0.79784587
Fdx1	0.04666858	5.38919564	0.03529341	0.85288363	0.79784587
Glp2r	-0.0999972	1.68436136	0.03528485	0.85290127	0.79784587
Arg2	0.06328705	2.92564472	0.0352654	0.8529413	0.79784587
Smcr8	0.02866015	5.78459802	0.03524828	0.85297657	0.79784587
Opn4	0.16018706	-0.0898355	0.03524471	0.85298393	0.79784587
Polr1e	0.05484955	1.80469132	0.03522354	0.85302754	0.79784587
Dhcr7	-0.0397715	3.72930431	0.03517237	0.85313303	0.79788908
Chd7	0.04579908	4.64904099	0.03509612	0.85329037	0.79798077
Rpap2	-0.0276265	4.70556522	0.03504524	0.85339545	0.79802358
Acbd3	0.02223576	6.49257134	0.03498408	0.85352188	0.79806036
Acbd4	0.05745667	2.3604109	0.03496679	0.85355764	0.79806036
Rpf1	-0.0321795	4.10670821	0.03494017	0.85361271	0.79806036
Tex26	0.13707312	-0.7564321	0.03483888	0.85382248	0.79810684
Fhod3	0.03796696	5.14905749	0.03483439	0.85383179	0.79810684
D030056L22I	0.03691976	4.86622	0.03479117	0.8539214	0.79810684
Bub3	0.02422769	5.8548539	0.03478277	0.85393881	0.79810684
Cutc	0.04246591	3.81524854	0.03477305	0.85395898	0.79810684
Sec16a	-0.0256996	6.43811088	0.03451909	0.85448681	0.79842891
Topbp1	0.03399671	5.30103597	0.03450238	0.85452163	0.79842891
Mchr1	0.0607897	2.58860554	0.03448058	0.85456702	0.79842891
Commd10	-0.0331162	4.67204562	0.03444192	0.85464761	0.79842891
Lsm1	-0.0428996	4.10099304	0.03442287	0.85468733	0.79842891
Ttn	-0.0541282	3.64854809	0.03441246	0.85470904	0.79842891

Ephb6	0.04826927	3.56772938	0.0343954	0.85474463	0.79842891
Gga2	0.03481352	4.47761348	0.03437928	0.85477827	0.79842891
2610020C07I	-0.0973608	0.53748742	0.03433622	0.85486814	0.79845744
Dpf2	0.02390277	5.81680931	0.03424598	0.85505671	0.79857814
Tbc1d2b	-0.0299683	4.3888856	0.03415648	0.85524397	0.79865272
Bhlhe40	0.0218703	7.52034272	0.03415082	0.85525584	0.79865272
Trmt13	0.06815708	1.35786916	0.03410085	0.85536051	0.79865272
Kank4	0.04690898	3.92980639	0.03409442	0.85537398	0.79865272
Serpinb6b	-0.0558111	5.19949551	0.03401706	0.85553621	0.79873359
Vgll4	-0.0547876	2.97093093	0.03397112	0.85563264	0.79873359
Gas1	0.04670161	5.78647272	0.03393401	0.85571057	0.79873359
Rps8	-0.0354203	7.08743797	0.03392025	0.8557395	0.79873359
Kif22	0.08445389	0.89356003	0.03391174	0.85575738	0.79873359
Mir690	-0.2479745	-2.00979	0.03387938	0.8558254	0.79874167
Luc7l3	-0.0234623	8.5707348	0.03382598	0.85593773	0.79875304
Sppl2a	0.02830915	6.38309585	0.03381716	0.8559563	0.79875304
Set	-0.0224707	8.09528691	0.03376945	0.85605675	0.79879139
Slc25a15	-0.0401152	3.41197835	0.03367002	0.85626632	0.79893154
Pafah1b2	-0.0193202	8.35178325	0.03360763	0.85639799	0.79895693
2310002F09I	0.29384689	-1.5177749	0.03357668	0.85646336	0.79895693
Nsun3	-0.0310067	4.6415673	0.03354832	0.85652328	0.79895693
Htra3	0.06171645	4.06121175	0.03354466	0.85653103	0.79895693
Fkbp10	-0.0894312	2.21110393	0.0335032	0.85661868	0.79896205
Gm5113	-0.0442238	4.18435604	0.0334859	0.85665527	0.79896205
Arpp19	0.01985474	9.06957208	0.03333435	0.85697623	0.79920538
Tmem59	-0.0310026	6.13848722	0.03330666	0.85703495	0.79920538
Zfp68	-0.0308324	6.18202329	0.03322361	0.85721125	0.79931439
L3mbtl2	-0.0352914	3.73725995	0.03310697	0.85745923	0.79938205
Usp35	0.06200771	1.87628475	0.033085	0.85750597	0.79938205
Tcf25	-0.0186532	8.4293522	0.03303959	0.85760267	0.79938205
Man1b1	0.03918288	3.68547036	0.03303002	0.85762305	0.79938205
Fndc4	0.04111912	3.68251726	0.03301444	0.85765627	0.79938205
Nudt13	-0.0450816	2.66711577	0.03299529	0.85769709	0.79938205
Antxr1	0.03176347	6.12795934	0.03299408	0.85769965	0.79938205
Cyb561	0.02436338	4.44562997	0.03291756	0.85786288	0.79941696
Zzz3	0.02129009	7.29131947	0.03287129	0.85796165	0.79941696
Recql5	0.05501956	2.43642197	0.03283703	0.85803484	0.79941696
2610507B11I	-0.0201374	8.00325485	0.03283067	0.85804845	0.79941696
Pcdhgb2	0.05762838	2.11965788	0.03264478	0.85844629	0.79941696
Sgms1	-0.0229877	5.94055763	0.03264195	0.85845236	0.79941696
Erc2	0.03080591	8.10052414	0.03258686	0.85857051	0.79941696
5530401A14I	0.11085643	0.01836977	0.032574	0.85859811	0.79941696
Psmd7	-0.0205434	6.76951316	0.03257223	0.85860189	0.79941696

Cfh	-0.0409248	7.13021819	0.03254131	0.85866828	0.79941696
Dixdc1	0.01858365	6.91295512	0.03251907	0.85871602	0.79941696
Gpr153	-0.0506059	3.00274264	0.03250821	0.85873935	0.79941696
Zfp442	0.03716092	3.50310262	0.03245682	0.85884979	0.79941696
Brf2	0.09425823	0.70218386	0.03245524	0.85885317	0.79941696
Nudt2	0.04422437	3.19216054	0.03245099	0.85886232	0.79941696
Lrfn1	0.1059373	0.21389315	0.03244192	0.85888183	0.79941696
Acsf2	0.03170914	4.8861984	0.03244029	0.85888532	0.79941696
Agpat5	-0.024391	5.497355	0.03243118	0.85890493	0.79941696
Agfg2	0.03413963	3.6596711	0.03241932	0.85893042	0.79941696
Chic1	-0.0232843	6.36591201	0.03240561	0.85895992	0.79941696
Trim25	0.03314094	6.41306615	0.03230858	0.85916886	0.79941696
Pan3	0.02113586	6.70390034	0.03229381	0.8592007	0.79941696
Tagln3	0.02795101	6.38412678	0.03224959	0.85929604	0.79941696
Smn1	0.03877814	4.31988517	0.03220565	0.85939085	0.79941696
Rpl7l1	-0.0266406	4.58990866	0.03218899	0.85942682	0.79941696
Acot10	-0.1703239	-0.5193226	0.03217252	0.85946237	0.79941696
Tbl2	-0.0349872	3.49257687	0.03214983	0.85951139	0.79941696
Slc38a2	0.04414882	9.37180083	0.03209821	0.85962295	0.79941696
Cep76	-0.0377668	3.92024491	0.03208343	0.85965493	0.79941696
Tmc2	-0.1119786	-0.2558401	0.03208265	0.8596566	0.79941696
Actr2	0.01930392	8.88066286	0.03205193	0.85972308	0.79941696
Pank2	0.02878192	5.50651976	0.03203511	0.85975948	0.79941696
St6galnac6	-0.0375447	4.07207896	0.03198639	0.85986497	0.79941696
Milr1	0.17641783	-0.3117248	0.03197714	0.85988499	0.79941696
Eif2b5	-0.0292155	4.56639285	0.03196565	0.85990989	0.79941696
Psmb2	0.03134692	5.36654514	0.03195424	0.85993461	0.79941696
Cryzl1	0.02836716	5.36087716	0.03195397	0.85993522	0.79941696
Adnp2	0.03345065	4.66110951	0.03179943	0.86027056	0.79967346
Crybg3	0.03696421	4.24807861	0.03174415	0.86039072	0.79972991
Socs2	0.03029644	5.3342442	0.03169613	0.86049518	0.79973303
Cacul1	0.02240588	6.85414444	0.03168797	0.86051294	0.79973303
Ubn1	-0.0239088	5.81141917	0.03163332	0.86063195	0.7997884
Crabp2	-0.0552207	6.22136604	0.03159004	0.86072629	0.79982083
Becn1	-0.0315802	5.24781277	0.03152246	0.86087369	0.79989278
Sowaha	-0.0283209	7.44815938	0.03147654	0.86097396	0.79989278
Llph	0.03085571	7.07072829	0.03145134	0.861029	0.79989278
Ndufs6	-0.0363847	5.1640407	0.03144563	0.86104149	0.79989278
Cc2d1b	0.03730718	3.62334227	0.03135398	0.86124192	0.80002374
Odf2l	0.04437995	3.49166624	0.03131325	0.86133109	0.80005134
Sod1	0.03615378	8.00215232	0.03122371	0.86152734	0.80008979
Tsen2	0.05348588	2.55053388	0.03115488	0.86167838	0.80008979
Shpk	-0.1125458	0.61399241	0.03114426	0.86170171	0.80008979

Dtnb	-0.0314123	4.6439669	0.03106929	0.86186646	0.80008979
Lamtor1	-0.0376787	5.52992674	0.03104716	0.86191513	0.80008979
Tut1	0.05706214	2.2972698	0.03103166	0.86194924	0.80008979
Rabl6	-0.0261327	5.81756979	0.03101141	0.86199381	0.80008979
Tmc8	-0.1981817	-1.0856689	0.03099451	0.862031	0.80008979
Wee1	-0.0255227	5.51696953	0.03097143	0.86208183	0.80008979
Ppp3ca	-0.0243184	9.63284311	0.03092015	0.86219483	0.80008979
Wfdc1	-0.0796709	2.89032144	0.0308669	0.86231226	0.80008979
Gnai2	0.0395151	7.06060397	0.03086263	0.86232168	0.80008979
Cytl1	-0.2070549	-0.5109423	0.03085806	0.86233177	0.80008979
Smim8	-0.0480991	3.79567374	0.03076853	0.86252949	0.80008979
Man1a2	0.02131817	7.88075799	0.03070817	0.86266294	0.80008979
Gm13034	0.15798233	-1.6862613	0.03069496	0.86269216	0.80008979
Abca7	0.07221875	1.81543376	0.03067819	0.86272927	0.80008979
Fbxo41	-0.034608	5.17505856	0.03067595	0.86273424	0.80008979
Gdf5	-0.174029	-1.6391325	0.03063561	0.86282357	0.80008979
Nexn	0.0369548	5.68358116	0.03059588	0.86291159	0.80008979
Exoc6b	0.0204993	6.79077474	0.03058597	0.86293356	0.80008979
Tifab	0.08754897	1.68083859	0.03057918	0.8629486	0.80008979
Rhbdd1	-0.0513743	3.65204601	0.03056231	0.86298602	0.80008979
Cln8	0.03229614	3.78794767	0.03055124	0.86301058	0.80008979
Med1	0.02295039	7.2607964	0.03052557	0.86306751	0.80008979
Ppcs	-0.0482721	2.41680731	0.0304524	0.86322999	0.80008979
Nr2f1	0.0273639	6.76098752	0.03043138	0.86327669	0.80008979
2610307P16I	0.11559741	0.88520349	0.03036713	0.86341958	0.80008979
A830018L16I	0.02728677	6.95865729	0.03034203	0.86347543	0.80008979
Aasdh	-0.0605343	2.09204686	0.03032894	0.86350459	0.80008979
Hmgcl	-0.0452068	2.57252918	0.03031046	0.86354574	0.80008979
Slc35d1	-0.0289645	4.13099089	0.03029334	0.86358387	0.80008979
Mpc2	-0.0253024	5.93669597	0.03029178	0.86358735	0.80008979
Bola3	0.04370485	3.33328221	0.03025343	0.86367281	0.80008979
Zfp654	0.03233034	5.43811125	0.03024989	0.86368071	0.80008979
Isoc2b	-0.083657	0.67709782	0.03024291	0.86369627	0.80008979
Suv39h2	-0.0439742	3.0955712	0.03022563	0.8637348	0.80008979
Pdzrn4	0.05186513	2.404402	0.0302229	0.8637409	0.80008979
Tmem127	0.02464705	6.90098997	0.03017847	0.86384003	0.80008979
Nhp2	0.04874745	3.21327063	0.03017812	0.86384082	0.80008979
Pcdha1	-0.1603812	-0.9128144	0.03010364	0.86400718	0.80008979
Fam26f	-0.1086552	1.22710449	0.03009842	0.86401885	0.80008979
U2af1l4	-0.0583882	2.09191891	0.03009022	0.86403717	0.80008979
Pmpcb	-0.0226032	5.02703198	0.03008609	0.8640464	0.80008979
Ppp2r5d	-0.0291022	5.52700385	0.03004698	0.86413388	0.80008979
Mns1	-0.0515926	2.41699082	0.03003908	0.86415155	0.80008979

Agr2	-0.107242	-0.1481052	0.03000521	0.86422735	0.80008979
Vdac1	-0.019402	8.05749857	0.02998641	0.86426946	0.80008979
Rbbp4	-0.0229341	5.94783195	0.02997905	0.86428594	0.80008979
3200001D21I	-0.1520626	0.18461242	0.02990995	0.8644408	0.80014506
Gtf2ird2	0.05266108	2.65349216	0.02987821	0.86451201	0.80014506
Prelid1	-0.0320611	5.79419782	0.02987285	0.86452403	0.80014506
Rps11	0.03534255	6.85286099	0.02973901	0.8648247	0.80036829
Lsm14a	0.02310357	8.08565336	0.02967765	0.86496279	0.80038727
Cldn5	0.15576697	-0.2046789	0.02962863	0.8650732	0.80038727
Acer2	-0.0545777	3.38377005	0.0296184	0.86509626	0.80038727
Ccdc36	0.20786995	-1.4751331	0.0296064	0.86512332	0.80038727
B230216G23	0.14170794	-0.2643548	0.02959784	0.86514261	0.80038727
Zscan25	-0.1822446	-0.360117	0.02952584	0.86530505	0.80039474
Gm5795	0.21921749	-2.0561598	0.02950425	0.8653538	0.80039474
Prcp	-0.0348575	3.71054934	0.02947018	0.86543075	0.80039474
Hpgds	-0.0614944	2.40122513	0.02946705	0.86543783	0.80039474
Arl6ip4	-0.0352922	4.90499571	0.02946251	0.86544809	0.80039474
Clec2l	-0.0691552	1.15729385	0.02941443	0.86555681	0.80044027
Chid1	-0.0335875	3.41051126	0.02930985	0.86579357	0.80053745
Foxk2	0.0170916	6.46145582	0.02930662	0.86580088	0.80053745
Polr3a	-0.0335237	3.97303105	0.0292892	0.86584036	0.80053745
Tmtc3	-0.033325	5.40573068	0.02926053	0.86590538	0.80054256
Ccdc17	0.06783537	1.07721992	0.02920836	0.86602376	0.800588
Sfxn4	-0.0512144	3.64508379	0.02918644	0.86607352	0.800588
Gng8	-0.1165401	-0.4881482	0.02913212	0.86619695	0.80063888
Tlk2	0.02077358	7.11573256	0.02910986	0.86624757	0.80063888
Mtfr2	0.20713386	-1.0937227	0.02907888	0.86631802	0.80064901
Nup133	-0.0257897	4.51006149	0.02903882	0.8664092	0.80067396
Polg2	-0.094759	0.97007582	0.02901475	0.86646402	0.80067396
Stxbp1	-0.0236081	10.0420214	0.02890518	0.86671387	0.80078424
Ttc25	-0.1494065	-0.1515874	0.02890109	0.8667232	0.80078424
Fhod1	0.05491199	2.36053804	0.02888414	0.8667619	0.80078424
4930563E18I	-0.2862944	-1.5944678	0.02875623	0.86705428	0.80092115
Zxdb	0.02413166	5.19819493	0.02875037	0.86706768	0.80092115
Rnaseh2a	0.0537291	2.75816186	0.02874121	0.86708865	0.80092115
Dctn5	-0.0259223	6.19143987	0.02863833	0.86732436	0.80098253
Tm9sf3	-0.0206954	7.28578233	0.02862134	0.86736333	0.80098253
Kbtbd11	0.02071759	7.70405204	0.02858313	0.86745102	0.80098253
6430503K07I	-0.1622291	-0.7179992	0.02856937	0.86748261	0.80098253
Tsga10	0.02723769	4.98707404	0.02855885	0.86750676	0.80098253
D5Ertd605e	-0.2360667	-1.4829157	0.02852885	0.86757569	0.80098253
Cwc22	-0.0246441	5.52773881	0.02851158	0.86761538	0.80098253
Mlh1	0.03975032	3.81828075	0.02850465	0.8676313	0.80098253

Fzd10	-0.074242	1.03771457	0.02847028	0.86771035	0.80100056
Syde1	-0.0431439	5.18608369	0.0283431	0.86800321	0.80110148
Col27a1	0.0672562	2.12821318	0.0283427	0.86800413	0.80110148
Fstl4	0.03564644	3.03605396	0.02833535	0.8680211	0.80110148
Mfn2	-0.0209457	6.63419644	0.02831943	0.86805781	0.80110148
Tnpo3	-0.0225852	6.1065511	0.02828242	0.86814321	0.80112535
Ivd	-0.0264371	4.16781787	0.02825566	0.86820499	0.80112742
Zpbp	0.12653429	0.39865645	0.02818451	0.8683694	0.80122418
Rab11fip3	-0.02217	6.19412059	0.02807681	0.86861866	0.80139922
Gm14403	0.02735146	3.69756188	0.0280428	0.86869749	0.801417
Zfp652	-0.0222594	6.34153792	0.02801189	0.86876916	0.80142818
Ubr5	-0.0254267	7.85059962	0.02794687	0.86892006	0.80151243
Rab3b	-0.0230456	5.12020472	0.02788993	0.86905237	0.80157953
Btk	-0.1436807	0.14467041	0.02782337	0.8692072	0.80166312
Neurod2	-0.0233957	5.81515841	0.02778698	0.86929194	0.80166312
Rgsl1	-0.0689062	1.52849034	0.02774975	0.86937867	0.80166312
Ttc27	0.03317804	3.37302631	0.02774863	0.86938129	0.80166312
Cd160	0.09087871	1.22639695	0.0277126	0.86946529	0.80168563
2010012O05	0.02315718	6.00612599	0.02759541	0.86973892	0.80178541
Plxna3	0.05865001	2.16951874	0.02756058	0.86982036	0.80178541
Trim52	0.18280086	-1.6565991	0.02755507	0.86983325	0.80178541
Egln2	-0.0381828	4.09706298	0.02754355	0.86986021	0.80178541
4932411N23	-0.1290359	-0.9359805	0.02753876	0.86987142	0.80178541
Agl	0.02894179	5.63069025	0.02751228	0.86993339	0.80178761
Fam120b	-0.0242855	6.59916245	0.02737846	0.87024707	0.80202012
Serpine3	-0.056824	1.95541913	0.02735384	0.87030487	0.80202012
4933408B17	-0.3215385	-1.1572245	0.02730014	0.87043102	0.80208145
R3hcc1	-0.0339994	4.26773556	0.02720509	0.87065464	0.80218372
Grpel1	0.03212427	4.23880688	0.02717096	0.87073502	0.80218372
Ppib	0.04324334	2.66338172	0.02717007	0.87073714	0.80218372
1700063D05	-0.0516291	1.83602218	0.02713486	0.87082012	0.80218372
Cap2	0.02810264	8.29989561	0.0271264	0.87084008	0.80218372
Sap30bp	0.03434051	3.65865418	0.02701243	0.87110912	0.80234252
Gyg	0.02087667	5.57171995	0.02700287	0.87113172	0.80234252
Rabepk	-0.0449761	2.99144991	0.02695056	0.87125544	0.80237785
Gm8801	0.0883346	-0.2353467	0.0269361	0.87128964	0.80237785
Tanc1	-0.0202707	5.909847	0.02688154	0.87141884	0.80237785
Gm9958	0.0601179	1.63163496	0.02680292	0.87160525	0.80237785
Dcaf4	0.03935573	3.15447989	0.02678842	0.87163967	0.80237785
Nfam1	0.10154946	1.15050389	0.02677993	0.87165981	0.80237785
Emx1	-0.0893598	0.52695818	0.02676643	0.87169187	0.80237785
Zcwpw1	-0.0893916	0.6780819	0.02675608	0.87171644	0.80237785
Gfpt1	0.02293089	6.60092891	0.02672075	0.87180037	0.80237785

D11Wsu47e	-0.0421576	2.51229817	0.02671443	0.87181539	0.80237785
Abhd15	0.16737191	-1.3237041	0.02669876	0.87185266	0.80237785
Atg13	0.01923342	5.92728263	0.02668091	0.8718951	0.80237785
Ercc3	0.02911141	4.2118526	0.02665983	0.87194524	0.80237785
Larp1	-0.0197159	7.30000833	0.02660276	0.87208111	0.80242823
Fam134c	-0.0512307	2.95746964	0.02658675	0.87211926	0.80242823
Degs1	0.03012484	6.56259852	0.02648941	0.8723514	0.80256262
Chadl	0.06232977	1.07999139	0.0264755	0.87238461	0.80256262
1500017E21I	-0.1792037	-1.8060662	0.02644001	0.87246939	0.80258575
Rhod	0.06797231	1.72168286	0.02639931	0.87256667	0.80259742
Bag4	-0.0226774	5.95107043	0.02634098	0.87270624	0.80259742
Lzic	0.03336325	4.1531194	0.02630322	0.87279667	0.80259742
Tcea1	0.01936232	8.06123568	0.02629453	0.8728175	0.80259742
Zfp850	0.04563207	2.65478176	0.02628299	0.87284515	0.80259742
Rab6a	-0.0177209	10.1161715	0.02626685	0.87288385	0.80259742
B3galt1	0.01923575	5.80671302	0.02626028	0.87289959	0.80259742
Sdc4	0.04897513	5.26521449	0.02617586	0.87310218	0.80272884
Gm16861	0.05421169	2.44946528	0.02613336	0.87320432	0.8027679
Wdr44	0.02526254	4.89162934	0.02601131	0.87349806	0.80285727
Dclk1	0.02712383	10.5375499	0.02600186	0.87352083	0.80285727
Tspan3	-0.0288927	7.0022232	0.02599468	0.87353814	0.80285727
Gdf11	0.07936367	1.80607405	0.02599067	0.8735478	0.80285727
Kbtbd3	0.03569562	3.29849578	0.025945	0.87365795	0.80285727
Khdrbs3	0.02092991	6.39263603	0.02593592	0.87367985	0.80285727
Tspan31	0.03234014	5.85510856	0.02591962	0.87371918	0.80285727
Tmem63c	0.04393669	4.14277226	0.02582439	0.87394929	0.80299149
Setd7	0.02003625	8.66298225	0.0258098	0.87398459	0.80299149
5830454E08I	-0.1066468	0.68021135	0.02571136	0.87422298	0.80311452
Nsdhl	-0.026806	4.56977349	0.02569796	0.87425547	0.80311452
Erp44	0.03072374	4.53609662	0.0256806	0.87429755	0.80311452
Exoc8	0.02540495	4.65866988	0.02563728	0.87440268	0.80315626
AV039307	-0.1000377	0.33744871	0.02555037	0.87461383	0.80321729
Zcchc16	0.04565558	2.91030763	0.02555015	0.87461438	0.80321729
Agbl5	0.04127168	2.76543721	0.02553625	0.87464819	0.80321729
Dkc1	0.01995423	6.20082846	0.02547801	0.87478993	0.80327732
Fam110a	0.07643757	1.23693417	0.02546035	0.87483295	0.80327732
Med7	-0.0310398	4.81836147	0.02540154	0.87497633	0.80332774
Coa7	-0.050508	2.48376687	0.02535043	0.87510104	0.80332774
2210416O15	-0.1166131	-0.6375409	0.02534764	0.87510786	0.80332774
Foxo3	-0.022426	6.59802806	0.02533995	0.87512665	0.80332774
Epb4.1	-0.021715	4.96364631	0.02525429	0.87533602	0.80344373
4933432I09R	0.21027638	-0.7107174	0.02521611	0.87542947	0.80344373
Dpysl5	-0.0392151	4.75965098	0.02521502	0.87543214	0.80344373

Gpr83	-0.0503136	2.73427723	0.02513273	0.87563378	0.80346546
Eif3e	-0.020938	7.15623584	0.02509199	0.87573374	0.80346546
2010107G12	0.26545673	-1.5028494	0.02506826	0.875792	0.80346546
C330027C09I	-0.0601893	1.86514799	0.02498659	0.87599272	0.80346546
Jade2	0.02109406	5.26676133	0.02495591	0.87606822	0.80346546
Mrps15	-0.0295589	4.23879358	0.02491777	0.87616214	0.80346546
Phc3	-0.0200263	8.00497733	0.02487812	0.87625985	0.80346546
Kiss1r	0.08848052	0.15055536	0.02487698	0.87626267	0.80346546
Atg12	-0.0296475	5.11943071	0.02486938	0.87628141	0.80346546
Rapgef4	0.02408137	7.31173449	0.02486444	0.87629358	0.80346546
Hhipl1	-0.1488872	-1.2036781	0.02486307	0.87629697	0.80346546
Gm16894	0.06295512	1.2872279	0.02483977	0.87635444	0.80346546
Esyt1	-0.0400447	3.45697832	0.02483589	0.87636401	0.80346546
Nck2	-0.0277672	4.64343953	0.02483423	0.87636811	0.80346546
Nrg3os	-0.1019178	0.39788992	0.02482425	0.87639274	0.80346546
Prkaa2	0.02082434	7.2938567	0.02481492	0.87641577	0.80346546
Dcaf12	-0.0319534	5.27104991	0.02478255	0.8764957	0.80346546
Tspyl4	0.01991361	8.38403276	0.02476073	0.87654962	0.80346546
Zfp953	0.04452792	2.98051226	0.02468501	0.87673689	0.80346546
Rad17	-0.0296962	4.17621479	0.02463937	0.87684991	0.80346546
1110032A03I	-0.0248984	6.65671181	0.02463402	0.87686315	0.80346546
Pigk	0.03052274	5.13219269	0.02462573	0.87688371	0.80346546
Elp6	-0.0568335	2.98309569	0.02460898	0.87692522	0.80346546
Tmem169	0.03420274	2.9231717	0.0246066	0.87693112	0.80346546
Fam114a1	0.03769086	6.31411309	0.02454495	0.87708406	0.80346546
Stk17b	0.02840091	5.75517255	0.02448817	0.87722512	0.80346546
B2m	0.03213289	9.02542822	0.02445178	0.87731559	0.80346546
Slc37a4	0.04529832	1.97965061	0.02443612	0.87735454	0.80346546
Tiam2	0.03145571	4.92606776	0.0244228	0.8773877	0.80346546
Rnf216	0.0189354	5.97522611	0.02440038	0.8774435	0.80346546
Mrps10	-0.0368231	2.80224304	0.02436802	0.87752411	0.80346546
Acacb	0.07844868	1.28171749	0.02436349	0.8775354	0.80346546
Cntn5	-0.0609327	2.35726734	0.02433529	0.87760568	0.80346546
Guf1	-0.0466754	3.90420422	0.02433357	0.87760999	0.80346546
Il2rg	0.12686096	0.43366703	0.02432241	0.87763781	0.80346546
3110021N24	0.0391493	2.41221087	0.02430143	0.87769014	0.80346546
Maneal	-0.0268085	4.31987879	0.02423792	0.87784872	0.80346546
Twf2	0.03311505	3.43721528	0.02421931	0.87789522	0.80346546
Plekhg2	0.03710021	4.20003844	0.02418465	0.87798189	0.80346546
Zfyve20	0.02366656	5.76894164	0.02416574	0.87802921	0.80346546
Pld4	0.09550672	-0.3204414	0.02411934	0.87814537	0.80346546
G0s2	-0.0740295	1.60032905	0.024109	0.87817127	0.80346546
Pidd1	0.17851339	-1.6530424	0.0241024	0.87818782	0.80346546

Speer8-ps1	-0.1075293	0.0963112	0.02409738	0.87820039	0.80346546
Hdgfrp2	0.02899397	5.05959439	0.02409102	0.87821632	0.80346546
Zfp74	-0.0278419	5.97278665	0.02403428	0.87835863	0.80346546
Gnal	0.02677928	8.43372102	0.02402503	0.87838183	0.80346546
Eif4g1	-0.0173177	7.06173756	0.02402346	0.87838579	0.80346546
Ccna2	0.04958021	1.98448009	0.02397792	0.87850014	0.80346546
Wac	-0.0215574	8.63974807	0.02393711	0.87860271	0.80346546
Clip4	-0.0244053	5.24150222	0.023907	0.87867845	0.80346546
3425401B19l	-0.0549504	3.75183628	0.02384097	0.87884473	0.80346546
Phka1	-0.0327463	4.255368	0.0237398	0.87909992	0.80346546
Pop5	-0.0304518	4.39829903	0.02373867	0.87910278	0.80346546
Lpin3	0.20850252	-1.554597	0.02372986	0.87912504	0.80346546
Atp13a2	0.03939977	4.17613048	0.02369599	0.8792106	0.80346546
Galk1	0.04868055	2.74754331	0.02368813	0.87923049	0.80346546
Phyhip	0.02465046	6.76786975	0.02367443	0.87926512	0.80346546
Cdipt	-0.0244853	4.67887572	0.0236465	0.87933577	0.80346546
Slc35d2	-0.0823307	0.16421298	0.02364518	0.87933911	0.80346546
Gsto2	-0.1550433	-0.5104815	0.02360836	0.87943232	0.80346546
Rpap1	0.03703603	2.8673749	0.0235645	0.87954344	0.80346546
Ddx39	-0.048961	2.37267461	0.02356286	0.87954761	0.80346546
BC100451	-0.2045003	-1.5581313	0.02354318	0.87959749	0.80346546
Nup62-il4i1	-0.1509486	-1.2666709	0.02350145	0.87970338	0.80346546
5830418K08l	0.03213133	5.57717409	0.02350049	0.87970582	0.80346546
Glrx2	0.01764565	6.7348839	0.02348731	0.87973928	0.80346546
Nthl1	0.09644271	0.3650133	0.02345687	0.87981659	0.80346546
Ampd2	-0.0275268	3.93877048	0.02344142	0.87985585	0.80346546
Mei1	-0.0712306	0.94362677	0.02342584	0.87989546	0.80346546
Lhx9	0.14419161	-1.07217	0.02339996	0.8799613	0.80346546
Tead4	0.14276941	0.04047983	0.02335388	0.88007858	0.80346546
Zfp943	0.03633241	3.03883785	0.02334713	0.88009577	0.80346546
P2rx3	0.06793948	1.27183356	0.02333773	0.8801197	0.80346546
Tmem86a	0.05382227	4.25573713	0.02332634	0.88014873	0.80346546
Cdk16	0.0184633	7.18779947	0.02331436	0.88017925	0.80346546
Ginm1	0.03747181	5.23454548	0.02331274	0.88018339	0.80346546
Gm10865	0.08518206	-0.4188497	0.02330307	0.88020803	0.80346546
Npat	0.02200353	5.99288499	0.02329375	0.8802318	0.80346546
Utp11l	-0.0326303	4.11218496	0.02329346	0.88023255	0.80346546
Pinx1	-0.0372628	3.43679909	0.02326475	0.88030576	0.80346735
Tprgl	-0.0207677	6.93458413	0.02324584	0.88035403	0.80346735
Rsph3b	0.02816759	4.40296319	0.02317388	0.88053786	0.80347926
Aox1	0.03733885	3.12249429	0.02316452	0.88056181	0.80347926
Sun2	-0.0291177	6.58459989	0.02316012	0.88057306	0.80347926
Immt	-0.0210861	6.62963615	0.02314727	0.88060592	0.80347926

Gpr62	-0.0884899	0.89676421	0.02308981	0.880753	0.80355896
Msc	-0.1955474	-0.979222	0.02306099	0.88082685	0.80356983
Cops7b	0.03890287	3.71950173	0.02302643	0.88091548	0.80356983
Zbtb5	0.04184454	2.58687091	0.02299191	0.88100404	0.80356983
Ncapd3	0.03520078	4.27461115	0.022981	0.88103204	0.80356983
Syde2	0.04383002	3.66367295	0.02296318	0.88107782	0.80356983
Fam3a	-0.0313771	3.56031125	0.02293843	0.8811414	0.80356983
Phf11c	-0.1435652	-1.5857981	0.02292228	0.88118292	0.80356983
B330016D10	0.10178687	0.44110558	0.02286587	0.88132805	0.80360318
Acta1	0.06763972	0.90019356	0.02286164	0.88133893	0.80360318
Rag1	-0.4554766	-1.9203626	0.02276365	0.8815915	0.803779
Tmem51	0.05446496	2.21709795	0.02268001	0.8818075	0.80392147
P2ry6	-0.069414	0.78746139	0.02261007	0.88198846	0.80398753
Sec61b	0.03130779	4.79413797	0.02259284	0.88203307	0.80398753
Dip2a	-0.0309378	5.45376591	0.02258275	0.88205921	0.80398753
Atcay	-0.0249368	5.5480024	0.02255109	0.88214126	0.80400786
Atoh8	-0.0481081	1.84341406	0.02243577	0.88244062	0.80405248
Tmem67	0.03750215	3.81149428	0.02241756	0.88248796	0.80405248
Kat6a	-0.0166121	8.11258679	0.02238171	0.88258124	0.80405248
Dusp12	-0.0479969	2.30696083	0.02233864	0.8826934	0.80405248
Safb	0.01968757	6.30017107	0.02232798	0.88272116	0.80405248
Bap1	-0.0249498	4.87571872	0.02232256	0.88273529	0.80405248
D830031N03	0.02314848	5.26126611	0.02229242	0.88281385	0.80405248
Ankrd12	-0.0227251	9.70283078	0.02227121	0.8828692	0.80405248
Mrpl9	0.02658859	4.48985531	0.02227099	0.88286975	0.80405248
Mrpl28	0.04411388	3.36146762	0.02226252	0.88289186	0.80405248
Alyref2	-0.045109	1.75103512	0.02225286	0.88291709	0.80405248
Lrrc14	-0.0387763	2.72830869	0.02222808	0.88298178	0.80405248
Tnfrsf22	0.05241352	1.74743848	0.02221617	0.88301288	0.80405248
LOC1026344	0.07296249	1.10325878	0.02217824	0.88311203	0.80405248
Lrp2bp	0.09663305	0.78292561	0.02217037	0.88313262	0.80405248
Ppp1r2	0.01875754	8.03802023	0.02216056	0.88315826	0.80405248
Clec2d	0.05549605	2.1306809	0.02214231	0.88320601	0.80405248
Adss	0.01567845	7.09678092	0.02206571	0.88340666	0.80413646
Casp8ap2	0.01672779	6.4656115	0.02204188	0.88346915	0.80413646
Rif1	-0.0283387	5.78944348	0.02202288	0.883519	0.80413646
Acbd6	-0.025879	4.71462232	0.02201142	0.88354907	0.80413646
9630028B13	-0.0625848	2.68441156	0.0219602	0.8836836	0.80413646
Lhfpl2	0.02788086	5.18302644	0.02195546	0.88369605	0.80413646
Slit2	0.02051588	5.82072371	0.02186608	0.88393121	0.80413646
Pcdhgb1	-0.0420017	4.09657467	0.02185019	0.88397308	0.80413646
Zswim7	0.04233307	2.12660425	0.02183325	0.88401772	0.80413646
Odf2	0.02872099	5.56183303	0.02183307	0.88401821	0.80413646

Cldn2	0.09017732	1.2896757	0.02183073	0.88402437	0.80413646
AW822252	0.1100774	0.07551761	0.02179395	0.88412136	0.80413646
Mafg	0.02837591	4.34596512	0.02177944	0.88415965	0.80413646
4933421O10	-0.0380837	3.5009679	0.02175388	0.88422712	0.80413646
Cyp2j12	0.10324203	-0.5687111	0.02174864	0.88424096	0.80413646
Snx24	-0.0307969	4.98465963	0.02174355	0.8842544	0.80413646
Kdm7a	0.01823524	7.30540922	0.02169557	0.88438122	0.80414722
Med30	-0.0384395	3.31133533	0.02163978	0.88452885	0.80414722
Anapc16	0.0349507	5.99677279	0.02162855	0.88455858	0.80414722
Pth1r	0.07555324	1.07751311	0.02157973	0.88468796	0.80414722
Dach1	0.05135692	2.04567433	0.02157518	0.88470004	0.80414722
Sfpq	-0.0196444	7.54784806	0.02157476	0.88470116	0.80414722
Klhdc7a	0.04936847	2.71863597	0.0215424	0.88478699	0.80414722
Lair1	-0.0470947	3.29310948	0.02153518	0.88480615	0.80414722
Fam193a	0.02045587	7.39228776	0.0215234	0.88483743	0.80414722
Rnf135	0.03820919	2.75832843	0.02151346	0.88486383	0.80414722
Fam21	-0.022946	6.01400352	0.02144846	0.88503659	0.80417497
Zfc3h1	-0.0287841	6.97548533	0.02144205	0.88505366	0.80417497
Limd2	-0.0261225	4.46135496	0.02140935	0.88514068	0.80417497
Gsk3b	0.01585125	9.51769032	0.02139319	0.8851837	0.80417497
Arl10	0.07312565	0.91584458	0.0213802	0.88521831	0.80417497
Gsap	0.04407333	2.23454519	0.0213672	0.88525294	0.80417497
Sh2d5	-0.0269768	4.63060511	0.02128969	0.88545971	0.8043085
Aup1	0.04308513	3.08808871	0.02126491	0.88552589	0.80431432
Evi2a-evi2b	0.12162318	-1.3671055	0.02118247	0.88574636	0.80440752
Tmsb10	-0.0355395	6.42642058	0.02116484	0.88579357	0.80440752
Serf2	-0.0328805	6.41739383	0.02115952	0.88580783	0.80440752
Zfml	0.02143589	7.89140434	0.02108975	0.88599485	0.80443875
9530068E07I	0.03132931	6.71850553	0.0210753	0.88603364	0.80443875
Gnat1	-0.1182127	-0.4817534	0.02106952	0.88604914	0.80443875
1110054M08	-0.067005	1.12950431	0.02103347	0.88614594	0.80443875
Ap2a1	-0.0291163	4.06991428	0.02099657	0.88624513	0.80443875
Uba7	-0.0767442	0.63843414	0.02098807	0.88626801	0.80443875
Ensa	0.01883575	8.89617653	0.02098556	0.88627474	0.80443875
Hscb	-0.0392553	4.15092299	0.02096856	0.88632048	0.80443875
Rph3al	0.06590366	1.23331176	0.02092096	0.88644864	0.80445696
Polr3b	-0.028114	4.43934886	0.02091671	0.88646011	0.80445696
4930426D05I	-0.1368379	-0.7480271	0.02080066	0.88677321	0.80462125
Pigh	0.05247887	2.03473925	0.02077206	0.88685051	0.80462125
Tmc6	0.08045226	0.25957733	0.02075904	0.88688572	0.80462125
AI662270	-0.0657302	0.58541783	0.02075649	0.88689261	0.80462125
Lsm3	0.02948954	4.10836897	0.02072007	0.88699117	0.80462125
Samm50	0.02786543	3.50730105	0.02071513	0.88700456	0.80462125

Rasa2	-0.0207162	5.06270366	0.02069477	0.88705971	0.80462125
Wdr78	-0.0446842	3.27238946	0.02060426	0.88730517	0.80474421
Rasgef1a	-0.0221225	6.81493714	0.0205872	0.88735149	0.80474421
Gys1	-0.0546451	1.99857252	0.02057867	0.88737467	0.80474421
Fbxo28	-0.0166956	6.1643748	0.02054857	0.88745648	0.80476415
Rab40c	-0.0378086	2.90796542	0.02048015	0.88764265	0.80487874
Stat5b	-0.0277942	4.00803531	0.0204381	0.88775724	0.80490127
Herpud1	-0.0257126	4.41628755	0.02039847	0.88786533	0.80490127
Igtp	-0.037951	3.72896167	0.02039143	0.88788456	0.80490127
Zfyve1	-0.0229488	4.19700661	0.02034588	0.88800895	0.80490127
Trub1	-0.0220828	4.67288102	0.0203431	0.88801655	0.80490127
Adar	0.02750644	6.00892083	0.0203395	0.88802639	0.80490127
Nars2	-0.0339791	3.83879504	0.02028989	0.88816205	0.80494076
Gm10638	-0.1120718	-0.6071244	0.02027983	0.8881896	0.80494076
Zfp825	0.0252719	4.52217882	0.02015697	0.88852641	0.80499338
5730508B09I	0.03799594	2.79684429	0.02012906	0.88860307	0.80499338
Klhdc4	-0.0327942	3.05653402	0.02012009	0.88862772	0.80499338
Zfp597	-0.0201732	4.89162682	0.02012	0.88862797	0.80499338
Cox7c	-0.0257306	7.31570746	0.02008345	0.88872846	0.80499338
Sardh	0.05112717	2.20150384	0.02007514	0.88875131	0.80499338
G630025P09	0.22046263	-1.6225258	0.02007387	0.88875482	0.80499338
Mier1	-0.0200069	6.44738241	0.02004179	0.88884311	0.80499338
Chchd1	0.03304654	3.59794453	0.02002844	0.88887988	0.80499338
Nudt11	0.02781152	4.15227388	0.02002807	0.88888091	0.80499338
Gm15698	-0.1917983	-1.4406106	0.02001906	0.88890571	0.80499338
Hdc	0.10403062	-0.2146941	0.01993861	0.88912762	0.80503949
Parl	0.03181409	3.34903701	0.0199251	0.88916494	0.80503949
Psma1	-0.0237753	5.5577464	0.01991475	0.88919351	0.80503949
Cox6b1	-0.0326014	5.47414806	0.019897	0.88924255	0.80503949
Rpp14	-0.0223638	5.40968028	0.01989223	0.88925576	0.80503949
Gm11413	-0.1471189	-1.251623	0.01984626	0.88938289	0.80508094
Dctn3	0.03094083	5.68725454	0.01983242	0.8894212	0.80508094
Srfbp1	-0.0275054	3.92073567	0.01978636	0.88954881	0.80514229
Trrap	-0.030552	6.51894758	0.01969823	0.88979336	0.80521961
Ndufaf6	-0.0487988	1.59740847	0.01966006	0.88989945	0.80521961
Tmem210	-0.1137559	0.06352342	0.0196564	0.88990963	0.80521961
Gm14446	-0.0306931	3.72874383	0.01958741	0.89010167	0.80521961
Myocd	0.19566291	-1.5673105	0.01953176	0.89025684	0.80521961
Scg5	0.02019785	6.32416838	0.01952652	0.89027146	0.80521961
1700007K13I	-0.0879287	0.10950649	0.01951556	0.89030205	0.80521961
Prkar2b	0.0193534	5.63127322	0.01950586	0.89032913	0.80521961
4930467D21I	-0.170064	-1.3164276	0.01949357	0.89036346	0.80521961
Gm15217	-0.1359374	-1.6360095	0.01948321	0.8903924	0.80521961

Ildr2	0.02134286	9.87099209	0.01947336	0.89041992	0.80521961
Tarsl2	0.03006417	4.5568024	0.01946687	0.89043806	0.80521961
Mettl22	-0.0450845	2.59116622	0.01946328	0.8904481	0.80521961
Eml1	-0.0166899	5.94191498	0.01941017	0.89059663	0.80521961
Nedd4	-0.0142071	9.5001887	0.01937645	0.89069107	0.80521961
2410131K14I	-0.0399319	1.90497599	0.01937395	0.89069806	0.80521961
Zbtb7b	-0.0431513	2.97171509	0.01936324	0.89072809	0.80521961
Stx17	0.02029149	5.51081247	0.01934513	0.89077885	0.80521961
Nop10	-0.0249631	4.74626895	0.01930811	0.8908827	0.80521961
Zfa-ps	-0.0547486	1.11562698	0.01927351	0.89097986	0.80521961
Dock6	-0.0273637	3.65249162	0.01926982	0.89099024	0.80521961
Fbxw8	-0.0233503	3.88757682	0.01926726	0.89099743	0.80521961
2900076A07	0.06810649	0.95672739	0.01921147	0.89115428	0.80521961
Daxx	0.02264982	3.87874798	0.01920418	0.8911748	0.80521961
Xlr4b	-0.1277183	-0.3542584	0.01916238	0.89129251	0.80521961
Hdac10	-0.0455867	1.601888	0.01914491	0.89134174	0.80521961
Rhov	0.08716853	1.2476218	0.01911835	0.89141663	0.80521961
Mrps11	0.05411033	1.41683712	0.01910804	0.89144574	0.80521961
Gpld1	-0.0224833	4.90241886	0.01910103	0.89146551	0.80521961
Pltp	-0.0393289	4.01039227	0.01908436	0.89151255	0.80521961
L3mbtl4	-0.089127	0.00633733	0.01908297	0.89151648	0.80521961
Mapk13	0.17804062	-1.634588	0.01907142	0.8915491	0.80521961
Insr	-0.0234971	5.99010779	0.01897453	0.89182311	0.80539026
Phf8	-0.0222187	5.6399736	0.01894367	0.89191053	0.80539026
2900056M20	-0.0235505	7.95961071	0.01894117	0.89191761	0.80539026
Anks3	0.0324824	2.94614511	0.01889858	0.8920384	0.80540298
Zfp454	0.03760435	2.31027199	0.018894	0.8920514	0.80540298
Palmd	-0.024398	5.6060739	0.01885569	0.89216017	0.80544714
Ush1g	0.24433554	-1.4019684	0.01879162	0.89234234	0.80555756
Il18	-0.0219243	4.1713392	0.01870199	0.89259772	0.80573405
Obsl1	0.03149092	3.21369127	0.01867724	0.89266835	0.80574375
Gal3st4	0.05794682	1.56458374	0.01864436	0.89276224	0.80576128
Kcnmb4	0.02454507	3.94159509	0.01862851	0.89280753	0.80576128
Sh3rf2	0.03184238	3.63581108	0.01860082	0.8928867	0.8057787
Suco	-0.0197445	6.12057899	0.01856615	0.89298595	0.80581422
Safb2	-0.0210311	5.15612092	0.01853511	0.89307485	0.8058404
Pcdhga2	0.05195071	2.1827769	0.01846605	0.89327295	0.80593536
Zeb2os	-0.0490755	1.51606069	0.01844862	0.89332301	0.80593536
Abl1	0.02555018	5.32165928	0.01843582	0.89335977	0.80593536
Abhd12	-0.0191066	6.33405157	0.01837812	0.89352573	0.80602762
Rapgef6	-0.0154796	6.7995315	0.01831488	0.89370788	0.80602762
Egln1	-0.0166164	6.86090147	0.0183037	0.89374013	0.80602762
C230091D08	0.02390168	6.46143414	0.01826728	0.89384523	0.80602762

4931408D14	0.08041739	0.71792381	0.01823964	0.89392505	0.80602762
Syt11	0.02141769	8.29352381	0.0182362	0.893935	0.80602762
Nap1l4	-0.0204457	5.59025974	0.01822212	0.8939757	0.80602762
Zfp869	0.02220357	5.11244838	0.01820439	0.89402694	0.80602762
Kif21a	0.02570888	8.49741799	0.01816577	0.89413873	0.80602762
Edrf1	-0.0299768	5.06486854	0.01815979	0.89415602	0.80602762
Zfp868	0.02593804	4.89066314	0.01814597	0.89419604	0.80602762
Cacnb1	-0.0198224	5.12663718	0.01814109	0.89421018	0.80602762
Atp11c	0.02658082	4.6841988	0.01813055	0.89424073	0.80602762
Zkscan14	-0.055805	2.42938013	0.01809222	0.89435189	0.80607382
Adprh	0.02017988	4.24486342	0.01802258	0.89455411	0.80618524
Pcbp2	0.01625881	6.942454	0.01800043	0.89461854	0.80618524
Irf8	-0.0531872	1.6282917	0.01797366	0.89469644	0.80618524
Selm	0.03578345	4.47033158	0.0179241	0.8948408	0.80618524
Cox6a1	0.01966666	6.82536447	0.01791792	0.89485883	0.80618524
Wnt3	0.11629515	-0.8614481	0.01785805	0.89503353	0.80618524
Sqrdl	0.039078	3.40384126	0.01782202	0.89513881	0.80618524
Crtc1	-0.020805	6.31936751	0.01781631	0.89515551	0.80618524
St8sia2	0.03970406	2.85033802	0.01781453	0.89516071	0.80618524
Map3k11	-0.0288379	3.78564312	0.01781252	0.89516658	0.80618524
Gpr173	-0.0361972	2.88554124	0.01780914	0.89517647	0.80618524
Armc10	-0.0295966	4.74546226	0.017803	0.89519444	0.80618524
Nudcd2	0.03110933	3.02746944	0.01774385	0.89536759	0.80627298
Xrcc5	-0.0264011	4.92129584	0.0177288	0.8954117	0.80627298
Edc4	0.03407173	3.93410082	0.0176274	0.89570939	0.80638466
Ikzf1	-0.0285165	4.34495436	0.01760686	0.89576979	0.80638466
Pias1	-0.0144835	6.84402528	0.01759916	0.89579246	0.80638466
Synpo	-0.0165937	6.50701661	0.01759845	0.89579454	0.80638466
Zfp938	-0.0267614	4.44416906	0.01758458	0.89583537	0.80638466
Nova2	0.02351196	7.26096089	0.01754645	0.89594766	0.80638847
Atp5sl	-0.0308424	3.21692517	0.01754245	0.89595945	0.80638847
Atmin	0.01584122	6.26839051	0.01751996	0.89602575	0.80639421
Trim8	0.01986177	6.14101651	0.01741621	0.8963322	0.80655797
Plagl1	-0.0254798	6.20316342	0.01741001	0.89635055	0.80655797
Lrrc23	-0.0796667	0.84062034	0.01739751	0.89638753	0.80655797
Tymp	0.04466753	2.53864225	0.01735733	0.89650653	0.80661111
Zfp329	0.0184359	6.15661559	0.01731066	0.89664489	0.80663734
Dlc1	-0.0184116	6.60840981	0.01730706	0.89665557	0.80663734
Sarm1	-0.0380996	3.71037135	0.01727815	0.89674141	0.80666063
Pcgf5	-0.0256473	4.11613292	0.01720437	0.89696079	0.80676147
Dgki	0.02938943	4.88385758	0.01719725	0.89698199	0.80676147
Nde1	-0.035072	3.81609371	0.01717999	0.89703337	0.80676147
Zfp422	0.01994122	6.39820939	0.01714408	0.89714041	0.80677403

Col4a5	-0.0397877	3.29532106	0.01713508	0.89716725	0.80677403
Lsr	0.03687766	3.45580772	0.01707767	0.89733863	0.80677512
Farp1	0.01642063	5.39111264	0.01707041	0.89736032	0.80677512
Dhx29	0.0309137	4.80532999	0.01706949	0.89736306	0.80677512
Nat14	0.04713645	1.17082962	0.01705437	0.89740829	0.80677512
Ppm1b	0.01360524	8.14640098	0.01698935	0.89760285	0.80686955
Gpr183	-0.0752672	-0.160354	0.01691342	0.89783058	0.80686955
Pqlc2	0.06494869	0.36876817	0.01689596	0.89788303	0.80686955
Cdkal1	-0.025778	4.0708894	0.01689478	0.89788658	0.80686955
Pawr	0.03635902	5.34760824	0.01687164	0.8979561	0.80686955
Tbrg4	-0.02416	3.84983377	0.01685508	0.8980059	0.80686955
Ap2m1	0.01523033	7.80336144	0.01684683	0.89803072	0.80686955
Nap1l3	0.0204366	5.81766145	0.01681757	0.8981188	0.80686955
Lef1	0.02645264	4.50686433	0.01681425	0.8981288	0.80686955
Zfand2a	0.01829002	7.00570716	0.01676236	0.89828519	0.80686955
Cmas	-0.0135025	6.76787663	0.01675681	0.89830192	0.80686955
Fntb	-0.0263242	4.03066677	0.01674789	0.89832885	0.80686955
Psmb10	-0.0341361	3.21934283	0.016713	0.89843419	0.80686955
Btbd9	-0.0241847	4.98296484	0.01670629	0.89845445	0.80686955
Rps18	-0.0347495	6.22385773	0.01670086	0.89847087	0.80686955
Zdhhc23	-0.0662438	1.06906349	0.01669118	0.8985001	0.80686955
Tbc1d12	0.02604765	3.91318309	0.01667737	0.89854186	0.80686955
Zfp954	-0.044761	1.98684143	0.01666059	0.89859264	0.80686955
Tpm3	-0.0161637	7.43066738	0.01658458	0.89882286	0.80702241
Npm1	0.01513548	9.66262949	0.016563	0.89888834	0.80702736
Gabpb1	0.0186929	5.10050839	0.01649199	0.89910406	0.80712102
Stag2	0.01604342	6.69822651	0.0164796	0.89914176	0.80712102
Mlxip	-0.0190967	5.16406634	0.01646695	0.89918024	0.80712102
Slmap	0.0154464	8.96692926	0.01644608	0.89924378	0.80712102
Setd1b	0.01943327	5.46467722	0.01641396	0.89934167	0.80712102
Ppip5k1	-0.0270598	5.49143345	0.01640597	0.89936604	0.80712102
Chmp4b	-0.0148953	6.43768416	0.01635558	0.89951981	0.80712102
Slain2	0.01642029	6.73373401	0.01633006	0.89959778	0.80712102
Sfi1	-0.0394144	3.39486103	0.01631846	0.89963325	0.80712102
Nrg4	0.09778797	-0.4053342	0.01631268	0.89965092	0.80712102
Sirt3	-0.0246476	3.59719105	0.01625121	0.89983909	0.80712102
Ercc2	0.05032631	1.24485289	0.01624472	0.89985897	0.80712102
2210039B01I	-0.0433317	1.77921097	0.0162306	0.89990227	0.80712102
Mark2	-0.0192576	6.32552604	0.01621881	0.89993841	0.80712102
Hist1h1a	-0.145504	-1.2556486	0.01621845	0.89993952	0.80712102
Fam183b	-0.1035087	0.14856088	0.01621427	0.89995235	0.80712102
Abcb1b	0.04037976	1.66778348	0.01617156	0.90008347	0.80718481
Sytl3	-0.0534351	0.95486318	0.01612739	0.90021925	0.80721015

Hook3	0.01452074	8.61231605	0.01612334	0.9002317	0.80721015
Gpr174	-0.1533286	-1.3478259	0.01609809	0.9003094	0.80721184
Prkcb	0.01957747	11.0318329	0.01607042	0.90039466	0.80721184
Zfp113	0.02898664	4.27672379	0.01601312	0.90057138	0.80721184
Tgm1	0.1549632	-1.4017659	0.01599127	0.90063885	0.80721184
Comp	-0.1284648	-1.3445254	0.01598806	0.90064877	0.80721184
Zfp383	-0.0345795	2.57221034	0.01597593	0.90068626	0.80721184
Trpm6	-0.0318055	3.12694538	0.0159548	0.9007516	0.80721184
Ehbp1	-0.0174032	6.77901696	0.01595064	0.90076447	0.80721184
Tmem252	-0.0692151	-0.111035	0.01591803	0.90086539	0.80721184
Atp6v1c1	-0.013036	7.37129224	0.01591204	0.90088395	0.80721184
Zfp467	0.03003757	4.00171468	0.0159039	0.90090917	0.80721184
Etohd2	-0.0554814	1.40307632	0.01587774	0.90099024	0.80721184
Ttc8	0.01792847	4.70920459	0.01585613	0.90105726	0.80721184
Fam114a2	-0.0244921	5.08308205	0.01585093	0.90107341	0.80721184
Gpr65	-0.0747848	0.52277596	0.01583034	0.90113731	0.80721535
Crebl2	-0.0241385	5.09833326	0.0157814	0.9012894	0.80725257
Bnip2	-0.0216146	7.06219684	0.01576532	0.90133943	0.80725257
Fuom	0.02899104	3.47949281	0.01575534	0.90137048	0.80725257
Gm13298	0.025047	5.02359113	0.01573662	0.9014288	0.80725257
As3mt	0.03093298	3.54657067	0.01564895	0.90170224	0.80725257
Ppic	-0.0371429	4.60365247	0.01563699	0.90173959	0.80725257
Golga1	0.01654651	5.51897548	0.01558228	0.9019107	0.80725257
Gfra4	-0.0218988	4.35583022	0.01548919	0.90220257	0.80725257
Spock2	-0.0206018	8.11714131	0.01546781	0.9022697	0.80725257
Col8a1	-0.0299898	5.5011309	0.01546376	0.90228245	0.80725257
Srrm4os	0.04915729	1.83106618	0.01546249	0.90228642	0.80725257
Pdzd7	-0.056717	1.33017718	0.01543868	0.90236128	0.80725257
Fam32a	-0.0200917	6.0705098	0.01541873	0.90242404	0.80725257
Cyp4f14	-0.1459562	-0.5174918	0.01541557	0.902434	0.80725257
4732416N19	-0.1382404	-1.9310366	0.01540273	0.90247441	0.80725257
Pydc3	0.07165846	0.73469597	0.01535941	0.90261091	0.80725257
Retsat	0.04288349	2.56790834	0.0153558	0.9026223	0.80725257
Plekhf2	-0.0265507	4.63531593	0.01534299	0.90266272	0.80725257
Gpbp1	0.01514745	7.99359467	0.01533036	0.90270256	0.80725257
Hus1	0.02194434	4.38056297	0.01531021	0.90276619	0.80725257
Tnfsf8	0.14182839	-0.2182772	0.01526513	0.90290868	0.80725257
Pcdha7	0.08991377	-0.6336059	0.01526073	0.9029226	0.80725257
Gabra5	-0.0177165	5.66006483	0.0152561	0.90293725	0.80725257
Gtl3	-0.0170806	4.59835817	0.01524474	0.9029732	0.80725257
Gpc4	0.02328785	4.23406779	0.01523104	0.90301657	0.80725257
Ncoa6	-0.016686	8.53285239	0.0151951	0.90313045	0.80725257
Pde4a	-0.0227036	6.32580238	0.01519044	0.90314523	0.80725257

Trmt11	0.04260594	1.67032125	0.01518096	0.90317532	0.80725257
Ankrd63	0.03006744	4.06143589	0.01517574	0.90319185	0.80725257
Gbp10	0.02538273	3.82201888	0.01513705	0.90331468	0.80725257
Ppp5c	-0.019505	4.63015193	0.01513443	0.90332302	0.80725257
BC005624	0.01984391	6.12337461	0.01511179	0.90339497	0.80725257
Dnase2a	-0.0781324	-0.3412987	0.01509993	0.90343271	0.80725257
Phldb2	-0.0241338	7.68725526	0.01507888	0.90349966	0.80725257
Tnrc6b	-0.0165875	8.72502466	0.01505376	0.90357966	0.80725257
1700034H15I	-0.0492865	2.06910052	0.01505215	0.90358478	0.80725257
4930563E22I	0.08100715	0.91985291	0.01504535	0.90360646	0.80725257
3010001F23I	-0.0508583	1.13950441	0.01501967	0.90368832	0.80725257
Pald1	0.04532242	1.79750332	0.01501688	0.90369722	0.80725257
Nbeal1	-0.0192156	6.22589735	0.01500502	0.90373506	0.80725257
Muc1	0.08492842	-0.6133025	0.01496531	0.90386187	0.80725257
Pigt	-0.0201569	5.31439876	0.01495728	0.90388752	0.80725257
B4galnt1	-0.0202699	5.39993322	0.01494987	0.90391122	0.80725257
Grem1	0.05622308	0.83619109	0.01492987	0.90397517	0.80725257
Fam20c	0.04075991	2.99503013	0.01492699	0.90398438	0.80725257
Ago4	0.04065092	3.22251351	0.01486513	0.9041825	0.80725257
Eftud2	0.01999604	5.01527971	0.01484744	0.90423925	0.80725257
Osgep	-0.0236452	3.93937832	0.01478063	0.90445382	0.80725257
Hebp2	-0.0249008	3.40226414	0.01477186	0.90448202	0.80725257
Men1	-0.0264558	4.03317558	0.01475643	0.90453166	0.80725257
Bex1	0.02471793	5.22081363	0.01474346	0.9045734	0.80725257
Vmn1r65	-0.0477117	1.14576224	0.0146954	0.90472828	0.80725257
Adat2	-0.0560281	1.78734387	0.01469237	0.90473805	0.80725257
Fkbp3	-0.0140782	6.9856642	0.01469104	0.90474235	0.80725257
Foxj2	-0.0168196	6.55270403	0.01468708	0.90475509	0.80725257
Alg9	-0.0374482	2.99543005	0.01468606	0.90475841	0.80725257
Trim68	-0.0557148	1.24116995	0.01468505	0.90476167	0.80725257
Gnb1l	-0.0828449	0.36464636	0.01468347	0.90476676	0.80725257
Fgfr1op2	0.01552377	7.74239464	0.01467636	0.90478969	0.80725257
Pfkfb4	-0.0508622	1.19847148	0.01465745	0.90485074	0.80725257
2410021H03I	-0.0968864	0.56287823	0.01465039	0.90487355	0.80725257
Dhx38	-0.0215107	4.56252447	0.01463451	0.90492486	0.80725257
Prr5	0.05014574	1.40299752	0.01462417	0.90495828	0.80725257
Cecr6	0.02910869	4.67755729	0.0145801	0.90510084	0.80729044
Spata17	-0.1601175	-1.2522356	0.01452738	0.90527169	0.80729044
Fam76b	-0.0254156	4.65486334	0.01448064	0.90542345	0.80729044
Nap1l1	-0.0131816	8.68607673	0.01447877	0.90542951	0.80729044
E2f5	-0.0202597	4.32218395	0.01447488	0.90544215	0.80729044
Pcdhb2	0.06279608	1.80948339	0.01444817	0.905529	0.80729044
Stxbp5	0.02278395	7.72459988	0.01443172	0.90558252	0.80729044

5830416I19R	0.16419559	-0.9376962	0.01441059	0.90565132	0.80729044
Dnmbp	-0.0266458	3.65661278	0.01439628	0.90569794	0.80729044
Gpatch8	0.01422374	8.04888298	0.01437672	0.9057617	0.80729044
1810043G02	0.02757734	2.74556179	0.01433605	0.90589443	0.80729044
Rnf7	0.02099079	7.98133013	0.01429188	0.90603879	0.80729044
Rbmx	0.01480556	6.76613628	0.01427498	0.90609409	0.80729044
Aard	-0.0480822	1.58846714	0.01427309	0.90610026	0.80729044
Capn7	-0.0153752	6.54872315	0.01426662	0.90612144	0.80729044
Nkx3-1	0.07515885	0.14710856	0.0142525	0.90616769	0.80729044
Arrdc2	-0.0456726	1.72076775	0.01425165	0.90617047	0.80729044
Letm2	0.02815354	3.25028569	0.0142384	0.90621389	0.80729044
Flot2	-0.0175458	4.8676707	0.0141829	0.90639592	0.80729044
Mrpl33	-0.0284005	4.31556903	0.01418108	0.90640191	0.80729044
Efhc2	0.05429228	2.12178194	0.0141694	0.90644026	0.80729044
Mif4gd	-0.0320827	3.18581163	0.01416809	0.90644457	0.80729044
Arih2	-0.0186091	4.72467566	0.01415529	0.90648662	0.80729044
Vil1	-0.0906392	-0.0528895	0.01415182	0.90649804	0.80729044
Zfr	0.01645639	8.62368606	0.01413641	0.90654871	0.80729044
Trp53rk	0.06020151	2.02455285	0.01411014	0.90663517	0.80729044
Wnt5b	-0.0505418	1.1476272	0.01410182	0.90666256	0.80729044
Pet112	-0.0297062	2.78670512	0.01409636	0.90668055	0.80729044
Trim63	0.05696974	1.2032021	0.01407344	0.90675605	0.80730425
Polr2c	-0.0239132	4.23038674	0.01401493	0.90694915	0.80732856
Lincrna-cox2	-0.1768318	-1.3891797	0.0139866	0.90704277	0.80732856
Hnrnph1	0.01139646	8.53090032	0.01398622	0.90704403	0.80732856
Ttc30a2	-0.0715308	-0.1179123	0.01398015	0.90706411	0.80732856
BC026585	0.07652771	0.725837	0.01397434	0.90708333	0.80732856
Dhx33	-0.0248421	4.90226004	0.01394216	0.90718985	0.80736996
Gm10778	-0.0265785	3.91403951	0.01388256	0.90738748	0.80738557
Zc3h12c	-0.0130739	6.25593924	0.01388164	0.90739052	0.80738557
Ndufb3	-0.0219062	5.49258129	0.01387808	0.90740234	0.80738557
Foxq1	-0.0834982	1.12475046	0.01384334	0.90751775	0.80738557
Tfpi	-0.0269105	6.01795099	0.01378741	0.90770387	0.80738557
Phldb1	0.01912695	4.12078655	0.01377102	0.90775847	0.80738557
Zfp760	-0.0187673	5.25327047	0.0137653	0.90777755	0.80738557
Rcor2	0.04580533	1.62983719	0.01376135	0.90779072	0.80738557
Trp53cor1	0.0706343	-0.0263745	0.01375667	0.90780631	0.80738557
Wdr77	0.01731256	5.07304581	0.0137318	0.90788928	0.80738557
Acaa2	-0.0235658	4.83542078	0.01372562	0.90790992	0.80738557
Nphs2	-0.0317772	3.92360048	0.01372039	0.90792739	0.80738557
Traip	0.07865126	0.84322021	0.01369219	0.90802161	0.807414
Tmem243	0.02590138	4.06310175	0.01367491	0.90807937	0.807414
AU040320	0.0255594	4.17222439	0.01362725	0.90823894	0.80742185

Gpx4	-0.0337959	4.24864188	0.01361264	0.90828789	0.80742185
Ccdc86	0.03338942	2.91362023	0.01358377	0.90838476	0.80742185
Trp53bp2	-0.0153516	5.93993044	0.01356868	0.90843539	0.80742185
Tmed8	0.01367307	6.24397623	0.01353211	0.90855831	0.80742185
Rgs7bp	-0.0165011	8.51584196	0.01352205	0.90859213	0.80742185
Dgkh	0.0294127	5.56521568	0.01350812	0.90863901	0.80742185
Crx	0.09958779	-1.2279969	0.01350527	0.9086486	0.80742185
Dgkq	0.03800268	3.8686968	0.01347264	0.90875852	0.80742185
Zfp326	-0.0152017	5.58681216	0.01346289	0.90879136	0.80742185
Slc38a3	-0.0268739	4.10921612	0.01346062	0.90879902	0.80742185
Sv2a	0.01965608	5.86958726	0.01345789	0.90880823	0.80742185
Ppie	-0.0411153	1.3935998	0.01339198	0.90903077	0.80756142
Mbtps1	0.01757286	5.43469457	0.01337583	0.90908535	0.80756142
Ccdc30	0.03166887	3.32439912	0.01335752	0.90914732	0.80756314
Srpk2	-0.0134586	7.79659098	0.01330868	0.9093128	0.80765434
Rbm26	-0.0147737	7.13250946	0.01329181	0.90937002	0.80765434
Cdh5	-0.0271194	5.44742373	0.01322507	0.90959681	0.80777901
Slc14a1	-0.0334432	2.67003188	0.01321518	0.90963046	0.80777901
Lsm14b	-0.0147938	5.55529305	0.0131678	0.90979185	0.80786903
Stmn2	-0.0121778	7.85579106	0.01314558	0.90986765	0.80788301
Emp2	-0.0240481	4.33314643	0.01312364	0.90994254	0.8078962
Sobp	0.01320472	7.44545726	0.01308238	0.91008357	0.80790879
Pusl1	0.04055149	2.33169202	0.01307296	0.91011582	0.80790879
Stam	0.01446221	6.06935406	0.01305567	0.91017498	0.80790879
Mau2	-0.01808	5.4300466	0.0130408	0.91022592	0.80790879
Tyro3	-0.0189349	4.93736118	0.01303176	0.91025692	0.80790879
Pcsk4	-0.0378073	1.48792254	0.0130054	0.91034731	0.80792119
Emc10	0.0220781	5.07381744	0.01298757	0.9104085	0.80792119
Il1bos	0.11162257	-2.0499981	0.01302267	0.91048914	0.80792119
Rtn4rl2	-0.0509374	0.92144409	0.0129499	0.91053792	0.80792119
Ap1g2	0.0984328	0.00113336	0.01293848	0.91057723	0.80792119
Zfp51	0.02862293	4.1864609	0.01289809	0.91071626	0.80792119
Cldn22	-0.172854	-1.6312496	0.01288633	0.91075679	0.80792119
Capn10	-0.0307865	2.36488744	0.01288286	0.91076876	0.80792119
Insl6	0.05513429	1.1793507	0.0128661	0.91082657	0.80792119
Ccdc137	-0.028669	4.23153916	0.01285314	0.91087129	0.80792119
Rfc3	-0.0391844	1.77971401	0.01281	0.91102032	0.80800012
Gm5801	-0.0704749	-0.3960546	0.01276462	0.91117734	0.80800213
Klhl35	0.12343779	-0.7426236	0.01274348	0.91125061	0.80800213
Pigq	-0.0156918	5.82510229	0.01274205	0.91125557	0.80800213
Pcdh12	-0.2638665	-1.9877816	0.01273096	0.91129403	0.80800213
1600023N17	0.08799287	-0.9773858	0.01272266	0.91132282	0.80800213
H2-M3	0.04382659	2.40479271	0.01268161	0.91146534	0.80807525

Rab11a	-0.0140252	6.56897151	0.01264795	0.91158242	0.80812581
Sp3os	0.0348928	3.10306267	0.01262044	0.9116782	0.80815747
Wbp5	0.02061666	6.62007551	0.01257133	0.91184946	0.80819456
Slc25a36	-0.015877	4.98566063	0.01255954	0.91189062	0.80819456
Rpl5	-0.0191528	8.2506935	0.01255571	0.91190399	0.80819456
Ceacam2	-0.039247	1.35594275	0.0125396	0.91196028	0.80819456
Uros	-0.0243375	3.53309665	0.01248997	0.9121339	0.8082533
Ell	0.02014685	3.49996243	0.01248347	0.91215669	0.8082533
Hist1h4i	-0.0733203	-0.2487635	0.01246918	0.91220676	0.8082533
Ctps2	0.0139664	5.66905144	0.01242239	0.91237092	0.80832875
Mir568	-0.0298954	2.11864824	0.01241068	0.91241205	0.80832875
Nelfcd	0.02304696	3.75889393	0.01239023	0.91248392	0.80833921
1700101I11R	-0.0905436	0.31317228	0.0123693	0.91255757	0.80835123
Ccdc105	0.11270151	-0.7318402	0.01232098	0.91272778	0.8083624
Stab1	-0.0570182	1.19212054	0.0123208	0.9127284	0.8083624
Naa60	0.01340477	6.11045487	0.01231457	0.9127504	0.8083624
Ube3c	0.01496885	6.07983139	0.01225701	0.91295367	0.80848921
Oxsr1	0.01446272	5.96237437	0.01223384	0.91303562	0.80850858
Lgmn	0.02481019	4.12100921	0.01217102	0.91325821	0.80865247
Taf2	0.01607647	5.91568039	0.01213564	0.91338387	0.80866616
Nol6	-0.0176404	5.48247362	0.01212816	0.91341044	0.80866616
B230118H07	-0.0176563	5.48858594	0.01210449	0.91349461	0.80866616
Tnnt3	-0.1451232	-1.5294982	0.01208545	0.9135624	0.80866616
Pmf1	0.04127187	3.35773183	0.01208215	0.91357415	0.80866616
Rnasek	-0.0234642	7.09958463	0.01203991	0.91372472	0.80874624
Insm1	0.04607338	2.14257829	0.01198611	0.9139169	0.80884213
Lrfn2	-0.0333888	1.80541065	0.01196683	0.91398584	0.80884213
Efna4	0.05333126	1.84046717	0.01193384	0.914104	0.80884213
Eri1	0.01556894	5.71435781	0.01191872	0.9141582	0.80884213
Fbll1	-0.0361549	2.23625039	0.01190939	0.91419169	0.80884213
Bcap31	-0.0221625	5.73327821	0.01188592	0.91427593	0.80884213
Foxp1	-0.0122729	8.77787499	0.01188456	0.91428081	0.80884213
Ube2e1	-0.0234608	4.09330134	0.01187534	0.91431393	0.80884213
Lmf2	-0.0296763	2.46143487	0.01184142	0.91443588	0.80889684
Snx2	0.012787	7.57439358	0.01181503	0.91453088	0.80892769
Zfp862-ps	0.03073679	2.31865458	0.01177251	0.91468419	0.80899117
Wscd2	-0.0252469	3.52687442	0.01176179	0.91472288	0.80899117
1700030K09I	-0.0485032	1.60509031	0.01173561	0.91481744	0.80902163
Rnd2	0.04421885	1.72906481	0.01165714	0.91510155	0.80918397
Fbxo10	-0.0269818	4.18832971	0.0116517	0.91512128	0.80918397
Ddx60	0.05283755	1.88541566	0.01157322	0.91540646	0.80938295
Ccdc90b	0.02157934	5.8537065	0.01154227	0.91551916	0.80942942
A830082K12	-0.0147334	6.61205122	0.01147609	0.91576076	0.80958982

Tmem234	-0.0192465	4.73707194	0.01144401	0.9158781	0.80963788
Bag1	0.01862637	7.80584386	0.01141875	0.91597061	0.80963788
Ric8b	0.02085339	5.65115964	0.01141193	0.91599562	0.80963788
Tkt	0.02089993	4.83904856	0.01138515	0.91609387	0.80967153
Rhbdf2	0.10506074	-1.7055015	0.01133754	0.91626876	0.80977292
Med11	0.03604313	2.92929786	0.01130227	0.91639861	0.80983449
Cd48	0.07176439	0.34257758	0.01127132	0.91651271	0.80984225
Ttc30b	-0.021012	3.5675271	0.01125808	0.91656154	0.80984225
Lrpprc	-0.0229793	5.97469776	0.01123961	0.91662975	0.80984225
Myh11	0.06593147	2.52897226	0.0112198	0.91670298	0.80984225
8030462N17	-0.0137403	5.47395335	0.01121836	0.91670831	0.80984225
Tdrd5	-0.057045	1.22862972	0.01120087	0.916773	0.80984624
Gm13242	0.09162513	-0.9866561	0.01117826	0.9168567	0.80986701
Spata1	0.04605747	1.26760515	0.0111584	0.91693032	0.80987888
Ube2j2	0.02396373	2.89842445	0.01112997	0.91703579	0.80991887
Cyb5	-0.0191803	5.88858328	0.01108083	0.91721844	0.81002702
Tlr8	0.13509949	-1.0531026	0.01101579	0.91746079	0.81017838
Gng2	0.01055008	7.99385618	0.01100254	0.91751025	0.81017838
Zhx1	-0.0127645	8.00743807	0.01096479	0.91765136	0.8101993
Zcchc14	-0.0140077	6.32427807	0.0109493	0.91770933	0.8101993
Ttll11	-0.0234308	4.20607825	0.01094084	0.917741	0.8101993
Hells	-0.0451671	1.48357957	0.01093182	0.91777478	0.8101993
Galnt5	-0.1123174	-1.0142029	0.01087285	0.91799599	0.81031094
Bora	-0.0435339	1.55862434	0.01086602	0.91802168	0.81031094
Bad	0.04253915	2.66095758	0.0108256	0.91817369	0.81039196
Foxn2	-0.0193917	4.77039956	0.01080111	0.91826595	0.81039658
Acsf3	0.04194629	1.50699388	0.01078691	0.9183195	0.81039658
Brd1	0.01312623	6.0043742	0.01077629	0.91835959	0.81039658
Ndufa3	-0.0203633	4.66141307	0.01071581	0.91858812	0.81054509
Itga1	0.01842534	4.19163823	0.01068669	0.91869843	0.81056046
Ern1	0.03836529	2.21275338	0.0106566	0.91881253	0.81056046
Acads	0.08535361	-0.6553317	0.0106291	0.91891695	0.81056046
Tspan6	-0.0276351	3.9849307	0.01061996	0.9189517	0.81056046
Tor1aip1	0.01545113	7.34527973	0.01061674	0.91896393	0.81056046
Gm5544	0.12221208	-0.6670952	0.01059529	0.91904552	0.81056046
Gm13031	-0.1084347	-0.4832155	0.01059503	0.91904653	0.81056046
Zfp946	-0.0277998	2.87394696	0.01057385	0.91912718	0.81056046
Tha1	0.0603249	0.05886685	0.01056847	0.91914767	0.81056046
Tshz2	-0.0125366	6.13773992	0.01054578	0.91923422	0.81058366
Zfp148	0.01170307	7.82606425	0.01048582	0.91946329	0.81071851
1700034I23R	-0.0865904	-0.3781941	0.01045427	0.9195841	0.81071851
Lrrtm4	-0.0170561	5.30938419	0.01043862	0.91964409	0.81071851
Rassf5	-0.0169057	4.01244149	0.01043659	0.91965189	0.81071851

Stox1	-0.0360221	1.31133111	0.01042552	0.91969434	0.81071851
Aldh1a3	0.07278116	-0.040302	0.01040724	0.91976454	0.81071851
Brcc3	0.01618147	5.43400162	0.01037426	0.91989129	0.81071851
5930430L01F	0.02346267	4.70600952	0.01036962	0.91990916	0.81071851
Plekhm3	-0.0165518	6.33117024	0.01036436	0.91992938	0.81071851
Chodl	-0.1264072	-1.4040797	0.0103346	0.92004401	0.81076643
Cyb5r4	0.01241005	5.78849428	0.01027273	0.92028284	0.81086239
Mfap4	-0.033568	3.76607458	0.01026696	0.92030518	0.81086239
Grhl2	-0.0676025	0.49224649	0.01025959	0.92033368	0.81086239
Gtf3c3	0.01609321	5.1667821	0.01023825	0.92041628	0.81087222
Tesc	-0.0242504	3.01370342	0.01018435	0.92062528	0.81087222
Smox	-0.0277606	2.88869692	0.01017439	0.92066397	0.81087222
Mtfr1	0.02742597	3.94927546	0.01015542	0.92073771	0.81087222
Padi4	-0.1262736	-2.0630303	0.01012941	0.92083889	0.81087222
Fam149b	-0.0143779	5.38865691	0.01012259	0.92086547	0.81087222
Gm17801	0.09534655	-0.6337183	0.0100953	0.92097183	0.81087222
Arhgef37	-0.0601984	0.28806604	0.01008555	0.92100989	0.81087222
Foxg1	0.01631986	6.13935789	0.01007788	0.9210398	0.81087222
Clcn1	0.06340594	0.05914403	0.01007514	0.92105049	0.81087222
Nlrp3	-0.0975096	-0.9564663	0.0100738	0.92105574	0.81087222
Gipc1	-0.0315843	3.03328594	0.01007067	0.92106795	0.81087222
Igsf8	0.0171554	5.06803153	0.01002968	0.9212282	0.81092449
Ikzf5	0.01469677	5.07149542	0.01002466	0.92124785	0.81092449
Armc4	-0.0769702	-0.9967115	0.0099579	0.9215096	0.81096075
9530082P21I	-0.0157386	5.19462914	0.00995162	0.92153424	0.81096075
Ifi205	0.08302668	-1.2619821	0.00994124	0.92157503	0.81096075
Rhot2	-0.0176783	5.09631847	0.00994031	0.9215787	0.81096075
Tbc1d20	-0.0228896	4.21044894	0.0099255	0.92163693	0.81096075
Gm4787	0.07426815	2.161135	0.00990064	0.92173481	0.81096075
Rnf138rt1	-0.1124407	-1.5642962	0.0098919	0.92176925	0.81096075
Lrp11	0.0154925	5.96147788	0.00988305	0.92180411	0.81096075
Tssc4	0.02481769	2.47401417	0.00986304	0.92188305	0.81096075
Gm12522	-0.0458923	1.18137555	0.0098453	0.9219531	0.81096075
Rpl24	-0.0164613	6.6964983	0.00976765	0.92226041	0.81096075
Hmgn1	-0.01982	8.11437481	0.0097615	0.92228482	0.81096075
Ift81	-0.0118159	5.57400414	0.00974967	0.92233177	0.81096075
Uggt1	-0.0223156	5.11580547	0.00973172	0.92240303	0.81096075
Tm2d1	0.02317678	2.9591863	0.00972853	0.92241574	0.81096075
Kansl1	0.01026568	6.95826193	0.00972766	0.92241918	0.81096075
Mmab	0.01621654	4.42183548	0.00972244	0.92243993	0.81096075
Ascc3	0.02212001	5.75611155	0.0097003	0.92252797	0.81096075
Mapk1ip1	-0.0152724	4.40151255	0.00969768	0.92253842	0.81096075
Bmpr2	-0.0137295	9.84150137	0.00968167	0.92260218	0.81096075

Oasl2	-0.0232355	7.17688143	0.00965415	0.92271187	0.81096075
Trim21	-0.0262116	3.83998389	0.00964162	0.92276186	0.81096075
Prrt1	0.01686255	4.88909322	0.00963578	0.92278517	0.81096075
Pparg	-0.037523	1.72385308	0.00962712	0.92281979	0.81096075
Mnd1	0.08755505	0.36796527	0.00961694	0.92286044	0.81096075
Sstr3	0.01857557	3.54620134	0.00961474	0.92286926	0.81096075
Mettl23	0.02760263	2.83220101	0.00960299	0.92291623	0.81096075
Poli	-0.0186849	3.37439859	0.00956461	0.92306993	0.81104284
Ankrd27	-0.0127107	5.38224598	0.00954864	0.92313397	0.81104616
Zbtb38	-0.0115189	6.89674946	0.00949439	0.92335188	0.8110907
Pou5f1	0.41254442	-2.4473957	0.10827656	0.92342074	0.8110907
Vsig10	-0.0500888	1.46708961	0.00947666	0.92342325	0.8110907
Slc13a3	-0.030887	6.93531399	0.00947604	0.92342577	0.8110907
Btf3l4	0.01071	7.05131846	0.00940553	0.92371023	0.8112876
Pisd-ps2	0.04796242	1.82561188	0.0093702	0.9238532	0.81130483
Fam57a	-0.0402177	1.66580717	0.00935515	0.92391414	0.81130483
Tnr	0.02360311	5.08287813	0.00935297	0.92392299	0.81130483
Dennd5a	-0.0100564	7.52315641	0.00934113	0.92397101	0.81130483
Psenen	-0.0258135	5.2136911	0.0092921	0.92417015	0.81142673
Fam217b	0.01649349	4.010407	0.0092645	0.92428249	0.81147242
Schip1	0.01928066	3.86375504	0.00923837	0.924389	0.81150745
Fgf13	0.01487213	6.45146223	0.00921711	0.92447578	0.81150745
Slco2b1	0.03096142	2.70595602	0.009195	0.92456613	0.81150745
H2-Q4	0.03661448	1.686645	0.0091875	0.92459679	0.81150745
Sirpb1b	-0.1594516	-1.704659	0.00918087	0.92462392	0.81150745
Actl6b	0.02620384	3.2826385	0.00914061	0.92478882	0.81159924
Kcnk6	0.0419119	1.35946233	0.00907752	0.925048	0.81174895
Dlgap4	0.0118608	7.19902997	0.0090658	0.92509628	0.81174895
Rab11b	0.01206407	9.08833055	0.00904714	0.92517313	0.81174895
Tmed1	0.04792118	1.04176317	0.0090151	0.92530533	0.81174895
Tpmt	-0.0235409	3.69758478	0.00899908	0.92537152	0.81174895
Lrrc8c	-0.0188492	4.05607155	0.0089984	0.92537433	0.81174895
Polr3k	-0.0163022	6.06279642	0.00898909	0.92541285	0.81174895
Dnajc6	0.01251889	8.53886935	0.0089693	0.92549472	0.81174895
Rbm45	-0.0200035	3.49260112	0.00896746	0.92550234	0.81174895
Usp28	-0.0248621	3.5545743	0.00891837	0.92570589	0.81183835
Upk1b	-0.0551878	1.27671909	0.00891379	0.92572492	0.81183835
Mpp1	0.01182871	5.83676708	0.00886602	0.92592359	0.81195693
Ppp1ca	0.02006392	5.51876175	0.00885228	0.92598082	0.81195693
Hey1	-0.0137247	6.35228255	0.00880573	0.92617512	0.81205872
Kif3b	0.01399447	6.59547066	0.00878794	0.92624949	0.81205872
Fam228b	-0.0447708	1.62913522	0.00878114	0.92627795	0.81205872
Ufl1	-0.0137409	5.79660196	0.00875845	0.92637296	0.81208911

Abcb4	0.05453511	0.37409322	0.0087317	0.92648512	0.81213452
Lhpp	-0.0250729	2.63005756	0.00871136	0.92657053	0.81215649
Phf5a	-0.020693	4.82766972	0.00869336	0.92664621	0.81216992
Nlgn3	0.01538076	5.98583432	0.00864898	0.92683314	0.81228085
Arhgap20	-0.011714	8.42146037	0.00863047	0.92691123	0.81228764
Amz2	-0.0158599	4.75288145	0.00861854	0.92696162	0.81228764
Cd163	-0.044728	1.35450107	0.008596	0.92705691	0.81230358
Rimkla	-0.0244923	3.67743877	0.00857342	0.92715246	0.81230358
6330419J24F	-0.0242445	3.50590208	0.00857143	0.92716091	0.81230358
Sh3rf1	-0.0124077	5.05297757	0.0085416	0.92728736	0.81232258
3110009E18I	-0.0466142	1.10227185	0.00851737	0.92739028	0.81232258
Tifa	-0.0249515	3.62939513	0.00850129	0.92745865	0.81232258
Mtfp1	-0.0190346	4.76527358	0.0084957	0.92748241	0.81232258
4931440F15I	-0.0855336	-0.2478327	0.00848538	0.92752636	0.81232258
Wdr20	-0.0173866	3.5679198	0.00847811	0.9275573	0.81232258
Olfr1372-ps1	0.05854211	-0.054429	0.00846066	0.92763167	0.81232258
Csrnp1	-0.0386264	1.76997254	0.00845272	0.92766553	0.81232258
C2cd3	0.01790109	5.22226321	0.0084108	0.92784461	0.81237515
Msx2	-0.0391864	2.29540939	0.0084104	0.92784631	0.81237515
Enpp5	-0.0114631	7.09987463	0.00834673	0.92811917	0.81241984
Tnfrsf18	0.03285082	1.90014686	0.00834048	0.92814599	0.81241984
Cenpn	-0.0488087	1.17111086	0.00833772	0.92815784	0.81241984
Uhmk1	0.01156422	6.47143497	0.00833596	0.92816541	0.81241984
Gm1141	-0.1736612	-1.6419073	0.00832084	0.92823039	0.81241984
Mpdu1	0.02599584	2.850041	0.00830921	0.92828041	0.81241984
Pkp2	0.0315809	2.23426187	0.0082961	0.92833685	0.81241984
Adam4	0.05676875	0.97597989	0.008286	0.92838035	0.81241984
Slc35e3	-0.0151958	4.481118	0.00826932	0.92845228	0.81242995
Dennd4c	0.01281481	6.04642157	0.00824265	0.92856742	0.81247787
Cnot4	-0.0134582	7.41731286	0.0082056	0.92872766	0.81256524
Mtmr6	0.01032521	7.40515423	0.00818341	0.92882382	0.81259654
Zfp758	0.01848874	3.79792439	0.00815293	0.92895613	0.81265946
Trdmt1	0.02904066	2.84535639	0.00807482	0.92929633	0.81283961
Aarsd1	-0.0201457	3.70563603	0.00806727	0.92932926	0.81283961
Mvb12a	0.02651735	1.88568296	0.00805324	0.92939057	0.81283961
Amfr	0.0134352	5.82024607	0.00804074	0.92944525	0.81283961
Lsm10	-0.0248598	2.36384022	0.00800599	0.92959745	0.81283961
LOC1012436	-0.0780267	-0.5993772	0.00798996	0.92966775	0.81283961
Fam171a1	0.01464962	5.50197085	0.00798502	0.92968944	0.81283961
Ddx54	-0.018514	3.98407135	0.00794051	0.92988514	0.81283961
C730002L08F	-0.0286552	2.30631274	0.00791301	0.93000634	0.81283961
Nufip1	0.01874277	4.1197829	0.00790914	0.93002339	0.81283961
Pard6a	-0.0353726	2.15301672	0.00790852	0.93002613	0.81283961

Tmem230	-0.0117955	6.66927818	0.00790263	0.93005214	0.81283961
Mcat	0.02605672	2.54081818	0.00789959	0.93006553	0.81283961
Gkn3	-0.109402	-1.1762182	0.00789773	0.93007375	0.81283961
1700003M07	-0.0163657	3.86364677	0.00785708	0.93025343	0.81283961
Gosr1	-0.0147078	5.27713659	0.00785004	0.93028461	0.81283961
Creb1	0.00874553	7.03094245	0.00780737	0.93047383	0.81283961
Ccnk	-0.011006	6.38171229	0.00779538	0.93052709	0.81283961
4930513N10	0.07063254	-0.4688363	0.00779209	0.9305417	0.81283961
Moxd1	-0.0235034	2.93073035	0.00778978	0.93055198	0.81283961
Gpr6	0.10483614	-1.4290837	0.00776865	0.93064597	0.81283961
Szrd1	-0.0189806	4.64014995	0.00774047	0.93077154	0.81283961
Osr1	-0.0269104	6.37133727	0.00771462	0.93088692	0.81283961
A830052D11	-0.0479779	0.26860189	0.00771104	0.93090293	0.81283961
Ly6c1	0.04397927	1.66203813	0.00770613	0.93092485	0.81283961
Fam150b	0.10432196	-1.1072825	0.00765421	0.93115731	0.81283961
1700049G17	-0.01996	3.46919692	0.00763842	0.93122819	0.81283961
Psph	-0.025144	3.65078526	0.0076141	0.93133743	0.81283961
Hars2	0.01613621	3.90856284	0.00760943	0.93135847	0.81283961
Ccdc9	-0.0200047	2.73401385	0.00760691	0.93136981	0.81283961
Zwilch	-0.0401592	1.4763998	0.0076054	0.9313766	0.81283961
Fam184a	0.01835465	4.85798108	0.00759244	0.93143493	0.81283961
Zbtb4	-0.00925	7.52108843	0.00756827	0.93154387	0.81283961
Cps1	0.11521933	-1.1991951	0.00756792	0.93154545	0.81283961
Sf3b3	-0.0125235	5.27586135	0.007556	0.93159924	0.81283961
Cpox	0.01593678	5.72688531	0.00755528	0.93160247	0.81283961
Kcnj6	0.01885414	5.88929856	0.00754698	0.93163994	0.81283961
Tnfaip8l2	-0.0823113	-0.6473809	0.00753735	0.93168345	0.81283961
Ofd1	-0.019878	4.18802212	0.00750459	0.93183169	0.81283961
Slamf7	-0.0518007	1.12128087	0.00746295	0.9320206	0.81283961
Slain1	-0.0179864	4.91292818	0.00744143	0.93211843	0.81283961
Gngt2	-0.0447727	0.72351475	0.00743885	0.93213017	0.81283961
Fastkd5	-0.0169107	3.34987176	0.00740305	0.93229323	0.81283961
Psmb3	-0.0156794	4.45844632	0.00737671	0.93241348	0.81283961
Ppig	0.0130955	9.2691446	0.00737576	0.93241782	0.81283961
9130401M01	-0.0140481	4.78490337	0.00736942	0.93244682	0.81283961
Manba	-0.0305646	2.7862483	0.00736476	0.93246814	0.81283961
Reln	0.02119977	5.25469557	0.00736397	0.93247174	0.81283961
Rps15a-ps4	-0.0312173	1.32191386	0.00734306	0.93256744	0.81283961
Tshb	-0.1032079	-1.2726407	0.0073331	0.93261306	0.81283961
Ogfod1	-0.0119148	7.29648385	0.00732718	0.93264018	0.81283961
Hars	-0.0100561	5.89879143	0.00731808	0.93268192	0.81283961
Cenpp	0.02631078	2.28834247	0.00730802	0.93272808	0.81283961
Opalin	-0.0392546	1.45829791	0.00730674	0.93273399	0.81283961

Ifi47	-0.0278968	3.77676149	0.00730106	0.93276008	0.81283961
Lama2	-0.016057	5.21919044	0.00728204	0.93284747	0.81283961
Casp4	-0.0382455	0.78416586	0.00726047	0.93294673	0.81283961
Zzef1	0.02199565	5.30413241	0.00723542	0.93306224	0.81283961
Mir6336	0.08956585	-1.5248579	0.00721945	0.93313595	0.81283961
Ccdc88b	0.04280116	0.13683675	0.00721233	0.93316884	0.81283961
Acox3	0.01511387	4.07254784	0.00720809	0.93318844	0.81283961
Gnaq	0.00881131	8.68177688	0.0072037	0.93320876	0.81283961
Ccpg1os	-0.0197866	2.81273087	0.00719502	0.93324889	0.81283961
Gm3716	-0.0703601	-0.725553	0.00718788	0.93328195	0.81283961
Sptbn4	0.03131955	3.60591124	0.00718658	0.93328798	0.81283961
Piezo1	-0.0352223	1.30366693	0.00716954	0.93336689	0.81283961
Rpl18a	-0.0163632	7.4643089	0.00716807	0.93337372	0.81283961
4930570G19	-0.0249304	3.53760825	0.00716329	0.93339586	0.81283961
Spdl1	-0.0617372	-0.1312964	0.00715503	0.93343417	0.81283961
Tpgs1	-0.0268964	3.14348779	0.00714407	0.93348508	0.81283961
Gstk1	-0.0224293	3.26041485	0.00713343	0.93353449	0.81283961
Rab2b	-0.0112441	5.9805312	0.00713041	0.93354852	0.81283961
Ms4a4c	-0.1172138	-1.6384002	0.00712309	0.93358257	0.81283961
Adh1	-0.0532911	0.18930139	0.00710901	0.9336481	0.81283961
Ntrk1	-0.0729466	-1.4088131	0.00709947	0.9336925	0.81283961
Ppan	-0.0312476	1.68015532	0.00707586	0.93380256	0.81288284
Bax	-0.0228899	2.61546348	0.00705705	0.93389041	0.81290672
Vip	-0.0223562	2.80295463	0.00700243	0.9341461	0.81300957
Sap30l	0.01806802	3.93973451	0.00698785	0.93421453	0.81300957
Gigyf1	0.02875033	2.40603753	0.00698624	0.9342221	0.81300957
Sec62	0.01076258	7.55522414	0.00696056	0.93434281	0.81300957
Mrpl23	0.01798934	3.13313811	0.00695023	0.93439144	0.81300957
Rap2b	0.01176767	5.93718849	0.00694523	0.93441497	0.81300957
Fech	0.01163524	7.02489319	0.00694172	0.9344315	0.81300957
Myd88	0.02662392	2.78060897	0.0068574	0.93482997	0.81325151
Echdc2	-0.0268169	2.26378607	0.00684873	0.93487109	0.81325151
Zdhhc16	0.02245367	2.26251779	0.00684456	0.93489087	0.81325151
Tulp4	0.00970415	8.14953768	0.00678896	0.93515523	0.81338161
Grcc10	-0.0276893	3.80079278	0.00676672	0.93526131	0.81338161
Slc2a6	-0.0388736	1.04842691	0.00676335	0.93527738	0.81338161
Bak1	0.02037565	3.90940784	0.00675256	0.9353289	0.81338161
Gm13582	-0.1141603	-1.3285864	0.0067375	0.93540091	0.81338161
Ubxn8	-0.0175918	4.33700244	0.00673657	0.93540532	0.81338161
Pilra	0.06009168	-0.5315319	0.0067244	0.93546356	0.81338161
Gm6815	0.05741511	-0.9340087	0.0066818	0.93566785	0.81340191
Nkain3	-0.0277814	1.994663	0.00667268	0.93571163	0.81340191
Ttyh3	0.01473182	6.04932733	0.00667008	0.93572417	0.81340191

Capn9	-0.160114	-2.3766796	0.00669907	0.9357287	0.81340191
Erich2	-0.0410311	0.43049639	0.00662521	0.93594019	0.81347269
Myadml2	-0.0516776	0.67170072	0.00659456	0.93608822	0.81347269
Vps37a	-0.0122876	7.7321129	0.00659219	0.93609968	0.81347269
Rgl2	0.02422616	2.1835214	0.00658068	0.93615536	0.81347269
Pou4f1	-0.1303398	-1.0522652	0.00659218	0.93624231	0.81347269
Fundc2	-0.0136987	7.72147033	0.00651969	0.93645121	0.81347269
Ccnt2	-0.0187367	4.8957623	0.00650209	0.93653686	0.81347269
Plekhd1	-0.0716077	-1.030758	0.00649596	0.93656669	0.81347269
5830432E09I	-0.0759917	-1.1119705	0.00649175	0.93658721	0.81347269
Trmt2b	-0.019863	5.21430636	0.00648301	0.93662983	0.81347269
Cpsf1	-0.0199745	4.0570391	0.0064742	0.9366728	0.81347269
Terf2	0.00966853	6.26750373	0.00647002	0.93669323	0.81347269
Fam210a	-0.0101149	6.44242624	0.0064646	0.93671965	0.81347269
Hspe1	-0.0120799	6.34454213	0.00646131	0.93673572	0.81347269
Nwd1	0.0150242	5.14178275	0.0064608	0.93673821	0.81347269
Pcdhgb6	0.02630166	2.14496782	0.00644803	0.93680063	0.81347269
Pdcd5	0.01231899	6.90457778	0.0064382	0.9368487	0.81347269
Perp	-0.025351	6.65280061	0.00642723	0.93690244	0.81347269
Slc10a7	-0.0159026	3.06877639	0.00641574	0.93695872	0.81347269
Rp2h	0.01515274	4.92032944	0.00639459	0.93706251	0.81347609
Lrch1	0.01689001	4.25291968	0.00638154	0.93712662	0.81347609
Mettl4	-0.0147004	5.08264826	0.00636341	0.93721576	0.81347609
Raf1	0.01219781	4.73108242	0.00635712	0.93724675	0.81347609
Manea	0.01226923	5.51538336	0.00633985	0.93733185	0.81347609
Slx4ip	0.0199504	3.03855891	0.00632161	0.93742187	0.81347609
H2-Ke6	-0.0317307	2.38317371	0.00631424	0.93745825	0.81347609
Supt3	-0.0222586	2.28573846	0.00630014	0.93752799	0.81347609
Rras2	0.01560964	4.65178085	0.00625522	0.93775061	0.81347609
Slc24a5	-0.0205152	2.28941633	0.00624221	0.93781526	0.81347609
Sypl	0.01400983	6.38862586	0.00623059	0.93787301	0.81347609
Slc39a1	-0.0184614	5.19620193	0.00621882	0.9379316	0.81347609
Cox17	-0.0128933	5.38108613	0.00621504	0.93795044	0.81347609
Clec4a3	-0.0943196	-0.2808366	0.00621503	0.93795047	0.81347609
Slc39a5	0.04830399	-0.5327547	0.0062123	0.9379641	0.81347609
Gm19557	0.07661347	-0.643446	0.006189	0.93808025	0.81347609
Ebpl	0.02757928	2.36077055	0.0061794	0.93812821	0.81347609
Fam124a	-0.0147077	4.4110444	0.00613439	0.93835348	0.81347609
Gm16062	-0.0487914	-0.2612787	0.00611272	0.93846219	0.81347609
Ina	0.01241162	6.3206916	0.00609561	0.93854823	0.81347609
Tmem150co:	-0.08185	-0.5281174	0.00609273	0.93856272	0.81347609
Kbtbd4	-0.0129197	4.48817103	0.00606782	0.93868814	0.81347609
Hiat1	-0.0102817	6.3790195	0.00606314	0.93871175	0.81347609

Map1s	0.01720613	3.04419634	0.00605081	0.93877395	0.81347609
Rps6ka6	-0.0229368	2.62959887	0.0060494	0.93878107	0.81347609
Gm10354	-0.1104997	-2.0395813	0.00604617	0.93879739	0.81347609
Fsd2	0.09656133	-1.7199672	0.00604292	0.93881381	0.81347609
Mblac1	0.02470759	2.67170348	0.00603657	0.9388459	0.81347609
Map2k6	-0.0145425	4.00294987	0.00603321	0.93886288	0.81347609
Elk1	-0.0132309	4.81294127	0.00603096	0.93887429	0.81347609
Atf1	0.01120922	7.58170167	0.00599031	0.93908018	0.81347609
1700026D08	0.03716409	0.68948357	0.00598231	0.93912079	0.81347609
Mthfs	0.02216984	2.33692126	0.00597346	0.93916574	0.81347609
Ccpg1	0.01059595	6.52446713	0.00595903	0.93923912	0.81347609
Extl2	0.01142581	6.08279579	0.0059583	0.93924285	0.81347609
Sephs2	-0.0151691	4.04139257	0.00595728	0.93924802	0.81347609
Dgcr14	-0.0159268	3.75036642	0.0059443	0.93931409	0.81347609
Psma2	-0.0122127	6.68666278	0.00594317	0.93931987	0.81347609
Gm10416	0.05502631	0.47683774	0.00594192	0.93932625	0.81347609
Arih1	-0.0083601	7.93081181	0.00593122	0.93938077	0.81347609
Aida	-0.0102333	6.45288865	0.00587463	0.93967003	0.81356248
Ovgp1	0.04837073	0.34036295	0.00585706	0.93976017	0.81356248
I830077J02R	0.0364494	0.98222716	0.00585512	0.93977012	0.81356248
Srsf7	0.00908268	5.89883115	0.00583911	0.93985234	0.81356248
Zfp324	0.02315597	3.0189689	0.00583134	0.93989227	0.81356248
Tbc1d2	0.05350823	-0.1847319	0.00579383	0.94008554	0.81356248
Tmem120a	-0.0566319	0.1265171	0.0057894	0.94010841	0.81356248
Car8	-0.0233914	2.79708353	0.0057799	0.94015744	0.81356248
Gm11127	0.01918127	3.78729841	0.00577652	0.9401749	0.81356248
Clk1	0.01613862	6.46913764	0.00577131	0.94020186	0.81356248
Kit	0.01090931	4.87311702	0.00576031	0.94025874	0.81356248
Agfg1	-0.0091762	5.82001065	0.00574966	0.9403139	0.81356248
Trip13	-0.0563343	0.72257965	0.00574368	0.94034486	0.81356248
Pnma3	0.01895079	2.98502679	0.0057347	0.94039142	0.81356248
Ric3	0.01080355	6.1222974	0.00573066	0.94041239	0.81356248
A730020E08	-0.0238401	2.69495924	0.00572382	0.94044788	0.81356248
Taf13	-0.0104836	6.57924145	0.00566205	0.94076945	0.81369897
Stx18	0.0179974	3.37978799	0.00566108	0.94077452	0.81369897
Klf7	0.0104285	6.5193186	0.00565868	0.94078707	0.81369897
Rpl7	0.01312254	6.91340087	0.00558063	0.94119602	0.81395624
Cox19	-0.0161283	3.39470768	0.00557883	0.94120549	0.81395624
Scand1	0.065173	-1.4286428	0.00553698	0.94142601	0.81406933
Cryl1	-0.0178604	3.57111571	0.00553106	0.94145725	0.81406933
Ahsa1	-0.0103887	5.77367489	0.00551143	0.94156103	0.81410675
Mcee	0.01523113	4.05548428	0.00548752	0.94168771	0.8141283
Plcl2	-0.0145735	5.54410284	0.00546635	0.94180007	0.8141283

Atxn2	0.0112732	8.07817741	0.00546246	0.94182076	0.8141283
Pex2	0.01706122	4.2015927	0.0054611	0.94182797	0.8141283
Snx33	-0.0172075	3.96125282	0.00543937	0.9419436	0.81413234
Abcc1	0.01926486	3.88169339	0.00543749	0.94195364	0.81413234
Rnf19a	0.00914016	6.03704394	0.00540706	0.94211597	0.81420043
Ubap1l	0.11843634	-1.8406109	0.00540005	0.94215344	0.81420043
Lin54	0.01199684	4.48532347	0.00534087	0.94247065	0.81425467
9030204H09	0.07041303	-1.183812	0.00532753	0.94254241	0.81425467
Spin1	0.00717354	8.82687423	0.00527476	0.94282717	0.81425467
Veph1	0.04888341	-0.1458469	0.00526761	0.94286587	0.81425467
Snrpn	-0.0281788	0.80614836	0.00526471	0.94288156	0.81425467
Zbtb45	0.01977273	2.41541769	0.00526391	0.9428859	0.81425467
Mybl2	-0.0615628	-1.316841	0.00525497	0.94293434	0.81425467
Cd109	0.02565153	3.07290153	0.005248	0.94297213	0.81425467
Dear1	-0.036445	1.65996176	0.00523537	0.94304066	0.81425467
Gm13498	-0.044158	-0.4467189	0.00522727	0.94308463	0.81425467
Tuft1	-0.0429606	0.44373696	0.00521683	0.94314141	0.81425467
Sec23b	-0.0133994	4.50824447	0.00520504	0.9432056	0.81425467
Mrpl19	0.01082993	5.02303377	0.00520264	0.94321865	0.81425467
Tceanc2	-0.01095	4.63240654	0.00519651	0.94325208	0.81425467
Mst1r	-0.0526657	-0.7294972	0.00517813	0.94335235	0.81425467
Tube1	-0.0430448	0.36225688	0.0051769	0.94335906	0.81425467
Col28a1	0.06735038	-0.372539	0.00516255	0.94343748	0.81425467
Hist1h4h	0.06477899	-1.3963942	0.00516056	0.94344833	0.81425467
Nckipsd	-0.0148187	4.12030892	0.00515035	0.94350423	0.81425467
Kcng1	-0.0513561	0.10079227	0.00514526	0.94353207	0.81425467
Srebf1	0.01629307	2.89281282	0.0051443	0.94353733	0.81425467
AY512931	-0.0323226	1.78850411	0.00514246	0.94354744	0.81425467
Slc25a30	-0.0335957	1.14282659	0.00513093	0.94361063	0.81425698
Rwdd2a	-0.0182968	2.99713776	0.00510242	0.94376724	0.81428887
Phactr4	0.0144445	6.13209661	0.00510217	0.94376861	0.81428887
Kcnj5	-0.0692969	-0.0883408	0.00508388	0.94386929	0.81432353
Fam187b	-0.0641837	0.85249679	0.00505311	0.94403911	0.81436502
Oas1c	0.03140656	1.37818016	0.00504849	0.94406468	0.81436502
Kdm2b	-0.0105125	4.37736772	0.00504229	0.94409895	0.81436502
Ttll1	-0.0089491	5.61818762	0.00502488	0.9441954	0.81439602
Prelp	0.0156313	8.14582054	0.00499449	0.94436413	0.81448934
Sall2	0.01034267	5.73749934	0.00497345	0.94448122	0.81449931
Kcns1	0.02437926	1.60185337	0.00497066	0.94449675	0.81449931
Tfdp1	0.01030289	6.37155299	0.00491816	0.94479013	0.8147001
Fgf18	-0.022635	2.47317753	0.00490039	0.9448898	0.81473384
Gm15880	0.09560791	-0.8696785	0.00486482	0.94508983	0.8148541
Zmat5	-0.0221313	2.44656038	0.00483137	0.94527863	0.81491257

Slc35g1	0.01881212	4.50834341	0.00480906	0.94540487	0.81491257
Gab2	0.01079131	6.02991746	0.00480716	0.94541569	0.81491257
Taok1	0.00739715	9.64326833	0.00480458	0.94543026	0.81491257
H19	-0.1321281	-1.5509996	0.01983952	0.94546044	0.81491257
Mtif3	-0.0152721	3.36360623	0.00478531	0.94553962	0.81492862
Zfp523	-0.0129637	4.07551606	0.0047725	0.94561247	0.81493921
4931429L15I	-0.0613304	-1.1998334	0.00475162	0.94573138	0.81498948
Nup85	0.01388067	3.70621767	0.00473237	0.94584124	0.81498959
Ddx47	0.00950107	5.91726426	0.00472698	0.94587201	0.81498959
Steap3	-0.0171827	4.09819426	0.00471612	0.94593414	0.81498959
Ccdc141	-0.0128895	4.28111604	0.00470253	0.94601196	0.81498959
Heatr2	-0.011391	4.21724217	0.00469863	0.94603433	0.81498959
Cd9	-0.0210029	4.85260339	0.00468268	0.94612587	0.81501628
BC031181	0.01294772	6.05618873	0.00463858	0.94637971	0.81508687
Fnbp4	0.01039603	5.76659596	0.00463439	0.94640388	0.81508687
Eif3l	-0.0099425	5.5720538	0.00462246	0.94647282	0.81508687
Edem3	-0.0089789	6.01959876	0.00462073	0.94648283	0.81508687
Mrpl44	-0.012874	3.56917656	0.00461591	0.94651069	0.81508687
Syt9	0.01582597	3.34875711	0.00460024	0.94660143	0.81511285
Pcdhgc3	0.01980878	4.17040003	0.00456305	0.94681737	0.81523714
Impdh1	-0.0204723	2.57882557	0.00455325	0.94687438	0.81523714
Max	0.0099497	8.08866805	0.00454318	0.9469331	0.81523714
Zfp13	-0.0272504	1.79674284	0.00453375	0.94698811	0.81523714
Slc39a14	-0.0151717	3.35867201	0.00451162	0.94711743	0.81525789
Cpxm1	0.01955239	3.30145207	0.0045089	0.94713337	0.81525789
Mff	0.00784814	7.56692729	0.00449542	0.94721229	0.81527366
Npsr1	0.03140809	1.3576542	0.00446594	0.94738544	0.81532104
Nkiras1	-0.0079229	6.11160246	0.00443472	0.94756938	0.81532104
Zbtbd6	0.0531911	0.54639363	0.00442655	0.9476176	0.81532104
9330162012I	-0.0427493	0.74118326	0.0044257	0.94762262	0.81532104
Fam69c	-0.0383398	0.11835775	0.00441552	0.94768281	0.81532104
Nfxl1	-0.015549	2.91886483	0.0044142	0.9476906	0.81532104
Scp2	0.01395023	7.10915731	0.00441406	0.94769147	0.81532104
Abcb7	-0.0114579	5.77587624	0.00440079	0.94777	0.81533648
Trib2	-0.0111523	6.19818176	0.00437011	0.9479521	0.81543825
Tada1	-0.0111829	5.26378133	0.00435735	0.94802805	0.81543825
2700062C07I	-0.0141213	3.96942133	0.00435029	0.9480701	0.81543825
Gm20139	0.04629971	-0.1396949	0.00433924	0.94813598	0.81544279
Gemin4	0.01809569	2.31786914	0.00431821	0.9482616	0.81549871
Spi1	-0.0367667	0.49073913	0.00428021	0.94848942	0.8155421
Hrsp12	-0.0136105	4.29304503	0.00427784	0.94850366	0.8155421
Parpbp	-0.0450104	0.83810134	0.00426992	0.94855126	0.8155421
Alg12	0.02275968	2.79775609	0.00426939	0.94855448	0.8155421

Gypc	0.01676366	5.39498822	0.00423152	0.94878284	0.81563699
Gm14634	-0.0455557	-0.1042854	0.00419453	0.94900685	0.81563699
Pcdh19	-0.0113854	6.24158074	0.00419182	0.9490233	0.81563699
Zmpste24	-0.0124273	5.50399362	0.00418523	0.94906329	0.81563699
Gins4	0.01437629	4.78379394	0.00418442	0.94906821	0.81563699
Chkb	-0.0218634	1.33850918	0.00417942	0.94909866	0.81563699
Tfec	0.0826934	-1.7338427	0.00416673	0.94917582	0.81563699
Gm12657	0.01523159	3.51445235	0.00415963	0.94921912	0.81563699
Ehd1	0.01590591	3.5760169	0.00415008	0.94927737	0.81563699
Nav2	-0.0145962	7.18840989	0.00414852	0.94928685	0.81563699
Polr3d	0.01590919	3.08164623	0.00413709	0.94935667	0.81563699
Psd2	0.01317984	4.92517248	0.00412301	0.94944281	0.81563699
Hsbp1l1	-0.0238937	1.59126542	0.00411867	0.94946939	0.81563699
Chfr	0.01831998	4.18743872	0.00408727	0.94966213	0.81563699
Usp46	0.00749665	7.22034114	0.00408676	0.94966523	0.81563699
Spcs2	-0.0121789	5.45084433	0.00407897	0.94971317	0.81563699
1810030O07	-0.0092909	6.11730603	0.00406004	0.94982983	0.81563699
Ctdspl	0.01066098	6.4620004	0.00404758	0.94990677	0.81563699
A630007B06	-0.0085131	7.27132585	0.00404686	0.9499112	0.81563699
Msh5	-0.1278116	-1.8124745	0.00404431	0.94992697	0.81563699
Smarcd2	-0.0198469	3.09274541	0.0040346	0.94998701	0.81563699
Gm711	0.11141165	-1.6191454	0.00403277	0.94999834	0.81563699
Cntnap4	-0.0189992	3.32376177	0.00400789	0.9501526	0.81571737
Zfp653	-0.0209603	2.40126424	0.00399586	0.95022738	0.81572953
Kcnc1	0.01110193	6.98292561	0.00396573	0.95041512	0.8157645
Syce2	-0.0268315	1.79897998	0.00396072	0.95044639	0.8157645
Kcne1l	-0.0752	-1.2949601	0.00396015	0.95044998	0.8157645
Gbp7	-0.0116887	4.86337133	0.00392867	0.95064703	0.81588158
Cd151	0.01926488	5.12328317	0.00389817	0.95083874	0.81594313
Klhdc3	0.00970519	4.44002084	0.00389796	0.95084001	0.81594313
Apof	0.04829611	-0.0834847	0.00388643	0.9509127	0.81595347
Armcx1	0.0082256	5.69029124	0.00386303	0.95106047	0.81602823
Zfp239	-0.0088744	5.66201513	0.00384807	0.9511552	0.81605747
Ppp1r3d	-0.0282755	1.13591682	0.00383215	0.95125623	0.81609213
Rabggta	0.01533001	3.21503695	0.00380641	0.95142002	0.81609475
Dnajc14	-0.0131125	4.30884159	0.003803	0.95144172	0.81609475
Fbxo47	-0.030463	1.25856779	0.00378909	0.95153053	0.81609475
Armcx3	-0.0069749	7.26398778	0.00378783	0.95153852	0.81609475
4933427D14	0.01806036	4.10754685	0.00377832	0.95159932	0.81609475
Nfkbil1	0.04225051	-0.2143319	0.00377459	0.95162318	0.81609475
Gucy1a3	-0.0110734	7.27725386	0.00376268	0.95169947	0.81610817
Gm16907	0.03809436	1.60754631	0.00374854	0.95179021	0.81613397
Shb	0.01842949	1.70974903	0.00368883	0.95217521	0.81625541

Creg1	-0.0139199	4.80450992	0.00367775	0.95224698	0.81625541
Egfem1	0.02656662	1.82446598	0.00366532	0.95232768	0.81625541
Donson	-0.0153409	3.37025423	0.00365159	0.95241692	0.81625541
Gm5	0.0583595	-0.5194814	0.00365011	0.95242658	0.81625541
Scarb1	0.02311419	2.48768877	0.00364559	0.952456	0.81625541
Sub1	0.00843813	8.79956942	0.00363137	0.95254869	0.81625541
R74862	-0.0103087	4.58028853	0.00363101	0.95255105	0.81625541
B130006D01	0.06557156	0.52011361	0.00362594	0.95258414	0.81625541
Bcas3	-0.010643	4.43762814	0.00360392	0.95272813	0.81625541
Rbpj	0.00715791	6.51270234	0.00359769	0.95276898	0.81625541
Pcmtd1	0.00706167	8.28762032	0.00359597	0.95278025	0.81625541
Ovol1	-0.0424214	-0.3744116	0.00359376	0.95279473	0.81625541
Prdm11	0.05610654	-0.5723408	0.00356527	0.95298201	0.81625541
Eqtn	0.04772097	0.10408834	0.00353209	0.95320102	0.81625541
Prpf40a	0.00789262	6.39276281	0.00351914	0.95328677	0.81625541
Gm4788	0.03127334	0.00703406	0.00351454	0.9533173	0.81625541
Asphd2	-0.0146942	2.68261618	0.00351186	0.95333508	0.81625541
Eef1g	-0.0105482	7.72429687	0.0035073	0.95336533	0.81625541
Nit1	-0.0136639	2.91999726	0.00350285	0.9533949	0.81625541
Slu7	0.00831349	5.97942903	0.00350204	0.95340029	0.81625541
Ccdc71l	-0.0097867	5.24140435	0.00350014	0.95341287	0.81625541
Cib1	0.0214136	1.9956303	0.00349774	0.95342882	0.81625541
Gm15708	0.05989659	-1.0342753	0.0034916	0.95346968	0.81625541
Pkn1	0.02152034	2.85347814	0.00348619	0.95350568	0.81625541
Rbm27	0.00646731	7.46391826	0.00348328	0.9535251	0.81625541
Bcl2l12	0.03573075	0.23537933	0.0034766	0.95356964	0.81625541
Oma1	0.01173166	3.22056231	0.00344898	0.95375421	0.81631047
Srek1ip1	0.00872199	6.02457811	0.00344882	0.9537553	0.81631047
Zfp668	0.01273085	3.70242168	0.00341395	0.95398934	0.81643721
AW011738	0.02023699	2.60334174	0.00339756	0.95409983	0.81643721
Mllt6	-0.0082241	6.01059421	0.00337983	0.9542196	0.81643721
Mettl7a3	-0.0527522	-1.7645304	0.00337138	0.9542768	0.81643721
Dlx5	0.01665629	2.04843014	0.003342	0.95447621	0.81643721
Pglyrp1	-0.0262663	0.74415633	0.00333822	0.95450196	0.81643721
Senp1	-0.0098154	5.22528024	0.00333745	0.95450719	0.81643721
Clec4g	-0.0574724	-1.5949193	0.00333376	0.95453229	0.81643721
Tab2	0.00669433	8.33770501	0.00332534	0.95458971	0.81643721
Cdkn2d	0.01123434	4.11351863	0.00331416	0.95466601	0.81643721
Cd14	-0.042131	-0.0168069	0.00330867	0.95470355	0.81643721
Ndufc1	0.01143191	5.67207683	0.00330802	0.954708	0.81643721
Ccnb1	0.05509376	-1.0194549	0.00330518	0.95472742	0.81643721
Casp2	0.0105122	3.86831317	0.00329898	0.95476987	0.81643721
9030624G23	-0.0126274	3.50587459	0.00329261	0.95481347	0.81643721

Slitrk5	-0.011187	5.86905806	0.0032757	0.95492955	0.81648458
Chst15	-0.009372	5.98406679	0.00322193	0.95530057	0.81665771
Mrpl24	-0.0163315	2.82661338	0.00321772	0.95532973	0.81665771
Paqr3	0.01848179	2.38706999	0.00321517	0.9553474	0.81665771
Gm11627	0.02429609	1.40812479	0.00321122	0.95537479	0.81665771
Ift52	-0.009874	4.94907835	0.00318499	0.95555724	0.81672083
Cep85l	-0.0123099	4.01500005	0.0031823	0.95557599	0.81672083
Paqr9	-0.0110463	5.44706084	0.00317445	0.95563072	0.81672083
Ano7	-0.0517544	-1.3843268	0.00316317	0.95570957	0.81672451
Cul7	0.0126282	3.0716944	0.00315302	0.95578059	0.81672451
Map4k1	0.02696525	0.43633808	0.00314781	0.95581711	0.81672451
Adipor1	-0.0093057	6.28407623	0.00312924	0.95594749	0.81675803
Fam53c	0.00785582	5.54866896	0.00308558	0.95625556	0.81675803
Ntrk2	0.01048097	8.36611345	0.00307832	0.956307	0.81675803
Heph	0.02856741	2.0407408	0.00307775	0.95631105	0.81675803
Atrnl1	0.00721454	6.22704218	0.00304628	0.95653469	0.81675803
Abca2	0.01137637	6.10425242	0.00304423	0.95654934	0.81675803
Lyplal1	-0.0149824	2.81065316	0.00303901	0.95658653	0.81675803
Polr2d	-0.0138856	3.84926725	0.00303581	0.95660941	0.81675803
Fcer2a	-0.029467	0.56786535	0.00302084	0.95671643	0.81675803
Raph1	-0.0112803	7.73997908	0.00301561	0.95675383	0.81675803
Poc5	0.00867098	3.97921674	0.00300195	0.95685179	0.81675803
Cep55	-0.0530517	-1.7708971	0.00300075	0.95686045	0.81675803
Wbp4	-0.0063513	6.16916851	0.00296947	0.95708564	0.81675803
Katna1	0.01510499	3.60927627	0.00296614	0.95710969	0.81675803
Sh3bp1	-0.0131337	3.23093993	0.00295891	0.95716193	0.81675803
1700037H04	-0.011095	4.31945239	0.00294804	0.95724056	0.81675803
Tvp23a	0.01524658	4.20461113	0.00294515	0.95726153	0.81675803
Pola2	0.01721913	1.80786801	0.00293779	0.9573149	0.81675803
Pik3r3	0.00612026	6.59840432	0.00293343	0.95734655	0.81675803
Eif2s3x	0.00667754	7.27309505	0.00291289	0.95749601	0.81675803
Atp6v0e	0.01420447	4.75238444	0.00291181	0.95750385	0.81675803
Ring1	-0.0096486	4.47181518	0.00290645	0.95754299	0.81675803
Mapkap1	0.00639625	6.5705397	0.00290468	0.95755591	0.81675803
Vmp1	-0.0080368	5.58892093	0.00289751	0.95760826	0.81675803
Hmox2	-0.0110969	5.03916106	0.00289474	0.9576285	0.81675803
Ppef2	-0.0589104	-0.8436793	0.00287372	0.95778247	0.81675803
Dpp4	-0.015858	5.50219731	0.00287113	0.95780147	0.81675803
Gabarapl2	0.00816957	6.7008753	0.00284494	0.95799418	0.81675803
Gng3	0.00670118	8.0666176	0.00282737	0.95812394	0.81675803
Thop1	-0.0159404	2.56012789	0.00282335	0.95815369	0.81675803
Zfp688	0.02447386	1.2095014	0.00282258	0.95815944	0.81675803
Ankrd17	0.00717993	8.9309109	0.00281307	0.95822989	0.81675803

S100a10	0.01445997	5.84680724	0.00281216	0.95823666	0.81675803
Mapk7	-0.0133098	2.64550853	0.00281007	0.95825215	0.81675803
Il1b	0.04742911	-1.7587399	0.00280691	0.95827561	0.81675803
Unc119	0.02086075	1.76904327	0.00280504	0.95828949	0.81675803
Tti1	-0.0095474	4.735674	0.00280359	0.95830027	0.81675803
Frg1	0.00743499	4.81586465	0.00279948	0.95833079	0.81675803
Dcun1d2	0.00841644	5.52819333	0.00278208	0.95846038	0.81675803
Pds5a	-0.0068633	6.86280437	0.00278208	0.95846039	0.81675803
Gm6525	-0.0722172	-1.3451882	0.0027687	0.95856028	0.81675803
2610305D13I	0.02976803	0.60259501	0.00275037	0.95869754	0.81675803
Mad1l1	0.01607077	2.26136408	0.00274207	0.95875985	0.81675803
Thap7	-0.0151657	2.81002998	0.00273635	0.95880287	0.81675803
Cd276	0.01630171	2.49201803	0.00272265	0.95890603	0.81675803
Nfia	0.00797085	9.13850662	0.00271792	0.95894172	0.81675803
Fam206a	0.00815429	5.91732222	0.00271331	0.9589765	0.81675803
Pdpk1	-0.0054658	7.65899046	0.00271207	0.95898587	0.81675803
Hmgcs2	-0.012126	4.07339553	0.00270526	0.95903738	0.81675803
Zkscan16	0.01152366	5.24616112	0.0027029	0.95905522	0.81675803
Nek8	0.03188632	1.27127554	0.00269935	0.95908208	0.81675803
Cep83	-0.0073073	5.98075362	0.00269866	0.95908729	0.81675803
Bcl7a	-0.009758	4.93507583	0.00269682	0.95910123	0.81675803
Dcaf11	-0.0073757	4.86651126	0.0026925	0.95913398	0.81675803
Bre	-0.0088429	4.14336878	0.00265473	0.95942135	0.81695105
Apbb2	-0.0060959	7.4120643	0.00262563	0.95964417	0.8169905
Cox7a2	-0.0099477	6.00398457	0.00262139	0.95967676	0.8169905
Tomm6os	0.03869109	-0.1003295	0.00261847	0.95969918	0.8169905
Rpl22l1	0.01005062	5.0394012	0.00261058	0.95975985	0.8169905
Ptk2	0.00943768	6.57805731	0.0026091	0.95977125	0.8169905
Rbmxl1	0.00761457	5.34589188	0.00258963	0.95992154	0.81705196
Ssr2	-0.0170273	3.49506716	0.00258321	0.95997121	0.81705196
Gm13251	-0.0163541	2.15003669	0.00256856	0.96008476	0.81705196
Srm	0.01028567	4.05505289	0.00256339	0.96012495	0.81705196
Gata3	0.04036313	-0.4203698	0.00255038	0.96022611	0.81705196
Hmg20b	-0.0212097	2.17156615	0.00254849	0.9602409	0.81705196
Ubr2	-0.00957	6.2756488	0.00254495	0.96026848	0.81705196
Cyb561d1	0.01018127	3.70922664	0.00252585	0.9604177	0.81712725
Rnf185	0.00736939	5.79909455	0.00251123	0.96053233	0.81717311
5330434G04	0.01191263	5.00213605	0.00250232	0.96060236	0.81718102
Xiap	0.00721315	7.12351358	0.00246425	0.96090291	0.817189
Kdm5d	0.01173214	4.88224008	0.00246172	0.96092299	0.817189
Mir350	0.07576776	-2.0322071	0.00243833	0.96110896	0.817189
Gm16845	0.03423546	0.69117642	0.0024231	0.96123045	0.817189
Gmnn	0.01458316	2.06183801	0.00240981	0.96133688	0.817189

Kif13b	-0.0094445	3.67690673	0.00240492	0.96137609	0.817189
Efcab4b	0.05330375	-1.4068119	0.00240448	0.96137959	0.817189
Fbxo4	-0.0154825	3.73373503	0.00240187	0.96140057	0.817189
Itgae	-0.0606385	-1.8146035	0.00239902	0.96142346	0.817189
Kdm4a	-0.0114383	3.03882422	0.00239705	0.96143923	0.817189
Plcl1	0.00801019	5.43543837	0.00239324	0.96146987	0.817189
Gpr75	0.01286187	3.63762434	0.00239068	0.96149048	0.817189
Golm1	-0.0093701	3.26257839	0.00238596	0.96152851	0.817189
Akap7	-0.0071005	6.50819349	0.00238592	0.96152883	0.817189
Tbp	-0.0059562	5.33457464	0.00238104	0.96156814	0.817189
Adhfe1	0.00924394	5.11547406	0.00237915	0.96158339	0.817189
AI839979	0.03053199	0.19634029	0.00236282	0.96171533	0.81719983
Bpgm	-0.0073638	6.8275735	0.0023607	0.96173252	0.81719983
Ttll5	0.00731961	5.3838044	0.00234683	0.96184503	0.81719983
4930430F08I	0.00995563	3.77965773	0.0023467	0.96184608	0.81719983
Zfp27	0.01038203	4.00866786	0.0023382	0.96191512	0.81719983
2700060E02I	-0.0067625	5.83392768	0.00232767	0.96200094	0.81719983
Elf2	0.00874936	6.74349939	0.00232518	0.96202125	0.81719983
Neurl3	-0.0242647	1.02709041	0.00231144	0.96213353	0.81724362
Kcnc4	0.00996866	4.33426305	0.00228972	0.9623117	0.81725039
Cant1	-0.0117184	3.30876225	0.00228585	0.96234353	0.81725039
Hnrnpm	-0.006158	6.77001369	0.0022804	0.96238841	0.81725039
Ccsap	-0.0118694	3.95722994	0.00227869	0.96240256	0.81725039
Alad	0.01241588	2.53834977	0.00226353	0.96252772	0.81725039
Mastl	0.02197008	1.51160158	0.00224837	0.96265335	0.81725039
Ttc29	-0.0844705	-2.3012411	0.00224717	0.96266327	0.81725039
Gna12	0.0070628	5.26986265	0.00224458	0.96268482	0.81725039
Rnf150	-0.0077723	7.33779721	0.00222847	0.96281884	0.81725039
Scyl2	-0.0077125	5.8534477	0.002227	0.96283111	0.81725039
Prrt2	-0.0058034	7.21379978	0.00222571	0.96284184	0.81725039
Ephb3	0.01779919	1.63705225	0.00222028	0.96288715	0.81725039
Ubac1	0.00927499	3.83876854	0.00220267	0.96303452	0.81725039
1600016N20	0.04031389	-0.9326504	0.00220095	0.96304895	0.81725039
Knstrn	0.02636694	0.64181907	0.00220053	0.9630525	0.81725039
Phf21a	0.00634358	6.60498082	0.00218222	0.96320642	0.81728335
G2e3	-0.0092408	4.38339843	0.00218146	0.96321283	0.81728335
Ikbkg	0.0055628	5.88354635	0.00214823	0.96349381	0.81728634
Epc2	-0.0051186	7.43690002	0.00214397	0.96353	0.81728634
Zfp382	-0.0096554	4.01271086	0.00214035	0.96356077	0.81728634
Ift20	0.00702013	6.78178911	0.00213508	0.96360565	0.81728634
Gm10474	0.08324172	-1.5017368	0.00213188	0.96363296	0.81728634
Ywhah	0.00464546	10.4947765	0.00212883	0.96365894	0.81728634
Cdkn2c	0.01697674	2.73098207	0.00212604	0.96368277	0.81728634

Phospho2	-0.0075248	5.00546569	0.00212376	0.96370223	0.81728634
Allc	-0.0811477	-1.1534135	0.00208894	0.96400079	0.81741392
Dlx1as	0.00967462	3.27813681	0.0020862	0.96402434	0.81741392
Ccdc121	0.02593942	0.68327863	0.00208498	0.96403491	0.81741392
Mettl7a1	-0.0093875	6.05892047	0.00205333	0.96430871	0.81759456
Mtg2	-0.0219197	0.41438466	0.00204371	0.96439231	0.81761393
Sp3	-0.0059946	6.67392446	0.00203025	0.96450971	0.81766194
D030047H15	-0.0225576	0.23058066	0.0020112	0.96467652	0.81775184
Gclm	-0.007635	5.32642741	0.00199917	0.96478223	0.81778653
C2cd2l	-0.0079957	5.27762797	0.00199226	0.96484308	0.81778653
Gmpr	0.01417409	2.07266291	0.0019766	0.96498141	0.81778653
Ppp1r11	0.00751218	4.56555625	0.00196475	0.96508648	0.81778653
Yars2	-0.0076552	4.04331491	0.00195924	0.9651355	0.81778653
Mdm2	-0.0047413	6.69822729	0.00195354	0.96518618	0.81778653
Zfp108	-0.010895	3.07925753	0.00195053	0.96521295	0.81778653
Slc27a4	0.00978562	3.48703901	0.0019505	0.96521323	0.81778653
Ppp2r1b	-0.0059569	5.52140425	0.00194325	0.96527792	0.81778653
Oas2	-0.0279948	0.13255888	0.00192335	0.96545609	0.81778653
1700007P06I	-0.0318393	-0.667829	0.00192044	0.96548222	0.81778653
Dapk3	-0.0160705	1.76013072	0.00191435	0.96553693	0.81778653
Prrc1	-0.0066136	6.03799627	0.00191005	0.96557563	0.81778653
Itpr2	-0.0062571	5.33781271	0.00190538	0.96561767	0.81778653
Rexo4	-0.0076351	4.59770965	0.00189054	0.96575171	0.81778653
Jmy	-0.0051966	7.18953639	0.00188546	0.96579773	0.81778653
9030617O03	0.01088136	2.99011174	0.00188473	0.96580436	0.81778653
Phactr2	-0.0062524	8.26110327	0.00187409	0.96590101	0.81778653
Mrpl40	0.01046511	3.57713701	0.00187282	0.96591251	0.81778653
Sumo1	0.00731338	7.58817477	0.00187058	0.96593292	0.81778653
Rpl27	0.00770398	6.01299865	0.00185053	0.96611586	0.81788996
Arrb1	0.00588944	7.40032448	0.0018426	0.9661885	0.81789999
Dapk2	-0.0206377	0.52057744	0.00183294	0.96627715	0.81791508
Tpd52	-0.0057657	5.82796118	0.0018206	0.96639083	0.81791508
Apol7d	0.0568945	-1.7446552	0.00181635	0.96643005	0.81791508
Kdelc1	0.01066488	2.8426093	0.00181252	0.96646547	0.81791508
Ctr9	-0.0063911	5.2111628	0.00179737	0.96660583	0.81791508
Mamstr	0.0238587	0.20664591	0.00179381	0.96663889	0.81791508
Egr2	-0.0199431	3.93185383	0.00178844	0.9666888	0.81791508
Zfp280b	-0.0070472	4.89230233	0.00178803	0.96669258	0.81791508
Tbc1d8	0.0102954	4.20495322	0.0017811	0.96675716	0.81791829
Tm2d2	-0.0126523	4.24607519	0.00173394	0.9672	0.81805579
Mfap2	-0.0250659	-0.0144527	0.00172855	0.96725096	0.81805579
Aif1l	-0.0132588	2.10260785	0.00172213	0.96731181	0.81805579
Nxph2	-0.0269556	0.00279407	0.00172211	0.96731196	0.81805579

Zfp251	-0.0087174	3.78981328	0.00171723	0.96735831	0.81805579
Cntfr	-0.0121592	4.14223416	0.00171524	0.96737718	0.81805579
Zfp119a	0.01651261	1.13560126	0.00170516	0.96747311	0.81805579
Ppp2r2d	-0.0067659	4.71076883	0.00167345	0.96777687	0.81805579
Clasp1	0.00573356	7.49695129	0.00165903	0.96791593	0.81805579
C78339	-0.0114118	2.77947753	0.00165697	0.96793576	0.81805579
Spint1	-0.0319209	-0.3384106	0.00165343	0.96797001	0.81805579
Klhdc8b	0.01598827	2.2070895	0.00165237	0.96798028	0.81805579
Wdr18	-0.0066355	3.64050136	0.00164775	0.96802511	0.81805579
Scrn3	0.00655078	4.81804462	0.00164642	0.96803797	0.81805579
Sox11	-0.008509	4.87642157	0.00164153	0.96808541	0.81805579
Phf3	-0.0046315	8.18853207	0.00163293	0.96816913	0.81805579
Cd2	0.03649789	-0.4858184	0.00162753	0.96822171	0.81805579
1700047M11	-0.0143026	1.0395494	0.00162086	0.96828688	0.81805579
Drd5	0.02545661	0.25089244	0.00161784	0.96831641	0.81805579
Rdh9	0.02495845	0.40008975	0.00161449	0.96834923	0.81805579
Fkbp8	0.00768543	5.10116843	0.00160552	0.96843727	0.81805579
Ccdc18	0.01794855	1.71139588	0.0015964	0.96852695	0.81805579
Tmem60	-0.0064239	5.36769151	0.00158897	0.96860028	0.81805579
Arpc3	0.00742413	4.43972326	0.00158807	0.96860914	0.81805579
Lrp1	-0.0051407	7.3421971	0.00158763	0.96861347	0.81805579
5730420D15I	-0.0507059	-0.8912027	0.00157828	0.96870597	0.81805579
Map7d2	0.00545466	7.71414111	0.00157143	0.96877393	0.81805579
Pef1	-0.0068687	4.475448	0.00156556	0.96883233	0.81805579
Gjc2	0.02328546	0.31923454	0.00155796	0.96890801	0.81805579
Rps13	0.00726962	5.90492291	0.00155378	0.96894967	0.81805579
Enpep	-0.0146522	1.97642137	0.00155185	0.96896903	0.81805579
Sec61g	0.00853635	4.7707202	0.00154623	0.9690252	0.81805579
Cc2d2a	-0.004954	5.78023341	0.00154429	0.96904458	0.81805579
Efcab12	0.0212925	0.82845375	0.00154196	0.96906792	0.81805579
Rpl6	0.00627057	7.75574068	0.00152908	0.96919737	0.81805579
Upf1	0.00713528	4.27540549	0.00151354	0.96935424	0.81805579
P2rx7	-0.0138352	2.03038646	0.00151134	0.9693765	0.81805579
Ppip5k2	0.00603498	5.30691516	0.00150962	0.96939386	0.81805579
2410016O06	-0.0094656	3.82287711	0.00150955	0.9693946	0.81805579
Lrrc8a	0.00488598	6.15744372	0.00150933	0.96939683	0.81805579
Tacr1	-0.009396	3.12697226	0.00150657	0.96942482	0.81805579
Pex11g	-0.0370694	-1.2928139	0.00149157	0.96957727	0.81805579
Gba2	-0.0096976	3.2358917	0.00149046	0.96958858	0.81805579
Rpgr	0.00878607	4.34552002	0.00148665	0.96962753	0.81805579
Ier5l	-0.0361853	-1.2465365	0.00148392	0.96965539	0.81805579
Xlr3b	0.01643083	1.1616973	0.00147449	0.96975187	0.8180859
Adi1	0.00704515	7.13727082	0.00143237	0.97018682	0.81835576

Kynu	-0.0477312	-0.5723934	0.0014265	0.97024795	0.81835576
Aldh3a2	0.00449977	6.15963536	0.00141753	0.97034161	0.81835576
Gstm7	-0.009677	4.40010318	0.00141663	0.97035099	0.81835576
Ccser1	0.01133644	3.31125484	0.00141426	0.97037585	0.81835576
Glo1	-0.0060759	6.44821502	0.00138148	0.97072103	0.81855138
Rps6ka2	0.0064495	5.22080832	0.00137637	0.97077514	0.81855138
Ptcd2	-0.0067318	4.69005806	0.00137206	0.97082094	0.81855138
Phf11a	0.02574462	-0.4520695	0.00135299	0.97102438	0.81855138
4930579K19I	0.05041737	-1.3326872	0.00135066	0.97104931	0.81855138
0610011F06I	0.00738166	3.61598656	0.00134826	0.97107495	0.81855138
Sf1	-0.0043109	6.7156002	0.00134273	0.97113431	0.81855138
Csgalnact1	0.00890114	3.77298761	0.00134001	0.9711636	0.81855138
Myof	-0.0090307	5.02471238	0.00132712	0.97130256	0.81855138
Gcc2	0.00595975	7.54554474	0.00132571	0.97131785	0.81855138
Atf2	-0.0037707	8.8748874	0.00131978	0.97138194	0.81855138
B930059L03I	-0.0631951	-1.1958104	0.00131971	0.97138279	0.81855138
Adh7	0.01791905	1.41363985	0.00131719	0.97141003	0.81855138
Bcdin3d	-0.0182228	0.61609728	0.00130761	0.9715142	0.81855138
Nagk	0.00911229	3.23250208	0.00130224	0.97157273	0.81855138
Dhx36	-0.0058074	6.26856195	0.00130147	0.97158109	0.81855138
Strada	0.00871284	3.65046623	0.00128637	0.97174641	0.81862476
Spg7	-0.0088052	3.55351131	0.00128241	0.97178985	0.81862476
Spen	0.0060518	6.87515701	0.00126308	0.97200316	0.81863932
Gm16880	-0.0448926	-0.2276483	0.00125885	0.9720501	0.81863932
Tdrd7	0.0053657	4.68204311	0.00124443	0.97221058	0.81863932
Ndufaf4	0.00524999	5.76558867	0.00124234	0.97223393	0.81863932
Nadsyn1	0.01225013	1.37774701	0.00122943	0.97237853	0.81863932
Eva1b	-0.0167856	0.88173957	0.00121995	0.97248509	0.81863932
Wnt2b	0.00831948	2.90172608	0.00121607	0.97252888	0.81863932
Cdk2	0.01344221	1.70180027	0.00121354	0.97255745	0.81863932
Lypla2	0.01007227	2.10910724	0.0012063	0.97263946	0.81863932
Pex26	-0.0073522	3.8248316	0.00119897	0.97272264	0.81863932
Peli2	-0.0054077	5.70732848	0.00119213	0.97280049	0.81863932
Rhbdf1	-0.0121309	1.5426094	0.00118305	0.97290433	0.81863932
Ldoc1l	-0.0056786	4.91591851	0.00118129	0.97292443	0.81863932
G730013B05	-0.0196328	0.35112344	0.0011757	0.97298853	0.81863932
Ints6	-0.005373	4.96000102	0.00116964	0.97305823	0.81863932
Vcpip1	0.00469163	7.35241349	0.00116877	0.9730682	0.81863932
4930414L22F	-0.0083007	3.17796153	0.00116121	0.97315548	0.81863932
AA388235	0.00777451	3.22421595	0.0011612	0.97315562	0.81863932
Ggn	0.02550126	-0.0259875	0.00116004	0.97316899	0.81863932
Pkmyt1	0.02267248	-0.4137615	0.00114264	0.97337083	0.81863932
Bahcc1	-0.0071691	3.98732664	0.00114229	0.973375	0.81863932

Snord91a	-0.0077273	3.26393971	0.00113821	0.9734225	0.81863932
Trdn	-0.0193804	0.16367472	0.00112075	0.97362709	0.81863932
Ccdc115	-0.0051998	4.90636218	0.00111877	0.97365036	0.81863932
Sntg2	-0.0174628	0.90102064	0.00111592	0.97368395	0.81863932
Tmem238	-0.0217016	-0.4145217	0.00111306	0.97371768	0.81863932
Phactr1	-0.0044466	7.94788212	0.00111213	0.97372868	0.81863932
Ficd	-0.0086256	2.89199918	0.00111053	0.97374753	0.81863932
Mettl10	-0.0070605	5.06836517	0.00111016	0.97375192	0.81863932
Asic1	-0.0061038	4.84553209	0.00110819	0.97377518	0.81863932
Gtf3c2	-0.0055279	6.34947437	0.00110513	0.97381143	0.81863932
Fam20a	-0.0080261	2.57563194	0.00110342	0.9738317	0.81863932
Atp6v1f	-0.0080172	4.75645672	0.00109905	0.97388354	0.81863932
Rassf8	-0.0105635	3.3170677	0.00109558	0.97392482	0.81863932
Tipin	-0.0099674	4.23054188	0.00109051	0.97398516	0.81863932
Asic2	-0.0054022	5.16739371	0.00108592	0.97403992	0.81863932
Tcta	-0.0070491	4.4087972	0.00108061	0.97410348	0.81863932
Sft2d3	-0.0074676	3.19211929	0.00107932	0.97411894	0.81863932
1700021F05I	0.00504001	4.52234889	0.00107411	0.97418147	0.81864074
Polr3f	-0.0052003	4.75486577	0.00106162	0.97433192	0.81866026
Hypk	0.00663912	6.55731281	0.00106162	0.974332	0.81866026
Papola	-0.0039302	8.23868163	0.00105705	0.97438721	0.81866026
Crk	0.0036225	7.7634951	0.00104763	0.97450154	0.81866519
Dsc3	0.03636869	-1.3625269	0.00104573	0.97452466	0.81866519
Tab3	0.00409601	6.0446983	0.00104155	0.97457559	0.81866519
Slc25a40	-0.0078754	3.38294368	0.00102789	0.97474281	0.81875454
Lsm11	0.00541063	4.47219831	0.0009982	0.97511011	0.81901194
Trmt112	0.00484159	5.06178834	0.00097392	0.97541458	0.81911231
Enox2	0.00843357	4.58699672	0.00097319	0.97542386	0.81911231
Pbxip1	0.00812425	5.41108187	0.00096983	0.97546627	0.81911231
Tsr1	-0.0050177	6.03519977	0.00096929	0.9754731	0.81911231
Adck4	-0.0158775	1.29627474	0.00096116	0.97557619	0.81914777
Zfp248	0.00626023	4.558211	0.00095548	0.97564847	0.81915734
B4galt4	0.00653306	4.72188672	0.00094648	0.9757634	0.81915962
Xylt1	-0.0093263	2.83464097	0.00094573	0.97577294	0.81915962
Hist1h4a	-0.0290447	-1.4203992	0.0009099	0.97623616	0.81935709
Arhgap26	0.00480403	7.57515888	0.00090832	0.97625673	0.81935709
2810055G20	-0.0089143	2.25090386	0.00090631	0.97628301	0.81935709
Zfp942	0.00618655	3.51107222	0.00090493	0.97630111	0.81935709
Trim12c	-0.0063552	5.55706008	0.00090405	0.97631261	0.81935709
Pdk1	-0.0038881	6.19472714	0.00089264	0.97646261	0.81943186
Zbtb44	-0.0036667	7.23538293	0.00088177	0.9766062	0.81950126
Rps6ka4	0.00879752	2.71149669	0.00087145	0.97674354	0.8195654
Map3k19	0.0097471	2.77347339	0.00085908	0.9769091	0.8196532

Mrps36	0.0048631	5.16856347	0.0008374	0.9772022	0.81979686
6330418K02I	0.01317014	1.11067126	0.00083173	0.97727952	0.81979686
Mpzl3	-0.0234055	-0.9425043	0.00082629	0.977354	0.81979686
Igf2bp1	0.03431779	-1.5499557	0.00082539	0.9773663	0.81979686
Adamts2	0.00747551	3.97568395	0.00081504	0.97750854	0.81979686
Rdm1	0.01124983	2.51132514	0.00081099	0.97756447	0.81979686
Tnfrsf21	0.00551809	6.54315861	0.00081001	0.97757812	0.81979686
Dhx35	-0.0065827	2.62896772	0.00080949	0.97758525	0.81979686
Ip6k1	-0.0044553	5.79046022	0.00079618	0.97777032	0.81979686
Tsen34	-0.0056871	4.22262638	0.00079442	0.97779483	0.81979686
Ltk	0.01218102	1.34450816	0.00079111	0.97784118	0.81979686
Tpr	-0.0034991	9.14637842	0.00077291	0.97809748	0.81979686
Prr14l	0.00421949	7.45751808	0.00076771	0.97817117	0.81979686
2610207O16	-0.0170124	0.22999278	0.0007657	0.97819976	0.81979686
Cpt1c	-0.0073108	3.36408473	0.00076376	0.97822739	0.81979686
Herc3	-0.0052916	7.65233924	0.00076304	0.97823774	0.81979686
Actr6	0.0067781	3.63828272	0.00075437	0.97836173	0.81979686
Ubp1	0.00410851	6.29825145	0.00075012	0.97842263	0.81979686
Lyg2	0.0664296	-1.660059	0.00074138	0.97859638	0.81979686
Dut	0.00516399	4.83592111	0.00073379	0.97865883	0.81979686
Ptms	0.00718168	6.61621914	0.0007327	0.97867468	0.81979686
Dna2	0.026616	-0.6860066	0.00072703	0.97875722	0.81979686
Stil	-0.0157356	0.88091538	0.00072669	0.97876223	0.81979686
Ppp1r3c	-0.0051304	6.08555093	0.00072329	0.97881196	0.81979686
Dnah7a	-0.0070761	2.08891377	0.00071477	0.97893706	0.81979686
Rab5b	0.0034922	7.01530066	0.00071136	0.97898738	0.81979686
Sh3bp5l	0.00535566	3.77831697	0.00070723	0.97904844	0.81979686
Pten	-0.0030728	9.22052376	0.00070413	0.97909439	0.81979686
Cbln1	0.00983655	1.84551883	0.00070323	0.97910776	0.81979686
Scube1	-0.003775	5.95682339	0.00070221	0.97912289	0.81979686
2610318N02	0.03410525	-2.0397248	0.00069807	0.97918456	0.81979686
Thoc7	0.00426099	6.34780559	0.00069743	0.97919409	0.81979686
Acsl4	0.00343566	6.37633032	0.00069734	0.97919539	0.81979686
15-Sep	-0.0055366	6.70943786	0.00069419	0.97924252	0.81979686
Scamp3	0.00611174	3.63923408	0.00069239	0.97926943	0.81979686
Nr1d1	0.00371057	8.75075476	0.00069211	0.97927354	0.81979686
Jmjd7	0.01924638	-0.6699958	0.0006866	0.97935622	0.81981507
Ube4a	-0.0038037	5.74196263	0.00067746	0.97949404	0.81987944
Epha3	-0.0063558	2.92841282	0.0006637	0.97970339	0.81990603
Cst7	0.03503506	-1.9428477	0.00065958	0.97976643	0.81990603
Gm4759	0.0370016	-0.9557459	0.00065896	0.97977586	0.81990603
Wdr83os	-0.0049891	5.70252191	0.00065616	0.97981899	0.81990603
Serpind1	0.00740538	4.92559936	0.00065272	0.97987195	0.81990603

Ctbs	-0.0061914	3.00363502	0.00065146	0.9798914	0.81990603
Frem3	0.01631545	0.105963	0.00063732	0.98011077	0.81992937
Rrp36	-0.008475	2.16322795	0.00063661	0.98012186	0.81992937
Ccdc62	0.01087549	1.89830832	0.00063502	0.98014668	0.81992937
4933408N05	-0.0210332	-1.0389726	0.00063397	0.98016302	0.81992937
4930488L21F	-0.0159123	-0.5601524	0.00062113	0.98036483	0.81996488
Rbm12b2	0.00509449	4.39119605	0.00061791	0.98041589	0.81996488
D630045J12F	-0.0035939	6.85489924	0.000616	0.98044612	0.81996488
Samsn1	0.01373863	0.62142411	0.00061581	0.9804492	0.81996488
Gm3258	0.01678791	-0.8824314	0.00060825	0.98056954	0.82001455
Mettl16	-0.0038928	5.66215822	0.00059839	0.98072767	0.82009583
C920025E04I	0.0275823	-1.1611135	0.00059018	0.98086024	0.82015572
Mrpl30	0.00401534	5.69190516	0.00057373	0.98112887	0.82024767
Mreg	-0.007769	2.40495326	0.0005719	0.98115897	0.82024767
Wtip	0.00839084	2.38718565	0.0005696	0.98119685	0.82024767
Nr2c2ap	-0.0070018	2.05884682	0.00056856	0.98121404	0.82024767
Rimbp3	-0.016003	0.85861884	0.00056044	0.98134868	0.82028626
9430038I01R	0.01553169	0.73306406	0.00055843	0.98138212	0.82028626
Smc1b	-0.0265028	-0.7178383	0.00055006	0.98152215	0.82030211
Sirt1	-0.004791	4.47245671	0.00053349	0.98180249	0.82030211
Stk16	0.00471747	4.51350464	0.0005264	0.98192378	0.82030211
Gm19522	0.00661107	2.40790063	0.00052264	0.98198843	0.82030211
Mir665	-0.017442	-0.6775952	0.0005202	0.9820305	0.82030211
C4a	0.01610804	-0.5173054	0.00051529	0.98211546	0.82030211
Atg4b	0.004464	3.8982455	0.00051006	0.98220654	0.82030211
Wdr47	0.00437195	6.50006207	0.00050967	0.98221339	0.82030211
Fbln7	0.00812171	4.07381577	0.00050636	0.98227113	0.82030211
1700124L16F	0.02567802	-1.713115	0.00050565	0.98228368	0.82030211
Evi2a	0.00711391	2.7935894	0.00050387	0.98231486	0.82030211
Arhgap42	0.00299922	5.70538165	0.00050062	0.98237184	0.82030211
Lcp2	-0.0050732	3.02345756	0.00049671	0.98244094	0.82030211
Hist2h2bb	0.0199793	-0.8157757	0.00049657	0.98244327	0.82030211
Gm10790	-0.0164426	-0.5039736	0.00049625	0.98244893	0.82030211
Dgcr6	-0.0057644	3.95728815	0.00049478	0.98247503	0.82030211
4933406I18R	-0.015212	-0.502122	0.00049353	0.98249724	0.82030211
Adamtsl3	0.00783903	4.39157337	0.00049165	0.98253045	0.82030211
Asb14	0.0150311	0.03318188	0.00048631	0.98262561	0.82030211
Nlrp1b	0.06109689	-1.6327494	0.00048624	0.98262688	0.82030211
Nabp1	0.0043035	3.64228668	0.0004832	0.98268125	0.82030211
Fbxw10	0.01120832	0.68857113	0.00046647	0.98298367	0.82031385
Rcc1	0.00829882	1.73423824	0.00046165	0.98307176	0.82031385
Rnf20	-0.0029105	6.44450563	0.00046074	0.98308842	0.82031385
Abca17	0.03344267	-0.2030973	0.00046025	0.98309754	0.82031385

A430107P09	-0.0135248	-0.089564	0.00045836	0.98313226	0.82031385
Hexa	-0.0064361	3.74109112	0.00045588	0.983178	0.82031385
Dus4l	0.00634742	2.58436004	0.00044426	0.98339363	0.82031385
1110008L16F	0.00318292	4.36276235	0.00043989	0.98347553	0.82031385
Ppp1r15b	-0.0026036	5.41122845	0.00043937	0.98348537	0.82031385
Bst1	0.02289304	-1.3908768	0.00043807	0.98350983	0.82031385
Ncoa1	0.00228901	8.10415337	0.00043668	0.98353583	0.82031385
Scarna3a	0.01854452	-0.9411287	0.00043172	0.98362966	0.82031385
0610040B10	-0.013631	0.65165979	0.00042884	0.98368443	0.82031385
Rnf5	0.00376028	5.68227105	0.00041232	0.98400161	0.82031385
Szt2	0.00506698	3.93196404	0.00040945	0.98405741	0.82031385
Gm13889	-0.0066642	1.54006208	0.00040473	0.98414964	0.82031385
Asah2	-0.0032276	5.7264065	0.0004021	0.98420114	0.82031385
Pcdhb5	-0.0054101	1.85880226	0.00039913	0.98425953	0.82031385
Nsg1	-0.0032075	5.26403443	0.00039856	0.98427082	0.82031385
Stat2	0.00304038	4.68576171	0.00039736	0.98429457	0.82031385
Slc38a7	-0.0055558	2.39339797	0.00039509	0.98433948	0.82031385
Rnft2	0.0040919	4.54258712	0.00039257	0.98438938	0.82031385
Prrc2b	-0.0025257	8.7073686	0.00039071	0.98442641	0.82031385
Zc3h12a	0.01502485	-0.5978045	0.00039057	0.98442927	0.82031385
Dtx3	-0.0033694	5.05564026	0.00038964	0.98444776	0.82031385
Klrb1c	-0.0193344	-0.3976589	0.0003866	0.98450859	0.82031385
Gse1	0.00364667	5.57081042	0.0003863	0.98451461	0.82031385
Pla2g15	-0.0077832	2.06800813	0.00038261	0.98458872	0.82031385
Id3	0.00537278	6.0191475	0.00038065	0.98462835	0.82031385
Ndufb2	0.00338231	3.99487828	0.00038004	0.98464056	0.82031385
Zfp791	-0.0055252	2.83005514	0.00037322	0.98477903	0.82031385
Kifap3	0.0033512	8.45919916	0.00036963	0.98485245	0.82031385
2610015P09I	-0.0038363	3.72388813	0.00036874	0.98487072	0.82031385
Psmb6	0.00365734	4.90056129	0.00036828	0.98488012	0.82031385
Tmem33	-0.002255	6.24306401	0.00036547	0.98493793	0.82031385
Mir344c	0.034429	-1.6073122	0.00036456	0.98495654	0.82031385
Akap2	-0.0022581	7.52593438	0.0003525	0.98520756	0.82031385
Zfp711	0.004236	4.20917401	0.00035236	0.98521039	0.82031385
Metap1	-0.0025628	6.1717032	0.00035189	0.98522025	0.82031385
Optc	-0.0152312	-1.6157922	0.00035045	0.98525048	0.82031385
Stk4	0.00210866	5.92312332	0.00034646	0.98533475	0.82031385
Snhg5	0.00364551	3.73901185	0.00034509	0.98536384	0.82031385
E130215H24	-0.028169	-1.8867216	0.00056015	0.98537522	0.82031385
Chl1	-0.0039591	7.47835662	0.00034444	0.98537761	0.82031385
Zfp773	-0.010054	1.1356325	0.00033927	0.98548769	0.8203207
Zeb2	-0.0025572	8.89773701	0.00033502	0.98557896	0.8203207
Dhps	-0.0061966	3.07884382	0.00033415	0.98559762	0.8203207

Dmtn	-0.0030974	6.22968677	0.00033006	0.98568612	0.8203207
Heca	0.0030275	4.53730829	0.00032812	0.98572823	0.8203207
Nudt15	0.00637241	1.94917808	0.00032704	0.98575161	0.8203207
5830415F09I	0.01594125	-0.7140421	0.00032248	0.98585141	0.82032968
Nceh1	0.00235903	6.21461893	0.0003198	0.98591015	0.82032968
Homer3	-0.00471	1.81476363	0.00031821	0.98594528	0.82032968
Axl	0.00483705	5.27439036	0.00030665	0.98620293	0.82049331
Cdc25c	-0.0229001	-1.6134283	0.00030116	0.98632686	0.8205112
Lyar	-0.0026877	4.22170673	0.00030031	0.98634638	0.8205112
Ccnc	-0.0025694	5.84716191	0.00029404	0.98648966	0.82057966
2810008D09I	-0.0086939	0.70757999	0.00028271	0.98675236	0.82071983
Esrp2	-0.0127181	-0.9592268	0.00027874	0.98684572	0.82071983
Zfp322a	-0.0023053	6.03840484	0.00027841	0.9868535	0.82071983
Ribc1	0.01093672	-0.2145785	0.00027635	0.98690213	0.82071983
Cnbd2	0.00488849	2.38608532	0.00027226	0.98699961	0.82072748
Hsd17b7	0.00244905	5.00823565	0.00027084	0.98703332	0.82072748
Nudcd1	-0.0031899	3.65886042	0.00026344	0.98721186	0.82082522
1700109H08I	0.01875373	-1.4188016	0.00025756	0.98735519	0.82084049
Pdzk1ip1	-0.0080958	1.63263449	0.00025603	0.9873928	0.82084049
Zfp692	0.00627774	2.60333688	0.00025521	0.98741323	0.82084049
Slc36a1	-0.0025174	4.99323155	0.0002501	0.98753975	0.82089495
Ifi203	0.00570779	3.12728401	0.00024695	0.98761833	0.82090956
Angptl6	0.00932539	-0.1215775	0.00024369	0.98770043	0.82092654
0610038B21I	-0.0181824	-1.8223318	0.00023988	0.987797	0.82092654
Rasgrp1	-0.0027185	9.64199137	0.00023525	0.98791531	0.82092654
Rufy2	-0.0033897	6.03410088	0.00023277	0.98797917	0.82092654
Phyhd1	0.00493169	2.26409937	0.00023124	0.98801876	0.82092654
Gm2011	-0.0059208	1.36350918	0.000227	0.98812904	0.82092654
Aak1	0.00249891	9.72626354	0.00022552	0.9881678	0.82092654
Cdk2ap1	-0.0022042	6.49375569	0.0002247	0.98818925	0.82092654
Hdac6	0.00406691	3.12401288	0.00022258	0.98824514	0.82092654
Dscaml1	0.00458008	3.82701036	0.00022022	0.98830775	0.82092654
Kxd1	-0.003048	4.23318675	0.00021734	0.98838439	0.82092654
Ly96	0.00370884	3.76427015	0.00021517	0.98844256	0.82092654
Nkx2-2	-0.0073863	0.88378594	0.00021498	0.98844755	0.82092654
Dio2	-0.0021768	5.92017166	0.00020745	0.98865185	0.82092654
Glg1	-0.0021139	6.66224155	0.00020593	0.98869344	0.82092654
Rgs1	0.00591308	-0.295751	0.00020504	0.98871774	0.82092654
Ranbp10	-0.0022842	4.71513143	0.00020434	0.9887371	0.82092654
S100a1	0.00411108	4.83217325	0.00020388	0.98874978	0.82092654
Zdhhc1	0.00320799	3.24992215	0.00019664	0.98895142	0.82092654
Gm6623	0.00394845	1.5751644	0.00019063	0.98912146	0.82092654
D730045A05	-0.01601	-0.3651192	0.00019028	0.9891316	0.82092654

Zscan26	-0.0014699	7.19874015	0.00018926	0.98916072	0.82092654
Scaf8	-0.0018671	5.88073503	0.00018793	0.98919884	0.82092654
Atp6v0c-ps2	-0.0077777	-0.3216039	0.00018693	0.98922769	0.82092654
Col8a2	0.00569774	2.09764553	0.00018155	0.98938355	0.82092654
Heatr6	0.00235956	4.8189271	0.00018052	0.9894139	0.82092654
Itgb1	-0.0026567	6.34118709	0.00017937	0.98944748	0.82092654
Smdt1	-0.0031143	5.06212921	0.00017905	0.98945693	0.82092654
Tacr3	-0.0055706	1.90532315	0.00017854	0.98947206	0.82092654
Hif1an	-0.0027909	4.74848081	0.00017762	0.98949934	0.82092654
Slc28a3	0.00763857	1.09393552	0.00017658	0.98952997	0.82092654
Gpr52	-0.0046673	1.71756204	0.00017198	0.98966734	0.82097571
Entpd5	0.00228377	4.33180174	0.00017052	0.98971125	0.82097571
Rhbdl3	-0.0038115	2.78035487	0.00016697	0.98981887	0.82101436
Mepce	0.00227903	4.31707559	0.00016479	0.98988549	0.82101902
Dyrk1a	0.00142838	7.22479528	0.00016151	0.98998665	0.82105231
Pgf	-0.0080643	1.21134176	0.00015416	0.99021719	0.82115317
Gm10433	-0.0377301	-1.6576177	0.00014965	0.99036121	0.82115317
Hsd17b13	-0.0127876	-1.215818	0.00014965	0.99036124	0.82115317
Acer1	0.00609793	0.2981237	0.00014751	0.99043045	0.82115317
Fbxo17	-0.004704	1.79926331	0.00014563	0.99049171	0.82115317
Dis3l2	-0.0025316	3.42555374	0.00014313	0.99057356	0.82115317
Ghr	0.00233255	5.29795125	0.0001429	0.99058126	0.82115317
A530013C23	0.01959872	-1.4641545	0.00014074	0.9906526	0.82115317
CK137956	-0.0067934	0.78151713	0.00014002	0.99067649	0.82115317
Nmi	0.00397275	3.19911829	0.00013877	0.99071849	0.82115317
Six1	0.00379332	5.37716334	0.00013628	0.99080211	0.82117189
Pck2	0.0028797	3.2418545	0.00013432	0.99086825	0.82117613
Arhgef33	0.00767003	-0.5838041	0.00012599	0.99115603	0.82129119
Clec2i	0.00686661	-1.1682639	0.00012557	0.99117092	0.82129119
Pabpc1	0.00132284	8.19210904	0.00012248	0.99128018	0.82129119
Cfb	-0.0040037	2.67106589	0.00012123	0.99132478	0.82129119
Mroh7	0.01108066	-0.4460366	0.00011994	0.99137088	0.82129119
Unc93b1	-0.0046507	1.36127615	0.00011787	0.99144592	0.82129119
Slc8a1	0.00220063	9.18535327	0.00011752	0.99145857	0.82129119
Kcnn3	-0.0028367	4.1629489	0.0001154	0.99153576	0.82129119
Enkd1	-0.0049196	0.55606555	0.00011484	0.99155638	0.82129119
Inhbe	-0.0216012	-1.9838091	0.00011055	0.99171546	0.82131206
Igdcc4	0.00141416	5.11809547	0.00010941	0.9917584	0.82131206
Casq2	0.00728784	-0.2411714	0.00010767	0.9918244	0.82131206
G3bp1	-0.0015603	5.14055908	0.00010763	0.99182573	0.82131206
Gga3	-0.0015426	5.38664136	0.00010294	0.99200573	0.82140767
Inppl1	-0.0021292	4.24130284	0.00010147	0.99206327	0.82140767
Rapgef2	0.00136333	7.49215571	9.65E-05	0.99226193	0.8215216

Gldn	-0.0052056	0.93540306	9.47E-05	0.99233268	0.82152963
4930526I15R	-0.0041191	1.65572054	9.22E-05	0.9924346	0.82153807
Diexf	0.0017559	4.40759709	9.09E-05	0.99248742	0.82153807
Lmo1	0.00809436	-0.3943841	8.98E-05	0.99253304	0.82153807
Svip	-0.0011588	5.08626736	8.76E-05	0.99262608	0.82153807
Sacm1l	0.0014067	6.00551482	8.71E-05	0.99264813	0.82153807
Zfp2	0.00163848	4.45091169	8.55E-05	0.99271389	0.82154196
Htr6	-0.0088686	-1.4043342	8.38E-05	0.99278599	0.8215511
Ankrd9	0.00452147	0.40616197	7.54E-05	0.99315882	0.821718
Adra2a	-0.0022102	4.09654235	7.39E-05	0.99322848	0.821718
Gatad1	-0.0011059	7.04557195	7.26E-05	0.99328703	0.821718
Spata9	-0.001897	3.51825626	7.22E-05	0.9933033	0.821718
Fam103a1	0.00116775	7.87600911	7.02E-05	0.99339899	0.821718
Galnt3	0.00967652	-0.752167	6.90E-05	0.99345381	0.821718
Rfx2	-0.0023571	2.14871108	6.83E-05	0.99348891	0.821718
Uba1y	0.00884577	-1.1650433	6.78E-05	0.99351003	0.821718
Stx7	-0.0010293	7.46022408	6.51E-05	0.99364396	0.821718
Fas	0.00628477	0.28238961	6.49E-05	0.99365002	0.821718
Kansl3	0.00111975	5.88337963	6.48E-05	0.99365938	0.821718
Shc4	0.00212714	2.54008987	6.21E-05	0.99379101	0.82177634
Gap43	0.0010358	7.23966044	5.52E-05	0.99414596	0.8219237
Kcnj13	-0.0016047	6.22314713	5.49E-05	0.99415991	0.8219237
Pcdhga7	-0.002117	2.71389991	5.31E-05	0.99425641	0.8219237
Fzr1	0.00220376	2.83729254	5.30E-05	0.99426471	0.8219237
Fam219b	-0.0016957	3.70312131	5.06E-05	0.9943953	0.8219237
Trim62	0.00127257	4.08115042	5.04E-05	0.99440527	0.8219237
Mpp4	-0.0042339	-1.0414371	4.97E-05	0.99444766	0.8219237
Srsf4	-0.0018608	2.37820875	4.79E-05	0.99454608	0.8219237
Mmp15	-0.0030492	1.11482654	4.72E-05	0.99458931	0.8219237
Sfxn2	0.00177207	2.52378903	4.62E-05	0.9946453	0.8219237
Slc7a7	-0.0035769	1.12585442	4.49E-05	0.99471848	0.8219237
Trappc5	-0.0013929	4.15310119	4.40E-05	0.99477435	0.8219237
Gm14327	0.00241019	2.97504976	4.38E-05	0.99478291	0.8219237
Fgf12	-0.0008464	7.94471617	4.29E-05	0.99484195	0.8219237
Armc1	-0.000766	7.59764091	4.21E-05	0.99488542	0.8219237
Cmip	0.00085736	9.08758755	3.61E-05	0.99526608	0.82193457
Ermp1	-0.0008685	5.74204652	3.52E-05	0.99532556	0.82193457
Prr24	0.00099979	4.53434013	3.47E-05	0.99536106	0.82193457
Camk1d	0.00070229	8.32294529	3.44E-05	0.99537617	0.82193457
Zfp511	0.0018521	2.57961167	3.32E-05	0.99545804	0.82193457
Amd1	-0.0031752	-0.1636389	3.32E-05	0.99546187	0.82193457
Kdsr	-0.0009757	4.62322859	3.30E-05	0.9954714	0.82193457
Lrp10	-0.0015368	4.25497654	3.10E-05	0.99561574	0.82193457

AI414108	-0.0014299	4.67017966	3.00E-05	0.99568164	0.82193457
6330403K07I	0.00063858	7.67692203	2.98E-05	0.99569932	0.82193457
Tfap2b	0.0010699	6.8869382	2.98E-05	0.99570067	0.82193457
Mir8091	0.00452649	-0.5753025	2.92E-05	0.9957395	0.82193457
Zfp958	0.00122918	3.44409452	2.78E-05	0.99584811	0.82193457
Ap5z1	0.00178428	2.61958372	2.77E-05	0.99585631	0.82193457
Gm10421	-0.0065621	0.08783032	2.73E-05	0.99588639	0.82193457
Ubald1	-0.0008847	4.48217382	2.60E-05	0.99597979	0.82193457
Dhx57	0.00120368	5.85111194	2.56E-05	0.99601157	0.82193457
Tacc2	-0.0007096	5.52330764	2.54E-05	0.99603025	0.82193457
Nid2	0.00129813	3.41703602	2.36E-05	0.99617475	0.82193457
Pygo1	0.00076378	4.86566756	2.25E-05	0.99626372	0.82193457
Slc35f1	0.00081924	7.28824329	2.20E-05	0.9963014	0.82193457
Uqcrq	0.00074649	4.16383216	2.03E-05	0.99644929	0.82193457
Msn	0.00075303	7.6261454	2.02E-05	0.99645941	0.82193457
Scn3a	-0.0011864	5.62374621	2.00E-05	0.99647682	0.82193457
Ube2z	0.00071518	6.14013848	1.97E-05	0.99650479	0.82193457
Gm1564	-0.0020541	1.12274621	1.96E-05	0.99651246	0.82193457
Gpsm2	0.00131346	2.5646379	1.92E-05	0.99654779	0.82193457
Ptk7	0.00182923	1.28696047	1.72E-05	0.99673309	0.82203702
Fam115a	-0.0006665	6.74609982	1.44E-05	0.99700792	0.82221328
Eif4b	0.00037565	7.59682671	1.27E-05	0.99718735	0.82231086
Slc7a8	-0.0006441	5.5378534	1.11E-05	0.9973781	0.82240433
Vps41	0.00047377	7.02432777	1.07E-05	0.99742293	0.82240433
Epn1	0.0004989	4.47923716	9.32E-06	0.99759417	0.82244453
Cdc42	0.00033404	8.71371659	8.25E-06	0.99773684	0.82244453
Dctn4	-0.0003273	7.41932633	8.20E-06	0.9977443	0.82244453
9930012K11I	0.00141359	1.30483916	7.86E-06	0.99779146	0.82244453
Tmem177	-0.0006501	3.68336884	7.58E-06	0.99783119	0.82244453
Btrc	0.00038566	6.32207989	7.53E-06	0.9978384	0.82244453
Ddx31	0.00105941	1.36652736	6.41E-06	0.99800579	0.82245895
Dnph1	0.00105108	1.62737352	6.35E-06	0.9980145	0.82245895
Dhx40	0.00031532	5.37711587	4.86E-06	0.99826381	0.82245895
Gfpt2	-0.0004365	4.19350323	4.68E-06	0.99829484	0.82245895
Numa1	-0.0002477	5.77399352	4.66E-06	0.9982986	0.82245895
Lifr	-0.0003824	5.94162796	4.42E-06	0.99834287	0.82245895
Ly6c2	-0.0038686	-0.5892945	4.21E-06	0.99838345	0.82245895
Mphosph6	0.00047376	4.50521503	4.14E-06	0.99839684	0.82245895
Mgme1	-0.0006317	2.92595142	4.07E-06	0.99841019	0.82245895
Tekt2	-0.0025779	-0.5607391	3.78E-06	0.9984671	0.82245895
Gabrg2	0.00025062	6.44501646	2.88E-06	0.99866392	0.82251012
Rfk	-0.0003601	9.48783152	2.37E-06	0.99878622	0.82251012
6820408C15I	-0.0044466	-0.5140302	2.27E-06	0.99881188	0.82251012

Mir376a	-0.0068367	0.06383784	2.22E-06	0.99882506	0.82251012
Cry2	-0.0002101	6.33826493	2.19E-06	0.99883484	0.82251012
Dram2	-0.0001962	5.47488685	1.25E-06	0.99912	0.8226321
Atp9a	0.0001875	4.80433389	1.06E-06	0.99918868	0.8226321
Nrxn3	-0.0001816	8.1011022	9.52E-07	0.99923109	0.8226321
Nr1h4	0.00153277	-0.4247243	8.84E-07	0.99925922	0.8226321
Slc7a15	0.00456205	-1.0209344	8.14E-07	0.99928919	0.8226321
Rnf2	9.49E-05	5.42801567	4.24E-07	0.99948678	0.8226321
Elfn1	0.000157	4.83375736	3.91E-07	0.99950753	0.8226321
4933409K07I	0.00010479	7.36388262	3.02E-07	0.99956701	0.8226321
Nek7	8.04E-05	6.94421238	2.71E-07	0.99958973	0.8226321
Emr1	-0.0007826	0.09863425	2.65E-07	0.99959431	0.8226321
Llgl2	0.00030697	-0.2605733	4.05E-08	0.99984137	0.82266409
Pigo	-6.23E-05	1.91965557	9.95E-09	0.99992139	0.82266409
Fgfr1	-1.93E-05	6.04436897	6.32E-09	0.99993737	0.82266409
Ccer1	-0.031994	-2.3162818	6.88E-11	0.9999935	0.82266409
Fam221a	-5.32E-05	0.13123574	-1.22E-09	1	0.82266409
Tmem259	2.56E-05	3.37626986	-1.30E-09	1	0.82266409
