# Supplementary material for: Mapping the physiological and molecular markers of stress and SSRI antidepressant treatment in S100a10 corticostriatal neurons
Source: Mol Psychiatry. 2019 Aug 20;25(5):1112–29. doi: 10.1038/s41380-019-0473-6 (PMC7031043; doi:10.1038/s41380-019-0473-6)
Supplement: Supplementary file 16 — Supplemental Table S1c [file 41380_2019_473_MOESM16_ESM.pdf]

Suppl Table 1c: Genelist representing the differentially expressed genes (287) between the Gh and Sh+Flx anxious groups, in the context of all the genes examined.

symbol	logFC	logCPM	F	PValue	FDR
Nptx2	2.1742852	4.03919698	95.8072544	4.73E-09	5.67E-05
Inhba	1.24148178	4.79140572	83.8707863	1.41E-08	6.09E-05
Rasl10a	1.7584531	3.35772303	81.2130874	1.84E-08	6.09E-05
Ecm1	2.20151573	3.89611697	80.1893556	2.03E-08	6.09E-05
Hpcal4	1.22625334	9.96877456	62.3341141	1.47E-07	0.00035299
Sccpdh	0.88937003	6.28810619	58.8285079	2.29E-07	0.00045673
Arl15	0.96726539	6.06996797	54.5878987	4.00E-07	0.00062765
Cstb	1.38155365	6.70738108	54.2511208	4.19E-07	0.00062765
Gcnt1	2.12135515	2.91051144	51.8683755	5.83E-07	0.0007055
C1ql3	1.12385551	6.79431107	51.8006066	5.89E-07	0.0007055
Cck	0.87726086	7.75430441	49.6170796	8.06E-07	0.00087724
Fam46a	1.00700696	5.75775238	48.7358225	9.17E-07	0.0009151
Wfs1	1.20233667	6.64987679	46.6753405	1.25E-06	0.00115052
Col5a2	1.5414895	2.99859565	43.0860054	2.19E-06	0.00175396
Sfrp4	1.6954151	3.4712962	43.0716442	2.20E-06	0.00175396
Vamp5	1.25557755	5.48863501	42.5492196	2.39E-06	0.00178954
Cemip	-1.2051445	3.50331555	41.9791334	2.62E-06	0.00184874
Hapln4	0.96815403	6.04615777	41.4864141	2.85E-06	0.00189381
Rspo1	-1.57059	3.09497021	41.1404636	3.01E-06	0.00190022
Aqp1	3.04640538	1.78608843	40.4903842	3.36E-06	0.00201281
Casr	2.6984852	1.89347907	39.454947	4.01E-06	0.00225561
Kcnip3	0.67526131	7.64498816	39.2610729	4.14E-06	0.00225561
Ppl	1.59051223	2.44147578	38.9490691	4.37E-06	0.00227681
Hrg	3.62815965	1.03122995	37.9076908	5.24E-06	0.00259319
Penk	1.23764466	7.08273198	37.7277479	5.41E-06	0.00259319
S100a6	1.7279191	5.94037353	36.8476339	6.33E-06	0.00291229
Filip1l	-0.7786908	5.30520595	36.6461626	6.57E-06	0.00291229
Ryr1	-2.5103012	2.63715206	35.7976409	7.66E-06	0.00323878
Dnajb7	-8.1811647	-1.1210456	39.9717093	7.84E-06	0.00323878
Slc2a12	1.35190446	4.38770554	34.895061	9.05E-06	0.00347985
Stra6	1.91219874	7.10475634	34.8471566	9.13E-06	0.00347985
Chga	1.2221897	4.36510179	34.639597	9.49E-06	0.00347985
Fbxl16	-0.9813851	7.27841856	34.5845128	9.59E-06	0.00347985
Nptx1	0.92470683	7.55312455	33.5309229	1.17E-05	0.00406461
Dgkz	-0.7432521	6.32633871	33.331603	1.22E-05	0.00406461
Smyd3	0.75110252	6.18558953	33.3063036	1.22E-05	0.00406461
Aldoa	0.66419429	9.15691313	33.0454427	1.28E-05	0.00408183
Sdpr	1.27685577	7.13197726	33.0035077	1.30E-05	0.00408183
Sulf2	0.73014732	6.53957386	32.4952535	1.43E-05	0.00428206
Zwint	0.60758791	6.89570744	32.4465889	1.44E-05	0.00428206
Thbs1	-1.7885046	2.76445923	32.3659942	1.47E-05	0.00428206
F5	-2.266976	2.6508564	31.8108491	1.64E-05	0.00458293

Cdkn1a	0.90623464	5.39687725	31.7279639	1.66E-05	0.00458293
Rgs13	3.66693149	0.46691348	31.662559	1.68E-05	0.00458293
Htra4	1.68456421	3.17792456	31.4878348	1.74E-05	0.00463937
Fetub	8.94444754	-0.5004889	40.5722986	1.79E-05	0.00466982
F2rl2	1.74643687	1.92100183	31.0276874	1.91E-05	0.00487004
Bmp3	1.21539537	5.23358809	30.8482925	1.98E-05	0.0049439
Cldn1	3.08728696	3.08447347	30.6930807	2.04E-05	0.00499721
Clmn	-0.7630129	5.98196232	30.5589112	2.10E-05	0.00503219
Nlrp6	5.05233576	0.89625499	30.2387171	2.24E-05	0.00519363
Uchl5	0.66122574	6.67267395	30.203212	2.26E-05	0.00519363
Slc39a6	0.6522707	6.58109972	30.1179952	2.30E-05	0.00519363
Stk38l	0.68356861	5.63816732	29.2191218	2.77E-05	0.00613798
Pde8a	-0.836403	4.07415265	29.0751716	2.85E-05	0.00621039
Gm11549	0.75854578	6.88483758	28.9512514	2.93E-05	0.00625995
Wipf3	0.8239457	7.87146541	28.5494897	3.19E-05	0.00669349
Fabp7	1.09544952	4.52045423	28.398988	3.29E-05	0.00679134
Acss1	-0.9955698	3.75449704	28.1669407	3.46E-05	0.00701429
Ifitm1	1.30373244	5.15849788	27.8165963	3.73E-05	0.00743498
Lancl2	0.61256834	8.49514295	27.5826328	3.92E-05	0.00769146
Stip1	0.60227368	7.04168692	27.2435574	4.22E-05	0.00814514
Unc13c	-0.7389801	5.92405696	26.8651386	4.58E-05	0.00870753
Rgs2	0.765834	7.30623261	26.501838	4.96E-05	0.00928653
Cytip	-1.6946934	2.74174815	26.2483456	5.25E-05	0.00967191
Grasp	0.91295397	4.23156831	26.1804579	5.33E-05	0.00967191
Rbp4	1.1918254	4.08437903	26.068599	5.47E-05	0.00967632
Syn2	0.74474434	7.70541142	26.044806	5.49E-05	0.00967632
Gm21119	-2.7491028	0.59824028	25.7867051	5.82E-05	0.01010481
Capn6	1.14276099	4.31627963	25.6099656	6.06E-05	0.01036557
Vimp	0.77289467	6.1270351	25.4057748	6.35E-05	0.01070347
Kcne4	-1.3505065	3.15263084	25.3160566	6.48E-05	0.0107723
Eno3	1.41148636	3.21469551	24.9189718	7.09E-05	0.01146595
Dhrs1	0.77495718	5.70162098	24.9141692	7.10E-05	0.01146595
Col3a1	1.17771693	5.0592517	24.8648735	7.18E-05	0.01146595
Csf2rb	-2.5467998	1.3256327	24.7286956	7.41E-05	0.01167568
Gprc5a	3.67280314	0.9928613	24.4267834	7.95E-05	0.01221808
Serpinf1	1.35586691	5.60135265	24.4204643	7.96E-05	0.01221808
Ppp1r1c	-3.4414815	1.07525846	24.2239788	8.33E-05	0.01249385
Mansc4	2.18382694	1.99202878	24.1865902	8.40E-05	0.01249385
Sulf1	1.12302268	5.8986324	24.1186149	8.54E-05	0.01249385
Isyna1	1.01852377	6.19626698	24.1010386	8.57E-05	0.01249385
Dcdc2a	-0.8286703	7.4805659	24.0587125	8.66E-05	0.01249385
Cab39l	0.71287486	5.7725496	23.9298607	8.93E-05	0.01272437
Rarb	0.7880988	5.04062113	23.7589498	9.29E-05	0.01299719

Chgb	0.80775713	9.20757245	23.7400548	9.33E-05	0.01299719
Col1a1	1.16163033	6.95686148	23.6499421	9.53E-05	0.01312459
Gm16523	-2.0569375	1.10422528	23.5582149	9.74E-05	0.0132607
Topaz1	3.96790506	0.39184056	23.4818472	9.92E-05	0.01335174
Scg3	1.00307854	6.66561104	22.9633151	0.00011233	0.01494768
Slc5a7	-0.9064481	4.15569526	22.8755814	0.00011474	0.01497704
Ubr3	0.76899735	9.67637616	22.8640265	0.00011506	0.01497704
Vsnl1	0.67260667	12.0201766	22.5652135	0.0001237	0.0157025
Sst	0.86832184	6.90303756	22.5457914	0.00012428	0.0157025
P2ry1	1.09099936	4.02734054	22.5365285	0.00012456	0.0157025
Aox3	0.9225256	6.1406988	21.9651941	0.00014329	0.01769838
Arhgap6	0.83559168	4.73769687	21.8939797	0.00014584	0.01769838
Mfsd3	3.28105328	0.54359466	21.8937907	0.00014584	0.01769838
Ccna1	-6.4701536	-1.0159597	21.8809701	0.00014631	0.01769838
Bcap29	0.76845343	5.17023279	21.3264164	0.000168	0.01994038
Tomm70a	0.47878145	7.12662431	21.3086644	0.00016875	0.01994038
Hspa13	0.81334953	4.88156484	21.2800972	0.00016996	0.01994038
Slc39a10	0.61773575	8.38104969	21.2226958	0.00017243	0.01994038
Slc34a2	2.49041965	0.83213554	21.2058201	0.00017317	0.01994038
Dkk3	0.73738802	8.69595645	21.0755122	0.00017895	0.02040414
Unc5c	0.74208967	6.71418233	21.0190657	0.00018153	0.02040414
Efhd2	0.59382311	6.73441745	20.9779103	0.00018343	0.02040414
D630023F18	1.39039414	2.00640674	20.9654144	0.00018401	0.02040414
Lrrc55	-0.7341335	4.49946179	20.890845	0.00018752	0.02060299
Fmn1	-0.5968548	6.50161144	20.8248167	0.0001907	0.02076115
Mall	2.64669468	0.83161331	20.7010746	0.0001968	0.02123028
Ucma	6.16793249	-0.7750345	20.6394631	0.00019993	0.02123028
D17Wsu92e	-0.4814285	7.63463128	20.6316708	0.00020032	0.02123028
Rassf3	-0.687637	5.51837471	20.3784988	0.00021376	0.02198168
Ccdc69	1.33289701	2.06926137	20.3654273	0.00021449	0.02198168
Glt8d2	0.95471801	3.02758039	20.3614911	0.0002147	0.02198168
E030003E18I	1.21196975	2.39681955	20.3385902	0.00021597	0.02198168
Cpped1	0.57475168	6.46665598	20.3241698	0.00021678	0.02198168
Gm10538	-4.693394	-0.6379455	20.2790265	0.00021932	0.02198168
Neat1	-0.8221053	5.07494143	20.2518616	0.00022086	0.02198168
Mdh2	0.51786088	7.24664761	20.2302071	0.0002221	0.02198168
Eprs	0.51562573	7.57236391	20.0648163	0.00023182	0.02275558
Vstm2a	0.64908245	6.5331679	19.9888967	0.00023644	0.0230202
Spp1	1.16472797	7.62210651	19.7980933	0.0002485	0.02399936
Synpr	1.02505072	5.17764605	19.7490181	0.00025171	0.02411497
Spef2	-2.4616277	0.09680215	19.5818539	0.00026299	0.02470293
Pou5f2	-4.6423558	-0.1208998	19.5741868	0.00026352	0.02470293
Shroom2	0.47445339	6.79998881	19.566848	0.00026403	0.02470293

Anp32a	0.46590439	7.60656272	19.5065251	0.00026826	0.02471413
Gjc3	-0.8752636	5.44559443	19.5062405	0.00026828	0.02471413
Wisp1	1.25885952	1.94884932	19.2252662	0.00028898	0.02623587
6330415B21	-1.2935846	3.14758581	19.1820033	0.00029232	0.02623587
Elavl4	0.5961395	6.90619641	19.1734619	0.00029299	0.02623587
Tmem44	-0.745204	4.61437698	19.1660741	0.00029356	0.02623587
1700007G11	-2.964535	0.29803362	19.0885251	0.00029969	0.02658487
Ints12	0.69674219	4.73342716	19.0090275	0.00030611	0.0269552
Gm21671	-2.3136027	0.9986675	18.9509296	0.0003109	0.02717734
Vangl1	1.22221817	3.81104559	18.8828327	0.00031663	0.02747701
Nnat	1.21857707	8.65677086	18.7112084	0.00033157	0.02856695
Inpp5d	-1.2474893	2.51991615	18.6378746	0.00033819	0.0287897
AA387883	-3.014875	0.28824158	18.6043926	0.00034127	0.0287897
Wdfy4	-1.8586166	1.56055721	18.6032707	0.00034137	0.0287897
Cyp2f2	2.02997289	4.94083326	18.4249975	0.00035826	0.0295092
Kctd1	-0.5232608	6.47743231	18.3649231	0.00036416	0.0295092
Xrcc4	0.90292645	3.43775911	18.3640335	0.00036424	0.0295092
Cartpt	-1.7164094	1.40408598	18.3473206	0.0003659	0.0295092
Zfp710	-0.8846278	3.6340195	18.317638	0.00036887	0.0295092
Jagn1	0.6934099	4.86171929	18.3159132	0.00036905	0.0295092
Stk10	-0.9210197	3.50792912	18.3068778	0.00036996	0.0295092
Theg	3.89307284	-0.8968574	18.301252	0.00037052	0.0295092
Omd	-0.6865589	5.17347677	18.2605869	0.00037466	0.0295092
Appl2	-0.5579534	6.30559206	18.2536808	0.00037536	0.0295092
Mterfd2	0.64166719	5.9146448	18.2376755	0.00037701	0.0295092
Snap25	0.47281801	14.1167379	18.2116266	0.0003797	0.02952689
Sdhb	0.51227285	5.60717895	18.1716431	0.00038387	0.02958607
B230217C12	-0.5044367	5.85137644	18.142577	0.00038693	0.02958607
Cd84	-1.4257366	2.46459589	18.1337686	0.00038787	0.02958607
Pgr15l	4.08288707	0.38897474	18.0583018	0.00039598	0.03001331
Nid1	0.74969724	7.06377941	18.015206	0.00040069	0.03001697
Tmem200c	1.45963949	1.70233929	18.0120481	0.00040104	0.03001697
Gm10220	-1.7583771	1.69414283	17.8305895	0.0004216	0.03135968
Tbrg3	-1.211102	4.19389579	17.7782903	0.00042773	0.03161993
Vwa1	0.86002907	4.20174091	17.740783	0.0004322	0.03171511
Snord19	-6.1462748	-1.6782624	18.979494	0.00043437	0.03171511
Ubl7	0.52014285	6.5759092	17.7011803	0.00043697	0.03171511
Creg2	0.64977996	7.62972907	17.6550443	0.0004426	0.03182748
Piga	-1.3262909	3.12116202	17.6450114	0.00044383	0.03182748
A230057D06	-1.3922867	3.28385481	17.5368847	0.00045738	0.03232182
Slc25a42	0.59420989	4.84202156	17.5329926	0.00045788	0.03232182
Capn1	0.69925901	4.19335936	17.525604	0.00045882	0.03232182
Gm10471	-1.7819331	1.85774165	17.4733434	0.00046556	0.03243202

Top1mt	1.02057424	3.13439273	17.471461	0.0004658	0.03243202
Baiap2	0.5936957	6.25207541	17.3877341	0.00047683	0.03280959
Trnp1	0.58761936	6.17704548	17.3780942	0.00047812	0.03280959
Slc44a3	6.31417505	-1.1805436	17.3681936	0.00047944	0.03280959
Ptprf	0.64039639	4.74980605	17.2998342	0.00048872	0.03308406
Uchl1	0.59245951	6.11240315	17.2853155	0.00049071	0.03308406
Cmah	-0.6893121	6.36639796	17.2697496	0.00049286	0.03308406
Mmp17	0.72429526	5.60588234	17.2578865	0.0004945	0.03308406
Wtap	0.49824942	6.35981409	17.2306935	0.0004983	0.0331526
Grem1	1.88820642	0.83619109	17.1767986	0.00050591	0.03347309
Ywhab	0.42678908	10.3409979	17.0625998	0.00052247	0.03421996
Nrp2	0.59935611	5.60289257	17.0403344	0.00052576	0.03421996
Pappa	1.91611942	2.78235808	17.0403001	0.00052577	0.03421996
Ccrn4l	0.63194497	5.44062537	16.9870075	0.00053376	0.03444022
Mobp	-0.8342357	5.81453183	16.9468534	0.00053986	0.03444022
Ccl19	-1.0607557	3.93998028	16.9289307	0.00054261	0.03444022
Kif4	-1.7417403	1.16208681	16.9177109	0.00054434	0.03444022
6030407O03	5.62231493	-0.8430597	16.910128	0.00054552	0.03444022
4930488L21F	-2.7621317	-0.5601524	16.8884954	0.00054888	0.03444022
Sh3gl2	0.62760783	9.33445628	16.8858656	0.00054929	0.03444022
Sod3	1.03457724	5.39044112	16.853365	0.00055438	0.03449835
Selenbp1	-1.3654645	3.05446523	16.8393802	0.00055659	0.03449835
Timp2	0.66702617	8.97279797	16.8251003	0.00055885	0.03449835
Fbxl13	6.94635643	-0.9624995	16.7847923	0.0005653	0.03459232
Clu	1.02672638	7.27932893	16.7734874	0.00056713	0.03459232
Ddit4l	-0.5955151	4.93093377	16.7456736	0.00057164	0.03459232
E530011L22F	-0.9503221	2.52860136	16.7438967	0.00057193	0.03459232
Sox2ot	-0.7414633	4.98179201	16.6978552	0.00057949	0.03469857
Sez6	0.77968867	4.84745636	16.6863075	0.00058141	0.03469857
1500015O10	1.51152636	5.14687595	16.6804697	0.00058238	0.03469857
Ttn	-1.2803658	3.64854809	16.6392087	0.00058929	0.03493649
Heyl	0.75720714	6.00162945	16.5996428	0.000596	0.03516035
Necab1	0.52678573	7.85856048	16.562407	0.0006024	0.03536334
Cdh13	1.01466162	4.87616683	16.4944199	0.00061427	0.03588429
Wdr52	-1.4893376	2.19941663	16.4749154	0.00061772	0.03591084
Phactr3	-0.488462	6.15735084	16.454399	0.00062137	0.03594883
Pdia3	0.6589186	7.51848694	16.4344164	0.00062496	0.03598231
Mturn	-0.5141529	6.37009665	16.4016002	0.00063089	0.03615017
Las1l	-0.7666754	4.97411249	16.3486043	0.00064061	0.03642701
Postn	-1.1133497	2.19193298	16.3421215	0.00064181	0.03642701
Ppp1r1b	0.87677322	5.39614067	16.2565345	0.00065788	0.0371632
Plxna4os1	-3.3946171	-0.4873521	16.2231687	0.00066427	0.0373478
Pde1a	0.40069261	9.4446545	16.1953992	0.00066964	0.03743703

Slc17a7	0.54043402	7.53302562	16.1826949	0.00067211	0.03743703
C130030K03I	-1.4023384	2.25501604	16.1549671	0.00067754	0.03756474
Cxx1c	0.53582015	5.91556429	16.1307859	0.00068231	0.03765513
Myo1b	-0.6568431	5.93592783	16.10919	0.00068661	0.03771838
Slc6a17	0.52607938	8.25018407	16.0415615	0.00070025	0.03829238
Gxylt1	-0.6283707	5.66834184	16.0148933	0.00070572	0.03841572
Myo6	-0.7071318	6.39830014	15.9222575	0.00072506	0.03919264
Vopp1	0.52790893	5.84434405	15.8963795	0.00073057	0.03919264
Muc5b	-6.6523505	-1.4645111	16.9220772	0.00073227	0.03919264
Cckar	-4.4387686	-0.7734753	15.8846743	0.00073308	0.03919264
Capn5	0.55905127	5.56500213	15.8319607	0.00074449	0.03952336
Zic4	0.83835745	6.26531762	15.7899015	0.00075373	0.03952336
Aspg	1.92825839	0.89639221	15.7671163	0.00075879	0.03952336
Adam3	-7.1218341	-1.935804	16.7848639	0.00075914	0.03952336
Tmem88b	-0.6448709	5.67365952	15.7633536	0.00075963	0.03952336
Zfp59	-0.9547828	3.33000166	15.7422561	0.00076436	0.03952336
Ablim1	-0.4640727	6.74637897	15.7386437	0.00076517	0.03952336
Npy	1.00374279	3.41690542	15.7364253	0.00076567	0.03952336
Trim2	0.58873922	9.04551566	15.7200504	0.00076936	0.03954373
Glra1	2.35603976	0.24685112	15.6935736	0.00077538	0.03968278
Caml	0.75848812	3.67011036	15.6095908	0.00079482	0.04048623
Celf4	0.45605621	9.32146305	15.5961336	0.00079799	0.04048623
Myrf	-0.9363143	3.72404626	15.5824282	0.00080122	0.04048623
Dock10	-0.9963993	5.74765679	15.526544	0.00081458	0.04098794
Slc25a17	0.71402888	5.69498941	15.5115058	0.00081821	0.04099857
Aspm	-1.8608941	1.19774706	15.4754549	0.000827	0.04122615
Prex2	-0.6076118	7.37348837	15.4647056	0.00082964	0.04122615
Gm5862	-2.0942525	0.79766452	15.3607735	0.00085565	0.04227836
Cxx1a	0.54073004	6.02202972	15.3461853	0.00085937	0.04227836
Cd33	-0.8257479	3.1598662	15.3382574	0.0008614	0.04227836
Lipa	0.6567918	4.63551706	15.3063437	0.00086963	0.04250799
Crygs	-6.1457501	-2.0379571	15.2836594	0.00087553	0.04255321
Spag17	-4.980504	0.24423401	15.2755125	0.00087766	0.04255321
Slc2a3	0.62939989	5.24964401	15.2456357	0.00088552	0.04274111
Soat1	0.5355295	5.47047829	15.233739	0.00088868	0.04274111
Nucb2	0.60411873	5.26267907	15.2114215	0.00089462	0.042855
Il17ra	-0.5806957	4.71235539	15.1573424	0.00090922	0.04338051
Catsper2	-1.3063412	1.9578967	15.074342	0.00093213	0.04423718
Thbd	1.03508792	8.3227459	15.0656663	0.00093456	0.04423718
Nomo1	0.65041471	5.3458177	15.0478425	0.00093958	0.04429958
Zfp446	0.92345355	3.31004203	15.0324903	0.00094392	0.04432993
Lancl1	0.56266481	6.54720907	15.0086519	0.00095071	0.04439941
Plxnb3	-1.5119025	2.04041531	15.0013097	0.00095282	0.04439941

Zfp963	-1.0488482	2.52966685	14.9818109	0.00095842	0.04448768
Phyhipl	0.52806656	7.4588456	14.871335	0.0009909	0.04575263
Gm1966	-1.1136543	2.95962697	14.8632825	0.00099332	0.04575263
Mphosph10	-0.6555724	5.16500049	14.8317998	0.00100282	0.04587557
Gm2115	0.9892991	3.08649973	14.829072	0.00100365	0.04587557
Cyp26b1	-1.0098882	5.38657079	14.8141534	0.00100819	0.04590796
Grik1	-1.1981735	2.80971496	14.7611249	0.00102452	0.04647484
Flrt1	0.49215513	6.61301072	14.7427412	0.00103025	0.04654813
Klf10	0.52741404	5.6640606	14.7310557	0.00103391	0.04654813
Caprin1	0.37831282	8.54069451	14.6671598	0.00105418	0.04726592
Schip1	0.75491043	3.86375504	14.6353414	0.00106444	0.04726592
Bmper	-0.8466861	3.17057497	14.6183062	0.00106997	0.04726592
Stmnd1	-3.8863004	-0.550832	14.6021986	0.00107524	0.04726592
1700026D08I	-2.0611886	0.68948357	14.6018877	0.00107534	0.04726592
Cyp4a12b	-1.6341205	3.11657346	14.5949144	0.00107763	0.04726592
Scml2	-2.4591508	-0.0223448	14.5833146	0.00108145	0.04726592
Dzip1	-0.4862775	6.54829099	14.5764936	0.0010837	0.04726592
Nefl	-0.5288899	8.72747445	14.5513074	0.00109206	0.04726592
Dnajc21	-0.5904065	7.02084378	14.550211	0.00109243	0.04726592
Sytl2	-0.6530787	5.78833679	14.5430844	0.00109481	0.04726592
Zfp703	0.64004271	4.68493457	14.535897	0.00109721	0.04726592
Tceal5	0.50081306	6.04172697	14.5056894	0.00110739	0.04753332
Ptpmt1	-0.6525393	4.1804834	14.4284876	0.00113388	0.04839788
Pogk	-0.4259273	6.21945239	14.4234899	0.00113561	0.04839788
Ankrd55	1.10759512	2.6483128	14.3729514	0.00115336	0.04878094
0610009L18F	-1.435442	0.8437656	14.3695925	0.00115455	0.04878094
Amph	0.61525498	6.92174511	14.3631969	0.00115682	0.04878094
Cenpf	-1.0183055	2.51014231	14.3221446	0.00117151	0.0492271
Rasl10b	-0.9370613	3.49792742	14.301065	0.00117914	0.0493513
Mlip	-0.713067	4.86704619	14.2912344	0.00118271	0.0493513
Ppp1r3b	-0.8601716	4.93530695	14.2143159	0.00121109	0.05036016
Rcan2	-0.3857798	7.45083841	14.1688408	0.00122823	0.05089591
Miat	-1.0502096	6.21309444	14.1567574	0.00123283	0.0509052
Slco2a1	1.13776748	4.43168881	14.1459513	0.00123695	0.0509052
Homer2	-0.4716399	7.54094242	14.1320539	0.00124229	0.05094952
Fat4	-0.5136552	5.96841046	14.0796822	0.00126261	0.05140818
Reep6	0.6000313	4.20138878	14.0791131	0.00126283	0.05140818
Pkia	0.40756237	8.02784986	14.0618783	0.0012696	0.05140818
Plekhh1	-0.8248725	4.58905436	14.0563179	0.00127179	0.05140818
Plk4	-0.8041928	3.65666331	14.0483646	0.00127493	0.05140818
Hcls1	-1.2002311	1.43159804	14.0343831	0.00128048	0.05145855
Capn11	-4.2777613	0.03771976	13.9994208	0.00129446	0.05161884
Tm4sf20	-5.5752293	-1.3584128	14.8164779	0.00129681	0.05161884

Snap91	0.65826055	8.93722681	13.9921407	0.0012974	0.05161884
Csnk1a1	0.3730616	7.62192174	13.9629996	0.00130921	0.0517393
Foxred2	-0.909022	2.99096199	13.9586975	0.00131097	0.0517393
Irs1	0.45571397	6.08048577	13.9527784	0.00131339	0.0517393
Epdr1	0.62881832	5.86306948	13.9166487	0.00132825	0.05215344
Clstn2	0.6742402	6.26457341	13.8926118	0.00133825	0.05237416
Vbp1	0.58259027	5.18967613	13.8816257	0.00134284	0.05238283
Arid3b	-1.3127151	2.17230131	13.8619954	0.0013511	0.05253374
Kctd6	0.61813965	5.85232124	13.832442	0.00136363	0.05284952
Jdp2	-0.712859	3.35051052	13.7960342	0.00137925	0.05318332
Itm2a	0.89825367	6.66213787	13.7916934	0.00138113	0.05318332
Ywhaz	0.36225131	12.3202423	13.776551	0.00138769	0.05326481
Atp6ap2	0.50328614	7.25392313	13.7516805	0.00139855	0.05351002
4930539N22	-3.5222728	0.71910845	13.6984248	0.00142211	0.05406945
4930570G19	-1.0719173	3.53760825	13.6982305	0.0014222	0.05406945
St8sia2	1.05590361	2.85033802	13.6306874	0.00145272	0.05505509
Scg2	0.63618094	6.44632555	13.5989842	0.0014673	0.05534632
Cd300lg	-3.6741276	-1.1975361	13.5939024	0.00146965	0.05534632
Ptger3	1.19753756	3.47970624	13.5822835	0.00147504	0.05537525
Hpse	5.39798155	-1.4409293	13.5574539	0.00148664	0.05560294
Cd24a	-0.7690534	5.29033458	13.5481888	0.00149099	0.05560294
Trim3	0.61322716	4.52986376	13.539607	0.00149504	0.05560294
Nes	-0.6457682	4.26178602	13.4861638	0.0015205	0.05611199
Cap1	0.46396775	7.01686267	13.4628609	0.00153175	0.05611199
A930012L18I	-1.207617	2.57284962	13.4624147	0.00153197	0.05611199
Cdk18	0.8584811	4.00573225	13.46237	0.00153199	0.05611199
4833422C13I	1.48277894	2.31396796	13.4620368	0.00153215	0.05611199
Prkcq	-1.2625092	2.94313215	13.4253015	0.00155008	0.05659561
AA413626	5.05572198	-0.6663741	13.407974	0.00155862	0.0567344
Col12a1	0.53294486	5.40904331	13.3965982	0.00156426	0.05676693
Ociad2	0.44358237	6.64314573	13.3718737	0.00157658	0.05704128
Hspa1b	1.08020226	2.12639653	13.3473139	0.00158893	0.05731486
Sptbn2	-0.864444	6.97958108	13.3182798	0.00160366	0.05759405
Paqr8	0.51494992	5.11849638	13.3131424	0.00160628	0.05759405
Sqle	0.54304948	5.75304706	13.2795071	0.00162358	0.05804025
Myo1d	-0.6273505	4.42317551	13.2627118	0.00163229	0.058178
Fyttd1	0.46596029	7.36104306	13.2446218	0.00164173	0.05834085
Insig2	0.42781071	6.13606135	13.2227919	0.0016532	0.05845006
4930444F02I	-4.9826489	-0.6026053	13.212447	0.00165867	0.05845006
Ptbp2	0.55417868	6.37537676	13.20675	0.00166169	0.05845006
Osmr	0.97432531	3.50329174	13.1983307	0.00166616	0.05845006
Plekhg4	2.29496328	1.36026031	13.1926191	0.0016692	0.05845006
Ehbp1l1	-0.6100247	4.25592144	13.1667509	0.00168306	0.05853208

Nupl1	0.48313059	6.08892636	13.1556738	0.00168903	0.05853208
Chek2	-1.2315387	2.34680415	13.1551067	0.00168934	0.05853208
Psmc6	0.50395938	5.85723152	13.1518592	0.0016911	0.05853208
Fam160a1	0.72090308	4.32997888	13.1366925	0.00169932	0.05864734
Col9a2	1.14637809	2.67963195	13.1276018	0.00170428	0.05864924
Adam12	0.66793071	5.7691662	13.1105532	0.00171361	0.05880138
Psap	0.57643052	8.49462902	13.0881219	0.00172597	0.05905448
Pafah1b3	1.47759696	2.03369673	13.067383	0.00173749	0.05905448
Ttc4	-0.5950226	3.91169362	13.066539	0.00173796	0.05905448
Dcc	-0.6509988	5.17733694	13.061615	0.00174071	0.05905448
Myt1	-1.0138272	2.81373741	13.0101345	0.00176973	0.05986946
Arhgap29	0.71363682	9.98480511	12.9842823	0.00178451	0.05996392
Ankrd44	-0.7074586	5.2431102	12.97741	0.00178846	0.05996392
Prrt2	0.44387797	7.21379978	12.9710321	0.00179213	0.05996392
Zfp804b	-1.2149931	2.52131327	12.9703057	0.00179255	0.05996392
Ptges	0.98353901	4.28233446	12.9390342	0.0018107	0.06018051
Gfap	0.7960226	4.21164272	12.9340883	0.00181359	0.06018051
08-Mar	-0.4537045	6.24265239	12.9299027	0.00181603	0.06018051
Luzp2	0.65810475	6.55836174	12.9246257	0.00181913	0.06018051
Cyp2t4	-3.2394032	-0.8172949	12.8998884	0.0018337	0.06026753
Col4a2	0.65616931	4.28926856	12.8964995	0.00183571	0.06026753
Nol10	-0.9115954	4.15900657	12.8945629	0.00183685	0.06026753
Hipk2	-0.4179532	7.03675887	12.8457695	0.00186604	0.06105772
Rab4b	0.72295495	3.46362841	12.8184516	0.0018826	0.06115132
Tspan2	-0.5782451	6.3946554	12.8060847	0.00189015	0.06115132
Gm20199	-1.8268674	2.13159792	12.8046634	0.00189102	0.06115132
Nxph1	0.649855	4.83149152	12.8017764	0.00189279	0.06115132
Gm17821	-0.6536488	9.70641566	12.7991014	0.00189443	0.06115132
Ptgs2	0.8271715	4.12798815	12.7503442	0.0019246	0.06184308
Ubc	0.53676588	6.71051976	12.7454571	0.00192766	0.06184308
Napb	0.4806397	9.75654075	12.7395575	0.00193135	0.06184308
Mybpc1	1.83616335	0.97446557	12.710237	0.00194982	0.06226813
LOC1010557(	-7.3265591	-1.8437998	13.3573821	0.00197594	0.0629343
Shisa3	-1.1465044	3.96110094	12.6583493	0.001983	0.06299159
Pkig	-0.518495	4.79792239	12.6400521	0.00199485	0.06306266
Kitl	-0.6431127	4.77964071	12.638633	0.00199577	0.06306266
BB031773	4.4141342	-1.0355317	12.6060759	0.00201705	0.0635675
4930572O13	3.46052703	0.43251936	12.5695269	0.00204125	0.06416116
Cox7a1	1.21203467	2.11222014	12.5326983	0.00206595	0.06465111
Ttr	-1.4162187	5.00354118	12.5030758	0.00208607	0.06465111
Golgb1	-0.4478291	7.98617775	12.5018969	0.00208687	0.06465111
Ly6g6e	4.38989523	-0.7640857	12.4966888	0.00209043	0.06465111
E130311K13I	0.86340251	3.48396889	12.4918756	0.00209373	0.06465111

Atp1a3	0.64951063	9.48253758	12.4917705	0.0020938	0.06465111
Prg4	0.94272342	8.48540854	12.4905649	0.00209462	0.06465111
Dynlrb2	-1.687609	0.69709176	12.4143728	0.0021476	0.06598919
Krt12	-0.619761	4.21129125	12.4119212	0.00214933	0.06598919
Cend1	0.42016443	7.05317087	12.3974651	0.00215955	0.06598919
Ywhag	0.40787792	10.2179561	12.3968121	0.00216002	0.06598919
Zkscan2	-0.5856146	4.76898938	12.3788414	0.00217281	0.06621106
Bmx	-1.6717398	1.85250892	12.3631509	0.00218405	0.06628786
9630001P10I	-2.081788	0.21611996	12.3540774	0.00219057	0.06628786
Fez1	-0.4985976	5.52476133	12.3521886	0.00219193	0.06628786
Atp6v1g2	0.43218082	9.45908367	12.3274758	0.00220984	0.06640746
Ptch1	-0.5429644	4.75473834	12.3247489	0.00221182	0.06640746
Rdh10	0.65875421	3.6843256	12.3237818	0.00221253	0.06640746
4930473A02I	-2.3957857	0.53604873	12.2842252	0.00224156	0.06678455
Nbas	-0.6932862	5.07413997	12.2814677	0.00224359	0.06678455
Jam2	0.47181831	5.78114302	12.266501	0.0022547	0.06678455
Alox5ap	-1.4559982	1.4941033	12.2619579	0.00225808	0.06678455
Il2ra	1.44282381	1.922807	12.2564408	0.0022622	0.06678455
Klhl25	-0.9870056	1.87790418	12.2513167	0.00226603	0.06678455
Oasl1	5.12099927	-1.023746	12.2472198	0.0022691	0.06678455
Ube2a	0.59890666	6.34368022	12.2309945	0.00228129	0.06678455
Prkg2	0.62277058	4.33622435	12.222938	0.00228737	0.06678455
Cdh19	-1.7174327	1.52153738	12.2224395	0.00228775	0.06678455
Slc25a35	0.65811983	4.99898898	12.2208702	0.00228893	0.06678455
Rab9b	0.42163435	6.46327609	12.2168083	0.00229201	0.06678455
2900097C17I	-0.6227919	10.8249655	12.1936719	0.00230961	0.0670369
C430049B03I	-1.65376	1.132541	12.1907267	0.00231186	0.0670369
Panx2	-0.7392731	4.05650636	12.1792044	0.0023207	0.06713047
Lgals3	1.51898092	1.77857783	12.1643736	0.00233212	0.06714649
Map6d1	-0.577895	5.02829765	12.1639284	0.00233246	0.06714649
Slfn5	-0.8534231	4.46679279	12.1512406	0.00234229	0.06716187
Camk2n2	-0.5603046	4.57960333	12.1487596	0.00234422	0.06716187
Hspa5	0.45336471	7.24254107	12.1254842	0.00236237	0.06747003
Phlda1	0.55553447	7.72269091	12.1205542	0.00236624	0.06747003
Atp6v1e1	0.41716715	6.40075552	12.1125078	0.00237256	0.06748969
Sh3rf1	0.46683286	5.05297757	12.0794033	0.00239878	0.0677848
Ldb2	0.45084659	6.02685681	12.0756401	0.00240179	0.0677848
Omg	0.65905255	7.44931293	12.0752138	0.00240213	0.0677848
Pcdhb16	-0.7682037	4.67358307	12.0708899	0.00240558	0.0677848
Cog7	-0.7409283	4.52669569	12.0500956	0.00242227	0.06783493
Syt5	0.57149165	5.40819515	12.0442489	0.00242698	0.06783493
Cycs	0.52028102	8.2438788	12.0381394	0.00243192	0.06783493
Kcnip2	-0.4446371	5.69882774	12.0344568	0.0024349	0.06783493

Stard13	0.48971822	5.39133096	12.0238327	0.00244352	0.06783493
Otof	-1.3550642	2.91644019	12.0147531	0.00245092	0.06783493
Cpxm2	-1.0934478	2.65931824	12.0132833	0.00245212	0.06783493
D030045P18	4.32165538	-0.5537525	11.9966411	0.00246575	0.06783493
Cd248	0.96216566	4.35918922	11.9953477	0.00246682	0.06783493
Dnaja1	0.34504047	9.6899873	11.9926241	0.00246905	0.06783493
Ret	1.4193874	1.82544934	11.9918808	0.00246967	0.06783493
Tmem194b	-1.7721456	2.10193843	11.9748492	0.00248373	0.06806505
Srpx2	2.30787587	0.24432946	11.9471768	0.00250676	0.06851751
Grin2c	-1.2236184	2.15088364	11.941307	0.00251168	0.06851751
Taf5l	-0.579483	4.1315046	11.9225702	0.00252745	0.06879091
Pik3c2a	-0.449643	6.15002745	11.8906896	0.00255453	0.06937028
Tacc1	0.36363356	7.88929376	11.8323088	0.00260495	0.07057961
Tusc5	-4.5136774	0.35486227	11.8063986	0.00262769	0.07094027
Vgf	1.0474556	3.63822286	11.8005652	0.00263283	0.07094027
Pnmal1	0.80549886	3.26626482	11.7968283	0.00263614	0.07094027
Ackr1	0.7122899	5.69606705	11.7902569	0.00264196	0.07094027
Rapgef5	0.43461421	6.3808839	11.7644463	0.00266496	0.07119864
Phf21b	-1.1684022	2.20298585	11.7608468	0.00266819	0.07119864
Adrbk2	-0.6272749	5.20441657	11.7529129	0.00267531	0.07119864
Eml6	-0.7765847	4.31941007	11.7528562	0.00267536	0.07119864
Gm15881	-2.5959304	0.39379139	11.7423465	0.00268483	0.07129225
Plekha7	-0.7715812	2.98852326	11.7119564	0.00271243	0.07186565
Kpna4	0.34703258	7.20475338	11.6938534	0.00272902	0.07214551
Kif5a	-0.5477489	9.49887627	11.679599	0.00274216	0.07233321
1700110K17I	-1.983672	0.8249802	11.6364477	0.00278236	0.07323251
Gm12060	-4.6328342	-1.0311945	11.6111831	0.00280621	0.07358283
Ebf1	-0.7907071	4.28809844	11.6093339	0.00280796	0.07358283
Klc1	0.46839348	7.49123601	11.602391	0.00281456	0.07359466
Klhl30	-1.6072613	0.76413979	11.5861856	0.00283002	0.07383781
Yipf3	0.52944951	4.45809091	11.5689677	0.00284656	0.074006
Mtpn	0.42842243	10.0811484	11.5666108	0.00284883	0.074006
Rbfox3	0.37296938	7.81275432	11.5382457	0.00287633	0.07455857
Brsk1	-0.7001788	3.49277298	11.519577	0.00289458	0.0746349
Dcp2	-0.4280087	7.18498446	11.5116526	0.00290237	0.0746349
Zfp960	0.52865464	4.46068238	11.5113867	0.00290263	0.0746349
Atp6v1b2	0.42191383	8.40068724	11.5046266	0.0029093	0.0746349
Paqr6	-1.1024692	2.09604064	11.5034787	0.00291043	0.0746349
Ak5	0.54715808	6.20518148	11.4902801	0.0029235	0.07480973
Exosc2	-0.7395378	3.39289145	11.4754494	0.00293825	0.07502706
Snx20	-1.4898422	0.86909136	11.4671386	0.00294656	0.07507907
Cd8a	-1.6401409	0.35350132	11.455572	0.00295816	0.0752147
Mamld1	0.50643416	6.24014348	11.4207245	0.00299343	0.07542666

Atp13a5	0.65455823	4.94737896	11.4194958	0.00299468	0.07542666
Flt1	-0.6934745	4.99645471	11.4194192	0.00299476	0.07542666
Cops5	0.37279113	6.0242677	11.4169605	0.00299727	0.07542666
Cnr1	0.50510332	6.5698156	11.4162497	0.00299799	0.07542666
Tanc2	-0.6948743	8.5132507	11.3948862	0.00301988	0.07570436
Rbp1	0.97852664	7.75562961	11.3931436	0.00302167	0.07570436
Erlec1	0.46367693	5.82466938	11.3866222	0.00302839	0.07571435
Ung	1.23269191	2.10678737	11.3404649	0.00307644	0.07663365
Kif18a	2.02414208	0.99561614	11.3327465	0.00308456	0.07663365
Dlat	0.41980867	8.21845619	11.326898	0.00309072	0.07663365
Arhgap12	-0.4184515	5.65828219	11.3268634	0.00309076	0.07663365
Gm14092	-4.4609662	-1.0279516	11.3124932	0.00310597	0.07672639
Cdr1	-0.8221162	9.91723274	11.3112258	0.00310731	0.07672639
Maged1	0.396697	8.26477941	11.3051949	0.00311372	0.07672648
BC061194	-2.043302	0.34113822	11.2878785	0.00313221	0.07702358
B3gntl1	-0.8100747	2.62906946	11.2677065	0.0031539	0.07714134
Htr7	-0.8615085	3.40319101	11.2669106	0.00315476	0.07714134
Tfcp2l1	0.72556462	7.02929852	11.2604651	0.00316173	0.07714134
Gdpd1	0.52532897	5.43781772	11.258096	0.0031643	0.07714134
Slc7a4	0.90934593	2.98030875	11.2535656	0.00316921	0.07714134
Gatsl2	-0.4425222	6.17021355	11.237136	0.00318709	0.07727195
Zfp207	0.36708719	7.90237065	11.2367837	0.00318748	0.07727195
Hecw2	-0.7195248	6.36028967	11.2125026	0.00321412	0.07776041
Srrm4os	-1.4351051	1.83106618	11.193372	0.00323529	0.07805871
Cadps2	-0.5814887	6.41826898	11.1895915	0.00323949	0.07805871
Myadm	0.3934864	7.63973607	11.1807137	0.00324938	0.07813976
Hmgcs2	-0.8086912	4.07339553	11.1539813	0.00327935	0.07859704
Klk11	-7.376048	-1.816411	11.6987797	0.00328152	0.07859704
Snapc2	0.72764823	4.22325126	11.1386405	0.0032967	0.07861797
Irs4	-1.3167703	1.99826185	11.1377367	0.00329772	0.07861797
Epm2a	1.54278919	1.61414237	11.1338924	0.00330209	0.07861797
Ypel2	-0.376948	6.38247528	11.0953913	0.00334614	0.07925283
Sec13	0.49297624	4.60747021	11.0939122	0.00334784	0.07925283
Dnase1l2	-4.4363478	-0.4876255	11.0929808	0.00334892	0.07925283
A730036I17F	5.40906865	-1.0930303	11.0862988	0.00335664	0.07925283
Slc40a1	-1.0459899	2.11802595	11.0818062	0.00336184	0.07925283
Gcnt2	0.51132521	5.3346145	11.0674133	0.00337856	0.07949057
Ehd3	0.42481095	7.70821092	11.0437245	0.00340629	0.07977358
Cd48	-2.4189027	0.34257758	11.0436601	0.00340636	0.07977358
Gfra2	0.54685102	7.57978057	11.04008	0.00341057	0.07977358
Serpina9	2.44627572	-0.6081491	11.0257801	0.00342746	0.08001218
Fzd4	-0.9822297	2.98805303	11.0033345	0.00345414	0.08047826
Kcnk10	1.42717221	0.71105858	10.9938702	0.00346546	0.08058526

Aplp1	0.49177682	8.34531989	10.9741552	0.00348918	0.08071043
Atad1	0.40276523	7.21366824	10.9700675	0.00349412	0.08071043
Ptprz1	-0.5706185	6.78132457	10.9679033	0.00349674	0.08071043
Fmo1	0.55802012	6.87527009	10.9575606	0.00350928	0.08071043
Dgkg	0.73086879	6.62842365	10.9558389	0.00351137	0.08071043
Crtac1	0.94672311	3.20349585	10.9536107	0.00351408	0.08071043
Ppp2r2b	0.4098267	6.76285514	10.9503767	0.00351802	0.08071043
Skil	0.38487514	7.34534605	10.9274153	0.00354613	0.08119963
Ccdc148	-0.6236933	4.10954772	10.9177839	0.00355799	0.08124932
Hapln1	0.82646892	3.8075648	10.9146442	0.00356187	0.08124932
Zfp746	-0.5140152	4.31556848	10.8962764	0.00358464	0.0812675
Mzt1	-0.4392855	5.93690151	10.8949325	0.00358631	0.0812675
Krt19	1.26258046	1.77903333	10.8923554	0.00358952	0.0812675
Emp3	1.03792642	3.79766243	10.8805363	0.00360428	0.0812675
Morf4l2	0.48156145	7.36995146	10.8803105	0.00360456	0.0812675
Fam163b	0.46265117	7.61194462	10.8724329	0.00361444	0.0812675
2310067B10I	0.74760777	4.10431381	10.87211	0.00361485	0.0812675
Hsd11b1	1.27776092	1.67588969	10.865308	0.0036234	0.0812675
Scn1a	-0.5952591	7.07931107	10.8638114	0.00362528	0.0812675
Pdia6	0.59022549	4.75854562	10.8525137	0.00363955	0.0812675
St8sia5	0.81216381	4.23302689	10.8518527	0.00364039	0.0812675
Kcnj2	-0.5102543	5.54481899	10.831483	0.00366627	0.0812675
Palm2	-0.6265315	7.15217427	10.8305873	0.00366741	0.0812675
Tfrc	-0.5863881	5.51718518	10.829195	0.00366919	0.0812675
Dhx37	-0.8310265	2.78793217	10.8290682	0.00366935	0.0812675
Dhrs11	2.09443353	0.67137026	10.8268119	0.00367223	0.0812675
4930592I03R	-6.7642062	-1.8628536	11.340179	0.00367802	0.0812675
Ythdf2	0.50056243	5.07872959	10.8133018	0.00368954	0.08129464
Msl2	0.42891645	7.27702736	10.8107461	0.00369283	0.08129464
Ace2	4.74090954	-0.716021	10.8002435	0.00370636	0.08136641
Aldh3b2	1.89512983	-0.2498659	10.797677	0.00370968	0.08136641
Ncoa5	-0.658122	4.6525009	10.7647168	0.00375255	0.08205738
Edil3	0.57822065	6.55862889	10.7629346	0.00375489	0.08205738
Ankmy2	0.37222612	6.26991117	10.7403962	0.00378454	0.08255477
Swap70	-0.5041091	4.83364218	10.7254979	0.00380428	0.08283458
Klf11	-0.6392426	4.21877842	10.7199017	0.00381173	0.08284609
Kifc2	-0.4772327	6.32616462	10.7048677	0.00383181	0.08313173
Gde1	0.52271409	5.24696666	10.6961665	0.00384349	0.08323428
Capg	0.92812085	2.58282	10.6786423	0.00386713	0.08359504
Serinc2	1.64363803	0.7617066	10.6727233	0.00387515	0.08361748
Anln	-0.4529237	4.85821024	10.6423282	0.00391663	0.08436052
A730090N16	-2.1849122	0.77945632	10.5680613	0.00402007	0.08643307
Ccdc42	3.30184311	-1.1815401	10.5381531	0.00406258	0.08708354

Prkab2	0.56475378	4.92458605	10.536554	0.00406487	0.08708354
Nrsn1	-0.4157798	6.50508988	10.5219685	0.00408579	0.08737553
Fam101b	0.6320555	4.20018008	10.5012584	0.00411571	0.08785844
Eif5	0.3373406	8.75249106	10.4714972	0.00415913	0.08835868
Ncdn	0.50359093	8.21970628	10.4643227	0.00416967	0.08835868
Ppp3r1	0.35580906	10.0948401	10.464198	0.00416986	0.08835868
Ezh1	-0.5717651	5.19034021	10.4615813	0.00417371	0.08835868
Aldoc	0.51099752	7.18074671	10.4600037	0.00417603	0.08835868
Hs3st2	0.67375912	4.34219434	10.4540352	0.00418484	0.08838889
Unc93a	4.17094882	-0.8055965	10.4475868	0.00419438	0.08843442
Hic2	-0.9028008	2.3305498	10.4425746	0.00420181	0.08843541
Myo18a	-0.4891942	6.30847376	10.4311493	0.00421881	0.08863734
Fndc9	1.17614527	2.94928762	10.4084793	0.00425276	0.08913483
9430041J12F	-1.4203735	1.8680772	10.4054126	0.00425738	0.08913483
Kctd4	0.69180189	5.06433096	10.3729243	0.00430662	0.09000843
A2m	1.25450129	1.67001198	10.3669294	0.00431577	0.09004263
Mbp	-0.6358408	7.0503577	10.336775	0.00436215	0.09049001
Atad5	-0.6730949	3.88604756	10.3309249	0.00437121	0.09049001
3110079O15	-4.3195492	-1.8125332	10.3273009	0.00437684	0.09049001
Ano3	0.57094325	7.88990707	10.32677	0.00437766	0.09049001
Crtc3	-0.5751034	4.93875182	10.3262178	0.00437852	0.09049001
Gpr115	1.15099472	1.61554284	10.3124237	0.00440001	0.09049001
Akap5	0.41514798	8.01809372	10.3122142	0.00440034	0.09049001
Grwd1	-1.2655023	1.41673485	10.3088663	0.00440557	0.09049001
4930509J09F	-3.3034371	-0.3195613	10.3048717	0.00441183	0.09049001
Fam101a	1.33888237	2.83924127	10.2998855	0.00441965	0.09049001
Ltbp1	0.83866544	4.37816882	10.2994488	0.00442033	0.09049001
Dnajc7	0.35313389	6.56262307	10.2859972	0.00444151	0.09076839
Dpm1	0.57795576	5.2982306	10.2712062	0.00446493	0.09077132
4932411E22I	-0.9948827	2.56302696	10.2710055	0.00446525	0.09077132
Lig1	0.57010069	4.07456712	10.2696946	0.00446733	0.09077132
Rsrc2	0.37345331	7.78253955	10.2667729	0.00447197	0.09077132
Ddah1	0.43738268	7.1291285	10.219086	0.00454854	0.0921693
Pacsin2	0.41849198	6.2208514	10.191284	0.00459385	0.09293024
Cul9	-0.790516	3.96622826	10.1842793	0.00460535	0.09300568
Pex19	0.44266793	6.27237414	10.1716722	0.00462612	0.09326786
Dnah9	-1.488845	1.50568372	10.1652958	0.00463666	0.09328674
Pqlc1	-0.5923171	3.33315234	10.1616921	0.00464264	0.09328674
Gm7120	1.08022543	2.94204316	10.1375031	0.00468294	0.09393894
Myo1f	-1.2727687	1.27857512	10.1263423	0.00470167	0.09415688
Scrt2	0.70396068	3.35000913	10.1038899	0.00473959	0.0946021
Pdk2	0.39032757	7.0969543	10.1006653	0.00474507	0.0946021
Dusp9	1.68113642	0.92120224	10.0896652	0.00476379	0.0946021

Hspb6	0.56776747	4.31120956	10.0875756	0.00476736	0.0946021
Npbwr1	-2.4540707	0.40904425	10.085409	0.00477106	0.0946021
Rffl	-0.7956994	3.08964374	10.0852737	0.00477129	0.0946021
Npy1r	-0.5323483	5.20521963	10.0628295	0.00480983	0.09520853
Gigyf1	-1.1242601	2.40603753	10.0563546	0.00482101	0.09527239
Vwa3a	-1.3829539	2.05989752	10.0263521	0.0048732	0.09562887
Cadm3	0.51226262	7.86896536	10.0231342	0.00487884	0.09562887
Dpf3	-2.1440631	0.0628116	10.0186889	0.00488663	0.09562887
Acsl1	-0.4120366	6.51345693	10.0185972	0.00488679	0.09562887
Susd4	0.48585999	4.42295152	10.0167583	0.00489002	0.09562887
Zfp131	-0.5289564	5.28940919	10.0166588	0.0048902	0.09562887
Osbpl1a	-0.3820573	8.12679639	10.010801	0.0049005	0.09562887
Tulp1	-5.4884001	-1.9917509	10.0062802	0.00490847	0.09562887
Arntl2	-1.1728641	2.04468903	9.99943624	0.00492056	0.09562887
Nek9	-0.3736782	6.73170943	9.98697969	0.00494264	0.09562887
Daam1	-0.4740632	6.59201576	9.98160622	0.00495221	0.09562887
Tars2	-0.7593838	2.52134656	9.9805767	0.00495404	0.09562887
Apobec2	-5.3853427	-1.4578593	9.97813979	0.00495839	0.09562887
Itih2	0.68613504	5.04283293	9.97455691	0.00496478	0.09562887
Ncam1	0.52669374	7.88637421	9.97365452	0.0049664	0.09562887
Rassf4	-0.8101788	3.13006963	9.97021632	0.00497254	0.09562887
Fdft1	0.42597773	6.27779223	9.9644527	0.00498287	0.09562887
Rpp25	0.64220709	3.94710413	9.96382646	0.00498399	0.09562887
Gm1979	-2.009775	0.01681008	9.94491988	0.00501805	0.09562887
Sv2b	0.53301587	9.37558707	9.94296139	0.00502159	0.09562887
Plagl2	-0.6034896	5.17042877	9.94237821	0.00502264	0.09562887
She	-1.6071606	1.34917998	9.93618547	0.00503386	0.09562887
Bdnf	0.62519337	4.26333758	9.92771677	0.00504925	0.09562887
Wdr92	0.383384	5.3582105	9.92716231	0.00505026	0.09562887
Tekt5	-2.1341693	0.32940631	9.92243786	0.00505887	0.09562887
Gm20752	-1.53464	0.53927889	9.91993805	0.00506344	0.09562887
Smarca2	-0.3775973	9.78810952	9.91819997	0.00506661	0.09562887
Chac2	0.72404854	3.90892416	9.91685428	0.00506907	0.09562887
Tox2	0.74339631	2.75127914	9.91349979	0.00507521	0.09562887
4933402D24I	-2.5436167	0.32881142	9.91149168	0.00507888	0.09562887
Itgbl1	0.65154649	6.1219165	9.90643915	0.00508815	0.09562887
Mtbp	-1.4552124	1.38346682	9.89754724	0.00510451	0.09562887
Armcx4	-0.5080152	6.06988026	9.89069333	0.00511715	0.09562887
Klhl40	2.16560619	0.1157573	9.88966915	0.00511904	0.09562887
Hsp90b1	0.4712335	9.12486298	9.88739069	0.00512326	0.09562887
Cmpk2	0.46727836	4.55212567	9.88562857	0.00512652	0.09562887
Trim62	0.56385495	4.08115042	9.86665943	0.00516177	0.09612188
B3galnt2	-0.5971056	3.82718944	9.86278665	0.005169	0.09612188

Myom3	2.9074782	-0.2197565	9.84626446	0.00519997	0.09654793
Tmco6	1.11541552	1.74919843	9.81703464	0.00525527	0.09742367
Speer4a	-1.7474377	0.36362606	9.81018759	0.00526832	0.09751461
Zfp873	-0.5935485	3.36059903	9.80279353	0.00528246	0.09757855
Insig1	0.38759383	6.28332118	9.79985974	0.00528807	0.09757855
Zfp106	-0.3174137	9.16699433	9.77298789	0.00533985	0.09838234
Ltbp3	0.65774561	4.64029279	9.76051732	0.00536407	0.09846814
Srxn1	0.3696472	6.28059447	9.75006898	0.00538445	0.09846814
Bche	-0.8579189	7.2717699	9.75003519	0.00538452	0.09846814
Golm1	0.59835889	3.26257839	9.74942627	0.00538571	0.09846814
Gnaz	0.47524257	5.82216852	9.74728061	0.00538991	0.09846814
Tnnt2	0.72023829	4.613484	9.7452727	0.00539384	0.09846814
Slco1c1	-0.802777	4.33303442	9.73634177	0.00541137	0.09863774
Ppm1h	0.39224461	6.75681679	9.7295838	0.00542467	0.09869029
Serbp1	0.32719669	9.37863843	9.7238977	0.00543589	0.09869029
Slc35e1	-0.5684124	4.92752732	9.72234044	0.00543897	0.09869029
Cabp7	3.71319158	-1.1968149	9.71352714	0.00545643	0.09870421
Cxxc5	0.44903237	6.44888195	9.70597519	0.00547144	0.09870421
Rap1gds1	0.30968215	8.5849631	9.70541838	0.00547255	0.09870421
Adamts15	0.92697988	2.60173174	9.70258266	0.00547819	0.09870421
Med15	0.3745674	6.51689822	9.70120047	0.00548095	0.09870421
Slc22a2	0.88950818	4.58244843	9.67872813	0.00552597	0.09903503
Mmp19	1.11390028	2.61996574	9.67681174	0.00552983	0.09903503
Farsa	1.28113932	1.36654708	9.67584467	0.00553177	0.09903503
Cfl1	0.38857778	8.59102715	9.67311694	0.00553727	0.09903503
1700008I05R	4.11894039	-1.3148425	9.66963182	0.00554431	0.09903503
Cntnap5c	-1.7277409	1.45542887	9.66385123	0.005556	0.09903503
Nudt10	0.62056988	3.41269048	9.66055888	0.00556267	0.09903503
Tpbg	0.95044848	3.35700631	9.65917339	0.00556548	0.09903503
Rprm	0.67335213	3.97565688	9.65433286	0.0055753	0.09906272
Folh1	-1.3426698	1.91520935	9.6497422	0.00558464	0.09908165
Cdh3	-2.0957001	0.55645402	9.64080722	0.00560287	0.09925797
Ppil6	-1.4907583	2.35318332	9.63226091	0.00562037	0.09942084
Qpctl	0.82808184	2.65051681	9.61667392	0.00565243	0.09969341
Cndp1	-5.4480606	-1.3929104	9.61589613	0.00565404	0.09969341
Hebp1	0.61760278	3.58687395	9.6124053	0.00566125	0.09969341
Cmya5	-0.8459195	3.28475375	9.60862297	0.00566907	0.09969341
Stx12	0.37303048	7.93683339	9.5989991	0.00568904	0.09982336
Arrdc3	-0.5584149	5.87080385	9.59660631	0.00569401	0.09982336
Krt1	1.47712843	1.74797374	9.59302558	0.00570147	0.09982336
Igfbp6	0.90441937	4.92640641	9.58250639	0.00572343	0.09997573
Slc24a4	1.13492826	2.83603818	9.58086597	0.00572687	0.09997573
AK010878	-0.6183126	3.75420995	9.57336114	0.00574261	0.10010456

Tpp1	0.54725337	6.58033688	9.5576227	0.00577577	0.10036732
2900026A02I	-0.4848066	5.8920017	9.55563071	0.00577998	0.10036732
D630041G03	-0.7292879	4.20906915	9.55190462	0.00578787	0.10036732
Zfp64	0.71723267	2.7789355	9.55033163	0.0057912	0.10036732
Mprip	-0.415213	7.49135185	9.52071038	0.00585439	0.10122319
Meg3	-0.8171613	10.8517456	9.51926294	0.00585749	0.10122319
Tnfsf10	-0.812962	3.01706064	9.49632774	0.00590697	0.1019311
Bcl2a1d	-1.2504517	1.18069673	9.47647568	0.00595017	0.10241918
5031434C07I	6.27701293	-1.6976484	9.87897652	0.00596036	0.10241918
Ell3	1.019776	2.29649561	9.47156527	0.00596091	0.10241918
Mppe1	-1.4220548	0.69121084	9.46417269	0.00597712	0.10255055
Taf6	0.49181522	4.52925267	9.45894517	0.00598861	0.10260073
Fgf16	5.43318643	-1.5914107	9.45186415	0.00600422	0.10272114
Eno1b	0.37593461	6.04849842	9.44535854	0.00601859	0.10275275
Zfp687	-0.684629	3.60778931	9.43989131	0.00603071	0.10275275
Per1	-0.5272548	5.52922724	9.43839216	0.00603403	0.10275275
Nup37	-1.3433716	1.22698112	9.43553152	0.00604038	0.10275275
Por	0.50169732	5.52808753	9.42322041	0.00606781	0.10307282
Ppifos	4.17868641	-1.5463159	9.41120197	0.00609471	0.1032897
Rab11fip4os	-3.4058057	-0.7582432	9.40424847	0.00611034	0.1032897
Ecm2	-0.7088271	5.03263301	9.39996361	0.00611999	0.1032897
Numbl	0.52261617	4.00453307	9.39476604	0.00613172	0.1032897
Fam84a	0.45741383	4.98378711	9.39405724	0.00613332	0.1032897
Zdbf2	-0.7751151	5.97330262	9.39291375	0.00613591	0.1032897
Exph5	-0.7608805	6.36395724	9.39068447	0.00614095	0.1032897
Ferd3l	3.65362339	-1.5862539	9.78558422	0.00615354	0.10335637
C1qtnf1	0.64036333	4.86034582	9.37554982	0.00617531	0.10340334
A230072E10	-2.001048	0.01454516	9.36589919	0.00619733	0.10340334
Gpi1	0.37108049	7.06928865	9.36520928	0.0061989	0.10340334
Atxn10	0.32772322	7.66352388	9.36489692	0.00619962	0.10340334
Ppp1r42	-2.5235657	-0.5472227	9.36234115	0.00620547	0.10340334
Serinc1	0.39185768	8.80303299	9.35756304	0.00621642	0.10340334
1700018G05	-3.908051	-0.9983156	9.35740424	0.00621678	0.10340334
2510039O18	0.90189321	3.40800812	9.35072132	0.00623213	0.10351494
1810058I24R	0.68735686	4.48426675	9.34370518	0.0062483	0.10363967
Pdlim1	0.74803793	3.85893549	9.33434752	0.00626993	0.10369392
Psip1	0.39153711	8.79468226	9.33161712	0.00627626	0.10369392
Vwc2	0.80152326	3.14969717	9.32917808	0.00628191	0.10369392
Zfyve27	-0.5492683	4.26992673	9.32516323	0.00629124	0.10369392
Tcap	-1.6190436	0.86915898	9.32360569	0.00629486	0.10369392
Batf2	-3.3468158	-0.9426477	9.31369126	0.00631797	0.1039052
Vwc2l	0.79406361	3.42519573	9.31066729	0.00632504	0.1039052
Rnf144b	-0.5534661	4.36927858	9.29625914	0.00635884	0.10422691

Pot1b	-0.7163084	3.44286884	9.29189288	0.00636912	0.10422691
Rnf19b	0.49962421	4.34190555	9.29120728	0.00637073	0.10422691
Atp13a4	-1.192068	2.28564418	9.28262158	0.00639101	0.104416
Cobll1	-0.5829945	4.89653014	9.27195236	0.00641631	0.10455281
Pcbp3	0.60261452	4.77316804	9.27160807	0.00641713	0.10455281
Etv3	0.48123563	3.91685719	9.26456688	0.00643388	0.10455281
Pcsk1	-0.7404849	3.26775986	9.26243435	0.00643897	0.10455281
Atp6ap1	0.50462979	7.63568664	9.25907908	0.00644698	0.10455281
Rgcc	0.81171363	2.86685262	9.25707589	0.00645177	0.10455281
8430431K14I	-1.4617296	0.78650113	9.24741054	0.00647492	0.10478625
Arf2	-0.5125431	6.06373611	9.24141494	0.00648933	0.10487773
Irf4	-0.7830143	5.17414265	9.22978154	0.00651739	0.10507567
Snord118	-4.1687102	-1.5551468	9.22724528	0.00652353	0.10507567
Fam71d	-3.4238552	-1.4505967	9.22543962	0.0065279	0.10507567
Esr1	1.55416631	1.10288743	9.22173881	0.00653687	0.10507884
F8	-0.6566696	3.0977305	9.19339021	0.00660605	0.10604853
Vill	1.23011619	1.24115167	9.18474903	0.0066273	0.10624722
Atg7	0.61703757	6.32718181	9.17743126	0.00664535	0.10637174
Lmod1	-0.7155605	5.0582383	9.17440664	0.00665283	0.10637174
Pdcd10	0.42436392	6.07404911	9.16575924	0.00667426	0.10657213
Tmem40	2.27897756	-0.055011	9.15222527	0.00670796	0.10667607
Pcdh1	0.50378332	7.22796233	9.14893312	0.00671619	0.10667607
5530601H04I	-0.7609277	3.42913105	9.14740797	0.00672	0.10667607
Dstn	0.50581751	8.25470472	9.14682142	0.00672147	0.10667607
Spag16	-1.5911425	0.42113633	9.14528603	0.00672531	0.10667607
Tmem26	4.77014337	-1.4309923	9.12838425	0.00676778	0.10720774
Sh3gl3	-0.5934417	5.61268157	9.11558319	0.00680015	0.10752762
Ednrb	0.74369504	3.98408025	9.11330188	0.00680593	0.10752762
Pex12	0.54537388	4.06742378	9.10290327	0.00683238	0.10761212
Pld5	-0.7164304	4.2995414	9.10264281	0.00683304	0.10761212
Plcxd3	0.55584009	5.01837522	9.09868949	0.00684312	0.10761212
Nab1	0.34140715	6.61814642	9.09316315	0.00685725	0.10761212
Wnt5b	-1.3221866	1.1476272	9.09095794	0.00686289	0.10761212
Adra1d	0.55471735	3.5059579	9.088845	0.00686831	0.10761212
Hmcn1	-0.6962647	3.75683083	9.08330885	0.00688251	0.10761212
Fam20a	0.72623455	2.57563194	9.08305453	0.00688317	0.10761212
1700007F19I	-1.6695615	0.05421021	9.07499184	0.00690392	0.10771162
Gm15319	-1.7354176	0.17664311	9.07359559	0.00690752	0.10771162
Asap2	-0.5083847	6.25243567	9.0662362	0.00692653	0.10786765
Pm20d2	-0.4905547	4.21869945	9.04989485	0.00696896	0.10826257
Gpr55	2.2668128	-0.1849099	9.04950684	0.00696997	0.10826257
Kpna2	0.38412321	5.1767939	9.0323582	0.00701482	0.10874859
Trpv3	-3.2939097	-0.520925	9.02941908	0.00702253	0.10874859

Ncf2	-1.1306059	2.47627398	9.02121035	0.00704414	0.10874859
Enkur	-0.9855596	2.14024953	9.0206648	0.00704558	0.10874859
Prom1	-1.0204768	2.67292899	9.02025176	0.00704667	0.10874859
Slc16a10	-0.8157967	2.28528914	9.00830857	0.00707825	0.10902963
Gdap10	-1.0457392	2.40450832	9.00541752	0.00708592	0.10902963
Emd	0.43027075	5.92569873	9.00305411	0.00709219	0.10902963
Gpnmb	1.99221372	1.08924575	8.99462666	0.00711462	0.10911232
Eef1a2	0.46269785	5.59721423	8.99418635	0.00711579	0.10911232
Iqub	-1.3156057	1.53412581	8.96716417	0.00718825	0.11001879
Crhr1	-0.9873394	1.89336019	8.96529988	0.00719328	0.11001879
Smyd1	1.92000656	1.45294208	8.94410796	0.00725073	0.11075596
Ankrd29	0.63108063	4.6037215	8.93601746	0.00727279	0.1109515
Gm3002	-1.0295926	3.60229734	8.91945273	0.00731821	0.11150195
Prdm5	0.78945743	3.03414188	8.91607783	0.0073275	0.11150195
Zc3h12d	2.58645184	-0.835249	8.9046423	0.00735908	0.11184038
Stard8	-0.3653745	5.63681122	8.89630764	0.00738219	0.11194218
Trp53inp1	-0.4843498	5.78981049	8.89109647	0.00739668	0.11194218
Pgk1	0.34164242	7.98381182	8.89010485	0.00739944	0.11194218
6430548M08	0.4472805	6.91741558	8.88876808	0.00740316	0.11194218
Akr1c14	-0.6549302	3.92824185	8.87747707	0.0074347	0.11227731
Prpf38a	-0.5448885	4.10992073	8.86913012	0.00745811	0.11243011
Cc2d1a	-0.8539299	2.91803253	8.86717915	0.0074636	0.11243011
Ube2d2a	0.4272792	9.04656971	8.85708546	0.00749204	0.1127168
Paqr5	-1.0159443	3.21834626	8.84902166	0.00751485	0.11291811
Terf2ip	0.40282559	6.07454767	8.84516236	0.0075258	0.11294085
4933432I09R	-5.8791297	-0.7107174	8.82018676	0.00759705	0.11382974
Car12	0.763666	3.31071986	8.81775247	0.00760404	0.11382974
Ahcyl2	-0.4408295	8.1213262	8.81310831	0.00761738	0.11388717
Ctsb	0.46365567	7.9144416	8.79756981	0.00766223	0.1144148
Ankrd13d	-0.7379662	3.01863563	8.79100731	0.00768125	0.11455609
Ptpn1	-0.4251708	4.77236984	8.78181547	0.00770799	0.1148119
Kcnh4	-1.2229273	1.83296226	8.76788371	0.00774872	0.11527516
Mtx2	0.34446624	5.6916001	8.74790466	0.00780755	0.11539152
Leprel1	-1.1755403	2.42918779	8.74775863	0.00780798	0.11539152
Pls3	0.30737409	7.5080234	8.74593024	0.00781339	0.11539152
Lingo1	0.38121854	7.59023862	8.74456174	0.00781744	0.11539152
Cecr2	-0.5848031	3.27743897	8.73995504	0.00783109	0.11539152
Mctp2	0.9773345	1.62389427	8.73984113	0.00783143	0.11539152
D5Ertd579e	-0.4055803	7.30516997	8.73685464	0.00784029	0.11539152
Mx2	1.93067923	0.06366285	8.73615626	0.00784237	0.11539152
Pnpla1	-1.4064739	0.93716736	8.73146948	0.00785631	0.11539152
P4ha3	1.23384182	2.42550254	8.73021947	0.00786003	0.11539152
Xbp1	0.41131874	6.19549155	8.72937978	0.00786253	0.11539152

Cgrrf1	0.5141155	3.86189549	8.71672177	0.00790035	0.11580466
Gm20172	-1.9297811	0.71115803	8.70611298	0.00793221	0.11612945
2010109A12	-4.8963752	-2.023385	8.70034989	0.00794957	0.11624156
Ccdc87	1.41357443	1.01600754	8.68281831	0.00800266	0.11659286
Gpr137b-ps	-0.610681	3.63692507	8.68266169	0.00800313	0.11659286
A330023F24	-1.1630927	4.37251926	8.68169891	0.00800606	0.11659286
Ttl	-0.3592028	5.9524659	8.67667955	0.00802134	0.11659286
Fcho1	-0.862903	2.7911638	8.67451709	0.00802793	0.11659286
Rhobtb2	-0.3724698	5.37579515	8.66921754	0.00804411	0.11659286
Mbd1	0.51258502	4.27586133	8.66912012	0.00804441	0.11659286
Vapa	0.38405015	7.18529711	8.66502372	0.00805694	0.11659286
Igf2	-0.6873137	10.4379258	8.66167804	0.00806719	0.11659286
Hnrnpll	0.36839059	6.34844981	8.66045193	0.00807096	0.11659286
Tmem72	-3.9317913	-1.1869953	8.65526796	0.00808688	0.11668212
Ggact	0.66180537	5.86444957	8.64425893	0.00812081	0.11703068
Gpd1	0.53123615	4.49827584	8.63604787	0.00814622	0.11724932
Tbc1d24	0.5104145	6.35733208	8.63133404	0.00816084	0.11724932
Csmd3	-0.7842493	5.37477156	8.62988391	0.00816535	0.11724932
Phex	1.17896403	1.95507507	8.62335167	0.00818568	0.11740049
Slc25a5	0.32934958	7.08642197	8.61579673	0.00820927	0.11759791
Iqcj	-3.4767365	-1.5324239	8.60665914	0.00823789	0.11772844
Ccdc89	1.99547621	0.33416161	8.60650192	0.00823839	0.11772844
Fign	-0.6116297	4.7871539	8.60348265	0.00824787	0.11772844
5031425F14I	4.63829524	-2.2204453	8.58188383	0.00831607	0.1184203
Apoo	0.44078474	4.83079787	8.57761421	0.00832963	0.1184203
Pola1	-0.8158546	2.66497059	8.57438296	0.0083399	0.1184203
Ccdc97	0.47806114	5.32492824	8.5733308	0.00834325	0.1184203
Nmb	-2.2789658	0.12625906	8.57253584	0.00834578	0.1184203
Clcn7	-0.7629818	3.02906878	8.55633988	0.00839754	0.1186196
Pirb	-1.9675027	-0.2529225	8.55599057	0.00839866	0.1186196
Snai1	0.87480677	2.27376053	8.5525429	0.00840973	0.1186196
Pycard	0.75754596	2.9570236	8.55186575	0.0084119	0.1186196
Espn	-1.9351192	-0.5472656	8.55164653	0.00841261	0.1186196
Camk2d	0.39015461	7.31796937	8.54957729	0.00841926	0.1186196
Hmgxb4	-0.4117317	5.2954056	8.52867265	0.00848679	0.11932975
A730043L09I	5.09148013	-2.1676103	8.52633112	0.00849439	0.11932975
Zfp474	-4.5968487	-1.4850592	8.52474133	0.00849956	0.11932975
Rxfp3	2.00460733	1.2141781	8.51326971	0.00853693	0.11971416
Phtf2	0.45902401	5.23459006	8.50369639	0.00856827	0.12001301
Erich1	0.77057786	2.96128345	8.48774942	0.00862074	0.12060698
Cx3cr1	-0.7834346	3.35088241	8.48213117	0.00863932	0.12065202
Scara3	0.88480828	6.56283487	8.48068279	0.00864411	0.12065202
Pcdhga10	-0.503511	3.54483059	8.46273846	0.00870377	0.12091455

Ift74	0.39178688	5.84316581	8.46227629	0.00870531	0.12091455
Ubl4b	-2.0281487	0.06162456	8.46066862	0.00871068	0.12091455
Smap2	0.30724994	7.69726981	8.45998038	0.00871298	0.12091455
Gen1	-1.7407721	0.53316377	8.45985399	0.0087134	0.12091455
Gm10421	-3.2384462	0.08783032	8.43986781	0.00878047	0.12170426
Col9a3	-1.0594481	2.69052244	8.4350012	0.00879689	0.12179088
Olfm2	0.50968478	5.18180106	8.41937523	0.00884984	0.12228234
Cops3	0.38582493	5.48155789	8.41750373	0.00885621	0.12228234
Anks1	-0.5494453	3.49404808	8.41550179	0.00886302	0.12228234
Fnbp1l	0.4357734	8.5932555	8.41012477	0.00888136	0.12236558
Cx3cl1	0.45321246	6.78151362	8.40774245	0.00888949	0.12236558
E330033B04I	-1.3531532	2.95242948	8.40396804	0.0089024	0.12238296
Wbp2	0.36852416	7.02579978	8.400259	0.0089151	0.12238296
Ndufv2	0.41069516	6.21579475	8.39841946	0.00892141	0.12238296
Capn15	0.86800189	1.8302036	8.39032881	0.00894922	0.12262391
Nek6	0.58748404	4.83374912	8.38729706	0.00895966	0.12262671
Gramd4	-0.5455204	4.03964658	8.37990095	0.0089852	0.1227709
Tmx3	0.45928023	5.80840114	8.37831006	0.0089907	0.1227709
Onecut2	-0.6823579	3.59482213	8.37298635	0.00900914	0.12288263
Dym	0.37016943	5.63051636	8.36624326	0.00903256	0.12306192
Nek5	-4.4823175	-1.245548	8.35679643	0.00906549	0.12337016
Plcg1	-0.4978058	4.07223723	8.35277226	0.00907956	0.12342134
Glrb	0.37367897	6.74614551	8.34529918	0.00910574	0.1235816
Morc2b	-1.6614428	1.68893426	8.34034836	0.00912314	0.1235816
Dffb	-1.5500361	1.45709339	8.34009528	0.00912403	0.1235816
Gabrd	-1.0652364	2.84095757	8.33765296	0.00913262	0.1235816
Dpm3	1.02605954	2.16360858	8.32618026	0.00917312	0.12398953
Gimap1	-0.8522276	3.12082073	8.31339183	0.0092185	0.12446244
LOC1008622(	0.91283746	2.56939459	8.30130808	0.00926161	0.12490367
Gas5	-0.3317902	6.98860921	8.28798768	0.00930939	0.12530451
Rasgef1b	-0.4404798	5.48781559	8.28719098	0.00931226	0.12530451
Hhip	-0.5456154	4.43848583	8.27822763	0.00934458	0.12548334
Efemp1	0.72779516	7.48332412	8.27769409	0.00934651	0.12548334
Gm4432	-1.7224403	-0.0696758	8.26076268	0.00940791	0.1261663
Sorbs2os	-1.036295	3.15431538	8.24255613	0.00947445	0.12676349
Hpcal1	0.52458336	4.60151312	8.24125137	0.00947923	0.12676349
Zfp366	2.45867596	-0.0905949	8.23520593	0.00950145	0.12676349
Zfp641	-0.9237166	3.02665455	8.23430274	0.00950478	0.12676349
Entpd1	-0.7154012	3.01205359	8.23312208	0.00950913	0.12676349
Ubtd1	1.64745688	-0.1061916	8.23126943	0.00951595	0.12676349
Gpc3	0.8136446	3.71078847	8.22538496	0.00953768	0.12691167
Cobl	0.39384952	7.98664521	8.21485217	0.00957669	0.12718739
Utp20	-0.6580806	4.51937359	8.21405959	0.00957964	0.12718739

Nsf	0.41173823	9.99472431	8.20989361	0.00959512	0.12725185
Aspa	0.4569032	6.03393762	8.20498899	0.00961339	0.12725185
Dlc1	-0.4014738	6.60840981	8.20419091	0.00961637	0.12725185
Lrp8	0.69602579	5.85580177	8.20036742	0.00963064	0.12730006
Plgrkt	0.64838654	4.07499512	8.19176651	0.00966284	0.1275848
Vtcn1	1.90914925	-0.1211964	8.18004667	0.0097069	0.12799192
Arf1	0.34941184	8.31488361	8.17788668	0.00971505	0.12799192
Nol8	0.357922	6.46452917	8.17217707	0.00973661	0.12807524
Ambra1	-0.5005448	5.77368778	8.16682946	0.00975686	0.12807524
4930405J17F	-1.935354	1.00072485	8.16558957	0.00976156	0.12807524
Ccdc3	0.42302914	5.39949026	8.1618818	0.00977564	0.12807524
Sptbn1	-0.6338717	10.099249	8.16104153	0.00977883	0.12807524
Fzd5	-1.1309272	2.67247355	8.15927767	0.00978554	0.12807524
Nptn	0.38802949	8.10613287	8.13454375	0.00988012	0.12917194
4930487H11	-3.3519949	-1.2748223	8.12505094	0.00991669	0.12945866
Sbspon	-1.5930933	0.73858241	8.12133008	0.00993106	0.12945866
Gm14378	-1.9862364	-0.2341426	8.1204467	0.00993448	0.12945866
Zfand5	0.30936855	8.01763302	8.11701577	0.00994776	0.12949082
Prkar1b	0.35703593	7.74528638	8.10454551	0.0099962	0.12998006
Gm19689	-4.4075243	-1.7117989	8.39507981	0.01005766	0.13063738
Chuk	-0.5356533	4.87758158	8.07470206	0.01011319	0.13121639
Mon1b	0.34601731	5.95988164	8.06024246	0.01017043	0.13181618
8430419L09F	0.43971762	5.70735474	8.04940363	0.01021357	0.13207543
Otub2	0.60166224	3.60956246	8.04907439	0.01021488	0.13207543
Map1a	-0.6491055	10.3099317	8.04495269	0.01023134	0.13207543
Apobec4	-2.7419503	-0.0917944	8.04415148	0.01023454	0.13207543
Evl	0.58410224	4.22198237	8.03475386	0.0102722	0.13229558
Cpsf4	-0.9244	1.62507283	8.03400991	0.01027519	0.13229558
Slc38a4	1.32237545	1.56652704	8.02966266	0.01029266	0.13229558
Zc3h7b	-0.4086845	6.02032441	8.02888535	0.01029579	0.13229558
Per3	-0.3595613	5.90032191	8.01607724	0.01034749	0.13256604
Sh2d4b	2.21018934	-0.1638562	8.01555499	0.01034961	0.13256604
Clpx	0.37917788	5.3907089	8.01530076	0.01035064	0.13256604
Zfp182	-0.4412975	4.90802302	8.01094693	0.01036828	0.13256604
Jup	0.44626108	5.22506196	8.00998506	0.01037219	0.13256604
Mir6414	-4.4928783	-1.7663391	8.00432051	0.01039521	0.13267108
Ssbp3	0.34079896	5.98619152	8.00251326	0.01040256	0.13267108
Metap1d	-1.060517	2.28054549	7.99430895	0.01043603	0.13282846
Uchl3	0.55670182	3.58882508	7.99351032	0.01043929	0.13282846
Nrp1	0.49823703	4.973746	7.98502507	0.01047405	0.13282846
Hspa1a	0.57381287	3.80987944	7.97868183	0.01050011	0.13282846
Wnt7b	1.22558473	1.64236323	7.97719406	0.01050624	0.13282846
Gm15910	-0.7483024	4.50043733	7.97513484	0.01051472	0.13282846

Rtl1	-0.8833223	2.9729589	7.96944063	0.01053821	0.13282846
Vdac1	0.31645812	8.05749857	7.9654888	0.01055455	0.13282846
Uqcrfs1	0.31043474	6.5995679	7.96521879	0.01055567	0.13282846
Als2cl	-1.345378	0.5495359	7.9651477	0.01055597	0.13282846
Notch3	-1.1696231	2.36530039	7.96360983	0.01056233	0.13282846
Lamc2	0.6761756	2.97337274	7.96251254	0.01056688	0.13282846
Fam192a	0.42644761	5.25633001	7.95531023	0.01059677	0.13282846
Gnao1	0.37783071	10.1861888	7.95109436	0.01061431	0.13282846
C630031E19I	-1.4427167	1.84095033	7.9478019	0.01062804	0.13282846
Col24a1	-2.0631031	0.43538325	7.94430006	0.01064265	0.13282846
Pak7	-0.5262493	6.03121147	7.94353958	0.01064583	0.13282846
Zfp651	-0.552957	4.59660151	7.94185682	0.01065286	0.13282846
Srsf1	0.322591	7.49633913	7.94038333	0.01065903	0.13282846
Olfr1417	6.06889517	-2.0114826	8.23502674	0.01066514	0.13282846
Neurl1b	-0.7111556	4.89012946	7.93826274	0.0106679	0.13282846
Fert2	0.3790779	4.96364727	7.9381929	0.0106682	0.13282846
Dcps	-0.7882124	2.22805824	7.93632695	0.01067601	0.13282846
Cela1	1.03790608	1.3190181	7.93488895	0.01068204	0.13282846
Cd86	-2.3115678	-0.1300237	7.93247156	0.01069219	0.13282846
Ldha	0.39770162	7.8797741	7.91767687	0.01075451	0.13346423
Lrrc45	-0.5829861	3.71480371	7.91403058	0.01076993	0.13347109
Top2a	-0.9046793	2.36370115	7.91227821	0.01077735	0.13347109
4933428G20	-1.3908401	1.69656661	7.90855843	0.01079312	0.13352831
Crip2	0.42044703	4.55658148	7.90480026	0.01080908	0.13358775
Zc2hc1a	0.3878991	7.30989653	7.89720787	0.01084141	0.1336846
Rab11fip4	-0.5748336	5.52632492	7.89390723	0.01085549	0.1336846
Lrrc3b	0.79259365	3.63449655	7.89135463	0.0108664	0.1336846
Cstl1	2.47584449	0.25753104	7.89078274	0.01086884	0.1336846
2410018L13F	-0.7971222	1.73556512	7.8883108	0.01087942	0.1336846
1700008O03	-0.760036	2.94146089	7.88373617	0.01089902	0.1336846
Ada	-1.1120354	1.31544401	7.88348555	0.0109001	0.1336846
Cyb5r1	0.72229188	2.67456624	7.8820583	0.01090622	0.1336846
Kcne2	-2.9289855	-0.9974753	7.87783113	0.01092439	0.13375707
Fyco1	0.39028059	6.72397002	7.87548706	0.01093447	0.13375707
Myh1	1.93280304	0.52930343	7.87042424	0.01095629	0.13376216
Zfp563	-0.4874953	4.894565	7.87020779	0.01095723	0.13376216
Brca2	-0.5672196	3.83286062	7.86682961	0.01097182	0.13380385
Dgka	0.48135865	4.79442651	7.85833833	0.01100858	0.13405304
Vstm2b	0.6584573	3.40517909	7.85285595	0.01103239	0.13405304
Cd7	3.47034124	-1.7326019	7.85101723	0.01104039	0.13405304
Tagln2	0.70515776	5.7784713	7.85008778	0.01104444	0.13405304
Akr1b8	0.93155433	2.14255392	7.84608995	0.01106186	0.13405304
Grn	0.74062088	5.16360744	7.84546732	0.01106457	0.13405304

M1ap	-4.2337028	-1.5333077	7.84240248	0.01107795	0.13405304
Asl	0.96911348	2.93065126	7.84131683	0.0110827	0.13405304
Tll1	0.67275436	3.55557792	7.83896176	0.01109299	0.13405304
Apip	0.58525669	4.17678292	7.8287337	0.01113784	0.13438761
Hcn3	1.05085617	1.66083011	7.82667688	0.01114689	0.13438761
D930048N14	1.64652908	0.71953324	7.82319377	0.01116222	0.13438761
Sat1	0.46290794	5.94072351	7.82163675	0.01116908	0.13438761
Syvn1	0.5750253	4.83708926	7.81988894	0.01117679	0.13438761
Hook1	0.68642338	5.07421021	7.81525589	0.01119725	0.1344986
Gtf2a2	0.45574249	4.82817	7.80859126	0.01122676	0.1346302
Nron	-2.1830772	0.25872648	7.80770454	0.01123069	0.1346302
Abi3	-0.9286343	1.67972369	7.79881544	0.0112702	0.1348491
Arhgef16	3.95689856	-0.9134374	7.79852946	0.01127147	0.1348491
Sprr1a	4.16644225	-0.2489927	8.08137809	0.01128772	0.13490867
Tex10	0.48202644	4.35355981	7.78587056	0.01132801	0.13519528
Pvt1	-0.9183421	2.41868138	7.7826549	0.01134242	0.13519528
Atp6v1d	0.28798712	8.33107629	7.77537811	0.01137511	0.13519528
Ubqln2	0.39136924	7.64015162	7.77533099	0.01137532	0.13519528
Dnajc5	0.29498319	7.92614819	7.77414283	0.01138067	0.13519528
Cog8	0.8257035	2.01771711	7.77069422	0.01139621	0.13519528
Rtn3	0.39778301	9.97579174	7.7697502	0.01140046	0.13519528
Klhl13	0.34574667	6.10968308	7.76940743	0.01140201	0.13519528
Atp13a1	-0.6561973	2.84849454	7.76131036	0.0114386	0.13535478
Adam19	0.48129365	4.64126525	7.76000717	0.01144451	0.13535478
Prcp	-0.5675212	3.71054934	7.75688734	0.01145865	0.13535478
Cdc14a	-0.7130673	4.04350205	7.75593571	0.01146297	0.13535478
Gm2109	-4.9004527	-2.2724261	7.75395197	0.01147197	0.13535478
Pifo	-3.0611636	-0.2393641	7.74749652	0.01150134	0.13544532
Gm7361	-2.2037753	-0.8434527	7.74729258	0.01150227	0.13544532
Zranb3	-0.8011221	2.7816883	7.73466673	0.01155995	0.13582835
Prps2	0.38655873	6.17915191	7.73353596	0.01156513	0.13582835
Sptb	-0.8298549	5.42552413	7.7327308	0.01156882	0.13582835
Calml4	-1.1286234	1.39227829	7.7291831	0.0115851	0.13587657
Zfp622	-0.5107524	4.68996095	7.72689377	0.01159562	0.13587657
Emid1	-1.4643378	0.7803174	7.72419016	0.01160805	0.13588933
Xdh	-0.6789898	3.2821717	7.72139664	0.01162092	0.13590708
Cd209a	-1.1322571	3.352711	7.71510773	0.01164994	0.13603218
AW495222	-0.9463679	1.74746184	7.71244078	0.01166227	0.13603218
Car7	1.07478125	1.42663876	7.70757818	0.01168479	0.13603218
2310007B03I	6.24551418	-1.8865621	8.41107008	0.01170162	0.13603218
Rpp40	0.74004187	2.58937608	7.70147372	0.01171314	0.13603218
Dnah5	-1.3688695	2.34714386	7.70041314	0.01171807	0.13603218
Atp5g1	0.50061382	5.41654017	7.70023743	0.01171889	0.13603218

Pdzd4	-0.4471136	5.59251781	7.69813724	0.01172866	0.13603218
Pcdhgb7	-0.8022357	2.21993231	7.69305199	0.01175236	0.13603218
Vps36	0.57226384	3.86772111	7.69266003	0.01175419	0.13603218
Agbl1	-2.4803653	-0.8698047	7.69084385	0.01176267	0.13603218
Dnaja4	0.44641892	4.57273453	7.68751788	0.01177822	0.13603218
Popdc2	-1.8699615	0.81035692	7.68606959	0.011785	0.13603218
Tgm2	0.53874267	4.09520623	7.68486398	0.01179064	0.13603218
Serpinb8	0.81813035	3.40522974	7.6795645	0.01181549	0.13618767
Snrpa1	0.44306714	4.24823877	7.6748837	0.01183749	0.13631002
Unc5a	0.52376484	4.9158948	7.66793743	0.01187022	0.13655559
Ftx	-0.7043277	4.04392197	7.66507292	0.01188374	0.13657999
Zc3h12c	-0.3077047	6.25593924	7.66143821	0.01190093	0.13663577
Lgr6	2.70889234	-0.8661721	7.65922381	0.01191142	0.13663577
Igsf11	0.40879399	5.86683619	7.65223929	0.01194455	0.13688478
Scamp2	1.12039945	2.70673317	7.64396026	0.01198397	0.13720517
Adam4	-1.9340794	0.97597989	7.63326678	0.01203509	0.13735262
Gm11744	-2.4949998	-0.4086737	7.63233945	0.01203954	0.13735262
Phf6	0.35659522	5.27930574	7.63194641	0.01204142	0.13735262
Dyrk2	0.80108622	3.24863198	7.62951483	0.01205309	0.13735262
Sned1	0.73969095	4.23200509	7.62928438	0.01205419	0.13735262
Enpp2	-0.4169623	8.24836656	7.62061348	0.0120959	0.13753982
Syne1	-0.6552297	8.5747803	7.61738085	0.01211149	0.13753982
Fancl	-0.6395888	2.71916364	7.61632332	0.01211659	0.13753982
Asah1	0.42640906	5.68220169	7.61529813	0.01212155	0.13753982
Ncoa4	0.31123423	7.81367737	7.61395296	0.01212805	0.13753982
Hip1	-0.543452	4.55084302	7.60771673	0.01215823	0.13775169
Rgs9	-0.6569714	5.09525429	7.60456913	0.0121735	0.1377943
Nceh1	0.35038183	6.21461893	7.60106775	0.01219051	0.13785653
Morc2a	-0.3304893	6.17560802	7.59295175	0.01223003	0.13807824
C2cd2	0.52695293	4.33129603	7.59230841	0.01223317	0.13807824
Ythdf1	0.28537549	6.7367696	7.5898088	0.01224538	0.13808587
Dcx	-0.4815058	5.69217413	7.58417912	0.01227292	0.13817402
2900052N01	-0.904027	2.81930191	7.58349484	0.01227627	0.13817402
Clip3	0.44112023	10.1860474	7.57786321	0.0123039	0.13835495
Ext2	0.93117729	5.05571587	7.57405287	0.01232263	0.13842885
Prps1l3	-0.4242699	4.70122548	7.57004554	0.01234237	0.13842885
BC052040	0.49964921	4.25305052	7.56754502	0.0123547	0.13842885
Rtp1	4.06109377	-1.5165248	7.56219266	0.01238114	0.13842885
Msx1os	-4.4711432	-1.7882486	7.56211599	0.01238152	0.13842885
Ptpn5	0.42364075	5.9042918	7.56172709	0.01238345	0.13842885
Me2	0.43841161	6.29207004	7.56012297	0.01239139	0.13842885
Aldob	2.39480744	0.34713287	7.55676372	0.01240803	0.13848561
Rspo4	-2.7822845	-1.1341276	7.54913924	0.0124459	0.138713

Hsd17b10	0.50858251	4.46645797	7.54800007	0.01245157	0.138713
Wnt9b	4.29738302	-1.9055539	7.54106288	0.01248616	0.13882523
Mta1	-0.4336828	4.54349994	7.54049918	0.01248897	0.13882523
Kcnh5	-0.5562558	5.78019818	7.53785345	0.01250219	0.13882523
Tmem176a	0.86191004	4.32210892	7.53669009	0.01250801	0.13882523
Nt5dc1	0.81322901	3.02824271	7.52340775	0.01257466	0.13943577
Hunk	-0.5496053	4.23948636	7.51320951	0.01262611	0.1398138
Sh2d3c	0.44254715	4.98566397	7.51202395	0.01263211	0.1398138
Sirpa	0.37682527	7.17435197	7.50229278	0.01268144	0.14023024
3110035E14I	0.31917925	7.87383963	7.4787987	0.01280144	0.14142661
Il1a	-2.7607298	-1.0177028	7.4669031	0.01286268	0.14197223
Kpna1	0.34114477	6.91615196	7.46362267	0.01287963	0.14202837
Tcte2	0.71986703	2.88138795	7.46109519	0.0128927	0.14204175
Hivep1	-0.3908856	7.4051704	7.45613623	0.0129184	0.14211402
Ggta1	-1.0764732	2.39992791	7.45524978	0.012923	0.14211402
Capzb	0.44187671	8.59633439	7.45113008	0.01294439	0.14221873
Setd2	-0.3858379	7.47981448	7.4299905	0.01305482	0.14330048
Zeb1	-0.2655302	7.53527288	7.42108944	0.01310163	0.14363213
Stac2	0.39341032	5.87709308	7.41968742	0.01310902	0.14363213
Olig2	0.57730229	3.43458375	7.41275559	0.01314563	0.14390157
Notch4	-1.1290795	1.02824809	7.40514605	0.01318594	0.14403734
Mpzl2	0.56604093	7.15656014	7.4011404	0.01320722	0.14403734
Gaa	0.48934518	5.861557	7.40099394	0.013208	0.14403734
Klf2	-0.5903433	4.01005213	7.39718847	0.01322826	0.14403734
Slc6a20a	-0.78074	5.60574727	7.39692988	0.01322963	0.14403734
Clic5	-0.9210525	2.94378902	7.39682439	0.01323019	0.14403734
Ap3b1	-0.3548125	6.59633929	7.39346665	0.0132481	0.14410123
Pate2	1.56603937	0.20605937	7.38164843	0.01331132	0.14454849
Rlf	-0.4195335	5.84215649	7.38126886	0.01331336	0.14454849
Trib3	4.3265145	-1.1817498	7.3770805	0.01333585	0.14466153
Kcnj12	0.67838315	3.19352967	7.37062673	0.01337059	0.14487126
Col19a1	-0.7755009	3.91312099	7.36899707	0.01337937	0.14487126
Pabpc4	0.44556992	3.89769323	7.36644673	0.01339314	0.14488931
Def8	-0.4677155	4.42867502	7.36118962	0.01342157	0.14506579
Pou6f1	-0.4554958	5.0576674	7.35204941	0.01347115	0.14547042
Arl6ip1	0.30273692	7.00630343	7.34823673	0.01349189	0.14556317
Gm16861	-0.9983462	2.44946528	7.34566651	0.0135059	0.1455831
Prima1	0.90502485	1.40377764	7.34258895	0.01352269	0.145633
Cyp2c44	3.31228092	-0.9963417	7.33805293	0.01354748	0.14576888
Tcp11l2	0.41489061	4.28628694	7.33551202	0.01356139	0.14578754
Echdc2	-0.8671773	2.26378607	7.32551853	0.01361624	0.14612283
Ppt2	1.06814307	1.23233552	7.32538476	0.01361698	0.14612283
Adamts10	-0.8323257	2.08207284	7.32297985	0.01363022	0.14613395

Anxa5	0.63527142	8.20189293	7.31552261	0.01367136	0.14644395
Jun	0.33365295	6.02552746	7.3112421	0.01369504	0.1465665
Atp8b2	-0.5727075	4.3200366	7.30644755	0.01372162	0.14671982
Nkd2	-0.6885332	4.51143517	7.29666326	0.01377603	0.14699733
Kif13a	-0.3744887	5.84859562	7.29625517	0.01377831	0.14699733
Dhfr	-0.4997661	3.80304629	7.29516361	0.01378439	0.14699733
Cox5a	0.36051035	6.35385492	7.28782966	0.01382537	0.14713622
2410007B07	3.47160503	-1.9733839	7.28634534	0.01383368	0.14713622
Narfl	0.72014794	2.64718879	7.28623844	0.01383428	0.14713622
Erbb3	-2.3919399	-0.3009485	7.27924553	0.0138735	0.1472526
Fanci	-0.8133838	2.90436969	7.27816803	0.01387956	0.1472526
Dach2	1.97456634	0.10560805	7.27771417	0.01388211	0.1472526
Ttk	-2.5061132	-0.5704396	7.27541184	0.01389506	0.14725954
Arhgef2	-0.6251075	5.56237743	7.26191257	0.01397126	0.14726383
D130017N08	-0.5746699	3.83627959	7.25954065	0.0139847	0.14726383
Dpep2	-4.2862942	-1.7038124	7.25916079	0.01398685	0.14726383
Foxo6	0.73321822	2.60702137	7.25895762	0.013988	0.14726383
AI593442	-0.3605977	8.70047291	7.25798715	0.01399351	0.14726383
Spata2	-0.4758568	5.49015829	7.2574215	0.01399672	0.14726383
Cyp19a1	-5.9662806	-1.5822901	7.5074099	0.01400695	0.14726383
Gmnc	-2.3256383	-0.5135682	7.25141258	0.01403086	0.14726383
Zswim6	0.35662356	6.10393689	7.25004777	0.01403863	0.14726383
Chrna5	-1.9854235	-0.3679875	7.24805473	0.01404998	0.14726383
Vac14	0.51694649	4.71468339	7.24747296	0.01405329	0.14726383
C030013G03	-0.6930027	2.95740626	7.24488396	0.01406806	0.14726383
Crb1	-3.0003689	-0.9575645	7.24214797	0.01408368	0.14726383
Tmem204	-0.6063703	3.94258334	7.23966916	0.01409785	0.14726383
Sh2d1a	-3.9104114	-1.5924025	7.23963735	0.01409803	0.14726383
Tmem104	0.58153508	3.95676381	7.23759436	0.01410972	0.14726383
Pramef8	-0.6232607	4.01107825	7.23717767	0.01411211	0.14726383
Srp72	0.27721568	7.21966283	7.23552287	0.01412159	0.14726383
Ide	0.29311451	7.24622988	7.23421208	0.0141291	0.14726383
Dera	1.04994399	2.55249595	7.21442724	0.01424306	0.14828051
Cited2	-0.3890732	6.39891047	7.21298457	0.01425141	0.14828051
Dnajb9	0.49188514	5.83039723	7.20276896	0.01431069	0.14876806
Prox1	-0.4689559	4.8871507	7.19810141	0.01433787	0.14892132
Mst1	-3.4612222	-1.388693	7.19063449	0.01438147	0.14924472
Chp2	-1.1728155	0.84874003	7.1849146	0.01441497	0.14944171
Arf4	0.37967257	8.85386897	7.18313472	0.01442541	0.14944171
Agt	1.70877877	1.29022148	7.17800955	0.01445552	0.14962424
Medag	0.30909622	6.21870146	7.17514232	0.0144724	0.14966958
Wdr41	-0.3764106	4.83009109	7.17293375	0.01448542	0.14967493
Dnmbp	-0.598034	3.65661278	7.16892425	0.01450908	0.14979018

Sun1	-0.4874388	4.71651478	7.16417722	0.01453715	0.14995072
Tcte1	1.69051035	0.71897727	7.16054226	0.01455868	0.15004362
Slc3a2	0.37225126	6.32439206	7.1531049	0.01460286	0.1502368
Xrcc2	-0.9176165	2.5538066	7.14909609	0.01462673	0.1502368
Tcf20	-0.4050211	8.10599558	7.1488296	0.01462832	0.1502368
Dner	0.56033735	5.47834739	7.14691003	0.01463977	0.1502368
Sumf2	0.69814394	2.65517025	7.14485017	0.01465207	0.1502368
Hpca	0.34856511	8.12687707	7.14432704	0.01465519	0.1502368
Fgf5	0.80931573	2.67938052	7.14264463	0.01466524	0.1502368
Klf14	-2.8193575	0.34924461	7.13885784	0.0146879	0.1502665
Yrdc	-0.5627091	3.48619489	7.13796672	0.01469324	0.1502665
Pcdhgb4	-0.8388496	2.29278833	7.13561805	0.01470732	0.15028215
Cyp1b1	0.51724655	7.03186445	7.12174848	0.01479076	0.15100596
Sytl1	2.20215645	0.01323853	7.119379	0.01480507	0.15102329
Tmprss5	-4.6663427	-1.6692367	7.11223451	0.01484831	0.15133545
Itpk1	0.50405999	3.82827909	7.11013613	0.01486103	0.15133636
Basp1	0.35766763	9.41422249	7.10527876	0.01489054	0.15150799
Dynlrb1	0.4283867	8.41788242	7.10106514	0.01491619	0.15153386
Lrrc19	-1.9399074	0.34240193	7.10070386	0.01491839	0.15153386
Taf9b	0.42848462	6.91473087	7.09255705	0.01496813	0.15191023
Pigs	0.48965099	4.61576132	7.08506214	0.01501405	0.15196496
Wdr31	0.98634256	1.78852451	7.08422966	0.01501916	0.15196496
1700001K19I	-1.7253019	0.15610211	7.08402504	0.01502042	0.15196496
Mkx	0.55748367	5.3746159	7.08339636	0.01502428	0.15196496
Rit2	0.33841439	6.72674347	7.07811723	0.01505674	0.15204789
Mapk13	-4.1860639	-1.634588	7.07793421	0.01505787	0.15204789
Myoc	-0.8243338	2.14626982	7.07105942	0.01510027	0.15224847
Phf14	-0.3108507	6.44424764	7.07043188	0.01510415	0.15224847
Rad23b	0.27702276	7.99286749	7.06783481	0.0151202	0.15224847
Met	0.57151492	3.05753534	7.06648018	0.01512859	0.15224847
Wdr74	0.72054795	2.90109075	7.06436794	0.01514167	0.15225217
Nmt1	0.32084031	5.26669908	7.05362503	0.0152084	0.15279484
Haus3	0.47712551	4.11729922	7.04418822	0.01526728	0.15310746
Dst	-0.7106432	9.14796673	7.04320947	0.01527341	0.15310746
Ptges2	-0.6669315	2.3907811	7.0424965	0.01527787	0.15310746
Rdh12	1.71822606	0.1413352	7.0319133	0.01534426	0.15364429
Adam1b	-1.881053	0.2342261	7.02267995	0.01540246	0.15409813
Lgi1	0.38056674	7.46443041	7.01710584	0.01543771	0.15419951
Palmd	-0.4602344	5.6060739	7.01524106	0.01544952	0.15419951
Cdca2	-3.2301814	-0.3892356	7.01407504	0.01545691	0.15419951
5430416N02	0.88833807	2.04785831	7.01294256	0.01546409	0.15419951
Fut10	-0.5793308	3.61978587	7.00929317	0.01548726	0.15430208
Abcc4	0.64349394	5.12241116	7.00373645	0.01552262	0.15452579

Tmc7	-0.4837773	4.52991918	6.99829501	0.01555733	0.1547427
Pink1	-0.2770874	7.52866259	6.99535068	0.01557615	0.15478974
Nop58	-0.3773651	6.14567815	6.99351245	0.01558791	0.15478974
Gm21284	-1.5054397	0.13904433	6.98890555	0.01561743	0.15490887
Idh3b	0.29302117	7.06719223	6.98685748	0.01563058	0.15490887
Col6a3	0.83229701	3.31318191	6.985269	0.01564078	0.15490887
Tcf12	0.40950312	7.35152925	6.98357772	0.01565165	0.15490887
Klhl1	-0.7817312	3.01700362	6.97953133	0.01567769	0.15503851
Tspan13	0.54755322	8.54940171	6.97740745	0.01569138	0.15504585
St8sia4	-0.7123286	3.27236278	6.96859799	0.01574831	0.15548004
Hmgb2	-0.5629203	4.01804394	6.96490979	0.01577221	0.15551466
Dyrk4	-3.620449	-2.242214	7.19793074	0.01577779	0.15551466
Meiob	3.37186516	-0.9393233	6.9578434	0.01581812	0.15566543
DQ267100	-1.8847368	0.15390844	6.95709713	0.01582297	0.15566543
Rtn4rl1	0.34276577	6.19990827	6.95569867	0.01583208	0.15566543
Gm3230	-1.1508366	1.29743143	6.95305042	0.01584934	0.1557073
Rab10	0.32329025	8.51286959	6.94659166	0.01589152	0.15597976
H2afy	0.40546958	5.91929454	6.94292279	0.01591554	0.15597976
Cabyr	-0.6869653	4.12377264	6.94283028	0.01591615	0.15597976
Zfp180	0.34822651	5.1210023	6.9281699	0.01601253	0.15675681
Ankzf1	0.94141221	1.79473831	6.92679309	0.01602162	0.15675681
Ehf	-1.5799225	0.60550469	6.9197772	0.016068	0.15708233
Camk2n1	-0.3036865	10.5133201	6.91623725	0.01609146	0.15714073
Pi16	2.45783954	-0.6008267	6.91491773	0.01610022	0.15714073
Nat8	-2.0179949	0.23578524	6.9060703	0.01615906	0.15758661
Stk33	-1.0741512	1.62331277	6.89177596	0.01625464	0.158296
Tmem132a	0.66915023	2.66849841	6.89123967	0.01625824	0.158296
Cdh2	0.47918484	6.47293761	6.88100799	0.01632706	0.1587928
Pcdh18	-0.6139153	3.19480884	6.87788172	0.01634815	0.1587928
Rnf111	0.27447255	7.07452193	6.87774989	0.01634904	0.1587928
Id4	-0.4420147	5.33668979	6.87463292	0.01637011	0.15886852
Serpinb6c	-1.2136671	2.72569528	6.87126788	0.01639288	0.15896071
Ppid	0.3446404	6.12373406	6.86876734	0.01640982	0.15899628
Eva1a	1.45973782	1.13258846	6.86395415	0.01644249	0.15918405
Uba3	0.35294464	6.23925471	6.86154837	0.01645885	0.15921371
Mecom	-1.3650868	1.34295964	6.84644151	0.01656199	0.16008208
Dclk3	0.46894998	5.48919709	6.84383089	0.01657988	0.16012582
Rimbp2	0.61649238	5.19509569	6.83813169	0.01661903	0.16037453
Calb1	0.39365731	6.05115069	6.83069451	0.01667027	0.16069584
Il27ra	3.13835019	-1.5039429	6.82940591	0.01667916	0.16069584
2810468N07	-0.7269937	2.59220216	6.82581559	0.01670397	0.16080554
Fancd2os	1.89156973	0.21805888	6.82372502	0.01671844	0.16081554
Sprn	0.44011586	5.91404279	6.8141772	0.01678469	0.16125494

Olfr464	-4.1472262	-1.9463999	6.81326306	0.01679105	0.16125494
2810047C21I	1.02068482	1.87145865	6.80300936	0.01686256	0.16159933
Gsdma	-5.1427252	-2.0985064	6.80232835	0.01686732	0.16159933
Ercc6l	2.19931912	-0.7930277	6.79927302	0.0168887	0.16159933
Mmp13	4.17422388	-1.9234618	6.79743102	0.01690161	0.16159933
Lzts3	-0.3471615	5.7471234	6.79724895	0.01690288	0.16159933
Baz1a	-0.4765897	4.11718974	6.79524634	0.01691693	0.16159933
Mtus2	-0.3135134	6.24056567	6.78938683	0.01695809	0.16159933
Gulp1	0.56768382	5.95941799	6.78898187	0.01696094	0.16159933
Sik1	-0.5460931	4.19170344	6.78588312	0.01698276	0.16159933
Apba2	-0.4605084	4.81600432	6.78482157	0.01699024	0.16159933
Brwd3	-0.3818437	6.15172761	6.78356325	0.01699912	0.16159933
App	0.32973236	8.66509883	6.78206452	0.01700969	0.16159933
1700029J07F	-0.8316393	2.15905679	6.7820592	0.01700973	0.16159933
Gpr22	0.43911225	5.80525701	6.78015359	0.01702319	0.16159933
Cep112	-0.5544469	3.64725574	6.77928562	0.01702932	0.16159933
P2rx5	3.06464209	-0.9221441	6.77038093	0.01709239	0.16202452
Arl8a	0.35434516	6.34525013	6.76873497	0.01710408	0.16202452
Wrn	-0.3357768	5.66555382	6.76723792	0.01711472	0.16202452
Mtap7d3	-1.0675179	1.90900847	6.76470452	0.01713273	0.16206698
Slco1a4	-0.6178608	4.88980651	6.75349714	0.0172127	0.16253736
Clvs1	0.44120957	4.16527292	6.7518207	0.0172247	0.16253736
2700038G22	-1.6053218	-0.1344479	6.75135907	0.017228	0.16253736
Bzw2	0.49919683	3.56638894	6.74920271	0.01724345	0.16253736
Ccdc80	1.0805845	2.41847797	6.74824416	0.01725032	0.16253736
Tusc1	0.52776304	3.18417392	6.74373208	0.01728271	0.1626241
Mrps36	0.42334931	5.16856347	6.74226864	0.01729323	0.1626241
Dynll1	0.38304804	8.0929737	6.74127421	0.01730039	0.1626241
1810019D21I	3.28512759	-0.5570724	6.73769163	0.01732618	0.1626241
Prkcsh	0.60238253	3.3401779	6.73751908	0.01732743	0.1626241
Fbxo8	0.38668712	4.84429171	6.72905978	0.01738851	0.16306963
Zfp930	0.529712	4.10431084	6.72508731	0.01741728	0.16321162
Acot11	-0.5577891	3.82662615	6.7225717	0.01743553	0.16325486
Snrpa	0.54227657	4.66726131	6.7197158	0.01745627	0.16325603
Arap2	-0.4302756	7.05856224	6.71819931	0.01746729	0.16325603
Mvk	0.57330014	2.941983	6.71584074	0.01748446	0.16325603
Itpr1	-0.4625844	8.99082868	6.71505425	0.01749018	0.16325603
Samd14	-0.9771271	2.17700552	6.71164071	0.01751506	0.16336095
Stap1	-1.6032282	0.31756925	6.70649566	0.01755264	0.16354004
Fam71e1	-1.4466947	0.25455267	6.70527375	0.01756158	0.16354004
Lst1	1.80821948	-0.2032257	6.69994744	0.0176006	0.16377604
Gfpt2	0.49385883	4.19350323	6.68713514	0.01769485	0.16452527
Capns1	0.48442791	9.23059355	6.67825128	0.01776054	0.16500792

Zcchc12	-0.5180235	4.29286974	6.67214862	0.01780582	0.16523531
Oc90	5.06076592	-2.3165054	6.88407555	0.01783836	0.16523531
Rbm11	0.57902451	3.26912102	6.66753507	0.01784014	0.16523531
Tmem181b-|	-0.730264	5.11164669	6.6675264	0.01784021	0.16523531
Itpkc	0.81055479	2.14887091	6.65538244	0.0179309	0.16575063
Casc4	0.35248079	8.2105552	6.65485198	0.01793487	0.16575063
Mrc1	-1.0834291	2.19715843	6.65408131	0.01794064	0.16575063
3110070M22	2.44755309	-0.8977719	6.65267241	0.01795121	0.16575063
2700069I18R	1.31242369	0.80104733	6.64904342	0.01797844	0.16580191
Isl1	-1.2836951	1.28366596	6.64824387	0.01798445	0.16580191
Pitpna	0.25619746	8.72835352	6.64495858	0.01800915	0.16590196
Mir872	-3.6123077	-1.3539564	6.64118035	0.01803761	0.16603642
Trim28	0.33179119	5.90610985	6.63231637	0.01810458	0.16651727
Themis2	-1.1968019	1.61244666	6.63058932	0.01811766	0.16651727
Lsg1	0.44408482	4.02144153	6.62444607	0.01816428	0.16676881
Cdo1	0.57646751	6.85151754	6.62264721	0.01817795	0.16676881
02-Mar	-0.4319068	4.75045565	6.62131838	0.01818806	0.16676881
Cmtm4	-0.3351074	6.3562398	6.61965374	0.01820073	0.16676881
Plcb4	-0.4267943	7.05152563	6.6126887	0.01825386	0.16712777
Dvl2	-0.7479708	2.37289552	6.60674182	0.01829937	0.16741639
Ap2a2	0.29766218	6.88755799	6.60390721	0.0183211	0.16748727
Ppp1cc	0.2935442	7.62011982	6.60167683	0.01833822	0.16750443
Gpatch4	-0.3858008	4.88868949	6.60002019	0.01835095	0.16750443
Ndufab1	0.36483763	5.61527553	6.59360664	0.01840032	0.16770816
Gatc	0.41763311	7.36425707	6.59348258	0.01840128	0.16770816
Thpo	0.88799679	2.09779978	6.58861636	0.01843884	0.1679227
Comtd1	1.04585292	1.05456235	6.58512529	0.01846584	0.16793597
Esr2	-1.2174	0.69385445	6.58421832	0.01847286	0.16793597
Kif6	-1.5821358	0.20610313	6.58299137	0.01848237	0.16793597
Cpsf3l	-0.6617372	2.63719254	6.57787094	0.01852209	0.16807728
Zfp438	0.47297539	3.94995783	6.57505612	0.01854397	0.16807728
D130040H23	-0.6546654	2.95254379	6.57414259	0.01855107	0.16807728
C920021L13F	0.95686611	1.96554996	6.57252494	0.01856366	0.16807728
Wnt2	2.36341958	-1.0513622	6.57195616	0.01856809	0.16807728
Zbtb3	2.54772828	-0.3909106	6.56706422	0.01860624	0.16829537
Wnt5a	-0.4823088	5.5511028	6.56328941	0.01863573	0.16836596
Sdcbp	0.34206121	9.43623157	6.56246768	0.01864216	0.16836596
5730455P16I	0.30203914	7.14297718	6.56036582	0.01865861	0.16838755
Gm10336	-0.5620307	4.45239221	6.55419295	0.01870703	0.16869733
Zranb2	0.30999162	8.01362559	6.54744052	0.01876014	0.16904904
Fam214a	-0.4213069	5.23806352	6.54195472	0.01880342	0.16927736
Mkrn1	0.32149935	6.7341484	6.53931487	0.01882429	0.16927736
Skap2	-0.4739197	4.03246123	6.53713844	0.01884151	0.16927736

Gpr85	0.50540589	3.9713865	6.53707414	0.01884202	0.16927736
Mansc1	0.56807146	4.31736911	6.53290091	0.0188751	0.16944739
B3galt5	-0.784241	3.22309558	6.53090852	0.01889091	0.16946233
Ptgr1	0.90539236	2.92611121	6.51330645	0.01903127	0.17059362
Ormdl1	0.78995113	3.70147365	6.51116841	0.0190484	0.17061945
Ubr1	-0.4754543	6.15641405	6.50132074	0.01912751	0.17120004
Smco3	-0.6213573	2.69433529	6.49607216	0.01916983	0.17145066
Ppp2ca	0.25885877	8.45582872	6.49059312	0.01921411	0.17171851
Grb10	-0.2919681	6.92938384	6.48601334	0.01925122	0.17184861
Mgp	0.78303857	8.28888369	6.48484269	0.01926072	0.17184861
Ncbp1	-0.3956385	4.95939243	6.48298082	0.01927584	0.17184861
Slc14a2	-2.6010033	-0.4003729	6.48172113	0.01928607	0.17184861
Ddx18	0.35721351	5.05537199	6.47687948	0.01932547	0.17204795
Cmtm8	-1.7330596	0.26693661	6.47494565	0.01934123	0.17204795
G530011O06	0.6934685	4.65973078	6.47368103	0.01935154	0.17204795
Cgn	-0.8184014	2.17936625	6.46894522	0.01939022	0.17218193
Sstr4	0.84033544	4.50972522	6.46831619	0.01939537	0.17218193
Mrs2	-0.6296689	3.96128606	6.46529085	0.01942013	0.17227406
AU023762	0.57040822	3.25929013	6.46271567	0.01944124	0.17233364
Cckbr	0.46771084	4.6516168	6.45575275	0.01949843	0.17251819
Rasgrp3	-0.5064614	4.97241042	6.4550018	0.01950461	0.17251819
Cd209c	-0.7114774	2.7033176	6.45492172	0.01950527	0.17251819
Clec11a	-0.6602646	2.21982313	6.45285109	0.01952232	0.17254158
Cct3	0.36289892	5.12571248	6.44090251	0.01962105	0.17292446
Bhlha15	-2.0880699	-0.1500511	6.44076225	0.01962222	0.17292446
Cse1l	0.2979895	6.72638057	6.44024842	0.01962648	0.17292446
Pcdhga1	0.94587344	1.75969594	6.43865668	0.01963967	0.17292446
Tjp2	0.37505758	5.62012645	6.4376411	0.0196481	0.17292446
Lamc3	0.74039134	2.59835371	6.43672757	0.01965568	0.17292446
Spata32	-1.7983142	-0.0808031	6.43422281	0.01967649	0.17292446
Mef2d	0.32876181	7.08971438	6.43349484	0.01968254	0.17292446
Gpr68	0.51536114	3.49668745	6.43102646	0.01970308	0.17292446
Nos1	0.67782138	3.24246223	6.43019067	0.01971004	0.17292446
Acat2	0.39887964	5.25145575	6.4264529	0.0197412	0.17307103
Ntsr1	1.06100843	1.74796493	6.42444554	0.01975796	0.17309122
Tmem30a	0.325064	7.94358369	6.42019939	0.01979345	0.17327543
Orai2	-0.544372	3.83792651	6.41758318	0.01981536	0.17334049
Fmnl2	0.34469005	8.09144302	6.41513371	0.01983589	0.17339347
Itpr3	-0.8339668	2.12556191	6.4103803	0.01987581	0.17360024
Ccnf	1.4469792	0.81005706	6.40886692	0.01988854	0.17360024
Slc35b1	-0.47186	3.70442567	6.40393138	0.01993011	0.17383518
Lats2	-0.4046991	6.82253489	6.40222752	0.01994449	0.17383518
Ptpn7	-0.684307	3.28926987	6.4004705	0.01995932	0.17383796

Yipf2	1.00358064	1.33348163	6.39792785	0.01998081	0.17389867
Gapdh	0.29998002	9.52446363	6.39275907	0.02002458	0.17406283
Zfp940	-0.5889858	3.31935635	6.39226787	0.02002874	0.17406283
Herc2	-0.5584886	7.52920546	6.39040172	0.02004458	0.1740741
Pgbd5	0.3248032	7.53473833	6.38450046	0.02009473	0.17417305
Rinl	-1.0472228	1.37320554	6.38400107	0.02009898	0.17417305
Fzd9	-2.5053046	-0.7379368	6.38392851	0.0200996	0.17417305
Igsf9	2.22026826	-0.311805	6.37444195	0.02018055	0.17469607
E030025P04I	-3.7115849	-1.4199404	6.37343851	0.02018913	0.17469607
Cntln	-0.3227692	6.57104022	6.36488293	0.02026249	0.17520421
Mrps25	0.34661523	4.61514581	6.36310377	0.02027778	0.17520993
Fip1l1	0.27992835	6.50639175	6.35594011	0.02033948	0.175341
Ttc23	0.71739655	2.80822105	6.35481762	0.02034917	0.175341
Fam228a	-1.4103085	1.31402514	6.35388268	0.02035724	0.175341
Arl4d	-0.8656615	2.09657324	6.35293133	0.02036546	0.175341
Prpf8	-0.3873932	8.26146319	6.35252301	0.02036899	0.175341
Zc3h15	0.25736608	7.05509728	6.35092967	0.02038276	0.175341
Sqstm1	0.67033678	8.74120663	6.34761681	0.02041144	0.175341
Pycr1	1.69722817	0.14120247	6.3463067	0.02042279	0.175341
Mpl	-1.4976531	1.52011049	6.34310493	0.02045056	0.175341
Zfp788	-0.2955684	6.23732821	6.34305505	0.020451	0.175341
Rgs17	0.3918505	8.03310869	6.34122051	0.02046693	0.175341
Zbtb39	-0.4280834	4.42513232	6.34102289	0.02046865	0.175341
Angel1	-1.1242952	1.42903695	6.33718668	0.02050201	0.17550128
Arl5c	2.19826166	-0.6989785	6.3284359	0.02057835	0.17602893
Fam173b	1.03690267	0.83055761	6.32342534	0.0206222	0.17627815
Srrm1	-0.2485151	7.56337242	6.31999638	0.02065228	0.17633964
Rapgef3	-0.6014713	2.66865458	6.31780981	0.02067148	0.17633964
Ap1s2	0.36945649	6.31247399	6.31384539	0.02070634	0.17633964
Cirh1a	0.36091314	4.83870112	6.31009137	0.02073942	0.17633964
Sdk1	0.61948812	4.14071295	6.30299404	0.02080212	0.17633964
Ctla2b	2.54119795	-0.6607534	6.30177349	0.02081292	0.17633964
2900092D14I	-0.5040206	5.46346693	6.30049362	0.02082426	0.17633964
Apol9a	2.64460997	-1.0042618	6.29968847	0.02083139	0.17633964
Nuak1	-0.4320904	5.19287171	6.29849838	0.02084194	0.17633964
Lcorl	-0.3794536	5.13712707	6.29802538	0.02084614	0.17633964
Spry1	0.66532339	3.19488316	6.29785106	0.02084768	0.17633964
Zc4h2	0.39538072	4.71566266	6.2958924	0.02086506	0.17633964
Ppp1r15b	-0.3121838	5.41122845	6.2934526	0.02088674	0.17633964
Prl	5.05921857	1.48097504	6.41497771	0.02088875	0.17633964
Esyt3	0.79099675	2.46429092	6.29276913	0.02089282	0.17633964
Cyp4a12a	-1.5781439	0.33010548	6.29214989	0.02089832	0.17633964
Minos1	0.39101067	6.48496504	6.29211324	0.02089865	0.17633964

Hemk1	-0.9080341	1.69722115	6.28782899	0.0209368	0.17633964
Ifitm2	0.7932629	7.3431538	6.28675832	0.02094634	0.17633964
Arl4c	-0.4557965	5.17834358	6.28623998	0.02095097	0.17633964
Khdrbs2	-0.7763399	1.68183347	6.28447015	0.02096676	0.17633964
Tnnc1	-1.0247689	1.94600666	6.28327093	0.02097747	0.17633964
Sema4a	0.62769145	3.73889398	6.28132248	0.02099488	0.17633964
Rassf2	-0.4800635	7.49587503	6.27945571	0.02101158	0.17633964
Prlr	-0.6249373	2.37048023	6.2774351	0.02102967	0.17633964
Sema3f	-1.3259293	0.80992883	6.27517096	0.02104996	0.17633964
Tmem109	0.47477473	3.48127732	6.27080087	0.02108919	0.17633964
Sc5d	0.43851213	4.4301661	6.27033097	0.02109342	0.17633964
Gm5607	-0.684292	3.8160498	6.26751694	0.02111873	0.17633964
Mog	-0.7628267	2.85453928	6.26664252	0.0211266	0.17633964
Gart	-0.4820111	4.37755038	6.26617586	0.0211308	0.17633964
Tctn3	-0.9949501	2.73399384	6.26577703	0.02113439	0.17633964
Fancd2	0.95408394	1.26540415	6.26262043	0.02116285	0.17633964
Pprc1	-0.5589357	3.91972088	6.26144611	0.02117345	0.17633964
Rab7	0.37024646	10.3187668	6.26133305	0.02117447	0.17633964
Agfg2	0.47534301	3.6596711	6.26059173	0.02118116	0.17633964
Lrrc8a	0.31464254	6.15744372	6.26021393	0.02118457	0.17633964
9130008F23I	-1.967645	-0.5012529	6.2597306	0.02118894	0.17633964
Hspg2	0.47637016	3.48405132	6.25029553	0.02127437	0.17692765
Actn2	-0.7061618	2.19011377	6.24514825	0.02132114	0.17719356
Akap8	-0.482467	5.82462808	6.23930899	0.02137433	0.17737304
Cntnap5b	-0.5108667	4.15925846	6.23737914	0.02139195	0.17737304
Sorcs3	0.66980735	4.18264428	6.23690472	0.02139628	0.17737304
Ranbp17	-0.5755311	3.12490601	6.23628105	0.02140198	0.17737304
Slain1os	-1.5733584	1.00212883	6.22883554	0.02147012	0.17758233
Camkk2	-0.4775117	6.00634976	6.22861152	0.02147218	0.17758233
Fam168a	0.25006625	8.13100082	6.22757316	0.0214817	0.17758233
Mcm3	-0.9432836	0.97874568	6.22704532	0.02148654	0.17758233
Shc2	-0.4914777	4.57055209	6.2212869	0.02153946	0.17779021
Lcp1	-0.5160698	4.76173276	6.22107748	0.02154139	0.17779021
Zfand1	-0.5588975	4.29971391	6.21356985	0.02161061	0.17810571
Rundc3b	0.44482282	4.81308097	6.21043548	0.02163958	0.17810571
Slc44a1	-0.3077851	5.88927923	6.20997576	0.02164383	0.17810571
Sptan1	-0.7075466	9.21986903	6.20623251	0.0216785	0.17810571
Ascl2	2.82843079	-0.7872353	6.20600525	0.02168061	0.17810571
Bag2	-0.7705956	3.17330684	6.20454673	0.02169413	0.17810571
Pkhd1l1	-3.1051532	-0.6430587	6.20349913	0.02170385	0.17810571
Nubp2	0.60869825	3.47215544	6.20275014	0.0217108	0.17810571
Chrdl1	0.72531798	2.68327976	6.20246337	0.02171347	0.17810571
Hbs1l	-0.3257779	5.48903497	6.1959692	0.02177387	0.17847889

Ythdf3	0.28210766	7.18928412	6.19385493	0.02179357	0.17850527
Car3	1.38158092	1.3697851	6.19242639	0.02180689	0.17850527
Scoc	0.34989773	8.74565605	6.18839275	0.02184457	0.17865254
Hps5	-0.5897862	3.60626407	6.18700796	0.02185752	0.17865254
Oprk1	-0.8210404	3.81311892	6.18453532	0.02188066	0.17865254
Nsl1	-0.7273746	4.58000397	6.18411975	0.02188456	0.17865254
Vmn2r18	-4.322011	-1.8672575	6.18041416	0.0219193	0.1788143
Srsf10	0.31096764	6.59195119	6.17758021	0.02194592	0.17890954
Acot13	0.38449273	5.31541963	6.17381593	0.02198133	0.17907629
Rad54l2	-0.2962483	5.63481962	6.1696129	0.02202094	0.17927704
Nup155	-0.4759872	5.24826961	6.16597919	0.02205525	0.17931693
Serpinb9b	-2.6365264	-0.7571018	6.16592246	0.02205578	0.17931693
Pdcd4	0.27396673	7.48491951	6.14492593	0.02225522	0.18065545
Gm12070	0.32833847	10.3711141	6.14392249	0.02226481	0.18065545
Nol3	0.5956057	2.90621969	6.14383148	0.02226568	0.18065545
Olfm4	3.6011644	-2.1604963	6.13866916	0.02231505	0.18093278
Tax1bp1	0.27637736	8.62016464	6.13710058	0.02233007	0.18093278
Smpd1	0.51539808	4.97641497	6.13526686	0.02234765	0.1809528
Klf15	-0.5836814	4.25353879	6.13367654	0.02236291	0.18095401
Slc25a22	0.37769979	4.94675892	6.12487175	0.02244761	0.18143202
Dnajc16	0.61939749	3.07893277	6.12312907	0.02246442	0.18143202
T2	-1.6386534	-0.1876592	6.12281595	0.02246744	0.18143202
Pvrl1	0.39618356	4.31616693	6.11885084	0.02250573	0.18161882
Ndufb6	0.43562706	5.63196151	6.10993408	0.02259213	0.18219322
Gpr116	-0.9704098	4.84174366	6.10695819	0.02262104	0.18226221
Tctn1	-1.2842501	2.90632425	6.10592206	0.02263112	0.18226221
Slc9b2	0.62543881	4.25861711	6.101699	0.02267225	0.1823663
Necap1	0.28727229	7.2058062	6.10135891	0.02267556	0.1823663
4930512B01	-1.3516214	0.91539927	6.09990642	0.02268973	0.1823663
Hectd1	-0.4024138	7.4322099	6.09801291	0.02270821	0.18239245
Inf2	0.33333493	6.07676718	6.09614335	0.02272648	0.18241682
Ivns1abp	0.3285005	7.54592213	6.09311373	0.02275611	0.18253235
Cenpo	-0.9395981	2.19298701	6.08510668	0.02283465	0.18300036
2010111I01R	-0.427374	4.66778239	6.08405118	0.02284502	0.18300036
Prom2	3.82879104	-1.9620922	6.0743014	0.02294111	0.1835493
Naip5	-0.9687146	1.51567196	6.07398826	0.0229442	0.1835493
Tmx4	0.34387805	8.3392014	6.06302511	0.02305281	0.18421691
Clec3b	-1.2671306	0.90492489	6.06246027	0.02305842	0.18421691
Ifi204	1.73057038	-0.3667039	6.05776153	0.02310516	0.18437745
Il12rb2	-0.9887106	2.58358439	6.05253105	0.02315731	0.18437745
Map2k5	-0.5599176	3.60652918	6.05033341	0.02317926	0.18437745
Gm20753	3.18322697	-1.593677	6.04856457	0.02319695	0.18437745
Lekr1	-0.7080099	2.74701292	6.04852381	0.02319736	0.18437745

Amy1	-0.4327701	4.31067599	6.04801157	0.02320248	0.18437745
Nacad	0.5801317	3.25361314	6.04736532	0.02320895	0.18437745
Naa50	0.28243141	7.71159215	6.04735869	0.02320901	0.18437745
Ccdc134	-0.9493062	0.62097452	6.0462873	0.02321974	0.18437745
Dag1	0.44045061	6.67986052	6.04373237	0.02324534	0.18437745
Gpc1	0.60560943	3.38315005	6.04284053	0.02325428	0.18437745
Casp9	-0.4125132	4.67728021	6.03901207	0.02329272	0.18437745
L3mbtl2	-0.4743718	3.73725995	6.03752357	0.02330769	0.18437745
9630013A20I	-0.9577868	1.48920394	6.03683526	0.02331461	0.18437745
Islr	0.79661828	6.83435633	6.03627925	0.0233202	0.18437745
Ercc1	0.76715539	2.21367991	6.03581734	0.02332485	0.18437745
Kpna3	0.32202539	7.16999286	6.02709888	0.02341279	0.18494338
Stxbp3a	-0.3407871	4.61286465	6.02440166	0.02344007	0.18494338
Fads2	0.68888671	3.20624724	6.02340192	0.02345019	0.18494338
Afap1l1	-0.4787343	5.54664804	6.02260914	0.02345822	0.18494338
Trim11	-0.5846239	2.75592502	6.01636289	0.02352159	0.18525445
Uqcrq	0.4089465	4.16383216	6.01555717	0.02352978	0.18525445
Ccp110	-0.3857986	5.8898063	6.0141501	0.02354408	0.18525445
Casp6	0.72011482	2.37330272	6.00806412	0.02360608	0.1856203
C130021I20R	1.38050457	1.23443208	6.00351588	0.02365253	0.18580073
Pacrg	0.69042522	2.80278156	6.00277994	0.02366005	0.18580073
Adcy10	-1.3763866	0.20138722	5.9977391	0.02371167	0.18608408
Fnip1	-0.4161208	5.8156585	5.99432678	0.02374669	0.18623683
Zmiz1	0.31287979	8.34258388	5.98829163	0.02380876	0.18653782
Adamts12	0.79389997	2.48891366	5.98742334	0.02381771	0.18653782
Klhdc4	-0.5802776	3.05653402	5.98523627	0.02384026	0.18653782
Eif2s2	0.27752193	7.44919119	5.98454649	0.02384737	0.18653782
Pim2	0.38533966	4.10662835	5.97734232	0.02392184	0.1869982
Pcdhb7	-0.9285924	2.24062595	5.96007143	0.02410145	0.18803706
Cenpk	1.25062239	1.17615186	5.95895834	0.02411307	0.18803706
Derl2	0.47891968	3.56033296	5.95635763	0.02414026	0.18803706
Rabgap1l	0.34935284	8.19784169	5.95632226	0.02414063	0.18803706
Abcb9	-0.9530209	1.61859793	5.95563953	0.02414778	0.18803706
Fam122b	0.42484463	4.12560287	5.95544583	0.02414981	0.18803706
Bex4	-0.5533674	4.0910986	5.9538885	0.02416611	0.18803706
Trappc9	-0.4956432	5.74787062	5.95054196	0.02420119	0.18803706
Rab32	-0.7194482	3.17081687	5.94984295	0.02420853	0.18803706
Dear1	-1.3230114	1.65996176	5.94486281	0.02426086	0.18803706
Zfp865	0.41932365	4.44992551	5.94435963	0.02426616	0.18803706
Pdk3	0.33620384	5.03255319	5.94427783	0.02426702	0.18803706
Kcna6	0.43441634	5.75091568	5.94390127	0.02427098	0.18803706
Abat	-0.3617285	6.69238941	5.94356096	0.02427456	0.18803706
Oscar	1.83396501	0.10059127	5.94069433	0.02430476	0.1881493

Psmd12	0.27944605	6.71043969	5.93729626	0.02434061	0.18830512
Nme6	-0.8581597	1.77490388	5.92548542	0.0244657	0.18907814
Ubxn4	0.26327938	7.28314811	5.92455053	0.02447563	0.18907814
Rrp1	0.35384877	7.75798968	5.92339569	0.0244879	0.18907814
Minpp1	0.39752782	4.91685676	5.92136636	0.02450949	0.18912288
Ntrk3	0.45601865	5.67850937	5.91671676	0.02455903	0.18938311
Dnah6	-1.5000023	0.7087383	5.91014759	0.02462921	0.18949805
Grb7	1.17539163	0.847069	5.90916021	0.02463978	0.18949805
Ddost	0.40195446	4.27040541	5.90639429	0.02466941	0.18949805
Rhpn1	-0.9806019	0.76521571	5.90546676	0.02467936	0.18949805
Tox	0.45668191	5.74876714	5.90503502	0.02468399	0.18949805
Pvrl3	-0.3785463	5.40676394	5.90496099	0.02468478	0.18949805
Col1a2	0.5456188	7.37951761	5.9037394	0.02469789	0.18949805
Elmo2	0.3177004	5.92396524	5.90318491	0.02470385	0.18949805
Tmem263	0.33982435	7.21113596	5.90166624	0.02472016	0.18949805
Canx	0.34094537	7.9635512	5.90033752	0.02473444	0.18949805
Mfap3l	0.46650097	5.63323855	5.89907763	0.02474799	0.18949805
Comt	0.32459734	5.68667028	5.8944688	0.02479763	0.18975685
Phyh	0.30534741	7.28550816	5.8926265	0.02481751	0.18978767
Rmi2	-0.7227038	2.19828046	5.88957732	0.02485044	0.18984665
Kcnip1	0.56265525	4.52694561	5.8888642	0.02485815	0.18984665
Ampd3	0.36580371	5.59985315	5.88444039	0.02490604	0.18984665
Zfp526	-0.6007893	3.07793303	5.88329428	0.02491846	0.18984665
Rrs1	0.34244388	4.79496528	5.88218538	0.02493049	0.18984665
Twsg1	0.5070963	7.87788273	5.88145461	0.02493842	0.18984665
Dusp26	-0.3237763	6.07584242	5.88072307	0.02494636	0.18984665
Cyp27a1	-0.950469	1.73152539	5.87966827	0.02495782	0.18984665
Lrrc34	-2.917432	-1.0279857	5.87874033	0.0249679	0.18984665
Gmfb	0.2947601	8.2222009	5.87440689	0.02501504	0.19005212
Saal1	-0.7677176	2.24950395	5.87334006	0.02502666	0.19005212
Chrna2	-2.0079761	-0.6586616	5.86600505	0.02510672	0.19053931
Mios	-0.4329399	4.46628771	5.85968267	0.02517597	0.1909438
Col25a1	0.43585465	5.59930305	5.85761541	0.02519865	0.19099491
Ccdc169	3.17125905	-2.2268421	5.85139289	0.02526708	0.19127593
Jag1	0.54038188	3.72956607	5.85133914	0.02526767	0.19127593
Erf	-0.6099788	3.33277045	5.84430777	0.02534525	0.19172478
Slc16a13	0.67473451	2.78603537	5.84306554	0.02535899	0.19172478
Larp1b	0.50596406	4.07434066	5.83843176	0.02541029	0.19180894
Hnmt	-0.4370081	5.1539492	5.83578975	0.02543959	0.19180894
Gcdh	-0.5290358	3.16647822	5.83565255	0.02544111	0.19180894
Gm14420	-0.3887553	5.40322491	5.8350919	0.02544734	0.19180894
Sox18	-0.9171457	1.74663565	5.83474965	0.02545114	0.19180894
Klhl5	-0.3619135	5.72844719	5.83123492	0.0254902	0.19180894

Cpm	0.53314196	4.42576325	5.830653	0.02549667	0.19180894
Mrps28	0.5904615	2.96171414	5.83051151	0.02549825	0.19180894
Zfp445	-0.3874312	6.82886788	5.8267301	0.02554037	0.19197266
Aldh1l2	-0.9817338	2.81231289	5.82568008	0.02555207	0.19197266
Abcd2	-0.5162859	4.77158187	5.82298551	0.02558215	0.19207811
Map2k3	-0.6037075	3.88687435	5.82060395	0.02560876	0.19215747
Slc30a9	-0.31218	6.51316511	5.81494116	0.02567217	0.19241475
Lrrc57	0.36482175	5.83911175	5.81341997	0.02568924	0.19241475
Arr3	-3.1148282	-1.161148	5.81250641	0.02569949	0.19241475
Pcdhb6	-1.6221036	0.85978822	5.81169239	0.02570863	0.19241475
Spats2	0.51042331	3.28971686	5.81006486	0.02572692	0.19241475
P2ry14	-0.9557535	1.47512137	5.80636899	0.0257685	0.19241475
Pcolce	0.67295949	4.76115866	5.80547186	0.0257786	0.19241475
Lsm2	0.65956269	4.23845865	5.80490487	0.02578499	0.19241475
Plekha5	-0.3877374	5.42071835	5.80357817	0.02579994	0.19241475
Gnptab	0.37922623	6.15644286	5.80239635	0.02581327	0.19241475
Marcks	0.38756083	9.44935108	5.80164134	0.02582179	0.19241475
Thy1	0.30633833	7.45923875	5.80039568	0.02583586	0.19241475
Cables1	-0.5996005	3.13166928	5.79441295	0.02590352	0.1927988
Nup107	-0.5199841	3.78393651	5.79056511	0.02594715	0.19300356
Gpr155	0.41105552	5.73513975	5.78540904	0.02600574	0.19321619
Ankrd50	-0.3582435	5.18671778	5.78386129	0.02602335	0.19321619
Prkch	-0.6857022	2.40953756	5.78194159	0.02604522	0.19321619
Dab2ip	-0.3046184	6.24974934	5.78148779	0.02605039	0.19321619
Rmi1	-0.3470595	5.67301753	5.78053066	0.02606131	0.19321619
Ccdc107	0.73694586	2.78089312	5.77954606	0.02607254	0.19321619
B230217O12	-0.8540829	1.77765777	5.77086675	0.02617179	0.19383173
Chst11	0.36342404	5.6926055	5.76525194	0.02623622	0.19418466
Gpr98	0.79308009	2.82382447	5.76389032	0.02625187	0.19418466
Lrch3	-0.4174664	4.78819514	5.76007	0.02629584	0.19434462
Cml3	-0.866806	1.66790898	5.75687832	0.02633264	0.19434462
Kdelr1	0.49457516	4.34912127	5.7562273	0.02634015	0.19434462
Six3	-0.6067961	3.04223827	5.75610827	0.02634153	0.19434462
Dlg1	-0.3124339	7.4792636	5.75497297	0.02635464	0.19434462
Pls1	-0.4993721	4.03669189	5.751865	0.02639056	0.19448977
Wls	0.5068577	5.22275416	5.74877685	0.02642631	0.19463346
Nme1	0.32063421	6.28486745	5.74484356	0.02647192	0.19484956
Ube2h	0.27629138	7.70872382	5.74154347	0.02651026	0.19496966
Srsf12	-0.5001664	4.02511157	5.74022027	0.02652565	0.19496966
Nfatc2	-0.5706567	3.35874337	5.7363194	0.02657107	0.19496966
Ptgs1	0.56312908	3.44608602	5.73315186	0.02660803	0.19496966
Tmem100	0.45658218	3.5969053	5.7320805	0.02662054	0.19496966
Cstf2	0.40949773	6.37257671	5.73178368	0.02662401	0.19496966

Adcyap1r1	0.42768375	6.44811207	5.73005478	0.02664421	0.19496966
Cyfip2	-0.4160245	9.89947236	5.72989683	0.02664606	0.19496966
Pop4	0.53427197	4.0052	5.72834405	0.02666422	0.19496966
S100pbp	-0.4028506	5.02257202	5.72704417	0.02667944	0.19496966
Map3k6	0.73923124	1.76105039	5.72610629	0.02669043	0.19496966
Trappc12	0.33727572	5.73215981	5.72599305	0.02669175	0.19496966
Mdm4	-0.2763933	6.15415626	5.72154244	0.02674396	0.19496966
Hsd17b2	1.42156999	1.4829574	5.72002791	0.02676175	0.19496966
Nup188	0.606925	3.73342481	5.71847097	0.02678005	0.19496966
Ocm	-1.894246	-0.9293338	5.7176194	0.02679007	0.19496966
2010012O05	0.3326697	6.00612599	5.7175033	0.02679143	0.19496966
Atp10b	-1.1264767	1.16803816	5.71588309	0.0268105	0.19496966
Mut	0.26293306	5.76690923	5.71585773	0.0268108	0.19496966
9330175M2(	-1.6084598	1.18172473	5.7141507	0.02683091	0.19496966
Pnpo	0.5207179	3.78994723	5.71359446	0.02683747	0.19496966
Tubb6	0.65623691	3.92177905	5.71197894	0.02685652	0.19496966
Kdelr2	0.58113099	5.18839106	5.71145647	0.02686269	0.19496966
Sdccag8	-0.3280712	6.24734401	5.70756629	0.02690864	0.19518489
Oas1a	-1.5841935	0.68271435	5.70127927	0.02698309	0.19556329
Vps18	-0.5335606	3.64210523	5.70040485	0.02699347	0.19556329
Hgf	0.90492804	2.0473698	5.69458624	0.02706261	0.19594568
Fbxl14	-0.3939318	5.24253951	5.69111839	0.02710391	0.19600942
Adck2	-0.6842774	2.23540847	5.69052958	0.02711093	0.19600942
Syne2	-0.3467447	5.49056442	5.68972632	0.02712051	0.19600942
BC029722	0.90150906	2.07928093	5.68492276	0.02717788	0.196096
Kremen1	0.52006423	3.34457656	5.6830804	0.02719992	0.196096
Frmpd1os	-3.324281	-1.7054424	5.68298451	0.02720107	0.196096
Elovl4	0.51876227	4.81666935	5.68269657	0.02720452	0.196096
Xrcc6bp1	1.00767163	1.74874407	5.68187402	0.02721437	0.196096
Pcnt	-0.4175467	5.11387486	5.67647862	0.02727907	0.19630559
Hnf1b	2.59309384	-0.7988781	5.67584507	0.02728667	0.19630559
Gm5148	-0.4970319	3.95471613	5.67314174	0.02731917	0.19630559
Mir690	-3.4104085	-2.00979	5.67309993	0.02731967	0.19630559
Trp53inp2	0.25183391	7.84324072	5.67262257	0.02732541	0.19630559
Gfod1	-0.3263473	6.81594251	5.66974445	0.02736006	0.19643668
B3galt1	0.28235944	5.80671302	5.65966842	0.02748176	0.19719223
Amer1	-0.4616305	4.17907539	5.65457215	0.02754355	0.19738519
Cdh18	0.64340487	3.95230194	5.653631	0.02755498	0.19738519
Vnn1	-1.1717821	1.71551037	5.65108239	0.02758595	0.19738519
Nefm	-0.5001246	9.24354979	5.65071392	0.02759043	0.19738519
Polr2b	-0.3045343	6.23667482	5.64995611	0.02759965	0.19738519
Calm2	0.24858756	12.25381	5.64874413	0.0276144	0.19738519
Plekhm2	-0.6007699	3.60509107	5.64795324	0.02762403	0.19738519

Noxred1	2.91419938	-0.8251038	5.64545288	0.0276545	0.19748509
Il31ra	0.73094586	2.40872718	5.64285576	0.02768619	0.19759357
Gm16617	-2.9068373	-0.99522	5.63549149	0.02777628	0.19793453
Rbak	-0.7205876	3.25545728	5.63537341	0.02777772	0.19793453
Spink10	-2.3516698	-0.9899243	5.63380084	0.027797	0.19793453
Ap4e1	-0.5390873	4.05886757	5.63280496	0.02780922	0.19793453
Rrbp1	0.36961888	6.08677972	5.63116443	0.02782936	0.19793453
Gm1123	-3.244483	-2.3133754	5.6297386	0.02784688	0.19793453
Slc35f2	-1.897685	0.09581704	5.6257282	0.02789622	0.19793453
Dph1	-2.8048036	-1.3555422	5.62361175	0.02792229	0.19793453
Ddx42	-0.3403364	6.02745186	5.62242404	0.02793694	0.19793453
Micall2	1.75343706	0.41934992	5.62195115	0.02794277	0.19793453
Pdxdc1	-0.2760988	6.24525789	5.62108333	0.02795348	0.19793453
Gm9199	1.62134066	-0.4350192	5.62008617	0.02796579	0.19793453
Slc29a4	0.96078013	1.10904848	5.61972179	0.02797029	0.19793453
Gm20125	-3.9316378	-1.3196063	5.61888206	0.02798067	0.19793453
Dot1l	-0.6407923	3.89011164	5.61878354	0.02798189	0.19793453
Ndufa8	0.35036266	5.2594952	5.61269195	0.02805728	0.19835067
4933424G05	-1.3805987	1.07474415	5.6067252	0.02813135	0.19870014
Tgfb2	0.43099075	4.28430239	5.60594034	0.02814111	0.19870014
Rbbp9	0.42419306	6.4796997	5.60336076	0.02817321	0.19870014
Ikbkb	-0.4817605	4.30039297	5.60290116	0.02817893	0.19870014
Zfp92	-0.8653378	2.40848391	5.60203914	0.02818967	0.19870014
Apc2	-0.5804366	4.60083919	5.59831657	0.0282361	0.19891034
Steap2	0.33744238	4.92439914	5.59408979	0.02828893	0.19905303
Ergic3	0.50103553	5.24047087	5.59382252	0.02829227	0.19905303
Dpf1	0.72357478	2.26390189	5.59183483	0.02831715	0.19905303
Gnb2l1	-0.3622104	5.840013	5.59138059	0.02832284	0.19905303
St3gal1	0.36221367	5.20677659	5.58761325	0.02837008	0.19916249
Pde2a	0.43150944	6.77089871	5.58748611	0.02837168	0.19916249
Cd3g	-3.3543824	-1.3715452	5.58322024	0.02842528	0.19942187
Romo1	0.70100484	4.01738062	5.57483749	0.02853095	0.1995774
Rtn1	0.2692906	10.4495925	5.57385082	0.02854341	0.1995774
Chd2	-0.318702	6.94922404	5.5733359	0.02854992	0.1995774
Parp14	-0.601342	4.15536348	5.57042519	0.02858674	0.1995774
Gtpbp3	-0.4972108	3.02973878	5.56822059	0.02861467	0.1995774
Gyk	-0.6743217	4.36426834	5.56693263	0.02863099	0.1995774
Rnf39	0.8204802	1.8296328	5.56662883	0.02863485	0.1995774
Flad1	0.78068399	2.04431352	5.56587638	0.02864439	0.1995774
F930015N05	-0.543001	3.60806078	5.56498081	0.02865576	0.1995774
Setd8	0.34615262	7.28392501	5.56406435	0.02866739	0.1995774
Pou2f3	1.51011991	1.17603717	5.56332724	0.02867676	0.1995774
Acot1	0.40060588	3.95154939	5.56302035	0.02868066	0.1995774

Prr3	0.37960238	4.200943	5.56137242	0.0287016	0.1995774
Fam134a	0.3362958	5.4206259	5.56095884	0.02870686	0.1995774
Vim	0.46216768	8.07016048	5.56033667	0.02871478	0.1995774
Ccdc108	-1.1219253	1.32172893	5.55941583	0.0287265	0.1995774
Gm527	-0.8382947	2.04063156	5.55868709	0.02873578	0.1995774
Bend3	-0.5516562	4.24324062	5.55777271	0.02874742	0.1995774
Rab26os	-1.2293672	0.98218832	5.55453365	0.02878872	0.19974832
Usp49	-0.5011936	3.96459954	5.55177717	0.02882392	0.19983359
Dcn	0.62269398	7.6761973	5.55095844	0.02883438	0.19983359
Endov	-0.4109722	4.5500775	5.53967296	0.02897906	0.20072008
Hspa8	0.261257	10.7212684	5.53496115	0.0290397	0.20094376
Gnai1	0.28318692	9.05039895	5.53443361	0.0290465	0.20094376
Gadd45a	0.49118653	4.16244323	5.53325574	0.02906169	0.20094376
Prss23	0.54381149	3.46917784	5.52545505	0.0291625	0.20136907
Kcnh3	-0.4646006	3.65536925	5.52496193	0.02916888	0.20136907
4930486L24I	3.91334253	-2.2845267	5.52459408	0.02917365	0.20136907
Tbl3	0.76178259	2.07913153	5.51989779	0.02923455	0.20141866
Clec7a	-2.1950877	0.80824858	5.5192605	0.02924283	0.20141866
Camkv	-0.381318	6.00284055	5.51866927	0.02925051	0.20141866
Pfn1	0.52933889	6.68741467	5.51864829	0.02925078	0.20141866
Cd37	2.04920558	-0.4761001	5.51750826	0.02926559	0.20141866
Zfp772	0.46654282	4.258458	5.51498657	0.0292984	0.20141866
Kdr	-0.5732192	2.63592826	5.51497376	0.02929856	0.20141866
Npy5r	1.23220283	0.98837721	5.51106775	0.02934945	0.20163838
Rhobtb3	-0.449476	4.45855426	5.50993743	0.0293642	0.20163838
Unc13a	-0.6432207	6.96026908	5.50848091	0.02938321	0.20165332
1700025F24I	-3.6406959	-2.177585	5.50401716	0.02944157	0.20193808
Sult5a1	-1.9277188	-0.535735	5.49840245	0.02951515	0.20214069
4931440P22I	2.28876939	-1.4315636	5.49805153	0.02951976	0.20214069
BC004004	0.52065408	5.27753471	5.49584252	0.02954878	0.20214069
6430573F11I	-0.6431683	2.57922385	5.49546041	0.0295538	0.20214069
Surf4	0.44448091	4.57231606	5.49435289	0.02956836	0.20214069
Mgat4b	-0.5621926	2.87867883	5.49404701	0.02957238	0.20214069
Mamdc4	-1.9910162	-0.0726255	5.48923772	0.02963572	0.20231428
Dock8	-0.7032526	2.85605871	5.48808076	0.02965098	0.20231428
Gm5065	-2.5951884	-1.8556745	5.48599089	0.02967856	0.20231428
Tbcel	0.40167604	5.2480881	5.4845966	0.02969698	0.20231428
Gm1045	3.72904045	-1.1786568	5.4831907	0.02971557	0.20231428
Hs3st4	-0.3766379	5.45313509	5.48307758	0.02971707	0.20231428
Srpk3	1.42288756	0.31649771	5.48299388	0.02971817	0.20231428
Tmem43	0.64223848	3.88559186	5.48187893	0.02973293	0.20231428
Pcdh20	-0.6453482	3.064212	5.47899134	0.02977117	0.20239685
Hps1	-0.9801536	1.40435611	5.47737039	0.02979266	0.20239685

5430402O13	-1.8696987	-0.5565172	5.47525888	0.02982068	0.20239685
Cbfb	-0.4298372	6.03301844	5.47155456	0.02986992	0.20239685
Cpne2	0.37209995	4.41297001	5.47153661	0.02987016	0.20239685
Gmds	-0.5334122	3.36496846	5.47143522	0.0298715	0.20239685
4921504A21I	0.60804538	3.35380676	5.47115328	0.02987526	0.20239685
AI467606	-2.5375012	-1.3976377	5.47077676	0.02988027	0.20239685
Dhrs4	0.53264759	3.26827748	5.46863862	0.02990874	0.20247518
Mns1	-0.6902859	2.41699082	5.46608072	0.02994284	0.2025915
Iffo2	-0.4064508	4.67135398	5.46130769	0.03000659	0.20280891
Capsl	-1.2283984	1.18901442	5.46031195	0.0300199	0.20280891
Lsm12	0.32010965	5.8603497	5.45987323	0.03002577	0.20280891
Gm4841	0.81723937	2.36667917	5.45731778	0.03005999	0.20292558
Pxn	-0.3760783	5.22610762	5.45116498	0.03014256	0.20293607
Mical3	-0.4969932	6.27076941	5.44960401	0.03016355	0.20293607
Ripk3	-2.4899647	-0.6316331	5.44901533	0.03017147	0.20293607
Med26	0.57968765	2.57431246	5.4474659	0.03019233	0.20293607
Lrrc61	0.35354861	5.11850472	5.44668773	0.03020281	0.20293607
Wdr6	-0.3829722	4.85577563	5.4466288	0.0302036	0.20293607
Htr2a	0.50948534	4.42755385	5.44609451	0.0302108	0.20293607
2610507I01R	-0.3442277	4.73203653	5.4458389	0.03021424	0.20293607
Dmrta1	-0.9127261	3.04450842	5.44525856	0.03022207	0.20293607
2310014L17R	1.66273399	-0.1128098	5.44447125	0.03023268	0.20293607
Stk19	-0.85134	2.00526309	5.44333951	0.03024795	0.20293607
Tbc1d22a	0.46441843	3.48816352	5.43631425	0.03034291	0.20345046
Adpgk	0.82155533	2.07154623	5.43476001	0.03036397	0.20345046
C2cd4c	0.77542399	3.3817538	5.43390294	0.03037558	0.20345046
Ncs1	0.36773505	6.14444869	5.42961245	0.03043382	0.20372656
Mc5r	-1.5189969	0.66592367	5.42731777	0.03046502	0.20382146
6430584L05R	0.59859513	3.02541422	5.42592629	0.03048395	0.20383427
Alg8	0.45270846	3.33045314	5.41983075	0.03056706	0.20392348
Zcwpw1	1.28889402	0.6780819	5.41980913	0.03056735	0.20392348
Apopt1	-0.3359823	4.12066254	5.41905862	0.0305776	0.20392348
Cdk19	-0.3017421	7.00391856	5.41885084	0.03058044	0.20392348
Slc30a1	0.31814241	5.11534121	5.41849054	0.03058536	0.20392348
Nxt1	0.77444118	2.54312673	5.4172356	0.03060251	0.20392348
Smap1	0.26072736	6.28610141	5.41621382	0.03061649	0.20392348
Sec14l5	-2.7289865	-1.0991668	5.40649815	0.03074972	0.20469705
Mylip	-0.5126976	4.11546844	5.40432017	0.03077968	0.20478264
Ndufc2	0.43160531	5.2850835	5.40181298	0.03081421	0.20489851
Igsf21	0.60916361	3.61207176	5.40014342	0.03083722	0.20493776
Pycr2	-0.6774115	2.68835934	5.39493532	0.03090914	0.2053018
Tmem66	0.35486896	5.98328088	5.3890305	0.03099091	0.20561923
Lrig3	-1.0481276	1.08464464	5.38830502	0.03100098	0.20561923

Spata33	2.23653733	-0.1889386	5.3870186	0.03101883	0.20561923
9330102E08I	-0.516653	4.04629306	5.38566698	0.0310376	0.20561923
Blnk	0.56512972	3.30553498	5.38529421	0.03104278	0.20561923
Baz2b	-0.2637617	6.99648662	5.3837087	0.03106482	0.20565147
Vps13b	-0.5180656	6.99017199	5.38218604	0.031086	0.20565738
Ncald	0.28253254	7.7043195	5.38117634	0.03110006	0.20565738
Ifnlr1	3.43532751	-0.4183534	5.37702154	0.03115797	0.20592664
Siglece	-2.4049349	-0.5955054	5.37278324	0.03121717	0.20620412
Tmem150co:	-2.8005742	-0.5281174	5.36497864	0.03132653	0.20670365
9030624G23	-0.5053271	3.50587459	5.36492221	0.03132732	0.20670365
Islr2	0.62598767	2.79239509	5.36176739	0.03137165	0.20688214
Depdc7	-1.0295755	1.14205109	5.35914665	0.03140852	0.20691464
Rnf14	0.2402859	8.3873547	5.35896152	0.03141113	0.20691464
Klhl34	-0.729306	5.45597322	5.35510346	0.03146551	0.2070707
Zdhhc4	-0.5105692	2.59386182	5.35482767	0.0314694	0.2070707
Prkg1	-0.2978249	5.8376894	5.35134818	0.03151855	0.20728018
P2ry13	0.84200504	2.42328425	5.3462684	0.03159045	0.20753084
Arxes1	0.6873956	2.41449458	5.34620685	0.03159132	0.20753084
Prpf19	0.29602773	7.10692766	5.34437317	0.03161732	0.20758778
Nrg2	-2.0647233	-0.8635565	5.33844799	0.0317015	0.20774176
Atraid	0.39810666	4.54314961	5.33842676	0.03170181	0.20774176
Cdkn2d	0.44307639	4.11351863	5.33556407	0.03174257	0.20774176
Ankrd46	0.29319353	6.77853635	5.33550648	0.03174339	0.20774176
Bid	-0.5820113	2.81864589	5.33546975	0.03174391	0.20774176
Ube2ql1	0.34691645	6.24484934	5.3350065	0.03175052	0.20774176
Cdc42bpa	-0.3227154	9.05411707	5.33418692	0.0317622	0.20774176
Ube2d3	0.29587369	9.02053854	5.33199152	0.03179353	0.20783312
Gm6787	2.84267865	-0.4742893	5.33060566	0.03181332	0.20784905
Gpm6a	0.36946793	9.42964172	5.32691722	0.03186606	0.2080594
Lgals1	0.77252484	4.62330975	5.32592521	0.03188026	0.2080594
Ppp4r2	0.27936372	8.3018626	5.32455196	0.03189993	0.20807439
Fig4	-0.3936896	4.3065797	5.32228006	0.03193251	0.20812774
Vps16	-0.6195757	4.44293353	5.31813511	0.03199204	0.20812774
Mfsd6	0.40755101	7.207852	5.3177484	0.0319976	0.20812774
Usp40	-0.4457794	4.56375978	5.31722013	0.03200519	0.20812774
Arhgap27	-0.602687	3.24383541	5.31616295	0.0320204	0.20812774
Serpina3n	0.86437783	2.68992215	5.31585209	0.03202488	0.20812774
Atp1b1	0.25349261	10.1417314	5.31489676	0.03203863	0.20812774
Aldh3a1	1.86489331	-0.7939376	5.31329859	0.03206166	0.20812774
Gm2762	5.17270049	-1.6727129	5.45664078	0.03207145	0.20812774
Fastkd3	-0.4004934	3.79209498	5.31094768	0.03209556	0.20812774
Tap1	-0.9515959	1.05045136	5.30988455	0.0321109	0.20812774
Kat2a	0.32695978	5.89910999	5.30948561	0.03211666	0.20812774

4632428N05	-0.9048319	2.15941038	5.30628411	0.03216293	0.20831485
Ppp1r9b	-0.3042283	6.66886217	5.30370797	0.03220021	0.2083295
Pabpc5	-0.728749	2.29010092	5.3028025	0.03221333	0.2083295
Sdhaf2	0.35248416	5.20430452	5.30252318	0.03221738	0.2083295
Fam213a	0.4913376	7.66914769	5.30117649	0.0322369	0.20834324
Cdsn	1.04279916	0.95293506	5.29743882	0.03229115	0.20837577
Armc8	0.30379591	7.05120599	5.29688096	0.03229926	0.20837577
Chit1	2.66544329	-1.5464265	5.43922261	0.03231203	0.20837577
Prex1	0.41825573	5.9249427	5.2948438	0.03232888	0.20837577
Klk6	-2.5307052	-1.3802806	5.29484022	0.03232893	0.20837577
Fank1	1.12940391	1.2666404	5.29071323	0.03238904	0.20865086
1600029O15	0.95524094	1.62135196	5.28908718	0.03241275	0.20869138
Zfp619	-0.5398086	2.83341509	5.28760635	0.03243437	0.20871833
Gm867	-3.1115128	-1.991753	5.2857459	0.03246155	0.20878105
Abhd13	0.30866122	5.20449293	5.28064169	0.03253624	0.20911101
Pcdhb3	0.71191983	2.11823708	5.27985525	0.03254777	0.20911101
Haus7	0.8194467	2.08117045	5.27814148	0.03257291	0.20916028
Dnajc13	-0.4265844	6.26013549	5.26850812	0.0327146	0.20951843
6330549D23I	-1.0369906	1.73944601	5.26823276	0.03271866	0.20951843
Ctsa	0.441253	5.4161235	5.2674992	0.03272948	0.20951843
Crhbp	0.86403037	3.05964508	5.26698664	0.03273704	0.20951843
Tmem159	0.56300506	4.62406698	5.26541963	0.03276017	0.20951843
Fbxo42	-0.3207117	5.14758796	5.26527449	0.03276232	0.20951843
Hrasls	-0.3930359	5.07368425	5.26437584	0.03277559	0.20951843
Inhbb	-1.0600909	1.70768754	5.26403839	0.03278058	0.20951843
Ltbp4	-0.3940233	4.59000141	5.26366224	0.03278614	0.20951843
Skida1	0.47981013	4.1254437	5.2608194	0.03282819	0.20967527
Gal3st3	0.37999137	6.01131671	5.25568215	0.03290434	0.21004959
Gm19619	-2.6139031	-1.2366144	5.25421457	0.03292612	0.2100767
Sh3bgrl	-0.3700303	8.852275	5.25023646	0.03298527	0.210342
Ryr3	-0.8088804	5.30691319	5.24749565	0.03302609	0.21049022
Ect2l	-2.7430437	-0.6733218	5.24308105	0.03309196	0.21079784
Mreg	0.7416877	2.40495326	5.24106423	0.0331221	0.21083753
Tmem130	0.45612157	5.16068055	5.24030874	0.0331334	0.21083753
Spin4	0.75694401	2.74782339	5.2377557	0.03317161	0.21089326
Eno2	0.37817471	8.48693367	5.23737094	0.03317738	0.21089326
Cnot10	-0.4501854	4.35910618	5.23551876	0.03320514	0.21090151
Zw10	0.88704603	2.49338319	5.23378649	0.03323113	0.21090151
Ntng1	-0.3896883	5.3774984	5.23376098	0.03323151	0.21090151
Smim18	0.70859364	2.12277284	5.23237543	0.03325231	0.21092176
Adam11	0.59834899	3.57860668	5.22944401	0.03329637	0.21103242
Dbhos	-0.7534214	3.35818867	5.22887053	0.033305	0.21103242
Ptgis	-0.7412174	2.96369474	5.2256557	0.03335341	0.21116058

Ssb	0.2804341	8.09090107	5.22489439	0.03336489	0.21116058
Sema3d	0.64200372	6.49635754	5.22401664	0.03337812	0.21116058
Large	0.35393522	6.42464331	5.21970995	0.03344316	0.21145648
Pak6	-0.4639218	3.74563752	5.21858211	0.03346021	0.21145648
Hsbp1	0.36578328	8.97240026	5.21716835	0.0334816	0.21146831
0610040F04I	-1.3354848	0.96442766	5.21612511	0.0334974	0.21146831
Prkce	0.32197199	8.83402021	5.21339493	0.03353878	0.21156364
Rfc4	-0.7389313	1.9149827	5.21279786	0.03354783	0.21156364
Fbn2	-0.9619051	1.50799282	5.21010713	0.03358868	0.21164964
Prkdc	-0.4924981	5.15821367	5.20957171	0.03359682	0.21164964
Hdac3	-0.4310382	4.7598794	5.20701805	0.03363565	0.21178285
Ap1s1	0.53522663	2.8383921	5.20499748	0.03366641	0.21186514
Dio3os	-3.3611525	-1.726407	5.20119856	0.03372432	0.21211816
Tmem215	0.82997522	3.3389055	5.19890396	0.03375936	0.21222708
Tns4	-1.1069253	1.63779668	5.19478572	0.03382235	0.21251151
Fibin	-0.6100021	4.43120011	5.19134597	0.03387507	0.2127311
St13	0.32574734	6.32199189	5.18844542	0.03391959	0.21282532
Arg1	2.47099629	-0.9245313	5.18781621	0.03392926	0.21282532
AI427809	-1.4570695	1.2099746	5.186897	0.03394338	0.21282532
C130060C02I	-1.4919135	0.02592742	5.18393695	0.03398892	0.21299935
09-Sep	0.46423999	5.57392956	5.18124679	0.03403037	0.21314755
Qprt	0.93250498	1.21650241	5.17993013	0.03405068	0.21316326
Fam196a	-0.4572875	4.27266502	5.17764694	0.03408593	0.21327242
Mirlet7bhg	-2.1456911	0.81513679	5.17648109	0.03410394	0.2132737
Bcl2	-0.3956552	6.6580827	5.17487765	0.03412873	0.21330687
Rassf10	0.66286052	2.070177	5.17383486	0.03414487	0.21330687
Tnfrsf19	0.36453825	5.27041797	5.17239575	0.03416715	0.21333478
Nkx6-1	2.2619308	-0.5665665	5.1621647	0.03432603	0.21412311
Dnmt3aos	-3.2574583	-1.8159098	5.16196311	0.03432916	0.21412311
Cyfip1	-0.3092791	6.22946683	5.15430479	0.03444865	0.21459491
Ezh2	-0.6526518	3.03078086	5.1538932	0.03445508	0.21459491
Zfp112	-0.8570208	2.14004169	5.15367078	0.03445856	0.21459491
4933413J09F	-2.9890662	-1.5657156	5.15060441	0.03450655	0.21478208
C5ar1	2.33446776	-0.0568141	5.14491254	0.03459583	0.21519247
A730020M07	0.55674806	3.94037188	5.14234463	0.0346362	0.21519247
Snx12	0.31621569	7.72627146	5.14198687	0.03464183	0.21519247
B3gnt8	-1.7973456	-0.1537182	5.14127532	0.03465302	0.21519247
Slc6a7	0.41191108	5.20504357	5.14068397	0.03466233	0.21519247
Cox20	0.37431397	5.02108414	5.13691172	0.03472177	0.21544982
Zfp507	-0.3663284	4.73466432	5.13497087	0.0347524	0.2155282
D330045A20	-1.5933809	-0.0948508	5.1258641	0.03489653	0.21631002
Plaa	0.24413808	6.40093827	5.12421855	0.03492264	0.21633737
Xpo4	-0.4261722	4.12366709	5.12331008	0.03493707	0.21633737

Hars	0.26589429	5.89879143	5.12041671	0.03498306	0.21646901
Fam98b	0.35467656	6.91903186	5.11885751	0.03500788	0.21646901
Cyb5b	0.2627633	7.53246763	5.11856359	0.03501256	0.21646901
Hipk1	-0.2572043	8.53399763	5.11701044	0.0350373	0.2165102
1700034G24	1.84576471	-0.0836493	5.11536041	0.0350636	0.21656101
Col26a1	-0.8530004	1.50329749	5.11294131	0.03510221	0.21664101
Lpar3	-1.0123141	2.27219078	5.1122823	0.03511274	0.21664101
Bend6	-0.3245857	6.64558195	5.11093281	0.0351343	0.21666244
Gucy2f	-1.9869521	0.43556857	5.10807045	0.03518009	0.21676842
Dusp28	-0.6172656	3.63802561	5.10759611	0.03518769	0.21676842
Sema4g	0.6807848	2.77395805	5.10597856	0.0352136	0.21681652
Spop	-0.2584918	7.72348367	5.10375274	0.03524929	0.21682751
Tnks1bp1	-0.4920153	3.62382755	5.10360922	0.0352516	0.21682751
Runx3	-1.0925387	0.86058091	5.09734521	0.03535227	0.21693167
Tmem106b	0.2953315	7.56207036	5.09589583	0.03537562	0.21693167
Fadd	0.45493296	4.30094282	5.09442415	0.03539933	0.21693167
Ndc1	0.59591951	2.89070847	5.0935909	0.03541277	0.21693167
Bak1	0.55981984	3.90940784	5.09352241	0.03541388	0.21693167
Adra1b	0.48076284	3.92520741	5.09285395	0.03542466	0.21693167
Msl1	-0.2433448	8.01020206	5.09269427	0.03542724	0.21693167
Tacr2	1.96690169	0.30130535	5.09171423	0.03544306	0.21693167
Cadm1	0.42821071	6.13708656	5.09146188	0.03544713	0.21693167
Snd1	0.30511433	5.02085632	5.08852956	0.03549451	0.21693167
Jpx	-0.6233025	2.7866681	5.08757474	0.03550996	0.21693167
Ctgf	0.43752916	5.68766544	5.0864972	0.0355274	0.21693167
Gprasp1	-0.5382276	8.9720936	5.08644368	0.03552827	0.21693167
Gca	-0.3726844	6.18271984	5.08573501	0.03553974	0.21693167
Zfp458	-0.5858331	4.61657535	5.08473919	0.03555587	0.21693167
Mrps2	0.31953011	4.70560518	5.0840676	0.03556676	0.21693167
Cat	0.35398111	8.12823621	5.08346815	0.03557647	0.21693167
Hsdl2	0.37086966	6.17222768	5.07894547	0.03564989	0.21726871
Trhr2	-1.4700248	0.03532587	5.07643455	0.03569072	0.21740694
Rpl12	-0.3327253	6.78598507	5.07343107	0.03573964	0.2174922
Bsg	0.60091389	7.36556229	5.07334487	0.03574104	0.2174922
Smarcad1	-0.4136449	5.2613754	5.07180616	0.03576613	0.21753434
Jak3	1.55733725	-0.2724256	5.06979684	0.03579893	0.21762327
Tlr2	2.76283305	-1.1053509	5.06811507	0.0358264	0.21767978
Sec22b	0.27965685	7.09426359	5.0653335	0.03587189	0.21784567
Fam229b	-0.601326	2.47483404	5.06295574	0.03591083	0.21797162
Mark3	0.25658471	6.40504925	5.05898756	0.03597593	0.21820129
5031426D15	-0.9579883	3.58407533	5.05842828	0.03598511	0.21820129
Dap	0.51152221	5.24106919	5.05565668	0.03603067	0.21836697
Cpt1a	-0.3768973	5.93109532	5.05326307	0.03607007	0.21849516

Doc2a	0.62081355	2.81656322	5.05145648	0.03609983	0.21852683
Clec18a	1.67067754	0.16717546	5.05029366	0.03611901	0.21852683
Cep162	-0.2784085	6.06887196	5.04962516	0.03613004	0.21852683
Bmpr1b	0.51104565	3.2590408	5.04533112	0.03620097	0.21874237
Tec	0.51365956	5.07150878	5.04525644	0.03620221	0.21874237
Mei4	1.47318714	0.42421138	5.04377719	0.03622668	0.2187677
Nudt17	-0.9624245	1.00296696	5.04279541	0.03624293	0.2187677
Ipo5	0.28953116	6.79794917	5.03879361	0.03630927	0.21877881
Man2c1	-0.7853428	2.65960172	5.03475023	0.03637643	0.21877881
Lztr1	0.26931204	5.7292613	5.03471389	0.03637703	0.21877881
Pycrl	0.54292708	2.65084745	5.03470506	0.03637718	0.21877881
Frmd3	-0.7311119	2.67448322	5.03460676	0.03637881	0.21877881
Lactb	-0.4315843	3.6918282	5.0340361	0.03638831	0.21877881
Rps15a-ps6	-0.578668	2.45714199	5.03258295	0.03641249	0.21877881
Pnldc1	1.94961845	-0.2701485	5.03219442	0.03641896	0.21877881
Prpf31	0.3773505	4.10967263	5.03185092	0.03642468	0.21877881
Ccdc92	0.29692713	6.70486455	5.03168385	0.03642746	0.21877881
Gstm7	-0.5805353	4.40010318	5.02885702	0.03647457	0.21895197
Gnl1	-0.2863044	5.89680834	5.02110749	0.03660409	0.21951497
Ube2v1	0.34846501	6.89310435	5.02018387	0.03661956	0.21951497
Mcts2	0.42896684	3.74713524	5.01935431	0.03663346	0.21951497
Mmp23	-2.0480271	-0.4723101	5.01886381	0.03664168	0.21951497
Gpr61	0.76800377	1.32576376	5.01104598	0.03677302	0.22010308
Stmn3	0.38066722	5.74413792	5.01083283	0.03677661	0.22010308
Osgin2	0.3729232	5.95443226	5.00739019	0.03683462	0.22034016
2700046G09	-0.951662	1.75503727	5.00480021	0.03687833	0.2204915
Ammecr1l	-0.2721779	5.73285666	5.0033894	0.03690217	0.22052296
Sncaip	0.50481361	5.65640111	5.0015308	0.03693359	0.22052296
Ptpn11	-0.2543936	7.67249814	5.00014731	0.03695701	0.22052296
Rars	0.32988885	5.45583611	5.00013303	0.03695725	0.22052296
Zak	-0.2952428	6.48262938	4.99743508	0.03700296	0.22068574
Umps	0.38316765	4.08953276	4.99166766	0.03710088	0.22115962
Tgm3	-0.5768089	2.62821885	4.9873302	0.03717472	0.22148952
Ccdc15	-0.5055271	4.26370537	4.98581264	0.03720059	0.22153346
Socs1	-2.8898569	-0.8725271	4.98184747	0.03726829	0.22182632
Mtf2	-0.3683708	5.55865667	4.97714245	0.03734881	0.22201072
1700019G17	1.02255339	1.04324127	4.97523017	0.03738159	0.22201072
Nrip2	0.87868475	2.07644589	4.97352926	0.03741077	0.22201072
Celf1	0.30756809	8.13798273	4.97230191	0.03743185	0.22201072
Sptlc1	-0.4067215	3.62416493	4.97212973	0.0374348	0.22201072
Gm684	0.50190737	3.50100373	4.97198709	0.03743725	0.22201072
Hdlbp	0.22697312	8.49839793	4.9716229	0.03744351	0.22201072
Plin4	1.16674867	0.99542139	4.97122049	0.03745043	0.22201072

Olfr856-ps1	-0.7487727	4.31338526	4.97024725	0.03746716	0.22201072
Sh3bp1	0.53759999	3.23093993	4.96853498	0.03749661	0.22201072
Tmem158	0.41569259	4.10897851	4.96792647	0.03750709	0.22201072
Slc35g2	-0.5063809	3.02421412	4.96707578	0.03752174	0.22201072
Lhx8	-0.7861708	2.6491906	4.95935084	0.03765505	0.22260362
Snx1	0.29334949	5.72601252	4.95911588	0.03765912	0.22260362
Thnsl1	-0.4871217	3.96352648	4.9560501	0.03771218	0.22280203
Exosc6	-0.4787193	3.34859601	4.95502804	0.03772989	0.22280203
Got1	0.30598895	8.34719757	4.95157835	0.03778973	0.22304543
Pcdh7	0.32053695	8.15354378	4.94872936	0.03783924	0.2232276
Lmnb1	0.70966222	1.82116979	4.94521008	0.03790049	0.22344131
Rrm1	-0.3752647	4.32761159	4.94450471	0.03791278	0.22344131
Ncan	0.37241158	8.37898818	4.94241144	0.03794928	0.22346908
Cxx1b	0.38713001	5.87807493	4.94209439	0.03795481	0.22346908
Cntn1	0.32407603	8.31121589	4.94077614	0.03797782	0.22349468
Gm8979	-0.932492	0.55509727	4.93880435	0.03801227	0.22358753
AW551984	-0.6888815	3.43531085	4.93633267	0.0380555	0.22373192
Usp48	-0.4960567	5.49429636	4.934861	0.03808127	0.22377355
Tomt	-1.0331682	0.54964426	4.92401457	0.03827178	0.22478277
Vipr2	1.8758125	0.13129878	4.91970593	0.03834776	0.22504129
Arhgap18	0.35637917	5.25575095	4.91938769	0.03835338	0.22504129
Pthlh	0.70441082	2.27733895	4.91613402	0.03841088	0.2252683
H2-Aa	-0.894385	4.46773061	4.91434248	0.03844258	0.22530212
Mael	-1.5884611	0.65600567	4.91368224	0.03845428	0.22530212
Nphp4	-0.7451408	2.08215996	4.91226285	0.03847942	0.22533921
Fam175b	-0.3453889	4.53151692	4.90728722	0.03856772	0.22574591
Gm10845	-0.6345073	5.57078128	4.90262903	0.0386506	0.22606459
Rb1	0.29468267	6.94098613	4.90210567	0.03865992	0.22606459
P4hb	0.49754904	5.58337131	4.89907356	0.03871399	0.22627027
Prdx6	0.31715178	6.75152676	4.89726449	0.03874629	0.22634858
Pdgfrb	-0.6272492	4.4274663	4.89540265	0.03877956	0.22643251
Rnf181	0.3658109	6.26670878	4.89383969	0.03880752	0.22648532
Ankef1	-1.5725544	0.39636914	4.89122875	0.03885427	0.22655019
Col23a1	0.56912371	5.23591138	4.89110629	0.03885647	0.22655019
Lipg	0.98585101	1.29673501	4.88909006	0.03889262	0.22665062
Olfr691	3.25454185	-2.0704792	4.88598336	0.0389484	0.22686527
Shisa4	0.45675254	4.60312327	4.87880156	0.03907769	0.22750098
Gm16702	-0.4851226	4.40077098	4.87781265	0.03909553	0.22750098
Cdh7	0.44321298	3.87185716	4.87207905	0.03919915	0.22796326
Retnla	1.29754775	1.0485688	4.87131184	0.03921304	0.22796326
Stx6	0.34985515	4.9700372	4.86702642	0.03929073	0.22830405
Nkain4	-0.9999041	1.74106904	4.8651528	0.03932475	0.2283741
Ylpm1	-0.5349936	8.12727214	4.8638008	0.03934932	0.2283741

Trpc3	-0.4927766	3.63334456	4.86321357	0.03935999	0.2283741
Lmtk2	-0.3463244	6.76082334	4.85980434	0.03942204	0.22854271
Msln	0.88615483	2.22083589	4.85732458	0.03946725	0.22854271
Man1a	0.44108034	5.19943443	4.85728383	0.03946799	0.22854271
Gng3	0.27774843	8.0666176	4.85659858	0.03948049	0.22854271
Tdp2	0.48522836	3.61974665	4.85635124	0.03948501	0.22854271
B4galnt2	-0.7713206	3.0710878	4.85438394	0.03952093	0.22854271
Abhd12	0.30629012	6.33405157	4.85429035	0.03952264	0.22854271
Fads3	0.45358347	3.57480128	4.85063731	0.03958944	0.22881852
L1td1	-0.5856123	2.81831236	4.84805657	0.03963672	0.22898124
Aff2	0.42137967	5.87645456	4.84589421	0.03967637	0.22909983
Slc9a8	-0.4494741	4.08268611	4.84187151	0.03975027	0.2293648
Trp63	-0.9029996	2.24132109	4.84067934	0.0397722	0.2293648
Tmem134	-0.5639405	2.61010811	4.84020356	0.03978096	0.2293648
2410006H16I	-0.6907595	2.57226497	4.83910177	0.03980124	0.2293648
Chdh	-0.9830625	1.47474422	4.83650931	0.03984902	0.2293648
Megf10	-0.5316422	4.61145867	4.83483758	0.03987986	0.2293648
Dcdc2c	-0.7033415	5.66716477	4.83433542	0.03988913	0.2293648
Cstad	-0.6793526	2.55510493	4.83405037	0.03989439	0.2293648
1700001D01I	-1.4924187	1.20263267	4.83302634	0.03991331	0.2293648
Ctc1	-0.3805565	4.13550488	4.83300038	0.03991379	0.2293648
Fam159b	2.96716549	-2.0502647	4.82845136	0.03999793	0.22965023
Mkrn2	0.36599567	5.14989952	4.82818234	0.04000292	0.22965023
Myh13	2.91254593	-1.4079411	4.82720705	0.04002099	0.22965023
Gm5531	-0.6633813	2.74342724	4.82334359	0.04009266	0.22991303
Pclo	-0.6198185	10.1329204	4.8226692	0.04010518	0.22991303
Jund	-0.3783026	5.02178906	4.81915508	0.04017052	0.23011895
Ebf2	0.67651076	3.16521469	4.81867097	0.04017953	0.23011895
Sap130	0.2867841	6.64404836	4.81683378	0.04021375	0.23019212
Eif2b1	0.44691492	4.79094889	4.81516949	0.04024477	0.23019212
Cyb561	0.29431851	4.44562997	4.81489076	0.04024997	0.23019212
Xpo6	0.24055159	6.87091952	4.81068347	0.04032854	0.23041476
Foxd2os	-0.6467003	2.51119551	4.80903424	0.04035938	0.23041476
Cplx1	0.23883918	8.44920453	4.80883665	0.04036308	0.23041476
Bicd2	-0.3062137	6.4485102	4.80868775	0.04036586	0.23041476
Bclaf1	0.22809143	9.51920161	4.80569235	0.04042196	0.23062506
Rabl2	-0.4765252	3.87155561	4.80010123	0.04052691	0.23111373
Hiat1	0.28946898	6.3790195	4.79796789	0.04056704	0.23123245
2510049J12F	-1.3328484	1.13682001	4.79686677	0.04058777	0.23124054
Nphp1	-0.4569146	3.83450692	4.79202814	0.040679	0.23143486
Spon1	0.37991761	5.35663672	4.79152851	0.04068843	0.23143486
Pan2	-0.5345545	3.5095207	4.79141223	0.04069063	0.23143486
Tet2	-0.4579409	6.67525259	4.79004548	0.04071645	0.23143486

Mettl2	-0.5388765	4.13577789	4.78948977	0.04072695	0.23143486
Trpc5	-0.4955816	4.12058133	4.7889145	0.04073783	0.23143486
Samd4	-0.3045582	7.26723062	4.78597145	0.04079352	0.23164139
Sp6	2.21982301	-0.8815398	4.7849148	0.04081354	0.23164522
Cpne5	0.40298066	4.94584608	4.77603081	0.04098228	0.23249277
Pafah2	0.47057541	3.40663434	4.773235	0.04103555	0.23268474
Tldc1	0.88138293	1.15750519	4.77044434	0.0410888	0.23287641
Tymp	-0.7487418	2.53864225	4.76799177	0.04113566	0.23291328
Paip2b	-0.2954762	5.25895448	4.76754602	0.04114418	0.23291328
Picalm	0.22610391	8.08333013	4.76426365	0.04120701	0.23291328
Disp2	0.35454711	7.01997622	4.76416643	0.04120887	0.23291328
Csf3r	-1.0320097	1.43917202	4.76365565	0.04121866	0.23291328
Tmprss6	3.60236814	-2.147482	4.76305626	0.04123015	0.23291328
Usp27x	-0.3390564	4.93859851	4.76298862	0.04123145	0.23291328
Trpm4	-0.8858927	2.0419583	4.76065605	0.04127619	0.2330417
Fbln1	0.61249966	4.89785489	4.75828565	0.04132172	0.2330417
Rrm2	1.12474823	1.42874558	4.75811075	0.04132508	0.2330417
Hmga2	0.41026251	3.850615	4.7577497	0.04133202	0.2330417
Actl6a	0.44353044	3.68241767	4.75642261	0.04135754	0.23307586
Cul5	0.24582486	7.02475883	4.75445658	0.04139538	0.23313477
Tmem95	3.60996427	-2.4731922	4.75385706	0.04140693	0.23313477
Elovl5	0.47797707	6.21453658	4.75194649	0.04144375	0.23323244
Serpinh1	0.52718815	4.25435092	4.7473905	0.04153171	0.23354094
Sytl3	-0.9347627	0.95486318	4.74544368	0.04156936	0.23354094
2610307P16I	-1.6328601	0.88520349	4.74536141	0.04157095	0.23354094
Aldh6a1	-0.3319793	5.93071237	4.74507082	0.04157657	0.23354094
Gpr63	-0.5539236	3.16893815	4.74390387	0.04159916	0.23355828
Wdr13	-0.2254882	6.73099886	4.73949721	0.0416846	0.23377227
Ddx55	-0.3625239	4.40733803	4.73877165	0.04169868	0.23377227
Nsdhl	0.3635599	4.56977349	4.73851654	0.04170364	0.23377227
Usp54	-0.2366342	6.65042022	4.73791313	0.04171536	0.23377227
Usp14	0.2844162	6.97105052	4.73663649	0.04174017	0.23378446
Slc35d2	1.13766127	0.16421298	4.73579253	0.04175658	0.23378446
Rfc1	0.25057121	5.87408846	4.73277216	0.04181536	0.23386901
Zfp689	-0.8079588	1.65049291	4.73224933	0.04182555	0.23386901
Tmem191c	-0.4851774	4.21084663	4.73200749	0.04183026	0.23386901
4932413F04I	-1.6346651	0.1333129	4.73066109	0.04185651	0.23390655
Ostm1	0.32189612	5.44644216	4.72293041	0.04200758	0.23439703
4930500J02F	-2.4619993	-0.6155177	4.7226162	0.04201373	0.23439703
AB124611	1.91665811	-0.6492218	4.72233032	0.04201933	0.23439703
Serpine2	0.33224087	5.98452131	4.72216493	0.04202257	0.23439703
Myo9b	-0.3829361	4.49957266	4.71813294	0.04210164	0.23472874
Tmem68	0.30571647	5.12180934	4.71435079	0.04217596	0.23484487

Olfr613	-0.6785409	5.43212935	4.7141505	0.0421799	0.23484487
4933428C19I	-2.293105	-0.7452506	4.71407946	0.0421813	0.23484487
Sparc	0.58813898	8.82825556	4.70967947	0.04226796	0.23516699
Ndnf	-0.3542582	5.19802815	4.70914881	0.04227843	0.23516699
Idh3g	0.27380076	6.41635125	4.70714049	0.04231806	0.23527817
Svop	0.41128378	5.76057809	4.70437696	0.04237267	0.23547247
Tecr	0.41624408	7.85813035	4.69989987	0.04246131	0.23582014
Pcdhb9	0.6834625	2.25524528	4.69843516	0.04249036	0.23582014
Prrx1	0.43283203	7.06862892	4.69823595	0.04249431	0.23582014
Grin1	0.40999963	6.12469084	4.6971628	0.04251561	0.23582905
Gnb2	0.46597892	3.61938385	4.69399549	0.04257854	0.23606221
Phax	0.3064172	5.38378733	4.69230881	0.04261209	0.23606221
Zfp964	-0.9487186	1.2599047	4.69155478	0.0426271	0.23606221
Polr1a	-0.606306	4.59391222	4.69108315	0.04263649	0.23606221
Igfbp4	0.50143472	5.14860724	4.68855408	0.04268689	0.23622149
Angpt2	0.46627189	3.99421813	4.6862911	0.04273205	0.23622149
Kif23	-0.9592917	0.84121658	4.68619092	0.04273405	0.23622149
Polr2k	-0.3717123	4.07922009	4.68568466	0.04274416	0.23622149
Slc35f3	0.30795	5.58481233	4.68417377	0.04277435	0.23624374
Dock4	-0.4666422	6.18838306	4.68328927	0.04279203	0.23624374
Cbln2	-0.548331	3.41947887	4.68252262	0.04280736	0.23624374
Hapln2	-1.0824001	1.13557707	4.68121767	0.04283348	0.23627898
Cyp20a1	0.71005018	2.77855337	4.68003614	0.04285714	0.23630066
Apobec3	-0.7221123	2.31423405	4.67729149	0.04291217	0.23649515
Acvr1	0.39251983	4.31727452	4.67427541	0.04297272	0.23671996
1700123L14F	-1.951605	0.3657633	4.67231854	0.04301206	0.23682774
Catip	0.69362007	2.46427014	4.66695655	0.04312008	0.23731335
Abcb1b	-0.6956307	1.66778348	4.66518504	0.04315583	0.23739917
Ly6a	-1.1762259	2.3561402	4.66422011	0.04317532	0.23739917
Arnt2	0.25923947	8.03920942	4.66092113	0.04324202	0.23765683
Vmn2r57	1.31876761	0.70186386	4.65845761	0.04329191	0.23772391
Lin28b	-0.6406997	3.02215074	4.65815517	0.04329804	0.23772391
Dhx34	0.72818776	1.42848274	4.65737878	0.04331378	0.23772391
Abr	-0.3391774	7.69705062	4.65396419	0.04338307	0.23793896
Arl6ip5	0.37075104	3.89534564	4.65349051	0.0433927	0.23793896
Tsg101	0.32027878	5.33394706	4.6524377	0.04341409	0.23794733
Fam46c	-0.597807	2.4252796	4.65075066	0.04344841	0.23802645
5730522E02I	-1.2758944	1.39875561	4.64954752	0.04347289	0.23805171
Ermap	-1.8385654	0.46988567	4.64839384	0.04349639	0.23807152
Caprin2	-0.6206265	2.7154879	4.6436731	0.04359269	0.23848959
Arsb	0.29459655	7.01737196	4.64230628	0.04362062	0.23849222
Slc14a1	0.62855609	2.67003188	4.6417006	0.043633	0.23849222
Mal	-0.4064179	6.0909116	4.63886424	0.04369103	0.23870049

Map3k9	-0.4797723	6.22637479	4.63743342	0.04372034	0.2387517
1810026B05I	-0.409643	3.78998523	4.63516779	0.0437668	0.23889646
Pik3r5	-0.5127671	2.80084635	4.63111669	0.04385001	0.23917276
Ghdc	0.61249375	2.22972725	4.63075908	0.04385736	0.23917276
Ppfia4	-0.4224524	3.62828696	4.62929915	0.0438874	0.23922762
Kcnab1	0.32207009	5.30295476	4.62662565	0.04394246	0.2394188
Camk1d	0.25571896	8.32294529	4.6228159	0.04402107	0.23971525
Ift172	-0.5935376	4.63791237	4.62093256	0.04405998	0.23971525
Zfp52	0.38425424	4.08566603	4.61946041	0.04409043	0.23971525
Rab3gap2	-0.4503245	6.46892476	4.61858826	0.04410848	0.23971525
Cpt2	0.45931978	3.28725638	4.61826187	0.04411524	0.23971525
Arpc3	0.40027082	4.43972326	4.61817799	0.04411697	0.23971525
Cblb	0.33686885	6.78147852	4.61617529	0.04415846	0.23971684
Trmt61a	0.3752862	4.52357401	4.61586541	0.04416488	0.23971684
Pou5f1	-3.1020895	-2.4473957	4.61526568	0.04417732	0.23971684
Akap11	-0.4172147	8.45941084	4.61306571	0.04422296	0.23985585
Pgam5	0.32499086	6.09809971	4.60833008	0.04432141	0.24028096
Fbxl18	-0.8065829	1.31069494	4.60571857	0.0443758	0.24046567
G6b	2.78932019	-1.0109044	4.6047671	0.04439564	0.24046567
Dld	0.25707364	7.47831163	4.60084538	0.0444775	0.24080019
Zfp189	0.45554505	3.26918649	4.59901477	0.04451578	0.24089849
Firre	-0.7465895	3.39928148	4.59711284	0.04455558	0.24100499
Psma4	0.25021215	6.88952147	4.59236199	0.04465519	0.24137493
Otop2	2.2499096	-0.9323722	4.59192864	0.04466428	0.24137493
Spata21	2.60290312	-1.3681654	4.58700798	0.04476774	0.24182491
Dcaf5	-0.2729135	7.07539042	4.58584138	0.04479231	0.24184853
Pebp4	2.09355898	-0.8767812	4.58401167	0.04483087	0.24188693
Tiparp	0.29290782	4.91605335	4.58308167	0.04485049	0.24188693
Slitrk1	0.29056642	7.08121807	4.58203807	0.04487251	0.24188693
Cuta	0.41239379	6.28943736	4.5807222	0.0449003	0.24188693
Ddx17	-0.3603303	8.05832796	4.58050437	0.0449049	0.24188693
9230110C19I	0.53475285	3.02451979	4.57976082	0.04492061	0.24188693
Micalcl	-0.8773196	1.66296183	4.57831532	0.04495117	0.24191904
Slc43a3	1.80222551	0.2159002	4.57674496	0.0449844	0.24191904
Chsy1	0.31669021	5.20745645	4.57661389	0.04498718	0.24191904
Neu3	-0.511457	3.12335179	4.57441543	0.04503374	0.24195979
Shank1	-0.4096212	8.94138459	4.5743486	0.04503516	0.24195979
Nup98	-0.2713343	6.39272742	4.57190013	0.04508709	0.24196187
Stk35	-0.3129254	5.14102998	4.57165148	0.04509237	0.24196187
Dock7	-0.3347007	6.05798925	4.57147292	0.04509616	0.24196187
Uso1	0.24422605	6.42363592	4.56922756	0.04514386	0.24207238
Rbks	-1.2200636	1.44680274	4.56822653	0.04516514	0.24207238
Nrk	0.95234416	1.47613572	4.56765011	0.0451774	0.24207238

Lhfp	0.45627983	6.11296999	4.561704	0.04530409	0.24256168
9530026P05I	1.0424994	0.5180559	4.56146331	0.04530922	0.24256168
Sox2	0.55410461	3.57343566	4.55916479	0.04535831	0.24263393
Sp7	1.07016519	0.83037531	4.55893414	0.04536324	0.24263393
Ikbkap	-0.4737107	5.38567574	4.55663193	0.04541248	0.24278883
Itpa	0.35077864	4.19649389	4.55213988	0.04550871	0.24309142
Spr	0.64799174	2.83179434	4.5520953	0.04550967	0.24309142
Smco1	-0.6005921	2.72570555	4.5488727	0.04557886	0.24329332
Timp1	1.2082589	1.52982041	4.54844262	0.0455881	0.24329332
Ccdc71	0.45393447	4.2507704	4.54289177	0.04570758	0.24379766
Col14a1	-1.0958954	0.85800448	4.54216195	0.04572332	0.24379766
Retnlg	-2.8800079	-1.3288722	4.53500321	0.045878	0.24450146
Asf1b	2.38671224	-1.5884799	4.53416516	0.04589615	0.24450146
Myct1	-3.0776066	-1.6149048	4.53088425	0.04596727	0.2446161
Dlgap1	-0.3746523	8.83633306	4.52892335	0.04600984	0.2446161
S100a11	0.47695685	9.5078464	4.52784677	0.04603323	0.2446161
Rwdd2b	0.77843536	2.25778466	4.52588906	0.04607579	0.2446161
Atrn	0.32281243	7.87994801	4.52541418	0.04608613	0.2446161
Pdzd2	-0.3626062	6.18780981	4.52503915	0.04609429	0.2446161
Mapk8ip2	-0.6913519	4.63969142	4.52440445	0.0461081	0.2446161
Slc9a4	-2.8267444	-1.2674155	4.52410719	0.04611458	0.2446161
Tufm	-0.415645	3.65762386	4.52350928	0.0461276	0.2446161
Doc2g	-2.2172883	-0.3671259	4.52264718	0.04614638	0.2446161
Trpm7	-0.2938613	6.58706459	4.52239213	0.04615194	0.2446161
Nipal4	-0.9194724	0.8634244	4.52189504	0.04616278	0.2446161
Vamp4	0.2319639	6.82262268	4.52018686	0.04620004	0.24470526
4930470H14I	-0.6864543	6.76646202	4.51800287	0.04624772	0.24483513
Snx13	-0.2159482	6.88848207	4.51632084	0.04628449	0.24483513
Tpra1	0.7365116	2.47467841	4.51598917	0.04629174	0.24483513
Adamts20	-0.958504	2.22272247	4.5153222	0.04630633	0.24483513
Hnrnpc	0.24555756	7.27085951	4.51423038	0.04633023	0.24485337
Focad	-0.3697441	5.28452876	4.51286128	0.04636021	0.24485816
Kirrel3	0.58310515	3.45483046	4.51191637	0.04638092	0.24485816
Zfyve19	-0.401695	3.28719945	4.51095725	0.04640195	0.24485816
Mrpl4	0.46855711	4.75516618	4.50934547	0.04643731	0.24485816
9130024F11I	0.46844066	4.23553446	4.50810405	0.04646457	0.24485816
Mir32	5.21738201	-1.9347568	4.61397146	0.04650094	0.24485816
Col4a3	1.49606625	0.00043083	4.50627972	0.04650466	0.24485816
Fam84b	0.48490665	2.78382697	4.50418316	0.04655078	0.24485816
Slc19a3	-1.6219425	0.32608447	4.50337735	0.04656852	0.24485816
Hspa12b	-0.5624138	3.62175018	4.50321478	0.0465721	0.24485816
Arhgef12	0.2139223	9.52684002	4.50239144	0.04659024	0.24485816
Ppapdc1b	0.37188668	4.27415139	4.50085751	0.04662405	0.24485816

Pvalb	0.61749203	2.39315365	4.5005963	0.04662981	0.24485816
Mfn1	-0.3828533	5.318936	4.49985385	0.04664619	0.24485816
Tagln3	0.32920278	6.38412678	4.49956529	0.04665255	0.24485816
Rph3al	0.9735721	1.23331176	4.49930601	0.04665827	0.24485816
Hsp90aa1	0.29612508	10.9285529	4.49779294	0.04669168	0.24492614
Vps51	0.52647066	3.72065299	4.49570456	0.04673783	0.24506089
Shroom4	-0.6221515	3.13879811	4.49398342	0.04677591	0.2451532
Znfx1	-0.3731787	5.36773482	4.49235801	0.0468119	0.24520301
Rom1	0.83705807	1.51486163	4.49170518	0.04682636	0.24520301
Il1rapl1	-0.4183249	3.91836958	4.49066213	0.04684948	0.24521686
Kcnd3os	-2.1935715	-1.0140629	4.48870079	0.04689299	0.24533737
Zfand6	0.38354919	5.61016138	4.48720706	0.04692616	0.24540369
Spata19	2.67136441	-2.0523574	4.48303459	0.04701895	0.24571196
Ly6e	-0.2832726	6.82283664	4.48271159	0.04702614	0.24571196
Gm5523	0.31692606	5.24483222	4.47773369	0.04713714	0.2461845
Setd1b	-0.3207265	5.46467722	4.47649516	0.0471648	0.24619304
Ak4	0.37651442	5.3752424	4.47525049	0.04719262	0.24619304
Coil	0.53749759	3.23445408	4.47445616	0.04721038	0.24619304
Lrrcc1	-0.3419787	5.61702841	4.47398133	0.04722101	0.24619304
Chd7	-0.5175171	4.64904099	4.46815917	0.04735147	0.24666055
Tram2	0.55576265	3.62401292	4.46814138	0.04735187	0.24666055
Gprin1	0.37656631	4.69339055	4.46571174	0.04740643	0.24675944
Mob3c	0.4226025	4.65589937	4.4654613	0.04741206	0.24675944
Gm5820	-0.8583418	1.83755461	4.46207732	0.04748819	0.24701029
Dnaaf1	-2.4449894	-1.8853075	4.46052301	0.04752321	0.24701029
Mbnl2	0.26245798	9.1115468	4.45992063	0.04753678	0.24701029
Prkag3	-0.9531243	1.82360284	4.45872975	0.04756364	0.24701029
Prpf4	0.30782046	5.00342337	4.45711885	0.04759999	0.24701029
Kynu	-2.5314391	-0.5723934	4.45632707	0.04761787	0.24701029
Sh3yl1	0.52245863	3.06946594	4.45555738	0.04763526	0.24701029
Dennd5a	-0.219633	7.52315641	4.45418816	0.04766622	0.24701029
Ubn2	-0.3196639	7.06862462	4.45378254	0.04767539	0.24701029
Snrnp200	-0.3995469	6.89140975	4.45344255	0.04768308	0.24701029
Slc25a43	2.92529094	-1.5902955	4.45276313	0.04769845	0.24701029
Syt16	-0.302122	6.11761101	4.45162625	0.04772419	0.24701029
AF357359	-0.6273067	2.62008413	4.4501715	0.04775715	0.24701029
Sp8	1.03892835	0.59072781	4.44987175	0.04776394	0.24701029
BC006965	-0.8425919	1.79044994	4.44961993	0.04776965	0.24701029
Trim39	-0.3594574	4.31409837	4.44707346	0.04782742	0.24720226
Ctps	-0.4480203	3.98879188	4.44320288	0.04791538	0.24755004
Serpinb9	-0.4133522	7.10563105	4.44171478	0.04794924	0.24755342
Tmem25	0.59374986	3.05454486	4.44135771	0.04795737	0.24755342
Gtf2e1	0.48445079	3.80428153	4.43807882	0.0480321	0.2477967

Kmt2a	-0.3856269	8.41968347	4.43667854	0.04806406	0.2477967
Plk2	0.34505166	7.04814951	4.43656833	0.04806657	0.2477967
Slc25a18	0.62438858	2.52180671	4.43506319	0.04810095	0.24786722
Gm16596	1.35164787	0.01562541	4.43343826	0.0481381	0.24789937
Slc26a7	0.69024349	4.94168083	4.43297944	0.04814859	0.24789937
Nkiras1	0.25045711	6.11160246	4.43185199	0.04817439	0.24792561
Psmc1	0.27642064	6.37600681	4.4294252	0.04822997	0.24810504
Klf4	-0.4918086	6.47978248	4.42810109	0.04826033	0.24813188
Tmx2	0.32355713	5.98579548	4.42739065	0.04827663	0.24813188
Slc22a17	0.34829392	5.86933989	4.42167486	0.04840798	0.24859696
Kdm2b	-0.3117436	4.37736772	4.42002495	0.04844597	0.24859696
Ubr5	-0.3203035	7.85059962	4.41907653	0.04846783	0.24859696
C330021F23I	-0.5473363	2.50168453	4.41873619	0.04847567	0.24859696
Psma6	0.28171989	6.42066675	4.41811296	0.04849004	0.24859696
2310009A05I	-0.6699263	2.69505631	4.41792789	0.04849431	0.24859696
Cda	2.3795316	-0.9011938	4.41714246	0.04851243	0.24859696
Vps13c	-0.4979246	6.48139958	4.41610362	0.0485364	0.24861344
Hid1	0.29449371	6.11254569	4.41433523	0.04857725	0.24871627
Lum	0.53468672	5.256301	4.40712515	0.04874418	0.24915237
Ccdc27	2.51426726	-1.0113339	4.40666703	0.04875481	0.24915237
Fam174b	0.48853522	6.56798464	4.40610397	0.04876788	0.24915237
Trank1	-0.6059291	6.22042844	4.40595679	0.04877129	0.24915237
Hrc	2.17722684	-0.3257219	4.4050613	0.04879209	0.24915237
Sgk2	-1.4941051	-0.36339	4.40506079	0.0487921	0.24915237
Gm14827	-0.8357146	2.66352071	4.40361334	0.04882573	0.24915237
Cml1	-0.9013231	1.30335737	4.40189676	0.04886564	0.24915237
Rnh1	0.4181393	4.45654197	4.40100344	0.04888643	0.24915237
Cacnb1	0.30854197	5.12663718	4.39994341	0.04891111	0.24915237
Vkorc1	0.56566001	5.37488519	4.39952722	0.04892081	0.24915237
Cebpa	-0.8743418	2.51636749	4.39798115	0.04895684	0.24915237
Anapc1	-0.2740202	6.17805781	4.39763157	0.04896499	0.24915237
Adap1	0.37831434	4.20432379	4.39662249	0.04898852	0.24915237
Nfatc4	0.81680709	3.20919778	4.39661285	0.04898875	0.24915237
Gpr126	0.47352971	3.94251813	4.39572549	0.04900946	0.24915237
4930511M06	-0.7189019	3.22956521	4.39473808	0.04903251	0.24915237
Fabp4	-2.6097681	-0.6828978	4.39454992	0.04903691	0.24915237
Tbc1d30	-0.3750167	6.6190001	4.39090211	0.0491222	0.24947987
Slc1a4	0.42906709	4.33679528	4.38922653	0.04916143	0.24957329
Gtpbp8	0.36363747	3.77131635	4.38634312	0.04922903	0.24971852
Zfp952	0.31546512	4.62373598	4.38559136	0.04924667	0.24971852
Nagk	0.51804094	3.23250208	4.38533891	0.04925259	0.24971852
Pgap2	-0.4507188	4.20578138	4.38386793	0.04928714	0.24977285
Tmem150b	-2.4482364	-0.9969211	4.38310689	0.04930502	0.24977285

Iqgap3	2.42434175	-0.6190251	4.38076246	0.04936016	0.24987226
5730405O15	-1.794213	-0.293786	4.37995547	0.04937916	0.24987226
Sfxn3	0.28589445	7.52997578	4.3792534	0.04939569	0.24987226
Clint1	0.22937297	6.88746452	4.37819483	0.04942063	0.24987226
Ninl	-0.5356695	3.10125781	4.37745256	0.04943812	0.24987226
Fanca	-3.5299832	-2.154117	4.37673773	0.04945498	0.24987226
Lancl3	0.59467325	3.95821715	4.37563965	0.04948089	0.24987226
Arhgef10	-0.3170764	5.27670448	4.37366063	0.04952761	0.24987226
Fkbp3	0.24281207	6.9856642	4.37338896	0.04953403	0.24987226
Bptf	-0.3555904	8.32255731	4.37309545	0.04954097	0.24987226
Mga	-0.371447	7.82664413	4.37251981	0.04955457	0.24987226
4930547E14I	-2.5918548	-0.8269126	4.37101421	0.04959018	0.24987226
Coq10b	0.34432892	5.61281997	4.37077293	0.04959589	0.24987226
Ube2j1	0.28726106	7.45386094	4.36853632	0.04964884	0.24995589
Thbs3	-0.9386248	1.10642805	4.36830896	0.04965423	0.24995589
Polk	-0.3449191	5.18978043	4.36562235	0.04971793	0.2501714
Esrp2	1.7635482	-0.9592268	4.36240094	0.04979444	0.25045113
Shq1	-0.958785	1.05411316	4.35810588	0.04989665	0.25083947
5530401A14I	-1.399363	0.01836977	4.35561485	0.04995605	0.25083947
Cdh9	0.6379899	3.91287303	4.35463687	0.04997939	0.25083947
Unc80	-0.4800138	8.33898979	4.3539998	0.0499946	0.25083947
Nos3	-1.046976	0.88492449	4.35380746	0.04999919	0.25083947
Atp5c1	0.28686042	8.18712895	4.3537136	0.05000143	0.25083947
Smarcc1	0.21420035	7.39862034	4.35180881	0.05004695	0.25083947
Zbbx	-1.8583738	-0.6191186	4.35129417	0.05005925	0.25083947
Mfge8	0.65591248	3.1495473	4.34982239	0.05009446	0.25083947
Abtb1	-0.7137923	1.96667002	4.34956115	0.05010072	0.25083947
Dffa	0.30166754	5.08935317	4.34950542	0.05010205	0.25083947
Atp5o	0.369185	6.67836527	4.34820271	0.05013325	0.25088693
4930405A21I	-1.0965617	1.03742886	4.34736029	0.05015343	0.25088693
Slc29a2	1.20823881	0.35312579	4.34303074	0.05025732	0.25130164
Syncrip	0.23672907	7.70853875	4.34063	0.05031503	0.2514852
Pdcd5	0.31847749	6.90457778	4.33912566	0.05035123	0.25153869
Mast3	-0.3062589	7.30129697	4.33828888	0.05037138	0.25153869
Asb14	-1.7409185	0.03318188	4.3375681	0.05038874	0.25153869
Gria4	-0.3368682	6.76190526	4.33580421	0.05043126	0.25164398
Dsn1	0.75595445	1.38617472	4.33495041	0.05045186	0.25164398
Tubg1	0.39246968	4.38928208	4.33348219	0.0504873	0.25171592
Elavl3	0.31463641	7.19794494	4.33231887	0.0505154	0.25175121
Nfrkb	-0.3712838	4.24815793	4.32970148	0.05057869	0.25196178
Ddx31	0.83592554	1.36652736	4.32795494	0.05062098	0.25206757
Zic5	0.79979533	1.62181319	4.32558332	0.05067846	0.2521458
Actr5	1.55925631	0.08199314	4.32488172	0.05069548	0.2521458

Tmem173	-0.7941578	1.61094865	4.32397681	0.05071744	0.2521458
Ccdc153	-0.7733059	1.80355475	4.32383393	0.05072091	0.2521458
Itm2b	0.43318288	8.98079647	4.32037025	0.05080507	0.25245941
Qtrtd1	-0.6826272	2.08839529	4.31910555	0.05083584	0.25246252
Acot9	0.39676501	3.80085597	4.31861186	0.05084786	0.25246252
Crim1	0.36989592	6.98334758	4.31636986	0.05090248	0.25254218
Fignl2	-0.9677117	1.1062992	4.31613739	0.05090814	0.25254218
Zbtb41	-0.2722923	6.45382066	4.31535724	0.05092717	0.25254218
Ccdc181	-0.3345019	5.18637453	4.31308952	0.05098251	0.25271197
2610035D17I	0.50832534	3.31067357	4.30834731	0.05109846	0.25318193
Trpc4ap	0.2860844	5.32545809	4.30530605	0.05117298	0.25344631
Gabpb2	-0.2812024	6.14422032	4.30055703	0.0512896	0.25382108
Snhg12	0.46142337	3.49717348	4.30049834	0.05129104	0.25382108
Hdac11	-0.3011841	5.42574727	4.29779973	0.05135744	0.25404471
Tceal7	-1.7059451	-0.1028315	4.29359351	0.05146114	0.25431353
Hint1	0.32441453	5.21577441	4.29342848	0.05146521	0.25431353
4430402I18R	-0.5256461	2.61787943	4.29245062	0.05148936	0.25431353
Tmem87b	0.37170893	4.94297907	4.29107861	0.05152326	0.25431353
Dnah11	-1.541637	-0.3051368	4.29051524	0.05153718	0.25431353
Ano1	-0.7134379	1.54957846	4.28998124	0.05155039	0.25431353
Fbxw17	-0.7336319	2.00307824	4.28957477	0.05156044	0.25431353
Bend4	-0.4004462	4.40579276	4.28693202	0.05162586	0.25443021
Snai2	0.71773165	3.53862021	4.28688827	0.05162694	0.25443021
Iqsec1	-0.3990073	7.84159001	4.28604522	0.05164783	0.25443021
Mroh8	1.54499841	-1.0031828	4.28350405	0.05171086	0.25463594
Dock2	-0.5805735	2.3581505	4.28258207	0.05173374	0.25464394
Mrpl17	0.28141046	5.65330269	4.28066089	0.05178147	0.25474887
Zbtb34	-0.3338675	6.30562841	4.27959859	0.05180789	0.25474887
Alyref2	-0.6307535	1.75103512	4.27872512	0.05182962	0.25474887
Zfp281	0.30772374	6.16581119	4.27779791	0.0518527	0.25474887
Nucb1	0.49922867	4.56765034	4.27695302	0.05187374	0.25474887
Ptges3l	-1.1657031	1.05137096	4.27659344	0.05188269	0.25474887
Ulk4	-1.1588571	1.39967079	4.27379037	0.05195258	0.2549434
Fam166b	-1.8268493	-0.4522952	4.27292597	0.05197415	0.2549434
Bbs5	0.46566195	3.73149981	4.2724444	0.05198618	0.2549434
Celf5	0.31129648	6.86974412	4.26877974	0.05207778	0.25503967
Bag3	0.35633736	5.08106989	4.26831502	0.05208941	0.25503967
Pno1	0.33327592	3.98519793	4.26825463	0.05209092	0.25503967
2410076I21R	-1.0840645	0.47192172	4.26776336	0.05210321	0.25503967
Zfp488	-0.6161653	7.72018288	4.26690445	0.05212472	0.25503967
Dcun1d1	0.23252761	6.8076592	4.26655046	0.05213359	0.25503967
01-Sep	0.71745394	2.38936797	4.26490183	0.05217491	0.25513758
Hprt	0.2439537	7.88764741	4.26158762	0.05225808	0.25534007

Elmo1	0.27607223	7.15388229	4.26025514	0.05229156	0.25534007
Tstd1	-2.2018498	-1.3567026	4.25954635	0.05230938	0.25534007
Ajuba	-0.4701824	2.82632005	4.25874502	0.05232954	0.25534007
Krt18	-2.7575651	-1.6566113	4.25757717	0.05235893	0.25534007
Gje1	-0.7996568	1.15646148	4.2573776	0.05236396	0.25534007
Trabd2b	0.48256743	6.3225332	4.25731372	0.05236556	0.25534007
Akap9	-0.3097878	8.44979472	4.25619617	0.05239371	0.25536
Phf11d	-0.8947019	1.52265086	4.25474802	0.05243021	0.25536
Zfp433	-0.3844473	3.82422455	4.25461272	0.05243362	0.25536
Ssfa2	-0.2771313	5.4928508	4.25148339	0.0525126	0.25562137
Tmem231	0.45998244	2.88553283	4.25079548	0.05252998	0.25562137
4932438A13	-0.4862175	7.50608328	4.24872988	0.0525822	0.25577157
Dexi	0.37017056	3.84167745	4.2468402	0.05263003	0.25586024
Ccnd2	-0.2715418	7.42819143	4.24632186	0.05264316	0.25586024
Myl9	-0.6280621	5.49638529	4.24455358	0.05268798	0.25588813
Dicer1	-0.3085897	6.2937579	4.24440926	0.05269164	0.25588813
Dusp3	-0.2409372	7.09315937	4.24306188	0.05272581	0.25594603
Btf3l4	0.22743217	7.05131846	4.2417378	0.05275943	0.25594603
Rpn1	0.33979433	5.65877509	4.24122845	0.05277236	0.25594603
Ckmt1	0.36081201	5.28012258	4.24057191	0.05278905	0.25594603
Mllt11	0.22291414	7.83720873	4.23818142	0.05284983	0.25600448
Slc30a7	-0.3681978	4.66352327	4.2362243	0.05289966	0.25600448
Adra2c	-0.6683331	2.43547941	4.23599399	0.05290552	0.25600448
Ldlrap1	-0.9632135	1.14273587	4.23597161	0.05290609	0.25600448
Adcy2	-0.3159252	5.76768634	4.2358974	0.05290798	0.25600448
Rnd1	-0.8043009	1.29585045	4.23476833	0.05293676	0.25604026
Tmem232	1.01616257	1.34682122	4.23118135	0.05302829	0.25637943
Snora23	-1.3759702	0.98583809	4.22950488	0.05307113	0.25648302
Slc7a15	-2.4372156	-1.0209344	4.22666892	0.0531437	0.2567301
Gnpda1	0.33084489	4.59177892	4.22513636	0.05318296	0.25676308
Med13l	-0.4137864	6.48937418	4.22472882	0.05319341	0.25676308
Cxxc1	0.28014101	5.98700297	4.22311628	0.05323476	0.25685917
Zfp184	-0.5672063	2.16563795	4.22102678	0.0532884	0.25701444
Acvr1c	0.4215073	4.78138108	4.21696329	0.0533929	0.25728495
Gm17644	-0.7417846	7.68606543	4.21557152	0.05342874	0.25728495
Cbr4	0.39261224	4.17034349	4.21539572	0.05343327	0.25728495
Tph2	2.61280224	-1.2085196	4.21496418	0.05344439	0.25728495
Ttc9c	0.33303752	6.09942812	4.21389214	0.05347203	0.25728495
Nphs2	0.55516394	3.92360048	4.21147644	0.05353437	0.25728495
BC005561	-0.4959274	5.15199396	4.2110094	0.05354643	0.25728495
Slc11a1	1.9770796	-0.4192487	4.20959298	0.05358303	0.25728495
Pcdhac2	0.42681192	4.03707919	4.20944228	0.05358692	0.25728495
Zfp871	-0.2702879	9.06013318	4.2077981	0.05362945	0.25728495

Ogg1	0.84471532	0.72943137	4.20754387	0.05363603	0.25728495
Zfp579	0.62953099	1.47293401	4.20745626	0.05363829	0.25728495
Arhgap23	-0.3631224	5.68416057	4.20618817	0.05367112	0.25728495
Mcts1	0.28930258	5.13383329	4.20601877	0.05367551	0.25728495
4930402H24I	-0.3694459	5.56917673	4.20521934	0.05369622	0.25728495
Sfrp2	1.07521608	0.51677032	4.20519436	0.05369687	0.25728495
Ak1	0.39542791	3.98628346	4.20469871	0.05370971	0.25728495
Far2	-0.496461	3.82220963	4.20184933	0.05378363	0.25752866
B3gat3	-0.4906509	3.19066001	4.20108012	0.0538036	0.25752866
Fkbp10	-1.0184544	2.21110393	4.19733547	0.05390095	0.25772936
Gm6994	-1.4100961	0.04688228	4.19728186	0.05390235	0.25772936
Esd	0.30281092	6.08734854	4.19622611	0.05392983	0.25772936
Sfrp1	-0.5014895	6.64119233	4.19615771	0.05393161	0.25772936
Rad51b	2.67428766	-1.3350402	4.19524747	0.05395533	0.25773982
Uggt2	-0.4901257	4.89630415	4.19091656	0.0540683	0.25817653
Peg10	0.34735562	6.36412456	4.18980236	0.05409741	0.25821257
Mkrn3	-2.1343393	-0.2692378	4.18506154	0.05422147	0.25858622
Rbbp8	0.4665177	4.13428988	4.18436467	0.05423973	0.25858622
Itga2	-2.1871994	0.13157257	4.18433646	0.05424047	0.25858622
Cramp1l	-0.267135	6.39451023	4.18325782	0.05426876	0.2586181
Epas1	-0.2582579	8.09369916	4.18003974	0.05435324	0.25885678
Gng4	-0.3122949	5.47719063	4.17970354	0.05436207	0.25885678
Tchp	-0.706091	2.32131566	4.17747738	0.05442061	0.25897772
Nt5m	0.43923967	3.91316768	4.17709331	0.05443072	0.25897772
AI182371	-4.0971872	-2.1088921	4.17504589	0.05448463	0.25913129
Dcaf4	0.48829317	3.15447989	4.1716643	0.05457382	0.25936818
Lrrfip1	0.28093162	5.98272944	4.17151497	0.05457776	0.25936818
Sox21	0.50820237	3.44466163	4.17026062	0.05461088	0.25942265
Chaf1a	-0.7697097	1.78037866	4.16897124	0.05464496	0.2594816
Lypd2	-1.2287189	1.82578607	4.16805802	0.05466911	0.25949338
Slc25a4	0.24740364	9.98946024	4.16192066	0.05483172	0.25972669
Prcc	0.4148499	4.29593695	4.16184849	0.05483363	0.25972669
Sipa1l3	0.30639728	5.34766959	4.16174395	0.05483641	0.25972669
Marf1	-0.267435	7.87549306	4.16142982	0.05484475	0.25972669
Sema5a	0.31601789	6.42910481	4.16061387	0.05486641	0.25972669
Angptl1	-1.5180581	0.5520539	4.16048028	0.05486996	0.25972669
Slc9a6	0.28651279	6.2789716	4.16047601	0.05487008	0.25972669
Il7	2.71650135	-0.8826703	4.15838456	0.05492566	0.2598467
6430503K07I	-1.9976139	-0.7179992	4.15788958	0.05493882	0.2598467
Rora	-0.2548755	8.37690505	4.15394191	0.05504395	0.26005131
Begain	-0.4728803	3.44830392	4.1535879	0.05505339	0.26005131
Snord71	-3.5635173	-1.3463177	4.15349549	0.05505585	0.26005131
Acp1	0.27268432	7.14125661	4.15300446	0.05506894	0.26005131

Qsox1	0.6563569	2.23119922	4.15216745	0.05509127	0.26005421
Adcy1	-0.3938282	8.75656607	4.14981506	0.05515409	0.26016755
Mvd	0.62456823	1.82124357	4.1496412	0.05515873	0.26016755
Atm	-0.3289226	5.99784166	4.14794254	0.05520415	0.26027924
Fignl1	1.87429416	-0.7257423	4.1457477	0.05526289	0.26045365
Supt16	-0.3874309	6.65731618	4.14258769	0.05534759	0.26072599
Ubxn7	0.21075831	7.31127239	4.14064477	0.05539974	0.26072599
9530080O11	0.60691758	2.39329389	4.14042137	0.05540574	0.26072599
Dxo	0.53242407	2.09703573	4.1401775	0.05541229	0.26072599
Atp5d	0.48793427	6.11576955	4.13953577	0.05542953	0.26072599
Vps4b	0.28426244	5.43696052	4.13767894	0.05547945	0.26085835
Aqp4	0.42507427	5.58475126	4.13681098	0.0555028	0.26086574
Gdap1l1	0.41589192	3.45144051	4.13385009	0.05558256	0.26109526
Gm4349	-1.060726	0.61440801	4.13337955	0.05559524	0.26109526
Tyrp1	4.63593041	-1.86409	4.13198446	0.05563287	0.26116958
Mrps17	0.42043137	4.15799816	4.1281993	0.05573512	0.26154707
Egfl6	1.29888184	0.88646447	4.12642547	0.05578312	0.26166974
Psmd4	0.36536219	5.3258604	4.12477367	0.05582785	0.26167539
Mcam	-0.8338234	1.72639642	4.1247673	0.05582802	0.26167539
Gm10677	-0.9644193	1.60301891	4.12236857	0.05589306	0.26185636
Col13a1	-0.7922517	2.28792226	4.12102792	0.05592944	0.26185636
Hnrnpd	0.2206459	8.29361653	4.12023388	0.055951	0.26185636
Fgf12	0.26259733	7.94471617	4.11934372	0.05597519	0.26185636
Slc18a2	-0.5737712	2.87449392	4.11931538	0.05597596	0.26185636
Sh3rf2	-0.4807998	3.63581108	4.1156493	0.05607569	0.2621119
Pwp1	-0.4444794	4.19205255	4.11333536	0.05613874	0.2621119
Spp2	-1.8515014	0.45542696	4.11145518	0.05619004	0.2621119
Pde6d	0.45715621	4.93270465	4.11068672	0.05621102	0.2621119
4921507L20R	-1.8539869	-0.5854421	4.11051315	0.05621576	0.2621119
Itfg1	0.25614769	7.75586571	4.11028472	0.05622199	0.2621119
Cyb5d1	0.78826288	1.39283836	4.10956769	0.05624158	0.2621119
Snx10	0.2702969	6.49559681	4.10923396	0.0562507	0.2621119
Trerf1	-0.4008644	5.40018908	4.1085136	0.05627039	0.2621119
Mfap1a	0.20891968	7.41490946	4.10775997	0.056291	0.2621119
Habp4	-0.2011018	7.39970962	4.10719715	0.0563064	0.2621119
Zfp568	-0.4570212	3.27424501	4.10687154	0.05631531	0.2621119
Vwa8	-0.3653587	5.28914095	4.10668716	0.05632035	0.2621119
Mtcp1	-0.5701995	3.03143394	4.10607884	0.056337	0.2621119
Dusp27	1.19308602	1.8053028	4.1048935	0.05636947	0.26216109
Aif1l	-0.660669	2.10260785	4.09969167	0.05651218	0.26272277
Ppp1r9a	-0.4993456	8.91038892	4.09815768	0.05655434	0.26281677
Zfhx2	-0.3741421	5.15266286	4.097058	0.05658459	0.26284848
Necab2	0.44247331	3.43692255	4.09631414	0.05660506	0.26284848

Dlst	0.24264119	6.53853897	4.09483456	0.05664581	0.26293572
Zfp429	-0.584114	2.01858964	4.09209872	0.05672123	0.26318383
Zswim5	-0.3262503	4.89871036	4.09098487	0.05675197	0.26322448
Mcf2	-0.6894903	3.13970048	4.08378219	0.05695123	0.26362119
Manbal	0.50102784	3.04796041	4.0828937	0.05697586	0.26362119
Engase	-0.8567267	0.75839057	4.08241981	0.05698901	0.26362119
Chac1	0.65211062	1.81672077	4.08148388	0.05701498	0.26362119
Tfg	0.23242398	6.88161521	4.0812641	0.05702108	0.26362119
Diap3	1.38918003	0.48127226	4.08036133	0.05704614	0.26362119
Cstf2t	0.2380595	6.61119104	4.08026557	0.0570488	0.26362119
Ccnl1	-0.5363	4.7158139	4.08013704	0.05705237	0.26362119
Pdhb	0.21189673	6.93228658	4.07852668	0.05709712	0.26362119
Acyp1	0.40515245	5.19441005	4.07845903	0.057099	0.26362119
Pard3b	-0.4523514	3.32297747	4.07782371	0.05711667	0.26362119
Pdcd1lg2	-1.3554986	0.07482019	4.07514904	0.05719111	0.26362119
Hp1bp3	0.19916056	8.1320659	4.07422617	0.05721683	0.26362119
Ppp2r1a	0.21603236	7.25493951	4.07410031	0.05722033	0.26362119
Tmem200a	0.56722012	3.68035073	4.07324345	0.05724422	0.26362119
Gm8787	-2.2090418	-1.3720546	4.07311834	0.05724771	0.26362119
Heatr1	-0.4948938	4.11923889	4.07248501	0.05726537	0.26362119
Ostf1	0.34220953	5.90732594	4.07219582	0.05727344	0.26362119
Cdc5l	0.2058313	6.18232915	4.07128165	0.05729895	0.26362119
Gspt2	0.37485387	4.58635054	4.07021525	0.05732873	0.26362119
Tjp1	0.24135761	9.48507631	4.06991657	0.05733707	0.26362119
Mir181b-2	-2.4621367	-1.1861624	4.06972957	0.05734229	0.26362119
Slco4c1	-0.7013201	2.13125651	4.0696755	0.0573438	0.26362119
Ovol1	-1.4514157	-0.3744116	4.06748833	0.05740495	0.26370093
4933413L06F	-1.2993785	-0.1724786	4.06619056	0.05744126	0.26370093
Fam134b	0.33508848	5.61864022	4.06610819	0.05744357	0.26370093
4632415L05F	0.29521878	5.48944619	4.06510724	0.0574716	0.26370093
Pld3	0.37980975	7.04304457	4.06438527	0.05749183	0.26370093
Sbk1	-0.3859908	4.16353769	4.06294944	0.05753208	0.26370093
4930506C21I	1.36284612	-0.2857268	4.0627918	0.0575365	0.26370093
Rab1	0.25768193	9.05096433	4.06276292	0.05753731	0.26370093
Ankrd1	-2.0187752	-1.3890658	4.06065798	0.05759638	0.26376905
Abhd16a	0.31601856	4.50715658	4.05786333	0.05767491	0.26376905
Armcx2	0.24297909	5.54431266	4.05769492	0.05767965	0.26376905
Alas1	0.29455779	4.46677621	4.05690274	0.05770194	0.26376905
Fam13b	-0.2281692	6.9242022	4.05625017	0.0577203	0.26376905
Cnpy4	-0.3514261	4.55218705	4.05579402	0.05773315	0.26376905
Shisa5	0.46671334	5.06994187	4.05522342	0.05774921	0.26376905
Plbd2	0.31405352	5.28181595	4.05474294	0.05776275	0.26376905
4933427I22R	-2.982741	-1.2003321	4.05407591	0.05778154	0.26376905

Kank1	-0.432327	3.58697731	4.05104	0.05786718	0.26376905
Brd8	-0.2680228	5.86129526	4.05027441	0.0578888	0.26376905
Dync1h1	-0.7791962	9.33827441	4.0497502	0.05790361	0.26376905
Fam178b	2.01891553	-1.1721613	4.04936869	0.05791439	0.26376905
9330133O14	0.43455233	4.07579386	4.04834353	0.05794336	0.26376905
Slc35b4	-0.2687983	5.64591633	4.04825881	0.05794576	0.26376905
Sbf2	-0.2792403	7.08779668	4.04824655	0.05794611	0.26376905
Sin3b	0.3706836	4.8061056	4.04792353	0.05795524	0.26376905
BC005764	-0.7079265	2.18317284	4.047538	0.05796614	0.26376905
Tmem255a	0.40874584	4.74803875	4.04737871	0.05797065	0.26376905
Rpap3	0.28699722	4.49697845	4.04489396	0.05804099	0.26389959
Kcnh7	-0.3936854	6.28135969	4.04466345	0.05804752	0.26389959
Cd34	-0.4793465	3.49869475	4.04334825	0.0580848	0.26389959
Spef1	-0.7425841	1.53809466	4.04325349	0.05808749	0.26389959
Pacs1	0.2551985	6.74546738	4.04119152	0.05814599	0.26406519
Atp5g3	0.2863652	8.20651786	4.03166393	0.05841718	0.26519621
Nup88	-0.3000203	5.69244822	4.02615996	0.0585745	0.2656947
Rgs6	-0.5065008	3.12285949	4.0259145	0.05858153	0.2656947
Tpm4	0.38460058	9.13505951	4.02549491	0.05859354	0.2656947
Snhg11	-0.743326	9.45745744	4.02263265	0.05867558	0.265966
Napg	0.24034862	6.73051535	4.02175816	0.05870067	0.26597905
Ubap2l	0.24498194	8.54563331	4.01506805	0.05889303	0.26662794
Casp3	0.41839527	4.02964314	4.01465639	0.05890489	0.26662794
Eif5b	0.29269029	9.35457205	4.01341698	0.05894061	0.26662794
Gm10125	-0.9369021	1.01722952	4.01227061	0.05897368	0.26662794
Igfn1	-0.6009904	4.3999423	4.01203858	0.05898037	0.26662794
6430531B16	2.05446271	-0.6821775	4.01029775	0.05903063	0.26662794
Ipmk	-0.2923802	5.56169021	4.00963199	0.05904986	0.26662794
Gjc2	-1.2295899	0.31923454	4.00918853	0.05906268	0.26662794
Zfp87	0.31010037	5.56435072	4.00744446	0.05911311	0.26662794
Gm19757	-0.548534	4.53340146	4.00682859	0.05913093	0.26662794
Gjd2	0.67370219	1.72208142	4.00488378	0.05918724	0.26662794
Aven	0.73818707	1.31727111	4.00414143	0.05920876	0.26662794
Ppp1r16b	-0.344538	6.6634523	4.00314783	0.05923756	0.26662794
Smc1a	-0.3120045	8.10839609	4.00282017	0.05924706	0.26662794
Bach1	0.31945169	5.05667399	4.00199702	0.05927095	0.26662794
Trim35	0.2381857	6.9522191	4.00187369	0.05927453	0.26662794
Ncstn	0.37796226	5.1058978	4.00079863	0.05930573	0.26662794
Gltpd2	2.92833482	-1.9420875	3.99941959	0.05934579	0.26662794
Heatr5b	-0.3976059	5.48811715	3.99857275	0.05937041	0.26662794
Mafb	0.28346415	5.48543859	3.99846163	0.05937364	0.26662794
Twistnb	-0.2696535	5.20112615	3.99786709	0.05939093	0.26662794
Lrrc56	-1.2404199	0.36716053	3.9977755	0.0593936	0.26662794

Gtf2a1	0.22855068	6.99718016	3.99763934	0.05939756	0.26662794
Asun	-0.305192	4.53145219	3.99613368	0.05944138	0.26662794
Cetn4	0.53059284	4.02178654	3.99592722	0.05944739	0.26662794
Neo1	0.2823435	7.54444732	3.99564029	0.05945574	0.26662794
Tie1	-1.6840765	0.39482434	3.99468359	0.05948361	0.26662794
Etaa1	0.4018868	4.87202741	3.99451746	0.05948845	0.26662794
Hsf2bp	-0.8163569	2.07785124	3.99448035	0.05948953	0.26662794
F2r	0.518289	2.66735324	3.99350237	0.05951804	0.26665592
Oas3	-1.5047674	0.6839544	3.99096982	0.05959194	0.26677519
Mb21d2	-0.337025	5.16656613	3.98972388	0.05962833	0.26677519
Zik1	0.42167916	3.46310341	3.98924448	0.05964234	0.26677519
Mpeg1	-0.6691207	2.86253962	3.98869274	0.05965847	0.26677519
Eya1	0.34546446	7.18135044	3.98815879	0.05967408	0.26677519
Actn4	-0.2265716	6.28219437	3.98801389	0.05967832	0.26677519
Tada1	0.3256749	5.26378133	3.98642275	0.05972488	0.2668837
Srp14	0.33798222	6.88944563	3.98393331	0.05979781	0.26709159
Gch1	-0.8691645	0.83721912	3.98331252	0.05981601	0.26709159
Cmpk1	0.27445084	7.47820594	3.981175	0.05987873	0.26727201
Fahd1	0.29738175	5.2997062	3.97844282	0.05995901	0.26738188
Rapgef1	-0.2865456	6.0110437	3.97842474	0.05995955	0.26738188
Adam9	0.27939504	4.9569587	3.97805808	0.05997033	0.26738188
Efcab6	0.86460011	1.7914979	3.97716161	0.0599967	0.26739992
Clstn1	0.29621926	8.08403304	3.97505591	0.06005871	0.26757667
Rorb	-0.3204262	8.50372588	3.9722023	0.06014285	0.2678519
Pgap3	-1.5746059	-0.2730341	3.9710737	0.06017616	0.26788991
Fiz1	0.41627818	3.76023	3.97039794	0.06019612	0.26788991
Gpr171	-0.9337641	1.52802902	3.96957491	0.06022044	0.26789857
Ndst4	0.59359559	3.24257886	3.96824809	0.06025967	0.26797354
Htr1b	-0.7022738	1.29237253	3.96605868	0.06032446	0.26812792
Cox6a1	0.2924193	6.82536447	3.96515276	0.06035129	0.26812792
Ssr1	0.2731372	7.3121515	3.96467412	0.06036547	0.26812792
Fkbp1a	0.28285943	9.78767574	3.96339594	0.06040337	0.26812792
Mzb1	-2.5657271	-1.791709	3.96329611	0.06040633	0.26812792
Fbxo36	0.7119015	2.9116626	3.96228623	0.06043629	0.26816151
A930013F10	-1.1090544	1.71661462	3.96032988	0.06049438	0.26831983
Akr1b10	0.33154517	3.77109963	3.95767759	0.06057323	0.26845072
Nrsn2	0.35957428	5.88585076	3.9576246	0.06057481	0.26845072
Tagap1	-0.3273458	4.77504619	3.95707588	0.06059113	0.26845072
Bcas1	-0.4169794	4.7132492	3.95486592	0.06065695	0.26857996
Laptm4b	0.27846304	5.34753559	3.95427898	0.06067444	0.26857996
A730017L22I	-0.5121627	4.89894303	3.95383816	0.06068759	0.26857996
Lama4	0.38737315	4.07808699	3.95210337	0.06073934	0.26870969
Calcr	1.44845189	0.29845713	3.95016398	0.06079725	0.26886271

Nxnl1	3.29549236	-1.2919197	3.9491158	0.06082858	0.26886271
Ccdc88a	0.31530315	9.01307016	3.94869115	0.06084128	0.26886271
Nhlrc4	-1.8766607	-0.4151963	3.94473767	0.06095963	0.26926683
Peak1	-0.3826655	6.77754492	3.94413514	0.0609777	0.26926683
Pdp2	-0.5454772	3.55228606	3.94296309	0.06101285	0.26932275
4933412E12I	-0.6760822	1.84800618	3.94015691	0.0610971	0.26950299
Arpp19	0.2161554	9.06957208	3.94003606	0.06110073	0.26950299
Usp47	0.19927627	7.1442615	3.93935535	0.06112119	0.26950299
Slc35a2	-0.5389474	3.20080837	3.93812657	0.06115814	0.26956668
Taz	0.46564638	3.1171464	3.93616166	0.06121729	0.26972809
Rassf9	-1.0209892	0.90130565	3.93483471	0.06125726	0.26980497
Calr	0.27686477	6.40600111	3.93324027	0.06130534	0.26991745
Ywhah	0.19970882	10.4947765	3.9324679	0.06132865	0.26992082
Med1	-0.2651801	7.2607964	3.93079397	0.06137919	0.27004402
Usp29	-0.3512584	5.56455857	3.92953697	0.06141717	0.2701119
Zfp560	-0.4338972	4.12025676	3.92549787	0.06153941	0.27026293
Rcbtb1	-0.3106173	6.01330375	3.92541963	0.06154178	0.27026293
Lrp11	0.30653438	5.96147788	3.92526472	0.06154648	0.27026293
Trem2	1.50629867	-0.4979594	3.92423215	0.06157778	0.27026293
Polr3g	-0.431209	3.14058005	3.9240269	0.061584	0.27026293
Macf1	-0.4843505	9.05836431	3.92329738	0.06160613	0.27026293
Itsn1	-0.3585743	7.01485215	3.92318663	0.06160949	0.27026293
Plekhb2	0.2310553	6.30581925	3.92015958	0.0617014	0.27051951
Ube2v2	0.24464487	6.88664878	3.91977281	0.06171316	0.27051951
Trim21	-0.5133143	3.83998389	3.91750595	0.06178211	0.27072266
BC018242	0.33639268	4.75332154	3.91657871	0.06181033	0.27074729
Angptl7	1.17956455	1.56096418	3.91383608	0.06189392	0.27101429
N4bp2	-0.4830039	3.4697598	3.91244662	0.06193631	0.27109967
H2-K2	-1.9563512	-0.367524	3.91061509	0.06199225	0.27109967
Camsap3	0.40931195	3.48513944	3.91037906	0.06199946	0.27109967
Herc1	-0.5475116	8.10533263	3.9095431	0.06202501	0.27109967
Wasl	0.23384735	8.11217707	3.90949097	0.0620266	0.27109967
Scnm1	0.42688536	4.02330932	3.90808348	0.06206965	0.2711405
Irf2bp2	-0.2199543	8.43053574	3.90732647	0.06209282	0.2711405
Pus1	0.65799564	1.66141706	3.90598274	0.06213397	0.2711405
Mafk	0.6114056	3.20017283	3.90560456	0.06214556	0.2711405
Scrg1	1.06936384	1.44732958	3.90477589	0.06217096	0.2711405
Mc4r	1.97974451	-0.7315664	3.90474868	0.06217179	0.2711405
Gprasp2	-0.3993977	6.17426828	3.90294956	0.06222698	0.27128238
Plb1	0.76398821	2.38062734	3.90115854	0.06228197	0.27142331
Eqtn	-1.6854755	0.10408834	3.89926181	0.06234026	0.27157637
Fuca2	0.30339386	4.54226025	3.89854075	0.06236244	0.27157637
Nanog	-1.3207789	-0.5025616	3.89299721	0.06253326	0.2721347

Spock1	0.28634236	8.18431266	3.89290508	0.0625361	0.2721347
Calm1	0.2001441	12.308041	3.89189903	0.06256716	0.27217096
Itgam	-0.5261143	3.55949503	3.88247481	0.06285897	0.27321535
Sox7	-1.0843201	0.68090057	3.88244223	0.06285998	0.27321535
C1qtnf6	-0.904491	1.04805475	3.88036872	0.0629244	0.27321535
2900056M2(	0.33735487	7.95961071	3.88016133	0.06293085	0.27321535
Tek	-0.5805154	2.9134999	3.87915309	0.0629622	0.27321535
Keap1	-0.3125619	4.39016593	3.87880258	0.0629731	0.27321535
Impad1	0.23568787	7.02690267	3.87772023	0.06300679	0.27321535
Syt15	-1.0071027	1.38226934	3.87731629	0.06301936	0.27321535
Ppef1	-1.7378027	-0.7102362	3.87657748	0.06304237	0.27321535
Cd52	-0.6679234	2.35416589	3.87627541	0.06305179	0.27321535
Cyc1	0.34764772	6.32295405	3.87606945	0.0630582	0.27321535
Tmco1	0.52939187	4.77673781	3.87295541	0.06315532	0.27337089
Npas1	1.42426211	-0.7981625	3.8727721	0.06316104	0.27337089
Erich3	-0.6461598	3.68636656	3.87247416	0.06317035	0.27337089
Triap1	-0.6953062	3.20084066	3.87199184	0.06318541	0.27337089
Maf	0.33751436	7.20796103	3.87062065	0.06322825	0.27343154
Ift46	0.43310661	4.57608285	3.87008196	0.06324509	0.27343154
Plcd1	-0.6052171	2.01051143	3.86588678	0.06337642	0.27390042
Rhbdl1	-0.8315385	0.61950411	3.86456514	0.06341785	0.27398063
Ltbp2	-2.8931183	-1.1453635	3.86295619	0.06346834	0.27409987
Dync1li1	0.34424425	5.04728039	3.85991361	0.06356394	0.27432572
Sh3pxd2b	-0.328252	4.59955708	3.85983382	0.06356645	0.27432572
Ndufa3	0.3852323	4.66141307	3.85800801	0.0636239	0.27447474
Cmklr1	0.81964409	1.08346113	3.8561603	0.0636821	0.27462689
Pcdhgb1	-0.5536595	4.09657467	3.85407094	0.06374799	0.27478626
Maged2	0.32179619	4.4644189	3.85353345	0.06376495	0.27478626
Rps6ka6	0.5760949	2.62959887	3.85040767	0.06386369	0.27502538
Pex10	0.52458151	2.67676415	3.8503231	0.06386637	0.27502538
Rbpms2	0.68185921	2.82537584	3.84925387	0.06390019	0.27507211
Ift80	-0.2801154	4.88958403	3.84664798	0.0639827	0.27532832
Taf7l	-1.1081342	1.07692648	3.84524003	0.06402733	0.27542141
Akap12	0.32651544	8.54412102	3.84200694	0.06412995	0.27570885
Szt2	-0.4933133	3.93196404	3.84168456	0.06414019	0.27570885
Gnpat	-0.3161226	5.40993141	3.83824328	0.06424965	0.27608027
Urm1	-0.5879294	2.19931317	3.83676285	0.06429681	0.27617827
Bahcc1	-0.434967	3.98732664	3.83600754	0.06432088	0.27617827
Hey2	-0.573211	2.26917077	3.83464151	0.06436445	0.27617827
Epb4.1l4a	0.64242764	1.53030088	3.83463339	0.06436471	0.27617827
C1galt1	0.39706376	4.66589561	3.83201299	0.06444837	0.27639002
Tcf7	0.36202117	4.87830236	3.83094201	0.0644826	0.27639002
Gdnf	1.49067244	-0.5461555	3.83072169	0.06448965	0.27639002

Pisd-ps1	-0.5523878	5.75833039	3.83019867	0.06450637	0.27639002
A4galt	0.54826746	2.77150386	3.82887474	0.06454873	0.27647261
E2f5	-0.3234356	4.32218395	3.82588517	0.06464451	0.276636
Plec	-0.3645797	6.34716249	3.82586519	0.06464515	0.276636
Myo10	-0.2732121	5.70474364	3.82552125	0.06465618	0.276636
Rab19	0.93622347	1.98296092	3.82198444	0.06476972	0.27700007
Nkx1-2	4.32780896	-1.9523449	4.02132772	0.06478753	0.27700007
Pbx3	0.40856145	7.274062	3.81982509	0.06483916	0.27703488
5031410I06R	-0.8027406	2.61793418	3.81973864	0.06484194	0.27703488
Mmgt2	0.48923238	3.31545724	3.8175604	0.06491207	0.27723561
Kcnj15	2.74484061	-0.7769083	3.81467541	0.0650051	0.27751584
AW112010	0.70368784	1.65671888	3.81385215	0.06503167	0.27751584
Psenen	0.5241175	5.2136911	3.81337097	0.06504721	0.27751584
Zcchc9	0.2645528	5.82683185	3.80875362	0.06519652	0.2780538
Abhd8	-0.2952185	4.56973272	3.80752333	0.06523637	0.27812471
Efna2	1.13711134	0.54814271	3.8062188	0.06527865	0.27820595
Ccnl2	-0.5273886	4.42696557	3.80149996	0.06543188	0.27875975
Nkain1	-0.3114027	4.40543864	3.80020186	0.0654741	0.27881315
Polr3c	0.39877168	3.20865398	3.79887726	0.06551722	0.27881315
Ago4	-0.6492484	3.22251351	3.79854974	0.06552788	0.27881315
Cps1	2.80099119	-1.1991951	3.79777245	0.0655532	0.27881315
Fam227a	-0.9064134	1.77176102	3.79753883	0.06556082	0.27881315
Cacng5	0.64864303	2.68079669	3.79615375	0.06560597	0.27890614
Haus1	0.54582291	2.88420026	3.79416474	0.06567088	0.279083
Mob3a	0.48993724	2.63458704	3.79278909	0.06571581	0.2791106
Foxg1	0.31711172	6.13935789	3.79216764	0.06573612	0.2791106
Slc12a5	-0.3467524	7.82459709	3.79182599	0.06574729	0.2791106
Cnrip1	0.25816108	6.07897393	3.78950542	0.06582321	0.27930073
Ddx26b	-0.5499577	4.50924521	3.78903176	0.06583872	0.27930073
Fam81a	-0.2917274	5.71032253	3.78372479	0.06601278	0.27993993
A630089N07	-0.6807215	8.10768397	3.78246744	0.06605409	0.28001598
Oxt	-1.1271765	0.36731434	3.77993307	0.06613746	0.28027018
Nudt11	0.38125157	4.15227388	3.77758566	0.06621479	0.28049861
Pid1	0.27806168	6.78804196	3.77456915	0.0663143	0.28076199
4930447C04I	-0.9395678	2.03600287	3.77424282	0.06632508	0.28076199
Dad1	0.51816572	5.04388937	3.77352296	0.06634886	0.28076199
Ift88	-0.3758889	4.21810903	3.77286098	0.06637074	0.28076199
Tpm1	0.29197545	9.356263	3.77027971	0.06645612	0.28095613
A430078G23	-0.4463997	3.71615754	3.77005517	0.06646355	0.28095613
Kif18b	2.32554983	-1.7836768	3.76878769	0.06650553	0.28103437
9330159M07	-0.5676847	2.13112621	3.76762817	0.06654395	0.28109757
Smoc2	0.54329163	3.40767044	3.76534599	0.06661966	0.28131815
Ccdc159	-1.3714596	0.35808225	3.76342761	0.06668338	0.28148795

Nme4	1.55784806	0.20736977	3.76092304	0.06676667	0.28174024
Iqca	-1.7982946	-0.2712999	3.7595936	0.06681093	0.2818277
Ift57	0.2318562	5.92742361	3.7574702	0.0668817	0.28202686
Ccl7	-1.8234649	-0.4119397	3.7555791	0.06694479	0.28219356
1810041L15F	-0.4044483	5.52631739	3.75230892	0.06705406	0.28252924
Cdk5r1	0.30918662	6.96452263	3.75178429	0.06707161	0.28252924
Gng5	0.38121005	6.39957695	3.74813829	0.06719371	0.28294407
Zfp384	0.27535862	5.17019792	3.74655116	0.06724695	0.2830687
Nicn1	0.2293189	6.46661073	3.74509574	0.06729581	0.28317483
Zfp385c	-0.6481216	1.90571192	3.74237865	0.06738713	0.28324075
Daam2	-0.4013708	5.00971729	3.74200959	0.06739954	0.28324075
Palld	-0.6811775	1.61944209	3.7418332	0.06740548	0.28324075
Akap7	-0.2815199	6.50819349	3.74181141	0.06740621	0.28324075
Spin2c	0.51168006	2.93583366	3.73999441	0.06746738	0.28324075
Prkcg	0.29299828	8.20544702	3.73876754	0.06750872	0.28324075
Ifitm10	1.31160565	0.45607829	3.73773835	0.06754342	0.28324075
Gabra3	0.2663682	5.47618171	3.73739469	0.06755501	0.28324075
Bcar3	0.51219827	2.67844132	3.73732096	0.0675575	0.28324075
Wdr5	-0.6221717	3.23638315	3.7369943	0.06756852	0.28324075
Kcnj9	-0.352266	5.02242823	3.73609302	0.06759894	0.28324075
Kmt2c	-0.3099574	8.17683856	3.73553914	0.06761764	0.28324075
Tcfl5	-2.6416296	-1.4827412	3.73490313	0.06763912	0.28324075
Them4	0.42072372	4.2544018	3.73480051	0.06764259	0.28324075
Smim22	-1.9831288	-0.4654635	3.73220423	0.06773037	0.28350918
Zfp60	-0.2597136	6.37575966	3.72949138	0.06782223	0.28379085
Eif4ebp2	-0.3290042	5.77871641	3.72881795	0.06784505	0.28379085
Myh10	-0.3726841	8.54003851	3.72513494	0.06797004	0.28418548
St3gal4	-0.6748288	3.96093969	3.72388748	0.06801244	0.28418548
Btbd17	1.02998352	0.43753418	3.72364287	0.06802075	0.28418548
Elovl7	-0.3786952	4.0967768	3.72167839	0.06808759	0.28418548
Taf11	0.30726696	5.26365295	3.7216436	0.06808877	0.28418548
Hey1	0.28221883	6.35228255	3.72065776	0.06812234	0.28418548
Dact3	-0.2827598	4.11706436	3.71983	0.06815054	0.28418548
Ptpn2	-0.4539423	4.93947843	3.7196169	0.0681578	0.28418548
Gm15545	-1.7566764	-0.7285073	3.71919156	0.0681723	0.28418548
Psmb5	0.31718721	5.10193767	3.71906254	0.0681767	0.28418548
Hagh	0.36778924	4.40177537	3.71701727	0.06824647	0.2843142
Fam120a	0.22515423	8.53748752	3.71676551	0.06825506	0.2843142
Oprd1	0.45040096	4.16299304	3.71533325	0.06830397	0.28437654
Zfyve28	-0.5265074	3.78121154	3.7149368	0.06831752	0.28437654
Ndufaf4	0.28355282	5.76558867	3.71288807	0.06838757	0.28456923
Clk1	-0.4124446	6.46913764	3.70937379	0.06850793	0.28497104
Morn2	0.54676809	2.89696583	3.70755108	0.06857045	0.28513207

Klhl15	-0.5331252	3.77533684	3.70599729	0.0686238	0.28525328
Dedd2	0.79724262	1.48902123	3.70468967	0.06866873	0.28525328
Lag3	2.16301872	-1.3344653	3.70462201	0.06867106	0.28525328
Rpp30	0.32514228	3.992085	3.70113514	0.06879105	0.28565262
Polr3d	0.48277654	3.08164623	3.69853049	0.06888084	0.28592633
Rraga	0.29863677	6.03591811	3.69653618	0.06894968	0.28600199
Tpo	-2.762783	-2.0661477	3.69625757	0.0689593	0.28600199
Map3k7	0.20796778	6.60263861	3.69526269	0.06899368	0.28600199
Tnrc6a	-0.2685813	6.73545539	3.69516183	0.06899716	0.28600199
Pigp	0.39245624	4.8019085	3.69454539	0.06901848	0.28600199
Clcn6	-0.3948382	3.55535968	3.69314103	0.06906705	0.28607162
Helz	-0.3503881	6.72444191	3.69267879	0.06908305	0.28607162
Acpp	-0.8165001	4.28697878	3.69099838	0.06914125	0.28618147
Pcsk2	0.30488719	7.2390638	3.6905331	0.06915737	0.28618147
Abi1	0.20976755	7.09800737	3.68889983	0.06921401	0.28631689
Ppp5c	0.30429534	4.63015193	3.68773	0.0692546	0.2863859
Pcdhgb8	-0.8628659	1.14866749	3.68663159	0.06929275	0.28644472
5830403L16F	1.64714902	-0.5332773	3.68576633	0.06932281	0.28647011
Tceal3	0.28594671	5.14347973	3.68206461	0.0694516	0.28690323
Thbs4	-1.0301394	0.57915578	3.68137749	0.06947553	0.28690323
Tspan33	-0.6851916	1.50651382	3.68012568	0.06951916	0.2869604
Nr2f2	0.29274332	7.60255619	3.67903139	0.06955733	0.2869604
A330070K13	2.18933575	-0.6852585	3.67891871	0.06956126	0.2869604
Mamdc2	1.34751171	0.03578776	3.67722332	0.06962045	0.2870411
Cfb	-0.6999999	2.67106589	3.67698532	0.06962876	0.2870411
Ebf3	-0.6526641	1.46044412	3.67604647	0.06966157	0.28707751
Zfp84	-0.2820442	5.15423552	3.67505854	0.0696961	0.28708151
Zbtb9	-0.6232518	2.9040012	3.67464747	0.06971048	0.28708151
Cerkl	1.29474337	-0.1444446	3.67101549	0.06983766	0.28740803
Dock9	-0.3371621	6.49843439	3.67036667	0.0698604	0.28740803
Prkca	-0.3260663	8.12929589	3.67032782	0.06986176	0.28740803
Rpl22	-0.3936032	6.20026759	3.66898574	0.06990884	0.28742611
Dhx15	-0.2061941	6.58366258	3.6688342	0.06991416	0.28742611
Plekhn1	1.44081113	-0.0644064	3.66750139	0.06996096	0.28747233
H2-T23	-0.3162012	4.24340416	3.66714677	0.06997342	0.28747233
08-Sep	-0.2762253	5.95391556	3.6663481	0.07000148	0.28748901
Tgs1	-0.3776801	6.52407515	3.66454709	0.07006481	0.28757208
Atp6v0c	1.98468526	-1.4626993	3.66392315	0.07008677	0.28757208
Gspt1	0.20838152	7.58456715	3.66372496	0.07009375	0.28757208
Med27	0.56208572	3.23350047	3.66255856	0.07013482	0.28764204
Rbm4b	-0.3534309	4.34630072	3.66125011	0.07018092	0.2877326
C1qbp	0.24597408	5.21647744	3.66001907	0.07022434	0.28779817
Gatad2b	0.20370426	7.58803378	3.65813134	0.07029096	0.28779817

Akap6	-0.4680996	8.14468972	3.65760766	0.07030946	0.28779817
Spdef	-2.9347827	-1.9795163	3.65739685	0.07031691	0.28779817
Sdcbp2	1.47784949	-0.7021209	3.6573921	0.07031708	0.28779817
Nadsyn1	-0.6781324	1.37774701	3.65591359	0.07036934	0.28787141
Galm	0.6866499	2.35655813	3.6540972	0.0704336	0.28787141
St8sia6	-1.1259247	0.86685262	3.65321361	0.07046488	0.28787141
Ctxn2	-0.8058525	1.98381935	3.6531342	0.0704677	0.28787141
Ccdc151	-0.8672328	0.63614155	3.65284869	0.07047781	0.28787141
Csmd2os	-1.5226723	-0.3659152	3.65191819	0.07051078	0.28787141
Bsdc1	0.29332533	5.28190997	3.65150086	0.07052557	0.28787141
Gm15413	2.13872062	-0.5351781	3.65145282	0.07052728	0.28787141
Sgcz	-0.9163718	1.8960049	3.65051641	0.07056048	0.28788865
Entpd3	-0.6501729	1.63353035	3.64997818	0.07057958	0.28788865
Xlr4a	1.36131401	0.03908689	3.64804504	0.07064821	0.28795184
Lphn2	-0.2561458	6.35521224	3.64766715	0.07066163	0.28795184
Zfp362	0.30775179	5.19322583	3.6475104	0.0706672	0.28795184
Polg	-0.4156452	3.26366115	3.6417265	0.07087307	0.28854617
Mir186	-2.0067765	-0.8361679	3.63991092	0.07093784	0.28854617
Sec23ip	-0.2961721	5.45952368	3.63986733	0.07093939	0.28854617
Coasy	0.41809377	3.65694836	3.63981913	0.07094111	0.28854617
Cdk5r2	-0.2442029	6.69943838	3.63851592	0.07098765	0.28854617
Thap4	0.38780105	4.20500576	3.63790035	0.07100964	0.28854617
Tmem97	-0.6680143	2.13363819	3.63694893	0.07104364	0.28854617
Tubgcp4	0.37073326	3.73131625	3.63624056	0.07106897	0.28854617
Mcm3ap	-0.469242	4.45572644	3.6354951	0.07109564	0.28854617
Sh2b2	-1.25527	0.01806364	3.6350853	0.07111031	0.28854617
St3gal6	-0.3848469	4.08416693	3.63432947	0.07113737	0.28854617
Clptm1	0.28220886	6.04131309	3.63372812	0.0711589	0.28854617
Mrpl30	0.31495535	5.69190516	3.63352697	0.07116611	0.28854617
Slc38a11	1.91246884	0.50440634	3.63264154	0.07119784	0.28854617
Atpaf1	0.25619638	6.0288518	3.63246156	0.07120429	0.28854617
Ago2	-0.2291266	7.24356027	3.63187955	0.07122515	0.28854617
Ndufa10	0.26309638	6.26299292	3.63172578	0.07123067	0.28854617
Ppapdc1a	1.37676713	-0.1787357	3.63053472	0.07127339	0.28854617
BC020402	-0.8953446	1.68090219	3.63029813	0.07128189	0.28854617
Gng8	-1.3679444	-0.4881482	3.62961194	0.07130652	0.28854617
Lage3	0.52185668	3.39926527	3.62926324	0.07131904	0.28854617
Snhg8	-0.5214071	2.70202649	3.62586922	0.07144105	0.28886025
Mif	0.34642283	5.21874154	3.62576185	0.07144491	0.28886025
Tmem91	0.76293801	1.95762266	3.62400846	0.07150804	0.28901792
Mpv17	0.321254	5.95198813	3.62256676	0.07156	0.28905267
Pced1b	-0.4427703	3.1020314	3.62232982	0.07156854	0.28905267
Lzts1	0.6278416	2.77788599	3.62176113	0.07158905	0.28905267

Smn1	-0.4135211	4.31988517	3.6204828	0.07163517	0.28914143
Aqp9	-2.247236	-1.2215807	3.61834679	0.07171232	0.28915613
Fgd2	1.00895687	0.37736609	3.61829637	0.07171415	0.28915613
Pcdh8	0.54033847	2.58289504	3.61772764	0.07173471	0.28915613
Cngb1	1.14182772	0.65399982	3.61770849	0.0717354	0.28915613
E130309D02I	0.36408764	4.011427	3.61467114	0.07184531	0.28940148
Krr1	0.20710477	6.82468627	3.61464928	0.0718461	0.28940148
Klrc1	1.49252624	-0.657688	3.61365288	0.0718822	0.28940148
Mrpl32	0.41805152	3.95919809	3.61335693	0.07189293	0.28940148
D4Ertd617e	-2.6248575	-1.1719346	3.61165425	0.07195468	0.28955271
Alg6	0.44465025	3.97261279	3.61019397	0.07200768	0.28955744
Bivm	0.26205976	4.9555411	3.61015763	0.072009	0.28955744
Timm17a	0.32816191	5.75091044	3.60916544	0.07204504	0.28955744
Pdzd9	-1.1686316	0.29976476	3.60895842	0.07205256	0.28955744
Lsm14b	0.24482532	5.55529305	3.60772943	0.07209724	0.28957244
Syt1	0.27389001	9.90610824	3.60752551	0.07210466	0.28957244
Nmur2	4.0232344	-1.6835497	3.60557403	0.07217567	0.28976047
Acss2os	-2.1826768	-1.5760205	3.60316275	0.07226353	0.28997994
Hmgb3	0.24415181	5.81681194	3.60274496	0.07227877	0.28997994
Gm20743	2.16987754	-1.8229197	3.59717811	0.07248213	0.29069844
Csnk2b	0.28984673	5.72714885	3.59559474	0.0725401	0.29083102
Ism1	0.64334183	3.28477602	3.59494876	0.07256376	0.29083102
Msantd4	0.22028686	7.36767747	3.59341437	0.07262	0.29086333
Gbp4	-0.5078773	2.90450644	3.59340361	0.0726204	0.29086333
Ctsd	0.46317635	7.20349076	3.5927315	0.07264505	0.29086478
Chchd10	0.29473939	5.60173779	3.59136777	0.0726951	0.29096789
Slc7a5	0.37265408	4.14321219	3.58904205	0.07278054	0.29121257
Wdr73	-0.3017724	4.41027299	3.58746881	0.07283841	0.29134676
Spaca1	-1.5022933	0.59477305	3.58544705	0.07291285	0.29149321
Syap1	0.45036924	5.92517928	3.58515256	0.0729237	0.29149321
Adhfe1	-0.3728421	5.11547406	3.58377463	0.0729745	0.29150433
Pon3	0.59537958	3.32299925	3.58375646	0.07297517	0.29150433
Gnas	0.22886753	10.8121039	3.58240979	0.07302485	0.29160552
Asic2	0.31000542	5.16739371	3.58121677	0.0730689	0.29162721
Specc1l	-0.2540587	6.43890441	3.58045205	0.07309714	0.29162721
Gpr34	0.55921049	2.07134329	3.58006194	0.07311156	0.29162721
Fmo2	0.68646997	2.18109682	3.57962568	0.07312769	0.29162721
Praf2	0.37701507	4.63199854	3.57637355	0.07324802	0.29183335
Atp8b5	-2.0516237	-0.868689	3.57473123	0.07330888	0.29183335
Tiam2	0.37224649	4.92606776	3.57440048	0.07332114	0.29183335
Emilin1	0.60570007	2.02283567	3.57419184	0.07332888	0.29183335
Col22a1	-1.9028125	-0.3577219	3.57332036	0.07336121	0.29183335
Cdh4	0.41155117	3.48979286	3.57288892	0.07337722	0.29183335

BC049635	-0.5860677	3.11311476	3.57245177	0.07339345	0.29183335
Elovl2	0.60666702	2.66654295	3.57215771	0.07340436	0.29183335
Ttbk1	-0.4403195	4.05638659	3.57190692	0.07341368	0.29183335
Zfp358	0.41314738	3.17915035	3.5716541	0.07342306	0.29183335
Kdm3b	-0.201394	7.03143235	3.57000122	0.07348448	0.29198056
Msra	0.49584598	3.48859937	3.56906533	0.07351929	0.29202195
Nudt3	-0.307433	6.12764947	3.56765316	0.07357184	0.29209902
Tuba4a	0.26230212	7.31249873	3.56696045	0.07359763	0.29209902
Gm16740	1.75463069	-0.806682	3.56657836	0.07361186	0.29209902
1810062G17	-3.190214	-1.2913555	3.56442701	0.07369205	0.2923045
Gss	0.47226779	3.45881164	3.56327411	0.07373507	0.2923045
Sssca1	0.51968861	2.92878654	3.56322585	0.07373687	0.2923045
Tppp3	-0.4094352	3.54507337	3.56165636	0.07379547	0.29244002
Nxph2	1.20794885	0.00279407	3.55986981	0.07386225	0.29256312
Ctla2a	-0.7645616	1.86945644	3.55951837	0.0738754	0.29256312
Sema6c	-1.0185811	0.69316727	3.55552378	0.07402499	0.29303789
9030612E09I	-1.0222378	1.32594339	3.55438985	0.07406752	0.29303789
1700112E06I	0.79433545	1.17197641	3.55435862	0.07406869	0.29303789
Apln	-0.6550906	2.58939571	3.55197641	0.07415813	0.29329485
Vps28	0.27925004	5.80975147	3.54929662	0.07425889	0.2935964
Atp6v0d1	0.25142333	7.16095009	3.54825836	0.07429797	0.29365397
Prmt1	0.57900526	2.8682413	3.54690759	0.07434885	0.29366412
Mre11a	0.65990043	2.75621419	3.54624377	0.07437387	0.29366412
1700109K24I	-1.3481184	0.84873822	3.54623756	0.07437411	0.29366412
Phyhip	0.30131851	6.76786975	3.54489369	0.07442479	0.29374156
Fam212b	0.25438561	6.76084735	3.544061	0.07445621	0.29374156
Lyz2	-0.5725283	3.56935062	3.54322831	0.07448765	0.29374156
Ndufc1	0.3739662	5.67207683	3.5426153	0.0745108	0.29374156
Tusc2	-0.2582138	4.92345459	3.54246824	0.07451636	0.29374156
Pidd1	-1.9723264	-1.6530424	3.53913097	0.07464257	0.29414225
Sox4	-0.4726423	2.90899654	3.53740532	0.07470793	0.29430296
Gm9159	-0.8100225	2.04989357	3.53573412	0.07477128	0.29439928
Brd1	-0.2380406	6.0043742	3.53546373	0.07478154	0.29439928
Cped1	0.39844973	7.25689517	3.53445567	0.07481979	0.29445308
Ap3s1	0.28936253	7.18832894	3.53181165	0.07492024	0.29475151
Crispld1	-0.4148066	3.89178035	3.53078797	0.07495917	0.29480783
Matr3	-0.278778	8.16150937	3.52942888	0.07501089	0.29491439
Pus10	-0.4889853	3.92504545	3.5271822	0.07509648	0.29508332
Gla	0.46021861	3.05440702	3.52700762	0.07510314	0.29508332
Trnt1	0.31209001	4.77161066	3.52577229	0.07515025	0.29510249
Gpld1	-0.3056903	4.90241886	3.52547105	0.07516175	0.29510249
Tnfsf13b	-2.2760256	-1.2214902	3.52494193	0.07518194	0.29510249
Arap3	0.8965669	1.59536031	3.52304914	0.07525423	0.29528946

Ogdh	-0.2785006	7.67834973	3.52144772	0.07531546	0.29532144
Casd1	0.28652523	5.80115426	3.52125962	0.07532266	0.29532144
Rabac1	0.629221	3.07237434	3.52081203	0.07533978	0.29532144
Serping1	0.48482538	7.04095034	3.52025695	0.07536102	0.29532144
Rnasel	-0.3293206	4.6797789	3.51868099	0.07542138	0.29544101
Gtf2b	0.32155091	4.83713545	3.51817212	0.07544087	0.29544101
Avp	1.8515345	-1.658286	3.51263671	0.07565336	0.2960768
Rrp9	-0.8229366	1.10828275	3.5117612	0.07568703	0.2960768
Slc15a2	-0.4993238	2.85421887	3.51142112	0.07570012	0.2960768
Xpnpep3	0.32810684	4.78115851	3.51136919	0.07570212	0.2960768
4930563F08I	-1.7634905	-0.1062883	3.51063484	0.07573038	0.29609063
Pmepa1	0.25814039	7.36000559	3.50898753	0.07579382	0.29620663
Ppp1r3f	-0.3386543	4.33312591	3.50835714	0.07581812	0.29620663
Riok2	0.25780875	5.09861726	3.50793874	0.07583425	0.29620663
Asic3	3.87473927	-2.0363089	3.57512326	0.07591222	0.29639215
Kcnt2	-0.4206605	5.43269242	3.50542487	0.07593124	0.29639215
Rassf6	-1.161233	0.63128141	3.50332216	0.07601249	0.2966126
Xpo1	0.25027826	6.55374024	3.50186433	0.07606887	0.29673593
LOC1005036	0.57064172	3.67730937	3.49904855	0.07617792	0.29703523
Lhx9	1.80101555	-1.07217	3.49860258	0.0761952	0.29703523
Pros1	0.43386536	5.1652713	3.49752455	0.07623701	0.2971015
Etnk1	0.20605417	8.62575492	3.49422346	0.0763652	0.29750423
Uck1	0.36865653	3.16796925	3.49165509	0.0764651	0.29779657
Ocln	-0.5690576	2.73975676	3.48891625	0.0765718	0.29811518
Nabp2	0.34114097	5.88498159	3.48531162	0.0767125	0.29856589
Dmrtc1a	0.87003303	0.89570495	3.48312978	0.07679781	0.29875486
Pbld1	-0.4896614	3.46365228	3.48279402	0.07681095	0.29875486
Moxd1	-0.5069704	2.93073035	3.4803969	0.07690482	0.29901855
Tbcb	0.38805371	4.55125127	3.47978804	0.07692868	0.29901855
Tecpr2	-0.4529449	5.55760619	3.47856293	0.07697673	0.29910821
Trex1	-0.6971492	1.23374519	3.47656901	0.07705499	0.29931522
Wdr90	-1.2857005	1.35133883	3.47535212	0.0771028	0.29937672
Myc	-0.4439544	3.6606208	3.47450327	0.07713618	0.29937672
Lrp6	-0.2411014	6.82316275	3.47405131	0.07715395	0.29937672
Dapp1	-0.4305918	4.05572574	3.47346142	0.07717716	0.29937672
Rab9	0.31647235	5.70015538	3.47298728	0.07719582	0.29937672
Cnksr3	0.56216486	1.78761728	3.47152099	0.07725356	0.29944692
Unc45b	-2.3093428	-0.6686464	3.47125768	0.07726393	0.29944692
Grin1os	1.00837181	0.24889645	3.46961607	0.07732864	0.29956242
Klhdc8a	0.40061674	3.8166038	3.4690732	0.07735005	0.29956242
Vcl	0.28646713	6.50105909	3.46859875	0.07736877	0.29956242
1700019A02I	-1.9023369	-1.7189132	3.46786597	0.0773977	0.29957755
Dnpep	0.45777143	2.95419845	3.4662931	0.07745982	0.29972114

Chpt1	0.34565525	5.44121006	3.46541104	0.07749468	0.29975919
Naa20	0.33308038	4.99462686	3.4634663	0.07757161	0.29977866
Rev3l	-0.314258	7.29469965	3.46341574	0.07757362	0.29977866
Hmbs	0.42960298	3.04214054	3.46338547	0.07757481	0.29977866
Tesc	0.44609186	3.01370342	3.46162551	0.07764452	0.29991743
Aurka	1.09136338	-0.188556	3.4612143	0.07766081	0.29991743
Gm16677	-2.3039071	-1.2552854	3.4602751	0.07769805	0.2999645
Arxes2	0.44524345	3.63078278	3.45818122	0.07778113	0.30018849
Cttnbp2	-0.3839239	6.72279741	3.45443083	0.07793021	0.30060672
Gns	0.356116	6.50749551	3.45419235	0.0779397	0.30060672
Nme7	0.4580954	3.52830339	3.45171094	0.07803853	0.30084657
Vamp8	0.79408586	5.10451775	3.45129816	0.07805499	0.30084657
Lmo7	-0.4675609	6.02077423	3.45040224	0.07809072	0.30084657
Tmem98	-0.6036964	3.79390521	3.45011	0.07810237	0.30084657
Celf2	0.26733199	9.78204798	3.44664743	0.07824066	0.30098355
Col7a1	-2.2126755	-1.240098	3.44607599	0.07826351	0.30098355
Dcakd	0.42529385	4.5004566	3.44602002	0.07826575	0.30098355
Podxl	-0.2664679	4.29086336	3.44532792	0.07829343	0.30098355
Lmbr1l	-0.8933008	0.32699274	3.44498308	0.07830723	0.30098355
Mief1	-0.3509907	4.4905079	3.44472919	0.07831739	0.30098355
Arfgef2	0.3243226	6.1855433	3.44447689	0.07832749	0.30098355
Csdc2	-0.2913512	6.84565661	3.44418931	0.078339	0.30098355
Tnfrsf13c	-1.2416878	0.45441152	3.44229651	0.07841482	0.30102896
Ddx51	-0.3014694	3.89237777	3.44220115	0.07841865	0.30102896
1700096K18I	0.8964383	1.18970557	3.44123	0.07845758	0.30102896
D10Jhu81e	0.37386013	3.58095012	3.44051827	0.07848613	0.30102896
Cpne1	0.25588186	5.90281002	3.4403018	0.07849482	0.30102896
Hexim1	0.26086616	5.41505595	3.43981849	0.07851422	0.30102896
Lrig1	0.41567666	3.267394	3.4395057	0.07852677	0.30102896
Tceb1	-0.2742406	6.28248636	3.43863184	0.07856187	0.30106711
Otx2	-1.3076468	1.57621485	3.43777129	0.07859644	0.30110326
Gclm	0.31698788	5.32642741	3.43608638	0.07866419	0.30118814
Malat1	-0.6323888	12.8518958	3.43596934	0.0786689	0.30118814
Prelid1	0.34322239	5.79419782	3.43492791	0.07871081	0.30121015
Csrnp2	0.29752456	5.41423429	3.43457671	0.07872495	0.30121015
Vcam1	-0.3312897	5.84331922	3.43306505	0.07878584	0.30134686
Gria3	-0.3208551	8.51083982	3.43229914	0.07881672	0.3013687
Ppp2r3d	-0.5027221	3.95125935	3.43059504	0.07888546	0.30153527
Cd9	0.57248433	4.85260339	3.42939309	0.07893399	0.3016245
Creb3l2	0.39310118	5.98241077	3.42640499	0.07905478	0.30190275
Baiap3	-0.8930687	1.53709293	3.42630037	0.07905902	0.30190275
Bud13	-0.4843443	2.07170836	3.42572167	0.07908244	0.30190275
Nufip2	0.21833782	7.74209213	3.42439189	0.07913628	0.30199479

Tbck	-0.3576383	4.68675334	3.42388105	0.07915698	0.30199479
Gm1673	0.94834236	0.4194867	3.42321223	0.07918409	0.302002
Pkm	0.25948164	8.20468187	3.42245477	0.0792148	0.30202295
Nlrp3	-1.6337761	-0.9564663	3.42085166	0.07927985	0.30217475
Edem1	0.24309029	4.94744188	3.41802036	0.07939488	0.30246478
Tyr	-0.6468862	2.55561045	3.41773573	0.07940645	0.30246478
Slc23a2	-0.2411531	7.23608384	3.41660823	0.07945233	0.30246666
Xcl1	2.0330984	-0.5193357	3.41599764	0.07947718	0.30246666
Ube2r2	0.29456592	8.34444785	3.41467764	0.07953094	0.30246666
Ino80	-0.3125596	5.08845141	3.41418367	0.07955107	0.30246666
Trpv6	0.64180593	1.64659565	3.4130808	0.07959603	0.30246666
Tle2	-0.6326932	1.5759767	3.41299301	0.07959961	0.30246666
Frmd4a	-0.3664265	5.54003423	3.4119661	0.07964151	0.30246666
Adora3	-2.0816139	-1.5513716	3.41169422	0.0796526	0.30246666
Pdlim4	0.53304329	2.4719041	3.41030319	0.0797094	0.30246666
Il5	2.60300898	-1.8544092	3.40927087	0.07975159	0.30246666
Ccrl2	-0.8691481	0.53143071	3.40923063	0.07975323	0.30246666
Gja1	0.53646201	7.63703982	3.40856167	0.07978058	0.30246666
Nckap5	-0.4746262	3.2134606	3.40821471	0.07979477	0.30246666
Cacng8	1.1035944	0.70465337	3.40772519	0.0798148	0.30246666
Egfl7	-0.5112403	2.41048046	3.40767914	0.07981668	0.30246666
G2e3	-0.3668321	4.38339843	3.40717992	0.07983711	0.30246666
4930452G13	-2.4972497	-1.7082756	3.40702448	0.07984347	0.30246666
Phpt1	0.43492786	3.28584852	3.40658239	0.07986157	0.30246666
Plac9b	-0.4930295	3.93696673	3.40464426	0.07994096	0.3025465
Cnn1	1.97624644	-0.3466643	3.40453104	0.0799456	0.3025465
2310009B15I	-0.6647352	2.1245251	3.40421791	0.07995844	0.3025465
Atp1b3	0.38990842	8.8426417	3.40262593	0.08002374	0.3026122
Tsku	0.76556672	2.14957255	3.40205351	0.08004723	0.3026122
Shprh	-0.3911011	5.96668018	3.40194689	0.08005161	0.3026122
Dnm1	0.2836834	8.86763299	3.40052003	0.08011021	0.30273817
Snora81	1.91693585	-1.7249084	3.39625021	0.08028588	0.30320614
Znrf1	-0.2314865	6.14635925	3.3958846	0.08030094	0.30320614
Fam43b	0.69805122	2.4659987	3.39566464	0.08031	0.30320614
Mospd2	0.29341319	5.1513818	3.39416728	0.08037173	0.30327795
Haghl	-0.7597261	0.89350603	3.3932739	0.08040859	0.30327795
Narf	-0.3168921	5.05031962	3.39269896	0.08043232	0.30327795
Nubpl	-0.5219957	2.80469616	3.3920134	0.08046062	0.30327795
Dgkb	0.34074291	7.92645672	3.39137935	0.08048681	0.30327795
Gm14446	0.40792064	3.72874383	3.39101303	0.08050195	0.30327795
Slc22a6	-0.4683961	7.15639669	3.39090796	0.08050629	0.30327795
2310039L15I	-0.6742983	2.85952207	3.38992668	0.08054685	0.30333533
Nfatc1	0.3448018	3.99991759	3.38872426	0.08059659	0.30342722

Retn	2.43974227	-1.5550005	3.38802967	0.08062534	0.30344006
Zfp61	0.5111381	3.04587609	3.3867703	0.08067749	0.30351882
Gga3	0.2791422	5.38664136	3.38630047	0.08069695	0.30351882
Eltd1	-0.7403688	2.92488026	3.38368204	0.08080554	0.30383182
Tmem141	-0.5907509	1.88506136	3.38171091	0.0808874	0.30390147
Cthrc1	0.43680429	2.88728204	3.38132622	0.08090339	0.30390147
Cast	0.38116794	6.45665256	3.38077513	0.08092629	0.30390147
Abca8a	-0.5777297	2.39900376	3.38023111	0.08094891	0.30390147
Pcdhgc5	0.40309196	4.78780458	3.38013247	0.08095302	0.30390147
Chat	-0.8488093	1.53177093	3.37830642	0.08102901	0.30390147
Zdhhc8	-0.3784117	4.78650998	3.37795393	0.08104368	0.30390147
Aptx	-0.2646067	5.00670793	3.37790762	0.08104561	0.30390147
Frmpd4	0.27944156	7.77636696	3.37774335	0.08105245	0.30390147
Gabbr2	0.31720175	7.12221704	3.37648232	0.081105	0.303923
Pnma1	0.72809444	1.58380208	3.37638736	0.08110895	0.303923
Cdk11b	0.19637189	7.39709748	3.37521104	0.08115801	0.30401168
Mllt6	-0.2599299	6.01059421	3.37106576	0.08133113	0.30456494
A130077B15	-0.5392424	7.65489008	3.3689159	0.08142109	0.3048065
Il1f9	-0.6470182	2.29455078	3.36560414	0.08155989	0.3052307
Bcar1	0.2968443	4.23651133	3.36373675	0.08163828	0.30535601
Man2a1	0.2284463	6.07197675	3.36359175	0.08164437	0.30535601
Actg1	0.18950688	8.97424616	3.36156975	0.08172935	0.30551684
Ttc5	-0.2785019	4.61761479	3.36082554	0.08176066	0.30551684
Speg	-0.2934626	5.58177323	3.35956118	0.08181388	0.30551684
Atxn7l3	-0.3168281	5.96367307	3.3593469	0.0818229	0.30551684
Psmc3ip	-0.7329638	2.02747276	3.35882448	0.0818449	0.30551684
Tti2	-0.3449549	4.5965	3.35871196	0.08184964	0.30551684
Cacna2d2	0.39475285	4.99637062	3.35797602	0.08188065	0.30551684
Ilvbl	-0.5627646	2.36640121	3.35771947	0.08189146	0.30551684
Fbxo30	-0.3601556	5.38115965	3.35439416	0.08203177	0.30594498
Mlph	0.64158841	2.44175312	3.35324974	0.08208012	0.30603
Fam109b	1.05836273	1.55754912	3.35217577	0.08212553	0.306104
Orc4	0.24735934	5.58400376	3.35057123	0.08219342	0.30626173
Panx1	0.66268482	2.45878713	3.34964427	0.08223267	0.30631268
Smad1	0.23456143	5.76445627	3.34607378	0.08238407	0.30670978
Slc2a5	-1.3239758	-0.6597049	3.34561468	0.08240356	0.30670978
Sparcl1	0.32751509	8.69193104	3.34531895	0.08241611	0.30670978
Adamts3	-0.3933772	4.12886583	3.34336546	0.08249912	0.30684402
Trove2	0.2371238	7.07330955	3.34211884	0.08255214	0.30684402
Klhdc7a	-0.6483882	2.71863597	3.3412698	0.08258827	0.30684402
Slc4a10	0.24491637	8.98850848	3.3409556	0.08260165	0.30684402
Tpd52	0.24710385	5.82796118	3.34077351	0.0826094	0.30684402
Hmgcr	0.29418873	5.79284207	3.34028341	0.08263027	0.30684402

Zfp93	-0.4626187	3.83897095	3.34025371	0.08263154	0.30684402
Fra10ac1	0.29031702	4.40983291	3.33864913	0.08269992	0.3068914
Slc22a14	-3.3533104	-1.9966081	3.33768727	0.08274094	0.3068914
Smoc1	0.41408905	4.56734441	3.33761642	0.08274396	0.3068914
Ankrd54	-0.529077	2.01306305	3.33754987	0.0827468	0.3068914
Slc43a2	-0.3240384	5.45789386	3.33538901	0.08283905	0.30713842
Pkd2l2	-0.3893742	3.72470118	3.33202112	0.08298307	0.30744653
Prrt3	0.75950166	1.74678824	3.33182411	0.08299151	0.30744653
Sema3e	0.69590158	4.19611162	3.33152108	0.08300448	0.30744653
Ppp3r2	-1.6890971	-0.420535	3.33104554	0.08302484	0.30744653
Rfx3	0.35992992	7.27365649	3.32990128	0.08307387	0.30753299
Cep152	-0.7810314	1.78541853	3.32867211	0.08312657	0.30760174
Grap2	-0.7816223	1.1940081	3.32827022	0.08314381	0.30760174
Tomm20	-0.1909428	7.75160618	3.32725235	0.0831875	0.30762241
Cyp46a1	0.30741606	4.74106673	3.32694303	0.08320078	0.30762241
Zfp521	0.32583155	5.60118123	3.32578478	0.08325052	0.30771135
Grsf1	0.21326088	6.82277294	3.32513653	0.08327838	0.30771934
Uprt	0.28871921	4.57891651	3.32372123	0.08333924	0.30782603
Spast	0.22010171	6.53538806	3.32326984	0.08335866	0.30782603
Samd8	-0.2455942	6.05743869	3.32208759	0.08340955	0.3079132
Slc25a12	0.22226414	7.49870517	3.32095225	0.08345846	0.3079132
Themis	-1.1419278	0.53572388	3.32088957	0.08346116	0.3079132
Acp2	0.2308783	5.57459214	3.32030102	0.08348653	0.3079132
Tmco5	0.97346944	0.61873959	3.31923606	0.08353245	0.3079132
Fry	-0.4448404	8.22700154	3.31870545	0.08355534	0.3079132
Pcdhb14	-0.4867462	3.02501865	3.31854539	0.08356225	0.3079132
Fndc7	2.66382564	-1.3887912	3.31667342	0.08364308	0.3080482
5930403L14F	-0.4954626	4.76609012	3.31650549	0.08365033	0.3080482
Elk4	-0.2169291	6.40798049	3.31327384	0.0837901	0.30843776
Card10	1.05692946	0.38311934	3.31242818	0.08382671	0.30843776
Tnfrsf11b	-0.7393437	3.89176417	3.31176781	0.08385532	0.30843776
Syndig1	-0.3958195	3.49911957	3.31167974	0.08385914	0.30843776
Senp8	0.3056083	4.61752894	3.30917752	0.08396765	0.30865007
Nin	-0.2506248	6.83042218	3.30892175	0.08397875	0.30865007
Ccdc82	-0.3570995	7.54247244	3.30853701	0.08399545	0.30865007
Wnt7a	-0.7470493	1.63757621	3.30797264	0.08401995	0.30865007
Lmln	-0.5162547	2.3864814	3.30681136	0.0840704	0.30874069
BC068157	0.39063395	5.92522908	3.30393718	0.08419542	0.30910502
Rps6ka1	-0.5391028	2.51458449	3.3027346	0.08424779	0.3091505
Tulp2	-1.0520322	0.43315833	3.30246728	0.08425944	0.3091505
Scn11a	2.87065156	-1.9906575	3.30182149	0.08428758	0.30915905
Lpl	0.49794286	4.20486379	3.2998215	0.08437481	0.30938425
Glb1l	0.47366738	3.33240544	3.29745166	0.08447831	0.30966894

Ddx52	0.2378586	4.67861184	3.29463205	0.08460165	0.30976679
Txndc9	0.23851491	5.80377906	3.29424481	0.0846186	0.30976679
Prmt7	-0.4908235	3.30379527	3.2939084	0.08463333	0.30976679
Piwil4	1.77587004	-0.897219	3.29390496	0.08463349	0.30976679
Cops4	0.23120913	6.02394846	3.29331493	0.08465933	0.30976679
Hipk3	-0.1854596	7.79073933	3.29206675	0.08471404	0.30976679
Nsun3	0.30737154	4.6415673	3.29200414	0.08471678	0.30976679
Brpf1	0.27850882	4.65622633	3.29160078	0.08473447	0.30976679
Crip1	0.68080979	2.42763612	3.29094059	0.08476343	0.30976679
Kifc1	-2.0953495	-1.4155674	3.29093523	0.08476367	0.30976679
Edn3	-0.3528922	6.20018642	3.28908515	0.08484489	0.30996901
Tmtc1	0.28091611	8.45845134	3.2881472	0.0848861	0.310025
Nqo2	0.2993547	5.67377076	3.28607272	0.08497733	0.31011197
Zc2hc1c	-0.7339516	2.21582011	3.28601806	0.08497974	0.31011197
Ncoa3	-0.2601348	6.64765796	3.28583942	0.0849876	0.31011197
Ddx24	-0.238603	5.66962142	3.28510607	0.08501988	0.31013527
Pcdhgc4	0.99980963	0.9926195	3.28272049	0.085125	0.31042415
Pcdh15	0.43960086	4.87020321	3.28133768	0.08518599	0.31046391
Pgam1	0.22259307	9.43813812	3.28107627	0.08519753	0.31046391
Polg2	-1.0677258	0.97007582	3.28071063	0.08521367	0.31046391
B930025P03	2.08224396	-1.1895272	3.2786926	0.08530282	0.31069417
Tor1aip2	0.29578062	7.06303032	3.27638165	0.08540504	0.31084938
Speer4b	-0.862929	0.58808674	3.27628722	0.08540922	0.31084938
Hyou1	0.37856444	5.17947452	3.27554694	0.08544199	0.31084938
Zgpat	0.66501778	1.82987523	3.27500112	0.08546617	0.31084938
Ssc5d	0.56162017	2.55307744	3.27479694	0.08547521	0.31084938
Ssr4	0.36991405	3.42232035	3.27374168	0.08552198	0.31092505
Gad2	0.35393426	8.8081103	3.27206365	0.08559641	0.31100773
Myb	-0.8718607	0.61825725	3.27152353	0.08562039	0.31100773
Pms1	-0.4663811	2.75300023	3.27147287	0.08562264	0.31100773
Peg3	-0.3733798	8.84130121	3.27024933	0.08567697	0.31111074
Catsperd	1.80650029	-0.7666412	3.26808455	0.08577321	0.3113658
Th	1.27740741	0.60102874	3.26529419	0.08589745	0.31160897
Anxa1	0.50014066	6.7899027	3.26528114	0.08589803	0.31160897
Tmod4	1.50247211	-0.5114358	3.26482717	0.08591826	0.31160897
Cct6a	0.20699083	8.24498773	3.26223105	0.08603407	0.31193336
Mxra7	-0.5556341	2.78163918	3.26143209	0.08606974	0.31193336
Dpp10	0.30884015	6.87344246	3.26107169	0.08608584	0.31193336
Tmem17	0.61923045	1.7627744	3.25776113	0.08623389	0.31230655
Supt6	-0.2862025	7.40632526	3.25760243	0.08624099	0.31230655
Zfat	-0.5425232	2.02412204	3.2569946	0.08626821	0.31231067
Cog2	0.3509682	3.48085586	3.256086	0.08630891	0.3123636
Yipf4	0.33350445	4.77633016	3.25494299	0.08636015	0.3124546

Usp10	0.29243954	5.20254236	3.25359212	0.08642074	0.31257672
AI413582	-0.373718	3.26757408	3.25302715	0.0864461	0.31257672
Actb	0.2679928	9.97274988	3.25108871	0.08653317	0.31279712
Zfp709	-0.393691	4.13899333	3.24962052	0.08659919	0.3129413
Lonrf3	0.27746615	5.72486877	3.24756742	0.08669161	0.31318075
Wdr18	-0.2951584	3.64050136	3.24668745	0.08673125	0.31322949
Dus2	-0.5153125	2.42156585	3.24584651	0.08676916	0.31327191
Rnf149	-0.3139714	5.59735815	3.24421527	0.08684274	0.31342693
Dennd4a	-0.2316133	7.418427	3.24348512	0.0868757	0.31342693
Dync2h1	-0.5194947	6.15787971	3.24315502	0.08689061	0.31342693
Adamts1	0.3764524	4.34942457	3.2406085	0.0870057	0.3134867
Rab2a	0.19307309	9.67092269	3.24034761	0.0870175	0.3134867
Rsu1	0.34117763	6.85772679	3.23930369	0.08706474	0.3134867
Recql5	-0.5510558	2.43642197	3.2383309	0.08710879	0.3134867
Frzb	-0.4544953	3.21545134	3.23812938	0.08711792	0.3134867
Ddx59	-0.5218584	2.5780165	3.23808389	0.08711998	0.3134867
Ppp2r1b	-0.2443405	5.52140425	3.23788795	0.08712886	0.3134867
5430416O09	-2.6305769	-1.8621924	3.23752082	0.08714549	0.3134867
Bves	-1.2820161	-0.6052803	3.23688149	0.08717447	0.3134867
Il12a	0.63483116	1.92491101	3.23678223	0.08717897	0.3134867
2610002M06	0.2018871	7.06876313	3.23622311	0.08720432	0.3134867
Tub	-0.3826844	6.11922784	3.23584876	0.0872213	0.3134867
Slc25a54	-1.9118227	-0.925918	3.23371268	0.08731826	0.31364839
Rnf182	-0.6089487	3.01037818	3.23302344	0.08734957	0.31364839
Traf3	-0.3008688	5.05773297	3.23266641	0.0873658	0.31364839
Oprm1	-0.8035205	1.91344994	3.23232598	0.08738127	0.31364839
Car10	0.28170609	8.35833458	3.23130186	0.08742784	0.31364839
Taf1	-0.2652969	6.83215299	3.23086514	0.08744771	0.31364839
Selenbp2	-1.2154767	-0.2173687	3.2308231	0.08744962	0.31364839
Axin2	-0.3545916	4.98891397	3.22909258	0.0875284	0.31383695
Rab3il1	0.4115687	4.12135723	3.22786716	0.08758424	0.31394316
Olfr692	2.95856404	-1.3157961	3.22516766	0.08770739	0.31429051
Hs3st3a1	-0.482168	4.59110995	3.2223815	0.0878347	0.31465258
Fkbp7	0.49396227	3.61304404	3.2193418	0.08797385	0.3150568
Rnf217	0.29686382	4.29072797	3.21466815	0.08818829	0.31573036
Akap10	-0.2793532	4.87047953	3.21229217	0.08829754	0.31593674
Trim67	-0.724226	1.22140215	3.212267	0.0882987	0.31593674
Ccz1	0.27443543	4.8060253	3.21083981	0.0883644	0.31602843
Gpc5	0.55107163	3.53972772	3.20984181	0.08841038	0.31602843
Adamtsl4	0.45529213	2.8491448	3.20954743	0.08842395	0.31602843
Acadsb	-0.2591949	6.82862526	3.20895888	0.08845108	0.31602843
Cd300a	-0.5055557	3.03887199	3.20884627	0.08845627	0.31602843
Gm16432	-1.0323849	1.11354933	3.20726543	0.0885292	0.31619466

Mphosph8	0.25723326	8.41146248	3.20614061	0.08858113	0.31628582
Acacb	-0.8949933	1.28171749	3.20521365	0.08862396	0.31634442
Lrrc7	-0.4609547	8.01311351	3.20253135	0.08874802	0.3165197
A330050F15	0.70370749	2.27249062	3.20250382	0.08874929	0.3165197
Enthd2	0.7369856	1.43722193	3.20243766	0.08875236	0.3165197
Lhfpl1	1.66539535	-0.7826807	3.20115347	0.08881183	0.31652849
Trim41	-0.2600106	4.34501675	3.20050516	0.08884187	0.31652849
Foxr2	-1.3316027	0.49524515	3.20037537	0.08884788	0.31652849
Dpysl3	-0.1965234	6.30285504	3.20010222	0.08886054	0.31652849
Mllt10	-0.2424554	6.2237758	3.19919988	0.08890239	0.31656421
Tgtp2	-0.4734728	3.77523968	3.19873877	0.08892378	0.31656421
Tom1l1	-0.3595645	3.91602837	3.1981764	0.08894987	0.31656421
Spata18	-0.9773753	0.3596755	3.19665913	0.08902032	0.31662773
A330009N23	0.84997691	1.21981126	3.19665321	0.0890206	0.31662773
Cr2	-1.1499683	1.0796989	3.19533683	0.08908178	0.31666271
Farsb	0.23930323	5.68963609	3.19479256	0.08910708	0.31666271
Plk1	-1.8014794	-0.9043404	3.19446475	0.08912233	0.31666271
Gm10409	-0.4409146	4.00369907	3.19393263	0.08914709	0.31666271
Carm1	0.27034044	5.24535522	3.19249119	0.08921419	0.31666271
Syngr3	0.28458542	5.08592985	3.1921203	0.08923147	0.31666271
Mcm6	0.33240741	3.64528183	3.19205955	0.0892343	0.31666271
Cds2	-0.2771733	7.73388419	3.19147578	0.0892615	0.31666271
Gdf9	-1.656027	-0.9128164	3.19132732	0.08926841	0.31666271
Ggh	0.49912339	3.23462189	3.18899129	0.08937736	0.31678737
Polr2e	0.34712738	4.39097372	3.18871215	0.08939039	0.31678737
Ybey	-0.4273105	4.3752205	3.18836149	0.08940676	0.31678737
Uba6	0.30464462	5.70518127	3.18830579	0.08940936	0.31678737
Gm7102	-0.5651136	5.46312547	3.18752562	0.0894458	0.31681931
Ddc	-0.7224726	1.34508419	3.18651014	0.08949325	0.31681931
Itpr2	-0.2563226	5.33781271	3.18641403	0.08949775	0.31681931
Nckap1l	-0.4362674	3.03439757	3.18315902	0.08965005	0.31726469
4933413G19	-2.7654711	-1.7289615	3.18166055	0.08972027	0.31734287
Foxm1	0.83950171	1.41921814	3.18155667	0.08972514	0.31734287
Hcfc1	-0.2746913	6.90610262	3.18022772	0.08978747	0.31746957
3830408C21I	-0.5645048	2.47583336	3.17782506	0.08990029	0.31777465
Zfp54	0.87968254	1.59679142	3.1770227	0.08993801	0.31781415
Zfp277	-0.4414023	6.31918205	3.17549591	0.09000982	0.3179741
Pgp	0.28484385	3.71346144	3.17376797	0.09009118	0.31816765
Acn9	-0.4298858	2.90800262	3.17273033	0.09014007	0.31824648
Nfkb1	-0.3219609	4.41632121	3.17147663	0.09019919	0.31836134
Chd6	-0.3022885	7.19872025	3.17022051	0.09025847	0.31847671
Klkb1	-2.7754162	-1.2638815	3.16951636	0.09029172	0.31850019
Ndufa4	0.28955625	7.34644343	3.16738006	0.09039268	0.31864613

Tet3	-0.2992014	6.03705093	3.16706879	0.09040741	0.31864613
Agmat	1.52330121	-0.0296026	3.16695225	0.09041292	0.31864613
Jph4	-0.2324965	7.22585029	3.1661421	0.09045125	0.31868745
Msantd1	-1.8313144	-0.632476	3.16365069	0.09056925	0.31886006
Plxdc1	0.38231441	2.91556988	3.16293705	0.09060309	0.31886006
Gm5136	-1.4067777	0.1255518	3.16040407	0.09072329	0.31886006
Shank3	-0.3488134	4.59092802	3.15993903	0.09074538	0.31886006
Nbea	-0.457288	8.50315609	3.15972292	0.09075565	0.31886006
Ifltd1	-0.4047919	5.40439857	3.15969827	0.09075682	0.31886006
Eif4b	-0.1871454	7.59682671	3.15962555	0.09076028	0.31886006
Rnf121	0.84681644	0.95704987	3.1592496	0.09077814	0.31886006
Mrps23	0.32600274	4.23293926	3.15912018	0.09078429	0.31886006
Ryr2	-0.5405182	8.03232556	3.15905018	0.09078762	0.31886006
Gtf3c5	-0.3989831	3.13958845	3.1589344	0.09079312	0.31886006
Tubb4b	0.20782629	7.02528068	3.15757781	0.09085764	0.31899309
Pla2g4a	0.3220264	4.59834427	3.15608524	0.09092868	0.31914895
Bnip3	0.24504346	6.08881487	3.15243639	0.09110263	0.3192847
3300002I08R	-1.2487128	-0.3547542	3.15228191	0.09111	0.3192847
Phf20	0.23722458	6.49914567	3.152083	0.0911195	0.3192847
Glt28d2	0.51385131	3.29985925	3.15187297	0.09112953	0.3192847
Gpr65	-1.0680069	0.52277596	3.15156581	0.09114419	0.3192847
Ptrhd1	-0.3550549	3.71099187	3.15113138	0.09116494	0.3192847
Arel1	-0.2381608	6.26592541	3.15076151	0.09118261	0.3192847
Hhipl1	-1.7827343	-1.2036781	3.15059933	0.09119036	0.3192847
Thra	-0.3170991	5.82827563	3.15024456	0.09120731	0.3192847
Pdha1	0.1838882	7.41076837	3.14883425	0.09127474	0.31942737
Prtg	-0.6543522	1.53552983	3.1480141	0.09131397	0.31947133
4930444P10I	-2.1415369	-1.494696	3.14559524	0.09142981	0.31959915
Nipsnap3b	0.36337128	3.68325496	3.14534112	0.09144199	0.31959915
Park7	0.3426226	4.76409095	3.14509395	0.09145384	0.31959915
Clk3	-0.2801696	4.70824977	3.14502272	0.09145726	0.31959915
Mrpl55	0.42278127	3.61984209	3.14266175	0.09157053	0.31990164
Trpv2	-0.4019358	3.07012746	3.14104038	0.09164842	0.32008035
Ccnh	0.2650909	4.97463151	3.13844191	0.0917734	0.32038271
Chrna4	-0.3142425	4.50706817	3.13812834	0.09178849	0.32038271
Ube2q1	0.19524419	7.10382136	3.13542089	0.09191895	0.32070066
Fbn1	0.40243605	3.99856872	3.13470688	0.09195339	0.32070066
Myo9a	-0.3267036	8.3603113	3.13411293	0.09198206	0.32070066
Vav3	0.40369759	3.29633522	3.13358021	0.09200777	0.32070066
Foxp3	-1.557097	-0.7855002	3.13346197	0.09201348	0.32070066
Zfp398	-0.3253526	4.49869841	3.13106592	0.09212926	0.3209271
Mill2	0.99862881	0.60879185	3.13100819	0.09213205	0.3209271
Mier2	0.73097408	1.225176	3.12950496	0.09220477	0.32105535

Crebrf	-0.2280241	7.2754805	3.12872119	0.09224272	0.32105535
Snrnp70	-0.3211008	5.73712397	3.12858541	0.09224929	0.32105535
Cnot11	0.27231865	4.38164142	3.12670328	0.09234049	0.32115469
Mtmr12	-0.2009623	6.44446954	3.12644818	0.09235286	0.32115469
Tgfa	-0.2281345	5.15283698	3.12594037	0.09237749	0.32115469
Fbxl12	-0.4947982	2.3507409	3.12532955	0.09240713	0.32115469
Rae1	0.32510915	3.83274446	3.12502841	0.09242174	0.32115469
Adam23	-0.2851242	7.15181729	3.12467823	0.09243874	0.32115469
Gadd45g	0.51721044	2.06850127	3.12231466	0.09255356	0.32129563
Wfdc18	1.43423404	-0.5678841	3.12219941	0.09255916	0.32129563
Cep72	-0.974823	1.07004551	3.12218644	0.09255979	0.32129563
Stk32a	-0.6174485	1.75903453	3.12089325	0.09262269	0.32140464
Zfp655	0.24891753	5.7401676	3.12043743	0.09264487	0.32140464
Accs	-0.5022078	2.8710364	3.11846901	0.09274074	0.32156791
Ddx43	-2.3678548	-2.0649255	3.11813206	0.09275716	0.32156791
Dnaic2	-2.3107927	-1.540072	3.11781752	0.09277249	0.32156791
Plxnc1	-0.2248007	6.4050276	3.11682821	0.09282073	0.32164204
Tcea1	0.21068241	8.06123568	3.11531589	0.09289454	0.32171304
Fem1c	-0.2680021	5.33679925	3.11530744	0.09289495	0.32171304
Emc4	0.21493809	6.33194098	3.1125521	0.09302959	0.32203825
Timeless	-0.8470049	2.14805561	3.1122854	0.09304264	0.32203825
AI661453	0.67561404	1.73037844	3.10889334	0.09320874	0.32251993
Atp5f1	0.20554785	7.46224354	3.10781575	0.09326157	0.32260955
Hmmr	-0.4085465	3.27664422	3.10516679	0.09339161	0.32285481
Rdm1	-0.7040399	2.51132514	3.10452425	0.09342319	0.32285481
BC030307	0.90420288	0.74594489	3.1038578	0.09345595	0.32285481
Usp35	0.59854332	1.87628475	3.1025737	0.09351911	0.32285481
Stamos	2.11527633	-1.1689577	3.10166084	0.09356405	0.32285481
Ngfr	-0.4107707	3.55633351	3.10161819	0.09356615	0.32285481
Snta1	0.47632512	2.78513284	3.10115477	0.09358897	0.32285481
Irgq	0.24024442	7.40639249	3.1009156	0.09360075	0.32285481
Lck	-1.1486538	0.1777984	3.10076176	0.09360833	0.32285481
Exosc5	0.74534129	0.96836672	3.1001965	0.09363618	0.32285481
Lama3	-0.6188091	2.5107146	3.09994139	0.09364875	0.32285481
Ccdc6	0.22815946	6.7624702	3.09962711	0.09366424	0.32285481
Metap1	0.2331878	6.1717032	3.09924779	0.09368294	0.32285481
Sgsm1	0.49646272	4.53170126	3.09713277	0.0937873	0.32288424
Dfna5	0.3610154	4.32154807	3.09708984	0.09378942	0.32288424
Kdm6b	-0.316903	5.61535267	3.0970076	0.09379348	0.32288424
Gpr146	-0.3651948	4.1900935	3.09641624	0.09382269	0.32288424
Fam24a	-3.0062228	-2.0965885	3.09634331	0.09382629	0.32288424
Kank4os	-2.2682649	-1.3756295	3.09435897	0.09392437	0.32305052
Intu	-0.3757792	4.51360505	3.09416107	0.09393416	0.32305052

Ctsz	0.42542267	3.99981656	3.09371492	0.09395623	0.32305052
4930480K15I	-0.8877047	2.7430116	3.09318391	0.09398251	0.32305052
Pcdhga6	0.51670634	2.36475453	3.09219594	0.09403142	0.32310373
Ogfod3	0.68166569	2.08932277	3.09153371	0.09406423	0.32310373
Fth1	-0.2073727	9.06580264	3.09103938	0.09408872	0.32310373
Kcnq1ot1	-0.6670533	8.05882548	3.09069258	0.09410591	0.32310373
B4galt2	-0.4055476	3.39230077	3.08969238	0.09415551	0.32316482
Pdlim7	0.31043968	4.32830393	3.08883907	0.09419784	0.32316482
Irf2bpl	0.24908629	5.74511218	3.08870173	0.09420466	0.32316482
Gm15987	-2.1796708	-1.7761642	3.08657588	0.09431024	0.32337076
Paip1	0.26144034	6.24463162	3.08640567	0.0943187	0.32337076
Ube2m	0.38875727	3.10543801	3.08563352	0.09435708	0.32340977
Sla	-0.4045988	3.91966931	3.08501063	0.09438806	0.32342339
Mterfd1	0.30071823	5.07451548	3.08394554	0.09444106	0.32351243
Maats1	-1.2795366	0.57079124	3.08232956	0.09452154	0.32369552
Ywhae	0.20988939	10.7332426	3.07881768	0.09469671	0.32411998
Ubap1l	2.70828447	-1.8406109	3.0787596	0.09469961	0.32411998
Ranbp2	-0.3210134	7.62672975	3.07670498	0.09480228	0.32426492
Rsph1	-0.6234237	2.13736563	3.07647584	0.09481374	0.32426492
Wdr11	-0.3436463	5.39380015	3.07595867	0.0948396	0.32426492
Myo5b	0.46473214	4.19877001	3.07551905	0.0948616	0.32426492
Med6	0.35474776	4.33643809	3.07520436	0.09487734	0.32426492
Arhgap15	-0.3597161	3.82737943	3.07392791	0.09494125	0.32435567
Gm20751	1.76576221	-1.2658381	3.07348321	0.09496353	0.32435567
Mcmdc2	-0.6778585	2.5289707	3.07305163	0.09498515	0.32435567
Glt1d1	0.72885084	1.04808471	3.07223018	0.09502633	0.32440377
Slc35f1	0.30230805	7.28824329	3.07086523	0.09509479	0.32451014
Cpa6	2.04838786	-1.1804844	3.06988529	0.09514398	0.32451014
Olfr1393	-2.5958369	-1.883936	3.06979382	0.09514857	0.32451014
Pappa2	0.68023422	2.28047774	3.06897419	0.09518974	0.32451014
Chrnb2	-0.3173751	3.9374046	3.06852475	0.09521232	0.32451014
Prr13	0.29709722	5.59039695	3.0683706	0.09522007	0.32451014
Kif2a	0.21305289	7.65851713	3.0665772	0.09531025	0.32465017
5330426P16I	0.56652748	2.64642788	3.06642663	0.09531783	0.32465017
Pus7l	0.76718138	1.59734635	3.06593668	0.09534248	0.32465017
Scube1	0.24925512	5.95682339	3.06324881	0.09547789	0.32500236
Poldip3	0.31035214	5.76822409	3.06280649	0.09550019	0.32500236
Tenm1	-0.452048	5.44457367	3.06086318	0.09559825	0.32524365
Pcdhb15	-0.5323595	2.72245539	3.0598679	0.09564852	0.32532225
Has1	-1.0596455	0.34808261	3.05827342	0.09572912	0.325493
Zmym6	-0.3973245	4.80463903	3.05752042	0.09576721	0.325493
Lyrm1	-0.4758525	2.71571946	3.05683187	0.09580205	0.325493
Rsad2	0.86485459	1.25882069	3.05672547	0.09580744	0.325493

Dbx2	0.50440734	1.98242717	3.05600876	0.09584373	0.32552394
Tubd1	-0.7410788	1.56401701	3.0537662	0.09595738	0.32579232
Tmem178	0.24967627	5.34075358	3.0531256	0.09598987	0.32579232
Pik3ap1	0.53680347	3.13275307	3.05284002	0.09600436	0.32579232
Ndufa4l2	1.1521362	-0.0866325	3.051953	0.09604938	0.32585277
Birc3	0.44920801	4.01387721	3.05053196	0.09612156	0.32600529
Dsg1c	-1.4669984	-0.8061699	3.04884623	0.09620727	0.32614955
Osbp2	-0.2717593	5.65116238	3.04843873	0.096228	0.32614955
Ptprn	0.32482108	6.91147551	3.04808888	0.0962458	0.32614955
Vdr	0.85776941	0.75906916	3.04727528	0.09628722	0.3261976
Slc26a4	1.01974472	1.05431985	3.04585138	0.09635975	0.32627451
Psapl1	-3.5945832	-1.7375642	3.04506515	0.09639983	0.32627451
Ntng2	0.62140384	1.99714029	3.04491983	0.09640724	0.32627451
Dram2	0.30811686	5.47488685	3.04438742	0.09643439	0.32627451
Aldh1a2	0.38881193	9.18687312	3.0441571	0.09644614	0.32627451
Eif1ad	0.35825544	4.17200908	3.04273252	0.09651885	0.32635531
Pik3ca	-0.1901374	7.01483984	3.04223093	0.09654447	0.32635531
Snx33	-0.4084661	3.96125282	3.04208775	0.09655178	0.32635531
Slc27a1	0.4623798	3.86559533	3.04129902	0.09659208	0.32635945
Psen1	0.28320853	5.05000366	3.04099715	0.09660751	0.32635945
Kcng2	-1.064506	0.29275489	3.03873041	0.09672346	0.32647162
Tmem218	0.42581226	3.03925079	3.0386883	0.09672561	0.32647162
Vti1a	0.22644286	6.91696689	3.0382527	0.09674792	0.32647162
Mink1	-0.3092591	5.58472022	3.03734816	0.09679424	0.32647162
Gba2	-0.4572507	3.2358917	3.03676391	0.09682418	0.32647162
Gjb2	0.36508349	9.09863828	3.03671282	0.0968268	0.32647162
Eif4g2	0.24779629	10.874292	3.0352439	0.09690212	0.32647162
Gucy2c	1.72394041	-0.8766283	3.0350074	0.09691425	0.32647162
Camk4	-0.2790624	9.29810961	3.03460394	0.09693496	0.32647162
Ttc1	0.28850498	6.00652084	3.03459233	0.09693555	0.32647162
Zc3h10	0.51854329	3.0586151	3.03336049	0.09699879	0.32647162
Adam17	-0.3099621	4.19544606	3.03301281	0.09701665	0.32647162
Gpm6b	0.28860015	8.47914724	3.03295064	0.09701985	0.32647162
Dusp10	-0.4366824	3.731895	3.03216198	0.09706037	0.32647162
Xkrx	0.59297142	2.04510831	3.0320848	0.09706434	0.32647162
Grik2	-0.4010093	5.18302285	3.03158793	0.09708988	0.32647162
Lemd3	-0.2793778	4.50277454	3.03128533	0.09710544	0.32647162
Lrrc10b	0.55794719	3.93809863	3.03078038	0.09713141	0.32647162
Rybp	-0.2280923	6.64734823	3.0281489	0.09726689	0.32652141
BC051142	-0.9385829	1.26833807	3.02806055	0.09727144	0.32652141
Prkrir	-0.2225968	5.34315048	3.02803798	0.09727261	0.32652141
Nudt4	-0.3127225	9.94797792	3.0276894	0.09729057	0.32652141
Phykpl	0.43078004	3.26191407	3.0274552	0.09730264	0.32652141

Fn3k	-0.4707307	3.23187119	3.02731598	0.09730982	0.32652141
Hes5	0.63795601	1.57656207	3.02599878	0.09737775	0.32665783
Sec62	0.22517953	7.55522414	3.02333028	0.09751555	0.32702846
9030025P20I	-0.4295691	3.21124724	3.02243434	0.09756186	0.32703388
Blcap	0.3487724	5.14326123	3.02224254	0.09757178	0.32703388
Galnt15	0.91273568	1.00556472	3.02146617	0.09761194	0.32707694
Wnt9a	-0.4233198	3.82353206	3.01989323	0.09769336	0.32709008
Tfap2a	0.40077274	3.94544385	3.01889885	0.09774488	0.32709008
Glcci1	-0.2075253	6.88902066	3.01889672	0.09774499	0.32709008
Bach2	-0.3719335	4.7164935	3.01776	0.09780392	0.32709008
Gm12505	-0.5027353	1.77653996	3.01738363	0.09782344	0.32709008
Tbx18	0.34709039	6.20995825	3.01681721	0.09785282	0.32709008
Sipa1l1	-0.3483671	8.88422936	3.01623402	0.09788309	0.32709008
Coro7	-0.3752829	4.16065774	3.01615098	0.0978874	0.32709008
Kmt2b	-0.344018	4.65995809	3.0160657	0.09789183	0.32709008
Yars2	0.30047883	4.04331491	3.01515773	0.09793898	0.32709008
Asb1	-0.3188817	4.51615356	3.01474838	0.09796024	0.32709008
Ccdc170	-0.8714895	0.66336915	3.01398296	0.09800002	0.32709008
Trappc3	0.27224491	5.00076725	3.0138783	0.09800546	0.32709008
9530027J09F	-2.8132755	-0.9858513	3.01354397	0.09802284	0.32709008
Ddn	-0.2301163	6.89353307	3.01349192	0.09802555	0.32709008
Sgcg	-1.758911	-0.352772	3.01254667	0.09807471	0.32712934
Arhgap1	0.27568339	5.65617668	3.01221545	0.09809195	0.32712934
Ccdc58	-0.4087518	3.0272777	3.01093686	0.09815851	0.32717599
Ptpn6	-0.8466931	0.89948557	3.01089719	0.09816058	0.32717599
Vpreb3	2.32781637	-1.2330033	3.00963432	0.09822637	0.32726042
Spred1	0.21162795	8.20895939	3.00936217	0.09824056	0.32726042
Txnl4b	0.42931287	3.25776878	3.00841089	0.09829017	0.32732934
Traf3ip2	-0.4530465	2.99233899	3.00791734	0.09831591	0.32732934
Kctd15	-0.6153162	2.12360408	3.00621894	0.09840458	0.32737513
Zswim1	-0.3161343	3.84826167	3.00611908	0.0984098	0.32737513
Sspo	-2.5278674	-1.2452705	3.00608305	0.09841168	0.32737513
Dnm1l	0.22370077	8.30233516	3.00445164	0.09849694	0.3274846
Crat	0.24400818	5.36198451	3.00440697	0.09849928	0.3274846
Nek11	0.93914788	0.02576182	3.00269252	0.09858898	0.32769185
Zbtb22	-0.3980068	3.3208382	3.00166994	0.09864252	0.32771166
Cttn	0.24415758	5.83338286	3.00153359	0.09864967	0.32771166
Icam5	0.45872384	2.88514723	2.99971764	0.09874485	0.32793689
Ranbp9	0.18852957	7.07801354	2.99882247	0.09879181	0.32800189
E030030I06R	-0.5725676	2.81872364	2.99812076	0.09882864	0.32803322
Irak4	0.45564037	3.52899172	2.9969531	0.09888996	0.32814581
Mchr1	0.56135758	2.58860554	2.99615423	0.09893194	0.32819417
Tbc1d31	-0.3675974	3.31663818	2.99405771	0.09904221	0.32841909

Lhcgr	-2.1941784	-1.0558066	2.99382254	0.09905459	0.32841909
Zfp319	0.39828938	3.1469779	2.99310141	0.09909255	0.32845404
Naa40	-0.4226472	2.94332388	2.99216446	0.09914191	0.3285267
Ddx3x	-0.1658153	8.72107366	2.98998965	0.09925658	0.32855611
Gtf2h3	-0.3879591	3.5832589	2.98992958	0.09925975	0.32855611
Astn2	-0.4018351	3.40841357	2.98894714	0.09931161	0.32855611
Camkk1	0.3605473	4.88035327	2.98876276	0.09932134	0.32855611
Casp1	-0.719422	1.62809504	2.9881767	0.0993523	0.32855611
E4f1	0.49160354	2.50487204	2.98785427	0.09936933	0.32855611
Fdps	0.40039057	3.83319565	2.98772863	0.09937597	0.32855611
Supt3	-0.479147	2.28573846	2.98766809	0.09937917	0.32855611
Uvrag	-0.2306337	5.56574296	2.98731734	0.0993977	0.32855611
Gpr108	-0.4746333	3.10882504	2.98653937	0.09943883	0.32860136
Ccbe1	0.36865663	3.84561048	2.98198774	0.09967985	0.32930207
Gm16973	-0.4639421	2.88073708	2.98149719	0.09970587	0.32930207
Sidt1	-0.323985	5.12946762	2.97921625	0.09982694	0.32959487
Robo4	-1.034279	0.64692429	2.97855116	0.09986228	0.32959487
Map2k1	0.1781834	7.71419502	2.97827255	0.09987709	0.32959487
Cbr3	-0.4847183	2.12842456	2.9759337	0.10000149	0.32989156
Cdkn1c	-0.4491167	5.69488742	2.97534536	0.10003281	0.32989156
Cdc27	0.23210167	7.83018118	2.97482968	0.10006028	0.32989156
Cysltr2	-1.8978017	-0.9832844	2.97431958	0.10008745	0.32989156
Cdh1	0.52537427	6.50236452	2.97386138	0.10011187	0.32989156
Rap1gap2	0.37086462	6.77560268	2.97347861	0.10013227	0.32989156
Slc39a8	-0.6674953	2.78541867	2.97227406	0.10019651	0.33001241
4933407K13I	-0.7914843	1.84513764	2.97160161	0.1002324	0.33003983
Tmem60	0.27743787	5.36769151	2.97108201	0.10026013	0.33004042
Bzrap1	-0.551166	4.8099904	2.97026125	0.10030397	0.33008886
AF529169	-0.7785442	1.49854143	2.96918487	0.10036148	0.33008886
Gbp6	-0.3187577	5.06947314	2.96889065	0.10037721	0.33008886
Ccdc68	-0.9490019	0.39708641	2.96874306	0.1003851	0.33008886
Nrip3	0.25712608	5.45211388	2.96749151	0.10045205	0.33012765
Cenpc1	0.26440864	5.59738244	2.96725477	0.10046472	0.33012765
Soga1	-0.2002575	7.13274724	2.9668518	0.10048629	0.33012765
Lin37	0.29709219	4.4352741	2.9664618	0.10050717	0.33012765
Usp39	0.3204527	4.1659872	2.96566248	0.10054998	0.33017771
Fhl3	0.70141605	2.04694674	2.96287046	0.10069968	0.33057865
Cept1	0.37610012	3.88413645	2.96163822	0.10076583	0.33066102
Coq9	0.31921019	4.37311304	2.96121975	0.10078831	0.33066102
Kdm5b	-0.3286683	5.90629015	2.96071895	0.10081522	0.33066102
Ptchd4	-0.3420581	4.03406526	2.9598322	0.10086289	0.33066102
Sdhc	0.30494194	6.84374404	2.95934158	0.10088927	0.33066102
Arhgap25	0.38868804	3.64359913	2.95874766	0.10092122	0.33066102

Agtr1b	-1.0865155	2.19031854	2.95866762	0.10092553	0.33066102
Nudt16	0.31548953	4.68686686	2.95809376	0.10095641	0.33066102
Il18	-0.2764538	4.1713392	2.95778054	0.10097327	0.33066102
Plk5	-0.9507061	1.09162456	2.95600867	0.10106871	0.33067941
Ap2b1	0.21821494	7.86662395	2.95545483	0.10109857	0.33067941
Rtn4	0.21976376	9.9353984	2.95437378	0.10115687	0.33067941
Chrna7	0.75250863	1.221174	2.95367045	0.10119483	0.33067941
Cdk2	-0.6960877	1.70180027	2.95339963	0.10120945	0.33067941
Fbxo46	-0.5041848	1.66258558	2.95329579	0.10121505	0.33067941
Cox7b	0.29095614	7.33657115	2.95283655	0.10123985	0.33067941
Smg8	-0.2632873	4.41716152	2.95255602	0.101255	0.33067941
Rorc	-0.8279007	1.15378792	2.95186106	0.10129255	0.33067941
Rab4a	0.26224325	4.98587144	2.95126882	0.10132456	0.33067941
Tmem260	-0.3827366	3.77287118	2.95110241	0.10133355	0.33067941
Stmn2	0.18248357	7.85579106	2.9508494	0.10134723	0.33067941
Gp5	2.04466555	-1.3607598	2.95049863	0.1013662	0.33067941
Ephb1	-0.367792	3.65570156	2.95043537	0.10136962	0.33067941
Tacstd2	-1.1657114	0.91133709	2.94998947	0.10139374	0.33067941
Rac1	0.20140352	10.032103	2.94842494	0.10147842	0.33067941
Impg1	-1.0985445	0.27161682	2.9469067	0.10156068	0.33067941
Slc45a3	1.37818889	0.25526345	2.94547776	0.10163817	0.33067941
Dnah7b	-0.3551877	3.50336842	2.94332338	0.10175513	0.33067941
Bhlhe40	-0.2031864	7.52034272	2.94324282	0.10175951	0.33067941
Nlrc5	-1.2471428	0.00570201	2.94269084	0.1017895	0.33067941
H2-Ab1	-0.6587811	4.25397424	2.94248496	0.10180069	0.33067941
Strip2	0.46290489	5.1919143	2.94212152	0.10182045	0.33067941
4930523C07I	-0.4176006	3.74449244	2.94171355	0.10184263	0.33067941
Zfp827	0.23832121	6.30042239	2.94146584	0.10185611	0.33067941
Atp6v1a	0.25232363	9.34063743	2.94146194	0.10185632	0.33067941
Pccb	-0.30284	4.70569849	2.9412566	0.10186749	0.33067941
Prpf6	-0.2462703	5.5425876	2.94105782	0.1018783	0.33067941
Sh2d2a	-0.7482794	1.19444342	2.9410305	0.10187979	0.33067941
Tox4	0.22029519	5.999696	2.94085611	0.10188928	0.33067941
Acmsd	-0.9001154	0.69188002	2.94034642	0.10191701	0.33067941
Zfp672	0.29091003	4.76502454	2.94003515	0.10193396	0.33067941
Acbd7	1.5698389	-1.2442013	2.93927284	0.10197547	0.33067941
Scarf1	-1.6050106	-0.7980304	2.93884169	0.10199895	0.33067941
2310003H01I	-0.723228	0.9227629	2.9377534	0.10205826	0.33067941
Tmem82	-0.8991708	0.41533048	2.93762767	0.10206512	0.33067941
Acox3	-0.306174	4.07254784	2.93757205	0.10206815	0.33067941
Slc37a3	0.36092102	4.04118976	2.93673925	0.10211357	0.33067941
Ngf	0.82539973	0.84562194	2.93660331	0.10212098	0.33067941
Gtf2i	-0.1941269	7.28734558	2.93624205	0.10214069	0.33067941

Kdelc2	0.31768466	4.49541638	2.93538361	0.10218755	0.33067941
Fam126b	0.26001645	8.29445503	2.93477622	0.10222072	0.33067941
Tle1	-0.2535235	5.53266061	2.93445349	0.10223835	0.33067941
D030056L22I	0.33610195	4.86622	2.93403801	0.10226105	0.33067941
Tgfbi	-0.9403646	2.29086337	2.9335288	0.10228888	0.33067941
Rnf215	0.49345054	2.67262694	2.93186539	0.10237985	0.33067941
Nell2	0.27361941	6.79545258	2.93013125	0.1024748	0.33067941
Eng	-0.3468672	3.29847141	2.9295694	0.10250558	0.33067941
Wdr59	-0.3865363	4.08083724	2.92902768	0.10253527	0.33067941
Cacnb4	-0.2383858	9.03744874	2.92888275	0.10254321	0.33067941
Nf1	-0.3181471	8.07663234	2.92735556	0.10262698	0.33067941
Scyl3	0.25451341	5.298139	2.9268094	0.10265695	0.33067941
Prmt8	-0.2408016	7.02981199	2.92679071	0.10265798	0.33067941
Ergic2	-0.2970702	4.64021062	2.92555506	0.10272583	0.33067941
Slamf1	-2.007055	-1.076657	2.92499155	0.1027568	0.33067941
Zfand3	0.26998235	5.53411354	2.9247755	0.10276867	0.33067941
Rims1	-0.2863756	6.48495215	2.92469178	0.10277327	0.33067941
Ankrd42	0.29945167	4.01704178	2.92394931	0.10281409	0.33067941
Slc19a2	-0.4046021	3.68181895	2.92340318	0.10284413	0.33067941
Opa3	0.34267725	5.05372894	2.9227018	0.10288272	0.33067941
Fars2	-0.4803573	2.72872315	2.92206932	0.10291754	0.33067941
Foxc1	0.31003272	8.36033334	2.92200711	0.10292096	0.33067941
Sugp1	0.30611413	4.58008137	2.92151339	0.10294815	0.33067941
Scfd2	-0.4397396	2.97274159	2.92098151	0.10297745	0.33067941
Usmg5	0.28953181	5.86410141	2.92052302	0.10300271	0.33067941
Smad7	-0.4166028	3.43137267	2.92011543	0.10302518	0.33067941
Fam198b	-0.3942433	3.62236405	2.92001444	0.10303075	0.33067941
Glrx	0.27875016	6.11625311	2.91996532	0.10303345	0.33067941
AF357425	-0.9260527	3.01202693	2.9197462	0.10304554	0.33067941
Rbm47	0.33737622	4.57140377	2.91968918	0.10304868	0.33067941
Shh	0.66398856	2.02679904	2.9184126	0.1031191	0.33067941
Usp28	-0.4516606	3.5545743	2.91808818	0.103137	0.33067941
Rbm6	-0.2669644	6.21619282	2.91771131	0.10315781	0.33067941
Acvr2b	-0.8960347	0.6247075	2.9177046	0.10315818	0.33067941
Entpd2	-0.9118945	0.55519252	2.91702956	0.10319545	0.33067941
Mmp16	-0.3097557	5.36699332	2.91640036	0.10323021	0.33067941
Sgsh	0.65342734	1.3039999	2.91634633	0.1032332	0.33067941
Tac2	0.77051236	1.01105244	2.91610742	0.1032464	0.33067941
Tph1	-2.4541143	-0.7652572	2.91589243	0.10325828	0.33067941
Myt1l	0.28078419	8.60644459	2.91572198	0.1032677	0.33067941
Oip5	-1.4479173	0.22054863	2.91566404	0.10327091	0.33067941
Pkhd1	-1.9625729	-0.6882453	2.9154241	0.10328417	0.33067941
Glra4	-2.0549737	-1.6182774	2.91504522	0.10330512	0.33067941

Snapc3	0.27446325	4.89748963	2.91469606	0.10332443	0.33067941
Cntn2	-0.3640469	5.70335049	2.91466867	0.10332595	0.33067941
Arpp21	-0.242024	8.75965728	2.91327029	0.10340333	0.33082328
Kremen2	-1.1171226	-0.7455648	2.91198459	0.10347454	0.33082328
Hint3	0.32263415	4.30640939	2.91171696	0.10348937	0.33082328
Bms1	-0.2344117	6.0653261	2.91143089	0.10350522	0.33082328
1700024B18I	-1.5838847	-0.3069596	2.91107713	0.10352483	0.33082328
Ankrd27	-0.2223655	5.38224598	2.91086402	0.10353665	0.33082328
Lyst	-0.3646328	6.33483125	2.90803923	0.10369341	0.33114514
Col6a1	-0.2791147	5.05740719	2.90795956	0.10369784	0.33114514
Rtn4rl2	0.76155775	0.92144409	2.90686596	0.10375861	0.33114514
Mir665	-1.6842942	-0.6775952	2.90654976	0.10377619	0.33114514
Kdm8	0.7828896	1.41305886	2.90631957	0.10378899	0.33114514
Man2a2	-0.240908	6.90128601	2.90606239	0.10380329	0.33114514
Bmyc	0.28618859	5.26490475	2.90282789	0.10398336	0.33163124
Iglon5	0.39592475	3.56600338	2.90081958	0.10409535	0.33190002
Arsa	-0.4937142	2.4348493	2.89982266	0.10415099	0.33194033
Lsm10	0.46948736	2.36384022	2.89960001	0.10416342	0.33194033
Bmp15	0.69163649	2.22693816	2.8987988	0.10420818	0.3319946
Spag6	0.58120971	1.99929427	2.8978027	0.10426385	0.33205344
Fgfr3	-0.3472471	3.90040749	2.89733796	0.10428983	0.33205344
BC003965	0.21751252	6.03354468	2.89698042	0.10430983	0.33205344
Fhl1	0.21062922	7.70582581	2.89647763	0.10433796	0.33205471
Mesdc1	-0.2845084	4.04770127	2.8932894	0.10451652	0.33248442
Gm9079	1.17908589	0.57744358	2.89293362	0.10453647	0.33248442
Zmynd19	-0.7138059	1.55240992	2.89210592	0.1045829	0.33248442
Acot6	0.47112628	2.70793181	2.89208577	0.10458403	0.33248442
Gm17769	-0.8128711	0.14104306	2.89047688	0.10467435	0.33261169
5031414D18I	1.67140625	-1.0537703	2.89038311	0.10467961	0.33261169
Rptoros	-2.2396596	-1.2315957	2.88844057	0.10478879	0.33286585
1110019D14I	0.44767263	2.68830163	2.88797116	0.10481519	0.33286585
Nox4	-0.7307884	1.22891877	2.88731562	0.10485207	0.33287803
Hecw1	-0.4629019	6.93926334	2.88691509	0.10487462	0.33287803
Gm13889	0.56296437	1.54006208	2.88530521	0.10496529	0.33297129
Ebi3	2.05012462	-1.0286193	2.88516545	0.10497316	0.33297129
Scd3	0.51431402	3.20431338	2.88491258	0.10498741	0.33297129
Galnt6	-0.6873512	1.98311809	2.88031122	0.10524713	0.33370662
Atg2a	-0.4504906	3.99208014	2.87882566	0.10533114	0.33383111
Tmed9	0.20726274	6.26273586	2.87863123	0.10534215	0.33383111
BC065397	-0.9803456	0.92368029	2.87748722	0.1054069	0.33394797
Taldo1	0.25944866	4.07313907	2.87699465	0.1054348	0.333948
Ank3	-0.4130307	9.13226804	2.87559165	0.10551431	0.33402676
Dcaf10	-0.2707137	5.20849913	2.87557152	0.10551545	0.33402676

Trim17	0.85212433	0.58160471	2.87329817	0.10564443	0.33421263
Nr4a3	0.50763407	4.23225603	2.87295402	0.10566398	0.33421263
Abhd17b	0.27115429	5.76779969	2.8726846	0.10567928	0.33421263
Clip2	-0.4573657	3.95451113	2.87256984	0.1056858	0.33421263
Bcl7b	-0.3013129	4.11000641	2.87185127	0.10572662	0.33425348
Srp68	0.23949469	5.94571397	2.87064385	0.10579527	0.33438222
Bbs2	-0.3189892	5.17881808	2.86975345	0.10584592	0.33445405
Tgfb3	-0.5102868	4.69475048	2.86919648	0.10587762	0.33446597
Spns1	-0.5913244	2.31003266	2.86705657	0.10599952	0.33456331
Lrrc24	0.69481211	0.76078617	2.86674529	0.10601726	0.33456331
Reps2	0.19325549	9.58647786	2.86673286	0.10601797	0.33456331
Ndst1	-0.230703	5.73500255	2.8662149	0.10604751	0.33456331
Pygl	-0.8162097	1.09311284	2.86620416	0.10604812	0.33456331
Kank3	-0.7042247	1.87037182	2.86537992	0.10609514	0.33462351
03-Mar	-1.3067427	0.04069443	2.86462156	0.10613843	0.33467189
Dbn1	-0.3179535	4.44211037	2.86226539	0.10627304	0.33500814
Pigt	0.28600132	5.31439876	2.8585416	0.1064862	0.33559177
Sdc3	0.26010101	5.2388551	2.8561944	0.10662082	0.33592762
Creb3l1	0.37283806	5.25308453	2.85462056	0.1067112	0.33608469
Slc43a1	-0.9793574	0.58700747	2.854349	0.10672681	0.33608469
Ngef	-0.2692598	5.79286165	2.85273601	0.10681954	0.33627337
Inmt	0.57243928	3.49614324	2.8519769	0.10686322	0.33627337
Prrt1	0.28934493	4.88909322	2.85184234	0.10687096	0.33627337
Dcaf15	-0.6877187	1.33317004	2.85104751	0.10691672	0.33632898
D630003M21	-1.2086852	0.44311259	2.8493159	0.10701649	0.3363691
Pik3r1	0.21331885	8.09936598	2.84924187	0.10702076	0.3363691
Arhgdib	0.44453186	7.44678784	2.84910102	0.10702888	0.3363691
Tm7sf3	-0.3270129	4.54772077	2.84887649	0.10704182	0.3363691
Rbm33	-0.311668	5.58997343	2.84835918	0.10707166	0.33637458
Slc38a10	0.36136827	3.43456733	2.8473632	0.10712913	0.33637705
Ap3d1	-0.3053399	6.40065025	2.84712633	0.1071428	0.33637705
Arhgef7	0.23599101	6.71719714	2.84687218	0.10715747	0.33637705
Cipc	0.19795418	6.68247745	2.84581461	0.10721855	0.33637705
Trim37	-0.2566074	8.21341836	2.84514209	0.10725742	0.33637705
Stat5a	-0.8227972	1.7590182	2.84494828	0.10726862	0.33637705
Abcc3	-1.4279891	-0.6679128	2.8446492	0.10728591	0.33637705
Spire2	0.41728015	2.52442626	2.84445486	0.10729715	0.33637705
Idi1	0.29939997	5.81647658	2.84344617	0.10735549	0.33647188
Gm19897	-1.3788489	-0.0053827	2.84250123	0.10741019	0.33655522
Wnk1	-0.1816507	10.008663	2.84199768	0.10743935	0.33655853
Plekhg6	1.83853461	0.0553454	2.8410228	0.10749582	0.33657987
Rgs5	-0.4139796	4.11792046	2.84090985	0.10750237	0.33657987
Gna12	-0.2515697	5.26986265	2.84002134	0.10755388	0.33665313

Gsto2	-1.5999933	-0.5104815	2.83828883	0.1076544	0.33687972
Cir1	0.20170528	6.95479119	2.83729128	0.10771233	0.33697294
Ghsr	-1.6480129	-0.3539409	2.83663695	0.10775035	0.33700384
A630075F10	-1.2715828	-0.7287085	2.83490088	0.10785129	0.33720322
Zbtb25	-0.3620833	3.35792443	2.83445705	0.10787712	0.33720322
Tmem8	0.44349508	2.32998663	2.83408851	0.10789857	0.33720322
Carhsp1	-0.4729407	5.37283522	2.83177176	0.10803352	0.33749299
Tapt1	-0.2301677	5.13774678	2.83124505	0.10806423	0.33749299
Il5ra	-1.8180579	-0.831976	2.8310461	0.10807583	0.33749299
Camk2a	-0.3572233	11.2091826	2.83033607	0.10811725	0.33753431
P2rx7	-0.602984	2.03038646	2.82960804	0.10815974	0.33757896
Bscl2	0.3577777	4.33923054	2.82815815	0.10824441	0.33768017
Dopey1	-0.3906503	5.58148673	2.82777928	0.10826655	0.33768017
B4galt6	0.1817176	7.17140345	2.82698249	0.10831313	0.33768017
Fbxw7	-0.205849	8.24940269	2.82686053	0.10832026	0.33768017
Six3os1	-0.792634	0.77632868	2.82664009	0.10833315	0.33768017
Aspscr1	0.57909094	2.7002338	2.82600261	0.10837044	0.33770851
Slc18b1	0.33373965	5.1384207	2.82412887	0.10848014	0.3379624
Snx16	0.28195729	4.76565689	2.82319356	0.10853494	0.3380452
Dctn2	0.20966568	5.87271617	2.82255833	0.10857218	0.33804872
Arhgef5	0.39249727	5.78450739	2.82210876	0.10859855	0.33804872
Dazl	0.70034095	1.50788797	2.82173021	0.10862075	0.33804872
Fam89a	-2.5268079	-1.4200627	2.8200853	0.10871731	0.3382613
Enpp6	-0.5135901	2.77992828	2.81912484	0.10877373	0.33832591
Diap2	0.24349019	7.4642199	2.81877023	0.10879457	0.33832591
Tfb2m	0.27418998	5.65396134	2.81788211	0.10884679	0.33840042
Ogfrl1	-0.2248695	7.93342895	2.81674566	0.10891365	0.3385204
Mgat1	-0.3412625	3.42055107	2.81601293	0.10895678	0.3385666
Calcoco1	0.1958953	7.47821036	2.81414808	0.10906666	0.33882009
1500009C09I	-0.2592885	5.50390501	2.8126539	0.10915478	0.33889365
BC016579	2.52058852	-1.2504356	2.81263697	0.10915578	0.33889365
Myo1a	-2.3597136	-1.8296905	2.81230745	0.10917523	0.33889365
Cyp2j12	-1.2012226	-0.5687111	2.8108346	0.1092622	0.33907572
Vmn1r65	-0.6658758	1.14576224	2.80991611	0.10931648	0.33908886
Dbpht2	-0.2304196	7.6516806	2.80972158	0.10932797	0.33908886
Nsun2	-0.2301557	5.15905274	2.8093258	0.10935138	0.33908886
Prkar1a	0.17304831	9.6161682	2.80858792	0.10939502	0.33913639
Cdk6	-0.7927905	2.18186163	2.8073985	0.10946541	0.33923814
Mgme1	-0.5290546	2.92595142	2.80707622	0.1094845	0.33923814
Pcdhb11	-0.9951072	0.96598835	2.80520621	0.1095953	0.33936582
Lrrc16a	0.31214105	4.29144179	2.80512743	0.10959997	0.33936582
Med12l	-0.4182146	5.30820352	2.8049462	0.10961072	0.33936582
Fcrls	-0.8443021	1.54344306	2.80381695	0.10967771	0.33948546

Mir6390	-2.1645576	0.08973674	2.80216951	0.10977552	0.33968687
Noxo1	-1.3486162	-0.2666399	2.80170619	0.10980305	0.33968687
Fxr2	-0.3559875	3.71798348	2.80128852	0.10982787	0.33968687
Sh3glb2	0.22754866	5.23675854	2.80024846	0.10988971	0.33979037
Fam19a4	-2.0342628	-1.4218659	2.79870836	0.10998136	0.33987119
Hexdc	0.62674085	1.95366144	2.79863339	0.10998582	0.33987119
Efcab1	0.38407349	3.56238764	2.7983787	0.11000098	0.33987119
Mfn2	-0.2082503	6.63419644	2.79732966	0.11006347	0.33997655
Elavl2	0.23842715	6.93896308	2.79500131	0.11020232	0.34031765
Mtmr9	-0.3334322	3.64425542	2.79297412	0.11032337	0.34060365
Gmppb	0.83183826	0.94314568	2.79139897	0.11041754	0.34066394
Myof	-0.4164486	5.02471238	2.7912364	0.11042727	0.34066394
Gm5617	0.8684042	1.57345453	2.79049059	0.11047189	0.34066394
Txn1	0.31295964	6.41714117	2.79046356	0.11047351	0.34066394
Csgalnact2	-0.3480205	3.38256503	2.7902694	0.11048513	0.34066394
Eno1	0.64837218	0.80270498	2.78917481	0.11055068	0.34073056
Pcbp1	0.24751385	5.75618372	2.78863898	0.11058278	0.34073056
01-Mar	-0.3046346	5.07859103	2.78848349	0.1105921	0.34073056
Lypla2	0.48818264	2.10910724	2.78581492	0.11075215	0.34113592
Fpgs	-0.8624982	0.54372643	2.78311247	0.11091452	0.34144297
Slc35f5	0.34342992	4.54118939	2.78301397	0.11092044	0.34144297
Gzmb	1.82034435	-0.3237874	2.78226685	0.11096538	0.34144297
Slc38a1	-0.2106336	7.61637541	2.78225853	0.11096588	0.34144297
LOC1026341(	-2.0787141	-1.4732897	2.78055868	0.11106821	0.34162795
Hnrnpm	-0.214976	6.77001369	2.78031225	0.11108305	0.34162795
Xkr6	-0.5764461	2.9998954	2.77858239	0.11118732	0.34176435
Loxl4	-1.7265995	-1.1594294	2.7784524	0.11119516	0.34176435
Map7d2	0.2294143	7.71414111	2.77815632	0.11121302	0.34176435
Cspp1	-0.30636	4.72589044	2.777487	0.1112534	0.34180074
Gpr37	-0.4020104	3.7003291	2.77614396	0.11133449	0.34192389
Cetn3	0.27187241	7.87997825	2.77587743	0.11135059	0.34192389
Sod2	0.20535355	8.45926204	2.77287546	0.11153212	0.34223518
Tmem206	-0.3612372	4.40141597	2.77287216	0.11153232	0.34223518
Naif1	0.95232287	0.03808639	2.77278338	0.11153769	0.34223518
Gas2l2	-1.8549646	-0.998051	2.77198941	0.11158576	0.34229498
Clspn	-1.3000749	-0.1388371	2.77012867	0.11169852	0.34249688
Arglu1	-0.1800942	6.85567336	2.76965722	0.11172712	0.34249688
Nipa1	-0.3039732	4.82904993	2.76924394	0.11175219	0.34249688
Sidt2	0.23103615	5.19264066	2.76784779	0.11183693	0.34249688
Dsp	-0.7230822	1.66527481	2.76758527	0.11185287	0.34249688
Slc36a2	-2.2681763	-1.7024372	2.76748817	0.11185877	0.34249688
A930006K02	-0.9013477	0.2417027	2.76716449	0.11187843	0.34249688
Vps13d	-0.3900045	6.62824952	2.76634761	0.11192807	0.34249688

Cux2	0.33877746	4.80386625	2.76629267	0.11193141	0.34249688
Enoph1	0.2319556	4.85653078	2.76619122	0.11193757	0.34249688
Zfp260	0.20001517	7.24177903	2.76428945	0.11205325	0.34276324
Lamc1	0.24258579	6.02517539	2.76237726	0.1121697	0.34301405
Aatk	-0.4328863	5.14712158	2.7615425	0.11222059	0.34301405
Akap2	-0.2001507	7.52593438	2.76050648	0.11228377	0.34301405
Parp10	0.58650125	1.9135946	2.76005465	0.11231134	0.34301405
Hmha1	-0.72081	1.25601341	2.76005385	0.11231139	0.34301405
Btn2a2	-2.1002791	-0.4733493	2.75967535	0.1123345	0.34301405
Drp2	-0.3815614	5.99951499	2.75965493	0.11233574	0.34301405
Uba5	0.20860561	5.87438216	2.75710753	0.11249137	0.34328062
Slc30a2	-0.474118	2.67361515	2.75687075	0.11250585	0.34328062
Txnl1	0.20408036	7.14529889	2.75681871	0.11250903	0.34328062
Scamp1	0.19213384	7.87224213	2.7554023	0.1125957	0.34342803
Gm17751	-2.1250347	-0.8407761	2.75437027	0.11265889	0.34342803
Slc25a33	0.41701996	3.31598457	2.75401972	0.11268036	0.34342803
Hist1h2bn	0.54496928	3.01491365	2.75383859	0.11269146	0.34342803
Lrpap1	0.32693376	5.78732057	2.75368722	0.11270074	0.34342803
Lrrc39	-0.4993614	2.13293728	2.75128698	0.11284793	0.34368737
Cts8	-0.8663765	0.00836896	2.75105311	0.11286229	0.34368737
Ccl27a	-0.2819414	5.16769628	2.75089594	0.11287194	0.34368737
Frmd4b	-0.2646264	5.2760679	2.75036193	0.11290472	0.34369982
Sf3b3	-0.2400779	5.27586135	2.74845323	0.11302201	0.34396942
D7Ertd443e	-0.5621537	1.54308926	2.74780493	0.11306188	0.34400333
Dclre1c	-0.4046003	5.24539351	2.74627959	0.11315575	0.34419398
Usp6nl	-0.2296855	5.91250472	2.74549797	0.11320389	0.34419398
A530046M15	-0.9230356	0.47076079	2.74532094	0.11321479	0.34419398
Ctsc	0.53360348	2.05099363	2.74491996	0.1132395	0.34419398
Samd5	-0.3561333	3.98939051	2.74380184	0.11330843	0.3442184
Sstr1	0.64333044	3.05387091	2.74341494	0.11333229	0.3442184
Nupr1	-0.4591529	6.67529031	2.74339108	0.11333377	0.3442184
Stk32b	0.48044365	2.80468229	2.7411273	0.11347351	0.34432163
Odc1	-0.1776274	6.42482973	2.74096924	0.11348328	0.34432163
Ppp2r5b	0.35762995	3.29609995	2.74089787	0.11348769	0.34432163
Pphln1	0.20113731	7.20904503	2.74077425	0.11349532	0.34432163
Gabarap	0.33149401	7.19834281	2.74030119	0.11352456	0.34432163
Usp30	0.30405491	4.18557355	2.73960301	0.11356772	0.34432163
Chmp7	0.26198334	5.62002362	2.7395821	0.11356902	0.34432163
Coq5	0.20118394	6.38058398	2.73834684	0.11364543	0.34442301
Hsf2	0.25609357	5.63223317	2.73777483	0.11368084	0.34442301
Trim23	0.23769786	6.39756976	2.7376473	0.11368873	0.34442301
5830444B04I	-0.8129776	1.32362467	2.73685493	0.1137378	0.34448453
1700001L05I	-0.4393454	4.34217344	2.73502332	0.11385133	0.34471306

Arpc5l	-0.2608622	5.82331496	2.73470904	0.11387083	0.34471306
5033406O09	2.48744962	-0.5531035	2.73368995	0.11393406	0.34481733
Rsrp1	-0.3695001	6.29280834	2.73304724	0.11397397	0.34485094
4933411K20I	0.21679299	6.72775489	2.73220549	0.11402625	0.34486845
Rin3	0.4166105	4.36278488	2.73202696	0.11403735	0.34486845
Map3k15	-0.8576926	0.18468205	2.73052095	0.11413097	0.34490776
Mpzl1	-0.3788864	3.20946085	2.73025329	0.11414762	0.34490776
Cacna2d1	0.2916316	7.62950505	2.72989774	0.11416974	0.34490776
Cd74	-0.5778804	6.73494248	2.72957594	0.11418977	0.34490776
S1pr2	-0.706905	1.73585486	2.72945836	0.11419709	0.34490776
Fastkd5	-0.3253148	3.34987176	2.72903973	0.11422315	0.34490776
Tmem63a	-0.3604469	4.55997756	2.72751553	0.11431809	0.34510631
Nsmce2	0.25306489	5.28630791	2.72705915	0.11434654	0.34510631
Tmcc1	-0.1656236	6.94096167	2.72651568	0.11438042	0.3451216
Nyap1	-0.4170482	2.96489102	2.72532333	0.11445481	0.34518115
Nnt	-0.2322466	5.08269873	2.72527528	0.11445781	0.34518115
Snord64	-0.9450629	0.11126049	2.72436527	0.11451462	0.34523722
Ppih	0.39504788	3.16833844	2.72354641	0.11456577	0.34523722
Sel1l	0.23602048	7.13736227	2.72270752	0.11461819	0.34523722
Slc28a3	-0.9940515	1.09393552	2.72158501	0.11468839	0.34523722
Naip1	-2.4203199	-1.843122	2.72157196	0.11468921	0.34523722
Nmrk1	-0.2943799	4.76005865	2.72130206	0.1147061	0.34523722
Scpep1	0.34572211	5.11264015	2.72116191	0.11471487	0.34523722
Tbx20	1.60495915	-1.4327561	2.72089464	0.11473159	0.34523722
Adrb3	-1.3141396	-0.894653	2.72071245	0.11474299	0.34523722
2310034G01	1.14651877	0.91151971	2.71954306	0.11481622	0.34523722
Rpl13a	-0.2451689	7.00463303	2.71940906	0.11482461	0.34523722
Ythdc1	0.20689647	7.24314683	2.71899981	0.11485026	0.34523722
Fezf1	2.38469305	-0.6692118	2.71898534	0.11485116	0.34523722
Hs6st2	0.37006911	4.82029221	2.71836303	0.11489017	0.3452678
Fbxw2	-0.2511196	5.59019633	2.71789844	0.1149193	0.3452687
Acsf2	-0.2908689	4.8861984	2.71730731	0.11495637	0.34527832
Parp6	-0.2618017	5.88867392	2.71692817	0.11498016	0.34527832
Ttc18	-1.003197	0.60106621	2.71626163	0.11502199	0.34531735
Rrp1b	-0.3267791	3.45718733	2.71448467	0.11513361	0.34551539
Qrsl1	0.41649169	2.65418748	2.71429287	0.11514566	0.34551539
Fbln5	-0.5994509	4.21843074	2.71318938	0.11521505	0.34563699
Gm11696	-0.6707117	1.18376986	2.71093564	0.11535691	0.34582731
Eif2b5	-0.2691597	4.56639285	2.71084909	0.11536236	0.34582731
Gbf1	-0.2572132	5.9862477	2.71043129	0.11538868	0.34582731
Arhgap30	-0.5499406	2.85648946	2.71003513	0.11541365	0.34582731
Tomm40l	-0.2715377	3.94998471	2.70977827	0.11542984	0.34582731
Immp2l	1.00242053	-0.0527315	2.70914124	0.11547001	0.34582731

Kcp	1.36773149	0.03723685	2.70863183	0.11550214	0.34582731
Cul3	0.17413718	8.90574059	2.70823846	0.11552696	0.34582731
Trip4	0.21168197	5.85807798	2.70805734	0.11553839	0.34582731
Oxct1	-0.1985071	8.33302897	2.70724282	0.1155898	0.34587499
Cnp	-0.2711792	6.61886777	2.70689011	0.11561208	0.34587499
Cyp2s1	0.56936718	3.8419522	2.70541728	0.11570514	0.34594762
Mterf1b	-0.8538498	0.95309795	2.70525549	0.11571537	0.34594762
Egr1	-0.3883384	9.14900846	2.70485266	0.11574084	0.34594762
Scarb2	0.42762696	4.27595107	2.7046777	0.1157519	0.34594762
Ubr7	0.22861468	4.90069223	2.70346156	0.11582885	0.34603815
Zfp9	0.23168943	5.92135413	2.70328568	0.11583998	0.34603815
4930556M19	-0.7234093	1.12915762	2.70193117	0.11592577	0.34620804
Igsf6	-0.748944	1.69934988	2.70144854	0.11595635	0.34621305
Phf19	1.05606829	0.71528443	2.7008176	0.11599635	0.34621816
Mrpl44	-0.3122187	3.56917656	2.70050952	0.11601589	0.34621816
Coro2b	0.26879997	6.52167001	2.69898774	0.11611244	0.34630512
LOC1010560	0.41559613	3.43306051	2.69833184	0.11615409	0.34630512
Lhpp	-0.4428922	2.63005756	2.69832897	0.11615427	0.34630512
Izumo4	-0.6362097	1.70919881	2.69822777	0.1161607	0.34630512
Fam50a	-0.3667791	4.12554342	2.69733807	0.11621722	0.34631363
Nsg2	0.21069843	7.8674914	2.69695837	0.11624135	0.34631363
Tspan31	0.3298783	5.85510856	2.69681755	0.1162503	0.34631363
Efr3a	-0.22172	7.52803135	2.69552833	0.11633229	0.34635104
Pcyt1b	0.29190596	5.32802086	2.69532726	0.11634509	0.34635104
Cdkl2	0.22657043	6.06379876	2.69525592	0.11634962	0.34635104
Trpm2	-0.5045915	3.5551595	2.69428852	0.1164112	0.34644823
Rttn	-0.5394057	2.57433777	2.69189082	0.11656399	0.34681676
Gfi1	-2.136533	-1.3103246	2.73506223	0.11661003	0.34686756
Il33	-0.2840998	5.16812239	2.69047955	0.11665404	0.34691229
Fam174a	-0.2925348	5.07082992	2.68962708	0.11670846	0.34698799
Ifngr1	0.40593672	3.18392937	2.68893513	0.11675267	0.34703325
D930015E06	-0.5953222	2.15529752	2.68826187	0.1167957	0.347075
Fh1	0.24014447	5.26825445	2.68736137	0.11685327	0.34715996
Zfp82	0.6046879	1.45211517	2.68616833	0.11692961	0.34719268
Cebpb	0.44373493	2.72160581	2.68574133	0.11695695	0.34719268
Ctnnd1	-0.1923817	8.01841914	2.68537566	0.11698036	0.34719268
Tmem161a	-0.450031	3.19158888	2.68523758	0.11698921	0.34719268
Pde7b	-0.1790579	6.60018981	2.68492475	0.11700925	0.34719268
Bpnt1	0.204427	6.08443902	2.68342169	0.11710558	0.34739246
Fgfr1op	-0.2606026	4.38467889	2.68231325	0.11717668	0.34751731
Eml5	-0.4583967	5.85855028	2.68130929	0.11724113	0.34757083
Asic1	0.29952733	4.84553209	2.6811279	0.11725278	0.34757083
Dbi	0.32277479	7.09650929	2.67936637	0.11736597	0.34764387

1810037I17R	0.30367456	5.63404425	2.67935987	0.11736639	0.34764387
Sox5	0.27399511	5.99055607	2.67927431	0.11737189	0.34764387
Zfp78	0.48749722	2.73225711	2.67893768	0.11739353	0.34764387
Stil	-0.8642366	0.88091538	2.67790526	0.11745995	0.34775457
Znrf2	0.33037515	4.47525774	2.67607854	0.11757758	0.34800538
Exoc5	0.19765582	6.2944493	2.67568735	0.11760279	0.34800538
Cd300ld	-1.4839019	0.25936695	2.67500161	0.11764699	0.34805018
Fblim1	0.63443196	3.58566952	2.67371988	0.11772967	0.34820875
Rpl13	-0.2821145	7.37476852	2.67287989	0.11778389	0.34824008
Zfp143	-0.4146771	3.39032296	2.67265483	0.11779842	0.34824008
Bloc1s5	0.47518533	3.3249666	2.67178401	0.11785467	0.34827705
Dennd3	-0.6511767	1.70226287	2.67156085	0.11786909	0.34827705
Impact	0.188265	7.83618296	2.67094598	0.11790883	0.34830854
Lrrc20	0.37830218	4.21284955	2.67044865	0.11794098	0.34831761
Mapre2	0.17729516	9.8021625	2.66975071	0.11798613	0.34836502
Ncbp2	0.21194614	6.54868575	2.66574185	0.11824582	0.34903681
Atf4	-0.1977583	6.67721885	2.66533926	0.11827194	0.34903681
Zfp874a	-0.2683793	4.88317352	2.66222148	0.11847444	0.34951754
Tmem33	0.19244786	6.24306401	2.66193273	0.11849321	0.34951754
Mrps6	0.34751475	4.25592822	2.65975568	0.11863488	0.34977496
Fggy	-0.5089436	2.85345833	2.65969397	0.1186389	0.34977496
Kif1c	-0.3311648	5.6443033	2.65857957	0.11871149	0.34990286
Inpp5f	-0.1769171	6.79477906	2.6581033	0.11874254	0.34990823
Tmc6	0.91592418	0.25957733	2.65729327	0.11879535	0.34997776
Mycbp2	-0.4074174	8.84042078	2.6562977	0.11886031	0.34999965
Scaf11	-0.1587906	7.60854134	2.65593092	0.11888425	0.34999965
6330403A02I	0.26386766	7.47245341	2.65583579	0.11889046	0.34999965
1700030L20F	-0.7101441	1.35267767	2.65394279	0.11901412	0.35027759
Adcy9	0.27419227	6.22779904	2.65343777	0.11904714	0.35028867
Tcea3	0.38254042	3.93148528	2.65280288	0.11908866	0.35032477
Cd164	0.37169086	7.83696566	2.65163219	0.11916527	0.35041566
Grm4	0.35904634	4.02745174	2.65137319	0.11918223	0.35041566
Myo5a	-0.4069486	9.77832343	2.6509897	0.11920734	0.35041566
Mir1954	4.19503908	-1.8444918	2.69257502	0.11928382	0.35045064
Kat2b	0.22546591	6.08005269	2.64949403	0.11930534	0.35045064
Chd3	-0.203728	7.99789304	2.64925918	0.11932074	0.35045064
Acyp2	0.25921333	4.62581168	2.64897637	0.11933928	0.35045064
1700030C10I	0.7749362	1.00427737	2.64857576	0.11936556	0.35045064
Ctcfl	-0.5449399	3.10142924	2.64730755	0.11944878	0.35053822
Matn2	0.4413267	3.70694898	2.64670347	0.11948844	0.35053822
Zfp287	-0.4033199	4.00793578	2.64656684	0.11949742	0.35053822
Dnajc12	0.39349081	3.61725097	2.64509279	0.11959428	0.35053822
Ubiad1	-0.5206192	1.89560882	2.64507414	0.11959551	0.35053822

Med17	-0.2669025	3.9562245	2.6450249	0.11959874	0.35053822
Aars2	-0.6283967	1.65531529	2.64500154	0.11960028	0.35053822
Dnm2	-0.2236848	5.25761461	2.64397729	0.11966764	0.35064984
Vps33b	0.27847791	5.18058997	2.64306717	0.11972754	0.35067577
Mrps5	-0.4560718	2.91157501	2.64295126	0.11973517	0.35067577
Gpr141	-2.1652355	-0.5865768	2.641585	0.11982516	0.35067577
Btaf1	-0.3005668	6.20105171	2.64121224	0.11984973	0.35067577
Tada2b	-0.2693718	4.39056583	2.64099081	0.11986432	0.35067577
Slc25a46	0.1920711	7.86801122	2.64091868	0.11986908	0.35067577
Pltp	-0.4735338	4.01039227	2.64068084	0.11988476	0.35067577
Ptpn23	0.26307924	4.86843946	2.63948572	0.11996359	0.35067577
Mn1	-0.3372804	4.58576522	2.63919978	0.11998246	0.35067577
Sh3pxd2a	-0.3031261	7.07643607	2.63878725	0.12000969	0.35067577
Phc3	-0.2063935	8.00497733	2.63877954	0.12001019	0.35067577
Mbd2	0.25096713	7.38678796	2.63825188	0.12004503	0.35067577
Pmpca	-0.1862033	6.05823535	2.63806825	0.12005716	0.35067577
Mycbpap	-0.7660017	0.72901751	2.63739502	0.12010164	0.35072013
A730020E08	0.50979842	2.69495924	2.6368259	0.12013925	0.35074444
Mvp	-0.4294899	3.7879624	2.63477969	0.12027459	0.35074593
Tnfrsf23	0.6336987	1.09561265	2.63477199	0.1202751	0.35074593
Mmp12	-1.729785	-1.5258281	2.63476987	0.12027524	0.35074593
Mrps18b	0.49240578	2.96801777	2.63429323	0.1203068	0.35074593
Igj	-0.7555582	1.01319306	2.63385442	0.12033585	0.35074593
Txnl4a	-0.2469891	6.03320287	2.63373679	0.12034364	0.35074593
Atp2a3	0.74796502	0.6266662	2.63338944	0.12036665	0.35074593
Gm15645	-0.575258	1.94279585	2.6331154	0.12038481	0.35074593
Spint2	0.48941625	2.87373047	2.63283561	0.12040335	0.35074593
Ube2s	0.38472344	3.69044708	2.63229293	0.12043932	0.35076539
Thsd1	-1.1261808	0.40548521	2.63142033	0.12049719	0.35084859
Sclt1	-0.2988861	4.66918407	2.630738	0.12054246	0.35087925
Ttc39c	0.48869791	2.23883921	2.6303786	0.12056631	0.35087925
Cyb5r2	2.57516339	-1.4312352	2.62938635	0.1206322	0.3509857
Tgfb1	0.51645809	2.17365205	2.62851725	0.12068994	0.35102117
Fam131c	1.75759281	-0.4741631	2.62824048	0.12070834	0.35102117
Csf2ra	-0.4027029	3.82945891	2.62787966	0.12073232	0.35102117
Hopx	-0.4734622	3.04404463	2.62350551	0.12102357	0.35134527
Gigyf2	-0.237032	6.95812281	2.62337454	0.1210323	0.35134527
1700110I01R	-0.8442573	1.59061764	2.62178453	0.12113838	0.35134527
Tex9	-0.2228131	4.98234399	2.62125957	0.12117343	0.35134527
Grap	-0.7639121	0.96999009	2.62124872	0.12117416	0.35134527
Myrip	0.28940556	6.38887838	2.62104767	0.12118758	0.35134527
Ppargc1b	-0.4700228	3.29665832	2.62101029	0.12119008	0.35134527
Ddx41	-0.3415082	3.8381311	2.62077612	0.12120572	0.35134527

Cdc40	0.19866094	6.87260442	2.62023744	0.12124171	0.35134527
Mir124a-2	-1.2758453	-0.8553492	2.61999774	0.12125773	0.35134527
Dnajc9	0.25377413	5.91835945	2.61934651	0.12130126	0.35134527
Tm9sf2	0.24313728	5.92029723	2.61921687	0.12130993	0.35134527
Slc25a45	1.38659144	-0.4163997	2.61830512	0.12137091	0.35134527
Cdk8	0.20015406	5.70955149	2.61782085	0.12140332	0.35134527
Agfg1	0.19578993	5.82001065	2.61746513	0.12142713	0.35134527
Exoc6	0.23717356	5.37342463	2.61745888	0.12142755	0.35134527
C130071C03I	-0.4732124	2.7042152	2.61724136	0.12144211	0.35134527
Prdm15	-0.4101683	3.40894954	2.61717978	0.12144623	0.35134527
Pla2g4e	-0.2942153	4.55148466	2.61678581	0.12147261	0.35134527
Fezf2	0.24660267	5.18090564	2.61613531	0.12151619	0.35134527
Pa2g4	0.17757595	6.38809118	2.61611736	0.12151739	0.35134527
Kcnd1	-0.5362369	2.40261102	2.61583828	0.12153609	0.35134527
Rere	0.17627904	8.29609131	2.61559368	0.12155248	0.35134527
Pcolce2	-0.8533114	1.14660412	2.61508811	0.12158637	0.35134527
Tom1l2	0.20649959	7.57538087	2.61453201	0.12162367	0.35134527
Tada3	-0.2673781	5.0122739	2.61438075	0.12163381	0.35134527
Tmem45b	-0.602831	2.23267171	2.61434922	0.12163593	0.35134527
Zfp719	0.26528554	5.71662256	2.61332979	0.12170433	0.35145809
Atic	0.30875773	4.23843957	2.6127061	0.12174621	0.35149425
Vmn2r85	-1.0688348	1.10954057	2.61191077	0.12179963	0.35156373
Lphn3	0.30401742	6.76500608	2.61103334	0.1218586	0.35161315
Phf23	0.30839753	4.64131352	2.61037527	0.12190284	0.35161315
Bcas1os2	-1.3633841	-0.1968734	2.61034573	0.12190483	0.35161315
Naa30	0.22373638	5.57942222	2.60984128	0.12193876	0.35162466
Rrm2b	0.20467012	6.74187979	2.60941355	0.12196754	0.35162466
Dmwd	-0.279236	4.86534183	2.60830678	0.12204205	0.3517282
C130074G19	-0.2525335	6.86910222	2.60800767	0.1220622	0.3517282
Snap29	0.23277905	6.39853214	2.6053527	0.12224119	0.35203323
Oxsm	-0.456738	3.16295024	2.6046126	0.12229114	0.35203323
Dcst1	-0.6360851	1.39971641	2.60441586	0.12230442	0.35203323
Fam210b	0.31088649	5.27692419	2.60423259	0.1223168	0.35203323
Srsf6	-0.1886863	5.68150112	2.6040887	0.12232651	0.35203323
Pcdhb20	-0.4368193	3.63432425	2.60382348	0.12234443	0.35203323
Hdgf	0.2485652	6.54387813	2.60298253	0.12240124	0.35211211
Slc36a1os	-0.5827388	2.78054286	2.60166506	0.12249032	0.35216241
AA415398	-0.5076212	2.98746953	2.6015144	0.12250051	0.35216241
Chrnb4	-0.8768061	0.14843537	2.60141923	0.12250695	0.35216241
Hdc	-1.2189854	-0.2146941	2.60027512	0.12258438	0.35224594
Il7r	-1.2057692	-0.0123462	2.60012071	0.12259483	0.35224594
Smim6	-2.5948838	-1.6185278	2.59874531	0.122688	0.35242908
Elfn1	0.36676655	4.83375736	2.59827745	0.12271971	0.35243563

Tyw3	-0.4600991	3.04954393	2.59670395	0.12282643	0.35257162
Gucd1	-0.4443536	3.58927075	2.59663648	0.12283101	0.35257162
Igdcc3	1.31886395	0.90123757	2.59623144	0.1228585	0.35257162
Hif3a	-0.4771887	2.85379318	2.59500911	0.12294151	0.35257162
Il11ra1	-0.266629	4.34286303	2.59498167	0.12294337	0.35257162
Nckap5l	0.66357824	1.45412957	2.59446855	0.12297824	0.35257162
Me3	0.38720109	4.21230339	2.59418134	0.12299776	0.35257162
Gm5544	-2.3199519	-0.6670952	2.59411037	0.12300259	0.35257162
Gatm	-0.200912	6.23725596	2.59264882	0.12310198	0.35270167
Dlg4	-0.2029153	7.45345862	2.59250819	0.12311155	0.35270167
Col2a1	-1.8567754	-1.1175281	2.59139756	0.12318716	0.35270167
Kif17	0.40978119	3.1648129	2.59103244	0.12321202	0.35270167
Polr2f	0.46949629	2.1390759	2.59082236	0.12322633	0.35270167
Gucy2e	-0.6655659	1.54458021	2.58967209	0.12330472	0.35270167
Mfsd4	-0.3619044	7.06259956	2.58913366	0.12334144	0.35270167
Npr1	-0.8122186	0.10392755	2.58912119	0.12334229	0.35270167
5930412G12	-0.8993524	0.82290441	2.58898915	0.12335129	0.35270167
Iffo1	-0.4479023	3.77255364	2.58891352	0.12335645	0.35270167
Alg10b	0.24261917	5.55240241	2.58868673	0.12337192	0.35270167
Mipol1	-0.3244948	3.67320862	2.58740422	0.12345945	0.35286767
Rbm20	-0.4970289	2.41221173	2.58431332	0.1236707	0.35332459
Arid1b	-0.3021095	6.24376872	2.5842019	0.12367833	0.35332459
Idh3a	0.19882771	6.62865407	2.58298571	0.12376157	0.35345879
Rfng	-0.3776422	3.51082264	2.5826534	0.12378433	0.35345879
Tmed10	0.20705099	6.82479277	2.57938049	0.12400873	0.35401516
Fbxo48	-1.3330291	-0.7738056	2.57781051	0.12411655	0.3542385
Miip	0.87316708	0.92175841	2.57730801	0.12415108	0.35425263
Dbndd2	-0.2029054	5.98223656	2.57443401	0.12434881	0.35470915
Armcx6	0.98283933	0.96305597	2.57412176	0.12437031	0.35470915
D10Bwg137(	-0.3955333	7.06518929	2.5727661	0.12446373	0.35489106
Wnt11	1.91857708	-1.0895182	2.57214294	0.12450669	0.35492776
Kansl1l	-0.2483776	5.50091386	2.57171989	0.12453588	0.35492776
Thumpd1	0.25552182	5.95277989	2.57071808	0.12460501	0.3550403
Mgl2	-0.9339868	0.39839275	2.56977308	0.12467026	0.35504186
Dennd6b	-0.6358221	2.8384596	2.56939364	0.12469648	0.35504186
Hivep2	-0.3947691	8.90992796	2.56926922	0.12470507	0.35504186
Acot7	0.22036277	6.14942926	2.56813121	0.12478374	0.35504186
Brox	0.19339271	5.69031614	2.56766262	0.12481614	0.35504186
Rab27a	-0.3301343	3.40232344	2.56758742	0.12482135	0.35504186
Adam8	-1.6517719	-0.6615818	2.56737944	0.12483573	0.35504186
Ptprs	0.2528041	6.88843335	2.56727829	0.12484273	0.35504186
Stard3	0.45640106	2.37085646	2.56548901	0.1249666	0.35530976
Eda	0.52551797	2.48147763	2.56380601	0.12508325	0.355557

Snora28	2.23590986	-1.4223794	2.56213454	0.12519922	0.35571087
4921507P07I	0.95484266	0.29850225	2.56174734	0.12522611	0.35571087
Cutc	0.36577184	3.81524854	2.5617419	0.12522649	0.35571087
Lta4h	-0.2536939	4.27326009	2.56113287	0.12526879	0.35574664
Bcl6b	-0.9228644	0.05208606	2.55959034	0.125376	0.3559667
Tnni1	1.96708912	-1.0624681	2.55741628	0.12552729	0.35631178
Stra13	0.39327789	4.00883163	2.55655536	0.12558726	0.35638789
Brk1	0.26410886	6.30129478	2.5561771	0.12561362	0.35638789
Utp14b	-0.3394954	4.91142659	2.55469845	0.12571673	0.35659595
Acot2	0.28993238	4.85835318	2.5539961	0.12576574	0.35665049
Dapk1	-0.2630701	5.9237312	2.5534811	0.1258017	0.35666176
Cyp2j6	0.31895437	4.00812163	2.55308608	0.12582928	0.35666176
Mier3	-0.2452562	5.08618564	2.55148361	0.12594126	0.3567
Nccrp1	-1.7571631	-1.1306751	2.55137117	0.12594912	0.3567
Slc46a1	0.57257292	1.24647663	2.55127035	0.12595617	0.3567
Pogz	-0.2064017	7.70921291	2.55118815	0.12596192	0.3567
Lrpprc	-0.3479557	5.97469776	2.54951849	0.12607873	0.35680902
Znf512b	0.2299294	4.93881231	2.54951643	0.12607888	0.35680902
Muc6	0.77736269	1.55308457	2.54928634	0.12609499	0.35680902
Ddr2	-0.3800939	6.27923198	2.54819219	0.12617162	0.35680902
Cxcl11	2.59981014	-2.3691744	2.54809835	0.1261782	0.35680902
Plekhf1	-0.514379	3.09606806	2.54808438	0.12617918	0.35680902
Zdhhc17	-0.2515404	6.78753137	2.54723089	0.126239	0.35683356
Ppip5k2	-0.2481729	5.30691516	2.54711036	0.12624745	0.35683356
Spryd7	0.21176029	5.47150062	2.54660848	0.12628265	0.35684882
Ssh2	-0.2201426	6.53010038	2.54601961	0.12632396	0.35687536
Piezo2	-0.491192	2.92854699	2.54529462	0.12637484	0.35687536
4930447N08	-1.4504367	-0.703037	2.54520066	0.12638144	0.35687536
Gcat	-0.8924436	0.83684208	2.54370212	0.12648669	0.35708838
Dclk1	0.26840589	10.5375499	2.54163833	0.12663183	0.35741385
Nktr	-0.2788957	7.07356696	2.5409625	0.1266794	0.35746387
Kcnn4	-2.4261133	-1.6284853	2.5395649	0.12677784	0.35753674
Tsc22d2	0.18498273	6.84533582	2.53946625	0.12678479	0.35753674
A830082N09	-0.3963739	4.43826558	2.53932437	0.12679479	0.35753674
Trim71	2.00549121	-0.8253599	2.53854006	0.12685008	0.35760845
Gpcpd1	-0.2230264	6.404355	2.53769108	0.12690996	0.35769307
Fam83h	-0.5625302	1.56831649	2.53679711	0.12697306	0.35777793
Lrif1	0.28142565	5.127569	2.53641802	0.12699982	0.35777793
Eif4e3	0.22974718	5.53897092	2.53514194	0.12708997	0.35794769
Uqcr11	0.43204745	4.69448431	2.53451924	0.12713399	0.35798747
Gm16907	0.95922744	1.60754631	2.53385587	0.1271809	0.35803539
Sephs1	-0.249832	5.67359731	2.53299902	0.12724153	0.35812188
Gm19705	0.6559385	1.55104948	2.53194516	0.12731614	0.35824768

Rrn3	0.19138854	5.81564163	2.53059011	0.12741215	0.35831691
Rbm43	0.28729066	4.53418318	2.53040117	0.12742555	0.35831691
Tcirg1	-0.5614088	1.52990941	2.53023519	0.12743732	0.35831691
Zcchc5	-1.4453588	-0.4091356	2.52990935	0.12746042	0.35831691
Wdr95	-2.3345301	-1.3915746	2.52938746	0.12749744	0.35832892
Ttc9	-0.2735745	4.23542392	2.52900557	0.12752454	0.35832892
Tlcd2	-0.539392	1.37738879	2.52835043	0.12757104	0.35836329
Cidea	2.22889779	-1.9543343	2.56689793	0.12763581	0.35836329
Msx2	-0.6815184	2.29540939	2.52717114	0.12765479	0.35836329
Tmem9b	0.21389518	5.44738389	2.5268837	0.12767522	0.35836329
Cdc42se2	-0.1822097	6.29738969	2.52576872	0.12775448	0.35836329
5031425E22I	-0.4059938	3.42439837	2.52569998	0.12775937	0.35836329
Nudt21	0.30818148	4.7723066	2.52554822	0.12777016	0.35836329
Krt222	-0.2229067	6.63809537	2.52473249	0.1278282	0.35836329
Tmub2	-0.3193021	3.40390632	2.52465016	0.12783406	0.35836329
Mex3d	-0.404275	3.9163	2.52462272	0.12783601	0.35836329
4931428F04I	0.67833758	1.13749693	2.52380995	0.12789387	0.3583892
Ndrg2	0.2641358	8.89316175	2.52365217	0.12790511	0.3583892
Asgr1	0.47605181	6.16241364	2.52088138	0.12810261	0.3587978
Pla2g16	0.33195177	6.73766589	2.52001452	0.12816448	0.3587978
Sirpb1a	-1.980624	-0.9216171	2.51984911	0.12817629	0.3587978
Igsf5	-3.1405655	-1.9997255	2.51920278	0.12822244	0.3587978
Gm5083	-1.8023464	-0.806053	2.51910999	0.12822907	0.3587978
2210015D19I	0.33822325	3.178976	2.51901271	0.12823602	0.3587978
Fut8	-0.3028546	6.99074698	2.51866788	0.12826066	0.3587978
Acaca	-0.3517907	5.67826567	2.51701625	0.12837873	0.35892421
4930414L22I	0.38564918	3.17796153	2.51698872	0.1283807	0.35892421
R3hcc1	-0.3274431	4.26773556	2.51677828	0.12839576	0.35892421
Obscn	0.86812108	0.39962601	2.51581078	0.12846499	0.35903395
Fam131b	-0.3085187	4.95506433	2.51530399	0.12850128	0.359043
Bbs9	0.221907	5.07388828	2.5149282	0.12852819	0.359043
Gm16023	-0.6641262	0.95694866	2.51342372	0.12863601	0.35917324
Gm9776	0.45071228	2.31677585	2.51317891	0.12865357	0.35917324
Ramp1	0.36839522	4.14651163	2.51302241	0.12866479	0.35917324
Pmp22	0.37169601	8.57001292	2.51104968	0.12880637	0.35930457
Fam167a	0.53235441	2.37068262	2.5100935	0.12887506	0.35930457
Veph1	-1.1012387	-0.1458469	2.51002978	0.12887964	0.35930457
Arsg	-0.3535704	3.08586952	2.50999238	0.12888233	0.35930457
A630023P12	1.85160321	-1.2681202	2.50995312	0.12888515	0.35930457
Grk4	-0.4448185	4.63754829	2.50985984	0.12889185	0.35930457
Ino80e	0.51158932	2.32870918	2.50923804	0.12893655	0.35930532
Pamr1	-0.3045495	4.03100599	2.50902143	0.12895213	0.35930532
Senp5	-0.1930606	5.58786185	2.50800258	0.12902542	0.359362

Ddit3	-0.3137221	3.40717023	2.50790437	0.12903249	0.359362
Plxna4	0.29457694	7.02214531	2.50575231	0.12918747	0.35965917
Slfn8	-0.508325	3.41249401	2.50442094	0.12928346	0.35965917
Kdm5a	-0.2246011	7.06546638	2.50384695	0.12932487	0.35965917
Slc1a1	0.2507789	6.01790734	2.50357035	0.12934484	0.35965917
Ugt8a	-0.2922074	5.3525216	2.50355031	0.12934628	0.35965917
Lrfn3	0.34441235	3.13777682	2.50330418	0.12936405	0.35965917
Atg9b	1.00428489	0.83379581	2.50310513	0.12937842	0.35965917
Gimap8	-0.611279	1.21229968	2.50309089	0.12937945	0.35965917
Fbxo11	0.17242536	8.26058227	2.50154887	0.12949083	0.35983233
Mtfmt	-0.4286512	3.0333309	2.50139668	0.12950183	0.35983233
Osr2	1.14498921	-0.06452	2.49902788	0.12967318	0.36020244
Gm10389	-0.386469	4.2490491	2.49804349	0.12974446	0.36020244
Rad9b	-0.9317834	0.48771744	2.49794635	0.1297515	0.36020244
Yap1	0.25977647	5.91019961	2.49778712	0.12976304	0.36020244
Scg5	0.23271457	6.32416838	2.49747818	0.12978542	0.36020244
Cap2	0.26935209	8.29989561	2.49416798	0.13002557	0.36070255
Aldh1a7	0.83312707	0.42021628	2.49399252	0.13003831	0.36070255
Gm16796	2.49257889	-2.0218992	2.49374933	0.13005598	0.36070255
Pde4d	-0.2601525	7.08529817	2.49162188	0.13021064	0.36104789
Has3	-0.7028216	1.97224757	2.49116058	0.1302442	0.36105321
Angptl2	-0.4833663	4.88653529	2.49076693	0.13027285	0.36105321
Rp2h	0.29828645	4.92032944	2.48898224	0.13040284	0.36132985
Mettl16	0.25057905	5.66215822	2.48753665	0.13050825	0.36149348
Usp31	-0.301317	7.46671707	2.48734447	0.13052227	0.36149348
Egfem1	-0.677575	1.82446598	2.48598712	0.13062134	0.36168423
Gm5176	-1.2564017	-0.4659472	2.48479307	0.13070857	0.36170491
Chrac1	-0.4237615	2.94502002	2.4842082	0.13075133	0.36170491
Trp53bp1	-0.3319804	5.47033742	2.48344006	0.1308075	0.36170491
Cd274	-0.4345111	3.76592191	2.48343349	0.13080798	0.36170491
Hectd3	-0.2987412	4.6912483	2.48309434	0.13083279	0.36170491
Rap2b	0.21842192	5.93718849	2.48285304	0.13085045	0.36170491
Atf2	0.16354116	8.8748874	2.48274374	0.13085845	0.36170491
Rnf40	-0.3637552	3.98065516	2.48257995	0.13087043	0.36170491
Bcl11a	0.22669258	6.56535469	2.48127351	0.1309661	0.36188578
Abtb2	-0.521865	2.54631764	2.48017292	0.13104675	0.36202511
Gpr107	0.32345179	3.80767005	2.47966839	0.13108374	0.36204379
Prpf4b	0.20381858	7.35039184	2.47885465	0.13114343	0.36212514
C030029H02I	-0.8325971	1.08253575	2.47797879	0.13120772	0.36218676
Fads1	0.23776202	6.22578949	2.47772654	0.13122624	0.36218676
Ptprd	-0.2726175	8.31428597	2.47725411	0.13126093	0.36219905
4930519F09I	-0.4266871	2.53184829	2.47432662	0.13147617	0.36263553
Gpr75	0.40801671	3.63762434	2.47427905	0.13147968	0.36263553

Ing2	-0.2360173	5.0945217	2.4737196	0.13152086	0.36266559
Lypd6	-0.2895887	4.9247082	2.47280469	0.13158824	0.36276786
04-Sep	-0.2159469	5.93010736	2.47181101	0.13166147	0.36287106
Ift81	-0.1882217	5.57400414	2.47147461	0.13168627	0.36287106
Rangap1	0.24477327	6.60553911	2.4708149	0.13173493	0.36291906
Cst3	0.36662714	7.90737531	2.47041674	0.1317643	0.36291906
Sall1	0.22149061	4.82359432	2.46976541	0.13181238	0.36296799
Creld1	0.39678607	3.59084989	2.46817647	0.13192974	0.36298776
Snapin	0.29476768	7.51446308	2.46812849	0.13193328	0.36298776
Xrn1	-0.2447716	6.04494522	2.46726766	0.13199692	0.36298776
Slc6a9	-0.3512266	4.07559452	2.46723693	0.13199919	0.36298776
Smurf2	-0.1933239	7.06923446	2.46646948	0.13205595	0.36298776
Gpr52	0.56870771	1.71756204	2.46630012	0.13206849	0.36298776
Mtmr3	-0.2163411	6.04740863	2.46603469	0.13208813	0.36298776
Setd6	-0.2350631	4.8372087	2.46576553	0.13210805	0.36298776
1700102P08I	-1.5968898	-1.0640683	2.46557188	0.13212238	0.36298776
Ppif	0.32080744	4.94539068	2.46556817	0.13212266	0.36298776
Ttll5	0.2372598	5.3838044	2.46509942	0.13215736	0.36299983
Sigirr	-2.2604213	-1.8461738	2.46385668	0.13224943	0.36316942
Hepacam2	-1.2045289	0.47403687	2.46199945	0.13238716	0.3634643
Impa1	0.20660808	5.28422292	2.46063852	0.1324882	0.36359341
Stat1	0.24941135	5.50301959	2.46054816	0.13249491	0.36359341
Trpa1	-1.5040226	-0.9427308	2.45979307	0.13255101	0.36366403
Galnt4	0.54948484	2.3252378	2.45936129	0.1325831	0.36366876
Mdm2	-0.1682429	6.69822729	2.45800777	0.13268377	0.36384449
Car8	0.46009165	2.79708353	2.45768303	0.13270793	0.36384449
Msmo1	0.32862057	4.99067555	2.45589294	0.13284123	0.3641266
Dcaf17	-0.2593792	4.46786314	2.45458936	0.13293841	0.36426729
Kl	-0.2537007	5.31075688	2.45438837	0.1329534	0.36426729
Clec14a	-0.6081485	1.55869742	2.45328574	0.13303567	0.36433772
Nfs1	-0.2733206	4.3159272	2.4532284	0.13303995	0.36433772
Ric3	0.22407441	6.1222974	2.45052042	0.13324227	0.36480836
Gan	-0.4316493	3.39614427	2.44977479	0.13329804	0.3648397
Apool	0.62375439	2.40628015	2.44894219	0.13336035	0.3648397
Upp1	2.01903314	-1.5056241	2.44841809	0.13339959	0.3648397
Naprt1	-0.8860471	0.45155741	2.44823966	0.13341296	0.3648397
U2af1	-0.3019401	4.10283801	2.4476026	0.13346068	0.3648397
Cep350	-0.2802586	6.68424156	2.44722005	0.13348934	0.3648397
Rpl5	-0.2672938	8.2506935	2.44704782	0.13350225	0.3648397
Gpd1l	0.17461725	7.02544809	2.44699138	0.13350648	0.3648397
Aimp1	0.33065655	4.50395021	2.44631937	0.13355687	0.3648397
Hnf1a	-1.3654017	-0.9538867	2.44629941	0.13355836	0.3648397
Map3k10	-0.3301857	3.26749762	2.44393967	0.13373547	0.36499967

Eef1d	-0.2420749	5.27726507	2.4434843	0.13376968	0.36499967
Rilpl2	-0.4179332	3.19208681	2.44311906	0.13379712	0.36499967
Invs	0.27337197	4.82609087	2.44297402	0.13380802	0.36499967
Hspa1l	0.45996424	2.37598543	2.4427973	0.13382131	0.36499967
Smndc1	0.27833091	5.33377647	2.44270838	0.13382799	0.36499967
Glt25d1	-0.2956984	3.99311375	2.44234405	0.13385538	0.36499967
Card14	-1.172105	0.21782031	2.44227261	0.13386075	0.36499967
Tex14	-1.5435477	-0.4598515	2.4414077	0.13392581	0.36508357
Zbed3	0.27135606	5.56461188	2.44055226	0.13399019	0.36508357
Msc	-1.7952355	-0.979222	2.44052214	0.13399246	0.36508357
Katnal1	0.2129858	6.85502897	2.44024316	0.13401346	0.36508357
Sik3	-0.2335705	7.39081185	2.43828469	0.13416103	0.36540246
Nipa2	0.27061477	4.76445489	2.43738771	0.13422868	0.36543151
Atp5sl	0.37293713	3.21692517	2.4369768	0.13425969	0.36543151
Tas1r1	-1.3397091	-0.3951764	2.43686987	0.13426776	0.36543151
Nr1i3	-1.6668429	-1.1087511	2.4365254	0.13429376	0.36543151
Trim59	-0.4181324	3.40018725	2.43553663	0.13436842	0.36548033
S100a9	-1.2271366	0.04202695	2.43522917	0.13439165	0.36548033
Adcy8	-0.4149084	3.85815397	2.43470663	0.13443113	0.36548033
Plp2	0.46262407	4.26104445	2.43467175	0.13443377	0.36548033
Ccdc63	2.36088698	-2.0957611	2.43417855	0.13447105	0.36549617
Gm11413	1.68633053	-1.251623	2.43378734	0.13450064	0.36549617
Adck3	0.37073054	2.71438383	2.43297268	0.13456226	0.36558068
Pacs2	-0.1884048	6.07020537	2.43255606	0.13459379	0.3655834
Mettl13	-0.4281951	2.10148209	2.42944978	0.13482915	0.36606993
Klf12	-0.1777244	7.35340552	2.42938521	0.13483405	0.36606993
Suclg2	0.2961078	5.58089658	2.42895537	0.13486666	0.36607547
Ccnt1	-0.2467089	6.30201614	2.42706706	0.13501002	0.36622816
Tspan7	0.22537096	8.1016807	2.42677155	0.13503247	0.36622816
AA987161	0.22461722	5.33446726	2.4263903	0.13506144	0.36622816
Cyp2u1	0.67409802	0.87259289	2.42607378	0.1350855	0.36622816
Tsen54	1.14461546	-0.256233	2.42595215	0.13509475	0.36622816
Pdcd11	-0.2430746	4.16139788	2.42553704	0.13512631	0.36622816
Pdrg1	0.33334811	5.4404089	2.42525329	0.13514789	0.36622816
Efemp2	0.54361165	2.75571536	2.42489674	0.13517502	0.36622816
Lap3	0.23646802	5.62660117	2.42459289	0.13519814	0.36622816
Ccpg1os	0.36030214	2.81273087	2.42277266	0.13533673	0.36651337
Cd59a	0.36558101	4.58348747	2.42169391	0.13541895	0.36651337
Cd79b	-1.9348272	-1.9913514	2.42165678	0.13542179	0.36651337
1700101E01I	1.63634665	-0.8806146	2.42075084	0.13549088	0.36651337
Tmbim4	0.33029018	4.59340577	2.42052023	0.13550848	0.36651337
Rev1	-0.2488673	4.38275609	2.42046617	0.1355126	0.36651337
Rgs8	0.27701351	6.68073307	2.42039992	0.13551766	0.36651337

Tmem126b	0.32944125	4.18323739	2.41921562	0.13560807	0.3666462
Atp6v0e2	0.24188192	5.29601481	2.41895463	0.13562801	0.3666462
Abcg2	-0.3136606	5.21468383	2.41789399	0.13570905	0.36665975
Ap1s3	-0.4066394	3.67176924	2.41788355	0.13570985	0.36665975
Pon2	0.40503183	5.932534	2.4176871	0.13572487	0.36665975
Smg9	0.43243447	2.90670721	2.41712024	0.13576821	0.36669413
Sec22a	-0.4021764	2.98563507	2.41607093	0.1358485	0.36679605
Nmd3	-0.2655383	5.08200007	2.41582648	0.13586721	0.36679605
Tshr	1.79616755	-0.4251946	2.41416879	0.13599417	0.36698696
Yes1	0.25949263	4.78123407	2.41410302	0.13599921	0.36698696
Atp2b2	-0.2765794	9.28140881	2.41333707	0.13605793	0.36706269
Gdpd2	-0.6042319	2.27646471	2.4128422	0.13609588	0.36708238
1110020A21I	0.72089461	1.0658075	2.412293	0.13613801	0.36711334
Lect1	-1.15245	-0.5496984	2.41083493	0.13624994	0.36715038
Plxna2	-0.1938569	7.727418	2.41074348	0.13625697	0.36715038
A430005L14I	0.42588515	2.80963571	2.41006609	0.13630901	0.36715038
Micu3	-0.2348888	7.58963231	2.40978618	0.13633052	0.36715038
Ahctf1	-0.2520769	6.60380263	2.408787	0.13640735	0.36715038
Zfp566	-0.559785	1.84360935	2.40855238	0.13642539	0.36715038
Zfp410	-0.290446	4.00281026	2.40829761	0.13644499	0.36715038
Rfx8	-2.2772437	-1.9345285	2.40826343	0.13644762	0.36715038
Crmp1	-0.2482645	6.06433348	2.40784341	0.13647994	0.36715038
Rnd3	-0.3071766	5.26196549	2.40751998	0.13650484	0.36715038
Emc3	0.29338048	5.9107534	2.40745114	0.13651014	0.36715038
Gp49a	1.2774594	0.04712162	2.40705964	0.13654028	0.36715038
Akr1c13	0.65900163	1.750315	2.40661411	0.13657459	0.36715038
Gm20748	-0.6262818	1.58141484	2.40653151	0.13658096	0.36715038
Mrpl34	0.44093598	3.32687172	2.40567956	0.1366466	0.36724441
Prr14	0.27543539	5.45232595	2.40434273	0.13674968	0.36743899
Mboat7	0.23172178	5.19985747	2.4034536	0.13681829	0.36754088
Selm	0.41414214	4.47033158	2.40226799	0.13690985	0.3675553
9330182L06I	-0.2858701	5.05696034	2.40211708	0.13692151	0.3675553
Mina	-0.4112199	2.58326857	2.40189515	0.13693866	0.3675553
Aff4	0.1712231	8.98795918	2.40153394	0.13696657	0.3675553
Rasgrp1	0.27502559	9.64199137	2.40139745	0.13697712	0.3675553
Gramd1b	-0.2388121	5.84688195	2.39885504	0.13717381	0.36800064
Mccc1os	0.78110415	0.88030954	2.39748362	0.13728005	0.36814309
Lgals3bp	0.38701731	4.89866269	2.39737598	0.1372884	0.36814309
Zfp473	1.5339812	-0.3005322	2.39661284	0.13734756	0.3682193
Rnf169	-0.172204	6.75839916	2.39589627	0.13740314	0.36828587
2310001H17I	1.71060438	-0.9587405	2.39504774	0.137469	0.36835037
2610008E11I	-0.2695926	5.29799621	2.39455199	0.13750749	0.36835037
Fam173a	0.38771616	3.09933044	2.3940194	0.13754886	0.36835037

Fbxl7	0.38733652	6.20230065	2.39363179	0.13757897	0.36835037
Amotl1	-0.2368681	6.88193783	2.39342276	0.13759522	0.36835037
Preb	-0.2971397	5.17850217	2.3925507	0.13766301	0.36835037
Cmas	0.16134027	6.76787663	2.39254251	0.13766365	0.36835037
Bsn	-0.3848347	9.30856583	2.39205879	0.13770127	0.36835037
Il34	-0.2943866	3.78935797	2.39130353	0.13776004	0.36835037
Gm13293	-0.7006771	0.84650605	2.39069625	0.13780731	0.36835037
Hirip3	0.22039133	5.06218827	2.38965423	0.13788847	0.36835037
Hsbp1l1	0.5654715	1.59126542	2.38955966	0.13789584	0.36835037
Wdr81	-0.4188965	3.02157766	2.3891603	0.13792696	0.36835037
Socs5	-0.2097235	6.54267267	2.38885287	0.13795093	0.36835037
Igsf3	-0.2522126	4.5003432	2.38884949	0.13795119	0.36835037
Pcdh19	0.27095935	6.24158074	2.38809954	0.13800967	0.36835037
Pofut1	0.32818472	3.36432094	2.38757954	0.13805023	0.36835037
Ccdc11	-1.0282469	-0.1982129	2.38747343	0.13805851	0.36835037
Tab3	0.19601214	6.0446983	2.38736084	0.1380673	0.36835037
Spock3	0.22373585	5.22420083	2.38715675	0.13808323	0.36835037
Dhcr24	0.3097555	5.09585216	2.38707358	0.13808972	0.36835037
C330018D20I	-0.3011044	3.75577288	2.38689204	0.13810389	0.36835037
Lyg2	-3.3973462	-1.660059	2.42158117	0.13819082	0.36850015
Scrt1	-0.3211678	5.3381451	2.38508543	0.138245	0.36856258
Mab21l3	2.24236261	-1.79967	2.38378782	0.13834647	0.36868969
Map4k4	-0.2102403	6.42734492	2.38368827	0.13835425	0.36868969
Prrx2	0.69210843	3.43939736	2.38314463	0.13839679	0.368721
Ube2l3	0.21258025	7.51291036	2.38262082	0.13843779	0.36873422
Olfr113	4.55370449	-2.0314591	2.4169351	0.13854539	0.36873422
Sppl2a	0.23824006	6.38309585	2.38101388	0.13856367	0.36873422
Wnt10a	0.49841764	1.65062776	2.38097273	0.13856689	0.36873422
Med13	-0.1759276	9.08293275	2.3808667	0.1385752	0.36873422
Helb	-0.3496181	3.42155083	2.38072263	0.13858649	0.36873422
Fgf1	0.2377332	7.40894278	2.3802091	0.13862676	0.36875941
Rbm5	-0.23047	6.94168735	2.37956636	0.13867717	0.36881159
Fam222b	0.18280316	6.75289279	2.37903833	0.1387186	0.36881777
Slbp	0.21608122	5.38558167	2.37875186	0.13874108	0.36881777
Lrrn4cl	-0.2374669	4.95697795	2.37797223	0.1388023	0.3688986
Pias4	0.49939698	1.41421414	2.37744581	0.13884365	0.36892663
Adamts13	-0.392005	4.12095028	2.37564398	0.13898529	0.36918983
Zrsr1	-0.1498348	6.95800016	2.37540161	0.13900436	0.36918983
Sharpin	0.31369606	3.8065174	2.37469895	0.13905965	0.3692548
Pafah1b2	0.16239768	8.35178325	2.37393745	0.13911961	0.3693321
Sf3b1	-0.191874	8.28905656	2.37325919	0.13917303	0.36939205
Tbxa2r	2.10009324	-2.105866	2.37279786	0.13920939	0.36940667
Rpp21	0.42935008	3.15334798	2.37174847	0.13929212	0.36943051

Hsf5	1.62256199	0.24239207	2.37171651	0.13929464	0.36943051
Pex7	-0.2636047	4.47396907	2.37151019	0.13931092	0.36943051
Mfap5	-0.8723704	2.15899869	2.3689311	0.13951454	0.36981508
Asb8	0.18698608	6.2702894	2.36880528	0.13952448	0.36981508
Aars	0.24132589	5.61114746	2.36850038	0.13954858	0.36981508
Mars	0.21383731	4.89170001	2.36656553	0.13970161	0.37011512
Trpm6	-0.3882412	3.12694538	2.3662877	0.13972361	0.37011512
Hsd3b2	-1.7017571	-1.284295	2.36563435	0.13977534	0.37017027
Slc30a10	-0.2944216	5.06742658	2.36515526	0.13981329	0.37018891
Mrpl51	0.22721383	5.05127658	2.36286893	0.13999456	0.37058694
Tmem41a	0.33012117	3.74697865	2.36106017	0.14013817	0.37088205
Purb	-0.1764437	9.71124071	2.36061263	0.14017373	0.37088205
Six5	-0.5321846	3.32592734	2.36022678	0.14020439	0.37088205
Bloc1s1	0.45479895	5.14775056	2.35976649	0.14024099	0.37088205
Bin3	0.40403587	2.93786398	2.35951623	0.14026089	0.37088205
Loxl2	-0.3675322	3.97633276	2.35866238	0.14032881	0.37097975
Gnptg	-0.2610038	4.63941865	2.35785075	0.14039342	0.37100111
Ermard	-0.3165711	3.92559189	2.35752802	0.14041912	0.37100111
Sfswap	-0.354622	4.61788931	2.35739343	0.14042983	0.37100111
Ktn1	-0.1888644	7.13143191	2.3558576	0.14055222	0.37124253
Med10	0.28905743	3.50898414	2.35501477	0.14061943	0.37130686
Lats1	0.18667192	7.15099515	2.35419638	0.14068474	0.37130686
Tgif1	0.43447529	2.7617842	2.35379772	0.14071656	0.37130686
Tmed5	0.27513331	5.54968377	2.35379587	0.14071671	0.37130686
Scaf1	-0.2638648	4.68206565	2.35360943	0.14073159	0.37130686
Txk	1.64069891	-1.7280901	2.35181186	0.14087522	0.37152985
Lbp	0.68169243	3.35016585	2.35150694	0.1408996	0.37152985
Grid1	-0.4075278	2.92400738	2.3511719	0.14092639	0.37152985
Gm5415	-0.7827457	2.10985562	2.35099917	0.14094021	0.37152985
Zfp169	-0.2888787	4.07761419	2.35029253	0.14099675	0.371588
Prc1	0.74423648	1.22386664	2.34994798	0.14102432	0.371588
Kcnk13	0.75512804	0.83313891	2.34923331	0.14108154	0.3716163
Cers3	-1.7091317	-1.6244005	2.34865693	0.14112772	0.3716163
Zbtb18	-0.205259	7.35838544	2.34865144	0.14112816	0.3716163
E130012A19I	-0.3946267	2.9411	2.34759174	0.14121309	0.3717582
Tdgf1	-1.6678453	-0.1595847	2.34704122	0.14125724	0.37179269
Man1c1	0.23576108	6.0539353	2.34447984	0.14146286	0.37221114
Col6a4	1.47575635	-0.2166068	2.34397851	0.14150315	0.37221114
Rrnad1	0.31137876	3.60625809	2.34389999	0.14150946	0.37221114
Gm11944	-0.9094237	0.61037529	2.34236278	0.14163308	0.3724545
Wipi2	-0.2820426	5.00363007	2.3417437	0.14168291	0.37250373
Il6st	0.25062815	7.38933824	2.33768423	0.14201014	0.37322379
Bcl7a	0.27997282	4.93507583	2.33757308	0.14201912	0.37322379

Epcam	-2.2298824	-0.4798403	2.33623122	0.14212749	0.37338649
Zscan25	-1.5446682	-0.360117	2.33603459	0.14214338	0.37338649
1700001K23I	2.12362346	-1.6193535	2.33474551	0.1422476	0.37357831
Kdm7a	-0.1892341	7.30540922	2.33417919	0.14229342	0.3736167
Sox11	0.31977009	4.87642157	2.33270456	0.1424128	0.37384819
A230103J11I	0.55518045	1.99749863	2.33208676	0.14246285	0.37385118
Psmb4	0.2745692	5.59698976	2.33159096	0.14250303	0.37385118
Eid2b	0.24170961	4.42914406	2.33153478	0.14250759	0.37385118
Tex30	-0.6473178	2.38532697	2.33002293	0.14263021	0.37404765
Unc79	-0.3965529	5.96216454	2.32984127	0.14264495	0.37404765
4933405O20	-2.1476612	-0.526677	2.32825605	0.14277367	0.37428438
Gm14164	1.64762349	-0.9193449	2.32707998	0.14286926	0.37428438
Pin4	-0.2525269	4.63656401	2.32695873	0.14287912	0.37428438
Cep250	-0.3021002	4.7744097	2.32673	0.14289772	0.37428438
Id1	-0.5038507	3.82219196	2.32659283	0.14290887	0.37428438
Htra3	-0.5171076	4.06121175	2.32642225	0.14292275	0.37428438
Rps19bp1	0.35736145	2.88831036	2.32589325	0.14296579	0.37431524
Ddx39b	0.19949037	5.43056837	2.32494029	0.14304336	0.3744258
Pkp2	0.49996303	2.23426187	2.32460651	0.14307055	0.3744258
Tktl2	-2.3354341	-1.1117292	2.32371361	0.14314329	0.37445696
Tsc22d3	-0.350063	6.56716066	2.32357234	0.14315481	0.37445696
Col28a1	1.46685927	-0.372539	2.32305056	0.14319734	0.37445696
Tsen15	-0.29574	3.62941797	2.32233831	0.14325543	0.37445696
Caly	0.34344936	3.47990388	2.32217845	0.14326847	0.37445696
Nyx	1.44958676	-0.5550092	2.32215894	0.14327006	0.37445696
Pop7	0.3755757	2.62452614	2.32112078	0.14335479	0.37459666
AI429214	0.48171292	3.37119213	2.32071609	0.14338784	0.37460127
Ribc1	-1.0130925	-0.2145785	2.31901379	0.14352694	0.3748348
Adss	-0.1607982	7.09678092	2.3185684	0.14356336	0.3748348
9530068E07I	0.32911974	6.71850553	2.31847347	0.14357112	0.3748348
AI317395	-1.6120966	-0.8851693	2.31699203	0.14369235	0.37506954
Cdk7	-0.2077935	6.25268145	2.31623252	0.14375455	0.37515013
Zfp866	-0.208366	5.11100105	2.31514652	0.14384355	0.3752123
Fbxo33	-0.2648581	4.8653221	2.31457145	0.1438907	0.3752123
Nup160	-0.3178377	4.50080403	2.31449388	0.14389706	0.3752123
Mavs	0.42344107	4.855431	2.31441294	0.1439037	0.3752123
Nebl	0.21201334	6.61374962	2.31255981	0.14405579	0.37552709
Fitm2	-0.9267581	0.590713	2.31212432	0.14409156	0.37553859
C78339	-0.4099805	2.77947753	2.31120418	0.14416717	0.37562749
Mir103-2	-1.1895924	0.14579881	2.31094593	0.1441884	0.37562749
Sh3bgr	-1.4175355	-0.4642722	2.30931359	0.14432268	0.37576799
Il18r1	1.46858431	-0.6174719	2.30921579	0.14433073	0.37576799
Drc1	-0.3501377	3.18557095	2.30889844	0.14435686	0.37576799

Mettl9	-0.2434079	4.83069938	2.30866728	0.14437589	0.37576799
Serpina10	2.89656864	-1.9734045	2.30838397	0.14439922	0.37576799
Mrpl35	0.19734341	5.08137465	2.30751618	0.14447071	0.37585395
Fmnl3	0.43230759	3.58003564	2.30722121	0.14449502	0.37585395
Drd3	-1.3253229	-1.0424144	2.30578851	0.14461317	0.37607959
Bdh2	-0.524919	3.09510841	2.30474454	0.14469934	0.37609136
Mcph1	0.32037292	5.21063922	2.30463075	0.14470873	0.37609136
Hsd3b4	-0.8639598	0.76477743	2.30384886	0.14477332	0.37609136
Ndufa13	0.38978678	5.844441	2.3036174	0.14479244	0.37609136
Tmem59	0.25810269	6.13848722	2.30327785	0.1448205	0.37609136
Cog4	0.25909952	4.60369643	2.3032121	0.14482593	0.37609136
Ube2k	0.16898297	8.11082422	2.30284264	0.14485647	0.37609136
Tspan1	-2.1989956	-1.3200596	2.30252776	0.14488251	0.37609136
Vapb	0.16716287	6.66791428	2.30205964	0.14492123	0.37609136
Gabrr2	-1.8234453	-0.870399	2.30193249	0.14493174	0.37609136
Asgr2	2.33901525	-2.1466861	2.30150175	0.14496738	0.37610235
Athl1	-0.387463	3.28005213	2.3010095	0.14500812	0.37610964
Asxl2	-0.2107593	6.49427482	2.29979	0.14510912	0.37610964
Med16	-0.2277917	5.29758617	2.29945995	0.14513646	0.37610964
Slc25a32	0.30974508	3.88956618	2.29912178	0.14516449	0.37610964
Prune	0.23159863	4.58506646	2.29883632	0.14518815	0.37610964
Slc25a23	-0.2078672	8.69692611	2.29871555	0.14519816	0.37610964
Mdh1	0.16880893	8.81871868	2.2986946	0.1451999	0.37610964
Sh3tc2	1.93341264	-1.284331	2.29834067	0.14522925	0.37610964
Jazf1	0.26098993	5.2823902	2.29775598	0.14527775	0.37610964
Cdh15	2.01042431	-1.396928	2.29767756	0.14528425	0.37610964
Allc	-2.406148	-1.1534135	2.29582775	0.14543783	0.37634893
Tmbim6	0.28104748	6.63596553	2.29580716	0.14543954	0.37634893
Krt80	-0.463339	3.54297797	2.29521334	0.14548888	0.37636727
Paip2	0.19842038	7.53393563	2.29362122	0.14562127	0.37636727
Gem	1.06291338	2.8642039	2.29239124	0.14572365	0.37636727
Purg	-0.2463717	6.01491215	2.29175145	0.14577694	0.37636727
Adra2b	-1.0185774	0.18659244	2.29171055	0.14578035	0.37636727
Gpank1	0.55043413	2.00662254	2.29096383	0.14584257	0.37636727
4933439C10I	-0.5914053	1.82049563	2.29065365	0.14586843	0.37636727
Gemin4	-0.4086747	2.31786914	2.29033534	0.14589497	0.37636727
Tmem53	0.77051213	1.62310313	2.29008363	0.14591596	0.37636727
Mak	-0.6773347	2.07930266	2.28994688	0.14592737	0.37636727
Palm	-0.2222445	4.89436797	2.28993798	0.14592811	0.37636727
Chrm2	0.55159673	1.96358718	2.28992632	0.14592908	0.37636727
Dok7	-1.3225804	-0.7094101	2.28972042	0.14594626	0.37636727
Tmem71	1.38310311	0.03806179	2.28965073	0.14595207	0.37636727
Hgs	0.31585764	4.12396268	2.28959273	0.14595691	0.37636727

Hk2	-0.4209364	2.80291057	2.28933801	0.14597817	0.37636727
1500017E21I	-1.8133483	-1.8060662	2.2893053	0.1459809	0.37636727
Slc39a13	0.36406983	5.17208541	2.28818144	0.14607472	0.3765281
Ints7	0.36675151	3.58778534	2.28540294	0.14630698	0.37704564
Pdyn	0.38590236	3.32308681	2.28468555	0.14636702	0.37711922
Cep128	-0.3687451	4.25132638	2.28413076	0.14641348	0.37715776
Ppp1r35	0.56633961	1.49108591	2.28302424	0.14650618	0.3773154
Klf9	0.20579516	8.08354523	2.28243159	0.14655586	0.37734617
Syt2	0.39950561	3.39364271	2.28204065	0.14658864	0.37734617
Ten1	-0.359519	3.27363178	2.28053243	0.1467152	0.37734617
Rexo2	0.1996407	6.99962684	2.28017955	0.14674483	0.37734617
Dock3	-0.3750464	7.93754843	2.28016893	0.14674572	0.37734617
Tbc1d10a	0.67021607	1.66524401	2.28004863	0.14675582	0.37734617
Lgr4	0.21524043	5.69464346	2.27991495	0.14676705	0.37734617
Zfp12	-0.2744131	4.74653812	2.27986506	0.14677124	0.37734617
Zfp459	-0.4879397	3.00448716	2.27950235	0.14680171	0.37734617
Diap1	-0.2279568	4.50604362	2.27853064	0.14688338	0.37747507
Cmtm3	0.36849419	3.14524451	2.2773018	0.14698673	0.37765964
Kctd7	0.48716606	2.79768907	2.2766475	0.1470418	0.37771748
Crx	1.28259627	-1.2279969	2.27628491	0.14707232	0.37771748
Rdh1	-0.565471	2.07293096	2.27532803	0.14715292	0.37783412
Glp1r	0.91911272	0.0642864	2.27465403	0.14720972	0.37783412
Ubqln1	0.16306198	7.10321703	2.27462241	0.14721239	0.37783412
Ccdc146	-0.6835842	1.56847198	2.27358263	0.14730007	0.37797816
Mok	0.4485871	2.36552259	2.27261235	0.14738195	0.37810725
E330020D12I	-0.4705895	3.84757115	2.2718928	0.14744271	0.3781821
2610507B11I	-0.167453	8.00325485	2.2704501	0.14756462	0.37831262
Ap5b1	-1.1466074	-0.0106222	2.27035601	0.14757257	0.37831262
Dnajc4	0.478562	2.40710364	2.27016924	0.14758836	0.37831262
Yars	0.2412125	5.01004981	2.26971728	0.14762659	0.37832962
Mrgbp	-0.7721991	0.49676204	2.26892434	0.14769368	0.37842057
Rala	0.16481753	6.48951825	2.2673336	0.14782838	0.37868468
Marcksl1	0.28146112	4.29698089	2.26589896	0.14794999	0.37889659
Tspan5	0.18549993	7.34070433	2.26561137	0.14797438	0.37889659
Gm13446	-0.4472338	2.27103668	2.2648963	0.14803505	0.37897092
Tmem221	1.22047618	-0.4644933	2.26401634	0.14810975	0.37901053
Ndfip2	-0.2162479	6.27191141	2.26396844	0.14811382	0.37901053
Ube2t	1.51069214	-1.0713827	2.26353606	0.14815055	0.37902352
Morn3	-1.9033323	-1.2840511	2.26204939	0.1482769	0.3791091
Pde6c	-2.0070855	-0.9940223	2.26142307	0.14833017	0.3791091
1700001J11F	-1.4707454	-1.5119809	2.26109443	0.14835813	0.3791091
A330021E22	-0.3041656	4.08752451	2.26094553	0.1483708	0.3791091
Ctxn3	0.34978442	7.22561565	2.26077212	0.14838556	0.3791091

9030204H09	1.48284586	-1.183812	2.26035394	0.14842116	0.3791091
Hnrnph2	0.16796377	7.21234144	2.25992347	0.14845781	0.3791091
Lipt2	0.50218646	2.11350335	2.25975559	0.14847211	0.3791091
Dnajb13	-1.7114802	-1.4063093	2.25963438	0.14848243	0.3791091
Gm12359	-1.0577898	0.15565247	2.25942155	0.14850056	0.3791091
Herc4	-0.2972938	4.61268462	2.25768114	0.14864892	0.37936141
Smpd5	1.90004873	-2.011353	2.25742329	0.14867091	0.37936141
Mylpf	1.23288179	-0.1585606	2.25714766	0.14869443	0.37936141
Ppap2a	0.33241932	3.7937364	2.25641083	0.14875731	0.37944101
Ccdc116	0.87710743	0.07424863	2.25575169	0.14881359	0.37947335
Gphn	0.2433665	5.91839685	2.25552022	0.14883337	0.37947335
Lrba	-0.2530826	5.84648866	2.2548637	0.14888946	0.37953557
A730017C20	-0.1968217	5.8893004	2.25422153	0.14894435	0.37959469
Cdhr2	-1.5561677	-0.9457568	2.2522743	0.14911095	0.37993341
Mcm5	-0.6491678	0.90631205	2.25192673	0.14914071	0.37993341
Tmem184c	0.21010174	6.04331967	2.2514004	0.14918579	0.37996743
Tbc1d10b	0.21480054	4.60840359	2.25079864	0.14923735	0.37997055
Nfkb2	0.99298423	0.95481219	2.25015485	0.14929253	0.37997055
Pcdhb21	-0.724278	1.24682171	2.25008407	0.1492986	0.37997055
Abcf1	0.18892713	6.76225071	2.24963136	0.14933742	0.37997055
Surf1	-0.3131228	4.01917473	2.24953539	0.14934566	0.37997055
Pcm1	-0.2713274	8.57560764	2.24791783	0.14948448	0.38008056
Pigr	-0.5418669	3.83540318	2.2478588	0.14948955	0.38008056
Gm10069	-0.6780222	0.87244405	2.24727757	0.14953947	0.38008056
Thoc5	0.32598355	3.55371757	2.24723131	0.14954345	0.38008056
Zdhhc9	0.22824158	6.30578159	2.24676109	0.14958385	0.38008056
Xrra1	1.53480054	-1.5196995	2.24653911	0.14960293	0.38008056
Zbtb7b	0.46521812	2.97171509	2.2464446	0.14961106	0.38008056
Zfp593	0.70769885	0.84499048	2.24565202	0.14967921	0.38012919
Tmem179b	0.46448162	3.23647778	2.24504329	0.14973157	0.38012919
Utp6	0.20646315	6.67201553	2.24473346	0.14975823	0.38012919
4831440E17I	-0.6567485	1.79053662	2.24451421	0.1497771	0.38012919
Tspo	0.53064888	2.02514548	2.24430985	0.1497947	0.38012919
C1rl	1.38797563	0.26255936	2.24394245	0.14982633	0.38012919
Lrrd1	-1.2447775	-0.3771125	2.24353396	0.14986151	0.38012919
Gxylt2	-0.3724706	3.90150015	2.24312903	0.14989639	0.38012919
Pip5kl1	-1.2785242	0.28413704	2.24201365	0.14999253	0.38012919
Ctr9	-0.226174	5.2111628	2.24188179	0.1500039	0.38012919
Hpgds	-0.533099	2.40122513	2.24137937	0.15004723	0.38012919
Bfsp2	1.28522755	-0.7331281	2.2413462	0.15005009	0.38012919
Stard3nl	0.25698567	4.46531517	2.24115621	0.15006648	0.38012919
Rfesd	-0.279476	4.17403698	2.24106233	0.15007458	0.38012919
Srfbp1	-0.2971378	3.92073567	2.24017949	0.15015078	0.3801591

Cntrl	-0.321588	4.31281987	2.24014072	0.15015413	0.3801591
9530059O14	-0.8776379	2.91368661	2.23982229	0.15018162	0.3801591
Atp5k	0.26051434	5.92428404	2.23924861	0.15023117	0.38020417
Snap47	0.17165143	7.28616584	2.23752441	0.15038022	0.38046223
Pde4c	-1.0525696	-0.1185333	2.23733408	0.15039668	0.38046223
Fxn	-0.4544101	2.26426653	2.23569787	0.15053831	0.38072869
Smarcc2	0.16777979	8.25320197	2.23538279	0.1505656	0.38072869
Stab1	-0.7733871	1.19212054	2.23074754	0.15096777	0.38155861
Mkl2	0.28371905	9.41262042	2.23043322	0.15099509	0.38155861
Fam71b	-1.7312564	-1.7554449	2.22998172	0.15103434	0.38155861
2810055G20	-0.4441216	2.25090386	2.22994221	0.15103777	0.38155861
Fcgr3	-0.728963	1.06475756	2.22942378	0.15108286	0.38155861
Ephb2	-0.5933409	2.12447497	2.22931621	0.15109222	0.38155861
Sycp3	-0.5400963	2.03633713	2.22903328	0.15111683	0.38155861
Barhl2	-1.304219	-0.0727471	2.22826986	0.15118327	0.38164591
Slc6a18	-0.6599173	1.13879394	2.22790315	0.1512152	0.38164606
Mpp7	0.25715078	4.60590607	2.2266191	0.15132706	0.38183775
Dhx35	-0.3466037	2.62896772	2.22629946	0.15135492	0.38183775
Eme2	-0.5168498	1.98866727	2.22504839	0.15146403	0.381939
Taf8	0.34438731	2.78515592	2.22452084	0.15151007	0.381939
Zswim3	0.59868945	2.11516617	2.22450287	0.15151164	0.381939
Rgl1	0.15592692	7.58694642	2.22401705	0.15155405	0.381939
Trim36	-0.3625207	3.36901537	2.22401164	0.15155452	0.381939
Iigp1	-0.381034	4.08486279	2.22358158	0.15159207	0.38195326
Pgd	0.29108182	5.24899362	2.22280096	0.15166027	0.38204471
Huwe1	-0.3156918	9.48061089	2.22189149	0.15173977	0.38216459
1700110C19I	-1.3171431	-0.1240952	2.22085891	0.15183009	0.38223961
Hccs	0.27664167	4.98550885	2.22082124	0.15183339	0.38223961
St3gal2	0.19136091	5.92266657	2.21963932	0.15193686	0.3824197
Cct2	0.15505832	7.0039922	2.21895581	0.15199674	0.38249001
A830018L16I	0.22750912	6.95865729	2.21683769	0.15218246	0.38287694
Fasn	-0.2825934	6.43402626	2.21558055	0.15229283	0.38307412
Snx15	0.22919539	4.50132427	2.21515367	0.15233033	0.38308798
Scamp4	0.33894897	3.4873528	2.21400101	0.15243163	0.38326227
Lgi3	-0.343271	3.63126066	2.21255359	0.15255896	0.38335743
Ensa	0.19341937	8.89617653	2.21225855	0.15258493	0.38335743
Gab1	-0.1936531	7.04320334	2.21214331	0.15259508	0.38335743
Insl5	-1.6726512	-1.3287275	2.24341545	0.15261873	0.38335743
Icam1	0.72596113	1.87701251	2.21175192	0.15262954	0.38335743
Pcdha8	-1.3324913	0.18925913	2.21029074	0.15275828	0.38360034
Srd5a1	-0.5315848	2.56381811	2.20971686	0.15280888	0.38364695
Tmem234	0.26703085	4.73707194	2.20801809	0.15295878	0.38394281
Smtn	0.52884207	2.3287142	2.20737626	0.15301546	0.38394535

4931403G20	-0.7963443	0.89301541	2.20649444	0.15309338	0.38394535
Commd6	0.30338485	5.30398125	2.20647946	0.1530947	0.38394535
Rpgrip1	-1.2829335	-0.9646415	2.20611047	0.15312732	0.38394535
Ubxn2b	0.21795211	6.02215154	2.20532424	0.15319685	0.38394535
Zfp354a	-0.2891506	3.36857815	2.20519362	0.15320841	0.38394535
Uevld	-0.2318867	5.20567585	2.20502251	0.15322354	0.38394535
Calr3	-0.9016803	-0.0376434	2.20459167	0.15326167	0.38394535
Trrap	-0.3254866	6.51894758	2.2045692	0.15326366	0.38394535
Xlr	0.81770987	0.44675069	2.20438012	0.15328039	0.38394535
1110057K04I	0.2083298	5.54252814	2.20280711	0.15341971	0.38405977
Frmd8	0.27912976	3.95943943	2.20270891	0.15342841	0.38405977
1700094D03I	-0.6566223	1.92607072	2.20244995	0.15345137	0.38405977
Cryba2	1.5292866	-1.2182359	2.20241623	0.15345435	0.38405977
Ccdc173	0.39452106	3.05128335	2.20177724	0.15351101	0.38412128
Nt5c3b	-0.3005502	3.23958664	2.19975173	0.15369075	0.38444151
Dnajc10	0.18357975	6.34267739	2.19961174	0.15370319	0.38444151
Gja6	-1.2553429	-1.1012232	2.19876613	0.15377831	0.3845491
Klrd1	-1.0013018	-0.5107385	2.19732314	0.15390661	0.38463735
Mtcl1	-0.3248766	5.72007567	2.19726691	0.15391161	0.38463735
Guf1	-0.444691	3.90420422	2.19708798	0.15392753	0.38463735
Dennd6a	-0.1991505	6.86793009	2.19692448	0.15394208	0.38463735
Ogdhl	-0.5524619	1.86515548	2.19628993	0.15399855	0.38464388
Dpy19l4	-0.2591809	4.18310181	2.19617339	0.15400893	0.38464388
Gm4461	-1.1308357	0.55151926	2.19323323	0.15427095	0.38510901
Nemf	-0.2322205	7.13975157	2.1926564	0.15432242	0.38510901
Letm2	-0.3720348	3.25028569	2.19164725	0.15441252	0.38510901
Gm19434	-0.6365023	0.9469362	2.19145549	0.15442965	0.38510901
Uri1	0.18654352	6.632009	2.19141628	0.15443315	0.38510901
3110057O12	-0.328738	3.00491508	2.19111614	0.15445996	0.38510901
Gm6938	1.51730681	-0.7268445	2.19074199	0.1544934	0.38510901
Gm5088	0.47198696	1.55621154	2.19029336	0.1545335	0.38510901
Gm6981	0.5333504	1.51002647	2.18994377	0.15456475	0.38510901
Alms1-ps2	-0.748031	0.45228043	2.18984682	0.15457342	0.38510901
Car11	0.22619555	5.14702599	2.189712	0.15458548	0.38510901
Fbxo45	-0.1846257	6.25555646	2.18856091	0.15468846	0.38510901
Capza2	0.17224524	8.06231364	2.18839521	0.15470329	0.38510901
Trnau1ap	-0.2662767	3.70772269	2.1882376	0.1547174	0.38510901
Atp11b	-0.2254413	6.31297214	2.18746059	0.15478698	0.38510901
Vtn	-0.3950195	6.16509271	2.18712891	0.1548167	0.38510901
Ccdc74a	0.42851607	2.01806722	2.18708023	0.15482106	0.38510901
Cdc37	0.24945898	5.08594465	2.18675134	0.15485053	0.38510901
Sufu	0.26040337	4.10405418	2.1866062	0.15486354	0.38510901
Gmnn	0.44321774	2.06183801	2.18598519	0.15491921	0.38510901

Epha5	0.2560944	6.13213742	2.18556374	0.15495701	0.38510901
Epha4	-0.2056865	7.2171443	2.18516525	0.15499275	0.38510901
Prickle1	-0.2651564	5.67967014	2.18498944	0.15500853	0.38510901
Iqgap2	-0.2628927	4.97802915	2.18480107	0.15502543	0.38510901
Pomgnt1	-0.3640327	2.95261862	2.18463869	0.15504	0.38510901
Cchcr1	-0.7600054	0.99538075	2.18418806	0.15508045	0.38510901
Rtfdc1	0.26860004	5.58179619	2.18314323	0.15517429	0.38510901
Lmo1	1.1931088	-0.3943841	2.18301987	0.15518538	0.38510901
Fancf	0.4773871	3.01588235	2.1827589	0.15520883	0.38510901
Coro1c	0.23795312	5.89259875	2.18194194	0.15528227	0.38510901
Crabp1	2.31559955	-1.4468871	2.18191959	0.15528428	0.38510901
Apc	-0.2712066	9.68081644	2.18173369	0.155301	0.38510901
Ica1	-0.3108593	4.14762539	2.18153915	0.15531849	0.38510901
AF357355	-2.0515863	-1.001034	2.18103986	0.15536341	0.38510901
4930404I05R	-1.8916346	-1.5477678	2.18085715	0.15537985	0.38510901
Was	-0.6708579	0.99596923	2.1806601	0.15539758	0.38510901
Chmp1b	0.26711881	5.41564145	2.18016043	0.15544256	0.38510901
Psat1	0.19988316	6.48892202	2.18011686	0.15544649	0.38510901
Cpsf7	-0.2483317	5.71114193	2.17917177	0.15553161	0.38510901
Spn	-1.0006301	-0.2228099	2.17909239	0.15553876	0.38510901
Ubr2	-0.2860972	6.2756488	2.17904098	0.15554339	0.38510901
Txndc11	0.28975702	3.87622766	2.17871868	0.15557244	0.38510901
Ppap2b	0.31973762	6.43322826	2.17783885	0.15565176	0.38510901
A830080D01	-0.2661977	4.31144439	2.17750966	0.15568145	0.38510901
Triobp	0.33007535	6.03592652	2.17746987	0.15568503	0.38510901
Akap1	-0.3563318	3.33678222	2.17727015	0.15570305	0.38510901
Tigd2	-0.2559961	4.43055657	2.17638484	0.15578295	0.38510901
Zfp462	-0.2513314	6.71344293	2.17558547	0.15585513	0.38510901
Stt3a	0.22810993	5.16260031	2.17557506	0.15585607	0.38510901
Slc22a23	-0.2016436	6.19523421	2.17541861	0.1558702	0.38510901
Nat9	0.45920097	1.99609858	2.17539282	0.15587253	0.38510901
Fam189b	0.26916497	4.60001513	2.1752766	0.15588303	0.38510901
Mir378b	-1.1789154	0.21148148	2.17509418	0.15589951	0.38510901
Etv1	0.2168178	7.73736449	2.17468112	0.15593684	0.38512178
Hfm1	-0.6759873	1.86536128	2.17372538	0.15602325	0.38519716
Hsph1	0.22434996	9.09662742	2.17341022	0.15605176	0.38519716
Myot	-1.74843	-2.0569196	2.17273213	0.15611311	0.38519716
Zfp947	0.43747129	2.25516998	2.17265696	0.15611991	0.38519716
Ak3	0.2254876	8.15985105	2.17256563	0.15612818	0.38519716
Eef1b2	-0.2549457	7.031599	2.17216932	0.15616406	0.38520631
Ifitm6	1.02682108	0.49823614	2.17169637	0.15620689	0.38523261
Vsig10l	-0.5221574	2.41814072	2.17126898	0.1562456	0.38524875
Gm15401	-0.7064646	0.45559154	2.16908102	0.15644398	0.38565849

Flnc	0.51242681	2.89884862	2.16816033	0.15652755	0.38571027
Fah	-0.3220307	3.18799279	2.16813992	0.1565294	0.38571027
Zfp420	-0.2331777	4.40160952	2.16632662	0.15669415	0.38594419
Tshz2	-0.1806365	6.13773992	2.16595251	0.15672817	0.38594419
2410004B18	0.21788626	5.13860267	2.16583678	0.15673869	0.38594419
Klhdc9	-0.2852284	3.65695043	2.16567682	0.15675324	0.38594419
Pdcd6ip	0.16210996	7.45387281	2.16433095	0.15687572	0.38616634
Gm12191	-0.5041415	1.2385392	2.16361733	0.1569407	0.38624692
Cbx2	-0.7210952	1.04972225	2.16114508	0.15716609	0.38672215
Slc35a4	0.24518738	5.95206393	2.15937617	0.1573276	0.38679143
Mas1	0.43130286	2.57691821	2.15924591	0.1573395	0.38679143
Phf20l1	-0.2456427	5.71701924	2.1587683	0.15738315	0.38679143
Dctn5	0.22502017	6.19143987	2.15874299	0.15738546	0.38679143
Ydjc	0.53245617	1.79528566	2.15856797	0.15740146	0.38679143
Cep83	0.20687605	5.98075362	2.15842087	0.1574149	0.38679143
Cmtr1	-0.3055918	5.59587889	2.15836152	0.15742033	0.38679143
Plxdc2	0.17933438	6.74706993	2.15708606	0.15753699	0.38696201
Cd180	-0.5016497	2.7357235	2.15626457	0.15761219	0.38696201
D430041D05	0.26367845	7.66574254	2.15612126	0.15762531	0.38696201
Oas1g	-1.1784841	-0.3560232	2.15559616	0.1576734	0.38696201
BB557941	2.02090668	-0.6396822	2.15558069	0.15767482	0.38696201
Tm9sf1	0.32340657	3.98937282	2.15548452	0.15768363	0.38696201
Cflar	-0.176726	6.86384183	2.15420539	0.15780087	0.38717038
Fryl	-0.2442043	7.5460087	2.15375642	0.15784204	0.38719208
Adat2	-0.6819989	1.78734387	2.15290669	0.15792001	0.38724626
Ticrr	1.37092677	-0.3232316	2.15281084	0.1579288	0.38724626
Napsa	-1.647795	-0.6509732	2.15059078	0.15813273	0.38761673
Ewsr1	-0.2862595	7.69583582	2.15046141	0.15814462	0.38761673
Elac1	-0.2220855	5.18459079	2.14971314	0.15821344	0.38770604
Akip1	0.43943907	2.97505457	2.14910703	0.1582692	0.38776335
Rps15a	-0.1860112	7.20599227	2.14745818	0.15842103	0.38803771
5330434G04	-0.3351497	5.00213605	2.1471873	0.15844599	0.38803771
Ctsk	0.55398604	2.38531761	2.14607455	0.15854857	0.38814463
Mfap1b	0.16950538	6.89563488	2.14601056	0.15855447	0.38814463
Slc30a3	0.28868874	4.31403031	2.14519885	0.15862935	0.38815942
Wbscr27	-0.2220399	4.58273635	2.14519756	0.15862947	0.38815942
Gtf2e2	0.22870352	4.51126659	2.14489118	0.15865775	0.38815942
Tm9sf4	0.24978896	4.70297856	2.14367769	0.1587698	0.38830295
Aard	0.57345969	1.58846714	2.14355361	0.15878126	0.38830295
Ppp1r12c	-0.1937042	5.32770825	2.14273357	0.15885705	0.38833562
Cenpe	-0.4535977	2.89436466	2.14188529	0.15893549	0.38833562
Spdl1	-1.1068897	-0.1312964	2.14187824	0.15893614	0.38833562
Med25	0.34219266	2.48573837	2.1416202	0.15896001	0.38833562

Rab20	-2.0113832	-2.0306466	2.14115782	0.15900279	0.38833562
Dis3l	-0.2571598	3.87216742	2.14109503	0.1590086	0.38833562
Slc6a20b	-0.4476704	1.71811984	2.14064617	0.15905015	0.38833562
Tex264	0.30397938	3.26062366	2.14060418	0.15905404	0.38833562
Gm12250	-0.8073831	0.08217832	2.1394399	0.15916187	0.38842481
Gm15698	2.01551369	-1.4406106	2.13925262	0.15917923	0.38842481
Tifab	-0.7457733	1.68083859	2.13890762	0.1592112	0.38842481
Rgs19	0.28830564	4.53633584	2.13846041	0.15925266	0.38842481
Snx30	-0.2344495	5.50898575	2.13811203	0.15928497	0.38842481
D930016D06	-0.3961813	4.25689725	2.13780557	0.15931339	0.38842481
Naa10	0.46185193	2.32544461	2.13776013	0.15931761	0.38842481
2610002J02F	0.47092767	3.24413391	2.13677886	0.15940867	0.38856772
Myo3b	0.81414998	0.40957379	2.13592477	0.15948798	0.38865184
Mrrf	0.26035208	3.972877	2.13570822	0.1595081	0.38865184
Ndufaf3	0.27030104	4.14631992	2.13535901	0.15954054	0.38865184
Trmt1l	0.20531381	4.88441546	2.13487782	0.15958526	0.38868172
Wbscr16	-0.5459673	1.98241993	2.13418948	0.15964927	0.38871876
A330102I10F	-0.7366459	1.29368866	2.13401616	0.15966539	0.38871876
Glra2	0.3143441	3.31837041	2.13354053	0.15970963	0.38874745
Reep4	1.0167834	0.28493167	2.13181009	0.15987074	0.3888869
Orc3	0.16685283	7.85503594	2.13180801	0.15987093	0.3888869
Lif	1.28299708	-0.9504071	2.13154752	0.1598952	0.3888869
Rnf31	-0.3679901	3.23321975	2.13103002	0.15994343	0.3888869
Slmo2	0.18559389	6.85782777	2.13083683	0.15996144	0.3888869
Nup153	-0.1997908	6.16442377	2.13048877	0.15999389	0.3888869
Fbxo21	0.23573583	5.66249624	2.13048511	0.15999423	0.3888869
Ipw	-0.6185113	4.05705094	2.12951444	0.16008478	0.38898242
Setx	-0.2909582	7.13268707	2.12936742	0.16009849	0.38898242
Efhb	-0.6551366	0.67924051	2.12899166	0.16013357	0.38898871
Dusp5	-0.8886717	-0.2021188	2.12726767	0.16029459	0.3892692
Zfp423	-0.214479	6.14028828	2.12705952	0.16031404	0.3892692
Frmpd1	-0.3177511	2.76514959	2.12548029	0.16046174	0.38954886
Psmb11	-0.6537161	1.33147065	2.12472292	0.16053264	0.38961838
Zfp317	-0.2750896	4.05380451	2.12447929	0.16055545	0.38961838
Gm7694	-0.4710562	1.83915516	2.12204814	0.16078331	0.38994408
Sipa1	-0.3861784	2.6000308	2.12200135	0.1607877	0.38994408
Fam115c	0.69225054	1.3926034	2.12189156	0.160798	0.38994408
Mdn1	-0.5054192	6.51506517	2.12108479	0.16087371	0.38994408
Kctd10	0.27977501	4.71801975	2.12089303	0.16089171	0.38994408
Grina	0.20865875	7.00624105	2.12087272	0.16089362	0.38994408
Pilrb2	-1.9744986	-0.5024789	2.12061735	0.16091759	0.38994408
Zfp101	-0.3461486	2.54349396	2.11933603	0.16103797	0.39012317
Bbox1	-0.8078701	0.58517396	2.11913727	0.16105665	0.39012317

Nr1h3	0.52590951	2.61585111	2.11815573	0.16114895	0.39023711
Lamp2	0.28701447	7.76757349	2.11761264	0.16120004	0.39023711
D7Ertd715e	-0.5071461	4.14583375	2.11682422	0.16127426	0.39023711
9430008C03I	-0.5934541	1.71279956	2.11671269	0.16128476	0.39023711
Pign	0.24952339	4.09877498	2.11659469	0.16129587	0.39023711
Lgals12	-1.055777	-0.2240564	2.11655321	0.16129978	0.39023711
C230004F18I	-0.4482393	3.88591467	2.11621333	0.16133179	0.39023711
Galnt12	0.71724355	0.82133625	2.11544377	0.1614043	0.39033366
Sat2	0.40981654	2.12702867	2.11418366	0.16152311	0.39051235
Wdr76	-0.4217144	2.1984456	2.11391442	0.16154851	0.39051235
Spg11	-0.2514219	5.11874808	2.11362298	0.16157601	0.39051235
Gtpbp10	-0.2794785	4.49151741	2.11272002	0.16166125	0.39063953
Tex261	0.38554666	1.98762786	2.11216409	0.16171376	0.39068758
Mtmr2	-0.2108811	6.65554123	2.11163876	0.1617634	0.39069458
Asna1	0.25299465	6.49108005	2.11144289	0.16178191	0.39069458
AW549542	-0.910553	-0.2372378	2.11034521	0.1618857	0.39086641
Cpxm1	-0.4275105	3.30145207	2.10910336	0.16200322	0.39099797
Dppa2	2.43583715	-1.8299176	2.1090794	0.16200548	0.39099797
Sgpp2	-0.2754641	3.73622858	2.10710327	0.16219271	0.39137096
Arl5b	0.31387936	4.18578629	2.10640084	0.16225932	0.39138165
Sntn	-1.0103053	-0.1784704	2.10636729	0.1622625	0.39138165
Trim32	0.20761252	7.59726018	2.10545204	0.16234935	0.39151228
Tgfb1i1	0.31502637	6.85124362	2.10240473	0.16263892	0.39213162
Ppm1d	0.24021744	5.13616871	2.10193722	0.1626834	0.39215991
Zfp677	0.46987385	3.72824083	2.10143065	0.16273161	0.39219718
Dgcr14	0.29571334	3.75036642	2.09714206	0.16314048	0.39289526
Maml2	0.2486867	5.76477574	2.09693185	0.16316055	0.39289526
AU041133	-0.4059634	2.83217733	2.09664247	0.16318819	0.39289526
C2cd2l	0.25823053	5.27762797	2.09637877	0.16321338	0.39289526
Isca2	0.21151112	5.90232956	2.09602079	0.16324758	0.39289526
Polr3b	-0.2788887	4.43934886	2.09566425	0.16328166	0.39289526
Ddah2	0.50396657	3.18176182	2.09552057	0.16329539	0.39289526
Wdr17	-0.3452219	5.11350837	2.09539049	0.16330783	0.39289526
Nckipsd	0.29825715	4.12030892	2.09498201	0.16334689	0.39289526
Atoh8	-0.4695198	1.84341406	2.09495641	0.16334934	0.39289526
1700020I14R	-0.2246007	6.32617778	2.09357853	0.16348117	0.39298347
Bag6	0.23059315	5.6876848	2.09357383	0.16348162	0.39298347
Dnmt3b	0.94589492	0.32546079	2.09354318	0.16348456	0.39298347
Gpr1	-2.1749902	-1.4864563	2.0932015	0.16351727	0.39298347
Amigo1	0.20128551	5.74003178	2.09218509	0.16361463	0.3930198
Qars	0.2723045	3.78645225	2.09106536	0.16372197	0.3930198
Parp8	-0.289865	4.80146209	2.0910373	0.16372466	0.3930198
Tmem170b	-0.2012006	7.50671763	2.09078505	0.16374885	0.3930198

Usp36	-0.2723579	4.61989909	2.09053464	0.16377287	0.3930198
Alg11	0.21406131	6.70173638	2.09022926	0.16380217	0.3930198
Spag5	-0.7167041	1.90163562	2.09021144	0.16380388	0.3930198
Shroom1	0.53882266	1.58595977	2.09018216	0.16380669	0.3930198
B930018H19	1.98608779	-0.9073337	2.08996277	0.16382775	0.3930198
Pwwp2a	-0.2726134	5.79995736	2.08889438	0.16393032	0.39318177
A230056P14	-0.2815919	4.00572703	2.0883931	0.16397848	0.39318177
Acvr1b	0.25133057	6.06409219	2.08823408	0.16399376	0.39318177
Atpif1	0.22369949	7.27261637	2.08678643	0.16413294	0.39330921
Xrcc3	0.36495516	3.43280101	2.08617945	0.16419134	0.39330921
Fam92a	0.193053	6.562983	2.0860166	0.16420702	0.39330921
Tor2a	0.45366824	1.89822862	2.08552395	0.16425444	0.39330921
Slc6a1	0.25157395	7.70563936	2.08522525	0.1642832	0.39330921
Ntn5	0.79520908	0.36897687	2.08518352	0.16428722	0.39330921
Tlr3	0.27117058	4.87896512	2.08510743	0.16429455	0.39330921
H60b	1.0335494	0.46473753	2.0849506	0.16430965	0.39330921
Arhgap39	0.23982021	4.98563479	2.08388003	0.16441281	0.39345931
Kdm6a	-0.2447189	6.12837456	2.08361802	0.16443807	0.39345931
Ppp1cb	0.16559762	9.23777984	2.08289578	0.16450772	0.39354734
Gm10432	0.70508335	1.42658681	2.08151485	0.16464099	0.39373711
Zfp472	-0.4986552	1.75869423	2.08139252	0.1646528	0.39373711
Fermt2	-0.1476155	7.13789339	2.08022877	0.16476523	0.39392729
Ankrd28	-0.1686375	6.58204557	2.07934908	0.16485027	0.39405195
D6Ertd474e	1.22192384	0.2199904	2.07853669	0.16492886	0.39416113
Acvrl1	-0.7012925	0.83253302	2.07742622	0.16503635	0.39430062
Haus8	-0.6473226	1.04829652	2.07725353	0.16505308	0.39430062
Tnc	0.55220362	1.7869043	2.07688662	0.16508862	0.39430687
Gata6	-2.1304535	-1.377922	2.07637981	0.16513772	0.39433805
D730045A05	-1.4793779	-0.3651192	2.07607232	0.16516753	0.39433805
4933406F09I	-2.5038322	-1.1412617	2.07407146	0.16536162	0.39472274
Gys1	0.54608874	1.99857252	2.07114143	0.16564634	0.39504666
Pdcd2l	0.32278841	3.02662668	2.07088473	0.16567131	0.39504666
Khdrbs1	0.16974391	6.94592177	2.07031968	0.1657263	0.39504666
Pfas	-0.3309179	3.81241102	2.06964988	0.16579151	0.39504666
Abhd11os	0.75465072	0.35444913	2.06950598	0.16580552	0.39504666
Strbp	-0.2341277	9.34693261	2.06911757	0.16584336	0.39504666
Diras2	-0.1745537	8.13275265	2.06895368	0.16585932	0.39504666
Med29	0.25877748	3.3883453	2.06882394	0.16587197	0.39504666
C4b	-0.4000542	2.77120039	2.06863524	0.16589035	0.39504666
Gm13807	1.75830523	-2.139352	2.06819538	0.16593323	0.39504666
Syt3	0.35379528	3.66352254	2.06810615	0.16594192	0.39504666
Cdh10	0.28377557	5.14094305	2.06737754	0.16601297	0.39504666
Slc16a4	0.60617412	1.73228008	2.06718991	0.16603128	0.39504666

Ncln	-0.4629916	2.44640941	2.06716716	0.1660335	0.39504666
Lgi4	-0.6116077	1.45022589	2.06691564	0.16605804	0.39504666
Gabrg2	0.21233438	6.44501646	2.06661551	0.16608732	0.39504666
Mcrs1	-0.3355954	3.23936336	2.06638791	0.16610954	0.39504666
Pyroxd2	0.55646191	1.1896043	2.06628843	0.16611925	0.39504666
Fbxo2	0.32977794	3.07285821	2.06582545	0.16616445	0.39504666
Plp1	-0.3036683	8.66461957	2.06573388	0.16617339	0.39504666
Gm6682	0.19274943	5.04837045	2.06512169	0.16623319	0.39504666
Mme	-0.277436	4.26111758	2.06461332	0.16628286	0.39504666
Micu2	0.2146348	5.20628011	2.06456682	0.16628741	0.39504666
Nob1	-0.3468222	3.0220439	2.06455043	0.16628901	0.39504666
Tex40	-0.6573862	2.10217503	2.06370148	0.16637201	0.39516545
Cmip	0.20551311	9.08758755	2.06278703	0.16646147	0.39524683
Pdia4	0.23487722	4.46957625	2.06267658	0.16647228	0.39524683
Sytl4	-0.5898656	2.35993316	2.06054159	0.16668139	0.39566486
Slc7a6os	0.33831975	3.9736553	2.06006093	0.16672851	0.39569829
Plxnb1	-0.3033665	4.42722131	2.05938729	0.16679458	0.39577666
Abca13	1.99857337	-1.7270547	2.05888543	0.16684382	0.39581507
Fbxl21	-0.2966259	3.14489174	2.05741226	0.16698847	0.39607977
Zswim4	0.38925496	2.37334394	2.0568551	0.16704322	0.39612611
Fzd1	0.31020144	6.13564224	2.05654026	0.16707416	0.39612611
Cox18	0.36917466	2.90177609	2.05529425	0.1671967	0.39622857
Spen	-0.2441005	6.87515701	2.05454577	0.16727037	0.39622857
2810442N19	-1.0636175	0.21262058	2.05445472	0.16727933	0.39622857
Tpst1	-0.2382717	5.01559497	2.05444887	0.16727991	0.39622857
Glyctk	-0.6858783	1.38324795	2.05441938	0.16728281	0.39622857
Cyth2	0.27921874	4.26547245	2.05372069	0.16735161	0.39629717
Fgd1	0.34349502	3.05365511	2.05312456	0.16741035	0.39629717
Rnf144a	0.25016411	4.77117646	2.05311749	0.16741104	0.39629717
Med19	-0.2574192	4.61208039	2.05260365	0.16746169	0.39633871
Gstm1	0.30864636	7.64972735	2.05192575	0.16752853	0.39634696
Zadh2	-0.242795	4.80412858	2.05189703	0.16753136	0.39634696
Gnai3	0.23156075	6.40500411	2.05122506	0.16759766	0.39639425
Snx19	-0.2406139	4.75984491	2.05102344	0.16761756	0.39639425
Lsm5	0.54868594	1.66590589	2.050334	0.16768561	0.39647691
Spred3	-0.2877387	5.07279754	2.04795999	0.16792023	0.39695327
Kti12	0.49108977	2.3447234	2.04747819	0.16796789	0.39697238
Pde12	0.33766814	3.23531426	2.04720818	0.16799461	0.39697238
Actr8	0.23712224	4.68296503	2.04635534	0.16807904	0.39709353
Ep400	-0.2589034	6.99752642	2.04599613	0.16811461	0.39709924
Slc25a21	-0.9503491	0.19478389	2.04484448	0.16822873	0.39714149
Plekhf2	-0.3065222	4.63531593	2.04468778	0.16824427	0.39714149
AI450353	-0.6619106	2.02107079	2.04459843	0.16825313	0.39714149

Cabp1	-0.3831671	2.88542464	2.04447715	0.16826515	0.39714149
Katnbl1	0.28837245	4.70717542	2.04338288	0.1683737	0.39731939
Trp73	-1.1268324	-0.2048053	2.04270879	0.16844061	0.39739898
Zfp37	0.23884795	6.80022442	2.04140505	0.16857012	0.39756158
Gmps	0.19632437	6.75866356	2.04134658	0.16857593	0.39756158
Cep44	0.35358154	2.75179763	2.03992251	0.16871753	0.39781719
Vps37d	0.62747027	0.98645161	2.03913302	0.1687961	0.39792409
Vmn1r58	-0.4630929	4.2997275	2.03630965	0.16907743	0.39850886
Ppp1r26	-0.2571758	4.18231138	2.03578771	0.1691295	0.39855315
Mccc2	-0.3062684	3.4833902	2.0352131	0.16918685	0.39856061
Mapk1	0.17355087	9.09375929	2.03508908	0.16919923	0.39856061
Ksr1	0.28308246	3.86342717	2.03444615	0.16926343	0.39863343
Gal	1.36273579	-0.4440306	2.0333898	0.16936898	0.39880358
Rps23	-0.2638035	7.24528783	2.03219928	0.16948803	0.39890066
Tspyl5	0.18265594	5.78190886	2.03216022	0.16949193	0.39890066
Man1a2	0.17346219	7.88075799	2.03172712	0.16953527	0.39890066
1700001L19F	-0.3846281	3.38356868	2.03164547	0.16954344	0.39890066
Bcl9l	0.24569159	5.44706175	2.03082142	0.16962594	0.39901637
Cdk5rap1	0.69596358	1.39925974	2.02799853	0.16990892	0.39954149
1110059G10	0.30489538	4.20899495	2.02792896	0.1699159	0.39954149
Osgepl1	0.27291559	4.77018591	2.02718149	0.16999093	0.39963945
Ap1ar	-0.1612152	6.76523956	2.02658729	0.1700506	0.39966634
Naaa	-0.3455654	3.92316073	2.02640302	0.17006911	0.39966634
Coq4	-0.258651	3.84372595	2.02529917	0.17018005	0.39978804
Scn10a	-1.8267015	-1.6089936	2.05247178	0.17018767	0.39978804
Gm4980	-0.6673514	0.8216005	2.02454615	0.17025578	0.39986961
Ap5s1	0.46543209	2.35698614	2.02284471	0.17042705	0.40001989
Rnasek	0.30430312	7.09958463	2.02276986	0.17043459	0.40001989
Cnksr2	-0.3178027	9.57153435	2.02249147	0.17046263	0.40001989
Pwwp2b	-0.5012227	2.38647075	2.0222645	0.1704855	0.40001989
Zfp668	0.30909502	3.70242168	2.02225179	0.17048678	0.40001989
Cops2	0.19215378	8.04261981	2.02087978	0.1706251	0.40019309
Slc12a8	-0.7974219	0.36052363	2.01945068	0.17076932	0.40019309
Ntn4	-0.5325761	1.3451484	2.01894557	0.17082033	0.40019309
Greb1l	-0.3906915	3.07983397	2.0186033	0.1708549	0.40019309
Erp44	0.27017654	4.53609662	2.01845751	0.17086963	0.40019309
Tspan14	0.25648293	3.50768409	2.01840344	0.1708751	0.40019309
Cys1	-0.3131541	5.15008941	2.01838051	0.17087741	0.40019309
Zfp46	-0.1828344	5.51714231	2.01835328	0.17088017	0.40019309
Loh12cr1	-0.3622312	2.95649361	2.01832606	0.17088292	0.40019309
Ndufs8	0.35583857	4.82298406	2.01820879	0.17089477	0.40019309
Cebpz	0.16371827	6.19603558	2.01760666	0.17095563	0.40020101
Slu7	-0.199225	5.97942903	2.01751416	0.17096498	0.40020101

Tert	1.1463462	-0.679246	2.01714089	0.17100273	0.40020928
Pcdhb22	0.38460898	3.03876864	2.0163385	0.17108391	0.40020928
1110037F02I	-0.2187145	5.5306821	2.01616412	0.17110155	0.40020928
Cln3	0.41368536	2.55672215	2.0161578	0.17110219	0.40020928
C630043F03I	-0.3048192	3.2595682	2.01528615	0.17119044	0.4002989
Gar1	-0.2292757	4.14274439	2.0151191	0.17120736	0.4002989
Tmem178b	-0.2239346	7.54212313	2.01347843	0.17137364	0.4005906
Ncaph	1.14598698	-0.3833775	2.01322813	0.17139902	0.4005906
Mylk4	-0.6504102	1.16968029	2.01231726	0.17149144	0.4007276
Tbccd1	0.24505601	4.58301558	2.01187349	0.17153648	0.4007276
Wbp4	0.16533719	6.16916851	2.01166137	0.17155802	0.4007276
Ap3m2	-0.1868871	6.61854869	2.01097038	0.1716282	0.40081335
Tmem168	-0.3353789	3.25320225	2.00974944	0.17175229	0.40100924
Ttll12	0.31064923	4.13670622	2.00948631	0.17177905	0.40100924
1700086O06	1.0100364	-0.332981	2.00873263	0.17185572	0.40111003
Pafah1b1	0.16518241	9.15130596	2.00833421	0.17189627	0.40112649
Nup85	-0.2860532	3.70621767	2.00798968	0.17193134	0.40113018
A230056J06F	-1.3211713	-0.1013602	2.00644179	0.17208902	0.40139234
Fhl2	0.20082025	5.79220638	2.005917	0.17214252	0.40139234
Amigo3	-1.5270532	-0.5059474	2.00589996	0.17214426	0.40139234
Fbxo34	-0.2389706	5.26287799	2.00500657	0.17223539	0.40149917
0610030E20I	0.20742514	5.46102833	2.00479348	0.17225713	0.40149917
Kcnk1	0.22667819	5.55611367	2.00414084	0.17232375	0.40154257
Cdk5	0.25916805	4.20561656	2.0039309	0.17234518	0.40154257
H2afx	0.29519913	3.05993906	2.00362582	0.17237634	0.40154257
Gtse1	1.36784134	-1.4834709	2.00256611	0.17248461	0.40166322
Sumo2	0.26141862	8.73781098	2.00235423	0.17250627	0.40166322
Got2	0.16942518	7.01775185	2.00203935	0.17253846	0.40166322
Plekhh2	-0.2190084	5.65818336	2.00180637	0.17256229	0.40166322
Gnl3	0.25806619	4.71939905	2.00132424	0.17261161	0.40169993
Dlx6os1	-0.3051481	3.48672029	2.0007908	0.17266619	0.40174889
2010107E04I	0.23261734	5.84260277	2.00041356	0.1727048	0.40176068
Vipr1	0.53053488	2.42237839	1.99940358	0.17280824	0.4018957
Itgb1	0.28089797	6.34118709	1.99919151	0.17282997	0.4018957
A630001G21	-0.8844188	0.62575873	1.99880948	0.17286912	0.4019087
2210408I21R	-0.3438048	4.27033096	1.99766506	0.17298646	0.40201657
Epb4.1l1	0.20149746	8.48495277	1.99747706	0.17300575	0.40201657
Adamts18	-0.8679074	0.25730253	1.99737493	0.17301622	0.40201657
Cercam	-0.4537843	1.93764476	1.99695909	0.1730589	0.40203772
Nutf2	0.33365675	3.14388551	1.996583	0.1730975	0.40204941
4732491K20I	-0.4974535	2.09539837	1.99521505	0.173238	0.40229773
Syt10	0.4252126	2.31250601	1.99451283	0.17331018	0.40231383
Rps4l	-0.3618056	1.95132353	1.99449394	0.17331212	0.40231383

Ppp2r2a	-0.1630331	7.10196112	1.99369852	0.17339393	0.40242572
Tnik	-0.279705	7.36927851	1.99289213	0.17347691	0.40251987
C230035I16R	-1.2182241	0.05992777	1.99264313	0.17350254	0.40251987
Ppcs	-0.3913444	2.41680731	1.9923247	0.17353533	0.40251987
Slc5a5	-0.2711983	4.92902346	1.99092934	0.17367909	0.40277533
Mettl20	-0.4047891	2.58159646	1.99015527	0.17375891	0.40280007
Selplg	-0.7215345	0.91735611	1.98990999	0.17378421	0.40280007
Tsc1	-0.2731357	5.94436256	1.98984741	0.17379067	0.40280007
Rgs11	-0.7795217	1.394385	1.9893786	0.17383904	0.40283422
Trappc1	0.23763807	5.99795277	1.98851125	0.17392858	0.40289553
Gm12709	-0.4855281	1.93978936	1.98816945	0.17396388	0.40289553
Gpr180	-0.319184	3.9525995	1.98814477	0.17396643	0.40289553
Tmem175	0.21788314	4.99426847	1.9877187	0.17401044	0.40291955
Pgm1	0.25777817	3.70359012	1.98675767	0.17410978	0.40297663
Eif3a	-0.2111785	9.48235141	1.98644294	0.17414232	0.40297663
Ola1	0.15865199	7.60669843	1.98626757	0.17416046	0.40297663
Entpd4	-0.2094511	6.04676775	1.98591068	0.17419738	0.40297663
Dopey2	-0.2826307	5.69919646	1.98534939	0.17425546	0.40297663
Abhd14b	-0.2931199	5.31205164	1.98518104	0.17427289	0.40297663
Ubxn6	0.18271792	5.56864892	1.98512379	0.17427881	0.40297663
Dap3	0.20623531	4.8301408	1.98467033	0.17432576	0.40297663
Yipf5	0.17804319	5.41711625	1.98436668	0.17435721	0.40297663
Cpne8	-0.1952793	6.41143721	1.98386131	0.17440957	0.40297663
4930581F22I	-0.807067	0.41831874	1.98377222	0.1744188	0.40297663
Bcas3	-0.2489717	4.43762814	1.98357835	0.17443889	0.40297663
Smc5	-0.2963858	5.74348268	1.98240006	0.17456105	0.40318107
Actl6b	-0.3870358	3.2826385	1.98104907	0.17470126	0.40342709
Erich6	-0.8620565	1.27272317	1.97952103	0.17486	0.40371581
Tonsl	0.84908915	0.46502871	1.97907963	0.17490589	0.40374134
Fmr1	-0.1657201	6.28502288	1.97876619	0.17493848	0.40374134
E130008D07I	-0.5644304	2.27454662	1.97790722	0.17502784	0.40382944
Fam188b	-0.5626516	1.34550415	1.97775106	0.17504409	0.40382944
Caskin1	0.25792314	5.76529907	1.97728116	0.17509301	0.40386449
Rarres1	1.77029585	-0.6069795	1.97584986	0.1752421	0.40396218
Vgll4	-0.4191362	2.97093093	1.97555501	0.17527284	0.40396218
Tmem181c-p	-0.4878915	4.26990538	1.9754917	0.17527944	0.40396218
Stx8	0.2565548	5.33836295	1.97509438	0.17532086	0.40396218
Pmfbp1	1.41139349	-1.3142859	1.97476529	0.17535519	0.40396218
Zdhhc6	-0.2741818	4.61483211	1.97462605	0.17536971	0.40396218
Zmat4	0.16912119	5.6454348	1.97432125	0.17540151	0.40396218
Klhl18	-0.2609075	4.45705032	1.97428572	0.17540522	0.40396218
Gcc1	0.23891975	4.93927588	1.97379745	0.17545617	0.40400184
Neurl2	1.11756735	-0.3121361	1.97340526	0.17549712	0.40401843

Lrrc48	0.44788675	2.39259325	1.97156615	0.17568927	0.40411865
Cfl2	0.24388156	7.7484807	1.97153001	0.17569304	0.40411865
Vma21	0.18989658	6.21773459	1.97116927	0.17573077	0.40411865
Mapkapk5	-0.2495365	4.18382528	1.9711271	0.17573518	0.40411865
Limch1	-0.3676541	5.83790073	1.97109349	0.17573869	0.40411865
Ypel4	0.49578179	2.45977121	1.97104847	0.1757434	0.40411865
Rdx	-0.1700503	7.03495233	1.97072857	0.17577686	0.40411865
Tyrobp	-0.5577424	1.37625052	1.969062	0.17595131	0.40434023
Prdx4	0.28438236	3.41192748	1.96896213	0.17596178	0.40434023
Idh1	0.22401892	5.41998055	1.96884035	0.17597453	0.40434023
Atp6v0a2	0.24168673	5.13639764	1.96796027	0.17606676	0.40436652
Rhox8	-0.589026	1.56502683	1.9679062	0.17607243	0.40436652
Rian	-0.3366399	6.84491912	1.96769484	0.17609458	0.40436652
Acr	1.11332481	-0.0750512	1.9674426	0.17612103	0.40436652
Sigmar1	0.27636206	3.01872859	1.96625566	0.17624556	0.40457485
Dctd	0.61482695	1.64541764	1.96577273	0.17629625	0.40461367
Lgi2	-0.4660317	2.70263887	1.96516914	0.17635964	0.40462121
Carns1	0.58742004	2.38018108	1.96492528	0.17638526	0.40462121
Tas1r3	-0.927332	-0.1782649	1.96427278	0.17645383	0.40462121
Zfp637	0.32763249	3.80381429	1.96421675	0.17645971	0.40462121
Utp15	-0.2480037	5.04699468	1.96400014	0.17648249	0.40462121
1700113A16I	0.29509898	3.91329545	1.96381206	0.17650226	0.40462121
Spsb4	-0.6949126	1.11843022	1.96297969	0.17658981	0.40474443
Tm7sf2	0.77163657	1.14682049	1.96232799	0.17665839	0.40482414
Tmeff2	0.18969331	6.92265816	1.96190027	0.17670342	0.40484985
Gng2	0.14085946	7.99385618	1.96094905	0.17680361	0.40488814
Pgr	-0.2425092	5.93249371	1.96070752	0.17682906	0.40488814
Htra2	0.60112973	1.29477846	1.96050521	0.17685038	0.40488814
St18	-0.38196	3.77041997	1.96034255	0.17686752	0.40488814
1700017G19	-0.8206315	0.70194423	1.96013724	0.17688917	0.40488814
Fibp	0.26348901	4.96051385	1.95951352	0.17695494	0.40496128
Casp4	-0.6274695	0.78416586	1.95897667	0.17701158	0.4050135
Cd79a	-0.5642011	2.13856601	1.95794067	0.17712094	0.40518631
S1pr3	-0.3119093	3.33109425	1.95743879	0.17717394	0.40523016
Fuk	-0.455059	1.56267441	1.95667033	0.17725515	0.40533847
Ppdpf	0.23347445	5.09338992	1.95550183	0.17737871	0.40554358
2610028E06I	1.13722888	-0.2727317	1.95457118	0.17747719	0.40563533
Bola3	0.34318436	3.33328221	1.95448249	0.17748658	0.40563533
Gm1943	0.30858884	3.5392695	1.95364279	0.1775755	0.40576113
Dtd1	0.22158261	6.04522579	1.95318739	0.17762375	0.40576613
Hdac10	-0.4625304	1.601888	1.95280857	0.1776639	0.40576613
Sos2	-0.1808809	7.66871831	1.95266293	0.17767934	0.40576613
Dcaf12	-0.2777928	5.27104991	1.95091313	0.17786495	0.40599258

Dpcr1	1.4338643	-1.0147611	1.95082708	0.17787409	0.40599258
Pet112	-0.3475863	2.78670512	1.95047845	0.1779111	0.40599258
Rnf114	-0.2627498	5.39363236	1.95000358	0.17796153	0.40599258
Dmc1	-1.6493914	-1.4697402	1.94991359	0.17797109	0.40599258
Sipa1l2	-0.3405023	4.53337432	1.94948658	0.17801645	0.40599258
Lrrc23	-0.8563821	0.84062034	1.94943368	0.17802207	0.40599258
Gda	-0.2688073	7.16852614	1.94903017	0.17806496	0.40599258
Scaf8	-0.1970182	5.88073503	1.94885468	0.17808361	0.40599258
Fhl4	-0.819584	0.87634716	1.94831533	0.17814096	0.4060091
Thap3	-0.5535955	2.29989503	1.94814885	0.17815866	0.4060091
Socs7	-0.2109582	6.96844492	1.9458125	0.17840737	0.40644455
Ear2	-1.2564634	-0.4067613	1.94556542	0.1784337	0.40644455
Fstl5	0.31266675	4.1514189	1.94509882	0.17848343	0.40644455
Utp3	0.1769945	6.12821502	1.94495162	0.17849912	0.40644455
Nsd1	-0.1900384	8.22829954	1.94425923	0.17857296	0.40644455
2700049A03I	-0.2567024	3.7645438	1.94413821	0.17858587	0.40644455
Tmx1	0.28496378	4.8957351	1.94412465	0.17858731	0.40644455
Rsf1	-0.2156553	7.58645342	1.94092067	0.1789295	0.40692002
Clic6	-0.4509395	2.60919011	1.9404733	0.17897735	0.40692002
Trappc11	-0.2100119	5.46142792	1.9404671	0.17897801	0.40692002
Emc9	0.3842694	2.43498504	1.94045385	0.17897943	0.40692002
Fxyd6	0.2523306	5.08938502	1.94025748	0.17900043	0.40692002
Mob4	0.15397269	7.02416311	1.94013862	0.17901315	0.40692002
Als2	-0.2628483	4.90617991	1.93994299	0.17903408	0.40692002
Myg1	0.36664792	3.22637071	1.9392612	0.17910705	0.40696109
Col4a6	0.42455145	2.4222693	1.93913919	0.17912012	0.40696109
Banp	0.26075246	4.65768493	1.93846374	0.17919245	0.40704822
Ndufs4	0.15571227	6.61749597	1.9379362	0.17924897	0.40709902
Gdpd5	0.28479166	3.75002037	1.93750373	0.17929533	0.40709902
Aasdh	-0.5109909	2.09204686	1.93730342	0.1793168	0.40709902
Atp5j2	0.25000483	5.43706898	1.9368376	0.17936675	0.40713525
Kif21b	-0.3054954	5.75344638	1.93634859	0.17941921	0.40717714
Megf11	0.22075456	5.52647012	1.93534778	0.17952662	0.40724572
Adnp	0.1708717	7.86024029	1.93529422	0.17953237	0.40724572
Celsr2	0.21442059	7.16649663	1.93488991	0.17957579	0.40724572
Epyc	-1.0749047	-0.2199656	1.93479994	0.17958545	0.40724572
Rnase6	-1.669362	-1.1019717	1.93422671	0.17964704	0.40726354
Garnl3	-0.3046744	5.19900696	1.93409375	0.17966133	0.40726354
Pdzd11	0.21937711	6.13967955	1.93351166	0.17972389	0.40732827
Zic3	0.62015103	1.36545791	1.93220312	0.17986465	0.40757014
Chst9	1.19995886	-0.8220098	1.93161715	0.17992772	0.4076121
Adcy3	0.46345168	2.3116347	1.93139873	0.17995124	0.4076121
Upf2	-0.1622623	6.89054025	1.92972941	0.1801311	0.40783662

Rpusd1	-0.2916506	2.98295012	1.92956932	0.18014836	0.40783662
Asb3	0.27505767	4.02289855	1.92887194	0.18022358	0.40783662
Sf3a3	0.22468931	4.70077619	1.92880461	0.18023084	0.40783662
Clock	-0.1727309	7.60340753	1.92837414	0.18027729	0.40783662
Ndufv3	0.30814213	5.13708218	1.92819635	0.18029648	0.40783662
Cpe	0.19627021	10.0402984	1.92812332	0.18030436	0.40783662
Efna5	-0.2015173	6.08444567	1.92791947	0.18032637	0.40783662
Asphd1	0.35454096	2.41463671	1.92728226	0.18039517	0.40783662
Cd151	0.42878881	5.12328317	1.92672986	0.18045485	0.40783662
Clgn	0.47256076	2.17288949	1.92654856	0.18047444	0.40783662
Uhmk1	0.17576991	6.47143497	1.92641056	0.18048935	0.40783662
Smim15	0.18407181	6.1523121	1.92637615	0.18049307	0.40783662
Nav1	-0.282032	7.3625303	1.92575147	0.1805606	0.40783843
Samd12	-0.3543851	3.24731974	1.92573869	0.18056199	0.40783843
Sema3a	0.24576071	6.40552977	1.92444259	0.1807022	0.40801375
Bop1	0.36454631	2.95513326	1.92426872	0.18072102	0.40801375
Syndig1l	0.41964975	2.34801023	1.92387383	0.18076377	0.40801375
Rnf5	0.25391427	5.68227105	1.92376199	0.18077589	0.40801375
Pex11b	-0.279209	5.43222602	1.92294058	0.18086486	0.40813765
Rab5c	0.18694695	6.19290957	1.91926692	0.18126346	0.40889535
Rad52	-0.2699096	3.57807302	1.91921665	0.18126892	0.40889535
BC030499	-0.6055384	2.4979529	1.91845531	0.18135167	0.40900496
Slc6a13	-0.3650065	6.53886407	1.91772396	0.1814312	0.40905503
Hdac7	-0.2728238	3.70229096	1.917623	0.18144218	0.40905503
Hsd17b12	0.36551744	3.64403364	1.91708226	0.18150101	0.40911065
Ugp2	0.1627464	6.43308734	1.91627806	0.18158856	0.40917112
Arrdc4	-0.4307326	2.72777521	1.91620811	0.18159617	0.40917112
Trpv4	1.01871833	-0.4879297	1.91575902	0.18164509	0.40920434
Klhl38	-1.0304034	0.04729006	1.9147636	0.18175356	0.40924224
Smr3a	-1.6876333	-0.5832651	1.91433597	0.18180018	0.40924224
Nfyb	-0.2054088	5.3894868	1.91429479	0.18180467	0.40924224
Elp6	-0.4951004	2.98309569	1.914051	0.18183126	0.40924224
B630005N14	0.16095213	6.47885509	1.91403714	0.18183277	0.40924224
Tbxas1	1.6047878	-1.1977504	1.91368434	0.18187126	0.40925194
Inip	0.2286732	5.15802191	1.91318505	0.18192574	0.40925887
Hdac6	-0.3776194	3.12401288	1.91302979	0.18194269	0.40925887
Rpa1	0.20995561	4.58334867	1.9121452	0.18203927	0.40930829
Igfbp5	0.30226418	8.64917915	1.91210213	0.18204398	0.40930829
Slc11a2	-0.2449512	4.99603711	1.9118896	0.18206719	0.40930829
06-Sep	0.15234384	6.97292922	1.91131422	0.18213006	0.40931367
Wdr35	-0.2788363	4.69647187	1.91124211	0.18213794	0.40931367
Pex26	0.29289141	3.8248316	1.9095025	0.1823282	0.40958438
Ptprm	-0.3018569	5.20958612	1.90948702	0.18232989	0.40958438

Kif9	-0.5685299	2.35054182	1.90920276	0.18236101	0.40958438
Scand1	1.21223803	-1.4286428	1.90838833	0.18245018	0.40964586
Nrbp2	-0.2066248	6.88218076	1.90825074	0.18246525	0.40964586
Trappc10	-0.1932951	5.8965905	1.90801574	0.182491	0.40964586
Gng7	-0.2646874	5.43551438	1.90724041	0.18257597	0.40975978
Dnmt1	-0.253478	5.72564765	1.90688571	0.18261485	0.40977026
Ndor1	-0.3602446	3.23268518	1.90649568	0.18265763	0.40978946
Gpr137	0.33835822	3.18893614	1.90603978	0.18270764	0.40979546
Extl1	-0.2931181	3.57333937	1.90559487	0.18275646	0.40979546
Jmjd1c	-0.2050379	8.21493046	1.90524204	0.18279519	0.40979546
Pi4k2b	0.47914305	2.02012472	1.90522395	0.18279718	0.40979546
Apmap	0.30249348	4.89839302	1.90456131	0.18286994	0.40984966
Rab21	0.18060589	7.62913437	1.90422617	0.18290676	0.40984966
Sertad3	-0.9223451	0.88470497	1.90406903	0.18292402	0.40984966
Pla2g6	0.3703143	2.71901809	1.9018824	0.18316449	0.41031166
Mtmr7	-0.3067579	5.04746558	1.90052752	0.18331368	0.4104446
Kcne1l	1.53964264	-1.2949601	1.90041784	0.18332576	0.4104446
5730508B09	-0.3819803	2.79684429	1.9000954	0.18336129	0.4104446
Chst2	-0.1727278	7.83723352	1.89970366	0.18340447	0.4104446
Tmem63c	0.37496624	4.14277226	1.89954216	0.18342227	0.4104446
Cacna1g	0.29209363	4.47879731	1.89947689	0.18342947	0.4104446
Tm2d3	-0.3072989	3.24777264	1.89904869	0.18347669	0.41047356
Ankrd52	-0.2267976	5.89362456	1.89799993	0.1835924	0.41065571
9330117O12	-0.5471835	1.48878023	1.89722765	0.18367766	0.41076971
Tpgs2	0.16491119	6.19132369	1.89647406	0.18376091	0.41087916
Cstf1	0.34287988	2.83750905	1.8958702	0.18382765	0.41095166
Apol9b	-1.1041836	-0.6994516	1.89397119	0.18403774	0.41128064
Dclre1b	-0.291392	3.22638578	1.89364653	0.18407368	0.41128064
Gcnt4	0.3118819	4.22489231	1.89360899	0.18407784	0.41128064
Svep1	-0.3870833	2.75200356	1.89234843	0.1842175	0.41145178
Zmynd10	-0.8132261	0.25024876	1.89229741	0.18422315	0.41145178
2610034B18	0.35005104	4.17684086	1.88892505	0.18459743	0.41209838
Uxs1	0.25205058	3.5382047	1.88881939	0.18460917	0.41209838
Rps3a1	-0.2386271	7.93110802	1.88875886	0.1846159	0.41209838
Tnrc18	-0.1786876	5.12993566	1.8882908	0.18466792	0.41210673
Ccdc157	-0.2926502	3.04729122	1.88804839	0.18469488	0.41210673
Ahcyl1	0.17393158	9.39081939	1.88778693	0.18472395	0.41210673
Pold4	0.50800728	2.12316132	1.88748727	0.18475728	0.41210673
Slc30a4	-0.1687081	6.40576099	1.88678549	0.18483537	0.41213991
Gm15800	-0.3925951	8.44724005	1.88638702	0.18487973	0.41213991
Porcn	0.26388659	4.36489916	1.88624695	0.18489532	0.41213991
Tmem136	0.30058217	3.41993937	1.88611679	0.18490982	0.41213991
Ccndbp1	0.21584454	6.68427437	1.88549991	0.18497852	0.41214863

Tpd52l2	0.27368365	6.30856718	1.88546369	0.18498256	0.41214863
C130083M11	-0.40154	2.78221445	1.88420173	0.18512322	0.41233919
Gm3500	-0.8082089	-0.3763363	1.88402518	0.18514291	0.41233919
Rreb1	-0.2826278	5.25007658	1.88376992	0.18517138	0.41233919
Arhgef33	1.00304419	-0.5838041	1.882788	0.18528095	0.41236972
Rbm24	-0.2940434	3.97278208	1.88273004	0.18528742	0.41236972
Ints10	-0.2769546	4.16093091	1.88272136	0.18528839	0.41236972
Gprin3	0.29610576	3.67896331	1.88210845	0.18535683	0.41242772
Tmem150a	0.38384407	2.52339698	1.88185155	0.18538553	0.41242772
Cyp2j9	-0.4748539	2.44796972	1.88127305	0.18545017	0.41242772
Alox15	-2.4439303	-1.1973716	1.88053176	0.18553303	0.41242772
Chd4	-0.1798023	7.44856066	1.88035911	0.18555234	0.41242772
Tspan11	-0.4407105	3.38958987	1.87969278	0.18562688	0.41242772
Enpp1	0.34280049	5.3449305	1.87967385	0.185629	0.41242772
Gm15055	1.5986066	-1.4464587	1.87939225	0.18566051	0.41242772
Lynx1	0.19191746	7.54082893	1.87936503	0.18566355	0.41242772
Ntrk1	-1.2506599	-1.4088131	1.87917432	0.1856849	0.41242772
Fndc8	1.68543257	-2.2316128	1.87909944	0.18569328	0.41242772
10-Sep	-0.3772326	2.8240973	1.87807868	0.18580759	0.41260508
Fdxr	0.53035284	1.38721774	1.87731955	0.18589265	0.41271392
1810010H24	0.56168549	1.16088715	1.87686358	0.18594377	0.41271392
D430036J16F	-0.4139796	2.35604874	1.87670177	0.18596191	0.41271392
9430018G01	-1.7096616	-1.5023933	1.87617521	0.18602097	0.41271392
0610031J06F	0.38008022	4.49173626	1.87507641	0.18614429	0.41271392
Ajap1	0.21296488	5.20149619	1.87471748	0.1861846	0.41271392
Lcat	0.47901298	2.85756413	1.87445301	0.1862143	0.41271392
Wnt1	-1.2310817	-1.3555042	1.87423391	0.18623891	0.41271392
Plekhb1	-0.2454391	5.81147658	1.87419272	0.18624354	0.41271392
Atp11a	0.20185059	6.90085178	1.87404048	0.18626065	0.41271392
Myh7b	0.58550889	2.32790488	1.87367608	0.1863016	0.41271392
Adar	-0.2576964	6.00892083	1.87342992	0.18632927	0.41271392
B4galt3	-0.4180891	1.94859098	1.87330404	0.18634342	0.41271392
Rnaseh2c	0.52018397	2.73847874	1.87292751	0.18638576	0.41271392
Cep41	0.4657157	2.89860501	1.8728319	0.18639651	0.41271392
Prkra	0.27301301	3.57028419	1.87191614	0.18649953	0.41271392
Odf3b	-1.0575158	0.00321497	1.87120073	0.18658007	0.41271392
Rad1	-0.3172714	4.79840405	1.87097886	0.18660505	0.41271392
Lrp1b	-0.3561428	5.97905963	1.87074577	0.18663131	0.41271392
Syngap1	-0.2205156	7.35111782	1.86997642	0.18671799	0.41271392
Npr3	-0.3210979	5.29409063	1.86993222	0.18672297	0.41271392
Lrrc3	0.31342531	3.66983848	1.86992983	0.18672324	0.41271392
Nhsl1	0.22713929	7.15377938	1.86974457	0.18674412	0.41271392
Dbt	-0.226595	5.64794495	1.86970071	0.18674907	0.41271392

Tmem30b	0.69251001	2.72185465	1.86949708	0.18677202	0.41271392
Thsd7a	-0.1921816	6.78848442	1.8693486	0.18678876	0.41271392
Snx2	-0.1608408	7.57439358	1.86852669	0.18688147	0.41271392
2610203C20I	-0.2661477	5.8366634	1.86851618	0.18688266	0.41271392
Neurl1a	-0.2972879	4.46459299	1.86848764	0.18688588	0.41271392
Cep85	0.28596678	3.329014	1.86762146	0.18698364	0.41271392
6330418K02I	0.59869108	1.11067126	1.86760902	0.18698504	0.41271392
Sema6a	-0.2348152	5.73162937	1.86745414	0.18700253	0.41271392
5031439G07	-0.1783499	5.64957202	1.86735004	0.18701429	0.41271392
Rps12	-0.3011592	6.21202793	1.86722569	0.18702833	0.41271392
Zfp664	-0.1950031	7.69577042	1.86641638	0.18711976	0.4128396
Cdr2l	0.28081109	3.23007911	1.86580305	0.18718908	0.41291648
Zswim8	-0.2302502	5.28010502	1.86408395	0.18738356	0.41325517
Rgmb	0.23834775	4.71335	1.86383598	0.18741164	0.41325517
A230073K19	-0.5892319	4.54489843	1.86321904	0.1874815	0.41327753
Fbxl5	-0.1777798	5.6184531	1.86243333	0.18757053	0.41327753
Ppargc1a	0.2046319	7.95652804	1.86222238	0.18759444	0.41327753
Arap1	-0.3238203	3.70806444	1.86182468	0.18763953	0.41327753
Map3k19	-0.433209	2.77347339	1.86180403	0.18764187	0.41327753
Rmnd1	0.4543648	1.31481141	1.86173412	0.1876498	0.41327753
Dtx3	0.23273699	5.05564026	1.86143404	0.18768384	0.41327753
Ubap2	0.16377252	6.09196296	1.86112744	0.18771862	0.41327753
Ccdc112	0.32848603	4.19145441	1.86058059	0.18778067	0.41327753
Acsl4	0.17741009	6.37633032	1.86051638	0.18778796	0.41327753
Map4k2	-0.2488988	4.85664931	1.8603981	0.18780139	0.41327753
Mkl1	0.17502322	5.55924494	1.86005354	0.1878405	0.41328767
Neurl4	-0.269145	4.54166249	1.85934628	0.18792083	0.41331458
Trim6	-1.3365983	-0.5497775	1.8593381	0.18792176	0.41331458
Cox6a2	0.47635945	1.09993149	1.85874376	0.18798929	0.41335096
Slc36a1	0.2165835	4.99323155	1.85858508	0.18800733	0.41335096
Mepce	-0.2416798	4.31707559	1.85760145	0.18811918	0.41345703
Mst1r	-1.050586	-0.7294972	1.85755358	0.18812463	0.41345703
Pold3	-0.2213626	4.88174881	1.85578446	0.18832601	0.41378233
Ccl3	2.26483637	-2.0153871	1.85564636	0.18834174	0.41378233
Gtf3c4	-0.2065836	5.27152828	1.85496109	0.18841983	0.41387796
Mib1	0.20440974	6.21587514	1.85349681	0.18858681	0.41392626
Calu	0.24550717	6.14089054	1.85303137	0.18863993	0.41392626
Exosc10	0.21909294	4.86434649	1.8527095	0.18867668	0.41392626
Gas2l1	0.31893153	3.8345885	1.85254038	0.18869599	0.41392626
Tagap	-0.3791081	2.53749694	1.85221565	0.18873307	0.41392626
Leap2	1.56042534	-0.4596987	1.85185278	0.18877452	0.41392626
Cdc42ep2	0.42440779	2.08049696	1.85164344	0.18879844	0.41392626
Usp50	1.95951654	-1.5371428	1.85154511	0.18880967	0.41392626

Eid2	0.1940889	5.24405361	1.85115943	0.18885375	0.41392626
Ubb	0.34370088	6.41461667	1.85086444	0.18888747	0.41392626
Gm11837	0.98471517	-0.748374	1.8508558	0.18888846	0.41392626
Zdhhc1	0.31141318	3.24992215	1.85064121	0.188913	0.41392626
P2rx6	-0.7104776	1.20179979	1.85046082	0.18893363	0.41392626
B3gnt7	1.65510051	-0.8629206	1.84971257	0.18901922	0.41392626
1110058L19R	0.23978255	4.25525354	1.84953039	0.18904007	0.41392626
Taok2	-0.2165111	5.310984	1.84941143	0.18905369	0.41392626
Smpd3	0.23090317	4.76558049	1.84933988	0.18906188	0.41392626
Ppp2r3a	-0.1874466	6.51542	1.84932163	0.18906396	0.41392626
C330024D21I	-0.8878504	0.122273	1.84824541	0.1891872	0.41392817
Wdhd1	-0.3997658	2.70306363	1.84818526	0.18919409	0.41392817
Lrrc36	-1.0394953	-0.0502323	1.8480357	0.18921123	0.41392817
B230219D22	0.17453187	8.00694298	1.84786036	0.18923132	0.41392817
Msx1	0.50659915	2.36231787	1.84780502	0.18923766	0.41392817
1700071M16	-1.1648673	0.65191251	1.84718545	0.18930867	0.41400789
BC017158	0.31675237	3.34202633	1.84683995	0.18934829	0.41401892
Trib1	0.21072645	5.66745826	1.84640647	0.18939801	0.41405071
Zfp119b	0.67612742	0.7155587	1.84611034	0.18943198	0.41405071
Cars	-0.3153717	3.58290147	1.84482309	0.18957975	0.41429808
Wwp2	-0.2704315	4.35375428	1.84405752	0.1896677	0.41441466
Dcun1d5	-0.1900568	5.95831292	1.84280563	0.18981163	0.41465349
Helq	-0.4409423	2.56920515	1.84239132	0.18985929	0.41468197
Cluh	-0.223744	5.1625154	1.8411842	0.18999824	0.41490979
Brms1l	0.18745509	5.94753582	1.84075667	0.19004749	0.41492055
Rad50	-0.2345444	6.08377612	1.84045246	0.19008254	0.41492055
Acaa1b	0.65698273	0.75719299	1.83918672	0.19022845	0.41492055
Ramp2	0.3456446	4.5868832	1.83869788	0.19028485	0.41492055
AU040972	-0.7479474	1.08626308	1.83850622	0.19030696	0.41492055
Stox1	-0.481838	1.31133111	1.83833098	0.19032718	0.41492055
Mapre3	0.17400666	7.32864478	1.83823712	0.19033802	0.41492055
Anapc13	0.29496284	5.13802789	1.83822542	0.19033937	0.41492055
Gm2897	-0.329644	3.6036603	1.83822096	0.19033988	0.41492055
Msh2	0.24161121	4.64569386	1.83813643	0.19034964	0.41492055
Eppk1	-1.4488953	-0.5770483	1.83749457	0.19042375	0.41500655
Erlin1	0.2358802	4.56877923	1.8367928	0.19050481	0.41510768
Kctd8	-0.5167041	2.1487354	1.83612062	0.1905825	0.41511054
05-Sep	-0.1769418	6.40078037	1.8361189	0.1905827	0.41511054
Gm9899	0.25845913	3.86221393	1.83559037	0.19064381	0.41511054
Sox9	0.24585455	5.66087096	1.835582	0.19064478	0.41511054
Vav1	-0.7432491	0.25682111	1.8341929	0.19080551	0.41534138
Mef2a	0.15052392	8.26170554	1.83356684	0.19087801	0.41534138
Ifitm3	0.39566414	7.69025273	1.83351337	0.1908842	0.41534138

Smpd2	-0.4461861	2.31042198	1.83336089	0.19090186	0.41534138
Ero1l	0.1798308	6.37695427	1.83296562	0.19094766	0.41534138
Dimt1	0.23680978	5.04268064	1.8325767	0.19099274	0.41534138
Pxdc1	-0.3793842	3.64415578	1.83256953	0.19099357	0.41534138
Lamtor2	0.33696409	4.07058306	1.83170393	0.19109393	0.4154842
Fus	-0.2661653	5.94252772	1.83025341	0.19126227	0.4157609
Tbc1d8b	-0.2634406	4.33544602	1.829724	0.19132375	0.4157609
Pnkd	0.17516154	6.53540609	1.82962593	0.19133515	0.4157609
Cnbd2	0.39824124	2.38608532	1.82880947	0.19143003	0.4157609
Pmvk	-0.2439626	4.37566919	1.82847682	0.1914687	0.4157609
Dpys	-1.4519909	-0.9644051	1.82835243	0.19148316	0.4157609
Ercc8	-0.47259	2.35817298	1.82817507	0.19150379	0.4157609
B230206H07	-1.8254461	-1.6970281	1.8279644	0.19152829	0.4157609
Myo1e	-0.2214744	4.55199139	1.82782042	0.19154504	0.4157609
Slc38a6	-0.4310046	3.17223248	1.82753629	0.19157809	0.4157609
Slc9a1	0.24093143	5.26187894	1.82732153	0.19160308	0.4157609
Alkbh8	0.28363667	4.91475563	1.82649191	0.19169966	0.41589511
Tmem125	0.69964731	0.65984188	1.82610359	0.19174489	0.41591787
Dcun1d4	0.18290504	7.403709	1.82567139	0.19179524	0.41592239
Mir350	-1.6012989	-2.0322071	1.82548953	0.19181643	0.41592239
Zfhx3	-0.1619834	7.2958918	1.82444964	0.19193766	0.4160928
Klc2	0.25263894	5.48248672	1.82379204	0.19201437	0.4160928
Napepld	0.24181659	6.43308313	1.82353383	0.1920445	0.4160928
Sdccag3	-0.2228303	4.74850971	1.82330773	0.19207089	0.4160928
Hdac5	-0.2665057	5.88492629	1.82318815	0.19208485	0.4160928
Cd81	0.3252116	7.79192516	1.82302852	0.19210349	0.4160928
Mzf1	0.72610151	0.2685717	1.8224268	0.19217375	0.41616973
Mid1	0.3097694	4.12698169	1.82179555	0.1922475	0.41617011
Son	-0.240522	8.07903712	1.82134692	0.19229994	0.41617011
Urb1	-0.3963951	2.76951926	1.82097383	0.19234356	0.41617011
Hyal1	0.44761706	3.56170097	1.82084245	0.19235892	0.41617011
Dmap1	-0.3198454	3.50122349	1.82065566	0.19238077	0.41617011
Golph3	0.18640989	8.57650586	1.82064138	0.19238244	0.41617011
Mcm2	-0.3832045	2.2388127	1.82032414	0.19241955	0.41617521
Sp9	0.46618651	1.30070133	1.81914917	0.19255707	0.41629371
Tcf4	0.1378826	9.26007733	1.81901247	0.19257308	0.41629371
Sh3d19	0.20960028	6.73968738	1.81865219	0.19261527	0.41629371
Rc3h2	0.16225648	7.92859234	1.81851155	0.19263175	0.41629371
Il4ra	0.89431719	0.15441314	1.81821781	0.19266616	0.41629371
Mnd1	1.15482197	0.36796527	1.81687924	0.19282309	0.41629371
Acss2	0.39992227	4.31701525	1.81675989	0.19283709	0.41629371
Gm13749	-0.8842556	0.63489644	1.81673863	0.19283958	0.41629371
Dact1	-0.2629668	6.05623153	1.81629	0.19289222	0.41629371

Mtx3	-0.2097685	5.99207355	1.81618624	0.1929044	0.41629371
9930014A18I	-0.7056186	0.8415086	1.81594294	0.19293295	0.41629371
Gm13157	0.31421753	3.36192233	1.81574261	0.19295647	0.41629371
Pou3f3os	-0.270295	3.71496871	1.8153406	0.19300367	0.41629371
Slc20a1	-0.2500458	5.34830117	1.81529203	0.19300937	0.41629371
Trmt12	-0.3207495	3.27438155	1.8152804	0.19301074	0.41629371
Nfyc	0.24249269	4.60424449	1.815112	0.19303052	0.41629371
Ntn1	0.35868421	3.73349064	1.81455882	0.1930955	0.41635076
Pcdha3	1.27289633	-0.4062687	1.814295	0.19312651	0.41635076
Fam102a	-0.2107256	5.70098183	1.8132848	0.19324527	0.41647461
Atox1	0.3506805	4.36596324	1.81290938	0.19328943	0.41647461
Gltscr1	-0.2450862	4.38183036	1.81280162	0.19330211	0.41647461
Atf6b	-0.2999909	3.13973333	1.81232082	0.19335869	0.41647461
Slamf9	-1.4244973	-0.3339572	1.81215439	0.19337828	0.41647461
B020004J07I	-0.8682215	0.1922254	1.81177818	0.19342257	0.41647461
Abra	-1.8285828	-0.3836444	1.81173729	0.19342739	0.41647461
Rnf113a2	0.23799716	4.72744651	1.81091673	0.19352404	0.41660781
Il11	-0.6622012	0.53524101	1.8090204	0.19374764	0.41681151
Mmd2	0.27019465	4.00550522	1.80877151	0.19377701	0.41681151
Zfp292	-0.2066462	7.57624967	1.80849282	0.1938099	0.41681151
Ermn	0.20421313	6.68337228	1.80848219	0.19381116	0.41681151
Phtf1os	0.49049071	1.14798045	1.80811171	0.1938549	0.41681151
Traf1	-0.8939523	0.98828572	1.80791614	0.19387799	0.41681151
Tenc1	-0.2630938	5.97022186	1.80789707	0.19388024	0.41681151
Rad23a	0.26732158	4.96103067	1.807503	0.19392679	0.41681151
Traf4	-0.323068	2.52163121	1.80707223	0.19397768	0.41681151
Garem	-0.2347676	4.79906231	1.80689976	0.19399806	0.41681151
Wdr34	0.32364251	3.15942228	1.80658522	0.19403524	0.41681151
Mms19	0.34488811	3.6826746	1.80657609	0.19403632	0.41681151
Clrn1	1.76487347	-1.0221017	1.8061455	0.19408723	0.4168461
Eif2b2	0.31683644	4.10672235	1.80557779	0.19415438	0.41691555
Sergef	-0.4931311	1.78404877	1.80371977	0.19437434	0.41727777
Ccdc136	-0.2104244	5.29502051	1.80335097	0.19441804	0.41727777
Ntpcr	0.37308738	2.61603098	1.80327034	0.1944276	0.41727777
Gpc6	0.27842308	7.3480851	1.80270009	0.19449519	0.41734147
Limk1	0.35826667	2.85508073	1.80243206	0.19452697	0.41734147
Bcat2	0.38969353	2.1273565	1.80099506	0.19469747	0.41742455
Igf1	-0.2479334	5.25462119	1.80088244	0.19471084	0.41742455
Rpl35	-0.2420718	4.96906104	1.80084317	0.19471551	0.41742455
Gm13483	-1.0510379	-0.7401471	1.80052576	0.1947532	0.41742455
AA986860	0.45778381	1.71718232	1.80050413	0.19475577	0.41742455
Rps6ka3	0.15721778	7.88393158	1.80034356	0.19477484	0.41742455
Aacs	-0.3435693	2.68904219	1.79920643	0.19490995	0.41763939

Gm4814	1.63255988	-1.5837911	1.79880371	0.19495784	0.41766726
4933434E20I	0.20034214	4.51298392	1.79823784	0.19502514	0.41773672
Dhcr7	0.28329266	3.72930431	1.79765376	0.19509464	0.41777485
Fbxw11	-0.1699811	7.36807432	1.79746716	0.19511685	0.41777485
Sox10	0.29264601	4.29225309	1.79650651	0.19523124	0.41777485
Usp1	0.20887009	5.3274602	1.79627928	0.19525831	0.41777485
Pdpr	0.3034728	3.89356158	1.7961137	0.19527804	0.41777485
Grik5	0.2876069	5.30620687	1.79605218	0.19528537	0.41777485
Asb6	-0.3354726	2.52934525	1.79593708	0.19529909	0.41777485
Pef1	0.23226434	4.475448	1.79574462	0.19532202	0.41777485
Ofd1	-0.2998362	4.18802212	1.7950222	0.19540815	0.41788444
Prkaca	0.19124573	7.24830415	1.79399286	0.19553096	0.41802571
Smim4	0.40260732	2.15559357	1.79388336	0.19554402	0.41802571
Slc32a1	0.30061285	4.04629258	1.79240208	0.19572093	0.41830942
Slit3	0.27845855	4.60546321	1.79218726	0.1957466	0.41830942
Uqcr10	0.28862416	5.90447573	1.7916548	0.19581025	0.41837078
Pcdhb10	-0.6224733	1.33394292	1.79056796	0.19594024	0.41849396
Klrg1	1.98166531	-2.0796857	1.79055341	0.19594199	0.41849396
Oat	0.22215953	8.127886	1.79021027	0.19598305	0.41849396
Tiprl	0.17199223	6.36973233	1.7900045	0.19600768	0.41849396
Dpp8	-0.1294429	8.3975238	1.78822915	0.19622036	0.41884462
Eif3g	0.23274847	5.42210614	1.78804973	0.19624187	0.41884462
Dph7	0.26101723	3.34348573	1.78727106	0.19633525	0.41889739
Slc6a15	0.24494427	4.70351203	1.78687776	0.19638243	0.41889739
Ogt	-0.2783794	7.68691781	1.78633213	0.19644792	0.41889739
Mycbp	0.25654804	5.63413358	1.78611606	0.19647386	0.41889739
Suco	-0.1934491	6.12057899	1.78606736	0.19647971	0.41889739
Dact2	0.38841073	2.14960065	1.78575438	0.19651729	0.41889739
Tnpo3	-0.179559	6.1065511	1.78554983	0.19654186	0.41889739
Hars2	-0.2444374	3.90856284	1.78482041	0.1966295	0.41889739
Mir6920	-1.3644031	-1.5890956	1.78476846	0.19663575	0.41889739
Lamp1	0.28565531	7.56325449	1.78474202	0.19663892	0.41889739
Shc3	-0.3547758	3.93974062	1.78427477	0.19669509	0.41889739
Dph5	-0.2639087	4.65440441	1.78414005	0.19671129	0.41889739
1500012F01I	-0.2786086	3.6609381	1.78405667	0.19672132	0.41889739
Gtf2ird2	-0.4338986	2.65349216	1.78304869	0.19684258	0.41908109
Dnajb14	-0.2202175	4.2317679	1.78070798	0.19712453	0.41956463
Nlgn1	0.23207694	6.61629113	1.78058161	0.19713977	0.41956463
Hnrnpk	0.15880976	7.92294978	1.78018374	0.19718775	0.41959217
Glul	0.210762	10.5718556	1.77986329	0.1972264	0.41959987
H2-T24	-0.2990637	4.91991878	1.7787495	0.19736082	0.41975636
Gbp3	-0.3219769	4.32332538	1.77867304	0.19737006	0.41975636
Dnajb11	0.17906327	5.90712065	1.77759665	0.19750008	0.41995831

Cdc7	-0.5043363	2.8060365	1.77640967	0.19764359	0.42008492
2210404O09	-0.4226457	2.13870663	1.77612317	0.19767825	0.42008492
Etohd2	-0.5888111	1.40307632	1.77599126	0.19769421	0.42008492
Mmab	0.21899042	4.42183548	1.77594395	0.19769994	0.42008492
Atg4a	0.44610585	3.5910716	1.77523124	0.1977862	0.42019366
0610040J01F	-0.8799247	0.12504341	1.77413618	0.19791883	0.42039905
Acad9	0.29613419	4.13075264	1.77364433	0.19797844	0.42039905
Ube2n	0.13536506	7.43722179	1.77344567	0.19800252	0.42039905
Prmt10	-0.4265852	3.33900613	1.77296618	0.19806066	0.42039905
Wdr16	-0.9680836	-0.2244289	1.77247758	0.19811993	0.42039905
E030019B06i	-1.3157608	-0.5614128	1.77245457	0.19812272	0.42039905
9130019O22	-0.4212735	2.12545968	1.77218745	0.19815513	0.42039905
Rilp	1.02713842	-0.4836868	1.77207502	0.19816877	0.42039905
Grm5	-0.2124375	6.71237517	1.77168496	0.19821612	0.42039905
Klrg2	-1.1025923	-0.5935712	1.77071427	0.198334	0.42039905
Mapk1ip1l	0.20086675	8.02239861	1.77068561	0.19833748	0.42039905
Polr2h	0.3366635	4.08275577	1.77059496	0.19834849	0.42039905
Hspbp1	0.37011865	2.63907772	1.77040494	0.19837158	0.42039905
Cntnap4	-0.3960857	3.32376177	1.7699582	0.19842588	0.42039905
Dnali1	-0.7963804	0.12482844	1.7696185	0.19846718	0.42039905
Ercc5	-0.2010021	5.02331105	1.76939274	0.19849463	0.42039905
Map7d1	0.20333298	6.98034278	1.76925199	0.19851175	0.42039905
Frmpd3	0.67915999	1.33049436	1.768976	0.19854532	0.42039905
Hsf1	0.19258484	5.47374936	1.76867548	0.19858188	0.42039905
Txndc17	0.22755021	6.32172748	1.76865013	0.19858496	0.42039905
Ankrd10	-0.2622443	3.86599986	1.76824775	0.19863393	0.42042839
Lxn	0.18303183	4.9324771	1.76700198	0.19878563	0.42053436
Cdca3	-1.1383879	-0.5375831	1.76699526	0.19878645	0.42053436
Exo5	-0.324121	3.74028073	1.76678319	0.19881229	0.42053436
Pfkfb1	-0.9950419	-0.0473381	1.76640554	0.19885831	0.42053436
Hist1h2bp	0.93722317	-0.7282023	1.76639521	0.19885957	0.42053436
Iqch	-1.0982679	-0.5441629	1.76581949	0.19892976	0.42060851
1500004A13I	0.29514096	4.96989979	1.76513483	0.19901327	0.42067411
Ltk	-0.5809539	1.34450816	1.76447025	0.19909437	0.42067411
Naip2	-0.7752196	0.32866889	1.7644257	0.19909981	0.42067411
Arih2	-0.2080145	4.72467566	1.76441355	0.1991013	0.42067411
Kdm4b	-0.326575	2.7623584	1.76367745	0.19919118	0.42078979
Ndnl2	0.29387216	3.46944245	1.76309546	0.19926228	0.42079299
5330417C22I	0.35033818	3.85966934	1.76289288	0.19928704	0.42079299
Pcdhb13	-0.3897546	3.50224641	1.76280229	0.19929811	0.42079299
Acsl6	-0.2080209	6.0861071	1.76166279	0.19943744	0.42101295
Zgrf1	-0.4358234	2.5509492	1.76129586	0.19948234	0.42103351
Deptor	0.16022657	7.41084037	1.76098047	0.19952093	0.42104077

Rogdi	0.2260922	4.61086762	1.76055138	0.19957346	0.42107741
Cdip1	0.1513466	6.7745802	1.76006099	0.19963351	0.42112992
A330076H08	-0.4438942	3.78922034	1.75974164	0.19967263	0.42113822
Cdc25a	0.32766103	2.73292837	1.75945479	0.19970778	0.42113822
Vegfb	-0.2252227	4.6599426	1.75837987	0.19983955	0.4213419
Mboat1	-0.8366706	0.97998995	1.75689477	0.20002178	0.42155045
Cnst	0.20582107	6.16085831	1.75650623	0.20006949	0.42155045
Itgae	1.66818342	-1.8146035	1.75642776	0.20007913	0.42155045
Gm7444	-0.9458519	0.29348424	1.75635864	0.20008762	0.42155045
Rnf26	-0.3497775	3.12694688	1.75614013	0.20011446	0.42155045
Macrod2	0.23752781	5.09283958	1.75499579	0.20025511	0.42160626
Col4a3bp	-0.1451947	7.25980068	1.75478192	0.20028141	0.42160626
Clcn1	-0.8586228	0.05914403	1.75462741	0.20030041	0.42160626
Tnfsf8	-1.5394682	-0.2182772	1.75392384	0.20038697	0.42160626
Cer1	1.83950485	-1.0477751	1.75386975	0.20039362	0.42160626
Hibadh	0.25574683	5.59095261	1.75373384	0.20041035	0.42160626
Fam227b	-0.6676199	0.68226812	1.75372793	0.20041108	0.42160626
Zfp286	-0.3568135	3.27813945	1.7528544	0.20051862	0.42160626
A930011O12	-0.5029219	4.98274965	1.75261852	0.20054768	0.42160626
Ccdc14	0.47300934	1.8649265	1.75196588	0.20062809	0.42160626
Atp5j	0.20233925	7.73128512	1.75196118	0.20062867	0.42160626
Map6	0.26340448	5.70222243	1.75190664	0.20063539	0.42160626
Nrxn3	0.24994392	8.1011022	1.75181938	0.20064615	0.42160626
6430571L13I	-0.9010239	0.33278228	1.75172306	0.20065802	0.42160626
6030419C18I	0.45595362	2.38184951	1.75162593	0.20066999	0.42160626
Nat6	0.36133332	3.18126994	1.75134817	0.20070424	0.42160626
Chek1	-0.6239377	0.84374277	1.74947564	0.20093529	0.42198725
H2afz	0.17804477	7.20723172	1.74919987	0.20096935	0.42198725
Fut4	0.88691981	-0.2588624	1.74902199	0.20099132	0.42198725
Usp16	-0.191185	6.12332536	1.74862848	0.20103993	0.42200208
Tmem59l	0.32307421	4.17803681	1.74827796	0.20108325	0.42200208
Ssr3	0.21294791	8.00002703	1.74810929	0.2011041	0.42200208
Naa35	0.17457661	6.08608385	1.74717954	0.20121906	0.42216935
AA388235	-0.3023127	3.22421595	1.74606819	0.20135659	0.42236367
5730420D15I	-1.6521524	-0.8912027	1.74586124	0.20138222	0.42236367
Cybrd1	0.4296551	2.11232125	1.74488431	0.20150323	0.42252332
Sorl1	0.24537524	6.15766545	1.74453501	0.20154652	0.42252332
1700030K09I	0.58209355	1.60509031	1.74439249	0.20156418	0.42252332
Numa1	-0.1515773	5.77399352	1.74386782	0.20162924	0.42258572
Pcdha10	0.95027722	-0.3966167	1.74298144	0.2017392	0.42274113
Homez	0.20391592	4.36385714	1.74270114	0.20177399	0.42274113
Rpe65	-1.0048121	0.67821647	1.74058722	0.20203659	0.42312888
Poldip2	0.21422514	5.13046729	1.74039942	0.20205994	0.42312888

Gga1	-0.2602457	3.54956689	1.74014229	0.20209192	0.42312888
Serf1	0.34593224	2.89627059	1.74003204	0.20210563	0.42312888
Btbd1	-0.1542958	7.23030801	1.73954542	0.20216617	0.42312888
4930578C19I	-1.7867695	-1.5017189	1.73950615	0.20217105	0.42312888
Akirin2	0.21527729	6.60891221	1.73880993	0.20225771	0.42317737
Rab36	0.21374076	3.97702559	1.73868248	0.20227358	0.42317737
Gmeb2	0.35776369	2.96301519	1.7384684	0.20230023	0.42317737
Rab30	-0.2056996	4.8877884	1.73816453	0.20233808	0.42318262
Tgm5	-1.2037083	0.06643321	1.73774115	0.20239082	0.42321902
Sorcs2	0.2820591	3.64157151	1.73744178	0.20242813	0.42322313
2310010J17F	-0.6382645	0.76878002	1.7366653	0.20252493	0.42335161
Pex1	-0.2561933	4.55242646	1.7359576	0.20261321	0.42346222
Ttll9	-1.8798191	-1.3124561	1.73502741	0.20272931	0.42363094
Cntn3	0.22722901	5.52149284	1.73187817	0.203123	0.42431404
Hps3	-0.3004163	4.08700997	1.7315779	0.20316059	0.42431404
Thap2	0.22315021	5.05056081	1.73156268	0.2031625	0.42431404
Men1	0.28567152	4.03317558	1.73016017	0.20333818	0.4246069
Ccdc106	-0.494039	1.77355217	1.72727054	0.20370074	0.42512161
E2f2	-0.4973704	3.00064518	1.72726585	0.20370133	0.42512161
Hexa	0.39615706	3.74109112	1.72707334	0.20372551	0.42512161
Slc2a9	1.61550725	-0.8794348	1.72706424	0.20372666	0.42512161
Cox6b1	0.30369988	5.47414806	1.72632953	0.20381899	0.425151
Rasip1	-0.3789803	2.4086378	1.72617604	0.20383828	0.425151
Ikzf4	-0.1918267	4.19381993	1.72591162	0.20387153	0.425151
Hyls1	0.36765494	2.25186842	1.7258224	0.20388275	0.425151
Nap1l4	0.19906882	5.59025974	1.7252696	0.20395228	0.42517725
Cdca8	-0.8486315	0.45926939	1.72515779	0.20396634	0.42517725
Tmem169	-0.287203	2.9231717	1.72401177	0.2041106	0.42540391
Fndc4	-0.290925	3.68251726	1.72311671	0.20422335	0.42556484
Prrc1	-0.2007667	6.03799627	1.72254176	0.20429582	0.42564179
Tmem37	-0.9642972	0.73722853	1.72161467	0.20441274	0.425761
Ncoa7	0.18500118	6.32087926	1.72151773	0.20442497	0.425761
Ror2	0.76486365	0.84523662	1.72124253	0.20445969	0.425761
Ciita	-0.5000861	1.62985973	1.72095007	0.2044966	0.42576383
Mat2a	-0.1534812	7.87146733	1.7199894	0.20461791	0.42592704
Zfp316	-0.3228679	3.73283924	1.71976607	0.20464613	0.42592704
Sh3bgrl3	0.21196086	6.73140754	1.71909396	0.20473106	0.42596001
Gale	-0.980229	-0.2678959	1.71895572	0.20474854	0.42596001
Cd209b	2.09632004	-1.5381916	1.71862435	0.20479044	0.42596001
Gpx8	0.35883384	5.63847575	1.7184371	0.20481412	0.42596001
2610035F20I	0.31292225	3.01354408	1.71776458	0.20489919	0.42596001
Bambi-ps1	1.13268298	-0.9567035	1.71769105	0.2049085	0.42596001
Rps19	-0.2913499	4.67855439	1.71767169	0.20491095	0.42596001

Pcf11	-0.172351	6.58616928	1.71725437	0.20496377	0.42599587
2610015P09I	-0.2597933	3.72388813	1.71617331	0.20510067	0.4261403
Syt7	-0.2478627	6.93971431	1.71614367	0.20510443	0.4261403
Atp6ap1l	0.72795617	1.11229166	1.71506074	0.20524169	0.42635152
Crnkl1	-0.2280058	4.56709497	1.71367107	0.205418	0.42658723
Fam185a	0.25387581	3.69657058	1.7136049	0.2054264	0.42658723
E030019B13I	1.67601892	-1.3835577	1.71252248	0.20556387	0.42678737
Aox4	-0.7998756	0.66151393	1.71228489	0.20559406	0.42678737
Manea	0.2014353	5.51538336	1.7103847	0.2058357	0.42718813
E530001F21I	-1.4619035	0.14723667	1.71020595	0.20585845	0.42718813
Oxsr1	-0.1772116	5.96237437	1.70954738	0.2059423	0.42728808
Yy2	-0.8462958	-0.0337332	1.70878647	0.20603923	0.42734471
Zfp62	0.19733781	6.19282385	1.70877284	0.20604096	0.42734471
Dsg2	0.36395392	3.13330013	1.70827398	0.20610454	0.42740256
Psme2b	-0.3617367	3.17254541	1.70657592	0.20632114	0.42775735
5033404E19I	-2.1017728	-1.5114324	1.70593698	0.20640272	0.42775735
Psmg3	0.4941465	2.00134747	1.70589784	0.20640771	0.42775735
Alg14	0.33497418	4.58603862	1.70581336	0.2064185	0.42775735
Gtf3a	0.37071594	2.55083537	1.70549736	0.20645887	0.42776697
Dnal4	0.31983216	2.43868295	1.70455851	0.20657885	0.42794152
Tnfsf12	-0.3139504	3.32585799	1.70400372	0.20664979	0.42801272
Rpain	0.30991677	2.84744177	1.70356795	0.20670553	0.42801272
Ccdc160	0.48154683	1.8488038	1.70345144	0.20672044	0.42801272
Rasl11a	-0.8980324	0.00434263	1.70248498	0.20684414	0.42819482
Lamp5	-0.2019267	5.75821944	1.70201394	0.20690447	0.42824567
Prodh	-0.4133548	2.14169972	1.70143068	0.2069792	0.42832632
Ccdc109b	0.50146914	1.96654742	1.7006905	0.20707408	0.42844863
Hdhd3	0.83350545	0.2830346	1.70036717	0.20711554	0.4284604
Esm1	0.47872537	2.24517017	1.69996079	0.20716767	0.42849422
2210016L21I	0.18478944	6.25796474	1.69949226	0.2072278	0.42852789
9130011E15I	0.27150194	3.63260374	1.69889029	0.20730507	0.42852789
Fzd6	0.36673767	3.99486454	1.69863884	0.20733737	0.42852789
Itgb5	0.33547141	4.05281799	1.69819681	0.20739415	0.42852789
Lca5l	-0.6621579	1.38066019	1.69773408	0.20745361	0.42852789
Tusc3	0.19868628	4.91653902	1.69769094	0.20745915	0.42852789
Slc39a3	0.29068627	3.21713689	1.69753911	0.20747867	0.42852789
Slc35b3	-0.3779821	2.18496086	1.69703093	0.207544	0.42852789
Gm14327	-0.4692858	2.97504976	1.69691726	0.20755862	0.42852789
Plch2	-0.4146087	3.25218423	1.6968411	0.20756841	0.42852789
Gm10825	-1.6458267	-1.3724712	1.69676993	0.20757757	0.42852789
Mesdc2	0.25140087	6.39054405	1.6963391	0.20763299	0.42856843
Metrn	0.90107351	-0.6406921	1.69598009	0.20767918	0.42858991
Wdr36	-0.2447552	3.83820002	1.69540792	0.20775284	0.42866804

Mis12	0.20937549	5.24682689	1.69483084	0.20782716	0.42872255
4933416I08R	-1.3689868	-1.0641654	1.69437167	0.20788631	0.42872255
Cd3eap	-0.2894224	3.59037109	1.69423834	0.20790349	0.42872255
2310045N01	0.38947286	4.79237446	1.69391644	0.20794498	0.42872255
Smc6	-0.1444798	7.65803915	1.69372787	0.20796929	0.42872255
Gm19395	-1.6779089	-0.4158174	1.69352658	0.20799525	0.42872255
Fam149a	-0.1750385	5.22175722	1.69325822	0.20802985	0.42872255
Scaf4	0.15630508	6.40873382	1.69285631	0.20808169	0.42875561
Phka1	-0.2767721	4.255368	1.69249278	0.2081286	0.42877848
Gimap6	-0.619268	1.78011228	1.69009796	0.20843793	0.42931724
Map4k5	-0.1562037	5.95929711	1.68991329	0.20846181	0.42931724
5330413P13I	0.51485505	1.58644939	1.68932318	0.20853813	0.42939287
Ganab	0.23872284	5.05845587	1.68907499	0.20857024	0.42939287
1700105P06I	3.08892759	-1.8469964	1.68822165	0.2086807	0.42943059
Ccl22	-1.4922189	0.31341969	1.68797473	0.20871267	0.42943059
Lztfl1	0.18560393	6.37156356	1.68781107	0.20873387	0.42943059
Gm14403	-0.2125823	3.69756188	1.68738176	0.20878948	0.42943059
Arhgef18	-0.2012568	4.8838218	1.68714814	0.20881975	0.42943059
Tyms	-0.2819492	3.66644618	1.68693884	0.20884687	0.42943059
Nacc1	0.15223391	6.99277199	1.6867324	0.20887363	0.42943059
Vmn2r29	-0.3058509	3.38591024	1.6867185	0.20887543	0.42943059
Cdc42bpb	-0.2048535	6.53365385	1.68640109	0.20891658	0.42944147
Cbfa2t3	-0.2247954	4.7943287	1.68544295	0.20904087	0.4296232
Tns3	-0.1598596	6.05708022	1.68512758	0.20908179	0.42963358
Ipo7	0.20015789	5.83228562	1.68357209	0.2092838	0.42990524
Tet1	-0.1905482	6.20291372	1.68355677	0.20928579	0.42990524
1190005I06R	-1.2492058	-1.2426018	1.68287149	0.20937487	0.42992432
Brms1	-0.338544	2.6401315	1.68276742	0.2093884	0.42992432
Arhgap8	-1.1679142	-0.3610325	1.68192785	0.20949761	0.42992432
Tmem170	-0.3319271	3.25063668	1.68191647	0.20949909	0.42992432
Rnf141	-0.2503382	4.23117867	1.68186464	0.20950583	0.42992432
Wdfy2	-0.5045064	1.70188876	1.68157622	0.20954336	0.42992432
Slfn3	1.44764629	-0.2510446	1.68155305	0.20954638	0.42992432
Tifa	-0.3564185	3.62939513	1.68054151	0.20967809	0.43000812
Tmem119	0.41718956	3.49084428	1.68047559	0.20968667	0.43000812
Psme3	0.14391225	6.7298075	1.6804121	0.20969495	0.43000812
Tceal8	0.27382512	7.27685964	1.67998246	0.20975092	0.43004927
Gfm1	-0.2685672	4.55150656	1.67932121	0.20983712	0.43009001
Tmcc2	-0.2255865	4.78003844	1.67916289	0.20985776	0.43009001
Rapsn	-1.9685018	-2.5007466	1.67900359	0.20987853	0.43009001
Ppp1r16a	-0.3612996	2.41050644	1.67838103	0.20995974	0.43018282
Cenpt	0.40759866	2.40118375	1.67801034	0.21000812	0.43020139
Zfp185	-0.4189127	3.37363519	1.67776108	0.21004065	0.43020139

Phf1	0.4295949	2.86487896	1.67722349	0.21011084	0.43027156
Soat2	-0.442084	1.76274704	1.6769285	0.21014937	0.43027689
Zfp385a	0.32399801	6.61742759	1.67591231	0.21028216	0.43031087
Sephs2	0.24709273	4.04139257	1.67561836	0.2103206	0.43031087
Lypd1	-0.2870158	4.83227986	1.67522832	0.21037161	0.43031087
Toe1	-0.3506548	2.66854513	1.67513134	0.21038429	0.43031087
Galns	-0.6456947	0.66042022	1.67501935	0.21039894	0.43031087
Tmem50b	0.16393057	5.85195413	1.67500193	0.21040122	0.43031087
Itgb3	0.38912714	2.3627299	1.67480608	0.21042684	0.43031087
Ranbp6	-0.2356543	6.39165545	1.67430493	0.21049243	0.43031087
Tep1	-0.4394551	2.96774896	1.67407418	0.21052263	0.43031087
Sugt1	0.19264416	5.59679068	1.67405391	0.21052529	0.43031087
Bcl2l15	-1.4369577	-0.0955266	1.67312857	0.21064648	0.43043772
LOC106740	-0.2686176	4.1747146	1.6730312	0.21065923	0.43043772
Rab5b	0.16925718	7.01530066	1.67245506	0.21073474	0.43051855
Arhgap27os3	-1.6674417	-1.6423363	1.67212142	0.21077848	0.43053446
3010026O09	0.34091847	3.29919568	1.67110087	0.21091235	0.43073007
Trim9	0.25084133	8.22979145	1.67056973	0.21098206	0.43073007
Xk	0.26184349	5.23281007	1.67037056	0.21100821	0.43073007
Ralgapb	-0.2008382	7.62537635	1.67029509	0.21101811	0.43073007
Pde8b	-0.1947095	6.1734771	1.66973991	0.21109103	0.43080548
Xcr1	-0.5279332	1.55130213	1.66902997	0.21118431	0.43087113
Gpn1	-0.2368018	3.93186967	1.66894747	0.21119516	0.43087113
Exd2	-0.214983	5.36403923	1.6683703	0.21127104	0.43092083
Scap	0.25933315	4.49942401	1.66814306	0.21130093	0.43092083
1700010I14R	0.72545721	-0.0902284	1.66779806	0.21134631	0.43092083
Lrrn2	0.28835799	4.65525531	1.66743282	0.21139437	0.43092083
Armc2	-0.4543728	1.77471484	1.66712871	0.21143439	0.43092083
1700048O20	0.38863855	1.87947772	1.66699582	0.21145189	0.43092083
Pqbp1	0.20143916	4.38254597	1.66674117	0.21148541	0.43092083
Sf3b2	0.18380411	6.8265091	1.66633448	0.21153897	0.43092083
Nxn	0.34316143	5.05717024	1.66585246	0.21160247	0.43092083
Dyx1c1	0.47351706	2.02333161	1.6657869	0.21161111	0.43092083
Lins	0.31179704	3.94188219	1.66575489	0.21161533	0.43092083
Podn	0.29293349	5.0364247	1.6653034	0.21167483	0.43096872
Dpm2	0.22405003	4.06808179	1.66497703	0.21171786	0.43098306
Aldh1a3	0.93056052	-0.040302	1.66333983	0.21193387	0.43134946
Casp7	-0.4510678	2.71536491	1.66260575	0.21203082	0.43147344
Gm6313	-0.5625565	1.20104026	1.66162711	0.21216015	0.43160006
Arhgef15	0.41420569	2.8955382	1.66158947	0.21216512	0.43160006
Asphd2	0.31811048	2.68261618	1.6609628	0.21224799	0.43167715
Hydin	-0.8615419	0.40792578	1.66074568	0.21227671	0.43167715
Pfn4	0.45066077	1.95490093	1.66010906	0.21236095	0.43167715

B2m	0.26512093	9.02542822	1.66009125	0.21236331	0.43167715
Arhgef25	-0.2109494	5.31720204	1.65986959	0.21239265	0.43167715
Dr1	0.1985931	5.49912096	1.65966836	0.21241929	0.43167715
A330040F15	-1.0319275	-0.8518091	1.65852223	0.21257112	0.43191239
Katnb1	0.37760261	2.94699057	1.65811084	0.21262564	0.4319499
Sdf2l1	0.32708143	2.66460887	1.65754295	0.21270094	0.43202959
4932443I19R	1.62812312	-1.4886467	1.65529623	0.21299918	0.4325002
Appl1	0.15505027	7.58721653	1.65520237	0.21301165	0.4325002
Cttnbp2nl	0.16704803	6.16218878	1.65477664	0.21306823	0.4325002
Plac8	1.74340393	-1.2201908	1.65463017	0.2130877	0.4325002
Hddc3	0.31727111	2.87659112	1.65421891	0.21314238	0.4325002
Ankrd34c	-0.2581394	4.32065125	1.65416662	0.21314933	0.4325002
H2-DMb2	1.73529374	-1.6585688	1.65368712	0.21321311	0.43255632
Atg9a	0.22434357	5.13802662	1.65329467	0.21326532	0.43258897
Camta2	-0.1882248	7.54902242	1.65120967	0.213543	0.43307886
Gtf2h2	0.19362916	4.9605787	1.6504246	0.21364768	0.43321778
Tbl1xr1	-0.1385145	7.63651991	1.65006827	0.21369521	0.43324081
9330158H04	1.70206639	-1.188079	1.64970481	0.2137437	0.43326578
Ptpn9	-0.1838322	5.88928776	1.64868034	0.21388047	0.43346964
Spag9	-0.1550142	8.37034525	1.64825965	0.21393667	0.43351017
Mmachc	0.21531631	4.6196112	1.64719231	0.21407932	0.43367555
Gm9767	1.07643208	-1.0617499	1.64686723	0.21412279	0.43367555
Vwf	-0.4917036	2.07504973	1.64671751	0.21414282	0.43367555
Tarsl2	0.27315062	4.5568024	1.64656566	0.21416313	0.43367555
Pcx	-0.4109845	2.01039584	1.64557756	0.21429537	0.43386996
Ptchd2	-0.3725771	2.78492461	1.64529389	0.21433335	0.43387028
Myo1h	0.98875271	-1.0196732	1.64503529	0.21436799	0.43387028
Mon1a	0.30940016	2.34691386	1.64265658	0.21468689	0.4344423
Hmg20a	0.24654083	5.9347233	1.64172282	0.21481224	0.43457074
Zfp748	-0.2146602	4.81917853	1.64164318	0.21482293	0.43457074
Pcbd1	0.37987691	1.94610971	1.64115967	0.21488788	0.43462871
Xylt2	-0.5332424	2.16298136	1.64088626	0.21492462	0.43462961
Pcdh12	-2.7233927	-1.9877816	1.64034427	0.21499747	0.43470352
Ldb3	-0.4686932	2.06945824	1.63998824	0.21504534	0.43472691
Mrps14	0.2156766	5.65995833	1.63890442	0.21519114	0.43486404
Spice1	-0.2865883	3.42151264	1.63847652	0.21524875	0.43486404
Tfr2	-0.6474591	0.97778747	1.63751784	0.21537787	0.43486404
Pkp1	0.64646948	1.20327792	1.63711664	0.21543193	0.43486404
Kcnn1	-0.4526411	1.94683146	1.63668347	0.21549032	0.43486404
Rps6ka2	-0.2224852	5.22080832	1.63664735	0.21549519	0.43486404
Cbl	0.15656781	6.56782542	1.63646526	0.21551975	0.43486404
Zkscan3	0.25065463	3.32695568	1.63641408	0.21552665	0.43486404
Vdac2	0.14782264	7.27146482	1.63606303	0.215574	0.43486404

Fgd4	0.20831831	4.93470296	1.63602676	0.21557889	0.43486404
Zdhhc14	0.24688654	4.30619587	1.63593626	0.2155911	0.43486404
Kalrn	-0.1862477	11.7778886	1.63584664	0.21560319	0.43486404
Fgfbp3	-0.3444258	2.68347118	1.63584499	0.21560341	0.43486404
Rnase1	-1.7864298	-0.8983568	1.63561671	0.21563421	0.43486404
Zfp457	-0.7795398	0.29169321	1.63544152	0.21565785	0.43486404
Prss22	1.22535791	-1.6286607	1.6349865	0.21571927	0.43491466
Pcyt2	-0.4766671	1.66845225	1.63468739	0.21575966	0.43492287
Cox7c	0.23184957	7.31570746	1.63423189	0.21582118	0.43492827
Ntn3	0.62536734	1.52422669	1.63391906	0.21586345	0.43492827
Tmem110	-0.2801705	2.80599648	1.633861	0.21587129	0.43492827
Gpr137c	0.23470247	4.87523066	1.63339363	0.21593446	0.43497846
Pabpn1	-0.1779297	5.54509059	1.633043	0.21598186	0.43497846
Syce2	0.52129038	1.79897998	1.63268492	0.21603029	0.43497846
Mex3b	-0.2925236	3.37780557	1.63260206	0.21604149	0.43497846
Kpnb1	0.14806339	8.29382347	1.63222948	0.2160919	0.43500681
8430427H17I	0.15642639	6.53254101	1.63046918	0.21633024	0.43534243
Raver2	-0.2864235	3.14227939	1.63027504	0.21635654	0.43534243
Tmc3	-0.6767257	0.99473456	1.63019291	0.21636767	0.43534243
Gstp1	0.21267765	4.93816257	1.62871832	0.21656763	0.43558448
Mynn	-0.1740495	5.5697216	1.62846746	0.21660167	0.43558448
Fam98c	1.00196214	-0.7052618	1.62845343	0.21660357	0.43558448
Rpl7l1	-0.1898547	4.58990866	1.62814919	0.21664486	0.43558448
2010106C02I	-2.017134	-1.616105	1.62793965	0.21667331	0.43558448
Tram1	0.24156039	5.97538072	1.62769733	0.21670621	0.43558448
Ubl4	0.14160192	6.74027419	1.6268863	0.21681637	0.43573278
Sertad1	0.3849676	2.92452952	1.62641485	0.21688044	0.43578841
Tbc1d2b	-0.2071298	4.3888856	1.62584576	0.21695782	0.43581776
Limk2	-0.2011236	5.20943889	1.62561682	0.21698895	0.43581776
Stab2	-1.519443	-1.1978547	1.62550453	0.21700423	0.43581776
Nol7	-0.2248341	5.77233861	1.62426231	0.21717328	0.43603663
Btrc	-0.1792336	6.32207989	1.62416871	0.21718603	0.43603663
Lcor	-0.3077044	3.98024282	1.62372423	0.21724656	0.43608506
Dmxl2	-0.3138234	8.52492844	1.62195118	0.21748825	0.43649695
Tecpr1	-0.3558812	3.46884039	1.62167072	0.21752651	0.43649695
Parm1	0.2272347	4.32365914	1.62138209	0.2175659	0.43649695
R3hdm1	-0.2211153	9.58790115	1.62115019	0.21759755	0.43649695
Gm960	-1.0433708	-0.5551087	1.62068128	0.21766157	0.43650884
Klhl14	0.5009838	1.43658351	1.62057284	0.21767637	0.43650884
Zfp937	-0.2169275	5.13090645	1.62027673	0.21771681	0.43651684
Fbxo5	0.73230379	0.50880187	1.61918721	0.2178657	0.43667732
Meis1	0.58049555	1.67791817	1.61915732	0.21786978	0.43667732
Zfp704	-0.1779337	6.73180824	1.61813689	0.21800934	0.43680704

Zfp51	-0.3271181	4.1864609	1.61813644	0.21800941	0.43680704
Lman2	0.19237917	5.61149008	1.61788417	0.21804393	0.43680704
Igf2os	-0.4361452	3.56174372	1.6173048	0.21812323	0.43689283
Chp1	0.14303356	7.55719185	1.61605868	0.21829393	0.43698604
1110008F13I	0.43094978	2.33546273	1.61583731	0.21832427	0.43698604
Zbtb37	-0.3739096	2.91738233	1.61564913	0.21835006	0.43698604
Fbxl3	0.14447518	7.46914678	1.61559948	0.21835687	0.43698604
Rpl27a	-0.1844579	6.97053072	1.61552835	0.21836662	0.43698604
Klhl6	-0.8523076	0.02189517	1.61511556	0.21842323	0.43698604
Speer8-ps1	-0.9133396	0.0963112	1.61510123	0.21842519	0.43698604
Atp2b4	0.23824248	7.45255742	1.61454257	0.21850183	0.43705429
Tomm6	0.26117697	5.29163217	1.61432056	0.2185323	0.43705429
Erg	-0.5958804	1.59530933	1.612965	0.21871844	0.43727332
E130308A19I	-0.2122217	5.17680017	1.61294606	0.21872104	0.43727332
Kcnq3	-0.1870954	5.53464675	1.61272538	0.21875136	0.43727332
Rgn	-2.1466852	-1.5682967	1.61202478	0.21884766	0.43732098
Msl3	0.17911117	5.48611517	1.6119033	0.21886436	0.43732098
Wdr83os	0.24694543	5.70252191	1.61175502	0.21888475	0.43732098
4930431F12I	-0.476371	2.63778753	1.61024449	0.2190926	0.43756922
Kctd12	0.14265809	7.42126274	1.61012785	0.21910866	0.43756922
Ppt1	0.17210095	6.44486495	1.61005561	0.21911861	0.43756922
Gm711	-2.0354526	-1.6191454	1.60974202	0.2191618	0.43758249
Cep170	0.19676075	7.48685712	1.60928423	0.21922487	0.43763545
Sp1	-0.1513849	7.09172639	1.60883585	0.21928666	0.43768585
Tmed3	0.3791492	2.97606323	1.60821059	0.21937287	0.43778495
Snapc1	0.15807932	5.51212667	1.60688014	0.21955644	0.4380783
Rpl23	-0.2070216	7.17334771	1.60570954	0.21971813	0.43832787
Sema7a	-0.1947619	5.34240719	1.60508351	0.21980465	0.43838286
9530091C08I	-0.5471895	4.62543707	1.60498046	0.2198189	0.43838286
Rif1	-0.2419214	5.78944348	1.60336285	0.2200427	0.43848708
Ears2	-0.7432497	0.22658193	1.6033039	0.22005086	0.43848708
A330041J22I	-0.895116	0.08516935	1.60327872	0.22005435	0.43848708
Xpo7	-0.1321776	7.18190454	1.60314807	0.22007244	0.43848708
Tenm2	-0.288987	7.00559318	1.60302422	0.22008959	0.43848708
Gm6484	-1.788927	-1.9123091	1.60240556	0.22017528	0.43848708
Mir8091	-0.9941891	-0.5753025	1.60238256	0.22017847	0.43848708
Scarna3a	-1.2513174	-0.9411287	1.60223408	0.22019904	0.43848708
Tfam	0.20879027	4.82690855	1.60206199	0.22022289	0.43848708
D830005E20	-0.6826211	-0.32603	1.60195795	0.22023731	0.43848708
Yeats2	0.17886463	5.57972497	1.60141655	0.22031236	0.43851968
Znf41-ps	0.35066363	2.72156312	1.60130622	0.22032766	0.43851968
Jrkl	-0.2555659	3.30938128	1.60104752	0.22036354	0.43851968
Rab31	-0.212815	5.56481474	1.59918294	0.22062234	0.43896176

Kcmf1	0.16503734	7.50021097	1.59723625	0.22089296	0.43923718
Mrpl43	0.23220967	5.11496143	1.59713546	0.22090698	0.43923718
Atrnl1	0.16501413	6.22704218	1.59710049	0.22091185	0.43923718
Zfp354c	0.18184171	5.84331025	1.59679832	0.22095389	0.43923718
Rpl30	-0.7315324	0.13246401	1.59676795	0.22095812	0.43923718
2900041M22	0.82332667	-0.0298824	1.5964893	0.2209969	0.43923718
Fam168b	-0.1655054	8.42329081	1.59587768	0.22108207	0.43923718
Jarid2	-0.174781	5.84144929	1.5958523	0.2210856	0.43923718
Iah1	0.34906847	3.18003593	1.59581453	0.22109086	0.43923718
Lrrk2	-0.2992407	5.84619947	1.59544787	0.22114194	0.43926578
Gps1	0.18491853	5.13884661	1.59517611	0.2211798	0.43926813
Arhgef10l	-0.2910453	3.02220245	1.5947649	0.22123711	0.4393091
Calhm2	-0.4610545	2.54101821	1.59401643	0.22134148	0.43934165
D830031N03	-0.1958558	5.26126611	1.5937127	0.22138385	0.43934165
Fam221b	0.94457639	-0.509092	1.59363469	0.22139473	0.43934165
Rnf115	0.17411524	5.90660914	1.59339903	0.22142762	0.43934165
Gtf3c1	-0.2558638	6.48782816	1.59333225	0.22143693	0.43934165
Rps27	-0.6958513	-0.165867	1.59242144	0.22156409	0.4394564
Atn1	0.16350934	7.89650302	1.5921694	0.22159929	0.4394564
Irgm2	-0.2751699	5.57099843	1.5919386	0.22163153	0.4394564
Fam3a	0.26090735	3.56031125	1.59186688	0.22164155	0.4394564
Slc39a11	0.42594077	1.72164444	1.59081121	0.22178912	0.43967618
Fhad1	0.42463091	2.56366346	1.59008795	0.22189028	0.43974912
Trim52	-1.3667661	-1.6565991	1.59002317	0.22189935	0.43974912
Egln3	0.24578027	7.45130752	1.58942764	0.2219827	0.43984151
Pik3r2	0.22482498	4.18510452	1.58909115	0.22202981	0.43986208
Ptpro	0.26646196	3.42849331	1.58869075	0.22208589	0.43990041
Wee1	0.18253378	5.51696953	1.58783581	0.22220568	0.44003959
Scrn1	0.23107622	5.45736724	1.58766489	0.22222964	0.44003959
Macc1	-1.5751302	-2.0982567	1.58568228	0.22250781	0.44039604
Sox6	0.26644698	5.00814424	1.58535024	0.22255444	0.44039604
Ptms	0.33460057	6.61621914	1.58513294	0.22258496	0.44039604
Mkln1os	-0.4890757	1.23498685	1.58480082	0.22263162	0.44039604
Pik3cb	-0.1969833	5.5200715	1.58465055	0.22265274	0.44039604
Spred2	-0.1673759	6.51431557	1.58433973	0.22269642	0.44039604
Snx4	0.16109287	6.79050908	1.58424094	0.22271031	0.44039604
1600020E01I	-0.6713446	1.1376363	1.58396985	0.22274842	0.44039604
Aldh1b1	0.71943498	0.04492689	1.58383089	0.22276796	0.44039604
Cyb5rl	-1.3074505	-0.4919336	1.58376379	0.2227774	0.44039604
Ppp1r12b	-0.3244616	5.67617441	1.58295815	0.22289073	0.44054736
Olfm3	0.22159566	4.62868668	1.58240985	0.2229679	0.4405837
Sfmbt1	-0.1605022	5.97509888	1.58214032	0.22300585	0.4405837
Rag1	-2.3226082	-1.9203626	1.58192889	0.22303563	0.4405837

Lefty2	1.52663267	-1.3405723	1.5817823	0.22305627	0.4405837
Ralbp1	0.15154597	6.77749448	1.58107022	0.2231566	0.44070918
Pum1	-0.1400729	6.90389033	1.58063087	0.22321853	0.4407588
A930004D18	-0.4488115	2.82111606	1.57968302	0.22335222	0.44079299
Uhrf1bp1l	0.21994716	7.76480923	1.57965651	0.22335596	0.44079299
Kif1b	-0.2170081	9.93094244	1.57956679	0.22336862	0.44079299
Sec14l1	-0.1663923	6.38234486	1.5794643	0.22338308	0.44079299
Stat5b	-0.2392503	4.00803531	1.57898208	0.22345114	0.44085465
Htr5b	1.40480986	-0.3098906	1.57865352	0.22349753	0.44086683
Mtss1l	0.17106419	7.05287022	1.57832252	0.22354427	0.44086683
Ppp1r3c	-0.2388054	6.08555093	1.57815631	0.22356775	0.44086683
Mtor	-0.260171	6.58603647	1.57744876	0.22366773	0.44088113
Gm6297	-0.7685489	1.00891899	1.57739367	0.22367552	0.44088113
Sod1	0.25714182	8.00215232	1.57700476	0.2237305	0.44088113
Nrbp1	0.17497887	5.94088008	1.57690823	0.22374415	0.44088113
Uqcrb	0.19694922	6.81664099	1.57680268	0.22375908	0.44088113
Strn4	-0.1775189	5.44574804	1.57599825	0.22387287	0.4410025
4931406P16I	-0.1803407	5.65674613	1.57582953	0.22389675	0.4410025
Mllt1	-0.2232194	4.59534888	1.57541364	0.22395562	0.4410025
Mad1l1	0.38383391	2.26136408	1.57532637	0.22396798	0.4410025
Rragd	-0.1371437	7.20974552	1.57401559	0.22415366	0.44129557
Gm20767	-0.5922801	1.1002341	1.57344455	0.22423462	0.44135115
Zfp7	0.40343224	2.73567721	1.57329656	0.2242556	0.44135115
Mir376a	-1.3528147	0.06383784	1.57248919	0.22437014	0.44150401
Hist1h2be	0.36278371	2.78253402	1.57212391	0.22442198	0.44153348
Aldh16a1	1.03169002	0.16075163	1.57093823	0.22459037	0.44179219
Atp5b	0.13288828	10.5802722	1.57053601	0.22464753	0.44183206
Lrrc49	-0.2508588	4.99706323	1.56672825	0.22518957	0.4428254
Mthfs	-0.3604704	2.33692126	1.56540568	0.22537823	0.44312362
Ifnar1	-0.1996582	5.75464592	1.56351755	0.22564791	0.44345987
Avl9	-0.1872622	6.00225198	1.56346683	0.22565516	0.44345987
Lclat1	0.17251909	6.05359353	1.56304715	0.22571516	0.44345987
Cntn4	0.23697398	5.2390942	1.56301732	0.22571942	0.44345987
Slc26a6	-0.9569737	-0.1905844	1.56291263	0.2257344	0.44345987
Hivep3	-0.3006517	6.88983087	1.56241238	0.22580595	0.44352768
Sap18	0.22517218	6.70105069	1.56054916	0.22607271	0.44397884
Scgn	1.73273327	-1.7738319	1.55978052	0.22618288	0.44412236
Ptplad1	0.14566897	6.68911998	1.55931441	0.22624972	0.44418078
Fam171a2	-0.3080633	2.68908278	1.55785883	0.22645861	0.444518
Zfhx2os	-0.7861202	0.39938693	1.55755066	0.22650286	0.44453201
Spata17	-1.6239498	-1.2522356	1.55700234	0.22658164	0.44461374
Gm20063	0.21755441	3.75008213	1.55672656	0.22662127	0.44461866
Stx11	0.53100125	2.10284213	1.55624209	0.22669091	0.44468245

Mlst8	0.27599226	2.91787372	1.55524102	0.22683491	0.44479087
A130010J15R	0.37833328	3.56718103	1.55494126	0.22687805	0.44479087
Zfp658	-0.3635472	2.26400875	1.5548861	0.22688599	0.44479087
Pcsk6	0.40300667	1.62600231	1.55482523	0.22689475	0.44479087
Rpl3	-0.217181	8.2228053	1.55375842	0.22704838	0.44485426
9430091E24R	-0.5330503	1.9031491	1.55367635	0.2270602	0.44485426
Zfyve21	-0.2635638	4.02987159	1.55337872	0.22710309	0.44485426
Itgb7	-1.1892057	-1.4779286	1.55328869	0.22711606	0.44485426
Adm	-0.3773912	2.24747852	1.55300173	0.22715742	0.44485426
Rs1	-1.5336816	-0.2740352	1.55269481	0.22720168	0.44485426
Mir8115	-1.0734091	-1.2524233	1.55263101	0.22721088	0.44485426
Myh11	0.78811974	2.52897226	1.55253822	0.22722426	0.44485426
Pacsin3	0.28245747	3.80814932	1.55134921	0.22739581	0.44504001
Ddr1	0.2315213	3.43016774	1.5512543	0.22740951	0.44504001
Colec12	-0.1478273	6.68445516	1.55109209	0.22743293	0.44504001
Amotl2	-0.3660851	3.80934103	1.55068208	0.22749214	0.44504001
Suv420h1	-0.1392099	6.72862008	1.5505934	0.22750494	0.44504001
Mrps21	0.23982889	5.03713123	1.54949064	0.2276643	0.445279
Ccdc114	-0.9086647	0.152478	1.54910206	0.22772048	0.44531616
Rbms3	0.19925872	7.34695524	1.54855561	0.22779952	0.445398
Sstr3	0.23535861	3.54620134	1.54804788	0.227873	0.44545823
Vegfa	-0.1978898	5.38698117	1.5478287	0.22790472	0.44545823
Kirrel2	1.43739272	-0.5200892	1.54738882	0.22796841	0.44551001
Zfp365	-0.2215697	9.10482669	1.54644429	0.22810525	0.44570469
Lrrc27	-0.3464061	3.21464008	1.54567813	0.22821632	0.44584897
AI464131	0.38112739	2.93921916	1.54391806	0.22847174	0.44626436
Dcbld2	-0.1807684	5.26479498	1.54369954	0.22850348	0.44626436
Dlgap5	-0.5671504	0.66188119	1.54339143	0.22854823	0.446279
Osbpl5	-0.2415039	3.92473795	1.54242113	0.22868926	0.4464445
Vat1	0.28295646	4.62141289	1.54229535	0.22870755	0.4464445
Cers4	0.28969079	5.54302222	1.54177075	0.22878385	0.44647616
Dnajb3	-1.0427202	-0.1025041	1.54167122	0.22879833	0.44647616
Dok1	-0.9687871	-0.2343909	1.54095429	0.22890266	0.44660698
Xist	-0.9033733	0.81075274	1.54059857	0.22895445	0.44663526
Musk	0.32047985	3.43332663	1.53955003	0.2291072	0.44676662
Oser1	0.27240723	4.12300263	1.53944822	0.22912204	0.44676662
Zfp781	-0.2067352	6.24306577	1.53936818	0.22913371	0.44676662
Obfc1	0.32216906	2.73504634	1.53883492	0.22921145	0.44678346
Cxxc4	0.20298009	6.13019003	1.53879714	0.22921696	0.44678346
Metap2	0.14139317	7.30908151	1.53847133	0.22926448	0.44680336
Fam53b	0.23399884	4.28924641	1.53821121	0.22930243	0.4468046
Hist1h2bc	0.23372992	5.43187797	1.53760234	0.22939128	0.44690503
Ppil4	0.1702034	5.95392326	1.53689346	0.22949479	0.44703395

Stoml3	-1.7058485	-1.6978164	1.53653735	0.2295468	0.44706256
Trim43b	-0.9767538	-0.7916784	1.53573677	0.2296638	0.44715939
Zfp513	0.41282132	1.85284122	1.5356862	0.2296712	0.44715939
Pum2	0.13287438	8.19361958	1.53471445	0.22981332	0.44736336
Tube1	-0.7481506	0.36225688	1.53418267	0.22989114	0.44744212
Hmces	0.36972618	2.01871036	1.53286883	0.23008356	0.44771858
Anxa3	-0.282686	5.81377732	1.53270237	0.23010795	0.44771858
Pcdhga12	-0.2961461	2.95173077	1.53217659	0.23018503	0.44779578
Sirpb1b	-1.8260195	-1.704659	1.53160495	0.23026886	0.44788611
Clip1	-0.2071482	7.59804925	1.53068296	0.23040415	0.44807648
Kcnip4	0.16455542	7.02681081	1.52953262	0.23057309	0.44820609
Timp4	-0.369339	2.45770989	1.52936921	0.2305971	0.44820609
Dtx3l	-0.1833331	4.87767254	1.52931742	0.23060471	0.44820609
Susd5	0.42079946	2.31888443	1.52921002	0.23062049	0.44820609
1700066M21	0.17585007	4.79759952	1.52807182	0.23078785	0.44845856
Nr2f6	0.34610236	2.59815813	1.52739452	0.23088752	0.44851963
Zc3h12b	0.19029491	5.05571314	1.5273492	0.23089419	0.44851963
Ncoa1	0.13526295	8.10415337	1.52656868	0.23100911	0.44865382
Fam73a	-0.1819784	6.03463599	1.52634071	0.23104269	0.44865382
Nqo1	0.41988664	4.40171669	1.52606061	0.23108396	0.44865382
Olfr55	-1.6604675	-1.2121881	1.52524395	0.23120433	0.44865382
Lrp2bp	0.79389351	0.78292561	1.52510987	0.2312241	0.44865382
Gm17660	-1.647469	-1.4513759	1.52502622	0.23123644	0.44865382
Aspn	-1.2490678	-0.6780731	1.52447782	0.23131733	0.44865382
Tfb1m	0.34905789	2.80023338	1.52447474	0.23131778	0.44865382
Spata13	0.19450915	4.58809525	1.52435398	0.2313356	0.44865382
Nrn1	0.15972492	7.30682903	1.52432447	0.23133995	0.44865382
Syn1	0.26767427	10.2351077	1.52388173	0.23140529	0.44865382
Dleu2	0.40514524	2.42882056	1.52359762	0.23144724	0.44865382
Rnf138rt1	-1.3352771	-1.5642962	1.52338155	0.23147914	0.44865382
Htt	-0.2631086	6.73840291	1.52332323	0.23148775	0.44865382
Clec5a	-0.8021393	-0.1262084	1.52242997	0.23161972	0.44881437
4833419F23I	0.89649853	-0.1610463	1.52225518	0.23164555	0.44881437
Afg3l2	0.18611605	5.7591495	1.52118806	0.23180334	0.44904745
Pglyrp3	1.71780109	-2.0173844	1.537854	0.23187072	0.44908875
Mrpl15	0.31567721	4.9439857	1.52043056	0.23191544	0.44908875
Otub1	0.26231816	5.7233998	1.51960559	0.2320376	0.44908875
Zfp954	-0.4283667	1.98684143	1.51957132	0.23204267	0.44908875
Scarna3b	-1.4467552	-1.0973364	1.51943143	0.2320634	0.44908875
1110001J03F	0.37164588	2.66001857	1.51930626	0.23208194	0.44908875
4933433G19	-0.5848532	0.90088046	1.51927102	0.23208716	0.44908875
Cherp	0.15845184	5.54780331	1.51888467	0.23214441	0.44912697
G630071F17	-0.8404015	-0.2688964	1.518526	0.23219758	0.44915727

Nudt14	0.56800968	1.06890462	1.5179905	0.23227699	0.44923831
P4ha2	-0.3686199	1.85672875	1.51764838	0.23232774	0.44926391
Lpar4	-0.4451021	2.27780024	1.51702329	0.23242051	0.44934528
Phlpp2	-0.2374331	5.35627201	1.51685922	0.23244486	0.44934528
Pias3	0.2981696	2.71943935	1.51620205	0.23254245	0.44942132
Fabp5	0.21251251	4.98497235	1.51608894	0.23255925	0.44942132
Il1rl2	-1.4239208	-1.0983278	1.51567487	0.23262077	0.44946768
Etl4	-0.1476757	7.93056652	1.51459729	0.23278097	0.44950083
Kif20b	-0.4584555	1.82200334	1.51457164	0.23278479	0.44950083
Abhd5	0.21024633	6.67456623	1.51439845	0.23281055	0.44950083
1700024G13	-2.1624067	-1.5267944	1.51431686	0.23282269	0.44950083
Gm4477	-0.8591641	-0.8045183	1.51406246	0.23286054	0.44950083
Rbm42	0.22435033	4.24635712	1.51404501	0.23286313	0.44950083
Dio2	0.18649031	5.92017166	1.51290909	0.23303224	0.44966032
Nrep	0.19315275	7.95133817	1.51282115	0.23304534	0.44966032
Fam83b	-1.0923009	-0.6156451	1.51273345	0.2330584	0.44966032
Cuedc1	-0.344907	2.60927741	1.51224775	0.23313077	0.44972748
Gabra5	0.17612294	5.66006483	1.51139243	0.23325827	0.44990097
Uba2	0.15732125	5.79945367	1.51003332	0.23346106	0.4502196
Mrpl42	0.19157264	5.90486063	1.50975225	0.23350302	0.45022802
Blm	-0.4881638	2.1997472	1.5083678	0.23370987	0.45039786
Pdcd2	0.23505987	3.87021714	1.50836123	0.23371086	0.45039786
Elmod2	-0.2415273	3.45349902	1.50823148	0.23373025	0.45039786
Nmnat2	-0.1668386	7.15343766	1.50815598	0.23374154	0.45039786
Tango2	0.23392987	3.743236	1.50710686	0.23389847	0.45060238
Ybx2	-0.8449769	-0.0781036	1.50682783	0.23394023	0.45060238
Tnip1	0.26444988	4.09255579	1.50666485	0.23396463	0.45060238
Atp6v1h	0.12919127	6.29465663	1.50636012	0.23401026	0.45060238
Arsj	0.56095917	0.86203898	1.5059996	0.23406425	0.45060238
Cenpi	-1.1116579	-0.5162075	1.50593825	0.23407344	0.45060238
Cd163l1	-1.4566984	-1.1743733	1.50513547	0.23419373	0.45070635
Fut2	-0.7577139	0.57040956	1.5050755	0.23420272	0.45070635
Daxx	0.20039497	3.87874798	1.50480593	0.23424313	0.45071169
Fam20b	-0.1865983	4.91662611	1.50430961	0.23431756	0.45078248
Cenph	-1.040077	-0.5446331	1.50372211	0.2344057	0.45087106
Abcc5	-0.3631738	4.93688186	1.50350086	0.23443891	0.45087106
Lurap1	0.41809705	1.9634037	1.50306843	0.23450382	0.45091435
Ddhd1	0.1929627	6.49345786	1.50270157	0.23455891	0.45091435
Top3b	-0.3022923	3.52638291	1.50259867	0.23457437	0.45091435
2310011J03F	0.29203141	2.93835107	1.50221986	0.23463128	0.45094028
Gcfc2	-0.4249898	2.09808493	1.5020076	0.23466317	0.45094028
Wdr20	-0.2316932	3.5679198	1.50134768	0.23476237	0.45105853
Gm128	-0.9745922	-0.8928464	1.50065375	0.23486674	0.45118667

Cfp	-0.6458599	1.41500791	1.50021797	0.23493231	0.45124025
4833412C05I	-0.7456582	0.25183559	1.49904044	0.23510961	0.45150838
Tmppe	-0.3486014	3.02552922	1.49858296	0.23517854	0.45156834
Hist2h2bb	-1.1167549	-0.8157757	1.49830987	0.23521969	0.45157496
Zfp449	0.26150399	5.20703437	1.49786497	0.23528677	0.45162783
Arl5a	-0.1581753	6.9303259	1.497627	0.23532266	0.45162783
Cox6b2	-0.5061709	2.88656334	1.49716423	0.23539246	0.45168942
Cd247	-1.7105195	-1.5631675	1.49644555	0.23550093	0.45182515
Cav1	-0.2813505	6.14573405	1.49579941	0.2355985	0.45189701
Slc41a3	0.48552978	2.46873405	1.49569775	0.23561385	0.45189701
Fkbp11	1.19870932	-1.4184632	1.4945906	0.23578117	0.45214551
Degs2	-0.5151912	1.57855019	1.49338639	0.23596333	0.45231907
Wdr44	0.19132774	4.89162934	1.4930415	0.23601554	0.45231907
Mpdu1	0.35123495	2.850041	1.49301003	0.2360203	0.45231907
Kcnj10	-0.2156329	5.29891954	1.49279701	0.23605255	0.45231907
Hmgcll1	0.27000724	3.35418801	1.49274438	0.23606052	0.45231907
Pde1c	-0.3074863	4.17786531	1.49182733	0.23619944	0.45247595
Vcan	-0.3602149	4.12263198	1.49170509	0.23621797	0.45247595
Myeov2	0.2116133	4.74117315	1.49116678	0.23629957	0.45255987
Marveld1	0.31736473	4.34164061	1.48999221	0.23647774	0.4526294
Mrm1	-0.2493077	3.09996865	1.48993511	0.23648641	0.4526294
Snapc5	-0.2235684	6.27476818	1.48972221	0.23651873	0.4526294
Trip13	0.91390425	0.72257965	1.48955306	0.2365444	0.4526294
Zfp738	0.19451366	5.23921593	1.48945023	0.23656002	0.4526294
4933400F21I	-0.8725767	1.22189907	1.48940763	0.23656649	0.4526294
Stx4a	0.20398286	6.39747626	1.48918403	0.23660044	0.4526294
5830416P10I	-0.6391269	0.35402887	1.48849125	0.23670568	0.45275841
Hpgd	-0.3195535	3.95251446	1.48809315	0.23676619	0.45276728
Zfp108	-0.3122729	3.07925753	1.48796326	0.23678593	0.45276728
Notch1	-0.3900787	2.82737551	1.48695641	0.23693906	0.45292918
Gm3219	-0.4825667	0.84330458	1.48666217	0.23698383	0.45292918
Fgfr4	-0.940437	-0.6665554	1.48666063	0.23698407	0.45292918
Gpkow	0.16353902	6.8147221	1.48622451	0.23705045	0.45298377
Lifr	0.21485276	5.94162796	1.48546534	0.23716607	0.45313239
Snca	0.1407792	7.68402649	1.48518285	0.2372091	0.45314232
Maml1	-0.1892427	5.19911972	1.48474982	0.2372751	0.4531961
Zbtb12	-0.6972956	-0.2350096	1.48432333	0.23734012	0.453248
Mybpc2	0.58542484	1.39858629	1.4837741	0.23742388	0.45326347
Inadl	-0.2130945	3.94804209	1.48364328	0.23744384	0.45326347
Lfng	-0.8727254	-0.3731663	1.48309245	0.2375279	0.45326347
B9d2	-0.4374338	1.8269852	1.48293437	0.23755203	0.45326347
Rcbtb2	-0.2296446	4.36717018	1.48278591	0.23757469	0.45326347
Slc25a1	0.3380721	3.82764877	1.48278187	0.23757531	0.45326347

Lhfpl4	-0.1563809	6.02255288	1.48159016	0.23775734	0.45353851
Tmem259	0.32210921	3.37626986	1.47921362	0.23812089	0.45415967
C77370	-0.3170829	6.42862139	1.47852182	0.23822686	0.45424679
Pde6a	-0.3905276	2.04928689	1.47842014	0.23824243	0.45424679
Commd4	0.33974784	3.50891298	1.47817022	0.23828073	0.45424749
Dgke	0.22053381	5.5125218	1.47770816	0.23835156	0.4543102
Lrrc71	-1.4381137	-1.4883196	1.47693039	0.23847084	0.45441395
Smad5	-0.1585088	6.179103	1.47685843	0.23848188	0.45441395
2810408A11I	1.07859973	-0.898105	1.47656564	0.23852681	0.45442725
Fyb	-0.3027282	4.4935111	1.47614066	0.23859204	0.45447922
Tmem245	0.20197548	5.51547541	1.47454476	0.2388372	0.45481062
Uty	-0.2025203	5.05926965	1.47451368	0.23884197	0.45481062
Ssrp1	-0.1996932	6.70649479	1.47371906	0.23896416	0.45497096
Psmb8	-0.4493944	2.65757263	1.47340763	0.23901208	0.45498984
B3galt4	1.15656623	-0.7880389	1.47177886	0.23926285	0.4553153
Dpf2	0.15665946	5.81680931	1.47165384	0.23928212	0.4553153
Aprt	0.28997942	3.50790968	1.47155658	0.2392971	0.4553153
Synm	-0.2076392	5.50732263	1.47089324	0.23939935	0.45543749
Rnf125	-0.5298997	1.24006064	1.4704608	0.23946604	0.455492
Rsbn1	-0.1578034	6.12056606	1.47008073	0.23952467	0.45553117
Piezo1	0.50479892	1.30366693	1.46857715	0.2397568	0.45590023
Eva1b	-0.5910977	0.88173957	1.46810571	0.23982964	0.45592514
Ttbk2	-0.1771945	7.80790211	1.46799606	0.23984658	0.45592514
Gchfr	-0.8948289	0.21337602	1.46756394	0.23991338	0.45592514
Zic1	0.25423651	8.07189603	1.46750707	0.23992218	0.45592514
Cul1	0.12879101	7.35300591	1.46654086	0.24007163	0.45613676
Mbtd1	-0.1818519	5.66318019	1.46607471	0.24014377	0.45620146
Eml3	0.3501532	2.79223939	1.46560249	0.24021689	0.45626798
Tmem243	0.26606821	4.06310175	1.4652058	0.24027833	0.45627345
Cebpg	-0.1549622	6.39864754	1.46509197	0.24029597	0.45627345
Tmem198	0.45052004	2.00437496	1.46417551	0.240438	0.4563965
Lrrc17	-0.7366722	0.06156484	1.46407439	0.24045368	0.4563965
Trak1	-0.1442662	7.52589125	1.46393625	0.2404751	0.4563965
Gypc	-0.3123324	5.39498822	1.46343227	0.24055328	0.45647252
Kcnmb1	-1.2280834	-1.0258641	1.46282286	0.24064785	0.45651851
Atp13a3	0.1384913	7.15130974	1.46278481	0.24065375	0.45651851
Cacna1b	-0.2383694	6.84283263	1.4609918	0.24093228	0.4569606
Uckl1	-0.263945	3.45904809	1.46079345	0.24096312	0.4569606
Snx27	0.13646386	7.29329358	1.46021008	0.24105384	0.45698852
Poll	-0.5082196	0.87699302	1.46020805	0.24105416	0.45698852
Psma8	-0.8554507	0.17721155	1.45975671	0.24112438	0.45702613
Plekhj1	0.4144131	2.19298864	1.45918932	0.2412127	0.45702613
2310047M10	-0.3385328	2.67892349	1.45903411	0.24123686	0.45702613

Gpr64	0.7546652	1.17481498	1.4589372	0.24125195	0.45702613
Lyl1	-0.8546488	-0.0769813	1.45874322	0.24128216	0.45702613
Slc35e2	0.20721393	5.59113456	1.4586096	0.24130297	0.45702613
Ovol2	-0.4115305	1.7981292	1.45818068	0.2413698	0.45704482
Dynlt3	0.18099	9.11236474	1.45805635	0.24138917	0.45704482
Padi4	1.46353191	-2.0630303	1.45639473	0.24164829	0.45746311
Ric8	-0.2061977	5.22884109	1.45573801	0.2417508	0.45748883
1700028P14I	-0.5084289	0.53112999	1.45571661	0.24175414	0.45748883
Zfp160	-0.2076582	4.46987999	1.45557355	0.24177648	0.45748883
Pim1	-0.6364954	0.33204306	1.45441052	0.24195819	0.45769064
4931403E22I	1.63436486	-1.295699	1.45440166	0.24195957	0.45769064
Tgoln1	0.19568505	6.81995248	1.45394423	0.24203109	0.45775362
Rdh14	0.21883033	5.61458882	1.45334183	0.24212531	0.45785951
Pdhx	0.17227485	5.1461394	1.45271961	0.24222268	0.45797132
Strip1	-0.195151	4.37981101	1.45228681	0.24229044	0.45802712
Xndc1	-0.2947786	3.95280623	1.45154602	0.24240647	0.45812906
Eea1	0.15148618	7.47743477	1.45145411	0.24242087	0.45812906
Elf3	-0.84663	-0.0623376	1.45040729	0.24258498	0.45829507
A530032D15	0.5621985	0.8682512	1.45032299	0.2425982	0.45829507
Igf1r	-0.1576502	6.90698274	1.44993531	0.24265901	0.45829507
Ntm	-0.1552076	7.47633413	1.44970571	0.24269504	0.45829507
Plscr3	0.52907064	1.25531177	1.44967373	0.24270006	0.45829507
Vwa5b2	-0.3491177	3.81022683	1.44913609	0.24278445	0.45838215
Grk1	-0.5212372	1.35031602	1.44797506	0.24296682	0.458641
Arpc2	0.16511458	8.49607501	1.44768828	0.24301189	0.458641
Itm2c	0.2620579	7.70303371	1.44729027	0.24307446	0.458641
Smarcd1	-0.1623513	5.84974477	1.44728849	0.24307474	0.458641
Fam120c	-0.2323029	6.59807918	1.44613212	0.24325666	0.45891195
Slc46a3	-0.474092	2.39258348	1.44568014	0.24332781	0.45891688
Helz2	-0.5817132	1.98281562	1.44562868	0.24333591	0.45891688
Klhl2	0.15410426	6.90275888	1.44487136	0.2434552	0.45898167
Slc16a12	0.3116365	4.08482467	1.44477743	0.24347	0.45898167
Tax1bp3	0.34389984	4.64836874	1.44468066	0.24348525	0.45898167
Actn1	0.31763161	4.61802019	1.44427078	0.24354985	0.45899787
Tysnd1	-0.3367646	2.27809315	1.44413981	0.2435705	0.45899787
Riiad1	0.69328729	0.2172966	1.44388409	0.24361082	0.45900163
Slc13a4	-0.274063	8.44182862	1.44330753	0.24370176	0.45902689
Tmem121	1.54892941	-1.3857168	1.44295924	0.24375671	0.45902689
Usp11	-0.1798539	7.40496028	1.44279622	0.24378244	0.45902689
Gng11	0.31428788	5.55179355	1.44265115	0.24380534	0.45902689
Hspb7	0.64322162	0.47307852	1.44258372	0.24381598	0.45902689
Psmd13	0.24664624	3.96068932	1.44218541	0.24387887	0.45902689
Slc35d1	0.19937284	4.13099089	1.44209887	0.24389254	0.45902689

Rela	-0.2388222	4.25629368	1.44184842	0.24393209	0.4590292
Dlec1	-1.3565626	-0.3980741	1.44144384	0.24399601	0.4590721
Necab3	0.24525251	5.96483057	1.44093292	0.24407676	0.4590721
Gbp9	-0.2997573	3.98239406	1.44072575	0.24410951	0.4590721
Fam26f	0.73289723	1.22710449	1.44028242	0.24417962	0.4590721
Mc1r	1.34786359	-1.8869924	1.44011427	0.24420622	0.4590721
Itprip	-0.9976739	-0.6277225	1.43987315	0.24424436	0.4590721
Pxmp4	0.24409681	3.31185349	1.43927153	0.24433957	0.4590721
Gm11437	1.43811729	-1.1134879	1.43906997	0.24437148	0.4590721
Sycp2	-0.5291367	2.48123862	1.43899727	0.24438299	0.4590721
Grhl2	0.79654034	0.49224649	1.43889155	0.24439973	0.4590721
Hc	1.69840818	-1.9135058	1.43879962	0.24441429	0.4590721
Tpk1	0.25947369	4.03306475	1.43869952	0.24443014	0.4590721
Ccdc23	0.39926603	3.16987757	1.43855377	0.24445323	0.4590721
Glyr1	0.18730001	5.78046531	1.43821486	0.24450692	0.45908263
Rpsa	-0.192051	6.48231852	1.43800978	0.24453941	0.45908263
Tmem246	-0.2072025	4.14919465	1.43779257	0.24457384	0.45908263
Leng9	0.50483445	1.11170176	1.43754575	0.24461296	0.45908411
Rnps1	0.13431661	6.25131145	1.43691145	0.24471354	0.45911893
Tal2	2.1237235	-2.0176317	1.43687204	0.24471979	0.45911893
Tmem9	0.25211666	4.96445114	1.43670351	0.24474652	0.45911893
Gm20597	-1.3906513	-0.6734254	1.43619534	0.24482716	0.45916053
Chrnb1	0.30731855	2.16982591	1.4351186	0.24499812	0.45916053
Top1	0.160574	8.05108416	1.43510147	0.24500084	0.45916053
D15Ertd621e	-0.1349356	7.09753655	1.43488789	0.24503477	0.45916053
Foxd1	0.30713328	5.52951661	1.43471929	0.24506156	0.45916053
Hnrnpa0	0.1351673	7.6352573	1.43435083	0.24512012	0.45916053
Tcf19	-0.4115954	2.42159714	1.43385912	0.2451983	0.45916053
Tmtc4	0.20367494	4.07511298	1.43351657	0.24525277	0.45916053
Fam89b	0.33590562	2.77397986	1.43318447	0.24530561	0.45916053
Tbc1d32	-0.2612556	4.73628723	1.43315029	0.24531105	0.45916053
Tatdn1	0.19312682	4.60363615	1.43310265	0.24531863	0.45916053
Isoc2a	-0.3579577	1.96736771	1.43304104	0.24532843	0.45916053
Casp12	0.36129287	4.19056482	1.4329044	0.24535017	0.45916053
Arhgef17	-0.1896433	6.61858914	1.43275597	0.2453738	0.45916053
Tmub1	0.34959415	2.31800996	1.43269914	0.24538284	0.45916053
Emc6	0.22935722	3.83980552	1.43264659	0.24539121	0.45916053
Bcas2	-0.1367214	5.77682026	1.43212588	0.24547411	0.45916053
Wdr45	0.21924539	4.46017267	1.43187361	0.24551429	0.45916053
Slc7a6	0.42770288	2.89527166	1.43182334	0.2455223	0.45916053
Akap8l	-0.3759383	3.41002979	1.43156836	0.24556292	0.45916053
Map2k7	-0.2346677	5.36639003	1.43149968	0.24557386	0.45916053
Plod3	0.39652491	2.62282767	1.43123349	0.24561628	0.45916815

Atf3	-0.959495	1.54733601	1.43072152	0.24569789	0.45923317
Nxf2	-1.4088255	-1.3173708	1.43024479	0.24577391	0.45923317
Phf10	0.16685499	5.32366642	1.43007993	0.24580021	0.45923317
Map3k8	-0.3925037	1.90323526	1.43005337	0.24580445	0.45923317
AU022754	-0.9185513	-0.2459675	1.42951727	0.24588999	0.45932133
Gm20324	-1.0254957	-0.5352857	1.42899957	0.24597263	0.45940405
Scube3	-0.7725595	1.4791475	1.42734448	0.24623708	0.45982625
Dok5	0.26939022	3.31033104	1.42688903	0.24630992	0.45989055
Csnk2a1	0.13242138	6.99510857	1.42637358	0.24639238	0.4599728
4930593A02l	1.45450792	-1.3030035	1.42611373	0.24643397	0.45997874
Dhrs7b	0.40924109	2.406399	1.42568238	0.24650302	0.46000453
Pard6a	-0.4678212	2.15301672	1.42519482	0.2465811	0.46000453
Hrh3	0.20927362	4.7445627	1.4251808	0.24658335	0.46000453
Fosb	0.43277886	4.38460971	1.42486186	0.24663444	0.46000453
Sirt5	-0.299688	2.29635344	1.42470558	0.24665948	0.46000453
Sar1b	0.16954168	5.62421325	1.42458845	0.24667825	0.46000453
Bcl3	0.54533125	0.8728901	1.4238891	0.24679036	0.46014194
Mrpl54	0.36249648	2.69776809	1.42330966	0.2468833	0.46024357
Pcdhgc3	0.3597176	4.17040003	1.42279161	0.24696643	0.46032689
11-Sep	0.13819491	7.5978951	1.42162267	0.24715415	0.46053326
Acad12	-0.907967	-0.9552592	1.42141647	0.24718728	0.46053326
Lims1	0.21305552	7.53140052	1.42090315	0.24726978	0.46053326
Insm1	-0.5039612	2.14257829	1.42063826	0.24731237	0.46053326
Cpeb2	-0.1361278	7.36638977	1.42026854	0.24737183	0.46053326
Sfr1	0.17619702	6.95960474	1.41964227	0.24747259	0.46053326
Trim65	-0.2681399	4.06082884	1.41952131	0.24749205	0.46053326
Aph1c	-0.2490693	3.93626938	1.41935076	0.2475195	0.46053326
Dtwd1	0.47709185	2.03221828	1.41920656	0.24754272	0.46053326
Foxj3	0.1725228	7.73374767	1.41918589	0.24754604	0.46053326
Prkag2	0.16856585	6.12234664	1.41917591	0.24754765	0.46053326
Card11	-0.9936049	-1.0961286	1.41905448	0.2475672	0.46053326
Fam110b	0.25931139	3.61497942	1.41892157	0.2475886	0.46053326
Rbm27	0.13054091	7.46391826	1.41867272	0.24762868	0.46053326
Cnnm4	0.37188279	2.88400049	1.41851559	0.24765399	0.46053326
Gabbr1	0.19165406	7.35813942	1.41810801	0.24771965	0.46058385
Fkbp15	-0.1873113	4.74414784	1.41754184	0.24781091	0.46062077
Mbip	-0.3193227	3.81516301	1.41739391	0.24783476	0.46062077
Raver1	0.22788911	6.00400555	1.417269	0.2478549	0.46062077
D8Ertd82e	-0.2511441	4.35499075	1.41672615	0.24794245	0.46063813
Hsd17b4	-0.2055757	5.79095532	1.41638804	0.24799701	0.46063813
Slc26a8	-0.6456387	0.88939182	1.41638609	0.24799732	0.46063813
Grrp1	-1.126522	-1.7128194	1.41625737	0.24801809	0.46063813
Patl2	-2.331618	-1.4006495	1.43113095	0.24808512	0.46069116

Paox	0.6345608	0.96696285	1.41538616	0.24815875	0.46075645
Gab2	-0.1856871	6.02991746	1.41489878	0.24823748	0.4608209
Sh3d21	-0.6105604	1.01929013	1.41455122	0.24829365	0.4608209
Il17rd	-0.3991192	2.50258226	1.41433128	0.2483292	0.4608209
Vps52	0.18914986	4.55164951	1.4142188	0.24834738	0.4608209
Mkks	0.21654869	5.11911302	1.41340065	0.24847969	0.46083404
Mapk11	0.2826157	3.69421599	1.41312377	0.24852449	0.46083404
Cdh8	0.19244342	5.4722849	1.41300005	0.24854451	0.46083404
Nr1h4	1.20037861	-0.4247243	1.41290921	0.24855922	0.46083404
Irak1bp1	-0.204954	5.9075469	1.41256774	0.24861449	0.46083404
Slc17a8	0.35898685	2.68306358	1.41252303	0.24862173	0.46083404
Pter	0.23995431	3.4557309	1.41251005	0.24862383	0.46083404
Tnfrsf1a	0.36202311	4.11588072	1.41225121	0.24866574	0.4608404
Ttc14	-0.3250439	6.58514574	1.41194885	0.24871471	0.46085983
Gpr150	-0.50581	0.66432843	1.41116128	0.24884232	0.46102496
Fopnl	-0.2225355	4.94606246	1.41078702	0.24890299	0.46106603
Bok	-0.2411676	4.24152508	1.41002439	0.24902668	0.46122382
Pbx2	-0.2112275	4.84810198	1.4096479	0.24908777	0.46126563
4930509E16I	-1.4789191	-1.2491604	1.40886753	0.24921446	0.46137699
Trim43a	-0.7892436	-0.2022028	1.40880288	0.24922496	0.46137699
Fam135b	0.36559617	4.6382602	1.40830857	0.24930526	0.4614038
Smyd4	0.31160706	2.62051006	1.4082394	0.2493165	0.4614038
Pisd-ps2	-0.5754595	1.82561188	1.407596	0.24942107	0.461526
Arhgap31	-0.191599	6.53570096	1.40671465	0.24956441	0.46164454
Cd38	0.31366832	2.81277897	1.40639537	0.24961636	0.46164454
Fxyd1	0.2917377	3.46058344	1.40636535	0.24962125	0.46164454
Map1lc3a	-0.2421992	4.80328015	1.40580805	0.24971196	0.46164454
Snord4a	0.5205137	0.58716041	1.40579526	0.24971405	0.46164454
Pnma3	-0.2973982	2.98502679	1.40578069	0.24971642	0.46164454
Rwdd4a	0.17621442	5.61345418	1.40495139	0.2498515	0.46174038
Cacng3	-0.1555697	5.64058481	1.40481556	0.24987363	0.46174038
Col4a1	0.25330336	3.65295433	1.40412228	0.24998664	0.46174038
1700013F07I	-0.6473289	-0.0736069	1.40387078	0.25002765	0.46174038
Agap1	-0.1803166	6.71017495	1.40329853	0.250121	0.46174038
Mdga1	-0.4168593	2.48382082	1.40327927	0.25012414	0.46174038
Tsen34	-0.23535	4.22262638	1.4032252	0.25013297	0.46174038
Spata2l	0.24042084	4.60710982	1.40307682	0.25015718	0.46174038
Gm4371	1.29043792	-0.7745846	1.40292562	0.25018186	0.46174038
Cul4b	0.13945467	7.03109806	1.40280637	0.25020132	0.46174038
Zfp652	-0.1575082	6.34153792	1.40273118	0.2502136	0.46174038
Yipf1	0.29622535	3.00966955	1.40254681	0.25024369	0.46174038
Ppcdc	-0.3243131	2.87627421	1.40177595	0.25036959	0.46174038
Nipal3	0.24681664	4.4565347	1.40173479	0.25037632	0.46174038

Vps35	0.14078236	6.730399	1.40139366	0.25043206	0.46174038
Dsc2	0.85459892	-0.1495148	1.4010139	0.25049414	0.46174038
Dclre1a	-0.2908701	3.56079589	1.40100638	0.25049537	0.46174038
Uba1	0.13909403	7.91621844	1.40094578	0.25050527	0.46174038
1700006F04l	-1.420838	-0.7678995	1.40091942	0.25050958	0.46174038
Ifitm7	-1.1140956	-1.6271057	1.40073712	0.25053939	0.46174038
Snx22	-0.759921	-0.2486315	1.39996956	0.25066494	0.46190069
Qtrt1	-0.3311677	2.71706356	1.39895023	0.25083181	0.46198471
Gm5796	-0.837644	-0.9775086	1.39888547	0.25084241	0.46198471
Ufsp1	0.42895574	1.89725205	1.39876792	0.25086167	0.46198471
Nup62	0.18994186	5.49699393	1.39874847	0.25086485	0.46198471
Gbx2	-1.5461176	-1.7696169	1.39821686	0.25095195	0.46207405
Nrd1	-0.1409828	7.74028781	1.39766984	0.25104161	0.46210682
Tmem205	0.43344468	1.69329535	1.39763746	0.25104692	0.46210682
Fam57b	0.40205535	2.79398044	1.39695943	0.25115811	0.46222172
Cox6c	0.19223829	7.55381088	1.39649863	0.25123372	0.46222172
Nnmt	-1.0666152	-0.2419865	1.39631354	0.2512641	0.46222172
Acot12	1.63442624	-1.5535456	1.39612492	0.25129506	0.46222172
Zfp820	0.49454515	1.39759634	1.39608068	0.25130232	0.46222172
Lrrc4c	-0.1887974	8.09940003	1.39559482	0.2513821	0.46229746
Nexn	-0.2404513	5.68358116	1.3946829	0.25153193	0.46250197
Zcchc18	0.16338149	7.20061704	1.39444322	0.25157133	0.4625034
Pak1	0.13450973	9.92302229	1.39314636	0.25178464	0.46282453
Gins3	0.55755265	1.21726451	1.39261147	0.2518727	0.46288667
Usp51	0.58873212	1.25592002	1.39247141	0.25189576	0.46288667
Arl8b	0.12572415	7.95545664	1.39206807	0.25196219	0.46293771
Loxl1	0.46015163	1.84589249	1.39156024	0.25204586	0.46302041
Sgcb	0.21089926	4.81756868	1.39000466	0.25230239	0.46328851
Gabrg3	-0.3454297	4.67302753	1.38996823	0.2523084	0.46328851
Ap2a1	0.24170831	4.06991428	1.38937023	0.25240711	0.46328851
Rmdn1	-0.2836941	3.73525051	1.3891973	0.25243566	0.46328851
9530077C05l	0.36235484	2.2458995	1.38859485	0.25253517	0.46328851
Zdhhc5	0.18019159	5.9280113	1.38849293	0.25255201	0.46328851
Tmem180	0.3476355	2.4419611	1.38840127	0.25256716	0.46328851
Gm1564	0.5331187	1.12274621	1.38816273	0.25260658	0.46328851
Adam32	-0.9689001	-0.4974968	1.38806961	0.25262197	0.46328851
Prkar2b	0.16309268	5.63127322	1.38794924	0.25264187	0.46328851
Galt	0.19252686	4.43305	1.38789742	0.25265043	0.46328851
Nit2	0.32390419	2.27151986	1.38731617	0.25274655	0.46328851
Vmac	-0.3176294	3.71146309	1.38728071	0.25275241	0.46328851
Glb1l2	-1.1171966	-0.4162008	1.38669965	0.25284854	0.46328851
Map2k2	-0.2367972	4.35852667	1.38641008	0.25289647	0.46328851
Klra2	-0.9546966	0.277556	1.38626506	0.25292047	0.46328851

Zc3hav1l	-0.2107631	6.00486285	1.38626372	0.2529207	0.46328851
Map4k3	-0.159498	6.59915635	1.38587254	0.25298546	0.46328851
Brinp3	-0.203279	4.53933013	1.38538707	0.25306587	0.46328851
Stk11ip	-0.3056116	3.48361789	1.38506359	0.25311947	0.46328851
Fam19a3	1.04676211	-0.4615225	1.3998261	0.25312054	0.46328851
Bcorl1	0.18052057	4.54898799	1.38495111	0.25313811	0.46328851
D19Bwg1357	0.16647181	5.51629324	1.38490153	0.25314633	0.46328851
Rab27b	0.19539723	5.41007318	1.38465345	0.25318745	0.46328851
Trim25	-0.2171527	6.41306615	1.38455557	0.25320368	0.46328851
Fendrr	-1.2436282	-0.3723177	1.38440086	0.25322933	0.46328851
Fam19a2	0.17907078	5.53212453	1.38403211	0.25329048	0.46328851
Mdk	0.35750442	3.66848016	1.38393388	0.25330677	0.46328851
05-Mar	0.12993773	6.50440038	1.38389218	0.25331369	0.46328851
Kctd3	-0.2079602	5.13243541	1.3833933	0.25339646	0.46336913
Fnta	-0.1953823	6.63782903	1.3828574	0.25348541	0.4634263
Smim12	0.35338489	2.10273062	1.38273872	0.25350512	0.4634263
Tbc1d10c	1.11877336	-0.871725	1.38197253	0.25363238	0.46358817
Mettl22	0.38829411	2.59116622	1.3812776	0.25374787	0.4637285
Kif7	-0.5428528	1.49510603	1.38035261	0.25390171	0.46388101
Kcnf1	-0.2314455	5.28870068	1.38031004	0.2539088	0.46388101
Epg5	-0.3008036	5.48609808	1.37975744	0.25400076	0.46391428
Tmem35	0.26284036	3.85577349	1.37973509	0.25400448	0.46391428
Acat3	-0.6401143	0.69937389	1.37920625	0.25409254	0.46396517
Cntnap3	-0.4355572	1.93386135	1.3788691	0.2541487	0.46396517
Fbxo47	0.53832768	1.25856779	1.378574	0.25419786	0.46396517
Tesk2	0.34274482	2.09730237	1.37813506	0.25427102	0.46396517
Pla2g12a	-0.2671143	3.72783429	1.37764954	0.25435197	0.46396517
Iscu	0.20718664	6.3829146	1.37741358	0.25439132	0.46396517
Ankrd11	-0.1319054	8.84875745	1.37705739	0.25445075	0.46396517
Exoc2	-0.188493	5.89155414	1.37681832	0.25449064	0.46396517
Polr3a	-0.2292106	3.97303105	1.37670976	0.25450876	0.46396517
Tnfaip3	-0.360538	2.85126605	1.37641418	0.25455809	0.46396517
Slc25a26	-0.2429608	3.09585486	1.37640395	0.2545598	0.46396517
Uspl1	0.15745656	5.10207478	1.37640241	0.25456006	0.46396517
Cenpl	-0.3916	1.72929489	1.37637329	0.25456492	0.46396517
Bcmo1	-1.6172132	-1.5031222	1.37631448	0.25457474	0.46396517
Vwa5b1	-0.6622229	0.4933153	1.37607486	0.25461475	0.46396749
Arl1	0.27205492	3.68099753	1.37550204	0.25471043	0.46402462
Dynlt1f	-0.4784293	0.61524014	1.375274	0.25474853	0.46402462
Cdkn2aipnl	0.19893074	4.48162468	1.37519132	0.25476235	0.46402462
Cdkl1	0.38781373	2.6430747	1.37488086	0.25481424	0.4640337
Zdhhc3	-0.159438	5.5882829	1.37455289	0.25486907	0.4640337
Wdr96	-0.5488562	1.22536066	1.37412899	0.25493996	0.4640337

Kctd21	0.23800067	3.31513064	1.37410827	0.25494342	0.4640337
Ccdc135	0.82977248	-0.2724766	1.37384295	0.25498781	0.4640337
Lat	-1.2878476	-1.0664694	1.37377118	0.25499982	0.4640337
Rftn1	0.50560586	1.5953295	1.37350614	0.25504417	0.4640439
Nup214	-0.1814728	6.07547627	1.37302201	0.25512521	0.46412083
Timm8b	0.17013788	5.55387668	1.37212672	0.25527516	0.46429998
4930452B06	0.28085013	3.51300428	1.37197116	0.25530123	0.46429998
4930447A16	1.68388026	-1.7228355	1.3707072	0.25551315	0.46455617
Nck2	0.20118978	4.64343953	1.3706683	0.25551968	0.46455617
Mtch2	0.14137655	5.59819807	1.36952481	0.25571161	0.46483454
Gm765	-0.3235306	2.83983776	1.36896795	0.25580514	0.46487874
Nmt2	0.14188809	6.98586461	1.36856952	0.25587209	0.46487874
Dus4l	0.3505345	2.58436004	1.3682917	0.25591879	0.46487874
Edaradd	1.67880751	-1.1186257	1.36822715	0.25592964	0.46487874
Syn3	0.22865539	6.89338645	1.36822495	0.25593001	0.46487874
Ythdc2	-0.2508883	5.51634512	1.36791111	0.25598278	0.46490407
Zdhhc22	0.32435773	2.47904651	1.36677456	0.25617399	0.46511914
Fam179a	1.06423874	-0.4688586	1.36650513	0.25621935	0.46511914
Bcl11b	0.25983261	6.10353451	1.36613044	0.25628244	0.46511914
Tmbim1	-0.2365672	5.07240249	1.36585935	0.25632811	0.46511914
Mfsd1	0.27407772	5.17209041	1.3658557	0.25632872	0.46511914
Spock2	0.19673589	8.11714131	1.36582299	0.25633423	0.46511914
Ccdc77	0.27545295	3.53799165	1.36506018	0.25646278	0.46528189
Ranbp10	-0.192805	4.71513143	1.36455966	0.25654717	0.4653645
Rab40c	-0.2967676	2.90796542	1.36420948	0.25660623	0.46540115
Agpat3	0.207527	4.96856857	1.36382878	0.25667047	0.46544715
Bhlhe41	0.18802993	7.69616051	1.36349078	0.25672751	0.46545001
Degs1	0.21620617	6.56259852	1.36286041	0.25683395	0.46545001
Chordc1	0.14258191	6.72134123	1.36209607	0.25696308	0.46545001
Dusp16	-0.2446309	4.92493437	1.36202955	0.25697432	0.46545001
Galnt9	0.21119419	4.68290137	1.36201564	0.25697667	0.46545001
2810032G03	0.40936076	2.61698762	1.36200755	0.25697804	0.46545001
Ptpn21	-0.2180281	4.8586225	1.36198324	0.25698215	0.46545001
Ypel1	0.31672919	3.12855469	1.36197837	0.25698297	0.46545001
Bccip	-0.2098609	5.57108292	1.36066832	0.25720452	0.46564732
Ncapg2	-0.3584167	2.56099685	1.36066517	0.25720505	0.46564732
Ptma	-0.2288689	9.04456798	1.36029495	0.2572677	0.46564732
Ptgs2os	0.59869407	0.78329448	1.36023024	0.25727866	0.46564732
Mum1	0.39715031	2.98551717	1.35955234	0.25739344	0.46564732
Chchd6	-0.2608579	3.43106469	1.35846672	0.2575774	0.46564732
Cnot4	0.17316997	7.41731286	1.35844647	0.25758083	0.46564732
Arid5b	-0.1574413	6.23806089	1.35837787	0.25759246	0.46564732
Frs3	0.38610544	2.42524315	1.3583616	0.25759522	0.46564732

Arhgap9	-0.8463675	0.95300811	1.3582249	0.2576184	0.46564732
Wdr82	-0.1339464	6.39638875	1.35817294	0.25762721	0.46564732
Slc22a21	0.94144436	0.03731727	1.35813533	0.25763358	0.46564732
Steap3	0.29100683	4.09819426	1.35768005	0.2577108	0.46564732
Akap17b	-0.17832	5.61061691	1.35754968	0.25773292	0.46564732
Zfp791	0.33067686	2.83005514	1.35725529	0.25778288	0.46564732
Stx7	0.14864242	7.46022408	1.35707534	0.25781342	0.46564732
Tcam1	2.0796631	-1.9937716	1.37130634	0.25782553	0.46564732
Slx4ip	0.2916396	3.03855891	1.35650424	0.25791037	0.46564732
Ttc22	-1.7264161	-1.6064773	1.35635158	0.2579363	0.46564732
Mir3473f	-1.3753396	-1.3801563	1.3562441	0.25795455	0.46564732
Nmi	-0.394214	3.19911829	1.35610291	0.25797854	0.46564732
Stc1	-0.3843451	2.90925401	1.35607615	0.25798308	0.46564732
Olfr558	-1.6397824	-1.0715801	1.35605776	0.25798621	0.46564732
Zc3h11a	-0.1298291	6.83947605	1.35526978	0.25812012	0.46574059
Mfi2	-0.976876	-0.3768667	1.35460634	0.25823293	0.46574059
Pla2g4c	0.72798179	0.09718778	1.354318	0.25828198	0.46574059
Clec4a2	-0.7950532	-0.2856982	1.35413372	0.25831333	0.46574059
Maoa	0.18102534	4.91687969	1.35403862	0.25832952	0.46574059
Satb2	-0.1979419	7.21935707	1.35401563	0.25833343	0.46574059
Gzmm	1.46204735	-1.6068137	1.35376992	0.25837525	0.46574059
Pitpnc1	0.13592959	6.72428963	1.3537609	0.25837678	0.46574059
Dlx4	-1.858761	-1.9372752	1.35369562	0.2583879	0.46574059
Hltf	-0.1947353	4.91250401	1.35270424	0.25855673	0.46590585
Slc35a5	0.18870375	5.44790196	1.35204269	0.25866947	0.46590585
Hist1h2bl	0.33170377	3.11037497	1.35203241	0.25867122	0.46590585
Tdrd7	-0.1770064	4.68204311	1.35183644	0.25870463	0.46590585
Stxbp5l	-0.3239933	8.44935517	1.35119341	0.2588143	0.46590585
Csrnp1	0.48813796	1.76997254	1.3510316	0.2588419	0.46590585
Hist3h2ba	0.95160921	0.02817008	1.35091098	0.25886248	0.46590585
Fsd2	1.52229605	-1.7199672	1.35019973	0.25898388	0.46590585
Nhlh2	-1.1349769	-0.6575748	1.35001592	0.25901527	0.46590585
Ube2f	0.20231541	4.74670963	1.34965834	0.25907634	0.46590585
Ptpn3	0.18694583	6.04739918	1.3496352	0.25908029	0.46590585
Dnah8	0.55129974	1.34852445	1.34959686	0.25908684	0.46590585
2610524H06I	0.3080863	2.15892431	1.34947937	0.25910692	0.46590585
H2-M3	-0.4524819	2.40479271	1.34943026	0.25911531	0.46590585
Ccno	1.27668438	-0.7397575	1.34914975	0.25916324	0.46590585
L1cam	0.2742665	6.82476182	1.34896242	0.25919525	0.46590585
Vstm2l	0.6386282	-0.1881095	1.34876545	0.25922892	0.46590585
Olfr287	0.94497604	0.25108927	1.34872	0.25923669	0.46590585
Aimp2	0.25913473	3.29249552	1.34861394	0.25925482	0.46590585
Slc4a1ap	0.1630147	5.65214781	1.34835491	0.25929912	0.46590585

Fjx1	0.20712464	4.08630098	1.34827002	0.25931363	0.46590585
Lpp	0.16391248	7.6917757	1.34814233	0.25933547	0.46590585
Rell1	0.28349147	5.182429	1.34731267	0.25947743	0.46609096
4921536K21I	-0.9087011	0.51703229	1.34666491	0.25958834	0.46620701
St3gal3	0.25676492	3.19153663	1.34648064	0.2596199	0.46620701
Sfxn2	0.30228934	2.52378903	1.346209	0.25966643	0.46622067
Eif4e2	0.17552335	5.28784106	1.3457152	0.25975105	0.46630269
Zbtb38	-0.1371518	6.89674946	1.34486009	0.25989767	0.46641488
Tyk2	0.34817289	2.82495666	1.3443057	0.25999279	0.46641488
Pik3cd	-0.3072145	2.8153947	1.34426789	0.25999928	0.46641488
Ssr2	0.39476496	3.49506716	1.34424928	0.26000247	0.46641488
Tpp2	0.18646697	6.74275554	1.34399101	0.2600468	0.46641488
Ubr4	-0.2573908	7.17668192	1.34398853	0.26004723	0.46641488
Meig1	-0.6836725	0.54987628	1.34305756	0.2602071	0.46657973
Cacna1h	-0.3145146	3.2704708	1.34299956	0.26021706	0.46657973
Clec10a	-1.1850024	-1.1430793	1.34173243	0.26043488	0.46682836
Nsun5	0.33342881	1.70862212	1.34157271	0.26046235	0.46682836
Adal	0.20950443	3.96032848	1.34151274	0.26047267	0.46682836
Fabp3	-0.2385169	3.7126582	1.34050592	0.26064594	0.467069
Sra1	0.20050454	5.14917602	1.33976283	0.26077392	0.46717821
Cers2	0.23972618	6.07253664	1.33960648	0.26080086	0.46717821
Bmp8b	-1.496365	-1.422884	1.33936327	0.26084277	0.46717821
Sema4f	-0.270858	4.77742819	1.33911526	0.26088552	0.46717821
Dnd1	1.17794978	-1.5052537	1.33902005	0.26090193	0.46717821
Ubox5	0.26410293	3.32960985	1.33875501	0.26094763	0.46719018
Plekha3	0.16956974	5.32988785	1.33769397	0.26113068	0.46744802
Eif2ak3	-0.2890643	3.26880954	1.33729471	0.2611996	0.46750151
Ccr7	1.53877533	-1.9770979	1.33663872	0.26131289	0.46752583
Cope	0.20357035	4.71358402	1.3365705	0.26132467	0.46752583
Ptrh2	0.17140327	4.96435401	1.33642627	0.26134959	0.46752583
Cs	0.11997516	7.85166602	1.33618987	0.26139044	0.46752583
Trib2	0.19210822	6.19818176	1.33608175	0.26140913	0.46752583
Rbm15	-0.1924554	3.93178982	1.33579773	0.26145823	0.46752583
Eif3c	0.12465488	8.03434266	1.3356344	0.26148646	0.46752583
Pcdh11x	0.25632602	4.67107763	1.33533518	0.26153821	0.46754854
Fbxl19	-0.270325	3.63288	1.33472944	0.26164299	0.46766606
Rab3gap1	-0.2074415	4.95786349	1.33439012	0.26170172	0.46767383
Gpc4	0.22271591	4.23406779	1.33425305	0.26172544	0.46767383
Rims2	-0.2325995	6.98291186	1.33386966	0.26179182	0.46769383
Trim33	0.12283478	7.7052077	1.33373731	0.26181474	0.46769383
Nus1	0.13250884	6.88160066	1.33173092	0.26216253	0.4681292
Gm11127	-0.2923507	3.78729841	1.33148686	0.26220487	0.4681292
Fam206a	0.18060863	5.91732222	1.33123541	0.26224851	0.4681292

Aldh1a1	0.26170788	7.64304832	1.33098181	0.26229253	0.4681292
1700123O21	1.22106742	-1.0875901	1.33095665	0.2622969	0.4681292
Eya2	0.26295389	6.41533242	1.33083843	0.26231742	0.4681292
Ankfy1	-0.1683326	6.00458421	1.33075392	0.2623321	0.4681292
Zfp397	-0.1243778	5.88909395	1.33046023	0.2623831	0.46815046
Fcgrt	0.38680499	3.55292364	1.32990389	0.26247975	0.46821366
Mrpl20	0.27759972	4.0552747	1.32951672	0.26254703	0.46821366
Eif3m	0.14387229	6.20702064	1.32945482	0.26255779	0.46821366
Cops6	0.178176	5.82043294	1.32924633	0.26259404	0.46821366
Rita1	0.46662372	0.99608685	1.32910643	0.26261836	0.46821366
Htr1f	-0.4933589	1.8051509	1.32890666	0.2626531	0.46821366
Ppp6r3	-0.133282	6.4274008	1.32813262	0.26278777	0.46832994
Lig3	0.20227019	4.99211735	1.32804802	0.26280249	0.46832994
Gbp8	0.49250949	1.80266192	1.3278575	0.26283565	0.46832994
Zfp68	-0.1944101	6.18202329	1.32742666	0.26291066	0.4683939
Spa17	0.44143846	2.41346414	1.32713768	0.26296099	0.46839391
Csrp2bp	-0.1777132	4.95271622	1.32697753	0.26298889	0.46839391
Vezt	-0.1864584	4.86885438	1.32641185	0.26308746	0.46847181
Tatdn3	-0.2799308	2.52536768	1.32623022	0.26311912	0.46847181
Tmem219	-0.4392075	2.53294204	1.32605316	0.26314998	0.46847181
Usp17la	-0.6839543	0.44794742	1.32576852	0.26319962	0.46849052
Vgll3	-0.3420673	2.95816261	1.32475979	0.2633756	0.46856042
Nlrp5-ps	0.76448147	0.46725446	1.32474606	0.263378	0.46856042
Msh6	-0.2256753	4.74445881	1.32473623	0.26337972	0.46856042
Cib1	0.41860534	1.9956303	1.32464647	0.26339538	0.46856042
Lamtor3	0.21627009	5.61417099	1.32377656	0.26354729	0.46876102
Fli1	0.27136501	4.25282813	1.32291523	0.26369782	0.46886053
Ccl25	0.572698	1.70971134	1.32289174	0.26370192	0.46886053
Jak2	0.14067334	6.25237217	1.32263158	0.26374741	0.46886053
Tigd3	0.74127369	-0.2656558	1.32240335	0.26378732	0.46886053
Abhd10	0.21627291	4.33952825	1.32233664	0.26379899	0.46886053
Gpr179	-1.3239687	-0.4016833	1.32153143	0.26393988	0.46897175
Il15	0.40797599	1.90573341	1.32153136	0.26393989	0.46897175
Gm20337	-0.4592492	2.04686625	1.32121019	0.26399612	0.46900206
Clca1	-1.1512167	-0.4968022	1.32047307	0.26412521	0.46916182
LOC1005034	-1.0108425	-1.3524867	1.32008489	0.26419323	0.46921304
Npas2	-0.2794612	5.29330213	1.31975087	0.26425178	0.46924743
Psd2	0.2282601	4.92517248	1.31941772	0.26431019	0.46925506
Chml	-0.1418953	6.24860913	1.31903609	0.26437712	0.46925506
Rnf19a	-0.1427581	6.03704394	1.31828206	0.26450943	0.46925506
1700102H20	-1.4878713	-1.2506463	1.3181989	0.26452403	0.46925506
Nrm	-0.5351216	0.49915898	1.31804384	0.26455125	0.46925506
Zan	1.81704233	-2.1385419	1.31786033	0.26458348	0.46925506

Tnfrsf10b	-0.2707643	3.26675785	1.31769941	0.26461173	0.46925506
Spaca6	-0.6141936	2.96142993	1.31769412	0.26461266	0.46925506
Tunar	0.33574933	3.21410415	1.31731205	0.26467977	0.46925506
Pick1	0.22584665	3.57902935	1.31729279	0.26468316	0.46925506
Sart1	0.18752645	4.71131357	1.31727034	0.2646871	0.46925506
Ephb3	0.43175135	1.63705225	1.31689248	0.26475349	0.4693033
Myo16	-0.3117954	4.34876829	1.31651091	0.26482056	0.46935271
Rabif	0.19706434	5.84337403	1.31559703	0.26498129	0.46956808
1810055G02	0.18321965	5.57403239	1.31536955	0.26502132	0.46956952
Ndufb9	0.2024882	6.52462575	1.31497235	0.26509123	0.46960355
Tomm34	0.16686269	5.83412048	1.31476349	0.265128	0.46960355
Scn2a1	-0.2401363	8.05753643	1.31459221	0.26515816	0.46960355
Birc6	-0.262622	7.93359802	1.31306558	0.26542717	0.469888
Bmpr2	0.15990123	9.84150137	1.31271518	0.26548896	0.469888
Ccdc13	1.0056836	-0.3812154	1.31245009	0.26553573	0.469888
Gna14	-0.7090828	1.3106898	1.31239959	0.26554464	0.469888
Med24	0.17996948	4.25752126	1.31232189	0.26555835	0.469888
Hmgn2	-0.2345178	6.50993329	1.31223584	0.26557353	0.469888
Fmn2	-0.2270108	6.65774574	1.31212309	0.26559343	0.469888
Asic4	0.6953313	-0.0935885	1.31184924	0.26564176	0.46990409
Srsf4	-0.3098994	2.37820875	1.31128933	0.26574062	0.47000954
Vta1	0.19348245	5.04032592	1.30998335	0.26597139	0.47030022
Pcgf3	0.15466584	5.67129604	1.30991479	0.26598351	0.47030022
Mad2l1bp	0.42632292	1.72148209	1.30944674	0.26606629	0.47037714
Il22ra1	0.38939727	1.58253079	1.3088082	0.26617927	0.47050742
Ushbp1	-0.7223495	0.5480966	1.30778905	0.26635973	0.47075692
Crct1	1.37205076	-1.9746755	1.32040835	0.26651338	0.47090725
Ttc30a1	0.56483437	1.65587813	1.30675265	0.2665434	0.47090725
Ggct	0.23785295	4.48865281	1.30664353	0.26656275	0.47090725
Gzf1	0.16806896	5.15141052	1.30606108	0.26666606	0.47102027
Pdcl	0.18697682	5.15656467	1.30573161	0.26672452	0.47105406
Zpr1	0.18608288	4.3602774	1.3046273	0.26692059	0.47133082
Havcr2	-0.5326487	1.70023594	1.30412514	0.26700982	0.47141886
Slc13a1	1.46044495	-1.3495689	1.30389566	0.2670506	0.47142137
Lhfpl5	-1.0310788	-0.401819	1.30183841	0.26741661	0.4719979
Nbeal2	-0.3412506	1.80362844	1.30127729	0.26751655	0.47207409
Mndal	-0.3158258	2.99456607	1.30115349	0.26753861	0.47207409
Wsb1	-0.3001674	4.11688101	1.29960985	0.26781384	0.47235709
S100a10	0.31033102	5.84680724	1.29942278	0.26784722	0.47235709
Mustn1	0.36541635	3.85996265	1.29894957	0.26793168	0.47235709
Actr2	0.12288495	8.88066286	1.29887567	0.26794487	0.47235709
Zfp786	0.70962921	0.13871326	1.29849682	0.26801252	0.47235709
Dsg3	-0.983166	-0.7353267	1.29839148	0.26803134	0.47235709

Cd93	-0.4871294	3.15480574	1.29796376	0.26810775	0.47235709
Rassf5	0.18812199	4.01244149	1.29766747	0.26816069	0.47235709
Spata3	-1.0774318	-0.8067021	1.29756616	0.2681788	0.47235709
Foxc2	0.3097837	6.72668038	1.29737417	0.26821312	0.47235709
Mapk12	0.37757603	1.78775596	1.29725608	0.26823423	0.47235709
Tgtp1	-0.2885214	3.77009538	1.29695796	0.26828754	0.47235709
Exosc7	0.26526202	3.35910013	1.29691546	0.26829514	0.47235709
Dazap1	0.24300679	3.48253625	1.29683066	0.26831031	0.47235709
Elmod1	0.17582521	7.65046911	1.29676437	0.26832217	0.47235709
Atg16l2	-0.5651591	1.46873458	1.29659302	0.26835282	0.47235709
Pdgfd	0.4121103	3.02315085	1.29636567	0.26839349	0.47235709
Cd99l2	0.20221692	5.75979085	1.29627916	0.26840897	0.47235709
Dok4	-0.4358006	2.3734439	1.29600663	0.26845774	0.47237351
Grpr	0.80535674	-0.1149348	1.29548439	0.26855124	0.47241528
Itga3	-0.2933692	2.92621062	1.29540143	0.2685661	0.47241528
Akr1c12	-1.4006265	-0.1706401	1.2952131	0.26859982	0.47241528
Park2	-0.2937695	3.05760267	1.29471483	0.26868909	0.47245855
Foxf1	-1.5510365	-1.9447467	1.29457637	0.2687139	0.47245855
3200001D21I	0.97303814	0.18461242	1.29441523	0.26874278	0.47245855
Fgf7	-0.4412303	1.59761899	1.29371004	0.26886922	0.47261145
F3	0.25685631	5.60082471	1.29317476	0.26896524	0.47266198
Mien1	0.22373922	5.3825051	1.29310976	0.2689769	0.47266198
Eaf2	-0.3906831	1.50594615	1.29220823	0.26913874	0.4728544
Gdi2	0.11992449	8.14845822	1.29202006	0.26917253	0.4728544
Cyp51	0.19849087	5.33022634	1.29184014	0.26920485	0.4728544
Figf	0.57486382	1.88956967	1.2903782	0.26946765	0.47316078
Sptlc2	-0.159041	5.09740509	1.29018775	0.26950191	0.47316078
Ctse	-0.9835042	-0.3797391	1.28994295	0.26954595	0.47316078
Pex5	-0.2154024	3.90300021	1.2895974	0.26960814	0.47316078
Ap2s1	0.28177965	3.45522275	1.28956024	0.26961483	0.47316078
Diablo	-0.2678011	4.26106158	1.28955184	0.26961634	0.47316078
Mff	0.13274413	7.56692729	1.28861616	0.26978484	0.47322995
Lrrc4	-0.1784035	5.96828391	1.28851142	0.26980371	0.47322995
Gm13315	-1.4889021	-1.3209303	1.28842025	0.26982014	0.47322995
U2surp	-0.1345758	7.73191875	1.28829902	0.26984198	0.47322995
Osbpl2	0.15082237	5.46258645	1.288236	0.26985334	0.47322995
Cep120	-0.1657153	6.33522438	1.2870844	0.27006098	0.47348605
Acap2	0.15735584	6.73780263	1.2865337	0.27016035	0.47348605
Efnb3	-0.2478289	4.91469848	1.28610554	0.27023764	0.47348605
Reep3	0.22133163	7.27510265	1.28585032	0.27028372	0.47348605
Nars	0.12500501	7.85975066	1.28579381	0.27029393	0.47348605
Sec11c	-0.2019189	4.53769326	1.28571116	0.27030886	0.47348605
Psmc2	0.1258293	6.27084943	1.28477325	0.27047833	0.47348605

Arhgap17	-0.220096	4.57259103	1.28474112	0.27048413	0.47348605
Rnls	0.50470774	1.0564206	1.28468207	0.27049481	0.47348605
Gm15412	-1.9361915	-1.650277	1.2846506	0.2705005	0.47348605
Cdc42ep1	-0.5077751	2.57236804	1.28462549	0.27050504	0.47348605
Optc	-1.2353335	-1.6157922	1.28416263	0.27058874	0.47348605
Timmdc1	0.20142922	4.29248554	1.28405763	0.27060773	0.47348605
Zdhhc24	0.21621412	5.97825576	1.28394773	0.27062761	0.47348605
Dtnbp1	-0.1971348	5.32169113	1.28369558	0.27067322	0.47348605
Dlg3	-0.1973807	6.55474967	1.28312288	0.27077687	0.47348605
Dph6	0.1395654	6.23839124	1.28310411	0.27078027	0.47348605
Fbxo28	0.13189119	6.1643748	1.28285534	0.27082531	0.47348605
Cmtm5	-0.3867409	2.42396211	1.28246936	0.27089521	0.47348605
Sil1	0.35926311	2.5779536	1.28238475	0.27091054	0.47348605
Stam2	0.16034131	4.84893538	1.28222001	0.27094038	0.47348605
Adamts6	-0.4323896	1.791435	1.28212665	0.2709573	0.47348605
Sgta	0.20110588	4.96266654	1.28187304	0.27100325	0.47348605
Nras	-0.1460665	7.27390837	1.28178883	0.27101852	0.47348605
Tm2d1	0.26536688	2.9591863	1.28177539	0.27102095	0.47348605
Prune2	-0.146067	6.64228343	1.28174015	0.27102734	0.47348605
Actrt3	-1.1398703	-0.9276285	1.28132406	0.27110277	0.47354875
Rhbdd2	0.20643421	4.33556615	1.27971248	0.27139518	0.47397137
Xpa	0.21046781	5.24649638	1.27939435	0.27145296	0.47397137
Snrpd2	0.30298642	4.07892904	1.27914215	0.27149877	0.47397137
Zfp609	-0.1344308	7.47356055	1.27896573	0.27153082	0.47397137
Araf	0.13875348	7.24708396	1.27848802	0.27161763	0.47397137
Rtbdn	-0.8500384	0.38635475	1.27840358	0.27163298	0.47397137
Hhex	-0.460985	1.7525512	1.27770498	0.27176002	0.47397137
A630007B06	0.15122324	7.27132585	1.27745984	0.27180461	0.47397137
Dgat1	0.50330345	1.99018369	1.27735581	0.27182354	0.47397137
Zfr2	-0.2566478	3.76353164	1.27726862	0.27183941	0.47397137
Klf8	-0.2326278	2.88497504	1.27719096	0.27185354	0.47397137
Lpcat4	0.18254273	5.66208883	1.2771559	0.27185992	0.47397137
Srek1	-0.1641731	6.65390786	1.2767754	0.27192918	0.47397137
Nrf1	0.21634513	3.87283049	1.27672816	0.27193777	0.47397137
Asprv1	-0.7082725	0.50430577	1.27672483	0.27193838	0.47397137
Atp6v0d2	1.29530016	0.3918407	1.27649712	0.27197984	0.47397465
Insl6	0.54011305	1.1793507	1.27589813	0.27208894	0.47409578
Zfp114	-0.6915929	0.38684237	1.27561794	0.27214	0.47409805
Gpr135	-1.0118592	-0.6005813	1.2752991	0.27219811	0.47409805
Ptprc	0.45845078	1.9383955	1.27517467	0.27222079	0.47409805
Tead1	0.16977175	7.27776457	1.27502217	0.27224859	0.47409805
Iars	-0.1818624	6.46595723	1.27463721	0.2723188	0.47414773
Maz	0.21543731	3.87511503	1.27442206	0.27235804	0.47414773

Ublcp1	0.14918439	6.44773241	1.27421453	0.2723959	0.47414773
Dck	0.23366327	4.99238985	1.27345189	0.2725351	0.47432109
Ppat	-0.1904973	4.38681869	1.27299953	0.27261771	0.47432187
Ip6k2	0.18830351	4.0375015	1.27297017	0.27262308	0.47432187
Syde2	-0.3088416	3.66367295	1.27279884	0.27265437	0.47432187
Rab13	-0.2881978	4.24254159	1.27212139	0.27277817	0.47446831
Fam3c	0.13635793	6.43511488	1.27079612	0.27302057	0.47482098
Loxl3	-0.4200953	1.77272486	1.26886512	0.27337428	0.47536709
Sarnp	0.18455161	5.53756592	1.26864839	0.27341401	0.47536716
Zmat2	0.13917804	7.1416039	1.26821061	0.2734943	0.47543773
Ucp3	1.09233456	-0.2060946	1.26785841	0.27355892	0.47548103
Cacna2d4	-0.5384529	1.19254656	1.26747216	0.2736298	0.47553523
Arfgap2	-0.2647969	4.17412182	1.26687324	0.27373977	0.4756573
Epn2	0.11814551	6.4669456	1.26557789	0.2739778	0.47600185
Brat1	0.34727036	2.28411883	1.2648914	0.27410406	0.47615213
Cep70	0.27694849	4.03986928	1.26371225	0.27432111	0.47646006
6530402F18I	0.5452814	1.42045997	1.26305529	0.27444213	0.47657703
Cic	-0.1500792	6.64039487	1.2629147	0.27446804	0.47657703
Pyhin1	-0.6559429	1.688976	1.26237406	0.27456771	0.47667014
Dos	0.17895636	6.40590854	1.2619915	0.27463826	0.47667014
Slc7a7	-0.6094411	1.12585442	1.26197621	0.27464108	0.47667014
BC028528	0.42881378	1.6431558	1.2615334	0.27472277	0.47667015
Wipi1	0.24172896	4.24351571	1.26148672	0.27473139	0.47667015
Gpr139	0.87686605	-0.5549748	1.26132902	0.27476049	0.47667015
Rnf113a1	0.3137887	1.95448335	1.26055265	0.27490383	0.47684974
Ldlr	0.19674305	3.69142439	1.25859702	0.27526532	0.47730335
Gripap1	0.1856599	4.91768759	1.25819388	0.27533992	0.47730335
Fam120aos	-0.2440844	4.61883601	1.25816431	0.27534539	0.47730335
Rec8	-0.6988315	0.39427074	1.25814206	0.27534951	0.47730335
Shisa2	-0.3779507	2.48056302	1.25780536	0.27541184	0.47730335
Psmd5	0.15507551	5.36568857	1.25691691	0.27557639	0.47730335
Frem1	-0.6392925	1.21713709	1.25668554	0.27561927	0.47730335
Rnft2	0.22608665	4.54258712	1.25668545	0.27561928	0.47730335
Grk6	-0.2756192	2.89637392	1.25668504	0.27561936	0.47730335
AI854517	-0.2308672	3.70653765	1.25665754	0.27562446	0.47730335
AI607873	-0.4189877	1.61003838	1.25656626	0.27564137	0.47730335
Vsx2	1.92591785	-1.7368724	1.25641267	0.27566984	0.47730335
Utp23	-0.2074263	4.38435016	1.2561536	0.27571787	0.47730335
Bmp2k	-0.1757474	5.06824754	1.25612423	0.27572332	0.47730335
Nedd4	-0.1143733	9.5001887	1.25526138	0.27588337	0.47748126
Amer3	-0.2592271	3.42676157	1.25499007	0.27593372	0.47748126
Mtdh	0.14597604	6.76294372	1.25477124	0.27597434	0.47748126
Zc3h7a	-0.2406281	5.52498518	1.25449562	0.27602551	0.47748126

Cdc45	0.59052803	0.92619424	1.25439467	0.27604426	0.47748126
Pla2g7	0.40211621	3.67048988	1.25409142	0.27610058	0.47748126
Ank	0.15585887	6.15385939	1.25406663	0.27610519	0.47748126
Samsn1	0.61739309	0.62142411	1.25376018	0.27616212	0.47749633
Slc48a1	0.18746743	4.84507852	1.25359053	0.27619364	0.47749633
Noc2l	0.22979475	4.00868273	1.25232522	0.27642892	0.47777043
Fat2	0.39079234	2.77609445	1.25230877	0.27643198	0.47777043
Reep1	0.17954112	6.96590109	1.25193861	0.27650086	0.47780404
Gm608	-0.1454019	8.24058658	1.25177549	0.27653122	0.47780404
Ankhd1	-0.1525275	7.51690378	1.25142411	0.27659664	0.47784812
2700099C18I	-0.4708072	1.75363503	1.25077284	0.27671794	0.47797
Rab35	0.16666454	4.52165069	1.25060186	0.2767498	0.47797
Pole2	-0.7208866	1.03558652	1.25040265	0.27678692	0.47797
Crls1	0.20993864	4.55231975	1.25010546	0.27684231	0.47799673
Rcl1	-0.3994815	2.11762534	1.24955422	0.2769451	0.47806087
Pecam1	-0.5099342	1.99038698	1.24947808	0.2769593	0.47806087
Ccbl2	-0.3652865	2.49897631	1.24901876	0.27704499	0.47813987
Stambpl1	-0.2204659	3.56400113	1.24871716	0.27710128	0.4781681
Mal2	-0.1344849	6.39667895	1.24808614	0.27721909	0.47820545
Arhgef19	0.45090978	1.47445845	1.24757001	0.2773155	0.47820545
Yif1b	0.39953215	1.7760898	1.24743584	0.27734057	0.47820545
Bhlha9	1.3552441	-1.2344932	1.24731807	0.27736258	0.47820545
Esco1	0.16633894	5.65472216	1.24723845	0.27737746	0.47820545
Btbd11	0.17536555	4.43944584	1.24711542	0.27740045	0.47820545
Nupl2	0.30298789	3.28139523	1.24683254	0.27745334	0.47820545
P2rx3	-0.4948113	1.27183356	1.24660175	0.27749649	0.47820545
Ncam2	0.18239069	7.16289986	1.24641613	0.2775312	0.47820545
Bcan	-0.286245	4.67204944	1.24613732	0.27758336	0.47820545
Hsp90ab1	0.13320051	10.3381856	1.24607338	0.27759532	0.47820545
Mrap	0.45322348	1.69198266	1.24600162	0.27760874	0.47820545
Ipo9	-0.1572937	7.01402493	1.24571599	0.27766219	0.47820545
Phxr4	-0.6438611	1.70294405	1.24557154	0.27768923	0.47820545
Cbln1	0.41919221	1.84551883	1.24539705	0.27772189	0.47820545
Fam216b	0.55282643	1.06230103	1.24509882	0.27777773	0.47823284
Slc25a16	0.16417052	5.57022558	1.24422821	0.27794082	0.47844484
Des	0.62600727	0.62764967	1.24393315	0.27799612	0.47847126
Nid2	-0.2981219	3.41703602	1.24321092	0.27813155	0.47851076
Magi3	-0.1448308	6.70809646	1.24310975	0.27815053	0.47851076
Oasl2	-0.261251	7.17688143	1.24262461	0.27824155	0.47851076
Atg5	-0.1721387	4.61398251	1.24149742	0.2784532	0.47851076
Bicc1	0.22425493	6.88745803	1.24130202	0.27848991	0.47851076
Bbs7	0.2100437	4.04676063	1.24124019	0.27850153	0.47851076
Adgb	1.38093489	-0.9356772	1.24116068	0.27851647	0.47851076

Slc38a7	0.30198187	2.39339797	1.23965408	0.27879978	0.47851076
Zfp580	0.20503378	3.72006209	1.23964618	0.27880126	0.47851076
Snrpb	0.19834131	3.74360166	1.23954883	0.27881958	0.47851076
Nmnat1	0.56687598	0.27411572	1.23948654	0.2788313	0.47851076
Zfp780b	0.16169153	5.39516929	1.23939066	0.27884935	0.47851076
Ikzf1	-0.2394778	4.34495436	1.23935023	0.27885696	0.47851076
Cdadc1	0.13713947	6.00419115	1.23934551	0.27885785	0.47851076
Zfp451	-0.2100748	5.17462745	1.23933367	0.27886008	0.47851076
Lysmd3	-0.2016562	4.76163054	1.23931443	0.2788637	0.47851076
Snord99	-1.5036189	-1.8805611	1.23912107	0.2789001	0.47851076
Zfp715	-0.2101888	4.43571062	1.23883067	0.27895477	0.47851076
Phkb	-0.1829908	6.1319598	1.23874921	0.27897012	0.47851076
Abca2	-0.2274369	6.10425242	1.2386679	0.27898543	0.47851076
Ash1l	-0.1465434	9.15914459	1.23850764	0.27901561	0.47851076
Nmrk2	-0.4408682	0.66187961	1.238451	0.27902628	0.47851076
1700012D14I	-0.9724193	-0.1883919	1.2382556	0.2790631	0.47851076
Lin52	-0.173797	4.53970168	1.23812332	0.27908802	0.47851076
Greb1	-0.6200821	0.39296859	1.23792876	0.27912468	0.47851076
Bcdin3d	-0.5649267	0.61609728	1.23791542	0.2791272	0.47851076
Txndc16	0.17460242	5.40969844	1.2378828	0.27913334	0.47851076
Gm10653	-0.5548457	0.53454069	1.2376182	0.27918322	0.47851076
Prss36	-0.5975161	0.86859551	1.23742971	0.27921876	0.47851076
Drd1a	0.20925677	4.35712398	1.23725095	0.27925246	0.47851076
Znhit6	0.16230137	5.17501458	1.23722303	0.27925773	0.47851076
Nkiras2	0.91195249	0.14816501	1.23687263	0.27932382	0.4785513
Slc16a3	1.01879158	-1.1228918	1.23667391	0.27936131	0.4785513
Abca8b	-0.3477297	3.33237728	1.23597434	0.27949334	0.47858215
Lacc1	0.22075483	4.5523204	1.23564873	0.27955482	0.47858215
Igsf8	0.19025876	5.06803153	1.23542495	0.27959709	0.47858215
Clnk	0.98081676	-0.6436547	1.23540636	0.2796006	0.47858215
Card6	0.2112478	4.85767612	1.23518265	0.27964286	0.47858215
Il1b	-1.4306865	-1.7587399	1.23458403	0.27975599	0.47858215
Hmgxb3	0.24451092	3.94378601	1.23444796	0.27978171	0.47858215
Mrpl36	0.22802453	5.37409144	1.23426658	0.279816	0.47858215
Casq2	-0.8511374	-0.2411714	1.23419788	0.279829	0.47858215
5830454E08I	0.74084713	0.68021135	1.23409891	0.27984771	0.47858215
Fahd2a	-0.3097715	2.7954796	1.23377219	0.27990951	0.47858215
Crabp2	-0.3444171	6.22136604	1.23365534	0.27993161	0.47858215
Tmem132c	-0.3072416	2.32488406	1.23321664	0.28001463	0.47858215
Rfxap	-0.2661182	4.03711884	1.23319737	0.28001827	0.47858215
Rnf13	0.18198497	7.06736367	1.23296994	0.28006132	0.47858215
BC018473	1.4138273	-1.5335454	1.23295774	0.28006363	0.47858215
Paxip1	-0.1323708	6.18251122	1.23285387	0.2800833	0.47858215

Ndufa7	0.25944925	4.6453274	1.23255789	0.28013934	0.47858215
Slc22a18	0.71854984	1.18189298	1.23235383	0.28017799	0.47858215
C1ql2	1.35045985	-1.1618038	1.23235075	0.28017857	0.47858215
Pi4ka	0.20336811	8.10487444	1.23188369	0.28026705	0.47866502
Ndst3	0.25508761	4.32585703	1.23072732	0.28048629	0.47897114
Dhdh	0.18324715	6.4983225	1.230451	0.28053871	0.47899236
Chd3os	0.11925704	6.56164557	1.22944493	0.28072969	0.47919514
Fstl1	0.33473835	7.22418471	1.22925205	0.28076632	0.47919514
P4htm	0.29685019	2.39224684	1.22919306	0.28077752	0.47919514
Ralyl	-0.1265054	7.08495102	1.22774429	0.2810529	0.47959676
Samm50	0.21464261	3.50730105	1.22679288	0.28123393	0.47978835
Coa3	0.17744548	4.39961295	1.22659228	0.28127212	0.47978835
Mmd	0.12962329	7.33839218	1.22652274	0.28128536	0.47978835
Mbnl3	-0.463853	1.24336771	1.2258844	0.28140694	0.4799074
Srgap1	-0.2739522	5.14765201	1.22573556	0.2814353	0.4799074
Gipc2	-0.4597424	1.46851857	1.22488751	0.28159695	0.48011469
1700124L16F	-1.2918032	-1.713115	1.22435085	0.28169931	0.48022084
Farp1	-0.1390519	5.39111264	1.22369229	0.28182499	0.48036671
Gm10649	-1.0022876	-1.0484694	1.22346475	0.28186843	0.48037238
Sepn1	0.32831774	3.45527712	1.22289254	0.28197771	0.48049025
Gm14015	-1.259703	-0.754935	1.22263661	0.28202661	0.4805052
Tnks	-0.1753946	5.86862362	1.22233943	0.2820834	0.48053359
Snrpf	-0.3359967	2.83226967	1.22196927	0.28215416	0.48055255
Gm16894	-0.4430287	1.2872279	1.22186141	0.28217479	0.48055255
Siae	0.20279661	4.5606751	1.22115138	0.2823106	0.48068945
Bex2	0.18431982	6.92183574	1.2210215	0.28233545	0.48068945
Sphk1	-0.3569354	4.58508224	1.22050237	0.28243481	0.48075628
B3gat1	-0.1993929	6.02154827	1.22039695	0.28245499	0.48075628
Chid1	0.21658284	3.41051126	1.21956187	0.28261494	0.48093814
Pik3c3	-0.1499695	5.50158072	1.21934712	0.28265609	0.48093814
Ndufa2	0.23050327	5.45859902	1.21921031	0.28268232	0.48093814
Syp	0.22478586	8.81758959	1.21877466	0.28276583	0.4810119
Ccdc60	-1.0467598	-0.8743788	1.21855405	0.28280814	0.48101553
Car2	0.13054516	7.0909454	1.21813925	0.28288771	0.48108254
Parp3	0.24599999	4.28637517	1.21729323	0.28305008	0.4811051
Ctdspl	0.18484776	6.4620004	1.21712828	0.28308175	0.4811051
Sap30	0.28589523	3.11921207	1.21699412	0.28310752	0.4811051
Shmt2	0.28449925	2.55247759	1.21660725	0.28318183	0.4811051
Mta3	-0.1829108	5.56696149	1.21660679	0.28318192	0.4811051
Spata45	-1.3380866	-0.5529667	1.21627345	0.28324597	0.4811051
Tanc1	-0.1363908	5.909847	1.21625321	0.28324986	0.4811051
Mrpl40	0.26572147	3.57713701	1.21606008	0.28328698	0.4811051
Hsd11b2	1.8269069	-1.1414281	1.21603247	0.28329229	0.4811051

Ptch2	-1.2689605	-1.929152	1.21592283	0.28331337	0.4811051
Xrcc5	-0.2164885	4.92129584	1.21552576	0.28338971	0.4811051
1110038F14l	0.29350415	3.35510242	1.21531863	0.28342955	0.4811051
AI839979	-0.7318511	0.19634029	1.21517489	0.2834572	0.4811051
Myo1c	0.23759232	5.19518825	1.21509411	0.28347274	0.4811051
1700047A11l	-1.4938407	-1.7041524	1.21493388	0.28350357	0.4811051
Xpnpep2	1.03112359	0.25433481	1.21435964	0.28361409	0.48114992
Pdzd8	-0.1245099	7.10026311	1.21429286	0.28362695	0.48114992
Btbd16	-0.945148	-0.8711988	1.21390384	0.28370186	0.48114992
Acnat1	-0.7199431	0.1652493	1.21366242	0.28374836	0.48114992
Gapvd1	-0.1641451	6.04140785	1.21362906	0.28375478	0.48114992
Twf1	0.16990255	7.41799396	1.21354467	0.28377104	0.48114992
Zfp133-ps	-0.5233541	0.55557483	1.21251942	0.28396866	0.48141682
Polb	-0.225883	4.32665146	1.21207954	0.2840535	0.48149249
Smchd1	-0.165765	6.18192511	1.21182497	0.28410261	0.48150759
6030458C11l	-0.2238648	4.92836648	1.21155703	0.28415432	0.48152708
AI846148	0.25226559	2.95781087	1.21055367	0.28434806	0.48176489
Slc18a3	-0.8457063	0.02036782	1.21000983	0.28445315	0.48176489
Arl4a	0.18903829	7.29655897	1.20981735	0.28449035	0.48176489
Senp7	-0.1228069	6.97076646	1.20981572	0.28449067	0.48176489
Zc3h8	0.27654889	2.7597881	1.20978917	0.2844958	0.48176489
Mad2l2	0.31869839	3.11655822	1.20923942	0.2846021	0.48187676
Usp24	-0.1768314	7.4004889	1.20848801	0.28474748	0.48195055
Cacng7	-0.1724987	5.83701289	1.2083483	0.28477452	0.48195055
Pex2	0.24540326	4.2015927	1.20833779	0.28477656	0.48195055
Sh3bp2	-0.4559437	1.47945493	1.20791423	0.28485856	0.48195055
Rbm3	-0.1987282	7.90721489	1.20789835	0.28486164	0.48195055
Ece1	0.18245091	5.98034349	1.20759439	0.2849205	0.48195055
Slc12a7	-0.4603772	3.82914986	1.20755887	0.28492739	0.48195055
Kif2c	1.21115695	-1.5242468	1.206752	0.28508374	0.48202197
Pstpip1	1.1680832	-0.5833353	1.20661809	0.2851097	0.48202197
Ddit4	0.32849746	2.67190027	1.20654928	0.28512304	0.48202197
Rbm28	0.18311668	4.98405403	1.20651023	0.28513061	0.48202197
Sh3bgrl2	0.20705219	4.30173649	1.20591595	0.28524587	0.48208583
Cirbp	-0.3241587	4.87862209	1.20582106	0.28526428	0.48208583
B930003M22	-0.7833393	-0.1766797	1.20569287	0.28528915	0.48208583
Wdr45b	-0.1877212	4.47916037	1.20509265	0.28540564	0.48221464
Slc4a3	-0.3093147	3.42105113	1.20451431	0.28551796	0.48233635
Hdgfrp3	0.13098859	8.00669921	1.20373671	0.28566905	0.48252354
Melk	0.85346736	0.17902448	1.20323775	0.28576606	0.48261933
Ankrd34b	0.24708052	4.5032415	1.20269852	0.28587095	0.48262292
Myom2	-0.8445476	0.39220815	1.20238128	0.28593269	0.48262292
Nrg3	0.14649049	6.24416023	1.20190847	0.28602472	0.48262292

Josd1	0.14756511	5.03477143	1.20179855	0.28604613	0.48262292
Plekha4	0.60670924	0.34010636	1.20167645	0.2860699	0.48262292
Tslp	1.4139893	-1.0755982	1.20166333	0.28607246	0.48262292
Herc3	0.21065939	7.65233924	1.20156654	0.28609131	0.48262292
Nt5dc2	-0.5636626	2.65514329	1.20151298	0.28610174	0.48262292
Efcab2	-0.1862855	3.97879449	1.20132247	0.28613885	0.48262292
Glb1	0.31942927	3.05622481	1.20115647	0.28617119	0.48262292
Pparg	0.42533226	1.72385308	1.19976686	0.28644212	0.48299432
6820408C15I	1.03047832	-0.5140302	1.19960696	0.28647331	0.48299432
6330416G13	-0.2285865	3.76233321	1.19940661	0.28651241	0.48299432
Btbd19	-0.3454678	2.62610308	1.19883083	0.2866248	0.48309524
Plxnd1	-0.1846228	4.68512081	1.19858667	0.28667248	0.48309524
Dtwd2	0.31263713	2.91413883	1.19827377	0.28673359	0.48309524
Ldoc1	-1.1302707	-1.3494484	1.1982736	0.28673363	0.48309524
Hmox1	0.30587677	2.83955747	1.19758407	0.28686837	0.48325427
Gfod2	-0.6896517	0.02465691	1.19717435	0.28694848	0.48332122
Mecr	0.50834871	1.30272389	1.19695427	0.28699152	0.48332574
Map2k4	0.14571582	7.68792526	1.19424378	0.28752231	0.48415157
Stat4	-0.3328341	1.82879885	1.19364065	0.2876406	0.48428267
Pip5k1b	-0.3369353	2.98888508	1.19337242	0.28769323	0.48430318
Gm2061	-0.4687772	1.29447359	1.19291455	0.2877831	0.48438638
Necap2	-0.2961619	3.41780728	1.19267576	0.28782998	0.4843972
Tmem203	0.2710836	2.72407557	1.19174363	0.28801308	0.48463725
Cks2	0.57789071	0.30209845	1.19127076	0.28810603	0.48465241
Dnajb5	0.18737568	5.88746748	1.19122928	0.28811419	0.48465241
Atpaf2	0.23867818	3.16296147	1.19077354	0.28820381	0.48465241
Rpn2	0.22649761	5.31139917	1.19061103	0.28823578	0.48465241
Lmx1b	1.14549545	0.19280357	1.19043576	0.28827026	0.48465241
Ccl5	-0.5291618	1.43448762	1.190278	0.2883013	0.48465241
B830017H08	0.68928302	-0.2125688	1.19025729	0.28830538	0.48465241
Rad51d	-0.201601	5.28271466	1.18981267	0.28839289	0.48473148
Drg2	0.20679575	3.88772029	1.18916015	0.28852139	0.48487941
Ipo8	-0.1742127	5.43226085	1.18878351	0.2885956	0.48493547
Gna15	1.55306508	-1.6031651	1.18857984	0.28863573	0.48493547
Mitd1	0.28577015	3.3516068	1.18820589	0.28870945	0.48499128
Nudt7	-0.2444063	2.42761993	1.18783864	0.28878187	0.48504489
Ndufb5	0.15765284	6.73413816	1.18679295	0.2889882	0.48532339
Gabpa	0.14072407	6.71949932	1.18656505	0.2890332	0.4853309
Kdm1b	-0.2192312	4.2157133	1.18573444	0.28919727	0.48544535
Eci1	-0.2125489	3.68755631	1.18563509	0.2892169	0.48544535
Cry1	0.19889116	4.34636312	1.18560441	0.28922297	0.48544535
Hscb	-0.2952556	4.15092299	1.18514006	0.28931476	0.48553136
H2-Q5	-0.7585512	-0.6068189	1.18424719	0.28949136	0.48555173

Gm4951	-0.4119497	3.36598933	1.18422389	0.28949597	0.48555173
Zswim7	0.3111814	2.12660425	1.18391139	0.28955782	0.48555173
Wdr43	-0.1737067	4.90443713	1.18360277	0.28961891	0.48555173
Zbed6	0.11725985	6.82936848	1.18358055	0.28962331	0.48555173
Crcp	-0.2117153	4.59667811	1.18352124	0.28963506	0.48555173
Lrrn4	-1.1022607	-0.7844833	1.18347369	0.28964447	0.48555173
A230070E04	-0.2326027	4.56051307	1.18343947	0.28965125	0.48555173
Sde2	-0.1665469	4.81445549	1.18316707	0.2897052	0.48557419
Pde11a	-0.7125628	-0.3706956	1.18281301	0.28977534	0.48562379
Hdac9	-0.1280991	6.45270884	1.18208333	0.28991996	0.48569308
Fkbp4	0.21944998	5.92966816	1.18195329	0.28994575	0.48569308
Zfp39	-0.1620591	5.36008907	1.18183457	0.28996929	0.48569308
Ttll13	-1.0586836	-0.9046798	1.18145818	0.29004395	0.48569308
Zfp40	-0.1991416	4.31869583	1.18108723	0.29011755	0.48569308
C920009B18	0.52813999	0.99448876	1.18097078	0.29014066	0.48569308
1700123O20	0.24370888	3.87720246	1.18079402	0.29017575	0.48569308
Brsk2	-0.1834568	5.66464878	1.18055104	0.29022398	0.48569308
Ppp2cb	0.11990221	7.09415398	1.18049864	0.29023439	0.48569308
Fam63a	0.27697651	4.35498773	1.18039808	0.29025436	0.48569308
Srd5a3	-0.3839065	2.96750846	1.18020945	0.29029182	0.48569308
Tmem57	-0.1263594	6.12425202	1.1801513	0.29030336	0.48569308
Dnmt3a	-0.1712938	6.59707257	1.17984847	0.29036352	0.48572587
Arl11	-1.5379289	-0.8594678	1.17907159	0.29051793	0.48585277
Myl4	0.41880412	2.24549213	1.17880053	0.29057182	0.48585277
Prkcdbp	0.29956029	5.17983032	1.17867635	0.29059652	0.48585277
Ppfibp2	-0.2094807	3.58271338	1.17842477	0.29064656	0.48585277
Gm19990	-1.0622585	-0.756335	1.17834434	0.29066256	0.48585277
Olfm1	0.13199024	9.19770912	1.17824264	0.2906828	0.48585277
Fam212a	0.94140744	-0.7309088	1.17680053	0.29096993	0.48619664
Klri2	0.71440927	0.40890264	1.17672645	0.29098469	0.48619664
Slitrk6	0.53385444	0.63049086	1.17659775	0.29101033	0.48619664
Nlk	-0.1476612	8.55890243	1.17608417	0.2911127	0.48625831
Wscd2	0.24827333	3.52687442	1.17579458	0.29117044	0.48625831
Hace1	-0.1568847	5.00898893	1.17536166	0.29125679	0.48625831
Rmdn3	-0.1925987	3.79942027	1.17524584	0.2912799	0.48625831
Snx32	0.19291461	4.27697155	1.17475957	0.29137694	0.48625831
Ficd	0.27932706	2.89199918	1.17466962	0.2913949	0.48625831
Gpbp1	0.13247383	7.99359467	1.17448323	0.29143211	0.48625831
Tmem116	-0.6188939	-0.0018565	1.17440138	0.29144845	0.48625831
Plcxd2	-0.1764849	7.79209282	1.17411813	0.29150502	0.48625831
Brpf3	-0.1437749	5.26307102	1.17394206	0.29154019	0.48625831
Tmsb10	0.26426759	6.42642058	1.17360678	0.29160717	0.48625831
Rcan1	-0.1726048	5.88676338	1.17326747	0.29167498	0.48625831

Ikzf2	0.19820465	4.23824318	1.17313228	0.29170201	0.48625831
Proser2	-0.4108297	2.69453176	1.17298932	0.29173059	0.48625831
F830016B08I	0.18837095	4.1638108	1.17284114	0.29176022	0.48625831
Otulin	-0.2842241	3.54519405	1.1727577	0.2917769	0.48625831
Opcml	0.17603104	8.17137437	1.17274396	0.29177965	0.48625831
C130050O18	-1.3618248	-2.0678571	1.17266098	0.29179625	0.48625831
Zscan29	-0.1827389	5.40459204	1.17254865	0.29181871	0.48625831
Taf1a	-0.2777375	3.23536216	1.17200689	0.2919271	0.48636615
Fam195b	0.32155677	2.5685986	1.17181923	0.29196466	0.48636615
Kdm4c	-0.222397	5.52623136	1.17141578	0.29204542	0.48639004
Eftud2	-0.1777981	5.01527971	1.17134184	0.29206023	0.48639004
Ctns	-0.2839182	2.65639179	1.17035251	0.29225842	0.48662636
Lipe	0.35921041	2.00487894	1.17022792	0.2922834	0.48662636
Rai1	-0.1750078	5.70124103	1.16942515	0.29244437	0.48682668
Spata5l1	0.93629861	-0.7576528	1.16877763	0.2925743	0.48697528
Gpn2	-0.3648814	1.66041057	1.16817808	0.29269467	0.48702825
Chd5	-0.220287	6.75626534	1.1680142	0.29272758	0.48702825
Mcmbp	0.13130961	5.68264481	1.1680115	0.29272812	0.48702825
Gm19557	-1.0200865	-0.643446	1.16774797	0.29278106	0.48703052
Pet100	0.3291881	3.54535669	1.16749037	0.29283282	0.48703052
Rhpn2	-0.3233415	4.0440724	1.16735251	0.29286053	0.48703052
Ints3	-0.158381	5.30231358	1.16711199	0.29290887	0.48703052
4930429F24I	0.60093145	0.47399518	1.16685818	0.2929599	0.48703052
Ccdc32	0.2136422	4.90145932	1.16679056	0.2929735	0.48703052
Arhgap21	-0.1976419	8.18769952	1.16519362	0.29329485	0.48727686
Fam96b	0.27622355	2.67795125	1.1651559	0.29330245	0.48727686
Zmynd11	0.12039612	7.87641286	1.16497955	0.29333797	0.48727686
Tra2a	0.15159569	6.08364828	1.16476694	0.29338079	0.48727686
Rbbp7	0.14021228	7.41912342	1.16451886	0.29343078	0.48727686
Dnajc25	-0.3632277	2.95692566	1.16444107	0.29344645	0.48727686
Foxk1	-0.1378664	7.16862433	1.16380507	0.29357466	0.48727686
Ctdnep1	0.16641778	5.82205095	1.16375228	0.2935853	0.48727686
Foxl1	-0.6187789	0.5607768	1.16373683	0.29358842	0.48727686
Cwc27	-0.158183	5.33572549	1.16349707	0.29363677	0.48727686
Ier3	-0.2749233	4.1511689	1.16344435	0.29364741	0.48727686
Slc2a2	0.89944378	-0.36637	1.16342629	0.29365105	0.48727686
Ndn	0.19072058	6.22948305	1.16326669	0.29368325	0.48727686
Arntl	-0.1852705	3.98379063	1.16322662	0.29369133	0.48727686
Lsm11	-0.1849555	4.47219831	1.1623607	0.2938661	0.48740166
4930451G09	-0.4185564	1.59307176	1.16212578	0.29391353	0.48740166
Gskip	0.21106427	4.39527717	1.16202519	0.29393385	0.48740166
Fchsd1	-0.3670052	2.02506794	1.16166145	0.29400733	0.48740166
Gstcd	0.40528321	1.73762496	1.16115635	0.2941094	0.48740166

Gm20594	-0.4449218	0.45296374	1.16106454	0.29412796	0.48740166
1600012H06	0.22517614	4.59940473	1.16092776	0.29415561	0.48740166
Acer2	0.34260203	3.38377005	1.16077508	0.29418648	0.48740166
Cdc73	0.14707379	6.15338114	1.16033942	0.2942746	0.48740166
Ddx39	-0.3432595	2.37267461	1.16032236	0.29427805	0.48740166
Ptges3	0.16063592	7.90167011	1.16004711	0.29433373	0.48740166
Gdf10	0.48862089	2.35877311	1.1599822	0.29434687	0.48740166
Msi1	-0.399416	1.91976697	1.15995963	0.29435144	0.48740166
Rnaseh2a	0.34366872	2.75816186	1.15959262	0.29442572	0.48740166
Actn3	0.93589108	-0.3285975	1.15936158	0.29447249	0.48740166
4932435O22	-1.8734833	-1.8514079	1.15933016	0.29447885	0.48740166
Dcxr	0.6957631	0.43505574	1.15895251	0.29455533	0.48740166
Prdm4	-0.217399	4.99589618	1.15859825	0.2946271	0.48740166
Prr24	0.18287004	4.53434013	1.158524	0.29464214	0.48740166
Rmnd5a	-0.1373974	7.57797669	1.15838001	0.29467132	0.48740166
Gm13102	-1.0471641	-1.4191695	1.15837508	0.29467232	0.48740166
Oxld1	0.47929108	1.12722432	1.15824823	0.29469803	0.48740166
Ttc25	0.92015528	-0.1515874	1.15822553	0.29470263	0.48740166
Txndc15	0.19615873	4.33369455	1.15784632	0.2947795	0.48740831
Hibch	-0.2122127	4.11210801	1.15774409	0.29480023	0.48740831
Tbx3	-0.2389191	3.70014722	1.15760347	0.29482875	0.48740831
Irak3	-0.3609463	2.79377996	1.15738053	0.29487397	0.48741578
Ncmap	-1.3632511	-0.974297	1.156536	0.29504534	0.48763175
Fam183b	-0.8882347	0.14856088	1.15621265	0.29511099	0.48767295
Dusp19	0.20225748	4.78410897	1.1559503	0.29516427	0.4876937
Strap	0.11265908	7.46723644	1.15564835	0.29522561	0.48772776
Gm6537	1.23099545	-1.9734939	1.1551284	0.29533128	0.48777492
Timm13	0.35775785	2.54144414	1.15508997	0.29533909	0.48777492
Sugct	-0.4147383	1.49156978	1.1548292	0.2953921	0.48777492
Anpep	0.27028336	6.47703909	1.15470637	0.29541708	0.48777492
Rnaseh2b	0.24397643	4.32882291	1.15441607	0.29547612	0.48780515
Cxcl5	-0.609049	0.65863524	1.15397962	0.29556491	0.48788448
Pik3c2b	-0.170143	5.11271293	1.15320688	0.29572221	0.48807203
Ano8	0.53039264	0.67743509	1.15296518	0.29577143	0.48807203
2310057M21	0.18826374	4.09603702	1.15282101	0.2958008	0.48807203
Lrguk	0.24681225	3.24587054	1.15253268	0.29585954	0.48810171
2610034M16	0.73513355	0.1506247	1.15177457	0.29601407	0.48828938
Rnmtl1	-0.3728126	2.06520423	1.15156501	0.2960568	0.48829261
Ptger4	-0.6551915	0.6562272	1.15120645	0.29612994	0.48834598
Chad	1.96067468	-1.747927	1.15084541	0.29620361	0.48840021
Rhoa	0.24198108	8.61857226	1.15042406	0.29628962	0.48847477
Msto1	0.44820534	1.83431399	1.15017188	0.29634111	0.48849242
Abracl	0.21778306	4.25687212	1.14934897	0.29650922	0.48870226

Rictor	-0.2267306	6.50234641	1.14911773	0.29655648	0.4887129
Ascl4	1.31036709	-1.2625005	1.14884017	0.29661322	0.48873915
Hcfc1r1	0.20460056	4.47149002	1.14786809	0.29681206	0.48893893
5930438M14	0.81327873	-0.5977789	1.14784827	0.29681612	0.48893893
Wnt6	-0.4051676	3.15703096	1.14735327	0.29691745	0.48903857
N4bp2l1	0.22199666	4.03004279	1.14701681	0.29698635	0.48906293
Prkaa1	0.13851862	5.69360873	1.1466695	0.29705749	0.48906293
Mtg1	0.31800408	2.77387314	1.14655465	0.29708102	0.48906293
Morf4l1	0.14791131	8.47922785	1.14648358	0.29709558	0.48906293
Zfp35	-0.1627791	4.4899564	1.14594364	0.29720625	0.48914358
Wibg	0.44234843	2.09290631	1.14584602	0.29722627	0.48914358
Dus3l	0.22559284	3.75938333	1.1451799	0.29736289	0.48920612
Plekha1	-0.1335697	6.73437233	1.14502898	0.29739385	0.48920612
Pirt	-1.4182971	-0.890339	1.14462032	0.29747772	0.48920612
Mapk8	0.15469238	7.5751734	1.14444418	0.29751388	0.48920612
Gm12504	-0.6331933	0.86210628	1.14444094	0.29751454	0.48920612
1700008F21I	0.64443206	0.94287761	1.14435004	0.29753321	0.48920612
Epn1	0.17379723	4.47923716	1.1442672	0.29755022	0.48920612
Lcn2	-1.8888497	-1.2419215	1.14373549	0.29765942	0.48928261
Gpr88	-0.190873	6.09706618	1.14357068	0.29769328	0.48928261
Mip	-0.9942822	0.37341419	1.14309172	0.29779172	0.48928261
Mageh1	0.20462798	4.38329685	1.14307509	0.29779513	0.48928261
Txn2	0.22086886	4.68308684	1.14264511	0.29788354	0.48928261
Gt(ROSA)26	-0.2851403	2.76247461	1.14257662	0.29789763	0.48928261
Snhg7	-0.4057274	1.28793666	1.14243126	0.29792752	0.48928261
Lbr	-0.2256426	3.4404604	1.14214377	0.29798666	0.48928261
Prkd1	-0.3040197	2.50520455	1.14200308	0.29801561	0.48928261
Pip4k2c	0.15824495	6.1114067	1.14175628	0.2980664	0.48928261
Pigz	-0.3304182	2.17195228	1.1416853	0.29808101	0.48928261
Dnajc15	-0.1953194	4.25157973	1.14137643	0.2981446	0.48928261
Cdk5rap3	-0.2644367	3.15904172	1.14116454	0.29818823	0.48928261
Fuca1	0.23358633	5.15110516	1.14115213	0.29819078	0.48928261
Aloxe3	0.37022053	1.83074161	1.14106081	0.29820959	0.48928261
Tbc1d16	0.18302512	4.73877911	1.1404566	0.29833406	0.4893552
Cdk9	-0.1939008	4.38479661	1.14044937	0.29833555	0.4893552
Eif4ebp1	0.3931002	1.68990599	1.13971303	0.29848734	0.48949775
Apoa1bp	0.22008331	3.85453329	1.13927488	0.29857771	0.48949775
C1qa	-1.0336245	-1.5955507	1.1392686	0.29857901	0.48949775
Cd200r1	-0.7480656	-0.6579988	1.13923492	0.29858595	0.48949775
Mrpl9	-0.1952915	4.48985531	1.13856667	0.29872386	0.48965023
Cox7a2l	-0.1787707	7.11294571	1.13804829	0.2988309	0.48965023
Nhs	-0.2314453	3.61151607	1.13803348	0.29883396	0.48965023
Sbno1	-0.1910066	8.41056419	1.13788204	0.29886523	0.48965023

Mir3473	-1.065816	-0.4214497	1.13765914	0.29891128	0.48965023
Stbd1	0.21149216	4.08856523	1.13759621	0.29892429	0.48965023
Pip5k1c	-0.1510041	6.37559619	1.1371995	0.29900627	0.48969279
Adcyap1	0.27229718	2.9020933	1.13707479	0.29903205	0.48969279
Gja4	-0.7194164	-0.5079181	1.13684983	0.29907856	0.48970198
A830009L08I	-0.4562501	0.87970727	1.1365844	0.29913344	0.4897249
Hras	-0.2412869	5.0526213	1.13637228	0.29917732	0.48972978
Samd4b	0.13792534	6.83146274	1.1351455	0.29943123	0.49005698
Krt20	-0.2939381	3.87469395	1.13490185	0.2994817	0.49005698
Hgsnat	0.15312556	5.17726335	1.13481365	0.29949997	0.49005698
Rc3h1	-0.1198138	7.36999408	1.13440711	0.2995842	0.49012784
Rplp2	-0.2141652	5.9945226	1.133844	0.29970093	0.49023198
Per2	-0.2081965	5.05396561	1.13370514	0.29972973	0.49023198
Fastkd1	-0.2856611	2.74592134	1.1333567	0.299802	0.49027553
Cd109	0.37901132	3.07290153	1.13318204	0.29983823	0.49027553
Nelfe	-0.2590964	3.95887873	1.13294995	0.29988639	0.49028733
Msh5	-1.904816	-1.8124745	1.13239789	0.30000098	0.49031992
Sema4c	0.51471728	0.62827725	1.13224363	0.30003301	0.49031992
Gm16287	0.42485283	1.40048103	1.13206697	0.3000697	0.49031992
Rab38	1.2196416	-1.447128	1.13206508	0.3000701	0.49031992
Tmie	-0.3737788	1.66140643	1.13168265	0.30014954	0.49038282
Smagp	0.64928356	0.54925906	1.13080288	0.3003324	0.49061465
H2-Q1	-0.2813907	6.0697146	1.1302224	0.30045314	0.4906614
Muc15	0.50312045	2.10105816	1.13014586	0.30046907	0.4906614
Cpsf6	-0.1917121	5.58266669	1.12975527	0.30055035	0.4906614
Nt5c3	-0.1436461	5.14275865	1.12970794	0.3005602	0.4906614
Cyp4f13	0.43178346	1.16738394	1.12968069	0.30056587	0.4906614
Dhx9	-0.1876201	7.76847638	1.12920261	0.30066541	0.4907246
Snw1	0.1420636	6.48926014	1.12910114	0.30068655	0.4907246
Prrc2c	-0.1819526	10.0574127	1.12787463	0.30094214	0.49107482
Ccdc64	-0.569406	0.49411901	1.12707552	0.30110883	0.49127988
Cdkal1	0.21355287	4.0708894	1.12659295	0.30120955	0.49132873
Gpr87	-1.1363407	-1.7229467	1.12653894	0.30122083	0.49132873
Cd28	0.64269955	0.22626198	1.12628661	0.30127351	0.49134775
Xlr3c	0.93480451	-1.4935788	1.12573824	0.30138806	0.49146762
Tfe3	0.16958806	5.30511724	1.12520887	0.30149869	0.49158109
Vrk3	-0.341545	2.8399183	1.12491227	0.3015607	0.49161526
Gm13051	-1.0645397	-1.3911024	1.12470284	0.30160449	0.49161973
Acsl5	0.1430228	5.63286945	1.12419595	0.30171052	0.49167932
Gnat2	2.13192767	-1.6727266	1.12413557	0.30172316	0.49167932
Selt	0.15779002	8.78450393	1.12331334	0.30189528	0.49189287
Akt1	-0.186301	5.08357645	1.1226313	0.30203815	0.49205871
C4bp-ps1	-1.7322691	-1.5809795	1.12240354	0.30208588	0.49206953

Pctp	-0.329247	2.59913689	1.12215342	0.30213831	0.49208273
Champ1	-0.1740539	4.80465221	1.12197287	0.30217616	0.49208273
Cyp4b1	-0.9966739	-1.0150473	1.11995902	0.30259882	0.49270401
Trmt2a	-0.2279611	3.786557	1.11884451	0.30283307	0.49295169
Mycl	-0.3082897	2.03148345	1.11872992	0.30285717	0.49295169
Spdya	-0.4986892	0.69594156	1.11864309	0.30287543	0.49295169
Kdsr	0.1793749	4.62322859	1.11845221	0.30291558	0.49295169
Entpd6	-0.256488	3.09505747	1.11770851	0.30307209	0.49303289
Ilf3	-0.1360162	5.74793852	1.11765194	0.303084	0.49303289
Eno4	-0.413248	1.34689556	1.11740975	0.303135	0.49303289
Morn4	-0.1761081	5.72266138	1.11730864	0.30315629	0.49303289
Zfp41	-0.3026461	1.96882306	1.11723724	0.30317133	0.49303289
4933427E11I	1.32438567	-1.2907902	1.11699966	0.30322137	0.49304732
Cacybp	0.13570437	6.42728372	1.11669966	0.30328458	0.49304838
Tmc8	-1.3701663	-1.0856689	1.11660576	0.30330437	0.49304838
Tnfrsf4	-1.9086822	-2.2440682	1.11617962	0.30339419	0.4931217
Gas2	0.22430164	2.92876743	1.11600113	0.30343182	0.4931217
Syna	0.35625877	1.95238682	1.11522163	0.30359625	0.49331359
Klrb1b	-0.9853672	0.72419676	1.11505088	0.30363228	0.49331359
Papss1	0.15265318	4.96505417	1.11474524	0.3036968	0.49335147
Wwox	0.19105791	4.0488127	1.11418844	0.30381438	0.49347219
Krba1	-0.1845231	3.86026108	1.11400313	0.30385352	0.49347219
2610001J05F	0.22990974	5.47852933	1.11366525	0.30392491	0.49352121
A330035P11I	0.41428331	1.93256336	1.11323082	0.30401674	0.49354722
Cox11	0.18006788	3.91883494	1.11306219	0.30405239	0.49354722
Rusc1	-0.2028936	5.8504456	1.11300462	0.30406457	0.49354722
B130024G19	0.49601143	1.1616731	1.11244457	0.30418303	0.49358801
Gm16039	-0.1501852	4.51962695	1.11241916	0.3041884	0.49358801
Rbm45	0.2243026	3.49260112	1.11230126	0.30421335	0.49358801
Stac	0.77709734	0.1183344	1.11199747	0.30427764	0.49362545
Klhl33	1.41344739	-1.944118	1.11161802	0.30435797	0.49368889
Zmynd12	1.1148729	-1.5953784	1.11128587	0.30442831	0.49371353
4930453N24	-0.1567062	4.68066758	1.11115695	0.30445561	0.49371353
Pank2	0.16958916	5.50651976	1.1105867	0.30457644	0.49373596
Lmcd1	0.39737875	2.10485348	1.11056464	0.30458112	0.49373596
Amer2	-0.1981129	4.18477025	1.11050797	0.30459313	0.49373596
4932416H05I	0.2695314	2.63823692	1.10949856	0.30480719	0.49401607
Jmy	-0.1261011	7.18953639	1.1091903	0.3048726	0.49402371
Tcn2	0.30832505	4.22294844	1.10908756	0.3048944	0.49402371
Tead2	-0.3472074	2.48379968	1.10876661	0.30496253	0.49406726
Kcnk3	0.29870073	1.8758333	1.10832338	0.30505665	0.49415289
Sox13	-0.3239812	3.36118298	1.1078716	0.30515263	0.49419354
St7	0.26383993	3.42973863	1.10731419	0.30527111	0.49419354

Cerk	-0.2257017	4.07824315	1.10717006	0.30530175	0.49419354
Lrig2	-0.1590298	5.05686369	1.10706111	0.30532492	0.49419354
Sdf2	0.21681949	4.44281333	1.10703629	0.3053302	0.49419354
Caps2	-1.141577	-1.2710401	1.10671104	0.30539938	0.49419354
Arl14ep	0.1303612	6.37628164	1.10657385	0.30542856	0.49419354
Pdpk1	0.11036762	7.65899046	1.10640316	0.30546488	0.49419354
Kcnj6	0.22657764	5.88929856	1.10633387	0.30547962	0.49419354
Rpgr	-0.2358231	4.34552002	1.10626438	0.30549441	0.49419354
Tagln	-0.3837493	3.30791348	1.10587544	0.3055772	0.4942607
Bnip3l	0.1503404	8.24542799	1.10492442	0.30577976	0.49449871
Msn	-0.1766245	7.6261454	1.10470899	0.30582567	0.49449871
Arhgdia	0.19668317	8.02589297	1.10460315	0.30584823	0.49449871
Pnrc2	0.21348469	7.27023271	1.10413966	0.30594704	0.49453049
Adh7	0.53199312	1.41363985	1.10401534	0.30597355	0.49453049
Vash1	0.25145749	3.42591804	1.10352457	0.30607824	0.49453049
Tmem70	-0.1789805	4.80019778	1.1034586	0.30609232	0.49453049
Slc35d3	0.32204346	1.68033819	1.10335757	0.30611388	0.49453049
Slc26a1	0.92611618	-0.6319388	1.10322297	0.30614261	0.49453049
Bola2	-0.3347985	2.45401443	1.10283662	0.30622508	0.49453049
Ifi44	0.30352798	3.33645629	1.10279065	0.30623489	0.49453049
Pak3	0.18359758	7.31845417	1.10276894	0.30623953	0.49453049
Copg1	-0.1394178	7.25418824	1.10231556	0.30633636	0.49462016
Daf2	0.46489961	0.64992593	1.10193772	0.30641709	0.4946838
Acvr2a	0.15864387	6.16874614	1.10073246	0.30667479	0.49496128
Nde1	-0.2811222	3.81609371	1.10055978	0.30671173	0.49496128
Atat1	0.14621024	5.68342495	1.10050867	0.30672267	0.49496128
Ctsh	0.24707944	4.97746503	1.10007086	0.30681637	0.49496128
C330007P06I	0.16988612	7.68773185	1.0999567	0.30684081	0.49496128
Srp54b	0.11640258	6.34362651	1.099945	0.30684331	0.49496128
1700007L15F	-0.652028	-0.0113701	1.09975125	0.3068848	0.49496128
Repin1	0.18888824	4.53713337	1.09931434	0.30697838	0.49496128
Gm9958	-0.4018518	1.63163496	1.09905039	0.30703493	0.49496128
AW146154	0.28251039	3.00440561	1.09886558	0.30707453	0.49496128
2610207O16	-0.6631916	0.22999278	1.0987607	0.30709701	0.49496128
Ssbp1	-0.1449088	5.47566653	1.09872721	0.30710419	0.49496128
Fam151b	0.40210474	1.36776781	1.0986243	0.30712625	0.49496128
Rcan3	-0.1570877	5.12241842	1.09795791	0.30726915	0.49506904
Cltb	0.1814142	5.79999966	1.09792692	0.3072758	0.49506904
Ankrd63	-0.2681097	4.06143589	1.0977332	0.30731736	0.49506939
Mcat	-0.3083203	2.54081818	1.09728622	0.30741328	0.49511631
Fgd5	-0.5976636	1.84175982	1.09720735	0.3074302	0.49511631
E330009J07F	-0.4700526	1.34869234	1.09701958	0.30747051	0.49511631
Selk	0.18486761	6.15916449	1.09636705	0.30761065	0.49525664

5031434O11	-0.8966507	0.19315469	1.09622875	0.30764036	0.49525664
Nell1	0.19636479	4.05279299	1.09560087	0.30777531	0.49527796
L3mbtl1	-0.5028709	1.58648883	1.09540199	0.30781807	0.49527796
Rpl9	-0.1870029	7.1941389	1.09525749	0.30784914	0.49527796
Akr1e1	0.15374075	5.1974539	1.09497832	0.30790919	0.49527796
Dab1	-0.1868566	6.61765701	1.09489746	0.30792658	0.49527796
Scx	-0.6717623	-0.0094432	1.09482195	0.30794283	0.49527796
Tekt1	0.84375668	-0.1937425	1.09482065	0.30794311	0.49527796
Galr1	1.45314678	-1.8175192	1.0932382	0.30828382	0.49572939
Fam220a	-0.1588236	4.2702704	1.09301725	0.30833144	0.49572939
Eif4g1	-0.1168306	7.06173756	1.09276535	0.30838573	0.49572939
Dock1	0.15577775	5.76329246	1.09269495	0.30840091	0.49572939
Fam47e	1.23016467	-1.255421	1.09255647	0.30843076	0.49572939
Mterfd3	0.26110167	2.97547655	1.09227157	0.3084922	0.4957616
Cenpm	-1.2182682	-1.5130958	1.09202046	0.30854636	0.49578211
Procr	-0.5955381	1.61350465	1.09157358	0.30864278	0.49581116
Fbxl8	1.13815006	-0.7816331	1.09130027	0.30870178	0.49581116
Fbxo24	-0.7170969	-0.1138298	1.09114766	0.30873472	0.49581116
Lamb1	0.18064664	4.17024606	1.09045756	0.30888377	0.49581116
Tbpl1	0.11210958	6.41403785	1.09023089	0.30893274	0.49581116
Fam219aos	0.31493668	3.09071208	1.09019793	0.30893986	0.49581116
5430435G22	0.26998249	4.44542886	1.09016195	0.30894764	0.49581116
Psmb1	0.13811328	6.01550234	1.09012413	0.30895581	0.49581116
Dydc2	1.01407804	-0.8900281	1.08950778	0.30908905	0.49581116
Asf1a	0.13599798	5.03613629	1.08941671	0.30910875	0.49581116
2610528A11	-1.2304582	-1.5100274	1.08939004	0.30911452	0.49581116
Lrrc14b	-0.2895509	2.80334172	1.08931668	0.30913038	0.49581116
Ppp1r13l	-0.3938126	1.34262441	1.08927693	0.30913898	0.49581116
Det1	-0.3612651	1.32187188	1.08900853	0.30919704	0.49581116
Zfp202	-0.3825812	1.45069473	1.08880625	0.3092408	0.49581116
Tbc1d9	-0.1626883	5.7477432	1.08871979	0.30925951	0.49581116
A430033K04	-0.1925239	5.56796504	1.08844075	0.30931991	0.49581116
Scn1b	0.13117229	5.60432202	1.08841966	0.30932447	0.49581116
l7Rn6	0.16885279	6.09145303	1.08829681	0.30935107	0.49581116
Hspd1	0.09981382	8.27695858	1.08794425	0.30942741	0.4958666
AW011738	-0.3615072	2.60334174	1.08775467	0.30946847	0.4958666
Aqp7	-0.6336992	0.05441588	1.08703053	0.30962537	0.49605164
Rrp12	0.21033724	3.92891724	1.08683779	0.30966715	0.49605221
Isg15	-0.3473922	1.64213574	1.08661056	0.30971642	0.49606478
Pp2d1	-0.4896975	0.70436595	1.08627274	0.30978969	0.49606724
Aldh18a1	-0.2429873	3.57618485	1.08591636	0.309867	0.49606724
Scly	0.28338466	3.52715264	1.08587024	0.30987701	0.49606724
Slc6a6	0.12597032	7.94764577	1.08577882	0.30989685	0.49606724

Ext1	0.12909632	5.68457115	1.08564879	0.30992507	0.49606724
Fam172a	0.14399802	5.60774182	1.08507767	0.31004906	0.49616622
Polr1c	0.22110277	4.05554449	1.08498229	0.31006977	0.49616622
Glipr1	1.08514835	-0.9652955	1.08452408	0.31016931	0.49625919
C530005A16I	0.25080353	3.24968215	1.08413841	0.31025312	0.49632698
Ctu1	0.3829885	1.55210896	1.08330794	0.3104337	0.49654953
Taf7	0.20324769	3.82423093	1.08283406	0.31053681	0.49664812
Sf3a2	0.21090349	3.0888591	1.08227704	0.31065806	0.4967757
Lyrm5	0.18321445	5.17912218	1.08188454	0.31074354	0.49679611
Pcdha11	0.55062847	0.37164766	1.08183747	0.3107538	0.49679611
Spcs3	-0.130079	6.30157083	1.08087695	0.31096313	0.49706441
Ptpn13	0.14968306	6.26099968	1.07955811	0.31125087	0.49724442
Fbxo38	-0.1305965	5.22070644	1.07955752	0.311251	0.49724442
Eif2d	0.23847145	3.93549472	1.07948546	0.31126673	0.49724442
Gm3893	-0.2411366	5.8712045	1.0794213	0.31128074	0.49724442
Tspan18	0.36350174	2.75603736	1.07940937	0.31128334	0.49724442
Uqcrh	0.18983075	7.05499761	1.07881968	0.31141213	0.49731919
Erv3	1.64839452	-1.2482462	1.07881476	0.31141321	0.49731919
Tnfaip8l1	0.31310321	2.81437686	1.07847673	0.31148707	0.49737082
Rpia	-0.2497441	2.92032853	1.07745439	0.3117106	0.49750233
Sgsm2	-0.198604	4.61831494	1.07708136	0.31179222	0.49750233
Rhou	0.16760125	6.75176283	1.07697623	0.31181523	0.49750233
Dennd1b	-0.1784545	4.59060003	1.07687814	0.3118367	0.49750233
Serpina3g	-1.0239248	-0.4356293	1.07665471	0.31188561	0.49750233
Nkapl	0.61620145	0.25498264	1.07625101	0.311974	0.49750233
Gm5803	0.79973077	-0.6594237	1.07613376	0.31199968	0.49750233
Nfkbil1	0.67323516	-0.2143319	1.07597632	0.31203417	0.49750233
Abcf3	-0.1782323	5.02089209	1.07580042	0.31207271	0.49750233
Plekho1	0.19499753	4.03851156	1.07580007	0.31207278	0.49750233
Grhl3	0.98326089	-0.2245876	1.07556231	0.31212489	0.49750233
Commd5	0.36205695	2.05512367	1.07536376	0.3121684	0.49750233
Ehd2	0.26598385	4.92766503	1.07528557	0.31218554	0.49750233
Eid3	1.50643028	-1.901409	1.07524766	0.31219385	0.49750233
Phospho2	-0.169267	5.00546569	1.07492793	0.31226396	0.49750233
Cdt1	0.71435776	-0.1894261	1.07455491	0.31234577	0.49750233
Gm6654	-0.4941273	0.80954439	1.07445808	0.31236701	0.49750233
Khdrbs3	0.13658203	6.39263603	1.07391144	0.31248697	0.49750233
4930481A15I	0.5012367	1.32952791	1.07383515	0.31250372	0.49750233
Hmbox1	-0.1586505	5.1907916	1.07377937	0.31251596	0.49750233
Pigg	0.20786348	3.45784024	1.07373043	0.31252671	0.49750233
Tasp1	-0.1491087	5.96691952	1.07369465	0.31253456	0.49750233
Pcdhb19	-0.2774413	2.92157149	1.07355232	0.31256581	0.49750233
Mterf1a	-0.5283941	1.18052134	1.07354941	0.31256645	0.49750233

Stam	0.13095024	6.06935406	1.07229764	0.31284148	0.49787392
Pcdh17	0.18798907	6.97977252	1.0720511	0.31289569	0.49789402
Klhl20	0.17272146	5.71641113	1.07180139	0.3129506	0.49791524
Grik3	-0.222617	5.57650509	1.07152116	0.31301225	0.49794717
Oxtr	0.30783456	3.55185533	1.07024577	0.31329302	0.49832763
Mlc1	0.35583295	3.17657548	1.06999556	0.31334815	0.49833303
Sema4b	0.25514798	2.77315186	1.06985263	0.31337964	0.49833303
Cd47	0.1582904	7.49962198	1.06866017	0.31364257	0.49857885
Fam49a	0.13255716	8.23706648	1.06839777	0.31370046	0.49857885
Mybbp1a	-0.1888694	4.39925614	1.06838392	0.31370352	0.49857885
Tmem132b	-0.2063423	7.49325511	1.06812025	0.31376171	0.49857885
Acsm3	0.99425021	-0.887876	1.06803484	0.31378057	0.49857885
Apol7b	-0.772525	-0.4814013	1.06797006	0.31379487	0.49857885
5430427O19	-0.8545026	0.2311527	1.06783063	0.31382565	0.49857885
Nr6a1	-0.2786617	3.0928936	1.06724276	0.31395549	0.49869936
Syt9	0.24352048	3.34875711	1.06711015	0.31398479	0.49869936
Cln6	-0.4962214	1.96632768	1.06597619	0.31423547	0.49903133
Sfn	-1.1832152	-1.1509467	1.06548995	0.31434305	0.49907415
Wdr12	0.20580629	4.64403414	1.06547759	0.31434579	0.49907415
1700080N15	-0.9358584	0.14757437	1.06489965	0.31447372	0.49921109
Kdm2a	-0.1233941	7.17003186	1.06413364	0.31464339	0.49930461
Zscan2	-0.4740539	1.3090685	1.06411049	0.31464852	0.49930461
Gdap1	-0.1909185	7.46345264	1.06388233	0.31469909	0.49930461
Ccdc88c	-0.2418325	4.2524516	1.06388089	0.31469941	0.49930461
Bbs12	-0.3989776	1.27863567	1.06349153	0.31478572	0.4993754
Klhl8	-0.1995511	4.31491677	1.06285544	0.3149268	0.49942478
Gstm6	-0.3347331	2.2532875	1.06266929	0.3149681	0.49942478
Stox2	-0.1768355	7.99509909	1.06254083	0.31499661	0.49942478
Ifih1	0.16168475	5.32452961	1.06237323	0.31503381	0.49942478
I830012O16F	0.26472277	4.05990536	1.06233547	0.31504219	0.49942478
Pbx1	0.11979072	9.2799897	1.06222341	0.31506707	0.49942478
Atad2b	-0.1636364	5.16211525	1.06180259	0.31516051	0.49950624
Gpr45	0.27128422	2.23163796	1.06161634	0.31520188	0.49950624
Sgol1	-0.5122054	0.8431864	1.06130344	0.31527139	0.49951951
Ssbp2	0.14093538	6.26453336	1.06120316	0.31529367	0.49951951
Strn3	0.12063122	8.17349927	1.06049153	0.31545186	0.49967293
Birc5	-0.5630507	0.28308296	1.06021326	0.31551375	0.49967293
Stag2	-0.1286172	6.69822651	1.05954429	0.3156626	0.49967293
Myzap	0.3089699	4.17181973	1.05925082	0.31572793	0.49967293
Rrp36	0.34513326	2.16322795	1.0590727	0.31576759	0.49967293
Tubb5	0.13130103	7.90547831	1.05899898	0.315784	0.49967293
Slc4a5	-0.8267805	0.04024916	1.05891987	0.31580162	0.49967293
Usp33	-0.147651	6.93210108	1.05869538	0.31585162	0.49967293

Amigo2	0.34268938	2.41715299	1.05866423	0.31585856	0.49967293
Whsc1	0.11689713	6.66122419	1.05865553	0.3158605	0.49967293
Gdi1	0.13158692	8.88966639	1.05856021	0.31588174	0.49967293
Uhrf1bp1	-0.2342863	3.87314258	1.05851775	0.3158912	0.49967293
Csmd1	-0.2714783	6.08621205	1.05809517	0.31598536	0.49970454
Lypla1	0.18436053	5.19566608	1.05805358	0.31599464	0.49970454
Alg1	-0.8117464	0.0254511	1.05751985	0.31611363	0.49974713
Zfp110	-0.2000294	4.42279014	1.05710706	0.31620571	0.49974713
2210018M11	0.15753412	6.75589325	1.05695386	0.31623989	0.49974713
Egflam	0.25720016	3.52709281	1.0569402	0.31624294	0.49974713
Pigo	-0.3817386	1.91965557	1.05667849	0.31630134	0.49974713
Bcl2l2	-0.117593	6.25153777	1.05664433	0.31630897	0.49974713
Ebp	0.28535807	2.6593953	1.05654007	0.31633224	0.49974713
Traf5	0.4239235	1.45352908	1.05643629	0.31635541	0.49974713
Dab2	0.17848765	8.40747023	1.05605059	0.31644153	0.49976558
2810025M15	-0.2814331	2.41788734	1.05601022	0.31645055	0.49976558
Arl2bp	-0.1714174	5.67878258	1.05576351	0.31650565	0.4997867
Kif3c	0.14399938	6.41499445	1.05553636	0.3165564	0.49979993
Rab3d	0.21192406	3.19566862	1.05535244	0.3165975	0.49979993
Mrap2	-0.5576065	1.52222856	1.05507538	0.31665943	0.4998318
Syne4	1.1683189	-1.3494945	1.05365879	0.31697632	0.50012839
Ncf1	-0.2622237	2.38759849	1.05354261	0.31700233	0.50012839
Resp18	0.2108986	3.1582388	1.05349838	0.31701223	0.50012839
Gm20362	-0.5495534	0.71752703	1.05348881	0.31701437	0.50012839
Magee2	0.20343015	4.78781903	1.05293702	0.31713794	0.50025744
Gpr133	-0.3209279	2.64997106	1.05245094	0.31724686	0.50030391
Cnot3	0.155077	5.42677765	1.05224924	0.31729206	0.50030391
Rslcan18	-0.3057935	2.38677572	1.05211996	0.31732104	0.50030391
Pfkl	0.18507308	4.18051187	1.0519004	0.31737027	0.50030391
Dbndd1	-0.3639305	1.76124318	1.05141426	0.31747931	0.50030391
Exoc8	0.16257043	4.65866988	1.05136057	0.31749135	0.50030391
Gltscr1l	-0.1352242	6.39227417	1.05097985	0.31757679	0.50030391
Serp2	0.23894151	3.78297655	1.05082422	0.31761172	0.50030391
Glis1	0.62179183	0.52950865	1.05076586	0.31762482	0.50030391
Mtag2	-1.0535379	-0.504034	1.05061363	0.31765899	0.50030391
Cd164l2	1.20970646	-1.0215828	1.05040348	0.31770618	0.50030391
Slc25a3	0.11202829	7.93988783	1.05035352	0.3177174	0.50030391
Chrnb3	-0.4948136	1.28994325	1.0502431	0.3177422	0.50030391
Pxylp1	0.22598268	3.30128163	1.04973998	0.31785523	0.50030391
Fam214b	0.19547676	4.40234151	1.04966659	0.31787172	0.50030391
Adprh	0.15390313	4.24486342	1.04960771	0.31788495	0.50030391
Kbtbd3	0.22663956	3.29849578	1.04935521	0.3179417	0.50030391
0610007P14l	0.20598814	4.25240262	1.04930744	0.31795244	0.50030391

Cep85l	-0.2291004	4.01500005	1.04926865	0.31796116	0.50030391
Smim20	0.25416851	3.66801231	1.04870365	0.31808822	0.50033488
Arhgap20os	0.92591563	0.33173042	1.04835816	0.31816594	0.50033488
Tdrd6	0.9135298	-0.5008325	1.04833003	0.31817227	0.50033488
Kntc1	-1.0179506	0.00439838	1.04823542	0.31819356	0.50033488
Mapk10	0.13892971	9.60904329	1.04815362	0.31821197	0.50033488
4930529M08	1.11271853	-0.9366219	1.04806677	0.31823152	0.50033488
2810013P06I	-0.1987609	4.65624148	1.04778791	0.31829429	0.50036788
Setd5	-0.1274009	8.40831928	1.04709352	0.31845068	0.50051366
Hist1h2bm	0.43704656	1.35635533	1.04700503	0.31847061	0.50051366
Kif22	0.45370688	0.89356003	1.04629396	0.31863088	0.50053763
Cox8a	0.16712872	7.4453694	1.04614498	0.31866447	0.50053763
Tmem74b	-0.8267292	-1.2658424	1.04611921	0.31867028	0.50053763
Tbc1d4	0.2778311	3.07730751	1.04589106	0.31872173	0.50053763
Acat1	0.13829234	7.12008456	1.04578085	0.31874659	0.50053763
D11Wsu47e	-0.2646357	2.51229817	1.04571841	0.31876068	0.50053763
Asb5	-0.5259905	0.5376072	1.0456397	0.31877844	0.50053763
Trdn	-0.6018924	0.16367472	1.04513745	0.31889178	0.50056506
0610010B08I	-0.2267782	4.54974306	1.0450415	0.31891344	0.50056506
Dsc3	-1.2544325	-1.3625269	1.04485323	0.31895594	0.50056506
Haus4	0.40361566	1.58054984	1.04482154	0.3189631	0.50056506
Amdhd2	0.36725449	1.22239547	1.04438595	0.31906147	0.50065383
Rxfp2	0.70599578	-0.5855475	1.04391516	0.31916784	0.50075513
Cdc37l1	-0.1204643	6.61550498	1.04348194	0.31926576	0.50084315
Eri3	0.18905089	4.21132991	1.04310765	0.3193504	0.50088893
Crebzf	-0.1775257	5.31094743	1.04286499	0.31940529	0.50088893
Pabpc1	-0.1220703	8.19210904	1.04279809	0.31942042	0.50088893
Nxph4	0.5724295	0.63504163	1.04240288	0.31950985	0.50096357
Kif11	-0.3560097	2.2910576	1.0417788	0.31965114	0.50107239
Zfp809	0.15808528	4.84571026	1.04172671	0.31966294	0.50107239
Rltpr	-0.3016545	1.80579924	1.04129384	0.31976099	0.50109641
Cstf3	-0.1932388	5.29501296	1.04128908	0.31976207	0.50109641
Fhl5	2.08319371	-2.2783668	1.04110496	0.31980379	0.50109641
Ufm1	0.14085468	6.00772638	1.04077789	0.31987792	0.50114699
Rundc1	0.17213354	5.45873072	1.03920877	0.32023389	0.50163905
Fam107a	0.22353918	7.39578337	1.03886137	0.32031277	0.501697
Zfp949	-0.1726672	4.59449231	1.03848025	0.32039935	0.50171157
Dlx5	0.29280824	2.04843014	1.03845156	0.32040586	0.50171157
D17Ertd648e	-0.4838776	0.72780669	1.03816129	0.32047182	0.50174925
Telo2	0.47711354	1.49145732	1.03747481	0.32062789	0.50187184
Dse	-0.3116022	3.90497036	1.03744823	0.32063394	0.50187184
3110043O21	-0.2493641	3.97870213	1.0371347	0.32070525	0.50191787
Aoc2	-0.458364	1.2423825	1.0368731	0.32076477	0.50194542

Gm10790	-0.7997638	-0.5039736	1.03651375	0.32084656	0.5020078
Zbtb44	0.12566145	7.23538293	1.03586509	0.32099426	0.5021179
Topbp1	-0.1816758	5.30103597	1.03568321	0.32103569	0.5021179
Wdr54	0.25685396	3.28234784	1.03550752	0.32107572	0.5021179
BC053749	0.37517354	2.84818733	1.03546841	0.32108464	0.5021179
Ring1	-0.182513	4.47181518	1.03508036	0.32117308	0.502175
Tox3	0.22493085	5.04677067	1.03487803	0.3212192	0.502175
Nprl2	0.28116856	2.467394	1.03475637	0.32124694	0.502175
Flt3l	-0.4074833	1.70226686	1.03444099	0.32131887	0.50222188
Fam213b	-0.191322	4.12437467	1.03380932	0.321463	0.50238158
Sord	-0.194452	4.3922817	1.03343416	0.32154864	0.50244985
Fam149b	0.14532712	5.38865691	1.03306038	0.321634	0.50250636
Mdfi	1.1009394	-1.4504208	1.03290838	0.32166872	0.50250636
Kat8	0.19726123	3.25460002	1.03242907	0.32177824	0.50255243
Ogfod1	0.1414293	7.29648385	1.0323909	0.32178696	0.50255243
Klhl21	-0.2144106	4.54310308	1.03222839	0.32182411	0.50255243
Chmp2b	0.18868183	6.34152136	1.03186802	0.3219065	0.50256516
Lbh	-0.2420173	6.3473381	1.03182568	0.32191619	0.50256516
Tomm6os	-0.8277974	-0.1003295	1.03151703	0.32198678	0.50260985
Bnc2	0.22151117	7.67740496	1.03105169	0.32209325	0.50266318
H13	-0.2020858	4.82160613	1.03082395	0.32214538	0.50266318
LOC1005050	0.64815805	0.48424062	1.03081745	0.32214687	0.50266318
Mycn	-0.3186165	2.05725625	1.03033482	0.32225737	0.50275251
Fbxo22	0.12370452	6.86914648	1.03014839	0.32230007	0.50275251
Acads	0.8432641	-0.6553317	1.03001748	0.32233006	0.50275251
Syt12	-0.185145	4.19268637	1.02973828	0.32239403	0.50278681
Dhx36	-0.1659778	6.26856195	1.0292899	0.32249681	0.50281827
Cct7	0.14749377	6.67429444	1.02913999	0.32253117	0.50281827
Ralgds	0.12164729	5.87226687	1.0290327	0.32255578	0.50281827
Ppp6r1	-0.1711414	4.81027619	1.02891767	0.32258216	0.50281827
Cenpw	-0.3948555	1.57348839	1.02831256	0.32272098	0.5029477
Rgs16	0.20391024	3.66457542	1.02816079	0.3227558	0.5029477
Wwc1	-0.1738525	4.29363113	1.02793254	0.3228082	0.5029477
2610018G03	0.41869235	1.93661506	1.02782372	0.32283318	0.5029477
Rfwd2	-0.1174999	7.26114381	1.0272654	0.3229614	0.50307406
9430020K01I	-0.1120037	8.95856324	1.02707343	0.3230055	0.50307406
Lrrc8d	-0.1569396	5.77363906	1.02692193	0.32304031	0.50307406
4930404H11	1.64762146	-1.2410107	1.02624794	0.32319524	0.50309434
Mkln1	-0.1591889	6.15447147	1.02621336	0.32320319	0.50309434
Cmtm7	-0.3342886	1.86623402	1.0261536	0.32321694	0.50309434
Gm5105	1.00015539	-0.6224718	1.02600241	0.32325171	0.50309434
Ranbp3l	-0.1921342	7.77261109	1.02586915	0.32328236	0.50309434
Clec4g	-1.0263292	-1.5949193	1.02568777	0.32332409	0.50309434

1110007C09I	0.33427837	2.33907643	1.02558643	0.3233474	0.50309434
Nudt9	-0.1919398	5.37527833	1.02490523	0.3235042	0.5031613
Cxcr5	0.59272777	-0.2027588	1.02470374	0.3235506	0.5031613
Tpgs1	0.32088862	3.14348779	1.02462543	0.32356863	0.5031613
Rsph4a	-0.3044309	2.93990736	1.02453975	0.32358837	0.5031613
Uba7	-0.543144	0.63843414	1.02448702	0.32360051	0.5031613
Atrip	-0.2118844	3.49551033	1.02383275	0.32375127	0.50333036
Xrcc1	0.24847988	2.99943505	1.02330379	0.32387322	0.50340941
Ywhaq	0.12096697	9.00817021	1.02324757	0.32388619	0.50340941
Blvrb	0.41132243	1.2397928	1.02216028	0.32413708	0.50373399
Gstt2	0.45318166	2.13162719	1.0217703	0.32422714	0.503764
Lsm7	0.2277013	3.70808593	1.02171236	0.32424052	0.503764
Hectd2	-0.1971083	4.47598569	1.02042905	0.32453712	0.50408038
Zfp932	0.23050522	4.46617735	1.02022668	0.32458393	0.50408038
Glis2	0.1936206	5.21343482	1.02011903	0.32460883	0.50408038
4930526I15R	-0.4362818	1.65572054	1.02010308	0.32461252	0.50408038
Pik3ip1	0.27330266	4.0136335	1.01960556	0.32472765	0.50416136
Syt4	0.16752336	7.23979806	1.01920193	0.32482109	0.50416136
Kif13b	-0.1949508	3.67690673	1.0191888	0.32482413	0.50416136
Hps6	0.40901729	1.04365059	1.01915022	0.32483307	0.50416136
Kat7	0.12735594	5.9074198	1.01862886	0.32495383	0.50428343
Hat1	0.15774641	5.05281116	1.01686009	0.32536398	0.50482413
Rnf2	0.14499006	5.42801567	1.01676285	0.32538655	0.50482413
Maf1	-0.1849052	4.57616593	1.01576861	0.32561744	0.5050243
Nkx6-2	-0.6318105	0.35293073	1.01569582	0.32563436	0.5050243
Nr4a1	-0.5130067	4.92205442	1.01566255	0.32564208	0.5050243
Tsta3	-0.3775562	2.29746459	1.01523553	0.32574133	0.5051128
Ccdc34os	1.39064581	-1.6641624	1.01498673	0.32579918	0.50512516
Ccdc137	-0.2484311	4.23153916	1.01472361	0.32586037	0.50512516
Ampd2	-0.1811617	3.93877048	1.01465708	0.32587584	0.50512516
Eif1a	0.16422492	6.62350168	1.01409854	0.32600579	0.5052612
Ccer1	1.65634571	-2.3162818	1.04608399	0.32615155	0.50540445
Unc5b	-0.2973977	2.36938693	1.01333898	0.32618263	0.50540445
Arfip2	0.14585739	5.19645534	1.01296184	0.32627048	0.50547518
Pced1a	0.20093579	4.13256654	1.01202044	0.32648992	0.50569335
Adra2a	-0.256222	4.09654235	1.0119809	0.32649914	0.50569335
C030018K13I	-0.4937031	1.52609964	1.01181437	0.32653798	0.50569335
4930519G04	-0.3324384	2.31728697	1.01118944	0.3266838	0.50582936
Speer7-ps1	-0.6042343	0.64711457	1.01107596	0.32671028	0.50582936
Rdh5	1.11062124	-1.2298068	1.0108253	0.3267688	0.50583135
Sorbs1	0.10264843	7.52415737	1.01061136	0.32681876	0.50583135
Adora2a	0.56054075	1.89798399	1.01044772	0.32685698	0.50583135
Pelo	0.24901655	3.1943276	1.01019019	0.32691714	0.50583135

Tmem47	0.17328977	7.50737928	1.01016613	0.32692276	0.50583135
Gyltl1b	-1.3031179	-1.9403739	1.00893038	0.32721166	0.50621294
Crlf2	-0.4944916	0.43132701	1.00863026	0.32728187	0.50622677
Aar2	0.25180923	3.78509195	1.00850077	0.32731218	0.50622677
Rnf130	0.11721807	7.06320133	1.00833506	0.32735096	0.50622677
Ulk2	-0.1173294	7.46675213	1.00816964	0.32738968	0.50622677
Aftph	-0.1268134	6.94774223	1.00791804	0.32744859	0.50625249
Lrrc14	-0.2612274	2.72830869	1.00768737	0.32750261	0.50627065
Fkbp9	0.23781829	5.31413582	1.00705402	0.32765099	0.50643466
Cdpf1	0.3157258	3.2397897	1.00660359	0.32775658	0.506471
Zfp750	-0.4636675	2.36164251	1.0065929	0.32775909	0.506471
Ctnnb1	-0.108939	9.22493966	1.00609045	0.32787693	0.50654503
Brf1	-0.164824	4.23692636	1.00602795	0.32789159	0.50654503
Gmpr2	0.24996581	3.44926098	1.00530241	0.32806187	0.50671738
Hist1h2ak	-1.0095809	-1.7770005	1.00514078	0.32809982	0.50671738
Rnf112	-0.2361866	4.83507087	1.0047619	0.3281888	0.50671738
Abca5	-0.1911336	5.65973686	1.00467704	0.32820873	0.50671738
Gstm4	0.32936477	1.67802119	1.00465158	0.32821471	0.50671738
E030013I19R	-0.9650857	-0.3495429	1.0044458	0.32826306	0.50672669
Rin2	0.159308	6.55233572	1.00405821	0.32835415	0.50680198
Atg101	0.29324994	2.8013093	1.00331482	0.32852896	0.50700315
Ergic1	0.13087414	6.53841747	1.00314396	0.32856916	0.50700315
Usp3	-0.162012	4.04294891	1.00256943	0.32870437	0.50702847
Tm6sf1	-0.3211463	1.87347446	1.00237597	0.32874992	0.50702847
Bex1	-0.2039023	5.22081363	1.00236866	0.32875164	0.50702847
Rpl41	-0.2045393	8.18340747	1.0019782	0.3288436	0.50702847
Ly75	-0.9479458	-0.5198203	1.00159603	0.32893363	0.50702847
Gm16576	0.50441623	0.70651927	1.00156815	0.32894021	0.50702847
Coq7	0.24622762	4.22424086	1.00120824	0.32902503	0.50702847
4933408N05	-0.8983345	-1.0389726	1.00091625	0.32909388	0.50702847
Cntfr	0.30402109	4.14223416	1.00089053	0.32909994	0.50702847
1700017B05I	0.25427919	2.86725022	1.00076693	0.32912909	0.50702847
Etv4	0.91844785	-1.3660121	1.00070678	0.32914327	0.50702847
Deaf1	-0.2036033	4.04081843	1.00066841	0.32915232	0.50702847
Cdh24	1.02054897	-1.4470112	1.000566	0.32917648	0.50702847
Pcif1	0.16682148	3.96278739	1.00055827	0.3291783	0.50702847
Bag5	0.13635856	6.29016191	1.00009438	0.32928776	0.50713184
Fzd2	0.2761308	4.20920458	0.9997026	0.32938023	0.50713965
Gng13	-0.3856376	1.38099842	0.9996962	0.32938174	0.50713965
Apol8	-0.6248252	-0.2681025	0.9995347	0.32941987	0.50713965
Ppp4r4	-0.2079554	4.90833308	0.99917087	0.3295058	0.50720673
Zkscan7	-0.4217657	1.50064791	0.99838067	0.32969253	0.50733502
Xpc	-0.1957765	3.9902574	0.99806969	0.32976606	0.50733502

Atp6v1c1	0.10323195	7.37129224	0.99797648	0.3297881	0.50733502
Strn	0.1069463	6.48418196	0.99783695	0.3298211	0.50733502
N4bp1	-0.1181083	7.19119929	0.99750405	0.32989986	0.50733502
Srsf7	-0.1187477	5.89883115	0.99744842	0.32991302	0.50733502
Txlng	0.1388779	5.65176157	0.99735781	0.32993446	0.50733502
Cacna1a	-0.1977296	6.4472544	0.99681595	0.33006273	0.50733502
Coch	0.17939664	7.79535415	0.99677909	0.33007145	0.50733502
Emc1	-0.1456982	4.6600872	0.99661851	0.33010948	0.50733502
Rps8	-0.1918742	7.08743797	0.9965282	0.33013086	0.50733502
Nradd	-0.6615793	0.29732932	0.99651577	0.33013381	0.50733502
4933404O12	0.17843866	3.94674766	0.99649019	0.33013987	0.50733502
Gpr20	-1.0561909	-1.386732	0.99627404	0.33019107	0.5073486
Parg	0.12687659	5.62381836	0.99606985	0.33023945	0.50735784
B3gnt2	0.17518721	5.73961934	0.99518314	0.33044964	0.50752986
Best1	0.38448027	1.46628805	0.99512847	0.33046261	0.50752986
Nol12	-0.2685291	2.80881033	0.99467156	0.330571	0.50752986
Zfp11	0.1657103	4.27418731	0.99445547	0.33062228	0.50752986
BC055402	-1.6191568	-2.4646339	0.99422956	0.33067591	0.50752986
Cyb5r3	0.25558856	7.41204742	0.99420525	0.33068168	0.50752986
Ccdc103	-0.7872993	-0.0614749	0.9941675	0.33069064	0.50752986
Smarca1	-0.1584739	5.63378935	0.99403273	0.33072264	0.50752986
Tmem101	-0.411259	2.47401716	0.99396181	0.33073947	0.50752986
Zfp942	-0.2057868	3.51107222	0.99366165	0.33081076	0.50752986
Fam63b	-0.1194303	7.76590666	0.99335019	0.33088475	0.50752986
A630020A06	-0.7476031	0.01746508	0.99322842	0.33091368	0.50752986
Myh3	-0.2733933	2.04655776	0.99313108	0.33093681	0.50752986
Trabd	-0.330891	2.25259154	0.99309774	0.33094474	0.50752986
Fam60a	0.21258624	3.5921575	0.99289556	0.33099279	0.50753857
Klhdc10	-0.1275119	6.89725649	0.99221081	0.33115562	0.50772323
Usf2	-0.1483103	4.98736857	0.99193191	0.33122197	0.50773785
Zfp444	0.23002573	3.28469872	0.99165639	0.33128753	0.50773785
Slc35e3	0.166355	4.481118	0.99163618	0.33129234	0.50773785
Skap1	-0.6576246	-0.0607999	0.99135667	0.33135888	0.50773829
Spsb2	0.48737776	0.96639361	0.99127875	0.33137743	0.50773829
Ccr9	-0.2608188	3.73349126	0.9909586	0.33145367	0.50779013
Fbxo9	-0.1178627	6.44977445	0.99049843	0.33156329	0.50783374
Cox19	0.2204417	3.39470768	0.99033363	0.33160256	0.50783374
Tbc1d12	0.20039367	3.91318309	0.99012354	0.33165263	0.50783374
Rbm4	-0.6594027	-0.5383219	0.98999055	0.33168434	0.50783374
Esam	-0.5440428	1.35258999	0.98986203	0.33171498	0.50783374
Fbxo40	0.8563795	-0.5305245	0.98977153	0.33173656	0.50783374
Wdr53	0.27659006	3.18469403	0.98934027	0.33183941	0.50792627
BC005537	0.13437801	7.01994452	0.98854756	0.33202859	0.50808394

Als2cr12	-0.7831568	-1.0692108	0.98853674	0.33203118	0.50808394
Neurl3	-0.5126535	1.02709041	0.98837538	0.33206971	0.50808394
Dcaf6	-0.1639332	6.91944124	0.98791005	0.33218085	0.50818906
Ccdc117	0.19217354	4.88265275	0.98756384	0.33226357	0.50823971
Mcf2l	-0.2847222	4.68359106	0.98741632	0.33229883	0.50823971
Pald1	-0.365168	1.79750332	0.9869847	0.33240201	0.50825415
Def6	-0.5071286	0.86126945	0.98690294	0.33242157	0.50825415
Star	0.28685114	2.52896689	0.9868443	0.33243559	0.50825415
Tmem81	0.54244976	0.54016435	0.98647218	0.3325246	0.50832534
Sprtn	0.33459233	1.57573087	0.98606461	0.33262213	0.50840953
Zfp874b	0.17673973	4.64430705	0.98581571	0.33268171	0.50843571
Rpl32	-0.1924735	6.79791779	0.98527629	0.33281089	0.50852127
Ipcef1	0.15153449	7.00043625	0.985119	0.33284856	0.50852127
Flnb	-0.1280171	6.1926105	0.98505006	0.33286508	0.50852127
Zfp455	-0.2322559	3.18800045	0.98466675	0.33295694	0.50855083
Sft2d1	-0.201695	3.36527521	0.9846149	0.33296936	0.50855083
Clcn3	-0.1308961	6.71782553	0.98325967	0.33329443	0.5087685
Ltf	-0.5979286	0.23885672	0.98323327	0.33330076	0.5087685
1200014J11F	-0.1328613	5.36314191	0.98295114	0.33336849	0.5087685
Fosl2	-0.3126686	6.03954939	0.98285936	0.33339053	0.5087685
Eml4	-0.1350705	6.15080929	0.98253696	0.33346796	0.5087685
Mief2	-0.4175849	1.96346521	0.98239904	0.33350108	0.5087685
Fam78a	0.5511966	0.64030231	0.98234082	0.33351507	0.5087685
Uvssa	-0.1845794	4.85422542	0.98226767	0.33353265	0.5087685
07-Sep	-0.1343036	9.11917513	0.98224025	0.33353923	0.5087685
Ccdc174	-0.1705227	4.70050512	0.98221322	0.33354573	0.5087685
Pqlc2	0.4963518	0.36876817	0.98207394	0.3335792	0.5087685
Phyhd1	-0.3165009	2.26409937	0.98177187	0.3336518	0.50881442
St8sia3	0.16113782	7.82621868	0.98076151	0.33389479	0.50904524
Gipc1	0.30972852	3.03328594	0.98038689	0.33398496	0.50904524
Lpin1	-0.1742248	4.6454142	0.98028336	0.33400988	0.50904524
Shc4	-0.2661614	2.54008987	0.98026809	0.33401355	0.50904524
Stx16	-0.1121749	6.02270895	0.98022201	0.33402465	0.50904524
Map3k5	-0.2328102	5.08153796	0.9800827	0.33405819	0.50904524
Hmgb1	0.14771461	8.09298652	0.97935937	0.33423243	0.50924595
9130019P16I	-0.5968097	0.2995342	0.97915275	0.33428222	0.50925702
Efcc1	-0.2819392	2.53421232	0.97886828	0.33435079	0.5092967
Crispld2	-0.4440949	2.2841983	0.97822106	0.33450688	0.5093923
Tcp11l1	0.15861926	5.22320502	0.97814919	0.33452422	0.5093923
Slc25a30	0.45330874	1.14282659	0.97805584	0.33454674	0.5093923
Plekhm1	0.14375442	5.38139561	0.97774857	0.33462089	0.5093923
2410089E03I	-0.2669389	5.88888922	0.97772647	0.33462623	0.5093923
Gap43	0.13785755	7.23966044	0.97745396	0.33469201	0.50942768

Hdac8	-0.3424587	1.90663571	0.97725529	0.33473998	0.50943595
S1pr1	0.25441146	5.64766175	0.97687544	0.33483173	0.50944709
Bco2	-0.6836122	0.95810225	0.97686594	0.33483403	0.50944709
Csmd2	-0.322366	4.84623264	0.97655166	0.33490996	0.50944709
Rara	0.23006902	5.15309412	0.97652061	0.33491746	0.50944709
Gpr50	1.36834003	-1.5457712	0.97623894	0.33498554	0.50947476
4930579G24	-0.2586958	2.52831405	0.97595346	0.33505456	0.50947476
Lyn	0.27133629	3.40651406	0.9759174	0.33506328	0.50947476
Ptgdr	0.23849836	5.28157991	0.97538958	0.33519095	0.5095279
Adap2	-0.2796224	3.73538247	0.97506212	0.33527019	0.5095279
St3gal5	0.14684313	6.4731257	0.97486906	0.33531691	0.5095279
Kras	-0.1413268	8.1423861	0.97484614	0.33532246	0.5095279
Gm14057	0.54739472	2.1774852	0.97473498	0.33534937	0.5095279
Tma16	-0.2280717	3.76072172	0.9747179	0.33535351	0.5095279
Hcrtr2	-0.5146935	0.50982421	0.97348075	0.33565321	0.50991857
1500015L24I	-1.0813938	-0.0251291	0.97302784	0.33576303	0.50997177
Dhx8	-0.1333794	5.10622165	0.97298507	0.3357734	0.50997177
Mfap2	-0.6003212	-0.0144527	0.97231089	0.33593696	0.5100023
Htr3a	-0.5243785	0.66237424	0.97224993	0.33595176	0.5100023
Ttpal	-0.1609152	5.12525269	0.97217846	0.33596911	0.5100023
Ppp1r14c	-0.2136706	3.61494466	0.97181345	0.33605772	0.5100023
C130036L24I	0.7474803	-0.1689288	0.97174102	0.33607531	0.5100023
F730043M19	-0.444294	0.97539026	0.97137127	0.33616511	0.5100023
Gls	0.16196801	9.46596455	0.97132831	0.33617555	0.5100023
Trpm3	-0.1371884	6.82614017	0.97111346	0.33622775	0.5100023
Rps6ka5	-0.275659	3.29476778	0.97098103	0.33625993	0.5100023
Mtrf1	-0.2197325	3.13216346	0.97088984	0.3362821	0.5100023
Tbc1d14	-0.1427526	5.17225701	0.97084077	0.33629402	0.5100023
Fam124a	-0.1773671	4.4110444	0.97079751	0.33630454	0.5100023
Mob1a	-0.2140596	3.39147199	0.9703497	0.33641341	0.51010281
Filip1	0.23199297	3.97005883	0.96997797	0.33650382	0.51011995
Ss18l1	0.14224972	6.11108159	0.96969872	0.33657177	0.51011995
Plekha6	-0.1312684	6.85142814	0.96965715	0.33658188	0.51011995
Churc1	-0.1446004	4.54674334	0.96960284	0.3365951	0.51011995
Gimap3	0.34096962	2.71471156	0.96924942	0.33668113	0.51015423
Endou	-0.339984	1.87124236	0.96910169	0.33671709	0.51015423
Ano6	0.19347057	6.43153776	0.96898498	0.33674551	0.51015423
Fgr	0.61183395	0.32425747	0.96864273	0.33682887	0.51015873
Ttc33	-0.1224093	6.57140083	0.96862296	0.33683369	0.51015873
Btbd7	-0.1588816	5.67035502	0.96842689	0.33688146	0.51016656
Kbtbd11	-0.1206241	7.70405204	0.96773358	0.33705044	0.51035793
Ppef2	-1.1820615	-0.8436793	0.96732198	0.33715082	0.51041994
Wipf1	0.2100809	6.02779989	0.96721616	0.33717664	0.51041994

A230050P20	-0.2789789	1.98851893	0.96605894	0.33745911	0.51075138
2610203C22I	-0.6946577	0.00997097	0.96589794	0.33749844	0.51075138
Nusap1	-0.3902866	2.16744972	0.96570176	0.33754637	0.51075138
Ces2g	0.29987492	3.49425668	0.96562069	0.33756618	0.51075138
Nrcam	0.16727757	7.77680341	0.96513638	0.33768455	0.51086594
Dcaf12l1	0.14404129	5.6089195	0.96468305	0.3377954	0.51091429
Pxdn	0.33709403	4.05497605	0.96465674	0.33780184	0.51091429
Rhbdf2	0.95783992	-1.7055015	0.96424573	0.33790239	0.51100184
Plin3	0.21366028	3.68225391	0.96343738	0.33810027	0.51120392
Dlg2	-0.1643026	9.50865062	0.96335115	0.33812139	0.51120392
Gm4013	-1.2200357	-1.6984571	0.96283342	0.33824822	0.51126552
Apba1	-0.1646584	6.80136776	0.96275045	0.33826855	0.51126552
St7l	-0.2053354	4.22717222	0.96258354	0.33830946	0.51126552
Fen1	0.25982481	2.73471219	0.96248792	0.3383329	0.51126552
Ccdc176	-0.4041397	2.36353421	0.96164711	0.3385391	0.5114565
Lmf2	0.26398321	2.46143487	0.96149042	0.33857754	0.5114565
Mtr	-0.3357663	2.80101481	0.96145024	0.3385874	0.5114565
Efcab14	0.14705337	7.24510589	0.96123697	0.33863974	0.51147105
Dthd1	-0.9023149	-0.4413479	0.96093636	0.33871354	0.51148044
Ube2g2	-0.1836548	4.67683069	0.96086368	0.33873139	0.51148044
Emx2os	-0.2744295	2.96091169	0.96041171	0.33884239	0.51158227
Creld2	-0.282249	3.22286523	0.96000756	0.33894169	0.51158227
C230029M16	1.45800105	-1.9497572	0.95977269	0.33899942	0.51158227
Mbd4	0.22327919	4.40401113	0.95972139	0.33901203	0.51158227
Fbxo17	0.37094657	1.79926331	0.95923545	0.33913151	0.51158227
Gm14393	-0.2645016	2.67963904	0.95909963	0.33916492	0.51158227
E430018J23F	0.33058485	2.49952801	0.95899603	0.33919041	0.51158227
Kmo	-0.5234169	0.99366397	0.9589636	0.33919838	0.51158227
Crym	0.18239453	3.43461405	0.9586746	0.33926949	0.51158227
Gabra2	0.19912852	4.85233987	0.95856836	0.33929564	0.51158227
Ints2	0.20207278	4.08922005	0.95852018	0.3393075	0.51158227
Sstr2	0.26779093	2.75647597	0.95850418	0.33931144	0.51158227
Med18	-0.4240685	1.08521293	0.95817474	0.33939254	0.51164012
Drd2	-0.3119441	3.84286227	0.95793174	0.33945237	0.51166592
Rab26	0.2759593	3.91471612	0.95702842	0.33967494	0.51191261
Gm10406	-0.6803489	-0.4766145	0.9569206	0.33970152	0.51191261
Zfp945	-0.1496598	5.18265876	0.95669703	0.33975665	0.51193126
Slc25a13	0.5076481	0.32074734	0.95604736	0.3399169	0.51203784
Aox2	-1.2857027	-0.919169	0.95582456	0.33997188	0.51203784
A830082K12	-0.1358186	6.61205122	0.95581291	0.33997476	0.51203784
Gm1653	-1.0631304	0.50863292	0.95571711	0.33999841	0.51203784
Vmp1	0.14633252	5.58892093	0.95554385	0.34004118	0.51203786
Disp1	-0.2472613	3.22651825	0.95451601	0.34029506	0.51235574

A330069E16	0.41825817	0.70240005	0.95432894	0.3403413	0.51236094
Cops7a	0.15985609	6.91236937	0.95365123	0.34050888	0.51254879
Top2b	-0.12489	8.14488493	0.9534411	0.34056086	0.51256261
E2f6	-0.1408869	5.50491294	0.95262482	0.34076291	0.51272934
Myh2	0.74916207	0.20706863	0.95246997	0.34080125	0.51272934
Kansl2	0.13465626	4.72657534	0.9521902	0.34087055	0.51272934
Entpd7	0.22619944	4.12638199	0.95203959	0.34090786	0.51272934
Sf1	0.11476037	6.7156002	0.95196562	0.34092619	0.51272934
Zmat3	-0.1389438	9.37056188	0.95169369	0.34099358	0.51272934
Fam161b	-0.2134343	3.87855137	0.95139011	0.34106884	0.51272934
Sowahc	0.16744588	4.15343982	0.95089928	0.34119056	0.51272934
Pdgfa	-0.1388018	5.81482077	0.95075226	0.34122703	0.51272934
Vps33a	-0.1373389	6.28335802	0.95072001	0.34123504	0.51272934
Dnajc28	0.16715102	3.87108935	0.95055084	0.34127701	0.51272934
Hsf4	0.38959563	1.34637394	0.95024792	0.34135219	0.51272934
Fsip1	1.01216765	-1.5380426	0.95018926	0.34136676	0.51272934
Nipbl	-0.1272825	7.96640291	0.95012075	0.34138376	0.51272934
Tbl2	0.1898507	3.49257687	0.95008634	0.3413923	0.51272934
Rasgrf2	-0.25904	6.84330007	0.95003085	0.34140608	0.51272934
Serinc5	-0.1889524	5.0789743	0.94974547	0.34147695	0.51272934
Nr2c2	-0.0989161	7.33174205	0.94950303	0.34153717	0.51272934
St6galnac5	0.16782619	4.74676532	0.94948954	0.34154052	0.51272934
Atp5a1	0.09761178	9.40853935	0.94915903	0.34162263	0.51272934
Ehhadh	0.39856293	1.10449656	0.94892455	0.34168091	0.51272934
Aktip	-0.1300506	6.09517602	0.94873049	0.34172915	0.51272934
Tle4	-0.1549449	6.40043195	0.94863074	0.34175395	0.51272934
Hsd17b13	-1.1377045	-1.215818	0.94858614	0.34176504	0.51272934
Zbtb42	0.56250656	0.59707261	0.9485472	0.34177472	0.51272934
Pi4kb	-0.1796383	4.34799035	0.9482777	0.34184175	0.51272934
Cecr6	0.23486005	4.67755729	0.94817504	0.34186728	0.51272934
Rpl37	-0.1799744	6.35124797	0.94816236	0.34187044	0.51272934
AA543186	0.89942063	-1.0680148	0.94779631	0.34196151	0.51275978
Flywch1	0.21040528	3.82844992	0.94773662	0.34197637	0.51275978
Scn3a	-0.2559756	5.62374621	0.9468977	0.34218524	0.51294423
Endod1	-0.1489451	4.92098218	0.94688394	0.34218866	0.51294423
Ccdc73	-0.2492414	3.33422071	0.94672652	0.34222788	0.51294423
Stoml2	0.1886234	4.28592508	0.94652892	0.34227711	0.51295382
Serinc3	0.15673701	8.71528027	0.94621374	0.34235566	0.51300394
Rmst	-0.6399575	0.98297744	0.946051	0.34239622	0.51300394
Pla1a	0.56901366	0.99689346	0.94532268	0.34257786	0.51316678
Snhg3	0.34047919	3.02494622	0.94527157	0.34259061	0.51316678
Ankrd39	0.35974459	1.7336555	0.9449878	0.34266142	0.51316697
Rprd1a	0.13142887	6.72617376	0.94492764	0.34267644	0.51316697

Brwd1	-0.172847	6.9745461	0.94406209	0.34289257	0.51335151
Khk	-0.4613401	2.21443771	0.94399766	0.34290866	0.51335151
Gsg1l	0.14817551	5.847959	0.94389332	0.34293473	0.51335151
Gsta4	0.20278182	6.83520109	0.94374764	0.34297113	0.51335151
Tmem108	0.28279811	3.46789091	0.94352886	0.34302581	0.51336918
Mrpl46	-0.2616402	3.48417152	0.9432639	0.34309204	0.51340415
Cpsf3	0.1487472	5.09229648	0.94307756	0.34313864	0.51340972
Emr4	-1.2458814	-1.2104886	0.94284604	0.34319654	0.51343221
4930539J05F	-0.500249	0.32804557	0.94230918	0.34333086	0.51351751
D17Wsu104e	0.35221403	2.07184343	0.94213595	0.34337421	0.51351751
Gm19461	-0.8959459	-0.8248495	0.94210406	0.3433822	0.51351751
Vps54	-0.1201073	6.89711108	0.94172494	0.34347711	0.51355566
Olfr239	-1.1376971	-1.2311747	0.94165961	0.34349347	0.51355566
Cnot1	-0.1246753	8.09961705	0.94141957	0.34355359	0.51356621
Fam69a	0.15452348	4.66369222	0.94112333	0.34362781	0.51356621
Ogn	-0.2302108	8.11652656	0.94100107	0.34365845	0.51356621
Kidins220	-0.1466604	8.28762378	0.94094673	0.34367207	0.51356621
Erbb2ip	-0.1055511	7.94454887	0.93992886	0.34392729	0.51379749
Zdhhc18	0.26405318	3.24459797	0.93985428	0.343946	0.51379749
Mir22hg	0.19881139	3.66681709	0.93975324	0.34397136	0.51379749
Scp2	0.20380589	7.10915731	0.93959612	0.34401079	0.51379749
Zfp428	-0.4655013	0.8567422	0.93947103	0.34404218	0.51379749
Best3	0.85814585	-0.0664537	0.93930341	0.34408426	0.51379749
Lars	-0.1417918	5.12564252	0.93849147	0.34428817	0.51403789
Slc31a1	0.1363137	5.88304645	0.93793277	0.34442859	0.51407326
Slc39a2	-0.7002345	0.47883261	0.93784083	0.3444517	0.51407326
Ralb	0.18388805	4.37147083	0.93769392	0.34448864	0.51407326
Elk3	-0.158836	4.99464242	0.93766768	0.34449524	0.51407326
Tor1aip1	-0.1452022	7.34527973	0.93754338	0.3445265	0.51407326
Pkn1	-0.3627489	2.85347814	0.93723674	0.34460363	0.51408873
Anxa7	0.12474236	6.45229665	0.93716087	0.34462272	0.51408873
Hdx	-0.3283548	2.92416688	0.93672455	0.34473252	0.51418848
Golph3l	0.16735764	5.99608675	0.93650802	0.34478703	0.51420574
Stk32c	-0.2638671	2.62635104	0.93611151	0.34488688	0.51422271
Ube2g1	0.13589541	6.83465287	0.93601063	0.34491229	0.51422271
Gm14436	0.15614979	5.06734564	0.93576143	0.34497507	0.51422271
Crocc	-0.413552	2.06079015	0.93570567	0.34498912	0.51422271
Hcst	-1.1462784	-1.5368851	0.9356105	0.3450131	0.51422271
Ppia	0.1550295	9.86341578	0.93456807	0.34527594	0.51438785
Chodl	-1.1870478	-1.4040797	0.93450488	0.34529188	0.51438785
Cdh11	0.11076972	6.72503246	0.93440257	0.34531769	0.51438785
9930012K11I	-0.4799461	1.30483916	0.93436651	0.34532679	0.51438785
Tnnt1	-0.4196993	1.24684882	0.93431944	0.34533867	0.51438785

Osgin1	-0.6804933	0.20312214	0.93387901	0.34544983	0.51445889
Pou3f3	-0.1486073	5.33868191	0.93377062	0.3454772	0.51445889
Nop9	-0.2426688	3.74602846	0.93361998	0.34551523	0.51445889
Bard1	-0.3752787	1.56742693	0.93314587	0.34563498	0.51457322
Ppbp	-0.9706497	-0.2778654	0.93284805	0.34571024	0.51462128
Osbpl11	0.17468626	4.16970836	0.93223498	0.34586523	0.51470208
Ykt6	-0.1631428	5.12626291	0.9319397	0.34593991	0.51470208
Mtrr	-0.1871003	4.01069066	0.93174871	0.34598823	0.51470208
Creb3	-0.2949273	3.08932762	0.93156423	0.34603491	0.51470208
Gm1604b	0.33967111	1.39017072	0.93140468	0.34607529	0.51470208
Ctif	0.13391093	7.02124935	0.93139945	0.34607661	0.51470208
Aurkaip1	0.18802867	4.92854804	0.93120403	0.34612608	0.51470208
Mapk8ip3	-0.1878133	6.77528462	0.93094219	0.34619237	0.51470208
Zfp119a	0.38877016	1.13560126	0.93082352	0.34622243	0.51470208
Myo5c	-0.5551294	1.43219009	0.93075044	0.34624094	0.51470208
AI462493	-0.2702413	3.34834797	0.93062063	0.34627382	0.51470208
Acsf3	0.3884085	1.50699388	0.93059515	0.34628027	0.51470208
Pagr1a	0.20240868	3.04353143	0.92970644	0.3465055	0.5149412
Zfp551	-0.3925696	2.48571282	0.9296211	0.34652714	0.5149412
Upf3a	0.18916965	5.8631165	0.92942212	0.3465776	0.51495229
Dlg5	-0.1518323	5.22165872	0.92910043	0.3466592	0.51500964
Mrps26	-0.1743912	3.85237009	0.92820937	0.34688538	0.51528173
D6Wsu163e	0.17446171	4.54406392	0.92795377	0.34695029	0.51531424
Gbp11	-0.8547221	-0.1374851	0.92755447	0.34705174	0.51533575
Gm8615	0.22967357	2.82423481	0.9275251	0.34705921	0.51533575
Nek2	0.5313005	1.56168566	0.92729768	0.34711701	0.51533575
Chtf18	-1.039754	-1.2665603	0.92721941	0.3471369	0.51533575
Eif2b4	0.23977595	3.19675848	0.92639297	0.34734708	0.51545985
Cish	0.44961841	1.17032559	0.92638647	0.34734873	0.51545985
Pex11g	-0.9693567	-1.2928139	0.92638297	0.34734962	0.51545985
Ezr	0.13035022	6.23592406	0.92593059	0.34746475	0.5154804
Mgrn1	0.11520524	6.53038613	0.92535332	0.34761174	0.5154804
Hkdc1	-0.3498486	1.82969467	0.92527457	0.3476318	0.5154804
Ccdc62	-0.4153587	1.89830832	0.92524307	0.34763982	0.5154804
10-Mar	-0.7453682	-0.6737566	0.9251534	0.34766266	0.5154804
Ppil1	-0.1901513	3.77002774	0.92497549	0.34770799	0.5154804
Khsrp	0.12903799	6.76865432	0.92497301	0.34770862	0.5154804
Nsmf	0.14456745	5.93049297	0.92488523	0.34773099	0.5154804
Ndufaf6	-0.3358144	1.59740847	0.92445977	0.34783943	0.5154804
Fundc2	0.16291886	7.72147033	0.92410855	0.34792899	0.5154804
Klhl41	-0.411724	1.5136362	0.92409892	0.34793144	0.5154804
Dnajc8	0.15090666	6.22569928	0.92408477	0.34793505	0.5154804
Ap1g2	-0.8537377	0.00113336	0.9240547	0.34794272	0.5154804

Smad6	0.33510316	2.58406296	0.92382089	0.34800236	0.5154804
Cd59b	0.63685594	-0.4724651	0.92379436	0.34800913	0.5154804
Pih1d2	0.58784521	0.08336365	0.92345019	0.34809695	0.51554672
Col6a5	-1.5124787	-1.8134956	0.92297507	0.34821823	0.51564492
Arfgap1	0.17661721	4.53177529	0.92273674	0.34827909	0.51564492
Lor	0.42568347	0.3638543	0.92268454	0.34829243	0.51564492
Snx25	-0.1375225	5.1798809	0.92182489	0.34851209	0.51588254
Syce1	-1.6075167	-2.0044352	0.92165386	0.34855582	0.51588254
Timp3	0.23006741	9.35869266	0.92155082	0.34858217	0.51588254
Crem	0.14416745	4.8377127	0.9208431	0.34876321	0.51605935
Dhx30	-0.2415935	4.70608379	0.92074692	0.34878782	0.51605935
Tjp3	-0.9025944	-0.8532734	0.9205212	0.3488456	0.51608108
Cant1	0.23483784	3.30876225	0.92030892	0.34889994	0.51609576
Pom121	0.13706315	6.32319514	0.9201458	0.34894171	0.51609576
Fmnl1	-0.1897161	5.94178596	0.91966101	0.34906589	0.51612384
9030624J02F	0.11856481	5.22420034	0.91955318	0.34909352	0.51612384
Rem1	-1.1419868	-0.5359513	0.91936956	0.34914058	0.51612384
Tdp1	0.27864976	2.43650373	0.91917035	0.34919164	0.51612384
Camkmt	0.15745036	4.00481931	0.91914023	0.34919936	0.51612384
Aip	0.19995529	5.1871287	0.91906254	0.34921928	0.51612384
C130046K22I	-0.291927	2.95526203	0.91882398	0.34928045	0.5161368
Cc2d2a	-0.1208926	5.78023341	0.91869219	0.34931425	0.5161368
Fgfbp1	0.34241422	4.61375719	0.91738396	0.34965001	0.51651029
Slc25a14	0.18493307	4.89905277	0.91730678	0.34966984	0.51651029
Syt13	0.1419781	6.10051852	0.91720334	0.34969641	0.51651029
Golga7	-0.1360201	7.26421945	0.91671646	0.34982151	0.51663135
Rnf167	0.229254	4.18757965	0.9163207	0.34992324	0.51671787
Itgav	0.13228788	5.95813429	0.91549005	0.35013691	0.51696964
Madd	0.17661149	6.89785884	0.91528106	0.3501907	0.51698003
Adamts17	0.42628296	2.62311081	0.91512728	0.35023029	0.51698003
Ilk	0.17400844	6.00172694	0.91419252	0.35047105	0.51721423
Dpt	0.76906919	0.73137947	0.91401975	0.35051557	0.51721423
Sema6d	-0.1527234	6.16563267	0.91400833	0.35051852	0.51721423
Dmrta2	0.70197675	-0.2709334	0.91349289	0.3506514	0.51729339
Ppp1r10	0.12136564	5.87724125	0.91316357	0.35073634	0.51729339
Yod1	-0.1632384	4.82945894	0.9130623	0.35076247	0.51729339
BC037704	0.46041849	1.27085945	0.91298029	0.35078363	0.51729339
Rpa2	0.24796787	3.4815477	0.91296281	0.35078814	0.51729339
Psmg1	0.25747373	3.13073301	0.91235379	0.35094533	0.51744808
Gm19522	0.2776778	2.40790063	0.91222164	0.35097945	0.51744808
Toporsos	0.343527	1.98353634	0.9114705	0.35117349	0.51767042
6430550D23I	-0.7726133	-0.2776649	0.911231	0.35123539	0.51769795
Gm12942	-0.3685653	2.89548821	0.91093163	0.35131279	0.51772365

9330179D12I	-0.4916489	1.07492761	0.91082914	0.35133929	0.51772365
Ccdc126	0.31024167	2.28289532	0.9098932	0.35158145	0.51801675
Mtmr10	-0.1578691	4.27423478	0.9096491	0.35164464	0.51804612
Api5	0.12101659	6.94753257	0.90937461	0.35171573	0.51807992
Rdh9	-0.6249104	0.40008975	0.90916777	0.3517693	0.51807992
Ak6	0.2791249	2.76185152	0.90893452	0.35182973	0.51807992
Afg3l1	0.25075262	3.28180885	0.90873126	0.35188241	0.51807992
Prss48	-1.0492961	-0.1717178	0.90869443	0.35189196	0.51807992
Kcnc2	-0.1690108	5.91234506	0.90855865	0.35192715	0.51807992
Rplp0	-0.1783129	6.63245504	0.90786373	0.35210735	0.5181994
Emc7	0.19264879	5.39308965	0.907769	0.35213193	0.5181994
Rab2b	0.12680736	5.9805312	0.90764101	0.35216514	0.5181994
Gas2l3	-0.2442941	4.64263568	0.90757835	0.3521814	0.5181994
2700070H01I	-0.7823134	-1.1984218	0.90721937	0.35227457	0.51822979
Ndp	0.60017292	1.01292183	0.90713401	0.35229672	0.51822979
2310030G06	0.33644443	2.80094313	0.90678269	0.35238795	0.51822979
Slc30a6	0.21148568	3.08987767	0.90669782	0.35240999	0.51822979
Arhgap4	0.89270424	-0.1392605	0.90648261	0.35246589	0.51822979
E030024N20	0.13078575	5.67388708	0.90616674	0.35254797	0.51822979
Jph3	-0.1478507	5.02951187	0.90600664	0.35258957	0.51822979
Skint3	-0.4597037	1.16596641	0.9059655	0.35260027	0.51822979
Lpar2	-0.3869603	1.631953	0.90577258	0.35265042	0.51822979
Insrr	-0.7005191	0.39472325	0.90575244	0.35265566	0.51822979
Arl6	0.14340418	5.70105526	0.90523936	0.35278909	0.51822979
Adora2b	-0.5303279	0.44978686	0.90516259	0.35280906	0.51822979
E2f4	-0.1708912	4.54122018	0.90510178	0.35282488	0.51822979
Ube2e3	0.14639688	7.65512624	0.90505905	0.352836	0.51822979
Sdc2	0.21303185	6.59430272	0.90500079	0.35285115	0.51822979
Tbk1	-0.1639991	5.39326555	0.90473393	0.3529206	0.51823634
Pdp1	0.15228611	7.11894952	0.90450855	0.35297927	0.51823634
Dak	-0.3206615	2.76656113	0.90448487	0.35298543	0.51823634
Calm3	0.10459381	8.7129131	0.90431127	0.35303063	0.51823916
Chn2	0.11794971	5.29458178	0.90401304	0.35310829	0.51825772
Rpl14-ps1	-0.2682	2.59739712	0.90393039	0.35312982	0.51825772
BC022687	0.3096816	1.99778136	0.90370707	0.353188	0.51827959
Ccdc163	-0.817096	0.00792254	0.90326577	0.35330301	0.51838484
Ndufa1	0.19154469	5.23904643	0.90277139	0.35343192	0.51847059
1700073E17I	-0.3742837	2.24183211	0.90247717	0.35350866	0.51847059
Atp8b4	-0.9052392	-0.8469891	0.90221322	0.35357753	0.51847059
D5Ertd605e	-1.0725207	-1.4829157	0.90218532	0.35358481	0.51847059
Tmem29	-0.1188324	4.81473657	0.9020632	0.35361669	0.51847059
Gata3	-0.7385863	-0.4203698	0.9017775	0.35369127	0.51847059
Tceb2	0.15742163	5.70765472	0.90175314	0.35369763	0.51847059

Foxn3	0.15282948	6.54156047	0.90158204	0.3537423	0.51847059
Ddx11	-0.7101691	0.10747502	0.90151405	0.35376006	0.51847059
Zfp532	-0.1288018	5.64739454	0.9012785	0.35382158	0.51847059
Rad18	0.23319967	4.2203029	0.90085112	0.35393324	0.51847059
Fam210a	-0.1193845	6.44242624	0.90079267	0.35394852	0.51847059
Tvp23b	-0.1795733	4.76558333	0.90077338	0.35395356	0.51847059
Psg23	-0.4948992	1.27596816	0.90071982	0.35396756	0.51847059
Tfcp2	0.1582275	3.79045747	0.89927306	0.354346	0.51891685
Dusp23	0.31889375	2.79903088	0.89922381	0.35435889	0.51891685
Dcaf8	-0.113986	5.70611161	0.89898966	0.35442019	0.51894316
Ppp1r15a	0.36382212	2.79105364	0.89880563	0.35446839	0.51895028
Fa2h	-0.2520532	4.01003315	0.8984918	0.35455059	0.51900718
Rnf219	0.12332602	5.20945198	0.89750281	0.35480984	0.51928027
Cftr	-0.4943043	1.59461546	0.89733997	0.35485255	0.51928027
Hbegf	0.35598507	1.72876483	0.897284	0.35486723	0.51928027
Ubxn1	-0.2276032	5.407365	0.89683405	0.35498529	0.51933367
Siah3	0.4398472	0.8095347	0.89681437	0.35499046	0.51933367
1600002H07I	0.24118052	3.04316158	0.89664753	0.35503425	0.5193343
E230029C05I	-0.5611433	0.54626243	0.8961568	0.3551631	0.5194003
Synpo	-0.1184187	6.50701661	0.89612081	0.35517255	0.5194003
Stk16	-0.1943702	4.51350464	0.89598021	0.35520949	0.5194003
4930528D03I	-0.6722086	-0.1350901	0.8954153	0.35535792	0.51946023
Psmb2	0.16461507	5.36654514	0.89535866	0.35537281	0.51946023
AI847159	1.2735421	-1.8989295	0.89532904	0.3553806	0.51946023
Lzic	-0.1994511	4.1531194	0.89501873	0.35546218	0.51951608
Galnt1	0.15481289	6.23301899	0.8948246	0.35551324	0.51952729
Dazap2	0.14039772	8.82880673	0.89443402	0.35561599	0.5195953
Siglech	-0.312277	2.13107819	0.89431784	0.35564656	0.5195953
Fam217b	0.16618266	4.010407	0.89392602	0.35574969	0.51968258
Rps6ka4	-0.2807163	2.71149669	0.8929889	0.35599652	0.51996602
C5ar2	-0.3095273	1.32805436	0.89272759	0.35606539	0.51996602
Cd2ap	-0.1411685	7.43091632	0.89241636	0.35614744	0.51996602
Zfp383	-0.2594614	2.57221034	0.89239376	0.3561534	0.51996602
Arhgdig	0.28174437	3.12759263	0.89236566	0.35616081	0.51996602
2510003E04I	-0.12992	7.09266894	0.8920722	0.35623821	0.52001562
BC048546	0.15342817	5.10503088	0.89186231	0.35629358	0.52003306
4833420G17	0.15692315	4.88925606	0.89152987	0.35638131	0.52008554
Naa60	-0.1140773	6.11045487	0.89139696	0.35641639	0.52008554
Zfp382	-0.2027134	4.01271086	0.89099608	0.35652223	0.52014341
Alkbh5	-0.1339257	6.397967	0.89091777	0.35654292	0.52014341
Ankrd32	-0.1835838	4.11258224	0.890573	0.35663399	0.52020566
A330032B11	-0.2792497	2.15244353	0.89042737	0.35667246	0.52020566
Gpr4	-0.2471372	5.56002445	0.88996715	0.35679411	0.52028213

Gpr84	-1.1450829	-1.0057645	0.88975644	0.35684982	0.52028213
Wrb	0.12489846	5.3922587	0.88948617	0.3569213	0.52028213
Arrdc2	0.35974777	1.72076775	0.88941454	0.35694024	0.52028213
Pex5l	0.15167621	6.82776309	0.88940746	0.35694212	0.52028213
E130006D01I	-1.0971695	-2.0148907	0.88881408	0.35709913	0.52044765
Btg2	0.30734097	3.76373562	0.88848275	0.35718685	0.52046027
I830077J02R	0.44570814	0.98222716	0.88841273	0.35720539	0.52046027
Ttf1	-0.1367048	4.43710407	0.88828895	0.35723817	0.52046027
Lig4	0.17555174	4.33541158	0.88770086	0.35739398	0.5205752
Usp21	0.13320073	5.1328123	0.88747102	0.35745489	0.5205752
Cds1	-0.1545167	5.48304788	0.88745507	0.35745912	0.5205752
Cd200	0.15117595	6.86633435	0.88733507	0.35749093	0.5205752
Liph	1.61439843	-1.6566254	0.88696033	0.3575903	0.52062084
Zbtb26	0.13887822	4.37235312	0.88677469	0.35763954	0.52062084
Dnaja3	-0.1386487	5.03685258	0.88665362	0.35767166	0.52062084
Actr1a	0.1153284	6.16861461	0.88647093	0.35772014	0.52062084
Rps14	-0.2995644	4.3577063	0.88639743	0.35773964	0.52062084
3110052M02	-0.1460188	4.84073189	0.88595309	0.35785759	0.52072921
Snx8	0.34314919	1.59947853	0.88530559	0.35802956	0.52085495
Mul1	0.20656728	3.14208276	0.88523579	0.35804811	0.52085495
Ccdc155	0.73514983	-0.6128863	0.88513654	0.35807448	0.52085495
Acbd4	0.2793938	2.3604109	0.88485992	0.358148	0.52089863
Amot	0.14940611	4.95629387	0.88446999	0.35825167	0.52091237
Clns1a	0.12824858	6.19127817	0.88437504	0.35827692	0.52091237
Trhde	-0.1908053	5.81450051	0.88432245	0.35829091	0.52091237
Lrrc59	0.11064187	6.71192555	0.88401027	0.35837395	0.52091237
Vldlr	-0.1578958	6.06539724	0.88400656	0.35837494	0.52091237
Apol6	-0.584162	-0.189787	0.88357259	0.35849043	0.521017
Ndufs1	0.1529268	6.10677345	0.88317759	0.35859559	0.52106658
Wnt3	-0.8303521	-0.8614481	0.88289479	0.35867091	0.52106658
BC018507	-0.1831504	6.72558247	0.88269841	0.35872323	0.52106658
Vsx1	-0.2818039	3.86348811	0.88258499	0.35875345	0.52106658
Mir28b	1.10697121	-0.4985451	0.88245745	0.35878744	0.52106658
Acly	-0.1444365	7.0689687	0.88222767	0.35884868	0.52106658
Batf	0.68847453	0.02424304	0.88160558	0.35901456	0.52106658
Itfg3	0.29809718	1.93936477	0.88160041	0.35901594	0.52106658
Zfp300	-0.2586657	2.71413456	0.88155638	0.35902769	0.52106658
Unc13b	-0.1602373	6.53909414	0.88132781	0.35908867	0.52106658
Fam193b	-0.2902731	3.14884989	0.88119934	0.35912295	0.52106658
4833417C18I	1.1005984	-1.3667908	0.88119489	0.35912414	0.52106658
Rcn3	0.3975186	3.12705558	0.88116503	0.3591321	0.52106658
Aifm3	-0.1593769	5.53542131	0.88115911	0.35913368	0.52106658
Zfa-ps	-0.371156	1.11562698	0.88025484	0.35937512	0.52132381

Mgll	-0.1391532	5.42694072	0.88015794	0.35940101	0.52132381
Sgol2	0.23449909	3.58539855	0.88000614	0.35944157	0.52132381
Mtx1	-0.255619	2.53885428	0.87945604	0.3595886	0.5214739
Mlxip	-0.1395177	5.16406634	0.87902109	0.35970491	0.52149526
H2-Ke6	-0.3782912	2.38317371	0.87894518	0.35972522	0.52149526
Otogl	0.93326621	-1.2053507	0.87887931	0.35974284	0.52149526
Psmd3	0.15212425	4.38659442	0.87874971	0.35977751	0.52149526
Kcng4	0.36818106	1.67984873	0.87810641	0.35994969	0.52168169
Ace	-0.2917674	5.32388205	0.87783172	0.36002325	0.52169675
Pigu	0.2189289	3.77721647	0.87774227	0.36004721	0.52169675
Irgm1	0.22436352	4.39098843	0.87734747	0.36015298	0.52178688
Tapbp	0.29729112	4.62292862	0.87701748	0.36024142	0.52185187
Slitrk5	0.18026124	5.86905806	0.87658028	0.36035864	0.52192378
Slmo1	-0.1899825	5.01944233	0.8762062	0.36045898	0.52192378
Grin2b	-0.1770687	7.05758381	0.87608206	0.36049229	0.52192378
Zfp72	-0.2717431	3.12617452	0.8760397	0.36050365	0.52192378
Mfsd9	0.50902034	1.13707931	0.87597637	0.36052065	0.52192378
Prr11	-0.227225	3.12267496	0.87577684	0.3605742	0.52192378
Trp53	0.17543096	5.99533336	0.87555912	0.36063265	0.52192378
Gpc2	-0.7840314	0.06735224	0.87533816	0.36069197	0.52192378
Rasa2	-0.1347597	5.06270366	0.87526876	0.36071061	0.52192378
9230112J17F	1.13573097	-1.563652	0.87520822	0.36072687	0.52192378
Dusp4	0.44829442	1.31826327	0.87493213	0.36080104	0.52196802
Cyth1	-0.2290244	3.89157414	0.87474734	0.36085069	0.52197679
Med7	0.18197479	4.81836147	0.87454108	0.36090612	0.52199393
Gm10754	-0.3456896	1.74058134	0.87427287	0.36097821	0.52203516
Tnfrsf22	0.32859389	1.74743848	0.87386806	0.36108707	0.52204088
Bcr	-0.1760591	6.07003854	0.87369661	0.36113319	0.52204088
Rgs7bp	0.13253674	8.51584196	0.87362139	0.36115343	0.52204088
Trps1	-0.1491904	6.29006397	0.87351367	0.36118241	0.52204088
4930429B21I	0.1562933	5.16830204	0.87344781	0.36120013	0.52204088
Ranbp1	-0.1224072	5.21532742	0.87300372	0.36131966	0.52215061
A230009B12	-0.8802335	0.00823792	0.87274663	0.36138888	0.52218764
Tubgcp3	-0.1474337	4.42504064	0.87219501	0.36153747	0.52233932
1700003M07	0.17314802	3.86364677	0.87097347	0.36186683	0.52272139
Igf2r	-0.1564257	4.7242819	0.8708905	0.36188922	0.52272139
Trim66	-0.2122945	5.4375356	0.8706657	0.36194988	0.52274596
Morc4	-0.2269369	2.89235091	0.87028905	0.36205156	0.52277247
Nts	-0.4822517	1.01467383	0.86987356	0.36216377	0.52277247
Ddx3y	-0.1151323	6.3508727	0.86987088	0.36216449	0.52277247
F13a1	0.43659562	1.59449827	0.86984247	0.36217217	0.52277247
Col16a1	0.29917627	1.53962728	0.86971567	0.36220642	0.52277247
Tnxb	-0.397081	1.28082218	0.86962785	0.36223015	0.52277247

Prx	0.72030919	-0.4765211	0.86924576	0.36233341	0.52283514
Nifk	0.1944331	4.61760274	0.86864216	0.36249662	0.52283514
Angptl4	-0.3434276	2.19976334	0.86835327	0.36257477	0.52283514
Mirg	-0.5842299	1.06503662	0.86829518	0.36259049	0.52283514
Psme1	0.19233142	7.09575098	0.8682702	0.36259725	0.52283514
Nfat5	0.12595326	8.73989418	0.8682459	0.36260382	0.52283514
Zfp706	0.12180263	7.48004342	0.86822919	0.36260834	0.52283514
Rbbp6	0.10572666	9.12289896	0.86798749	0.36267376	0.52283514
Dtx4	0.14226765	5.31482988	0.86796489	0.36267988	0.52283514
Gm10778	0.2122514	3.91403951	0.86785305	0.36271015	0.52283514
Dnaja2	0.09265867	7.78861547	0.86738248	0.36283757	0.52293444
Ppard	-0.1414028	5.18174367	0.86701593	0.36293687	0.52293444
Mrpl48	0.16071938	4.45737419	0.86699645	0.36294215	0.52293444
Arid3a	-0.3114275	2.96326975	0.8669538	0.36295371	0.52293444
Abca12	1.05714891	-1.4656762	0.86666635	0.36303161	0.52298377
Rragb	0.13491325	4.62507117	0.86647678	0.363083	0.52299489
Ncapg	-0.8243767	-0.3341187	0.86570121	0.36329336	0.52323496
Gpr157	-0.3437077	2.14962224	0.8655334	0.3633389	0.52323762
Soga3	0.16297641	6.51710607	0.864704	0.36356409	0.52347892
Pyurf	0.19567631	5.11563781	0.86459434	0.36359388	0.52347892
BC048403	-0.1703485	4.04730673	0.86434197	0.36366246	0.52349785
Pi4k2a	0.1573175	4.91082753	0.86422421	0.36369446	0.52349785
P2ry10	-1.2295215	-1.3492971	0.86387275	0.36378999	0.52354647
Btf3	0.1779521	6.23544685	0.86375192	0.36382285	0.52354647
Plcb1	-0.1810867	8.78715465	0.863468	0.36390006	0.52354647
Stxbp3b	-0.673861	-0.9580444	0.86313027	0.36399194	0.52354647
Zfp944	0.18686979	3.91730998	0.86312552	0.36399323	0.52354647
Ripk4	-0.7328794	-0.0521389	0.86294029	0.36404364	0.52354647
Ptgr2	-0.1587044	6.42291844	0.86261701	0.36413164	0.52354647
Usp32	-0.1321198	7.80025164	0.86255765	0.3641478	0.52354647
Oas1c	0.39566125	1.37818016	0.86248576	0.36416737	0.52354647
Srgn	0.19887296	5.14252766	0.862367	0.36419971	0.52354647
Nostrin	-0.5435937	0.87042759	0.86233243	0.36420913	0.52354647
Sh2d7	-0.4271732	1.00357488	0.86194947	0.36431344	0.52358711
Ighmbp2	-0.3251099	1.95229387	0.86168783	0.36438474	0.52358711
Slc16a9	0.22545925	4.83671912	0.86161533	0.36440449	0.52358711
Nkx2-2os	-1.1726308	-0.6508866	0.86157841	0.36441456	0.52358711
Ltbr	0.33456931	2.37578847	0.86131015	0.36448768	0.52358711
Trmt13	-0.3452564	1.35786916	0.86126599	0.36449972	0.52358711
Slamf7	-0.5301175	1.12128087	0.85991893	0.36486727	0.52405221
Caln1	0.16361592	4.57744271	0.85971547	0.36492283	0.52406916
Mtrf1l	-0.1884292	4.04188522	0.85954133	0.36497039	0.52407462
Mrps34	0.22807454	3.02931867	0.85909902	0.36509124	0.5241647

Morn5	-1.2898126	-1.4756517	0.85899137	0.36512066	0.5241647
Ror1	0.5226223	0.88849533	0.85847399	0.36526212	0.52419092
Rusc2	0.16346512	6.58476708	0.85834207	0.36529819	0.52419092
Gins4	0.20575913	4.78379394	0.8581538	0.36534969	0.52419092
Zfp808	0.15981486	3.85726606	0.85813272	0.36535546	0.52419092
Armc1	0.10928504	7.59764091	0.85802559	0.36538477	0.52419092
Tln1	-0.1703265	6.04945015	0.85785802	0.36543063	0.52419092
Ndufaf1	0.24898077	3.33368147	0.85774194	0.36546239	0.52419092
Irf5	0.47957085	0.96066234	0.85764439	0.36548909	0.52419092
Pcmtd2	-0.1566816	4.73890337	0.85746423	0.36553841	0.52419887
Irf6	-0.2527371	3.74546148	0.85700418	0.3656644	0.52425459
Lsamp	0.21764464	5.43219533	0.85700263	0.36566482	0.52425459
4931406H21I	0.89188202	0.24026653	0.85684053	0.36570923	0.52425549
Nrg1	-0.2388857	3.36162218	0.85649727	0.36580328	0.52432756
Gorasp2	0.12583128	7.01602837	0.85601423	0.3659357	0.5244546
Ngrn	-0.1719909	4.21976295	0.85501475	0.36620992	0.5247848
Wac	0.12876054	8.63974807	0.85445056	0.36636484	0.5249122
Edc3	0.23556012	3.0896471	0.85437174	0.36638649	0.5249122
Mocs2	0.13541977	6.84766251	0.8540836	0.36646565	0.52496282
Cbwd1	0.17292445	4.13995644	0.85389516	0.36651744	0.52497421
Gtf2f1	-0.1559646	5.9128178	0.85369727	0.36657184	0.52498933
Atp5h	0.16967199	6.99955852	0.85323606	0.36669865	0.52505481
Eomes	0.59751024	0.41617846	0.85303288	0.36675454	0.52505481
Tnks2	-0.1067007	8.33239635	0.85285197	0.36680431	0.52505481
Gm13238	-0.5050851	0.09036922	0.85268634	0.36684989	0.52505481
Mxd4	-0.327296	2.4850677	0.85239319	0.36693058	0.52505481
Mir9-2	-1.0530762	-0.9702704	0.85214381	0.36699924	0.52505481
Csrp1	0.20924359	7.56769806	0.85195423	0.36705145	0.52505481
Pik3r4	-0.1540925	5.1104775	0.85187693	0.36707275	0.52505481
Cd300lb	-1.3682544	-1.3241389	0.8517409	0.36711022	0.52505481
Chpf	0.21334477	3.44592117	0.85170013	0.36712145	0.52505481
Coro6	-0.5232218	1.88178598	0.85152377	0.36717004	0.52505481
Olfr78	0.83407015	-0.7827399	0.85147997	0.36718211	0.52505481
Ntrk2	0.17094955	8.36611345	0.85146033	0.36718752	0.52505481
Fam65a	0.17173683	7.33207848	0.85116619	0.36726859	0.52510579
Commd2	0.17785253	4.30203494	0.85101284	0.36731087	0.52510579
Zfp456	-0.2472969	3.08223601	0.8507249	0.36739027	0.52515661
Rad9a	-0.1983809	3.49725422	0.84998385	0.36759473	0.52538616
Meaf6	0.14265117	5.01797315	0.84891622	0.36788958	0.52570298
Sntg1	0.1558057	5.58941701	0.84881221	0.36791833	0.52570298
Hn1l	0.25072469	4.36652087	0.84849395	0.3680063	0.52570298
Tnrc6b	-0.1245327	8.72502466	0.84847846	0.36801058	0.52570298
Dgkd	-0.1764531	5.44293754	0.84838694	0.36803589	0.52570298

G6pc3	0.20230762	3.9418678	0.84798942	0.36814582	0.52570469
Proca1	-0.2980671	1.88584222	0.8478547	0.36818309	0.52570469
Ido2	-0.3264585	2.00854124	0.84784683	0.36818527	0.52570469
Col11a2	-0.5212447	0.10976493	0.84774779	0.36821267	0.52570469
Ankle2	-0.1249541	5.23852826	0.84756917	0.3682621	0.52571259
Gorasp1	0.21944295	3.03344982	0.84710693	0.36839005	0.52582418
Cdyl2	0.16641321	5.28059106	0.8469353	0.36843758	0.52582418
Parl	-0.2073472	3.34903701	0.8466893	0.36850572	0.52582418
Spg7	-0.2278117	3.55351131	0.84665252	0.3685159	0.52582418
2310061J03F	-0.353705	2.3589709	0.84648758	0.3685616	0.52582674
Mob3b	0.15875016	6.71471211	0.84594173	0.36871289	0.52597991
Amdhd1	-1.0791937	-1.7095124	0.84545304	0.36884841	0.52605797
Camk1	-0.1823142	3.80136889	0.84542762	0.36885546	0.52605797
Rcsd1	-0.2205498	4.59087504	0.84495413	0.36898684	0.52618268
Srrd	0.3883888	1.36859612	0.84454615	0.36910009	0.52628151
Zmym3	-0.1589518	6.02195042	0.84416764	0.36920521	0.52636873
Trh	1.44837092	-1.6101563	0.84378276	0.36931215	0.52645708
Rnf122	0.54315549	0.02224621	0.84348534	0.36939481	0.52645708
Mrps31	0.19273631	4.56028874	0.84340096	0.36941827	0.52645708
Prmt3	0.1656955	4.30951432	0.84331193	0.36944302	0.52645708
Kcnh1	0.16531922	6.07146337	0.84256212	0.36965159	0.52669161
Snurf	-0.3393229	1.5189843	0.84197273	0.36981565	0.52681912
Gpsm3	-0.4228475	2.31143864	0.84192457	0.36982906	0.52681912
Eif2s1	0.11168506	6.78122622	0.84156126	0.36993024	0.52690058
3110062M04	0.42415753	0.70944072	0.84031373	0.37027801	0.52731949
Dalrd3	-0.241459	3.33754619	0.84004939	0.37035176	0.52731949
Usp38	-0.1566261	5.03510708	0.83970252	0.37044857	0.52731949
C1ql1	-0.3092659	2.81366461	0.83954549	0.37049241	0.52731949
Ppox	0.45531295	1.015352	0.83953622	0.37049499	0.52731949
Cecr5	0.47835971	0.6165469	0.83941563	0.37052867	0.52731949
Aco1	-0.160625	4.27600672	0.83915612	0.37060114	0.52731949
Camk1g	0.23709169	4.06859878	0.83908861	0.37062	0.52731949
Vmn2r118	1.21400618	-1.2699865	0.83908629	0.37062064	0.52731949
Psmd2	0.13195287	5.80504036	0.8388697	0.37068115	0.52734293
Tcof1	-0.1671332	4.23575164	0.83862042	0.37075081	0.52737257
Slc10a4	-0.2441238	2.53480028	0.83839936	0.3708126	0.52737257
Sel1l3	-0.2377423	4.96079136	0.83814455	0.37088384	0.52737257
Plau	0.56327907	-0.1551436	0.83802585	0.37091703	0.52737257
Fgf23	-1.0114349	-1.0142725	0.83793075	0.37094363	0.52737257
9130221H12I	-0.196666	3.48044409	0.83785003	0.37096621	0.52737257
Smu1	0.13632108	5.43062054	0.83654933	0.37133029	0.5278275
Ttc38	-0.1808949	3.77982838	0.8363747	0.37137921	0.52783439
Mmgt1	0.14868806	4.66020871	0.83561288	0.37159274	0.52807519

Ano2	0.67855088	0.14009161	0.83524501	0.37169591	0.52815104
Edf1	0.22615105	6.41286507	0.83510811	0.37173431	0.52815104
Gli2	0.21861535	3.37357665	0.83369801	0.37213024	0.52865085
Acta2	-0.2477677	3.71239408	0.83275295	0.37239594	0.52896491
Nat1	0.39346254	2.09121257	0.83259753	0.37243966	0.52896491
Tmem42	-0.2620418	2.41919743	0.83216407	0.37256164	0.52902495
Ddx56	0.26111208	3.08038876	0.83192191	0.37262981	0.52902495
Nsfl1c	0.15253602	5.06797912	0.83175273	0.37267744	0.52902495
Pipox	-0.458253	0.57711512	0.83170585	0.37269064	0.52902495
Syt14	-0.4171831	1.37972022	0.83166268	0.3727028	0.52902495
Map1s	-0.2021805	3.04419634	0.83149988	0.37274865	0.52902686
Extl2	-0.1350513	6.08279579	0.83134424	0.3727925	0.52902686
Slc29a1	0.20559409	2.59628195	0.83106493	0.3728712	0.52907585
Ankrd23	0.70012482	-0.7013477	0.83071742	0.37296915	0.52915214
Mpv17l	-0.1360554	5.64672728	0.83031694	0.37308208	0.52920118
Anks1b	0.15172473	8.50588551	0.83022173	0.37310894	0.52920118
Gm8234	-0.2340625	3.69496776	0.83012479	0.37313628	0.52920118
Taco1	-0.3242459	1.58711343	0.82987377	0.37320711	0.52923895
Pip4k2a	0.09766882	7.90645861	0.8295879	0.37328779	0.52929068
Gm3435	-0.2512444	2.79609154	0.82914461	0.37341295	0.52940547
Nav3	-0.2080078	6.74141912	0.82847555	0.37360197	0.52945865
Emx1	0.49649912	0.52695818	0.82835215	0.37363685	0.52945865
Stat6	-0.1611868	5.05259947	0.82826225	0.37366226	0.52945865
Fam110a	-0.4347358	1.23693417	0.82804617	0.37372335	0.52945865
Rasl11b	-0.1364581	5.61450664	0.82802899	0.37372821	0.52945865
Wbscr17	-0.1949256	3.82824717	0.82788347	0.37376936	0.52945865
Qpct	0.37957865	1.21858287	0.82777996	0.37379864	0.52945865
2610020H08I	-0.4632918	0.73171678	0.82776047	0.37380415	0.52945865
Ecscr	-0.7582344	-0.6788505	0.82706507	0.37400092	0.52964196
Ablim2	0.14914402	6.07377197	0.8269857	0.37402339	0.52964196
Alg13	-0.2801764	3.17797422	0.82676401	0.37408615	0.52964196
Vti1b	-0.1349026	5.76983741	0.82660604	0.37413089	0.52964196
2200002D01I	-0.456439	0.63943445	0.82652195	0.3741547	0.52964196
Slc25a20	-0.2032134	4.78265158	0.82633986	0.37420628	0.52965237
Bckdhb	-0.2908759	2.62445648	0.82569533	0.37438894	0.52979728
Nfasc	0.14740144	7.70953215	0.82566639	0.37439714	0.52979728
Srsf11	-0.1213436	6.73223638	0.8254209	0.37446675	0.52983318
Camsap2	-0.140003	8.77325357	0.82523244	0.3745202	0.5298462
Ttc7	0.27121708	3.14285201	0.82500113	0.37458581	0.52987644
Nfic	0.14256315	7.69138183	0.82463202	0.37469056	0.52990904
Mfsd7b	-0.2376062	2.61069559	0.82460807	0.37469736	0.52990904
Cwc25	-0.1518347	4.51326967	0.82431734	0.37477989	0.52996317
Zfp605	-0.1425137	4.69555872	0.82412871	0.37483345	0.52996743

Gbas	0.13057168	6.53615095	0.82399508	0.37487141	0.52996743
Itgal	-0.5067114	0.64269499	0.82369153	0.37495764	0.53002677
Phip	-0.1432836	7.35489708	0.82305958	0.37513726	0.53015444
1700001O22	-0.4629288	0.38566742	0.82302218	0.37514789	0.53015444
Cadps	-0.1831647	8.58472785	0.82290655	0.37518077	0.53015444
Wasf3	-0.1328456	5.95092442	0.82128734	0.37564165	0.53074307
Magoh	-0.151392	5.17826882	0.82087013	0.37576053	0.53084841
3110040N11	-0.2340915	3.1539725	0.82070066	0.37580884	0.53085403
Lsm6	-0.1319038	5.28972953	0.82045364	0.37587927	0.5308909
Sesn3	-0.121327	6.09215675	0.82023992	0.37594022	0.53091437
Flt4	-0.6653571	1.00648881	0.81993101	0.37602834	0.5309762
Zfp354b	0.31003552	2.41234248	0.81970799	0.37609198	0.53100346
Cisd2	0.1221305	6.17756167	0.819405	0.37617847	0.53103912
Cryz	-0.2461143	2.82523574	0.81925546	0.37622116	0.53103912
Ralgapa1	-0.1965798	7.7368995	0.81897464	0.37630136	0.53103912
Rspo2	0.25765995	4.39083096	0.81887371	0.37633019	0.53103912
Rps16	-0.1776264	6.26269801	0.81866729	0.37638916	0.53103912
Upf3b	-0.149358	5.6288383	0.8185322	0.37642776	0.53103912
Unc45a	0.25171315	2.75728444	0.8183704	0.376474	0.53103912
AW554918	-0.1310406	4.9547317	0.81829945	0.37649428	0.53103912
Mrps18c	0.19133116	4.53628145	0.81822232	0.37651633	0.53103912
Angpt1	0.20365462	4.59662105	0.81793317	0.376599	0.53109317
Trim44	0.08884396	9.18709954	0.81765304	0.37667911	0.53114361
Mettl21a	0.25771941	2.89583709	0.81742941	0.37674309	0.53117128
D630045J12F	0.12898222	6.85489924	0.81718616	0.3768127	0.53120688
Tmco3	0.18487222	3.76678227	0.81692762	0.3768867	0.53124866
Gins1	0.65714904	0.15266599	0.81645792	0.3770212	0.53137571
Fgl2	0.21812214	4.78953892	0.81624295	0.37708278	0.53139996
Cd4	-0.377385	3.24034563	0.81546645	0.37730534	0.53152294
Cage1	-0.4431785	1.19686726	0.81537359	0.37733197	0.53152294
Dkc1	-0.112903	6.20082846	0.81532059	0.37734717	0.53152294
Glod4	0.11897009	5.51592075	0.81510723	0.37740836	0.53152294
Zfp111	-0.165139	5.16737656	0.81505836	0.37742238	0.53152294
Dll4	-1.0661008	-1.5188964	0.81500969	0.37743635	0.53152294
Lhx6	0.16438481	4.21620547	0.81460291	0.37755307	0.5316248
Ubac1	0.1778449	3.83876854	0.8139528	0.37773974	0.53175633
Lgals9	0.2091082	4.40799855	0.81386405	0.37776523	0.53175633
Zfp322a	0.12454757	6.03840484	0.8138137	0.37777969	0.53175633
Shcbp1l	0.60806154	0.19433113	0.81355982	0.37785263	0.53176064
Unc119b	0.21529969	4.03047117	0.81345087	0.37788394	0.53176064
Gmcl1	-0.1505014	4.31263932	0.81333946	0.37791596	0.53176064
Sdr39u1	0.14909464	4.98892235	0.81314994	0.37797044	0.53177482
Smyd5	0.22834902	3.00264503	0.81259484	0.37813008	0.53185635

Exoc3l	-0.3751754	1.39302069	0.81238755	0.37818971	0.53185635
Mapk6	-0.1002461	7.5119382	0.81234631	0.37820158	0.53185635
Ddx19a	0.15581186	5.4407088	0.81233082	0.37820604	0.53185635
Pcdha5	0.45983866	0.8341099	0.8121518	0.37825756	0.53186065
Ebf4	-0.5525354	1.13461649	0.81201156	0.37829792	0.53186065
Nfu1	0.16192194	4.48020055	0.81117456	0.37853897	0.53206863
Ston2	-0.2040941	4.6411271	0.81107888	0.37856654	0.53206863
Ccar2	-0.2068042	4.23538832	0.81103516	0.37857913	0.53206863
Zfp213	0.43819785	1.09284585	0.81063087	0.37869567	0.53215838
Tspan12	0.27688911	3.01681319	0.81025754	0.37880332	0.53215838
Chd1	-0.0982212	6.5805177	0.81021197	0.37881646	0.53215838
Tmem198b	-0.4230016	0.79725695	0.81012814	0.37884064	0.53215838
Aldh7a1	-0.1249903	4.98129847	0.80997738	0.37888414	0.53215838
Gm16845	0.64317929	0.69117642	0.80988909	0.37890961	0.53215838
Slc35g3	-1.1749333	-1.5128325	0.80930237	0.37907896	0.53232472
Lysmd1	0.20942799	3.29837465	0.80910235	0.37913672	0.53232472
BC068281	-0.2253596	2.29279265	0.80901687	0.3791614	0.53232472
Jag2	0.38623147	0.88017764	0.80845094	0.37932491	0.53247076
Xylt1	-0.2719353	2.83464097	0.80815445	0.37941061	0.53247076
Lrrc4b	0.17214501	4.57026457	0.80814198	0.37941422	0.53247076
Atp2c1	-0.1151025	6.56727985	0.80795692	0.37946772	0.53247076
Gm3086	-0.6990525	0.26584689	0.80788772	0.37948773	0.53247076
Mvb12b	-0.1178992	6.44063794	0.80760823	0.37956857	0.53249195
1810022K09I	0.14342092	4.99892239	0.80731409	0.37965367	0.53249195
Sema4d	-0.2222653	3.84624337	0.80710692	0.37971363	0.53249195
Nfkbia	-0.2445417	2.74707365	0.80698927	0.37974769	0.53249195
Cyyr1	-0.8545676	0.36582894	0.80689749	0.37977426	0.53249195
Imp4	-0.1680785	4.57065773	0.80678698	0.37980625	0.53249195
2310022B05I	0.12870008	6.31292069	0.80666329	0.37984207	0.53249195
Il15ra	0.23174824	2.60517085	0.80650025	0.37988929	0.53249195
Ccdc88b	0.46654597	0.13683675	0.80645284	0.37990302	0.53249195
Adamtsl3	0.32768643	4.39157337	0.80584068	0.3800804	0.53267823
Mir128-1	-0.5097906	1.50571919	0.80501011	0.38032126	0.53289846
Cdc23	0.13768766	5.00918478	0.80486825	0.38036241	0.53289846
Rspo3	-0.2427288	7.1031934	0.80483855	0.38037103	0.53289846
Sugp2	-0.2472679	3.99885619	0.80433131	0.38051827	0.53298183
Ucp2	0.24326048	7.46890025	0.80420896	0.38055379	0.53298183
Usp34	-0.1545735	8.49626078	0.80415778	0.38056866	0.53298183
Cxadr	0.19905468	6.56603292	0.80402039	0.38060856	0.53298183
Ints9	0.13990123	4.3612064	0.80386308	0.38065425	0.53298349
Grb14	-0.2181624	3.48487065	0.8037035	0.38070061	0.53298609
Ube2c	0.45925659	0.65337984	0.80301845	0.38089974	0.53311178
Ngb	0.40699089	0.84514126	0.80274892	0.38097812	0.53311178

Usp22	0.10989845	7.05458543	0.80257555	0.38102855	0.53311178
Inpp4b	0.19404707	3.81977761	0.80245778	0.38106282	0.53311178
Parp12	-0.175604	4.35770763	0.80236595	0.38108954	0.53311178
Col8a2	-0.3895598	2.09764553	0.80235488	0.38109276	0.53311178
Idh2	-0.2032312	4.04339643	0.80232311	0.381102	0.53311178
Slc8a2	0.16640248	5.55719983	0.80196049	0.38120755	0.53319715
Mrps11	0.34718876	1.41683712	0.80122978	0.38142037	0.53335006
4930483K19I	0.6597268	-0.4326204	0.80101935	0.38148169	0.53335006
Atp5g2	0.21958037	4.07447845	0.80099417	0.38148902	0.53335006
Slc38a5	-0.9321565	-1.1945764	0.80097361	0.38149502	0.53335006
Snip1	0.22256596	3.26961063	0.80026755	0.38170088	0.53357557
Itga6	-0.2279555	3.55203255	0.80005416	0.38176313	0.53360031
2310040G24	0.68387726	0.08428143	0.79958783	0.38189922	0.53367449
Cspg5	-0.1566509	5.25798228	0.79945968	0.38193663	0.53367449
E2f3	0.15076046	5.37305131	0.79927311	0.3819911	0.53367449
Rps29	-0.1405834	6.7774653	0.79913348	0.38203188	0.53367449
Apbb1	0.13343534	5.52168404	0.79910903	0.38203902	0.53367449
Fam132b	0.72516697	-0.102842	0.79884526	0.38211607	0.53370475
Lmbrd2	0.13506007	6.12720119	0.7984709	0.38222546	0.53370475
Casz1	-0.3674032	2.11849461	0.79844472	0.38223311	0.53370475
Trit1	-0.2317556	3.53345895	0.79842478	0.38223894	0.53370475
3632451O06	0.20630946	4.23193063	0.79763215	0.38247073	0.5338974
Fsd1l	0.16086366	6.17601251	0.79741937	0.38253298	0.5338974
Fgfrl1	-0.2585514	2.25860153	0.79727172	0.38257619	0.5338974
Ier5	0.13614024	6.19845767	0.79721283	0.38259343	0.5338974
Ap4b1	0.28981677	2.03441604	0.79714972	0.3826119	0.5338974
Ttc13	-0.2256625	3.19742116	0.79703866	0.38264441	0.5338974
1810024B03I	-0.8842785	-0.6292529	0.79616157	0.3829013	0.53394303
Gpsm1	-0.2160845	2.66469454	0.79610511	0.38291785	0.53394303
Zcchc2	-0.1295816	6.13430018	0.79609704	0.38292022	0.53394303
Slc25a39	0.20802264	4.37736588	0.7960148	0.38294432	0.53394303
Cd44	0.20586646	3.13111967	0.79588718	0.38298172	0.53394303
Hs6st1	0.15814343	4.62727952	0.79575164	0.38302146	0.53394303
Heatr6	-0.1536096	4.8189271	0.7957211	0.38303041	0.53394303
Mfng	-0.5994149	-0.3038503	0.79560605	0.38306415	0.53394303
Bckdha	-0.3070389	1.13284598	0.79555678	0.38307859	0.53394303
Cyp4f17	1.16536258	-1.7790445	0.79540547	0.38312297	0.53394303
B3gnt1	0.1457402	4.84392782	0.79485951	0.38328314	0.53410031
Nae1	-0.1435396	5.057229	0.79457622	0.3833663	0.53410031
Lrrc75a	-0.1982945	3.25761938	0.7945649	0.38336962	0.53410031
Flrt3	-0.1240784	5.81551562	0.79440674	0.38341605	0.53410287
Psmg4	-0.3763246	1.72007138	0.79400373	0.38353441	0.53417179
Rufy1	0.14556116	4.69976452	0.79392894	0.38355638	0.53417179

Pacsin1	0.12843857	6.57730162	0.79378273	0.38359934	0.53417179
Taf4a	0.13683199	4.74494453	0.79329468	0.38374279	0.53430941
Chst10	0.17915206	3.87552992	0.79219579	0.38406606	0.53469735
Nanos2	-1.3615697	-2.2041108	0.79169567	0.38421331	0.53483233
Dmgdh	-0.5512416	0.06703914	0.79150417	0.38426972	0.53483233
Arhgap36	0.57735753	0.91719473	0.79129905	0.38433016	0.53483233
Fam111a	0.18820409	3.34294009	0.79126005	0.38434165	0.53483233
Rin1	0.2097419	3.80022868	0.79066858	0.384516	0.53501278
Igf2bp1	-1.3682142	-1.5499557	0.79034989	0.38460999	0.53508138
Ccdc57	0.28489406	1.83468668	0.79000729	0.38471107	0.53510746
Pam	0.12652979	6.3274475	0.78972747	0.38479366	0.53510746
Slc38a2	0.21982459	9.37180083	0.78970793	0.38479942	0.53510746
Susd1	0.36187381	1.90334626	0.78968071	0.38480746	0.53510746
2610005L07I	-0.153925	6.35267353	0.7892216	0.38494302	0.53523382
Gmppa	0.14763436	3.73672678	0.78881535	0.38506304	0.53533854
Tmem255b	-1.0657914	-0.9751836	0.78853469	0.38514599	0.53539171
Pole4	0.14526764	5.26961281	0.78822123	0.38523866	0.53545838
Nos1ap	0.23488205	3.69490422	0.78784261	0.38535064	0.53555187
Ccdc34	0.1440617	6.08041037	0.78678648	0.38566326	0.53589376
Dnajb2	-0.1526814	5.41496715	0.78660227	0.38571782	0.53589376
Gucy1a3	-0.1592607	7.27725386	0.78655816	0.38573089	0.53589376
Htr2c	0.17918878	4.57506655	0.78626428	0.38581797	0.5359213
Mir3064	0.61644696	-0.6415369	0.78593627	0.38591519	0.5359213
Cyth3	-0.1521581	6.99938131	0.78590432	0.38592467	0.5359213
Qrfpr	-0.6717473	0.13814898	0.78566737	0.38599493	0.5359213
Nsmce4a	-0.1728991	4.58308296	0.78559159	0.3860174	0.5359213
Kcnv1	-0.1925527	6.74877182	0.78544339	0.38606136	0.5359213
Apbb1ip	-0.2164427	3.19513467	0.7854346	0.38606397	0.5359213
Timm44	0.19803903	3.40871495	0.78470638	0.38628007	0.53601713
Shf	-0.411609	1.94867076	0.78464404	0.38629858	0.53601713
Etfb	-0.2041098	4.37938077	0.78442034	0.38636501	0.53601713
Cpne7	0.2733806	2.63343277	0.7842718	0.38640912	0.53601713
2900079G21	0.31467144	1.16559202	0.78427166	0.38640917	0.53601713
Zfp326	-0.1160211	5.58681216	0.78426791	0.38641028	0.53601713
E130215H24I	-1.0442923	-1.8867216	0.78407421	0.38646782	0.53601713
Mysm1	-0.128831	6.25552941	0.78399596	0.38649107	0.53601713
Mt3	0.69584778	-1.0707301	0.78383283	0.38653955	0.53602228
Utp14a	0.17730168	3.98157565	0.78309871	0.3867578	0.5361563
Slc7a1	0.13415617	5.28969118	0.7830644	0.38676801	0.5361563
Hjurp	-0.2567224	2.44062211	0.78294535	0.38680342	0.5361563
4933430I17R	1.03515931	-1.6878358	0.78290551	0.38681528	0.5361563
Abcb8	-0.1584402	4.05750279	0.78274381	0.38686338	0.53616093
Tbp	0.10796019	5.33457464	0.78218836	0.38702871	0.53632799

Acadl	0.16802837	5.29580048	0.78200254	0.38708404	0.53634261
Gtf2h4	-0.3568954	1.44532771	0.78175354	0.3871582	0.53638331
Pfkfb4	-0.3834845	1.19847148	0.78138941	0.3872667	0.53647156
Gemin6	-0.1936059	2.96628022	0.78108157	0.38735846	0.53649252
Lrrk1	0.20412049	5.08688029	0.78103696	0.38737175	0.53649252
Kctd18	-0.1438031	4.36837927	0.78088781	0.38741622	0.53649252
Hck	-0.772382	-0.4803346	0.78027994	0.38759754	0.53668155
Mcu	0.18933802	4.64332139	0.77974251	0.38775796	0.53684159
Pstpip2	-0.1653103	3.88083727	0.77948287	0.38783549	0.53688687
3110039I08R	-0.6245515	-0.0970578	0.77879685	0.38804046	0.53710853
Grhl1	-0.2283848	2.97171947	0.7784586	0.38814158	0.53718641
Zfp592	-0.1311087	5.65613648	0.77730571	0.38848655	0.53753563
Tbc1d8	-0.2220368	4.20495322	0.77721725	0.38851303	0.53753563
Rab8a	0.24119114	4.84206691	0.77716536	0.38852857	0.53753563
Ccdc171	0.20636111	3.20507222	0.77661567	0.38869323	0.53760212
Pax6	0.28432401	2.24170717	0.77659099	0.38870063	0.53760212
Slc6a12	-0.3005656	3.69420656	0.77655538	0.3887113	0.53760212
Caap1	-0.2005618	2.76923956	0.77572658	0.38895978	0.53788366
Nr2c2ap	0.25701054	2.05884682	0.77521918	0.38911203	0.53803207
Carkd	-0.1690422	3.50228562	0.77489398	0.38920965	0.53810492
Crh	-0.6046474	0.14931193	0.77443517	0.38934744	0.53823328
B230208H11	-0.848388	-0.0817268	0.77407861	0.38945457	0.53823992
Glrx2	0.10124843	6.7348839	0.77393601	0.38949743	0.53823992
Sepp1	0.18193431	8.0461809	0.77387487	0.38951581	0.53823992
Ccbl1	-0.3852827	1.5529635	0.77371137	0.38956496	0.53823992
Abcc6	-0.9009627	-0.8892767	0.77367144	0.38957696	0.53823992
Tmem64	0.16242263	8.87291316	0.77342222	0.38965191	0.53828137
Abcb6	0.45055259	1.61675515	0.77308125	0.38975447	0.53835997
C530008M17	0.17157053	5.06620149	0.77277228	0.38984745	0.53835997
Sdc1	-0.3482609	2.17858721	0.77275375	0.38985303	0.53835997
2610316D01	0.32035708	2.20637587	0.7726355	0.38988862	0.53835997
Ppp1r2	0.11077924	8.03802023	0.77238458	0.38996417	0.5384022
Scmh1	0.12991611	6.11744616	0.77214881	0.39003517	0.53843816
Ogfod2	0.21367535	3.487368	0.77086636	0.39042172	0.53890763
Sdha	-0.0995242	8.6038196	0.77066789	0.39048159	0.53890763
Jmjd6	-0.1817672	2.98096008	0.77057293	0.39051025	0.53890763
Isca1	0.09442318	6.6628127	0.77028616	0.39059679	0.53896495
Shfm1	0.15607253	4.75682789	0.77012195	0.39064636	0.53897125
Efnb1	0.29200018	3.42047178	0.76966853	0.39078328	0.53909805
Lhx2	0.13886413	5.46482425	0.76945637	0.39084738	0.53912437
Kdelc1	0.22135565	2.8426093	0.76900736	0.39098307	0.53920939
4833424O15	0.15622674	4.66209956	0.76886278	0.39102678	0.53920939
Fam73b	-0.1779288	3.66081619	0.76880552	0.39104409	0.53920939

Stx1b	-0.1309235	6.40618772	0.76845751	0.39114934	0.53929242
Whrn	-0.2241001	3.21545349	0.76821061	0.39122403	0.53933331
Ces5a	0.93336562	-1.1757707	0.76792943	0.39130912	0.53933976
Lrrc40	0.16612122	5.10632569	0.76770753	0.39137629	0.53933976
Zpbp	0.66191811	0.39865645	0.76749745	0.3914399	0.53933976
Cbx7	-0.1798777	4.92502466	0.76732074	0.39149341	0.53933976
1110008L16I	0.13266395	4.36276235	0.76730127	0.39149931	0.53933976
Rai14	0.15102826	6.49851665	0.76728886	0.39150307	0.53933976
Yaf2	0.12312877	7.42070674	0.76713945	0.39154833	0.53933976
Uqcrc1	-0.1184655	5.02004495	0.76688914	0.39162417	0.53933976
Rpl36a	-0.1864575	6.48678995	0.76685658	0.39163404	0.53933976
0610037L13I	0.17244075	4.97435407	0.76638642	0.39177655	0.53943755
Abcb10	0.19368529	3.68773731	0.76626892	0.39181218	0.53943755
Ooep	0.76453721	-1.0041709	0.76614675	0.39184923	0.53943755
Tnfrsf1b	-0.4916486	1.11957083	0.76593799	0.39191255	0.53943755
Pnisr	-0.1659273	7.66635585	0.7658796	0.39193027	0.53943755
Spata1	0.37945526	1.26760515	0.76452971	0.39234012	0.53982993
Pradc1	-0.2469593	2.72955504	0.76429521	0.39241139	0.53982993
Prok2	-0.8957885	-1.3584056	0.76422461	0.39243285	0.53982993
Lmf1	0.25017753	2.93702703	0.76421483	0.39243582	0.53982993
Snn	-0.1079776	6.63171243	0.76419866	0.39244073	0.53982993
Ubqln4	0.12259659	6.15936586	0.76342459	0.39267614	0.53994549
Eps8l2	-0.4239005	1.20284362	0.76329413	0.39271583	0.53994549
Olfml1	-0.1905376	4.58663499	0.76323383	0.39273418	0.53994549
Trmt61b	-0.2014813	3.3221804	0.7630665	0.39278511	0.53994549
Arsi	0.54262024	0.61766923	0.76304829	0.39279065	0.53994549
Ctnnbl1	-0.1828868	3.28848364	0.76284386	0.39285289	0.53994549
Raly	0.2605053	2.78369417	0.76281451	0.39286182	0.53994549
Nudt12	-0.3143956	2.84423276	0.76273696	0.39288543	0.53994549
Plxna1	-0.1850305	5.51998092	0.76229224	0.39302088	0.54006553
Nrarp	-0.2826729	2.7117854	0.76215408	0.39306298	0.54006553
4632427E13I	0.4469663	1.56758032	0.76156861	0.39324143	0.54024874
Rnf11	-0.1054099	6.86253353	0.76116968	0.39336309	0.5403539
Unc93b1	-0.3730336	1.36127615	0.76062828	0.39352829	0.54045764
Scpep1os	-0.7091249	-1.5246449	0.76056693	0.39354702	0.54045764
Cbfa2t2	0.09525048	6.40881928	0.76003825	0.39370846	0.54045764
Thap6	0.18896924	3.86927998	0.75997105	0.39372898	0.54045764
2610306M01	0.22723995	2.61735373	0.75994487	0.39373698	0.54045764
1700009P17I	-0.3375139	1.70603759	0.75977407	0.39378916	0.54045764
Nelfa	0.1348776	4.89453732	0.75966176	0.39382348	0.54045764
Enox1	0.18157134	3.98225846	0.75965938	0.39382421	0.54045764
Rps4x	-0.1679473	7.89493547	0.75947562	0.39388037	0.54045764
Scin	0.93580062	-0.5378997	0.75932584	0.39392615	0.54045764

Atp6v0c-ps2	0.51388528	-0.3216039	0.75929678	0.39393504	0.54045764
Trp53cor1	0.53548931	-0.0263745	0.75878413	0.39409181	0.54050661
9430083A17	0.50339054	1.31944819	0.75878059	0.39409289	0.54050661
Dbp	-0.2224239	4.08973715	0.7587373	0.39410613	0.54050661
Stk39	0.15090757	7.75126051	0.7582731	0.39424818	0.54057514
Map4k1	-0.4099979	0.43633808	0.75808185	0.39430672	0.54057514
Ttc37	0.21136948	3.70231221	0.75802695	0.39432353	0.54057514
Dctpp1	0.26143824	1.63545479	0.75798406	0.39433666	0.54057514
1700028K03	0.41029055	2.08229983	0.75766359	0.3944348	0.54064779
Plekho2	0.20106906	4.99004047	0.75733742	0.39453472	0.54068882
2310015A10	-0.2779368	2.92595868	0.75720257	0.39457604	0.54068882
Zfpl1	0.23956881	2.51937251	0.75712382	0.39460018	0.54068882
Bri3bp	-0.1361076	5.72851111	0.75692792	0.39466023	0.54070923
D930020B18	-1.1753857	-2.0613081	0.75654427	0.39477786	0.54075056
Slc12a4	0.45094213	1.46995527	0.75653502	0.3947807	0.54075056
Fzd10	0.38133108	1.03771457	0.75630443	0.39485143	0.54078559
Zfp783	-0.3740745	1.36514797	0.75609718	0.39491502	0.54081083
Ncoa6	0.1174731	8.53285239	0.75536108	0.39514099	0.54105842
Tmem88	-0.3031547	2.02488157	0.75510263	0.39522038	0.54110525
Nap1l2	-0.171272	5.39456633	0.75455339	0.39538917	0.54127446
Gm5086	-0.8981463	-0.7664952	0.75421426	0.39549344	0.54135532
Ralgps2	0.10802188	6.33811271	0.7538005	0.39562071	0.54143562
Patl1	0.12134212	6.17278137	0.75372962	0.39564252	0.54143562
Med12	-0.1661914	4.99139276	0.75338959	0.39574717	0.54151651
Rnf38	0.09636132	7.97757377	0.75310293	0.39583543	0.54151651
Trmt10a	-0.2103421	2.92068667	0.75309689	0.39583729	0.54151651
Thsd7b	0.37000722	1.27825354	0.75285276	0.39591247	0.5415575
Bap1	-0.1448772	4.87571872	0.75253208	0.39601127	0.5415717
Chrm3	-0.2025026	3.59287046	0.75242081	0.39604556	0.5415717
Kcnb2	-0.1518441	5.28418829	0.75237874	0.39605852	0.5415717
Mrps22	0.12571233	4.4030088	0.75220472	0.39611216	0.54158321
Acan	-0.878035	-0.8003149	0.75151765	0.39632404	0.54176603
Ctnna1	0.14404442	7.91023598	0.75113768	0.39644129	0.54176603
Cyp4f15	-0.3887804	1.15049786	0.75097867	0.39649037	0.54176603
Pianp	0.12142298	6.45296759	0.75093077	0.39650516	0.54176603
Ociad1	0.10040766	7.1434542	0.75092292	0.39650758	0.54176603
Nek3	-0.4665078	0.95365832	0.75089142	0.3965173	0.54176603
AK129341	-0.1994268	5.21366025	0.75043201	0.39665917	0.54187053
Vdac3	0.11312015	6.33344942	0.75026335	0.39671128	0.54187053
Blzf1	-0.1512346	4.90929891	0.75020426	0.39672953	0.54187053
Erap1	-0.2714564	3.54311035	0.74996021	0.39680495	0.54191173
Psmb3	0.15614743	4.45844632	0.74942169	0.39697144	0.54199054
Eif2ak4	0.26211879	3.93746764	0.74940542	0.39697647	0.54199054

Clic3	0.68145421	-0.5019365	0.74933443	0.39699843	0.54199054
Sdhaf1	0.24613649	2.31989015	0.7488461	0.39714951	0.542135
Ptrf	0.2174332	6.57759735	0.74869713	0.39719561	0.54213614
Nudt18	-0.2082265	3.81344971	0.74800775	0.39740908	0.54236569
Pomgnt2	0.27434294	2.41069811	0.7475871	0.39753942	0.54248174
Ddb2	-0.3468397	1.05610744	0.74726102	0.3976405	0.54255786
Crlf1	0.61519526	-0.4769437	0.74677396	0.39779156	0.54264899
Akr1c21	1.43704685	-1.1791494	0.74675346	0.39779792	0.54264899
Alkbh3	-0.2243099	3.75199751	0.74630729	0.39793637	0.54277603
Nagpa	-0.2676662	2.8786944	0.74613038	0.39799129	0.54278911
Adamts16	0.53502596	0.94222066	0.74588242	0.39806828	0.5428323
BC024978	0.15402593	4.79122258	0.74561293	0.39815198	0.54285046
Srebf1	-0.1992575	2.89281282	0.74554767	0.39817225	0.54285046
Fat1	-0.1341835	6.13438627	0.7453442	0.39823547	0.54287428
6330408A02	0.14645139	3.61196677	0.74519967	0.39828039	0.54287428
Cd40	0.7756723	-1.0778425	0.74460908	0.39846401	0.54306276
Egln2	-0.1987697	4.09706298	0.74417069	0.39860039	0.54314266
Lrfn5	0.164139	5.23599728	0.74412906	0.39861335	0.54314266
Ubash3b	-0.1325328	5.78563294	0.74387964	0.39869098	0.54318663
Pgpep1	0.19011737	4.21898306	0.74320768	0.39890023	0.5434099
2810001G20	-0.2337799	4.81931109	0.7428896	0.39899934	0.54344572
F10	-0.6927361	-0.8615185	0.74283203	0.39901728	0.54344572
Ruvbl2	-0.1459573	4.49916799	0.74255812	0.39910266	0.5435002
Eif2a	0.10019391	6.74759483	0.74231726	0.39917776	0.54350904
Tmem256	-0.2430517	2.86839731	0.74217543	0.399222	0.54350904
Txndc12	0.20345104	3.96438473	0.7421007	0.39924531	0.54350904
Chd8	-0.135801	6.62880664	0.74154246	0.3994195	0.54363676
Bmp2	0.19638716	4.62517624	0.7413028	0.39949432	0.54363676
Gm7609	0.6114627	-0.6325316	0.74127387	0.39950336	0.54363676
Prrg4	-0.3803275	1.78377809	0.74121829	0.39952071	0.54363676
Ifit1	0.20132042	6.03124522	0.74078272	0.39965675	0.5437601
Gnpnat1	0.14997043	4.78318396	0.74047967	0.39975145	0.54382716
Mfhas1	-0.1287985	5.25303798	0.73997216	0.39991011	0.54398121
Csf1r	-0.1916376	3.45706931	0.73969461	0.39999693	0.5440375
Gpx1	0.22786853	6.58973415	0.73875236	0.40029185	0.54428572
Atp1b2	0.14621412	9.04784506	0.73869206	0.40031073	0.54428572
Ctss	-0.2413339	3.74244017	0.73867597	0.40031577	0.54428572
9630028B13	-0.3658948	2.68441156	0.73823714	0.40045325	0.54434162
Serpini1	0.12829673	7.56108133	0.7382227	0.40045778	0.54434162
Pds5b	-0.1090551	7.92641985	0.73810952	0.40049325	0.54434162
Lmbrd1	0.10060899	6.75262406	0.73773858	0.40060953	0.54440871
Ap3m1	0.11214694	5.66474037	0.73756122	0.40066514	0.54440871
Inpp5b	0.13508421	4.90277006	0.73736693	0.40072608	0.54440871

Zfp386	0.12226137	5.38364077	0.73724196	0.40076529	0.54440871
Fn3krp	0.14720628	4.38382354	0.73722723	0.40076991	0.54440871
Nsa2	0.11783074	6.72256089	0.73684767	0.40088902	0.54449335
Epm2aip1	0.1579259	7.97617275	0.73673103	0.40092563	0.54449335
Serpinb10	-0.6717095	-0.6672712	0.73655419	0.40098115	0.54449335
Ankrd24	-0.2443909	2.49941167	0.73635839	0.40104264	0.54449335
Add3	0.11082885	8.2735249	0.73630455	0.40105955	0.54449335
Slc7a14	0.13374909	7.01975861	0.73515066	0.40142221	0.54484321
Armc4	0.66381521	-0.9967115	0.73514957	0.40142255	0.54484321
Twist1	0.23864522	4.73255054	0.73491463	0.40149646	0.54484321
Gm5464	0.54433533	0.45420489	0.73490582	0.40149923	0.54484321
DQ267102	-0.7877978	-0.3130751	0.73457575	0.40160309	0.54487522
Lpcat3	0.2059792	3.53870214	0.73405943	0.40176564	0.54487522
Alkbh7	-0.2921976	1.95290312	0.73390907	0.401813	0.54487522
Acad11	0.14589614	4.50550654	0.73388857	0.40181945	0.54487522
Smim11	-0.1969033	3.79796202	0.73386954	0.40182545	0.54487522
Emr1	0.54326821	0.09863425	0.7337298	0.40186947	0.54487522
3110045C21I	-0.8383139	-0.5811746	0.73371494	0.40187415	0.54487522
Cln8	0.15803462	3.78794767	0.73367478	0.4018868	0.54487522
Car9	0.70825357	-0.7352023	0.73233275	0.40230997	0.5453872
Scarb1	0.33649201	2.48768877	0.73207208	0.40239224	0.54543699
Cxcl14	0.16903201	3.20959099	0.73183518	0.40246703	0.54547663
Rap1a	0.13711519	8.59777155	0.73158278	0.40254674	0.54549478
Pgm2l1	0.16562059	10.1785393	0.73140593	0.4026026	0.54549478
Sall3	-0.2648058	1.87691425	0.73136012	0.40261707	0.54549478
Gpr124	-0.2001423	4.91586024	0.73079526	0.40279559	0.54567491
Gck	0.71491622	-0.9810828	0.73050734	0.40288663	0.54573651
R3hcc1l	-0.1371064	4.57641189	0.7297718	0.40311935	0.54590123
Tpcn2	-0.7358093	-0.307709	0.72969693	0.40314305	0.54590123
Psmd9	0.16892279	4.39542341	0.7296908	0.40314499	0.54590123
Slit1	0.24146916	2.95992299	0.72893249	0.40338515	0.54609531
Pcdhb5	0.23073634	1.85880226	0.72883306	0.40341666	0.54609531
Deb1	0.13547476	4.91334652	0.72864946	0.40347484	0.54609531
Ing5	0.16702688	4.683819	0.72856331	0.40350215	0.54609531
Naa38	0.21263171	3.65795244	0.72851863	0.40351631	0.54609531
Flot2	-0.1258778	4.8676707	0.7282775	0.40359276	0.54611737
Arvcf	-0.1769135	4.0585454	0.72810812	0.40364648	0.54611737
Srpx	-0.6956491	0.14940974	0.72796709	0.40369121	0.54611737
Wif1	0.72770765	-1.1356309	0.72788893	0.40371601	0.54611737
Gdf5	0.84291963	-1.6391325	0.72773148	0.40376596	0.54611737
Flna	0.13812859	5.69101565	0.72760459	0.40380622	0.54611737
Ddx5	-0.1073677	9.43719994	0.72709388	0.40396835	0.54621512
Prdm16	-0.2412737	3.06366429	0.72708954	0.40396973	0.54621512

Klhdc2	0.08827301	7.86071972	0.72633067	0.40421081	0.546473
Aspdh	-0.6328897	0.11307525	0.72613394	0.40427335	0.546473
Itgax	-0.7410114	-0.4385055	0.72605847	0.40429734	0.546473
Trio	-0.1677198	7.7197757	0.72576474	0.40439074	0.54653756
Lpxn	0.94588509	-1.4889475	0.72517073	0.40457973	0.54670423
Fam198a	0.35306421	1.78053797	0.72509021	0.40460536	0.54670423
Kif14	-0.9718949	-1.3150309	0.72477643	0.40470526	0.5467087
Igf2bp2	0.30330826	1.32033371	0.7245489	0.40477772	0.5467087
Enpp4	-0.1606507	4.28764148	0.72442762	0.40481635	0.5467087
Cybb	-0.5342641	0.85518632	0.72441801	0.40481941	0.5467087
Fbl	-0.1769546	4.29211646	0.72436303	0.40483693	0.5467087
Fn1	0.20561425	8.00392047	0.72405868	0.40493391	0.5467412
Dstyk	-0.1124514	5.49522802	0.72387559	0.40499227	0.5467412
Prkcd	-0.206751	4.08542494	0.72385772	0.40499796	0.5467412
Ddx46	0.09639226	7.32957545	0.72355228	0.40509534	0.54681103
Hist1h4a	0.79913121	-1.4203992	0.72318914	0.40521117	0.54690573
Rnf25	-0.2866135	2.08902688	0.72288942	0.40530681	0.54694365
Ints1	-0.1747921	4.56946716	0.7227425	0.4053537	0.54694365
AI115009	0.45335241	0.65848687	0.72235083	0.40547875	0.54694365
Fos	0.58540659	3.49210614	0.72235028	0.40547893	0.54694365
Tmem51	0.30765958	2.21709795	0.72232146	0.40548813	0.54694365
Rnf185	-0.1249645	5.79909455	0.7221404	0.40554596	0.54694365
Mat2b	-0.1001996	7.44759251	0.72209971	0.40555896	0.54694365
Zbtb21	0.13669165	5.05564308	0.72191179	0.405619	0.54695794
Tcp1	-0.088218	7.08689799	0.72175897	0.40566783	0.54695794
Mrpl19	0.12615431	5.02303377	0.72163773	0.40570658	0.54695794
Adam33	-0.5905344	-0.0202813	0.72136834	0.4057927	0.54695969
Dctn6	0.15609329	6.37633636	0.72134794	0.40579922	0.54695969
Slc13a5	-0.2578686	2.26735821	0.72120263	0.40584568	0.54696076
Gramd1c	0.4086558	1.68330996	0.72068176	0.40601231	0.54711017
Gls2	0.27202736	2.43157506	0.72045953	0.40608343	0.54711017
Fam216a	0.11424949	5.4381747	0.72030617	0.40613252	0.54711017
Caskin2	-0.2206306	2.60278845	0.72020095	0.40616621	0.54711017
Smarce1	0.11925323	6.41595894	0.7200769	0.40620592	0.54711017
Pisd	0.14357726	4.45308824	0.71998911	0.40623404	0.54711017
Kcnh6	0.49583456	-0.1846566	0.71985702	0.40627635	0.54711017
Atp8a2	0.26639217	3.82763017	0.71941489	0.406418	0.54721566
Ier5l	0.83113595	-1.2465365	0.71908301	0.40652438	0.54721566
Atg14	-0.174853	4.2719661	0.71887241	0.40659191	0.54721566
Oaz2	0.13038356	6.79732827	0.71872602	0.40663886	0.54721566
Ntsr2	-0.2370551	3.21728665	0.71870707	0.40664494	0.54721566
Sh3tc1	-0.8652603	-1.176216	0.71862851	0.40667014	0.54721566
Map2k3os	0.73333069	-0.8604769	0.71861479	0.40667454	0.54721566

Myh9	-0.1174641	6.86419916	0.71788088	0.40691007	0.54745973
Tinf2	0.23609711	2.67584235	0.71776479	0.40694735	0.54745973
Wdpcp	0.14846256	3.98123373	0.71740655	0.40706241	0.54751423
Scd1	0.1183066	6.0379921	0.71724827	0.40711326	0.54751423
Fam132a	-0.2886618	1.84484177	0.71721167	0.40712502	0.54751423
Tle6	-0.5465185	0.31341144	0.71702349	0.4071855	0.54753408
Hfe	0.34370185	3.40174727	0.71682534	0.40724919	0.54755824
Zbtb48	0.35559685	1.61841727	0.7166088	0.40731881	0.54756006
Sh3glb1	0.0992541	8.20721644	0.71653471	0.40734264	0.54756006
Nlrp1a	-0.2589353	1.62889049	0.71639457	0.40738771	0.54756006
2510009E07I	-0.1118858	6.8544813	0.71591021	0.40754355	0.547627
1700008J07F	0.24265713	3.20091997	0.7157942	0.40758089	0.547627
Bloc1s3	-0.1912344	3.16661035	0.71529812	0.40774061	0.547627
Pet2	-0.8279581	-0.6324574	0.71529534	0.4077415	0.547627
Sirt4	0.2998812	1.73272007	0.71519421	0.40777408	0.547627
Nop14	-0.1222884	4.94818265	0.71510679	0.40780224	0.547627
Rwdd1	0.12570014	5.23543726	0.71509721	0.40780533	0.547627
Tsr2	-0.1271592	4.95762152	0.71501235	0.40783266	0.547627
Eid1	0.10020763	8.03895077	0.71460832	0.40796287	0.547627
Tspan15	-0.2804693	1.86957769	0.71446633	0.40800864	0.547627
Dnah7a	0.22287909	2.08891377	0.7144265	0.40802148	0.547627
Pex16	0.39532502	0.74110707	0.7142709	0.40807165	0.547627
Hspb1	0.23623406	5.07250514	0.71405076	0.40814265	0.547627
Rilpl1	0.1436138	5.94586796	0.71388083	0.40819747	0.547627
Ugcg	0.1435423	6.93736904	0.71388056	0.40819756	0.547627
Vrk1	-0.16939	3.86179314	0.71381527	0.40821862	0.547627
Mir684-1	-0.4127785	0.23407638	0.71377039	0.4082331	0.547627
C230052I12R	0.2134148	2.85948988	0.7136851	0.40826062	0.547627
Ssu72	0.14057394	5.2620379	0.71337839	0.40835962	0.54769845
Polr3k	0.142307	6.06279642	0.7128165	0.40854106	0.54788045
Adipor2	-0.1463158	4.69808897	0.71218742	0.40874436	0.54803068
Bmp5	0.2253707	5.86013317	0.71214029	0.4087596	0.54803068
Ksr2	-0.1595724	6.0655444	0.712032	0.40879461	0.54803068
Slc12a9	-0.3777518	1.25442533	0.71190358	0.40883613	0.54803068
Kat6a	-0.0936763	8.11258679	0.71156128	0.40894685	0.54811774
Maml3	-0.1425158	4.82223032	0.71125064	0.40904737	0.54819112
Tctex1d2	0.17456993	4.3958505	0.71093634	0.40914912	0.54821655
Gm1821	0.16761894	4.9269643	0.71085018	0.40917701	0.54821655
Prr32	-0.6856429	-0.3942978	0.71068279	0.40923122	0.54821655
Tril	-0.1671604	3.67077525	0.71062628	0.40924952	0.54821655
Gstp2	0.14933427	4.53982424	0.70999662	0.40945355	0.54821655
Il6ra	0.27377865	2.57813041	0.70995979	0.40946548	0.54821655
Lrrc18	-0.2308012	2.93618998	0.70987824	0.40949192	0.54821655

4933432I03R	-0.7711074	0.10659377	0.70986391	0.40949656	0.54821655
Lrrn1	0.16350714	6.20171724	0.70980488	0.4095157	0.54821655
Naglu	-0.4139789	1.20985482	0.70975886	0.40953062	0.54821655
Kcnj5	-0.7495944	-0.0883408	0.70963773	0.4095699	0.54821655
Dpy30	0.18244514	4.81348672	0.70937356	0.40965558	0.54826995
Gm561	0.15257613	3.43496853	0.70905375	0.40975934	0.54834754
Rnpep	0.20353264	3.44437432	0.70849496	0.40994074	0.54836349
Gm3383	-0.483819	-0.2915731	0.70834974	0.4099879	0.54836349
Zfp688	0.39416933	1.2095014	0.7083338	0.40999308	0.54836349
Pkd1l3	-0.4316246	0.8831859	0.70822096	0.41002973	0.54836349
Rab17	0.93825892	-1.5520423	0.70819565	0.41003795	0.54836349
Ino80dos	-0.2656067	2.98122524	0.70817088	0.410046	0.54836349
Trmt5	-0.1894656	3.47428555	0.7079703	0.41011117	0.54837908
Rasgrf1	-0.1761617	8.33628821	0.70785315	0.41014924	0.54837908
Igfbp3	-0.252226	3.83518722	0.70671015	0.41052095	0.54876716
Ppfibp1	-0.1000536	6.07535407	0.70667883	0.41053114	0.54876716
Tlk2	0.10230657	7.11573256	0.70631583	0.4106493	0.54886385
Gcsh	0.14703606	5.68617672	0.70595287	0.41076751	0.54896057
Slc24a3	-0.1313855	6.88660705	0.70559411	0.41088439	0.5490555
Prap1	-1.2353267	-1.6095757	0.70544431	0.41093321	0.54905948
Polr2c	-0.1696876	4.23038674	0.70454104	0.41122779	0.54937589
Phkg2	0.24886306	2.60356202	0.70443689	0.41126177	0.54937589
Alms1	-0.1755916	5.05209571	0.70402358	0.41139668	0.54945153
Tbc1d1	-0.1281386	4.55994656	0.70388194	0.41144293	0.54945153
Gtpbp4	-0.0944092	6.31664662	0.70384181	0.41145604	0.54945153
Zdhhc21	-0.1247572	6.37299455	0.70336185	0.41161283	0.54951877
Rnf208	0.12099694	4.92523072	0.7032351	0.41165424	0.54951877
Csnk1d	0.09305413	6.50616897	0.70316185	0.41167819	0.54951877
Slc37a2	-0.6760634	-0.1409012	0.70305905	0.41171179	0.54951877
Cers6	0.16475538	5.97498481	0.70290211	0.41176309	0.54951877
Kcnb1	-0.1627251	8.01709243	0.70260055	0.41186171	0.54951877
Eps8	0.12536205	5.3883578	0.70257215	0.41187099	0.54951877
Fbxo44	0.1540895	4.46392844	0.70256454	0.41187348	0.54951877
Nif3l1	0.19533451	3.38042311	0.70216664	0.41200366	0.54953971
Mmrn2	-0.7895382	-0.3197743	0.70171007	0.41215311	0.54953971
Kctd2	-0.1266021	5.52235383	0.70167377	0.41216499	0.54953971
Smarcb1	0.14295259	4.78304177	0.7015796	0.41219583	0.54953971
Fes	-0.6828156	-0.3101794	0.70137319	0.41226344	0.54953971
Mir568	-0.2250598	2.11864824	0.701362	0.4122671	0.54953971
Mto1	0.17811491	3.51939617	0.70123808	0.4123077	0.54953971
Dyrk1a	-0.0941243	7.22479528	0.70120048	0.41232001	0.54953971
Olfr920	-1.0420442	-0.1309598	0.70115686	0.41233431	0.54953971
Spint1	0.6378058	-0.3384106	0.70111489	0.41234806	0.54953971

Fzr1	-0.2552036	2.83729254	0.69995136	0.41272957	0.54998695
Frat1	0.33126138	1.57041128	0.69963022	0.41283496	0.55006619
Gng12	0.14383587	6.58084897	0.69938222	0.41291638	0.55011303
Ripply2	0.59539347	-0.3361722	0.69924335	0.41296199	0.55011303
2700089E24I	0.11555481	8.43300585	0.69826905	0.41328215	0.55013258
Gfra1	-0.1802056	3.28474915	0.69811373	0.41333322	0.55013258
Bcl9	-0.1099981	6.42996014	0.69796196	0.41338314	0.55013258
Cyb5r4	0.10221882	5.78849428	0.69792416	0.41339557	0.55013258
1700019D03I	-0.3169334	1.69475413	0.69781765	0.41343061	0.55013258
Pld1	-0.175354	5.52894528	0.69781166	0.41343258	0.55013258
Pms2	-0.1975945	3.62544284	0.69773319	0.41345839	0.55013258
Otx1	0.25272243	2.08237612	0.69760098	0.4135019	0.55013258
Ltv1	0.1285883	4.79175737	0.69758863	0.41350596	0.55013258
Pias1	0.09118854	6.84402528	0.69755611	0.41351667	0.55013258
Prr5	-0.3476291	1.40299752	0.6974951	0.41353674	0.55013258
Abi3bp	0.17523622	4.18280323	0.69740978	0.41356483	0.55013258
Parvg	0.35760776	1.48542068	0.69738237	0.41357385	0.55013258
Slc4a7	-0.170453	3.91386654	0.69718153	0.41363996	0.55015941
Zfp961	0.11351227	5.09700489	0.69680951	0.41376247	0.55023008
Pcdha1	0.76223152	-0.9128144	0.69674118	0.41378498	0.55023008
Vezf1	0.12405714	7.58884059	0.69652207	0.41385717	0.55025691
Kirrel	0.21323398	4.57305455	0.69640104	0.41389705	0.55025691
Art3	-0.7659815	-0.4051055	0.69612657	0.41398752	0.55031609
Mettl1	0.74101889	-0.7518971	0.69596884	0.41403953	0.55032413
Acp5	0.71505518	-0.7192985	0.69493443	0.41438082	0.55062665
9930111J21F	0.25328541	2.9542156	0.69482538	0.41441682	0.55062665
Gm12429	0.33871638	0.85949264	0.69472574	0.41444972	0.55062665
Herpud2	0.1245489	6.21592798	0.69454253	0.41451023	0.55062665
6820431F20I	-0.132542	8.79642571	0.69452864	0.41451482	0.55062665
Cdh6	-0.1821029	2.79517801	0.69434412	0.41457578	0.55062665
Cdnf	0.31477174	2.31471702	0.69430415	0.41458898	0.55062665
Creb5	-0.1382568	4.20964926	0.69368947	0.41479215	0.5508354
Zfpm1	0.25257452	1.76314135	0.69345181	0.41487074	0.55087868
Csnk1g2	-0.1618989	5.59108536	0.69316457	0.41496577	0.55094377
Gpx7	0.32413332	2.44494039	0.69293934	0.4150403	0.55094401
Abhd4	0.16584269	5.30739511	0.69288597	0.41505796	0.55094401
Maff	0.23696413	3.06441849	0.69198512	0.41535629	0.55127891
Gm6710	-0.1966884	2.44380817	0.69180489	0.41541601	0.55129708
Lin9	-0.2241891	3.01299053	0.6915031	0.41551605	0.55131105
Copb1	0.1049297	6.10022922	0.6913465	0.41556798	0.55131105
Scn9a	-0.318817	2.68024145	0.6912339	0.41560532	0.55131105
Bst2	-0.4842874	1.61087395	0.69121773	0.41561068	0.55131105
Racgap1	0.20087785	3.21053084	0.69096836	0.4156934	0.5513257

Gnpda2	0.13717603	5.96309501	0.69079671	0.41575035	0.5513257
Mov10l1	-1.4793602	-2.089242	0.6907544	0.41576439	0.5513257
Chmp4b	0.096697	6.43768416	0.69062941	0.41580587	0.5513257
Lrrc38	0.3447147	1.44435448	0.68988664	0.41605251	0.55159164
Pomk	-0.1440452	4.76491231	0.68950967	0.41617777	0.55163847
Gpaa1	0.33939435	1.39963622	0.6895031	0.41617995	0.55163847
Scd4	0.53767021	-0.175425	0.68915038	0.41629721	0.55173282
Nov	0.26110853	7.77909032	0.68836386	0.41655886	0.55187587
Pitpnm2os1	0.77744026	-0.1977303	0.688347	0.41656447	0.55187587
Dnah10	0.39845066	1.13140033	0.68819854	0.41661388	0.55187587
Ikbkg	-0.0996001	5.88354635	0.68818972	0.41661682	0.55187587
Esrrb	0.68468287	-0.3614553	0.68813345	0.41663556	0.55187587
Gm6260	-0.3699694	1.52096462	0.68770341	0.41677876	0.55190153
Mob1b	0.11636195	5.1918482	0.68767972	0.41678665	0.55190153
Xiap	-0.1205063	7.12351358	0.68766012	0.41679318	0.55190153
Pole	-0.3414339	1.08411934	0.68743229	0.41686909	0.55194101
Capza1	0.11769921	6.23692652	0.68708685	0.41698421	0.55203241
Ttyh3	0.14916447	6.04932733	0.68664958	0.41713002	0.5521644
4930579K19l	1.1567039	-1.3326872	0.6864596	0.41719339	0.55217321
Cdc6	0.44281257	0.74257693	0.6862368	0.41726773	0.55217321
Rbbp5	-0.1071486	5.69643705	0.6861689	0.41729039	0.55217321
Gatad2a	0.14097408	5.71910595	0.68587503	0.41738848	0.55217321
Smim3	-0.2373989	3.12589515	0.68583032	0.41740341	0.55217321
Tmem253	-0.9070417	-1.077582	0.68566922	0.4174572	0.55217321
Gm14379	0.75592027	-0.7781729	0.68566254	0.41745943	0.55217321
Ccl28	0.33605404	1.68767328	0.6853206	0.41757364	0.55226328
Rbm34	0.11095748	5.32933223	0.68515183	0.41763003	0.55227686
Zfp36l2	-0.1487336	6.13442197	0.68500303	0.41767976	0.55228164
Zkscan17	-0.1848887	3.86490782	0.68457725	0.4178221	0.55240885
Rad54b	-0.6870106	-0.1526747	0.68436883	0.4178918	0.55241083
Brap	0.13163672	5.40008628	0.68429693	0.41791585	0.55241083
Tbce	-0.1444415	4.40846817	0.68378692	0.4180865	0.55257542
Elmsan1	0.11197217	5.92131118	0.683428	0.41820666	0.55267324
Tgif2	0.28262538	2.2958135	0.68320774	0.41828043	0.55270974
Ncapd3	0.18871399	4.27461115	0.68253249	0.41850671	0.55294772
Mecp2	-0.1047646	8.93917506	0.68206236	0.41866436	0.55306884
Fam219a	-0.1681043	3.871757	0.6819837	0.41869074	0.55306884
Prob1	-0.2876839	1.69995626	0.68143015	0.4188765	0.55318991
Aldh2	-0.1719775	5.30454069	0.68123317	0.41894264	0.55318991
Ccdc25	0.10396042	5.86118626	0.68118967	0.41895724	0.55318991
Fbxo3	-0.09583	6.50548621	0.68116011	0.41896717	0.55318991
Senp1	-0.1402116	5.22528024	0.6804711	0.41919865	0.55343452
D130043K22	0.21814292	3.37003417	0.68019901	0.41929011	0.55347046

Wdsub1	-0.204352	3.07045806	0.68002768	0.41934772	0.55347046
Taf6l	-0.4117761	0.66481131	0.67997773	0.41936451	0.55347046
Rft1	-0.3119062	2.59796167	0.67962489	0.4194832	0.5535661
Casp8	0.17032845	5.33351205	0.67915169	0.41964246	0.55369764
Fam21	-0.1291961	6.01400352	0.67905397	0.41967536	0.55369764
Chchd3	0.13379853	5.11662928	0.6785382	0.41984907	0.55383796
Mir344b	-1.0789415	-2.0874716	0.67846361	0.4198742	0.55383796
Etfa	0.12108688	6.60591641	0.67814728	0.4199808	0.55387705
Zfp946	0.22092609	2.87394696	0.67800247	0.42002961	0.55387705
Gpr83	-0.253003	2.73427723	0.67788731	0.42006844	0.55387705
Mlxipl	-0.3834979	0.95267483	0.67782682	0.42008884	0.55387705
4930469G21	-0.5297508	-0.1709537	0.67757629	0.42017333	0.55390601
BC002163	0.14119816	3.58141497	0.67723767	0.42028758	0.55390601
Plscr4	0.24806629	3.14324998	0.67713319	0.42032284	0.55390601
Cdr2	-0.2441995	2.74416149	0.6770533	0.4203498	0.55390601
Cbx3	0.12031646	8.36237764	0.67701032	0.42036431	0.55390601
Mutyh	0.47251983	-0.0979963	0.67693919	0.42038832	0.55390601
Zc3hav1	0.13501713	5.52843251	0.6765788	0.42051	0.55400539
Ost4	0.18619398	7.07732292	0.67639773	0.42057116	0.55402502
Tulp4	-0.0968672	8.14953768	0.67586117	0.42075247	0.5542029
Nol11	0.14786422	4.28240854	0.67538632	0.42091304	0.55426587
4930507D05I	0.82770032	-1.1882022	0.6752278	0.42096666	0.55426587
Szrd1	-0.1772731	4.64014995	0.67517524	0.42098444	0.55426587
4933424G06	-0.5643416	0.47933401	0.67510167	0.42100933	0.55426587
Tbc1d15	0.12228678	5.20688553	0.67503559	0.42103169	0.55426587
Kcnq5	-0.1530171	6.25302514	0.67381906	0.42144365	0.55469037
Wfdc17	-0.4222193	0.93154725	0.6738098	0.42144679	0.55469037
Dnajc14	-0.1747303	4.30884159	0.67348032	0.42155847	0.55477589
Paf1	-0.1214006	5.95108344	0.67334482	0.42160442	0.55477589
Gm20257	-0.3008651	2.45904281	0.67232327	0.42195104	0.55506967
Sypl2	0.55083115	0.14248168	0.67226939	0.42196933	0.55506967
4930404N11	0.47737511	0.04994917	0.67225756	0.42197335	0.55506967
Magel2	-0.7003675	0.39993086	0.6720971	0.42202784	0.55506967
C3ar1	-0.27491	2.03882037	0.67200412	0.42205942	0.55506967
Bean1	-0.1426689	3.92752223	0.67178733	0.42213307	0.55510557
Tor1a	0.20477089	2.96947977	0.67138374	0.42227023	0.55517675
Ms4a6d	-1.0003638	-1.2205621	0.67135524	0.42227991	0.55517675
Qdpr	-0.106173	5.53356808	0.67116716	0.42234386	0.55519986
Mid2	0.12362594	5.67538724	0.67096386	0.42241299	0.5552298
B230319C09I	0.78350055	-1.0365313	0.67077736	0.42247643	0.55525224
Clip4	0.1292861	5.24150222	0.67032839	0.4226292	0.55539207
Fbxo4	-0.2499113	3.73373503	0.67005751	0.42272142	0.55545231
Mrpl28	0.24325984	3.36146762	0.66933332	0.42296812	0.55557417

Zmat5	0.25981693	2.44656038	0.66923344	0.42300216	0.55557417
Dgat2	0.17192511	3.75689684	0.66909829	0.42304823	0.55557417
Eml1	-0.0979592	5.94191498	0.66902674	0.42307262	0.55557417
Zfp248	0.16420841	4.558211	0.66901051	0.42307815	0.55557417
Il17rc	0.30068652	1.90977284	0.66886065	0.42312925	0.55557417
Mr1	0.1905729	4.41173809	0.66872742	0.42317469	0.55557417
Thoc2	-0.1384727	7.06080005	0.6686772	0.42319182	0.55557417
Parp2	0.11813933	5.19107671	0.66856031	0.42323169	0.55557417
Rhof	0.20793643	3.497237	0.66760722	0.42355702	0.5557988
Nrxn1	-0.1322473	9.77366721	0.66749801	0.42359432	0.5557988
Grid2	-0.2708571	2.47251112	0.66731939	0.42365534	0.5557988
Fam26e	0.22437677	4.62726743	0.66729688	0.42366303	0.5557988
Tmem50a	0.14537953	5.6736444	0.66721934	0.42368952	0.5557988
Taf3	-0.1055543	6.44110544	0.66713447	0.42371853	0.5557988
Papss2	0.14973058	5.79153678	0.66710769	0.42372768	0.5557988
Mlec	-0.1060472	6.89116658	0.66681204	0.42382874	0.55587047
Lin54	0.13404926	4.48532347	0.6665185	0.42392912	0.55589648
Myo1g	0.70725805	0.13617966	0.66636016	0.42398327	0.55589648
Dnal1	-0.1059691	7.03713148	0.66634684	0.42398783	0.55589648
Pcmt1	0.09308291	6.42797914	0.66574429	0.42419403	0.55610596
Ccdc129	0.53728834	-0.2345757	0.66523107	0.42436979	0.55627506
Gas8	-0.2147723	3.60001775	0.66492069	0.42447613	0.55627506
Nfx1	0.0980626	6.44274509	0.66471661	0.42454608	0.55627506
Cnn2	-0.22463	6.26796327	0.66459232	0.42458869	0.55627506
Gm12657	0.19224529	3.51445235	0.66454725	0.42460414	0.55627506
Galntl6	0.13696945	5.12805334	0.66444013	0.42464087	0.55627506
Gm5795	1.04221294	-2.0561598	0.66438822	0.42465867	0.55627506
Ptdss2	0.18089527	3.24280538	0.66422483	0.42471471	0.55627506
Fam162a	0.17619351	5.00443547	0.66414796	0.42474108	0.55627506
Fkbpl	0.42385735	0.62039627	0.66377047	0.4248706	0.55638385
Opn1mw	-0.8024585	-1.6765362	0.66330471	0.42503049	0.5565161
1700084C01I	-0.2935384	1.65505672	0.66313882	0.42508746	0.5565161
Tfap2d	-0.7310118	-0.1952972	0.66307029	0.425111	0.5565161
Adh5	0.11914827	6.35142183	0.66215393	0.42542596	0.55685395
Plxnb2	-0.1305641	4.78627518	0.66204891	0.42546207	0.55685395
Fam114a1	0.19546462	6.31411309	0.66179086	0.42555084	0.55689364
Pkdcc	-0.4409803	0.20160882	0.66169041	0.42558541	0.55689364
Lox	0.23641672	3.27472047	0.66122249	0.42574646	0.55701283
Paxbp1	-0.1730643	5.67637254	0.66115554	0.42576952	0.55701283
Aoc3	-0.4729815	3.18181364	0.6607551	0.42590744	0.55711177
Dcaf11	-0.1156486	4.86651126	0.66066586	0.42593818	0.55711177
Slc16a14	0.22109873	4.04172305	0.66022504	0.42609011	0.55724962
Slc39a14	-0.1842899	3.35867201	0.66000102	0.42616735	0.55726991

Dis3	0.139727	3.97753571	0.65991013	0.42619869	0.55726991
Hspb2	-0.3553799	1.09885574	0.65966086	0.42628467	0.55732148
Ino80b	0.50671434	-0.0544744	0.65936323	0.42638737	0.5573254
Farp2	-0.1776555	2.80240206	0.65912719	0.42646884	0.5573254
Cd302	0.16506823	4.11724662	0.6589209	0.42654006	0.5573254
Pde10a	-0.1708101	7.06493354	0.65890661	0.426545	0.5573254
Wdfy3	-0.2011557	7.86625625	0.65890474	0.42654564	0.5573254
Slitrk4	0.13483979	6.29654105	0.65884321	0.42656689	0.5573254
Rpl36al	0.17436529	7.29476133	0.65864986	0.42663367	0.55735184
Braf	-0.1039591	8.58365271	0.6585011	0.42668506	0.55735818
Slc25a51	-0.093235	6.87552165	0.65802721	0.42684884	0.55745747
Gm3558	0.60459867	-1.2723541	0.65800909	0.4268551	0.55745747
Cdkl3	-0.1710337	3.66734289	0.65787713	0.42690072	0.55745747
Dcun1d3	-0.1324238	4.96451194	0.65698106	0.42721073	0.55780146
Rps3	-0.1830617	6.97730527	0.65670964	0.4273047	0.55786334
Irs3	0.30459391	2.20963821	0.65621993	0.42747433	0.55791903
Gper1	-0.2986641	1.96462985	0.65621611	0.42747565	0.55791903
Pcbd2	0.20696314	4.06968922	0.65618301	0.42748712	0.55791903
Wdr61	0.14916968	4.33102401	0.6558044	0.42761835	0.55802948
Bzw1	0.13792796	8.32615048	0.65547257	0.42773341	0.55803058
Sos1	0.09932464	7.58363665	0.65536907	0.4277693	0.55803058
Insc	0.45502899	0.14504822	0.65535211	0.42777519	0.55803058
BC064078	-0.4821746	0.67834504	0.65519239	0.4278306	0.55803058
Zfp558	0.30275708	2.74543963	0.6551302	0.42785217	0.55803058
Dvl3	0.10565881	6.09227167	0.65466392	0.42801401	0.55815752
Tmem216	0.20045973	2.85697965	0.65415592	0.42819043	0.55815752
Lepr	0.16480699	5.63711491	0.65413819	0.42819659	0.55815752
Col18a1	0.28613557	1.82900347	0.65412588	0.42820087	0.55815752
Errfi1	-0.1024682	6.88726728	0.6541253	0.42820107	0.55815752
Whsc1l1	0.08948674	7.71998622	0.65404449	0.42822915	0.55815752
Gm6525	-1.1315055	-1.3451882	0.65369227	0.42835155	0.55825631
Plcd3	-0.1921908	3.08169908	0.65300301	0.42859125	0.55850792
Alk	0.43745345	0.73476148	0.65271006	0.42869319	0.55852172
Gpr56	0.21538337	2.97073981	0.65259484	0.4287333	0.55852172
Pold1	-0.4116779	0.57114693	0.65251174	0.42876223	0.55852172
Rwdd3	-0.25451	2.62408244	0.65239411	0.42880318	0.55852172
Tnip2	0.17675858	3.05890154	0.65230262	0.42883504	0.55852172
Abcb1a	0.19344985	5.71532328	0.65204306	0.42892544	0.55855503
Slc4a11	0.73841341	-0.2079535	0.65196137	0.42895389	0.55855503
Cab39	0.08992614	7.71350092	0.65167906	0.42905226	0.55856493
Ankrd13a	0.12831053	4.90859285	0.65164603	0.42906377	0.55856493
Tnfrsf21	0.15505495	6.54315861	0.65153801	0.42910142	0.55856493
Lrrc8b	-0.1303866	5.96179813	0.65113602	0.42924158	0.55868665

Lsm1	0.18374445	4.10099304	0.65065134	0.42941066	0.55873915
4921511H03I	-1.0248178	-1.1054533	0.65063123	0.42941768	0.55873915
Ttc30a2	0.47585198	-0.1179123	0.65061918	0.42942188	0.55873915
Lrp10	0.22345707	4.25497654	0.65014622	0.42958698	0.558844
Fam208a	-0.134961	6.76686734	0.65012099	0.42959579	0.558844
Csgalnact1	-0.1929149	3.77298761	0.64983333	0.42969626	0.55891398
Kndc1	-0.203588	6.21769581	0.64966158	0.42975626	0.55891539
Sag	0.60828933	-0.7599543	0.64956308	0.42979068	0.55891539
Mtmr14	-0.2045253	3.11934636	0.6493125	0.42987826	0.55895567
Kmt2d	-0.1495875	7.22961619	0.64920737	0.42991501	0.55895567
Shroom3	-0.1917254	2.89460344	0.64901035	0.4299839	0.55898455
Crbn	0.10225897	6.44617945	0.64876144	0.43007095	0.55903704
Zhx3	0.11110713	6.21195891	0.6485606	0.43014122	0.55906769
Plk3	0.33476262	1.70869438	0.64816121	0.43028099	0.55915772
Kcna2	-0.1541442	8.75410394	0.64790938	0.43036917	0.55915772
Cntd1	0.21040486	2.33775579	0.64775798	0.43042219	0.55915772
Cdk2ap1	-0.1157468	6.49375569	0.64763108	0.43046664	0.55915772
A730098P11I	0.10748788	6.70378136	0.64754594	0.43049647	0.55915772
Psmd8	0.11690079	5.10244073	0.64745636	0.43052785	0.55915772
Ankub1	-0.5446653	0.64183141	0.64742934	0.43053732	0.55915772
Krt73	-0.7729228	-1.1365842	0.64708959	0.4306564	0.55925172
Brinp1	-0.1165531	6.39757904	0.64648273	0.43086921	0.55942018
Peo1	0.26266951	2.5381301	0.64644315	0.4308831	0.55942018
Pgm5	-0.190473	6.55807452	0.64632015	0.43092626	0.55942018
Hn1	0.16416516	4.32365813	0.64502331	0.43138169	0.55995072
Trim7	-0.4506961	0.29043078	0.64474205	0.43148057	0.55999767
Gm10548	-0.312276	1.74627685	0.64465441	0.43151139	0.55999767
Cnih4	0.11765126	5.19353871	0.64394945	0.4317594	0.56025882
4932411N23	0.61399985	-0.9359805	0.64351224	0.43191333	0.56039783
Tln2	-0.1124188	6.80998264	0.64313454	0.43204637	0.56043387
Klf5	-0.12618	6.62739111	0.64302066	0.4320865	0.56043387
Smc3	-0.1093075	8.16643801	0.64300293	0.43209275	0.56043387
Ankrd66	1.5901576	-2.1740184	0.64281348	0.43215952	0.56043387
Usp5	-0.1474848	4.66250852	0.64265178	0.43221652	0.56043387
Rtkn	0.20055593	3.44422276	0.64263656	0.43222188	0.56043387
Tmem127	-0.117988	6.90098997	0.64231407	0.43233561	0.56052064
Tuba8	0.19983493	2.26077848	0.64167349	0.43256164	0.56057805
Aqr	-0.1578988	5.22653794	0.64158997	0.43259112	0.56057805
Mxd1	-0.1147377	6.02346775	0.64154833	0.43260582	0.56057805
Slc1a5	-0.2662069	2.10216484	0.64153606	0.43261016	0.56057805
2210039B01I	-0.275648	1.77921097	0.64152535	0.43261394	0.56057805
E330011O21	-0.5281657	-0.1778107	0.64111649	0.43275833	0.56070448
Stard10	0.17610355	3.3328825	0.64043311	0.43299984	0.56095671

Gfy	1.3084025	-1.9307506	0.63997257	0.43316272	0.56109424
Flt3	0.44671168	0.51524891	0.63986803	0.43319971	0.56109424
Anxa8	0.2823199	3.46734733	0.63973522	0.4332467	0.56109442
Leprel2	0.20933472	3.23305607	0.6393713	0.43337552	0.56120056
Nrxn2	0.11716165	5.40005457	0.63892237	0.43353451	0.56125845
Cc2d1b	-0.1683446	3.62334227	0.63845432	0.43370036	0.56125845
Grik4	-0.3064153	1.91714354	0.63826748	0.4337666	0.56125845
Grin3a	0.1650556	4.72129811	0.63821056	0.43378678	0.56125845
Syt6	0.19708699	4.7974429	0.63817888	0.43379802	0.56125845
Cacng4	-0.4287654	1.11388818	0.6381704	0.43380103	0.56125845
Nol9	0.19029474	3.57969393	0.63813171	0.43381475	0.56125845
Lrrc8c	0.15731973	4.05607155	0.63806624	0.43383796	0.56125845
Gpr176	-0.256178	2.61658925	0.63805481	0.43384202	0.56125845
Foxq1	-0.5724254	1.12475046	0.63778681	0.43393708	0.56132079
Vprbp	-0.1090307	6.75123308	0.63739656	0.43407556	0.56140966
3110082I17R	0.25193854	2.68629787	0.63732902	0.43409954	0.56140966
Thop1	0.23448583	2.56012789	0.63694825	0.43423474	0.56147152
Creg1	0.18385815	4.80450992	0.6368314	0.43427625	0.56147152
Cbx6	0.1099292	6.62613416	0.63679825	0.43428802	0.56147152
Ccdc96	-0.3436285	1.74268235	0.63646038	0.43440807	0.5615661
Ldhd	0.31008221	1.85823546	0.63631299	0.43446045	0.56157183
Tmem145	-0.2827119	2.44946133	0.63618047	0.43450756	0.56157183
Car5b	0.24021543	1.96853498	0.63605216	0.43455318	0.56157183
Slc25a24	0.18162213	5.32740564	0.63580884	0.43463971	0.56162305
Nrgn	-0.1028818	7.23116509	0.63559227	0.43471675	0.56163139
Apol7e	-0.5229882	-0.2586059	0.6355069	0.43474713	0.56163139
Siah1b	0.29746179	1.8626382	0.63506033	0.43490608	0.56163139
Pcdhgb5	0.26426391	2.4519966	0.63505468	0.43490809	0.56163139
Mir1188	-0.5878743	-0.5347815	0.6350032	0.43492641	0.56163139
Ash2l	0.11252171	5.00464747	0.63500001	0.43492755	0.56163139
Bcl7c	-0.3459613	1.35263335	0.63453412	0.43509348	0.56173258
Zscan26	-0.0851222	7.19874015	0.63447411	0.43511486	0.56173258
Pzp	0.77830903	-0.8954508	0.63438495	0.43514663	0.56173258
Prkci	0.09210501	7.35728916	0.63424588	0.43519619	0.56173601
1110002L01R	-0.3380917	1.11753927	0.63397283	0.43529353	0.56180109
Thoc1	-0.1455103	4.76711734	0.63359236	0.43542921	0.56186291
4930520O04	-0.9160744	-1.0839657	0.6335754	0.43543526	0.56186291
Mef2c	-0.124245	10.0758779	0.63298988	0.4356442	0.56205854
Rab33a	-0.182988	3.31455884	0.63288755	0.43568074	0.56205854
Gm14325	-0.1225293	4.34187906	0.63240134	0.43585439	0.562222
Nolc1	0.11386966	5.18570524	0.63208513	0.43596738	0.5622321
Atp7b	-0.7709368	-0.6278342	0.63204913	0.43598024	0.5622321
Tmc4	-0.1776963	3.05233319	0.6319853	0.43600306	0.5622321

Emb	0.22259271	6.18088226	0.63152116	0.43616902	0.56233235
Fam204a	-0.1615835	5.54557337	0.63134813	0.43623091	0.56233235
LOC1026324	-1.3881086	-1.9097647	0.63132634	0.43623871	0.56233235
Zfp618	-0.2764037	1.48784735	0.63124271	0.43626863	0.56233235
Mki67	-0.1617932	3.76884703	0.63088167	0.43639784	0.56243106
Pramel5	-0.9384787	-1.7145602	0.6306519	0.4364801	0.56243106
Cyp3a13	-0.7503917	-1.1165901	0.63063514	0.4364861	0.56243106
Lrrc75b	-0.1691086	4.44886064	0.63027784	0.43661408	0.56253543
Mpdz	-0.1052037	6.41183289	0.62995673	0.43672914	0.56262315
Rpl28	0.18919445	5.33779006	0.62934552	0.43694828	0.56279522
Atad3a	0.20350894	3.12139672	0.62929543	0.43696625	0.56279522
Bora	0.32717198	1.55862434	0.62919106	0.43700369	0.56279522
Smarca5-ps	-0.2534649	1.23453082	0.62897274	0.43708202	0.56283558
Rpl10a	0.18642899	8.07832466	0.62854796	0.4372345	0.56286161
Pus7	-0.1620058	3.84631136	0.62852502	0.43724274	0.56286161
Nxf7	-0.5732055	0.83819956	0.62852362	0.43724324	0.56286161
Tbc1d5	-0.0970216	6.45387232	0.62815589	0.43737531	0.56297111
Sez6l2	0.13438141	5.43861925	0.62792523	0.43745819	0.56298979
A230046K03	-0.1496457	6.37811693	0.62779289	0.43750575	0.56298979
Ell	-0.1432352	3.49996243	0.62752982	0.43760031	0.56298979
Atg16l1	-0.1805949	3.88649107	0.62735296	0.43766391	0.56298979
Gjb6	0.17342074	8.86000288	0.62733839	0.43766915	0.56298979
Cct6b	1.22209348	-1.4905165	0.62733077	0.43767189	0.56298979
Cad	-0.2805872	1.781203	0.62682582	0.43785354	0.56316296
Zfp599	0.23163656	2.83120872	0.62661127	0.43793076	0.5632018
AI504432	0.12662442	5.6203491	0.62614201	0.43809973	0.5633586
Snrpb2	-0.1275686	5.68008037	0.62588271	0.43819314	0.56341822
Pcdhgb2	0.24425382	2.11965788	0.62500805	0.43850847	0.56366235
Wtip	0.2742687	2.38718565	0.62499997	0.43851139	0.56366235
Faf1	-0.1362243	4.73011911	0.62496442	0.43852421	0.56366235
Hoxd11	-0.585392	-0.3836331	0.6247668	0.43859551	0.56369349
Tmem129	-0.1902193	3.06683303	0.6244585	0.43870678	0.56377599
Mapk3	0.13971986	7.23936848	0.62418309	0.43880621	0.56378203
Ppp1r12a	-0.0958769	7.45763336	0.62416928	0.4388112	0.56378203
Aes	0.13000509	6.35405243	0.62405436	0.43885271	0.56378203
Mir6336	-0.8236834	-1.5248579	0.62355583	0.43903282	0.56392137
LOC1005047	0.62250375	-0.317366	0.62344073	0.43907442	0.56392137
Ttyh2	-0.2328793	2.6258952	0.62336323	0.43910244	0.56392137
Nup210l	-0.8716731	-0.3714807	0.6231962	0.43916282	0.56393844
Kif27	-0.2463895	2.20180061	0.62288318	0.43927603	0.56398489
Gars	0.09051818	6.01948736	0.62270189	0.43934161	0.56398489
Ntf3	-1.0556535	-1.2165419	0.62249566	0.43941624	0.56398489
Rab6a	0.08627024	10.1161715	0.62249346	0.43941704	0.56398489

4933416C03I	-1.2075293	-1.228785	0.62239659	0.4394521	0.56398489
AW549877	-0.1010904	7.65307398	0.62223039	0.43951226	0.56398489
Asb2	0.58260521	0.02670531	0.62218511	0.43952865	0.56398489
Acaa1a	-0.1511224	3.87713101	0.62202924	0.43958509	0.56399688
Wdr91	-0.1970324	3.08481468	0.62184553	0.43965163	0.56402182
1700040L02I	-0.2675666	1.83594238	0.62141172	0.43980881	0.56415996
Dbil5	0.94020761	-1.4337472	0.62128837	0.43985352	0.56415996
Gm17296	-0.3496809	2.16490273	0.62071969	0.44005974	0.56436401
Man2b1	0.16816442	4.0911535	0.62023236	0.44023657	0.56453034
2810403D21I	0.50431335	0.25113302	0.61962405	0.44045747	0.56475313
Ivd	-0.1236565	4.16781787	0.61930184	0.44057455	0.56484278
Adsl	-0.1676186	4.44688596	0.61901735	0.44067796	0.56489168
Gemin2	-0.2392422	2.12116911	0.61893738	0.44070704	0.56489168
Srp54a	0.08490309	7.11641345	0.61873747	0.44077974	0.5649244
Pfkfb3	0.1287678	4.54080087	0.61847319	0.44087587	0.56498715
Gm13152	-0.375077	1.28296953	0.61816477	0.44098811	0.56502299
Taf1c	-0.3826156	1.20429894	0.61813704	0.4409982	0.56502299
Fbxw9	-0.3006131	2.04056221	0.61796387	0.44106124	0.56504331
CK137956	0.46504099	0.78151713	0.61761909	0.4411868	0.5651437
Nucks1	0.10001875	8.85000621	0.61742164	0.44125872	0.56517539
1110032F04I	0.24728829	2.18019869	0.61695062	0.44143039	0.56525596
Gm15708	0.73719844	-1.0342753	0.61693402	0.44143644	0.56525596
Usp43	-0.5570783	1.0019832	0.6168042	0.44148377	0.56525596
Gm4262	-0.1932141	3.20968272	0.61666978	0.44153279	0.56525596
Tenm4	-0.169461	5.97639143	0.61651385	0.44158966	0.56525596
Epha7	0.13611106	6.56712493	0.61647227	0.44160483	0.56525596
Chrm4	-0.2603377	2.00723448	0.61597759	0.44178535	0.56542658
Hmox2	0.16165764	5.03916106	0.61576911	0.44186146	0.56545631
Nphs1	-0.2575106	2.0604557	0.61561733	0.44191688	0.56545631
Fbxl2	0.16698381	4.66631756	0.61552603	0.44195023	0.56545631
Aebp1	0.21287353	7.19862456	0.61512578	0.44209646	0.56553091
Elovl6	-0.1225916	6.01508993	0.61510793	0.44210298	0.56553091
Plscr2	-0.2046516	4.4211804	0.6148602	0.44219353	0.56558633
Prpf40b	-0.2040071	3.81498405	0.61416803	0.44244668	0.56583076
Grtp1	0.23250784	3.25590908	0.61407935	0.44247913	0.56583076
Tex15	-0.2473582	2.37812707	0.61354074	0.44267631	0.56596934
Ggt5	0.41697353	1.43336164	0.61352165	0.4426833	0.56596934
Gm6402	-0.2272005	1.77517024	0.6133961	0.44272928	0.56596934
Trim47	-0.3551311	2.25336164	0.61295659	0.44289031	0.5659778
Rd3	-1.3015463	-1.8484215	0.61291994	0.44290374	0.5659778
2210416O15	-0.5711659	-0.6375409	0.61285165	0.44292877	0.5659778
Tspyl2	0.12363686	6.06718009	0.61283222	0.44293589	0.5659778
Lpo	-0.943209	-0.8109951	0.61273319	0.4429722	0.5659778

Mks1	-0.5281147	0.20709525	0.61235212	0.44311193	0.56605557
Eif2ak2	-0.1507762	5.80721038	0.61230941	0.4431276	0.56605557
Rps5	-0.1393149	5.76664419	0.61213311	0.44319228	0.56607781
1700034J05F	1.11837469	-1.6206311	0.61193239	0.44326593	0.566092
Nisch	-0.1073347	7.3684988	0.61170611	0.44334898	0.566092
Vat1l	0.17236882	5.41946063	0.61160986	0.44338432	0.566092
Seh1l	-0.1382175	5.64471097	0.61158766	0.44339247	0.566092
6330403K07I	0.09152447	7.67692203	0.61124861	0.44351698	0.5661543
Dars	0.10956739	5.23673454	0.61108317	0.44357776	0.5661543
Mcm4	-0.1475092	4.37157769	0.61102233	0.44360011	0.5661543
Shank2	-0.199627	6.98539914	0.61081769	0.44367532	0.5661543
Rps7	-0.1117942	6.62288406	0.61081136	0.44367764	0.5661543
AU040320	-0.1700825	4.17222439	0.61012165	0.44393125	0.56641757
Supt5	0.1313011	5.64203307	0.60997679	0.44398455	0.56642522
Eef1e1	0.14844946	3.33863197	0.60963278	0.44411115	0.56643309
Aldh5a1	-0.1179733	6.74500979	0.60946728	0.44417208	0.56643309
Pttg1ip	0.19893743	6.25434244	0.60939478	0.44419878	0.56643309
Nlgn3	0.1254455	5.98583432	0.60913511	0.44429441	0.56643309
Mau2	-0.1234914	5.4300466	0.60909971	0.44430745	0.56643309
Chl1	0.16210296	7.47835662	0.60898439	0.44434994	0.56643309
Tnfaip1	-0.1289822	6.03251067	0.608949	0.44436298	0.56643309
Gm13375	0.25667352	2.74227121	0.60874718	0.44443735	0.56643309
Plod1	0.21123881	3.01885443	0.60861552	0.44448587	0.56643309
Gatsl3	0.35133166	1.40357407	0.60859239	0.4444944	0.56643309
Prpf3	-0.1346988	4.35903595	0.60854303	0.4445126	0.56643309
Gpr161	-0.5225422	-0.3219541	0.60841907	0.4445583	0.56643309
Smim24	0.29541974	2.27280505	0.60814755	0.44465843	0.56650039
Spats2l	-0.1108185	5.09041211	0.60799274	0.44471553	0.56651288
Tmem69	0.13697247	4.43179247	0.60779457	0.44478865	0.56654576
Fam96a	-0.1482314	5.17674394	0.60738692	0.44493912	0.56667715
Cyb561a3	-0.1625011	3.48963815	0.60704908	0.44506389	0.56677578
Gjc1	-0.3381987	1.80271383	0.60691619	0.44511298	0.56677802
Reep2	0.14666039	4.64945687	0.60609604	0.44541614	0.56708976
Tcea2	-0.2144086	2.74191191	0.60599773	0.44545251	0.56708976
Gnai2	0.17520581	7.06060397	0.60567922	0.44557035	0.56717949
0610040B10I	0.52433347	0.65165979	0.6055439	0.44562043	0.56718295
Lpcat2	0.26498968	2.55061214	0.60533065	0.44569937	0.56722314
Slc35a1	-0.1702431	5.25560159	0.60483631	0.44588245	0.56737024
Kcnma1	-0.145874	7.25180016	0.60468326	0.44593916	0.56737024
Smg1	-0.1698912	7.69714299	0.60463488	0.44595709	0.56737024
Slc12a2	-0.1041698	6.5705165	0.60432446	0.44607214	0.56739178
Itga2b	-0.4875761	-0.091508	0.60424202	0.44610271	0.56739178
Prosc	0.1064396	5.53997079	0.60382101	0.44625885	0.56739178

Thoc7	0.12154357	6.34780559	0.60345383	0.4463951	0.56739178
Smug1	-0.1191468	4.24447993	0.60332742	0.44644202	0.56739178
Cldn25	0.13637419	5.89007369	0.60330458	0.4464505	0.56739178
Bub1b	0.50108096	0.47634977	0.60310555	0.4465244	0.56739178
Atad2	-0.184558	4.80944559	0.60292525	0.44659136	0.56739178
Clic1	0.20045706	4.48526169	0.6028906	0.44660422	0.56739178
Tank	0.14233469	6.08627914	0.60274023	0.44666009	0.56739178
Myh14	-0.292324	2.29748057	0.60251649	0.44674322	0.56739178
Rnf187	0.11574433	6.80386205	0.60241672	0.4467803	0.56739178
4930590J08F	-0.7313632	-0.7086782	0.60229983	0.44682375	0.56739178
Pacrgl	0.206044	3.30867818	0.60225774	0.44683939	0.56739178
Zfand2a	-0.107389	7.00570716	0.60216234	0.44687486	0.56739178
H2-Ob	-0.8540684	-0.2005902	0.6020509	0.4469163	0.56739178
Cd2bp2	0.14331604	4.98529416	0.60183298	0.44699735	0.56739178
Nadk	-0.1336145	5.45393474	0.60178506	0.44701517	0.56739178
Zfp389	-0.8955576	-1.5357067	0.60178225	0.44701622	0.56739178
Spsb1	-0.2339218	2.8969923	0.60172465	0.44703764	0.56739178
Akr7a5	0.22577388	2.3865428	0.60163921	0.44706943	0.56739178
Klhdc3	-0.1205865	4.44002084	0.60155995	0.44709892	0.56739178
Ccdc175	-1.3160661	-1.7965656	0.60153394	0.4471086	0.56739178
Fastkd2	-0.2482454	2.91665665	0.60152724	0.4471111	0.56739178
Pigk	0.14853178	5.13219269	0.60129751	0.4471966	0.56741296
Gm16701	-0.4527399	0.69909148	0.60120988	0.44722922	0.56741296
Zfp955a	-0.1163652	5.05285549	0.60110052	0.44726993	0.56741296
Nlrc4	-1.2313934	-1.284615	0.60093038	0.44733329	0.56743323
Dhx32	0.16391957	4.10411335	0.60033385	0.44755554	0.56765502
Synb	-0.6046374	-0.6087097	0.60010899	0.44763935	0.5677012
Gm6568	0.33383154	0.79574085	0.59989352	0.4477197	0.56774298
Tcaim	0.15030352	4.43598128	0.59927154	0.44795176	0.5679771
Agpat4	0.14371852	5.19971773	0.5983364	0.44830102	0.56835976
Rel	0.12573562	4.52771932	0.59786867	0.44847587	0.56846617
Sgk1	0.1047722	6.58128598	0.59785793	0.44847988	0.56846617
Zfp804a	-0.1557961	5.92882127	0.5974494	0.4486327	0.56854684
Tnfaip2	-0.4054924	0.47359873	0.59743397	0.44863848	0.56854684
Lrrc28	0.1279843	4.49518742	0.59698734	0.44880564	0.5686028
8430408G22	-1.1564038	-1.1148148	0.59690354	0.44883702	0.5686028
Armc7	-0.2453224	2.3542711	0.59682769	0.44886542	0.5686028
1810013L24F	0.09284948	7.41829365	0.59677199	0.44888628	0.5686028
Dusp7	0.0914239	6.05121561	0.59668188	0.44892003	0.5686028
Ldoc1l	0.1236348	4.91591851	0.59640393	0.44902416	0.56867454
1700028J19F	-0.6832256	-1.2807606	0.59619113	0.4491039	0.56871539
Pxmp2	-0.2101933	2.49324457	0.59559053	0.4493291	0.56893275
Uimc1	0.13794941	4.62464259	0.59541538	0.4493948	0.56893275

Mpp3	-0.1605097	3.41134994	0.59522988	0.44946441	0.56893275
Dhx58	-0.4285524	0.55327898	0.59508264	0.44951967	0.56893275
Cnnm3	0.12404049	4.18017385	0.5950336	0.44953808	0.56893275
Trmu	0.29264629	2.08059847	0.59490132	0.44958774	0.56893275
1700023F06I	0.96383414	-1.5503012	0.59480899	0.44962241	0.56893275
Mpc2	0.11197359	5.93669597	0.59450546	0.44973641	0.56893275
Lama2	-0.1451372	5.21919044	0.59445379	0.44975582	0.56893275
Stk17b	-0.1359052	5.75517255	0.59444564	0.44975888	0.56893275
Zdhhc16	-0.210343	2.26251779	0.59432836	0.44980294	0.56893275
Fzd7	-0.2233779	5.93664349	0.59396349	0.44994007	0.56893275
Olfr1033	-0.1613102	3.91660529	0.59385814	0.44997968	0.56893275
Setd3	0.07817242	6.79583478	0.59372652	0.45002917	0.56893275
Gm6086	-0.8045724	-1.5354807	0.59365401	0.45005644	0.56893275
8030462N17	0.09993805	5.47395335	0.59364151	0.45006114	0.56893275
Ticam2	0.48433004	0.92254392	0.5935298	0.45010316	0.56893275
Foxo1	0.10160001	7.15529798	0.59345666	0.45013067	0.56893275
Armc6	-0.2459235	1.69641095	0.59326514	0.45020273	0.56896377
Rab11b	0.09762968	9.08833055	0.59254068	0.45047546	0.56920048
Sfxn5	-0.1463724	4.82337831	0.59247929	0.45049859	0.56920048
Abcg1	-0.1427033	4.94718893	0.59238896	0.45053262	0.56920048
Wdr24	0.24867575	2.33608061	0.59225958	0.45058136	0.56920201
Psmc5	0.10361954	6.68950131	0.59211407	0.45063619	0.56921124
Timm23	0.12144119	6.17913277	0.59192534	0.45070733	0.56921912
Brd9	-0.0923266	5.50512023	0.59170045	0.45079212	0.56921912
N4bp3	0.36253388	1.3835988	0.59161322	0.45082501	0.56921912
Coq3	0.16349221	3.37844803	0.59136049	0.45092034	0.56921912
Ccl17	0.34102895	1.45136625	0.59123827	0.45096645	0.56921912
Eif2b3	0.19139586	2.84573605	0.59120698	0.45097826	0.56921912
Cdh5	0.18117096	5.44742373	0.59104752	0.45103844	0.56921912
Chil1	0.51240054	0.532544	0.59097513	0.45106576	0.56921912
Hspb8	-0.1804861	6.19497759	0.5909587	0.45107196	0.56921912
Ddx49	0.35624054	0.76089997	0.59064991	0.45118854	0.56921912
Lsp1	-0.2051341	3.52750994	0.59052366	0.45123622	0.56921912
Osr1	0.23198165	6.37133727	0.59033227	0.45130851	0.56921912
Dok3	0.76424608	-1.3432992	0.59016138	0.45137308	0.56921912
Erbb4	-0.1507129	4.58491755	0.59007078	0.45140731	0.56921912
Polr1d	0.13216866	6.27355112	0.5898217	0.45150146	0.56921912
Spag7	0.14374941	5.28595099	0.58962134	0.45157722	0.56921912
4930430F08I	0.15839189	3.77965773	0.58956925	0.45159691	0.56921912
Pbxip1	0.20244785	5.41108187	0.58946603	0.45163595	0.56921912
Ccdc28a	0.21682548	2.50024667	0.58945228	0.45164115	0.56921912
Gm5595	0.21785911	2.83552425	0.58932171	0.45169054	0.56921912
A730056A06	0.31225206	1.59917993	0.58918704	0.45174149	0.56921912

Tug1	0.08216299	8.05181296	0.58902412	0.45180315	0.56921912
Sox1	0.15223863	4.72724917	0.58875123	0.45190644	0.56921912
6230400D17	0.52747298	0.25397357	0.58867139	0.45193667	0.56921912
Dnajb6	0.08347983	7.95725082	0.58862911	0.45195268	0.56921912
Znrf3	0.09759012	5.9800087	0.58859498	0.4519656	0.56921912
Bet1	0.16327398	4.8666255	0.58847924	0.45200943	0.56921912
Nalcn	-0.1155465	6.39924881	0.5884744	0.45201127	0.56921912
Slc25a11	0.11390393	5.65167589	0.58844914	0.45202083	0.56921912
Arhgef3	-0.1392754	5.69113974	0.58817084	0.45212626	0.56926194
Zfp418	-0.1657757	3.63764889	0.58810843	0.45214991	0.56926194
Tmem161b	-0.24078	3.57042843	0.58796132	0.45220566	0.56927228
Adprhl2	0.74257274	-0.4313696	0.58762513	0.4523331	0.56934107
Smpdl3a	0.17094102	4.81997081	0.58753272	0.45236814	0.56934107
Rgs22	-0.4889006	0.3420577	0.58733198	0.45244428	0.56934107
Irf2bp1	-0.1386551	3.91958908	0.58731567	0.45245046	0.56934107
Arfip1	0.1425689	6.0988811	0.58703044	0.45255868	0.56941741
Cry2	-0.107416	6.33826493	0.58623727	0.45285984	0.56973647
Sec1	-0.513332	-0.0709414	0.58595136	0.45296847	0.56976963
Mrpl22	0.18691679	3.03106697	0.58581714	0.45301949	0.56976963
Lmtk3	-0.3763849	1.61875562	0.58579232	0.45302892	0.56976963
Rcc2	0.09815974	5.31857421	0.58549833	0.45314069	0.56984791
Gdap2	0.10667247	4.7954107	0.58537834	0.45318633	0.56984791
Alx4	0.18742581	5.70558227	0.58522753	0.4532437	0.56986021
Cox4i2	-0.811965	-1.5290112	0.58502682	0.45332006	0.56989638
Tmem214	0.14659958	4.44956222	0.58418165	0.45364185	0.57023309
Itgb4	0.32125854	3.19530324	0.58407332	0.45368312	0.57023309
Xirp2	-0.3241762	2.38411212	0.58369402	0.45382767	0.57035492
Klhl11	-0.1430776	4.45954021	0.58350179	0.45390096	0.57036086
Zfp341	0.21678933	3.38565362	0.58343181	0.45392765	0.57036086
Hsdl1	-0.155486	4.27791316	0.58311893	0.45404699	0.57036103
Senp3	-0.1278211	4.80653401	0.58307624	0.45406328	0.57036103
Dennd5b	-0.1562902	6.59461121	0.58305687	0.45407067	0.57036103
Klhl9	0.08646444	7.39308747	0.58275766	0.45418486	0.57044138
Ubap1	-0.1139588	5.09841286	0.58247457	0.45429293	0.57044138
Zfp212	0.20710399	2.92232215	0.58246854	0.45429524	0.57044138
Comp	0.78845488	-1.3445254	0.58239016	0.45432517	0.57044138
Zfp867	-0.1383188	4.15322404	0.58220611	0.45439546	0.57046983
2610044O15	-0.1385508	4.55994414	0.58171618	0.45458267	0.57062543
Spc25	-0.3382037	1.57807401	0.5816324	0.4546147	0.57062543
Alox12	-0.3457034	1.23960461	0.5814329	0.45469098	0.57064932
2310036O22	-0.1529688	4.55739908	0.58122124	0.45477193	0.57064932
Akr1c18	0.41616443	1.31564521	0.5812088	0.45477668	0.57064932
Qrich1	0.08782059	6.48425595	0.58085476	0.45491214	0.57070796

Rps27a	-0.1201518	6.62252119	0.58083755	0.45491873	0.57070796
Pcyox1	0.13427056	5.62445195	0.58061718	0.45500307	0.57071534
Ube2w	0.09042573	6.24687381	0.58057318	0.45501992	0.57071534
Ctso	0.17327518	5.03282715	0.58015488	0.45518011	0.57085647
Nudt1	-0.5582225	0.61450683	0.57986414	0.4552915	0.57091936
Itgb8	-0.1680156	4.16989927	0.57977516	0.45532561	0.57091936
Cldn22	1.19144371	-1.6312496	0.57954644	0.45541328	0.57096067
Urah	-0.5239536	0.09704359	0.57944049	0.4554539	0.57096067
Rps24	-0.1323192	7.28769221	0.57889196	0.45566431	0.57113584
Clcn5	-0.2920263	2.3199123	0.57882758	0.45568901	0.57113584
Rab10os	0.1817543	3.09165259	0.57820485	0.4559281	0.57137569
Gm5124	0.11740052	5.01792414	0.57791831	0.45603818	0.57145384
Nelfcd	0.15405532	3.75889393	0.57755677	0.45617713	0.57154969
Cacnb2	-0.1024498	6.33615313	0.57747098	0.45621011	0.57154969
Abca9	0.14164948	6.25692304	0.57721734	0.45630765	0.57157919
Phtf1	0.09672966	5.56154343	0.57693168	0.45641754	0.57157919
Olfr539	0.7289946	-0.4667982	0.57673937	0.45649154	0.57157919
Srl	0.23694024	1.87903088	0.5766261	0.45653514	0.57157919
Krt7	-1.0751989	-1.709268	0.57657133	0.45655623	0.57157919
Ube4b	-0.0909614	7.77348714	0.5764646	0.45659732	0.57157919
Ppp3ca	0.10493323	9.63284311	0.57635348	0.4566401	0.57157919
Ly6c1	-0.3855624	1.66203813	0.57628154	0.4566678	0.57157919
Lrp5	0.282959	3.28031446	0.57621788	0.45669232	0.57157919
1700015F17I	-0.8584242	-0.8664059	0.57616953	0.45671094	0.57157919
Ctdsp2	-0.1362817	8.00564391	0.57576459	0.45686696	0.57171277
Rnf126	0.11741407	3.99171202	0.57564472	0.45691316	0.57171277
Tnfsf9	-1.4739483	-2.0132422	0.57550822	0.45696578	0.57171887
Cep78	0.21418956	2.62711095	0.57536636	0.45702047	0.57172757
Pcgf5	-0.1521295	4.11613292	0.5746015	0.45731555	0.57201928
Usp12	-0.0918775	5.81733712	0.57451439	0.45734918	0.57201928
Lime1	-0.3598424	2.74346106	0.57419047	0.45747426	0.57211596
AA414768	-0.1673839	3.09144475	0.57371317	0.45765866	0.57228682
St6gal2	0.14618498	4.83107162	0.57354215	0.45772476	0.57230972
Ptprb	-0.2881753	5.31799751	0.57330064	0.45781814	0.57233975
Eri1	0.10777997	5.71435781	0.57323283	0.45784436	0.57233975
Pcp4	-0.099043	8.83186666	0.57275068	0.45803089	0.5724791
Papolg	0.08948332	5.32489383	0.57258931	0.45809335	0.5724791
Apold1	-0.329687	1.93923786	0.57257405	0.45809925	0.5724791
Slc16a7	-0.1417895	4.68758201	0.57232965	0.45819387	0.57248839
Kcnh2	0.24504135	2.32121856	0.5722185	0.45823691	0.57248839
2410015M2C	-0.2123355	2.73795877	0.57218447	0.4582501	0.57248839
Pld4	0.46403599	-0.3204414	0.57200906	0.45831804	0.57251354
Micu1	-0.1084544	4.45383813	0.57180365	0.45839762	0.57255324

Sash1	0.09484515	6.31869525	0.57164216	0.45846021	0.57257169
Dynlt1a	0.12017065	4.34694742	0.5715004	0.45851516	0.5725806
Trip12	-0.0924037	8.84253983	0.57124337	0.45861482	0.57264534
Peg13	-0.1009814	8.15164275	0.57095467	0.4587268	0.57272545
Shcbp1	-0.6543127	-0.5415224	0.5703345	0.4589675	0.57291929
Gm15706	-0.2346302	1.90207161	0.57020896	0.45901625	0.57291929
Zyx	0.10465784	5.44226379	0.57008431	0.45906466	0.57291929
Zfp414	0.1535207	4.50877681	0.56992721	0.45912569	0.57291929
Zfp773	-0.4193321	1.1356325	0.56992015	0.45912843	0.57291929
AY512931	-0.354188	1.78850411	0.5698155	0.45916909	0.57291929
Prkaa2	0.09979388	7.2938567	0.56968974	0.45921796	0.57292058
Psmd6	0.10360634	5.94318755	0.56943219	0.45931807	0.57295965
Atf1	0.10926441	7.58170167	0.5692535	0.45938755	0.57295965
Cacng2	0.10422358	5.27518469	0.56923998	0.45939281	0.57295965
Kcnc4	0.1592606	4.33426305	0.56911124	0.45944288	0.57296242
Fdxacb1	-0.3498363	1.52243624	0.5685503	0.45966114	0.57317493
Spata5	0.15239653	4.23811925	0.56821241	0.4597927	0.57323396
Mapk7	0.1887102	2.64550853	0.56818133	0.4598048	0.57323396
Zdhhc20	-0.12093	5.43041885	0.5678835	0.45992082	0.57323396
Setdb1	-0.1655227	4.51143675	0.56772335	0.45998322	0.57323396
Nt5c1a	-0.3687015	0.79955439	0.56770752	0.45998939	0.57323396
Sgpp1	-0.094145	6.5640417	0.56769074	0.45999593	0.57323396
Naga	0.29412528	3.0896614	0.56755183	0.46005007	0.57323396
Npc1l1	-0.5718088	0.04696487	0.56733646	0.46013403	0.57323396
Trim8	0.11317403	6.14101651	0.56720504	0.46018528	0.57323396
Trmt1	-0.2193278	2.8026084	0.56720025	0.46018715	0.57323396
Cxcl1	1.44581135	-1.9014253	0.56681613	0.46033699	0.57336097
Snx3	0.10154827	6.82295888	0.56662735	0.46041066	0.5733931
Rnf138	-0.1511592	3.49491587	0.56607643	0.46062576	0.57360133
Ppp6c	0.13716173	6.26254495	0.56549759	0.46085194	0.57382332
Cit	0.11993838	7.31494479	0.56499768	0.46104743	0.5739782
Egr3	0.12072538	7.83055763	0.56483962	0.46110927	0.5739782
Prph	-0.9165396	-0.8824467	0.5648119	0.46112012	0.5739782
Atp1a2	-0.1661631	11.2666227	0.56467124	0.46117516	0.57398706
Afap1l2	0.19155947	2.61867956	0.56441109	0.46127699	0.57405413
Ptcd2	0.13772535	4.69005806	0.56385543	0.46149462	0.57417791
Nbeal1	-0.1178681	6.22589735	0.56375103	0.46153552	0.57417791
Ghitm	0.08337453	8.70164854	0.56350486	0.46163201	0.57417791
Zfp296	-0.5472733	-0.1268593	0.56339026	0.46167693	0.57417791
Oxr1	0.08659226	8.62710565	0.56334542	0.46169452	0.57417791
Grip2	-0.2662695	2.18847827	0.56334357	0.46169524	0.57417791
Pvrl4	-0.2862242	1.55218341	0.56305969	0.46180657	0.57417791
Gm4759	0.79518098	-0.9557459	0.5630438	0.4618128	0.57417791

Azi2	0.10327594	6.72420063	0.56296731	0.46184281	0.57417791
Rps10	-0.1712485	4.92802873	0.56293394	0.4618559	0.57417791
Fap	-0.3343464	1.52176877	0.56257053	0.46199851	0.5742045
Kin	0.14527934	4.30831348	0.56245323	0.46204455	0.5742045
Gmfg	-0.4337682	1.11371591	0.56226124	0.46211993	0.5742045
Asb4	0.51153154	0.34542722	0.56221661	0.46213746	0.5742045
Baz1b	-0.0886124	7.83348427	0.5621258	0.46217312	0.5742045
Fam129c	-0.7460032	-0.3552634	0.56199514	0.46222444	0.5742045
Gltp	-0.1573386	5.52220233	0.56172049	0.46233235	0.5742045
Slc38a3	0.17456288	4.10921612	0.56169068	0.46234407	0.5742045
Ttc8	0.10664502	4.70920459	0.5616628	0.46235502	0.5742045
Tmem222	0.24240746	4.53161946	0.56165838	0.46235676	0.5742045
BC035044	-0.3953166	0.39691978	0.56152878	0.4624077	0.57420822
Ip6k1	-0.1182607	5.79046022	0.56117927	0.46254513	0.57431193
Rps13	-0.1355194	5.90492291	0.56107245	0.46258714	0.57431193
Ackr3	0.17038551	3.93527382	0.56090357	0.46265357	0.57433286
Samd15	0.27320094	2.41371152	0.5607858	0.46269991	0.57433286
Zfp142	-0.133992	5.2626512	0.56038348	0.46285827	0.57446988
Mettl15	-0.2809828	1.63250249	0.55956122	0.46318219	0.57478355
Mapk4	-0.1052701	7.79315389	0.55942777	0.4632348	0.57478355
Msrb2	0.13230981	4.28512107	0.55937656	0.46325499	0.57478355
Psmb7	0.11017815	6.85311655	0.55872283	0.46351285	0.574876
Tmem176b	0.2007378	5.40405704	0.55816861	0.46373165	0.574876
Prss16	-0.7703034	-1.0446287	0.5581557	0.46373675	0.574876
Ermp1	0.10986843	5.74204652	0.55809313	0.46376146	0.574876
5430421F17I	0.43935125	0.8712816	0.55799873	0.46379876	0.574876
2610301B20I	0.1061888	5.3130088	0.55797532	0.463808	0.574876
Msrb1	-0.1512533	4.22744188	0.55788092	0.4638453	0.574876
Pcgf2	-0.1471972	5.09123752	0.55787938	0.46384591	0.574876
Tspyl1	0.07716468	7.44783897	0.55776229	0.46389217	0.574876
Lrrc47	0.23841142	2.66498574	0.5577406	0.46390075	0.574876
Rpl17	-0.1229636	6.86589986	0.5577387	0.4639015	0.574876
Afap1	-0.1333039	7.61847143	0.55772848	0.46390553	0.574876
AA474331	-1.1350831	-1.6410653	0.55754736	0.46397712	0.57489173
Cd276	-0.2343886	2.49201803	0.55745346	0.46401424	0.57489173
Smpdl3b	0.6955755	-0.2309417	0.55727701	0.46408401	0.57491869
Tro	-0.1625793	6.41530895	0.557066	0.46416746	0.5749626
Nudcd2	-0.1733549	3.02746944	0.55678122	0.46428013	0.57504269
4930563E22I	0.4848066	0.91985291	0.55609132	0.46455327	0.57524794
Pdap1	0.0958404	6.04391655	0.55606385	0.46456415	0.57524794
Mettl24	-0.5009777	-0.0698367	0.5559987	0.46458996	0.57524794
Cdan1	-0.1605718	3.37040138	0.55568613	0.46471381	0.57534181
Tubb2a	0.13154729	7.72927064	0.55526133	0.46488222	0.57535646

Chst13	0.87110247	-0.7207541	0.55525741	0.46488377	0.57535646
Evc2	-0.2861329	2.24314415	0.55520914	0.46490292	0.57535646
Pard3	0.10519428	5.12882172	0.55517158	0.46491782	0.57535646
Brdt	-0.153287	4.23195234	0.55494029	0.46500956	0.57537736
Ngly1	0.11720246	5.55152088	0.55488676	0.4650308	0.57537736
Tmcc3	0.113214	6.34903671	0.5542531	0.46528233	0.57561969
Mms22l	0.3859582	0.73336835	0.55415126	0.46532278	0.57561969
Emc2	0.11486996	6.48024617	0.55374811	0.46548295	0.5757061
Erich2	0.37272841	0.43049639	0.55356614	0.46555528	0.5757061
Olfml2a	0.26185826	3.54799309	0.55355476	0.4655598	0.5757061
Prdm6	0.1923689	5.62563107	0.55333072	0.46564888	0.5757061
E130309F12I	-0.2092394	2.72267869	0.55331825	0.46565384	0.5757061
Malsu1	0.12513636	4.73281261	0.55315763	0.46571771	0.5757061
Plat	0.19832493	4.7598382	0.55312889	0.46572915	0.5757061
B630019K06	-0.1591668	3.26317026	0.5528361	0.46584563	0.57579066
Gad1	0.17097	7.57420596	0.55251254	0.46597441	0.57588819
Ube2j2	-0.1690573	2.89842445	0.55239625	0.46602072	0.57588819
2010015L04I	0.17336824	3.55873562	0.55209008	0.46614265	0.57593792
Edn1	-0.5247962	0.28649331	0.55205371	0.46615714	0.57593792
Pigx	-0.2128172	2.84092419	0.55128276	0.46646443	0.57616865
Rassf1	-0.218497	3.92843638	0.55125476	0.46647559	0.57616865
Npdc1	0.12741058	4.46292027	0.55111943	0.46652957	0.57616865
Ralgapa2	-0.1829447	4.62057579	0.55093503	0.46660314	0.57616865
Rbl1	-0.3133524	1.59039662	0.55092068	0.46660886	0.57616865
Gm20939	-0.1547068	3.27695277	0.55086129	0.46663256	0.57616865
Slc45a1	0.2089467	2.61914513	0.55046733	0.46678981	0.57629825
Tmem254a	0.33969994	1.45185985	0.55035725	0.46683377	0.57629825
Dkkl1	0.2449557	2.23243727	0.55001501	0.46697047	0.57630564
Egr2	-0.3638845	3.93185383	0.54991822	0.46700914	0.57630564
Amn1	-0.151092	4.58958325	0.54990168	0.46701575	0.57630564
P4ha1	-0.127265	3.95493008	0.54971699	0.46708956	0.57630564
Pus3	0.18210194	3.36515777	0.54963272	0.46712324	0.57630564
Vmn2r46	-0.6647825	-1.6613097	0.54961959	0.46712849	0.57630564
Socs2	-0.1261165	5.3342442	0.54939424	0.46721858	0.57635742
Mpg	-0.2411196	1.96183919	0.54912621	0.46732578	0.57643028
Kcng3	-0.2788152	3.24075379	0.54859158	0.46753973	0.57663478
Plekhg1	-0.1197767	5.36137989	0.54843308	0.46760319	0.57665366
Tsnaxip1	-1.0726178	-1.2806416	0.54798947	0.46778088	0.57674376
Gpr101	0.27178293	2.41118593	0.54793324	0.46780341	0.57674376
Nova1	-0.1005008	7.15070647	0.54757063	0.46794875	0.57674376
Crhr2	0.9565936	-1.7099164	0.54743505	0.46800311	0.57674376
Nwd2	0.24948185	5.78589024	0.54735871	0.46803372	0.57674376
Lnp	0.1258387	6.97294153	0.54725148	0.46807673	0.57674376

F630111L10F	-0.2750778	2.56004193	0.54702036	0.46816944	0.57674376
Neil1	-0.4829085	-0.0590332	0.54699278	0.46818051	0.57674376
Mtmr4	-0.1362376	5.94243077	0.54699227	0.46818071	0.57674376
Vash2	-0.2600469	1.75752496	0.54696959	0.46818981	0.57674376
Slc25a40	-0.1800178	3.38294368	0.54685453	0.46823598	0.57674376
Map3k13	-0.1693418	5.16798129	0.54680924	0.46825416	0.57674376
Tmem115	0.22456372	2.20816434	0.54662936	0.46832636	0.57677337
Hif1a	-0.1063299	6.51471301	0.54634711	0.4684397	0.57685363
Fgf18	0.22994072	2.47317753	0.54610919	0.46853527	0.57685633
Dnajb1	0.09034847	6.61552097	0.5458963	0.46862081	0.57685633
Zmym2	0.11544074	8.1176782	0.54585327	0.4686381	0.57685633
Hist1h4j	0.32427595	0.67386973	0.54576266	0.46867452	0.57685633
Sema6b	-0.2102178	2.90012105	0.54573514	0.46868558	0.57685633
Drd5	-0.4591195	0.25089244	0.54551637	0.46877353	0.57685633
Tst	-0.1731647	4.00671627	0.54534358	0.46884302	0.57685633
Golim4	0.12879496	5.93506605	0.54527232	0.46887168	0.57685633
Zmynd15	-0.4645351	0.33496792	0.54526303	0.46887542	0.57685633
Cnot8	0.11871434	4.59883218	0.54479682	0.469063	0.57702784
Stx1a	-0.1204038	5.57076194	0.5445713	0.46915379	0.5770581
Glipr2	-0.2693811	4.05857951	0.54434311	0.46924568	0.5770581
Fkbp5	0.2175436	5.97414013	0.54429375	0.46926556	0.5770581
Ulk1	0.11454556	6.46626402	0.54418332	0.46931005	0.5770581
Lin7c	0.08833923	7.8688952	0.54410554	0.46934138	0.5770581
Ercc6	-0.1557552	5.60019108	0.54401785	0.46937671	0.5770581
Lpar1	0.17592929	6.85129751	0.54366303	0.46951973	0.57709229
Dennd4c	-0.1039374	6.04642157	0.54359284	0.46954803	0.57709229
Gpatch1	-0.1013672	5.0819513	0.5435902	0.46954909	0.57709229
Slc26a2	0.21022901	6.09247466	0.54318765	0.46971145	0.57710726
Dhx16	-0.2078026	2.95949162	0.54314151	0.46973006	0.57710726
Zbtb11	-0.113733	6.58427433	0.54309921	0.46974712	0.57710726
Ak2	0.15830399	4.85332912	0.5430821	0.46975403	0.57710726
Ubtf	-0.0987147	5.83029552	0.54294137	0.46981082	0.57711782
Pkd1	-0.1435734	5.17481873	0.54279439	0.46987014	0.5771315
Fbll1	0.2440481	2.23625039	0.54245444	0.4700074	0.57713199
Tceanc	-0.1999137	3.2960577	0.54243696	0.47001446	0.57713199
Arid4a	-0.1128804	7.65718291	0.54243533	0.47001512	0.57713199
Pde5a	0.14884262	6.52878451	0.54193206	0.47021844	0.5772553
A930011G23	0.53867043	-0.3035177	0.54191856	0.4702239	0.5772553
Fibcd1	-0.4941205	0.43586962	0.54174744	0.47029307	0.5772553
Lrrfip2	-0.0922659	5.83690377	0.54170963	0.47030835	0.5772553
Fam207a	-0.1875818	3.37033488	0.54113945	0.47053896	0.57747917
Cox4i1	0.17281634	6.74873163	0.54074312	0.47069937	0.57761684
S100a4	0.316407	3.08503307	0.54057254	0.47076844	0.5776424

Sspn	-0.2021051	4.45629155	0.54026657	0.47089237	0.57773527
Poc5	-0.1163693	3.97921674	0.53975065	0.47110145	0.57793259
1700063D05I	-0.2302476	1.83602218	0.5395902	0.47116651	0.5779532
Mrpl27	-0.1432653	5.32071459	0.53940514	0.47124157	0.57798606
Parp11	-0.1832531	3.76435497	0.53927627	0.47129384	0.57799098
Gltpd1	-0.1304873	3.73848941	0.53895117	0.47142577	0.57806775
0610011F06I	0.14745977	3.61598656	0.53886413	0.4714611	0.57806775
Slco2b1	0.23932546	2.70595602	0.53876524	0.47150125	0.57806775
Mcm9	-0.2842461	2.0178278	0.53857722	0.47157759	0.57808113
Trim43c	0.75430055	-1.17469	0.53845395	0.47162766	0.57808113
Cyp2b10	1.02823245	-1.7136659	0.53830549	0.47168797	0.57808113
Smim5	0.54913236	-0.0767975	0.53826295	0.47170525	0.57808113
Lgr5	0.22188986	2.48091856	0.53790344	0.47185135	0.57814858
Tmem18	-0.1390427	4.31066498	0.53788994	0.47185684	0.57814858
Cystm1	-0.2442687	2.13882184	0.53751621	0.47200881	0.57819346
Myoz3	-0.363638	1.1393035	0.5374665	0.47202903	0.57819346
Ero1lb	0.16522755	3.76443278	0.53744367	0.47203831	0.57819346
Pigq	0.10190531	5.82510229	0.53702447	0.47220888	0.57826077
Llgl1	-0.1249475	3.95670421	0.53696527	0.47223298	0.57826077
Fgd3	0.65285714	-0.2428206	0.53695264	0.47223812	0.57826077
A430035B10	-0.1888219	2.99546973	0.53662628	0.472371	0.57831776
Epc1	-0.0891693	6.38698738	0.53634939	0.47248379	0.57831776
Tmem189	-0.2021538	2.5608425	0.5361296	0.47257334	0.57831776
Ttc9b	-0.1962936	3.14900253	0.53608447	0.47259174	0.57831776
Ctage5	0.08882686	5.84547173	0.53607281	0.47259649	0.57831776
Zbed5	0.16738934	3.8677752	0.53603792	0.47261071	0.57831776
Rbm12b2	-0.1506596	4.39119605	0.53600852	0.4726227	0.57831776
Sult4a1	0.09118934	7.03888384	0.53564088	0.47277259	0.57844208
Scel	0.17526102	3.33749651	0.53543579	0.47285625	0.57848533
Casc1	0.56118191	-0.6692017	0.53472231	0.47314746	0.57878247
Pir	-0.1380683	3.46972287	0.53456857	0.47321025	0.57880016
Peli1	-0.0999192	5.95279181	0.53419249	0.47336391	0.57892551
Phrf1	-0.1373518	4.78880025	0.53408113	0.47340942	0.57892551
Creb3l4	0.93666463	-0.9489596	0.53387221	0.47349483	0.57896375
Apbb3	-0.249187	2.08151078	0.53376816	0.47353738	0.57896375
Mmp9	0.67772625	-0.7822995	0.53320154	0.47376919	0.57904388
Abcc9	-0.269091	3.11914776	0.53309726	0.47381187	0.57904388
Rabgap1	0.0853784	7.271955	0.53303909	0.47383568	0.57904388
Layn	0.28946629	1.44309849	0.53286794	0.47390576	0.57904388
Smyd2	0.12722724	5.5721474	0.53281681	0.47392669	0.57904388
Zfp36	0.20613441	4.95134142	0.53278438	0.47393997	0.57904388
Zxdb	0.1038674	5.19819493	0.53278095	0.47394138	0.57904388
Vmn2r1	-0.9923505	-1.1488416	0.53221934	0.47417146	0.57926589

4931406C07I	0.13660613	6.65583506	0.53198027	0.47426947	0.57928599
Krt77	-0.6717953	-0.5571166	0.53172978	0.47437219	0.57928599
Slc35a3	-0.112698	4.86209558	0.53171003	0.47438029	0.57928599
Pde7a	0.1317888	5.13023311	0.53159755	0.47442643	0.57928599
Arx	-0.2166386	2.59868068	0.53153364	0.47445265	0.57928599
Anks3	-0.1724586	2.94614511	0.53143009	0.47449513	0.57928599
9130023H24I	-0.1628949	3.76212086	0.53124987	0.47456909	0.57928599
Slc25a37	-0.1126777	5.36740566	0.5312276	0.47457823	0.57928599
Nkain3	0.24720969	1.994663	0.53103692	0.47465651	0.57928599
Tacr3	0.2948724	1.90532315	0.53100006	0.47467164	0.57928599
Micall1	-0.1061068	5.22167441	0.53073287	0.47478137	0.57936086
Zkscan14	-0.3106508	2.42938013	0.53052248	0.4748678	0.57938009
Htr5a	0.17677065	5.37112254	0.530459	0.47489388	0.57938009
Sh3gl1	-0.1772294	3.29642853	0.53009566	0.47504321	0.57950324
Kpna6	-0.1050893	7.46646261	0.52985688	0.4751414	0.57956398
Rhoq	-0.120336	6.22370793	0.52963733	0.47523171	0.57961511
Glud1	-0.0741834	8.41518438	0.52940108	0.47532892	0.57967463
Rpgrip1l	-0.1458599	5.86432918	0.5292141	0.47540588	0.57970945
Hcn1	0.14466262	7.36017206	0.52899069	0.47549786	0.57976258
Yipf6	0.08828727	7.26621431	0.52856316	0.47567397	0.57984584
C530044C16I	0.91118595	-1.6134224	0.52850499	0.47569794	0.57984584
Clca2	-0.7900045	-1.5911448	0.52797352	0.47591703	0.57984584
Oraov1	-0.166709	3.70779662	0.52790138	0.47594678	0.57984584
Relb	0.31500481	0.4167856	0.52788006	0.47595557	0.57984584
3110007F17I	-0.3552511	1.17181934	0.5278602	0.47596376	0.57984584
Wrap53	-0.3095137	1.41100641	0.52779892	0.47598904	0.57984584
Tcf25	0.07456017	8.4293522	0.52779696	0.47598985	0.57984584
Atl3	0.09804839	7.4225482	0.52776772	0.47600191	0.57984584
Zkscan4	0.2266804	2.2997498	0.52685823	0.47637734	0.58007641
Rps6kb1	0.08508998	7.12851419	0.52672317	0.47643314	0.58007641
1700085C21I	-1.0280138	-1.704339	0.52668531	0.47644878	0.58007641
Apoe	-0.2277431	6.97856543	0.52668415	0.47644926	0.58007641
Fam19a1	0.09499633	5.87262786	0.52660724	0.47648104	0.58007641
Cep135	0.17676701	4.32391521	0.52635625	0.47658478	0.58007641
4931408D14I	0.42888197	0.71792381	0.52627161	0.47661976	0.58007641
Psma3	0.0965395	6.99917801	0.52625798	0.4766254	0.58007641
Fam203a	0.23927442	2.59822023	0.52606589	0.47670483	0.58007641
Tmem79	-0.7871155	-0.2191697	0.52602065	0.47672353	0.58007641
Mtmr11	0.15338015	3.419479	0.52601951	0.47672401	0.58007641
Dirc2	-0.1235845	4.94217804	0.52565933	0.47687301	0.58016698
Zfp334	0.10221964	4.89297415	0.52557454	0.4769081	0.58016698
Nans	0.24398186	2.40515802	0.52548833	0.47694378	0.58016698
Ccdc18	0.34522524	1.71139588	0.5248157	0.47722231	0.58016927

Ptp4a1	0.13549371	4.59349163	0.52480352	0.47722736	0.58016927
Cdon	-0.1705653	5.1561861	0.52459588	0.4773134	0.58016927
Cplx3	0.28455604	1.32700046	0.52458523	0.47731781	0.58016927
Dnajc18	0.10324241	6.93329218	0.5245024	0.47735214	0.58016927
Tex12	0.54085909	-0.1499423	0.52446805	0.47736638	0.58016927
Wdr83	0.18463847	2.6962975	0.52446027	0.4773696	0.58016927
Zbtb20	0.09350451	6.86078302	0.52442135	0.47738574	0.58016927
Tspan8	0.24270345	4.35901052	0.52426948	0.4774487	0.58016927
Dnajc6	0.09571844	8.53886935	0.52416512	0.47749198	0.58016927
Suv39h2	0.18275929	3.0955712	0.5241266	0.47750795	0.58016927
Txndc5	0.11941393	4.95852922	0.52397461	0.47757099	0.58016927
Larp6	0.14221371	3.96276884	0.52394691	0.47758248	0.58016927
Gm5577	-0.5718729	1.02627357	0.52375565	0.47766183	0.58016927
Map2k6	0.13533484	4.00294987	0.5237219	0.47767584	0.58016927
Pilra	-0.5339799	-0.5315319	0.5236136	0.47772079	0.58016927
Gm10190	-0.5862674	-0.2077484	0.52312821	0.47792231	0.58035516
Tpcn1	0.18471852	5.97215689	0.52289505	0.47801917	0.58041392
Krt10	0.22003257	2.23718246	0.52277578	0.47806873	0.58041525
Nmnat3	0.23295877	2.54700516	0.52223568	0.47829325	0.58054614
Ano5	0.49084061	0.82467018	0.52222206	0.47829891	0.58054614
Rps6kb2	0.20064828	2.55862419	0.52211186	0.47834475	0.58054614
Dock6	-0.1407687	3.65249162	0.52205008	0.47837045	0.58054614
Snord42a	0.57485381	-1.1470432	0.52185667	0.47845092	0.58058497
A330076C08	-0.6028003	0.12744797	0.5215721	0.47856935	0.58065091
Trim30d	-0.1603802	3.62302109	0.52149312	0.47860223	0.58065091
Adam5	-0.5591836	0.8451942	0.52116631	0.47873832	0.58075019
Gclc	0.09560918	6.26031202	0.52106374	0.47878105	0.58075019
Ubac2	0.17660745	2.53218994	0.52080536	0.47888871	0.58077165
Scara5	0.26190924	1.61318864	0.52078851	0.47889573	0.58077165
Cygb	-0.1543671	4.10908569	0.51964771	0.47937157	0.58128985
Ankrd34a	0.10652277	6.32298013	0.51930675	0.47951395	0.58133389
Crybg3	-0.15306	4.24807861	0.51914857	0.47958002	0.58133389
Ap5z1	0.25492302	2.61958372	0.51902151	0.47963311	0.58133389
Vars2	0.24260365	2.25968597	0.5189115	0.47967908	0.58133389
Bambi	-0.3219466	1.23573943	0.51889315	0.47968675	0.58133389
Serpinb6b	-0.2154354	5.19949551	0.51886347	0.47969915	0.58133389
Smtnl2	-0.2675622	1.14257697	0.51862229	0.47979997	0.58139724
Rph3a	-0.1144111	8.88862341	0.51847307	0.47986237	0.58141402
Tubgcp6	-0.1713597	3.40036445	0.51803196	0.48004689	0.58154552
Grp	0.45406102	0.11469407	0.51798147	0.48006802	0.58154552
4930579G18	-0.5671167	0.33076995	0.51785576	0.48012063	0.58155043
Rpl4	-0.1033523	8.80304344	0.51773343	0.48017184	0.58155363
Plekhm3	-0.1166364	6.33117024	0.51749354	0.48027229	0.58161647

Slc9a3r2	-0.1714149	5.10276259	0.51723737	0.48037959	0.58162936
Slain1	-0.1478806	4.91292818	0.51720992	0.48039109	0.58162936
Sox12	-0.1771139	2.99499347	0.51710804	0.48043378	0.58162936
Syt11	0.11380013	8.29352381	0.51700441	0.4804772	0.58162936
Rasa3	-0.1158776	5.2610341	0.51685643	0.48053923	0.58163854
Zer1	-0.124821	4.95266359	0.51675459	0.48058192	0.58163854
Gpatch11	0.12971949	5.43093597	0.51643465	0.48071609	0.58169774
Zglp1	0.83539947	-1.4615656	0.51633797	0.48075664	0.58169774
Tspyl4	-0.0908455	8.38403276	0.51629052	0.48077655	0.58169774
Eef2	-0.0860128	8.35795737	0.51607765	0.48086587	0.58174703
Stap2	-0.314819	1.64210429	0.51552939	0.48109605	0.58194555
Gdpgp1	-0.1404675	4.8161016	0.51545535	0.48112715	0.58194555
Agpat9	0.28420698	1.27578134	0.51524424	0.48121584	0.5819635
Cyhr1	-0.1084477	5.5707049	0.51507323	0.48128771	0.5819635
Ccdc67	0.79104273	-1.3627201	0.5150235	0.48130861	0.5819635
Supt4a	0.18666216	4.73820163	0.51495746	0.48133637	0.5819635
Morn1	0.42746195	0.458594	0.51476419	0.48141763	0.5819906
Gm10474	-0.9832401	-1.5017368	0.51467298	0.48145598	0.5819906
Pcnx	0.10967189	7.32151792	0.51391983	0.48177289	0.58199241
Ap1m1	-0.1645937	3.73255191	0.51391496	0.48177494	0.58199241
Sec24b	0.10036012	7.02039347	0.51386777	0.48179481	0.58199241
Tgfbr1	0.1245272	5.32494421	0.51374159	0.48184794	0.58199241
Wnt16	0.34752497	1.69469413	0.51371584	0.48185879	0.58199241
Tatdn2	-0.158377	4.03065392	0.51371569	0.48185885	0.58199241
Bhlhe22	0.11856183	5.19943466	0.51371168	0.48186054	0.58199241
Cnppd1	-0.1723207	4.12454066	0.51364407	0.48188902	0.58199241
Agps	0.0851308	7.19752054	0.51363019	0.48189486	0.58199241
Cpeb3	0.10206423	7.55417114	0.5130544	0.48213749	0.58215663
Pbx4	0.39288747	0.13138919	0.51290163	0.48220189	0.58215663
Papd7	-0.1302744	4.69030791	0.5124635	0.48238669	0.58215663
Clec2l	-0.2892046	1.15729385	0.51244482	0.48239457	0.58215663
Epb4.1	-0.0978028	4.96364631	0.51243472	0.48239884	0.58215663
Alg3	0.16304879	3.44367456	0.51241804	0.48240587	0.58215663
Qser1	0.10813459	6.91275708	0.51235907	0.48243076	0.58215663
Dtl	0.13186817	4.00306842	0.51225879	0.48247308	0.58215663
Igfbp7	-0.175255	5.10106368	0.51216683	0.48251189	0.58215663
Scaper	-0.1242479	6.38242996	0.51215485	0.48251695	0.58215663
Zfp324	0.22026025	3.0189689	0.51176345	0.48268222	0.58229736
Dusp15	0.17549096	2.85404897	0.51133047	0.48286515	0.58245937
Slc26a10	-0.3301071	0.66412926	0.51109462	0.48296485	0.58249592
Pex14	0.20406304	2.54592797	0.51094956	0.48302619	0.58249592
Eif4a2	-0.0775008	8.98994268	0.51091366	0.48304137	0.58249592
Galnt3	-0.7955423	-0.752167	0.51072804	0.48311988	0.58253194

Ist1	0.10461108	5.83351638	0.51039783	0.48325959	0.58255142
Hspb11	0.18890393	3.06169139	0.51039521	0.48326071	0.58255142
Cxcl12	-0.1112704	6.01762405	0.51034496	0.48328197	0.58255142
Tdo2	0.82860704	-0.9958907	0.51018843	0.48334823	0.58257266
Prdm1	0.31974866	2.00380264	0.50990464	0.4834684	0.58265885
Skiv2l2	0.09501564	6.77451449	0.50943941	0.48366552	0.58275234
Lrfn1	-0.4275488	0.21389315	0.50938968	0.48368659	0.58275234
Kcnn2	0.1353661	3.8382123	0.50928922	0.48372918	0.58275234
Kcng1	0.51714082	0.10079227	0.50926222	0.48374062	0.58275234
Nub1	0.09853055	5.30271563	0.5084267	0.48409506	0.58298492
Pik3c2g	-1.0174573	-2.0787659	0.50829133	0.48415253	0.58298492
Tmod3	-0.1391995	7.80433048	0.50828265	0.48415621	0.58298492
Jakmip2	0.11082459	6.83067057	0.50827871	0.48415788	0.58298492
Iqsec2	-0.1172598	5.69733138	0.50823349	0.48417709	0.58298492
Eif4h	-0.0805856	7.04645059	0.50806488	0.48424869	0.58299583
AW822252	-0.5434315	0.07551761	0.50798289	0.48428351	0.58299583
Eps8l1	0.17173983	2.75832042	0.5078383	0.48434493	0.58301117
Tollip	0.09138401	6.59682381	0.50769343	0.48440648	0.58301958
Rsl1	0.20716693	2.89096568	0.5075927	0.48444929	0.58301958
Cort	-0.844345	-1.6899519	0.50735574	0.48455002	0.58302847
Ido1	0.33131707	1.31686761	0.50722544	0.48460542	0.58302847
Col6a6	-0.9724534	-1.4085711	0.50700809	0.48469786	0.58302847
Pydc4	-0.8751315	-1.4334036	0.50695585	0.48472008	0.58302847
Lyrm4	0.102657	5.02712425	0.50689564	0.4847457	0.58302847
BC030500	0.15234415	3.90057048	0.50688841	0.48474878	0.58302847
1700071K01I	-0.7855018	-0.3649096	0.50632554	0.48498835	0.58308525
Akap14	-0.6178654	-0.9875609	0.50630376	0.48499763	0.58308525
Gli1	-0.3354847	1.65310861	0.5061899	0.48504612	0.58308525
Tmem209	0.11738519	4.22701157	0.50618145	0.48504972	0.58308525
Cers5	0.09915711	4.92314804	0.50602461	0.48511652	0.58308525
Stc2	0.43984508	-0.1603115	0.50600909	0.48512313	0.58308525
Slk	0.09653696	8.16449595	0.50574255	0.4852367	0.58308525
Gmpr	0.23574148	2.07266291	0.50568674	0.48526049	0.58308525
Hilpda	-0.2044194	2.63433833	0.50563522	0.48528245	0.58308525
Tmem164	0.10964904	4.58664058	0.50563422	0.48528288	0.58308525
Rpp14	0.11265741	5.40968028	0.50539846	0.48538339	0.58314011
Tbx21	-0.9592976	-0.7649604	0.50519787	0.48546894	0.58314011
Sdhd	0.14513392	6.88424588	0.50518455	0.48547462	0.58314011
Abca7	-0.287077	1.81543376	0.50457095	0.48573646	0.58339612
Psmd7	-0.0808759	6.76951316	0.50411668	0.48593047	0.58357061
Zfp868	0.13486629	4.89066314	0.50383513	0.48605079	0.58363768
Anxa6	0.12416669	5.87471101	0.5037579	0.4860838	0.58363768
Fam53a	-0.1578154	3.55264989	0.50343047	0.48622379	0.58374725

Ccdc115	-0.1097484	4.90636218	0.5030499	0.4863866	0.58387776
Gm5468	0.3583814	1.88273939	0.50294846	0.48643001	0.58387776
Tfpt	0.20396188	2.7330613	0.50268279	0.48654373	0.58393192
Ulk3	-0.1501864	3.94958032	0.50244565	0.48664528	0.58393192
Gm2518	0.58384395	-0.6249179	0.50242586	0.48665376	0.58393192
Klhl28	-0.1695076	3.90429028	0.50238755	0.48667017	0.58393192
Bmp1	0.20656683	2.83587107	0.50203968	0.48681922	0.58405224
Dpp9	0.11610628	4.59523009	0.50185288	0.48689928	0.58408978
Zfp598	0.11140948	4.07853737	0.50094592	0.48728835	0.58449796
Sucla2	-0.0795614	6.74457014	0.50082537	0.4873401	0.5845015
Snhg6	0.25756108	1.68394643	0.49996224	0.48771093	0.58476653
Smurf1	0.11133577	5.13253771	0.49965408	0.48784344	0.58476653
Myh7	0.20105998	2.56956059	0.49963384	0.48785215	0.58476653
Agbl2	0.27649219	1.56639521	0.49962851	0.48785444	0.58476653
A830019L24I	0.71663169	-0.6944655	0.49950506	0.48790755	0.58476653
Rnf216	0.08563288	5.97522611	0.49943351	0.48793833	0.58476653
Rab1b	0.15691999	4.50357591	0.49932406	0.48798543	0.58476653
Ctnnd2	0.11600081	9.36130243	0.49931895	0.48798763	0.58476653
Vps13a	-0.1305485	7.32010642	0.49928893	0.48800054	0.58476653
Tceal6	0.09710885	4.72825426	0.4987801	0.4882196	0.58497049
Letmd1	0.11935896	4.8416147	0.49851353	0.48833444	0.58501814
Ttc39d	-1.0887413	-1.637163	0.49830016	0.48842638	0.58501814
Hddc2	0.13902915	3.66227842	0.49828853	0.48843139	0.58501814
Pnrc1	0.09272818	7.15483839	0.49821641	0.48846248	0.58501814
Pdf	0.11844971	5.08672564	0.49812096	0.48850362	0.58501814
AW046200	-0.5701745	0.14785655	0.49779534	0.48864404	0.58512779
Eps15l1	-0.1051137	5.75893508	0.49709871	0.48894467	0.58531513
Zfp81	-0.1330218	4.91312818	0.49689824	0.48903125	0.58531513
Mettl6	-0.1847404	3.84319082	0.49677283	0.48908542	0.58531513
Dennd1c	-0.7078177	-0.2605778	0.49667227	0.48912886	0.58531513
Mtfr2	-0.9806895	-1.0937227	0.49666249	0.48913309	0.58531513
Prps1	0.08423696	5.87724468	0.49664552	0.48914042	0.58531513
Ltn1	-0.1120752	6.60022805	0.49664043	0.48914262	0.58531513
Bckdk	-0.1143685	4.30818593	0.49623077	0.48931968	0.58542426
Fgfr2	-0.1329695	6.83047451	0.49607314	0.48938784	0.58542426
Ncor1	-0.1055853	9.19656886	0.49583234	0.48949199	0.58542426
4930505A04I	-0.5814216	-0.1434764	0.49581321	0.48950027	0.58542426
Pih1d1	-0.2086339	3.36208392	0.49574709	0.48952888	0.58542426
Ehmt2	0.10879558	5.08226054	0.4957415	0.4895313	0.58542426
Slc4a9	-1.2507262	-1.3931945	0.49563818	0.489576	0.58542426
Ninj1	0.33883911	2.11751895	0.495263	0.48973841	0.58551824
Zfp27	-0.1504265	4.00866786	0.49523073	0.48975238	0.58551824
Ssh1	-0.1836224	2.40579911	0.49493333	0.48988118	0.58561376

Map1b	-0.1827649	12.0341584	0.49466045	0.48999942	0.58565633
Epb4.1l3	0.09136406	7.79596914	0.49462544	0.4900146	0.58565633
Pram1	-0.3412189	0.92672815	0.49448758	0.49007435	0.58566929
Zfp94	-0.2024213	2.68639115	0.49431391	0.49014965	0.58570083
Prelid2	-0.7230179	-0.7033293	0.49374881	0.49039479	0.58591201
Arhgap32	-0.13766	9.3418549	0.49360656	0.49045653	0.58591201
P2ry6	0.32277745	0.78746139	0.49356828	0.49047315	0.58591201
Zfp846	-0.1634921	3.9051803	0.49299699	0.49072126	0.58614993
Ttll7	-0.104981	7.62182125	0.49284556	0.49078706	0.58615535
Ccdc166	0.16873959	3.31457511	0.49276128	0.49082369	0.58615535
2010107G12	-1.1371766	-1.5028494	0.49260266	0.49089264	0.58617924
Hnrnpl	0.10791944	5.22655387	0.49213157	0.49109752	0.5862906
2700054A10	0.18752856	2.52735864	0.49208519	0.4911177	0.5862906
Kazald1	-0.2908911	1.58188185	0.49197559	0.49116539	0.5862906
Fnip2	-0.1248241	4.74590785	0.49185805	0.49121654	0.5862906
Gm15663	-0.1784992	3.1623083	0.49182556	0.49123068	0.5862906
Rp9	-0.1357764	4.0520469	0.49149779	0.49137339	0.58640248
Thrsp	0.23516591	3.17040787	0.49120014	0.49150304	0.58649876
Cd160	0.37284506	1.22639695	0.49079699	0.49167874	0.58664443
Hmgcl	0.17881941	2.57252918	0.49069527	0.49172308	0.58664443
Fcgr2b	0.547806	0.11290006	0.49046986	0.49182139	0.58665127
2700029M09	-0.0959725	5.45364033	0.49045745	0.4918268	0.58665127
Ing4	0.12472868	3.88740656	0.48972981	0.49214436	0.58690266
Synj2	0.12487354	6.45483524	0.48966964	0.49217063	0.58690266
Mettl10	-0.1446253	5.06836517	0.48954318	0.49222586	0.58690266
Cxcl9	-0.7039076	-0.5124598	0.48943912	0.49227132	0.58690266
Sec14l2	0.21117556	2.46746972	0.48941332	0.49228259	0.58690266
Nlgn2	0.09872573	6.3200892	0.48922828	0.49236344	0.58694062
Mei1	0.32179888	0.94362677	0.48898412	0.49247016	0.58697378
Tcerg1	-0.0905509	6.61335519	0.48881021	0.49254619	0.58697378
1700018A04	-0.86057	-1.9461086	0.48869259	0.49259763	0.58697378
Jade2	-0.093389	5.26676133	0.48866193	0.49261104	0.58697378
Hist1h1c	0.29390105	2.87540411	0.48856091	0.49265523	0.58697378
Bhlhb9	0.08841019	5.47364275	0.48843583	0.49270995	0.58697378
Nek7	-0.1074292	6.94421238	0.48829927	0.49276971	0.58697378
Tead4	-0.655646	0.04047983	0.48826806	0.49278337	0.58697378
Aamp	-0.1356235	4.84802461	0.48765136	0.49305339	0.58723701
Sobp	-0.0805826	7.44545726	0.48748675	0.49312551	0.5872645
Slc6a8	0.09277291	6.34157848	0.48664413	0.49349496	0.58764604
Pepd	-0.1371714	3.62132166	0.48612196	0.49372415	0.5878034
Pip5k1a	0.12692248	3.99435861	0.48611941	0.49372527	0.5878034
Gas7	0.0836255	9.7921324	0.48589171	0.49382527	0.58786401
1110065P20	-0.4269605	0.87558483	0.48519194	0.49413281	0.58810061

Impa2	0.53406808	-0.1342386	0.48519014	0.4941336	0.58810061
Ndufb11	0.13411265	5.59865569	0.4851043	0.49417135	0.58810061
Lin7b	0.13417894	3.68429884	0.48467418	0.49436057	0.58822006
Coro1b	0.11834639	5.32204427	0.48458239	0.49440097	0.58822006
Thsd4	0.19848011	6.29216156	0.48454126	0.49441907	0.58822006
Bcl2l1	-0.1571221	4.44907231	0.48432907	0.49451248	0.58827275
Pde9a	0.28996213	2.03889486	0.48388039	0.49471011	0.58842904
Mtfr1l	0.12281679	5.6179435	0.48380773	0.49474213	0.58842904
Sfmbt2	0.23715873	2.5418789	0.48368252	0.49479731	0.58843622
Galnt10	-0.27531	1.9081187	0.48338615	0.49492796	0.58850699
Gpatch8	-0.0824143	8.04888298	0.48332462	0.49495509	0.58850699
Nudt6	0.16135438	3.7359988	0.48277709	0.49519665	0.58873129
Stat2	-0.1060436	4.68576171	0.48258225	0.49528266	0.58873129
9430015G10	0.2424788	2.45774709	0.48251367	0.49531294	0.58873129
Slc2a1	-0.1543294	4.64273172	0.48245153	0.49534038	0.58873129
Pkp4	-0.0809829	8.59015621	0.48200269	0.49553865	0.5889085
Slitrk3	-0.135985	6.08726695	0.48161001	0.49571223	0.58902585
Wdr46	0.17838382	4.10368222	0.48155679	0.49573577	0.58902585
4933412O06	-0.4100441	1.25724567	0.48098926	0.49598684	0.58913507
Atg3	0.08221799	6.65030074	0.48090459	0.49602431	0.58913507
Dpp3	-0.1496342	3.80938584	0.48079251	0.49607393	0.58913507
C130060K24I	0.58629093	0.00503325	0.48055716	0.49617815	0.58913507
Cst6	0.58342713	-0.7388505	0.48044983	0.49622568	0.58913507
AI506816	0.6738137	-1.375879	0.48034687	0.4962713	0.58913507
Chic2	-0.1543751	3.94294952	0.48034197	0.49627347	0.58913507
Ccdc142	-0.4852296	-0.4755545	0.47990481	0.49646721	0.58913507
Gm10433	-1.046623	-1.6576177	0.47987874	0.49647877	0.58913507
Meox1	-0.6413171	-1.373733	0.47982924	0.49650072	0.58913507
Tmem210	0.51491612	0.06352342	0.47969452	0.49656046	0.58913507
Zfp345	-0.6119269	-0.7265107	0.47966371	0.49657412	0.58913507
Hps4	-0.2086802	2.44925576	0.47966131	0.49657518	0.58913507
Nosip	0.11700957	4.60528241	0.47964454	0.49658262	0.58913507
Dsel	0.11721761	5.03122799	0.47954514	0.49662671	0.58913507
Med31	-0.1470977	3.37068714	0.47942895	0.49667826	0.58913507
Capn10	0.18812849	2.36488744	0.47931777	0.49672759	0.58913507
Mastl	0.31231462	1.51160158	0.4792513	0.49675709	0.58913507
Acin1	0.08046083	5.86499961	0.47923937	0.49676238	0.58913507
Terf1	-0.1414488	3.52866775	0.47874398	0.49698232	0.58933755
Srsf2	0.0881919	8.34931977	0.47830403	0.49717778	0.58946574
Ephx4	-0.1083698	5.18556437	0.47823032	0.49721055	0.58946574
Htr1d	0.36477099	1.15101983	0.47816836	0.49723809	0.58946574
Kdm5c	-0.1255394	5.20889389	0.47800155	0.49731225	0.5894953
AF357426	-0.5426424	-0.8974646	0.47756767	0.49750524	0.5896657

Gpt	0.43494937	0.46254604	0.47698927	0.49776272	0.58991249
Coro1a	0.14696484	4.02559701	0.47677311	0.49785901	0.58994248
Snrnp25	0.22099472	2.33329262	0.4767113	0.49788655	0.58994248
Syngr1	-0.092724	6.81639169	0.47620094	0.49811403	0.59015363
Nbl1	0.23603225	6.51471903	0.4760678	0.4981734	0.59016166
Galc	-0.1661725	3.76757953	0.47589803	0.49824913	0.59016166
Tmem179	-0.145013	3.41467723	0.47585428	0.49826864	0.59016166
Tti1	0.12521571	4.735674	0.4755926	0.49838542	0.59024159
Cdhr3	-0.6619536	-0.2814062	0.4753803	0.49848019	0.59029546
Sorcs1	0.15384643	4.97864272	0.47471092	0.49877921	0.59053202
Cenpu	-0.3655661	-0.0263099	0.47461038	0.49882415	0.59053202
Usp46	0.08083047	7.22034114	0.47460202	0.49882789	0.59053202
Samd10	-0.1803025	3.49672755	0.47431222	0.49895746	0.5905465
Igf2bp3	-0.1705153	4.2010688	0.47429876	0.49896348	0.5905465
Dcdc2b	0.15810997	3.77536492	0.47424382	0.49898805	0.5905465
Lrrc41	-0.1236096	4.24108537	0.47392755	0.49912955	0.5905833
Dohh	-0.1825422	3.27307213	0.47389594	0.49914369	0.5905833
Rbm26	0.08816596	7.13250946	0.47370792	0.49922785	0.5905833
Tmed7	0.10712893	5.69417952	0.47368578	0.49923776	0.5905833
Foxr1	-0.5179995	-1.0863952	0.47361563	0.49926917	0.5905833
Arhgap11a	0.18523048	3.16691669	0.47351316	0.49931505	0.5905833
Fhod3	0.13994366	5.14905749	0.47294927	0.49956766	0.59080248
Lipo1	-0.1530302	4.94977586	0.47287931	0.49959901	0.59080248
Tmem65	0.08341427	8.11504766	0.47238889	0.49981892	0.59090528
Zdhhc2	-0.0971072	5.21262082	0.47211789	0.49994051	0.59090528
E130112N10	0.73306266	-0.6904843	0.47192761	0.50002592	0.59090528
Gm10354	-0.9657941	-2.0395813	0.47181147	0.50007806	0.59090528
Oma1	0.13642268	3.22056231	0.47177391	0.50009492	0.59090528
Fto	-0.0815351	7.00755629	0.47174693	0.50010704	0.59090528
Pcna	-0.1056617	6.30910801	0.47170005	0.50012809	0.59090528
Tulp3	0.14985608	5.2594078	0.47169772	0.50012913	0.59090528
Nudt13	0.17043378	2.66711577	0.47164057	0.5001548	0.59090528
Ddx27	-0.110221	4.1812579	0.47158586	0.50017937	0.59090528
Atp5e	0.16758789	5.23526752	0.4714731	0.50023002	0.59090683
Dach1	-0.2401742	2.04567433	0.4711218	0.50038788	0.59094065
Mast4	0.09758445	6.6848335	0.47105854	0.50041631	0.59094065
Cdc42bpg	-0.2666777	1.33986723	0.47090274	0.50048636	0.59094065
Hyi	0.26923291	1.56627172	0.4707943	0.50053511	0.59094065
Pknox1	0.12509785	3.85227558	0.47075766	0.50055159	0.59094065
Smad9	0.10648075	5.40048518	0.47075071	0.50055472	0.59094065
Sike1	0.082049	6.60274024	0.47048895	0.50067247	0.5910214
Tbcd	-0.1350542	3.95613517	0.47036411	0.50072864	0.59102945
Pgbd1	0.1777042	2.33747517	0.47012685	0.50083543	0.59108772

Nop2	-0.1981957	2.78938159	0.47003512	0.50087673	0.59108772
Ano4	-0.1328102	3.81478993	0.46979337	0.50098559	0.59112556
Exoc6b	0.08026638	6.79077474	0.46974472	0.50100751	0.59112556
Tmem135	0.0753263	6.07846201	0.46952962	0.50110442	0.5911421
Kcnu1	0.36281074	0.58323831	0.46949447	0.50112025	0.5911421
2310069B03I	-1.1643871	-1.3945449	0.47217528	0.50130446	0.59123302
Eif5a	0.1551851	7.19992216	0.46902566	0.50133159	0.59123302
Fermt1	-0.6060899	0.35393899	0.46899411	0.50134582	0.59123302
Ddx6	0.07063214	8.14171641	0.4688855	0.50139481	0.59123302
Slc45a4	-0.1420976	3.61257026	0.46865558	0.50149853	0.59124562
Hipk4	-0.1686307	3.97058021	0.46859523	0.50152576	0.59124562
Masp1	-0.2349565	1.67919418	0.46853355	0.5015536	0.59124562
Polr1b	-0.2594857	2.23088517	0.46818439	0.50171122	0.59124661
Bfar	0.11489972	5.04974187	0.46813843	0.50173198	0.59124661
D8Ertd738e	0.21505572	3.18612086	0.46807292	0.50176156	0.59124661
Mgat5b	-0.1258816	3.71645702	0.46798019	0.50180345	0.59124661
Prkd2	-0.2128943	2.88515912	0.46795108	0.50181659	0.59124661
Kptn	-0.2539832	1.85330009	0.46787567	0.50185066	0.59124661
Trappc4	0.16107248	3.52625341	0.46761459	0.50196864	0.59132743
Tprgl	0.09306737	6.93458413	0.46735357	0.50208663	0.5913547
Mospd3	0.18010644	5.64086596	0.46734491	0.50209055	0.5913547
Igdcc4	0.09232743	5.11809547	0.46687354	0.50230376	0.59150068
Wbscr25	0.70200514	-0.9108051	0.46675309	0.50235827	0.59150068
Irs2	0.16477032	4.35157986	0.466742	0.50236329	0.59150068
Opn3	0.17338084	3.12974357	0.46663427	0.50241206	0.59150068
Phkg1	-0.2484458	1.630539	0.46627618	0.5025742	0.5916334
C2	0.26081756	2.81686402	0.46565614	0.50285516	0.59180439
Ybx3	0.13615989	7.39449024	0.465625	0.50286929	0.59180439
1700109H08I	0.85787289	-1.4188016	0.46557313	0.50289281	0.59180439
Setbp1	-0.0805901	6.75198855	0.46539348	0.50297427	0.59180439
Sypl	0.12292043	6.38862586	0.46508305	0.50311511	0.59180439
Tmem192	-0.2053978	2.30239886	0.46505088	0.50312971	0.59180439
Kcnq1	-0.8023366	-1.3330321	0.46491912	0.5031895	0.59180439
Kcnab2	-0.1193674	5.95193359	0.46472947	0.5032756	0.59180439
Wdr25	-0.1875976	1.99011313	0.46472787	0.50327633	0.59180439
H2-Q4	-0.2609408	1.686645	0.46468369	0.50329639	0.59180439
Thyn1	0.16455973	3.4539141	0.46466604	0.5033044	0.59180439
Cdk20	-0.3510142	1.18876467	0.4645502	0.50335701	0.59180439
Kctd11	0.22516302	3.32432119	0.46453952	0.50336186	0.59180439
Emcn	-0.4985641	0.59230906	0.46412815	0.50354875	0.59188928
Ppa1	0.10070783	5.77835253	0.46402672	0.50359486	0.59188928
Spns2	0.15042428	3.5192407	0.46400068	0.50360669	0.59188928
Dbr1	-0.1336146	3.96110004	0.46394552	0.50363177	0.59188928

Gm10033	0.16687471	4.59439636	0.46379399	0.50370066	0.59191216
Dip2c	-0.106114	6.83602647	0.46363122	0.50377469	0.59191721
Fam115a	-0.1198412	6.74609982	0.46342098	0.50387033	0.59191721
Faah	0.14055555	4.14335949	0.46316535	0.50398666	0.59191721
Ipo13	-0.1117663	5.39730335	0.46300568	0.50405935	0.59191721
Cndp2	-0.1132404	4.36161415	0.4628805	0.50411634	0.59191721
Bola1	0.24292812	1.69514703	0.462759	0.50417168	0.59191721
Ireb2	-0.1035738	6.4449494	0.46267084	0.50421183	0.59191721
Bmp4	-0.1952045	7.4127385	0.46249873	0.50429024	0.59191721
Nsmce1	-0.2517189	3.55079185	0.46245287	0.50431114	0.59191721
Trim26	-0.1181689	4.99294878	0.46233492	0.50436489	0.59191721
Pld6	0.86984398	-1.921622	0.46217468	0.50443794	0.59191721
Phb	0.13364858	3.81060631	0.46208785	0.50447753	0.59191721
Tmem150c	0.15771385	4.08315618	0.46206472	0.50448807	0.59191721
Peli3	-0.4761154	-0.7958979	0.46206138	0.50448959	0.59191721
Frmd6	-0.1080126	5.61707454	0.46205378	0.50449306	0.59191721
Prdm2	-0.0927857	7.02812682	0.46192762	0.50455059	0.59191721
BC029214	0.14699293	3.49030025	0.46180332	0.50460729	0.59191721
Fam160b1	0.11376451	5.00786603	0.46172335	0.50464377	0.59191721
Zfp862-ps	-0.1920566	2.31865458	0.46172271	0.50464406	0.59191721
Slc7a3	0.27044527	1.30527275	0.46159293	0.50470327	0.59192869
Agpat2	0.32095056	1.41287467	0.46143339	0.50477609	0.59195611
Gm10509	0.19259943	2.66833887	0.46131098	0.50483196	0.59196367
Cnpy3	0.16820677	3.44903708	0.46095134	0.50499619	0.59207879
1500011B03I	0.121376	5.03776438	0.4605167	0.5051948	0.59207879
Cdc25b	-0.1779508	2.97562223	0.46049668	0.50520395	0.59207879
Cryl1	0.1657482	3.57111571	0.46033893	0.50527606	0.59207879
Poc1a	0.20072354	2.80503987	0.46031147	0.50528862	0.59207879
Renbp	0.31401805	2.99842113	0.46009008	0.50538987	0.59207879
Aatf	-0.1316738	3.91732597	0.45975561	0.5055429	0.59207879
Ephx1	-0.1869349	4.19076574	0.45969378	0.5055712	0.59207879
Ddx10	-0.0820829	5.55496738	0.45951792	0.5056517	0.59207879
Ttc21b	-0.1385274	4.37167452	0.45950112	0.5056594	0.59207879
Rdh18-ps	0.32315244	0.35944236	0.45947372	0.50567194	0.59207879
Sp100	-0.1470736	5.07670591	0.45927474	0.50576306	0.59207879
Oxa1l	0.1164419	5.17887572	0.45901818	0.50588059	0.59207879
Gm7904	-1.1887769	-1.4868777	0.45894064	0.50591612	0.59207879
Hykk	-0.1365632	5.34662653	0.4589252	0.5059232	0.59207879
Tmod1	0.11615148	4.73124694	0.45866666	0.5060417	0.59207879
Gemin7	-0.1773391	3.2202262	0.45864871	0.50604993	0.59207879
Ube2q2	-0.0907608	6.36693787	0.45862604	0.50606032	0.59207879
Zfp654	-0.1223089	5.43811125	0.45851395	0.50611172	0.59207879
Cep57l1	0.17516774	2.90026587	0.45832597	0.50619793	0.59207879

Fam109a	-0.2843965	0.94421152	0.45829773	0.50621089	0.59207879
Pan3	0.07960551	6.70390034	0.45829528	0.50621201	0.59207879
Fam217a	0.66580864	-0.6141347	0.45824175	0.50623657	0.59207879
Slc16a8	0.88092244	-1.1791877	0.45818697	0.5062617	0.59207879
Fbxw5	-0.120327	5.41476069	0.45810675	0.50629851	0.59207879
Dok2	-0.5336844	-0.7678923	0.45786231	0.5064107	0.59207879
A430090L17I	0.69134106	0.76348656	0.45784599	0.50641819	0.59207879
Slco3a1	0.12917316	4.96501028	0.45778492	0.50644622	0.59207879
Arg2	-0.2165962	2.92564472	0.45778161	0.50644774	0.59207879
Lims2	-0.1916849	2.67256742	0.45776136	0.50645704	0.59207879
Ndufaf7	-0.1015006	4.55722598	0.45774887	0.50646278	0.59207879
Dph3	-0.0929878	5.71694722	0.45709874	0.50676142	0.5923181
Zp3r	0.87151325	-1.4150992	0.4569339	0.5068372	0.5923181
Gm14405	0.33584332	0.15617204	0.45692467	0.50684144	0.5923181
Csnk1g1	-0.0822158	6.24794993	0.45687273	0.50686532	0.5923181
Klhl24	-0.0756473	6.75247327	0.45674359	0.5069247	0.5923297
Slfn1	-0.6297997	-1.0410111	0.45653422	0.507021	0.59238442
Dusp18	0.11150593	5.35682692	0.45630309	0.50712735	0.59239867
Oaz3	0.24793611	2.23339133	0.45629269	0.50713213	0.59239867
Glrx5	0.13821186	3.73372452	0.45605857	0.5072399	0.59246676
Tex35	1.0200118	-1.7940402	0.45576126	0.50737681	0.59256888
Ppp3cb	0.07537615	8.65277141	0.45530649	0.50758635	0.5927558
Psme4	-0.1178261	6.43544393	0.45476967	0.5078339	0.59298706
8430429K09I	0.13710543	4.21523201	0.45440343	0.50800291	0.59311189
Ctu2	0.25114117	1.72551479	0.45423604	0.50808019	0.59311189
Spc24	-0.2245686	2.43892939	0.45405183	0.50816525	0.59311189
Cep83os	-0.1016582	5.37662296	0.45394907	0.50821272	0.59311189
Dhx38	-0.1169991	4.56252447	0.45393483	0.5082193	0.59311189
Nav2	0.15290877	7.18840989	0.45389444	0.50823796	0.59311189
Ankib1	-0.0767554	6.52754493	0.45378077	0.50829048	0.59311538
Nrg4	0.5137189	-0.4053342	0.45337131	0.50847974	0.59318757
Frmd5	-0.1351969	5.14701112	0.45306605	0.50862092	0.59318757
Ccdc162	0.42270778	0.22297356	0.45296111	0.50866947	0.59318757
Zfp583	0.22258384	3.39265592	0.45293645	0.50868088	0.59318757
Ppp1r1a	0.14141756	7.06598969	0.45285399	0.50871904	0.59318757
1110046J04F	-0.290418	1.55816368	0.45279129	0.50874806	0.59318757
1700003M02	-0.4396769	0.2590926	0.4527346	0.5087743	0.59318757
Lrrtm4	0.11215795	5.30938419	0.45256206	0.50885417	0.59318757
Rab11fip3	-0.0878359	6.19412059	0.45252402	0.50887178	0.59318757
Kdm1a	-0.1070747	5.91142414	0.45252247	0.5088725	0.59318757
Adrb1	-0.182636	3.6706366	0.45246912	0.5088972	0.59318757
Smox	0.18319512	2.88869692	0.45222776	0.50900899	0.59323805
Med30	0.17558233	3.31133533	0.45199388	0.50911735	0.59323805

Gm9833	0.22126454	1.15406897	0.45195999	0.50913305	0.59323805
E130317F20I	-0.3210556	1.16468983	0.45183272	0.50919204	0.59323805
Lca5	0.14056124	4.18042977	0.45173283	0.50923835	0.59323805
Gm15787	0.34041834	0.71033111	0.45170839	0.50924968	0.59323805
Kif26a	0.19113666	2.41192325	0.45162732	0.50928726	0.59323805
Fam20c	-0.2288421	2.99503013	0.45142519	0.509381	0.59326506
Rps9	-0.151524	6.45473868	0.45136371	0.50940952	0.59326506
Cacna1e	-0.1540219	8.22203066	0.4510461	0.5095569	0.59337898
Grin2d	0.21727221	1.80761077	0.45092788	0.50961177	0.59338518
Nxf1	-0.0886107	5.93111729	0.45027538	0.50991483	0.59366438
Suv39h1	-0.1705515	3.57991777	0.45001749	0.5100347	0.59366438
Bbs4	-0.0835743	5.56476616	0.449904	0.51008746	0.59366438
Slx4	-0.1161721	5.16651281	0.44987587	0.51010055	0.59366438
Rpl23a	-0.1177036	7.25485524	0.44980804	0.51013209	0.59366438
Cilp	-0.5246673	-0.1917374	0.4497042	0.51018038	0.59366438
Ascc3	-0.1515954	5.75611155	0.449558	0.51024839	0.59366438
5830417I10R	-0.1190075	6.11099523	0.44944215	0.5103023	0.59366438
Anp32e	-0.0895523	8.05480532	0.44915926	0.51043397	0.59366438
Fnbp1	-0.0765944	6.73205691	0.44909221	0.51046518	0.59366438
Coa7	0.21292953	2.48376687	0.44902156	0.51049808	0.59366438
Esrrg	-0.1378867	6.04331139	0.44899817	0.51050897	0.59366438
Tgfbrap1	0.09063322	5.59622484	0.44895833	0.51052753	0.59366438
Nppc	-0.5399066	-0.4988418	0.4488667	0.5105702	0.59366438
Car4	0.17639468	3.1795533	0.44873184	0.51063302	0.59366438
Ube2l6	-0.1552877	5.58533511	0.44870676	0.5106447	0.59366438
Gabra1	-0.0969153	8.20508337	0.44810925	0.51092321	0.59390397
Misp	0.76143389	-1.123436	0.44784103	0.51104833	0.59390397
Slc7a11	0.13186636	9.83007909	0.44779298	0.51107074	0.59390397
Rabl6	-0.0993081	5.81756979	0.4477875	0.5110733	0.59390397
Pdzk1ip1	-0.3417972	1.63263449	0.44766895	0.51112862	0.59390397
Atp2a1	0.88884603	-0.7557197	0.44762667	0.51114835	0.59390397
Hira	-0.1091587	4.13802974	0.44736615	0.51126996	0.59398765
Snx6	0.1016101	6.40291174	0.44720257	0.51134635	0.59400017
Vps39	0.08759077	5.87363614	0.44713064	0.51137995	0.59400017
Kri1	0.17754921	2.89361133	0.44700269	0.51143972	0.59401199
Fndc3a	0.08445924	8.08988479	0.4468086	0.51153041	0.5940597
Nudt22	0.22650772	1.45155025	0.44664545	0.51160667	0.59409066
Dennd2d	-0.5548956	-0.2058797	0.44633959	0.51174968	0.59414476
Kcnh8	-0.8164766	-1.0813013	0.44633359	0.51175248	0.59414476
Gm14295	-0.0732012	5.87168386	0.4458786	0.51196536	0.59428538
Gm3716	0.54003741	-0.725553	0.44568048	0.51205811	0.59428538
Slc9b1	0.77994797	-0.7893292	0.44567107	0.51206251	0.59428538
Clic4	0.13098898	8.64253682	0.44548712	0.51214865	0.59428538

Rsl24d1	-0.1161172	5.39693246	0.44547296	0.51215528	0.59428538
Ptn	0.18701115	10.3823205	0.44543866	0.51217135	0.59428538
Rhod	-0.2798985	1.72168286	0.44468279	0.5125256	0.5944868
Gpr123	0.11708492	6.43854164	0.44438595	0.51266484	0.5944868
Mbtps1	-0.1013251	5.43469457	0.4442557	0.51272596	0.5944868
Zfr	0.09222513	8.62368606	0.44408585	0.51280568	0.5944868
Mical1	0.19358584	2.58725045	0.443934	0.51287697	0.5944868
Tlr6	0.98613046	-1.3341172	0.4438403	0.51292097	0.5944868
Cdkn1b	0.13412828	4.09793414	0.44380102	0.51293942	0.5944868
Crip3	-1.244939	-1.9973046	0.443568	0.51304888	0.5944868
Hif1an	-0.1402606	4.74848081	0.44354046	0.51306182	0.5944868
Dmkn	0.5067896	-0.2987898	0.44353466	0.51306454	0.5944868
Klhdc1	-0.1166148	4.04135567	0.44345982	0.5130997	0.5944868
Tfpi	0.15461509	6.01795099	0.44343437	0.51311167	0.5944868
Ccs	-0.2111034	2.13602256	0.44343084	0.51311332	0.5944868
Zcchc3	-0.1368105	5.00157565	0.44340037	0.51312764	0.5944868
Dmtn	-0.1141557	6.22968677	0.44332421	0.51316344	0.5944868
Zfp692	-0.2702827	2.60333688	0.44318199	0.51323029	0.5944868
Fktn	-0.0971432	5.73022151	0.44305659	0.51328925	0.5944868
Gm4925	0.55057705	-0.0737922	0.44299522	0.51331811	0.5944868
Armc10	0.14463292	4.74546226	0.44298067	0.51332495	0.5944868
Slc16a11	0.17376281	3.31827081	0.44289176	0.51336676	0.5944868
Slc29a3	-0.1194873	5.26561223	0.44277752	0.5134205	0.5944868
Kctd17	-0.1551735	4.42842515	0.44266353	0.51347413	0.5944868
4921534H16I	1.04358784	0.0222078	0.44254711	0.51352891	0.5944868
Lrrc58	-0.1084101	10.1415831	0.44253136	0.51353632	0.5944868
Ccnb2	0.52717651	-0.2245179	0.44196363	0.51380364	0.59468629
Htr6	0.72924818	-1.4043342	0.44191036	0.51382873	0.59468629
Ndst2	-0.1571342	3.30347432	0.44174956	0.51390449	0.59468629
Ankrd49	0.14212539	4.93883628	0.44172208	0.51391744	0.59468629
Gm12338	0.13864941	4.54261356	0.44163828	0.51395694	0.59468629
Esyt2	-0.0805535	5.89773321	0.44119375	0.51416652	0.59486726
Gosr2	-0.076848	6.62556428	0.4410169	0.51424995	0.59486726
Ccdc51	0.30891825	0.54766976	0.44099059	0.51426236	0.59486726
Rps21	-0.1314411	5.20132058	0.4403221	0.51457794	0.59516273
Parp9	0.15886245	3.39208783	0.44016095	0.51465407	0.59516273
Pold2	0.25766276	2.57042042	0.44004174	0.51471039	0.59516273
Elac2	-0.1954743	2.88312398	0.43999919	0.5147305	0.59516273
Uxt	0.18614395	3.52008913	0.43989316	0.51478061	0.59516273
Lrriq3	-0.4358511	0.57315114	0.43981344	0.5148183	0.59516273
Mier1	-0.093528	6.44738241	0.43971323	0.51486568	0.59516273
Otud4	-0.0861863	6.54656309	0.4394872	0.51497257	0.59522884
Taf2	-0.0959106	5.91568039	0.43904643	0.51518112	0.59541243

Mtmr1	0.09686509	5.14613504	0.43885833	0.51527017	0.59543159
Gm12185	-0.3226265	0.86068717	0.43872158	0.51533492	0.59543159
Igflr1	-0.7274933	-0.7968711	0.43869637	0.51534686	0.59543159
BC049352	-0.7653702	-1.7776477	0.43838434	0.51549468	0.59551328
D030040B21	0.39097741	-0.4700405	0.43825631	0.51555535	0.59551328
Hnrnpr	0.07216288	7.8987067	0.43805969	0.51564856	0.59551328
Ppip5k1	0.13968635	5.49143345	0.43787959	0.51573395	0.59551328
Mrpl45	0.16158426	3.64461514	0.43787781	0.5157348	0.59551328
Gjb1	-0.5472025	-0.6787146	0.43787149	0.5157378	0.59551328
2210013O21	-0.1282703	5.29079884	0.43781276	0.51576565	0.59551328
Npas3	0.14845178	3.93110127	0.43757224	0.51587975	0.59557217
Vps4a	0.09337665	5.55781122	0.43725752	0.51602912	0.59557217
Ankra2	0.13320049	3.44615038	0.43721553	0.51604905	0.59557217
Syngr2	0.28172794	1.68918734	0.43709094	0.51610821	0.59557217
Dscr3	0.12508597	4.05739242	0.43698971	0.51615629	0.59557217
Nlrc3	0.71325709	-0.9379213	0.4369378	0.51618094	0.59557217
Fbxo31	0.16698937	3.94589921	0.43692887	0.51618518	0.59557217
Eftud1	-0.1695049	3.10423985	0.43670188	0.51629302	0.59557217
Ahr	0.13926397	4.19486177	0.43667628	0.51630519	0.59557217
Aim1l	-0.5151355	-0.2877581	0.43653531	0.51637219	0.59557217
Btc	0.96923296	-1.977228	0.43643378	0.51642045	0.59557217
Lef1	-0.1294948	4.50686433	0.43619019	0.51653627	0.59557217
Rac2	-0.1699684	2.78924291	0.436176	0.51654302	0.59557217
Ipo11	0.09812677	6.18942871	0.43612938	0.51656519	0.59557217
2810429I04R	0.66201707	-1.2896192	0.43609823	0.51658001	0.59557217
Cnn3	0.12381428	6.27032767	0.43599567	0.5166288	0.59557217
1700018L02R	0.43298727	0.78479279	0.43589919	0.5166747	0.59557217
Btbd9	-0.1199406	4.98296484	0.43579905	0.51672235	0.59557217
Taf10	0.1300557	4.15935946	0.4356493	0.51679362	0.59557217
Phldb2	-0.129716	7.68725526	0.43555047	0.51684067	0.59557217
Ophn1	-0.1204454	4.9296589	0.43550773	0.51686102	0.59557217
Map3k1	0.1054497	5.10802838	0.4345663	0.5173096	0.59601734
Rab12	0.07790502	7.08669075	0.43445498	0.51736269	0.59601734
Gm15446	-0.2735165	1.42491026	0.43431082	0.51743145	0.59601734
Snupn	-0.1603811	3.39514449	0.43427943	0.51744643	0.59601734
Ptp4a3	0.16137112	3.47659249	0.43404194	0.51755975	0.59606428
Anxa9	0.48501993	-0.5916334	0.43398542	0.51758673	0.59606428
1600002K03I	0.44348954	0.25287878	0.43384437	0.51765406	0.59607524
Spata6	0.12536655	3.91402258	0.43370928	0.51771857	0.59607524
Vamp1	-0.0860719	6.0370303	0.43365275	0.51774556	0.59607524
Phlpp1	-0.0986088	6.22097017	0.43347925	0.51782844	0.59611335
Lonrf2	0.10202548	8.07291777	0.43330509	0.51791165	0.59613824
Cdca7	-0.458094	0.33717584	0.43319108	0.51796614	0.59613824

Cox7a2	0.12643026	6.00398457	0.43312149	0.5179994	0.59613824
Gm6583	-0.7815202	-1.0788076	0.43270052	0.5182007	0.59626449
Lrmp	-0.5655764	-0.0754907	0.43268383	0.51820869	0.59626449
Upb1	0.54586372	-0.4929598	0.4325361	0.51827936	0.59628852
Ctps2	-0.0824187	5.66905144	0.43240878	0.51834029	0.59630133
BC023829	-0.1502712	3.99356688	0.4319862	0.5185426	0.59640688
Kif5c	-0.1112383	9.59216499	0.43186504	0.51860063	0.59640688
Ndfip1	0.10538247	7.96191732	0.43183095	0.51861696	0.59640688
Rrp7a	-0.1488257	4.01122213	0.4317563	0.51865272	0.59640688
Sass6	-0.1399249	3.77705377	0.4316972	0.51868104	0.59640688
Ccdc65	-0.2169972	2.03665474	0.4315611	0.51874626	0.5964246
Bcl2l12	0.39251856	0.23537933	0.43108852	0.51897284	0.59658205
Nxpe2	-0.3633672	1.13167238	0.4310308	0.51900052	0.59658205
Pspc1	0.11732221	5.2308124	0.43094158	0.51904332	0.59658205
Cnih1	0.13337204	5.24449197	0.43086	0.51908246	0.59658205
Dnajc2	0.09296702	6.43593183	0.43034338	0.51933046	0.5966881
Gm10584	-0.5872075	-1.1520838	0.4303096	0.51934668	0.5966881
Gmeb1	-0.0873679	5.29855396	0.4301246	0.51943554	0.5966881
Tomm5	0.12831583	4.59282901	0.42998618	0.51950205	0.5966881
Ints4	-0.0917769	5.51097562	0.42997225	0.51950874	0.5966881
Bbc3	0.37358259	-0.1095236	0.42988801	0.51954923	0.5966881
Foxd2	-0.2349318	2.72874182	0.42979432	0.51959426	0.5966881
Tmem143	-0.1962979	2.62157586	0.4297741	0.51960398	0.5966881
Eef1g	-0.1166866	7.72429687	0.42973419	0.51962316	0.5966881
Abcc1	-0.1679709	3.88169339	0.429426	0.51977136	0.59680105
Etohi1	-0.1262206	4.21814731	0.4289586	0.51999625	0.59688945
2610100L16F	0.21149866	2.15137763	0.42887656	0.52003574	0.59688945
LOC171588	1.21417395	-1.1518178	0.43147823	0.52010508	0.59688945
Phf7	-0.4020008	0.91577903	0.42872576	0.52010835	0.59688945
Mrpl57	0.10846434	4.6920637	0.42867097	0.52013474	0.59688945
Phf8	-0.1056637	5.6399736	0.42864469	0.52014739	0.59688945
Srf	-0.1111614	4.85046671	0.42835712	0.52028592	0.59693618
Il10rb	-0.1740101	3.04627877	0.4283532	0.52028781	0.59693618
E230016M11	-0.4396597	0.20227686	0.42808433	0.52041739	0.59702765
Ccnt2	-0.1603595	4.8957623	0.42795772	0.52047844	0.59704049
Rsad1	0.14856896	3.20766424	0.42760416	0.52064896	0.59710815
Car15	-0.2946144	2.46758281	0.42757136	0.52066478	0.59710815
Stk11	-0.1555481	5.51305633	0.42745829	0.52071934	0.59710815
Zbtb4	-0.0695135	7.52108843	0.42738297	0.52075569	0.59710815
Fam184b	-0.2241618	2.75541386	0.4273187	0.52078671	0.59710815
Vps29	0.09769402	5.86087869	0.42714533	0.52087041	0.59714694
Il13ra1	0.13426933	4.87857504	0.42694735	0.52096602	0.59719937
Gm8179	-0.7914146	-0.1915981	0.42606227	0.52139381	0.59757547

Treml2	-0.7275106	-0.6834042	0.42599826	0.52142478	0.59757547
Sin3a	0.08059778	6.53073228	0.4258452	0.52149883	0.59757547
Abca17	0.72462124	-0.2030973	0.4257921	0.52152453	0.59757547
Slc35f6	-0.2125253	1.93306923	0.42555197	0.52164076	0.59757547
Phf12	0.07613664	6.74279914	0.42554943	0.52164199	0.59757547
Nr1d2	-0.0828609	7.42943897	0.42554652	0.52164339	0.59757547
Mapkbp1	0.11239396	5.2659979	0.42530531	0.5217602	0.59762936
1810043H04	-0.268529	1.96213509	0.42524326	0.52179025	0.59762936
BC025920	-0.2551585	1.46221459	0.4250997	0.52185979	0.59765186
Sp140	0.10409845	4.89215056	0.42490096	0.5219561	0.59770484
3425401B19	-0.2348893	3.75183628	0.42479826	0.52200588	0.59770484
Jph1	0.13544446	4.82841873	0.42445449	0.52217257	0.5978134
Pex6	-0.1533039	3.79924889	0.42438709	0.52220526	0.5978134
Srcin1	-0.1635996	6.08474988	0.42413485	0.52232764	0.5978134
Tbkbp1	0.20247252	2.13818558	0.42403001	0.52237852	0.5978134
Gareml	0.24462577	2.21577455	0.42402073	0.52238303	0.5978134
Katna1	-0.1775357	3.60927627	0.42398534	0.5224002	0.5978134
Fbrsl1	-0.1202362	4.62818827	0.42384946	0.52246617	0.59783177
Rbx1	0.10954675	6.58727791	0.42360506	0.52258486	0.59785107
Sbf1	0.15254255	4.72245381	0.42357699	0.52259849	0.59785107
Sub1	0.09107547	8.79956942	0.423461	0.52265484	0.59785107
Cilp2	0.446253	0.61710162	0.42337166	0.52269825	0.59785107
Nploc4	-0.0924634	5.5117451	0.42329248	0.52273672	0.59785107
Acer1	-0.3374602	0.2981237	0.42319815	0.52278257	0.59785107
Fam180a	-0.2168945	5.14193717	0.42300444	0.52287674	0.59785522
Exosc4	-0.1518342	3.40495254	0.42297795	0.52288962	0.59785522
Nup35	-0.1469716	3.28593471	0.42288263	0.52293597	0.59785522
Itgb3bp	0.20237247	3.3353098	0.42218797	0.52327399	0.59810889
Mettl4	-0.1200704	5.08264826	0.42203997	0.52334606	0.59810889
Cacna1d	-0.1704797	5.10948348	0.42198555	0.52337257	0.59810889
Celsr3	-0.2465947	4.73290508	0.42193525	0.52339707	0.59810889
Tm2d2	0.19772404	4.24607519	0.42169892	0.52351221	0.59810889
Krt9	0.12497283	3.95812675	0.42163524	0.52354324	0.59810889
Lgals8	-0.0952742	6.12087827	0.42152513	0.52359691	0.59810889
Lingo3	0.17880598	3.01449084	0.42146966	0.52362394	0.59810889
Arih1	-0.0704437	7.93081181	0.42130406	0.52370469	0.59810889
Fsbp	-0.7409604	-1.8040217	0.42128381	0.52371456	0.59810889
Ankrd9	-0.3479032	0.40616197	0.4212399	0.52373597	0.59810889
Ccdc141	-0.1259219	4.28111604	0.42119644	0.52375717	0.59810889
Exo1	-0.5419327	-0.6416641	0.42108289	0.52381255	0.5981151
Ccnb1	0.57436186	-1.0194549	0.4207574	0.52397137	0.59815871
Rhno1	0.15875837	2.51360383	0.42066439	0.52401677	0.59815871
Fam186b	-0.765828	-0.8546847	0.42063108	0.52403303	0.59815871

Pddc1	0.12938399	3.60516068	0.42059522	0.52405054	0.59815871
Sertad2	-0.0973969	5.37338932	0.42003636	0.52432351	0.59839321
Tmem131	0.09552482	7.23294098	0.41994584	0.52436775	0.59839321
Npepl1	0.22258069	2.06684462	0.41986372	0.52440789	0.59839321
Fam133b	0.0890913	5.13952641	0.41976561	0.52445585	0.59839321
Ptprj	0.10914973	6.83767348	0.41917493	0.52474478	0.59859509
Slc16a6	0.15087934	3.11581184	0.41909409	0.52478434	0.59859509
U2af1l4	0.21238583	2.09191891	0.4189617	0.52484915	0.59859509
Trim13	0.19369876	2.67528309	0.41886588	0.52489607	0.59859509
Sgk3	0.10036053	5.20054935	0.41871873	0.52496813	0.59859509
Snrpd1	-0.1022481	5.26247952	0.41870112	0.52497676	0.59859509
Fxyd5	0.1760958	6.75997088	0.41852641	0.52506234	0.59859509
Ccnyl1	0.10247539	5.32079068	0.41850197	0.52507431	0.59859509
Svil	0.12584296	4.74877453	0.41848496	0.52508265	0.59859509
Sfi1	-0.2028471	3.39486103	0.41822624	0.52520944	0.59868265
Pm20d1	-0.2135628	2.06002401	0.41789294	0.52537287	0.59881194
Mus81	0.18586803	2.15460591	0.41770345	0.52546582	0.59886089
Ttc12	0.36952134	1.39047069	0.41734734	0.52564059	0.59898498
Slc7a8	0.11701021	5.5378534	0.4172778	0.52567473	0.59898498
Pvr	0.17825284	2.53603465	0.4170171	0.52580276	0.59907386
Pxk	0.09428878	5.46625676	0.41687172	0.52587418	0.59909824
Ptprn2	0.08769167	7.30390433	0.41673927	0.52593926	0.59911539
1700012D01I	0.54362808	0.14562904	0.41662533	0.52599526	0.59912219
Arrdc1	0.29710949	0.91740947	0.41631931	0.52614572	0.59923657
Cntnap5a	-0.1500614	4.3575796	0.41621293	0.52619805	0.59923918
Rbm15b	-0.1181349	3.8137189	0.41587123	0.52636617	0.59928111
Mdm1	-0.1646676	3.14417576	0.41579843	0.526402	0.59928111
Atl2	-0.1009915	6.17203477	0.41578384	0.52640919	0.59928111
1700028E10I	-0.5532839	-0.7948921	0.41564166	0.52647918	0.59928111
Btd	0.19011943	3.78710427	0.41562968	0.52648508	0.59928111
Cnot7	-0.0852909	6.74951294	0.41543431	0.52658129	0.59933366
Crnde	-1.0198258	-1.7775101	0.41512488	0.52673373	0.59941792
6330419J24F	-0.1686804	3.50590208	0.41481653	0.52688573	0.59941792
Gm13031	-0.6822958	-0.4832155	0.41477642	0.52690551	0.59941792
9430037G07	0.2191162	2.05071542	0.4146489	0.5269684	0.59941792
Mzt2	0.16515679	2.97104028	0.41456842	0.52700809	0.59941792
Ccdc59	-0.1229011	5.04935982	0.41453632	0.52702393	0.59941792
Rbm10	0.10057075	4.92040676	0.41452517	0.52702943	0.59941792
Prmt5	-0.0939839	4.74281688	0.41444229	0.52707032	0.59941792
Abcc8	-0.1955247	2.69792828	0.41426001	0.52716026	0.59941792
Slc22a5	-0.1814354	2.58385577	0.41415389	0.52721264	0.59941792
Lpgat1	0.08799661	7.83784343	0.41410691	0.52723583	0.59941792
B3galt2	0.12235808	5.41767912	0.41398308	0.52729697	0.59941792

Cep290	0.09459019	7.10751982	0.41396478	0.52730601	0.59941792
Rasd1	0.38294389	0.3412436	0.41331092	0.52762905	0.59972822
Fzd3	-0.0861222	7.99007841	0.41300142	0.52778209	0.59977753
Mylk	-0.1104252	6.02107429	0.41292482	0.52781998	0.59977753
Slc2a10	-0.3211579	0.93217711	0.41291937	0.52782268	0.59977753
Magohb	-0.1834671	2.81648979	0.41255267	0.52800414	0.59978411
Ap4m1	-0.2814547	1.14471779	0.41250098	0.52802973	0.59978411
Sec22c	-0.1111172	4.63441973	0.41241808	0.52807077	0.59978411
B130006D01	0.74206158	0.52011361	0.41231225	0.52812317	0.59978411
Nfam1	-0.3934018	1.15050389	0.41221601	0.52817083	0.59978411
Epn3	-0.3605425	0.59555436	0.41210715	0.52822475	0.59978411
Tmem67	-0.1618724	3.81149428	0.41203201	0.52826197	0.59978411
Nfil3	0.21665347	2.54597835	0.41200478	0.52827547	0.59978411
Sall2	-0.094129	5.73749934	0.41190032	0.52832722	0.59978411
Ccdc71l	0.10615334	5.24140435	0.41189611	0.52832931	0.59978411
Ndufa11	0.1196845	3.88211188	0.41174215	0.52840562	0.59981388
Nr2c1	0.12023646	4.14095446	0.4115918	0.52848015	0.59984163
Kiz	0.12229135	4.925296	0.41143606	0.52855738	0.59987243
Mlycd	0.13211898	3.16265208	0.41122661	0.52866128	0.59993349
Ptpn4	-0.1050126	7.20235562	0.4110659	0.52874102	0.59996713
Med21	0.1473186	5.66338597	0.41052315	0.52901049	0.60009196
Zfp367	0.1433009	3.52014188	0.41041503	0.52906421	0.60009196
Tbc1d22b	-0.1283664	4.46307118	0.41032388	0.5291095	0.60009196
Rnf34	0.1080131	5.2729918	0.41028609	0.52912828	0.60009196
Ppp1r7	-0.1039673	7.31260704	0.41021718	0.52916252	0.60009196
Mark1	0.0837944	5.97795323	0.41015527	0.52919329	0.60009196
Slc25a47	-0.4844286	-0.6245567	0.41013816	0.5292018	0.60009196
Plcl2	-0.1261505	5.54410284	0.41002116	0.52925996	0.60010109
1700021F05I	0.0983715	4.52234889	0.40989221	0.52932408	0.60011698
Fbln7	-0.2322407	4.07381577	0.40939156	0.52957314	0.6002958
Cldn11	0.14734409	6.53876508	0.40933228	0.52960264	0.6002958
Rnf207	-0.427231	0.9727215	0.40927294	0.52963218	0.6002958
Rasal3	0.60494434	-0.2015139	0.40861594	0.52995943	0.60056569
Gbp10	-0.1306426	3.82201888	0.40854264	0.52999596	0.60056569
Arhgap20	0.08059502	8.42146037	0.40849292	0.53002075	0.60056569
Zfp369	-0.1128056	5.24517541	0.40829988	0.53011699	0.60057182
Cdc42ep4	-0.1618037	6.00259451	0.40825354	0.5301401	0.60057182
Atp6v0a1	0.09324223	7.21774793	0.40818034	0.53017661	0.60057182
Add2	-0.1133174	7.61685825	0.40774783	0.5303924	0.60072533
Ccdc102a	-0.2712621	1.41072203	0.40770768	0.53041245	0.60072533
Fgd6	0.10593611	6.33846098	0.40745944	0.53053639	0.60080888
Ap1b1	0.09464981	5.70390084	0.40723562	0.53064818	0.60087866
Atp6v1f	0.1527112	4.75645672	0.40663814	0.53094682	0.60115998

Etv6	0.09289351	5.18970227	0.40582645	0.53135303	0.60152089
9930111J21F	-0.2107682	3.80923068	0.40567355	0.53142962	0.60152089
Zfp948	0.14764899	3.61939955	0.40561919	0.53145685	0.60152089
Prpsap1	0.08231955	4.70373033	0.40559995	0.53146649	0.60152089
Hs3st3b1	-0.1699561	3.71015302	0.40541134	0.53156099	0.601571
Nxt2	0.10841039	6.76471218	0.40484365	0.53184565	0.60183627
Ccdc12	0.14250753	3.63339538	0.4047126	0.5319114	0.6018538
Mrps30	-0.1580731	3.23911452	0.40415366	0.532192	0.60207501
BC033916	0.32451212	0.31825089	0.4040438	0.53224719	0.60207501
Lin7a	0.08426377	7.6292927	0.40396863	0.53228495	0.60207501
Inpp5k	0.09880075	4.16728098	0.40392276	0.532308	0.60207501
Cntnap1	0.11595517	5.86954492	0.40360123	0.53246961	0.60216971
Mical2	0.08922588	8.91771607	0.40351615	0.53251239	0.60216971
Crebbp	-0.0778353	8.16522246	0.40345612	0.53254257	0.60216971
Plxna3	-0.2340426	2.16951874	0.40334308	0.53259943	0.60217714
Atp8a1	0.10570922	7.56623088	0.40302612	0.5327589	0.60230058
Twist2	-0.369644	0.8075385	0.40270315	0.53292148	0.60235381
Mrpl16	-0.0906731	5.6404914	0.40260706	0.53296987	0.60235381
Glra3	-0.2710666	1.20109565	0.40245368	0.53304713	0.60235381
Wfdc2	0.57462928	-0.6818079	0.40230725	0.53312091	0.60235381
Fam122a	0.11481769	4.73155212	0.40225755	0.53314595	0.60235381
Zfp91	0.06930492	7.46042978	0.40224137	0.53315411	0.60235381
Mapt	0.0780974	6.89262027	0.40214443	0.53320297	0.60235381
Sdk2	0.16925289	3.60874328	0.40213373	0.53320836	0.60235381
Dll3	0.70613675	-1.1968643	0.40167876	0.53343778	0.60255614
Irf3	-0.1846075	2.69589221	0.40123664	0.5336609	0.6026771
Hist1h1d	0.55080244	-1.3350052	0.40121792	0.53367035	0.6026771
Sash3	-0.3102227	1.90364415	0.40116744	0.53369584	0.6026771
Dolk	-0.1865797	2.47522989	0.4007033	0.53393028	0.60288499
Adarb2	-0.1246137	4.9735665	0.40015482	0.53420758	0.60314123
St6gal1	0.13613425	6.19199686	0.40002626	0.53427261	0.6031578
Alkbh1	0.14545512	4.29498988	0.3998249	0.53437451	0.60320008
Efcab7	-0.2094846	2.55634321	0.39972784	0.53442363	0.60320008
Rbm41	-0.1186724	4.52288136	0.39943636	0.53457122	0.60320008
Psma7	0.0866145	6.45837861	0.39937086	0.53460439	0.60320008
Smok4a	-0.282916	1.7706538	0.39933483	0.53462264	0.60320008
Cfdp1	0.11262995	6.92855852	0.39926196	0.53465956	0.60320008
Tmem19	-0.1275864	3.56691785	0.39925587	0.53466264	0.60320008
Wash	-0.1239924	3.84877299	0.39904881	0.53476756	0.60321333
Sptssb	0.15084611	4.63872135	0.3990328	0.53477568	0.60321333
Gm17019	-0.5854066	-1.2206057	0.39893451	0.5348255	0.60321333
Anxa11	-0.1035081	3.947233	0.39881673	0.53488521	0.60322386
Snhg5	-0.1242957	3.73901185	0.39844914	0.53507164	0.60332117

Ccdc78	-0.3873799	-0.2894292	0.39837494	0.53510929	0.60332117
Plch1	-0.1618188	3.10887711	0.39825442	0.53517045	0.60332117
Rgs3	-0.1454742	4.10032418	0.39811103	0.53524323	0.60332117
Slc39a1	0.14762576	5.19620193	0.39806709	0.53526554	0.60332117
Mettl18	0.27250623	2.00591038	0.39805088	0.53527377	0.60332117
Rrp8	0.12984043	4.19096313	0.39756257	0.53552179	0.60354392
Cdk5rap2	-0.1326025	3.57249977	0.39725219	0.53567955	0.60366491
Src	-0.1551834	3.27587709	0.39705947	0.53577755	0.60371854
Mettl7a1	-0.1286504	6.05892047	0.39667518	0.53597307	0.60386701
Osgep	-0.1239796	3.93937832	0.39660228	0.53601017	0.60386701
1810020O05	-0.4251912	-0.8442492	0.39638624	0.53612015	0.60393411
Glce	-0.0970873	6.45973608	0.39598978	0.5363221	0.60409365
Cyb5	0.11427758	5.88858328	0.39588561	0.53637519	0.60409365
Jak1	-0.083533	7.64637337	0.39580849	0.5364145	0.60409365
Tigit	-0.7504739	-1.5637393	0.39570495	0.53646728	0.60409365
Upk1b	-0.3970619	1.27671909	0.39544218	0.53660128	0.60409365
Clstn3	0.1393921	4.49061883	0.39539028	0.53662775	0.60409365
Rps6	-0.1226182	8.46228235	0.3953495	0.53664855	0.60409365
Prickle2	-0.1069344	7.94042736	0.3952069	0.53672131	0.60409365
Frem3	-0.4831223	0.105963	0.39517177	0.53673924	0.60409365
Gbp2	0.18273093	4.51041072	0.39511828	0.53676653	0.60409365
Cgref1	0.18132437	2.1814467	0.39492894	0.53686318	0.60409365
Ly86	-0.236644	2.08681599	0.39475244	0.5369533	0.60409365
Csk	-0.1716079	2.98253537	0.39460934	0.53702639	0.60409365
BC026585	0.39687602	0.725837	0.3945094	0.53707744	0.60409365
Isg20	0.22618357	1.67087983	0.39450078	0.53708185	0.60409365
Fbxo16	0.19123073	2.08289641	0.39449174	0.53708647	0.60409365
4930594C11I	-0.5907338	1.31140351	0.3944159	0.53712522	0.60409365
Atg4c	-0.0936815	6.30646857	0.39432315	0.53717262	0.60409365
Tmem165	0.11308126	4.66271088	0.3941639	0.53725402	0.60409365
Cmss1	-0.2934577	2.41005536	0.39407875	0.53729755	0.60409365
Ngdn	-0.1374947	3.72143171	0.39403272	0.53732109	0.60409365
Igsf10	0.15276761	3.27417852	0.39373019	0.53747582	0.60421089
Akt2	-0.1079747	5.97679673	0.39348543	0.53760107	0.60429497
Galnt14	-0.2252976	2.27094978	0.39321602	0.537739	0.60439328
Gins2	-0.2383878	2.15849279	0.39301349	0.53784273	0.60445314
Dlgap4	-0.078049	7.19902997	0.39257771	0.53806606	0.60460412
Trim34b	-0.4370575	-0.8356453	0.39255432	0.53807805	0.60460412
Macrod1	-0.4026555	0.16466757	0.39238926	0.53816269	0.60464249
Lpin2	0.09598606	6.58671134	0.39228993	0.53821363	0.60464301
Eln	0.19845286	2.43103705	0.39209266	0.53831484	0.60464761
Nck1	-0.1137189	4.98985231	0.39208511	0.53831871	0.60464761
Slc44a5	-0.236008	1.67814149	0.3916945	0.53851921	0.60478698

Zfp120	0.14160294	4.37521183	0.39158122	0.53857739	0.60478698
Fxyd4	0.60226247	-0.3788599	0.3915483	0.53859429	0.60478698
Exd1	-0.4426631	-0.3507734	0.39111782	0.53881548	0.60495903
1810014B01I	0.18446546	2.62046531	0.39093906	0.53890738	0.60495903
Slc27a3	0.74976191	-1.6827004	0.39092079	0.53891678	0.60495903
Prepl	0.11690935	7.5494485	0.390724	0.53901798	0.60495903
Trip10	0.26569135	2.25482018	0.39064374	0.53905927	0.60495903
Zdhhc12	-0.3722888	0.66138768	0.39045806	0.5391548	0.60495903
Acsbg1	0.10340746	4.57552639	0.39039654	0.53918647	0.60495903
Tmtc3	0.12149906	5.40573068	0.39032696	0.53922228	0.60495903
A730046J19I	-0.4093577	0.59754944	0.39030069	0.5392358	0.60495903
Cass4	-0.3872333	0.49765302	0.3902135	0.53928069	0.60495903
L3hypdh	-0.200666	2.99052929	0.39005544	0.53936208	0.60495903
Anapc4	-0.0973007	5.48691874	0.39005465	0.53936249	0.60495903
H2-Eb1	-0.2718384	3.08413101	0.38990493	0.5394396	0.60495903
Enpep	0.22956182	1.97642137	0.38985921	0.53946315	0.60495903
Itgb6	0.71646208	-1.8874354	0.38977751	0.53950525	0.60495903
As3mt	-0.1545418	3.54657067	0.38904424	0.53988331	0.60532629
Pdlim5	-0.1213806	7.39530716	0.38867175	0.54007556	0.60548515
A930001C03I	0.85770575	-1.796291	0.38829363	0.54027084	0.60563355
Pcgf1	-0.1754753	3.18836724	0.38821963	0.54030907	0.60563355
Bend5	-0.1483409	3.27714719	0.38799259	0.54042641	0.60570838
Polr2l	0.11910954	3.98022412	0.38788217	0.54048349	0.60571167
Reep5	0.07841627	8.46075093	0.38779125	0.5405305	0.60571167
Il1bos	0.89943432	-2.0499981	0.39009967	0.54058355	0.60571444
Fam129b	0.14608009	5.3393645	0.38745827	0.54070274	0.6057913
Ptdss1	-0.0955377	4.8909108	0.38717036	0.54085174	0.60584468
Paics	-0.1045631	8.66683901	0.38711301	0.54088143	0.60584468
1810034E14I	0.31619104	1.11510821	0.3869248	0.54097889	0.60584468
Enox2	0.16637686	4.58699672	0.38689009	0.54099687	0.60584468
Car13	0.13727638	7.61595465	0.38687763	0.54100332	0.60584468
Ccng2	-0.127208	4.33906316	0.38651744	0.54118994	0.6059835
Brix1	0.09754222	4.72605738	0.38644308	0.54122849	0.6059835
Ets1	-0.1054513	4.0399327	0.38603152	0.5414419	0.60609662
Ocrl	0.10063271	6.61188684	0.38595933	0.54147935	0.60609662
Gtsf1	0.68447671	-1.7485368	0.38592916	0.54149501	0.60609662
Ccnk	0.07702392	6.38171229	0.38585794	0.54153196	0.60609662
Tbc1d25	-0.1438166	3.07336258	0.38552689	0.5417038	0.60623229
Dctn3	0.13383971	5.68725454	0.38514623	0.54190151	0.60634188
Narg2	0.13977065	3.98398958	0.38499586	0.54197965	0.60634188
4931430N09	0.40001722	1.7189984	0.38498758	0.54198395	0.60634188
Rpa3	-0.16999	3.1345464	0.38483447	0.54206354	0.60634188
Gsr	0.0816135	5.88993082	0.38475937	0.54210259	0.60634188

Sorbs2	-0.14992	7.36528757	0.38475374	0.54210552	0.60634188
Tmem194	-0.2304025	2.91223127	0.3842977	0.54234273	0.60653713
Pex3	0.09711711	5.12396252	0.38422344	0.54238138	0.60653713
Zfp148	0.07077797	7.82606425	0.38383569	0.54258325	0.60663197
Slfn2	-0.4459251	0.01257306	0.38379799	0.54260288	0.60663197
Proz	-0.2593574	1.46770786	0.38376868	0.54261815	0.60663197
E130102H24I	-0.5109932	-1.0345136	0.38356619	0.54272364	0.6066404
Dock11	0.10200343	5.48991872	0.38355974	0.542727	0.6066404
1700003E16I	0.30713238	0.61505747	0.38337258	0.54282455	0.60669281
Olfr316	-0.6930087	-0.6026175	0.38323747	0.54289498	0.60671183
L3mbtl3	0.11530784	4.1829686	0.3831456	0.54294289	0.60671183
Kcnk12	-0.5557489	-1.2942537	0.38270882	0.54317075	0.60690982
Erich5	0.53613132	-0.7867221	0.38210355	0.5434868	0.60720631
Zfp266	-0.0685628	6.88720034	0.38163999	0.5437291	0.60742034
Cabin1	0.09201526	5.24959159	0.38133338	0.54388947	0.60753013
Efcab10	-0.4612718	-0.4047209	0.38125815	0.54392883	0.60753013
Tgfbr2	-0.1302027	5.70117988	0.38097091	0.54407917	0.60764137
Rgs1	0.48278571	-0.295751	0.38069133	0.54422558	0.60770238
Ifi47	-0.1994776	3.77676149	0.38060132	0.54427273	0.60770238
Baalc	-0.0729684	7.07479323	0.38036925	0.54439434	0.60770238
Cacna1i	-0.1360336	4.05638113	0.38034333	0.54440793	0.60770238
Kctd16	0.14883142	3.81506031	0.38032049	0.5444199	0.60770238
Itga8	-0.1922864	2.59275693	0.38028546	0.54443826	0.60770238
Golga3	-0.0634223	6.79705468	0.38006509	0.5445538	0.60772887
Gpr3	0.27420609	1.08837726	0.37994168	0.54461852	0.60772887
Slc16a5	0.4577871	-0.6751443	0.37992008	0.54462986	0.60772887
Dusp6	0.13267261	5.38548791	0.37969375	0.5447486	0.60772887
Tbc1d22bos	-0.7294772	-1.0139922	0.37967762	0.54475706	0.60772887
Taf4b	0.14070187	2.90213099	0.37964662	0.54477334	0.60772887
C1qb	0.23292497	2.39464549	0.37941607	0.54489435	0.60772887
6030440G07	-0.474252	-1.2399311	0.37926133	0.54497561	0.60772887
Msl3l2	-0.1434765	4.12858652	0.37912947	0.54504487	0.60772887
Eif4ebp3	0.28016006	0.94907495	0.37903492	0.54509454	0.60772887
1700088E04I	0.9421764	-1.5563058	0.37900921	0.54510805	0.60772887
Perp	0.19477769	6.65280061	0.37891473	0.5451577	0.60772887
Antxr1	-0.1078418	6.12795934	0.37876247	0.54523772	0.60772887
Rai2	0.14944593	3.16974792	0.37873546	0.54525192	0.60772887
Yy1	0.08223437	5.38509177	0.37872802	0.54525583	0.60772887
Sp5	0.44453054	1.50954197	0.37869251	0.5452745	0.60772887
Gramd3	0.12249407	4.51568435	0.37859706	0.54532469	0.60772887
Pfdn4	0.14908667	3.92869864	0.37792371	0.54567897	0.60802437
Adck5	-0.276742	1.01876887	0.37790012	0.54569139	0.60802437
Riok1	-0.1332908	3.87879186	0.37775152	0.54576964	0.60805498

Rn4.5s	-0.2875067	0.52362576	0.37753835	0.54588193	0.60812351
Pgm3	-0.1145366	4.00884134	0.3772586	0.54602935	0.60813452
Col10a1	0.47230899	-0.5031777	0.37710596	0.54610982	0.60813452
Atxn1	-0.0936983	8.6929335	0.37704423	0.54614237	0.60813452
Naalad2	0.23326178	1.33162238	0.37704249	0.54614329	0.60813452
Nudcd3	-0.0693985	6.41496489	0.37695038	0.54619186	0.60813452
Mthfsd	-0.2353355	1.83132044	0.3769416	0.54619649	0.60813452
Mgea5	0.09239798	8.6334026	0.37684433	0.5462478	0.60813511
Sac3d1	-0.2124559	2.31542067	0.37660816	0.54637241	0.60816571
Cep95	-0.1127158	3.92792727	0.37659975	0.54637685	0.60816571
Grin2a	-0.1141127	6.61728008	0.37628493	0.54654305	0.60829416
Lrrc8e	-0.3459891	-0.1319681	0.37614981	0.54661441	0.60831705
Slc25a34	-0.3274607	0.06385238	0.3757366	0.54683275	0.60844204
Lad1	0.88221057	-1.4804742	0.37566999	0.54686796	0.60844204
Syde1	0.15657103	5.18608369	0.37564885	0.54687914	0.60844204
Mir384	-0.8029697	-1.205553	0.37549837	0.54695871	0.60845716
Chst12	-0.1995597	1.74990168	0.37543098	0.54699435	0.60845716
Ppm1k	-0.0959741	6.66579069	0.37524949	0.54709036	0.60850744
Gadl1	0.45899712	-0.5549758	0.37515306	0.54714137	0.60850767
Adam18	-0.6741744	-0.7034302	0.37489304	0.547279	0.60860421
Kcnc3	0.14890198	5.24132334	0.37475096	0.54735424	0.60863136
Pdik1l	-0.1152708	3.96548955	0.37461923	0.547424	0.60865242
Gm10012	0.11044391	3.8279026	0.37451673	0.5474783	0.60865629
Uhrf2	-0.0912913	5.29468683	0.37398721	0.54775898	0.6089118
Dnajc1	-0.097897	5.08351019	0.37357332	0.54797855	0.60898765
Ei24	-0.098634	6.08275607	0.37351665	0.54800863	0.60898765
Timm9	0.09707174	4.92435511	0.37345183	0.54804304	0.60898765
Apex1	0.54383094	-1.045947	0.37335086	0.54809664	0.60898765
3110002H16	0.17468142	3.3694138	0.37331673	0.54811476	0.60898765
Ppie	-0.2175665	1.3935998	0.37318067	0.54818702	0.60898765
A330049N07	-0.3887915	-0.2488455	0.37314544	0.54820573	0.60898765
9330151L19	0.13277361	4.78112007	0.37300433	0.54828068	0.60898765
Pole3	-0.1670866	2.9234239	0.37298659	0.54829011	0.60898765
Ptprr	0.10175396	4.20917954	0.37290073	0.54833573	0.60898765
Xpr1	0.08058859	7.89515667	0.37259349	0.54849904	0.6090253
Bcap31	0.12528059	5.73327821	0.37257001	0.54851152	0.6090253
Kdm4d	-0.4750792	-0.1753256	0.37254995	0.54852219	0.6090253
Commd1	0.15369267	4.15830275	0.37222173	0.54869677	0.60916265
Ddx54	0.12500948	3.98407135	0.37189064	0.54887298	0.60923245
Trim34a	-0.1866104	3.15628048	0.37187379	0.54888195	0.60923245
Sync	-0.3345496	1.07307096	0.37178623	0.54892857	0.60923245
Zcchc11	-0.0859312	6.83603884	0.37161103	0.54902188	0.60923245
D17H6S53E	-0.137562	2.75255333	0.37160968	0.5490226	0.60923245

Immp1l	0.0933856	5.4472159	0.37149622	0.54908304	0.60923245
Mtpap	-0.1029662	4.90302838	0.37143484	0.54911574	0.60923245
Pigm	-0.1267656	4.17321797	0.37087241	0.54941558	0.60950864
Mrpl38	0.13726177	3.37361832	0.37072929	0.54949193	0.60953688
Hnrnpab	0.10995427	7.5806322	0.37053284	0.54959676	0.60955276
Rabggtb	-0.0897301	6.33194334	0.37051169	0.54960805	0.60955276
Cgnl1	0.10294949	6.07090284	0.36978713	0.54999505	0.60974723
Sec11a	0.15699254	4.0758586	0.36976009	0.5500095	0.60974723
Tom1	-0.1352314	4.10098346	0.36965143	0.55006758	0.60974723
Klhl29	-0.1470191	5.07510909	0.36957189	0.55011011	0.60974723
Spty2d1	-0.0920518	4.96492595	0.36947277	0.55016311	0.60974723
Abhd15	0.5856713	-1.3237041	0.3694033	0.55020026	0.60974723
Zdhhc7	0.16059819	3.13165854	0.36940234	0.55020078	0.60974723
Sytl5	0.13256655	4.73836367	0.36932829	0.55024039	0.60974723
Epor	0.30001939	0.39059471	0.36932597	0.55024163	0.60974723
Vps9d1	0.12150736	3.70412847	0.36915625	0.55033243	0.60979143
Muc2	0.95855168	-1.8954559	0.36897337	0.55043031	0.60984345
Ccdc43	-0.0998431	4.71901413	0.36855316	0.55065533	0.60998262
Cxcr2	-0.137259	4.02308333	0.36854857	0.55065779	0.60998262
Rfc2	-0.1436659	3.34450161	0.36815419	0.55086914	0.61016031
Ccdc47	0.07190016	7.07371652	0.36765855	0.55113498	0.61039831
Zbed4	0.13194055	4.47113745	0.36749548	0.5512225	0.61042339
Tigd5	0.34849127	0.66060187	0.36742555	0.55126004	0.61042339
Tmem14c	0.16395736	3.11534299	0.36733146	0.55131055	0.61042339
Rpl37a	-0.1271665	5.95519467	0.36716165	0.55140174	0.61046792
Smim14	-0.116432	7.54290449	0.36701386	0.55148113	0.61049938
Rsbn1l	-0.0854287	5.5308702	0.36685595	0.55156599	0.61053041
Pmaip1	0.14595592	3.4226659	0.36677197	0.55161112	0.61053041
Adrb2	-0.2590671	2.24218647	0.36657934	0.55171468	0.61058859
Ik	-0.0633102	8.56539055	0.36632068	0.55185379	0.61068611
Bcam	0.1985782	3.57616704	0.36613439	0.55195403	0.61069166
Spata24	-0.3229443	0.34077621	0.366057	0.55199567	0.61069166
Cxcl17	0.74524465	-1.1080661	0.36602707	0.55201178	0.61069166
Pomt1	0.2623535	1.47726047	0.36580229	0.5521328	0.61069482
Map3k3	0.13497429	3.27514667	0.36574011	0.55216628	0.61069482
C2cd5	-0.1218787	5.79741466	0.36573762	0.55216763	0.61069482
Ttf2	-0.2801037	1.79361035	0.36547308	0.55231013	0.61077322
Celrr	0.43408917	0.30316582	0.36517011	0.55247342	0.61077322
Mknk1	0.10969918	4.31864868	0.36511321	0.5525041	0.61077322
Gm5441	-0.478835	0.35213005	0.36499936	0.55256549	0.61077322
Rubie	-0.734757	-1.4883069	0.36494638	0.55259406	0.61077322
Tef	-0.0642293	6.79325453	0.36489013	0.5526244	0.61077322
Smad3	0.08695367	6.75745112	0.3648715	0.55263445	0.61077322

Cars2	-0.1963016	2.19304104	0.36484912	0.55264652	0.61077322
Larp7	-0.0843223	5.74968368	0.364515	0.55282682	0.61091611
Cyp2e1	-0.2317175	1.04891713	0.36362022	0.55331022	0.61118915
Fbxo6	0.16502481	2.55592837	0.36351474	0.55336726	0.61118915
Dnajc11	0.11416362	3.98636206	0.36348489	0.55338341	0.61118915
Fam49b	0.0768423	6.79964942	0.36345322	0.55340053	0.61118915
S100a8	0.76967706	-1.1417502	0.36333971	0.55346194	0.61118915
Ubn1	-0.0833921	5.81141917	0.36333889	0.55346238	0.61118915
Alg9	0.18464281	2.99543005	0.36330788	0.55347916	0.61118915
Zfp518b	-0.075212	5.59134857	0.36330227	0.55348219	0.61118915
Tbx2	0.59411781	-1.4237405	0.36276401	0.55377355	0.61145451
Eif2s3x	-0.0744564	7.27309505	0.3625625	0.5538827	0.61151865
Tmem5	-0.0972686	4.60960499	0.36245665	0.55394006	0.6115256
Cables2	-0.1016069	5.03028034	0.36195767	0.55421058	0.61167595
Tmem251	-0.1216294	3.7918645	0.3618378	0.55427561	0.61167595
Cdk4	0.14062735	4.37868643	0.36178672	0.55430332	0.61167595
Osbpl7	0.17981092	2.31354449	0.36177799	0.55430806	0.61167595
Kcns1	-0.2129809	1.60185337	0.36172594	0.5543363	0.61167595
Pard6b	0.15564987	3.34604039	0.36164042	0.55438272	0.61167595
Oplah	-0.1774058	1.8057182	0.3613485	0.55454119	0.61179444
Cox17	0.09823372	5.38108613	0.36123741	0.55460153	0.61180464
H2-K1	0.14121993	5.06587241	0.3609033	0.55478305	0.61194853
Lhfpl2	0.1136372	5.18302644	0.36062108	0.55493648	0.61202826
9830147E19I	0.18641446	1.87966411	0.36058234	0.55495755	0.61202826
Ccnj	-0.1491363	2.89291347	0.36041954	0.5550461	0.61206955
Traf6	-0.0829442	6.18016976	0.35991106	0.55532284	0.61231834
Vps8	-0.1639179	4.47332854	0.35961539	0.55548389	0.61243953
Cryab	-0.1321174	7.16970998	0.35919394	0.5557136	0.6126234
Eif3k	-0.1199472	4.33275932	0.3591028	0.55576329	0.6126234
Impdh1	0.1758988	2.57882557	0.35899014	0.55582474	0.6126234
Orai1	0.26814612	1.85472513	0.35885058	0.55590088	0.6126234
Ndufa9	0.09908342	6.21260257	0.35874496	0.55595852	0.6126234
Malt1	-0.1368184	3.60673066	0.3587	0.55598305	0.6126234
Neb	-0.2756949	2.55395156	0.35865291	0.55600875	0.6126234
Spink8	-0.407855	-0.1941397	0.35853709	0.55607198	0.6126367
Jtb	-0.1045897	4.13897678	0.35815593	0.55628015	0.61280967
Zfp239	-0.0843913	5.66201513	0.35780355	0.55647274	0.61284798
Ino80c	0.111613	5.69305021	0.35777732	0.55648708	0.61284798
Sncg	-0.5121402	-0.3725225	0.35775158	0.55650115	0.61284798
2010315B03I	-0.1075923	4.22427496	0.3577178	0.55651962	0.61284798
Snhg4	-0.1427109	3.32764924	0.35742207	0.55668137	0.61296974
Gm13251	0.19101021	2.15003669	0.35716702	0.55682095	0.61299752
Slc25a28	-0.1058261	3.8932425	0.35696089	0.5569338	0.61299752

Clk4	-0.0992303	5.76616854	0.35690538	0.55696421	0.61299752
Nedd4l	0.11609293	8.72859637	0.35684616	0.55699664	0.61299752
Pawr	-0.1650307	5.34760824	0.35678562	0.5570298	0.61299752
Zfp617	0.08831284	5.81358953	0.35673166	0.55705936	0.61299752
Arid1a	-0.1023474	8.05983056	0.35668089	0.55708717	0.61299752
Enho	0.26333061	0.55148826	0.3566281	0.5571161	0.61299752
Adcy6	-0.1081536	4.09255721	0.35648564	0.55719417	0.6130271
Six4	0.16967298	3.45258233	0.35633357	0.55727753	0.6130625
Tpi1	0.09006629	6.96436296	0.35612212	0.55739349	0.613115
Sfxn1	0.0772172	5.91828744	0.35592354	0.55750242	0.613115
Muc1	0.43093231	-0.6133025	0.3558377	0.55754953	0.613115
Chpf2	0.18839971	2.28687468	0.35580817	0.55756573	0.613115
Scyl2	-0.0974315	5.8534477	0.35577991	0.55758124	0.613115
Tbc1d2	-0.4272518	-0.1847319	0.35562379	0.55766694	0.61315294
Tmed2	0.10480199	5.71179949	0.35549573	0.55773726	0.61317395
Polr2i	0.1692579	3.0562357	0.3552946	0.55784773	0.6132391
Psd	-0.1075896	5.56008766	0.35500442	0.55800718	0.61335809
Ccr10	0.74364922	-1.0131679	0.35485494	0.55808935	0.61337711
Elf2	0.10644189	6.74349939	0.35476495	0.55813884	0.61337711
Magi1	-0.1011009	6.35945009	0.35469348	0.55817814	0.61337711
Itfg2	0.12645838	3.37979046	0.35448504	0.55829281	0.61342171
Gpr132	-0.6106591	-1.4548746	0.35435604	0.55836379	0.61342171
Cnpy2	0.13339845	5.15196504	0.35434042	0.55837239	0.61342171
Egr4	-0.1841319	3.23099371	0.35384636	0.55864444	0.61356226
Topors	-0.0765545	6.03630215	0.35376087	0.55869154	0.61356226
Zhx1	-0.072437	8.00743807	0.35364396	0.55875596	0.61356226
Spg21	0.1332053	5.13850679	0.35357386	0.55879459	0.61356226
2410016O06	-0.1482985	3.82287711	0.35356818	0.55879772	0.61356226
Lrriq1	-0.2030297	3.09680899	0.35355002	0.55880773	0.61356226
Unc50	-0.0897013	5.64596372	0.35342133	0.55887867	0.61358389
Dgcr8	-0.0982982	4.13811439	0.35324795	0.55897428	0.61363261
Ctsl	0.14243594	6.74069323	0.35302223	0.5590988	0.61367402
Zfp2	0.10326745	4.45091169	0.35295699	0.5591348	0.61367402
Ube4a	0.08685196	5.74196263	0.35290093	0.55916574	0.61367402
Pbld2	-0.3348824	0.65689591	0.35267876	0.55928838	0.61374567
Dusp14	0.08649902	5.93030232	0.352597	0.55933352	0.61374567
Cyp7b1	0.14548832	2.44135494	0.35227781	0.55950984	0.61380237
Gm3696	-0.3037735	0.04726077	0.35223647	0.55953269	0.61380237
Cpne3	0.09072994	7.19702496	0.35222512	0.55953895	0.61380237
Zfp280b	0.09888157	4.89230233	0.35212115	0.55959642	0.61380918
Eny2	0.07803424	7.31050281	0.35189804	0.55971976	0.61388825
Tpd52l1	0.11682665	5.00453111	0.35170068	0.55982892	0.61392336
Rarres2	0.48055986	0.59490737	0.35165479	0.5598543	0.61392336

Ankle1	0.84255172	-1.8448087	0.35148436	0.55994861	0.61397055
Pear1	0.19034299	3.42299803	0.35101859	0.56020649	0.61419708
Tubgcp2	0.11008863	3.66953994	0.35084847	0.56030073	0.61424417
D16Ertd472e	0.10575491	6.32211038	0.35048132	0.56050424	0.61436406
Cuedc2	0.12227715	4.43643921	0.35044043	0.56052691	0.61436406
Tbc1d13	0.12328217	4.34928635	0.35037357	0.56056399	0.61436406
Chrd	0.17419235	2.10372602	0.35025566	0.5606294	0.61437951
Phldb1	-0.0964931	4.12078655	0.34991052	0.56082092	0.61449761
Ppp2r2cos	0.35954731	0.74501721	0.34982464	0.5608686	0.61449761
9430016H08I	0.12835788	3.49549773	0.34978411	0.5608911	0.61449761
Cdkl4	-0.1162886	4.17292672	0.34952696	0.56103392	0.61459785
Blvra	0.16784541	2.55710238	0.34917872	0.56122743	0.61475361
Gm266	-0.2953512	0.6425385	0.3490721	0.56128671	0.61476231
Ctnna2	0.08039876	6.28819644	0.34890114	0.56138178	0.6147962
C030046E11I	0.09107619	5.47917695	0.34883184	0.56142033	0.6147962
Uhrf1	-0.3748893	0.39544064	0.34863996	0.56152709	0.61485689
Gpr111	0.86004579	-2.0538352	0.34845903	0.56162779	0.6148638
Pllp	0.14350714	2.36712096	0.34841461	0.56165252	0.6148638
Ncapd2	0.16122292	2.67232371	0.34803252	0.56186531	0.6148638
Pfkfb2	-0.0777546	5.92260936	0.34802733	0.56186821	0.6148638
Mrps35	0.14134183	3.99390383	0.34802212	0.56187111	0.6148638
D430020J02I	-0.4074312	0.56245737	0.34798333	0.56189272	0.6148638
Prdx3	0.10206083	5.17439889	0.34798319	0.5618928	0.6148638
Ap5m1	-0.138144	3.37570823	0.34784457	0.56197005	0.61489215
Dcp1a	0.08209197	5.44539344	0.3476567	0.56207478	0.61493272
Trpt1	0.13553061	3.12327704	0.34755164	0.56213336	0.61493272
Rapgef2	0.08191885	7.49215571	0.34749563	0.5621646	0.61493272
Gimap9	0.49236663	0.14078881	0.34736835	0.56223559	0.61493272
Arhgap5	-0.0774262	8.64702615	0.34717804	0.56234178	0.61493272
Alas2	0.31579351	1.07308465	0.34703278	0.56242286	0.61493272
Mrpl18	0.11094699	5.44431311	0.34701813	0.56243104	0.61493272
Eif2s3y	0.14824472	5.08863465	0.34693582	0.56247699	0.61493272
Pbdc1	0.10426614	4.59042554	0.34691515	0.56248853	0.61493272
Ppm1g	0.09142554	4.91335133	0.34685769	0.56252061	0.61493272
Serp1	0.14007885	7.92018947	0.34655843	0.56268777	0.61505931
Fam222a	0.22342279	2.20401045	0.34645111	0.56274775	0.61506873
Elovl1	0.20365191	3.3782113	0.34630558	0.56282909	0.6151015
Ttc39a	-0.3142894	1.32521746	0.34614315	0.56291991	0.61514461
Kifap3	-0.1026114	8.45919916	0.34585152	0.56308304	0.61523697
Rock2	0.08302366	10.0961384	0.34580838	0.56310718	0.61523697
Gm13298	0.11734159	5.02359113	0.34521664	0.56343849	0.6155428
Zmynd8	0.08236645	6.82734109	0.34485434	0.56364153	0.61565732
Ints5	0.16499582	3.12011438	0.34484612	0.56364613	0.61565732

Armcx1	-0.0777023	5.69029124	0.34466325	0.56374868	0.61567268
Ppm1e	0.09979962	8.03567188	0.34463768	0.56376302	0.61567268
1700034F02I	-0.838621	-1.1505467	0.34429048	0.56395781	0.61580465
Scamp3	0.13595805	3.63923408	0.34411306	0.56405741	0.61580465
Hdac4	0.11534088	5.07433914	0.3440934	0.56406844	0.61580465
Pomp	0.09132525	7.36047553	0.3440204	0.56410944	0.61580465
Prdx5	0.10978988	5.90269109	0.34391387	0.56416926	0.61580465
Mfsd12	0.58644378	-1.2930054	0.34387271	0.56419238	0.61580465
Pitpnm2	0.09873224	6.50109081	0.34357757	0.56435821	0.61590835
Sympk	-0.1284117	4.57832281	0.34352057	0.56439025	0.61590835
Slc22a8	-0.1302531	8.06097155	0.34327649	0.56452748	0.61600197
Pigb	-0.1754793	2.54584533	0.34309074	0.56463196	0.61604966
Acbd3	-0.0686045	6.49257134	0.3429837	0.56469218	0.61604966
Arhgef9	-0.0823366	9.43024303	0.34283648	0.56477503	0.61604966
Furin	0.1746781	2.94407601	0.34283308	0.56477695	0.61604966
Gm17762	-0.4553453	-0.4665116	0.34263219	0.56489004	0.61607461
Copz2	0.15969803	6.78348118	0.34260969	0.56490271	0.61607461
Limd2	-0.1044963	4.46135496	0.34246393	0.5649848	0.61610803
Erc2	0.09982698	8.10052414	0.34198761	0.56525322	0.61634462
Sntb1	0.19001508	1.79795292	0.34188183	0.56531287	0.61635353
Decr1	0.11949101	5.39052888	0.34117226	0.56571329	0.61662523
Capn7	-0.0751839	6.54872315	0.3410625	0.56577528	0.61662523
Fuz	-0.3388516	0.47719722	0.34105499	0.56577952	0.61662523
Gm10789	0.72886142	-1.9141048	0.34100362	0.56580854	0.61662523
Irf7	0.37228668	0.15226717	0.3409842	0.56581951	0.61662523
Spats1	-0.2972518	0.64257121	0.34076058	0.56594586	0.61670681
Cysltr1	-0.399623	-0.4593609	0.34056214	0.56605804	0.61677292
Nkd1	0.12225059	4.85827505	0.34041318	0.56614227	0.61679962
Mest	-0.1330306	6.37721065	0.34020626	0.56625932	0.61679962
Rnf103	0.07233716	6.23756175	0.34013367	0.56630039	0.61679962
Prr14l	-0.0881035	7.45751808	0.34004714	0.56634937	0.61679962
Abcg3	-0.9180238	-1.7768559	0.33988762	0.56643966	0.61679962
Leprot	0.15222565	5.01098668	0.33983409	0.56646997	0.61679962
Slc2a4	-0.3907263	0.01525547	0.33974645	0.5665196	0.61679962
Heatr3	0.10305512	4.64907755	0.33957308	0.5666178	0.61679962
Tha1	-0.3495587	0.05886685	0.33956503	0.56662236	0.61679962
Cyth4	-0.204959	1.85872667	0.33952723	0.56664377	0.61679962
Lmo4	0.09628926	9.51337193	0.33951784	0.56664909	0.61679962
Gsto1	0.13277491	4.19893539	0.33935019	0.56674409	0.61684697
Slc26a5	-0.7339679	-1.149165	0.33923422	0.56680982	0.61686245
Sap30bp	-0.121623	3.65865418	0.33889585	0.56700171	0.61694137
Mypn	-0.2643389	1.79093751	0.33887803	0.56701182	0.61694137
Xxylt1	-0.1279222	3.52555456	0.33882369	0.56704265	0.61694137

Fkbp2	-0.108928	4.37876841	0.33867589	0.56712651	0.61694137
Dgkk	0.18428386	3.76416739	0.33865226	0.56713992	0.61694137
Sema3c	0.10959283	3.8203494	0.33816509	0.56741655	0.61710441
Dlgap3	-0.1287805	4.07743113	0.33811763	0.56744351	0.61710441
Gbp7	0.10766512	4.86337133	0.33811608	0.56744439	0.61710441
Zfp575	0.16446808	2.81930145	0.33799124	0.56751533	0.61712552
0610038B21	0.68172527	-1.8223318	0.33781042	0.56761811	0.61718124
Stx2	0.16914762	2.63517198	0.33758515	0.5677462	0.61726447
Stau1	-0.0704842	6.3092757	0.33714137	0.56799872	0.61745337
D430042O09	-0.1422733	3.42769263	0.33708987	0.56802804	0.61745337
Fbln2	-0.3026639	0.80247329	0.33700805	0.56807462	0.61745337
Gopc	0.07091882	6.07453978	0.33660423	0.56830465	0.61760343
Abcb7	-0.0999966	5.77587624	0.33658463	0.56831582	0.61760343
Neto2	0.1129671	5.74021452	0.33637868	0.56843322	0.61764079
Sowaha	-0.0904015	7.44815938	0.33634336	0.56845335	0.61764079
Mtf1	0.07137928	5.99906572	0.33610438	0.56858965	0.61773283
Myh8	-0.5756493	-1.5295231	0.33589164	0.56871103	0.61780866
Cp	0.09963395	7.00285072	0.33556896	0.56889523	0.61794792
Rxra	-0.1381808	5.7142349	0.33540575	0.56898845	0.61794792
Nfkbib	0.11991543	3.71176623	0.335396	0.56899402	0.61794792
Pcdhb17	-0.1166645	4.75954171	0.3352683	0.56906698	0.61797111
Klrb1f	-0.433323	-0.1107929	0.33512224	0.56915045	0.61797481
Ftsj3	0.09542829	5.00058319	0.33498984	0.56922614	0.61797481
Tmem200b	-0.4172826	-0.2351335	0.33472752	0.56937615	0.61797481
Runx1	-0.1822563	3.56880583	0.3347248	0.56937771	0.61797481
1700030J22F	0.15108867	3.65914995	0.33469027	0.56939746	0.61797481
Wnt4	0.09056391	5.58221254	0.33466517	0.56941182	0.61797481
Srpk1	-0.0708799	6.0743028	0.33463059	0.5694316	0.61797481
Nop56	-0.0902391	6.54885014	0.33443945	0.56954098	0.6180375
Gpr26	0.18014948	3.43552285	0.33424219	0.5696539	0.61810288
Lincrna-cox2	-0.7977593	-1.3891797	0.33410067	0.56973495	0.61810288
Slc25a38	0.13313616	2.90799022	0.3340638	0.56975607	0.61810288
Lingo4	0.72573703	-1.408254	0.33358298	0.57003161	0.61828616
Prkag2os1	-0.4629113	-0.1707102	0.33351819	0.57006876	0.61828616
Psmg2	-0.1457309	3.21681785	0.33349283	0.5700833	0.61828616
Lphn1	-0.0945387	8.35124724	0.33337911	0.57014852	0.61828616
Atp5l	0.09166848	6.48418274	0.33331873	0.57018315	0.61828616
Nudt16l1	-0.0926012	4.1446376	0.33322598	0.57023636	0.61828788
Nrbf2	-0.0851086	4.25797767	0.33257287	0.57061133	0.61856696
Sirt7	0.14373752	3.02721932	0.33244144	0.57068685	0.61856696
Surf2	-0.1362212	5.00252869	0.33242713	0.57069508	0.61856696
Dennd4b	-0.1461704	3.90586378	0.33241792	0.57070037	0.61856696
Pgs1	-0.1057775	4.41509529	0.33224766	0.57079823	0.61861705

Heg1	-0.091021	6.24919506	0.33206994	0.57090042	0.61863357
Mbtps2	0.09827614	4.92830405	0.33204148	0.57091679	0.61863357
2210016F16I	0.11315584	5.04382769	0.33177883	0.57106788	0.6187413
Ranbp3	0.09956461	3.79785599	0.33154178	0.57120432	0.61883315
Lct	-0.4399724	-0.2845821	0.33137542	0.57130012	0.61885323
Drg1	-0.1084139	5.31877549	0.33133011	0.57132621	0.61885323
Arhgef28	0.10119484	4.27869738	0.33106876	0.57147678	0.61891763
Timm21	0.13002244	4.15049091	0.33104751	0.57148902	0.61891763
2900011O08	0.07457406	7.725197	0.3309381	0.57155208	0.61891784
Zbtb8b	-0.2275121	1.87284961	0.33086784	0.57159258	0.61891784
Gpr37l1	0.16680575	3.0098003	0.33072575	0.57167451	0.61895059
Inpp4a	-0.1010487	6.53634804	0.33017701	0.57199112	0.61900088
Hbp1	0.07352786	6.72167701	0.33017398	0.57199287	0.61900088
Ado	-0.086761	5.37027576	0.33012213	0.5720228	0.61900088
Zkscan6	-0.1579929	2.9109064	0.33002846	0.57207689	0.61900088
Ackr4	-0.696638	-1.4397916	0.32999416	0.5720967	0.61900088
Tubb2b	0.08389259	5.27879931	0.32998424	0.57210243	0.61900088
Serpind1	-0.1675987	4.92559936	0.32989277	0.57215526	0.61900088
Gsta3	-0.3165755	0.88015063	0.32983681	0.57218759	0.61900088
Ndrg4	0.07699675	10.805371	0.32977262	0.57222467	0.61900088
Megf9	-0.0887996	5.961198	0.32974983	0.57223784	0.61900088
Clec2d	-0.208981	2.1306809	0.3295252	0.57236766	0.61908539
R3hdm2	-0.0726311	9.00287395	0.32937875	0.57245233	0.61910218
Trpc1	-0.1075136	4.59398598	0.32931953	0.57248658	0.61910218
Adrm1	0.08691676	4.16478739	0.32898617	0.57267943	0.61925482
Arpc4	0.13835811	5.34592983	0.32882188	0.57277452	0.61930173
Nsun6	0.15354477	2.75050169	0.32868648	0.57285291	0.61933057
Trpc7	-0.1828293	2.40421593	0.32832645	0.57306147	0.61950012
Arsk	0.15399953	3.59236562	0.32818928	0.57314097	0.61953013
Pou3f4	0.25317572	0.57012501	0.32791387	0.57330066	0.61964682
Stim2	0.09730533	5.92475523	0.32776535	0.57338681	0.619684
Mccc1	-0.1264429	3.58728736	0.32729805	0.57365803	0.61991547
Pmpcb	-0.0746051	5.02703198	0.32721803	0.57370451	0.61991547
Fance	-0.1446259	2.70443339	0.32711667	0.57376338	0.61992315
Slx1b	-0.103504	3.6325023	0.3269139	0.5738812	0.61995909
Mfsd11	0.12711862	3.18360502	0.32688124	0.57390018	0.61995909
Grem2	0.09297572	5.2695346	0.32626107	0.57426085	0.62026972
Agpat1	0.10420534	4.8021746	0.32620871	0.57429133	0.62026972
Mpp6	-0.1119679	8.59069649	0.32605768	0.57437924	0.62029001
Cd163	0.2725938	1.35450107	0.32599849	0.5744137	0.62029001
3110009E18I	0.28849364	1.10227185	0.32582484	0.57451482	0.62034327
Abca6	-0.2153038	1.65908555	0.32565965	0.57461106	0.62039125
Tfap4	-0.2272584	1.05252867	0.32550668	0.5747002	0.62043156

Smarca5	0.06845546	7.57245339	0.32540267	0.57476082	0.62044107
Ankrd33b	0.07557482	6.30615254	0.32521422	0.57487071	0.62049238
Clec4a3	0.60509006	-0.2808366	0.32514345	0.57491198	0.62049238
Ms4a4b	-0.5114417	-0.5138973	0.32485148	0.57508233	0.62051063
Zfp759	0.11391822	3.76817556	0.32484832	0.57508417	0.62051063
Rgs20	0.11983498	5.70952215	0.32484805	0.57508433	0.62051063
Fhdc1	0.29794991	0.87332126	0.32455833	0.57525347	0.62056752
Emc8	-0.07578	5.95088228	0.32454278	0.57526255	0.62056752
Zap70	-0.634475	-1.6527262	0.32430646	0.57540059	0.62056752
1700034H15I	0.23673767	2.06910052	0.32428924	0.57541065	0.62056752
Lpar6	0.16730423	2.23537527	0.32422005	0.57545108	0.62056752
Atxn7l2	0.25275993	2.34763128	0.32411398	0.57551308	0.62056752
Dgkh	-0.1445628	5.56521568	0.32403467	0.57555943	0.62056752
Map3k14	0.25542472	1.77007305	0.32399774	0.57558102	0.62056752
Ahrr	0.84912899	-1.6587342	0.32388493	0.57564699	0.62056752
Fzd8	0.24852587	1.75858584	0.32376211	0.57571882	0.62056752
Zfp74	0.10073659	5.97278665	0.32375491	0.57572303	0.62056752
Rnft1	0.10328715	4.55113215	0.32369362	0.57575888	0.62056752
Rcn2	0.07629799	6.30450958	0.3234822	0.57588259	0.62063587
Xkr8	-0.2201248	2.41378249	0.32340814	0.57592594	0.62063587
B3glct	-0.0846763	5.47446189	0.32319357	0.57605157	0.62066019
Zfp697	-0.0964451	4.89969601	0.32319256	0.57605217	0.62066019
Dcun1d2	0.090001	5.52819333	0.32264952	0.57637036	0.62089294
Map3k4	-0.1010075	4.98220469	0.32264693	0.57637188	0.62089294
Clmp	0.10854883	6.01158504	0.32242902	0.57649966	0.62097473
Kifc5b	-0.6372388	-1.1644293	0.32227274	0.57659134	0.62097969
Kctd13	0.11363826	6.17116268	0.32223537	0.57661326	0.62097969
Thumpd2	-0.1922263	1.67259557	0.32211165	0.57668587	0.62097969
Hist1h1e	0.19394484	2.92887352	0.32176347	0.5768903	0.62097969
4930426L09I	-0.2675083	0.84824692	0.32174235	0.57690271	0.62097969
Ndufb4	0.12434764	5.90445898	0.32171674	0.57691775	0.62097969
Nckap1	-0.0801695	9.44861355	0.32170001	0.57692758	0.62097969
Sdr42e1	-0.130472	2.65229571	0.32163202	0.57696752	0.62097969
Rest	-0.1215437	4.90144498	0.3216262	0.57697094	0.62097969
Vmn2r-ps12!	-0.3577702	0.85826362	0.32107724	0.57729365	0.62127118
Ttc30b	0.11205161	3.5675271	0.32051501	0.57762454	0.621531
Aagab	0.08956925	4.71518943	0.3202788	0.57776367	0.621531
Slc35b2	0.20572761	2.09245304	0.3200682	0.57788777	0.621531
Kcna1	0.09087312	7.66046982	0.32006804	0.57788787	0.621531
Rhov	0.35904763	1.2476218	0.3199751	0.57794266	0.621531
Selo	-0.1908699	1.26997923	0.31976949	0.57806389	0.621531
Hlcs	-0.105687	4.42716132	0.31966821	0.57812363	0.621531
Med9os	-0.5913569	-1.163347	0.31955749	0.57818896	0.621531

Map7	-0.0827682	5.79155177	0.31953144	0.57820433	0.621531
Galk2	0.11667039	3.52257411	0.31944822	0.57825344	0.621531
Thtpa	0.09195526	4.55589553	0.3192063	0.57839625	0.621531
Cenpa	-0.3234119	1.06374212	0.31919006	0.57840584	0.621531
Spidr	0.17241499	2.25997588	0.31912368	0.57844504	0.621531
Gucy1b3	-0.08985	6.61329345	0.31900787	0.57851345	0.621531
1110054M08	-0.2613083	1.12950431	0.31891033	0.57857107	0.621531
5830415F09I	-0.5092005	-0.7140421	0.31883191	0.57861741	0.621531
Acot4	-0.4134211	0.0646272	0.31869199	0.5787001	0.621531
Cwh43	0.63686424	-1.0775898	0.31860728	0.57875018	0.621531
Banf1	0.11314134	4.45274061	0.31860115	0.5787538	0.621531
Scn7a	0.09962238	4.22866684	0.31856407	0.57877573	0.621531
Tesk1	0.10681521	4.95553184	0.31854164	0.57878899	0.621531
Gtpbp1	0.13011949	4.45862357	0.31854096	0.5787894	0.621531
Arhgap33	-0.1377979	5.35412079	0.31849789	0.57881486	0.621531
Stt3b	0.07843658	5.87106778	0.31840941	0.57886719	0.621531
Cd83	0.14593613	2.18761984	0.31836812	0.57889161	0.621531
Mroh1	-0.1417542	4.29708474	0.31835371	0.57890014	0.621531
Lcmt1	0.09792438	4.85914656	0.31824104	0.57896679	0.621531
Mthfd1	-0.1328906	3.54331546	0.31813203	0.5790313	0.621531
Zfp574	0.11259439	4.88659637	0.31811706	0.57904015	0.621531
Ccl6	-0.321309	0.33841368	0.31785449	0.57919559	0.62161524
Tceb3	0.08541311	6.00143541	0.31777971	0.57923988	0.62161524
Exog	-0.1433137	2.92209593	0.31768964	0.57929322	0.62161524
Zfyve26	0.1139357	4.09534786	0.31763387	0.57932626	0.62161524
Exoc7	0.09566288	4.63607834	0.31750174	0.57940455	0.62164354
Tmem185b	-0.1877775	3.21262042	0.31726544	0.57954461	0.62173811
Tstd2	0.14724663	2.78070103	0.31712549	0.5796276	0.62177144
Acbd5	0.05677252	7.15738997	0.31698107	0.57971326	0.62180764
Paqr9	-0.1096604	5.44706084	0.31684513	0.57979391	0.62183845
Kbtbd2	-0.0693856	5.97260635	0.31644202	0.58003322	0.6220394
Cnih2	0.10680129	3.54137883	0.31621412	0.5801686	0.62211865
Tut1	-0.1831937	2.2972698	0.31611828	0.58022555	0.62211865
Dpysl2	-0.075354	8.72760806	0.31604777	0.58026746	0.62211865
Mid1ip1	-0.1270833	6.08785117	0.31596796	0.58031491	0.62211865
Rad21	0.064475	7.13610302	0.31575628	0.58044078	0.62219789
Draxin	0.71788524	-1.4922165	0.31537184	0.58066951	0.62228307
Anks6	-0.1561128	2.0881777	0.31524079	0.58074753	0.62228307
Plekhg2	-0.133635	4.20003844	0.31520536	0.58076863	0.62228307
Zcchc10	-0.1430345	3.6333882	0.31516934	0.58079007	0.62228307
Aldh3a2	-0.0671015	6.15963536	0.31505857	0.58085604	0.62228307
4930563E18I	0.90718206	-1.5944678	0.31504704	0.58086292	0.62228307
Msantd3	0.16233152	2.9884941	0.31494381	0.58092441	0.62228307

D3Ertd254e	-0.108265	6.52483606	0.31467553	0.58108429	0.62228307
Hmgn1	-0.1111102	8.11437481	0.31457798	0.58114245	0.62228307
Slc38a9	0.13578592	4.58404155	0.31451395	0.58118063	0.62228307
Gk5	0.1546957	1.99776092	0.31432583	0.58129282	0.62228307
Nol4	0.08698413	6.37124719	0.31407689	0.58144137	0.62228307
Cetn2	0.11404996	5.26854616	0.31407261	0.58144392	0.62228307
Tmf1	0.06065694	7.01464471	0.31406011	0.58145138	0.62228307
Gpr89	-0.123972	3.42303375	0.31355408	0.58175359	0.62228307
Chm	-0.0810559	6.37748255	0.31352809	0.58176911	0.62228307
Sp4	0.06902691	5.9452757	0.31350912	0.58178045	0.62228307
Zfp263	-0.1190979	4.16060069	0.313505	0.58178291	0.62228307
C2cd3	0.11034558	5.22226321	0.31346899	0.58180443	0.62228307
Nhej1	0.39484858	1.11955873	0.31342034	0.58183351	0.62228307
Vwa5a	0.09424311	5.49992937	0.3133907	0.58185123	0.62228307
Arhgap44	0.07243122	6.68127894	0.31336349	0.58186749	0.62228307
Tubgcp5	-0.1376557	4.13761734	0.31320362	0.58196307	0.62228307
Tppp	-0.0666082	8.81381514	0.31318277	0.58197554	0.62228307
Hadhb	0.07516592	6.50866812	0.31314678	0.58199706	0.62228307
Ccdc149	-0.1018841	4.44351434	0.31299154	0.58208991	0.62228307
Rbm46	-0.3252812	1.57546975	0.31293364	0.58212455	0.62228307
Ankk1	-0.3923044	-0.2694108	0.31291373	0.58213647	0.62228307
Cep57	0.09306483	4.61047186	0.31274257	0.58223889	0.62228307
Usp45	-0.0778647	6.85723407	0.31265962	0.58228855	0.62228307
Zfp639	0.1273896	4.46191698	0.31265658	0.58229037	0.62228307
Phactr2	-0.0807561	8.26110327	0.31256215	0.5823469	0.62228307
Trpc6	0.17470842	2.85249854	0.31254689	0.58235604	0.62228307
Gm10536	-0.5224328	-1.3984401	0.31248187	0.58239498	0.62228307
Ctdsp1	0.10509755	6.68530634	0.31237792	0.58245724	0.62228307
Htr4	0.26115957	0.69245636	0.31235859	0.58246882	0.62228307
Krtcap3	-0.4294525	-0.9806215	0.31233648	0.58248206	0.62228307
Stoml1	0.14239578	3.03885234	0.31231522	0.5824948	0.62228307
Appbp2	-0.0848716	5.28876882	0.3122273	0.58254748	0.62228383
Zfp109	-0.13497	3.49465105	0.31204132	0.58265894	0.62230325
4930577N17	0.27384541	0.10867021	0.31196997	0.58270171	0.62230325
Hist1h2bh	0.47796629	-0.7848318	0.31191334	0.58273566	0.62230325
Mrvi1	0.12211212	5.83222849	0.31182122	0.5827909	0.62230325
Agr2	0.35154068	-0.1481052	0.3117278	0.58284694	0.62230325
Slit2	0.07865521	5.82072371	0.31167695	0.58287744	0.62230325
Ptgfr	-0.1621353	4.72271228	0.31153247	0.58296413	0.62234032
Gdf3	-0.404596	-0.750508	0.31124105	0.58313905	0.62245668
Tshb	0.66105711	-1.2726407	0.31117773	0.58317708	0.62245668
Dip2a	-0.1166456	5.45376591	0.31095256	0.58331233	0.62254556
Psph	0.15871448	3.65078526	0.31082664	0.583388	0.62257083

Map4	0.06231552	9.19688123	0.31070965	0.58345832	0.6225904
Tiam1	0.09059701	6.45558787	0.31049174	0.58358934	0.62267473
Nudc	-0.0938697	5.63005536	0.31032292	0.58369089	0.62267628
Ifrd2	-0.2502444	1.23966364	0.31031643	0.58369479	0.62267628
Npnt	0.09780229	3.77911532	0.31013724	0.58380262	0.62269166
Rnf145	0.07542987	5.73026321	0.31010881	0.58381973	0.62269166
Smarca4	-0.0993245	7.01594341	0.31001176	0.58387815	0.62269166
Litaf	0.11349905	4.59247002	0.30986414	0.58396704	0.62269166
9330020H09	-0.4372705	-1.0428225	0.30986058	0.58396918	0.62269166
Dynlt1b	0.13380801	4.20983588	0.30975962	0.58402999	0.62270105
Extl3	0.07406743	6.75161783	0.30964378	0.58409977	0.62271869
Fbxw10	-0.2918844	0.68857113	0.30955706	0.58415203	0.62271869
Eml2	-0.1340648	2.7595467	0.30947326	0.58420253	0.62271869
1700052N19	-0.1013602	3.84405497	0.30929174	0.58431196	0.6227799
Rsl1d1	0.07338499	6.66736682	0.30910993	0.5844216	0.62284133
Ppp4r1	-0.1037441	4.32499418	0.30887773	0.58456169	0.62293519
Pitpnb	-0.069175	6.1491041	0.30876936	0.5846271	0.62294946
Zfp113	-0.1224757	4.27672379	0.30838735	0.58485778	0.62306227
Hells	-0.238212	1.48357957	0.30831108	0.58490386	0.62306227
Lysmd4	-0.114316	3.30306928	0.30824343	0.58494474	0.62306227
Gm13826	-0.1290044	2.96121639	0.30821859	0.58495975	0.62306227
Raf1	0.08469251	4.73108242	0.30816341	0.5849931	0.62306227
Zfp330	-0.0912532	6.1249446	0.30807525	0.5850464	0.6230631
Esyt1	-0.1385423	3.45697832	0.30795434	0.58511949	0.6230631
Zfp146	0.13195426	4.37017683	0.30790396	0.58514996	0.6230631
Bag4	0.07756384	5.95107043	0.30765904	0.58529811	0.62315517
Prdm12	-0.5191429	-0.1195421	0.30758899	0.5853405	0.62315517
Maea	0.06747572	5.48426659	0.30743667	0.58543269	0.62316701
Epha2	0.66645819	-1.6460887	0.30737925	0.58546745	0.62316701
Abcb4	-0.329347	0.37409322	0.30731273	0.58550773	0.62316701
Gm19466	0.41641173	-0.2042499	0.30703512	0.58567588	0.62323822
Anapc15	0.15350283	2.43938327	0.30703043	0.58567872	0.62323822
Zfp523	0.10392847	4.07551606	0.30682689	0.58580206	0.62324823
Dync1i2	0.07272827	7.48767004	0.30667627	0.58589338	0.62324823
Snhg18	0.13887867	4.28839592	0.30664603	0.58591171	0.62324823
Fgf11	0.0817701	6.14600193	0.30661277	0.58593188	0.62324823
Atp6v0a4	-0.3368245	0.14184209	0.30650909	0.58599476	0.62324823
Il18rap	-0.5197774	-0.4302662	0.30643823	0.58603775	0.62324823
Pigv	-0.1652815	2.81035164	0.30641403	0.58605242	0.62324823
Kcnn3	-0.1413096	4.1629489	0.30580725	0.58642078	0.62358459
Oas1b	0.40012851	0.04731173	0.30541462	0.5866594	0.62372858
Ppp1r2-ps3	0.34585363	-0.4670166	0.30535941	0.58669296	0.62372858
Rab15	-0.065198	6.31468885	0.30532739	0.58671244	0.62372858

Nphp3	-0.1574959	2.49082718	0.30512565	0.58683514	0.62380364
Ppan	0.20484419	1.68015532	0.30486663	0.58699276	0.62390044
Sh3bp5l	0.11263872	3.77831697	0.30480482	0.58703039	0.62390044
Gc	0.74259623	-2.7059228	0.3063638	0.58716482	0.62394942
09-Mar	-0.1910122	1.83379373	0.30449769	0.58721743	0.62394942
Maneal	-0.0947564	4.31987879	0.30437767	0.58729055	0.62394942
5830416I19R	-0.8014193	-0.9376962	0.30436714	0.58729697	0.62394942
Gpr19	-0.1379783	3.45953435	0.30415449	0.58742658	0.62394942
Nap1l3	0.08684066	5.81766145	0.3041079	0.58745498	0.62394942
Ypel5	0.07988862	7.68390628	0.30406599	0.58748053	0.62394942
Nek8	-0.3448557	1.27127554	0.30404508	0.58749329	0.62394942
Ndufs5	0.49508741	-1.4944173	0.30383651	0.5876205	0.62402918
Zfand2b	0.23702249	1.87465094	0.30366779	0.58772344	0.62408317
Tmpo	-0.0978725	5.86284667	0.30335911	0.58791189	0.62416639
Zfp646	0.1073052	3.74038985	0.30328845	0.58795504	0.62416639
Apod	0.16427475	10.3568368	0.3032275	0.58799227	0.62416639
Amz2	-0.09413	4.75288145	0.30311709	0.58805972	0.62416639
Gstm2	-0.1727749	5.29499627	0.3031127	0.58806241	0.62416639
Dnajc17	-0.2035986	2.30473884	0.30300127	0.5881305	0.62418334
Gimap4	-0.2033177	3.0918841	0.30266454	0.58833637	0.62423583
Idnk	0.1438105	3.48742722	0.30257564	0.58839075	0.62423583
Mettl25	-0.1927995	2.64327942	0.3025599	0.58840038	0.62423583
Ube2e2	-0.0703338	6.58122371	0.30255554	0.58840305	0.62423583
Tchh	-0.1426258	3.01158791	0.30249417	0.58844059	0.62423583
Fancc	0.17967442	1.8744647	0.30234196	0.58853374	0.62427935
Ctnnal1	0.0934974	4.46199785	0.30205802	0.58870757	0.62431077
Nyap2	-0.0936709	5.85461771	0.30201163	0.58873599	0.62431077
Cald1	0.1003511	10.2790295	0.30200445	0.58874038	0.62431077
Zfp105	-0.1618941	3.11122438	0.30187391	0.58882035	0.62431077
C1qtnf3	0.9481122	-2.2217414	0.30186792	0.58882402	0.62431077
Pcmtd1	-0.0647116	8.28762032	0.30177504	0.58888092	0.62431583
Ptpra	-0.0724564	6.44475606	0.30162434	0.58897329	0.62435848
Trp53i13	0.43422812	-0.2615405	0.30150825	0.58904446	0.62437866
Galk1	0.17144336	2.74754331	0.30125996	0.58919674	0.6244848
Trim16	-0.1674352	2.64917315	0.30104141	0.58933085	0.62451308
Ifngr2	0.09968059	5.14388699	0.30101812	0.58934514	0.62451308
Mipep	0.12336115	4.032367	0.30084936	0.58944875	0.62451308
Rab43	-0.0994468	5.4568672	0.30078082	0.58949083	0.62451308
Rpl11	-0.1028916	7.046423	0.30058776	0.58960942	0.62451308
C730002L08R	0.18105952	2.30631274	0.30049107	0.58966883	0.62451308
AI118078	-0.3939543	-0.438439	0.30038906	0.58973152	0.62451308
Sell	0.77731499	-1.0459632	0.30038097	0.5897365	0.62451308
Nop10	0.09824056	4.74626895	0.300328	0.58976906	0.62451308

Eif4g3	0.07897489	8.94854397	0.30031391	0.58977772	0.62451308
Rab22a	-0.0744157	5.64374601	0.30001703	0.58996029	0.62451308
Zfp14	-0.1452428	3.10914981	0.29996562	0.58999191	0.62451308
C920006O11	0.1896251	1.74888489	0.29989198	0.59003722	0.62451308
Usp25	-0.0652662	7.47059909	0.2998206	0.59008115	0.62451308
Timm10	0.1472326	3.84783903	0.29973496	0.59013385	0.62451308
Pnp	0.09807635	4.58401959	0.2996806	0.59016732	0.62451308
Epha3	-0.1375684	2.92841282	0.29949806	0.5902797	0.62451308
Eci2	-0.1016842	5.4609683	0.29943731	0.59031712	0.62451308
Hip1r	-0.1586426	3.19870087	0.29941606	0.59033021	0.62451308
Akt3	-0.072276	8.0782817	0.29933464	0.59038037	0.62451308
Eed	-0.0956048	4.49116874	0.29927307	0.5904183	0.62451308
Commd8	0.11292246	5.76987155	0.29925465	0.59042965	0.62451308
Usp8	0.05860251	7.66784425	0.29923912	0.59043922	0.62451308
Gm11517	-0.5526056	-1.0909567	0.29908849	0.59053205	0.62451308
Ethe1	0.17561056	2.6815575	0.29902551	0.59057087	0.62451308
Terf2	0.06571393	6.26750373	0.29894897	0.59061806	0.62451308
Lamtor4	0.24371068	1.98318415	0.29890164	0.59064724	0.62451308
Ckap5	-0.1010029	7.87263961	0.2987334	0.59075101	0.62451308
Atcay	0.09046735	5.5480024	0.29872695	0.59075499	0.62451308
Ddx4	0.25368456	0.98895315	0.29867365	0.59078787	0.62451308
Fpgt	-0.0879831	4.93554178	0.29818267	0.59109095	0.62471782
Tcf7l1	0.12913551	5.36479086	0.29804918	0.5911734	0.62471782
BC021891	0.21617568	1.57565952	0.29786952	0.59128442	0.62471782
Usp13	-0.1573472	3.87018205	0.29785058	0.59129612	0.62471782
Primpol	0.21081776	2.5564363	0.29780974	0.59132137	0.62471782
Iqce	0.13316778	3.35711486	0.29777671	0.59134179	0.62471782
2410004P03I	-0.1737198	2.3157078	0.29774823	0.59135939	0.62471782
Cnep1r1	0.07377481	5.90933978	0.29761194	0.59144366	0.62471782
Csad	-0.0959939	4.22252035	0.29760001	0.59145104	0.62471782
Acot8	0.22287014	1.57010583	0.2974427	0.59154834	0.62476548
Kctd12b	0.10356926	4.98080591	0.29735473	0.59160276	0.62476786
Gm4961	0.54879326	-1.7273926	0.29718165	0.59170987	0.62477595
Gm7008	-0.3622376	-0.366684	0.29713613	0.59173805	0.62477595
BC037032	-0.3095074	0.53871092	0.29708948	0.59176693	0.62477595
Cdkn3	-0.458982	-0.6948635	0.29682556	0.59193037	0.62489342
Cox10	0.0890097	4.25664074	0.29660668	0.592066	0.6249815
Gzma	-0.6662655	-0.5829634	0.29640965	0.59218813	0.62505533
Phf11a	-0.3798084	-0.4520695	0.29620117	0.59231743	0.62513671
Gbe1	0.09924803	3.96139429	0.29595185	0.59247212	0.62519347
Socs3	0.20763689	1.04320367	0.29594622	0.59247562	0.62519347
Glp2r	0.2915256	1.68436136	0.29572345	0.59261392	0.62528431
Ska1	1.0925952	-1.8990028	0.29544629	0.59278608	0.62536252

Pkp3	0.58500729	-1.3205244	0.29539024	0.59282091	0.62536252
Gm5	0.56651965	-0.5194814	0.29535196	0.5928447	0.62536252
Sf3b5	-0.1395705	3.86018362	0.29525259	0.59290646	0.62536934
Nsmaf	-0.0944429	4.77577922	0.29516779	0.59295918	0.62536934
Zkscan1	0.06213357	7.6698238	0.29508955	0.59300783	0.62536934
Zfp174	0.10891332	3.76830348	0.29488677	0.59313396	0.62544728
Grid2ip	-0.2996321	1.10690443	0.29459014	0.59331856	0.62558685
Rab7l1	0.21401509	4.22114935	0.29447236	0.59339189	0.62559452
R74862	-0.0928928	4.58028853	0.29441067	0.59343031	0.62559452
Snord47	0.58125588	-1.5171434	0.29403653	0.59366341	0.62578091
Homer1	-0.0767921	8.40981841	0.29392949	0.59373013	0.62578091
Vcpip1	-0.0743992	7.35241349	0.29387536	0.59376388	0.62578091
Slc9a9	0.16604242	3.46094576	0.29341466	0.59405128	0.62600944
BC005624	0.08720207	6.12337461	0.29318595	0.59419407	0.62600944
Alg5	-0.1712307	3.24676587	0.29308994	0.59425403	0.62600944
Antxr2	0.2133005	3.3378226	0.29307329	0.59426443	0.62600944
Hhat	0.53675784	-0.7515552	0.2930577	0.59427416	0.62600944
Ttpa	0.24213605	1.48140709	0.29281473	0.59442598	0.62600944
Sptbn4	-0.2006826	3.60591124	0.29280281	0.59443343	0.62600944
Ppil2	0.08655137	4.47776369	0.29267426	0.59451378	0.62600944
Gria2	-0.1121212	9.08586185	0.2926127	0.59455227	0.62600944
Psmb9	-0.202301	3.32181791	0.29260093	0.59455964	0.62600944
Tmc2	-0.336698	-0.2558401	0.29254419	0.59459512	0.62600944
Rxrb	0.12647259	3.23806349	0.29251455	0.59461365	0.62600944
Wdtc1	0.08480735	5.20720695	0.29244002	0.59466027	0.62600944
Nono	0.05555579	7.47503312	0.29201652	0.59492529	0.62617831
Wdr8	-0.1740297	2.26818252	0.29198592	0.59494445	0.62617831
AI987944	0.11727669	3.80950383	0.29193308	0.59497754	0.62617831
Lyplal1	0.14308589	2.81065316	0.29163203	0.59516613	0.62632174
Tmem87a	-0.095823	4.44738721	0.29137031	0.59533018	0.62639414
Wdfy1	0.09846339	4.67529189	0.29131402	0.59536547	0.62639414
Pnpla2	0.12931289	4.47056317	0.29123991	0.59541195	0.62639414
Gpr6	0.7183287	-1.4290837	0.29115506	0.59546517	0.62639414
Pura	0.05552912	6.97518795	0.2911052	0.59549645	0.62639414
E230016K23I	0.53383078	-0.708949	0.29095465	0.59559092	0.62643848
2900009J06F	-0.5253133	-0.6726362	0.29074284	0.59572388	0.62649296
9330159F19I	-0.1015589	6.85919471	0.29059326	0.59581782	0.62649296
Trappc6a	0.30882844	1.50051565	0.29057301	0.59583053	0.62649296
Cdkl5	0.09254021	8.25420541	0.29041493	0.59592985	0.62649296
Phf2	-0.0561336	6.53371212	0.29039987	0.5959393	0.62649296
Slc24a1	0.47327676	-0.0401307	0.29030669	0.59599786	0.62649296
Rbms1	0.07843345	6.26706979	0.29028913	0.5960089	0.62649296
Chmp2a	0.11384003	5.63235119	0.29006839	0.59614767	0.62653549

Rps6kl1	0.17178424	2.37169747	0.28997968	0.59620346	0.62653549
Ptger2	-0.4296915	-0.470083	0.28997515	0.59620631	0.62653549
Nubp1	0.17007419	2.29977372	0.28985709	0.59628058	0.62654386
Gm2a	0.143151	5.00654987	0.28979615	0.59631892	0.62654386
Gm9866	0.15054256	2.57794585	0.28916839	0.59671419	0.62684295
Lrch2	0.11642566	4.19729802	0.28905092	0.59678822	0.62684295
Wdyhv1	-0.1016963	4.05496611	0.28900086	0.59681978	0.62684295
Cacna1f	0.53030174	-0.5969001	0.2889803	0.59683274	0.62684295
Cryzl1	0.08522243	5.36087716	0.28890232	0.5968819	0.62684295
Ank1	0.12276114	5.05992668	0.28878192	0.59695782	0.62684295
Zfp790	0.10319466	4.40422388	0.28876264	0.59696997	0.62684295
04-Mar	-0.0815457	4.92876459	0.28850926	0.59712983	0.62695582
Gpr25	-0.1254646	3.66236568	0.28833952	0.59723695	0.62701191
Opn1sw	0.45753873	0.40682071	0.28817521	0.5973407	0.62701191
Snx14	0.08641401	5.44398163	0.28816759	0.59734552	0.62701191
D330050G23	-0.2340775	1.95972551	0.28809292	0.59739267	0.62701191
Zbtb49	-0.1999978	1.51435636	0.28786307	0.59753789	0.62704832
Tlr8	0.75362442	-1.0531026	0.28778284	0.5975886	0.62704832
Mrpl1	-0.0965951	4.24105416	0.2877187	0.59762914	0.62704832
Vps41	-0.0777736	7.02432777	0.28763533	0.59768185	0.62704832
Optn	0.09669606	4.13149135	0.28762129	0.59769073	0.62704832
Hist2h2be	0.10426717	6.00580263	0.28754097	0.59774152	0.62704832
Klrb1c	0.57655084	-0.3976589	0.28741685	0.59782002	0.62707574
Commd3	0.07678278	5.0746158	0.28729021	0.59790015	0.62710486
Rnf170	-0.0779057	5.90689747	0.28676871	0.59823034	0.62732455
Zcchc16	-0.1523083	2.91030763	0.28674793	0.5982435	0.62732455
BC003331	0.06477787	6.91312747	0.28671122	0.59826676	0.62732455
Ptprt	-0.1110355	6.91884679	0.28661076	0.59833042	0.62733638
Sh2d5	0.098899	4.63060511	0.28651624	0.59839033	0.62734427
2610305D13I	-0.3046828	0.60259501	0.28607639	0.59866928	0.62736558
Ggps1	0.09243337	6.34114367	0.28590572	0.59877759	0.62736558
Pcdhga9	0.15160168	2.52573333	0.28589961	0.59878147	0.62736558
Mettl23	0.15490707	2.83220101	0.28586601	0.5988028	0.62736558
1810043G02	0.12289707	2.74556179	0.2857078	0.59890325	0.62736558
Mrps27	0.12517417	3.35582252	0.28550865	0.59902975	0.62736558
Pdss2	-0.1078747	3.1149329	0.28549821	0.59903638	0.62736558
Ckap2	0.36509636	0.36365987	0.28546882	0.59905506	0.62736558
Ccdc61	-0.2474926	0.48748189	0.28546472	0.59905766	0.62736558
Wdr26	0.05954819	8.56156612	0.2852614	0.59918688	0.62736558
Tada2a	-0.1039256	3.75529382	0.2851805	0.59923831	0.62736558
Pcsk2os1	0.14552474	3.00792967	0.28517068	0.59924455	0.62736558
Ppil3	0.13468365	3.79873045	0.28512975	0.59927058	0.62736558
Klf6	-0.0663427	7.42428216	0.28512505	0.59927357	0.62736558

Gm1715	-0.6174531	-1.7361002	0.28472929	0.59952534	0.62736558
Ccdc124	0.1308241	4.3329808	0.28467762	0.59955823	0.62736558
Gm5535	0.899117	-1.7777729	0.28464974	0.59957597	0.62736558
Fam154b	-0.2355752	0.89675354	0.28462359	0.59959262	0.62736558
Nfe2	-0.7896688	-1.1396766	0.28452891	0.5996529	0.62736558
Fgf9	-0.0994249	4.84515337	0.28442172	0.59972117	0.62736558
A530054K11	0.07770641	5.33622178	0.28432628	0.59978196	0.62736558
Tinagl1	-0.3521387	0.32428798	0.28427263	0.59981613	0.62736558
Chmp1a	0.10679396	4.57431843	0.28416538	0.59988447	0.62736558
Plscr1	0.13289712	4.17838695	0.28400869	0.59998435	0.62736558
Gabrq	0.17899727	1.88100598	0.28392721	0.60003629	0.62736558
Rnaseh1	0.16788791	2.79486791	0.28391563	0.60004368	0.62736558
Impg2	-0.5725081	-0.3888372	0.28374635	0.60015164	0.62736558
Kcnj3	-0.1037308	5.04747371	0.28362057	0.60023188	0.62736558
Ceacam1	0.16187371	2.96410889	0.28356459	0.6002676	0.62736558
Nipal2	-0.1640067	2.85416739	0.28354205	0.60028199	0.62736558
Mxra8	0.13180458	5.68300485	0.28337542	0.60038835	0.62736558
Bcor	0.07371328	5.37892961	0.2833559	0.60040081	0.62736558
Slc5a6	-0.1255869	4.25431609	0.28329923	0.60043699	0.62736558
G630025P09	-0.7496146	-1.6225258	0.28328367	0.60044693	0.62736558
Traf2	-0.2202991	1.32158657	0.28327657	0.60045147	0.62736558
A930009A15	-0.708238	-1.9983995	0.28308317	0.60057499	0.62736558
Plekhd1	-0.4738935	-1.030758	0.28307814	0.6005782	0.62736558
Swsap1	-0.1192378	3.36427758	0.28304151	0.60060161	0.62736558
Gm10560	0.71641074	-1.0415094	0.28292826	0.60067397	0.62736558
Abca4	-0.1971989	3.09333688	0.28285765	0.6007191	0.62736558
Paqr7	0.08043032	4.56034292	0.28279235	0.60076084	0.62736558
Zfp90	-0.1026034	3.80356774	0.28278849	0.60076331	0.62736558
Pde4a	0.09747443	6.32580238	0.2827772	0.60077053	0.62736558
Tdg	0.24626095	0.31915329	0.28273362	0.60079839	0.62736558
Kcna4	0.11522166	5.66132831	0.2826839	0.60083018	0.62736558
Rgs10	0.09542234	4.32710585	0.28267326	0.60083699	0.62736558
Cadm4	0.14686524	3.23482238	0.28261219	0.60087604	0.62736558
Glg1	0.07548742	6.66224155	0.28251488	0.60093828	0.62736558
2310002F09I	0.73042408	-1.5177749	0.28245343	0.60097759	0.62736558
Colgalt2	-0.1850114	1.4849493	0.28207659	0.60121879	0.62754241
Spon2	0.62979744	-0.6560705	0.28202505	0.60125179	0.62754241
Gm11201	0.57140947	-0.2656289	0.28189378	0.60133586	0.62757546
Papd5	-0.0810938	6.11240003	0.28142018	0.60163942	0.62762682
Chst15	-0.0876819	5.98406679	0.28132619	0.60169969	0.62762682
Dip2b	-0.0879528	7.80946321	0.28129191	0.60172168	0.62762682
Epc2	0.05857614	7.43690002	0.28116482	0.60180322	0.62762682
Ern1	-0.1978544	2.21275338	0.2810651	0.60186722	0.62762682

Megf8	0.1118947	5.0083475	0.28101522	0.60189923	0.62762682
Nr1h2	-0.1275334	4.09397517	0.2808904	0.60197936	0.62762682
Tspan4	-0.1843243	2.48387872	0.28082979	0.60201828	0.62762682
Gm5643	0.07475736	5.48793776	0.28075411	0.60206688	0.62762682
Kcns3	0.17756096	1.97479653	0.28066537	0.60212388	0.62762682
Zfp869	-0.0871702	5.11244838	0.28062445	0.60215017	0.62762682
Rufy3	0.07754943	8.36759982	0.28053108	0.60221017	0.62762682
Negr1	0.07015969	8.24486592	0.28035959	0.60232039	0.62762682
Pcnxl3	0.08342614	4.69194894	0.28034682	0.60232859	0.62762682
Sec61a1	0.09926538	4.9649272	0.28020693	0.60241854	0.62762682
Col4a5	-0.1645534	3.29532106	0.28004542	0.60252242	0.62762682
BC037034	0.10556586	3.84047215	0.28003647	0.60252818	0.62762682
Fam199x	-0.0836219	4.77628233	0.28000775	0.60254666	0.62762682
E030011O05	0.45072009	-0.8651926	0.27994574	0.60258655	0.62762682
Hist1h4i	0.34234123	-0.2487635	0.27989946	0.60261634	0.62762682
Tsga10	0.08648677	4.98707404	0.27982319	0.60266542	0.62762682
Kcnmb2	-0.2875174	0.84059901	0.27980079	0.60267984	0.62762682
Dhx29	-0.1246293	4.80532999	0.27976313	0.60270408	0.62762682
Ska2	-0.1474596	2.75900867	0.27976065	0.60270568	0.62762682
Aarsd1	0.1185023	3.70563603	0.27971196	0.60273703	0.62762682
Tbc1d7	-0.1023868	4.04487966	0.27956876	0.60282923	0.62762682
Pusl1	0.18733573	2.33169202	0.27955796	0.60283619	0.62762682
Ablim3	-0.0623503	5.35289374	0.27946212	0.60289792	0.62762682
Lrrc29	-0.4857886	-0.596089	0.27942348	0.60292281	0.62762682
Ccdc38	-0.3319767	-0.1987506	0.27932803	0.60298431	0.62762682
Lonp2	-0.0673853	5.557762	0.27903706	0.60317187	0.62762682
Ube2d1	0.08555756	6.92626022	0.27902917	0.60317696	0.62762682
Itpka	-0.0979752	4.18836864	0.27901655	0.6031851	0.62762682
Rab11fip1	0.1153224	3.66126826	0.27899844	0.60319678	0.62762682
Clec16a	0.08137506	6.3092255	0.27880688	0.60332034	0.62762682
Tmem39a	-0.0900187	4.96808589	0.27879396	0.60332867	0.62762682
Cited4	0.21982364	0.97690506	0.27878919	0.60333175	0.62762682
Stk40	0.07948058	4.15938639	0.2787197	0.60337658	0.62762682
Carf	-0.086102	4.65339293	0.27850357	0.60351608	0.62768111
Dyrk1b	-0.1180931	3.09395663	0.27847643	0.6035336	0.62768111
4931429L15R	-0.5306739	-1.1998334	0.27835128	0.60361442	0.62771065
Hlf	0.0745501	10.0220648	0.27802631	0.60382438	0.62777363
Bbs1	0.09806448	5.65451516	0.27801819	0.60382963	0.62777363
Cpz	-0.6661427	-1.0697219	0.2778798	0.60391909	0.62777363
Hlx	0.61828735	-1.893623	0.27787739	0.60392065	0.62777363
Polm	-0.1314189	3.19492597	0.2776469	0.60406971	0.62777363
Ccdc110	0.4157462	0.36733429	0.27758144	0.60411206	0.62777363
Tnfrsf18	0.18456012	1.90014686	0.27746098	0.60419001	0.62777363

Idua	0.12214026	3.57886831	0.27735298	0.6042599	0.62777363
Palm3	0.1428794	2.25433235	0.27730114	0.60429347	0.62777363
Fam184a	-0.1080975	4.85798108	0.27729711	0.60429607	0.62777363
Them6	-0.1547013	2.62308151	0.27729275	0.6042989	0.62777363
Scn5a	-0.2907396	1.37007776	0.277226	0.60434212	0.62777363
Dynll2	-0.06598	7.03477523	0.27717981	0.60437203	0.62777363
Ybx1	0.08052915	7.17213892	0.27703858	0.60446351	0.62777363
Tmem147	0.1185851	3.44846783	0.27700034	0.60448828	0.62777363
Letm1	0.09196282	5.94169306	0.2768976	0.60455484	0.62777363
Kcnj8	-0.3776305	0.66783399	0.27688017	0.60456614	0.62777363
Olig1	0.09375705	4.07031576	0.2764408	0.60485102	0.62793449
Vasn	-0.0949173	3.78869828	0.27636582	0.60489967	0.62793449
Specc1	0.07230297	6.56145607	0.27636201	0.60490213	0.62793449
Cep104	0.08108688	4.65266436	0.27631786	0.60493078	0.62793449
Tmem220	-0.1519553	2.86846777	0.27592883	0.60518333	0.62814219
Obsl1	0.12108315	3.21369127	0.27584538	0.60523754	0.62814401
Tmem14a	0.09221716	4.84503277	0.27514002	0.60569612	0.6284875
Setd4	-0.3039682	1.04697933	0.27510301	0.6057202	0.6284875
Tsen2	0.16198136	2.55053388	0.27504585	0.6057574	0.6284875
Fam76b	0.10945103	4.65486334	0.2750061	0.60578328	0.6284875
Nutm1	-0.8663641	-1.5031532	0.27493295	0.60583089	0.6284875
Fam65b	-0.0968271	5.89319166	0.27425734	0.60627106	0.6288666
Mrpl33	0.1219522	4.31556903	0.27412751	0.60635572	0.6288666
Agap2	0.07670131	6.9295941	0.27410385	0.60637115	0.6288666
Myo19	-0.1626184	2.91947592	0.27404984	0.60640638	0.6288666
Clpp	0.09814439	3.49623974	0.27376693	0.60659099	0.62900358
Plcz1	0.54731787	-0.6400122	0.27366503	0.60665752	0.62901629
Htatsf1	0.05546997	8.39789574	0.27357562	0.6067159	0.62901629
Lrp3	0.0994185	4.92686059	0.27350685	0.60676082	0.62901629
2900076A07I	0.24526247	0.95672739	0.27279812	0.60722412	0.6294421
Prrg3	-0.0603376	6.63317947	0.27265779	0.60731596	0.6294595
Bbs10	0.1366158	2.99343135	0.27261184	0.60734603	0.6294595
Decr2	-0.0737194	5.53858173	0.27241429	0.60747536	0.6294741
Adssl1	0.15087997	2.68975469	0.27239666	0.60748691	0.6294741
Adck4	0.25616047	1.29627474	0.2722798	0.60756345	0.6294741
1700037H04I	0.1065708	4.31945239	0.27226923	0.60757037	0.6294741
Isg20l2	-0.1173374	4.04315154	0.2721519	0.60764724	0.6294765
2010300C02I	-0.08627	5.50339108	0.27203853	0.60772154	0.6294765
Sesn1	0.06935157	6.4168834	0.27202504	0.60773038	0.6294765
Gps2	0.08751639	4.02309499	0.27181258	0.60786967	0.62950659
Gabra6	0.98935868	-2.0502817	0.27329559	0.60791194	0.62950659
Usp2	-0.085761	5.72951967	0.27174023	0.60791712	0.62950659
Psmd11	0.09210626	5.94030805	0.27165852	0.60797072	0.62950766

Wdr7	-0.0996995	7.54758209	0.27119292	0.60827633	0.62956926
Apbb2	0.06223479	7.4120643	0.27118849	0.60827923	0.62956926
Fbxw15	0.47879335	-0.9967257	0.27114345	0.60830881	0.62956926
Chchd5	0.13888243	2.63589755	0.27108686	0.60834598	0.62956926
Nhp2	0.14203368	3.21327063	0.27100298	0.60840109	0.62956926
Gm5431	0.25853642	0.93549401	0.27097992	0.60841625	0.62956926
Frat2	-0.1829826	3.01946885	0.27088245	0.6084803	0.62956926
Mcidas	0.48831525	-1.6433173	0.27079844	0.60853551	0.62956926
Cpd	-0.083726	6.32169833	0.27079317	0.60853898	0.62956926
Eif3h	0.08049277	6.19535446	0.27076741	0.60855591	0.62956926
Tal1	-0.3644776	0.26914581	0.27057509	0.60868237	0.62960278
Hist1h4k	0.17807278	1.94575363	0.270542	0.60870414	0.62960278
Slc37a1	0.21839104	0.7991249	0.27038724	0.60880595	0.62960278
Eefsec	-0.1510687	2.69284834	0.27036693	0.60881931	0.62960278
Spire1	-0.0846385	7.44840204	0.2703185	0.60885118	0.62960278
Hs3st6	0.43629115	-0.5819177	0.26994741	0.6090955	0.62976175
Prpf38b	0.06582708	7.32605818	0.26992527	0.60911008	0.62976175
Fndc1	-0.1854644	1.61004126	0.26954968	0.6093576	0.62996188
Ndufaf5	0.08887613	4.79767237	0.26943981	0.60943005	0.62996188
Rcn1	0.12972941	4.76303695	0.26939217	0.60946147	0.62996188
B230312C02	0.84254397	-1.0641934	0.26908447	0.60966448	0.63007569
A630033H20	0.41540813	-0.6315604	0.2690658	0.6096768	0.63007569
Ctnnbip1	0.09006379	3.99076024	0.26888493	0.60979621	0.63014473
Strada	-0.1281631	3.65046623	0.26878836	0.60985999	0.63014934
Iqcg	-0.1250813	2.95988689	0.26871882	0.60990592	0.63014934
Esf1	-0.0604946	7.18536144	0.26861903	0.60997185	0.63016309
Tmem38b	-0.124001	3.18982367	0.26845779	0.61007842	0.63021882
Dtna	0.07336035	7.13003161	0.26826127	0.61020834	0.63023737
Parpbp	-0.3586316	0.83810134	0.26815687	0.61027739	0.63023737
Zfp516	0.08100164	5.28253629	0.26797715	0.6103963	0.63023737
Hnrnpul1	-0.0690839	6.59340048	0.26794984	0.61041438	0.63023737
Usp15	0.07579999	6.41798032	0.26786215	0.61047242	0.63023737
Pfdn1	0.12783079	3.51727609	0.26784968	0.61048067	0.63023737
Cmc1	0.11599595	3.90903451	0.2677239	0.61056395	0.63023737
Sfpq	-0.0692334	7.54784806	0.26754483	0.61068254	0.63023737
Katnal2	0.46376156	-0.4732178	0.26740236	0.61077694	0.63023737
Bmp6	-0.1317278	6.6331763	0.2673359	0.61082099	0.63023737
D130020L05I	-0.1377343	2.63523436	0.26726161	0.61087023	0.63023737
Rpl35a	-0.1055432	5.46905397	0.26714835	0.61094531	0.63023737
Atp6v1g1	0.09048849	6.90417305	0.2670892	0.61098454	0.63023737
Mettl14	0.08474055	5.82265542	0.26696508	0.61106686	0.63023737
Fubp1	0.07463712	7.1259254	0.26674132	0.61121534	0.63023737
Ppm1m	0.12052897	3.80344664	0.2665595	0.61133604	0.63023737

B3galt6	0.17999999	2.33602443	0.26655772	0.61133722	0.63023737
Slc10a7	0.10245909	3.06877639	0.2665023	0.61137403	0.63023737
Pptc7	0.0588189	6.66629707	0.26648882	0.61138298	0.63023737
Mt2	0.11938177	5.17961798	0.2664687	0.61139634	0.63023737
Rnf7	0.08931064	7.98133013	0.26642792	0.61142343	0.63023737
Tango6	-0.1510879	2.27746456	0.2663853	0.61145174	0.63023737
Ggcx	0.11503104	3.1388994	0.266352	0.61147386	0.63023737
Sostdc1	0.33899939	0.73496236	0.26629383	0.61151251	0.63023737
Pam16	0.11470788	3.18915383	0.26625975	0.61153515	0.63023737
Itgb2	0.22523135	1.44270914	0.26610597	0.61163736	0.63023737
Rab3c	-0.0795833	9.29562799	0.26608501	0.61165129	0.63023737
Aifm1	0.11140602	4.10845366	0.26603974	0.61168139	0.63023737
Trappc6b	-0.0832302	7.79666568	0.26601035	0.61170092	0.63023737
Ifi27	0.11148371	4.907542	0.26598421	0.61171831	0.63023737
Boc	-0.1001181	3.53156235	0.26596995	0.61172779	0.63023737
Ctbp2	-0.0922996	4.21634869	0.26533582	0.61214977	0.63061787
B330016D10	-0.3487503	0.44110558	0.26499281	0.6123783	0.63077608
Lpcat1	0.15697086	2.86572616	0.26494722	0.61240869	0.63077608
1700003D09I	0.47761599	-0.5231101	0.26462978	0.61262037	0.63093368
Prss35	0.27040055	0.77546363	0.26455979	0.61266707	0.63093368
Rps6kc1	-0.077835	4.98005424	0.26428409	0.61285107	0.6310689
Tubb4a	0.08382508	10.3898448	0.26419687	0.61290931	0.63107461
Etfdh	-0.0884161	5.16765696	0.26399421	0.61304468	0.63114632
Otud5	0.070237	5.85476213	0.26377807	0.61318913	0.63114632
Rab18	-0.0715405	7.34877302	0.26376306	0.61319916	0.63114632
Pcp4l1	0.09539377	6.03176788	0.26372165	0.61322684	0.63114632
Kif1a	-0.0983698	9.884119	0.26369827	0.61324247	0.63114632
Cdc42ep5	-0.1849705	2.49276455	0.26341881	0.61342938	0.63125492
Evi2a	-0.1633708	2.7935894	0.26338289	0.61345341	0.63125492
Nfe2l1	0.06927566	7.82225763	0.26304108	0.61368221	0.63141248
Tpr	-0.064518	9.14637842	0.26299665	0.61371197	0.63141248
Mif4gd	0.13644371	3.18581163	0.26284962	0.61381046	0.63145956
Pigh	0.18776108	2.03473925	0.26263683	0.61395305	0.6315472
Apitd1	-0.4801816	-0.4734113	0.26256513	0.61400112	0.6315472
Gsg1	0.55331141	-0.8084375	0.26233637	0.61415453	0.63160067
Gon4l	-0.0860539	5.89696095	0.26233033	0.61415859	0.63160067
Pak4	-0.1667733	4.81414536	0.2621392	0.61428682	0.63161854
Brd4	0.06259086	8.73212684	0.26204585	0.61434948	0.63161854
Ttc28	-0.0782306	5.76228808	0.26194788	0.61441526	0.63161854
Gm4922	0.46409409	-1.3059537	0.26184457	0.61448463	0.63161854
Pwp2	0.17042333	2.67198387	0.26180309	0.61451249	0.63161854
Palb2	0.27638371	0.75081152	0.26172734	0.61456337	0.63161854
Rbpj	-0.0610694	6.51270234	0.26168797	0.61458982	0.63161854

Simc1	-0.0830113	4.18232201	0.26162416	0.6146327	0.63161854
Tmem229b	-0.1299565	3.13060807	0.26159745	0.61465064	0.63161854
Ccdc86	0.14713734	2.91362023	0.26118189	0.61493005	0.63169034
Fam131a	-0.0797342	5.2288787	0.26116302	0.61494274	0.63169034
Pcsk5	0.12136842	4.26563116	0.26113373	0.61496244	0.63169034
Foxp1	-0.0575245	8.77787499	0.26108122	0.61499777	0.63169034
Tmem2	0.0795567	4.21362306	0.26101684	0.61504109	0.63169034
Zfp784	0.12767436	3.96359625	0.26095296	0.61508408	0.63169034
Ppfia2	0.07783354	7.76153196	0.26094455	0.61508974	0.63169034
Emx2	-0.1254395	2.96208256	0.26081496	0.61517698	0.63171452
Mbd3	-0.0843227	4.4107566	0.26075287	0.61521879	0.63171452
Fam57a	-0.2012651	1.66580717	0.26043217	0.61543483	0.63179559
Pcdhb8	0.23679046	1.09888164	0.26041706	0.61544502	0.63179559
Gstk1	-0.1350841	3.26041485	0.26040074	0.61545601	0.63179559
Trip11	-0.0569489	7.25368334	0.2601528	0.61562316	0.63179603
Alpk1	0.15604735	2.5294278	0.26013261	0.61563678	0.63179603
D430019H16	0.0892148	6.30631517	0.26008769	0.61566708	0.63179603
Ccar1	0.06414381	8.06170419	0.26002607	0.61570864	0.63179603
Xab2	-0.0969575	3.85964232	0.2600089	0.61572022	0.63179603
Mrpl24	0.14632398	2.82661338	0.25985153	0.61582641	0.63185085
Tmem56	-0.0812787	6.25923531	0.25955904	0.61602387	0.63199162
Ccna2	0.16000727	1.98448009	0.259492	0.61606915	0.63199162
Pcdhga3	-0.1082476	3.43958644	0.25931701	0.61618738	0.63204588
Gm14204	-0.172067	2.45307692	0.25917945	0.61628035	0.63204588
Armc9	-0.1218217	3.36893747	0.2591794	0.61628038	0.63204588
Gm13582	-0.6973632	-1.3285864	0.25891753	0.61645746	0.63214048
Slc24a2	0.09055733	9.69693903	0.25875694	0.6165661	0.63214048
Pnck	0.08864647	3.71850949	0.25868491	0.61661485	0.63214048
Parp4	-0.0714758	5.70580261	0.2586647	0.61662853	0.63214048
Znhit2	0.12630071	3.10195257	0.25862853	0.61665301	0.63214048
L3mbtl4	0.3017427	0.00633733	0.25849636	0.61674249	0.63214048
Gal3st1	0.30537975	0.20452388	0.2584945	0.61674375	0.63214048
Mybpc3	0.7802338	-2.0274697	0.25841896	0.6167949	0.63214048
Tmem62	-0.1369187	3.23541605	0.25821943	0.61693007	0.63221073
Noc3l	0.08632324	4.93042067	0.25816192	0.61696903	0.63221073
Tcp10b	0.64619682	-1.9858622	0.25803276	0.61705658	0.63224634
Arfrp1	-0.1083338	3.99464723	0.25785443	0.61717749	0.63231613
Polr2m	-0.0700321	8.6440255	0.25770904	0.6172761	0.63236307
Ldhb	0.07133325	8.96055698	0.25751953	0.61740471	0.63244071
1810032O08	-0.3689244	-0.4284775	0.25733922	0.61752712	0.632512
Ust	0.13599129	5.5251111	0.25710717	0.61768473	0.63261336
Fam107b	-0.1212769	5.28728601	0.25703801	0.61773172	0.63261336
Ppp2r5c	-0.0658034	7.0535404	0.25681538	0.61788305	0.63270643

Ank2	0.08671606	10.1838387	0.25661201	0.61802136	0.63270643
Ppp1r3fos	-0.5576795	0.05384402	0.25660228	0.61802798	0.63270643
Zfp831	0.13406998	5.24283615	0.25641274	0.61815695	0.63270643
Slc22a4	0.11298052	3.02269968	0.2561892	0.61830913	0.63270643
Camk2b	0.07587965	8.06092179	0.25609475	0.61837346	0.63270643
Tmem107	-0.1670503	1.71680392	0.25594243	0.61847723	0.63270643
Mbnl1	0.07445334	9.06154523	0.25590396	0.61850344	0.63270643
Gpam	0.11940223	3.29092738	0.25586928	0.61852707	0.63270643
Psmd14	0.06016383	6.24716733	0.25586579	0.61852945	0.63270643
Ubald1	0.0878348	4.48217382	0.25584195	0.6185457	0.63270643
D330050I16F	-0.3298544	-0.4734021	0.25571457	0.61863253	0.63270643
2700081O15	-0.0696371	6.20960645	0.25541806	0.61883475	0.63270643
Acot10	-0.4800945	-0.5193226	0.25537396	0.61886484	0.63270643
Pts	-0.0877324	5.2535485	0.2553479	0.61888263	0.63270643
Plcl1	-0.0827427	5.43543837	0.25532571	0.61889777	0.63270643
Ap2m1	0.05929477	7.80336144	0.25525409	0.61894665	0.63270643
Psrc1	0.19566026	1.47407062	0.25514255	0.61902279	0.63270643
Celf3	0.08931391	5.51005772	0.25514139	0.61902358	0.63270643
Supt7l	0.07957122	5.59171236	0.25512442	0.61903517	0.63270643
Vhl	-0.0750661	4.8722494	0.25500927	0.6191138	0.63270643
Chrna3	0.29984832	0.04976638	0.25498072	0.6191333	0.63270643
Ppapdc3	-0.1589311	2.36618951	0.25487632	0.61920461	0.63270643
Fis1	0.09433773	3.99027107	0.25487373	0.61920638	0.63270643
Mcm8	0.10589505	3.21512786	0.25470496	0.61932171	0.63270643
Pcdh10	0.08268886	7.10637105	0.25469086	0.61933134	0.63270643
Taok1	-0.0538396	9.64326833	0.25467055	0.61934523	0.63270643
Zfp941	-0.0920844	4.53019868	0.25461168	0.61938547	0.63270643
Rpp38	0.1449971	2.56177142	0.25459192	0.61939898	0.63270643
Tmem208	-0.1553312	2.06204217	0.25457934	0.61940758	0.63270643
1500011K16I	0.12853224	3.942228	0.25433391	0.61957543	0.63282391
Ppp1r14b	0.14597162	3.52403494	0.25419103	0.61967319	0.63286979
Alkbh6	-0.1240191	3.12318257	0.2538463	0.61990921	0.63302509
Cml5	0.28726256	0.85791997	0.25378346	0.61995225	0.63302509
Eif1b	0.07031174	5.81897077	0.25372547	0.61999198	0.63302509
Rbm3os	-0.3721193	-0.0460533	0.25362828	0.62005858	0.63302509
Lyar	-0.0780631	4.22170673	0.25348208	0.62015879	0.63302509
Hsd17b7	-0.0748442	5.00823565	0.25343731	0.62018948	0.63302509
Nespas	0.66109336	-1.9246916	0.25342886	0.62019527	0.63302509
Dhx57	-0.1233984	5.85111194	0.25321646	0.62034095	0.63311982
Mrpl21	0.09044646	4.23155519	0.25308258	0.6204328	0.63312276
Pln	-0.3276407	0.61041831	0.25305815	0.62044957	0.63312276
6030443J06F	-0.1955881	1.76003475	0.25286022	0.62058544	0.63320746
Ccr5	0.24865673	1.19262425	0.25270566	0.62069159	0.63326181

Copa	-0.0732152	7.7765974	0.25251585	0.62082199	0.63330473
Rhobtb1	-0.1492574	2.61378833	0.25249049	0.62083942	0.63330473
Itih5	-0.0894778	6.2904773	0.25234239	0.62094122	0.63331906
Cbln4	0.09136749	4.04057016	0.25230632	0.62096602	0.63331906
Gm2011	-0.1995416	1.36350918	0.25223927	0.62101212	0.63331906
Frem2	0.17839922	1.81353757	0.25216059	0.62106624	0.63332032
H2-Ke2	0.12829573	3.77446252	0.25175276	0.6213469	0.63341009
Pik3r6	0.246393	1.61009317	0.25173811	0.62135698	0.63341009
Dut	-0.0956883	4.83592111	0.25172784	0.62136405	0.63341009
Stard6	-0.1500718	2.41474258	0.25172525	0.62136584	0.63341009
Ganc	0.09737631	4.43255697	0.25156727	0.62147464	0.63344851
Neurod6	0.08239613	6.14249421	0.25151693	0.62150932	0.63344851
Ndufb8	0.086663	4.70216124	0.25141159	0.6215819	0.63346642
Rasal2	-0.0819298	7.19857233	0.25128383	0.62166995	0.63346642
Slc52a3	0.20180684	1.79376373	0.25106999	0.6218174	0.63346642
Anapc16	0.11875994	5.99677279	0.25098592	0.62187539	0.63346642
Ifi27l2a	0.15143872	2.32156448	0.25096898	0.62188708	0.63346642
Cpb2	-0.7714164	-1.7620521	0.25096162	0.62189215	0.63346642
Oscp1	-0.1161693	3.51831819	0.25083678	0.6219783	0.63346642
Nol6	-0.0800247	5.48247362	0.25067602	0.62208926	0.63346642
Gfm2	0.15370781	3.58539059	0.25063238	0.62211938	0.63346642
Sacs	-0.2113337	3.7593415	0.25061023	0.62213468	0.63346642
Mettl17	0.18878885	1.69387304	0.25042256	0.6222643	0.63346642
Plin2	0.17669587	3.42219361	0.25042152	0.62226502	0.63346642
D10Wsu102e	-0.0692992	5.43301154	0.25030922	0.62234261	0.63346642
Bcl2l13	-0.0853924	5.02911328	0.2502742	0.62236681	0.63346642
Ldlrad3	-0.1273185	5.77201379	0.25026151	0.62237558	0.63346642
Sri	0.07164958	6.57914909	0.2501902	0.62242486	0.63346642
Prdx1	0.08778841	7.18344903	0.25014326	0.62245732	0.63346642
Gm14326	-0.112961	4.52824376	0.25007145	0.62250696	0.63346642
Ppp1r21	-0.0633878	5.54478482	0.25003536	0.62253191	0.63346642
Mir1191	-0.6808788	-1.5833267	0.24988852	0.62263348	0.63351594
Camsap1	0.07908707	6.94819669	0.24970318	0.62276172	0.63354245
Leng1	-0.1504481	4.27470386	0.24969796	0.62276533	0.63354245
Spag8	0.54050504	-1.5179381	0.24955893	0.62286156	0.63356858
Wnt2b	0.11969175	2.90172608	0.24947885	0.62291701	0.63356858
Stxbp4	0.07012658	7.33360469	0.24943161	0.62294973	0.63356858
Cd14	0.3519831	-0.0168069	0.24904479	0.62321775	0.63378735
Dlx1	0.13417353	3.88112312	0.24885585	0.62334876	0.63384034
Zbtb14	0.08267177	5.14679904	0.24862713	0.62350744	0.63384034
Mrps15	-0.0928389	4.23879358	0.24848937	0.62360305	0.63384034
4933407L21R	0.29264935	0.14756745	0.24836629	0.62368851	0.63384034
Tmem63b	0.0861031	5.59252783	0.24828762	0.62374315	0.63384034

4930545L23F	-0.4198069	0.66038367	0.24824973	0.62376947	0.63384034
Zfp623	0.11162193	3.4205748	0.24824406	0.6237734	0.63384034
Fam135a	0.07867047	5.76038231	0.24822858	0.62378415	0.63384034
Tekt2	0.51691785	-0.5607391	0.24820675	0.62379932	0.63384034
Unk	-0.1134703	3.96633081	0.24806017	0.62390117	0.63384034
Prr16	0.09771292	4.14023859	0.24791691	0.62400075	0.63384034
Zcchc24	0.10350508	7.2701703	0.24790421	0.62400957	0.63384034
Ets2	-0.0717237	5.6900743	0.24779096	0.62408833	0.63384034
Pak2	-0.0658795	6.79178155	0.24773409	0.62412787	0.63384034
B3gat2	-0.1802781	2.95666919	0.2476896	0.62415882	0.63384034
Rpap2	0.07338168	4.70556522	0.24768232	0.62416388	0.63384034
Hadh	0.09971801	4.85452117	0.24764626	0.62418897	0.63384034
1700016K19I	-0.2508602	1.06684404	0.24753398	0.6242671	0.63384034
Zmym4	-0.0664662	7.67947104	0.24744705	0.62432759	0.63384034
Rsrc1	-0.0646527	5.32700455	0.24744589	0.6243284	0.63384034
Satb1	0.08334513	7.71689747	0.24728268	0.62444203	0.6338516
Itga10	0.13150413	2.96327098	0.24727789	0.62444536	0.6338516
Prkab1	0.12456123	3.39809455	0.24678035	0.62479204	0.63414975
Prdm8	0.10036781	5.55077021	0.24655894	0.62494645	0.63425273
Dlx6	0.21997808	0.88559198	0.24647062	0.62500807	0.63426151
Ripk1	-0.1121023	3.73532796	0.24623324	0.62517376	0.6343759
Ascl1	0.12887479	3.37908203	0.24606437	0.62529169	0.63444181
Klf7	0.0688207	6.5193186	0.24589918	0.6254071	0.63450515
Hmgn5	0.0763496	7.16198763	0.24542234	0.62574051	0.63478963
2310035C23I	0.08384737	6.63927903	0.24506933	0.6259876	0.63498651
2810442I21R	0.86928731	-2.1376449	0.24457784	0.62633199	0.63518377
Gadd45b	-0.1248898	2.14979483	0.24455881	0.62634534	0.63518377
Gm15328	-0.4370724	-0.036985	0.24455443	0.62634841	0.63518377
2310039H08I	0.17732966	2.04685104	0.24444826	0.62642287	0.63518377
Dmtf1	0.06413191	6.44770029	0.24441347	0.62644727	0.63518377
Gdf11	-0.2381088	1.80607405	0.24430426	0.62652389	0.63519216
Notch2	0.10726813	7.07450311	0.24424212	0.62656749	0.63519216
Sec61b	0.10301366	4.79413797	0.24417491	0.62661466	0.63519216
2410021H03I	0.36556075	0.56287823	0.24405614	0.62669803	0.6352229
Slc39a9	-0.077037	4.89213791	0.24391148	0.62679962	0.6352721
Mgmt	0.31902957	-0.09654	0.24369644	0.62695069	0.63537145
E430025E21I	0.06395914	6.34156917	0.24360007	0.62701842	0.63538632
Ebag9	-0.0571484	5.98741643	0.24347498	0.62710637	0.63542167
Agbl3	-0.1126119	3.1280662	0.24323348	0.62727623	0.63547707
9430021M05	0.10904817	4.43883393	0.24320461	0.62729654	0.63547707
Tlk1	-0.0502354	7.60864649	0.24317094	0.62732024	0.63547707
Arc	-0.186241	5.37353445	0.2430601	0.62739824	0.63550234
Dnajc30	-0.0788849	4.96880301	0.24281913	0.62756791	0.63559443

Arl3	0.08997528	5.64605501	0.24278023	0.62759531	0.63559443
Prnp	0.07121713	8.00642486	0.24255071	0.62775704	0.63560223
Rbm22	0.07770026	4.93733979	0.24248264	0.62780501	0.63560223
Cdhr1	0.13105735	2.46622717	0.24240233	0.62786164	0.63560223
Pygo2	-0.092281	3.87133083	0.24236095	0.62789081	0.63560223
Fut9	0.09331669	7.1307687	0.24233823	0.62790684	0.63560223
Fntb	0.09809645	4.03066677	0.24229306	0.62793869	0.63560223
Poln	0.45921369	-1.1123698	0.2421803	0.62801823	0.63560223
Ttc34	-0.3561673	-0.1334546	0.24215485	0.62803619	0.63560223
Ciart	0.13226823	3.12972536	0.24209179	0.62808068	0.63560223
Cd36	0.42618692	-0.390192	0.24180696	0.62828175	0.63575198
2300009A05	-0.1496796	2.43271049	0.2414938	0.62850298	0.63590912
Mgat5	0.09233823	4.25700485	0.24136577	0.62859348	0.63590912
Inppl1	0.1040595	4.24130284	0.24129449	0.62864388	0.63590912
Josd2	-0.1652614	2.23009201	0.24128663	0.62864944	0.63590912
Rtcb	-0.0866132	4.1718067	0.24112691	0.6287624	0.63596967
Pcdhb12	-0.1609923	2.49227365	0.24089756	0.6289247	0.63608011
Riok3	-0.0659888	6.33207179	0.24051056	0.62919877	0.6363029
Adprm	-0.1262344	3.70174473	0.24032269	0.62933192	0.6363029
Aplf	0.08611927	4.27157919	0.24012686	0.62947077	0.6363029
Atp2b3	0.09595481	6.97569938	0.24008785	0.62949844	0.6363029
Zfp606	-0.0834206	4.59360883	0.24004706	0.62952738	0.6363029
Fbxo10	-0.1188289	4.18832971	0.23996294	0.62958706	0.6363029
Klhl3	0.150873	2.05259147	0.23994762	0.62959793	0.6363029
Vstm4	0.12985081	4.68068533	0.23988024	0.62964575	0.6363029
Zfp652os	0.35344355	-0.6744618	0.23984014	0.62967421	0.6363029
Naa25	-0.0878713	4.53536187	0.23983718	0.62967631	0.6363029
Ptpn14	-0.1107007	6.35989005	0.23969886	0.62977451	0.63634843
Abhd11	-0.1090973	3.15532683	0.23909797	0.6302015	0.63672616
Capn12	-0.4173587	-0.7080199	0.23896248	0.63029788	0.63676981
Mphosph9	-0.0993978	4.87189749	0.23878568	0.63042368	0.63678735
A330074K22	-0.2195878	1.28954745	0.23875213	0.63044756	0.63678735
Nln	0.08365719	4.43820929	0.23865095	0.63051959	0.63678735
Vangl2	0.15364757	1.96473114	0.2386099	0.63054882	0.63678735
Cdh12	0.09383791	6.34900079	0.23856456	0.63058111	0.63678735
Zcchc8	-0.0938706	3.8751708	0.238484	0.63063849	0.6367916
Gm15713	-0.4971971	-0.6500398	0.23836583	0.63072267	0.63680735
Ap3b2	-0.1061405	5.0760068	0.23831284	0.63076043	0.63680735
Stag3	0.8744736	-1.6816123	0.23820947	0.6308341	0.63682804
Pelp1	-0.1123814	3.28073169	0.23809778	0.63091373	0.63683035
Zfp467	-0.1164561	4.00171468	0.23805709	0.63094274	0.63683035
Sv2a	0.08023199	5.86958726	0.23768718	0.63120665	0.6370087
Ikzf3	0.14942917	2.779429	0.23764106	0.63123958	0.6370087

Mpp1	0.06118932	5.83676708	0.23758581	0.63127903	0.6370087
Plek	-0.0787415	4.63025579	0.23658323	0.63199579	0.63767824
Pcdha7	-0.3491243	-0.6336059	0.23635503	0.6321592	0.63777273
Elp3	-0.067773	5.3579593	0.23630373	0.63219595	0.63777273
Rsph3a	0.08186088	4.43966848	0.23596012	0.63244222	0.63791237
Cradd	0.11320705	4.87140784	0.235913	0.63247601	0.63791237
Cenpq	0.19402149	2.30652887	0.23588769	0.63249416	0.63791237
Commd9	0.15964653	2.15963691	0.23575054	0.63259253	0.63791845
Tex2	-0.0547036	6.54955136	0.23570812	0.63262298	0.63791845
Trappc5	0.1035208	4.15310119	0.23565653	0.63265999	0.63791845
Haus2	0.06722269	5.4932092	0.23532389	0.63289881	0.63804641
Rdh11	0.24699254	1.0106902	0.23528981	0.63292329	0.63804641
Ptpn22	0.1518983	2.35806896	0.23524972	0.63295209	0.63804641
Abcd4	0.16897324	1.46587845	0.23518301	0.63300001	0.63804641
Glrx3	-0.069479	5.19640005	0.23509193	0.63306546	0.63805867
Prorsd1	0.08332698	3.89631622	0.23477885	0.63329056	0.63823183
Uck2	0.10324619	3.46702241	0.23458933	0.63342691	0.63831553
Tmtc2	0.12552327	2.97770247	0.23439735	0.6335651	0.63840107
Srm	-0.099667	4.05505289	0.23418728	0.6337164	0.6384998
Cnnm2	0.11208751	3.86824534	0.23384208	0.6339652	0.63869674
Bcat1	0.07942157	5.79615957	0.23375341	0.63402914	0.63870743
Rab42	0.48398888	-1.7234385	0.23354035	0.63418286	0.63879349
Vmn2r86	-0.279733	0.46302815	0.23348716	0.63422125	0.63879349
Ctdp1	-0.0836621	3.88503483	0.2334001	0.63428409	0.63880306
Dhps	-0.1561863	3.07884382	0.23332307	0.63433971	0.63880534
Iqcb1	-0.0818496	4.50208082	0.233213	0.63441919	0.63883167
Cox16	0.0969002	4.60842813	0.2328887	0.63465353	0.63901391
Rep15	-0.5967908	-1.3864379	0.23277484	0.63473585	0.63904307
Wfikkn2	-0.2107975	2.97892266	0.23261064	0.63485462	0.63910891
Ammecr1	0.17463398	2.35776292	0.23242546	0.63498862	0.6391583
Setd7	0.06010394	8.66298225	0.23237353	0.63502621	0.6391583
Mdfic	0.14135776	5.61031651	0.23232162	0.63506379	0.6391583
Usp9x	-0.08712	9.85788988	0.23196904	0.63531918	0.63934274
Zfp595	-0.0878927	3.35783293	0.23192124	0.63535382	0.63934274
Vps25	0.09784891	5.12728745	0.23174862	0.63547896	0.63941493
Gnb4	-0.0783616	4.79186828	0.23149278	0.63566453	0.63954792
BC039771	-0.2050786	0.57098261	0.23124436	0.63584485	0.63964021
Ier2	-0.2059865	1.23049555	0.23121925	0.63586308	0.63964021
Edrf1	-0.107649	5.06486854	0.23095456	0.63605535	0.63977988
Marveld2	0.37469424	0.62572504	0.23080033	0.63616745	0.63983889
2810029C07I	0.22346399	2.23293347	0.23069321	0.63624533	0.63986348
Zkscan16	-0.105564	5.24616112	0.23011525	0.63666593	0.64023271
Zfml	-0.0708079	7.89140434	0.23002844	0.63672916	0.64023403

Ptprv	-0.4917443	-0.9318184	0.22996667	0.63677417	0.64023403
Msrb3	0.09479188	5.39694695	0.22968368	0.63698042	0.64038765
Dusp8	-0.0872736	5.57658294	0.22950384	0.63711159	0.64046575
Puf60	0.06461883	6.5038711	0.22942585	0.63716849	0.64046918
Hoga1	-0.3687373	-0.0458098	0.22930858	0.63725406	0.64050144
H2afy2	0.10208085	4.52595508	0.22921811	0.6373201	0.64050211
Rps17	-0.0774291	7.26328492	0.22914299	0.63737495	0.64050211
Nt5c2	-0.0639028	5.54623857	0.22898163	0.63749281	0.64050211
Zfp329	-0.0670508	6.15661559	0.228932	0.63752906	0.64050211
Faim2	0.06568395	6.72865198	0.22887167	0.63757315	0.64050211
Rpl15	-0.0765122	7.96298966	0.22886828	0.63757563	0.64050211
Usp37	-0.0849517	5.63517387	0.22871809	0.6376854	0.64055865
2810006K23l	0.08933606	4.99404715	0.228603	0.63776955	0.64058239
Epb4.1l2	0.06491279	7.5391637	0.22849454	0.63784888	0.64058239
Angptl6	-0.2986342	-0.1215775	0.22840886	0.63791156	0.64058239
Zscan20	-0.1395191	2.05490218	0.22839323	0.63792299	0.64058239
Pdk1	0.06215344	6.19472714	0.22827642	0.63800848	0.64061451
Gata2	-0.3360524	0.77249264	0.22815743	0.63809558	0.64062996
Actr3b	-0.0717068	4.85592865	0.22805259	0.63817235	0.64062996
Zfp939	-0.173241	2.6746555	0.22803622	0.63818434	0.64062996
Fcrl6	0.2815879	0.28566598	0.22763776	0.63847633	0.64086934
Atg10	0.10514346	4.0415969	0.22726182	0.63875211	0.64104872
Rfx5	-0.0743926	4.85278337	0.22718388	0.63880932	0.64104872
Tprkb	0.05893375	5.39818019	0.22717529	0.63881562	0.64104872
Efcab4a	-0.2283551	0.98823308	0.22704981	0.63890775	0.64108746
Rabep2	0.15605163	2.27559331	0.22687988	0.63903257	0.64111272
Gnal	0.08098437	8.43372102	0.22686977	0.63904	0.64111272
Gp1ba	0.43876788	0.3865583	0.22676917	0.63911393	0.64111456
Ippk	-0.0888426	3.8158183	0.22666552	0.63919011	0.64111456
Leng8	-0.1169755	6.48428617	0.22660028	0.63923807	0.64111456
Fam160b2	0.06554998	6.58432787	0.22657594	0.63925597	0.64111456
Pja1	0.05683077	6.81192113	0.22630897	0.63945235	0.64125781
Tceanc2	0.07128786	4.63240654	0.22607098	0.63962753	0.64137978
Scn8a	-0.1142227	8.83649402	0.22569833	0.63990206	0.64158032
Hnrnpu	-0.0600898	8.63388312	0.22562997	0.63995246	0.64158032
Unkl	0.08468078	5.40387752	0.22558141	0.63998825	0.64158032
Zfp821	-0.1013776	3.53873423	0.22530461	0.64019242	0.64173128
Tram1l1	-0.0877885	4.25816909	0.22508294	0.64035603	0.64181204
Chsy3	0.1223332	2.91210775	0.22505024	0.64038018	0.64181204
Dhx33	-0.0996553	4.90226004	0.22457088	0.64073436	0.64211329
Pvrl2	0.23433198	1.42364104	0.22438641	0.64087079	0.64219589
Casc3	-0.0779653	5.49386002	0.22428466	0.64094607	0.64219589
Snrnp48	0.07472317	4.90671398	0.22423484	0.64098293	0.64219589

Zfp275	0.07665322	5.65090821	0.22401401	0.64114641	0.64219589
Bad	0.19314334	2.66095758	0.22396165	0.64118518	0.64219589
Fam45a	0.07267384	4.9588035	0.22388988	0.64123834	0.64219589
Gm3414	0.1176749	3.33853117	0.22385608	0.64126338	0.64219589
Klre1	-0.5063463	-1.0160264	0.22368516	0.64139003	0.64219589
Itga4	-0.097237	4.8409161	0.22368106	0.64139307	0.64219589
Exosc8	-0.0833688	3.93105156	0.2236376	0.64142528	0.64219589
Chfr	0.13279451	4.18743872	0.2235502	0.64149007	0.64219589
Mmadhc	0.08579251	5.8456984	0.22353901	0.64149837	0.64219589
Babam1	-0.0782173	4.48843025	0.22344634	0.64156709	0.64219589
Fgf10	-0.2169469	2.46889666	0.22340705	0.64159623	0.64219589
B3gnt3	0.40703694	-0.0407022	0.22318022	0.64176453	0.64219589
Mtif3	0.10486545	3.36360623	0.22317758	0.64176649	0.64219589
Gnb5	-0.0710971	6.22872661	0.22310233	0.64182234	0.64219589
Zfp938	0.09466827	4.44416906	0.22304434	0.6418654	0.64219589
Zfp30	0.09685394	3.69679721	0.22302622	0.64187885	0.64219589
2810410L24F	-0.2773891	-0.0493362	0.22301216	0.64188929	0.64219589
Rabep1	-0.0544566	7.91370563	0.22278905	0.64205501	0.64230803
Orc1	-0.1985335	1.46819534	0.22259631	0.64219825	0.64232136
Apcdd1	-0.1402954	3.98764952	0.22255039	0.64223239	0.64232136
Exosc3	-0.0919202	4.95860279	0.22241455	0.64233341	0.64232136
Rpl10	-0.0970311	7.8633684	0.22236766	0.64236828	0.64232136
Vps53	0.05849124	6.21974496	0.22236083	0.64237337	0.64232136
Rbm12	-0.0658457	5.25852923	0.22233827	0.64239014	0.64232136
Cited1	-0.2019429	1.57350636	0.22219727	0.64249506	0.64237263
Lmo2	-0.1000018	4.39602433	0.22191664	0.64270399	0.64252788
Rngtt	0.0698258	5.27968148	0.22156054	0.64296934	0.6427395
Mrps10	-0.1109399	2.80224304	0.22138673	0.64309895	0.64281541
Mboat2	0.12445619	4.15070648	0.22082918	0.64351514	0.64317773
Gm16982	-0.3602414	0.00829823	0.22069345	0.64361656	0.64322542
Gm5801	0.29415126	-0.3960546	0.22054653	0.64372638	0.64328149
Rgs9bp	-0.4661953	-0.2906914	0.22035053	0.64387296	0.6433313
Thoc6	-0.2095101	1.21006605	0.22033621	0.64388367	0.6433313
Isoc1	-0.0795817	5.90672511	0.220083	0.64407316	0.64335416
Sbsn	-0.1688921	1.79275705	0.2200548	0.64409426	0.64335416
Agbl5	-0.1211235	2.76543721	0.21984151	0.644254	0.64335416
Amica1	-0.1977949	1.3615346	0.21983575	0.64425831	0.64335416
Atf7	-0.1047097	4.26147139	0.21982439	0.64426682	0.64335416
Gm15417	0.19806485	1.17185303	0.21972175	0.64434372	0.64335416
Gm6277	0.15311997	2.28629519	0.21968809	0.64436895	0.64335416
Evc	0.13275605	2.46743737	0.21960558	0.64443079	0.64335416
Dpp6	0.08901885	6.35339394	0.21960545	0.64443089	0.64335416
Atad3aos	-0.3208308	0.04760216	0.21958828	0.64444376	0.64335416

Ttc7b	0.08114739	8.19378473	0.21950527	0.64450599	0.64336265
Ptov1	0.12066787	5.14555831	0.21939253	0.64459054	0.64339342
Arhgef26	0.09468478	4.33599906	0.2191628	0.64476291	0.64351183
Egfr	-0.0930001	5.96327822	0.21901652	0.64487271	0.64354509
Gnl2	-0.0618737	5.97776156	0.21897525	0.64490371	0.64354509
Slc10a3	0.15524392	2.47682987	0.21877944	0.64505078	0.64363822
Wbp1	-0.1137463	3.50318168	0.21859762	0.64518743	0.64366225
Gm11346	-0.4817641	-0.647839	0.21852416	0.64524265	0.64366225
Trip6	-0.1339162	3.17019165	0.21839708	0.64533822	0.64366225
Desi2	-0.0636284	6.81310931	0.21833274	0.64538661	0.64366225
Magee1	-0.0872817	6.91827672	0.2183029	0.64540906	0.64366225
Arpc1b	0.14095487	6.03862312	0.21822867	0.64546492	0.64366225
Lemd2	-0.1560442	2.11383672	0.21807729	0.64557886	0.64366225
Col4a4	0.47087199	-0.8139241	0.21807607	0.64557977	0.64366225
Snrpd3	0.14229139	2.63607777	0.21798097	0.64565137	0.64366225
Iba57	-0.2020696	0.816488	0.21795701	0.64566942	0.64366225
Gm13629	0.16764683	1.52851678	0.21788993	0.64571994	0.64366225
Slmap	-0.0562156	8.96692926	0.21783252	0.64576319	0.64366225
Reck	0.10155886	5.35084657	0.21781304	0.64577786	0.64366225
Tial1	-0.0628186	5.9119415	0.21774739	0.64582732	0.64366225
Itsn2	0.05679161	7.26278222	0.21765504	0.64589692	0.64367805
Git1	-0.0642117	6.87270412	0.21755171	0.64597482	0.64370211
Slc16a2	0.06875605	5.39027333	0.21730439	0.64616134	0.64378772
2810004N23	-0.1258739	4.55543889	0.21729523	0.64616825	0.64378772
Tdrd3	-0.0633084	5.51620388	0.21718535	0.64625117	0.64381677
Mir325	-0.5049462	-1.4886438	0.2169737	0.64641095	0.64387349
Bnip2	-0.0796182	7.06219684	0.21692229	0.64644978	0.64387349
Lmnb2	0.11370125	3.01885766	0.21677774	0.64655898	0.64387349
3110082J24F	0.39653888	-0.6274529	0.21677461	0.64656134	0.64387349
Rad54l	0.23070081	0.81036565	0.21672385	0.64659969	0.64387349
Lrrc73	-0.1474611	2.43181403	0.21668284	0.64663069	0.64387349
Inpp5j	-0.1154837	3.41493072	0.2165592	0.64672415	0.64391302
Myef2	0.07828648	5.63887936	0.21626557	0.64694624	0.64395066
Pla2r1	0.28075801	-0.1361902	0.21624968	0.64695827	0.64395066
Cox14	-0.0714872	5.326743	0.2162389	0.64696642	0.64395066
Gpatch3	0.35100985	-0.187732	0.21622486	0.64697705	0.64395066
Rab3a	0.05245453	6.72958893	0.216106	0.64706701	0.64398669
Ddt	-0.1209741	3.74680858	0.21585492	0.64725715	0.64407896
Chd9	-0.072614	7.98540391	0.21579738	0.64730075	0.64407896
Rapgefl1	0.06493552	6.31641623	0.21572019	0.64735923	0.64407896
Fstl4	0.09818443	3.03605396	0.21569958	0.64737485	0.64407896
Rhbdd1	0.13527652	3.65204601	0.21555354	0.64748557	0.64413559
Pitpnm1	-0.1192832	3.35286185	0.21523358	0.64772827	0.64421948

Rnf24	0.06677552	5.58235448	0.21521391	0.64774319	0.64421948
Rfk	-0.1074583	9.48783152	0.21498475	0.64791717	0.64421948
F630042J09F	-0.4683415	-0.9915842	0.21494328	0.64794867	0.64421948
Otud1	-0.0808254	6.12233648	0.2148856	0.64799248	0.64421948
Fam179b	-0.0594342	6.53940217	0.2148602	0.64801178	0.64421948
Elf1	0.0801233	5.89231624	0.21482704	0.64803697	0.64421948
Polr3h	-0.0988846	2.96034667	0.21480516	0.6480536	0.64421948
Serpina1d	0.53145341	-1.5769784	0.21477696	0.64807502	0.64421948
Ccdc36	0.52767577	-1.4751331	0.21468876	0.64814205	0.64421948
Adam21	-0.2577842	0.29743149	0.21466301	0.64816162	0.64421948
Ehbp1	0.06379291	6.77901696	0.21452918	0.64826337	0.6442563
Traf3ip1	0.06483609	4.88522269	0.214426	0.64834184	0.6442563
St6galnac2	-0.1817847	1.27839149	0.21440206	0.64836005	0.6442563
D3Ertd751e	0.07285074	5.72744736	0.21424196	0.64848187	0.64428579
Bet1l	-0.1403543	3.14726464	0.21415736	0.64854626	0.64428579
Tnr	-0.1132961	5.08287813	0.21413169	0.6485658	0.64428579
Brd3	0.06077403	6.20578828	0.21404438	0.64863228	0.64428579
Nfkbiz	-0.1621893	2.15685519	0.21399884	0.64866696	0.64428579
Pibf1	-0.1016848	4.56124547	0.21389145	0.64874875	0.64428579
Naa16	-0.0979841	4.04612996	0.21386837	0.64876634	0.64428579
Ccdc105	0.4613999	-0.7318402	0.21363377	0.64894513	0.6443945
Ccdc152	-0.1458722	1.90916393	0.21358355	0.64898342	0.6443945
Flrt2	0.05705245	7.45037819	0.21335657	0.64915654	0.64451297
Wdr78	-0.144513	3.27238946	0.212856	0.64953874	0.64483897
Hspb3	-0.2557231	0.34003333	0.21277082	0.64960383	0.6448406
Ppp2r5d	0.0785324	5.52700385	0.21271293	0.64964807	0.6448406
Pnma2	0.06743496	6.40633816	0.21259107	0.64974124	0.64485265
Il21r	-0.4595093	-0.8992308	0.21255619	0.64976791	0.64485265
Gldc	0.12118796	2.46772824	0.21225306	0.64999982	0.6449607
Pim3	0.11701853	2.92316168	0.21207226	0.65013824	0.6449607
AW209491	0.07791164	4.72485102	0.21200166	0.65019231	0.6449607
Prkrip1	0.10890591	3.70442885	0.21186837	0.65029442	0.6449607
Kat6b	-0.0525205	7.17037169	0.21186536	0.65029673	0.6449607
Zbtb45	0.13006348	2.41541769	0.2118487	0.65030949	0.6449607
Mthfd2l	-0.1705953	2.19025458	0.21177751	0.65036405	0.6449607
Entpd5	-0.0804991	4.33180174	0.21177635	0.65036494	0.6449607
Itga5	0.11381205	2.85314034	0.21175814	0.6503789	0.6449607
Fam98a	0.06368679	5.72445129	0.21169121	0.6504302	0.6449607
Mex3a	0.12848786	2.55651255	0.21159564	0.65050348	0.6449607
Btk	-0.4235982	0.14467041	0.21157011	0.65052305	0.6449607
Mast2	0.05715632	6.29563596	0.21143256	0.65062857	0.64497714
Epsti1	-0.2288876	1.15801116	0.21132413	0.65071177	0.64497714
Sgce	-0.0820837	4.38630861	0.21131246	0.65072073	0.64497714

Zfp850	-0.1309965	2.65478176	0.21123388	0.65078104	0.64497714
Cbln3	-0.1954888	1.1983502	0.21119757	0.65080891	0.64497714
Cd27	0.53935327	-1.5497778	0.21089674	0.65103997	0.64515273
Rnpc3	-0.1143954	4.82241866	0.21070167	0.65118989	0.64524791
Ercc3	-0.081692	4.2118526	0.21024012	0.65154499	0.64539556
3110056K07I	-0.118136	2.46016357	0.2101968	0.65157834	0.64539556
Luzp1	0.05507549	8.22082905	0.21001984	0.65171462	0.64539556
Lrrc1	0.10297872	4.36593242	0.21001137	0.65172115	0.64539556
Sar1a	0.08027741	7.05937469	0.2099934	0.65173499	0.64539556
Emc10	0.08885644	5.07381744	0.20995235	0.65176662	0.64539556
Rap1gap	-0.1001906	4.12132355	0.20991916	0.65179219	0.64539556
Aqp6	-0.5283998	-1.3654759	0.20989518	0.65181067	0.64539556
AI662270	0.20867368	0.58541783	0.20987088	0.6518294	0.64539556
Prokr1	-0.5253715	-0.1820214	0.20973781	0.65193197	0.64539556
Dpy19l1	-0.0562474	6.7235325	0.20959964	0.65203852	0.64539556
Rnf128	-0.1754157	1.67447049	0.20940959	0.65218514	0.64539556
Ccl9	0.15125899	1.82352796	0.20929831	0.65227103	0.64539556
Sec24c	-0.061454	6.32172526	0.20926168	0.6522993	0.64539556
Nr3c2	0.06820275	5.95236095	0.20916694	0.65237246	0.64539556
Cct8l1	-0.7566126	-1.1835794	0.20912231	0.65240693	0.64539556
C030023E24I	-0.1914975	2.42704436	0.20903381	0.65247529	0.64539556
Uba1y	0.41480997	-1.1650433	0.20896304	0.65252997	0.64539556
Zfand4	-0.206839	1.73462481	0.208931	0.65255473	0.64539556
Plekhg5	0.08031287	4.25616956	0.20890781	0.65257265	0.64539556
Lrrc26	0.50798381	-1.5248642	0.20877583	0.65267467	0.64539556
Olfml3	0.11034268	4.9645008	0.20861049	0.65280253	0.64539556
Mcm7	0.12098819	2.98666999	0.20854869	0.65285034	0.64539556
Urod	-0.0655942	5.0701814	0.2084394	0.6529349	0.64539556
Psd4	-0.31087	0.02147733	0.20839455	0.65296961	0.64539556
Tmem167	-0.0612767	6.42597284	0.20831926	0.65302789	0.64539556
Kcnk6	0.19538433	1.35946233	0.20824356	0.6530865	0.64539556
Knop1	-0.07301	5.63143563	0.20817945	0.65313615	0.64539556
Stx5a	0.08680388	4.58266496	0.20811428	0.65318663	0.64539556
Col8a1	0.11055833	5.5011309	0.20808236	0.65321136	0.64539556
1700007K13I	-0.2858697	0.10950649	0.2079265	0.65333213	0.64539556
Arhgap19	-0.1094617	3.4055371	0.20786562	0.65337932	0.64539556
Sae1	0.06558909	5.57085668	0.2077811	0.65344485	0.64539556
Galnt2	-0.0891425	3.84837964	0.20776623	0.65345637	0.64539556
Vps37b	0.10434186	3.12214722	0.20771241	0.65349811	0.64539556
Prmt2	0.09460039	4.78853725	0.20769872	0.65350873	0.64539556
Trpc4	0.10349301	3.39938902	0.2075759	0.653604	0.64539556
Lonp1	-0.0774675	4.27239562	0.20742258	0.65372298	0.64539556
Rfxank	0.12996887	2.94693999	0.2074135	0.65373003	0.64539556

Usf1	-0.1332086	2.76170238	0.20737218	0.65376211	0.64539556
Prpsap2	0.08094057	4.20561193	0.20733405	0.65379171	0.64539556
Wscd1	-0.0933871	3.74854037	0.20731509	0.65380643	0.64539556
Kcnj14	-0.5552448	-1.0669554	0.207276	0.65383678	0.64539556
Rab8b	-0.0605264	6.34753689	0.20727004	0.65384141	0.64539556
Lrrtm1	0.08828868	4.64472738	0.20725475	0.65385328	0.64539556
Fbxo18	-0.0589278	5.50909359	0.20724377	0.65386181	0.64539556
Myocd	0.70580617	-1.5673105	0.20723087	0.65387183	0.64539556
Myom1	-0.3073428	0.4210617	0.20709626	0.65397639	0.64544557
2700060E02I	-0.0618344	5.83392768	0.20697774	0.65406849	0.64548327
Polh	0.11963594	2.48231141	0.20687675	0.65414699	0.64550453
Clec2f	-0.3689348	-0.7657779	0.20679047	0.65421408	0.64550453
Max	0.06709679	8.08866805	0.20674205	0.65425174	0.64550453
Ipo4	-0.0799532	4.14921411	0.2064433	0.65448419	0.64568068
Slc17a6	-0.0765063	4.81099216	0.20621826	0.65465942	0.64580036
Gpsm2	0.13558371	2.5646379	0.20600489	0.65482568	0.64581475
Lnpep	0.05046485	6.88376445	0.20597235	0.65485105	0.64581475
Gm4788	-0.2385105	0.00703406	0.2059696	0.65485319	0.64581475
Sec24d	0.0884631	4.40749598	0.20592274	0.65488972	0.64581475
Mdp1	0.0784175	4.40685737	0.20567438	0.65508342	0.64587624
Faim	-0.0762984	5.76111039	0.2056139	0.65513061	0.64587624
Srgap2	-0.0566523	6.07483362	0.20560391	0.65513841	0.64587624
Dnaaf2	0.13096531	3.59941112	0.20556624	0.65516781	0.64587624
Tubb3	-0.0791766	4.68233189	0.2050781	0.65554904	0.64617973
4921511C10I	-0.7680353	-1.6615581	0.20503393	0.65558357	0.64617973
Thap11	-0.0682043	4.58550204	0.20493819	0.65565842	0.64619835
Reps1	-0.0608515	6.00264591	0.20480704	0.65576099	0.64619835
Slc27a4	-0.1003521	3.48703901	0.20478321	0.65577962	0.64619835
Evi2a-evi2b	-0.3855005	-1.3671055	0.20471366	0.65583404	0.64619835
Lamb2	0.13577877	4.32199266	0.20466481	0.65587226	0.64619835
P2rx1	0.6584976	-2.3316039	0.20429559	0.65616135	0.6464119
Prmt6	-0.1059527	3.52929591	0.20425013	0.65619696	0.6464119
Nkx2-2	0.22935085	0.88378594	0.20406325	0.65634342	0.64650299
Snf8	0.09784703	4.26851453	0.20381869	0.65653521	0.64663872
Lrrc32	0.10892637	4.80677595	0.20372587	0.65660804	0.64665727
Adamts2	-0.1207635	3.97568395	0.20355132	0.65674504	0.64673901
Ccne1	0.15315103	1.77384286	0.20344103	0.65683165	0.64677111
Trp53i11	0.11431774	8.13256173	0.20332354	0.65692394	0.64680881
Lrrtm2	-0.0738345	6.66013688	0.20313453	0.65707247	0.64689123
Samd1	0.13454662	2.79565482	0.20303474	0.65715093	0.64689123
Efcab5	-0.1708481	2.66556156	0.20301087	0.6571697	0.64689123
Znhit3	0.0979933	3.91894958	0.20284268	0.657302	0.64696828
Uchl4	-0.3373508	-0.6099704	0.20256735	0.65751871	0.64707475

Gm8580	-0.2647852	-0.6333046	0.20253334	0.65754549	0.64707475
Bloc1s2	0.1099347	3.29573558	0.20241745	0.65763678	0.64707475
Pdss1	-0.1501031	2.13099445	0.20236476	0.65767828	0.64707475
Atxn7l3b	0.0555512	7.34105172	0.20236216	0.65768033	0.64707475
Wdr5b	-0.2021807	1.16772343	0.20225951	0.65776122	0.64708267
Hypk	-0.0903688	6.55731281	0.20212393	0.65786811	0.64708267
Gas6	-0.0814316	4.3991346	0.20209368	0.65789196	0.64708267
Thbs2	0.11605586	5.06590599	0.20195574	0.65800075	0.64708267
Amn	0.30330878	-0.2705762	0.2019535	0.65800252	0.64708267
Elmod3	-0.1379709	3.29131426	0.20194076	0.65801257	0.64708267
Tfdp1	0.06595576	6.37155299	0.2016238	0.65826274	0.64727553
Il2rb	0.26244806	0.95630689	0.20143331	0.6584132	0.64730532
Ccdc53	-0.0910815	3.93266763	0.20142058	0.65842326	0.64730532
Lsmem1	0.49054017	-1.4809773	0.20138017	0.6584552	0.64730532
Pof1b	-0.267811	0.09221184	0.20124701	0.65856044	0.64735565
4931414P19I	0.27542204	1.11634917	0.2010405	0.65872375	0.64746303
Trmt11	-0.1539125	1.67032125	0.20094265	0.65880117	0.64748598
Uox	-0.3418295	-0.8076337	0.20082871	0.65889134	0.64748936
Ncl	-0.0518954	8.89214441	0.20080167	0.65891274	0.64748936
Zc3h3	0.13641629	2.62834499	0.20052049	0.65913542	0.6476334
Mib2	-0.1016659	3.61288825	0.20048004	0.65916748	0.6476334
Xaf1	-0.1044595	3.44495522	0.20040901	0.65922376	0.64763556
Nova2	0.07729654	7.26096089	0.20033383	0.65928335	0.64764098
Mcemp1	0.33576156	0.11998169	0.20017694	0.65940775	0.64771005
2010320M18	-0.1857851	1.75692754	0.19983556	0.65967864	0.64792299
Homer3	-0.1183117	1.81476363	0.19952328	0.65992668	0.64809565
Tac1	0.10483869	4.18556843	0.19947051	0.65996862	0.64809565
Gpr27	0.4271049	-1.2313857	0.19924048	0.66015151	0.64809565
Zfp787	-0.1865638	1.59554729	0.19921997	0.66016782	0.64809565
Fam169b	-0.1593133	1.94048087	0.1991471	0.66022579	0.64809565
Hsd17b14	-0.2155534	0.86079391	0.19914291	0.66022913	0.64809565
Wwtr1	0.07705422	6.61329783	0.19911715	0.66024962	0.64809565
4930451C15I	0.27848581	0.31137663	0.19906971	0.66028737	0.64809565
1110032A03I	-0.0707357	6.65671181	0.19897344	0.66036399	0.64811773
Mast1	-0.0877771	4.88121181	0.19855435	0.66069779	0.6483728
Nsun4	-0.0938097	2.77881446	0.19851124	0.66073215	0.6483728
Pde4dip	-0.0797151	7.48586352	0.19838058	0.66083632	0.64842189
Fndc5	-0.0655441	5.00331875	0.19811447	0.66104861	0.6485628
Zhx2	0.08275942	6.38843228	0.19800333	0.66113733	0.6485628
Fyn	0.05297037	6.00425844	0.19798493	0.66115202	0.6485628
Taf1b	0.06832518	4.16744644	0.1978724	0.66124187	0.6485628
Ppig	-0.0678591	9.2691446	0.19786133	0.66125072	0.6485628
Nelfb	0.0765826	5.02264515	0.19778056	0.66131524	0.64857297

Slc31a2	-0.122606	5.11246442	0.19749807	0.66154101	0.64874126
Xpo5	-0.0798929	4.68001948	0.19719507	0.6617834	0.64886238
Rqcd1	0.07704113	4.95419579	0.19714624	0.66182248	0.64886238
Tmem151b	0.11273784	4.64442946	0.19713437	0.66183198	0.64886238
Zfp493	0.16496234	2.60744916	0.19703952	0.66190791	0.64886238
Ciz1	0.06497434	5.70076852	0.19694177	0.66198619	0.64886238
4930412C18I	0.23754127	1.24851585	0.19693751	0.66198961	0.64886238
Tmem183a	0.06292719	5.63463001	0.19648342	0.66235356	0.6491471
Mad2l1	0.10937094	4.07790964	0.19639306	0.66242604	0.6491471
Fam69c	-0.2673795	0.11835775	0.19637229	0.6624427	0.6491471
D3Bwg0562e	0.07160991	7.1409987	0.19614562	0.66262463	0.64927225
Rpusd3	-0.2341456	0.91871578	0.1960166	0.66272824	0.64932064
Tomm40	0.10063313	3.08970157	0.19593677	0.66279237	0.64933036
Alox5	-0.4633722	-1.3258855	0.19578322	0.66291576	0.64939811
Tmem229a	0.07024931	6.08972277	0.19553998	0.66311135	0.64945617
Retsat	-0.153962	2.56790834	0.19550924	0.66313608	0.64945617
Fam181b	-0.1326399	2.12299327	0.19547346	0.66316487	0.64945617
E130309D14I	-0.0870414	4.98591316	0.19543979	0.66319195	0.64945617
Gm5860	0.12576979	2.07446429	0.19524114	0.66335184	0.64955963
Bhmt2	0.62253896	-0.5181588	0.19509743	0.66346758	0.64961984
Gm14169	0.14269959	1.90935333	0.19485645	0.66366175	0.64975684
Dscaml1	0.13560073	3.82701036	0.19473748	0.66375767	0.64979763
Zc3h14	-0.0556684	6.41769269	0.19456792	0.66389444	0.64987839
Clec2i	-0.4803833	-1.1682639	0.19442891	0.66400661	0.64988388
Rad17	0.0846376	4.17621479	0.19442648	0.66400858	0.64988388
Flii	-0.0660543	5.38782624	0.19414988	0.66423193	0.65004936
Crot	0.06316531	5.34776257	0.19382796	0.66449212	0.65025085
Ikbke	0.2417084	0.5804378	0.19358446	0.6646891	0.6503171
Robo1	0.07310474	7.25073871	0.19346916	0.66478243	0.6503171
Arhgap24	-0.1014519	3.94176612	0.19341257	0.66482824	0.6503171
Ankrd40	-0.048842	7.06114299	0.19328923	0.66492813	0.6503171
Kbtbd4	-0.0729663	4.48817103	0.1932811	0.66493472	0.6503171
Elp4	0.06921261	4.2881464	0.19321791	0.6649859	0.6503171
Zcchc14	-0.0582731	6.32427807	0.19312996	0.66505717	0.6503171
Prox2	-0.1799082	1.61354989	0.1930875	0.66509158	0.6503171
Higd1a	0.05890691	6.64605834	0.1930767	0.66510034	0.6503171
Ndufb7	0.11316863	4.23432189	0.1930736	0.66510285	0.6503171
Pdgfc	-0.1245469	2.48770178	0.19287158	0.66526665	0.65042415
C1qc	-0.2094048	2.26937786	0.19280142	0.66532356	0.6504267
Ddx28	-0.1099965	2.5404257	0.19265514	0.66544225	0.65048963
Ttll11	-0.0992695	4.20607825	0.19258247	0.66550124	0.65049419
Ift43	-0.1385019	3.32495798	0.19240392	0.66564623	0.65056984
Serhl	0.33602585	-0.4036211	0.19231402	0.66571926	0.65056984

Hist1h2bg	0.22974068	0.15143316	0.19225251	0.66576923	0.65056984
Taf9	0.0615543	5.52362682	0.19220032	0.66581165	0.65056984
Surf6	-0.0855609	3.73763035	0.19214371	0.66585766	0.65056984
Fez2	0.06877286	5.48917661	0.19202212	0.66595652	0.65056984
Eif5a2	-0.0659043	6.5726775	0.19186644	0.66608315	0.65056984
Snrpg	0.0874516	5.054598	0.19186483	0.66608446	0.65056984
5730480H06I	0.38105464	0.11818548	0.19178896	0.6661462	0.65056984
Rapgef6	-0.0500518	6.7995315	0.19161178	0.66629043	0.65056984
Khnyn	0.10128433	2.936284	0.19153662	0.66635163	0.65056984
Gprc5c	-0.1325507	3.07802592	0.19152094	0.6663644	0.65056984
Fam169a	-0.0936602	5.76125406	0.19151762	0.66636711	0.65056984
Htr1a	-0.094506	3.55770126	0.19150596	0.66637661	0.65056984
Rps15a-ps4	-0.1594421	1.32191386	0.19148523	0.66639349	0.65056984
Mcoln1	-0.1108405	2.92515908	0.19137843	0.6664805	0.65060175
Fam126a	-0.0746676	4.10421944	0.19130073	0.66654382	0.65061053
Dixdc1	0.0450154	6.91295512	0.19099553	0.6667927	0.65075298
Amt	-0.172458	1.03484184	0.19098849	0.66679844	0.65075298
Arid2	0.05111486	6.70487632	0.19087045	0.66689476	0.65079394
Rpl14	-0.0971104	6.55012026	0.1907937	0.66695741	0.65080205
Hcn4	0.1620727	1.30114906	0.19069013	0.66704197	0.65083153
Tpt1	0.08327801	9.33039457	0.19059118	0.66712278	0.6508345
Ect2	0.43183421	-0.849832	0.19049529	0.66720112	0.6508345
Cdk12	0.05269545	7.26615932	0.19048682	0.66720805	0.6508345
Tmco4	0.1722707	2.26886504	0.19034622	0.66732296	0.65089357
Uqcc1	0.09147369	3.87512325	0.19025594	0.66739677	0.65091255
Rmnd5b	0.05987692	4.74085418	0.18996283	0.66763657	0.6510934
S100a13	0.1731509	3.41422825	0.18988148	0.66770316	0.65110532
Pnpla8	0.05347349	6.88366748	0.18980332	0.66776715	0.6511081
Pnpt1	0.10868693	3.92011407	0.18961312	0.66792296	0.6511081
Zbtb7a	-0.0809269	6.03367033	0.18960851	0.66792673	0.6511081
Rbmx	-0.0541116	6.76613628	0.18944655	0.66805948	0.6511081
Ept1	0.05793164	4.98612583	0.18941251	0.66808738	0.6511081
Rnf165	0.07702953	6.31500496	0.18940445	0.668094	0.6511081
Bcl10	-0.1106053	4.85800539	0.18923894	0.66822973	0.6511081
Clcn2	-0.087373	3.90367884	0.18917968	0.66827836	0.6511081
Dgcr2	0.06109907	4.64335063	0.18913158	0.66831782	0.6511081
Pfkp	0.08257323	7.31963642	0.18906442	0.66837294	0.6511081
Dnah2	0.17398479	1.78466754	0.18900933	0.66841816	0.6511081
Arhgap26	-0.0692892	7.57515888	0.18895701	0.66846112	0.6511081
Ubxn8	0.09318004	4.33700244	0.18892531	0.66848715	0.6511081
Ntan1	0.07608058	5.48359563	0.18890892	0.66850061	0.6511081
Sumo1	-0.0727944	7.58817477	0.18888343	0.66852155	0.6511081
Mab21l1	-0.1938461	1.21648583	0.18858885	0.66876358	0.65129087

Slc6a11	0.11100515	4.54546055	0.18832778	0.66897828	0.65136139
Lias	-0.0688956	4.59285949	0.18831797	0.66898636	0.65136139
Dbnl	0.07285791	5.18181118	0.18830239	0.66899917	0.65136139
Rps20	-0.078106	6.34851348	0.1879489	0.66929019	0.65159176
Wdr47	0.08335933	6.50006207	0.18771234	0.66948512	0.65172856
Lcmt2	0.12166286	2.52533972	0.18749326	0.66966578	0.65176962
Sec16a	-0.0602746	6.43811088	0.18748223	0.66967488	0.65176962
Thrb	0.05938952	7.02512197	0.18746321	0.66969057	0.65176962
Tnfrsf12a	0.29784104	-0.8392961	0.18725887	0.66985921	0.65178292
1700052K11I	-0.1458458	2.85164241	0.18724737	0.6698687	0.65178292
Recql	-0.0814574	3.96390845	0.18720493	0.66990374	0.65178292
Zfp956	-0.1688077	2.29736313	0.18718288	0.66992194	0.65178292
Sphkap	-0.1050156	7.2290455	0.18687687	0.67017475	0.6517937
Ascc1	0.12240297	3.49080484	0.18671354	0.67030978	0.6517937
Fam188a	0.06559969	6.11642959	0.18660684	0.67039803	0.6517937
Il16	0.18173207	1.13578219	0.18653518	0.67045732	0.6517937
Etnppl	-0.143085	2.95248482	0.18651186	0.67047662	0.6517937
Brd7	-0.053603	5.86924557	0.18646544	0.67051504	0.6517937
Orc5	0.09946242	3.3294651	0.18645552	0.67052324	0.6517937
Slc22a15	-0.1090251	2.70741686	0.18642445	0.67054896	0.6517937
Rnf43	-0.1263801	3.23811628	0.18635913	0.67060303	0.6517937
Mmaa	-0.1135165	3.75448196	0.18620542	0.67073032	0.6517937
Hdgfrp2	0.07924598	5.05959439	0.18618186	0.67074984	0.6517937
Pcdhga11	0.13419542	3.13527731	0.18618169	0.67074998	0.6517937
Fhit	-0.2522262	1.2402851	0.18616577	0.67076317	0.6517937
Ankfn1	-0.1687818	2.48226336	0.18611373	0.67080628	0.6517937
Fhod1	-0.1390843	2.36053804	0.18607667	0.67083699	0.6517937
Capn9	-0.6018999	-2.3766796	0.18705754	0.67084229	0.6517937
Anxa2	0.12212883	6.18191341	0.1859438	0.67094712	0.6517937
Trhr	0.17536453	1.50417131	0.18592471	0.67096295	0.6517937
Cyp4x1	0.18129887	0.99608208	0.18591967	0.67096712	0.6517937
Asxl3	-0.1423804	4.1941141	0.18572299	0.67113023	0.65189927
Dpy19l3	0.07424013	5.02698243	0.18559879	0.67123329	0.65194649
C2cd4b	-0.2582861	-0.0762654	0.18552301	0.67129619	0.65195471
Tardbp	0.04998719	7.45746874	0.18536651	0.67142614	0.65198087
Far1	0.05899822	6.28201656	0.18534347	0.67144528	0.65198087
Mthfd2	-0.1927734	1.46340259	0.18529391	0.67148645	0.65198087
Zfp69	-0.2052383	0.82551149	0.18518194	0.67157948	0.65201833
Cr1l	-0.0782919	5.40021269	0.18503817	0.67169899	0.65207676
Apba3	0.1254827	2.53870786	0.18493227	0.67178705	0.65207676
Aqp11	-0.133979	1.77804299	0.18487001	0.67183884	0.65207676
Bc1	0.76030563	-1.8486001	0.18484762	0.67185746	0.65207676
Mlh3	-0.0595922	5.77549953	0.18471009	0.67197191	0.65213499

Plekhg3	0.08537926	3.33748503	0.18460543	0.67205903	0.65216669
Sox30	0.42375652	-1.0289941	0.18424934	0.67235568	0.65224102
Bdh1	-0.0882571	3.63196056	0.18419177	0.67240367	0.65224102
Snrpc	0.11342544	3.22601681	0.18418223	0.67241162	0.65224102
Cln5	0.10040414	6.00046903	0.18415204	0.6724368	0.65224102
Ndufb3	0.07888058	5.49258129	0.18410529	0.67247577	0.65224102
Ggnbp1	-0.5083663	-0.8377932	0.18407796	0.67249857	0.65224102
Rrp15	0.11380285	2.82371295	0.18405601	0.67251687	0.65224102
Psg16	-0.1897182	1.38269931	0.18376496	0.67275975	0.65239781
Gm12216	-0.3624204	-0.754129	0.18373173	0.67278749	0.65239781
Aggf1	-0.0568436	6.57041674	0.18360377	0.67289436	0.65244861
Vamp2	0.05143118	10.2441072	0.18339946	0.67306507	0.6525508
Fbxo43	-0.2634923	-0.5212013	0.18334722	0.67310873	0.6525508
Smpd4	0.07724253	5.12357101	0.1831592	0.67326597	0.6526504
Cd2	-0.4420587	-0.4858184	0.18295169	0.67343961	0.65276589
Wnk2	0.08522927	6.04766164	0.18270836	0.67364337	0.65278227
Arv1	-0.2070432	0.87145989	0.18268359	0.67366412	0.65278227
Polr3f	-0.0672353	4.75486577	0.18263898	0.6737015	0.65278227
Dnttip1	0.12082101	2.98877728	0.18260351	0.67373122	0.65278227
Nme2	-0.087131	6.80607066	0.18257735	0.67375315	0.65278227
Prss12	0.12316363	1.85980198	0.18254106	0.67378356	0.65278227
Ube3b	-0.0648007	6.09056482	0.18211748	0.67413884	0.65307364
Zfc3h1	-0.0840545	6.97548533	0.18191715	0.67430704	0.65314802
Slc4a8	-0.0908013	6.84351407	0.1818693	0.67434723	0.65314802
Npm1	-0.0501143	9.66262949	0.18176679	0.67443337	0.65314802
Ccdc22	-0.2093904	0.91836404	0.18173682	0.67445855	0.65314802
Tm4sf1	-0.088354	4.66416052	0.1817014	0.67448832	0.65314802
4930432K21I	0.24786821	-0.3257549	0.18162795	0.67455006	0.65315499
Psd3	0.06635741	9.82749616	0.18144864	0.67470085	0.65318882
Krit1	-0.0746752	5.84090534	0.18138564	0.67475385	0.65318882
Vkorc1l1	-0.0465199	6.22933451	0.18136409	0.67477197	0.65318882
Cnga2	0.57581779	-2.1500629	0.18228521	0.67480317	0.65318882
Cklf	-0.1934556	0.98049407	0.18119906	0.67491088	0.65324028
Zkscan5	-0.1253031	3.89448709	0.18110418	0.67499076	0.65326481
Pml	0.06447977	4.59802592	0.18099699	0.67508105	0.65329337
Gm16938	-0.1425944	2.17302732	0.18088665	0.67517401	0.65329337
Ifrd1	-0.0695166	5.97236501	0.18087489	0.67518393	0.65329337
Sdsl	0.4332821	-0.6387176	0.18071174	0.67532146	0.65337365
Pomt2	-0.0825077	3.59827126	0.18040723	0.67557836	0.6535694
Pin1	0.09866828	3.03710293	0.18013234	0.67581048	0.65374115
Gm13242	-0.3645205	-0.9866561	0.1799965	0.67592527	0.65377166
Hr	0.14463374	3.0286007	0.17996582	0.67595121	0.65377166
Calb2	0.15796109	1.07864787	0.1797828	0.67610596	0.6538071

Fastk	-0.0871622	3.68472314	0.17975693	0.67612784	0.6538071
Prss53	-0.5787845	-1.4968797	0.1797288	0.67615163	0.6538071
Kctd20	0.11587392	3.14629532	0.17957415	0.67628249	0.65388084
Mx1	0.38802208	0.01692365	0.17916571	0.67662843	0.65416251
Tmem252	-0.2327572	-0.111035	0.17909117	0.67669161	0.65417078
Dao	-0.7299668	-1.5808403	0.17893915	0.67682052	0.65424258
Nrip1	-0.0648579	6.16891455	0.17878005	0.67695549	0.65432024
Pdia5	-0.2922168	0.10203084	0.17870738	0.67701717	0.65432704
Eps15	-0.0519321	8.62271066	0.17858659	0.67711972	0.65437335
Jsrp1	0.35955895	-0.75672	0.17828776	0.6773736	0.65456588
Chrm5	0.18246348	1.61380987	0.17818995	0.67745675	0.65459341
1700049G17	-0.0971758	3.46919692	0.17803131	0.67759168	0.65462327
Npr2	0.0725426	4.07112397	0.17802507	0.67759698	0.65462327
Wdr62	0.22521773	1.01093731	0.17784884	0.67774695	0.65466209
Etf1	0.05195469	6.47593528	0.17778849	0.67779833	0.65466209
Rpl19	0.07623447	7.38560878	0.17778517	0.67780116	0.65466209
Sgip1	-0.0673759	8.10724872	0.17750589	0.67803906	0.65472353
Hook2	0.17891436	1.55443116	0.17743185	0.67810217	0.65472353
Slc25a25	0.07073434	5.0207656	0.17741836	0.67811367	0.65472353
Kif16b	-0.0556377	4.96535644	0.17733184	0.67818743	0.65472353
Pmm1	0.07118225	4.66168759	0.17729623	0.6782178	0.65472353
Aasdhppt	-0.0595341	5.24523466	0.17727902	0.67823247	0.65472353
Pmf1	-0.1584607	3.35773183	0.17726144	0.67824747	0.65472353
Ar	-0.0876382	4.30558559	0.17706975	0.67841101	0.65482862
Acot3	-0.3257277	0.33841072	0.17694873	0.67851432	0.65487556
Rps28	-0.090974	5.54608439	0.17629441	0.6790736	0.65525256
Polr3gl	0.09677012	2.88161822	0.17626217	0.67910118	0.65525256
Zcchc7	-0.0689147	5.64380246	0.17622292	0.67913477	0.65525256
Zscan12	0.07398497	4.26561548	0.17620389	0.67915106	0.65525256
9530036O11	-0.2686825	-0.2627716	0.17611279	0.67922905	0.65525256
Exosc9	-0.0722035	4.0254313	0.17610791	0.67923323	0.65525256
Psmc4	0.07366998	4.92455703	0.17598202	0.67934104	0.65530378
Neu2	-0.2012896	0.65232574	0.17570027	0.6795825	0.65538033
Ecsit	-0.0651696	3.81187568	0.17568424	0.67959624	0.65538033
Ston1	-0.1225257	4.79793078	0.17565136	0.67962444	0.65538033
Hs1bp3	-0.0768232	4.68705527	0.17560978	0.6796601	0.65538033
Hexb	-0.0692306	4.74106074	0.17552976	0.67972874	0.65538033
Alpl	0.14234067	3.38132943	0.17547339	0.6797771	0.65538033
Rnaset2b	0.10301408	4.08648288	0.17544265	0.67980348	0.65538033
Wdr89	0.15214047	1.09939631	0.17513605	0.68006672	0.65558134
Adam10	0.05824357	5.75542415	0.17469847	0.68044288	0.65586515
Pbrm1	0.04573901	7.48916556	0.17466618	0.68047067	0.65586515
Gm609	0.48052954	-1.8422175	0.17450274	0.68061133	0.65594793

Nle1	-0.2511269	-0.0880185	0.17415448	0.68091131	0.65608914
Rhbdd3	-0.2364085	-0.0468391	0.17414856	0.68091641	0.65608914
Atp2a2	0.06046836	9.64115715	0.17414184	0.6809222	0.65608914
Gm12992	0.15720843	1.37021767	0.17407019	0.68098396	0.65609587
Hyal3	0.21748214	0.5348794	0.17382647	0.68119417	0.65624181
Smc2	-0.0782348	4.51070417	0.17372855	0.68127868	0.65624181
Gpr153	0.11435655	3.00274264	0.17361862	0.68137358	0.65624181
Rbbp4	0.05517184	5.94783195	0.17361436	0.68137725	0.65624181
Rcc1	0.15718062	1.73423824	0.1735771	0.68140943	0.65624181
2310061I04R	-0.0789528	4.20453282	0.17331266	0.6816379	0.65628665
Rbck1	0.07866922	4.11843858	0.17328919	0.68165819	0.65628665
Idi2	-0.2913322	-0.0722269	0.17328192	0.68166447	0.65628665
Anapc7	0.09077927	3.5193806	0.17326951	0.6816752	0.65628665
Zbtb10	-0.0564172	5.46634052	0.17313291	0.68179331	0.65632319
Tfdp2	-0.0464744	6.23340646	0.17309886	0.68182276	0.65632319
Nanp	-0.0974022	4.00038521	0.17300533	0.68190368	0.65633711
Fut11	-0.0871308	3.90062084	0.17293549	0.68196412	0.65633711
Ccdc167	0.0554266	5.38308184	0.17289213	0.68200164	0.65633711
Gm3258	0.28794064	-0.8824314	0.17279302	0.68208745	0.65636694
Taf5	-0.1028476	3.93774759	0.17258674	0.68226613	0.65636786
Ehd1	-0.1022556	3.5760169	0.17253247	0.68231316	0.65636786
Efs	0.13277087	2.17687059	0.17249631	0.6823445	0.65636786
Phgdh	-0.1318599	2.53464317	0.17248696	0.6823526	0.65636786
Efhc2	-0.1870672	2.12178194	0.17229746	0.68251691	0.65636786
Ckb	-0.0623593	7.92797394	0.1722705	0.68254029	0.65636786
Gm6251	-0.1935942	0.42859106	0.1721949	0.68260588	0.65636786
Ankrd37	-0.1655206	1.64700432	0.17215412	0.68264127	0.65636786
Zfp1	-0.0661515	4.28516805	0.17204337	0.68273739	0.65636786
Wbp11	0.05073783	6.05145539	0.17195413	0.68281487	0.65636786
Ndufa5	0.07542013	5.61427803	0.17193882	0.68282817	0.65636786
Plcb2	-0.1707597	1.39988177	0.17193852	0.68282843	0.65636786
Slc52a2	0.24302355	0.70323119	0.17191574	0.68284821	0.65636786
Chchd2	0.06105928	7.6487621	0.1719016	0.68286049	0.65636786
Tceal1	0.06734417	5.42049778	0.171844	0.68291053	0.65636786
Plrg1	0.06609424	4.51438789	0.17146195	0.68324263	0.65656122
Gli3	-0.0590346	5.12944884	0.17141053	0.68328736	0.65656122
Aim1	-0.1154831	3.96012799	0.17137779	0.68331585	0.65656122
Prrc2b	-0.0528899	8.7073686	0.17132102	0.68336525	0.65656122
Zc3h6	-0.0748737	4.89306762	0.17129737	0.68338583	0.65656122
Kdm3a	0.05668039	5.9982817	0.17123293	0.68344192	0.65656244
AI854703	-0.1183024	3.07551616	0.17116659	0.68349967	0.65656525
Kcnc1	0.07265108	6.98292561	0.17103776	0.68361188	0.65659482
Abhd2	0.05733276	6.17205916	0.17098901	0.68365435	0.65659482

Cyp39a1	-0.1139829	3.46184472	0.17094243	0.68369493	0.65659482
Ccdc94	0.14863621	1.59061397	0.17086348	0.68376374	0.65660825
Chchd4	0.07921507	4.78100268	0.17063923	0.68395928	0.65670174
Tmeff1	-0.0744774	5.11783559	0.17062605	0.68397078	0.65670174
Fnbp4	0.06292177	5.76659596	0.170522	0.68406156	0.65673625
Aco2	0.05095341	7.41179208	0.17039988	0.68416815	0.65674464
Ppp6r2	-0.0775788	4.59010303	0.17038357	0.68418239	0.65674464
Ppp2r2d	-0.0681975	4.71076883	0.17032353	0.68423481	0.65674464
Trf	-0.0913438	5.62323471	0.1701996	0.68434306	0.65678364
Gm14440	-0.0644074	5.28812743	0.17012238	0.68441053	0.65678364
Zfp608	0.06832135	5.16319009	0.1700887	0.68443997	0.65678364
Sult2b1	-0.2257774	0.36922458	0.16997245	0.68454158	0.65682851
Abi2	0.04973277	8.54721792	0.16946474	0.68498588	0.65711563
2810008D09I	-0.2122197	0.70757999	0.16945434	0.68499499	0.65711563
Leo1	-0.0524535	6.10134011	0.16944242	0.68500543	0.65711563
Tlr7	0.15760162	1.59115268	0.16932199	0.68511094	0.65715387
Polr2d	-0.0983886	3.84926725	0.16919406	0.68522307	0.65715387
Ankrd17	0.05567932	8.9309109	0.16912817	0.68528085	0.65715387
Dync2li1	-0.0937639	3.59751117	0.16911381	0.68529344	0.65715387
Ccdc127	-0.0477902	6.5235289	0.16900736	0.6853868	0.65715387
Nfe2l2	-0.0664906	7.84925387	0.16891966	0.68546375	0.65715387
Slc10a1	0.4773315	-1.138606	0.16887842	0.68549994	0.65715387
H3f3a	0.0936367	8.1858611	0.16884945	0.68552537	0.65715387
4933426M11	-0.0750931	5.71371529	0.16883374	0.68553916	0.65715387
Glmn	-0.1122664	3.94395078	0.16873676	0.6856243	0.65716092
Tmem80	-0.1022886	2.82179066	0.16870036	0.68565626	0.65716092
Coprs	-0.0995585	3.63211372	0.16863374	0.68571478	0.65716441
Rnf8	-0.0837792	4.25082204	0.16850028	0.68583203	0.65722419
Mob2	0.12992487	2.48552716	0.16829108	0.68601594	0.65723264
Mars2	-0.1636048	2.29555454	0.16818577	0.68610858	0.65723264
Tab1	0.11551863	2.84188839	0.16818356	0.68611052	0.65723264
Adck1	0.08950915	3.05850564	0.16817411	0.68611883	0.65723264
4932441J04F	0.34932455	-0.8893644	0.1681183	0.68616794	0.65723264
Ccdc120	-0.1270492	2.46031896	0.16811581	0.68617013	0.65723264
Rccd1	-0.1533957	1.48306808	0.16793932	0.68632549	0.65732661
Pja2	0.05914309	8.93374676	0.16783837	0.6864144	0.65732661
Tmem51os1	0.30931575	-0.5113356	0.16779986	0.68644832	0.65732661
Heph	-0.213832	2.0407408	0.16771143	0.68652623	0.65732661
Slc37a4	-0.1210058	1.97965061	0.16769277	0.68654268	0.65732661
Trim63	-0.1959303	1.2032021	0.16754903	0.68666939	0.65738746
Rprd2	-0.0573085	7.26401555	0.16739487	0.68680535	0.65738746
Slc1a3	0.07135066	7.23936215	0.1673764	0.68682165	0.65738746
C030034I22R	-0.1484307	1.65412909	0.16737168	0.68682581	0.65738746

Tlr5	0.39540155	-1.2240123	0.1672181	0.68696135	0.65746464
Chst8	0.21451301	0.65193605	0.16709883	0.68706666	0.6575116
Fan1	0.13731395	1.65864258	0.16703819	0.68712022	0.6575116
Tprn	0.15522664	2.34731961	0.16681694	0.68731573	0.65763908
Eif3l	0.05923505	5.5720538	0.16671394	0.6874068	0.65763908
Ndufa6	-0.0622695	5.13130613	0.16663603	0.6874757	0.65763908
Mfsd7c	0.21164417	1.40256348	0.16658589	0.68752006	0.65763908
Mtfp1	0.08427164	4.76527358	0.16657691	0.68752801	0.65763908
Uqcc2	0.10159298	3.50855036	0.16642602	0.68766154	0.65771427
Fam155a	0.06107402	6.69012916	0.16625	0.68781741	0.65781081
Gm10658	-0.3129705	-0.5900214	0.16604713	0.68799716	0.65793018
Cox15	0.06509204	5.47204878	0.16589299	0.68813383	0.65795456
Gm14288	0.15240516	0.84512697	0.16587178	0.68815264	0.65795456
Sox17	-0.1929705	1.86351752	0.16581595	0.68820217	0.65795456
Sec24a	-0.0578595	5.49204325	0.16577059	0.68824242	0.65795456
Gm16386	-0.135612	2.00126101	0.16549377	0.68848814	0.65813694
Prep	0.0557556	4.39806506	0.16525694	0.68869858	0.65820972
Pcdha9	0.36204919	0.22596979	0.16518857	0.68875936	0.65820972
Rpf1	0.06986645	4.10670821	0.16515449	0.68878966	0.65820972
1700024P16I	0.26496406	0.72847317	0.16513095	0.68881059	0.65820972
Mir1b	0.74215393	-1.1700818	0.16504617	0.68888599	0.65820972
Mapkapk2	-0.087161	4.27647673	0.16503711	0.68889406	0.65820972
Ninj2	-0.3692092	-0.9280136	0.16486848	0.68904411	0.65822246
Adam28	-0.249685	0.2058532	0.16483769	0.68907152	0.65822246
Nhlrc2	-0.0789982	3.76158255	0.16483181	0.68907676	0.65822246
Synj1	-0.0918563	9.53680777	0.1647751	0.68912725	0.65822246
Mcl1	0.0588334	8.67175862	0.16468114	0.68921092	0.65824012
4921511I17R	-0.6472585	-2.4337037	0.16457712	0.68930358	0.65824012
Dmxl1	-0.0790278	7.30485153	0.16456922	0.68931063	0.65824012
Pla2g2d	0.29784242	-0.6830274	0.16418353	0.68965453	0.65849012
Fxr1	0.05010993	6.88681768	0.16399538	0.68982248	0.65849012
Rgs4	-0.0539922	9.56276024	0.16398999	0.68982729	0.65849012
Rassf8	0.12726308	3.3170677	0.16394681	0.68986585	0.65849012
Morc3	-0.0902352	4.5466549	0.16391101	0.68989783	0.65849012
Hist1h2ai	0.33575635	-1.5684036	0.16390051	0.6899072	0.65849012
Reln	0.09967775	5.25469557	0.16372811	0.69006124	0.65849012
Slc44a2	-0.0675349	5.08209519	0.16372042	0.69006811	0.65849012
Nudcd1	-0.0794823	3.65886042	0.16371182	0.6900758	0.65849012
Rpl26	-0.3110157	-1.291403	0.16364098	0.69013911	0.65849012
Flcn	0.05867501	4.56344802	0.16359494	0.69018028	0.65849012
Isoc2b	0.19952213	0.67709782	0.16353683	0.69023225	0.65849012
Rpl7	-0.0710019	6.91340087	0.16337258	0.69037919	0.65851185
Bhmt	0.5130365	-1.2367822	0.16330754	0.6904374	0.65851185

Lactb2	0.09164996	3.85021558	0.16329417	0.69044936	0.65851185
Tdrp	0.074789	5.87769403	0.16318013	0.69055147	0.65851185
5430417L22F	-0.0482321	6.02537351	0.16316715	0.69056309	0.65851185
Serf2	0.09083288	6.41739383	0.16314274	0.69058495	0.65851185
Ppfia1	0.04438496	6.96011551	0.16305339	0.69066498	0.65853573
Dnajc5b	0.37847835	-0.5513479	0.16293979	0.69076679	0.658542
Dpysl5	0.10235556	4.75965098	0.16279736	0.69089447	0.658542
Scarf2	0.22425634	1.58556916	0.16268469	0.69099553	0.658542
Efhd1	-0.0773411	4.60422175	0.16257172	0.69109689	0.658542
4930578E11I	-0.2789156	-0.6153435	0.1625599	0.6911075	0.658542
S1pr5	0.14282487	2.05751533	0.16255642	0.69111062	0.658542
Plekha8	-0.0811829	4.32360688	0.16248956	0.69117063	0.658542
Utp11l	-0.0865309	4.11218496	0.16245537	0.69120133	0.658542
Zfp346	0.06283835	4.78813054	0.16243428	0.69122027	0.658542
Nynrin	0.06559724	5.67264852	0.16243296	0.69122145	0.658542
Rxfp1	0.18367022	2.35363465	0.16226956	0.69136821	0.65859582
Cd6	-0.3331056	-0.0356959	0.16224761	0.69138794	0.65859582
Scyl1	-0.0637637	4.19231876	0.16202357	0.69158932	0.65873525
Ptpn18	-0.468828	-1.6842723	0.16194961	0.69165583	0.65874621
Cdc42	-0.0467928	8.71371659	0.16172329	0.69185948	0.65884634
1810030O07	0.05858223	6.11730603	0.16167183	0.69190581	0.65884634
Slc1a2	0.07456572	10.9655463	0.1616494	0.69192601	0.65884634
Zfyve20	-0.0612085	5.76894164	0.16153468	0.69202933	0.65889233
Thap1	0.09964893	3.74336405	0.1613714	0.69217646	0.65898003
Cd80	-0.1809301	1.40087992	0.16121735	0.69231534	0.65905986
Timm8a1	-0.0759174	5.39448299	0.16109889	0.69242221	0.65910919
Rfx2	0.11545664	2.14871108	0.16085619	0.69264128	0.65925853
Smad2	0.04503016	6.52510052	0.16079137	0.69269982	0.65925853
Srrm4	0.07833981	6.13290984	0.1607422	0.69274423	0.65925853
Agtr1a	0.55721882	-1.2617721	0.16066069	0.69281787	0.65927621
Ccdc150	0.57533138	-1.6023388	0.16046015	0.69299917	0.65935579
Aff1	0.06045102	7.01573603	0.16044639	0.69301161	0.65935579
Chia1	-0.3620954	-0.1819364	0.16017672	0.69325562	0.65944929
Rab5a	-0.0502354	5.49876439	0.16014846	0.6932812	0.65944929
Dlx2	0.15704874	0.88539876	0.16008914	0.69333491	0.65944929
Fermt3	0.25741277	0.28907828	0.16007349	0.69334909	0.65944929
Gm6225	0.37903713	-1.1707404	0.1600336	0.69338521	0.65944929
Mageb16-ps	0.36680201	-0.6955082	0.15980467	0.69359265	0.65952269
Nap1l1	0.04378718	8.68607673	0.15978224	0.69361298	0.65952269
Tdrd5	0.21299095	1.22862972	0.15976611	0.6936276	0.65952269
Ap3s2	0.05218808	6.62475984	0.15961292	0.69376653	0.65956115
Pank1	-0.0528467	6.29469563	0.1595348	0.6938374	0.65956115
Ccdc39	-0.0939256	4.3535668	0.15943613	0.69392695	0.65956115

Adc	-0.1465636	1.78714451	0.15942917	0.69393327	0.65956115
Usp53	-0.0525276	6.92540021	0.15941798	0.69394343	0.65956115
Rgag4	-0.1165683	2.68256884	0.15930262	0.69404817	0.65958457
1190007I07R	0.13467489	2.18462635	0.15925493	0.69409148	0.65958457
Nwd1	-0.0736649	5.14178275	0.15912843	0.69420642	0.65958457
C1s1	-0.2402316	0.94045248	0.15903776	0.69428882	0.65958457
Stmn1	-0.0527216	8.45410907	0.15896363	0.69435622	0.65958457
Neurod2	0.05596713	5.81515841	0.15894438	0.69437373	0.65958457
Atp1a4	0.39233698	-0.6699236	0.24546392	0.69440349	0.65958457
Tbcc	0.08263598	3.28109105	0.15888064	0.69443169	0.65958457
Dnajb12	0.06657871	3.90776884	0.15882677	0.6944807	0.65958457
Nod1	0.13144774	2.07213642	0.15876317	0.69453856	0.65958457
Pank3	0.04934784	7.493322	0.15872432	0.69457392	0.65958457
Snx7	-0.0945121	4.97341229	0.1586012	0.69468599	0.6596387
Rtn2	0.1273157	2.61842012	0.15850729	0.69477151	0.6596676
Ccdc9	0.091222	2.73401385	0.15836532	0.69490086	0.65968945
Gm16897	-0.1980668	0.57425721	0.15833692	0.69492674	0.65968945
Bphl	-0.0949866	4.07017953	0.15830066	0.69495979	0.65968945
Zfp511	0.13026176	2.57961167	0.15814105	0.69510531	0.6597753
Gna11	0.06754138	5.92150648	0.15803802	0.6951993	0.65981221
Klhl32	0.15510525	0.85298259	0.15781432	0.69540349	0.65990853
Gm13034	0.35841308	-1.6862613	0.15779937	0.69541714	0.65990853
Samd9l	0.08339636	6.92484062	0.15774575	0.6954661	0.65990853
Add1	0.05189672	7.6835705	0.15765661	0.69554754	0.65993352
Map3k12	-0.0710044	5.62385011	0.15743854	0.69574686	0.66005837
Gstz1	-0.0599607	4.65541876	0.15735218	0.69582585	0.66005837
Sncb	0.07507269	4.66624454	0.15733181	0.69584448	0.66005837
Tes	-0.0804519	3.71867944	0.15710836	0.69604897	0.66011369
Il1rn	-0.45149	-1.1083452	0.15700159	0.69614674	0.66011369
Bin1	-0.0642886	5.36717275	0.15698594	0.69616107	0.66011369
Snapc4	-0.1264384	3.72206953	0.15689772	0.69624189	0.66011369
Srsf9	-0.0793935	4.93587337	0.1568315	0.69630257	0.66011369
Klhl7	0.05425982	6.31339067	0.15680561	0.6963263	0.66011369
Bysl	-0.0797948	3.83428023	0.15679621	0.69633491	0.66011369
Agk	0.06912028	4.41723715	0.15670317	0.69642021	0.66011369
Ndrg1	-0.0831424	7.57194374	0.15661763	0.69649865	0.66011369
Acadm	0.08011711	5.06558011	0.15655968	0.69655181	0.66011369
Cotl1	0.08580647	3.14023198	0.15652298	0.69658548	0.66011369
Rell2	-0.1474149	1.68782175	0.15651295	0.69659468	0.66011369
Ptplb	-0.0641923	5.00768767	0.15646518	0.69663851	0.66011369
Tubg2	0.07733561	4.31544614	0.15642598	0.69667449	0.66011369
Jam3	-0.111175	2.89043088	0.15627257	0.69681535	0.66019492
Gm6548	0.10946331	3.51256874	0.1561475	0.69693023	0.66025153

Pecr	0.1569162	1.25200424	0.15599752	0.69706807	0.66029803
Poli	-0.0754687	3.37439859	0.15597129	0.69709218	0.66029803
9030617O03	-0.0991482	2.99011174	0.15585245	0.69720147	0.66029803
Ddx23	0.05794472	5.43142983	0.15580373	0.69724628	0.66029803
Sntg2	-0.2056191	0.90102064	0.15574023	0.69730471	0.66029803
Mrpl53	-0.0847467	3.73890533	0.15563246	0.6974039	0.66029803
Nfya	0.05290624	5.71546218	0.15557626	0.69745564	0.66029803
Ndufs2	0.06085184	6.6411601	0.15556637	0.69746475	0.66029803
Ppap2c	0.15388651	1.53986906	0.1554476	0.69757414	0.66029803
Sntb2	-0.058255	5.67243718	0.15541925	0.69760025	0.66029803
Nup43	-0.1220652	2.47967835	0.15538999	0.69762721	0.66029803
Marveld3	-0.5266289	-0.9875168	0.15534361	0.69766995	0.66029803
Txnrd2	-0.1792207	1.2978111	0.15528925	0.69772005	0.66029803
Oas2	-0.2468842	0.13255888	0.1551792	0.69782151	0.66029803
Kif5b	-0.0487254	8.37481273	0.15517734	0.69782322	0.66029803
Zfpm2	0.10837223	3.7385624	0.15513584	0.6978615	0.66029803
Tsn	0.06090305	6.2732336	0.15505383	0.69793715	0.66031744
St5	-0.0888213	4.99487358	0.15499219	0.69799401	0.66031907
Aup1	-0.116131	3.08808871	0.15492233	0.69805849	0.6603279
Cldn2	-0.2424934	1.2896757	0.15481271	0.69815969	0.66036428
Rsph3b	-0.0708341	4.40296319	0.15476124	0.69820722	0.66036428
Adh1	-0.2456972	0.18930139	0.15463875	0.69832038	0.66041915
Ovgp1	-0.2397744	0.34036295	0.15438278	0.69855701	0.66055285
Rasgrp2	0.09427372	2.56032541	0.15436649	0.69857207	0.66055285
Sptssa	-0.0734767	6.40935421	0.15412813	0.69879264	0.66069943
Oacyl	-0.3116116	0.29856542	0.15406536	0.69885076	0.66069943
Nf2	-0.0542884	6.09246586	0.15387941	0.69902301	0.66069943
Cisd3	-0.1061151	2.82960951	0.15385423	0.69904634	0.66069943
Prrg2	-0.2678384	-0.1605994	0.15383199	0.69906695	0.66069943
Nedd9	-0.0592982	4.36676939	0.15379821	0.69909826	0.66069943
Zfp422	0.05971329	6.39820939	0.15378201	0.69911328	0.66069943
Cubn	-0.0914431	5.30480592	0.15358778	0.69929339	0.6608175
Gnl3l	0.04769162	8.04128811	0.15342242	0.69944684	0.66087635
Ptgfrn	0.05561528	5.24899347	0.15332567	0.69953666	0.66087635
Zscan22	0.06121332	5.38400301	0.15325617	0.69960121	0.66087635
Evpl	-0.2012419	1.2386368	0.15320219	0.69965135	0.66087635
Fau	-0.0893001	5.68970486	0.15318077	0.69967125	0.66087635
4930506M07	-0.0527917	5.40685335	0.15312296	0.69972496	0.66087635
Cbll1	0.06090912	6.17443958	0.15310467	0.69974196	0.66087635
Sec31a	-0.0405344	7.07424304	0.15297818	0.69985954	0.66093527
C920025E04I	0.42299615	-1.1611135	0.15277762	0.7000461	0.66097476
2310065F04I	-0.7378811	-1.6420184	0.15276975	0.70005343	0.66097476
Ccdc91	0.06172246	5.37046407	0.15275523	0.70006694	0.66097476

Tspyl3	0.05626171	4.6678116	0.15265571	0.70015956	0.6609778
Zfp454	0.10688035	2.31027199	0.15263318	0.70018054	0.6609778
Rab14	-0.0434135	7.96281547	0.15243499	0.70036512	0.66109462
Pask	0.21588674	0.83317781	0.1523818	0.70041469	0.66109462
Frrs1l	0.0590428	7.44286109	0.15211034	0.7006678	0.66120925
Uaca	-0.0824802	7.66103414	0.15202576	0.70074671	0.66120925
Nat14	0.14098864	1.17082962	0.1520227	0.70074956	0.66120925
Lrrn3	0.05214183	5.76530635	0.15201474	0.70075699	0.66120925
Cenpj	-0.0737273	4.00138366	0.15192177	0.70084377	0.66123903
4933427D14I	-0.1192744	4.10754685	0.15178925	0.70096751	0.66130368
Apol7d	0.49013852	-1.7446552	0.15168602	0.70106395	0.66134257
3110021N24	-0.0977831	2.41221087	0.15157148	0.701171	0.66139145
Pik3cg	-0.1055738	2.45335659	0.1514474	0.70128702	0.66144878
Zeb2	0.05435171	8.89773701	0.15127874	0.7014448	0.66151302
Erdr1	0.10098313	3.40101231	0.15116138	0.70155466	0.66151302
4933431E20I	-0.0495435	6.76028562	0.15104313	0.7016654	0.66151302
Pfdn5	-0.0973952	5.7391927	0.15097584	0.70172843	0.66151302
Rnf44	-0.040002	6.73438254	0.1509471	0.70175536	0.66151302
Csnk2a2	0.06943909	5.19443205	0.15087892	0.70181926	0.66151302
Agl	0.06814389	5.63069025	0.15070627	0.70198113	0.66151302
Eri2	0.0784153	4.18640994	0.15064371	0.70203981	0.66151302
Cnot6	0.06008457	6.42197688	0.15061421	0.70206749	0.66151302
Foxj1	0.21772269	0.73725854	0.1506113	0.70207022	0.66151302
Gna13	-0.0484269	7.53995271	0.15060289	0.70207811	0.66151302
Enah	-0.0604162	8.35869406	0.15053353	0.70214319	0.66151302
Ckap4	-0.0782223	5.1576854	0.15048968	0.70218436	0.66151302
Prokr2	0.18185945	1.92358666	0.15048834	0.70218561	0.66151302
Mtfr1	-0.1054189	3.94927546	0.15047464	0.70219847	0.66151302
Pgm2	0.06942706	4.06380993	0.15035533	0.7023105	0.66151302
Cyp2d22	0.09520171	3.38887593	0.15033293	0.70233154	0.66151302
Frrs1	-0.2196918	1.18737088	0.15031391	0.7023494	0.66151302
Hic1	-0.1168425	3.26674791	0.15021131	0.7024458	0.66155178
Zbtb16	-0.0523683	4.67160739	0.14997712	0.70266596	0.66170709
Rab39b	0.06026779	6.74306171	0.14987138	0.70276542	0.66174872
Il1rl1	0.33527277	-0.1980598	0.14968186	0.70294381	0.66186466
Scube2	0.21081254	1.10588406	0.14950511	0.7031103	0.66191366
Mag	-0.1052639	2.55410134	0.14949755	0.70311742	0.66191366
4931429I11R	0.27638112	-0.0218343	0.14943935	0.70317226	0.66191366
Hk1	-0.0777202	5.81242358	0.14937062	0.70323705	0.66191366
Rnf10	-0.0567834	5.83996032	0.14922938	0.70337024	0.66191366
Tgm4	0.17197717	1.23746859	0.1491798	0.70341701	0.66191366
Hist1h4d	0.11674362	2.81423744	0.14917806	0.70341865	0.66191366
Lrfn2	0.11752544	1.80541065	0.14913807	0.70345638	0.66191366

Cox8b	-0.4956248	-1.4825481	0.14909896	0.7034933	0.66191366
Pnp2	0.25553315	-0.4451936	0.14894405	0.70363953	0.66194298
Lrtm1	-0.2307659	3.1295136	0.14887423	0.70370547	0.66194298
Rufy2	-0.0864456	6.03410088	0.14886853	0.70371085	0.66194298
Apaf1	0.0802513	3.97683856	0.14883179	0.70374556	0.66194298
Cltc	-0.0630956	8.89667839	0.14870374	0.70386655	0.66200479
Cenpb	-0.0894297	4.12701979	0.148624	0.70394193	0.6620237
Ddx25	0.08875118	4.1774863	0.1485442	0.7040174	0.66204268
H2-DMa	0.17093159	2.02322094	0.14826879	0.70427801	0.66223575
Aplnr	0.43341246	-1.2725046	0.14818667	0.70435577	0.66225687
Rpl8	0.07219999	5.66174501	0.14773157	0.70478717	0.66257832
Spin1	0.03796002	8.82687423	0.14769355	0.70482325	0.66257832
Zkscan8	-0.0571924	6.10581544	0.14765099	0.70486363	0.66257832
D230025D16	0.04988222	5.65065119	0.14740546	0.70509677	0.66262942
Tnfrsf25	0.55747933	-1.3089208	0.14735036	0.70514911	0.66262942
Zbtb43	0.08211655	4.51846627	0.14730737	0.70518997	0.66262942
Fkrp	-0.0555485	5.29122087	0.14729236	0.70520423	0.66262942
Apobec1	0.09911067	3.35042293	0.14726341	0.70523175	0.66262942
Nup93	-0.0933295	4.53506346	0.14720879	0.70528366	0.66262942
Slc24a5	0.0993873	2.28941633	0.14712422	0.70536408	0.66262942
Ran	0.04215593	7.71307405	0.14711373	0.70537405	0.66262942
Rplp1	-0.0753809	5.20971012	0.14706966	0.70541598	0.66262942
Gm6498	0.2895243	-1.0240939	0.14698537	0.70549617	0.66265277
Psen2	0.14089836	1.61387442	0.1469141	0.70556399	0.6626645
Sgms1	0.04871854	5.94055763	0.14675552	0.70571497	0.6627033
Cd244	0.44026027	-0.6517929	0.14673853	0.70573116	0.6627033
Gm11627	0.16224751	1.40812479	0.14669637	0.70577132	0.6627033
Cd46	-0.2548889	1.16625754	0.14657536	0.70588662	0.66275499
Mfsd2a	0.17186168	1.27973298	0.1464474	0.7060086	0.66275499
Sp3os	0.11890554	3.10306267	0.14639045	0.70606291	0.66275499
Orc2	-0.0547042	5.78244805	0.14637523	0.70607743	0.66275499
Pf4	-0.3703732	-1.4000512	0.14629751	0.70615157	0.66275499
Tlr1	-0.4566825	-0.9829122	0.14629034	0.70615841	0.66275499
Shd	0.12413366	2.34245362	0.14614196	0.70630003	0.66283596
Mon2	0.05986655	6.62046016	0.14594033	0.7064926	0.66294458
Prrc2a	0.04903292	7.7199155	0.1458657	0.70656392	0.66294458
Suv420h2	0.22885562	-0.0499833	0.14575884	0.70666607	0.66294458
Cand1	0.05826969	7.71060483	0.14574373	0.70668052	0.66294458
A530013C23	-0.3820362	-1.4641545	0.14573114	0.70669256	0.66294458
St6galnac4	-0.1195718	2.46563388	0.14564066	0.70677909	0.66295662
Gm11149	-0.346007	0.11160232	0.14555267	0.70686328	0.66295662
2610318N02	0.4722561	-2.0397248	0.14548382	0.70692917	0.66295662
Zwilch	0.17919851	1.4763998	0.14546955	0.70694283	0.66295662

Elfn2	-0.0677789	5.01552476	0.14542843	0.70698219	0.66295662
Ccpg1	0.05262721	6.52446713	0.14531006	0.70709555	0.66301101
Tmsb15b1	0.16066887	1.17472549	0.14519334	0.70720737	0.66306394
Trappc2	0.06783829	3.89742879	0.1450972	0.70729952	0.66309842
Gtf2h5	0.07211694	5.19964753	0.14495957	0.7074315	0.66314903
Tnfaip8l2	0.34277323	-0.6473809	0.14492542	0.70746425	0.66314903
Rxrg	0.14910767	0.91500745	0.1445136	0.70785964	0.66331607
Rb1cc1	-0.056281	8.50531799	0.14444893	0.70792179	0.66331607
Kif26b	0.12202525	1.966585	0.1444288	0.70794113	0.66331607
Cdh23	-0.3383337	-1.1264785	0.14440807	0.70796106	0.66331607
B4galt5	0.06971482	4.63843676	0.14440285	0.70796607	0.66331607
Ptcra	-0.2794118	-0.9409011	0.14432059	0.70804517	0.66331607
Gp1bb	-0.1245962	1.60154465	0.14424199	0.70812076	0.66331607
Gemin5	-0.0970549	3.93796006	0.14417642	0.70818384	0.66331607
Baz2a	-0.0578892	6.12712443	0.14416487	0.70819496	0.66331607
Gm15217	0.3417379	-1.6360095	0.14416344	0.70819633	0.66331607
Fancm	-0.0736392	4.04748168	0.14408357	0.70827319	0.66333618
Ccdc113	0.18869018	1.09575397	0.14389497	0.70845479	0.66342977
Slco4a1	0.17501716	1.80494302	0.14386474	0.70848392	0.66342977
Sirt6	-0.1039158	2.94757241	0.14367371	0.70866802	0.6635087
Afmid	-0.1708907	1.12223607	0.1436085	0.7087309	0.6635087
Chchd7	-0.0830143	3.63145787	0.14334924	0.70898104	0.6635087
Nom1	-0.0613954	4.52773846	0.1433186	0.70901063	0.6635087
Pcdhgb6	-0.1251241	2.14496782	0.14328612	0.70904199	0.6635087
2610020C07I	0.19745722	0.53748742	0.14328145	0.70904649	0.6635087
Abca1	0.05848835	5.05854337	0.14322481	0.7091012	0.6635087
Zfp955b	-0.0514156	5.83765315	0.14306961	0.70925114	0.6635087
Hmgb1-rs17	-0.1656527	0.38820929	0.14300853	0.70931018	0.6635087
Cactin	-0.0947301	3.17297834	0.14300724	0.70931143	0.6635087
A930005H10	-0.1084525	3.04340925	0.14294807	0.70936863	0.6635087
Pdlim3	0.32678807	-0.6166203	0.14294023	0.70937621	0.6635087
Pdxp	0.08315453	4.93016259	0.14288115	0.70943334	0.6635087
Rbm17	0.05150743	6.6248111	0.14287248	0.70944173	0.6635087
Dysf	0.28273872	0.33250589	0.14280729	0.70950479	0.6635087
1700027H10	-0.2412532	0.39054886	0.14280294	0.70950899	0.6635087
Ubald2	-0.1101257	2.32642476	0.14280181	0.70951009	0.6635087
Atp1a1	0.05986028	7.63614666	0.14273026	0.70957933	0.66352163
Rnf157	-0.0640342	7.48577637	0.14262146	0.70968464	0.66354585
Gadd45gip1	0.10922324	2.62818782	0.1425013	0.70980101	0.66354585
Tnfaip6	-0.1279961	1.74255489	0.14247245	0.70982895	0.66354585
Cask	-0.0446126	7.6195269	0.14241568	0.70988395	0.66354585
Edc4	0.09653601	3.93410082	0.14229383	0.71000205	0.66354585
Gsn	0.07645993	5.4563857	0.14226691	0.71002815	0.66354585

Hes7	-0.5139149	-1.7035648	0.1422607	0.71003417	0.66354585
Trp53rk	-0.1882202	2.02455285	0.1421817	0.71011079	0.66354585
Bai1	-0.0685066	6.13424862	0.14214813	0.71014334	0.66354585
Ttc3	-0.0655868	9.98771488	0.14207136	0.71021783	0.66354585
Med11	0.12883957	2.92929786	0.14201726	0.71027032	0.66354585
Bdp1	0.05601799	7.43458905	0.14186815	0.71041509	0.66354585
Ago1	0.03996458	6.22133921	0.14186599	0.71041718	0.66354585
Ict1	0.09113053	4.30702121	0.14177622	0.71050438	0.66354585
Pkn2	-0.0507988	6.06826145	0.14166404	0.71061339	0.66354585
Crebl2	0.07229391	5.09833326	0.14163033	0.71064616	0.66354585
Gas1	0.09554662	5.78647272	0.1416158	0.71066028	0.66354585
Tbc1d17	-0.12581	2.57286383	0.14146837	0.71080364	0.66354585
Wdr70	-0.0791009	3.54999649	0.14146583	0.71080612	0.66354585
Dll1	-0.25099	0.10744602	0.14143585	0.71083528	0.66354585
Rex2	-0.3583495	-1.6257951	0.14134396	0.71092469	0.66354585
Ifi30	0.15508643	2.01187958	0.14132092	0.71094711	0.66354585
Pithd1	-0.0672774	4.77159265	0.14131045	0.7109573	0.66354585
Exoc4	0.0495533	5.87037846	0.14130406	0.71096352	0.66354585
Ccdc40	-0.2489218	0.06775867	0.14127643	0.71099041	0.66354585
Ramp3	-0.2833471	-0.3875601	0.1410076	0.71125226	0.66366264
4930525G20	0.12612994	2.28997565	0.14095798	0.71130061	0.66366264
Tspan17	0.07244457	3.27720702	0.14089914	0.71135798	0.66366264
Pld2	-0.1279306	2.40034598	0.14088876	0.7113681	0.66366264
Cabp4	0.45262236	-0.8660315	0.14086359	0.71139264	0.66366264
Rtn4ip1	-0.102845	3.29227625	0.14075265	0.71150084	0.66371188
Xlr3b	-0.1617097	1.1616973	0.14053502	0.71171323	0.6638583
Klk14	-0.223189	-0.0143907	0.14026825	0.71197385	0.66402434
Napa	0.0525945	6.07574235	0.14023931	0.71200215	0.66402434
Enkd1	0.17298247	0.55606555	0.14004397	0.71219318	0.66415078
Paqr4	0.08999495	3.91087184	0.13994788	0.71228721	0.66415713
Slc9a3r1	-0.1031299	4.2153837	0.13988748	0.71234634	0.66415713
Pfn2	0.04479235	7.71573022	0.13986703	0.71236636	0.66415713
Armcx5	-0.0504265	5.36847734	0.13931564	0.71290682	0.66455452
Asph	-0.0487542	6.75367526	0.13926445	0.71295706	0.66455452
Lat2	-0.2435749	-0.025745	0.1392624	0.71295907	0.66455452
Gprc5b	-0.0715776	4.83589244	0.13919924	0.71302107	0.66456058
Mrto4	0.09207558	3.32261323	0.1390394	0.71317804	0.66465516
Ttyh1	0.06602738	7.60305799	0.1389122	0.71330304	0.66468116
Rgs7	0.05359315	7.1722786	0.13889806	0.71331694	0.66468116
Tor4a	-0.0894466	3.20994434	0.1386418	0.71356897	0.66470514
Adat1	0.1260204	1.8778403	0.1385677	0.7136419	0.66470514
Abl2	-0.0661174	5.655913	0.1385311	0.71367793	0.66470514
Trmt6	-0.0601321	4.64719553	0.13849928	0.71370925	0.66470514

Mtmr6	-0.0424777	7.40515423	0.13847518	0.71373299	0.66470514
1700125H03I	0.36546829	-0.4861726	0.13843877	0.71376884	0.66470514
Gabarapl2	0.05625574	6.7008753	0.13831524	0.71389053	0.66470514
Hnrnpf	-0.0695902	6.78931311	0.13827141	0.71393372	0.66470514
Zxda	-0.0606024	5.37995996	0.13826712	0.71393795	0.66470514
9430076C15I	0.4603051	-1.4963144	0.13822722	0.71397728	0.66470514
Bub3	0.04903686	5.8548539	0.1382247	0.71397976	0.66470514
Gm10046	0.17688386	0.33357784	0.1381953	0.71400874	0.66470514
Rhot1	0.05092886	6.11619502	0.13798636	0.71421482	0.66478807
Pard6g	0.07966911	5.59374964	0.13797081	0.71423016	0.66478807
Fbxo25	-0.0626972	4.92372084	0.13793616	0.71426435	0.66478807
Zrsr2	-0.0476675	6.20356364	0.13747935	0.7147156	0.66514363
Gcn1l1	0.083576	4.54736474	0.13738498	0.71480893	0.66514363
Cd63	0.11694989	4.98247981	0.13738087	0.71481299	0.66514363
Tspan9	-0.0918784	3.49513706	0.13726692	0.71492574	0.66516569
Pglyrp1	0.17210114	0.74415633	0.13720174	0.71499025	0.66516569
Tarbp2	-0.1009286	2.33320718	0.13718853	0.71500334	0.66516569
Eya4	0.14804827	2.43857973	0.13709818	0.7150928	0.66519725
Foxn2	-0.0676299	4.77039956	0.13689062	0.71529843	0.66526941
Fam35a	-0.1221071	2.54139424	0.13688465	0.71530435	0.66526941
Pyroxd1	-0.0722325	3.35080755	0.13682194	0.71536652	0.66526941
Cdk2ap2	0.08563108	3.39186092	0.1367288	0.71545888	0.66526941
Acrbp	-0.2781408	0.21321377	0.13668337	0.71550395	0.66526941
Sacm1l	-0.0556054	6.00551482	0.13656098	0.7156254	0.66526941
Ubxn2a	0.04928185	6.98286005	0.13651862	0.71566745	0.66526941
Bloc1s6	-0.0553536	5.60075722	0.13649465	0.71569124	0.66526941
Gm6815	0.25992232	-0.9340087	0.13648882	0.71569703	0.66526941
Arhgap42	-0.049548	5.70538165	0.13645976	0.71572589	0.66526941
Adcy4	-0.2495727	0.47949154	0.13636166	0.71582331	0.66530238
AI414108	0.09702829	4.67017966	0.1362371	0.71594708	0.66530238
Lamb3	0.4604579	-1.016154	0.13620448	0.71597951	0.66530238
Elp2	-0.0633134	5.93591872	0.13594219	0.71624038	0.66530238
Ece2	-0.0931291	2.54732572	0.13591581	0.71626664	0.66530238
Madcam1	0.46013207	-2.0561187	0.13580464	0.7163773	0.66530238
Ssx2ip	0.04244534	6.28551564	0.13576811	0.71641368	0.66530238
Zmiz2	0.04774245	6.4725955	0.1357076	0.71647395	0.66530238
Cdc42se1	-0.0622948	5.51171644	0.13560624	0.71657494	0.66530238
Dmrt3	0.79576436	-1.5248204	0.13556973	0.71661132	0.66530238
Zfp235	0.09569923	3.30221274	0.13554235	0.71663862	0.66530238
Akt1s1	0.1339033	1.7893999	0.13550939	0.71667147	0.66530238
Mgat2	0.08986709	3.28423238	0.13549836	0.71668248	0.66530238
Becn1	-0.0655637	5.24781277	0.13549325	0.71668756	0.66530238
Inpp5e	0.05343304	5.0308167	0.13546152	0.7167192	0.66530238

Trmt112	0.05708228	5.06178834	0.13544762	0.71673306	0.66530238
4930515G01	0.38439196	-0.7787779	0.13544522	0.71673546	0.66530238
Acbd6	-0.0642013	4.71462232	0.13540404	0.71677653	0.66530238
Lsr	0.10595531	3.45580772	0.13536358	0.71681689	0.66530238
Fam193a	0.05126818	7.39228776	0.13519784	0.71698228	0.66540432
Ubp1	-0.0551685	6.29825145	0.13513126	0.71704876	0.66541444
B230216G23	-0.3018846	-0.2643548	0.13498301	0.71719683	0.66550029
Sox8	0.0960626	3.86742114	0.13478228	0.71739748	0.66561677
Eif3i	-0.051109	5.80342661	0.13474624	0.71743352	0.66561677
Lemd1	0.4787595	-1.7102109	0.13434134	0.71783884	0.66590329
Ube2o	0.07568914	4.91134424	0.13427164	0.71790868	0.66590329
Tmem132d	-0.0856711	3.96671457	0.13427116	0.71790917	0.66590329
Pex13	-0.0493118	5.35870662	0.13418654	0.71799398	0.66593039
Pgls	-0.1396847	1.35967136	0.13397372	0.71820745	0.66594241
Snrnp40	-0.0757863	4.37384869	0.1339166	0.71826477	0.66594241
Trak2	-0.0485223	6.83064815	0.13389814	0.7182833	0.66594241
Tbl1x	0.03814389	6.44132166	0.13386573	0.71831584	0.66594241
Gm14305	-0.092287	2.61102588	0.13384753	0.7183341	0.66594241
Mitf	0.07815217	3.67275615	0.13384107	0.71834059	0.66594241
Gfpt1	-0.0512717	6.60092891	0.13358366	0.71859918	0.66613057
4930513N10	0.30520391	-0.4688363	0.13346559	0.71871788	0.66618904
5730409E04I	0.03681984	6.93327288	0.13329799	0.71888649	0.66626644
Trim24	0.05103405	5.55712033	0.13327198	0.71891266	0.66626644
Slc7a2	-0.0805832	6.92220245	0.13308626	0.71909966	0.6663613
Gpbp1l1	-0.055828	6.29620693	0.13303975	0.71914651	0.6663613
Tomm22	-0.0641628	5.96992969	0.13296078	0.71922609	0.6663613
Gramd2	-0.2927607	-0.4528712	0.13285768	0.71933001	0.6663613
Prkacb	0.04096099	9.17765633	0.13279341	0.71939482	0.6663613
Naaladl1	0.21950977	0.48937503	0.13269974	0.71948931	0.6663613
Erc1	0.04276348	7.67618624	0.13266557	0.71952378	0.6663613
Smcr8	0.0556492	5.78459802	0.13256947	0.71962078	0.6663613
Zfx	-0.0549268	6.22463205	0.13251468	0.71967609	0.6663613
Cct4	0.04846654	5.9909585	0.13248147	0.71970963	0.6663613
Net1	-0.0512997	5.59464004	0.13243994	0.71975158	0.6663613
Zfp217	0.10636264	3.61460677	0.13236923	0.71982301	0.6663613
Prdx2	0.07145433	6.20444139	0.13235018	0.71984226	0.6663613
A530072M11	0.25607997	0.62588716	0.13232734	0.71986533	0.6663613
B3gnt9	0.13514932	1.8098825	0.1322615	0.71993188	0.6663613
Dpyd	0.08873406	3.2659172	0.13225527	0.71993817	0.6663613
Kxd1	-0.0753203	4.23318675	0.13223276	0.71996094	0.6663613
Tars	0.0613309	4.55001334	0.13217769	0.72001661	0.66636132
B3gnt5	-0.283298	0.09588602	0.13195757	0.7202393	0.66648247
Fam69b	0.09649142	3.04810245	0.13193829	0.72025882	0.66648247

Ccdc30	-0.0971613	3.32439912	0.13171633	0.72048361	0.66663897
Slc15a4	-0.106728	2.35216293	0.13156407	0.72063792	0.66673024
Papln	0.36934779	-0.9964692	0.13146215	0.72074129	0.66676433
Gabrg1	0.09331413	3.40402134	0.13137686	0.72082781	0.66676433
Slc2a8	-0.1331891	1.62197619	0.13132534	0.72088009	0.66676433
Dgkq	0.11803536	3.8686968	0.13125536	0.72095113	0.66676433
Zbtb24	0.0610148	5.10803084	0.13125337	0.72095315	0.66676433
Anxa4	0.07629591	6.49079403	0.13109752	0.72111143	0.66685921
Cdc123	0.04860578	6.25246	0.13101016	0.7212002	0.66688981
LOC1012436	-0.2924635	-0.5993772	0.1308094	0.72140432	0.66702705
Gtf2a1l	0.55839444	-1.665669	0.13042871	0.72179188	0.66732822
Pitrm1	-0.0548501	5.2643786	0.13038002	0.72184149	0.66732822
Cdk15	0.18277598	0.70685136	0.13025669	0.72196721	0.66739293
Sult1a1	-0.0889215	5.7934373	0.12990844	0.72232257	0.66762738
Tmem8b	-0.0640641	5.07765794	0.12983775	0.72239477	0.66762738
Kcnq2	0.06546351	5.52989196	0.12979533	0.72243811	0.66762738
Psg29	0.23363864	0.02160437	0.1297082	0.72252715	0.66762738
Fam163a	-0.1628252	1.81468194	0.1296681	0.72256814	0.66762738
Zscan21	0.07476232	4.60120424	0.1296531	0.72258348	0.66762738
Cggbp1	0.05216521	7.33132985	0.12959762	0.7226402	0.66762738
Slpi	-0.4678664	-1.3967674	0.12957158	0.72266683	0.66762738
Gm3985	0.29467174	-0.8746755	0.12939417	0.72284833	0.66773377
Bst1	-0.421161	-1.3908768	0.12932844	0.72291563	0.66773377
Bai2	0.08015816	4.57330281	0.12929559	0.72294925	0.66773377
Fam117b	0.03644374	7.00148895	0.12912184	0.72312723	0.66779709
Casp2	0.06558256	3.86831317	0.12911979	0.72312934	0.66779709
Timm17b	0.15547207	2.74288703	0.12905722	0.72319346	0.66780481
Dido1	-0.0520194	6.59201609	0.128792	0.72346547	0.66794956
Ankrd45	-0.0526421	6.27744958	0.12875441	0.72350405	0.66794956
Xkr4	0.08653758	4.24244498	0.12869225	0.72356787	0.66794956
Jmjd8	-0.0815126	3.88038761	0.12865312	0.72360804	0.66794956
Ino80d	0.04901973	7.35145253	0.12863262	0.72362909	0.66794956
4921524J17F	-0.1079782	4.04418156	0.12850372	0.7237615	0.66802029
Rnase4	-0.0923124	5.15514776	0.12843769	0.72382936	0.66802632
Gba	-0.0661381	3.90078188	0.12838882	0.7238796	0.66802632
Gpr62	0.1963663	0.89676421	0.12823744	0.72403527	0.6681185
Gltscr2	-0.0805617	5.54829414	0.12816472	0.72411009	0.66813606
Ski	-0.0396637	8.16273402	0.12757841	0.72471422	0.66864197
Hs6st3	0.08957016	2.87786613	0.12749943	0.72479572	0.66866565
Igtp	0.09400166	3.72896167	0.12723093	0.72507301	0.66886994
Fkbp1b	0.07547897	4.2158302	0.12716735	0.72513871	0.66887903
Ttll3	0.12717178	1.83886483	0.12699635	0.72531553	0.6689906
Slc22a12	-0.3622338	0.35222828	0.12659022	0.72573601	0.66932688

Mfsd7a	0.47562569	-0.7476039	0.12643563	0.72589627	0.66942312
Myl6	0.06983118	7.87742737	0.12612275	0.72622096	0.6696401
Pcid2	0.0906216	4.26634617	0.12610116	0.72624338	0.6696401
Samhd1	0.04763386	5.59678699	0.12566152	0.72670042	0.66996661
Iws1	-0.0430329	6.44071015	0.12565292	0.72670937	0.66996661
Spo11	0.32269364	0.07250482	0.12558814	0.7267768	0.66997402
Npl	0.16677259	1.59114051	0.12553648	0.72683058	0.66997402
Gcm1	-0.3502065	-1.3960054	0.12542519	0.72694648	0.66997402
Birc2	0.04778927	5.4885812	0.12539794	0.72697487	0.66997402
Krt26	-0.4712585	-2.0467071	0.12536782	0.72700625	0.66997402
Rps26	-0.0769613	5.54799771	0.12532288	0.72705309	0.66997402
Wdr86	-0.2684827	1.31056023	0.12522932	0.72715061	0.67001234
Rtf1	0.038024	8.31775041	0.12516491	0.72721778	0.67002268
Rnd2	-0.1444942	1.72906481	0.12499697	0.727393	0.67006328
Zfp408	-0.0559111	4.68741714	0.12496481	0.72742658	0.67006328
Zfp597	0.05026721	4.89162682	0.12490831	0.72748555	0.67006328
Ky	-0.1918982	0.5283509	0.12490822	0.72748566	0.67006328
Sepsecs	-0.1234737	2.25079891	0.12482854	0.72756886	0.67006984
Nenf	0.06821168	4.26834623	0.12474474	0.72765641	0.67006984
Megf6	0.1513913	1.38357605	0.1247407	0.72766063	0.67006984
Olfr1372-ps1	0.21837346	-0.054429	0.12463962	0.72776628	0.6700771
Gucy1a2	-0.0708919	7.95296243	0.12462609	0.72778043	0.6700771
Kif3b	-0.0526367	6.59547066	0.12446071	0.7279534	0.67016734
Rab3b	0.04865591	5.12020472	0.12441515	0.72800107	0.67016734
Kif15	0.17788915	1.06210072	0.12416172	0.72826644	0.67016734
Armc3	0.31901841	-0.9834486	0.12409874	0.72833243	0.67016734
Nudt8	0.19523104	0.48689699	0.12404071	0.72839325	0.67016734
Cpeb4	-0.0539189	8.15948239	0.12402061	0.72841432	0.67016734
Pygo1	-0.0570502	4.86566756	0.12401511	0.72842009	0.67016734
Lsm14a	-0.0472087	8.08565336	0.12392652	0.72851299	0.67016734
Ssbp4	0.08850635	2.9201445	0.12389815	0.72854275	0.67016734
Mri1	0.13612672	1.14525126	0.12383202	0.72861213	0.67016734
Kif19a	-0.3431686	-1.1562717	0.12382856	0.72861576	0.67016734
Brcc3	-0.055743	5.43400162	0.12380615	0.72863927	0.67016734
Rras2	-0.0698295	4.65178085	0.12370501	0.72874543	0.67016734
Rce1	-0.232654	0.41775303	0.12367551	0.72877641	0.67016734
Ptger1	-0.0756941	3.11132064	0.12366579	0.72878661	0.67016734
Prkcb	-0.0541999	11.0318329	0.12362896	0.72882529	0.67016734
Ptcd1	-0.0716239	3.44479309	0.12362469	0.72882977	0.67016734
Ikbip	0.0941281	4.11084961	0.12335688	0.72911122	0.67037467
Uros	-0.0764753	3.53309665	0.1232839	0.72918797	0.67039336
Me1	0.04474415	5.34578068	0.12323111	0.72924351	0.67039336
Gm17801	-0.347832	-0.6337183	0.12304744	0.72943684	0.67051962

Skp2	0.10437192	2.8285553	0.12283919	0.72965625	0.67058818
Arid4b	-0.051446	7.72302548	0.12281959	0.7296769	0.67058818
Mnt	0.05364199	5.01566522	0.1228172	0.72967942	0.67058818
Trim15	-0.3685711	-1.2295396	0.12272721	0.72977431	0.67062392
Fbxw8	0.0588445	3.88757682	0.12265569	0.72984975	0.67063251
Ppme1	-0.0551868	5.17759303	0.12258692	0.72992231	0.67063251
Tyw5	-0.0652804	4.84978132	0.12255911	0.72995165	0.67063251
Gm2027	-0.2128068	0.23977782	0.1224638	0.73005226	0.6706735
Svopl	-0.2802502	-0.2633265	0.12229921	0.73022612	0.67069991
Chst1	-0.0530871	5.89299389	0.12224651	0.73028181	0.67069991
Scfd1	-0.058382	4.64103708	0.12219675	0.73033441	0.67069991
Aass	0.23096704	0.83297659	0.12217301	0.73035951	0.67069991
Srsf5	-0.0372497	8.0887758	0.12217156	0.73036104	0.67069991
Hepacam	0.08129154	4.23256353	0.12202339	0.73051775	0.67079238
Pttg1	-0.049188	4.90101036	0.12176445	0.73079188	0.67094415
Ypel3	0.04263894	5.84479946	0.12161005	0.7309555	0.67094415
Naca	0.05080639	7.84367929	0.12160606	0.73095973	0.67094415
Slc22a3	0.16730875	0.82570762	0.1215527	0.7310163	0.67094415
Stpg1	-0.1526922	0.68737858	0.1215512	0.73101789	0.67094415
Spcs1	0.06301728	5.36136485	0.12154997	0.73101919	0.67094415
Hcar1	-0.1249443	4.37220881	0.12134127	0.7312406	0.67103453
Snx24	0.07265723	4.98465963	0.12129896	0.73128551	0.67103453
Hnrnpa1	0.0448243	5.07049439	0.12124853	0.73133905	0.67103453
5730507C01I	0.08874545	3.12580485	0.12120162	0.73138886	0.67103453
Ate1	0.04807642	6.22399955	0.12105493	0.73154471	0.67103453
Exoc1	0.07066233	5.66903012	0.12097823	0.73162624	0.67103453
Gpr17	-0.1030782	2.74550711	0.1209041	0.73170506	0.67103453
Fam189a1	0.05414799	5.77277244	0.12089064	0.73171938	0.67103453
Tmod2	-0.0406165	9.94559003	0.12087545	0.73173554	0.67103453
Dnlz	-0.0709842	3.76247593	0.12073246	0.73188768	0.67103453
Aph1a	0.10277793	2.94250574	0.12070604	0.7319158	0.67103453
Adcy5	-0.0389273	6.29168631	0.12064731	0.73197833	0.67103453
Itk	-0.2896152	-0.1746427	0.12062834	0.73199854	0.67103453
Fbxl22	-0.2550866	-0.6497519	0.12060382	0.73202465	0.67103453
Rpl34	-0.1314812	0.76180846	0.12059516	0.73203387	0.67103453
Rasgef1c	0.08345028	3.5194525	0.12056238	0.73206878	0.67103453
Tjap1	0.15701528	1.35414695	0.12055219	0.73207964	0.67103453
Nr2e1	0.0925341	3.69179722	0.12036661	0.73227742	0.67103453
Pth1r	0.17694534	1.07751311	0.120332	0.73231432	0.67103453
Slc1a6	0.18679667	0.37276094	0.12030982	0.73233798	0.67103453
Zmpste24	0.06952875	5.50399362	0.12025296	0.73239863	0.67103453
Derl1	0.05960059	5.09007702	0.12024477	0.73240736	0.67103453
Hebp2	-0.0705581	3.40226414	0.12008813	0.73257453	0.67103453

Gm13710	0.18439096	0.50755034	0.12007497	0.73258858	0.67103453
Sla2	0.21544035	-0.1584206	0.1200683	0.7325957	0.67103453
Fcf1	-0.0623414	4.74357637	0.12000631	0.7326619	0.67103453
C130026L21F	-0.1640836	1.06076622	0.11999958	0.73266908	0.67103453
Dek	-0.0540641	8.40392104	0.11994459	0.73272782	0.67103453
Nat10	0.07167696	3.42472653	0.11990808	0.73276683	0.67103453
Klra9	0.41588688	-0.9669091	0.11986169	0.7328164	0.67103453
Nbr1	0.04665756	7.89776255	0.11982587	0.73285468	0.67103453
Irak1	0.03794494	6.53281379	0.11973413	0.73295277	0.67107304
Ankrd6	-0.0657268	5.51365967	0.11941356	0.73329583	0.67133581
Cdyl	-0.0556034	4.10914746	0.11922952	0.73349303	0.67143605
1700123M08	0.14340536	1.25179356	0.11916545	0.73356171	0.67143605
Rpl18	-0.0861144	5.80811809	0.11913972	0.7335893	0.67143605
Cebpd	-0.1546898	1.35921377	0.11905605	0.73367905	0.67143605
Kcnj11	0.07148394	3.39176252	0.11904989	0.73368565	0.67143605
Ptcd3	-0.0693669	5.21562764	0.11890203	0.73384433	0.67152995
Gm10791	-0.2357284	0.77777269	0.11873489	0.73402383	0.67164289
Daglb	-0.0786429	3.13088302	0.11864491	0.73412054	0.67168005
Dand5	0.07077231	3.55729578	0.11855739	0.73421462	0.67169438
6330409D20	-0.1664076	0.73553757	0.11851364	0.73426168	0.67169438
Plag1	0.10170013	2.70955177	0.11847387	0.73430446	0.67169438
Tmsb15b2	0.23357723	-0.3630723	0.11826428	0.73453003	0.67176716
2810049E08I	0.22892724	0.86852892	0.11825097	0.73454436	0.67176716
Mthfr	-0.1050958	2.89904496	0.11824359	0.73455231	0.67176716
Zc3h13	-0.0470778	8.06484843	0.11814355	0.73466007	0.67177377
Prdm10	0.09590412	2.96049665	0.11813274	0.73467173	0.67177377
Gm13498	0.20839048	-0.4467189	0.11785215	0.73497426	0.67195129
Phactr4	-0.0694208	6.13209661	0.1178486	0.73497808	0.67195129
A930007I19F	-0.4599985	-1.0315165	0.11775806	0.73507579	0.67197796
H2-DMb1	0.22085244	0.53285769	0.11771759	0.73511948	0.67197796
Msi2	0.05516477	9.31989194	0.11766479	0.73517649	0.67197878
Tnfaip8l3	-0.0421237	6.59912069	0.11746127	0.73539638	0.67204577
Arhgap10	0.06662179	4.4409268	0.11745407	0.73540416	0.67204577
Kcna3	0.14760655	1.38186323	0.11730327	0.73556723	0.67204577
Pcp2	-0.5129675	-1.5685669	0.11729674	0.7355743	0.67204577
Zfp85	0.08652707	2.44957893	0.11729238	0.73557902	0.67204577
Irf2	0.05870826	5.24195731	0.11728548	0.73558648	0.67204577
1700020L24F	0.34670055	-1.6620476	0.11717544	0.73570556	0.67210328
Chchd1	0.07903748	3.59794453	0.11711749	0.7357683	0.67210933
Nhlrc1	-0.064055	4.10563353	0.11699011	0.73590625	0.67213096
Fam13c	0.06136224	4.49680051	0.11691317	0.73598962	0.67213096
Klhl4	-0.0540173	4.81035219	0.11685729	0.73605019	0.67213096
Zfyve9	0.07403458	3.85483059	0.11684886	0.73605933	0.67213096

Acss3	-0.1088744	2.36379029	0.11683662	0.7360726	0.67213096
Fcer2a	0.18119436	0.56786535	0.116779	0.73613508	0.67213676
4933406I18R	-0.2361305	-0.502122	0.11662156	0.73630589	0.67224147
Hspa9	-0.0396656	7.17277212	0.11647889	0.73646078	0.67233163
Lyrm2	-0.061011	4.85568116	0.11642364	0.73652079	0.67233516
Jade1	-0.0443095	6.43364774	0.11601662	0.73696339	0.67243133
Rrad	-0.3304334	-1.1122327	0.11584861	0.73714633	0.67243133
Akna	-0.0719495	3.34321702	0.11578465	0.73721602	0.67243133
Etnk2	0.10044098	2.60113181	0.11578393	0.7372168	0.67243133
Edem3	0.04497109	6.01959876	0.11577585	0.73722561	0.67243133
Cpb1	-0.1431154	2.22765188	0.11566887	0.73734223	0.67243133
Hist1h4b	0.29792738	-1.8082194	0.11565232	0.73736027	0.67243133
Echdc1	0.10966175	1.77405329	0.11563282	0.73738153	0.67243133
Wdr4	0.11199031	2.39994244	0.11561477	0.73740121	0.67243133
C87436	-0.0927696	3.60586379	0.11545454	0.73757603	0.67243133
Meis2	0.04906417	6.62560199	0.11543738	0.73759476	0.67243133
Cep89	-0.0942146	3.02045372	0.11539297	0.73764324	0.67243133
Zfp931	-0.0807039	2.57628972	0.11531043	0.73773338	0.67243133
Zdhhc15	-0.0916499	3.45046656	0.11520443	0.73784917	0.67243133
Ubxn10	-0.206672	0.82229865	0.11520064	0.73785332	0.67243133
Dlk1	0.0988339	3.41940784	0.11519711	0.73785717	0.67243133
Fam150b	0.41359874	-1.1072825	0.11511797	0.73794368	0.67243133
Tnfrsf14	-0.5018847	-1.8318768	0.11509805	0.73796546	0.67243133
Zfp644	-0.0477472	7.40811311	0.11496136	0.73811495	0.67243133
Gm10865	-0.1902572	-0.4188497	0.11493962	0.73813874	0.67243133
Dnajc19	0.05647947	4.74616253	0.11492489	0.73815486	0.67243133
Cd1d1	-0.405477	-1.2586938	0.11492189	0.73815814	0.67243133
Tnfrsf9	-0.3623835	-1.4474965	0.11488334	0.73820033	0.67243133
Epha8	-0.1816339	0.67208975	0.11486377	0.73822174	0.67243133
Copg2	-0.065025	5.85953571	0.11486198	0.7382237	0.67243133
Foxj2	0.04733949	6.55270403	0.11483256	0.73825591	0.67243133
Tma7	0.05795968	6.81927028	0.11479346	0.73829872	0.67243133
Immt	-0.0471242	6.62963615	0.11476155	0.73833367	0.67243133
Lrrc51	-0.1368976	2.09673844	0.114731	0.73836712	0.67243133
Sf3a1	-0.0592252	5.62274131	0.11472687	0.73837165	0.67243133
Cntn6	-0.1252687	3.21774274	0.11466729	0.73843691	0.67243133
Siah2	-0.0610085	4.21540597	0.11459459	0.73851658	0.67243133
Arl6ip4	-0.0683735	4.90499571	0.11456836	0.73854533	0.67243133
Tnk2	-0.0558847	5.09369377	0.11454363	0.73857244	0.67243133
4732471J01F	-0.1275707	1.65435426	0.11450695	0.73861265	0.67243133
Chst14	-0.1413814	1.60237469	0.11447515	0.73864752	0.67243133
Wdr60	0.06452914	5.41442641	0.1142557	0.73888832	0.67247921
Lnx1	-0.0692633	4.01709519	0.11422601	0.73892092	0.67247921

Camta1	-0.0687021	9.37254189	0.11421892	0.73892869	0.67247921
Ina	-0.0537241	6.3206916	0.11417769	0.73897397	0.67247921
Svip	0.0418234	5.08626736	0.11413261	0.7390235	0.67247921
Trim45	-0.1017139	2.49172665	0.11412028	0.73903704	0.67247921
Clvs2	0.07904676	4.43268842	0.11405981	0.73910348	0.67248857
Gm13139	0.11161301	2.32441717	0.11386587	0.7393167	0.672593
Prr5l	-0.0926868	2.40754669	0.11385147	0.73933254	0.672593
Shisa9	0.06053293	6.09755279	0.1138022	0.73938675	0.672593
Alyref	0.08468349	2.80293546	0.11370056	0.73949861	0.67264152
Spryd4	0.11628964	2.31508092	0.11365168	0.73955242	0.67264152
Tmed8	0.03959039	6.24397623	0.11357759	0.73963401	0.67266464
Sppl3	-0.0440207	5.25228808	0.11347551	0.73974649	0.67271585
Abcd1	0.09674202	3.7141478	0.11340148	0.73982808	0.67273896
Xlr3a	-0.1856618	0.44850432	0.11310198	0.74015851	0.67294391
Clk2	0.05935034	5.15025855	0.11309533	0.74016585	0.67294391
Col6a2	-0.1065669	5.41390763	0.11300873	0.74026149	0.67297977
Triqk	0.08716678	3.52130228	0.11279886	0.74049344	0.67313953
Ift22	0.06902584	4.09852226	0.11269752	0.74060551	0.6731494
Fam72a	-0.2716169	0.51817619	0.11268546	0.74061886	0.6731494
Nkrf	-0.0628705	5.84414015	0.11261447	0.74069742	0.6731494
Hist1h1a	-0.3490821	-1.2556486	0.11258582	0.74072913	0.6731494
Wdr3	-0.0838898	3.22778779	0.11252084	0.74080106	0.67315143
Pcdhga8	0.09274684	2.67989961	0.11242698	0.74090501	0.67315143
Ttc17	-0.0539953	4.84486367	0.11236924	0.74096899	0.67315143
Ndc80	-0.3017538	-0.3951648	0.11233131	0.74101102	0.67315143
Pot1a	0.05533386	5.1867932	0.11226497	0.74108456	0.67315143
Cct8	0.04124324	6.86970819	0.11224104	0.74111109	0.67315143
Fam129a	0.08351129	3.7238136	0.11222866	0.74112483	0.67315143
Rgl2	-0.1002031	2.1835214	0.11213063	0.74123356	0.67317426
Phf3	0.03837261	8.18853207	0.11210464	0.74126239	0.67317426
Zbtb5	0.09216036	2.58687091	0.11191627	0.74147149	0.67327774
C1galt1c1	-0.076035	4.60541063	0.11190071	0.74148877	0.67327774
Pigl	-0.0839727	4.09399198	0.1118499	0.74154522	0.67327794
Stxbp2	-0.0849971	4.19154777	0.11174269	0.74166436	0.67333506
Fxyd7	0.10190606	2.00153272	0.11164401	0.74177408	0.67334942
Tob2	-0.0580075	5.13169507	0.11162734	0.74179262	0.67334942
Map3k2	0.04383811	6.20693347	0.11145541	0.74198392	0.67345276
Dtnb	-0.0622664	4.6439669	0.11137406	0.74207449	0.67345276
Pcyox1l	0.11762044	1.9727679	0.11134707	0.74210455	0.67345276
Plekhs1	-0.259165	-0.1763468	0.11128192	0.74217713	0.67345276
Sumo3	-0.0634082	6.45768741	0.11121021	0.74225704	0.67345276
Trp53bp2	0.04457758	5.93993044	0.1111591	0.74231401	0.67345276
Rgma	0.08007813	4.29821178	0.11115043	0.74232368	0.67345276

Atg12	0.06180685	5.11943071	0.11108476	0.7423969	0.67345276
Plcb3	-0.0747144	4.33601191	0.11104002	0.7424468	0.67345276
Irf9	0.0776889	3.88034125	0.11102029	0.74246882	0.67345276
Pygm	-0.1005028	3.78732599	0.1108835	0.74262147	0.67349919
Ddx60	-0.1669584	1.88541566	0.1108421	0.74266769	0.67349919
Man2b2	0.09094206	3.67084702	0.11072809	0.74279504	0.67349919
Ccne2	-0.1187339	1.99831562	0.11068462	0.7428436	0.67349919
Zfp647	0.11858936	1.3500515	0.1106435	0.74288956	0.67349919
Ift27	-0.0870027	2.67768703	0.11063785	0.74289588	0.67349919
Atp7a	0.05277512	5.55625978	0.11062193	0.74291367	0.67349919
Gm15816	0.21215374	0.7008866	0.11044379	0.7431129	0.6736288
Aff3	-0.0446656	7.61446663	0.11033045	0.74323974	0.67367901
Mblac2	-0.0589865	6.02592494	0.11029378	0.74328079	0.67367901
Nfib	0.03837226	8.11049038	0.11007729	0.74352333	0.67381604
Phf5a	-0.07371	4.82766972	0.10998562	0.74362611	0.67381604
Rbfox1	-0.0451775	9.25183504	0.10993092	0.74368746	0.67381604
Mlx	0.07860283	3.4619609	0.10993014	0.74368834	0.67381604
Ncr1	-0.2263811	-0.1231929	0.10986079	0.74376614	0.67381604
Bcas3os1	-0.2944216	0.45882069	0.10985083	0.74377732	0.67381604
Ormdl3	0.08024379	4.20754267	0.10980761	0.74382583	0.67381604
Gosr1	-0.0550435	5.27713659	0.10973421	0.74390823	0.67383971
Kif21a	-0.0630866	8.49741799	0.10956011	0.7441038	0.67388933
Padi2	0.07716662	4.03186539	0.10955871	0.74410538	0.67388933
2310069G16	-0.1501322	1.60202341	0.10948993	0.7441827	0.67388933
B430319G15	-0.1168399	1.96980397	0.10948514	0.74418809	0.67388933
Gemin8	-0.0737567	3.45202186	0.10939811	0.74428595	0.67389599
Nt5e	0.08894695	4.27306299	0.10937851	0.74430799	0.67389599
Psmd1	-0.034824	7.50243881	0.1093194	0.7443745	0.67390526
Nuf2	-0.3136547	-0.0548779	0.10926112	0.74444009	0.6739137
1700034I23R	-0.2915094	-0.3781941	0.10918671	0.74452387	0.67393859
F8a	0.06339069	3.41659898	0.10897013	0.74476786	0.67408922
Bbx	-0.0417602	7.92283414	0.10893909	0.74480285	0.67408922
Tomm7	-0.0512756	4.6452699	0.10874037	0.74502701	0.67418774
BC051226	0.3376664	-1.6089049	0.10871536	0.74505524	0.67418774
H19	-0.3184121	-1.5509996	0.10869291	0.74508059	0.67418774
Klk10	0.26621503	-0.2302173	0.10855753	0.74523346	0.67424805
2010107G23	-0.0619732	4.05042054	0.10853063	0.74526386	0.67424805
Mrpl50	-0.0623707	5.7014081	0.10848435	0.74531614	0.67424805
Cyb561d2	-0.1360722	1.76477958	0.10841614	0.74539324	0.67426686
Arl6ip6	-0.0601314	4.59308749	0.10833519	0.74548477	0.67427746
Adarb1	-0.0542706	6.5564066	0.10826128	0.74556837	0.67427746
Alx1	0.16315995	2.53238733	0.10823252	0.74560092	0.67427746
Cnot2	0.0411416	6.21927661	0.10820666	0.74563018	0.67427746

Rhot2	-0.058205	5.09631847	0.10811652	0.74573221	0.67431881
Lingo2	0.05798466	5.23399232	0.10802444	0.74583648	0.67436218
Zfp426	0.04407825	6.10522924	0.10779906	0.74609193	0.67449497
Ttc29	0.53936343	-2.3012411	0.1077183	0.74618354	0.67449497
Laptm5	-0.1207288	3.30029559	0.10766348	0.74624574	0.67449497
Rtn4r	0.05948353	4.37400648	0.10764592	0.74626567	0.67449497
Zfp53	0.07945071	2.75478349	0.10754651	0.74637852	0.67449497
Mthfd1l	0.08516744	3.49817884	0.1075336	0.74639318	0.67449497
2810454H06l	-0.2741902	-0.9132212	0.10752031	0.74640828	0.67449497
Exoc3	-0.0446078	6.57584827	0.10744886	0.74648944	0.67449497
Fgf2	-0.1143692	1.59855045	0.10744815	0.74649025	0.67449497
Rhbdl3	0.09531483	2.78035487	0.10733045	0.74662401	0.67454321
Wdr27	-0.3970556	-0.7713851	0.10730206	0.74665628	0.67454321
Hnrnpa2b1	0.0310319	8.6580106	0.10711129	0.74687328	0.67457206
Atg13	-0.0385537	5.92728263	0.10709136	0.74689597	0.67457206
Med14	-0.0544203	7.67537787	0.10706052	0.74693108	0.67457206
Ly6h	0.13850953	0.87209353	0.10702378	0.7469729	0.67457206
Ufc1	-0.055236	5.14966962	0.10697993	0.74702284	0.67457206
Acsl3	0.03946169	7.2432512	0.1069341	0.74707504	0.67457206
Dgki	0.07322144	4.88385758	0.10692754	0.74708251	0.67457206
Tsr3	0.0576376	4.06406222	0.10669442	0.74734825	0.67471978
G3bp1	-0.0489991	5.14055908	0.10668516	0.7473588	0.67471978
Ldlrad4	0.05333988	4.36462641	0.10654836	0.7475149	0.67478921
Plcg2	-0.0969188	1.88534038	0.10651901	0.7475484	0.67478921
Ankdd1b	-0.0673528	3.20222513	0.10608891	0.74803996	0.67514439
I730030J21R	-0.3994641	-1.3284042	0.10604906	0.74808556	0.67514439
Ppm1l	-0.0664051	4.72174536	0.1059933	0.74814938	0.67514439
Osbpl10	0.13285351	2.18542351	0.10596555	0.74818115	0.67514439
Tssk2	0.49327186	-1.7611136	0.10592834	0.74822375	0.67514439
Popdc3	-0.3277953	-0.7530781	0.10579223	0.74837967	0.67521519
Setd1a	-0.0522916	5.5857919	0.10576143	0.74841498	0.67521519
Znrd1as	-0.0703707	3.55287635	0.10569403	0.74849224	0.67523402
Scrn3	-0.0534397	4.81804462	0.1056002	0.74859985	0.67528023
Rtel1	-0.1028013	2.53186171	0.10553911	0.74866994	0.67529259
1600023N17	-0.3284456	-0.9773858	0.10542787	0.74879762	0.67530195
Gm17066	-0.0699635	4.80154573	0.1054024	0.74882687	0.67530195
Plaur	0.31904566	-1.2438699	0.10538271	0.74884948	0.67530195
Creb1	0.03209558	7.03094245	0.10517018	0.74909369	0.67532423
Syt17	0.09662105	3.80397589	0.1051339	0.74913541	0.67532423
Ripk2	0.06909679	4.3644406	0.10513068	0.74913911	0.67532423
Ddx1	0.03941661	6.99049267	0.10511439	0.74915784	0.67532423
Mpp5	-0.0410206	6.90625661	0.1050698	0.74920913	0.67532423
G630090E17	0.47609546	-1.1328509	0.10501211	0.74927551	0.67532423

Arpc5	0.05188367	7.34556386	0.10497997	0.74931249	0.67532423
Stk38	-0.050494	4.80460911	0.10491329	0.74938924	0.67532423
Matk	-0.0728069	3.61966561	0.10484505	0.74946782	0.67532423
Tgds	-0.0890622	2.74649204	0.10484168	0.7494717	0.67532423
Galnt7	-0.0707857	3.06937041	0.10479424	0.74952634	0.67532423
Ccnd1	-0.059409	6.16639767	0.10477192	0.74955206	0.67532423
Hrct1	-0.434468	-2.1066727	0.10470202	0.74963261	0.67532423
Gabarapl1	0.04098924	8.09279506	0.10462201	0.74972484	0.67532423
Ankrd13c	-0.038497	6.39659034	0.10456772	0.74978746	0.67532423
9530052E02I	0.36417231	-0.9840896	0.10455412	0.74980314	0.67532423
Mt1	0.06949968	6.01750431	0.10449245	0.74987429	0.67532423
Socs4	-0.0524085	5.019161	0.10446199	0.74990944	0.67532423
Pdgfrl	-0.1166869	2.57959627	0.1043974	0.74998399	0.67532423
Grin3b	0.35735896	-1.7901405	0.10436974	0.75001592	0.67532423
Hnrnph3	-0.0474867	4.49209724	0.10433295	0.75005841	0.67532423
Npc2	-0.0886283	4.68547146	0.10426838	0.75013299	0.67534061
Cmtr2	-0.1030159	2.13066756	0.10409698	0.75033107	0.67541566
Zeb2os	-0.1164581	1.51606069	0.10406428	0.75036889	0.67541566
Tmem123	-0.0733596	5.06646632	0.10404988	0.75038554	0.67541566
Cenpn	-0.1695479	1.17111086	0.10386928	0.75059453	0.67551019
Ahsa1	0.0450059	5.77367489	0.10366954	0.75082588	0.67551019
Olfml2b	-0.1821425	0.63137112	0.10365902	0.75083808	0.67551019
Cdk14	0.04390244	6.91946638	0.10364056	0.75085947	0.67551019
B4galnt4	-0.0907001	3.61833574	0.10361326	0.75089112	0.67551019
Mfsd5	0.07869164	3.64305993	0.10352252	0.75099633	0.67551019
Col17a1	0.44231185	-1.226709	0.10334096	0.75120702	0.67551019
Cpt1c	0.08490141	3.36408473	0.10332872	0.75122122	0.67551019
Myo18b	0.22124812	-0.6149514	0.10330235	0.75125184	0.67551019
Phf21a	0.04363826	6.60498082	0.10329069	0.75126538	0.67551019
Usp18	0.11599667	1.69808751	0.10325901	0.75130217	0.67551019
Osbpl6	-0.0440678	7.06424395	0.1032136	0.75135492	0.67551019
Lair1	-0.1027519	3.29310948	0.10320759	0.75136191	0.67551019
Calr4	-0.3955825	-1.2051204	0.1032061	0.75136364	0.67551019
Fam208b	-0.0436733	6.44869303	0.10320517	0.75136472	0.67551019
Asap1	0.04597688	7.9708348	0.10314589	0.7514336	0.67551019
Lepre1	0.08907416	2.22410944	0.10309983	0.75148714	0.67551019
Lrrc9	-0.155829	1.59318661	0.10304116	0.75155535	0.67551019
Snx5	-0.0370806	7.0692407	0.10301881	0.75158134	0.67551019
Cdkn2aip	-0.0586057	4.20203269	0.10281981	0.7518129	0.67551019
Gcc2	0.05247806	7.54554474	0.10281202	0.75182197	0.67551019
Tmem201	0.07656308	3.96789872	0.1027685	0.75187263	0.67551019
Hist1h2bj	0.2917074	-1.5692539	0.10271651	0.75193319	0.67551019
Zfp607	0.11066694	2.48139595	0.10270748	0.75194371	0.67551019

Tpmt	0.07941432	3.69758478	0.10267398	0.75198274	0.67551019
1810044D09I	-0.320679	-1.5291417	0.10267071	0.75198654	0.67551019
4930455C13I	0.27596478	-0.7454366	0.10260489	0.75206325	0.67551019
Tmsb15l	0.15346664	1.11771483	0.10257356	0.75209978	0.67551019
Nlrx1	0.14430143	1.86726911	0.10255075	0.75212636	0.67551019
Nlrp10	-0.2487176	-0.7884599	0.10240942	0.75229121	0.67551277
Zufsp	0.10188252	3.44116249	0.10240561	0.75229565	0.67551277
Nvl	-0.0543794	5.01649047	0.10237444	0.75233203	0.67551277
Zfp935	0.06092784	4.49689751	0.10233727	0.75237541	0.67551277
15-Sep	0.06694541	6.70943786	0.10225846	0.75246742	0.67551277
Atp10d	-0.0936265	2.36718858	0.10219146	0.75254567	0.67551277
Kcnmb4	-0.0574988	3.94159509	0.10217725	0.75256227	0.67551277
Fbxl17	0.04275621	8.00944252	0.10216165	0.75258049	0.67551277
Bpgm	-0.048414	6.8275735	0.1020334	0.75273039	0.67559668
Rnf17	-0.1829628	0.73325128	0.10187829	0.75291181	0.67561209
Rab23	-0.0450917	5.37717903	0.10187283	0.75291821	0.67561209
Ankrd26	0.04678197	5.96875239	0.10184109	0.75295535	0.67561209
Esrra	0.11535998	2.04728223	0.10181401	0.75298705	0.67561209
Spata4	-0.4034844	-1.0776967	0.1017257	0.75309045	0.67561209
Map3k11	0.06895824	3.78564312	0.1017256	0.75309056	0.67561209
C2cd4d	-0.4963161	-2.1321454	0.10164416	0.75318595	0.67561209
Gab3	0.08424976	2.77288416	0.10163314	0.75319887	0.67561209
Mrpl49	0.07180107	4.77436411	0.10152992	0.75331985	0.67566999
Rbmxl1	0.04763608	5.34589188	0.10137592	0.75350047	0.67573617
Fam117a	-0.125145	3.77824039	0.1013708	0.75350648	0.67573617
Rer1	0.0588576	5.71875699	0.10124316	0.7536563	0.67577882
Hist1h1b	-0.3285146	-1.8051552	0.10118189	0.75372825	0.67577882
Gng10	-0.0744571	4.81533876	0.10114886	0.75376705	0.67577882
B930041F14	0.05475881	5.11013948	0.10113805	0.75377975	0.67577882
Igip	0.04792189	5.87735053	0.10093877	0.75401402	0.67590412
Cebpzos	-0.0705136	2.99218171	0.10072165	0.75426954	0.67590412
Setmar	0.10645664	2.36982812	0.10071911	0.75427253	0.67590412
Fam196b	0.11470511	1.52133932	0.10071437	0.75427812	0.67590412
Ptrh1	0.4163843	-1.2887047	0.10067415	0.75432548	0.67590412
Ccser1	0.09547658	3.31125484	0.10061834	0.75439123	0.67590412
Zfp763	-0.0668849	4.04657361	0.10060975	0.75440135	0.67590412
Fam154a	0.48303285	-1.9889816	0.10058977	0.75442489	0.67590412
Cd55	0.07427989	5.98307271	0.10052141	0.75450546	0.67590412
Rasd2	0.05506518	4.98101848	0.10050421	0.75452574	0.67590412
Swt1	0.06264743	4.57133182	0.10047135	0.75456449	0.67590412
Zzef1	-0.082537	5.30413241	0.10044396	0.75459679	0.67590412
Tbrg4	0.05901044	3.84983377	0.10028721	0.75478174	0.67597617
Klrk1	0.19563374	0.53259997	0.1002801	0.75479012	0.67597617

Cntf	0.30468305	-1.5165918	0.10019171	0.75489449	0.67599559
Btg1	-0.0646128	5.37506462	0.10016613	0.7549247	0.67599559
Gabpb1	0.04601757	5.10050839	0.10007455	0.7550329	0.67604192
Snx9	-0.057803	4.81473648	0.1000154	0.75510281	0.67605397
Ecd	0.05719482	4.1883553	0.09989432	0.75524599	0.67607464
C330027C09I	0.12139341	1.86514799	0.09982831	0.75532409	0.67607464
Rwdd2a	0.07809461	2.99713776	0.09980168	0.75535562	0.67607464
Mcee	0.06488362	4.05548428	0.09978023	0.755381	0.67607464
Ccm2	-0.0614487	3.74234732	0.09975729	0.75540816	0.67607464
Thrap3	0.03787872	7.46786872	0.09967066	0.75551074	0.67611592
Utp18	0.0521153	4.20392868	0.09949203	0.75572241	0.67625018
Klhl42	-0.037766	6.43862941	0.09944877	0.75577371	0.67625018
Papd4	-0.0583577	5.05321007	0.09936146	0.75587727	0.67629232
Clasp2	0.04059433	8.0401354	0.0992906	0.75596136	0.67631703
Strc	0.27259995	-1.2857172	0.09923937	0.75602217	0.6763209
Aaas	-0.0773439	3.0916257	0.09909195	0.75619727	0.67638546
Nup50	0.04314993	5.45782005	0.09907525	0.75621711	0.67638546
Cdc16	-0.0523019	4.94816157	0.09897017	0.75634201	0.67638546
Ep300	-0.0430592	8.10979494	0.09896799	0.75634461	0.67638546
Rpl39	-0.0698405	7.34060567	0.09892166	0.7563997	0.67638546
Abca3	0.06705438	4.11377564	0.09872024	0.7566394	0.67638546
Shisa7	-0.05984	6.36471315	0.09866122	0.75670969	0.67638546
Prelp	-0.0699562	8.14582054	0.09865352	0.75671886	0.67638546
Mnat1	0.06008791	4.19867211	0.09862579	0.75675189	0.67638546
Pdzrn3	0.03928098	7.30350977	0.09860819	0.75677286	0.67638546
Gm166	0.14233505	0.64345671	0.09860767	0.75677348	0.67638546
2810408M09	0.06599787	3.2236678	0.09839682	0.75702484	0.67638546
Lypd6b	-0.0678467	3.94970159	0.0983442	0.75708762	0.67638546
Nars2	-0.074933	3.83879504	0.09834334	0.75708864	0.67638546
Supv3l1	-0.0752499	3.17870659	0.09833771	0.75709536	0.67638546
Rn45s	-0.069539	14.5621267	0.09832727	0.75710782	0.67638546
Tcerg1l	-0.0827917	2.69450517	0.09828461	0.75715873	0.67638546
Baiap2l2	-0.2798085	-1.0733073	0.09828198	0.75716188	0.67638546
Pydc3	0.17875867	0.73469597	0.09824049	0.75721139	0.67638546
Nhlh1	0.2559339	-0.2689233	0.09819283	0.75726831	0.67638546
Eda2r	-0.2973792	0.18129697	0.0981827	0.7572804	0.67638546
Adcy7	0.14337104	1.81924374	0.09801376	0.75748227	0.6765153
Acd	0.06185428	3.94948651	0.09773661	0.75781383	0.67675688
Shisa6	-0.0676512	5.30408921	0.09769322	0.75786578	0.67675688
N28178	-0.0565017	7.04077347	0.09746481	0.7581395	0.67695083
Ccr2	0.10933407	2.65618563	0.09739897	0.75821846	0.67697086
Gm12522	0.14445496	1.18137555	0.09702387	0.7586689	0.67728267
Set	0.03808817	8.09528691	0.09701397	0.7586808	0.67728267

Pcdha2	-0.1942676	0.19844634	0.09689447	0.75882451	0.67735622
Tmem55a	-0.0431215	6.17654235	0.09680945	0.75892682	0.67735622
Atp6v1c2	0.27882576	-0.1751808	0.09675919	0.75898732	0.67735622
Gsx1	0.46571405	-1.7004673	0.09675743	0.75898944	0.67735622
4932418E24I	-0.2276768	-0.0372914	0.09660778	0.75916968	0.67741089
Phlda3	-0.0929209	2.61215372	0.09656789	0.75921776	0.67741089
Rps2	-0.0480202	6.30221728	0.0965657	0.75922039	0.67741089
Pigc	0.10400105	3.10018614	0.0965041	0.75929465	0.67742668
Grk5	0.04760763	5.33355787	0.09640762	0.759411	0.67746042
Nup133	-0.0469356	4.51006149	0.09634372	0.75948811	0.67746042
Gm715	-0.1234166	1.23912547	0.09621074	0.75964865	0.67746042
Med9	0.0624912	4.76136036	0.09620199	0.75965921	0.67746042
Nr5a2	-0.4707467	-2.0178375	0.09613158	0.75974426	0.67746042
Wdr75	0.06274114	4.66345154	0.09612606	0.75975093	0.67746042
AI314180	0.03998238	7.16951713	0.09606516	0.75982453	0.67746042
P2rx4	0.07944205	3.21501847	0.0960546	0.7598373	0.67746042
Tmem120b	-0.2480146	0.44603953	0.09599017	0.75991519	0.67746042
Cldn8	0.4697859	-2.2535441	0.09598324	0.75992357	0.67746042
Dram1	-0.1665118	0.62220117	0.09591552	0.76000548	0.67746042
Polr2g	-0.0548768	5.38000688	0.09589944	0.76002493	0.67746042
Rhoc	-0.0830831	3.48230513	0.09586395	0.76006787	0.67746042
Ptprg	-0.0426635	6.91792331	0.09580408	0.76014034	0.67747333
Snord91a	0.07287913	3.26393971	0.09575852	0.7601955	0.67747333
Ocel1	-0.1203922	1.7625037	0.09566175	0.76031271	0.67752384
Commd7	0.05291044	4.6837121	0.09561832	0.76036533	0.67752384
Itga11	-0.0886003	2.90705307	0.09546832	0.76054718	0.6775815
Tgm1	0.41875132	-1.4017659	0.09543401	0.7605888	0.6775815
2700062C07I	-0.0652613	3.96942133	0.09534515	0.76069661	0.6775815
Mov10	0.08434169	2.92325618	0.09532015	0.76072696	0.6775815
Arl13b	-0.053902	4.2966268	0.09527315	0.76078402	0.6775815
Pnoc	-0.2253761	-0.2246849	0.09521645	0.76085288	0.6775815
Aox1	-0.0754938	3.12249429	0.0952065	0.76086496	0.6775815
B430212C06	0.46028445	-1.5430371	0.09518575	0.76089017	0.6775815
Mtch1	0.0407522	6.41998609	0.09514535	0.76093925	0.6775815
Rbmx2	0.06363405	4.45926901	0.09506373	0.76103845	0.67761944
Klhl17	-0.1050967	3.50189595	0.09490983	0.76122563	0.67772413
Cspg4	-0.0829177	2.74830297	0.09487403	0.7612692	0.67772413
Npsr1	0.1416026	1.3576542	0.09463013	0.76156624	0.67793817
Ube3c	0.04177014	6.07983139	0.09456866	0.76164117	0.67795448
Ccdc93	0.04855452	5.01806247	0.09446829	0.76176358	0.67796524
Ap1g1	-0.0479413	7.3243594	0.09446591	0.76176649	0.67796524
Tapbpl	-0.0590174	3.28314003	0.09430159	0.76196703	0.67802001
Mpp4	-0.2299358	-1.0414371	0.09421628	0.76207123	0.67802001

Pikfyve	-0.0503974	6.77056476	0.09420952	0.76207949	0.67802001
H2afj	0.08127299	3.5416245	0.09417844	0.76211746	0.67802001
Trdmt1	-0.1003825	2.84535639	0.094123	0.76218522	0.67802001
Opa1	-0.0441545	7.78864065	0.0940956	0.76221871	0.67802001
Itch	0.0338336	7.05379418	0.09406404	0.7622573	0.67802001
Fgf14	-0.0398595	6.28372255	0.09400058	0.76233491	0.67802001
Dmpk	0.07340404	3.50546501	0.0939984	0.76233758	0.67802001
Pdzd7	-0.1361838	1.33017718	0.09381972	0.76255627	0.67814051
Actr10	0.03546434	7.66079374	0.09378259	0.76260173	0.67814051
Gm13308	-0.298143	-1.3918595	0.09374895	0.76264294	0.67814051
Tab2	-0.0355247	8.33770501	0.09367303	0.76273597	0.67817288
Dusp11	-0.0395775	6.38920136	0.09358836	0.76283976	0.67821481
Dhrs3	0.07777815	4.64623871	0.09348013	0.76297252	0.67823944
Gm5141	0.08607862	3.06213134	0.09343467	0.7630283	0.67823944
BC017643	-0.098614	2.48039427	0.09342728	0.76303736	0.67823944
G0s2	0.14719438	1.60032905	0.09325419	0.76324992	0.67837802
Ptplad2	0.07278632	3.7755559	0.09304229	0.76351042	0.67854779
Sema5b	0.11003536	3.53938769	0.09297527	0.76359288	0.67854779
Nxpe3	0.08098961	4.07112781	0.09296062	0.7636109	0.67854779
Zfp708	0.07031619	3.49910848	0.0927149	0.76391354	0.67873001
Ccdc37	-0.1676919	0.91688166	0.09270209	0.76392932	0.67873001
9430038I01R	-0.1923874	0.73306406	0.09261917	0.76403155	0.67877048
Igfals	0.46711989	-2.2123969	0.09251575	0.76415913	0.67878964
Smim1	0.15449898	1.65134295	0.09246845	0.76421752	0.67878964
Abhd17a	-0.0644497	3.66080097	0.09246387	0.76422316	0.67878964
Pcbp4	0.07872683	2.70007356	0.09238083	0.76432569	0.67883036
Sf3b6	0.05251262	5.00952642	0.09228162	0.76444825	0.67888886
Il1rapl2	-0.0898183	3.23313246	0.0921788	0.76457533	0.67890217
Rpe	0.05124352	5.42631599	0.09215318	0.76460701	0.67890217
Fam228b	0.14840019	1.62913522	0.09213193	0.7646333	0.67890217
Ttll1	0.03828013	5.61818762	0.09185551	0.76497545	0.67891917
Mrpl3	-0.0401782	5.42029151	0.09180895	0.76503314	0.67891917
Tuba1a	-0.0331001	9.67220925	0.09179904	0.76504542	0.67891917
Eral1	0.06690969	3.54601251	0.09179358	0.76505218	0.67891917
Hist2h4	0.17612189	0.30175153	0.0917777	0.76507186	0.67891917
Fam175a	0.0794971	2.6092575	0.09167076	0.76520444	0.67891917
Nek10	-0.2118187	0.42378789	0.09163361	0.76525053	0.67891917
Mroh7	-0.4186347	-0.4460366	0.09160964	0.76528025	0.67891917
Ica1l	0.04362897	5.02285201	0.09160891	0.76528117	0.67891917
Rbm38	-0.2334344	0.8031753	0.09157277	0.765326	0.67891917
Gsdmd	-0.1074152	2.29812276	0.09153749	0.76536979	0.67891917
A230077H06	-0.1341412	2.28415411	0.0915278	0.76538182	0.67891917
Brinp2	-0.0507961	4.4590988	0.09152166	0.76538943	0.67891917

Lrrc16b	-0.1694076	2.98873128	0.0913099	0.76565246	0.67907963
Gapt	0.26907024	-0.1591237	0.09128156	0.76568768	0.67907963
Olfr99	-0.3745352	-1.5113581	0.09123912	0.76574045	0.67907963
Nhsl2	0.03620579	8.08117747	0.09104497	0.76598199	0.67924354
Arhgap35	-0.0392769	8.93178848	0.09095423	0.76609498	0.67926122
Rbm39	-0.0331858	7.64977397	0.09083675	0.76624136	0.67926122
Wrnip1	-0.0497617	5.40301787	0.09083595	0.76624236	0.67926122
6720489N17	-0.0636219	3.66694827	0.09081021	0.76627444	0.67926122
Ckmt2	-0.3462996	-1.0422931	0.09080131	0.76628553	0.67926122
D630024D03	0.39293819	-1.7869437	0.09072925	0.76637539	0.67929059
Rexo1	-0.0510719	4.84694613	0.09064565	0.76647968	0.67933275
Gm16853	0.30352921	-0.9169055	0.09050128	0.76665991	0.67938063
Secisbp2l	-0.0359735	7.76977179	0.09042626	0.76675363	0.67938063
Nab2	-0.0613378	5.10920248	0.09039707	0.76679011	0.67938063
Hist1h2bk	0.26366865	-1.1580995	0.09037829	0.76681358	0.67938063
Abcd3	-0.0376868	6.90680663	0.0903176	0.76688945	0.67938063
Gid4	-0.037444	5.84590795	0.09029059	0.76692322	0.67938063
Rasgef1a	-0.0462165	6.81493714	0.09025117	0.76697252	0.67938063
Fkbp14	0.07399427	4.22152797	0.09023518	0.76699252	0.67938063
Itga1	-0.053574	4.19163823	0.09015209	0.76709649	0.67938063
Myl12b	0.05242037	7.8337359	0.09014246	0.76710855	0.67938063
Rhoj	0.06530597	5.38522687	0.09010317	0.76715773	0.67938063
Dhdds	0.04845915	4.89381283	0.09000208	0.76728433	0.6794425
Kcnab3	0.04569726	5.49972719	0.08986825	0.76745204	0.67952635
Astn1	0.05581154	8.03624661	0.08983598	0.7674925	0.67952635
Rundc3a	-0.049572	5.33651374	0.08970922	0.76765152	0.6796169
3110039M2(	-0.1343783	2.13060271	0.08964894	0.76772718	0.67963364
Tcf24	0.15658396	1.26987938	0.08937034	0.76807725	0.67967864
Rbm25	-0.0416881	8.74047397	0.08926886	0.7682049	0.67967864
Plekha2	0.05685267	5.21607089	0.08925503	0.7682223	0.67967864
Mrps7	-0.0486005	5.25942909	0.08923599	0.76824626	0.67967864
Skp1a	0.0419839	8.04762672	0.08922789	0.76825646	0.67967864
Grcc10	0.10019295	3.80079278	0.08913381	0.76837491	0.67967864
Nudt19	0.05196595	4.87249555	0.08909218	0.76842734	0.67967864
Kifc3	-0.0713874	3.01247194	0.08907754	0.76844578	0.67967864
Ppfia3	-0.0592513	4.67632354	0.08905163	0.76847844	0.67967864
Gm14322	0.05307986	4.29984094	0.08903393	0.76850074	0.67967864
Arhgef1	-0.0642505	4.30203653	0.08901296	0.76852716	0.67967864
Agap3	0.03901896	5.85847907	0.0889716	0.76857929	0.67967864
Gm10768	0.52686986	-2.1390661	0.09029976	0.76858095	0.67967864
Thoc3	0.07200281	4.17805033	0.08895264	0.7686032	0.67967864
Fat3	-0.0787257	8.06281523	0.08893191	0.76862934	0.67967864
Htra1	0.0646132	3.91348664	0.08886982	0.76870764	0.6796977

Ldb1	0.05468325	3.97055761	0.08875884	0.76884768	0.67976765
Rnf166	-0.0621676	4.71944735	0.08871718	0.76890028	0.67976765
Cript	0.04829296	7.53137225	0.08858276	0.76907006	0.67983012
Ccdc55	0.0365079	6.97211916	0.08857136	0.76908447	0.67983012
Tbc1d9b	-0.0371171	6.59204366	0.08849503	0.76918096	0.67986522
Cdc20	0.2336359	-0.3598917	0.08839628	0.76930585	0.67992543
D630013N20	-0.4662361	-0.8906889	0.08824276	0.76950016	0.67995623
Zfp273	-0.0691032	3.24774517	0.08818562	0.76957253	0.67995623
Ndufs7	0.05371542	5.10040801	0.08815566	0.76961049	0.67995623
Krcc1	0.07000849	7.09047023	0.08810063	0.76968022	0.67995623
Fam120b	-0.0428842	6.59916245	0.08808553	0.76969936	0.67995623
Gnaq	0.03080022	8.68177688	0.08801504	0.76978872	0.67995623
Ccr4	-0.1914171	0.09339256	0.08787541	0.76996586	0.67995623
Mpc1	-0.0369328	6.49887657	0.08786374	0.76998068	0.67995623
Sfrp5	-0.4049853	-2.1405377	0.08783445	0.77001786	0.67995623
Ccdc101	0.05735611	4.2368717	0.08781791	0.77003886	0.67995623
Cxcl16	0.10296972	3.95304305	0.08778875	0.77007588	0.67995623
B230118H07	-0.0475048	5.48858594	0.0877451	0.77013132	0.67995623
Gramd1a	0.08496036	3.06794535	0.08773206	0.77014789	0.67995623
Serpinb1a	0.1250672	2.21914967	0.08767999	0.77021404	0.67995623
Csnk1e	-0.0434491	6.14590417	0.08766882	0.77022824	0.67995623
Vsig2	-0.0627932	3.29637463	0.08765237	0.77024915	0.67995623
Csrp2	-0.0534582	4.57851133	0.08752865	0.77040645	0.67998796
Gm14005	0.18106514	0.45134043	0.0875234	0.77041313	0.67998796
Pgrmc1	0.05965797	7.58892138	0.08749015	0.77045543	0.67998796
Clcn4-2	0.03646621	6.19279501	0.08729978	0.77069777	0.6801155
9930104L06I	0.08235118	3.10313452	0.08722491	0.77079315	0.6801155
Rap2c	-0.0325748	6.85442395	0.0872208	0.77079839	0.6801155
Ptgds	-0.1029069	12.2504338	0.08718308	0.77084646	0.6801155
Rab24	-0.0489288	5.10408853	0.08715372	0.77088389	0.6801155
Fam103a1	-0.0412411	7.87600911	0.08703365	0.77103704	0.68014094
5830432E09I	0.27709697	-1.1119705	0.08699388	0.77108778	0.68014094
Mctp1	0.04567382	5.38954545	0.08691995	0.77118216	0.68014094
Gatad1	-0.0381384	7.04557195	0.08687966	0.7712336	0.68014094
Tyro3	-0.0485531	4.93736118	0.0868757	0.77123865	0.68014094
Pla2g4d	0.35167802	-2.0625272	0.08726659	0.77127788	0.68014094
Slc19a1	-0.0793249	3.08317596	0.08677864	0.77136266	0.68014094
Zmym1	-0.0711249	3.92520281	0.08673754	0.77141518	0.68014094
Rpp25l	-0.108869	2.05738718	0.08673075	0.77142387	0.68014094
Lace1	-0.0680654	3.18675385	0.08658779	0.7716067	0.68025205
Echdc3	0.12035524	1.11695065	0.08632858	0.77193862	0.6804471
Cog1	0.0570828	4.11567653	0.08626896	0.77201504	0.6804471
Tmem167b	0.06151498	6.20567732	0.08626676	0.77201786	0.6804471

Mrpl12	-0.0672646	3.85456759	0.08618956	0.77211685	0.6804471
Pcyt1a	-0.0452242	5.75957855	0.08615273	0.7721641	0.6804471
Ftsj2	-0.1187996	2.03862433	0.08614902	0.77216886	0.6804471
Rims3	0.15006562	0.8032777	0.08605176	0.77229368	0.68047739
Cwf19l2	0.03946429	5.62558779	0.0860337	0.77231687	0.68047739
Kdelr3	-0.1363261	1.07251434	0.08581135	0.77260255	0.68067902
Sertad4	-0.0729106	5.75548275	0.08573474	0.77270108	0.68071575
Sqrdl	-0.0850465	3.40384126	0.08526476	0.77330656	0.6811775
Mettl5	-0.0633005	3.45748939	0.08516028	0.77344141	0.6811775
Vegfc	0.09065604	2.74930106	0.0851503	0.77345429	0.6811775
Rbfa	-0.0841681	2.63680944	0.08514233	0.77346459	0.6811775
Lrrtm3	-0.0462823	4.97338521	0.08505255	0.77358055	0.6811775
Zfp58	0.06837996	3.40158186	0.08503777	0.77359965	0.6811775
Aurkb	0.27886515	-1.0478296	0.0850194	0.77362339	0.6811775
Bri3	-0.0585253	4.3199298	0.08488392	0.77379853	0.68127064
Rgs14	-0.0924461	2.19227833	0.08482692	0.77387227	0.68127064
Dctn1	-0.0537628	5.19929677	0.08480562	0.77389984	0.68127064
Uggt1	-0.0660869	5.11580547	0.08470815	0.774026	0.68132466
Dhtkd1	-0.1688535	1.49539204	0.08465566	0.77409398	0.68132466
Gm10408	0.19597895	-0.4186977	0.08452501	0.77426328	0.68132466
BC030867	0.33020341	-1.5114321	0.08452459	0.77426383	0.68132466
Fam134c	-0.0926212	2.95746964	0.08450738	0.77428613	0.68132466
Ccdc33	0.17361491	0.10981215	0.08444521	0.77436676	0.68132466
Orai3	-0.0652567	5.00577325	0.08443543	0.77437944	0.68132466
Nfatc2ip	0.07675102	2.6767404	0.08433488	0.77450991	0.68132466
Ankrd16	-0.1059833	2.54010471	0.0843075	0.77454545	0.68132466
Rab11a	0.03616714	6.56897151	0.08421768	0.77466209	0.68132466
Actr3	-0.0296302	8.04242918	0.08421443	0.77466631	0.68132466
Ptar1	-0.0574172	3.79022026	0.08419558	0.7746908	0.68132466
Sgtb	-0.0393942	8.05187458	0.08418789	0.77470079	0.68132466
Nr1d1	-0.0408765	8.75075476	0.08397714	0.77497478	0.68150226
Cbs	0.0606194	3.17066703	0.08394504	0.77501655	0.68150226
Tacr1	0.06834893	3.12697226	0.08387441	0.77510848	0.68151269
Atp2b1	0.05063717	9.27705514	0.08384475	0.7751471	0.68151269
Cdk17	0.04197617	7.87426799	0.08380479	0.77519914	0.68151269
1190002N15	0.04576138	5.34921163	0.08364192	0.77541138	0.68164924
Tmem120a	-0.2065617	0.1265171	0.08346899	0.77563697	0.68179751
Cd200r4	-0.407281	-2.3478976	0.08336813	0.77576866	0.68186322
Hexim2	0.08060973	2.30209558	0.08330514	0.77585095	0.6818855
Epha1	-0.4469548	-1.3427968	0.08323294	0.77594532	0.68191839
Sh2b3	-0.0651406	3.5835136	0.08315881	0.77604225	0.68195353
Heatr9	0.38265974	-1.4993693	0.08305597	0.77617679	0.68197029
Angptl3	-0.2205831	0.11043517	0.08304969	0.77618502	0.68197029

2810403A07I	0.0475347	6.56468175	0.08301368	0.77623216	0.68197029
Gmip	0.09532949	1.52538376	0.08293263	0.77633829	0.6819814
Tor1b	-0.0608306	3.76440609	0.08291705	0.77635869	0.6819814
Ccdc122	0.09647054	2.24255344	0.08286323	0.77642922	0.68199332
Nabp1	0.05559134	3.64228668	0.08276101	0.7765632	0.68206099
Cplx2	0.0456134	9.63292106	0.08262505	0.77674158	0.68214691
Stxbp1	0.03987944	10.0420214	0.08259963	0.77677495	0.68214691
Mroh5	-0.2156272	-0.7387819	0.0824737	0.77694032	0.68214726
Slc6a14	-0.4371378	-1.0287029	0.08245622	0.77696329	0.68214726
Neu4	-0.0856405	2.46054325	0.08243761	0.77698775	0.68214726
Pkmyt1	0.191656	-0.4137615	0.08241007	0.77702394	0.68214726
Sim1	0.2322722	-0.605711	0.08238252	0.77706015	0.68214726
Tefm	-0.0690832	3.32562014	0.08223092	0.77725954	0.68219687
E130307A14I	-0.0757421	2.29238303	0.08218324	0.77732229	0.68219687
Golga4	-0.0321299	7.9148528	0.08215821	0.77735525	0.68219687
Ints8	-0.0466822	5.49096989	0.08215313	0.77736194	0.68219687
Unc13d	-0.2495688	-0.8819921	0.08212309	0.77740149	0.68219687
Ano7	0.27994698	-1.3843268	0.08196105	0.77761499	0.68229848
Aifm2	0.09937232	2.59545827	0.08194873	0.77763123	0.68229848
Zfp395	0.05531356	6.06510323	0.0818309	0.77778663	0.68238483
Eif4a1	0.03250924	7.37071226	0.08178739	0.77784405	0.68238522
Slc50a1	-0.0743779	3.05410095	0.08167971	0.77798621	0.68243875
Alkbh4	0.11601232	1.04714267	0.08165486	0.77801904	0.68243875
Kansl3	-0.0397113	5.88337963	0.08149867	0.77822546	0.68256982
Bud31	0.04746447	4.69115838	0.08145392	0.77828465	0.68257174
Zfp810	0.04620865	4.65836076	0.08140828	0.77834502	0.6825747
Psmf1	-0.054469	4.76051962	0.08134406	0.77843	0.68258487
Snrpe	0.05417027	4.52125606	0.08131339	0.77847061	0.68258487
Csrnp3	-0.0534936	7.29463756	0.08124298	0.77856385	0.68261665
R3hdm4	-0.0405608	7.68370037	0.08116866	0.77866232	0.682653
2410002F23I	0.0661387	3.20414754	0.08096524	0.77893209	0.68273826
Snhg1	-0.0586165	4.49501652	0.08095665	0.77894349	0.68273826
Gpr158	0.04545333	8.18522937	0.0809456	0.77895814	0.68273826
9630033F20I	0.05497233	4.43530924	0.08092341	0.77898761	0.68273826
Kcnd2	-0.0462754	6.04117674	0.08081344	0.77913362	0.68281626
Pten	0.03286525	9.22052376	0.08053126	0.77950882	0.68304324
Abhd6	0.05300549	4.18091732	0.08051054	0.77953641	0.68304324
Pomc	-0.269506	0.35685037	0.0804359	0.77963579	0.68304324
Arf5	0.07584503	3.03196978	0.08043097	0.77964235	0.68304324
Meis3	0.07303406	3.60716697	0.08040436	0.7796778	0.68304324
Zfp653	0.09509339	2.40126424	0.08029987	0.77981702	0.68308835
Sirt1	-0.058387	4.47245671	0.08028011	0.77984337	0.68308835
Apoa2	0.266284	-1.2367279	0.08014835	0.78001911	0.68312882

Robo2	-0.04532	6.52649214	0.08010433	0.78007785	0.68312882
4930428E07I	-0.2342356	-0.2484161	0.08004103	0.78016236	0.68312882
Nlrp1b	-0.4442999	-1.6327494	0.08001175	0.78020146	0.68312882
Tm9sf3	-0.0346235	7.28578233	0.07998275	0.7802402	0.68312882
Vmn2r87	-0.1517814	1.17231692	0.07997449	0.78025123	0.68312882
Atp9a	-0.0520025	4.80433389	0.0798675	0.78039421	0.68312882
Raph1	0.05823066	7.73997908	0.07986656	0.78039547	0.68312882
D330023K18	0.13880189	1.2343076	0.07984055	0.78043024	0.68312882
Pou3f1	0.07972321	2.01277333	0.07980481	0.78047804	0.68312882
Icmt	-0.0408379	4.61061729	0.07971222	0.78060191	0.68312882
Pabpc4l	-0.1212095	2.23419676	0.0797016	0.78061614	0.68312882
Slc41a2	0.0571453	4.01969231	0.07969039	0.78063113	0.68312882
Rgsl1	0.12048661	1.52849034	0.07960937	0.78073961	0.68317383
Fam86	-0.0794651	3.38812708	0.07955524	0.78081212	0.68317649
Gpt2	0.0423801	4.97022842	0.07950161	0.78088397	0.68317649
Mtus1	-0.0349979	6.68441201	0.07947802	0.7809156	0.68317649
Slc33a1	0.06114997	3.78701826	0.07940526	0.78101315	0.68317649
Perm1	-0.2416793	-0.4116374	0.0793735	0.78105575	0.68317649
Dync1li2	-0.029646	8.01244482	0.07931154	0.78113888	0.68317649
Eaf1	-0.0432711	5.06304878	0.07929944	0.78115511	0.68317649
Secisbp2	0.05805194	3.83289147	0.07925459	0.78121532	0.68317649
Atp13a2	-0.068351	4.17613048	0.07917985	0.78131568	0.68317649
Pnkp	-0.0927679	1.65228763	0.07905617	0.78148188	0.68317649
Pstk	-0.0631314	4.18438685	0.07897585	0.78158988	0.68317649
Mogs	0.11128513	1.72178949	0.07897459	0.78159157	0.68317649
Srrm3	-0.0728738	2.95902668	0.07890823	0.78168086	0.68317649
Pck2	-0.0692097	3.2418545	0.07887802	0.78172152	0.68317649
Myh4	0.29566032	-0.373539	0.07887228	0.78172924	0.68317649
4933431G14	-0.1737176	0.30495939	0.07885085	0.78175808	0.68317649
Sft2d3	0.0638424	3.19211929	0.07880127	0.78182484	0.68317649
Cldn10	0.10535405	1.85072512	0.07878749	0.78184339	0.68317649
Gabrb1	0.05996546	3.55491588	0.07878267	0.78184989	0.68317649
Tdrkh	0.05919895	4.42683345	0.0787242	0.78192865	0.68317649
U2af2	0.06264272	4.69481803	0.07871531	0.78194063	0.68317649
Rcor1	0.04368035	5.10840281	0.07850881	0.78221908	0.68332448
Tmem238	-0.1941785	-0.4145217	0.07850506	0.78222414	0.68332448
Pdzk1	-0.074704	2.85859714	0.07843821	0.78231436	0.68335345
Cog5	0.06061012	4.63452983	0.07835536	0.78242625	0.68337721
Sco1	0.06468673	3.29472571	0.07833356	0.78245569	0.68337721
Tfeb	-0.1247438	1.47424651	0.07818935	0.78265062	0.68345578
Zcchc6	-0.0292289	7.22987234	0.07818257	0.78265979	0.68345578
Sh2d1b1	0.22988139	0.8331285	0.07803653	0.78285739	0.68351377
Mlf2	0.03188175	6.45549964	0.07801458	0.78288711	0.68351377

Supt20	-0.0458808	4.95552167	0.07800199	0.78290416	0.68351377
Lyrm7	0.04188967	4.50608836	0.07796482	0.7829545	0.68351377
Wars	0.04327663	5.11435786	0.07786352	0.78309176	0.68356785
Senp6	0.02930392	8.47980854	0.07781704	0.78315477	0.68356785
Trmt10c	0.05137952	4.78226766	0.07779276	0.78318768	0.68356785
Fsd1	0.09214573	2.29276353	0.0777072	0.78330375	0.68360901
Tbc1d23	-0.0506511	4.34883226	0.07767385	0.78334901	0.68360901
4930503L19I	0.07731944	3.03546724	0.07757375	0.78348491	0.68367779
Gpatch2l	-0.0400697	5.15068383	0.07751192	0.7835689	0.68370126
Ecel1	0.09488254	1.9455997	0.07734087	0.78380145	0.68374079
Pde3b	0.09786155	2.68835774	0.07728347	0.78387954	0.68374079
Srsf3	0.03850849	7.78346233	0.07728259	0.78388074	0.68374079
H2-T10	0.09713172	1.18483638	0.0772325	0.78394893	0.68374079
Col5a3	0.33857377	-1.0560599	0.07722879	0.78395398	0.68374079
Usb1	0.10553182	1.54034789	0.07722674	0.78395677	0.68374079
4930427A07I	-0.2509689	-0.0174891	0.07714695	0.78406543	0.68378513
Tpx2	0.10477367	1.79765562	0.07710557	0.7841218	0.68378513
Med4	0.05894289	3.6371704	0.0770275	0.78422821	0.68382813
Trim46	0.05721145	4.42013848	0.07698383	0.78428776	0.68383027
Tcta	-0.0594216	4.4087972	0.0768755	0.78443555	0.68390933
Rbm8a	-0.0529266	5.27386236	0.07671433	0.78465563	0.68405141
Tns1	-0.0330443	6.61314246	0.076652	0.78474082	0.68407587
Sema3b	-0.1259309	2.712754	0.07654958	0.78488087	0.68411875
Cmc2	0.05499734	3.25622659	0.07643269	0.78504084	0.68411875
Dkk2	-0.3429244	-0.8060264	0.0764294	0.78504533	0.68411875
A930015D03	0.11914337	1.37320484	0.07642397	0.78505277	0.68411875
Rangrf	-0.0770864	2.89797601	0.07638435	0.78510702	0.68411875
Psmb10	0.07289069	3.21934283	0.07631314	0.78520457	0.68411875
Gpn3	0.06395081	4.49154742	0.07627194	0.78526104	0.68411875
Gabrb2	0.04538137	8.24512873	0.07625188	0.78528854	0.68411875
Slc8b1	-0.0926547	1.35538975	0.0762405	0.78530413	0.68411875
Stim1	0.05187214	4.00960311	0.07613448	0.78544954	0.68415539
Csnk1g3	0.02892614	7.45974778	0.07612652	0.78546045	0.68415539
Bax	0.07509523	2.61546348	0.0760348	0.78558634	0.68421528
Hinfp	0.08699978	1.97206335	0.07574241	0.78598818	0.68449518
Dnah17	0.2519453	-1.109053	0.07571782	0.78602202	0.68449518
Clta	-0.0407319	6.84018885	0.07560445	0.78617808	0.68452909
0610009O20	-0.0487931	4.90817008	0.07559139	0.78619607	0.68452909
Mettl7a3	-0.2347034	-1.7645304	0.07553405	0.78627506	0.68452909
Arpc1a	-0.0401668	6.16045961	0.07549907	0.78632326	0.68452909
Zfp276	-0.0789598	2.6787564	0.07548202	0.78634676	0.68452909
Ctsf	0.08686386	3.07466741	0.07531329	0.78657946	0.68466971
S100a16	-0.0890657	3.25723789	0.07516376	0.78678592	0.68466971

Spry4	0.0657308	3.46186275	0.07515012	0.78680476	0.68466971
Oprl1	0.05761797	3.3758433	0.07513953	0.78681941	0.68466971
Npm3	0.18313102	-0.8634113	0.07507118	0.78691386	0.68466971
Kcnj4	-0.0648863	3.36991447	0.07506611	0.78692088	0.68466971
1700047M11	-0.0975366	1.0395494	0.07497838	0.7870422	0.68466971
AI197445	-0.1655508	0.52961767	0.0749448	0.78708865	0.68466971
Cyba	-0.142619	0.14828041	0.07492841	0.78711134	0.68466971
Siah1a	0.03881521	5.65534911	0.07491528	0.78712951	0.68466971
Myd88	-0.0881137	2.78060897	0.07490973	0.78713718	0.68466971
Ginm1	0.06712577	5.23454548	0.07472949	0.7873868	0.68478082
Myl6b	-0.0861873	3.78639404	0.07469495	0.78743468	0.68478082
Fam178a	0.03044754	7.06139048	0.07465981	0.78748339	0.68478082
Mrpl52	0.05277914	3.85555016	0.07465241	0.78749364	0.68478082
Tstd3	-0.0438564	5.42823205	0.07458947	0.78758093	0.684807
Slc8a1	-0.0551556	9.18535327	0.07450816	0.78769376	0.68485538
Inhbe	-0.4431523	-1.9838091	0.07445633	0.78776572	0.68485672
Nhlrc3	-0.1186331	0.51559575	0.07442079	0.78781507	0.68485672
Rhoh	-0.1638995	-0.0962766	0.07437217	0.78788262	0.68485672
Lrp4	-0.0546293	3.95895007	0.07434235	0.78792405	0.68485672
Sesn2	-0.095498	2.55196552	0.07421389	0.78810265	0.68492886
Evi5l	-0.0589797	3.32774126	0.07419203	0.78813306	0.68492886
Hspa14	-0.0437811	4.2484401	0.07413572	0.78821141	0.68492886
Gpr173	0.07329546	2.88554124	0.07406611	0.78830833	0.68492886
Smad4	0.03400487	5.86879899	0.07400907	0.78838777	0.68492886
Zar1l	-0.1455702	0.48899529	0.07397886	0.78842986	0.68492886
Bnip1	0.09569487	2.16630286	0.07396079	0.78845504	0.68492886
Robo3	-0.1864644	2.01714321	0.07395393	0.7884646	0.68492886
Fer1l5	0.10087365	1.38580258	0.07383053	0.78863664	0.68498606
Dbf4	-0.0910419	1.82206078	0.07382466	0.78864484	0.68498606
Qsox2	0.069429	2.93619289	0.07357701	0.7889906	0.68515146
Ugdh	0.04012995	4.62494384	0.0735625	0.78901088	0.68515146
Stmn1-rs1	0.38536825	-1.1613984	0.07350614	0.78908967	0.68515146
Rad51	0.13920318	0.79535411	0.07350088	0.78909702	0.68515146
Casq1	0.25123895	0.15794126	0.0734835	0.78912132	0.68515146
Kif20a	-0.0855884	1.644675	0.07341343	0.78921934	0.68518688
Actr1b	-0.0398937	7.25929002	0.07330329	0.7893735	0.68527105
4930449E18l	-0.2428539	-0.8202355	0.07323999	0.78946215	0.68529833
Bace2	0.07782862	3.59463179	0.0731933	0.78952758	0.68530545
Ifit2	-0.0529031	5.61040824	0.07310794	0.78964723	0.68535963
Zc3h4	0.04124108	5.87777576	0.0730095	0.78978533	0.68538357
Spry2	0.03777778	5.95328064	0.07295771	0.78985802	0.68538357
Cmtm6	0.08085529	4.86642247	0.07294273	0.78987905	0.68538357
Sars	-0.0452136	4.81691384	0.07292074	0.78990993	0.68538357

Lmbr1	-0.0423454	5.0770825	0.07283046	0.79003674	0.68538357
C030006K11I	-0.0756688	2.96509353	0.07280054	0.79007879	0.68538357
Cux1	-0.026197	8.04491016	0.07274344	0.79015906	0.68538357
Wdr48	-0.0395162	5.5655483	0.07264941	0.79029131	0.68538357
Osbpl8	-0.0297208	7.70354913	0.07263252	0.79031508	0.68538357
2310015B20I	-0.1147237	1.96852753	0.07249218	0.79051267	0.68538357
Atxn2l	0.0357577	6.83832865	0.07248265	0.79052609	0.68538357
Sys1	0.06585733	3.2962455	0.07241452	0.7906221	0.68538357
Snap23	0.04642823	7.16619775	0.07236179	0.79069644	0.68538357
Ptafr	0.26872525	-1.0445953	0.07228961	0.79079824	0.68538357
Fam136a	0.05068389	3.84049672	0.07227937	0.79081269	0.68538357
Slc41a1	0.0559986	7.95331276	0.07227224	0.79082276	0.68538357
Cep131	-0.0663857	2.86455836	0.07214847	0.79099747	0.68538357
Htatip2	-0.0946505	2.04140005	0.07209375	0.79107476	0.68538357
Ifnar2	0.05632859	5.47723388	0.07208588	0.79108589	0.68538357
2900055J20F	0.07841206	3.12873871	0.07201702	0.79118321	0.68538357
Mfap4	-0.0888915	3.76607458	0.0720061	0.79119864	0.68538357
Scn2b	0.04071645	6.88841078	0.07200409	0.79120149	0.68538357
Zxdc	0.040095	5.4891973	0.07193689	0.79129652	0.68538357
Rgl3	0.15882787	0.42338172	0.07193209	0.7913033	0.68538357
Snord104	-0.2655336	-1.7075835	0.07191359	0.79132947	0.68538357
Gm16062	0.17586254	-0.2612787	0.07190641	0.79133963	0.68538357
Rab33b	0.03554182	5.90779528	0.07185866	0.7914072	0.68538357
Il18bp	-0.0827417	3.28860402	0.07181572	0.79146799	0.68538357
Vps37c	0.07494229	3.47069224	0.07179142	0.79150239	0.68538357
Asah2	0.04443075	5.7264065	0.07174724	0.79156497	0.68538357
Alpk2	-0.3409752	-1.4775081	0.07171537	0.79161012	0.68538357
Zfp771	0.14486048	0.69994245	0.07168498	0.79165318	0.68538357
2410127L17F	0.05558598	4.94433774	0.07166963	0.79167493	0.68538357
Fam118a	-0.0478273	4.55151073	0.07166615	0.79167986	0.68538357
Mpv17l2	0.0905269	2.69491878	0.07164024	0.7917166	0.68538357
Spcs2	-0.0517034	5.45084433	0.07160143	0.79177162	0.68538357
Efr3b	-0.0535206	6.81278274	0.07157995	0.79180209	0.68538357
Spaca5	0.33065615	-1.6395404	0.07154645	0.7918496	0.68538357
Xrcc6	-0.0659201	3.3703194	0.07148815	0.79193233	0.68540564
Bai3	0.05209571	6.39756936	0.07137084	0.7920989	0.68550027
Snx21	-0.1015556	1.94297433	0.07130301	0.79219529	0.68553414
Gpr151	0.38541873	-1.6206207	0.07122145	0.79231124	0.68558339
Alad	-0.0702791	2.53834977	0.07118246	0.79236669	0.68558339
Wisp2	-0.3234181	-1.02055	0.07108293	0.79250833	0.68561695
Agpat6	-0.0468494	4.13480936	0.07107474	0.79251999	0.68561695
Zfp768	0.08319735	2.42165946	0.07086949	0.79281243	0.68569945
2900005J15F	0.11114057	2.24729623	0.07086524	0.7928185	0.68569945

Mrpl39	-0.0415433	5.38481267	0.07083883	0.79285616	0.68569945
Nup62-il4i1	0.2667216	-1.2666709	0.07073532	0.79300385	0.68569945
Rad51ap2	-0.1387109	1.2965391	0.0706959	0.79306013	0.68569945
Gm996	-0.0524887	4.57695993	0.07066172	0.79310893	0.68569945
Hrsp12	0.05359843	4.29304503	0.07065694	0.79311576	0.68569945
Trap1	-0.0486871	4.50678277	0.07062558	0.79316056	0.68569945
Ppm1a	-0.0348226	6.77648494	0.07059648	0.79320212	0.68569945
Mgst2	-0.3154695	-1.4875532	0.07051731	0.79331529	0.68569945
Ephb6	0.06837257	3.56772938	0.07049832	0.79334244	0.68569945
Papola	0.03208755	8.23868163	0.07045211	0.79340853	0.68569945
Plbd1	-0.1511113	0.95484593	0.07042084	0.79345326	0.68569945
Smg7	0.02805146	8.02274237	0.07041464	0.79346214	0.68569945
Ankrd13b	-0.065853	3.86267914	0.0704062	0.79347421	0.68569945
Gm6623	-0.0760538	1.5751644	0.07031542	0.79360416	0.68576227
Cxcr4	0.28708721	-1.3127137	0.07021962	0.79374139	0.68578609
Nrros	-0.1399616	1.47144779	0.07018586	0.79378978	0.68578609
Mab21l2	-0.2817375	-0.8035876	0.07017114	0.79381087	0.68578609
Gnb1l	0.18069232	0.36464636	0.07013632	0.79386079	0.68578609
Tmem132e	-0.1096725	1.29276568	0.0700579	0.79397328	0.68583379
C330013E15I	0.14575141	0.41328429	0.06995617	0.79411929	0.68586768
Pebp1	0.04233455	8.27988098	0.06992509	0.79416392	0.68586768
Pola2	0.08543268	1.80786801	0.06991089	0.79418432	0.68586768
2700046A07I	0.08718079	2.66778136	0.06984653	0.79427679	0.68586871
Gimap5	-0.1605739	1.56612691	0.06983034	0.79430006	0.68586871
Exosc1	0.05185188	4.68225896	0.06963633	0.79457911	0.68600843
Agtpbp1	0.03742861	8.9554073	0.06962744	0.7945919	0.68600843
Lyg1	0.26224854	-1.5297551	0.06958968	0.79464628	0.68600843
Smco4	-0.0930256	1.93044032	0.06951386	0.79475547	0.68600843
Sun2	0.05171176	6.58459989	0.06939707	0.79492383	0.68600843
Alox12b	-0.1321254	2.01014161	0.06935297	0.79498743	0.68600843
Tuba1c	0.03024222	5.81028806	0.06933833	0.79500855	0.68600843
Xlr4b	0.24122018	-0.3542584	0.06930894	0.79505096	0.68600843
Gm8801	-0.1429513	-0.2353467	0.06929697	0.79506824	0.68600843
Mpst	0.15512419	0.44164435	0.06927811	0.79509545	0.68600843
Agbl4	-0.1003651	2.42252129	0.06923823	0.79515303	0.68600843
5930430L01I	-0.0611101	4.70600952	0.06919107	0.79522114	0.68600843
Gm5126	-0.0786461	2.50523899	0.06916862	0.79525355	0.68600843
Ccdc66	0.03429513	5.59627486	0.06916151	0.79526383	0.68600843
Angel2	-0.0362017	5.77778424	0.06905887	0.79541218	0.68608697
Med20	-0.0420825	5.04033689	0.06892499	0.79560585	0.68613972
Hist1h4h	0.24308395	-1.3963942	0.0689135	0.79562248	0.68613972
Cadm2	0.04032668	8.70551438	0.0688978	0.79564521	0.68613972
Coq2	-0.0426522	4.82113311	0.06884607	0.7957201	0.6861549

Polr2a	0.03337178	7.30718596	0.06878662	0.79580623	0.68617587
Klhl22	-0.0422682	5.05935465	0.06870292	0.79592755	0.68617587
Mapk9	-0.0314076	8.50240878	0.06868388	0.79595515	0.68617587
Nipsnap1	-0.0423408	4.73071935	0.06867115	0.79597361	0.68617587
Pigf	0.09794376	1.56171039	0.06862098	0.79604638	0.68618921
Nt5c	0.06092669	3.6139377	0.06854549	0.79615594	0.68622369
Amd1	0.14236872	-0.1636389	0.06851446	0.79620099	0.68622369
Ric8b	-0.0511125	5.65115964	0.06844383	0.79630357	0.68623707
E2f1	0.08021061	1.94559375	0.06842487	0.79633112	0.68623707
Fbxw4	0.07007479	2.72014757	0.06829963	0.79651318	0.68626094
Tor3a	-0.0818124	3.71345958	0.06829451	0.79652063	0.68626094
Rfwd3	-0.034726	5.39806734	0.06828757	0.79653073	0.68626094
Nadk2	-0.0420473	4.75716011	0.06805316	0.79687201	0.68650558
Morc1	0.36448774	-1.4927498	0.06790729	0.79708471	0.68663943
Rbm48	-0.0489489	3.43229954	0.067766	0.79729096	0.6867677
Gsk3b	-0.0281821	9.51769032	0.06762457	0.79749765	0.68682523
Cenpv	-0.057094	2.83724603	0.06758904	0.79754961	0.68682523
Tnfrsf11a	0.07604172	2.63779918	0.06758507	0.79755541	0.68682523
Cep19	0.03182115	5.80327152	0.06748569	0.79770083	0.68682523
Cdc14b	-0.0522499	4.35642075	0.06746543	0.79773048	0.68682523
Mettl8	-0.0654937	3.77776576	0.0674508	0.79775191	0.68682523
Ppp3cc	0.04016408	5.04839543	0.06743237	0.79777889	0.68682523
Tmem237	0.06872655	3.40213193	0.0674007	0.79782529	0.68682523
Rbm14	0.07529591	3.06542224	0.06734809	0.79790236	0.68682523
Gm973	0.07438864	2.46480324	0.06732837	0.79793126	0.68682523
Mcur1	0.05603712	5.21029881	0.06713197	0.79821934	0.68690965
Ang	-0.1259102	2.28939294	0.06707915	0.79829689	0.68690965
Xlr4c	0.29326279	-1.3636002	0.06707807	0.79829848	0.68690965
Rasa1	-0.0300502	6.61581885	0.06706813	0.79831307	0.68690965
Hacl1	-0.0818329	2.44761928	0.06706605	0.79831613	0.68690965
Lcp2	-0.0591049	3.02345756	0.06701507	0.79839102	0.68692473
Sap30l	0.05582692	3.93973451	0.06692997	0.7985161	0.68698299
Imp3	-0.0556148	4.80297023	0.06675102	0.79877941	0.6870735
Haus6	-0.0524685	3.95219475	0.06669435	0.79886287	0.6870735
Mgat4a	-0.0499787	5.48766177	0.06669247	0.79886563	0.6870735
Btbd3	-0.0366853	8.37597415	0.06662399	0.79896654	0.6870735
Eef2k	-0.0497535	4.34373641	0.06661152	0.79898493	0.6870735
Slc39a7	-0.0533978	4.19422238	0.06656114	0.7990592	0.6870735
Sv2c	-0.0580433	4.80415195	0.06653704	0.79909475	0.6870735
Dlx1as	0.05576619	3.27813681	0.06651114	0.79913295	0.6870735
Dgcr6	-0.0646006	3.95728815	0.06650796	0.79913765	0.6870735
Tbc1d19	0.0408878	5.83751372	0.06640393	0.79929118	0.68712512
Cacnb3	-0.0329905	6.89215654	0.0663605	0.79935532	0.68712512

Zcchc4	0.19939425	-0.1139491	0.06635068	0.79936983	0.68712512
Pde3a	0.13496971	1.06521131	0.06621503	0.79957032	0.68724814
Mypop	0.06447208	2.50815487	0.06616343	0.79964663	0.6872644
Hdhd2	-0.0362944	5.52610197	0.06609114	0.7997536	0.68730702
Agtr2	-0.246672	-1.1446577	0.06598177	0.79991559	0.68739689
Grip1	0.05744107	4.08404271	0.06587683	0.80007113	0.68748122
Gpx4	0.07420437	4.24864188	0.06582157	0.80015308	0.68750231
Ctsw	-0.3510446	-2.2378359	0.06569561	0.80034004	0.68761362
Tcf3	-0.0614665	3.66483439	0.06558151	0.80050956	0.68770993
Gpr35	-0.188972	0.11747477	0.06548603	0.80065153	0.68774343
Galnt13	-0.0396797	5.74392475	0.06547804	0.80066342	0.68774343
Tlr4	0.06593921	4.43886686	0.06536031	0.80083865	0.6877802
Eef1a1	-0.0415755	10.3156722	0.06534104	0.80086735	0.6877802
Arhgef37	-0.1405453	0.28806604	0.06525808	0.80099094	0.6877802
Ttc16	-0.3345123	-1.5557674	0.06524603	0.8010089	0.6877802
Fmo5	0.05513652	3.72328381	0.0652191	0.80104904	0.6877802
Wdr33	0.04624557	5.10123031	0.06521791	0.80105082	0.6877802
Chtop	-0.0338551	7.48466396	0.06507591	0.80126263	0.68789322
Bmpr1a	-0.0467537	7.88324888	0.06502446	0.80133945	0.68789322
1700019L03F	-0.1824004	-0.6529311	0.0650142	0.80135476	0.68789322
Hmg20b	-0.1056631	2.17156615	0.06481833	0.8016475	0.68798706
Hfe2	0.32776217	-1.659473	0.0647891	0.80169122	0.68798706
Nrde2	0.10042101	2.0474504	0.06466104	0.80188291	0.68798706
Gfra4	-0.0453598	4.35583022	0.06464334	0.80190942	0.68798706
Wwp1	-0.0339282	7.5347871	0.06463676	0.80191928	0.68798706
Pop1	-0.0847039	2.24893073	0.06459277	0.80198519	0.68798706
Acot5	-0.2098575	0.37298916	0.06458566	0.80199584	0.68798706
Zfp799	-0.03634	5.66244101	0.06455347	0.80204409	0.68798706
Ilf2	-0.0437289	5.70695281	0.06454949	0.80205005	0.68798706
Cand2	0.05548862	3.67553313	0.06451666	0.80209928	0.68798706
Urgcp	0.04789131	4.39058297	0.06450198	0.80212129	0.68798706
Jakmip1	0.04124265	4.70511181	0.06446871	0.80217119	0.68798706
B3gnt6	-0.3380745	-1.3576428	0.06444224	0.80221091	0.68798706
Gpr160	0.17246912	0.01767484	0.06436611	0.80232517	0.68801827
AU022252	-0.0679221	2.42007903	0.06431835	0.80239688	0.68801827
Whamm	0.08352031	3.09783913	0.06425463	0.80249261	0.68801827
Lnx2	-0.0421458	4.43629105	0.06420586	0.80256591	0.68801827
Dtx1	-0.0462881	4.62920153	0.06419363	0.8025843	0.68801827
Tacc2	-0.0357258	5.52330764	0.0641885	0.80259201	0.68801827
2810417H13I	-0.1542	0.64624972	0.064091	0.80273867	0.68809473
Vps45	-0.0488038	3.79900705	0.06401551	0.80285229	0.68809705
3010001F23I	-0.1051846	1.13950441	0.06401286	0.80285628	0.68809705
Fbxo27	0.05253856	3.83541768	0.06388088	0.80305512	0.68821821

Gm4285	0.09258924	1.33931549	0.06381956	0.80314758	0.68823982
Fam53c	0.03552361	5.54866896	0.06374452	0.80326079	0.68823982
Cyb5d2	-0.0523708	3.33322216	0.06373807	0.80327052	0.68823982
Mrpl41	-0.0414697	4.74946959	0.06368534	0.80335012	0.68823982
Knstrn	0.14465488	0.64181907	0.06367371	0.80336769	0.68823982
Igsf9b	-0.0708542	3.48985487	0.0634873	0.80364938	0.68840459
Itpripl1	0.14148732	1.21223054	0.06344542	0.80371273	0.68840459
Styx	-0.0532133	4.25906461	0.06343238	0.80373246	0.68840459
Fv1	0.13979791	0.54461146	0.06330785	0.80392098	0.68844511
Dcaf12l2	-0.1889294	-0.1083279	0.06329899	0.8039344	0.68844511
Pggt1b	-0.0459914	4.10302833	0.06324132	0.80402178	0.68844511
3000002C10I	0.06086997	2.14423524	0.06321092	0.80406785	0.68844511
Mrps33	0.03022575	6.8965535	0.0631587	0.80414704	0.68844511
Cyb561d1	0.05091711	3.70922664	0.06314519	0.80416753	0.68844511
Gse1	0.04677572	5.57081042	0.06313553	0.80418218	0.68844511
1110008P14I	-0.0381571	4.56807897	0.06294457	0.80447207	0.68863141
Pnpla3	0.13132385	1.33916254	0.06291452	0.80451773	0.68863141
Lamtor5	0.04589065	5.97963691	0.06287862	0.8045723	0.68863141
Rps25	-0.0568088	7.66263212	0.0628131	0.80467193	0.68866745
Atf6	0.03533687	6.94906502	0.06272059	0.80481269	0.68871611
Osbp	-0.0308614	5.35473339	0.06265362	0.80491467	0.68871611
Ppic	0.07459984	4.60365247	0.06262067	0.80496485	0.68871611
Gtpbp2	0.04995408	4.27138484	0.06255527	0.80506451	0.68871611
Cdk1	0.32447908	-1.4653054	0.06251758	0.80512198	0.68871611
Amz1	0.08334223	1.78497787	0.06248122	0.80517743	0.68871611
Tmprss7	-0.1499015	0.24829116	0.06245161	0.8052226	0.68871611
Mmp15	-0.111867	1.11482654	0.06240799	0.80528916	0.68871611
AI837181	-0.044754	4.19362542	0.06239297	0.80531209	0.68871611
Smek2	0.02992163	6.69850331	0.06233807	0.80539592	0.68871611
Actg2	-0.2399298	-0.8348818	0.06224168	0.80554319	0.68871611
Copz1	0.0455796	6.13808659	0.06223788	0.80554899	0.68871611
Lrfn4	0.09028155	2.51329453	0.06223028	0.80556061	0.68871611
Gria1	0.04723073	6.49915439	0.06221159	0.80558918	0.68871611
Hcrtr1	0.31320191	-1.4360629	0.06210964	0.80574511	0.68871611
Golga5	-0.0468012	4.18905184	0.06208308	0.80578575	0.68871611
Vmn2r84	0.17367398	0.21344119	0.06206478	0.80581377	0.68871611
Derl3	-0.2467812	-1.2662247	0.06201383	0.80589178	0.68871611
Wdr55	-0.0428333	4.11983613	0.06191009	0.80605072	0.68871611
Trim30a	0.05567328	3.79956859	0.06190976	0.80605122	0.68871611
Mapk8ip1	0.03593703	5.53140948	0.06189062	0.80608056	0.68871611
Tmem138	-0.090101	1.17105868	0.06188908	0.80608292	0.68871611
Pcsk7	0.06042452	2.68672644	0.06185794	0.80613067	0.68871611
Zfp65	0.0480191	5.05369381	0.06183963	0.80615874	0.68871611

Eif3f	-0.0397852	5.99474716	0.06183457	0.80616651	0.68871611
Srprb	0.05347895	3.49806931	0.06176715	0.80626994	0.68875534
Slc30a5	-0.042726	3.98366903	0.06164304	0.80646051	0.68886899
H2-D1	-0.0486039	5.17764564	0.06159329	0.80653697	0.68888517
Tbx22	-0.2780703	-1.4582454	0.06154524	0.80661082	0.68889912
D630032N06	0.32290848	-0.9869433	0.06138193	0.80686212	0.68894133
Ddo	-0.065669	3.49965367	0.06135042	0.80691064	0.68894133
Pak1ip1	0.03939125	4.15962948	0.06134963	0.80691186	0.68894133
Id3	0.0681815	6.0191475	0.06133653	0.80693204	0.68894133
Tspan32	0.28514127	-1.3717372	0.06132623	0.8069479	0.68894133
Adamts9	0.08899898	2.69377646	0.06123046	0.8070955	0.68901823
Depdc5	-0.0476487	5.25652569	0.06118216	0.80716999	0.68903271
Frk	0.05975216	4.02607557	0.06114361	0.80722946	0.68903436
Nkap	-0.0434651	5.26887566	0.06110624	0.80728713	0.68903447
Fgf13	-0.0383294	6.45146223	0.061059	0.80736006	0.6890476
Elmo3	0.09595189	1.38740745	0.0610038	0.80744532	0.6890685
Pgam2	0.12814217	0.82968011	0.06096866	0.80749961	0.6890685
Trub1	0.03813388	4.67288102	0.06082795	0.80771719	0.68920505
Gpr82	-0.3734007	-1.234143	0.06078314	0.80778652	0.68921511
Cdkn2c	-0.090775	2.73098207	0.06072418	0.80787782	0.68922342
Slfn9	-0.0915453	1.62630599	0.06070252	0.80791137	0.68922342
Irak2	-0.0455405	3.65460377	0.06061919	0.80804049	0.6892363
Mrps12	0.06303013	3.59818794	0.06061849	0.80804157	0.6892363
Zmat1	-0.0388917	5.17673557	0.06053017	0.80817853	0.68924294
Pmel	0.25099587	-1.8486004	0.06050725	0.80821409	0.68924294
Eif4a3	-0.0395085	5.00288545	0.06045847	0.80828979	0.68924294
Atp9b	-0.0609776	3.99564467	0.06030629	0.80852617	0.68924294
Sardh	-0.0912767	2.20150384	0.06030377	0.80853009	0.68924294
Zfp629	-0.0483568	4.52124725	0.06028933	0.80855253	0.68924294
2610037D02I	-0.1581524	0.16203569	0.06019152	0.80870465	0.68924294
Zfp800	0.04236099	5.95938566	0.06018065	0.80872156	0.68924294
Tenm3	-0.0488967	6.36800306	0.06015395	0.80876311	0.68924294
Tmem160	-0.1183852	1.07426555	0.06012193	0.80881296	0.68924294
Xpnpep1	-0.045725	4.48513859	0.06011321	0.80882654	0.68924294
Phf13	0.05704759	3.5556069	0.06011241	0.80882779	0.68924294
Gpr165	-0.060876	3.639751	0.06005803	0.80891246	0.68924294
Emp2	-0.0516387	4.33314643	0.06004707	0.80892955	0.68924294
Rhob	0.0324952	7.36944358	0.06002153	0.80896934	0.68924294
Mrgpre	0.06568492	3.56459171	0.06002097	0.80897021	0.68924294
2210408F21I	-0.0782156	2.4430017	0.05996627	0.80905547	0.68926655
Tra2b	0.04530975	6.4036067	0.05982069	0.80928258	0.68931937
Fbxl12os	0.09489891	2.29522123	0.05980373	0.80930906	0.68931937
Rbms2	-0.0451958	5.91312169	0.05976469	0.80937001	0.68931937

Kank4	-0.0621264	3.92980639	0.05975414	0.80938649	0.68931937
Rpl31	0.0471316	8.64150413	0.05973317	0.80941925	0.68931937
Txlna	-0.0369645	5.13475204	0.05970527	0.80946284	0.68931937
Gldn	0.13816861	0.93540306	0.05965847	0.80953598	0.68932017
Ppp2r5a	0.03621006	5.75642456	0.05963103	0.8095789	0.68932017
Rgp1	0.03897993	4.49069946	0.05953055	0.80973608	0.68940499
Slirp	-0.0412991	4.7971432	0.05937604	0.80997806	0.68952
Hnrnph1	-0.0234789	8.53090032	0.05937077	0.80998632	0.68952
Rfc5	-0.0719163	2.67648638	0.05930719	0.810086	0.68955584
Brip1	-0.0687612	3.21142349	0.0591993	0.81025528	0.68963208
Fam221a	0.16350522	0.13123574	0.05917671	0.81029074	0.68963208
Os9	0.05027995	4.71647211	0.0588592	0.81078995	0.68996437
Auts2	0.02963444	6.88281406	0.05880689	0.81087233	0.68996437
Pnn	-0.0396139	7.701744	0.05872897	0.81099512	0.68996437
Sarm1	0.07004344	3.71037135	0.05868723	0.81106093	0.68996437
2510002D24I	-0.0632167	3.33183668	0.0586524	0.81111587	0.68996437
Mpped2	-0.0381472	6.62221731	0.05864716	0.81112413	0.68996437
Mybl2	0.20279286	-1.316841	0.05863814	0.81113836	0.68996437
Midn	0.0390436	5.84324131	0.05863579	0.81114207	0.68996437
Ppp1r36	-0.2008495	-0.2099402	0.05854345	0.81128782	0.68998465
5430405H02I	-0.0937388	1.41100039	0.05848869	0.8113743	0.68998465
Paqr3	0.07717568	2.38706999	0.0584801	0.81138787	0.68998465
Fgfr1op2	0.03038372	7.74239464	0.05847472	0.81139638	0.68998465
Dda1	-0.0397818	4.55962534	0.05837794	0.81154935	0.69006573
Klc4	0.07342865	2.29237103	0.05821508	0.81180709	0.69021477
Wwc2	0.04241058	4.8312372	0.05819437	0.8118399	0.69021477
Ercc6l2	-0.0354891	5.12584143	0.05811432	0.81196675	0.69026281
Krt25	0.38443599	-1.5438074	0.05797257	0.81219159	0.69026281
Mgst3	-0.0542543	3.55588685	0.05796735	0.81219987	0.69026281
2700097O09	0.06627423	2.8756526	0.05795529	0.81221903	0.69026281
Zfp934	0.05475191	3.61369777	0.05784437	0.8123952	0.69026281
Ccny	-0.0304581	8.53740711	0.05783287	0.81241348	0.69026281
Chrm1	-0.0358743	5.75229557	0.05774619	0.81255128	0.69026281
Slco5a1	-0.0821374	2.19075446	0.05766737	0.8126767	0.69026281
2810474O19	-0.0328179	6.6693074	0.05762508	0.81274403	0.69026281
Pnpla6	-0.0567974	4.04739104	0.05759115	0.81279806	0.69026281
Brf2	0.12563509	0.70218386	0.05758942	0.81280081	0.69026281
Cfh	0.0544956	7.13021819	0.05756444	0.81284061	0.69026281
Plod2	0.06782173	3.8469129	0.05754637	0.81286941	0.69026281
Kcna5	0.11297924	0.85548491	0.05750267	0.81293905	0.69026281
Zfp553	0.06463499	3.70563064	0.05746527	0.81299868	0.69026281
1110004E09I	0.04912448	5.77998357	0.05738623	0.81312476	0.69026281
Fbxl6	0.11198426	0.94416263	0.05736552	0.81315783	0.69026281

Ogfr	0.08449608	1.96457029	0.05731939	0.81323147	0.69026281
Ppm1f	-0.0392326	4.58566477	0.05730379	0.81325639	0.69026281
Cog6	-0.0310136	5.57264426	0.05726477	0.8133187	0.69026281
Col15a1	-0.1154952	1.46629283	0.05725692	0.81333126	0.69026281
1700029I15R	-0.1840411	-0.841606	0.05712895	0.81353584	0.69026281
Zfp536	0.03156835	4.61730141	0.05711063	0.81356514	0.69026281
Ing3	0.04408838	4.2107681	0.0570575	0.81365016	0.69026281
Pcbp2	0.02892524	6.942454	0.05698349	0.81376868	0.69026281
Ppp2r5e	-0.032645	6.40327889	0.05698059	0.81377333	0.69026281
Arhgef4	0.04222242	5.08822958	0.05692596	0.81386084	0.69026281
Iqgap1	0.02920316	6.34663726	0.05691867	0.81387253	0.69026281
Polr3e	0.05751824	3.83577343	0.05691827	0.81387317	0.69026281
Vars	0.07140529	2.45858868	0.05691434	0.81387947	0.69026281
B230216N24	0.07744957	2.24691743	0.05685782	0.81397009	0.69026281
Zfp811	0.05255105	3.43051123	0.05684329	0.81399339	0.69026281
Slc5a3	-0.0355704	5.78479516	0.0568106	0.81404584	0.69026281
Gak	0.03111584	6.70953113	0.05680736	0.81405103	0.69026281
Hist1h3f	-0.2119174	-1.7081125	0.0568056	0.81405385	0.69026281
Pabpc1l	0.45329335	-1.9515663	0.05679606	0.81406916	0.69026281
Gkn3	0.29374251	-1.1762182	0.05677796	0.81409821	0.69026281
Cbx8	-0.1511111	0.15420196	0.05675922	0.81412828	0.69026281
Cops8	-0.0298786	5.82412212	0.05673977	0.81415951	0.69026281
Phb2	0.03891036	5.0191574	0.05671334	0.81420194	0.69026281
Orc6	-0.0405545	4.67622612	0.05657617	0.81442236	0.69037185
A930017M01	-0.065047	2.61294087	0.056516	0.81451914	0.69037185
Gpatch2	-0.0377109	4.12318121	0.0564988	0.81454681	0.69037185
Ggnbp2	0.02677804	7.36108644	0.05648989	0.81456114	0.69037185
Coq10a	-0.0463274	4.11376662	0.05643283	0.814653	0.69040083
Wdr1	-0.0298867	6.28277704	0.05639143	0.81471966	0.69040847
D930015M05	0.21588772	0.26353852	0.05628459	0.81489184	0.69050552
Memo1	0.0383897	4.53854241	0.05624172	0.81496097	0.69051259
Acy1	0.11449415	0.78264564	0.05620721	0.81501663	0.69051259
Rims4	-0.2617589	-1.1305353	0.0561686	0.81507894	0.69051259
Dennd2a	-0.0442731	4.23267622	0.05612327	0.81515213	0.69051259
Slc25a29	-0.1162453	0.83597894	0.05610076	0.81518848	0.69051259
0610010F05I	-0.0402076	5.99731277	0.05603582	0.81529339	0.69055261
Use1	-0.0646058	4.73330947	0.05593382	0.81545832	0.69064346
Gm5089	0.06001015	4.78982062	0.05576959	0.81572419	0.6907094
Magt1	0.04679737	5.22966798	0.05576623	0.81572963	0.6907094
9830166K06I	-0.1255452	0.88567121	0.05571505	0.81581257	0.6907094
Gm8773	0.30296849	-1.5269507	0.05571362	0.81581489	0.6907094
Cpne9	-0.0637854	4.22777363	0.05568209	0.81586602	0.6907094
Asxl1	0.03982742	5.35960439	0.05567208	0.81588224	0.6907094

Abhd17c	-0.0362013	5.2820418	0.05561811	0.81596978	0.69073373
Commd10	-0.0420181	4.67204562	0.05558326	0.81602633	0.69073373
Lyz1	-0.1457314	1.04164161	0.05552655	0.81611841	0.69076285
Arhgap22	0.17520694	0.14062593	0.05546385	0.81622026	0.69080023
Prss57	-0.3569016	-1.3924371	0.05532027	0.81645372	0.69094899
Stx3	-0.0361204	4.78234578	0.05523997	0.81658442	0.69101077
Slc26a11	-0.0866615	2.08885462	0.05510905	0.81679775	0.6911321
Zcchc17	0.03426121	5.21882703	0.05508116	0.81684322	0.6911321
Ccdc50	-0.0335252	7.37499426	0.05498665	0.81699742	0.69121373
Efna1	-0.1517853	0.04723327	0.05487935	0.81717267	0.69124886
Galnt5	0.25194383	-1.0142029	0.05487691	0.81717666	0.69124886
Pgap1	0.04345215	5.42461788	0.05485522	0.8172121	0.69124886
Cdk10	-0.044872	4.11816307	0.05481563	0.81727681	0.69125477
Ak7	-0.0908146	1.73166403	0.05474789	0.81738761	0.69126321
Notum	0.07450943	1.64644829	0.05473895	0.81740224	0.69126321
Zfp764	-0.0790616	2.98678652	0.05462109	0.81759519	0.69136541
Piwil2	-0.2492322	-0.4778187	0.05457069	0.81767778	0.69136541
Vit	-0.1122313	2.21496262	0.0545594	0.81769627	0.69136541
Mapk1ip1	0.03684399	4.40151255	0.0544904	0.8178094	0.69141224
Nat2	0.07237596	3.0417629	0.05439858	0.81796008	0.69149081
Pkib	0.03483283	5.513234	0.05436113	0.81802157	0.69149398
Stradb	0.0350595	5.42089571	0.05431387	0.8180992	0.6915108
Rspry1	-0.0366905	4.75303855	0.05410097	0.81844935	0.69174818
Sapcd2	0.29448684	-1.1384531	0.0540729	0.81849557	0.69174818
Snrnp35	0.06154462	3.60208092	0.05396051	0.81868077	0.69184754
Il17rb	-0.1738384	-0.0002551	0.05393145	0.81872868	0.69184754
Dancr	-0.1545795	-0.0093493	0.05385085	0.81886165	0.69191109
Tmem86b	-0.1240228	0.33394382	0.05377475	0.8189873	0.69194179
AI597479	-0.0354062	5.87756521	0.05374668	0.81903366	0.69194179
Zfp777	0.0694181	2.08717096	0.05372389	0.81907132	0.69194179
Mrpl13	0.03892768	4.72842033	0.05354932	0.81936004	0.69205816
Trappc8	-0.02999	6.26284445	0.05352234	0.81940472	0.69205816
Ctcf	0.02764623	6.37279785	0.05351179	0.81942218	0.69205816
Grhpr	-0.0792189	2.24002637	0.05350089	0.81944023	0.69205816
Ifi203	0.08238026	3.12728401	0.05344478	0.81953318	0.69206919
Atr	-0.0453242	4.55086409	0.05342325	0.81956887	0.69206919
Bin2	0.06771762	3.02808699	0.0533521	0.81968683	0.69212
Nop16	-0.0721413	2.38297078	0.05330953	0.81975744	0.69213082
Bank1	-0.0915208	1.7660714	0.05326117	0.81983772	0.6921498
Cby1	-0.0794594	3.21791179	0.0531642	0.81999877	0.69219729
Siva1	-0.0773933	2.66080766	0.05315676	0.82001113	0.69219729
Chmp3	-0.0346693	6.51614272	0.05311462	0.82008117	0.69219729
Cnbp	-0.0343225	9.17261251	0.05308816	0.82012517	0.69219729

Nudt15	0.08151017	1.94917808	0.05303479	0.82021395	0.69222343
Klhl26	-0.0433711	3.63165044	0.05285672	0.82051047	0.69228789
Lrp12	0.03648897	4.10002591	0.05281453	0.82058081	0.69228789
Ptp4a2	0.03939395	9.07797715	0.05281358	0.8205824	0.69228789
Ppp1r3d	0.10374767	1.13591682	0.05280775	0.82059211	0.69228789
Tshz3	-0.0319771	6.13421341	0.05277984	0.82063866	0.69228789
Jade3	0.06492201	4.31488406	0.05276223	0.82066803	0.69228789
Fndc3b	0.0250785	5.7564573	0.05274608	0.82069498	0.69228789
Manba	-0.081803	2.7862483	0.05260434	0.82093165	0.69239916
Gm16515	0.05419446	5.5106867	0.05259784	0.82094252	0.69239916
Chmp4c	0.30138414	-1.6063499	0.05254312	0.82103399	0.69240715
Rasal1	-0.0756063	3.28396186	0.05251148	0.8210869	0.69240715
Zfp512	0.03262093	5.07894091	0.05245922	0.82117433	0.69240715
Milr1	0.21930759	-0.3117248	0.05232733	0.82139517	0.69240715
Spata7	-0.0407913	4.05031783	0.05230798	0.8214276	0.69240715
Gpx3	-0.066019	3.06524729	0.05230414	0.82143404	0.69240715
Lzts2	-0.0635345	2.82548724	0.05229279	0.82145307	0.69240715
Ppib	0.05992428	2.66338172	0.05228673	0.82146323	0.69240715
Tmem184b	-0.0350997	4.72598387	0.05228129	0.82147234	0.69240715
Prickle4	0.30417677	-1.8831369	0.05224477	0.82153358	0.69241003
Nudt2	0.05624183	3.19216054	0.05219466	0.82161762	0.69241604
Apeh	-0.0817848	2.13324778	0.0521621	0.82167227	0.69241604
Hist3h2a	0.12119411	0.54765609	0.0521364	0.82171541	0.69241604
Frmd7	-0.1578658	-0.1446833	0.05210271	0.82177199	0.69241604
Galnt11	-0.0513529	3.29136292	0.05205699	0.82184878	0.69243203
Hk1os	-0.1233014	0.80907124	0.05198249	0.82197401	0.6924486
Zfp959	0.06963032	1.73695519	0.05195871	0.82201401	0.6924486
Dapk2	0.10985971	0.52057744	0.05193299	0.82205726	0.6924486
Git2	0.0314411	5.44319972	0.05187661	0.82215214	0.6924486
Ripply3	-0.1041371	1.46341984	0.05182875	0.82223273	0.6924486
AA465934	0.11736598	0.237487	0.05178966	0.82229857	0.6924486
Sbk3	-0.1382077	0.45675442	0.05173906	0.82238384	0.6924486
Abcc10	0.09782762	0.96648343	0.0517031	0.82244446	0.6924486
Pou2f2	0.0661311	3.6192154	0.05169663	0.82245537	0.6924486
1600016N20	-0.1840457	-0.9326504	0.0516776	0.82248748	0.6924486
Poc1b	0.04257749	4.59320195	0.05166177	0.82251417	0.6924486
Rdh13	0.04368511	3.81621794	0.05163324	0.8225623	0.6924486
Slc39a12	-0.0937161	2.63789016	0.05145238	0.82286779	0.6926383
Dennd1a	-0.0320355	5.51187402	0.05143136	0.82290332	0.6926383
Eif3b	0.04078827	3.89030819	0.05138213	0.82298659	0.69265826
Adam15	-0.040736	4.21539987	0.05134896	0.82304271	0.69265826
Sec61a2	-0.0497813	4.92170337	0.05118631	0.82331822	0.69284143
Tsc22d4	-0.0483141	3.17516185	0.05111413	0.82344062	0.69284414

Aida	-0.030177	6.45288865	0.05108408	0.8234916	0.69284414
Ndufaf2	-0.0511533	4.81039689	0.05108208	0.823495	0.69284414
Ccdc184	0.04730733	3.20706414	0.05102126	0.82359824	0.69288222
Il10ra	0.17289516	0.14749932	0.05098667	0.82365699	0.69288222
Pex11a	0.10107231	1.16454539	0.05095322	0.82371382	0.69288222
Mblac1	-0.0706834	2.67170348	0.05083129	0.82392114	0.69299014
Nt5dc3	-0.0397618	5.92130142	0.05078916	0.82399284	0.69299014
Rab39	-0.1219091	0.57454384	0.05077572	0.82401572	0.69299014
Zbtb2	0.03356344	4.67072501	0.0507141	0.82412066	0.69302972
Otud7b	-0.0265644	7.25464734	0.05051858	0.82445406	0.69326141
Zranb1	-0.0324998	4.76917413	0.05037826	0.82469377	0.69338994
Tbx15	-0.049278	6.7955854	0.0503584	0.82472773	0.69338994
Mir1931	-0.2667255	-1.7815643	0.05032747	0.82478062	0.69338994
Suds3	-0.0404773	5.04632402	0.0502233	0.82495889	0.69348587
Ccdc125	0.06917455	2.84858073	0.05019314	0.82501053	0.69348587
Arf6	0.03400243	6.84791232	0.05011614	0.82514247	0.69354809
Csf1	-0.0480806	4.87426401	0.049911	0.82549449	0.69375803
Sestd1	0.04023845	7.06403076	0.04990308	0.8255081	0.69375803
Synj2bp	0.02556687	7.03416657	0.04981374	0.82566167	0.6938384
Mir6236	0.0877434	4.65422758	0.04968884	0.82587659	0.69395207
Gm20139	0.16857088	-0.1396949	0.0496678	0.82591283	0.69395207
Rragc	-0.0293448	5.81121884	0.0495208	0.8261662	0.69411626
Phka2	0.05918149	4.89890177	0.04943906	0.82630725	0.69411979
Dnase1l3	-0.2678088	-0.8624302	0.04942194	0.82633682	0.69411979
Ccnjl	0.11791518	1.08242256	0.04941761	0.82634429	0.69411979
Prpf18	-0.03322	4.94499553	0.04917466	0.8267644	0.69440755
Ccni	-0.0290612	8.52286496	0.04913361	0.82683548	0.69440755
Dusp12	0.07109528	2.30696083	0.04911898	0.82686082	0.69440755
Tctn2	-0.0537076	2.69486415	0.04902343	0.82702643	0.69445611
Dnttip2	0.02677054	6.01952394	0.04900594	0.82705676	0.69445611
A330093E20	0.16085403	0.23437656	0.04898528	0.82709261	0.69445611
Xylb	-0.0532506	3.45145933	0.04893225	0.82718464	0.69448469
Spopl	-0.0463451	3.63922646	0.04888371	0.82726891	0.69450675
Hnrnpdl	-0.0235271	7.47776317	0.0488287	0.82736449	0.69451797
Asns	-0.0417759	5.25667555	0.04880926	0.82739826	0.69451797
Rprml	0.06221969	3.00857413	0.04873227	0.82753215	0.69454474
Ssna1	0.0627664	3.00827153	0.04872422	0.82754615	0.69454474
Lax1	-0.2251393	-0.2643545	0.04866167	0.82765501	0.69458743
Myh6	-0.1296185	0.26047312	0.04848519	0.82796255	0.69477409
Nfxl1	-0.0514931	2.91886483	0.04843127	0.82805662	0.69477409
Capn3	-0.0881907	0.85097629	0.04843088	0.8280573	0.69477409
Srpk2	-0.0256707	7.79659098	0.04840098	0.82810949	0.69477409
Tpst2	-0.0611472	2.1402579	0.0483276	0.82823766	0.69483294

Igbp1	-0.040015	5.79648607	0.04814816	0.82855148	0.69497501
Celf6	-0.0552696	2.68095841	0.04808927	0.82865462	0.69497501
Polrmt	-0.0657778	2.23954961	0.04808613	0.82866012	0.69497501
Dnajb4	0.02598478	8.41229707	0.04807233	0.82868429	0.69497501
Lurap1l	-0.0557253	2.60578454	0.04806499	0.82869716	0.69497501
Barx2	-0.0792523	1.91715805	0.04799193	0.82882523	0.6950058
Acaa2	0.04399108	4.83542078	0.04797562	0.82885383	0.6950058
Limd1	-0.0490698	5.16136736	0.04791926	0.82895271	0.6950058
Pcnxl2	0.04898336	4.8164733	0.04790483	0.82897804	0.6950058
Hnrnpa3	-0.0248674	9.05026182	0.04787863	0.82902404	0.6950058
4930426D05I	0.21122041	-0.7480271	0.04773955	0.82926842	0.69516201
Zbtb1	-0.0340691	4.59540125	0.0477057	0.82932796	0.69516325
Cnih3	-0.0325675	5.92678748	0.04767036	0.82939014	0.69516672
Msantd2	-0.0749808	3.04456159	0.04748901	0.82970961	0.69538581
Rpl21	0.04146548	7.82122317	0.0474002	0.82986629	0.69544166
Mta2	0.03650061	4.80843552	0.04735282	0.82994995	0.69544166
Ostc	0.04045052	4.63120836	0.04734653	0.82996105	0.69544166
Crkl	0.02595154	6.05458741	0.04731966	0.83000852	0.69544166
Inpp5a	0.037157	4.56294931	0.04710395	0.83039006	0.69567869
Hist1h2bf	0.17286831	-0.8437802	0.04702508	0.8305298	0.69567869
Med8	-0.0330985	3.81984149	0.04700917	0.830558	0.69567869
Vcp	-0.0242545	7.68924044	0.04699092	0.83059036	0.69567869
Sgcd	-0.0610301	2.93559348	0.04697575	0.83061726	0.69567869
Sorbs3	0.04999487	7.07168681	0.04696295	0.83063996	0.69567869
Syf2	-0.0463256	5.63618597	0.04690151	0.83074897	0.6956863
Zbp1	0.09346468	1.02666579	0.04689235	0.83076524	0.6956863
Sult6b1	0.10585869	0.88888743	0.04674381	0.83102914	0.69582526
Rab3ip	0.03248356	5.14672072	0.04673274	0.83104882	0.69582526
Mrfap1	0.03731023	9.51155094	0.04670088	0.83110548	0.69582526
Ncor2	-0.0332692	6.83000484	0.04662156	0.83124665	0.69587156
Ptbp3	0.03019245	7.19156415	0.04660452	0.831277	0.69587156
Nuak2	-0.2461733	-0.498086	0.04648172	0.83149584	0.6960061
Epb4.1l5	-0.0364115	4.6767128	0.04637049	0.83169432	0.69612359
BC055324	-0.1094764	0.65282369	0.04622366	0.83195672	0.69629454
Cd320	-0.0590974	2.81476072	0.04618329	0.83202893	0.69630632
Luc7l	-0.0354706	5.4931452	0.04614442	0.8320985	0.69631588
Adk	0.03610436	4.8192347	0.04609144	0.83219337	0.69632729
C1qtnf4	0.06842133	2.03375182	0.04607188	0.83222842	0.69632729
0610043K17I	-0.0639057	1.52594547	0.04602846	0.83230622	0.69634374
5730403I07R	-0.3555034	-1.970672	0.04592398	0.83249361	0.69642507
P2ry12	-0.0498908	3.67863473	0.04587672	0.83257845	0.69642507
Gm1987	-0.2409095	-1.4241787	0.04582241	0.832676	0.69642507
Sgpl1	0.04543084	4.29909087	0.04575959	0.83278891	0.69642507

Gin1	0.05200874	3.43491305	0.04575886	0.83279023	0.69642507
Pcdhga5	-0.0506858	3.68672093	0.04574116	0.83282205	0.69642507
Ctbs	0.05076179	3.00363502	0.04570659	0.83288423	0.69642507
LOC381967	-0.1655767	-0.4098663	0.04567048	0.83294921	0.69642507
Rad51ap1	-0.1926189	-0.3231284	0.04561914	0.83304165	0.69642507
Rab6b	-0.02603	10.4152573	0.0456186	0.83304261	0.69642507
Get4	0.04270204	3.89752722	0.04558876	0.83309636	0.69642507
Kdm4a	-0.0499119	3.03882422	0.04553964	0.83318488	0.69642507
Ccdc183	0.12023599	0.60674389	0.04552972	0.83320277	0.69642507
Luc7l3	-0.0271891	8.5707348	0.04545232	0.83334237	0.69642507
Tbca	0.0416498	6.43366701	0.0454435	0.83335828	0.69642507
Nap1l5	0.02422242	7.1916082	0.04536133	0.83350663	0.69642507
Stambp	0.03490417	4.3459198	0.04535924	0.83351041	0.69642507
Ifi205	-0.1792922	-1.2619821	0.04533022	0.83356283	0.69642507
Scai	0.03366131	7.0689649	0.04531933	0.83358252	0.69642507
C1qtnf2	0.1107434	1.96562314	0.04529076	0.83363415	0.69642507
Ceacam2	-0.0739458	1.35594275	0.04525715	0.83369492	0.69642507
Larp1	0.02569837	7.30000833	0.04523505	0.83373489	0.69642507
Sp3	-0.0282908	6.67392446	0.0452317	0.83374095	0.69642507
Mcfd2	0.04492957	4.96654256	0.04507026	0.83403328	0.69662065
Wdr37	-0.0253951	7.17723915	0.04500577	0.8341502	0.69664274
1700026L06F	-0.2357398	-1.4318368	0.04498465	0.83418851	0.69664274
Ormdl2	0.11057117	1.43733539	0.04495722	0.83423827	0.69664274
Abce1	0.02852922	6.26132422	0.04492494	0.83429686	0.69664274
Flot1	-0.0300745	5.46448358	0.04489536	0.83435058	0.69664274
Nme5	0.05011423	3.39963312	0.04485619	0.83442172	0.69665357
Glis3	-0.0525046	3.09215531	0.04462897	0.83483511	0.69687396
Dguok	-0.062186	2.97350383	0.04458014	0.83492409	0.69687396
Elavl1	0.02438203	6.63422728	0.04457338	0.83493641	0.69687396
Polq	0.09068745	2.63055267	0.04456423	0.8349531	0.69687396
Mis18bp1	-0.0906732	1.0692987	0.04455132	0.83497664	0.69687396
Gpd2	0.02476404	6.9886585	0.04445378	0.83515459	0.69695848
Amacr	0.04429466	3.65200596	0.04443203	0.83519431	0.69695848
Ch25h	-0.2408418	-1.5622507	0.04438215	0.8352854	0.69698593
Sfxn4	-0.063905	3.64508379	0.04431944	0.83540002	0.69702095
F11r	-0.0616176	2.71136474	0.0442874	0.83545861	0.69702095
Kcnk5	-0.0850526	2.33406875	0.04422643	0.83557017	0.69702095
Tmem106a	0.12070603	1.6543206	0.04419914	0.83562012	0.69702095
Ppara	-0.0504555	4.0984941	0.04414364	0.83572179	0.69702095
C230091D08I	0.03768399	6.46143414	0.04411279	0.83577831	0.69702095
Gm16532	-0.1414096	1.04913494	0.0441056	0.83579149	0.69702095
Thg1l	-0.0675562	2.94982883	0.04410367	0.83579502	0.69702095
Zic2	0.04413725	6.54778614	0.04404269	0.83590685	0.69702095

Cisd1	-0.0308517	5.07555563	0.0440413	0.8359094	0.69702095
Clec1a	0.09256915	1.93760054	0.04395166	0.83607393	0.69704764
Unc5d	-0.0398699	6.21275361	0.04393147	0.836111	0.69704764
Plekhh3	0.08646905	1.54505035	0.04392143	0.83612946	0.69704764
AI606473	-0.1731535	-0.0018368	0.04387189	0.83622048	0.69704764
Ube3a	-0.0309073	6.7638615	0.04386539	0.83623243	0.69704764
1500009L16F	-0.0375464	4.68452919	0.04372172	0.83649676	0.69719793
Tpm2	0.05290131	6.33843715	0.04370412	0.83652917	0.69719793
Stom	-0.0898721	5.20970168	0.0435722	0.83677232	0.69726593
Dtd2	-0.0527038	4.09246295	0.04357027	0.83677589	0.69726593
Mettl11b	0.29839497	-2.3411546	0.04355473	0.83680455	0.69726593
Coa5	-0.0308127	7.00338621	0.04353355	0.83684366	0.69726593
Rab11fip5	-0.0319138	5.95716067	0.04348923	0.83692547	0.69728559
Gsdmc4	0.29923208	-1.6809568	0.04343993	0.83701652	0.69731294
Lrp1	-0.0268712	7.3421971	0.04339211	0.8371049	0.69731785
Cntn5	-0.0845358	2.35726734	0.04333311	0.83721401	0.69731785
Cib2	0.08558168	2.27751293	0.04329511	0.83728434	0.69731785
Lrsam1	0.05377043	3.61904986	0.0432832	0.83730638	0.69731785
Mrpl2	-0.05802	3.1418661	0.04327932	0.83731356	0.69731785
Swi5	0.0385622	5.71478352	0.04316904	0.83751785	0.69738979
Prkx	0.03999706	4.57559272	0.0431368	0.83757762	0.69738979
Pik3r3	-0.0234521	6.59840432	0.04307949	0.83768394	0.69738979
Lima1	0.03880322	7.28145281	0.043058	0.83772383	0.69738979
Gm9839	0.26936574	-0.419658	0.04302963	0.83777649	0.69738979
Acadvl	0.03838319	4.90350757	0.04299674	0.83783759	0.69738979
Ddrgk1	0.03946153	4.10669404	0.04296823	0.83789055	0.69738979
Tssc4	0.05205161	2.47401417	0.04294281	0.83793779	0.69738979
4933409K07I	-0.0379027	7.36388262	0.04291409	0.83799119	0.69738979
Anapc11	0.04087849	4.06063091	0.04291065	0.83799759	0.69738979
Serpinb6a	-0.0496086	5.68020965	0.04276011	0.83827777	0.69738979
Samd3	0.10086843	1.32817497	0.04275787	0.83828195	0.69738979
Ptk2	-0.0381602	6.57805731	0.0426464	0.83848976	0.69738979
Parp16	-0.109956	1.90198188	0.04264496	0.83849244	0.69738979
Tmem196	-0.047527	3.16291533	0.04263276	0.83851521	0.69738979
Tyw1	-0.0844792	2.0133922	0.04259325	0.83858894	0.69738979
Hmga2-ps1	-0.1286518	0.57436353	0.04255254	0.83866495	0.69738979
A530058N18	0.08781521	1.14834101	0.04254059	0.83868727	0.69738979
Ipp	-0.046477	3.30453463	0.04251369	0.83873753	0.69738979
Ttc27	-0.0408834	3.37302631	0.04251246	0.83873982	0.69738979
06-Mar	-0.0278177	8.79093608	0.04249784	0.83876715	0.69738979
9530082P21I	0.03191636	5.19462914	0.04243218	0.83888991	0.69738979
Ddx50	-0.0257042	6.27247617	0.04238582	0.83897666	0.69738979
Ms4a6b	-0.185685	0.48023595	0.04237904	0.83898934	0.69738979

Srrm2	0.03520438	10.1848417	0.04232581	0.839089	0.69738979
Scd2	-0.0256239	8.33435945	0.04229884	0.83913952	0.69738979
Herc6	0.03359296	5.29591099	0.04229251	0.83915139	0.69738979
Nup54	0.04078888	3.41473095	0.04222734	0.83927353	0.69738979
Gtf2f2	0.04190868	3.1438691	0.04220058	0.83932373	0.69738979
Mrpl37	0.03708004	4.02283892	0.0421816	0.83935934	0.69738979
Pars2	-0.0763004	1.84573629	0.04216749	0.83938581	0.69738979
Gpr162	0.08510187	2.14572844	0.04213478	0.83944721	0.69738979
LOC1016697(	0.20316607	-0.978259	0.04213316	0.83945024	0.69738979
Pskh1	-0.0534464	3.80027565	0.04212534	0.83946494	0.69738979
Mdc1	0.03047004	5.55897763	0.04210647	0.83950035	0.69738979
Rbm18	0.03065017	6.24774405	0.04208566	0.83953944	0.69738979
Spg20	0.02969009	5.84138243	0.04204863	0.83960902	0.69738979
Zfp335	-0.0494214	3.92791599	0.04204661	0.83961283	0.69738979
Phf11c	-0.1977561	-1.5857981	0.04195422	0.83978654	0.69745183
Hist2h3b	0.16208913	-1.2253731	0.04194495	0.839804	0.69745183
Trim12a	-0.052001	3.76202107	0.04179581	0.84008491	0.69762825
Tmem230	0.02711265	6.66927818	0.04177034	0.84013293	0.69762825
Grm2	0.03798769	3.64352703	0.04172742	0.84021389	0.6976471
Tbata	0.14098856	-0.0328198	0.04161672	0.84042291	0.69772063
Axin1	-0.0467593	3.37018017	0.04157654	0.84049885	0.69772063
Iqcc	-0.0648034	2.65551747	0.04154598	0.84055664	0.69772063
Mmp24	0.06790205	2.48675089	0.04153117	0.84058465	0.69772063
Dhrs7	0.05385926	3.8290547	0.04150822	0.84062805	0.69772063
Azin1	-0.0229402	7.21388816	0.04149556	0.84065201	0.69772063
Rasl12	0.14262489	-0.0654377	0.04124845	0.84112036	0.69796197
Rnf168	0.02711642	6.53820425	0.04123577	0.84114443	0.69796197
Ptprh	0.17712404	-0.9851058	0.0412329	0.84114989	0.69796197
C230037L18F	-0.098589	1.60175458	0.04119899	0.84121429	0.69796197
Elk1	0.03440732	4.81294127	0.04118851	0.84123419	0.69796197
Mtif2	0.02906266	5.36245845	0.04114408	0.84131862	0.69798366
Hist1h2bb	-0.1680195	-1.2061748	0.04105605	0.84148605	0.6980742
2010002M12	-0.0553552	2.29318535	0.04081817	0.84193939	0.69832416
Cox5b	-0.0305929	6.31383891	0.0408092	0.8419565	0.69832416
Hes1	0.04264282	3.68862672	0.04080617	0.84196229	0.69832416
Pdcd6	0.03162027	5.94969641	0.04066083	0.84224	0.69844602
1110006O24	0.17614081	-0.6314302	0.04062859	0.84230168	0.69844602
Aebp2	-0.0231879	6.84929857	0.04061011	0.84233704	0.69844602
Gm15421	0.06151959	1.85128343	0.04058858	0.84237825	0.69844602
Zfp524	0.12490597	-0.0109418	0.04057357	0.84240698	0.69844602
Hnrnpul2	-0.0211213	8.048024	0.04054633	0.84245915	0.69844602
Tmem181a	-0.0466139	3.45736147	0.04047382	0.84259809	0.69848971
Atmin	0.02405357	6.26839051	0.04041495	0.842711	0.69848971

Btla	-0.1884189	-0.3808945	0.0404143	0.84271224	0.69848971
Cenpp	0.06172588	2.28834247	0.04038266	0.84277297	0.69848971
Tmem38a	-0.0380561	5.49186598	0.04035717	0.84282189	0.69848971
Dmrt2	-0.203551	-0.9402289	0.04033639	0.84286179	0.69848971
Clec4a1	-0.1883859	-0.821454	0.04029589	0.8429396	0.69850585
Inpp1	0.05655144	3.03063513	0.04022674	0.84307254	0.69854615
Nfix	-0.0252752	8.37197493	0.04020992	0.84310489	0.69854615
Kcns2	-0.0396228	3.87739497	0.04016166	0.84319775	0.69856429
Tmem236	0.28281706	-1.7102304	0.04013793	0.84324345	0.69856429
Kcne3	0.1541217	-0.4643361	0.04007801	0.84335886	0.69857533
Frs2	-0.0222644	7.4298957	0.04003967	0.84343275	0.69857533
Hrh1	0.0494005	2.61130733	0.04003246	0.84344666	0.69857533
C8g	-0.1401955	0.37226714	0.03994251	0.84362019	0.69857533
Ccdc104	0.02658831	7.65613033	0.03993819	0.84362854	0.69857533
Clpb	0.04516742	3.74008951	0.03992687	0.84365038	0.69857533
Top3a	0.08006672	0.99311995	0.03991925	0.8436651	0.69857533
B130034C11	0.12316914	0.87810322	0.03981808	0.84386058	0.69861595
Cct5	0.02389417	6.33837543	0.03979522	0.84390478	0.69861595
Dhodh	0.06583776	2.11767985	0.03979181	0.84391139	0.69861595
Tbc1d20	-0.0446722	4.21044894	0.03975743	0.84397789	0.69861595
Tmem241	-0.050265	3.00809008	0.0397229	0.84404472	0.69861595
Metrnl	0.06143416	2.14349993	0.03971286	0.84406417	0.69861595
Rpf2	0.0318172	4.36084234	0.039635	0.84421499	0.69864403
Tmsb4x	0.03733203	8.89562074	0.03960256	0.84427788	0.69864403
Hook3	0.02274643	8.61231605	0.03956154	0.84435745	0.69864403
Vps11	-0.0332835	4.10357047	0.03955114	0.84437762	0.69864403
Oaz1	-0.0330322	5.13371728	0.0395411	0.84439711	0.69864403
Slc4a4	-0.0255175	7.86424389	0.03950157	0.84447382	0.69864403
1300002E11	-0.0321737	5.25378338	0.03948476	0.84450647	0.69864403
Lrrc42	0.04530006	3.25022116	0.03942722	0.84461826	0.69868824
Cep164	0.04310193	3.31652975	0.0393477	0.84477288	0.69876789
Tacc3	0.06197734	2.22085714	0.0392705	0.84492315	0.69884391
Med28	-0.0393695	5.31342009	0.0392011	0.84505838	0.698844
Podxl2	-0.0683647	2.17688717	0.03918311	0.84509344	0.698844
Gtf3c2	0.03281597	6.34947437	0.03918061	0.84509832	0.698844
Ncoa2	-0.0276824	8.34814056	0.03909348	0.84526831	0.69893631
Zfp280d	-0.0360086	6.75746108	0.03904247	0.84536791	0.6989704
A230072C01	0.05808247	3.64761711	0.03874859	0.84594312	0.69937983
Nufip1	0.04249269	4.1197829	0.03872984	0.84597989	0.69937983
Ms4a6c	-0.1155241	0.37829737	0.0386863	0.84606533	0.69940218
Rheb	-0.0284596	7.10790637	0.03860322	0.84622848	0.69948877
Timm50	0.07386247	1.68706968	0.0384507	0.84652848	0.69953143
A630072M18	0.04473714	3.50751564	0.03844957	0.8465307	0.69953143

Adamts8	-0.1257515	-0.3330181	0.03839262	0.84664288	0.69953143
Atg2b	-0.0414592	6.04259617	0.03834684	0.84673313	0.69953143
Clptm1l	-0.0387279	4.51045933	0.03827256	0.84687967	0.69953143
Gm14391	-0.0998111	1.93452359	0.03826852	0.84688765	0.69953143
Snhg10	-0.0928547	0.7763282	0.03825839	0.84690765	0.69953143
Poglut1	0.02691803	5.22591774	0.03825199	0.84692028	0.69953143
Dhx40	0.02765361	5.37711587	0.03822827	0.84696712	0.69953143
Gm4944	0.03387662	4.17769705	0.03822739	0.84696885	0.69953143
Wbp5	-0.0359144	6.62007551	0.03820636	0.84701039	0.69953143
Dok6	-0.0677743	2.04157033	0.03820514	0.8470128	0.69953143
Wfdc15b	0.23530091	-2.0026989	0.03818199	0.84705854	0.69953143
Mfsd10	-0.0841504	0.81582909	0.03816209	0.84709787	0.69953143
Dus1l	-0.0553078	3.06335656	0.03805021	0.8473192	0.69965946
Dpysl4	-0.0549	2.8588484	0.03801419	0.84739054	0.69965946
Sec23b	-0.0376455	4.50824447	0.03798767	0.84744307	0.69965946
Fam13a	-0.0319779	3.99578644	0.03795469	0.84750843	0.69965946
Ddx58	-0.0412507	5.01473816	0.03793623	0.84754503	0.69965946
Aaed1	-0.0478362	4.32597343	0.03782104	0.84777361	0.69973556
Ehmt1	0.04711715	4.40796228	0.03780993	0.84779568	0.69973556
Pds5a	-0.0253077	6.86280437	0.03779952	0.84781636	0.69973556
Kctd9	-0.031442	4.95379289	0.03773557	0.84794345	0.69973556
Herpud1	-0.034939	4.41628755	0.03766878	0.84807631	0.69973556
Otud3	0.06911835	1.74259567	0.03763244	0.84814865	0.69973556
Pcgf6	-0.0510847	3.32218114	0.03761594	0.84818151	0.69973556
Gpr149	-0.1102461	0.73379147	0.03761184	0.84818967	0.69973556
Pcca	-0.0294311	5.77101254	0.03760285	0.84820758	0.69973556
B4galt4	0.04130953	4.72188672	0.03758482	0.84824351	0.69973556
Mdga2	0.03260956	6.06321093	0.03756654	0.84827993	0.69973556
Mrps18a	0.05314701	3.38640429	0.03751339	0.84838591	0.69975522
Slc35g1	0.05257683	4.50834341	0.03748069	0.84845113	0.69975522
Ap4s1	-0.0252161	5.86757563	0.03746669	0.84847906	0.69975522
Fbxo32	-0.0349183	5.02528935	0.03736391	0.84868433	0.69983945
4833427F10I	0.1465323	-0.7880059	0.03733146	0.84874921	0.69983945
Clec12a	-0.190235	-0.500665	0.03732004	0.84877204	0.69983945
Fam105a	0.05004267	3.79205502	0.03729858	0.84881495	0.69983945
Dolpp1	0.06263962	1.23214382	0.03725643	0.8488993	0.69985842
Tmem248	-0.027834	5.76332469	0.03722869	0.84895483	0.69985842
Blmh	0.03021538	5.01121896	0.03716791	0.84907658	0.69987631
Zfp661	-0.0459606	3.17375051	0.03715951	0.84909342	0.69987631
Rsph9	-0.0874656	2.03446966	0.03713028	0.84915201	0.69987644
Mphosph6	0.04489959	4.50521503	0.03707576	0.84926139	0.69989276
Akirin1	-0.0255482	5.78929013	0.03706215	0.8492887	0.69989276
Ppwd1	-0.0364552	3.72394038	0.03700853	0.84939636	0.69990029

Ufd1l	0.02167434	6.36786093	0.03699938	0.84941472	0.69990029
Rhog	0.05011289	3.23796966	0.03678686	0.84984228	0.70018059
Ggn	0.15008882	-0.0259875	0.03675648	0.8499035	0.70018059
Scn3b	0.02978353	5.74038972	0.03672906	0.84995877	0.70018059
Harbi1	-0.0726969	2.1471248	0.03671419	0.84998877	0.70018059
Ing1	-0.0362814	4.90266404	0.03664734	0.85012366	0.70022883
Nefh	-0.0487974	5.51403652	0.03662722	0.85016427	0.70022883
Wapal	-0.0198823	7.56975924	0.03657163	0.85027658	0.70023261
4632434I11R	0.08853715	0.48499813	0.03656706	0.85028581	0.70023261
Htr2b	-0.0968678	0.19008035	0.03648706	0.85044759	0.70030295
Zfp691	-0.0678463	2.53282717	0.03646195	0.85049841	0.70030295
Gm16675	0.56565525	-1.3070885	0.4113865	0.85054665	0.70030295
Cep170b	-0.0277596	7.77688155	0.03637315	0.85067826	0.70036316
Dnm3	0.03822631	8.50630383	0.03633618	0.85075321	0.70037309
Slc27a2	0.06915582	1.809053	0.03630952	0.85080728	0.70037309
Urb2	-0.0394959	4.20458844	0.03627245	0.85088249	0.70038685
Gm20750	0.21534782	-1.2381917	0.03623498	0.85095855	0.70040132
2610027K06I	0.1716947	-0.8805224	0.03617528	0.85107984	0.70045301
Ppp1r18	-0.0536038	3.13725075	0.03614374	0.85114396	0.70045764
Iqck	0.07347535	2.81758821	0.03609808	0.85123683	0.70048594
Kmt2e	0.02384465	9.52673944	0.03596657	0.85150466	0.70064911
Mafg	0.03661344	4.34596512	0.03591509	0.85160966	0.70064911
Kiss1r	-0.1083312	0.15055536	0.0359146	0.85161064	0.70064911
Tnnt3	-0.263392	-1.5294982	0.03583115	0.85178098	0.70072541
Dnase1	0.10261913	1.03143966	0.03581186	0.8518204	0.70072541
Lix1	0.02759557	4.84179322	0.03576271	0.85192085	0.70075991
Gm7457	-0.2092777	-0.9471554	0.03567633	0.85209756	0.70085712
1110038B12I	-0.0539354	2.45169573	0.03564058	0.85217077	0.7008692
Pdcd7	0.03862394	3.61848309	0.0356068	0.85223997	0.70087798
Aif1	0.08488569	2.15680055	0.03550241	0.85245406	0.70093934
A430105I19F	-0.052618	3.33664494	0.03549738	0.85246437	0.70093934
0610009B22I	-0.041474	5.56179021	0.03548481	0.85249017	0.70093934
Arf3	0.02880576	10.0859814	0.03543118	0.85260032	0.70097476
Cbx4	-0.0307582	4.43718425	0.03540685	0.85265031	0.70097476
Drosha	0.02285954	6.89887873	0.03536809	0.85272999	0.70099214
Cdk13	-0.0206716	7.17706312	0.03533091	0.85280646	0.70100418
Rem2	0.11064966	0.43792714	0.03530355	0.85286278	0.70100418
Tex26	0.13407739	-0.7564321	0.03527563	0.85292024	0.70100418
Klhdc8b	0.07396595	2.2070895	0.0351981	0.85307997	0.70108735
Gm4787	-0.1463548	2.161135	0.03516884	0.85314031	0.70108882
Traip	0.12326603	0.84322021	0.03508693	0.85330935	0.70114904
Otud6b	-0.0288912	6.33504162	0.0350766	0.85333068	0.70114904
Alox8	-0.0436929	3.29180948	0.03497624	0.85353809	0.70116823

Mtg2	-0.0909051	0.41438466	0.03497388	0.85354297	0.70116823
Eya3	0.02967718	5.04768282	0.03495377	0.85358457	0.70116823
Smg6	-0.0266864	5.6233263	0.034952	0.85358824	0.70116823
Coa4	-0.0627239	2.07474742	0.03484895	0.8538016	0.70124507
Cxcl10	0.07903095	2.81711053	0.03484539	0.85380899	0.70124507
Slc25a53	0.06236299	2.57101255	0.03478981	0.85392421	0.70124507
Ptpru	-0.0426044	3.24593205	0.03476779	0.85396989	0.70124507
Elp5	0.0342818	4.81554388	0.03476554	0.85397456	0.70124507
Zfp758	0.03810367	3.79792439	0.03470191	0.85410663	0.70130544
Dusp1	-0.053129	6.28480359	0.03466936	0.85417425	0.70131287
Crlf3	-0.0530916	3.11838493	0.03462814	0.85425992	0.70133513
Mir425	-0.2116205	-1.6929276	0.03451706	0.85449105	0.70135767
Rps19-ps3	0.08112168	0.61789326	0.03449909	0.85452847	0.70135767
LOC1026344	-0.0901225	1.10325878	0.03447389	0.85458098	0.70135767
Il17re	-0.0996864	0.83772249	0.03443007	0.85467231	0.70135767
C230024C17I	0.24598288	-1.9188972	0.03439846	0.85473825	0.70135767
Dnase1l1	-0.0612601	2.18178417	0.03439801	0.85473918	0.70135767
Agmo	-0.0925486	1.67511286	0.03439547	0.85474447	0.70135767
Klhl36	0.11139418	0.6160963	0.03439	0.85475589	0.70135767
Glo1	-0.0302239	6.44821502	0.03420917	0.85513371	0.70161961
Hmgn3	0.03077147	6.929729	0.03410717	0.85534725	0.70164885
Spryd3	0.04494867	4.21771774	0.03410511	0.85535157	0.70164885
Smim13	-0.0235983	8.64473928	0.0340844	0.85539499	0.70164885
2310022A10I	0.0394174	3.59599026	0.03408024	0.85540371	0.70164885
Prr18	-0.0426749	3.12852725	0.03397088	0.85563314	0.70177499
Lrch1	0.03971114	4.25291968	0.03395109	0.85567469	0.70177499
Guk1	0.0399586	4.85729885	0.03392219	0.85573542	0.70177674
Zfp760	-0.0293577	5.25327047	0.0338122	0.85596673	0.70189106
Gm10416	-0.1349246	0.47683774	0.03380018	0.85599204	0.70189106
Bahd1	0.04565431	4.09485007	0.03374973	0.85609828	0.70190061
E330023G01	-0.073342	1.42806035	0.03372153	0.85615771	0.70190061
Runx1t1	-0.0226133	7.80809386	0.03371118	0.85617953	0.70190061
Hrk	-0.044264	4.09783662	0.03358002	0.8564563	0.70200433
Avpi1	0.03813147	3.24214639	0.03357391	0.85646921	0.70200433
Yeats4	0.02850526	5.23072457	0.03351997	0.8565832	0.70200433
Tlr13	-0.1287925	0.9213886	0.03351049	0.85660326	0.70200433
Mettl7a2	-0.0569515	1.73110516	0.03349772	0.85663025	0.70200433
Prrg1	0.0541022	2.50080234	0.03347578	0.85667666	0.70200433
Llgl2	-0.1084966	-0.2605733	0.0334331	0.85676701	0.70200433
Smarcd3	0.03850041	4.1281128	0.03342933	0.85677499	0.70200433
Car5a	-0.1246795	-0.2991194	0.03335531	0.8569318	0.70205187
Flywch2	0.06543371	1.0455833	0.0333466	0.85695025	0.70205187
Sftpc	-0.2490738	-1.19407	0.0332821	0.85708707	0.70207712

Ildr2	-0.0279464	9.87099209	0.03325736	0.85713957	0.70207712
Ncf4	-0.1522032	-0.9556413	0.03324917	0.85715696	0.70207712
Gpr18	-0.1581724	-0.4416102	0.03314053	0.85738782	0.70220563
Slc36a4	-0.030736	4.97063688	0.03312018	0.85743112	0.70220563
Cd53	-0.0479044	2.34394497	0.03300352	0.85767953	0.70236104
Chkb	-0.0611462	1.33850918	0.03281186	0.85808864	0.7025399
Efnb2	0.02671211	6.13005427	0.03281017	0.85809225	0.7025399
Zfp775	0.05970557	3.00237761	0.03273716	0.85824843	0.7025399
Sec61g	0.03930478	4.7707202	0.03273025	0.85826321	0.7025399
Wnk3	-0.0330827	6.92545557	0.03269614	0.85833626	0.7025399
Rras	-0.0535123	4.72190055	0.03268648	0.85835694	0.7025399
Rbl2	0.02059679	7.24597562	0.03263219	0.8584733	0.7025399
Runx2	-0.0382934	4.70215214	0.03262296	0.85849307	0.7025399
Gstm3	0.06303065	1.53275135	0.03259308	0.85855716	0.7025399
Kif3a	0.02967006	8.10931872	0.03256986	0.85860698	0.7025399
Syne3	0.11967014	0.83033734	0.03249341	0.85877114	0.7025399
Foxl2	-0.1874105	-0.4672663	0.03248632	0.85878637	0.7025399
Alcam	0.03735839	9.49713943	0.03247403	0.85881278	0.7025399
Tia1	0.03779773	5.2628024	0.03245812	0.85884698	0.7025399
Med22	0.03639413	3.78866602	0.03244598	0.8588731	0.7025399
Nsun7	0.07459536	2.25496462	0.03243269	0.85890168	0.7025399
Cyld	0.02239496	7.32138492	0.03242877	0.85891009	0.7025399
Ralgps1	-0.0328199	6.58154748	0.03236876	0.85903923	0.7025399
Tk1	-0.1822126	-0.8897527	0.03236121	0.8590555	0.7025399
Alg12	-0.062185	2.79775609	0.0323539	0.85907123	0.7025399
Dpp7	-0.0689459	1.49041866	0.0323139	0.8591574	0.7025624
Cmbl	-0.0476551	6.17617277	0.03228433	0.85922113	0.70256654
Cep68	-0.0325271	5.0034212	0.03214404	0.8595239	0.70276613
Smarcal1	-0.0341186	6.14881675	0.03209681	0.85962599	0.70278427
Gm7173	-0.1354099	-0.6906791	0.03206162	0.8597021	0.70278427
Hyal2	0.07384663	1.18618416	0.0320345	0.85976079	0.70278427
Tktl1	-0.2938789	-1.6232973	0.03202524	0.85978083	0.70278427
Mak16	-0.0290669	4.98461037	0.03194729	0.85994968	0.70285013
Snora30	0.20669086	-1.4576189	0.03193387	0.85997878	0.70285013
Nedd8	0.04016633	5.60706888	0.03185763	0.86014417	0.70293353
Slc25a27	0.03633683	4.58888176	0.03183273	0.86019822	0.70293353
Snora31	-0.1858403	-1.2004144	0.03180181	0.86026538	0.70294045
Wfdc1	-0.0829329	2.89032144	0.03173899	0.86040195	0.70299818
Fam195a	0.15482551	-1.2383846	0.03171493	0.86045427	0.70299818
Mylk3	0.1203636	0.35676851	0.03166497	0.86056303	0.70299818
Mark4	0.03679426	3.63180136	0.03166138	0.86057084	0.70299818
Msh3	-0.0365583	4.12936455	0.03162168	0.86065731	0.70302086
Rcor2	-0.0699842	1.62983719	0.031525	0.86086816	0.70312303

Ppp1r13b	-0.0275551	5.6077684	0.0315105	0.86089981	0.70312303
Evi5	0.02034632	7.79948001	0.03142446	0.86108776	0.70322858
Cd82	0.10821763	1.51331837	0.03135826	0.86123255	0.70329886
Gm15408	0.1595277	-0.4333354	0.0312299	0.86151376	0.70347264
Iqsec3	0.03458656	6.55451205	0.03118654	0.8616089	0.70347264
Prkag1	0.02689345	4.21440019	0.03117651	0.86163089	0.70347264
Trappc3l	0.22429234	-2.1751454	0.03113227	0.86172804	0.70347264
Pkd2	-0.0386383	6.20222856	0.0311149	0.86176621	0.70347264
Fech	0.02462663	7.02489319	0.03110052	0.8617978	0.70347264
Osbpl3	0.03952854	5.09364473	0.03098967	0.86204166	0.70361319
Trmt2b	-0.0434015	5.21430636	0.03096042	0.86210609	0.70361319
Oxnad1	0.04723809	3.78928905	0.03088782	0.8622661	0.70361319
Gm10516	0.06378647	2.08014304	0.03084882	0.86235216	0.70361319
Aig1	0.04992241	3.24048573	0.03084225	0.86236666	0.70361319
Rpl22l1	-0.0337274	5.0394012	0.03081688	0.86242266	0.70361319
1110012L19R	-0.0553754	3.3695072	0.03080182	0.86245592	0.70361319
Pnpla7	0.06243863	2.12670054	0.03078653	0.8624897	0.70361319
Fem1b	-0.0210163	7.94531779	0.03078243	0.86249877	0.70361319
Slc25a15	0.03814135	3.41197835	0.03074467	0.86258222	0.70363333
Pcsk9	0.10147683	-0.0783968	0.03069162	0.86269956	0.70366826
Pgrmc2	0.02514035	5.49575319	0.0306722	0.86274254	0.70366826
Gm15441	0.18161534	-1.1953439	0.03051259	0.86309633	0.70390887
Jkamp	-0.0319385	4.43764506	0.03044758	0.8632407	0.70397867
Gm4532	-0.2739034	-1.3773558	0.03037899	0.86339319	0.70405508
Nlrp4f	-0.2327574	-1.9884153	0.03033566	0.86348963	0.70408578
Stk36	-0.0736878	1.23416764	0.03029161	0.86358772	0.70411783
Cpq	0.04748221	4.27189071	0.03014837	0.86390723	0.70425262
Zfp711	-0.0391548	4.20917401	0.03014564	0.86391333	0.70425262
Slco1a5	0.17847915	-0.5127274	0.03013842	0.86392947	0.70425262
Osbpl9	0.0252141	6.74841289	0.03009524	0.86402595	0.70428333
Ccng1	-0.0221765	8.15001711	0.02998862	0.8642645	0.70439797
Fbxo7	0.03705392	3.37786508	0.02997982	0.86428422	0.70439797
Lsm4	-0.0453553	3.33172149	0.02989318	0.86447842	0.70443971
Vasp	-0.0584647	3.48114641	0.02989125	0.86448274	0.70443971
Rpl29	0.03191829	6.10243951	0.02987825	0.86451191	0.70443971
Zbtb17	-0.0500308	2.67988355	0.02973599	0.86483151	0.70456606
Parn	-0.0268986	4.67553841	0.02973088	0.86484299	0.70456606
1110015O18	0.13330789	-0.9283855	0.02973067	0.86484347	0.70456606
Snph	-0.0331562	5.88306559	0.0296483	0.86502888	0.70466917
Irf1	0.05439274	2.9745514	0.02953475	0.86528493	0.70482981
Kctd14	-0.0982984	-0.0124869	0.02948644	0.86539402	0.70483481
2810428I15R	0.05661552	2.82113477	0.02947991	0.86540877	0.70483481
D030047H15	0.08613911	0.23058066	0.02945307	0.86546944	0.70483628

Foxk2	-0.0171135	6.46145582	0.02938251	0.86562903	0.70491832
Ano10	0.04675387	2.49026703	0.02933084	0.865746	0.70493804
Gpr12	-0.0388991	4.05832662	0.02930389	0.86580708	0.70493804
Zfyve1	0.02751388	4.19700661	0.02929237	0.86583319	0.70493804
Wiz	-0.0275584	4.43720815	0.02926788	0.86588871	0.70493804
4933421O10	-0.0449316	3.5009679	0.02920472	0.86603201	0.70494841
Gm13212	-0.0549869	1.56577892	0.02918848	0.86606889	0.70494841
Gm19710	-0.1361044	0.31012239	0.02918446	0.86607803	0.70494841
Coro2a	0.03687928	5.32555707	0.02915021	0.86615584	0.70496382
Vamp3	0.03096556	7.50340041	0.02907718	0.86632191	0.70505107
Slc8a3	-0.0345111	3.92640813	0.02903714	0.86641305	0.70507732
Rhbdf1	-0.0601063	1.5426094	0.02900082	0.86649576	0.70509672
Ppp4c	-0.0620257	2.05232108	0.02884437	0.86685273	0.70533255
Kcnj13	0.0368454	6.22314713	0.02882222	0.86690336	0.70533255
B9d1	-0.0469344	2.37419088	0.0287502	0.86706808	0.70538816
Slc9a7	-0.0412436	4.25515932	0.02870966	0.8671609	0.70538816
Atp11c	0.033929	4.6841988	0.02866629	0.86726026	0.70538816
Cdc34	-0.0482864	3.16329513	0.02866426	0.86726493	0.70538816
Kcnk9	-0.0577492	2.87097366	0.02865265	0.86729153	0.70538816
9230114K14I	0.06027235	1.94223052	0.0286157	0.86737627	0.70538816
Clybl	0.03127184	4.35151724	0.02848124	0.86768514	0.70538816
Il1rap	-0.0339314	3.72863637	0.02847711	0.86769462	0.70538816
Fscn1	-0.0291795	5.37348676	0.02844739	0.867763	0.70538816
Mex3c	0.02345525	5.21447884	0.02842112	0.86782348	0.70538816
Tfip11	0.04241401	3.79497696	0.02841927	0.86782773	0.70538816
Ccdc8	-0.1405917	-0.2951193	0.02841091	0.86784698	0.70538816
Uba52	0.03293135	6.7196023	0.02840854	0.86785243	0.70538816
Prr12	0.02611844	5.7528392	0.02838474	0.86790726	0.70538816
Srpr	-0.0349686	6.37658832	0.02836305	0.86795723	0.70538816
Acad8	0.02631651	4.24425617	0.02835662	0.86797205	0.70538816
Gucy2g	0.06646361	1.35849486	0.02835619	0.86797304	0.70538816
Mro	0.02840601	4.44287072	0.02831659	0.86806435	0.7054145
Rtp3	-0.1286384	0.44595136	0.02824998	0.8682181	0.7054667
Ly6c2	-0.1595033	-0.5892945	0.02823772	0.86824641	0.7054667
Cluap1	0.02153245	5.50407573	0.02814443	0.8684621	0.70548865
Ppp1r3g	0.08656268	0.59363059	0.02812071	0.86851699	0.70548865
Cdh22	0.07166661	1.76906801	0.02812036	0.86851781	0.70548865
Rad51c	0.07078293	1.97713349	0.02811803	0.8685232	0.70548865
Fkbp8	-0.0321457	5.10116843	0.02809869	0.86856797	0.70548865
Gm101	-0.1754552	-1.1663423	0.02798405	0.86883376	0.70554699
Ppp1ca	-0.0356475	5.51876175	0.02797629	0.86885176	0.70554699
Scml4	0.038251	4.00497164	0.02796797	0.86887108	0.70554699
Mageb16	-0.11574	1.09658276	0.02796608	0.86887546	0.70554699

Eif1ax	-0.0305569	6.23929247	0.02790288	0.86902227	0.7055592
Ascc2	0.03600352	3.30241568	0.02788785	0.8690572	0.7055592
Polr1e	0.04860182	1.80469132	0.0278357	0.86917851	0.7055592
Trappc13	0.01956601	6.39477302	0.02782663	0.86919961	0.7055592
Gm10941	-0.1578533	-1.0465899	0.02781127	0.86923536	0.7055592
Timm10b	0.04727285	2.6479208	0.02777304	0.86932439	0.7055592
Pdcl3	0.03679491	4.225778	0.0277624	0.8693492	0.7055592
B4galt7	0.08039735	1.05384379	0.02775698	0.86936182	0.7055592
Taf1d	0.0309494	3.99055053	0.02765268	0.86960512	0.70570884
Socs6	0.0261957	4.27814036	0.02762384	0.86967249	0.70571568
Csde1	-0.0166527	9.14077136	0.02752369	0.86990669	0.70578387
Sppl2b	-0.0662361	1.50331952	0.02751704	0.86992226	0.70578387
Atp6v0e	-0.0436963	4.75238444	0.02751231	0.86993332	0.70578387
Npepps	-0.0264569	6.32023894	0.02741985	0.87014997	0.70591182
Phf11b	0.11001717	0.71371584	0.02736087	0.87028838	0.70593265
Fcer1g	0.04770761	3.20022187	0.02732566	0.87037106	0.70593265
Utrn	-0.0200888	7.75997577	0.02729153	0.87045127	0.70593265
Alg2	0.01812123	7.44663579	0.02728525	0.87046604	0.70593265
B930059L03I	-0.2051126	-1.1958104	0.02724194	0.8705679	0.70593265
Tmem45a	-0.0613026	2.20741099	0.02721695	0.87062671	0.70593265
Crybb1	0.18246386	-1.8920045	0.0271612	0.87075803	0.70593265
Cwf19l1	-0.0428917	3.90492963	0.02713	0.87083159	0.70593265
Ppp1r11	0.02791852	4.56555625	0.02712677	0.87083919	0.70593265
Kcnt1	-0.0383677	5.40537708	0.02710266	0.87089607	0.70593265
Mpnd	-0.0410181	3.83602864	0.02709857	0.87090573	0.70593265
Nr3c1	0.01745023	7.2766846	0.02708459	0.87093871	0.70593265
Bag1	-0.0286275	7.80584386	0.02708321	0.87094196	0.70593265
Ckap2l	0.07034032	0.9087401	0.02699607	0.87114781	0.70600681
Ssh3	-0.058681	1.67855805	0.02699456	0.87115137	0.70600681
5830418P13I	-0.1047224	0.17613779	0.02695556	0.8712436	0.70603379
Lpar5	0.15998237	-0.7769448	0.02692354	0.87131937	0.70604741
Arhgap28	-0.0393915	4.00882004	0.02680941	0.87158985	0.70612221
Dnph1	0.06865327	1.62737352	0.02680527	0.87159968	0.70612221
Mgat3	0.02239376	6.48403666	0.02679168	0.87163192	0.70612221
Six1	-0.0532922	5.37716334	0.02678511	0.87164753	0.70612221
Gm14634	-0.1157709	-0.1042854	0.02673856	0.87175807	0.70616399
Txlnb	-0.0591287	2.24854528	0.02669351	0.87186513	0.70617102
Ddx21	-0.023451	4.80739924	0.02666476	0.87193351	0.70617102
Rfc3	-0.0561029	1.77971401	0.0266605	0.87194365	0.70617102
Actr6	-0.0403923	3.63828272	0.02657618	0.87214443	0.70623865
Dlgap2	-0.032776	6.60868622	0.0265759	0.87214511	0.70623865
Gm10638	-0.1243187	-0.6071244	0.0265154	0.87228938	0.7062709
2900008C10I	-0.1157127	0.21761927	0.02650897	0.87230473	0.7062709

Tlcd1	-0.0655364	2.1458999	0.02646486	0.87241001	0.7062709
Chn1os3	-0.1200412	-0.0760087	0.02646033	0.87242083	0.7062709
Nkx2-1	-0.0638693	1.90266197	0.0263947	0.8725777	0.70635015
Rgag1	0.07084875	0.93509014	0.02633671	0.87271646	0.70641246
Rpl6	-0.0259942	7.75574068	0.02627414	0.87286636	0.70641246
Gm5512	-0.0508884	1.38961734	0.02627344	0.87286805	0.70641246
Gamt	0.07197583	1.99468364	0.02624137	0.87294495	0.70641246
Zfp870	-0.0342902	3.74149517	0.02622995	0.87297235	0.70641246
Larp4b	0.01604324	7.45689149	0.02618267	0.87308584	0.70641246
Lix1l	0.04129192	6.40813005	0.02616225	0.8731349	0.70641246
Tfec	0.23503637	-1.7338427	0.02614197	0.87318361	0.70641246
Avil	0.20957381	-1.4523535	0.02614116	0.87318556	0.70641246
Kif24	-0.0883716	1.04121101	0.02611479	0.87324895	0.70641602
Ppp1r37	-0.0292883	4.26330119	0.02604145	0.87342545	0.70642196
Lrrc2	0.06378846	1.76997162	0.02603681	0.87343663	0.70642196
Tradd	0.07785079	0.67360552	0.02600715	0.87350807	0.70642196
4930419G24	-0.0722361	1.826446	0.02598148	0.87356995	0.70642196
Cbr1	0.02630572	4.80828654	0.0259435	0.87366156	0.70642196
Arl9	-0.2091727	-1.9153914	0.02593661	0.87367819	0.70642196
Gm11186	0.20139145	-1.441342	0.02591773	0.87372374	0.70642196
G6pdx	0.02780329	5.75914343	0.02589906	0.87376883	0.70642196
Npy2r	0.04355169	2.84536466	0.02589146	0.87378719	0.70642196
Gnat1	-0.1301078	-0.4817534	0.02583382	0.87392648	0.70647262
Eogt	-0.0254749	5.15256979	0.02581348	0.87397569	0.70647262
Cd226	0.086744	0.44264566	0.02579147	0.87402894	0.70647262
3110001I22R	0.06956401	1.10874887	0.02576517	0.87409261	0.70647262
1600014C10I	-0.0302491	4.83188295	0.02572544	0.87418885	0.70647262
Pcsk2os2	0.11512729	1.71436286	0.02569615	0.87425986	0.70647262
Cacna2d3	-0.0318022	5.15526663	0.02569494	0.87426279	0.70647262
1810026J23F	-0.023358	5.9224383	0.02565565	0.87435808	0.70647272
Shkbp1	0.07625269	0.80269498	0.02564625	0.8743809	0.70647272
Aim2	-0.0679898	2.19248865	0.0255725	0.87456005	0.70653936
Dars2	0.04548016	3.49596305	0.02556373	0.87458137	0.70653936
Gm10767	0.04896863	1.92261135	0.02541935	0.87493289	0.70677316
Tnrc6c	-0.0222062	6.9612226	0.02539284	0.87499755	0.70677316
Gfer	-0.0423108	3.00280689	0.02534497	0.87511439	0.70677316
Zbtb33	0.02465533	5.58479729	0.02534137	0.87512318	0.70677316
Mrpl23	-0.0343145	3.13313811	0.02530438	0.87521355	0.70677316
Sbno2	-0.0508785	1.71964168	0.0252787	0.87527631	0.70677316
F2	0.15871083	-1.044684	0.02516357	0.87555817	0.70677316
Frg1	0.02228316	4.81586465	0.02514139	0.87561254	0.70677316
Gm10635	0.095427	0.85486743	0.0250966	0.87572242	0.70677316
Smim7	-0.0315975	6.30717233	0.02509285	0.87573162	0.70677316

Tead3	0.04406868	2.73021835	0.02505775	0.87581781	0.70677316
Ptpre	0.03049071	5.24251314	0.02502753	0.87589207	0.70677316
Wdr77	0.02368657	5.07304581	0.02501797	0.87591556	0.70677316
Zfp503	-0.0332556	3.92476051	0.02499636	0.8759687	0.70677316
Gm1141	0.21479708	-1.6419073	0.02496611	0.87604312	0.70677316
Tdrd9	0.18517472	-1.9587224	0.02496596	0.87604349	0.70677316
Itgb1bp1	0.02414822	4.68775085	0.0249633	0.87605004	0.70677316
Aph1b	-0.0359927	4.15526441	0.02496221	0.8760527	0.70677316
Ftl1	-0.0299055	6.19724188	0.02496032	0.87605737	0.70677316
Cdc26	-0.0408289	4.58922415	0.02490817	0.87618579	0.70677316
Tspan3	0.02869328	7.0022232	0.02486002	0.87630448	0.70677316
Gm20110	-0.1651926	-1.7276857	0.02485984	0.87630493	0.70677316
Tmem199	-0.0256683	4.14860616	0.02485123	0.87632616	0.70677316
Hap1	0.03409598	4.20512542	0.02485001	0.87632917	0.70677316
Kcnk4	-0.0809142	0.31919566	0.0248431	0.8763462	0.70677316
Phactr1	-0.0209598	7.94788212	0.02475693	0.87655899	0.70687516
Rprd1b	0.02742615	5.10640068	0.02472746	0.87663185	0.70687516
Npas4	-0.1207879	3.17988758	0.02472022	0.87664976	0.70687516
Wars2	-0.0431442	3.67473032	0.02465878	0.87680182	0.70691537
1810021B22I	-0.0934415	0.54550243	0.02465238	0.87681769	0.70691537
Sybu	-0.0276705	5.28269704	0.02458632	0.8769814	0.70699608
Ubfd1	-0.0186466	6.94136484	0.02456201	0.87704174	0.70699608
A530064D06	0.16974308	-1.7685004	0.02454059	0.87709489	0.70699608
Pcdha12	-0.0794708	1.20786734	0.02449984	0.8771961	0.70703006
Larp4	0.01862387	7.14921979	0.02446116	0.87729226	0.70705999
Prkd3	-0.0244418	5.66350149	0.0243582	0.87754858	0.70714125
Fam160a2	-0.0246642	6.65594544	0.02434941	0.87757048	0.70714125
Prrt4	-0.1216908	-0.6122405	0.02433003	0.87761881	0.70714125
Rnf146	-0.0224135	5.45688731	0.02432583	0.87762928	0.70714125
Psmb6	0.02969596	4.90056129	0.02427517	0.87775569	0.70719552
LOC1008616	0.10181333	-0.5326978	0.02424411	0.87783327	0.70721045
Zzz3	-0.0182684	7.29131947	0.02420341	0.87793497	0.7072448
Mrpl14	-0.0477207	3.03606529	0.02409668	0.87820215	0.70729006
Heatr2	0.02577762	4.21724217	0.02407648	0.87825279	0.70729006
Sphk2	-0.029643	4.07618968	0.02406457	0.87828263	0.70729006
S100a1	-0.044655	4.83217325	0.0240574	0.87830063	0.70729006
Rnf150	-0.0254215	7.33779721	0.02402482	0.87838238	0.70729006
Klhl35	-0.1481019	-0.7426236	0.02401872	0.87839767	0.70729006
Dio3	0.13704458	-0.9980248	0.02400288	0.87843744	0.70729006
Hint2	-0.0407557	3.50760824	0.02396547	0.87853143	0.70729006
Slc17a5	-0.0286346	3.89423497	0.02394266	0.87858877	0.70729006
Eapp	0.02331702	5.25186188	0.02389797	0.87870119	0.70729006
Klf3	-0.0270294	7.62085981	0.02386281	0.87878971	0.70729006

Ndufv1	0.02849849	4.74009569	0.02384483	0.878835	0.70729006
Tmem39b	0.05983793	1.82656269	0.02384123	0.87884406	0.70729006
Mplkip	0.02396794	4.05352396	0.02383356	0.87886338	0.70729006
Asb15	-0.1082439	0.58360885	0.02382439	0.8788865	0.70729006
Ahdc1	-0.0254237	4.66728877	0.02380457	0.87893647	0.70729006
Slc12a6	-0.0188269	6.78989424	0.02377725	0.87900539	0.70729006
Jakmip3	-0.0423895	4.95956322	0.02374329	0.87909112	0.70729006
Hiatl1	0.02827269	4.0000402	0.02371707	0.87915733	0.70729006
Asb11	-0.0751516	0.90161594	0.02371113	0.87917236	0.70729006
Gm10440	-0.2506348	-1.531503	0.02365485	0.87931464	0.70735701
Cacna1c	-0.0312093	6.3128366	0.02352618	0.87964063	0.70752783
Rabgef1	0.0316484	4.26776228	0.0235244	0.87964515	0.70752783
Sp110	-0.0409354	3.39387382	0.02341751	0.87991664	0.70769866
Celsr1	0.04568976	2.35191803	0.0233594	0.88006453	0.70770885
Ppapdc2	-0.0184125	6.28486106	0.02335024	0.88008784	0.70770885
Stxbp6	-0.0227645	6.56505081	0.02334288	0.88010659	0.70770885
Tuft1	-0.0911953	0.44373696	0.02331394	0.88018033	0.70772062
Zfp36l1	0.02605249	7.72852485	0.02320833	0.88044981	0.70781672
Ankrd35	-0.0369924	2.76425955	0.02320208	0.88046579	0.70781672
Efcab9	0.13075273	-0.5491644	0.02319763	0.88047715	0.70781672
Slc16a1	-0.0285507	5.03789291	0.0231473	0.88060584	0.70784988
Avpr1a	0.14621372	-0.6701335	0.02312543	0.88066179	0.70784988
4930467D21I	0.17743452	-1.3164276	0.02310637	0.8807106	0.70784988
Odf2l	-0.0381199	3.49166624	0.02308124	0.88077495	0.70784988
Rabl3	0.02732913	4.40385949	0.02306306	0.88082154	0.70784988
Pcdhac1	0.10626517	-0.0962579	0.02301939	0.88093351	0.70784988
N4bp2l2	-0.0175627	6.28193823	0.02301536	0.88094386	0.70784988
Sgms2	-0.0580327	3.45145195	0.02299689	0.88099125	0.70784988
Acer3	0.03885987	3.65386616	0.02290464	0.88122829	0.70798768
B430010I23F	0.15021473	-0.1740072	0.02287511	0.88130427	0.70798768
Ppp2r4	-0.021448	5.70209698	0.02286118	0.88134012	0.70798768
Lrrc6	0.03361235	3.88873187	0.02278289	0.88154187	0.70802207
Rpusd2	0.02675404	4.33296508	0.0227519	0.88162181	0.70802207
Zfp280c	0.02505966	5.37018434	0.02274672	0.88163519	0.70802207
Ahnak	0.0310109	9.0753298	0.0227335	0.8816693	0.70802207
Nrg3os	0.09709986	0.39788992	0.02272993	0.88167854	0.70802207
Mttp	0.04905777	2.01336206	0.02267279	0.88182616	0.70809314
Tsnax	0.01783301	7.46016546	0.02258067	0.88206458	0.70823709
Tnip3	0.07431691	1.79841068	0.0225358	0.88218091	0.70824998
Fxyd2	-0.0982976	-0.1409644	0.02252886	0.88219891	0.70824998
G730013B05	0.08699761	0.35112344	0.02241401	0.88249719	0.7082551
Emilin2	-0.1672992	-1.0549577	0.02240435	0.88252234	0.7082551
Hcn2	-0.056212	1.3127917	0.02239609	0.88254382	0.7082551

Agpat5	-0.0205438	5.497355	0.02237674	0.88259418	0.7082551
Incenp	0.03328378	3.47791435	0.0223754	0.88259766	0.7082551
6720483E21I	0.17122901	-1.7520889	0.02236946	0.88261313	0.7082551
Zfp236	-0.0270426	5.532951	0.0223671	0.88261927	0.7082551
Hs3st5	-0.0637826	1.6804255	0.02219539	0.88306718	0.70849022
Ndufb2	0.02583202	3.99487828	0.022186	0.88309173	0.70849022
Sepw1	0.04273681	5.57534825	0.02214025	0.88321142	0.70849022
1700120C14I	-0.1280989	-1.1689078	0.02213627	0.88322182	0.70849022
Itih3	0.06031101	1.81272516	0.02210344	0.88330778	0.70849022
Artn	0.1986331	-2.0795552	0.02208663	0.88335183	0.70849022
Cyp4v3	0.03779648	4.68104747	0.02207679	0.8833776	0.70849022
Dcp1b	-0.0277717	4.21256879	0.02205163	0.88344356	0.70849022
Foxo3	-0.0208872	6.59802806	0.02203164	0.88349601	0.70849022
Dis3l2	0.03206469	3.42555374	0.02198146	0.88362774	0.70849022
2310068J16F	0.13793817	-0.8088933	0.02197597	0.88364216	0.70849022
Hsd3b1	-0.3174686	-1.4652277	0.02197263	0.88365095	0.70849022
A4gnt	0.15886578	-1.4684835	0.0219539	0.88370017	0.70849022
Acta1	0.06739036	0.90019356	0.02189742	0.88384872	0.70849022
Ptbp1	-0.0317757	6.00333761	0.02184791	0.8839791	0.70849022
Ephb4	-0.0554422	3.24174514	0.02176972	0.8841853	0.70849022
Myl12a	0.03938941	6.85744348	0.02175182	0.88423258	0.70849022
Ddx19b	0.02523472	5.11197622	0.02174072	0.88426189	0.70849022
Smc4	0.0244413	6.28417346	0.02173807	0.88426888	0.70849022
Emp1	0.048156	3.82778592	0.02173652	0.88427298	0.70849022
BC031361	0.04040689	2.8200458	0.0217358	0.88427488	0.70849022
Bend7	-0.0632386	0.64875533	0.02172761	0.88429652	0.70849022
Zfp518a	0.02075226	5.61763445	0.02172352	0.88430734	0.70849022
Zfp825	0.02615837	4.52217882	0.02170447	0.88435769	0.70849022
Wdr65	-0.0855529	1.0782569	0.02167657	0.88443149	0.70849022
Ndrg3	-0.0147714	8.73840103	0.02162547	0.88456674	0.70849022
H1f0	0.02182612	6.9773006	0.0215952	0.88464696	0.70849022
Itpkb	-0.0364083	3.35426576	0.0215851	0.88467374	0.70849022
Med23	-0.0231679	4.19630727	0.02157764	0.88469351	0.70849022
Dedd	0.0246962	4.20891729	0.02155825	0.88474495	0.70849022
Pou6f2	-0.0600741	1.93296602	0.02155775	0.88474626	0.70849022
Vav2	0.0487285	1.76348009	0.02150295	0.88489174	0.70850081
Adi1	-0.0271357	7.13727082	0.02149016	0.88492574	0.70850081
Gja3	0.2103392	-1.8770498	0.02148593	0.88493697	0.70850081
Aoah	-0.133222	-0.6572058	0.02138479	0.88520609	0.7086689
Tmem154	0.04636046	3.39839812	0.02134379	0.88531535	0.70870899
Nek1	0.02435081	6.39445689	0.02130062	0.88543052	0.70871301
Mb21d1	0.06890556	0.81745462	0.02129755	0.88543873	0.70871301
Fbxl20	-0.017166	6.62663552	0.02126673	0.88552103	0.70873152

Cd8b1	0.08963475	-0.329234	0.02119078	0.88572414	0.70880533
Grm8	0.05189725	2.66462651	0.02116684	0.88578821	0.70880533
Efcab12	-0.0791212	0.82845375	0.02116587	0.88579082	0.70880533
Fancb	0.08713948	0.97425989	0.02112184	0.88590878	0.70881745
Gm13547	0.22408639	-2.4282955	0.02106617	0.88605813	0.70881745
Aak1	0.02411717	9.72626354	0.02105899	0.88607743	0.70881745
Shoc2	0.01686774	8.6086196	0.02105129	0.88609809	0.70881745
Usp19	0.02336531	4.7584314	0.02102369	0.88617224	0.70881745
D030028A08	0.05247387	1.33502954	0.02101601	0.88619288	0.70881745
Dnaic1	0.0642613	0.56456003	0.02100582	0.88622027	0.70881745
Gm11992	-0.1095855	-0.7542537	0.02089094	0.88652954	0.70896208
Nxph3	0.09541987	-0.3782904	0.02087955	0.88656025	0.70896208
St6galnac6	-0.0306596	4.07207896	0.02087271	0.88657871	0.70896208
4930479D17	0.06598079	1.0864378	0.0208254	0.88670638	0.70900454
Mettl21c	0.16542373	-0.5973106	0.0207963	0.88678498	0.70900454
Impdh2	0.02534082	3.73965062	0.02078726	0.88680941	0.70900454
07-Mar	-0.0228257	4.80727516	0.02076266	0.88687592	0.70901038
Ccser2	0.01574027	8.27618302	0.02073812	0.88694233	0.70901614
Stk24	0.02340645	7.31064576	0.02067043	0.88712565	0.70901849
Gm3604	-0.0345122	2.73309904	0.02065519	0.88716698	0.70901849
Cdca5	0.16576645	-1.4969449	0.02062273	0.88725502	0.70901849
Magi2	-0.0239128	7.42040035	0.0206154	0.88727492	0.70901849
Lefty1	-0.0568427	1.93851202	0.02060293	0.88730878	0.70901849
Spag4	-0.1551968	-1.6186552	0.02060229	0.8873105	0.70901849
1500015A07	0.05324779	2.19845466	0.02058366	0.8873611	0.70901849
Eif4e	-0.0155006	7.4070904	0.02056239	0.8874189	0.70901849
Ifi35	-0.0665177	2.04781192	0.0205265	0.8875165	0.70902547
Ubl3	-0.0163691	7.21135999	0.02051563	0.88754606	0.70902547
Gm11128	0.11667756	-0.8059278	0.02037931	0.88791764	0.70920277
Tsc22d1	0.0212579	9.67766436	0.02033734	0.88803228	0.70920277
Ppp1r3e	-0.0315583	4.20352793	0.02031935	0.88808146	0.70920277
Gal3st4	0.06191162	1.56458374	0.02029334	0.88815262	0.70920277
Enc1	-0.0299758	8.26105106	0.02029125	0.88815835	0.70920277
Fmod	0.03777242	7.83887884	0.0202354	0.88831126	0.70920277
Anapc10	-0.0274406	4.5033661	0.02021399	0.88836997	0.70920277
Pdgfra	0.025496	5.49716989	0.02020216	0.88840239	0.70920277
Snx11	-0.0324933	3.09313506	0.02017958	0.88846435	0.70920277
Mia3	0.02253418	6.29115697	0.02016318	0.88850935	0.70920277
Stau2	0.01772472	7.12925765	0.02015894	0.88852099	0.70920277
Col20a1	-0.0673685	1.24662046	0.02015702	0.88852626	0.70920277
Gm20300	-0.0184123	7.24327955	0.02013046	0.88859923	0.70920277
Mlf1	-0.1042028	-0.3012403	0.02011214	0.88864956	0.70920277
Pdxk	-0.0198063	7.60065812	0.02010969	0.88865629	0.70920277

Dnaaf3	-0.1267903	-1.1739159	0.02007944	0.88873948	0.70922026
Gkap1	0.02703552	5.17658871	0.02005863	0.88879676	0.70922026
Pdpn	0.0418797	5.22471167	0.02003716	0.88885588	0.70922026
Copb2	0.01458256	7.23618966	0.01991977	0.88917965	0.70926229
Car14	0.07167799	2.80967489	0.01991515	0.88919241	0.70926229
Speer4e	-0.1282458	-1.3641308	0.01990967	0.88920754	0.70926229
Nme3	-0.0316518	3.44429198	0.01990435	0.88922224	0.70926229
Taf13	-0.0196477	6.57924145	0.01988295	0.88928141	0.70926229
Pou2f1	0.01941066	5.80107812	0.0198797	0.88929039	0.70926229
Yif1a	-0.0570423	1.70687004	0.01986787	0.88932312	0.70926229
Fam43a	-0.0285888	6.20468166	0.01977579	0.8895781	0.7094184
9930021J03F	0.02136526	8.19119709	0.01973363	0.88969507	0.70946034
Atp5s	-0.0208582	4.66930354	0.01970872	0.88976424	0.70946034
Epha6	-0.0266664	4.797951	0.01967687	0.88985273	0.70946034
Zfp933	-0.0244436	4.53456843	0.01966367	0.88988942	0.70946034
Mfsd8	-0.0292227	3.26371644	0.01964173	0.88995044	0.70946034
Leprotl1	0.0170525	6.11791754	0.0196289	0.88998615	0.70946034
Neil2	-0.1166964	0.05266386	0.0195965	0.89007636	0.70948503
Llph	-0.0241951	7.07072829	0.0195325	0.89025478	0.70958002
Efna3	-0.0491747	1.5606198	0.01947261	0.89042201	0.70962148
Gtf3c3	-0.0221788	5.1667821	0.01947143	0.89042531	0.70962148
C1d	0.02335147	4.9718572	0.01943353	0.89053129	0.70965871
Brca1	-0.0672115	1.08808677	0.01939911	0.89062761	0.70968825
Trim14	-0.0698268	2.15536847	0.01936523	0.89072252	0.70971666
Vip	-0.037042	2.80295463	0.01930238	0.89089878	0.70980987
Crtc1	0.02128478	6.31936751	0.01924571	0.89105797	0.70987341
Trim12c	-0.0293267	5.55706008	0.01922918	0.89110447	0.70987341
Prickle3	0.04947292	2.54428717	0.01921073	0.89115637	0.70987341
Alkbh2	-0.0573984	1.16747695	0.01915985	0.89129963	0.70992324
02-Sep	-0.0192829	7.89735262	0.01914643	0.89133747	0.70992324
Akr1a1	-0.0269345	7.81607609	0.01910306	0.89145977	0.70997342
Tnfsf15	-0.207131	-1.6671084	0.01899088	0.89177681	0.71017869
Rlbp1	0.05840882	1.98670262	0.0189391	0.89192348	0.71022873
Kcnrg	0.08328608	1.30895024	0.01892683	0.89195826	0.71022873
Mnda	0.0949683	-0.6338785	0.01890328	0.89202506	0.7102347
Clasp1	0.01929107	7.49695129	0.01883182	0.892228	0.71034485
Map10	-0.0643801	1.696296	0.01881282	0.89228203	0.71034485
Rpl24	-0.0227648	6.6964983	0.01876575	0.89241599	0.71040087
Pgf	0.08911094	1.21134176	0.01874224	0.89248296	0.71040087
Echs1	-0.0260337	4.38260883	0.01866936	0.89269084	0.71040087
Mrps9	-0.0292469	3.40868777	0.01865376	0.89273538	0.71040087
Ccdc19	0.04664189	2.03876038	0.01864065	0.89277284	0.71040087
Erbb2	0.07792661	1.57463106	0.01864026	0.89277397	0.71040087

Papolb	0.09488566	0.72027379	0.01859433	0.89290527	0.71040087
Atl1	0.02714509	6.2395318	0.01856726	0.89298277	0.71040087
Efna4	0.06691021	1.84046717	0.01854838	0.89303684	0.71040087
Lamtor1	-0.029093	5.52992674	0.01852138	0.8931142	0.71040087
Cntnap2	0.02862689	6.26469547	0.0185132	0.89313765	0.71040087
Rab28	0.01898937	5.36756956	0.01850836	0.89315155	0.71040087
Sertm1	0.02345606	4.58370972	0.01849962	0.89317661	0.71040087
Aen	0.02700999	4.24432563	0.01849299	0.89319563	0.71040087
Ech1	0.03625521	3.948323	0.01845971	0.89329117	0.71040087
Adamtsl2	-0.1032284	-0.4198794	0.01844477	0.89333406	0.71040087
Mapk14	0.01584488	6.53290444	0.01843545	0.89336084	0.71040087
Vil1	0.09944849	-0.0528895	0.01836033	0.89357694	0.71045933
Ciao1	-0.019557	5.39363612	0.01834811	0.89361214	0.71045933
Slc20a2	-0.0283446	6.03436327	0.01834804	0.89361233	0.71045933
Acy3	0.04705864	1.57757437	0.01832573	0.89367662	0.71046328
Rftn2	0.02295257	4.75878567	0.01824268	0.89391627	0.71055238
Zfp282	0.05000649	1.90740085	0.01824089	0.89392146	0.71055238
Wasf2	-0.020645	6.44383348	0.01820832	0.89401558	0.71055238
Igfbp2	-0.0368682	4.96392241	0.01820471	0.89402604	0.71055238
4921515E04I	0.13044086	-0.8939193	0.01811565	0.89428391	0.71071017
1700007J10F	0.08358464	0.06999481	0.0180114	0.89458663	0.71090357
Ttc32	0.0424348	2.57335124	0.01795647	0.89474649	0.71098343
Rab40b	0.02607639	4.03718663	0.01787084	0.89499619	0.71107539
Yae1d1	-0.0181772	6.50183287	0.017868	0.89500446	0.71107539
Txnip	0.03069933	6.72781444	0.01785572	0.89504034	0.71107539
Adam1a	0.07505302	1.28166211	0.01782833	0.89512038	0.71109179
Cnr2	-0.0612894	0.95599685	0.01777899	0.89526468	0.71115526
Pou4f1	-0.1918813	-1.0522652	0.01784106	0.89531903	0.71115526
Lama1	0.03145421	3.50150878	0.01769338	0.8955156	0.71126422
Tbr1	-0.023754	6.77187326	0.01764385	0.89566103	0.71128115
Diras1	-0.0260774	5.13546591	0.01763165	0.8956969	0.71128115
Gm8300	0.09641893	-0.6615759	0.0175865	0.89582971	0.71128115
Tmem128	-0.0308319	3.40362018	0.01756355	0.89589728	0.71128115
9130401M01	-0.021599	4.78490337	0.01755713	0.8959162	0.71128115
Ephx2	-0.024508	3.85688729	0.01752957	0.89599741	0.71128115
Akap13	-0.0165778	7.25756423	0.01751353	0.89604472	0.71128115
Mgst1	0.04757029	3.65503979	0.01750472	0.89607071	0.71128115
Nfkbie	-0.0527504	2.13805308	0.01750447	0.89607146	0.71128115
Arid5a	0.04957501	1.19531805	0.01748312	0.89613447	0.71128402
Man1b1	0.02921983	3.68547036	0.01746006	0.89620256	0.71129092
Ago3	0.02005032	6.0504347	0.01737811	0.89644498	0.71139201
1700011I03R	0.09035755	-0.0163869	0.01737684	0.89644873	0.71139201
Grb2	0.01834457	5.1641288	0.01729744	0.89668415	0.7114711

Pi15	-0.096346	0.75635518	0.01729566	0.89668943	0.7114711
Airn	0.04533507	1.75210693	0.01728313	0.89672663	0.7114711
Foxo4	0.04621819	1.66540675	0.01724997	0.89682515	0.71150213
Cd3e	-0.0581076	2.30550467	0.01718367	0.89702244	0.71150381
Nup205	0.02728604	3.99104559	0.01711155	0.89723746	0.71150381
Stk25	-0.0136978	6.73281195	0.01710777	0.89724874	0.71150381
Tamm41	0.04325172	2.28639379	0.0171029	0.89726329	0.71150381
Dph2	0.03955895	1.76580914	0.01710287	0.89726337	0.71150381
Ube2e1	-0.0281111	4.09330134	0.01708479	0.89731737	0.71150381
Bmp7	-0.0323174	7.77650848	0.01707154	0.89735696	0.71150381
Ahi1	-0.0236501	6.88549225	0.01706609	0.89737325	0.71150381
Plcd4	0.03777461	2.1931038	0.01705584	0.89740387	0.71150381
C330006A16	-0.0188061	5.98893754	0.01703568	0.89746418	0.71150381
Otud7a	-0.0430628	3.09168606	0.01701152	0.89753648	0.71150381
Ccdc121	0.07456579	0.68327863	0.01700769	0.89754795	0.71150381
Heca	0.0217798	4.53730829	0.01699043	0.89759962	0.71150381
Slc35e4	0.05779538	1.114314	0.0169623	0.89768394	0.71152355
Crb2	0.10641968	-0.6515591	0.01687325	0.89795128	0.7115827
Itpripl2	0.02384934	5.91294504	0.01683884	0.89805478	0.7115827
Fras1	-0.0402683	4.82325395	0.01682932	0.89808343	0.7115827
Dmp1	0.08875016	0.28825488	0.01682562	0.89809456	0.7115827
Rit1	-0.0255741	4.74407317	0.01681188	0.89813594	0.7115827
Ticam1	0.02793939	4.60652184	0.01679354	0.89819118	0.7115827
Grpel1	-0.0251893	4.23880688	0.01678703	0.89821079	0.7115827
Serpine1	0.0923939	-0.2823831	0.01677641	0.89824282	0.7115827
Camk2g	-0.0204236	7.23186606	0.01674222	0.89834596	0.7115827
Clcc1	0.02407456	4.36557929	0.01673996	0.89835276	0.7115827
Lenep	-0.0590587	1.41408675	0.01671334	0.89843315	0.71159932
Fst	-0.074761	0.61537621	0.01656568	0.89888019	0.71175539
B4galt1	0.02908559	4.19173818	0.01653606	0.89897012	0.71175539
Safb	-0.0169257	6.30017107	0.01652128	0.89901502	0.71175539
Chic1	0.01662013	6.36591201	0.01649381	0.89909852	0.71175539
Srgap3	0.02273143	9.0810211	0.01646072	0.89919922	0.71175539
Vrk2	0.08293575	0.45857261	0.01645639	0.89921239	0.71175539
Mtss1	0.02096226	6.68507177	0.01642375	0.89931182	0.71175539
Slc23a1	0.05757296	0.69192188	0.01639326	0.8994048	0.71175539
Smarcd2	0.03993684	3.09274541	0.01639213	0.89940826	0.71175539
Sec63	-0.0138981	7.07591059	0.01637567	0.89945848	0.71175539
Snrpn	-0.0498247	0.80614836	0.01635592	0.89951876	0.71175539
Nmral1	-0.0424559	2.8431498	0.01634118	0.89956381	0.71175539
Arhgef11	0.01638999	6.85563668	0.01633571	0.89958052	0.71175539
Neurod1	0.0318598	3.51486705	0.01633413	0.89958535	0.71175539
Ikzf5	0.01840247	5.07149542	0.01631716	0.89963722	0.71175539

Abcf2	0.02001417	5.35137765	0.01631004	0.89965899	0.71175539
Kat5	-0.0189774	4.68628449	0.01629774	0.89969663	0.71175539
Plce1	-0.0168295	4.90128955	0.01628083	0.89974839	0.71175539
Plcxd1	-0.0528196	1.75679054	0.01626069	0.89981007	0.71175539
Spi1	0.0709957	0.49073913	0.01625781	0.89981886	0.71175539
Tmem240	-0.0486847	0.89522004	0.01621048	0.89996399	0.71182317
Tmem261	0.02451846	3.81384098	0.01618355	0.90004663	0.71184152
Kcnd3	0.02671123	5.96922766	0.01615083	0.90014717	0.71187358
Sdad1	0.01767013	4.79160737	0.01612327	0.90023191	0.71187358
Abhd14a	-0.0388802	2.80306502	0.01611235	0.9002655	0.71187358
Lsm3	0.0246813	4.10836897	0.01604747	0.9004654	0.71194532
Trim68	0.05746732	1.24116995	0.01603233	0.90051208	0.71194532
4930524B15I	0.18602399	-1.5930791	0.01602504	0.90053458	0.71194532
Ccnc	0.01892836	5.84716191	0.01596289	0.90072658	0.71198139
Naip6	0.19338997	-2.0934829	0.01594291	0.90078838	0.71198139
Ptprk	-0.0162573	6.4903299	0.01590452	0.90090724	0.71198139
Prss54	0.13791063	-1.2922153	0.01590121	0.90091748	0.71198139
Ccdc158	0.10882792	-0.7985472	0.01589946	0.90092292	0.71198139
Ibtk	-0.0148078	5.74726595	0.01589227	0.90094518	0.71198139
Fam161a	0.04525832	1.83373933	0.01587576	0.90099637	0.71198139
Pofut2	0.03323433	3.73010993	0.01585243	0.90106876	0.71199161
Zmym5	0.01656691	6.59672674	0.01578569	0.90127605	0.71210379
A830052D11	-0.0677064	0.26860189	0.01575861	0.90136032	0.71210379
Pqlc3	0.02866531	3.54648936	0.01574936	0.90138911	0.71210379
Chd1l	-0.0294606	3.10993716	0.01571388	0.90149964	0.71214413
Peli2	-0.0196037	5.70732848	0.01567977	0.901606	0.71218118
Ceacam20	-0.0481914	1.81222087	0.01565907	0.90167064	0.71218526
Rpl36	-0.0326684	5.25284376	0.01563544	0.90174445	0.71219658
Mertk	0.02955247	3.7656962	0.01560329	0.90184496	0.712229
Sdf4	0.01333795	7.98580178	0.01556717	0.90195802	0.71227131
Ghr	-0.0243222	5.29795125	0.01548843	0.90220496	0.71241934
D830030K20	-0.0810627	0.44318328	0.01546879	0.90226664	0.71242108
Mapkap1	-0.0147004	6.5705397	0.01533671	0.90268253	0.71250983
Ppm1b	0.01291465	8.14640098	0.01530954	0.90276832	0.71250983
Ruvbl1	-0.0235213	4.59810199	0.01529713	0.90280752	0.71250983
Zfp882	0.02054031	4.53737201	0.01528979	0.9028307	0.71250983
Pdgfb	0.04158792	2.23334722	0.01528487	0.90284626	0.71250983
Wipf2	-0.0152607	7.56041348	0.01527509	0.90287717	0.71250983
Ptk2b	-0.0196445	7.47935581	0.01527179	0.90288762	0.71250983
Clec9a	-0.1098861	-0.0266248	0.01526357	0.90291361	0.71250983
Rnf220	0.01640465	6.94879055	0.01526329	0.90291452	0.71250983
Eif2ak1	0.01617385	6.22664605	0.01520065	0.90311287	0.71256514
Gngt2	-0.063173	0.72351475	0.01515918	0.90324441	0.71256514

Skiv2l	-0.0235184	5.25710978	0.01515892	0.90324525	0.71256514
BC049715	-0.1372783	-0.4258807	0.01511092	0.90339773	0.71256514
Dzip1l	-0.0256976	3.47305147	0.01508676	0.9034746	0.71256514
Ccdc24	-0.129038	-0.9110974	0.01508378	0.90348407	0.71256514
Murc	-0.1254834	-0.1710877	0.01507786	0.9035029	0.71256514
Aldh3b1	-0.0628139	0.86317787	0.01507589	0.90350917	0.71256514
Zfp712	0.02444617	3.87900977	0.01501265	0.90371073	0.71256514
Snx29	-0.0396103	2.1760274	0.01501006	0.90371898	0.71256514
Plagl1	0.02364968	6.20316342	0.01500772	0.90372646	0.71256514
Zfp879	-0.0460355	2.11439961	0.01500227	0.90374384	0.71256514
Krtcap2	0.02651588	3.33774937	0.01499779	0.90375812	0.71256514
Ctnna3	0.1062855	-0.1813405	0.01492377	0.90399469	0.71266438
A930003A15	-0.1645266	-1.9242753	0.01492117	0.90400301	0.71266438
Cops7b	-0.0319121	3.71950173	0.01486886	0.90417056	0.71273946
Mum1l1	0.06831965	1.70224745	0.01484326	0.90425265	0.71273946
Kars	0.01902195	5.13841216	0.01482187	0.9043213	0.71273946
Coq6	-0.0526062	1.27354156	0.01481338	0.90434857	0.71273946
Crk	0.01359955	7.7634951	0.01478619	0.90443593	0.71273946
Parva	-0.0203189	7.42564118	0.01474526	0.90456762	0.71273946
Pde1b	-0.0187447	5.52007197	0.01471594	0.90466204	0.71273946
Dtx2	-0.0445977	1.76024627	0.01470609	0.90469381	0.71273946
Rasa4	-0.0954135	-0.4240529	0.01469716	0.9047226	0.71273946
Rnf214	-0.0142738	6.33644603	0.01469163	0.90474044	0.71273946
Donson	-0.0306017	3.37025423	0.01467209	0.90480349	0.71273946
Sft2d2	-0.0245933	6.40335609	0.01466805	0.90481651	0.71273946
Cdh20	0.0312853	3.92881565	0.01465061	0.90487282	0.71273946
Mir6369	0.10271028	0.57777275	0.01463246	0.90493146	0.71273946
4933408B17I	0.2642288	-1.1572245	0.01455691	0.90517596	0.71285715
Slc39a5	-0.0747847	-0.5327547	0.01454952	0.90519992	0.71285715
Pcdhb18	0.02885164	4.15419096	0.01448266	0.90541687	0.71298111
4921531C22I	-0.0369792	2.74290352	0.01445461	0.90550804	0.71300602
Zfp57	-0.0477497	1.89585509	0.01434956	0.90585032	0.71319993
Stat3	0.01422155	5.61785731	0.01434249	0.90587341	0.71319993
Junb	-0.0647227	1.53956335	0.01431332	0.9059687	0.71321058
Cldn5	-0.1170146	-0.2046789	0.01429588	0.9060257	0.71321058
Crtap	0.04147185	4.08574639	0.01423131	0.90623712	0.71321058
Zfp770	-0.0147089	6.22912452	0.01422706	0.90625106	0.71321058
Rabggta	0.02959061	3.21503695	0.01422504	0.90625767	0.71321058
Hspa2	-0.022443	4.93156623	0.01420431	0.90632566	0.71321058
Radil	-0.0447069	2.13082272	0.01415249	0.90649584	0.71321058
Senp2	0.01256841	6.40988616	0.01415125	0.90649992	0.71321058
Asap3	0.03948861	2.87495429	0.01415009	0.90650373	0.71321058
Zfp318	0.02423845	6.73134452	0.0141262	0.90658231	0.71321058

Tnfaip8	-0.0277562	5.03713846	0.01411907	0.90660577	0.71321058
Ctdspl2	0.01506184	6.57527525	0.01408026	0.90673356	0.71321058
Elf4	-0.0373634	3.91616552	0.01402796	0.90690609	0.71321058
Bmi1	0.01764506	6.05149084	0.01402189	0.90692616	0.71321058
Mtm1	-0.0424023	2.68765722	0.01402122	0.90692836	0.71321058
Tpm3	-0.0148584	7.43066738	0.01401487	0.90694933	0.71321058
Usp42	0.02138432	4.91321096	0.01401459	0.90695026	0.71321058
Cdca7l	0.0726341	0.60647303	0.01401197	0.90695893	0.71321058
Zfp958	-0.0275106	3.44409452	0.01398003	0.90706451	0.71324677
Tkt	0.02312022	4.83904856	0.01395437	0.90714941	0.7132667
Arid3c	0.13810913	-1.2824569	0.01392621	0.9072427	0.71327388
Ell2	-0.0150025	6.02742888	0.01391566	0.90727766	0.71327388
Rap2a	0.01197273	7.59364331	0.01388398	0.90738277	0.71330969
Prr15	-0.0627929	0.78841463	0.01385883	0.90746627	0.71331119
Cnnm1	0.01743664	6.37053827	0.01383299	0.90755218	0.71331119
Map9	-0.0186043	7.90118263	0.01380698	0.9076387	0.71331119
Nrap	-0.1276256	-1.1094914	0.01378401	0.9077152	0.71331119
Taf15	-0.0199167	5.09488274	0.01376803	0.90776842	0.71331119
Grm7	0.03234443	4.58101982	0.01375294	0.90781874	0.71331119
Asb13	-0.0192844	4.60256161	0.01372396	0.90791545	0.71331119
Ackr2	0.07359795	0.49083056	0.01371663	0.90793994	0.71331119
Efhc1	-0.0426078	1.78039606	0.01370258	0.90798688	0.71331119
C230079O03	-0.1836709	-2.0034868	0.01365613	0.90814221	0.71331119
Dlk2	0.0414018	1.71967125	0.01364787	0.90816986	0.71331119
Ccdc138	-0.0345408	2.60096319	0.0136393	0.90819855	0.71331119
Serinc4	-0.1522068	-1.0261286	0.01360162	0.90832486	0.71331119
Nxpe4	-0.0270524	2.96070174	0.01358488	0.90838102	0.71331119
Drap1	-0.0287956	3.96958289	0.01356087	0.90846162	0.71331119
Col11a1	0.02826153	3.3666283	0.01354723	0.90850745	0.71331119
Pias2	-0.0150191	6.01353074	0.01353749	0.90854021	0.71331119
Rnf139	0.0169306	5.09145467	0.01352882	0.90856936	0.71331119
6720468P15I	0.18707445	-0.4914859	0.01350795	0.90863958	0.71331119
Arl2	0.02829487	3.71063299	0.01350743	0.90864132	0.71331119
Gsap	0.0335623	2.23454519	0.01350039	0.90866504	0.71331119
Atxn1l	0.01551029	6.29541844	0.01349147	0.90869508	0.71331119
Hdac1	-0.0223702	5.29501835	0.01342055	0.90893421	0.71338375
Slc25a36	-0.0164155	4.98566063	0.0134172	0.90894556	0.71338375
N6amt2	0.02642581	3.86164223	0.01339324	0.90902649	0.71338375
Pdlim2	0.03313904	3.29156799	0.01339017	0.90903687	0.71338375
Slc35c2	-0.0436832	1.83053402	0.01337584	0.90908535	0.71338375
Dusp22	-0.0175492	4.8711312	0.01334017	0.90920606	0.71343173
Rnf41	0.01596813	5.35339329	0.0133011	0.90933851	0.71345675
Fcho2	0.01640166	6.99913835	0.01329562	0.90935711	0.71345675

Elof1	-0.0348164	3.1789434	0.0132616	0.90947261	0.71349121
Mmp14	0.03760978	3.91261797	0.01319457	0.9097006	0.71349121
Inha	-0.0391867	1.64997007	0.01318513	0.90973277	0.71349121
Armcx3	0.01300111	7.26398778	0.01317722	0.90975973	0.71349121
B230209E15	0.01922105	6.31479577	0.01316696	0.90979469	0.71349121
Zfp667	-0.0261212	4.79433515	0.01316616	0.90979744	0.71349121
Pdk4	-0.0302185	2.80876838	0.01316011	0.90981807	0.71349121
Cxcr6	-0.0814537	0.03419362	0.01313847	0.9098919	0.71350238
C130026I21R	-0.0501804	1.06014838	0.01311262	0.90998018	0.71351568
Serpinb1b	0.07699468	-0.1128303	0.01309701	0.91003355	0.71351568
Cyr61	-0.0688776	2.43826021	0.01307793	0.9100988	0.71351568
Zfp740	-0.0212638	4.79995904	0.01306379	0.91014718	0.71351568
Gstt1	0.02859947	4.87976353	0.01304404	0.91021482	0.713522
Hcfc2	0.0239766	4.10084685	0.01300171	0.91035997	0.7135397
2010109I03R	-0.0960463	-1.33369	0.01299418	0.91038582	0.7135397
Aldh9a1	0.01724797	5.52377629	0.01297406	0.91045491	0.7135397
Rgs18	0.08085756	-0.0944499	0.012968	0.91047573	0.7135397
Heatr5a	-0.0203784	4.46749891	0.01289481	0.91072758	0.71364109
Rap1b	-0.0169734	7.25010556	0.01289233	0.9107361	0.71364109
Pigw	-0.0474058	1.51908733	0.01287093	0.9108099	0.71364109
Itga9	0.02411818	3.28035532	0.0128612	0.91084347	0.71364109
Spata9	0.02639738	3.51825626	0.01281421	0.91100575	0.71365445
Sms	-0.0134635	7.09061847	0.01281411	0.91100611	0.71365445
Slc25a10	-0.0507772	1.47873183	0.01279236	0.91108131	0.71365445
Cbr2	-0.0515437	1.3138646	0.01277963	0.91112539	0.71365445
Axl	0.03133632	5.27439036	0.01275669	0.91120483	0.71365445
Fam189a2	-0.0589241	0.55392825	0.01267734	0.9114802	0.71365445
Vps37a	0.01702123	7.7321129	0.01265175	0.9115692	0.71365445
Trappc2l	0.03233539	3.37912869	0.01265065	0.91157302	0.71365445
Mtap	0.025964	4.5968512	0.01264671	0.91158672	0.71365445
Emg1	-0.0335301	2.94861394	0.01263353	0.91163262	0.71365445
Ercc2	-0.0441124	1.24485289	0.01263053	0.91164306	0.71365445
Cep97	-0.0175074	4.58543126	0.01262714	0.91165484	0.71365445
Kcnq4	0.11059954	-1.0620554	0.01261718	0.91168954	0.71365445
Cdc25c	0.11484487	-1.6134283	0.01261567	0.91169481	0.71365445
Egf	0.18135652	-1.2277752	0.01259123	0.91178	0.71365504
Gm2381	0.11415652	-1.2024585	0.01258127	0.91181474	0.71365504
Lsm8	-0.0222227	5.66105013	0.01255997	0.91188912	0.71366662
Erh	0.02155575	5.18294234	0.01251928	0.91203132	0.71373126
Ilkap	-0.0199609	4.44686146	0.012467	0.91221439	0.71382788
Ppm1j	-0.1259629	-1.8285793	0.01236945	0.91255701	0.71390032
Unc119	0.04534265	1.76904327	0.01236792	0.9125624	0.71390032
A230001M1(	0.06700444	0.24697136	0.01236063	0.91258808	0.71390032

Dnm3os	-0.0540711	1.87406229	0.01235954	0.91259192	0.71390032
Smdt1	-0.025761	5.06212921	0.01228158	0.91286682	0.71390032
Hs3st1	-0.0221336	4.20816129	0.01227339	0.91289575	0.71390032
Tap2	-0.0671854	0.2777487	0.01226129	0.91293853	0.71390032
Srek1ip1	0.0164082	6.02457811	0.01222413	0.91307	0.71390032
Mlkl	-0.0673053	0.73842643	0.01220577	0.91313502	0.71390032
Gm15880	0.15982709	-0.8696785	0.01219781	0.91316324	0.71390032
Arnt	0.01487333	6.02241584	0.01218499	0.91320868	0.71390032
Dmd	-0.0243717	7.88618675	0.01218466	0.91320985	0.71390032
Glt8d1	0.02110534	4.23810645	0.01211151	0.91346964	0.71390032
Arhgef6	0.01555345	6.45736664	0.01207942	0.91358388	0.71390032
Cep63	-0.0215931	5.47835818	0.01207789	0.91358932	0.71390032
Dnah1	0.02758083	3.06537276	0.01207725	0.9135916	0.71390032
Psma5	-0.0163749	5.72397389	0.01207328	0.91360574	0.71390032
Timd4	0.18740578	-2.1091428	0.01205763	0.91366151	0.71390032
Cinp	-0.0219635	4.26238492	0.01203791	0.91373186	0.71390032
Denr	-0.0156241	5.7057541	0.01203781	0.91373221	0.71390032
Nfia	0.01676677	9.13850662	0.012029	0.91376365	0.71390032
Mmp11	0.06422697	1.13783293	0.01202247	0.91378695	0.71390032
Mapkapk3	-0.049604	2.24862019	0.0120145	0.91381544	0.71390032
Vipas39	0.01511441	4.82685102	0.01201151	0.9138261	0.71390032
Rimklb	0.02178931	3.68555585	0.01200224	0.91385922	0.71390032
Fbxl15	-0.073645	-0.4412051	0.011997	0.91387795	0.71390032
Rpl7a	0.01924502	7.31122517	0.0119706	0.91397236	0.71390032
Nacc2	-0.0129867	6.50946676	0.01196419	0.91399533	0.71390032
Zfp229	-0.0237866	3.67899273	0.0119529	0.91403572	0.71390032
4930565N06	0.06648158	1.61358252	0.01191819	0.91416013	0.71392422
Ednra	-0.0208396	5.01863621	0.01190968	0.91419065	0.71392422
Tmem242	0.02431694	4.09529041	0.01189233	0.9142529	0.71392422
Grm3	-0.0161695	5.65836054	0.01186608	0.9143472	0.71392422
4833418N02	-0.0413565	1.55173817	0.01184953	0.91440671	0.71392422
Rnf6	-0.0115548	6.81073045	0.01182013	0.91451252	0.71392422
Stag1	-0.0128431	7.40633933	0.01177461	0.91467661	0.71392422
Mir344c	-0.1279893	-1.6073122	0.01176631	0.91470656	0.71392422
Ska3	-0.0382487	1.45807061	0.01173379	0.91482402	0.71392422
Hspa4	0.0156807	8.78386391	0.01172601	0.91485213	0.71392422
Sumf1	0.02230461	5.03265388	0.0117203	0.91487281	0.71392422
Atxn2	0.01625317	8.07817741	0.01171866	0.91487874	0.71392422
Tirap	-0.0263034	4.4284544	0.01171539	0.91489056	0.71392422
Zfp951	-0.0279838	2.46295741	0.01170257	0.91493693	0.71392422
Fam171b	-0.0163457	7.11323488	0.01169605	0.91496054	0.71392422
Atrx	0.01662277	9.86601145	0.01164845	0.91513305	0.71400505
Mgat4c	0.0283897	2.88416955	0.01159972	0.91531005	0.71400505

Noa1	-0.016651	4.42358664	0.01159281	0.91533518	0.71400505
Tuba1b	0.01228763	9.23594797	0.01157811	0.91538865	0.71400505
Safb2	0.01661675	5.15612092	0.01157493	0.91540022	0.71400505
AU019823	-0.0191973	5.02484557	0.01155539	0.91547138	0.71400505
3830406C13I	-0.0159582	6.13628386	0.01153972	0.91552847	0.71400505
Adrbk1	0.01802952	5.09070677	0.01153626	0.91554109	0.71400505
Sltm	-0.0137758	7.70883421	0.01149964	0.9156747	0.71406275
Btbd10	0.01539417	5.98224862	0.01139898	0.91604309	0.71430351
Map3k7cl	0.07172347	0.50452359	0.01124698	0.91660253	0.71464399
Sf3b4	-0.0181244	4.65157292	0.01123429	0.91664941	0.71464399
Rpl18a	-0.0204287	7.4643089	0.01121316	0.91672752	0.71464399
Stard5	-0.0325115	3.55374453	0.0112104	0.91673773	0.71464399
E130310I04R	-0.1883515	-1.5761338	0.01118985	0.91681378	0.71464399
Zfp943	-0.0250391	3.03883785	0.01117821	0.9168569	0.71464399
Tmem126a	0.01818282	4.47075064	0.01116726	0.91689745	0.71464399
Zbtb8a	0.03906685	2.95987828	0.01114124	0.91699394	0.71467268
Mmp28	0.07979453	0.01821936	0.01107904	0.91722508	0.71474149
Prkar2a	-0.0135947	6.40143471	0.01107557	0.917238	0.71474149
4930583P06I	-0.1378779	-1.6649089	0.01106932	0.91726128	0.71474149
Sgsm3	-0.0233119	3.69861639	0.01093744	0.91775373	0.71507869
Fdx1	0.0253934	5.38919564	0.01090072	0.91789139	0.71510451
Ddx20	0.02747258	3.71303709	0.01089674	0.91790629	0.71510451
Atg4d	-0.0373181	2.16866913	0.0108512	0.91807737	0.71519126
Ebpl	-0.0367584	2.36077055	0.01081999	0.91819484	0.71519309
Zfp28	-0.0299032	2.80136131	0.01081884	0.91819916	0.71519309
Hhatl	-0.0576602	0.58083269	0.01079379	0.91829355	0.71520755
Rpl31-ps12	0.01995453	4.06261921	0.01078223	0.91833717	0.71520755
Adam22	0.01726329	7.72163475	0.01071631	0.91858624	0.71526864
Cytl1	-0.1060589	-0.5109423	0.01070321	0.91863583	0.71526864
Bcs1l	0.0247867	2.77376128	0.01070154	0.91864214	0.71526864
Coa6	-0.0186119	3.73919613	0.01069828	0.91865451	0.71526864
Faf2	-0.0177324	4.40507578	0.01067357	0.91874814	0.71529503
Bcl6	0.0104602	6.10404496	0.01064077	0.91887262	0.71530388
Naf1	-0.0191881	4.34017599	0.0106391	0.91887896	0.71530388
Traf3ip3	0.0958134	-0.8302029	0.01061458	0.91897215	0.71532993
Nup210	-0.0329992	3.04798879	0.01058709	0.91907675	0.71536485
Zscan18	0.02043762	3.71227459	0.01055358	0.91920445	0.71541093
H1fx	0.08095686	-1.4640314	0.01051989	0.91933306	0.71541093
1810062O18	0.06443651	0.63893738	0.01049999	0.9194091	0.71541093
Ryk	-0.0217448	4.46530085	0.01047439	0.91950703	0.71541093
Lyve1	-0.0348808	3.43353616	0.01046593	0.91953943	0.71541093
Timm22	-0.0206744	3.87699275	0.01046094	0.91955854	0.71541093
4930528A17I	-0.1326528	-1.4834842	0.01043826	0.91964547	0.71541093

Sirt3	0.01970832	3.59719105	0.01042525	0.91969538	0.71541093
Rbm12b1	0.02169064	4.6326872	0.01041097	0.91975022	0.71541093
Smo	0.02776778	6.95353589	0.01040522	0.91977228	0.71541093
Mocos	0.04418057	1.42942326	0.01039981	0.91979308	0.71541093
B3galnt1	-0.0138629	4.8280408	0.01032553	0.920079	0.71556746
Prdm11	0.09860083	-0.5723408	0.0103165	0.92011383	0.71556746
Zc3hc1	-0.0302026	2.28311213	0.01028104	0.92025073	0.71562745
Sdc4	-0.0312809	5.26521449	0.01025069	0.9203681	0.7156611
Gm5547	-0.1591573	-1.9888501	0.01023896	0.92041352	0.7156611
Zfp747	0.01869094	3.70442029	0.01018749	0.9206131	0.71574231
Stub1	0.04771718	0.86634592	0.01018121	0.92063749	0.71574231
Ccdc81	0.07399901	0.12320534	0.01013076	0.92083366	0.71581268
Mapre1	0.01456846	7.8572816	0.01012719	0.92084755	0.71581268
Acad10	-0.0464791	0.81067931	0.01011077	0.92091153	0.71581595
Phc1	0.01265942	6.3147553	0.01007769	0.92104054	0.71586977
4833411C07I	-0.1217022	-1.3355056	0.01005385	0.92113365	0.71588459
5830418K08I	-0.0210567	5.57717409	0.01004221	0.92117917	0.71588459
Jmjd7	-0.0769016	-0.6699958	0.0100063	0.92131973	0.71594736
Pdzrn4	0.02877697	2.404402	0.00997038	0.92146061	0.71598006
Mettl3	-0.0210537	3.85454975	0.00996509	0.92148137	0.71598006
1700101I11R	-0.078771	0.31317228	0.00993326	0.92160643	0.71603077
Ubl5	0.02049756	5.95117824	0.00985243	0.92192492	0.71606616
Rpusd4	-0.026193	2.7187492	0.00983365	0.9219991	0.71606616
Gm2694	0.06441513	0.04466497	0.00983226	0.92200461	0.71606616
Scamp5	-0.0168019	6.44709612	0.00981534	0.92207154	0.71606616
Tmed1	0.05023251	1.04176317	0.00980059	0.92212988	0.71606616
Zim1	-0.0421773	1.09994664	0.00976592	0.92226728	0.71606616
Snrk	-0.0118603	6.38919124	0.0097553	0.92230941	0.71606616
Fbf1	-0.0185148	4.05102096	0.00974758	0.92234006	0.71606616
Rarg	0.02625879	2.85799831	0.00974611	0.92234588	0.71606616
Cpne4	-0.0159087	6.36856692	0.0097124	0.92247984	0.71606616
Ier3ip1	-0.0164122	5.53606747	0.00970261	0.9225188	0.71606616
2500004C02I	-0.0212196	3.8040238	0.00970208	0.92252089	0.71606616
2010005H15I	0.12018515	-1.5641879	0.0097015	0.9225232	0.71606616
Vcpkmt	-0.0330225	1.7494766	0.00968497	0.92258903	0.71606616
2810433D01I	0.02779037	3.1447098	0.00968373	0.92259396	0.71606616
Dcbld1	-0.0344264	1.98688471	0.00968004	0.92260867	0.71606616
Gm15760	0.03396209	2.13793902	0.00965073	0.92272551	0.71611044
4930538K18I	-0.0623888	0.49385641	0.00961656	0.92286199	0.71616006
Ccdc130	0.03564486	2.27195659	0.00960479	0.92290906	0.71616006
Higd2a	0.01667661	5.32361296	0.00955654	0.92310226	0.71617868
1700128F08I	-0.0795688	-0.8445133	0.00954551	0.92314652	0.71617868
Maob	0.01748884	4.16422355	0.00949731	0.92334013	0.71617868

Cul4a	-0.0110021	7.55684241	0.00949535	0.92334802	0.71617868
Cnot6l	0.01173283	7.06788096	0.00949124	0.92336458	0.71617868
Uckl1os	0.07042611	-0.4792258	0.00948993	0.92336982	0.71617868
Vsig10	0.05059326	1.46708961	0.0094801	0.92340942	0.71617868
Iqcd	-0.1035549	-1.7489565	0.00946243	0.92348059	0.71617868
Slc9a5	-0.0295784	2.60183647	0.00945196	0.92352279	0.71617868
Tmem186	0.02654517	2.68750825	0.0094499	0.92353109	0.71617868
Cpa2	-0.0667045	-0.4612285	0.00942354	0.92363747	0.7162148
Atg4b	0.01957269	3.8982455	0.00940591	0.92370871	0.71622367
Spag1	-0.0223877	2.86066119	0.00937152	0.92384783	0.71626324
Bfsp1	-0.0583887	0.95059007	0.00935449	0.92391683	0.71626324
Lilrb4	-0.059824	0.43424165	0.00934333	0.92396208	0.71626324
Cacul1	0.01215823	6.85414444	0.00933423	0.92399899	0.71626324
Rimbp3	0.06338356	0.85861884	0.00928461	0.92420062	0.71637318
Msr1	-0.109519	-1.3220509	0.00924807	0.92434946	0.71640019
Adra1a	-0.0177467	4.48599076	0.00924202	0.92437409	0.71640019
Tmem223	0.01863431	3.72335312	0.00922089	0.92446034	0.71640019
Fam32a	0.01531615	6.0705098	0.00921736	0.92447476	0.71640019
Mknk2	-0.0171182	3.96354222	0.00914352	0.92477691	0.71658797
Lars2	0.01557111	13.4159353	0.00909129	0.92499139	0.71666794
4933406C10I	-0.0916943	-1.0047284	0.00908924	0.9249998	0.71666794
Asnsd1	-0.0197318	5.29360696	0.0090331	0.92523103	0.7167741
Tk2	-0.026804	3.29112277	0.00901671	0.92529869	0.7167741
Catsperg1	0.09187298	-1.0719641	0.00901243	0.92531638	0.7167741
Mir17hg	0.13084223	-1.6099372	0.00895548	0.92555197	0.71689555
Prdm9	0.03313162	1.62547008	0.00893252	0.92564718	0.71689555
Rab34	-0.0234228	4.25800237	0.00893117	0.92565275	0.71689555
4930599N23	-0.118636	-2.0599011	0.00889659	0.9257964	0.7169528
Il13ra2	-0.0451491	1.98597913	0.00886968	0.92590836	0.7169528
E130114P18I	-0.0442763	0.83679261	0.00886659	0.92592121	0.7169528
Dna2	0.09209723	-0.6860066	0.0088383	0.92603911	0.7169528
4833439L19F	0.01522027	7.79754832	0.00882053	0.92611326	0.7169528
Aldh4a1	0.0201297	4.20524868	0.00881629	0.92613101	0.7169528
Sec23a	-0.0125795	6.49396383	0.00879482	0.92622071	0.7169528
Hvcn1	-0.0658992	-0.3066528	0.00879398	0.92622424	0.7169528
Ccdc17	0.03709819	1.07721992	0.00875033	0.92640698	0.7169528
Foxp4	0.02107585	4.89409226	0.0087389	0.92645494	0.7169528
Il20rb	0.10372212	-0.6847299	0.00872948	0.92649445	0.7169528
Acox1	0.01139091	6.99373077	0.00872772	0.92650183	0.7169528
Gorab	0.01829353	3.49205678	0.00871148	0.92657004	0.7169528
Ubxn11	-0.0573097	1.41000936	0.00870976	0.92657726	0.7169528
Pmm2	-0.0214133	3.40285091	0.00868879	0.92666542	0.7169528
Baiap2l1	0.01898368	3.464308	0.00868425	0.92668455	0.7169528

Cog3	-0.0137914	5.52673204	0.00862921	0.92691655	0.71701512
Trmt10b	-0.0189336	3.70171068	0.00861967	0.92695684	0.71701512
Ppp2r2c	0.01351586	9.02177401	0.00860412	0.92702254	0.71701512
C7	0.04928793	0.35357518	0.00860286	0.92702788	0.71701512
Cav2	0.01738013	5.18438214	0.00859421	0.92706446	0.71701512
Ccdc85c	-0.0357718	0.96337472	0.00849085	0.92750305	0.7172272
Mbd5	0.01381516	7.79862583	0.00848646	0.92752174	0.7172272
Hspa12a	-0.0135687	8.26681948	0.00848353	0.92753421	0.7172272
Sp2	0.0211999	5.31463402	0.00844627	0.92769305	0.7172272
Agtrap	-0.0292184	4.03293785	0.00844402	0.92770269	0.7172272
Srrt	0.01620104	4.88344828	0.00843735	0.92773113	0.7172272
Slc7a10	-0.053709	0.19958365	0.00839997	0.92789093	0.7172272
Trmt44	-0.0516966	0.66664518	0.00839149	0.92792723	0.7172272
Gtf3c6	0.02045699	5.50182977	0.00838188	0.92796841	0.7172272
Armc5	0.0221098	2.58306319	0.00837598	0.92799369	0.7172272
Mxi1	-0.0119364	6.32122359	0.00837055	0.92801694	0.7172272
C1ra	-0.0425276	0.72250136	0.00836113	0.92805735	0.7172272
Wsb2	-0.009663	7.7269333	0.00825132	0.92852996	0.71754614
Casc5	-0.0654224	0.48233608	0.00819537	0.92877201	0.71767667
Mir143hg	-0.0928981	-1.3937044	0.00815255	0.9289578	0.71767667
Cpsf2	0.01145181	6.35806903	0.00815223	0.92895916	0.71767667
D1Ertd622e	-0.0114485	5.78632366	0.00814492	0.92899095	0.71767667
Gabra4	0.01490639	5.85303945	0.00813832	0.92901965	0.71767667
Rps18	-0.0242322	6.22385773	0.00812774	0.92906564	0.71767667
L2hgdh	-0.0106946	6.11921602	0.00811563	0.92911837	0.71767667
Cdk16	0.01087103	7.18779947	0.00808362	0.92925792	0.71773818
Mrps16	0.02068712	3.20787223	0.00801578	0.92955454	0.71780588
Il2rg	-0.0701279	0.43366703	0.00801522	0.92955698	0.71780588
Zfp219	-0.0284915	1.95613144	0.00799593	0.92964156	0.71780588
Usp4	-0.013272	5.49972037	0.0079955	0.92964345	0.71780588
Ift122	-0.0180034	3.72749146	0.00798157	0.9297046	0.71780588
Alx3	0.02889417	3.2206397	0.00795126	0.92983782	0.71780588
Parp1	-0.0102414	6.13886078	0.00794862	0.92984943	0.71780588
Tshz1	-0.0104639	6.00307208	0.00794579	0.92986192	0.71780588
Gm20187	0.07065875	0.42513865	0.00792994	0.92993169	0.71780588
E2f7	-0.1022332	-1.079622	0.00792693	0.92994496	0.71780588
Gpr125	-0.0249895	3.35494586	0.00790896	0.93002421	0.71782078
Jrk	-0.0286207	2.19497887	0.00787723	0.9301643	0.71783109
9530051G07	0.0460399	0.85505439	0.0078707	0.93019319	0.71783109
Cyp4f14	0.10870746	-0.5174918	0.00785499	0.9302627	0.71783109
Tmem202	0.07192375	0.48555398	0.00785168	0.93027733	0.71783109
Ush1g	0.15762951	-1.4019684	0.00783678	0.93034337	0.7178358
Henmt1	-0.080798	-0.5878007	0.00779645	0.93052233	0.71790255

S100b	0.01769342	6.07697975	0.00779027	0.93054978	0.71790255
Slc25a19	0.01965581	3.33440775	0.00777332	0.93062519	0.71791077
Usp20	0.01953656	3.79233137	0.00776094	0.93068033	0.71791077
Rab37	0.05048131	0.41174114	0.00769236	0.93098642	0.71810063
11-Mar	-0.0347011	1.34585352	0.00766029	0.93113007	0.71816518
Btbd8	0.04300308	0.87581466	0.00762872	0.93127174	0.71821064
Gyg	0.0110915	5.57171995	0.00761911	0.93131493	0.71821064
Usp7	-0.0103436	7.09946502	0.0075972	0.93141349	0.71821064
Dhrs13	-0.0609407	-0.3668726	0.00759378	0.9314289	0.71821064
Gm9962	0.041385	0.50648119	0.00753703	0.93168494	0.71834505
Sart3	-0.018603	4.68295842	0.00752815	0.93172506	0.71834505
Cpeb1	-0.0162135	5.01021217	0.00748769	0.93190832	0.71834505
Insr	-0.0147007	5.99010779	0.00748596	0.93191616	0.71834505
Smek1	-0.0120154	5.62499511	0.00748061	0.93194044	0.71834505
Arfgap3	-0.0182101	4.63360754	0.00745532	0.93205528	0.71834505
1700021K19I	0.01220437	5.53596705	0.00744587	0.93209823	0.71834505
Myh15	-0.0991428	-1.1483054	0.00744019	0.93212408	0.71834505
Mia	-0.0540901	-0.1530321	0.00743601	0.93214307	0.71834505
Rnf32	-0.0200819	3.43782625	0.00739925	0.93231058	0.71837858
St6galnac3	-0.0202454	3.5054826	0.00736292	0.93247654	0.71837858
Rlim	0.00990387	7.70261019	0.00733392	0.93260932	0.71837858
Gbp5	0.03988339	2.13971357	0.00732857	0.9326338	0.71837858
Slc5a12	0.05239991	0.11531533	0.00732601	0.93264555	0.71837858
Cwc15	-0.014269	6.787145	0.00732313	0.93265876	0.71837858
Zbtb6	-0.011809	5.09024405	0.00732306	0.93265907	0.71837858
Gm5069	-0.0142761	4.42178415	0.00728503	0.93283373	0.71837858
Cpox	-0.0156109	5.72688531	0.00728179	0.9328486	0.71837858
Dapk3	-0.0312807	1.76013072	0.00725996	0.93294911	0.71837858
Itga7	-0.0687185	-0.3260729	0.00724667	0.93301032	0.71837858
AV039307	-0.0531559	0.33744871	0.00723368	0.93307024	0.71837858
Yme1l1	0.01119136	6.23404001	0.00719578	0.93324537	0.71837858
9330162012I	0.05598983	0.74118326	0.00719541	0.93324709	0.71837858
Lhfpl3	-0.0178018	3.85531894	0.00718305	0.93330432	0.71837858
Pnmal2	-0.0127736	6.34505682	0.00717187	0.93335609	0.71837858
E2f8	-0.0518974	0.8932315	0.00717145	0.93335804	0.71837858
Sh2b1	0.01451354	4.06126179	0.00717088	0.93336068	0.71837858
Erlin2	-0.011573	5.40469007	0.00716884	0.93337016	0.71837858
BC100451	-0.1068558	-1.5581313	0.00715277	0.93344469	0.71837858
Apof	-0.0644811	-0.0834847	0.00715242	0.93344629	0.71837858
Mdh1b	0.09284035	-1.1226519	0.00713153	0.93354333	0.71840709
Rpl38	0.01514453	6.22195774	0.00710828	0.93365149	0.71844417
Pea15a	0.01143518	7.66623059	0.0070822	0.93377299	0.71849149
Gne	-0.0117142	5.51174018	0.00705343	0.93390733	0.71849596

Sirt2	0.01544697	6.66112863	0.00703726	0.93398296	0.71849596
Acp6	-0.0286361	2.46566331	0.00703646	0.93398668	0.71849596
6430562O15	0.11407163	-1.7198949	0.0070296	0.93401878	0.71849596
Nfkbid	0.08186877	-1.1082854	0.00699318	0.93418953	0.71856084
Nr2f1	-0.0129469	6.76098752	0.00698602	0.93422313	0.71856084
Rfx7	0.01061531	7.87409775	0.00687075	0.93476674	0.71892248
AF251705	0.06512008	-0.5359717	0.0068609	0.93481337	0.71892248
Odf2	0.0156532	5.56183303	0.00679061	0.93514735	0.71904442
Fancg	0.02380685	2.75922905	0.00678599	0.93516938	0.71904442
Lmna	-0.0184673	3.66476007	0.00677993	0.93519827	0.71904442
2410004N09	-0.0365925	0.54396546	0.00677068	0.93524239	0.71904442
Taf12	-0.0209598	4.10431954	0.00675527	0.93531596	0.71904442
Pcnp	-0.0117555	7.86009019	0.00670844	0.93554003	0.71904442
Exoc3l4	0.06941035	-0.9109404	0.00669199	0.93561892	0.71904442
Pcdhga2	-0.0313687	2.1827769	0.00667837	0.93568433	0.71904442
Pcdha6	-0.0500522	0.30558676	0.00667282	0.93571097	0.71904442
Tmem41b	0.01221758	4.8261981	0.00666108	0.93576742	0.71904442
Mamstr	0.04718719	0.20664591	0.00662648	0.93593409	0.71904442
Tmem106c	-0.027695	3.83407144	0.00662342	0.93594885	0.71904442
Pknox2	-0.0108764	7.21215062	0.00661893	0.93597048	0.71904442
Pde4b	-0.0131666	7.9733752	0.00661228	0.93600259	0.71904442
E030018B13I	0.13392195	-1.4538829	0.00658286	0.93614479	0.71904442
Pcdhb4	0.03194845	2.06915542	0.00653406	0.93638141	0.71904442
Fam118b	-0.016373	5.23855314	0.00651472	0.93647541	0.71904442
Txnrd1	0.01387778	4.89554205	0.00650091	0.93654263	0.71904442
Npc1	-0.0133607	4.96851897	0.00649386	0.93657693	0.71904442
Acap3	0.01328074	4.76959602	0.00647062	0.93669029	0.71904442
2410131K14I	0.02306955	1.90497599	0.0064561	0.93676119	0.71904442
Ick	-0.0111109	6.31242919	0.00644595	0.93681083	0.71904442
Sh3bp5	-0.0113784	6.78191508	0.00643383	0.9368701	0.71904442
Patz1	-0.0132814	4.85691423	0.00643242	0.93687699	0.71904442
Myo7a	-0.0286342	2.71220591	0.00640804	0.93699646	0.71904442
Sez6l	-0.0120399	6.13618277	0.00640479	0.93701241	0.71904442
0610010K14I	0.01857067	3.74429142	0.00637485	0.93715949	0.71904442
4930550C14I	-0.0416058	1.73402426	0.00636325	0.93721654	0.71904442
Susd2	-0.0161127	5.00079841	0.00635989	0.9372331	0.71904442
Pla2g5	0.04395189	1.43780082	0.00635765	0.93724415	0.71904442
Ache	0.02474524	2.62982925	0.00635629	0.93725083	0.71904442
Tmem28	-0.031948	1.70191866	0.00633993	0.93733144	0.71904442
A330048O09	0.05801549	0.44010957	0.00633985	0.93733187	0.71904442
Erp29	0.01787861	4.632178	0.00633585	0.93735157	0.71904442
Hax1	-0.0142945	5.40342421	0.00633565	0.93735254	0.71904442
Klhl12	0.01252714	4.79415184	0.00633397	0.93736083	0.71904442

Rps15	-0.017725	6.36645525	0.00633223	0.93736942	0.71904442
Gm16712	0.3026039	-1.9720096	0.08029921	0.93738524	0.71904442
Pnlip	-0.1214871	-1.7420603	0.00631697	0.9374448	0.71904442
Plvap	-0.041764	0.6205832	0.00631065	0.93747603	0.71904442
Chmp5	0.01493367	6.60861862	0.00630905	0.93748395	0.71904442
Prss8	0.13997796	-2.1549414	0.00628583	0.93759883	0.71904442
Sort1	-0.0113274	7.68974787	0.00628513	0.93760229	0.71904442
Pfdn2	-0.0145007	5.53679415	0.00628281	0.93761377	0.71904442
Mcm10	-0.1068888	-1.3005088	0.00625611	0.93774621	0.71909994
Mocs1	0.02112713	4.31887356	0.00620475	0.93800171	0.71924981
Nsg1	-0.0126412	5.26403443	0.0061759	0.93814569	0.71929237
4732416N19	-0.0897566	-1.9310366	0.00615462	0.93825212	0.71929237
Cdca4	-0.0207437	2.88013428	0.00614808	0.93828486	0.71929237
Psma2	0.01224327	6.68666278	0.00614556	0.93829747	0.71929237
Cdv3	-0.0090885	7.74731071	0.00611299	0.93846087	0.71937159
Cdkn2b	-0.0963947	-1.6133933	0.00608752	0.93858893	0.71940331
Pes1	0.01448302	4.47611042	0.00606962	0.9386791	0.71940331
Pcdhga7	0.02219413	2.71389991	0.00606895	0.93868247	0.71940331
Fubp3	-0.0114998	5.88427179	0.0060487	0.93878462	0.71941697
Arrb1	-0.010646	7.40032448	0.00603343	0.9388618	0.71941697
Klhl23	0.0150739	5.1496477	0.00602973	0.93888052	0.71941697
Gtf2ird1	0.01482555	3.59592891	0.00600072	0.9390274	0.7194314
Mrps24	0.01829323	3.95201043	0.0059991	0.93903562	0.7194314
Rtp4	0.02678305	3.93435672	0.00599043	0.93907957	0.7194314
Chst3	-0.0602703	0.66964206	0.00596442	0.93921171	0.71948168
Gm7854	0.05825957	0.00976066	0.00594991	0.93928554	0.71948168
Phc2	-0.0159809	4.79911662	0.005915	0.93946353	0.71948168
Psmd10	-0.020541	4.10335909	0.0059141	0.93946811	0.71948168
Shmt1	0.0808427	-0.4443665	0.00590539	0.93951264	0.71948168
Slitrk2	-0.0168239	5.37782692	0.00590445	0.93951746	0.71948168
1110004F10I	-0.0131645	6.35477478	0.00587571	0.93966451	0.71948168
Nprl3	-0.0171395	3.06488781	0.00587434	0.93967154	0.71948168
Dscam	-0.012941	5.64786648	0.00587154	0.93968591	0.71948168
Ndufb10	0.01186985	5.91536809	0.0058276	0.93991152	0.71960842
4930539E08I	-0.0191283	3.15092154	0.00578268	0.94014308	0.7196718
Arfgef1	0.01088836	7.73294247	0.00577414	0.94018724	0.7196718
Uap1	-0.0100415	5.8001539	0.00575367	0.9402931	0.7196718
Pop5	-0.0147637	4.39829903	0.00574638	0.94033086	0.7196718
Tdrd1	-0.0423228	0.84020245	0.00573058	0.94041282	0.7196718
Ccdc90b	0.01496469	5.8537065	0.00572695	0.94043166	0.7196718
Srebf2	-0.0143899	5.20482012	0.00572655	0.94043375	0.7196718
A630066F11I	-0.0303782	1.4922324	0.00567425	0.9407058	0.7196718
LOC1000389	0.09582732	-1.5510624	0.00567053	0.94072521	0.7196718

Ddi2	-0.0222601	2.7742584	0.00566898	0.9407333	0.7196718
Zfp97	0.01249456	4.12311113	0.00565195	0.9408222	0.7196718
Gabrb3	-0.0117896	8.62854624	0.00564928	0.94083616	0.7196718
Bcl2a1a	0.03792639	0.16812951	0.00564596	0.94085352	0.7196718
Fuom	0.0172975	3.47949281	0.00564441	0.94086159	0.7196718
Gbgt1	0.04492751	0.52653388	0.00563789	0.94089572	0.7196718
Ccdc79	-0.0418211	0.91589966	0.00560957	0.94104407	0.7197393
Six2	0.01703798	5.88949627	0.00557896	0.94120482	0.71981627
Kcnj16	-0.0158697	4.19006785	0.00553713	0.94142524	0.71986139
Cldn12	0.01092187	5.41596748	0.00552164	0.94150705	0.71986139
Relt	-0.0328295	1.18477798	0.00550761	0.94158124	0.71986139
Bub1	-0.0525732	0.38533996	0.0054717	0.94177163	0.71986139
Smim8	-0.020041	3.79567374	0.0054659	0.94180248	0.71986139
Slc35f4	0.01982291	2.69234322	0.00544205	0.94192931	0.71986139
Gapdhs	-0.0411558	1.29571558	0.0054346	0.94196902	0.71986139
Fam102b	-0.0087449	5.84785971	0.00543265	0.94197939	0.71986139
Atp10a	0.01739075	3.72381248	0.00542205	0.94203591	0.71986139
Cyp4f16	0.02883807	1.73480182	0.0053999	0.94215424	0.71986139
Syk	-0.0324642	1.46868149	0.0053978	0.94216548	0.71986139
2810002D19I	0.01902118	3.26031947	0.0053954	0.9421783	0.71986139
Ppp1r8	-0.0250513	3.99769124	0.00539433	0.942184	0.71986139
Nos2	0.09799869	-1.9593026	0.00538744	0.94222088	0.71986139
Suclg1	-0.0112577	5.19103964	0.00537196	0.94230378	0.71986139
Rnf135	-0.019454	2.75832843	0.00536829	0.94232348	0.71986139
Prpf40a	-0.0097445	6.39276281	0.00536546	0.94233863	0.71986139
Dcaf7	0.00865133	7.9744213	0.00534837	0.94243036	0.71986139
Chka	0.01348778	4.53753609	0.00533318	0.94251202	0.71986139
Atxn7l1	-0.0092974	6.14296575	0.00531981	0.94258399	0.71986139
Eif3e	0.00955776	7.15623584	0.00531259	0.94262292	0.71986139
Calcrl	-0.0146359	4.34254882	0.00530302	0.94267452	0.71986139
Spata22	-0.0867427	-0.4635355	0.00530043	0.94268849	0.71986139
Mark2	0.01098597	6.32552604	0.00529538	0.94271574	0.71986139
Ctbp1	-0.0132259	5.30065741	0.0052814	0.94279127	0.71986139
Pfkm	-0.0121916	5.87960746	0.00527247	0.94283958	0.71986139
Cacfd1	-0.0121106	4.71033613	0.00526375	0.94288679	0.71986139
Trim27	-0.0109929	4.48871746	0.0052509	0.94295642	0.71986866
Sh3bp4	-0.0156151	2.8722618	0.0052082	0.9431884	0.71999986
Ndufs3	0.01034999	6.44442752	0.00518302	0.94332566	0.72005874
Nmbr	-0.0488106	0.19861684	0.00517031	0.94339506	0.72006582
Mfsd2b	0.07111983	-0.5072159	0.00515533	0.94347696	0.72008244
Anp32b	-0.0115893	8.28371329	0.00514046	0.94355837	0.72009868
Ntmt1	0.01980669	2.14595237	0.00511748	0.94368446	0.72013382
Mpzl3	-0.0661437	-0.9425043	0.00511016	0.94372468	0.72013382

Upp2	0.01644667	3.38280111	0.00506289	0.9439851	0.72025793
Oaf	0.03031542	1.012557	0.00505881	0.94400761	0.72025793
Zfp953	0.01971972	2.98051226	0.00504439	0.94408737	0.7202729
Golga7b	-0.0146392	4.36180067	0.0050154	0.94424795	0.72034952
Mir377	-0.078143	-0.8233461	0.00500072	0.94432949	0.72036584
Fam187b	0.06468487	0.85249679	0.00498392	0.94442292	0.72036982
Gpr182	0.02307524	4.10662129	0.00497815	0.94445502	0.72036982
Cryga	-0.1033813	-1.8631762	0.00493019	0.94472279	0.72049391
Mpp2	-0.0089405	6.71920698	0.00491768	0.94479285	0.72049391
Tnpo2	-0.0085917	5.98827346	0.00490681	0.94485375	0.72049391
Wbp1l	-0.0141435	5.04040967	0.00490429	0.94486788	0.72049391
Zbtb46	0.0310112	1.31475956	0.00487961	0.94500655	0.72049391
Mcc	0.01038745	6.54266634	0.00487372	0.94503969	0.72049391
Rtca	0.01061438	5.33134198	0.00486632	0.94508138	0.72049391
Ccdc132	0.01008689	6.47996057	0.00486319	0.94509901	0.72049391
Zbtbd6	-0.055994	0.54639363	0.00483343	0.94526698	0.7205041
Ccsap	0.01652041	3.95722994	0.00483149	0.94527793	0.7205041
Hmgcs1	-0.0112545	7.21088302	0.00482331	0.94532418	0.7205041
Map2	-0.01381	8.3176101	0.00481821	0.94535304	0.7205041
Mrgprf	0.03981264	2.03239922	0.00479398	0.94549039	0.72056293
Pth2r	0.07241312	-1.3312473	0.00477452	0.94560097	0.72060027
Shpk	-0.044005	0.61399241	0.00476158	0.94567465	0.72060027
Gpr156	0.03612121	1.13406632	0.00474359	0.94577718	0.72060027
Vps26b	0.00737727	7.41283833	0.00474308	0.94578007	0.72060027
Lasp1	-0.0075557	6.52538605	0.00470014	0.94602566	0.72069617
Nkx3-1	-0.0419926	0.14710856	0.00470003	0.94602631	0.72069617
Ahsa2	-0.0113186	4.73143672	0.00468575	0.94610819	0.72071271
Gpalpp1	-0.009832	5.76288545	0.00465466	0.94628699	0.72079364
Gm10814	-0.0515701	0.76249945	0.00464637	0.94633481	0.72079364
Ttll4	-0.0198883	2.29826772	0.00459946	0.94660594	0.72092886
Trafd1	0.01504833	5.08967615	0.00459243	0.94664669	0.72092886
Gnb1	0.00740282	9.34935257	0.00458446	0.94669294	0.72092886
Mllt3	0.00898378	7.72199041	0.00455408	0.94686958	0.72101753
Ube2cbp	0.05646893	0.2999942	0.00453165	0.94700038	0.72107127
Mrc2	0.02122446	4.96143287	0.00451574	0.94709336	0.72107405
Eif3d	0.01115609	4.54766849	0.00449361	0.94722293	0.72107405
Slain2	0.00860144	6.73373401	0.004482	0.94729107	0.72107405
Gsk3a	-0.0073123	7.32149468	0.00446855	0.94737008	0.72107405
Dennd2c	-0.0410252	-0.3429158	0.00445701	0.94743794	0.72107405
Slc25a44	0.00826491	5.90625202	0.00445689	0.94743866	0.72107405
Dagla	0.01030281	5.43864015	0.00443739	0.9475536	0.72107405
Fbxl4	0.01309848	3.57468846	0.0044307	0.9475931	0.72107405
Mras	-0.0072905	6.99979418	0.0044287	0.9476049	0.72107405

Mesp2	0.03983554	1.96370812	0.00442204	0.94764426	0.72107405
Myadml2	-0.0414641	0.67170072	0.0044183	0.94766635	0.72107405
Tmem74	-0.0262952	1.24584302	0.00439665	0.94779456	0.72109513
C1qtnf7	-0.0239341	4.36202515	0.00439329	0.94781447	0.72109513
Tle3	0.01001043	5.22576563	0.00434355	0.94811027	0.72127234
Ccdc85a	-0.010293	5.81768813	0.0043339	0.94816785	0.72127234
Synrg	0.0080477	6.70088302	0.0042993	0.94837486	0.72132693
Mlh1	0.01593216	3.81828075	0.00428745	0.94844597	0.72132693
Mis18a	-0.018653	1.95977578	0.00428503	0.94846048	0.72132693
Zfp830	0.01135935	5.20672535	0.00428169	0.94848055	0.72132693
Dusp2	-0.057517	-1.032968	0.00425227	0.94865759	0.72141576
B4galnt1	0.01073133	5.39993322	0.004227	0.94881012	0.72146674
Rapgef4	-0.0099162	7.31173449	0.00422122	0.94884512	0.72146674
1700023L04R	0.05608265	-0.0254087	0.0042052	0.94894209	0.72149466
Rac3	0.05611077	-0.9383691	0.00416849	0.94916513	0.72161843
2810408I11R	-0.0436939	0.38143534	0.00411358	0.94950055	0.72182762
Apobr	-0.0444884	0.34536625	0.00408795	0.94965789	0.72183663
Cd68	0.02164208	2.3580621	0.00406365	0.94980756	0.72183663
Hes6	-0.0168066	2.20106607	0.00405093	0.94988607	0.72183663
S1pr4	0.04229213	-0.8166348	0.00404934	0.94989589	0.72183663
Mea1	0.01507079	4.7171691	0.00404344	0.94993231	0.72183663
Ebna1bp2	0.00998498	5.39896302	0.00402754	0.95003073	0.72183663
Fam19a5	-0.0088051	4.98488575	0.00402111	0.95007058	0.72183663
Ms4a4c	-0.0903111	-1.6384002	0.00400759	0.95015447	0.72183663
Ppp4r1l-ps	-0.0255665	1.82378909	0.0040039	0.95017743	0.72183663
A430107P09	0.04259977	-0.089564	0.0040023	0.95018733	0.72183663
Ak8	0.05111296	-0.7773796	0.00399087	0.95025841	0.72183663
Naa15	0.00731374	6.62462264	0.00398501	0.95029492	0.72183663
H6pd	-0.0150102	3.62020881	0.00398484	0.95029599	0.72183663
Dclk2	-0.0134976	4.01234502	0.00395055	0.95050999	0.72193363
Pcnxl4	0.01212925	4.64560216	0.00394341	0.95055465	0.72193363
Chrna1	-0.0225226	2.07646178	0.00393545	0.95060454	0.72193363
Nkg7	-0.0736213	0.02728111	0.00389777	0.95084125	0.72199089
Rnpepl1	-0.0126975	4.18973917	0.00388359	0.95093061	0.72199089
Cbx5	0.00726383	9.15622761	0.00388192	0.95094114	0.72199089
Serpinc1	0.04024221	0.12159231	0.00388077	0.95094838	0.72199089
Arhgef40	-0.0119527	3.63309355	0.00386714	0.95103454	0.72199089
2310033P09I	0.01591368	3.12529055	0.00386601	0.95104167	0.72199089
Cep192	0.01566363	4.46641486	0.00384782	0.95115683	0.72203255
Pcsk4	-0.0203205	1.48792254	0.00380852	0.95140657	0.7221489
Pitpnm3	-0.0096517	6.08220485	0.00380473	0.9514307	0.7221489
Gpr174	-0.0721241	-1.3478259	0.0037664	0.95167564	0.72220777
Spsb3	-0.0165761	2.64510332	0.00376077	0.95171175	0.72220777

Lrtm2	-0.011436	5.89053661	0.00375622	0.95174091	0.72220777
1110034G24	0.02465681	1.72551301	0.00374748	0.95179702	0.72220777
5730559C18I	0.08979489	-0.9736511	0.00374067	0.95184074	0.72220777
Slc35c1	-0.0140578	3.33152428	0.00373472	0.95187904	0.72220777
BC031181	0.01158514	6.05618873	0.00371416	0.95201153	0.72220777
Cyp2r1	-0.0303697	0.36074522	0.00371146	0.95202896	0.72220777
Fam76a	-0.00901	5.87616487	0.00370804	0.95205101	0.72220777
Twf2	-0.0128912	3.43721528	0.00369331	0.95214623	0.72221061
Fbrs	-0.0100189	4.54391855	0.00368355	0.95220942	0.72221061
Rptor	0.00985647	5.34640575	0.00367556	0.95226119	0.72221061
Sars2	0.02087874	1.56896706	0.0036702	0.95229598	0.72221061
Rps27l	0.01800503	4.62792645	0.00363428	0.95252971	0.72226711
Bmf	-0.0191099	2.81806401	0.00363268	0.95254013	0.72226711
Rassf7	0.04157412	0.14261191	0.00362123	0.9526149	0.72226711
Adamtsl5	-0.0143231	2.76696844	0.00361992	0.95262342	0.72226711
Manf	-0.0154284	3.8192679	0.00361249	0.95267204	0.72226711
Atf5	0.01504772	3.26731139	0.00359307	0.95279926	0.72228567
Tsr1	0.00963444	6.03519977	0.00359034	0.95281714	0.72228567
Abl1	-0.0115126	5.32165928	0.00357696	0.95290506	0.72230659
BC030336	0.00838538	5.66464111	0.00355979	0.95301809	0.72231919
C77080	0.01193627	5.81398474	0.00355612	0.95304231	0.72231919
Abt1	0.01342608	3.38623331	0.0035045	0.95338395	0.72238258
Mrpl10	-0.0100439	4.71819044	0.00350372	0.95338912	0.72238258
Espl1	0.05697039	-1.4992261	0.00349909	0.95341988	0.72238258
Gtdc1	-0.0098292	6.26817212	0.00347849	0.95355703	0.72238258
Serac1	0.01174767	5.45659715	0.00347739	0.95356435	0.72238258
Suox	-0.0127742	4.24986719	0.00346543	0.9536442	0.72238258
Wnk4	0.01674499	4.36271897	0.00344179	0.95380235	0.72238258
Ccdc28b	-0.0126397	3.40580795	0.00343965	0.9538167	0.72238258
Pglyrp2	-0.1054922	-1.8629835	0.0034341	0.95385396	0.72238258
Fam171a1	0.00956395	5.50197085	0.00343395	0.95385498	0.72238258
Ncaph2	-0.0094431	4.2766549	0.00342795	0.95389522	0.72238258
Ntf5	0.0867275	-1.8553363	0.0034147	0.95398431	0.72238258
Nasp	0.01107015	4.75297334	0.00341457	0.95398518	0.72238258
Pcdhga4	0.02006266	1.68857709	0.00340468	0.95405177	0.72238258
Leprel4	0.0173355	2.95741642	0.00339037	0.95414834	0.72238258
Igsf1	-0.0474692	0.70347853	0.00338019	0.95421714	0.72238258
Psme2	0.0135488	5.28007112	0.00336935	0.95429057	0.72238258
Rfx1	-0.0161006	3.37654182	0.0033663	0.95431118	0.72238258
Sik2	-0.0079079	5.84896474	0.00336592	0.95431382	0.72238258
A930024E05	-0.0351799	0.66919933	0.0033556	0.9543838	0.72238258
Ndufs6	-0.011872	5.1640407	0.00335198	0.95440836	0.72238258
Inca1	0.02298755	0.79195085	0.00334541	0.954453	0.72238258

Eif4enif1	0.00648526	6.6647732	0.00332019	0.95462481	0.72244827
Psma1	-0.0097171	5.5577464	0.00331468	0.95466245	0.72244827
Arl16	-0.0089699	4.2914429	0.00330611	0.95472105	0.72244827
Prkcz	-0.0076728	6.3385358	0.00329733	0.9547811	0.72244827
Rhebl1	0.03567993	0.05855139	0.00325256	0.95508884	0.72262069
Smc1b	-0.0558514	-0.7178383	0.0032404	0.95517274	0.72262069
Scnn1a	0.01592671	2.58433615	0.0032379	0.95518999	0.72262069
Hdac2	-0.0066241	7.14680496	0.00321554	0.95534482	0.72267403
Thada	-0.0107149	5.05442858	0.0032103	0.95538119	0.72267403
Thumpd3	0.0092242	4.77480916	0.00317714	0.95561195	0.72280293
1700012B09I	-0.0429349	-0.4327784	0.00316173	0.95571963	0.72283872
Mpi	-0.0101713	4.47507128	0.00314715	0.95582172	0.72287028
Rnf123	-0.0091702	4.46546386	0.00312817	0.955955	0.72292543
Hsd17b11	-0.0106843	5.02271974	0.00311746	0.9560304	0.72293679
Snx17	0.01185262	4.55007244	0.00310645	0.95610802	0.72294984
Aamdc	0.01068548	4.22658645	0.00308244	0.95627777	0.72296474
Nit1	-0.0132059	2.91999726	0.00307252	0.95634811	0.72296474
Fbxo41	-0.0108609	5.17505856	0.00305843	0.95644824	0.72296474
Smg5	-0.0094179	4.39297691	0.00305183	0.95649516	0.72296474
Sowahb	-0.0121572	3.51482256	0.0030435	0.9565545	0.72296474
Ms4a1	-0.0767669	-1.7458976	0.0030408	0.95657377	0.72296474
Epha10	0.02869084	2.34216075	0.00303785	0.95659487	0.72296474
Zfp191	-0.0100018	5.01105649	0.00302783	0.95666642	0.72296474
Pank4	0.01363759	2.77003552	0.00302718	0.95667104	0.72296474
Gle1	-0.0083228	4.91011353	0.00298517	0.95697241	0.72311442
Zfp385b	-0.0080411	6.65665764	0.00297699	0.95703135	0.72311442
Laptm4a	-0.0107209	8.0527121	0.00297437	0.95705025	0.72311442
Asb7	0.00577937	6.12224714	0.00295142	0.95721615	0.72316298
Ptpn12	0.00825516	6.02226526	0.00294877	0.95723529	0.72316298
Fchsd2	-0.0064116	6.70699461	0.00291837	0.95745609	0.72324037
Ggt7	0.01425127	2.63432091	0.00291459	0.95748363	0.72324037
Cul7	0.01222564	3.0716944	0.00290975	0.95751892	0.72324037
Bod1	-0.0086526	6.10494432	0.00290062	0.95758551	0.72324505
Dleu7	0.02167245	1.26264563	0.00285367	0.95792983	0.7234422
Enpp3	0.02780369	0.61980347	0.00284858	0.95796736	0.7234422
1700007P06I	0.0372721	-0.667829	0.00283	0.95810455	0.72346768
Cst7	0.0748479	-1.9428477	0.00282318	0.95815501	0.72346768
Eva1c	-0.0197736	2.79785056	0.00281909	0.95818529	0.72346768
Kazn	0.0078362	6.12045193	0.00280812	0.95826664	0.72346768
Mss51	-0.0596478	-0.9823114	0.0028032	0.95830315	0.72346768
Zfp128	-0.0145728	2.6900895	0.00279293	0.95837952	0.72347973
Tmem86a	-0.0186634	4.25573713	0.00277	0.95855057	0.72356324
Tfap2b	0.01011592	6.8869382	0.00275792	0.95864095	0.72358586

1110051M20	0.00950736	4.1589489	0.00273165	0.95883822	0.72364391
Nudt5	-0.0155514	2.90762845	0.00273159	0.95883872	0.72364391
Nhp2l1	-0.0073522	6.06380056	0.00272002	0.95892589	0.7236641
Col5a1	0.01138592	3.40369357	0.00267568	0.95926173	0.72383728
Gm10785	0.02569348	0.94385693	0.00267377	0.95927626	0.72383728
Gpr183	0.02986498	-0.160354	0.00266122	0.95937181	0.72385115
Gstm5	-0.0101411	5.97796216	0.00264305	0.95951067	0.72385115
Rbm7	0.00930542	5.12838101	0.00263805	0.95954894	0.72385115
Dnajc27	0.00782326	6.17751237	0.00263013	0.95960966	0.72385115
Ube2i	0.0070276	7.37592641	0.0026295	0.9596145	0.72385115
Wbscr22	0.01120146	3.15028174	0.00262392	0.9596573	0.72385115
Ciapin1	-0.0079804	4.72236937	0.00261006	0.95976387	0.72385416
Rnf4	0.00606532	6.47763243	0.00260769	0.95978217	0.72385416
BC052688	-0.0158589	2.15406068	0.00259742	0.95986133	0.72386827
BC027231	0.01091737	3.97245081	0.00256192	0.96013635	0.723932
Pou2af1	0.01652568	4.8134834	0.0025503	0.96022677	0.723932
Rbpms	0.01364145	5.27075179	0.00254405	0.96027546	0.723932
Icosl	0.01881084	1.45258805	0.00251887	0.9604724	0.723932
Dnase2a	-0.0316075	-0.3412987	0.00251001	0.96054194	0.723932
Bbip1	-0.0068272	5.93859455	0.00250897	0.96055006	0.723932
Dyrk3	0.01996088	1.85283218	0.00249607	0.96065157	0.723932
Zfp839	-0.0075107	5.26943064	0.00249425	0.96066591	0.723932
Col27a1	-0.0208585	2.12821318	0.00249288	0.96067669	0.723932
Slc2a4rg-ps	0.0229896	1.80381614	0.00247865	0.96078895	0.723932
Neurod4	0.03164003	0.44780469	0.0024737	0.96082813	0.723932
Stard4	0.00925637	3.99959699	0.00247327	0.96083152	0.723932
H3f3b	-0.0103687	9.00360196	0.00247241	0.96083833	0.723932
Zfp407	-0.0086394	4.98637286	0.00246758	0.96087655	0.723932
Tbrg1	0.00985393	4.80640048	0.00245974	0.9609387	0.723932
Vps26a	0.00571661	7.2527483	0.00245837	0.9609496	0.723932
Cd22	-0.0874295	-2.1449166	0.00244171	0.96108202	0.723932
Srp9	0.00815249	6.79681905	0.0024372	0.96111789	0.723932
Noc4l	0.01153058	3.21503517	0.00243323	0.96114961	0.723932
Fam114a2	-0.0095951	5.08308205	0.00243257	0.96115484	0.723932
D2hgdh	0.00911869	4.1262795	0.00242332	0.96122872	0.72393696
Nkain2	0.00886127	6.31843164	0.00241228	0.96131705	0.72393696
Stard7	0.00595864	6.2672211	0.0023984	0.96142838	0.72393696
C030037D09I	0.0243808	1.34915979	0.00239357	0.96146724	0.72393696
Zbtb8os	0.01313231	4.06613915	0.0023866	0.96152332	0.72393696
Foxred1	-0.0121496	3.05111748	0.00238313	0.96155132	0.72393696
Dapl1	-0.0107033	5.38142757	0.002379	0.96158457	0.72393696
Lgmn	-0.0106991	4.12100921	0.00231927	0.96206947	0.72417215
Etv5	-0.0082022	6.16652977	0.00231771	0.96208229	0.72417215

Setdb2	0.01327152	2.9904605	0.00231715	0.9620868	0.72417215
Adamts5	0.01089027	3.69374067	0.00229411	0.96227568	0.72417215
Gm15455	0.03024748	-0.8764265	0.00228819	0.96232427	0.72417215
Id2	-0.0099797	6.09138023	0.00228395	0.96235918	0.72417215
Ccm2l	-0.0480339	-0.5703278	0.00228172	0.96237758	0.72417215
Vwa9	0.00792555	4.48041734	0.00227957	0.96239525	0.72417215
Ttc39b	0.00804887	7.09488054	0.002274	0.9624412	0.72417215
Srbd1	0.01065108	3.49013912	0.00223907	0.96273056	0.72432539
Bgn	0.01153267	8.30310633	0.00223483	0.96276582	0.72432539
Fam78b	0.0076015	6.81848028	0.00222616	0.96283808	0.72433003
Nog	-0.0308387	-0.0920429	0.00221959	0.96289296	0.72433003
Ift52	0.00809922	4.94907835	0.00219523	0.96309696	0.72441697
Polr2j	-0.0170583	3.17793511	0.00219135	0.96312951	0.72441697
Ccdc84	-0.0276004	1.12289914	0.00218059	0.9632201	0.72443961
8030423F21I	0.03049503	-0.8160297	0.00217002	0.96330931	0.7244612
Tnni2	-0.0515895	-1.3371784	0.00214955	0.9634826	0.72454602
Nr4a2	-0.0181437	6.88908107	0.00213513	0.96360525	0.72459276
F420014N23	-0.0207426	0.69867719	0.0021271	0.96367372	0.72459875
Dnajc24	0.00868648	3.81975444	0.0020965	0.9639357	0.72473568
Nfe2l3	-0.0141548	2.48759013	0.0020824	0.96405714	0.72473568
9330188P03I	0.03149296	0.9897814	0.00208186	0.96406174	0.72473568
Tgfbr3	0.01043366	6.77981759	0.00207767	0.9640979	0.72473568
Atp8b1	0.01265789	2.4639012	0.00205569	0.96428824	0.72483326
Adamtsl1	-0.0108084	3.07913631	0.00204169	0.96440997	0.72487927
Golt1b	-0.0075662	5.46758268	0.00201355	0.96465589	0.72496339
Slfn10-ps	-0.036686	-0.7631627	0.00201209	0.96466866	0.72496339
4932438H23I	0.03279889	-0.1891643	0.00200538	0.96472758	0.72496339
Chtf8	0.00787371	5.11956091	0.00200124	0.96476404	0.72496339
Ftsj1	-0.0081885	3.73079047	0.00198182	0.9649353	0.72504659
0610012G03	0.01014329	3.83728121	0.00195264	0.96519423	0.72514319
Tsfm	-0.0096448	2.62494558	0.00192949	0.96540096	0.72514319
Ubash3a	-0.0433695	-1.2589354	0.00192072	0.96547962	0.72514319
Cep55	-0.0396534	-1.7708971	0.00191844	0.96550015	0.72514319
Parvb	0.01127273	3.54574773	0.00191777	0.96550612	0.72514319
Shb	0.01326521	1.70974903	0.00191352	0.96554439	0.72514319
Eci3	0.02235508	0.66836361	0.00191175	0.96556033	0.72514319
Kdm5d	-0.0102779	4.88224008	0.00191042	0.9655723	0.72514319
Ddhd2	0.00560675	6.00364545	0.00190573	0.96561452	0.72514319
Mvb12a	-0.0128612	1.88568296	0.00189965	0.96566936	0.72514319
Pigyl	0.01930872	3.02094125	0.00187665	0.96587772	0.72525417
Eif6	0.01022113	4.38628276	0.00186708	0.96596478	0.7252736
Fundc1	0.00663669	6.44940792	0.0018605	0.96602472	0.7252736
Mbd6	-0.0125789	3.97290676	0.00184854	0.96613406	0.7252835

Dpagt1	0.01180168	3.3175558	0.00184581	0.96615904	0.7252835
Lmo3	-0.006169	7.13282891	0.00183063	0.96629846	0.7253427
Saysd1	0.0117475	2.32347982	0.00181388	0.96645284	0.72535268
Uap1l1	0.01732726	4.09503862	0.00181358	0.96645565	0.72535268
Gid8	0.0049852	6.55454777	0.0018058	0.96652762	0.72535268
Zfp251	0.00892995	3.78981328	0.00179776	0.96660215	0.72535268
Taok3	-0.0064071	5.86871677	0.00178414	0.96672886	0.72535268
2700094K13l	0.01174464	3.19657688	0.00178383	0.96673174	0.72535268
Arl10	-0.0205785	0.91584458	0.0017834	0.96673574	0.72535268
Ghrl	0.02860045	-0.638868	0.00175081	0.96704086	0.72553616
Galnt18	0.01235807	2.29504213	0.00174091	0.96713412	0.72553795
Slc13a3	0.01326597	6.93531399	0.0017377	0.96716441	0.72553795
Stk3	0.00618366	5.66440243	0.00171888	0.96734265	0.7256262
Dctn4	-0.004683	7.41932633	0.00167966	0.96771716	0.72574172
Rimkla	-0.0104859	3.67743877	0.00167913	0.96772221	0.72574172
Ccdc177	0.01313804	2.74825824	0.00167838	0.9677294	0.72574172
Cul2	0.00745052	5.27435675	0.00166715	0.96783755	0.72574172
Zfyve16	0.00696245	4.90161948	0.00166017	0.96790483	0.72574172
Trim56	0.00867753	3.52885382	0.00165974	0.96790902	0.72574172
Lyrm9	-0.0057193	5.68993301	0.00165787	0.96792712	0.72574172
Tsacc	0.03027589	0.05827724	0.00164621	0.96804003	0.72574172
Cnga4	-0.0250783	0.3023142	0.00163823	0.96811753	0.72574172
Egln1	0.00496618	6.86090147	0.00163554	0.9681437	0.72574172
Adipor1	0.0067243	6.28407623	0.00163353	0.96816325	0.72574172
Anapc2	0.00539545	4.74039092	0.00162232	0.96827263	0.72575168
Cks1b	0.01489242	3.42281938	0.00161975	0.96829775	0.72575168
Efcab11	0.03135795	-0.5475158	0.00161192	0.9683744	0.72576371
Stxbp5	0.00758512	7.72459988	0.00159712	0.96851987	0.72578639
Sav1	0.005443	6.59991401	0.00159015	0.96858862	0.72578639
Lman2l	-0.0090807	3.23296751	0.001589	0.96859993	0.72578639
Golga2	0.00576616	5.49769387	0.00157581	0.96873046	0.72578639
Neto1	0.00756258	7.50462645	0.00157501	0.96873845	0.72578639
Ropn1l	0.04393186	-1.0281018	0.001572	0.96876829	0.72578639
Gm15133	0.05623577	-1.759902	0.00156064	0.96888131	0.72582565
Dzank1	-0.0064638	8.55865494	0.00153482	0.96913963	0.72597376
Steap4	-0.0424208	0.17579235	0.00151392	0.96935031	0.72608616
Icam4	0.02921801	-0.0270766	0.00149794	0.96951243	0.72616217
Tmem258	0.01081044	2.43606167	0.00147435	0.96975336	0.72623365
Cntrob	0.01341105	2.14754063	0.00146113	0.96988918	0.72623365
Aldh1l1	0.00780216	3.6319835	0.00145503	0.96995204	0.72623365
Zbtb40	-0.0144434	2.50864861	0.00142682	0.97024463	0.72623365
Cbx1	0.00701696	4.65570054	0.00142589	0.9702543	0.72623365
Zfp26	-0.0056339	6.27134914	0.00142261	0.97028856	0.72623365

H2afv	-0.0088237	5.31528394	0.00141245	0.97039476	0.72623365
Wdr19	-0.0077048	4.6043891	0.00140925	0.97042835	0.72623365
Tc2n	0.01892943	0.86585266	0.00140611	0.97046125	0.72623365
Pinx1	0.00875204	3.43679909	0.00140356	0.97048806	0.72623365
Ptx3	-0.0370688	-1.326802	0.00140126	0.97051225	0.72623365
Ss18	0.00551705	5.34131109	0.00139758	0.97055095	0.72623365
Rcor3	0.00540735	5.44193821	0.00139679	0.97055928	0.72623365
2010204K13I	-0.0101345	2.63947415	0.00138666	0.9706662	0.72623365
Atxn3	-0.0053889	5.32775843	0.00138523	0.97068126	0.72623365
Stx17	-0.0054197	5.51081247	0.00138079	0.97072827	0.72623365
Rasgrp4	0.024888	-0.1143058	0.00137686	0.97076992	0.72623365
Zfhx4	0.00532259	8.17332463	0.00137034	0.97083925	0.72623365
Sec16b	0.03210775	-0.2236277	0.00136572	0.97088836	0.72623365
Rbm19	-0.0114999	2.26735387	0.00136469	0.97089936	0.72623365
4930486F22I	-0.0465496	-1.2626018	0.00136437	0.97090278	0.72623365
Bcl2l11	0.0086973	5.05409116	0.00136069	0.97094199	0.72623365
Scrib	0.01142064	2.10448195	0.00135077	0.97104813	0.72626768
Rpap1	0.00878811	2.8673749	0.00133305	0.97123853	0.72633147
Sh3rf3	0.0069375	5.61462486	0.00132824	0.97129042	0.72633147
Mybl1	-0.0074107	4.22013849	0.00132593	0.97131537	0.72633147
Kit	0.00522136	4.87311702	0.00131407	0.97144398	0.72638229
Cpne6	-0.0082176	5.07405814	0.00130406	0.97155286	0.72641834
Rpl27	-0.0064384	6.01299865	0.0012923	0.97168137	0.7264516
Prim1	-0.0111907	2.94450573	0.0012889	0.97171866	0.7264516
Qk	-0.0043118	9.3229642	0.00127373	0.97188542	0.72653092
C030034L19I	0.04210578	-1.2749103	0.00125142	0.97213268	0.72667039
Zfp442	0.00725469	3.50310262	0.00124236	0.97223369	0.72670054
4930478L05I	0.04718769	-1.0812398	0.00122452	0.97243362	0.72680461
Nampt	0.00445711	7.31073047	0.00120929	0.97260562	0.7268878
Pcdh9	-0.0054657	7.3123863	0.0012026	0.97268138	0.72689906
Kcnk2	0.00433491	7.24702257	0.00119099	0.9728135	0.726914
Stmn4	0.00706282	6.7672225	0.00119018	0.97282276	0.726914
Wasf1	-0.0059667	7.99226552	0.00118236	0.97291219	0.72693546
Dzip3	-0.0056643	6.90177053	0.00116442	0.9731184	0.72704418
Mrpl11	0.00643562	4.64254819	0.00115743	0.97319921	0.72705919
Ndel1	0.00549421	4.91685716	0.00114708	0.97331915	0.72709409
Zfp3	-0.0083017	3.98973026	0.00114294	0.97336736	0.72709409
Thap7	-0.0098048	2.81002998	0.00113399	0.97347177	0.72709573
Opalin	-0.0149959	1.45829791	0.00112738	0.97354924	0.72709573
Brd2	0.00354736	7.92473855	0.00112559	0.97357017	0.72709573
Uqcrc2	-0.0034177	6.7286354	0.001122	0.9736124	0.72709573
N6amt1	0.00444207	5.66789646	0.00110867	0.97376952	0.72712797
Tmem11	-0.0079552	3.33295823	0.00110688	0.97379073	0.72712797

Adnp2	0.0063913	4.66110951	0.00110291	0.97383772	0.72712797
Gm6588	-0.0400117	-0.4709742	0.00109572	0.97392315	0.72714642
Map1lc3b	0.00536503	7.87064155	0.00107928	0.97411938	0.7272084
Desi1	0.00430932	5.52702697	0.0010786	0.97412762	0.7272084
Klf13	-0.0040992	7.23167154	0.00106792	0.97425597	0.72724269
Pcsk1n	0.00546713	4.4277672	0.00106328	0.97431194	0.72724269
Fcgr1	-0.0262937	-0.627529	0.00105155	0.97445391	0.72724269
3632454L22F	-0.0181031	0.7864583	0.00104972	0.97447611	0.72724269
Ddx47	0.00446509	5.91726426	0.00104623	0.97451861	0.72724269
Doc2b	0.00831119	4.21046912	0.00104465	0.9745379	0.72724269
Irf8	-0.0129473	1.6282917	0.00103278	0.97468286	0.72730554
Zbtb7c	0.00625554	4.09881819	0.00099464	0.97515454	0.72741317
Cpsf1	-0.0081076	4.0570391	0.00099368	0.97516654	0.72741317
Ccdc85b	0.00571708	4.38375005	0.00099046	0.97520682	0.72741317
Aplp2	0.0033171	8.46249735	0.00098678	0.97525292	0.72741317
Sbds	0.0042374	6.7129318	0.00098635	0.9752583	0.72741317
1110059E24I	0.00388417	5.63787582	0.0009835	0.97529411	0.72741317
Fas	0.02454347	0.28238961	0.00098222	0.97531019	0.72741317
C030039L03F	-0.0080526	4.05436571	0.00098199	0.97531302	0.72741317
1810011O10	0.00955521	4.30148064	0.00096269	0.97555673	0.72744164
Ppa2	-0.0043936	5.40210486	0.00096228	0.975562	0.72744164
Pla2g3	-0.0248044	-0.0423008	0.00096003	0.97559046	0.72744164
Bod1l	0.00407639	8.59520974	0.00094989	0.97571967	0.72744164
Gimap7	0.0220318	0.15483927	0.00094759	0.97574912	0.72744164
Iars2	-0.0045767	5.53871229	0.00094561	0.97577443	0.72744164
Dnajc3	0.005926	6.76679914	0.00094125	0.97583037	0.72744164
Neu1	-0.0065585	3.96564936	0.00094072	0.97583714	0.72744164
Anapc5	-0.0035857	6.32639075	0.00093468	0.9759148	0.72745425
Vps72	-0.0108328	2.61179427	0.00091882	0.97611998	0.72751716
Pkdrej	0.01973887	0.50668192	0.00091877	0.97612069	0.72751716
Il17d	-0.0111686	0.95456877	0.00090521	0.9762974	0.72759642
Gm2382	0.00856318	2.975679	0.00090131	0.97634855	0.72759642
Dpcd	-0.0085896	3.13768392	0.00088973	0.9765009	0.72766467
Traf7	-0.0054361	3.73534693	0.00086955	0.97676891	0.7278191
D2Wsu81e	-0.0079024	2.04981063	0.00085656	0.976943	0.72788592
Mocs3	0.01931863	-0.9356207	0.00085164	0.97700925	0.72788592
Arcn1	0.0028712	7.30276195	0.0008493	0.97704093	0.72788592
Gstt3	-0.0063694	5.20221588	0.00084152	0.97714623	0.72791909
Isy1	0.00517379	4.96121102	0.0008276	0.97733595	0.72801513
Znrd1	0.00580219	4.5581472	0.00081819	0.97746523	0.72805108
Ints6	0.00448338	4.96000102	0.00081524	0.9775058	0.72805108
Slc47a1	0.00976919	5.38233735	0.00079577	0.97777594	0.72813129
Dcaf13	-0.0041249	4.45411103	0.00079358	0.97780656	0.72813129

Tm6sf2	-0.0310206	-1.4528194	0.00079125	0.97783913	0.72813129
Amfr	-0.0042143	5.82024607	0.00079	0.97785669	0.72813129
Fcrlb	-0.0224944	-1.4451806	0.00077687	0.97804138	0.72822176
Tmed4	0.00606589	5.53251403	0.00076734	0.97817645	0.72822176
Grpel2	0.00522362	4.21739814	0.00076651	0.97818835	0.72822176
Atxn7	0.00404933	5.79439811	0.00076018	0.97827852	0.72822176
Ptpla	-0.0075818	2.93398082	0.0007534	0.97837556	0.72822176
Kbtbd8	0.00653987	3.16545921	0.00074999	0.97842458	0.72822176
Csf2rb2	-0.0209237	-0.1943771	0.00074343	0.97851911	0.72822176
Hus1	-0.0048193	4.38056297	0.00074024	0.97856521	0.72822176
Rint1	0.00494242	4.06276788	0.00073982	0.97857135	0.72822176
Ercc4	0.00555562	3.83463509	0.00073803	0.97859718	0.72822176
Txnrd3	0.00663668	3.37677223	0.00073074	0.97870316	0.72822176
Golga1	0.00357252	5.51897548	0.00072533	0.9787822	0.72822176
Lrwd1	0.01066655	1.80651946	0.000722	0.97883084	0.72822176
Lrp2	-0.0864357	-1.6730941	0.00070564	0.97907198	0.72822176
Gga2	-0.0051024	4.47761348	0.00070522	0.97907824	0.72822176
Edem2	-0.0095635	1.59219095	0.00070393	0.97909739	0.72822176
Prpf39	0.00589831	5.45336515	0.00070249	0.97911883	0.72822176
Kansl1	-0.0027464	6.95826193	0.00069675	0.97920419	0.72822176
Smim19	-0.005294	4.67526929	0.00069288	0.97926205	0.72822176
Olr1	0.01797937	-0.3912774	0.00068761	0.97934113	0.72822176
Ifit3	0.00641019	5.94234365	0.00068625	0.97936152	0.72822176
Gtl3	0.00361884	4.59835817	0.00068539	0.97937449	0.72822176
Pou3f2	-0.0045029	4.41732911	0.00068523	0.97937678	0.72822176
Ovca2	0.00574511	3.35314287	0.00068116	0.9794381	0.72822214
Numb	0.00429492	5.65734998	0.00067601	0.97951607	0.7282349
Zfp612	-0.0043509	7.4305425	0.00065877	0.97977883	0.72838504
2810021J22F	0.00486479	4.0634388	0.00064804	0.97994411	0.72845844
Ttc19	0.00369278	6.26122451	0.00064449	0.97999923	0.72845844
Gm5113	-0.0061435	4.18435604	0.00064054	0.98006053	0.7284588
Zfp707	0.01124125	1.37092506	0.00062995	0.98022605	0.72851696
Chaf1b	-0.0260533	-1.7793604	0.00062901	0.98028467	0.72851696
Chn1	-0.0028021	9.71703491	0.00062389	0.98032128	0.72851696
Stx18	0.00597717	3.37978799	0.00061789	0.98041619	0.72852706
Epb4.1l4b	0.00585357	3.33579708	0.00061487	0.98046412	0.72852706
Endog	-0.0196288	-0.8303844	0.00061152	0.98051738	0.72852706
Rabepk	-0.0067144	2.99144991	0.00059684	0.98075255	0.72865659
Slc2a6	-0.011421	1.04842691	0.00058429	0.98095593	0.72873536
Tspan6	0.00646371	3.9849307	0.0005828	0.98098028	0.72873536
Adamts4	-0.0091724	1.78413118	0.00057269	0.98114596	0.72881323
Aga	-0.007575	3.47617596	0.00056316	0.9813034	0.72886286
Hrh2	0.00818259	2.16821105	0.00056041	0.98134919	0.72886286

Eif1	0.00391724	7.99043545	0.0005537	0.98146114	0.72886286
Opn4	-0.0214098	-0.0898355	0.00055091	0.98150781	0.72886286
Cep76	-0.0048729	3.92024491	0.00055036	0.98151709	0.72886286
Klhl10	-0.0287896	-0.8904987	0.00053266	0.98181669	0.72894173
Kank2	-0.0043503	7.56540758	0.00053173	0.98183259	0.72894173
Fgfr1	-0.0049008	6.04436897	0.00052517	0.98194492	0.72894173
Sec14l3	-0.0225821	-0.5228344	0.00052415	0.98196242	0.72894173
Rgs12	0.0058945	3.26343381	0.00052408	0.9819636	0.72894173
Tnfsf13	0.0231385	-1.1613654	0.00052264	0.9819885	0.72894173
Abcg4	-0.0050731	4.08949237	0.00050946	0.98221695	0.72903584
Oard1	-0.0047282	4.57408272	0.00050629	0.9822724	0.72903584
Srp19	0.00297048	6.44334797	0.00050429	0.98230739	0.72903584
Ufl1	-0.0032812	5.79660196	0.00050137	0.98235879	0.72903584
Hist1h4c	-0.0161609	-0.8188294	0.00048984	0.98256277	0.72914204
Ddb1	0.0029784	6.36962205	0.0004759	0.98281259	0.72918378
Cachd1	0.00497705	3.39907885	0.00047389	0.98284885	0.72918378
Pbk	0.01729116	0.03747487	0.00047162	0.98289	0.72918378
Zcrb1	-0.0028188	6.95352091	0.00046118	0.98308047	0.72918378
Ahcy	-0.0044699	3.87366617	0.00045766	0.98314518	0.72918378
Lama5	-0.0065044	1.76974396	0.00045755	0.98314713	0.72918378
02-Mar	0.0048912	5.33229017	0.00045428	0.9832075	0.72918378
Cdipt	0.003385	4.67887572	0.00045233	0.9832435	0.72918378
C4a	-0.0191665	-0.5173054	0.00045232	0.98324381	0.72918378
Thnsl2	0.00663608	1.59740363	0.00044845	0.98331546	0.72918378
Gtf2h1	0.00388099	5.09621696	0.00044801	0.98332375	0.72918378
Pip4k2b	0.00288101	7.02044146	0.00044445	0.98339008	0.72918378
Pcdhb2	-0.0106602	1.80948339	0.00044336	0.98341057	0.72918378
Btbd6	0.0035361	4.22448594	0.00043827	0.98350591	0.72920932
Bcl2a1b	0.00778587	1.45049734	0.00043159	0.98363205	0.72923293
Tmem177	-0.0046457	3.68336884	0.0004279	0.98370223	0.72923293
Gm4890	-0.0102896	-1.3919237	0.00042695	0.98372043	0.72923293
Rchy1	-0.0030372	5.50211776	0.00041843	0.98388363	0.72926826
Scn4b	-0.004298	4.78443793	0.00041215	0.98400496	0.72926826
Proser1	0.0033086	5.5896054	0.00041151	0.98401744	0.72926826
Diexf	0.00371438	4.40759709	0.00041117	0.98402405	0.72926826
Rmdn2	-0.0042536	3.71864916	0.00040762	0.98409306	0.72926826
Capn2	-0.0030616	7.15307409	0.00040311	0.98418141	0.72926826
D730005E14	-0.0189888	-1.8001265	0.00040245	0.98419436	0.72926826
Mrpl47	0.00339034	3.55239049	0.00039212	0.98439842	0.72927755
Pla2g15	-0.0077936	2.06800813	0.00039029	0.98443488	0.72927755
Enpp5	0.00246141	7.09987463	0.00038491	0.98454239	0.72927755
Sh3kbp1	-0.0027018	6.38425475	0.00038296	0.98458177	0.72927755
Sdr9c7	0.03297518	-1.6837212	0.00038149	0.98461121	0.72927755

Mospd1	0.00330555	6.13396769	0.00037883	0.98466502	0.72927755
Gm20754	-0.0101148	0.72571659	0.00037753	0.9846914	0.72927755
Ankrd12	-0.0029397	9.70283078	0.0003726	0.98479174	0.72927755
Gpr21	-0.0098403	0.86234498	0.00036715	0.98490318	0.72927755
1700003F12I	0.00995113	-2.0074229	0.00036672	0.98491217	0.72927755
Ccnd3	-0.0070212	4.56294001	0.00036431	0.98496183	0.72927755
Rnf152	-0.0025655	6.19103377	0.00036264	0.98499631	0.72927755
Zdhhc13	-0.0042806	4.23415247	0.00035814	0.98508969	0.72927755
Haus5	-0.0067697	1.62049443	0.00035619	0.98513028	0.72927755
Upf1	0.00344635	4.27540549	0.00035174	0.98522337	0.72927755
Zfp85os	0.00523842	2.59106711	0.00035131	0.98523258	0.72927755
Folr2	-0.0138665	-0.8304092	0.00035085	0.98524213	0.72927755
Pygb	0.00244587	6.4839507	0.0003423	0.98542295	0.72936631
2810459M11	0.0071035	1.84804127	0.00033666	0.98554359	0.72936961
Chadl	0.00704795	1.07999139	0.000334	0.98560075	0.72936961
Dchs1	0.00513362	2.92556451	0.00033357	0.98561012	0.72936961
Zdhhc23	0.00912654	1.06906349	0.00032893	0.98571056	0.72939886
2310002D06I	0.02307518	-1.6989495	0.00032094	0.98588516	0.72943928
03-Sep	-0.0023134	8.9122498	0.00032086	0.985887	0.72943928
Ids	0.00237116	9.64471167	0.00031409	0.98603664	0.72944373
Hsd3b3	0.00938992	0.3184857	0.0003109	0.98610761	0.72944373
Lss	0.00352215	4.11244123	0.00030649	0.98620642	0.72944373
Slc2a13	0.00251606	6.84437649	0.00030536	0.98623207	0.72944373
Tvp23a	-0.0046042	4.20461113	0.00030402	0.98626229	0.72944373
Atp6v0b	-0.0032478	4.83149849	0.00030371	0.98626918	0.72944373
Klf16	0.00429152	2.45963519	0.00029947	0.98636547	0.72944373
Rars2	-0.0033503	3.90870699	0.00029846	0.98638843	0.72944373
Tmem151a	0.00345314	4.63055063	0.00029175	0.98654234	0.72944373
Tipin	0.00511447	4.23054188	0.00029171	0.98654325	0.72944373
Mfap3	0.00273927	4.95890308	0.00029085	0.98656302	0.72944373
Fam219b	-0.0041069	3.70312131	0.00028263	0.98675418	0.72950053
Cth	0.0085445	0.94286867	0.00027998	0.98681642	0.72950053
Slc4a2	0.00429577	3.65489648	0.00027764	0.98687161	0.72950053
Lgalsl	-0.001904	6.23959162	0.0002757	0.98691772	0.72950053
Hspbap1	0.00534508	2.22163898	0.00027449	0.98694648	0.72950053
Ttc26	0.00525525	2.9379197	0.00026472	0.98718081	0.72950053
Bace1	0.00194642	5.79319228	0.00026386	0.98720157	0.72950053
Suz12	0.00205984	6.88359314	0.00026317	0.98721841	0.72950053
Ly96	-0.0040646	3.76427015	0.00026266	0.9872308	0.72950053
Vstm5	0.00533208	2.30334241	0.00026191	0.98724899	0.72950053
Pag1	-0.0023881	5.64446304	0.00025441	0.98743281	0.72958792
Ptk7	0.00661567	1.28696047	0.00025214	0.9874891	0.72958792
Clp1	0.00393592	2.44874484	0.00024594	0.98764383	0.72961588

Npat	-0.0022574	5.99288499	0.00024522	0.9876619	0.72961588
Ndufa12	-0.0023285	5.67767073	0.00023963	0.98780339	0.72961588
Agrn	0.00310814	5.00625141	0.00023937	0.98780995	0.72961588
Auh	-0.0023464	5.11797038	0.00023852	0.98783158	0.72961588
Psmc3	-0.0022237	5.5806721	0.0002334	0.98796281	0.72966781
Cd72	0.01218427	-0.3622226	0.00021923	0.98833403	0.72989696
Bre	0.0025257	4.14336878	0.0002169	0.98839606	0.72989777
Xpot	-0.0017171	7.00853197	0.00021378	0.98847991	0.72991467
A830010M2(	-0.0026205	8.6443168	0.00021137	0.98854504	0.72991776
Zc3h18	-0.0027805	3.91174905	0.00020687	0.98866751	0.72996318
Jmjd4	-0.0024935	4.63418793	0.00020131	0.98882085	0.73003139
M6pr	0.00250433	6.22491652	0.00019802	0.9889126	0.73004353
Snrnp27	-0.0028762	5.43105277	0.00019472	0.98900543	0.73004353
Rnf180	-0.0043454	2.8396008	0.0001942	0.98902017	0.73004353
Dpp4	0.00395114	5.50219731	0.00017889	0.98946181	0.73032451
Ptpdc1	0.00180252	5.41754893	0.00017479	0.98958332	0.7303679
Cd97	-0.0036251	3.10544041	0.00017035	0.98971626	0.7303679
Ispd	-0.003202	3.40984449	0.00016895	0.98975877	0.7303679
Znhit1	0.00303264	3.24479417	0.00016876	0.98976454	0.7303679
Ift20	0.00194067	6.78178911	0.00016342	0.98992767	0.73044327
Ubtd2	-0.0019617	5.27685835	0.00015932	0.99005494	0.73046999
Lysmd2	-0.0020066	4.50476051	0.00015833	0.99008588	0.73046999
Rtkn2	-0.0062427	1.19225338	0.00014996	0.99035145	0.73062091
Nim1k	0.00260726	4.19746749	0.00014536	0.99050037	0.73068576
Clasrp	-0.003239	2.54147042	0.00013529	0.9908356	0.73073933
Kctd5	-0.0030974	2.84732475	0.00013442	0.99086491	0.73073933
Ctf1	-0.0041509	2.64594289	0.00013325	0.9909048	0.73073933
Grm1	-0.0021187	5.38932871	0.00013282	0.99091933	0.73073933
Ufsp2	-0.0018162	5.28542917	0.00012998	0.99101709	0.73073933
Prim2	0.00297604	2.69583079	0.00012736	0.9911081	0.73073933
Mmp2	-0.005138	1.32979393	0.0001266	0.99113471	0.73073933
Rerg	0.00424053	2.91998122	0.00012474	0.99120013	0.73073933
Tsc2	0.00217546	5.85466584	0.00012378	0.99123395	0.73073933
Zc3h12a	-0.0134543	-0.5978045	0.00012202	0.99129656	0.73073933
Mllt4	-0.001134	7.44020408	0.00012184	0.99130304	0.73073933
Mir5119	0.01129912	-1.4890308	0.00012178	0.9913052	0.73073933
Nip7	0.00160306	5.02357649	0.0001178	0.99144842	0.73079934
Tmem144	0.00299092	2.21110874	0.00011479	0.9915582	0.73079934
Aste1	-0.0033408	2.36355824	0.00011372	0.99159787	0.73079934
Asrgl1	-0.0018983	5.83523342	0.00011277	0.99163279	0.73079934
Kbtbd7	-0.0016657	5.2378738	0.00011119	0.99169173	0.73079934
Atp2c2	-0.0059382	0.00134164	0.00010511	0.99192208	0.73086195
Nanos1	-0.0013053	5.23537647	0.00010202	0.9920418	0.73086195

Dock5	0.00129263	6.84778138	1.00E-04	0.99212086	0.73086195
Bicd1	0.00146582	6.59984116	9.77E-05	0.9922136	0.73086195
Tcf7l2	0.00191696	6.41243746	9.73E-05	0.9922299	0.73086195
4931440F15I	0.00429119	-0.2478327	9.69E-05	0.99224554	0.73086195
Cyp11a1	-0.0063817	-0.1870064	9.51E-05	0.99231719	0.73086195
Rexo4	0.00171259	4.59770965	9.49E-05	0.99232626	0.73086195
Ppp2r3c	-0.0013067	4.97086748	9.34E-05	0.99238597	0.73086195
Atf7ip	0.0010189	6.92989395	9.33E-05	0.99239002	0.73086195
1110017D15I	-0.006104	-0.1132766	8.96E-05	0.9925407	0.73086195
Rfx4	-0.00246	3.75945816	8.70E-05	0.99265183	0.73086195
Fam104a	0.00184082	5.23095913	8.64E-05	0.99267492	0.73086195
Nthl1	-0.0064021	0.3650133	8.55E-05	0.99271605	0.73086195
Bloc1s4	-0.0024645	4.26340494	8.41E-05	0.99277251	0.73086195
Hadha	-0.0013404	4.94884333	8.36E-05	0.99279413	0.73086195
Nek4	0.00171439	4.4182294	8.19E-05	0.99286983	0.73086195
Casp8ap2	0.0010195	6.4656115	8.18E-05	0.99287521	0.73086195
AB041803	0.0038801	2.42440817	7.94E-05	0.99297829	0.73087068
Trub2	-0.0022858	3.0142733	7.69E-05	0.99308981	0.73087068
Il1r1	-0.0018303	4.30797735	7.56E-05	0.99314774	0.73087068
Dtymk	-0.0014646	4.20043631	7.48E-05	0.9931875	0.73087068
Foxp2	-0.0014503	7.30765241	7.47E-05	0.99319221	0.73087068
Adora1	0.00123412	7.10715741	7.32E-05	0.99325755	0.73087385
Foxf2	0.00250149	2.76111386	6.87E-05	0.99346826	0.73098398
Zfp600	0.00569288	-1.1369565	6.56E-05	0.9936194	0.73105027
Nat8l	0.001497	6.37905119	6.23E-05	0.99377892	0.73107113
Eepd1	0.00207577	2.48443429	6.17E-05	0.99381096	0.73107113
Btbd2	0.00150971	4.50350605	6.04E-05	0.99387804	0.73107113
Snx18	0.00106768	6.1685314	5.98E-05	0.99390455	0.73107113
Tmem117	0.00194076	3.28497422	5.85E-05	0.99397244	0.73107113
Hsd3b7	-0.0018731	2.68274283	5.77E-05	0.99401402	0.73107113
Ngfrap1	-0.0011186	6.52437935	5.00E-05	0.99442726	0.73133014
Nbn	0.00104788	4.74914227	4.78E-05	0.99455481	0.73136879
Gpr137b	-0.0014587	2.9335845	4.50E-05	0.99471311	0.73136879
Rbfox2	-0.0008086	7.5197972	4.47E-05	0.99473485	0.73136879
Cwc22	-0.0009915	5.52773881	4.39E-05	0.99477905	0.73136879
St8sia1	-0.0010589	6.67540381	4.30E-05	0.99483124	0.73136879
Galnt16	0.00128837	4.41989815	4.28E-05	0.99484625	0.73136879
1700086L19I	0.00153981	3.09670287	3.81E-05	0.99513775	0.73152762
1700025G04	0.00082196	6.95265813	3.74E-05	0.99518446	0.73152762
Gm1976	-0.0010994	4.49603841	3.61E-05	0.99526295	0.73154042
Zfp13	-0.0023029	1.79674284	3.20E-05	0.99554297	0.73167572
Ccl2	-0.004518	-0.4998618	3.12E-05	0.99559909	0.73167572
Lonrf1	0.00082904	6.32971245	2.99E-05	0.99569097	0.73167572

Cfhr2	-0.0032279	0.69676622	2.99E-05	0.99569142	0.73167572
Pkd2l1	0.00899425	-1.3225536	2.90E-05	0.99575969	0.73168099
Luc7l2	0.00070864	7.39714737	2.59E-05	0.99599154	0.73180115
Serpine3	0.00171038	1.95541913	2.52E-05	0.99604765	0.73180115
Tlr9	-0.0032648	-0.3613039	2.44E-05	0.99610653	0.73180115
Synpo2	-0.0012155	3.37274146	2.28E-05	0.99624046	0.73185464
Ehd4	-0.0008231	4.01675613	1.97E-05	0.99650122	0.7320013
Lipt1	-0.0017917	1.47774105	1.89E-05	0.99657646	0.73201166
Tob1	0.00055704	6.21399761	1.54E-05	0.99690704	0.73207208
Ube2z	0.00062074	6.14013848	1.48E-05	0.99696712	0.73207208
Zfp628	0.0011069	2.2903361	1.38E-05	0.99706963	0.73207208
Fem1a	0.00050138	5.55688316	1.35E-05	0.9971097	0.73207208
Mpped1	-0.0005456	6.04425152	1.31E-05	0.99714879	0.73207208
Chmp6	-0.0009419	2.23547176	1.28E-05	0.99717634	0.73207208
Shc1	-0.0007324	6.42383923	1.19E-05	0.99727691	0.73207208
Pemt	-0.0048897	-0.981126	1.09E-05	0.99739315	0.73207208
Rab11fip2	-0.0003853	6.93761623	1.08E-05	0.997415	0.73207208
Lman1	-0.0007815	5.02057679	1.08E-05	0.99741604	0.73207208
Tnpo1	-0.0003943	6.64202774	1.03E-05	0.9974772	0.73207208
Hspa4l	0.00057674	7.12134736	9.05E-06	0.99762987	0.73207208
Apex2	-0.0017826	1.18741274	8.97E-06	0.99764004	0.73207208
Rps11	0.00059647	6.85286099	8.48E-06	0.9977054	0.73207208
Cdc42ep3	0.00055989	3.36695661	8.33E-06	0.99772643	0.73207208
Calcoco2	-0.0013595	-0.0155403	8.05E-06	0.99776454	0.73207208
Ift140	0.00093638	3.26109083	8.04E-06	0.99776598	0.73207208
Rnf20	0.00037811	6.44450563	7.75E-06	0.997807	0.73207208
Efcab4b	0.01151863	-1.4068119	7.65E-06	0.99782018	0.73207208
Tmem55b	0.00043626	4.99186399	6.41E-06	0.99800489	0.73213466
Xrn2	-0.0003019	5.65336846	5.17E-06	0.99820906	0.73213466
Nfatc3	0.00038703	6.91576641	5.12E-06	0.99821736	0.73213466
Dync1i1	-0.0004194	5.12427395	4.11E-06	0.9984034	0.73213466
Slc9a2	0.00065438	5.21957124	4.01E-06	0.99842314	0.73213466
Arrb2	0.00041284	4.08125527	3.98E-06	0.99842833	0.73213466
Gm16880	-0.0021288	-0.2276483	3.95E-06	0.99843453	0.73213466
Ube2b	-0.0002382	7.64249085	3.88E-06	0.99844849	0.73213466
Faxc	-0.0002469	7.8893427	2.66E-06	0.99871537	0.73213466
Nedd1	0.00048731	2.72720241	2.62E-06	0.99872533	0.73213466
Srr	0.00022693	6.94514693	2.36E-06	0.99878978	0.73213466
BC021785	0.00021474	-1.6363028	2.33E-06	0.9987964	0.73213466
1700037C18I	-0.0007549	0.04865188	2.10E-06	0.99885892	0.73213466
Rnmt	0.00018193	5.92020427	2.06E-06	0.9988695	0.73213466
Fam58b	-0.0003465	4.09447951	1.74E-06	0.99896013	0.73213466
Rock1	-0.0001952	8.00893157	1.58E-06	0.99900995	0.73213466

Crtc2	0.00017616	4.0438307	9.59E-07	0.99922854	0.73213466
Stk4	0.000109	5.92312332	9.31E-07	0.99923974	0.73213466
Tex13	0.00056046	-0.5693191	7.08E-07	0.99933722	0.73213466
Abhd3	0.0002255	3.71128761	4.62E-07	0.99946447	0.73213466
G3bp2	9.36E-05	9.47815298	4.61E-07	0.99946495	0.73213466
Tssc1	-0.0001298	3.27212638	4.47E-07	0.99947296	0.73213466
Hs2st1	8.49E-05	6.7575588	4.07E-07	0.99949755	0.73213466
Zyg11b	-0.0001003	8.98696616	3.56E-07	0.99952976	0.73213466
Gusb	-0.0001057	2.80361293	1.69E-07	0.99967602	0.73213466
Tmem163	9.17E-05	2.75960425	1.62E-07	0.99968253	0.73213466
Lpin3	0.00374529	-1.554597	1.55E-07	0.99969023	0.73213466
Ctxn1	3.95E-05	7.61037203	1.48E-07	0.9996965	0.73213466
Ptchd1	8.37E-05	4.63115282	1.24E-07	0.99972204	0.73213466
Hspe1	-3.55E-05	6.34454213	5.76E-08	0.99981098	0.73213466
Gm11974	0.00015527	0.35866908	4.87E-08	0.99982612	0.73213466
Zfp157	1.89E-05	4.72711089	1.41E-08	0.99990641	0.73213466
Dvl1	9.85E-06	4.07621373	2.90E-09	0.99995756	0.73213466
Tas2r137	0.00731363	-1.9346366	4.14E-10	0.99998405	0.73213466
