# Supplementary material for: Mapping the physiological and molecular markers of stress and SSRI antidepressant treatment in S100a10 corticostriatal neurons
Source: Mol Psychiatry. 2019 Aug 20;25(5):1112–29. doi: 10.1038/s41380-019-0473-6 (PMC7031043; doi:10.1038/s41380-019-0473-6)
Supplement: Supplementary file 17 — Supplemental Table S2a [file 41380_2019_473_MOESM17_ESM.pdf]

Suppl Table S2a: Genelist representing the differentially expressed genes (3463) between the Sh and Sh+Flx groups in the context of all genes examined.

symbol	logFC	logCPM	F	PValue	FDR
Emd	1.89852729	5.92569873	164.27461	4.48E-11	3.68E-07
Cstb	2.46424586	6.70738108	140.136178	1.83E-10	7.51E-07
Medag	1.03924865	6.21870146	79.0524953	2.28E-08	6.25E-05
Mvk	1.96993244	2.941983	72.8784165	4.37E-08	8.35E-05
Maff	2.54874781	3.06441849	71.4936117	5.08E-08	8.35E-05
Cdkn1a	1.37878238	5.39687725	66.7721367	8.67E-08	0.00010631
Tmcc3	1.25079508	6.34903671	66.4014042	9.05E-08	0.00010631
Sdpr	1.88182634	7.13197726	61.5466929	1.62E-07	0.00016686
Ftx	-2.0566827	4.04392197	59.1236051	2.20E-07	0.00019272
Rgs13	6.37380702	0.46691348	58.6388396	2.34E-07	0.00019272
Ecm1	1.84533065	3.89611697	57.5589168	2.70E-07	0.00020151
Rgs2	1.14701662	7.30623261	54.6712503	3.96E-07	0.00027122
Wdr92	0.89574415	5.3582105	53.4678	4.67E-07	0.00029515
Nptx2	1.66818959	4.03919698	50.9175489	6.68E-07	0.00034486
Crem	1.09140519	4.8377127	50.6121904	6.98E-07	0.00034486
Usp29	-1.2850458	5.56455857	50.5646199	7.03E-07	0.00034486
Cycs	1.08183473	8.2438788	50.4587491	7.13E-07	0.00034486
Atp11b	-1.1072117	6.31297214	49.6377408	8.03E-07	0.00035149
Fam46a	1.01580735	5.75775238	49.4866196	8.21E-07	0.00035149
Errfi1	0.90258081	6.88726728	49.2088677	8.55E-07	0.00035149
Ryr1	-2.5119642	2.63715206	48.1862173	9.95E-07	0.00038935
Aox3	1.37590194	6.1406988	47.7318087	1.06E-06	0.00039775
Nr4a3	2.21035485	4.23225603	45.1998157	1.57E-06	0.00055628
Rtl1	-2.2139707	2.9729589	44.5947978	1.72E-06	0.00055628
Sccpdh	0.77387292	6.28810619	44.4672844	1.76E-06	0.00055628
Akr1b10	1.12837988	3.77109963	44.463053	1.76E-06	0.00055628
Capn6	1.52918134	4.31627963	43.5518748	2.03E-06	0.00059103
Pgk1	0.75980104	7.98381182	43.5299746	2.04E-06	0.00059103
Cdk11b	0.70731883	7.39709748	43.395527	2.09E-06	0.00059103
Hrg	4.6498267	1.03122995	43.1596465	2.17E-06	0.00059341
Med23	-1.0369405	4.19630727	42.6407791	2.36E-06	0.00059923
Erbb4	-1.3211361	4.58491755	42.6091309	2.37E-06	0.00059923
F8	-1.4268063	3.0977305	42.5106326	2.41E-06	0.00059923
Dnaja1	0.63926026	9.6899873	40.9215747	3.13E-06	0.00075589
Topaz1	5.77852627	0.39184056	40.4935785	3.36E-06	0.00076852
Tpm4	1.35902119	9.13505951	40.4821787	3.37E-06	0.00076852
A230057D06	-2.0323638	3.28385481	40.0544979	3.62E-06	0.00078931
Acot13	0.99739283	5.31541963	40.0045306	3.65E-06	0.00078931
Vimp	0.9734469	6.1270351	39.8403553	3.75E-06	0.00079084
Sqstm1	1.6605749	8.74120663	39.6872655	3.85E-06	0.00079146
Tfrc	-1.0833257	5.51718518	39.4042436	4.04E-06	0.00081048
Pcsk2os1	-1.7148554	3.00792967	38.4101331	4.80E-06	0.00093971
Mapre3	0.79946101	7.32864478	37.9985044	5.16E-06	0.0009865
Aldoa	0.72086249	9.15691313	37.7149872	5.43E-06	0.0010135

Zyx	0.86820347	5.44226379	37.4530358	5.68E-06	0.00102512
Tnnt2	1.52009855	4.613484	37.3998821	5.74E-06	0.00102512
Omd	-0.9889284	5.17347677	36.6995736	6.50E-06	0.00113722
Stip1	0.69594447	7.04168692	36.3294218	6.95E-06	0.00118869
Ptprz1	-1.1103113	6.78132457	36.2243044	7.09E-06	0.00118869
Ppfia4	-1.1539128	3.62828696	35.9996591	7.38E-06	0.00121353
Tbrg3	-1.7723055	4.19389579	35.8062192	7.65E-06	0.00123253
Meg3	-1.7027657	10.8517456	35.5029655	8.09E-06	0.00125904
Abi1	0.65315798	7.09800737	35.4506679	8.16E-06	0.00125904
Rcan1	0.95812169	5.88676338	35.3600808	8.30E-06	0.00125904
Zfp804b	-1.9730042	2.52131327	35.2799643	8.43E-06	0.00125904
Rbp4	1.45035345	4.08437903	34.9358511	8.98E-06	0.00129874
Ppp2ca	0.60118165	8.45582872	34.761057	9.28E-06	0.00129874
Bdnf	1.13112024	4.26333758	34.6530102	9.47E-06	0.00129874
Prl	-6.912935	1.48097504	37.0533248	9.64E-06	0.00129874
Ano1	-2.0586612	1.54957846	34.5055093	9.73E-06	0.00129874
Bcas1	-1.3294619	4.7132492	34.4780425	9.78E-06	0.00129874
Bmyc	1.0131749	5.26490475	34.4700798	9.80E-06	0.00129874
Gm561	1.09378038	3.43496853	34.3831682	9.96E-06	0.00129916
Osgin2	1.02270799	5.95443226	33.7701275	1.12E-05	0.00143606
Scn1a	-1.1002948	7.07931107	33.5718934	1.16E-05	0.00146841
Hace1	-0.8389366	5.00898893	33.2176189	1.24E-05	0.00154774
Insig2	0.67704714	6.13606135	32.8815451	1.33E-05	0.00160879
Nqo2	0.98180168	5.67377076	32.7950555	1.35E-05	0.00160879
Ptgs2	1.39846751	4.12798815	32.7181027	1.37E-05	0.00160879
Akap12	0.99157898	8.54412102	32.6596591	1.38E-05	0.00160879
Fmo1	1.01187242	6.87527009	32.5263336	1.42E-05	0.00160879
Igf1r	-0.7760637	6.90698274	32.4776504	1.43E-05	0.00160879
Tial1	-0.7887165	5.9119415	32.4724679	1.44E-05	0.00160879
Idh3b	0.64447983	7.06719223	32.4275755	1.45E-05	0.00160879
Capg	1.63841555	2.58282	32.1784972	1.52E-05	0.0016475
6330403K07I	0.66748371	7.67692203	32.1699529	1.52E-05	0.0016475
Pdlim1	1.48106773	3.85893549	32.0887893	1.55E-05	0.00164851
Kcnj10	-1.0046463	5.29891954	31.8859749	1.61E-05	0.00164851
Gabrq	-2.0247022	1.88100598	31.8734778	1.61E-05	0.00164851
4933424G05	-3.6405382	1.07474415	31.867133	1.62E-05	0.00164851
Aqp1	2.63281416	1.78608843	31.8433387	1.62E-05	0.00164851
Vwa5b2	-1.6987385	3.81022683	31.7472989	1.66E-05	0.00165026
Actg1	0.58337023	8.97424616	31.6542936	1.69E-05	0.00165026
Agrn	-1.2005034	5.00625141	31.547821	1.72E-05	0.00165026
Mrps33	0.68900573	6.8965535	31.5431755	1.72E-05	0.00165026
Gng3	0.73415861	8.0666176	31.5357891	1.73E-05	0.00165026
Ndufab1	0.80660545	5.61527553	31.4776282	1.75E-05	0.00165027
Snrnp70	-1.021971	5.73712397	31.3202856	1.80E-05	0.00165131
Snx2	0.6631142	7.57439358	31.2938383	1.81E-05	0.00165131

Cab39l	0.84360756	5.7725496	31.2717761	1.82E-05	0.00165131
Dnah7b	-1.1405666	3.50336842	31.2491945	1.83E-05	0.00165131
Dcx	-1.002046	5.69217413	31.172658	1.86E-05	0.00165855
Stra6	2.02214324	7.10475634	31.1187819	1.88E-05	0.00165855
Gm10471	-2.2553774	1.85774165	31.0653287	1.90E-05	0.0016586
Gdpd2	-2.2592873	2.27646471	30.8617384	1.98E-05	0.00170974
Irs4	-2.345881	1.99826185	30.6500428	2.06E-05	0.00176586
Homer2	-0.7185874	7.54094242	30.4069153	2.17E-05	0.00182716
Gm14827	-2.2510097	2.66352071	30.3804003	2.18E-05	0.00182716
Casr	2.1906284	1.89347907	30.2403076	2.24E-05	0.00184826
Pak6	-1.1315576	3.74563752	30.2250348	2.25E-05	0.00184826
Cox5b	0.87574177	6.31383891	29.9992229	2.36E-05	0.00191655
Pik3c2b	-0.8640759	5.11271293	29.935357	2.39E-05	0.00191865
Hpcal4	0.8423993	9.96877456	29.8984318	2.40E-05	0.00191865
Shank1	-1.0864595	8.94138459	29.7849382	2.46E-05	0.00193775
Sntb1	1.89492974	1.79795292	29.756971	2.48E-05	0.00193775
Fabp7	1.08403238	4.52045423	29.6413847	2.54E-05	0.00195394
S100a6	1.67188349	5.94037353	29.6253264	2.54E-05	0.00195394
Htra4	1.67263469	3.17792456	29.5479195	2.58E-05	0.00196712
Cnrip1	0.72666542	6.07897393	29.4473157	2.64E-05	0.00199016
Srxn1	0.64311525	6.28059447	29.2711674	2.74E-05	0.00204219
Gjd2	-1.8801903	1.72208142	29.2359148	2.76E-05	0.00204219
Zc3h15	0.5529369	7.05509728	29.1220991	2.82E-05	0.00204577
Csrp2	1.03113512	4.57851133	29.1115708	2.83E-05	0.00204577
Grin2c	-1.897458	2.15088364	29.0584051	2.86E-05	0.00204577
Wisp1	1.56973811	1.94884932	29.0542991	2.86E-05	0.00204577
Il1rap	-1.0921632	3.72863637	28.9768742	2.91E-05	0.00204577
Efhd2	0.7310736	6.73441745	28.9390437	2.93E-05	0.00204577
Rhou	0.92881222	6.75176283	28.935242	2.94E-05	0.00204577
Jpx	-1.5757512	2.7866681	28.8181488	3.01E-05	0.00205636
Kcnj16	-1.1618086	4.19006785	28.8170998	3.01E-05	0.00205636
Atp8b2	-1.1474124	4.3200366	28.7490026	3.05E-05	0.00205636
Zmym3	-0.9556661	6.02195042	28.7210374	3.07E-05	0.00205636
Ccndbp1	0.86479802	6.68427437	28.6948112	3.09E-05	0.00205636
Arl15	0.69890841	6.06996797	28.6521035	3.12E-05	0.00205636
Uqcrfs1	0.59064477	6.5995679	28.6260018	3.13E-05	0.00205636
Tbc1d30	-0.9793494	6.6190001	28.5993424	3.15E-05	0.00205636
Mdh2	0.61458367	7.24664761	28.4595268	3.25E-05	0.00209179
Bag5	0.74271462	6.29016191	28.4443062	3.26E-05	0.00209179
Nup155	-0.9764435	5.24826961	28.3185726	3.35E-05	0.00213175
Tmc7	-0.9597414	4.52991918	28.1927618	3.44E-05	0.00216027
Rad23b	0.55523532	7.99286749	28.1839701	3.44E-05	0.00216027
Gm15910	-1.4445214	4.50043733	28.1203771	3.49E-05	0.00216832
Pcdhga3	-1.1977627	3.43958644	28.060753	3.53E-05	0.00216832
Coa3	0.86474094	4.39961295	28.0605622	3.54E-05	0.00216832

Pisd-ps1	-1.6165333	5.75833039	27.9889334	3.59E-05	0.00217686
Eprs	0.60972129	7.57236391	27.9730139	3.60E-05	0.00217686
Leng8	-1.3466465	6.48428617	27.9181125	3.64E-05	0.00218656
Lancl2	0.61634199	8.49514295	27.8160645	3.73E-05	0.00221089
Gm2115	1.42882695	3.08649973	27.7991544	3.74E-05	0.00221089
Brox	0.63852113	5.69031614	27.6472287	3.86E-05	0.00223816
Tagln2	1.43735346	5.7784713	27.6430129	3.87E-05	0.00223816
Car3	3.28750824	1.3697851	27.6257341	3.88E-05	0.00223816
Coq10b	0.92029879	5.61281997	27.6106874	3.89E-05	0.00223816
Ddn	-0.715591	6.89353307	27.5372294	3.96E-05	0.0022582
Ets1	0.90407478	4.0399327	27.461986	4.02E-05	0.0022641
Anapc1	-0.6877778	6.17805781	27.4614809	4.02E-05	0.0022641
Emc1	-0.7617746	4.6600872	27.3836399	4.09E-05	0.00228121
Lmcd1	2.11976745	2.10485348	27.3553179	4.12E-05	0.00228121
Iqub	-2.2532842	1.53412581	27.3159201	4.15E-05	0.00228121
Dhrs1	0.83811115	5.70162098	27.3023291	4.16E-05	0.00228121
Hpcal1	0.9805704	4.60151312	27.0895963	4.36E-05	0.00237368
Mbp	-1.0741185	7.0503577	26.9030261	4.54E-05	0.00245641
Col24a1	-4.5729138	0.43538325	26.8321684	4.61E-05	0.00246511
Ywhaz	0.50612538	12.3202423	26.8274077	4.62E-05	0.00246511
2310022B05I	0.74920929	6.31292069	26.7908586	4.66E-05	0.00246898
Gm16523	-2.1230209	1.10422528	26.7069781	4.74E-05	0.00249889
Zc3hav1	0.86594129	5.52843251	26.5783568	4.88E-05	0.00255448
Adrbk2	-0.9717229	5.20441657	26.5057452	4.96E-05	0.00257243
Unc13c	-0.7417801	5.92405696	26.4635489	5.01E-05	0.00257243
Gramd3	1.06444401	4.51568435	26.4612128	5.01E-05	0.00257243
Snhg11	-2.0259147	9.45745744	26.4113035	5.06E-05	0.0025748
Kifc2	-0.7732603	6.32616462	26.4010556	5.08E-05	0.0025748
Dstn	0.88595201	8.25470472	26.3654382	5.12E-05	0.00257933
Lin28b	-1.5867505	3.02215074	26.2572296	5.24E-05	0.00262605
Ankrd13d	-1.3712148	3.01863563	26.2263982	5.28E-05	0.00262812
Cck	0.63491171	7.75430441	26.1578017	5.36E-05	0.00263143
Cd209c	-1.4529004	2.7033176	26.1396972	5.38E-05	0.00263143
Anp32a	0.53959635	7.60656272	26.1247037	5.40E-05	0.00263143
Zswim5	-0.8504824	4.89871036	26.1134043	5.41E-05	0.00263143
AA413626	-8.1134264	-0.6663741	25.9214487	5.65E-05	0.00271372
Glt8d1	-0.9815966	4.23810645	25.9070166	5.67E-05	0.00271372
Fam192a	0.77803944	5.25633001	25.8954399	5.68E-05	0.00271372
Ptchd4	-1.0711297	4.03406526	25.8634547	5.72E-05	0.00271372
Phlda1	0.81947935	7.72269091	25.8279602	5.77E-05	0.00271372
9330175M2(	-3.2277356	1.18172473	25.8147918	5.79E-05	0.00271372
Ccdc69	1.50569878	2.06926137	25.784623	5.82E-05	0.00271372
Tubb6	1.5117851	3.92177905	25.769785	5.84E-05	0.00271372
H1f0	0.76181914	6.9773006	25.7328535	5.89E-05	0.00271372
Enoph1	0.71206873	4.85653078	25.7200702	5.91E-05	0.00271372

Prkd1	-1.3982802	2.50520455	25.6679652	5.98E-05	0.00273055
Mall	3.55397734	0.83161331	25.4239741	6.32E-05	0.00286966
Arf4	0.73574837	8.85386897	25.3244317	6.46E-05	0.00291019
Vps28	0.76910739	5.80975147	25.3139318	6.48E-05	0.00291019
Dthd1	-5.9973973	-0.4413479	25.2084649	6.64E-05	0.00292422
Hist1h2bc	1.00737306	5.43187797	25.1815438	6.68E-05	0.00292422
Il17ra	-0.7489272	4.71235539	25.1732378	6.69E-05	0.00292422
Baiap2l1	1.04142293	3.464308	25.1549573	6.72E-05	0.00292422
Lrba	-0.8774048	5.84648866	25.0949339	6.81E-05	0.00292422
F5	-1.9457724	2.6508564	25.0651439	6.86E-05	0.00292422
Uggt2	-1.2612611	4.89630415	25.0515703	6.88E-05	0.00292422
Klhl18	-0.9633387	4.45705032	25.0484468	6.88E-05	0.00292422
Actn2	-1.4060013	2.19011377	25.0238845	6.92E-05	0.00292422
Atp5g3	0.73085065	8.20651786	25.0138514	6.94E-05	0.00292422
Jph3	-0.7863886	5.02951187	24.9951554	6.97E-05	0.00292422
Heyl	0.97639382	6.00162945	24.9945354	6.97E-05	0.00292422
Eml6	-1.146118	4.31941007	24.9924782	6.97E-05	0.00292422
Col19a1	-1.5118781	3.91312099	24.9241528	7.08E-05	0.00294822
Ttll4	-1.5082979	2.29826772	24.8987036	7.13E-05	0.00294822
Fosb	1.89538043	4.38460971	24.8907091	7.14E-05	0.00294822
A530046M15	-3.2716377	0.47076079	24.8133706	7.27E-05	0.00298511
E330023G01	-2.1845639	1.42806035	24.7829308	7.32E-05	0.00298511
Wsb1	-1.2956838	4.11688101	24.7716846	7.34E-05	0.00298511
Kbtbd8	-1.1734703	3.16545921	24.6114253	7.61E-05	0.0030824
Plekhg5	-0.8793031	4.25616956	24.5610339	7.70E-05	0.00310329
Pnpla1	-2.3064759	0.93716736	24.4617559	7.88E-05	0.00315002
Dnase1l2	-5.4309171	-0.4876255	24.4545445	7.90E-05	0.00315002
Malat1	-1.8588746	12.8518958	24.4162709	7.97E-05	0.00315078
Csnk1a1	0.4939272	7.62192174	24.4119262	7.97E-05	0.00315078
Pigm	-1.0556192	4.17321797	24.3735804	8.05E-05	0.00316379
Cap1	0.62279787	7.01686267	24.3125747	8.16E-05	0.00319339
Atp5g1	0.95184255	5.41654017	24.2927231	8.20E-05	0.00319339
Uchl1	0.73540742	6.11240315	24.1526612	8.47E-05	0.0032569
Hrh1	-1.2377313	2.61130733	24.1282238	8.52E-05	0.0032569
Gapdh	0.58426337	9.52446363	24.1214296	8.53E-05	0.0032569
Kcnh3	-0.973207	3.65536925	24.1127195	8.55E-05	0.0032569
AF357359	-1.5025464	2.62008413	24.1082129	8.56E-05	0.0032569
Ndufa11	0.95187615	3.88211188	24.0376807	8.70E-05	0.00327785
Sfr1	0.73516624	6.95960474	24.0360969	8.71E-05	0.00327785
Capzb	0.83237888	8.59633439	24.0119162	8.75E-05	0.00327785
Cdan1	-1.0532647	3.37040138	24.0026208	8.77E-05	0.00327785
Epha6	-0.9761161	4.797951	23.9391001	8.91E-05	0.00331208
F420014N23	-2.1908294	0.69867719	23.8637697	9.07E-05	0.00333841
Cyb561a3	-1.0251226	3.48963815	23.8459551	9.10E-05	0.00333841
1110059E24I	0.60982545	5.63787582	23.8399196	9.12E-05	0.00333841

1810058I24R	1.18821875	4.48426675	23.8278466	9.14E-05	0.00333841
Med12	-0.9422797	4.99139276	23.7723619	9.26E-05	0.00333841
Mtpn	0.61610124	10.0811484	23.7470663	9.32E-05	0.00333841
Atf4	0.5941986	6.67721885	23.7467744	9.32E-05	0.00333841
Trank1	-1.4740328	6.22042844	23.736753	9.34E-05	0.00333841
Gm16702	-1.0851119	4.40077098	23.7361714	9.34E-05	0.00333841
Nbas	-0.9674245	5.07413997	23.6853333	9.46E-05	0.00335982
Ufd1l	0.55075987	6.36786093	23.6725278	9.48E-05	0.00335982
Klhdc1	-0.8723027	4.04135567	23.6255674	9.59E-05	0.00338281
Ppp1r16b	-0.8471872	6.6634523	23.5728725	9.71E-05	0.00341025
Gabarapl1	0.63451458	8.09279506	23.5554655	9.75E-05	0.00341025
Pcnxl3	-0.763236	4.69194894	23.3348304	0.00010276	0.00357887
H2-T23	-0.7972272	4.24340416	23.2931435	0.00010379	0.00359665
Klra2	-4.385162	0.277556	23.2729306	0.00010429	0.00359665
Slc4a4	-0.6150324	7.86424389	23.2612033	0.00010459	0.00359665
Dcaf17	-0.8149271	4.46786314	23.1595497	0.00010716	0.00366313
Miat	-1.4115845	6.21309444	23.1498826	0.00010741	0.00366313
Gm20199	-2.2330505	2.13159792	23.0654134	0.00010961	0.00372269
Cpm	1.09573803	4.42576325	23.0432045	0.0001102	0.00372721
Ubxn6	0.63766224	5.56864892	22.978082	0.00011194	0.00377054
Psmd6	0.6584438	5.94318755	22.9002586	0.00011406	0.00382624
Tjp2	0.69737154	5.62012645	22.8774126	0.00011469	0.00383175
Stard13	0.67884146	5.39133096	22.7384457	0.0001186	0.00394663
Rgs9	-1.2165427	5.09525429	22.6576969	0.00012095	0.0039949
Lmtk2	-0.7639281	6.76082334	22.6550285	0.00012103	0.0039949
Mdm1	-1.2660079	3.14417576	22.6007101	0.00012263	0.0040069
Trappc3	0.76392662	5.00076725	22.5834389	0.00012315	0.0040069
Bora	-1.9062773	1.55862434	22.5772872	0.00012333	0.0040069
Zic4	1.09163144	6.26531762	22.5770882	0.00012334	0.0040069
Csmd3	-1.3022334	5.37477156	22.5081888	0.00012543	0.00405165
Pgbd5	0.6300911	7.53473833	22.49385	0.00012586	0.00405165
Pou5f2	-4.3837447	-0.1208998	22.4830393	0.0001262	0.00405165
Morc2a	-0.5689697	6.17560802	22.4232135	0.00012805	0.00407456
Dlg5	-0.7845216	5.22165872	22.4079654	0.00012853	0.00407456
Tpm1	0.74299974	9.356263	22.3987913	0.00012882	0.00407456
Atp2b2	-0.8767556	9.28140881	22.3963947	0.00012889	0.00407456
Cebpb	1.31966449	2.72160581	22.3355422	0.00013083	0.00409969
Cntn3	-0.8607696	5.52149284	22.3336428	0.00013089	0.00409969
Map3k15	-2.5513534	0.18468205	22.3169056	0.00013142	0.00409969
Dlst	0.5681147	6.53853897	22.3088163	0.00013168	0.00409969
Cacna1a	-0.9889978	6.4472544	22.2271478	0.00013435	0.0041503
Col11a1	-1.1450617	3.3666283	22.2125742	0.00013483	0.0041503
Tmcc2	-0.8051503	4.78003844	22.2017744	0.00013518	0.0041503
Pcdhb20	-1.3365431	3.63432425	22.1974263	0.00013533	0.0041503
Psmd12	0.54161225	6.71043969	22.1646597	0.00013642	0.00416828

Gprc5a	3.29044788	0.9928613	22.11144	0.00013822	0.004193
Clcn2	-0.9527796	3.90367884	22.0925138	0.00013886	0.004193
Phyh	0.59458043	7.28550816	22.0882684	0.00013901	0.004193
Gosr2	0.54730719	6.62556428	22.0806101	0.00013927	0.004193
Gm20752	-2.3074658	0.53927889	22.006759	0.00014183	0.00425443
Rbm34	0.63180075	5.32933223	21.9829731	0.00014267	0.00425445
Myh3	-1.3014744	2.04655776	21.9772682	0.00014287	0.00425445
Atp13a4	-1.9041004	2.28564418	21.9287205	0.00014459	0.00427412
Cd300lg	-6.4282418	-1.1975361	21.9279989	0.00014462	0.00427412
Ndufa8	0.70105042	5.2594952	21.9004325	0.0001456	0.00427412
Abca5	-0.922719	5.65973686	21.9003746	0.00014561	0.00427412
Rab7	0.69631974	10.3187668	21.8645136	0.0001469	0.00429688
Clmn	-0.6528327	5.98196232	21.7702274	0.00015038	0.00438289
Ankrd13c	0.55751067	6.39659034	21.7375505	0.0001516	0.00439118
Chchd10	0.74520553	5.60173779	21.7235872	0.00015213	0.00439118
Strn	-0.4995764	6.48418196	21.7200071	0.00015227	0.00439118
Cotl1	1.03558677	3.14023198	21.6807624	0.00015376	0.00439154
Vcl	0.72874784	6.50105909	21.6800655	0.00015379	0.00439154
Sfi1	-1.4507279	3.39486103	21.6775628	0.00015388	0.00439154
Isyna1	1.02554699	6.19626698	21.6559244	0.00015471	0.00439997
Klhl13	0.57744236	6.10968308	21.6029966	0.00015677	0.00443456
Plxnd1	-0.7858965	4.68512081	21.5859764	0.00015743	0.00443456
A330023F24I	-1.8089262	4.37251926	21.5740674	0.0001579	0.00443456
Pkd1	-0.9413311	5.17481873	21.5693395	0.00015809	0.00443456
Leo1	0.59378266	6.10134011	21.4848464	0.00016146	0.00448623
Pcdhb15	-1.4471386	2.72245539	21.4827693	0.00016154	0.00448623
A930011O12	-1.8981777	4.98274965	21.4423144	0.00016318	0.00448623
Cntn2	-1.013584	5.70335049	21.4331174	0.00016356	0.00448623
Impdh2	0.82043102	3.73965062	21.4237462	0.00016395	0.00448623
Unc80	-1.1636899	8.33898979	21.422526	0.000164	0.00448623
Thap11	0.70903455	4.58550204	21.4144055	0.00016433	0.00448623
6330415B21I	-1.2585687	3.14758581	21.4061043	0.00016467	0.00448623
Nat8l	-0.907996	6.37905119	21.39433	0.00016516	0.00448623
Notch1	-1.5083239	2.82737551	21.388794	0.00016539	0.00448623
Cyp1b1	0.97832247	7.03186445	21.3687347	0.00016622	0.00449402
Adcy2	-0.7147027	5.76768634	21.3552599	0.00016678	0.00449445
Pcna	0.73040098	6.30910801	21.3022193	0.00016902	0.0045398
Rinl	1.97653249	1.37320554	21.2425344	0.00017157	0.00459342
Hapln2	-2.1994081	1.13557707	21.2193023	0.00017258	0.00460536
Kctd21	0.94621595	3.31513064	21.176219	0.00017446	0.00464054
Fetub	7.32912949	-0.5004889	25.6255236	0.00017685	0.0046798
Cd34	-1.0971377	3.49869475	21.1143964	0.0001772	0.0046798
Snrnp25	1.63363966	2.33329262	21.1013065	0.00017779	0.0046798
Pcdhb22	-1.3091133	3.03876864	21.0918491	0.00017822	0.0046798
Nrbf2	0.68927437	4.25797767	21.0685678	0.00017927	0.00469241

Gm10220	-1.8969621	1.69414283	21.0477883	0.00018021	0.00470215
Gpr165	-1.1800138	3.639751	21.0263553	0.00018119	0.00471275
Cend1	0.5554224	7.05317087	20.9785056	0.0001834	0.00471828
Galk2	0.9627068	3.52257411	20.9612305	0.0001842	0.00471828
Adam23	-0.768752	7.15181729	20.9549242	0.0001845	0.00471828
Eya1	0.83617577	7.18135044	20.9538295	0.00018455	0.00471828
Pnrc1	0.61964761	7.15483839	20.9410502	0.00018515	0.00471828
Ndufs4	0.51436153	6.61749597	20.9380202	0.00018529	0.00471828
Camk2a	-1.03446	11.2091826	20.9325534	0.00018555	0.00471828
4933413L06F	-2.8770482	-0.1724786	20.9230455	0.000186	0.00471828
Sbds	0.62590234	6.7129318	20.8570136	0.00018914	0.00478329
Insl5	-7.3496455	-1.3287275	22.4947241	0.00019092	0.00481344
Rheb	0.66744531	7.10790637	20.7739353	0.00019318	0.00484538
Ap1s3	-1.2732854	3.67176924	20.7702147	0.00019337	0.00484538
Ccdc25	0.57611635	5.86118626	20.733145	0.0001952	0.0048765
Lima1	0.86433782	7.28145281	20.6879768	0.00019746	0.00491807
Pdzd11	0.71634057	6.13967955	20.6155688	0.00020115	0.00494905
Sprr1a	8.95882488	-0.2489927	22.2481299	0.00020173	0.00494905
Ik	0.47589057	8.56539055	20.6019679	0.00020185	0.00494905
Nicn1	0.54029906	6.46661073	20.5949819	0.00020221	0.00494905
Slc34a2	2.46634594	0.83213554	20.5853622	0.00020271	0.00494905
Mfsd4	-1.082906	7.06259956	20.5839579	0.00020278	0.00494905
Tagln3	0.70992745	6.38412678	20.581302	0.00020292	0.00494905
Gpsm1	-1.1111243	2.66469454	20.5597112	0.00020405	0.00496176
Car13	1.08858337	7.61595465	20.5379951	0.00020519	0.00497473
Irs1	0.55313356	6.08048577	20.5104754	0.00020664	0.00499522
Picalm	0.46971041	8.08333013	20.4834428	0.00020808	0.00500612
Bzw2	-0.8659407	3.56638894	20.479118	0.00020831	0.00500612
Fanci	-1.3910236	2.90436969	20.3736025	0.00021403	0.00511473
Smarce1	0.64025642	6.41595894	20.3636323	0.00021458	0.00511473
Aldh1a2	1.12835379	9.18687312	20.3526495	0.00021519	0.00511473
Med12l	-1.1428341	5.30820352	20.3504182	0.00021532	0.00511473
Mpp7	0.76654394	4.60590607	20.2984974	0.00021822	0.0051687
E030003E18I	1.22668087	2.39681955	20.2573758	0.00022054	0.00518714
Trim56	0.95944658	3.52885382	20.2571737	0.00022056	0.00518714
Slc12a6	-0.5516399	6.78989424	20.2513469	0.00022089	0.00518714
Psma7	0.63436712	6.45837861	20.2116674	0.00022316	0.00522567
Bmp1	-1.3403047	2.83587107	20.1987653	0.00022391	0.00522823
Dnajb7	-6.5031068	-1.1210456	21.7511442	0.00022566	0.00525423
Trmt61b	1.07724007	3.3221804	20.0738611	0.00023127	0.0053528
Ucma	4.69110817	-0.7750345	20.0677479	0.00023164	0.0053528
Hprt	0.53164547	7.88764741	20.0542088	0.00023246	0.0053528
Kif26b	-1.5393832	1.966585	20.0534635	0.0002325	0.0053528
Prkdc	-1.0140107	5.15821367	20.040723	0.00023327	0.0053544
Sav1	0.61531072	6.59991401	20.020827	0.00023448	0.0053544

Penk	0.84483032	7.08273198	20.0140729	0.00023489	0.0053544
1700028P14I	2.04027298	0.53112999	20.0094196	0.00023518	0.0053544
Pik3c2a	-0.5913261	6.15002745	19.9833511	0.00023678	0.00537593
Gapvd1	-0.6655101	6.04140785	19.9514755	0.00023875	0.00540335
Ktn1	-0.5670946	7.13143191	19.9426313	0.0002393	0.00540335
Dock3	-1.1978348	7.93754843	19.8810525	0.00024317	0.00547456
Fat4	-0.6316269	5.96841046	19.87138	0.00024379	0.00547456
Dlgap1	-0.8181663	8.83633306	19.8252639	0.00024674	0.00552108
Dock10	-1.1051987	5.74765679	19.800404	0.00024835	0.00552108
Tmem194b	-2.4577675	2.10193843	19.7856486	0.00024931	0.00552108
Smad1	0.57283068	5.76445627	19.7851345	0.00024934	0.00552108
DQ267100	-3.1284104	0.15390844	19.7827125	0.0002495	0.00552108
Bcan	-1.266298	4.67204944	19.7767493	0.00024989	0.00552108
Grik4	-1.7455728	1.91714354	19.7462151	0.00025189	0.00552822
Ppp4r2	0.53936966	8.3018626	19.7415477	0.0002522	0.00552822
Bmpr1b	-1.0269393	3.2590408	19.7172018	0.00025381	0.00552822
Mllt11	0.48227842	7.83720873	19.7074843	0.00025446	0.00552822
Srp54b	0.49429927	6.34362651	19.6833139	0.00025608	0.00552822
Psmb1	0.59719669	6.01550234	19.6709759	0.00025691	0.00552822
Larp7	0.61559098	5.74968368	19.6686914	0.00025706	0.00552822
Litaf	0.93848107	4.59247002	19.6632834	0.00025743	0.00552822
Pex12	0.78175097	4.06742378	19.6620266	0.00025751	0.00552822
Gem	3.30962149	2.8642039	19.6551355	0.00025798	0.00552822
Gm10389	-1.1249256	4.2490491	19.6467402	0.00025855	0.00552822
Celsr3	-1.8511623	4.73290508	19.636142	0.00025927	0.00552822
Map1a	-1.1099705	10.3099317	19.6337662	0.00025943	0.00552822
Tpi1	0.69896879	6.96436296	19.6208903	0.00026031	0.00552822
Fgf1	0.6931933	7.40894278	19.61745	0.00026054	0.00552822
Gm12070	0.60813131	10.3711141	19.6111844	0.00026097	0.00552822
Lrp1b	-1.2181198	5.97905963	19.5685705	0.00026391	0.00557614
Aen	0.89877545	4.24432563	19.5139125	0.00026774	0.00563054
Herc2	-1.0361917	7.52920546	19.5122111	0.00026786	0.00563054
Folh1	-1.7890897	1.91520935	19.4920829	0.00026928	0.00563488
Tmem100	0.84560388	3.5969053	19.4899328	0.00026943	0.00563488
Tuba1c	0.50906776	5.81028806	19.4735236	0.0002706	0.00564495
Ehd3	0.56347548	7.70821092	19.3745681	0.00027777	0.00577975
Taf10	0.90069307	4.15935946	19.3638423	0.00027856	0.00578153
Galnt7	-0.9720778	3.06937041	19.3436068	0.00028005	0.00579066
Glt28d2	1.34165545	3.29985925	19.3325406	0.00028088	0.00579066
Erdr1	-1.2070727	3.40101231	19.3214138	0.0002817	0.00579066
Cxx1b	0.78191723	5.87807493	19.3199136	0.00028182	0.00579066
Hfm1	-2.2496536	1.86536128	19.3034927	0.00028304	0.00579218
B4galnt2	-1.5824292	3.0710878	19.2883312	0.00028418	0.00579218
Myl4	1.76771338	2.24549213	19.2804704	0.00028478	0.00579218
Mirg	-3.0935292	1.06503662	19.2748044	0.00028521	0.00579218

Syne1	-1.1272542	8.5747803	19.2720535	0.00028541	0.00579218
Galnt16	-0.9149283	4.41989815	19.2198143	0.0002894	0.00582732
Exph5	-1.1315345	6.36395724	19.2145636	0.0002898	0.00582732
Gadd45a	0.98668099	4.16244323	19.2142131	0.00028983	0.00582732
4930579G24	-1.1423568	2.52831405	19.205423	0.00029051	0.00582732
Cox5a	0.60311921	6.35385492	19.2030516	0.00029069	0.00582732
Ubr1	-0.8085569	6.15641405	19.1879139	0.00029186	0.00583388
2310015B20	2.07768166	1.96852753	19.180511	0.00029244	0.00583388
Nup205	-0.9371664	3.99104559	19.1576145	0.00029422	0.00584969
Acsl6	-0.7185153	6.0861071	19.1462078	0.00029512	0.00584969
Lhfpl3	-0.9228633	3.85531894	19.1430627	0.00029536	0.00584969
Med16	-0.6650413	5.29758617	19.114167	0.00029765	0.0058807
Tiparp	0.59863256	4.91605335	19.0900906	0.00029956	0.00590435
Utp20	-1.0208252	4.51937359	19.0687683	0.00030127	0.00592381
Lyst	-0.9664158	6.33483125	19.0497968	0.0003028	0.00593966
Gdap10	-1.4584999	2.40450832	19.0132203	0.00030577	0.00598366
Ccdc134	-1.6919928	0.62097452	19.0026976	0.00030663	0.00598626
Gfap	0.94266738	4.21164272	18.984743	0.0003081	0.00598774
Gm16675	6.57680953	-1.3070885	18.9775036	0.0003087	0.00598774
Simc1	-0.7064212	4.18232201	18.9747459	0.00030893	0.00598774
Frem2	-1.5206147	1.81353757	18.9649434	0.00030974	0.00598774
Med31	0.97737793	3.37068714	18.9576127	0.00031035	0.00598774
Glt25d1	-0.8042497	3.99311375	18.9284589	0.00031278	0.00602051
Bmp2	1.01028807	4.62517624	18.898379	0.00031531	0.00604186
Fam19a3	7.90354952	-0.4615225	20.3071739	0.00031536	0.00604186
Gm14378	-3.2643075	-0.2341426	18.881454	0.00031674	0.00605427
Akap8	-0.826443	5.82462808	18.8719938	0.00031755	0.00605557
Ogt	-0.9331911	7.68691781	18.8632488	0.00031829	0.00605574
Zbtb16	-0.588631	4.67160739	18.8482671	0.00031958	0.00606608
0610010K14	1.01964007	3.74429142	18.8223185	0.00032181	0.0060929
Pdcd10	0.63765709	6.07404911	18.8081808	0.00032303	0.0060929
Intu	-0.9116608	4.51360505	18.7997701	0.00032376	0.0060929
Aurkb	5.90775746	-1.0478296	18.795646	0.00032412	0.0060929
Thap1	1.12943251	3.74336405	18.7890976	0.00032469	0.0060929
Kctd10	0.85438784	4.71801975	18.7763231	0.00032581	0.00609805
Panx2	-0.9783954	4.05650636	18.769005	0.00032645	0.00609805
Gabpa	0.57288385	6.71949932	18.7551576	0.00032767	0.00610693
Serpinc1	-2.908917	0.12159231	18.7458207	0.0003285	0.00610844
Nnat	1.38968701	8.65677086	18.718468	0.00033092	0.0061397
Ascl1	-1.1025649	3.37908203	18.6961624	0.00033292	0.0061628
Tenm2	-1.0671151	7.00559318	18.682532	0.00033414	0.00617158
Gatad2b	0.46114625	7.58803378	18.6660123	0.00033564	0.00618523
Neurl1b	-1.0525715	4.89012946	18.6427779	0.00033775	0.00619695
Clta	0.65020464	6.84018885	18.6403283	0.00033797	0.00619695
Creb3l1	1.02607747	5.25308453	18.6341579	0.00033853	0.00619695

Psmb5	0.73856534	5.10193767	18.5669193	0.00034474	0.00627647
9830147E19I	-1.3407233	1.87966411	18.5558138	0.00034578	0.00627647
Dnajb5	0.7759582	5.88746748	18.5552687	0.00034583	0.00627647
2410089E03I	-1.2402887	5.88888922	18.5470414	0.0003466	0.00627647
Clic4	0.91385045	8.64253682	18.5460105	0.0003467	0.00627647
Gm14295	0.47451132	5.87168386	18.5232868	0.00034884	0.00629639
Usp24	-0.7026992	7.4004889	18.518072	0.00034933	0.00629639
4932438A13I	-1.0850143	7.50608328	18.4987839	0.00035116	0.00631553
Coa4	1.51780419	2.07474742	18.4824356	0.00035272	0.00632973
Eno3	1.18797603	3.21469551	18.4400451	0.0003568	0.00638899
Lcmt1	0.74222275	4.85914656	18.3866487	0.00036201	0.00645697
Coro7	-0.9210941	4.16065774	18.38508	0.00036217	0.00645697
Ampd3	0.66126843	5.59985315	18.3676465	0.00036389	0.00646054
Ddx26b	-1.1875555	4.50924521	18.339184	0.00036672	0.00646054
Dock5	0.56907019	6.84778138	18.334948	0.00036714	0.00646054
Mdh1	0.47781406	8.81871868	18.3324478	0.00036739	0.00646054
Atp7a	-0.6921817	5.55625978	18.3308699	0.00036755	0.00646054
A330032B11	-1.2736583	2.15244353	18.3291211	0.00036772	0.00646054
Rhobtb2	-0.5427207	5.37579515	18.3276583	0.00036787	0.00646054
Vegfa	-0.6652747	5.38698117	18.2959781	0.00037106	0.00648185
Blnk	1.08237345	3.30553498	18.2926025	0.0003714	0.00648185
Mrpl16	0.62648926	5.6404914	18.2905384	0.00037161	0.00648185
Pcdhb16	-0.9212141	4.67358307	18.2779931	0.00037288	0.00648185
Rbbp5	-0.5531588	5.69643705	18.2765795	0.00037302	0.00648185
Ran	0.47104311	7.71307405	18.2416204	0.0003766	0.00653016
Pvrl3	-0.674593	5.40676394	18.224621	0.00037835	0.00654673
Sde2	0.65006904	4.81445549	18.1961449	0.00038131	0.00657287
Zgrf1	-1.4651331	2.5509492	18.1945091	0.00038148	0.00657287
Gm4371	8.46120634	-0.7745846	18.1869991	0.00038226	0.00657287
Srp68	0.60411654	5.94571397	18.1711243	0.00038392	0.00658769
Pcdh11x	-0.9793817	4.67107763	18.1598785	0.00038511	0.00659422
Ttc17	-0.7281125	4.84486367	18.1417283	0.00038702	0.00661328
Ahi1	-0.8261286	6.88549225	18.1222288	0.0003891	0.00662364
Isy1	0.78694666	4.96121102	18.1208566	0.00038924	0.00662364
Comt	0.57856672	5.68667028	18.0900953	0.00039254	0.00666591
Plcg1	-0.7312201	4.07223723	18.0788357	0.00039375	0.00667274
Psmd4	0.80516963	5.3258604	18.0632635	0.00039544	0.00667686
Sdhb	0.51205848	5.60717895	18.0615866	0.00039562	0.00667686
Grik2	-1.0022011	5.18302285	18.0243343	0.00039969	0.00673167
Brip1	-1.1992984	3.21142349	18.0049057	0.00040183	0.00675386
Atg3	0.50504513	6.65030074	17.98816	0.00040368	0.00677117
Dynll1	0.63416972	8.0929737	17.9794795	0.00040464	0.00677353
Atp5c1	0.59722333	8.18712895	17.9714419	0.00040554	0.00677473
A330021E22I	-0.8809732	4.08752451	17.9302994	0.00041016	0.00683799
Rap1gds1	0.42104517	8.5849631	17.8974461	0.00041389	0.0068862

Adam10	-0.593878	5.75542415	17.8836303	0.00041547	0.00689852
Akap11	-0.8590644	8.45941084	17.842541	0.00042021	0.00691621
Ptprd	-0.7608685	8.31428597	17.838474	0.00042068	0.00691621
Il31ra	1.3239565	2.40872718	17.8365247	0.0004209	0.00691621
Adamts16	-2.7698049	0.94222066	17.8340603	0.00042119	0.00691621
Acot7	0.59921091	6.14942926	17.8308315	0.00042157	0.00691621
AI450353	-1.8936572	2.02107079	17.830698	0.00042158	0.00691621
Vim	0.89549366	8.07016048	17.822428	0.00042255	0.00691705
Ccp110	-0.6741331	5.8898063	17.8089186	0.00042413	0.00691705
Foxf2	1.20186628	2.76111386	17.8086489	0.00042416	0.00691705
Ebf3	-1.4658999	1.46044412	17.7782908	0.00042773	0.00694978
Zc3h7b	-0.6142781	6.02032441	17.7772568	0.00042786	0.00694978
Anp32e	0.57137842	8.05480532	17.7676327	0.000429	0.00695309
Cadps2	-0.7502792	6.41826898	17.7568957	0.00043027	0.00695309
Paxbp1	-0.8981164	5.67637254	17.7479294	0.00043134	0.00695309
Kmt2a	-0.8013685	8.41968347	17.7405175	0.00043223	0.00695309
Nrk	1.89867123	1.47613572	17.7338129	0.00043303	0.00695309
A230073K19	-1.8402557	4.54489843	17.7329526	0.00043314	0.00695309
Fbxo22	0.52180528	6.86914648	17.7142462	0.00043539	0.00697559
Tnrc18	-0.5459709	5.12993566	17.6750037	0.00044015	0.00702366
Kcnma1	-0.8403124	7.25180016	17.6742188	0.00044025	0.00702366
Gm5523	0.64438437	5.24483222	17.6684607	0.00044095	0.00702366
Lyrm7	0.63943999	4.50608836	17.6321014	0.00044542	0.00706268
Zfp563	-0.7285179	4.894565	17.6266893	0.0004461	0.00706268
Thbs1	-1.3516386	2.76445923	17.6259647	0.00044619	0.00706268
Unc45a	1.27648022	2.75728444	17.6207018	0.00044684	0.00706268
Trappc9	-0.8691481	5.74787062	17.6024583	0.00044911	0.00708281
Man2a2	-0.5999938	6.90128601	17.5949011	0.00045005	0.00708281
1500015O10	1.6609727	5.14687595	17.5897762	0.0004507	0.00708281
A630089N07	-1.5153755	8.10768397	17.5649599	0.00045382	0.00711827
Kcnip3	0.45093698	7.64498816	17.5403704	0.00045694	0.00715353
Bzw1	0.74551796	8.32615048	17.5054702	0.0004614	0.00720971
Fam199x	-0.6900077	4.77628233	17.49349	0.00046295	0.00722011
Sugp2	-1.1728798	3.99885619	17.4814497	0.0004645	0.0072283
Ferd3l	6.88649985	-1.5862539	18.701557	0.00046523	0.0072283
Psmd13	0.880687	3.96068932	17.4597515	0.00046733	0.00724716
Igf2r	-0.6863459	4.7242819	17.4450519	0.00046925	0.00726326
Dynlt3	0.63674776	9.11236474	17.4331126	0.00047082	0.00727383
Prlr	-1.0462715	2.37048023	17.4060952	0.00047439	0.00731522
Vmn2r86	-2.5180843	0.46302815	17.377319	0.00047822	0.00735204
Ddx18	0.58690525	5.05537199	17.3747619	0.00047856	0.00735204
Ncald	0.51237069	7.7043195	17.3562695	0.00048105	0.00737475
Kif15	-2.2070139	1.06210072	17.3504236	0.00048184	0.00737475
Mtx2	0.48669253	5.6916001	17.3387867	0.00048341	0.00737801
Txndc17	0.72762941	6.32172748	17.3315602	0.00048439	0.00737801

Sugt1	0.62914919	5.59679068	17.3289615	0.00048474	0.00737801
Slc29a1	0.9549509	2.59628195	17.3211894	0.0004858	0.00738043
Aldh7a1	0.58053875	4.98129847	17.3122823	0.00048701	0.00738524
Atp6v1b2	0.53869024	8.40068724	17.3018633	0.00048844	0.00739321
Adamts3	-0.9431456	4.12886583	17.2696862	0.00049287	0.00740694
2310047M10	1.22774511	2.67892349	17.267003	0.00049324	0.00740694
Gnas	0.50451279	10.8121039	17.2623542	0.00049388	0.00740694
Tanc2	-0.9045524	8.5132507	17.2610891	0.00049406	0.00740694
Snx9	0.77552008	4.81473648	17.2561157	0.00049475	0.00740694
Hp1bp3	0.41042295	8.1320659	17.241794	0.00049675	0.00740694
Slc2a4rg-ps	-1.8600179	1.80381614	17.2364661	0.00049749	0.00740694
Scn10a	-7.4600717	-1.6089936	18.4218854	0.00049879	0.00740694
Rbms1	0.61217684	6.26706979	17.2221246	0.0004995	0.00740694
Ube2a	0.69458084	6.34368022	17.2215963	0.00049957	0.00740694
Arhgap29	0.8762881	9.98480511	17.2153078	0.00050046	0.00740694
Slc38a6	-1.3347137	3.17223248	17.2147819	0.00050053	0.00740694
1110004E09I	0.85459371	5.77998357	17.2110209	0.00050106	0.00740694
Fancb	-2.9101837	0.97425989	17.2006789	0.00050252	0.00741518
9330182L06F	-0.803583	5.05696034	17.1672473	0.00050727	0.00745603
Fam160b2	-0.5813916	6.58432787	17.1661269	0.00050743	0.00745603
Mrps6	0.92011436	4.25592822	17.1573083	0.00050869	0.00745603
Ndrg2	0.68986623	8.89316175	17.1557487	0.00050892	0.00745603
Zfp704	-0.5892582	6.73180824	17.1345	0.00051197	0.00748746
Ifitm1	1.09998908	5.15849788	17.125714	0.00051324	0.00749269
Pitpna	0.41141075	8.72835352	17.1001022	0.00051696	0.00751788
Lyg1	5.35525329	-1.5297551	17.0990476	0.00051712	0.00751788
Rnf181	0.713768	6.26670878	17.0949784	0.00051771	0.00751788
Tfcp2l1	1.00092315	7.02929852	17.0494215	0.00052442	0.00760179
Appl2	-0.5527942	6.30559206	17.0355084	0.00052648	0.00761299
Slc25a17	0.75094339	5.69498941	17.0317588	0.00052704	0.00761299
Unc79	-1.1235887	5.96216454	17.0080749	0.00053058	0.00764805
5031410I06R	-1.7603315	2.61793418	17.0031079	0.00053133	0.00764805
Ltv1	0.63911784	4.79175737	16.9714443	0.00053611	0.00770343
Ndufb6	0.76426022	5.63196151	16.9410422	0.00054075	0.00774449
Gm5464	-2.4949858	0.45420489	16.9403677	0.00054086	0.00774449
Trpm7	-0.5773557	6.58706459	16.9084878	0.00054577	0.00779051
Mansc4	1.76643046	1.99202878	16.9072169	0.00054597	0.00779051
Ifrd1	0.67950946	5.97236501	16.8894846	0.00054872	0.00780353
4932418E24I	-2.7833893	-0.0372914	16.8845413	0.00054949	0.00780353
Mrfap1	0.74696498	9.51155094	16.8805947	0.00055011	0.00780353
Il1rapl1	-0.8319977	3.91836958	16.874795	0.00055101	0.00780353
Snip1	1.07240034	3.26961063	16.8708965	0.00055163	0.00780353
Zfp488	-1.2638286	7.72018288	16.8594389	0.00055342	0.00780787
Ugt8a	-0.7458472	5.3525216	16.8568444	0.00055383	0.00780787
Plekha5	-0.6723862	5.42071835	16.8342026	0.00055741	0.00784057

Ptprk	-0.5266955	6.4903299	16.8264964	0.00055863	0.00784057
Gm16982	-3.435831	0.00829823	16.8216695	0.0005594	0.00784057
Pde1a	0.40835851	9.4446545	16.8181047	0.00055997	0.00784057
Rmnd5b	0.56645552	4.74085418	16.8047919	0.00056209	0.0078438
Chrd	-1.2109223	2.10372602	16.8047066	0.00056211	0.0078438
Ifi27	0.95306413	4.907542	16.7938475	0.00056385	0.00785475
Cxxc5	0.60105033	6.44888195	16.782547	0.00056566	0.00786674
Atp5b	0.43507778	10.5802722	16.7672367	0.00056814	0.00788778
Gm19466	-3.1778786	-0.2042499	16.7575216	0.00056971	0.00789631
AF357425	-2.3000212	3.01202693	16.7466023	0.00057149	0.00789715
Clasrp	-1.152687	2.54147042	16.7453403	0.0005717	0.00789715
Tspan2	-0.6820188	6.3946554	16.722246	0.00057547	0.0079294
1110037F02I	-0.6298369	5.5306821	16.7082838	0.00057777	0.0079294
Paics	0.6969231	8.66683901	16.7078395	0.00057784	0.0079294
Rapgefl1	-0.5732589	6.31641623	16.6947274	0.00058001	0.0079294
Cntnap1	-0.7399775	5.86954492	16.6861483	0.00058144	0.0079294
Snx13	-0.4154413	6.88848207	16.6840057	0.00058179	0.0079294
Fndc1	-1.5126652	1.61004126	16.679419	0.00058255	0.0079294
Zfp445	-0.667523	6.82886788	16.6754389	0.00058322	0.0079294
Hmox1	1.15350329	2.83955747	16.6743631	0.0005834	0.0079294
Ctcfl	-1.4286522	3.10142924	16.6726787	0.00058368	0.0079294
Gm21671	-1.9109512	0.9986675	16.6474026	0.00058791	0.00797373
Ppp2r2cos	-2.5462122	0.74501721	16.6401148	0.00058914	0.00797721
Slc22a2	1.29659435	4.58244843	16.6243453	0.0005918	0.0080001
Dnajc11	0.81255488	3.98636206	16.6127731	0.00059377	0.00801347
Haus2	0.56328871	5.4932092	16.5881595	0.00059797	0.00805693
Ext2	1.3664192	5.05571587	16.578328	0.00059965	0.00806164
Cfl1	0.51106781	8.59102715	16.5746981	0.00060028	0.00806164
Arhgef28	-0.7306703	4.27869738	16.5601749	0.00060278	0.00808206
Ppp2r1a	0.43625389	7.25493951	16.5391691	0.00060642	0.0081112
Otof	-1.7351942	2.91644019	16.536276	0.00060693	0.0081112
Rpap3	0.58209083	4.49697845	16.5277495	0.00060841	0.00811787
Mdn1	-1.5167286	6.51506517	16.5153261	0.00061059	0.0081228
Slbp	0.57130983	5.38558167	16.5075906	0.00061195	0.0081228
Inip	0.68953269	5.15802191	16.5050082	0.0006124	0.0081228
Lrrtm4	-0.7006593	5.30938419	16.5030917	0.00061274	0.0081228
Ube2j1	0.5634081	7.45386094	16.4696476	0.00061866	0.00818805
Fgf7	1.56985135	1.59761899	16.4550876	0.00062125	0.00820919
1700008O03	-1.1089822	2.94146089	16.4370638	0.00062448	0.00823863
Adcy1	-0.8234036	8.75656607	16.4284349	0.00062603	0.00824588
Osbpl6	-0.5591041	7.06424395	16.4156284	0.00062835	0.00826056
Lemd3	-0.6765751	4.50277454	16.4111455	0.00062916	0.00826056
Rilpl2	1.1301477	3.19208681	16.4046108	0.00063035	0.00826292
Zfp28	-1.1405585	2.80136131	16.3929877	0.00063246	0.00827744
Pes1	0.74981819	4.47611042	16.3705134	0.00063657	0.0082934

Vasn	-0.7239459	3.78869828	16.3700196	0.00063666	0.0082934
Ercc4	-0.8260217	3.83463509	16.3697789	0.00063671	0.0082934
Ccer1	-7.2239289	-2.3162818	19.1554905	0.00064192	0.00834806
Sertad1	1.27446008	2.92452952	16.3317629	0.00064373	0.00835839
Dcun1d1	0.45629551	6.8076592	16.3156789	0.00064673	0.00838406
Rfx1	-1.1261696	3.37654182	16.2967113	0.00065028	0.00841322
Ezr	0.54986756	6.23592406	16.2898441	0.00065157	0.00841322
Slc12a2	-0.5602939	6.5705165	16.2873304	0.00065205	0.00841322
Parp2	0.58430399	5.19107671	16.2725418	0.00065484	0.00843603
Dync2h1	-1.2639852	6.15787971	16.2333366	0.00066231	0.00850347
Eif3k	0.85051126	4.33275932	16.2329196	0.00066239	0.00850347
Atp5f1	0.47136059	7.46224354	16.2248329	0.00066395	0.00850347
Nckap5	-1.0904477	3.2134606	16.223444	0.00066421	0.00850347
Ept1	-0.5360364	4.98612583	16.2055923	0.00066766	0.00852542
Eif1b	0.57895931	5.81897077	16.2038136	0.000668	0.00852542
Stmn1	0.537529	8.45410907	16.1810408	0.00067243	0.0085686
Depdc5	-0.8052633	5.25652569	16.1618153	0.00067619	0.00859566
5430417L22I	-0.4825806	6.02537351	16.154917	0.00067755	0.00859566
Ccdc142	-2.9210098	-0.4755545	16.1541843	0.00067769	0.00859566
Pgap1	-0.778872	5.42461788	16.1388497	0.00068071	0.00861267
Orc1	-1.610011	1.46819534	16.1367627	0.00068113	0.00861267
Chit1	6.76079635	-1.5464265	17.1734458	0.00068576	0.0086579
Gm12359	-2.8813158	0.15565247	16.0832434	0.00069181	0.00871679
Mnt	-0.6079603	5.01566522	16.0795885	0.00069254	0.00871679
4930473A02I	-2.5031061	0.53604873	16.0733282	0.00069381	0.00871933
Kcnn2	-0.7849356	3.8382123	16.0619502	0.00069611	0.00872189
Dopey2	-0.8225593	5.69919646	16.0610056	0.0006963	0.00872189
Tmem206	-0.8802704	4.40141597	16.0547695	0.00069756	0.00872189
Snx4	0.51454982	6.79050908	16.0504949	0.00069843	0.00872189
Ep400	-0.7529655	6.99752642	16.0384443	0.00070089	0.00872189
Fut8	-0.8070711	6.99074698	16.0320882	0.00070219	0.00872189
Firre	-1.4540075	3.39928148	16.0319017	0.00070223	0.00872189
Dlat	0.49938674	8.21845619	16.0260799	0.00070342	0.00872189
A130077B15	-1.2342791	7.65489008	16.0253906	0.00070356	0.00872189
Trerf1	-0.8211613	5.40018908	16.0184639	0.00070498	0.00872636
Ccpg1os	0.9546497	2.81273087	16.0046873	0.00070782	0.00874699
Commd6	0.85966173	5.30398125	15.9960988	0.00070959	0.00874699
Cd84	-1.2946033	2.46459589	15.9949072	0.00070984	0.00874699
Hsd17b10	0.75698042	4.46645797	15.9746139	0.00071406	0.0087732
Midn	0.67678328	5.84324131	15.974395	0.0007141	0.0087732
Hnf1b	-5.5580245	-0.7988781	15.9566292	0.00071782	0.00880566
Ccdc91	0.65670155	5.37046407	15.9436213	0.00072055	0.00882114
Cops4	0.51101959	6.02394846	15.9404143	0.00072123	0.00882114
Swsap1	0.90392179	3.36427758	15.9296576	0.0007235	0.00883577
Tmem251	0.83314242	3.7918645	15.9245654	0.00072457	0.0088358

Cxx1c	0.53576555	5.91556429	15.9132522	0.00072698	0.00884297
Speg	-0.664883	5.58177323	15.9116604	0.00072731	0.00884297
Ndufa6	0.60651256	5.13130613	15.9051822	0.00072869	0.00884665
Atp5o	0.75970391	6.67836527	15.8921178	0.00073148	0.00886744
Tle1	-0.606743	5.53266061	15.8827482	0.00073349	0.0088709
Ctdnep1	0.64648155	5.82205095	15.8793404	0.00073422	0.0088709
Ppia	0.6745255	9.86341578	15.8757	0.00073501	0.0088709
Ift27	1.10554636	2.67768703	15.8579369	0.00073884	0.0089041
Gbp10	0.83831804	3.82201888	15.850107	0.00074054	0.00891148
A230046K03I	-0.7591243	6.37811693	15.7954343	0.00075251	0.00899376
Prpf19	0.53378589	7.10692766	15.7942841	0.00075276	0.00899376
Aldh2	0.85651913	5.30454069	15.7857353	0.00075465	0.00899376
Dph5	-0.8106851	4.65440441	15.7814925	0.00075559	0.00899376
Vsnl1	0.58040718	12.0201766	15.7751792	0.000757	0.00899376
Lgals3bp	0.99946064	4.89866269	15.7725594	0.00075758	0.00899376
E330020D12I	-1.2671336	3.84757115	15.7712525	0.00075787	0.00899376
Rab3gap1	-0.7141293	4.95786349	15.7651132	0.00075924	0.00899376
Las1l	-0.7179335	4.97411249	15.7557209	0.00076134	0.00899376
Ywhab	0.40995257	10.3409979	15.7542692	0.00076166	0.00899376
Tpd52l1	0.83578649	5.00453111	15.7465612	0.00076339	0.00899376
Dpysl2	-0.5424646	8.72760806	15.7404869	0.00076475	0.00899376
1810011O10	1.39513414	4.30148064	15.7395815	0.00076496	0.00899376
Thbd	1.17339071	8.3227459	15.7350862	0.00076597	0.00899376
Cct6a	0.46307592	8.24498773	15.735012	0.00076599	0.00899376
Glrx	0.67031204	6.11625311	15.7350026	0.00076599	0.00899376
Hscb	1.13277553	4.15092299	15.7330653	0.00076642	0.00899376
Arl6	0.61223056	5.70105526	15.7266765	0.00076787	0.00899376
Hydin	-2.6129139	0.40792578	15.7253453	0.00076817	0.00899376
Rlf	-0.6389156	5.84215649	15.7134454	0.00077086	0.00900646
Por	0.67048546	5.52808753	15.7037861	0.00077306	0.00900646
Exoc4	-0.524216	5.87037846	15.7017747	0.00077351	0.00900646
Pea15a	0.54495192	7.66623059	15.7012437	0.00077363	0.00900646
Ercc1	1.27068076	2.21367991	15.6648506	0.00078197	0.00908526
Ipw	-1.7568841	4.05705094	15.6618098	0.00078267	0.00908526
Chmp4b	0.46264829	6.43768416	15.6533138	0.00078464	0.00908526
Ankrd44	-0.7585829	5.2431102	15.6524945	0.00078483	0.00908526
Ppp2r3a	-0.5662543	6.51542	15.6393088	0.00078788	0.00910783
Abcg1	-0.7503665	4.94718893	15.6127638	0.00079408	0.00915825
2210016L21I	0.58640668	6.25796474	15.6045519	0.00079601	0.00915825
Ttc14	-1.1588995	6.58514574	15.6018683	0.00079664	0.00915825
Tyms	-0.893903	3.66644618	15.5986858	0.00079739	0.00915825
1700110K17I	-2.57098	0.8249802	15.5929549	0.00079874	0.00915825
Sorbs2os	-1.4209729	3.15431538	15.5866015	0.00080024	0.00915825
Asgr1	1.37142215	6.16241364	15.5813242	0.00080149	0.00915825
Mme	-0.7895213	4.26111758	15.5775007	0.00080239	0.00915825

Ensa	0.5250632	8.89617653	15.5694166	0.00080431	0.00915825
Arhgef12	0.39827465	9.52684002	15.5662174	0.00080507	0.00915825
Ranbp17	-0.9491857	3.12490601	15.5654154	0.00080526	0.00915825
Smarcb1	0.67622895	4.78304177	15.5635273	0.00080571	0.00915825
Ubr4	-0.9020283	7.17668192	15.5592596	0.00080673	0.00915825
Aatk	-1.0933596	5.14712158	15.549663	0.00080902	0.00916299
Zfp961	0.5393071	5.09700489	15.5481822	0.00080938	0.00916299
Bud31	0.66916551	4.69115838	15.5323629	0.00081317	0.00918555
Pir	0.75465431	3.46972287	15.5305724	0.00081361	0.00918555
5031426D15I	-1.7351233	3.58407533	15.5187975	0.00081645	0.00920498
Plekhh1	-0.8849436	4.58905436	15.510301	0.0008185	0.00921552
Heatr5b	-0.7725111	5.48811715	15.5011323	0.00082073	0.00922794
AI593442	-0.5460223	8.70047291	15.4868573	0.00082421	0.00924524
Gm16907	-2.4333332	1.60754631	15.4795317	0.000826	0.00924524
Psmb7	0.61208655	6.85311655	15.4716828	0.00082792	0.00924524
Slc25a25	0.67126713	5.0207656	15.4684007	0.00082873	0.00924524
Ppif	0.82805036	4.94539068	15.4677039	0.0008289	0.00924524
Pbld1	-1.0638004	3.46365228	15.4672328	0.00082902	0.00924524
Nsl1	-1.1765326	4.58000397	15.4626008	0.00083016	0.0092454
Paip2	0.517404	7.53393563	15.4360828	0.00083671	0.00929078
Sez6l	-0.6035799	6.13618277	15.4349078	0.000837	0.00929078
Ankrd11	-0.4428607	8.84875745	15.4324284	0.00083762	0.00929078
Usp25	0.46983979	7.47059909	15.4167665	0.00084153	0.00931963
9330102E08I	-0.8857447	4.04629306	15.4117731	0.00084277	0.00931963
Xpo4	-0.7621455	4.12366709	15.4045589	0.00084458	0.00931963
Olfr613	-1.2693389	5.43212935	15.4038619	0.00084476	0.00931963
Hnrnpab	0.73987569	7.5806322	15.3824215	0.00085016	0.00936664
Stmn2	0.41794422	7.85579106	15.369973	0.00085331	0.00937639
Rbp1	1.24678845	7.75562961	15.3699184	0.00085332	0.00937639
Rap2a	0.39916227	7.59364331	15.3531522	0.00085759	0.00940834
Ncapd3	-0.8953499	4.27461115	15.3495129	0.00085852	0.00940834
Zfp759	-0.7772543	3.76817556	15.3130782	0.00086789	0.00949831
Siah2	0.71734407	4.21540597	15.2963742	0.00087222	0.00952179
Fry	-1.0258983	8.22700154	15.2956117	0.00087242	0.00952179
Cspp1	-0.737208	4.72589044	15.2914216	0.00087351	0.00952179
Gm3002	-1.3317928	3.60229734	15.2706951	0.00087893	0.00955999
Ube4a	-0.5927852	5.74196263	15.2691196	0.00087934	0.00955999
Fam160a1	0.78561525	4.32997888	15.2414358	0.00088664	0.00962277
Larp1b	0.83155835	4.07434066	15.2299087	0.00088969	0.00962277
0610012G03	0.92343078	3.83728121	15.2274227	0.00089035	0.00962277
Pdzd2	-0.6900928	6.18780981	15.2268482	0.00089051	0.00962277
Spata32	-3.4115192	-0.0808031	15.2251204	0.00089097	0.00962277
Pcdhb6	-2.6101432	0.85978822	15.2175973	0.00089297	0.00963177
Mmp17	0.72884557	5.60588234	15.2009888	0.00089742	0.00966703
Cxcr2	-0.9053639	4.02308333	15.1797278	0.00090314	0.00971011

Plk5	-2.2182692	1.09162456	15.1773736	0.00090378	0.00971011
Amy1	-0.6858065	4.31067599	15.1545609	0.00090997	0.00976389
Tsc22d1	0.59250411	9.67766436	15.1499967	0.00091122	0.00976449
Plxna2	-0.487519	7.727418	15.1432039	0.00091307	0.00976741
11-Sep	0.45154016	7.5978951	15.1403081	0.00091387	0.00976741
Cyfip2	-0.705674	9.89947236	15.0922067	0.00092714	0.0098322
Stxbp2	-0.9956695	4.19154777	15.0919396	0.00092722	0.0098322
Trnp1	0.54932502	6.17704548	15.0906193	0.00092758	0.0098322
Ptpn13	0.56639452	6.26099968	15.0872031	0.00092854	0.0098322
Bai1	-0.7262738	6.13424862	15.0826986	0.00092979	0.0098322
Nfrkb	-0.6677405	4.24815793	15.079812	0.0009306	0.0098322
Sh3bgr	5.77272995	-0.4642722	15.0755976	0.00093178	0.0098322
Ift20	0.62407317	6.78178911	15.0746249	0.00093205	0.0098322
Pcgf3	0.52535814	5.67129604	15.0705342	0.00093319	0.0098322
Cttnbp2	-0.860054	6.72279741	15.0695328	0.00093347	0.0098322
Pcyt1a	0.59408524	5.75957855	15.0680207	0.0009339	0.0098322
Mir181b-2	-5.5899125	-1.1861624	15.0648198	0.0009348	0.0098322
Myo6	-0.7028135	6.39830014	15.0623835	0.00093548	0.0098322
Tmem181b-ı	-1.1601947	5.11164669	15.046837	0.00093986	0.00984823
A730017L22I	-1.0454776	4.89894303	15.0426684	0.00094104	0.00984823
Hecw2	-0.8526378	6.36028967	15.0395298	0.00094193	0.00984823
Nelfe	0.94624547	3.95887873	15.0394977	0.00094193	0.00984823
Chchd3	0.63998989	5.11662928	15.0357514	0.000943	0.00984823
Mpv17	0.66244416	5.95198813	15.0126212	0.00094958	0.00988682
Gm5531	-1.1997953	2.74342724	15.0114147	0.00094992	0.00988682
Dnah6	-2.6313736	0.7087383	15.0100959	0.0009503	0.00988682
Rab5c	0.53704288	6.19290957	15.0021059	0.00095259	0.00989448
Nron	-3.0397774	0.25872648	14.9991191	0.00095344	0.00989448
Dnajc28	0.66652718	3.87108935	14.9884136	0.00095652	0.0099139
Gm11549	0.53305781	6.88483758	14.9726131	0.00096108	0.00994019
Eif1ax	0.74846708	6.23929247	14.9702121	0.00096178	0.00994019
Ndufb10	0.6398698	5.91536809	14.967079	0.00096269	0.00994019
Smg1	-0.8966646	7.69714299	14.9598133	0.0009648	0.00994948
Ier5	0.60698593	6.19845767	14.9548718	0.00096623	0.00995182
Serpinf1	1.14028675	5.60135265	14.9399377	0.00097059	0.00998421
Fam92a	0.52199453	6.562983	14.9317693	0.00097299	0.00999526
Herc1	-1.1350073	8.10533263	14.9276599	0.00097419	0.00999526
2610001J05F	0.87906083	5.47852933	14.9238453	0.00097532	0.00999526
Hnrnpc	0.44696103	7.27085951	14.8685869	0.00099173	0.01013846
Mylk4	-1.7938675	1.16968029	14.8684838	0.00099176	0.01013846
Irak4	1.0326826	3.52899172	14.862292	0.00099361	0.01014483
Kmt2d	-0.7402883	7.22961619	14.8559035	0.00099554	0.01015184
Rprm	0.82490752	3.97565688	14.8493496	0.00099751	0.01015326
Lrp4	-0.7470669	3.95895007	14.8472426	0.00099815	0.01015326
Sumo1	0.67049808	7.58817477	14.837516	0.00100109	0.01017059

Acot2	0.72110387	4.85835318	14.8316251	0.00100287	0.01017615
Relb	1.73003758	0.4167856	14.8272707	0.00100419	0.01017701
Ikzf4	-0.5626684	4.19381993	14.8189286	0.00100673	0.01019017
Trrap	-0.8775699	6.51894758	14.7807499	0.00101844	0.01028441
Lrrc55	-0.6296046	4.49946179	14.7770413	0.00101959	0.01028441
Cdk7	-0.5265271	6.25268145	14.7763615	0.0010198	0.01028441
Rassf10	1.1142611	2.070177	14.7667803	0.00102276	0.01029447
Fndc3b	0.42070167	5.7564573	14.7650313	0.00102331	0.01029447
Klf14	-3.9867836	0.34924461	14.7610194	0.00102455	0.01029447
Atxn7	-0.5621024	5.79439811	14.7568038	0.00102586	0.01029505
Vwa1	0.80982887	4.20174091	14.7526373	0.00102716	0.0102955
Gfi1	7.71726915	-1.3103246	15.6507045	0.00102906	0.01030197
Pgpep1	0.89748279	4.21898306	14.7323428	0.00103351	0.01033389
Bai3	-0.776086	6.39756936	14.7187485	0.00103778	0.01033684
Kif21b	-0.8736784	5.75344638	14.7162083	0.00103858	0.01033684
Palld	1.35156469	1.61944209	14.7149492	0.00103898	0.01033684
Ranbp2	-0.7305002	7.62672975	14.714792	0.00103903	0.01033684
Slc8a2	-0.7440112	5.55719983	14.7113083	0.00104013	0.01033684
Nwd1	-0.7378343	5.14178275	14.7074527	0.00104135	0.01033684
Nalcn	-0.5912165	6.39924881	14.6883149	0.00104742	0.01038459
Astn2	-0.9274051	3.40841357	14.6827143	0.0010492	0.01038975
Apc2	-0.9654538	4.60083919	14.6742074	0.00105192	0.01040412
Arhgap18	0.62643297	5.25575095	14.6564482	0.00105762	0.01044791
C1qbp	0.49503415	5.21647744	14.6370436	0.00106389	0.01049718
Ttn	-1.1295328	3.64854809	14.6291404	0.00106645	0.01050556
Smim15	0.51437058	6.1523121	14.6188816	0.00106979	0.01050556
Grasp	0.68054879	4.23156831	14.61698	0.00107041	0.01050556
Fam111a	0.8198535	3.34294009	14.6152586	0.00107097	0.01050556
Rgsl1	-1.583073	1.52849034	14.6097696	0.00107276	0.01050556
Hsd11b1	1.52198071	1.67588969	14.6095265	0.00107284	0.01050556
Gm5069	0.64284018	4.42178415	14.6010442	0.00107562	0.01050556
Ephb2	-1.6186022	2.12447497	14.6005409	0.00107578	0.01050556
Myo1b	-0.6025444	5.93592783	14.5991529	0.00107624	0.01050556
Txnl1	0.46337117	7.14529889	14.5881055	0.00107987	0.01052111
D630045J12R	-0.5691148	6.85489924	14.5838296	0.00108128	0.01052111
Hmgb3	0.49362475	5.81681194	14.5825739	0.00108169	0.01052111
Prrc1	0.58144325	6.03799627	14.5787597	0.00108295	0.01052111
Zfand3	0.60718432	5.53411354	14.5731385	0.00108481	0.01052673
Anxa5	1.02368186	8.20189293	14.5691884	0.00108612	0.01052699
Olfr78	-3.9503908	-0.7827399	14.5601054	0.00108913	0.01054378
Slc44a1	-0.4715672	5.88927923	14.5526179	0.00109163	0.01055547
Gria4	-0.6536904	6.76190526	14.5477941	0.00109323	0.01055861
Nrn1	0.49521259	7.30682903	14.5319267	0.00109855	0.01056244
Bri3bp	-0.6171121	5.72851111	14.5287974	0.0010996	0.01056244
Unc13a	-1.118895	6.96026908	14.5285851	0.00109967	0.01056244

Ryr2	-1.218946	8.03232556	14.5277847	0.00109994	0.01056244
Uck1	0.75648798	3.16796925	14.5274293	0.00110006	0.01056244
Smco1	-1.0943567	2.72570555	14.5148306	0.0011043	0.01059081
Nup214	-0.5986442	6.07547627	14.5078483	0.00110666	0.01059709
Tcf7	0.74276975	4.87830236	14.505267	0.00110753	0.01059709
Cnbp	0.57583326	9.17261251	14.493964	0.00111137	0.01062142
Fnip2	-0.6793717	4.74590785	14.4819432	0.00111546	0.01064818
Tmem67	-0.9723911	3.81149428	14.4624398	0.00112214	0.01068844
Serpinh1	0.96311041	4.25435092	14.462035	0.00112228	0.01068844
Kcnh4	-1.6899793	1.83296226	14.4408704	0.00112958	0.0107455
Rassf3	-0.5775865	5.51837471	14.4340348	0.00113195	0.0107475
Acbd3	0.45317887	6.49257134	14.4327193	0.00113241	0.0107475
4930444F02I	-4.767662	-0.6026053	14.4191251	0.00113714	0.01077344
Col3a1	0.96711426	5.0592517	14.4173301	0.00113776	0.01077344
Zfp804a	-0.8329635	5.92882127	14.4030454	0.00114276	0.01080481
Prom1	-1.2773532	2.67292899	14.4003482	0.0011437	0.01080481
Inhba	0.50612106	4.79140572	14.3620998	0.00115721	0.01091989
Cd24a	-0.8192578	5.29033458	14.3541905	0.00116003	0.0109339
Sp100	0.86752819	5.07670591	14.3477614	0.00116232	0.01093838
Ppl	0.95053745	2.44147578	14.3454027	0.00116317	0.01093838
Sra1	0.66921839	5.14917602	14.3272756	0.00116967	0.01098493
4930511M06	-1.3115391	3.22956521	14.3241582	0.00117079	0.01098493
Utp15	-0.6928497	5.04699468	14.3169025	0.0011734	0.01099513
Cyb5b	0.44082729	7.53246763	14.3107583	0.00117563	0.01099513
4930519F09I	-1.0485423	2.53184829	14.2995208	0.0011797	0.01099513
Caprin2	-1.1035552	2.7154879	14.2966507	0.00118074	0.01099513
Kcnip2	-0.4855292	5.69882774	14.293327	0.00118195	0.01099513
Lhpp	1.02088063	2.63005756	14.2912677	0.0011827	0.01099513
Mospd1	0.65087799	6.13396769	14.2904257	0.00118301	0.01099513
Ppp2cb	0.41835809	7.09415398	14.288425	0.00118374	0.01099513
Gm7173	-3.058656	-0.6906791	14.2862289	0.00118454	0.01099513
Tspan4	-1.3723131	2.48387872	14.2842655	0.00118525	0.01099513
Mir3064	-2.7570081	-0.6415369	14.2739394	0.00118903	0.01100397
Usp33	-0.5589103	6.93210108	14.2704331	0.00119031	0.01100397
Dnajc30	0.62054243	4.96880301	14.2689398	0.00119086	0.01100397
Lrrc8b	-0.6308846	5.96179813	14.2670338	0.00119156	0.01100397
Cyp26b1	-1.052299	5.38657079	14.2464976	0.00119913	0.01104951
Gfod1	-0.5071462	6.81594251	14.2463545	0.00119918	0.01104951
Tmem181c-p	-1.3416962	4.26990538	14.2416746	0.00120091	0.01105308
2410076I21R	-2.008583	0.47192172	14.237685	0.00120239	0.01105431
Atg7	0.76910598	6.32718181	14.231707	0.00120461	0.01106234
Calm3	0.41580162	8.7129131	14.2251528	0.00120705	0.01107236
Chsy3	-0.975757	2.91210775	14.2159925	0.00121047	0.01109133
Hexim2	1.06068108	2.30209558	14.2086433	0.00121322	0.01109862
Minos1	0.59732609	6.48496504	14.2046563	0.00121471	0.01109862

Grem2	0.60633882	5.2695346	14.1997798	0.00121654	0.01109862
Pdk2	0.46340941	7.0969543	14.1979619	0.00121723	0.01109862
BC002163	0.65496393	3.58141497	14.1881367	0.00122093	0.01109862
Rdh10	0.71153935	3.6843256	14.1879159	0.00122101	0.01109862
Htt	-0.8713783	6.73840291	14.1877379	0.00122108	0.01109862
Slmo2	0.48375199	6.85782777	14.184427	0.00122233	0.01109862
Eya2	0.99956034	6.41533242	14.1779141	0.00122479	0.01109862
Dock4	-0.8580073	6.18838306	14.1742486	0.00122618	0.01109862
Sema4g	-1.1964079	2.77395805	14.169559	0.00122796	0.01109862
Ank3	-0.9997549	9.13226804	14.1694152	0.00122801	0.01109862
Olfr1033	0.81681605	3.91660529	14.1582132	0.00123227	0.01109862
Selo	-1.265288	1.26997923	14.1499086	0.00123544	0.01109862
Gm20748	1.57692236	1.58141484	14.14894	0.00123581	0.01109862
Vps13d	-0.9299448	6.62824952	14.145084	0.00123729	0.01109862
Frzb	-0.9812527	3.21545134	14.1448632	0.00123737	0.01109862
Sncaip	0.91500632	5.65640111	14.1437544	0.0012378	0.01109862
Dok7	-3.3396975	-0.7094101	14.1407805	0.00123893	0.01109862
Zpr1	0.62206018	4.3602774	14.1367398	0.00124048	0.01109862
Mrpl12	0.8894313	3.85456759	14.1365678	0.00124055	0.01109862
Utp11l	0.8160056	4.11218496	14.1354753	0.00124097	0.01109862
Taf1	-0.5631806	6.83215299	14.1267828	0.00124431	0.01111643
Eif4g2	0.54966831	10.874292	14.1228298	0.00124584	0.01111795
Eif1a	0.6316443	6.62350168	14.1135815	0.00124941	0.01113775
Magee1	-0.7476898	6.91827672	14.0933137	0.00125728	0.01118596
Stat3	0.44691732	5.61785731	14.0926468	0.00125754	0.01118596
Bzrap1	-1.3280104	4.8099904	14.085205	0.00126045	0.01119629
Gabrg1	-0.9983375	3.40402134	14.0826942	0.00126143	0.01119629
Ptprm	-0.8803305	5.20958612	14.0773231	0.00126353	0.01120285
Rit2	0.47821581	6.72674347	14.065346	0.00126823	0.011226
Pcdhb19	-1.0685218	2.92157149	14.0637207	0.00126887	0.011226
Grin1os	-2.024988	0.24889645	14.0568958	0.00127156	0.01122817
Birc6	-0.9026344	7.93359802	14.0561666	0.00127185	0.01122817
Vmn2r84	-2.9732381	0.21344119	14.0479428	0.0012751	0.01122977
Hilpda	1.12705339	2.63433833	14.04693	0.0012755	0.01122977
Arx	-1.209004	2.59868068	14.0451976	0.00127619	0.01122977
Crip2	0.56315592	4.55658148	14.0418925	0.0012775	0.01122977
Ttpa	-1.6362668	1.48140709	14.0216802	0.00128554	0.01126623
Cdo1	0.89746654	6.85151754	14.0204562	0.00128603	0.01126623
Slc25a5	0.4208077	7.08642197	14.0176782	0.00128714	0.01126623
Eml5	-1.1392863	5.85855028	14.0166712	0.00128754	0.01126623
Gm4814	-5.2175847	-1.5837911	14.014287	0.0012885	0.01126623
H19	4.84803761	-1.5509996	14.0000363	0.00129422	0.01130421
Drp2	-0.9416385	5.99951499	13.994596	0.00129641	0.01131133
Kctd12b	0.7404651	4.98080591	13.9886985	0.00129879	0.01132008
Tmem44	-0.6238645	4.61437698	13.9810624	0.00130188	0.01133497

Myo10	-0.5102499	5.70474364	13.9672893	0.00130747	0.01136175
Polr2m	0.51907781	8.6440255	13.9637382	0.00130891	0.01136175
Arntl	-0.6389777	3.98379063	13.9618833	0.00130967	0.01136175
Ssbp3	0.45349846	5.98619152	13.9598932	0.00131048	0.01136175
D130017N08	-0.7819996	3.83627959	13.9463321	0.00131603	0.01138768
06-Sep	0.41564919	6.97292922	13.9458026	0.00131624	0.01138768
2610035F20I	0.87686168	3.01354408	13.9366467	0.00132	0.01139737
Prdx5	0.75537736	5.90269109	13.9363203	0.00132014	0.01139737
Thpo	-1.2504009	2.09779978	13.9310243	0.00132232	0.01140422
Pirt	8.70921054	-0.890339	13.9250186	0.00132479	0.01140512
D930016D06	-1.0521424	4.25689725	13.9240433	0.0013252	0.01140512
Eif2s2	0.42440994	7.44919119	13.9159331	0.00132855	0.01141973
1700021F05I	0.57947445	4.52234889	13.9132243	0.00132967	0.01141973
4932411E22I	-1.1630842	2.56302696	13.9090959	0.00133139	0.01142251
Per3	-0.4743316	5.90032191	13.9029107	0.00133396	0.01143263
Ywhah	0.37573394	10.4947765	13.8859354	0.00134104	0.01148136
1110019D14I	0.97540567	2.68830163	13.8732464	0.00134636	0.01151493
2900092D14I	-0.7927805	5.46346693	13.8649796	0.00134984	0.01153269
Nat10	-0.7673608	3.42472653	13.8556866	0.00135377	0.0115542
Msl3	0.52868631	5.48611517	13.8421332	0.00135951	0.01158364
Gm19434	-1.6086046	0.9469362	13.839526	0.00136062	0.01158364
Arl4a	0.68752211	7.29655897	13.8375896	0.00136144	0.01158364
Zwint	0.39645345	6.89570744	13.8312425	0.00136415	0.01159464
Nosip	0.65443749	4.60528241	13.8188896	0.00136943	0.01162749
Anxa1	1.1766421	6.7899027	13.808997	0.00137367	0.01165149
Grm5	-0.6180332	6.71237517	13.7675641	0.00139161	0.01179145
Fam179a	-4.055714	-0.4688586	13.7560475	0.00139664	0.0118189
Kcnt1	-0.9154884	5.40537708	13.7535707	0.00139772	0.0118189
Srfbp1	0.73661333	3.92073567	13.7471401	0.00140054	0.01182035
Polr2g	0.69127088	5.38000688	13.7466234	0.00140077	0.01182035
Cyb5	0.70512883	5.88858328	13.7308462	0.00140772	0.0118668
Trmt112	0.58187929	5.06178834	13.7109716	0.00141652	0.01191328
Rab12	0.43942853	7.08669075	13.706268	0.00141862	0.01191328
Rasgrf1	-0.8337086	8.33628821	13.7042193	0.00141953	0.01191328
Me2	0.59226009	6.29207004	13.702181	0.00142044	0.01191328
Dzip1l	-0.7615949	3.47305147	13.7020914	0.00142048	0.01191328
Scube3	-2.4907657	1.4791475	13.697642	0.00142246	0.01191504
Pls1	-0.7695872	4.03669189	13.6951293	0.00142359	0.01191504
Uck2	0.80016024	3.46702241	13.685089	0.00142808	0.01194051
Jmjd6	0.77413591	2.98096008	13.6814684	0.00142971	0.01194196
Rad1	-0.8944505	4.79840405	13.6726527	0.00143368	0.01195581
Sdhaf2	0.57366911	5.20430452	13.6711468	0.00143435	0.01195581
Arf1	0.45700816	8.31488361	13.6680929	0.00143573	0.01195581
Arhgap19	0.93308106	3.4055371	13.6617962	0.00143858	0.01196738
Rnf185	0.54859975	5.79909455	13.655291	0.00144152	0.01196866

Kidins220	-0.5722768	8.28762378	13.655023	0.00144164	0.01196866
A330076H08	-1.3166795	3.78922034	13.6480724	0.0014448	0.01198275
Rab22a	0.50456235	5.64374601	13.6357041	0.00145043	0.01199045
Clns1a	0.52292474	6.19127817	13.6356811	0.00145044	0.01199045
Etfa	0.55328261	6.60591641	13.633869	0.00145127	0.01199045
Pld6	-5.0382369	-1.921622	13.6332289	0.00145156	0.01199045
Cuedc2	0.79895476	4.43643921	13.6261894	0.00145478	0.01200487
Pelo	0.91252367	3.1943276	13.6230311	0.00145623	0.01200487
Ccdc181	0.60061021	5.18637453	13.6134845	0.00146061	0.01202106
Wipi1	0.79360926	4.24351571	13.6123869	0.00146112	0.01202106
4930447C04l	-1.8548688	2.03600287	13.6067396	0.00146372	0.01203042
Cast	0.81161174	6.45665256	13.6014612	0.00146616	0.01203201
Ap5s1	1.22036972	2.35698614	13.5999771	0.00146684	0.01203201
Dgkz	-0.4838733	6.32633871	13.5935808	0.0014698	0.0120355
Pnisr	-0.7476703	7.66635585	13.5927282	0.0014702	0.0120355
Gm16287	-1.5057047	1.40048103	13.5738253	0.00147898	0.01209539
Phax	0.5229667	5.38378733	13.5600591	0.00148542	0.01212986
Gprasp1	-0.9503387	8.9720936	13.5585019	0.00148615	0.01212986
Fhl2	0.5358535	5.79220638	13.5471218	0.0014915	0.01216142
Cln8	-0.6777008	3.78794767	13.5426709	0.00149359	0.01216645
Cnn1	4.60381094	-0.3466643	13.5391531	0.00149525	0.01216791
Plag1	-1.0863873	2.70955177	13.527127	0.00150094	0.01218759
Gon4l	-0.6229779	5.89696095	13.5250676	0.00150192	0.01218759
Sgcg	-3.8653586	-0.352772	13.5227826	0.001503	0.01218759
Pkd2l2	-0.7698495	3.72470118	13.5196338	0.0015045	0.01218759
Actb	0.57155635	9.97274988	13.5183986	0.00150509	0.01218759
Ttll3	-1.3606797	1.83886483	13.5095599	0.00150929	0.01220965
Gdap1l1	0.79744888	3.45144051	13.5043055	0.0015118	0.01221792
A4gnt	5.3162954	-1.4684835	13.4926189	0.0015174	0.01224481
Pcdhga12	-0.8782394	2.95173077	13.4881461	0.00151955	0.01224481
Dusp14	0.57582399	5.93030232	13.4880354	0.0015196	0.01224481
Acot9	0.72533701	3.80085597	13.4832566	0.0015219	0.01225132
Stk39	0.66345395	7.75126051	13.4640279	0.00153119	0.01231403
Ywhae	0.44664914	10.7332426	13.4581867	0.00153402	0.01232476
Egln3	0.78123478	7.45130752	13.4472957	0.00153932	0.01235526
Zfp866	-0.5022065	5.11100105	13.4369507	0.00154437	0.01236211
Ifi27l2a	1.16653024	2.32156448	13.4364054	0.00154464	0.01236211
Cox7b	0.64153582	7.33657115	13.4363114	0.00154468	0.01236211
Yap1	0.62575371	5.91019961	13.4292874	0.00154813	0.0123776
Nxf7	3.24837361	0.83819956	13.4190676	0.00155315	0.01240341
Pcnx	-0.583315	7.32151792	13.4165832	0.00155437	0.01240341
Ccnl2	-0.9586748	4.42696557	13.4081942	0.00155851	0.01242312
Cnot11	0.56697208	4.38164142	13.4054567	0.00155987	0.01242312
Zbtb6	-0.5054478	5.09024405	13.4004211	0.00156236	0.01243093
Iqsec1	-0.7353775	7.84159001	13.395122	0.00156499	0.01243981

Snrpb	0.66194252	3.74360166	13.3843267	0.00157036	0.01247043
Hcn2	-1.3811086	1.3127917	13.3804271	0.0015723	0.01247383
Cul9	-0.9684215	3.96622826	13.3632216	0.00158092	0.01253006
Rpgr	-0.8501392	4.34552002	13.3571131	0.00158399	0.0125423
Mical3	-0.7807947	6.27076941	13.3511507	0.00158699	0.01254686
Spata6	0.70463495	3.91402258	13.3499097	0.00158762	0.01254686
Etf1	0.45181659	6.47593528	13.3398484	0.0015927	0.01256518
Mob3c	0.75377355	4.65589937	13.3309409	0.00159722	0.01256518
Kmt2c	-0.6102501	8.17683856	13.3288077	0.0015983	0.01256518
Mlxip	-0.5492463	5.16406634	13.3252018	0.00160013	0.01256518
Slc2a12	0.81966219	4.38770554	13.3248215	0.00160033	0.01256518
Samd4b	0.48130568	6.83146274	13.3220115	0.00160176	0.01256518
Cc2d1a	-1.0901704	2.91803253	13.3206414	0.00160246	0.01256518
Dapp1	0.86834431	4.05572574	13.3205674	0.0016025	0.01256518
Pbx3	0.85040208	7.274062	13.3182172	0.00160369	0.01256518
Gldc	-0.9898371	2.46772824	13.3147169	0.00160548	0.0125672
Smap2	0.38580004	7.69726981	13.3075856	0.00160913	0.01258377
Hbegf	1.40448514	1.72876483	13.2998917	0.00161307	0.0125932
Tubb4b	0.42704835	7.02528068	13.2951242	0.00161552	0.0125932
Grik1	-1.0514433	2.80971496	13.2933525	0.00161643	0.0125932
Nob1	0.95169365	3.0220439	13.2932962	0.00161646	0.0125932
Cand2	0.80797412	3.67553313	13.2893898	0.00161847	0.01259693
Zbtb34	-0.6172966	6.30562841	13.2834959	0.00162151	0.01260511
Grm2	-0.6702347	3.64352703	13.2814093	0.00162259	0.01260511
Fam214a	-0.6005163	5.23806352	13.2724529	0.00162723	0.01262919
Hsbp1	0.6007082	8.97240026	13.259299	0.00163406	0.01266044
Zfp619	-0.8541045	2.83341509	13.2587759	0.00163434	0.01266044
Fosl2	1.29955988	6.03954939	13.241927	0.00164314	0.01271665
Nbea	-0.9969551	8.50315609	13.2250475	0.00165201	0.01276024
Rbm42	0.67004939	4.24635712	13.2236898	0.00165273	0.01276024
Snap25	0.40494432	14.1167379	13.2223587	0.00165343	0.01276024
Pdlim4	1.06209455	2.4719041	13.2119805	0.00165892	0.01278574
Zfp712	-0.7474027	3.87900977	13.2102275	0.00165984	0.01278574
Ctgf	0.75524797	5.68766544	13.2018074	0.00166431	0.01279684
Pou4f1	7.29714314	-1.0522652	13.9426517	0.0016644	0.01279684
Ttyh1	-0.6674713	7.60305799	13.1918177	0.00166963	0.01282507
Ube2r2	0.58359739	8.34444785	13.1869759	0.00167222	0.01282993
Ift88	-0.6949491	4.21810903	13.1847882	0.00167339	0.01282993
6230400D17I	-2.6101653	0.25397357	13.1727441	0.00167984	0.0128452
Mgrn1	0.43738725	6.53038613	13.1704428	0.00168108	0.0128452
Tle3	0.5596245	5.22576563	13.1670417	0.00168291	0.0128452
Pla2g4a	0.68165409	4.59834427	13.1663113	0.0016833	0.0128452
Senp7	-0.4052731	6.97076646	13.1565249	0.00168857	0.0128452
Fryl	-0.6224938	7.5460087	13.1551565	0.00168931	0.0128452
Ahctf1	-0.5965307	6.60380263	13.1540198	0.00168993	0.0128452

Tmem87a	-0.637528	4.44738721	13.1507524	0.0016917	0.0128452
Rsbn1l	-0.5111752	5.5308702	13.1498722	0.00169217	0.0128452
Wfs1	0.65341857	6.64987679	13.1471959	0.00169362	0.0128452
AU040972	-2.0778517	1.08626308	13.1395091	0.00169779	0.0128452
Zfp292	-0.5648609	7.57624967	13.1381155	0.00169855	0.0128452
Atg2b	-0.7638722	6.04259617	13.1352138	0.00170013	0.0128452
Spata19	-6.0166518	-2.0523574	13.134559	0.00170049	0.0128452
Pdia3	0.5693212	7.51848694	13.1337167	0.00170094	0.0128452
Dlx6os1	-0.7982062	3.48672029	13.1327471	0.00170147	0.0128452
Nop9	0.88762458	3.74602846	13.1318759	0.00170195	0.0128452
1700110C19I	-3.436635	-0.1240952	13.1169953	0.00171008	0.01288595
E030019B06I	-4.4077907	-0.5614128	13.1102429	0.00171378	0.01288595
Asph	-0.4781256	6.75367526	13.1099383	0.00171395	0.01288595
Slco2a1	1.08116396	4.43168881	13.1058714	0.00171618	0.01288595
Naa50	0.41654551	7.71159215	13.1036276	0.00171741	0.01288595
Sh2b3	0.86523715	3.5835136	13.1028477	0.00171784	0.01288595
Sod3	0.95392075	5.39044112	13.1019818	0.00171832	0.01288595
Ociad2	0.43922085	6.64314573	13.0927518	0.00172341	0.01288825
Sik2	-0.4943876	5.84896474	13.0908759	0.00172445	0.01288825
Ciita	-1.3669982	1.62985973	13.0875207	0.0017263	0.01288825
9530080O11	1.16654645	2.39329389	13.0866206	0.0017268	0.01288825
Shcbp1l	-2.3977981	0.19433113	13.0841814	0.00172815	0.01288825
Slc15a2	-0.9849857	2.85421887	13.0834095	0.00172858	0.01288825
Eri1	0.51910373	5.71435781	13.0801192	0.00173041	0.01288825
Tmem5	0.60744062	4.60960499	13.07874	0.00173117	0.01288825
Zfp277	-0.9287046	6.31918205	13.0747078	0.00173341	0.01289325
Trnau1ap	0.65872613	3.70772269	13.0702015	0.00173592	0.01290023
Ttc1	0.61835534	6.00652084	13.0580261	0.00174271	0.01293902
Capn1	0.6094989	4.19335936	13.0424019	0.00175148	0.01298367
Cul3	0.38246064	8.90574059	13.0416735	0.00175189	0.01298367
Csmd1	-1.0431588	6.08621205	13.0375949	0.00175418	0.01298898
Rnf113a2	0.65110736	4.72744651	13.0316602	0.00175753	0.01299308
Hax1	0.67993034	5.40342421	13.0310069	0.0017579	0.01299308
Atp6v1g2	0.44884803	9.45908367	13.0235667	0.00176211	0.01301248
Ndufaf3	0.67598996	4.14631992	13.0104462	0.00176955	0.01305573
Mcmdc2	-1.4398771	2.5289707	13.0045076	0.00177293	0.01306895
C030018K13I	-1.9139072	1.52609964	12.9982779	0.00177649	0.01308343
4930431F12I	-1.3768048	2.63778753	12.9777951	0.00178823	0.01315567
Gck	-3.3854875	-0.9810828	12.9755971	0.0017895	0.01315567
Ccdc43	0.61566714	4.71901413	12.9693414	0.00179311	0.01317041
C130030K03I	-1.2678952	2.25501604	12.9610069	0.00179793	0.01319401
Gpr88	-0.6226834	6.09706618	12.948828	0.00180499	0.01322266
Shisa7	-0.7333465	6.36471315	12.9471399	0.00180597	0.01322266
Dopey1	-0.8662308	5.58148673	12.9456211	0.00180686	0.01322266
Cxxc1	0.50069749	5.98700297	12.940472	0.00180986	0.01322266

Kdm5b	-0.6699795	5.90629015	12.9404445	0.00180987	0.01322266
Ccl2	2.76885216	-0.4998618	12.9322593	0.00181466	0.01324405
Slc35b1	0.68858569	3.70442567	12.9276755	0.00181734	0.01324405
Myd88	1.25387575	2.78060897	12.9269216	0.00181778	0.01324405
Fgfr3	-0.7789907	3.90040749	12.9244194	0.00181925	0.01324405
Fam101a	1.5879081	2.83924127	12.9181623	0.00182292	0.01325906
Crct1	6.42485668	-1.9746755	13.6217164	0.00182777	0.01328256
Dhx37	-0.919611	2.78793217	12.9022692	0.00183229	0.01329364
Vps13c	-0.9121734	6.48139958	12.9015809	0.0018327	0.01329364
Rad54l2	-0.4282968	5.63481962	12.8984173	0.00183457	0.01329364
Amer3	-0.8276159	3.42676157	12.8964033	0.00183576	0.01329364
Hipk2	-0.4341167	7.03675887	12.8910187	0.00183896	0.01330504
Syt7	-0.7277645	6.93971431	12.8836366	0.00184334	0.01332506
Car11	-0.5506795	5.14702599	12.8712927	0.00185071	0.01336167
Pag1	-0.5306478	5.64446304	12.8689386	0.00185212	0.01336167
Shroom2	0.38422731	6.79998881	12.8669834	0.00185329	0.01336167
Chd8	-0.5711327	6.62880664	12.8574394	0.00185901	0.01339119
Myzap	1.1489412	4.17181973	12.8512981	0.0018627	0.01340605
Fam208b	-0.5044095	6.44869303	12.8381918	0.00187061	0.0134512
Vdac2	0.41510506	7.27146482	12.8340632	0.00187311	0.0134574
Lrrc19	-2.3259367	0.34240193	12.8307444	0.00187513	0.01346009
4931440P22I	-4.5487287	-1.4315636	12.8233301	0.00187963	0.01348064
Trpc1	-0.6827991	4.59398598	12.8176633	0.00188308	0.01348965
Hspa8	0.40755629	10.7212684	12.8158756	0.00188417	0.01348965
2610301B20I	0.50996962	5.3130088	12.8065322	0.00188988	0.01351874
Zic1	0.85145311	8.07189603	12.8019291	0.00189269	0.01352387
Nphs1	-1.1835149	2.0604557	12.798521	0.00189478	0.01352387
Palm3	-0.966029	2.25433235	12.7973078	0.00189553	0.01352387
Tacc3	1.19164071	2.22085714	12.7908581	0.00189949	0.0135404
Adcy5	-0.4005768	6.29168631	12.7872232	0.00190173	0.0135446
Snrpb2	0.59462017	5.68008037	12.7787458	0.00190696	0.0135701
Wdr33	-0.6673966	5.10123031	12.7702182	0.00191224	0.01359589
1110008L16I	0.5458726	4.36276235	12.7654776	0.00191518	0.01359857
Cyp7b1	-0.8767036	2.44135494	12.764278	0.00191593	0.01359857
Chaf1b	5.8319899	-1.7793604	13.4563761	0.00191892	0.01360557
Eif5	0.37247281	8.75249106	12.7550905	0.00192164	0.01360557
Ndufa5	0.6868129	5.61427803	12.7547106	0.00192188	0.01360557
Gpd1l	0.39911196	7.02544809	12.7447773	0.00192808	0.01363774
Pcdh20	-1.0155435	3.064212	12.7302215	0.00193721	0.01369053
Gtpbp4	0.4022018	6.31664662	12.7196834	0.00194385	0.01371233
Nf1	-0.6855703	8.07663234	12.7143184	0.00194724	0.01371233
Slco1c1	-0.8443857	4.33303442	12.7131404	0.00194799	0.01371233
Klhl17	-1.2036631	3.50189595	12.7126022	0.00194833	0.01371233
Lif	-3.3382672	-0.9504071	12.7121103	0.00194864	0.01371233
Hspd1	0.34155434	8.27695858	12.7078755	0.00195132	0.01371946

Kcnb1	-0.7259372	8.01709243	12.701146	0.00195559	0.01373227
Mrps21	0.72748497	5.03713123	12.6997422	0.00195648	0.01373227
Ywhaq	0.42721993	9.00817021	12.6888351	0.00196343	0.01375501
Cadm2	-0.5728691	8.70551438	12.6867921	0.00196474	0.01375501
Pak2	0.47377734	6.79178155	12.6867805	0.00196474	0.01375501
Psme1	0.82096444	7.09575098	12.6832975	0.00196697	0.01375888
Timm23	0.59175993	6.17913277	12.6759546	0.00197167	0.01378004
Adam1a	-2.2001016	1.28166211	12.6721443	0.00197412	0.01378541
Lrrc7	-0.9852462	8.01311351	12.6687359	0.00197631	0.01378899
Tex13	-2.2814299	-0.5693191	12.6654293	0.00197844	0.01379212
Gcdh	0.80608834	3.16647822	12.6626781	0.00198021	0.01379277
Ltbp4	-0.6020582	4.59000141	12.6575001	0.00198355	0.01380384
Pik3cd	-0.963836	2.8153947	12.6550091	0.00198516	0.01380384
Iqsec3	-0.7492755	6.55451205	12.649718	0.00198858	0.01381595
Bach1	0.57724289	5.05667399	12.6402521	0.00199472	0.01384689
0610043K17I	-1.0697132	1.52594547	12.6317998	0.00200021	0.0138589
Klf4	0.84330488	6.47978248	12.6290128	0.00200203	0.0138589
Exosc1	0.71772312	4.68225896	12.6277438	0.00200286	0.0138589
Pgam1	0.44108834	9.43813812	12.6272358	0.00200319	0.0138589
Shc2	-0.7119809	4.57055209	12.6229697	0.00200598	0.0138665
Abcd4	-1.2562542	1.46587845	12.6164919	0.00201022	0.01388413
Akr1e1	0.52939434	5.1974539	12.6135938	0.00201212	0.01388558
Smok4a	-1.6342296	1.7706538	12.6027734	0.00201923	0.01392296
Daxx	0.58609264	3.87874798	12.5984138	0.0020221	0.01393108
Dst	-1.0140023	9.14796673	12.5900084	0.00202765	0.01394733
Cgnl1	0.62528958	6.07090284	12.5897039	0.00202785	0.01394733
AB041803	-1.5513425	2.42440817	12.5856037	0.00203057	0.01395432
Cnga4	2.49596238	0.3023142	12.5801328	0.00203419	0.01395998
Kcnt2	-0.8506993	5.43269242	12.5788511	0.00203504	0.01395998
Vash1	-0.8533312	3.42591804	12.5766845	0.00203648	0.01395998
Vamp5	0.70259479	5.48863501	12.568646	0.00204183	0.01398499
Eif2b2	0.84577902	4.10672235	12.546193	0.00205686	0.01407619
Ppp1r9b	-0.4818351	6.66886217	12.5436093	0.0020586	0.01407636
Ankrd55	1.03658678	2.6483128	12.5325797	0.00206603	0.01411545
Pygb	0.49829346	6.4839507	12.5224923	0.00207286	0.01415031
Trib1	0.55830128	5.66745826	12.5023173	0.00208658	0.01423218
Prr11	-0.8757371	3.12267496	12.4840168	0.00209912	0.01430582
Fam103a1	0.50002542	7.87600911	12.4809385	0.00210124	0.01430839
Ccdc82	-0.7158992	7.54247244	12.472806	0.00210684	0.01431642
Ttc13	-0.9043851	3.19742116	12.4682058	0.00211002	0.01431642
Dlk2	-1.2668408	1.71967125	12.4654425	0.00211193	0.01431642
Ryr3	-1.3209278	5.30691319	12.4653434	0.002112	0.01431642
Dgka	0.60730602	4.79442651	12.4641278	0.00211284	0.01431642
Mpped2	0.56277928	6.62221731	12.4640865	0.00211287	0.01431642
Cyb5r4	0.43386968	5.78849428	12.4467784	0.00212489	0.01438601

Ppp1r11	0.62611058	4.56555625	12.4371947	0.00213158	0.01441942
Ptch1	-0.535819	4.75473834	12.4298234	0.00213674	0.01442253
Cops3	0.47040521	5.48155789	12.4294138	0.00213702	0.01442253
Sptbn4	-1.3918105	3.60591124	12.4287541	0.00213749	0.01442253
Atp6v1d	0.36471872	8.33107629	12.4265184	0.00213906	0.01442253
Chp1	0.3974333	7.55719185	12.4140053	0.00214786	0.01447
Wdr13	-0.3649098	6.73099886	12.4064437	0.0021532	0.01449409
Idi1	0.63263487	5.81647658	12.4023567	0.00215609	0.01450168
Ccdc160	1.30702753	1.8488038	12.3998101	0.00215789	0.01450194
Ppfibp2	0.68277361	3.58271338	12.3754733	0.00217522	0.01460267
Gm10845	-1.0460502	5.57078128	12.3737707	0.00217643	0.01460267
Gm10785	-1.9339733	0.94385693	12.3683088	0.00218034	0.01461698
Mrpl21	0.6480825	4.23155519	12.3623775	0.0021846	0.01463187
Zfp459	-1.1592166	3.00448716	12.3602558	0.00218613	0.01463187
Gnpda1	0.56926399	4.59177892	12.3523959	0.00219179	0.01465781
Lrrk2	-0.8627135	5.84619947	12.3388608	0.00220157	0.01471127
Uqcrh	0.68561796	7.05499761	12.325994	0.00221091	0.01476171
4930470H14I	-1.20848	6.76646202	12.3216238	0.0022141	0.01477097
Fam212b	0.50130003	6.76084735	12.316442	0.00221788	0.0147842
Wasf2	0.54189506	6.44383348	12.3124669	0.00222079	0.01479158
Atp6v1g1	0.63195541	6.90417305	12.3068279	0.00222492	0.01480709
Asah2	-0.5652775	5.7264065	12.3009904	0.0022292	0.01482359
2010107E04I	0.59818504	5.84260277	12.295036	0.00223358	0.01484071
Praf2	0.72133663	4.63199854	12.2832459	0.00224228	0.01488444
Zhx2	0.67189618	6.38843228	12.2810637	0.00224389	0.01488444
Mgat5	-0.6870535	4.25700485	12.2787662	0.00224559	0.01488444
Tatdn3	-0.8490285	2.52536768	12.2696639	0.00225235	0.01491717
Peg3	-0.7710484	8.84130121	12.2599187	0.0022596	0.01495317
A630075F10I	-2.8192042	-0.7287085	12.2510366	0.00226624	0.01498502
Arhgdia	0.69146022	8.02589297	12.2429548	0.00227229	0.01501057
Rian	-0.9139757	6.84491912	12.2392745	0.00227506	0.01501057
Brpf3	-0.4650069	5.26307102	12.238578	0.00227558	0.01501057
Zar1l	2.07030241	0.48899529	12.23407	0.00227897	0.01502088
Atm	-0.5803826	5.99784166	12.2265629	0.00228463	0.01504612
Txndc5	0.58169981	4.95852922	12.2180712	0.00229105	0.01507633
Atp8b4	-4.4337582	-0.8469891	12.2139429	0.00229418	0.01508484
Slitrk6	-1.7825581	0.63049086	12.2112858	0.0022962	0.01508603
Ak3	0.55883297	8.15985105	12.2037298	0.00230194	0.01511169
Fam89b	1.03834123	2.77397986	12.1968118	0.00230722	0.01513422
Ankfy1	-0.5231563	6.00458421	12.1851646	0.00231612	0.01518054
Aspa	0.56681929	6.03393762	12.1778678	0.00232172	0.01520512
Esd	0.52246772	6.08734854	12.1749506	0.00232397	0.01520769
Cacna1b	-0.7376683	6.84283263	12.1678733	0.00232942	0.01523124
Grk1	-1.5616274	1.35031602	12.1653388	0.00233138	0.01523192
Mrpl9	0.62610859	4.48985531	12.1596445	0.00233578	0.01524855

Cxcl17	-4.8421265	-1.1080661	12.1560175	0.00233858	0.01525477
Tyr	-1.2439546	2.55561045	12.1523989	0.00234139	0.01526095
Stx8	0.65801381	5.33836295	12.1389607	0.00235184	0.01531623
1500015L24I	-3.9116122	-0.0251291	12.1333875	0.00235619	0.01531623
Stbd1	0.69782591	4.08856523	12.1316383	0.00235756	0.01531623
Nrep	0.55483543	7.95133817	12.1314972	0.00235767	0.01531623
Ndnf	-0.5455642	5.19802815	12.129553	0.00235919	0.01531623
Tcte2	0.87785523	2.88138795	12.1138557	0.0023715	0.01538403
Gria2	-0.7833114	9.08586185	12.1063461	0.00237742	0.01541025
Mir128-1	-2.0089453	1.50571919	12.0988167	0.00238337	0.01542733
Ppa1	0.52473968	5.77835253	12.0982618	0.00238381	0.01542733
Nt5c3	0.47115431	5.14275865	12.0952651	0.00238618	0.01543053
Lamtor3	0.67838464	5.61417099	12.0853913	0.00239402	0.01546169
Ube2h	0.40182513	7.70872382	12.0798579	0.00239842	0.01546169
Mcm3ap	-0.8567241	4.45572644	12.0766415	0.00240099	0.01546169
Ubqln1	0.37664315	7.10321703	12.0755875	0.00240183	0.01546169
Polrmt	-1.0261755	2.23954961	12.0752111	0.00240213	0.01546169
Sulf1	0.80763486	5.8986324	12.0750117	0.00240229	0.01546169
Rhoa	0.84206595	8.61857226	12.0640037	0.00241109	0.01550621
Ptpn7	-0.9713328	3.28926987	12.0373289	0.00243257	0.01561908
Rab1	0.44771238	9.05096433	12.0366103	0.00243316	0.01561908
Tcf12	0.54508106	7.35152925	12.034512	0.00243485	0.01561908
Cdk19	-0.450624	7.00391856	12.0317827	0.00243707	0.01561908
Bag3	0.6070018	5.08106989	12.0304566	0.00243814	0.01561908
Ephb1	-0.733105	3.65570156	12.0202264	0.00244646	0.01566015
Cyp2f2	1.72324462	4.94083326	12.0166458	0.00244938	0.01566663
Bbs12	-1.3379449	1.27863567	12.0122527	0.00245296	0.01567736
Ubl7	0.42886972	6.5759092	12.001365	0.00246187	0.01572054
Ulk3	-0.7694525	3.94958032	11.9993298	0.00246354	0.01572054
Yeats4	0.54495249	5.23072457	11.9969643	0.00246549	0.01572073
D10Bwg1379	-0.9168843	7.06518929	11.9831931	0.00247683	0.0157808
Gm17644	-1.272152	7.68606543	11.9716629	0.00248637	0.01582932
Cntn5	-1.3841084	2.35726734	11.9653743	0.00249159	0.01584546
Npc1	-0.6023707	4.96851897	11.9627017	0.00249381	0.01584546
Pogz	-0.4602202	7.70921291	11.9585787	0.00249724	0.01584546
Lphn1	-0.5787313	8.35124724	11.9526517	0.00250219	0.01584546
Adra1a	-0.6474661	4.48599076	11.9524004	0.0025024	0.01584546
Ralgapa2	-0.8362797	4.62057579	11.9520919	0.00250266	0.01584546
Ostf1	0.61066845	5.90732594	11.9515529	0.00250311	0.01584546
1110038B12I	-0.9977123	2.45169573	11.9478308	0.00250622	0.01584546
Ift22	0.7321182	4.09852226	11.9477858	0.00250625	0.01584546
Mcee	0.75980737	4.05548428	11.9438324	0.00250956	0.01585272
Muc6	-1.6009398	1.55308457	11.9399979	0.00251278	0.01585272
Ybey	-0.856162	4.3752205	11.939508	0.00251319	0.01585272
Adarb2	-0.6906675	4.9735665	11.9301804	0.00252103	0.01587807

Ccdc88c	-0.7685251	4.2524516	11.9301319	0.00252107	0.01587807
Stk32a	-1.2065862	1.75903453	11.927065	0.00252366	0.01588217
1600029O15	1.5206886	1.62135196	11.9088606	0.00253905	0.01593528
Ubp1	-0.528323	6.29825145	11.9084218	0.00253943	0.01593528
Dnm3os	-1.7763153	1.87406229	11.9056165	0.00254181	0.01593528
Nploc4	0.49276272	5.5117451	11.904652	0.00254263	0.01593528
Pkm	0.51518444	8.20468187	11.9015792	0.00254524	0.01593528
Dock7	-0.5546681	6.05798925	11.8996107	0.00254692	0.01593528
Prorsd1	0.5958706	3.89631622	11.8992518	0.00254722	0.01593528
Cntnap3	-1.3528968	1.93386135	11.8988022	0.00254761	0.01593528
Cmpk1	0.48301139	7.47820594	11.8954903	0.00255043	0.01594081
Tenm3	-0.7260526	6.36800306	11.8892927	0.00255572	0.01596175
Gria3	-0.6266608	8.51083982	11.8839908	0.00256026	0.01597275
Atrnl1	-0.4519501	6.22704218	11.8826943	0.00256137	0.01597275
Srp54a	0.3725544	7.11641345	11.8756668	0.0025674	0.01599129
Svep1	-0.987585	2.75200356	11.874696	0.00256823	0.01599129
Chst11	0.52165386	5.6926055	11.8704203	0.00257191	0.01600207
B3gnt9	1.34983814	1.8098825	11.8614969	0.00257961	0.01603304
Mapre1	0.50487757	7.8572816	11.860126	0.00258079	0.01603304
Pebp1	0.57992365	8.27988098	11.8548014	0.0025854	0.01604864
Mfi2	3.72725178	-0.3768667	11.8500584	0.00258951	0.01604864
Rin2	0.56268784	6.55233572	11.8498337	0.0025897	0.01604864
Ica1l	-0.500196	5.02285201	11.8449241	0.00259396	0.01604864
Plxna1	-0.7780199	5.51998092	11.8438633	0.00259489	0.01604864
Casp4	1.63508882	0.78416586	11.8419089	0.00259659	0.01604864
Lgr5	-1.0561039	2.48091856	11.8414671	0.00259697	0.01604864
Magee2	-0.6731397	4.78781903	11.8381045	0.0025999	0.01605467
Zfp40	-0.6372953	4.31869583	11.8298925	0.00260706	0.01606435
Pycrl	0.84384214	2.65084745	11.8280963	0.00260863	0.01606435
Mrpl17	0.46990281	5.65330269	11.827833	0.00260886	0.01606435
Snx1	0.45444517	5.72601252	11.8260676	0.00261041	0.01606435
Prkg1	-0.4422121	5.8376894	11.8244908	0.00261179	0.01606435
Azin1	0.38633095	7.21388816	11.8228873	0.00261319	0.01606435
Hspa5	0.44671773	7.24254107	11.8127007	0.00262214	0.01608837
Agk	-0.5910928	4.41723715	11.812003	0.00262275	0.01608837
Ubtf	0.46289145	5.83029552	11.8112068	0.00262345	0.01608837
Polr3a	-0.6739178	3.97303105	11.8094987	0.00262495	0.01608837
Txndc11	0.67451278	3.87622766	11.8046198	0.00262926	0.01608837
Vps13b	-0.7971878	6.99017199	11.8038805	0.00262991	0.01608837
Met	0.74386736	3.05753534	11.800866	0.00263257	0.01608837
Cd300a	-0.9907691	3.03887199	11.8006493	0.00263276	0.01608837
Atp6v0d1	0.47849507	7.16095009	11.7891181	0.00264297	0.01612508
Prkag1	0.5250926	4.21440019	11.7888997	0.00264316	0.01612508
Nfasc	-0.5803927	7.70953215	11.7869882	0.00264486	0.01612508
Rangap1	0.58254007	6.60553911	11.7832723	0.00264816	0.01612508

Tenm4	-0.7676504	5.97639143	11.7828046	0.00264858	0.01612508
Slc25a35	0.67244819	4.99898898	11.774188	0.00265625	0.0161519
Ttbk1	-0.8139406	4.05638659	11.772554	0.00265771	0.0161519
Pcca	0.53115518	5.77101254	11.7712363	0.00265889	0.0161519
Klhl26	-0.6416518	3.63165044	11.7674608	0.00266226	0.0161519
A730020M07	0.87300744	3.94037188	11.7664006	0.00266321	0.0161519
Hint1	0.54321721	5.21577441	11.7643471	0.00266505	0.0161519
Hecw1	-1.0071533	6.93926334	11.760083	0.00266887	0.0161519
Atp13a1	-0.8284309	2.84849454	11.7588638	0.00266997	0.0161519
Eid2b	0.54630869	4.42914406	11.7580798	0.00267067	0.0161519
Abcc5	-1.0504916	4.93688186	11.7513823	0.00267669	0.0161764
Ufl1	-0.5050496	5.79660196	11.7460051	0.00268153	0.01619377
Nyap1	-0.9054943	2.96489102	11.7380205	0.00268874	0.01622539
Slc26a11	-1.2935053	2.08885462	11.7331791	0.00269312	0.01623991
Slfn5	-0.8290899	4.46679279	11.721498	0.00270373	0.01629191
Fstl4	-0.7259762	3.03605396	11.7125055	0.00271193	0.01632275
Atad2b	-0.5631994	5.16211525	11.7115265	0.00271282	0.01632275
AI504432	-0.589341	5.6203491	11.7014104	0.00272208	0.01636647
Abcg4	-0.7855581	4.08949237	11.6990451	0.00272425	0.01636719
Bicc1	0.76265812	6.88745803	11.6969393	0.00272618	0.01636719
Tiprl	0.4494584	6.36973233	11.6934229	0.00272941	0.01636981
Nup93	-0.8148732	4.53506346	11.6921304	0.0027306	0.01636981
Son	-0.6312446	8.07903712	11.6837834	0.00273829	0.01639631
Paqr7	-0.5200593	4.56034292	11.6827419	0.00273925	0.01639631
Strn4	-0.4849632	5.44574804	11.680843	0.00274101	0.01639631
Tmem136	-0.766002	3.41993937	11.677462	0.00274413	0.01640307
Rpl36al	0.78163841	7.29476133	11.6711574	0.00274997	0.01642603
Nrp2	0.49650811	5.60289257	11.6670767	0.00275376	0.01643118
Kntc1	-3.207484	0.00439838	11.6659192	0.00275483	0.01643118
Vmn1r58	-1.1705398	4.2997275	11.6555656	0.00276447	0.01646719
Fam160a2	-0.5588003	6.65594544	11.6551295	0.00276488	0.01646719
Cxx1a	0.48808426	6.02202972	11.6484965	0.00277107	0.01649198
Spag5	-1.8545847	1.90163562	11.6463796	0.00277305	0.01649198
Pigr	-1.3334804	3.83540318	11.6390525	0.00277992	0.01651932
Fnbp4	-0.5346311	5.76659596	11.633049	0.00278556	0.01651932
Dnah1	-0.8830323	3.06537276	11.6329709	0.00278563	0.01651932
Myo9b	-0.6134442	4.49957266	11.6329097	0.00278569	0.01651932
Frmd8	0.64297682	3.95943943	11.6229196	0.0027951	0.01653292
Brsk1	-0.7273725	3.49277298	11.6227733	0.00279524	0.01653292
Scn8a	-0.880929	8.83649402	11.6217173	0.00279624	0.01653292
Pomp	0.56096635	7.36047553	11.619856	0.002798	0.01653292
1810026B05I	-0.6463458	3.78998523	11.6198108	0.00279804	0.01653292
D930015E06I	-1.2603452	2.15529752	11.6168708	0.00280082	0.01653746
5830418K08I	-0.7488589	5.57717409	11.5970435	0.00281965	0.01663447
Scly	0.95485381	3.52715264	11.5953174	0.0028213	0.01663447

Ntng1	-0.573463	5.3774984	11.5902756	0.00282611	0.01665091
Gm15881	-2.6874689	0.39379139	11.5852382	0.00283093	0.01666735
Tgfb1i1	0.78514831	6.85124362	11.582668	0.00283339	0.01666741
Fam193b	-1.0355575	3.14884989	11.5809949	0.002835	0.01666741
Cacna1h	-0.9657835	3.2704708	11.5751481	0.00284061	0.01667191
Piwil4	3.47905987	-0.897219	11.5726162	0.00284304	0.01667191
Pitpnm2os1	-2.6571824	-0.1977303	11.5708297	0.00284476	0.01667191
4931406P16I	-0.5012788	5.65674613	11.5704889	0.00284509	0.01667191
Atp2b1	-0.6242936	9.27705514	11.5696468	0.0028459	0.01667191
Gata3	3.14272702	-0.4203698	11.5317179	0.0028827	0.01687466
Pdzd9	-2.1644932	0.29976476	11.5297501	0.00288462	0.01687466
Rsu1	0.67688765	6.85772679	11.5245182	0.00288974	0.01689259
Mycbp2	-0.8945046	8.84042078	11.507321	0.00290664	0.01697551
Col11a2	-1.9727452	0.10976493	11.5058113	0.00290813	0.01697551
Acyp2	0.54394292	4.62581168	11.5009318	0.00291295	0.01697551
Sipa1l1	-0.7218709	8.88422936	11.4997993	0.00291407	0.01697551
Snord64	-1.8765229	0.11126049	11.4996123	0.00291425	0.01697551
Dbhos	-1.1336894	3.35818867	11.4975186	0.00291632	0.01697555
Taldo1	0.52212576	4.07313907	11.4933422	0.00292046	0.01698257
Aldh1a1	0.81716804	7.64304832	11.4921292	0.00292166	0.01698257
Cfl2	0.60731856	7.7484807	11.4853328	0.00292841	0.01698649
Mrps16	0.80106274	3.20787223	11.4847059	0.00292903	0.01698649
Xpo5	-0.6110837	4.68001948	11.4846135	0.00292912	0.01698649
Dennd2c	2.20975865	-0.3429158	11.4817308	0.00293199	0.01698649
Zfp418	-0.7492924	3.63764889	11.4798037	0.00293391	0.01698649
Ube3a	-0.5261705	6.7638615	11.4789763	0.00293474	0.01698649
Chd6	-0.5909281	7.19872025	11.474876	0.00293883	0.01699819
Metap1	0.4631481	6.1717032	11.4702673	0.00294343	0.01701285
Nt5m	0.72946247	3.91316768	11.4656618	0.00294804	0.01702751
Cdhr3	-3.7666272	-0.2814062	11.4495346	0.00296424	0.01710906
Higd2a	0.59457816	5.32361296	11.4385995	0.00297528	0.01715979
Ecsit	0.52674477	3.81187568	11.4317378	0.00298223	0.01715979
Pnpla3	-1.8151567	1.33916254	11.4314963	0.00298248	0.01715979
Ntan1	0.5971087	5.48359563	11.4306238	0.00298336	0.01715979
Csmd2	-1.1690942	4.84623264	11.43002	0.00298398	0.01715979
Gpr137c	-0.6248656	4.87523066	11.4284655	0.00298555	0.01715979
Itga8	-1.0461309	2.59275693	11.4215436	0.00299259	0.01717854
Plcd4	-1.0051762	2.1931038	11.4211473	0.002993	0.01717854
Cramp1l	-0.4547201	6.39451023	11.413409	0.00300089	0.01721028
Dpp3	0.77331068	3.80938584	11.4097642	0.00300462	0.01721028
Cd28	2.11855391	0.22626198	11.4095767	0.00300481	0.01721028
Fam184b	-1.140223	2.75541386	11.4033033	0.00301123	0.0172349
Acot1	0.57715838	3.95154939	11.4012868	0.0030133	0.0172349
4930509J09F	-3.1855831	-0.3195613	11.3946234	0.00302015	0.01726205
Spg21	0.79480376	5.13850679	11.3880454	0.00302692	0.01728875

Fam213b	0.664007	4.12437467	11.37711	0.00303823	0.01734125
Dynlt1b	0.86138656	4.20983588	11.3627799	0.00305311	0.01741043
Ttll1	0.42764543	5.61818762	11.3613622	0.00305458	0.01741043
Igsf21	-0.9047068	3.61207176	11.3542405	0.00306201	0.01742117
Bcl2l11	0.81643061	5.05409116	11.3540455	0.00306222	0.01742117
Dpm2	0.59054612	4.06808179	11.3534624	0.00306282	0.01742117
Pde6d	0.80605632	4.93270465	11.3446151	0.00307208	0.01745591
Tomm7	0.54133696	4.6452699	11.3435696	0.00307318	0.01745591
Sptssb	0.83944274	4.63872135	11.3411099	0.00307576	0.01745851
Gm19990	4.34228196	-0.756335	11.3365417	0.00308056	0.01747369
Birc3	0.89977503	4.01387721	11.3242039	0.00309357	0.01753336
Rpp30	0.5736342	3.992085	11.3196328	0.0030984	0.01753336
Ces5a	5.25610036	-1.1757707	11.3176902	0.00310046	0.01753336
Cdc37	0.59025114	5.08594465	11.3174293	0.00310073	0.01753336
Kalrn	0.49051995	11.7778886	11.3164721	0.00310175	0.01753336
Tgif1	0.93839055	2.7617842	11.314325	0.00310402	0.01753416
Cobl	0.48341245	7.98664521	11.3079915	0.00311075	0.01756008
Vmn2r-ps12	-2.2254936	0.85826362	11.3053889	0.00311352	0.01756364
Fancm	-0.6746184	4.04748168	11.3021931	0.00311692	0.01757078
Ift74	0.44816112	5.84316581	11.295221	0.00312436	0.01760064
Cacna1e	-0.8190725	8.22203066	11.2925039	0.00312726	0.01760493
Tnip3	1.6384447	1.79841068	11.2782044	0.00314259	0.01767119
Sycp2	-1.4709604	2.48123862	11.2750223	0.00314602	0.01767119
Stc1	-1.1340534	2.90925401	11.2736435	0.0031475	0.01767119
Plcb4	-0.5794139	7.05152563	11.2734549	0.0031477	0.01767119
Tcf3	0.81069891	3.66483439	11.2695514	0.00315191	0.01767119
Zzef1	-0.8762773	5.30413241	11.2678657	0.00315373	0.01767119
Kcnq1ot1	-1.3139011	8.05882548	11.2647066	0.00315714	0.01767119
Efhc1	1.24818552	1.78039606	11.2631119	0.00315887	0.01767119
Themis	2.18722781	0.53572388	11.2602483	0.00316196	0.01767119
Tsc1	-0.6719855	5.94436256	11.257405	0.00316504	0.01767119
Hapln4	0.50867433	6.04615777	11.2569182	0.00316557	0.01767119
Ddx3x	0.32195905	8.72107366	11.2567904	0.00316571	0.01767119
Hcls1	1.07793563	1.43159804	11.255617	0.00316698	0.01767119
Exoc2	-0.5405469	5.89155414	11.2365802	0.0031877	0.01777473
Dnajb4	0.40226506	8.41229707	11.2333745	0.0031912	0.0177822
Zfp157	-0.5566452	4.72711089	11.2162164	0.00321003	0.01785977
Naa40	-0.7926945	2.94332388	11.2154907	0.00321083	0.01785977
BC018507	-0.6837611	6.72558247	11.2143644	0.00321207	0.01785977
Nop56	-0.5431655	6.54885014	11.2127784	0.00321382	0.01785977
Btaf1	-0.6293947	6.20105171	11.2075813	0.00321955	0.01787843
Slc12a5	-0.6215681	7.82459709	11.2057947	0.00322152	0.01787843
Mrpl27	0.68442572	5.32071459	11.2031241	0.00322448	0.01788274
Inpp5a	0.57581804	4.56294931	11.1974243	0.00323079	0.01790567
Hmbs	0.79260472	3.04214054	11.1925353	0.00323622	0.01792365

Tet3	-0.5656208	6.03705093	11.1901689	0.00323884	0.01792421
Ido2	-1.2114436	2.00854124	11.1885211	0.00324068	0.01792421
Ypel5	0.49597473	7.68390628	11.1800405	0.00325013	0.0179331
Ylpm1	-0.8527266	8.12727214	11.1798463	0.00325034	0.0179331
Pnp	0.60869583	4.58401959	11.1776226	0.00325283	0.0179331
Atp5j2	0.63553343	5.43706898	11.1774874	0.00325298	0.0179331
H2-Q5	-2.3916492	-0.6068189	11.1772931	0.00325319	0.0179331
Anapc13	0.77602983	5.13802789	11.1735013	0.00325743	0.01794443
Sat1	0.56540524	5.94072351	11.1711995	0.00326001	0.0179466
Dscam	-0.5790338	5.64786648	11.1670613	0.00326465	0.01794819
Gpr37	-0.8617383	3.7003291	11.1670444	0.00326467	0.01794819
Tmem121	7.12205011	-1.3857168	11.1527375	0.00328076	0.01801541
Akap6	-0.8723076	8.14468972	11.1522755	0.00328128	0.01801541
Igsf6	-1.6829574	1.69934988	11.1480663	0.00328603	0.01802744
Slc36a1os	-1.1614387	2.78054286	11.1464503	0.00328786	0.01802744
Slitrk2	-0.7461553	5.37782692	11.1381881	0.00329721	0.01806668
Dyrk1b	-0.7499217	3.09395663	11.1308761	0.00330551	0.01810011
Rrp7a	0.78159663	4.01122213	11.1248981	0.00331232	0.0181253
Pou3f3	-0.5333006	5.33868191	11.1205041	0.00331733	0.01814065
Hnrnph3	0.49145157	4.49209724	11.1167528	0.00332162	0.01815201
Lrrc32	0.81383043	4.80677595	11.1095543	0.00332986	0.01818495
Uty	-0.5719258	5.05926965	11.1070253	0.00333276	0.01818871
Mfap1b	0.38560691	6.89563488	11.0979199	0.00334323	0.01822489
Emr4	-6.0019387	-1.2104886	11.0957723	0.0033457	0.01822489
Fam174b	0.8454336	6.56798464	11.0954771	0.00334604	0.01822489
Mettl2	-0.8166793	4.13577789	11.0918526	0.00335022	0.01823557
Fgf16	-4.3933147	-1.5914107	11.0878118	0.00335489	0.01824134
B230206H07	-4.5083859	-1.6970281	11.0870929	0.00335572	0.01824134
Pak7	-0.6432953	6.03121147	11.0680401	0.00337783	0.01831035
Nphp4	-1.1146332	2.08215996	11.0675728	0.00337838	0.01831035
Med13l	-0.6867569	6.48937418	11.0665519	0.00337957	0.01831035
Gadd45g	0.99202805	2.06850127	11.0663234	0.00337983	0.01831035
Snf8	0.76468148	4.26851453	11.0651875	0.00338116	0.01831035
Gemin6	0.73777067	2.96628022	11.0646514	0.00338178	0.01831035
Ppp4r4	-0.6991609	4.90833308	11.0566967	0.00339107	0.01834857
Erlin1	-0.5563093	4.56877923	11.0471344	0.00340228	0.01839395
Slc1a2	-0.6354557	10.9655463	11.0457256	0.00340393	0.01839395
Bsn	-0.8849749	9.30856583	11.0412018	0.00340925	0.01840506
Aida	0.44601763	6.45288865	11.0387399	0.00341215	0.01840506
Cdc42ep2	1.10289914	2.08049696	11.0381722	0.00341282	0.01840506
Psmb2	0.58836762	5.36654514	11.0356161	0.00341583	0.01840506
Dnaja4	0.53950657	4.57273453	11.0344693	0.00341719	0.01840506
Fkbp15	-0.523316	4.74414784	11.0309754	0.00342131	0.01841521
Gstt3	0.74640813	5.20221588	11.0277226	0.00342516	0.0184221
Sec13	0.49185052	4.60747021	11.0261017	0.00342708	0.0184221

1700123M08	1.46472483	1.25179356	11.0176199	0.00343713	0.01845919
Ddx39b	0.45292912	5.43056837	11.0150402	0.0034402	0.01845919
Eif3m	0.41837299	6.20702064	11.0145954	0.00344073	0.01845919
Zbtb7c	0.65236329	4.09881819	11.0127171	0.00344296	0.01845919
Scn2a1	-0.756299	8.05753643	11.0065171	0.00345034	0.01848673
Dusp15	-0.8210011	2.85404897	11.0022183	0.00345547	0.01850216
Sptbn1	-0.7342806	10.099249	10.9973363	0.00346131	0.01852134
Cox7a1	1.17579152	2.11222014	10.9948309	0.00346431	0.01852534
Mrps15	0.62792498	4.23879358	10.9904639	0.00346955	0.01852983
Sema6d	-0.5681616	6.16563267	10.9898782	0.00347025	0.01852983
Cox8a	0.58629307	7.4453694	10.9884918	0.00347192	0.01852983
Oat	0.57266311	8.127886	10.985718	0.00347525	0.01853087
Tmem26	-4.7181379	-1.4309923	10.9829534	0.00347857	0.01853087
Aldh5a1	-0.5213031	6.74500979	10.9817334	0.00348004	0.01853087
Shc1	0.74388463	6.42383923	10.9793811	0.00348287	0.01853087
BC030499	-1.568862	2.4979529	10.9770223	0.00348572	0.01853087
Lmo7	-0.8506014	6.02077423	10.976715	0.00348609	0.01853087
Ythdc2	-0.7208366	5.51634512	10.9752204	0.00348789	0.01853087
Lrrc4	-0.5341251	5.96828391	10.9715089	0.00349238	0.0185427
Gm14092	-5.0168258	-1.0279516	10.9675965	0.00349711	0.01854739
Pla2g4e	-0.6196353	4.55148466	10.9670471	0.00349777	0.01854739
Impg2	-4.0440684	-0.3888372	10.9592768	0.0035072	0.01857984
4930519G04	-1.1207675	2.31728697	10.9582741	0.00350841	0.01857984
Fat3	-0.9494523	8.06281523	10.9552098	0.00351214	0.01858759
Spop	-0.3786215	7.72348367	10.9423577	0.00352781	0.01865851
Smtnl2	-1.2693902	1.14257697	10.9402005	0.00353045	0.01866046
Hipk4	-0.8177049	3.97058021	10.9254226	0.00354858	0.01874423
Tpd52l2	0.69043986	6.30856718	10.921345	0.0035536	0.01875869
Vps13a	-0.6298811	7.32010642	10.9163221	0.00355979	0.01877934
Patl2	7.95412703	-1.4006495	11.4396924	0.00356283	0.01877945
Atr	-0.6426017	4.55086409	10.9110814	0.00356627	0.01877945
Tax1bp1	0.36833557	8.62016464	10.9103254	0.00356721	0.01877945
Decr1	0.69499586	5.39052888	10.9076372	0.00357053	0.01877945
Cmya5	-0.8846588	3.28475375	10.9070687	0.00357124	0.01877945
Cpt2	0.73015037	3.28725638	10.8922896	0.0035896	0.01885243
Shoc2	0.3882255	8.6086196	10.8922067	0.00358971	0.01885243
Pcdhb17	-0.660987	4.75954171	10.8867686	0.00359649	0.01886787
Abhd5	0.59376227	6.67456623	10.8860619	0.00359737	0.01886787
Aprt	0.82485293	3.50790968	10.8843336	0.00359953	0.01886787
Sufu	0.58161825	4.10405418	10.8808945	0.00360383	0.01887838
D8Ertd82e	-0.7000983	4.35499075	10.8770565	0.00360864	0.01889152
4930469G21	-2.3484122	-0.1709537	10.8698663	0.00361767	0.01891329
Slc25a27	-0.6887286	4.58888176	10.8685358	0.00361934	0.01891329
Nfatc1	0.63648625	3.99991759	10.866953	0.00362133	0.01891329
Rgcc	0.88615551	2.86685262	10.8664164	0.003622	0.01891329

Eogt	-0.5229403	5.15256979	10.8552561	0.00363608	0.01897474
Dgke	-0.6223096	5.5125218	10.8509664	0.00364151	0.01899101
Psma3	0.44279624	6.99917801	10.8453967	0.00364857	0.01901576
Dnah11	-2.4694589	-0.3051368	10.8410027	0.00365415	0.01903212
Tet2	-0.7169314	6.67525259	10.8392794	0.00365634	0.01903212
Sgpp2	-0.6274741	3.73622858	10.8281653	0.0036705	0.01907616
Pon3	1.08675115	3.32299925	10.827421	0.00367146	0.01907616
D17H6S53E	-0.741061	2.75255333	10.8263174	0.00367287	0.01907616
Nudt6	0.78269823	3.7359988	10.8253656	0.00367408	0.01907616
Zdhhc17	-0.5319727	6.78753137	10.8185756	0.00368278	0.01910923
Poll	1.40557411	0.87699302	10.8088299	0.00369529	0.01913097
Fbrsl1	-0.6071091	4.62818827	10.8060602	0.00369886	0.01913097
Zfp280c	-0.551537	5.37018434	10.8058021	0.00369919	0.01913097
Slc39a2	-2.842508	0.47883261	10.8052991	0.00369984	0.01913097
Pik3r5	-0.7874165	2.80084635	10.8048204	0.00370046	0.01913097
Athl1	0.81978461	3.28005213	10.8044528	0.00370093	0.01913097
Bcas1os2	-2.6519386	-0.1968734	10.7916176	0.00371752	0.01918733
Epg5	-0.9016971	5.48609808	10.7908859	0.00371847	0.01918733
Klf10	0.45782955	5.6640606	10.7905984	0.00371884	0.01918733
Rftn2	0.55184255	4.75878567	10.7887598	0.00372122	0.01918759
Zmat2	0.42000479	7.1416039	10.7830191	0.00372868	0.01921397
Fam84a	-0.5061072	4.98378711	10.7790858	0.00373379	0.01921697
Myeov2	0.5744062	4.74117315	10.778947	0.00373397	0.01921697
Pou2f2	-1.0021751	3.6192154	10.7743555	0.00373996	0.01921697
Angel1	-1.3868132	1.42903695	10.7739926	0.00374043	0.01921697
Tor1b	-0.7057691	3.76440609	10.7712423	0.00374402	0.01921697
Arhgef3	-0.6023062	5.69113974	10.7711099	0.00374419	0.01921697
Lrrn3	-0.4398024	5.76530635	10.7691714	0.00374672	0.01921697
Psmc1	0.43548541	6.37600681	10.7682228	0.00374796	0.01921697
Pcdh18	-0.75619	3.19480884	10.7605679	0.00375799	0.01923948
Mccc1	-0.7301933	3.58728736	10.7564573	0.00376338	0.01923948
Alms1	-0.7134541	5.05209571	10.7559087	0.0037641	0.01923948
Exd2	-0.5664278	5.36403923	10.7527632	0.00376824	0.01923948
Dmxl2	-0.861367	8.52492844	10.7504244	0.00377131	0.01923948
4930449E18I	-2.7856798	-0.8202355	10.7499495	0.00377194	0.01923948
Cnot6l	-0.3973624	7.06788096	10.7493328	0.00377275	0.01923948
Lrp6	-0.4171297	6.82316275	10.7475942	0.00377504	0.01923948
Wdr53	0.96094052	3.18469403	10.7453732	0.00377797	0.01923948
Mogs	-1.3488608	1.72178949	10.7446177	0.00377896	0.01923948
Llgl1	-0.5598428	3.95670421	10.7444877	0.00377914	0.01923948
Ambra1	-0.5829434	5.77368778	10.7434968	0.00378044	0.01923948
Isoc2a	1.02151444	1.96736771	10.7392172	0.0037861	0.01924992
Map3k10	-0.6933908	3.26749762	10.7383979	0.00378718	0.01924992
Itpr1	-0.6092775	8.99082868	10.7323674	0.00379517	0.01927858
Mga	-0.604794	7.82664413	10.7213343	0.00380982	0.01932951

Tmem40	2.87335869	-0.055011	10.7212806	0.00380989	0.01932951
Sema4f	-0.7713633	4.77742819	10.7130353	0.00382089	0.01936226
5530601H04l	-0.8051855	3.42913105	10.7129063	0.00382106	0.01936226
Dcdc2c	-1.0750799	5.66716477	10.7106124	0.00382413	0.01936585
Arpc2	0.45504427	8.49607501	10.7008343	0.00383722	0.0194202
Grm3	-0.4909184	5.65836054	10.6964567	0.0038431	0.01943799
C630031E19l	-1.5392388	1.84095033	10.686191	0.00385693	0.01949592
Pappa	1.4831658	2.78235808	10.6809107	0.00386406	0.01951998
Map3k9	-0.7503173	6.22637479	10.6770734	0.00386925	0.0195342
Baiap3	-1.7191907	1.53709293	10.6740315	0.00387338	0.01954301
Mrpl48	0.57521615	4.45737419	10.6713167	0.00387706	0.01954959
Llph	0.60417648	7.07072829	10.6677606	0.00388189	0.01955032
Sphkap	-0.8693898	7.2290455	10.6677091	0.00388196	0.01955032
Cdk5r1	-0.5552289	6.96452263	10.6658724	0.00388446	0.01955091
Clca1	-3.8723215	-0.4968022	10.6634341	0.00388777	0.01955163
Cidea	6.48540758	-1.9543343	11.1664321	0.00388936	0.01955163
Dock9	-0.5747884	6.49843439	10.6572468	0.00389621	0.01957411
Neat1	-0.5622523	5.07494143	10.652908	0.00390214	0.0195914
Eif4enif1	0.36852148	6.6647732	10.6512388	0.00390442	0.0195914
AI597479	0.51116146	5.87756521	10.6470701	0.00391013	0.019599
Zfp169	-0.6279471	4.07761419	10.6466489	0.0039107	0.019599
Ahsa1	0.4764222	5.77367489	10.6430898	0.00391558	0.01961151
Adat1	-1.1855926	1.8778403	10.6367037	0.00392436	0.01964349
Nova1	-0.4498809	7.15070647	10.628329	0.0039359	0.01968314
Mrpl44	0.62645699	3.56917656	10.6272638	0.00393737	0.01968314
Dpy19l1	-0.408194	6.7235325	10.624987	0.00394052	0.01968314
Atg5	0.50255417	4.61398251	10.6239943	0.00394189	0.01968314
Gpaa1	-1.3133684	1.39963622	10.6214344	0.00394543	0.01968314
Cybrd1	-1.0916226	2.11232125	10.6204215	0.00394683	0.01968314
1700112E06l	1.38896423	1.17197641	10.6171464	0.00395137	0.01968314
Atp2c1	-0.4344824	6.56727985	10.6170945	0.00395144	0.01968314
Emc4	0.39416429	6.33194098	10.6039843	0.00396966	0.01975665
Fam131c	4.29154055	-0.4741631	10.6030205	0.003971	0.01975665
Ssh3	-1.1229857	1.67855805	10.5960492	0.00398074	0.01979308
Tnrc6a	-0.4630999	6.73545539	10.5928236	0.00398525	0.01980353
Slc26a8	-1.9046795	0.88939182	10.5894421	0.00398998	0.01981509
Pot1b	-0.7723175	3.44286884	10.5839545	0.00399768	0.01984133
Supt6	-0.5207466	7.40632526	10.5808358	0.00400206	0.01985109
Acsl4	-0.4337971	6.37633032	10.5603875	0.00403093	0.01998222
Phf20l1	-0.5684828	5.71701924	10.5447042	0.00405323	0.02008063
Abcc10	-1.4051591	0.96648343	10.539409	0.00406079	0.02009927
Tomm22	0.60311831	5.96992969	10.5383905	0.00406224	0.02009927
Zfand6	0.6112296	5.61016138	10.5369328	0.00406433	0.02009927
1700047M11	-1.1468847	1.0395494	10.5324171	0.00407079	0.02011914
Dzip1	-0.4186554	6.54829099	10.5282699	0.00407674	0.02013355

Macf1	-0.8184032	9.05836431	10.5269694	0.0040786	0.02013355
Puf60	0.4563285	6.5038711	10.5218025	0.00408603	0.0201581
Pacsin2	0.41846622	6.2208514	10.5008805	0.00411626	0.02029249
4930512B01l	-1.8776508	0.91539927	10.4978289	0.00412069	0.02029249
1500004A13l	-0.7356738	4.96989979	10.4972331	0.00412155	0.02029249
1110002L01l	1.44810987	1.11753927	10.4961366	0.00412315	0.02029249
Gm15401	-1.5507839	0.45559154	10.4941858	0.00412598	0.02029429
Nhs	-0.7243242	3.61151607	10.4895682	0.00413271	0.0203152
Ncdn	0.52353757	8.21970628	10.4870533	0.00413637	0.02032107
Rtfdc1	0.61379767	5.58179619	10.4852345	0.00413903	0.02032124
Hmmr	-0.7614777	3.27664422	10.4825661	0.00414292	0.02032124
Scrib	-1.0154457	2.10448195	10.4819237	0.00414386	0.02032124
Sipa1l3	-0.4971355	5.34766959	10.4802586	0.0041463	0.02032124
Rel	0.50940959	4.52771932	10.4772519	0.0041507	0.02032502
Ints6	-0.5146165	4.96000102	10.4763522	0.00415201	0.02032502
Itgam	-0.8702136	3.55949503	10.4689396	0.00416289	0.02036611
Gm11437	-4.1120719	-1.1134879	10.4663412	0.0041667	0.02037267
Rbm47	0.67430296	4.57140377	10.4548145	0.00418369	0.02044015
Ero1l	-0.4318233	6.37695427	10.4536048	0.00418548	0.02044015
Setd2	-0.4722376	7.47981448	10.4463182	0.00419626	0.02047956
Urb1	-0.9598535	2.76951926	10.4435219	0.00420041	0.02047956
Hdgf	0.51268582	6.54387813	10.4418784	0.00420285	0.02047956
Gm21284	-1.8566992	0.13904433	10.441428	0.00420352	0.02047956
Cep350	-0.6013444	6.68424156	10.4388773	0.00420731	0.02048588
Tas1r3	-2.2308069	-0.1782649	10.4345388	0.00421376	0.02050516
Fam53b	0.64036132	4.28924641	10.4313298	0.00421854	0.02051628
AI414108	-0.8747545	4.67017966	10.4282169	0.00422318	0.02052142
Usp19	-0.5310043	4.7584314	10.4224153	0.00423185	0.02052142
Cltb	0.59373075	5.79999966	10.4205604	0.00423463	0.02052142
Bag1	0.58758002	7.80584386	10.4199572	0.00423553	0.02052142
Cts8	-1.6939329	0.00836896	10.4191752	0.0042367	0.02052142
Sgk1	0.44056721	6.58128598	10.4189579	0.00423703	0.02052142
Arhgap39	-0.5377883	4.98563479	10.418377	0.0042379	0.02052142
Rn4.5s	-1.5167701	0.52362576	10.4155121	0.0042422	0.02052142
Zfp60	-0.4288493	6.37575966	10.4146925	0.00424342	0.02052142
Zmynd15	-2.2949598	0.33496792	10.4138995	0.00424462	0.02052142
Nme1	0.43452871	6.28486745	10.4122693	0.00424706	0.02052142
Sub1	0.45553008	8.79956942	10.4097017	0.00425092	0.02052799
Atp1a4	-2.4166723	-0.6699236	10.3983383	0.00426804	0.02059858
Top1mt	0.79416111	3.13439273	10.3939997	0.0042746	0.02061812
Loxl3	-1.2120585	1.77272486	10.3894595	0.00428148	0.02063917
Atp5j	0.51213858	7.73128512	10.36972	0.00431151	0.02077175
Hic2	-0.9063765	2.3305498	10.3627837	0.00432212	0.02080697
Fam71b	-5.178783	-1.7554449	10.3609478	0.00432493	0.02080697
Mybpc2	1.50496726	1.39858629	10.3599792	0.00432641	0.02080697

1700123O20	0.75548514	3.87720246	10.3544904	0.00433484	0.02083431
Ghsr	-3.0032509	-0.3539409	10.3529747	0.00433717	0.02083431
Carm1	0.4984818	5.24535522	10.3476758	0.00434532	0.02086129
Hes6	0.86192681	2.20106607	10.344845	0.00434969	0.02086135
Stx4a	0.57029058	6.39747626	10.3437818	0.00435133	0.02086135
Cps1	-4.3547066	-1.1991951	10.3419685	0.00435413	0.02086135
Capn2	0.49343824	7.15307409	10.3394628	0.004358	0.02086135
Rfc2	0.82299675	3.34450161	10.3394434	0.00435803	0.02086135
Commd10	0.58655315	4.67204562	10.3377115	0.0043607	0.02086202
Med29	0.58142912	3.3883453	10.3326631	0.00436852	0.02087868
Ptges	0.85422797	4.28233446	10.3321819	0.00436927	0.02087868
Ddit4l	-0.4672914	4.93093377	10.3264208	0.00437821	0.02089591
Plekhm2	-0.8399705	3.60509107	10.3263118	0.00437838	0.02089591
Fbxo36	1.2141601	2.9116626	10.3242176	0.00438163	0.02089591
Ndufa1	0.67270599	5.23904643	10.3233104	0.00438304	0.02089591
Pdha1	0.33318954	7.41076837	10.3151765	0.00439571	0.02094417
Scn4b	-0.6821831	4.78443793	10.3111929	0.00440193	0.02096166
4930419G24	-1.5034158	1.826446	10.3087755	0.00440571	0.02096752
Slc8b1	-1.0810731	1.35538975	10.304619	0.00441222	0.02098634
Eif4a3	0.50998298	5.00288545	10.3021585	0.00441608	0.02098956
Pcdh10	-0.5514592	7.10637105	10.3009299	0.00441801	0.02098956
Tmem218	0.79676845	3.03925079	10.2975807	0.00442327	0.02099174
Gabrb2	-0.5543922	8.24512873	10.2973844	0.00442358	0.02099174
Map7	-0.4716011	5.79155177	10.2927976	0.00443079	0.02101385
Arhgef2	-0.787697	5.56237743	10.2903954	0.00443457	0.02101966
Herc6	-0.5464263	5.29591099	10.2874158	0.00443927	0.0210298
Skida1	0.74176834	4.1254437	10.282483	0.00444706	0.02105457
Fcf1	0.58032473	4.74357637	10.2808254	0.00444968	0.02105485
Snx15	0.49722581	4.50132427	10.2761686	0.00445706	0.02106764
Lin37	0.5562919	4.4352741	10.2758808	0.00445751	0.02106764
Ctc1	-0.5583165	4.13550488	10.2632727	0.00447754	0.02115015
Krt20	-0.9675939	3.87469395	10.2603745	0.00448216	0.02115981
Golph3	0.45468186	8.57650586	10.2526645	0.00449448	0.02120576
Mocs2	0.48862025	6.84766251	10.2498292	0.00449901	0.02121499
Col10a1	-2.7237784	-0.5031777	10.24297	0.00451001	0.02124294
Pclo	-0.9315994	10.1329204	10.2429089	0.00451011	0.02124294
Ptcd1	0.6769351	3.44479309	10.2403831	0.00451417	0.02124987
Aldh1l1	0.6855808	3.6319835	10.2328684	0.00452626	0.02129107
Prdx2	0.66510282	6.20444139	10.2317296	0.0045281	0.02129107
Cebpd	1.45329261	1.35921377	10.2276591	0.00453467	0.02129181
Rgs19	0.64113203	4.53633584	10.2270888	0.00453559	0.02129181
Cntnap5b	-0.6398237	4.15925846	10.2268175	0.00453603	0.02129181
Myl12b	0.59206484	7.8337359	10.2182324	0.00454993	0.02134132
Mmadhc	0.6113371	5.8456984	10.2170958	0.00455177	0.02134132
Grm7	-0.9436965	4.58101982	10.2144699	0.00455603	0.02134912

Mpc1	0.40482298	6.49887657	10.20783	0.00456683	0.02137842
Slc4a11	3.82201456	-0.2079535	10.2074263	0.00456748	0.02137842
2700060E02I	0.44946327	5.83392768	10.2038626	0.00457329	0.02138754
Gm5415	-1.7382368	2.10985562	10.202308	0.00457583	0.02138754
Fam124a	-0.6034895	4.4110444	10.2014426	0.00457724	0.02138754
Aurkaip1	0.66037614	4.92854804	10.1867083	0.00460136	0.02148422
Uba3	0.42245331	6.23925471	10.1848688	0.00460438	0.02148422
Taf4a	-0.4908626	4.74494453	10.1819426	0.00460919	0.02148422
Acat1	0.43865756	7.12008456	10.1813361	0.00461019	0.02148422
Adcy8	-0.8874756	3.85815397	10.1808423	0.004611	0.02148422
Anapc15	0.89893403	2.43938327	10.1786313	0.00461464	0.021489
Dzank1	-0.5412461	8.55865494	10.1735799	0.00462297	0.02149495
Ttbk2	-0.478863	7.80790211	10.1732828	0.00462346	0.02149495
Rprd2	-0.45593	7.26401555	10.1730987	0.00462376	0.02149495
Nsun6	-0.8256225	2.75050169	10.1678217	0.00463248	0.02151948
Ttc21b	-0.6765307	4.37167452	10.166737	0.00463428	0.02151948
Hspb6	0.59022042	4.31120956	10.1650239	0.00463711	0.02152049
Slc23a1	-1.4364701	0.69192188	10.1630626	0.00464036	0.02152342
Grsf1	0.37843341	6.82277294	10.1557947	0.00465243	0.0215672
Tmem55b	0.57695652	4.99186399	10.1371113	0.00468359	0.02169803
Itga10	-0.8386233	2.96327098	10.1351802	0.00468683	0.02169803
Zfp316	-0.8187902	3.73283924	10.133114	0.00469029	0.02169803
Grhl1	-0.8255609	2.97171947	10.1325684	0.00469121	0.02169803
Mrpl38	0.72644085	3.37361832	10.1304192	0.00469481	0.02170203
Ap5m1	-0.7965244	3.37570823	10.1289069	0.00469735	0.02170203
Mcf2l	-0.9716841	4.68359106	10.117944	0.00471581	0.02176339
Nop10	0.57874117	4.74626895	10.1175622	0.00471646	0.02176339
Elovl7	-0.6416632	4.0967768	10.1163036	0.00471858	0.02176339
Cadm1	-0.6133718	6.13708656	10.1128275	0.00472445	0.02177826
Gm20110	-4.4748395	-1.7276857	10.1008996	0.00474467	0.02185918
E130309F12I	-0.8696147	2.72267869	10.0922375	0.00475941	0.02191192
Naa60	0.38548971	6.11045487	10.0910407	0.00476145	0.02191192
Fam169b	-1.1280916	1.94048087	10.0863198	0.00476951	0.02193673
Tmem117	-0.8301757	3.28497422	10.0829025	0.00477535	0.02195131
Wdfy3	-0.8514089	7.86625625	10.0795515	0.00478109	0.0219654
Gtf2e2	0.49740579	4.51126659	10.0761307	0.00478695	0.02198006
Dzip3	-0.5545359	6.90177053	10.0637443	0.00480825	0.02206554
Myo5a	-0.8607135	9.77832343	10.0617163	0.00481175	0.02206927
Ncapd2	-0.9121929	2.67232371	10.0567358	0.00482035	0.02209597
Ermard	-0.6402068	3.92559189	10.0549186	0.00482349	0.02209597
Kctd20	0.94572812	3.14629532	10.0536809	0.00482564	0.02209597
Pkp2	-1.1123638	2.23426187	10.0521069	0.00482836	0.02209614
Ybx1	0.50600753	7.17213892	10.0498573	0.00483226	0.02210168
LOC1010560	0.81049772	3.43306051	10.0473118	0.00483668	0.02210957
Krt12	-0.5522549	4.21129125	10.0457056	0.00483946	0.02211001

Ankmy2	0.35955645	6.26991117	10.0402271	0.00484899	0.02213309
Rsrp1	-0.7105378	6.29280834	10.039703	0.0048499	0.02213309
Tm6sf2	-3.8058907	-1.4528194	10.034554	0.00485887	0.02215777
Add3	0.41078539	8.2735249	10.0335056	0.0048607	0.02215777
Rpe65	-2.6710462	0.67821647	10.0307557	0.0048655	0.02216736
Gje1	-1.2401179	1.15646148	10.0228452	0.00487934	0.0222181
Mnat1	0.61448281	4.19867211	10.0209935	0.00488259	0.02222057
Gabrg2	-0.4856848	6.44501646	10.0125438	0.00489743	0.02227369
Lama3	-1.075536	2.5107146	10.0112667	0.00489968	0.02227369
Degs2	-1.4472658	1.57855019	10.0044284	0.00491173	0.02231615
Cd7	4.34740694	-1.7326019	10.0008457	0.00491806	0.02231884
Tsg101	0.4738813	5.33394706	9.99797841	0.00492314	0.02231884
Cops7a	0.53745489	6.91236937	9.99766999	0.00492368	0.02231884
Chtop	0.4289738	7.48466396	9.99602191	0.0049266	0.02231884
Grm1	-0.6033809	5.38932871	9.99590005	0.00492681	0.02231884
1700009P17l	1.27057956	1.70603759	9.99488103	0.00492862	0.02231884
Rims2	-0.6756566	6.98291186	9.98992787	0.00493741	0.02234632
Spns1	-1.0616218	2.31003266	9.97958481	0.00495581	0.0223819
Gm6583	-4.0020031	-1.0788076	9.97867914	0.00495742	0.0223819
Nkx1-2	7.44225759	-1.9523449	11.1142912	0.0049584	0.0223819
Cyfip1	-0.4346837	6.22946683	9.97640288	0.00496149	0.0223819
Rev3l	-0.548601	7.29469965	9.97636922	0.00496155	0.0223819
Usp51	-1.5270917	1.25592002	9.97588737	0.00496241	0.0223819
Cs	0.32829277	7.85166602	9.97481016	0.00496433	0.0223819
Zfp518a	-0.4446922	5.61763445	9.96437795	0.004983	0.02245378
Pcolce2	-1.9030402	1.14660412	9.95945547	0.00499184	0.02246353
Pddc1	0.64402099	3.60516068	9.95678858	0.00499664	0.02246353
Nrg3os	-2.1868057	0.39788992	9.95579792	0.00499842	0.02246353
Acsf2	0.5845375	4.8861984	9.95570835	0.00499858	0.02246353
Eci2	0.61307876	5.4609683	9.95443093	0.00500088	0.02246353
Mtor	-0.6869642	6.58603647	9.95205318	0.00500517	0.02246353
Smap1	0.35429351	6.28610141	9.95143225	0.00500629	0.02246353
Mettl17	-1.1839187	1.69387304	9.95101855	0.00500703	0.02246353
Tceal6	0.43722099	4.72825426	9.94487468	0.00501813	0.02250102
Dicer1	-0.4793978	6.2937579	9.94181694	0.00502366	0.02251354
Rnpc3	-0.8283123	4.82241866	9.93859711	0.00502949	0.02252739
Tmem145	-1.2234206	2.44946133	9.93593638	0.00503432	0.02252869
Pnpt1	-0.8041786	3.92011407	9.93486647	0.00503626	0.02252869
D030040B21	-1.8621657	-0.4700405	9.93390381	0.005038	0.02252869
Hnrnpa2b1	0.29907456	8.6580106	9.93133037	0.00504268	0.02253734
Tmem170b	-0.4554995	7.50671763	9.92893902	0.00504703	0.02254452
Gm1943	0.72235877	3.5392695	9.90901913	0.00508342	0.02268552
Mtap7d3	1.29275034	1.90900847	9.90830081	0.00508473	0.02268552
Fam73a	-0.4652389	6.03463599	9.90713407	0.00508688	0.02268552
Wdr54	0.8302788	3.28234784	9.89677785	0.00510592	0.02275071

Gm20337	-1.3106611	2.04686625	9.89534247	0.00510857	0.02275071
Brwd1	-0.5656465	6.9745461	9.89319935	0.00511252	0.02275071
Parp3	0.74129637	4.28637517	9.89317671	0.00511257	0.02275071
Grin2b	-0.6215771	7.05758381	9.88885818	0.00512054	0.02277388
Fam72a	2.89644261	0.51817619	9.8872178	0.00512358	0.02277505
Cdt1	2.63501206	-0.1894261	9.8814238	0.00513431	0.02280794
Mdga2	-0.5529257	6.06321093	9.88022814	0.00513653	0.02280794
Sned1	0.85946466	4.23200509	9.87725897	0.00514204	0.02280968
Sh3gl2	0.50817988	9.33445628	9.8757279	0.00514488	0.02280968
Gjb2	0.73321243	9.09863828	9.87553401	0.00514524	0.02280968
Me3	-0.7795972	4.21230339	9.86967956	0.00515614	0.02284079
Insr	-0.5382251	5.99010779	9.86877783	0.00515782	0.02284079
Olfr856-ps1	-1.0927505	4.31338526	9.86658531	0.00516191	0.02284659
Ube2b	0.38138207	7.64249085	9.86312528	0.00516837	0.02286287
Mapkapk2	0.68787686	4.27647673	9.85824582	0.00517749	0.02289091
Dnah9	-1.6530469	1.50568372	9.84584422	0.00520076	0.02297225
Idh1	0.50524008	5.41998055	9.84546403	0.00520148	0.02297225
Wdr59	-0.6784004	4.08083724	9.8417558	0.00520846	0.02299073
E2f5	0.53335235	4.32218395	9.83455478	0.00522205	0.02301715
Fbxl12	-0.8666525	2.3507409	9.83447761	0.0052222	0.02301715
Eif3f	0.5063134	5.99474716	9.83413178	0.00522285	0.02301715
Pglyrp3	6.36215174	-2.0173844	10.2676203	0.00522673	0.0230219
Fras1	-1.0467522	4.82325395	9.82505969	0.00524003	0.02306812
Slc30a7	-0.5787399	4.66352327	9.8229193	0.00524409	0.02307364
Mst1	-4.2081227	-1.388693	9.81845567	0.00525257	0.0230986
Cox6c	0.53972265	7.55381088	9.81455778	0.00525999	0.02311886
Enpp6	-0.9777979	2.77992828	9.80213681	0.00528371	0.0232107
Pfn1	0.77015129	6.68741467	9.79727497	0.00529303	0.02322532
Tspan1	-5.177398	-1.3200596	9.79653279	0.00529445	0.02322532
Ankrd46	0.41059458	6.77853635	9.79597908	0.00529552	0.02322532
Isg15	1.06053016	1.64213574	9.79427067	0.0052988	0.02322731
Sox4	0.7870528	2.90899654	9.78995794	0.00530709	0.02324221
P2ry1	0.74820631	4.02734054	9.78955859	0.00530786	0.02324221
Tia1	-0.654083	5.2628024	9.78809149	0.00531068	0.02324221
2610307P16I	2.27847687	0.88520349	9.78230644	0.00532183	0.02327862
Chd9	-0.5132453	7.98540391	9.78010542	0.00532608	0.02328482
Abracl	0.65096874	4.25687212	9.77708067	0.00533193	0.02329293
Frk	0.77064014	4.02607557	9.77621297	0.00533361	0.02329293
1110012L19I	1.04899115	3.3695072	9.77017932	0.00534529	0.02333158
Ints2	-0.6487463	4.08922005	9.75954232	0.00536597	0.02340938
Atp5k	0.56537507	5.92428404	9.75791143	0.00536914	0.02341082
Tnk2	-0.5229262	5.09369377	9.73808335	0.00540794	0.0235675
Npnt	-0.5631169	3.77911532	9.73588435	0.00541227	0.02357383
Lrrc39	-0.9300191	2.13293728	9.73192678	0.00542005	0.02359525
Gabra5	-0.4516913	5.66006483	9.72875618	0.0054263	0.02360054

Ndufb5	0.47317753	6.73413816	9.72839636	0.00542701	0.02360054
Mx2	1.86832283	0.06366285	9.72416456	0.00543537	0.02362437
Pla2g16	0.68917599	6.73766589	9.71293123	0.00545761	0.02370852
Vezf1	0.47774151	7.58884059	9.70909035	0.00546524	0.02370968
Cpne2	0.49730667	4.41297001	9.70859016	0.00546623	0.02370968
Gm16386	-1.0561768	2.00126101	9.7084165	0.00546658	0.02370968
Sorcs2	-0.6734127	3.64157151	9.70619019	0.00547101	0.02370968
Syndig1l	-0.9321017	2.34801023	9.70554007	0.0054723	0.02370968
Adh5	0.45868973	6.35142183	9.697674	0.00548799	0.02376511
Scai	-0.5141981	7.0689649	9.68894066	0.00550546	0.02382821
Rbm5	-0.4748386	6.94168735	9.68468418	0.005514	0.02385261
Ldb2	0.39826254	6.02685681	9.683216	0.00551694	0.02385281
Adam17	-0.5604667	4.19544606	9.67914114	0.00552514	0.02387566
Gbp8	1.40819855	1.80266192	9.66884419	0.0055459	0.02391629
Ube2l3	0.43843159	7.51291036	9.6682685	0.00554706	0.02391629
Pitpnm3	-0.4946672	6.08220485	9.66736246	0.00554889	0.02391629
Chmp3	0.47817585	6.51614272	9.66619372	0.00555126	0.02391629
Spc24	-1.0807355	2.43892939	9.66571148	0.00555223	0.02391629
U2surp	-0.3701759	7.73191875	9.66417839	0.00555533	0.02391629
Ccdc28a	0.92611296	2.50024667	9.66368925	0.00555632	0.02391629
Ppdpf	0.52936334	5.09338992	9.66066301	0.00556246	0.02391629
Sox11	-0.6547736	4.87642157	9.65995349	0.00556389	0.02391629
Arpp19	0.33842605	9.06957208	9.657693	0.00556848	0.02391629
Grin2a	-0.6086821	6.61728008	9.65741675	0.00556904	0.02391629
Rab43	0.5910243	5.4568672	9.65721272	0.00556946	0.02391629
Polr2e	0.62160452	4.39097372	9.65428215	0.00557541	0.02392409
Vmn2r87	-1.8482612	1.17231692	9.65345227	0.00557709	0.02392409
Ralgapa1	-0.7078008	7.7368995	9.65108967	0.0055819	0.02393222
Shisa6	-0.7253105	5.30408921	9.64661915	0.00559101	0.02395876
Lgals12	-2.395162	-0.2240564	9.64384867	0.00559666	0.02397048
1810055G02	0.48783482	5.57403239	9.64218436	0.00560006	0.02397254
Setmar	1.0733628	2.36982812	9.63986616	0.00560479	0.02398033
Myoz3	-1.619744	1.1393035	9.63577571	0.00561316	0.02400364
Rabggtb	0.47541106	6.33194334	9.63353807	0.00561775	0.02401003
Abcc9	-1.1090831	3.11914776	9.63219575	0.0056205	0.02401003
Prkd3	0.49810439	5.66350149	9.62856773	0.00562794	0.02402084
Tec	0.75579243	5.07150878	9.62811478	0.00562887	0.02402084
Gm1821	0.65067422	4.9269643	9.62630527	0.00563259	0.02402169
Slc46a1	-1.0998028	1.24647663	9.62517383	0.00563492	0.02402169
Serp1	0.80278728	7.92018947	9.62076201	0.005644	0.02404794
B3gntl1	-0.743428	2.62906946	9.61613822	0.00565354	0.02406557
Bdh1	0.64209852	3.63196056	9.61591531	0.005654	0.02406557
Pdzd8	-0.3538904	7.10026311	9.61162553	0.00566286	0.02408208
1700028K03I	-1.4765295	2.08229983	9.60958864	0.00566707	0.02408208
Casq1	-2.9974212	0.15794126	9.6094558	0.00566735	0.02408208

Kdm5a	-0.4454626	7.06546638	9.60837056	0.0056696	0.02408208
Ddi2	0.99194557	2.7742584	9.60598875	0.00567453	0.02408294
Gm10677	-1.5111929	1.60301891	9.60544493	0.00567566	0.02408294
Lix1l	0.84906176	6.40813005	9.60155123	0.00568374	0.02410477
Ace2	4.30926822	-0.716021	9.59956731	0.00568786	0.02410981
AI661453	1.2473284	1.73037844	9.59675028	0.00569371	0.0241222
Chst2	-0.3888236	7.83723352	9.59357442	0.00570033	0.02413398
Kcnj9	-0.5616827	5.02242823	9.59259451	0.00570237	0.02413398
Oaz2	0.49617007	6.79732827	9.58672439	0.00571461	0.02416521
Vti1b	0.45377175	5.76983741	9.58523777	0.00571772	0.02416521
Hnrnpk	0.3727812	7.92294978	9.58483297	0.00571857	0.02416521
Arsi	2.04462762	0.61766923	9.5766595	0.00573568	0.02421936
Nsmaf	-0.5553452	4.77577922	9.57590067	0.00573728	0.02421936
Gabarap	0.67406534	7.19834281	9.57044826	0.00574873	0.02425525
Lphn3	-0.5973341	6.76500608	9.56524587	0.00575968	0.02425992
Ppp1r7	-0.5233181	7.31260704	9.56387447	0.00576257	0.02425992
Tagap1	-0.5234941	4.77504619	9.56329082	0.0057638	0.02425992
Gm16023	-1.3859142	0.95694866	9.56310468	0.00576419	0.02425992
Ccdc102a	1.35388092	1.41072203	9.56291476	0.00576459	0.02425992
Ddx55	-0.5101176	4.40733803	9.55443588	0.00578251	0.02432287
Casp8	0.65473054	5.33351205	9.55087664	0.00579005	0.02434212
Ipo9	-0.4371602	7.01402493	9.54771154	0.00579676	0.02434653
D330045A20	-2.1081349	-0.0948508	9.54758882	0.00579702	0.02434653
4930486F22I	-4.4006839	-1.2626018	9.54452498	0.00580353	0.02436141
Pcdhgb4	-0.9433879	2.29278833	9.54154821	0.00580986	0.02437553
Sclt1	-0.5811819	4.66918407	9.53388589	0.00582619	0.02442663
E330033B04I	-1.324952	2.95242948	9.53304554	0.00582798	0.02442663
Cisd1	0.46484029	5.07555563	9.52140266	0.0058529	0.02451265
Rab13	0.80993017	4.24254159	9.52067096	0.00585447	0.02451265
Wnt5b	1.35852935	1.1476272	9.51907967	0.00585789	0.02451446
Daam2	-0.6492313	5.00971729	9.50761229	0.00588257	0.02460522
Adam22	-0.5300594	7.72163475	9.50394942	0.00589048	0.0246203
Ophn1	-0.5845526	4.9296589	9.50316798	0.00589216	0.0246203
Slc22a15	-0.8027142	2.70741686	9.49699975	0.00590551	0.02464675
Slc29a3	-0.5586075	5.26561223	9.49660345	0.00590637	0.02464675
Inpp5f	-0.3347672	6.79477906	9.49608792	0.00590749	0.02464675
Chrac1	0.83966903	2.94502002	9.49412864	0.00591174	0.02465196
Ubc	0.48946587	6.71051976	9.49151119	0.00591742	0.02466314
Bod1	0.52125762	6.10494432	9.48842671	0.00592412	0.02467324
Gm10409	-0.7656449	4.00369907	9.48763267	0.00592585	0.02467324
Prr13	0.54183755	5.59039695	9.48482238	0.00593196	0.02467643
Ahcyl2	0.46904085	8.1213262	9.48452205	0.00593262	0.02467643
Spry1	0.83491085	3.19488316	9.48000329	0.00594247	0.0247049
Slc5a7	-0.5978655	4.15569526	9.4780155	0.00594681	0.02471044
Gm7457	-3.9470659	-0.9471554	9.47059612	0.00596303	0.02476534

Igdcc4	-0.4155143	5.11809547	9.46830507	0.00596805	0.02477367
Hspa2	-0.6139214	4.93156623	9.46579405	0.00597356	0.02478402
Hspa1b	0.89376367	2.12639653	9.46349724	0.0059786	0.02478997
A330035P11I	1.22512903	1.93256336	9.46239354	0.00598103	0.02478997
Gm8234	-0.771798	3.69496776	9.45822502	0.0059902	0.02481546
Cir1	0.36969006	6.95479119	9.45536778	0.00599649	0.02482197
Cdk2ap1	0.46537558	6.49375569	9.45333565	0.00600097	0.02482197
Med26	0.76688224	2.57431246	9.44833523	0.00601201	0.02482197
C77370	-0.8665257	6.42862139	9.4479901	0.00601277	0.02482197
Adamts20	-1.4187181	2.22272247	9.44755301	0.00601374	0.02482197
BC005624	0.50217258	6.12337461	9.44704236	0.00601487	0.02482197
Rab35	0.46085876	4.52165069	9.44643726	0.00601621	0.02482197
Recql5	1.02012812	2.43642197	9.44392679	0.00602176	0.02482197
Lnp	-0.5412345	6.97294153	9.44383748	0.00602196	0.02482197
Rtca	0.46622684	5.33134198	9.44383439	0.00602197	0.02482197
C030046E11I	-0.4646355	5.47917695	9.44215965	0.00602568	0.02482481
Xrcc2	-1.0717079	2.5538066	9.43798327	0.00603494	0.02484367
Nsun3	0.5249673	4.6415673	9.43622413	0.00603885	0.02484367
Plekhb1	-0.5636083	5.81147658	9.4360094	0.00603932	0.02484367
Map2	-0.6588452	8.3176101	9.43249255	0.00604714	0.02486052
Ahcyl1	0.38971415	9.39081939	9.43144638	0.00604947	0.02486052
Lgals9	0.76156505	4.40799855	9.42569632	0.00606228	0.02490072
Eif3g	0.55147343	5.42210614	9.42389636	0.0060663	0.02490477
Golgb1	-0.394104	7.98617775	9.41760399	0.00608036	0.02493995
Abca8b	-1.0015072	3.33237728	9.41614976	0.00608362	0.02493995
Dusp8	-0.5510207	5.57658294	9.41599376	0.00608397	0.02493995
Agmat	2.90798541	-0.0296026	9.40975727	0.00609795	0.02497422
Fbxo8	0.45892659	4.84429171	9.40955649	0.00609841	0.02497422
Lca5l	-1.5605075	1.38066019	9.40813403	0.0061016	0.02497486
Hunk	-0.6139532	4.23948636	9.40261592	0.00611401	0.02501321
Bbip1	0.4159412	5.93859455	9.39992226	0.00612008	0.02502558
Mrps36	0.51848154	5.16856347	9.39797931	0.00612446	0.02503105
1810037I17R	0.58915987	5.63404425	9.39624236	0.00612839	0.02503462
Lysmd4	0.65504135	3.30306928	9.39268346	0.00613643	0.02505502
Nudcd2	0.7244499	3.02746944	9.38398381	0.00615613	0.025123
Pcdh9	-0.4975251	7.3123863	9.37663843	0.00617283	0.02517863
Anxa4	0.70939821	6.49079403	9.3746596	0.00617733	0.02518451
Uaca	0.71747827	7.66103414	9.3716258	0.00618425	0.0252002
Lct	-2.5400068	-0.2845821	9.36923937	0.00618969	0.02520989
Anapc4	-0.47716	5.48691874	9.36659541	0.00619573	0.02521341
Gm4922	-2.8918564	-1.3059537	9.36392068	0.00620185	0.02521341
Pctp	1.00423126	2.59913689	9.36363641	0.0062025	0.02521341
Rbbp7	0.39982061	7.41912342	9.36347551	0.00620287	0.02521341
A530058N18	-1.3179721	1.14834101	9.36162741	0.0062071	0.02521341
Nudt16l1	0.49872893	4.1446376	9.36081342	0.00620896	0.02521341

Sptbn2	-0.7736759	6.97958108	9.35817673	0.00621501	0.0252194
Itga7	-2.6384175	-0.3260729	9.35749354	0.00621658	0.0252194
Bambi-ps1	2.87927818	-0.9567035	9.35537726	0.00622143	0.02522665
Ptk2b	-0.5054363	7.47935581	9.35181497	0.00622962	0.02524404
Dcun1d5	0.43042968	5.95831292	9.35083855	0.00623186	0.02524404
Clcn3	-0.4163244	6.71782553	9.34653932	0.00624176	0.02527168
Nav1	-0.6753079	7.3625303	9.34393953	0.00624776	0.0252835
Opa3	0.64072518	5.05372894	9.34145736	0.00625349	0.02529154
Cacna1i	-0.7299149	4.05638113	9.3404124	0.0062559	0.02529154
Rnf14	0.31752098	8.3873547	9.33115557	0.00627733	0.02536062
Notum	-1.0028205	1.64644829	9.33036641	0.00627916	0.02536062
Rin1	-0.7481029	3.80022868	9.32822399	0.00628413	0.02536823
AF357426	-2.4287698	-0.8974646	9.3188558	0.00630592	0.02543593
Med24	-0.4774346	4.25752126	9.31744362	0.00630921	0.02543593
Fhl1	0.38254632	7.70582581	9.31702884	0.00631018	0.02543593
Ifitm3	0.97771339	7.69025273	9.31401462	0.00631722	0.0254518
Chrdl1	-0.86808	2.68327976	9.31175836	0.00632249	0.02546056
Nsun5	-0.8889338	1.70862212	9.30855202	0.00632999	0.02547278
Has3	-1.3552692	1.97224757	9.30781197	0.00633172	0.02547278
Mis18bp1	-1.3099278	1.0692987	9.30630497	0.00633525	0.02547451
Nrgn	0.39553488	7.23116509	9.3040601	0.00634051	0.0254832
Mettl6	0.79285778	3.84319082	9.30248727	0.0063442	0.02548557
Fat1	-0.4883944	6.13438627	9.29843662	0.00635372	0.02551132
Aars	0.49958958	5.61114746	9.29296978	0.00636658	0.02555049
Mycn	-0.9773289	2.05725625	9.28706494	0.00638051	0.02559388
Klf15	-0.7306744	4.25353879	9.28344584	0.00638906	0.02561569
Tceb2	0.52429895	5.70765472	9.28174589	0.00639308	0.02561932
Ciart	-0.8331539	3.12972536	9.27955149	0.00639828	0.02562764
Poc1b	0.57161732	4.59320195	9.27646106	0.0064056	0.02563989
4632427E13I	-1.5024498	1.56758032	9.27562967	0.00640758	0.02563989
Sdf2l1	0.79217235	2.66460887	9.26987717	0.00642124	0.02568207
Tbc1d9	-0.4856622	5.7477432	9.26589627	0.00643072	0.02570746
Tulp4	-0.362393	8.14953768	9.25927766	0.0064465	0.02574578
Dnajc7	0.33528208	6.56262307	9.25925151	0.00644657	0.02574578
Rrp36	1.10689728	2.16322795	9.25580107	0.00645481	0.0257662
Gjc3	-0.616139	5.44559443	9.245936	0.00647846	0.02584804
Zfp182	-0.4749036	4.90802302	9.24455394	0.00648178	0.02584874
Thbs3	-1.4815087	1.10642805	9.2395339	0.00649386	0.02588434
Hivep2	-0.8112626	8.90992796	9.23517674	0.00650436	0.02591364
Rsrc2	0.35404855	7.78253955	9.23064149	0.00651531	0.0259447
Pml	0.46260326	4.59802592	9.22510086	0.00652872	0.0259855
Trim66	-0.6982997	5.4375356	9.22358769	0.00653239	0.0259862
Ralgps1	-0.5810895	6.58154748	9.22241988	0.00653522	0.0259862
2310039L15F	-1.1341693	2.85952207	9.2186391	0.0065444	0.0260046
Zufsp	-1.001857	3.44116249	9.21790715	0.00654618	0.0260046

Nadk2	0.49735087	4.75716011	9.21097252	0.00656305	0.02605903
Tnc	-1.1360746	1.7869043	9.19226705	0.00660881	0.02620617
Tnip1	0.64085226	4.09255579	9.19167962	0.00661025	0.02620617
Ube2d3	0.39966534	9.02053854	9.18867795	0.00661763	0.02620617
A330049N07	-1.9559068	-0.2488455	9.1885758	0.00661788	0.02620617
Ccdc141	-0.5680182	4.28111604	9.18794612	0.00661943	0.02620617
Ica1	-0.6547765	4.14762539	9.18688385	0.00662204	0.02620617
Phlpp1	-0.4560779	6.22097017	9.18665198	0.00662261	0.02620617
B230118H07	0.50154607	5.48858594	9.18543375	0.00662561	0.02620617
Elp5	0.57970074	4.81554388	9.1806376	0.00663744	0.0262403
Col5a1	-0.6677221	3.40369357	9.17819997	0.00664345	0.02625031
Ppard	0.44658811	5.18174367	9.17528798	0.00665065	0.02625031
Prkx	0.60447159	4.57559272	9.173627	0.00665476	0.02625031
Kndc1	-0.7506815	6.21769581	9.17332344	0.00665551	0.02625031
Dync1h1	-1.1768531	9.33827441	9.17315163	0.00665594	0.02625031
Serbp1	0.31755126	9.37863843	9.16869688	0.00666697	0.02628123
Rab3gap2	-0.6216919	6.46892476	9.16699763	0.00667119	0.02628524
Zfp58	0.72706852	3.40158186	9.16213425	0.00668327	0.02631818
Pole4	0.5115686	5.26961281	9.16105524	0.00668595	0.02631818
Trp53inp2	0.31988003	7.84324072	9.15927388	0.00669039	0.02631987
Dynlrb1	0.50309239	8.41788242	9.15830965	0.00669279	0.02631987
Mrps23	0.57225902	4.23293926	9.15124044	0.00671042	0.02637529
Tmem260	-0.6892158	3.77287118	9.15008812	0.0067133	0.02637529
Ppp1r9a	-0.7881439	8.91038892	9.14520017	0.00672553	0.02639265
Gm10538	-2.8191305	-0.6379455	9.14484763	0.00672641	0.02639265
Efcab2	0.51564143	3.97879449	9.1430091	0.00673102	0.02639265
Eif4a1	0.34452605	7.37071226	9.14276051	0.00673164	0.02639265
Ube2l6	0.74916066	5.58533511	9.14087157	0.00673638	0.02639265
Pou2af1	0.97981928	4.8134834	9.13866681	0.00674191	0.02639265
Gm13547	-4.3332622	-2.4282955	9.1384945	0.00674234	0.02639265
Lipt2	1.00378547	2.11350335	9.13807077	0.00674341	0.02639265
Pprc1	-0.6929232	3.91972088	9.13673999	0.00674675	0.02639316
2900026A02	-0.4813633	5.8920017	9.13541329	0.00675008	0.02639365
Lzts3	-0.402777	5.7471234	9.12290705	0.00678161	0.02650431
Irgm2	0.67018781	5.57099843	9.11834026	0.00679316	0.02653684
G630090E17	-6.1685162	-1.1328509	9.11425603	0.00680351	0.02654597
Mgat5b	-0.5570172	3.71645702	9.11377826	0.00680472	0.02654597
Fam171b	-0.469421	7.11323488	9.10951946	0.00681554	0.02654597
Anxa7	0.3920482	6.45229665	9.10828654	0.00681867	0.02654597
Mfap3	-0.4758338	4.95890308	9.10774591	0.00682005	0.02654597
Srp72	0.31113781	7.21966283	9.10755758	0.00682053	0.02654597
Zfp202	-1.1293682	1.45069473	9.1073831	0.00682097	0.02654597
Crebrf	-0.390042	7.2754805	9.10723823	0.00682134	0.02654597
Macrod2	0.54496315	5.09283958	9.1001843	0.00683931	0.02657676
Trub1	-0.4673225	4.67288102	9.09951404	0.00684102	0.02657676

Mlx	0.72228967	3.4619609	9.09911857	0.00684203	0.02657676
Atp5d	0.77759533	6.11576955	9.09905807	0.00684218	0.02657676
Kctd6	0.48352438	5.85232124	9.09710897	0.00684716	0.02658353
Sptb	-0.914201	5.42552413	9.08940426	0.00686687	0.02661191
Rnf217	0.51184991	4.29072797	9.0891792	0.00686745	0.02661191
Ntn4	-1.1275882	1.3451484	9.08911462	0.00686762	0.02661191
Fnta	0.51745017	6.63782903	9.08821887	0.00686991	0.02661191
Nfkbie	1.25604689	2.13805308	9.08792771	0.00687066	0.02661191
Gsk3a	0.33020721	7.32149468	9.08320366	0.00688279	0.02664632
Cdc14b	-0.6285901	4.35642075	9.07596263	0.00690142	0.02670587
Katnbl1	-0.6300748	4.70717542	9.06964739	0.00691771	0.02674333
Rmi2	-0.9176514	2.19828046	9.069276	0.00691867	0.02674333
Qprt	1.27817353	1.21650241	9.06843084	0.00692086	0.02674333
Prdx6	0.43065164	6.75152676	9.0666206	0.00692554	0.02674884
Rab5a	0.37970455	5.49876439	9.06331567	0.0069341	0.02675046
Sh3bp5	0.42924456	6.78191508	9.06290374	0.00693516	0.02675046
Dpysl5	-0.7532586	4.75965098	9.06268767	0.00693572	0.02675046
Pcdh7	-0.4544016	8.15354378	9.06071449	0.00694084	0.02675763
Mapk3	0.5678203	7.23936848	9.05668438	0.0069513	0.02677652
Akr7a5	0.88716842	2.3865428	9.05631685	0.00695225	0.02677652
Wipf3	0.47411415	7.87146541	9.04606084	0.00697896	0.02686679
Sp110	0.84601698	3.39387382	9.04316693	0.00698652	0.02688147
Lime1	-1.4038213	2.74346106	9.04209548	0.00698932	0.02688147
Arhgap28	0.72328977	4.00882004	9.03783537	0.00700046	0.02691174
Cacng7	-0.4942088	5.83701289	9.02808801	0.00702603	0.02699192
Per2	-0.5954757	5.05396561	9.02716194	0.00702847	0.02699192
Timm17a	0.54603376	5.75091044	9.02613512	0.00703117	0.02699192
Cep19	0.3694119	5.80327152	9.02121446	0.00704413	0.02702169
Slc25a53	-0.9869523	2.57101255	9.02069545	0.0070455	0.02702169
Sipa1	0.81014581	2.6000308	9.01548538	0.00705925	0.02706181
Nhp2	0.86529804	3.21327063	9.01307421	0.00706563	0.02706443
Gm14391	-1.4860882	1.93452359	9.01273621	0.00706652	0.02706443
Sp7	1.50462018	0.83037531	9.01094503	0.00707126	0.02706998
Tfam	0.50756877	4.82690855	9.00935002	0.00707549	0.02707354
Inpp1	-0.8788257	3.03063513	9.00731117	0.00708089	0.02708161
Tnfaip8	0.74710046	5.03713846	9.00483352	0.00708747	0.02709415
Znfx1	-0.5339636	5.36773482	9.00026529	0.0070996	0.02712793
Caprin1	0.29604086	8.54069451	8.99204556	0.0071215	0.02719896
Stox2	-0.5396931	7.99509909	8.98919686	0.00712911	0.02720916
Upp2	-0.7381758	3.38280111	8.98856733	0.00713079	0.02720916
Uqcr10	0.69769252	5.90447573	8.98426585	0.0071423	0.02723854
Focad	-0.5239671	5.28452876	8.98321234	0.00714512	0.02723854
Hist1h2bg	1.6845113	0.15143316	8.98043371	0.00715257	0.02724839
Tars2	-0.7058381	2.52134656	8.97977496	0.00715433	0.02724839
Myl6	0.62689509	7.87742737	8.97839772	0.00715803	0.02724983

Slx1b	-0.5395271	3.6325023	8.97522267	0.00716656	0.02726305
Rps6ka5	-0.8411467	3.29476778	8.97463563	0.00716814	0.02726305
Mei1	-1.3738806	0.94362677	8.96650046	0.00719004	0.02733372
Gng2	0.30149898	7.99385618	8.96264678	0.00720045	0.02736061
Chid1	-0.5802134	3.41051126	8.96138294	0.00720386	0.02736094
Gm15800	-0.9264146	8.44724005	8.9590169	0.00721026	0.0273726
Fli1	0.72800613	4.25282813	8.95445008	0.00722263	0.02740573
2310007B03I	6.81577762	-1.8865621	9.88384484	0.00722719	0.02740573
Gm20172	-1.982317	0.71115803	8.95210429	0.00722899	0.02740573
Fdft1	0.40411605	6.27779223	8.94515742	0.00724787	0.0274599
Dennd6b	-1.1996879	2.8384596	8.94438939	0.00724996	0.0274599
Oc90	-5.6494483	-2.3165054	9.30357221	0.00727022	0.02751433
Gtf2f2	0.62073491	3.1438691	8.93666396	0.00727103	0.02751433
Snx10	0.41756547	6.49559681	8.92892021	0.00729221	0.02756958
Zcchc9	0.41682882	5.82683185	8.92887444	0.00729234	0.02756958
Sfmbt2	-1.0536418	2.5418789	8.9255908	0.00730134	0.02759093
Mfsd8	-0.6280827	3.26371644	8.9217308	0.00731194	0.02760054
Odc1	0.32044465	6.42482973	8.92068057	0.00731483	0.02760054
Prex2	-0.4824152	7.37348837	8.91995394	0.00731683	0.02760054
Smc5	-0.6575958	5.74348268	8.91977644	0.00731732	0.02760054
Melk	-2.6940651	0.17902448	8.91678901	0.00732554	0.02761888
Fkbp3	0.34815083	6.9856642	8.91189714	0.00733902	0.02765704
Cdh11	-0.3416538	6.72503246	8.90610635	0.00735502	0.02769281
Fbxl17	-0.4103558	8.00944252	8.90602292	0.00735526	0.02769281
4930570G19	-0.8847647	3.53760825	8.9034791	0.0073623	0.02770663
Ano4	-0.5917219	3.81478993	8.8963304	0.00738212	0.02776853
Pcdhb18	-0.7361825	4.15419096	8.8833184	0.00741837	0.0278921
Ubxn2a	0.40021747	6.98286005	8.87782915	0.00743372	0.02793703
Fam178a	-0.3295083	7.06139048	8.87287925	0.00744759	0.02797637
C230004F18I	-0.9717614	3.88591467	8.86893204	0.00745867	0.0280052
Smim14	0.6116253	7.54290449	8.86470548	0.00747056	0.02803702
E130215H24I	5.21465029	-1.8867216	8.86184837	0.0074786	0.02805441
Hnrnpr	0.32497205	7.8987067	8.85968653	0.0074847	0.02806239
Trp53bp1	-0.657444	5.47033742	8.85867267	0.00748756	0.02806239
Setdb1	-0.671244	4.51143675	8.8551963	0.00749738	0.02808638
Sgcb	-0.5543217	4.81756868	8.85233121	0.00750548	0.02810026
Rela	0.60950435	4.25629368	8.85042322	0.00751088	0.02810026
Ttc18	-1.9159412	0.60106621	8.85021621	0.00751147	0.02810026
Actr1a	0.37037755	6.16861461	8.848747	0.00751563	0.02810026
Mast1	-0.5995423	4.88121181	8.84741988	0.00751939	0.02810026
Vps25	0.59847709	5.12728745	8.84587902	0.00752376	0.02810026
Hivep3	-0.7664627	6.88983087	8.84483726	0.00752672	0.02810026
Gtf3c1	-0.6262459	6.48782816	8.84390008	0.00752938	0.02810026
Serpina3n	1.15780665	2.68992215	8.84226338	0.00753403	0.02810026
Ankrd13b	-0.7594656	3.86267914	8.83998142	0.00754052	0.02810026

Thsd7b	-1.2987393	1.27825354	8.83969963	0.00754132	0.02810026
Ptpla	-0.8436484	2.93398082	8.83942078	0.00754211	0.02810026
Ubl4	0.33083846	6.74027419	8.83716412	0.00754853	0.02811145
Lphn2	-0.3998954	6.35521224	8.8354572	0.0075534	0.02811681
Neb	-1.4043464	2.55395156	8.82403933	0.00758601	0.02822374
Adam3	-5.0174498	-1.935804	9.17969983	0.00759304	0.02822374
Zrsr1	0.28904508	6.95800016	8.82017035	0.0075971	0.02822374
Spin2c	0.81919204	2.93583366	8.81967617	0.00759852	0.02822374
Gabra4	-0.5095615	5.85303945	8.81940639	0.00759929	0.02822374
Bcl10	0.81268414	4.85800539	8.81483063	0.00761243	0.02825977
Xcr1	-1.2414146	1.55130213	8.81152326	0.00762194	0.02827655
Snrnp200	-0.5532119	6.89140975	8.81086826	0.00762383	0.02827655
Vps26a	0.34785643	7.2527483	8.80523356	0.00764007	0.02832401
Nat8	-2.2400416	0.23578524	8.80311564	0.00764619	0.0283339
St13	0.42723493	6.32199189	8.80138968	0.00765118	0.02833961
Tnfsf9	-4.5012518	-2.0132422	8.79570347	0.00766763	0.02838777
1810026J23F	0.44007748	5.9224383	8.79312189	0.00767512	0.02839809
Abca17	-3.1558386	-0.2030973	8.79213663	0.00767798	0.02839809
Ppef2	-3.0577015	-0.8436793	8.79035349	0.00768315	0.02839809
Slc38a1	-0.3802929	7.61637541	8.78997877	0.00768424	0.02839809
Fam189a1	-0.4641961	5.77277244	8.78703312	0.0076928	0.02841696
Ptprn	-0.5809564	6.91147551	8.785686	0.00769672	0.02841866
Fam227a	-1.4096509	1.77176102	8.78116323	0.0077099	0.02844583
Mrps25	0.40835953	4.61514581	8.77978435	0.00771392	0.02844583
Gm7102	-0.9694565	5.46312547	8.77877228	0.00771687	0.02844583
Bambi	1.43357644	1.23573943	8.77841134	0.00771792	0.02844583
Rhoj	0.70696404	5.38522687	8.77126481	0.00773882	0.02850782
Pmaip1	0.74866869	3.4226659	8.77028661	0.00774168	0.02850782
Ubox5	0.70945718	3.32960985	8.76713755	0.00775091	0.02851869
Wdr17	-0.7507875	5.11350837	8.76691139	0.00775157	0.02851869
Ptplad2	-0.7188175	3.7755559	8.76413896	0.00775971	0.02853586
Rarb	0.48613902	5.04062113	8.7628027	0.00776363	0.02853752
Fam228a	-1.856173	1.31402514	8.75808474	0.00777751	0.02857575
Rasal2	-0.4965122	7.19857233	8.75676052	0.00778141	0.0285773
Huwe1	-0.6626657	9.48061089	8.75413665	0.00778914	0.02858099
Ikbkb	0.60093227	4.30039297	8.75339288	0.00779134	0.02858099
Tuba1b	0.34239089	9.23594797	8.75288108	0.00779285	0.02858099
9030025P20I	-0.7183985	3.21124724	8.74259744	0.00782326	0.02867972
Dnajc13	-0.5687481	6.26013549	8.74003588	0.00783085	0.02869476
Ypel3	0.36308445	5.84479946	8.73848161	0.00783546	0.02869637
Slc25a12	0.36684652	7.49870517	8.73753494	0.00783827	0.02869637
Arid1b	-0.5671465	6.24376872	8.73564689	0.00784388	0.02870412
Ube2c	-1.5424546	0.65337984	8.73182316	0.00785526	0.02872151
Cnot7	0.38856776	6.74951294	8.73170009	0.00785562	0.02872151
D130020L05I	-0.7624824	2.63523436	8.72811858	0.00786629	0.02874773

Sumo2	0.5707185	8.73781098	8.72590577	0.00787289	0.02875907
Xirp2	-1.2375912	2.38411212	8.71892606	0.00789375	0.02882246
Nt5c	0.71316191	3.6139377	8.71704882	0.00789937	0.02883017
Ubqln4	0.41904456	6.15936586	8.71411347	0.00790817	0.02884947
Pdcd6	0.48005227	5.94969641	8.710937	0.00791771	0.02887144
Pomc	-3.4214614	0.35685037	8.70926231	0.00792274	0.02887697
Bc1	-5.3596842	-1.8486001	8.70665769	0.00793057	0.02888441
Stoml2	0.59930513	4.28592508	8.70624643	0.00793181	0.02888441
Kcnh7	-0.6113077	6.28135969	8.70259094	0.00794282	0.02891169
Cplx1	0.32164781	8.44920453	8.69939408	0.00795246	0.02893397
Mir872	4.55666677	-1.3539564	8.69778086	0.00795733	0.02893795
Ndufa12	0.45248132	5.67767073	8.69613264	0.00796231	0.02893795
Iscu	0.55470249	6.3829146	8.69553523	0.00796411	0.02893795
Mrpl34	0.90433639	3.32687172	8.69324211	0.00797105	0.02895035
Tpt1	0.58864259	9.33039457	8.69072465	0.00797867	0.02896523
Prkch	0.8207237	2.40953756	8.68193311	0.00800535	0.02904925
Itpk1	0.55891547	3.82827909	8.67908858	0.008014	0.02906782
Dnajb6	0.32102043	7.95725082	8.67581458	0.00802397	0.02909115
Zswim8	-0.5138396	5.28010502	8.6744141	0.00802824	0.0290938
Sap18	0.5540183	6.70105069	8.67038859	0.00804053	0.02912549
Psma4	0.34471867	6.88952147	8.66787043	0.00804823	0.02912888
Gas2l3	-0.7505561	4.64263568	8.66776349	0.00804856	0.02912888
Dctpp1	0.90477724	1.63545479	8.6661979	0.00805335	0.02913339
Spg11	-0.5142032	5.11874808	8.66490784	0.0080573	0.02913486
Otud4	-0.3955179	6.54656309	8.66249723	0.00806468	0.02914874
Akip1	0.90423012	2.97505457	8.65675704	0.0080823	0.02919388
Sf3b6	0.51346416	5.00952642	8.65611453	0.00808427	0.02919388
Setd8	0.4412905	7.28392501	8.65238843	0.00809574	0.02922243
Itih3	-1.2766245	1.81272516	8.65060467	0.00810123	0.02922942
Reln	-0.7754914	5.25469557	8.64681071	0.00811293	0.02925878
Calcoco2	-1.6527622	-0.0155403	8.6436053	0.00812283	0.02927891
3110035E14I	0.3433638	7.87383963	8.64269761	0.00812563	0.02927891
Gpr171	-1.4509201	1.52802902	8.6411535	0.00813041	0.02928327
Arhgap33	-0.7559256	5.35412079	8.6399704	0.00813407	0.02928362
Itgav	-0.4010136	5.95813429	8.63463728	0.00815059	0.02933026
Mut	0.32282006	5.76690923	8.63167524	0.00815979	0.02934715
Amer1	-0.5773974	4.17907539	8.62906609	0.00816789	0.02934715
Arntl2	-1.0862219	2.04468903	8.62906172	0.00816791	0.02934715
Ncf2	-1.0478599	2.47627398	8.6285276	0.00816957	0.02934715
Dbi	0.61790312	7.09650929	8.62009109	0.00819585	0.02942871
Asf1a	0.38376852	5.03613629	8.61561579	0.00820983	0.02945633
Psmd3	0.48566978	4.38659442	8.61477855	0.00821245	0.02945633
Gsto1	0.70644556	4.19893539	8.6141887	0.0082143	0.02945633
Atp8a1	-0.5130287	7.56623088	8.60782395	0.00823424	0.02951497
C430049B03I	1.37339229	1.132541	8.60520716	0.00824245	0.02953153

2610203C20I	-0.5700333	5.8366634	8.59533031	0.00827354	0.02961276
Gcnt1	0.8547238	2.91051144	8.59457988	0.00827591	0.02961276
Stx11	1.247914	2.10284213	8.59309787	0.00828058	0.02961276
Epha4	-0.4164571	7.2171443	8.59238109	0.00828285	0.02961276
Ankib1	-0.3338575	6.52754493	8.59228906	0.00828314	0.02961276
Cecr2	-0.5766617	3.27743897	8.59101234	0.00828717	0.02961429
Gm15645	-1.0717176	1.94279585	8.58805931	0.00829651	0.02963477
Mmd	-0.3430206	7.33839218	8.58134319	0.00831779	0.02969787
Chga	0.62497475	4.36510179	8.57866394	0.00832629	0.02971341
Wdr52	-1.0623655	2.19941663	8.57769597	0.00832937	0.02971341
Snrpa1	0.46996722	4.24823877	8.57135034	0.00834956	0.02977252
Ephb3	-1.0939731	1.63705225	8.56534938	0.0083687	0.02982785
Klhl22	-0.4735644	5.05935465	8.56326204	0.00837538	0.02983868
Fgf18	0.96454929	2.47317753	8.55467084	0.0084029	0.02992376
Cdc6	1.72725691	0.74257693	8.55198313	0.00841153	0.02994152
Lyrm9	-0.4169436	5.68993301	8.54650402	0.00842915	0.02999126
Mid1ip1	0.70515375	6.08785117	8.54117198	0.00844634	0.03002263
Cyp4f15	-1.3038654	1.15049786	8.54034611	0.008449	0.03002263
Dido1	-0.4320994	6.59201609	8.53961009	0.00845138	0.03002263
Cela1	1.09401086	1.3190181	8.53923989	0.00845258	0.03002263
Rimklb	-0.6024585	3.68555585	8.53788485	0.00845696	0.03002521
Ak6	0.88416272	2.76185152	8.53675331	0.00846061	0.03002522
Atpaf1	0.39389299	6.0288518	8.53129955	0.00847827	0.03007229
Baz2b	-0.333273	6.99648662	8.52876591	0.00848649	0.03007229
Efr3b	-0.6001703	6.81278274	8.52828579	0.00848804	0.03007229
Pcdhb3	-0.8878214	2.11823708	8.52814173	0.00848851	0.03007229
Ndfip1	0.49650014	7.96191732	8.5254301	0.00849732	0.03009052
Slco5a1	-1.0201402	2.19075446	8.52294237	0.00850541	0.03010619
Tspan14	-0.5280871	3.50768409	8.51964473	0.00851614	0.03011927
Trio	-0.593518	7.7197757	8.51878795	0.00851893	0.03011927
Slc7a10	-1.9468957	0.19958365	8.51840374	0.00852018	0.03011927
B3galnt2	-0.5374963	3.82718944	8.5170573	0.00852457	0.03011927
Kcnc3	-0.7328542	5.24132334	8.51618283	0.00852742	0.03011927
Zfp429	0.84931005	2.01858964	8.51231397	0.00854006	0.0301506
Tmem131	-0.4568332	7.23294098	8.51121969	0.00854363	0.0301506
Mkx	0.60696585	5.3746159	8.49632101	0.00859249	0.03029315
Zfp457	-1.842739	0.29169321	8.49605657	0.00859336	0.03029315
Acp5	2.87803525	-0.7192985	8.49553369	0.00859508	0.03029315
Itih2	0.66433474	5.04283293	8.49243295	0.00860529	0.03031614
Uqcrb	0.48450408	6.81664099	8.4888809	0.00861701	0.03033725
Ctnna1	0.49998498	7.91023598	8.48819412	0.00861927	0.03033725
Strc	-2.9565533	-1.2857172	8.48726018	0.00862236	0.03033725
St8sia3	-0.4957937	7.82621868	8.48429861	0.00863215	0.03035869
Magel2	-2.3391385	0.39993086	8.47899137	0.00864972	0.03040747
2010204K13I	0.80101294	2.63947415	8.47640592	0.00865829	0.0304246

Cdh15	-4.6648494	-1.396928	8.47382129	0.00866687	0.03044173
Ctdsp1	0.579498	6.68530634	8.47235337	0.00867175	0.03044585
Rsf1	-0.466894	7.58645342	8.47034796	0.00867842	0.03045565
Fam189a2	-1.7795345	0.55392825	8.46928652	0.00868195	0.03045565
Pard6b	0.7484475	3.34604039	8.46803157	0.00868612	0.0304573
Grhl2	-2.02269	0.49224649	8.46039953	0.00871158	0.03053353
Ovgp1	-1.7444142	0.34036295	8.45053064	0.00874462	0.0306296
Nt5c3b	0.59893322	3.23958664	8.4492803	0.00874882	0.0306296
Bnip3l	0.42310303	8.24542799	8.44887705	0.00875017	0.0306296
Gm15408	-2.5120249	-0.4333354	8.44686575	0.00875692	0.0306402
Zfp709	-0.6475163	4.13899333	8.44352681	0.00876815	0.03065255
Srrm4os	-1.1402048	1.83106618	8.44290472	0.00877025	0.03065255
Cbr1	0.46326347	4.80828654	8.43999963	0.00878003	0.03065255
Btbd8	-1.3821915	0.87581466	8.43937365	0.00878214	0.03065255
Hist1h2bl	0.88847546	3.11037497	8.43922681	0.00878263	0.03065255
Terf1	-0.6089888	3.52866775	8.43769021	0.00878782	0.03065255
Vezt	-0.4818409	4.86885438	8.43697956	0.00879021	0.03065255
Xkr4	-0.7556543	4.24244498	8.43679958	0.00879082	0.03065255
Aph1c	-0.6152616	3.93626938	8.43585183	0.00879402	0.03065255
Akirin2	0.48438952	6.60891221	8.43408941	0.00879997	0.0306603
Dctn2	0.36338351	5.87271617	8.42904716	0.00881703	0.03070294
Dagla	-0.4586803	5.43864015	8.42826313	0.00881968	0.03070294
C030023E24I	-1.3356597	2.42704436	8.42351844	0.00883577	0.03072314
Myh10	-0.5951212	8.54003851	8.42324521	0.0088367	0.03072314
Snca	0.34029077	7.68402649	8.42324386	0.0088367	0.03072314
Aff1	0.43622645	7.01573603	8.41952132	0.00884935	0.0307541
Luc7l	-0.4911863	5.4931452	8.41651	0.00885959	0.03076436
Kif20a	-0.9427836	1.644675	8.41645313	0.00885978	0.03076436
Znf512b	-0.4174348	4.93881231	8.41502815	0.00886464	0.03076821
Kif16b	0.3840756	4.96535644	8.41168628	0.00887603	0.03079474
Glis1	1.74126802	0.52950865	8.41035959	0.00888056	0.03079481
Gpr56	-0.7930757	2.97073981	8.4094853	0.00888354	0.03079481
Stat6	0.53973381	5.05259947	8.40830206	0.00888758	0.03079583
Zfp64	0.67420938	2.7789355	8.40507927	0.0088986	0.03080918
Neto2	-0.5977849	5.74021452	8.40498152	0.00889893	0.03080918
Recql	-0.562635	3.96390845	8.4033744	0.00890443	0.03081524
Gcnt4	-0.6970141	4.22489231	8.40070374	0.00891358	0.03083392
Tm2d1	-0.6827276	2.9591863	8.39632567	0.0089286	0.03085402
Nktr	-0.5243609	7.07356696	8.39628548	0.00892874	0.03085402
Mfhas1	-0.4292635	5.25303798	8.3957273	0.00893065	0.03085402
Pate2	-1.7058759	0.20605937	8.39398768	0.00893663	0.0308617
Cnga2	-5.2115646	-2.1500629	8.72014335	0.00894066	0.03086266
Thoc2	-0.5050763	7.06080005	8.38953402	0.00895195	0.03088866
Gprasp2	-0.5897715	6.17426828	8.38478216	0.00896833	0.03093219
Slc35f3	-0.4158093	5.58481233	8.37983096	0.00898544	0.03097818

Gdpd5	0.58806649	3.75002037	8.37540248	0.00900077	0.03101802
Hist1h1a	3.46789731	-1.2556486	8.3654165	0.00903544	0.03111155
Acaca	-0.6465748	5.67826567	8.36540542	0.00903548	0.03111155
Gna11	0.51320851	5.92150648	8.36063811	0.00905208	0.03114629
Lsm3	0.59621982	4.10836897	8.35950624	0.00905603	0.03114629
Lpl	-0.8090045	4.20486379	8.35924714	0.00905693	0.03114629
Prss12	-0.8379465	1.85980198	8.35528042	0.00907079	0.03117664
Tprgl	0.39647926	6.93458413	8.3545473	0.00907335	0.03117664
Gm14327	-1.0960575	2.97504976	8.35175565	0.00908311	0.03119715
Gm16039	0.41505672	4.51962695	8.3470587	0.00909957	0.03123883
Tmem191c	-0.6875881	4.21084663	8.34510725	0.00910642	0.03123883
Gadd45b	0.73228132	2.14979483	8.34504061	0.00910665	0.03123883
Timm8b	0.43099244	5.55387668	8.34339136	0.00911244	0.03124566
Rnf157	-0.4974898	7.48577637	8.33955746	0.00912592	0.03127882
Smpd4	-0.5258822	5.12357101	8.33550921	0.00914018	0.03128632
Ppp1cb	0.33190292	9.23777984	8.33449062	0.00914377	0.03128632
Tspan33	1.14685324	1.50651382	8.33404189	0.00914535	0.03128632
Trmt44	-1.8326094	0.66664518	8.33358875	0.00914695	0.03128632
AI854517	-0.6025028	3.70653765	8.33273155	0.00914997	0.03128632
Myo9a	-0.5571664	8.3603113	8.3324557	0.00915095	0.03128632
Paqr6	-0.9816867	2.09604064	8.32848301	0.00916498	0.03132126
Ccdc129	2.10874882	-0.2345757	8.3266636	0.00917141	0.03133023
Snx3	0.39037623	6.82295888	8.31679436	0.0092064	0.0314367
Gm11517	-3.6144674	-1.0909567	8.31288909	0.00922029	0.03144669
Zfp746	0.45124157	4.31556848	8.31268595	0.00922101	0.03144669
Ankrd63	-0.7012558	4.06143589	8.31086448	0.0092275	0.03144669
Fut4	2.13020707	-0.2588624	8.30960835	0.00923198	0.03144669
Acsl3	-0.3483987	7.2432512	8.30940342	0.00923271	0.03144669
Dgkk	-0.9491027	3.76416739	8.30875258	0.00923503	0.03144669
1110001J03F	0.91026235	2.66001857	8.3084477	0.00923611	0.03144669
Krr1	0.31462024	6.82468627	8.30592032	0.00924513	0.03146436
Mcf2	-1.041515	3.13970048	8.30364065	0.00925327	0.03147904
Clvs1	0.50458327	4.16527292	8.29601309	0.00928057	0.03155885
Cemip	-0.5459517	3.50331555	8.29450769	0.00928597	0.03156415
Nudt10	0.56697199	3.41269048	8.28923753	0.0093049	0.03159317
Zkscan2	-0.4695753	4.76898938	8.28846868	0.00930766	0.03159317
Chmp2a	0.64621646	5.63235119	8.2884054	0.00930789	0.03159317
Dnaja2	0.2866511	7.78861547	8.28552371	0.00931826	0.03159317
Lrp2	-4.3536026	-1.6730941	8.2846125	0.00932155	0.03159317
Gfra4	0.51137496	4.35583022	8.28416605	0.00932315	0.03159317
Fbxl16	-0.4891966	7.27841856	8.28379201	0.0093245	0.03159317
Pomt2	-0.5450593	3.59827126	8.28358105	0.00932526	0.03159317
Lrrtm2	-0.5061067	6.66013688	8.27775765	0.00934628	0.03164141
Rnasel	-0.500183	4.6797789	8.27750253	0.0093472	0.03164141
Tmem179	-0.6054118	3.41467723	8.2755113	0.0093544	0.03165274

Kat7	0.36465284	5.9074198	8.27023684	0.0093735	0.03168196
Pom121	-0.4177745	6.32319514	8.26994097	0.00937457	0.03168196
Cdc42	0.33576102	8.71371659	8.26986534	0.00937484	0.03168196
Ncf4	3.21760772	-0.9556413	8.2688703	0.00937845	0.03168196
Klf13	-0.3612224	7.23167154	8.26544003	0.0093909	0.03171099
Grk4	-0.8413103	4.63754829	8.26408265	0.00939584	0.03171462
Fam208a	-0.5014551	6.76686734	8.25885005	0.00941488	0.03174317
Drap1	0.76826975	3.96958289	8.25851517	0.0094161	0.03174317
Cerkl	-1.9464011	-0.1444446	8.25812263	0.00941753	0.03174317
Il13ra1	0.61421544	4.87857504	8.25751457	0.00941974	0.03174317
Iqsec2	-0.4829998	5.69733138	8.25421676	0.00943177	0.03177068
Elmo3	1.1503234	1.38740745	8.25131947	0.00944236	0.03178631
Rps2	0.4636835	6.30221728	8.25082886	0.00944415	0.03178631
Tbccd1	0.49571873	4.58301558	8.2483798	0.00945311	0.03180344
Armc1	0.33863539	7.59764091	8.24464115	0.0094668	0.03183647
Npr2	-0.4976093	4.07112397	8.23555348	0.00950017	0.03193005
Nfkbia	0.81645071	2.74707365	8.23494981	0.0095024	0.03193005
Shprh	-0.620159	5.96668018	8.23239041	0.00951182	0.03194866
Brca2	-0.5991806	3.83286062	8.22720618	0.00953095	0.0319872
Lmbr1	-0.4373231	5.0770825	8.22717065	0.00953108	0.0319872
Cers3	4.86536801	-1.6244005	8.22495021	0.00953928	0.03200166
Csnk1e	-0.4326228	6.14590417	8.22130515	0.00955277	0.03203383
Ces2g	-0.8908559	3.49425668	8.22002295	0.00955752	0.03203668
Necab1	0.38482494	7.85856048	8.21756921	0.00956661	0.03204315
Dnajb2	0.518266	5.41496715	8.21739878	0.00956724	0.03204315
Map3k7cl	-1.9483683	0.50452359	8.21457702	0.00957772	0.0320594
Hagh	0.56175187	4.40177537	8.21390595	0.00958021	0.0320594
Kcnc1	-0.5276403	6.98292561	8.21294011	0.0095838	0.0320594
Irf3	0.84981657	2.69589221	8.20639973	0.00960813	0.03212774
Efemp1	0.78406389	7.48332412	8.2046684	0.00961459	0.03212846
Grip2	-1.1007422	2.18847827	8.20424437	0.00961617	0.03212846
Plekha2	0.57010231	5.21607089	8.20035218	0.0096307	0.03216394
Lrp1	-0.3785722	7.3421971	8.19551902	0.00964878	0.03221122
Fam65b	-0.5354429	5.89319166	8.19364354	0.0096558	0.03222158
Zfp580	0.53383259	3.72006209	8.18725348	0.00967978	0.03228849
Mrpl2	0.79563147	3.1418661	8.18326946	0.00969476	0.03232535
Etnk2	0.85607723	2.60113181	8.17976361	0.00970797	0.03234379
Tecpr2	-0.7444124	5.55760619	8.17954738	0.00970878	0.03234379
Spns2	-0.6355865	3.5192407	8.17866779	0.0097121	0.03234379
Zdhhc1	-0.6540714	3.24992215	8.17745625	0.00971667	0.03234591
Atp9b	-0.7217697	3.99564467	8.17332459	0.00973228	0.03238474
Gm6682	0.3847038	5.04837045	8.17015593	0.00974426	0.03240483
BC048546	0.47919353	5.10503088	8.16964408	0.0097462	0.03240483
Snrpe	0.55025099	4.52125606	8.1672277	0.00975535	0.03242215
Gm15441	3.81230039	-1.1953439	8.16582536	0.00976067	0.0324252

Slc18a2	-0.7899973	2.87449392	8.16434696	0.00976628	0.0324252
Fbxl21	-0.5936297	3.14489174	8.16386599	0.0097681	0.0324252
Hist1h2be	0.83484459	2.78253402	8.1609283	0.00977926	0.03244913
Eed	0.50155574	4.49116874	8.15941312	0.00978502	0.03245514
Hsd17b11	0.55071474	5.02271974	8.15627882	0.00979695	0.0324816
Slc6a17	0.38574667	8.25018407	8.15260428	0.00981096	0.03250747
Pcmtd2	-0.4968805	4.73890337	8.15194998	0.00981345	0.03250747
Kdm6a	-0.5007205	6.12837456	8.15111978	0.00981662	0.03250747
9030204H09	-2.8269349	-1.183812	8.14857484	0.00982634	0.03252655
A330093E20	-2.2376933	0.23437656	8.14329168	0.00984655	0.03258033
Osr2	2.35489093	-0.06452	8.14043738	0.00985749	0.0326034
Ppp1r10	0.36323499	5.87724125	8.13919107	0.00986227	0.03260609
Il16	1.18647131	1.13578219	8.1366296	0.0098721	0.03262547
Map6d1	-0.4922895	5.02829765	8.13291806	0.00988637	0.03265949
Cxcl14	-0.566392	3.20959099	8.12816082	0.00990469	0.03270687
Gm16617	-3.5986878	-0.99522	8.12588973	0.00991345	0.03271222
Ptpro	-0.6048726	3.42849331	8.12567682	0.00991427	0.03271222
Hsd3b4	-1.731973	0.76477743	8.12424434	0.0099198	0.03271733
Map1lc3a	0.61911301	4.80328015	8.12187508	0.00992896	0.03273439
Dennd5b	-0.6279958	6.59461121	8.12071956	0.00993342	0.03273599
E130112N10	-3.7899516	-0.6904843	8.1190787	0.00993977	0.03274378
Ppfia1	0.31315367	6.96011551	8.11415192	0.00995886	0.03278601
Fbxo27	0.61031889	3.83541768	8.11291615	0.00996365	0.03278601
Nip7	0.42154222	5.02357649	8.11268321	0.00996456	0.03278601
Gpr155	-0.5143225	5.73513975	8.10510862	0.009994	0.03286974
Setx	-0.571577	7.13268707	8.10360351	0.00999987	0.0328756
Dnajc1	-0.4636147	5.08351019	8.10259765	0.01000379	0.0328756
C230035I16R	2.74732987	0.05992777	8.10067945	0.01001127	0.03288704
Nr4a2	1.29137255	6.88908107	8.09913553	0.01001729	0.03289368
Pak1	0.32446767	9.92302229	8.0962534	0.01002855	0.0329052
Pikfyve	-0.4884887	6.77056476	8.09618841	0.01002881	0.0329052
Stmn1-rs1	-3.8998029	-1.1613984	8.09423598	0.01003644	0.03290805
Thsd7a	-0.4189403	6.78848442	8.09391834	0.01003768	0.03290805
Nipsnap1	0.45941633	4.73071935	8.09005592	0.01005281	0.03293262
Tfap2a	0.69745144	3.94544385	8.08995877	0.01005319	0.03293262
Ube2k	0.31700035	8.11082422	8.08719317	0.01006404	0.03294492
Mfn1	-0.4952386	5.318936	8.08530033	0.01007147	0.03294492
Rgs11	-1.7429322	1.394385	8.08482775	0.01007333	0.03294492
Epcam	-4.3634356	-0.4798403	8.08343451	0.0100788	0.03294492
Scrt1	-0.6095888	5.3381451	8.08228064	0.01008334	0.03294492
Col1a1	0.7186763	6.95686148	8.08197461	0.01008454	0.03294492
Dap	0.69330467	5.24106919	8.08185618	0.01008501	0.03294492
Nmur2	-4.6538508	-1.6835497	8.0721629	0.01012322	0.0330566
Ebna1bp2	0.46686256	5.39896302	8.06643727	0.01014586	0.03311739
Mettl21a	0.84180217	2.89583709	8.06318679	0.01015874	0.03313642

Kcnq2	-0.5188267	5.52989196	8.06293148	0.01015975	0.03313642
Chchd6	0.64088476	3.43106469	8.05935228	0.01017396	0.03315769
Rp9	0.5613254	4.0520469	8.05925607	0.01017434	0.03315769
Bhmt	4.28584289	-1.2367822	8.05620913	0.01018646	0.03316425
Ltn1	-0.4548901	6.60022805	8.05590789	0.01018765	0.03316425
Fam196b	-1.0877003	1.52133932	8.05442004	0.01019357	0.03316425
Ppp2r3d	-0.807323	3.95125935	8.05393143	0.01019552	0.03316425
Zc3h6	-0.530429	4.89306762	8.05327733	0.01019812	0.03316425
Tmsb4x	0.55670882	8.89562074	8.05148993	0.01020525	0.03316425
Naip5	-1.0876296	1.51567196	8.05070696	0.01020837	0.03316425
Acadvl	0.56784448	4.90350757	8.05063955	0.01020864	0.03316425
Gtsf1	-3.3904816	-1.7485368	8.04957314	0.01021289	0.03316496
A430078G23	-0.6778972	3.71615754	8.04712064	0.01022268	0.03318363
Stx12	0.34161394	7.93683339	8.04589597	0.01022757	0.03318641
Snapin	0.55349258	7.51446308	8.04107751	0.01024684	0.0332203
Slc25a20	0.67150873	4.78265158	8.04079243	0.01024798	0.0332203
Map1b	-0.803368	12.0341584	8.0402536	0.01025014	0.0332203
Sptan1	-0.8299677	9.21986903	8.03685049	0.01026378	0.0332514
Pop4	0.63931449	4.0052	8.0328376	0.0102799	0.03329048
Grwd1	1.1167158	1.41673485	8.03060861	0.01028886	0.03330638
Stxbp5l	-0.8211734	8.44935517	8.02764587	0.01030078	0.03333185
Zcchc2	-0.4119888	6.13430018	8.02517159	0.01031075	0.03335098
Fads3	-0.5888253	3.57480128	8.02362037	0.01031701	0.03335809
Ralgds	0.339572	5.87226687	8.02248606	0.01032159	0.03335977
Rasgrf2	-0.8059069	6.84330007	8.01827428	0.0103386	0.03340164
Zfp46	-0.3654533	5.51714231	8.01326355	0.01035889	0.03345402
Psma6	0.38330372	6.42066675	8.012109	0.01036357	0.03345599
D7Ertd443e	-0.9522054	1.54308926	8.00782854	0.01038094	0.03348516
Enpep	-1.0521397	1.97642137	8.00657775	0.01038603	0.03348516
Mfap1a	0.29159295	7.41490946	8.00610892	0.01038793	0.03348516
Fhl3	1.18891239	2.04694674	8.00572333	0.0103895	0.03348516
Pnma1	1.17040826	1.58380208	8.00364406	0.01039796	0.03348516
Morf4l2	0.41961294	7.36995146	8.00281948	0.01040132	0.03348516
Psg29	-1.7832277	0.02160437	8.00274816	0.01040161	0.03348516
8430419L09I	0.46025225	5.70735474	8.00186631	0.0104052	0.03348516
Cnn3	0.55507408	6.27032767	7.99982266	0.01041352	0.03349884
Mrpl23	0.63531485	3.13313811	7.99714832	0.01042443	0.03352082
Cct7	0.42987019	6.67429444	7.99459119	0.01043488	0.0335303
Usp9x	-0.5323724	9.85788988	7.99442869	0.01043554	0.0335303
Dcc	-0.4956067	5.17733694	7.99233192	0.01044411	0.03354473
Abca8a	-0.9113281	2.39900376	7.98580286	0.01047086	0.03361199
Dmgdh	1.86053444	0.06703914	7.98411912	0.01047777	0.03361199
Hectd1	-0.4690878	7.4322099	7.98228417	0.0104853	0.03361199
Rab36	-0.4553672	3.97702559	7.98155761	0.01048829	0.03361199
Lpar3	-1.3048728	2.27219078	7.98093874	0.01049083	0.03361199

Inha	-0.9698414	1.64997007	7.98021437	0.01049381	0.03361199
1700047A11I	-3.9197467	-1.7041524	7.97925807	0.01049774	0.03361199
Ndufv2	0.40426391	6.21579475	7.97925015	0.01049777	0.03361199
Gm11413	-3.2950663	-1.251623	7.97795678	0.0105031	0.03361594
Gbp6	0.54614335	5.06947314	7.97509856	0.01051487	0.03363311
Emilin1	0.9094308	2.02283567	7.97466716	0.01051665	0.03363311
Prkar1b	0.35935742	7.74528638	7.97218398	0.01052689	0.03364774
Gm19757	-0.8027831	4.53340146	7.97157358	0.01052941	0.03364774
Hiatl1	-0.5259027	4.0000402	7.96761331	0.01054577	0.03368216
Clcn4-2	-0.3484078	6.19279501	7.96640363	0.01055077	0.03368216
Leap2	-3.3060083	-0.4596987	7.96599169	0.01055247	0.03368216
Mief1	0.55073099	4.4905079	7.96254539	0.01056674	0.03371264
Gja6	-2.4121353	-1.1012232	7.96170505	0.01057023	0.03371264
09-Mar	1.03609455	1.83379373	7.95824182	0.01058459	0.03374537
Eif2b5	0.47118465	4.56639285	7.95423347	0.01060125	0.03378537
Micalcl	-1.1508564	1.66296183	7.95226636	0.01060943	0.03379835
Pign	-0.4869069	4.09877498	7.95071884	0.01061588	0.03380577
Cyc1	0.5235551	6.32295405	7.94946429	0.0106211	0.03380932
Gm15133	4.16851645	-1.759902	7.94771557	0.0106284	0.03381282
Klc1	0.3992275	7.49123601	7.94722688	0.01063043	0.03381282
Eif1	0.48194116	7.99043545	7.94219462	0.01065145	0.03386657
Myo18a	-0.4416553	6.30847376	7.94009609	0.01066023	0.03388137
Phka2	-0.8121264	4.89890177	7.93860917	0.01066645	0.03388622
Gulp1	0.66462724	5.95941799	7.93776233	0.01067	0.03388622
Micu2	0.42760674	5.20628011	7.93479899	0.01068242	0.03391256
Kcnd2	-0.4802866	6.04117674	7.93324165	0.01068896	0.03392021
Hivep1	-0.4102699	7.4051704	7.93206481	0.0106939	0.03392279
Man2c1	-0.9976271	2.65960172	7.9280583	0.01071074	0.0339631
Ndrg1	0.64920136	7.57194374	7.92526949	0.01072248	0.03398721
Prkcd	0.72535376	4.08542494	7.91951711	0.01074674	0.03405098
Zfp781	-0.4933222	6.24306577	7.91557305	0.01076341	0.03409065
Apbb1	0.43244554	5.52168404	7.91163308	0.01078009	0.03413033
9530036O11	-1.7891289	-0.2627716	7.90868624	0.01079258	0.03415674
Atp10d	-0.8604572	2.36718858	7.90340855	0.010815	0.0341953
Gm12709	-0.9519434	1.93978936	7.90182905	0.01082172	0.0341953
C1qb	1.08229917	2.39464549	7.90178338	0.01082191	0.0341953
Zbtb48	1.25529306	1.61841727	7.90152821	0.010823	0.0341953
Phlpp2	-0.5513655	5.35627201	7.9009244	0.01082557	0.0341953
Chrna4	-0.5019984	4.50706817	7.89734312	0.01084083	0.03421731
Tbck	-0.542876	4.68675334	7.89733536	0.01084086	0.03421731
Yae1d1	0.38409801	6.50183287	7.88994653	0.01087242	0.03430374
Wfdc18	2.71269452	-0.5678841	7.88721587	0.01088411	0.03431084
Tns1	0.33624418	6.61314246	7.88685791	0.01088564	0.03431084
Msx1os	-3.9826137	-1.7882486	7.88590643	0.01088972	0.03431084
Ctsa	0.52412332	5.4161235	7.88421101	0.01089699	0.03431084

Ndufb4	0.65853262	5.90445898	7.8815486	0.01090841	0.03431084
Bub1	-2.1243171	0.38533996	7.88115718	0.01091009	0.03431084
Ccdc101	0.55586706	4.2368717	7.87954365	0.01091702	0.03431084
Aimp2	0.63271212	3.29249552	7.8791122	0.01091888	0.03431084
Ccdc47	-0.3312129	7.07371652	7.87894603	0.01091959	0.03431084
Dmxl1	-0.5752999	7.30485153	7.87775851	0.0109247	0.03431084
Lmo2	0.57209189	4.39602433	7.87687868	0.01092848	0.03431084
Myo1a	-3.9631691	-1.8296905	7.87672308	0.01092915	0.03431084
Pwwp2a	-0.557288	5.79995736	7.87540788	0.01093481	0.03431084
Galnt6	-1.1983338	1.98311809	7.87493143	0.01093687	0.03431084
Erlin2	-0.3821372	5.40469007	7.87456657	0.01093844	0.03431084
Snx25	-0.4026411	5.1798809	7.87365214	0.01094238	0.03431084
Rnf141	0.54589977	4.23117867	7.87289497	0.01094564	0.03431084
Pkia	0.30487741	8.02784986	7.87106364	0.01095354	0.03432251
Akap9	-0.4348247	8.44979472	7.86829726	0.01096548	0.03434683
Fut9	-0.5621197	7.1307687	7.86429906	0.01098276	0.03438529
Zmym1	-0.6974677	3.92520281	7.86352167	0.01098612	0.03438529
Gucy2f	-2.3031698	0.43556857	7.85945723	0.01100373	0.03441626
Isg20	1.03057522	1.67087983	7.85840904	0.01100828	0.03441626
Rttn	-0.9388233	2.57433777	7.85833884	0.01100858	0.03441626
Trim43a	-1.8197691	-0.2022028	7.85507863	0.01102273	0.0344474
Mbnl2	0.34913413	9.1115468	7.84936245	0.01104759	0.03450471
Chm	-0.4136077	6.37748255	7.84893265	0.01104947	0.03450471
Asun	-0.426423	4.53145219	7.84682815	0.01105864	0.03452023
Pvr	-0.8002967	2.53603465	7.84386368	0.01107157	0.03454748
Ikbkap	-0.6179957	5.38567574	7.84144896	0.01108212	0.03456457
Cldn1	1.33480853	3.08447347	7.84068476	0.01108546	0.03456457
Tnfaip3	0.88675536	2.85126605	7.83822287	0.01109623	0.03457375
Zfp280b	-0.4658626	4.89230233	7.83808859	0.01109681	0.03457375
Nxf1	-0.3668604	5.93111729	7.83573128	0.01110714	0.0345928
Runx3	1.37229704	0.86058091	7.8337399	0.01111587	0.03459662
Mmab	0.46337045	4.42183548	7.83353055	0.01111678	0.03459662
Clic3	2.33661335	-0.5019365	7.83190524	0.01112391	0.03460165
Zglp1	-3.6867564	-1.4615656	7.83124317	0.01112682	0.03460165
4930455C13I	-2.5984175	-0.7454366	7.82954438	0.01113428	0.03461176
Gdi2	0.29573299	8.14845822	7.8250747	0.01115394	0.03465975
Abcf1	0.35201044	6.76225071	7.82236883	0.01116585	0.03468367
Xylb	-0.6592195	3.45145933	7.81983533	0.01117703	0.03470526
P2ry6	-1.2986477	0.78746139	7.81819316	0.01118427	0.03471465
Cep164	-0.6198208	3.31652975	7.81235052	0.0112101	0.0347813
Zcchc24	0.63104637	7.2701703	7.81119147	0.01121523	0.0347813
Naa16	0.58263367	4.04612996	7.80981212	0.01122135	0.0347813
Cdh7	-0.5910565	3.87185716	7.80915529	0.01122426	0.0347813
Gtf2h5	0.57050714	5.19964753	7.80855751	0.01122691	0.0347813
Cdc42bpa	-0.399331	9.05411707	7.80671707	0.01123507	0.03479348

Lrrc45	-0.597983	3.71480371	7.80510907	0.01124221	0.03479677
Gm9899	0.53353832	3.86221393	7.80457033	0.0112446	0.03479677
Acvr2b	-1.483912	0.6247075	7.80343964	0.01124963	0.03479922
Tmem45b	-1.0627946	2.23267171	7.79045619	0.01130749	0.03496505
Rpe	0.48272644	5.42631599	7.78250611	0.01134309	0.03506193
Kif1a	-0.5548568	9.884119	7.77827158	0.0113621	0.03510749
Mia3	-0.45437	6.29115697	7.77305957	0.01138555	0.03516673
Mink1	-0.4946357	5.58472022	7.7669705	0.01141301	0.03523125
Fbxo24	-1.9847462	-0.1138298	7.7665276	0.01141501	0.03523125
Dgki	-0.648239	4.88385758	7.76551138	0.0114196	0.03523219
Atpif1	0.43665531	7.27261637	7.76406511	0.01142614	0.03523821
Ak1	0.54413249	3.98628346	7.76318354	0.01143013	0.03523821
Ssh2	-0.390051	6.53010038	7.75577708	0.01146369	0.03532519
Nsa2	0.3909022	6.72256089	7.75488181	0.01146775	0.03532519
Batf2	3.0656528	-0.9426477	7.75411487	0.01147123	0.03532519
Chrnb4	-1.5959521	0.14843537	7.7528081	0.01147717	0.03533023
5730405O15	-2.3517419	-0.293786	7.75041789	0.01148804	0.03535045
Slc35d2	-1.4974332	0.16421298	7.74655828	0.01150561	0.03539128
Malsu1	0.47183751	4.73281261	7.74527724	0.01151145	0.03539599
Gramd1a	-0.7994165	3.06794535	7.74425196	0.01151613	0.03539633
Chrnb2	-0.518218	3.9374046	7.74336533	0.01152017	0.03539633
Ndufb11	0.55763483	5.59865569	7.74054796	0.01153304	0.03542261
Slc6a18	-1.2617329	1.13879394	7.73947314	0.01153795	0.03542446
Pcsk7	-0.6986881	2.68672644	7.73713788	0.01154863	0.03542578
Neu3	-0.6873104	3.12335179	7.73704817	0.01154904	0.03542578
Kcne4	0.75829231	3.15263084	7.73655295	0.01155131	0.03542578
Ankrd10	-0.5637922	3.86599986	7.7318278	0.01157296	0.03547894
Il1bos	5.58277912	-2.0499981	8.01130097	0.01158527	0.03550342
Csnk2b	0.44450317	5.72714885	7.72677585	0.01159616	0.03552356
Stard4	0.54493914	3.99959699	7.72343908	0.01161151	0.03555059
Ctla2a	-1.10477	1.86945644	7.72293367	0.01161384	0.03555059
Gas5	-0.3201425	6.98860921	7.72156777	0.01162013	0.03555059
Rsrc1	0.36303086	5.32700455	7.72110005	0.01162229	0.03555059
Atf2	0.28881265	8.8748874	7.71791008	0.011637	0.03557761
Spp1	0.80684775	7.62210651	7.71730864	0.01163978	0.03557761
Zbtb24	-0.4786373	5.10803084	7.71619085	0.01164494	0.03558015
Gpr183	-1.610928	-0.160354	7.714789	0.01165141	0.03558671
Glod4	0.36637894	5.51592075	7.71178273	0.01166532	0.03561594
4833420G17	-0.4557776	4.88925606	7.70935131	0.01167658	0.03563708
Dok2	2.34328085	-0.7678923	7.70534819	0.01169514	0.03567821
Slc9a9	-0.8800188	3.46094576	7.70457366	0.01169873	0.03567821
Ptprj	-0.4883546	6.83767348	7.70283288	0.01170682	0.03568962
Astn1	-0.5484265	8.03624661	7.69935082	0.01172301	0.03572573
Tomm70a	0.28745387	7.12662431	7.69737468	0.01173221	0.03574052
Jakmip3	-0.8211658	4.95956322	7.69588764	0.01173914	0.03574837

Akap8l	-0.8541373	3.41002979	7.69426067	0.01174672	0.03575823
Snai1	0.85565606	2.27376053	7.68452325	0.01179224	0.03587992
Lzic	0.57603905	4.1531194	7.68360771	0.01179653	0.03587992
Cbx1	0.52242305	4.65570054	7.68290998	0.0117998	0.03587992
Bnc2	0.63481414	7.67740496	7.68107447	0.01180841	0.03589282
Rwdd1	0.40843886	5.23543726	7.67696069	0.01182772	0.0359284
D17Ertd648e	-1.33071	0.72780669	7.67671945	0.01182886	0.0359284
Uxs1	-0.5058171	3.5382047	7.67377958	0.01184268	0.03595712
Numa1	-0.3177771	5.77399352	7.67043748	0.01185843	0.03598189
Bckdhb	-0.9025439	2.62445648	7.66783595	0.0118707	0.03598189
Arhgap21	-0.5182734	8.18769952	7.66702162	0.01187454	0.03598189
Thoc1	-0.5283062	4.76711734	7.66644407	0.01187727	0.03598189
Znrd1	0.57879526	4.5581472	7.66608604	0.01187896	0.03598189
Tmod4	-2.0841052	-0.5114358	7.66567072	0.01188092	0.03598189
Tm7sf3	-0.5316409	4.54772077	7.6655499	0.01188149	0.03598189
Snrpg	0.57209092	5.054598	7.66051495	0.0119053	0.03604072
Tbc1d7	0.54307811	4.04487966	7.65793435	0.01191753	0.03606445
Uqcrq	0.46702351	4.16383216	7.65550461	0.01192905	0.03606616
Mapre2	0.30054825	9.8021625	7.65543447	0.01192938	0.03606616
Myof	0.71429749	5.02471238	7.65503937	0.01193126	0.03606616
Tpm2	0.77842037	6.33843715	7.65072065	0.01195177	0.0361149
Sorbs2	-0.7098387	7.36528757	7.64779154	0.01196571	0.03614372
Spag1	-0.6359707	2.86066119	7.64613285	0.01197361	0.0361543
3110040N11	0.72319324	3.1539725	7.64247906	0.01199103	0.03619361
Stat2	-0.4275466	4.68576171	7.63176927	0.01204227	0.03633492
Rars	0.40282141	5.45583611	7.63031604	0.01204924	0.03634102
Apba1	-0.4769024	6.80136776	7.62926504	0.01205428	0.03634102
Skap2	0.51800564	4.03246123	7.62823149	0.01205925	0.03634102
Itpka	-0.5123622	4.18836864	7.62734847	0.01206349	0.03634102
Clybl	0.53581335	4.35151724	7.62674266	0.0120664	0.03634102
Nfil3	0.92933992	2.54597835	7.6243476	0.01207792	0.03636239
Armcx2	0.33371879	5.54431266	7.62111611	0.01209348	0.0363959
Bop1	0.74108219	2.95513326	7.61956867	0.01210094	0.03640501
Rora	-0.3539936	8.37690505	7.61274531	0.01213388	0.03649078
Rnf187	0.43251505	6.80386205	7.60887079	0.01215264	0.03653381
Vav2	0.89739875	1.76348009	7.60182676	0.01218682	0.03661158
Cep170b	-0.415086	7.77688155	7.60170358	0.01218742	0.03661158
Ttc3	-0.4971134	9.98771488	7.59578803	0.0122162	0.03668465
Snx6	0.43514022	6.40291174	7.59324273	0.01222861	0.03670391
Glmn	-0.762805	3.94395078	7.59251285	0.01223217	0.03670391
Raver1	0.55083014	6.00400555	7.59054724	0.01224177	0.03670391
Zdhhc21	-0.4268838	6.37299455	7.58975313	0.01224565	0.03670391
Dpm3	1.04210914	2.16360858	7.58853801	0.01225159	0.03670391
Mmp9	2.93654298	-0.7822995	7.58845095	0.01225201	0.03670391
Rpl7	0.5052286	6.91340087	7.5879956	0.01225424	0.03670391

Samd9l	0.61158444	6.92484062	7.58715718	0.01225834	0.03670391
Trappc2	0.49440035	3.89742879	7.58063711	0.01229028	0.03678614
Cds1	-0.4640432	5.48304788	7.57607238	0.0123127	0.03683983
Apln	-0.9868537	2.58939571	7.57368666	0.01232443	0.03685166
Rnf34	0.46941131	5.2729918	7.57344512	0.01232562	0.03685166
Uhrf1bp1	-0.6111423	3.87314258	7.57104061	0.01233746	0.03687365
Cbln3	-1.1777809	1.1983502	7.56569558	0.01236383	0.03692323
D7Ertd715e	-1.0370645	4.14583375	7.56528663	0.01236585	0.03692323
Gm8787	-2.9972338	-1.3720546	7.56454174	0.01236953	0.03692323
Prdx1	0.51293918	7.18344903	7.56367406	0.01237382	0.03692323
Mog	-0.8620053	2.85453928	7.56244513	0.01237989	0.03692323
9430021M05	-0.6314086	4.43883393	7.56199851	0.0123821	0.03692323
Megf10	-0.6787861	4.61145867	7.56131188	0.0123855	0.03692323
Foxm1	1.28187844	1.41921814	7.55880822	0.0123979	0.03694679
Slc2a8	-1.0290427	1.62197619	7.55650689	0.0124093	0.03696738
Skp2	-0.8312564	2.8285553	7.55406862	0.0124214	0.03699001
Tuba4a	0.391142	7.31249873	7.5520574	0.01243139	0.03700635
Maged2	0.45517502	4.4644189	7.55024248	0.01244041	0.0370198
Gkap1	0.53026216	5.17658871	7.54876033	0.01244779	0.03702833
Slc35d1	-0.4556444	4.13099089	7.54388533	0.01247207	0.03708715
Gins3	1.3263515	1.21726451	7.53867913	0.01249807	0.03712818
Arap2	-0.4697382	7.05856224	7.53778861	0.01250252	0.03712818
Kcnj5	3.17525255	-0.0883408	7.53766032	0.01250316	0.03712818
Tekt5	-1.7859301	0.32940631	7.53707399	0.01250609	0.03712818
Ltbp2	-4.3539392	-1.1453635	7.53610072	0.01251096	0.03712818
Tbx18	0.56755853	6.20995825	7.534079	0.01252108	0.03712818
Vwa5a	0.47351167	5.49992937	7.53396943	0.01252163	0.03712818
Slc6a8	0.37690595	6.34157848	7.53312006	0.01252589	0.03712818
Dusp22	-0.4143024	4.8711312	7.53299337	0.01252652	0.03712818
Emc2	0.43137086	6.48024617	7.52977367	0.01254267	0.0371549
Ksr2	-0.5458564	6.0655444	7.52852646	0.01254893	0.0371549
Smc6	-0.3061314	7.65803915	7.52650131	0.01255911	0.0371549
Mapkap1	0.33097741	6.5705397	7.52577095	0.01256278	0.0371549
Shank2	-0.7391089	6.98539914	7.52573347	0.01256297	0.0371549
Dus3l	-0.5690067	3.75938333	7.52461436	0.01256859	0.0371549
Igfn1	-0.8568494	4.3999423	7.52326637	0.01257538	0.0371549
Scin	3.5276251	-0.5378997	7.52258588	0.0125788	0.0371549
BC005764	-0.997341	2.18317284	7.52179324	0.01258279	0.0371549
Dhx57	-0.6972845	5.85111194	7.52165996	0.01258346	0.0371549
Nono	0.2821966	7.47503312	7.52130221	0.01258527	0.0371549
Tpk1	0.60450318	4.03306475	7.51826411	0.01260058	0.03717872
Nefh	-0.7530719	5.51403652	7.51790766	0.01260238	0.03717872
Mrap	1.172841	1.69198266	7.51598932	0.01261206	0.03718286
Cnot2	0.34367637	6.21927661	7.51583745	0.01261283	0.03718286
Catsper2	-0.9248935	1.9578967	7.51492897	0.01261742	0.03718305

Slc26a1	-2.2922281	-0.6319388	7.51139899	0.01263527	0.03721081
Maob	-0.4966161	4.16422355	7.51127528	0.01263589	0.03721081
Zcwpw1	-1.4333873	0.6780819	7.5054135	0.0126656	0.03727478
Vmn2r85	-2.0089953	1.10954057	7.50519853	0.01266669	0.03727478
Stx1a	0.47224262	5.57076194	7.5009378	0.01268833	0.03732509
Zfp287	-0.6575189	4.00793578	7.49781968	0.01270419	0.03735839
4933428G20	-1.3303602	1.69656661	7.49572092	0.01271488	0.03737645
Fbn2	-1.1512853	1.50799282	7.49307456	0.01272837	0.03740274
Sdk2	-0.7385199	3.60874328	7.48773725	0.01275564	0.03746111
Fam198a	-1.1505245	1.78053797	7.48740161	0.01275735	0.03746111
Klk14	-1.6975443	-0.0143907	7.48604975	0.01276427	0.03746803
Gorasp2	0.38154158	7.01602837	7.48278532	0.01278099	0.0374921
Ift57	0.32725725	5.92742361	7.4826675	0.01278159	0.0374921
Arid1a	-0.4887202	8.05983056	7.48078632	0.01279124	0.03749557
Loxl2	-0.676883	3.97633276	7.4802547	0.01279397	0.03749557
Cetn2	0.58011424	5.26854616	7.4785425	0.01280276	0.03749557
Mdm2	0.29389467	6.69822729	7.47845457	0.01280321	0.03749557
Ccdc12	0.64178752	3.63339538	7.47799205	0.01280558	0.03749557
Naaa	-0.6700913	3.92316073	7.47641719	0.01281368	0.0375059
Cntn4	-0.539661	5.2390942	7.47304138	0.01283104	0.0375301
Itgb8	-0.592851	4.16989927	7.47239453	0.01283437	0.0375301
Trim15	-2.8356681	-1.2295396	7.47214771	0.01283564	0.0375301
Csmd2os	-2.5351604	-0.3659152	7.4695114	0.01284923	0.03754554
4930429B21I	0.44500748	5.16830204	7.46935001	0.01285006	0.03754554
Lamp5	-0.4139155	5.75821944	7.46139837	0.01289113	0.03765217
Cct4	0.37010437	5.9909585	7.4586422	0.01290541	0.03768046
Myef2	-0.4736384	5.63887936	7.45763917	0.0129106	0.03768226
Khdrbs3	0.35625879	6.39263603	7.45469551	0.01292587	0.03771343
Myoc	-0.8241668	2.14626982	7.45121316	0.01294396	0.0377528
Mreg	-0.8973591	2.40495326	7.44912357	0.01295483	0.0377711
Ngfrap1	0.45133772	6.52437935	7.44450241	0.0129789	0.03780966
Chchd2	0.40975645	7.6487621	7.44395619	0.01298175	0.03780966
Etl4	-0.3284146	7.93056652	7.44393546	0.01298186	0.03780966
Gm5089	0.73392076	4.78982062	7.44210483	0.01299141	0.03782408
Kcnj2	-0.4293106	5.54481899	7.43952352	0.01300489	0.03784632
E030030I06R	-0.9204997	2.81872364	7.43815849	0.01301203	0.03784632
Sae1	0.40155855	5.57085668	7.43799867	0.01301286	0.03784632
Vtcn1	1.79188085	-0.1211964	7.4364652	0.01302089	0.03785626
Gfra2	0.46736637	7.57978057	7.43363028	0.01303573	0.03788186
Prkcdbp	0.85530196	5.17983032	7.43269854	0.01304061	0.03788186
Lyplal1	0.75596345	2.81065316	7.43214441	0.01304352	0.03788186
Phtf1	-0.3476714	5.56154343	7.42982427	0.01305569	0.03789393
Vldlr	-0.4818073	6.06539724	7.42895415	0.01306026	0.03789393
Cnnm3	-0.4524748	4.18017385	7.42871667	0.01306151	0.03789393
Xbp1	0.38817932	6.19549155	7.42487601	0.01308169	0.0379391

Slc30a2	-0.8190055	2.67361515	7.41859121	0.0131148	0.03801698
Npas3	-0.6057623	3.93110127	7.41600509	0.01312845	0.03801698
Mbd3	0.45387318	4.4107566	7.41585412	0.01312925	0.03801698
Kcmf1	0.35703952	7.50021097	7.41574648	0.01312982	0.03801698
Gm10791	-1.7930758	0.77777269	7.41272157	0.01314581	0.03801698
Rad23a	0.53510383	4.96103067	7.41222684	0.01314842	0.03801698
Blmh	0.42206135	5.01121896	7.41172685	0.01315107	0.03801698
Ppfia2	-0.429069	7.76153196	7.41098479	0.013155	0.03801698
Auh	0.42082622	5.11797038	7.41088558	0.01315552	0.03801698
Gm16853	-2.8909972	-0.9169055	7.40958793	0.01316239	0.03801698
Rbbp9	0.49539693	6.4796997	7.40826196	0.01316942	0.03801698
Tmem56	-0.4264867	6.25923531	7.40805144	0.01317053	0.03801698
Atad3a	-0.698132	3.12139672	7.40745732	0.01317368	0.03801698
Slco1a4	-0.608422	4.88980651	7.40673871	0.01317749	0.03801698
H3f3b	0.59746395	9.00360196	7.406079	0.01318099	0.03801698
Gpr3	1.27421364	1.08837726	7.40578501	0.01318255	0.03801698
Hs1bp3	-0.5025368	4.68705527	7.40481763	0.01318769	0.03801845
Ldhd	1.18338781	1.85823546	7.40352567	0.01319455	0.03802489
Ywhag	0.31563881	10.2179561	7.39986571	0.013214	0.03805671
Tbc1d25	0.66751964	3.07336258	7.39970634	0.01321485	0.03805671
Cyp3a13	-2.6736644	-1.1165901	7.39746741	0.01322677	0.03807769
Zbtb8b	-1.066265	1.87284961	7.39622263	0.0132334	0.03808344
Cabin1	-0.4047178	5.24959159	7.39421171	0.01324412	0.03809948
Dnah7a	-0.7154672	2.08891377	7.39343907	0.01324824	0.03809948
Slco1a5	2.92366968	-0.5127274	7.39217239	0.013255	0.03810559
Fbxw8	0.46097663	3.88757682	7.39128163	0.01325976	0.03810593
Plxna4os1	-2.0122816	-0.4873521	7.38746041	0.01328019	0.03814342
Ube2v2	0.33722021	6.88664878	7.38564274	0.01328991	0.03814342
Nr6a1	-0.7432485	3.0928936	7.38550199	0.01329067	0.03814342
A230056P14I	-0.5520847	4.00572703	7.38537093	0.01329137	0.03814342
Atf3	2.03999417	1.54733601	7.38385325	0.0132995	0.0381502
5830416P10I	-1.4277574	0.35402887	7.38261963	0.01330611	0.0381502
Pard6g	0.60651017	5.59374964	7.38233152	0.01330766	0.0381502
Kat2b	0.38293818	6.08005269	7.37860327	0.01332766	0.03819424
Fam118a	0.4917296	4.55151073	7.37714319	0.01333551	0.0382034
Shroom3	0.65231117	2.89460344	7.37620651	0.01334055	0.03820451
9530091C08I	-1.1990372	4.62543707	7.37294679	0.01335809	0.03824142
A930017M01	-0.745244	2.61294087	7.3678162	0.01338575	0.03830726
Asb8	0.33060083	6.2702894	7.36491119	0.01340144	0.03833881
Prrx1	0.57658658	7.06862892	7.36373107	0.01340782	0.03834372
Klhl34	-0.9349616	5.45597322	7.35884794	0.01343425	0.03840595
Bptf	-0.4767515	8.32255731	7.35639687	0.01344754	0.03841872
Rexo2	0.36272568	6.99962684	7.35547484	0.01345254	0.03841872
Mybbp1a	-0.5008217	4.39925614	7.35528813	0.01345356	0.03841872
Fkbp1a	0.39997015	9.78767574	7.35401303	0.01346048	0.03841872

Slitrk4	-0.4885177	6.29654105	7.35371755	0.01346209	0.03841872
Rai1	-0.4392767	5.70124103	7.3503272	0.01348052	0.03845796
Ggct	0.57590794	4.48865281	7.34922691	0.0134865	0.03846169
Sft2d3	-0.5942761	3.19211929	7.34621123	0.01350293	0.03848614
6820431F20I	-0.4471716	8.79642571	7.34593359	0.01350444	0.03848614
Asna1	0.49813463	6.49108005	7.34492262	0.01350995	0.0384885
Fzd3	-0.3777822	7.99007841	7.34293043	0.01352082	0.03850612
Ndufaf1	0.70438902	3.33368147	7.33253956	0.01357768	0.03865464
Epb4.1l2	0.36949957	7.5391637	7.33159408	0.01358286	0.03865601
Slain1os	-1.6370747	1.00212883	7.33045157	0.01358913	0.03866047
Elfn2	-0.501423	5.01552476	7.32947864	0.01359447	0.03866229
Cnot1	-0.356069	8.09961705	7.31789265	0.01365827	0.038828
Mir186	-3.0932075	-0.8361679	7.31718203	0.01366219	0.038828
Zfhx2	-0.5012752	5.15266286	7.3145159	0.01367692	0.03885644
Zfp523	-0.4856959	4.07551606	7.31323168	0.01368403	0.03886021
Usp17la	-1.6294094	0.44794742	7.31256637	0.01368771	0.03886021
Terf2	0.32604395	6.26750373	7.31040974	0.01369965	0.03887189
Mir8091	-1.9804708	-0.5753025	7.31011479	0.01370128	0.03887189
Haus1	0.80019766	2.88420026	7.30496033	0.01372987	0.03892322
Sun1	-0.4874072	4.71651478	7.30360817	0.01373738	0.03892322
0610009L18I	1.04349145	0.8437656	7.30230398	0.01374463	0.03892322
Fibp	0.51284604	4.96051385	7.30219029	0.01374526	0.03892322
4933421O10	-0.6970016	3.5009679	7.30115873	0.013751	0.03892322
Pdzrn4	-0.8063914	2.404402	7.30097777	0.01375201	0.03892322
Arpp21	-0.3887037	8.75965728	7.30088477	0.01375252	0.03892322
Ap4b1	0.94813245	2.03441604	7.29880822	0.01376408	0.03894252
Gabra2	-0.5803088	4.85233987	7.2919433	0.01380237	0.03902729
Tnfaip8l1	0.8527564	2.81437686	7.29173334	0.01380354	0.03902729
Chdh	-1.165063	1.47474422	7.28989191	0.01381383	0.03903457
Nek6	0.57891701	4.83374912	7.28957358	0.01381561	0.03903457
Zcchc14	-0.3620326	6.32427807	7.27797455	0.01388064	0.03920483
2410007B07I	3.49626185	-1.9733839	7.27633722	0.01388985	0.03921566
Klhl41	-1.1411816	1.5136362	7.27559591	0.01389402	0.03921566
Rictor	-0.5819745	6.50234641	7.27474408	0.01389882	0.03921573
Cep95	-0.490909	3.92792727	7.27234166	0.01391235	0.03924044
Vegfb	0.4668498	4.6599426	7.26712039	0.01394181	0.03931004
Atf5	0.70876964	3.26731139	7.26479518	0.01395495	0.0393336
Gpr19	-0.6758114	3.45953435	7.26260597	0.01396734	0.03935502
Rhox8	-1.1746512	1.56502683	7.26050479	0.01397924	0.03937505
Ndufa4	0.46417438	7.34644343	7.25668855	0.01400088	0.03942249
Dusp9	1.44623472	0.92120224	7.2511477	0.01403237	0.03947109
Banf1	0.57782045	4.45274061	7.25100264	0.01403319	0.03947109
Dio2	-0.423852	5.92017166	7.2502994	0.01403719	0.03947109
Strbp	-0.4555901	9.34693261	7.25027238	0.01403735	0.03947109
Vkorc1l1	-0.2944345	6.22933451	7.24300951	0.01407876	0.03956531

Tfg	0.30995523	6.88161521	7.24270756	0.01408048	0.03956531
Rpl27	0.50475944	6.01299865	7.24094709	0.01409054	0.03958005
Nhp2l1	0.394462	6.06380056	7.23916802	0.01410072	0.03958307
Mmp16	-0.5054775	5.36699332	7.23907501	0.01410125	0.03958307
Ddb2	-1.0804668	1.05610744	7.23171693	0.01414342	0.03968788
Cct2	0.2801546	7.0039922	7.22886292	0.01415981	0.03971629
Pcsk1	-0.6687589	3.26775986	7.2282725	0.01416321	0.03971629
Pan2	-0.684938	3.5095207	7.22162367	0.01420149	0.03978934
Fuca2	0.41403308	4.54226025	7.22127791	0.01420349	0.03978934
Hdac9	-0.3204702	6.45270884	7.2212273	0.01420378	0.03978934
Becn1	0.50382538	5.24781277	7.21907951	0.01421617	0.03981049
Ccl7	2.61275245	-0.4119397	7.21799469	0.01422244	0.03981447
Chmp1a	0.55441272	4.57431843	7.21700699	0.01422814	0.03981688
Chmp2b	0.51505511	6.34152136	7.21202674	0.01425696	0.03988393
Rhoq	0.45725211	6.22370793	7.2083087	0.01427851	0.03990979
Rdh1	-1.0424848	2.07293096	7.20821488	0.01427905	0.03990979
Tor1aip2	0.4523712	7.06303032	7.20709061	0.01428558	0.03990979
Taf8	0.62397973	2.78515592	7.20708317	0.01428562	0.03990979
Zfp385a	0.71326238	6.61742759	7.20352838	0.01430628	0.039952
Pxdn	-0.9586799	4.05497605	7.20232529	0.01431327	0.039952
Lims1	0.50354388	7.53140052	7.20197473	0.01431531	0.039952
Mpzl2	0.58696017	7.15656014	7.20031026	0.014325	0.03995472
Ascl2	2.77513255	-0.7872353	7.19959139	0.01432919	0.03995472
Lingo2	-0.4973997	5.23399232	7.19836837	0.01433631	0.03995472
Samhd1	0.35662144	5.59678699	7.19799283	0.0143385	0.03995472
Fmo5	0.6049215	3.72328381	7.19763414	0.01434059	0.03995472
Cnot10	-0.5208438	4.35910618	7.19563586	0.01435225	0.03997364
Cnot3	0.40840613	5.42677765	7.1942389	0.0143604	0.03998281
Tenm1	-0.7165657	5.44457367	7.19081359	0.01438042	0.04001567
Lyg2	7.10896155	-1.660059	7.43829486	0.01438195	0.04001567
Pcdhga7	-0.7790237	2.71389991	7.18885279	0.01439189	0.04002558
Rad51	1.4199857	0.79535411	7.18802898	0.01439672	0.04002558
C130071C03I	-0.7719378	2.7042152	7.18744842	0.01440012	0.04002558
Hn1	0.56131584	4.32365813	7.1842447	0.0144189	0.04006423
Smoc1	0.61455304	4.56734441	7.17986642	0.0144446	0.0401221
Tek	-0.7472417	2.9134999	7.17358057	0.0144816	0.04021128
Jph4	-0.361127	7.22585029	7.17146545	0.01449408	0.04023232
Ubl4b	1.93730328	0.06162456	7.17059854	0.01449919	0.04023293
Nmt1	0.32345988	5.26669908	7.16867862	0.01451053	0.0402508
Agap1	-0.4139263	6.71017495	7.16471787	0.01453395	0.04030216
2010015L04I	-0.6261387	3.55873562	7.16200419	0.01455002	0.04033312
8430431K14I	-1.2783605	0.78650113	7.15899281	0.01456787	0.040369
Dapk1	-0.4344176	5.9237312	7.15655383	0.01458236	0.04039551
Ccdc34os	-3.6584767	-1.6641624	7.15317218	0.01460246	0.04043757
Cmtr2	-0.8635039	2.13066756	7.14954032	0.01462408	0.04048382

Gm17821	-0.5091031	9.70641566	7.14842251	0.01463075	0.04048862
Zfp12	-0.4733264	4.74653812	7.14430987	0.01465529	0.04053929
Speer4b	-1.2969494	0.58808674	7.14370285	0.01465892	0.04053929
Mrpl39	0.41880824	5.38481267	7.14192878	0.01466952	0.04054714
Calcr	-1.846127	0.29845713	7.14157706	0.01467163	0.04054714
Fndc7	3.71798873	-1.3887912	7.13893236	0.01468745	0.04057009
Plin4	-1.3576765	0.99542139	7.13854026	0.0146898	0.04057009
Slc25a11	0.3995199	5.65167589	7.13241326	0.01472655	0.04065792
Zfp36l1	0.4827906	7.72852485	7.13140654	0.0147326	0.04066096
Vwa8	-0.4931447	5.28914095	7.12618619	0.01476401	0.0407168
Prdx3	0.47252714	5.17439889	7.12599177	0.01476518	0.0407168
Ntpcr	0.73220118	2.61603098	7.1255626	0.01476776	0.0407168
Dnajc9	0.42144831	5.91835945	7.12399827	0.01477719	0.0407168
Mrpl30	0.45207508	5.69190516	7.12393043	0.0147776	0.0407168
Scarb1	-1.0242902	2.48768877	7.12273468	0.01478481	0.04072301
Enpp2	-0.3918845	8.24836656	7.11940479	0.01480492	0.04074941
Gm10069	-1.2009002	0.87244405	7.1193568	0.01480521	0.04074941
Rps6	0.55006774	8.46228235	7.11868441	0.01480927	0.04074941
Hic1	0.83684779	3.26674791	7.11624507	0.01482402	0.04077635
Plcb1	-0.5479235	8.78715465	7.11436371	0.01483541	0.04078352
Sult5a1	2.22311541	-0.535735	7.11417515	0.01483655	0.04078352
Rabif	0.47110572	5.84337403	7.10337308	0.01490213	0.04093936
C230091D08I	-0.4959409	6.46143414	7.10319677	0.01490321	0.04093936
Cep72	-1.4363546	1.07004551	7.1006271	0.01491886	0.04096866
Mcts2	0.52217089	3.74713524	7.09716544	0.01493997	0.04100353
Mzt2	0.69926694	2.97104028	7.09690983	0.01494153	0.04100353
Gtf2b	0.46322485	4.83713545	7.09506813	0.01495278	0.0410078
Zfyve27	-0.4670544	4.26992673	7.09502113	0.01495306	0.0410078
D130043K22I	-0.7235962	3.37003417	7.09229681	0.01496972	0.04103978
Ewsr1	-0.5166396	7.69583582	7.09004616	0.0149835	0.04106385
Rasgrp4	-1.6886098	-0.1143058	7.08916196	0.01498891	0.04106499
Psmc2	0.2960634	6.27084943	7.08816726	0.01499501	0.041068
Skap1	1.82627901	-0.0607999	7.08721058	0.01500087	0.04107038
Slc5a12	-1.6727516	0.11531533	7.07886217	0.01505216	0.04119707
Ccdc148	-0.5024868	4.10954772	7.07131138	0.01509871	0.04131074
Zfp260	0.31991539	7.24177903	7.0696148	0.0151092	0.04132566
Plrg1	0.42594289	4.51438789	7.06685353	0.01512628	0.04133861
Sestd1	-0.4991882	7.06403076	7.06653972	0.01512822	0.04133861
Map3k6	0.84190615	1.76105039	7.06641045	0.01512902	0.04133861
Sirt7	-0.6802714	3.02721932	7.06147002	0.01515964	0.0414085
Anxa2	0.87159399	6.18191341	7.05927294	0.01517327	0.04143199
Cryl1	0.65527931	3.57111571	7.04734829	0.01524754	0.04160866
Psap	0.41985234	8.49462902	7.04725765	0.0152481	0.04160866
Bpgm	0.42304618	6.8275735	7.04433055	0.01526639	0.04164475
Cd6	2.72122811	-0.0356959	7.04246172	0.01527808	0.04166282

Dtwd2	-0.7324655	2.91413883	7.04110886	0.01528655	0.04167209
Irgm1	0.66846512	4.39098843	7.0392942	0.01529792	0.04168925
Acy3	0.94287711	1.57757437	7.03234495	0.01534155	0.04178388
Ogfod1	-0.3739187	7.29648385	7.03214342	0.01534282	0.04178388
Lsm2	0.77906313	4.23845865	7.0279689	0.01536909	0.04183676
Ints1	-0.5614944	4.56946716	7.02744184	0.01537241	0.04183676
Drg2	0.50533216	3.88772029	7.02229234	0.0154049	0.04191131
Stx3	-0.4016971	4.78234578	7.01859896	0.01542825	0.04193977
Tceal5	0.34814077	6.04172697	7.01813151	0.01543121	0.04193977
Epha10	-1.4838069	2.34216075	7.01761422	0.01543449	0.04193977
Rasl11b	0.41405461	5.61450664	7.01741075	0.01543578	0.04193977
Ccdc64	-1.3794151	0.49411901	7.01630672	0.01544277	0.0419449
Lrtm1	-1.5469451	3.1295136	7.0110025	0.01547641	0.04202238
4933402D24I	-2.019264	0.32881142	7.00884234	0.01549013	0.0420411
Psmd8	0.39358339	5.10244073	7.00775524	0.01549704	0.0420411
Rpl12	0.40704309	6.78598507	7.00750292	0.01549865	0.0420411
Gm5862	-1.4099844	0.79766452	7.00580658	0.01550944	0.04204115
Gm19461	-2.7521772	-0.8248495	7.00540216	0.01551201	0.04204115
Blvra	0.76313288	2.55710238	7.00394213	0.01552131	0.04204115
Sin3b	0.48127588	4.8061056	7.00374798	0.01552255	0.04204115
Smpd3	-0.4447838	4.76558049	7.00324032	0.01552578	0.04204115
Pura	0.27273914	6.97518795	7.0026799	0.01552935	0.04204115
Kcnn3	-0.6813779	4.1629489	7.00110134	0.01553942	0.04204504
1700001K19I	-1.6402502	0.15610211	7.00085009	0.01554102	0.04204504
Nolc1	0.38868755	5.18570524	6.99881095	0.01555404	0.0420664
Far2	-0.6765711	3.82220963	6.99174339	0.01559924	0.04216766
Taok3	0.40624852	5.86871677	6.99135384	0.01560174	0.04216766
Igf2bp3	-0.6300309	4.2010688	6.98835417	0.01562097	0.04220576
Zfp57	-1.1186407	1.89585509	6.98348457	0.01565225	0.04227637
Slc22a5	-0.7857972	2.58385577	6.98244575	0.01565893	0.04228053
Dcbld1	-0.9164389	1.98688471	6.98024249	0.01567311	0.04230492
Coch	0.49355413	7.79535415	6.97803927	0.01568731	0.04232934
Dnajb3	2.19976021	-0.1025041	6.97014278	0.01573831	0.04245302
Ssfa2	-0.361023	5.4928508	6.96850514	0.01574891	0.04246767
Large	0.42580349	6.42464331	6.96523176	0.01577012	0.04249099
Mmp19	0.94483441	2.61996574	6.9644794	0.015775	0.04249099
1700003F12I	-3.9826405	-2.0074229	6.96399133	0.01577817	0.04249099
Josd1	0.35616615	5.03477143	6.96383201	0.0157792	0.04249099
Zfp65	-0.5335045	5.05369381	6.96318423	0.01578341	0.04249099
Cetn3	0.44462271	7.87997825	6.9619572	0.01579137	0.04249658
Cops8	0.33179685	5.82412212	6.96108723	0.01579702	0.04249658
Snap23	0.4716195	7.16619775	6.96047625	0.01580099	0.04249658
3110057O12	-0.5648659	3.00491508	6.95861144	0.01581312	0.04251435
Kif5a	-0.4249639	9.49887627	6.95786959	0.01581794	0.04251435
Spcs3	-0.3260192	6.30157083	6.95193375	0.01585662	0.04259474

Ntrk2	-0.5161173	8.36611345	6.95168873	0.01585822	0.04259474
Gm10754	-1.0516533	1.74058134	6.94834272	0.01588007	0.04262698
Aga	0.83369112	3.47617596	6.9478212	0.01588348	0.04262698
Pard3b	0.58039423	3.32297747	6.94746896	0.01588578	0.04262698
Akr1a1	0.54798405	7.81607609	6.9439572	0.01590876	0.04265826
Psmb4	0.48383132	5.59698976	6.94278479	0.01591644	0.04265826
Paqr8	-0.3716449	5.11849638	6.94262998	0.01591746	0.04265826
Doc2a	0.767248	2.81656322	6.94207598	0.01592109	0.04265826
Ankhd1	-0.3747611	7.51690378	6.94126649	0.0159264	0.04265826
Ssb	0.33132217	8.09090107	6.94048987	0.01593149	0.04265826
Mif	0.50569771	5.21874154	6.9391314	0.0159404	0.04265826
Ccdc71	0.59365724	4.2507704	6.9387534	0.01594288	0.04265826
Plk1	-2.9936033	-0.9043404	6.93802784	0.01594765	0.04265826
B4galt1	0.60054647	4.19173818	6.93777014	0.01594934	0.04265826
Parp1	0.30470035	6.13886078	6.93664959	0.0159567	0.04266406
Sncg	2.43525034	-0.3725225	6.93363018	0.01597656	0.04270325
Serping1	0.75339619	7.04095034	6.93257542	0.0159835	0.04270792
Atp9a	-0.4881438	4.80433389	6.92961827	0.01600298	0.04273629
Adamts6	-1.045302	1.791435	6.92866059	0.01600929	0.04273629
Ppid	0.3468704	6.12373406	6.92794074	0.01601404	0.04273629
Pcbd2	0.71570939	4.06968922	6.92705641	0.01601988	0.04273629
Dear1	-1.3467552	1.65996176	6.92668206	0.01602235	0.04273629
Armc5	0.63779612	2.58306319	6.92522966	0.01603194	0.04273629
Tpm3	0.34215063	7.43066738	6.92498487	0.01603356	0.04273629
Cmc1	0.61106372	3.90903451	6.92465889	0.01603571	0.04273629
Ubr5	-0.4065527	7.85059962	6.92341756	0.01604391	0.04274429
Zfp462	-0.4600802	6.71344293	6.9204298	0.01606368	0.042765
Exosc3	0.51254411	4.95860279	6.92028133	0.01606466	0.042765
Dkc1	0.32991191	6.20082846	6.91910887	0.01607243	0.042765
Mir8115	-2.2510822	-1.2524233	6.91909782	0.0160725	0.042765
Sparc	0.76784244	8.82825556	6.91563978	0.01609543	0.04281214
Arl16	0.41411557	4.2914429	6.91370851	0.01610825	0.04283002
Mrpl11	0.52272847	4.64254819	6.91305752	0.01611257	0.04283002
Zfp641	-0.9182028	3.02665455	6.91203874	0.01611934	0.04283416
Rbm17	0.36505165	6.6248111	6.91091838	0.01612679	0.04283588
Wnt10a	0.86509626	1.65062776	6.90925569	0.01613785	0.04283588
Haus7	0.93505728	2.08117045	6.90790562	0.01614684	0.04283588
Ipo5	0.35048575	6.79794917	6.90778172	0.01614766	0.04283588
Tmsb15b2	1.94732637	-0.3630723	6.90774574	0.0161479	0.04283588
Fam69c	-1.5543288	0.11835775	6.90657076	0.01615573	0.04283588
Myt1	-0.7744613	2.81373741	6.90645924	0.01615647	0.04283588
Clpp	0.50117137	3.49623974	6.90067992	0.01619503	0.04292427
Nudcd1	-0.514204	3.65886042	6.8979906	0.01621301	0.04295807
Pcbd1	0.79117036	1.94610971	6.89710611	0.01621893	0.0429599
Pram1	-1.2240349	0.92672815	6.89551355	0.01622959	0.04297193

Gm684	0.59894141	3.50100373	6.89486607	0.01623393	0.04297193
Naip2	-1.5363726	0.32866889	6.89252707	0.0162496	0.04298906
Hdx	-0.8960551	2.92416688	6.89211108	0.01625239	0.04298906
Gpank1	1.04458384	2.00662254	6.8915605	0.01625609	0.04298906
Greb1l	-0.6879294	3.07983397	6.88777143	0.01628153	0.0430425
Dpy19l3	-0.473879	5.02698243	6.8832291	0.01631209	0.04308526
Snapc5	0.49939464	6.27476818	6.88192338	0.01632089	0.04308526
Scara3	0.83396793	6.56283487	6.88179012	0.01632179	0.04308526
Frmd4b	0.40953566	5.2760679	6.88066994	0.01632934	0.04308526
Nup133	-0.3969508	4.51006149	6.88062747	0.01632963	0.04308526
Dazl	-1.0781831	1.50788797	6.88047556	0.01633065	0.04308526
Crocc	-1.1910019	2.06079015	6.87900653	0.01634056	0.04308526
Ccdc104	0.35639291	7.65613033	6.87889511	0.01634131	0.04308526
Cped1	0.60491905	7.25689517	6.87836595	0.01634488	0.04308526
Fap	-1.1539557	1.52176877	6.87519226	0.01636632	0.04312794
Gfra1	-0.5843148	3.28474915	6.87377993	0.01637587	0.04313928
2410021H03I	-2.1666717	0.56287823	6.87168746	0.01639004	0.04315069
Chgb	0.42669199	9.20757245	6.87108593	0.01639411	0.04315069
Impa2	-2.0734502	-0.1342386	6.87081329	0.01639596	0.04315069
Tro	-0.6082959	6.41530895	6.86975045	0.01640316	0.04315582
Ccdc90b	0.551557	5.8537065	6.86870376	0.01641025	0.04316067
Cep85l	-0.5751486	4.01500005	6.86157512	0.01645867	0.04327417
Prrt4	-2.1610359	-0.6122405	6.85855131	0.01647926	0.04330168
Mphosph9	-0.5515047	4.87189749	6.85849042	0.01647967	0.04330168
Elmo1	0.35749431	7.15388229	6.85105545	0.01653041	0.04341326
Mtmr3	-0.3515284	6.04740863	6.85072063	0.0165327	0.04341326
Oxt	1.73017073	0.36731434	6.84909048	0.01654385	0.04342867
Cnnm2	-0.5896279	3.86824534	6.84732018	0.01655597	0.0434466
Tmem38b	-0.6280194	3.18982367	6.84635957	0.01656255	0.04344778
Zbtb42	1.52356698	0.59707261	6.84571167	0.01656699	0.04344778
Tapt1	-0.3578283	5.13774678	6.8426094	0.01658827	0.04347768
Nav3	-0.6491662	6.74141912	6.84250657	0.01658897	0.04347768
Coil	0.66494319	3.23445408	6.83713453	0.01662589	0.04354742
Ncan	-0.4627405	8.37898818	6.83709272	0.01662618	0.04354742
Noc4l	0.61215716	3.21503517	6.83593361	0.01663415	0.04355443
Ptgr2	0.46508856	6.42291844	6.83382404	0.01664868	0.04357859
Parpbp	2.11309103	0.83810134	6.83285826	0.01665534	0.04358214
Kdm3b	-0.2785236	7.03143235	6.82800041	0.01668887	0.04365598
Sppl2b	-1.0741061	1.50331952	6.82635471	0.01670025	0.04367184
Gbf1	-0.412241	5.9862477	6.82544518	0.01670654	0.04367439
Scaper	-0.4684022	6.38242996	6.81680437	0.01676644	0.04379952
Mrpl1	-0.4768739	4.24105416	6.81588745	0.01677281	0.04379952
Fam47e	3.22990734	-1.255421	6.81585918	0.016773	0.04379952
Cox7a2l	0.46350964	7.11294571	6.81546857	0.01677572	0.04379952
Ech1	0.74581339	3.948323	6.81457826	0.01678191	0.04380176

Cacna1d	-0.7031027	5.10948348	6.81357458	0.01678889	0.04380203
Serac1	-0.5451778	5.45659715	6.81283684	0.01679402	0.04380203
Twf1	0.41479403	7.41799396	6.8122652	0.016798	0.04380203
Polr2f	0.77134249	2.1390759	6.81062172	0.01680944	0.04380824
Folr2	2.89076293	-0.8304092	6.81039245	0.01681104	0.04380824
Fam132b	-2.10475	-0.102842	6.8068368	0.01683583	0.04384044
Igfbp4	0.62379804	5.14860724	6.80637795	0.01683903	0.04384044
Ifi35	1.30731612	2.04781192	6.8059408	0.01684208	0.04384044
Btc	-4.55932	-1.977228	6.80517441	0.01684743	0.04384044
Glo1	0.44024099	6.44821502	6.80385938	0.01685662	0.04384044
Akr1b8	0.90027051	2.14255392	6.80285957	0.01686361	0.04384044
Nup160	-0.5158004	4.50080403	6.80206212	0.01686919	0.04384044
Nfkbib	0.5454678	3.71176623	6.80179159	0.01687108	0.04384044
Gm16712	4.09864396	-1.9720096	6.80028574	0.01688161	0.04384044
Prdm8	0.54679767	5.55077021	6.80026284	0.01688177	0.04384044
Coro1b	0.46571734	5.32204427	6.80022052	0.01688207	0.04384044
Adcy9	-0.4633831	6.22779904	6.79849269	0.01689417	0.04385801
Clip1	-0.452349	7.59804925	6.79593163	0.01691212	0.04389075
2700062C07I	0.56666804	3.96942133	6.79312976	0.01693178	0.04392426
Ifitm2	0.90206695	7.3431538	6.79256898	0.01693572	0.04392426
Nmrk2	-1.0394326	0.66187961	6.78880145	0.01696221	0.04397182
Epm2aip1	-0.4985952	7.97617275	6.78843962	0.01696476	0.04397182
Als2	-0.4950409	4.90617991	6.7846213	0.01699166	0.04402765
Tmem209	-0.4265406	4.22701157	6.78382576	0.01699727	0.0440283
Spata17	4.1392627	-1.2522356	6.78265964	0.01700549	0.04403379
Ppwd1	-0.4831635	3.72394038	6.78200705	0.0170101	0.04403379
Pde10a	-0.5647954	7.06493354	6.78037716	0.01702161	0.04404971
Rgs18	1.93573499	-0.0944499	6.77758255	0.01704137	0.04407068
1700007G11	-1.6566717	0.29803362	6.77701762	0.01704536	0.04407068
Isca1	0.28051758	6.6628127	6.77695622	0.0170458	0.04407068
Tmem170	0.68307167	3.25063668	6.77602817	0.01705236	0.04407379
Gm20594	-1.0790504	0.45296374	6.77449761	0.0170632	0.04407924
Dlgap5	-1.2175006	0.66188119	6.77421611	0.0170652	0.04407924
Syap1	0.6434281	5.92517928	6.77270685	0.01707589	0.04409301
Tti2	-0.4694896	4.5965	6.76933449	0.01709982	0.04414093
Atp2b3	-0.5446389	6.97569938	6.76398075	0.01713789	0.0442253
Tfe3	0.43263844	5.30511724	6.76232022	0.01714971	0.04424192
Tktl1	-3.3121712	-1.6232973	6.76073981	0.01716097	0.04425709
Eny2	0.34324177	7.31050281	6.75981237	0.01716759	0.04425822
Hypk	0.56141515	6.55731281	6.75916846	0.01717218	0.04425822
Igsf3	-0.4231233	4.5003432	6.75676147	0.01718936	0.04428861
Ino80dos	-0.8449808	2.98122524	6.75419657	0.0172077	0.0443131
Phip	-0.4299513	7.35489708	6.75368089	0.01721138	0.0443131
Tubg1	0.50198451	4.38928208	6.75316964	0.01721504	0.0443131
Pkp1	1.40852613	1.20327792	6.74901706	0.01724478	0.04435706

Hn1l	0.74386058	4.36652087	6.7489101	0.01724555	0.04435706
Masp1	-0.8966267	1.67919418	6.74852468	0.01724831	0.04435706
2310015A10I	-0.8426338	2.92595868	6.7460622	0.01726598	0.0443886
Wnt4	0.4077403	5.58221254	6.74285178	0.01728904	0.0444174
A330102I10F	-1.3857084	1.29368866	6.74281874	0.01728928	0.0444174
Capn9	5.3952918	-2.3766796	6.96274569	0.01729449	0.0444174
2610507I01R	-0.3807094	4.73203653	6.74149509	0.0172988	0.0444174
Gucy2c	2.6397406	-0.8766283	6.73741151	0.0173282	0.04447901
Kat2a	0.36661737	5.89910999	6.72486729	0.01741888	0.0446978
Colec12	-0.3094389	6.68445516	6.72058419	0.01744996	0.04476359
Rpl6	0.43133191	7.75574068	6.71875683	0.01746324	0.04478368
Wdr7	-0.5120779	7.54758209	6.71742897	0.0174729	0.04479447
Wwtr1	0.46732614	6.61329783	6.71543376	0.01748742	0.04481308
E530011L22F	-0.6062634	2.52860136	6.71460861	0.01749343	0.04481308
Acnat1	-1.6820639	0.1652493	6.71418502	0.01749651	0.04481308
Sgsm2	-0.487439	4.61831494	6.71188106	0.01751331	0.04482586
4931430N09	-1.5131731	1.7189984	6.7118192	0.01751376	0.04482586
Spire2	-0.6436431	2.52442626	6.7112563	0.01751787	0.04482586
Atf6	-0.3752819	6.94906502	6.70990374	0.01752774	0.04483716
Unc13d	2.66566513	-0.8819921	6.7071388	0.01754794	0.04486593
0610009B22I	0.61348815	5.56179021	6.70566867	0.01755869	0.04486593
Ehd2	0.73486725	4.92766503	6.70543344	0.01756041	0.04486593
Stag3	-3.559596	-1.6816123	6.70537758	0.01756082	0.04486593
Pla2g4d	-6.2814493	-2.0625272	6.91617122	0.01761415	0.04498821
Aoc2	-1.1755902	1.2423825	6.69386816	0.01764525	0.04504445
Acbd4	-0.8061846	2.3604109	6.69361172	0.01764714	0.04504445
Rptor	-0.4208593	5.34640575	6.69018553	0.01767236	0.04509483
Cds2	-0.4047753	7.73388419	6.68888062	0.01768198	0.04510185
Wdr4	-0.865176	2.39994244	6.68790459	0.01768918	0.04510185
Ecel1	0.88745232	1.9455997	6.68757945	0.01769157	0.04510185
Gm4532	-3.1938937	-1.3773558	6.68601484	0.01770312	0.0451173
Ykt6	0.4435971	5.12626291	6.68445868	0.01771461	0.04512153
Twf2	0.55342786	3.43721528	6.68430341	0.01771576	0.04512153
Gnptab	-0.4245035	6.15644286	6.67400659	0.01779202	0.04530172
9330159M07	-0.7702012	2.13112621	6.66750265	0.01784038	0.04538775
Alox8	-0.605489	3.29180948	6.66734532	0.01784155	0.04538775
Hap1	-0.581471	4.20512542	6.66631729	0.01784921	0.04538775
Sytl3	1.12525414	0.95486318	6.66565911	0.01785412	0.04538775
Diap1	-0.3955384	4.50604362	6.66558602	0.01785466	0.04538775
Elf3	-1.7964336	-0.0623376	6.66501191	0.01785894	0.04538775
Phka1	-0.5646877	4.255368	6.66325216	0.01787207	0.04539462
Camk2g	-0.4190472	7.23186606	6.66316853	0.01787269	0.04539462
Kxd1	0.55320524	4.23318675	6.66124868	0.01788702	0.04541699
Itga4	-0.5529467	4.8409161	6.66038918	0.01789344	0.04541926
Csf3r	-1.1594275	1.43917202	6.65736032	0.01791609	0.04544884

Nid1	0.47488428	7.06377941	6.65735155	0.01791616	0.04544884
Fkbp1b	0.56516125	4.2158302	6.65571818	0.01792838	0.04545641
Smndc1	0.47405635	5.33377647	6.65476016	0.01793556	0.04545641
H2-T24	-0.610898	4.91991878	6.654737	0.01793573	0.04545641
Mir3473f	-3.3740503	-1.3801563	6.65156513	0.01795951	0.04550264
Anapc11	0.5175419	4.06063091	6.64677953	0.01799546	0.04557966
F630111L10F	-0.9733101	2.56004193	6.64461399	0.01801175	0.04560687
Smim3	0.7223023	3.12589515	6.6434386	0.0180206	0.0456119
1810013L24F	0.3145402	7.41829365	6.64146413	0.01803548	0.0456119
Plekha7	-0.5782307	2.98852326	6.64099632	0.018039	0.0456119
Atp2a2	-0.3763773	9.64115715	6.64073048	0.01804101	0.0456119
Taz	-0.6036488	3.1171464	6.64000475	0.01804648	0.0456119
Pex19	0.35805035	6.27237414	6.63993177	0.01804703	0.0456119
Nsmce4a	0.51394458	4.58308296	6.63697058	0.01806938	0.04565436
Trp53	0.49730439	5.99533336	6.63219	0.01810554	0.04570907
Mrpl24	0.76769973	2.82661338	6.63175438	0.01810884	0.04570907
Gabra1	-0.3855398	8.20508337	6.63159519	0.01811004	0.04570907
Tsc22d4	0.5570478	3.17516185	6.63116746	0.01811328	0.04570907
Adam1b	-1.7275557	0.2342261	6.62616127	0.01815125	0.04578962
Narf	-0.4442666	5.05031962	6.62548977	0.01815635	0.04578962
Nr4a1	1.44279329	4.92205442	6.62418364	0.01816627	0.04579863
Atp6v1c1	0.26632656	7.37129224	6.62355321	0.01817106	0.04579863
4931406C07I	0.49940008	6.65583506	6.61893616	0.0182062	0.04587312
Mcm6	0.48107167	3.64528183	6.61736313	0.01821819	0.04588926
Camk4	-0.4227176	9.29810961	6.61591476	0.01822923	0.04590301
Rnf112	-0.6289202	4.83507087	6.61207195	0.01825858	0.04596282
Nid2	0.71090408	3.41703602	6.60859968	0.01828514	0.04601559
D630023F18I	0.76709367	2.00640674	6.60653613	0.01830094	0.04604127
Lrrc24	-1.0393221	0.76078617	6.60292589	0.01832863	0.04608423
C1qtnf6	1.24338925	1.04805475	6.60116014	0.01834219	0.04608423
Prcp	0.52593453	3.71054934	6.60091645	0.01834406	0.04608423
Vgll4	0.80348186	2.97093093	6.60035598	0.01834837	0.04608423
Ralyl	0.30290581	7.08495102	6.59981898	0.0183525	0.04608423
Tceal3	0.39346693	5.14347973	6.59956814	0.01835443	0.04608423
A2m	1.01635382	1.67001198	6.59880047	0.01836033	0.04608423
Dbnl	0.43202019	5.18181118	6.598183	0.01836508	0.04608423
2610020C07I	-1.3046411	0.53748742	6.59774039	0.01836848	0.04608423
Ptges3	0.3907841	7.90167011	6.59363281	0.01840012	0.04614952
Nrg4	-1.8822166	-0.4053342	6.59280665	0.01840649	0.04615141
Fcer2a	-1.3842533	0.56786535	6.58609007	0.01845838	0.04625677
Gabrb1	-0.5457617	3.55491588	6.58591012	0.01845977	0.04625677
Mageb16	-1.9760605	1.09658276	6.5838645	0.0184756	0.04626254
AI462493	0.77730527	3.34834797	6.58377214	0.01847632	0.04626254
Ino80	-0.4441562	5.08845141	6.58343184	0.01847896	0.04626254
Relt	1.15109197	1.18477798	6.58266203	0.01848492	0.04626338

Actr3b	-0.3869078	4.85592865	6.58137027	0.01849493	0.04627435
Ppp1r1a	0.57817721	7.06598969	6.58026979	0.01850347	0.04628162
BC005561	-0.652223	5.15199396	6.57889895	0.01851411	0.04629414
Cdca3	2.89657469	-0.5375831	6.57509346	0.01854368	0.04633142
Dbn1	-0.5003806	4.44211037	6.57482291	0.01854578	0.04633142
Col9a3	-0.9445603	2.69052244	6.57480391	0.01854593	0.04633142
Pphln1	0.31820929	7.20904503	6.57358427	0.01855542	0.04634104
Scrn3	0.41553329	4.81804462	6.5708781	0.01857649	0.04637182
Slc35e1	-0.4608744	4.92752732	6.57008558	0.01858267	0.04637182
Fbxo7	-0.5439624	3.37786508	6.56969114	0.01858574	0.04637182
Dera	1.06856796	2.55249595	6.56910587	0.01859031	0.04637182
Gabrb3	-0.421796	8.62854624	6.5671644	0.01860546	0.04639552
Adat2	1.24331675	1.78734387	6.56229822	0.01864349	0.04647626
Rara	0.64390149	5.15309412	6.55989752	0.01866228	0.04650353
Carf	-0.4207507	4.65339293	6.55945567	0.01866574	0.04650353
Srcin1	-0.682467	6.08474988	6.55842037	0.01867386	0.04650964
Kcnq3	-0.3818836	5.53464675	6.55746662	0.01868133	0.04651417
Gprin1	0.47055191	4.69339055	6.55582519	0.01869421	0.04652964
Gm13157	0.59429235	3.36192233	6.55523195	0.01869887	0.04652964
Fmn1	-0.3406257	6.50161144	6.55410969	0.01870768	0.04653748
Igfbp6	0.80011005	4.92640641	6.55099692	0.01873215	0.04658424
Tnik	-0.5475358	7.36927851	6.54699765	0.01876363	0.04664843
Hist1h2bn	0.89694098	3.01491365	6.54449518	0.01878337	0.04667597
Gna13	0.32677777	7.53995271	6.54338169	0.01879215	0.04667597
Atp5l	0.43329343	6.48418274	6.54314671	0.01879401	0.04667597
Grid1	-0.6865395	2.92400738	6.54271411	0.01879742	0.04667597
Poc1a	0.8339153	2.80503987	6.53603044	0.01885029	0.04679309
Hrh2	-0.9479379	2.16821105	6.5312451	0.01888824	0.04687314
Hrct1	4.52286468	-2.1066727	6.53011004	0.01889725	0.04688136
Gpr75	-0.6511731	3.63762434	6.5279984	0.01891403	0.04690883
Kcnj15	2.8583288	-0.7769083	6.52362098	0.01894888	0.04698107
Timeless	1.33905177	2.14805561	6.52275237	0.0189558	0.04698406
Tmem80	-0.6393882	2.82179066	6.52119906	0.01896819	0.04699626
DQ267102	-2.3678742	-0.3130751	6.52018408	0.01897629	0.04699626
Mustn1	0.90496753	3.85996265	6.51998479	0.01897788	0.04699626
AI837181	0.47698498	4.19362542	6.51681417	0.0190032	0.04703846
Tnr	-0.6364721	5.08287813	6.51638735	0.01900662	0.04703846
Plxna3	-0.911439	2.16951874	6.51570314	0.01901209	0.04703846
Fopnl	0.46620077	4.94606246	6.51333514	0.01903104	0.04707118
Daam1	-0.3964903	6.59201576	6.51250817	0.01903766	0.04707339
Cort	-3.1749927	-1.6899519	6.50996475	0.01905805	0.04710963
Cspg5	-0.4592989	5.25798228	6.50807982	0.01907317	0.04713283
Lrriq1	-0.8994395	3.09680899	6.50559771	0.0190931	0.04715458
Fkbp4	0.54861528	5.92966816	6.50499209	0.01909797	0.04715458
Nlgn3	-0.42363	5.98583432	6.50484166	0.01909918	0.04715458

Notch4	1.06064794	1.02824809	6.50278991	0.01911568	0.04717415
Ergic2	-0.4432023	4.64021062	6.50242939	0.01911858	0.04717415
Cox14	0.40257684	5.326743	6.4996842	0.01914069	0.04721452
Slc35a2	-0.6677404	3.20080837	6.4973525	0.01915949	0.04724672
Slc2a13	-0.3833616	6.84437649	6.49505028	0.01917808	0.04727733
4930481A15I	1.27369912	1.32952791	6.49208178	0.01920207	0.04727733
Sult1a1	0.6669802	5.7934373	6.49177891	0.01920452	0.04727733
Elf4	0.86242813	3.91616552	6.49128984	0.01920848	0.04727733
Dlx1as	-0.5384514	3.27813681	6.49073872	0.01921294	0.04727733
Mettl24	-1.8796025	-0.0698367	6.49035738	0.01921602	0.04727733
Atad2	-0.608638	4.80944559	6.48997116	0.01921915	0.04727733
Telo2	-1.0992186	1.49145732	6.48987859	0.0192199	0.04727733
Emc9	0.70987751	2.43498504	6.4891911	0.01922547	0.04727733
Gc	-4.8809293	-2.7059228	6.69376388	0.01923647	0.04727733
Mapk8ip3	-0.5288173	6.77528462	6.48764338	0.019238	0.04727733
Nt5dc3	-0.4501166	5.92130142	6.48728231	0.01924093	0.04727733
Npepps	-0.41565	6.32023894	6.48550183	0.01925537	0.04729867
4931403E22I	-3.8025135	-1.295699	6.484705	0.01926184	0.04730041
Sall1	0.35976503	4.82359432	6.48297196	0.01927591	0.04731917
Mob1b	0.35789914	5.1918482	6.48234662	0.01928099	0.04731917
Ssna1	0.74546705	3.00827153	6.48140935	0.0192886	0.04732373
St6galnac3	-0.6320412	3.5054826	6.47862584	0.01931125	0.04736514
Ankub1	-1.7368438	0.64183141	6.47359866	0.01935221	0.04745146
Prrc2c	-0.4530445	10.0574127	6.47011732	0.01938064	0.04750699
Atp6v0d2	3.02122583	0.3918407	6.46608351	0.01941364	0.04756272
BC023829	0.60565799	3.99356688	6.46592328	0.01941495	0.04756272
Arhgap6	0.46299855	4.73769687	6.46423489	0.01942878	0.04757525
Sorcs3	-0.6823597	4.18264428	6.46388619	0.01943164	0.04757525
Lrrc20	0.62492432	4.21284955	6.46261454	0.01944207	0.0475758
Lgals3	1.12671759	1.77857783	6.46244673	0.01944344	0.0475758
Yipf3	0.39742489	4.45809091	6.46085776	0.01945648	0.04759353
Stra13	0.64896955	4.00883163	6.45914255	0.01947056	0.04761381
Nrbp2	-0.4009318	6.88218076	6.45813289	0.01947886	0.04761993
Birc2	0.3473733	5.4885812	6.45222329	0.0195275	0.0477108
Lpgat1	-0.3572059	7.83784343	6.45220646	0.01952764	0.0477108
Slc20a1	-0.4863344	5.34830117	6.45081645	0.0195391	0.04772461
P2ry14	-1.0136397	1.47512137	6.44974121	0.01954797	0.04773209
Sri	0.37932616	6.57914909	6.44823506	0.0195604	0.04773988
Asnsd1	0.56218354	5.29360696	6.4474534	0.01956686	0.04773988
Tcea1	0.30404011	8.06123568	6.44724437	0.01956858	0.04773988
Sord	0.49437958	4.3922817	6.44591155	0.0195796	0.04775258
Peli1	0.34554676	5.95279181	6.44149573	0.01961614	0.04782751
Myh7	0.74585989	2.56956059	6.43932922	0.0196341	0.04785709
Dnajc12	0.64495655	3.61725097	6.43498332	0.01967017	0.04793081
Taf12	0.69004239	4.10431954	6.42372449	0.01976398	0.04813141

G630071F17	-1.8700418	-0.2688964	6.42369711	0.01976421	0.04813141
Hlf	0.36007497	10.0220648	6.4220359	0.01977809	0.04814907
Rac1	0.29741374	10.032103	6.4212628	0.01978456	0.04814907
Tcam1	6.06091445	-1.9937716	6.62222326	0.01979401	0.04814907
Zfp939	-0.8956654	2.6746555	6.42002709	0.01979489	0.04814907
2700029M09	0.34973203	5.45364033	6.41848145	0.01980783	0.04816629
Kif23	-1.0760542	0.84121658	6.41644164	0.01982493	0.0481936
Spty2d1	0.3891992	4.96492595	6.41546934	0.01983308	0.04819916
Adi1	0.48834909	7.13727082	6.41403	0.01984515	0.04820383
Mir325	-2.7122788	-1.4886438	6.41384235	0.01984673	0.04820383
Iglon5	-0.5788935	3.56600338	6.41047122	0.01987505	0.04825835
Bnip3	0.35127125	6.08881487	6.40905167	0.01988698	0.04827258
Pcdhb5	-0.6821609	1.85880226	6.40813558	0.01989469	0.04827258
Myo7a	-0.8974844	2.71220591	6.40767979	0.01989853	0.04827258
Edem3	-0.3394347	6.01959876	6.40611215	0.01991173	0.04829035
Fam185a	0.49787082	3.69657058	6.4052847	0.0199187	0.04829301
Kcnmb1	-3.102216	-1.0258641	6.40424294	0.01992748	0.04829726
Abcc8	-0.7986124	2.69792828	6.40349895	0.01993376	0.04829726
Pigg	-0.5078013	3.45784024	6.40298617	0.01993808	0.04829726
Prmt6	0.60605725	3.52929591	6.40163137	0.01994952	0.04831072
Adamts12	0.80944332	2.48891366	6.40068683	0.01995749	0.0483158
Xrcc4	0.53303905	3.43775911	6.39607607	0.01999648	0.04839593
Atoh8	0.83274923	1.84341406	6.39426084	0.02001185	0.04841635
Ppp1r16a	-0.7123706	2.41050644	6.39368863	0.0200167	0.04841635
Prkag3	-1.2504285	1.82360284	6.39136261	0.02003642	0.0484498
Basp1	0.34620784	9.41422249	6.3888533	0.02005772	0.04848704
Fsip1	4.12591939	-1.5380426	6.38562059	0.0200852	0.04853919
Usmg5	0.43479856	5.86410141	6.38312935	0.02010641	0.04857615
Parva	0.4379827	7.42564118	6.38140066	0.02012114	0.04859745
Pcdhga10	-0.4348232	3.54483059	6.37794993	0.02015057	0.04864579
Mtap	0.59972034	4.5968512	6.37766746	0.02015299	0.04864579
Ndufb9	0.47302329	6.52462575	6.36763858	0.02023883	0.04883865
Denr	0.36739715	5.7057541	6.36566856	0.02025574	0.04884848
Pip5k1b	-0.7593626	2.98888508	6.36507076	0.02026087	0.04884848
Arhgap20os	-2.2305007	0.33173042	6.36310556	0.02027776	0.04884848
Hadhb	0.3455355	6.50866812	6.36302203	0.02027848	0.04884848
Dolpp1	-0.8180258	1.23214382	6.36293245	0.02027925	0.04884848
B3glct	-0.375962	5.47446189	6.36264838	0.0202817	0.04884848
H2afy	0.39272836	5.91929454	6.36215894	0.02028591	0.04884848
Pafah1b3	1.07043962	2.03369673	6.36163086	0.02029045	0.04884848
Hyal2	-1.0298305	1.18618416	6.35933368	0.02031023	0.04888178
Ifnar1	-0.4161054	5.75464592	6.35192402	0.02037417	0.04902131
Sgsm1	-0.7348798	4.53170126	6.35037535	0.02038756	0.04903918
Cpne4	-0.41582	6.36856692	6.34806894	0.02040752	0.04906033
Prickle1	-0.464327	5.67967014	6.34753815	0.02041212	0.04906033

Sf3b5	0.65602225	3.86018362	6.34599881	0.02042546	0.04906033
Hist1h4k	0.87144579	1.94575363	6.3459322	0.02042604	0.04906033
Gm20063	0.4428408	3.75008213	6.34571995	0.02042788	0.04906033
Il1f9	-0.9069667	2.29455078	6.34488356	0.02043513	0.04906033
Aig1	0.73065081	3.24048573	6.34437746	0.02043952	0.04906033
C230029M16	5.66308565	-1.9497572	6.34384876	0.02044411	0.04906033
Kbtbd3	-0.5777658	3.29849578	6.3427986	0.02045322	0.04906788
Agl	-0.4438651	5.63069025	6.33621851	0.02051044	0.0491772
D830005E20	-1.3722318	-0.32603	6.33618252	0.02051075	0.0491772
Eif2s1	0.30970732	6.78122622	6.33284668	0.02053983	0.04922199
Pcyox1l	-0.9141825	1.9727679	6.3326654	0.02054141	0.04922199
Nupl1	0.33786275	6.08892636	6.33154678	0.02055117	0.04923103
Tdrd6	-2.2016582	-0.5008325	6.33080001	0.02055769	0.04923229
Dcn	0.75386143	7.6761973	6.3226867	0.02062868	0.0493879
Ism1	0.84465566	3.28477602	6.31985543	0.02065351	0.04943296
1700063D05	-0.7979594	1.83602218	6.31829503	0.02066722	0.0494469
Hspa12a	-0.382054	8.26681948	6.31782213	0.02067137	0.0494469
Pi4ka	-0.4824396	8.10487444	6.31182875	0.0207241	0.04952115
Phyhipl	0.34908176	7.4588456	6.3117838	0.0207245	0.04952115
Wnk3	-0.4694172	6.92545557	6.31092041	0.02073211	0.04952115
Rbfox3	0.27613753	7.81275432	6.31011466	0.02073921	0.04952115
Lifr	-0.4686368	5.94162796	6.30955344	0.02074416	0.04952115
Clec1a	1.13561501	1.93760054	6.30930019	0.0207464	0.04952115
Rasl10b	-0.618549	3.49792742	6.30921888	0.02074712	0.04952115
Trpm2	-0.7541134	3.5551595	6.30881804	0.02075065	0.04952115
Sgta	0.46652994	4.96266654	6.30813984	0.02075664	0.04952115
Tnfrsf23	1.03065716	1.09561265	6.30605641	0.02077504	0.04955067
Atp6v1e1	0.30141586	6.40075552	6.30361946	0.02079658	0.04957407
Pcdhac1	-1.8948065	-0.0962579	6.3035823	0.02079691	0.04957407
Arhgap32	-0.5164644	9.3418549	6.30171605	0.02081343	0.04958608
Nap1l5	0.28583464	7.1916082	6.30164932	0.02081402	0.04958608
D5Ertd579e	-0.3461135	7.30516997	6.30043061	0.02082481	0.04959742
Slc35b3	-0.705757	2.18496086	6.29839955	0.02084282	0.04962592
Rufy2	-0.5793174	6.03410088	6.29759244	0.02084998	0.04962859
Ctxn3	0.64409293	7.22561565	6.29436869	0.0208786	0.04966947
LOC106740	-0.5433529	4.1747146	6.29429668	0.02087924	0.04966947
Cxcl1	6.45301927	-1.9014253	6.2923428	0.02089661	0.04969641
Adh1	1.64868213	0.18930139	6.28714341	0.02094291	0.04979212
Med10	0.47510467	3.50898414	6.28351966	0.02097525	0.04985458
Hcfc1r1	0.50009379	4.47149002	6.28211912	0.02098776	0.04986884
Ppp1cc	0.2865517	7.62011982	6.28140753	0.02099412	0.04986884
Spef1	0.99732359	1.53809466	6.28042905	0.02100287	0.04986884
Zcchc12	-0.4796821	4.29286974	6.27988687	0.02100772	0.04986884
Arpc1b	0.83480698	6.03862312	6.27945536	0.02101158	0.04986884
Dnmt3a	-0.397764	6.59707257	6.27173094	0.02108084	0.05001876

Sf3b4	0.44412256	4.65157292	6.26862914	0.02110872	0.05007046
LOC171588	-6.1888253	-1.1518178	6.46044704	0.02112405	0.05009237
Slc35e2	-0.4499154	5.59113456	6.26548178	0.02113705	0.0501024
D630041G03	-0.5989635	4.20906915	6.2651026	0.02114047	0.0501024
Agbl1	2.33684911	-0.8698047	6.25675967	0.0212158	0.05025899
Rasgrp3	-0.4872849	4.97241042	6.25643054	0.02121877	0.05025899
Rbl2	0.28650766	7.24597562	6.25527805	0.02122921	0.05026921
Gnl3	0.44651455	4.71939905	6.25327828	0.02124732	0.05029004
Sdhc	0.47530382	6.84374404	6.25295612	0.02125024	0.05029004
Tmem91	1.05370557	1.95762266	6.25188258	0.02125997	0.05029011
Bbs9	0.35002191	5.07388828	6.25021669	0.02127508	0.05029011
Prss22	2.57811234	-1.6286607	6.25020162	0.02127522	0.05029011
Gan	-0.6678827	3.39614427	6.24937444	0.02128273	0.05029011
Rpl7l1	0.37329218	4.58990866	6.24893735	0.0212867	0.05029011
Mrps12	0.67309519	3.59818794	6.24890594	0.02128698	0.05029011
Nol10	-0.607356	4.15900657	6.24740655	0.0213006	0.05030783
Sv2c	-0.6157901	4.80415195	6.23852397	0.0213815	0.05048438
Tomm34	0.36468463	5.83412048	6.23466789	0.02141672	0.05055303
C2	0.96432485	2.81686402	6.22998848	0.02145955	0.05063959
Snrpf	0.8113201	2.83226967	6.22717303	0.02148537	0.05068596
Map4k2	-0.4472414	4.85664931	6.22385915	0.02151581	0.05074319
Dnah10	-1.2020148	1.13140033	6.22154023	0.02153713	0.05076867
Ube2d2a	0.36961378	9.04656971	6.22091095	0.02154292	0.05076867
Gpr137b	0.55809984	2.9335845	6.2206698	0.02154514	0.05076867
Plekha8	-0.4901862	4.32360688	6.21801061	0.02156963	0.05081182
Trabd2b	0.6140813	6.3225332	6.21623786	0.02158598	0.05083576
Ctnnbl1	0.53032927	3.28848364	6.21484431	0.02159884	0.05085147
Hint3	-0.4742924	4.30640939	6.2140641	0.02160604	0.05085386
Eno1b	0.30508401	6.04849842	6.21325465	0.02161352	0.0508569
3010026O09	0.70112788	3.29919568	6.21231423	0.02162221	0.05086058
Dnajc2	0.34334528	6.43593183	6.211746	0.02162746	0.05086058
Slc19a2	-0.5830581	3.68181895	6.21091403	0.02163515	0.05086412
Pde4c	-1.7144443	-0.1185333	6.2068391	0.02167288	0.05093823
4933439C10I	-0.9511332	1.82049563	6.2047815	0.02169195	0.05096849
Ap3b2	-0.5589156	5.0760068	6.20331259	0.02170558	0.05098594
Stx7	0.31624209	7.46022408	6.20243952	0.02171369	0.05099041
Eif2b3	-0.5976494	2.84573605	6.19595439	0.021774	0.05111744
Pcdh15	-0.6391843	4.87020321	6.19342529	0.02179758	0.05115817
Kmo	1.3849289	0.99366397	6.19238097	0.02180732	0.05116643
2010002M12	0.68411863	2.29318535	6.18875561	0.02184118	0.05123124
L2hgdh	0.29584355	6.11921602	6.18736428	0.02185419	0.05124713
Ralgapb	-0.3994504	7.62537635	6.18449994	0.021881	0.05129349
Kcnj8	-1.6982212	0.66783399	6.18391937	0.02188643	0.05129349
Fam96a	0.50240783	5.17674394	6.18077875	0.02191588	0.05134786
Gm5176	-2.065754	-0.4659472	6.17665862	0.02195458	0.05142387

1700030L20F	-1.1178877	1.35267767	6.17472489	0.02197277	0.05143826
Tagap	-0.6921308	2.53749694	6.1740437	0.02197918	0.05143826
Med20	0.41025241	5.04033689	6.17400973	0.0219795	0.05143826
2310002D06I	3.30572871	-1.6989495	6.17239932	0.02199467	0.05143968
Cacna2d3	-0.5064897	5.15526663	6.1718277	0.02200005	0.05143968
Parl	0.56846437	3.34903701	6.17163562	0.02200186	0.05143968
Rnf7	0.44914151	7.98133013	6.17128777	0.02200514	0.05143968
Dlgap3	-0.5416477	4.07743113	6.16984561	0.02201874	0.05145684
Ccnt2	-0.578646	4.8957623	6.16831107	0.02203322	0.05146766
5830417I10R	-0.4430223	6.11099523	6.16768523	0.02203913	0.05146766
Srp19	0.33027675	6.44334797	6.16598157	0.02205522	0.05146766
Socs2	0.4317882	5.3342442	6.16579351	0.022057	0.05146766
Srrm4	-0.5027494	6.13290984	6.16564148	0.02205844	0.05146766
Ccdc27	-2.678649	-1.0113339	6.16514892	0.0220631	0.05146766
Hmgn2	0.53986444	6.50993329	6.1647139	0.02206721	0.05146766
Zfp235	-0.6430174	3.30221274	6.1623693	0.02208939	0.05150479
Ranbp3l	0.50427091	7.77261109	6.15971835	0.0221145	0.05154872
Ptrf	0.6993774	6.57759735	6.15869316	0.02212422	0.05155675
C3ar1	-0.86542	2.03882037	6.15771321	0.02213352	0.05156379
Abcc1	-0.6504344	3.88169339	6.15617111	0.02214816	0.0515688
Ust	0.67094738	5.5251111	6.15616502	0.02214822	0.0515688
Nt5c1a	1.22978974	0.79955439	6.15197812	0.02218801	0.05162998
Echdc3	1.08760201	1.11695065	6.15147934	0.02219276	0.05162998
Fkbp8	0.50505507	5.10116843	6.15141827	0.02219334	0.05162998
9330159F19I	-0.4906836	6.85919471	6.14968267	0.02220986	0.0516538
Plch1	-0.6691901	3.10887711	6.1484808	0.02222131	0.05166582
4930539J05F	-1.3352482	0.32804557	6.14738667	0.02223175	0.05167545
Pet2	-2.565934	-0.6324574	6.1455856	0.02224893	0.05170076
Deptor	0.30184084	7.41084037	6.1438601	0.0222654	0.05172442
Rcbtb1	-0.4124188	6.01330375	6.14086952	0.02229399	0.0517762
B3gnt5	-2.1636775	0.09588602	6.13759625	0.02232532	0.05183433
Mir1931	-3.2130654	-1.7815643	6.13553063	0.02234512	0.05186565
Pbdc1	0.45044626	4.59042554	6.13254399	0.02237379	0.05191576
Ajap1	-0.4050775	5.20149619	6.13196495	0.02237935	0.05191576
Icosl	0.93817828	1.45258805	6.12689847	0.02242808	0.05201413
Gm14403	0.4067972	3.69756188	6.12475403	0.02244874	0.05204737
Srrt	-0.4335142	4.88344828	6.12252475	0.02247025	0.05208253
Ttc9c	0.40180297	6.09942812	6.12059556	0.02248887	0.05209553
Cep97	-0.3846532	4.58543126	6.12027437	0.02249198	0.05209553
Msantd4	0.28775669	7.36767747	6.1199749	0.02249487	0.05209553
Msn	0.43856801	7.6261454	6.11742705	0.0225195	0.05213789
Cd8a	-1.1992397	0.35350132	6.11645597	0.0225289	0.05214495
Slc11a2	-0.4469443	4.99603711	6.11436081	0.02254919	0.05217722
6030443J06F	-0.9723264	1.76003475	6.1090334	0.02260087	0.0522821
Tatdn1	0.40035332	4.60363615	6.10759989	0.0226148	0.05229424

Capza1	0.3556095	6.23692652	6.10718389	0.02261885	0.05229424
Eif5a	0.60081608	7.19992216	6.10648601	0.02262564	0.05229522
Fabp5	0.4272643	4.98497235	6.10504407	0.02263966	0.05231293
Csnk2a2	0.45020645	5.19443205	6.10133674	0.02267578	0.0523795
Isca2	0.3705168	5.90232956	6.10077907	0.02268122	0.0523795
Bcl3	1.12437679	0.8728901	6.09765177	0.02271174	0.05243525
Pkd2l1	-2.869995	-1.3225536	6.09585161	0.02272933	0.05245144
Psg23	-1.2617646	1.27596816	6.09562808	0.02273152	0.05245144
Tctn3	-0.9238891	2.73399384	6.09282031	0.02275899	0.05248427
Bcl2a1b	0.94370811	1.45049734	6.09229015	0.02276418	0.05248427
Idh3a	0.31077345	6.62865407	6.09221655	0.0227649	0.05248427
Trpm6	0.63925058	3.12694538	6.09046514	0.02278206	0.05249332
Slco4c1	-0.8457177	2.13125651	6.08952983	0.02279123	0.05249332
Arhgap35	-0.3233611	8.93178848	6.08819803	0.02280429	0.05249332
Pttg1	0.35005809	4.90101036	6.08809194	0.02280533	0.05249332
Gpr37l1	-0.7158119	3.0098003	6.08803646	0.02280588	0.05249332
Glra4	-3.6628385	-1.6182774	6.08757406	0.02281042	0.05249332
Rbm12	0.34740641	5.25852923	6.08725654	0.02281353	0.05249332
Eif4e	0.26739396	7.4070904	6.08585085	0.02282734	0.05251038
Stat1	0.39468115	5.50301959	6.08205311	0.02286468	0.05258156
Utp14b	-0.5584078	4.91142659	6.07934434	0.02289135	0.05261444
Ccdc109b	0.95311155	1.96654742	6.07923122	0.02289247	0.05261444
Fh1	0.36190681	5.26825445	6.07865211	0.02289818	0.05261444
Iqgap2	-0.4575334	4.97802915	6.07798162	0.02290479	0.05261492
Vps36	0.51077893	3.86772111	6.07723355	0.02291217	0.05261716
1700019G17	1.15590728	1.04324127	6.0758564	0.02292576	0.05262033
Akirin1	0.33752654	5.78929013	6.0754864	0.02292941	0.05262033
Ly6g6e	2.75794101	-0.7640857	6.07507357	0.02293348	0.05262033
Tob2	0.44459601	5.13169507	6.07449947	0.02293915	0.05262033
Tcea3	0.60434836	3.93148528	6.0725563	0.02295836	0.05264968
Serpinb10	-1.9256455	-0.6672712	6.06845209	0.02299898	0.05270772
Usp34	-0.4458658	8.49626078	6.06817594	0.02300171	0.05270772
Nsmce2	0.371534	5.28630791	6.06765929	0.02300683	0.05270772
Immp2l	1.54486339	-0.0527315	6.0671189	0.02301219	0.05270772
Ldhb	0.36114558	8.96055698	6.06676165	0.02301573	0.05270772
Atrn	-0.4000022	7.87994801	6.06525937	0.02303063	0.05272715
Dip2c	-0.4001048	6.83602647	6.0640102	0.02304303	0.05274085
Tmem132c	-0.707026	2.32488406	6.06293891	0.02305367	0.05274272
Copz2	0.77240995	6.78348118	6.06263543	0.02305668	0.05274272
Pcm1	-0.4663436	8.57560764	6.06102087	0.02307273	0.05276474
Gucy1a3	-0.4606402	7.27725386	6.05894219	0.02309341	0.05279734
Ap3d1	-0.4568028	6.40065025	6.05534723	0.02312922	0.05286089
Ldha	0.36215753	7.8797741	6.05486077	0.02313407	0.05286089
Gm5607	-0.7054236	3.8160498	6.0540991	0.02314166	0.05286355
Gm20767	-1.1898796	1.1002341	6.05045008	0.0231781	0.05292866

1700071M16	-2.3766709	0.65191251	6.04995538	0.02318304	0.05292866
Alkbh1	0.57810566	4.29498988	6.04714203	0.02321118	0.05297819
Psip1	0.32445182	8.79468226	6.04642846	0.02321833	0.05297978
Csgalnact1	-0.5993108	3.77298761	6.04054683	0.0232773	0.05309962
Rab40b	-0.4899362	4.03718663	6.03520409	0.02333103	0.05320739
Polr3h	0.52924136	2.96034667	6.03411903	0.02334195	0.05321755
AY512931	-1.1419062	1.78850411	6.0299016	0.02338448	0.05329972
Immp1l	0.38127966	5.4472159	6.02842332	0.02339941	0.05331896
Asap2	-0.4088762	6.25243567	6.02429359	0.02344116	0.05339288
Fmn2	-0.5251448	6.65774574	6.02393013	0.02344484	0.05339288
A430107P09	-1.6134017	-0.089564	6.02316762	0.02345256	0.05339567
Dek	0.39653965	8.40392104	6.02138424	0.02347063	0.05342201
Acp2	-0.3111681	5.57459214	6.01934983	0.02349126	0.05345416
Naca	0.3659315	7.84367929	6.01829559	0.02350196	0.05346371
Osbp2	-0.3936671	5.65116238	6.01755378	0.02350949	0.05346604
Piezo1	-0.9961995	1.30366693	6.0140757	0.02354484	0.05353162
Mrps18b	0.78295486	2.96801777	6.01259077	0.02355995	0.05355116
Cdk2ap2	0.59084665	3.39186092	6.00947837	0.02359165	0.05356136
Arhgap24	0.59408186	3.94176612	6.009436	0.02359209	0.05356136
Ankrd35	0.60246659	2.76425955	6.00887125	0.02359784	0.05356136
Galnt13	-0.3964225	5.74392475	6.00830928	0.02360358	0.05356136
Gm1564	-1.0787821	1.12274621	6.00791492	0.0236076	0.05356136
Nkain3	-0.8328074	1.994663	6.00783294	0.02360844	0.05356136
4930402H24	-0.4493691	5.56917673	6.00767419	0.02361006	0.05356136
Ypel2	-0.2776463	6.38247528	6.00421549	0.02364538	0.05362669
Pop7	0.61702501	2.62452614	6.00235965	0.02366435	0.05364842
Taf2	-0.3570931	5.91568039	6.00200204	0.02366801	0.05364842
Cd46	-1.7005096	1.16625754	6.00112353	0.023677	0.053654
Ccl25	-1.1307743	1.70971134	5.99969725	0.02369161	0.0536723
Psmd7	0.27937533	6.76951316	5.99667345	0.0237226	0.05371734
Med21	0.60811276	5.66338597	5.9961877	0.02372759	0.05371734
Hars	0.28845017	5.89879143	5.99584555	0.0237311	0.05371734
Anxa3	0.60190316	5.81377732	5.99375611	0.02375255	0.0537511
Frrs1l	-0.3764391	7.44286109	5.99033095	0.02378777	0.05381598
Gpn1	0.44791511	3.93186967	5.9876252	0.02381563	0.05386418
Rrp1	0.35756488	7.75798968	5.98641896	0.02382806	0.05387197
Ska3	-0.8883639	1.45807061	5.98601917	0.02383218	0.05387197
Xk	-0.5074068	5.23281007	5.98382289	0.02385484	0.05390837
Rrm2	-1.214409	1.42874558	5.98121003	0.02388183	0.0539508
Plscr1	0.64321118	4.17838695	5.98047225	0.02388946	0.0539508
Plxnc1	-0.3178991	6.4050276	5.98009957	0.02389331	0.0539508
Ppp2r5a	0.35146757	5.75642456	5.97906716	0.02390399	0.05396009
Srp9	0.42526161	6.79681905	5.97665804	0.02392893	0.05400024
Capns1	0.49263943	9.23059355	5.97608025	0.02393492	0.05400024
Tgtp1	0.64971495	3.77009538	5.97407497	0.02395571	0.05403231

Rbm12b2	-0.5189896	4.39119605	5.97341615	0.02396254	0.0540329
Msrb3	0.50710279	5.39694695	5.97235871	0.02397351	0.05404282
Gm5441	-1.8466404	0.35213005	5.96905003	0.02400789	0.05410547
Cadps	-0.5212158	8.58472785	5.96822136	0.02401651	0.05411005
Hspa1a	0.49514033	3.80987944	5.96592504	0.0240404	0.05414905
Txndc9	0.32113087	5.80377906	5.9615224	0.0240863	0.0542075
1810034E14I	1.25629477	1.11510821	5.96114282	0.02409026	0.0542075
0610040F04I	-1.4003	0.96442766	5.9609179	0.02409261	0.0542075
Morc2b	-1.371637	1.68893426	5.9609058	0.02409273	0.0542075
Wdr70	-0.5209687	3.54999649	5.95486796	0.02415586	0.05432148
Cryba2	3.00943188	-1.2182359	5.95426307	0.02416219	0.05432148
A230050P20I	-0.7226946	1.98851893	5.95416442	0.02416322	0.05432148
Cenpf	-0.6516374	2.51014231	5.95305667	0.02417483	0.05433271
A630066F11I	0.99823242	1.4922324	5.94345563	0.02427567	0.05452269
1700029J07F	-0.7733995	2.15905679	5.94314311	0.02427896	0.05452269
Rpl10	0.53626891	7.8633684	5.94311476	0.02427926	0.05452269
Sema5a	-0.394381	6.42910481	5.94214756	0.02428945	0.05452489
Srsf6	-0.2848546	5.68150112	5.94176213	0.02429351	0.05452489
Cdr2l	0.50355063	3.23007911	5.93849049	0.02432801	0.05455317
Rhbdd1	-0.7332627	3.65204601	5.93813914	0.02433172	0.05455317
Nudc	0.42934246	5.63005536	5.93806954	0.02433245	0.05455317
Psma1	0.42902856	5.5577464	5.93805036	0.02433265	0.05455317
Psmb11	-1.1112133	1.33147065	5.93598534	0.02435446	0.05458717
Ndufa2	0.53437068	5.45859902	5.93320609	0.02438385	0.05463814
Pdap1	0.31507757	6.04391655	5.93195118	0.02439713	0.054653
Exoc6b	-0.2847592	6.79077474	5.9305672	0.02441179	0.05466048
Ttll7	-0.3759672	7.62182125	5.93038001	0.02441377	0.05466048
Mtfmt	-0.6425153	3.0333309	5.92886053	0.02442988	0.05468165
Pdcd4	0.26929353	7.48491951	5.92706312	0.02444895	0.05469801
Echdc1	0.79307614	1.77405329	5.92670334	0.02445276	0.05469801
Zfp653	0.83858841	2.40126424	5.92628962	0.02445716	0.05469801
Pgd	0.49612278	5.24899362	5.92532291	0.02446742	0.05470092
Kank1	-0.5380944	3.58697731	5.92491411	0.02447176	0.05470092
Leprel2	-0.6243165	3.23305607	5.92144229	0.02450868	0.05476684
Zfp658	-0.7081155	2.26400875	5.92088782	0.02451458	0.05476684
Gusb	-0.636766	2.80361293	5.91840402	0.02454104	0.05480569
Rnf165	-0.457335	6.31500496	5.91800303	0.02454531	0.05480569
BC061194	-1.5662749	0.34113822	5.91634287	0.02456302	0.05483033
Psme3	0.27018237	6.7298075	5.91475199	0.02457999	0.05484768
Gm4262	-0.5965154	3.20968272	5.91436403	0.02458414	0.05484768
Zcchc8	-0.4547254	3.8751708	5.91147505	0.02461501	0.05489847
Dpysl3	-0.2670642	6.30285504	5.91098419	0.02462026	0.05489847
Hadh	0.5063384	4.85452117	5.9100387	0.02463038	0.05490195
Csnk1g1	-0.3003589	6.24794993	5.90958968	0.02463518	0.05490195
Racgap1	0.59789499	3.21053084	5.90823094	0.02464973	0.05491123

Agfg2	0.46539881	3.6596711	5.90769091	0.02465552	0.05491123
Mgp	0.85165663	8.28888369	5.90673083	0.0246658	0.05491123
Mctp1	-0.3881493	5.38954545	5.90670644	0.02466607	0.05491123
Arpc5	0.39594266	7.34556386	5.90258156	0.02471032	0.05499486
Brk1	0.42415085	6.30129478	5.90066083	0.02473096	0.05502589
Nav2	-0.5753683	7.18840989	5.89810699	0.02475844	0.05507211
Thyn1	0.61264206	3.4539141	5.89668327	0.02477377	0.0550913
Epha7	-0.4446368	6.56712493	5.89035061	0.02484209	0.05522829
Tgm2	0.49325782	4.09520623	5.88905813	0.02485606	0.0552444
Sh3bgrl3	0.41395826	6.73140754	5.8870309	0.02487799	0.0552782
Gabbr1	-0.3980317	7.35813942	5.88468255	0.02490342	0.05531975
Mgat4c	-0.6729004	2.88416955	5.88321931	0.02491928	0.05534002
Abca1	-0.3730464	5.05854337	5.88195007	0.02493304	0.05535564
Tnfaip8l3	0.30186698	6.59912069	5.87593517	0.0249984	0.05548575
Necab2	0.52551248	3.43692255	5.8724931	0.02503589	0.05555078
Slc16a4	-1.0277245	1.73228008	5.87200437	0.02504122	0.05555078
Ndufa9	0.4085671	6.21260257	5.86882911	0.02507586	0.05558659
Leng1	0.7016144	4.27470386	5.86877219	0.02507649	0.05558659
Wdr27	-3.5176884	-0.7713851	5.86866556	0.02507765	0.05558659
Tmem82	-1.2715382	0.41533048	5.86611441	0.02510553	0.05562452
Tmem151b	-0.6523526	4.64442946	5.8658614	0.02510829	0.05562452
Spag9	-0.2955158	8.37034525	5.86482397	0.02511964	0.05563466
Zfp236	-0.4524192	5.532951	5.86311593	0.02513834	0.05565489
Psmd9	0.50484634	4.39542341	5.86275254	0.02514232	0.05565489
Gm15417	1.06957876	1.17185303	5.86122407	0.02515906	0.05565582
Ovol1	1.92834714	-0.3744116	5.86023713	0.02516988	0.05565582
Twist1	0.74406478	4.73255054	5.86001314	0.02517234	0.05565582
Usp31	-0.4763879	7.46671707	5.85968355	0.02517596	0.05565582
Fastkd1	-0.6469688	2.74592134	5.8596252	0.0251766	0.05565582
Arf6	0.37384174	6.84791232	5.85594679	0.02521698	0.05571675
Zmym6	-0.571391	4.80463903	5.85587986	0.02521772	0.05571675
Vamp3	0.46838387	7.50340041	5.85495453	0.02522789	0.05572425
Proca1	0.78473325	1.88584222	5.84966838	0.02528608	0.05583778
Cdh6	-0.5321717	2.79517801	5.84886736	0.02529492	0.05584228
Snap47	0.27812524	7.28616584	5.84788868	0.02530571	0.05585111
2700099C18I	-0.9844383	1.75363503	5.84626699	0.02532361	0.05586707
Emc8	0.31417691	5.95088228	5.84600196	0.02532654	0.05586707
Asxl3	-0.8730293	4.1941141	5.843797	0.0253509	0.0559058
Tspan13	0.50639317	8.54940171	5.84101995	0.02538162	0.05595645
Ecd	0.45135894	4.1883553	5.83995314	0.02539343	0.05595645
Cpt1c	-0.6606221	3.36408473	5.83987579	0.02539429	0.05595645
Eml2	-0.5945433	2.7595467	5.83845395	0.02541004	0.05597616
Oprk1	-0.8213932	3.81311892	5.83697177	0.02542648	0.05599623
Crx	-2.100233	-1.2279969	5.83616496	0.02543543	0.05599623
Pgm1	0.4422152	3.70359012	5.8357899	0.02543959	0.05599623

Sbk3	-1.4998754	0.45675442	5.83516136	0.02544657	0.05599659
Gm609	-3.1987708	-1.8422175	5.82730569	0.02553395	0.05617384
Cdkn1b	0.50337606	4.09793414	5.82464544	0.02556362	0.05622405
Ddx43	-3.5572253	-2.0649255	5.82244686	0.02558817	0.05625146
Wdr76	-0.7216738	2.1984456	5.8223035	0.02558977	0.05625146
St8sia1	-0.4170682	6.67540381	5.81894979	0.02562727	0.05630076
1700007L15F	-1.5824694	-0.0113701	5.81838331	0.02563361	0.05630076
Hmgcs2	0.5719385	4.07339553	5.81797264	0.02563821	0.05630076
Hells	-1.0280839	1.48357957	5.81775469	0.02564065	0.05630076
Abca2	-0.5127378	6.10425242	5.81723683	0.02564645	0.05630076
Phxr4	-1.4237489	1.70294405	5.81602562	0.02566002	0.05631551
Pou2f3	1.48440867	1.17603717	5.81078541	0.02571882	0.0564295
Zfp575	-0.6761842	2.81930145	5.8095887	0.02573227	0.05644394
Mpnd	0.64504946	3.83602864	5.80740589	0.02575682	0.0564794
Lamc3	0.71926932	2.59835371	5.80648607	0.02576718	0.0564794
Tango6	0.70768932	2.27746456	5.80620061	0.02577039	0.0564794
Api5	0.29938658	6.94753257	5.80570971	0.02577592	0.0564794
Xkr6	-0.84857	2.9998954	5.80462304	0.02578816	0.0564848
Hjurp	-0.7097438	2.44062211	5.80427103	0.02579213	0.0564848
Csf2ra	-0.645547	3.82945891	5.8028554	0.02580809	0.05650471
Atp13a5	0.45117659	4.94737896	5.80078711	0.02583144	0.05651176
Rcor1	0.37685061	5.10840281	5.80058656	0.0258337	0.05651176
Tmem201	-0.5825666	3.96789872	5.80049619	0.02583472	0.05651176
Gabrg3	-0.7188981	4.67302753	5.79956728	0.02584521	0.05651176
Rapgef6	-0.2753142	6.7995315	5.79929088	0.02584834	0.05651176
Itga9	-0.505665	3.28035532	5.79853704	0.02585686	0.05651176
Unc13b	-0.4178643	6.53909414	5.79830801	0.02585945	0.05651176
Entpd3	-0.8190563	1.63353035	5.79557847	0.02589032	0.0565642
Dnajb1	0.29892354	6.61552097	5.79296684	0.02591991	0.05661379
Gtf2h2	0.3571195	4.9605787	5.79068977	0.02594573	0.05665514
Thtpa	0.3927558	4.55589553	5.78913313	0.02596341	0.05667095
Wdr5b	1.09627962	1.16772343	5.7874734	0.02598226	0.05667095
Eef2k	-0.4725636	4.34373641	5.78740798	0.02598301	0.05667095
Slc8a3	-0.4912333	3.92640813	5.78730377	0.02598419	0.05667095
2610306M01	0.64057282	2.61735373	5.78701674	0.02598745	0.05667095
Igf2bp2	0.86253763	1.32033371	5.78563657	0.02600315	0.05669014
2700038G22	-1.4623084	-0.1344479	5.78472388	0.02601353	0.05669774
Rhoc	0.69141445	3.48230513	5.78282964	0.0260351	0.05671603
Evi2a	-0.7625109	2.7935894	5.78270045	0.02603657	0.05671603
Aip	0.53014488	5.1871287	5.78216938	0.02604263	0.05671603
2310068J16F	2.35462943	-0.8088933	5.77612988	0.02611155	0.05685107
Med15	0.2892568	6.51689822	5.77422504	0.02613333	0.05688343
Wnt7a	-0.9939028	1.63757621	5.77075627	0.02617305	0.0569548
Ncbp2	0.31734957	6.54868575	5.76849613	0.02619897	0.0569961
Coq5	0.29238416	6.38058398	5.76230489	0.02627011	0.05713088

Gde1	0.37945781	5.24696666	5.76189539	0.02627482	0.05713088
D3Ertd254e	-0.469617	6.52483606	5.76116518	0.02628323	0.05713404
Trappc8	-0.3194568	6.26284445	5.75930919	0.02630461	0.0571654
Trappc11	-0.3614531	5.46142792	5.75526589	0.02635125	0.05725163
Sdr39u1	0.41119247	4.98892235	5.75357625	0.02637077	0.0572789
Gm2897	-0.5609888	3.6036603	5.75276616	0.02638014	0.0572841
Rcn1	0.60173615	4.76303695	5.75167229	0.02639279	0.05729644
Pfn2	0.29755699	7.71573022	5.74668804	0.02645052	0.0574066
Camta1	-0.5185401	9.37254189	5.74555078	0.02646371	0.05742007
Uqcr11	0.70084779	4.69448431	5.74336359	0.02648911	0.05746
Helq	-0.8025082	2.56920515	5.73934443	0.02653584	0.05752567
Tbl1x	0.25016343	6.44132166	5.73910679	0.02653861	0.05752567
A630023P12	-3.2070817	-1.2681202	5.73866032	0.0265438	0.05752567
Erh	0.48548429	5.18294234	5.73792824	0.02655233	0.05752567
Snx18	0.33977394	6.1685314	5.73729659	0.02655969	0.05752567
Mrpl54	0.76440713	2.69776809	5.73682374	0.0265652	0.05752567
Mpp6	0.49908016	8.59069649	5.73655129	0.02656837	0.05752567
Xrn1	-0.3874044	6.04494522	5.73351775	0.02660376	0.0575777
Flot1	0.35281812	5.46448358	5.73315817	0.02660795	0.0575777
Ngf	1.18890941	0.84562194	5.73269008	0.02661342	0.0575777
Kdsr	-0.4061932	4.62322859	5.73052667	0.0266387	0.05761722
Prr15	1.51216017	0.78841463	5.72987673	0.02664629	0.0576185
Tnfaip6	-0.8287704	1.74255489	5.72795248	0.02666881	0.05764975
Lyar	0.37311572	4.22170673	5.72720022	0.02667761	0.05764975
Sirpa	-0.3374112	7.17435197	5.72684361	0.02668179	0.05764975
Gbas	0.35459252	6.53615095	5.7257786	0.02669427	0.05766155
Trpc6	-0.7713656	2.85249854	5.72348279	0.02672118	0.05770452
Pggt1b	0.42789505	4.10302833	5.72277398	0.0267295	0.05770732
Ftsj2	1.01361397	2.03862433	5.71610046	0.02680794	0.05785522
Anxa8	0.88209758	3.46734733	5.71542709	0.02681587	0.05785522
Cc2d2a	-0.3015734	5.78023341	5.71449809	0.02682682	0.05785522
Tjp3	2.31405944	-0.8532734	5.71442946	0.02682763	0.05785522
Six4	-0.6967136	3.45258233	5.71395642	0.0268332	0.05785522
Znhit1	0.58019192	3.24479417	5.71239864	0.02685157	0.05786486
Dyrk3	0.99876262	1.85283218	5.71238307	0.02685175	0.05786486
Pqbp1	0.37248767	4.38254597	5.70946266	0.02688623	0.05792396
Gimap3	0.87105171	2.71471156	5.70600798	0.02692707	0.05799444
Syn2	0.34881813	7.70541142	5.70550249	0.02693305	0.05799444
Map1lc3b	0.41118713	7.87064155	5.70075943	0.02698926	0.05810025
Snapc3	0.37816416	4.89748963	5.69710756	0.02703262	0.05816993
Vcan	-0.7355712	4.12263198	5.69684179	0.02703578	0.05816993
Pkd1l3	-1.1957196	0.8831859	5.69502191	0.02705742	0.05818589
Trim62	-0.4296767	4.08115042	5.69453974	0.02706316	0.05818589
Ccs	0.78315693	2.13602256	5.69443228	0.02706444	0.05818589
Usp42	-0.4289609	4.91321096	5.69369296	0.02707324	0.05818959

Zdhhc8	-0.502034	4.78650998	5.69251167	0.02708731	0.05818992
Gadd45gip1	0.73882824	2.62818782	5.6921568	0.02709154	0.05818992
2010107G23	0.4540702	4.05042054	5.69189708	0.02709463	0.05818992
Rpl4	0.35189234	8.80304344	5.69001253	0.0271171	0.05821381
Fbl	0.51032145	4.29211646	5.68963456	0.02712161	0.05821381
A330050F15I	1.01337535	2.27249062	5.68918199	0.02712701	0.05821381
Gins4	0.55723873	4.78379394	5.68629514	0.02716148	0.05827257
St3gal1	0.3648945	5.20677659	5.68439462	0.0271842	0.05829153
Efhb	-1.0570305	0.67924051	5.68436964	0.0271845	0.05829153
Ccdc176	-1.0403887	2.36353421	5.68255288	0.02720624	0.05832293
Chrnb3	-1.1893838	1.28994325	5.67938804	0.02724416	0.05836694
AW551984	-0.7598035	3.43531085	5.67906412	0.02724804	0.05836694
Plp1	-0.5219662	8.66461957	5.67830037	0.0272572	0.05836694
Gm19705	1.01398503	1.55104948	5.67770622	0.02726433	0.05836694
Lamtor5	0.46302361	5.97963691	5.6764302	0.02727965	0.05836694
Tubb5	0.317611	7.90547831	5.6762452	0.02728187	0.05836694
Fzd1	0.53291169	6.13564224	5.6761413	0.02728312	0.05836694
Timp2	0.39344624	8.97279797	5.6761029	0.02728358	0.05836694
Mpeg1	-0.8378014	2.86253962	5.67491505	0.02729785	0.05838227
Psmc3	0.35497295	5.5806721	5.67393936	0.02730958	0.05839216
Cep152	-0.9968203	1.78541853	5.6715428	0.02733841	0.0584386
Fbxl13	-2.5976414	-0.9624995	5.66932728	0.02736509	0.05848043
Rit1	0.48185033	4.74407317	5.66683559	0.02739513	0.0584925
8430427H17I	-0.2922027	6.53254101	5.6667134	0.02739661	0.0584925
Drd1a	-0.4387696	4.35712398	5.66671275	0.02739662	0.0584925
Tmem42	-0.705455	2.41919743	5.66649838	0.0273992	0.0584925
Glis2	0.47672843	5.21343482	5.66425396	0.0274263	0.05852205
Ost4	0.59348572	7.07732292	5.6641724	0.02742729	0.05852205
Sytl2	-0.4137664	5.78833679	5.6634683	0.0274358	0.05852501
Eef1d	0.37755654	5.27726507	5.66133273	0.02746162	0.05855076
1700071K01I	-2.6855516	-0.3649096	5.66129172	0.02746212	0.05855076
Hnrnph2	0.26606704	7.21234144	5.66001336	0.02747759	0.05856856
Itga6	-0.5769081	3.55203255	5.65742164	0.02750898	0.05861787
AW046200	-1.9068554	0.14785655	5.65692647	0.02751499	0.05861787
F11r	-0.7240757	2.71136474	5.65480079	0.02754077	0.0586576
Ticam2	-1.5569323	0.92254392	5.65362473	0.02755505	0.05867281
Arhgdib	0.69746477	7.44678784	5.64525549	0.02765691	0.05887444
Pkhd1l1	-2.7154541	-0.6430587	5.64439969	0.02766735	0.05888141
Nudt14	1.16286336	1.06890462	5.64179932	0.02769909	0.0589289
Lsmem1	4.04137261	-1.4809773	5.6413862	0.02770414	0.0589289
Ankle1	-4.0765656	-1.8448087	5.64081086	0.02771117	0.0589289
Nradd	1.65081684	0.29732932	5.63947558	0.0277275	0.05894837
C130026L21F	-1.0819217	1.06076622	5.63305514	0.02780615	0.0591003
Ercc6l	-1.9450578	-0.7930277	5.63074798	0.02783448	0.05913331
Arl3	0.45888554	5.64605501	5.63061807	0.02783607	0.05913331

Atp5a1	0.23785445	9.40853935	5.6297833	0.02784633	0.05913981
Dctn3	0.54640768	5.68725454	5.62790972	0.02786937	0.05917345
Ripk1	0.56008786	3.73532796	5.62648941	0.02788684	0.05919527
Sowahb	-0.5437313	3.51482256	5.62523804	0.02790225	0.05920006
E030013I19R	2.63191116	-0.3495429	5.62504443	0.02790464	0.05920006
Scd1	-0.3262706	6.0379921	5.62455146	0.02791071	0.05920006
Ppih	0.60014955	3.16833844	5.62352951	0.02792331	0.059201
Snord4a	-1.0321829	0.58716041	5.62327612	0.02792643	0.059201
L3mbtl1	-1.1948921	1.58648883	5.62276264	0.02793276	0.059201
Dsc3	3.49603634	-1.3625269	5.62131669	0.0279506	0.05921811
BC039771	-1.0246176	0.57098261	5.62094048	0.02795525	0.05921811
Spata21	3.64325785	-1.3681654	5.61924121	0.02797623	0.05924018
Mag	-0.6531343	2.55410134	5.61877419	0.027982	0.05924018
Cyp2j9	-0.8690375	2.44796972	5.61834661	0.02798729	0.05924018
Rbak	-0.7595059	3.25545728	5.61736281	0.02799945	0.05924956
Tbrg1	0.49892216	4.80640048	5.61682226	0.02800613	0.05924956
Sbno1	-0.4429977	8.41056419	5.61598245	0.02801652	0.05925629
Dnm3	-0.5045164	8.50630383	5.61539479	0.0280238	0.05925642
Arid3c	-3.0625084	-1.2824569	5.61394668	0.02804173	0.05927848
l7Rn6	0.39245227	6.09145303	5.61338777	0.02804865	0.05927848
Gk5	0.66191846	1.99776092	5.61021807	0.02808796	0.05934629
Adam12	0.44211031	5.7691662	5.60822746	0.02811268	0.05938324
Lrch1	-0.5001307	4.25291968	5.60635634	0.02813593	0.05938634
Ifnar2	0.5204404	5.47723388	5.60585818	0.02814213	0.05938634
Ap5z1	-0.8288223	2.61958372	5.60568516	0.02814428	0.05938634
Vprbp	-0.331979	6.75123308	5.60550824	0.02814648	0.05938634
Clec9a	-2.4102156	-0.0266248	5.60458347	0.02815799	0.05938634
E130008D07	-0.9567358	2.27454662	5.60349815	0.0281715	0.05938634
Xpnpep1	0.43116413	4.48513859	5.60309671	0.0281765	0.05938634
Tmem181a	-0.5491585	3.45736147	5.60255951	0.02818319	0.05938634
Fam76a	0.36106662	5.87616487	5.60244706	0.02818459	0.05938634
Tra2b	0.45357067	6.4036067	5.60206116	0.0281894	0.05938634
Oprl1	-0.5231252	3.3758433	5.60172212	0.02819362	0.05938634
Plp2	0.7355205	4.26104445	5.60099257	0.02820272	0.05939027
Boc	-0.4605126	3.53156235	5.59881626	0.02822986	0.05941152
Mettl21c	-2.7892879	-0.5973106	5.59752116	0.02824603	0.05941152
Epha8	-1.35869	0.67208975	5.59735187	0.02824815	0.05941152
Herc3	-0.464793	7.65233924	5.59709131	0.0282514	0.05941152
Cacna1c	-0.5266697	6.3128366	5.59677085	0.02825541	0.05941152
Tcf7l1	0.60902043	5.36479086	5.59670909	0.02825618	0.05941152
Tmem159	0.61375084	4.62406698	5.59320689	0.02829997	0.05948839
Robo2	-0.387791	6.52649214	5.59197708	0.02831537	0.05950554
Wdr11	-0.4582036	5.39380015	5.58947556	0.02834672	0.05954827
Hepacam	-0.5469188	4.23256353	5.58801215	0.02836508	0.05954827
Samd4	-0.3399902	7.26723062	5.58782085	0.02836748	0.05954827

Frmd3	-0.7946595	2.67448322	5.58778235	0.02836796	0.05954827
Prdm10	-0.6447234	2.96049665	5.58746588	0.02837193	0.05954827
4833424O15	-0.4293495	4.66209956	5.58625021	0.0283872	0.0595651
Alg11	-0.3627569	6.70173638	5.58390407	0.02841668	0.05961175
Xrcc6	0.56393092	3.3703194	5.58179223	0.02844325	0.05963635
Apoo	0.36388239	4.83079787	5.58079009	0.02845587	0.05963635
5031425F14I	3.54268107	-2.2204453	5.58066882	0.02845739	0.05963635
Pde9a	-1.0236605	2.03889486	5.58066596	0.02845743	0.05963635
Wnt9a	-0.5992204	3.82353206	5.57865288	0.0284828	0.059662
Itm2a	0.64142877	6.66213787	5.57849707	0.02848476	0.059662
Pcdhga2	-0.8673601	2.1827769	5.57795091	0.02849165	0.059662
Kl	0.38835505	5.31075688	5.57696201	0.02850412	0.059662
Dcaf12	0.4853467	5.27104991	5.5768161	0.02850597	0.059662
Rad50	-0.4194526	6.08377612	5.57418928	0.02853913	0.05969367
2810408M09	0.51103056	3.2236678	5.57228088	0.02856326	0.05969367
Ttll12	-0.5182371	4.13670622	5.57186277	0.02856855	0.05969367
Hpca	0.3182319	8.12687707	5.57153456	0.0285727	0.05969367
4933406F09I	-4.4418181	-1.1412617	5.57093894	0.02858024	0.05969367
Gstt1	0.64637135	4.87976353	5.56969838	0.02859594	0.05969367
Saysd1	-0.6516815	2.32347982	5.56956838	0.02859759	0.05969367
Nog	-1.5755939	-0.0920429	5.56947053	0.02859883	0.05969367
Ptp4a1	0.43866214	4.59349163	5.56874666	0.028608	0.05969367
Tmem161a	-0.6901602	3.19158888	5.56799249	0.02861756	0.05969367
Lepre1	-0.6545328	2.22410944	5.56785382	0.02861931	0.05969367
Gm12504	-1.3398364	0.86210628	5.56780117	0.02861998	0.05969367
Cyb5r3	0.663023	7.41204742	5.56741296	0.0286249	0.05969367
Lcp2	0.54305733	3.02345756	5.56710094	0.02862886	0.05969367
Snurf	-0.8921314	1.5189843	5.56668139	0.02863418	0.05969367
Ephb6	-0.622032	3.56772938	5.56643509	0.0286373	0.05969367
Slc22a12	-2.2853249	0.35222828	5.56060638	0.02871135	0.05983284
Twsg1	0.54126822	7.87788273	5.56000236	0.02871903	0.05983369
Ctsb	0.36960357	7.9144416	5.55796818	0.02874493	0.05985986
Slc6a11	-0.5909716	4.54546055	5.55787165	0.02874616	0.05985986
Lrig2	-0.3600236	5.05686369	5.55707006	0.02875638	0.05986596
Crim1	-0.4256417	6.98334758	5.55331173	0.02880432	0.05995058
Tmem132b	-0.4967297	7.49325511	5.55215117	0.02881914	0.05996625
Pdcd2l	0.53154869	3.02662668	5.55076024	0.02883692	0.05998805
Rnaseh2a	0.79014946	2.75816186	5.54893823	0.02886022	0.06002134
Prkrip1	0.59380383	3.70442885	5.54767485	0.02887639	0.06002909
Palm2	-0.4758656	7.15217427	5.54750613	0.02887855	0.06002909
Gcsh	0.43432931	5.68617672	5.54670933	0.02888876	0.06003512
Atp5s	0.35011638	4.66930354	5.54405693	0.02892276	0.06009058
Sfrp1	-0.5931402	6.64119233	5.54138779	0.02895702	0.06014656
Pak1ip1	0.37551863	4.15962948	5.53868952	0.02899171	0.06020339
Prdm9	-0.8550376	1.62547008	5.53545386	0.02903336	0.06026002

Cope	0.43453865	4.71358402	5.53543186	0.02903364	0.06026002
Nfe2l2	0.39532009	7.84925387	5.53432801	0.02904786	0.06027432
Ift52	0.42427845	4.94907835	5.53298562	0.02906517	0.06029502
Gemin8	0.53443782	3.45202186	5.5312247	0.0290879	0.06031548
Coa6	0.4259254	3.73919613	5.53107506	0.02908983	0.06031548
Sdcbp	0.31996434	9.43623157	5.53021216	0.02910097	0.06031548
Nemf	-0.3807277	7.13975157	5.52959784	0.02910891	0.06031548
Sirt4	-0.8844069	1.73272007	5.52937936	0.02911173	0.06031548
Hoxd11	-1.8294763	-0.3836331	5.52839651	0.02912444	0.0603266
1700003M02	-1.5016247	0.2590926	5.52719926	0.02913992	0.06034346
Nbeal1	-0.3857232	6.22589735	5.52537024	0.02916359	0.0603748
Gprin3	-0.5352025	3.67896331	5.52489513	0.02916975	0.0603748
Tdrp	0.45896487	5.87769403	5.52306913	0.02919341	0.06039641
Myrf	-0.5813576	3.72404626	5.52295512	0.02919488	0.06039641
Lage3	0.64918753	3.39926527	5.52016319	0.0292311	0.06045612
Vdr	1.14560063	0.75906916	5.51862041	0.02925114	0.06048234
Ezh2	-0.6906832	3.03078086	5.51683171	0.02927439	0.06051276
Abat	-0.35776	6.69238941	5.51635603	0.02928058	0.06051276
Cox6a1	0.35687963	6.82536447	5.51468459	0.02930233	0.06054249
Itga3	-0.5875311	2.92621062	5.5136535	0.02931575	0.06054289
Cyp4f13	-0.9174981	1.16738394	5.51353829	0.02931725	0.06054289
Lrrc57	0.35573643	5.83911175	5.51271639	0.02932796	0.06054979
H2-Q4	-0.9076692	1.686645	5.51010109	0.02936206	0.06060497
Stc2	-1.4419786	-0.1603115	5.50899106	0.02937655	0.06061965
Kcnc2	-0.438782	5.91234506	5.50807532	0.02938851	0.06062911
Hsd3b7	0.58379429	2.68274283	5.5066153	0.02940758	0.06064306
Usp48	-0.5247408	5.49429636	5.5064284	0.02941003	0.06064306
Cpd	-0.3810606	6.32169833	5.50394286	0.02944254	0.06068251
Arhgef10l	-0.569171	3.02220245	5.50383694	0.02944393	0.06068251
Fstl5	-0.5220808	4.1514189	5.5023178	0.02946382	0.06070828
Zfyve28	-0.6586362	3.78121154	5.50032134	0.02948998	0.06074696
3110007F17I	-1.1963331	1.17181934	5.49859088	0.02951268	0.06076511
Aaed1	0.61540008	4.32597343	5.49818011	0.02951807	0.06076511
AW146154	0.63420245	3.00440561	5.49773329	0.02952394	0.06076511
Kmt2b	-0.4533118	4.65995809	5.49739616	0.02952837	0.06076511
Pik3ap1	0.74908731	3.13275307	5.49566823	0.02955107	0.06078839
Pxn	0.38517067	5.22610762	5.49540917	0.02955447	0.06078839
Gbp3	0.5893476	4.32332538	5.4938917	0.02957442	0.06081422
Top2b	-0.3041256	8.14488493	5.48839936	0.02964677	0.06094774
Rab3il1	0.56328853	4.12135723	5.48731879	0.02966103	0.0609618
Ucp2	0.70010345	7.46890025	5.48629959	0.02967449	0.06097421
Zeb2os	-0.8506885	1.51606069	5.48474059	0.02969508	0.06100128
Amica1	1.0443314	1.3615346	5.48371724	0.02970861	0.06100535
Maea	0.28617579	5.48426659	5.48346761	0.02971191	0.06100535
Mvp	0.66071746	3.7879624	5.48236085	0.02972655	0.06102017

Atp10b	-1.0831137	1.16803816	5.47963645	0.02976262	0.06107895
Xpa	0.45541647	5.24649638	5.4786271	0.029776	0.06109115
Galnt15	1.27048798	1.00556472	5.47774877	0.02978764	0.06109979
Gm12185	-1.1395559	0.86068717	5.47611099	0.02980937	0.06112911
Crb2	2.34364034	-0.6515591	5.47537094	0.0298192	0.061134
Mon1b	0.28501506	5.95988164	5.47315429	0.02984864	0.06117912
Ptprb	-0.7775135	5.31799751	5.47132764	0.02987294	0.06119927
Aqp7	-1.4802621	0.05441588	5.47030531	0.02988654	0.06119927
B3galt2	-0.4766271	5.41767912	5.46965111	0.02989525	0.06119927
Nefl	-0.3320618	8.72747445	5.46924726	0.02990063	0.06119927
C130026I21R	1.07640958	1.06014838	5.46877761	0.02990689	0.06119927
Tmem198b	1.19346343	0.79725695	5.46838762	0.02991208	0.06119927
Fahd1	0.35338007	5.2997062	5.46816774	0.02991501	0.06119927
Capn11	2.97041638	0.03771976	5.46794	0.02991805	0.06119927
Cox7c	0.45532547	7.31570746	5.46481664	0.02995971	0.06125473
Dach2	1.65596407	0.10560805	5.46478993	0.02996006	0.06125473
Jmjd1c	-0.3595677	8.21493046	5.46370271	0.02997458	0.06126917
Hsd17b2	1.35821948	1.4829574	5.46267232	0.02998834	0.06128206
4831440E17I	-1.0057704	1.79053662	5.46199487	0.0299974	0.06128533
Ncmap	-3.1264224	-0.974297	5.45910475	0.03003606	0.06134906
Ppcs	0.65825414	2.41680731	5.4573147	0.03006003	0.06138278
Spaca6	-1.2630277	2.96142993	5.45537101	0.03008609	0.06140689
C330024D21I	-1.6430349	0.122273	5.45491451	0.03009221	0.06140689
Megf6	-1.0007342	1.38357605	5.45463225	0.030096	0.06140689
Erich2	1.25660543	0.43049639	5.45420557	0.03010173	0.06140689
Pin4	0.3938597	4.63656401	5.4506732	0.03014917	0.06148075
Pim2	0.37068438	4.10662835	5.44978853	0.03016107	0.06148075
Tubgcp3	-0.368507	4.42504064	5.44959313	0.0301637	0.06148075
Wbp4	0.27263266	6.16916851	5.44928399	0.03016786	0.06148075
Sik1	0.50105057	4.19170344	5.44849995	0.03017841	0.06148701
1700001J11F	-2.266566	-1.5119809	5.44751925	0.03019161	0.06149866
Cox6b1	0.57939729	5.47414806	5.44562485	0.03021713	0.06152779
N4bp2l1	-0.499399	4.03004279	5.44534654	0.03022088	0.06152779
Gart	0.45018932	4.37755038	5.44433227	0.03023456	0.06153445
Lin7c	0.28001593	7.8688952	5.44399348	0.03023912	0.06153445
Neurl2	2.08343491	-0.3121361	5.43976665	0.0302962	0.06163534
Il6st	0.37824706	7.38933824	5.43205946	0.03040059	0.0618324
Lrrc9	-1.1236561	1.59318661	5.42995196	0.03042921	0.06187529
Acer1	-1.1866355	0.2981237	5.42747076	0.03046293	0.06191959
Ccdc108	-1.1109672	1.32172893	5.42692525	0.03047036	0.06191959
Rnaset2b	0.61120069	4.08648288	5.42668732	0.03047359	0.06191959
A330070K13I	-2.7855826	-0.6852585	5.42537281	0.03049149	0.06194064
Snx19	-0.4031427	4.75984491	5.42467664	0.03050097	0.06194459
Car15	-1.04787	2.46758281	5.42123046	0.03054795	0.06202468
Tmem132d	-0.5682725	3.96671457	5.42046281	0.03055843	0.06203062

Zfp451	-0.4354133	5.17462745	5.41888682	0.03057995	0.06205898
Emp1	0.80038824	3.82778592	5.4138531	0.0306488	0.06218336
Gm19395	-2.7343716	-0.4158174	5.41251576	0.03066712	0.06220089
Kcng2	-1.3399851	0.29275489	5.41211799	0.03067258	0.06220089
Eef1a2	0.37361211	5.59721423	5.40991747	0.03070276	0.06224674
Rdh12	-1.3212457	0.1413352	5.40848342	0.03072244	0.06227129
Cyth2	0.47090882	4.26547245	5.40679967	0.03074558	0.06230281
A630020A06	-1.872028	0.01746508	5.40535734	0.03076541	0.06232763
Scml2	-1.4664035	-0.0223448	5.40452403	0.03077687	0.06233549
Srsf3	0.33327543	7.78346233	5.40157404	0.0308175	0.0624024
Ubxn1	0.6057488	5.407365	5.40035978	0.03083424	0.06242091
Slc8a1	-0.4959414	9.18535327	5.39825774	0.03086324	0.0624589
Asic1	-0.4298942	4.84553209	5.39789843	0.0308682	0.0624589
Ndufc2	0.45161263	5.2850835	5.39572268	0.03089826	0.06250433
Hexim1	0.32723533	5.41505595	5.39499635	0.0309083	0.06250926
Slc37a1	-0.9772832	0.7991249	5.39392476	0.03092312	0.06252385
Tcf4	0.23745639	9.26007733	5.39273189	0.03093963	0.06253703
4930539N22	-2.1634527	0.71910845	5.39235417	0.03094486	0.06253703
Tnfrsf12a	1.73847274	-0.8392961	5.38959299	0.03098311	0.06259895
Nr1h2	0.59158026	4.09397517	5.38813569	0.03100333	0.0626244
Zscan12	0.4099966	4.26561548	5.38679167	0.03102198	0.06263549
Kif2c	-2.7903761	-1.5242468	5.38664186	0.03102406	0.06263549
Tapbpl	-0.4474825	3.28314003	5.38550579	0.03103984	0.06265196
Mcts1	0.32761665	5.13383329	5.38349715	0.03106776	0.06269292
Rps13	0.44325981	5.90492291	5.37851873	0.03113709	0.0628174
Cacna2d1	-0.4340077	7.62950505	5.37619695	0.03116948	0.06285694
Kcna2	-0.4662952	8.75410394	5.3760175	0.03117199	0.06285694
Gstp1	0.39914257	4.93816257	5.37234102	0.03122336	0.06294509
Mertk	-0.5717749	3.7656962	5.37112038	0.03124044	0.06296408
Trip10	1.00679221	2.25482018	5.36925797	0.03126651	0.06300118
9130221H12	0.48853103	3.48044409	5.36816457	0.03128184	0.06301661
Trim12a	0.61016129	3.76202107	5.36686402	0.03130007	0.06303789
Rrad	2.50706879	-1.1122327	5.36561843	0.03131755	0.06305763
Txn1	0.45974078	6.41714117	5.36505675	0.03132543	0.06305806
Hdac3	-0.425611	4.7598794	5.36363621	0.03134538	0.06307323
Rpl10a	0.601979	8.07832466	5.36281763	0.03135688	0.06307323
Lrrc4b	-0.4438721	4.57026457	5.36270697	0.03135844	0.06307323
Gpi1	0.29335271	7.06928865	5.36196196	0.03136891	0.06307323
Pdk3	0.32394114	5.03255319	5.36174704	0.03137193	0.06307323
Atl1	-0.4871576	6.2395318	5.3608899	0.03138399	0.06307323
2610005L07	-0.4077669	6.35267353	5.36049593	0.03138953	0.06307323
Ppp1r13l	0.8991085	1.34262441	5.36015275	0.03139436	0.06307323
Alkbh3	0.63691771	3.75199751	5.35960517	0.03140207	0.0630733
Ly96	0.61144348	3.76427015	5.35664454	0.03144378	0.06313588
Sap30	0.64430875	3.11921207	5.35630339	0.03144859	0.06313588

Sppl3	-0.302784	5.25228808	5.3557452	0.03145646	0.06313626
Emp3	0.77462548	3.79766243	5.35102609	0.0315231	0.06325457
Tmem210	-1.8438193	0.06352342	5.34946633	0.03154516	0.0632805
Sox6	-0.5148763	5.00814424	5.34887283	0.03155356	0.0632805
4932435O22	4.55037198	-1.8514079	5.34848003	0.03155912	0.0632805
Dynll2	0.29137305	7.03477523	5.34780559	0.03156867	0.06328421
Vamp8	1.0769647	5.10451775	5.34653907	0.03158661	0.06330474
Plcd1	0.73225902	2.01051143	5.34491239	0.03160967	0.06333551
Dand5	0.48420341	3.55729578	5.34342252	0.03163081	0.06334396
Cript	0.38090353	7.53137225	5.34267135	0.03164147	0.06334396
Zfp318	-0.486856	6.73134452	5.34266056	0.03164163	0.06334396
4933409K07I	-0.4270314	7.36388262	5.34244319	0.03164471	0.06334396
Eva1b	1.15328315	0.88173957	5.3410498	0.03166451	0.06335898
Gsta4	0.51720759	6.83520109	5.34082963	0.03166764	0.06335898
4930594C11I	-2.0765786	1.31140351	5.33575703	0.03173982	0.06348795
Dcdc2b	0.53014144	3.77536492	5.33496466	0.03175111	0.06349508
Ranbp3	0.40215725	3.79785599	5.33367288	0.03176953	0.06351646
Hdlbp	0.23509153	8.49839793	5.33305053	0.03177841	0.06351876
Rnf144b	-0.4181005	4.36927858	5.33043634	0.03181574	0.06356206
Dhps	0.79022831	3.07884382	5.32973503	0.03182576	0.06356206
Pcgf1	0.66077699	3.18836724	5.32952683	0.03182873	0.06356206
Cdkn2aip	0.42293144	4.20203269	5.32936786	0.03183101	0.06356206
Bclaf1	0.24015438	9.51920161	5.32683221	0.03186728	0.06361903
Trappc4	0.56508352	3.52625341	5.32519074	0.03189078	0.06364137
Nup85	-0.4638492	3.70621767	5.32411552	0.03190619	0.06364137
Wdr81	-0.6238055	3.02157766	5.32409505	0.03190648	0.06364137
Ccdc115	0.36020049	4.90636218	5.32388855	0.03190944	0.06364137
Trim35	0.28448021	6.9522191	5.32325604	0.03191851	0.06364401
Spock3	-0.3473088	5.22420083	5.32130288	0.03194653	0.06367277
Cdc123	0.32053065	6.25246	5.32117099	0.03194842	0.06367277
Zfp830	0.4073788	5.20672535	5.31758827	0.0319999	0.0637599
Cab39	0.2574731	7.71350092	5.31495577	0.03203778	0.06381991
Cndp2	0.37895597	4.36161415	5.31293914	0.03206684	0.06386231
Limch1	-0.6195577	5.83790073	5.30949843	0.03211648	0.06394567
Suclg2	0.46536125	5.58089658	5.30892351	0.03212478	0.06394671
Anln	-0.3213472	4.85821024	5.30188925	0.03222657	0.06413379
Hid1	0.33196198	6.11254569	5.30064311	0.03224464	0.06415422
Sertm1	0.38389254	4.58370972	5.30007601	0.03225286	0.06415506
4930480K15I	-1.2144548	2.7430116	5.29718566	0.03229483	0.064223
Zbtb11	-0.3693851	6.58427433	5.29483718	0.03232898	0.06427535
Serinc5	-0.4581163	5.0789743	5.29097185	0.03238527	0.06437169
Nmrk1	-0.4162655	4.76005865	5.2895888	0.03240543	0.0643962
Nat9	0.70017009	1.99609858	5.28811654	0.03242692	0.06442332
Zfp692	-0.9326221	2.60333688	5.28447991	0.03248006	0.0645133
Psmg1	0.65803641	3.13073301	5.28210571	0.0325148	0.06456379

D130040H23	-0.6264986	2.95254379	5.28166952	0.03252119	0.06456379
4932411N23	-1.7904182	-0.9359805	5.28094297	0.03253183	0.06456932
Cinp	0.46743963	4.26238492	5.27924697	0.03255669	0.06459665
Ppp1r42	-1.9765617	-0.5472227	5.27893124	0.03256132	0.06459665
Frmpd1	-0.5048172	2.76514959	5.27252012	0.0326555	0.06476787
Osbpl1a	-0.2769365	8.12679639	5.26672012	0.03274097	0.06492172
1700026D08	1.09974998	0.68948357	5.26580109	0.03275454	0.06493296
Heg1	-0.3731915	6.24919506	5.26021059	0.0328372	0.06506755
Urod	0.33145608	5.0701814	5.2601388	0.03283827	0.06506755
Pde3b	-0.8425534	2.68835774	5.25908528	0.03285387	0.06508278
Sema4d	-0.5701157	3.84624337	5.25834985	0.03286477	0.06508868
Ubb	0.62072553	6.41461667	5.25732531	0.03287996	0.06510307
Dcbld2	-0.3311844	5.26479498	5.25540017	0.03290852	0.06513461
Cry1	0.413115	4.34636312	5.2548521	0.03291666	0.06513461
Hey1	0.34712054	6.35228255	5.25464985	0.03291966	0.06513461
Adam28	-1.3313838	0.2058532	5.25380338	0.03293223	0.0651438
Xpr1	-0.31748	7.89515667	5.25190335	0.03296047	0.06517748
Eno1	0.89962754	0.80270498	5.25159095	0.03296512	0.06517748
Ndufs3	0.34491803	6.44442752	5.24901677	0.03300343	0.06523753
Cdhr2	-2.4174863	-0.9457568	5.24745829	0.03302665	0.06526773
Sbk1	-0.4394435	4.16353769	5.24468727	0.03306798	0.06532726
2610035D17	0.57844848	3.31067357	5.24437304	0.03307267	0.06532726
A730090N16	-1.4154277	0.77945632	5.24157845	0.03311441	0.065394
Net1	0.33387487	5.59464004	5.23959297	0.03314411	0.06543089
Mapkapk3	1.05235134	2.24862019	5.23926515	0.03314901	0.06543089
Acvr1b	-0.4170631	6.06409219	5.23710359	0.03318138	0.06547906
Ogg1	0.95723312	0.72943137	5.23310043	0.03324142	0.0655818
Slc39a12	-1.0035108	2.63789016	5.23210946	0.03325631	0.06558683
Serpinb6a	0.59147975	5.68020965	5.23186797	0.03325993	0.06558683
Jun	0.28224272	6.02552746	5.23127544	0.03326884	0.06558865
Rilpl1	0.39330067	5.94586796	5.23062228	0.03327865	0.06559227
Peak1	-0.4523969	6.77754492	5.22769682	0.03332267	0.06566327
Vps54	-0.2869952	6.89711108	5.22680967	0.03333602	0.06567385
Klrg1	4.53104035	-2.0796857	5.22405709	0.03337751	0.06573983
Brwd3	-0.348376	6.15172761	5.22303615	0.03339292	0.06575441
Vdac3	0.30924056	6.33344942	5.22010871	0.03343713	0.0658257
Lace1	-0.518586	3.18675385	5.21861084	0.03345978	0.06585451
2900052N01	-0.7209047	2.81930191	5.21546779	0.03350736	0.06592559
Pum2	-0.2451971	8.19361958	5.21516542	0.03351194	0.06592559
Tox4	0.2847893	5.999696	5.21162679	0.0335656	0.06599344
Pygl	-1.0453998	1.09311284	5.21143362	0.03356854	0.06599344
Rpl7a	0.42607348	7.31122517	5.2111307	0.03357313	0.06599344
Ube2e2	0.29348996	6.58122371	5.21052618	0.03358231	0.06599344
Gpm6b	-0.3843071	8.47914724	5.21024583	0.03358657	0.06599344
Tmf1	0.24752815	7.01464471	5.20938794	0.03359961	0.06600327

Eef1a1	0.38475293	10.3156722	5.20667165	0.03364092	0.06606848
Wdr19	-0.4807158	4.6043891	5.20614858	0.03364888	0.06606848
Elf1	0.41018743	5.89231624	5.20346101	0.03368982	0.06612578
Zc3h8	-0.570162	2.7597881	5.20317674	0.03369415	0.06612578
Tmod3	0.47023441	7.80433048	5.20204695	0.03371138	0.0661306
Cnksr2	-0.5461517	9.57153435	5.20196054	0.0337127	0.0661306
Dmap1	0.54963039	3.50122349	5.19973237	0.03374671	0.06618152
Rsph4a	0.70963687	2.93990736	5.19754716	0.0337801	0.0662312
Gm6313	-0.9973639	1.20104026	5.19646497	0.03379665	0.06623702
Kpna4	0.23128174	7.20475338	5.19629937	0.03379919	0.06623702
Tll1	0.55694642	3.55557792	5.19245846	0.03385801	0.06633647
Rxrg	-0.8974742	0.91500745	5.1916336	0.03387065	0.06633793
Smim18	0.76727403	2.12277284	5.19135719	0.03387489	0.06633793
Cybb	-1.5829878	0.85518632	5.18872047	0.03391536	0.06638597
Os9	-0.4556444	4.71647211	5.18870634	0.03391558	0.06638597
Usp35	-0.779376	1.87628475	5.18669729	0.03394645	0.06643058
Rnf11	0.27714371	6.86253353	5.18100976	0.03403403	0.06657399
Ctu2	0.88576224	1.72551479	5.1808861	0.03403593	0.06657399
Ccna2	-0.7225324	1.98448009	5.17916291	0.03406252	0.06661014
Gm10125	-1.0660329	1.01722952	5.17558501	0.03411779	0.06670235
Alms1-ps2	-1.1470548	0.45228043	5.17361754	0.03414823	0.06674599
Hhip	-0.4279238	4.43848583	5.17272667	0.03416202	0.06675707
Fam21	-0.359716	6.01400352	5.17208674	0.03417193	0.06676056
Tceal8	0.52210188	7.27685964	5.17116803	0.03418617	0.0667725
Mir378b	-1.9856833	0.21148148	5.1690282	0.03421935	0.06680757
Psd	-0.4353324	5.56008766	5.16896174	0.03422038	0.06680757
Eif2b4	0.57079014	3.19675848	5.16680118	0.03425392	0.06685717
Nbeal2	-0.6824789	1.80362844	5.16605081	0.03426558	0.06686405
Stx18	0.55861914	3.37978799	5.16537962	0.03427601	0.06686853
Airn	0.83842596	1.75210693	5.16335265	0.03430753	0.06689444
Ampd2	-0.4075819	3.93877048	5.16246367	0.03432137	0.06689444
Gm21119	-1.1774528	0.59824028	5.16234666	0.03432319	0.06689444
Rbpms2	0.83928464	2.82537584	5.16178157	0.03433199	0.06689444
Zfp747	0.42229568	3.70442029	5.16172491	0.03433287	0.06689444
Btbd19	-0.7248989	2.62610308	5.16138751	0.03433813	0.06689444
Grik3	-0.5146377	5.57650509	5.16000423	0.03435968	0.06692057
Mina	0.61030018	2.58326857	5.15618833	0.03441922	0.06702065
Il1a	2.26369484	-1.0177028	5.15454313	0.03444492	0.06705481
Tollip	0.29701653	6.59682381	5.15099701	0.0345004	0.06712605
Acsm3	-2.2093105	-0.887876	5.15078297	0.03450376	0.06712605
Ctsf	-0.7431219	3.07466741	5.15037074	0.03451021	0.06712605
Bcl2a1d	0.92501759	1.18069673	5.15011719	0.03451418	0.06712605
Ctso	0.53593139	5.03282715	5.14802388	0.034547	0.06717397
Mov10l1	-3.3867081	-2.089242	5.14385009	0.03461253	0.06727623
Prpf18	0.34667839	4.94499553	5.14363171	0.03461596	0.06727623

Cog4	0.38814539	4.60369643	5.14200639	0.03464152	0.06729421
Smarcd3	0.5047232	4.1281128	5.14200197	0.03464159	0.06729421
Strap	0.23775006	7.46723644	5.14080664	0.0346604	0.06730255
Phf5a	0.52605908	4.82766972	5.14068862	0.03466226	0.06730255
Rab32	0.71782823	3.17081687	5.14001771	0.03467282	0.06730716
Begain	-0.5298331	3.44830392	5.13871045	0.03469341	0.06733123
Mlxipl	-1.0762733	0.95267483	5.13801339	0.0347044	0.06733666
Surf6	0.43237868	3.73763035	5.13730324	0.0347156	0.06734248
Sptlc2	-0.3178148	5.09740509	5.13617592	0.03473338	0.06736108
Rps19bp1	0.54529529	2.88831036	5.13503949	0.03475132	0.06737997
Stam2	0.32147262	4.84893538	5.13377202	0.03477134	0.06739321
Pno1	0.36705187	3.98519793	5.13356897	0.03477454	0.06739321
Edrf1	-0.5120226	5.06486854	5.13245464	0.03479216	0.06740509
Adam4	-1.4280623	0.97597989	5.13214326	0.03479708	0.06740509
Toe1	0.5968143	2.66854513	5.12893488	0.03484785	0.06747431
Ccne2	-0.8426914	1.99831562	5.12884805	0.03484923	0.06747431
S100a11	0.55957705	9.5078464	5.12754494	0.03486988	0.06747589
Szt2	-0.5893708	3.93196404	5.12737402	0.03487258	0.06747589
Entpd7	-0.536072	4.12638199	5.12671655	0.03488301	0.06747589
Scmh1	0.33585501	6.11744616	5.12667661	0.03488364	0.06747589
5730522E02I	-1.2745179	1.39875561	5.12620659	0.03489109	0.06747589
Nkd2	0.59813512	4.51143517	5.12564014	0.03490008	0.06747739
Acsbg1	-0.3928741	4.57552639	5.12459754	0.03491663	0.0674935
Kcnd1	-0.7847997	2.40261102	5.12288399	0.03494384	0.0675233
Mlkl	1.43495767	0.73842643	5.12259241	0.03494847	0.0675233
Lrp3	-0.4477706	4.92686059	5.12160203	0.03496421	0.06753783
1110032A03I	0.36772565	6.65671181	5.11993133	0.03499078	0.06755732
Ptger4	-1.3701212	0.6562272	5.11969477	0.03499455	0.06755732
B9d2	0.82235612	1.8269852	5.11941784	0.03499896	0.06755732
Pias3	-0.5474588	2.71943935	5.11788987	0.03502329	0.0675884
Slc25a4	0.27495594	9.98946024	5.11711307	0.03503566	0.06759642
Pyroxd1	-0.4376177	3.35080755	5.11176431	0.03512101	0.06773691
Uba1y	-2.0490821	-1.1650433	5.11042917	0.03514235	0.06773691
Ube2n	0.23001198	7.43722179	5.11037046	0.03514329	0.06773691
Lcor	-0.5386477	3.98024282	5.11030084	0.03514441	0.06773691
Nabp2	0.43271137	5.88498159	5.10997053	0.03514969	0.06773691
Creb3l2	0.51206142	5.98241077	5.10941828	0.03515852	0.06773805
Mok	-0.6416925	2.36552259	5.10766549	0.03518658	0.06777622
Tgm5	-2.1370652	0.06643321	5.1050889	0.03522786	0.06783984
Dpy30	0.52164064	4.81348672	5.10258385	0.03526805	0.06788894
Aars2	-0.8456166	1.65531529	5.10247043	0.03526988	0.06788894
Mrpl42	0.35486503	5.90486063	5.099019	0.03532534	0.06797978
Spryd3	-0.5743523	4.21771774	5.09739404	0.03535149	0.06798277
Polm	0.57048395	3.19492597	5.09703941	0.0353572	0.06798277
Afmid	1.0429439	1.12223607	5.09695488	0.03535856	0.06798277

Dhx35	-0.5199748	2.62896772	5.09686642	0.03535998	0.06798277
Rgs10	0.4177929	4.32710585	5.09499203	0.03539018	0.06801219
Fbxo43	-1.4008782	-0.5212013	5.09488426	0.03539192	0.06801219
Gga1	0.44826016	3.54956689	5.09437617	0.03540011	0.06801219
Slc4a8	-0.5159102	6.84351407	5.09358964	0.03541279	0.06802066
Gm12250	1.34540347	0.08217832	5.09271086	0.03542697	0.06803199
Spred3	-0.4750697	5.07279754	5.09102265	0.03545422	0.06806016
Ccdc39	-0.5579462	4.3535668	5.0907765	0.0354582	0.06806016
Scfd2	-0.5755552	2.97274159	5.0900671	0.03546966	0.06806626
Slc27a2	-0.7878067	1.809053	5.08917499	0.03548408	0.06806914
Nxpe3	-0.6213218	4.07112781	5.08894959	0.03548772	0.06806914
LOC1005036	0.7102083	3.67730937	5.08754655	0.03551042	0.06809677
Nlrp6	1.62319504	0.89625499	5.08587255	0.03553751	0.0680999
Cntnap5c	-1.2088707	1.45542887	5.08580216	0.03553865	0.0680999
Ebp	0.62160078	2.6593953	5.08563458	0.03554137	0.0680999
Wbp11	0.27628151	6.05145539	5.08539868	0.03554519	0.0680999
Bcl9	-0.3070486	6.42996014	5.08297792	0.03558442	0.06815918
Trpc5	-0.5365664	4.12058133	5.08237991	0.03559412	0.06816188
Zik1	-0.4919513	3.46310341	5.08109374	0.035615	0.06816438
Gsap	-0.6805752	2.23454519	5.08095318	0.03561728	0.06816438
Eapp	0.34685183	5.25186188	5.0807664	0.03562031	0.06816438
Btbd17	-1.2215086	0.43753418	5.07899873	0.03564903	0.06820345
Gm608	-0.3062178	8.24058658	5.07649144	0.0356898	0.06825955
Kif20b	-0.9099754	1.82200334	5.07617442	0.03569496	0.06825955
Cuta	0.45537444	6.28943736	5.07078782	0.03578275	0.06841152
Rab21	0.29586528	7.62913437	5.06925007	0.03580786	0.0684436
Fam89a	-3.4738278	-1.4200627	5.06616138	0.03585835	0.06851373
Elovl2	0.68970667	2.66654295	5.06598563	0.03586122	0.06851373
Atp6v0a4	-1.3638149	0.14184209	5.06391248	0.03589516	0.06856263
Ddt	0.62600203	3.74680858	5.06223965	0.03592257	0.06857994
Ndufv3	0.52334287	5.13708218	5.06206469	0.03592544	0.06857994
Rtkn2	-1.1083175	1.19225338	5.06183204	0.03592925	0.06857994
Mrps2	0.31937097	4.70560518	5.06072785	0.03594736	0.06859858
Ptpre	-0.4589168	5.24251314	5.05807854	0.03599086	0.06866564
Fbxw15	-1.983128	-0.9967257	5.05588005	0.036027	0.06870327
Jmjd4	-0.3807664	4.63418793	5.0556063	0.0360315	0.06870327
Dguok	0.6462876	2.97350383	5.0550021	0.03604144	0.06870327
Gm11744	-1.8561915	-0.4086737	5.05436117	0.03605199	0.06870327
Rgs6	-0.5634914	3.12285949	5.0540925	0.03605641	0.06870327
Rapgef5	0.28502993	6.3808839	5.05382965	0.03606074	0.06870327
BC051142	-1.1617942	1.26833807	5.05277087	0.03607817	0.06871357
Cntnap4	-0.6802406	3.32376177	5.05248616	0.03608286	0.06871357
Snx14	-0.3747561	5.44398163	5.05197342	0.03609131	0.06871374
Pcdhb14	-0.6131259	3.02501865	5.05003742	0.03612324	0.06875859
Ppef1	-1.9899917	-0.7102362	5.04567899	0.03619522	0.0688663

Xdh	0.55187711	3.2821717	5.0455965	0.03619658	0.0688663
Vamp4	0.24542464	6.82262268	5.04415357	0.03622045	0.06886963
Smox	0.62833935	2.88869692	5.04339856	0.03623295	0.06886963
Plce1	0.29662552	4.90128955	5.0429764	0.03623993	0.06886963
Chchd4	0.45373665	4.78100268	5.04247955	0.03624816	0.06886963
Ing1	0.4448535	4.90266404	5.04235952	0.03625015	0.06886963
Dnmt1	-0.4244001	5.72564765	5.0416989	0.03626109	0.06886963
Prkab1	0.58334603	3.39809455	5.04150388	0.03626432	0.06886963
Ccnj	-0.5585959	2.89291347	5.04144126	0.03626536	0.06886963
Ldoc1	2.81127159	-1.3494484	5.04062893	0.03627883	0.06887928
Lsm6	0.32800024	5.28972953	5.03826504	0.03631804	0.0689378
Npepl1	0.79851375	2.06684462	5.03546382	0.03636457	0.06901018
Ppa2	0.32284975	5.40210486	5.03233378	0.03641664	0.0690754
Ndufs7	0.42731332	5.10040801	5.03173571	0.03642659	0.0690754
Man2b1	0.50689627	4.0911535	5.03145047	0.03643134	0.0690754
Sel1l3	-0.5916912	4.96079136	5.03137818	0.03643255	0.0690754
Abra	-3.1682105	-0.3836444	5.03071049	0.03644367	0.06908055
Cd59b	1.56485271	-0.4724651	5.03007323	0.03645429	0.06908157
Csrnp3	-0.4467094	7.29463756	5.02966967	0.03646102	0.06908157
Sbf2	-0.3152012	7.08779668	5.02911546	0.03647026	0.06908316
Mettl10	0.48021752	5.06836517	5.0251144	0.03653706	0.06919367
Slc12a8	-1.2206931	0.36052363	5.02461259	0.03654544	0.06919367
Ddx51	-0.3664163	3.89237777	5.0231808	0.03656939	0.06922306
Ino80c	0.43685718	5.69305021	5.02079749	0.03660928	0.06926993
Zfp706	0.29416615	7.48004342	5.0201571	0.03662001	0.06926993
Capza2	0.26122448	8.06231364	5.01973735	0.03662704	0.06926993
Mex3b	-0.532968	3.37780557	5.01968858	0.03662786	0.06926993
Fmnl2	0.30985228	8.09144302	5.01893237	0.03664053	0.06927796
Acyp1	0.46937323	5.19441005	5.01537922	0.03670016	0.06937473
Fsd2	-2.8766539	-1.7199672	5.01394843	0.0367242	0.06938181
Slc24a5	-0.5974338	2.28941633	5.01366179	0.03672902	0.06938181
Ndst3	-0.5439345	4.32585703	5.01330551	0.03673501	0.06938181
Rfx4	-0.6271965	3.75945816	5.01314715	0.03673767	0.06938181
Bcas2	0.25650936	5.77682026	5.01012805	0.03678848	0.06946181
Cox7a2	0.45859373	6.00398457	5.00755248	0.03683188	0.06952779
Kdm4c	-0.4556042	5.52623136	4.99966748	0.03696513	0.06975123
Spsb3	0.60603465	2.64510332	4.99954415	0.03696722	0.06975123
Pdlim5	0.47373959	7.39530716	4.99810051	0.03699168	0.06978136
Ppp3r2	-1.8873871	-0.420535	4.99693459	0.03701144	0.06978668
Grin3b	2.61888316	-1.7901405	4.99667256	0.03701589	0.06978668
Thoc7	0.37106266	6.34780559	4.99615772	0.03702462	0.06978668
Rbm7	0.41711874	5.12838101	4.99559001	0.03703425	0.06978668
Prrg1	-0.666113	2.50080234	4.99543071	0.03703696	0.06978668
Mknk1	0.39982004	4.31864868	4.99470782	0.03704923	0.0697938
Hint2	0.62778207	3.50760824	4.99266833	0.03708387	0.06984306

Wdr3	-0.5577767	3.22778779	4.99095968	0.03711292	0.06985402
Hmcn1	-0.4884311	3.75683083	4.99037862	0.03712281	0.06985402
Apobec4	-1.9413928	-0.0917944	4.99018042	0.03712618	0.06985402
Skil	0.25993733	7.34534605	4.98977536	0.03713307	0.06985402
Ctage5	-0.2709833	5.84547173	4.98934503	0.0371404	0.06985402
Smug1	0.344827	4.24447993	4.98911821	0.03714426	0.06985402
Aamdc	0.45139305	4.22658645	4.98867996	0.03715172	0.06985402
Sort1	-0.3273494	7.68974787	4.98833012	0.03715768	0.06985402
Gpr116	-0.8290948	4.84174366	4.98767579	0.03716883	0.069859
Mon2	-0.3580774	6.62046016	4.98224677	0.03726147	0.07000961
Prpf6	0.32195232	5.5425876	4.98198184	0.037266	0.07000961
Pcdhb2	-1.1618198	1.80948339	4.98133449	0.03727706	0.07001439
Nwd2	-0.7639878	5.78589024	4.97898565	0.03731724	0.07007384
Git2	0.30863868	5.44319972	4.9783805	0.0373276	0.07007729
Lrrc16a	-0.4268985	4.29144179	4.97705685	0.03735027	0.07009112
Abt1	0.52089828	3.38623331	4.97695475	0.03735202	0.07009112
Rab26os	-1.1082937	0.98218832	4.97624336	0.03736422	0.07009272
B3galt5	-0.7057237	3.22309558	4.97590971	0.03736993	0.07009272
Unc5d	-0.4471839	6.21275361	4.97529829	0.03738042	0.07009397
D930015M05	-2.0166104	0.26353852	4.97402438	0.03740227	0.07009397
Rif1	-0.4484148	5.78944348	4.97394252	0.03740368	0.07009397
Col9a2	0.72411489	2.67963195	4.97388221	0.03740471	0.07009397
Cdkn2aipnl	0.3837823	4.48162468	4.97198641	0.03743727	0.07013898
Polr3d	0.54809798	3.08164623	4.96961313	0.03747806	0.07014943
Stx6	0.35325678	4.9700372	4.96908257	0.03748719	0.07014943
BC030336	-0.3127017	5.66464111	4.96907075	0.03748739	0.07014943
Coro6	-1.3952463	1.88178598	4.96883872	0.03749139	0.07014943
Ivns1abp	-0.2899114	7.54592213	4.96865824	0.03749449	0.07014943
Slc25a37	-0.342227	5.36740566	4.96845685	0.03749796	0.07014943
Vapa	0.29256386	7.18529711	4.9681879	0.03750259	0.07014943
Rnf219	0.29100132	5.20945198	4.96657287	0.0375304	0.07018547
Ahnak	0.49639764	9.0753298	4.96319234	0.03758869	0.07027
Gm5643	0.31540125	5.48793776	4.96296024	0.03759269	0.07027
E130309D02	0.42529026	4.011427	4.96062971	0.03763294	0.07032924
Hebp1	0.44514628	3.58687395	4.95788565	0.0376804	0.07040192
Plch2	-0.7307228	3.25218423	4.95513373	0.03772806	0.07047494
Soga1	-0.2614354	7.13274724	4.95348614	0.03775662	0.07051104
2410015M20	0.65862131	2.73795877	4.95302975	0.03776454	0.07051104
Sec14l2	0.67412509	2.46746972	4.95158715	0.03778958	0.07054177
Rpl3	0.40638183	8.2228053	4.95056741	0.03780729	0.0705588
Ccnd3	0.86380755	4.56294001	4.94807707	0.03785058	0.07061501
Cdh19	-1.041604	1.52153738	4.94739847	0.03786239	0.07061501
Scn3a	-0.6265581	5.62374621	4.94708622	0.03786782	0.07061501
Apc	-0.4296509	9.68081644	4.9468591	0.03787177	0.07061501
Atp6v1h	0.23460113	6.29465663	4.94609409	0.03788509	0.07062382

Hiat1	-0.293542	6.3790195	4.94347659	0.0379307	0.07069281
Gm10336	-0.503078	4.45239221	4.94260621	0.03794588	0.07070507
Prdm6	0.65577383	5.62563107	4.94016085	0.03798857	0.07076856
Bche	-0.6106258	7.2717699	4.93916797	0.03800591	0.07078483
Fgfr4	-1.7446755	-0.6665554	4.93534403	0.03807281	0.07088087
Cdkl4	-0.4260997	4.17292672	4.93523429	0.03807473	0.07088087
Nucb2	0.3406854	5.26267907	4.93267248	0.03811962	0.07094838
Pcbp3	0.45176414	4.77316804	4.93195107	0.03813227	0.07095586
AI847159	2.90161202	-1.8989295	4.9292927	0.03817894	0.07102661
5430402O13	-1.9602456	-0.5565172	4.92619552	0.03823339	0.07110858
Fam120b	-0.3318556	6.59916245	4.92577889	0.03824072	0.07110858
Golga2	0.32046038	5.49769387	4.92531099	0.03824895	0.07110858
Ehd1	0.57680098	3.5760169	4.92458371	0.03826176	0.0711163
Aldh1a7	1.25086453	0.42021628	4.92286574	0.03829203	0.07115646
0610040J01F	-1.6303821	0.12504341	4.92230416	0.03830192	0.07115877
Gm13749	-1.4620604	0.63489644	4.92169512	0.03831266	0.07116154
AA415398	-0.6693933	2.98746953	4.92123778	0.03832073	0.07116154
Fgf2	0.81101452	1.59855045	4.92016726	0.03833962	0.07116224
Nkain4	-1.0385781	1.74106904	4.9199703	0.0383431	0.07116224
Lbr	-0.4655702	3.4404604	4.91974448	0.03834708	0.07116224
Sfswap	-0.5361777	4.61788931	4.91705056	0.03839467	0.07123447
Cmtr1	-0.483072	5.59587889	4.91645122	0.03840527	0.07123805
Dnajc10	-0.2760105	6.34267739	4.91488722	0.03843294	0.07127329
Zbp1	0.98327402	1.02666579	4.91363805	0.03845506	0.07127451
6330409D20I	-1.0888843	0.73553757	4.91328642	0.03846129	0.07127451
Dedd	0.37905253	4.20891729	4.9125295	0.0384747	0.07127451
Galnt5	-2.4920147	-1.0142029	4.91246856	0.03847578	0.07127451
Ccdc158	2.04349243	-0.7985472	4.91240182	0.03847696	0.07127451
Usp11	-0.3344902	7.40496028	4.91157043	0.0384917	0.07128575
Polq	-0.9447443	2.63055267	4.90943112	0.03852965	0.07133996
Uvssa	-0.4228422	4.85422542	4.90815496	0.03855231	0.07136051
Ube2g1	0.31262194	6.83465287	4.90782793	0.03855811	0.07136051
Zfp422	0.34578457	6.39820939	4.90577518	0.0385946	0.07139944
Ankrd16	-0.8479106	2.54010471	4.90566699	0.03859652	0.07139944
Abhd4	0.44935028	5.30739511	4.90367074	0.03863204	0.07144907
Zfp128	0.62593865	2.6900895	4.90276266	0.03864822	0.0714629
Kctd7	0.7377665	2.79768907	4.90223369	0.03865764	0.07146425
Fastk	0.44928704	3.68472314	4.90105875	0.03867858	0.07148684
Pdzrn3	0.28213379	7.30350977	4.90022006	0.03869353	0.07148684
Rwdd3	-0.7035649	2.62408244	4.89983788	0.03870035	0.07148684
Rgs12	-0.6186335	3.26343381	4.89959671	0.03870465	0.07148684
Mta1	-0.3491256	4.54349994	4.89437045	0.03879802	0.07164319
Rras2	0.423363	4.65178085	4.89153788	0.03884873	0.07166518
Ung	0.76555601	2.10678737	4.89099163	0.03885852	0.07166518
Emp2	-0.4676591	4.33314643	4.89083198	0.03886138	0.07166518

Kdelr1	0.45522004	4.34912127	4.89075826	0.03886271	0.07166518
Clk1	-0.4782537	6.46913764	4.89066355	0.0388644	0.07166518
Gm9159	-0.9746377	2.04989357	4.89040204	0.03886909	0.07166518
Bcas3os1	-2.0327885	0.45882069	4.88986313	0.03887875	0.07166518
Prmt2	0.47708156	4.78853725	4.88981117	0.03887968	0.07166518
Zc3h12d	1.83675806	-0.835249	4.88928972	0.03888904	0.07166635
D8Ertd738e	0.74904246	3.18612086	4.88851022	0.03890302	0.07167314
Rab11a	0.27652656	6.56897151	4.88811241	0.03891016	0.07167314
Synb	-1.8878655	-0.6087097	4.88537171	0.03895939	0.07174774
Tsen34	0.44908862	4.22262638	4.88452434	0.03897462	0.0717575
Hspbap1	-0.72049	2.22163898	4.88397389	0.03898452	0.0717575
Pard3	0.3130278	5.12882172	4.8834666	0.03899365	0.0717575
Ccdc53	0.46483727	3.93266763	4.88313514	0.03899961	0.0717575
Otulin	0.61732394	3.54519405	4.88227511	0.03901509	0.07176992
Orc5	-0.4921016	3.3294651	4.88034127	0.03904993	0.07181792
Tex264	0.46112495	3.26062366	4.87768586	0.03909782	0.07188991
Cops6	0.35854622	5.82043294	4.87650064	0.03911921	0.0719102
Fam102b	0.26237766	5.84785971	4.87588188	0.03913039	0.0719102
Zkscan3	-0.4345286	3.32695568	4.87562108	0.0391351	0.0719102
Hspe1	0.33715605	6.34454213	4.87493339	0.03914753	0.07191696
Aox1	0.5472447	3.12249429	4.87395189	0.03916527	0.07193347
Snrpa	0.4832165	4.66726131	4.87324239	0.0391781	0.07194097
Evc2	-0.8837542	2.24314415	4.87082995	0.03922177	0.07199368
Dennd1c	-2.1990446	-0.2605778	4.87068867	0.03922433	0.07199368
Uap1	0.29304166	5.8001539	4.86985058	0.03923951	0.07200547
Atl2	-0.3524992	6.17203477	4.86909218	0.03925326	0.07201374
Ttll5	-0.3422318	5.3838044	4.86863506	0.03926155	0.07201374
Arl14ep	0.27843557	6.37628164	4.86728324	0.03928607	0.07204264
Lman2l	0.49687284	3.23296751	4.86379598	0.0393494	0.07214269
Camsap2	-0.3563225	8.77325357	4.85995168	0.03941936	0.07224533
Pdss2	0.44863969	3.1149329	4.85936969	0.03942996	0.07224533
Get4	0.4503024	3.89752722	4.8592714	0.03943175	0.07224533
Apol6	-1.3674711	-0.189787	4.85433667	0.03952179	0.07239416
Dhrs3	0.59337906	4.64623871	4.85203918	0.03956379	0.07245494
Acot12	-2.8096957	-1.5535456	4.84929768	0.03961397	0.07253068
Nmnat2	-0.3003751	7.15343766	4.84679508	0.03965985	0.07259067
B3galt4	-2.051471	-0.7880389	4.84654665	0.0396644	0.07259067
Ikzf2	0.42570524	4.23824318	4.84417842	0.03970787	0.07265405
Nat1	0.95904026	2.09121257	4.84306236	0.03972838	0.07267539
Ap2m1	0.26374799	7.80336144	4.84095653	0.0397671	0.0727165
Spryd7	0.29202333	5.47150062	4.84087788	0.03976855	0.0727165
Plec	-0.4091401	6.34716249	4.83607532	0.03985702	0.07286207
Lum	0.61505045	5.256301	4.8334698	0.03990512	0.07289797
Gm1979	-1.3152998	0.01681008	4.8334252	0.03990594	0.07289797
Commd9	0.76488531	2.15963691	4.83324407	0.03990929	0.07289797

Tmem47	-0.3808761	7.50737928	4.83308961	0.03991214	0.07289797
Cebpzos	0.49632607	2.99218171	4.83214581	0.03992958	0.07291362
Lrrc4c	-0.3680307	8.09940003	4.83115794	0.03994784	0.07292892
Rasgef1b	-0.3362281	5.48781559	4.83065072	0.03995723	0.07292892
Mfsd2b	-2.2130373	-0.5072159	4.82978143	0.03997331	0.07292892
Ipo13	-0.372218	5.39730335	4.82977389	0.03997345	0.07292892
Spaca5	-3.0164041	-1.6395404	4.82547414	0.04005311	0.07305192
Pcdhb9	-0.7232706	2.25524528	4.8251761	0.04005864	0.07305192
Junb	1.23481063	1.53956335	4.8216759	0.04012364	0.07315421
Rrm1	-0.370127	4.32761159	4.81913264	0.04017094	0.07320109
Dusp3	-0.256899	7.09315937	4.81885164	0.04017617	0.07320109
Cdc5l	0.22405913	6.18232915	4.81863702	0.04018016	0.07320109
Csdc2	0.33647119	6.84565661	4.81837877	0.04018497	0.07320109
Pigt	-0.3613829	5.31439876	4.81733493	0.04020441	0.07322027
Atp6v0a1	-0.3279812	7.21774793	4.81658961	0.0402183	0.07322692
Sec23b	-0.4067354	4.50824447	4.81618274	0.04022588	0.07322692
Edc4	-0.5776369	3.93410082	4.81502166	0.04024753	0.07324247
Hs2st1	-0.3041839	6.7575588	4.81476891	0.04025225	0.07324247
Kif1b	-0.3927467	9.93094244	4.81362627	0.04027357	0.07326504
Prr12	-0.3533643	5.7528392	4.81232891	0.04029779	0.07329289
Nsd1	-0.3068971	8.22829954	4.80981715	0.04034473	0.07335246
Anp32b	0.35897722	8.28371329	4.80943687	0.04035185	0.07335246
Rragc	0.2938866	5.81121884	4.80865815	0.04036642	0.07335246
Rdh11	1.14032862	1.0106902	4.80834515	0.04037227	0.07335246
Mak	-0.9479667	2.07930266	4.80819073	0.04037517	0.07335246
Rnps1	0.24600644	6.25131145	4.80616282	0.04041314	0.07340523
Jarid2	-0.30834	5.84144929	4.80548545	0.04042584	0.07341207
Adam15	-0.395481	4.21539987	4.80417027	0.0404505	0.07344063
Gng11	0.64632995	5.55179355	4.80164017	0.04049799	0.07351061
Ubxn4	0.23687874	7.28314811	4.80005588	0.04052776	0.07354841
Kcnh8	-3.308996	-1.0813013	4.7986844	0.04055356	0.07357897
Zfp831	-0.6114378	5.24283615	4.79549356	0.04061363	0.07367171
Pcdha6	-1.357944	0.30558676	4.79215182	0.04067666	0.0737235
Kptn	-0.8245017	1.85330009	4.79181167	0.04068308	0.0737235
Kdelr3	-1.0052019	1.07251434	4.79169518	0.04068528	0.0737235
Rpgrip1l	-0.4537053	5.86432918	4.79145819	0.04068976	0.0737235
Slc16a13	0.60202582	2.78603537	4.79144973	0.04068992	0.0737235
Kcns2	-0.4368106	3.87739497	4.7911273	0.04069601	0.0737235
Ctsw	-3.750817	-2.2378359	4.79007847	0.04071582	0.07374315
Smchd1	-0.3403915	6.18192511	4.78933184	0.04072993	0.07375246
D430019H16	-0.3884924	6.30631517	4.78876974	0.04074056	0.07375545
Snapc4	-0.733667	3.72206953	4.78321993	0.04084567	0.07392945
Ccdc28b	0.48011373	3.40580795	4.78185549	0.04087156	0.07396002
Xlr4b	2.40684861	-0.3542584	4.78018126	0.04090335	0.07398836
Tmem177	0.52008566	3.68336884	4.78001592	0.04090649	0.07398836

Galm	0.80845682	2.35655813	4.77960891	0.04091422	0.07398836
2310003H01l	-0.9716368	0.9227629	4.77768317	0.04095084	0.07403828
Ndufaf2	0.50553317	4.81039689	4.77656915	0.04097203	0.07403838
Pacsin3	0.5172468	3.80814932	4.77632354	0.04097671	0.07403838
Scamp4	0.51086543	3.4873528	4.77625991	0.04097792	0.07403838
Ppp2r3c	0.29726207	4.97086748	4.77564579	0.04098961	0.07404323
Baz1b	-0.2606382	7.83348427	4.77028163	0.04109191	0.07421137
Bhlhe40	0.25881219	7.52034272	4.7698184	0.04110075	0.07421137
Pip5kl1	-1.751578	0.28413704	4.76874586	0.04112125	0.07423207
Rasa2	-0.3142073	5.06270366	4.76702472	0.04115416	0.07427516
4932413F04l	-1.4947054	0.1333129	4.76407722	0.04121058	0.07436068
4933431G14	-1.2906529	0.30495939	4.76098384	0.0412699	0.07445136
Syt5	0.37551297	5.40819515	4.76006199	0.04128759	0.07446694
Brd7	0.27169183	5.86924557	4.75858981	0.04131587	0.07450159
Fam43b	0.87288227	2.4659987	4.75702793	0.0413459	0.07453569
Olfr1417	-3.8564689	-2.0114826	4.87498388	0.04135292	0.07453569
Pxdc1	0.64593014	3.64415578	4.7548117	0.04138854	0.07458354
Itga2	-2.0054077	0.13157257	4.754221	0.04139992	0.07458769
Zdhhc20	-0.3612245	5.43041885	4.74788573	0.04152213	0.07479149
Ralb	0.42655426	4.37147083	4.74737387	0.04153203	0.07479291
Rbm33	-0.4111538	5.58997343	4.74285468	0.04161949	0.07493399
Lrrtm3	-0.3487297	4.97338521	4.7417481	0.04164093	0.07495619
Calb1	0.32111871	6.05115069	4.74104816	0.0416545	0.0749642
Nomo1	0.35957185	5.3458177	4.73863618	0.04170131	0.07502715
Slc2a2	-1.882073	-0.36637	4.73810163	0.0417117	0.07502715
Dscaml1	-0.7272785	3.82701036	4.73783547	0.04171687	0.07502715
Nxn	0.64868259	5.05717024	4.73585807	0.0417553	0.07507984
Bbs2	-0.4083943	5.17881808	4.73490715	0.0417738	0.07509667
Nqo1	0.80034827	4.40171669	4.73342828	0.04180259	0.07513199
Bcr	-0.4134602	6.07003854	4.73141102	0.04184189	0.07518619
Arhgef4	-0.3826409	5.08822958	4.72980928	0.04187312	0.07522587
A930005H10	0.64306748	3.04340925	4.72796293	0.04190917	0.075255
Swi5	0.42035581	5.71478352	4.72791163	0.04191017	0.075255
Ggt7	-0.5931438	2.63432091	4.72757156	0.04191681	0.075255
Cmtm7	0.73827141	1.86623402	4.72571739	0.04195304	0.07530361
Cat	0.36072614	8.12823621	4.72381083	0.04199034	0.07533773
Mtrf1l	0.43117246	4.04188522	4.72380853	0.04199039	0.07533773
Slc9a7	-0.5441836	4.25515932	4.72024489	0.0420602	0.07544652
Cdc42se1	0.37832999	5.51171644	4.7188025	0.0420885	0.0754808
Dnajb11	0.29217199	5.90712065	4.71741612	0.04211571	0.0754993
Gm15446	-0.8884285	1.42491026	4.71734119	0.04211718	0.0754993
Cdv3	0.25697979	7.74731071	4.71602733	0.042143	0.0755291
Wls	0.45678096	5.22275416	4.70848743	0.04229148	0.07577869
Cdh22	-0.9572706	1.76906801	4.70750833	0.0423108	0.07579679
Pitpnm2	-0.3777007	6.50109081	4.70603198	0.04233996	0.07583249

Gm5431	-1.0524892	0.93549401	4.70430406	0.04237412	0.07587713
Rbm24	-0.4997341	3.97278208	4.70280788	0.04240372	0.0759136
Tbce	-0.388699	4.40846817	4.70179169	0.04242383	0.07593307
Fbln2	-1.0705064	0.80247329	4.69657416	0.0425273	0.07610168
Apol9a	2.13585379	-1.0042618	4.6959803	0.04253909	0.07610622
Ccdc146	1.00646085	1.56847198	4.69217635	0.04261473	0.07622495
Commd5	0.78713412	2.05512367	4.6909122	0.0426399	0.07625337
Fnbp1	-0.2476295	6.73205691	4.68493082	0.04275922	0.07645012
Capn7	-0.2803482	6.54872315	4.6797808	0.04286226	0.07661769
9330179D12I	1.14536191	1.07492761	4.67889387	0.04288003	0.07663078
A930013F10I	-1.1243935	1.71661462	4.67789394	0.04290008	0.07663078
Chl1	-0.485619	7.47835662	4.67763302	0.04290531	0.07663078
Tet1	-0.3253225	6.20291372	4.677555	0.04290688	0.07663078
Idh3g	0.27860092	6.41635125	4.67591672	0.04293976	0.07667284
Tle4	-0.3592079	6.40043195	4.67365638	0.04298516	0.07673305
Dact3	-0.3166416	4.11706436	4.67330257	0.04299228	0.07673305
Nek10	-1.6404007	0.42378789	4.67284453	0.04300149	0.07673305
Fam217b	-0.3690267	4.010407	4.67100596	0.04303848	0.07678239
Gm17066	-0.4823986	4.80154573	4.66964161	0.04306595	0.07681473
Tnks2	-0.2538394	8.33239635	4.66693623	0.04312049	0.07689532
Zfp266	-0.2398248	6.88720034	4.66419911	0.04317574	0.07697715
Prodh	-0.6886606	2.14169972	4.65972497	0.04326624	0.07709935
St6galnac5	-0.3696914	4.74676532	4.65945856	0.04327163	0.07709935
Pfdn5	0.58128986	5.7391927	4.65941941	0.04327242	0.07709935
Kcnrg	-1.4440655	1.30895024	4.65801407	0.0433009	0.07713336
Atp8b5	-2.1164515	-0.868689	4.65620346	0.04333762	0.07718204
Msln	0.90585211	2.22083589	4.65505367	0.04336095	0.07720687
Cpsf7	-0.3603623	5.71114193	4.65422787	0.04337772	0.07722
Pla2g4c	1.37957298	0.09718778	4.64937324	0.04347644	0.0773578
C7	-1.1531514	0.35357518	4.64909501	0.04348211	0.0773578
Zhx1	0.26360378	8.00743807	4.64903344	0.04348336	0.0773578
Vps4a	0.31712101	5.55781122	4.64561607	0.04355302	0.07744878
Hykk	0.43766706	5.34662653	4.64560012	0.04355335	0.07744878
Golga3	0.2218605	6.79705468	4.6447368	0.04357097	0.07745637
Asah1	-0.3271739	5.68220169	4.64446748	0.04357647	0.07745637
Cabyr	-0.5462159	4.12377264	4.64123449	0.04364253	0.07755689
4931403G20	-1.1352334	0.89301541	4.64077661	0.04365189	0.07755689
Ccdc97	0.34809153	5.32492824	4.6395616	0.04367676	0.0775843
Pcp4l1	0.41967101	6.03176788	4.63811996	0.04370628	0.07761996
Syt10	-0.6419131	2.31250601	4.6362432	0.04374474	0.07766134
Med27	0.63970259	3.23350047	4.63606123	0.04374847	0.07766134
Mzf1	-1.146185	0.2685717	4.6338725	0.04379338	0.07772428
Bmf	0.67063328	2.81806401	4.63270226	0.04381742	0.07775014
Repin1	-0.3739799	4.53713337	4.62896854	0.0438942	0.07786958
Trappc10	-0.2935173	5.8965905	4.62723088	0.04392999	0.07791625

Scaf8	-0.2927126	5.88073503	4.62514696	0.04397295	0.07797562
Klf8	-0.4457496	2.88497504	4.62388449	0.043999	0.07798983
Plxnb1	-0.4606545	4.42722131	4.62346045	0.04400776	0.07798983
Mff	0.25179988	7.56692729	4.62323923	0.04401232	0.07798983
Acp1	0.29150027	7.14125661	4.62291979	0.04401892	0.07798983
Oxsr1	-0.2809268	5.96237437	4.61952797	0.04408903	0.07808914
Ptn	0.63776041	10.3823205	4.61906181	0.04409868	0.07808914
Rgl1	0.22459966	7.58694642	4.61882984	0.04410348	0.07808914
Nudt18	0.54636047	3.81344971	4.61821687	0.04411617	0.07809479
Gpbp1	0.2637378	7.99359467	4.61749109	0.0441312	0.07810457
Med14	-0.3739607	7.67537787	4.61696562	0.04414208	0.07810702
Wif1	1.97378127	-1.1356309	4.61529213	0.04417677	0.07814016
Gpr63	-0.5864096	3.16893815	4.61463002	0.0441905	0.07814016
Igf2	-0.529172	10.4379258	4.61460716	0.04419098	0.07814016
Hsd17b14	1.04973533	0.86079391	4.61383959	0.0442069	0.07814016
Pomgnt1	-0.5299512	2.95261862	4.61343691	0.04421526	0.07814016
Add2	-0.3923201	7.61685825	4.61316247	0.04422096	0.07814016
1810041L15I	-0.4694661	5.52631739	4.6124322	0.04423612	0.07814016
Ppil6	-0.9572633	2.35318332	4.61239593	0.04423687	0.07814016
4930578C19I	-2.9713315	-1.5017189	4.6116549	0.04425227	0.07815056
St8sia4	-0.5743664	3.27236278	4.61069722	0.04427217	0.07816891
Wdr6	-0.3580789	4.85577563	4.60887474	0.04431007	0.07821082
Noc2l	-0.4418683	4.00868273	4.60858712	0.04431606	0.07821082
Plxnb2	-0.3434109	4.78627518	4.60818381	0.04432445	0.07821082
Aqp6	-3.2468276	-1.3654759	4.60662173	0.04435698	0.07825142
Dmc1	-2.5269812	-1.4697402	4.60581713	0.04437374	0.07826419
Gm6537	-2.5207858	-1.9734939	4.6044144	0.04440299	0.07826632
Ccnf	1.19873451	0.81005706	4.60428587	0.04440567	0.07826632
Scel	0.52786352	3.33749651	4.60371984	0.04441748	0.07826632
Suds3	0.40971286	5.04632402	4.60348475	0.04442239	0.07826632
Ticrr	-2.0235974	-0.3232316	4.60347626	0.04442256	0.07826632
Gm6654	1.10552243	0.80954439	4.60167343	0.0444602	0.07831585
Uqcrc2	0.21897842	6.7286354	4.60054705	0.04448374	0.07834051
Hs6st2	-0.4902671	4.82029221	4.59990364	0.04449719	0.07834741
Nbn	0.32591339	4.74914227	4.59888304	0.04451853	0.07836821
Eif6	0.53698328	4.38628276	4.59664085	0.04456546	0.07843403
Ppp1r15a	0.85278624	2.79105364	4.59420387	0.04461654	0.07849851
Vsig10	-1.1146435	1.46708961	4.59398138	0.04462121	0.07849851
Nacad	-0.4956743	3.25361314	4.59314157	0.04463882	0.07850321
BC022687	0.7240734	1.99778136	4.59294359	0.04464298	0.07850321
Alx3	0.74782534	3.2206397	4.5888825	0.0447283	0.07863641
Sdad1	-0.2973	4.79160737	4.58819828	0.04474269	0.07864441
Pyurf	0.4800018	5.11563781	4.58775665	0.04475198	0.07864441
Ptov1	0.56466463	5.14555831	4.58620971	0.04478455	0.07868482
Prph	3.06784519	-0.8824467	4.58569481	0.0447954	0.07868705

Abhd17b	0.35827595	5.76779969	4.58486004	0.04481299	0.07868986
Sfrp4	0.55757845	3.4712962	4.58457155	0.04481907	0.07868986
Paip2b	0.29053565	5.25895448	4.58425631	0.04482571	0.07868986
Atg4d	-0.7381965	2.16866913	4.58251608	0.04486242	0.07873748
Ntmt1	0.60091973	2.14595237	4.58039111	0.04490729	0.07879654
Scarna3a	-1.9998995	-0.9411287	4.57969501	0.044922	0.07879654
Tesc	0.5396823	3.01370342	4.57956107	0.04492483	0.07879654
Hnrnpul2	0.22449254	8.048024	4.5783759	0.04494989	0.07882367
Casd1	-0.3352572	5.80115426	4.57697542	0.04497952	0.0788452
Smarcad1	-0.4050028	5.2613754	4.57688888	0.04498135	0.0788452
Dpysl4	-0.6198311	2.8588484	4.57418262	0.04503868	0.07892885
Sfxn2	-0.5569734	2.52378903	4.57245921	0.04507523	0.0789667
Mis12	0.34740262	5.24682689	4.57225813	0.0450795	0.0789667
Impa1	0.28190426	5.28422292	4.56983832	0.04513088	0.07903089
Mir124a-2	-1.7038272	-0.8553492	4.56962685	0.04513537	0.07903089
Srsf4	0.57939394	2.37820875	4.56847338	0.04515989	0.07904571
09-Sep	0.45311501	5.57392956	4.5683239	0.04516307	0.07904571
Clint1	0.23472822	6.88746452	4.56746056	0.04518143	0.07906101
Lmbr1l	-1.0123617	0.32699274	4.56500798	0.04523364	0.0791302
Aldh3a2	0.25629374	6.15963536	4.56469889	0.04524023	0.0791302
Prrt1	0.37080291	4.88909322	4.5642384	0.04525004	0.07913053
Hist1h2bm	0.96748979	1.35635533	4.56358698	0.04526392	0.07913797
Them6	-0.648248	2.62308151	4.56252682	0.04528653	0.07916066
Acr	-1.7513091	-0.0750512	4.561701	0.04530415	0.07917327
Pcmt1	0.24395707	6.42797914	4.561097	0.04531704	0.07917327
Mpc2	0.32151377	5.93669597	4.5608345	0.04532265	0.07917327
Bckdk	0.34788238	4.30818593	4.55970236	0.04534683	0.07918606
Robo3	-1.6543273	2.01714321	4.55958945	0.04534924	0.07918606
Rnd1	-0.8630413	1.29585045	4.5589088	0.04536378	0.07919464
Prmt1	0.67219652	2.8682413	4.55746079	0.04539474	0.07922049
Kpnb1	0.25794902	8.29382347	4.55731464	0.04539787	0.07922049
9430076C15I	2.56487228	-1.4963144	4.55433647	0.04546163	0.0793149
Gdf3	-1.6115366	-0.750508	4.55344107	0.04548081	0.07933154
Gm266	-1.0962201	0.6425385	4.55205072	0.04551063	0.0793667
Samd1	0.66184676	2.79565482	4.55153723	0.04552164	0.07936907
Pts	0.38179542	5.2535485	4.55014069	0.04555162	0.07939034
Arnt2	-0.2582305	8.03920942	4.55006909	0.04555316	0.07939034
Tmem11	0.502772	3.33295823	4.54899118	0.04557631	0.07941297
Tsr3	0.3782336	4.06406222	4.54856512	0.04558547	0.07941297
1700028E10I	-1.8330212	-0.7948921	4.54666993	0.04562622	0.07946712
Ppip5k1	-0.4600244	5.49143345	4.54575556	0.0456459	0.07947875
Tmem64	0.41476381	8.87291316	4.54546091	0.04565224	0.07947875
Lnx1	-0.4336628	4.01709519	4.54477294	0.04566705	0.0794877
Susd4	-0.3270466	4.42295152	4.54344468	0.04569567	0.07952067
Lrrk1	0.53473433	5.08688029	4.54289312	0.04570755	0.07952452

Lrrc27	0.58408008	3.21464008	4.54149635	0.04573768	0.07956008
Samd15	-0.7706872	2.41371152	4.54018172	0.04576605	0.07958111
E030024N20	0.29572235	5.67388708	4.54003898	0.04576913	0.07958111
Wfdc2	1.98259984	-0.6818079	4.53806444	0.04581178	0.07963843
2810442N19	-1.5755945	0.21262058	4.53707542	0.04583316	0.07965875
Sall3	-0.6612417	1.87691425	4.53497484	0.04587861	0.07971985
Prc1	-1.0178892	1.22386664	4.53455414	0.04588772	0.07971985
Scarna3b	-2.6471523	-1.0973364	4.53387404	0.04590245	0.07972859
5430435G22	0.57986046	4.44542886	4.5327782	0.0459262	0.07975298
Igbp1	0.39594402	5.79648607	4.53200344	0.04594299	0.0797582
Slitrk3	-0.4315282	6.08726695	4.53174447	0.04594861	0.0797582
Nkain1	-0.3360198	4.40543864	4.53128067	0.04595867	0.07975881
Sin3a	-0.2626135	6.53073228	4.52960811	0.04599497	0.07980496
5830415F09I	2.17427395	-0.7140421	4.52882591	0.04601195	0.07981758
Poldip3	0.3780245	5.76822409	4.52837321	0.04602179	0.07981779
Hs3st3b1	0.58531167	3.71015302	4.52676665	0.04605671	0.07985888
Arrdc2	0.89821023	1.72076775	4.52626829	0.04606754	0.07985888
Selm	0.61174121	4.47033158	4.52594273	0.04607463	0.07985888
Plaur	2.33130093	-1.2438699	4.52418387	0.04611291	0.07990838
Pgp	0.34189965	3.71346144	4.52252892	0.04614896	0.07994193
Sbf1	-0.5050501	4.72245381	4.52240241	0.04615172	0.07994193
Emc3	0.40559204	5.9107534	4.5214813	0.0461718	0.07994392
Znhit2	0.52892093	3.10195257	4.52145718	0.04617233	0.07994392
Prpf8	-0.329214	8.26146319	4.52006839	0.04620262	0.07997874
Gm6994	-1.3916506	0.04688228	4.51964346	0.0462119	0.07997874
Zfp472	-0.736129	1.75869423	4.51786245	0.04625079	0.08002288
Zfand1	0.49311106	4.29971391	4.51758414	0.04625687	0.08002288
Fam129a	0.54060546	3.7238136	4.51639847	0.04628279	0.08005087
Snrnp40	0.44027254	4.37384869	4.51204553	0.04637809	0.0801857
Slc24a2	-0.4030336	9.69693903	4.51174367	0.0463847	0.0801857
Chrna2	-1.7444851	-0.6586616	4.51150154	0.04639001	0.0801857
Vps29	0.31377443	5.86087869	4.51033709	0.04641555	0.08021297
Olig1	-0.3809251	4.07031576	4.50969502	0.04642964	0.08022045
Hibadh	0.42315328	5.59095261	4.50916373	0.0464413	0.08022374
Dip2a	-0.4381822	5.45376591	4.50581925	0.04651479	0.08030192
Klrk1	1.38673874	0.53259997	4.50579547	0.04651531	0.08030192
Gdap1	-0.4066315	7.46345264	4.50564376	0.04651864	0.08030192
Brd8	-0.2773914	5.86129526	4.50532563	0.04652564	0.08030192
Hyls1	0.60615844	2.25186842	4.50387054	0.04655766	0.08034032
Ddx59	0.63004044	2.5780165	4.50319316	0.04657258	0.08034919
Dimt1	-0.3849247	5.04268064	4.49873733	0.04667083	0.08049994
C1s1	-1.2848718	0.94045248	4.4983422	0.04667955	0.08049994
BC052688	-0.6538621	2.15406068	4.49684761	0.04671257	0.08052983
Itpa	0.35004617	4.19649389	4.49667074	0.04671647	0.08052983
Bre	0.36666229	4.14336878	4.4959951	0.04673141	0.08053868

Mapk6	-0.2357626	7.5119382	4.49306174	0.04679631	0.08063363
Ddah1	0.29745208	7.1291285	4.49180201	0.04682422	0.08066183
Ifngr1	0.528902	3.18392937	4.4914368	0.04683231	0.08066183
Map3k13	-0.5070462	5.16798129	4.48965903	0.04687173	0.08070916
Dlg4	0.2794841	7.45345862	4.48931221	0.04687942	0.08070916
Sart1	0.37190086	4.71131357	4.487725	0.04691466	0.0807529
1190007I07R	-0.745543	2.18462635	4.48605035	0.04695186	0.08080002
Celsr2	-0.3442076	7.16649663	4.48347158	0.04700922	0.0808818
B430319G15	0.78567778	1.96980397	4.48277263	0.04702478	0.08088436
Rbm18	0.31974565	6.24774405	4.48236142	0.04703394	0.08088436
Gm7854	-1.5550221	0.00976066	4.48207886	0.04704024	0.08088436
Wibg	0.9141758	2.09290631	4.48027291	0.04708048	0.08090367
Scml4	0.4968383	4.00497164	4.48027197	0.0470805	0.08090367
Cecr6	-0.5196228	4.67755729	4.48024984	0.047081	0.08090367
Hspb11	0.57681136	3.06169139	4.47931142	0.04710193	0.08092272
Mrpl41	0.35122562	4.74946959	4.47842989	0.0471216	0.08093959
Tmem71	1.80632288	0.03806179	4.47700756	0.04715335	0.08096847
Fgf9	-0.4072415	4.84515337	4.47679448	0.04715811	0.08096847
Ap4m1	-0.9719123	1.14471779	4.47552602	0.04718646	0.08098988
Ppargc1a	-0.332734	7.95652804	4.47535465	0.04719029	0.08098988
Gltp	0.45016647	5.52220233	4.47258936	0.04725216	0.08107913
1700011I03R	1.51186203	-0.0163869	4.47115563	0.04728427	0.08111627
Map4k3	-0.2925435	6.59915635	4.47074194	0.04729354	0.08111627
Prnp	-0.315912	8.00642486	4.4674476	0.04736744	0.08121241
Tab3	-0.2681639	6.0446983	4.46729858	0.04737079	0.08121241
Mpp1	0.26504839	5.83676708	4.46692236	0.04737924	0.08121241
Tlcd2	-0.7264571	1.37738879	4.46275349	0.04747297	0.08135611
Faf2	-0.3759135	4.40507578	4.46207548	0.04748823	0.0813653
Tspyl1	0.21859672	7.44783897	4.46115038	0.04750907	0.08136544
Napb	0.29264514	9.75654075	4.46106029	0.0475111	0.08136544
Cyp11a1	-1.3785357	-0.1870064	4.46043925	0.04752509	0.08136544
Nol3	0.50011593	2.90621969	4.4603143	0.04752791	0.08136544
Hs3st1	0.41020886	4.20816129	4.45878497	0.04756239	0.08139949
Tmem200b	1.49812219	-0.2351335	4.45855367	0.04756761	0.08139949
Hebp2	0.43693752	3.40226414	4.45697107	0.04760333	0.08140641
Mir377	-2.4440907	-0.8233461	4.45679211	0.04760737	0.08140641
Braf	-0.2807655	8.58365271	4.45601863	0.04762484	0.08140641
Dr1	0.33513036	5.49912096	4.45601002	0.04762504	0.08140641
Iqck	-0.8429961	2.81758821	4.45586597	0.04762829	0.08140641
Polr2d	0.54116807	3.84926725	4.45544618	0.04763778	0.08140641
Ap2a2	0.24958702	6.88755799	4.45530437	0.04764098	0.08140641
Cmbl	0.64197609	6.17617277	4.45395892	0.0476714	0.08144145
Slc30a5	-0.3625579	3.98366903	4.45237516	0.04770723	0.08147588
Tcp1	0.21928384	7.08689799	4.45219202	0.04771138	0.08147588
Gm9839	-2.4100971	-0.419658	4.45062323	0.04774691	0.08151962

Flt3l	-0.8755381	1.70226686	4.44978867	0.04776582	0.08153497
Ilk	0.40753014	6.00172694	4.44801342	0.04780608	0.0815726
Prkag2	0.30775268	6.12234664	4.4479414	0.04780772	0.0815726
Htr2b	-1.0492526	0.19008035	4.44742803	0.04781937	0.08157555
Gprc5c	0.65677517	3.07802592	4.446577	0.04783869	0.08159157
Ctcf	0.25245413	6.37279785	4.44487569	0.04787734	0.08164055
Ufc1	0.37134381	5.14966962	4.44397675	0.04789777	0.08165845
Kirrel3	-0.5606103	3.45483046	4.44154643	0.04795307	0.08172487
Ube2e3	0.34089736	7.65512624	4.4413909	0.04795662	0.08172487
Prrc2b	-0.2712484	8.7073686	4.44051101	0.04797666	0.08174207
Aox2	3.36773935	-0.919169	4.43865141	0.04801904	0.08179733
Etfb	0.50874269	4.37938077	4.43404858	0.04812414	0.08195937
Zfp239	0.30178882	5.66201513	4.43329432	0.04814139	0.08197176
Bmp3	0.47971069	5.23358809	4.43267837	0.04815548	0.08197877
Cpsf1	-0.5240656	4.0570391	4.43199814	0.04817104	0.08198829
Haus3	0.38809676	4.11729922	4.43084589	0.04819742	0.08200468
Tspan17	-0.4092952	3.27720702	4.43070587	0.04820063	0.08200468
Cspg4	-0.5659411	2.74830297	4.42946686	0.04822902	0.082036
Snap29	0.30880073	6.39853214	4.42902241	0.04823921	0.08203635
Nhlrc2	-0.4057553	3.76158255	4.42485731	0.0483348	0.082127
Ppp1r3b	0.50062705	4.93530695	4.42460396	0.04834062	0.082127
Car5a	-1.445649	-0.2991194	4.42422436	0.04834934	0.082127
Aasdhppt	-0.3045729	5.24523466	4.42409751	0.04835226	0.082127
Lpcat1	-0.6487724	2.86572616	4.42324663	0.04837182	0.082127
Usp39	0.40709261	4.1659872	4.42318361	0.04837327	0.082127
Nbr1	0.27865921	7.89776255	4.42316743	0.04837364	0.082127
Rpl18a	0.43248906	7.4643089	4.42300295	0.04837742	0.082127
Dtna	-0.3015802	7.13003161	4.42268579	0.04838472	0.082127
Stambpl1	-0.4198953	3.56400113	4.42235036	0.04839244	0.082127
Tex15	-0.6957875	2.37812707	4.41809957	0.04849035	0.08225528
Smarca2	-0.2524281	9.78810952	4.41746684	0.04850494	0.08225528
Plcb2	-0.8793805	1.39988177	4.41731258	0.0485085	0.08225528
Chchd5	0.56395953	2.63589755	4.41729292	0.04850895	0.08225528
Cyp2c44	-2.4490789	-0.9963417	4.41689825	0.04851806	0.08225528
1810043G02	0.48906232	2.74556179	4.41523091	0.04855655	0.08229395
Lrrc8c	-0.4176704	4.05607155	4.41504282	0.0485609	0.08229395
Foxo1	0.28503431	7.15529798	4.41447989	0.0485739	0.08229902
Hgsnat	-0.2992101	5.17726335	4.41350978	0.04859632	0.08232004
Sar1a	0.38316031	7.05937469	4.41189352	0.04863371	0.08236639
Hcfc1	-0.3271307	6.90610262	4.40956654	0.04868758	0.08244065
Col23a1	0.54135039	5.23591138	4.40894772	0.04870192	0.08244768
Zfp1	0.33657961	4.28516805	4.40852156	0.0487118	0.08244768
Snx27	-0.2374869	7.29329358	4.4015271	0.04887424	0.0827056
Dmtf1	0.2783238	6.44770029	4.40035562	0.04890151	0.08272326
Rrp8	0.44454212	4.19096313	4.40021396	0.04890481	0.08272326

Foxred2	-0.5053296	2.99096199	4.39721355	0.04897474	0.08282449
Cradd	0.50623735	4.87140784	4.39150826	0.04910801	0.08301966
Kdm4a	-0.4904328	3.03882422	4.39140866	0.04911034	0.08301966
Zfp365	-0.3872821	9.10482669	4.39064823	0.04912814	0.08301969
Fbxo38	-0.2630353	5.22070644	4.3905448	0.04913056	0.08301969
Rpl34	0.81840499	0.76180846	4.38985283	0.04914676	0.08302042
Gpbp1l1	0.33222067	6.29620693	4.38966351	0.04915119	0.08302042
Ntn3	-1.055353	1.52422669	4.38892158	0.04916857	0.08302863
Spata1	-0.9260282	1.26760515	4.38859342	0.04917626	0.08302863
2810047C21I	0.83477473	1.87145865	4.38539582	0.04925126	0.08313818
Marf1	-0.2819061	7.87549306	4.3843399	0.04927605	0.08316295
Fbxl14	-0.345247	5.24253951	4.38343474	0.04929732	0.08318176
Gda	-0.4246945	7.16852614	4.3824429	0.04932063	0.08320402
Gm5105	-2.1166285	-0.6224718	4.38074241	0.04936063	0.08323253
Lmbrd1	-0.2453107	6.75262406	4.38057814	0.0493645	0.08323253
Mecr	0.97846526	1.30272389	4.38027648	0.0493716	0.08323253
Slc35e3	-0.349396	4.481118	4.37982939	0.04938212	0.08323253
Pcolce	0.62975306	4.76115866	4.37957281	0.04938817	0.08323253
Gpr149	-1.1678443	0.73379147	4.37911554	0.04939894	0.08323361
Lrrtm1	0.41636609	4.64472738	4.37700498	0.04944868	0.08330035
Mllt6	-0.2987016	6.01059421	4.37530427	0.0494888	0.08335086
Cdsn	1.01198947	0.95293506	4.37382279	0.04952378	0.08339269
Acn9	0.49194156	2.90800262	4.37225309	0.04956088	0.08343806
Tmed2	-0.3745962	5.71179949	4.37072428	0.04959704	0.08348057
Ngdn	0.48182986	3.72143171	4.36947112	0.0496267	0.08348057
Ndufa10	0.30256117	6.26299292	4.36935934	0.04962935	0.08348057
Olfr920	-2.6011184	-0.1309598	4.36916004	0.04963407	0.08348057
Zfp609	-0.2530213	7.47356055	4.36887697	0.04964078	0.08348057
Rapgef3	-0.4972454	2.66865458	4.36861113	0.04964707	0.08348057
Wdr34	-0.4790268	3.15942228	4.3668845	0.049688	0.0835227
Gtdc1	-0.3542955	6.26817212	4.36669647	0.04969245	0.0835227
Cblb	0.32938155	6.78147852	4.3650353	0.04973187	0.08357186
Egfr	-0.4308846	5.96327822	4.36198019	0.04980444	0.08367671
Cbx3	0.31513993	8.36237764	4.36053926	0.04983872	0.08371718
Yy2	-1.3291828	-0.0337332	4.35814587	0.0498957	0.08379577
Dctn5	0.33624454	6.19143987	4.35523461	0.04996512	0.08389521
Gm10406	-1.5354158	-0.4766145	4.35445767	0.04998366	0.08390255
Tmem229a	-0.3386437	6.08972277	4.35404908	0.04999342	0.08390255
Dnah5	-1.0755928	2.34714386	4.35376884	0.05000011	0.08390255
Kctd15	-0.7517938	2.12360408	4.35322093	0.0500132	0.08390738
9430041J12F	-0.9843658	1.8680772	4.35150056	0.05005432	0.08394674
Larp6	0.4011194	3.96276884	4.35114905	0.05006272	0.08394674
R74862	0.35940397	4.58028853	4.35061551	0.05007548	0.08394674
Cep192	-0.5247433	4.46641486	4.35053056	0.05007752	0.08394674
Exosc6	0.45305228	3.34859601	4.34986384	0.05009347	0.08395636

Arsj	0.99608925	0.86203898	4.34669898	0.05016928	0.08405932
Zscan18	-0.4272024	3.71227459	4.34644562	0.05017536	0.08405932
Kif13b	-0.4046376	3.67690673	4.34589432	0.05018858	0.08406434
Jmy	-0.253868	7.18953639	4.34350036	0.05024604	0.08414343
Kars	0.33755239	5.13841216	4.34008668	0.0503281	0.08426368
Chtf18	-2.2971655	-1.2665603	4.33759508	0.05038809	0.08434694
Etohd2	-0.9140005	1.40307632	4.33701891	0.05040197	0.08435301
Park7	0.42491355	4.76409095	4.33400691	0.05047463	0.0844574
Myh15	-2.8497128	-1.1483054	4.33155972	0.05053375	0.08453911
1700008F21I	-1.3666602	0.94287761	4.33058921	0.05055722	0.08456116
Copg2	-0.4192139	5.85953571	4.32942148	0.05058547	0.0845912
Klhl9	-0.2356946	7.39308747	4.32886921	0.05059884	0.08459635
Asb11	-0.9727801	0.90161594	4.32822281	0.05061449	0.0846053
Atp6ap1l	-1.1440113	1.11229166	4.32535146	0.05068408	0.08470441
Cwc15	0.35689065	6.787145	4.32258312	0.05075128	0.08479947
Ptprg	-0.2993216	6.91792331	4.3218595	0.05076886	0.08480334
Shank3	-0.4354101	4.59092802	4.32115163	0.05078607	0.08480334
LOC1005050	-1.4073859	0.48424062	4.32095038	0.05079096	0.08480334
Cchcr1	-1.0499981	0.99538075	4.32078983	0.05079487	0.08480334
1700034H15I	-0.8616523	2.06910052	4.32009913	0.05081167	0.08481415
Rhno1	0.51904721	2.51360383	4.31734304	0.05087876	0.08490828
Llgl2	1.27162985	-0.2605733	4.31607491	0.05090967	0.08490828
Itpr2	-0.2977617	5.33781271	4.31602316	0.05091093	0.08490828
Cmtm4	-0.2737426	6.3562398	4.31595275	0.05091264	0.08490828
Ccdc132	-0.3041016	6.47996057	4.31566292	0.05091971	0.08490828
Gpr162	-0.8588061	2.14572844	4.3152279	0.05093032	0.08490875
Mipol1	0.41847995	3.67320862	4.31400153	0.05096024	0.0849414
Atg9a	-0.3591864	5.13802662	4.31242042	0.05099885	0.08496634
Klhl14	-0.8639553	1.43658351	4.31224998	0.05100301	0.08496634
Psme2	0.49396837	5.28007112	4.31211878	0.05100622	0.08496634
Islr	0.71270195	6.83435633	4.3110729	0.05103178	0.0849917
Sec16a	-0.2878413	6.43811088	4.3101585	0.05105414	0.08501171
Ptprs	-0.3378689	6.88843335	4.30920874	0.05107737	0.08503282
Tspan11	-0.6596739	3.38958987	4.30879465	0.05108751	0.08503282
Zfpm2	-0.6062503	3.7385624	4.30818575	0.05110242	0.08504041
Mrps34	0.52587766	3.02931867	4.30579231	0.05116106	0.08512077
Zfp398	-0.3659424	4.49869841	4.30185278	0.05125775	0.08526438
Rps21	0.43897222	5.20132058	4.30108512	0.05127661	0.08527373
Hif1a	-0.3123599	6.51471301	4.30077965	0.05128412	0.08527373
Nkx2-1	-0.8777166	1.90266197	4.29913977	0.05132446	0.085306
S1pr4	-1.3780433	-0.8166348	4.29874632	0.05133414	0.085306
Heca	0.34804688	4.53730829	4.29794726	0.05135381	0.085306
Vill	-0.8295393	1.24115167	4.29790614	0.05135482	0.085306
Klrd1	-1.3641065	-0.5107385	4.29788183	0.05135542	0.085306
Sik3	-0.3151502	7.39081185	4.29667672	0.05138511	0.08533488

Tmem178b	-0.3394152	7.54212313	4.29633316	0.05139357	0.08533488
Pck2	0.52609964	3.2418545	4.29584595	0.05140558	0.08533757
Mbd2	0.3348332	7.38678796	4.29520689	0.05142134	0.08534649
Ola1	0.2334484	7.60669843	4.29357999	0.05146147	0.08538538
Cdc40	-0.2562566	6.87260442	4.29341502	0.05146555	0.08538538
Gcfc2	-0.7846881	2.09808493	4.29284263	0.05147968	0.08539159
Trp53bp2	-0.273067	5.93993044	4.29157297	0.05151104	0.08542637
Triobp	0.51298734	6.03592652	4.29101683	0.05152478	0.08543193
Gm5577	-1.8472764	1.02627357	4.28957259	0.05156049	0.08547389
Krt10	0.62876649	2.23718246	4.28820132	0.05159443	0.0855129
Lcorl	-0.3237027	5.13712707	4.28444448	0.05168752	0.08564993
Dnaja3	0.30611971	5.03685258	4.28400997	0.0516983	0.08565052
Akap14	-1.9130665	-0.9875609	4.28349509	0.05171108	0.08565271
Nelfb	0.35758007	5.02264515	4.28265794	0.05173186	0.08565271
Kcnh5	-0.4326443	5.78019818	4.28257465	0.05173393	0.08565271
Gabrd	-0.7723632	2.84095757	4.28212557	0.05174508	0.08565271
Max	0.30800679	8.08866805	4.28176975	0.05175392	0.08565271
Cacng5	-0.6699379	2.68079669	4.28143839	0.05176215	0.08565271
Nek11	1.12757295	0.02576182	4.27732037	0.05186459	0.08580494
Gstm7	0.56317205	4.40010318	4.27659522	0.05188265	0.08581755
Pitpnc1	-0.2415275	6.72428963	4.27592613	0.05189932	0.08582785
Tmem72	2.7259482	-1.1869953	4.27473068	0.05192913	0.0858456
Bpnt1	0.25819594	6.08443902	4.27465782	0.05193094	0.0858456
Xpot	-0.2429998	7.00853197	4.27403763	0.05194641	0.0858539
Tmem230	0.28081645	6.66927818	4.27093965	0.05202377	0.08596446
Sema4c	1.08169169	0.62827725	4.27022365	0.05204166	0.08597675
C030039L03R	-0.5219105	4.05436571	4.26951218	0.05205945	0.08598886
Higd1a	0.28291471	6.64605834	4.26806032	0.05209578	0.08603157
Slc40a1	-0.6507003	2.11802595	4.266148	0.05214367	0.08608692
Ccdc125	0.65562935	2.84858073	4.26558931	0.05215767	0.08608692
Henmt1	-1.9234743	-0.5878007	4.26546782	0.05216072	0.08608692
Cenpn	1.19445498	1.17111086	4.26473102	0.05217919	0.08610012
Aspm	-0.999612	1.19774706	4.26163712	0.05225684	0.0861863
Ninj2	-1.8239463	-0.9280136	4.26161618	0.05225736	0.0861863
Tarbp2	-0.5631495	2.33320718	4.26139658	0.05226288	0.0861863
Fgd1	0.49065877	3.05365511	4.25664587	0.05238238	0.08636605
Bbx	-0.264629	7.92283414	4.25474656	0.05243025	0.08640869
Ocrl	-0.3441086	6.61188684	4.25446559	0.05243733	0.08640869
Tex9	-0.283345	4.98234399	4.25436836	0.05243978	0.08640869
Clk4	-0.3567686	5.76616854	4.25345627	0.05246279	0.08642927
Gm10653	-1.0384833	0.53454069	4.2520607	0.05249802	0.08645636
Nme2	0.45265434	6.80607066	4.25197148	0.05250027	0.08645636
Nucb1	0.52128958	4.56765034	4.25100235	0.05252475	0.08647934
Hdac1	0.40817629	5.29501835	4.25053972	0.05253644	0.08648127
Adamtsl1	-0.4742678	3.07913631	4.24902274	0.0525748	0.08651889

Fgf23	-2.6472439	-1.0142725	4.24880311	0.05258035	0.08651889
Mir350	-2.5002086	-2.0322071	4.24775219	0.05260694	0.08653772
Aebp1	0.61905953	7.19862456	4.24751868	0.05261285	0.08653772
Ndufs2	0.33295268	6.6411601	4.24650187	0.0526386	0.08656275
Tmtc3	-0.4167427	5.40573068	4.24551353	0.05266364	0.08657342
BC030867	-2.3574084	-1.5114321	4.24541423	0.05266616	0.08657342
Uba5	0.26055392	5.87438216	4.24466987	0.05268503	0.0865754
Acad8	0.32352049	4.24425617	4.24453567	0.05268843	0.0865754
Atf6b	0.47543052	3.13973333	4.24249719	0.05274015	0.08662756
Zfp362	0.32574155	5.19322583	4.24245375	0.05274125	0.08662756
Gpd1	0.38750447	4.49827584	4.23916563	0.05282479	0.08672023
Emx2	0.50585292	2.96208256	4.23868242	0.05283708	0.08672023
Lrpprc	-0.4694471	5.97469776	4.23816698	0.0528502	0.08672023
Fam109a	-0.8847588	0.94421152	4.23799132	0.05285467	0.08672023
Shb	0.63475776	1.70974903	4.2379526	0.05285565	0.08672023
Ttc37	-0.4834948	3.70231221	4.23774336	0.05286098	0.08672023
Ube2e1	0.46861014	4.09330134	4.23683895	0.052884	0.08674069
Ano2	-1.5959876	0.14009161	4.23592255	0.05290734	0.08675207
Ndufa3	0.42182481	4.66141307	4.23562528	0.05291492	0.08675207
Edf1	0.5466092	6.41286507	4.23532356	0.05292261	0.08675207
Cst6	1.83315992	-0.7388505	4.23368219	0.05296446	0.08680336
Gm16861	-0.6878456	2.44946528	4.23024973	0.0530521	0.08692172
Kcnj12	0.54170103	3.19352967	4.22975009	0.05306487	0.08692172
2310001H17I	2.38670776	-0.9587405	4.22900522	0.05308391	0.08692172
Aldh9a1	0.30743139	5.52377629	4.22899923	0.05308406	0.08692172
Gm6710	0.48870641	2.44380817	4.22878461	0.05308955	0.08692172
Ppapdc2	0.24845663	6.28486106	4.22824054	0.05310347	0.08692719
Crybb1	2.74134386	-1.8920045	4.22731113	0.05312726	0.08694881
Ankrd1	-2.0327568	-1.3890658	4.22627402	0.05315381	0.08697495
Prr14l	-0.3267324	7.45751808	4.22525046	0.05318004	0.08697569
Alg9	-0.616729	2.99543005	4.22508897	0.05318417	0.08697569
Cdk5rap2	-0.4294179	3.57249977	4.22462861	0.05319597	0.08697569
Mal2	-0.2480445	6.39667895	4.22460455	0.05319659	0.08697569
BC016579	-3.1721454	-1.2504356	4.22373777	0.05321882	0.08698388
1700020I14R	-0.3182793	6.32617778	4.2231764	0.05323322	0.08698388
Glg1	-0.3032892	6.66224155	4.22291427	0.05323994	0.08698388
Lacc1	0.42589767	4.5523204	4.22275885	0.05324393	0.08698388
Usp3	0.3327026	4.04294891	4.22071593	0.05329639	0.08705227
Cxcl5	-1.2087457	0.65863524	4.21922577	0.05333469	0.08709411
Smim11	0.49915817	3.79796202	4.21889471	0.0533432	0.08709411
Rpl23	0.34358917	7.17334771	4.21721334	0.05338646	0.08714743
4930451C15I	1.40484607	0.31137663	4.21664669	0.05340105	0.08715394
Shfm1	0.38027629	4.75682789	4.21341012	0.05348446	0.08726276
Rtp3	-1.5088706	0.44595136	4.21323561	0.05348896	0.08726276
Zdbf2	-0.5648719	5.97330262	4.21240907	0.05351029	0.08728023

Gltscr1l	-0.2732111	6.39227417	4.21188721	0.05352376	0.08728488
Fau	0.50530183	5.68970486	4.20992579	0.05357443	0.08735018
Tmem186	-0.5649848	2.68750825	4.20836756	0.05361472	0.08739853
Fxn	-0.6238845	2.26426653	4.20725635	0.05364347	0.08742806
9430018G01	-2.9995332	-1.5023933	4.20635681	0.05366676	0.087444
Ebpl	0.74179563	2.36077055	4.20605665	0.05367453	0.087444
Nipbl	-0.2815242	7.96640291	4.20489931	0.05370451	0.08747551
Sdc3	-0.3225726	5.2388551	4.20410291	0.05372516	0.0874918
Tstd1	-2.1090766	-1.3567026	4.20304434	0.05375261	0.08751917
Arl4c	-0.3671095	5.17834358	4.20126389	0.05379883	0.08757707
1700125H03	-2.3684684	-0.4861726	4.19938342	0.05384769	0.08763924
Rnaseh2b	0.45101314	4.32882291	4.19739564	0.05389939	0.08770602
Morf4l1	0.28699709	8.47922785	4.19682239	0.05391431	0.08771294
Slc35a3	-0.3260009	4.86209558	4.19582858	0.05394019	0.08773633
Atad5	-0.4484181	3.88604756	4.19509808	0.05395922	0.08773633
Nuak2	2.41701631	-0.498086	4.19504068	0.05396071	0.08773633
Slc6a7	0.39795274	5.20504357	4.19449793	0.05397486	0.08774197
Chp2	-0.8951202	0.84874003	4.1935222	0.0540003	0.08776597
Lmbrd2	-0.321383	6.12720119	4.19291924	0.05401603	0.08777418
Nim1k	0.45591836	4.19746749	4.19053782	0.0540782	0.08783817
Hps1	-0.8811746	1.40435611	4.19053735	0.05407821	0.08783817
Rgl2	-0.6092911	2.1835214	4.19018283	0.05408747	0.08783817
Capn5	0.29052467	5.56500213	4.18901239	0.05411806	0.08785333
Fcho1	-0.6184073	2.7911638	4.1890078	0.05411818	0.08785333
Clspn	-1.4690033	-0.1388371	4.18841868	0.05413359	0.08785638
Alkbh7	0.73575225	1.95290312	4.18811836	0.05414144	0.08785638
Nnmt	1.85467409	-0.2419865	4.18754576	0.05415642	0.08786291
Vmn1r65	-0.8150416	1.14576224	4.1871475	0.05416685	0.08786291
Arhgap31	0.33794525	6.53570096	4.18575921	0.05420319	0.08790452
Lims2	-0.5912446	2.67256742	4.18474419	0.05422979	0.08791398
Hlcs	0.37360858	4.42716132	4.18472008	0.05423042	0.08791398
Inpp5j	-0.5299319	3.41493072	4.18371151	0.05425686	0.0879395
Necap1	0.23932296	7.2058062	4.18309628	0.05427299	0.08794831
Pzp	-2.1078481	-0.8954508	4.18229041	0.05429414	0.08796523
Tnfrsf10b	-0.4990681	3.26675785	4.18159453	0.0543124	0.08797748
Card6	0.397116	4.85767612	4.17963631	0.05436384	0.08802987
Ppp1r3g	-1.0962769	0.59363059	4.17954766	0.05436617	0.08802987
Psmg4	0.88255393	1.72007138	4.17725949	0.05442635	0.08810995
Ccr2	0.73909182	2.65618563	4.17631728	0.05445115	0.08813116
Gal3st1	-1.1883175	0.20452388	4.17594728	0.05446089	0.08813116
Rwdd2b	0.75462381	2.25778466	4.17403658	0.05451123	0.08819526
Dmrtc1a	0.9277308	0.89570495	4.17284747	0.05454259	0.08822863
Cr2	-1.2741517	1.0796989	4.17030607	0.05460968	0.08829898
Nol8	0.26213567	6.46452917	4.16936507	0.05463455	0.08829898
Fyttd1	0.26765745	7.36104306	4.16931923	0.05463576	0.08829898

Mbtd1	-0.3123561	5.66318019	4.16895176	0.05464547	0.08829898
Pcdha1	-2.07529	-0.9128144	4.16881061	0.05464921	0.08829898
Actn3	2.12903205	-0.3285975	4.16836708	0.05466093	0.08829898
1110017D15I	-1.3044488	-0.1132766	4.16835369	0.05466129	0.08829898
Vta1	0.34266852	5.04032592	4.16759814	0.05468127	0.088312
Sidt1	-0.393148	5.12946762	4.16675505	0.05470358	0.088312
Gm20362	-1.1171899	0.71752703	4.16668392	0.05470547	0.088312
Ubn2	-0.3237879	7.06862462	4.16642491	0.05471232	0.088312
1700085C21I	-2.5255253	-1.704339	4.16567576	0.05473216	0.08832667
Ndufc1	0.42369307	5.67207683	4.16267834	0.05481161	0.08843753
9530059O14	-1.2097926	2.91368661	4.15838584	0.05492563	0.08860409
Ppp2r5d	0.34724331	5.52700385	4.15714239	0.0549587	0.08863312
Ube2i	0.28461656	7.37592641	4.15689879	0.05496519	0.08863312
Ap1s2	0.30518579	6.31247399	4.15620334	0.0549837	0.08864529
Dusp4	1.03109118	1.31826327	4.15580502	0.05499431	0.08864529
Epor	-1.0206382	0.39059471	4.15487104	0.05501918	0.088668
Trmt10c	0.38591363	4.78226766	4.1531982	0.05506378	0.08871285
Avpi1	0.43588856	3.24214639	4.15301731	0.0550686	0.08871285
Ankrd32	-0.4134649	4.11258224	4.1525219	0.05508182	0.08871675
Asb5	-1.0811448	0.5376072	4.15117147	0.05511786	0.08874496
Eci1	0.40013168	3.68755631	4.15105659	0.05512093	0.08874496
Vps8	-0.5721115	4.47332854	4.14805857	0.05520104	0.08885654
Apaf1	-0.4202838	3.97683856	4.14388465	0.0553128	0.08901901
Ddx47	0.28262052	5.91726426	4.14291596	0.05533878	0.08904338
Fam20a	-0.4869155	2.57563194	4.14227331	0.05535602	0.08905005
Mir6369	-1.6677765	0.57777275	4.14195386	0.05536459	0.08905005
Glul	0.32226897	10.5718556	4.13669065	0.05550604	0.08926009
Ssu72	0.34896069	5.2620379	4.13588447	0.05552775	0.08927753
Rnf40	-0.4785791	3.98065516	4.13480078	0.05555693	0.08930699
Kitl	-0.3720859	4.77964071	4.1332971	0.05559747	0.08935466
Mcm8	-0.4266517	3.21512786	4.13255411	0.0556175	0.08936249
Ash1l	-0.278116	9.15914459	4.13231027	0.05562408	0.08936249
Vars2	0.71696491	2.25968597	4.13162708	0.05564252	0.08937464
Rfk	0.52495351	9.48783152	4.12802259	0.0557399	0.08950674
Scd2	-0.2610121	8.33435945	4.12777718	0.05574654	0.08950674
Hip1	-0.4077793	4.55084302	4.12502858	0.05582094	0.0896087
Pdrg1	0.46091722	5.4404089	4.12444138	0.05583685	0.08961673
Ap3s1	0.32163581	7.18832894	4.12321166	0.05587019	0.08965273
Pip5k1c	-0.2898089	6.37559619	4.12262411	0.05588612	0.08965368
Rcsd1	0.49764425	4.59087504	4.1222436	0.05589645	0.08965368
Sim1	-1.8214616	-0.605711	4.12198346	0.0559035	0.08965368
Nr1h3	0.79223318	2.61585111	4.12137209	0.0559201	0.08966279
Zfp105	0.60457262	3.11122438	4.12072841	0.05593757	0.08967108
Gpx1	0.5937526	6.58973415	4.12037809	0.05594709	0.08967108
Metap2	0.23444034	7.30908151	4.11971551	0.05596509	0.08968244

Brca1	-1.0053557	1.08808677	4.11763004	0.05602178	0.0897558
Narg2	-0.4500441	3.98398958	4.11711425	0.05603581	0.08976078
Timp1	1.11367499	1.52982041	4.11600033	0.05606613	0.08977814
Sdk1	0.50800903	4.14071295	4.1159135	0.0560685	0.08977814
Nsmf	-0.3144261	5.93049297	4.1149016	0.05609606	0.08979081
Cd8b1	-1.2574354	-0.329234	4.11398356	0.05612107	0.08979081
Calhm2	-0.7374112	2.54101821	4.11374805	0.05612749	0.08979081
Abcb6	-0.9647545	1.61675515	4.11366897	0.05612965	0.08979081
Tmem69	-0.3556956	4.43179247	4.11361822	0.05613103	0.08979081
B3gat2	-0.7151574	2.95666919	4.11124623	0.05619574	0.08984872
Slc25a33	0.52279982	3.31598457	4.11081575	0.05620749	0.08984872
Tspan15	-0.6792414	1.86957769	4.11037513	0.05621952	0.08984872
Myh1	1.25349355	0.52930343	4.11030275	0.0562215	0.08984872
Celsr1	-0.6273081	2.35191803	4.11028836	0.05622189	0.08984872
Prpf39	-0.4731986	5.45336515	4.10895139	0.05625842	0.08988962
Rnh1	0.42083302	4.45654197	4.10769682	0.05629273	0.08992695
Mcam	-0.870616	1.72639642	4.10670431	0.05631988	0.08993752
D17Wsu92e	0.21446739	7.63463128	4.10665507	0.05632123	0.08993752
2210408I21R	-0.4949782	4.27033096	4.1051767	0.05636171	0.08998468
Tcte1	1.37127028	0.71897727	4.10444596	0.05638173	0.08999916
Phf1	-0.7128236	2.86487896	4.1028181	0.05642635	0.09004214
Aif1	0.96071676	2.15680055	4.1026646	0.05643056	0.09004214
Kif21a	-0.4074634	8.49741799	4.10003041	0.05650287	0.09014001
Acan	-2.1134104	-0.8003149	4.09872529	0.05653874	0.09016466
Psmb10	0.55699528	3.21934283	4.09837292	0.05654842	0.09016466
2810004N23	0.58192432	4.55543889	4.09765786	0.05656809	0.09016466
Wtap	0.24230221	6.35981409	4.09672436	0.05659377	0.09016466
Adamts1	0.44177548	4.34942457	4.09657315	0.05659793	0.09016466
Ncor2	-0.3124556	6.83000484	4.09654892	0.0565986	0.09016466
Thada	-0.3906193	5.05442858	4.09634633	0.05660417	0.09016466
Ppp3r1	0.22237947	10.0948401	4.09627708	0.05660608	0.09016466
Chadl	0.79504407	1.07999139	4.09583728	0.05661819	0.09016647
Inpp4b	-0.4601005	3.81977761	4.09496453	0.05664222	0.09018727
Alpl	0.70266263	3.38132943	4.0939865	0.05666917	0.09019983
Csde1	0.20324479	9.14077136	4.09381758	0.05667383	0.09019983
Prkaa1	0.26198348	5.69360873	4.09348376	0.05668303	0.09019983
Ccdc51	0.96410465	0.54766976	4.09240224	0.05671286	0.09021661
Map2k1	0.2089636	7.71419502	4.09195072	0.05672532	0.09021661
Krba1	-0.3522429	3.86026108	4.09190755	0.05672651	0.09021661
Fuom	0.47438907	3.47949281	4.0859976	0.05688986	0.0904535
Gabbr2	-0.3655211	7.12221704	4.08572259	0.05689747	0.0904535
C4a	-1.7275345	-0.5173054	4.08480068	0.05692301	0.09047659
Dak	0.70098324	2.76656113	4.08419688	0.05693974	0.09048569
Clec4g	2.1685058	-1.5949193	4.08287307	0.05697644	0.0905265
D19Bwg1357	0.29071463	5.51629324	4.0824054	0.05698941	0.09052961

Trim67	-0.8530921	1.22140215	4.08126314	0.05702111	0.09056246
Lnpep	-0.2245969	6.88376445	4.08049809	0.05704235	0.0905787
Dennd4b	-0.5249127	3.90586378	4.07903675	0.05708295	0.09060833
Adk	0.34333664	4.8192347	4.0790327	0.05708306	0.09060833
Lhx2	0.32135116	5.46482425	4.07830797	0.0571032	0.09062281
AB124611	1.60800974	-0.6492218	4.07757131	0.05712369	0.09063782
Dhdds	-0.333211	4.89381283	4.07693462	0.0571414	0.09064843
Oip5	-1.697442	0.22054863	4.07651773	0.05715301	0.09064933
Zfp354a	-0.3936333	3.36857815	4.07581402	0.0571726	0.09066291
Bet1	0.45120797	4.8666255	4.07493291	0.05719713	0.09068433
Ascc3	-0.4761119	5.75611155	4.07384072	0.05722757	0.09070443
Tubgcp4	-0.3944952	3.73131625	4.07368581	0.05723189	0.09070443
AI467606	3.14995945	-1.3976377	4.07325937	0.05724378	0.09070578
Rpl41	0.44171952	8.18340747	4.07269932	0.05725939	0.09071304
Dazap2	0.30635094	8.82880673	4.07158036	0.05729061	0.09074501
Col5a2	0.46265804	2.99859565	4.06937752	0.05735213	0.09081317
Zp3r	-3.4174113	-1.4150992	4.06924827	0.05735574	0.09081317
Barx2	0.72329264	1.91715805	4.06710645	0.05741563	0.09089048
Tgtp2	0.56596989	3.77523968	4.06654654	0.0574313	0.09089778
Bloc1s6	0.30462297	5.60075722	4.06337647	0.0575201	0.0910208
Papolg	-0.2380923	5.32489383	4.06203645	0.05755769	0.09106274
Tmprss7	1.20970112	0.24829116	4.06100776	0.05758656	0.09109089
Cyp2e1	-0.7623643	1.04891713	4.06022799	0.05760845	0.09110799
Gga2	0.39766748	4.47761348	4.05875181	0.05764993	0.09115605
Rap1b	0.31143004	7.25010556	4.05769014	0.05767979	0.09117753
Ppp1ca	0.45728964	5.51876175	4.05732082	0.05769017	0.09117753
Trappc12	0.28397555	5.73215981	4.05699976	0.05769921	0.09117753
Zfp507	-0.3254049	4.73466432	4.05614691	0.05772321	0.09117753
Lrrc16b	-1.1601499	2.98873128	4.05610999	0.05772425	0.09117753
Fcrl6	-1.2953697	0.28566598	4.05590284	0.05773008	0.09117753
Nme7	-0.5183391	3.52830339	4.05450888	0.05776934	0.09122201
Slc25a3	0.22013549	7.93988783	4.05344534	0.05779932	0.09123991
Slc16a9	0.50690192	4.83671912	4.05331897	0.05780288	0.09123991
Gm6484	-2.760528	-1.9123091	4.05022006	0.05789033	0.09136041
Rab2a	0.21588593	9.67092269	4.0495424	0.05790948	0.09136888
Rs1	-2.9083668	-0.2740352	4.04924309	0.05791794	0.09136888
Syt16	-0.2960701	6.11761101	4.04556884	0.05802188	0.09151529
Zfp410	-0.3687462	4.00281026	4.04318341	0.05808947	0.09160432
Nhsl1	0.34970471	7.15377938	4.04068344	0.05816041	0.09168143
Ulk4	-1.1688426	1.39967079	4.04067416	0.05816067	0.09168143
Dmd	-0.4721505	7.88618675	4.04012734	0.0581762	0.09168832
Ptcd3	-0.4101516	5.21562764	4.03950028	0.05819402	0.09168881
Dusp23	0.70697677	2.79903088	4.03875814	0.05821511	0.09168881
Mrpl55	0.50162772	3.61984209	4.03832737	0.05822736	0.09168881
Wdr37	-0.2430973	7.17723915	4.03830943	0.05822787	0.09168881

Htr1f	-0.8268808	1.8051509	4.03815376	0.05823229	0.09168881
Hsf4	-0.7908669	1.34637394	4.03680789	0.05827058	0.09173152
Reps2	0.23660172	9.58647786	4.03433548	0.05834099	0.09182477
Ankrd13a	0.3201203	4.90859285	4.03326638	0.05837146	0.09183891
Dhx9	-0.3687574	7.76847638	4.03301966	0.0583785	0.09183891
Znhit3	0.45615715	3.91894958	4.03284461	0.05838349	0.09183891
Gpatch8	-0.2386365	8.04888298	4.0316	0.058419	0.09187718
Rab9b	0.25138798	6.46327609	4.03082319	0.05844118	0.09188024
Mrpl50	0.39431502	5.7014081	4.03015761	0.05846018	0.09188024
Sostdc1	1.44030092	0.73496236	4.02983499	0.0584694	0.09188024
Gm1045	-2.6532035	-1.1786568	4.02978448	0.05847084	0.09188024
Zfp956	0.80623394	2.29736313	4.0295747	0.05847684	0.09188024
Kdm2a	-0.2419894	7.17003186	4.02911143	0.05849007	0.09188347
Lhfpl4	-0.2578788	6.02255288	4.02817919	0.05851673	0.09189462
Nxph3	-1.3205478	-0.3782904	4.02808106	0.05851953	0.09189462
Atp5e	0.5234088	5.23526752	4.02709986	0.0585476	0.09192113
H3f3a	0.49255264	8.1858611	4.02647325	0.05856553	0.09193172
Ssbp2	-0.2804045	6.26453336	4.02543975	0.05859512	0.09196061
Ubtd1	1.17696566	-0.1061916	4.0240462	0.05863505	0.09200106
Camkk2	-0.3977244	6.00634976	4.02325482	0.05865774	0.09200106
Dbndd2	-0.2538426	5.98223656	4.02285548	0.05866919	0.09200106
Notch3	-0.8365197	2.36530039	4.02274617	0.05867232	0.09200106
Kif3c	0.2916472	6.41499445	4.02258791	0.05867686	0.09200106
Zfp960	-0.3119922	4.46068238	4.02095866	0.05872362	0.09204221
R3hdm1	-0.358853	9.58790115	4.02089288	0.05872551	0.09204221
1190002N15	-0.3295616	5.34921163	4.01834447	0.05879873	0.09213795
A330048O09	-1.5503079	0.44010957	4.01798701	0.05880901	0.09213795
Psme4	-0.3637347	6.43544393	4.01739037	0.05882617	0.09214727
D6Wsu163e	-0.3504946	4.54406392	4.0150066	0.0588948	0.09223719
Pbk	1.69789764	0.03747487	4.01396504	0.05892481	0.09226134
G2e3	-0.4144086	4.38339843	4.01355298	0.05893669	0.09226134
Scd3	0.64246683	3.20431338	4.01330321	0.05894389	0.09226134
Nek3	-1.0220984	0.95365832	4.01120845	0.05900433	0.09233835
Cabp1	-0.5549109	2.88542464	4.0070896	0.05912338	0.09250704
Zfp652	0.27350931	6.34153792	4.0055977	0.05916656	0.09254311
Micall2	-1.3834836	0.41934992	4.00551531	0.05916895	0.09254311
Gapdhs	-1.0604131	1.29571558	4.00120889	0.05929382	0.09272077
Mrap2	1.16971168	1.52222856	3.99950735	0.05934324	0.0927804
Adc	-0.7534827	1.78714451	3.99752664	0.05940084	0.09285278
Eri2	-0.4096948	4.18640994	3.99699365	0.05941634	0.09285936
Ap4e1	-0.4700236	4.05886757	3.99511865	0.05947094	0.09292701
Nefm	-0.4482358	9.24354979	3.99387799	0.05950709	0.09296583
Hist1h2bb	1.63832694	-1.2061748	3.99163187	0.05957261	0.09304598
Vgf	0.60524982	3.63822286	3.99134337	0.05958103	0.09304598
Naa38	0.51327346	3.65795244	3.99043227	0.05960764	0.09306984

Klhl6	-1.3691194	0.02189517	3.9894386	0.05963667	0.09309749
Pafah1b2	0.21072042	8.35178325	3.98793	0.05968078	0.09314865
Rpl35	0.37768619	4.96906104	3.98502584	0.05976579	0.09326363
Nin	-0.2811487	6.83042218	3.98374149	0.05980343	0.09330465
Hexdc	-0.752956	1.95366144	3.98058502	0.05989606	0.09343143
Procr	1.21573707	1.61350465	3.97839504	0.05996042	0.09350507
Zbtb8os	0.56861012	4.06613915	3.97820458	0.05996602	0.09350507
5031434O11	-1.7582848	0.19315469	3.97735325	0.05999106	0.09352638
Gpx7	0.82286805	2.44494039	3.97543666	0.06004749	0.09359659
Derl2	-0.3904633	3.56033296	3.97168011	0.06015826	0.09375147
Opcml	-0.3440209	8.17137437	3.96919122	0.06023178	0.09384309
Bysl	0.40175292	3.83428023	3.96891697	0.06023989	0.09384309
Madcam1	3.18908603	-2.0561187	3.9676789	0.0602765	0.09388234
Gm16796	3.89962898	-2.0218992	3.96722848	0.06028983	0.09388531
Pdcd5	0.31277534	6.90457778	3.96491627	0.0603583	0.09397412
Donson	-0.5255514	3.37025423	3.96428641	0.06037697	0.09398538
Soat2	-0.6885414	1.76274704	3.96331249	0.06040584	0.09401253
A230001M1(	-1.148217	0.24697136	3.96277956	0.06042165	0.09401933
Sacm1l	-0.2990302	6.00551482	3.96138724	0.06046297	0.09405149
Slc38a4	0.89174895	1.56652704	3.96131199	0.06046521	0.09405149
Tax1bp3	0.60497536	4.64836874	3.95946302	0.06052013	0.09411912
Rai14	0.3582453	6.49851665	3.95807432	0.06056143	0.09414887
Padi2	-0.4629134	4.03186539	3.95788039	0.0605672	0.09414887
U2af1	-0.384474	4.10283801	3.95746049	0.06057969	0.09414887
Cnksr3	-0.6089172	1.78761728	3.95708293	0.06059092	0.09414887
Rnf114	0.39776313	5.39363236	3.95689423	0.06059654	0.09414887
Ephx2	0.36769921	3.85688729	3.95605893	0.06062141	0.09416971
Ctsh	0.5049816	4.97746503	3.95369094	0.06069198	0.09426151
Dock6	-0.3911018	3.65249162	3.95022165	0.06079553	0.0944045
4930488L21I	-1.2075654	-0.5601524	3.94932843	0.06082222	0.09442811
Ddx56	0.57727052	3.08038876	3.94572173	0.06093015	0.0945778
C920025E04I	2.32331718	-1.1611135	3.94471793	0.06096023	0.09459296
Ranbp9	0.21643893	7.07801354	3.94462776	0.06096293	0.09459296
Fra10ac1	0.31488948	4.40983291	3.94303259	0.06101076	0.09464607
Sh3rf3	-0.399864	5.61462486	3.94271823	0.06102019	0.09464607
Fundc2	0.34503834	7.72147033	3.94083494	0.06107673	0.09471589
Ubap1l	-3.0219136	-1.8406109	3.94027838	0.06109345	0.09472395
B630005N14	0.23458855	6.47885509	3.9393177	0.06112232	0.09475084
1700008J07I	0.55790837	3.20091997	3.93809298	0.06115915	0.09478171
Mtch1	0.26596026	6.41998609	3.93762729	0.06117317	0.09478171
Eif4e3	0.29094847	5.53897092	3.93750541	0.06117683	0.09478171
Zkscan4	-0.633511	2.2997498	3.93695706	0.06119334	0.09478942
Ndufs6	0.43288531	5.1640407	3.93607661	0.06121985	0.09481261
Hexb	-0.3313388	4.74106074	3.93566118	0.06123236	0.09481413
Cdkn3	1.89918534	-0.6948635	3.93502043	0.06125167	0.09482616

Bend5	0.48470078	3.27714719	3.93348346	0.06129801	0.09488002
Crnkl1	-0.334791	4.56709497	3.93278808	0.06131898	0.09489447
1700052N19	-0.3618007	3.84405497	3.93208993	0.06134005	0.09489447
Epha3	-0.4851769	2.92841282	3.93141271	0.0613605	0.09489447
9530077C05I	0.64837782	2.2458995	3.93129982	0.06136391	0.09489447
Lag3	2.21547038	-1.3344653	3.93104854	0.0613715	0.09489447
Stpg1	0.87978053	0.68737858	3.93087927	0.06137661	0.09489447
Zfp821	0.42993075	3.53873423	3.92991347	0.06140579	0.09491098
Abtb2	0.68683478	2.54631764	3.92976146	0.06141039	0.09491098
Socs3	0.76597354	1.04320367	3.92777895	0.06147034	0.09498578
Ncoa4	0.22337261	7.81367737	3.92665435	0.06150438	0.09502052
A230103J11F	0.73753925	1.99749863	3.92559518	0.06153646	0.0950305
Gm3435	-0.5398088	2.79609154	3.92524321	0.06154713	0.0950305
Brsk2	-0.3361761	5.66464878	3.92515657	0.06154975	0.0950305
Abcd2	-0.4254309	4.77158187	3.92482484	0.06155981	0.0950305
Trim33	-0.2107543	7.7052077	3.92445411	0.06157105	0.0950305
Ccpg1	-0.2717646	6.52446713	3.92390276	0.06158776	0.0950305
3000002C10I	0.48152637	2.14423524	3.92355843	0.06159821	0.0950305
Dtx1	0.37044808	4.62920153	3.92311511	0.06161166	0.0950305
Gabra6	5.72662316	-2.0502817	4.00723179	0.0616149	0.0950305
Gtpbp8	0.34452532	3.77131635	3.92208632	0.06164288	0.09503508
Sprtn	-0.6567528	1.57573087	3.92183617	0.06165047	0.09503508
Fam195a	1.94187453	-1.2383846	3.92176723	0.06165257	0.09503508
Papss1	0.28696325	4.96505417	3.92104259	0.06167457	0.09505118
D030047H15	-1.0005376	0.23058066	3.91953065	0.06172052	0.09510416
Slit2	-0.2746232	5.82072371	3.91783445	0.06177211	0.09516581
Mrpl49	0.4635489	4.77436411	3.91743622	0.06178423	0.09516664
Zfp871	-0.2724561	9.06013318	3.91620133	0.06182183	0.09519839
BC031181	0.39966704	6.05618873	3.9157984	0.0618341	0.09519839
Dlgap2	-0.4136469	6.60868622	3.91561828	0.06183959	0.09519839
2310010J17F	0.97339947	0.76878002	3.9142885	0.06188012	0.09523277
Gtf3c3	-0.3178774	5.1667821	3.91412547	0.06188509	0.09523277
Inpp4a	-0.3580041	6.53634804	3.91349965	0.06190418	0.09524431
Gm10033	-0.5140239	4.59439636	3.91162263	0.06196147	0.09531461
Pcdha12	-0.9614074	1.20786734	3.91032878	0.06200099	0.09535757
Trim25	0.38331796	6.41306615	3.90980805	0.06201691	0.0953642
Abi3bp	-0.4310421	4.18280323	3.9083561	0.06206131	0.09541161
Ifi44	0.60647847	3.33645629	3.90804082	0.06207096	0.09541161
Tdp2	0.44825413	3.61974665	3.90652653	0.06211732	0.09546501
Mrps24	0.49246155	3.95201043	3.90548496	0.06214923	0.0954962
Mir344c	-2.4964241	-1.6073122	3.90320386	0.06221917	0.0955858
Dlx2	-0.7858926	0.88539876	3.90187725	0.06225989	0.09563049
Pdcd2	0.378211	3.87021714	3.90062012	0.06229851	0.09566503
Tex26	1.48857477	-0.7564321	3.90038746	0.06230566	0.09566503
Kank4	0.54287969	3.92980639	3.89923176	0.06234119	0.0957017

Gpr101	-0.6994151	2.41118593	3.89807247	0.06237685	0.09573857
Gm17660	-2.8374209	-1.4513759	3.89741504	0.06239709	0.09575175
Top1	0.2737638	8.05108416	3.89671292	0.06241871	0.09576704
Larp4b	0.19577608	7.45689149	3.89355833	0.06251594	0.09588872
Atp13a2	-0.5136439	4.17613048	3.89253299	0.06254759	0.09588872
Dscr3	0.37448972	4.05739242	3.89248161	0.06254917	0.09588872
Ptgdr	0.48638792	5.28157991	3.89245329	0.06255005	0.09588872
Palmd	0.35980891	5.6060739	3.89223902	0.06255666	0.09588872
2810429I04R	-1.98599	-1.2896192	3.89187154	0.06256801	0.09588872
Sdhd	0.43386355	6.88424588	3.89115632	0.0625901	0.09590409
Gucd1	0.56622001	3.58927075	3.89079144	0.06260138	0.09590409
Igtp	0.52069312	3.72896167	3.88901427	0.06265632	0.09593987
Khdrbs1	0.2345554	6.94592177	3.88897106	0.06265766	0.09593987
Ptpn23	-0.3258866	4.86843946	3.88866746	0.06266705	0.09593987
Lrrc61	0.29858817	5.11850472	3.888526	0.06267143	0.09593987
Cmah	-0.3341182	6.36639796	3.88544244	0.06276692	0.09606816
Thra	-0.3579699	5.82827563	3.88458997	0.06279334	0.09609071
Fbxo45	0.24622783	6.25555646	3.88403231	0.06281064	0.09609929
Bivm	0.27353598	4.9555411	3.88282222	0.06284819	0.09613884
Rbx1	0.34686934	6.58727791	3.88111361	0.06290125	0.0962021
Col12a1	0.28610297	5.40904331	3.88035143	0.06292494	0.09620383
Mttp	-0.6305386	2.01336206	3.88032402	0.06292579	0.09620383
Eif4b	0.20752147	7.59682671	3.87944043	0.06295326	0.09622793
LOC1026344	-0.9778661	1.10325878	3.87844212	0.06298432	0.0962575
Rab10	0.24195597	8.51286959	3.87763064	0.06300958	0.0962782
Pcbp1	0.29265345	5.75618372	3.87689395	0.06303252	0.09629535
Ddx23	0.28936015	5.43142983	3.87636376	0.06304903	0.09630268
Plxna4	-0.3852	7.02214531	3.87592058	0.06306284	0.09630588
Pde8b	-0.3017171	6.1734771	3.87386455	0.06312695	0.09638587
Nrxn3	-0.3895091	8.1011022	3.87191313	0.06318787	0.09646096
Serpini1	-0.3050183	7.56108133	3.87052953	0.0632311	0.09650904
Lpcat4	-0.3314582	5.66208883	3.86775732	0.06331782	0.09662346
Pxmp4	0.39852953	3.31185349	3.86610592	0.06336955	0.09668444
Lrp12	-0.3104059	4.10002591	3.86455148	0.06341828	0.09674084
Slc4a7	-0.4188871	3.91386654	3.86352788	0.0634504	0.09677187
4930528D03	-1.4470226	-0.1350901	3.86214653	0.06349377	0.09681411
Pnn	-0.3343791	7.701744	3.86189535	0.06350166	0.09681411
Amotl2	-0.6093848	3.80934103	3.86038782	0.06354903	0.09685857
Dact2	0.57676424	2.14960065	3.86021758	0.06355438	0.09685857
Impad1	-0.2351344	7.02690267	3.85916175	0.06358759	0.09688089
Fam228b	-0.9526853	1.62913522	3.8587719	0.06359986	0.09688089
Fam161a	0.75176507	1.83373933	3.85862788	0.06360439	0.09688089
Coro1c	0.33945161	5.89259875	3.85815119	0.06361939	0.09688579
Dcakd	0.47413804	4.5004566	3.85494806	0.06372032	0.09701237
Eif3a	-0.3061533	9.48235141	3.85476404	0.06372612	0.09701237

Asb14	-1.4704053	0.03318188	3.85418856	0.06374428	0.09701565
Igflr1	-1.9715018	-0.7968711	3.85394745	0.06375188	0.09701565
Zdhhc3	-0.2671978	5.5882829	3.85286209	0.06378614	0.09704982
2810433D01I	0.56879629	3.1447098	3.85227116	0.0638048	0.09706024
Hist1h1b	2.26677836	-1.8051552	3.8506956	0.06385459	0.097118
Tmem252	-1.065986	-0.111035	3.85000809	0.06387633	0.09713309
Mamld1	0.29761281	6.24014348	3.84882848	0.06391365	0.09717186
Pbld2	1.11540343	0.65689591	3.84669549	0.06398119	0.09725656
Hsdl2	0.33568262	6.17222768	3.84520155	0.06402855	0.09729912
Mien1	0.41182137	5.3825051	3.8450654	0.06403287	0.09729912
Chd5	-0.423136	6.75626534	3.84294882	0.06410004	0.09738318
Cpne8	-0.2748141	6.41143721	3.84154612	0.06414459	0.09743287
Hist3h2ba	1.79190898	0.02817008	3.8406724	0.06417237	0.09745704
Btbd16	-1.8036165	-0.8711988	3.8397246	0.06420251	0.09747126
Slc35a5	-0.3134457	5.44790196	3.83939558	0.06421298	0.09747126
Serp2	0.4584999	3.78297655	3.83916251	0.06422039	0.09747126
Ppp1r1b	0.42758602	5.39614067	3.83888683	0.06422917	0.09747126
Gm3383	-1.1224333	-0.2915731	3.83670827	0.06429855	0.09755854
Pcnxl2	-0.4660899	4.8164733	3.8349588	0.06435433	0.09762514
Zc3h14	0.24724545	6.41769269	3.83411479	0.06438125	0.09764797
Spc25	0.89429687	1.57807401	3.83308147	0.06441424	0.09767998
Gcc1	0.33421178	4.93927588	3.83218623	0.06444284	0.09770531
Podxl	-0.2806217	4.29086336	3.83081304	0.06448673	0.09773454
Fpgs	-1.0576518	0.54372643	3.83050022	0.06449673	0.09773454
Sp140	0.31829176	4.89215056	3.83046716	0.06449779	0.09773454
Armc4	-1.5692638	-0.9967115	3.82947315	0.06452958	0.09775275
Olfm3	-0.3534073	4.62868668	3.82934801	0.06453359	0.09775275
Gm19710	-1.5208193	0.31012239	3.82794132	0.06457862	0.09780294
Dbx2	0.58834991	1.98242717	3.82598732	0.06464124	0.09785278
Gnai1	0.24150496	9.05039895	3.82579615	0.06464737	0.09785278
Lamtor1	0.44457429	5.52992674	3.82553035	0.06465589	0.09785278
Gm11127	-0.50017	3.78729841	3.8250587	0.06467102	0.09785278
BC033916	-1.0113642	0.31825089	3.82505734	0.06467106	0.09785278
Enpp4	-0.387774	4.28764148	3.82414565	0.06470031	0.09787903
Cdh10	-0.3926778	5.14094305	3.82094278	0.06480321	0.09796729
Ankle2	-0.2651167	5.23852826	3.82087587	0.06480536	0.09796729
Tmem185b	-0.6431108	3.21262042	3.82066359	0.06481219	0.09796729
Wipf1	0.45242977	6.02779989	3.82054129	0.06481612	0.09796729
Park2	-0.490646	3.05760267	3.82020378	0.06482698	0.09796729
Pramel5	-2.225997	-1.7145602	3.82010422	0.06483018	0.09796729
Rest	0.43253559	4.90144498	3.81778705	0.06490477	0.09803252
Ndfip2	-0.2837042	6.27191141	3.8176452	0.06490934	0.09803252
Cggbp1	0.28536882	7.33132985	3.81761428	0.06491034	0.09803252
Dnah2	-0.7550834	1.78466754	3.8172818	0.06492105	0.09803252
Htr4	-0.9160239	0.69245636	3.81552265	0.06497776	0.09810013

Stag1	0.23115355	7.40633933	3.81474675	0.06500279	0.0981199
Anks1	-0.3757417	3.49404808	3.81345919	0.06504436	0.09816461
Prkag2os1	-1.5308166	-0.1707102	3.80886472	0.06519292	0.09836469
Sh2d3c	0.31554765	4.98566397	3.80861924	0.06520087	0.09836469
Slc13a1	-2.5020148	-1.3495689	3.80783395	0.0652263	0.09837645
Tril	-0.3877327	3.67077525	3.80763962	0.0652326	0.09837645
Frg1	0.27510867	4.81586465	3.80700942	0.06525302	0.09838919
Cd226	-0.9802539	0.44264566	3.80649934	0.06526956	0.09839607
Leprel1	-0.8487781	2.42918779	3.8050314	0.06531717	0.09844812
Metrnl	0.61152054	2.14349993	3.80448962	0.06533475	0.09844812
Stk17b	0.36202454	5.75517255	3.8038396	0.06535586	0.09844812
Ide	0.21343416	7.24622988	3.80330904	0.06537309	0.09844812
Nlrp1a	-0.5951564	1.62889049	3.80302956	0.06538216	0.09844812
Ppm1e	-0.3421104	8.03567188	3.80277914	0.0653903	0.09844812
Flrt3	-0.2795267	5.81551562	3.80223992	0.06540782	0.09844812
Fig4	-0.3308877	4.3065797	3.80215711	0.06541051	0.09844812
Mdm4	-0.2253509	6.15415626	3.80205424	0.06541386	0.09844812
Lrrcc1	-0.3331996	5.61702841	3.80145959	0.06543319	0.09844812
Sec24d	0.39602531	4.40749598	3.80137814	0.06543584	0.09844812
Ceacam20	-0.7964448	1.81222087	3.80080221	0.06545457	0.09845557
Cldn12	-0.2870948	5.41596748	3.80048923	0.06546475	0.09845557
Fnip1	-0.3443906	5.8156585	3.79974088	0.0654891	0.09847417
Tmem202	1.84176444	0.48555398	3.7968585	0.06558299	0.09859732
Olfml3	0.49291212	4.9645008	3.79606675	0.06560881	0.09860877
Slc45a4	-0.4032178	3.61257026	3.79588902	0.06561461	0.09860877
Prkca	-0.3474242	8.12929589	3.79125015	0.06576612	0.09881841
Mex3c	0.27101204	5.21447884	3.79029509	0.06579737	0.09884729
Cenpk	-1.0315155	1.17615186	3.78748476	0.06588941	0.09896747
Kdm8	0.84365344	1.41305886	3.78612709	0.06593392	0.09901624
Tmem151a	-0.3828574	4.63055063	3.78567698	0.06594869	0.09902032
Oasl2	0.49013584	7.17688143	3.78479824	0.06597753	0.09903571
Atp2c2	1.2038312	0.00134164	3.7843878	0.065991	0.09903571
Pnlip	-2.2707873	-1.7420603	3.78426347	0.06599509	0.09903571
Gtpbp10	0.36762113	4.49151741	3.78146383	0.06608709	0.09914676
Pnpla7	-0.7305193	2.12670054	3.78127768	0.06609321	0.09914676
Kdm4b	-0.4977075	2.7623584	3.78060528	0.06611534	0.09916185
Qsox2	-0.4903879	2.93619289	3.77877597	0.06617556	0.09923407
1700001O22	-1.0254046	0.38566742	3.77767813	0.06621174	0.09924486
Ctxn2	0.85690303	1.98381935	3.77745105	0.06621922	0.09924486
Gm16576	-1.0182378	0.70651927	3.77726725	0.06622528	0.09924486
Usp18	0.70360039	1.69808751	3.77709209	0.06623106	0.09924486
Cbfb	0.37026803	6.03301844	3.77646998	0.06625157	0.0992575
Hey2	-0.5726098	2.26917077	3.77420021	0.06632649	0.0993416
Mblac1	0.63869034	2.67170348	3.77364041	0.06634498	0.0993416
Suco	-0.2769364	6.12057899	3.77344252	0.06635152	0.0993416

Agtpbp1	-0.2853714	8.9554073	3.7727123	0.06637565	0.0993416
Cyb5r1	0.50096728	2.67456624	3.77220272	0.0663925	0.0993416
Opn4	-1.6291393	-0.0898355	3.77211025	0.06639556	0.0993416
Akt2	0.3493482	5.97679673	3.77209007	0.06639622	0.0993416
Nup54	0.38305046	3.41473095	3.77181909	0.06640518	0.0993416
Dnttip2	0.23590907	6.01952394	3.77147729	0.06641649	0.0993416
Snx17	0.40747817	4.55007244	3.77095572	0.06643374	0.09934933
Mprip	-0.2604916	7.49135185	3.76999029	0.0664657	0.09937904
Mrps18c	0.43036789	4.53628145	3.76842261	0.06651762	0.09943422
1110007C09I	0.64608928	2.33907643	3.76814561	0.0665268	0.09943422
Magoh	0.33699464	5.17826882	3.76556028	0.06661255	0.09954428
Zfand2a	0.27459642	7.00570716	3.76480554	0.06663761	0.09954653
Popdc2	1.27925666	0.81035692	3.76430358	0.06665428	0.09954653
Neurod2	0.27364599	5.81515841	3.76423939	0.06665641	0.09954653
Man1b1	-0.4160912	3.68547036	3.76405594	0.0666625	0.09954653
Dennd1b	-0.3294674	4.59060003	3.76338267	0.06668487	0.09954776
Pde7a	-0.3601289	5.13023311	3.76330222	0.06668755	0.09954776
Izumo4	-0.718198	1.70919881	3.76279584	0.06670438	0.0995548
Tmem119	0.62559272	3.49084428	3.76080267	0.06677068	0.09961382
Ccne1	-0.6605875	1.77384286	3.76077387	0.06677164	0.09961382
Fam126b	0.31007526	8.29445503	3.7605142	0.06678028	0.09961382
Sars2	0.66693198	1.56896706	3.75886274	0.06683528	0.09967777
Sdr42e1	0.44678926	2.65229571	3.75743325	0.06688293	0.09973073
Cers5	-0.2707114	4.92314804	3.75268494	0.06704148	0.09994903
Cirh1a	0.27851329	4.83870112	3.75188411	0.06706827	0.09997083
Calm2	0.20271044	12.25381	3.75120803	0.06709089	0.09998029
Ercc6l2	-0.2878514	5.12584143	3.75096736	0.06709895	0.09998029
Slc17a6	-0.33196	4.81099216	3.75047817	0.06711532	0.09998656
Fam196a	-0.4020201	4.27266502	3.74940919	0.06715112	0.10002177
Wapal	0.2015147	7.56975924	3.74891556	0.06716766	0.10002828
Hspb1	0.60810973	5.07250514	3.74709099	0.06722884	0.10008126
Clec2f	1.61345426	-0.7657779	3.74689797	0.06723531	0.10008126
Mrrf	0.34585591	3.972877	3.74676505	0.06723977	0.10008126
Rab1b	0.45606417	4.50357591	3.74342534	0.06735193	0.10023005
Hectd2	-0.3874687	4.47598569	3.74108379	0.0674307	0.10032911
Tank	0.37205546	6.08627914	3.73930672	0.06749055	0.10039998
3110082J24F	-1.6103263	-0.6274529	3.73730307	0.0675581	0.10048229
Slc25a24	0.48116145	5.32740564	3.7337077	0.06767952	0.10063486
Tlr6	-3.3405663	-1.3341172	3.73321016	0.06769634	0.10063486
Prdx4	-0.3951067	3.41192748	3.73317849	0.06769741	0.10063486
Tmem163	0.4890835	2.75960425	3.73276168	0.06771151	0.10063761
1700096K18I	0.94849489	1.18970557	3.73205349	0.06773547	0.10065502
Ccdc162	-1.2363275	0.22297356	3.7307419	0.06777987	0.10070279
Rac2	0.51198082	2.78924291	3.73026471	0.06779603	0.10070859
Slc5a6	-0.4576349	4.25431609	3.72972705	0.06781424	0.10071745

Lrrc8a	-0.2429276	6.15744372	3.72812112	0.06786868	0.10075294
Ccrn4l	0.29613883	5.44062537	3.72778941	0.06787993	0.10075294
Lsm12	0.26320419	5.8603497	3.72767223	0.06788391	0.10075294
Cops7b	0.51314006	3.71950173	3.72757601	0.06788717	0.10075294
Tmem41a	0.42455736	3.74697865	3.72713524	0.06790213	0.10075341
Prkar1a	0.1994236	9.6161682	3.72684414	0.067912	0.10075341
Golga7b	0.42584789	4.36180067	3.72617612	0.06793468	0.10076886
Kcnj3	-0.3977966	5.04747371	3.72475645	0.0679829	0.10082219
1810022K09I	0.31210651	4.99892239	3.72354287	0.06802415	0.10085007
Psmc5	0.27087182	6.68950131	3.72334445	0.0680309	0.10085007
Dpcd	0.58290032	3.13768392	3.72270855	0.06805253	0.10085007
Fam166b	1.69086052	-0.4522952	3.72240216	0.06806295	0.10085007
Sytl5	-0.440136	4.73836367	3.72239926	0.06806305	0.10085007
Slc25a38	0.4449373	2.90799022	3.7216655	0.06808803	0.10086765
Faxc	-0.3056314	7.8893427	3.72132956	0.06809946	0.10086765
Prosc	0.26718572	5.53997079	3.72055363	0.06812589	0.10088861
Cnn2	0.58902668	6.26796327	3.72011332	0.06814089	0.10089099
BC003965	0.25070634	6.03354468	3.71947762	0.06816255	0.10089099
Rpl24	0.33272155	6.6964983	3.71912404	0.0681746	0.10089099
Rph3a	-0.3174991	8.88862341	3.71906559	0.06817659	0.10089099
Gm12060	-2.1345487	-1.0311945	3.71710938	0.06824332	0.10097156
Aldh3b2	1.11261659	-0.2498659	3.71566924	0.0682925	0.10102613
Lrrc2	-0.7974543	1.76997162	3.71471402	0.06832513	0.10105622
Fam60a	-0.3952232	3.5921575	3.71312063	0.06837962	0.10111263
Gab1	0.25117017	7.04320334	3.71287921	0.06838788	0.10111263
Lamtor2	0.51055392	4.07058306	3.71197594	0.06841879	0.10112448
S100a10	0.59471168	5.84680724	3.71192587	0.0684205	0.10112448
Gng12	0.3519458	6.58084897	3.71115854	0.06844678	0.10114513
Hnrnpf	0.37810147	6.78931311	3.70855309	0.06853607	0.10125888
4921515E04I	-1.899925	-0.8939193	3.70794324	0.06855699	0.1012619
Hmg20b	0.85180247	2.17156615	3.70777519	0.06856276	0.1012619
Ccdc42	-1.8891973	-1.1815401	3.70683117	0.06859516	0.1012766
Fsd1l	-0.3631892	6.17601251	3.70676723	0.06859736	0.1012766
Otud5	0.27017403	5.85476213	3.70608908	0.06862065	0.10129279
Mcfd2	0.40952271	4.96654256	3.70516579	0.06865237	0.10132142
Cenpp	-0.5978116	2.28834247	3.70457699	0.06867261	0.10133309
Flnc	0.71775069	2.89884862	3.70299546	0.068727	0.10138175
Aak1	-0.3303448	9.72626354	3.70290104	0.06873025	0.10138175
AW495222	0.66987625	1.74746184	3.7023493	0.06874924	0.10139156
Xndc1	0.44442645	3.95280623	3.70176573	0.06876933	0.101403
Tmem258	0.53604908	2.43606167	3.70097112	0.0687967	0.10142515
Dhrs4	0.43703359	3.26827748	3.700121	0.06882599	0.10145014
Gtf2a1l	3.19035095	-1.665669	3.69880984	0.0688712	0.10149858
Naif1	1.10999619	0.03808639	3.69796567	0.06890033	0.1015233
Tmem68	-0.2698802	5.12180934	3.69594438	0.06897012	0.10159602

Celf3	-0.3526341	5.51005772	3.69582055	0.0689744	0.10159602
Oser1	0.42529877	4.12300263	3.69309318	0.06906871	0.1017167
Tns3	-0.2363499	6.05708022	3.68717447	0.06927389	0.10200059
Pofut1	-0.4086666	3.36432094	3.68601759	0.06931408	0.10204149
Inpp5d	-0.5245502	2.51991615	3.68384648	0.06938957	0.10211205
Mlec	0.25179589	6.89116658	3.68348297	0.06940222	0.10211205
Cst3	0.47624845	7.90737531	3.6834165	0.06940453	0.10211205
Eln	-0.6208078	2.43103705	3.68321039	0.0694117	0.10211205
Pabpc5	-0.6036693	2.29010092	3.68196052	0.06945522	0.10215778
Gng5	0.39938937	6.39957695	3.6795676	0.06953863	0.10226216
Mtfr2	-2.4210123	-1.0937227	3.67803971	0.06959194	0.10230929
Trpv2	0.43950973	3.07012746	3.67786808	0.06959793	0.10230929
Slc25a30	-0.9128377	1.14282659	3.67757912	0.06960802	0.10230929
Tyk2	-0.5618763	2.82495666	3.67641109	0.06964882	0.10235096
Zfp874b	0.35352014	4.64430705	3.67387002	0.06973768	0.10244843
Hs6st1	-0.3371339	4.62727952	3.67365938	0.06974505	0.10244843
Txnl4b	0.49140468	3.25776878	3.67344524	0.06975255	0.10244843
Mzb1	-3.0010962	-1.791709	3.67299821	0.0697682	0.10245311
Htatsf1	0.20331288	8.39789574	3.6720736	0.06980058	0.10248235
Ptbp1	0.43238221	6.00333761	3.67109455	0.06983488	0.10251441
Rbm26	-0.2502923	7.13250946	3.66873554	0.06991763	0.10261754
Dis3	-0.3299504	3.97753571	3.66800301	0.06994334	0.10263695
Tmprss5	-2.8066375	-1.6692367	3.66759459	0.06995768	0.10263968
Nrxn1	-0.3272029	9.77366721	3.66720659	0.06997131	0.10264135
Necap2	0.55505285	3.41780728	3.66642419	0.06999881	0.10266336
Kcnab2	-0.3500984	5.95193359	3.66603008	0.07001266	0.10266535
Usp54	-0.207976	6.65042022	3.66488012	0.0700531	0.10270633
Napsa	-2.0929939	-0.6509732	3.66277793	0.07012709	0.1027793
Clec14a	-0.7257298	1.55869742	3.66275563	0.07012788	0.1027793
Eif2b1	0.4105186	4.79094889	3.66238271	0.07014101	0.10278022
P2rx7	-0.6899848	2.03038646	3.65829436	0.07028521	0.10297316
Tecpr1	-0.5508742	3.46884039	3.65717103	0.07032489	0.10298754
Vhl	0.28617201	4.8722494	3.65716384	0.07032514	0.10298754
Tlr2	2.16735094	-1.1053509	3.65695221	0.07033262	0.10298754
Fam13c	-0.3512981	4.49680051	3.65581686	0.07037276	0.10302077
Tyw1	-0.803874	2.0133922	3.6552306	0.07039349	0.10302077
9630033F20I	0.3757826	4.43530924	3.65483657	0.07040743	0.10302077
Kcnb2	-0.3471556	5.28418829	3.65471118	0.07041187	0.10302077
Yars2	0.33306126	4.04331491	3.65428216	0.07042705	0.10302077
Mgst3	0.43786134	3.55588685	3.65418416	0.07043052	0.10302077
Apobec2	3.60257034	-1.4578593	3.65365838	0.07044913	0.10302759
Angptl4	0.70984077	2.19976334	3.65334439	0.07046025	0.10302759
Nrcam	-0.3348231	7.77680341	3.65190707	0.07051117	0.10306836
Filip1l	0.24531096	5.30520595	3.6518496	0.07051321	0.10306836
Chrnb1	-0.4843494	2.16982591	3.65063707	0.0705562	0.10311286

Scamp2	0.7309162	2.70673317	3.64950055	0.07059653	0.10315345
St3gal5	-0.29983	6.4731257	3.64870501	0.07062477	0.10316899
Prmt8	-0.2667082	7.02981199	3.6484939	0.07063227	0.10316899
Slc7a4	0.52225569	2.98030875	3.64795859	0.07065128	0.10317842
5031425E22I	0.47487561	3.42439837	3.64737476	0.07067202	0.10318215
Ccdc163	-1.5373474	0.00792254	3.64718008	0.07067894	0.10318215
Cdh20	-0.4813967	3.92881565	3.64304038	0.07082624	0.10337884
Pptc7	0.21952434	6.66629707	3.64224938	0.07085443	0.10339315
Adhfe1	0.36260973	5.11547406	3.64205914	0.07086121	0.10339315
Mrpl51	0.28627124	5.05127658	3.64108839	0.07089583	0.1034253
Spag16	0.9772125	0.42113633	3.63947423	0.07095342	0.10347493
Usp16	0.27915362	6.12332536	3.6394293	0.07095503	0.10347493
Camk1g	-0.5169315	4.06859878	3.63800218	0.071006	0.10353089
Musk	0.50419158	3.43332663	3.63663204	0.07105497	0.10358392
Snx5	0.22088934	7.0692407	3.63502846	0.07111234	0.10363266
Aqp4	-0.4060343	5.58475126	3.63499264	0.07111362	0.10363266
Rccd1	-0.724252	1.48306808	3.63381615	0.07115575	0.10365747
Marveld2	1.45383446	0.62572504	3.63381276	0.07115587	0.10365747
Dstyk	-0.2518795	5.49522802	3.63295698	0.07118653	0.10366494
Wbp2	0.24253629	7.02579978	3.63263189	0.07119818	0.10366494
Gnpda2	0.32143391	5.96309501	3.63261367	0.07119883	0.10366494
Nup210	-0.6229933	3.04798879	3.63081703	0.07126326	0.10374037
Cfp	-1.0404181	1.41500791	3.62963457	0.07130571	0.10377293
Msrb2	0.34019638	4.28512107	3.6294903	0.07131089	0.10377293
Tmem141	0.61619119	1.88506136	3.62876314	0.071337	0.10379256
Slc17a7	0.26719831	7.53302562	3.62545808	0.07145584	0.1039308
0610011F06I	0.38716252	3.61598656	3.62541748	0.0714573	0.1039308
Bcl11b	-0.452777	6.10353451	3.62473559	0.07148185	0.10393961
Napa	0.27566704	6.07574235	3.62454668	0.07148866	0.10393961
Gm1966	-0.5703695	2.95962697	3.62338963	0.07153034	0.10398182
Rpl8	0.38205675	5.66174501	3.61986912	0.07165733	0.1041318
Zfp113	-0.4359013	4.27672379	3.61982714	0.07165885	0.1041318
Fmo2	0.70887743	2.18109682	3.61931194	0.07167745	0.10414042
Zfp949	-0.3231158	4.59449231	3.61886041	0.07169376	0.10414571
Aoc3	-1.0949387	3.18181364	3.61827493	0.07171492	0.10415408
Zfp438	0.35092157	3.94995783	3.6169564	0.0717626	0.10415408
Hltf	-0.3278533	4.91250401	3.61693345	0.07176343	0.10415408
Myadm	0.22356004	7.63973607	3.61648509	0.07177965	0.10415408
Gpr89	0.43535594	3.42303375	3.61593932	0.0717994	0.10415408
Snw1	0.25647007	6.48926014	3.61579134	0.07180475	0.10415408
Gm16062	1.26283736	-0.2612787	3.6157328	0.07180687	0.10415408
Meox1	-1.9188174	-1.373733	3.61496438	0.07183469	0.10415408
Arhgap1	0.3109192	5.65617668	3.61479433	0.07184085	0.10415408
Mob1a	0.40990054	3.39147199	3.61472214	0.07184346	0.10415408
Syn3	-0.3914211	6.89338645	3.61441111	0.07185473	0.10415408

Ppp2r2d	0.30912861	4.71076883	3.61420675	0.07186213	0.10415408
S100a9	1.55981722	0.04202695	3.61414793	0.07186426	0.10415408
Serpina3g	1.97316532	-0.4356293	3.61338679	0.07189185	0.10415951
Chst1	-0.3007027	5.89299389	3.61334513	0.07189336	0.10415951
Bend6	-0.283911	6.64558195	3.61201056	0.07194175	0.10421125
Klhl3	-0.5560842	2.05259147	3.61033256	0.07200265	0.10428109
Amacr	0.40471488	3.65200596	3.60923344	0.07204257	0.10432053
Alg10b	-0.2844103	5.55240241	3.60883995	0.07205687	0.10432285
Rpl32	0.39463164	6.79791779	3.6069297	0.07212633	0.10440503
Tgfbrap1	-0.2650732	5.59622484	3.60613413	0.07215528	0.10442854
Birc5	-0.9829677	0.28308296	3.60278021	0.07227748	0.10457263
Khdrbs2	-0.5971157	1.68183347	3.60219099	0.07229898	0.10457263
Nhlrc1	0.35790194	4.10563353	3.60216628	0.07229988	0.10457263
Thnsl2	-0.6030353	1.59740363	3.60200574	0.07230574	0.10457263
Kcnk1	-0.3007846	5.55611367	3.60147625	0.07232506	0.10457657
Plod1	0.50613635	3.01885443	3.60110633	0.07233856	0.10457657
Mthfd1	-0.4654512	3.54331546	3.60088538	0.07234663	0.10457657
4930592I03R	-3.1168344	-1.8628536	3.67244127	0.07238008	0.10460652
Pcnxl4	-0.3863257	4.64560216	3.59959529	0.07239375	0.10460789
Abca7	-0.7856743	1.81543376	3.5983896	0.07243782	0.10465317
Zbtb9	-0.6001214	2.9040012	3.59712378	0.07248412	0.10470166
Uhrf2	-0.2829183	5.29468683	3.59610908	0.07252126	0.1047369
N4bp3	0.86564338	1.3835988	3.59379692	0.07260597	0.10484082
Dhtkd1	-1.0422767	1.49539204	3.59222057	0.07266379	0.10490588
Alg13	-0.597721	3.17797422	3.59123732	0.07269989	0.10493955
Zfp763	-0.416786	4.04657361	3.59072107	0.07271884	0.10494849
Gm13298	-0.3956364	5.02359113	3.58990776	0.07274872	0.10496499
Phf21a	-0.2603329	6.60498082	3.58971474	0.07275582	0.10496499
Trim43b	-1.5236795	-0.7916784	3.58870349	0.07279299	0.10499372
Pja1	0.22652814	6.81192113	3.58839749	0.07280424	0.10499372
Grina	0.27474673	7.00624105	3.58813069	0.07281406	0.10499372
Slitrk5	-0.3769553	5.86905806	3.58712317	0.07285113	0.10501913
Scn2b	-0.2972196	6.88841078	3.58695724	0.07285724	0.10501913
Dgkh	-0.519258	5.56521568	3.58657378	0.07287135	0.10502106
Nek7	0.29534916	6.94421238	3.58554826	0.07290912	0.10505707
1700037C18I	1.16241606	0.04865188	3.58493908	0.07293157	0.105071
Sepp1	0.42741613	8.0461809	3.58373995	0.07297577	0.10510457
Abca6	-0.7167019	1.65908555	3.58330824	0.0729917	0.10510457
E2f4	0.34680582	4.54122018	3.58299781	0.07300315	0.10510457
3110039I08R	-1.2914213	-0.0970578	3.58291994	0.07300602	0.10510457
Dynlrb2	0.96704859	0.69709176	3.58249076	0.07302186	0.10510875
1700003D09I	-1.993501	-0.5231101	3.58196948	0.0730411	0.10510875
Cnpy2	0.45040493	5.15196504	3.58129614	0.07306596	0.10510875
Ddhd2	-0.2433228	6.00364545	3.58110996	0.07307284	0.10510875
Gfm1	-0.4077621	4.55150656	3.5811092	0.07307287	0.10510875

Mir1191	-2.5182567	-1.5833267	3.58038727	0.07309954	0.10511169
Opa1	-0.2758124	7.78864065	3.58032376	0.07310189	0.10511169
Cdip1	0.21729599	6.7745802	3.57992319	0.07311669	0.10511169
Cd2ap	0.28674611	7.43091632	3.57966954	0.07312607	0.10511169
Hmgcr	-0.3105045	5.79284207	3.57748909	0.07320672	0.10520357
Itsn1	-0.3467103	7.01485215	3.57724945	0.07321559	0.10520357
Myom2	1.54930997	0.39220815	3.57637671	0.07324791	0.1052226
Leprel4	-0.5912362	2.95741642	3.57608626	0.07325867	0.1052226
Piwil2	-2.1575849	-0.4778187	3.57585486	0.07326724	0.1052226
Cdc16	0.32741106	4.94816157	3.57503542	0.07329761	0.10523815
Polr2c	0.40405125	4.23038674	3.5748716	0.07330368	0.10523815
Adamtsl4	-0.4816555	2.8491448	3.57356942	0.07335197	0.10526614
Traf2	-0.7933081	1.32158657	3.57355967	0.07335233	0.10526614
Ccdc166	0.46068397	3.31457511	3.57331003	0.07336159	0.10526614
Ift122	-0.3729593	3.72749146	3.57294273	0.07337522	0.10526732
Dip2b	-0.3218218	7.80946321	3.57240517	0.07339518	0.10527428
Rbm11	0.43466289	3.26912102	3.57212184	0.07340569	0.10527428
Clmp	0.37824991	6.01158504	3.57097909	0.07344814	0.10529352
Pramef8	0.41883433	4.01107825	3.57076679	0.07345603	0.10529352
Dck	0.38062472	4.99238985	3.57069954	0.07345853	0.10529352
Sesn2	0.68135728	2.55196552	3.57038136	0.07347035	0.10529352
Gatad1	0.24558381	7.04557195	3.56999952	0.07348455	0.1052955
Plekho2	0.46981468	4.99004047	3.56935755	0.07350842	0.10531135
Ep300	-0.2692038	8.10979494	3.56812747	0.07355418	0.10535855
Il1rn	2.68381035	-1.1083452	3.56738014	0.073582	0.10538003
Nsun7	-0.8271921	2.25496462	3.56655649	0.07361267	0.10538963
Lipo1	0.43821392	4.94977586	3.56628152	0.07362292	0.10538963
Zfyve21	0.40696356	4.02987159	3.56616734	0.07362717	0.10538963
Mob3a	0.48236277	2.63458704	3.5654655	0.07365333	0.10540871
Chchd7	-0.4206776	3.63145787	3.56428166	0.07369747	0.10545353
Camkv	-0.3229257	6.00284055	3.56356438	0.07372423	0.10547346
Asb4	-1.25379	0.34542722	3.56186367	0.07378773	0.10554593
Slc9b1	-2.6932513	-0.7893292	3.56105143	0.07381808	0.10557096
Zdhhc22	0.52747569	2.47904651	3.55907638	0.07389193	0.10561851
Ptma	0.3885542	9.04456798	3.55883706	0.07390088	0.10561851
Gpatch11	0.34392096	5.43093597	3.55882441	0.07390136	0.10561851
Rpl13	0.34260511	7.37476852	3.55842721	0.07391622	0.10561851
Pik3r2	0.33830023	4.18510452	3.55788195	0.07393664	0.10561851
Fam122b	0.33391706	4.12560287	3.55782285	0.07393885	0.10561851
Ddx31	-0.7641325	1.36652736	3.55775795	0.07394128	0.10561851
Parp11	-0.4825067	3.76435497	3.55655227	0.07398644	0.10563414
Slfn8	0.64672103	3.41249401	3.55623349	0.07399839	0.10563414
Cd160	-1.0110346	1.22639695	3.55622955	0.07399853	0.10563414
Hist1h1d	1.82946548	-1.3350052	3.55609368	0.07400362	0.10563414
Srgap3	-0.3480883	9.0810211	3.5556354	0.0740208	0.10564031

Fam115a	-0.3438093	6.74609982	3.55516396	0.07403848	0.10564719
Snapc1	0.23520543	5.51212667	3.55390369	0.07408576	0.10569631
Alas2	-0.9626358	1.07308465	3.55207723	0.07415434	0.10577579
Scap	-0.3808035	4.49942401	3.55127449	0.07418451	0.10579104
Lyrm1	0.53413193	2.71571946	3.55096494	0.07419614	0.10579104
Lrfn2	-0.5733476	1.80541065	3.55076524	0.07420365	0.10579104
Fam104a	0.39125082	5.23095913	3.55002945	0.07423132	0.10580255
Gpsm2	-0.5436986	2.5646379	3.54986611	0.07423746	0.10580255
Slc30a4	-0.2381647	6.40576099	3.54742074	0.07432952	0.10591538
Cbx7	0.37002827	4.92502466	3.54696133	0.07434683	0.10592168
Gtf2ird1	-0.3573949	3.59592891	3.54572885	0.07439329	0.1059695
Srgap2	-0.2350508	6.07483362	3.54381	0.07446568	0.10605425
Ypel1	-0.5127195	3.12855469	3.54262911	0.07451028	0.10609549
Dcaf5	-0.2443566	7.07539042	3.54190328	0.07453771	0.10609549
Mdp1	0.33361094	4.40685737	3.54177569	0.07454253	0.10609549
Trmt61a	-0.335462	4.52357401	3.54167646	0.07454628	0.10609549
Gtse1	-1.6423589	-1.4834709	3.54130553	0.0745603	0.10609708
Gm6588	-2.1237763	-0.4709742	3.53930435	0.074636	0.10618642
Pvalb	0.55653302	2.39315365	3.53551338	0.07477966	0.10637238
Acot4	-1.4604964	0.0646272	3.53417319	0.07483052	0.10641712
Lgals8	0.27703987	6.12087827	3.53400234	0.07483701	0.10641712
Pabpc4	0.30808425	3.89769323	3.53144927	0.07493402	0.10653664
Mafg	-0.3627467	4.34596512	3.5309057	0.07495469	0.1065476
Rdh18-ps	-0.9098303	0.35944236	3.53039973	0.07497394	0.10655653
Tmem25	-0.4960041	3.05454486	3.52906092	0.0750249	0.10661053
Mtmr4	-0.3563596	5.94243077	3.52849309	0.07504653	0.10662283
Dkk3	0.297051	8.69595645	3.52680918	0.0751107	0.10668167
Hps3	-0.4437107	4.08700997	3.52668635	0.07511539	0.10668167
Rbm6	-0.2974309	6.21619282	3.52618549	0.07513449	0.10668167
Acss3	0.6260541	2.36379029	3.52604455	0.07513987	0.10668167
Reep5	0.23909433	8.46075093	3.52517725	0.07517296	0.10671022
Sergef	-0.6851293	1.78404877	3.52472304	0.0751903	0.1067164
Btbd1	0.22230356	7.23030801	3.52396602	0.0752192	0.10672565
Dach1	-0.6519687	2.04567433	3.52387246	0.07522278	0.10672565
Gm15413	-1.8811703	-0.5351781	3.52336575	0.07524214	0.10673468
Gmfg	1.12368981	1.11371591	3.52268295	0.07526823	0.10675327
Fam115c	0.89863452	1.3926034	3.52048164	0.07535242	0.10683976
Serpine2	0.28115484	5.98452131	3.52040899	0.0753552	0.10683976
04-Mar	0.28649812	4.92876459	3.51916691	0.07540276	0.10688875
Dph3	0.26058259	5.71694722	3.51686065	0.07549116	0.10698091
Fkbpl	0.98352167	0.62039627	3.51679152	0.07549381	0.10698091
Foxo4	0.67968926	1.66540675	3.51578063	0.07553259	0.10701742
Mms22l	-0.983203	0.73336835	3.51377882	0.07560946	0.10710787
F3	0.42612717	5.60082471	3.51279663	0.07564721	0.10711206
Ntm	-0.2418363	7.47633413	3.5124847	0.07565921	0.10711206

Jag2	-0.8021247	0.88017764	3.51244186	0.07566085	0.10711206
Elovl6	-0.3049676	6.01508993	3.512263	0.07566773	0.10711206
Ormdl3	0.46827746	4.20754267	3.51192625	0.07568068	0.10711206
Sh2b1	0.3230896	4.06126179	3.51166804	0.07569062	0.10711206
Fez2	0.30873588	5.48917661	3.50805599	0.07582973	0.10728705
Cox6a2	0.66738988	1.09993149	3.50777979	0.07584038	0.10728705
Ggact	0.45356603	5.86444957	3.50482316	0.07595448	0.10740161
Nedd8	0.45609748	5.60706888	3.50446905	0.07596816	0.10740161
Zfp511	0.64564359	2.57961167	3.50435204	0.07597268	0.10740161
Cd2bp2	0.35653791	4.98529416	3.5043276	0.07597363	0.10740161
Lsm14b	0.24202393	5.55529305	3.502466	0.07604559	0.10743439
Hdac11	-0.2776891	5.42574727	3.50238614	0.07604868	0.10743439
Serpina1d	2.56970831	-1.5769784	3.50214722	0.07605793	0.10743439
Cish	0.9117916	1.17032559	3.50214143	0.07605815	0.10743439
Tmem70	0.32394162	4.80019778	3.50196771	0.07606487	0.10743439
Hnrnpul1	0.25441649	6.59340048	3.50169966	0.07607524	0.10743439
Adarb1	-0.3109083	6.5564066	3.49964632	0.07615475	0.10752819
4933433G19	-0.8873291	0.90088046	3.49922889	0.07617093	0.1075285
Tiam1	-0.3179584	6.45558787	3.4989655	0.07618114	0.1075285
Kin	-0.3686541	4.30831348	3.49784666	0.07622452	0.10757126
Zfp354c	-0.2684353	5.84331025	3.49730684	0.07624546	0.10757839
Abhd17c	-0.2930611	5.2820418	3.49704171	0.07625574	0.10757839
Ptprt	-0.4159188	6.91884679	3.49375044	0.07638358	0.10772688
Msl1	-0.201659	8.01020206	3.49365702	0.07638722	0.10772688
Derl1	-0.3248821	5.09007702	3.49212466	0.07644682	0.10776686
Plod3	-0.5973676	2.62282767	3.49210632	0.07644754	0.10776686
Phactr3	-0.2250706	6.15735084	3.49171002	0.07646296	0.10776686
Zfp11	0.31183965	4.27418731	3.49158031	0.07646801	0.10776686
Heatr2	0.31751742	4.21724217	3.49026	0.07651943	0.10782084
Ctsz	0.47806657	3.99981656	3.48894363	0.07657074	0.10787242
Rab11b	0.2372295	9.08833055	3.48864736	0.07658229	0.10787242
Eid1	0.22194748	8.03895077	3.48794932	0.07660952	0.10789228
Epdr1	0.30855161	5.86306948	3.48627446	0.07667489	0.10796585
Copz1	0.35965197	6.13808659	3.4856172	0.07670056	0.1079835
Lrrfip2	0.23475715	5.83690377	3.4828412	0.0768091	0.10811779
Rgag1	0.85823024	0.93509014	3.48094684	0.07688327	0.10820366
Vmp1	-0.2785275	5.58892093	3.47998561	0.07692094	0.10823814
Tmem39b	-0.722922	1.82656269	3.47910968	0.07695528	0.10826793
Gm15328	-1.4938589	-0.036985	3.47845706	0.07698088	0.10828541
Sfxn5	-0.3658088	4.82337831	3.47676665	0.07704723	0.10836019
Aqr	-0.3606391	5.22653794	3.47491196	0.07712011	0.10844413
Dpy19l4	-0.3262818	4.18310181	3.47449895	0.07713635	0.10844841
Rpp14	0.29782919	5.40968028	3.47382572	0.07716283	0.10845716
Pip5k1a	-0.3410777	3.99435861	3.47356732	0.07717299	0.10845716
Slc7a14	-0.3011621	7.01975861	3.47333445	0.07718216	0.10845716

Thumpd1	0.29921789	5.95277989	3.47291676	0.07719859	0.10846172
Tsnaxip1	-3.1009571	-1.2806416	3.47231462	0.0772223	0.10847648
Rnf207	-1.3120121	0.9727215	3.47195394	0.0772365	0.10847789
Stard10	0.41515196	3.3328825	3.4702916	0.077302	0.10855114
Ccdc169	-2.2818736	-2.2268421	3.4699601	0.07731507	0.10855114
Gmpr2	0.46143536	3.44926098	3.46684532	0.077438	0.10870517
Tmsb10	0.48999343	6.42642058	3.4622857	0.07761836	0.10892411
Prdm15	-0.4844233	3.40894954	3.46192614	0.0776326	0.10892411
Cd22	-2.8986335	-2.1449166	3.46189785	0.07763373	0.10892411
Txnl4a	0.28742289	6.03320287	3.45927732	0.07773763	0.10905127
Ggta1	0.74912045	2.39992791	3.45864938	0.07776255	0.10906762
Zmiz1	0.23847789	8.34258388	3.45812185	0.07778349	0.10907643
2610203C22I	-1.3149292	0.00997097	3.45782267	0.07779537	0.10907643
Cryab	0.44142232	7.16970998	3.45643463	0.07785052	0.10913513
Naa30	0.25728803	5.57942222	3.45422101	0.07793856	0.10922493
Grb7	0.90784527	0.847069	3.45360971	0.0779629	0.10922493
Traf7	0.34397269	3.73534693	3.4534664	0.0779686	0.10922493
Ccnt1	-0.2861207	6.30201614	3.45240973	0.07801069	0.10922493
Fam107b	0.4873674	5.28728601	3.45235438	0.07801289	0.10922493
Rapgef2	-0.2677266	7.49215571	3.45231413	0.0780145	0.10922493
Tmem263	0.26442561	7.21113596	3.4519242	0.07803003	0.10922493
Wasf1	-0.3301671	7.99226552	3.45161887	0.0780422	0.10922493
Phpt1	0.4525443	3.28584852	3.45153599	0.07804551	0.10922493
Glrx2	0.21409953	6.7348839	3.4514109	0.07805049	0.10922493
Isl1	-0.8809607	1.28366596	3.45115352	0.07806075	0.10922493
St3gal6	-0.3825639	4.08416693	3.44835927	0.07817226	0.10936232
Afg3l2	0.27800177	5.7591495	3.44665564	0.07824033	0.10943893
Cysltr2	-2.1657139	-0.9832844	3.44587867	0.0782714	0.10946376
Ckmt2	-2.6163175	-1.0422931	3.44531887	0.07829379	0.10947106
Mtr	-0.6810373	2.80101481	3.44508232	0.07830326	0.10947106
Cxcr6	-1.2288402	0.03419362	3.4438459	0.07835275	0.10950619
Tinagl1	-1.2891036	0.32428798	3.44378898	0.07835503	0.10950619
Spef2	-0.9686882	0.09680215	3.44344477	0.07836882	0.10950683
Tubgcp6	-0.4540217	3.40036445	3.44112026	0.07846198	0.10961359
Rnd3	-0.3645875	5.26196549	3.44083342	0.07847349	0.10961359
Cyp20a1	-0.6218046	2.77855337	3.44051626	0.07848622	0.10961359
Rbm43	0.33730622	4.53418318	3.44020862	0.07849856	0.10961359
Arhgef19	0.77021025	1.47445845	3.43945153	0.07852895	0.10963739
Rps5	0.34318111	5.76664419	3.43801897	0.07858649	0.10969909
Zfp873	0.35720491	3.36059903	3.43763323	0.07860199	0.1097021
Ccdc85c	-0.7170053	0.96337472	3.43662079	0.0786427	0.10973421
Arhgef37	1.10055649	0.28806604	3.43614539	0.07866182	0.10973421
Adcy3	-0.5985161	2.3116347	3.43606498	0.07866505	0.10973421
Rrp15	0.50653648	2.82371295	3.43461103	0.07872357	0.10977915
Dyrk4	3.39433808	-2.242214	3.50137702	0.07872398	0.10977915

1110004F10I	0.33576838	6.35477478	3.43412601	0.0787431	0.10978719
Pcdhb13	-0.5738017	3.50224641	3.43308099	0.0787852	0.1098153
Trpt1	0.43917224	3.12327704	3.43296234	0.07878998	0.1098153
Abhd10	-0.3554949	4.33952825	3.43203818	0.07882724	0.1098486
Rbm46	1.08373245	1.57546975	3.43082876	0.07887603	0.10989795
Grip1	-0.4261716	4.08404271	3.42967174	0.07892274	0.10994439
Rragd	-0.2023606	7.20974552	3.42888277	0.0789546	0.10996187
Abhd8	-0.2810009	4.56973272	3.42869878	0.07896204	0.10996187
Rpl27a	0.27948992	6.97053072	3.42764074	0.0790048	0.11000206
E2f6	0.27149637	5.50491294	3.42690674	0.07903449	0.11000206
Snx12	0.25873953	7.72627146	3.42668448	0.07904348	0.11000206
Dpcr1	2.00199678	-1.0147611	3.42665666	0.0790446	0.11000206
Adrb3	-1.5271831	-0.894653	3.42633001	0.07905782	0.11000206
Zbtb45	-0.502166	2.41541769	3.42576109	0.07908084	0.11000718
Fam83h	0.66406205	1.56831649	3.42557769	0.07908827	0.11000718
1700086O06	1.31715972	-0.332981	3.42429	0.07914041	0.11003995
Wnt2b	-0.4395567	2.90172608	3.42359535	0.07916856	0.11003995
Lars2	-0.3062342	13.4159353	3.42348272	0.07917312	0.11003995
Pcdhga6	-0.5392149	2.36475453	3.42340626	0.07917622	0.11003995
Snx22	-1.2450226	-0.2486315	3.42299249	0.079193	0.11003995
Ovol2	-0.6273024	1.7981292	3.42290677	0.07919647	0.11003995
Chmp5	0.36464031	6.60861862	3.4226829	0.07920555	0.11003995
Tdrd5	-1.0155658	1.22862972	3.42175421	0.07924322	0.11007368
Kynu	2.14526667	-0.5723934	3.42084274	0.07928021	0.11009252
Npy1r	-0.3111429	5.20521963	3.42075996	0.07928357	0.11009252
Ccdc157	-0.400348	3.04729122	3.41930564	0.07934264	0.11015593
Fendrr	-2.0474591	-0.3723177	3.41874962	0.07936523	0.11016315
Gm20139	-1.2991177	-0.1396949	3.41851807	0.07937464	0.11016315
Pigp	0.38300588	4.8019085	3.41755993	0.0794136	0.11019861
Smpd5	2.67465951	-2.011353	3.41605917	0.07947467	0.11025486
Taf13	0.26466775	6.57924145	3.41590463	0.07948097	0.11025486
Dnase1	-1.0208144	1.03143966	3.41505479	0.07951558	0.11028425
Mier1	0.26698579	6.44738241	3.41454113	0.0795365	0.11029467
Neurl4	-0.3651863	4.54166249	3.41363988	0.07957324	0.11030924
Mrps14	0.32556685	5.65995833	3.41362473	0.07957385	0.11030924
1700113A16	-0.4109039	3.91329545	3.41271916	0.07961078	0.1103358
Bbs7	-0.3521055	4.04676063	3.41249659	0.07961986	0.1103358
E2f1	0.5689622	1.94559375	3.41129003	0.0796691	0.11037248
Ptk2	-0.365044	6.57805731	3.41092458	0.07968402	0.11037248
Gtf2f1	0.31571933	5.9128178	3.41086103	0.07968662	0.11037248
Mirlet7bhg	-1.5639177	0.81513679	3.40926251	0.07975193	0.11044433
Katnb1	0.55257538	2.94699057	3.40746786	0.07982532	0.11051301
Rnf166	0.39225799	4.71944735	3.40739241	0.07982841	0.11051301
Kcnq5	-0.3535154	6.25302514	3.40608593	0.0798819	0.11056842
Igdcc3	-1.3381206	0.90123757	3.404318	0.07995433	0.11065006

Umps	0.31791535	4.08953276	3.40224248	0.08003947	0.11074923
Gstm6	0.59111426	2.2532875	3.40158657	0.0800664	0.11076785
Enc1	-0.4179342	8.26105106	3.40099334	0.08009077	0.11078291
Slc25a22	0.28204563	4.94675892	3.39982043	0.08013896	0.11083092
Zc3h7a	-0.3844682	5.52498518	3.39735291	0.08024047	0.11095263
Mtm1	-0.6263084	2.68765722	3.39569994	0.08030855	0.11102809
Cenpu	-1.0184644	-0.0263099	3.39534297	0.08032326	0.11102821
Mkrn2	0.31036586	5.14989952	3.39500035	0.08033738	0.11102821
Txk	2.05985602	-1.7280901	3.39446267	0.08035955	0.11102821
Samd8	-0.2480527	6.05743869	3.39438695	0.08036267	0.11102821
Cndp1	2.98771507	-1.3929104	3.39395151	0.08038063	0.11103296
Mthfd1l	-0.4700071	3.49817884	3.39353977	0.08039762	0.11103296
Echdc2	0.60555048	2.26378607	3.39309989	0.08041577	0.11103296
Fcgr3	0.89263161	1.06475756	3.39299381	0.08042015	0.11103296
Camsap3	-0.3881053	3.48513944	3.39237825	0.08044556	0.11104564
Pak3	-0.3436491	7.31845417	3.3916336	0.08047631	0.11104564
Top2a	-0.6076098	2.36370115	3.39155036	0.08047975	0.11104564
Gpx8	0.5413834	5.63847575	3.39146264	0.08048337	0.11104564
Hmbox1	-0.2832634	5.1907916	3.39013612	0.08053819	0.11110263
Itpkc	0.57424598	2.14887091	3.38932602	0.08057169	0.11113019
Zfp521	0.33805771	5.60118123	3.38883836	0.08059187	0.11113696
Pla2r1	1.11264093	-0.1361902	3.38840102	0.08060997	0.11113696
Angpt2	0.39985587	3.99421813	3.38822697	0.08061717	0.11113696
Tbc1d10b	0.26439641	4.60840359	3.38759925	0.08064316	0.11115414
Dpp7	-0.6715724	1.49041866	3.38676025	0.08067791	0.11118339
Aplp2	-0.194435	8.46249735	3.38633314	0.0806956	0.11118914
Pcbp4	-0.488272	2.70007356	3.38455308	0.0807694	0.11127217
Smg8	-0.2811277	4.41716152	3.38412384	0.08078721	0.11127805
Morc1	-2.557234	-1.4927498	3.38209195	0.08087157	0.11137559
Fank1	-0.8580373	1.2666404	3.38172447	0.08088684	0.11137795
Vac14	0.33710205	4.71468339	3.38103931	0.08091531	0.1113966
Slc44a2	-0.295395	5.08209519	3.38074647	0.08092748	0.1113966
Kit	-0.2638847	4.87311702	3.3778826	0.08104665	0.11154195
Riiad1	1.0396277	0.2172966	3.37684772	0.08108977	0.1115826
Slc7a6os	0.4168812	3.9736553	3.37526882	0.08115559	0.11163788
Ramp2	0.50735283	4.5868832	3.37523261	0.08115711	0.11163788
Atp5h	0.35774085	6.99955852	3.37209918	0.08128793	0.11179913
Arv1	-0.9383883	0.87145989	3.37160299	0.08130867	0.11180895
Rassf1	0.53498689	3.92843638	3.37072089	0.08134556	0.11181048
Adra1d	0.33908915	3.5059579	3.37063022	0.08134935	0.11181048
Cpne9	-0.5016932	4.22777363	3.37060038	0.0813506	0.11181048
Elof1	0.59256409	3.1789434	3.36999383	0.08137597	0.11182666
B4galt5	-0.3417129	4.63843676	3.36965366	0.08139021	0.11182752
Cyyr1	-1.6122793	0.36582894	3.36912191	0.08141247	0.11182783
Lrrc42	0.42975624	3.25022116	3.36899824	0.08141764	0.11182783

Mlip	-0.3601742	4.86704619	3.36800368	0.0814593	0.11186635
Pkig	-0.2679081	4.79792239	3.36694253	0.08150377	0.11190872
Wnt1	1.70466647	-1.3555042	3.36601621	0.08154261	0.11194335
Ptchd1	-0.4490296	4.63115282	3.36555534	0.08156194	0.11195119
Fam179b	-0.2365088	6.53940217	3.36430587	0.08161438	0.11200446
Trpv3	-1.6036458	-0.520925	3.36112039	0.08174825	0.11216946
Sst	0.32762955	6.90303756	3.36020272	0.08178687	0.11220371
Prr14	0.33291444	5.45232595	3.35821994	0.08187037	0.11229228
Crk	0.20543104	7.7634951	3.35734342	0.08190732	0.11229228
Arl6ip4	0.39470483	4.90499571	3.35733412	0.08190771	0.11229228
Gstp2	0.33628302	4.53982424	3.35728448	0.0819098	0.11229228
Dis3l	-0.3220632	3.87216742	3.35704868	0.08191974	0.11229228
9930014A18I	0.97295391	0.8415086	3.35625541	0.0819532	0.11231942
Tcf7l2	0.37537745	6.41243746	3.35546661	0.08198649	0.1123463
Zfp286	-0.5171517	3.27813945	3.35456461	0.08202457	0.11237975
Pcsk5	-0.4153796	4.26563116	3.35367824	0.08206202	0.11240432
Gjb6	0.42158398	8.86000288	3.35349274	0.08206985	0.11240432
Adora2b	-1.0447985	0.44978686	3.35291175	0.08209441	0.11241921
Kpna2	0.23427285	5.1767939	3.35205807	0.08213051	0.11243519
Chd2	-0.2475268	6.94922404	3.35198886	0.08213343	0.11243519
Rarg	0.4923869	2.85799831	3.35098251	0.08217601	0.11247474
Limd1	0.42243701	5.16136736	3.34996648	0.08221903	0.11251488
Mllt10	-0.253281	6.2237758	3.34879657	0.08226859	0.11256396
Gnb4	-0.2966493	4.79186828	3.34775743	0.08231264	0.11260548
Rimbp2	-0.4384418	5.19509569	3.34580293	0.08239556	0.11265725
Rhpn1	0.7456516	0.76521571	3.34572949	0.08239868	0.11265725
Armcx4	-0.3002737	6.06988026	3.34557523	0.08240523	0.11265725
Trappc3l	-3.2576949	-2.1751454	3.34557348	0.0824053	0.11265725
Ufsp2	0.29485129	5.28542917	3.34277595	0.08252419	0.11280101
Rsbn1	-0.2376761	6.12056606	3.34152964	0.08257721	0.11282295
Asic2	-0.3136389	5.16739371	3.34149481	0.08257869	0.11282295
Scaf11	-0.1780622	7.60854134	3.34143077	0.08258142	0.11282295
Pa2g4	0.20091393	6.38809118	3.34099439	0.0826	0.11282958
Med13	-0.2140713	9.08293275	3.3405272	0.08261989	0.112838
Spink8	1.31019088	-0.1941397	3.3389451	0.0826873	0.11289937
2610507B11I	-0.203327	8.00325485	3.33882787	0.0826923	0.11289937
Akap5	0.23683071	8.01809372	3.33748317	0.08274965	0.11295891
Mrpl52	0.35736587	3.85555016	3.33678697	0.08277936	0.1129807
Rxfp1	-0.9234679	2.35363465	3.33624418	0.08280253	0.11299356
Phf13	0.42793213	3.5556069	3.33435371	0.08288329	0.113085
Casc1	-1.4273368	-0.6692017	3.33125776	0.08301576	0.11322735
Iffo1	-0.5033193	3.77255364	3.33121839	0.08301744	0.11322735
Pilrb2	-2.7186285	-0.5024789	3.32982719	0.08307705	0.11322735
Phf23	0.36259779	4.64131352	3.32961856	0.08308599	0.11322735
Lars	-0.2664374	5.12564252	3.32961133	0.0830863	0.11322735

Foxl1	1.10697742	0.5607768	3.32949641	0.08309123	0.11322735
Fbxl6	-0.8689017	0.94416263	3.32938147	0.08309615	0.11322735
Rchy1	0.27763456	5.50211776	3.32934226	0.08309784	0.11322735
Olfm4	3.06496713	-2.1604963	3.32899276	0.08311282	0.11322811
9430091E24I	0.84141433	1.9031491	3.32868671	0.08312595	0.11322811
Vegfc	-0.5780216	2.74930106	3.3273021	0.08318536	0.11329026
Exoc5	-0.2201777	6.2944493	3.32682249	0.08320595	0.11329953
Jam3	-0.5240109	2.89043088	3.32456392	0.083303	0.11339864
Cobll1	-0.3372918	4.89653014	3.32437309	0.0833112	0.11339864
Klhl25	0.51176828	1.87790418	3.32416558	0.08332013	0.11339864
Mir665	-1.6178344	-0.6775952	3.32295906	0.08337204	0.1134505
Gtf2e1	0.40157831	3.80428153	3.32250448	0.0833916	0.11345835
Ubr2	-0.3544849	6.2756488	3.32081466	0.08346439	0.1135093
Asrgl1	0.33314659	5.83523342	3.32054563	0.08347598	0.1135093
Arf2	0.31655983	6.06373611	3.32051422	0.08347734	0.1135093
H13	-0.3737731	4.82160613	3.32005966	0.08349693	0.1135093
Gm14204	-0.6745247	2.45307692	3.31981825	0.08350734	0.1135093
Rps25	0.44238591	7.66263212	3.31971205	0.08351192	0.1135093
Arhgef9	-0.2596542	9.43024303	3.31888679	0.08354752	0.11353891
Plekhm3	-0.3087371	6.33117024	3.3175176	0.08360662	0.11360044
Ints12	0.2883528	4.73342716	3.31600647	0.0836719	0.11367035
Apmap	-0.3940771	4.89839302	3.31490618	0.08371947	0.11371618
Actl6a	-0.3742293	3.68241767	3.31399595	0.08375884	0.11372882
6820408C15I	1.88829038	-0.5140302	3.31399041	0.08375908	0.11372882
Bace1	-0.2183528	5.79319228	3.3137315	0.08377029	0.11372882
Acvr2a	-0.2853179	6.16874614	3.31283267	0.0838092	0.11376286
Rps16	0.37924526	6.26269801	3.31226419	0.08383382	0.11377748
Zfpl1	0.51595189	2.51937251	3.31138464	0.08387193	0.11381041
Crnde	3.21145775	-1.7775101	3.31037169	0.08391584	0.11385121
Wdr47	-0.3643583	6.50006207	3.31001739	0.08393121	0.11385326
Zfc3h1	-0.3755477	6.97548533	3.30969492	0.0839452	0.11385344
Bace2	0.52740731	3.59463179	3.30911406	0.0839704	0.11386884
Rab10os	-0.4401481	3.09165259	3.30711494	0.08405721	0.11396776
Ar	-0.3989141	4.30558559	3.30595467	0.08410764	0.11401341
D4Ertd617e	-2.3934227	-1.1719346	3.30570215	0.08411862	0.11401341
Lmln	-0.5287464	2.3864814	3.30319114	0.08422791	0.1141427
Gpnmb	1.19952472	1.08924575	3.30172508	0.08429179	0.11421044
Nuf2	-1.7806644	-0.0548779	3.30090296	0.08432763	0.11424018
1700024B18I	-1.790041	-0.3069596	3.30017213	0.08435951	0.11425588
Gm527	-0.6417119	2.04063156	3.29938668	0.08439379	0.11425588
Slc22a21	-1.4807743	0.03731727	3.29929357	0.08439786	0.11425588
Epb4.1	-0.2479955	4.96364631	3.29904879	0.08440854	0.11425588
E130311K13I	0.44016759	3.48396889	3.29904446	0.08440873	0.11425588
Ssbp1	0.25134137	5.47566653	3.29859673	0.08442829	0.11426353
Esrp2	-1.5479006	-0.9592268	3.29825321	0.08444329	0.11426502

Caml	0.34631036	3.67011036	3.29660709	0.08451523	0.11434355
Xpo7	-0.1895299	7.18190454	3.29454713	0.08460537	0.11444665
Tmco3	-0.3785132	3.76678227	3.29419285	0.08462088	0.1144488
Dgcr8	-0.3006006	4.13811439	3.29066852	0.08477537	0.11462611
Sel1l	-0.2639927	7.13736227	3.29056596	0.08477987	0.11462611
Upf3a	0.37544343	5.8631165	3.29008678	0.08480091	0.11463569
Plxnb3	-0.73956	2.04041531	3.28897927	0.08484954	0.11468227
1700109K24I	-1.4136545	0.84873822	3.28866684	0.08486327	0.11468227
Foxc1	0.34402257	8.36033334	3.28657348	0.0849553	0.11478777
Nufip1	0.39470743	4.1197829	3.28599285	0.08498085	0.11480341
Pstpip2	0.34125593	3.88083727	3.28528292	0.08501209	0.11482675
Txn2	0.40622695	4.68308684	3.28380481	0.0850772	0.11488913
Myl12a	0.52849954	6.85744348	3.28333157	0.08509806	0.11488913
Txlnb	-0.6564948	2.24854528	3.28328262	0.08510021	0.11488913
Nap1l1	0.19849523	8.68607673	3.2793589	0.08527337	0.115104
1700049G17	-0.4100221	3.46919692	3.27783741	0.08534063	0.11516173
Elmsan1	0.2433761	5.92131118	3.27775738	0.08534417	0.11516173
Rabgap1	-0.2121769	7.271955	3.27734312	0.08536249	0.11516755
Cd274	-0.4689203	3.76592191	3.27460189	0.08548386	0.11531236
Slc4a10	-0.2448977	8.98850848	3.27236136	0.0855832	0.11541417
Klhl11	-0.335585	4.45954021	3.27224356	0.08558843	0.11541417
Npas2	-0.4562358	5.29330213	3.27195013	0.08560145	0.11541417
Ndufaf5	0.29994136	4.79767237	3.27072563	0.08565582	0.11545468
Sppl2a	-0.2810792	6.38309585	3.27064064	0.08565959	0.11545468
Zcrb1	0.23890213	6.95352091	3.26981238	0.08569639	0.11548534
Gstcd	-0.7179319	1.73762496	3.26846124	0.08575646	0.11553092
Zfp664	0.26206302	7.69577042	3.26841924	0.08575832	0.11553092
Ubl3	0.20715956	7.21135999	3.267843	0.08578396	0.11554651
Dab1	-0.3358855	6.61765701	3.26746744	0.08580067	0.11555008
Nprl3	-0.4026539	3.06488781	3.26699193	0.08582183	0.11555965
Prtg	-0.6532922	1.53552983	3.26548848	0.08588879	0.11563086
Tpgs2	0.21784343	6.19132369	3.26410498	0.08595046	0.11569493
Ptchd2	-0.5509291	2.78492461	3.2623243	0.0860299	0.11577768
Pnpo	0.40611226	3.78994723	3.26209584	0.0860401	0.11577768
Lmnb2	0.43253361	3.01885766	3.2591648	0.08617108	0.11593495
Cacng2	-0.2556311	5.27518469	3.25786387	0.08622929	0.11599427
Chodl	2.40985391	-1.4040797	3.25731905	0.08625368	0.11599754
Zfp217	0.54667863	3.61460677	3.2568483	0.08627476	0.11599754
Col1a2	0.43858763	7.37951761	3.25661458	0.08628523	0.11599754
Nod1	-0.5876773	2.07213642	3.25654896	0.08628817	0.11599754
Oard1	0.39446369	4.57408272	3.2556053	0.08633045	0.11603398
Fbxo40	1.6836286	-0.5305245	3.25531394	0.08634351	0.11603398
Pdzd4	-0.2913607	5.59251781	3.25264769	0.08646314	0.11615563
Zkscan16	-0.4101814	5.24616112	3.25244632	0.08647218	0.11615563
Ythdf3	0.21044781	7.18928412	3.25235168	0.08647643	0.11615563

Pik3r4	-0.3007264	5.1104775	3.25190254	0.08649661	0.1161577
Pold4	0.67744076	2.12316132	3.25168815	0.08650624	0.1161577
Ireb2	-0.2818296	6.4449494	3.24982022	0.08659021	0.11624513
Ado	-0.2725046	5.37027576	3.24961057	0.08659964	0.11624513
Smarcal1	-0.3563765	6.14881675	3.24913649	0.08662097	0.11625478
2010109A12l	-2.5159655	-2.023385	3.24850396	0.08664944	0.116274
Hspa4l	-0.3744518	7.12134736	3.24786472	0.08667822	0.11628783
Adamts8	-1.2174528	-0.3330181	3.24764654	0.08668804	0.11628783
Dtd1	0.30022431	6.04522579	3.24697838	0.08671814	0.11630923
Gm4890	2.4537535	-1.3919237	3.24609726	0.08675785	0.1163435
Gm20597	2.15178646	-0.6734254	3.24420941	0.08684301	0.1164387
Ptpn22	0.5926352	2.35806896	3.24103096	0.0869866	0.11661145
Atxn10	0.19273834	7.66352388	3.24072963	0.08700022	0.11661145
Ndufb7	0.49685056	4.23432189	3.23989216	0.08703811	0.11664321
Mrpl32	0.41182588	3.95919809	3.23786246	0.08713001	0.11674733
Crygs	2.88488952	-2.0379571	3.23599744	0.08721456	0.11681058
2810013P06l	0.36386735	4.65624148	3.23581343	0.0872229	0.11681058
Fscn1	0.32076729	5.37348676	3.23493593	0.08726272	0.11681058
Mapk9	-0.2178595	8.50240878	3.23475592	0.08727089	0.11681058
Synj1	-0.4295441	9.53680777	3.2346807	0.08727431	0.11681058
Pfdn1	0.47218269	3.51727609	3.23431148	0.08729107	0.11681058
Trappc1	0.3210026	5.99795277	3.23423595	0.0872945	0.11681058
Tnrc6c	-0.2529759	6.9612226	3.23405574	0.08730268	0.11681058
Cyp4x1	-0.7933056	0.99608208	3.2340019	0.08730513	0.11681058
Pfas	-0.42411	3.81241102	3.23303957	0.08734884	0.11685005
Agbl2	-0.6835593	1.56639521	3.23216672	0.08738851	0.11688051
Mmachc	0.30416675	4.6196112	3.23191282	0.08740006	0.11688051
Zfp783	-0.7977225	1.36514797	3.23121614	0.08743174	0.11690386
Arfip1	0.35427422	6.0988811	3.23010828	0.08748215	0.11693902
Fgd2	0.93251612	0.37736609	3.22975814	0.08749809	0.11693902
Ddx28	0.45511867	2.5404257	3.22970046	0.08750072	0.11693902
2010012O05	0.25130135	6.00612599	3.2291897	0.08752398	0.11695109
Mad2l2	0.53427889	3.11655822	3.22803948	0.08757638	0.11700209
Gps1	0.27000141	5.13884661	3.22680848	0.08763251	0.11705805
Bsg	0.50122656	7.36556229	3.22590291	0.08767383	0.11708921
Dancr	1.46026462	-0.0093493	3.22567281	0.08768433	0.11708921
Snapc2	0.40331888	4.22325126	3.22475189	0.08772637	0.11712632
Hccs	0.33379341	4.98550885	3.22372118	0.08777346	0.11717015
Cftr	-0.9083484	1.59461546	3.22332769	0.08779144	0.11717513
Pop5	0.36003847	4.39829903	3.22207213	0.08784885	0.11723168
Cdh24	-1.763697	-1.4470112	3.22177735	0.08786234	0.11723168
Sec23a	-0.2409896	6.49396383	3.22109707	0.08789347	0.11725418
Polr3gl	-0.4001463	2.88161822	3.21925885	0.08797765	0.11734743
Camk2b	-0.2794714	8.06092179	3.21870254	0.08800314	0.11736239
Ube2q1	0.1980396	7.10382136	3.2183443	0.08801956	0.11736525

Epb4.1l1	-0.2652485	8.48495277	3.21793851	0.08803817	0.11737102
Zfp146	0.42978151	4.37017683	3.21720357	0.08807188	0.11739692
Rsad2	0.87028209	1.25882069	3.2095853	0.0884222	0.11784477
Yrdc	0.38228749	3.48619489	3.2087665	0.08845995	0.11787597
Pcbp2	0.22327475	6.942454	3.2082049	0.08848585	0.11789137
Peli3	1.35530097	-0.7958979	3.20624963	0.0885761	0.11799248
Ift172	-0.5289739	4.63791237	3.20559171	0.08860649	0.11801384
Zdhhc6	0.36479596	4.61483211	3.20518094	0.08862547	0.11802
Cluh	-0.2947557	5.1625154	3.20362442	0.08869744	0.1180967
Soat1	-0.2447632	5.47047829	3.20293202	0.08872948	0.11810192
Ppil3	0.47622047	3.79873045	3.20291852	0.0887301	0.11810192
Foxr1	-1.3569633	-1.0863952	3.20102876	0.0888176	0.11819925
Gab2	0.28065512	6.02991746	3.19976526	0.08887617	0.11825804
Evl	0.37664953	4.22198237	3.19771172	0.08897144	0.11836565
Syne2	-0.2565371	5.49056442	3.19674163	0.08901649	0.11840641
Tprn	-0.6442327	2.34731961	3.19587595	0.08905672	0.11844075
Trim2	0.27366645	9.04551566	3.19525819	0.08908543	0.11845977
Ghitm	0.19854988	8.70164854	3.19298434	0.08919123	0.11858127
Usp53	0.24101906	6.92540021	3.19239479	0.08921868	0.11859858
Mtf2	-0.3001926	5.55865667	3.19103583	0.089282	0.11866356
Slc13a5	-0.5694515	2.26735821	3.19003226	0.0893288	0.11870656
Fam183b	1.59768613	0.14856088	3.1891369	0.08937057	0.11874287
Med22	0.36296206	3.78866602	3.18876592	0.08938788	0.11874668
5730507C01I	0.44963031	3.12580485	3.18809328	0.08941929	0.11875279
Slc39a9	-0.2827089	4.89213791	3.18804851	0.08942138	0.11875279
Cyp4a12a	-1.1438018	0.33010548	3.18534324	0.08954782	0.11890149
Gstm2	0.60201415	5.29499627	3.18460006	0.08958259	0.11890949
1500012F01I	-0.3718415	3.6609381	3.18409848	0.08960606	0.11890949
Crls1	0.33198371	4.55231975	3.18403737	0.08960892	0.11890949
Mt2	0.40955744	5.17961798	3.18397782	0.08961171	0.11890949
Stap1	-1.157568	0.31756925	3.18232854	0.08968896	0.11899279
Gm9962	-0.8594527	0.50648119	3.1810275	0.08974996	0.11905449
1700048O20	0.5418737	1.87947772	3.18043407	0.08977779	0.1190722
B230219D22	0.23405424	8.00694298	3.1787085	0.08985879	0.1191604
Nebl	0.25748849	6.61374962	3.17800073	0.08989204	0.11918526
Anapc16	0.45286591	5.99677279	3.17584487	0.0899934	0.11928188
Dram2	-0.32301	5.47488685	3.17583341	0.08999394	0.11928188
Lpin3	2.7191161	-1.554597	3.1751771	0.09002483	0.11929242
Egflam	0.46805999	3.52709281	3.17504764	0.09003092	0.11929242
Btbd11	-0.2787051	4.43944584	3.17473315	0.09004572	0.11929281
AI607873	0.69680425	1.61003838	3.17342543	0.09010731	0.11935516
Soga3	-0.320923	6.51710607	3.17303332	0.09012579	0.1193604
P4ha1	-0.3081316	3.95493008	3.17214772	0.09016754	0.11939645
Zmynd8	-0.2586507	6.82734109	3.16996063	0.09027074	0.11951385
Nhej1	1.31084918	1.11955873	3.16911198	0.09031082	0.11953017

Whamm	-0.5800577	3.09783913	3.1690839	0.09031215	0.11953017
Pex7	0.3137543	4.47396907	3.16752642	0.09038576	0.11960833
Gosr1	0.3005964	5.27713659	3.16711038	0.09040544	0.11961511
Fbln1	0.53003677	4.89785489	3.16435593	0.09053583	0.11976762
Ppm1k	-0.2815138	6.66579069	3.16406012	0.09054985	0.11976762
Slc25a21	-1.1555474	0.19478389	3.16363214	0.09057013	0.11977518
Nop16	0.58444343	2.38297078	3.16246843	0.09062531	0.11981459
Bloc1s4	0.49500815	4.26340494	3.16238868	0.09062909	0.11981459
Lamb1	0.30874313	4.17024606	3.16207796	0.09064383	0.11981481
F730043M19	-0.8247749	0.97539026	3.16164642	0.09066431	0.11982261
Col6a1	-0.2894432	5.05740719	3.16039074	0.09072393	0.11986927
Muc15	-0.8845835	2.10105816	3.16028838	0.09072879	0.11986927
Adsl	-0.3725284	4.44688596	3.15864362	0.09080695	0.11995325
Slc35c2	0.67665861	1.83053402	3.15747523	0.09086252	0.12000737
Fam134b	-0.2993887	5.61864022	3.15677505	0.09089584	0.12001056
Trim34a	0.54634514	3.15628048	3.15630974	0.09091799	0.12001056
Med18	0.84005854	1.08521293	3.15615227	0.09092549	0.12001056
2010106C02I	-2.8558177	-1.616105	3.15613444	0.09092634	0.12001056
Klc4	0.58682836	2.29237103	3.15589081	0.09093794	0.12001056
Psmd11	0.32271199	5.94030805	3.15510324	0.09097546	0.1200408
Dcp2	-0.2234753	7.18498446	3.15429411	0.09101402	0.1200724
9630013A20I	-0.7014953	1.48920394	3.15391768	0.09103197	0.12007681
1110058L19I	0.32477052	4.25525354	3.15351729	0.09105106	0.12008272
Tcerg1l	-0.4645167	2.69450517	3.15251805	0.09109873	0.12012321
D430041D05	-0.3348975	7.66574254	3.15226115	0.091111	0.12012321
Esco1	-0.2719783	5.65472216	3.14963008	0.09123668	0.12025238
Zak	-0.2373542	6.48262938	3.14959779	0.09123822	0.12025238
Rbm14	0.50969493	3.06542224	3.14828217	0.09130115	0.12030799
Gm4461	-1.3727287	0.55151926	3.14810353	0.09130969	0.12030799
Jup	0.28215814	5.22506196	3.14700895	0.09136209	0.12035773
Des	0.97708148	0.62764967	3.14545037	0.09143676	0.12040207
Zfyve9	0.39258701	3.85483059	3.14542476	0.09143799	0.12040207
Zfp738	-0.2915472	5.23921593	3.14467135	0.09147411	0.12040207
Zyg11b	-0.2930989	8.98696616	3.14443113	0.09148563	0.12040207
Cnr2	-0.7793231	0.95599685	3.14411188	0.09150094	0.12040207
Rnf32	-0.4369455	3.43782625	3.14395377	0.09150852	0.12040207
Grid2ip	-1.0144266	1.10690443	3.14388521	0.09151181	0.12040207
Myh6	-1.0918191	0.26047312	3.14386174	0.09151294	0.12040207
B3gat1	-0.3310193	6.02154827	3.14330332	0.09153973	0.12041805
Polr2l	0.3513065	3.98022412	3.1405543	0.09167178	0.12057245
Pdgfrb	-0.5271778	4.4274663	3.13870744	0.09176062	0.12066998
Rbm48	-0.3347235	3.43229954	3.13826246	0.09178204	0.12067224
Plekhg3	-0.3516499	3.33748503	3.13806182	0.0917917	0.12067224
Insl6	0.86031863	1.1793507	3.13696297	0.09184462	0.12070883
Ccng1	0.23388627	8.15001711	3.13687412	0.0918489	0.12070883

Arel1	-0.2434813	6.26592541	3.13646177	0.09186877	0.12071564
Mavs	0.52657105	4.855431	3.13522981	0.09192817	0.12076193
Zdhhc2	-0.2498018	5.21262082	3.13512161	0.09193339	0.12076193
4930451G09	-0.7154122	1.59307176	3.13462308	0.09195744	0.12077422
Copa	-0.2619858	7.7765974	3.13307058	0.09203238	0.12085334
Rprd1b	0.31039842	5.10640068	3.13050813	0.09215623	0.12096957
Trh	-2.3489039	-1.6101563	3.1302255	0.0921699	0.12096957
Clic1	0.4981945	4.48526169	3.12980236	0.09219038	0.12096957
Ccl9	-0.5986867	1.82352796	3.12958531	0.09220088	0.12096957
Ahcy	0.38041865	3.87366617	3.12958432	0.09220093	0.12096957
Coq7	0.46095566	4.22424086	3.12941338	0.0922092	0.12096957
Tsen2	-0.5309783	2.55053388	3.12862706	0.09224727	0.1210002
Iigp1	0.46952473	4.08486279	3.12677002	0.09233726	0.12108965
Hnrnpa0	0.19964863	7.6352573	3.12661179	0.09234493	0.12108965
2410002F23I	-0.4297437	3.20414754	3.12579131	0.09238472	0.1211225
Gspt2	0.33650991	4.58635054	3.1232829	0.0925065	0.12126282
Rbm10	0.27302744	4.92040676	3.12293164	0.09252357	0.12126585
Zfp799	-0.2647688	5.66244101	3.12249725	0.09254468	0.12127419
Atp6v0e2	0.28211138	5.29601481	3.12096316	0.09261929	0.12135261
Tmc2	-1.1277027	-0.2558401	3.11814347	0.0927566	0.12151315
Spryd4	0.58246168	2.31508092	3.11592165	0.09286497	0.12163572
Rnf149	-0.298578	5.59735815	3.11472109	0.09292358	0.1216931
2310014L17I	-1.1613576	-0.1128098	3.11416317	0.09295084	0.12170452
Lonrf1	0.27421922	6.32971245	3.11383125	0.09296706	0.12170452
Dtnbp1	0.31993367	5.32169113	3.11363341	0.09297673	0.12170452
Tnfrsf14	-3.0167566	-1.8318768	3.1131942	0.0929982	0.12171324
Eif3e	0.23616303	7.15623584	3.11261412	0.09302656	0.12173098
Kdelc2	-0.3340459	4.49541638	3.11211533	0.09305096	0.12174352
Aim1	0.5289189	3.96012799	3.11165788	0.09307334	0.12175342
Ankrd39	-0.6579898	1.7336555	3.11075211	0.09311767	0.12179204
Phf11b	1.30830417	0.71371584	3.10979228	0.09316468	0.12181997
Bloc1s1	0.58968861	5.14775056	3.10955546	0.09317629	0.12181997
Cd163	-0.8593773	1.35450107	3.10940838	0.09318349	0.12181997
Ak2	0.39366249	4.85332912	3.10873514	0.09321649	0.12183561
Hist1h2bk	1.69559902	-1.1580995	3.10855943	0.0932251	0.12183561
Pja2	-0.2583697	8.93374676	3.10692063	0.09330549	0.12192128
Ddo	0.50380456	3.49965367	3.10634675	0.09333366	0.12193277
Polr3b	-0.3436678	4.43934886	3.10492132	0.09340367	0.12193277
3632451O06	0.41547982	4.23193063	3.10489378	0.09340503	0.12193277
Rrp9	0.76741641	1.10828275	3.10487804	0.0934058	0.12193277
Apol8	-1.0986941	-0.2681025	3.10472783	0.09341318	0.12193277
Clasp2	-0.2312991	8.0401354	3.1046518	0.09341692	0.12193277
Pold1	-0.8825114	0.57114693	3.10447817	0.09342545	0.12193277
Eif2ak3	-0.4376848	3.26880954	3.10432518	0.09343297	0.12193277
Sdha	-0.1997487	8.6038196	3.10377503	0.09346002	0.12194531

Gja3	3.59705317	-1.8770498	3.10352632	0.09347225	0.12194531
02-Mar	0.42821556	5.33229017	3.10302518	0.0934969	0.12195811
BB557941	2.23780015	-0.6396822	3.10210071	0.09354239	0.12199547
Tmem143	0.54967356	2.62157586	3.10167848	0.09356318	0.12199547
Lypla1	0.31803593	5.19566608	3.10126199	0.09358369	0.12199547
Sugp1	0.30989268	4.58008137	3.1012371	0.09358491	0.12199547
Xkrx	-0.6288062	2.04510831	3.10076795	0.09360802	0.12200624
Acat2	0.28068494	5.25145575	3.10040134	0.09362608	0.12201044
Hsp90b1	0.25620763	9.12486298	3.09969081	0.0936611	0.12203672
Ppp1r37	0.32093604	4.26330119	3.09868585	0.09371066	0.12208194
H2-Aa	-0.7749123	4.46773061	3.0980657	0.09374125	0.12210244
Tas2r137	-3.5423641	-1.9346366	3.23543562	0.09378912	0.12211603
C2cd4c	0.61182141	3.3817538	3.09709271	0.09378928	0.12211603
Zfp820	0.71458067	1.39759634	3.09695135	0.09379626	0.12211603
Josd2	-0.5913241	2.23009201	3.09535785	0.09387499	0.12219518
Zfp948	-0.4293828	3.61939955	3.09511905	0.09388679	0.12219518
Fam221a	1.27893455	0.13123574	3.09434515	0.09392506	0.12222563
Gfpt2	-0.3466339	4.19350323	3.09368377	0.09395777	0.1222401
Tmppe	-0.5222099	3.02552922	3.09291091	0.09399602	0.1222401
B3galt1	-0.2086871	5.80671302	3.09291073	0.09399603	0.1222401
H2-D1	0.36238882	5.17764564	3.09289142	0.09399699	0.1222401
Ppp1r1c	-1.0958329	1.07525846	3.09261778	0.09401054	0.1222401
Cdpf1	0.58023797	3.2397897	3.0905988	0.09411056	0.1223508
Ccdc32	0.36511196	4.90145932	3.08683673	0.09429727	0.12257415
Wbp1	0.4310255	3.50318168	3.08488563	0.09439428	0.12268085
Ncln	-0.5878841	2.44640941	3.0836319	0.09445667	0.12274253
Engase	-0.7261343	0.75839057	3.08312592	0.09448187	0.12275586
Lrp10	0.51651799	4.25497654	3.08262535	0.0945068	0.12276885
Ero1lb	-0.406195	3.76443278	3.08229759	0.09452313	0.12277066
Ube2g2	0.34610188	4.67683069	3.08128269	0.09457372	0.12281695
Slc9b2	0.43435179	4.25861711	3.08000462	0.09463746	0.12288032
Rps7	0.25426083	6.62288406	3.07966191	0.09465457	0.12288311
Klhl1	-0.5275269	3.01700362	3.07931781	0.09467174	0.122886
Rfx7	-0.2353575	7.87409775	3.07845244	0.09471495	0.12292268
4932438H23I	-1.3408353	-0.1891643	3.07646808	0.09481412	0.12303196
Iah1	0.50744195	3.18003593	3.07569848	0.09485262	0.12306248
Rb1cc1	-0.2729432	8.50531799	3.07394621	0.09494033	0.12313424
Tmem14c	0.46536784	3.11534299	3.07380041	0.09494764	0.12313424
Prdm5	-0.4443643	3.03414188	3.07336118	0.09496964	0.12313424
Sh2d4b	1.21704828	-0.1638562	3.07331249	0.09497208	0.12313424
Dsel	-0.3031315	5.03122799	3.07309773	0.09498284	0.12313424
Selk	0.30992255	6.15916449	3.07273637	0.09500095	0.12313717
Htatip2	0.62564021	2.04140005	3.07236715	0.09501946	0.12313717
Hcst	-2.2570488	-1.5368851	3.07215606	0.09503004	0.12313717
Cav2	0.34815119	5.18438214	3.0704711	0.09511457	0.12321063

Ngfr	0.42196723	3.55633351	3.07042828	0.09511672	0.12321063
Wwox	0.32293855	4.0488127	3.06805061	0.09523615	0.1233459
Zfp846	-0.4105264	3.9051803	3.06705802	0.09528606	0.12337691
Zbbx	-1.5305965	-0.6191186	3.0669775	0.09529011	0.12337691
Mtbp	-0.7664198	1.38346682	3.06631711	0.09532334	0.12339508
Abcf2	0.27074519	5.35137765	3.06610179	0.09533417	0.12339508
Gpr18	-1.5001543	-0.4416102	3.06543587	0.0953677	0.12341903
Uggt1	-0.4032529	5.11580547	3.06306726	0.09548704	0.12354444
Kcna5	-0.817911	0.85548491	3.06279563	0.09550074	0.12354444
Kif4	0.70886987	1.16208681	3.06237405	0.095522	0.12354444
Lpp	0.25750906	7.6917757	3.06231998	0.09552473	0.12354444
Lrtm2	-0.3487647	5.89053661	3.06193179	0.09554431	0.12355033
Wbscr25	-1.9845066	-0.9108051	3.06115522	0.09558351	0.12356487
Naf1	0.32075963	4.34017599	3.06111327	0.09558562	0.12356487
Ptger3	0.59410838	3.47970624	3.06038917	0.09562219	0.12358215
Gucy1a2	-0.3727533	7.95296243	3.06025294	0.09562907	0.12358215
1110032F04I	0.56128118	2.18019869	3.05906018	0.09568934	0.12363201
Nfkb2	1.16160435	0.95481219	3.05889415	0.09569773	0.12363201
Lpar4	-0.6442615	2.27780024	3.05823784	0.09573092	0.12365544
Sparcl1	-0.3082451	8.69193104	3.05782371	0.09575186	0.12366307
Hbp1	0.22429617	6.72167701	3.05746396	0.09577006	0.12366714
Ppp1r13b	-0.2673981	5.6077684	3.05690097	0.09579855	0.1236845
Zfp617	0.25955202	5.81358953	3.05618982	0.09583456	0.12370181
Fance	0.43135215	2.70443339	3.05599303	0.09584452	0.12370181
Sema6c	-1.0135372	0.69316727	3.05574449	0.09585711	0.12370181
Pam16	0.38933336	3.18915383	3.05509145	0.0958902	0.12371731
Hadha	0.25792709	4.94884333	3.05491316	0.09589923	0.12371731
Nabp1	0.34368524	3.64228668	3.05323325	0.09598441	0.12380776
Rhod	0.75869257	1.72168286	3.05217871	0.09603792	0.12385735
Nck2	0.30911472	4.64343953	3.05116054	0.09608963	0.12388863
Rgag4	-0.5195462	2.68256884	3.05110741	0.09609232	0.12388863
Iars	-0.292818	6.46595723	3.05059786	0.09611821	0.12390257
Tead4	-1.7541575	0.04047983	3.04794986	0.09625288	0.12405671
Fkbp7	0.4939374	3.61304404	3.0465095	0.09632622	0.12413177
4933430I17R	1.98334042	-1.6878358	3.04571471	0.09636672	0.12416449
Cdc25a	0.44200045	2.73292837	3.04489598	0.09640846	0.1241988
Pcdha8	-1.3807589	0.18925913	3.04450871	0.09642821	0.12420478
Lipg	0.75805593	1.29673501	3.04381425	0.09646364	0.1242095
Cd37	1.57617303	-0.4761001	3.04380996	0.09646385	0.1242095
Barhl2	-1.6634856	-0.0727471	3.04354815	0.09647721	0.1242095
Zfyve16	-0.3057094	4.90161948	3.04240455	0.0965356	0.12421922
Bcat2	0.517311	2.1273565	3.04201115	0.09655569	0.12421922
Mrpl4	0.40390691	4.75516618	3.04146865	0.09658341	0.12421922
Rell2	-0.6419019	1.68782175	3.04139068	0.0965874	0.12421922
Susd5	-0.6011544	2.31888443	3.04135508	0.09658922	0.12421922

Ssr3	0.28586979	8.00002703	3.04135325	0.09658931	0.12421922
Gpalpp1	0.25484293	5.76288545	3.04132888	0.09659055	0.12421922
Arhgef6	0.25558951	6.45736664	3.0409901	0.09660787	0.12422205
Ncbp1	-0.2709315	4.95939243	3.03932718	0.09669292	0.12431196
Gm20753	-2.0397324	-1.593677	3.03886036	0.09671681	0.12432323
Zc3h11a	-0.1943417	6.83947605	3.03843173	0.09673875	0.12433199
Mark4	-0.3649844	3.63180136	3.03678142	0.09682329	0.12442118
Cytip	0.55978476	2.74174815	3.03600382	0.09686315	0.12444081
Pycr1	-1.1214293	0.14120247	3.03575151	0.09687608	0.12444081
Usp36	-0.3255903	4.61989909	3.03545796	0.09689114	0.12444081
Mus81	0.51687692	2.15460591	3.03530227	0.09689913	0.12444081
B4galnt1	0.29568679	5.39993322	3.03498613	0.09691535	0.1244422
Ttc19	-0.257788	6.26122451	3.03284009	0.09702553	0.12456421
Usp13	-0.5118844	3.87018205	3.03221641	0.09705757	0.12456848
Whsc1l1	-0.1932745	7.71998622	3.0321856	0.09705916	0.12456848
Eif2s3y	-0.4674522	5.08863465	3.03076264	0.09713233	0.12464292
Fgl2	0.4250717	4.78953892	3.02952603	0.09719596	0.12470511
Atp6v1f	0.44836005	4.75645672	3.02796908	0.09727616	0.1247334
Il7r	-1.2882202	-0.0123462	3.0279676	0.09727623	0.1247334
Clic5	-0.5765425	2.94378902	3.02795179	0.09727705	0.1247334
Kif22	-0.8033852	0.89356003	3.02776359	0.09728675	0.1247334
AI987944	0.37738994	3.80950383	3.02762488	0.0972939	0.1247334
Ybx2	-1.2998193	-0.0781036	3.02615018	0.09736994	0.12480365
Mlph	0.59728629	2.44175312	3.02575228	0.09739047	0.12480365
Tshb	-2.0928334	-1.2726407	3.02562097	0.09739725	0.12480365
Grk5	0.27610413	5.33355787	3.02538492	0.09740943	0.12480365
Abhd11	0.39316788	3.15532683	3.02480754	0.09743924	0.12482118
Kcnk6	-0.7644352	1.35946233	3.02453154	0.09745349	0.12482118
Spint1	-1.3206403	-0.3384106	3.02310461	0.09752721	0.12489614
Gm3893	-0.3953352	5.8712045	3.02279828	0.09754304	0.12489696
Gamt	0.81919864	1.99468364	3.02197469	0.09758563	0.12493203
Fus	-0.3568266	5.94252772	3.02071816	0.09765065	0.1249958
Gtf2h3	-0.375774	3.5832589	3.01890583	0.09774452	0.12509647
2310061J03F	0.67696005	2.3589709	3.0185737	0.09776173	0.12509902
Dcaf6	-0.2993454	6.91944124	3.01658254	0.097865	0.12521167
Psenen	0.49770386	5.2136911	3.01501348	0.09794647	0.12529424
Slc30a9	-0.2274909	6.51316511	3.01475261	0.09796002	0.12529424
Ypel4	0.61157407	2.45977121	3.01355092	0.09802248	0.12535462
Casz1	0.74956136	2.11849461	3.01308049	0.09804695	0.12536485
Flt3	-0.9846011	0.51524891	3.0128106	0.09806098	0.12536485
Nbl1	0.63643028	6.51471903	3.01178211	0.0981145	0.12541376
Cmtm3	0.42895668	3.14524451	3.01136803	0.09813606	0.12541665
Kcna1	-0.2893075	7.66046982	3.01115241	0.09814729	0.12541665
Gm12429	-0.7009003	0.85949264	3.01077483	0.09816695	0.12542228
Spopl	-0.3530499	3.63922646	3.01030873	0.09819123	0.12542792

Gstm4	0.58135093	1.67802119	3.01006659	0.09820385	0.12542792
Tmem66	0.26466449	5.98328088	3.00975787	0.09821993	0.12542792
Cnbd2	-0.5121092	2.38608532	3.00951857	0.09823241	0.12542792
1700128F08I	-1.4993493	-0.8445133	3.00773917	0.09832521	0.12551651
9130401M01	0.28325874	4.78490337	3.00746163	0.0983397	0.12551651
Slc9a5	-0.5533232	2.60183647	3.00731022	0.0983476	0.12551651
Mrpl36	0.36901528	5.37409144	3.00504435	0.09846595	0.12564805
Msx2	0.77876031	2.29540939	3.00299395	0.0985732	0.12576537
Sh3d19	0.2793886	6.73968738	3.00263921	0.09859177	0.12576954
Jph1	-0.3599201	4.82841873	3.00204788	0.09862273	0.12578951
Olfr1393	2.59711949	-1.883936	3.00091564	0.09868204	0.12583171
Kcnj11	-0.3594592	3.39176252	3.00083186	0.09868644	0.12583171
Rap2b	0.24469378	5.93718849	2.99993272	0.09873357	0.12587229
Has1	-1.1148454	0.34808261	2.9983993	0.09881402	0.12595386
Tnnt3	2.79043126	-1.5294982	2.99812898	0.09882821	0.12595386
Chmp1b	0.31792525	5.41564145	2.99744913	0.09886391	0.12596948
Mir6236	-0.7080268	4.65422758	2.99712513	0.09888092	0.12596948
Papolb	-1.2863186	0.72027379	2.99702014	0.09888644	0.12596948
Ptcra	-1.279914	-0.9409011	2.9961061	0.09893447	0.12599301
Mir384	-2.6846662	-1.205553	2.99603517	0.0989382	0.12599301
Fam206a	0.2886506	5.91732222	2.99579361	0.0989509	0.12599301
Pvt1	-0.5940892	2.41868138	2.99494459	0.09899554	0.12603033
Ggcx	-0.3852539	3.1388994	2.99407326	0.09904139	0.12606917
Oacyl	-1.4793999	0.29856542	2.99317776	0.09908853	0.12610965
Grin1	-0.3390467	6.12469084	2.99143597	0.0991803	0.12617424
Gm13315	2.38756937	-1.3209303	2.99137679	0.09918342	0.12617424
Mab21l2	-1.8453923	-0.8035876	2.99120208	0.09919263	0.12617424
Zfp329	-0.2519767	6.15661559	2.99066533	0.09922094	0.12617424
Slc25a28	0.31775454	3.8932425	2.99061776	0.09922345	0.12617424
Plekhg4	1.17105699	1.36026031	2.99046705	0.0992314	0.12617424
Prdm12	-1.7578374	-0.1195421	2.98980609	0.09926627	0.12619103
CK137956	1.01933246	0.78151713	2.98940328	0.09928753	0.12619103
Mrpl45	0.42702705	3.64461514	2.98883205	0.09931768	0.12619103
Armcx6	0.96678469	0.96305597	2.98881076	0.09931881	0.12619103
Gm7609	-1.2019664	-0.6325316	2.98876229	0.09932137	0.12619103
Amotl1	0.27230175	6.88193783	2.98808199	0.0993573	0.12620273
Rgmb	-0.3041182	4.71335	2.98779272	0.09937258	0.12620273
Ndc1	-0.4682695	2.89070847	2.98771594	0.09937664	0.12620273
C030029H02I	-0.9773143	1.08253575	2.98687402	0.09942113	0.12623973
Eri3	0.31803995	4.21132991	2.98655742	0.09943787	0.12624148
Ilkap	0.31064164	4.44686146	2.98451745	0.09954581	0.126359
Pcsk2	-0.2842771	7.2390638	2.98256248	0.09964938	0.12647081
Klk11	3.09831575	-1.816411	3.03450733	0.09966909	0.12647081
Armcx5	0.23515391	5.36847734	2.98198372	0.09968006	0.12647081
Kcnk5	0.72963864	2.33406875	2.98148961	0.09970627	0.12647097

Gper1	0.6250829	1.96462985	2.98140118	0.09971096	0.12647097
Inmt	0.63128355	3.49614324	2.98029231	0.0997698	0.1264978
Arl6ip5	0.30143121	3.89534564	2.98005958	0.09978216	0.1264978
Xlr4c	2.12708375	-1.3636002	2.98002088	0.09978421	0.1264978
Gnb1	0.19024172	9.34935257	2.97940385	0.09981698	0.1264978
Tm2d2	0.56561076	4.24607519	2.97929639	0.09982269	0.1264978
Smad3	0.24698415	6.75745112	2.97926299	0.09982446	0.1264978
2010111I01R	-0.2992718	4.66778239	2.97833631	0.0998737	0.12653393
Kctd2	0.2717345	5.52235383	2.97814694	0.09988376	0.12653393
Mea1	0.43884868	4.7171691	2.97782359	0.09990095	0.12653621
Tk1	-1.8759633	-0.8897527	2.97681218	0.09995474	0.12658483
Acadm	0.36279905	5.06558011	2.97454533	0.10007542	0.12669838
5830444B04I	-0.9093465	1.32362467	2.97426616	0.1000903	0.12669838
Ccni	0.22954306	8.52286496	2.97425953	0.10009065	0.12669838
Ooep	1.52155345	-1.0041709	2.97377199	0.10011663	0.12671176
Lsm14a	0.23531765	8.08565336	2.97333561	0.1001399	0.12672168
Cldn10	-0.6526868	1.85072512	2.97259914	0.10017917	0.12673637
Ubr3	0.27604739	9.67637616	2.9722708	0.10019669	0.12673637
Ap3s2	-0.2286739	6.62475984	2.97225066	0.10019776	0.12673637
Ggps1	0.31202163	6.34114367	2.97016642	0.10030903	0.12685759
Gm20751	-1.6575955	-1.2658381	2.96934262	0.10035305	0.12688617
Tmem183a	0.25413088	5.63463001	2.96916568	0.10036251	0.12688617
Emilin2	2.23050305	-1.0549577	2.96793969	0.10042807	0.12694905
Tada3	0.28604991	5.0122739	2.96765811	0.10044313	0.12694905
AA387883	-1.0808513	0.28824158	2.9671864	0.10046838	0.12696143
Meis1	-0.8063961	1.67791817	2.96588866	0.10053786	0.1270297
Abca12	-1.8792281	-1.4656762	2.96342895	0.10066971	0.12716727
Cog6	-0.2230419	5.57264426	2.96328042	0.10067768	0.12716727
Nudt9	0.34292693	5.37527833	2.96271408	0.10070807	0.12718611
Chfr	-0.5129508	4.18743872	2.96198979	0.10074696	0.12721567
Pi16	1.42765339	-0.6008267	2.96132624	0.10078259	0.12724112
Ndst2	-0.4023168	3.30347432	2.9602697	0.10083937	0.12728479
Zfp85	0.43364134	2.44957893	2.96010617	0.10084816	0.12728479
Gpr158	-0.2851291	8.18522937	2.95897889	0.10090878	0.12734175
Cd59a	0.41624708	4.58348747	2.95855886	0.10093138	0.12735072
Zfp26	-0.2713955	6.27134914	2.95590586	0.10107426	0.12749239
Tldc1	-0.7170742	1.15750519	2.95569916	0.1010854	0.12749239
Znhit6	0.25148645	5.17501458	2.9556101	0.1010902	0.12749239
Hdac8	-0.5775436	1.90663571	2.95474299	0.10113696	0.1275318
Tinf2	0.48575808	2.67584235	2.95363872	0.10119654	0.12758736
Scn11a	3.41940626	-1.9906575	2.95319043	0.10122074	0.12759829
Ints10	-0.3658443	4.16093091	2.95185013	0.10129314	0.12766997
Reep4	-1.2298588	0.28493167	2.95097411	0.10134049	0.12771007
Ctsc	0.55535622	2.05099363	2.95049689	0.10136629	0.12772301
Thy1	0.22238271	7.45923875	2.95008757	0.10138843	0.12773132

Dph6	0.21167183	6.23839124	2.94737801	0.10153514	0.12789654
Plekhg2	0.42064736	4.20003844	2.94662254	0.10157608	0.12790363
Rpl13a	0.26689066	7.00463303	2.94645057	0.10158541	0.12790363
Txnip	0.42203817	6.72781444	2.94641296	0.10158745	0.12790363
Mex3d	-0.4385208	3.9163	2.94473591	0.10167843	0.12799857
Orc4	-0.2320762	5.58400376	2.94384788	0.10172664	0.12803965
05-Sep	-0.2192245	6.40078037	2.94311285	0.10176657	0.12806922
Usb1	0.66432331	1.54034789	2.94284175	0.1017813	0.12806922
Tgs1	-0.357545	6.52407515	2.94084933	0.10188964	0.12816335
Psmc6	0.23795116	5.85723152	2.94069664	0.10189795	0.12816335
Ptbp3	0.24925979	7.19156415	2.94060599	0.10190289	0.12816335
Ubald2	0.50924603	2.32642476	2.93907145	0.10198643	0.1282488
Mmp2	0.80986822	1.32979393	2.9383135	0.10202773	0.12828111
Pou6f1	-0.2963816	5.0576674	2.93698547	0.10210014	0.12835251
Gm13807	2.16411989	-2.139352	2.93644914	0.1021294	0.12836965
4930550C14I	0.89709032	1.73402426	2.93551948	0.10218013	0.12841379
H2-M3	0.66976594	2.40479271	2.93509946	0.10220307	0.12842298
Adra2c	-0.5424073	2.43547941	2.93400707	0.10226274	0.12847832
Senp6	0.18004224	8.47980854	2.93284024	0.10232653	0.12848988
Dnah17	-1.5923644	-1.109053	2.93268648	0.10233493	0.12848988
Pde6c	-2.3742664	-0.9940223	2.9326816	0.1023352	0.12848988
Klhl23	-0.3315886	5.1496477	2.93256636	0.1023415	0.12848988
Sftpc	2.80988652	-1.19407	2.9323854	0.1023514	0.12848988
Pan3	-0.2048447	6.70390034	2.93212333	0.10236574	0.12848988
Ak5	0.28296243	6.20518148	2.93140031	0.10240531	0.12851991
Gm14057	-0.9124049	2.1774852	2.930805	0.1024379	0.12854119
Ddx5	-0.216729	9.43719994	2.92960199	0.10250379	0.12860424
A530013C23I	1.83273017	-1.4641545	2.92928185	0.10252134	0.12860662
Tmem74	-0.6729338	1.24584302	2.92755236	0.10261618	0.12870595
Cpeb4	-0.2747981	8.15948239	2.92701381	0.10264573	0.12872337
Rplp0	0.340519	6.63245504	2.92646548	0.10267583	0.12874148
Ralbp1	0.20644098	6.77749448	2.92504879	0.10275365	0.12879739
Dna2	-1.5766263	-0.6860066	2.92477019	0.10276896	0.12879739
Aff4	0.19105259	8.98795918	2.92451401	0.10278304	0.12879739
Sirt3	0.33594411	3.59719105	2.92441422	0.10278853	0.12879739
Dpt	1.42320788	0.73137947	2.92408626	0.10280656	0.12879739
Pacs2	-0.2062209	6.07020537	2.9239429	0.10281445	0.12879739
Knop1	0.27947004	5.63143563	2.92296058	0.10286848	0.12881823
Cenpi	-1.5672445	-0.5162075	2.92286334	0.10287383	0.12881823
Gsg1l	-0.2716595	5.847959	2.92278569	0.10287811	0.12881823
Prdm11	-1.7239001	-0.5723408	2.92244776	0.1028967	0.1288219
Lrrc75a	-0.3865994	3.25761938	2.91999616	0.10303175	0.12894212
Osmr	0.46502541	3.50329174	2.91991816	0.10303605	0.12894212
Rxra	0.43105783	5.7142349	2.91985031	0.1030398	0.12894212
Tmem8b	-0.3038741	5.07765794	2.91945446	0.10306162	0.1289498

Spred2	0.21967221	6.51431557	2.91838185	0.10312079	0.1290042
Gad1	-0.4301449	7.57420596	2.91764743	0.10316133	0.12903527
Ctps	-0.3534767	3.98879188	2.91736134	0.10317713	0.12903539
Got2	0.20465209	7.01775185	2.91694068	0.10320036	0.12904481
Adck2	-0.4752201	2.23540847	2.91574806	0.10326626	0.12910757
Ppp1r26	0.31589642	4.18231138	2.9150924	0.10330251	0.12913325
Lrrc8e	-0.9908684	-0.1319681	2.91444068	0.10333856	0.12915688
Nae1	-0.2742165	5.057229	2.9138789	0.10336964	0.12915688
Ddit3	0.34275524	3.40717023	2.91370654	0.10337918	0.12915688
Ogdh	-0.2532623	7.67834973	2.91361452	0.10338428	0.12915688
Fxyd4	-1.6138184	-0.3788599	2.91310221	0.10341264	0.12917268
Hbs1l	-0.2233534	5.48903497	2.91162699	0.10349436	0.12923883
Sox12	-0.4283794	2.99499347	2.91157852	0.10349704	0.12923883
Rmdn1	-0.4128788	3.73525051	2.90959778	0.10360689	0.12935634
Bhlha15	1.46066401	-0.1500511	2.90914842	0.10363183	0.12936242
Slc4a3	-0.4661013	3.42105113	2.90893702	0.10364356	0.12936242
Rpl26	1.37255279	-1.291403	2.90864975	0.10365951	0.12936242
E130308A19I	0.29384276	5.17680017	2.90837588	0.10367472	0.12936242
A330074K22I	-0.8024567	1.28954745	2.90750614	0.10372303	0.1294027
Ahsa2	0.28277532	4.73143672	2.90722804	0.10373848	0.1294027
Sdf4	-0.1823492	7.98580178	2.90594039	0.10381007	0.12943397
D830030K20I	-1.0812318	0.44318328	2.90571336	0.1038227	0.12943397
Fxyd2	1.24838682	-0.1409644	2.9056086	0.10382853	0.12943397
Chrm1	-0.2558667	5.75229557	2.90543432	0.10383822	0.12943397
Manbal	0.4241046	3.04796041	2.90515913	0.10385354	0.12943397
Trim41	-0.2483999	4.34501675	2.90502994	0.10386073	0.12943397
Mrps22	0.24708968	4.4030088	2.90479525	0.10387379	0.12943397
Taf7l	-0.8943993	1.07692648	2.90417265	0.10390845	0.12945753
Cux1	-0.1654831	8.04491016	2.90291918	0.10397827	0.12951672
2010300C02I	-0.286197	5.50339108	2.90275403	0.10398747	0.12951672
Pon2	0.47194051	5.932534	2.90205316	0.10402654	0.12954575
Tsga10	-0.2818241	4.98707404	2.90055079	0.10411035	0.12963048
Zfp788	-0.1997621	6.23732821	2.89961734	0.10416246	0.12967571
9130019P16I	1.09594608	0.2995342	2.89847726	0.10422614	0.12973535
Sh3rf2	0.39853288	3.63581108	2.89729056	0.10429248	0.12979827
Tbl3	0.57330771	2.07913153	2.89668583	0.10432631	0.12982071
Taf9	0.24132625	5.52362682	2.89598787	0.10436536	0.12984965
Prex1	-0.3114621	5.9249427	2.89467765	0.10443873	0.12991766
Zkscan6	-0.4784681	2.9109064	2.8944471	0.10445164	0.12991766
Aox4	-1.057142	0.66151393	2.89260165	0.10455509	0.13002491
Nfya	0.22891177	5.71546218	2.89234453	0.10456951	0.13002491
Cbr4	0.3352003	4.17034349	2.89006902	0.10469726	0.13014513
Tspyl5	0.21826376	5.78190886	2.89005828	0.10469786	0.13014513
A230072E10I	-1.1024471	0.01454516	2.88872033	0.10477306	0.13021891
B3gnt3	1.71903915	-0.0407022	2.88800991	0.10481301	0.13024887

Fbxl3	0.19343551	7.46914678	2.88678939	0.10488169	0.13031452
Casp3	0.3646539	4.02964314	2.88398663	0.10503962	0.13049101
Loh12cr1	0.44791069	2.95649361	2.88317066	0.10508564	0.13051856
Zbed3	0.30535806	5.56461188	2.88303042	0.10509356	0.13051856
Snx11	-0.3931548	3.09313506	2.88156338	0.10517638	0.13060169
Extl1	-0.3579468	3.57333937	2.88100373	0.105208	0.13061124
Wfdc15b	-2.1057491	-2.0026989	2.88056046	0.10523304	0.13061124
Pafah2	0.36437215	3.40663434	2.88046227	0.10523859	0.13061124
Tmem242	0.40435343	4.09529041	2.8803023	0.10524764	0.13061124
Rabep2	0.57166738	2.27559331	2.87990018	0.10527037	0.13061973
6330549D23I	0.84713593	1.73944601	2.87883213	0.10533078	0.13063923
Syt12	-0.3143218	4.19268637	2.87880577	0.10533227	0.13063923
Fam20b	-0.2607762	4.91662611	2.87877919	0.10533377	0.13063923
P4ha2	-0.5099217	1.85672875	2.87779161	0.10538967	0.13068884
Lrp8	-0.4475188	5.85580177	2.87747472	0.10540761	0.13069137
Lrrc41	0.3087512	4.24108537	2.87686612	0.10544208	0.13071439
Limd2	0.30569458	4.46135496	2.87397064	0.10560626	0.13089817
Gpr111	-2.8378408	-2.0538352	2.87350597	0.10563264	0.13091112
Efs	0.54872202	2.17687059	2.87295915	0.10566368	0.13092986
Oaz3	-0.6167741	2.23339133	2.87098992	0.10577559	0.13103601
1700080N15	-1.3488535	0.14757437	2.87089062	0.10578123	0.13103601
Psmb6	0.33917188	4.90056129	2.87025327	0.10581748	0.1310373
Drc1	-0.4002632	3.18557095	2.87022084	0.10581933	0.1310373
Rnf169	-0.1886637	6.75839916	2.87003132	0.10583011	0.1310373
Gm7008	1.11914768	-0.366684	2.86937821	0.10586728	0.13106358
Ndufb8	0.30549543	4.70216124	2.86842432	0.10592159	0.13109835
Mapk8ip1	-0.2505586	5.53140948	2.86832466	0.10592726	0.13109835
Popdc3	-1.7437697	-0.7530781	2.86645588	0.10603377	0.1312104
Trim21	0.4569968	3.83998389	2.8659671	0.10606164	0.13122514
Psmd14	0.20141252	6.24716733	2.86546885	0.10609007	0.13122915
A830082N09	-0.4287127	4.43826558	2.86521905	0.10610432	0.13122915
Aph1a	-0.4990654	2.94250574	2.86507076	0.10611279	0.13122915
Sirt5	-0.4337185	2.29635344	2.86404305	0.10617146	0.13128196
Ifit1	0.42310121	6.03124522	2.86333007	0.10621219	0.13131257
Tcf24	0.87049242	1.26987938	2.86164573	0.10630848	0.13139831
Tacr2	1.34408715	0.30130535	2.86155765	0.10631352	0.13139831
Adipor1	0.28906277	6.28407623	2.86113934	0.10633745	0.13140813
AI314180	-0.2274612	7.16951713	2.85901927	0.10645883	0.13153484
Chrm3	-0.4085761	3.59287046	2.85818664	0.10650655	0.13153484
Slc27a1	-0.4558298	3.86559533	2.85818334	0.10650674	0.13153484
Zim1	-0.7509381	1.09994664	2.8580009	0.1065172	0.13153484
C1qtnf3	2.6722541	-2.2217414	2.85794484	0.10652041	0.13153484
Snd1	-0.2280412	5.02085632	2.85767284	0.10653601	0.13153484
2810049E08I	-1.2023708	0.86852892	2.85711226	0.10656816	0.13154044
Exoc8	-0.2689874	4.65866988	2.85703572	0.10657255	0.13154044

Parp6	-0.270652	5.88867392	2.85489318	0.10669554	0.13164909
Pcdhgb2	-0.5371125	2.11965788	2.85456852	0.10671419	0.13164909
Fundc1	0.2669058	6.44940792	2.8543948	0.10672417	0.13164909
Glrx3	0.24292541	5.19640005	2.85438651	0.10672465	0.13164909
Polr2h	0.45294864	4.08275577	2.85394132	0.10675024	0.1316609
Zfp719	-0.286469	5.71662256	2.85306653	0.10680053	0.13169994
Ntsr2	-0.5013084	3.21728665	2.85283338	0.10681394	0.13169994
Olfr558	-3.2395497	-1.0715801	2.85243539	0.10683684	0.13170841
Cdk10	-0.3345646	4.11816307	2.8512417	0.10690554	0.13177196
Rabggta	-0.419763	3.21503695	2.85098274	0.10692045	0.13177196
Gm5136	-1.3478837	0.1255518	2.84984723	0.10698586	0.13183281
Arfgap1	0.32136949	4.53177529	2.8488903	0.10704103	0.13187123
L3mbtl3	0.31627298	4.1829686	2.84874977	0.10704913	0.13187123
Ctps2	-0.2113193	5.66905144	2.84713081	0.10714254	0.13194812
Fdx1	0.44385634	5.38919564	2.84711146	0.10714366	0.13194812
Etfdh	0.28774447	5.16765696	2.84442479	0.10729889	0.13211949
Pet112	-0.4203656	2.78670512	2.84314492	0.10737293	0.13217859
Btg1	0.3521732	5.37506462	2.84303915	0.10737905	0.13217859
AU022252	0.46035748	2.42007903	2.84180177	0.10745069	0.13222654
Meis2	-0.2444279	6.62560199	2.84168697	0.10745734	0.13222654
Med17	-0.2756914	3.9562245	2.84142583	0.10747247	0.13222654
Gimap1	-0.4993068	3.12082073	2.84099018	0.10749771	0.13222654
Farp1	-0.2116644	5.39111264	2.84086001	0.10750526	0.13222654
Mt1	0.39763866	6.01750431	2.84070012	0.10751453	0.13222654
Haus8	0.75993732	1.04829652	2.84028404	0.10753865	0.13223642
B020004J07I	-1.0598501	0.1922254	2.8394009	0.10758986	0.13227961
Psma2	0.2715396	6.68666278	2.83875493	0.10762735	0.1323059
Mpp5	0.21325296	6.90625661	2.83756017	0.10769671	0.13237137
Rxfp3	1.0806312	1.2141781	2.83703115	0.10772744	0.13238934
Dab2	0.30614968	8.40747023	2.83593619	0.10779108	0.13244775
Cldn11	-0.3965429	6.53876508	2.83361449	0.10792616	0.13259391
Vps26b	0.18038329	7.41283833	2.83242797	0.10799527	0.1326505
Apba3	-0.5023351	2.53870786	2.83212899	0.1080127	0.1326505
Tbca	0.35148354	6.43366701	2.83198883	0.10802087	0.1326505
Prrg3	-0.1969884	6.63317947	2.8316941	0.10803805	0.1326505
Gm20754	-0.7736816	0.72571659	2.83143888	0.10805293	0.1326505
Ifit3	0.43985725	5.94234365	2.83112703	0.10807111	0.13265301
Mir17hg	-3.045876	-1.6099372	2.83036127	0.10811578	0.13268429
Tubb2b	0.25952348	5.27879931	2.83013673	0.10812888	0.13268429
Ostc	0.32322058	4.63120836	2.82923354	0.1081816	0.13272917
Phldb1	-0.2736054	4.12078655	2.82777225	0.10826696	0.13281407
Scara5	0.63240949	1.61318864	2.82749563	0.10828313	0.13281408
Agbl3	0.38647658	3.1280662	2.82611654	0.10836378	0.13287399
2310069B03I	4.16653085	-1.3945449	2.87382035	0.1083643	0.13287399
Gas2l2	-1.8005321	-0.998051	2.824317	0.10846912	0.13298267

Pnrc2	0.36585522	7.27023271	2.82383496	0.10849735	0.13298975
Prr18	-0.3975651	3.12852725	2.82366604	0.10850725	0.13298975
Hmgb2	0.36867514	4.01804394	2.82277971	0.1085592	0.13302225
Pomgnt2	0.52378222	2.41069811	2.82255329	0.10857248	0.13302225
Crabp2	0.57020554	6.22136604	2.82217966	0.10859439	0.13302225
Kctd12	0.19033766	7.42126274	2.82210949	0.1085985	0.13302225
Gigyf2	-0.2519814	6.95812281	2.82158081	0.10862952	0.13304041
Unc5c	0.27428364	6.71418233	2.82076649	0.10867731	0.13307911
Mid2	-0.2602343	5.67538724	2.81958249	0.10874684	0.13314442
Xpo6	-0.1839144	6.87091952	2.81835763	0.10881883	0.13317057
Uxt	0.4895405	3.52008913	2.81830251	0.10882207	0.13317057
Tbc1d10a	0.71326338	1.66524401	2.81795128	0.10884272	0.13317057
Fibcd1	-1.1557738	0.43586962	2.81795089	0.10884274	0.13317057
Ckap2l	0.7140109	0.9087401	2.81777153	0.10885329	0.13317057
Calm1	0.17032267	12.308041	2.8175654	0.10886542	0.13317057
Gmnc	-1.3963533	-0.5135682	2.81663022	0.10892044	0.13318549
Pms1	-0.4364468	2.75300023	2.81661068	0.10892159	0.13318549
Nfx1	-0.201824	6.44274509	2.81653197	0.10892623	0.13318549
Serinc4	-2.1227704	-1.0261286	2.81607504	0.10895313	0.13319856
Lhfp	0.36929513	6.11296999	2.81556963	0.10898289	0.13321513
Fam120c	-0.3385419	6.59807918	2.81456177	0.10904227	0.13324937
Oxnad1	0.45241417	3.78928905	2.81454387	0.10904333	0.13324937
Zfp36l2	0.31217642	6.13442197	2.81409424	0.10906983	0.13326195
Socs1	-2.2399199	-0.8725271	2.81361617	0.10909802	0.13327658
Bccip	0.31680787	5.57108292	2.80949203	0.10934155	0.13354947
Snora81	-1.7032817	-1.7249084	2.80928308	0.1093539	0.13354947
Cdh13	0.40986372	4.87616683	2.80891279	0.1093758	0.13355637
Nat2	0.53637591	3.0417629	2.80795513	0.10943246	0.13360571
Ern1	-0.6350565	2.21275338	2.80568376	0.10956699	0.13373853
Ndufa13	0.46973873	5.844441	2.80542294	0.10958245	0.13373853
Vit	-0.7780141	2.21496262	2.80522458	0.10959421	0.13373853
4930404N11	0.97091813	0.04994917	2.80502003	0.10960634	0.13373853
Arhgap26	-0.2772803	7.57515888	2.80312584	0.10971873	0.13382989
9430020K01I	-0.1851449	8.95856324	2.80299395	0.10972656	0.13382989
Pask	-0.9628741	0.83317781	2.80293496	0.10973006	0.13382989
2610028E06I	-1.2134799	-0.2727317	2.79944743	0.10993736	0.13406283
Zfp787	0.76228711	1.59554729	2.79809883	0.11001765	0.13414083
Dlec1	-2.0261368	-0.3980741	2.79721337	0.1100704	0.13418524
Mettl20	0.49840157	2.58159646	2.79330709	0.11030348	0.13444944
Vstm2b	-0.3944025	3.40517909	2.79301304	0.11032105	0.13445092
Dcaf10	-0.2768758	5.20849913	2.79202097	0.11038035	0.13450324
4933400F21I	-1.0522713	1.22189907	2.79140861	0.11041697	0.13452792
Nol12	0.44636233	2.80881033	2.79101413	0.11044057	0.13453673
Enah	-0.2721501	8.35869406	2.79044082	0.11047487	0.13455858
Dmrta1	0.68207983	3.04450842	2.78919359	0.11054955	0.13462959

2700046A07I	0.57453151	2.66778136	2.78891033	0.11056652	0.1346303
Pik3cb	-0.2589027	5.5200715	2.78798508	0.11062197	0.13465747
Rpl19	0.31804555	7.38560878	2.78774102	0.1106366	0.13465747
Casp7	0.61839018	2.71536491	2.78771802	0.11063798	0.13465747
Ublcp1	0.22307413	6.44773241	2.78618159	0.11073014	0.13473986
Mterfd3	0.41982306	2.97547655	2.78604287	0.11073847	0.13473986
Specc1	-0.2335125	6.56145607	2.78539258	0.11077751	0.13476741
Fgd3	-1.6936646	-0.2428206	2.78454944	0.11082815	0.13480907
Cct5	0.20041253	6.33837543	2.7823388	0.11096105	0.13495076
Kcnip1	0.40588879	4.52694561	2.78171173	0.11099878	0.13496219
Cmpk2	0.24776676	4.55212567	2.78156106	0.11100785	0.13496219
Ankrd24	-0.4664937	2.49941167	2.78109106	0.11103615	0.13496219
Slc22a4	0.3738487	3.02269968	2.78105591	0.11103826	0.13496219
Nans	0.58582056	2.40515802	2.78081861	0.11105255	0.13496219
Gcm1	-1.7077838	-1.3960054	2.78007307	0.11109746	0.1349968
Zfp120	-0.386148	4.37521183	2.77927764	0.1111454	0.13503224
Hnrnpd	0.1810233	8.29361653	2.77904407	0.11115948	0.13503224
Ceacam2	-0.5793787	1.35594275	2.77696364	0.11128499	0.13516473
Chpf2	-0.5129602	2.28687468	2.77610129	0.11133707	0.13520799
Pgm2	-0.297809	4.06380993	2.77579184	0.11135576	0.13521072
Pcsk6	-0.5404415	1.62600231	2.77517023	0.11139332	0.13523635
A230072C01I	-0.4797462	3.64761711	2.77463931	0.11142542	0.13525533
Ntn1	0.43838758	3.73349064	2.77228592	0.11156781	0.13540818
Zfp850	0.46756497	2.65478176	2.77148578	0.11161627	0.135447
Timm8a1	0.32115254	5.39448299	2.77030679	0.11168772	0.1355137
Syngap1	-0.280347	7.35111782	2.7692099	0.11175425	0.1355744
Rrs1	0.23503422	4.79496528	2.76791453	0.11183287	0.13562205
Zfp622	0.31464642	4.68996095	2.76776928	0.11184169	0.13562205
Npm1	0.19574196	9.66262949	2.76774736	0.11184303	0.13562205
Brat1	-0.5106432	2.28411883	2.76627409	0.11193254	0.13571057
Mrps31	0.35062896	4.56028874	2.76412675	0.11206315	0.13584889
Hsd17b4	0.28393507	5.79095532	2.76270308	0.11214985	0.13593313
Mt3	-1.3318184	-1.0707301	2.76244265	0.11216572	0.13593313
Arhgap12	-0.2071498	5.65828219	2.76200023	0.11219268	0.13594039
Nyap2	-0.2909677	5.85461771	2.76180156	0.11220479	0.13594039
Ndrg4	-0.2255463	10.805371	2.7610114	0.11225297	0.1359636
1700040L02I	0.55094888	1.83594238	2.76069513	0.11227226	0.1359636
Tmem63a	-0.3731263	4.55997756	2.7606737	0.11227357	0.1359636
BC021785	-1.7134891	-1.6363028	2.7596571	0.11233561	0.13598837
Gm2061	0.72953096	1.29447359	2.75959616	0.11233933	0.13598837
Tmem115	0.53087168	2.20816434	2.75944388	0.11234863	0.13598837
Gbp2	0.52792628	4.51041072	2.75925419	0.11236021	0.13598837
Pxylp1	-0.369423	3.30128163	2.75820203	0.11242447	0.13602647
Zfr2	-0.3914199	3.76353164	2.7580942	0.11243106	0.13602647
Zzz3	0.19551896	7.29131947	2.75792596	0.11244134	0.13602647

Ppm1b	0.17343982	8.14640098	2.75753992	0.11246494	0.13603499
E330011O21	-1.1879868	-0.1778107	2.75682636	0.11250857	0.13606774
Mau2	-0.2657002	5.4300466	2.75636313	0.1125369	0.13608198
Zfp52	0.2951627	4.08566603	2.75494341	0.11262379	0.13616702
Pip4k2a	0.17818596	7.90645861	2.75387183	0.11268942	0.13622633
Uspl1	0.22287037	5.10207478	2.75220216	0.11279178	0.13633002
Cdk8	0.20581308	5.70955149	2.74994459	0.11293036	0.13647744
Zbtb1	0.25722536	4.59540125	2.74827576	0.11303292	0.13658131
Thumpd3	0.27065638	4.77480916	2.74724381	0.1130964	0.13663793
Mir6414	2.5626999	-1.7663391	2.74624771	0.11315771	0.13669191
Gm3696	-0.8622289	0.04726077	2.74587916	0.11318041	0.13669923
Vcp	0.1859561	7.68924044	2.74407624	0.11329151	0.13679556
Hist2h3b	-1.3313366	-1.2253731	2.74404473	0.11329345	0.13679556
Dpf2	0.21469455	5.81680931	2.74354218	0.11332445	0.13681289
Ston2	-0.3798818	4.6411271	2.74312333	0.11335028	0.13681561
Gmds	0.39414167	3.36496846	2.74296597	0.11335999	0.13681561
Rtn4rl1	0.21534665	6.19990827	2.7426517	0.11337939	0.13681892
Sorcs1	-0.3886606	4.97864272	2.73780979	0.11367867	0.13715995
Mrpl13	0.28130288	4.72842033	2.73744593	0.1137012	0.13716699
Rpn2	0.32925432	5.31139917	2.73487489	0.11386054	0.13733906
Nfatc4	0.68789881	3.20919778	2.73433938	0.11389376	0.13735897
Ddah2	0.60287973	3.18176182	2.73349083	0.11394642	0.13740232
Cit	0.26728849	7.31494479	2.73249806	0.11400808	0.13744001
Hddc3	0.41027759	2.87659112	2.73244906	0.11401112	0.13744001
Tshz3	-0.2375314	6.13421341	2.7310696	0.11409685	0.13752319
Sarnp	0.27912695	5.53756592	2.73003653	0.11416111	0.13755206
Tlr13	-1.2197459	0.9213886	2.72989057	0.11417019	0.13755206
Myo1d	-0.2867329	4.42317551	2.72918595	0.11421405	0.13755206
Phlda3	0.51816978	2.61215372	2.72902226	0.11422424	0.13755206
Frem3	-1.2146964	0.105963	2.72891621	0.11423084	0.13755206
Slc17a8	0.51403783	2.68306358	2.72882628	0.11423644	0.13755206
Tie1	-1.2964812	0.39482434	2.72880192	0.11423796	0.13755206
Plb1	0.65348632	2.38062734	2.72748993	0.11431969	0.13761284
Acin1	-0.192048	5.86499961	2.72745405	0.11432192	0.13761284
H2-Ab1	-0.6795218	4.25397424	2.72688359	0.11435748	0.13763549
Atat1	0.23811683	5.68342495	2.72611511	0.11440541	0.13767301
Perm1	-1.4459763	-0.4116374	2.72315216	0.1145904	0.13787544
4921507P07I	-1.0259234	0.29850225	2.72267882	0.11461999	0.13789085
4930507D05I	1.64089233	-1.1882022	2.72210702	0.11465574	0.13791368
Wdhd1	-0.4738986	2.70306363	2.72109921	0.11471879	0.13796932
Erap1	-0.5166711	3.54311035	2.72005375	0.11478423	0.1380265
Acrbp	-1.1799903	0.21321377	2.71959372	0.11481305	0.1380265
Bend4	-0.3209967	4.40579276	2.71953525	0.11481671	0.1380265
Bet1l	0.53560505	3.14726464	2.71873292	0.11486698	0.13806674
Fzd4	-0.5180801	2.98805303	2.71447283	0.11513435	0.13836787

Myg1	0.45155962	3.22637071	2.7129809	0.11522816	0.13846037
Nhsl2	-0.1978888	8.08117747	2.71245095	0.1152615	0.13848019
Pmvk	0.31100122	4.37566919	2.71191286	0.11529537	0.13849323
Zfp955a	-0.2564455	5.05285549	2.71174304	0.11530606	0.13849323
Inf2	-0.2232347	6.07676718	2.71128664	0.1153348	0.13850751
Rab9	0.29513481	5.70015538	2.71025956	0.11539951	0.13856497
Bloc1s3	0.39218383	3.16661035	2.70978912	0.11542916	0.13858033
Sspo	-2.089725	-1.2452705	2.70911626	0.11547158	0.13861101
Ndufs8	0.44411776	4.82298406	2.70742452	0.11557833	0.13871108
Gm13139	0.5610412	2.32441717	2.70718209	0.11559364	0.13871108
Syndig1	0.35531744	3.49911957	2.70699311	0.11560557	0.13871108
Esyt3	-0.5099063	2.46429092	2.70493292	0.11573576	0.13884702
Cd82	1.07551115	1.51331837	2.70446345	0.11576546	0.13886237
Lysmd3	0.29799655	4.76163054	2.70393644	0.1157988	0.13886282
Ctxn1	-0.1690829	7.61037203	2.70375549	0.11581025	0.13886282
Zfp212	0.43038064	2.92232215	2.70365648	0.11581651	0.13886282
Pcp4	0.21195319	8.83186666	2.70290146	0.11586431	0.13889987
Map3k3	0.36626768	3.27514667	2.70255964	0.11588596	0.13890555
Mtx3	-0.2617552	5.99207355	2.70191417	0.11592685	0.1389343
Apol7e	1.12905021	-0.2586059	2.700621	0.11600882	0.13901227
Sh3pxd2a	0.31705347	7.07643607	2.69965239	0.11607026	0.13905407
LOC1016697(	-1.6751311	-0.978259	2.69953766	0.11607754	0.13905407
Sec1	-1.1956272	-0.0709414	2.69661715	0.11626304	0.13923958
Thrsp	0.57291498	3.17040787	2.69656635	0.11626627	0.13923958
Purb	-0.1936387	9.71124071	2.69605343	0.11629889	0.13925835
Obfc1	0.44079526	2.73504634	2.69541672	0.11633939	0.13928656
Kcnmb2	-0.8787152	0.84059901	2.69395244	0.11643261	0.13937785
Gspt1	0.17857542	7.58456715	2.69358507	0.116456	0.13938024
Ube3c	-0.2254559	6.07983139	2.69332091	0.11647283	0.13938024
Atox1	0.46331621	4.36596324	2.6931226	0.11648547	0.13938024
Heatr1	-0.419153	4.11923889	2.69242544	0.11652991	0.13941311
A230009B12	-1.5324565	0.00823792	2.69076801	0.11663562	0.13951928
Ddx58	0.36024008	5.01473816	2.68888792	0.11675568	0.13964257
Gm14393	0.44425783	2.67963904	2.6882748	0.11679487	0.13966781
Kcnh2	-0.5216404	2.32121856	2.68797284	0.11681417	0.13966781
1190005I06R	1.70920404	-1.2426018	2.6877602	0.11682777	0.13966781
Nle1	-0.9353755	-0.0880185	2.68564575	0.11696307	0.13980923
Ist1	0.25326011	5.83351638	2.68505912	0.11700064	0.13981687
Fbxl5	-0.2135097	5.6184531	2.68501468	0.11700348	0.13981687
Fbxw10	0.85139471	0.68857113	2.68463635	0.11702772	0.1398255
2210016F16I	0.33243267	5.04382769	2.684171	0.11705754	0.13984081
5430427O19	1.57524965	0.2311527	2.68316167	0.11712226	0.13989778
Pqlc3	-0.364557	3.54648936	2.6824393	0.1171686	0.1399328
Pgls	0.65534994	1.35967136	2.68130634	0.11724132	0.13999931
Thsd1	-1.1511004	0.40548521	2.6806017	0.11728658	0.140033

Supt20	-0.2720519	4.95552167	2.67981536	0.11733711	0.14006897
Clk2	-0.2860299	5.15025855	2.67897627	0.11739105	0.14006897
Sh3tc1	1.69298903	-1.176216	2.6789639	0.11739185	0.14006897
2810403D21I	-0.9766794	0.25113302	2.67871946	0.11740757	0.14006897
Rpl15	0.27341765	7.96298966	2.6787041	0.11740856	0.14006897
Ermp1	-0.2399004	5.74204652	2.67854251	0.11741895	0.14006897
Ugp2	0.19253434	6.43308734	2.67825721	0.11743731	0.14007053
Gars	0.18767499	6.01948736	2.67636489	0.11755913	0.14019549
Pcdhb10	-0.7743697	1.33394292	2.67516531	0.11763644	0.14022772
4930577N17	0.8053505	0.10867021	2.67501228	0.1176463	0.14022772
Erich1	0.45171105	2.96128345	2.67499666	0.11764731	0.14022772
Gm16894	0.67745619	1.2872279	2.67488665	0.1176544	0.14022772
Maf	0.29410607	7.20796103	2.67408904	0.11770585	0.1402687
Rad9b	-0.9790416	0.48771744	2.67275124	0.11779219	0.14035124
1700123L14I	-1.2714331	0.3657633	2.6722491	0.11782462	0.14036953
Sec61b	0.35181442	4.79413797	2.67181671	0.11785255	0.14037777
Naa15	-0.1895732	6.62462264	2.67161338	0.11786569	0.14037777
Cul4a	0.18461017	7.55684241	2.6712211	0.11789104	0.14038762
Polr1d	0.2912023	6.27355112	2.67045862	0.11794034	0.14042597
Gmeb1	-0.2179264	5.29855396	2.66917375	0.11802346	0.14050459
Pkhd1	2.1576298	-0.6882453	2.66795043	0.11810267	0.14057116
1110008F13I	0.59185403	2.33546273	2.66765262	0.11812196	0.14057116
Zfp952	-0.2444429	4.62373598	2.66751794	0.11813068	0.14057116
St6gal2	-0.3301026	4.83107162	2.66718646	0.11815216	0.14057147
Armc9	-0.3970098	3.36893747	2.66698595	0.11816516	0.14057147
Slfn9	0.62201087	1.62630599	2.66636478	0.11820543	0.14059903
Skp1a	0.2327817	8.04762672	2.66523197	0.1182789	0.14066607
Car5b	-0.4894914	1.96853498	2.6647158	0.1183124	0.14067504
Gm10408	-1.1069376	-0.4186977	2.66458832	0.11832068	0.14067504
Syce2	-0.6817271	1.79897998	2.6637654	0.11837411	0.14071821
1110046J04I	-0.7080431	1.55816368	2.66295253	0.11842692	0.14076063
Atg16l2	-0.8108038	1.46873458	2.66137134	0.11852972	0.14086245
Klf3	0.29559557	7.62085981	2.6603363	0.11859707	0.14092211
Abca3	-0.3547914	4.11377564	2.65958968	0.11864569	0.1409595
Wbscr22	-0.3563247	3.15028174	2.65852251	0.11871521	0.14102171
Ly6e	0.21843656	6.82283664	2.65806927	0.11874475	0.14103642
Strada	-0.402199	3.65046623	2.65757418	0.11877703	0.14105193
Eng	0.33098647	3.29847141	2.65734273	0.11879213	0.14105193
Csrp1	0.38853003	7.56769806	2.65694726	0.11881792	0.14106218
Pde5a	0.35693562	6.52878451	2.65571353	0.11889844	0.14113738
Acmsd	-0.8725591	0.69188002	2.6550764	0.11894005	0.14116638
Esm1	-0.6006608	2.24517017	2.65353134	0.11904102	0.14126582
Faim	0.28482602	5.76111039	2.65305374	0.11907225	0.14128249
Fam132a	-0.557163	1.84484177	2.6524255	0.11911335	0.14131085
Tcp11l2	0.24881885	4.28628694	2.65176857	0.11915634	0.14134145

Tmc4	-0.3652033	3.05233319	2.65052459	0.1192378	0.14141767
Prg4	0.43671757	8.48540854	2.65010624	0.11926521	0.14142086
Rrbp1	0.26050387	6.08677972	2.64995834	0.11927491	0.14142086
Klrb1b	-1.5858247	0.72419676	2.64954582	0.11930194	0.14143252
Efcab5	-0.6087465	2.66556156	2.64840998	0.11937643	0.14150041
Serpinb9	0.32014936	7.10563105	2.64770845	0.11942246	0.14153456
Timp4	-0.500186	2.45770989	2.64225245	0.11978119	0.14192237
Srgn	0.35599139	5.14252766	2.64220651	0.11978421	0.14192237
Cp	0.28987938	7.00285072	2.63990536	0.1199359	0.14208161
2610207O16	-1.0444251	0.22999278	2.63899054	0.11999627	0.14212184
Pcdha10	-1.1516372	-0.3966167	2.63886664	0.12000444	0.14212184
Ttc23	0.44909201	2.80822105	2.63857984	0.12002338	0.14212378
Rad51d	0.30559118	5.28271466	2.63734037	0.12010525	0.14218685
Kcnk4	-0.8518337	0.31919566	2.63724969	0.12011124	0.14218685
Nt5e	0.43881099	4.27306299	2.63614599	0.1201842	0.14225273
Orai1	0.74968705	1.85472513	2.63578557	0.12020804	0.14226046
Mettl16	0.26965636	5.66215822	2.63413783	0.12031708	0.14236901
Zrsr2	0.20630591	6.20356364	2.63245148	0.12042881	0.1424807
Emc6	0.30946525	3.83980552	2.63094801	0.12052852	0.14257815
Stard8	-0.1983757	5.63681122	2.63045085	0.12056152	0.14259666
Jund	0.28671595	5.02178906	2.62971049	0.12061067	0.14263002
Zbtb41	-0.2152355	6.45382066	2.62950326	0.12062443	0.14263002
Sec63	-0.1760676	7.07591059	2.62721773	0.12077634	0.1427891
Ank1	-0.3954681	5.05992668	2.62665617	0.1208137	0.14280422
Lox	0.50548994	3.27472047	2.62650323	0.12082388	0.14280422
Sqrdl	0.47156932	3.40384126	2.62350506	0.1210236	0.14301971
Ranbp6	-0.3075441	6.39165545	2.621857	0.12113355	0.14307391
Hpgds	-0.5775076	2.40122513	2.62184848	0.12113412	0.14307391
Vash2	-0.5737114	1.75752496	2.62144378	0.12116113	0.14307391
3110001I22R	0.67527931	1.10874887	2.62127366	0.12117249	0.14307391
Idua	-0.3695192	3.57886831	2.62120716	0.12117693	0.14307391
Fbxo16	-0.4992235	2.08289641	2.6210624	0.1211866	0.14307391
Asprv1	-1.0260862	0.50430577	2.62099176	0.12119132	0.14307391
Kcnab3	-0.2534928	5.49972719	2.61971737	0.12127647	0.14315387
Cnot8	0.25910793	4.59883218	2.61885678	0.12133401	0.14320123
Mras	0.17749428	6.99979418	2.61793094	0.12139595	0.14325376
Zfp607	-0.5502031	2.48139595	2.61696801	0.12146041	0.14328534
Rab39b	-0.2501704	6.74306171	2.6168436	0.12146874	0.14328534
Zfp628	0.48576971	2.2903361	2.61674998	0.12147501	0.14328534
Gm5544	-2.2172484	-0.6670952	2.61633706	0.12150267	0.14329571
1700008I05R	2.01288467	-1.3148425	2.61609821	0.12151867	0.14329571
Lrrc75b	-0.3628382	4.44886064	2.61467237	0.12161425	0.14338785
Xlr	-0.8485379	0.44675069	2.614013	0.12165848	0.14340877
Yif1b	0.56429354	1.7760898	2.6138876	0.1216669	0.14340877
Zfp296	-1.184383	-0.1268593	2.61208952	0.12178762	0.14353048

Dock1	-0.238124	5.76329246	2.61168439	0.12181484	0.1435352
Setd1b	-0.2445181	5.46467722	2.61151011	0.12182655	0.1435352
Rnf103	-0.200251	6.23756175	2.61116492	0.12184975	0.14354196
Klf6	0.20107891	7.42428216	2.60991949	0.1219335	0.14362004
Ercc6	-0.3522944	5.60019108	2.6080749	0.12205767	0.14374569
3110021N24	-0.4049925	2.41221087	2.60662683	0.12215525	0.14383083
Cep76	-0.3393053	3.92024491	2.60648277	0.12216496	0.14383083
Clcc1	-0.2913415	4.36557929	2.60512086	0.12225683	0.14389293
Med6	0.33741479	4.33643809	2.60494666	0.12226859	0.14389293
Rtn4rl2	-0.7211498	0.92144409	2.60492239	0.12227023	0.14389293
Speer7-ps1	-0.9845948	0.64711457	2.60348127	0.12236754	0.14398683
Samd5	-0.3372207	3.98939051	2.60287064	0.12240881	0.14401477
Cdca2	1.82454105	-0.3892356	2.60036548	0.12257826	0.14419349
Miip	-0.8529597	0.92175841	2.59965573	0.12262632	0.14422938
Fam134a	0.23580984	5.4206259	2.59860701	0.12269737	0.14429231
Rusc1	-0.3148094	5.8504456	2.59819347	0.1227254	0.14430462
Parp9	0.40253934	3.39208783	2.59739422	0.1227796	0.1443477
Gatad2a	0.27261317	5.71910595	2.59598989	0.1228749	0.14442209
Nars	0.18138062	7.85975066	2.59594401	0.12287802	0.14442209
Prelid2	1.63560199	-0.7033293	2.5950601	0.12293805	0.14445056
Cage1	-0.7843226	1.19686726	2.59500124	0.12294205	0.14445056
Cyp2j12	1.26474742	-0.5687111	2.59481108	0.12295497	0.14445056
BC018473	-2.4773625	-1.5335454	2.59427385	0.12299147	0.14445719
2010320M18	0.69519982	1.75692754	2.59388319	0.12301803	0.14445719
Dld	0.1930752	7.47831163	2.59380756	0.12302317	0.14445719
Hsd3b3	-0.8744683	0.3184857	2.59353734	0.12304155	0.14445719
Tnip2	-0.3574016	3.05890154	2.59328722	0.12305856	0.14445719
Prss57	2.70480507	-1.3924371	2.59293616	0.12308244	0.14445719
Gm14326	-0.383714	4.52824376	2.59291842	0.12308364	0.14445719
Mapk8ip2	-0.5286687	4.63969142	2.59218637	0.12313346	0.14449502
B3gnt1	0.26619305	4.84392782	2.59162531	0.12317165	0.14451921
Vps51	0.40352712	3.72065299	2.59012025	0.12327418	0.14460518
Ufm1	0.22784271	6.00772638	2.58984602	0.12329287	0.14460518
2810408I11R	-1.1150994	0.38143534	2.58965733	0.12330573	0.14460518
Ppp1r3c	0.31514501	6.08555093	2.589517	0.1233153	0.14460518
Arhgef17	-0.2559047	6.61858914	2.58835532	0.12339453	0.14466492
Scpep1os	-1.2781647	-1.5246449	2.58825407	0.12340144	0.14466492
Stk3	0.24216021	5.66440243	2.58784233	0.12342954	0.14466622
Pcx	-0.5136671	2.01039584	2.58751372	0.12345198	0.14466622
Gucy1b3	-0.2604332	6.61329345	2.58746411	0.12345536	0.14466622
Hist2h2be	0.3287218	6.00580263	2.58631535	0.12353382	0.14473753
Hnrnpm	-0.2080001	6.77001369	2.58587072	0.12356421	0.14475249
Nrg3	-0.2234412	6.24416023	2.58341283	0.12373233	0.14492805
Mir376a	-1.7292059	0.06383784	2.58316449	0.12374933	0.14492805
Frmd7	-1.0395951	-0.1446833	2.58242024	0.1238003	0.14496708

Necab3	-0.3241811	5.96483057	2.58187006	0.12383799	0.14499057
Disp2	-0.2710906	7.01997622	2.58047585	0.12393358	0.14503515
Uba52	0.33500258	6.7196023	2.58034716	0.12394241	0.14503515
Atxn7l3	-0.2816019	5.96367307	2.58026814	0.12394783	0.14503515
Galc	-0.3806811	3.76757953	2.58004249	0.12396331	0.14503515
Lst1	1.11735425	-0.2032257	2.5800194	0.12396489	0.14503515
Adora3	-2.2636109	-1.5513716	2.57971364	0.12398587	0.14503515
Yipf6	0.19364369	7.26621431	2.57901567	0.12403378	0.14503515
Wdtc1	-0.2549674	5.20720695	2.57900678	0.12403439	0.14503515
Rasa3	-0.2642304	5.2610341	2.57899952	0.12403489	0.14503515
H2afx	0.33968923	3.05993906	2.57844969	0.12407264	0.14505866
Mdk	0.48603327	3.66848016	2.57811321	0.12409575	0.14506505
Tmem168	-0.3865579	3.25320225	2.57781988	0.12411591	0.14506797
Pygo2	0.29942344	3.87133083	2.57722947	0.12415648	0.14509476
Dlx5	-0.4603695	2.04843014	2.57676506	0.12418841	0.14510597
Ehbp1l1	0.26912411	4.25592144	2.57653252	0.1242044	0.14510597
Lsm1	0.37161964	4.10099304	2.57589098	0.12424852	0.14510597
9530027J09F	-2.3517798	-0.9858513	2.57540668	0.12428185	0.14510597
Pgap3	1.41719228	-0.2730341	2.57525256	0.12429245	0.14510597
Rpl22	0.35378837	6.20026759	2.57516662	0.12429837	0.14510597
Mast4	-0.2285928	6.6848335	2.57504361	0.12430684	0.14510597
Tmem116	-0.9370512	-0.0018565	2.57503671	0.12430731	0.14510597
AA474331	-2.5082571	-1.6410653	2.57397638	0.12438033	0.14515248
Ppp2r4	0.2345551	5.70209698	2.57394512	0.12438248	0.14515248
Gramd1b	-0.251229	5.84688195	2.57270155	0.12446818	0.14523187
Slc23a2	-0.2066099	7.23608384	2.57189125	0.12452405	0.14524571
Rnf111	0.16780365	7.07452193	2.57180151	0.12453024	0.14524571
Rnf216	0.19454581	5.97522611	2.57176079	0.12453305	0.14524571
Fibin	-0.4501571	4.43120011	2.57067786	0.12460779	0.14531225
Ttr	-0.6246207	5.00354118	2.57021856	0.1246395	0.14532861
Chkb	-0.5426619	1.33850918	2.56982517	0.12466667	0.14533967
Frmpd1os	-2.4826391	-1.7054424	2.56931187	0.12470213	0.14536039
Aasdh	-0.5556022	2.09204686	2.56899626	0.12472394	0.1453652
Gse1	-0.3025305	5.57081042	2.56832208	0.12477054	0.14536834
Cercam	0.52156308	1.93764476	2.56811897	0.12478458	0.14536834
Neurod6	0.27854454	6.14249421	2.56806475	0.12478833	0.14536834
Alox15	-2.6763003	-1.1973716	2.56793401	0.12479737	0.14536834
Trex1	0.62164636	1.23374519	2.56718927	0.12484889	0.14540774
Prrc2a	-0.2086238	7.7199155	2.56666462	0.1248852	0.14542942
Reep1	0.26336022	6.96590109	2.56558675	0.12495983	0.14548649
Pllp	-0.3897026	2.36712096	2.56544563	0.12496961	0.14548649
AW209491	0.27179756	4.72485102	2.56479102	0.12501496	0.14551868
Epn3	0.94939045	0.59555436	2.56414417	0.1250598	0.14553954
1110015O18	-1.3440391	-0.9283855	2.56402161	0.1250683	0.14553954
Grb2	0.22382333	5.1641288	2.56355283	0.12510081	0.14555676

Btla	-1.657993	-0.3808945	2.5632845	0.12511942	0.14555781
Cpne3	0.25375381	7.19702496	2.56267248	0.12516188	0.14558612
Ccdc81	-1.2598499	0.12320534	2.56232056	0.12518631	0.14558612
Cdc23	0.24256199	5.00918478	2.562168	0.1251969	0.14558612
Exosc9	-0.2734417	4.0254313	2.56159084	0.12523698	0.14559272
Cnih3	0.24127253	5.92678748	2.56157604	0.125238	0.14559272
Abcb10	-0.3495897	3.68773731	2.56049149	0.12531335	0.14564694
Usf2	-0.2387073	4.98736857	2.56039472	0.12532008	0.14564694
Wdr45	0.30497002	4.46017267	2.55948927	0.12538303	0.14569949
Fbxl12os	-0.6341352	2.29522123	2.55702595	0.12555448	0.1458781
Ermap	-1.1899869	0.46988567	2.55659416	0.12558456	0.14589243
Gm3558	-1.2142834	-1.2723541	2.55622392	0.12561036	0.14590178
Cyp2t4	-1.3057335	-0.8172949	2.55401621	0.12576434	0.14605999
Med19	0.29236317	4.61208039	2.55342513	0.1258056	0.14608727
A930007I19F	-1.7962026	-1.0315165	2.55211367	0.12589722	0.146173
Akna	0.34278518	3.34321702	2.55112113	0.1259666	0.1462329
H2-Eb1	-0.752991	3.08413101	2.55049122	0.12601066	0.14624485
Ccdc84	-0.8920647	1.12289914	2.5504653	0.12601247	0.14624485
Myh14	-0.593783	2.29748057	2.54981329	0.1260581	0.14627714
Rilp	1.26345595	-0.4836868	2.54859004	0.12614375	0.14635587
Slc35a4	0.27991105	5.95206393	2.54805899	0.12618096	0.14637837
6430531B16I	-1.5970883	-0.6821775	2.54765239	0.12620945	0.14639077
Ybx3	0.3342365	7.39449024	2.54701061	0.12625444	0.14640627
Usp49	-0.3502447	3.96459954	2.54695354	0.12625845	0.14640627
Ppp1r12b	-0.417769	5.67617441	2.54669459	0.12627661	0.14640668
Crebbp	-0.198886	8.16522246	2.5460918	0.12631889	0.14643505
Lrfn5	-0.3164313	5.23599728	2.54518216	0.12638274	0.14648839
Vangl1	0.42352735	3.81104559	2.54239172	0.12657882	0.14669499
Dusp2	1.44864721	-1.032968	2.54166932	0.12662965	0.1467332
Rbm22	-0.2612487	4.93733979	2.54118895	0.12666345	0.14675168
Eif2ak2	0.31670253	5.80721038	2.53829722	0.1268672	0.14696703
Cnnm1	-0.2409775	6.37053827	2.53710285	0.12695147	0.14702797
Dxo	0.41654571	2.09703573	2.53704453	0.12695559	0.14702797
Zdhhc15	-0.4267114	3.45046656	2.53613164	0.12702005	0.1470819
Pde7b	0.17395364	6.60018981	2.53558513	0.12705865	0.14710055
Tnfsf15	2.88049754	-1.6671084	2.53539696	0.12707195	0.14710055
Abi2	-0.1964888	8.54721792	2.53511369	0.12709197	0.147103
Rab33a	0.37413416	3.31455884	2.53484095	0.12711124	0.1471046
Nacc2	-0.1942408	6.50946676	2.53407194	0.12716562	0.14714504
Pmepa1	0.22015289	7.36000559	2.53384044	0.12718199	0.14714504
Cpsf4	-0.5169356	1.62507283	2.53214753	0.12730181	0.14726293
4921511C10I	-2.6926478	-1.6615581	2.53146842	0.12734991	0.14729784
Nkx6-1	1.58341644	-0.5665665	2.5306257	0.12740963	0.14734467
Pp2d1	-0.789945	0.70436595	2.5303913	0.12742625	0.14734467
Cox16	-0.3372795	4.60842813	2.52907948	0.12751929	0.14743152

Adamts4	-0.5972295	1.78413118	2.52838128	0.12756885	0.14746807
Tnfrsf1b	-0.9025282	1.11957083	2.5280763	0.1275905	0.14747149
Ripk2	0.33750071	4.3644406	2.52783421	0.12760769	0.14747149
C130074G19	0.25897152	6.86910222	2.52631936	0.12771533	0.14757513
Gpd2	-0.1865949	6.9886585	2.52571406	0.12775837	0.14760411
Fuk	-0.5065352	1.56267441	2.52509531	0.12780238	0.14763421
Glipr2	0.64156	4.05857951	2.52392339	0.12788579	0.1477098
Msrb1	0.33697272	4.22744188	2.52354013	0.12791309	0.14772057
Zxdb	-0.2255979	5.19819493	2.52187734	0.12803158	0.14783663
Sardh	0.58013816	2.20150384	2.52046012	0.12813267	0.14793258
Fam213a	0.35279221	7.66914769	2.51935858	0.12821131	0.14800259
Map3k19	-0.5351163	2.77347339	2.5189272	0.12824213	0.14801737
Plekho1	0.30610484	4.03851156	2.51783484	0.12832019	0.14808668
Tbk1	-0.2751148	5.39326555	2.51689905	0.12838712	0.14814311
Fam109b	0.95271578	1.55754912	2.51571446	0.12847189	0.14822012
Ddx46	0.17975611	7.32957545	2.51511618	0.12851473	0.14823422
Gm14440	0.25008961	5.28812743	2.51504002	0.12852019	0.14823422
Zfp426	0.21795198	6.10522924	2.51443655	0.12856342	0.14826328
Atic	-0.3047581	4.23843957	2.51388883	0.12860267	0.14827704
Nuak1	-0.2742019	5.19287171	2.51376658	0.12861143	0.14827704
Hcn1	-0.3329732	7.36017206	2.51148892	0.12877483	0.148439
Ccdc55	0.1992671	6.97211916	2.5113051	0.12878803	0.148439
Tspo	0.58424743	2.02514548	2.51061069	0.1288379	0.14847566
Fermt2	0.16214816	7.13789339	2.50976028	0.12889901	0.14852525
Dsc2	-1.2794465	-0.1495148	2.50950619	0.12891727	0.14852548
Grm4	-0.352171	4.02745174	2.50818649	0.12901219	0.14861399
Foxj2	0.22029934	6.55270403	2.50740004	0.12906879	0.14865836
2610034B18I	0.41437588	4.17684086	2.50548867	0.12920647	0.1487948
Blm	-0.6083825	2.1997472	2.50519253	0.12922782	0.1487948
Dcp1a	0.21859671	5.44539344	2.5047933	0.12925661	0.1487948
Krtcap3	-1.2507078	-0.9806215	2.50460962	0.12926985	0.1487948
Ssc5d	0.48490614	2.55307744	2.50449999	0.12927776	0.1487948
Fbxo42	-0.2208306	5.14758796	2.50388712	0.12932198	0.14882484
Ldlrap1	0.7572587	1.14273587	2.5036341	0.12934023	0.14882502
Zfp867	-0.2917943	4.15322404	2.50313426	0.12937631	0.1488457
Tmpo	0.28686584	5.86284667	2.50248252	0.12942338	0.14885958
Aes	0.274262	6.35405243	2.50246554	0.1294246	0.14885958
H6pd	-0.3785017	3.62020881	2.5019895	0.12945899	0.14886125
1700006F04I	-1.6105248	-0.7678995	2.50194395	0.12946228	0.14886125
Slc25a26	0.32753433	3.09585486	2.49983239	0.12961495	0.14901242
Adam32	-1.2615211	-0.4974968	2.49962426	0.12963001	0.14901242
Zfp661	-0.3942358	3.17375051	2.49879054	0.12969036	0.14906094
Sesn3	-0.2137848	6.09215675	2.49809653	0.12974062	0.14909403
Rasl10a	0.29324834	3.35772303	2.49782271	0.12976046	0.14909403
Ifi47	0.53571852	3.77676149	2.49753947	0.12978098	0.14909403

Nubpl	0.43513269	2.80469616	2.49739146	0.12979171	0.14909403
Mrpl3	0.21351965	5.42029151	2.4959828	0.12989384	0.1491905
Kif17	0.42494666	3.1648129	2.49569479	0.12991474	0.14919365
Fzd6	0.45644647	3.99486454	2.49465755	0.12999002	0.14925925
Cmtm8	1.07400317	0.26693661	2.49434325	0.13001284	0.14926159
Tmem208	-0.4845666	2.06204217	2.49412937	0.13002837	0.14926159
Rundc3b	-0.2806701	4.81308097	2.49327167	0.13009068	0.14931226
AU022754	-1.2318833	-0.2459675	2.49060123	0.13028492	0.14951432
Siah3	-0.74576	0.8095347	2.49034639	0.13030347	0.14951473
Zxda	-0.269564	5.37995996	2.48875231	0.1304196	0.1496271
Tnfrsf11a	-0.4661472	2.63779918	2.48787584	0.13048351	0.14964208
Caly	0.36343378	3.47990388	2.48777999	0.1304905	0.14964208
Nfkbil1	-1.0168915	-0.2143319	2.48776443	0.13049163	0.14964208
F630042J09F	-1.5905696	-0.9915842	2.4875744	0.13050549	0.14964208
Pik3r6	0.75092394	1.61009317	2.48649899	0.13058397	0.14971118
Fbxo28	0.18391989	6.1643748	2.48605934	0.13061607	0.14971215
Casc4	-0.2189623	8.2105552	2.48578287	0.13063626	0.14971215
Sec22c	-0.27116	4.63441973	2.4857391	0.13063946	0.14971215
L1td1	0.4431564	2.81831236	2.48313185	0.13083005	0.14989875
Rpl22l1	0.3138006	5.0394012	2.4828592	0.13085	0.14989875
Inpp5e	0.23973446	5.0308167	2.48276346	0.130857	0.14989875
Speer4a	-0.8585781	0.36362606	2.4821768	0.13089994	0.14991433
Etv4	-1.3588587	-1.3660121	2.48207936	0.13090708	0.14991433
Mpped1	-0.2387851	6.04425152	2.48159537	0.13094252	0.14991662
Dtymk	0.26745165	4.20043631	2.48155386	0.13094556	0.14991662
Usf1	0.45818666	2.76170238	2.48069756	0.1310083	0.14996755
Mcm10	-2.208096	-1.3005088	2.47979113	0.13107474	0.15002272
Ccdc92	0.20858525	6.70486455	2.47862823	0.13116005	0.15009946
Palb2	-0.8337427	0.75081152	2.47837348	0.13117874	0.15009995
Cd109	-0.5777747	3.07290153	2.47804434	0.1312029	0.1501067
Smurf1	0.25190452	5.13253771	2.47696438	0.13128222	0.15014774
Map3k5	-0.3699758	5.08153796	2.47693971	0.13128403	0.15014774
Asf1b	1.56644035	-1.5884799	2.47662978	0.1313068	0.15014774
Zfp69	-0.7563323	0.82551149	2.47656115	0.13131185	0.15014774
Zfp97	-0.2631585	4.12311113	2.4735259	0.13153512	0.15038212
Prrg4	-0.7175796	1.78377809	2.47106532	0.13171646	0.15056849
Alx1	0.83515661	2.53238733	2.47052287	0.13175647	0.15059329
Agpat2	0.78200058	1.41287467	2.47022769	0.13177825	0.15059724
Lrrc23	0.98728998	0.84062034	2.4696119	0.13182371	0.15062824
Fgfr1op2	0.20120141	7.74239464	2.46843177	0.13191087	0.15070689
Ift46	0.36900226	4.57608285	2.46613476	0.13208072	0.15087997
Ppp3cc	0.25072267	5.04839543	2.46534177	0.13213942	0.15091096
2810417H13I	-0.9678424	0.64624972	2.46527221	0.13214457	0.15091096
Flt4	-1.1123805	1.00648881	2.46472675	0.13218496	0.15093612
Aldh4a1	0.34163255	4.20524868	2.46395058	0.13224247	0.1509808

Parp16	0.9070629	1.90198188	2.46335232	0.13228682	0.15100504
Stx2	-0.4548802	2.63517198	2.46316849	0.13230045	0.15100504
Fdps	0.36507148	3.83319565	2.4607937	0.13247667	0.15118519
Nol7	0.28296666	5.77233861	2.46053617	0.1324958	0.15118603
Sgce	0.28040944	4.38630861	2.45997795	0.13253727	0.15121236
Extl2	0.2324149	6.08279579	2.45901227	0.13260905	0.15127325
Erg	-0.7263186	1.59530933	2.45787998	0.13269328	0.15134832
Icmt	-0.2273487	4.61061729	2.45716424	0.13274655	0.15138808
Lonp1	-0.2679063	4.27239562	2.4559076	0.13284014	0.15147379
Pdhb	0.16439073	6.93228658	2.45403264	0.13297993	0.15161216
Nr2f2	0.25118689	7.60255619	2.45286404	0.13306715	0.15168525
Pdf	0.27617572	5.08672564	2.45263111	0.13308454	0.15168525
Zbtb18	-0.2129728	7.35838544	2.45233624	0.13310656	0.15168525
F10	-1.261204	-0.8615185	2.45218493	0.13311787	0.15168525
Tomm5	0.316928	4.59282901	2.45175143	0.13315025	0.15170113
Tub	-0.3384256	6.11922784	2.4512559	0.13318728	0.15172229
Ppp6c	0.2976158	6.26254495	2.45033725	0.13325597	0.15176754
Armc8	0.20834811	7.05120599	2.45023061	0.13326394	0.15176754
Galnt2	-0.3008787	3.84837964	2.44921649	0.13333982	0.15183292
Dus2	-0.4573388	2.42156585	2.44895517	0.13335938	0.15183415
Ogfod3	0.60419405	2.08932277	2.44858548	0.13338706	0.15183461
Ripply3	-0.7562737	1.46341984	2.44845635	0.13339673	0.15183461
AI606473	1.24831982	-0.0018368	2.44791375	0.13343737	0.15185984
Lonrf3	0.25240147	5.72486877	2.44749923	0.13346842	0.15186764
Rnasek	0.35524136	7.09958463	2.44732903	0.13348118	0.15186764
Ckap4	0.31289944	5.1576854	2.44637513	0.13355269	0.15192797
6330408A02I	-0.2643389	3.61196677	2.44600544	0.13358041	0.15193848
Trpv6	0.5735009	1.64659565	2.44565762	0.1336065	0.15194713
9530026P05I	0.75645602	0.5180559	2.44472091	0.1336768	0.15200604
Pmm2	0.36013805	3.40285091	2.44360667	0.13376048	0.15205998
Gins1	1.1467533	0.15266599	2.44357916	0.13376255	0.15205998
Mtcl1	-0.3637711	5.72007567	2.44323732	0.13378824	0.15205998
Foxp4	0.36128714	4.89409226	2.44310421	0.13379824	0.15205998
Tnfrsf1a	0.51779837	4.11588072	2.44198548	0.13388235	0.15213453
Syt4	-0.2680517	7.23979806	2.44050246	0.13399394	0.15221031
Chn1os3	-1.0887522	-0.0760087	2.44041175	0.13400077	0.15221031
Irak2	-0.2883441	3.65460377	2.44036098	0.13400459	0.15221031
Hdgfrp3	0.19040311	8.00669921	2.43930967	0.13408378	0.15226453
Gfm2	-0.4976834	3.58539059	2.43916918	0.13409436	0.15226453
Svip	0.19372112	5.08626736	2.43878486	0.13412333	0.15226453
Stub1	0.79785588	0.86634592	2.43874371	0.13412643	0.15226453
Psmg3	-0.6031069	2.00134747	2.4379496	0.1341863	0.15231146
Gm9767	1.53701829	-1.0617499	2.43762006	0.13421115	0.15231864
Wdr62	0.82152365	1.01093731	2.43734281	0.13423207	0.15232134
Camsap1	-0.2491765	6.94819669	2.43691204	0.13426457	0.15233719

Stmnd1	1.49039878	-0.550832	2.43643037	0.13430093	0.15235741
Smim20	0.39994799	3.66801231	2.43375126	0.13450336	0.152566
Ehhadh	0.65960492	1.10449656	2.43335318	0.13453347	0.1525791
Cpeb1	-0.2929396	5.01021217	2.43238002	0.13460712	0.15264156
Zfp110	0.30615151	4.42279014	2.43113918	0.13470109	0.15270928
Pebp4	-1.3839434	-0.8767812	2.43110076	0.134704	0.15270928
Mn1	-0.3366347	4.58576522	2.43056215	0.13474481	0.15273448
C530044C16I	2.05771901	-1.6134224	2.42938955	0.13483372	0.15281418
Nudt15	0.55597819	1.94917808	2.42904495	0.13485986	0.15282274
Pcsk2os2	-1.1538177	1.71436286	2.42838764	0.13490974	0.15285819
Ammecr1l	0.19007747	5.73285666	2.42662651	0.13504349	0.15298864
Tbp	0.1904423	5.33457464	2.42607175	0.13508566	0.15301532
Gm19689	3.24216059	-1.7117989	2.46242641	0.13512057	0.15303378
Prkcg	-0.2414553	8.20544702	2.42415686	0.13523132	0.1531182
Rpl11	0.31164073	7.046423	2.42414314	0.13523237	0.1531182
Scnm1	0.34755927	4.02330932	2.42388882	0.13525173	0.15311902
Cngb1	0.9197735	0.65399982	2.42331233	0.13529562	0.15314258
Gnb1l	1.11328137	0.36464636	2.42312624	0.1353098	0.15314258
N28178	-0.2997696	7.04077347	2.42255424	0.13535338	0.15317081
Smad2	0.17484503	6.52510052	2.42197807	0.13539729	0.15319941
Akt1s1	0.59638388	1.7893999	2.42169994	0.13541849	0.15320231
Scoc	0.22481084	8.74565605	2.42109645	0.13546452	0.15323329
Clip3	0.26284208	10.1860474	2.41958691	0.13557972	0.15334249
Ptpn2	-0.3810528	4.93947843	2.4192846	0.1356028	0.1533475
Scand1	1.47036765	-1.4286428	2.41878602	0.13564089	0.15336946
Myo16	-0.4340223	4.34876829	2.41785444	0.13571208	0.15341677
Ict1	0.39511836	4.30702121	2.417684	0.13572511	0.15341677
Zscan21	0.32681895	4.60120424	2.41750593	0.13573872	0.15341677
Optc	1.61137141	-1.6157922	2.41703024	0.1357751	0.15343678
Impg1	-1.04895	0.27161682	2.41653955	0.13581263	0.15344483
Mrto4	0.39154479	3.32261323	2.41622147	0.13583697	0.15344483
Zfp385c	-0.5243781	1.90571192	2.41591702	0.13586028	0.15344483
Eps8l1	0.37866559	2.75832042	2.41577138	0.13587142	0.15344483
Osbpl9	0.23177566	6.74841289	2.41564479	0.13588111	0.15344483
Aplnr	1.92843882	-1.2725046	2.41526531	0.13591017	0.15344483
Fam126a	-0.2645558	4.10421944	2.4152296	0.1359129	0.15344483
Slc4a1ap	0.21848892	5.65214781	2.4140942	0.13599989	0.15352195
Pros1	0.37744151	5.1652713	2.41005479	0.13630988	0.15385075
Plekha3	0.23044883	5.32988785	2.4091337	0.13638068	0.15390953
Lrrc56	-0.9820383	0.36716053	2.40761878	0.13649723	0.15401991
Smim19	0.3202012	4.67526929	2.40728442	0.13652297	0.15402781
Kcng4	0.60735502	1.67984873	2.40653556	0.13658064	0.15407172
Klhl28	-0.3611176	3.90429028	2.40603151	0.13661948	0.15409438
Il33	-0.2689089	5.16812239	2.40578627	0.13663838	0.15409455
Cox19	0.33761169	3.39470768	2.40519238	0.13668415	0.15412147

Diras2	-0.1920402	8.13275265	2.40499016	0.13669975	0.15412147
Cyp4a12b	-0.6324852	3.11657346	2.40324727	0.13683422	0.15425192
A230056J06F	1.6412893	-0.1013602	2.40275219	0.13687245	0.15426787
Mapk13	2.22723569	-1.634588	2.402552	0.13688791	0.15426787
Zfp599	-0.4387301	2.83120872	2.40233492	0.13690468	0.15426787
Msh2	0.28029117	4.64569386	2.40186867	0.1369407	0.15428731
Map7d1	0.24132446	6.98034278	2.4013058	0.13698421	0.15431517
2700046G09	-0.6694836	1.75503727	2.40096431	0.13701061	0.15432212
Snrpn	0.61386074	0.80614836	2.40055167	0.13704252	0.15432212
Ctif	0.22085535	7.02124935	2.40049754	0.1370467	0.15432212
Mfsd10	-0.6656313	0.81582909	2.39810996	0.13723152	0.15450907
E130102H24I	-1.3266829	-1.0345136	2.39732508	0.13729234	0.15455136
S100a1	0.47182689	4.83217325	2.39713998	0.13730669	0.15455136
Acer3	-0.4198387	3.65386616	2.3960409	0.13739192	0.15460969
5830454E08I	1.09163915	0.68021135	2.39578061	0.13741212	0.15460969
Rita1	-0.6276759	0.99608685	2.39574417	0.13741495	0.15460969
Gm13238	-0.8315125	0.09036922	2.39547018	0.13743621	0.15461245
Chuk	-0.3029417	4.87758158	2.39223969	0.1376872	0.15485038
Ddost	0.25512044	4.27040541	2.39203724	0.13770295	0.15485038
Lrrc28	-0.2562474	4.49518742	2.39202067	0.13770424	0.15485038
Zfp414	0.3250361	4.50877681	2.39150254	0.13774455	0.15487453
Cdk5r2	-0.2034188	6.69943838	2.38952104	0.13789885	0.15502681
Pex11b	0.33494334	5.43222602	2.3886289	0.13796839	0.15508377
Tmem65	0.18789483	8.11504766	2.38829418	0.13799449	0.1550919
Sorbs3	0.38076943	7.07168681	2.38785526	0.13802872	0.15510917
Zfp14	-0.420129	3.10914981	2.38710051	0.13808762	0.15515414
Allc	-2.0057707	-1.1534135	2.38612441	0.13816383	0.15521855
Zfp941	-0.2821159	4.53019868	2.38481895	0.13826583	0.15527117
Ankrd49	0.34309906	4.93883628	2.38481608	0.13826606	0.15527117
Rnaseh1	0.50242796	2.79486791	2.3847996	0.13826734	0.15527117
Ddx1	0.1877583	6.99049267	2.38372765	0.13835117	0.15534408
Lrrc40	0.31316552	5.10632569	2.38343533	0.13837404	0.15534854
Gm17296	-0.7101496	2.16490273	2.38281234	0.1384228	0.15538205
Lig4	-0.2818642	4.33541158	2.38236039	0.13845818	0.1553946
Plekhg1	0.24599921	5.36137989	2.38146843	0.13852805	0.1553946
Gm12338	0.33795315	4.54261356	2.38129941	0.13854129	0.1553946
Psmd10	0.44176533	4.10335909	2.38114719	0.13855322	0.1553946
Epc1	-0.1882024	6.38698738	2.3808348	0.1385777	0.1553946
Zfp341	0.44084571	3.38565362	2.38036406	0.13861461	0.1553946
Ttc25	-1.3390919	-0.1515874	2.38022922	0.13862518	0.1553946
5930430L01F	0.36153994	4.70600952	2.38010016	0.1386353	0.1553946
Rars2	-0.2915248	3.90870699	2.3798689	0.13865344	0.1553946
Fam114a2	0.30716964	5.08308205	2.37985159	0.13865479	0.1553946
Wwp2	0.29404785	4.35375428	2.3798456	0.13865526	0.1553946
2810002D19I	-0.4187488	3.26031947	2.37977433	0.13866085	0.1553946

1700028J19F	-1.3975298	-1.2807606	2.37780177	0.13881569	0.1555469
Pisd	-0.2605563	4.45308824	2.37703508	0.13887592	0.15559319
Ccdc174	0.25353002	4.70050512	2.37568099	0.13898238	0.15566014
Ss18	0.2329436	5.34131109	2.37567511	0.13898284	0.15566014
Jak3	-1.0110785	-0.2724256	2.37555237	0.1389925	0.15566014
Dnal4	0.3795971	2.43868295	2.37498267	0.13903732	0.15566853
Calml4	0.64337957	1.39227829	2.37497571	0.13903787	0.15566853
Scyl3	-0.2404111	5.298139	2.37427601	0.13909295	0.15570899
Galnt14	-0.5298487	2.27094978	2.37335273	0.13916566	0.15576917
Mknk2	0.28189217	3.96354222	2.37162092	0.13930218	0.15590075
Klf9	0.21230671	8.08354523	2.37120276	0.13933517	0.15591644
Myo19	-0.4621474	2.91947592	2.37061852	0.13938127	0.1559468
Olfr113	-4.2632684	-2.0314591	2.40567588	0.13940926	0.15595687
Mobp	-0.3157967	5.81453183	2.36990608	0.13943752	0.15596726
Flot2	0.22731011	4.8676707	2.36946461	0.13947239	0.1559682
Fcgrt	0.5284556	3.55292364	2.36941489	0.13947631	0.1559682
Pfkfb1	-1.163971	-0.0473381	2.36874008	0.13952963	0.15599669
Dmkn	1.13429274	-0.2987898	2.36861214	0.13953974	0.15599669
Rpusd2	-0.2705907	4.33296508	2.36749919	0.13962774	0.15605383
Trp53i11	0.43009784	8.13256173	2.36748541	0.13962883	0.15605383
Sike1	0.18395534	6.60274024	2.36612804	0.13973625	0.15615265
Ntrk3	-0.2951149	5.67850937	2.36566493	0.13977292	0.15616024
Gng7	-0.2917304	5.43551438	2.36556232	0.13978104	0.15616024
Rpl36	0.43333991	5.25284376	2.36529802	0.13980198	0.1561624
Onecut2	-0.3668055	3.59482213	2.36441898	0.13987163	0.15621898
Oasl1	1.464072	-1.023746	2.36286315	0.13999502	0.15632833
Layn	0.62025393	1.44309849	2.36270481	0.14000758	0.15632833
Rnf24	-0.2267771	5.58235448	2.36245795	0.14002717	0.15632897
Rbmx	-0.1904147	6.76613628	2.3617749	0.1400814	0.15636827
Slu7	0.21548891	5.97942903	2.35768218	0.14040684	0.1567081
Clcn1	-1.011587	0.05914403	2.35746712	0.14042397	0.1567081
Aldh1l2	-0.646862	2.81231289	2.35709414	0.14045367	0.15671995
Gm4980	-0.7335039	0.8216005	2.35671694	0.14048372	0.15671995
Zcchc11	-0.2194379	6.83603884	2.35661574	0.14049179	0.15671995
Ddx20	0.41346212	3.71303709	2.35577763	0.14055859	0.15677319
Rps15	0.36659458	6.36645525	2.35482449	0.14063461	0.1568367
Ppbp	-1.768607	-0.2778654	2.35349214	0.14074096	0.15689913
Gpam	-0.3550807	3.29092738	2.35349161	0.140741	0.15689913
Upp1	1.68406059	-1.5056241	2.35340565	0.14074787	0.15689913
Gm6251	0.70896232	0.42859106	2.35316427	0.14076714	0.15689934
Spin1	0.15159581	8.82687423	2.35281732	0.14079486	0.15690895
Rps6ka4	0.47187969	2.71149669	2.35208309	0.14085353	0.15693943
Grb14	0.40038609	3.48487065	2.35183063	0.14087372	0.15693943
Gm14322	0.28258804	4.29984094	2.35175835	0.14087949	0.15693943
4833439L19F	0.26063193	7.79754832	2.35124392	0.14092063	0.15696213

Kdr	-0.3793349	2.63592826	2.35059096	0.14097286	0.15696213
Fgfr1op	0.23801417	4.38467889	2.35056536	0.14097491	0.15696213
Bai2	-0.3565999	4.57330281	2.35054855	0.14097626	0.15696213
Mrps7	0.26233493	5.25942909	2.34961912	0.14105065	0.15700299
Gna12	0.23487815	5.26986265	2.34942085	0.14106653	0.15700299
Dact1	0.31254853	6.05623153	2.34927541	0.14107817	0.15700299
Apol7d	2.33939747	-1.7446552	2.34885705	0.14111168	0.15700299
1700088E04I	2.18851475	-1.5563058	2.34860269	0.14113206	0.15700299
Efcab4b	-1.4293887	-1.4068119	2.34842584	0.14114623	0.15700299
9630001P10I	-0.8804396	0.21611996	2.34842041	0.14114667	0.15700299
Mlst8	-0.333009	2.91787372	2.34759196	0.14121307	0.1570438
Neurl3	0.80971723	1.02709041	2.34748591	0.14122157	0.1570438
Hcrtr1	-1.8948192	-1.4360629	2.3470914	0.14125321	0.15705773
4930565N06	-0.9142936	1.61358252	2.34656758	0.14129523	0.15708321
Wdr82	0.17630997	6.39638875	2.34592916	0.14134647	0.15708471
Ppip5k2	-0.2458376	5.30691516	2.34581063	0.14135598	0.15708471
Mcm5	-0.6816012	0.90631205	2.34563077	0.14137042	0.15708471
Col4a6	0.46238571	2.4222693	2.34559816	0.14137304	0.15708471
Tac2	0.68245112	1.01105244	2.34483777	0.1414341	0.15713132
Mbd1	0.2646476	4.27586133	2.34391176	0.14150851	0.15719186
Zbed5	0.36352281	3.8677752	2.34368377	0.14152684	0.15719186
Ptger1	0.33073694	3.11132064	2.34286098	0.141593	0.15722832
Stxbp5	-0.3055231	7.72459988	2.34279977	0.14159793	0.15722832
4933412O06	-0.9464275	1.25724567	2.34215103	0.14165012	0.15724467
Acaa1a	-0.3011101	3.87713101	2.34214113	0.14165092	0.15724467
Mmp12	-1.9780064	-1.5258281	2.34155481	0.14169812	0.15727582
Gramd4	-0.2888189	4.03964658	2.34064193	0.14177163	0.15733617
Sgol1	-0.75707	0.8431864	2.34011543	0.14181406	0.15736201
Zfp458	-0.397028	4.61657535	2.33963998	0.14185238	0.15738328
Fktn	-0.2259627	5.73022151	2.33933526	0.14187694	0.15738929
Nfatc3	0.27459327	6.91576641	2.33906506	0.14189873	0.15739222
Rnf8	0.31722238	4.25082204	2.33826746	0.14196307	0.1574281
Igsf1	-1.2083575	0.70347853	2.33818918	0.14196939	0.1574281
BC004004	0.36449949	5.27753471	2.33766841	0.14201142	0.15745347
Dsg2	-0.428099	3.13330013	2.33434226	0.14228022	0.15770938
2610020H08I	-0.7622053	0.73171678	2.33432056	0.14228198	0.15770938
Slc43a2	-0.2736453	5.45789386	2.33410027	0.1422998	0.15770938
Prickle2	-0.2746009	7.94042736	2.33345266	0.14235222	0.15774621
Zfp790	0.29520102	4.40422388	2.33231257	0.14244455	0.15781286
Bicd1	-0.2330323	6.59984116	2.33223234	0.14245106	0.15781286
Spred1	0.19310489	8.20895939	2.33199888	0.14246997	0.15781286
Ugcg	-0.2620819	6.93736904	2.33144128	0.14251517	0.15783647
Col16a1	-0.4822972	1.53962728	2.33126211	0.14252969	0.15783647
Zfp773	-0.8033667	1.1356325	2.3310088	0.14255023	0.15783795
Tmem184b	-0.2321968	4.72598387	2.32961167	0.14266358	0.15794217

Tmsb15b1	-0.6446396	1.17472549	2.32888332	0.14272272	0.15798636
Sulf2	0.1946046	6.53957386	2.3281151	0.14278512	0.15803415
Cyb5d1	0.59631032	1.39283836	2.32630161	0.14293256	0.15817069
Phb	0.30960946	3.81060631	2.32612456	0.14294697	0.15817069
1700034F02I	-1.9734721	-1.1505467	2.3252654	0.14301689	0.15822676
Wdyhv1	0.28877801	4.05496611	2.32456945	0.14307356	0.15824542
Nmb	-0.9853039	0.12625906	2.32439753	0.14308757	0.15824542
Sh3bgrl2	0.2932139	4.30173649	2.32434898	0.14309152	0.15824542
Flna	0.25183799	5.69101565	2.32394904	0.14312411	0.15825621
Blzf1	0.27327035	4.90929891	2.32375668	0.14313978	0.15825621
C130060C02I	-1.0613984	0.02592742	2.32283596	0.14321484	0.15831789
Ppp4r1l-ps	-0.616773	1.82378909	2.32249811	0.14324239	0.15832706
Wscd1	-0.3114613	3.74854037	2.32216325	0.14326971	0.15833596
Pde12	0.36184305	3.23531426	2.32153324	0.14332112	0.15835274
Tnnc1	0.61205184	1.94600666	2.32133831	0.14333703	0.15835274
Chac1	-0.4830922	1.81672077	2.32118203	0.14334979	0.15835274
Aldob	-1.2649495	0.34713287	2.321033	0.14336196	0.15835274
Zfp142	-0.2631464	5.2626512	2.32068872	0.14339007	0.15836251
Acta1	0.69269519	0.90019356	2.32019462	0.14343043	0.1583858
Sat2	0.42762128	2.12702867	2.31903	0.14352561	0.15845436
Gm8179	-1.5548582	-0.1915981	2.31873894	0.14354941	0.15845436
Otx1	-0.4720199	2.08237612	2.31866931	0.14355511	0.15845436
Msra	-0.3916219	3.48859937	2.31843132	0.14357457	0.15845436
Lrrc58	0.25659902	10.1415831	2.31825601	0.14358891	0.15845436
Mettl11b	2.34300805	-2.3411546	2.31789361	0.14361856	0.1584658
Cacnb4	-0.21849	9.03744874	2.3175643	0.14364551	0.15847426
1500009L16I	0.28044141	4.68452919	2.31292324	0.14402595	0.15887265
H2-Ke2	0.40708577	3.77446252	2.31254503	0.14405701	0.15888558
Rbm3	0.29185379	7.90721489	2.31131247	0.14415827	0.15897594
Setd5	-0.1959502	8.40831928	2.31038317	0.14423468	0.15903886
Fam26e	0.4398247	4.62726743	2.31006341	0.14426098	0.15904653
Zfp263	-0.3335038	4.16060069	2.30940767	0.14431494	0.15905778
Ckmt1	0.28346774	5.28012258	2.30940613	0.14431507	0.15905778
Rpl18	0.40672038	5.80811809	2.30912557	0.14433816	0.15905778
Slc25a51	-0.1763487	6.87552165	2.30847322	0.14439187	0.15905778
Mta2	0.25690625	4.80843552	2.30815439	0.14441813	0.15905778
Nf2	0.21852018	6.09246586	2.30813512	0.14441972	0.15905778
Efcab14	-0.2303129	7.24510589	2.30809809	0.14442277	0.15905778
Golph3l	0.27477499	5.99608675	2.30798552	0.14443204	0.15905778
AK129341	-0.373365	5.21366025	2.30782391	0.14444536	0.15905778
Nup153	-0.2121401	6.16442377	2.30669093	0.14453874	0.15913929
Zic3	-0.6783876	1.36545791	2.30573995	0.14461718	0.15920124
Pars2	0.57394831	1.84573629	2.30553915	0.14463375	0.15920124
Arhgef18	-0.2342611	4.8838218	2.30515564	0.1446654	0.15920167
Tmem216	0.38537326	2.85697965	2.30506502	0.14467288	0.15920167

Fzd8	0.67509878	1.75858584	2.30448399	0.14472085	0.15923314
Adrm1	0.23044113	4.16478739	2.30368913	0.14478651	0.15928406
Ttc33	0.1899813	6.57140083	2.30342059	0.1448087	0.15928715
2700070H01I	-1.3046486	-1.1984218	2.3002484	0.14507114	0.15955448
Fam114a1	0.41185063	6.31411309	2.29986256	0.1451031	0.15956828
St8sia5	0.38766228	4.23302689	2.29870886	0.14519872	0.15964251
Tbc1d22a	0.29925947	3.48816352	2.29857944	0.14520945	0.15964251
Pcdh19	-0.273446	6.24158074	2.29723461	0.14532101	0.15974379
Slx4ip	0.3762826	3.03855891	2.29640474	0.1453899	0.15978292
Adamts10	-0.4820135	2.08207284	2.2963375	0.14539549	0.15978292
Clgn	-0.4903638	2.17288949	2.29583516	0.14543721	0.1598074
Dlx6	-0.6770186	0.88559198	2.29529014	0.1454825	0.1598358
Trpc3	-0.3370825	3.63334456	2.29461115	0.14553894	0.15985686
Gm4432	-0.8783509	-0.0696758	2.29459154	0.14554057	0.15985686
Lman1	-0.3407191	5.02057679	2.29405611	0.1455851	0.1598844
Spata4	-1.6677961	-1.0776967	2.29252344	0.14571264	0.1600031
2900041M22	0.99692907	-0.0298824	2.29221379	0.14573843	0.16001003
Gm10433	2.49425473	-1.6576177	2.29051737	0.14587979	0.16011248
Bok	0.32219987	4.24152508	2.29021706	0.14590483	0.16011248
Zbtb2	0.22610302	4.67072501	2.29019259	0.14590688	0.16011248
Abcd3	0.19238789	6.90680663	2.2900894	0.14591548	0.16011248
C2cd5	-0.3144466	5.79741466	2.28992565	0.14592914	0.16011248
5330426P16I	0.47327093	2.64642788	2.28818607	0.14607433	0.16025039
B4galt3	-0.464659	1.94859098	2.28769885	0.14611503	0.16026966
Parp4	0.22089192	5.70580261	2.28750889	0.1461309	0.16026966
Itgb6	1.7969426	-1.8874354	2.28682396	0.14618814	0.16031105
Ptar1	-0.3036523	3.79022026	2.28569912	0.1462822	0.16037051
Flywch2	-0.5474615	1.0455833	2.28565777	0.14628566	0.16037051
Gm11992	1.16288617	-0.7542537	2.28547558	0.1463009	0.16037051
Zfp503	0.32985314	3.92476051	2.28483113	0.14635484	0.16037586
Prpf3	0.26651751	4.35903595	2.28482388	0.14635544	0.16037586
Rhobtb1	-0.4700274	2.61378833	2.28471787	0.14636432	0.16037586
Kifc5b	-1.7166892	-1.1644293	2.28444238	0.14638738	0.16037642
Csnk1d	0.16780428	6.50616897	2.28413432	0.14641318	0.16037642
5330417C22I	-0.4171202	3.85966934	2.28401266	0.14642337	0.16037642
Ankrd9	0.81726797	0.40616197	2.28313712	0.14649672	0.16041405
Nudt8	0.90297994	0.48689699	2.28313671	0.14649675	0.16041405
Traf3	-0.2626468	5.05773297	2.28281778	0.14652348	0.16041736
Dennd1a	-0.2168434	5.51187402	2.28242444	0.14655646	0.16041736
Vmn2r118	-2.0701466	-1.2699865	2.28240208	0.14655833	0.16041736
Nsg1	0.24835734	5.26403443	2.28179204	0.14660949	0.16043972
Gfpt1	-0.2139524	6.60092891	2.28169303	0.1466178	0.16043972
Pex5l	-0.2531588	6.82776309	2.28108474	0.14666884	0.16045534
Fbxw17	0.54370196	2.00307824	2.28105749	0.14667113	0.16045534
Efna1	-0.9966768	0.04723327	2.28045234	0.14672192	0.16047138

Hnrnpdl	0.16078425	7.47776317	2.28041774	0.14672483	0.16047138
Rtf1	0.16237346	8.31775041	2.27958183	0.14679503	0.1605268
Ranbp1	0.19869178	5.21532742	2.27818705	0.14691226	0.16063362
Efcab12	-0.8043489	0.82845375	2.27781059	0.14694393	0.16064687
Mafk	0.48912212	3.20017283	2.27743073	0.14697588	0.16066044
Trip6	0.45230371	3.17019165	2.27617775	0.14708135	0.16075434
Kdm7a	-0.1952201	7.30540922	2.27367842	0.14729199	0.16095742
Sp3os	0.48370658	3.10306267	2.2733648	0.14731845	0.16095742
Zan	-2.2339646	-2.1385419	2.27317547	0.14733443	0.16095742
Fam154a	-3.0128319	-1.9889816	2.27284646	0.14736219	0.16095742
Appbp2	-0.2364225	5.28876882	2.27281232	0.14736507	0.16095742
Edil3	0.28316087	6.55862889	2.27257242	0.14738532	0.16095815
Tnfrsf18	-0.5426101	1.90014686	2.27130837	0.14749208	0.16105334
Pcdha9	-1.2878427	0.22596979	2.27090504	0.14752616	0.16106916
Ap1ar	0.17135739	6.76523956	2.27037669	0.14757082	0.16108156
Tmem57	-0.1751777	6.12425202	2.27030693	0.14757672	0.16108156
1700052K11I	0.5342109	2.85164241	2.26726146	0.14783449	0.16134149
Pde6a	0.47152685	2.04928689	2.26698359	0.14785804	0.16134576
Spdl1	1.19281599	-0.1312964	2.26665172	0.14788616	0.16135504
Got1	0.2090775	8.34719757	2.26572324	0.14796489	0.16141951
Rab37	-0.9420834	0.41174114	2.26398558	0.14811237	0.16153786
Rpa1	0.22817878	4.58334867	2.2639818	0.14811269	0.16153786
Gm4013	2.09008171	-1.6984571	2.26227403	0.1482578	0.16167437
Dtl	0.28007106	4.00306842	2.26204589	0.1482772	0.16167437
Tulp2	-0.827499	0.43315833	2.26157124	0.14831757	0.16169694
Dlg2	-0.2618412	9.50865062	2.25996082	0.14845463	0.16181707
Cyth3	0.27230056	6.99938131	2.25981401	0.14846713	0.16181707
Ten1	0.37356237	3.27363178	2.25955678	0.14848904	0.16181949
Trmt1l	-0.2112635	4.88441546	2.25876222	0.14855674	0.16186423
Rbm8a	0.3038608	5.27386236	2.25861272	0.14856949	0.16186423
Snhg1	0.3246941	4.49501652	2.25641231	0.14875719	0.16204725
Sod1	0.32659172	8.00215232	2.25604524	0.14878853	0.16205991
Mapk14	0.17513809	6.53290444	2.25320014	0.14903171	0.16226983
Pcdha2	-0.9703482	0.19844634	2.25317624	0.14903375	0.16226983
Mtmr11	0.31718147	3.419479	2.25295074	0.14905305	0.16226983
Slc38a11	-1.3418232	0.50440634	2.25286685	0.14906023	0.16226983
Fezf1	-2.0209743	-0.6692118	2.25238962	0.14910107	0.16227399
Antxr2	0.626277	3.3378226	2.25236085	0.14910354	0.16227399
Gm6297	-0.8810112	1.00891899	2.25201838	0.14913286	0.16227529
Syt2	-0.4204777	3.39364271	2.25188573	0.14914422	0.16227529
Hnrnpu	-0.1939265	8.63388312	2.25147849	0.1491791	0.16229175
Tmem150c	-0.3440765	4.08315618	2.25083544	0.14923419	0.16233021
BC035044	0.83446761	0.39691978	2.25022047	0.14928691	0.1623582
Clcn6	-0.3094283	3.55535968	2.25007437	0.14929943	0.1623582
Map7d2	0.21653144	7.71414111	2.2495757	0.1493422	0.16238322

Hnrnpa1	-0.1924276	5.07049439	2.24903462	0.14938862	0.16241078
Timp3	0.38862972	9.35869266	2.24865304	0.14942136	0.16241078
Nars2	-0.3709986	3.83879504	2.24858936	0.14942683	0.16241078
Zfyve19	0.28467252	3.28719945	2.2472351	0.14954312	0.16250326
Mcat	0.43395566	2.54081818	2.2471381	0.14955146	0.16250326
Dhcr7	-0.3062608	3.72930431	2.24565051	0.14967934	0.16262071
Satb2	-0.2613151	7.21935707	2.24509503	0.14972712	0.16263832
Tmem231	0.33342948	2.88553283	2.24500206	0.14973512	0.16263832
Agtr2	1.65790255	-1.1446577	2.24418238	0.14980567	0.16267149
Zfp330	0.25405287	6.1249446	2.24398319	0.14982282	0.16267149
Lrrn1	-0.2870619	6.20171724	2.24395758	0.14982503	0.16267149
Sowahc	0.25660958	4.15343982	2.24326813	0.14988441	0.16271446
Tbc1d13	0.31023104	4.34928635	2.24262382	0.14993993	0.16275324
St3gal2	0.19202098	5.92266657	2.24184355	0.1500072	0.16279508
Ccdc57	-0.4717226	1.83468668	2.2417173	0.15001808	0.16279508
Pdpr	-0.3401488	3.89356158	2.24144847	0.15004127	0.16279874
Cpped1	0.19040334	6.46665598	2.24095662	0.15008371	0.16280152
Meig1	0.92858443	0.54987628	2.24092856	0.15008613	0.16280152
Tymp	0.53646044	2.53864225	2.24073008	0.15010325	0.16280152
Iqgap1	0.185914	6.34663726	2.24040019	0.15013173	0.16281092
Suclg1	0.23749702	5.19103964	2.23897839	0.15025452	0.16292259
Ap2a1	-0.3063033	4.06991428	2.23810313	0.15033017	0.16298312
Bbs10	-0.397109	2.99343135	2.23737724	0.15039295	0.16302967
Klra9	1.78729176	-0.9669091	2.23662331	0.15045818	0.16307888
Pde4b	-0.2567754	7.9733752	2.23624728	0.15049073	0.16309265
Acad10	-0.6730232	0.81067931	2.23532839	0.15057031	0.16315737
Dclre1c	-0.3914997	5.24539351	2.23493606	0.1506043	0.1631727
3300002I08R	-1.092253	-0.3547542	2.23402604	0.15068318	0.16323664
Gm10635	-0.9281376	0.85486743	2.23347324	0.15073112	0.16326705
Crhr1	-0.5166374	1.89336019	2.23322137	0.15075297	0.1632692
Pcdh12	3.25026639	-1.9877816	2.23146238	0.15090566	0.16339209
Fam175b	0.23596447	4.53151692	2.23145623	0.1509062	0.16339209
4933427I22R	-1.9600785	-1.2003321	2.23087258	0.1509569	0.16341525
Neurod1	-0.3889098	3.51486705	2.23059253	0.15098124	0.16341525
Ttc28	0.23328703	5.76228808	2.23052362	0.15098723	0.16341525
Sdhaf1	0.43020357	2.31989015	2.22991772	0.1510399	0.16345073
M1ap	2.40571107	-1.5333077	2.22714305	0.15128141	0.16369053
Myh9	0.20789149	6.86419916	2.22577448	0.1514007	0.16379804
Chrna3	-0.9087347	0.04976638	2.22533156	0.15143933	0.16381827
Eif4h	0.16887446	7.04645059	2.22417635	0.15154014	0.16390575
Tgif2	0.52691825	2.2958135	2.2226351	0.15167477	0.16402978
Mynn	-0.2092831	5.5697216	2.2218639	0.15174218	0.16406205
Ppp1r14b	0.45571494	3.52403494	2.2218371	0.15174453	0.16406205
Traip	-1.0078696	0.84322021	2.22116322	0.15180347	0.16408369
Tpo	-1.9391528	-2.0661477	2.2211517	0.15180448	0.16408369

Pdxk	0.21736608	7.60065812	2.22062248	0.15185079	0.16411217
Acox3	-0.2648933	4.07254784	2.2195297	0.15194646	0.16417088
Gm12657	0.36352388	3.51445235	2.21944023	0.1519543	0.16417088
Tbx21	1.71402457	-0.7649604	2.21931766	0.15196503	0.16417088
Hook1	-0.3874414	5.07421021	2.21860439	0.15202753	0.16420815
Vwa3a	-0.6766964	2.05989752	2.21846789	0.15203949	0.16420815
Cnih4	0.21494611	5.19353871	2.21793719	0.15208602	0.16422027
Styx	-0.314236	4.25906461	2.21788408	0.15209068	0.16422027
Ankfn1	-0.5934757	2.48226336	2.21708849	0.15216046	0.16426568
Fzd5	-0.6065804	2.67247355	2.21694888	0.15217271	0.16426568
Rfwd2	0.17491776	7.26114381	2.21654923	0.15220778	0.16428197
Notch2	0.34203613	7.07450311	2.21589949	0.15226482	0.16432077
Pter	0.30250998	3.4557309	2.21568431	0.15228372	0.16432077
5730508B09I	0.39881628	2.79684429	2.21505609	0.1523389	0.16435874
Rpl31	0.30468184	8.64150413	2.21373489	0.15245503	0.16446245
Cops5	0.16405804	6.0242677	2.21306815	0.15251368	0.16450128
Hnmt	0.2638556	5.1539492	2.2128116	0.15253625	0.16450128
Ccdc86	0.42673436	2.91362023	2.21256891	0.15255761	0.16450128
Psmb3	0.27555841	4.45844632	2.21241574	0.15257109	0.16450128
Kcnv1	-0.3462117	6.74877182	2.21085672	0.1527084	0.16462773
Rps4x	0.29804756	7.89493547	2.21042409	0.15274652	0.16464723
Vwa5b1	-0.8754845	0.4933153	2.21017617	0.15276838	0.1646492
AA543186	1.40688872	-1.0680148	2.20897132	0.15287464	0.16470286
Zfp184	-0.4059473	2.16563795	2.20874145	0.15289493	0.16470286
Znrf1	-0.1869403	6.14635925	2.20872559	0.15289633	0.16470286
Snx30	-0.2417091	5.50898575	2.20870299	0.15289832	0.16470286
Ripk4	1.14377094	-0.0521389	2.20821963	0.15294099	0.16472074
Cdc7	-0.5702701	2.8060365	2.20806081	0.15295501	0.16472074
Sec22b	0.18446626	7.09426359	2.20757055	0.1529983	0.16474578
Bahd1	0.36154303	4.09485007	2.20729559	0.15302259	0.16475035
Scn9a	-0.577437	2.68024145	2.20705532	0.15304381	0.16475162
Gltpd1	0.26904201	3.73848941	2.20655963	0.15308762	0.16477719
Rptoros	-1.7078593	-1.2315957	2.20592484	0.15314373	0.1648042
Gpcpd1	-0.209328	6.404355	2.20582216	0.15315281	0.1648042
Vps9d1	-0.2909166	3.70412847	2.2050842	0.15321809	0.16485286
Set	0.18165592	8.09528691	2.20460926	0.15326011	0.16487649
Prep	-0.2033117	4.39806506	2.20437536	0.15328082	0.16487718
Fars2	0.42900873	2.72872315	2.20411231	0.1533041	0.16488065
Mrpl18	0.28802168	5.44431311	2.20300957	0.15340177	0.16495291
Syf2	0.33738206	5.63618597	2.2029006	0.15341143	0.16495291
Zbtb3	1.31179213	-0.3909106	2.20260254	0.15343784	0.16495973
Gpr141	-2.0762949	-0.5865768	2.20169748	0.15351808	0.16502441
Wbp1l	0.30244642	5.04040967	2.20124005	0.15355865	0.16504644
Myo3b	0.75451679	0.40957379	2.2002082	0.15365022	0.16510104
Sox5	-0.2535055	5.99055607	2.20015225	0.15365519	0.16510104

Slc38a7	-0.4137743	2.39339797	2.19998863	0.15366972	0.16510104
Gm7361	-1.1257368	-0.8434527	2.19942843	0.15371947	0.16511779
Cdk9	-0.2596891	4.38479661	2.19924782	0.15373551	0.16511779
Zmym2	-0.2426669	8.1176782	2.19913457	0.15374557	0.16511779
Phldb2	0.3104845	7.68725526	2.19797815	0.15384836	0.16520658
Cdk5rap1	-0.7146182	1.39925974	2.19704203	0.15393162	0.1652677
Gm14305	0.39318058	2.61102588	2.19688621	0.15394548	0.1652677
Wdr60	-0.2874033	5.41442641	2.19483205	0.1541284	0.16540349
Bend7	0.63412025	0.64875533	2.19479907	0.15413134	0.16540349
Nenf	0.29498423	4.26834623	2.19456969	0.15415178	0.16540349
Pik3c2g	-2.2422559	-2.0787659	2.19456198	0.15415247	0.16540349
Tfb2m	0.24260222	5.65396134	2.19190458	0.15438954	0.16560282
Gnpat	-0.247601	5.40993141	2.1918033	0.15439859	0.16560282
Cebpz	0.17052637	6.19603558	2.19180219	0.15439868	0.16560282
Maged1	0.17439527	8.26477941	2.19081026	0.15448729	0.16567624
1700101E01I	-1.8429276	-0.8806146	2.19039381	0.15452452	0.16569453
Zfhx2os	-0.9267319	0.39938693	2.18931375	0.1546211	0.16577647
Casc5	-1.039808	0.48233608	2.18845228	0.15469819	0.16583072
Rassf6	-0.9104508	0.63128141	2.18829743	0.15471205	0.16583072
Ift43	0.49166542	3.32495798	2.18691925	0.15483548	0.16590678
Apip	0.31212499	4.17678292	2.18652834	0.15487052	0.16590678
Smn1	-0.3235672	4.31988517	2.1863118	0.15488993	0.16590678
Wdsub1	0.38186826	3.07045806	2.18626204	0.15489439	0.16590678
Vipr1	-0.5611999	2.42237839	2.18626006	0.15489457	0.16590678
Fhl5	3.22073503	-2.2783668	2.18615342	0.15490413	0.16590678
Map3k12	-0.2700881	5.62385011	2.18585231	0.15493113	0.16591408
Bmp15	0.60013593	2.22693816	2.18523294	0.15498668	0.16594041
Pfkfb2	-0.2000352	5.92260936	2.18505846	0.15500233	0.16594041
Rpl17	0.25615001	6.86589986	2.18484773	0.15502124	0.16594041
Tomm40l	0.24487492	3.94998471	2.18467805	0.15503647	0.16594041
Mrm1	0.30387437	3.09996865	2.18356303	0.15513658	0.16602594
Cgn	-0.4782418	2.17936625	2.18266819	0.15521698	0.16609036
Klhl21	0.31497394	4.54310308	2.1811335	0.15535498	0.16621639
Igip	-0.2312653	5.87735053	2.17959035	0.1554939	0.16634336
Neto1	-0.3024533	7.50462645	2.17785862	0.15564997	0.16648865
Tnnt1	-0.64558	1.24684882	2.17754384	0.15567836	0.16649735
Fam178b	1.64538868	-1.1721613	2.17704931	0.15572298	0.16651733
Ankk1	-1.0886837	-0.2694108	2.17688766	0.15573756	0.16651733
Gap43	0.20878421	7.23966044	2.17582961	0.15583308	0.16657911
Cnot4	0.23214137	7.41731286	2.17579863	0.15583588	0.16657911
Marveld1	0.41823213	4.34164061	2.17513668	0.15589567	0.16662136
Ccdc126	-0.4676767	2.28289532	2.1735347	0.15604049	0.16675446
Ggnbp1	-1.7138619	-0.8377932	2.17306682	0.15608282	0.16676539
Pus10	-0.3882513	3.92504545	2.17297313	0.1560913	0.16676539
Crtap	0.53699836	4.08574639	2.17110419	0.15626053	0.16691616

A930015D03	0.63629699	1.37320484	2.17064281	0.15630234	0.16691616
Atg12	0.27932786	5.11943071	2.17038921	0.15632533	0.16691616
Adora1	-0.2167624	7.10715741	2.17031625	0.15633195	0.16691616
Zfp30	0.29574621	3.69679721	2.17029401	0.15633396	0.16691616
Rpusd1	-0.3107001	2.98295012	2.16975728	0.15638263	0.16693544
Atp6ap2	0.20442406	7.25392313	2.16964687	0.15639265	0.16693544
Rundc1	0.25347121	5.45873072	2.1693141	0.15642283	0.16694598
Hspg2	0.28067345	3.48405132	2.1688947	0.15646089	0.16696492
Stk33	0.63349945	1.62331277	2.16794454	0.15654714	0.16703528
Pdk4	-0.3863077	2.80876838	2.16605218	0.15671911	0.16719705
Tnfsf12	0.35331676	3.32585799	2.16546589	0.15677243	0.16723224
Adamts18	-0.8806656	0.25730253	2.16432642	0.15687613	0.16732114
Ccdc149	-0.266673	4.44351434	2.16350788	0.15695067	0.16737892
Tradd	0.72993499	0.67360552	2.16307037	0.15699053	0.1673865
Tex40	0.69484433	2.10217503	2.16287637	0.15700821	0.1673865
Kpna6	-0.2203209	7.46646261	2.16275938	0.15701887	0.1673865
Traf3ip1	-0.2070533	4.88522269	2.16241946	0.15704986	0.16739782
Msantd2	-0.5250126	3.04456159	2.16147851	0.15713567	0.16746757
Ppm1h	0.18685182	6.75681679	2.15956161	0.15731066	0.16762885
Srrm3	0.40431851	2.95902668	2.15937405	0.15732779	0.16762885
Ms4a6b	1.22210692	0.48023595	2.15904384	0.15735796	0.16763926
Slc36a4	-0.2578572	4.97063688	2.15793063	0.15745973	0.16772594
Zmiz2	-0.1958745	6.4725955	2.15673014	0.15756957	0.1677931
Nedd9	0.2223995	4.36676939	2.15662242	0.15757943	0.1677931
Phtf2	-0.2302978	5.23459006	2.15657224	0.15758402	0.1677931
Rps3a1	0.26651871	7.93110802	2.15632639	0.15760653	0.16779532
Prima1	0.49019931	1.40377764	2.15601129	0.15763538	0.16780431
Grn	0.39588456	5.16360744	2.15394665	0.15782459	0.16798397
2810055G20	-0.4306398	2.25090386	2.15181909	0.15801986	0.16815529
Zfp944	0.29880811	3.91730998	2.15165641	0.15803481	0.16815529
Bms1	-0.2033537	6.0653261	2.15152437	0.15804694	0.16815529
Nvl	-0.2601294	5.01649047	2.1509458	0.1581001	0.16819008
Oas1g	1.24306926	-0.3560232	2.15069426	0.15812322	0.16819291
2900011O08	0.19253268	7.725197	2.14906837	0.15827276	0.16831484
Slc6a1	-0.2450695	7.70563936	2.14900265	0.15827881	0.16831484
Megf11	-0.2446627	5.52647012	2.14822385	0.1583505	0.1683693
Polr1a	-0.4388719	4.59391222	2.14661027	0.15849917	0.16850557
Capn10	0.40205306	2.36488744	2.14582507	0.15857158	0.16853729
Nln	-0.2450307	4.43820929	2.14575425	0.15857811	0.16853729
Trim12c	0.31847533	5.55706008	2.14561973	0.15859052	0.16853729
D2Wsu81e	0.39588996	2.04981063	2.14368235	0.15876937	0.16870554
Fbxl19	-0.3401963	3.63288	2.1429685	0.15883533	0.16874007
Elk1	-0.24923	4.81294127	2.14271845	0.15885844	0.16874007
Coprs	0.35494018	3.63211372	2.14252791	0.15887606	0.16874007
Slc35a1	-0.3278633	5.25560159	2.14244225	0.15888398	0.16874007

2610037D02I	-0.9307645	0.16203569	2.14176721	0.15894641	0.16878151
Tcap	-0.7337859	0.86915898	2.14157624	0.15896408	0.16878151
Slc3a2	0.20332983	6.32439206	2.14133821	0.1589861	0.16878309
Fam84b	-0.3316534	2.78382697	2.14081091	0.1590349	0.1688131
Nfat5	-0.2049493	8.73989418	2.14058175	0.15905612	0.16881381
Mmgt2	0.37210143	3.31545724	2.14001993	0.15910814	0.16881775
Nr2f6	0.411689	2.59815813	2.13992091	0.15911731	0.16881775
Zfp703	0.24257472	4.68493457	2.13987625	0.15912145	0.16881775
D730045A05	-1.3870848	-0.3651192	2.1394834	0.15915784	0.16883457
Vpreb3	1.73906505	-1.2330033	2.13850437	0.15924858	0.16890903
Fat2	0.5094143	2.77609445	2.13768256	0.1593248	0.16896807
Yipf2	-0.5615561	1.33348163	2.13664521	0.15942108	0.16904836
Abcg3	-2.0704796	-1.7768559	2.1341611	0.15965191	0.16927129
Rangrf	-0.4173708	2.89797601	2.13359284	0.15970477	0.16929347
Steap3	0.38219773	4.09819426	2.13334435	0.15972789	0.16929347
Mrps9	0.32158844	3.40868777	2.133272	0.15973462	0.16929347
Sfmbt1	-0.1896659	5.97509888	2.13268772	0.15978901	0.16932927
Rtbdn	-1.1400296	0.38635475	2.13245528	0.15981065	0.16933038
Atp2a3	-0.6542539	0.6266662	2.13161081	0.15988931	0.16939188
Clec2d	0.57244315	2.1306809	2.13072778	0.15997161	0.16945723
Dio3os	1.99510587	-1.726407	2.130129	0.16002744	0.16949453
Ccdc14	-0.512173	1.8649265	2.12969405	0.16006802	0.16951566
Vmn2r18	-2.1480243	-1.8672575	2.12873708	0.16015733	0.1695884
Scpep1	0.30445374	5.11264015	2.12812029	0.16021493	0.16962061
Bmp7	0.39653502	7.77650848	2.12783697	0.16024139	0.16962061
Cep120	-0.2105832	6.33522438	2.12774849	0.16024966	0.16962061
B930059L03I	1.80853549	-1.1958104	2.12704858	0.16031507	0.16966799
Morn4	0.25318802	5.72266138	2.12640827	0.16037493	0.16970949
Il1rl1	-1.5415015	-0.1980598	2.12551192	0.16045878	0.16977636
Itgb3	-0.4143404	2.3627299	2.12341096	0.16065553	0.16996266
Gmnn	0.44026099	2.06183801	2.12286314	0.16070688	0.1699951
Rnf139	0.21167593	5.09145467	2.12178186	0.16080829	0.17008048
Sox2ot	-0.2655579	4.98179201	2.12149627	0.16083509	0.17008694
Apopt1	0.21114675	4.12066254	2.12084459	0.16089626	0.17009741
Snhg8	0.39742499	2.70202649	2.12079819	0.16090061	0.17009741
Pgbd1	0.37018059	2.33747517	2.1207294	0.16090707	0.17009741
Triqk	0.39344081	3.52130228	2.11806795	0.16115721	0.17032152
Nudt11	0.29214297	4.15227388	2.11803262	0.16116053	0.17032152
Kcne3	1.2785646	-0.4643361	2.11660562	0.16129484	0.17043971
Klhl40	1.02070013	0.1157573	2.11640393	0.16131384	0.17043971
Gmps	-0.2000913	6.75866356	2.11610606	0.16134189	0.17044745
Rps29	0.23329904	6.7774653	2.1154256	0.16140601	0.17049327
Ahr	-0.3184873	4.19486177	2.11499351	0.16144674	0.17051438
Fbxo41	-0.298182	5.17505856	2.11467036	0.16147721	0.17052465
Ramp1	-0.3533558	4.14651163	2.11423415	0.16151835	0.17054618

Incenp	0.3369776	3.47791435	2.11384099	0.16155544	0.17056343
B3gnt6	2.35591799	-1.3576428	2.11245709	0.16168608	0.17067943
Atf7	0.34654164	4.26147139	2.11173108	0.16175467	0.17072991
Mdfic	0.48343316	5.61031651	2.11119814	0.16180504	0.17075115
Tmem259	-0.3988212	3.37626986	2.1110786	0.16181634	0.17075115
Arhgdig	-0.4492271	3.12759263	2.11036821	0.16188352	0.17080011
Xrcc3	0.36153643	3.43280101	2.10981695	0.16193568	0.1708332
Ttc39b	-0.2547589	7.09488054	2.10837174	0.1620725	0.1709556
C4bp-ps1	-1.9538864	-1.5809795	2.10770612	0.16213556	0.17100018
Zfp280d	-0.2830007	6.75746108	2.10741933	0.16216275	0.1710069
Apex2	-0.9025675	1.18741274	2.10710207	0.16219282	0.17101668
P4htm	-0.3791695	2.39224684	2.10666705	0.16223407	0.17103823
5730409E04I	0.14660776	6.93327288	2.10588099	0.16230864	0.1710949
Mad2l1bp	0.55202322	1.72148209	2.10484325	0.16240715	0.17117679
Cyth1	-0.3519078	3.89157414	2.10391153	0.16249566	0.17124811
Zfp677	0.49977094	3.72824083	2.10267401	0.1626133	0.17135012
Siae	-0.2724357	4.5606751	2.10187015	0.16268978	0.17138864
Elmod1	-0.2361418	7.65046911	2.1018514	0.16269156	0.17138864
Gimap8	-0.5615508	1.21229968	2.10145312	0.16272947	0.17140661
Commd3	0.21069959	5.0746158	2.09796851	0.16306159	0.17173442
Zfp101	0.35251942	2.54349396	2.09698422	0.16315555	0.17181137
Tmem194	-0.5391907	2.91223127	2.09666056	0.16318646	0.1718219
Tcf20	-0.220514	8.10599558	2.0957614	0.16327237	0.17188474
4933416I08R	-1.5706787	-1.0641654	2.09559827	0.16328797	0.17188474
Fbxo6	0.38581607	2.55592837	2.09474494	0.16336956	0.17194414
Nrg2	1.33040459	-0.8635565	2.09457066	0.16338623	0.17194414
Uox	1.1542932	-0.8076337	2.09336892	0.16350124	0.17204314
Rgs16	0.30214536	3.66457542	2.09166239	0.16366473	0.17219312
Angptl3	-1.0942867	0.11043517	2.09131195	0.16369832	0.17220643
AF357355	-2.1960239	-1.001034	2.09107682	0.16372087	0.1722081
Prss23	0.32739524	3.46917784	2.09050934	0.1637753	0.17224331
Spag17	-1.4312586	0.24423401	2.09006076	0.16381834	0.17224569
Wbp5	0.28014216	6.62007551	2.0896352	0.16385919	0.17224569
Rgs3	0.34713239	4.10032418	2.0893885	0.16388287	0.17224569
Pstk	-0.3422688	4.18438685	2.08935215	0.16388636	0.17224569
Vps39	-0.1893276	5.87363614	2.08922067	0.16389899	0.17224569
Murc	-1.3549585	-0.1710877	2.08917575	0.1639033	0.17224569
Dctn1	-0.2809402	5.19929677	2.08820125	0.16399691	0.17232204
Pglyrp1	-0.6550795	0.74415633	2.08737668	0.16407618	0.17238328
Med4	0.3153163	3.6371704	2.0861995	0.16418941	0.1724802
Nagpa	0.46187819	2.8786944	2.08594224	0.16421417	0.17248417
Tsc2	-0.2844716	5.85466584	2.08544075	0.16426245	0.17250502
Urb2	-0.3046252	4.20458844	2.08514056	0.16429136	0.17250502
Ebf2	0.41391299	3.16521469	2.08508207	0.16429699	0.17250502
Brpf1	-0.2212754	4.65622633	2.08458911	0.16434448	0.17253284

Alyref	0.37158994	2.80293546	2.08397116	0.16440403	0.17257331
Wdr25	0.40148362	1.99011313	2.08340004	0.16445908	0.17260906
Eif4a2	0.15655757	8.98994268	2.08285984	0.16451118	0.17263919
Slc1a6	-0.7745029	0.37276094	2.08249027	0.16454684	0.17263919
Dut	0.27988352	4.83592111	2.08244919	0.1645508	0.17263919
Rasgef1c	-0.3586266	3.5194525	2.08138208	0.16465381	0.17272521
Rrp12	0.30850915	3.92891724	2.08053387	0.16473574	0.17278836
Epb4.1l4a	-0.4600518	1.53030088	2.08032367	0.16475605	0.17278836
Ndst1	-0.1895379	5.73500255	2.07972682	0.16481375	0.17282681
Ankrd28	-0.1704824	6.58204557	2.07926104	0.16485879	0.17285199
Rnase6	-1.7154857	-1.1019717	2.07763506	0.16501613	0.17299489
Trim37	-0.2273204	8.21341836	2.07726469	0.165052	0.17301043
Acap3	-0.2376171	4.76959602	2.07670879	0.16510585	0.17303292
Efhc2	-0.6577132	2.12178194	2.07660856	0.16511556	0.17303292
4933427D14I	-0.4324101	4.10754685	2.07563097	0.16521031	0.17311015
Zfp395	0.28961547	6.06510323	2.07460418	0.16530991	0.17319243
Cntn1	-0.2151569	8.31121589	2.07402092	0.16536652	0.17322965
Nudt16	0.27587834	4.68686686	2.07318751	0.16544745	0.17327165
Alg3	0.33670501	3.44367456	2.07314322	0.16545175	0.17327165
Fam172a	0.19959191	5.60774182	2.0728715	0.16547815	0.17327165
Cand1	-0.2213207	7.71060483	2.07273988	0.16549094	0.17327165
Trim7	-0.8768028	0.29043078	2.07207248	0.1655558	0.17331748
Tha1	0.8658824	0.05886685	2.07056605	0.16570232	0.17344878
Bex2	0.25523301	6.92183574	2.06980168	0.16577673	0.17350148
Gpkow	0.19320298	6.8147221	2.0696152	0.16579489	0.17350148
Ciao1	-0.2079178	5.39363612	2.06936404	0.16581935	0.17350499
Ptp4a2	0.25690991	9.07797715	2.06867195	0.16588678	0.17355345
Ube2cbp	-1.3078219	0.2999942	2.06827971	0.16592501	0.17357135
Nfkbiz	-0.4912919	2.15685519	2.06723167	0.1660272	0.17364196
Phc3	-0.1918142	8.00497733	2.06715418	0.16603476	0.17364196
Gm3086	-1.1989155	0.26584689	2.06671636	0.16607748	0.17366454
Nup98	-0.182596	6.39272742	2.06580703	0.16616625	0.17373526
Gm2381	-1.4920377	-1.2024585	2.06556886	0.16618951	0.17373748
Dirc2	0.2443991	4.94217804	2.06511955	0.1662334	0.17376126
Fas	-1.1494237	0.28238961	2.06470815	0.1662736	0.17378118
Med30	0.38168482	3.31133533	2.06374017	0.16636823	0.17385797
Sft2d2	0.30253967	6.40335609	2.06272818	0.16646723	0.17391869
Ebf4	-0.8517907	1.13461649	2.06271363	0.16646865	0.17391869
Pnmal1	0.33992001	3.26626482	2.06238261	0.16650105	0.17393044
Gorab	0.28636644	3.49205678	2.06148017	0.16658942	0.17400063
C5ar2	-0.4676163	1.32805436	2.06114893	0.16662187	0.17401241
Smyd5	0.35797129	3.00264503	2.05840033	0.16689144	0.17427179
Mypn	-0.7043033	1.79093751	2.05801428	0.16692934	0.17428923
Pef1	0.25086044	4.475448	2.05730685	0.16699883	0.17433963
Apobec3	0.4919936	2.31423405	2.05672384	0.16705612	0.17437729

Mrps17	0.30386309	4.15799816	2.05574271	0.16715259	0.17442821
Vsx1	0.45012129	3.86348811	2.05571003	0.1671558	0.17442821
Rbm12b1	-0.29678	4.6326872	2.05558025	0.16716857	0.17442821
Asphd1	-0.3605095	2.41463671	2.05452984	0.16727193	0.17451391
L3hypdh	0.46063704	2.99052929	2.05429812	0.16729475	0.17451556
Myadml2	-0.9001715	0.67170072	2.05363228	0.16736032	0.17456181
4933427E11I	2.00441105	-1.2907902	2.05140674	0.16757973	0.17476174
Icam4	-1.0999496	-0.0270766	2.05125678	0.16759453	0.17476174
Gch1	0.6315417	0.83721912	2.05045356	0.16767381	0.17482223
Guf1	-0.4517486	3.90420422	2.05020142	0.16769871	0.17482601
Brms1	0.39006068	2.6401315	2.04966021	0.16775216	0.17485956
Fastkd3	0.24924037	3.79209498	2.04909972	0.16780754	0.17489511
Ttyh3	-0.2537992	6.04932733	2.04877666	0.16783948	0.17490134
Parg	-0.1891825	5.62381836	2.04860866	0.16785608	0.17490134
Six3os1	-0.6659748	0.77632868	2.04759977	0.16795586	0.17497844
AI317395	-1.6501748	-0.8851693	2.04743	0.16797266	0.17497844
Dcaf13	0.20926883	4.45411103	2.04562861	0.16815102	0.17514205
Yes1	0.23976124	4.78123407	2.04510256	0.16820315	0.1751602
Shq1	-0.6592977	1.05411316	2.04496174	0.16821711	0.1751602
Slc37a2	-1.165096	-0.1409012	2.04468765	0.16824428	0.1751602
Hdac10	0.49850992	1.601888	2.04453826	0.16825909	0.1751602
Trappc5	0.29983899	4.15310119	2.04437781	0.168275	0.1751602
Mpg	0.48151802	1.96183919	2.04266643	0.16844482	0.17531476
AI115009	0.77818359	0.65848687	2.04222743	0.16848842	0.17532103
Ctnnd1	-0.1679107	8.01841914	2.0421761	0.16849351	0.17532103
Rps8	0.29154393	7.08743797	2.04181596	0.16852929	0.17533606
Actr1b	0.2167235	7.25929002	2.04112007	0.16859844	0.17538581
Dclre1a	0.36147108	3.56079589	2.04072873	0.16863735	0.17540408
Zfp456	-0.3932553	3.08223601	2.04045325	0.16866474	0.17541037
Fam118b	0.29839798	5.23855314	2.04003583	0.16870626	0.17543135
Iqcj	-1.6274786	-1.5324239	2.03915553	0.16879386	0.17549132
Dnaaf1	1.87249641	-1.8853075	2.03875812	0.16883342	0.17549132
Lrrc49	-0.2994312	4.99706323	2.03855358	0.16885379	0.17549132
Mycbpap	-0.6744678	0.72901751	2.03815702	0.16889328	0.17549132
Rapgef1	-0.2133141	6.0110437	2.03813517	0.16889546	0.17549132
Smyd4	-0.3798351	2.62051006	2.03805338	0.16890361	0.17549132
Zfp652os	1.05474829	-0.6744618	2.03795519	0.16891339	0.17549132
Rps17	0.23949397	7.26328492	2.03648585	0.16905985	0.17556859
Man1a2	-0.1771127	7.88075799	2.03625359	0.16908302	0.17556859
Reep2	0.28628203	4.64945687	2.036225	0.16908587	0.17556859
1810044D09I	-1.4606486	-1.5291417	2.0359994	0.16910838	0.17556859
Nfyc	0.27114076	4.60424449	2.03597901	0.16911041	0.17556859
Cytl1	1.73420612	-0.5109423	2.0359237	0.16911593	0.17556859
Adssl1	0.41807958	2.68975469	2.03520409	0.16918775	0.17558092
Gss	0.36024164	3.45881164	2.0350455	0.16920358	0.17558092

2700054A10I	-0.3770963	2.52735864	2.03496044	0.16921207	0.17558092
Spen	-0.2473327	6.87515701	2.03494856	0.16921326	0.17558092
Cdk18	0.33878629	4.00573225	2.03469605	0.16923847	0.17558492
Rgma	-0.3524737	4.29821178	2.03418118	0.1692899	0.1756161
Letmd1	-0.2391679	4.8416147	2.03394545	0.16931345	0.17561837
Srm	0.29130332	4.05505289	2.03258204	0.16944974	0.17573448
Gpr85	-0.272426	3.9713865	2.03239795	0.16946815	0.17573448
Igsf10	-0.3421328	3.27417852	2.03176071	0.16953191	0.17577842
Hmga2-ps1	-0.8961235	0.57436353	2.02943359	0.16976499	0.17598845
Nelfa	-0.2261046	4.89453732	2.02911188	0.16979724	0.17598845
4930452G13	-2.0664129	-1.7082756	2.02909718	0.16979871	0.17598845
1700018A04I	1.89176332	-1.9461086	2.02879982	0.16982853	0.17599716
Nceh1	-0.1878545	6.21461893	2.02828505	0.16988017	0.17600658
Pgm3	-0.2776865	4.00884134	2.02828223	0.16988045	0.17600658
Exosc4	-0.3451091	3.40495254	2.02788143	0.16992067	0.17602606
Gm5088	0.45388268	1.55621154	2.02682887	0.17002634	0.17611333
Gdf11	-0.6629706	1.80607405	2.02570422	0.17013933	0.17620816
Trim17	0.69740511	0.58160471	2.02543041	0.17016686	0.17621446
Slc14a1	-0.4111142	2.67003188	2.02501375	0.17020875	0.17623564
Prpf38b	-0.1888756	7.32605818	2.02444975	0.17026548	0.17627217
Tbcb	0.31574966	4.55125127	2.02357728	0.17035328	0.17634086
Pla2g12a	-0.3369162	3.72783429	2.02267887	0.17044375	0.17640203
4930578E11I	-1.0055024	-0.6153435	2.02240697	0.17047114	0.17640203
Phrf1	-0.2639037	4.78880025	2.02235121	0.17047676	0.17640203
Dalrd3	0.38328443	3.33754619	2.02200624	0.17051152	0.17641579
Il17rb	-1.0487084	-0.0002551	2.02172634	0.17053974	0.17642277
Ppm1j	1.5642655	-1.8285793	2.02119365	0.17059344	0.17645612
Rragb	0.20611458	4.62507117	2.02032233	0.17068134	0.17650731
Gm4349	-0.7964316	0.61440801	2.02015715	0.170698	0.17650731
Eef1b2	0.26074631	7.031599	2.02006448	0.17070736	0.17650731
Plgrkt	0.32079649	4.07499512	2.01968973	0.17074518	0.17652421
BC017158	0.34049632	3.34202633	2.01945518	0.17076886	0.17652649
Fbxo5	-0.8026525	0.50880187	2.01849983	0.17086536	0.17660403
Stk16	0.30531722	4.51350464	2.01785404	0.17093062	0.17664927
Tomm6	0.30796637	5.29163217	2.01741333	0.17097518	0.17666123
Hdhd2	0.2006001	5.52610197	2.01731435	0.17098519	0.17666123
Gm14405	-0.7024001	0.15617204	2.01690704	0.17102638	0.17666662
Tmem150a	0.39956257	2.52339698	2.0168378	0.17103339	0.17666662
Slc37a3	-0.3039629	4.04118976	2.01576633	0.17114182	0.1767564
Cdkl3	-0.2936068	3.66734289	2.01535125	0.17118385	0.17677064
Pex5	-0.2722177	3.90300021	2.01520542	0.17119862	0.17677064
Zfp951	-0.365773	2.46295741	2.0146874	0.17125109	0.17679248
G730013B05	-0.8396341	0.35112344	2.01457198	0.17126279	0.17679248
A730043L09I	1.79651062	-2.1676103	2.01401014	0.17131973	0.17681959
Gca	-0.251189	6.18271984	2.01374454	0.17134665	0.17681959

Lrrc59	0.17158559	6.71192555	2.01367612	0.17135359	0.17681959
Arr3	-1.6357065	-1.161148	2.01281906	0.17144052	0.17688708
Osr1	0.48655224	6.37133727	2.01111992	0.17161301	0.17702942
Mctp2	-0.4590458	1.62389427	2.01103582	0.17162155	0.17702942
Ogfr	0.50077955	1.96457029	2.01047143	0.1716789	0.17706635
Naip6	-1.6285353	-2.0934829	2.01018965	0.17170754	0.17707367
Tnfrsf9	-1.5995834	-1.4474965	2.0095644	0.17177111	0.1771032
Adra2a	0.37435065	4.09654235	2.00913023	0.17181527	0.1771032
Pmpcb	0.18515315	5.02703198	2.00906742	0.17182166	0.1771032
Ccdc88b	0.72992696	0.13683675	2.00906045	0.17182237	0.1771032
Smim24	0.54314456	2.27280505	2.00880921	0.17184793	0.17710734
Ppp6r2	-0.2730899	4.59010303	2.00751204	0.17197998	0.1772021
Klrg2	1.12442104	-0.5935712	2.00748248	0.17198299	0.1772021
Six2	0.35994334	5.88949627	2.00694231	0.17203802	0.17723657
Klhdc3	0.22116286	4.44002084	2.00642147	0.17209109	0.17726904
B230217C12I	-0.1674517	5.85137644	2.00583622	0.17215076	0.17730828
Klc2	0.29018998	5.48248672	2.00483253	0.17225314	0.17738114
Zfp109	0.34284758	3.49465105	2.00471963	0.17226467	0.17738114
Cdc26	0.39904045	4.58922415	2.00309234	0.17243083	0.17753
Vmn2r46	-1.2553702	-1.6613097	2.00210684	0.17253156	0.17761146
Ackr2	0.88871319	0.49083056	2.00183084	0.17255979	0.17761827
2900076A07I	-0.6930907	0.95672739	1.99968044	0.17277988	0.17782254
2210013O21	0.27926482	5.29079884	1.99932286	0.17281651	0.1778338
Wnt7b	0.64213907	1.64236323	1.99915124	0.17283409	0.1778338
Zfp592	-0.2112013	5.65613648	1.99818485	0.17293315	0.17791345
Nfe2	2.09085232	-1.1396766	1.99745851	0.17300765	0.17796782
2010109I03R	-1.2057306	-1.33369	1.9967278	0.17308264	0.17802267
Slc16a10	-0.3766454	2.28528914	1.99574261	0.1731838	0.17809658
Ank2	-0.2540047	10.1838387	1.99560606	0.17319783	0.17809658
Mex3a	0.39386591	2.55651255	1.99535018	0.17322412	0.17810133
Bin2	0.4126313	3.02808699	1.99484231	0.17327631	0.17812251
Lta4h	-0.2270698	4.27326009	1.99447858	0.1733137	0.17812251
Rfx8	-1.9981481	-1.9345285	1.99420999	0.17334132	0.17812251
Rpf2	0.22088134	4.36084234	1.99392449	0.17337068	0.17812251
Creb1	0.13981756	7.03094245	1.99352099	0.17341219	0.17812251
Fam129b	0.34239184	5.3393645	1.99339058	0.17342561	0.17812251
Card11	-1.2334028	-1.0961286	1.99334943	0.17342984	0.17812251
Cnpy4	0.24027855	4.55218705	1.99324339	0.17344076	0.17812251
Ddc	-0.5533927	1.34508419	1.9932261	0.17344254	0.17812251
4933432I03R	1.25731557	0.10659377	1.99304244	0.17346144	0.17812251
Sephs1	0.22142955	5.67359731	1.99200365	0.17356839	0.17821008
Minpp1	-0.2261935	4.91685676	1.99158241	0.17361179	0.17823237
Steap4	-1.9428194	0.17579235	1.98864406	0.17391486	0.17852121
Pip4k2c	0.21613095	6.1114067	1.98770246	0.17401212	0.17859874
Pacrgl	0.39834203	3.30867818	1.9871365	0.17407061	0.17862052

Slc45a1	-0.3974591	2.61914513	1.98692813	0.17409215	0.17862052
Stab1	-0.7589951	1.19212054	1.98686631	0.17409854	0.17862052
Mir344b	-1.9607783	-2.0874716	1.98632352	0.17415467	0.17865581
Rdh14	0.25158326	5.61458882	1.98418797	0.17437572	0.17886025
Pabpc1	0.17210844	8.19210904	1.98325886	0.174472	0.17893667
Qtrt1	-0.3999209	2.71706356	1.98196352	0.17460634	0.17903904
Ifnlr1	1.71391353	-0.4183534	1.98187643	0.17461538	0.17903904
Snora23	-0.9634193	0.98583809	1.9816424	0.17463966	0.17903931
Cers6	-0.2989298	5.97498481	1.9814541	0.17465921	0.17903931
Gm14436	0.23581569	5.06734564	1.9811394	0.17469188	0.17905046
Cnppd1	-0.3381714	4.12454066	1.98031903	0.17477707	0.17911545
Kctd4	0.31561461	5.06433096	1.97967774	0.17484371	0.1791614
Zdhhc7	0.38226976	3.13165854	1.97919509	0.17489388	0.17918464
Chrna1	0.52201372	2.07646178	1.97904017	0.17490999	0.17918464
Sptssa	0.27369333	6.40935421	1.97867364	0.17494811	0.17920135
Mgat4a	-0.2852357	5.48766177	1.97755282	0.17506473	0.17929846
Mettl7a3	-1.2056537	-1.7645304	1.97663726	0.17516006	0.17937375
Acbd6	0.24669803	4.71462232	1.97594113	0.17523259	0.1794124
Btf3l4	0.15557325	7.05131846	1.97585234	0.17524185	0.1794124
C2cd2	0.26878209	4.33129603	1.97556881	0.1752714	0.1794124
Mical1	0.41823566	2.58725045	1.97543722	0.17528512	0.1794124
Serinc2	-0.6443853	0.7617066	1.97312499	0.17552638	0.17963697
Pcdhb12	-0.4487706	2.49227365	1.97241981	0.17560004	0.1796857
Pcsk9	-0.8258977	-0.0783968	1.97225068	0.17561772	0.1796857
Kcnk12	-1.1982969	-1.2942537	1.97120897	0.17572661	0.17977474
Fez1	-0.2023851	5.52476133	1.97021634	0.17583046	0.17985859
Clu	0.34640422	7.27932893	1.96953549	0.17590173	0.1799091
Traf6	0.19415767	6.18016976	1.96765271	0.176099	0.18008846
Tbc1d31	-0.2984309	3.31663818	1.96604981	0.17626716	0.18020219
Kctd9	0.22755278	4.95379289	1.96601215	0.17627112	0.18020219
Klhdc2	0.14526978	7.86071972	1.96596573	0.17627599	0.18020219
Crbn	-0.1857092	6.44617945	1.96545115	0.17633002	0.18023501
Cap2	-0.2496982	8.29989561	1.96444387	0.17643584	0.18031441
Snrpd1	0.22181203	5.26247952	1.96417927	0.17646365	0.18031441
Pawr	0.42047026	5.34760824	1.96408546	0.17647352	0.18031441
Slc16a6	0.32341112	3.11581184	1.96325071	0.17656129	0.18038167
Irs2	0.35066937	4.35157986	1.96230727	0.17666057	0.18046066
Gpr156	0.7352205	1.13406632	1.96124038	0.17677291	0.18054289
Hsf2	0.21883	5.63223317	1.9611257	0.176785	0.18054289
Sipa1l2	-0.3413215	4.53337432	1.96038934	0.17686259	0.18059969
Tslp	1.96814133	-1.0755982	1.95979582	0.17692517	0.18064115
Cux2	-0.2846729	4.80386625	1.95805068	0.17710932	0.1807895
Fam135b	-0.4301263	4.6382602	1.95800198	0.17711446	0.1807895
LOC1008616	-0.9301193	-0.5326978	1.95742411	0.1771755	0.18081793
Phc2	0.30598122	4.79911662	1.95723613	0.17719535	0.18081793

Adcy10	-0.7723523	0.20138722	1.95711346	0.17720832	0.18081793
4931429L15F	-1.2500961	-1.1998334	1.95650418	0.17727271	0.18086119
Kcnn1	-0.5104646	1.94683146	1.95558339	0.17737008	0.18093807
BC037704	0.73181334	1.27085945	1.95517958	0.1774128	0.18095919
Dcaf12l1	-0.20497	5.6089195	1.95458893	0.17747531	0.18100049
Stradb	-0.218283	5.42089571	1.95319058	0.17762341	0.18110861
Med7	0.28528954	4.81836147	1.95289055	0.17765521	0.18110861
Mettl15	0.52736361	1.63250249	1.95219391	0.17772907	0.18110861
Bcl2l1	0.33057224	4.44907231	1.9521779	0.17773076	0.18110861
Tomt	0.65231943	0.54964426	1.95201858	0.17774766	0.18110861
Dgkq	-0.4701851	3.8686968	1.95181978	0.17776875	0.18110861
Wash	-0.2791307	3.84877299	1.9518041	0.17777041	0.18110861
Usp32	-0.2049015	7.80025164	1.95151517	0.17780106	0.18110861
Gstm1	0.31860721	7.64972735	1.95137874	0.17781554	0.18110861
Spn	0.95989417	-0.2228099	1.95132649	0.17782108	0.18110861
Nat6	0.38383568	3.18126994	1.95110098	0.17784501	0.18110861
Lysmd2	-0.225134	4.50476051	1.95109402	0.17784575	0.18110861
Sbspon	-0.82056	0.73858241	1.95085867	0.17787073	0.18111161
Tceb3	0.21646587	6.00143541	1.95055356	0.17790312	0.18112215
Gdap2	0.1951528	4.7954107	1.95033904	0.1779259	0.18112291
Pex1	-0.2748989	4.55242646	1.94929579	0.17803673	0.18120101
Zfp189	0.30144185	3.26918649	1.94920173	0.17804672	0.18120101
Yaf2	0.19968192	7.42070674	1.94888436	0.17808045	0.18121291
Fastkd5	-0.2840241	3.34987176	1.94790531	0.17818457	0.1812964
Lingo3	0.39186468	3.01449084	1.94764743	0.178212	0.18130188
Flii	0.21153598	5.38782624	1.94723031	0.17825639	0.18132459
Snta1	0.37467146	2.78513284	1.94564292	0.17842544	0.18147409
Mlh1	-0.3282897	3.81828075	1.94532855	0.17845894	0.18147611
Apod	0.45236287	10.3568368	1.94520994	0.17847158	0.18147611
Tipin	0.45160972	4.23054188	1.94368046	0.1786347	0.18160986
Faah	-0.3013581	4.14335949	1.94356231	0.17864731	0.18160986
9330133O14	0.31309506	4.07579386	1.94285924	0.17872236	0.18166368
Ccnd1	0.26902049	6.16639767	1.9421992	0.17879286	0.18169592
Dffa	0.20164695	5.08935317	1.94214827	0.1787983	0.18169592
Myom3	-1.1682775	-0.2197565	1.94163285	0.17885337	0.18172942
D3Bwg0562e	-0.2313739	7.1409987	1.94044864	0.17897998	0.18183559
Ilvbl	-0.4368934	2.36640121	1.93980485	0.17904886	0.18188309
Rxfp2	-0.9873065	-0.5855475	1.93851047	0.17918745	0.18199314
Mgea5	-0.2157886	8.6334026	1.93837943	0.17920149	0.18199314
Mrpl20	0.33991334	4.0552747	1.93765659	0.17927894	0.18203038
Tex35	-1.7733802	-1.7940402	1.93762382	0.17928245	0.18203038
Nfs1	0.24296055	4.3159272	1.93727769	0.17931956	0.18204557
Plod2	-0.4083284	3.8469129	1.93659077	0.17939323	0.18209786
Gm15698	-2.2114686	-1.4406106	1.93585519	0.17947215	0.18215548
Bag4	-0.199912	5.95107043	1.93475884	0.17958987	0.18225245

Ell3	0.48038618	2.29649561	1.93391122	0.17968094	0.18232237
Qtrtd1	-0.4670993	2.08839529	1.93313749	0.17976413	0.18237834
Mfsd5	0.35296986	3.64305993	1.93298541	0.17978048	0.18237834
Samd10	0.37654058	3.49672755	1.93271766	0.17980928	0.18238504
Ocln	-0.4204001	2.73975676	1.9322302	0.17986173	0.18239903
Tlr9	-1.0835147	-0.3613039	1.93202961	0.17988332	0.18239903
Top3a	-0.5635823	0.99311995	1.93197079	0.17988965	0.18239903
Stoml1	0.37072489	3.03885234	1.93111576	0.17998171	0.18244985
Slamf1	-1.8081208	-1.076657	1.93109293	0.17998417	0.18244985
Ly6a	-0.7138339	2.3561402	1.93083352	0.18001211	0.18245568
Ythdf2	0.20931821	5.07872959	1.93057782	0.18003966	0.1824611
Agtr1b	-0.8512643	2.19031854	1.92857788	0.1802553	0.18265712
Arl4d	-0.4518972	2.09657324	1.92828543	0.18028686	0.18266658
4930405J17F	-0.9500204	1.00072485	1.92786832	0.18033189	0.18268968
Lsm10	0.39014048	2.36384022	1.92619267	0.18051291	0.18282194
Rps20	0.25493155	6.34851348	1.92615731	0.18051673	0.18282194
Dram1	-0.7596509	0.62220117	1.92589027	0.1805456	0.18282194
Rpl5	0.24322303	8.2506935	1.92562335	0.18057446	0.18282194
Foxc2	0.42704853	6.72668038	1.92555413	0.18058194	0.18282194
Mthfr	-0.4190381	2.89904496	1.925425	0.18059591	0.18282194
Ric8	0.23026606	5.22884109	1.9250442	0.1806371	0.18282934
Mier2	-0.5854418	1.225176	1.9249462	0.1806477	0.18282934
Pcnt	-0.250042	5.11387486	1.92433205	0.18071417	0.18287166
Srsf12	0.30616384	4.02511157	1.92414869	0.18073402	0.18287166
Grap2	-0.5840048	1.1940081	1.9235099	0.18080319	0.18290661
Parvb	0.3689804	3.54574773	1.92341869	0.18081307	0.18290661
Axin2	-0.2835367	4.98891397	1.92291147	0.18086802	0.18293968
Snord19	-1.4424495	-1.6782624	1.94705832	0.18096067	0.18301087
Jrk	0.44826785	2.19497887	1.92124466	0.18104874	0.1830743
Ghdc	-0.3941174	2.22972725	1.92087624	0.18108871	0.1830743
Dhrs7	0.38113226	3.8290547	1.92077776	0.1810994	0.1830743
Olfr1372-ps1	0.96313004	-0.054429	1.92065723	0.18111248	0.1830743
Lsr	0.38197747	3.45580772	1.91951713	0.18123628	0.18316165
Gpr157	-0.5218081	2.14962224	1.91945091	0.18124347	0.18316165
Ints8	-0.2239714	5.49096989	1.91828788	0.18136987	0.18326686
Chia1	-1.2176485	-0.1819364	1.91720893	0.18148723	0.1833629
Six3	0.36370322	3.04223827	1.9165617	0.18155768	0.18341153
Eva1c	0.52860441	2.79785056	1.91581493	0.181639	0.18347113
Limk1	0.37673943	2.85508073	1.91488697	0.18174011	0.1835507
Zbed4	-0.3016691	4.47113745	1.91291722	0.18195497	0.18374513
Idh2	0.33235954	4.04339643	1.91249651	0.18200091	0.18376893
Sssca1	0.3956334	2.92878654	1.91211622	0.18204244	0.18377572
Trim9	-0.2816169	8.22979145	1.91202548	0.18205235	0.18377572
Chac2	0.32949089	3.90892416	1.91112079	0.1821512	0.18383929
Cyp39a1	0.39946423	3.46184472	1.91084437	0.18218142	0.18383929

Kctd5	0.37216695	2.84732475	1.91083523	0.18218242	0.18383929
Tpst1	-0.2342826	5.01559497	1.90923549	0.18235742	0.18398422
Aatf	0.27639283	3.91732597	1.90911318	0.18237081	0.18398422
Sync	0.79249701	1.07307096	1.90862818	0.18242392	0.18400281
Sugct	0.5518245	1.49156978	1.90853597	0.18243401	0.18400281
Rfc4	0.43681989	1.9149827	1.90738082	0.18256057	0.18410786
Gpr45	0.37247943	2.23163796	1.90579382	0.18273463	0.1842291
Col25a1	-0.2548438	5.59930305	1.90571165	0.18274364	0.1842291
Cep70	-0.3485638	4.03986928	1.90549912	0.18276697	0.1842291
Efhd1	0.2705125	4.60422175	1.90546741	0.18277045	0.1842291
Ptx3	1.55214007	-1.326802	1.90517109	0.18280298	0.18423929
Myo5b	-0.3774661	4.19877001	1.9048768	0.18283529	0.18424927
Cacnb2	-0.191015	6.33615313	1.90449093	0.18287767	0.18426938
Htr1a	-0.2967625	3.55770126	1.90223808	0.18312534	0.18449632
Cd99l2	-0.2514238	5.75979085	1.90192624	0.18315966	0.18450827
L3mbtl4	-0.8724738	0.00633733	1.90138995	0.18321869	0.18454512
Cacna2d2	-0.3082243	4.99637062	1.90109046	0.18325167	0.18455572
Rps15a	0.17562191	7.20599227	1.90061217	0.18330435	0.18458616
Phf14	-0.1610467	6.44424764	1.90001297	0.18337037	0.18463002
Zfp526	0.34678273	3.07793303	1.89954318	0.18342216	0.18465954
Npsr1	-0.6630234	1.3576542	1.89877942	0.18350639	0.18472021
9330162012I	0.98506115	0.74118326	1.89858923	0.18352737	0.18472021
Ncoa2	-0.206135	8.34814056	1.8983488	0.1835539	0.18472429
Npas1	-0.9793356	-0.7981625	1.89758288	0.18363844	0.18476439
Cmklr1	0.53581342	1.08346113	1.89758049	0.1836387	0.18476439
Ganc	0.27760718	4.43255697	1.89728042	0.18367184	0.18477511
Trip11	-0.1562644	7.25368334	1.89628486	0.18378182	0.18484113
Sytl4	0.58866894	2.35993316	1.89618764	0.18379256	0.18484113
Gm15319	-0.7529552	0.17664311	1.89607572	0.18380493	0.18484113
C330006A16I	0.1989865	5.98893754	1.89570275	0.18384617	0.18485998
Dsn1	-0.5055352	1.38617472	1.89439771	0.18399053	0.1849825
Rpl23a	0.24938111	7.25485524	1.89397288	0.18403755	0.18500715
2210404O09	-0.4385216	2.13870663	1.89290305	0.18415603	0.18507696
Zfp839	0.2111852	5.26943064	1.892851	0.1841618	0.18507696
Setd6	-0.2059346	4.8372087	1.89273596	0.18417455	0.18507696
Hhipl1	1.48569677	-1.2036781	1.89191357	0.1842657	0.18514592
Ppifos	-1.4521785	-1.5463159	1.89165195	0.18429471	0.18515244
Frem1	-0.7925744	1.21713709	1.88916013	0.18457131	0.18538501
Stard3	-0.4104742	2.37085646	1.88915999	0.18457132	0.18538501
Mbip	-0.3810505	3.81516301	1.88870341	0.18462206	0.18541332
Ppp1r18	0.40464834	3.13725075	1.88841359	0.18465427	0.18542301
Scp2	0.31136551	7.10915731	1.88810602	0.18468847	0.18543469
Gmfb	0.16718677	8.2222009	1.88777022	0.18472581	0.18544953
Fjx1	0.25032584	4.08630098	1.88747834	0.18475828	0.18545947
1700110I01R	-0.7057403	1.59061764	1.8867291	0.18484165	0.1854904

Tsc22d2	0.16432609	6.84533582	1.88659652	0.1848564	0.1854904
Rps27l	0.45714897	4.62792645	1.88645244	0.18487244	0.1854904
Traf3ip3	-1.4814542	-0.8302029	1.88619691	0.18490089	0.1854904
Lama1	-0.3263249	3.50150878	1.88618762	0.18490193	0.1854904
1700001D01I	-0.8591501	1.20263267	1.88588271	0.18493588	0.18550182
Adam19	0.23567308	4.64126525	1.8856089	0.18496638	0.18550977
Emc7	0.29351234	5.39308965	1.88475952	0.18506103	0.18558205
Mmd2	0.28619242	4.00550522	1.88425851	0.18511689	0.18561542
Spag8	1.52058649	-1.5179381	1.88394431	0.18515193	0.18562791
Gpc3	0.38749826	3.71078847	1.88263078	0.18529851	0.18575221
Cryz	0.37036344	2.82523574	1.88230977	0.18533435	0.18576548
Mafb	0.19482969	5.48543859	1.88120193	0.18545811	0.18585535
Crtc1	-0.2150356	6.31936751	1.88110242	0.18546924	0.18585535
Yme1l1	-0.1806245	6.23404001	1.88057036	0.18552872	0.18587052
Cntf	-1.3165946	-1.5165918	1.88056242	0.18552961	0.18587052
Tcta	0.3064843	4.4087972	1.87987752	0.18560621	0.1859246
Etv6	0.20194735	5.18970227	1.87963931	0.18563286	0.18592864
Snrpd3	0.42857296	2.63607777	1.87829354	0.18578352	0.18605687
Pcsk1n	-0.2349992	4.4277672	1.87793727	0.18582343	0.18607416
Cwc25	0.23170758	4.51326967	1.87685188	0.18594508	0.1861733
Arfgap3	0.2940826	4.63360754	1.87571906	0.18607216	0.18623438
Sgcd	-0.3971071	2.93559348	1.87541469	0.18610632	0.18623438
Gadl1	1.00190255	-0.5549758	1.87523442	0.18612655	0.18623438
Zfp768	0.44961232	2.42165946	1.87502438	0.18615013	0.18623438
Ptrhd1	0.27697092	3.71099187	1.87497303	0.1861559	0.18623438
Tlcd1	-0.5799311	2.1458999	1.87483833	0.18617103	0.18623438
Ttc32	-0.4510312	2.57335124	1.87472541	0.18618371	0.18623438
Ptpn18	1.66884172	-1.6842723	1.87469289	0.18618736	0.18623438
Gm8979	-0.5742485	0.55509727	1.87438979	0.1862214	0.18624577
Mrps28	0.33110558	2.96171414	1.8741639	0.18624678	0.18624848
Rc3h2	0.17334042	7.92859234	1.87354877	0.18631591	0.18629054
Gm13582	-1.6243208	-1.3285864	1.87338632	0.18633417	0.18629054
Cd209a	-0.5755262	3.352711	1.87115935	0.18658473	0.18651835
Psd3	-0.2230092	9.82749616	1.87042742	0.18666717	0.18657698
Klhl36	0.89354883	0.6160963	1.87023562	0.18668878	0.18657698
Gbp9	0.35193357	3.98239406	1.86884432	0.18684564	0.18671104
Tesk2	0.39306185	2.09730237	1.868295	0.18690761	0.18674187
Dad1	0.38221072	5.04388937	1.86816816	0.18692193	0.18674187
Slc7a15	-1.4933941	-1.0209344	1.86783157	0.18695992	0.18675712
AI662270	-0.62404	0.58541783	1.86749486	0.18699793	0.1867724
Smc1b	-1.348386	-0.7178383	1.86726384	0.18702402	0.18677576
Mtg1	-0.4163195	2.77387314	1.86687813	0.18706759	0.18678618
Tbc1d9b	0.17047951	6.59204366	1.86676905	0.18707991	0.18678618
Fam32a	0.22186377	6.0705098	1.86626563	0.18713679	0.18679967
Adam33	-0.9777551	-0.0202813	1.86624718	0.18713888	0.18679967

Snora30	-1.7242885	-1.4576189	1.86566941	0.18720419	0.18684218
Ccdc8	1.15183678	-0.2951193	1.86539459	0.18723527	0.1868505
Sumo3	0.27504176	6.45768741	1.86483882	0.18729813	0.18687928
Pwp1	-0.3084487	4.19205255	1.86473768	0.18730958	0.18687928
Otud6b	0.21813232	6.33504162	1.86427062	0.18736243	0.18690932
Mir690	-1.8620133	-2.00979	1.86255948	0.18755623	0.18707995
Lyrm2	0.25574286	4.85568116	1.86069282	0.18776793	0.18726838
C230079O03	-2.0712032	-2.0034868	1.86003692	0.18784239	0.18731991
Mterfd2	0.19971983	5.9146448	1.85981238	0.18786789	0.18732261
Mr1	0.3297723	4.41173809	1.85906725	0.18795253	0.18738228
Wrb	-0.1783118	5.3922587	1.85888422	0.18797333	0.18738228
Ctnnal1	-0.2325623	4.46199785	1.85836744	0.18803207	0.18739685
4921511H03I	-1.7921985	-1.1054533	1.85835449	0.18803354	0.18739685
Pabpc1l	2.24372952	-1.9515663	1.85673808	0.18821742	0.18754386
AW554918	-0.2010113	4.9547317	1.85665669	0.18822669	0.18754386
Pknox1	0.25104731	3.85227558	1.85539716	0.18837013	0.18766403
Zfp654	-0.2628334	5.43811125	1.85489917	0.18842688	0.18769782
Nipal2	-0.429767	2.85416739	1.85389886	0.18854095	0.18778868
Gm6402	-0.3903578	1.77517024	1.85324677	0.18861535	0.18784002
Drg1	0.26633193	5.31877549	1.85172904	0.18878866	0.18796037
Tufm	0.25699604	3.65762386	1.85162268	0.18880081	0.18796037
Fbxl2	-0.2966916	4.66631756	1.85154315	0.1888099	0.18796037
Sp1	0.16457383	7.09172639	1.85138768	0.18882766	0.18796037
Gm8773	1.70616239	-1.5269507	1.85098951	0.18887318	0.1879829
Ctsd	0.34709766	7.20349076	1.85075075	0.18890047	0.18798731
08-Mar	-0.1743732	6.24265239	1.85009195	0.18897582	0.18803952
Mrpl22	0.3380681	3.03106697	1.84869373	0.18913585	0.18817598
Cxcr5	-0.7793071	-0.2027588	1.84634837	0.18940467	0.18842063
Itm2c	0.30153476	7.70303371	1.84578048	0.18946983	0.18844059
Plagl1	-0.2883849	6.20316342	1.84577387	0.18947059	0.18844059
Sf3b2	0.20020558	6.8265091	1.84526715	0.18952876	0.18847563
Mxra7	-0.4157631	2.78163918	1.84417039	0.18965473	0.18857489
Ccdc22	-0.6766188	0.91836404	1.84392327	0.18968313	0.18857489
Lmna	0.31649666	3.66476007	1.84379908	0.1896974	0.18857489
Gm14164	-1.292029	-0.9193449	1.84314482	0.18977262	0.18861427
Gga3	-0.205919	5.38664136	1.84305527	0.18978291	0.18861427
1700007J10F	-0.7995375	0.06999481	1.84262785	0.18983208	0.18862273
Wdr20	-0.2626633	3.5679198	1.84258228	0.18983732	0.18862273
Fam219a	0.27599639	3.871757	1.842106	0.18989212	0.18864492
Arpc3	0.25353872	4.43972326	1.84198926	0.18990556	0.18864492
08-Sep	-0.1967045	5.95391556	1.84129487	0.1899855	0.18870152
Slc1a3	-0.230415	7.23936215	1.84076774	0.19004621	0.18873902
Snord91a	-0.3195981	3.26393971	1.83989977	0.19014623	0.18880141
Pofut2	-0.3710885	3.73010993	1.8398239	0.19015498	0.18880141
Ada	0.54038567	1.31544401	1.83943829	0.19019944	0.18881377

Cdk16	0.16674735	7.18779947	1.83931749	0.19021337	0.18881377
H2-Ke6	0.55460949	2.38317371	1.83840015	0.1903192	0.188896
Capsl	0.67959426	1.18901442	1.83776856	0.19039211	0.18894555
Shroom4	-0.3852611	3.13879811	1.83731196	0.19044484	0.18897325
Tspyl4	0.1717674	8.38403276	1.8371287	0.190466	0.18897325
Mon1a	0.32810471	2.34691386	1.83663219	0.19052337	0.18900735
Commd2	0.26858705	4.30203494	1.83390369	0.190839	0.18929762
Mecom	-0.7005244	1.34295964	1.83353908	0.19088122	0.18931666
C130021I20R	0.76833037	1.23443208	1.83292295	0.19095261	0.1893646
Tmem108	-0.4153125	3.46789091	1.83264766	0.19098451	0.18937339
Txndc16	-0.2118056	5.40969844	1.83213626	0.1910438	0.18940451
Psen2	0.4887545	1.61387442	1.83197939	0.19106199	0.18940451
Katna1	0.37569725	3.60927627	1.83133285	0.19113698	0.18945601
Ptprc	-0.5100848	1.9383955	1.83089711	0.19118754	0.18948327
Rbm28	0.2312322	4.98405403	1.83008536	0.19128178	0.18953343
Nckap1	-0.1973648	9.44861355	1.83006394	0.19128427	0.18953343
Ankrd50	-0.2037268	5.18671778	1.82940867	0.19136039	0.18956538
Pld1	0.29036223	5.52894528	1.82938921	0.19136265	0.18956538
Liph	-2.274671	-1.6566254	1.82774753	0.19155352	0.18973159
Actr5	-0.9710116	0.08199314	1.82754598	0.19157697	0.18973195
Cox4i1	0.34297819	6.74873163	1.8272877	0.19160702	0.18973886
Arnt	0.18990842	6.02241584	1.82659836	0.19168726	0.18979545
Snord118	-1.5028932	-1.5551468	1.82414885	0.19197274	0.19005521
Nusap1	-0.5181559	2.16744972	1.8238597	0.19200648	0.19006572
Slc35e4	-0.5985292	1.114314	1.82301754	0.19210477	0.19011429
Tmem50a	0.24515955	5.6736444	1.82300113	0.19210668	0.19011429
Chpf	0.32855273	3.44592117	1.82267085	0.19214525	0.19011429
Pigf	-0.5126177	1.56171039	1.82246413	0.19216939	0.19011429
Atg2a	-0.3519585	3.99208014	1.82244865	0.1921712	0.19011429
Pcdhga5	-0.3113699	3.68672093	1.82214834	0.19220628	0.19012611
Gad2	-0.2737075	8.8081103	1.82185195	0.19224091	0.19013749
AF251705	1.11492057	-0.5359717	1.82144612	0.19228834	0.19016151
Dusp26	0.18532628	6.07584242	1.82072506	0.19237265	0.190222
Dmrta2	-0.9195544	-0.2709334	1.82041115	0.19240937	0.19023542
3110079O15	-1.4588622	-1.8125332	1.81994818	0.19246354	0.19026609
Eno2	0.24401205	8.48693367	1.8191896	0.19255233	0.19033098
Zfp707	0.61662414	1.37092506	1.81780156	0.19271494	0.19046725
Msi2	0.22469358	9.31989194	1.81761728	0.19273655	0.19046725
Npr3	-0.3286122	5.29409063	1.81702641	0.19280583	0.19051282
Wdr77	-0.198975	5.07304581	1.81637703	0.19288201	0.19056518
Gpr52	-0.4835142	1.71756204	1.81606998	0.19291804	0.19057787
Acad11	0.22739819	4.50550654	1.81577921	0.19295217	0.19058868
Pole3	0.38821579	2.9234239	1.81551497	0.19298319	0.19059642
Eif5b	0.20603585	9.35457205	1.81511308	0.19303039	0.19062012
Znrf3	-0.1739826	5.9800087	1.81480804	0.19306622	0.19062411

Tchp	-0.4773082	2.32131566	1.81468385	0.19308081	0.19062411
Sp4	-0.1657275	5.9452757	1.81361958	0.1932059	0.1907247
Copb2	0.13917429	7.23618966	1.81336418	0.19323594	0.19073144
Appl1	-0.1650079	7.58721653	1.81299979	0.1932788	0.19074106
Tor1a	0.34833117	2.96947977	1.81278217	0.1933044	0.19074106
Smad9	0.21212211	5.40048518	1.81268945	0.19331531	0.19074106
Aggf1	0.18457364	6.57041674	1.81229656	0.19336155	0.19076378
BC037032	-0.7634371	0.53871092	1.81135826	0.19347203	0.19081824
Tbcc	0.28628235	3.28109105	1.81127152	0.19348224	0.19081824
Rbms3	0.21310288	7.34695524	1.81123633	0.19348639	0.19081824
Gm9079	-0.9019004	0.57744358	1.80968056	0.19366976	0.19097616
Pf4	-1.2984838	-1.4000512	1.80846172	0.19381357	0.19109505
Csrp2bp	0.20998162	4.95271622	1.80758227	0.19391742	0.19117451
Gm14288	0.50795038	0.84512697	1.80727911	0.19395324	0.19118688
Ccdc59	0.26409383	5.04935982	1.8066832	0.19402366	0.19121584
Itpkb	0.33428981	3.35426576	1.80663679	0.19402915	0.19121584
Myh11	-0.8396386	2.52897226	1.80599951	0.1941045	0.1912163
Entpd5	-0.2346098	4.33180174	1.80591534	0.19411445	0.1912163
Gyg	0.17090074	5.57171995	1.8058843	0.19411812	0.1912163
Lnx2	0.22294878	4.43629105	1.80584587	0.19412267	0.1912163
Zfp61	-0.3707113	3.04587609	1.80558859	0.1941531	0.19121678
Elavl4	0.18917784	6.90619641	1.80538823	0.19417681	0.19121678
Ptch2	1.60374274	-1.929152	1.80525181	0.19419295	0.19121678
Romo1	0.41691303	4.01738062	1.80385397	0.19435844	0.19126426
Scfd1	-0.2286028	4.64103708	1.80374039	0.1943719	0.19126426
Atp1b3	0.29280398	8.8426417	1.80344812	0.19440653	0.19126426
Ehd4	-0.2441706	4.01675613	1.80338992	0.19441343	0.19126426
Anks1b	-0.2338718	8.50588551	1.80334197	0.19441911	0.19126426
Farp2	0.29664431	2.80240206	1.80314709	0.1944422	0.19126426
Mrvi1	0.31191074	5.83222849	1.80267655	0.19449798	0.19126426
C2cd3	-0.2724737	5.22226321	1.80254539	0.19451353	0.19126426
Vps37a	0.21530057	7.7321129	1.80249092	0.19451999	0.19126426
Zfp937	-0.232067	5.13090645	1.80243997	0.19452603	0.19126426
Rhof	0.33709658	3.497237	1.80242746	0.19452752	0.19126426
Sdf2	0.29053065	4.44281333	1.80237432	0.19453382	0.19126426
4921536K21l	-1.0311378	0.51703229	1.80229112	0.19454369	0.19126426
Dvl1	-0.2464685	4.07621373	1.80191178	0.19458868	0.19128561
A930004D18	-0.5361115	2.82111606	1.80084538	0.19471524	0.19138713
Angptl2	-0.4074092	4.88653529	1.79975797	0.1948444	0.19149119
Col6a3	0.43068466	3.31318191	1.79914751	0.19491696	0.19153959
Pigl	-0.3392294	4.09399198	1.79847627	0.19499678	0.1915923
Paqr3	-0.4364342	2.38706999	1.79830444	0.19501722	0.1915923
Ftsj1	-0.2469015	3.73079047	1.7979425	0.19506028	0.1916117
Cers4	-0.3338313	5.54302222	1.79564725	0.19533363	0.19183604
Ablim1	-0.1565648	6.74637897	1.79544524	0.19535771	0.19183604

Mageb16-ps	-1.2479462	-0.6955082	1.7954371	0.19535868	0.19183604
Sybu	-0.2400103	5.28269704	1.79474278	0.19544148	0.19189442
Dnpep	0.33555063	2.95419845	1.79367641	0.19556873	0.19199643
Cyb5d2	0.28143494	3.33322216	1.79315222	0.19563132	0.19203493
Rcn2	0.17965402	6.30450958	1.79280865	0.19567235	0.192051
Id3	0.40359671	6.0191475	1.79262394	0.19569442	0.192051
Slc7a2	-0.2979539	6.92220245	1.79202908	0.1957655	0.19209783
Pitpnb	0.16681424	6.1491041	1.79086028	0.19590527	0.19221202
Prcc	0.2836421	4.29593695	1.79050187	0.19594815	0.19223115
Col26a1	-0.503317	1.50329749	1.7897469	0.19603852	0.19229605
Calb2	-0.5028918	1.07864787	1.78955839	0.1960611	0.19229605
Gdf10	0.59439451	2.35877311	1.7887273	0.19616065	0.19237074
C1qtnf7	0.51659259	4.36202515	1.78580007	0.19651181	0.19265388
Vangl2	-0.4208783	1.96473114	1.78573446	0.19651969	0.19265388
Ahdc1	-0.2265276	4.66728877	1.78573438	0.19651969	0.19265388
Hlx	-1.6782518	-1.893623	1.78482634	0.19662879	0.19273784
Mtdh	-0.1757664	6.76294372	1.7835361	0.19678393	0.19286691
Wdr78	-0.433499	3.27238946	1.7822234	0.19694193	0.19299875
Cacybp	0.17549659	6.42728372	1.78187222	0.19698423	0.19301719
Slc25a44	0.16525272	5.90625202	1.78141399	0.19703943	0.19304827
Ddx4	-0.6029358	0.98895315	1.78076579	0.19711756	0.19308103
Dbf4	0.47238272	1.82206078	1.78074673	0.19711986	0.19308103
Spats2	0.2824418	3.28971686	1.78043284	0.1971577	0.19309509
Gm6225	1.32248692	-1.1707404	1.78003025	0.19720626	0.19311963
Ccdc50	0.1961487	7.37499426	1.77965381	0.19725167	0.19314109
Taok1	-0.1424324	9.64326833	1.77933537	0.1972901	0.19315571
Ctf1	0.50759712	2.64594289	1.77804805	0.19744554	0.19326635
Gm20743	1.42565236	-1.8229197	1.77800996	0.19745014	0.19326635
Skiv2l2	0.17595384	6.77451449	1.77776859	0.19747931	0.19327188
4933404O12	0.24071011	3.94674766	1.77739833	0.19752405	0.19329266
Adap2	-0.3766266	3.73538247	1.777124	0.19755721	0.19329576
Ppap2c	0.5442224	1.53986906	1.77698302	0.19757426	0.19329576
Zfp825	0.23922885	4.52217882	1.77654829	0.19762683	0.19332418
Pus1	0.44236675	1.66141706	1.7761471	0.19767536	0.19334864
Mfsd3	-0.7831209	0.54359466	1.77569832	0.19772966	0.19335861
Hmg20a	0.25876284	5.9347233	1.77557124	0.19774504	0.19335861
Chaf1a	-0.5147995	1.78037866	1.77547961	0.19775613	0.19335861
Zfp710	-0.2611801	3.6340195	1.77421421	0.19790938	0.19347287
E2f2	0.52137878	3.00064518	1.77412598	0.19792007	0.19347287
Zfp85os	0.3863654	2.59106711	1.77248263	0.19811931	0.19364461
Mpzl3	-1.2845197	-0.9425043	1.7717218	0.19821165	0.19371182
Sypl	-0.2380746	6.38862586	1.77084926	0.1983176	0.19378031
Mettl1	1.19647949	-0.7518971	1.77075645	0.19832888	0.19378031
Hmgn1	0.28312091	8.11437481	1.77048158	0.19836227	0.1937899
2310033P09I	0.34479245	3.12529055	1.76985428	0.19843851	0.19384134

Ryk	-0.2896009	4.46530085	1.76959728	0.19846976	0.19384882
Cisd2	-0.1828277	6.17756167	1.76922757	0.19851471	0.1938697
Dedd2	0.56141017	1.48902123	1.76847639	0.1986061	0.1939359
Snrnp35	0.36920532	3.60208092	1.76810208	0.19865166	0.19395079
Srebf1	-0.3008892	2.89281282	1.76796339	0.19866854	0.19395079
Cd14	0.96944037	-0.0168069	1.7671926	0.19876241	0.19401938
Osgep	0.25976999	3.93937832	1.76636678	0.19886304	0.19409437
A730056A06	0.54743541	1.59917993	1.76617465	0.19888646	0.19409437
Adamts5	0.30906522	3.69374067	1.76550228	0.19896845	0.19415132
Serf2	0.31971223	6.41739383	1.76516845	0.19900917	0.19416801
Lat	-1.5078866	-1.0664694	1.76438139	0.19910522	0.19423867
Cdh3	-0.9255931	0.55645402	1.76365876	0.19919346	0.19426707
Avl9	-0.2045406	6.00225198	1.76361145	0.19919924	0.19426707
Fubp3	-0.197277	5.88427179	1.76356233	0.19920524	0.19426707
Dok5	-0.2900272	3.31033104	1.76243262	0.1993433	0.19436257
Cald1	0.25432341	10.2790295	1.76237394	0.19935047	0.19436257
Acot10	-1.3342898	-0.5193226	1.76174687	0.19942716	0.19441428
Pcyt1b	0.23714778	5.32802086	1.76141668	0.19946755	0.1944306
Lysmd1	0.31887043	3.29837465	1.76083512	0.19953872	0.19445906
Slc25a47	-1.0118344	-0.6245567	1.76079144	0.19954407	0.19445906
Taf11	0.21528001	5.26365295	1.76005397	0.19963437	0.194524
Rnf128	-0.504994	1.67447049	1.75967822	0.1996804	0.19452936
Phf10	0.19116289	5.32366642	1.7596226	0.19968721	0.19452936
Ngef	-0.2165566	5.79286165	1.75942574	0.19971134	0.1945298
Cep83os	-0.2008798	5.37662296	1.75793122	0.19989458	0.19468522
Mkrn3	-1.304774	-0.2692378	1.75700233	0.20000858	0.19477317
1700094D03I	-0.601732	1.92607072	1.7566745	0.20004883	0.19478929
Sema3d	0.39191709	6.49635754	1.75591436	0.2001422	0.19485712
Axin1	0.3055547	3.37018017	1.75524365	0.20022463	0.19491429
Fgfrl1	-0.3799317	2.25860153	1.75454673	0.20031033	0.19495842
Plcxd2	-0.2240588	7.79209282	1.75448921	0.20031741	0.19495842
Tshz2	-0.1615165	6.13773992	1.75310767	0.20048743	0.19510079
Rap1gap	-0.2981375	4.12132355	1.75229964	0.20058696	0.19517454
Rae1	0.24209175	3.83274446	1.74974315	0.20090226	0.19545819
Fbn1	-0.2924757	3.99856872	1.748699	0.20103122	0.1955605
Ugdh	0.19591771	4.62494384	1.74785267	0.20113582	0.19561249
Tex12	-1.0195427	-0.1499423	1.74772599	0.20115148	0.19561249
Slfn1	1.3056297	-1.0410111	1.74755962	0.20117205	0.19561249
Ccdc3	0.19780701	5.39949026	1.74685831	0.2012588	0.19561249
Slc22a8	0.32718188	8.06097155	1.74662603	0.20128755	0.19561249
Rab4b	0.2672026	3.46362841	1.74643127	0.20131165	0.19561249
Smtn	0.48837675	2.3287142	1.74634939	0.20132178	0.19561249
Nsfl1c	0.23032433	5.06797912	1.74632075	0.20132533	0.19561249
Sapcd2	1.78397853	-1.1384531	1.74624841	0.20133428	0.19561249
Mapk12	0.44925281	1.78775596	1.74611361	0.20135097	0.19561249

Lactb2	0.31289686	3.85021558	1.7461131	0.20135103	0.19561249
Msh6	-0.2626495	4.74445881	1.74595784	0.20137026	0.19561249
Esyt2	-0.1600009	5.89773321	1.74526014	0.20145666	0.1956733
Etohi1	0.25649607	4.21814731	1.74497361	0.20149216	0.19568466
Tyro3	-0.2263332	4.93736118	1.74477316	0.201517	0.19568566
Maml2	0.22746621	5.76477574	1.74414068	0.2015954	0.19573448
Aimp1	0.28401763	4.50395021	1.74395654	0.20161824	0.19573448
P2ry13	-0.464166	2.42328425	1.74363515	0.20165809	0.19573448
Nxph2	-0.8484198	0.00279407	1.74359933	0.20166254	0.19573448
Eps15l1	-0.2001631	5.75893508	1.7420055	0.20186036	0.19588533
Fam49b	-0.1683396	6.79964942	1.7419631	0.20186562	0.19588533
Pcdhb21	-0.5897632	1.24682171	1.74136449	0.20193998	0.19592962
Adamts15	0.38487897	2.60173174	1.74104474	0.20197972	0.19592962
Ctdp1	0.23084646	3.88503483	1.74088114	0.20200005	0.19592962
Ube2z	0.22153948	6.14013848	1.74082829	0.20200662	0.19592962
Ccm2l	1.30149156	-0.5703278	1.74062209	0.20203226	0.19593136
Gucy2e	0.51155921	1.54458021	1.74003304	0.20210551	0.19597928
Ucp3	1.34852386	-0.2060946	1.73952366	0.20216887	0.19599115
Itgae	1.93096429	-1.8146035	1.73947214	0.20217528	0.19599115
Tmem63c	-0.371915	4.14277226	1.73935961	0.20218929	0.19599115
Nr1d1	0.18675553	8.75075476	1.7388553	0.20225206	0.19602888
Tefm	0.31647236	3.32562014	1.73807865	0.20234878	0.19609949
Cdyl2	0.24049219	5.28059106	1.73777969	0.20238602	0.19611246
Osgin1	0.95489559	0.20312214	1.73745526	0.20242645	0.19612851
Il18bp	-0.4071566	3.28860402	1.73705389	0.20247648	0.19614556
Tacc1	0.13910215	7.88929376	1.73693119	0.20249178	0.19614556
Gm7904	2.60815723	-1.4868777	1.73666381	0.20252512	0.19615474
Prune2	-0.1705782	6.64228343	1.73494313	0.20273983	0.19633957
Ecscr	1.22722728	-0.6788505	1.73411313	0.20284351	0.1964026
Efna3	0.48027837	1.5606198	1.73403944	0.20285271	0.1964026
5830416I19R	1.99855381	-0.9376962	1.73371414	0.20289337	0.19641883
Nr1h4	-1.4925706	-0.4247243	1.73314981	0.20296392	0.19646398
4930563E18I	-2.1805712	-1.5944678	1.73189819	0.2031205	0.19657852
Ccr4	-0.911424	0.09339256	1.73182167	0.20313008	0.19657852
Ankef1	-0.9344918	0.39636914	1.73142183	0.20318013	0.19659192
Gm12522	-0.5994889	1.18137555	1.7313289	0.20319177	0.19659192
Grhpr	0.46869084	2.24002637	1.73090597	0.20324473	0.19662003
Eif2ak4	-0.3891839	3.93746764	1.73053632	0.20329104	0.19664168
Gm2694	-0.8070856	0.04466497	1.73019743	0.20333351	0.19665961
Parm1	0.2357718	4.32365914	1.7299989	0.20335839	0.19666054
Ccdc34	-0.2193539	6.08041037	1.72959132	0.20340949	0.19666616
Psat1	0.18167036	6.48892202	1.72957079	0.20341206	0.19666616
Cml1	0.60959193	1.30335737	1.72935972	0.20343853	0.19666862
Wdr24	-0.4292703	2.33608061	1.72911915	0.2034687	0.19667465
Slc44a3	-1.2896407	-1.1805436	1.72796159	0.20361396	0.19676804

Rps9	0.31723608	6.45473868	1.72780991	0.20363301	0.19676804
Dos	-0.2207954	6.40590854	1.72777703	0.20363713	0.19676804
Usp22	-0.1618505	7.05458543	1.72628771	0.20382424	0.19690606
Bcor	0.18210468	5.37892961	1.72625867	0.20382789	0.19690606
Pola1	-0.3751375	2.66497059	1.72553642	0.20391871	0.19695121
E2f8	0.88170052	0.8932315	1.7254261	0.20393259	0.19695121
Ing4	0.23967477	3.88740656	1.72531542	0.20394651	0.19695121
Ctla2b	1.35453818	-0.6607534	1.72442602	0.20405844	0.19703614
Zscan29	-0.227056	5.40459204	1.72370512	0.20414922	0.19710064
Rpp38	-0.3605935	2.56177142	1.72270349	0.20427543	0.19718937
Tmem8	-0.3426569	2.32998663	1.72254554	0.20429534	0.19718937
9930111J21F	-0.419884	2.9542156	1.72240475	0.20431309	0.19718937
Sec23ip	-0.1939269	5.45952368	1.7221897	0.20434021	0.19719238
Ufsp1	0.48359463	1.89725205	1.72178477	0.20439128	0.19721474
Ckap5	-0.2578991	7.87263961	1.72157394	0.20441787	0.19721474
Flrt1	0.17254104	6.61301072	1.72143535	0.20443536	0.19721474
2410018L13F	-0.3734891	1.73556512	1.72094963	0.20449666	0.19725072
Zfp386	0.18692994	5.38364077	1.72013585	0.20459941	0.19732668
Gm5803	-0.9935365	-0.6594237	1.71931706	0.20470286	0.19740329
Pianp	-0.1896528	6.45296759	1.71895255	0.20474894	0.19742456
Gm15217	1.27937274	-1.6360095	1.71769576	0.2049079	0.1975343
Fam86	0.37414622	3.38812708	1.71767272	0.20491082	0.1975343
Gstt2	0.61833104	2.13162719	1.71692235	0.2050058	0.19760269
Ccdc17	0.5504913	1.07721992	1.71614776	0.20510391	0.19767146
Thbs4	-0.7387604	0.57915578	1.71588666	0.20513699	0.19767146
Zfp36	0.39406225	4.95134142	1.71578952	0.2051493	0.19767146
Gli1	0.58199352	1.65310861	1.71553628	0.2051814	0.19767921
Gpr20	1.39324855	-1.386732	1.71532816	0.20520778	0.19768146
Gm12191	0.45623154	1.2385392	1.71507804	0.2052395	0.19768546
2410004B18I	0.19439647	5.13860267	1.71491605	0.20526004	0.19768546
Trmt13	-0.4804753	1.35786916	1.71353307	0.20543552	0.19783128
Rnf6	0.13916692	6.81073045	1.71283492	0.20552418	0.19786573
Ddx49	0.63607226	0.76089997	1.71261019	0.20555272	0.19786573
Cenpe	-0.3887755	2.89436466	1.71258745	0.20555561	0.19786573
C730002L08F	0.43733047	2.30631274	1.71249315	0.20556759	0.19786573
Atg9b	-0.7893576	0.83379581	1.71210733	0.20561662	0.19788975
2010005H15I	1.84906811	-1.5641879	1.71186539	0.20564737	0.19789165
Bdp1	-0.2033735	7.43458905	1.71171297	0.20566675	0.19789165
Dnajb12	0.21937457	3.90776884	1.71014665	0.205866	0.19806018
Nrd1	-0.1603937	7.74028781	1.70975957	0.20591528	0.1980844
Pbx1	0.15197814	9.2799897	1.7090899	0.20600057	0.19812346
4933413J09F	1.99345563	-1.5657156	1.70906224	0.20600409	0.19812346
Mak16	0.20852916	4.98461037	1.70870666	0.2060494	0.19814385
Wdr48	-0.2002015	5.5655483	1.70836892	0.20609244	0.19816206
E130012A19I	-0.3460206	2.9411	1.707945	0.20614648	0.19819083

Bcorl1	-0.2005684	4.54898799	1.70698443	0.20626901	0.19828543
Zfp433	-0.2421882	3.82422455	1.70531857	0.20648171	0.19845671
Dhx16	-0.3646038	2.95949162	1.70521086	0.20649547	0.19845671
Fam135a	-0.2119679	5.76038231	1.70426442	0.20661645	0.19854976
Ccdc121	0.78978594	0.68327863	1.70391814	0.20666073	0.1985691
Thoc6	0.57815117	1.21006605	1.70330782	0.20673881	0.19861904
Nxpe4	-0.3010082	2.96070174	1.70301747	0.20677597	0.19861904
Trim39	-0.2207453	4.31409837	1.7028592	0.20679623	0.19861904
Fam65a	-0.2585022	7.33207848	1.70275661	0.20680936	0.19861904
Cacul1	0.16430386	6.85414444	1.70179075	0.20693306	0.19871461
Zfp248	-0.2638176	4.558211	1.7006603	0.20707795	0.19883052
Prss54	1.70622919	-1.2922153	1.69928475	0.20725443	0.19893129
Lilrb4	-0.8054764	0.43424165	1.69920835	0.20726424	0.19893129
Calr4	1.43436607	-1.2051204	1.69917924	0.20726798	0.19893129
Gxylt1	-0.2145753	5.66834184	1.69908778	0.20727972	0.19893129
Wdfy2	-0.5130901	1.70188876	1.69877921	0.20731934	0.19894609
Rsl1d1	0.16929704	6.66736682	1.69846263	0.20736	0.19896187
Krt25	-1.9188998	-1.5438074	1.6979591	0.20742469	0.19897724
Vps53	0.16535873	6.21974496	1.69792541	0.20742902	0.19897724
Wdr61	0.23816538	4.33102401	1.69777267	0.20744865	0.19897724
Dnajc27	0.20295763	6.17751237	1.69750607	0.20748291	0.19898689
Dhx36	-0.2169915	6.26856195	1.69731652	0.20750728	0.19898704
Fancf	0.43124224	3.01588235	1.69662343	0.20759641	0.19904929
Odf2l	-0.336233	3.49166624	1.69597265	0.20768014	0.19910635
Nsg2	0.16713349	7.8674914	1.69556996	0.20773198	0.19913281
Ptpn9	0.18901591	5.88928776	1.69479355	0.20783196	0.19917072
Zfp13	-0.5348489	1.79674284	1.69467485	0.20784725	0.19917072
R3hcc1l	0.20865185	4.57641189	1.69455079	0.20786323	0.19917072
Akr1c14	-0.2896777	3.92824185	1.69451031	0.20786845	0.19917072
BC025920	0.52182135	1.46221459	1.69288615	0.20807784	0.19934811
Tbc1d8	-0.3224556	4.20495322	1.69247241	0.20813123	0.19936409
Arhgap30	-0.4384457	2.85648946	1.69238095	0.20814303	0.19936409
Lrrc8d	-0.204106	5.77363906	1.6919659	0.20819661	0.19939217
Zfand2b	-0.604725	1.87465094	1.69122785	0.20829192	0.19945124
Ms4a4b	1.28574331	-0.5138973	1.69108688	0.20831013	0.19945124
Wsb2	-0.1382795	7.7269333	1.69092459	0.20833109	0.19945124
Gm10778	-0.2855834	3.91403951	1.69058116	0.20837547	0.1994705
Foxd1	0.3668498	5.52951661	1.69013856	0.20843268	0.19950203
Arhgap11a	0.34389937	3.16691669	1.68936406	0.20853285	0.19957465
LOC1005047(	1.06068869	-0.317366	1.68900026	0.20857991	0.19959646
Gnaq	0.13495592	8.68177688	1.6882194	0.20868099	0.19965706
Enpp3	0.68369073	0.61980347	1.6881357	0.20869183	0.19965706
Nup188	-0.3288355	3.73342481	1.68746746	0.20877838	0.19970959
Lrch3	-0.2342381	4.78819514	1.68716737	0.20881726	0.19970959
Tram1l1	-0.2403148	4.25816909	1.68714906	0.20881963	0.19970959

Gm8615	0.30979975	2.82423481	1.6863912	0.20891787	0.19972702
Pdcd1lg2	-0.8059961	0.07482019	1.68634414	0.20892397	0.19972702
9030624J02F	0.16055512	5.22420034	1.68633958	0.20892456	0.19972702
Gtpbp2	-0.2656472	4.27138484	1.68625868	0.20893505	0.19972702
Nhlh1	1.10172768	-0.2689233	1.68461349	0.20914853	0.19990784
Cox8b	-1.8708312	-1.4825481	1.68306682	0.20934947	0.20007664
C1galt1	-0.2520931	4.66589561	1.68255035	0.20941663	0.20011755
Crcp	0.25440576	4.59667811	1.68224435	0.20945643	0.20013231
Alpk1	-0.4103787	2.5294278	1.68159466	0.20954096	0.20018981
Fut2	-0.8049459	0.57040956	1.6813122	0.20957773	0.20020166
Asgr2	-2.1632185	-2.1466861	1.68105567	0.20961113	0.2002103
Wnt3	-1.1339466	-0.8614481	1.68073086	0.20965343	0.20022743
Lgals1	0.46358982	4.62330975	1.67956976	0.20980471	0.20034478
D10Jhu81e	0.26180253	3.58095012	1.67941373	0.20982505	0.20034478
Slc38a5	-1.36686	-1.1945764	1.67877792	0.20990797	0.20040067
Surf4	0.25180707	4.57231606	1.67829799	0.20997058	0.20043716
Aqp9	1.70923977	-1.2215807	1.67742376	0.21008469	0.20048528
Zhx3	0.18227655	6.21195891	1.67741484	0.21008586	0.20048528
Tmem18	-0.2557885	4.31066498	1.67723946	0.21010876	0.20048528
Alg1	-1.0682065	0.0254511	1.67716441	0.21011856	0.20048528
C230037L18F	-0.6509536	1.60175458	1.67675311	0.21017228	0.20051327
Sall2	-0.1939903	5.73749934	1.67462405	0.21045066	0.20075016
Apol7b	1.02595789	-0.4814013	1.67448058	0.21046944	0.20075016
Rab19	0.64101946	1.98296092	1.67413726	0.21051438	0.2007642
Tpx2	-0.4934864	1.79765562	1.67399488	0.21053302	0.2007642
2310035C23I	-0.2285666	6.63927903	1.67271044	0.21070127	0.20090076
Nanog	-0.8649032	-0.5025616	1.6725286	0.2107251	0.20090076
Fdxacb1	-0.6186191	1.52243624	1.67215909	0.21077354	0.20092363
Usp21	0.18369274	5.1328123	1.67115273	0.21090554	0.20102615
Slc25a36	-0.1828946	4.98566063	1.67043805	0.21099934	0.20108715
Slc25a39	0.31969871	4.37736588	1.67029236	0.21101847	0.20108715
Spdef	1.90622222	-1.9795163	1.6692329	0.21115764	0.20119645
Eaf2	0.44629047	1.50594615	1.66845608	0.21125976	0.20127041
Elmo2	0.16909294	5.92396524	1.66775434	0.21135206	0.20132593
Gria1	-0.2517392	6.49915439	1.66733286	0.21140752	0.20132593
Actr10	-0.1501278	7.66079374	1.66733162	0.21140769	0.20132593
Prkrir	-0.1653811	5.34315048	1.66726837	0.21141601	0.20132593
Zfp655	0.18165228	5.7401676	1.66644802	0.21152402	0.201373
Rgp1	0.20479244	4.49069946	1.66642315	0.21152729	0.201373
Gnb2l1	0.20420535	5.840013	1.6663347	0.21153894	0.201373
Supt7l	0.20595424	5.59171236	1.66591619	0.21159408	0.20137885
Gt(ROSA)26S	-0.363348	2.76247461	1.66591606	0.21159409	0.20137885
Pde3a	-0.6635969	1.06521131	1.66571882	0.21162008	0.20138027
BC006965	0.56093118	1.79044994	1.66425971	0.21181247	0.20154001
Dbr1	-0.2552142	3.96110004	1.66384425	0.21186729	0.20156884

Apcdd1	0.40672922	3.98764952	1.66357052	0.21190342	0.20157988
Epb4.1l5	0.21788619	4.6767128	1.66290329	0.21199152	0.20164034
Ddx11	-0.9384214	0.10747502	1.66247341	0.2120483	0.20165261
Prmt5	0.19497391	4.74281688	1.66243421	0.21205348	0.20165261
Slc5a5	0.25387478	4.92902346	1.6621254	0.21209428	0.20165874
Fam216a	0.17434499	5.4381747	1.66201403	0.212109	0.20165874
LOC1005034	-1.0810383	-1.3524867	1.66144091	0.21218476	0.20166594
Cipc	0.15132676	6.68247745	1.6613358	0.21219866	0.20166594
Sec14l3	-1.3056455	-0.5228344	1.66130153	0.21220319	0.20166594
Rab8a	0.37473383	4.84206691	1.66103955	0.21223784	0.20166594
Dnlz	0.27761873	3.76247593	1.66090074	0.2122562	0.20166594
Mxi1	0.16915817	6.32122359	1.66080797	0.21226847	0.20166594
Cnih2	-0.2462695	3.54137883	1.66065787	0.21228833	0.20166594
Cd74	-0.4712338	6.73494248	1.65998472	0.21237741	0.20172725
Pim1	0.68353091	0.33204306	1.65936887	0.21245895	0.20175764
Sirt1	-0.2665697	4.47245671	1.6591933	0.21248221	0.20175764
Ccdc138	-0.394782	2.60096319	1.65918693	0.21248305	0.20175764
Ninj1	0.65781494	2.11751895	1.6585301	0.21257007	0.20181696
Hsd3b2	-1.4237649	-1.284295	1.65758819	0.21269495	0.20191219
Tbcd	-0.2596355	3.95613517	1.65695063	0.21277952	0.20196915
Zfp623	0.30006284	3.4205748	1.65641545	0.21285055	0.20200859
Ocel1	0.49870299	1.7625037	1.65626721	0.21287023	0.20200859
Asxl2	-0.1818938	6.49427482	1.65601456	0.21290377	0.2020171
Elfn1	-0.3125596	4.83375736	1.65488122	0.21305433	0.20213662
Fip1l1	0.14276558	6.50639175	1.65463401	0.21308719	0.20214128
Prkcb	-0.2069282	11.0318329	1.65437646	0.21312143	0.20214128
Ptp4a3	0.32359544	3.47659249	1.65411043	0.2131568	0.20214128
Tmem179b	0.40684574	3.23647778	1.65410431	0.21315762	0.20214128
Wdr1	-0.1664608	6.28277704	1.65350858	0.21323686	0.2021576
Chmp7	0.20720525	5.62002362	1.65349078	0.21323923	0.2021576
Cd2	1.30096954	-0.4858184	1.65342024	0.21324861	0.2021576
Srrm1	-0.1270232	7.56337242	1.65251462	0.21336916	0.20224854
Trim6	1.29275306	-0.5497775	1.65232561	0.21339432	0.20224908
Prps2	0.18364352	6.17915191	1.65146708	0.2135087	0.20231648
Mael	-0.9459658	0.65600567	1.65142224	0.21351467	0.20231648
4933406I18R	-0.9075823	-0.502122	1.65051112	0.21363614	0.20240824
D3Ertd751e	0.20750268	5.72744736	1.65002221	0.21370135	0.20244669
Alkbh5	0.1864863	6.397967	1.64918345	0.21381329	0.20252939
Tmem17	-0.4610956	1.7627744	1.64866415	0.21388263	0.20255486
Atrx	-0.2079987	9.86601145	1.64861298	0.21388947	0.20255486
Rp2h	0.24895265	4.92032944	1.64829541	0.21393189	0.20257169
Arl2	0.31489097	3.71063299	1.64749985	0.21403821	0.20264901
Gpr35	-0.8952692	0.11747477	1.64717169	0.21408208	0.20266721
Nck1	-0.2437769	4.98985231	1.64668899	0.21414664	0.20270497
Amdhd2	0.45747046	1.22239547	1.64649989	0.21417193	0.20270557

Slc9a3r1	0.37855068	4.2153837	1.64594783	0.21424581	0.20275214
Samd3	-0.6285291	1.32817497	1.6456809	0.21428154	0.20275495
Angptl1	-0.9861931	0.5520539	1.64555715	0.2142981	0.20275495
Nab1	0.14638872	6.61814642	1.64407352	0.21449686	0.20290194
Krt19	0.48942423	1.77903333	1.64402886	0.21450284	0.20290194
Spi1	0.75556754	0.49073913	1.64285832	0.21465982	0.20302707
Tbkbp1	-0.404153	2.13818558	1.64211874	0.21475908	0.20307558
9430015G10	-0.4456072	2.45774709	1.64195758	0.21478072	0.20307558
Ctnna2	-0.1805237	6.28819644	1.64183197	0.21479758	0.20307558
Xab2	0.24008541	3.85964232	1.64173992	0.21480994	0.20307558
Ap4s1	0.17141862	5.86757563	1.64105837	0.21490149	0.20313876
R3hdm4	0.18967587	7.68370037	1.63980898	0.21506944	0.20327414
Cnep1r1	-0.1774592	5.90933978	1.63960993	0.21509622	0.20327607
Ttc22	-1.8026883	-1.6064773	1.63938953	0.21512587	0.20328072
Gas8	0.34839526	3.60001775	1.63880926	0.21520395	0.20333113
Cdkn2d	0.25139443	4.11351863	1.63833942	0.2152672	0.20334647
Zfp748	-0.2243136	4.81917853	1.63819763	0.2152863	0.20334647
Foxk2	0.12786756	6.46145582	1.63813734	0.21529442	0.20334647
Fignl1	1.06222946	-0.7257423	1.63774561	0.21534718	0.20337294
Akap7	0.18653597	6.50819349	1.63724059	0.21541523	0.20341383
D10Wsu102e	0.18329957	5.43301154	1.6368133	0.21547282	0.20344484
Nipal4	-0.5460299	0.8634244	1.63654033	0.21550962	0.20345621
Mettl13	-0.3470423	2.10148209	1.63579689	0.2156099	0.2035275
Rnf145	-0.1731965	5.73026321	1.63539087	0.21566469	0.20355584
Kdm5d	-0.3083091	4.88224008	1.63377676	0.21588268	0.20373819
Pacs1	0.16244929	6.74546738	1.63283171	0.21601043	0.20383536
Hist1h2bp	0.92000921	-0.7282023	1.63215676	0.21610174	0.20389811
Stk11	0.3105121	5.51305633	1.63154168	0.21618498	0.20393416
Wdr90	-0.8208722	1.35133883	1.63141563	0.21620205	0.20393416
Ppfia3	-0.2614072	4.67632354	1.63132454	0.21621438	0.20393416
Bcl2l12	-0.7654213	0.23537933	1.62908807	0.21651747	0.20418829
Ciz1	0.18814811	5.70076852	1.62896983	0.21653351	0.20418829
Phospho2	0.21596898	5.00546569	1.62856366	0.21658861	0.20420457
Kcnk2	0.16158111	7.24702257	1.62835814	0.2166165	0.20420457
Atad1	-0.1548867	7.21366824	1.62829329	0.2166253	0.20420457
Tma7	0.22845545	6.81927028	1.62718806	0.21677538	0.20431029
Sgol2	0.32253687	3.58539855	1.62669757	0.21684202	0.20431029
Bcam	0.42744469	3.57616704	1.62659411	0.21685608	0.20431029
Efnb1	0.4439608	3.42047178	1.62656023	0.21686068	0.20431029
Abca9	0.24278057	6.25692304	1.62655243	0.21686174	0.20431029
Cd48	0.88512239	0.34257758	1.62612487	0.21691987	0.20434162
Smad6	0.44604708	2.58406296	1.6255769	0.21699438	0.20438839
Sstr2	0.3558645	2.75647597	1.62490096	0.21708635	0.20445159
Rnf122	0.72611983	0.02224621	1.62393779	0.21721747	0.20454654
Lmo4	0.21722851	9.51337193	1.62379489	0.21723694	0.20454654

Gm5148	0.28112419	3.95471613	1.62302709	0.21734155	0.2046216
Exoc3	-0.1765891	6.57584827	1.62192851	0.21749134	0.20473917
Gm11944	-0.7353689	0.61037529	1.62145178	0.21755639	0.20477695
Itgb1bp1	0.19534894	4.68775085	1.62110103	0.21760426	0.20479855
Nmnat1	0.65773611	0.27411572	1.62011124	0.21773942	0.2049023
Siah1a	-0.1816358	5.65534911	1.61958894	0.21781079	0.20493007
Fgf12	0.17377025	7.94471617	1.61953033	0.2178188	0.20493007
Hyal3	0.71139956	0.5348794	1.61911476	0.2178756	0.20496005
Ppan	0.49053131	1.68015532	1.61826109	0.21799235	0.20504586
Papln	-1.439318	-0.9964692	1.61797902	0.21803095	0.20504586
C920021L13F	-0.4688767	1.96554996	1.61790069	0.21804167	0.20504586
Slc7a11	0.26308546	9.83007909	1.61723518	0.21813276	0.20510806
Fam222b	0.15077645	6.75289279	1.61651241	0.21823175	0.20517767
Ror1	0.72286992	0.88849533	1.61612177	0.21828528	0.20518146
Tktl2	-1.9303883	-1.1117292	1.61606798	0.21829265	0.20518146
Sh2d1b1	-1.0789844	0.8331285	1.61578154	0.21833191	0.20518146
Pparg	-0.5049239	1.72385308	1.61575436	0.21833564	0.20518146
Trip12	-0.1602835	8.84253983	1.61481462	0.21846451	0.20527909
Adrb1	-0.3551172	3.6706366	1.61453731	0.21850256	0.20529137
Retnlg	2.20906343	-1.3288722	1.6141244	0.21855922	0.20532114
Rbm20	-0.3914032	2.41221173	1.61315453	0.2186924	0.20542277
Phactr2	0.19111487	8.26110327	1.61212327	0.21883412	0.20550373
AW011738	0.44605438	2.60334174	1.61188449	0.21886695	0.20550373
Cltc	-0.2162268	8.89667839	1.6116158	0.2189039	0.20550373
Gm12942	-0.5054001	2.89548821	1.6115896	0.2189075	0.20550373
Ttc29	-2.2933917	-2.3012411	1.61157291	0.2189098	0.20550373
Rpp25	0.26137958	3.94710413	1.61143614	0.21892861	0.20550373
Limk2	-0.2024885	5.20943889	1.61114761	0.2189683	0.20551752
Zdhhc16	0.34687903	2.26251779	1.61084763	0.21900958	0.20553279
Tmem29	0.15860672	4.81473657	1.60892483	0.21927439	0.20575709
Ccdc124	0.33471825	4.3329808	1.60874886	0.21929865	0.20575709
Foxo6	0.34224682	2.60702137	1.60850782	0.21933188	0.20576279
Gm8801	0.69463472	-0.2353467	1.60834162	0.2193548	0.20576279
Mb21d1	-0.6326891	0.81745462	1.60798681	0.21940373	0.20578494
Pde4a	-0.2382876	6.32580238	1.60780731	0.21942849	0.20578494
Exd1	0.93683758	-0.3507734	1.60725966	0.21950406	0.20583233
Klrc1	0.96874403	-0.657688	1.60642952	0.21961866	0.2059163
Tfap2d	1.16993269	-0.1952972	1.60615091	0.21965715	0.20592889
Tlr4	0.34225981	4.43886686	1.60457436	0.21987506	0.20610968
Arfip2	0.18382078	5.19645534	1.60200123	0.22023131	0.20642008
Leprot	0.3330047	5.01098668	1.60127245	0.22033234	0.20649123
Tbc1d23	-0.2335222	4.34883226	1.6008013	0.22039769	0.20652892
Usp43	-0.9524909	1.0019832	1.6006056	0.22042484	0.20653082
Fignl2	-0.5858876	1.1062992	1.60031689	0.2204649	0.20654481
Dexi	0.2276122	3.84167745	1.59995898	0.22051458	0.2065678

Desi2	0.16936764	6.81310931	1.59929778	0.22060639	0.20663026
Gxylt2	-0.3223907	3.90150015	1.59841548	0.22072898	0.20672152
A530054K11I	-0.1888372	5.33622178	1.59799183	0.22078787	0.20674891
Tor2a	0.3976997	1.89822862	1.59784323	0.22080853	0.20674891
Ctns	-0.3360834	2.65639179	1.59660563	0.22098071	0.20688656
Sh2d1a	1.89072368	-1.5924025	1.59607663	0.22105436	0.20691267
Kcna4	-0.2898575	5.66132831	1.59604361	0.22105896	0.20691267
Nop58	-0.1884141	6.14567815	1.59572824	0.22110288	0.20691483
Fam195b	0.36778241	2.5685986	1.59551032	0.22113324	0.20691483
AU023762	-0.2857569	3.25929013	1.59548483	0.22113679	0.20691483
Maml1	0.19350026	5.19911972	1.59432808	0.22129802	0.20704212
Efcab1	0.29543299	3.56238764	1.59387645	0.221361	0.20707748
Arid2	-0.1522345	6.70487632	1.59266774	0.22152969	0.2072117
Hrh3	0.22567646	4.7445627	1.59169291	0.22166586	0.20731547
Srrm2	-0.2264469	10.1848417	1.59084491	0.2217844	0.20740192
Gid8	0.14811445	6.55454777	1.59051141	0.22183104	0.20740192
Fam98b	0.19912043	6.91903186	1.59049027	0.221834	0.20740192
Gsdma	-1.9838386	-2.0985064	1.59027021	0.22186478	0.20740711
Scarb2	0.33418723	4.27595107	1.59003926	0.2218971	0.20741373
Erp29	0.29607744	4.632178	1.58981993	0.22192779	0.20741883
Fgf11	0.18105702	6.14600193	1.58960923	0.22195728	0.2074228
Gm5141	-0.3684375	3.06213134	1.58812668	0.22216492	0.20759324
Brdt	-0.2576534	4.23195234	1.58742483	0.2222633	0.20766156
Dcp1b	-0.2463323	4.21256879	1.58693104	0.22233255	0.2076807
Pus3	0.30889346	3.36515777	1.58691844	0.22233432	0.2076807
Cntnap2	-0.277968	6.26469547	1.58660745	0.22237795	0.20769785
Pdcl3	0.28582053	4.225778	1.58611049	0.22244769	0.20773938
Agfg1	0.15250494	5.82001065	1.58571972	0.22250255	0.20776701
A730020E08I	-0.4046494	2.69495924	1.58503476	0.22259875	0.20783322
Kif3a	-0.2126313	8.10931872	1.58415404	0.22272253	0.20792517
Fam46c	0.33400165	2.4252796	1.58359978	0.22280046	0.20797431
Kif5c	-0.2232074	9.59216499	1.58279402	0.22291383	0.2080565
Cystm1	0.44214137	2.13882184	1.58240343	0.22296881	0.20806125
4430402I18R	0.31762269	2.61787943	1.58239816	0.22296955	0.20806125
Pde11a	-0.869654	-0.3706956	1.58191345	0.2230378	0.20808901
Zfp473	-1.1920898	-0.3005322	1.58182734	0.22304993	0.20808901
AA388235	0.28775042	3.22421595	1.58150865	0.22309482	0.20809559
Mrpl53	0.28272678	3.73890533	1.58141777	0.22310763	0.20809559
Zfp326	-0.1703453	5.58681216	1.58057661	0.22322618	0.20818254
Slc35f6	0.42239927	1.93306923	1.58016882	0.22328369	0.20819746
Samsn1	-0.7346368	0.62142411	1.57986422	0.22332665	0.20819746
Rhbdl3	-0.3587164	2.78035487	1.57977786	0.22333884	0.20819746
Utp6	0.1747827	6.67201553	1.5793795	0.22339504	0.20819746
Plekhd1	1.26579534	-1.030758	1.57936548	0.22339702	0.20819746
Prelid1	0.2467277	5.79419782	1.57915405	0.22342686	0.20819746

Milr1	-1.0975145	-0.3117248	1.57899343	0.22344954	0.20819746
Ptplb	-0.206013	5.00768767	1.57893183	0.22345823	0.20819746
3110039M20	-0.5442524	2.13060271	1.5788474	0.22347015	0.20819746
Eif2d	0.28337147	3.93549472	1.57809404	0.22357655	0.20827297
5530401A14	-0.7532623	0.01836977	1.57507327	0.22400382	0.20864735
Pard6a	0.506399	2.15301672	1.57332058	0.2242522	0.20885503
Ldlrad4	-0.2050686	4.36462641	1.57134665	0.22453235	0.20909226
Slc31a2	0.35371477	5.11246442	1.57113383	0.22456258	0.20909672
Kirrel2	-1.2868726	-0.5200892	1.57001681	0.22472134	0.20922084
1700030K09	-0.557789	1.60509031	1.56959705	0.22478104	0.20925272
Frmpd4	0.19830736	7.77636696	1.56884166	0.22488852	0.20930931
Cacna1g	-0.2748854	4.47879731	1.56881187	0.22489276	0.20930931
Msantd3	0.37697927	2.9884941	1.56758356	0.22506767	0.20944838
Supv3l1	0.31609465	3.17870659	1.56622918	0.22526074	0.2095963
04-Sep	-0.177296	5.93010736	1.56609339	0.2252801	0.2095963
Rai2	0.30187337	3.16974792	1.565932	0.22530313	0.2095963
Tmed10	-0.1637375	6.82479277	1.56497761	0.22543933	0.20969248
Upf3b	-0.2180889	5.6288383	1.56485011	0.22545754	0.20969248
1700012B09	1.08082421	-0.4327784	1.56417208	0.22555438	0.20975881
Mgst2	-1.3862907	-1.4875532	1.56388108	0.22559595	0.20977179
Cdr2	0.36708195	2.74416149	1.56371713	0.22561938	0.20977179
Fancg	-0.3442655	2.75922905	1.5626996	0.22576486	0.20988331
Tcf25	0.1283115	8.4293522	1.5624013	0.22580754	0.20989924
Kcnk3	-0.3602477	1.8758333	1.56114957	0.22598671	0.21004204
Cdkl5	-0.2151135	8.25420541	1.55995746	0.22615751	0.21017702
Tom1	0.27369145	4.10098346	1.5596999	0.22619444	0.2101848
Ftl1	0.24720344	6.19724188	1.55952766	0.22621914	0.2101848
Porcn	-0.2417609	4.36489916	1.55916227	0.22627154	0.2101848
Auts2	-0.1545758	6.88281406	1.559064	0.22628564	0.2101848
Coro2b	-0.2092191	6.52167001	1.55900748	0.22629375	0.2101848
Shc3	-0.3392779	3.93974062	1.55871257	0.22633606	0.21020035
3110062M04	-0.5944388	0.70944072	1.55748697	0.22651201	0.21033999
Hfe2	-1.6725102	-1.659473	1.55700276	0.22658158	0.21037554
Sh3glb1	0.14976391	8.20721644	1.55673137	0.22662058	0.21037554
Oxtr	-0.3986836	3.55185533	1.55650826	0.22665265	0.21037554
Slc6a20b	-0.383905	1.71811984	1.55650799	0.22665269	0.21037554
Atp11c	-0.2422172	4.6841988	1.55396797	0.22701819	0.21069101
Nifk	0.26280281	4.61760274	1.55337635	0.22710343	0.21074632
Ptpn4	-0.2092197	7.20235562	1.55211614	0.22728514	0.21086107
Rgs17	0.20379701	8.03310869	1.55201834	0.22729924	0.21086107
Kcnd3	-0.2766928	5.96922766	1.55198502	0.22730405	0.21086107
Jade3	0.37408112	4.31488406	1.54994786	0.22759821	0.21110833
Haghl	-0.5441581	0.89350603	1.54977656	0.22762297	0.21110833
Arl9	-2.1504464	-1.9153914	1.54960579	0.22764765	0.21110833
LOC1008622	0.39223061	2.56939459	1.54936408	0.22768259	0.21111692

Amz1	-0.4278772	1.78497787	1.54835547	0.22782848	0.21121695
Phf21b	-0.4418321	2.20298585	1.54826296	0.22784187	0.21121695
Cops2	0.17179062	8.04261981	1.54747786	0.22795552	0.21129847
Kctd1	-0.1519069	6.47743231	1.54561115	0.22822603	0.21150841
Mir1954	2.91377884	-1.8444918	1.56311196	0.22823586	0.21150841
Fam210a	-0.1625979	6.44242624	1.54538236	0.22825921	0.21150841
Mrpl46	-0.3518851	3.48417152	1.54500592	0.22831383	0.21153517
Fech	0.17506098	7.02489319	1.54471868	0.22835551	0.21154976
Slc26a5	-1.4958349	-1.149165	1.54454271	0.22838105	0.21154976
4833417C18I	-1.4136136	-1.3667908	1.54377397	0.22849267	0.2116293
Shd	-0.3963233	2.34245362	1.54355176	0.22852494	0.21163534
Gimap9	1.03212802	0.14078881	1.54337093	0.22855121	0.21163583
Lsp1	0.35333264	3.52750994	1.54314736	0.2285837	0.21164206
Agpat3	-0.2158206	4.96856857	1.54148125	0.22882597	0.21184251
6530402F18I	-0.5527403	1.42045997	1.54098488	0.22889821	0.21188553
Dapk2	0.61146798	0.52057744	1.53973682	0.22907998	0.21201273
Dpyd	0.30441727	3.2659172	1.53968717	0.22908722	0.21201273
Vgll3	-0.3775834	2.95816261	1.53884237	0.22921037	0.21209745
Eif4ebp3	-0.5521043	0.94907495	1.5387052	0.22923037	0.21209745
Fam203a	0.41173432	2.59822023	1.53679357	0.22950938	0.2123317
Lats2	0.20243188	6.82253489	1.53570481	0.22966848	0.21245498
Klhl30	0.57716774	0.76413979	1.5351316	0.2297523	0.21249291
Hspb2	0.5685362	1.09885574	1.53507076	0.22976119	0.21249291
B230216G23	-0.9947417	-0.2643548	1.5344545	0.22985136	0.21255238
C5ar1	1.15822608	-0.0568141	1.53417255	0.22989262	0.21256269
Kifc3	-0.2930149	3.01247194	1.53391506	0.22993032	0.21256269
C330007P06I	0.20519516	7.68773185	1.53384832	0.22994009	0.21256269
Arhgef7	0.17325667	6.71719714	1.53315065	0.23004227	0.21263323
G530011O06	-0.3668856	4.65973078	1.5322731	0.23017088	0.21272818
Lurap1	-0.4256974	1.9634037	1.53084806	0.23037991	0.21286907
Myo18b	0.87988499	-0.6149514	1.53070495	0.23040092	0.21286907
Inpp5k	-0.1917228	4.16728098	1.53070427	0.23040102	0.21286907
Vps18	-0.2853429	3.64210523	1.53048247	0.23043358	0.21287523
Tsen15	0.24422312	3.62941797	1.52986988	0.23052354	0.21288819
Rps19	0.28877777	4.67855439	1.52980682	0.2305328	0.21288819
Tor4a	-0.289752	3.20994434	1.52971437	0.23054639	0.21288819
Cbln4	0.22384743	4.04057016	1.52968148	0.23055122	0.21288819
1700019A02I	-1.2285759	-1.7189132	1.52905789	0.23064285	0.21294888
Syt15	-0.642233	1.38226934	1.52855212	0.23071721	0.21296485
Trmu	-0.4464962	2.08059847	1.52850122	0.2307247	0.21296485
Banp	-0.2369145	4.65768493	1.52841154	0.23073788	0.21296485
Cpsf2	-0.1568321	6.35806903	1.52808413	0.23078604	0.21298538
Slc16a8	1.47770101	-1.1791877	1.52745966	0.23087793	0.21304626
Ptpn12	-0.1940432	6.02226526	1.52669332	0.23099075	0.21310496
Ubash3b	-0.1889187	5.78563294	1.52644335	0.23102757	0.21310496

Ncor1	-0.1947792	9.19656886	1.52641038	0.23103243	0.21310496
Gm13446	0.37129953	2.27103668	1.52615092	0.23107065	0.21310496
Atp6v0c	1.25380433	-1.4626993	1.52593555	0.23110239	0.21310496
Ppap2a	0.28322684	3.7937364	1.52586422	0.2311129	0.21310496
Fam19a4	1.53947836	-1.4218659	1.52579539	0.23112304	0.21310496
Rab3a	-0.1397212	6.72958893	1.52547886	0.2311697	0.21312407
Itga5	-0.3023391	2.85314034	1.52465749	0.23129082	0.21321183
Mapk1ip1l	0.19267462	8.02239861	1.52379375	0.23141828	0.21330227
Foxd2os	0.3716123	2.51119551	1.52357132	0.23145112	0.21330227
Ttc30a1	-0.5747177	1.65587813	1.52346522	0.23146679	0.21330227
Gm9199	-0.795691	-0.4350192	1.52265348	0.23158669	0.21338883
Tal2	-2.1933546	-2.0176317	1.52224192	0.23164751	0.21342095
Srrd	-0.5209209	1.36859612	1.52127496	0.23179049	0.21351571
Reps1	0.16656696	6.00264591	1.52119501	0.23180231	0.21351571
4930430F08I	0.25281035	3.77965773	1.5209459	0.23183917	0.21352572
Polr2j	0.48253553	3.17793511	1.52065343	0.23188245	0.21354166
Lrrc48	-0.3967173	2.39259325	1.52018304	0.23195208	0.21358185
Gjb1	-0.9863527	-0.6787146	1.51955905	0.23204449	0.213643
Rapgef4	-0.1927185	7.31173449	1.51856657	0.23219157	0.21375447
3010001F23I	-0.5218133	1.13950441	1.51735307	0.23237156	0.21389621
Eci3	0.65002224	0.66836361	1.5165667	0.23248829	0.2139797
Alox5ap	-0.5264355	1.4941033	1.51574683	0.23261008	0.21406782
Rnf146	0.17731069	5.45688731	1.51552896	0.23264246	0.21407365
Kbtbd7	-0.1945629	5.2378738	1.51403587	0.23286449	0.21423345
Nucks1	0.15703162	8.85000621	1.51401067	0.23286825	0.21423345
C530008M17	-0.2430484	5.06620149	1.51311089	0.23300219	0.21433268
Specc1l	0.17029032	6.43890441	1.51221987	0.23313492	0.2144184
Ano3	-0.2318625	7.88990707	1.51213509	0.23314755	0.2144184
Hs6st3	-0.3107964	2.87786613	1.51180357	0.23319697	0.21443985
Erlec1	-0.1714482	5.82466938	1.51159967	0.23322737	0.21444382
Glyr1	0.18864531	5.78046531	1.51073258	0.2333567	0.21453811
Dcps	-0.3423514	2.22805824	1.51056213	0.23338213	0.21453811
E130309D14	-0.2455161	4.98591316	1.51033685	0.23341575	0.21454502
Arxes2	-0.2906105	3.63078278	1.50914984	0.233593	0.21468393
Atp1a1	-0.1980063	7.63614666	1.50826788	0.23372481	0.21478106
Trim34b	0.85528212	-0.8356453	1.5072164	0.23388208	0.21490156
Ube3b	0.18610514	6.09056482	1.5059532	0.2340712	0.215031
Cep85	-0.2556869	3.329014	1.50592593	0.23407528	0.215031
Npr1	-0.6151493	0.10392755	1.50498923	0.23421565	0.21513131
Fhdc1	-0.6020693	0.87332126	1.50475637	0.23425056	0.21513131
Cep63	0.25158493	5.47835818	1.50467336	0.23426301	0.21513131
Mlc1	-0.4046069	3.17657548	1.50399592	0.23436462	0.21518122
Sf3a1	0.22002024	5.62274131	1.50396195	0.23436971	0.21518122
Adamts13	-0.3173486	4.12095028	1.50326361	0.23447452	0.2152534
Def6	0.64140572	0.86126945	1.50252134	0.23458598	0.2153309

Ifit2	0.24433439	5.61040824	1.50235258	0.23461134	0.2153309
Npbwr1	-0.9586099	0.40904425	1.50201534	0.23466201	0.21533547
Ptgr1	0.46435089	2.92611121	1.50197071	0.23466871	0.21533547
Spa17	-0.4974902	2.41346414	1.50139377	0.23475544	0.215391
Gm16701	-0.723217	0.69909148	1.50022892	0.23493066	0.21551366
Arid5b	-0.1715543	6.23806089	1.50015643	0.23494157	0.21551366
Nme3	0.28559376	3.44429198	1.49946524	0.23504563	0.21558505
Flt1	-0.2393921	4.99645471	1.4990828	0.23510323	0.21561382
6720483E21I	-1.4655027	-1.7520889	1.49867054	0.23516534	0.21564672
Hhex	0.48419257	1.7525512	1.49819546	0.23523694	0.21566488
Ivd	0.1931544	4.16781787	1.49819101	0.23523761	0.21566488
Dnase1l1	0.41977934	2.18178417	1.4963571	0.23551428	0.21588569
Gm17769	0.58777674	0.14104306	1.49624639	0.235531	0.21588569
Gm11201	-1.3715061	-0.2656289	1.49535551	0.23566556	0.21598494
Nedd4l	-0.2503931	8.72859637	1.49410293	0.23585492	0.21613439
B4galt6	-0.1322051	7.17140345	1.49336358	0.23596678	0.21621279
Gm17019	-1.2053814	-1.2206057	1.49285748	0.2360434	0.21623547
Fam83b	-1.0044739	-0.6156451	1.49285248	0.23604416	0.21623547
Hook3	-0.1438126	8.61231605	1.49234146	0.23612155	0.21626342
Svil	0.24329033	4.74877453	1.49220123	0.23614279	0.21626342
Lamtor4	-0.5620729	1.98318415	1.49209455	0.23615895	0.21626342
D630024D03	-1.7442795	-1.7869437	1.49195616	0.23617992	0.21626342
Diexf	-0.2248097	4.40759709	1.49118848	0.23629628	0.21634587
Scn7a	0.21835722	4.22866684	1.49046363	0.23640621	0.21641378
Snhg18	0.32536922	4.28839592	1.49035221	0.23642312	0.21641378
Amot	-0.1965703	4.95629387	1.48921315	0.23659602	0.21654793
Arfgef1	-0.1819479	7.73294247	1.48871431	0.23667179	0.21659317
Psmd5	0.16892174	5.36568857	1.48826124	0.23674064	0.21663205
Pigk	-0.2389129	5.13219269	1.48748205	0.2368591	0.21670823
Nkg7	1.2411636	0.02728111	1.48736689	0.23687662	0.21670823
Mrpl28	0.36361113	3.36146762	1.48703474	0.23692714	0.21673033
Clec11a	-0.3179673	2.21982313	1.48673579	0.23697263	0.21674781
Dnali1	-0.7536516	0.12482844	1.48631457	0.23703674	0.2167688
Serpine3	-0.4351214	1.95541913	1.4862385	0.23704832	0.2167688
4930547E14I	-1.4561811	-0.8269126	1.48596833	0.23708946	0.2167823
Ccdc96	0.53344586	1.74268235	1.48540273	0.2371756	0.21683694
Slc12a4	-0.6438201	1.46995527	1.48457054	0.23730243	0.21690544
Mto1	0.26260434	3.51939617	1.48456476	0.23730331	0.21690544
Nudt3	-0.2032009	6.12764947	1.48423515	0.23735356	0.21692725
M6pr	0.21791834	6.22491652	1.48251192	0.23761653	0.21712737
Stard6	-0.3682756	2.41474258	1.4824541	0.23762536	0.21712737
Kif9	-0.4899036	2.35054182	1.48219266	0.23766529	0.21713971
Ipo8	-0.1909749	5.43226085	1.48192917	0.23770554	0.21715235
Cdkn2b	1.58623662	-1.6133933	1.48168916	0.23774221	0.21716172
P2rx5	1.40276576	-0.9221441	1.4807247	0.23788965	0.21727225

Rsph1	0.44213233	2.13736563	1.47904663	0.23814647	0.21748263
Adnp2	-0.223719	4.66110951	1.47867272	0.23820374	0.21748936
Ccdc40	0.81278282	0.06775867	1.47865301	0.23820676	0.21748936
1700013F07I	-0.6649799	-0.0736069	1.47836241	0.23825128	0.21750585
Mylk	0.2196447	6.02107429	1.47750877	0.23838213	0.21760114
Lyrm4	0.17672418	5.02712425	1.47674521	0.23849926	0.21768131
Psmg2	0.30847812	3.21681785	1.47659093	0.23852293	0.21768131
Slitrk1	-0.1664352	7.08121807	1.47573854	0.23865378	0.21775521
Mtch2	0.14687287	5.59819807	1.47571825	0.2386569	0.21775521
Slc10a3	0.3938791	2.47682987	1.47528161	0.23872396	0.21776219
9030617O03	0.30548231	2.99011174	1.47517579	0.23874022	0.21776219
2310039H08I	0.46025316	2.04685104	1.47515104	0.23874402	0.21776219
Tnfsf10	-0.3266274	3.01706064	1.47479944	0.23879805	0.2177873
Pkp4	-0.1452326	8.59015621	1.47432453	0.23887105	0.21781807
Ppfibp1	-0.1443276	6.07535407	1.47423514	0.2388848	0.21781807
Dusp27	0.74813976	1.8053028	1.47387775	0.23893976	0.21784402
Snx32	0.22547744	4.27697155	1.47353103	0.23899309	0.21785995
Mtag2	-1.2336729	-0.504034	1.47341953	0.23901024	0.21785995
Havcr2	-0.5703205	1.70023594	1.47270909	0.23911959	0.21793544
Wdr86	0.94573451	1.31056023	1.47233444	0.23917727	0.21796385
Bmp4	0.39087433	7.4127385	1.47200513	0.23922799	0.2179859
Abr	-0.1994458	7.69705062	1.47144961	0.23931359	0.21803384
Eif3i	0.17142534	5.80342661	1.47125507	0.23934357	0.21803384
Ppm1a	0.16005254	6.77648494	1.47114726	0.23936019	0.21803384
Dsg3	1.07505501	-0.7353267	1.46992728	0.23954834	0.21818105
Smyd2	0.21451009	5.5721474	1.46895034	0.23969915	0.21829422
Hgs	0.25686642	4.12396268	1.46839751	0.23978455	0.21834329
Clca2	-1.2695471	-1.5911448	1.46825758	0.23980617	0.21834329
Trmt5	0.28542185	3.47428555	1.46757602	0.23991151	0.21839871
Cabp7	-1.1090423	-1.1968149	1.46721123	0.23996792	0.21839871
Elf2	0.22651798	6.74349939	1.46713889	0.23997911	0.21839871
Nkx2-2	-0.6051011	0.88378594	1.46710931	0.23998369	0.21839871
Unc50	0.18967528	5.64596372	1.46696402	0.24000616	0.21839871
Terf2ip	0.16534274	6.07454767	1.46666465	0.24005247	0.21839871
Fam71e1	-0.6317551	0.25455267	1.46666098	0.24005304	0.21839871
Cep78	-0.333927	2.62711095	1.46639069	0.24009487	0.21841259
Ydjc	-0.4569485	1.79528566	1.46533926	0.24025766	0.21853649
Smg5	-0.2066481	4.39297691	1.46462959	0.24036761	0.21861231
Sgpl1	-0.2623487	4.29909087	1.46417664	0.24043783	0.21865198
Fam107a	0.26507911	7.39578337	1.46392691	0.24047655	0.218663
Stx1b	0.18252673	6.40618772	1.46340498	0.24055751	0.21868865
Pcdh17	-0.2359247	6.97977252	1.463402	0.24055797	0.21868865
Dpp10	-0.2145009	6.87344246	1.46313487	0.24059942	0.21870214
Stim2	-0.2188102	5.92475523	1.46224749	0.24073717	0.21878389
Smco4	0.44228599	1.93044032	1.46221254	0.2407426	0.21878389

Sbno2	0.38757224	1.71964168	1.46151636	0.24085075	0.21885783
Kif27	-0.3758477	2.20180061	1.4612708	0.24088891	0.21885783
Aste1	-0.3681325	2.36355824	1.46104331	0.24092427	0.21885783
Slc35f1	-0.2228518	7.28824329	1.46071594	0.24097517	0.21885783
Glp2r	-0.6452253	1.68436136	1.46041557	0.24102188	0.21885783
Tcea2	-0.3483717	2.74191191	1.46029257	0.24104101	0.21885783
Sertad3	-0.8147562	0.88470497	1.46025788	0.24104641	0.21885783
4930506M07	0.16316937	5.40685335	1.46018147	0.24105829	0.21885783
Tns4	0.58301229	1.63779668	1.46014728	0.24106361	0.21885783
Slc19a1	-0.3215376	3.08317596	1.45948606	0.2411665	0.21892706
Zfp119a	0.4772544	1.13560126	1.45883884	0.24126727	0.21899435
Ror2	0.66690449	0.84523662	1.45842068	0.2413324	0.21902928
Atg4c	0.18964713	6.30646857	1.45762751	0.24145601	0.21911727
Sgsm3	-0.2633661	3.69861639	1.45700144	0.24155364	0.21918166
Kif2a	0.15182741	7.65851713	1.45658184	0.24161909	0.21921261
Shh	0.48647937	2.02679904	1.45644087	0.24164109	0.21921261
4930426D05	-1.2310708	-0.7480271	1.45596433	0.24171547	0.21923496
Irf4	0.3179437	5.17414265	1.45594124	0.24171907	0.21923496
Tac1	-0.294032	4.18556843	1.45516587	0.24184015	0.21932058
Ptprf	-0.1822392	4.74980605	1.45498269	0.24186877	0.21932233
Zbtb5	-0.3307069	2.58687091	1.45447301	0.24194842	0.21937035
Ccbl2	-0.4044785	2.49897631	1.45348983	0.24210215	0.21948553
Accs	-0.3514163	2.8710364	1.45276383	0.24221576	0.2195643
Lrpap1	0.23359864	5.78732057	1.45228066	0.2422914	0.2196067
Cpeb2	-0.1376162	7.36638977	1.45212376	0.24231597	0.2196067
Star	-0.3573796	2.52896689	1.45159965	0.24239807	0.21965688
Mybpc3	2.28351039	-2.0274697	1.4514238	0.24242562	0.21965763
Fbxo47	-0.5858518	1.25856779	1.45099108	0.24249344	0.21968431
Hnrnpll	0.15057618	6.34844981	1.45089486	0.24250852	0.21968431
Arih1	-0.1307617	7.93081181	1.45057788	0.24255822	0.21970512
Lrrc6	-0.2649352	3.88873187	1.44987686	0.24266818	0.2197805
Aarsd1	0.28061997	3.70563603	1.44962032	0.24270844	0.21979274
Tmem167	-0.1685925	6.42597284	1.44834507	0.24290868	0.21992827
Rpl14-ps1	0.36026828	2.59739712	1.44828763	0.2429177	0.21992827
Cyp4f16	0.4760048	1.73480182	1.44815607	0.24293837	0.21992827
Sez6	-0.2355742	4.84745636	1.44769331	0.2430111	0.21996988
Rnf19b	0.19655722	4.34190555	1.44737882	0.24306054	0.2199904
Ddx60	-0.6028852	1.88541566	1.44643928	0.24320832	0.22007971
Slirp	0.20939813	4.7971432	1.44641099	0.24321277	0.22007971
9230110C19	0.29207217	3.02451979	1.44609232	0.24326292	0.22010086
Oprm1	0.58431697	1.91344994	1.44531726	0.24338496	0.22018601
Lypd2	-0.7755312	1.82578607	1.44515437	0.24341061	0.22018601
St3gal3	0.26536124	3.19153663	1.44242335	0.2438413	0.22055133
Myo1f	-0.48236	1.27857512	1.44182305	0.2439361	0.22059973
Sv2a	-0.2112313	5.86958726	1.44174464	0.24394849	0.22059973

Nptx1	0.19443371	7.55312455	1.44063405	0.24412401	0.22073417
Zfp646	0.23748313	3.74038985	1.44015847	0.24419922	0.22077789
Pmm1	0.20285735	4.66168759	1.43968744	0.24427375	0.22082097
Cenpb	0.27563963	4.12701979	1.43877989	0.24441741	0.22092655
Nsun2	-0.1644637	5.15905274	1.43821741	0.24450651	0.22098278
Chst12	0.39266888	1.74990168	1.43777236	0.24457704	0.22102222
Rps18	0.34581992	6.22385773	1.43623795	0.2448204	0.22119585
Rab3d	0.24860431	3.19566862	1.43622149	0.24482301	0.22119585
Wdfy4	0.49587002	1.56055721	1.43559136	0.24492304	0.22126191
Gm20939	0.25593678	3.27695277	1.43409989	0.24516001	0.22145165
Cd300lb	1.76812193	-1.3241389	1.43353752	0.24524944	0.22150809
Mal	-0.2194176	6.0909116	1.43239314	0.24543156	0.22164822
Il1rapl2	-0.3552004	3.23313246	1.43217215	0.24546674	0.22165564
Lpcat3	-0.2949636	3.53870214	1.43199389	0.24549513	0.22165692
Slc52a2	-0.7015184	0.70323119	1.43158091	0.24556092	0.22169196
Neurod4	-0.8239142	0.44780469	1.43102329	0.24564978	0.22174783
Smu1	0.17952807	5.43062054	1.43063871	0.24571109	0.22177882
Abl2	-0.2127901	5.655913	1.42988791	0.24583084	0.22186254
Cyp2d22	-0.291602	3.38887593	1.42967808	0.24586433	0.2218684
Raly	0.36646638	2.78369417	1.42912967	0.24595186	0.22192302
Pdcd6ip	0.13377997	7.45387281	1.4287631	0.24601039	0.22195147
Smagp	0.78759366	0.54925906	1.42831679	0.24608168	0.22196947
Aph1b	-0.2686926	4.15526441	1.42823994	0.24609396	0.22196947
Tes	0.24428957	3.71867944	1.42813098	0.24611137	0.22196947
Phkb	-0.1981385	6.1319598	1.42780335	0.24616372	0.22199234
Il22ra1	-0.4237507	1.58253079	1.42671465	0.24633781	0.22210769
Acta2	-0.336032	3.71239408	1.42661636	0.24635353	0.22210769
B430212C06I	-1.6860152	-1.5430371	1.42649654	0.24637271	0.22210769
Grk6	-0.2934858	2.89637392	1.42614098	0.24642961	0.22213462
Hist1h1e	0.42842153	2.92887352	1.42508718	0.24659834	0.22226234
Tada2a	-0.2295035	3.75529382	1.42391465	0.24678627	0.22236625
Fsd1	0.41381132	2.29276353	1.42382175	0.24680116	0.22236625
Oas1a	0.8416898	0.68271435	1.4236874	0.24682271	0.22236625
Vps37b	0.27361286	3.12214722	1.42344501	0.24686159	0.22236625
Neu1	0.26427776	3.96564936	1.42339301	0.24686993	0.22236625
Gja4	-0.8739162	-0.5079181	1.42335547	0.24687595	0.22236625
Spsb4	-0.6002291	1.11843022	1.42296797	0.24693813	0.22238862
Zfp764	0.42763369	2.98678652	1.42268818	0.24698304	0.22238862
Zfp612	-0.21019	7.4305425	1.42266873	0.24698616	0.22238862
Zfp82	-0.4401376	1.45211517	1.42252628	0.24700902	0.22238862
Clip2	-0.3234893	3.95451113	1.42218234	0.24706425	0.2224063
Mkrn1	0.15221739	6.7341484	1.42204636	0.24708609	0.2224063
Wnk2	-0.2490279	6.04766164	1.42189849	0.24710984	0.2224063
Cdr1	-0.2988569	9.91723274	1.41914562	0.24755253	0.22275824
A930011G23	0.93860046	-0.3035177	1.4191298	0.24755507	0.22275824

2010315B03I	0.20930894	4.22427496	1.41879654	0.24760874	0.22278214
Zfp687	0.26800384	3.60778931	1.41697053	0.24790303	0.22302251
Abcf3	0.21131923	5.02089209	1.41604394	0.24805254	0.22313259
Esrrb	1.0217344	-0.3614553	1.41494827	0.24822948	0.22324786
Gm19619	1.47711952	-1.2366144	1.4149141	0.24823501	0.22324786
Rtp4	0.43379402	3.93435672	1.41390026	0.24839888	0.22334496
Morn3	-1.5553366	-1.2840511	1.4136678	0.24843648	0.22334496
Mrpl37	-0.2153235	4.02283892	1.41352209	0.24846005	0.22334496
Cox17	0.19768242	5.38108613	1.41350922	0.24846213	0.22334496
Slc41a1	0.25674503	7.95331276	1.4133707	0.24848454	0.22334496
Lpo	-1.3369998	-0.8109951	1.41323793	0.24850602	0.22334496
Ppm1m	0.28665599	3.80344664	1.41185572	0.2487298	0.22351637
Synm	-0.1995577	5.50732263	1.41172404	0.24875113	0.22351637
Sycp3	-0.4550212	2.03633713	1.41037078	0.24897049	0.22368902
Cfhr2	0.63591247	0.69676622	1.40999923	0.24903076	0.22371872
Olfml2a	0.42300919	3.54799309	1.40960291	0.24909508	0.22374761
Dapl1	0.26905233	5.38142757	1.40933924	0.24913787	0.22374761
Smdt1	0.29509624	5.06212921	1.40926553	0.24914984	0.22374761
Krt7	2.04391725	-1.709268	1.40892223	0.24920558	0.22374761
Brinp3	-0.2054358	4.53933013	1.40884852	0.24921755	0.22374761
Tdrd9	-1.5512184	-1.9587224	1.40879489	0.24922626	0.22374761
2610528A11I	-1.6279082	-1.5100274	1.406717	0.24956403	0.22402638
Gm20125	-2.1441475	-1.3196063	1.40651797	0.24959641	0.22403098
Pgr15l	0.88309382	0.38897474	1.40626081	0.24963826	0.22404407
Desi1	-0.1559294	5.52702697	1.40567693	0.24973332	0.22410491
Acss1	-0.2244915	3.75449704	1.40511709	0.2498245	0.22416188
9230114K14I	0.39981669	1.94223052	1.40495236	0.24985134	0.22416188
Il18	-0.1899962	4.1713392	1.40458496	0.24991121	0.22419112
Lmo3	0.18109344	7.13282891	1.40405826	0.24999708	0.22424367
Tdgf1	-1.2576861	-0.1595847	1.40329952	0.25012084	0.2243302
Btf3	0.23927037	6.23544685	1.40308724	0.25015548	0.22433679
Nudt7	0.26766905	2.42761993	1.40237297	0.25027208	0.22441687
Gsg1	-1.282829	-0.8084375	1.40204525	0.2503256	0.22444025
Ncaph	-0.9796901	-0.3833775	1.40187893	0.25035277	0.22444025
Adck3	-0.282741	2.71438383	1.40146296	0.25042074	0.22444425
Myot	1.49028742	-2.0569196	1.40142471	0.25042699	0.22444425
Cog7	-0.2681886	4.52669569	1.40135029	0.25043915	0.22444425
Rps15a-ps6	-0.3085623	2.45714199	1.40104191	0.25048956	0.22446495
Sh3bp5l	0.23977694	3.77831697	1.4004587	0.25058492	0.2245216
Thap3	-0.4919504	2.29989503	1.40032124	0.25060741	0.2245216
Ccdc87	0.57455087	1.01600754	1.39959137	0.25072684	0.2245867
Ptpn3	0.20036478	6.04739918	1.39954317	0.25073473	0.2245867
Pmf1	0.48074452	3.35773183	1.39903772	0.25081748	0.22463634
Phactr4	0.25963545	6.13209661	1.39870573	0.25087185	0.22465147
Gm4787	-0.9069644	2.161135	1.39854987	0.25089738	0.22465147

Cul7	-0.2647193	3.0716944	1.39843402	0.25091636	0.22465147
Setd3	0.12005433	6.79583478	1.39799291	0.25098865	0.2246854
Pdss1	-0.4031682	2.13099445	1.39786909	0.25100894	0.2246854
Gm3985	-0.9653387	-0.8746755	1.39652656	0.25122914	0.22485802
Pax6	-0.3682095	2.24170717	1.3962995	0.2512664	0.22486688
Ppp1r12a	-0.147939	7.45763336	1.39610814	0.25129781	0.22487051
Mllt4	0.12138907	7.44020408	1.39563895	0.25137485	0.22490979
Zscan25	1.24322175	-0.360117	1.3954748	0.25140181	0.22490979
Cdkn1c	0.34348692	5.69488742	1.39534093	0.2514238	0.22490979
Rprml	0.35127221	3.00857413	1.39465741	0.25153612	0.22498577
Prepl	-0.2372339	7.5494485	1.39442152	0.25157489	0.22499597
Fn3krp	-0.2111972	4.38382354	1.39421112	0.25160949	0.22500242
Ccz1	0.18682519	4.8060253	1.3934844	0.25172902	0.22508483
Tanc1	-0.1459009	5.909847	1.39287722	0.25182894	0.22514968
Tbpl1	0.12681043	6.41403785	1.39240534	0.25190664	0.22519465
Pold2	0.47996469	2.57042042	1.39189692	0.25199038	0.22524501
Alpk2	1.56956964	-1.4775081	1.39082315	0.25216737	0.2253787
Gna15	1.57407797	-1.6031651	1.38963219	0.25236386	0.2255298
Wdr35	-0.2329405	4.69647187	1.38933619	0.25241273	0.22554895
Pydc3	-0.6993312	0.73469597	1.38869811	0.25251811	0.22559815
Dyx1c1	-0.4305405	2.02333161	1.38867041	0.25252269	0.22559815
Rps26	0.27520538	5.54799771	1.38833305	0.25257843	0.22562342
Lins	0.28706256	3.94188219	1.38789601	0.25265067	0.22566342
Foxp2	0.2047645	7.30765241	1.38745092	0.25272426	0.22570463
Ctbp1	0.22596093	5.30065741	1.38613437	0.25294211	0.2258693
Nisch	-0.1641765	7.3684988	1.38600456	0.2529636	0.2258693
Slk	-0.16559	8.16449595	1.38528016	0.25308359	0.22595188
Xrcc5	0.24170076	4.92129584	1.38492077	0.25314314	0.2259805
Hectd3	-0.2179083	4.6912483	1.38461084	0.25319451	0.22599411
Cpox	0.22842331	5.72688531	1.38449709	0.25321337	0.22599411
Catsperg1	-1.1023783	-1.0719641	1.38328429	0.25341455	0.22614158
Aaas	0.29840947	3.0916257	1.38316932	0.25343363	0.22614158
Comp	-1.1393976	-1.3445254	1.38262943	0.25352326	0.22619695
Gm10825	-1.5077161	-1.3724712	1.38246405	0.25355073	0.22619695
N6amt1	0.15748303	5.66789646	1.38187911	0.2536479	0.22624342
Sharpin	0.24507918	3.8065174	1.38176525	0.25366682	0.22624342
Cd93	-0.4689562	3.15480574	1.38165344	0.2536854	0.22624342
Tmed8	-0.1381787	6.24397623	1.38004993	0.25395208	0.22645668
D330023K18	0.58594278	1.2343076	1.37944874	0.25405216	0.22652135
Wbscr17	-0.2640695	3.82824717	1.3792668	0.25408246	0.22652379
Arrdc3	-0.2178395	5.87080385	1.37854657	0.25420244	0.22658238
Fxr2	-0.2462175	3.71798348	1.37824591	0.25425254	0.22658238
Trap1	-0.2110697	4.50678277	1.37822337	0.2542563	0.22658238
2310045N01	0.37527832	4.79237446	1.37821046	0.25425845	0.22658238
Eya4	-0.4530131	2.43857973	1.37753631	0.25437085	0.22665798

Fam212a	1.01900174	-0.7309088	1.37712933	0.25443874	0.22669389
Selplg	-0.5851857	0.91735611	1.37632271	0.25457337	0.22678925
Tacr3	-0.4984123	1.90532315	1.37578669	0.25466288	0.2268444
Fgf10	-0.5784103	2.46889666	1.37551358	0.2547085	0.22685332
Zbtb26	0.17305791	4.37235312	1.37539636	0.25472808	0.22685332
Tmc3	-0.6250403	0.99473456	1.3749548	0.25480188	0.22689445
Tmcc1	0.11758965	6.94096167	1.37249458	0.25521353	0.2272364
Pou6f2	0.51339406	1.93296602	1.37145436	0.25538785	0.22736698
Rorb	-0.1911601	8.50372588	1.37093625	0.25547473	0.22741969
Sacs	-0.5194919	3.7593415	1.37047667	0.25555183	0.22746369
Il13ra2	0.59571828	1.98597913	1.36986658	0.25565422	0.22753019
Sdc2	0.27793773	6.59430272	1.36944074	0.25572572	0.22756918
Bfsp2	0.98049529	-0.7331281	1.36818334	0.25593701	0.22773254
Pi15	-0.90523	0.75635518	1.36762023	0.2560317	0.22775509
Wars	0.18705113	5.11435786	1.36756926	0.25604027	0.22775509
Map3k7	0.12644558	6.60263861	1.36753833	0.25604548	0.22775509
Chrm4	-0.397071	2.00723448	1.36639212	0.25623838	0.22790201
Ubald1	0.21070542	4.48217382	1.36622482	0.25626655	0.2279024
Pqlc1	0.21696714	3.33315234	1.36604053	0.25629759	0.22790535
Gm13212	0.37483583	1.56577892	1.36571532	0.25635237	0.2279294
Szrd1	0.261109	4.64014995	1.36547932	0.25639213	0.2279401
Kcnk10	-0.5025773	0.71105858	1.3646834	0.2565263	0.22803471
Ikbkg	0.14023851	5.88354635	1.36448312	0.25656008	0.22804007
AI839979	-0.7249291	0.19634029	1.36415204	0.25661592	0.22806505
Dhdh	0.19931988	6.4983225	1.36385196	0.25666655	0.22808538
Dgcr6	0.30532515	3.95728815	1.36320284	0.25677612	0.22815808
Mir6920	-1.258167	-1.5890956	1.36293982	0.25682053	0.22817288
Gm13152	-0.5525368	1.28296953	1.36231709	0.25692573	0.22824166
Mir22hg	-0.24646	3.66681709	1.36176271	0.25701942	0.22830022
2900005J15F	0.4770535	2.24729623	1.36116631	0.25712027	0.22833389
Riok1	0.25818135	3.87879186	1.36106685	0.25713709	0.22833389
Rnf10	0.17618634	5.83996032	1.36104574	0.25714066	0.22833389
Nppc	-0.9494616	-0.4988418	1.36068658	0.25720143	0.22836317
Paip1	-0.1761672	6.24463162	1.35879651	0.2575215	0.22858346
Rsad1	0.27994236	3.20766424	1.35862888	0.25754991	0.22858346
Crispld1	0.25746037	3.89178035	1.3585473	0.25756374	0.22858346
Chst3	-0.9723536	0.66964206	1.35851321	0.25756951	0.22858346
Ajuba	-0.266006	2.82632005	1.35812943	0.25763459	0.22858346
Pole	-0.4712572	1.08411934	1.35809606	0.25764024	0.22858346
Rnf26	0.30707429	3.12694688	1.35793813	0.25766703	0.22858346
Mettl8	-0.2976858	3.77776576	1.357759	0.25769741	0.22858346
Tmem19	0.24016791	3.56691785	1.35774472	0.25769983	0.22858346
Scamp1	0.13478751	7.87224213	1.3571074	0.25780797	0.22863064
Apoa2	-1.1557455	-1.2367279	1.35710337	0.25780866	0.22863064
Mtg2	-0.5698302	0.41438466	1.356579	0.25789768	0.22867375

Epsti1	0.59001107	1.15801116	1.35634893	0.25793675	0.22867375
Atrip	0.24738078	3.49551033	1.35632542	0.25794074	0.22867375
Ankrd45	-0.1792109	6.27744958	1.35579295	0.2580312	0.2287229
Mroh1	-0.2948879	4.29708474	1.35567148	0.25805184	0.2287229
Mef2a	0.12957603	8.26170554	1.35527605	0.25811905	0.2287578
Mtif2	-0.1710492	5.36245845	1.35489712	0.25818348	0.22879023
Utp3	0.15141768	6.12821502	1.35409286	0.25832029	0.22887737
Arl8a	-0.1630918	6.34525013	1.3538159	0.25836742	0.22887737
Rapsn	1.69696082	-2.5007466	1.35378327	0.25837298	0.22887737
Slc14a2	-1.2142411	-0.4003729	1.35366444	0.2583932	0.22887737
Vcpip1	-0.1650633	7.35241349	1.35307206	0.25849407	0.22894205
Zfp9	0.16399467	5.92135413	1.35256601	0.25858028	0.22899372
Zfp862-ps	0.33289709	2.31865458	1.35200629	0.25867567	0.22905352
Mastl	-0.5368251	1.51160158	1.35117718	0.25881706	0.22909812
Atp2b4	-0.2227035	7.45255742	1.35117334	0.25881772	0.22909812
Wnk4	0.35592245	4.36271897	1.3510455	0.25883953	0.22909812
Tmtc4	-0.1974107	4.07511298	1.35093162	0.25885896	0.22909812
Acap2	-0.1680918	6.73780263	1.35089386	0.2588654	0.22909812
Alg14	0.31219551	4.58603862	1.35041272	0.25894752	0.22914612
Tlr5	-1.2977484	-1.2240123	1.34947379	0.25910787	0.22924592
Uhmk1	-0.1476666	6.47143497	1.34942568	0.25911609	0.22924592
Zfp760	0.18638578	5.25327047	1.34896755	0.25919438	0.2292905
Ormdl1	0.36243596	3.70147365	1.34849909	0.25927446	0.22931272
Snrpd2	0.32045363	4.07892904	1.34849427	0.25927529	0.22931272
D630013N20	-2.1483778	-0.8906889	1.34785942	0.25938387	0.22938407
Arpc5l	0.19017601	5.82331496	1.34750846	0.25944392	0.22940497
Zfp385b	0.1807933	6.65665764	1.34739505	0.25946333	0.22940497
Bcl2	-0.2054114	6.6580827	1.34593924	0.25971265	0.22960071
Hmgb1	0.17866222	8.09298652	1.34548158	0.2597911	0.22963229
Rdh5	-1.3426724	-1.2298068	1.34540485	0.25980425	0.22963229
Cd248	0.32593519	4.35918922	1.34469134	0.25992662	0.22971575
Garnl3	-0.2621718	5.19900696	1.34425676	0.26000119	0.22975694
Syce1	-1.6210086	-2.0044352	1.34390772	0.2600611	0.22977852
Pdgfc	0.32884136	2.48770178	1.34378873	0.26008153	0.22977852
Slc33a1	-0.2535672	3.78701826	1.34259038	0.26028737	0.22993567
Mtrr	-0.2199609	4.01069066	1.34235009	0.26032867	0.22994744
Slc22a17	-0.193823	5.86933989	1.34187549	0.26041028	0.2299948
Nme6	0.40922145	1.77490388	1.34121859	0.26052328	0.23006988
Rtp1	1.35714783	-1.5165248	1.34051583	0.26064423	0.23015197
Ankrd17	-0.1630133	8.9309109	1.34024748	0.26069044	0.23016805
Plekhh3	-0.490061	1.54505035	1.33943924	0.26082968	0.23026625
Cog3	0.1742601	5.52673204	1.33859928	0.26097449	0.23036934
Srek1	-0.1769418	6.65390786	1.33814795	0.26105234	0.23041331
1110038F14l	0.32481012	3.35510242	1.33768247	0.26113266	0.23045946
Spink10	-1.0459513	-0.9899243	1.33734134	0.26119155	0.23046219

Mboat1	-0.7875506	0.97998995	1.33723955	0.26120912	0.23046219
Ighmbp2	-0.4026112	1.95229387	1.33712197	0.26122943	0.23046219
Pvrl4	-0.4435432	1.55218341	1.33701491	0.26124791	0.23046219
Rbpms	0.34195238	5.27075179	1.33673843	0.26129567	0.23047958
D430036J16F	-0.355181	2.35604874	1.33503407	0.26159029	0.23070726
Rab6a	0.12831853	10.1161715	1.33480579	0.26162978	0.23070726
Helz2	0.55533729	1.98281562	1.3347583	0.261638	0.23070726
Tusc5	1.19252376	0.35486227	1.33457977	0.26166889	0.23070975
Inpp5b	-0.1863287	4.90277006	1.33440743	0.26169872	0.2307113
Ccdc167	-0.1560931	5.38308184	1.33413105	0.26174656	0.23072873
Otud7a	-0.3940386	3.09168606	1.3337537	0.26181191	0.23076158
Nmd3	-0.1920561	5.08200007	1.33323397	0.26190193	0.23081618
Zfp959	-0.3499725	1.73695519	1.3329116	0.2619578	0.23084066
Rdh13	-0.2222926	3.81621794	1.33105368	0.26228005	0.23109986
Chmp6	0.31430288	2.23547176	1.33072043	0.26233791	0.23112606
Cilp2	0.779392	0.61710162	1.33005529	0.26245344	0.23120306
4930478L05F	-1.2293787	-1.0812398	1.32938063	0.26257069	0.23126817
Ppp3cb	0.12891891	8.65277141	1.32930619	0.26258363	0.23126817
Plcb3	0.26901552	4.33601191	1.3288553	0.26266204	0.23131244
Usp28	-0.3122773	3.5545743	1.32754803	0.26288953	0.23148798
Col28a1	-1.0553873	-0.372539	1.32713638	0.26296122	0.2315263
Uchl5	0.13792647	6.67267395	1.32662763	0.26304985	0.23156679
Rhebl1	-0.7833472	0.05855139	1.32654903	0.26306355	0.23156679
2500004C02I	-0.2434448	3.8040238	1.32603879	0.26315249	0.23157505
Stxbp6	-0.1797143	6.56505081	1.32603221	0.26315364	0.23157505
Pds5b	-0.1497403	7.92641985	1.32600088	0.2631591	0.23157505
Misp	1.2860392	-1.123436	1.32584866	0.26318564	0.23157505
Swap70	0.17798469	4.83364218	1.32558509	0.26323161	0.23158507
Spata45	-1.6881714	-0.5529667	1.32546027	0.26325338	0.23158507
Zc3h3	0.35128699	2.62834499	1.32507878	0.26331994	0.23161883
Naaladl1	-0.7460236	0.48937503	1.32478481	0.26337124	0.2316239
Faf1	-0.1978851	4.73011911	1.3247228	0.26338206	0.2316239
Polr1b	-0.4384227	2.23088517	1.32436374	0.26344474	0.23164477
Tacr1	-0.2776038	3.12697226	1.324264	0.26346216	0.23164477
Camkmt	0.19496688	4.00481931	1.32343522	0.26360693	0.23174726
Vma21	0.15274441	6.21773459	1.32314383	0.26365786	0.23176724
Apol9b	-0.8942811	-0.6994516	1.32281768	0.26371487	0.23179257
Usp50	-1.4322377	-1.5371428	1.32213088	0.26383499	0.23187335
Mc1r	-1.2743162	-1.8869924	1.32178136	0.26389614	0.2319023
AA465934	0.60187592	0.237487	1.3205758	0.26410722	0.23205826
Zfp114	-0.7060425	0.38684237	1.3204453	0.26413008	0.23205826
Hspa12b	0.30739048	3.62175018	1.32021219	0.26417092	0.23206405
1110057K04I	0.16116847	5.54252814	1.32008536	0.26419315	0.23206405
Fyb	0.30666909	4.4935111	1.3198872	0.26422788	0.23206853
Kcnj6	-0.2546398	5.88929856	1.31973411	0.26425472	0.23206853

Smim4	0.35168791	2.15559357	1.31852467	0.26446685	0.23223002
Speer8-ps1	-0.7790632	0.0963112	1.31834554	0.26449829	0.2322312
Gm10560	-1.4331823	-1.0415094	1.31804353	0.26455131	0.2322312
Dohh	0.30191775	3.27307213	1.31803407	0.26455297	0.2322312
Zcchc5	1.04026925	-0.4091356	1.31763349	0.26462331	0.23226814
Alx4	0.30145914	5.70558227	1.31640029	0.26484001	0.23243352
Hmga2	0.2161469	3.850615	1.31526924	0.26503897	0.23255974
Nmi	0.40444776	3.19911829	1.31526106	0.26504041	0.23255974
Ggn	0.88240363	-0.0259875	1.31495345	0.26509455	0.23258241
Klhdc8b	0.46941124	2.2070895	1.31469716	0.26513968	0.23259717
Rad54l	-0.5476123	0.81036565	1.31410355	0.26524423	0.23265402
Ubfd1	0.1394734	6.94136484	1.31400784	0.26526109	0.23265402
Pigs	0.21009213	4.61576132	1.31249741	0.26552738	0.23285316
S100b	0.24347553	6.07697975	1.31239863	0.26554481	0.23285316
Slc35g2	-0.2612999	3.02421412	1.31192513	0.26562836	0.23290159
Mturn	-0.1424534	6.37009665	1.3112482	0.26574788	0.23298153
Pacsin1	-0.1720245	6.57730162	1.31073478	0.26583858	0.23298945
Mis18a	-0.33486	1.95977578	1.31072122	0.26584097	0.23298945
Rpl29	0.22445403	6.10243951	1.31070016	0.26584469	0.23298945
Fyn	0.13632973	6.00425844	1.3105552	0.26587031	0.23298945
Dynlt1f	-0.4662789	0.61524014	1.31027257	0.26592026	0.23300838
Fes	0.93809067	-0.3101794	1.30949652	0.26605748	0.23309006
Snhg4	0.2726808	3.32764924	1.30942463	0.2660702	0.23309006
Dvl3	0.15090384	6.09227167	1.309202	0.26610958	0.23309972
Ube2s	0.28408162	3.69044708	1.30858137	0.26621942	0.23313169
Rrm2b	0.14509749	6.74187979	1.30845969	0.26624096	0.23313169
Sgcz	-0.5425161	1.8960049	1.30835626	0.26625927	0.23313169
Mterfd1	0.20032572	5.07451548	1.30835476	0.26625954	0.23313169
Tdrd7	-0.1763351	4.68204311	1.30807905	0.26630836	0.2331496
Thnsl1	0.26315873	3.96352648	1.30787942	0.26634372	0.23315572
Gm5124	0.18351836	5.01792414	1.30754525	0.26640292	0.23318271
Trmt2a	0.26032833	3.786557	1.30684594	0.26652686	0.23324572
Xlr3b	0.53456118	1.1616973	1.30681882	0.26653167	0.23324572
Tmem129	0.26866792	3.06683303	1.30655114	0.26657914	0.23326243
1700034I23R	-0.9729427	-0.3781941	1.3058804	0.26669812	0.23332888
Cpb2	-1.5794788	-1.7620521	1.30580295	0.26671186	0.23332888
Rps24	0.20744905	7.28769221	1.30505887	0.26684395	0.23341959
Wiz	0.18509258	4.43720815	1.30437941	0.26696463	0.23348702
2900056M20	-0.20641	7.95961071	1.304305	0.26697785	0.23348702
Fam163a	-0.5027802	1.81468194	1.30387383	0.26705448	0.23352919
Elk4	-0.1383428	6.40798049	1.30370982	0.26708364	0.23352984
Dlg1	-0.1469717	7.4792636	1.30314538	0.26718401	0.23355434
Pum1	-0.1277626	6.90389033	1.30309819	0.2671924	0.23355434
Il2rb	0.72878858	0.95630689	1.30307284	0.26719691	0.23355434
Fkbp9	0.28191073	5.31413582	1.30219467	0.26735318	0.23366609

Slc44a5	-0.4208541	1.67814149	1.30130448	0.26751171	0.23377978
Sowaha	-0.1904762	7.44815938	1.30096865	0.26757155	0.23380149
Fam161b	-0.2626053	3.87855137	1.3008458	0.26759344	0.23380149
Blvrb	0.46932421	1.2397928	1.30059705	0.26763778	0.23381537
Mars	0.15840275	4.89170001	1.30025402	0.26769894	0.23384395
1500017E21I	1.35244587	-1.8060662	1.29996317	0.26775081	0.2338644
Tmem200a	0.32881496	3.68035073	1.2997714	0.26778502	0.23386943
Rabl3	0.21172263	4.40385949	1.29904136	0.26791529	0.23395834
Mphosph10	0.19455289	5.16500049	1.29862824	0.26798906	0.23399789
Tmem60	0.18602787	5.36769151	1.2979661	0.26810733	0.23405166
Dnal1	0.14843673	7.03713148	1.29796468	0.26810758	0.23405166
Tenc1	0.24074241	5.97022186	1.29761174	0.26817066	0.23408186
Kcnu1	0.62118364	0.58323831	1.29722743	0.26823936	0.23411696
Tmem123	0.26884304	5.06646632	1.29566084	0.26851965	0.23432644
Uprt	0.18104548	4.57891651	1.2954614	0.26855536	0.23432644
Ddx24	-0.1530299	5.66962142	1.29540811	0.2685649	0.23432644
Nipa2	0.19107294	4.76445489	1.29518566	0.26860474	0.23433633
Metap1d	-0.4163261	2.28054549	1.29404308	0.26880949	0.23443721
Actr6	0.2806423	3.63828272	1.29398793	0.26881938	0.23443721
Shisa5	0.2779657	5.06994187	1.29393495	0.26882888	0.23443721
Cluap1	0.14574137	5.50407573	1.29374983	0.26886208	0.23443721
Rasal1	-0.3944955	3.28396186	1.29374472	0.268863	0.23443721
Znrd1as	0.24796699	3.55287635	1.29281302	0.26903016	0.23455809
Catsperd	-1.0231228	-0.7666412	1.29264692	0.26905997	0.2345592
Rcan3	0.16983532	5.12241842	1.29204068	0.26916883	0.23460357
Nek9	0.13199534	6.73170943	1.2919507	0.26918499	0.23460357
Hes5	-0.4266791	1.57656207	1.29188664	0.2691965	0.23460357
Gm5126	0.33412026	2.50523899	1.29031103	0.26947973	0.2348255
Cass4	0.70765597	0.49765302	1.28997833	0.26953959	0.23485276
Lxn	0.15650369	4.9324771	1.28979007	0.26957347	0.23485739
Tmem110	-0.2485574	2.80599648	1.28953039	0.2696202	0.23487321
Hsd17b12	0.28439227	3.64403364	1.289152	0.26968833	0.23490766
1810014B01I	0.34964726	2.62046531	1.28891395	0.2697312	0.2349201
Mroh7	-1.6605127	-0.4460366	1.2876462	0.26995966	0.23509416
Naga	0.44959214	3.0896614	1.2860705	0.27024396	0.23530124
Dcun1d3	0.184749	4.96451194	1.28601103	0.2702547	0.23530124
Raph1	-0.2445903	7.73997908	1.28580785	0.27029139	0.23530166
Rnf208	0.16458835	4.92523072	1.28565771	0.27031851	0.23530166
Nsf	0.16641981	9.99472431	1.28553279	0.27034108	0.23530166
Evpl	0.57683199	1.2386368	1.28425985	0.27057115	0.23544754
Armc2	-0.4093631	1.77471484	1.28417107	0.27058721	0.23544754
Adck5	-0.5053177	1.01876887	1.28403084	0.27061257	0.23544754
Gas7	0.13688188	9.7921324	1.28397173	0.27062326	0.23544754
Cxcl9	-1.2922908	-0.5124598	1.28340256	0.27072625	0.23551221
Aspn	-1.0933238	-0.6780731	1.28283719	0.2708286	0.23557631

Dnajb14	0.18748184	4.2317679	1.28210651	0.27096095	0.23566583
Taf1d	-0.2110457	3.99055053	1.28195251	0.27098885	0.23566583
Tmem132e	-0.4648346	1.29276568	1.28157161	0.27105789	0.23570093
Cyp2u1	0.50799442	0.87259289	1.28023622	0.27130011	0.23582329
Metrn	0.79689112	-0.6406921	1.28020224	0.27130627	0.23582329
Zbtb4	0.12034695	7.52108843	1.28016668	0.27131273	0.23582329
Thrb	0.16091101	7.02512197	1.27986512	0.27136747	0.23582329
Gm11974	0.57672187	0.35866908	1.27985484	0.27136934	0.23582329
Mrps5	-0.3273249	2.91157501	1.27984697	0.27137076	0.23582329
Cited1	0.51374522	1.57350636	1.27959942	0.27141571	0.23583742
Ifih1	0.18242304	5.32452961	1.27940105	0.27145174	0.23584379
Arhgef16	1.18886317	-0.9134374	1.27844127	0.27162613	0.23597036
Cdk17	-0.1693512	7.87426799	1.27747516	0.27180183	0.23609804
Tnks	-0.185647	5.86862362	1.27730511	0.27183277	0.23609996
Smarcd1	-0.154725	5.84974477	1.27696392	0.27189486	0.23612894
Zfp319	-0.2489828	3.1469779	1.27658925	0.27196307	0.23614307
6030458C11I	-0.2360163	4.92836648	1.2765589	0.27196859	0.23614307
Trnt1	0.1894937	4.77161066	1.27512158	0.27223047	0.23634548
Lcn2	-1.976278	-1.2419215	1.27474283	0.27229953	0.23636214
Fpgt	-0.182678	4.93554178	1.27470096	0.27230717	0.23636214
Tfip11	0.2781493	3.79497696	1.27398369	0.27243803	0.23645075
Gabarapl2	0.17571071	6.7008753	1.27362566	0.27250338	0.23647663
Ndufaf7	-0.169462	4.55722598	1.27327722	0.272567	0.23647663
Cgrrf1	0.19693148	3.86189549	1.27322754	0.27257607	0.23647663
Nol6	-0.1863475	5.48247362	1.27306966	0.2726049	0.23647663
Rala	0.12357721	6.48951825	1.27303241	0.27261171	0.23647663
Il17rd	-0.3836244	2.50258226	1.27228026	0.27274913	0.23657088
Nsmce1	0.43428857	3.55079185	1.27196929	0.27280598	0.23659521
Cbr3	0.31751887	2.12842456	1.27069064	0.27303988	0.23677308
Lmtk3	-0.6040487	1.61875562	1.27045632	0.27308277	0.2367853
Usp40	-0.2235729	4.56375978	1.26924835	0.27330403	0.23695215
P2ry10	1.5047334	-1.3492971	1.2689713	0.27335481	0.23696308
Kcnj13	-0.2526198	6.22314713	1.26886496	0.27337431	0.23696308
Rnf38	-0.1250751	7.97757377	1.26804044	0.27352552	0.23706915
Fbxo44	0.20787954	4.46392844	1.26726541	0.27366776	0.23715375
Elp6	0.41199484	2.98309569	1.26707401	0.2737029	0.23715375
Pdpk1	-0.1181729	7.65899046	1.26703704	0.27370969	0.23715375
Rhoh	0.68817713	-0.0962766	1.26638639	0.2738292	0.23719204
Luc7l3	-0.1494735	8.5707348	1.26636328	0.27383344	0.23719204
Vps16	-0.3048615	4.44293353	1.26632511	0.27384046	0.23719204
1810010H24I	-0.4462801	1.16088715	1.26556352	0.27398044	0.23728829
Lin52	0.1794478	4.53970168	1.26480825	0.27411936	0.23738358
Arhgef5	0.27428311	5.78450739	1.26409866	0.27424995	0.2374632
Zfp389	1.33041888	-1.5357067	1.26398982	0.27426999	0.2374632
D430020J02I	0.77819896	0.56245737	1.2638379	0.27429797	0.2374632

Ppt2	0.45334209	1.23233552	1.26256328	0.27453282	0.23762472
A230077H06	-0.4944806	2.28415411	1.26251147	0.27454237	0.23762472
Mapk7	-0.2866755	2.64550853	1.26217628	0.27460418	0.23764615
1700073E17I	-0.4579675	2.24183211	1.26204392	0.27462859	0.23764615
Slc26a6	0.85173221	-0.1905844	1.26190689	0.27465387	0.23764615
Mrpl15	0.30897772	4.9439857	1.26148573	0.27473157	0.23768836
Gprc5b	-0.2120315	4.83589244	1.26109739	0.27480324	0.23772535
Nlrp1b	-1.6845422	-1.6327494	1.26062341	0.27489076	0.23777224
Asphd2	-0.2786726	2.68261618	1.2604905	0.27491531	0.23777224
Lin7b	-0.2185508	3.68429884	1.26011514	0.27498465	0.23780718
Cd80	-0.537561	1.40087992	1.25991714	0.27502123	0.2378138
Tubgcp2	-0.2053519	3.66953994	1.25956153	0.27508696	0.23784561
Phkg1	-0.4138453	1.630539	1.25940387	0.2751161	0.23784579
Snph	-0.2234528	5.88306559	1.25910891	0.27517064	0.23786792
Tjp1	0.1343029	9.48507631	1.25893426	0.27520294	0.23787082
Zfp207	0.12269519	7.90237065	1.25826456	0.27532684	0.23795289
Ttc4	-0.1850069	3.91169362	1.25789733	0.27539481	0.23798661
Slc48a1	-0.1925945	4.84507852	1.25690257	0.27557905	0.23812079
Pik3ca	-0.1221317	7.01483984	1.2565889	0.27563718	0.23814598
Tnrc6b	-0.1568376	8.72502466	1.25605557	0.27573605	0.23820636
Cks1b	0.44292721	3.42281938	1.25487212	0.27595561	0.23837098
Kctd16	-0.2834977	3.81506031	1.2522886	0.27643573	0.23875189
Podn	0.25310648	5.0364247	1.25218678	0.27645468	0.23875189
Cdon	-0.2694048	5.1561861	1.25192018	0.27650429	0.23876409
Uchl4	-0.8358801	-0.6099704	1.25179869	0.2765269	0.23876409
Coq9	0.21242198	4.37311304	1.25147976	0.27658628	0.23877901
2210416O15	0.85210692	-0.6375409	1.25139375	0.27660229	0.23877901
BC048403	0.21037121	4.04730673	1.25088309	0.2766974	0.23883603
Slc2a1	-0.2509598	4.64273172	1.24955664	0.27694465	0.23902435
9130019O22	-0.3465152	2.12545968	1.24908595	0.27703245	0.23904647
A4galt	0.30921923	2.77150386	1.24892649	0.27706221	0.23904647
Nudt2	0.27982486	3.19216054	1.24891626	0.27706412	0.23904647
Plbd2	-0.1720868	5.28181595	1.24867345	0.27710943	0.23904647
Gm867	-1.4923992	-1.991753	1.2486399	0.2771157	0.23904647
Mbd4	-0.2661749	4.40401113	1.24835039	0.27716974	0.239068
Nrbp1	0.15953966	5.94088008	1.2477805	0.27727618	0.2391347
Pdk1	0.14564518	6.19472714	1.24682451	0.27745484	0.23925368
Fam131a	0.18137854	5.2288787	1.24673086	0.27747235	0.23925368
Bcl6b	-0.63878	0.05208606	1.24585246	0.27763666	0.23935064
Krt222	-0.1573725	6.63809537	1.24581837	0.27764304	0.23935064
Bves	-0.7999208	-0.6052803	1.24539508	0.27772226	0.23939383
Ccr7	1.53086347	-1.9770979	1.24412124	0.27796087	0.23957438
Helz	-0.2163812	6.72444191	1.24307198	0.27815761	0.23971399
Cutc	0.2555707	3.81524854	1.24294633	0.27818118	0.23971399
Gm16880	-1.2320984	-0.2276483	1.24264439	0.27823784	0.23971804

Six1	0.4107665	5.37716334	1.2426104	0.27824422	0.23971804
Gpr6	-1.3580467	-1.4290837	1.24227186	0.27830776	0.23974766
Olfr691	-1.4871701	-2.0704792	1.24181564	0.27839343	0.23979632
Nupr1	0.33502452	6.67529031	1.24109273	0.27852924	0.23988816
BC021891	-0.4721915	1.57565952	1.2408068	0.27858298	0.23990931
Fam149a	0.14994841	5.22175722	1.24032014	0.27867448	0.23996296
D730005E14I	-1.6826246	-1.8001265	1.23973852	0.27878389	0.24002408
4933411K20I	0.14971217	6.72775489	1.23951602	0.27882576	0.24002408
Narfl	0.31408416	2.64718879	1.23947716	0.27883307	0.24002408
Lgmn	0.24544416	4.12100921	1.23831308	0.27905227	0.24018762
Bach2	-0.2369288	4.7164935	1.23809858	0.27909268	0.24019725
Cyb561	0.14970473	4.44562997	1.23788389	0.27913314	0.24020537
Atp1a3	0.20601883	9.48253758	1.23773835	0.27916057	0.24020537
Trim26	0.19234244	4.99294878	1.237517	0.2792023	0.24021613
Gm20300	-0.147698	7.24327955	1.23735824	0.27923223	0.24021674
Rab2b	0.14641696	5.9805312	1.23680501	0.27933658	0.24028135
Pmfbp1	-1.183356	-1.3142859	1.2365057	0.27939305	0.24030478
Zfp579	0.34010296	1.47293401	1.23632762	0.27942665	0.24030854
Chic1	-0.1438896	6.36591201	1.23545875	0.27959071	0.24041669
Cttn	0.15208121	5.83338286	1.23535173	0.27961092	0.24041669
Nfe2l3	-0.3255813	2.48759013	1.23480876	0.27971351	0.24047975
Kdm1b	0.21891339	4.2157133	1.23402026	0.27986258	0.24058274
Gabpb1	0.16151351	5.10050839	1.23236485	0.2801759	0.24081168
Dnaaf3	1.02248226	-1.1739159	1.2323036	0.2801875	0.24081168
Rngtt	-0.1595331	5.27968148	1.23145468	0.28034837	0.24092475
C330013E15I	0.60509997	0.41328429	1.23071616	0.28048841	0.2410199
Fam207a	0.28721102	3.37033488	1.22949249	0.28072065	0.24117218
Fam122a	0.20265635	4.73155212	1.22947322	0.28072431	0.24117218
Chd1l	0.26364117	3.10993716	1.22744927	0.28110902	0.24147744
Krit1	-0.1879974	5.84090534	1.22668123	0.28125519	0.24157775
Sgip1	-0.1830832	8.10724872	1.2262253	0.281342	0.24162707
BC049635	-0.3612792	3.11311476	1.2254898	0.28148213	0.2416632
Polr2a	-0.1430773	7.30718596	1.22538949	0.28150125	0.2416632
Trmt6	0.18150617	4.64719553	1.22538799	0.28150154	0.2416632
Emid1	-0.5610729	0.7803174	1.22538723	0.28150168	0.2416632
BC065397	-0.5963277	0.92368029	1.22509199	0.28155796	0.24168627
Rint1	-0.2074082	4.06276788	1.22488795	0.28159687	0.24169443
Pcdhb8	-0.4754753	1.09888164	1.22380686	0.28180312	0.2418462
Mta3	-0.1846424	5.56696149	1.22333473	0.28189326	0.24188758
Sos1	-0.1409263	7.58363665	1.223246	0.2819102	0.24188758
Agtrap	0.35376443	4.03293785	1.22253729	0.28204559	0.24197849
Klf12	0.1260941	7.35340552	1.2217594	0.28219429	0.2420808
Nkapl	-0.6635747	0.25498264	1.22053818	0.28242796	0.24225596
Hip1r	-0.3158365	3.19870087	1.21992202	0.28254594	0.24233187
C030034L19I	-1.2307297	-1.2749103	1.21961482	0.2826048	0.24235706

Eno4	-0.4322818	1.34689556	1.21939293	0.28264732	0.24236823
A530032D15	0.53848657	0.8682512	1.21872529	0.2827753	0.24243419
Lyl1	-0.7836364	-0.0769813	1.21860063	0.2827992	0.24243419
Psmc4	0.1954847	4.92455703	1.21853014	0.28281272	0.24243419
Dlg3	-0.1945565	6.55474967	1.2180038	0.28291369	0.24247548
Sgms1	-0.1425831	5.94055763	1.2179715	0.28291989	0.24247548
Wdr43	0.17456534	4.90443713	1.21699754	0.28310686	0.24261043
B830017H08	0.72952892	-0.2125688	1.21582893	0.28333142	0.24277755
Prmt3	0.20047019	4.30951432	1.21524451	0.28344381	0.24284853
Nkrf	-0.2156124	5.84414015	1.21480095	0.28352915	0.24289633
Hapln1	-0.2833391	3.8075648	1.21449991	0.28358709	0.24292064
Unc93a	1.21852156	-0.8055965	1.21294448	0.2838867	0.24310743
Itprip	-0.9392881	-0.6277225	1.21287057	0.28390095	0.24310743
Adamts2	-0.289419	3.97568395	1.21284777	0.28390535	0.24310743
Nox4	0.4762641	1.22891877	1.21275386	0.28392345	0.24310743
Arhgap23	-0.2019209	5.68416057	1.21255146	0.28396248	0.24311551
Def8	0.18636508	4.42867502	1.21145469	0.28417407	0.2432546
Gm13889	0.36930202	1.54006208	1.21121551	0.28422024	0.2432546
Zfp354b	-0.3878076	2.41234248	1.21111771	0.28423913	0.2432546
Spp2	-0.9917779	0.45542696	1.21088854	0.28428338	0.2432546
Slc30a10	-0.2102803	5.06742658	1.21084301	0.28429217	0.2432546
Mamdc4	0.89590671	-0.0726255	1.21067248	0.28432511	0.2432546
Ccdc184	0.23185205	3.20706414	1.21063629	0.2843321	0.2432546
Cebpg	0.14138667	6.39864754	1.21038403	0.28438083	0.24327097
5730455P16I	0.13099421	7.14297718	1.21008013	0.28443956	0.2432767
Esam	-0.5975655	1.35258999	1.21001837	0.2844515	0.2432767
Msi1	0.41690659	1.91976697	1.20988986	0.28447634	0.2432767
Slc35c1	-0.2488174	3.33152428	1.20893096	0.28466177	0.24340995
Tmem106a	-0.6091369	1.6543206	1.20855572	0.28473438	0.24344671
Twist2	0.66203335	0.8075385	1.20834398	0.28477536	0.24345642
Tmem50b	0.13935708	5.85195413	1.20629391	0.28517256	0.24374991
4930545L23I	-0.8713946	0.66038367	1.206266	0.28517797	0.24374991
E130310I04R	-1.914038	-1.5761338	1.20545949	0.28533444	0.24383599
Klhdc4	-0.2532806	3.05653402	1.20544105	0.28533802	0.24383599
Zmynd19	-0.4703595	1.55240992	1.20500542	0.28542258	0.2438829
Lekr1	-0.2965587	2.74701292	1.20480961	0.2854606	0.24389003
Alk	-0.6566109	0.73476148	1.20458903	0.28550344	0.24390128
Snrpc	0.30251958	3.22601681	1.20335253	0.28574374	0.2440812
Als2cl	-0.5217878	0.5495359	1.20297021	0.2858181	0.24411934
Sag	-0.8212118	-0.7599543	1.20258234	0.28589356	0.24415087
Etnk1	-0.120933	8.62575492	1.20247513	0.28591442	0.24415087
Gbgt1	0.65298236	0.52653388	1.20208636	0.28599009	0.24419012
Atg13	0.12924226	5.92728263	1.20189735	0.28602689	0.24419617
Sec14l1	-0.1482621	6.38234486	1.20141762	0.28612032	0.24425056
Ube2f	0.19138423	4.74670963	1.20023913	0.28635	0.24442125

Homer1	0.16362461	8.40981841	1.19965391	0.28646415	0.24449329
Dll4	-1.2525824	-1.5188964	1.19939875	0.28651394	0.24451039
Mtmr10	-0.1813358	4.27423478	1.19892029	0.28660733	0.24453947
Prpf40a	0.1545616	6.39276281	1.19891933	0.28660752	0.24453947
Foxd2	0.42311003	2.72874182	1.19757899	0.28686936	0.24473284
2900009J06F	-1.1308965	-0.6726362	1.19745448	0.2868937	0.24473284
Rhobtb3	-0.2115206	4.45855426	1.19707948	0.28696703	0.24474515
Pisd-ps2	-0.5543959	1.82561188	1.19694146	0.28699402	0.24474515
Rpl28	0.27818577	5.33779006	1.19692384	0.28699747	0.24474515
Lrrc29	-1.1260898	-0.596089	1.19627683	0.28712406	0.24481788
Ptafr	-1.168241	-1.0445953	1.19609478	0.28715969	0.24481788
Ndst4	0.34407434	3.24257886	1.1960313	0.28717211	0.24481788
Tmem161b	-0.3396798	3.57042843	1.19582135	0.28721322	0.24482753
Tfap2b	0.22582683	6.8869382	1.1954637	0.28728325	0.24486183
E2f7	1.2479003	-1.079622	1.19507596	0.28735921	0.24489449
Abhd14a	-0.3435692	2.80306502	1.19496389	0.28738116	0.24489449
Lamb2	0.33953515	4.32199266	1.19336593	0.2876945	0.24513609
Hmgxb3	-0.2469936	3.94378601	1.19281753	0.28780214	0.24520239
Rsph3b	0.20350808	4.40296319	1.19248394	0.28786764	0.24523277
Hsd3b1	-2.7758989	-1.4652277	1.19176698	0.28800849	0.24532733
Fyco1	-0.1583257	6.72397002	1.19138826	0.28808293	0.24536531
Trmt1	-0.3291706	2.8026084	1.19109965	0.28813968	0.24538622
Nfu1	0.20010299	4.48020055	1.19095974	0.28816719	0.24538622
Stac2	0.1615226	5.87709308	1.18994965	0.28836593	0.24553001
Htr5b	-1.0209025	-0.3098906	1.18973703	0.28840778	0.24554022
Bhmt2	-1.5612371	-0.5181588	1.18891324	0.28857003	0.2456529
Mettl25	-0.3811991	2.64327942	1.18842415	0.28866642	0.24567558
Taf1a	-0.2772288	3.23536216	1.18818426	0.28871371	0.24567558
Fn3k	0.29454752	3.23187119	1.1881276	0.28872489	0.24567558
Mtmr12	-0.1239674	6.44446954	1.18802998	0.28874413	0.24567558
Myo1c	0.24551237	5.19518825	1.18801987	0.28874613	0.24567558
Sun2	-0.2104621	6.58459989	1.18722369	0.28890319	0.24578377
Chd1	-0.1187648	6.5805177	1.18634535	0.28907659	0.24590583
01-Sep	0.39750302	2.38936797	1.18617671	0.28910989	0.24590872
Glra3	-0.5002956	1.20109565	1.18579484	0.28918533	0.24594743
Usp1	0.16781603	5.3274602	1.18525173	0.28929268	0.24601327
Pomt1	-0.4883461	1.47726047	1.18503554	0.28933542	0.24602416
Dlx1	-0.2770798	3.88112312	1.18448426	0.28944446	0.24609142
Golga7	0.15534459	7.26421945	1.18422849	0.28949506	0.24610898
Tpbg	0.31930286	3.35700631	1.18387277	0.28956546	0.24614337
Prss48	-1.1173415	-0.1717178	1.18317201	0.28970422	0.24623264
4921531C22I	0.34154651	2.74290352	1.18303989	0.28973039	0.24623264
Suv39h2	-0.2886253	3.0955712	1.18237259	0.28986262	0.24631955
Pias2	-0.1375672	6.01353074	1.18213998	0.28990873	0.24633326
Bcl9l	-0.1940152	5.44706175	1.18182956	0.28997028	0.24636009

Apbb3	-0.3797583	2.08151078	1.18161568	0.2900127	0.24636743
Calcoco1	0.12695	7.47821036	1.18142728	0.29005008	0.24636743
Mtfr1l	0.18984552	5.6179435	1.18133269	0.29006884	0.24636743
Bmp5	0.325555	5.86013317	1.18076312	0.29018188	0.24641218
Med11	0.36290939	2.92929786	1.1807031	0.29019379	0.24641218
Tmem45a	-0.4099055	2.20741099	1.18061405	0.29021147	0.24641218
Rnmt	-0.1381682	5.92020427	1.18034477	0.29026494	0.24641573
Tmod2	0.12694221	9.94559003	1.18021217	0.29029127	0.24641573
Bub1b	-0.7325913	0.47634977	1.18014009	0.29030559	0.24641573
Rsl1	0.32420067	2.89096568	1.17972466	0.29038812	0.24646032
Alyref2	0.33206969	1.75103512	1.17928384	0.29047573	0.24650923
P2rx1	-1.6586693	-2.3316039	1.17900225	0.29053171	0.24653128
Mapk11	-0.2604293	3.69421599	1.17810394	0.2907104	0.24665744
Ddx21	-0.1576774	4.80739924	1.17765827	0.2907991	0.24670723
Gm3604	-0.2624027	2.73309904	1.17618528	0.29109254	0.24690176
2310034G01	0.74864541	0.91151971	1.17611832	0.29110589	0.24690176
Cic	-0.1484173	6.64039487	1.17605498	0.29111852	0.24690176
Stard3nl	-0.1894215	4.46531517	1.1755215	0.29122491	0.24696651
Ccdc116	-0.6662156	0.07424863	1.17515068	0.29129889	0.24700376
Zmpste24	0.20833163	5.50399362	1.17452212	0.29142434	0.24707593
Msx1	0.43191589	2.36231787	1.17442307	0.29144412	0.24707593
Gm711	-1.5062385	-1.6191454	1.17369297	0.29158995	0.24711755
Rhbdf2	-1.0304241	-1.7055015	1.17362754	0.29160302	0.24711755
Chn1	0.12181844	9.71703491	1.17361723	0.29160508	0.24711755
Mxd1	0.16546117	6.02346775	1.17357521	0.29161348	0.24711755
Yipf5	0.13742147	5.41711625	1.17316642	0.29169518	0.24714836
Pdxp	0.24854745	4.93016259	1.17309241	0.29170998	0.24714836
Emx2os	-0.3038277	2.96091169	1.17277263	0.29177392	0.24716379
Slc30a1	-0.1477429	5.11534121	1.17270056	0.29178833	0.24716379
Kdm6b	-0.2064134	5.61535267	1.17166027	0.29199648	0.24730557
Pgrmc2	0.15526423	5.49575319	1.1715633	0.29201589	0.24730557
Mcrs1	0.26231645	3.23936336	1.16997524	0.29233405	0.24754951
Gng4	0.16458343	5.47719063	1.16981071	0.29236704	0.24755194
Rps4l	0.28301247	1.95132353	1.16959044	0.29241121	0.24756384
Tph2	1.44797164	-1.2085196	1.16895055	0.29253959	0.24762867
Epc2	-0.1194118	7.43690002	1.16890842	0.29254805	0.24762867
Htr3a	-0.6033329	0.66237424	1.16831981	0.29266621	0.24770318
Zc2hc1c	-0.4286576	2.21582011	1.16655756	0.29302035	0.24797738
Eif1ad	-0.2182964	4.17200908	1.16553332	0.29322645	0.24812625
Vrk1	-0.218935	3.86179314	1.16492346	0.29334926	0.24819861
2210018M11	-0.1732783	6.75589325	1.16480878	0.29337237	0.24819861
AI413582	-0.2260832	3.26757408	1.1645455	0.29342541	0.2482001
Isoc2b	0.52600297	0.67709782	1.16445469	0.29344371	0.2482001
Zfp27	-0.2308086	4.00866786	1.16424919	0.29348513	0.2482001
Ephb4	-0.4094742	3.24174514	1.16408348	0.29351853	0.2482001

Wdr5	0.35299306	3.23638315	1.16405078	0.29352512	0.2482001
Ssr2	0.38157593	3.49506716	1.16384451	0.29356671	0.24820973
Fam154b	0.494704	0.89675354	1.16345473	0.29364531	0.24825066
Tmem125	-0.5731135	0.65984188	1.16314025	0.29370875	0.24827875
Asic4	-0.6649349	-0.0935885	1.16298408	0.29374027	0.24827986
Kiz	0.21098161	4.925296	1.16261842	0.29381407	0.24829384
Xylt2	0.44018907	2.16298136	1.16260273	0.29381723	0.24829384
Lrrc18	-0.3021139	2.93618998	1.16207425	0.29392394	0.24835848
Stk40	0.16217898	4.15938639	1.161428	0.2940545	0.24844326
4833427F10I	0.79809781	-0.7880059	1.16103387	0.29413416	0.24848502
Rfng	-0.2446629	3.51082264	1.16065713	0.29421034	0.24852383
Zfp39	-0.163487	5.36008907	1.16044984	0.29425226	0.2485337
Slain1	-0.2321928	4.91292818	1.16006063	0.294331	0.24857466
Slmap	0.13140285	8.96692926	1.15963796	0.29441654	0.24862135
1700017G19	0.66240894	0.70194423	1.15937795	0.29446918	0.24864026
Eif4g3	-0.1592862	8.94854397	1.15914401	0.29451655	0.24865471
Exo5	0.2765688	3.74028073	1.15869231	0.29460804	0.24870401
Abcb7	-0.1860263	5.77587624	1.15855698	0.29463546	0.24870401
Calr	0.14959428	6.40600111	1.15833415	0.29468061	0.24871659
Igfbp5	0.24256821	8.64917915	1.15795217	0.29475804	0.24875639
Eqtn	-0.8674444	0.10408834	1.15752863	0.29484393	0.24880333
A930024E05I	0.61936158	0.66919933	1.15665432	0.29502132	0.24892513
Efcab10	-0.9133061	-0.4047209	1.15642547	0.29506778	0.24892513
Nek4	-0.2034493	4.4182294	1.15636954	0.29507914	0.24892513
Plin3	0.23999984	3.68225391	1.15526189	0.29530414	0.24905857
Dapk3	0.40865975	1.76013072	1.1551625	0.29532434	0.24905857
Mterf1b	0.56153667	0.95309795	1.15514343	0.29532822	0.24905857
Tab2	0.12696525	8.33770501	1.15410424	0.29553955	0.24921122
Fgfr2	0.2109175	6.83047451	1.15380624	0.29560019	0.24923679
Ebi3	1.3154772	-1.0286193	1.15301648	0.29576098	0.24934293
Kras	-0.1602391	8.1423861	1.15288997	0.29578675	0.24934293
0610010B08I	0.24276682	4.54974306	1.15158418	0.29605289	0.24954168
Crebzf	-0.1903916	5.31094743	1.15106083	0.29615965	0.24958467
Bcl2l15	-1.3816385	-0.0955266	1.15103644	0.29616463	0.24958467
Smurf2	0.13329841	7.06923446	1.1505502	0.29626386	0.24964271
Ipo11	-0.1651719	6.18942871	1.15019711	0.29633596	0.24967786
1700015F17I	-1.3059757	-0.8664059	1.14863147	0.2966559	0.2499218
Cd200	0.17704499	6.86633435	1.14845913	0.29669114	0.24992588
Arhgap8	-1.0570642	-0.3610325	1.14805218	0.29677439	0.24996795
Frrs1	-0.6305124	1.18737088	1.14791767	0.29680192	0.24996795
Usp8	0.11478431	7.66784425	1.14751589	0.29688415	0.2500116
Pemt	0.97927128	-0.981126	1.14689223	0.29701186	0.25005098
Ankrd34b	0.25137576	4.5032415	1.14686739	0.29701695	0.25005098
Wars2	-0.2958074	3.67473032	1.14684181	0.29702219	0.25005098
Mbnl1	0.16289681	9.06154523	1.1459997	0.29719476	0.25017063

C330027C09I	-0.4058037	1.86514799	1.14554107	0.2972888	0.25022417
Spata3	1.00956053	-0.8067021	1.14529104	0.29734008	0.25024171
Ccdc85a	-0.173114	5.81768813	1.14445406	0.29751185	0.25033623
Rin3	0.28372387	4.36278488	1.14444694	0.29751331	0.25033623
Unc93b1	-0.4647482	1.36127615	1.14385357	0.29763517	0.25036649
Rab3ip	0.16152634	5.14672072	1.14355644	0.29769621	0.25036649
Galns	0.53094446	0.66042022	1.14346247	0.29771552	0.25036649
Slc15a4	-0.3242563	2.35216293	1.14343382	0.2977214	0.25036649
Nkd1	0.23323467	4.85827505	1.14343195	0.29772179	0.25036649
Tmem86b	-0.5679791	0.33394382	1.14338205	0.29773204	0.25036649
Morc4	0.25588971	2.89235091	1.14265311	0.29788189	0.25046687
Smek2	-0.1280951	6.69850331	1.14245601	0.29792243	0.25047533
Gpc6	-0.2204423	7.3480851	1.14194418	0.29802773	0.25053824
Parvg	0.46617772	1.48542068	1.14167933	0.29808224	0.25055843
Gpr107	-0.2239728	3.80767005	1.14109246	0.29820307	0.25059316
Adh7	0.54390404	1.41363985	1.14097434	0.2982274	0.25059316
Rimbp3	-0.6776838	0.85861884	1.14090202	0.29824229	0.25059316
Arpc1a	-0.164299	6.16045961	1.14083516	0.29825607	0.25059316
Nyx	-0.9508314	-0.5550092	1.140684	0.29828721	0.25059316
Kazn	-0.161998	6.12045193	1.14051638	0.29832174	0.25059316
Smarcc1	0.10966542	7.39862034	1.14044242	0.29833698	0.25059316
Snx21	-0.423667	1.94297433	1.14002169	0.2984237	0.25064039
Art3	-0.9855878	-0.4051055	1.13969307	0.29849146	0.25067168
Map2k4	-0.1465999	7.68792526	1.13941068	0.2985497	0.25067767
Bmp6	0.30148731	6.6331763	1.1393627	0.29855959	0.25067767
Mbd6	-0.3158418	3.97290676	1.1390933	0.29861517	0.25069873
Abcc4	0.25408326	5.12241116	1.13831137	0.29877657	0.25080861
Poc5	0.16925705	3.97921674	1.13791442	0.29885855	0.25085155
3830406C13I	0.1613768	6.13628386	1.13776814	0.29888876	0.25085155
Ddx39	0.34390162	2.37267461	1.13757016	0.29892967	0.25086026
Ccdc159	-0.8158552	0.35808225	1.13661896	0.2991263	0.2509612
Chordc1	0.1303612	6.72134123	1.13660896	0.29912837	0.2509612
Fam45a	0.16058015	4.9588035	1.13654523	0.29914155	0.2509612
Pagr1a	-0.2245381	3.04353143	1.13614004	0.29922537	0.2510059
Csad	0.18804011	4.22252035	1.13555011	0.29934746	0.25108269
Plekhb2	0.12620336	6.30581925	1.13486069	0.29949022	0.25116324
Gys1	-0.4085399	1.99857252	1.13479122	0.29950461	0.25116324
AA987161	-0.1562387	5.33446726	1.13396076	0.29967672	0.25128193
Pogk	-0.119306	6.21945239	1.13340684	0.29979159	0.25135261
Klhl38	0.78068784	0.04729006	1.1320951	0.30006386	0.25155522
Cmc2	0.21169725	3.25622659	1.13186575	0.3001115	0.2515695
Herpud2	0.1592965	6.21592798	1.13141874	0.30020438	0.25159636
Rnf168	0.14167285	6.53820425	1.13141686	0.30020477	0.25159636
Agr2	0.66741953	-0.1481052	1.13123945	0.30024164	0.25160161
Aurka	-0.6274209	-0.188556	1.130943	0.30030327	0.25160384

Sfn	-1.13383	-1.1509467	1.1308609	0.30032034	0.25160384
Piezo2	-0.3232973	2.92854699	1.13078493	0.30033613	0.25160384
Tmem120a	0.81087539	0.1265171	1.13008918	0.30048086	0.25169943
Zfp551	-0.4381034	2.48571282	1.12967032	0.30056803	0.25174679
Spcs1	0.19764062	5.36136485	1.1295133	0.30060072	0.25174851
Serpinb8	0.31004307	3.40522974	1.12892433	0.30072337	0.25180642
Zkscan8	-0.1598223	6.10581544	1.12888708	0.30073113	0.25180642
Cd79b	-1.366291	-1.9913514	1.12780847	0.30095594	0.25196898
Elavl3	0.16633899	7.19794494	1.12745192	0.3010303	0.25198409
Ms4a6d	1.46569199	-1.2205621	1.1272813	0.30106589	0.25198409
Lsg1	0.18334558	4.02144153	1.12728096	0.30106597	0.25198409
Psph	0.31513487	3.65078526	1.12644242	0.30124098	0.2521049
4732471J01F	-0.3901139	1.65435426	1.12608811	0.30131497	0.25214115
Rwdd4a	0.16280679	5.61345418	1.12582062	0.30137084	0.25216223
Olfm2	0.18550309	5.18180106	1.12556051	0.30142519	0.25218203
Fxr1	0.13037614	6.88681768	1.12443322	0.30166089	0.25234965
Frmd6	-0.1720515	5.61707454	1.12430868	0.30168694	0.25234965
Zbtb7b	-0.3355132	2.97171509	1.12389708	0.30177307	0.252396
Sf3b1	0.13231496	8.28905656	1.1234093	0.30187518	0.25245417
Ccar1	0.13996507	8.06170419	1.12327144	0.30190405	0.25245417
Ndc80	-0.9470717	-0.3951648	1.12276364	0.30201042	0.25251743
Erich3	-0.3679744	3.68636656	1.1223544	0.30209618	0.25256344
Pla2g7	-0.3752205	3.67048988	1.12201377	0.30216759	0.25259745
Gm8300	0.79888123	-0.6615759	1.12170097	0.30223318	0.25262659
Frmd4a	-0.2156809	5.54003423	1.12129431	0.30231849	0.2526509
Zfp672	0.17763908	4.76502454	1.12126926	0.30232374	0.2526509
Psmb9	-0.4238656	3.32181791	1.12079977	0.30242227	0.252699
Col4a1	0.23736765	3.65295433	1.12070196	0.3024428	0.252699
Icam5	-0.2789069	2.88514723	1.11970638	0.3026519	0.25282317
Srsf9	0.21778754	4.93587337	1.11969859	0.30265353	0.25282317
Osbpl5	0.20516679	3.92473795	1.1195551	0.30268369	0.25282317
Timm10	0.30224266	3.84783903	1.11887588	0.30282648	0.25291673
Dpep2	-1.591882	-1.7038124	1.11871432	0.30286045	0.25291941
Bcl7c	0.48869887	1.35263335	1.11772929	0.30306772	0.25306679
E230016K23I	1.03893263	-0.708949	1.11703165	0.30321463	0.25316374
4930581F22I	-0.6546099	0.41831874	1.11658633	0.30330846	0.25321636
Mylip	0.2325751	4.11546844	1.11571193	0.30349281	0.25334453
Rpl35a	0.22759834	5.46905397	1.11538447	0.30356189	0.25337646
Zfp131	-0.1756862	5.28940919	1.1147748	0.30369056	0.25344239
G6pdx	0.19075086	5.75914343	1.11471797	0.30370256	0.25344239
Tmem150b	-1.3579109	-0.9969211	1.11455657	0.30373663	0.2534451
2900008C10I	-0.7754228	0.21761927	1.11429531	0.3037918	0.2534654
Kif14	1.35837391	-1.3150309	1.11361924	0.30393464	0.25354491
Ctse	-0.8718093	-0.3797391	1.11352313	0.30395495	0.25354491
Cask	-0.1280024	7.6195269	1.11340628	0.30397965	0.25354491

Stat4	-0.3406626	1.82879885	1.11310503	0.30404333	0.2535723
E130114P18I	-0.4939531	0.83679261	1.11170751	0.30433902	0.25379315
Cfdp1	0.19849701	6.92855852	1.11148206	0.30438676	0.25379977
Gm10474	-1.5324051	-1.5017368	1.11137837	0.30440872	0.25379977
Luzp2	0.20028053	6.55836174	1.11113772	0.30445969	0.25379985
Dot1l	-0.2868974	3.89011164	1.11108634	0.30447057	0.25379985
Elac1	0.16005819	5.18459079	1.1104103	0.30461383	0.25389352
Edem1	-0.1383413	4.94744188	1.10948724	0.30480959	0.25401818
Ubash3a	1.37294985	-1.2589354	1.10916745	0.30487745	0.25401818
Shisa4	0.22704287	4.60312327	1.10912239	0.30488701	0.25401818
Mysm1	-0.1585805	6.25552941	1.10912235	0.30488702	0.25401818
Trmt12	0.24880067	3.27438155	1.10847131	0.30502524	0.25408384
Hsd17b7	-0.1546988	5.00823565	1.10845992	0.30502766	0.25408384
Cd36	-0.8628437	-0.390192	1.10830114	0.30506138	0.25408618
Pdlim2	0.31428707	3.29156799	1.10790117	0.30514635	0.2541312
Fbxl4	-0.2016125	3.57468846	1.1068658	0.30536646	0.25427792
Syt13	0.15713724	6.10051852	1.10676338	0.30538824	0.25427792
Thap7	0.31323033	2.81002998	1.10663602	0.30541534	0.25427792
Rplp2	0.22519584	5.9945226	1.10499678	0.30576434	0.25449556
Hat1	0.16826417	5.05281116	1.1049576	0.30577269	0.25449556
9130008F23I	0.84433219	-0.5012529	1.10489051	0.30578698	0.25449556
Efcab7	0.34917533	2.55634321	1.104792	0.30580798	0.25449556
Uimc1	-0.1859033	4.62464259	1.10456167	0.30585707	0.25449556
Pcdhgb7	-0.3195904	2.21993231	1.10453603	0.30586253	0.25449556
Rbfox1	-0.1480777	9.25183504	1.10415174	0.30594446	0.25453796
Arcn1	0.10353167	7.30276195	1.10364346	0.30605288	0.25460239
Cenpm	1.32761307	-1.5130958	1.10323706	0.3061396	0.25463398
Itfg2	-0.2267147	3.37979046	1.10317515	0.30615281	0.25463398
Mx1	-0.9569434	0.01692365	1.10275807	0.30624185	0.2546436
D330050I16F	-0.6452588	-0.4734021	1.10266916	0.30626084	0.2546436
Nlk	-0.1497294	8.55890243	1.10261495	0.30627241	0.2546436
Tirap	0.26793339	4.4284544	1.10249579	0.30629786	0.2546436
Spire1	-0.1783462	7.44840204	1.10239543	0.3063193	0.2546436
Best1	-0.410498	1.46628805	1.10153432	0.3065033	0.2547708
Arg2	-0.349075	2.92564472	1.10132537	0.30654798	0.25478217
Dtwd1	0.41622776	2.03221828	1.1008339	0.30665309	0.25483993
Insig1	0.129603	6.28332118	1.10071049	0.30667949	0.25483993
Gm13102	-0.9869949	-1.4191695	1.1003945	0.3067471	0.25487035
Zbtb22	0.25015058	3.3208382	1.10010433	0.30680921	0.25487781
Syne4	-1.1304144	-1.3494945	1.10004698	0.30682148	0.25487781
9030624G23	-0.2303251	3.50587459	1.09991793	0.30684911	0.25487781
Frmpd3	-0.5627737	1.33049436	1.0987419	0.30710104	0.25506129
Il12rb2	-0.4091578	2.58358439	1.09846085	0.30716129	0.25508556
Hdgfrp2	0.19727951	5.05959439	1.09761584	0.30734254	0.25515082
Dkkl1	0.36257216	2.23243727	1.0975677	0.30735287	0.25515082

Atl3	0.14356886	7.4225482	1.09753322	0.30736027	0.25515082
Clpx	0.139916	5.3907089	1.09751555	0.30736406	0.25515082
Cacng3	0.1378086	5.64058481	1.09709145	0.30745508	0.25518989
Clstn2	-0.1968724	6.26457341	1.097007	0.30747322	0.25518989
Mesdc1	0.17577891	4.04770127	1.09639299	0.30760508	0.25527355
Zbtb12	-0.6100872	-0.2350096	1.09445915	0.3080209	0.25559282
Mark1	0.13715493	5.97795323	1.09399117	0.30812164	0.2556506
2410127L17F	0.22113741	4.94433774	1.09370301	0.30818369	0.25567628
Rpl39	0.25407826	7.34060567	1.09311736	0.30830986	0.25575514
Pgm5	0.27146867	6.55807452	1.0918308	0.30858728	0.25595943
Paqr4	-0.2429871	3.91087184	1.09166669	0.30862269	0.25596298
Chst10	-0.2025473	3.87552992	1.0909516	0.30877706	0.25606516
Mrps18a	0.30415892	3.38640429	1.09051907	0.30887048	0.25611679
Rem1	-1.1991669	-0.5359513	1.09018018	0.3089437	0.25615167
0610031J06F	0.29663308	4.49173626	1.08864653	0.30927537	0.25639767
Ccl3	1.73484065	-2.0153871	1.08851984	0.30930279	0.25639767
Siah1b	-0.4094637	1.8626382	1.08826269	0.30935845	0.25641795
Hrsp12	0.21334082	4.29304503	1.08788184	0.30944092	0.25645669
Nos3	-0.4962802	0.88492449	1.0877587	0.30946759	0.25645669
Hmgxb4	0.15149437	5.2954056	1.0873009	0.30956678	0.25651302
10-Sep	0.29127589	2.8240973	1.08663548	0.30971102	0.25660667
Usp20	-0.2263253	3.79233137	1.08645948	0.30974918	0.25661242
Smg6	-0.1483264	5.6233263	1.085975	0.30985428	0.25667361
Nek1	-0.1825252	6.39445689	1.08581295	0.30988944	0.25667687
Mettl22	-0.3416414	2.59116622	1.08465992	0.3101398	0.25685835
3110056K07I	0.26769009	2.46016357	1.08409199	0.31026321	0.25692554
Tmem95	-1.3696151	-2.4731922	1.08399482	0.31028433	0.25692554
Cyr61	0.57043008	2.43826021	1.08378263	0.31033046	0.25692554
Itgbl1	0.21912108	6.1219165	1.08359751	0.31037072	0.25692554
Acot6	0.28992102	2.70793181	1.08350999	0.31038975	0.25692554
Nfxl1	-0.2434055	2.91886483	1.08342389	0.31040848	0.25692554
Dclre1b	0.21506342	3.22638578	1.08302979	0.31049421	0.25697062
Trpv4	-0.7530036	-0.4879297	1.0827399	0.3105573	0.25699079
Rab28	0.14599875	5.36756956	1.08263051	0.31058111	0.25699079
Mef2c	-0.1676578	10.0758779	1.08125963	0.31087971	0.25718996
3110009E18I	0.53978661	1.10227185	1.08123809	0.3108844	0.25718996
Lig3	0.18581738	4.99211735	1.08036108	0.31107564	0.25732226
Hmgcll1	-0.2295158	3.35418801	1.07987742	0.31118117	0.25738366
Tdp1	0.30693919	2.43650373	1.07898333	0.31137639	0.25751921
Srsf1	-0.1161038	7.49633913	1.07761179	0.31167618	0.25774121
Mns1	0.31236918	2.41699082	1.07708501	0.31179142	0.25781058
Eif3d	-0.1718486	4.54766849	1.07668667	0.31187861	0.25785673
Radil	-0.3987923	2.13082272	1.07622018	0.31198076	0.25791524
Etv1	0.16052511	7.73736449	1.07579686	0.31207349	0.25796595
Zc3hc1	0.31108015	2.28311213	1.07525948	0.31219126	0.25803273

Casp12	0.33677987	4.19056482	1.07514179	0.31221706	0.25803273
Tm7sf2	-0.568834	1.14682049	1.07486901	0.31227688	0.25805622
Rasal3	0.9403099	-0.2015139	1.07352077	0.31257274	0.25825803
Kazald1	0.44464895	1.58188185	1.07346975	0.31258394	0.25825803
Glb1l2	-0.9147271	-0.4162008	1.07295688	0.3126966	0.25830137
Avil	-1.2914002	-1.4523535	1.07294477	0.31269926	0.25830137
Crhbp	0.39845886	3.05964508	1.07252802	0.31279084	0.25835106
Dixdc1	-0.1066816	6.91295512	1.07213314	0.31287765	0.2583968
Snhg10	-0.5003989	0.7763282	1.07170932	0.31297086	0.2584478
Ap1g2	-0.9477425	0.00113336	1.07045526	0.31324688	0.25864975
Ppp2r2c	-0.1528566	9.02177401	1.06978615	0.31339429	0.25872492
Luzp1	-0.1297565	8.22082905	1.06975631	0.31340087	0.25872492
Abcg2	0.21234189	5.21468383	1.06954397	0.31344767	0.25872934
Slain2	0.1335548	6.73373401	1.06944639	0.31346918	0.25872934
Prrt3	0.42499927	1.74678824	1.06918251	0.31352736	0.25875137
4930529M08	-1.2679055	-0.9366219	1.06898488	0.31357094	0.25875988
Npat	-0.1546722	5.99288499	1.06885029	0.31360063	0.25875988
Trpc4	-0.2438683	3.39938902	1.06807765	0.31377112	0.25884816
Zfp454	0.2842891	2.31027199	1.06804432	0.31377848	0.25884816
Srp14	0.18499463	6.88944563	1.06793732	0.3138021	0.25884816
Gpld1	-0.1645169	4.90241886	1.0676454	0.31386655	0.2588545
Impact	0.11918294	7.83618296	1.06756627	0.31388403	0.2588545
Eml4	-0.1413667	6.15080929	1.06744236	0.3139114	0.2588545
Nr2c2ap	0.3101356	2.05884682	1.06733205	0.31393576	0.2588545
Gpr65	-0.5998319	0.52277596	1.06696797	0.3140162	0.25887861
Cdc37l1	0.12287484	6.61550498	1.06691459	0.314028	0.25887861
Tars	0.16942156	4.55001334	1.06668089	0.31407965	0.25888176
D6Ertd474e	0.857291	0.2199904	1.06661228	0.31409482	0.25888176
BC003331	0.1265874	6.91312747	1.06591678	0.31424862	0.25898255
Rap1a	0.17117175	8.59777155	1.06576081	0.31428312	0.25898502
Ap3b1	0.13495349	6.59633929	1.06501564	0.31444804	0.25908769
Kri1	-0.2862964	2.89361133	1.06491304	0.31447075	0.25908769
Capn12	-0.8159017	-0.7080199	1.06400525	0.31467185	0.2592108
Zfp963	-0.2831984	2.52966685	1.06384741	0.31470683	0.2592108
Fbxo9	0.12443837	6.44977445	1.06381146	0.3147148	0.2592108
Evi5l	-0.2240134	3.32774126	1.06352417	0.31477848	0.25921727
Ear2	-0.9207712	-0.4067613	1.06344365	0.31479634	0.25921727
Psma8	0.68202968	0.17721155	1.06334927	0.31481727	0.25921727
Lhx9	-1.0129368	-1.07217	1.06110882	0.31531464	0.25960079
Hps5	0.23201345	3.60626407	1.06084293	0.31537374	0.25960457
Men1	-0.229858	4.03317558	1.06080399	0.31538239	0.25960457
Mpdz	-0.1363804	6.41183289	1.06038209	0.3154762	0.25964173
AW549542	-0.6409687	-0.2372378	1.06031681	0.31549072	0.25964173
Syt11	-0.1641667	8.29352381	1.05982999	0.31559902	0.25968897
Hsbp1l1	-0.3816326	1.59126542	1.05977475	0.31561131	0.25968897

Haus6	-0.2025462	3.95219475	1.059474	0.31567825	0.25971804
Trhde	-0.2148815	5.81450051	1.05905085	0.31577245	0.25976954
Pmpca	-0.1180323	6.05823535	1.05876363	0.31583642	0.25979616
Smim7	0.21723429	6.30717233	1.05825175	0.31595047	0.25986397
Acbd5	0.10374147	7.15738997	1.05798912	0.316009	0.25988611
Tm9sf2	-0.1527668	5.92029723	1.0573538	0.31615067	0.2599766
Vmac	0.2963695	3.71146309	1.05601298	0.31644993	0.26019666
Arl10	-0.512346	0.91584458	1.05564099	0.31653303	0.26023894
Rab23	0.14437278	5.37717903	1.05522073	0.31662694	0.26029012
Zcchc10	0.27352735	3.6333882	1.05482526	0.31671535	0.26033676
Prim2	0.26729648	2.69583079	1.05406949	0.3168844	0.26043536
Ppie	0.3675139	1.3935998	1.0540057	0.31689867	0.26043536
Gyk	-0.2903368	4.36426834	1.05328004	0.31706112	0.26052425
Ppm1l	-0.2227613	4.72174536	1.05321855	0.31707489	0.26052425
H2afz	0.14098694	7.20723172	1.05292827	0.3171399	0.26052425
Tnni2	1.10923519	-1.3371784	1.05274771	0.31718036	0.26052425
Iqcb1	-0.1696164	4.50208082	1.05256862	0.31722048	0.26052425
Cklf	0.47297258	0.98049407	1.0524693	0.31724274	0.26052425
Mfn2	-0.1319578	6.63419644	1.05245064	0.31724692	0.26052425
Slc11a1	-0.8886793	-0.4192487	1.05239042	0.31726042	0.26052425
Lgi4	-0.4451596	1.45022589	1.05208185	0.31732959	0.26055502
Fabp4	-1.4070874	-0.6828978	1.05150867	0.31745813	0.26062558
Gm10790	0.76323534	-0.5039736	1.05141589	0.31747894	0.26062558
C1qc	0.49164331	2.26937786	1.05087118	0.31760118	0.26069988
Syngr2	0.45226463	1.68918734	1.05052804	0.31767821	0.26073708
Cdc20	0.80402786	-0.3598917	1.05027513	0.31773501	0.26075765
Snai2	0.36754875	3.53862021	1.04963343	0.31787917	0.26084992
Tlr3	-0.1916976	4.87896512	1.04906283	0.31800744	0.26091189
Tpp1	-0.1780578	6.58033688	1.04901506	0.31801818	0.26091189
Tkt	-0.2090722	4.83904856	1.04863232	0.31810426	0.26095647
Eif3b	0.18531879	3.89030819	1.04838338	0.31816027	0.26097343
Ogfrl1	-0.1431146	7.93342895	1.04825816	0.31818844	0.26097343
Klf11	-0.2056179	4.21877842	1.04778426	0.31829511	0.26103487
Lypd6b	-0.2303842	3.94970159	1.04729759	0.3184047	0.26109869
Erf	-0.2667496	3.33277045	1.046274	0.31863538	0.261217
Tgfbr2	0.22070358	5.70117988	1.04624961	0.31864088	0.261217
Vat1	0.23724957	4.62141289	1.04597844	0.31870203	0.261217
Cyth4	-0.3607952	1.85872667	1.04574836	0.31875392	0.261217
Ddx52	0.13412768	4.67861184	1.04559857	0.31878772	0.261217
Gm10012	0.18632478	3.8279026	1.04559742	0.31878798	0.261217
05-Mar	0.11300898	6.50440038	1.04554469	0.31879987	0.261217
Zbtb43	-0.2121285	4.51846627	1.04539867	0.31883282	0.261217
Ssr4	0.21207578	3.42232035	1.04538895	0.31883502	0.261217
Bmpr2	-0.1498163	9.84150137	1.04510295	0.31889957	0.26122935
Cldn22	-1.4642912	-1.6312496	1.04504054	0.31891365	0.26122935

Gid4	-0.1274992	5.84590795	1.0441362	0.31911789	0.26134554
Dchs1	-0.2867957	2.92556451	1.04413086	0.3191191	0.26134554
BC052040	0.19085161	4.25305052	1.04368315	0.31922028	0.26140236
Coq6	0.44709335	1.27354156	1.04342024	0.31927971	0.26142498
Ppm1f	-0.167937	4.58566477	1.04323405	0.31932181	0.26143341
Lpcat2	-0.3533689	2.55061214	1.04220919	0.31955369	0.26159719
Eef1g	0.1924444	7.72429687	1.04181643	0.31964262	0.26160299
Zdhhc5	0.15540379	5.9280113	1.04174512	0.31965877	0.26160299
Spock2	-0.1693267	8.11714131	1.04173109	0.31966194	0.26160299
Ddrgk1	0.20418787	4.10669404	1.04161566	0.31968809	0.26160299
Wdpcp	0.17996498	3.98123373	1.04118438	0.31978579	0.26163584
Nos1	-0.2741509	3.24246223	1.04103362	0.31981996	0.26163584
Arl2bp	-0.1754383	5.67878258	1.04101695	0.31982373	0.26163584
Pthlh	0.32974989	2.27733895	1.04083858	0.31986416	0.26164287
Zbtb49	-0.364394	1.51435636	1.04050355	0.31994011	0.26167896
Emx1	0.57023791	0.52695818	1.03930001	0.32021317	0.26186991
Otub2	0.21624943	3.60956246	1.03919374	0.3202373	0.26186991
Bdh2	0.37196225	3.09510841	1.03777573	0.32055947	0.26210728
Nutf2	0.25038293	3.14388551	1.03700516	0.32073472	0.26222449
Clpb	-0.2333721	3.74008951	1.03651453	0.32084638	0.26228969
Gm15880	-1.5297512	-0.8696785	1.03632815	0.32088881	0.26229828
Safb	0.13512779	6.30017107	1.03604844	0.3209525	0.26232426
Adrbk1	0.16801368	5.09070677	1.03585352	0.3209969	0.26233446
Rpl14	0.24059786	6.55012026	1.03544635	0.32108966	0.26234517
Tprkb	-0.1260418	5.39818019	1.03541733	0.32109628	0.26234517
Klhl10	1.06263698	-0.8904987	1.03537569	0.32110576	0.26234517
Zfp276	-0.2880864	2.6787564	1.0352262	0.32113983	0.26234693
Sec61g	0.23343864	4.7707202	1.03504851	0.32118034	0.26235394
Gm11186	1.22732448	-1.441342	1.03436877	0.32133534	0.26244954
Zfp811	0.22760026	3.43051123	1.03404061	0.32141021	0.26244954
2810468N07	-0.2711745	2.59220216	1.03396196	0.32142816	0.26244954
Tmem88	-0.3614222	2.02488157	1.03385196	0.32145326	0.26244954
Fndc9	0.38944091	2.94928762	1.03383546	0.32145703	0.26244954
Rbm19	0.31695186	2.26735387	1.03355713	0.32152056	0.26247534
Akt3	0.13589404	8.0782817	1.03289874	0.32167092	0.26253741
Btbd10	-0.1529627	5.98224862	1.03289248	0.32167235	0.26253741
Rpl9	0.18916885	7.1941389	1.03280458	0.32169243	0.26253741
Arglu1	-0.1099456	6.85567336	1.03242598	0.32177894	0.26257115
Gtf2a1	0.11811687	6.99718016	1.03230203	0.32180728	0.26257115
Lias	0.16176143	4.59285949	1.03198155	0.32188054	0.26257115
Ttc39d	-1.7372626	-1.637163	1.03197023	0.32188313	0.26257115
Diap3	-0.6669041	0.48127226	1.03192488	0.3218935	0.26257115
Lrrc36	0.76494021	-0.0502323	1.03116502	0.32206732	0.26265986
Mboat7	-0.1516214	5.19985747	1.03100538	0.32210385	0.26265986
Suox	0.23000885	4.24986719	1.03084526	0.3221405	0.26265986

Abcb8	0.18259679	4.05750279	1.03079775	0.32215138	0.26265986
Pcnp	0.14863145	7.86009019	1.03066317	0.32218219	0.26265986
Coq10a	0.20764851	4.11376662	1.03057926	0.3222014	0.26265986
Rab11fip2	-0.1209904	6.93761623	1.03047198	0.32222596	0.26265986
1700086L19I	-0.2558085	3.09670287	1.03019084	0.32229035	0.26266546
Ablim3	0.11982507	5.35289374	1.03016291	0.32229675	0.26266546
Fndc5	-0.1497366	5.00331875	1.02981344	0.32237681	0.26270466
Aspg	-0.4483879	0.89639221	1.02944702	0.32246079	0.26274704
Ppp1r2	0.13091123	8.03802023	1.02907642	0.32254575	0.26279022
Itm2b	0.21478733	8.98079647	1.02810739	0.32276806	0.26294528
Klhl24	-0.1134249	6.75247327	1.02789557	0.32281668	0.26295883
Slco4a1	0.46533009	1.80494302	1.02679496	0.32306949	0.26313868
Igj	-0.485004	1.01319306	1.02636573	0.32316816	0.26319296
Lin7a	-0.1393178	7.6292927	1.02621903	0.32320189	0.26319436
Sla2	0.63803128	-0.1584206	1.02529459	0.32341457	0.26334145
Ruvbl2	0.17671253	4.49916799	1.02495212	0.3234934	0.26337955
Hcrtr2	-0.526978	0.50982421	1.02453546	0.32358935	0.26343158
Acss2	0.31697254	4.31701525	1.02436119	0.3236295	0.26343817
Dazap1	-0.2194034	3.48253625	1.02376375	0.32376717	0.26349852
Ccdc120	-0.317116	2.46031896	1.0236836	0.32378565	0.26349852
Zfp809	0.15583474	4.84571026	1.02362215	0.32379982	0.26349852
Nap1l4	0.15392314	5.59025974	1.02278703	0.32399243	0.26359787
St8sia2	0.31548906	2.85033802	1.02268827	0.32401521	0.26359787
Rtn2	-0.3355503	2.61842012	1.02267565	0.32401813	0.26359787
Cox4i2	-1.0174207	-1.5290112	1.02232704	0.32409858	0.26363723
Apobec1	0.26493686	3.35042293	1.02189395	0.32419858	0.26369248
P2rx4	-0.2699608	3.21501847	1.02134174	0.32432614	0.26377013
Ercc3	-0.1810146	4.2118526	1.02107036	0.32438885	0.26379503
Tifa	0.27687085	3.62939513	1.02007598	0.32461879	0.26394593
Babam1	0.16818713	4.48843025	1.01999017	0.32463865	0.26394593
Lrp2bp	-0.7122821	0.78292561	1.01964588	0.32471832	0.26398459
Zfp715	-0.1813139	4.43571062	1.01908658	0.32484781	0.26406374
Clec4a1	-1.0100672	-0.821454	1.01855141	0.32497177	0.26412389
Rslcan18	-0.3189045	2.38677572	1.01835429	0.32501745	0.26412389
Cdkl2	0.14140476	6.06379876	1.01818548	0.32505657	0.26412389
Tceb1	0.15521407	6.28248636	1.01816431	0.32506148	0.26412389
Tmx4	-0.1409484	8.3392014	1.01807377	0.32508247	0.26412389
Kcnh1	-0.1862706	6.07146337	1.01776387	0.32515432	0.26415041
Cox20	-0.1694264	5.02108414	1.01765573	0.3251794	0.26415041
Exoc7	0.17034973	4.63607834	1.01677862	0.32538289	0.2642896
Larp1	-0.1221992	7.30000833	1.01662765	0.32541794	0.26429194
Scrn1	-0.1876765	5.45736724	1.01639215	0.32547261	0.26431023
Gstm5	0.20741323	5.97796216	1.01583129	0.32560288	0.2643899
Pkn2	0.13720051	6.06826145	1.01567818	0.32563845	0.26439266
Scd4	-0.6119903	-0.175425	1.01492693	0.32581308	0.26450832

Eda2r	-0.9984806	0.18129697	1.01432844	0.32595229	0.2645952
Inadl	0.1745109	3.94804209	1.0139527	0.32603973	0.26463601
Ncoa7	-0.1444096	6.32087926	1.01371854	0.32609424	0.26463601
Mab21l3	-1.4635559	-1.79967	1.0136974	0.32609917	0.26463601
Car10	0.16712931	8.35833458	1.01345289	0.3261561	0.26465609
Ergic1	0.1318417	6.53841747	1.01311862	0.32623396	0.26469313
Gpr4	-0.2689241	5.56002445	1.0125452	0.32636758	0.2647754
Syngr3	-0.1664934	5.08592985	1.01214988	0.32645974	0.26482344
4932441J04F	0.86466285	-0.8893644	1.01191579	0.32651433	0.26482344
Inca1	-0.4017144	0.79195085	1.01180037	0.32654125	0.26482344
Atp6ap1	0.16327674	7.63568664	1.01173858	0.32655566	0.26482344
Klhl42	0.12133197	6.43862941	1.0114902	0.32661361	0.2648443
Adprhl2	-0.9675552	-0.4313696	1.01120521	0.32668012	0.26485298
Ablim2	-0.1684944	6.07377197	1.01113816	0.32669576	0.26485298
Sart3	0.22642853	4.68295842	1.01103007	0.326721	0.26485298
Hist1h4i	0.68839262	-0.2487635	1.01046921	0.32685196	0.26489912
Camkk1	0.20854241	4.88035327	1.01024539	0.32690424	0.26489912
Ears2	-0.5747807	0.22658193	1.01022498	0.32690901	0.26489912
Oxld1	0.46660763	1.12722432	1.0102071	0.32691319	0.26489912
Fchsd1	-0.3472499	2.02506794	1.00989856	0.32698528	0.26489912
Rnf135	0.26219367	2.75832843	1.00973929	0.32702251	0.26489912
Guk1	0.23171271	4.85729885	1.009631	0.32704782	0.26489912
Gm2382	0.28271965	2.975679	1.00962289	0.32704972	0.26489912
Nub1	0.13896443	5.30271563	1.00954479	0.32706797	0.26489912
5430416O09	1.3338063	-1.8621924	1.00939424	0.32710317	0.26490152
Endou	0.34054787	1.87124236	1.00912047	0.32716719	0.26492727
Thap4	0.21070433	4.20500576	1.00869217	0.32726738	0.26498229
Ccdc11	-0.6592442	-0.1982129	1.00836602	0.32734371	0.26501798
Acvrl1	-0.4870082	0.83253302	1.00815373	0.3273934	0.26503211
Tifab	0.53508727	1.68083859	1.00731786	0.32758917	0.26516447
Gm2762	1.48682301	-1.6727129	1.0158472	0.32771181	0.265215
Scyl1	0.15930321	4.19231876	1.00677614	0.32771613	0.265215
Trim16	0.30833443	2.64917315	1.00636507	0.32781251	0.26526688
D11Wsu47e	0.26625559	2.51229817	1.00615126	0.32786266	0.26528134
Ccdc68	-0.5560731	0.39708641	1.00587153	0.32792829	0.26530832
1110059G10	0.22425884	4.20899495	1.00554607	0.32800467	0.265344
4930513N10	0.81353164	-0.4688363	1.00488054	0.32816093	0.26537795
Pcdha7	-0.7642706	-0.6336059	1.00485762	0.32816631	0.26537795
Tmem196	-0.2296228	3.16291533	1.00484436	0.32816943	0.26537795
Oscp1	0.24219303	3.51831819	1.00481728	0.32817579	0.26537795
Ehmt1	-0.2461307	4.40796228	1.00396288	0.32837656	0.26551418
H2-Ob	-1.1090589	-0.2005902	1.00359734	0.32846251	0.26555755
App	-0.1268047	8.66509883	1.00342892	0.32850212	0.26556345
Vnn1	-0.4771146	1.71551037	1.00262592	0.32869107	0.2656761
Uchl3	0.20378952	3.58882508	1.00256203	0.32870611	0.2656761

Nrm	0.47368231	0.49915898	1.00226573	0.32877588	0.26569499
F2r	-0.2697524	2.66735324	1.0021882	0.32879414	0.26569499
Pabpn1	-0.1386495	5.54509059	1.00184305	0.32887543	0.2656975
Thoc5	0.22637846	3.55371757	1.00182491	0.32887971	0.2656975
Ccnb2	-0.7868041	-0.2245179	1.00176327	0.32889423	0.2656975
Map6	0.21039729	5.70222243	1.00131225	0.32900052	0.26575724
Trib2	0.16986255	6.19818176	1.00103284	0.32906638	0.26578433
Gm2518	-0.7498694	-0.6249179	1.00008797	0.32928927	0.26591191
Adm	-0.3137567	2.24747852	0.99999894	0.32931028	0.26591191
Exosc2	-0.2156801	3.39289145	0.99995181	0.3293214	0.26591191
Myo1h	0.75028338	-1.0196732	0.9994725	0.32943456	0.26597715
Galt	-0.1608096	4.43305	0.99896668	0.32955404	0.26604748
Abcb9	0.40363273	1.61859793	0.99847921	0.32966924	0.26609607
Ubl5	0.21917843	5.95117824	0.99833661	0.32970295	0.26609607
Bckdha	0.34704352	1.13284598	0.99830104	0.32971136	0.26609607
Gm13710	0.56639459	0.50755034	0.99801739	0.32977843	0.26612407
Gm15545	-0.8907675	-0.7285073	0.99761967	0.3298725	0.26616681
Slc22a14	-1.7123116	-1.9966081	0.99751969	0.32989616	0.26616681
Slc25a46	0.12319826	7.86801122	0.99706563	0.33000361	0.26622738
Lypla2	-0.2873526	2.10910724	0.99663512	0.33010554	0.26628347
Zfp282	-0.3718055	1.90740085	0.99647027	0.33014459	0.26628883
Slfn10-ps	-0.9265781	-0.7631627	0.99415592	0.33069339	0.26670531
Dgcr2	-0.1402527	4.64335063	0.99327176	0.33090338	0.26683464
Smarcc2	0.11195818	8.25320197	0.99320738	0.33091868	0.26683464
Ccdc24	1.10883241	-0.9110974	0.99304769	0.33095663	0.26683907
C1ql3	0.15511283	6.79431107	0.99283604	0.33100694	0.26685345
Tmem107	0.3303445	1.71680392	0.99182588	0.3312472	0.2669997
Ptpn11	0.11326748	7.67249814	0.99180017	0.33125332	0.2669997
Dnttip1	-0.2763149	2.98877728	0.99139404	0.33134998	0.26705142
Pgrmc1	0.20124996	7.58892138	0.9910309	0.33143645	0.26709492
Ttc39a	-0.5372992	1.32521746	0.99063659	0.33153037	0.26714442
Gm2a	0.28271522	5.00654987	0.99027676	0.33161611	0.26717612
S1pr1	-0.2476126	5.64766175	0.99019866	0.33163473	0.26717612
Aagab	0.15777238	4.71518943	0.98991339	0.33170273	0.26719303
Opn3	0.24893443	3.12974357	0.98983788	0.33172074	0.26719303
Lhfpl2	0.18846097	5.18302644	0.98882824	0.33196159	0.26736083
Slc6a9	-0.2233318	4.07559452	0.9865426	0.33250776	0.26777447
Fbxo10	-0.2536304	4.18832971	0.98633457	0.33255753	0.26778831
Efemp2	0.36268344	2.75571536	0.9860939	0.33261512	0.26780845
Mmp28	0.77090769	0.01821936	0.98584737	0.33267413	0.26782973
Maz	-0.1878605	3.87511503	0.98548195	0.33276163	0.26786159
Mroh5	0.72286644	-0.7387819	0.98536819	0.33278887	0.26786159
Glb1	0.28134529	3.05622481	0.98516963	0.33283643	0.26786159
Slc26a7	0.35707356	4.94168083	0.9850365	0.33286833	0.26786159
Tmco6	0.35743335	1.74919843	0.98500173	0.33287666	0.26786159

4930412C18I	0.5206647	1.24851585	0.98430823	0.33304288	0.26796911
Pdik1l	-0.184348	3.96548955	0.98356662	0.33322076	0.26808599
Pkp3	1.07400465	-1.3205244	0.98326076	0.33329416	0.2681188
Gsr	-0.1301954	5.88993082	0.9830927	0.33333451	0.2681213
Chst8	0.51360457	0.65193605	0.98297604	0.33336251	0.2681213
Megf8	-0.2215486	5.0083475	0.98274041	0.33341909	0.26814057
Susd1	-0.3951612	1.90334626	0.98211314	0.33356978	0.2682355
Rnf2	0.14653102	5.42801567	0.98164471	0.33368237	0.26829783
Il15	0.36140383	1.90573341	0.98151905	0.33371258	0.26829783
Ddx50	-0.1249291	6.27247617	0.98081327	0.33388234	0.26840806
Pidd1	0.98902843	-1.6530424	0.97992868	0.33409528	0.26855298
Tap1	-0.4091302	1.05045136	0.97951323	0.33419535	0.26858634
Ace	-0.326654	5.32388205	0.97948509	0.33420213	0.26858634
Sar1b	0.14112553	5.62421325	0.97930589	0.33424531	0.26859477
Ctu1	-0.3606804	1.55210896	0.97891478	0.33433958	0.26864426
Ccl27a	-0.1774934	5.16769628	0.9783596	0.33447346	0.26872556
Uevld	-0.159689	5.20567585	0.97818593	0.33451536	0.26873295
Srsf11	-0.1320234	6.73223638	0.97770413	0.33463162	0.26878295
Catip	0.32039831	2.46427014	0.97757562	0.33466264	0.26878295
Rbpj	0.11817573	6.51270234	0.97752152	0.3346757	0.26878295
Trim65	0.22786325	4.06082884	0.97702088	0.3347966	0.26885377
Gtf2a2	0.16355898	4.82817	0.97649563	0.3349235	0.26892941
1700016K19I	0.54079058	1.06684404	0.97541359	0.33518514	0.26907854
C130083M11	-0.3041091	2.78221445	0.97533152	0.33520499	0.26907854
Mir425	1.10525221	-1.6929276	0.97532139	0.33520744	0.26907854
Hdac7	-0.2019659	3.70229096	0.97455975	0.3353918	0.26920023
B3gnt2	0.17444148	5.73961934	0.97428607	0.33545808	0.26921075
St6galnac2	-0.3819882	1.27839149	0.97423513	0.33547042	0.26921075
Slc31a1	0.14410821	5.88304645	0.97364639	0.33561306	0.26929894
Preb	0.19597661	5.17850217	0.97338146	0.33567728	0.26932417
Galr1	1.20480851	-1.8175192	0.97256107	0.33587626	0.26943808
Tonsl	-0.6448504	0.46502871	0.9725258	0.33588481	0.26943808
Tspan12	0.28462835	3.01681319	0.9716654	0.33609367	0.26957931
Gpr84	-1.0971509	-1.0057645	0.97133002	0.33617513	0.26961247
R3hcc1	0.21312752	4.26773556	0.97122509	0.33620063	0.26961247
Rps6ka1	-0.3048468	2.51458449	0.97096286	0.33626435	0.26963726
Psg16	0.48719572	1.38269931	0.97048704	0.33638002	0.2697037
Rps10	0.23957801	4.92802873	0.96976144	0.33655651	0.26981889
Hexa	-0.3074367	3.74109112	0.96941997	0.33663961	0.26985919
Creld1	-0.2434756	3.59084989	0.96827657	0.33691808	0.27005608
Baz2a	-0.1553283	6.12712443	0.96796047	0.33699513	0.2700802
Patl1	0.13756151	6.17278137	0.96788347	0.3370139	0.2700802
Sp2	0.24488465	5.31463402	0.96748623	0.33711076	0.27013149
Trp53i13	-0.8341377	-0.2615405	0.96672663	0.33729609	0.27024936
Fam219aos	0.29580902	3.09071208	0.96661382	0.33732363	0.27024936

Tmx3	-0.1625813	5.80840114	0.96618227	0.33742899	0.27030743
Tef	-0.1045148	6.79325453	0.96583989	0.33751262	0.27033576
Kcnj4	-0.2344711	3.36991447	0.96576818	0.33753014	0.27033576
Usp10	0.15765244	5.20254236	0.96502889	0.33771083	0.27042084
Nat14	-0.3536943	1.17082962	0.96486186	0.33775167	0.27042084
Zfp770	0.12203175	6.22912452	0.96480895	0.33776461	0.27042084
Mcl1	0.14771488	8.67175862	0.96479519	0.33776798	0.27042084
Usp46	-0.1151313	7.22034114	0.96444368	0.33785395	0.27046333
Cdh12	-0.2046689	6.34900079	0.9638854	0.33799057	0.27052752
Endog	0.87066545	-0.8303844	0.96384704	0.33799996	0.27052752
Vps37c	0.28263549	3.47069224	0.96307774	0.33818836	0.27065195
Rabac1	0.34199484	3.07237434	0.96254855	0.33831804	0.27072937
Shf	-0.4514592	1.94867076	0.96234304	0.33836842	0.27074333
Nfkb1	-0.1786701	4.41632121	0.96190394	0.33847609	0.27078796
8030423F21I	-0.6520648	-0.8160297	0.96184689	0.33849009	0.27078796
Fam162a	0.21865213	5.00443547	0.96098065	0.33870267	0.27093165
Hgf	-0.3827541	2.0473698	0.96026309	0.3388789	0.27097777
Pnma2	0.14701504	6.40633816	0.96025377	0.33888119	0.27097777
Lcat	-0.3485505	2.85756413	0.96011695	0.33891481	0.27097777
Sac3d1	0.33780624	2.31542067	0.96004029	0.33893365	0.27097777
Ddb1	-0.1364936	6.36962205	0.95998609	0.33894697	0.27097777
Pld5	-0.2415063	4.2995414	0.95991758	0.3389638	0.27097777
Ttc9b	0.27515052	3.14900253	0.95980649	0.33899111	0.27097777
Sntg2	-0.5152409	0.90102064	0.9589931	0.33919113	0.27108575
Lsm7	0.23161551	3.70808593	0.95898891	0.33919216	0.27108575
Rgs4	0.1338228	9.56276024	0.9580963	0.33941185	0.27123495
Cntrl	-0.2077385	4.31281987	0.95790489	0.33945899	0.27124625
Xpc	-0.191638	3.9902574	0.95766483	0.33951812	0.27126713
Alg2	-0.1072242	7.44663579	0.95702412	0.339676	0.27135707
Pth1r	-0.5093904	1.07751311	0.95694007	0.33969673	0.27135707
C2cd4b	0.58524059	-0.0762654	0.95630949	0.33985223	0.27145491
Npm3	0.66141094	-0.8634113	0.95589289	0.33995502	0.27151063
Rmnd5a	0.12879905	7.57797669	0.95533036	0.34009389	0.27159515
Morn1	-0.5673947	0.458594	0.95494806	0.34018831	0.27162515
Sdc1	-0.3949694	2.17858721	0.95464906	0.34026218	0.27162515
4930426L09F	0.48911895	0.84824692	0.95458968	0.34027686	0.27162515
Cox18	-0.2407001	2.90177609	0.95456786	0.34028225	0.27162515
6030440G07	-0.7416482	-1.2399311	0.9545094	0.3402967	0.27162515
Cdc45	0.51653908	0.92619424	0.95361644	0.34051749	0.27177499
Foxf1	1.35021044	-1.9447467	0.95283241	0.34071151	0.27190344
2310065F04I	1.86503525	-1.6420184	0.95245663	0.34080456	0.27195129
Hsph1	0.15095061	9.09662742	0.95225908	0.34085349	0.27196393
Ift140	-0.3363845	3.26109083	0.95208147	0.34089749	0.27197263
15-Sep	0.20955751	6.70943786	0.95170738	0.34099019	0.27198227
Timmdc1	0.18064337	4.29248554	0.95166581	0.34100049	0.27198227

Cdh8	-0.1587914	5.4722849	0.95163216	0.34100884	0.27198227
Slc25a15	0.21748345	3.41197835	0.95101746	0.34116125	0.27207742
Ncstn	0.18735483	5.1058978	0.95086203	0.3411998	0.27208177
Gm9833	-0.3204919	1.15406897	0.95067292	0.34124672	0.27209279
Ncoa1	-0.1068696	8.10415337	0.95038769	0.3413175	0.27212282
Prr3	-0.1564539	4.200943	0.9496631	0.3414974	0.27223985
Rpl21	0.1961173	7.82122317	0.94947946	0.34154302	0.2722498
Sumf2	-0.2584669	2.65517025	0.94892853	0.34167992	0.27233252
Canx	-0.1359812	7.9635512	0.9482973	0.34183687	0.2724312
Fam131b	-0.1990607	4.95506433	0.9480572	0.3418966	0.27243385
Ipp	0.22092947	3.30453463	0.94790642	0.34193411	0.27243385
Tpgs1	0.31674903	3.14348779	0.94787472	0.341942	0.27243385
Med1	0.12786306	7.2607964	0.94775101	0.34197279	0.27243385
Glrx5	0.20902987	3.73372452	0.94755789	0.34202085	0.27244105
Myh4	-1.0946758	-0.373539	0.94744835	0.34204812	0.27244105
Kremen1	-0.2080841	3.34457656	0.94712882	0.34212767	0.27247801
Ethe1	0.32347976	2.6815575	0.94666092	0.34224422	0.27254442
Gtf2h4	0.41223223	1.44532771	0.94588317	0.34243807	0.27267237
Trim30a	-0.2255709	3.79956859	0.94530646	0.34258191	0.27270645
Arhgef10	0.14744096	5.27670448	0.94516718	0.34261666	0.27270645
Plin2	0.36677619	3.42219361	0.94498835	0.34266129	0.27270645
Aptx	-0.1403863	5.00670793	0.94492947	0.34267598	0.27270645
Micall1	0.1440817	5.22167441	0.94487564	0.34268942	0.27270645
Scn3b	-0.1563446	5.74038972	0.944782	0.34271279	0.27270645
Zbtb44	-0.1227719	7.23538293	0.94475356	0.34271989	0.27270645
Ky	-0.4953394	0.5283509	0.94455943	0.34276836	0.27270645
Crat	0.13821895	5.36198451	0.94451489	0.34277948	0.27270645
Csgalnact2	-0.2055446	3.38256503	0.94388137	0.34293772	0.27280593
Cntrob	-0.3662377	2.14754063	0.94351441	0.34302942	0.27285247
Pdzd7	-0.4438677	1.33017718	0.94319715	0.34310873	0.27288915
Mblac2	-0.1819584	6.02592494	0.94258143	0.34326273	0.27298521
Rspo2	0.28867571	4.39083096	0.94183469	0.34344963	0.27304818
Fa2h	-0.2577177	4.01003315	0.94180264	0.34345766	0.27304818
Il27ra	-1.0733941	-1.5039429	0.94179708	0.34345905	0.27304818
Snap91	0.17850559	8.93722681	0.94173419	0.3434748	0.27304818
Pkd2	-0.2240686	6.20222856	0.94133273	0.34357535	0.27304996
Cib1	0.36210918	1.9956303	0.94112767	0.34362673	0.27304996
Ctbs	-0.2341694	3.00363502	0.94111103	0.34363089	0.27304996
Gpr1	-1.4348549	-1.4864563	0.94110059	0.34363351	0.27304996
Ap2s1	0.25302696	3.45522275	0.94106215	0.34364314	0.27304996
Samd14	0.35888283	2.17700552	0.94077558	0.34371496	0.27307946
Traf1	0.66935676	0.98828572	0.9406489	0.34374672	0.27307946
Dpf1	-0.2969451	2.26390189	0.9402182	0.34385472	0.27313886
Rexo1	0.16815708	4.84694613	0.93995932	0.34391965	0.27316404
Actg2	0.9266802	-0.8348818	0.93937664	0.34406587	0.27325377

Nova2	-0.1758298	7.26096089	0.93922466	0.34410403	0.27325767
Tubb3	0.17666136	4.68233189	0.93901358	0.34415703	0.27327335
Naip1	-1.433281	-1.843122	0.9379576	0.34442235	0.27345761
3110052M02	-0.1512167	4.84073189	0.93656308	0.34477317	0.27370446
Cd244	1.17823546	-0.6517929	0.93645703	0.34479987	0.27370446
Eif3c	0.10446819	8.03434266	0.93631739	0.34483503	0.27370594
Gm13483	-0.7168792	-0.7401471	0.935946	0.34492857	0.27375375
Fzd9	-0.941142	-0.7379368	0.93499529	0.34516818	0.27388732
Rcn3	0.43692936	3.12705558	0.93488511	0.34519597	0.27388732
Eid3	-1.3321728	-1.901409	0.93488165	0.34519684	0.27388732
Gtf2h1	0.18105395	5.09621696	0.93387166	0.34545169	0.27403969
Pus7l	-0.407949	1.59734635	0.93385629	0.34545557	0.27403969
Slc24a4	-0.3522333	2.83603818	0.93367664	0.34550093	0.27404923
Ankrd34a	0.14869305	6.32298013	0.93342757	0.34556383	0.27407267
Jtb	-0.1724842	4.13897678	0.93210368	0.34589843	0.27425268
Sec24a	-0.1376242	5.49204325	0.93197056	0.3459321	0.27425268
Tnfsf8	-1.0727238	-0.2182772	0.93183648	0.34596602	0.27425268
B3gnt8	-0.8037703	-0.1537182	0.93183292	0.34596692	0.27425268
Zc3h18	-0.1856295	3.91174905	0.93178346	0.34597944	0.27425268
Epha5	-0.1737363	6.13213742	0.93173775	0.345991	0.27425268
Dtx2	0.36857967	1.76024627	0.93127616	0.34610782	0.27431882
Fam150b	1.30319549	-1.1072825	0.93008244	0.34641018	0.27453199
9330151L19I	-0.2091237	4.78112007	0.92844666	0.34682513	0.27483434
Ccny	0.12490477	8.53740711	0.92824774	0.34687563	0.27484786
Ddx19b	-0.1704784	5.11197622	0.92767565	0.34702095	0.2749151
Erc1	-0.1131086	7.67618624	0.92759801	0.34704068	0.2749151
Trim43c	-1.0075036	-1.17469	0.9274146	0.34708729	0.2749151
Mest	-0.234895	6.37721065	0.92738707	0.34709429	0.2749151
Zcchc4	-0.7404409	-0.1139491	0.92563593	0.34753977	0.27522204
Amn1	-0.2014532	4.58958325	0.9256005	0.34754879	0.27522204
Icam1	0.46654236	1.87701251	0.92478149	0.34775743	0.27536073
Cyba	0.52038952	0.14828041	0.92462688	0.34779683	0.27536541
Fxyd7	-0.3070216	2.00153272	0.92427095	0.34788757	0.27541072
Rarres1	0.96736965	-0.6069795	0.92379253	0.3480096	0.27547497
Gm10440	1.7260922	-1.531503	0.92368997	0.34803576	0.27547497
Cdc34	0.28985567	3.16329513	0.92240733	0.34836324	0.27570762
Clic6	-0.3097629	2.60919011	0.92152788	0.34858803	0.27583417
Lgi3	-0.2308573	3.63126066	0.92139571	0.34862183	0.27583417
Ddx54	0.20660882	3.98407135	0.92138793	0.34862382	0.27583417
Txlng	0.13543261	5.65176157	0.92095403	0.34873482	0.27589544
Nol9	-0.2279082	3.57969393	0.9207742	0.34878084	0.27590529
Wdr91	0.25160283	3.08481468	0.9203607	0.34888668	0.27595364
Trit1	-0.2545333	3.53345895	0.92014234	0.3489426	0.27595364
Zcchc6	0.10027843	7.22987234	0.92014197	0.34894269	0.27595364
Ccdc150	-1.1392716	-1.6023388	0.91922738	0.34917702	0.27611239

Erbb3	-0.8447336	-0.3009485	0.91858148	0.34934265	0.27620272
Usp15	-0.138941	6.41798032	0.9185198	0.34935847	0.27620272
Erich6	-0.5558848	1.27272317	0.91793029	0.34950974	0.27629574
S100a16	0.33102473	3.25723789	0.91765481	0.34958046	0.27631323
Spata22	1.18511297	-0.4635355	0.91758221	0.3495991	0.27631323
Ina	0.15675734	6.3206916	0.91668309	0.34983009	0.27646921
Dym	0.1224063	5.63051636	0.91646059	0.34988728	0.27648782
B4galt7	-0.4485271	1.05384379	0.9153453	0.35017416	0.27668792
Camk2n2	-0.1587703	4.57960333	0.91519429	0.35021304	0.27669204
Dnajc25	-0.3004765	2.95692566	0.91445009	0.35040468	0.27681684
Cdc42se2	0.10992431	6.29738969	0.91402007	0.35051549	0.27687513
Dtx3l	0.13972794	4.87767254	0.91370565	0.35059654	0.27687513
Thrap3	0.11578173	7.46786872	0.91368492	0.35060188	0.27687513
Dctn6	0.1831978	6.37633636	0.91358591	0.35062741	0.27687513
Pde4dip	-0.1799931	7.48586352	0.91351034	0.3506469	0.27687513
Rras	0.30369855	4.72190055	0.91292078	0.35079898	0.27696861
G3bp1	0.14362198	5.14055908	0.91271804	0.3508513	0.27698331
Gpr176	-0.3053278	2.61658925	0.91165872	0.35112485	0.27717264
Cwc22	-0.1393904	5.52773881	0.91095193	0.35130754	0.27729022
Btg2	0.29452633	3.76373562	0.91061604	0.35139441	0.27729728
Vmn2r29	-0.229936	3.38591024	0.91042519	0.35144378	0.27729728
Mtf1	-0.1167373	5.99906572	0.91039397	0.35145185	0.27729728
Chek2	0.33740815	2.34680415	0.91026589	0.35148499	0.27729728
Aldh6a1	-0.152851	5.93071237	0.91017805	0.35150772	0.27729728
Nedd1	0.2851716	2.72720241	0.91013481	0.35151891	0.27729728
Gpatch3	0.68486794	-0.187732	0.90846945	0.35195027	0.27761091
Mrc1	-0.3893695	2.19715843	0.90827862	0.35199975	0.2776233
Fbxo21	-0.1595936	5.66249624	0.90803376	0.35206325	0.27764674
Esyt1	-0.2464201	3.45697832	0.90785501	0.35210962	0.27765666
Kansl2	0.13175904	4.72657534	0.90769349	0.35215152	0.27766307
Epha1	-1.3993594	-1.3427968	0.90654248	0.35245034	0.27783312
Ccnk	0.1187204	6.38171229	0.90646925	0.35246936	0.27783312
Odf3b	0.78725945	0.00321497	0.90635023	0.35250028	0.27783312
Cirbp	0.29977563	4.87862209	0.90618854	0.3525423	0.27783312
Atpaf2	-0.2069228	3.16296147	0.90613007	0.35255749	0.27783312
Pnoc	0.68733302	-0.2246849	0.90596726	0.35259981	0.27783312
6330419J24F	0.25094184	3.50590208	0.90595185	0.35260382	0.27783312
Senp8	0.16392471	4.61752894	0.9052914	0.35277555	0.27792833
Mir6336	-0.9941053	-1.5248579	0.90522706	0.35279229	0.27792833
Brinp1	-0.1459662	6.39757904	0.9040449	0.3531	0.27814409
Snhg7	-0.3517182	1.28793666	0.90324164	0.3533093	0.27825734
Spata33	0.85676162	-0.1889386	0.90323327	0.35331148	0.27825734
2700081O15	-0.1308921	6.20960645	0.90298286	0.35337677	0.27828209
Brms1l	0.13155254	5.94753582	0.90257934	0.35348201	0.2783383
Commd4	0.28516893	3.50891298	0.90219722	0.35358171	0.27839014

Ppp4c	0.36416549	2.05232108	0.90176512	0.3536945	0.27845227
4933412E12l	0.32765812	1.84800618	0.90027976	0.35408261	0.27873111
Ddx41	0.20249844	3.8381311	0.89989184	0.35418407	0.27876732
Suv420h1	-0.1060691	6.72862008	0.89971928	0.35422921	0.27876732
Zc3h10	0.30422463	3.0586151	0.89971494	0.35423035	0.27876732
Gm4944	0.16047912	4.17769705	0.89828811	0.35460396	0.27903462
Dpys	-1.0415446	-0.9644051	0.89797471	0.3546861	0.27907254
Trps1	-0.1564378	6.29006397	0.89750945	0.3548081	0.2791418
Egr3	0.15335567	7.83055763	0.8972508	0.35487594	0.27916845
Tfeb	0.42606621	1.47424651	0.89702862	0.35493423	0.27918759
Gm1987	-1.0841603	-1.4241787	0.89663623	0.35503722	0.27924187
Rps28	0.22013965	5.54608439	0.8956268	0.35530234	0.27941861
Pigyl	0.44482653	3.02094125	0.89550431	0.35533453	0.27941861
Suv39h1	0.24994272	3.57991777	0.89527664	0.35539437	0.27941861
Tigd5	-0.54427	0.66060187	0.89526318	0.35539791	0.27941861
Fbxw11	0.11886522	7.36807432	0.8944204	0.35561957	0.27956614
Ell	-0.1699948	3.49996243	0.89308572	0.355971	0.27980572
Gal3st4	-0.4057079	1.56458374	0.89300448	0.35599241	0.27980572
Farsa	-0.3718662	1.36654708	0.89274322	0.35606127	0.27983308
Fam71d	-0.9728851	-1.4505967	0.89222608	0.35619762	0.27991347
Cd68	0.32549143	2.3580621	0.89158201	0.35636755	0.28002023
Sntg1	-0.1688891	5.58941701	0.89131223	0.35643876	0.28004566
A630001G21	-0.6134703	0.62575873	0.89120129	0.35646805	0.28004566
Pdlim7	0.16699501	4.32830393	0.89092015	0.35654228	0.28007174
Cbfa2t3	-0.1641616	4.7943287	0.89068122	0.3566054	0.28007174
Ate1	-0.1290545	6.22399955	0.89051882	0.3566483	0.28007174
Mms19	-0.2489124	3.6826746	0.89043961	0.35666923	0.28007174
Nudt19	0.16671972	4.87249555	0.89041275	0.35667633	0.28007174
Med9	0.20076221	4.76136036	0.89030157	0.35670571	0.28007174
Them4	0.21627617	4.2544018	0.89002315	0.3567793	0.28009503
Foxr2	-0.6762086	0.49524515	0.88993155	0.35680352	0.28009503
Atp8b1	0.26865014	2.4639012	0.88842277	0.35720273	0.28036747
Zfp605	0.14859526	4.69555872	0.88836211	0.3572188	0.28036747
Nt5dc2	0.52829171	2.65514329	0.88735088	0.35748674	0.28055098
Rreb1	-0.1855415	5.25007658	0.88715902	0.35753761	0.28055211
Fam216b	-0.4651766	1.06230103	0.88699868	0.35758013	0.28055211
Bcdin3d	0.49603488	0.61609728	0.88695925	0.35759059	0.28055211
Zdhhc13	-0.2224266	4.23415247	0.88637502	0.35774558	0.28064693
Luc7l2	-0.1351962	7.39714737	0.8853215	0.35802533	0.28083958
Nfkbid	0.91777749	-1.1082854	0.88503206	0.35810224	0.2808731
Knstrn	0.53065528	0.64181907	0.88479257	0.3581659	0.28089623
Ogdhl	-0.353381	1.86515548	0.88421083	0.3583206	0.28098855
Tnfaip1	0.16111863	6.03251067	0.88409258	0.35835205	0.28098855
Stxbp3b	0.68609398	-0.9580444	0.88396431	0.35838618	0.28098855
Rpl38	0.17878174	6.22195774	0.88355192	0.35849593	0.28102719

Dhx34	-0.3199106	1.42848274	0.88352218	0.35850385	0.28102719
Wdr12	0.19563292	4.64403414	0.88326293	0.35857287	0.28104556
Stox1	0.33615154	1.31133111	0.88317728	0.35859567	0.28104556
Hif3a	-0.2799461	2.85379318	0.88249558	0.35877727	0.28113592
Pkdcc	-0.5164426	0.20160882	0.8824877	0.35877937	0.28113592
Fxyd5	0.27904996	6.75997088	0.88195128	0.35892237	0.28122115
Dleu7	0.37714569	1.26264563	0.88164696	0.35900352	0.28125793
Ddhd1	-0.1514821	6.49345786	0.88145576	0.35905453	0.28126075
Proser1	-0.1605048	5.5896054	0.88137691	0.35907557	0.28126075
Nedd4	-0.095777	9.5001887	0.88067435	0.35926308	0.28136387
Serpine1	-0.696977	-0.2823831	0.88062718	0.35927568	0.28136387
Mief2	0.40801273	1.96346521	0.88043562	0.35932684	0.28137712
Fam102a	-0.152989	5.70098183	0.87955894	0.35956109	0.28151965
Lonrf2	-0.1501935	8.07291777	0.87949807	0.35957736	0.28151965
Gnal	-0.168198	8.43372102	0.87890967	0.35973472	0.28161603
Slc17a5	0.17429141	3.89423497	0.87829342	0.35989963	0.28171829
4932443I19R	-1.046755	-1.4886467	0.87801787	0.3599734	0.28172369
Timm10b	0.27692773	2.6479208	0.87801163	0.35997507	0.28172369
Lincrna-cox2	-1.2655651	-1.3891797	0.87777727	0.36003783	0.28172653
Npc1l1	-0.7496801	0.04696487	0.87774207	0.36004726	0.28172653
Trem2	0.69038413	-0.4979594	0.87749173	0.36011432	0.28175218
Bcl6	0.09492786	6.10404496	0.87725356	0.36017814	0.28177529
Zfp273	-0.2205007	3.24774517	0.87684354	0.36028805	0.2818149
Ccdc110	0.84325013	0.36733429	0.87680885	0.36029735	0.2818149
Tppp3	0.20653363	3.54507337	0.87595796	0.36052559	0.28196659
Gjc1	-0.4337864	1.80271383	0.87570412	0.36059372	0.28197315
Mvd	0.28319917	1.82124357	0.87567109	0.36060259	0.28197315
Cd320	-0.2535006	2.81476072	0.87502228	0.36077682	0.28208255
Vps33b	0.16353423	5.18058997	0.87460133	0.36088992	0.28213219
Slc7a5	-0.1816108	4.14321219	0.87453049	0.36090896	0.28213219
Tspan18	0.34223712	2.75603736	0.87360027	0.36115911	0.28230089
Heph	0.49162009	2.0407408	0.87265736	0.36141292	0.28246275
Ptk7	-0.379099	1.28696047	0.87253002	0.36144722	0.28246275
Trp73	-0.7448694	-0.2048053	0.87225965	0.36152005	0.28246275
4933413G19	-1.4265216	-1.7289615	0.87224202	0.3615248	0.28246275
Msl3l2	0.20729995	4.12858652	0.87219296	0.36153802	0.28246275
Dpp8	-0.090367	8.3975238	0.87158515	0.36170185	0.28253277
Gle1	0.14139565	4.91011353	0.87150824	0.36172259	0.28253277
Rab11fip4os	-0.8604208	-0.7582432	0.87147791	0.36173077	0.28253277
Isg20l2	0.21986347	4.04315154	0.87113843	0.36182233	0.28257743
Rhot1	-0.1280874	6.11619502	0.87088409	0.36189095	0.28260417
Ube2d1	-0.1529268	6.92626022	0.87063046	0.36195939	0.28263077
Kif18a	-0.4725817	0.99561614	0.8704512	0.36200778	0.2826417
Rsph3a	0.16157189	4.43966848	0.86969658	0.36221158	0.28276295
Smc1a	-0.1508975	8.10839609	0.86962142	0.36223189	0.28276295

Rimkla	-0.2513583	3.67743877	0.86912069	0.36236722	0.28281966
Crmp1	-0.150265	6.06433348	0.86909797	0.36237336	0.28281966
Spon2	-1.2527557	-0.6560705	0.86890059	0.36242673	0.28283446
St6gal1	0.21133787	6.19199686	0.86855193	0.36252102	0.28288119
Serpinb6b	0.30465931	5.19949551	0.86826489	0.36259869	0.28289314
Scaf4	0.11183221	6.40873382	0.86824088	0.36260518	0.28289314
Gm6548	0.26153851	3.51256874	0.86720423	0.36288586	0.28304461
Pcdhga4	-0.327496	1.68857709	0.86711438	0.3629102	0.28304461
Pced1a	-0.1835215	4.13256654	0.86703838	0.36293079	0.28304461
Zbtb20	0.12046446	6.86078302	0.86701517	0.36293708	0.28304461
Gm10046	-0.4329155	0.33357784	0.8668421	0.36298398	0.28305433
Harbi1	0.3677714	2.1471248	0.86610975	0.36318253	0.28318229
Mettl3	0.19542711	3.85454975	0.86526813	0.3634109	0.28333348
Resp18	0.18917063	3.1582388	0.86507128	0.36346435	0.28334827
Gm15787	-0.4681514	0.71033111	0.86478538	0.36354199	0.28338192
Tnxb	-0.3927076	1.28082218	0.86401614	0.36375101	0.28351796
Fam229b	-0.2580562	2.47483404	0.86330519	0.36394435	0.28364176
Dhodh	-0.3216393	2.11767985	0.86272249	0.36410292	0.28373844
Zfp947	0.27912482	2.25516998	0.86229543	0.3642192	0.28380214
Fgd5	-0.5256146	1.84175982	0.86103416	0.36456294	0.28404306
Tnfsf13	-0.984853	-1.1613654	0.86079272	0.36462879	0.28406744
Usp4	-0.1374606	5.49972037	0.86066417	0.36466386	0.28406783
Enox1	0.19822868	3.98225846	0.86040391	0.36473488	0.28409623
Tfdp2	0.10367589	6.23340646	0.8601601	0.36480142	0.28411177
Clip4	-0.1433341	5.24150222	0.86007751	0.36482397	0.28411177
Egfem1	-0.4066851	1.82446598	0.85976383	0.36490962	0.28415155
Nup50	-0.1251153	5.45782005	0.85940772	0.36500689	0.28420037
Rep15	1.19660633	-1.3864379	0.85918255	0.36506842	0.28422135
Slc1a4	0.19859879	4.33679528	0.85887699	0.36515193	0.28424736
Mtmr6	0.10587704	7.40515423	0.85874514	0.36518797	0.28424736
Vamp1	-0.1213753	6.0370303	0.85868075	0.36520558	0.28424736
BC029214	0.20393953	3.49030025	0.85851454	0.36525103	0.28425581
Fam175a	-0.231584	2.6092575	0.85824467	0.36532484	0.28428634
Clp1	0.2309059	2.44874484	0.85792166	0.36541321	0.28432545
Fam171a1	-0.1538641	5.50197085	0.85780809	0.36544429	0.28432545
Snx20	0.4127023	0.86909136	0.85756551	0.36551069	0.28435019
Krt9	0.18321146	3.95812675	0.8573063	0.36558165	0.28437849
Supt4a	0.26111611	4.73820163	0.85715557	0.36562293	0.28438368
Sema5b	-0.3244613	3.53938769	0.85645054	0.36581609	0.28448088
Ctr9	-0.1393011	5.2111628	0.85611997	0.36590671	0.28448088
Tomm20	-0.0968537	7.75160618	0.85584183	0.36598298	0.28448088
2210039B01	-0.3225452	1.77921097	0.85582343	0.36598803	0.28448088
Cul1	0.09841112	7.35300591	0.8558157	0.36599015	0.28448088
Jdp2	-0.1775042	3.35051052	0.85581463	0.36599044	0.28448088
Plvap	-0.4846204	0.6205832	0.85576524	0.36600399	0.28448088

Adam5	0.73770821	0.8451942	0.85568937	0.3660248	0.28448088
Mvb12b	-0.1213093	6.44063794	0.85528364	0.36613612	0.28454049
Fam160b1	-0.1547041	5.00786603	0.85503161	0.36620529	0.2845562
Churc1	0.13595233	4.54674334	0.85495774	0.36622557	0.2845562
Agpat1	-0.1706542	4.8021746	0.85479952	0.36626901	0.28456305
Ppapdc1b	0.16212838	4.27415139	0.8537852	0.36654766	0.28475263
Bag6	0.15637971	5.6876848	0.85341964	0.36664817	0.28479205
Cep135	-0.215224	4.32391521	0.85334857	0.36666771	0.28479205
BB031773	-0.8727296	-1.0355317	0.8530011	0.36676328	0.28481733
9930021J03F	-0.1485853	8.19119709	0.85297826	0.36676957	0.28481733
Fbxl7	0.24165774	6.20230065	0.85247924	0.36690689	0.28489386
Dennd6a	0.1266306	6.86793009	0.85236827	0.36693744	0.28489386
Stxbp3a	-0.1280104	4.61286465	0.85200479	0.36703753	0.28494465
Bst2	0.57979777	1.61087395	0.85179653	0.36709489	0.28496227
Gpr12	0.21748143	4.05832662	0.85107055	0.36729496	0.28509065
Ido1	0.42540615	1.31686761	0.8507633	0.36737968	0.28512324
Tom1l2	0.11785114	7.57538087	0.85066666	0.36740633	0.28512324
6430503K07I	-0.8986622	-0.7179992	0.85048138	0.36745744	0.28513598
Tob1	0.12914168	6.21399761	0.85022515	0.36752813	0.28516391
Sf1	0.10849089	6.7156002	0.84949554	0.36772954	0.28529326
Atxn3	-0.139061	5.32775843	0.84926793	0.36779241	0.2853151
Smad7	-0.2300986	3.43137267	0.84890269	0.36789332	0.28536644
Atp13a3	-0.1052362	7.15130974	0.84819531	0.36808888	0.28549119
Kctd11	0.3203692	3.32432119	0.84783656	0.36818811	0.28554121
Pald1	0.34225506	1.79750332	0.84732233	0.36833042	0.28562463
Nup37	-0.4210991	1.22698112	0.8466933	0.36850461	0.28573274
Rmdn2	-0.1967751	3.71864916	0.84644451	0.36857354	0.28575923
Zic2	0.20392078	6.54778614	0.84476152	0.3690403	0.28609413
Parp10	0.32424311	1.9135946	0.84420325	0.36919532	0.28616205
Gal3st3	-0.1620077	6.01131671	0.84419523	0.36919755	0.28616205
Fgf5	0.29779094	2.67938052	0.84383668	0.36929716	0.28619471
S1pr3	-0.2111889	3.33109425	0.84379289	0.36930933	0.28619471
Tbc1d1	0.14223788	4.55994656	0.84365548	0.36934752	0.28619732
Arc	-0.3564228	5.37353445	0.8430234	0.36952326	0.28630084
Gne	0.12598558	5.51174018	0.84291679	0.36955291	0.28630084
Ilf2	0.16366112	5.70695281	0.84279922	0.36958561	0.28630084
Faim2	-0.1271936	6.72865198	0.84259415	0.36964267	0.28631806
Krtcap2	0.20278068	3.33774937	0.84246239	0.36967934	0.28631947
Klhl4	-0.1446436	4.81035219	0.8419311	0.36982724	0.28640703
Spr	0.28892151	2.83179434	0.84099336	0.3700885	0.28653275
Cul2	-0.1670314	5.27435675	0.84093296	0.37010533	0.28653275
Iqcc	0.29053949	2.65551747	0.84091484	0.37011039	0.28653275
Zmym4	-0.1270809	7.67947104	0.84084799	0.37012902	0.28653275
Ankrd40	0.10203687	7.06114299	0.84057423	0.37020535	0.28655911
Brd1	0.11600854	6.0043742	0.84047583	0.3702328	0.28655911

Ubqln2	0.13488997	7.64015162	0.84010708	0.37033566	0.28661174
Wnk1	-0.0987254	10.008663	0.83963474	0.37046749	0.28668677
Bcar1	0.14861518	4.23651133	0.83928919	0.37056397	0.28673443
Vsig10l	-0.3144522	2.41814072	0.83867911	0.37073441	0.28679999
Oxa1l	0.16365396	5.17887572	0.83864802	0.3707431	0.28679999
Pilra	0.67981928	-0.5315319	0.83861124	0.37075338	0.28679999
Ttc7b	-0.16386	8.19378473	0.83796297	0.37093462	0.28688855
Mcemp1	0.6725616	0.11998169	0.83795204	0.37093767	0.28688855
Creb3	-0.2936075	3.08932762	0.83778395	0.37098469	0.28689792
Slmo1	-0.1935703	5.01944233	0.83746348	0.37107435	0.28691453
Pex11a	0.42961475	1.16454539	0.83745765	0.37107599	0.28691453
A730046J19I	0.63103848	0.59754944	0.8364402	0.37136086	0.28710778
Tacc2	-0.1288253	5.52330764	0.83601901	0.37147888	0.28717201
4930479D17I	-0.4591223	1.0864378	0.8356015	0.37159593	0.28723548
Kdm3a	-0.12913	5.9982817	0.83525905	0.37169197	0.28726132
Peli2	0.14836932	5.70732848	0.8352072	0.37170652	0.28726132
1200014J11I	-0.1225223	5.36314191	0.83505076	0.37175041	0.28726132
Gm5547	-1.4101113	-1.9888501	0.83471176	0.37184554	0.28726132
Trip4	0.12097847	5.85807798	0.83462095	0.37187103	0.28726132
5033406O09	-1.2973259	-0.5531035	0.83448444	0.37190935	0.28726132
Prrt2	0.11245453	7.21379978	0.83443314	0.37192376	0.28726132
Grem1	-0.4183051	0.83619109	0.83440048	0.37193293	0.28726132
Shpk	0.58537491	0.61399241	0.83408752	0.37202081	0.28726132
Senp5	0.11214465	5.58786185	0.83400007	0.37204538	0.28726132
Fut11	-0.1961528	3.90062084	0.83394038	0.37206214	0.28726132
Tmem176b	0.25564641	5.40405704	0.83392397	0.37206675	0.28726132
Prkra	0.17914545	3.57028419	0.83386361	0.37208371	0.28726132
Elp4	0.1438193	4.2881464	0.8337296	0.37212136	0.2872634
Gm11346	-0.9721762	-0.647839	0.83350875	0.37218343	0.28727179
Magohb	0.26732721	2.81648979	0.8334422	0.37220213	0.28727179
Cbl	0.1129546	6.56782542	0.83313484	0.37228854	0.2873115
Cbwd1	0.17375458	4.13995644	0.83269388	0.37241255	0.28738022
Bbc3	0.52749493	-0.1095236	0.83254765	0.37245369	0.28738499
Fgd4	-0.1484231	4.93470296	0.83210665	0.3725778	0.28745376
2810474O19	-0.124581	6.6693074	0.83086935	0.37292632	0.28766746
Nudt21	0.17611378	4.7723066	0.8306897	0.37297697	0.28766746
Svop	0.17785387	5.76057809	0.83068347	0.37297872	0.28766746
Naa25	-0.1658046	4.53536187	0.83062652	0.37299478	0.28766746
Laptm4b	-0.1269935	5.34753559	0.83049808	0.373031	0.2876684
B3gat3	0.21936599	3.19066001	0.83005797	0.37315513	0.28773713
Mtus1	0.11648537	6.68441201	0.82982338	0.37322133	0.2877547
Acd	0.1822397	3.94948651	0.8297291	0.37324794	0.2877547
4931429I11R	-0.6856414	-0.0218343	0.82945323	0.37332581	0.28778774
Fam78a	0.5300425	0.64030231	0.8293108	0.37336602	0.28779174
H2-K1	0.22264	5.06587241	0.82905778	0.37343747	0.28781983

Mical2	0.13162223	8.91771607	0.82884292	0.37349817	0.28783676
Uqcc2	0.23984993	3.50855036	0.82861324	0.37356306	0.28783676
4930404H11I	1.18806385	-1.2410107	0.82860811	0.37356451	0.28783676
Rbfox2	0.11016788	7.5197972	0.82833724	0.37364106	0.28786669
Rnpep	0.22057097	3.44437432	0.82815335	0.37369305	0.28786669
Rasgrp1	-0.1686332	9.64199137	0.82798861	0.37373963	0.28786669
Psma5	0.13957134	5.72397389	0.82797514	0.37374344	0.28786669
Hmgn3	0.15494767	6.929729	0.82756899	0.37385831	0.28792818
Bcl11a	-0.1314048	6.56535469	0.82729604	0.37393555	0.28796068
Caap1	-0.20902	2.76923956	0.82707186	0.373999	0.2879793
Rtn4	-0.1192802	9.9353984	0.82696306	0.37402979	0.2879793
Agbl4	0.36982227	2.42252129	0.82657261	0.37414035	0.28803744
Tead3	0.26880329	2.73021835	0.82643673	0.37417884	0.28804009
Mbnl3	-0.3751496	1.24336771	0.82613046	0.37426561	0.2880799
Rab6b	-0.1110791	10.4152573	0.82571617	0.37438303	0.28814329
Acsl1	-0.1184829	6.51345693	0.82550896	0.37444178	0.2881555
Vip	-0.236244	2.80295463	0.82531089	0.37449795	0.2881555
Aard	-0.3650785	1.58846714	0.8252893	0.37450407	0.2881555
B230208H11	-0.9880519	-0.0817268	0.82492658	0.37460697	0.28820769
Colgalt2	-0.3144778	1.4849493	0.82471353	0.37466742	0.28822373
1700101I11R	0.73929284	0.31317228	0.824606	0.37469794	0.28822373
Ms4a6c	-0.5364915	0.37829737	0.82433912	0.3747737	0.28825174
Usp27x	-0.1456864	4.93859851	0.8241245	0.37483465	0.28825174
Gm13826	0.21792139	2.96121639	0.82410716	0.37483957	0.28825174
Car12	0.23569358	3.31071986	0.82391391	0.37489446	0.28826698
D030056L22I	-0.1806762	4.86622	0.82353459	0.37500223	0.28829902
Celf6	0.23413286	2.68095841	0.82352032	0.37500629	0.28829902
Tex2	0.10228628	6.54955136	0.8222562	0.37536578	0.28854841
Akap1	-0.2124032	3.33678222	0.82199265	0.37544079	0.28857908
Dclk2	-0.2024481	4.01234502	0.8210492	0.3757095	0.28870628
Asb13	-0.1492169	4.60256161	0.82100238	0.37572284	0.28870628
Tbc1d32	-0.209931	4.73628723	0.82099364	0.37572533	0.28870628
Grhl3	0.81706221	-0.2245876	0.82091835	0.37574679	0.28870628
Frat2	-0.3349822	3.01946885	0.81995088	0.37602267	0.28889125
Aif1l	0.2933532	2.10260785	0.8195842	0.37612731	0.28894463
Memo1	0.15340473	4.53854241	0.81927827	0.37621465	0.28898472
Rims1	-0.1544701	6.48495215	0.81914904	0.37625155	0.28898605
Hddc2	0.18001137	3.66227842	0.81873265	0.37637048	0.28905039
Grap	0.43896031	0.96999009	0.81859123	0.37641089	0.28905441
Cth	-0.4868612	0.94286867	0.81843091	0.37645671	0.28906259
4930526I15R	0.39688361	1.65572054	0.81792248	0.37660205	0.28914718
Cactin	0.23540188	3.17297834	0.81767379	0.37667318	0.28917478
1810019D21I	1.1003338	-0.5570724	0.81739858	0.37675191	0.28920821
Gsn	0.19032516	5.4563857	0.81699397	0.37686771	0.28927008
Lrch2	-0.2079273	4.19729802	0.81683697	0.37691265	0.28927756

Srsf2	0.11555042	8.34931977	0.81640443	0.37703652	0.28934561
Tmem221	0.76822219	-0.4644933	0.81564235	0.3772549	0.28948617
Dtnb	-0.1606323	4.6439669	0.81525858	0.37736495	0.28954359
Vopp1	0.11798232	5.84434405	0.81499655	0.37744011	0.28957422
Dcaf11	0.12847128	4.86651126	0.81449684	0.37758352	0.28965721
Otub1	0.20111323	5.7233998	0.81415617	0.37768133	0.2897052
Fam96b	0.23100379	2.67795125	0.81394433	0.37774217	0.28972483
Fam210b	0.17860356	5.27692419	0.81300806	0.37801123	0.28990415
Heatr6	-0.1592246	4.8189271	0.81277925	0.37807703	0.28992756
Setd1a	-0.1530855	5.5857919	0.81149914	0.37844547	0.29018301
Zfp568	0.20339167	3.27424501	0.81103699	0.37857861	0.29025371
Sema6a	-0.166452	5.73162937	0.81093399	0.37860829	0.29025371
A830082K12I	0.12983193	6.61205122	0.81072023	0.37866991	0.29027279
Spint2	0.26313903	2.87373047	0.81060259	0.37870382	0.29027279
Oaz1	0.15230122	5.13371728	0.81047962	0.37873927	0.2902729
A430105I19F	0.25119989	3.33664494	0.81006053	0.37886015	0.29033846
Ei24	-0.1518229	6.08275607	0.80968073	0.37896974	0.29038703
Ccdc38	-0.5597167	-0.1987506	0.80955526	0.37900595	0.29038703
Nrde2	0.33748636	2.0474504	0.80947361	0.37902952	0.29038703
Fam58b	0.23960209	4.09447951	0.809011	0.3791631	0.29044019
Mplkip	0.13940282	4.05352396	0.80898859	0.37916957	0.29044019
Itfg1	0.1124369	7.75586571	0.80844684	0.3793261	0.29052584
Tnfrsf4	1.62933612	-2.2440682	0.8083569	0.37935209	0.29052584
Mkl1	0.11556641	5.55924494	0.80781663	0.37950829	0.29059025
Rpgrip1	0.79385724	-0.9646415	0.80766162	0.37955313	0.29059025
Zfp629	-0.1768328	4.52124725	0.80762025	0.3795651	0.29059025
Mocs3	0.59846974	-0.9356207	0.80757696	0.37957762	0.29059025
C4b	-0.2470666	2.77120039	0.80735587	0.37964159	0.29061216
Hmgcs1	-0.1486002	7.21088302	0.80715494	0.37969973	0.2906296
Nt5dc1	-0.2587901	3.02824271	0.80693814	0.37976249	0.29065057
Cx3cr1	-0.2457144	3.35088241	0.80633953	0.37993584	0.29075617
Slc18a3	0.66759572	0.02036782	0.80614761	0.37999145	0.29077165
Fam187b	-0.8465812	0.85249679	0.80558994	0.38015309	0.29086825
Fxyd1	-0.2267565	3.46058344	0.80522132	0.38025999	0.29090922
Elavl1	0.10370471	6.63422728	0.80504033	0.38031249	0.29090922
Pdgfra	0.16010853	5.49716989	0.8050392	0.38031281	0.29090922
Mgat3	0.12280042	6.48403666	0.80471725	0.38040623	0.29091332
Tex10	-0.1523704	4.35355981	0.80465829	0.38042335	0.29091332
Trp53cor1	-0.5394764	-0.0263745	0.80465481	0.38042436	0.29091332
Zfp930	-0.1757438	4.10431084	0.80428647	0.38053129	0.29096802
Ergic3	0.19922971	5.24047087	0.80382331	0.38066581	0.2910438
Gm10536	-0.8757978	-1.3984401	0.80340384	0.3807877	0.29110992
Nfia	0.14268626	9.13850662	0.80324766	0.3808331	0.29111754
Sos2	-0.1188012	7.66871831	0.80309824	0.38087654	0.29112368
Rnf152	0.1180373	6.19103377	0.80257438	0.38102889	0.29121305

Wdr31	0.3278551	1.78852451	0.80176939	0.38126319	0.29136502
Arfgef2	-0.1566543	6.1855433	0.80100468	0.38148596	0.29148302
Nudt13	-0.2210292	2.66711577	0.80088	0.3815223	0.29148302
Herc4	-0.1699837	4.61268462	0.80087419	0.38152399	0.29148302
Tmem106c	0.31619638	3.83407144	0.80074412	0.38156191	0.2914849
Rps3	0.21512309	6.97730527	0.80052409	0.38162606	0.29150681
Atp5sl	0.20883505	3.21692517	0.80005397	0.38176318	0.29158445
Tmem120b	0.71417314	0.44603953	0.79959483	0.38189717	0.29165969
Tlk1	-0.0910899	7.60864649	0.79938156	0.38195944	0.29168014
Rps6kl1	0.30440088	2.37169747	0.79876965	0.38213816	0.29178312
Cr1l	0.16894859	5.40021269	0.79851092	0.38221377	0.29178312
A730017C20	0.11921605	5.8893004	0.79845374	0.38223048	0.29178312
Itga11	0.24788524	2.90705307	0.79836061	0.3822577	0.29178312
Strip1	-0.1447663	4.37981101	0.79831236	0.3822718	0.29178312
Fam171a2	0.22535659	2.68908278	0.79788663	0.38239629	0.29185104
Vasp	0.31515755	3.48114641	0.79740159	0.38253818	0.29193223
Mdga1	-0.3199652	2.48382082	0.7972528	0.38258173	0.29193524
Man1a	-0.1816553	5.19943443	0.79714536	0.38261318	0.29193524
Arsa	0.26357793	2.4348493	0.79691885	0.38267949	0.29195874
Mepce	0.15709905	4.31707559	0.79670244	0.38274286	0.29197998
Fancl	0.20744457	2.71916364	0.79641326	0.38282756	0.29201749
Vsx2	1.40665946	-1.7368724	0.79566204	0.38304773	0.29215832
Stk36	-0.3793829	1.23416764	0.79524192	0.38317094	0.29222298
Hmgcl	0.23241681	2.57252918	0.79513054	0.38320362	0.29222298
Hyi	-0.3544982	1.56627172	0.79464923	0.38334486	0.29228087
Tbx3	0.20168481	3.70014722	0.79462954	0.38335064	0.29228087
Myh7b	0.38415279	2.32790488	0.79440985	0.38341514	0.29230293
Mrpl14	0.28863613	3.03606529	0.7934371	0.38370092	0.29244599
Rps19-ps3	0.40893021	0.61789326	0.79342663	0.383704	0.29244599
Tead2	0.30626224	2.48379968	0.79324129	0.38375848	0.29244599
Asic3	1.23056782	-2.0363089	0.79958831	0.38376747	0.29244599
Dnph1	0.37669521	1.62737352	0.79304659	0.38381573	0.29244599
Ctnnd2	-0.1531827	9.36130243	0.79304472	0.38381628	0.29244599
Dnmt3b	-0.5707593	0.32546079	0.79284874	0.38387392	0.29246279
Zfml	-0.1369678	7.89140434	0.79247139	0.38398494	0.29251281
Tmem135	0.09791566	6.07846201	0.79227299	0.38404333	0.29251281
Gstk1	0.23987673	3.26041485	0.79226275	0.38404635	0.29251281
Ric8b	-0.1787094	5.65115964	0.79206963	0.3841032	0.29252901
Usp38	-0.150579	5.03510708	0.79065065	0.38452129	0.29282029
Nkx3-1	0.52875353	0.14710856	0.79030047	0.38462457	0.2928293
Rgs7	-0.1320854	7.1722786	0.79029678	0.38462565	0.2928293
Tra2a	0.12932232	6.08364828	0.79024815	0.38464	0.2928293
Tmem246	-0.1528849	4.14919465	0.79008262	0.38468884	0.29283935
Mef2d	0.11646757	7.08971438	0.78952721	0.38485278	0.29293701
Serpinb9b	-0.7982943	-0.7571018	0.78868557	0.3851014	0.29308213

Rgs7bp	0.13235804	8.51584196	0.78862254	0.38512002	0.29308213
Tarsl2	-0.1931021	4.5568024	0.78851976	0.3851504	0.29308213
Rell1	0.22137171	5.182429	0.78788185	0.38533903	0.29319853
Wdr44	-0.1395412	4.89162934	0.78723964	0.38552908	0.29331597
Wasf3	0.13338102	5.95092442	0.78662706	0.38571048	0.29342682
Impdh1	-0.2712444	2.57882557	0.78612935	0.38585796	0.29351185
Zbtb46	0.38990959	1.31475956	0.78599424	0.38589801	0.29351515
Ppp1r2-ps3	-0.5485501	-0.4670166	0.78575094	0.38597015	0.29354285
Foxp1	0.09993772	8.77787499	0.78527637	0.38611091	0.29362274
Slc24a1	-0.7326485	-0.0401307	0.78514444	0.38615005	0.29362534
Cad	-0.3229402	1.781203	0.78407603	0.38646728	0.29383937
Polg	-0.1950233	3.26366115	0.78381235	0.38654563	0.29386451
Dennd3	-0.3482388	1.70226287	0.78372414	0.38657185	0.29386451
Sgk2	-0.5981775	-0.36339	0.7833991	0.38666847	0.29391078
Klrb1c	-1.0513582	-0.3976589	0.78310792	0.38675507	0.29394009
Fam129c	-0.8659448	-0.3552634	0.782925	0.38680948	0.29394009
Cables1	-0.2203575	3.13166928	0.78282037	0.3868406	0.29394009
Trim27	0.13466039	4.48871746	0.78278847	0.38685009	0.29394009
Fam173b	0.36201643	0.83055761	0.78249372	0.38693781	0.29397956
1700027H10I	-0.5731519	0.39054886	0.78225454	0.38700901	0.29400648
Zfp758	-0.1871424	3.79792439	0.78178422	0.38714907	0.2940857
Ccdc30	0.24221152	3.32439912	0.78116649	0.38733314	0.29419834
Ednra	0.16786399	5.01863621	0.78095019	0.38739763	0.29422012
2610015P09I	-0.1787183	3.72388813	0.78037939	0.38756787	0.29432223
LOC1012436	-0.7186123	-0.5993772	0.77948587	0.3878346	0.29449182
Aim2	0.39198953	2.19248865	0.77939127	0.38786285	0.29449182
Golga5	0.16668295	4.18905184	0.77899789	0.38798038	0.29453851
Hspa1l	-0.2576288	2.37598543	0.77894553	0.38799603	0.29453851
Nipa1	-0.1610712	4.82904993	0.77874587	0.3880557	0.29454192
Rbfa	0.25966611	2.63680944	0.77869072	0.38807219	0.29454192
Tbc1d24	-0.1582155	6.35733208	0.77771794	0.38836315	0.29473404
Clec4a3	-1.0201451	-0.2808366	0.77760473	0.38839703	0.29473404
Pvrl1	0.14621573	4.31616693	0.77743497	0.38844785	0.29474539
Myrip	-0.1610077	6.38887838	0.77715794	0.38853079	0.29478111
Cbx6	-0.1214246	6.62613416	0.77687565	0.38861534	0.29479138
Mbtps1	-0.1353249	5.43469457	0.77687325	0.38861606	0.29479138
Magi2	-0.1514503	7.42040035	0.77630413	0.3887866	0.29489225
Mtmr7	-0.2009144	5.04746558	0.77619006	0.3888208	0.29489225
Efnb3	0.18424987	4.91469848	0.77540195	0.38905718	0.29501039
Ccdc74a	-0.2541096	2.01806722	0.77537936	0.38906395	0.29501039
Grpel2	-0.1677652	4.21739814	0.77531177	0.38908424	0.29501039
Nufip2	0.10721498	7.74209213	0.77476367	0.38924877	0.29510792
Dnajc5	0.09309257	7.92614819	0.77453308	0.38931803	0.2951332
Seh1l	0.15646718	5.64471097	0.77434922	0.38937326	0.29514378
Tmub1	0.25825591	2.31800996	0.77424754	0.38940381	0.29514378

Sox13	-0.2811563	3.36118298	0.7729111	0.38980567	0.29542112
Fcrls	0.41228768	1.54344306	0.77262962	0.38989039	0.29545761
Clec5a	0.58290057	-0.1262084	0.77248247	0.38993469	0.29545761
Fhad1	0.29691788	2.56366346	0.7723929	0.38996166	0.29545761
Nab2	0.17893057	5.10920248	0.77220964	0.39001685	0.29547219
Zfp583	-0.2862734	3.39265592	0.7717294	0.39016152	0.29555455
Col13a1	0.35668486	2.28792226	0.77126721	0.39030084	0.29561085
Mccc2	-0.1865342	3.4833902	0.77124418	0.39030778	0.29561085
07-Sep	-0.1236231	9.11917513	0.77002	0.39067714	0.29586334
Pskh1	0.24052245	3.80027565	0.76986109	0.39072513	0.29586811
Zfp946	-0.2418008	2.87394696	0.76971494	0.39076926	0.29586811
Abcb1b	-0.2777078	1.66778348	0.76964152	0.39079144	0.29586811
C8g	0.62546997	0.37226714	0.76942451	0.390857	0.29589049
Comtd1	0.34583677	1.05456235	0.76875575	0.39105914	0.29594982
Ftsj3	0.14440402	5.00058319	0.76872401	0.39106874	0.29594982
Gtf3c6	0.19806569	5.50182977	0.76862785	0.39109782	0.29594982
F2	-0.9966614	-1.044684	0.76858316	0.39111133	0.29594982
Ptgs2os	0.41516857	0.78329448	0.76856967	0.39111541	0.29594982
Mrpl43	0.16854778	5.11496143	0.76824325	0.39121415	0.29595625
Aoah	0.7516961	-0.6572058	0.7681943	0.39122897	0.29595625
Tmem164	0.13385804	4.58664058	0.76818451	0.39123193	0.29595625
Il5	-1.2716296	-1.8544092	0.76772254	0.39137174	0.29603477
Tmem192	-0.2643972	2.30239886	0.76740097	0.39146911	0.29605813
Gfer	-0.2256089	3.00280689	0.76738259	0.39147468	0.29605813
Rabl6	-0.1309262	5.81756979	0.76704454	0.39157708	0.29610833
Cul5	0.09866043	7.02475883	0.7665472	0.39172781	0.29619284
Zfp964	-0.3641853	1.2599047	0.76643799	0.39176092	0.29619284
Ppig	0.1422879	9.2691446	0.76592922	0.39191521	0.29628225
Tbx22	-1.1323358	-1.4582454	0.76575404	0.39196836	0.29629517
Creg2	0.14108621	7.62972907	0.7655707	0.392024	0.29630998
Phf11a	0.62953869	-0.4520695	0.76510488	0.39216541	0.29638961
Tmem198	0.33170653	2.00437496	0.76461577	0.39231397	0.29647463
Ppp6r3	-0.1001211	6.4274008	0.76439265	0.39238177	0.2964986
Slc16a7	-0.1706998	4.68758201	0.76416489	0.392451	0.29652365
Tmem167b	0.19896269	6.20567732	0.76389592	0.39253278	0.29654253
Pth2r	1.10723093	-1.3312473	0.76376102	0.3925738	0.29654253
Gpr21	-0.4503865	0.86234498	0.76372673	0.39258423	0.29654253
Hnrnph1	-0.0842012	8.53090032	0.76354148	0.39264058	0.29654388
9430008C03I	-0.3493335	1.71279956	0.76348363	0.39265818	0.29654388
Pnldc1	-0.6415153	-0.2701485	0.76301102	0.392802	0.29660811
Tspyl2	-0.1406795	6.06718009	0.76291263	0.39283195	0.29660811
Pigc	0.30510531	3.10018614	0.76272038	0.39289048	0.29660811
Zmynd12	0.92523344	-1.5953784	0.76261228	0.3929234	0.29660811
Sstr4	0.30356963	4.50972522	0.76260613	0.39292527	0.29660811
Cml3	0.31953328	1.66790898	0.76249292	0.39295975	0.29660811

Polr3c	-0.1783932	3.20865398	0.76207829	0.39308607	0.29667252
Dock2	-0.2432704	2.3581505	0.76185966	0.3931527	0.29667252
Cd44	-0.2063944	3.13111967	0.76185748	0.39315336	0.29667252
Sf3b3	0.12943164	5.27586135	0.76136779	0.39330267	0.29675793
Fbxw7	-0.1069809	8.24940269	0.76103044	0.39340557	0.29680833
Ago1	-0.0924874	6.22133921	0.76051536	0.39356277	0.29689967
Snx24	0.19035374	4.98465963	0.76026774	0.39363837	0.29692946
Zfp455	-0.2087727	3.18800045	0.75984501	0.39376749	0.29699959
Prrx2	0.42781157	3.43939736	0.75926682	0.3939442	0.29710561
Ccnyl1	0.13849652	5.32079068	0.75904147	0.3940131	0.29711029
Bcap31	0.1838759	5.73327821	0.75901008	0.3940227	0.29711029
Wdr55	0.15113057	4.11983613	0.75887382	0.39406437	0.29711446
Sh3tc2	-1.1003797	-1.284331	0.75818263	0.39427587	0.29722402
Slc39a5	-0.5295283	-0.5327547	0.75797927	0.39433813	0.29722402
Plscr2	0.24869362	4.4211804	0.75787556	0.39436988	0.29722402
Megf9	-0.1326542	5.961198	0.75783557	0.39438213	0.29722402
Sod2	0.10735423	8.45926204	0.75780824	0.3943905	0.29722402
Emc10	0.16864233	5.07381744	0.75749293	0.39448708	0.29726954
Nudt1	-0.6517394	0.61450683	0.75707248	0.39461591	0.29733937
Hepacam2	-0.6042621	0.47403687	0.7568872	0.39467271	0.29734411
Mipep	0.19328851	4.032367	0.75681593	0.39469456	0.29734411
Spsb2	0.43325109	0.96639361	0.75668154	0.39473577	0.2973479
9130024F11I	-0.1940939	4.23553446	0.75619567	0.3948848	0.2974329
Yif1a	0.36399892	1.70687004	0.75580356	0.39500514	0.29749628
Rnls	0.38786693	1.0564206	0.75557724	0.39507462	0.29751785
Smg7	-0.0919628	8.02274237	0.75545749	0.39511138	0.29751785
Rspry1	0.13670483	4.75303855	0.7552078	0.39518807	0.29751785
Nlrc4	-0.9903461	-1.284615	0.755199	0.39519078	0.29751785
Rnf5	0.1687099	5.68227105	0.75512089	0.39521477	0.29751785
Arl6ip6	-0.1580486	4.59308749	0.75499205	0.39525435	0.2975204
Ccdc61	0.4066932	0.48748189	0.75485296	0.39529709	0.29752532
Smc3	-0.1235338	8.16643801	0.75446127	0.39541749	0.29758868
Brix1	0.13675773	4.72605738	0.75383838	0.39560906	0.2977056
Mcoln1	-0.2289591	2.92515908	0.7533104	0.39577155	0.29780061
Srsf10	0.10967232	6.59195119	0.75234981	0.39606744	0.29799598
Myocd	1.48907108	-1.5673105	0.75210842	0.39614185	0.29800545
Slc19a3	-0.6069678	0.32608447	0.7520737	0.39615255	0.29800545
Hist1h2bj	0.79601842	-1.5692539	0.75187279	0.3962145	0.29802478
2310067B10I	0.20468051	4.10431381	0.75144668	0.39634593	0.29809636
Col4a5	-0.2664882	3.29532106	0.75122392	0.39641467	0.29812078
Npl	0.40377033	1.59114051	0.75099454	0.39648547	0.29812918
Slpi	1.0138861	-1.3967674	0.75081362	0.39654132	0.29812918
Mycbp	0.16731216	5.63413358	0.75077309	0.39655384	0.29812918
Fbxo3	-0.1005764	6.50548621	0.75071772	0.39657093	0.29812918
Phf6	0.1107348	5.27930574	0.75039744	0.39666985	0.29817086

Prokr2	0.42028096	1.92358666	0.7503033	0.39669893	0.29817086
Usp47	0.0868856	7.1442615	0.75008363	0.39676681	0.29819461
Jag1	0.20121702	3.72956607	0.74996131	0.39680461	0.29819575
Zfp934	0.20213949	3.61369777	0.74960891	0.39691355	0.29824929
Dmwd	-0.1519008	4.86534183	0.74949611	0.39694843	0.29824929
Jazf1	0.15255389	5.2823902	0.7491307	0.39706145	0.29830694
Scarf2	-0.4774308	1.58556916	0.7487847	0.39716851	0.2983601
Plscr3	-0.3876525	1.25531177	0.74828198	0.39732415	0.29844974
Apoe	-0.2860125	6.97856543	0.7480603	0.3973928	0.29847403
Map2k3	0.22771649	3.88687435	0.747653	0.397519	0.29854153
Ankrd27	-0.1123211	5.38224598	0.74723323	0.39764912	0.29861197
Trim68	-0.396013	1.24116995	0.74705397	0.3977047	0.29862642
Tmem86a	0.32528145	4.25573713	0.74659227	0.39784793	0.29870668
Nadk	0.15335432	5.45393474	0.74633333	0.39792828	0.29873844
Mark3	0.09850442	6.40504925	0.74622174	0.39796292	0.29873844
Hspb7	-0.4569951	0.47307852	0.74587027	0.39807205	0.29878819
Ptms	0.24410871	6.61621914	0.74576449	0.3981049	0.29878819
AW822252	-0.6532617	0.07551761	0.74565712	0.39813825	0.29878819
Sptlc1	-0.1562699	3.62416493	0.74551673	0.39818187	0.29879364
Ndel1	0.14651855	4.91685716	0.74533679	0.39823778	0.29880831
Primpol	0.3209706	2.5564363	0.74503804	0.39833063	0.2988507
Nfic	0.13535169	7.69138183	0.74489204	0.39837602	0.29885747
2700069I18R	0.40551968	0.80104733	0.74465582	0.39844947	0.29888529
0610007P14I	0.17130961	4.25240262	0.74409296	0.39862458	0.29898936
Slc4a2	0.22255162	3.65489648	0.74380897	0.39871297	0.29902296
1500015A07I	-0.311494	2.19845466	0.74371529	0.39874214	0.29902296
Shmt1	-0.8601251	-0.4443665	0.74270029	0.39905834	0.29923278
Ispd	0.21675214	3.40984449	0.74249308	0.39912294	0.29924408
Parp12	0.17728477	4.35770763	0.7424184	0.39914623	0.29924408
Gatsl3	0.39836733	1.40357407	0.7422165	0.39920919	0.29924496
Sox18	0.34981163	1.74663565	0.74218113	0.39922022	0.29924496
3110002H16I	-0.2481313	3.3694138	0.74203219	0.39926668	0.29925249
Cog1	-0.1691331	4.11567653	0.74096346	0.3996003	0.29946582
Fto	-0.103302	7.00755629	0.740887	0.39962418	0.29946582
LOC1000389	1.21179765	-1.5510624	0.74073526	0.39967158	0.29947404
Pfkl	0.16091242	4.18051187	0.74061071	0.3997105	0.2994759
BC068157	-0.1981877	5.92522908	0.74045038	0.39976061	0.29948614
Naa10	0.27168485	2.32544461	0.73995091	0.39991676	0.29950339
Gpr125	-0.2334646	3.35494586	0.739804	0.39996271	0.29950339
Smpd2	-0.2718538	2.31042198	0.73979977	0.39996403	0.29950339
Tardbp	0.09979325	7.45746874	0.73967925	0.40000173	0.29950339
Ptrh2	-0.1272966	4.96435401	0.7396526	0.40001007	0.29950339
Slc39a3	-0.1894902	3.21713689	0.73934175	0.40010733	0.29950339
5830418P13I	0.57338111	0.17613779	0.73929912	0.40012067	0.29950339
Pex13	-0.1158079	5.35870662	0.73921406	0.4001473	0.29950339

Mtss1l	-0.1163381	7.05287022	0.7391315	0.40017314	0.29950339
Psapl1	-1.5242912	-1.7375642	0.73903104	0.40020459	0.29950339
Nnt	0.12085101	5.08269873	0.73891734	0.40024019	0.29950339
Sass6	-0.1811893	3.77705377	0.73887319	0.40025401	0.29950339
Nrip1	0.13401249	6.16891455	0.73875004	0.40029258	0.29950339
Xcl1	0.85308844	-0.5193357	0.73866166	0.40032025	0.29950339
Prox1	0.1495766	4.8871507	0.73862981	0.40033023	0.29950339
Vapb	0.09462193	6.66791428	0.7383065	0.40043152	0.2995519
Kcnq4	0.86309872	-1.0620554	0.737953	0.4005423	0.29958139
Nlrc3	-0.9137149	-0.9379213	0.73794811	0.40054384	0.29958139
Arid5a	0.33220144	1.19531805	0.73737559	0.40072337	0.2996793
Crlf3	-0.2537659	3.11838493	0.73729812	0.40074767	0.2996793
Amdhd1	1.04629468	-1.7095124	0.73694938	0.4008571	0.29971238
Prap1	1.58834406	-1.6095757	0.73692297	0.40086539	0.29971238
Glrb	-0.1132163	6.74614551	0.73680855	0.4009013	0.29971238
Fcgr2b	-0.6444377	0.11290006	0.7363494	0.40104546	0.29979289
Rab27b	0.13884363	5.41007318	0.73583507	0.40120705	0.299879
Tmco4	0.35498235	2.26886504	0.73575048	0.40123363	0.299879
Fam117a	-0.3686112	3.77824039	0.73490522	0.40149942	0.30005036
Prpf31	0.14416425	4.10967263	0.73450139	0.40162649	0.30011804
4632434I11R	0.40367	0.48499813	0.73416122	0.40173359	0.30014972
Fbxl20	0.10096819	6.62663552	0.73413476	0.40174192	0.30014972
Lingo4	-1.0869697	-1.408254	0.73374984	0.40186315	0.30019255
Prom2	-1.115949	-1.9620922	0.73372082	0.4018723	0.30019255
Nynrin	-0.1419945	5.67264852	0.73347942	0.40194836	0.30022208
Echs1	0.17156608	4.38260883	0.73304701	0.40208466	0.3002966
Sil1	0.27898458	2.5779536	0.73268556	0.40219865	0.30035444
Bax	0.23486221	2.61546348	0.73226316	0.40233193	0.30041684
Nptn	0.11580703	8.10613287	0.73218904	0.40235532	0.30041684
Zfp133-ps	-0.4168006	0.55557483	0.73191423	0.40244207	0.30044687
Nkiras2	-0.7431831	0.14816501	0.73183008	0.40246864	0.30044687
Neu2	-0.4141066	0.65232574	0.73168943	0.40251305	0.30045273
Gm15706	-0.267112	1.90207161	0.73135867	0.40261753	0.30050343
4930483K19I	0.63617571	-0.4326204	0.73106618	0.40270995	0.30054512
Aass	-0.5322992	0.83297659	0.73055892	0.40287032	0.30063217
Xlr4a	-0.5645349	0.03908689	0.73046587	0.40289975	0.30063217
Tango2	0.16391092	3.743236	0.72996772	0.40305734	0.30072246
Slc35g1	0.24251694	4.50834341	0.72951152	0.40320175	0.30074926
Adcyap1r1	-0.1605009	6.44811207	0.72943805	0.40322501	0.30074926
E030019B13I	-0.9119086	-1.3835577	0.72941691	0.40323171	0.30074926
Trpm4	-0.3408991	2.0419583	0.72939189	0.40323963	0.30074926
Il18rap	0.75786406	-0.4302662	0.72837113	0.40356308	0.30096318
Det1	-0.3024874	1.32187188	0.72799237	0.40368319	0.30102545
Trim47	0.4113054	2.25336164	0.72781463	0.40373958	0.30104018
Aldh1b1	-0.4799721	0.04492689	0.72760759	0.40380527	0.30106185

Msl2	-0.1119142	7.27702736	0.7272052	0.403933	0.30111141
4931428F04I	0.36875136	1.13749693	0.72711142	0.40396278	0.30111141
Plk3	0.37849828	1.70869438	0.72705198	0.40398165	0.30111141
Neurl1a	-0.1932686	4.46459299	0.72677828	0.40406859	0.3011489
Tm2d3	-0.1915568	3.24777264	0.72630644	0.40421851	0.30123332
Prpf4b	0.11378111	7.35039184	0.72590203	0.40434708	0.30130182
1700025F24I	1.27693631	-2.177585	0.72573031	0.40440169	0.30131519
U2af2	-0.1969098	4.69481803	0.72548803	0.40447877	0.3013453
H2afj	0.24004197	3.5416245	0.72520377	0.40456922	0.30138537
Tigd2	-0.147606	4.43055657	0.72475187	0.40471308	0.30146522
Amph	0.13979708	6.92174511	0.72460597	0.40475954	0.30147251
Mpzl1	-0.1945394	3.20946085	0.72442266	0.40481793	0.30148868
Ccdc89	-0.5349005	0.33416161	0.72425643	0.40487089	0.3015008
Prr24	-0.1468266	4.53434013	0.72396185	0.40496477	0.30153334
Gm16740	-0.779844	-0.806682	0.72380632	0.40501435	0.30153334
Gpr61	-0.2885202	1.32576376	0.723774	0.40502465	0.30153334
Nes	-0.148376	4.26178602	0.72298709	0.40527564	0.30168352
Pou2f1	-0.1173888	5.80107812	0.72275755	0.4053489	0.30168352
Gm5617	0.45725172	1.57345453	0.72266942	0.40537703	0.30168352
Spata5l1	0.74103466	-0.7576528	0.72261508	0.40539438	0.30168352
Pifo	-0.8940287	-0.2393641	0.72256646	0.4054099	0.30168352
Mir9-2	-1.0043533	-0.9702704	0.72213128	0.40554887	0.30175961
Sntn	0.64967933	-0.1784704	0.72144444	0.40576837	0.3018956
C130036L24I	0.66537139	-0.1689288	0.72091994	0.4059361	0.3019842
B130024G19	-0.3924255	1.1616731	0.7208423	0.40596094	0.3019842
Lyz2	0.2758076	3.56935062	0.72042153	0.40609559	0.30205703
Tmem215	0.29613521	3.3389055	0.71985603	0.40627666	0.30216437
Dnajc15	0.1603698	4.25157973	0.71966851	0.40633673	0.3021817
1500011B03I	0.15691623	5.03776438	0.71873598	0.40663567	0.30237665
Gm128	0.69142778	-0.8928464	0.71763456	0.40698917	0.30257075
Cpsf6	-0.1586639	5.58266669	0.71758758	0.40700426	0.30257075
Taf15	0.14581708	5.09488274	0.71751265	0.40702833	0.30257075
Cyb561d1	-0.1719174	3.70922664	0.71746404	0.40704394	0.30257075
P4hb	0.19657467	5.58337131	0.71653544	0.4073424	0.30276522
Fam193a	-0.1227603	7.39228776	0.71620137	0.40744986	0.30281771
Usp6nl	0.12246831	5.91250472	0.71601637	0.40750938	0.30283456
1700084C01I	-0.3129162	1.65505672	0.71581967	0.40757269	0.30285422
2700089E24I	0.11920873	8.43300585	0.71564289	0.40762959	0.30286913
4930414L22I	0.20698653	3.17796153	0.71501653	0.40783132	0.30298749
Kctd3	-0.1536324	5.13243541	0.71491934	0.40786263	0.30298749
Rpl37	0.16562124	6.35124797	0.71402077	0.40815232	0.30316177
Serf1	0.23340304	2.89627059	0.71396288	0.408171	0.30316177
B4galnt4	-0.2433375	3.61833574	0.71380294	0.4082226	0.30317113
Adap1	0.15831994	4.20432379	0.71357419	0.40829641	0.30317113
Fen1	-0.2243013	2.73471219	0.71348138	0.40832637	0.30317113

Sertad2	-0.1274906	5.37338932	0.71346657	0.40833115	0.30317113
Tmed3	-0.2649336	2.97606323	0.71310807	0.40844689	0.3032063
Dusp11	-0.1141974	6.38920136	0.7130054	0.40848005	0.3032063
Maneal	0.14583836	4.31987879	0.71297709	0.40848919	0.3032063
Fam120a	0.09852712	8.53748752	0.71275402	0.40856125	0.30322735
Ralgps2	-0.1050555	6.33811271	0.71266089	0.40859134	0.30322735
Cntnap5a	-0.2079013	4.3575796	0.71196695	0.40881564	0.3033493
Lrfn1	-0.4971295	0.21389315	0.71192416	0.40882948	0.3033493
Srl	0.27688779	1.87903088	0.71175008	0.40888578	0.30335109
Gpx3	-0.2378886	3.06524729	0.71159189	0.40893695	0.30335109
Sarm1	-0.2544859	3.71037135	0.7115744	0.40894261	0.30335109
Hs3st4	-0.1337128	5.45313509	0.71098132	0.40913456	0.30346556
Irak3	0.30588106	2.79377996	0.71086947	0.40917077	0.30346556
Mmgt1	0.13580739	4.66020871	0.7107092	0.40922267	0.30347666
9530051G07	-0.42569	0.85505439	0.7105349	0.40927912	0.30349115
C87436	0.22582052	3.60586379	0.71008763	0.40942405	0.30357122
Selt	0.12965062	8.78450393	0.70994056	0.40947172	0.30357918
Gpr82	-1.1601736	-1.234143	0.70916979	0.40972169	0.30371979
Th	0.58502542	0.60102874	0.70906636	0.40975525	0.30371979
Pou3f2	-0.1489973	4.41732911	0.70891465	0.40980449	0.30371979
Polb	-0.1787546	4.32665146	0.70881896	0.40983555	0.30371979
Lsamp	-0.19831	5.43219533	0.70878632	0.40984614	0.30371979
Pm20d1	0.27868832	2.06002401	0.70849849	0.40993959	0.30374822
Rpp25l	0.32588603	2.05738718	0.70843325	0.40996078	0.30374822
Rnf214	-0.0991428	6.33644603	0.70832674	0.40999537	0.30374822
Il17rc	-0.3247847	1.90977284	0.70812204	0.41006186	0.3037701
Zeb1	-0.0819349	7.53527288	0.70732942	0.41031949	0.30393014
Dok6	-0.3004428	2.04157033	0.70722989	0.41035186	0.30393014
Enpp5	-0.1055726	7.09987463	0.70653153	0.41057908	0.30407104
Pcdhgb8	-0.3575677	1.14866749	0.70593824	0.41077227	0.3041867
AA986860	0.28250514	1.71718232	0.70554411	0.41090069	0.30425438
Tm9sf1	-0.1789254	3.98937282	0.70534801	0.4109646	0.3042743
Kdm4d	-0.6793432	-0.1753256	0.70511661	0.41104005	0.30428336
Irf2bp1	0.15270694	3.91958908	0.70508335	0.41105089	0.30428336
2310040G24	0.68846577	0.08428143	0.70445244	0.4112567	0.30440829
Noa1	0.13017469	4.42358664	0.70416039	0.41135202	0.30445143
Tmem245	-0.1426593	5.51547541	0.70384611	0.41145463	0.30446147
Olfr99	-1.1086755	-1.5113581	0.70379971	0.41146979	0.30446147
2810025M15	0.23540583	2.41788734	0.7037785	0.41147672	0.30446147
Pdhx	0.1197408	5.1461394	0.70333707	0.41162092	0.30454076
A330040F15	0.66921987	-0.8518091	0.70314409	0.41168399	0.30456
Ulk1	0.12592582	6.46626402	0.70291708	0.4117582	0.30457279
Sncb	-0.1645379	4.66624454	0.70286451	0.41177539	0.30457279
9330158H04	1.05389185	-1.188079	0.70254233	0.41188075	0.30462331
Il34	0.16179865	3.78935797	0.7021842	0.41199791	0.30468255

9530082P21I	-0.1326116	5.19462914	0.70192943	0.4120813	0.3047022
Srebf2	0.16376715	5.20482012	0.70187647	0.41209863	0.3047022
Mthfs	0.24633191	2.33692126	0.70148791	0.41222586	0.30472688
Clvs2	-0.1972868	4.43268842	0.70146711	0.41223267	0.30472688
Ccdc73	-0.2289355	3.33422071	0.70123735	0.41230793	0.30472688
2610002J02F	0.28463295	3.24413391	0.70117807	0.41232736	0.30472688
Hsd17b13	0.98799796	-1.215818	0.70106094	0.41236574	0.30472688
Il1rl2	-0.9117868	-1.0983278	0.70099631	0.41238691	0.30472688
Cacfd1	-0.1404318	4.71033613	0.70094844	0.4124026	0.30472688
Aven	0.30690588	1.31727111	0.70086905	0.41242862	0.30472688
Gm15455	0.53701492	-0.8764265	0.70060277	0.41251591	0.30476398
Pxmp2	-0.2247844	2.49324457	0.70012345	0.41267311	0.30485271
Pmp22	0.20201922	8.57001292	0.69982766	0.41277016	0.304897
Acat3	-0.4433568	0.69937389	0.69767574	0.4134773	0.30539189
Iba57	0.36593684	0.816488	0.69707878	0.41367379	0.30544507
Olfr316	-1.0014822	-0.6026175	0.6970596	0.41368011	0.30544507
Jakmip2	-0.1345262	6.83067057	0.69701016	0.41369639	0.30544507
Txnrd3	0.20521283	3.37677223	0.69689655	0.41373381	0.30544507
Casp6	0.25122778	2.37330272	0.69689258	0.41373511	0.30544507
AU019823	0.15061636	5.02484557	0.69641305	0.4138931	0.30552926
Gm9776	-0.2344412	2.31677585	0.69632081	0.41392349	0.30552926
Zkscan14	0.35444011	2.42938013	0.69577846	0.41410231	0.30561959
Ppme1	-0.1357929	5.17759303	0.69572409	0.41412024	0.30561959
Unc119b	0.21184879	4.03047117	0.69480629	0.41442312	0.30578702
Tmem37	0.65113355	0.73722853	0.69476722	0.41443603	0.30578702
Zc3h12b	0.12866665	5.05571314	0.69462832	0.4144819	0.30578702
C920006O11	-0.3010847	1.74888489	0.69458584	0.41449593	0.30578702
Gpr137	0.20178024	3.18893614	0.69400812	0.41468681	0.30590038
Commd8	0.18362499	5.76987155	0.69360757	0.41481923	0.30594756
Cdkal1	-0.1692316	4.0708894	0.69358947	0.41482522	0.30594756
Tpd52	0.11276792	5.82796118	0.69337281	0.41489688	0.30596953
3200001D21I	-0.7527755	0.18461242	0.69327434	0.41492945	0.30596953
Eef2	-0.1010245	8.35795737	0.69313509	0.41497552	0.30597604
Lrp11	-0.1332169	5.96147788	0.6929125	0.41504918	0.30600291
Chpt1	-0.1586619	5.44121006	0.69277207	0.41509566	0.30600973
6430548M08	0.13005614	6.91741558	0.69236573	0.4152302	0.30606084
B930003M22	0.60420529	-0.1766797	0.69233774	0.41523947	0.30606084
Aktip	-0.1121474	6.09517602	0.69217477	0.41529346	0.30607318
Irak1	0.09116989	6.53281379	0.69200836	0.41534859	0.30607755
Rpp40	0.21837393	2.58937608	0.69193208	0.41537386	0.30607755
Itih5	-0.1514393	6.2904773	0.69134201	0.41556946	0.30619423
Pbx2	0.15178533	4.84810198	0.69089998	0.41571608	0.30627481
4930572O13	-0.6286956	0.43251936	0.69000389	0.41601356	0.30643983
Gpr50	-1.2092484	-1.5457712	0.69000062	0.41601465	0.30643983
Zfp84	-0.1242117	5.15423552	0.68977692	0.41608896	0.3064476

Mtmr2	-0.1182979	6.65554123	0.68974443	0.41609976	0.3064476
Isoc1	0.14613588	5.90672511	0.6891278	0.41630472	0.30657107
Ino80e	-0.2774356	2.32870918	0.68829174	0.41658286	0.30673095
Smyd1	0.48477087	1.45294208	0.68825089	0.41659646	0.30673095
Fubp1	-0.1230394	7.1259254	0.68747505	0.41685484	0.30687616
Ccdc36	-0.938342	-1.4751331	0.68743449	0.41686835	0.30687616
Rab34	0.21696817	4.25800237	0.68714831	0.41696373	0.30691888
Plcg2	-0.2465499	1.88534038	0.68662253	0.41713904	0.30702043
Sema3b	-0.3891135	2.712754	0.68650021	0.41717984	0.30702297
Frat1	0.31908781	1.57041128	0.68496887	0.41769118	0.30737176
Hmgb1-rs17	-0.3618056	0.38820929	0.68447799	0.41785529	0.307465
Tpcn2	-0.6877398	-0.307709	0.68412623	0.41797295	0.30752362
Pdcl	0.1392556	5.15656467	0.68401615	0.41800978	0.30752362
Paqr9	-0.1654391	5.44706084	0.68373739	0.41810308	0.30755806
Tunar	0.24194424	3.21410415	0.68365267	0.41813144	0.30755806
Zfp689	-0.3155257	1.65049291	0.68266118	0.41846357	0.30775949
Krcc1	0.21785421	7.09047023	0.6826116	0.41848019	0.30775949
Casp1	-0.3515598	1.62809504	0.68222967	0.41860824	0.30782613
Il2ra	0.33053696	1.922807	0.68202479	0.41867696	0.30784911
Tmem232	0.38336846	1.34682122	0.68175351	0.41876798	0.30788849
Ttc16	-1.1014928	-1.5557674	0.68153424	0.41884156	0.30791505
Camk2d	0.1120949	7.31796937	0.68141278	0.41888233	0.30791748
Napg	0.09993487	6.73051535	0.68060816	0.41915258	0.30803339
Acot11	-0.1688588	3.82662615	0.68042171	0.41921524	0.30803339
Gba2	-0.2145437	3.2358917	0.68041514	0.41921745	0.30803339
Csf1r	-0.1818398	3.45706931	0.68039081	0.41922563	0.30803339
Yeats2	0.11487635	5.57972497	0.68038556	0.4192274	0.30803339
Zfp281	0.12686237	6.16581119	0.68001224	0.41935291	0.30809807
Gm10416	-0.6237238	0.47683774	0.67961794	0.41948554	0.30816796
Fam173a	0.21477316	3.09933044	0.67920235	0.4196254	0.30824316
Srpx	0.71429715	0.14940974	0.67827033	0.41993933	0.30844619
Fam101b	0.16393767	4.20018008	0.67801424	0.42002565	0.30847742
Ino80d	-0.1136025	7.35145253	0.67792153	0.4200569	0.30847742
Rpl31-ps12	0.16010319	4.06261921	0.67769266	0.42013408	0.30850653
Cxcl11	1.35887448	-2.3691744	0.67755788	0.42017954	0.3085099
Zbtb7a	-0.1579875	6.03367033	0.67745649	0.42021375	0.3085099
Ccr9	-0.2204904	3.73349126	0.67722113	0.42029316	0.30854064
A930009A15	-1.0021912	-1.9983995	0.67693839	0.42038859	0.30858314
Ier3	0.22127644	4.1511689	0.6764387	0.42055732	0.3086322
N4bp2l2	-0.0951098	6.28193823	0.67641489	0.42056537	0.3086322
Ltk	-0.3739274	1.34450816	0.67640685	0.42056808	0.3086322
Pitrm1	-0.1243276	5.2643786	0.67603593	0.42069341	0.30868644
Tmem253	-0.9723356	-1.077582	0.67596583	0.4207171	0.30868644
Cenpt	-0.2620473	2.40118375	0.67571257	0.42080271	0.30872169
Ifi204	0.59304979	-0.3667039	0.67534995	0.42092534	0.30878409

Slc10a1	-1.0154958	-1.138606	0.67517612	0.42098414	0.30879967
5031414D18I	-0.7407014	-1.0537703	0.67499507	0.4210454	0.30881704
Supt16	-0.1634751	6.65731618	0.67455962	0.4211928	0.30889759
Btd	-0.2497579	3.78710427	0.67397676	0.42139021	0.3089945
Pcdhgc3	-0.2460818	4.17040003	0.67394747	0.42140014	0.3089945
Slc6a6	-0.0991587	7.94764577	0.6736508	0.42150068	0.3090199
Stab2	0.88578947	-1.1978547	0.67358555	0.4215228	0.3090199
Arhgap17	0.1626534	4.57259103	0.6735125	0.42154756	0.3090199
Cwc27	0.12351153	5.33572549	0.67274936	0.42180641	0.30918207
Glud1	-0.0835945	8.41518438	0.67194692	0.42207885	0.30934816
Enho	-0.3652838	0.55148826	0.67186032	0.42210827	0.30934816
Spry2	-0.114395	5.95328064	0.6716674	0.42217382	0.30936861
Tbx2	0.82538727	-1.4237405	0.67125776	0.42231305	0.30944305
Orc2	-0.1176422	5.78244805	0.67112579	0.42235792	0.30944496
Prps1	-0.0980223	5.87724468	0.67087336	0.42244377	0.30944496
Ccdc117	0.16640837	4.88265275	0.67084558	0.42245322	0.30944496
Vkorc1	0.24251932	5.37488519	0.67080728	0.42246625	0.30944496
Fbf1	-0.1490651	4.05102096	0.67044531	0.42258941	0.30946073
Zfp369	-0.1487108	5.24517541	0.67042532	0.42259622	0.30946073
Erich5	-0.7015625	-0.7867221	0.67025557	0.42265399	0.30946073
Dsp	-0.3746156	1.66527481	0.67019444	0.4226748	0.30946073
Anpep	0.22493428	6.47703909	0.67019078	0.42267605	0.30946073
Gm2109	1.43369022	-2.2724261	0.66988342	0.42278071	0.30948315
Grrp1	-0.7880526	-1.7128194	0.66985917	0.42278896	0.30948315
Rnf138	-0.1662754	3.49491587	0.66976915	0.42281963	0.30948315
Arhgef11	0.10614871	6.85563668	0.66889277	0.4231183	0.30967419
Gata2	-0.5596492	0.77249264	0.66846082	0.42326563	0.30973933
Pcdh1	-0.1409218	7.22796233	0.66841083	0.42328269	0.30973933
Amigo3	0.80723466	-0.5059474	0.66819078	0.42335778	0.30974687
Gchfr	-0.6424071	0.21337602	0.66815978	0.42336836	0.30974687
Tbl1xr1	-0.0881048	7.63651991	0.66795425	0.42343851	0.30977062
Ddx19a	0.14355799	5.4407088	0.66769805	0.42352599	0.30980705
Utp23	-0.1520567	4.38435016	0.6674671	0.42360488	0.30983717
Gm1976	0.15292961	4.49603841	0.66704427	0.42374935	0.30990018
Snora28	-1.0991965	-1.4223794	0.6669943	0.42376644	0.30990018
Npc2	0.2379687	4.68547146	0.66664998	0.42388415	0.30995869
Cenpa	-0.4858313	1.06374212	0.66626818	0.42401474	0.3100266
Zranb1	-0.1183229	4.76917413	0.66601719	0.42410062	0.31006181
Ankrd37	-0.3337736	1.64700432	0.6652933	0.42434847	0.31021542
Tti1	-0.153848	4.735674	0.66492081	0.42447609	0.31028112
Zfp958	-0.1869641	3.44409452	0.66460447	0.42458452	0.31031598
Cflar	-0.100839	6.86384183	0.6645614	0.42459929	0.31031598
Trim44	0.08008503	9.18709954	0.66444992	0.42463751	0.31031632
Mettl7a1	0.17439681	6.05892047	0.66388536	0.42483117	0.31043024
Lrrc73	-0.2620455	2.43181403	0.66354834	0.42494684	0.31045928

Ptpn14	0.19215953	6.35989005	0.6635205	0.4249564	0.31045928
Lurap1l	0.20717477	2.60578454	0.66343944	0.42498423	0.31045928
Tctex1d2	0.17291203	4.3958505	0.66303107	0.42512448	0.31051696
Slc12a9	-0.3614371	1.25442533	0.66298952	0.42513875	0.31051696
Zfp524	0.5085676	-0.0109418	0.66263766	0.42525965	0.31057766
4930432K21I	0.47279521	-0.3257549	0.66200728	0.42547639	0.31070835
Fermt3	-0.4804404	0.28907828	0.6618286	0.42553786	0.31072562
Sult6b1	0.40676254	0.88888743	0.66168108	0.42558862	0.31073508
Tmem223	0.15654132	3.72335312	0.6612122	0.42575001	0.31080629
Asb3	-0.1675642	4.02289855	0.66117802	0.42576178	0.31080629
Chd4	-0.1066868	7.44856066	0.65958875	0.42630955	0.31117853
Xpo1	-0.1077939	6.55374024	0.65942639	0.42636557	0.31119178
E030018B13I	1.24455676	-1.4538829	0.65864458	0.4266355	0.31136114
Rpf1	0.13972241	4.10670821	0.65829595	0.42675595	0.3114214
Sox8	0.21978838	3.86742114	0.65796872	0.42686906	0.31147628
Tbc1d5	-0.1015995	6.45387232	0.65778099	0.42693397	0.31149599
Dcun1d4	0.11485509	7.403709	0.65745232	0.42704765	0.31153708
Pcgf5	0.15885683	4.11613292	0.65739902	0.42706609	0.31153708
Cxxc4	-0.1286261	6.13019003	0.65709223	0.42717225	0.31156956
Sdcbp2	0.58277305	-0.7021209	0.65705124	0.42718644	0.31156956
Zfhx3	0.09725905	7.2958918	0.65680707	0.42727096	0.31160356
Qser1	0.1298424	6.91275708	0.65654659	0.42736117	0.3116417
Glyctk	-0.4052829	1.38324795	0.65622119	0.42747389	0.3116896
Klhdc8a	-0.1734755	3.8166038	0.65613804	0.4275027	0.3116896
Gas2	-0.1703873	2.92876743	0.65600133	0.42755008	0.31169649
Cpsf3l	0.20981544	2.63719254	0.65571773	0.42764839	0.31172534
1300002E11I	-0.1349814	5.25378338	0.65559332	0.42769153	0.31172534
9330117O12	0.33115901	1.48878023	0.65555901	0.42770343	0.31172534
Ttyh2	-0.2400787	2.6258952	0.65538052	0.42776533	0.31174281
Pank1	-0.1095407	6.29469563	0.65472371	0.42799325	0.31188126
Antxr1	-0.1451106	6.12795934	0.65431537	0.42813504	0.31195285
Pik3c3	-0.1096332	5.50158072	0.65422221	0.42816741	0.31195285
Col2a1	-0.9309474	-1.1175281	0.65373246	0.42833758	0.31204918
Gcn1l1	-0.1796656	4.54736474	0.65343262	0.42844183	0.31209745
Med25	-0.1882407	2.48573837	0.65236468	0.42881343	0.31234046
Tmem199	0.13224699	4.14860616	0.65181644	0.42900439	0.31245186
AV039307	-0.5020573	0.33744871	0.65138143	0.429156	0.31250141
Fgf14	-0.1073279	6.28372255	0.65130428	0.4291829	0.31250141
Tmem88b	-0.1304961	5.67365952	0.65129404	0.42918647	0.31250141
Polr3f	0.1285985	4.75486577	0.65101981	0.42928211	0.31254335
Sec24c	0.11179741	6.32172526	0.65063682	0.42941572	0.31261294
Rabepk	0.22089515	2.99144991	0.65001387	0.4296332	0.31270679
Ncam1	-0.1417298	7.88637421	0.64987104	0.42968309	0.31270679
Tomm6os	-0.6250311	-0.1003295	0.64979164	0.42971082	0.31270679
Asb2	0.61953857	0.02670531	0.64956325	0.42979062	0.31270679

Fam184a	-0.1733936	4.85798108	0.64950882	0.42980964	0.31270679
Ankrd54	0.22870831	2.01306305	0.64948025	0.42981963	0.31270679
Pou5f1	-1.0368436	-2.4473957	0.64946608	0.42982458	0.31270679
Pde2a	-0.1529213	6.77089871	0.64939617	0.42984901	0.31270679
Fbxo17	0.29957938	1.79926331	0.64920365	0.42991631	0.31272807
4930487H11	-1.0120062	-1.2748223	0.64818328	0.43027327	0.31291045
Zfp647	0.29183395	1.3500515	0.64811482	0.43029724	0.31291045
Gm996	-0.1669332	4.57695993	0.64810676	0.43030006	0.31291045
Tsku	0.34760745	2.14957255	0.64796327	0.4303503	0.31291045
Trappc2l	0.24260229	3.37912869	0.64790175	0.43037184	0.31291045
1700109H08	-1.0322068	-1.4188016	0.6478343	0.43039546	0.31291045
Dusp6	0.173348	5.38548791	0.6474257	0.4305386	0.31298683
Stmn4	0.17636284	6.7672225	0.64695715	0.43070283	0.31306063
Gm12505	0.23329029	1.77653996	0.64691875	0.43071629	0.31306063
4921524J17F	-0.2610306	4.04418156	0.64680498	0.43075619	0.31306194
Rps6kb1	-0.0959203	7.12851419	0.6465914	0.43083109	0.31308869
Zfp810	-0.12948	4.65836076	0.64619031	0.43097182	0.31316327
Ifi205	0.67480836	-1.2619821	0.64599203	0.43104142	0.31318616
Pwp2	-0.2714394	2.67198387	0.64560861	0.43117605	0.31321698
Ccdc78	0.4935148	-0.2894292	0.64558392	0.43118472	0.31321698
Zfp600	-0.6423246	-1.1369565	0.64554563	0.43119817	0.31321698
Ubap2l	0.10058995	8.54563331	0.64503894	0.4313762	0.31330357
Slc25a42	0.11354927	4.84202156	0.64496434	0.43140243	0.31330357
Exosc7	0.18845102	3.35910013	0.64488097	0.43143173	0.31330357
Imp3	0.17997701	4.80297023	0.6446094	0.43152722	0.31334523
Pfkp	-0.150908	7.31963642	0.64382936	0.43180167	0.31351682
Rps14	-0.2725727	4.3577063	0.64356895	0.43189336	0.31354974
Pkn1	0.29377361	2.85347814	0.64348389	0.43192331	0.31354974
Igfals	1.3780242	-2.2123969	0.64272411	0.43219102	0.31371637
Ppp1r8	0.28905473	3.99769124	0.64243922	0.43229147	0.31376157
Mss51	-0.9080005	-0.9823114	0.64219356	0.43237812	0.31377055
Abhd3	-0.2403087	3.71128761	0.64209521	0.43241281	0.31377055
Nxt2	0.14126402	6.76471218	0.64207947	0.43241836	0.31377055
Oxr1	0.09500703	8.62710565	0.64185142	0.43249884	0.31377979
Sirt2	0.15045731	6.66112863	0.64182699	0.43250746	0.31377979
Txndc12	0.18536217	3.96438473	0.64159268	0.43259017	0.3138121
Foxj1	0.49300648	0.73725854	0.64122261	0.43272085	0.31387919
Csf1	-0.1778857	4.87426401	0.64082924	0.43285982	0.31392598
Abhd17a	0.17593608	3.66080097	0.64082381	0.43286174	0.31392598
BC018242	0.13949499	4.75332154	0.64057946	0.4329481	0.31393906
Ldlr	0.14042321	3.69142439	0.64055664	0.43295617	0.31393906
Lig1	0.13781898	4.07456712	0.63992967	0.4331779	0.31407213
Cryga	-1.1711207	-1.8631762	0.63973154	0.433248	0.31409525
Dmp1	0.53190068	0.28825488	0.63932572	0.43339166	0.31417169
Tulp3	0.18007282	5.2594078	0.63735163	0.43409151	0.31465127

Dock8	-0.2218813	2.85605871	0.63686159	0.43426552	0.31474964
Zmat4	0.09636024	5.6454348	0.63644619	0.43441311	0.31482885
Tdrd1	0.43676197	0.84020245	0.63595365	0.43458821	0.31490917
Mphosph8	0.12100854	8.41146248	0.6358686	0.43461846	0.31490917
Pole2	-0.4992274	1.03558652	0.63581118	0.43463888	0.31490917
Oaf	0.32761226	1.012557	0.63561024	0.43471036	0.31491495
2810403A07I	-0.133088	6.56468175	0.63557332	0.43472349	0.31491495
Col4a3	-0.5292064	0.00043083	0.63524676	0.43483971	0.31496768
Setd4	-0.4639553	1.04697933	0.63506198	0.43490549	0.31496768
Derl3	0.84557357	-1.2662247	0.63504111	0.43491292	0.31496768
Acad12	-0.645304	-0.9552592	0.63490234	0.43496233	0.31496768
Scrt2	-0.1814214	3.35000913	0.63483057	0.43498789	0.31496768
Dner	0.17408845	5.47834739	0.63468221	0.43504073	0.31497819
Ttl	-0.097181	5.9524659	0.63412534	0.43523916	0.3150941
Larp4	0.09810005	7.14921979	0.6334175	0.43549159	0.31524802
9130023H24I	0.18436295	3.76212086	0.63325063	0.43555114	0.31524802
Fkbp10	0.39804081	2.21110393	0.63320664	0.43556683	0.31524802
Zfp317	-0.1589403	4.05380451	0.63272424	0.43573905	0.3153295
Map4k1	-0.3647355	0.43633808	0.63264812	0.43576624	0.3153295
Htra1	0.17058949	3.91348664	0.63251285	0.43581455	0.3153295
Cdk4	0.19193078	4.37868643	0.63246155	0.43583288	0.3153295
Rfwd3	-0.1058367	5.39806734	0.63235262	0.43587179	0.3153299
A830009L08I	-0.3466983	0.87970727	0.63188958	0.43603728	0.31542186
Lrrc71	-0.9340467	-1.4883196	0.63158995	0.43614442	0.3154716
Gpr98	0.25773778	2.82382447	0.63120312	0.43628279	0.31554392
Rab7l1	0.33979304	4.22114935	0.63095154	0.43637283	0.31558127
Gm15713	0.79130865	-0.6500398	0.63063693	0.43648546	0.31563495
Gemin7	-0.2108657	3.2202262	0.63033627	0.43659314	0.31568504
Zfp2	-0.1405317	4.45091169	0.62975664	0.43680086	0.31580745
Evi2a-evi2b	0.67014935	-1.3671055	0.62930841	0.43696159	0.31585342
03-Mar	-0.6161744	0.04069443	0.62919966	0.4370006	0.31585342
Gm19897	-0.6504785	-0.0053827	0.62915317	0.43701728	0.31585342
Zfp428	-0.3808201	0.8567422	0.62911977	0.43702926	0.31585342
Slc39a11	-0.2749518	1.72164444	0.62904363	0.43705658	0.31585342
Pcsk4	-0.2637439	1.48792254	0.62849227	0.4372545	0.31595916
Bloc1s2	0.19317334	3.29573558	0.62842183	0.43727979	0.31595916
Eif3h	0.1279723	6.19535446	0.62759337	0.43757747	0.31612709
Uqcc1	-0.1718413	3.87512325	0.62749892	0.43761142	0.31612709
Hnrnpl	-0.1261178	5.22655387	0.62745397	0.43762759	0.31612709
Il10rb	-0.2107794	3.04627877	0.6271048	0.43775317	0.31617244
Rmdn3	-0.1427021	3.79942027	0.62697083	0.43780136	0.31617244
Dctd	-0.3663275	1.64541764	0.62695859	0.43780577	0.31617244
C230024C17I	1.15731194	-1.9188972	0.62649419	0.43797291	0.31623987
Eml3	0.23408832	2.79223939	0.62648536	0.43797609	0.31623987
Ripk3	0.80266153	-0.6316331	0.62596005	0.43816528	0.31634868

Bmi1	-0.1207204	6.05149084	0.62562814	0.43828488	0.31640724
Prim1	0.24051555	2.94450573	0.624248	0.43878278	0.31673886
Tmem39a	-0.1308044	4.96808589	0.62406896	0.43884743	0.31675771
Cdc42ep3	0.15237585	3.36695661	0.62372662	0.4389711	0.3167817
Upf2	-0.0926237	6.89054025	0.62365998	0.43899518	0.3167817
Il12a	0.27921086	1.92491101	0.62352656	0.4390434	0.3167817
Fam43a	0.16711138	6.20468166	0.62344076	0.43907441	0.3167817
Vcam1	-0.135623	5.84331922	0.62336458	0.43910195	0.3167817
Brf1	0.12982022	4.23692636	0.62333699	0.43911192	0.3167817
Prkci	-0.0916382	7.35728916	0.62283719	0.43929267	0.31688427
Ribc1	-0.5261668	-0.2145785	0.62252477	0.4394057	0.316938
Lck	0.52993756	0.1777984	0.62211761	0.43955309	0.31696801
Fam136a	0.1498117	3.84049672	0.62207735	0.43956767	0.31696801
Smcr8	-0.1234909	5.78459802	0.62201353	0.43959078	0.31696801
Adcy6	-0.142217	4.09255721	0.62198374	0.43960157	0.31696801
Bcl2a1a	0.38917907	0.16812951	0.62176619	0.43968037	0.31699702
Tsfm	0.17510414	2.62494558	0.62024334	0.44023259	0.31736731
Coa5	0.11796107	7.00338621	0.61947515	0.44051157	0.31754058
Gm4841	0.28041131	2.36667917	0.61925589	0.44059125	0.31757016
Ino80b	0.4962571	-0.0544744	0.61902479	0.44067526	0.31760286
Gm6568	-0.3384552	0.79574085	0.61891149	0.44071645	0.3176047
Me1	-0.1003836	5.34578068	0.61877843	0.44076484	0.31761173
Fam214b	0.15303097	4.40234151	0.61822387	0.4409666	0.31771088
Itgb1	-0.1601094	6.34118709	0.61818774	0.44097975	0.31771088
Neil1	-0.5205419	-0.0590332	0.61795833	0.44106326	0.31774213
Eif2ak1	0.10285371	6.22664605	0.61785621	0.44110044	0.31774213
Hnf1a	0.76918796	-0.9538867	0.61771799	0.44115078	0.31775054
Fam168a	0.07877211	8.13100082	0.61758449	0.4411994	0.31775771
Synpr	-0.1753352	5.17764605	0.61711158	0.44137171	0.31785396
Cd97	0.21231485	3.10544041	0.61700124	0.44141193	0.31785508
Tmem106b	-0.1025184	7.56207036	0.61658414	0.44156403	0.31793674
Acot3	-0.6143675	0.33841072	0.61553162	0.44194819	0.31817661
Apold1	0.33995152	1.93923786	0.61545932	0.44197459	0.31817661
Zw10	-0.3009011	2.49338319	0.61514673	0.4420888	0.31823096
Habp4	0.07788945	7.39970962	0.61499935	0.44214266	0.31824186
Ccdc113	0.38787734	1.09575397	0.61454991	0.44230699	0.31833225
Bbs4	0.09771946	5.56476616	0.61424662	0.44241793	0.31838422
1700105P06I	-1.3784269	-1.8469964	0.61401268	0.44250353	0.3184135
Aloxe3	-0.2825026	1.83074161	0.61392371	0.44253609	0.3184135
Tal1	-0.5474625	0.26914581	0.61363456	0.44264195	0.31845492
Fam188a	-0.1213653	6.11642959	0.6135548	0.44267116	0.31845492
Fastkd2	-0.2590826	2.91665665	0.61319448	0.44280314	0.31852199
Marveld3	-1.173165	-0.9875168	0.61306177	0.44285177	0.31852909
Phc1	-0.0976842	6.3147553	0.61270432	0.44298278	0.31859545
2310061I04R	-0.1450328	4.20453282	0.61250152	0.44305714	0.31862105

2310022A10I	0.17104196	3.59599026	0.61222228	0.44315956	0.31866092
Mir568	-0.2103521	2.11864824	0.61213895	0.44319013	0.31866092
Psmd1	-0.0824202	7.50243881	0.61184488	0.44329805	0.31870457
Tbx15	0.18133329	6.7955854	0.61171939	0.44334411	0.31870457
Obsl1	-0.1804705	3.21369127	0.61148368	0.44343065	0.31870457
Siglech	-0.2672665	2.13107819	0.61144606	0.44344446	0.31870457
Tmco1	0.20971067	4.77673781	0.61144539	0.44344471	0.31870457
Hkdc1	-0.2927121	1.82969467	0.61125187	0.44351578	0.31871227
Gatsl2	0.10331292	6.17021355	0.61120503	0.44353299	0.31871227
Dync2li1	0.17464605	3.59751117	0.61093635	0.44363171	0.31875534
Zkscan7	-0.3431479	1.50064791	0.61080603	0.4436796	0.31876189
Ddx27	0.12561512	4.1812579	0.609769	0.44406101	0.31900734
Gpx4	0.24055494	4.24864188	0.60954874	0.44414209	0.31900734
Mcm3	-0.2928944	0.97874568	0.60946161	0.44417417	0.31900734
Armc10	0.17362534	4.74546226	0.60945529	0.4441765	0.31900734
Zdhhc18	0.2022898	3.24459797	0.60913391	0.44429486	0.31906447
Brd2	0.08252134	7.92473855	0.60872054	0.44444717	0.31914571
Nudt12	0.29348085	2.84423276	0.60861612	0.44448565	0.31914571
Riok2	0.10726019	5.09861726	0.60815912	0.44465416	0.31923881
Angptl6	0.49807331	-0.1215775	0.60802876	0.44470224	0.31924545
Nme5	0.19074749	3.39963312	0.60782593	0.44477708	0.31927129
Lsm5	-0.3040831	1.66590589	0.60751129	0.44489321	0.31932676
Pcdhb11	-0.4526064	0.96598835	0.60718269	0.44501454	0.31937677
Tmem254a	-0.3339183	1.45185985	0.60705423	0.44506198	0.31937677
Entpd6	-0.1936407	3.09505747	0.60700692	0.44507946	0.31937677
Bcl7b	0.13829961	4.11000641	0.60671602	0.44518694	0.31940807
Lsm8	0.16099317	5.66105013	0.6066785	0.44520081	0.31940807
Gtf3c4	-0.118335	5.27152828	0.60610624	0.44541237	0.31953197
Zbtb17	-0.2288252	2.67988355	0.60585789	0.44550424	0.31956998
Spo11	0.74417405	0.07250482	0.6054306	0.44566237	0.3196307
Arhgap42	-0.1053614	5.70538165	0.60541899	0.44566667	0.3196307
Asxl1	0.13545214	5.35960439	0.60527581	0.44571968	0.31964083
Tekt1	0.62965562	-0.1937425	0.60503365	0.44580935	0.31967724
Wdr89	0.28300598	1.09939631	0.60446087	0.44602158	0.3197935
C030037D09I	-0.3979989	1.34915979	0.60438607	0.4460493	0.3197935
Commd1	0.2050776	4.15830275	0.60382954	0.44625569	0.31989498
Magt1	-0.1562483	5.22966798	0.60379449	0.44626869	0.31989498
Adra2b	-0.4870119	0.18659244	0.60334769	0.4464345	0.31998559
Camk1	0.15521482	3.80136889	0.6032441	0.44647295	0.31998559
Dcaf7	-0.0919956	7.9744213	0.60231581	0.44681781	0.32020483
Dok4	0.30789972	2.3734439	0.60177148	0.44702022	0.32032195
Slc27a3	1.07395231	-1.6827004	0.60092094	0.4473368	0.32052086
Hrc	-0.7135305	-0.3257219	0.60051881	0.4474866	0.32060025
Sphk2	-0.1479879	4.07618968	0.600196	0.44760692	0.32064505
Srek1ip1	0.12363159	6.02457811	0.60014168	0.44762717	0.32064505

AA414768	0.16946266	3.09144475	0.59999066	0.44768347	0.32065744
Igf2os	-0.2738286	3.56174372	0.59981172	0.44775021	0.32067195
Lenep	0.36752875	1.41408675	0.59972713	0.44778176	0.32067195
D1Ertd622e	0.09903239	5.78632366	0.5993132	0.44793621	0.3207293
Gstm3	0.27146091	1.53275135	0.59930333	0.44793989	0.3207293
Dhx40	-0.1108738	5.37711587	0.59902574	0.44804351	0.32075137
G6b	-0.9277708	-1.0109044	0.59891448	0.44808506	0.32075137
0610037L13I	0.16018087	4.97435407	0.59890718	0.44808779	0.32075137
Agpat4	0.14807038	5.19971773	0.59869608	0.44816663	0.32077987
Poldip2	-0.1279342	5.13046729	0.59855567	0.44821908	0.32078947
Cant1	-0.1892861	3.30876225	0.59756159	0.44859073	0.32102751
Hyal1	0.26780878	3.56170097	0.59654307	0.44897202	0.3212724
Nxnl1	-1.0318479	-1.2919197	0.59555149	0.44934374	0.32148164
Gpr68	0.15562756	3.49668745	0.5954927	0.44936579	0.32148164
2210015D19I	0.16304788	3.178976	0.59537706	0.44940918	0.32148164
Caln1	-0.140182	4.57744271	0.59529346	0.44944055	0.32148164
Ppox	0.39307569	1.015352	0.59524161	0.44946001	0.32148164
Ctsl	0.19371159	6.74069323	0.59493191	0.44957626	0.3215162
Naalad2	0.31020491	1.33162238	0.59488949	0.44959218	0.3215162
Snx7	0.19270843	4.97341229	0.5948003	0.44962567	0.3215162
Fhl4	-0.4589279	0.87634716	0.59434176	0.44979791	0.32161138
Mrpl57	0.12869214	4.6920637	0.59368499	0.45004479	0.32175991
Gpr150	-0.3264594	0.66432843	0.59324067	0.45021193	0.32185141
Lpar5	0.7830914	-0.7769448	0.59287089	0.45035112	0.32191385
Gm1123	-1.1721581	-2.3133754	0.59280055	0.4503776	0.32191385
Sema3c	-0.1495139	3.8203494	0.59251877	0.45048372	0.3219617
Kcns3	0.25453256	1.97479653	0.59238083	0.45053568	0.32197084
Tpr	-0.1013129	9.14637842	0.59167063	0.45080336	0.32213167
Phf8	-0.1298717	5.6399736	0.5915758	0.45083912	0.32213167
H2afv	0.19463213	5.31528394	0.59136569	0.45091838	0.32216029
Dyrk2	-0.2163576	3.24863198	0.59014832	0.45137801	0.32246065
Hist1h2bf	0.62307788	-0.8437802	0.58979861	0.45151019	0.32252613
Slc24a3	0.12486401	6.88660705	0.58969823	0.45154814	0.32252613
Vps33a	-0.1098271	6.28335802	0.58933136	0.45168689	0.32259335
Pdgfd	0.2890718	3.02315085	0.58924183	0.45172077	0.32259335
Zfp90	0.14804708	3.80356774	0.58818232	0.45212191	0.32285178
Cd302	0.15937824	4.11724662	0.58785744	0.45224503	0.32286489
Pou3f4	-0.336391	0.57012501	0.58773702	0.45229068	0.32286489
Zdhhc24	-0.1430376	5.97825576	0.5877214	0.4522966	0.32286489
Glt8d2	0.16049505	3.02758039	0.58771927	0.45229741	0.32286489
Kcna6	-0.1344806	5.75091568	0.58737998	0.45242607	0.32292869
9430016H08I	0.17321577	3.49549773	0.5872228	0.4524857	0.3229432
Irak1bp1	-0.1338792	5.9075469	0.58673229	0.45267185	0.32302127
Scyl2	-0.1269813	5.8534477	0.58672745	0.45267369	0.32302127
5031439G07	0.09984275	5.64957202	0.58655284	0.45273998	0.32304054

Arpc4	0.19615942	5.34592983	0.5862214	0.45286587	0.32308451
Timm22	-0.1576704	3.87699275	0.58618361	0.45288023	0.32308451
Tmem97	0.26029651	2.13363819	0.58604173	0.45293413	0.32309492
6330403A02I	0.12804932	7.47245341	0.58562058	0.45309421	0.32318106
Rnf220	0.10495506	6.94879055	0.58480241	0.45340546	0.32337501
Gp49a	0.59162202	0.04712162	0.58433399	0.45358382	0.32347414
A630033H20	-0.589538	-0.6315604	0.58410142	0.45367241	0.32348334
Nxt1	-0.2639909	2.54312673	0.5840145	0.45370553	0.32348334
Ano10	-0.2047204	2.49026703	0.58399019	0.45371479	0.32348334
Eftud1	-0.1958898	3.10423985	0.58344606	0.45392222	0.32360316
Efcab4a	0.37053325	0.98823308	0.58293192	0.45411835	0.32368948
Fam13b	0.08653112	6.9242022	0.58292218	0.45412206	0.32368948
Glra2	-0.1619478	3.31837041	0.58279444	0.45417081	0.32369615
Arid3a	0.25720909	2.96326975	0.58264816	0.45422665	0.3236995
Furin	-0.2349827	2.94407601	0.58255309	0.45426295	0.3236995
Wrnip1	0.12835692	5.40301787	0.58247267	0.45429366	0.3236995
Manf	0.20711332	3.8192679	0.58180633	0.45454821	0.32384619
Glcci1	-0.0894405	6.88902066	0.58172752	0.45457834	0.32384619
Zfp869	-0.1314556	5.11244838	0.58134203	0.45472572	0.32389079
Prss36	-0.4276742	0.86859551	0.58129242	0.4547447	0.32389079
Sema3f	-0.4148778	0.80992883	0.58122279	0.45477133	0.32389079
Ddit4	0.22585529	2.67190027	0.5811516	0.45479856	0.32389079
Tbl2	-0.1487734	3.49257687	0.58098531	0.45486218	0.32390803
Slc25a1	0.22385862	3.82764877	0.58086423	0.45490852	0.32391296
Rims3	-0.4024046	0.8032777	0.58052893	0.45503686	0.3239582
Ednrb	-0.1782458	3.98408025	0.58049229	0.45505089	0.3239582
A430005L14I	0.21275663	2.80963571	0.5803149	0.45511882	0.32396678
Srd5a3	-0.2663923	2.96750846	0.58025498	0.45514177	0.32396678
Gm10658	0.61050065	-0.5900214	0.5800652	0.45521447	0.32398666
Exoc6	0.11186166	5.37342463	0.57997628	0.45524853	0.32398666
Sesn1	0.10198324	6.4168834	0.57964187	0.45537669	0.32402382
Vmn2r1	-0.8514571	-1.1488416	0.5796343	0.4553796	0.32402382
Polr3k	0.13194794	6.06279642	0.57930747	0.45550491	0.32405978
Tmed4	-0.1666192	5.53251403	0.57929684	0.45550899	0.32405978
Slc1a5	-0.2613795	2.10216484	0.57899117	0.45562624	0.32411514
Utp14a	0.15155152	3.98157565	0.57888028	0.45566879	0.32411735
Casp9	-0.1310628	4.67728021	0.5782931	0.4558942	0.32424963
Rmi1	0.11268447	5.67301753	0.57807475	0.45597807	0.3242732
Pla2g2d	0.56638039	-0.6830274	0.57800139	0.45600626	0.3242732
Plcl2	-0.1535983	5.54410284	0.57788949	0.45604925	0.32427572
Tmeff1	-0.1369006	5.11783559	0.57755812	0.45617661	0.32433822
Ptplad1	0.08786331	6.68911998	0.57708506	0.45635853	0.3244395
Dlk1	-0.2237534	3.41940784	0.57648103	0.45659099	0.32457669
Lipe	0.24982218	2.00487894	0.5763726	0.45663274	0.32457829
Stap2	0.34624989	1.64210429	0.57555906	0.45694618	0.324773

Prpsap1	0.09813668	4.70373033	0.57499577	0.45716341	0.32486527
Tcp11l1	-0.1216946	5.22320502	0.57492721	0.45718986	0.32486527
Igsf11	-0.1105949	5.86683619	0.57491498	0.45719458	0.32486527
Tspan5	0.09333562	7.34070433	0.57454541	0.45733721	0.32493392
Gdnf	0.57069178	-0.5461555	0.57445979	0.45737026	0.32493392
Gbx2	1.18294583	-1.7696169	0.57386082	0.4576016	0.32505159
BC031361	-0.2121759	2.8200458	0.57374831	0.45764508	0.32505159
Plbd1	-0.4381311	0.95484593	0.57361864	0.4576952	0.32505159
Cdc42bpb	-0.1212666	6.53365385	0.57353833	0.45772624	0.32505159
Manea	-0.1224075	5.51538336	0.5735192	0.45773364	0.32505159
Cwh43	-0.740915	-1.0775898	0.57332407	0.45780908	0.32507708
Ngly1	0.11820309	5.55152088	0.57164171	0.45846038	0.32551143
Odf2	-0.149215	5.56183303	0.57152593	0.45850526	0.32551518
Grin3a	-0.1656943	4.72129811	0.57116441	0.45864544	0.32558657
Zfp513	-0.248807	1.85284122	0.5707973	0.45878786	0.32565954
Blcap	-0.1570323	5.14326123	0.57068849	0.45883008	0.32566139
Flrt2	-0.0937356	7.45037819	0.57050157	0.45890263	0.32568476
St6galnac4	-0.2353258	2.46563388	0.5701507	0.45903888	0.32575333
Lhfpl5	0.80815207	-0.401819	0.56948277	0.45929841	0.32590936
Pla1a	0.46222065	0.99689346	0.56919335	0.45941094	0.32593669
Ptpru	-0.1783423	3.24593205	0.56917975	0.45941623	0.32593669
Nom1	-0.1221295	4.52773846	0.56840743	0.45971676	0.32612175
Phex	-0.297936	1.95507507	0.56805332	0.45985466	0.32619142
5930412G12	0.46358893	0.82290441	0.56765539	0.4600097	0.32627324
Hspa9	0.08757455	7.17277212	0.56720937	0.46018359	0.32636841
Gnai3	0.12418465	6.40500411	0.56710357	0.46022485	0.32636951
Hist3h2a	0.41967676	0.54765609	0.5668501	0.46032373	0.32641147
Mospd3	0.21733132	5.64086596	0.56649094	0.4604639	0.3264827
Ptrh1	1.0206372	-1.2887047	0.56632278	0.46052955	0.32650108
Klhl15	0.21339274	3.77533684	0.56522053	0.46096027	0.32677826
Gltscr2	0.17855346	5.54829414	0.56433253	0.46130775	0.32699638
Tmem176a	0.24121745	4.32210892	0.56423005	0.46134788	0.32699662
Tmem241	-0.1947028	3.00809008	0.5641137	0.46139344	0.32700072
Wdr96	0.35326776	1.22536066	0.56320319	0.46175029	0.32717154
Riok3	0.10545318	6.33207179	0.56320069	0.46175127	0.32717154
Sec31a	-0.0777333	7.07424304	0.56311265	0.4617858	0.32717154
Spice1	-0.1668844	3.42151264	0.56300561	0.46182778	0.32717154
Trhr2	-0.4899194	0.03532587	0.56299106	0.46183349	0.32717154
Nudt4	0.14015523	9.94797792	0.56245871	0.4620424	0.32727043
Rnf126	0.11658256	3.99171202	0.56237883	0.46207376	0.32727043
Mast3	-0.1148238	7.30129697	0.562331	0.46209254	0.32727043
Gna14	-0.4817955	1.3106898	0.56191243	0.46225694	0.32735578
Otud3	-0.2633356	1.74259567	0.56181076	0.46229688	0.32735578
Rev1	-0.1204652	4.38275609	0.56162955	0.46236809	0.32735578
Ubn1	-0.1007527	5.81141917	0.56161868	0.46237236	0.32735578

Ccdc13	-0.6242591	-0.3812154	0.56117961	0.46254499	0.32744979
Mfap5	-0.448499	2.15899869	0.56106399	0.46259047	0.32745378
1700102H20I	1.05601084	-1.2506463	0.56046818	0.46282492	0.32759153
Golga1	0.099254	5.51897548	0.56016982	0.4629424	0.32764646
Plcl1	-0.1238366	5.43543837	0.55889491	0.46344495	0.3279739
Fam19a2	0.11389888	5.53212453	0.55874523	0.46350401	0.32798746
Dnajc24	-0.1441575	3.81975444	0.55839135	0.4636437	0.32805806
Ano6	0.15398044	6.43153776	0.55786421	0.4638519	0.32817712
Slc47a1	0.2687412	5.38233735	0.557492	0.463999	0.32823079
Dppa2	1.17124026	-1.8299176	0.55747019	0.46400763	0.32823079
Gm7694	-0.238728	1.83915516	0.5572939	0.46407733	0.32825184
Ninl	-0.1936055	3.10125781	0.55706039	0.46416968	0.32826098
Bbox1	-0.421284	0.58517396	0.55705927	0.46417012	0.32826098
Trmt2b	0.19601107	5.21430636	0.55678354	0.46427921	0.32830988
Rpp21	0.21113698	3.15334798	0.55645102	0.46441083	0.32834936
Cpz	-0.9833684	-1.0697219	0.55643381	0.46441764	0.32834936
Gpr180	-0.1689843	3.9525995	0.55633974	0.46445489	0.32834936
Invs	0.13214394	4.82609087	0.55594991	0.46460929	0.32842795
Tmub2	-0.1502549	3.40390632	0.55580426	0.464667	0.32842795
Aifm3	0.12636243	5.53542131	0.55575645	0.46468594	0.32842795
Rps11	0.16213718	6.85286099	0.55553086	0.46477535	0.3284629
Gyltl1b	0.88918806	-1.9403739	0.55534911	0.46484741	0.32848558
Dnajc16	-0.1857839	3.07893277	0.55516513	0.46492037	0.32850833
Pdzk1	-0.2094037	2.85859714	0.55506637	0.46495955	0.32850833
BC055402	1.35310743	-2.4646339	0.55457669	0.46515385	0.32861185
9530052E02I	-0.7992851	-0.9840896	0.55449567	0.46518602	0.32861185
Syde1	0.2047239	5.18608369	0.55369778	0.46550296	0.32876112
Mdfi	0.88783997	-1.4504208	0.55367405	0.46551239	0.32876112
Sox21	-0.1846825	3.44466163	0.55358405	0.46554816	0.32876112
Shisa3	-0.2438655	3.96110094	0.553561	0.46555732	0.32876112
Slco2b1	-0.248092	2.70595602	0.5533213	0.46565262	0.32878568
BC030307	0.36730491	0.74594489	0.55327229	0.46567211	0.32878568
Flad1	0.24221209	2.04431352	0.55306276	0.46575545	0.32881198
Tmem255a	0.14686255	4.74803875	0.55297749	0.46578937	0.32881198
Cherp	0.09558546	5.54780331	0.55258258	0.46594653	0.32889467
Zmynd10	-0.4524165	0.25024876	0.5519777	0.46618742	0.32903645
Oscar	-0.5492633	0.10059127	0.55173764	0.46628308	0.3290757
Ctnnbip1	0.12930988	3.99076024	0.55037775	0.46682558	0.3294063
Itch	0.08184481	7.05379418	0.55036251	0.46683167	0.3294063
Myt1l	0.12743438	8.60644459	0.54945822	0.467193	0.32960902
Tssc1	-0.1481548	3.27212638	0.54926484	0.46727034	0.32960902
Cdca8	0.50155023	0.45926939	0.54922341	0.4672869	0.32960902
Lss	-0.1501399	4.11244123	0.54915755	0.46731325	0.32960902
Bin1	-0.1266856	5.36717275	0.54905504	0.46735426	0.32960902
Prelp	0.17493838	8.14582054	0.54904173	0.46735958	0.32960902

Pik3r1	-0.0963093	8.09936598	0.54864179	0.46751963	0.32969361
Stat5b	0.1443294	4.00803531	0.54849335	0.46757906	0.32970722
Tvp23a	0.21148245	4.20461113	0.54838799	0.46762125	0.32970869
Cdc42ep4	0.20080863	6.00259451	0.54828581	0.46766217	0.32970925
Tbata	0.5111701	-0.0328198	0.54785311	0.46783552	0.32980318
L3mbtl2	0.14434228	3.73725995	0.54764876	0.46791742	0.32983263
Tmbim4	-0.1578211	4.59340577	0.54722537	0.4680872	0.32992401
Jade2	-0.1014367	5.26676133	0.54651891	0.46837071	0.33009553
4930563F08I	-0.7007384	-0.1062883	0.54640109	0.46841802	0.33010057
Zgpat	0.28281416	1.82987523	0.5457156	0.46869343	0.33026634
Arl5c	0.63252879	-0.6989785	0.54548982	0.46878421	0.33030198
Prkg2	-0.1306089	4.33622435	0.5452347	0.46888681	0.33034596
Casc3	-0.1284684	5.49386002	0.54507623	0.46895056	0.33035452
Fgfbp3	-0.2094605	2.68347118	0.54500467	0.46897935	0.33035452
Alkbh6	0.1879025	3.12318257	0.54391883	0.46941662	0.3306342
Bex1	0.15597347	5.22081363	0.54354139	0.46956877	0.33071302
Gas6	-0.1312863	4.3991346	0.54330159	0.46966548	0.33075115
Fezf2	0.11554884	5.18090564	0.54315707	0.46972378	0.33075115
Ascc2	-0.1598838	3.30241568	0.5431079	0.46974362	0.33075115
Opalin	-0.3430943	1.45829791	0.54231451	0.47006391	0.33094831
Spata9	-0.1649004	3.51825626	0.54199854	0.47019158	0.33100984
Dgkb	-0.14458	7.92645672	0.54188477	0.47023756	0.33101386
Htra2	0.29758621	1.29477846	0.54161976	0.47034469	0.33106092
Rbck1	0.14435153	4.11843858	0.5414808	0.47040088	0.33107212
Ccdc112	0.18930932	4.19145441	0.54124339	0.47049691	0.33111135
Sh3rf1	-0.0988249	5.05297757	0.54113599	0.47054036	0.33111358
Timm13	0.25579714	2.54144414	0.54047931	0.47080619	0.33127228
Etv3	0.11562371	3.91685719	0.54033077	0.47086636	0.33128625
BC049715	-0.9077819	-0.4258807	0.53971046	0.47111775	0.33143475
Mxra8	0.18979234	5.68300485	0.53925748	0.47130147	0.33153562
Slc39a8	-0.2734139	2.78541867	0.53911217	0.47136043	0.33154872
Arrb1	-0.102508	7.40032448	0.5387922	0.4714903	0.33161169
Pim3	0.1945267	2.92316168	0.5383727	0.47166066	0.33170313
Tubb4a	-0.1234139	10.3898448	0.53826234	0.4717055	0.33170627
Pepd	0.14982951	3.62132166	0.53795219	0.47183154	0.33176652
Rhpn2	-0.2356454	4.0440724	0.5377538	0.47191219	0.33179484
Fblim1	0.30072007	3.58566952	0.53763201	0.47196171	0.33179725
Kctd14	-0.4103895	-0.0124869	0.53749476	0.47201753	0.33179725
Ube4b	-0.0879985	7.77348714	0.53744759	0.47203672	0.33179725
Samm50	-0.1365767	3.50730105	0.53692623	0.47224887	0.33191799
Smc4	-0.1250092	6.28417346	0.53674224	0.47232378	0.33194225
Cryzl1	0.11546612	5.36087716	0.53650782	0.47241925	0.33198096
Atg4b	-0.1443463	3.8982455	0.53601221	0.47262119	0.33209448
Slc25a32	0.14605348	3.88956618	0.53560631	0.47278669	0.33214163
Smyd3	0.09797183	6.18558953	0.53556939	0.47280175	0.33214163

Slc2a5	0.51475255	-0.6597049	0.53551386	0.4728244	0.33214163
Oas3	0.52146095	0.6839544	0.53545123	0.47284995	0.33214163
Gm20187	-0.5805616	0.42513865	0.53523821	0.47293686	0.3321743
Zfp697	-0.1278995	4.89969601	0.53488978	0.47307908	0.33223472
Ubxn11	0.45741217	1.41000936	0.53473517	0.47314221	0.33223472
Caskin2	0.18443254	2.60278845	0.53473039	0.47314416	0.33223472
Mycl	-0.2158564	2.03148345	0.53427838	0.47332881	0.33232763
Homez	-0.1129044	4.36385714	0.53416555	0.47337492	0.33232763
Pycr2	-0.2139119	2.68835934	0.53401637	0.4734359	0.33232763
Tbrg4	-0.1358257	3.84983377	0.5340107	0.47343821	0.33232763
Vps35	0.08698712	6.730399	0.53376963	0.47353678	0.33236844
4933426M11	-0.1332635	5.71371529	0.5333159	0.47372239	0.33247018
AI464131	-0.224013	2.93921916	0.53321755	0.47376264	0.33247018
Erp44	0.1405972	4.53609662	0.53235153	0.47411729	0.33269065
Ckb	0.11369636	7.92797394	0.53084623	0.47473481	0.33309553
Ppp1r36	0.59083315	-0.2099402	0.53024043	0.4749837	0.33324172
Celf1	-0.1035496	8.13798273	0.52941631	0.47532265	0.33344472
Stk35	0.10645398	5.14102998	0.52933968	0.47535419	0.33344472
Cdk15	-0.3631035	0.70685136	0.52893938	0.47551899	0.33353186
Gucy2g	-0.2861054	1.35849486	0.52792689	0.47593626	0.33379605
Prss8	-1.0634096	-2.1549414	0.52769343	0.47603256	0.3338351
Hspa4	0.10856317	8.78386391	0.52698808	0.47632371	0.33401078
Map3k4	-0.1290336	4.98220469	0.52669516	0.47644471	0.33404347
Cdh1	0.23044558	6.50236452	0.52662779	0.47647255	0.33404347
Nup62	0.12048726	5.49699393	0.52652613	0.47651456	0.33404347
Cbll1	0.11957777	6.17443958	0.52645621	0.47654345	0.33404347
Ghrl	-0.4959135	-0.638868	0.52638341	0.47657355	0.33404347
Zfp397	-0.0781161	5.88909395	0.52625187	0.47662793	0.33404851
Aspdh	-0.5352616	0.11307525	0.52616942	0.47666201	0.33404851
Rbm41	-0.1314544	4.52288136	0.52601176	0.47672721	0.33406572
Arf3	-0.1153031	10.0859814	0.52532529	0.47701126	0.33423627
Slc32a1	-0.1627116	4.04629258	0.52515335	0.47708245	0.33425765
Slc38a9	-0.185646	4.58404155	0.52487248	0.47719879	0.33431066
Skint3	-0.355388	1.16596641	0.52448309	0.47736015	0.3343952
Edc3	0.19029939	3.0896471	0.52392471	0.47759169	0.33452456
Srsf7	-0.0860184	5.89883115	0.5238415	0.47762622	0.33452456
Uckl1os	-0.5373931	-0.4792258	0.52344813	0.47778947	0.33461039
Dbpht2	0.10381883	7.6516806	0.52299666	0.47797695	0.33471317
Pnkd	-0.0967331	6.53540609	0.52278404	0.47806529	0.33473537
Spata5	-0.1524317	4.23811925	0.52272434	0.4780901	0.33473537
Insc	0.40347449	0.14504822	0.52254516	0.47816457	0.33475899
9430083A17	-0.4263291	1.31944819	0.52223639	0.47829295	0.33481704
Tmtc2	-0.1860747	2.97770247	0.52208683	0.47835516	0.33481704
Ifitm6	0.46638355	0.49823614	0.52205189	0.47836969	0.33481704
Acaa2	0.14821308	4.83542078	0.52111036	0.47876163	0.33505425

Plekhs1	-0.5422565	-0.1763468	0.52104192	0.47879014	0.33505425
Jrkl	0.1456259	3.30938128	0.52088271	0.47885648	0.33507214
Hinfp	0.23657982	1.97206335	0.51990519	0.4792641	0.33532882
Cdk2	0.2842485	1.70180027	0.51978811	0.47931297	0.33533447
Git1	0.10371949	6.87270412	0.51932695	0.47950551	0.33544062
S100a8	0.88030877	-1.1417502	0.51905298	0.47961996	0.33549213
Pwwp2b	0.25673965	2.38647075	0.51882828	0.47971386	0.33552925
Ahrr	-0.8519672	-1.6587342	0.51867659	0.47977727	0.33554505
Ttc26	-0.2366692	2.9379197	0.5183277	0.47992316	0.33560034
Ctdspl	0.12546125	6.4620004	0.51829225	0.47993799	0.33560034
Fbln5	-0.2902479	4.21843074	0.51762964	0.48021529	0.33576568
Pdxdc1	0.08374811	6.24525789	0.51726215	0.48036921	0.33582551
Rnf170	-0.1097951	5.90689747	0.51722451	0.48038498	0.33582551
Nfix	-0.0906988	8.37197493	0.51713271	0.48042344	0.33582551
Sfrp2	0.38482986	0.51677032	0.51653899	0.48067233	0.33597091
Tcfl5	-1.1593231	-1.4827412	0.51569732	0.48102553	0.3361892
Acadsb	0.10549888	6.82862526	0.51548722	0.48111376	0.33622228
Gtf3c5	0.16242591	3.13958845	0.51464985	0.48146571	0.3364362
Csf2rb	-0.3267434	1.3256327	0.51456417	0.48150175	0.3364362
Brap	0.11319403	5.40008628	0.51403658	0.48172374	0.3365627
Gpr173	-0.194744	2.88554124	0.51389072	0.48178515	0.336577
Cstf2t	0.08454584	6.61119104	0.513622	0.48189831	0.33661078
Wrap53	-0.3093913	1.41100641	0.51357352	0.48191873	0.33661078
Tcp10b	0.82008549	-1.9858622	0.51348416	0.48195637	0.33661078
Rpl37a	0.15853689	5.95519467	0.51302861	0.48214836	0.33670972
Rab17	0.89208177	-1.5520423	0.5128743	0.48221342	0.33670972
F8a	0.13847721	3.41659898	0.51285647	0.48222094	0.33670972
Gm14005	-0.4286455	0.45134043	0.51267104	0.48229914	0.33672142
1700034G24	0.6528333	-0.0836493	0.51262246	0.48231963	0.33672142
Gm6981	0.25078245	1.51002647	0.51187543	0.48263492	0.3368337
Med8	0.11006275	3.81984149	0.51187144	0.48263661	0.3368337
Dcaf4	0.17256381	3.15447989	0.51168266	0.48271634	0.3368337
Arsk	-0.1999254	3.59236562	0.51164975	0.48273024	0.3368337
Sp6	0.71331094	-0.8815398	0.51153982	0.48277668	0.3368337
Olig2	-0.15784	3.43458375	0.51150465	0.48279155	0.3368337
Rft1	-0.2714423	2.59796167	0.51135043	0.48285672	0.3368337
Btn2a2	-0.9558209	-0.4733493	0.51133437	0.4828635	0.3368337
Dffb	0.36949106	1.45709339	0.51128988	0.48288231	0.3368337
B330016D10	-0.4782747	0.44110558	0.51122544	0.48290955	0.3368337
Hc	-0.9626017	-1.9135058	0.51117407	0.48293126	0.3368337
Map3k14	-0.3162239	1.77007305	0.51099013	0.48300903	0.33685231
Cbx5	-0.0839661	9.15622761	0.51081474	0.4830832	0.33685231
Col8a1	0.17558268	5.5011309	0.51073832	0.48311553	0.33685231
Renbp	-0.34125	2.99842113	0.51072329	0.48312188	0.33685231
Rn45s	-0.1664486	14.5621267	0.51022629	0.48333221	0.33693515

Tbr1	-0.1329298	6.77187326	0.51002036	0.4834194	0.33693515
Rps12	0.16772542	6.21202793	0.51001007	0.48342375	0.33693515
Mki67	-0.1453517	3.76884703	0.50997443	0.48343885	0.33693515
Rbm4	-0.454686	-0.5383219	0.50991839	0.48346258	0.33693515
Spidr	0.21939076	2.25997588	0.5098582	0.48348807	0.33693515
Rab14	0.07939541	7.96281547	0.50976478	0.48352765	0.33693515
Igf2bp1	1.01396362	-1.5499557	0.50947845	0.48364897	0.33699112
Btbd7	-0.1175717	5.67035502	0.50913207	0.48379581	0.33706485
Alg6	-0.1765932	3.97261279	0.50895881	0.48386928	0.33708747
Pik3ip1	-0.1927525	4.0136335	0.50827979	0.48415743	0.33725962
Nus1	-0.0817819	6.88160066	0.50814292	0.48421554	0.3372621
Paf1	-0.1091864	5.95108344	0.50807816	0.48424305	0.3372621
Hsf1	0.10403548	5.47374936	0.50775682	0.48437955	0.33732858
Zkscan5	-0.2192835	3.89448709	0.50736347	0.48454673	0.33741642
Tmem214	0.136473	4.44956222	0.50724984	0.48459505	0.33742148
Arid4b	-0.1102332	7.72302548	0.50704734	0.48468117	0.33745285
Myb	0.33737587	0.61825725	0.50668992	0.48483324	0.33749229
She	0.38020886	1.34917998	0.50666361	0.48484444	0.33749229
Akap10	-0.1097074	4.87047953	0.50662469	0.484861	0.33749229
Rnf20	-0.0979028	6.44450563	0.50650438	0.48491221	0.33749936
2310011J03F	0.17230519	2.93835107	0.50622391	0.48503163	0.33755389
Rnf167	0.17622977	4.18757965	0.50593581	0.48515435	0.33761071
Ramp3	-0.5251172	-0.3875601	0.50581262	0.48520684	0.33761865
Ccl5	-0.353737	1.43448762	0.50563628	0.485282	0.33763995
Slc4a9	-1.4553823	-1.3931945	0.50552936	0.48532758	0.33763995
Chil1	0.45498318	0.532544	0.50545171	0.48536069	0.33763995
AK010878	0.14309049	3.75420995	0.50464819	0.48570349	0.33784982
Panx1	0.26536799	2.45878713	0.50422437	0.48588447	0.33794108
Chst13	-0.8832862	-0.7207541	0.50414842	0.48591692	0.33794108
Chml	-0.0899988	6.24860913	0.50400087	0.48597996	0.33795632
Rexo4	0.12789599	4.59770965	0.50384806	0.48604526	0.33797314
Pam	-0.1026783	6.3274475	0.50374702	0.48608845	0.33797458
Arhgef1	0.15540673	4.30203653	0.50353545	0.4861789	0.33800888
Stac	0.54797823	0.1183344	0.50341289	0.48623131	0.33801672
Kcnk9	-0.2522622	2.87097366	0.50331176	0.48627457	0.3380182
Clec2l	-0.2868742	1.15729385	0.50306721	0.48637919	0.33806233
Rnf41	-0.0983353	5.35339329	0.5029444	0.48643175	0.33807028
Cdk13	-0.0790352	7.17706312	0.50266744	0.4865503	0.33812408
Gbp4	-0.1960257	2.90450644	0.50241956	0.48665646	0.33815823
Clec16a	-0.1043708	6.3092255	0.50236055	0.48668173	0.33815823
6430562O15	-1.0450914	-1.7198949	0.50217766	0.48676009	0.33817803
Gm2011	-0.2797114	1.36350918	0.50210197	0.48679252	0.33817803
4921534H16I	-1.0196434	0.0222078	0.50195117	0.48685715	0.33819434
Zfp771	0.38249379	0.69994245	0.50116699	0.48719347	0.33832097
Gm5820	0.28148737	1.83755461	0.50116515	0.48719426	0.33832097

Wfdc17	-0.3993881	0.93154725	0.50115708	0.48719772	0.33832097
Pamr1	-0.1328024	4.03100599	0.50114225	0.48720409	0.33832097
Adam11	0.19085558	3.57860668	0.50084474	0.48733179	0.33837423
Tmem243	-0.157943	4.06310175	0.50077177	0.48736312	0.33837423
Rnf215	-0.1934927	2.67262694	0.50062945	0.48742423	0.33838003
Slc16a2	-0.1055644	5.39027333	0.50054256	0.48746155	0.33838003
Ctsk	0.27906823	2.38531761	0.50046473	0.48749499	0.33838003
A730036I17F	-0.7968756	-1.0930303	0.50023888	0.48759203	0.33841816
S100a13	0.30440762	3.41422825	0.50014525	0.48763226	0.33841816
Ccnh	0.10313253	4.97463151	0.50001737	0.48768723	0.33842773
Pdcd7	-0.1498673	3.61848309	0.49957674	0.48787671	0.33851886
Slc39a13	0.16802358	5.17208541	0.49949531	0.48791174	0.33851886
Crlf2	-0.3430421	0.43132701	0.49942471	0.48794212	0.33851886
Apoa1bp	0.1485439	3.85453329	0.49915573	0.48805787	0.33856858
Gm16532	-0.4969283	1.04913494	0.49897531	0.48813554	0.33856858
Gps2	0.11937303	4.02309499	0.4989706	0.48813757	0.33856858
Ccdc58	0.16728147	3.0272777	0.49875721	0.48822946	0.33856858
Itgb7	-0.6890399	-1.4779286	0.49869471	0.48825638	0.33856858
Ccdc67	-0.7889976	-1.3627201	0.49851638	0.48833321	0.33856858
Neu4	-0.2163377	2.46054325	0.49849592	0.48834202	0.33856858
Leng9	0.29514038	1.11170176	0.49849288	0.48834333	0.33856858
Fam155a	-0.1118348	6.69012916	0.49822906	0.48845703	0.33859113
Nlrc5	0.50601885	0.00570201	0.49822623	0.48845825	0.33859113
Tmem101	-0.3008205	2.47401716	0.49799267	0.48855894	0.3386212
Smg9	0.20372646	2.90670721	0.4979345	0.48858402	0.3386212
Fbxo46	-0.2055322	1.66258558	0.4977938	0.4886447	0.3386347
2900079G21	-0.2524525	1.16559202	0.49758728	0.4887338	0.33864625
Gm6525	-1.0589342	-1.3451882	0.49756414	0.48874378	0.33864625
Hist1h2bh	0.62963593	-0.7848318	0.49732504	0.48884696	0.3386892
Sp3	0.09612674	6.67392446	0.49688617	0.48903646	0.33878874
Lamp1	0.15452003	7.56325449	0.4968014	0.48907308	0.33878874
Pipox	-0.3543105	0.57711512	0.49666641	0.48913139	0.33880058
Pced1b	-0.1624311	3.1020314	0.49622609	0.4893217	0.33890384
Ccdc85b	-0.1288379	4.38375005	0.49556315	0.48960847	0.33903197
Bbs1	-0.1357442	5.65451516	0.49554192	0.48961766	0.33903197
Mndal	-0.2009427	2.99456607	0.49551236	0.48963046	0.33903197
Kctd13	0.1420103	6.17116268	0.49521359	0.4897598	0.33908326
Entpd4	-0.1045515	6.04676775	0.49515068	0.48978704	0.33908326
Arhgap5	-0.0967147	8.64702615	0.49474256	0.48996384	0.33917709
Wdr16	0.5407283	-0.2244289	0.49450455	0.49006699	0.33919797
Galnt12	-0.3257741	0.82133625	0.49448253	0.49007654	0.33919797
Emcn	-0.4922231	0.59230906	0.49373853	0.49039925	0.33939275
2610002M06	0.07876485	7.06876313	0.49324419	0.49061387	0.3395127
Gtpbp1	-0.1689788	4.45862357	0.49298399	0.49072691	0.33956233
1700066M21	-0.0999003	4.79759952	0.4928836	0.49077053	0.33956392

Hist1h1c	0.32066656	2.87540411	0.49272823	0.49083805	0.33958206
Gpr25	0.17275091	3.66236568	0.49261408	0.49088768	0.3395878
Fbxl8	0.67083004	-0.7816331	0.49249006	0.4909416	0.33959652
Artn	0.99084921	-2.0795552	0.49224248	0.49104927	0.33963588
Pi4k2a	0.12314029	4.91082753	0.49216922	0.49108114	0.33963588
Ttc30a2	0.42666581	-0.1179123	0.49206026	0.49112855	0.33964009
Gm4951	0.2774728	3.36598933	0.49172152	0.49127597	0.33969429
Timm44	-0.1545391	3.40871495	0.49168149	0.4912934	0.33969429
Kcnh6	0.40919415	-0.1846566	0.49159531	0.49133092	0.33969429
Runx1	-0.2288846	3.56880583	0.49142288	0.49140601	0.33971763
Mllt1	-0.1285875	4.59534888	0.49122792	0.49149094	0.33973565
Nell1	-0.1281606	4.05279299	0.49108885	0.49155153	0.33973565
Tmem144	0.20129154	2.21110874	0.4910611	0.49156362	0.33973565
5330413P13I	0.30836157	1.58644939	0.49089592	0.49163561	0.33973565
Dpagt1	0.19888954	3.3175558	0.49088874	0.49163874	0.33973565
Gnai2	0.16869502	7.06060397	0.49061718	0.49175714	0.33974063
Akap17b	-0.1120325	5.61061691	0.49054811	0.49178726	0.33974063
BC028528	0.27170444	1.6431558	0.4905368	0.49179219	0.33974063
Iws1	0.0872833	6.44071015	0.49049298	0.4918113	0.33974063
Spats2l	0.10088258	5.09041211	0.49017601	0.49194958	0.3398076
6030407O03	-0.6608527	-0.8430597	0.4900009	0.492026	0.33983182
Drd3	-0.6129753	-1.0424144	0.48890212	0.49250601	0.34013477
Ing5	0.13609322	4.683819	0.48862238	0.49262834	0.34017554
Osbpl3	-0.1643229	5.09364473	0.48857785	0.49264782	0.34017554
Zbtb33	0.10896432	5.58479729	0.48826269	0.49278572	0.34024217
Scarf1	-0.6564927	-0.7980304	0.4880739	0.49286835	0.34026698
Snord47	-0.6912397	-1.5171434	0.48799147	0.49290444	0.34026698
Tmem9	-0.1526949	4.96445114	0.48783955	0.49297096	0.34028432
Cc2d1b	-0.1531665	3.62334227	0.48742713	0.49315163	0.34034114
Celf5	-0.1074511	6.86974412	0.48741049	0.49315892	0.34034114
Actn4	-0.0801586	6.28219437	0.4872826	0.49321497	0.34034114
Cadm4	0.19158812	3.23482238	0.48727362	0.49321891	0.34034114
Dcun1d2	0.11117057	5.52819333	0.48689928	0.49338304	0.34040438
L1cam	-0.1712497	6.82476182	0.48684885	0.49340516	0.34040438
9430038I01R	-0.4670045	0.73306406	0.48678123	0.49343481	0.34040438
Socs7	-0.1072104	6.96844492	0.48643122	0.49358838	0.34046769
Dars	0.09775143	5.23673454	0.48638326	0.49360944	0.34046769
Lrrn2	-0.1518859	4.65525531	0.48599601	0.49377946	0.34055639
Clnk	0.66029598	-0.6436547	0.48571437	0.49390317	0.34061313
Zfand4	-0.3096039	1.73462481	0.48560338	0.49395194	0.34061818
Fbxw4	-0.1813424	2.72014757	0.48532176	0.49407573	0.34065617
Bmper	0.15314887	3.17057497	0.48526332	0.49410142	0.34065617
Gimap5	-0.4334214	1.56612691	0.48519521	0.49413137	0.34065617
Dmpk	0.16819333	3.50546501	0.48493755	0.49424469	0.34070571
Atp1b2	0.11637348	9.04784506	0.4844614	0.49445422	0.34081588

Eral1	-0.1550739	3.54601251	0.48438598	0.49448743	0.34081588
4732416N19	0.7998399	-1.9310366	0.48416357	0.49458536	0.34083298
Gm2027	-0.4410451	0.23977782	0.48414128	0.49459518	0.34083298
Dbil5	-0.7873524	-1.4337472	0.48365531	0.4948093	0.34095195
Zfp566	-0.2536382	1.84360935	0.48339641	0.49492344	0.34100201
Atp8a2	-0.224441	3.82763017	0.4828904	0.49514665	0.3411272
Gm1141	1.03680767	-1.6419073	0.48274156	0.49521234	0.34114386
1810062G17	-0.8812794	-1.2913555	0.48251968	0.49531029	0.34118274
Tapbp	0.21738839	4.62292862	0.48221693	0.495444	0.34122198
Zfp750	0.33348231	2.36164251	0.48214734	0.49547474	0.34122198
Gm3414	-0.1827668	3.33853117	0.48210872	0.4954918	0.34122198
Hsdl1	-0.1461445	4.27791316	0.48200972	0.49553555	0.34122352
Cbx2	-0.3515749	1.04972225	0.48166306	0.49568878	0.34124918
Rmst	-0.4972433	0.98297744	0.48152361	0.49575044	0.34124918
Moxd1	-0.1842813	2.93073035	0.48146769	0.49577517	0.34124918
Hook2	0.32262654	1.55443116	0.48146075	0.49577824	0.34124918
Baiap2l2	-0.6422431	-1.0733073	0.48136084	0.49582243	0.34124918
Rpa3	-0.1976504	3.1345464	0.48130818	0.49584572	0.34124918
Plxdc2	-0.0846516	6.74706993	0.4812681	0.49586345	0.34124918
Ttc34	-0.5011546	-0.1334546	0.48099451	0.49598451	0.34128809
Rfc5	-0.2074902	2.67648638	0.48095267	0.49600303	0.34128809
Gm6498	-0.540899	-1.0240939	0.48052839	0.49619089	0.34138877
Whsc1	-0.0787119	6.66122419	0.47998229	0.49643287	0.34146348
Ppp1r35	0.25634587	1.49108591	0.47997876	0.49643443	0.34146348
Znrf2	0.14176209	4.47525774	0.47997037	0.49643815	0.34146348
Eaf1	0.10749758	5.06304878	0.47990829	0.49646567	0.34146348
Arfrp1	-0.1514163	3.99464723	0.47950783	0.49664326	0.34155705
Acot5	0.64267291	0.37298916	0.47936342	0.49670733	0.34157253
Lect1	0.48221171	-0.5496984	0.47899199	0.49687219	0.34161479
Rad9a	-0.1491342	3.49725422	0.47897438	0.49688001	0.34161479
Zbtb39	0.11797215	4.42513232	0.47881951	0.49694877	0.34161479
Sfxn1	-0.0899565	5.91828744	0.47878704	0.49696319	0.34161479
Grpel1	0.13470394	4.23880688	0.47875686	0.4969766	0.34161479
Slc46a3	-0.2784661	2.39258348	0.47840926	0.49713102	0.34167902
Enpp1	0.1855371	5.3449305	0.47835938	0.49715319	0.34167902
Zfp423	0.10118674	6.14028828	0.47803379	0.49729792	0.34173563
Elovl4	0.15854449	4.81666935	0.47798702	0.49731871	0.34173563
Mapkapk5	0.12131249	4.18382528	0.47756338	0.49750715	0.34183068
Arhgef15	-0.2276621	2.8955382	0.47748907	0.49754022	0.34183068
Ano8	-0.3316208	0.67743509	0.47704916	0.49773605	0.34190712
Pcdhgb5	-0.2184806	2.4519966	0.47702199	0.49774815	0.34190712
Cep128	-0.1651849	4.25132638	0.47695885	0.49777627	0.34190712
Slc6a20a	-0.2106863	5.60574727	0.47678156	0.49785524	0.34192391
Snx29	-0.2215294	2.1760274	0.47671717	0.49788393	0.34192391
Slfn3	-0.7344806	-0.2510446	0.47649048	0.49798495	0.34196472

Svopl	0.55896083	-0.2633265	0.47631624	0.49806262	0.34198948
Mtpap	0.11715239	4.90302838	0.47619241	0.49811783	0.34199882
Rybp	0.09391789	6.64734823	0.47589743	0.49824939	0.34206057
Taf1c	-0.3182589	1.20429894	0.47543368	0.49845636	0.34217408
Kpna1	0.08608769	6.91615196	0.47507966	0.49861445	0.34225402
Tcerg1	-0.091642	6.61335519	0.47448055	0.49888219	0.3424092
Rgl3	-0.3897853	0.42338172	0.47391049	0.49913718	0.34255561
Gltpd2	0.89397292	-1.9420875	0.47370492	0.49922919	0.34259015
Tbc1d2	0.51588256	-0.1847319	0.4728452	0.4996143	0.34282581
Pcdhga9	-0.1858118	2.52573333	0.47245711	0.49978832	0.34291659
Adcyap1	0.17213031	2.9020933	0.47230244	0.49985771	0.34293556
Zfp536	-0.0906256	4.61730141	0.47193191	0.50002399	0.34302101
A930012L18I	-0.2340176	2.57284962	0.47169905	0.50012854	0.34305427
Slc50a1	0.17454611	3.05410095	0.471638	0.50015595	0.34305427
Ctnnb1	0.07451203	9.22493966	0.47062905	0.50060944	0.34332699
Slit1	0.20209782	2.95992299	0.4704824	0.50067541	0.34332699
Mc4r	0.64061885	-0.7315664	0.47047472	0.50067887	0.34332699
Frs3	-0.224595	2.42524315	0.4703397	0.50073963	0.34333411
Ankzf1	-0.2441802	1.79473831	0.47026598	0.5007728	0.34333411
Gli2	-0.164192	3.37357665	0.4691627	0.5012698	0.34364159
Gm4788	0.36865728	0.00703406	0.46908484	0.5013049	0.34364159
Ring1	0.12401349	4.47181518	0.46881999	0.50142436	0.34369481
Zc3h4	-0.1048163	5.87777576	0.46868928	0.50148332	0.34370657
Zfp954	0.25326476	1.98684143	0.4684822	0.50157678	0.34374195
Gdf9	0.61651851	-0.9128164	0.46802884	0.50178147	0.34385356
Hspbp1	0.19567038	2.63907772	0.46789891	0.50184016	0.34386511
Spag6	-0.2322894	1.99929427	0.46775045	0.50190724	0.3438824
Chchd1	0.16235124	3.59794453	0.46757651	0.50198585	0.34388743
Trappc6b	0.11444493	7.79666568	0.46754907	0.50199825	0.34388743
Prkaa2	-0.0945291	7.2938567	0.46699663	0.50224807	0.34402292
S100a4	0.31386057	3.08503307	0.46692661	0.50227975	0.34402292
Pi4kb	-0.1292261	4.34799035	0.4667311	0.50236823	0.34405485
Glipr1	0.75703903	-0.9652955	0.46650805	0.50246919	0.34409532
Med9os	-0.7445821	-1.163347	0.46625254	0.5025849	0.34412701
Ntn5	-0.3711646	0.36897687	0.46622097	0.5025992	0.34412701
Rnf180	-0.2161163	2.8396008	0.46572491	0.50282399	0.34425224
Pdgfa	-0.0984895	5.81482077	0.46544957	0.50294884	0.34430904
Tmem205	0.2601626	1.69329535	0.46519585	0.50306393	0.34435551
Hes1	-0.1486846	3.68862672	0.46504727	0.50313135	0.34435551
Tsr2	0.10418766	4.95762152	0.46502287	0.50314242	0.34435551
Clptm1	-0.0985282	6.04131309	0.46470698	0.50328581	0.34442497
Vwc2	0.18119124	3.14969717	0.46427946	0.50348	0.34451538
Cln5	0.16969038	6.00046903	0.4641726	0.50352855	0.34451538
Calr3	-0.4030417	-0.0376434	0.46413933	0.50354367	0.34451538
Stk24	0.11539285	7.31064576	0.46382373	0.50368714	0.34458486

Ppil1	0.13890652	3.77002774	0.46357337	0.503801	0.34463406
Ubac1	0.13839945	3.83876854	0.46344007	0.50386164	0.34464686
Jkamp	0.12765417	4.43764506	0.46291726	0.5040996	0.34478094
Mbd5	-0.106491	7.79862583	0.46256152	0.50426164	0.34486306
Qpctl	0.18334656	2.65051681	0.46239291	0.50433847	0.34488691
Tmem180	-0.2078271	2.4419611	0.46162209	0.50468997	0.34506455
Grm8	-0.2520411	2.66462651	0.4616196	0.5046911	0.34506455
Acot8	0.28676728	1.57010583	0.4615471	0.50472419	0.34506455
Brf2	0.36474991	0.70218386	0.46090186	0.50501879	0.34523725
Mapt	-0.0866607	6.89262027	0.46071628	0.50510358	0.34526649
Ascc1	0.19522667	3.49080484	0.45999367	0.50543397	0.3454636
Zbtb25	-0.1462935	3.35792443	0.45987435	0.50548856	0.34547218
Abhd6	0.12765814	4.18091732	0.45917055	0.50581078	0.34566366
Fam117b	0.06878137	7.00148895	0.45897504	0.50590036	0.34569613
Lrig3	-0.3008778	1.08464464	0.45864263	0.50605272	0.34577149
Pank3	-0.0859856	7.493322	0.45847213	0.5061309	0.34577743
Reep6	-0.1077919	4.20138878	0.4584402	0.50614554	0.34577743
Pbrm1	0.0740756	7.48916556	0.4583239	0.50619888	0.34578513
Myh8	-0.8417594	-1.5295231	0.45807205	0.50631443	0.34583532
Tspan31	0.14467274	5.85510856	0.45784625	0.50641807	0.34587736
Hhat	-0.6601707	-0.7515552	0.4571775	0.50672523	0.34604047
Gatc	0.1136827	7.36425707	0.45714297	0.5067411	0.34604047
Pcdhac2	-0.1414731	4.03707919	0.45676505	0.50691483	0.34613036
Dab2ip	-0.0869072	6.24974934	0.4566065	0.50698775	0.34615139
Tm9sf4	-0.1150214	4.70297856	0.45645789	0.50705612	0.34616931
2810032G03	-0.2354785	2.61698762	0.45633178	0.50711415	0.34618017
Tcn2	0.20570579	4.22294844	0.45613798	0.50720334	0.34621231
Zfp92	-0.2575244	2.40848391	0.45598919	0.50727184	0.34623031
Fam63b	-0.0817983	7.76590666	0.45576723	0.50737406	0.34625884
Tmem160	0.3418351	1.07426555	0.45571547	0.5073979	0.34625884
Mrc2	0.22266799	4.96143287	0.45556353	0.50746789	0.34627785
Zfp174	-0.1346217	3.76830348	0.45534794	0.50756724	0.3463169
Spata18	-0.3590464	0.3596755	0.45513921	0.50766347	0.34634304
Tsta3	-0.2568034	2.29746459	0.45506934	0.50769568	0.34634304
Bag2	-0.215189	3.17330684	0.45499063	0.50773198	0.34634304
Edaradd	0.86387431	-1.1186257	0.45475945	0.50783861	0.34638703
Hdac6	-0.1824836	3.12401288	0.45446647	0.50797381	0.3464505
Ppp2r5e	0.09332693	6.40327889	0.45428804	0.50805618	0.34646203
Hars2	-0.1246337	3.90856284	0.4541616	0.50811456	0.34646203
Polr1e	-0.1955806	1.80469132	0.45415592	0.50811718	0.34646203
Clec4a2	-0.4721954	-0.2856982	0.45343656	0.50844957	0.34663985
Fmnl1	0.13618388	5.94178596	0.45338934	0.5084714	0.34663985
Tmem28	0.26920942	1.70191866	0.45331776	0.5085045	0.34663985
Csnk2a1	0.07518519	6.99510857	0.45301938	0.50864251	0.34670518
Mthfsd	-0.2474102	1.83132044	0.45236931	0.50894342	0.34688152

Ccdc103	-0.5450768	-0.0614749	0.45208438	0.50907541	0.34694129
Rpl36a	0.15253041	6.48678995	0.45199777	0.50911554	0.34694129
Nif3l1	0.15527986	3.38042311	0.45181073	0.50920223	0.34697053
Alg8	-0.1301246	3.33045314	0.45165584	0.50927404	0.34697053
Slc9a8	0.14359044	4.08268611	0.45163198	0.5092851	0.34697053
Nmnat3	-0.21744	2.54700516	0.45121036	0.50948067	0.34705715
Fam186b	0.79808601	-0.8546847	0.45117584	0.50949669	0.34705715
Tmem150co:	-0.7217042	-0.5281174	0.45075309	0.50969292	0.34716205
Zfp942	-0.1377699	3.51107222	0.45034135	0.50988417	0.34726354
BC051226	0.69032714	-1.6089049	0.45018001	0.50995915	0.34728582
Osbpl8	-0.0744068	7.70354913	0.45005906	0.51001537	0.34729533
B130006D01	-0.7246079	0.52011361	0.44995723	0.51006271	0.3472988
Zfp518b	0.08374486	5.59134857	0.44893048	0.51054049	0.34759532
Coasy	-0.1434377	3.65694836	0.44842482	0.51077609	0.34765709
Eif4g1	-0.0748503	7.06173756	0.44836037	0.51080613	0.34765709
Nup210l	-0.8261481	-0.3714807	0.44831663	0.51082652	0.34765709
Smim22	0.67054162	-0.4654635	0.4483076	0.51083073	0.34765709
Hmox2	0.14572719	5.03916106	0.44828189	0.51084271	0.34765709
Slc7a6	-0.2403418	2.89527166	0.44811316	0.51092139	0.34768184
Scg5	0.09680161	6.32416838	0.44770656	0.51111107	0.34778212
Lman2	-0.1030122	5.61149008	0.44708952	0.51139915	0.34793319
Ptprr	-0.1110727	4.20917954	0.44704974	0.51141774	0.34793319
Clec18a	0.51191379	0.16717546	0.44690523	0.51148525	0.34795032
Cd9	-0.2133289	4.85260339	0.44667251	0.51159401	0.3479955
Gpr126	0.14845189	3.94251813	0.44652834	0.51166141	0.34801255
Cfb	-0.2342138	2.67106589	0.44600922	0.51190423	0.34814889
Ints3	0.10192558	5.30231358	0.44587269	0.51196813	0.34816354
Spag7	-0.1325322	5.28595099	0.44542	0.51218009	0.34827886
Gm3258	0.4727083	-0.8824314	0.44501927	0.51236785	0.3483703
Tex30	0.29347609	2.38532697	0.44495211	0.51239933	0.3483703
Mpl	-0.3764425	1.52011049	0.4445672	0.51257982	0.34841098
Gm15421	-0.2013491	1.85128343	0.44456495	0.51258087	0.34841098
Hcar1	0.251728	4.37220881	0.44455331	0.51258633	0.34841098
Hs3st5	0.30118016	1.6804255	0.44438357	0.51266596	0.34843629
BC026585	0.4614262	0.725837	0.44423377	0.51273626	0.34845422
B3galt6	-0.2348989	2.33602443	0.44409519	0.5128013	0.34845422
Pola2	-0.2162052	1.80786801	0.44399375	0.51284892	0.34845422
Tex14	-0.6794424	-0.4598515	0.44396606	0.51286192	0.34845422
A530064D06	-0.7352311	-1.7685004	0.44379336	0.51294301	0.34848051
Pcdhgc4	0.38166104	0.9926195	0.44340419	0.51312585	0.34853145
Thumpd2	-0.2266777	1.67259557	0.44335913	0.51314702	0.34853145
2810410L24I	0.38376979	-0.0493362	0.44335186	0.51315044	0.34853145
Klhl35	0.64859939	-0.7426236	0.44327276	0.51318762	0.34853145
Afap1l1	0.13403218	5.54664804	0.4426441	0.51348327	0.34868111
Zdhhc4	0.14753229	2.59386182	0.44262379	0.51349283	0.34868111

Mmrn2	0.56346415	-0.3197743	0.44233783	0.51362742	0.34874369
Dennd4c	0.09856936	6.04642157	0.44224224	0.51367242	0.34874544
Ttll13	-0.663539	-0.9046798	0.44150911	0.51401782	0.3488948
Rabgap1l	-0.1004261	8.19784169	0.44145305	0.51404425	0.3488948
Alas1	0.09693263	4.46677621	0.44142413	0.51405788	0.3488948
A430035B10	-0.1716453	2.99546973	0.44141493	0.51406222	0.3488948
Ficd	0.17202382	2.89199918	0.44126808	0.51413147	0.34891299
Galntl6	-0.1168629	5.12805334	0.4409962	0.51425971	0.3489712
Tnpo1	0.08173409	6.64202774	0.44089983	0.51430519	0.34897325
Ppapdc1a	-0.4887033	-0.1787357	0.4403092	0.51458403	0.34913363
Amigo2	0.22167739	2.41715299	0.44015716	0.51465586	0.34915354
Prdm16	-0.1878598	3.06366429	0.43999408	0.51473292	0.349177
Tmem203	-0.165174	2.72407557	0.43987239	0.51479043	0.3491872
Dusp18	0.11529343	5.35682692	0.43907107	0.51516946	0.34941545
N4bp2	-0.1577751	3.4697598	0.43888323	0.51525838	0.34944693
Pycard	0.17414442	2.9570236	0.43841091	0.51548209	0.3495698
4930428E07l	-0.5862642	-0.2484161	0.43811615	0.51562179	0.3496122
S1pr5	-0.2367754	2.05751533	0.43809952	0.51562968	0.3496122
Ptbp2	0.10558214	6.37537676	0.43768404	0.51582671	0.34971694
Mib1	0.09922025	6.21587514	0.43747408	0.51592633	0.34975021
Mir143hg	-0.6843877	-1.3937044	0.43740127	0.51596089	0.34975021
Zfp185	-0.2125051	3.37363519	0.43728585	0.51601567	0.3497585
Arhgef40	0.12698653	3.63309355	0.43686444	0.51621579	0.34985155
Akr1c13	0.27235746	1.750315	0.43681751	0.51623809	0.34985155
Dgkd	-0.1278622	5.44293754	0.43656635	0.51635744	0.34988291
Atf1	-0.0998578	7.58170167	0.43652579	0.51637671	0.34988291
Zfp667	0.15476513	4.79433515	0.43642187	0.51642611	0.34988291
Zfyve20	0.10280937	5.76894164	0.43636185	0.51645464	0.34988291
Ankrd12	-0.1067334	9.70283078	0.4359858	0.51663349	0.34997523
Gmpr	0.22017238	2.07266291	0.43572259	0.51675874	0.35003123
Gm10865	-0.3660417	-0.4188497	0.43542537	0.51690023	0.35006647
Gdi1	0.08776112	8.88966639	0.4353863	0.51691884	0.35006647
Wdr45b	0.1140869	4.47916037	0.43531717	0.51695176	0.35006647
Tubgcp5	-0.1647508	4.13761734	0.43525549	0.51698114	0.35006647
C1d	0.11089244	4.9718572	0.43475733	0.51721852	0.35019836
Capn15	-0.1936701	1.8302036	0.43463894	0.51727496	0.35020772
Csrnp2	-0.1063605	5.41423429	0.43400319	0.51757825	0.35038218
Rab42	-0.6704326	-1.7234385	0.43392012	0.5176179	0.35038218
Kif6	-0.3813546	0.20610313	0.43372423	0.51771143	0.35041663
Tnpo3	0.09096729	6.1065511	0.43362939	0.51775672	0.35041843
Sf3a3	0.10655431	4.70077619	0.4334576	0.51783878	0.35042478
6430550D23l	-0.5242087	-0.2776649	0.43343123	0.51785138	0.35042478
Ano7	0.660072	-1.3843268	0.4332408	0.51794238	0.35043289
Mmp14	0.22870336	3.91261797	0.43322771	0.51794863	0.35043289
Bcas3	0.12068472	4.43762814	0.43311362	0.51800316	0.35044094

Zfp106	-0.0667488	9.16699433	0.43294099	0.5180857	0.35046792
Nfyb	0.09798902	5.3894868	0.43279377	0.5181561	0.3504867
Tmem126b	0.13585436	4.18323739	0.43181552	0.51862435	0.3507378
A330009N23	0.31803889	1.21981126	0.43179278	0.51863525	0.3507378
1700019D03I	-0.2619807	1.69475413	0.43175083	0.51865534	0.3507378
Ankrd34c	-0.1320529	4.32065125	0.43161787	0.51871905	0.35075202
Map2k2	0.13549899	4.35852667	0.43109915	0.51896774	0.3508692
E230016M11	-0.4959219	0.20227686	0.43107833	0.51897772	0.3508692
Nlrp3	0.5796244	-0.9564663	0.43097849	0.51902561	0.35087272
Ccbl1	-0.2727341	1.5529635	0.43072197	0.5191487	0.35092707
Map10	-0.3014052	1.696296	0.43030481	0.51934898	0.35103358
Brd9	-0.0786968	5.50512023	0.43000728	0.51949191	0.35108769
Dclk3	-0.1235083	5.48919709	0.42996033	0.51951447	0.35108769
Pcdhb7	0.25459808	2.24062595	0.42961586	0.51968005	0.35117071
Serhl	-0.4740079	-0.4036211	0.42942416	0.51977224	0.35120414
Hsp90ab1	0.07833742	10.3381856	0.42932122	0.51982176	0.35120872
Kbtbd2	-0.0806794	5.97260635	0.42891746	0.52001605	0.35130291
Smarcd2	-0.2143865	3.09274541	0.42878206	0.52008124	0.35130291
H2-Q1	0.1922633	6.0697146	0.42876512	0.5200894	0.35130291
Tgm1	-0.8914283	-1.4017659	0.42864587	0.52014683	0.35131283
Fert2	-0.0876535	4.96364727	0.42847101	0.52023105	0.35134085
Fn1	0.17429102	8.00392047	0.42789079	0.52051071	0.35150083
Cbx8	-0.4236962	0.15420196	0.4277578	0.52057485	0.35151526
Asb7	-0.0696336	6.12224714	0.42745445	0.5207212	0.3515852
Zswim4	0.1784482	2.37334394	0.42698248	0.52094905	0.35168536
Zfp800	0.11945743	5.95938566	0.42696992	0.52095511	0.35168536
Tmem147	0.14678513	3.44846783	0.426366	0.52124694	0.35185346
Idi2	-0.4696762	-0.0722269	0.42614147	0.52135551	0.35189785
AI429214	-0.202319	3.37119213	0.4258372	0.5215027	0.3519683
Cetn4	0.16856468	4.02178654	0.42568407	0.52157681	0.35198941
Pigh	-0.2383168	2.03473925	0.42489198	0.52196045	0.35221939
Matr3	-0.0983452	8.16150937	0.4246519	0.52207683	0.352269
Efna5	-0.0993947	6.08444567	0.42440721	0.5221955	0.35229371
Rfxank	0.18041248	2.94693999	0.42439964	0.52219917	0.35229371
Elovl5	0.14878378	6.21453658	0.42410764	0.52234084	0.35232855
Copb1	-0.0834281	6.10022922	0.42404373	0.52237186	0.35232855
Frs2	-0.0724794	7.4298957	0.42402815	0.52237943	0.35232855
Slc45a3	0.49791546	0.25526345	0.42350064	0.52263558	0.3524724
Slc41a2	-0.1354942	4.01969231	0.42329861	0.52273374	0.35250967
Drosha	0.08193253	6.89887873	0.42301323	0.52287246	0.35255198
Tbx20	-0.6170643	-1.4327561	0.42299306	0.52288227	0.35255198
Zfp560	0.14418529	4.12025676	0.42288678	0.52293395	0.35255791
1600002H07I	-0.1670709	3.04316158	0.42263281	0.52305748	0.35259702
Mrpl33	0.15849622	4.31556903	0.42259112	0.52307777	0.35259702
Cubn	0.16689802	5.30480592	0.42230066	0.52321913	0.35263414

Atxn1	-0.1036417	8.6929335	0.42225877	0.52323952	0.35263414
Far1	-0.0929884	6.28201656	0.42213015	0.52330215	0.35263414
Nop2	-0.192834	2.78938159	0.42197763	0.52337642	0.35263414
Pgam5	-0.0971485	6.09809971	0.42196913	0.52338057	0.35263414
Fam227b	-0.3134062	0.68226812	0.42194922	0.52339026	0.35263414
Fbxl18	-0.2518321	1.31069494	0.42174173	0.52349135	0.35264991
Ly75	-0.6009631	-0.5198203	0.42169869	0.52351232	0.35264991
Baiap2	0.0944093	6.25207541	0.42163699	0.52354238	0.35264991
Gm11627	0.28205258	1.40812479	0.42147538	0.52362116	0.35266565
Orai2	-0.1383636	3.83792651	0.421413	0.52365157	0.35266565
Spry4	-0.1571231	3.46186275	0.42117585	0.52376721	0.35270792
Dolk	-0.1868614	2.47522989	0.42105603	0.52382565	0.35270792
Phf19	0.42192339	0.71528443	0.42102033	0.52384307	0.35270792
Gm14446	-0.1445748	3.72874383	0.42085385	0.5239243	0.352727
Aldh18a1	0.14629073	3.57618485	0.42078634	0.52395725	0.352727
Tug1	0.06944994	8.05181296	0.42060249	0.52404699	0.35275853
Gm14634	-0.4494014	-0.1042854	0.41990321	0.52438859	0.35293266
Mir28b	-0.683079	-0.4985451	0.41989711	0.52439156	0.35293266
Adamts17	-0.3139241	2.62311081	0.41967328	0.52450099	0.35297741
Lgi2	-0.2228566	2.70263887	0.41910712	0.52477797	0.35313489
Mboat2	-0.1744184	4.15070648	0.41886316	0.5248974	0.35318408
Lamc1	0.09419935	6.02517539	0.41878227	0.52493701	0.35318408
Ms4a4c	0.85495275	-1.6384002	0.41842786	0.52511063	0.35327197
Ccser1	-0.2001925	3.31125484	0.41802085	0.52531014	0.35335133
Maml3	0.10895342	4.82223032	0.41801183	0.52531456	0.35335133
Rps23	0.12641357	7.24528783	0.41760918	0.52551208	0.35345526
Slc2a3	-0.1068016	5.24964401	0.4173123	0.52565779	0.35352026
Mul1	-0.144781	3.14208276	0.41723705	0.52569474	0.35352026
Tspan7	-0.0932678	8.1016807	0.41708413	0.52576984	0.35354183
Tnfrsf21	-0.1294287	6.54315861	0.41620059	0.52620411	0.35379863
Gm5595	-0.1845122	2.83552425	0.41613205	0.52623783	0.35379863
Ube2v1	0.10475582	6.89310435	0.41598034	0.52631248	0.35381419
Mob2	0.21029448	2.48552716	0.41591005	0.52634707	0.35381419
Zfp41	0.18431992	1.96882306	0.415754	0.52642388	0.35383688
Fgf13	-0.105168	6.45146223	0.41552223	0.52653799	0.35388464
Fbll1	0.21526353	2.23625039	0.41524732	0.52667341	0.35394671
Bnip2	0.11212948	7.06219684	0.41512353	0.5267344	0.35395876
Cacng8	-0.369391	0.70465337	0.4148716	0.52685858	0.35401326
Cdk20	-0.328009	1.18876467	0.41458139	0.5270017	0.35408048
Rnase1	-0.7560808	-0.8983568	0.41443061	0.52707608	0.35410151
Kif11	-0.2203831	2.2910576	0.41415719	0.52721101	0.35416321
Mettl4	-0.1159583	5.08264826	0.41371707	0.52742835	0.35428026
Mkks	0.12198076	5.11911302	0.41344179	0.52756437	0.35434267
Raf1	0.09825288	4.73108242	0.41315652	0.52770539	0.35440842
Cyp2s1	0.22281658	3.8419522	0.41291557	0.52782456	0.35445949

Pecam1	-0.2767303	1.99038698	0.41275335	0.52790482	0.35448443
4933428C19I	0.67387168	-0.7452506	0.41236376	0.52809766	0.35455864
Rab27a	0.13605143	3.40232344	0.41232795	0.52811539	0.35455864
Prss53	0.81767752	-1.4968797	0.41226865	0.52814476	0.35455864
Gpt	0.43198266	0.46254604	0.41213942	0.52820876	0.35457265
Ccdc155	0.48640068	-0.6128863	0.41163349	0.52845948	0.35471198
Tgfb3	0.18715214	4.69475048	0.41114155	0.52870348	0.35481854
Nup62-il4i1	0.70200777	-1.2666709	0.41100958	0.52876897	0.35481854
Arrdc1	-0.2924482	0.91740947	0.41089577	0.52882546	0.35481854
BC053749	0.23364179	2.84818733	0.41089475	0.52882597	0.35481854
Lclat1	-0.0927585	6.05359353	0.41079947	0.52887327	0.35481854
Zdhhc9	0.09322554	6.30578159	0.41079145	0.52887725	0.35481854
4930509E16I	0.71262677	-1.2491604	0.41059345	0.52897558	0.35485553
Nxf2	-0.7384884	-1.3173708	0.41039682	0.52907325	0.35488688
BC068281	-0.1601807	2.29279265	0.41032556	0.52910866	0.35488688
Fst	-0.3897626	0.61537621	0.41015342	0.52919421	0.35489992
Arid3b	-0.2326229	2.17230131	0.41011268	0.52921446	0.35489992
Tnni1	-0.6976584	-1.0624681	0.40958022	0.52947926	0.35504853
Smim8	0.18376939	3.79567374	0.40935584	0.52959092	0.35509443
Wnt6	-0.2573306	3.15703096	0.40859769	0.52996853	0.35531864
4933434E20I	-0.0961704	4.51298392	0.40824796	0.53014288	0.35540654
Fam78b	-0.1089009	6.81848028	0.40790048	0.53031622	0.35547955
Rgs5	-0.1506177	4.11792046	0.40772869	0.53040196	0.35547955
Gm16677	0.68912667	-1.2552854	0.40771985	0.53040637	0.35547955
Rbbp4	-0.0889743	5.94783195	0.40763546	0.5304485	0.35547955
Xylt1	-0.1997688	2.83464097	0.40759632	0.53046804	0.35547955
Eid2	0.09352782	5.24405361	0.40712807	0.53070191	0.35559595
Ebf1	-0.1550179	4.28809844	0.40707534	0.53072826	0.35559595
Scamp5	-0.1097771	6.44709612	0.40689349	0.53081915	0.35562785
Vwa9	-0.1061965	4.48041734	0.40672329	0.53090424	0.35564102
Otud1	0.1123733	6.12233648	0.40658814	0.53097183	0.35564102
Sgtb	0.08953018	8.05187458	0.40653807	0.53099687	0.35564102
Unc119	0.25808058	1.76904327	0.40650806	0.53101188	0.35564102
Nop14	0.09179804	4.94818265	0.40631553	0.5311082	0.35567654
Ubac2	-0.1561668	2.53218994	0.40581475	0.53135889	0.35581544
Lef1	0.1300349	4.50686433	0.40566766	0.53143256	0.35583578
Mgme1	0.20965131	2.92595142	0.40541566	0.53155883	0.35589133
Ldlrad3	-0.1762434	5.77201379	0.40491606	0.53180932	0.35603004
Scg3	0.13160873	6.66561104	0.40451731	0.53200941	0.35613498
Ttc27	-0.126396	3.37302631	0.40429546	0.53212079	0.35618053
1810043H04I	0.27745207	1.96213509	0.40348269	0.53252921	0.35639818
Sgsh	0.24190083	1.3039999	0.40347581	0.53253267	0.35639818
4833411C07I	0.684198	-1.3355056	0.40323349	0.53265455	0.35645072
Bnip1	0.22595229	2.16630286	0.40287415	0.53283539	0.35652719
Il15ra	0.16210264	2.60517085	0.40283406	0.53285557	0.35652719

Wwc1	-0.1101114	4.29363113	0.40247877	0.53303449	0.35655838
Xpnpep2	-0.6066474	0.25433481	0.40247679	0.53303549	0.35655838
Tmem173	0.23901385	1.61094865	0.40247244	0.53303768	0.35655838
Atp2a1	-0.7170439	-0.7557197	0.4023221	0.53311342	0.35655838
Rffl	-0.1670921	3.08964374	0.40224858	0.53315047	0.35655838
Tsacc	0.49387567	0.05827724	0.40222477	0.53316247	0.35655838
Tmem138	-0.2358508	1.17105868	0.40208892	0.53323094	0.35657515
Nespas	-0.964819	-1.9246916	0.40177449	0.53338949	0.35662317
Ddx17	-0.1107924	8.05832796	0.40173112	0.53341137	0.35662317
Aar2	0.16275177	3.78509195	0.40164838	0.5334531	0.35662317
4930404I05R	-0.7797527	-1.5477678	0.40150768	0.5335241	0.35662317
Hspa13	-0.1119425	4.88156484	0.4014862	0.53353494	0.35662317
Anxa6	0.11409589	5.87471101	0.40143042	0.53356309	0.35662317
Phkg2	-0.2037804	2.60356202	0.4012732	0.53364244	0.35664721
Tln2	-0.0901594	6.80998264	0.40083428	0.53386411	0.35676634
Tgfbr3	0.15032241	6.77981759	0.40028571	0.53414138	0.35692261
Cdh5	0.16377698	5.44742373	0.40000605	0.53428284	0.35698811
Gabrr2	-0.8366218	-0.870399	0.3998738	0.53434976	0.35700381
Kif18b	0.75074282	-1.7836768	0.39975659	0.53440908	0.35701442
Cnpy3	0.15692813	3.44903708	0.39926294	0.53465906	0.35711272
Mrps11	0.24902413	1.41683712	0.39926078	0.53466016	0.35711272
Hirip3	-0.0899839	5.06218827	0.39920289	0.53468949	0.35711272
Mroh8	-0.4581447	-1.0031828	0.3991229	0.53473002	0.35711272
Npy2r	-0.1820815	2.84536466	0.39816375	0.53521647	0.35740855
Zcchc18	0.08763973	7.20061704	0.3978996	0.53535059	0.35746906
Qk	-0.0770355	9.3229642	0.39766968	0.53546737	0.357518
Gak	-0.0838151	6.70953113	0.39723794	0.5356868	0.35763545
Nckipsd	-0.1338086	4.12030892	0.39687503	0.53587137	0.35769668
Aup1	-0.1929896	3.08808871	0.39685303	0.53588256	0.35769668
Apeh	0.23146625	2.13324778	0.39680095	0.53590906	0.35769668
Cwf19l1	-0.1612776	3.90492963	0.39644758	0.53608892	0.35778767
Sgms2	-0.2519112	3.45145195	0.39630697	0.53616052	0.35779568
Stk38	-0.0977149	4.80460911	0.39620966	0.53621009	0.35779568
AI506816	-0.5967381	-1.375879	0.39606275	0.53628492	0.35779568
C030034I22R	-0.2270896	1.65412909	0.39601798	0.53630774	0.35779568
Gm1715	-0.7793904	-1.7361002	0.39599668	0.53631859	0.35779568
Nos1ap	-0.1639567	3.69490422	0.39539505	0.53662532	0.35797125
Ipo4	-0.1124044	4.14921411	0.3952248	0.53671218	0.35800014
4933416C03I	-1.0595968	-1.228785	0.39495798	0.53684836	0.35806192
Bahcc1	-0.1330507	3.98732664	0.39477398	0.5369423	0.35809493
4933408N05	-0.5852971	-1.0389726	0.39462297	0.53701943	0.35809493
Ndufs5	0.56817145	-1.4944173	0.39460508	0.53702857	0.35809493
Pcdha11	0.31981923	0.37164766	0.39448833	0.53708821	0.35810565
Wdr83	0.16402732	2.6962975	0.39414687	0.53726272	0.35818969
Coq2	0.10409082	4.82113311	0.39398554	0.53734521	0.35818969

Serpina10	-1.0452026	-1.9734045	0.39395091	0.53736292	0.35818969
Taf9b	0.10380935	6.91473087	0.39390076	0.53738857	0.35818969
Apof	0.55173112	-0.0834847	0.39371886	0.53748162	0.35822266
Srbd1	0.14101263	3.49013912	0.39358012	0.53755261	0.35824092
Sash1	0.07973055	6.31869525	0.39324423	0.53772456	0.35832646
Gnl3l	-0.0766024	8.04128811	0.39309696	0.53779998	0.35834766
4930525G20	0.20668972	2.28997565	0.39289302	0.53790445	0.35838822
Rgs9bp	-0.6685005	-0.2906914	0.39233908	0.53818842	0.35854116
Ube2m	0.1407842	3.10543801	0.3922751	0.53822124	0.35854116
Rab8b	0.08380306	6.34753689	0.39187924	0.53842437	0.35862447
Pex2	0.14182725	4.2015927	0.39186132	0.53843357	0.35862447
Zscan26	-0.0668375	7.19874015	0.39159207	0.53857181	0.35868748
Slc16a11	0.17197353	3.31827081	0.39114809	0.53879992	0.35877852
A830080D01	0.11281748	4.31144439	0.39105081	0.53884993	0.35877852
Robo4	-0.374901	0.64692429	0.3909953	0.53887846	0.35877852
Myl9	0.20566069	5.49638529	0.39093538	0.53890927	0.35877852
Mob4	0.06908631	7.02416311	0.39090135	0.53892677	0.35877852
Cdc73	0.08607321	6.15338114	0.39066469	0.53904849	0.35883049
Mia	-0.3967229	-0.1530321	0.39033581	0.53921773	0.35891408
Taf5	0.15256583	3.93774759	0.38995387	0.53941439	0.35901248
Tcf19	0.2212255	2.42159714	0.38987907	0.53945292	0.35901248
Mrps26	0.11832251	3.85237009	0.38955441	0.53962022	0.35909474
1110008P14I	-0.0957167	4.56807897	0.38928905	0.53975704	0.35915671
Nrxn2	0.09547923	5.40005457	0.38906632	0.53987192	0.35920407
Tc2n	-0.3082142	0.86585266	0.38875927	0.54003037	0.35923164
Pitpnm1	-0.1627394	3.35286185	0.38875056	0.54003487	0.35923164
Tmsb15l	0.31145037	1.11771483	0.38873194	0.54004448	0.35923164
Cyb5rl	-0.6299154	-0.4919336	0.38851271	0.54015768	0.35926296
Snx16	0.10864074	4.76565689	0.38847145	0.54017899	0.35926296
Pbx4	-0.3434555	0.13138919	0.388294	0.54027065	0.35929485
Rbmx2	0.1336536	4.45926901	0.38790362	0.5404724	0.35939994
Pank2	0.09801004	5.50651976	0.38763544	0.54061108	0.35940382
Ak8	-0.5564298	-0.7773796	0.38763346	0.54061211	0.35940382
Arhgap44	-0.0805006	6.68127894	0.38760298	0.54062787	0.35940382
0610030E20I	-0.0935919	5.46102833	0.38755412	0.54065315	0.35940382
Pygo1	0.10058314	4.86566756	0.3874404	0.54071198	0.35941386
Tnks1bp1	-0.1400966	3.62382755	0.38694766	0.54096706	0.35955433
Ctdsp2	0.11550795	8.00564391	0.3867398	0.54107472	0.35956442
Kcnf1	0.1225414	5.28870068	0.38668472	0.54110326	0.35956442
Uhrf1bp1l	-0.1126473	7.76480923	0.38666499	0.54111348	0.35956442
Eif3l	0.09115786	5.5720538	0.38650631	0.54119571	0.35958999
Fam63a	0.17070677	4.35498773	0.38631212	0.54129638	0.35962781
Zcchc16	-0.1713009	2.91030763	0.38616223	0.5413741	0.35965038
Mylk3	-0.3860079	0.35676851	0.38583449	0.54154413	0.3596766
Cdc42ep1	-0.2944024	2.57236804	0.38580301	0.54156047	0.3596766

Cd40	0.57637087	-1.0778425	0.3857575	0.54158408	0.3596766
Tfpi	0.15028364	6.01795099	0.38568486	0.54162179	0.3596766
B2m	0.1363289	9.02542822	0.38566444	0.54163239	0.3596766
Tk2	-0.1873482	3.29112277	0.38549163	0.5417221	0.35970712
Rftn1	-0.2679805	1.5953295	0.38530679	0.5418181	0.35974179
Kbtbd4	0.10614603	4.48817103	0.38491092	0.5420238	0.3598493
Aifm2	0.2182341	2.59545827	0.38425335	0.54236581	0.36002071
Kansl1	0.06456179	6.95826193	0.38424607	0.5423696	0.36002071
Cacnb3	-0.0784849	6.89215654	0.38389806	0.54255077	0.36011189
Gcat	-0.3459528	0.83684208	0.38347377	0.5427718	0.36022951
Grp	0.4488367	0.11469407	0.383351	0.5428358	0.36024289
Tnfrsf19	-0.1020195	5.27041797	0.38305816	0.54298848	0.36030014
Pex3	-0.0969603	5.12396252	0.38294489	0.54304757	0.36030014
Dnaic1	-0.2825309	0.56456003	0.38293338	0.54305358	0.36030014
Tjap1	-0.2763764	1.35414695	0.38253677	0.54326055	0.36038162
Timm9	0.1000329	4.92435511	0.38250461	0.54327734	0.36038162
4930515G01	0.63136072	-0.7787779	0.38236267	0.54335145	0.36038162
Dnajc17	0.24720627	2.30473884	0.38236207	0.54335177	0.36038162
Slc25a23	-0.0863356	8.69692611	0.38144307	0.54383208	0.3606241
Vipr2	-0.5243213	0.13129878	0.38141238	0.54384814	0.3606241
Ccl19	0.15829736	3.93998028	0.38141075	0.54384899	0.3606241
Slc9a2	0.21929416	5.21957124	0.38129646	0.54390878	0.36063465
Igfbp3	0.19831805	3.83518722	0.38117078	0.54397455	0.36064917
Rlbp1	0.26514907	1.98670262	0.38066226	0.54424081	0.36074215
Sys1	-0.1572199	3.2962455	0.38051715	0.54431683	0.36074215
Gnao1	0.0858449	10.1861888	0.38050375	0.54432385	0.36074215
Socs5	-0.0838732	6.54267267	0.38049961	0.54432602	0.36074215
Ttc5	0.09405766	4.61761479	0.38048392	0.54433425	0.36074215
1110054M08	-0.2810703	1.12950431	0.38033936	0.54441001	0.36074438
Gnaz	0.09556028	5.82216852	0.38031	0.5444254	0.36074438
Zfp7	0.19231874	2.73567721	0.38015728	0.54450546	0.36076667
Pcdha5	-0.3374176	0.8341099	0.38007839	0.54454682	0.36076667
Bcar3	0.16358255	2.67844132	0.37961576	0.54478953	0.36089837
Mgst1	0.25021932	3.65503979	0.3792528	0.54498009	0.36099551
Steap2	0.08770698	4.92439914	0.37903819	0.54509282	0.36100779
A830018L16I	0.10094254	6.95865729	0.37901422	0.54510542	0.36100779
Slit3	0.13661885	4.60546321	0.3789667	0.54513039	0.36100779
Ltf	0.37954884	0.23885672	0.37883647	0.54519883	0.36102402
Hcn3	-0.2251965	1.66083011	0.37874957	0.54524451	0.36102518
Lyrm5	0.11299832	5.17912218	0.37845939	0.54539709	0.36109712
Zeb2	-0.0902999	8.89773701	0.37832581	0.54546736	0.36111455
Gtf3c2	-0.1021913	6.34947437	0.37802671	0.54562475	0.36117385
Cml5	0.3416385	0.85791997	0.37798859	0.54564482	0.36117385
Fbxo4	0.20167993	3.73373503	0.37749351	0.54590555	0.36130068
Gatm	-0.0768517	6.23725596	0.37738399	0.54596326	0.36130068

Clstn3	-0.141605	4.49061883	0.37737442	0.5459683	0.36130068
Spon1	-0.1069285	5.35663672	0.37670511	0.54632125	0.36150515
Ush1g	-1.1775141	-1.4019684	0.37656033	0.54639766	0.3615266
Anks6	0.17075597	2.0881777	0.37640955	0.54647725	0.36152711
4930556M19	-0.2735863	1.12915762	0.37639221	0.5464864	0.36152711
Ccna1	-0.6392687	-1.0159597	0.37629378	0.54653838	0.36153239
Ddr1	-0.1144781	3.43016774	0.37580099	0.54679872	0.36165888
Vti1a	0.08446355	6.91696689	0.37563254	0.54688776	0.36165888
Psd2	0.12923338	4.92517248	0.37562019	0.54689429	0.36165888
Adam9	-0.0854776	4.9569587	0.3755988	0.5469056	0.36165888
6430584L05R	-0.1630132	3.02541422	0.37501515	0.54721436	0.36178315
Fiz1	0.13279571	3.76023	0.37501151	0.54721629	0.36178315
Ccm2	0.12002743	3.74234732	0.37499398	0.54722557	0.36178315
Srd5a1	-0.2211399	2.56381811	0.37487787	0.54728703	0.36179468
Mat2a	-0.071772	7.87146733	0.3746694	0.54739743	0.36183856
Ccr5	-0.3129214	1.19262425	0.37377924	0.54786928	0.36212134
Smad4	0.0760033	5.86879899	0.37359697	0.547966	0.36214697
Nrip2	0.24557177	2.07644589	0.37354009	0.54799619	0.36214697
Foxo3	0.08545838	6.59802806	0.37325736	0.54814629	0.36221704
Nol4	-0.0965241	6.37124719	0.3729283	0.54832108	0.36230164
Nipsnap3b	0.1269295	3.68325496	0.37285041	0.54836247	0.36230164
Zfp35	0.09304139	4.4899564	0.37265514	0.54846626	0.36234108
Utrn	-0.0740819	7.75997577	0.37237208	0.54861678	0.36236553
Idnk	0.16508303	3.48742722	0.37236422	0.54862096	0.36236553
Kifap3	-0.1105607	8.45919916	0.37233684	0.54863553	0.36236553
Laptm4a	-0.1259964	8.0527121	0.37216559	0.54872664	0.36239658
Amfr	0.09149086	5.82024607	0.37197293	0.54882917	0.36239916
Slc25a13	-0.3148457	0.32074734	0.37193825	0.54884763	0.36239916
Tmem240	-0.2358228	0.89522004	0.37187516	0.54888122	0.36239916
Pcmtd1	-0.0720209	8.28762032	0.37182691	0.54890691	0.36239916
Crkl	0.0736822	6.05458741	0.37173749	0.54895452	0.36240148
Cd209b	-0.802438	-1.5381916	0.37159013	0.54903301	0.36240688
Ppp1r14c	0.1335543	3.61494466	0.37153475	0.54906251	0.36240688
Mtfp1	0.13162747	4.76527358	0.37141793	0.54912475	0.36240688
Bcs1l	-0.1416005	2.77376128	0.37139106	0.54913907	0.36240688
Snord71	-0.9033418	-1.3463177	0.37098199	0.54935714	0.36252169
Rpusd3	-0.3320421	0.91871578	0.37024022	0.54975299	0.36272696
Zfp935	-0.1217813	4.49689751	0.3702337	0.54975647	0.36272696
Mrgbp	0.31571509	0.49676204	0.3695979	0.5500962	0.36292198
Pappa2	0.2404148	2.28047774	0.36924446	0.55028524	0.36300381
Lcmt2	-0.171857	2.52533972	0.36914495	0.55033848	0.36300381
Ermn	-0.0919865	6.68337228	0.36911831	0.55035273	0.36300381
Senp2	-0.0642485	6.40988616	0.36898321	0.55042504	0.36302237
Tuba8	-0.1529461	2.26077848	0.36867658	0.55058922	0.36308582
Fbxo33	0.10528129	4.8653221	0.36860704	0.55062647	0.36308582

8430408G22	-0.9445213	-1.1148148	0.36855605	0.55065378	0.36308582
4930523C07I	0.15135658	3.74449244	0.36809118	0.55090292	0.3631976
Gm12216	-0.5396165	-0.754129	0.36807484	0.55091168	0.3631976
Six5	0.22436352	3.32592734	0.36759902	0.55116693	0.36333673
Phgdh	0.19247664	2.53464317	0.36741518	0.55126561	0.36335125
Thop1	0.18367211	2.56012789	0.36739326	0.55127737	0.36335125
Tnfaip2	-0.3150955	0.47359873	0.36721174	0.55137484	0.36338636
Hif1an	-0.1286083	4.74848081	0.36700397	0.55148645	0.36343077
Gm15055	0.60016043	-1.4464587	0.36691711	0.55153312	0.36343239
5730480H06I	-0.5285975	0.11818548	0.36664409	0.55167986	0.36348402
Sh3bp1	0.14840543	3.23093993	0.36646588	0.55177569	0.36348402
Fam3c	0.07329954	6.43511488	0.36639114	0.55181589	0.36348402
Lemd1	-0.7757475	-1.7102109	0.36636344	0.55183079	0.36348402
Parp8	-0.118629	4.80146209	0.36628574	0.55187259	0.36348402
Fbxo32	-0.1105477	5.02528935	0.36627787	0.55187682	0.36348402
Rab11fip4	-0.1256918	5.52632492	0.36616608	0.55193697	0.36348555
Galk1	0.20064663	2.74754331	0.36610917	0.5519676	0.36348555
Nxph1	-0.1088255	4.83149152	0.3657887	0.55214012	0.36354253
Stmn3	0.10773356	5.74413792	0.36578411	0.55214259	0.36354253
Hdc	0.45893761	-0.2146941	0.36550909	0.55229073	0.36358312
Rassf2	-0.1255558	7.49587503	0.36550541	0.55229271	0.36358312
Bcat1	-0.1008846	5.79615957	0.36467374	0.55274115	0.36384575
Theg	0.46704784	-0.8968574	0.36456128	0.55280184	0.36384575
Araf	0.07376386	7.24708396	0.36451938	0.55282445	0.36384575
Btbd2	-0.1221364	4.50350605	0.36398046	0.55311551	0.36400816
Ppm1d	-0.096786	5.13616871	0.36278549	0.55376192	0.36440439
Fam163b	0.0886789	7.61194462	0.36256231	0.55388281	0.36445476
2810008D09I	-0.2969354	0.70757999	0.36217832	0.55409093	0.36453883
Mettl14	0.09760425	5.82265542	0.36216291	0.55409928	0.36453883
Cd164	0.14159175	7.83696566	0.36186642	0.55426008	0.36461051
Numbl	0.10390043	4.00453307	0.36175423	0.55432095	0.36461051
Nek5	0.80137607	-1.245548	0.3617167	0.55434131	0.36461051
Vps4b	0.08482011	5.43696052	0.36157202	0.55441984	0.36463298
Ston1	0.19692822	4.79793078	0.36118711	0.55462885	0.36474125
Polr3e	-0.1446622	3.83577343	0.36103611	0.55471088	0.36476602
Iqch	-0.4781851	-0.5441629	0.36088147	0.55479492	0.36479209
Crtc3	-0.1079883	4.93875182	0.36037108	0.55507246	0.36494539
Rab11fip3	-0.0794967	6.19412059	0.36024366	0.55514179	0.36496177
Slc10a7	0.11927066	3.06877639	0.36006156	0.5552409	0.36499774
Amd1	-0.3251524	-0.1636389	0.3599752	0.55528792	0.36499945
Nek2	-0.3422035	1.56168566	0.35982447	0.55536999	0.36501305
Spg20	0.08916486	5.84138243	0.35977409	0.55539743	0.36501305
Hpgd	0.16745317	3.95251446	0.35954052	0.55552468	0.36506749
Clock	0.07668693	7.60340753	0.35914484	0.55574037	0.36514714
Rhot2	0.10878545	5.09631847	0.35908358	0.55577378	0.36514714

Crebl2	0.11867921	5.09833326	0.3590737	0.55577916	0.36514714
C920009B18I	-0.2902548	0.99448876	0.35870826	0.55597854	0.36524893
Gm5860	-0.172419	2.07446429	0.35854215	0.55606921	0.36527749
Ankrd6	-0.1180738	5.51365967	0.35842956	0.55613069	0.36527749
Figf	0.27783697	1.88956967	0.35832564	0.55618744	0.36527749
Rab26	0.16941409	3.91471612	0.35805407	0.5563358	0.36527749
Slc16a12	0.16387371	4.08482467	0.358049	0.55633858	0.36527749
Pccb	0.10655906	4.70569849	0.35803841	0.55634436	0.36527749
Kcp	-0.49102	0.03723685	0.35801382	0.5563578	0.36527749
Rasa1	-0.0694128	6.61581885	0.35793984	0.55639823	0.36527749
Suz12	-0.0746269	6.88359314	0.35767687	0.556542	0.36527749
Wrn	0.07724596	5.66555382	0.35763838	0.55656305	0.36527749
Cxcl16	0.21798083	3.95304305	0.35762744	0.55656904	0.36527749
Ppat	-0.1035782	4.38681869	0.35758955	0.55658976	0.36527749
Ccdc65	-0.197986	2.03665474	0.35757127	0.55659976	0.36527749
Fstl1	0.18919969	7.22418471	0.35728542	0.55675615	0.36535071
Fam24a	0.88521303	-2.0965885	0.35720486	0.55680024	0.36535071
Shroom1	-0.2155312	1.58595977	0.35676601	0.55704054	0.36546401
Cep55	-0.6843518	-1.7708971	0.35667167	0.55709223	0.36546401
Angpt1	0.13970148	4.59662105	0.35664597	0.55710631	0.36546401
Ostm1	0.08762941	5.44644216	0.35635514	0.55726571	0.3655394
Tpmt	0.1477253	3.69758478	0.35622232	0.55733853	0.365558
Zfp879	0.23085194	2.11439961	0.35574638	0.55759965	0.36567332
Mir103-2	-0.4558401	0.14579881	0.35566089	0.55764658	0.36567332
Etaa1	0.12147397	4.87202741	0.35565861	0.55764782	0.36567332
Anapc7	0.13189083	3.5193806	0.35539324	0.55779354	0.36571227
Ube2w	0.07096157	6.24687381	0.35538837	0.55779622	0.36571227
Heatr3	-0.1051841	4.64907755	0.35517857	0.55791148	0.36575866
Hspb3	0.33022031	0.34003333	0.35499156	0.55801425	0.36579614
Zbtb38	-0.0704522	6.89674946	0.3549126	0.55805766	0.36579614
Zfp72	0.17440806	3.12617452	0.3545472	0.55825861	0.36589868
Rps6ka6	-0.1816512	2.62959887	0.3536087	0.55877539	0.36620819
Ptprv	-0.5456158	-0.9318184	0.35321363	0.55899321	0.36628639
Pfkfb4	-0.2506463	1.19847148	0.35317804	0.55901284	0.36628639
Ube2o	-0.1288516	4.91134424	0.35311827	0.55904581	0.36628639
Trpm3	-0.0830563	6.82614017	0.35306904	0.55907297	0.36628639
Dtx4	0.09064752	5.31482988	0.35247613	0.55940028	0.36642761
Hist2h4	0.36756139	0.30175153	0.35240034	0.55944214	0.36642761
Rogdi	0.10110328	4.61086762	0.35232624	0.55948308	0.36642761
Gcc2	-0.1022151	7.54554474	0.35227039	0.55951394	0.36642761
Zfp874a	0.1006254	4.88317352	0.35218185	0.55956287	0.36642761
Arxes1	-0.172683	2.41449458	0.35216409	0.55957269	0.36642761
Pigu	0.13935233	3.77721647	0.35211359	0.5596006	0.36642761
Mansc1	-0.1300161	4.31736911	0.35203031	0.55964663	0.36642856
Elk3	-0.0990902	4.99464242	0.35189355	0.55972225	0.36644888

2200002D01I	0.30760406	0.63943445	0.35163525	0.55986512	0.36651322
Siva1	0.21322749	2.66080766	0.35148539	0.55994804	0.36653831
Aim1l	-0.4525289	-0.2877581	0.3508677	0.56029008	0.366733
Mid1	0.14041652	4.12698169	0.35074656	0.56035721	0.36674773
Padi4	-0.7531451	-2.0630303	0.3500967	0.56071759	0.36695205
Irf5	-0.3057141	0.96066234	0.35002266	0.56075868	0.36695205
Slc9a1	-0.1067703	5.26187894	0.34960445	0.56099087	0.36707476
Hvcn1	0.43501103	-0.3066528	0.34912088	0.56125959	0.367202
Foxl2	0.65680574	-0.4672663	0.34909375	0.56127467	0.367202
Plac8	-0.8580815	-1.2201908	0.34889297	0.56138633	0.36722284
Rock1	0.09713367	8.00893157	0.34887577	0.56139589	0.36722284
Prok2	-0.6244909	-1.3584056	0.34831791	0.56170636	0.36739669
Papss2	0.10921829	5.79153678	0.34784012	0.56197253	0.36747345
Tgfb1	0.18419547	2.17365205	0.34783468	0.56197556	0.36747345
Znf41-ps	-0.1611032	2.72156312	0.34782299	0.56198208	0.36747345
Sult4a1	0.07293944	7.03888384	0.34778626	0.56200255	0.36747345
A830010M2(	-0.1117241	8.6443168	0.34766063	0.56207259	0.36749
Atg101	0.18076537	2.8013093	0.34747814	0.56217435	0.36752236
Stamos	0.65530613	-1.1689577	0.34741153	0.56221151	0.36752236
Pxk	-0.0888002	5.46625676	0.34723018	0.56231269	0.36755927
Tmem98	0.18787157	3.79390521	0.34703612	0.56242099	0.36757371
Homer3	0.15677074	1.81476363	0.34703034	0.56242422	0.36757371
D17Wsu104e	0.21953028	2.07184343	0.34689081	0.56250212	0.36759184
Zfp933	-0.1060726	4.53456843	0.34682045	0.56254141	0.36759184
Gm5535	0.90184748	-1.7777729	0.34647031	0.56273702	0.36767683
Mgl2	-0.3484907	0.39839275	0.34642748	0.56276095	0.36767683
Ticam1	0.13207639	4.60652184	0.34611322	0.56293664	0.36775136
Sqle	0.08995662	5.75304706	0.34606339	0.56296451	0.36775136
Mxd4	0.2230246	2.4850677	0.34550588	0.5632765	0.36789641
Gm3500	-0.3518468	-0.3763363	0.34550576	0.56327656	0.36789641
Ccng2	-0.1202752	4.33906316	0.34542668	0.56332084	0.36789641
2310030G06	0.21617206	2.80094313	0.34521255	0.56344078	0.36793403
Nxpe2	-0.3012955	1.13167238	0.34513213	0.56348584	0.36793403
Ctss	0.1673241	3.74244017	0.3450841	0.56351275	0.36793403
C1qtnf1	0.12634118	4.86034582	0.34477451	0.56368629	0.36795511
Tssc4	0.1470001	2.47401417	0.34471598	0.56371911	0.36795511
Car2	0.06938353	7.0909454	0.34471276	0.56372091	0.36795511
Nup43	-0.1854451	2.47967835	0.34470706	0.56372411	0.36795511
Armcx3	-0.066484	7.26398778	0.34454241	0.56381646	0.36798617
Tm9sf3	-0.0718544	7.28578233	0.344307	0.56394854	0.36799606
Lzts1	-0.1801199	2.77788599	0.34428906	0.56395861	0.36799606
Sh2b2	-0.3714121	0.01806364	0.34426245	0.56397354	0.36799606
Rab18	0.08465025	7.34877302	0.34418408	0.56401753	0.36799606
Gsta3	0.34607344	0.88015063	0.34411648	0.56405549	0.36799606
Ccsap	-0.1474117	3.95722994	0.34376853	0.56425091	0.36809434

Creb5	-0.0974334	4.20964926	0.34338874	0.56446436	0.36820223
Dhx29	-0.1395394	4.80532999	0.34326138	0.56453598	0.36820223
Insm1	0.2481552	2.14257829	0.3431542	0.56459626	0.36820223
E130307A14I	-0.154392	2.29238303	0.34312234	0.56461418	0.36820223
Zranb3	-0.1701572	2.7816883	0.34307594	0.56464029	0.36820223
Fkbp14	0.14867023	4.22152797	0.34282249	0.56478291	0.36826602
Cstf3	-0.1138315	5.29501296	0.34262281	0.56489532	0.36830859
Cyp4f14	-0.8365559	-0.5174918	0.34254735	0.56493782	0.36830859
Enkur	0.19187804	2.14024953	0.34143521	0.56556483	0.36866094
Chd3os	0.06291745	6.56164557	0.34142962	0.56556799	0.36866094
Traf4	0.14074611	2.52163121	0.34105231	0.56578103	0.36877056
Sfpq	-0.0812554	7.54784806	0.34070164	0.56597918	0.36887046
Spaca1	-0.4272382	0.59477305	0.34058825	0.56604327	0.36888298
Hfe	0.22919168	3.40174727	0.34024464	0.56623761	0.36898037
Pten	0.06804964	9.22052376	0.34014237	0.56629547	0.36898883
Ythdf1	0.06037629	6.7367696	0.34005484	0.56634501	0.36899185
Tsc22d3	0.14435208	6.56716066	0.33990149	0.56643181	0.36899702
Rad51c	0.252076	1.97713349	0.33988219	0.56644274	0.36899702
Hus1	-0.1032477	4.38056297	0.33957963	0.56661408	0.36907939
Prkar2a	0.07625734	6.40143471	0.33934941	0.56674454	0.36913511
Grik5	-0.1282717	5.30620687	0.33917773	0.56684185	0.36915295
Polg2	0.32303687	0.97007582	0.33912629	0.56687102	0.36915295
Ppara	-0.1448245	4.0984941	0.3390634	0.56690668	0.36915295
Slc9a6	-0.0821925	6.2789716	0.33888469	0.56700804	0.36918971
Rtcb	-0.1029798	4.1718067	0.33871517	0.56710422	0.36920969
Greb1	0.34021826	0.39296859	0.33867227	0.56712857	0.36920969
Spata2	0.10592395	5.49015829	0.33833176	0.56732188	0.36930629
Ash2l	-0.08181	5.00464747	0.33785147	0.56759477	0.36945467
Keap1	-0.0940546	4.39016593	0.33773524	0.56766085	0.36946842
Atxn7l1	0.07411987	6.14296575	0.33704605	0.56805299	0.36968095
Adck1	-0.130994	3.05850564	0.33700325	0.56807735	0.36968095
Tctn2	0.14192687	2.69486415	0.33660075	0.56830664	0.36980088
Hhatl	0.33002629	0.58083269	0.33630767	0.5684737	0.36988031
Rab24	0.09877365	5.10408853	0.33571979	0.56880912	0.37006925
Snord99	0.8490854	-1.8805611	0.33532365	0.56903536	0.37015695
Pcp2	0.96864629	-1.5685669	0.33527062	0.56906566	0.37015695
Crispld2	0.25248276	2.2841983	0.33524722	0.56907902	0.37015695
Syt1	0.08386138	9.90610824	0.33515556	0.56913141	0.37016173
Fbxl15	-0.3951604	-0.4412051	0.33466674	0.56941092	0.37028637
Col4a3bp	-0.0633593	7.25980068	0.33466284	0.56941315	0.37028637
AI427809	-0.3938068	1.2099746	0.33456836	0.56946721	0.37029223
Dhx58	0.32428712	0.55327898	0.3341966	0.56968001	0.37035686
Fbxo31	0.15577701	3.94589921	0.33406895	0.56975311	0.37035686
Imp4	0.11237075	4.57065773	0.33400061	0.56979226	0.37035686
Mars2	0.2457922	2.29555454	0.33396254	0.56981407	0.37035686

Atp7b	-0.5800408	-0.6278342	0.33395902	0.56981609	0.37035686
1600016N20	0.47706871	-0.9326504	0.33386735	0.56986861	0.37035686
D15Ertd621e	0.06520694	7.09753655	0.33384395	0.56988202	0.37035686
Cdc42bpg	-0.2259133	1.33986723	0.33368329	0.5699741	0.37036858
Qrich1	0.06670516	6.48425595	0.33365524	0.56999018	0.37036858
Slc5a3	-0.0874663	5.78479516	0.33342844	0.57012023	0.37042379
Iqcd	0.68551341	-1.7489565	0.33330269	0.57019235	0.37044116
Ammecr1	-0.2097098	2.35776292	0.33322195	0.57023867	0.37044116
Rnf4	0.06882116	6.47763243	0.33314615	0.57028217	0.37044116
Dlx4	-0.8776042	-1.9372752	0.33286955	0.57044094	0.37051501
Il20rb	-0.7052216	-0.6847299	0.33268103	0.5705492	0.37055604
Myl6b	-0.1829968	3.78639404	0.33235114	0.57073875	0.37059775
Tcof1	-0.1053672	4.23575164	0.33234501	0.57074227	0.37059775
Mitf	-0.1216952	3.67275615	0.33233386	0.57074868	0.37059775
Tfdp1	0.08527876	6.37155299	0.33211826	0.57087263	0.37060335
Ccdc93	-0.0924336	5.01806247	0.33209943	0.57088346	0.37060335
Cers2	0.12546728	6.07253664	0.33208356	0.57089258	0.37060335
Spcs2	0.11106528	5.45084433	0.33198929	0.57094681	0.37060928
Ltbr	-0.2165116	2.37578847	0.33164397	0.5711455	0.37070897
Sez6l2	-0.1024908	5.43861925	0.33153484	0.57120832	0.37070901
Rgs1	-0.4799659	-0.295751	0.33148718	0.57123576	0.37070901
Irs3	-0.2196688	2.20963821	0.33118713	0.57140857	0.37079188
Draxin	-0.8295727	-1.4922165	0.33094724	0.57154681	0.37085231
Apool	0.22764903	2.40628015	0.3304784	0.57181718	0.37099845
Tmem158	0.1087956	4.10897851	0.32962331	0.57231096	0.37128951
Stk19	-0.2175712	2.00526309	0.32942741	0.5724242	0.37133366
Cthrc1	0.13590899	2.88728204	0.32890401	0.57272698	0.37149186
Myc	0.13821004	3.6606208	0.32879116	0.57279231	0.37149186
Sh3bp4	0.12235425	2.8722618	0.32865214	0.5728728	0.37149186
Nlrx1	-0.2564964	1.86726911	0.32864139	0.57287903	0.37149186
Atp1a2	-0.1291413	11.2666227	0.32861544	0.57289406	0.37149186
Cd247	0.79459772	-1.5631675	0.32824305	0.5731098	0.37160244
Flcn	-0.0830725	4.56344802	0.32744454	0.57357298	0.37187343
Retsat	0.1965614	2.56790834	0.3267908	0.57395275	0.3720903
Rassf9	0.30372684	0.90130565	0.32616638	0.57431596	0.3722964
Syde2	0.16554079	3.66367295	0.32602166	0.57440021	0.37232165
Usp5	0.11041434	4.66250852	0.32585326	0.57449827	0.37235584
Lpxn	0.6433984	-1.4889475	0.32570551	0.57458434	0.37235978
Fam69b	0.16302202	3.04810245	0.32563928	0.57462292	0.37235978
Slc35g3	-0.699734	-1.5128325	0.32560953	0.57464026	0.37235978
4930486L24F	0.87163649	-2.2845267	0.32543866	0.57473984	0.37239495
Ttf1	0.08264658	4.43710407	0.32517215	0.57489524	0.37243948
Rap1gap2	0.12435956	6.77560268	0.32516154	0.57490143	0.37243948
Mettl23	-0.1596259	2.83220101	0.32508769	0.5749445	0.37243948
C230052I12R	0.14404195	2.85948988	0.32466014	0.57519402	0.37253911

Pla2g3	0.47879472	-0.0423008	0.32455813	0.57525359	0.37253911
1700001K23I	0.72116727	-1.6193535	0.32454348	0.57526214	0.37253911
Gigyf1	-0.19289	2.40603753	0.32451355	0.57527962	0.37253911
Tdg	-0.2611244	0.31915329	0.32414408	0.57549549	0.37264954
Slc39a7	-0.1221551	4.19422238	0.32371573	0.57574595	0.37275542
Zbtb40	0.23380929	2.50864861	0.32368617	0.57576324	0.37275542
Cdk1	-0.8309104	-1.4653054	0.32363179	0.57579506	0.37275542
Pfn4	-0.1968104	1.95490093	0.32348892	0.57587866	0.37278019
Phyhd1	-0.1844564	2.26409937	0.32263484	0.57637897	0.37307466
Gipc2	0.22545089	1.46851857	0.32227524	0.57658987	0.37318178
Klkb1	-0.7835527	-1.2638815	0.32213338	0.57667312	0.37320628
Speer4e	0.57348799	-1.3641308	0.32179803	0.57687	0.37329767
Fbxw9	0.21474439	2.04056221	0.32173815	0.57690517	0.37329767
Zfp229	0.12819213	3.67899273	0.32152167	0.57703236	0.37335057
Kat6a	0.06293859	8.11258679	0.32138187	0.57711452	0.37337434
Csk	0.1605739	2.98253537	0.3212382	0.57719899	0.37338504
Camk2n1	-0.0654434	10.5133201	0.32119919	0.57722192	0.37338504
Spata13	0.08917133	4.58809525	0.32099234	0.57734359	0.37338956
Tmem30a	-0.0738796	7.94358369	0.32095125	0.57736777	0.37338956
Emg1	0.18085447	2.94861394	0.32093573	0.57737689	0.37338956
Zkscan17	0.12893672	3.86490782	0.32087841	0.57741062	0.37338956
Cenpc1	-0.0858057	5.59738244	0.3206066	0.5775706	0.37346363
Gp5	-0.6403097	-1.3607598	0.32042733	0.57767617	0.37347383
Klhl29	-0.1458162	5.07510909	0.32042548	0.57767726	0.37347383
Wdr95	0.81217667	-1.3915746	0.32013495	0.57784843	0.37355511
Sbsn	0.20541986	1.79275705	0.32001355	0.57791999	0.37357199
Spag4	-0.6648584	-1.6186552	0.31979361	0.57804967	0.37362643
Vstm5	0.19128733	2.30334241	0.31926822	0.57835969	0.37378425
Iqce	-0.1390177	3.35711486	0.3192257	0.5783848	0.37378425
Zfp53	-0.1364783	2.75478349	0.31908686	0.57846679	0.37380784
Pusl1	0.20478188	2.33169202	0.31880355	0.57863417	0.37388661
Apobr	0.40090106	0.34536625	0.31867798	0.57870838	0.37390517
4930599N23	0.71982078	-2.0599011	0.31844321	0.5788472	0.37396546
Tbc1d8b	0.11363357	4.33544602	0.31823015	0.57897324	0.37400786
Syncrip	0.06403601	7.70853875	0.31817844	0.57900383	0.37400786
Adar	-0.1111241	6.00892083	0.31804651	0.57908191	0.3740289
Fdxr	-0.212684	1.38721774	0.31795773	0.57913446	0.37403345
Cyb561d2	-0.2370958	1.76477958	0.31785724	0.57919397	0.37403502
Dcaf12l2	0.44958246	-0.1083279	0.3177999	0.57922792	0.37403502
Cox6b2	-0.243702	2.88656334	0.31771167	0.57928017	0.3740366
Psmd2	0.08106965	5.80504036	0.31764212	0.57932137	0.3740366
Ly86	-0.2136517	2.08681599	0.31745456	0.57943251	0.37407897
Ppp1r3e	-0.1211687	4.20352793	0.31731874	0.57951301	0.37410156
Asb6	0.14139441	2.52934525	0.31719524	0.57958623	0.37410299
Lrsam1	-0.1444728	3.61904986	0.31716147	0.57960626	0.37410299

Alox12b	-0.2627989	2.01014161	0.31703103	0.57968362	0.37412354
Hspb8	0.14041228	6.19497759	0.31659971	0.57993958	0.37425935
Cpne5	0.10805341	4.94584608	0.31632806	0.58010091	0.37427433
LOC1026324:	-0.9639476	-1.9097647	0.31612431	0.58022197	0.37427433
Zfp300	-0.1572788	2.71413456	0.31609568	0.58023898	0.37427433
Mlycd	-0.116824	3.16265208	0.31607597	0.5802507	0.37427433
Hist2h2bb	0.52034619	-0.8157757	0.31601267	0.58028832	0.37427433
AI846148	-0.1300864	2.95781087	0.31599483	0.58029893	0.37427433
Ipcef1	-0.0896562	7.00043625	0.3159835	0.58030566	0.37427433
Tmem175	0.08677071	4.99426847	0.31594745	0.5803271	0.37427433
Dnajc5b	0.50716735	-0.5513479	0.3158461	0.58038736	0.37428382
Ndufs1	-0.0932427	6.10677345	0.31506513	0.58085214	0.37455416
Sox9	0.09865539	5.66087096	0.3148242	0.58099568	0.37461733
Gba	-0.1027881	3.90078188	0.31440696	0.58124443	0.37474832
Tmc8	-0.636301	-1.0856689	0.31421185	0.58136083	0.37479397
Slc29a2	0.3183867	0.35312579	0.31390734	0.58154258	0.37488174
Trim36	-0.1395337	3.36901537	0.31380489	0.58160376	0.37489177
Adra1b	0.12164889	3.92520741	0.313368	0.5818648	0.37502628
Zfp595	-0.1023117	3.35783293	0.31324862	0.58193616	0.37502628
Shkbp1	0.28371327	0.80269498	0.31321889	0.58195394	0.37502628
Eea1	0.0731835	7.47743477	0.3131503	0.58199495	0.37502628
Lrrc51	0.23419331	2.09673844	0.31301198	0.58207768	0.37505019
B430010I23F	0.57439932	-0.1740072	0.31285862	0.58216944	0.37507991
Man2a1	-0.0696211	6.07197675	0.31274282	0.58223875	0.37509516
Prkcz	-0.0748687	6.3385358	0.31221123	0.5825571	0.37527084
Agap3	0.07366669	5.85847907	0.31211577	0.58261432	0.37527828
Ly6h	0.24248033	0.87209353	0.31179839	0.5828046	0.37536089
Dpm1	0.09749412	5.2982306	0.31172088	0.58285108	0.37536089
Rhbdd3	-0.3132347	-0.0468391	0.31167338	0.58287958	0.37536089
Ank	-0.0786586	6.15385939	0.31114705	0.5831955	0.37551051
Cav1	0.13881558	6.14573405	0.31106891	0.58324244	0.37551051
Rbm25	-0.082042	8.74047397	0.31097419	0.58329934	0.37551051
Carhsp1	0.16152553	5.37283522	0.3109679	0.58330312	0.37551051
Chka	-0.1027163	4.53753609	0.31090592	0.58334036	0.37551051
5930403L14F	-0.1512623	4.76609012	0.31073835	0.58344107	0.37554593
Peg13	-0.0759173	8.15164275	0.31025936	0.58372913	0.37570193
Cyp46a1	-0.0973294	4.74106673	0.30972542	0.58405059	0.37587143
Prmt10	0.18091658	3.33900613	0.30967004	0.58408395	0.37587143
Mcm2	0.15916922	2.2388127	0.30955428	0.5841537	0.37587549
Tyrp1	-1.2755188	-1.86409	0.3094355	0.58422529	0.37587549
Aldh3b1	0.29442412	0.86317787	0.3094319	0.58422746	0.37587549
Zmat5	0.18371348	2.44656038	0.30926219	0.58432978	0.37591189
Col6a6	0.6641306	-1.4085711	0.30883433	0.58458789	0.37604851
Ndnl2	-0.1283264	3.46944245	0.30857446	0.58474477	0.37608121
Trim14	0.28839988	2.15536847	0.30846663	0.58480989	0.37608121

Robo1	0.09028382	7.25073871	0.30841525	0.58484093	0.37608121
Uvrag	0.07415282	5.56574296	0.30832742	0.584894	0.37608121
Tmem238	0.37481928	-0.4145217	0.30831199	0.58490331	0.37608121
Foxk1	-0.0727114	7.16862433	0.30829381	0.5849143	0.37608121
Lrriq3	0.36023148	0.57315114	0.30821288	0.5849632	0.37608121
Otogl	0.54718022	-1.2053507	0.30806957	0.58504983	0.37608121
Ret	-0.2349385	1.82544934	0.30806839	0.58505054	0.37608121
Smad5	0.07358852	6.179103	0.30786152	0.58517563	0.37612078
Pof1b	0.33230573	0.09221184	0.30781524	0.58520362	0.37612078
Msantd1	-0.5112232	-0.632476	0.3076565	0.58529965	0.37615308
Ercc5	-0.0833536	5.02331105	0.30681393	0.58580992	0.3764363
Dsg1c	0.46752392	-0.8061699	0.30676178	0.58584153	0.3764363
Mapk10	0.07548534	9.60904329	0.30662426	0.58592491	0.3764363
Hist1h4c	0.39757208	-0.8188294	0.30655351	0.58596782	0.3764363
Stau2	-0.0712567	7.12925765	0.30655102	0.58596933	0.3764363
Gm10354	0.81962564	-2.0395813	0.30644108	0.58603602	0.37644971
Mfap4	0.19699666	3.76607458	0.30630099	0.58612101	0.37647489
Zfp574	0.11270503	4.88659637	0.30580791	0.58642038	0.37663774
Sh3yl1	0.14312795	3.06946594	0.30546777	0.58662708	0.37674106
1700007F19I	0.30069144	0.05421021	0.30526238	0.58675197	0.37679182
2300009A05I	-0.1730561	2.43271049	0.30506216	0.58687377	0.37684059
Tmem59	-0.0954419	6.13848722	0.30491015	0.58696627	0.37687055
Dnajc8	0.08758884	6.22569928	0.30483446	0.58701234	0.37687069
Ghr	0.11216361	5.29795125	0.30467962	0.58710662	0.37690177
Ifltd1	0.13125735	5.40439857	0.30418639	0.58740713	0.37706524
Lbp	-0.2558785	3.35016585	0.30371599	0.58769403	0.37721995
Celrr	-0.3876446	0.30316582	0.30316707	0.58802919	0.3774056
Ago3	-0.0880647	6.0504347	0.30266842	0.588334	0.37757175
Mpv17l	-0.0837974	5.64672728	0.30250942	0.58843127	0.37759522
Uba7	0.30066963	0.63843414	0.30245844	0.58846246	0.37759522
Opn1sw	0.52639436	0.40682071	0.30217603	0.58863531	0.37767665
Trmt10b	0.11457399	3.70171068	0.30204234	0.58871718	0.37769969
Ccl17	0.23912668	1.45136625	0.30167214	0.58894399	0.37779395
Dusp5	0.33616341	-0.2021188	0.3016525	0.58895603	0.37779395
Cct8l1	-0.8245559	-1.1835794	0.30134588	0.58914403	0.37788505
Gen1	-0.3119252	0.53316377	0.30107897	0.5893078	0.3779606
Rnmtl1	0.19881343	2.06520423	0.30075864	0.58950446	0.3779946
Cpne6	-0.1340227	5.07405814	0.30074344	0.5895138	0.3779946
Acvr1	-0.1035312	4.31727452	0.30069206	0.58954535	0.3779946
Dda1	0.09074445	4.55962534	0.30061968	0.58958981	0.3779946
Klhdc10	-0.0702118	6.89725649	0.30055511	0.58962949	0.3779946
Dnaaf2	0.1595289	3.59941112	0.30054329	0.58963674	0.3779946
Fam120aos	0.12460441	4.61883601	0.30035486	0.58975255	0.37803935
Gngt2	-0.2874045	0.72351475	0.29991987	0.59002006	0.37815467
Rcc1	0.211641	1.73423824	0.299784	0.59010367	0.37815467

Spock1	0.08130402	8.18431266	0.29977919	0.59010663	0.37815467
Cpsf3	0.08187042	5.09229648	0.29976315	0.5901165	0.37815467
4930506C21I	-0.3639331	-0.2857268	0.29966165	0.59017898	0.37816523
Mcidas	-0.5091274	-1.6433173	0.29948478	0.59028788	0.37820553
Cyb5r2	-0.6630527	-1.4312352	0.29927521	0.59041698	0.37825706
Eps8l2	-0.2544447	1.20284362	0.29919313	0.59046756	0.37825706
Wdr83os	0.11184752	5.70252191	0.29913012	0.59050639	0.37825706
Sdsl	-0.5839906	-0.6387176	0.29864291	0.59080684	0.37841933
Coro2a	-0.1265473	5.32555707	0.29857005	0.59085179	0.37841933
Kmt2e	-0.0711425	9.52673944	0.29803554	0.59118183	0.3785723
Bst1	0.65388744	-1.3908768	0.29803405	0.59118275	0.3785723
Slc2a9	-0.6037312	-0.8794348	0.29784544	0.5912993	0.37858562
Slc26a2	-0.1599539	6.09247466	0.29779493	0.59133052	0.37858562
Plekha1	0.06839015	6.73437233	0.2977768	0.59134173	0.37858562
Man1c1	0.08537677	6.0539353	0.29733159	0.59161708	0.3787324
Rps6kb2	-0.1561387	2.55862419	0.29710991	0.59175428	0.37876349
Aplp1	0.08202492	8.34531989	0.29710421	0.59175781	0.37876349
Celf4	0.06290371	9.32146305	0.296849	0.59191586	0.37881277
Erv3	-0.8952678	-1.2482462	0.29683102	0.59192699	0.37881277
Als2cr12	0.44415983	-1.0692108	0.29621325	0.59230994	0.37898394
Qdpr	0.07027074	5.53356808	0.29620399	0.59231568	0.37898394
Nfam1	0.35366391	1.15050389	0.29617642	0.59233278	0.37898394
Snord104	0.56395691	-1.7075835	0.29597067	0.59246044	0.37901941
Xrcc1	0.13239625	2.99943505	0.29593844	0.59248045	0.37901941
Ankrd66	0.90478105	-2.1740184	0.29575959	0.59259148	0.37906093
07-Mar	-0.087129	4.80727516	0.29522941	0.59292087	0.37920451
Igf1	-0.1029974	5.25462119	0.29519347	0.59294322	0.37920451
Kif5b	-0.069149	8.37481273	0.29514652	0.59297241	0.37920451
Ccdc151	0.24446069	0.63614155	0.29510137	0.59300048	0.37920451
1500011K16I	0.1469974	3.942228	0.29502472	0.59304815	0.37920548
1700120C14I	0.46342066	-1.1689078	0.29490102	0.59312509	0.37922518
Zfp324	-0.1590058	3.0189689	0.29447909	0.59338769	0.37936357
Ptpn6	0.27179052	0.89948557	0.29425855	0.59352506	0.37942188
9530068E07I	0.12385093	6.71850553	0.29396124	0.59371034	0.3795108
Pink1	0.05679224	7.52866259	0.29388398	0.5937585	0.37951207
Pou3f1	-0.1552435	2.01277333	0.29378798	0.59381837	0.37951707
Osbpl2	0.07276777	5.46258645	0.29370202	0.59387198	0.37951707
Chrna7	-0.2413919	1.221174	0.29362407	0.59392061	0.37951707
Atg16l1	-0.1278246	3.88649107	0.29353488	0.59397625	0.37951707
Rb1	0.07211751	6.94098613	0.29350133	0.59399719	0.37951707
Strip2	-0.1592525	5.1919143	0.29337792	0.59407421	0.37952761
Negr1	-0.0741385	8.24486592	0.29332693	0.59410604	0.37952761
Stk4	0.0613825	5.92312332	0.29306588	0.59426906	0.37959405
Adam21	-0.2918478	0.29743149	0.29301248	0.59430241	0.37959405
Mpst	0.30735803	0.44164435	0.2928393	0.59441062	0.37963366

Rab20	0.78892039	-2.0306466	0.29262632	0.59454375	0.37968918
Pfdn2	0.10268378	5.53679415	0.29252829	0.59460506	0.37969883
Lpar6	0.16647235	2.23537527	0.29203103	0.59491621	0.37983305
Olfm1	0.06631474	9.19770912	0.2920007	0.5949352	0.37983305
Il5ra	-0.6552371	-0.831976	0.29190261	0.59499662	0.37983305
Ankrd23	-0.4029413	-0.7013477	0.29189707	0.59500009	0.37983305
Fmod	0.15433202	7.83887884	0.29154905	0.59521813	0.37994272
03-Sep	-0.0713503	8.9122498	0.29135996	0.59533666	0.37994402
St8sia6	-0.3132828	0.86685262	0.29134561	0.59534566	0.37994402
Wdr46	-0.1408588	4.10368222	0.2913246	0.59535884	0.37994402
Actr3	0.05513227	8.04242918	0.29109001	0.59550598	0.37999068
Il11	0.26001495	0.53524101	0.29106062	0.59552442	0.37999068
Efcab6	-0.22349	1.7914979	0.2907222	0.59573684	0.38009671
Fam13a	0.08856855	3.99578644	0.29050176	0.59587529	0.38014765
Zfp382	0.115591	4.01271086	0.29044782	0.59590918	0.38014765
Klf5	0.08765916	6.62739111	0.29016616	0.5960862	0.38021874
Uap1l1	0.23361384	4.09503862	0.29008402	0.59613785	0.38021874
Ehf	0.33478605	0.60550469	0.29004975	0.5961594	0.38021874
Adrb2	-0.238317	2.24218647	0.28975912	0.59634222	0.38025976
Phf11c	-0.5045599	-1.5857981	0.28973286	0.59635875	0.38025976
Mnda	0.37685065	-0.6338785	0.28968805	0.59638695	0.38025976
Tacstd2	0.37632489	0.91133709	0.28965186	0.59640972	0.38025976
Efna4	0.27479393	1.84046717	0.28951278	0.59649728	0.38025976
Glis3	-0.1352027	3.09215531	0.28950637	0.59650132	0.38025976
Gpsm3	-0.2643998	2.31143864	0.28884335	0.59691908	0.38049657
Wnt5a	-0.1041653	5.5511028	0.28861351	0.59706405	0.38055946
Fam98a	-0.0742897	5.72445129	0.28837914	0.59721194	0.38059927
Fam169a	-0.1206757	5.76125406	0.28824643	0.59729573	0.38059927
Gm10516	-0.1931401	2.08014304	0.2882332	0.59730408	0.38059927
Snora31	-0.609633	-1.2004144	0.28822109	0.59731173	0.38059927
Cd3eap	0.12182599	3.59037109	0.28776564	0.59759947	0.3807531
4930524B15	0.81072064	-1.5930791	0.28763075	0.59768475	0.38077416
Ext1	-0.0665249	5.68457115	0.28753323	0.59774641	0.38077416
Gm10767	-0.1649751	1.92261135	0.2874434	0.59780323	0.38077416
Ofd1	-0.1288617	4.18802212	0.28742029	0.59781785	0.38077416
Irf2bpl	0.07986201	5.74511218	0.28702262	0.59806952	0.38090495
Oas1c	-0.2414581	1.37818016	0.28694912	0.59811606	0.38090507
Sucla2	-0.0603582	6.74457014	0.28687133	0.59816533	0.38090693
Fam188b	-0.2030295	1.34550415	0.28670005	0.59827383	0.38094651
Zfx	0.08203284	6.22463205	0.28638787	0.59847171	0.38104299
Slc52a3	0.22025093	1.79376373	0.28615084	0.59862204	0.38110526
Khsrp	-0.0719129	6.76865432	0.28608746	0.59866226	0.38110526
Cox15	0.0856016	5.47204878	0.28580953	0.59883865	0.38112903
Slc43a3	-0.4103127	0.2159002	0.28576039	0.59886986	0.38112903
Saal1	0.16164994	2.24950395	0.285698	0.59890948	0.38112903

Pde1b	-0.0842329	5.52007197	0.28569542	0.59891111	0.38112903
Fam110b	0.12140903	3.61497942	0.28564756	0.59894151	0.38112903
Cpxm1	0.16106544	3.30145207	0.28559039	0.59897782	0.38112903
Map3k11	0.11780548	3.78564312	0.28526997	0.59918143	0.38122405
Mapk4	0.07721466	7.79315389	0.28516428	0.59924862	0.38122405
Ncoa5	0.10548238	4.6525009	0.28513649	0.5992663	0.38122405
Hspa14	-0.0855679	4.2484401	0.28455623	0.59963551	0.38140522
1700037H04I	0.11415593	4.31945239	0.28448913	0.59967823	0.38140522
Gm20750	-0.6157541	-1.2381917	0.28447019	0.5996903	0.38140522
Smarca1	-0.0854689	5.63378935	0.28433357	0.59977731	0.38142301
Actn1	-0.1416334	4.61802019	0.28428055	0.59981109	0.38142301
Clcn5	-0.2128209	2.3199123	0.28413488	0.59990391	0.38145253
Gm16973	-0.1361317	2.88073708	0.28402452	0.59997426	0.38146774
Pcdhb4	-0.1974442	2.06915542	0.28389737	0.60005533	0.38148977
Wdr41	0.07488876	4.83009109	0.28370668	0.60017694	0.38153758
C130046K22I	-0.1586106	2.95526203	0.28309703	0.60056614	0.38175547
Vps11	-0.0910662	4.10357047	0.28269213	0.60082492	0.38189043
Ip6k2	-0.088175	4.0375015	0.2824756	0.60096341	0.38190956
Palm	-0.0781798	4.89436797	0.28236302	0.60103544	0.38190956
Ptdss1	0.08081594	4.8909108	0.28230964	0.6010696	0.38190956
Usp37	-0.1001871	5.63517387	0.2822206	0.60112659	0.38190956
Efr3a	-0.0729359	7.52803135	0.28221376	0.60113097	0.38190956
Hipk3	0.05426326	7.79073933	0.28214162	0.60117715	0.38190956
Pkib	0.07765497	5.513234	0.28213674	0.60118027	0.38190956
Cep89	0.15644997	3.02045372	0.28186727	0.60135285	0.3819597
Dse	0.17739095	3.90497036	0.28185966	0.60135773	0.3819597
Gm13629	-0.1960082	1.52851678	0.28179582	0.60139862	0.3819597
Anapc5	-0.0622178	6.32639075	0.2814649	0.60161074	0.38206489
Rbl1	0.23426524	1.59039662	0.28126122	0.60174137	0.38211833
Tpst2	0.14763344	2.1402579	0.28100888	0.6019033	0.38214805
4931408D14I	-0.2992919	0.71792381	0.28094032	0.60194731	0.38214805
Mitd1	0.14593856	3.3516068	0.28090495	0.60197002	0.38214805
Setd7	0.06938992	8.66298225	0.2808985	0.60197416	0.38214805
Nap1l3	0.08799528	5.81766145	0.28023029	0.60240352	0.38239109
9930111J21I	0.17957337	3.80923068	0.27999165	0.60255701	0.38243418
Nup35	-0.1188082	3.28593471	0.27998008	0.60256446	0.38243418
BC037034	-0.1059012	3.84047215	0.2791965	0.60306908	0.38267227
Adora2a	-0.2732754	1.89798399	0.27918601	0.60307584	0.38267227
Cmtm6	-0.1648058	4.86642247	0.27918067	0.60307928	0.38267227
Qrfpr	-0.4238265	0.13814898	0.27903988	0.60317005	0.38270032
S1pr2	0.23689984	1.73585486	0.27896344	0.60321935	0.38270206
Ackr4	-0.669787	-1.4397916	0.27842322	0.60356796	0.3828743
Irf2	-0.0912443	5.24195731	0.27837119	0.60360157	0.3828743
Epas1	-0.0680582	8.09369916	0.27832624	0.60363059	0.3828743
Tbcel	0.08944497	5.2480881	0.27799056	0.60384749	0.38298232

2410131K14I	-0.1513715	1.90497599	0.277786	0.60397974	0.38302305
Oprd1	0.12318008	4.16299304	0.27774707	0.60400491	0.38302305
Laptm5	-0.1957916	3.30029559	0.27760101	0.6040994	0.38305341
Syt17	0.16224823	3.80397589	0.27714396	0.60439524	0.38321144
Rbm39	0.05794479	7.64977397	0.27697989	0.60450152	0.38324664
Sec11a	0.13762973	4.0758586	0.27691431	0.60454402	0.38324664
Rpa2	0.1418107	3.4815477	0.27654014	0.60478658	0.38334823
Pyhin1	-0.3174975	1.688976	0.27652324	0.60479755	0.38334823
Asap3	0.16735231	2.87495429	0.27629029	0.60494867	0.38341445
Phb2	0.08670696	5.0191574	0.27619352	0.60501148	0.38342414
Ocm	-0.442191	-0.9293338	0.27607619	0.60508764	0.38342414
Mier3	0.07893101	5.08618564	0.27605113	0.60510391	0.38342414
Stx17	0.07766712	5.51081247	0.2759212	0.60518829	0.38344804
Igsf9b	0.15097508	3.48985487	0.27540541	0.6055235	0.38363086
Igsf8	-0.0901672	5.06803153	0.27490744	0.6058475	0.38379073
Prrg2	0.37613226	-0.1605994	0.27487406	0.60586923	0.38379073
B230312C02I	-0.7860084	-1.0641934	0.27477433	0.60593417	0.38380229
Pnpla2	0.1328076	4.47056317	0.27460974	0.60604138	0.38384061
Klhdc7a	-0.1750911	2.71863597	0.27450515	0.60610952	0.38385419
Rnft1	-0.0962205	4.55113215	0.2743573	0.60620589	0.38386663
Sec61a2	-0.1198433	4.92170337	0.27433172	0.60622256	0.38386663
Sec11c	0.0988214	4.53769326	0.27424849	0.60627682	0.38387141
Gm715	0.21314308	1.23912547	0.27399877	0.6064397	0.38394496
Oplah	-0.1561473	1.8057182	0.2733158	0.60688564	0.38419769
Plagl2	0.09675868	5.17042877	0.27299579	0.60709483	0.38428868
Ints7	0.1241891	3.58778534	0.27289548	0.60716044	0.38428868
Ptprh	0.45398609	-0.9851058	0.27288142	0.60716963	0.38428868
Ap1g1	-0.0851862	7.3243594	0.27275113	0.60725487	0.38429156
Egfl6	0.31175864	0.88646447	0.27273153	0.6072677	0.38429156
Lactb	-0.1011428	3.6918282	0.27263016	0.60733404	0.38430066
1700020L24I	-0.5265905	-1.6620476	0.27254995	0.60738654	0.38430066
Trafd1	-0.1240871	5.08967615	0.27249527	0.60742233	0.38430066
Gsx1	-0.6491899	-1.7004673	0.27229689	0.60755225	0.38432549
Ttk	0.48104385	-0.5704396	0.2721075	0.60767634	0.38432549
Nit2	-0.1460403	2.27151986	0.27208793	0.60768916	0.38432549
4933432I09R	-0.7365607	-0.7107174	0.27208092	0.60769375	0.38432549
Insrr	-0.3738183	0.39472325	0.27207843	0.60769539	0.38432549
Gm973	-0.1506977	2.46480324	0.27190605	0.60780838	0.38435645
Pigw	-0.2316222	1.51908733	0.27183848	0.60785268	0.38435645
Brinp2	-0.087913	4.4590988	0.27178976	0.60788464	0.38435645
Mcu	-0.1150447	4.64332139	0.27129475	0.60820946	0.38453225
Vamp2	0.06265319	10.2441072	0.27113259	0.60831595	0.38455818
Pold3	0.08383368	4.88174881	0.27108981	0.60834405	0.38455818
Fbxw2	-0.0805357	5.59019633	0.27076259	0.60855908	0.38466452
Gar1	0.08428305	4.14274439	0.27048302	0.60874293	0.38475114

Srr	0.0784888	6.94514693	0.27013992	0.60896873	0.38484706
Gm17762	0.43099594	-0.4665116	0.27011015	0.60898833	0.38484706
Zfp606	-0.0905013	4.59360883	0.26968619	0.60926761	0.38497535
Enox2	0.14530708	4.58699672	0.26965977	0.60928502	0.38497535
Rabgef1	-0.1091523	4.26776228	0.26951073	0.60938328	0.38500784
Rab39	-0.2803441	0.57454384	0.26907682	0.60966953	0.38515908
Plekha4	-0.2863265	0.34010636	0.26899518	0.60972342	0.38516352
Anxa11	-0.0851499	3.947233	0.26846786	0.61007176	0.38535395
5031434C07I	0.7336829	-1.6976484	0.26982245	0.61018351	0.38537767
Trim30d	0.11572453	3.62302109	0.26826922	0.61020309	0.38537767
Baz1a	-0.0971722	4.11718974	0.26800235	0.61037963	0.38545955
Mkl2	-0.10277	9.41262042	0.26788241	0.610459	0.38548006
Ndor1	-0.1393282	3.23268518	0.26756527	0.610669	0.38558304
Snord42a	-0.3994042	-1.1470432	0.26728677	0.61085355	0.38566055
Decr2	-0.0736386	5.53858173	0.26721844	0.61089885	0.38566055
Tmem41b	0.0775694	4.8261981	0.26716765	0.61093252	0.38566055
Camta2	-0.0772844	7.54902242	0.26700698	0.61103907	0.38569818
Tpcn1	0.13700171	5.97215689	0.26693207	0.61108877	0.38569993
Tnfrsf25	0.79407525	-1.3089208	0.26685718	0.61113845	0.38570167
Tbc1d2b	0.08416948	4.3888856	0.26674751	0.61121123	0.38571799
Grid2	-0.1773792	2.47251112	0.26640431	0.61143911	0.38575041
Wdr75	-0.1046056	4.66345154	0.2663785	0.61145626	0.38575041
Hsf5	-0.6486154	0.24239207	0.26637823	0.61145644	0.38575041
Rassf5	0.0854656	4.01244149	0.26634906	0.61147581	0.38575041
1600023N17	0.49018869	-0.9773858	0.26631674	0.61149729	0.38575041
Adipor2	0.08768701	4.69808897	0.26564336	0.61194504	0.38600324
Papola	-0.0626413	8.23868163	0.26548743	0.61204883	0.38603908
Pih1d1	0.15505521	3.36208392	0.26536072	0.61213319	0.38605778
Rbm15	-0.0858856	3.93178982	0.26530182	0.61217242	0.38605778
Tuft1	-0.3064485	0.44373696	0.26505764	0.6123351	0.38613075
Pcyt2	0.19409879	1.66845225	0.2649674	0.61239524	0.38613905
Lztfl1	0.07594791	6.37156356	0.26487147	0.61245919	0.38614975
Wipf2	-0.0644734	7.56041348	0.26480059	0.61250644	0.38614992
Olfr539	-0.4933064	-0.4667982	0.26469486	0.61257696	0.38616475
Zadh2	0.08927295	4.80412858	0.26451746	0.61269531	0.38619954
Iars2	-0.0796882	5.53871229	0.26442149	0.61275936	0.38619954
Vbp1	0.08017247	5.18967613	0.26437768	0.61278859	0.38619954
Mif4gd	-0.1369272	3.18581163	0.26433049	0.6128201	0.38619954
Tstd3	0.0842946	5.42823205	0.26419479	0.6129107	0.38622703
Gpr115	-0.183011	1.61554284	0.26410466	0.6129709	0.38623535
Irf1	0.16701832	2.9745514	0.26388925	0.61311482	0.38629642
Mapk1ip1	0.07980093	4.40151255	0.26378528	0.6131843	0.38631058
Slc9a3r2	0.12832919	5.10276259	0.26360071	0.61330771	0.38635871
Ccdc71l	0.08409811	5.24140435	0.2634572	0.6134037	0.38638957
Urah	0.33649642	0.09704359	0.26327545	0.61352532	0.38643656

Tubg2	0.1025998	4.31544614	0.26307998	0.61365617	0.38648936
Foxred1	0.13238233	3.05111748	0.2629574	0.61373826	0.38651144
Ikbke	0.2817699	0.5804378	0.26269694	0.61391277	0.38659172
C030006K11I	-0.1437909	2.96509353	0.26182517	0.61449766	0.38689175
Coa7	0.16111185	2.48376687	0.26180507	0.61451116	0.38689175
Avp	-0.4785376	-1.658286	0.26177638	0.61453043	0.38689175
Gm4477	0.35873507	-0.8045183	0.26157862	0.6146633	0.38694576
Synj2	-0.0926559	6.45483524	0.26146579	0.61473914	0.38696386
Mlf1	-0.3795397	-0.3012403	0.26139477	0.61478688	0.38696428
Eif2a	0.05951151	6.74759483	0.26088266	0.6151314	0.38713064
Fam168b	0.06826696	8.42329081	0.26077385	0.61520466	0.38713064
Agbl5	-0.1319126	2.76543721	0.26072898	0.61523488	0.38713064
Wasl	0.06034043	8.11217707	0.26072199	0.61523959	0.38713064
Dmtn	0.08898399	6.22968677	0.26055175	0.61535426	0.38717315
Slc39a6	0.06030421	6.58109972	0.26015503	0.61562166	0.3872697
Gramd1c	-0.2423348	1.68330996	0.26013233	0.61563697	0.3872697
Nanp	0.11824179	4.00038521	0.26008751	0.6156672	0.3872697
Asl	0.16907738	2.93065126	0.26002402	0.61571002	0.3872697
Rusc2	0.09226428	6.58476708	0.25997471	0.61574329	0.3872697
Rmnd1	0.16936574	1.31481141	0.2598832	0.61580503	0.38727889
LOC381967	-0.4046729	-0.4098663	0.25958719	0.61600486	0.38735998
5730559C18I	-0.6309753	-0.9736511	0.25943808	0.61610558	0.38735998
Cd163l1	-0.603137	-1.1743733	0.25941674	0.61611999	0.38735998
Gzmm	-0.6621989	-1.6068137	0.25941305	0.61612249	0.38735998
Acp6	0.17857912	2.46566331	0.25913804	0.61630834	0.38742632
Ccar2	-0.1139074	4.23538832	0.25906651	0.6163567	0.38742632
Myo1g	0.45912862	0.13617966	0.25904771	0.61636942	0.38742632
Ptpdc1	-0.0703289	5.41754893	0.2582291	0.61692351	0.38774495
Ccdc171	-0.1175904	3.20507222	0.25805534	0.61704127	0.38778931
Muc5b	0.56522721	-1.4645111	0.25935948	0.61714183	0.38782285
Fam98c	-0.3999084	-0.7052618	0.2578231	0.61719874	0.38782896
Ccdc130	-0.1875801	2.27195659	0.25764232	0.61732137	0.38784724
Cep131	-0.1289604	2.86455836	0.25764111	0.6173222	0.38784724
Runx2	-0.1109865	4.70215214	0.25749451	0.61742169	0.38788009
Pacrg	0.14979095	2.80278156	0.25654779	0.61806505	0.38825459
Gm10768	-0.7634949	-2.1390661	0.26080603	0.61818737	0.38827952
C1galt1c1	0.11182813	4.60541063	0.25635065	0.61819921	0.38827952
Ccdc107	0.15939858	2.78089312	0.25620005	0.61830174	0.38831424
Dll1	-0.3293628	0.10744602	0.25578402	0.61858519	0.3884525
Prr32	-0.399077	-0.3942978	0.25573821	0.61861641	0.3884525
Gm4961	-0.5093907	-1.7273926	0.25562184	0.61869576	0.38847265
Vps52	-0.0799314	4.55164951	0.25540209	0.61884565	0.3884964
Pex14	-0.1468148	2.54592797	0.2553905	0.61885356	0.3884964
Ndufb3	0.09520459	5.49258129	0.25535849	0.6188754	0.3884964
Pelp1	-0.1183785	3.28073169	0.25479657	0.61925911	0.38870651

Ano5	-0.3367134	0.82467018	0.25469465	0.61932875	0.38870651
Fermt1	0.51480329	0.35393899	0.2545543	0.6194247	0.38870651
2900055J20F	-0.1475847	3.12873871	0.25449953	0.61946215	0.38870651
Ppp1r15b	0.06267264	5.41122845	0.25448635	0.61947117	0.38870651
E330009J07F	-0.2185123	1.34869234	0.2543619	0.61955628	0.38870651
Ddx25	-0.1188954	4.1774863	0.25432794	0.61957951	0.38870651
Yars	0.08135916	5.01004981	0.25426737	0.61962096	0.38870651
Cd1d1	0.57673384	-1.2586938	0.25424576	0.61963574	0.38870651
Fhod1	-0.1687343	2.36053804	0.25385312	0.61990454	0.38884545
Nexn	-0.1120188	5.68358116	0.25330141	0.62028268	0.38905296
1810030O07	0.07498795	6.11730603	0.25289	0.62056499	0.38920033
Tmem130	-0.1002702	5.16068055	0.25264117	0.62073588	0.3892778
Cd33	-0.102635	3.1598662	0.2525004	0.62083261	0.38930876
Tram2	0.13397587	3.62401292	0.25225284	0.6210028	0.389376
Magi1	-0.0878946	6.35945009	0.25220661	0.62103459	0.389376
Kat8	-0.0972236	3.25460002	0.25205798	0.62113682	0.38941039
Ap3m1	-0.0654668	5.66474037	0.25158176	0.62146466	0.38942377
Galnt11	-0.1128627	3.29136292	0.25155799	0.62148103	0.38942377
Chst15	-0.0853977	5.98406679	0.25155703	0.62148169	0.38942377
Trim63	-0.2540999	1.2032021	0.25155526	0.62148291	0.38942377
Tma16	0.11317575	3.76072172	0.25155031	0.62148632	0.38942377
Slamf9	0.62239946	-0.3339572	0.25154907	0.62148717	0.38942377
Xlr3c	0.47481482	-1.4935788	0.25144887	0.62155621	0.38942377
Smoc2	0.13893794	3.40767044	0.25141755	0.62157779	0.38942377
Pomk	-0.0850573	4.76491231	0.25140769	0.62158459	0.38942377
Cplx3	0.19398261	1.32700046	0.25124474	0.6216969	0.38943987
Hps6	0.20308535	1.04365059	0.2511504	0.62176195	0.38943987
Eif2s3x	-0.0647429	7.27309505	0.25100865	0.62185971	0.38943987
Mrps30	0.12710662	3.23911452	0.25098111	0.62187871	0.38943987
Gm10432	-0.2444024	1.42658681	0.25092035	0.62192062	0.38943987
Pcdhgb6	-0.1638244	2.14496782	0.25090029	0.62193447	0.38943987
Smr3a	-0.616767	-0.5832651	0.25088943	0.62194196	0.38943987
Zfp407	-0.090713	4.98637286	0.25064761	0.62210887	0.38951471
Sntb2	-0.0745534	5.67243718	0.25048395	0.62222189	0.38954069
Wnt2	0.44266658	-1.0513622	0.25040453	0.62227676	0.38954069
Adamtsl3	0.18224249	4.39157337	0.25038165	0.62229256	0.38954069
Smim5	0.34623374	-0.0767975	0.24992003	0.62261168	0.38968419
Sdr9c7	-0.697927	-1.6837212	0.24991288	0.62261662	0.38968419
Eltd1	-0.188581	2.92488026	0.24970289	0.62276191	0.38974545
Gpr160	0.33428404	0.01767484	0.24960832	0.62282738	0.38975674
Nr2c1	-0.0911226	4.14095446	0.24952983	0.62288171	0.38976106
Ube2j2	0.11903388	2.89842445	0.2494413	0.62294302	0.38976975
Crybg3	0.10345667	4.24807861	0.24935098	0.62300557	0.38977922
Htr1d	-0.2543191	1.15101983	0.24924718	0.62307749	0.38979454
Unk	0.11718416	3.96633081	0.24907344	0.62319789	0.38979704

Trim11	-0.119759	2.75592502	0.2490653	0.62320354	0.38979704
Pear1	-0.1670547	3.42299803	0.24903612	0.62322376	0.38979704
Gmcl1	0.08280536	4.31263932	0.24886316	0.62334369	0.38981853
Pcdhgb1	-0.1417988	4.09657467	0.24884977	0.62335298	0.38981853
Fancd2	-0.1868791	1.26540415	0.248609	0.62352002	0.38989333
Nudt17	-0.2191521	1.00296696	0.24843306	0.62364215	0.38994003
Ss18l1	-0.0709312	6.11108159	0.24824007	0.62377617	0.38999416
Lix1	0.07270397	4.84179322	0.24777158	0.6241018	0.39014179
Daglb	-0.1180317	3.13088302	0.24769486	0.62415516	0.39014179
Slc35b2	-0.1905733	2.09245304	0.24766461	0.6241762	0.39014179
Abl1	0.09383642	5.32165928	0.24758583	0.62423101	0.39014179
Esf1	-0.0580629	7.18536144	0.24745095	0.62432488	0.39014179
Ythdc1	-0.0641152	7.24314683	0.24742319	0.6243442	0.39014179
Taok2	0.0815659	5.310984	0.24742265	0.62434458	0.39014179
Olr1	-0.3433602	-0.3912774	0.2472539	0.62446207	0.39018554
Rabl2	0.10475609	3.87155561	0.24715036	0.62453418	0.39020094
Ttc39c	0.15011102	2.23883921	0.2466025	0.62491607	0.39040986
Dnm2	-0.068416	5.25761461	0.24649657	0.62498997	0.39041684
A330041J22F	-0.3372387	0.08516935	0.2464122	0.62504884	0.39041684
Egr1	-0.1227482	9.14900846	0.24638225	0.62506974	0.39041684
Lyve1	-0.172878	3.43353616	0.24626035	0.62515483	0.39044031
Cbln2	-0.1288991	3.41947887	0.24592815	0.62538685	0.39055554
Zfp51	-0.1246119	4.1864609	0.24538062	0.6257697	0.39076494
Gipc1	-0.1639404	3.03328594	0.2452522	0.62585957	0.39079137
Cln3	-0.1478418	2.55672215	0.24504275	0.62600622	0.39085325
Fam35a	-0.1688433	2.54139424	0.24489436	0.62611015	0.39086037
2810428I15R	-0.1716629	2.82113477	0.24489067	0.62611274	0.39086037
Rps27a	0.08122766	6.62252119	0.24480863	0.62617022	0.39086657
Prob1	-0.1734143	1.69995626	0.24468915	0.62625396	0.39088915
Gpr62	-0.2856991	0.89676421	0.24450618	0.62638224	0.39093953
Crtac1	0.14119275	3.20349585	0.24415828	0.62662633	0.39106218
Fth1	0.05823563	9.06580264	0.24400589	0.62673331	0.3910801
Rer1	0.097203	5.71875699	0.2439239	0.62679089	0.3910801
Efcc1	-0.141329	2.53421232	0.24391407	0.6267978	0.3910801
Extl3	-0.0657945	6.75161783	0.24377114	0.6268982	0.39111306
Tmem237	-0.135638	3.40213193	0.24361431	0.62700841	0.39115213
Arih2	0.07709511	4.72467566	0.24306269	0.62739642	0.39132003
9330188P03I	0.32528207	0.9897814	0.24302733	0.62742131	0.39132003
Fgfbp1	0.17653135	4.61375719	0.24295658	0.62747112	0.39132003
Dcxr	-0.3371259	0.43505574	0.2429134	0.62750152	0.39132003
Inppl1	-0.1090315	4.24130284	0.24289338	0.62751562	0.39132003
Tmem63b	-0.0871813	5.59252783	0.2427943	0.6275854	0.39133218
Mc5r	-0.3150122	0.66592367	0.24269519	0.62765522	0.39133218
Tmed1	0.25705551	1.04176317	0.24266295	0.62767793	0.39133218
Itk	0.43577244	-0.1746427	0.24256676	0.62774572	0.3913396

Rundc3a	-0.0840044	5.33651374	0.24251094	0.62778507	0.3913396
Rasip1	-0.1438748	2.4086378	0.24235751	0.62789323	0.3913498
Actr2	-0.053079	8.88066286	0.24235266	0.62789666	0.3913498
Katnal2	0.4001032	-0.4732178	0.24189696	0.6282182	0.39146546
Srgap1	-0.1269455	5.14765201	0.24189442	0.62821999	0.39146546
Cysltr1	-0.3319494	-0.4593609	0.24188716	0.62822511	0.39146546
Bloc1s5	0.14896994	3.3249666	0.24170575	0.62835322	0.3914985
Wdr8	-0.1594028	2.26818252	0.24163486	0.6284033	0.3914985
Ndufv1	-0.094324	4.74009569	0.24160811	0.6284222	0.3914985
Mir3473	-0.4826301	-0.4214497	0.24154236	0.62846866	0.3914985
Ap5b1	0.38279808	-0.0106222	0.24137319	0.62858824	0.39154331
Nrip3	0.07556872	5.45211388	0.2412139	0.62870087	0.39158379
Taf5l	-0.0819026	4.1315046	0.24106406	0.62880686	0.39162013
Btbd6	-0.084213	4.22448594	0.24065879	0.62909376	0.39176912
Lipa	0.08050185	4.63551706	0.24007584	0.62950696	0.39197496
Cdipt	0.07813027	4.67887572	0.2400274	0.62954133	0.39197496
Mocs1	0.13903219	4.31887356	0.23999071	0.62956736	0.39197496
Ccdc62	0.22667417	1.89830832	0.23976945	0.62972439	0.39204303
Uba1	-0.0576138	7.91621844	0.23957993	0.62985897	0.39209711
Cse1l	-0.0590642	6.72638057	0.23944766	0.62995293	0.39210973
Gm960	0.39156858	-0.5551087	0.23940377	0.62998411	0.39210973
Bend3	0.11370593	4.24324062	0.23934996	0.63002235	0.39210973
Trim32	-0.0721103	7.59726018	0.23916064	0.63015694	0.39215129
Kcnc4	0.10595657	4.33426305	0.23912179	0.63018456	0.39215129
Madd	-0.0945345	6.89785884	0.23878002	0.63042771	0.39222925
Ccdc66	-0.0652963	5.59627486	0.23868043	0.63049861	0.39222925
Tyw3	-0.1461896	3.04954393	0.23864151	0.63052631	0.39222925
Mcm4	-0.0927571	4.37157769	0.23863131	0.63053357	0.39222925
Fam204a	0.1007694	5.54557337	0.23861041	0.63054846	0.39222925
Dnajc6	-0.0651312	8.53886935	0.23844926	0.63066323	0.39224662
Hist1h4a	0.47434508	-1.4203992	0.23843721	0.63067182	0.39224662
Wee1	-0.0724443	5.51696953	0.23827118	0.63079012	0.39229051
Itgal	0.26596724	0.64269499	0.2380366	0.63095735	0.39236384
Lrrc34	-0.5367727	-1.0279857	0.23797189	0.63100351	0.39236384
Pfkm	0.08589061	5.87960746	0.2379018	0.63105351	0.39236525
Nlgn2	0.07040454	6.3200892	0.23755841	0.63129858	0.39248605
Mad1l1	-0.1464926	2.26136408	0.2374958	0.6313433	0.39248605
Spata24	-0.2820078	0.34077621	0.23738586	0.63142182	0.39249688
Prkcsh	-0.1144244	3.3401779	0.23733768	0.63145624	0.39249688
Tmem165	-0.0893777	4.66271088	0.23718891	0.63156255	0.39253327
Smc2	-0.0884993	4.51070417	0.23698452	0.63170867	0.39259441
Bfar	0.08292195	5.04974187	0.23681713	0.6318284	0.39263913
Klhdc9	-0.0937329	3.65695043	0.2362604	0.63222699	0.39285712
Fam217a	0.49452794	-0.6141347	0.23617453	0.63228852	0.39286565
Cuedc1	0.13377622	2.60927741	0.23563778	0.63267345	0.39307415

Tubd1	0.21219328	1.56401701	0.23557329	0.63271974	0.39307415
Spats1	-0.2606224	0.64257121	0.23533586	0.63289021	0.39314306
Rerg	-0.1819567	2.91998122	0.23528559	0.63292632	0.39314306
Nipal3	-0.1018693	4.4565347	0.23520778	0.63298221	0.39314807
Col5a3	0.60532423	-1.0560599	0.2349382	0.63317597	0.39321802
Oxsm	0.13999437	3.16295024	0.23491795	0.63319053	0.39321802
Cep170	-0.0773556	7.48685712	0.23464599	0.63338614	0.39330978
Prdm2	-0.0680011	7.02812682	0.2345238	0.63347408	0.39333467
Cmss1	0.23108353	2.41005536	0.23434732	0.63360113	0.39333611
Supt5	-0.0842996	5.64203307	0.23430626	0.6336307	0.39333611
Zfp180	-0.0639774	5.1210023	0.23426805	0.63365822	0.39333611
Zc4h2	0.07596114	4.71566266	0.23412673	0.63376003	0.39333611
Rnf19a	0.0601444	6.03704394	0.23409242	0.63378475	0.39333611
Plekhm1	-0.0702997	5.38139561	0.23409186	0.63378515	0.39333611
Tamm41	-0.1639798	2.28639379	0.23405546	0.63381139	0.39333611
Satb1	-0.0836412	7.71689747	0.2339644	0.63387702	0.39333661
2810459M11	-0.1947013	1.84804127	0.23387988	0.63393795	0.39333661
Fis1	0.0949717	3.99027107	0.23380348	0.63399304	0.39333661
Ogn	-0.1194958	8.11652656	0.23378879	0.63400363	0.39333661
D430042O09	-0.1170189	3.42769263	0.23350372	0.6342093	0.39340839
Pithd1	0.08812538	4.77159265	0.23345619	0.6342436	0.39340839
Snrnp48	0.07812851	4.90671398	0.23342944	0.63426291	0.39340839
Erbb2	-0.2793206	1.57463106	0.2332056	0.63442454	0.39347894
Plcz1	-0.4856901	-0.6400122	0.23301553	0.63456186	0.39353442
Htr2a	0.10897329	4.42755385	0.23267136	0.6348107	0.3936428
Smco3	0.1186799	2.69433529	0.23264133	0.63483242	0.3936428
Gpr139	-0.3563241	-0.5549748	0.2324914	0.63494089	0.39368037
Coq4	0.08879683	3.84372595	0.23242301	0.63499039	0.39368136
Hist1h4h	0.46907262	-1.3963942	0.23202136	0.63528126	0.39383198
Exoc3l	0.20421198	1.39302069	0.23191564	0.63535788	0.39384977
Sema3a	-0.083441	6.40552977	0.23184834	0.63540666	0.39385031
Cnot6	-0.0763364	6.42197688	0.23172611	0.63549528	0.39387553
Mapk8	0.07348466	7.5751734	0.23162975	0.63556517	0.39388915
Ncoa3	-0.071065	6.64765796	0.23151442	0.63564883	0.3939113
Ifitm7	-0.4505071	-1.6271057	0.23067728	0.63625692	0.39420079
4930563E22I	-0.3004964	0.91985291	0.23066994	0.63626225	0.39420079
Lpar1	0.12277372	6.85129751	0.23066389	0.63626665	0.39420079
Actrt3	0.47943358	-0.9276285	0.23060726	0.63630784	0.39420079
Diap2	-0.0729566	7.4642199	0.23043005	0.63643676	0.39422494
Rbmxl1	-0.073773	5.34589188	0.23038009	0.63647312	0.39422494
Krt26	-0.5517819	-2.0467071	0.23035116	0.63649417	0.39422494
Mrpl10	0.08289248	4.71819044	0.23029003	0.63653867	0.39422494
G0s2	0.23059169	1.60032905	0.23022072	0.63658913	0.39422648
9430037G07	-0.1637026	2.05071542	0.23001959	0.63673561	0.39428554
Ssh1	0.12611961	2.40579911	0.22990042	0.63682244	0.39428554

Fadd	0.09626747	4.30094282	0.22984382	0.63686368	0.39428554
Mmp15	-0.2114254	1.11482654	0.2298264	0.63687638	0.39428554
Usp7	-0.0569155	7.09946502	0.22946773	0.63713793	0.39441775
Slc26a10	-0.2285661	0.66412926	0.22912817	0.63738578	0.39454147
Lgr6	0.44529179	-0.8661721	0.22875468	0.63765865	0.39468065
Mtss1	0.08083833	6.68507177	0.22865031	0.63773496	0.39469816
Chrna5	-0.3177164	-0.3679875	0.2285429	0.6378135	0.39471705
Hdhd3	0.30057425	0.2830346	0.22846559	0.63787005	0.39472232
Srpk3	-0.3041859	0.31649771	0.2283937	0.63792265	0.39472515
Rhbdf1	0.16837063	1.5426094	0.22813253	0.63811381	0.39481371
C2cd2l	0.08902032	5.27762797	0.22795372	0.63824477	0.39485369
Tmem87b	0.08708679	4.94297907	0.22791312	0.63827451	0.39485369
Ece1	-0.0789311	5.98034349	0.22772969	0.63840893	0.39490713
Lonp2	-0.060881	5.557762	0.22764146	0.63847361	0.39490989
Rrnad1	-0.0980746	3.60625809	0.22758849	0.63851246	0.39490989
Muc1	-0.3310136	-0.6133025	0.22750576	0.63857312	0.39490989
Sox30	0.49024323	-1.0289941	0.22743295	0.63862654	0.39490989
Bank1	-0.2065769	1.7660714	0.227396	0.63865365	0.39490989
Plaa	0.05137255	6.40093827	0.22715173	0.63883292	0.39499103
Ccr10	0.62069684	-1.0131679	0.22705914	0.6389009	0.39500335
Ebag9	0.05524062	5.98741643	0.22692016	0.63900298	0.3950168
4921504A21I	0.13110782	3.35380676	0.22689864	0.63901879	0.3950168
Oma1	-0.0950214	3.22056231	0.22672581	0.63914579	0.39506341
Mfsd2a	-0.2095559	1.27973298	0.22666524	0.63919032	0.39506341
Igsf9	0.4360154	-0.311805	0.22637342	0.63940493	0.39512912
Pls3	0.04938514	7.5080234	0.22632881	0.63943775	0.39512912
Lsm11	0.08148428	4.47219831	0.22632459	0.63944086	0.39512912
Zfp54	0.24050774	1.59679142	0.22615463	0.63956594	0.39516252
Zfp865	0.08287085	4.44992551	0.22610769	0.6396005	0.39516252
Npdc1	-0.0814434	4.46292027	0.22605521	0.63963914	0.39516252
4833422C13I	-0.1902325	2.31396796	0.22563804	0.63994651	0.39532269
Dnajc3	0.09090392	6.76679914	0.22542546	0.64010327	0.39537186
Ppcdc	0.13806456	2.87627421	0.22538575	0.64013255	0.39537186
Pde8a	0.0730099	4.07415265	0.22517741	0.6402863	0.39537186
Lrmp	-0.3970886	-0.0754907	0.22512812	0.64032268	0.39537186
Ap2b1	0.06172387	7.86662395	0.22505572	0.64037613	0.39537186
Chn2	-0.0590246	5.29458178	0.22500498	0.6404136	0.39537186
AW112010	0.17111474	1.65671888	0.22496069	0.64044631	0.39537186
N4bp1	-0.0561224	7.19119929	0.22495063	0.64045374	0.39537186
D030028A08	0.17348987	1.33502954	0.22483646	0.64053808	0.39537186
Crb1	0.51808053	-0.9575645	0.22479389	0.64056953	0.39537186
AU041133	-0.1298199	2.83217733	0.22479272	0.6405704	0.39537186
Lca5	-0.0993595	4.18042977	0.22469971	0.64063913	0.39537186
Gm11149	-0.4590732	0.11160232	0.22468304	0.64065145	0.39537186
Naa35	0.0626631	6.08608385	0.22457332	0.64073256	0.39538259

Gm15412	-0.7801213	-1.650277	0.22451952	0.64077234	0.39538259
Adnp	-0.0597401	7.86024029	0.22446434	0.64081315	0.39538259
Rbbp8	0.11113305	4.13428988	0.22436654	0.64088549	0.39539754
Ache	0.1508924	2.62982925	0.22410395	0.64107981	0.39540428
Sgpp1	-0.0591689	6.5640417	0.22404144	0.64112609	0.39540428
Mgat2	-0.1171811	3.28423238	0.22384987	0.64126798	0.39540428
Tbc1d14	0.06931117	5.17225701	0.22384653	0.64127046	0.39540428
Cdk5rap3	0.11541777	3.15904172	0.22384178	0.64127397	0.39540428
Tmem154	0.15750927	3.39839812	0.22381776	0.64129177	0.39540428
Smek1	-0.0686901	5.62499511	0.22378561	0.64131559	0.39540428
Slc25a14	-0.0937512	4.89905277	0.22376291	0.64133241	0.39540428
Mcph1	-0.1083032	5.21063922	0.22369775	0.64138069	0.39540428
Lrrn4	-0.5390475	-0.7844833	0.22362181	0.64143699	0.39540428
Pcgf6	0.12822676	3.32218114	0.22357719	0.64147006	0.39540428
Krt73	0.48056396	-1.1365842	0.22357227	0.64147371	0.39540428
Ezh1	-0.0855674	5.19034021	0.22349391	0.64153181	0.39541043
Srprb	0.10516734	3.49806931	0.22331622	0.64166361	0.39543387
Pld2	0.16724643	2.40034598	0.22331292	0.64166606	0.39543387
Gripap1	-0.0784813	4.91768759	0.22306316	0.64185143	0.39549148
LOC1010557(	-0.6799606	-1.8437998	0.22423961	0.64188429	0.39549148
Sh3glb2	-0.0650657	5.23675854	0.22299105	0.64190497	0.39549148
Rxrb	0.11206248	3.23806349	0.22283818	0.64201851	0.39549148
Ipmk	-0.0685452	5.56169021	0.22281011	0.64203936	0.39549148
Clec12a	0.46219311	-0.500665	0.22279815	0.64204825	0.39549148
Gpm6a	-0.0770714	9.42964172	0.22266592	0.64214651	0.39552237
Grin2d	0.14828921	1.80761077	0.22258663	0.64220545	0.39552902
Dhx15	-0.0507268	6.58366258	0.22240706	0.64233897	0.39554814
Abhd11os	0.24982978	0.35444913	0.22239332	0.6423492	0.39554814
Wisp2	0.57947097	-1.02055	0.22227285	0.64243882	0.39554814
Ankrd52	-0.0772734	5.89362456	0.222223	0.64247592	0.39554814
Bmp2k	-0.0738876	5.06824754	0.2222214	0.64247711	0.39554814
Zfand5	0.05113022	8.01763302	0.22206126	0.6425963	0.39558451
Jam2	0.06330507	5.78114302	0.22200544	0.64263786	0.39558451
Slc22a6	-0.1341444	7.15639669	0.2219045	0.64271303	0.39558451
Ecm2	0.11164463	5.03263301	0.22188345	0.64272871	0.39558451
Upb1	-0.3713902	-0.4929598	0.22158412	0.64295176	0.39566895
Marcksl1	0.08898714	4.29698089	0.22157014	0.64296219	0.39566895
9930012K11I	-0.2342869	1.30483916	0.22142087	0.64307349	0.39568826
Sv2b	0.08377571	9.37558707	0.22139894	0.64308985	0.39568826
Mtrf1	0.1058957	3.13216346	0.22125484	0.64319735	0.39572478
Deb1	0.07512131	4.91334652	0.22104006	0.64335765	0.39578299
Gm10421	-0.4710691	0.08783032	0.22099906	0.64338827	0.39578299
Mybpc1	0.21488498	0.97446557	0.22075409	0.64357125	0.39585797
Gpr146	-0.0993146	4.1900935	0.22070695	0.64360648	0.39585797
Hsf2bp	-0.2016042	2.07785124	0.22054769	0.64372552	0.39590156

D830031N03	0.07287355	5.26126611	0.22038846	0.64384458	0.39594516
Cacnb1	-0.0698523	5.12663718	0.22016483	0.64401191	0.39601842
Arl5a	0.06188926	6.9303259	0.2197844	0.64429678	0.39616396
Mpi	-0.0846748	4.47507128	0.21961848	0.64442112	0.39620647
Chmp4c	0.61145828	-1.6063499	0.21956351	0.64446233	0.39620647
Dnajc4	-0.1490572	2.40710364	0.21882244	0.64501848	0.39651872
Gm10548	-0.1810415	1.74627685	0.21851059	0.64525286	0.39663314
5830432E09I	0.44245766	-1.1119705	0.21838768	0.64534529	0.3966541
Acsf3	-0.1878873	1.50699388	0.21833692	0.64538347	0.3966541
Gsdmc4	-0.6517759	-1.6809568	0.21817478	0.64550547	0.39669942
Nde1	0.12935957	3.81609371	0.21806638	0.64558707	0.3967199
Sphk1	0.16121951	4.58508224	0.21779783	0.64578932	0.39681452
2610027K06I	-0.406929	-0.8805224	0.21767192	0.6458842	0.39684314
Mmp23	-0.3999326	-0.4723101	0.21717484	0.6462591	0.39704381
Mtfr1	0.13108087	3.94927546	0.21696379	0.64641843	0.39711202
Nov	0.14911045	7.77909032	0.21687616	0.64648462	0.39712299
Unc5a	-0.088399	4.9158948	0.21676319	0.64656996	0.39714574
Snhg5	0.09190309	3.73901185	0.21649435	0.64677319	0.3972301
A230070E04I	0.10228401	4.56051307	0.21645364	0.64680397	0.3972301
Immt	-0.0645446	6.62963615	0.21621921	0.64698132	0.39729396
4933405O20	-0.8095226	-0.526677	0.21618841	0.64700463	0.39729396
Rasl12	0.32640611	-0.0654377	0.21601239	0.64713788	0.3973461
P2rx6	-0.2380119	1.20179979	0.21581741	0.64728557	0.39740709
Edem2	0.17012227	1.59219095	0.2157146	0.64736347	0.39742523
3110045C21I	0.51472888	-0.5811746	0.21561426	0.64743953	0.39744224
Gm11128	-0.3379518	-0.8059278	0.21550682	0.64752099	0.39746256
Glra1	0.26525721	0.24685112	0.21539729	0.64760406	0.39748386
Stk10	-0.0954494	3.50792912	0.2151835	0.64776627	0.39753022
Farsb	0.06206337	5.68963609	0.21517027	0.64777632	0.39753022
St3gal4	-0.162518	3.96093969	0.2150192	0.64789101	0.39757092
Kremen2	0.31240912	-0.7455648	0.21487307	0.648002	0.39759614
Gnat1	0.39258356	-0.4817534	0.21483772	0.64802886	0.39759614
D630003M21	0.34082502	0.44311259	0.2147354	0.6481066	0.39759705
Nr2c2	0.04703391	7.33174205	0.21470844	0.6481271	0.39759705
Tyw5	0.08711697	4.84978132	0.21406388	0.64861743	0.39781523
Mfsd12	0.45445276	-1.2930054	0.2140204	0.64865054	0.39781523
Spast	0.05622055	6.53538806	0.21400252	0.64866416	0.39781523
Amt	0.18286516	1.03484184	0.21392695	0.64872171	0.39781523
Dnmbp	0.10272277	3.65661278	0.21392295	0.64872476	0.39781523
Ect2	0.46560765	-0.849832	0.21353434	0.64902094	0.39794368
Maats1	0.35866433	0.57079124	0.21352107	0.64903106	0.39794368
Amn	-0.3187615	-0.2705762	0.21344848	0.64908642	0.39794794
Shisa2	-0.1520799	2.48056302	0.21333552	0.64917261	0.39796248
Kcnn4	-0.8149198	-1.6284853	0.21329047	0.64920698	0.39796248
Olfr692	-0.6033211	-1.3157961	0.21293336	0.64947964	0.39809215

Rcor2	-0.18263	1.62983719	0.21288657	0.64951538	0.39809215
Usp30	0.08630556	4.18557355	0.21263354	0.64970877	0.39818098
Hnrnpa3	0.05237567	9.05026182	0.21235053	0.64992523	0.39826718
Zfp882	-0.0765917	4.53737201	0.21232296	0.64994633	0.39826718
Rnaseh2c	0.18960073	2.73847874	0.21204738	0.65015729	0.39836676
Lsm4	0.12659226	3.33172149	0.21176287	0.65037527	0.39847061
Ggh	0.13277917	3.23462189	0.2116965	0.65042615	0.39847208
Rraga	-0.0764966	6.03591811	0.21150695	0.6505715	0.39853142
Golm1	0.08827651	3.26257839	0.21084599	0.65107896	0.3988053
Lrrc47	0.15115596	2.66498574	0.21079829	0.65111563	0.3988053
Snhg6	-0.1685345	1.68394643	0.2107303	0.65116789	0.39880759
Islr2	-0.1242112	2.79239509	0.21038027	0.65143711	0.39894274
Fsbp	0.51663856	-1.8040217	0.21007167	0.6516747	0.39905851
Tlr7	-0.1790438	1.59115268	0.2096988	0.65196205	0.39920473
Sh2d5	-0.0877605	4.63060511	0.20962772	0.65201686	0.39920855
Gclc	-0.0606131	6.26031202	0.20933531	0.65224247	0.39930951
Ginm1	0.11550214	5.23454548	0.20928188	0.65228372	0.39930951
Bcl2l13	-0.0773756	5.02911328	0.20922516	0.6523275	0.39930951
Cyp2b10	-0.5931245	-1.7136659	0.20907935	0.65244011	0.39931937
Nutm1	-0.8006365	-1.5031532	0.2090662	0.65245027	0.39931937
10-Mar	-0.3586993	-0.6737566	0.20899374	0.65250625	0.39931937
Hdac4	0.08724689	5.07433914	0.20891642	0.65256599	0.39931937
Ppp4r1	-0.0819478	4.32499418	0.20884829	0.65261865	0.39931937
Nrg1	-0.1233081	3.36162218	0.20882698	0.65263512	0.39931937
Cckbr	-0.0845542	4.6516168	0.20869979	0.65273346	0.39934981
E130006D01I	-0.5896388	-2.0148907	0.2086044	0.65280724	0.39936522
Tmc6	-0.2536293	0.25957733	0.20842839	0.65294343	0.39941881
Brd4	0.05793413	8.73212684	0.20807542	0.65321673	0.39955467
Sepw1	-0.1396888	5.57534825	0.20801602	0.65326275	0.39955467
Iqgap3	0.51003797	-0.6190251	0.20773815	0.65347815	0.39961669
Ciapin1	-0.0715043	4.72236937	0.20771834	0.65349351	0.39961669
Abcc6	-0.4474326	-0.8892767	0.20769706	0.65351002	0.39961669
Edn3	0.09051657	6.20018642	0.20738404	0.6537529	0.39972893
Gm10190	-0.3728428	-0.2077484	0.20733518	0.65379083	0.39972893
Sec22a	0.11492547	2.98563507	0.20680227	0.65420491	0.39995234
Zfyve26	-0.0940443	4.09534786	0.20657651	0.65438052	0.40002995
Slc35f2	-0.3454104	0.09581704	0.2063977	0.65451969	0.40003082
Wfikkn2	0.20972726	2.97892266	0.20636678	0.65454377	0.40003082
Dock11	0.07307646	5.48991872	0.20634473	0.65456093	0.40003082
Ppp2r5b	0.09816451	3.29609995	0.20632458	0.65457662	0.40003082
Pradc1	-0.1342939	2.72955504	0.2062257	0.65465363	0.40004813
Gimap7	-0.335746	0.15483927	0.20611982	0.65473611	0.40006365
Rwdd2a	-0.1154872	2.99713776	0.20606817	0.65477637	0.40006365
Fbln7	-0.17714	4.07381577	0.20551929	0.65520445	0.40029544
Prkaca	-0.0676471	7.24830415	0.20539635	0.65530043	0.40032432

1810024B03I	0.45911157	-0.6292529	0.20518021	0.65546925	0.4003977
Sell	-0.5863725	-1.0459632	0.20457877	0.6559396	0.40065524
1500009C09I	0.07308783	5.50390501	0.20419552	0.65623975	0.40080878
Wdr74	0.12735101	2.90109075	0.20401147	0.65638402	0.4008652
Nfatc2ip	0.11947499	2.6767404	0.20395326	0.65642966	0.4008652
Ttc7	-0.1400231	3.14285201	0.20374137	0.65659587	0.40093691
Rph3al	0.21155184	1.23331176	0.20361712	0.65669338	0.40096666
BC100451	0.58216852	-1.5581313	0.2035308	0.65676115	0.4009745
Olfr239	0.55482634	-1.2311747	0.20347651	0.65680379	0.4009745
Psmb8	0.17190701	2.65757263	0.20329531	0.65694611	0.4010316
Agps	-0.0536735	7.19752054	0.20268326	0.65742746	0.40127995
Xlr3a	-0.2421605	0.44850432	0.20265388	0.65745058	0.40127995
Synj2bp	-0.0515419	7.03416657	0.20251358	0.65756105	0.40131757
Fam53a	-0.1002864	3.55264989	0.20181571	0.65811124	0.40162353
Akr1c21	-0.6669291	-1.1791494	0.20175178	0.6581617	0.40162451
Gpr137b-ps	-0.0891332	3.63692507	0.20136899	0.65846403	0.40177917
Cbs	0.09360816	3.17066703	0.20109497	0.65868067	0.40188152
Dhx8	-0.0606041	5.10622165	0.20096701	0.65878189	0.40190442
Gm13293	0.20127936	0.84650605	0.20087189	0.65885716	0.40190442
Cenpv	0.09943539	2.83724603	0.20086211	0.6588649	0.40190442
Ctnna3	-0.388195	-0.1813405	0.20062868	0.65904972	0.40198732
Alcam	0.09467594	9.49713943	0.20041447	0.65921943	0.40203232
Bard1	-0.1769908	1.56742693	0.20041209	0.65922132	0.40203232
Gtf3a	0.12758916	2.55083537	0.20022439	0.65937012	0.40207718
Cdadc1	-0.0556497	6.00419115	0.20019589	0.65939272	0.40207718
Vstm2l	-0.2451245	-0.1881095	0.19992072	0.65961104	0.40218047
Khk	-0.221028	2.21443771	0.19942772	0.66000263	0.40238938
Fam69a	0.06903969	4.66369222	0.19905903	0.66029587	0.4025383
Slc2a4	-0.2910716	0.01525547	0.19859468	0.66066565	0.40265661
Doc2g	-0.4459229	-0.3671259	0.19853237	0.66071531	0.40265661
Slc6a15	-0.0843453	4.70351203	0.19852205	0.66072354	0.40265661
Snhg3	0.16338841	3.02494622	0.19846744	0.66076707	0.40265661
Htr1b	-0.1558351	1.29237253	0.19831712	0.66088693	0.40265661
Pnma3	0.1176273	2.98502679	0.19827067	0.66092398	0.40265661
Dpf3	0.29177	0.0628116	0.19826992	0.66092458	0.40265661
Hrk	0.11380438	4.09783662	0.19823606	0.6609516	0.40265661
Pgs1	0.07989109	4.41509529	0.19819319	0.6609858	0.40265661
Hk2	0.11992011	2.80291057	0.1981661	0.66100742	0.40265661
Ulk2	-0.0526342	7.46675213	0.19813925	0.66102884	0.40265661
Ndufb2	0.07736104	3.99487828	0.1979649	0.66116801	0.40271154
Il1b	0.49387918	-1.7587399	0.1973264	0.66167831	0.4029925
Zfp598	-0.0697933	4.07853737	0.19720862	0.66177255	0.40302003
4930505A04I	0.36857197	-0.1434764	0.19706946	0.66188394	0.403058
Hist1h4d	0.14036933	2.81423744	0.19678808	0.66210932	0.40316538
P2ry12	0.10607845	3.67863473	0.19649639	0.66234316	0.40325448

Pick1	-0.0871398	3.57902935	0.19648314	0.66235378	0.40325448
Pip4k2b	-0.061492	7.02044146	0.19594378	0.66278674	0.40348819
Dbndd1	-0.1603965	1.76124318	0.19573815	0.662952	0.4035589
Ccl22	0.51347788	0.31341969	0.19544494	0.66318781	0.40367255
Zbtb37	0.14154177	2.91738233	0.19526732	0.66333077	0.40370428
Dnajc21	-0.0685351	7.02084378	0.1952575	0.66333868	0.40370428
Gtf2ird2	-0.1339253	2.65349216	0.19519712	0.66338729	0.40370428
Slfn2	-0.3142776	0.01257306	0.19512695	0.6634438	0.40370877
Alkbh2	0.18712072	1.16747695	0.19451122	0.66394019	0.40398092
Gm15760	-0.1621881	2.13793902	0.19444131	0.6639966	0.40398534
Mgat4b	-0.1120482	2.87867883	0.1942784	0.66412813	0.40403545
0610040B10I	-0.2950168	0.65165979	0.19411413	0.66426081	0.40405384
Pld3	0.08225616	7.04304457	0.19406357	0.66430167	0.40405384
4930405A21I	-0.2408192	1.03742886	0.19405103	0.6643118	0.40405384
Lrrd1	-0.3941589	-0.3771125	0.1939854	0.66436484	0.40405384
9330020H09I	0.34248955	-1.0428225	0.19393677	0.66440415	0.40405384
Alox5	0.46428892	-1.3258855	0.19385804	0.6644678	0.40406127
4632428N05	-0.1790634	2.15941038	0.19380006	0.66451468	0.40406127
Dph1	-0.4952699	-1.3555422	0.19363531	0.66464795	0.40410348
Aco1	-0.0750562	4.27600672	0.19359269	0.66468244	0.40410348
Nadsyn1	0.15406065	1.37774701	0.19346376	0.6647868	0.40413703
Tusc2	-0.0605215	4.92345459	0.19333361	0.66489218	0.40414199
Rhbdd2	0.08233806	4.33556615	0.19333223	0.6648933	0.40414199
T2	-0.2874244	-0.1876592	0.1931751	0.6650206	0.40418947
Pank4	-0.1122998	2.77003552	0.19305912	0.66511458	0.40421671
Btk	0.38109919	0.14467041	0.19295536	0.6651987	0.40423342
Zc3h13	-0.0621859	8.06484843	0.19290389	0.66524044	0.40423342
Ruvbl1	0.08240414	4.59810199	0.19270957	0.66539808	0.4042974
Tmem53	-0.2340749	1.62310313	0.19265284	0.66544412	0.4042974
Pid1	0.0665906	6.78804196	0.19244575	0.66561225	0.40436966
Myh2	0.34871566	0.20706863	0.19199712	0.66597685	0.40456125
2410004P03I	-0.1427194	2.3157078	0.1919232	0.66603697	0.40456788
Fzd10	-0.1947803	1.03771457	0.19182321	0.66611832	0.40458739
Arhgap36	-0.2782838	0.91719473	0.19166361	0.66624823	0.40461156
Kcnj14	-0.5147837	-1.0669554	0.19165336	0.66625657	0.40461156
Tmtc1	0.06837399	8.45845134	0.19146291	0.66641168	0.40466986
Mesp2	0.27124259	1.96370812	0.19139361	0.66646813	0.40466986
Col4a2	0.0802387	4.28926856	0.19135417	0.66650028	0.40466986
Tmem104	-0.0945251	3.95676381	0.19126564	0.66657243	0.40468377
Selenbp1	-0.1481669	3.05446523	0.19105985	0.66674023	0.40475575
Arvcf	-0.0868477	4.0585454	0.19083692	0.66692212	0.40483627
Tmbim6	0.08317046	6.63596553	0.19073033	0.66700914	0.40485589
Wbscr16	-0.1640858	1.98241993	0.19067666	0.66705297	0.40485589
Arl5b	-0.0968348	4.18578629	0.19055669	0.66715096	0.40486991
Arhgef33	0.31886951	-0.5838041	0.19050477	0.66719338	0.40486991

Gm15663	0.11817379	3.1623083	0.19040541	0.66727458	0.40486991
Rab33b	0.05697476	5.90779528	0.19037578	0.6672988	0.40486991
1600012H06I	0.09323665	4.59940473	0.19028738	0.66737106	0.40486991
9030612E09I	0.22059357	1.32594339	0.19025595	0.66739676	0.40486991
Sprn	-0.0762127	5.91404279	0.19022645	0.66742089	0.40486991
Txlna	0.0657784	5.13475204	0.19007298	0.66754643	0.40491618
1700001L19I	0.1247983	3.38356868	0.18987615	0.66770752	0.40498401
Mbtps2	0.07620072	4.92830405	0.18976519	0.66779839	0.40499559
Commd7	0.075765	4.6837121	0.18973248	0.66782517	0.40499559
St6galnac6	0.09244493	4.07207896	0.18966402	0.66788126	0.40499972
Abhd2	-0.06121	6.17205916	0.18907281	0.66836606	0.4052638
Mospd2	0.07059284	5.1513818	0.18900611	0.66842081	0.4052671
Tulp1	0.56890359	-1.9917509	0.18882072	0.66857305	0.40532951
Gm13051	0.44922217	-1.3911024	0.1884798	0.66885325	0.40546947
3425401B19I	0.15946508	3.75183628	0.18830391	0.66899793	0.40546987
Tfap4	0.16807866	1.05252867	0.18827128	0.66902477	0.40546987
Stt3b	-0.0595808	5.87106778	0.1882678	0.66902764	0.40546987
Spin4	-0.1442765	2.74782339	0.18823913	0.66905124	0.40546987
Rpl30	0.2660747	0.13246401	0.18806655	0.66919329	0.40552606
2610018G03	-0.1701286	1.93661506	0.18767297	0.66951757	0.40569266
Mfap3l	-0.090157	5.63323855	0.18746762	0.66968693	0.40573999
Numb	0.07159905	5.65734998	0.18745854	0.66969442	0.40573999
Retnla	-0.2475164	1.0485688	0.18679542	0.67024208	0.40604187
Klrb1f	-0.3170575	-0.1107929	0.18636813	0.67059558	0.40622608
Tgm3	-0.1116102	2.62821885	0.18619834	0.67073619	0.40625415
Haus5	0.15706114	1.62049443	0.1861928	0.67074078	0.40625415
Rnf17	0.24610807	0.73325128	0.18601858	0.67088513	0.4062974
Fam19a1	0.05789234	5.87262786	0.18598731	0.67091105	0.4062974
Tmed7	-0.066947	5.69417952	0.18591952	0.67096725	0.4063015
Ephx4	0.06700093	5.18556437	0.18559187	0.67123903	0.40643613
Caskin1	-0.0829567	5.76529907	0.18526545	0.67151009	0.4065362
Orc3	-0.0503183	7.85503594	0.18512931	0.67162322	0.4065362
Mrps27	0.09728863	3.35582252	0.18507668	0.67166697	0.4065362
Arg1	-0.4076425	-0.9245313	0.18498518	0.67174305	0.4065362
Slc25a29	-0.2142557	0.83597894	0.18492816	0.67179047	0.4065362
Arrdc4	0.1387539	2.72777521	0.18485609	0.67185041	0.4065362
1700001L05I	0.1174919	4.34217344	0.1848331	0.67186955	0.4065362
Zfp367	0.09826925	3.52014188	0.18482383	0.67187726	0.4065362
Atp1b1	0.04719762	10.1417314	0.18480798	0.67189044	0.4065362
Cdca5	0.51505374	-1.4969449	0.18479779	0.67189893	0.4065362
Fam220a	-0.0653237	4.2702704	0.18469829	0.67198173	0.40655637
Cdc27	-0.0599114	7.83018118	0.18451259	0.67213634	0.40659234
Pigz	-0.1395706	2.17195228	0.18450805	0.67214012	0.40659234
Tep1	0.14252666	2.96774896	0.18364819	0.67285725	0.40696972
4933406C10I	-0.4095519	-1.0047284	0.18364132	0.67286299	0.40696972

Arfgap2	0.09966746	4.17412182	0.18354799	0.67294095	0.4069798
Tpp2	-0.0729142	6.74275554	0.18350281	0.6729787	0.4069798
Cldn8	-0.683618	-2.2535441	0.18334227	0.67311287	0.40702037
Maf1	0.08020744	4.57616593	0.18330406	0.67314482	0.40702037
A330076C08I	-0.3770608	0.12744797	0.18286098	0.67351555	0.40721457
Syp	0.08887263	8.81758959	0.18267333	0.67367272	0.40727964
Trak2	-0.0581073	6.83064815	0.18235032	0.67394349	0.40739408
Evc	0.12353821	2.46743737	0.18232926	0.67396115	0.40739408
Fgd6	-0.074268	6.33846098	0.18224394	0.67403273	0.40740738
Rsl24d1	-0.0765502	5.39693246	0.18217924	0.67408701	0.40741024
Slc10a4	0.11494864	2.53480028	0.18168166	0.67450491	0.40761763
Wwp1	0.05783474	7.5347871	0.18165258	0.67452935	0.40761763
Pex16	0.19853388	0.74110707	0.18146222	0.67468942	0.40766337
Lrwd1	-0.1612667	1.80651946	0.18144461	0.67470424	0.40766337
Ganab	0.0812925	5.05845587	0.1812791	0.67484349	0.40770182
Fuz	0.24458411	0.47719722	0.18125108	0.67486708	0.40770182
Osbpl11	-0.0746009	4.16970836	0.18117056	0.67493486	0.4077128
Egr4	-0.1409968	3.23099371	0.1810165	0.67506461	0.40776121
Gli3	-0.0606731	5.12944884	0.18077617	0.67526714	0.40785357
Zfp148	-0.0488267	7.82606425	0.18067897	0.6753491	0.40786616
Klk10	0.32724849	-0.2302173	0.18063375	0.67538723	0.40786616
Emb	0.11929113	6.18088226	0.18048306	0.67551436	0.40790498
Osbpl10	0.17081955	2.18542351	0.18043991	0.67555078	0.40790498
Kcnk13	0.19959687	0.83313891	0.18032278	0.67564965	0.40793421
Otop2	-0.4112602	-0.9323722	0.1802193	0.67573703	0.40793421
Mrs2	0.1095077	3.96128606	0.18020622	0.67574808	0.40793421
Tmx2	0.06713124	5.98579548	0.18008222	0.67585283	0.40794516
Thap2	0.0722255	5.05056081	0.18005097	0.67587924	0.40794516
Cwf19l2	0.0571225	5.62558779	0.18000851	0.67591512	0.40794516
1700030J22F	-0.1088468	3.65914995	0.17987399	0.67602885	0.40798384
Lhx6	-0.0771223	4.21620547	0.17970265	0.67617376	0.40804133
Rcbtb2	-0.0818814	4.36717018	0.17914301	0.67664766	0.40829733
Cln6	0.20107515	1.96632768	0.17899547	0.67677275	0.40833944
Eme2	-0.1489011	1.98866727	0.17884106	0.67690373	0.40833944
C130060K24I	0.36829269	0.00503325	0.17881202	0.67692836	0.40833944
Pbxip1	0.11578411	5.41108187	0.17876726	0.67696635	0.40833944
Ttc9	-0.0732344	4.23542392	0.1786525	0.67706375	0.40833944
Srpr	0.09235241	6.37658832	0.17864217	0.67707253	0.40833944
Elmod2	-0.0834065	3.45349902	0.17863517	0.67707847	0.40833944
Zfp108	-0.1046144	3.07925753	0.17854784	0.67715262	0.40833944
Ncapg2	-0.1308167	2.56099685	0.17853377	0.67716457	0.40833944
Whrn	0.10723335	3.21545349	0.17841659	0.67726411	0.4083695
Ackr3	0.09853515	3.93527382	0.17833024	0.6773375	0.40838379
Sltm	-0.0567798	7.70883421	0.17808438	0.67754653	0.40847985
Gm6277	0.13766335	2.28629519	0.17777804	0.67780723	0.40859891

Cdnf	-0.1555994	2.31471702	0.17773552	0.67784344	0.40859891
Irf8	0.17203732	1.6282917	0.17735705	0.67816593	0.40875646
Snx33	0.10253784	3.96125282	0.17729306	0.6782205	0.40875646
Ier3ip1	-0.072598	5.53606747	0.17725378	0.678254	0.40875646
Car14	-0.2276722	2.80967489	0.17684529	0.67860266	0.4089366
Gm14169	0.13626703	1.90935333	0.17667407	0.67874893	0.40899476
Stxbp4	0.06083107	7.33360469	0.17652624	0.6788753	0.40904091
Acadl	0.08311594	5.29580048	0.17634378	0.67903136	0.40910495
Mutyh	0.24182637	-0.0979963	0.17622533	0.67913272	0.40912503
Samd12	0.1132883	3.24731974	0.176163	0.67918607	0.40912503
Ntf5	-0.6693709	-1.8553363	0.17613036	0.67921401	0.40912503
C330021F23I	-0.1119689	2.50168453	0.17591078	0.67940207	0.40918831
Malt1	-0.1003906	3.60673066	0.17589144	0.67941864	0.40918831
Cmas	-0.0437262	6.76787663	0.17566352	0.67961401	0.40925236
Reck	0.08731946	5.35084657	0.17562567	0.67964647	0.40925236
Xkr8	-0.1611782	2.41378249	0.17559313	0.67967437	0.40925236
Epn2	0.04399733	6.4669456	0.17536497	0.67987015	0.40934025
Cpxm2	0.13154332	2.65931824	0.17529508	0.67993014	0.40934639
Clec3b	-0.2221003	0.90492489	0.17517872	0.68003006	0.40937656
Mecp2	-0.0549195	8.93917506	0.1751127	0.68008678	0.40938072
Zfp442	-0.0887302	3.50310262	0.17501505	0.68017068	0.40940124
Papd7	-0.0768645	4.69030791	0.1749529	0.68022409	0.40940341
Nrap	0.51194687	-1.1094914	0.17462839	0.68050319	0.40954139
Prkd2	0.12776522	2.88515912	0.17445746	0.68065031	0.40959769
Hibch	-0.082797	4.11210801	0.17440395	0.68069639	0.40959769
Tmem229b	0.1064263	3.13060807	0.17431198	0.6807756	0.40961536
Sobp	0.04819985	7.44545726	0.17381454	0.68120447	0.40981464
Add1	-0.0553182	7.6835705	0.17376049	0.68125111	0.40981464
Zfp593	0.19220363	0.84499048	0.17375437	0.68125639	0.40981464
Hira	-0.0681083	4.13802974	0.1736608	0.68133716	0.40983323
Rab4a	-0.0623845	4.98587144	0.17340094	0.6815616	0.40988941
Nap1l2	-0.0867059	5.39456633	0.17338866	0.68157222	0.40988941
Kcnip4	0.05750302	7.02681081	0.17332589	0.68162646	0.40988941
Pmel	0.48419498	-1.8486004	0.17331916	0.68163228	0.40988941
Fign	0.089806	4.7871539	0.17326407	0.6816799	0.40988941
Pet100	0.1329513	3.54535669	0.17319398	0.6817405	0.40989586
Chat	0.17982118	1.53177093	0.17311018	0.68181297	0.40990945
Slc38a3	-0.0978125	4.10921612	0.17297122	0.68193319	0.40995174
Fcer1g	0.12412729	3.20022187	0.17267797	0.68218709	0.41003965
Gpr133	0.13645114	2.64997106	0.17264387	0.68221663	0.41003965
Cbx4	-0.0705024	4.43718425	0.17262948	0.68222909	0.41003965
Brcc3	0.06477289	5.43400162	0.17243097	0.68240113	0.41011306
Mpp2	-0.053926	6.71920698	0.17227468	0.68253667	0.41015167
Mcm9	-0.1549691	2.0178278	0.17221752	0.68258626	0.41015167
Rarres2	-0.3471827	0.59490737	0.17218429	0.68261509	0.41015167

Pigq	0.05818077	5.82510229	0.17201548	0.6827616	0.41017464
Ttc12	0.22455697	1.39047069	0.17200197	0.68277333	0.41017464
Tuba1a	0.04534749	9.67220925	0.17196777	0.68280303	0.41017464
Trove2	-0.0561653	7.07330955	0.17184044	0.68291362	0.41019939
Zfp651	0.08225182	4.59660151	0.17176092	0.68298271	0.41019939
Supt3	0.11658994	2.28573846	0.17173457	0.6830056	0.41019939
Mvb12a	0.12313452	1.88568296	0.17164743	0.68308134	0.41019939
Phf7	0.2640336	0.91577903	0.17163314	0.68309376	0.41019939
Lyz1	0.26219672	1.04164161	0.17151431	0.68319708	0.4102113
Cnnm4	-0.1317601	2.88400049	0.17149553	0.68321342	0.4102113
Nampt	-0.0538667	7.31073047	0.1713386	0.68334994	0.41025329
Cib2	0.17323322	2.27751293	0.17130039	0.6833832	0.41025329
Hmgn5	0.06660404	7.16198763	0.17105487	0.68359697	0.41035165
Vav1	0.22588915	0.25682111	0.17095603	0.68368308	0.41037337
Slx4	-0.0715008	5.16651281	0.17069384	0.68391165	0.41047141
B930018H19	0.61036127	-0.9073337	0.17065413	0.68394629	0.41047141
Btbd3	-0.0614817	8.37597415	0.17056709	0.68402221	0.410487
Naglu	-0.1922974	1.20985482	0.17013463	0.68439983	0.41068363
1700018G05	-0.4258485	-0.9983156	0.17004756	0.68447592	0.4106993
Rltpr	-0.1281927	1.80579924	0.16983236	0.6846641	0.41078223
Lefty2	-0.4829692	-1.3405723	0.16952032	0.68493721	0.41091406
Dph2	0.12243391	1.76580914	0.16946709	0.68498382	0.41091406
Gbe1	0.07559514	3.96139429	0.16935871	0.68507877	0.41094102
Itpr3	-0.1317559	2.12556191	0.16923491	0.68518726	0.41097611
Rbm38	-0.3188263	0.8031753	0.16916298	0.68525032	0.41098394
Igfbp7	-0.1028963	5.10106368	0.16905645	0.68534374	0.41100998
1700030C10I	-0.193335	1.00427737	0.16895232	0.68543509	0.41102607
Fzr1	0.12125627	2.83729254	0.1688845	0.68549461	0.41102607
1700023F06I	-0.5017664	-1.5503012	0.16877481	0.68559089	0.41102607
Plcxd3	-0.0780343	5.01837522	0.16876126	0.68560278	0.41102607
Zranb2	-0.0506591	8.01362559	0.16874095	0.68562062	0.41102607
Klhl20	0.07066654	5.71641113	0.16864793	0.68570231	0.41104506
Ptgfr	-0.1291939	4.72271228	0.16857101	0.68576989	0.41105559
Gm13034	-0.3635379	-1.6862613	0.16826822	0.68603605	0.41116739
Qars	-0.078905	3.78645225	0.16819366	0.68610164	0.41116739
Ipo7	-0.0645723	5.83228562	0.16815849	0.68613258	0.41116739
Cbfa2t2	0.04479988	6.40881928	0.16813129	0.68615651	0.41116739
Zfp791	-0.1176468	2.83005514	0.1678205	0.68643014	0.41130137
AU040320	-0.0902824	4.17222439	0.16738462	0.6868144	0.41148094
Zc2hc1a	0.05697052	7.30989653	0.16736698	0.68682996	0.41148094
Zfp691	-0.1530889	2.53282717	0.16718947	0.68698662	0.41152087
Sdccag3	-0.0689689	4.74850971	0.16717801	0.68699674	0.41152087
Cstl1	0.35730316	0.25753104	0.16692343	0.68722161	0.41158878
Serpinb6c	0.19996767	2.72569528	0.16689839	0.68724373	0.41158878
Galnt9	0.07564516	4.68290137	0.16687959	0.68726035	0.41158878

Tlr1	0.50740836	-0.9829122	0.16678121	0.68734731	0.41161087
Pcdhga11	-0.1300172	3.13527731	0.16656687	0.68753688	0.41164853
Thbs2	0.10805601	5.06590599	0.16654923	0.6875525	0.41164853
Sorbs1	0.04165233	7.52415737	0.16652295	0.68757575	0.41164853
Rsph9	-0.1920311	2.03446966	0.16648365	0.68761053	0.41164853
Tlk2	0.05172503	7.11573256	0.16620978	0.68785303	0.41176371
Tram1	-0.0807194	5.97538072	0.16601692	0.68802395	0.4118077
Sox17	0.19314642	1.86351752	0.16601379	0.68802672	0.4118077
Cplx2	-0.0673776	9.63292106	0.16582833	0.68819119	0.41187438
Hck	0.33162953	-0.4803346	0.16577516	0.68823836	0.41187438
Ankrd26	0.06257305	5.96875239	0.16568079	0.6883221	0.41189451
Fads1	0.05973877	6.22578949	0.16548315	0.68849758	0.41196952
Eif4ebp2	-0.0692949	5.77871641	0.16536936	0.68859866	0.41199626
Cks2	0.21905279	0.30209845	0.16532001	0.68864252	0.41199626
Jagn1	0.06370201	4.86171929	0.16500167	0.68892558	0.41212857
Elac2	-0.1207993	2.88312398	0.16492486	0.68899394	0.41212857
Zfp932	-0.0899961	4.46617735	0.16488127	0.68903272	0.41212857
Txnrd2	0.19005922	1.2978111	0.16481099	0.68909529	0.41212857
Plac9b	0.11006915	3.93696673	0.16478954	0.68911439	0.41212857
Tmem236	-0.6102053	-1.7102304	0.16439394	0.68946686	0.41230937
Klhl33	0.51286484	-1.944118	0.16419116	0.68964773	0.41234666
Ppp1r12c	-0.0536837	5.32770825	0.16418689	0.68965154	0.41234666
Ncam2	-0.0693569	7.16289986	0.1640763	0.68975024	0.41234666
Tmem14a	-0.0708302	4.84503277	0.16405167	0.68977222	0.41234666
1600014C10I	-0.0794061	4.83188295	0.16399036	0.68982696	0.41234666
Tmem38a	0.08147164	5.49186598	0.16398669	0.68983024	0.41234666
Smarca4	0.0745602	7.01594341	0.16391453	0.68989468	0.41235519
Tmod1	0.0705904	4.73124694	0.16375712	0.6900353	0.41240925
Gzf1	-0.0593536	5.15141052	0.16369853	0.69008767	0.41241056
Gm5113	-0.1003726	4.18435604	0.16362411	0.6901542	0.41241145
Macrod1	0.269205	0.16466757	0.16358461	0.69018952	0.41241145
Cacna2d4	0.19775141	1.19254656	0.16343057	0.6903273	0.41244452
Syna	0.13843895	1.95238682	0.16341054	0.69034522	0.41244452
Sdc4	0.12989244	5.26521449	0.16330142	0.69044287	0.41247288
Acsl5	-0.0559515	5.63286945	0.16322136	0.69051454	0.41248571
Ccdc106	0.16129181	1.77355217	0.16285146	0.69084596	0.41258727
Calu	0.07107579	6.14089054	0.16283495	0.69086076	0.41258727
Galnt3	0.48637903	-0.752167	0.1628262	0.69086861	0.41258727
Sepn1	0.11559379	3.45527712	0.16276186	0.69092631	0.41258727
Zfp251	0.08494028	3.78981328	0.16275156	0.69093554	0.41258727
Gm16432	-0.2302355	1.11354933	0.16238626	0.69126339	0.41275305
Tspyl3	0.05803224	4.6678116	0.16214872	0.6914768	0.41281402
Gypc	0.11646388	5.39498822	0.16205868	0.69155775	0.41281402
Rad21	0.04618049	7.13610302	0.16203459	0.69157941	0.41281402
BC029722	0.15860527	2.07928093	0.16195987	0.6916466	0.41281402

Sh3kbp1	0.05626214	6.38425475	0.16194985	0.69165562	0.41281402
Ube2ql1	0.0618994	6.24484934	0.16193736	0.69166685	0.41281402
Ip6k1	0.06567196	5.79046022	0.16161082	0.69196075	0.41292284
Tmem74b	-0.3330997	-1.2658424	0.16153789	0.69202643	0.41292284
Dmrt3	-0.8151392	-1.5248204	0.16148757	0.69207177	0.41292284
Pde1c	-0.1056284	4.17786531	0.16141514	0.69213704	0.41292284
Heatr9	-0.5610329	-1.4993693	0.16140314	0.69214785	0.41292284
Epn1	-0.0653904	4.47923716	0.16140007	0.69215061	0.41292284
Abhd12	0.05664637	6.33405157	0.16133982	0.69220492	0.41292526
Agap2	-0.0612843	6.9295941	0.16118114	0.692348	0.41298064
Aff2	-0.0815461	5.87645456	0.16102524	0.69248866	0.41303457
6330416G13	-0.0829604	3.76233321	0.16080257	0.6926897	0.4131245
Zfp637	0.09548435	3.80381429	0.16072237	0.69276215	0.41313773
4931414P19I	-0.238248	1.11634917	0.16052152	0.69294367	0.41320261
4930427A07I	-0.3675746	-0.0174891	0.16049075	0.69297149	0.41320261
Rap2c	0.04418776	6.85442395	0.16039898	0.69305449	0.41322212
Rpap1	-0.0997364	2.8673749	0.16010785	0.69331797	0.41334923
2510009E07I	0.05426603	6.8544813	0.15995391	0.6934574	0.41337562
Fbrs	0.06629082	4.54391855	0.15993974	0.69347023	0.41337562
Mfsd11	-0.0911237	3.18360502	0.15989242	0.69351311	0.41337562
Ccnl1	-0.1113079	4.7158139	0.15962735	0.69375344	0.41348888
Gm10584	-0.3723335	-1.1520838	0.15945742	0.69390763	0.41355079
Zfp74	0.07282837	5.97278665	0.15906298	0.69426589	0.4137227
Anxa9	0.30145696	-0.5916334	0.15902903	0.69429676	0.4137227
Dync1i1	-0.0873137	5.12427395	0.15887553	0.69443634	0.41377588
Crip3	0.69376639	-1.9973046	0.15870244	0.69459383	0.41383066
Setbp1	0.04868658	6.75198855	0.15866383	0.69462898	0.41383066
Trabd	-0.1363652	2.25259154	0.15856235	0.69472137	0.4138557
Cntd1	-0.104211	2.33775579	0.15846215	0.69481263	0.41388007
1700018L02F	-0.2777412	0.78479279	0.15835106	0.69491385	0.41391037
Diablo	-0.0979436	4.26106158	0.15825861	0.69499812	0.41393056
Rnpepl1	0.07951962	4.18973917	0.15811288	0.69513101	0.4139644
2010107G12	0.56410485	-1.5028494	0.15808585	0.69515567	0.4139644
Apbb1ip	0.09477957	3.19513467	0.15790686	0.695319	0.41402908
Gbp5	0.18258427	2.13971357	0.1578396	0.6953804	0.41402908
Naa20	-0.0717047	4.99462686	0.15780126	0.69541541	0.41402908
Nanos1	0.05157974	5.23537647	0.15761589	0.69558474	0.4140999
Smim12	0.12036287	2.10273062	0.15744485	0.69574109	0.41416298
4930467D21I	-0.4861617	-1.3164276	0.15726432	0.69590622	0.41420194
Msh5	-0.6157104	-1.8124745	0.15726311	0.69590733	0.41420194
Oraov1	-0.0950527	3.70779662	0.1570687	0.69608528	0.41427786
Ksr1	-0.0786477	3.86342717	0.15685943	0.69627697	0.4143367
Cul4b	0.04715398	7.03109806	0.15685071	0.69628497	0.4143367
Sema4b	-0.0994844	2.77315186	0.1567167	0.69640781	0.41436436
Ly6c1	-0.1970571	1.66203813	0.15668075	0.69644077	0.41436436

Ropn1l	0.40284972	-1.0281018	0.15663503	0.6964827	0.41436436
Pln	-0.2552901	0.61041831	0.15644882	0.69665353	0.414436
Dtx3	-0.0695004	5.05564026	0.15635474	0.69673989	0.41445737
Asb15	0.26905764	0.58360885	0.15619355	0.69688792	0.41450233
Ppapdc3	-0.1285864	2.36618951	0.15616262	0.69691633	0.41450233
B230209E15I	0.0693253	6.31479577	0.15605299	0.69701708	0.41453226
Paox	-0.2155769	0.96696285	0.15595202	0.6971099	0.41455746
Zfp143	0.09718905	3.39032296	0.15577993	0.69726818	0.41462159
Hs3st6	0.34224771	-0.5819177	0.1554272	0.69759293	0.41470999
Xaf1	-0.092217	3.44495522	0.15537848	0.69763782	0.41470999
Ippk	0.07323122	3.8158183	0.15536446	0.69765074	0.41470999
Trib3	0.46045059	-1.1817498	0.15535751	0.69765714	0.41470999
Stoml3	0.61314845	-1.6978164	0.15530669	0.69770398	0.41470999
1700024G13	-0.7309802	-1.5267944	0.15528976	0.69771959	0.41470999
Dcaf15	-0.1617524	1.33317004	0.15514592	0.6978522	0.41475881
Zfp94	-0.1132754	2.68639115	0.15479031	0.69818038	0.41492386
Skiv2l	-0.0783925	5.25710978	0.15450036	0.69844829	0.41503118
Loxl1	0.15698444	1.84589249	0.15448558	0.69846195	0.41503118
Apitd1	-0.3581935	-0.4734113	0.15441938	0.69852316	0.41503754
Wfdc1	0.18481602	2.89032144	0.15429456	0.69863861	0.41504772
Pde4d	0.06749455	7.08529817	0.15429168	0.69864128	0.41504772
A130010J15I	0.12096871	3.56718103	0.15419682	0.69872906	0.41505114
Ube2t	0.41537704	-1.0713827	0.15416271	0.69876063	0.41505114
2700097O09	0.10912354	2.8756526	0.15412177	0.69879853	0.41505114
Morn2	0.11307332	2.89696583	0.15406684	0.69884939	0.41505135
Gas2l1	0.09698048	3.8345885	0.15398374	0.69892635	0.41506707
Akap2	-0.0485005	7.52593438	0.15384401	0.69905582	0.41510236
Stam	0.05037204	6.06935406	0.15379216	0.69910387	0.41510236
Dbt	0.06683271	5.64794495	0.15375383	0.6991394	0.41510236
Nostrin	0.24968479	0.87042759	0.15370163	0.6991878	0.41510236
Tbc1d17	0.12674755	2.57286383	0.1534029	0.69946496	0.41523691
Vwc2l	-0.1054232	3.42519573	0.15327926	0.69957976	0.41524404
Gls	-0.0668765	9.46596455	0.15324854	0.69960829	0.41524404
E230029C05I	-0.2240086	0.54626243	0.15319884	0.69965446	0.41524404
0610010F05I	0.06943188	5.99731277	0.15317237	0.69967905	0.41524404
Fan1	0.13368865	1.65864258	0.15298345	0.69985465	0.41529692
Aqp11	-0.1219797	1.77804299	0.15296779	0.69986921	0.41529692
Tmem126a	-0.067161	4.47075064	0.15287192	0.69995837	0.41531984
Pih1d2	0.24262042	0.08336365	0.15269731	0.70012084	0.41534575
Gpr34	0.11425217	2.07134329	0.152684	0.70013322	0.41534575
Trip13	-0.2931894	0.72257965	0.15266207	0.70015364	0.41534575
Id2	-0.0857822	6.09138023	0.15254124	0.70026615	0.41538251
Tpra1	0.13383349	2.47467841	0.15222944	0.70055672	0.41548151
Ak4	0.07151598	5.3752424	0.15221881	0.70056663	0.41548151
AW549877	0.05101374	7.65307398	0.15219943	0.7005847	0.41548151

Crabp1	0.58178795	-1.4468871	0.15213492	0.70064487	0.41548722
Sspn	-0.1115886	4.45629155	0.15201206	0.7007595	0.41552521
Ccdc18	-0.1839439	1.71139588	0.15193117	0.700835	0.41554
Btbd9	-0.0728458	4.98296484	0.15160403	0.70114058	0.4156912
C77080	-0.0823122	5.81398474	0.15127748	0.70144599	0.41584085
Psd4	0.25833544	0.02147733	0.15122598	0.70149419	0.41584085
Phyhip	0.06387578	6.76786975	0.15101809	0.70168885	0.41592625
Acaa1b	0.18828955	0.75719299	0.1508828	0.70181563	0.4159714
Cpeb3	-0.0559741	7.55417114	0.15060943	0.70207198	0.41609333
4921507L20I	0.32741361	-0.5854421	0.15053535	0.70214149	0.41610453
Fcrlb	-0.4789266	-1.4451806	0.15046608	0.70220651	0.41611306
Ttc30b	-0.076986	3.5675271	0.15032526	0.70233874	0.41614764
Cartpt	-0.1520497	1.40408598	0.15029609	0.70236614	0.41614764
Ncapg	-0.3538878	-0.3341187	0.15021704	0.70244041	0.41615207
Dpp9	0.06392186	4.59523009	0.15018035	0.70247488	0.41615207
Dll3	0.40965934	-1.1968643	0.14984824	0.7027872	0.41630709
Klhl5	0.05913147	5.72844719	0.14974567	0.70288373	0.41631628
4930452B06I	0.09728552	3.51300428	0.14970223	0.70292463	0.41631628
Gm11696	0.15470214	1.18376986	0.14967031	0.70295469	0.41631628
Rnf130	-0.0452103	7.06320133	0.14956166	0.70305702	0.41634689
A330069E16I	0.16311294	0.70240005	0.14941749	0.70319287	0.41639734
Noxred1	-0.3839551	-0.8251038	0.14930847	0.70329565	0.41640204
Drd2	-0.1245842	3.84286227	0.14930159	0.70330214	0.41640204
Gm1653	-0.3921814	0.50863292	0.14908973	0.703502	0.41649037
Gemin2	-0.1199006	2.12116911	0.14878434	0.70379038	0.41662735
1700026L06I	-0.4567339	-1.4318368	0.14873739	0.70383476	0.41662735
D2hgdh	-0.0691059	4.1262795	0.14839815	0.70415557	0.41676903
Arhgap4	0.37071257	-0.1392605	0.14836761	0.70418446	0.41676903
Mmp11	0.23563869	1.13783293	0.14832348	0.70422624	0.41676903
Aebp2	-0.0443319	6.84929857	0.14824234	0.70430305	0.41678449
Rcor3	-0.0563504	5.44193821	0.14817061	0.70437098	0.41679467
Npas4	-0.2997089	3.17988758	0.14809807	0.7044397	0.41680533
Espl1	-0.3728079	-1.4992261	0.14794615	0.70458367	0.4168605
Kank2	0.07731488	7.56540758	0.14782193	0.70470146	0.41689993
5430421F17I	0.20176537	0.8712816	0.1477689	0.70475175	0.41689993
Zc3h12a	0.3030269	-0.5978045	0.14771289	0.70480489	0.41690136
Fndc8	0.56107672	-2.2316128	0.14762887	0.70488462	0.41691851
Fam167a	0.13074497	2.37068262	0.14750271	0.7050044	0.41695935
Bub3	0.04986419	5.8548539	0.14739083	0.70511066	0.41697113
Lefty1	-0.1639393	1.93851202	0.14737491	0.70512578	0.41697113
Elovl1	0.13720088	3.3782113	0.14728284	0.70521328	0.4169732
Sh3bgrl	0.0651646	8.852275	0.14726446	0.70523075	0.4169732
Kat6b	-0.0438371	7.17037169	0.14718464	0.70530663	0.41698807
Zcchc7	-0.0650356	5.64380246	0.14687453	0.70560166	0.41712035
Mapk1	-0.0472454	9.09375929	0.14678969	0.70568244	0.41712035

Cd151	0.12854499	5.12328317	0.1467895	0.70568261	0.41712035
4933424G06	-0.2897262	0.47933401	0.14669545	0.70577219	0.41714329
Nsdhl	-0.063901	4.56977349	0.14624464	0.70620202	0.41736733
D030045P18I	0.38821305	-0.5537525	0.14611789	0.70632301	0.41740882
Ddx42	-0.0537728	6.02745186	0.14596446	0.70646955	0.41746541
Gpr64	0.22584967	1.17481498	0.14547844	0.70693432	0.41767908
E030011O05	-0.3163962	-0.8651926	0.1453584	0.70704925	0.41767908
Col18a1	-0.1346409	1.82900347	0.14535454	0.70705295	0.41767908
Fam181b	-0.1211679	2.12299327	0.14534635	0.70706079	0.41767908
Noxo1	-0.2839545	-0.2666399	0.14532081	0.70708525	0.41767908
B230217O12	-0.1303115	1.77765777	0.14462163	0.70775585	0.41802452
Taf7	0.07654849	3.82423093	0.14452806	0.70784575	0.41802452
Krt1	-0.1718195	1.74797374	0.14452207	0.7078515	0.41802452
Slc41a3	0.1406182	2.46873405	0.14449491	0.7078776	0.41802452
Gsk3b	-0.0415121	9.51769032	0.1444148	0.70795459	0.41802452
Psen1	0.06159986	5.05000366	0.14439337	0.70797519	0.41802452
Diras1	0.07709729	5.13546591	0.14429132	0.70807331	0.41805242
Kcnd3os	-0.3645797	-1.0140629	0.14423764	0.70812494	0.41805287
Meiob	0.41738905	-0.9393233	0.14415581	0.70820368	0.41806933
Il7	-0.4733555	-0.8826703	0.14400351	0.70835027	0.41812583
Gm10814	0.2959141	0.76249945	0.14386129	0.70848724	0.41817665
Gpr26	-0.1237633	3.43552285	0.14364037	0.70870016	0.41827229
Treml2	-0.4428384	-0.6834042	0.14356543	0.70877243	0.41828491
Kdm1a	-0.0612432	5.91142414	0.14335449	0.70897598	0.41837499
4933431E20I	0.04924684	6.76028562	0.14320563	0.70911972	0.41842977
Coro1a	-0.0838727	4.02559701	0.14314088	0.70918227	0.41843664
Plekhf2	0.08213535	4.63531593	0.14284948	0.70946397	0.4185728
Atp10a	-0.0896818	3.72381248	0.14277702	0.70953407	0.41858411
Cep250	-0.076766	4.7744097	0.14242531	0.70987462	0.41874159
Dleu2	-0.1238057	2.42882056	0.14233314	0.70996394	0.41874159
Sorl1	-0.0728501	6.15766545	0.14232392	0.70997289	0.41874159
Acads	-0.3128067	-0.6553317	0.14228139	0.71001412	0.41874159
2810442I21R	-0.5213001	-2.1376449	0.14223758	0.71005659	0.41874159
Pstpip1	0.39630776	-0.5833353	0.14218592	0.71010669	0.41874159
Nras	0.04835627	7.27390837	0.14213332	0.71015771	0.41874163
Eef1e1	-0.0721855	3.33863197	0.14206987	0.71021927	0.41874296
Efnb2	-0.0545891	6.13005427	0.14202599	0.71026185	0.41874296
C630043F03I	0.08303987	3.2595682	0.14191704	0.71036761	0.41877527
Rex2	0.39027946	-1.6257951	0.14182485	0.71045714	0.41879801
Brd3	0.05087809	6.20578828	0.14173962	0.71053994	0.41881678
Slc27a4	-0.0837729	3.48703901	0.14156739	0.71070735	0.41888541
Noc3l	0.06564331	4.93042067	0.14144599	0.71082541	0.41890883
Ikzf3	0.11348971	2.779429	0.14142173	0.71084901	0.41890883
Vdac1	0.04212953	8.05749857	0.14135623	0.71091274	0.41891635
Zdhhc14	0.07279407	4.30619587	0.14118402	0.71108039	0.4189656

Tmem222	0.12662532	4.53161946	0.14116566	0.71109827	0.4189656
Galnt18	0.1134422	2.29504213	0.1410025	0.71125722	0.41902921
Unc5b	-0.1085006	2.36938693	0.14094943	0.71130895	0.41902965
Mrpl40	0.09235688	3.57713701	0.14071218	0.71154033	0.41911864
Slc35b4	-0.0502711	5.64591633	0.14068997	0.71156199	0.41911864
Twistnb	-0.0505359	5.20112615	0.14038363	0.7118611	0.41926477
Mtmr14	-0.0940544	3.11934636	0.14027989	0.71196248	0.41926554
5330434G04	-0.0941244	5.00213605	0.1402779	0.71196443	0.41926554
Cpb1	0.16487711	2.22765188	0.14016544	0.71207438	0.41927495
Pla2g15	0.15330791	2.06800813	0.14015719	0.71208244	0.41927495
Gm6815	-0.2656208	-0.9340087	0.13989257	0.71234135	0.41939589
Kif13a	-0.0518032	5.84859562	0.13984299	0.71238989	0.41939589
Gabra3	-0.051474	5.47618171	0.13936431	0.71285907	0.41964204
Tmco5	-0.2171033	0.61873959	0.13923287	0.71298806	0.41966878
Ikzf1	0.08134643	4.34495436	0.13921398	0.7130066	0.41966878
Lat2	0.23655756	-0.025745	0.13910187	0.71311668	0.41970351
Zfr	-0.0519176	8.62368606	0.13881495	0.71339865	0.41982691
Sytl1	0.28182993	0.01323853	0.13878459	0.7134285	0.41982691
Ccnc	0.05586363	5.84716191	0.13804768	0.71415431	0.42022394
Csf2rb2	-0.28952	-0.1943771	0.13769465	0.71450282	0.42039891
H60b	0.24777505	0.46473753	0.1372517	0.7149408	0.42061037
Wipi2	-0.0704357	5.00363007	0.13719108	0.71500081	0.42061037
Fam149b	0.05420783	5.38865691	0.137176	0.71501573	0.42061037
Itgax	-0.3193017	-0.4385055	0.13707333	0.7151174	0.42062245
B230216N24	-0.1172824	2.24691743	0.1370519	0.71513862	0.42062245
A430033K04	-0.0684595	5.56796504	0.13698488	0.71520503	0.4206314
Trhr	0.15148852	1.50417131	0.13684981	0.71533889	0.42066833
Pirb	0.24499663	-0.2529225	0.13679919	0.71538907	0.42066833
Fzd7	-0.1124965	5.93664349	0.13676663	0.71542137	0.42066833
Snupn	0.0926621	3.39514449	0.1366463	0.71554073	0.42068834
Gm20257	0.14245246	2.45904281	0.13662914	0.71555775	0.42068834
Zfp943	-0.0875031	3.03883785	0.13657287	0.7156136	0.42069108
Atg4a	-0.1212852	3.5910716	0.13648746	0.71569839	0.42071083
Mir5119	-0.4304482	-1.4890308	0.13621903	0.71596505	0.420826
Zmat3	0.053487	9.37056188	0.13618718	0.71599671	0.420826
8030462N17	0.04786168	5.47395335	0.13604177	0.71614131	0.42088089
Arhgap27	0.0952322	3.24383541	0.13581867	0.71636334	0.42097383
Letm1	0.06336873	5.94169306	0.13577994	0.7164019	0.42097383
Pinx1	-0.0898011	3.43679909	0.1355894	0.71659172	0.42105528
Mgll	-0.0547856	5.42694072	0.13521221	0.71696794	0.42124622
Dennd4a	-0.048943	7.418427	0.13506626	0.71711367	0.42130173
Il11ra1	-0.0606866	4.34286303	0.13499291	0.71718694	0.42130791
Cd81	0.09278913	7.79192516	0.1349531	0.71722672	0.42130791
B3gnt7	-0.4454841	-0.8629206	0.13470455	0.71747523	0.42142377
Rasd2	-0.0643388	4.98101848	0.13456083	0.71761904	0.4214652

Tmem220	-0.110084	2.86846777	0.13453158	0.71764832	0.4214652
Klre1	-0.4393225	-1.0160264	0.13400361	0.71817745	0.42174582
Prkab2	0.06541554	4.92458605	0.13368358	0.71849877	0.42182943
Alkbh4	0.1431696	1.04714267	0.13366967	0.71851275	0.42182943
Nelfcd	-0.0757209	3.75889393	0.13362595	0.71855668	0.42182943
Mageh1	-0.0720309	4.38329685	0.13360199	0.71858076	0.42182943
Card10	-0.2089086	0.38311934	0.13357139	0.71861151	0.42182943
Gapt	0.34930302	-0.1591237	0.1335552	0.71862778	0.42182943
Kif1c	-0.0768615	5.6443033	0.13338513	0.71879881	0.4218855
Rnf138rt1	-0.3894469	-1.5642962	0.13335815	0.71882596	0.4218855
Ldb3	0.14286926	2.06945824	0.13326874	0.71891593	0.42190818
Spdya	-0.173609	0.69594156	0.13307996	0.71910601	0.4219896
Rab11fip1	0.07772325	3.66126826	0.13270676	0.71948223	0.42218023
Ccdc60	0.3426036	-0.8743788	0.13262282	0.71956693	0.42219979
Abi3	-0.1195254	1.67972369	0.13250978	0.71968105	0.42223661
Omg	-0.0693395	7.44931293	0.13239917	0.71979275	0.422272
Nubp1	0.11415309	2.29977372	0.13214714	0.72004751	0.42238861
Rnf123	-0.0617991	4.46546386	0.13210087	0.7200943	0.42238861
Tmie	0.1292368	1.66140643	0.13193744	0.72025968	0.42243721
Lamb3	0.46335385	-1.016154	0.13191741	0.72027995	0.42243721
Lmod1	0.0914368	5.0582383	0.13186374	0.7203343	0.42243894
Rfx5	0.05680708	4.85278337	0.13154271	0.72065958	0.42256212
Lrguk	0.08245803	3.24587054	0.13151771	0.72068493	0.42256212
Atp11a	-0.0537365	6.90085178	0.13150425	0.72069859	0.42256212
Flnb	-0.0472097	6.1926105	0.1313903	0.72081417	0.42259975
Nlrp10	0.25886742	-0.7884599	0.13122699	0.72097993	0.42266175
Dgat2	0.07762032	3.75689684	0.13115575	0.72105228	0.42266175
Csnk1g2	0.07439179	5.59108536	0.13109824	0.72111069	0.42266175
Ccdc170	0.19591042	0.66336915	0.13108354	0.72112563	0.42266175
Nr2f1	0.05884589	6.76098752	0.13073999	0.72147493	0.42282579
Rgn	-0.5634478	-1.5682967	0.13070711	0.7215084	0.42282579
Abce1	0.05013311	6.26132422	0.13063699	0.72157976	0.42283747
G3bp2	0.05038663	9.47815298	0.13049677	0.72172254	0.42289098
5430405H02l	0.14306601	1.41100039	0.13038067	0.72184082	0.42292536
2510002D24l	0.09732141	3.33183668	0.13023328	0.72199108	0.42292536
Cyp2r1	0.17971473	0.36074522	0.13019148	0.72203372	0.42292536
Gm4925	-0.3004863	-0.0737922	0.13017018	0.72205543	0.42292536
2810029C07l	-0.1842723	2.23293347	0.13016622	0.72205948	0.42292536
Cd72	0.30774344	-0.3622226	0.13013635	0.72208995	0.42292536
Mchr1	0.12042144	2.58860554	0.12969451	0.72254114	0.42315946
Pgr	-0.0658661	5.93249371	0.12961888	0.72261845	0.42317459
Zfyve1	-0.0578469	4.19700661	0.12949967	0.72274038	0.42321584
Swt1	0.06756101	4.57133182	0.12917636	0.72307137	0.42335142
Tfb1m	0.09946594	2.80023338	0.12915036	0.72309801	0.42335142
Elp3	-0.0501581	5.3579593	0.1290879	0.72316202	0.42335142

Adprm	0.09259267	3.70174473	0.12907235	0.72317795	0.42335142
Gata6	0.48305245	-1.377922	0.1287447	0.72351402	0.42346129
Tekt2	0.39042833	-0.5607391	0.12874216	0.72351663	0.42346129
Rd3	0.70739739	-1.8484215	0.12873868	0.7235202	0.42346129
Tmem2	-0.0555433	4.21362306	0.12850364	0.72376158	0.42354145
Tgfbr1	-0.0628122	5.32494421	0.12845186	0.72381479	0.42354145
Rab31	0.06137795	5.56481474	0.12844536	0.72382148	0.42354145
Sigmar1	-0.070812	3.01872859	0.12840468	0.72386328	0.42354145
2610318N02	-0.439405	-2.0397248	0.12829114	0.72398003	0.42357961
Rnf13	-0.059228	7.06736367	0.12806976	0.72420783	0.42368272
Tubb2a	0.06648038	7.72927064	0.12793153	0.72435018	0.42373584
Slc12a7	-0.1526995	3.82914986	0.12778759	0.7244985	0.42377707
Ccrl2	0.16818835	0.53143071	0.12776307	0.72452378	0.42377707
Scn5a	0.18922803	1.37007776	0.12752245	0.72477196	0.42389206
Clptm1l	-0.0723596	4.51045933	0.12733128	0.72496933	0.42397461
Ifngr2	-0.0657599	5.14388699	0.12728585	0.72501626	0.42397461
Usp12	0.04326782	5.81733712	0.12713608	0.72517103	0.42403495
Cep83	-0.0512966	5.98075362	0.12688357	0.72543222	0.42414631
Slc35f4	-0.0945236	2.69234322	0.12683881	0.72547855	0.42414631
Stt3a	0.05539698	5.16260031	0.12680235	0.7255163	0.42414631
Nkx6-2	0.2083351	0.35293073	0.12671018	0.72561174	0.42416768
Lrrc17	0.21853106	0.06156484	0.12662074	0.72570439	0.42416768
Lpin2	-0.0546778	6.58671134	0.12661756	0.72570768	0.42416768
Tsen54	-0.2489932	-0.256233	0.12644586	0.72588567	0.42422168
Ccnjl	-0.1973912	1.08242256	0.12642886	0.72590329	0.42422168
Kcna3	-0.1484818	1.38186323	0.12629451	0.72604266	0.42427296
Tagln	-0.1412385	3.30791348	0.12616995	0.72617195	0.42431834
Slc25a40	0.08510314	3.38294368	0.12597367	0.72637582	0.4243836
Sh2d2a	0.15590034	1.19444342	0.12592314	0.72642834	0.4243836
2310069G16	-0.170587	1.60202341	0.12591334	0.72643853	0.4243836
Cnp	-0.058078	6.61886777	0.12580367	0.72655254	0.42440873
Anks3	-0.0864561	2.94614511	0.12574425	0.72661435	0.42440873
Gnat2	-0.7683408	-1.6727266	0.12562192	0.72674163	0.42440873
Cnih1	0.07128105	5.24449197	0.12554408	0.72682267	0.42440873
Aplf	-0.0630964	4.27157919	0.12553453	0.72683261	0.42440873
Cpt1a	0.06099099	5.93109532	0.12551167	0.72685641	0.42440873
Ddx6	0.03652593	8.14171641	0.12550733	0.72686093	0.42440873
Pnck	-0.0618801	3.71850949	0.12547496	0.72689464	0.42440873
Phf3	-0.0421488	8.18853207	0.12541497	0.72695713	0.42441506
Prss16	0.42286331	-1.0446287	0.125354	0.72702066	0.42442201
Gramd2	-0.2907589	-0.4528712	0.12514763	0.7272358	0.42449045
Taco1	0.12858962	1.58711343	0.12514247	0.72724119	0.42449045
Ociad1	0.04095491	7.1434542	0.12503738	0.72735083	0.42450707
Zxdc	0.0520306	5.4891973	0.12501617	0.72737297	0.42450707
1810021B22	-0.2215255	0.54550243	0.12495205	0.72743989	0.42451599

Itfg3	0.11415873	1.93936477	0.12471421	0.72768832	0.42463081
1700025G04	-0.0476782	6.95265813	0.12442407	0.72799174	0.42477771
Ect2l	-0.3894422	-0.6733218	0.124206	0.72822005	0.42488077
4930447A16I	-0.4822621	-1.7228355	0.12403069	0.72840376	0.42495778
Pydc4	0.38902709	-1.4334036	0.12356812	0.72888919	0.42521081
Klhl12	0.0568781	4.79415184	0.12350237	0.72895827	0.42522092
Ppp2r2b	0.04479082	6.76285514	0.12319924	0.72927705	0.42533611
D16Ertd472e	0.06559561	6.32211038	0.12319783	0.72927853	0.42533611
Bap1	0.0610544	4.87571872	0.12316698	0.72931099	0.42533611
Dnajc14	0.07680549	4.30884159	0.12305481	0.72942908	0.4253748
2610034M16	0.23001373	0.1506247	0.1229985	0.72948838	0.4253792
Pkmyt1	0.22570687	-0.4137615	0.12290277	0.72958924	0.42540783
Syt14	-0.1632498	1.37972022	0.1228268	0.72966931	0.42540954
Tmed5	0.06593625	5.54968377	0.12280177	0.72969569	0.42540954
Cstf1	0.08538553	2.83750905	0.12268986	0.72981369	0.42544816
Bid	0.08788533	2.81864589	0.1225887	0.72992043	0.4254802
Ankrd33b	-0.0464571	6.30615254	0.12214624	0.73038781	0.42571809
Nlrp5-ps	0.23468203	0.46725446	0.12205206	0.73048743	0.42571809
Ndp	0.23185865	1.01292183	0.1220102	0.73053171	0.42571809
Pcdhgc5	-0.0805239	4.78780458	0.12200642	0.73053571	0.42571809
Mtmr9	-0.0676191	3.64425542	0.12189997	0.73064838	0.42572246
Zswim3	-0.1336859	2.11516617	0.12185838	0.73069241	0.42572246
Asap1	-0.0515045	7.9708348	0.12185253	0.7306986	0.42572246
Retn	-0.4423877	-1.5550005	0.12171751	0.73084161	0.42572378
Stim1	-0.0664202	4.00960311	0.12171708	0.73084207	0.42572378
Mphosph6	0.08546605	4.50521503	0.12170369	0.73085626	0.42572378
Ppp1r3f	0.06163255	4.33312591	0.12160473	0.73096113	0.42573796
Arl1	-0.0845682	3.68099753	0.12158298	0.7309842	0.42573796
2610100L16I	-0.1175752	2.15137763	0.12150218	0.73106987	0.42575768
Lmo1	-0.276052	-0.3943841	0.12134305	0.7312387	0.42582583
Pabpc4l	-0.1637114	2.23419676	0.12120228	0.73138816	0.4258827
Il2rg	0.26008147	0.43366703	0.1211074	0.73148896	0.42591121
Filip1	0.08135286	3.97005883	0.12072097	0.73189992	0.42605801
Dnajb13	0.38698791	-1.4063093	0.12067147	0.73195261	0.42605801
Herpud1	-0.0626137	4.41628755	0.12060731	0.73202092	0.42605801
Slc13a3	0.12275003	6.93531399	0.12056157	0.73206964	0.42605801
Azi2	0.05013103	6.72420063	0.12054721	0.73208494	0.42605801
Gpr83	-0.1145795	2.73427723	0.12054569	0.73208656	0.42605801
Acy1	0.16812028	0.78264564	0.12052938	0.73210394	0.42605801
Zscan20	-0.1019455	2.05490218	0.12023958	0.73241289	0.42620763
4930593A02I	0.38085251	-1.3030035	0.1199759	0.73269437	0.42634125
Phf20	0.04635693	6.49914567	0.11990583	0.73276924	0.42635462
Npy	-0.0879281	3.41690542	0.11982465	0.73285598	0.42637491
Vipas39	0.04771812	4.82685102	0.11968893	0.7330011	0.42642916
Sars	0.06078369	4.81691384	0.11960582	0.73309002	0.4264507

Tsnax	0.04102591	7.46016546	0.11932803	0.73338746	0.42659353
Kank3	-0.1367527	1.87037182	0.11914112	0.7335878	0.42667661
P2rx3	-0.1562302	1.27183356	0.11909794	0.73363411	0.42667661
Tsr1	-0.0587754	6.03519977	0.11899434	0.73374525	0.42671106
Cdyl	0.05562786	4.10914746	0.11864492	0.73412053	0.42689909
Trp53inp1	0.05753115	5.78981049	0.11839472	0.73438961	0.42700023
Fancd2os	0.24667094	0.21805888	0.1183866	0.73439836	0.42700023
Pkdrej	0.22504339	0.50668192	0.11820738	0.73459131	0.42704948
Syn1	-0.0788008	10.2351077	0.1180274	0.73478526	0.42704948
Gbp7	0.06485591	4.86337133	0.11801173	0.73480214	0.42704948
F13a1	-0.1628978	1.59449827	0.11801089	0.73480305	0.42704948
Vav3	-0.0772652	3.29633522	0.11799373	0.73482156	0.42704948
Raver2	0.07699044	3.14227939	0.11792651	0.73489404	0.42704948
Rag1	0.58849536	-1.9203626	0.11791721	0.73490408	0.42704948
Slc9a4	0.56632401	-1.2674155	0.11791365	0.73490791	0.42704948
Celf2	-0.0516249	9.78204798	0.117874	0.73495068	0.42704948
Arhgap22	0.24443573	0.14062593	0.1177279	0.73510835	0.4271109
Mylpf	0.27147032	-0.1585606	0.11761645	0.7352287	0.42713424
Plcd3	-0.0836305	3.08169908	0.11752822	0.73532402	0.42713424
1600002K03I	-0.21907	0.25287878	0.11750187	0.7353525	0.42713424
Sh2d7	0.16261273	1.00357488	0.11749827	0.7353564	0.42713424
Rad51ap2	-0.1911136	1.2965391	0.11733877	0.73552884	0.42720369
Kat5	0.05161092	4.68628449	0.11729154	0.73557992	0.42720369
Zfp91	-0.037391	7.46042978	0.11693555	0.73596537	0.42739444
AI118078	-0.254108	-0.438439	0.1168922	0.73601235	0.42739444
Mfsd9	-0.1859578	1.13707931	0.1167302	0.73618801	0.42742949
Ndrg3	-0.0343174	8.73840103	0.1166931	0.73622826	0.42742949
Scube2	0.17150196	1.10588406	0.11669266	0.73622874	0.42742949
Tesk1	0.06751472	4.95553184	0.11647247	0.73646776	0.42753329
Tada1	-0.0577795	5.26378133	0.11643215	0.73651155	0.42753329
Nrp1	0.06117673	4.973746	0.11631452	0.73663936	0.42757728
4732491K20I	-0.1260029	2.09539837	0.11610773	0.73686425	0.42767761
Slc36a2	-0.4610192	-1.7024372	0.11596019	0.73702482	0.4277406
Mocos	-0.1515549	1.42942326	0.11570705	0.7373006	0.42784712
Pcf11	-0.0457721	6.58616928	0.11569615	0.73731248	0.42784712
Atxn7l3b	-0.0421364	7.34105172	0.11562859	0.73738615	0.42785967
9630028B13I	-0.1535427	2.68441156	0.11540404	0.73763116	0.42797162
Trim59	-0.0907082	3.40018725	0.11526214	0.73778612	0.42802249
Ch25h	0.41389307	-1.5622507	0.11522838	0.737823	0.42802249
A930003A15	0.48084064	-1.9242753	0.11505416	0.73801345	0.42810276
Endov	-0.0602907	4.5500775	0.11447258	0.73865034	0.42844197
Gnptg	-0.0581597	4.63941865	0.11440995	0.73871904	0.42845158
Slc25a45	0.25753373	-0.4163997	0.11393452	0.7392412	0.42872417
Atmin	-0.0404392	6.26839051	0.11382372	0.73936306	0.42876459
Ier5l	-0.3223399	-1.2465365	0.11354796	0.73966665	0.42891038

Cd79a	0.1409976	2.13856601	0.11318804	0.74006351	0.42911024
06-Mar	-0.0451785	8.79093608	0.11299132	0.74028073	0.42920591
Fads2	-0.0923223	3.20624724	0.11292465	0.74035439	0.42921575
1810020O05	0.23189569	-0.8442492	0.11283336	0.74045529	0.42921575
Kiss1r	0.19224177	0.15055536	0.11280529	0.74048632	0.42921575
Stard5	-0.1048483	3.55374453	0.11278697	0.74050659	0.42921575
Ago4	-0.1139033	3.22251351	0.11262143	0.74068971	0.4292913
Tmem127	-0.0479794	6.90098997	0.11256687	0.74075011	0.4292913
Ppil4	-0.0467336	5.95392326	0.11252755	0.74079364	0.4292913
Zap70	-0.3833159	-1.6527262	0.11243075	0.74090084	0.42932316
Edn1	0.23925242	0.28649331	0.11222791	0.74112565	0.42940099
Dkk2	0.45683112	-0.8060264	0.11221529	0.74113965	0.42940099
Sympk	0.0761891	4.57832281	0.11211895	0.74124651	0.42942307
Chic2	0.07651476	3.94294952	0.11207495	0.74129533	0.42942307
Serpind1	0.10279579	4.92559936	0.11202267	0.74135335	0.42942307
Fmnl3	0.09773749	3.58003564	0.1119926	0.74138674	0.42942307
Gpr179	0.3570117	-0.4016833	0.11182939	0.74156801	0.42949779
Gopc	-0.0408524	6.07453978	0.11177993	0.74162297	0.42949936
Prdm1	0.14262759	2.00380264	0.11170975	0.74170098	0.42951427
Yod1	-0.0600913	4.82945894	0.11164144	0.74177693	0.42952799
Tmem79	-0.4325639	-0.2191697	0.11146312	0.74197534	0.42959361
Tnfsf13b	-0.387154	-1.2214902	0.11144565	0.74199479	0.42959361
Dusp12	-0.1077226	2.30696083	0.11121069	0.7422565	0.42971487
Dvl2	-0.0989133	2.37289552	0.11115496	0.74231862	0.42971564
Pcyox1	-0.0587472	5.62445195	0.11111569	0.74236241	0.42971564
Urgcp	0.06182591	4.39058297	0.11094602	0.74255168	0.42979493
9830166K06I	-0.1837297	0.88567121	0.11078738	0.7427288	0.42986718
Crip1	0.13524926	2.42763612	0.11046813	0.74308566	0.43004343
Lmf1	-0.0925075	2.93702703	0.11033942	0.74322969	0.43009651
Ldb1	0.05927641	3.97055761	0.11020701	0.74337797	0.43015203
Armc7	0.10739898	2.3542711	0.110109	0.74348779	0.43018529
Armc6	-0.1063828	1.69641095	0.1100002	0.74360976	0.43022557
Gphn	-0.0546076	5.91839685	0.1099045	0.7437171	0.4302334
Gmppb	0.17113169	0.94314568	0.1098948	0.74372798	0.4302334
Pafah1b1	-0.0389126	9.15130596	0.10980987	0.74382329	0.43025825
Cyp4v3	0.08626685	4.68104747	0.10957484	0.74408726	0.43038065
Foxp3	-0.2790816	-0.7855002	0.10940261	0.74428089	0.43046235
Serpina9	0.23051909	-0.6081491	0.10929289	0.74440434	0.43050175
Cry2	0.04846969	6.33826493	0.10924789	0.74445498	0.43050175
Fam49a	-0.0434283	8.23706648	0.10920246	0.74450613	0.43050175
Gp1bb	-0.1102936	1.60154465	0.10903398	0.7446959	0.43058119
Tshz1	0.03878612	6.00307208	0.10889513	0.74485242	0.43062098
Akr1c12	-0.3592972	-0.1706401	0.10887999	0.7448695	0.43062098
Cda	0.34790287	-0.9011938	0.10878084	0.74498135	0.43065534
Neil2	-0.2870825	0.05266386	0.10873425	0.74503392	0.43065544

Lhfpl1	0.29500652	-0.7826807	0.10856121	0.74522931	0.43073809
Dlc1	-0.0474051	6.60840981	0.10832994	0.7454907	0.43085888
Rfxap	0.08086297	4.03711884	0.10822658	0.74560764	0.43089616
Zfp62	-0.0515965	6.19282385	0.10815503	0.74568862	0.43091266
Tmem55a	0.04621173	6.17654235	0.10806657	0.74578877	0.43092688
Rcan2	0.03365534	7.45083841	0.10801632	0.74584569	0.43092688
Slc25a16	0.04847175	5.57022558	0.10799441	0.74587051	0.43092688
Ikzf5	0.04819911	5.07149542	0.10786057	0.74602219	0.43098365
Bhlha9	-0.4480896	-1.2344932	0.10781519	0.74607364	0.43098365
Gal	-0.309408	-0.4440306	0.10772511	0.74617581	0.43101237
D5Ertd605e	0.37796538	-1.4829157	0.10757572	0.74634536	0.43108001
Hpse	0.4298844	-1.4409293	0.10703147	0.74696415	0.4313776
Pms2	-0.0783887	3.62544284	0.10701484	0.74698309	0.4313776
Rnf25	-0.1120604	2.08902688	0.10698414	0.74701804	0.4313776
Cog5	-0.0708314	4.63452983	0.10692789	0.74708212	0.4313843
Kcnab1	-0.0489777	5.30295476	0.10678688	0.74724281	0.43144677
Ube2q2	0.0439766	6.36693787	0.10667881	0.74736605	0.43148762
Nfe2l1	0.04404196	7.82225763	0.10652556	0.74754092	0.4315444
Gm4285	0.12020359	1.33931549	0.1065006	0.74756942	0.4315444
Ltbp3	-0.0697651	4.64029279	0.10638667	0.74769954	0.4315892
Magi3	-0.0430424	6.70809646	0.10613068	0.74799217	0.4317278
Dars2	0.09228391	3.49596305	0.10588359	0.74827501	0.43186072
Scg2	0.05541788	6.44632555	0.10578823	0.74838426	0.43189345
Glce	-0.0482839	6.45973608	0.10555507	0.74865162	0.43201741
1700024P16l	-0.2300947	0.72847317	0.10545676	0.74876446	0.43205219
Cd55	0.07849373	5.98307271	0.10521599	0.74904103	0.4321777
02-Sep	0.04589743	7.89735262	0.10517587	0.74908714	0.4321777
Trim45	0.09511789	2.49172665	0.10512466	0.74914603	0.43218134
Txndc15	-0.0598853	4.33369455	0.10495496	0.74934127	0.43224087
Pex6	0.07857373	3.79924889	0.10494355	0.74935441	0.43224087
Trub2	0.08970543	3.0142733	0.1048596	0.74945107	0.43226629
Zc3h12c	0.03591726	6.25593924	0.10479625	0.74952403	0.43227804
4931440F15l	-0.2752458	-0.2478327	0.10454507	0.74981358	0.43241469
Ppp6r1	0.05492862	4.81027619	0.1044546	0.74991797	0.43244455
Cyhr1	0.05064814	5.5707049	0.10440471	0.74997555	0.43244741
Cog2	0.06252516	3.48085586	0.1043138	0.75008052	0.4324776
Rad17	0.06353659	4.17621479	0.10407659	0.75035466	0.43260531
Eppk1	0.29840953	-0.5770483	0.10402871	0.75041003	0.43260689
Fhit	-0.1963253	1.2402851	0.10393028	0.75052392	0.43261781
Lin9	-0.0901768	3.01299053	0.10392136	0.75053424	0.43261781
Tmem128	0.07613829	3.40362018	0.10377668	0.75070175	0.43268402
Gm5801	0.20183861	-0.3960546	0.10343766	0.75109478	0.43285459
Armc3	-0.2834778	-0.9834486	0.10341843	0.7511171	0.43285459
1810062O18	0.21149551	0.63893738	0.10338518	0.75115568	0.43285459
Col17a1	-0.4858853	-1.226709	0.10306405	0.75152874	0.43301344

1110006O24	-0.2737256	-0.6314302	0.10305719	0.75153671	0.43301344
Tspan3	0.05804492	7.0022232	0.10295666	0.75165363	0.43305044
2700094K13I	0.09198445	3.19657688	0.10287166	0.75175254	0.43305419
Foxq1	0.24864483	1.12475046	0.10286052	0.75176551	0.43305419
Tasp1	-0.0463529	5.96691952	0.10276416	0.75187769	0.43308846
Shisa9	-0.0610856	6.09755279	0.1025147	0.75216839	0.43317128
Efna2	-0.1844835	0.54814271	0.10247122	0.75221911	0.43317128
Hs3st3a1	0.08795762	4.59110995	0.10242074	0.752278	0.43317128
Zfp420	-0.0526137	4.40160952	0.10229943	0.75241958	0.43317128
Fgr	-0.1919609	0.32425747	0.10229802	0.75242123	0.43317128
Zfp160	-0.0579613	4.46987999	0.10229563	0.75242402	0.43317128
Prickle4	-0.3876783	-1.8831369	0.10228443	0.75243709	0.43317128
Mrps10	-0.0763338	2.80224304	0.10227928	0.7524431	0.43317128
Ece2	0.08254362	2.54732572	0.10222949	0.75250125	0.43317442
Lepr	0.0676233	5.63711491	0.10205332	0.7527071	0.43326257
Fitm2	-0.2004103	0.590713	0.10197743	0.75279584	0.4332833
Atp5g2	0.08308088	4.07447845	0.10163483	0.75319689	0.43342713
Mpp4	0.24931358	-1.0414371	0.10163354	0.7531984	0.43342713
Klf7	-0.0466111	6.5193186	0.10162882	0.75320393	0.43342713
Yipf1	0.07978091	3.00966955	0.10155617	0.75328908	0.43344578
Zfp516	-0.0501079	5.28253629	0.10130033	0.75358918	0.43353807
B930025P03I	-0.3438454	-1.1895272	0.10128533	0.7536068	0.43353807
Creg1	-0.0735994	4.80450992	0.10128454	0.75360772	0.43353807
Mmp13	0.41777278	-1.9234618	0.10117509	0.75373625	0.43355644
Zfat	0.09762053	2.02412204	0.10116752	0.75374514	0.43355644
Ccdc37	-0.175893	0.91688166	0.10080031	0.75417694	0.43375805
4930583P06I	0.38523769	-1.6649089	0.1007797	0.75420119	0.43375805
Actr8	0.0533164	4.68296503	0.1007308	0.75425876	0.43376081
Hcn4	0.11692231	1.30114906	0.10052578	0.75450032	0.43386855
Grcc10	0.11253582	3.80079278	0.10048221	0.75455168	0.43386855
Il17d	-0.1185237	0.95456877	0.1003867	0.75466433	0.43390296
Enkd1	0.14754778	0.55606555	0.10030695	0.75475843	0.43391945
Surf2	0.07913893	5.00252869	0.10027292	0.7547986	0.43391945
Wnt9b	0.44232339	-1.9055539	0.1001315	0.75496561	0.43398511
Trak1	-0.0377016	7.52589125	0.10008525	0.75502026	0.43398617
Mri1	-0.1283224	1.14525126	0.09999884	0.75512239	0.43401452
Rnf144a	-0.0558817	4.77117646	0.09985607	0.75529124	0.43402427
Abtb1	-0.108951	1.96667002	0.09981704	0.75533743	0.43402427
Pfkfb3	-0.0517619	4.54080087	0.09981608	0.75533857	0.43402427
Cd47	-0.0517119	7.49962198	0.09980593	0.75535058	0.43402427
Tnfaip8l2	-0.2909714	-0.6473809	0.09973717	0.75543198	0.43403547
Acpp	-0.1360395	4.28697878	0.09970026	0.75547568	0.43403547
Agt	-0.191947	1.29022148	0.09954936	0.75565445	0.43410783
H1fx	0.26131246	-1.4640314	0.09939833	0.75583353	0.43411184
Cnr1	-0.0481058	6.5698156	0.09938866	0.755845	0.43411184

Ckap2	0.22265733	0.36365987	0.09938553	0.75584871	0.43411184
Gpr161	-0.2113624	-0.3219541	0.09933746	0.75590575	0.43411184
Pex11g	-0.325972	-1.2928139	0.09929149	0.75596031	0.43411184
Pigv	-0.0909603	2.81035164	0.0992763	0.75597834	0.43411184
Pdia4	-0.0514922	4.46957625	0.09921135	0.75605544	0.43412578
Fam159b	-0.3925749	-2.0502647	0.09891878	0.75640313	0.43424236
Ccdc94	-0.1150072	1.59061397	0.09884668	0.7564889	0.43424236
Rasd1	0.19409815	0.3412436	0.09875142	0.75660228	0.43424236
Kdm2b	0.04647971	4.37736772	0.09862042	0.75675829	0.43424236
Olfr464	0.39716166	-1.9463999	0.09856526	0.75682402	0.43424236
Jakmip1	0.05343009	4.70511181	0.09853797	0.75685653	0.43424236
Eva1a	-0.1635818	1.13258846	0.0985301	0.75686591	0.43424236
Sertad4	0.08440711	5.75548275	0.09852123	0.75687649	0.43424236
Cdc25c	0.33651609	-1.6134283	0.09852013	0.7568778	0.43424236
Dnm1l	-0.0418446	8.30233516	0.09848886	0.75691507	0.43424236
Syvn1	-0.0622825	4.83708926	0.09848487	0.75691984	0.43424236
Gm16515	0.08068086	5.5106867	0.09847665	0.75692964	0.43424236
2310057M21	0.05527089	4.09603702	0.09846352	0.7569453	0.43424236
Nanos2	0.50379661	-2.2041108	0.09839333	0.757029	0.43426007
Rab40c	0.08316968	2.90796542	0.09820173	0.75725768	0.43433748
Rufy3	0.04611795	8.36759982	0.09819171	0.75726965	0.43433748
Cdc25b	0.0828534	2.97562223	0.09796009	0.75754643	0.43439825
Col20a1	-0.1546805	1.24662046	0.09794425	0.75756537	0.43439825
Slc39a1	0.08014108	5.19620193	0.09793285	0.75757901	0.43439825
Nkain2	-0.05962	6.31843164	0.09788607	0.75763495	0.43439825
Jak1	-0.0413553	7.64637337	0.09788197	0.75763986	0.43439825
Nthl1	0.18986818	0.3650133	0.09769203	0.75786721	0.43449829
Hist1h2ai	0.25899462	-1.5684036	0.09763321	0.75793767	0.43450838
BC030500	0.06898478	3.90057048	0.09746272	0.75814201	0.4345857
Rassf8	0.10409609	3.3170677	0.09743246	0.75817829	0.4345857
Amer2	0.06157006	4.18477025	0.09730677	0.75832909	0.43464183
Clasp1	-0.045647	7.49695129	0.09715786	0.75850788	0.43467427
Zfp597	-0.0443377	4.89162682	0.0971405	0.75852875	0.43467427
Mip	0.30333231	0.37341419	0.09704818	0.75863968	0.43467427
E130317F20I	0.15088899	1.16468983	0.09702992	0.75866163	0.43467427
Mllt3	0.04227977	7.72199041	0.096991	0.75870842	0.43467427
Pnmal2	0.04827166	6.34505682	0.09697056	0.75873299	0.43467427
Batf	0.21797987	0.02424304	0.09695151	0.7587559	0.43467427
Proser2	-0.1233031	2.69453176	0.09686203	0.75886354	0.43470564
Zbed6	-0.0335235	6.82936848	0.09681786	0.75891669	0.43470579
Ang	0.15639338	2.28939294	0.09650426	0.75929446	0.43488199
5830403L16I	0.27593945	-0.5332773	0.09647467	0.75933014	0.43488199
Tm6sf1	0.10242079	1.87347446	0.09640998	0.75940816	0.43489637
Tada2b	-0.0513862	4.39056583	0.09629083	0.75955193	0.43492002
Dnd1	0.30608641	-1.5052537	0.09628806	0.75955528	0.43492002

Lrfn3	0.06835548	3.13777682	0.09610045	0.75978188	0.43500274
1110034G24	-0.1326707	1.72551301	0.09608082	0.75980561	0.43500274
Traf5	0.13284362	1.45352908	0.09597921	0.75992845	0.43504277
St7	-0.0777287	3.42973863	0.09587192	0.76005824	0.43505296
Egr2	-0.1560226	3.93185383	0.09585352	0.7600805	0.43505296
Tmem59l	-0.0766599	4.17803681	0.09583324	0.76010505	0.43505296
Hps4	0.09347789	2.44925576	0.09561828	0.76036537	0.43517165
Slc30a3	0.06461699	4.31403031	0.09545587	0.76056228	0.43525404
Topors	-0.0393384	6.03630215	0.09524008	0.76082418	0.43535429
Gm765	-0.0875788	2.83983776	0.09522426	0.7608434	0.43535429
Htra3	0.11078034	4.06121175	0.09515955	0.760922	0.43536896
Zfp827	0.04289633	6.30042239	0.09498157	0.76113837	0.43546244
Zfp938	0.06061288	4.44416906	0.0949019	0.76123529	0.43548758
Ptgfrn	-0.0449146	5.24899347	0.09475714	0.76141151	0.43554876
Tmed9	0.03753405	6.26273586	0.094727	0.76144822	0.43554876
Sh3pxd2b	0.05122194	4.59955708	0.09448547	0.76174262	0.43568684
Lrrc3	-0.0735094	3.66983848	0.09427925	0.76199431	0.43575957
Eif5a2	-0.0479015	6.5726775	0.09424465	0.76203658	0.43575957
C130050O18	0.38150003	-2.0678571	0.09421686	0.76207052	0.43575957
Sepsecs	0.10392598	2.25079891	0.09420759	0.76208185	0.43575957
6030419C18I	0.10613686	2.38184951	0.09410944	0.76220179	0.43577785
Nrsn2	-0.0549031	5.88585076	0.09409466	0.76221987	0.43577785
Sec61a1	0.05919362	4.9649272	0.09402584	0.76230402	0.43579043
Syngr1	-0.0420803	6.81639169	0.09398996	0.76234791	0.43579043
Pcdhga8	-0.0862095	2.67989961	0.09385954	0.7625075	0.43585134
Afap1l2	0.08135994	2.61867956	0.09376035	0.76262897	0.4358731
Golt1b	-0.052384	5.46758268	0.09374186	0.76265162	0.4358731
Fnbp1l	-0.0476486	8.5932555	0.09366972	0.76274003	0.43589331
Hr	0.10306471	3.0286007	0.09343829	0.76302386	0.4360252
Lin54	0.05014906	4.48532347	0.09326621	0.76323515	0.43608734
Tecr	0.05916982	7.85813035	0.09324219	0.76326466	0.43608734
Trpa1	0.27875083	-0.9427308	0.09322013	0.76329177	0.43608734
Cdc14a	-0.0807283	4.04350205	0.09308129	0.76346245	0.43615453
Zfp668	0.06699734	3.70242168	0.09298841	0.76357671	0.43618933
Csnk1g3	0.03196894	7.45974778	0.09292099	0.76365968	0.43618933
Ndufa4l2	0.20542598	-0.0866325	0.09284228	0.76375661	0.43618933
Gng13	-0.1176781	1.38099842	0.09282725	0.76377511	0.43618933
Plk2	-0.0508879	7.04814951	0.09281621	0.76378872	0.43618933
Kcnmb4	0.05492846	3.94159509	0.09272565	0.76390029	0.43622274
Nmt2	0.03691949	6.98586461	0.09267043	0.76396836	0.43622771
Acly	0.04757078	7.0689687	0.09263248	0.76401515	0.43622771
Lax1	0.32618049	-0.2643545	0.09248447	0.76419773	0.43630165
Fgfr1	0.06753177	6.04436897	0.09232375	0.76439619	0.43633515
Lyn	-0.0865718	3.40651406	0.09230148	0.76442371	0.43633515
Frmd5	-0.0627335	5.14701112	0.09228916	0.76443892	0.43633515

Dus1l	0.08583352	3.06335656	0.09221888	0.76452579	0.43633515
Ercc8	-0.104665	2.35817298	0.09218989	0.76456163	0.43633515
Ctdspl2	-0.0390641	6.57527525	0.09217912	0.76457494	0.43633515
Krt80	0.09694321	3.54297797	0.09192692	0.76488701	0.43648294
Rem2	0.17457947	0.43792714	0.09183602	0.76499959	0.43649672
Postn	-0.084057	2.19193298	0.09182167	0.76501737	0.43649672
Apba2	-0.0549283	4.81600432	0.09170358	0.76516374	0.43654993
Rpia	0.07342163	2.92032853	0.09158129	0.76531543	0.43658518
Pspc1	-0.0546903	5.2308124	0.09156813	0.76533176	0.43658518
Tox3	-0.0694387	5.04677067	0.09148487	0.76543511	0.43659733
Cyld	-0.0391008	7.32138492	0.09146291	0.76546237	0.43659733
Dag1	0.05455839	6.67986052	0.09136944	0.76557848	0.43659733
Klhl32	0.11862133	0.85298259	0.09136156	0.76558826	0.43659733
Gm13375	0.10545411	2.74227121	0.09131915	0.76564096	0.43659733
Ttc8	-0.0430274	4.70920459	0.09127474	0.76569617	0.43659733
2310036O22	0.06156792	4.55739908	0.09125163	0.7657249	0.43659733
Zfp808	0.05236632	3.85726606	0.09087502	0.76619366	0.43680901
Chd7	-0.0712945	4.64904099	0.09084443	0.76623179	0.43680901
Gm16596	-0.179162	0.01562541	0.09082534	0.76625559	0.43680901
Pla2g6	0.08091096	2.71901809	0.09074858	0.76635128	0.43683271
Grtp1	0.08779642	3.25590908	0.09067372	0.76644466	0.43683271
Itgb5	0.08099728	4.05281799	0.09064337	0.76648253	0.43683271
Fam57b	-0.1067391	2.79398044	0.09056975	0.76657442	0.43683271
Gm15816	-0.2054313	0.7008866	0.09050401	0.7666565	0.43683271
Zfpm1	-0.0912767	1.76314135	0.0904584	0.76671347	0.43683271
Sh3gl1	0.07264607	3.29642853	0.0904547	0.7667181	0.43683271
Ackr1	-0.0656909	5.69606705	0.09045129	0.76672236	0.43683271
Obscn	0.1601082	0.39962601	0.09032391	0.76688155	0.43689312
Rplp1	-0.0620705	5.20971012	0.08967622	0.76769294	0.43732506
C1ql1	-0.1010935	2.81366461	0.08958582	0.76780644	0.4373594
Hist1h2ak	-0.2912488	-1.7770005	0.0894305	0.7680016	0.43744026
Tor3a	-0.1009947	3.71345958	0.08938219	0.76806235	0.43744454
Osgepl1	0.05714511	4.77018591	0.08921365	0.76827438	0.4375251
Rnf121	0.14299081	0.95704987	0.08916858	0.76833112	0.4375251
Sirpb1a	-0.3508327	-0.9216171	0.08914287	0.7683635	0.4375251
Nckap1l	-0.0728323	3.03439757	0.08879219	0.76880559	0.43774651
Acox1	0.03626517	6.99373077	0.08837781	0.76932921	0.43796638
Srf	0.05094497	4.85046671	0.08837538	0.76933228	0.43796638
Mei4	0.19168061	0.42421138	0.08835395	0.7693594	0.43796638
Zdhhc12	0.17623671	0.66138768	0.08831802	0.76940488	0.43796638
Mfsd1	0.06881829	5.17209041	0.08822162	0.76952693	0.43800552
5730420D15	-0.3797971	-0.8912027	0.08804595	0.76974954	0.43810188
Ntsr1	-0.1229475	1.74796493	0.08791145	0.76992012	0.43816863
Ccdc63	0.37340932	-2.0957611	0.08780978	0.77004918	0.4382057
Rspo4	0.34677372	-1.1341276	0.08777615	0.77009189	0.4382057

Toporsos	-0.1082903	1.98353634	0.08747339	0.77047675	0.43839435
Esrrg	0.06466054	6.04331139	0.08739973	0.7705705	0.43841733
Coq3	0.06365665	3.37844803	0.08731424	0.77067935	0.43844891
Sec24b	-0.0425674	7.02039347	0.08724318	0.77076987	0.43847006
Kdm5c	-0.056921	5.20889389	0.08702361	0.77104985	0.43859898
Trp53rk	0.14663743	2.02455285	0.0869614	0.77112923	0.43861378
Camk1d	0.03534946	8.32294529	0.08689931	0.77120851	0.43861486
Sh3d21	0.14838843	1.01929013	0.08682185	0.77130745	0.43861486
Eya3	-0.0488536	5.04768282	0.08681473	0.77131655	0.43861486
Ick	-0.0408812	6.31242919	0.08679278	0.77134459	0.43861486
Mir6390	-0.3230236	0.08973674	0.08674024	0.77141173	0.43862269
Lrrc10b	-0.0995184	3.93809863	0.08666476	0.77150824	0.43864722
Abca4	0.11370846	3.09333688	0.08659113	0.77160242	0.43867042
Arhgap20	-0.0384832	8.42146037	0.08642206	0.77181885	0.43876311
Gm101	-0.2960934	-1.1663423	0.08637956	0.77187328	0.43876371
6720468P15I	0.49998492	-0.4914859	0.0862647	0.77202049	0.43881704
Lad1	-0.4184032	-1.4804742	0.08581385	0.77259935	0.4391157
Gm13308	-0.2870415	-1.3918595	0.0857438	0.77268943	0.43911874
Gm6086	-0.2958679	-1.5354807	0.0857266	0.77271156	0.43911874
Man2b2	-0.083409	3.67084702	0.08560342	0.77287008	0.43917846
Kif19a	-0.2646204	-1.1562717	0.08546969	0.77304232	0.43921233
Id1	0.10280723	3.82219196	0.0854474	0.77307105	0.43921233
Gjc2	0.17779375	0.31923454	0.0854327	0.77308999	0.43921233
Rrn3	-0.0350874	5.81564163	0.08537614	0.7731629	0.43922339
Nr3c2	0.04426215	5.95236095	0.08529846	0.77326309	0.43924994
Jak2	-0.0356056	6.25237217	0.08510846	0.77350832	0.43935888
Rnf125	-0.1264577	1.24006064	0.08500488	0.77364215	0.43937938
Purg	-0.0498795	6.01491215	0.08499778	0.77365132	0.43937938
Rnase4	-0.0780935	5.15514776	0.08493232	0.77373595	0.43939708
Zfp644	0.04313904	7.40811311	0.08475527	0.773965	0.43949679
Asns	0.05701367	5.25667555	0.08464643	0.77410594	0.43954645
Ankrd42	-0.0500492	4.01704178	0.0845634	0.77421352	0.43957717
Mfsd6	-0.0528024	7.207852	0.08450926	0.7742837	0.43958665
Cerk	-0.0620272	4.07824315	0.08441368	0.77440766	0.43961369
Ift81	-0.0347592	5.57400414	0.08439006	0.77443831	0.43961369
Nr1i3	-0.2986235	-1.1087511	0.08425875	0.77460875	0.43968008
Dcaf8	-0.0348752	5.70611161	0.0841837	0.77470623	0.43970504
Ap1m1	-0.0697513	3.73255191	0.08413764	0.77476608	0.43970865
Kif24	-0.1504815	1.04121101	0.08406631	0.77485881	0.43972212
Vstm4	0.0841188	4.68068533	0.08399941	0.77494581	0.43972212
Ppm1g	0.04512553	4.91335133	0.08399595	0.77495032	0.43972212
Tvp23b	-0.0544609	4.76558333	0.08375741	0.77526085	0.43986795
Mb21d2	0.05097943	5.16656613	0.08353031	0.77555694	0.43996968
Loxl4	0.28444174	-1.1594294	0.08351744	0.77557374	0.43996968
Ptpn5	0.04469312	5.9042918	0.08349676	0.77560072	0.43996968

Polr2b	0.03793628	6.23667482	0.08345447	0.77565591	0.43997062
Exosc5	0.12612608	0.96836672	0.08326286	0.7759062	0.44005758
Lrrn4cl	0.0445307	4.95697795	0.08325512	0.77591632	0.44005758
Fam110a	0.13927134	1.23693417	0.08315849	0.77604267	0.44009887
Zfp345	0.23470449	-0.7265107	0.08299422	0.77625763	0.44016251
Bola3	0.07234662	3.33328221	0.08293736	0.77633209	0.44016251
Topbp1	0.0520394	5.30103597	0.08292493	0.77634838	0.44016251
1110020A21I	0.13382185	1.0658075	0.08290911	0.7763691	0.44016251
Fmr1	-0.0341133	6.28502288	0.08268459	0.77666344	0.44027386
Atn1	0.03697988	7.89650302	0.08267759	0.77667263	0.44027386
Sc5d	0.04913941	4.4301661	0.08254418	0.77684775	0.44034276
Lztr1	-0.0344133	5.7292613	0.08240812	0.7770265	0.44041371
Vmn2r57	0.18601924	0.70186386	0.08236554	0.77708248	0.44041507
Trim71	-0.3167855	-0.8253599	0.08225704	0.77722518	0.44046557
Clec2i	-0.3559602	-1.1682639	0.0821962	0.77730524	0.44048057
Ccdc105	-0.2954901	-0.7318402	0.08203934	0.77751181	0.44055894
Tbc1d22bos	-0.3719511	-1.0139922	0.08200979	0.77755075	0.44055894
Fcho2	0.04258235	6.99913835	0.08155136	0.7781558	0.44081631
Phactr1	0.03904609	7.94788212	0.08154908	0.77815882	0.44081631
Unkl	0.05240488	5.40387752	0.08154374	0.77816588	0.44081631
4930538K18I	-0.1908454	0.49385641	0.08141484	0.77833634	0.44087576
Gm5	0.30132885	-0.5194814	0.08138327	0.77837811	0.44087576
Bco2	-0.2019897	0.95810225	0.08118583	0.77863956	0.44099346
H2-DMb2	-0.3709891	-1.6585688	0.08111587	0.77873229	0.44101559
Map3k1	0.04562189	5.10802838	0.08098249	0.77890919	0.44108538
Cep57l1	-0.0727088	2.90026587	0.08068038	0.77931046	0.44128221
Was	-0.133451	0.99596923	0.08056158	0.77946848	0.44134128
Aldh16a1	0.21725893	0.16075163	0.08047659	0.7795816	0.44137492
Mppe1	0.13127358	0.69121084	0.08042813	0.77964613	0.44138105
Rad51ap1	0.24684028	-0.3231284	0.08038507	0.77970349	0.44138312
Tube1	-0.1695907	0.36225688	0.08013399	0.78003826	0.44154052
Tst	0.06608352	4.00671627	0.080096	0.78008897	0.44154052
Btrc	-0.0412928	6.32207989	0.08005533	0.78014327	0.44154084
Mat2b	0.03333346	7.44759251	0.0799016	0.78034863	0.44162288
Ogfod2	0.06796961	3.487368	0.0798664	0.78039568	0.44162288
Gnl1	-0.0368863	5.89680834	0.07982077	0.7804567	0.441627
Gm14420	0.04615934	5.40322491	0.07969218	0.78062874	0.44169395
Cpa2	0.19150903	-0.4612285	0.07960526	0.78074512	0.44169852
Golga4	-0.0316396	7.9148528	0.07959351	0.78076085	0.44169852
Ptgis	0.09363669	2.96369474	0.07956575	0.78079804	0.44169852
Alkbh8	0.05907875	4.91475563	0.07931958	0.78112809	0.44184287
Gdpgp1	-0.0541043	4.8161016	0.07929526	0.78116073	0.44184287
Cdk12	0.03511092	7.26615932	0.07922393	0.78125648	0.44184976
Hopx	-0.0867396	3.04404463	0.0792061	0.78128042	0.44184976
Rab30	0.04527148	4.8877884	0.07895452	0.78161857	0.44201058

Galnt10	-0.1111542	1.9081187	0.07852979	0.78219077	0.44230373
Htr7	-0.0679879	3.40319101	0.07842001	0.78233894	0.44232937
Slc30a6	-0.0633921	3.08987767	0.07841644	0.78234375	0.44232937
Zmym5	-0.0378218	6.59672674	0.0783106	0.78248672	0.44237548
Bod1l	-0.0385483	8.59520974	0.07823484	0.78258912	0.44237548
S100pbp	-0.0466319	5.02257202	0.07820385	0.78263102	0.44237548
Med28	0.05811755	5.31342009	0.07819676	0.7826406	0.44237548
Ppap2b	-0.0579578	6.43322826	0.07804654	0.78284385	0.44239806
Sirpb1b	-0.4411595	-1.704659	0.0780372	0.78285649	0.44239806
Ercc2	0.111239	1.24485289	0.0780178	0.78288275	0.44239806
Kif3b	-0.0423523	6.59547066	0.07800814	0.78289584	0.44239806
Bak1	0.0722826	3.90940784	0.07773376	0.78326771	0.4425559
1700003E16I	0.13816833	0.61505747	0.07770982	0.7833002	0.4425559
Cx3cl1	0.04339045	6.78151362	0.07764395	0.78338959	0.4425559
Kank4os	0.29696694	-1.3756295	0.07764325	0.78339054	0.4425559
BC027231	0.06110864	3.97245081	0.07739756	0.78372435	0.44271404
Dusp28	0.07617684	3.63802561	0.07693849	0.7843496	0.44303298
Psme2b	-0.0796898	3.17254541	0.07690392	0.78439676	0.44303298
Sigirr	-0.4284944	-1.8461738	0.07680735	0.78452859	0.44303303
Bfsp1	0.18095929	0.95059007	0.07679554	0.78454471	0.44303303
Hmces	-0.0810398	2.01871036	0.07675064	0.78460604	0.44303303
Fkbp2	0.0534504	4.37876841	0.07671136	0.7846597	0.44303303
Col27a1	-0.1098943	2.12821318	0.07670648	0.78466637	0.44303303
Lfng	-0.1912147	-0.3731663	0.07658072	0.78483829	0.44309077
Sema6b	0.08395986	2.90012105	0.07655281	0.78487645	0.44309077
Tmem204	0.06543625	3.94258334	0.07638407	0.78510742	0.44319072
Hacl1	0.08767979	2.44761928	0.07619267	0.78536971	0.44330833
Ceacam1	-0.0837584	2.96410889	0.07607543	0.78553057	0.44336574
Gp1ba	-0.2383655	0.3865583	0.07603992	0.7855793	0.44336574
Akt1	0.05088336	5.08357645	0.07594029	0.78571614	0.44341252
Foxj3	0.04202279	7.73374767	0.07578306	0.78593226	0.44349659
Ddx10	0.03330869	5.55496738	0.07575344	0.78597301	0.44349659
Zfp775	0.09479418	3.00237761	0.07568629	0.78606541	0.44351827
A730098P11I	0.03725371	6.70378136	0.07556574	0.7862314	0.44358148
Xxylt1	-0.0600243	3.52555456	0.07551157	0.78630603	0.44359313
Rbm27	0.03009821	7.46391826	0.07541379	0.78644082	0.44363872
Sidt2	-0.0380237	5.19264066	0.07529257	0.78660806	0.44367647
Nrarp	0.08831678	2.7117854	0.07528704	0.78661569	0.44367647
Il17re	-0.1573013	0.83772249	0.07517756	0.78676686	0.4437124
Pcdha3	0.28469545	-0.4062687	0.07513764	0.78682201	0.4437124
Slc4a5	0.23697479	0.04024916	0.07509446	0.78688168	0.4437124
Tspan32	0.30217973	-1.3717372	0.07508458	0.78689535	0.4437124
Mrpl47	-0.0469036	3.55239049	0.07498841	0.78702832	0.44375694
Srpk2	0.03220382	7.79659098	0.07482915	0.78724874	0.4438312
Rfesd	-0.0526824	4.17403698	0.07481523	0.78726802	0.4438312

Mdh1b	-0.2840509	-1.1226519	0.07455276	0.78763187	0.44400587
Adpgk	0.10131883	2.07154623	0.07416043	0.78817703	0.44428271
Flywch1	0.06087581	3.82844992	0.07402245	0.78836913	0.44433723
Timm21	-0.0627871	4.15049091	0.07400714	0.78839045	0.44433723
Cmip	-0.0402494	9.08758755	0.07397416	0.7884364	0.44433723
Casp2	-0.049723	3.86831317	0.07389306	0.78854945	0.44433723
Sla	-0.0651483	3.91966931	0.07386122	0.78859384	0.44433723
Trim13	0.08183076	2.67528309	0.07385792	0.78859845	0.44433723
Zpbp	0.21858085	0.39865645	0.07381603	0.78865688	0.44433723
Zfp366	-0.2062722	-0.0905949	0.07376061	0.78873421	0.44433723
Lmf2	-0.0746174	2.46143487	0.07374191	0.7887603	0.44433723
Fkbp5	0.08938765	5.97414013	0.07366772	0.78886388	0.44434353
Fabp3	-0.0584545	3.7126582	0.07365646	0.78887961	0.44434353
Socs4	0.04321225	5.019161	0.07352211	0.78906734	0.44439172
Arsg	0.0593391	3.08586952	0.07351785	0.78907329	0.44439172
Syt9	0.06351925	3.34875711	0.07335482	0.78930136	0.4444897
Morn5	-0.4727843	-1.4756517	0.0733013	0.78937629	0.44450144
Pnpla8	-0.0345039	6.88366748	0.07324093	0.78946084	0.4445186
Cst7	-0.440108	-1.9428477	0.07289939	0.78993991	0.44470046
Ccbe1	0.05911886	3.84561048	0.07288531	0.78995968	0.44470046
Dnah8	-0.1290148	1.34852445	0.07287549	0.78997348	0.44470046
Gm19522	0.08035751	2.40790063	0.07285643	0.79000025	0.44470046
Dlgap4	0.03364831	7.19902997	0.07275893	0.79013728	0.44474714
Eepd1	-0.0711055	2.48443429	0.07270246	0.79021668	0.44475402
Slc36a1	0.0445141	4.99323155	0.0726028	0.79035691	0.44475402
Zfp358	0.05877993	3.17915035	0.07259463	0.79036841	0.44475402
Synpo	0.03420807	6.50701661	0.07258209	0.79038607	0.44475402
Usp14	0.03644734	6.97105052	0.07255793	0.79042008	0.44475402
Nphp3	0.07998458	2.49082718	0.07248013	0.79052965	0.44476524
Lasp1	0.02969163	6.52538605	0.07246693	0.79054824	0.44476524
E430025E21I	0.03580511	6.34156917	0.07222358	0.79089142	0.44492786
Fam219b	-0.0637085	3.70312131	0.07201984	0.79117921	0.44505216
Ccser2	-0.0293276	8.27618302	0.07199051	0.79122068	0.44505216
Gorasp1	0.06570588	3.03344982	0.07181573	0.79146797	0.4451608
Plat	-0.075814	4.7598382	0.0717404	0.79157466	0.44519034
Vrk3	0.08983311	2.8399183	0.07161457	0.791753	0.44526017
Gm7444	-0.1929645	0.29348424	0.07157027	0.79181582	0.44526503
Cenpj	-0.0509976	4.00138366	0.0712934	0.79220894	0.44543098
Hipk1	-0.0303613	8.53399763	0.07125816	0.79225903	0.44543098
Lrp5	0.10627098	3.28031446	0.07124798	0.79227351	0.44543098
Mettl9	0.04282013	4.83069938	0.07087819	0.79280002	0.44569651
Fam221b	0.20547833	-0.509092	0.0706912	0.79306684	0.44581601
Plekhf1	0.09144341	3.09606806	0.07046503	0.79339004	0.4459672
Ccl28	0.11010082	1.68767328	0.07030414	0.79362031	0.44602048
F830016B08I	0.0458314	4.1638108	0.07022955	0.79372717	0.44602048

Chrm2	0.10206145	1.96358718	0.07020326	0.79376484	0.44602048
Mthfd2	0.11422747	1.46340259	0.07018041	0.79379759	0.44602048
Cxadr	0.05894344	6.56603292	0.07017176	0.79380999	0.44602048
4930503L19F	-0.0784302	3.03546724	0.07015197	0.79383836	0.44602048
Lair1	-0.0866493	3.29310948	0.07011452	0.79389206	0.44602048
Crhr2	0.36934556	-1.7099164	0.07009577	0.79391896	0.44602048
Fntb	0.05380355	4.03066677	0.06993278	0.79415288	0.4461214
Gm14325	0.04144201	4.34187906	0.06975724	0.79440515	0.44618825
Cep44	-0.0649086	2.75179763	0.06975647	0.79440625	0.44618825
Prps1l3	0.04034977	4.70122548	0.06972448	0.79445227	0.44618825
Cdca7l	0.15832113	0.60647303	0.06969893	0.79448902	0.44618825
C1ra	-0.127807	0.72250136	0.06959044	0.79464518	0.44624545
Fggy	0.07953198	2.85345833	0.06954033	0.79471734	0.44625549
Pgf	0.17478378	1.21134176	0.06945356	0.79484238	0.44629521
Gpatch2	0.04193387	4.12318121	0.06939109	0.79493244	0.44629689
Nup107	-0.056659	3.78393651	0.06935817	0.79497993	0.44629689
Alad	0.06862545	2.53834977	0.06933852	0.79500827	0.44629689
Letm2	0.06213593	3.25028569	0.06924319	0.79514587	0.44634364
Ccnd2	-0.0355652	7.42819143	0.06915285	0.79527634	0.44636954
Vps37d	-0.1211487	0.98645161	0.06907041	0.79539549	0.44636954
4930590J08F	-0.241487	-0.7086782	0.06906575	0.79540223	0.44636954
C1qtnf2	-0.148023	1.96562314	0.0690609	0.79540924	0.44636954
Fndc4	0.05971675	3.68251726	0.0690022	0.79549414	0.44638671
Mrgpre	0.0717971	3.56459171	0.06893141	0.79559656	0.4464137
Nudcd3	-0.029682	6.41496489	0.06885278	0.79571039	0.4464471
Bri3	0.05328961	4.3199298	0.06880909	0.79577367	0.44645212
Acss2os	0.29072497	-1.5760205	0.06872334	0.79589794	0.44649136
Lgi1	-0.0386075	7.46443041	0.06865993	0.79598989	0.44651247
Tox	0.05089697	5.74876714	0.06840216	0.79636412	0.44665501
Zfp772	-0.0542022	4.258458	0.06837952	0.79639703	0.44665501
Doc2b	0.06992935	4.21046912	0.06837264	0.79640703	0.44665501
Tfec	-0.5095806	-1.7338427	0.0683324	0.79646552	0.44665734
Cdca4	-0.0716785	2.88013428	0.06828583	0.79653326	0.44665784
Trappc13	0.03065767	6.39477302	0.06819098	0.79667128	0.44665784
4921511I17R	0.43297859	-2.4337037	0.06818125	0.79668545	0.44665784
Ttc38	-0.0514903	3.77982838	0.06816637	0.79670711	0.44665784
Rbm45	-0.0552267	3.49260112	0.06814506	0.79673814	0.44665784
Arhgap9	-0.1894552	0.95300811	0.06808955	0.79681899	0.4466727
Stom	0.11878396	5.20970168	0.06804685	0.79688122	0.44667712
Ptgs1	-0.0622229	3.44608602	0.06790838	0.79708311	0.44673907
D930020B18	-0.3202453	-2.0613081	0.0678965	0.79710045	0.44673907
Nell2	-0.0426262	6.79545258	0.06779607	0.79724704	0.44679076
Gnpnat1	0.04541076	4.78318396	0.06767023	0.79743089	0.44686333
Rrp1b	-0.0513557	3.45718733	0.06757657	0.79756785	0.44690961
Cyp19a1	0.16029332	-1.5822901	0.06763864	0.79794672	0.44709142

Tmem9b	0.03495206	5.44738389	0.06722713	0.7980797	0.44713545
Gm9866	0.07374381	2.57794585	0.06715291	0.79818861	0.44716599
Pgap2	0.05554622	4.20578138	0.06687433	0.79859793	0.4473648
Wdr36	0.04909933	3.83820002	0.06681676	0.79868264	0.44738176
Lemd2	0.0880745	2.11383672	0.06675488	0.79877372	0.44740229
Plek	0.04192467	4.63025579	0.06639744	0.79930076	0.44764395
Mob3b	0.04660806	6.71471211	0.06636163	0.79935365	0.44764395
Ccdc80	0.1035912	2.41847797	0.06633612	0.79939134	0.44764395
Rlim	-0.0297708	7.70261019	0.06631467	0.79942303	0.44764395
Chek1	-0.1275446	0.84374277	0.06589729	0.80004079	0.44792564
Gdpd1	0.0399345	5.43781772	0.06583125	0.80013873	0.44792564
Smpd1	0.05497445	4.97641497	0.06580881	0.80017201	0.44792564
Zfp112	-0.095896	2.14004169	0.06579447	0.80019329	0.44792564
Marcks	0.04252927	9.44935108	0.06579091	0.80019857	0.44792564
Mtmr1	-0.0378452	5.14613504	0.06560795	0.80047027	0.44804721
B130034C11	0.16507289	0.87810322	0.06537744	0.80081314	0.4482086
Yipf4	-0.0485973	4.77633016	0.06533405	0.80087776	0.44821425
Vps45	-0.0511304	3.79900705	0.06518181	0.80110465	0.44824801
AF529169	-0.1149317	1.49854143	0.06516866	0.80112426	0.44824801
1700124L16	0.29813759	-1.713115	0.06513994	0.80116709	0.44824801
Gm5468	-0.1346672	1.88273939	0.06511944	0.80119768	0.44824801
Lats1	0.03108611	7.15099515	0.06507828	0.8012591	0.44824801
Cdh18	-0.0711777	3.95230194	0.06507412	0.80126531	0.44824801
Rad18	0.06384595	4.2203029	0.06499655	0.80138113	0.44825273
Zfp553	0.06797978	3.70563064	0.06499541	0.80138283	0.44825273
Rnft2	0.05508537	4.54258712	0.06485726	0.80158928	0.44831106
Tbc1d15	0.0364012	5.20688553	0.06485264	0.80159619	0.44831106
Tbxas1	-0.2657482	-1.1977504	0.06480697	0.80166449	0.44831875
Lama4	-0.0478164	4.07808699	0.06460725	0.80196349	0.44845545
Evi5	-0.0292362	7.79948001	0.06439711	0.80227862	0.44860115
Adcy4	-0.1679263	0.47949154	0.06430895	0.802411	0.44864465
Sh3bp2	0.10026672	1.47945493	0.06423888	0.80251628	0.44866508
Prmt7	-0.0681412	3.30379527	0.06421198	0.80255671	0.44866508
Shcbp1	-0.2214289	-0.5415224	0.06414771	0.80265335	0.44868858
Nrros	-0.1345597	1.47144779	0.06409033	0.80273967	0.44870632
Serinc3	-0.0417337	8.71528027	0.06401628	0.80285114	0.4487381
Pcgf2	-0.051581	5.09123752	0.06392027	0.80299575	0.44877048
Fchsd2	-0.0304551	6.70699461	0.06390533	0.80301827	0.44877048
Kctd17	-0.0604297	4.42842515	0.06384044	0.8031161	0.44879464
3110082I17R	-0.0799809	2.68629787	0.06360966	0.80346442	0.44895876
Use1	0.07271643	4.73330947	0.06350748	0.80361886	0.44901453
Dgcr14	0.05195472	3.75036642	0.06326247	0.80398974	0.44916447
Dctn4	0.02976252	7.41932633	0.06325528	0.80400062	0.44916447
4933408B17	-0.5387933	-1.1572245	0.06322194	0.80405116	0.44916447
Aifm1	0.05466174	4.10845366	0.06317679	0.8041196	0.44917218

Rfc3	0.08629394	1.77971401	0.06303054	0.80434151	0.44921069
Pot1a	-0.0437564	5.1867932	0.06301747	0.80436135	0.44921069
Hcfc2	0.05234935	4.10084685	0.06301392	0.80436674	0.44921069
Mthfd2l	0.09610828	2.19025458	0.0629873	0.80440717	0.44921069
Ell2	0.0328854	6.02742888	0.06278157	0.80471989	0.44935479
Slc43a1	-0.1332625	0.58700747	0.06265169	0.8049176	0.44940806
Kirrel	-0.0656019	4.57305455	0.06264708	0.80492463	0.44940806
B930041F14	-0.0430739	5.11013948	0.06252644	0.80510847	0.44947438
Oxct1	-0.0301224	8.33302897	0.06249738	0.80515279	0.44947438
Zfp688	0.11611658	1.2095014	0.06216175	0.80566539	0.44972999
Car4	-0.0646993	3.1795533	0.06200255	0.80590906	0.44982574
Mpp3	0.05173429	3.41134994	0.06195688	0.80597901	0.44982574
Fam134c	-0.0781218	2.95746964	0.06190987	0.80605105	0.44982574
Adcy7	-0.1138534	1.81924374	0.06190675	0.80605583	0.44982574
Nts	-0.1302128	1.01467383	0.06183412	0.8061672	0.4498404
Pglyrp2	-0.4710829	-1.8629835	0.06181823	0.80619157	0.4498404
Rab15	-0.0297962	6.31468885	0.06176487	0.80627345	0.44985555
Fahd2a	0.06877662	2.7954796	0.06142556	0.80679496	0.45010075
Ubiad1	-0.0828553	1.89560882	0.06140769	0.80682245	0.45010075
Iffo2	0.04359884	4.67135398	0.0612744	0.80702777	0.45018473
Paqr5	-0.0819042	3.21834626	0.06104891	0.80737564	0.45034822
Ccdc153	0.09026526	1.80355475	0.06086599	0.80765833	0.45044455
2410004N09	-0.1109198	0.54396546	0.06084436	0.8076918	0.45044455
Arrb2	0.05306082	4.08125527	0.06083082	0.80771275	0.45044455
Cep290	-0.0374977	7.10751982	0.06076834	0.80780945	0.45046791
Chst9	0.20643765	-0.8220098	0.06059116	0.80808394	0.45059041
Prokr1	0.25905592	-0.1820214	0.06027741	0.80857106	0.45083144
Ing3	0.04551781	4.2107681	0.06022883	0.80864661	0.45084147
Rabep1	0.02907688	7.91370563	0.06018061	0.80872162	0.45084147
Zmat1	0.04067484	5.17673557	0.06016006	0.8087536	0.45084147
Sec14l5	0.24749317	-1.0991668	0.05998632	0.80902421	0.45093729
Tas1r1	-0.2322163	-0.3951764	0.05997925	0.80903523	0.45093729
Tm4sf1	0.05233758	4.66416052	0.05992668	0.80911719	0.4509524
Ccdc6	0.03170247	6.7624702	0.05967942	0.80950324	0.45106787
Dfna5	0.05264536	4.32154807	0.05965397	0.80954303	0.45106787
BC049352	-0.2650965	-1.7776477	0.05963621	0.8095708	0.45106787
Gemin4	-0.0673297	2.31786914	0.05961013	0.80961157	0.45106787
Pdpn	0.07558697	5.22471167	0.05959786	0.80963076	0.45106787
Mypop	0.06206813	2.50815487	0.05958322	0.80965366	0.45106787
Nme4	0.19536226	0.20736977	0.05951703	0.80975724	0.451095
Cbr2	0.10984146	1.3138646	0.05941461	0.80991763	0.45111289
Jsrp1	-0.219739	-0.75672	0.05939368	0.80995042	0.45111289
Meis3	-0.0613399	3.60716697	0.05939138	0.80995402	0.45111289
Cabp4	0.29768107	-0.8660315	0.05935106	0.81001722	0.45111752
Tle2	-0.0848905	1.5759767	0.05925643	0.81016563	0.45116107

Inhbe	-0.4452222	-1.9838091	0.05919677	0.81025924	0.45116107
Rpain	0.05797478	2.84744177	0.05919624	0.81026008	0.45116107
Arsb	-0.0331336	7.01737196	0.0591313	0.81036204	0.45117391
Slc2a10	0.11542399	0.93217711	0.05911163	0.81039294	0.45117391
Timd4	0.38913504	-2.1091428	0.05891943	0.81069514	0.45130716
Pex26	0.05195691	3.8248316	0.05887942	0.81075812	0.45130716
Mmp24	-0.0788051	2.48675089	0.05878374	0.8109088	0.45130716
Tigit	-0.2956381	-1.5637393	0.05876955	0.81093117	0.45130716
Mtus2	-0.0297088	6.24056567	0.05875969	0.81094671	0.45130716
Kif26a	0.07021098	2.41192325	0.05875015	0.81096173	0.45130716
Wdr73	0.03864135	4.41027299	0.05871168	0.81102237	0.45131035
Ncaph2	0.03898138	4.2766549	0.05831718	0.81164546	0.4516265
Mib2	0.05582132	3.61288825	0.05828053	0.81170346	0.4516282
Ccdc183	-0.1343009	0.60674389	0.0582208	0.81179803	0.45165024
Map2k3os	-0.206793	-0.8604769	0.0581727	0.81187423	0.45166206
Afap1	-0.0451133	7.61847143	0.05807234	0.81203331	0.45170778
4930453N24	-0.0358611	4.68066758	0.05804407	0.81207814	0.45170778
Tmem234	-0.0445265	4.73707194	0.05801688	0.81212128	0.45170778
Kif7	-0.1179446	1.49510603	0.05785778	0.81237389	0.45181771
Stx16	-0.0272494	6.02270895	0.05779271	0.81247731	0.45184465
Gm166	0.10936273	0.64345671	0.05764625	0.81271031	0.45192739
Gm10789	-0.2929433	-1.9141048	0.05763008	0.81273606	0.45192739
Mmaa	0.06183654	3.75448196	0.05747708	0.81297985	0.45201429
Kdelc1	-0.0599261	2.8426093	0.05746298	0.81300234	0.45201429
Fah	-0.0527584	3.18799279	0.05739078	0.81311751	0.45204775
Cdh2	-0.0462463	6.47293761	0.05733304	0.81320967	0.45206841
Slc16a3	0.20437903	-1.1228918	0.0572114	0.81340399	0.45213571
Rnf150	-0.0411023	7.33779721	0.05716928	0.81347133	0.45213571
Piga	-0.0717817	3.12116202	0.05712836	0.81353678	0.45213571
Pdp1	0.03890352	7.11894952	0.05709159	0.8135956	0.45213571
Reep3	-0.0475908	7.27510265	0.05708523	0.81360578	0.45213571
Cyp4b1	0.22980414	-1.0150473	0.05701527	0.81371778	0.45214876
Emr1	-0.1513635	0.09863425	0.05700183	0.81373929	0.45214876
Lcp1	-0.050958	4.76173276	0.0569011	0.8139007	0.45220787
Setdb2	0.06801654	2.9904605	0.05653827	0.81448332	0.45250099
Tgfbi	0.13503673	2.29086337	0.05634985	0.81478664	0.45263891
Dhx33	-0.0524917	4.90226004	0.05628542	0.81489049	0.452666
Ccdc9	-0.0544426	2.73401385	0.05623531	0.81497131	0.4526803
Zbtb21	0.03739851	5.05564308	0.05614317	0.81512	0.4527323
Patz1	-0.0397597	4.85691423	0.05604228	0.81528296	0.45279221
Slc22a23	0.03226672	6.19523421	0.05599634	0.81535722	0.45280285
6430573F11I	0.06299886	2.57922385	0.05595016	0.81543188	0.45280637
Rce1	-0.1553423	0.41775303	0.05592429	0.81547374	0.45280637
Gm4759	0.25908094	-0.9557459	0.05584223	0.81560655	0.45284952
Galnt4	0.08082257	2.3252378	0.05520542	0.81664069	0.45336184

Papd5	-0.0369375	6.11240003	0.055163	0.8167098	0.45336184
Stk32c	0.06560043	2.62635104	0.05514512	0.81673895	0.45336184
Bhlhe41	0.03904942	7.69616051	0.0551384	0.81674989	0.45336184
Itga2b	-0.1509135	-0.091508	0.05502499	0.81693486	0.45342573
F930015N05	-0.0530813	3.60806078	0.05500019	0.81697533	0.45342573
5430416N02	0.0793025	2.04785831	0.05485334	0.81721517	0.45346283
Ptpra	0.03085604	6.44475606	0.05483164	0.81725063	0.45346283
Ephx1	-0.0669569	4.19076574	0.05478502	0.81732688	0.45346283
Syne3	0.15811769	0.83033734	0.05476407	0.81736114	0.45346283
Ppp2r5c	0.03066662	7.0535404	0.05476401	0.81736123	0.45346283
Zer1	-0.0410744	4.95266359	0.05475669	0.81737321	0.45346283
Cd200r4	0.327688	-2.3478976	0.05466356	0.81752563	0.45351678
Lrig1	-0.0517985	3.267394	0.05462674	0.81758594	0.45351963
Secisbp2	0.04771418	3.83289147	0.05455885	0.81769717	0.45355072
Themis2	-0.1131855	1.61244666	0.05446872	0.81784497	0.45359955
Bcl2l2	0.02670674	6.25153777	0.05443786	0.8178956	0.45359955
Socs6	-0.0367706	4.27814036	0.05435602	0.81802996	0.45363693
Crot	-0.0338987	5.34776257	0.05431484	0.8180976	0.45363693
Pdia6	0.04258452	4.75854562	0.05429599	0.81812858	0.45363693
Gpr135	-0.2233449	-0.6005813	0.0542371	0.81822538	0.45363807
Uqcrc1	-0.0314762	5.02004495	0.05419745	0.81829058	0.45363807
Efcab11	0.20028744	-0.5475158	0.05419136	0.8183006	0.45363807
Zfp384	0.03304378	5.17019792	0.05416048	0.81835141	0.45363807
1700019L03F	-0.1678612	-0.6529311	0.05411055	0.81843358	0.45364521
Scgn	-0.3063769	-1.7738319	0.0540856	0.81847466	0.45364521
Mkln1os	-0.089675	1.23498685	0.05384001	0.81887954	0.45383901
4933407K13I	-0.1087412	1.84513764	0.05373838	0.81904738	0.45390142
Pdyn	0.05577204	3.32308681	0.0536767	0.81914932	0.45392731
Cd27	0.27006695	-1.5497778	0.05364231	0.8192062	0.45392822
9930104L06F	-0.0667499	3.10313452	0.05357923	0.81931055	0.45394071
Nccrp1	0.2809735	-1.1306751	0.05356192	0.81933919	0.45394071
2810021J22F	0.04486585	4.0634388	0.05337688	0.81964574	0.45407099
Prdm4	-0.045118	4.99589618	0.0533248	0.81973211	0.45407099
Gemin5	-0.0590264	3.93796006	0.05329753	0.81977736	0.45407099
Ppp2r2a	0.02666637	7.10196112	0.05328671	0.81979533	0.45407099
Clec10a	0.24689347	-1.1430793	0.0532045	0.81993181	0.45411598
Bscl2	0.0490217	4.33923054	0.05306416	0.82016508	0.4542071
Eml1	0.02758491	5.94191498	0.05303905	0.82020685	0.4542071
Taf1b	-0.0351872	4.16744644	0.05297845	0.82030771	0.45422122
0610038B21I	-0.256129	-1.8223318	0.05292481	0.82039703	0.45422122
Mrpl35	0.02993383	5.08137465	0.05292413	0.82039815	0.45422122
Hsd11b2	-0.3429324	-1.1414281	0.05288779	0.8204587	0.45422415
Gng8	-0.1558067	-0.4881482	0.05272648	0.82072769	0.45434247
Tap2	-0.133827	0.2777487	0.05256549	0.82099659	0.45445619
Mrgprf	-0.1325726	2.03239922	0.0525373	0.82104372	0.45445619

Sec16b	-0.217575	-0.2236277	0.05237963	0.82130757	0.45457162
Slc25a18	0.06516116	2.52180671	0.05221595	0.82158192	0.45469285
Polr2k	0.03993996	4.07922009	0.05213377	0.82171983	0.45473223
Tceanc	0.06168745	3.2960577	0.05210763	0.82176372	0.45473223
Atxn2	-0.0361	8.07817741	0.05204367	0.82187116	0.4547514
Cd86	-0.1575442	-0.1300237	0.05202114	0.82190903	0.4547514
Zfp446	-0.0538845	3.31004203	0.05193443	0.82205484	0.45480146
Gm3716	-0.1944434	-0.725553	0.05183803	0.82221709	0.45486061
Slc7a3	-0.0963446	1.30527275	0.05167452	0.82249267	0.45498244
Nol11	0.0415413	4.28240854	0.05158539	0.82264307	0.45500284
Gstz1	-0.0350191	4.65541876	0.0515384	0.82272242	0.45500284
Fam151b	-0.0863493	1.36776781	0.05152885	0.82273856	0.45500284
Zfp111	-0.0425863	5.16737656	0.0515215	0.82275098	0.45500284
Smarca5-ps	-0.0725195	1.23453082	0.05140785	0.82294308	0.45505718
Cd52	-0.0791739	2.35416589	0.05139786	0.82295998	0.45505718
1110065P20I	0.13572743	0.87558483	0.05132074	0.82309049	0.45508771
1700023L04F	0.21465273	-0.0254087	0.0512998	0.82312593	0.45508771
Dusp7	-0.0268012	6.05121561	0.0511988	0.82329704	0.45515169
Elavl2	0.03326557	6.93896308	0.05116555	0.82335341	0.45515224
Poglut1	-0.031156	5.22591774	0.05109262	0.82347711	0.45519001
A630007B06	-0.0316798	7.27132585	0.05088773	0.82382515	0.45532482
Fbxo30	-0.0468104	5.38115965	0.05088383	0.82383178	0.45532482
Wbscr27	0.0339504	4.58273635	0.05055219	0.8243967	0.45560641
Slc37a4	-0.0648249	1.97965061	0.05029449	0.82483704	0.45578967
Zfa-ps	-0.0897544	1.11562698	0.05026608	0.82488566	0.45578967
Mkln1	-0.0366175	6.15447147	0.05026081	0.82489467	0.45578967
4930500J02F	0.23222304	-0.6155177	0.04997948	0.8253769	0.45602546
Pnpla6	-0.0549277	4.04739104	0.0499018	0.82551031	0.45605861
Ndufa7	0.05528448	4.6453274	0.04985946	0.82558306	0.45605861
Gpr174	0.27074285	-1.3478259	0.04984765	0.82560337	0.45605861
Orc6	0.03834943	4.67622612	0.04971032	0.82583962	0.45614589
Mtif3	0.04921976	3.36360623	0.0496913	0.82587236	0.45614589
Clcn7	0.05485825	3.02906878	0.04954245	0.82612887	0.4562569
Atp6v0a2	-0.0368351	5.13639764	0.04950485	0.82619372	0.45626206
Cox10	-0.0366323	4.25664074	0.0493524	0.82645694	0.45636649
Akap13	-0.0284115	7.25756423	0.04933103	0.82649387	0.45636649
Pou3f3os	-0.0445361	3.71496871	0.04903358	0.82700884	0.45662017
Nrsn1	0.02818736	6.50508988	0.04894347	0.82716516	0.4566758
Nhlrc3	0.09701104	0.51559575	0.04887986	0.82727561	0.4567061
Zbtb14	0.03706855	5.14679904	0.04880108	0.82741248	0.45675098
Mpdu1	0.06297062	2.850041	0.0486627	0.82765322	0.45683779
Ago2	0.02655409	7.24356027	0.0486468	0.82768089	0.45683779
Cbln1	-0.0815845	1.84551883	0.04859253	0.82777542	0.45685928
Ibtk	-0.0258327	5.74726595	0.04855353	0.82784338	0.45686611
Src	-0.0548176	3.27587709	0.04846202	0.82800297	0.4569235

Smpdl3b	0.19118258	-0.2309417	0.04829626	0.82829243	0.45705255
Tfcp2	0.03657259	3.79045747	0.04815601	0.82853774	0.45715722
Atcay	0.03822665	5.5480024	0.04805383	0.8287167	0.45722527
Sf3a2	0.04465686	3.0888591	0.04790119	0.82898443	0.45734228
Ier2	-0.0894753	1.23049555	0.04786153	0.82905407	0.45735
Asb1	-0.0388647	4.51615356	0.04780644	0.82915084	0.45737269
Pigo	0.07957012	1.91965557	0.04768368	0.82936671	0.45743117
Slc2a6	-0.1111099	1.04842691	0.04768285	0.82936816	0.45743117
Zfp119b	-0.1054114	0.7155587	0.04753308	0.82963191	0.4575219
Dpp6	0.04234771	6.35339394	0.04752623	0.82964399	0.4575219
Tmem30b	-0.1132173	2.72185465	0.04748776	0.82971181	0.45752859
Tmem33	-0.0256455	6.24306401	0.04737337	0.82991365	0.45760257
Slc16a5	0.15709882	-0.6751443	0.04734865	0.82995732	0.45760257
3632454L22F	0.10865197	0.7864583	0.0471588	0.83029297	0.45775693
Zfp275	-0.0343298	5.65090821	0.04710179	0.83039389	0.45775849
Anapc2	0.02907006	4.74039092	0.04709428	0.83040719	0.45775849
Itga1	0.03864057	4.19163823	0.04705529	0.83047626	0.45776586
Gm12992	-0.0835325	1.37021767	0.04690882	0.83073601	0.45785662
Dhrs13	0.15520937	-0.3668726	0.04689962	0.83075233	0.45785662
Qrsl1	0.05570417	2.65418748	0.04683074	0.83087464	0.45786694
Gm6938	-0.1988884	-0.7268445	0.04682633	0.83088247	0.45786694
Rab3b	-0.0297718	5.12020472	0.04647697	0.8315043	0.45817889
Gm14379	-0.1814631	-0.7781729	0.04632673	0.83177248	0.45824954
Ppil2	-0.0351868	4.47776369	0.04632484	0.83177585	0.45824954
Plekha6	0.02875925	6.85142814	0.04631145	0.83179978	0.45824954
Baalc	-0.0254951	7.07479323	0.04623506	0.83193632	0.45829405
Tgds	-0.0566844	2.74649204	0.04617781	0.83203874	0.45831975
Pcdhga1	0.08085692	1.75969594	0.04606511	0.83224053	0.45840018
Ppp1r3d	-0.0979696	1.13591682	0.04602898	0.83230529	0.45840513
Taf3	0.02893545	6.44110544	0.04594228	0.83246078	0.45846004
Rgs8	-0.0394258	6.68073307	0.0458744	0.83258262	0.45849642
Bicd2	0.02939046	6.4485102	0.04570153	0.83289334	0.4586368
Prss35	-0.109554	0.77546363	0.045652	0.83298249	0.45865516
Lrrc14b	-0.0618625	2.80334172	0.04557906	0.83311384	0.45869676
Lypd1	0.04458612	4.83227986	0.04549833	0.83325937	0.45874616
4930579K19I	-0.2914667	-1.3326872	0.04536867	0.83349337	0.45884425
Apbb2	0.02556517	7.4120643	0.04524496	0.83371697	0.458888
Dusp19	0.04061328	4.78410897	0.04523143	0.83374144	0.458888
I830077J02R	-0.1011141	0.98222716	0.04518153	0.83383173	0.458888
Zfp449	-0.0451473	5.20703437	0.04515095	0.8338871	0.458888
Kpna3	0.02883856	7.16999286	0.04514756	0.83389323	0.458888
Zfp78	0.0611215	2.73225711	0.0451395	0.83390783	0.458888
Gpr151	-0.2950615	-1.6206207	0.04495871	0.83423556	0.45903761
D630032N06	-0.2574131	-0.9869433	0.04478415	0.83455267	0.45918135
Tssk2	-0.3693885	-1.7611136	0.04471544	0.83467767	0.45919823

Tgfb2	0.03938267	4.28430239	0.04470587	0.83469508	0.45919823
Matn2	-0.0603516	3.70694898	0.04454687	0.83498474	0.45932684
Tmem51	0.07914283	2.21709795	0.04435835	0.83532889	0.45942325
4930447N08	0.18305448	-0.703037	0.04435583	0.8353335	0.45942325
Map2k5	-0.0476091	3.60652918	0.04435287	0.83533891	0.45942325
Lrif1	0.03920275	5.127569	0.04432843	0.83538358	0.45942325
Fam133b	0.02933526	5.13952641	0.04419376	0.83562999	0.45950497
Ppp3ca	0.03049158	9.63284311	0.0441365	0.83573486	0.45950497
Olfml1	-0.0473622	4.58663499	0.04407676	0.83584436	0.45950497
Dennd2a	-0.0405465	4.23267622	0.04407585	0.83584604	0.45950497
Xrra1	-0.2123219	-1.5196995	0.04404123	0.83590952	0.45950497
Mir32	-0.4040898	-1.9347568	0.044191	0.83601077	0.45950497
Mamstr	-0.1194867	0.20664591	0.04398252	0.83601726	0.45950497
Gfy	0.39008204	-1.9307506	0.04396422	0.83605085	0.45950497
Zwilch	-0.096229	1.4763998	0.04394601	0.83608431	0.45950497
Cacna1f	-0.2196304	-0.5969001	0.04394222	0.83609126	0.45950497
Pknox2	-0.0289662	7.21215062	0.04386539	0.83623242	0.45955183
Npy5r	0.11515006	0.98837721	0.0438325	0.83629289	0.45955433
Pdlim3	0.20667549	-0.6166203	0.04377782	0.83639348	0.45957888
Gpr153	-0.0584944	3.00274264	0.04359577	0.83672887	0.45973243
Sms	-0.0248666	7.09061847	0.04353428	0.8368423	0.45976402
Phykpl	0.05151367	3.26191407	0.04347652	0.83694894	0.45976991
Bola1	0.07608232	1.69514703	0.04343812	0.83701986	0.45976991
Macc1	-0.2975983	-2.0982567	0.04343759	0.83702085	0.45976991
2610305D13	0.12374196	0.60259501	0.04326657	0.83733717	0.45991294
Gpr123	-0.0384986	6.43854164	0.04313075	0.83758884	0.45999114
Tnpo2	0.02582753	5.98827346	0.04311289	0.83762198	0.45999114
1700003M07	0.03834591	3.86364677	0.04309915	0.83764746	0.45999114
1810032O08	-0.1544191	-0.4284775	0.0430461	0.83774591	0.46001447
Dhx32	-0.0443276	4.10411335	0.04294207	0.83793917	0.46006568
Slc25a19	0.04626882	3.33440775	0.04293564	0.83795112	0.46006568
Etv5	-0.0362954	6.16652977	0.04283788	0.83813295	0.46013478
Glt1d1	-0.0850606	1.04808471	0.04276681	0.8382653	0.46017669
Csrnp1	0.08547503	1.76997254	0.04264257	0.83849689	0.46027309
Timm50	0.08063829	1.68706968	0.04248568	0.83878987	0.46040316
Cys1	0.04757361	5.15008941	0.04235397	0.83903627	0.46048065
Stag2	-0.0252532	6.69822651	0.04235033	0.83904308	0.46048065
Mum1l1	0.11196655	1.70224745	0.04225982	0.83921265	0.46054272
Prr16	-0.0400489	4.14023859	0.04223015	0.83926826	0.46054272
Angptl7	0.11178922	1.56096418	0.04217894	0.83936432	0.46056469
Creld2	-0.0582464	3.22286523	0.04214644	0.83942532	0.46056741
Cpa6	-0.2399686	-1.1804844	0.04208669	0.8395375	0.46059821
Pi4k2b	0.07129758	2.02012472	0.04199558	0.83970875	0.46066142
2610316D01	-0.0750752	2.20637587	0.04188819	0.83991085	0.46074153
Otud7b	0.02420402	7.25464734	0.04185029	0.83998223	0.46074994

Adamtsl5	0.04871412	2.76696844	0.04176747	0.84013833	0.46080481
Fam174a	0.03678637	5.07082992	0.04173724	0.84019537	0.46080534
4930429F24I	-0.1130096	0.47399518	0.04158636	0.84048029	0.46088216
Prpf38a	0.0376768	4.10992073	0.04155524	0.84053912	0.46088216
Prr5l	-0.0546763	2.40754669	0.0415544	0.84054072	0.46088216
Pqlc2	-0.1037739	0.36876817	0.04150823	0.84062804	0.46088216
Zfp191	0.03758321	5.01105649	0.04149715	0.84064901	0.46088216
Rad52	-0.0408243	3.57807302	0.04148506	0.84067188	0.46088216
Parn	-0.0321492	4.67553841	0.04145267	0.84073319	0.46088372
Zc3hav1l	0.03756142	6.00486285	0.04142433	0.84078688	0.46088372
Gpt2	-0.0305344	4.97022842	0.04130449	0.84101402	0.46097749
Gm6623	0.05828558	1.5751644	0.04124375	0.84112929	0.46100993
Slc13a4	-0.0507642	8.44182862	0.04114229	0.84132202	0.46108481
Cenpw	0.08261196	1.57348839	0.04103288	0.84153014	0.4611374
Rc3h1	-0.0227636	7.36999408	0.04103285	0.84153018	0.4611374
Fbxw5	0.03699967	5.41476069	0.04100113	0.84159058	0.46113975
Rprd1a	-0.0279852	6.72617376	0.04089533	0.84179218	0.46121947
Tead1	0.03180698	7.27776457	0.04082111	0.84193378	0.4612663
Tmx1	0.04007212	4.8957351	0.04070076	0.84216365	0.46136148
Fam81a	0.03050179	5.71032253	0.04062274	0.84231287	0.46141248
Cstf2	0.03597002	6.37257671	0.04023558	0.84305552	0.46177108
Rtn4ip1	0.05743353	3.29227625	0.04022293	0.84307986	0.46177108
Ccdc173	0.05665678	3.05128335	0.04012872	0.84326118	0.46183961
Zswim7	-0.0574149	2.12660425	0.04007473	0.84336518	0.46186579
Cachd1	-0.0456255	3.39907885	0.04001333	0.84348355	0.46187632
Arl6ip1	-0.0222079	7.00630343	0.03996107	0.84358437	0.46187632
Pfdn4	0.05035909	3.92869864	0.03995933	0.84358773	0.46187632
Pds5a	0.0270388	6.86280437	0.03994821	0.8436092	0.46187632
1700029I15R	-0.1629719	-0.841606	0.03974423	0.84400344	0.46201033
Gm16938	-0.0677094	2.17302732	0.03973359	0.84402404	0.46201033
Smo	-0.0588887	6.95353589	0.03971159	0.84406662	0.46201033
Gtf2i	0.02256327	7.28734558	0.03964973	0.84418645	0.46201033
Cxcl10	0.0899581	2.81711053	0.03964453	0.84419653	0.46201033
Pdgfb	-0.0661956	2.23334722	0.03964171	0.84420199	0.46201033
Nacc1	-0.0233178	6.99277199	0.03961826	0.84424743	0.46201033
Rock2	0.02954654	10.0961384	0.03949376	0.844489	0.4620847
C1ql2	-0.231336	-1.1618038	0.03944737	0.84457909	0.4620847
Il6ra	-0.0652615	2.57813041	0.03943481	0.8446035	0.4620847
Mybl2	-0.1641302	-1.316841	0.03943239	0.84460821	0.4620847
Dync1li2	0.02088464	8.01244482	0.03931697	0.84483267	0.46217632
Serpinb1b	0.12990407	-0.1128303	0.03928848	0.84488814	0.46217632
Cygb	0.04137963	4.10908569	0.03912662	0.84520363	0.46231812
Cox11	0.03354078	3.91883494	0.03904678	0.8453595	0.46237261
Mcm7	-0.0537489	2.98666999	0.03900545	0.84544025	0.46238141
Clrn1	0.22570556	-1.0221017	0.03898097	0.84548811	0.46238141

Mettl7a2	-0.0619659	1.73110516	0.0387875	0.84586682	0.46255775
Scrg1	0.10253765	1.44732958	0.03856866	0.8462964	0.46273937
Tspan8	-0.0684928	4.35901052	0.03855713	0.84631908	0.46273937
Sdccag8	0.02699759	6.24734401	0.03852409	0.84638405	0.46273937
Pygm	0.0621273	3.78732599	0.03850371	0.84642414	0.46273937
Rasa4	0.16828969	-0.4240529	0.03845737	0.84651536	0.46275845
Alg5	-0.0650599	3.24676587	0.03838044	0.8466669	0.46280355
Atad3aos	-0.134118	0.04760216	0.03833598	0.84675455	0.46280355
Dydc2	-0.1838034	-0.8900281	0.03831948	0.84678708	0.46280355
Ctbp2	0.03532993	4.21634869	0.03826092	0.84690265	0.46280355
Cxcr4	-0.2073027	-1.3127137	0.03824735	0.84692944	0.46280355
Gm13251	0.06311764	2.15003669	0.03824313	0.84693777	0.46280355
Gsto2	0.18700801	-0.5104815	0.03819545	0.84703195	0.46280355
Gldn	-0.1129243	0.93540306	0.03818031	0.84706186	0.46280355
Etnppl	-0.0661943	2.95248482	0.03815867	0.84710463	0.46280355
Tlr8	0.25092822	-1.0531026	0.03803886	0.84734167	0.46290228
Proz	0.0824815	1.46770786	0.03796507	0.84748785	0.46295137
BC024978	-0.0339592	4.79122258	0.03785021	0.84771569	0.46304505
Atp6v1a	0.02874342	9.34063743	0.03780746	0.84780058	0.46306065
Paxip1	-0.0231004	6.18251122	0.03756147	0.84829003	0.46329719
Aldh1a3	-0.1448515	-0.040302	0.03744546	0.84852145	0.46339279
Itgb4	0.07967815	3.19530324	0.0374049	0.84860243	0.46340623
Il4ra	-0.1206554	0.15441314	0.03732096	0.8487702	0.46346705
Slc22a3	-0.0892931	0.82570762	0.03727969	0.84885276	0.46348032
Mettl5	0.04207514	3.45748939	0.03725244	0.84890729	0.46348032
Gm7120	0.06916694	2.94204316	0.03716078	0.84909088	0.46354977
4930444P10I	-0.217416	-1.494696	0.03702777	0.84935771	0.46359414
Sash3	0.09862223	1.90364415	0.03700614	0.84940116	0.46359414
Timm17b	0.08589638	2.74288703	0.03699625	0.84942101	0.46359414
2810408A11I	-0.1717484	-0.898105	0.03698737	0.84943886	0.46359414
Ints9	0.03003235	4.3612064	0.03697974	0.84945418	0.46359414
Rbks	0.10774448	1.44680274	0.03689865	0.84961721	0.46365233
Egf	-0.2334592	-1.2277752	0.03683329	0.84974875	0.46366079
Galnt1	-0.031324	6.23301899	0.036821	0.8497735	0.46366079
Tmem255b	-0.2183875	-0.9751836	0.03679059	0.84983476	0.46366079
Tmbim1	0.0404308	5.07240249	0.03677887	0.84985837	0.46366079
Chst14	-0.0778593	1.60237469	0.03668954	0.85003849	0.46371072
Rnf182	0.06352739	3.01037818	0.03667753	0.85006272	0.46371072
Trpc4ap	0.02641941	5.32545809	0.03664594	0.85012649	0.46371473
Smim1	0.10632021	1.65134295	0.0365361	0.8503484	0.46378813
Ccdc77	-0.0451746	3.53799165	0.03650965	0.85040189	0.46378813
Mfsd7a	-0.2295707	-0.7476039	0.03649559	0.85043034	0.46378813
Synrg	0.02300723	6.70088302	0.03644216	0.85053847	0.46380157
Nkap	-0.0353312	5.26887566	0.03641799	0.85058741	0.46380157
Prpsap2	0.03350208	4.20561193	0.03639979	0.85062429	0.46380157

Wdr26	0.02123071	8.56156612	0.03626173	0.85090426	0.46387334
Rps27	0.10831196	-0.165867	0.03624925	0.85092959	0.46387334
Enthd2	0.07856656	1.43722193	0.03621451	0.85100014	0.46387334
Usp2	-0.0313324	5.72951967	0.03620235	0.85102484	0.46387334
Tnfrsf11b	-0.0753048	3.89176417	0.03619582	0.8510381	0.46387334
Cep41	0.0651942	2.89860501	0.03614901	0.85113325	0.46389444
Dynlt1a	0.03009408	4.34694742	0.03611211	0.85120829	0.46390458
Dus4l	0.05524611	2.58436004	0.03602072	0.85139433	0.46394586
Gmppa	0.03156716	3.73672678	0.03601943	0.85139695	0.46394586
Slc35d3	0.05620938	1.68033819	0.0358333	0.85177659	0.46412197
Aldoc	0.02984692	7.18074671	0.03579581	0.85185319	0.46413294
Ttf2	-0.0860914	1.79361035	0.0357625	0.85192128	0.46413598
Plekhj1	0.06700889	2.19298864	0.03573784	0.8519717	0.46413598
Sirt6	-0.0528411	2.94757241	0.03568551	0.85207879	0.46416355
Cep68	-0.0348841	5.0034212	0.03552722	0.85240314	0.46430946
Gpc2	-0.157435	0.06735224	0.03524852	0.85297608	0.46459076
Vtn	-0.0533465	6.16509271	0.03518871	0.85309933	0.4646271
Epm2a	-0.0796387	1.61414237	0.03512642	0.85322783	0.46466629
Rcl1	-0.0654151	2.11762534	0.03507641	0.85333107	0.46469172
Myh13	-0.2237713	-1.4079411	0.03499269	0.85350407	0.46475514
Mfap2	0.11218352	-0.0144527	0.03494924	0.85359395	0.46477329
Rspo3	0.05501819	7.1031934	0.03492166	0.85365102	0.46477358
Slc38a2	0.04997881	9.37180083	0.03475618	0.85399397	0.4649295
Gnl2	0.02459423	5.97776156	0.0345848	0.85435004	0.46508355
Rasgef1a	-0.028919	6.81493714	0.03456554	0.85439012	0.46508355
2410016O06	0.04609962	3.82287711	0.03450157	0.85452331	0.46512525
Pus7	-0.0379617	3.84631136	0.03444792	0.8546351	0.46512874
Thsd4	0.05357364	6.29216156	0.03444418	0.8546429	0.46512874
Fxyd6	-0.0347454	5.08938502	0.03431826	0.85490565	0.46521808
Ms4a1	-0.2288328	-1.7458976	0.03430592	0.85493143	0.46521808
Ccdc137	0.04724276	4.23153916	0.03426932	0.85500792	0.46521808
Ankrd29	0.04095629	4.6037215	0.0342571	0.85503346	0.46521808
Stat5a	-0.092784	1.7590182	0.03418009	0.85519456	0.46522614
Nfatc2	0.0442855	3.35874337	0.03417945	0.85519589	0.46522614
Avpr1a	-0.182167	-0.6701335	0.03416884	0.8552181	0.46522614
Cct8	-0.0227752	6.86970819	0.0341307	0.85529797	0.46522853
Tspan6	0.0506103	3.9849307	0.03408096	0.85540219	0.46522853
Zfp322a	-0.0251509	6.03840484	0.03407901	0.85540628	0.46522853
Clk3	0.03065622	4.70824977	0.03405868	0.85544891	0.46522853
2700049A03	-0.0353811	3.7645438	0.03399217	0.85558844	0.46527363
Tmem261	0.03671745	3.81384098	0.03392555	0.85572835	0.46531893
Chrm5	0.08272416	1.61380987	0.03385254	0.85588185	0.46535409
Rbm4b	-0.0335414	4.34630072	0.03381289	0.85596528	0.46535409
Agmo	0.08969624	1.67511286	0.03376921	0.85605724	0.46535409
Dusp1	-0.0503772	6.28480359	0.033762	0.85607244	0.46535409

Tor1aip1	-0.0288102	7.34527973	0.03375163	0.85609428	0.46535409
C1rl	0.15817458	0.26255936	0.03369845	0.85620636	0.46535409
Arhgef26	-0.0379093	4.33599906	0.03369528	0.85621305	0.46535409
Stk32b	-0.0548317	2.80468229	0.03365622	0.85629543	0.46535409
Uhrf1	0.12496194	0.39544064	0.03365284	0.85630258	0.46535409
Scaf1	-0.0324016	4.68206565	0.03359772	0.85641893	0.46538655
Meaf6	0.0289386	5.01797315	0.03354384	0.85653275	0.46539297
Qpct	-0.0762993	1.21858287	0.03353852	0.856544	0.46539297
Mgat1	0.03673383	3.42055107	0.03344114	0.85674997	0.46541304
Safb2	0.0291081	5.15612092	0.03339413	0.85684953	0.46541304
Kctd18	0.02969659	4.36837927	0.03338145	0.85687641	0.46541304
Zcchc3	0.03823376	5.00157565	0.03337033	0.85689997	0.46541304
Ptpn21	-0.0343589	4.8586225	0.0333562	0.85692991	0.46541304
Hdac2	0.02152418	7.14680496	0.03334235	0.85695926	0.46541304
Gm1673	0.09371793	0.4194867	0.03332426	0.85699763	0.46541304
Gls2	0.05911914	2.43157506	0.03330714	0.85703394	0.46541304
Fam76b	-0.0408216	4.65486334	0.03326098	0.85713189	0.46543548
Cars2	0.05852766	2.19304104	0.03317196	0.857321	0.46548921
Zfp512	-0.026118	5.07894091	0.03316109	0.85734411	0.46548921
Rfc1	-0.0209455	5.87408846	0.03305784	0.85756381	0.46557312
Hsp90aa1	0.02662378	10.9285529	0.03303524	0.85761195	0.46557312
6720489N17	-0.0389572	3.66694827	0.03292144	0.85785459	0.46565308
Rps6ka3	0.02202969	7.88393158	0.03291303	0.85787255	0.46565308
Hrasls	-0.0310497	5.07368425	0.03281709	0.85807746	0.46567598
Scube1	-0.0261927	5.95682339	0.03279004	0.85813529	0.46567598
Prkar2b	0.02506475	5.63127322	0.03277715	0.85816288	0.46567598
1700021K19I	0.02559031	5.53596705	0.03276442	0.8581901	0.46567598
Ilf3	0.02328393	5.74793852	0.03276071	0.85819803	0.46567598
Polk	0.02988932	5.18978043	0.03272613	0.85827205	0.4656854
Trmt11	-0.0627482	1.67032125	0.03268257	0.85836532	0.46570527
Dnajc19	0.0305617	4.74616253	0.03252469	0.85870395	0.46583501
Rnf39	0.06294442	1.8296328	0.03249484	0.85876807	0.46583501
2310002F09I	-0.2497586	-1.5177749	0.03249186	0.85877448	0.46583501
Senp3	-0.0299066	4.80653401	0.03245081	0.85886272	0.46584028
Adam18	-0.1968367	-0.7034302	0.0324346	0.85889756	0.46584028
Fam3a	-0.0374807	3.56031125	0.03237578	0.85902412	0.46587818
C330018D20I	-0.035152	3.75577288	0.03222483	0.85934946	0.46597796
Calcrl	0.03672422	4.34254882	0.03221409	0.85937264	0.46597796
Zfp868	0.03433614	4.89066314	0.03221151	0.8593782	0.46597796
Map4k5	0.02148521	5.95929711	0.03202802	0.85977481	0.46614443
Ska1	0.28608275	-1.8990028	0.03201702	0.85979863	0.46614443
Dhfr	0.03421577	3.80304629	0.03193143	0.85998406	0.46616224
Rab11fip5	0.02731979	5.95716067	0.03188679	0.86008087	0.46616224
B4galt2	-0.0435212	3.39230077	0.03188529	0.86008414	0.46616224
Ppargc1b	-0.0529962	3.29665832	0.03188403	0.86008687	0.46616224

Anapc10	0.03428807	4.5033661	0.03187103	0.86011507	0.46616224
Ubr7	0.02475962	4.90069223	0.03172464	0.86043315	0.46629007
Thoc3	-0.0451147	4.17805033	0.03171028	0.86046439	0.46629007
Pcid2	0.0442998	4.26634617	0.03155044	0.86081264	0.46644803
Tmem189	0.04948441	2.5608425	0.03140203	0.86113679	0.46656903
Egln2	-0.0429118	4.09706298	0.03139188	0.86115899	0.46656903
Ccdc152	-0.0548324	1.90916393	0.03137029	0.86120623	0.46656903
Dusp16	-0.0386956	4.92493437	0.03133858	0.86127563	0.46657587
Plxdc1	-0.0382961	2.91556988	0.03130494	0.86134928	0.46658502
Ccdc177	-0.058875	2.74825824	0.03122928	0.86151512	0.46664409
Tstd2	0.04518812	2.78070103	0.03118566	0.86161082	0.46666075
B630019K06I	0.0379216	3.26317026	0.0311616	0.86166364	0.46666075
Sfxn3	-0.025612	7.52997578	0.03112946	0.86173422	0.46666075
Synpo2	-0.0473743	3.37274146	0.03111182	0.86177298	0.46666075
Rfx3	-0.0367687	7.27365649	0.031055	0.86189789	0.46667038
Kdelr2	-0.0445576	5.18839106	0.03105207	0.86190432	0.46667038
Kcne2	-0.1738742	-0.9974753	0.03093708	0.8621575	0.46677671
Pdia5	-0.1279357	0.10203084	0.03085599	0.86233632	0.46684278
Slc6a14	0.22640023	-1.0287029	0.03082724	0.86239979	0.46684639
Fam26f	-0.1129445	1.22710449	0.03076807	0.8625305	0.4668864
Slc16a1	0.03396899	5.03789291	0.03072907	0.86261672	0.4668977
Bola2	0.059152	2.45401443	0.03070724	0.862665	0.4668977
Jmjd8	-0.040573	3.88038761	0.03068118	0.86272266	0.46689816
LOC1026341(	0.20212556	-1.4732897	0.03064013	0.86281355	0.4669166
Cby1	0.06373913	3.21791179	0.03058898	0.86292688	0.46694719
Sft2d1	0.03559966	3.36527521	0.03053119	0.86305505	0.4669858
C2cd4d	0.22737188	-2.1321454	0.0304349	0.86326888	0.467063
Itpripl1	-0.0987067	1.21223054	0.03041578	0.86331138	0.467063
Id4	-0.0290017	5.33668979	0.03037621	0.86339939	0.46707987
Bcl7a	-0.0327057	4.93507583	0.03033709	0.86348643	0.46709621
Exog	0.04506529	2.92209593	0.03030561	0.86355655	0.46710324
Plekhg6	-0.1860098	0.0553454	0.03025659	0.86366578	0.46710324
Zfp532	-0.0236346	5.64739454	0.03025178	0.86367648	0.46710324
Il18r1	-0.1727986	-0.6174719	0.03020589	0.86377885	0.46710324
Naprt1	0.0996366	0.45155741	0.03017139	0.86385584	0.46710324
Map2k6	0.03291054	4.00294987	0.03012482	0.86395985	0.46710324
Uri1	-0.021872	6.632009	0.03011443	0.86398306	0.46710324
Yy1	-0.0236568	5.38509177	0.03009343	0.86403001	0.46710324
Gm17801	0.1821057	-0.6337183	0.0300824	0.86405465	0.46710324
Tbc1d16	0.02947822	4.73877911	0.03007655	0.86406774	0.46710324
1700007P06I	-0.1286099	-0.667829	0.03004832	0.86413088	0.46710665
Mettl18	0.07134055	2.00591038	0.02993646	0.86438137	0.46721133
Prkacb	-0.019432	9.17765633	0.02977824	0.8647365	0.46737
01-Mar	-0.0338063	5.07859103	0.02973563	0.8648323	0.46737
Rnf113a1	0.04796807	1.95448335	0.02971371	0.86488162	0.46737

Col22a1	-0.1524728	-0.3577219	0.02970448	0.86490238	0.46737
Lmnb1	-0.0548064	1.82116979	0.02966636	0.86498821	0.46738565
Mcc	0.02573907	6.54266634	0.02957263	0.86519947	0.46744704
Trdn	-0.1010065	0.16367472	0.02956549	0.86521557	0.46744704
9130011E15I	0.03591918	3.63260374	0.02951066	0.86533931	0.46744747
Rqcd1	0.02991669	4.95419579	0.0295025	0.86535774	0.46744747
Dennd2d	-0.1371699	-0.2058797	0.02948956	0.86538698	0.46744747
2510049J12F	0.11191059	1.13682001	0.02944102	0.86549667	0.46747599
Matk	-0.0406756	3.61966561	0.02929558	0.8658259	0.46762309
Tgm4	0.0743082	1.23746859	0.02916679	0.86611816	0.46775019
Vps72	-0.0648577	2.61179427	0.02912447	0.86621433	0.4677714
Xist	0.12992444	0.81075274	0.02904005	0.86640642	0.46781018
Ubap1	-0.0255824	5.09841286	0.02902531	0.86643998	0.46781018
Alg12	0.06110571	2.79775609	0.02901788	0.86645689	0.46781018
Fv1	0.09515628	0.54461146	0.02895261	0.86660565	0.46783819
Gnb2	0.03763452	3.61938385	0.02894518	0.86662261	0.46783819
Ttll9	-0.2257274	-1.3124561	0.02889749	0.86673142	0.46784203
Dok1	0.14645217	-0.2343909	0.02889216	0.86674358	0.46784203
Unc45b	0.21544442	-0.6686464	0.02881747	0.8669142	0.4679034
Gmeb2	-0.0446328	2.96301519	0.02875451	0.8670582	0.46795039
Fem1a	0.02319241	5.55688316	0.02858275	0.8674519	0.46810976
Phf11d	-0.0713578	1.52265086	0.02856464	0.86749347	0.46810976
Gm3230	-0.0741519	1.29743143	0.0285512	0.86752435	0.46810976
1700034J05F	0.25776441	-1.6206311	0.02848912	0.867667	0.46815599
Parp14	0.04368254	4.15536348	0.02846126	0.86773109	0.4681587
Gkn3	0.20603077	-1.1762182	0.02843742	0.86778594	0.4681587
Gm5065	-0.1613635	-1.8556745	0.02838983	0.86789552	0.46818709
Atraid	0.02879232	4.54314961	0.02818653	0.86836472	0.46840946
Kcns1	-0.0581736	1.60185337	0.02815566	0.8684361	0.46841722
Lgr4	0.02449023	5.69464346	0.02810288	0.86855828	0.46845238
Mdc1	0.02512226	5.55897763	0.02789131	0.86904916	0.46868637
Fam20c	0.05588459	2.99503013	0.02781001	0.86923831	0.46873433
Erbb2ip	0.01816207	7.94454887	0.02777211	0.86932656	0.46873433
Krt77	0.14726729	-0.5571166	0.02776807	0.86933599	0.46873433
Rtn4r	-0.0309529	4.37400648	0.02771715	0.86945468	0.46873433
Ripply2	-0.1214285	-0.3361722	0.02770929	0.86947302	0.46873433
Nagk	-0.0425101	3.23250208	0.02770618	0.86948026	0.46873433
Lama5	0.04957774	1.76974396	0.02765438	0.86960117	0.46874079
Gin1	-0.0394304	3.43491305	0.0276422	0.8696296	0.46874079
Cntln	-0.0220253	6.57104022	0.02761304	0.86969772	0.46874079
Irf6	0.04721672	3.74546148	0.02760335	0.86972036	0.46874079
Lamc2	0.03954865	2.97337274	0.02757325	0.86979074	0.46874798
Rab5b	-0.0217914	7.01530066	0.02742525	0.8701373	0.46888743
Slc29a4	-0.0658764	1.10904848	0.02740813	0.87017746	0.46888743
Manba	0.05830719	2.7862483	0.02736887	0.87026959	0.46888743

Cdhr1	0.04379085	2.46622717	0.02736542	0.87027768	0.46888743
Zbtb10	-0.0223524	5.46634052	0.02724625	0.87055777	0.46900758
Crlf1	0.11668577	-0.4769437	0.02714397	0.87079864	0.4691066
Zfp955b	-0.0232103	5.83765315	0.02703158	0.87106388	0.46918613
Tyrobp	0.06566373	1.37625052	0.02702622	0.87107653	0.46918613
Mterf1a	0.08250693	1.18052134	0.02698539	0.87117304	0.46918613
2810001G20	0.0489345	4.81931109	0.02698473	0.8711746	0.46918613
Ly6c2	0.16811318	-0.5892945	0.02693714	0.87128719	0.46921602
Cd3g	-0.2210359	-1.3715452	0.02690141	0.87137179	0.46923083
Tmem134	0.04125115	2.61010811	0.02677912	0.87166175	0.46933513
Mybl1	0.03321283	4.22013849	0.02677157	0.87167967	0.46933513
Pias1	0.01785035	6.84402528	0.02673069	0.87177677	0.46935118
Senp1	-0.027474	5.22528024	0.02671094	0.87182369	0.46935118
Tmem184c	0.02327771	6.04331967	0.02668677	0.87188115	0.46935137
Sema7a	-0.025811	5.34240719	0.0265877	0.87211699	0.46944184
Mamdc2	-0.1170759	0.03578776	0.02656821	0.87216344	0.46944184
Cntn6	-0.0649115	3.21774274	0.0264765	0.87238221	0.46949291
Mpv17l2	0.0571318	2.69491878	0.02645817	0.87242599	0.46949291
Fos	-0.1122029	3.49210614	0.02645662	0.87242969	0.46949291
Phf2	-0.0169297	6.53371212	0.02641151	0.87253749	0.46952018
Kansl1l	-0.0245984	5.50091386	0.02637153	0.87263313	0.4695409
Rdx	0.01978589	7.03495233	0.0262733	0.87286839	0.46963674
Tph1	0.19815857	-0.7652572	0.02621202	0.87301537	0.46967708
St5	-0.0380425	4.99487358	0.02619441	0.87305764	0.46967708
Rps15a-ps4	0.06064202	1.32191386	0.02610701	0.87326766	0.46973049
Ttll11	-0.0364298	4.20607825	0.02610553	0.87327123	0.46973049
Gm13242	0.14576015	-0.9866561	0.02602749	0.87345908	0.46980078
Lpin1	-0.0291504	4.6454142	0.02595109	0.87364326	0.4698691
Rbbp6	-0.0183757	9.12289896	0.0258748	0.87382744	0.46991704
Cdh23	0.13917761	-1.1264785	0.02586681	0.87384675	0.46991704
Slco3a1	0.02973002	4.96501028	0.02577849	0.87406036	0.46997433
Drd5	0.09985408	0.25089244	0.02577548	0.87406764	0.46997433
Lap3	-0.0238164	5.62660117	0.02570789	0.87423139	0.47002504
BC017643	-0.0526971	2.48039427	0.02568935	0.87427633	0.47002504
Ccdc23	0.05510744	3.16987757	0.02561923	0.87444649	0.47006627
Cgref1	-0.04651	2.1814467	0.02561062	0.87446741	0.47006627
Tdrkh	0.03472677	4.42683345	0.02558249	0.87453575	0.47007227
Gm10941	0.15497775	-1.0465899	0.02548765	0.87476647	0.47016213
Fzd2	0.04477331	4.20920458	0.02544691	0.87486571	0.47016213
Nkiras1	-0.0189686	6.11160246	0.02542163	0.87492733	0.47016213
Pias4	-0.0521087	1.41421414	0.02539706	0.87498726	0.47016213
Gale	-0.1235838	-0.2678959	0.02537713	0.87503589	0.47016213
Pgm2l1	-0.0327203	10.1785393	0.02537198	0.87504844	0.47016213
Cited2	-0.0239257	6.39891047	0.02533355	0.87514228	0.47016213
Gpatch1	0.02185554	5.0819513	0.02532519	0.87516271	0.47016213

Ccdc127	0.01903923	6.5235289	0.02530265	0.87521777	0.47016213
0610009O20	-0.028757	4.90817008	0.02526405	0.87531215	0.47017664
Champ1	0.02607522	4.80465221	0.02523389	0.87538595	0.47017664
Htr6	0.17780489	-1.4043342	0.02522145	0.8754164	0.47017664
8430429K09I	-0.0316742	4.21523201	0.02517419	0.87553214	0.47018791
Nudt5	0.0487104	2.90762845	0.02510792	0.87569465	0.47018791
Helb	-0.0357452	3.42155083	0.02509266	0.87573208	0.47018791
Kcnq1	-0.1924897	-1.3330321	0.02509002	0.87573857	0.47018791
Irf7	-0.0994508	0.15226717	0.02508136	0.87575983	0.47018791
Serinc1	-0.0201538	8.80303299	0.02507166	0.87578364	0.47018791
A830052D11	-0.0894883	0.26860189	0.02503236	0.8758802	0.47018791
4930520O04	0.18588703	-1.0839657	0.02502632	0.87589503	0.47018791
Dio3	0.14204375	-0.9980248	0.024967	0.87604092	0.47023551
Fam53c	0.02233778	5.54866896	0.02492054	0.87615532	0.4702662
Upf1	-0.02932	4.27540549	0.02478049	0.87650078	0.47036395
Susd2	0.03046405	5.00079841	0.02475894	0.87655402	0.47036395
Ifrd2	-0.0735017	1.23966364	0.02472043	0.87664924	0.47036395
Ccdc88a	0.02609263	9.01307016	0.02470266	0.87669321	0.47036395
Epha2	-0.1796442	-1.6460887	0.02467526	0.87676101	0.47036395
Bmp8b	0.20505616	-1.422884	0.02466103	0.87679625	0.47036395
Nckap5l	0.06403463	1.45412957	0.02464871	0.87682677	0.47036395
Gzma	-0.2009188	-0.5829634	0.02464699	0.87683104	0.47036395
Mtx1	0.0437879	2.53885428	0.02463833	0.87685248	0.47036395
BC005537	-0.0212891	7.01994452	0.02460996	0.87692279	0.47037097
Ssr1	0.0211909	7.3121515	0.02447655	0.87725399	0.47051791
Gzmb	-0.1421773	-0.3237874	0.02436772	0.87752485	0.4705532
Armcx1	0.02094223	5.69029124	0.0243436	0.87758498	0.4705532
Mre11a	-0.0495886	2.75621419	0.02433242	0.87761285	0.4705532
1700102P08I	0.15200007	-1.0640683	0.02432742	0.87762532	0.4705532
Peg10	0.02802672	6.36412456	0.02432015	0.87764344	0.4705532
Kctd8	-0.0619566	2.1487354	0.02431219	0.87766329	0.4705532
Trmt10a	0.03683162	2.92068667	0.02423812	0.87784824	0.47062166
Cyp51	-0.0277411	5.33022634	0.02414174	0.87808927	0.47072017
Nhlrc4	0.15210917	-0.4151963	0.02411765	0.8781496	0.47072181
Zfp931	0.03735808	2.57628972	0.02409111	0.87821612	0.47072677
Shc4	-0.0416116	2.54008987	0.02405837	0.8782982	0.47074007
Vwf	0.05681929	2.07504973	0.02402239	0.87838846	0.47075774
Cntfr	0.04564003	4.14223416	0.02377284	0.87901651	0.47098353
Bean1	-0.0269516	3.92752223	0.023762	0.87904387	0.47098353
Rac3	-0.1387388	-0.9383691	0.02374494	0.87908695	0.47098353
Xrn2	-0.0204549	5.65336846	0.02370664	0.8791837	0.47098353
Tbc1d19	0.02400282	5.83751372	0.02370518	0.87918738	0.47098353
Gbp11	0.12444247	-0.1374851	0.0237004	0.87919945	0.47098353
Fam105a	0.03873688	3.79205502	0.02369588	0.87921088	0.47098353
Chad	-0.2039672	-1.747927	0.02366401	0.87929148	0.47099601

Hk1os	0.086189	0.80907124	0.02363866	0.8793556	0.47099966
Rdh9	0.09867012	0.40008975	0.0236089	0.87943094	0.47100932
Aacs	-0.0409325	2.68904219	0.02350032	0.87970625	0.47112607
Mcur1	0.03451292	5.21029881	0.02342285	0.87990307	0.47116022
Plekhn1	-0.1067229	-0.0644064	0.02339005	0.87998649	0.47116022
Cpne1	0.02091733	5.90281002	0.02338702	0.8799942	0.47116022
Tusc3	-0.0239651	4.91653902	0.02337055	0.88003614	0.47116022
Zfp940	-0.0372284	3.31935635	0.02334785	0.88009394	0.47116022
Zcchc17	0.02242548	5.21882703	0.02333998	0.88011397	0.47116022
Perp	0.0552148	6.65280061	0.02330712	0.88019772	0.47117222
Cer1	0.21952155	-1.0477751	0.02328621	0.88025103	0.47117222
Lypd6	-0.0281207	4.9247082	0.02322844	0.88039847	0.471186
Amz2	-0.0266492	4.75288145	0.02321257	0.880439	0.471186
Urm1	0.04763684	2.19931317	0.02320874	0.88044878	0.471186
Exoc1	-0.0307029	5.66903012	0.02315443	0.8805876	0.47122961
Bhlhb9	-0.01918	5.47364275	0.02309037	0.88075158	0.47125457
Fbxo48	0.13234777	-0.7738056	0.02308741	0.88075916	0.47125457
Ddx3y	-0.0187328	6.3508727	0.02306903	0.88080624	0.47125457
2610044O15	-0.0276033	4.55994414	0.02303	0.88090631	0.47127743
Epb4.1l4b	0.03675597	3.33579708	0.02278937	0.88152514	0.47157781
Sap30l	-0.0321933	3.93973451	0.02275869	0.88160429	0.47158945
Atp6v0e	0.04179406	4.75238444	0.02272639	0.88168767	0.47160336
02-Mar	0.02554916	4.75045565	0.02264174	0.88190647	0.47168306
I730030J21R	-0.1988078	-1.3284042	0.02261396	0.88197836	0.47168306
Zfp777	0.04533105	2.08717096	0.0226022	0.88200883	0.47168306
Cyp27a1	-0.0579289	1.73152539	0.02248899	0.88230236	0.47180607
Eefsec	-0.0444929	2.69284834	0.02246923	0.88235367	0.47180607
Gab3	0.03915392	2.77288416	0.02242133	0.88247815	0.47183816
Cpq	-0.0444278	4.27189071	0.02240198	0.88252849	0.47183816
Gm8580	-0.0891339	-0.6333046	0.02227805	0.88285134	0.47198006
Adamtsl2	0.12260136	-0.4198794	0.02218356	0.88309811	0.47208129
R3hdm2	-0.0190345	9.00287395	0.02209441	0.88333143	0.4721753
Nr2e1	-0.0423349	3.69179722	0.02205482	0.8834352	0.47220006
Atxn1l	-0.0198114	6.29541844	0.02201647	0.88353581	0.47221977
Gm16897	0.07425456	0.57425721	0.02199698	0.88358699	0.47221977
Mzt1	-0.0199886	5.93690151	0.02195204	0.88370505	0.47223448
Abhd14b	-0.0321384	5.31205164	0.02194277	0.88372942	0.47223448
Crh	-0.0964852	0.14931193	0.02185659	0.88395623	0.4723072
Map2k7	-0.0290896	5.36639003	0.02184599	0.88398415	0.4723072
Fkbp11	0.14236071	-1.4184632	0.02182559	0.88403791	0.4723072
Ggnbp2	-0.0166237	7.36108644	0.02179489	0.88411889	0.47231977
4933407L21I	0.08999062	0.14756745	0.02174828	0.88424192	0.47235479
Tceal7	0.11544204	-0.1028315	0.02170192	0.88436442	0.47238953
Dync1li1	-0.0256121	5.04728039	0.02162117	0.88457813	0.47245305
Uba6	-0.0262	5.70518127	0.02160881	0.88461088	0.47245305

4930539E08I	0.03619182	3.15092154	0.02158387	0.884677	0.47245305
Cd38	-0.038898	2.81277897	0.02155282	0.88475935	0.47245305
Bsdc1	0.02274927	5.28190997	0.02154852	0.88477075	0.47245305
Lancl1	0.02160173	6.54720907	0.0215183	0.88485097	0.47246518
Ntng2	-0.0535339	1.99714029	0.02145022	0.88503192	0.47252024
Rgs20	0.03158569	5.70952215	0.02143626	0.88506905	0.47252024
Amigo1	0.02071334	5.74003178	0.02136284	0.88526457	0.47259224
Ttpal	-0.0246476	5.12525269	0.02134245	0.88531893	0.47259224
Ids	0.02046579	9.64471167	0.02129144	0.88545504	0.4726199
Rnf43	-0.0431942	3.23811628	0.02127994	0.88548575	0.4726199
Trim52	0.17647491	-1.6565991	0.02113962	0.88586112	0.47278956
Arap1	0.03599306	3.70806444	0.02111589	0.88592475	0.47279281
Tln1	0.0280457	6.04945015	0.02108486	0.88600797	0.47280652
Disp1	-0.0366189	3.22651825	0.02106298	0.8860667	0.47280717
Cited4	-0.0612066	0.97690506	0.02082013	0.88672062	0.47312538
Kcng1	0.10413981	0.10079227	0.02076575	0.88686759	0.47317308
Nr1d2	0.0188021	7.42943897	0.02070412	0.88703438	0.47318997
Degs1	0.02718818	6.56259852	0.02069771	0.88705174	0.47318997
Esr1	-0.0683802	1.10288743	0.02069024	0.88707196	0.47318997
Trpc7	0.04751826	2.40421593	0.02060712	0.8872974	0.47323436
Cdh4	-0.0323146	3.48979286	0.02060613	0.88730009	0.47323436
Tatdn2	-0.0324766	4.03065392	0.02059588	0.88732791	0.47323436
Atxn2l	-0.0192673	6.83832865	0.02057108	0.8873953	0.4732396
1110051M20	0.02633979	4.1589489	0.02037647	0.88792539	0.47349157
Papd4	0.02715737	5.05321007	0.02029004	0.88816164	0.47358682
Lbh	0.03658637	6.3473381	0.02025418	0.88825983	0.4736015
Esr2	0.06949412	0.69385445	0.0201895	0.88843713	0.4736015
Mark2	0.02160018	6.32552604	0.02018417	0.88845174	0.4736015
Vrk2	0.08943111	0.45857261	0.02017979	0.88846377	0.4736015
Mfsd7c	-0.0694918	1.40256348	0.02017487	0.88847728	0.4736015
Nkx2-2os	0.18115993	-0.6508866	0.02003789	0.88885386	0.47377151
Ssx2ip	0.01626045	6.28551564	0.01986316	0.88933614	0.47387662
Adam8	-0.1560103	-0.6615818	0.01983896	0.88940312	0.47387662
Gimap6	-0.0692513	1.78011228	0.01983412	0.88941651	0.47387662
Poli	0.02692879	3.37439859	0.01982464	0.88944275	0.47387662
Selenbp2	-0.0927461	-0.2173687	0.01981678	0.88946451	0.47387662
Tbc1d4	-0.0379453	3.07730751	0.01980845	0.88948758	0.47387662
Erc2	0.02520317	8.10052414	0.01979154	0.88953446	0.47387662
Arhgap27os3	0.18088901	-1.6423363	0.01978301	0.88955809	0.47387662
Ormdl2	0.07561978	1.43733539	0.01977873	0.88956996	0.47387662
Ddr2	-0.0360504	6.27923198	0.0196382	0.88996026	0.47405381
Tmem51os1	0.10615921	-0.5113356	0.01961204	0.89003307	0.47406187
Cebpa	0.06158844	2.51636749	0.01956095	0.89017543	0.47408317
Oas2	0.0877067	0.13255888	0.01955629	0.89018842	0.47408317
Stard7	-0.0170086	6.2672211	0.01950781	0.8903237	0.47412449

Dusp10	-0.0360964	3.731895	0.01937757	0.89068794	0.47422654
Ccdc114	-0.1100408	0.152478	0.0193756	0.89069344	0.47422654
Sema4a	0.03551834	3.73889398	0.01936234	0.89073062	0.47422654
Best3	0.12278048	-0.0664537	0.01934528	0.89077843	0.47422654
Cdk6	0.06483427	2.18186163	0.01933623	0.89080382	0.47422654
Bad	0.05997728	2.66095758	0.01928376	0.89095108	0.47427422
Glb1l	-0.0357304	3.33240544	0.01914571	0.8913395	0.47445025
Aspscr1	-0.0491819	2.7002338	0.01908558	0.89150913	0.47448715
Pop1	0.04550165	2.24893073	0.01904723	0.89161744	0.47448715
Zfp3	-0.0333191	3.98973026	0.0190181	0.89169979	0.47448715
Gpatch4	0.02062789	4.88868949	0.01899157	0.89177488	0.47448715
Pdzk1ip1	0.07178022	1.63263449	0.01896528	0.89184929	0.47448715
Ntrk1	-0.1216183	-1.4088131	0.01895409	0.89188102	0.47448715
Taf6	0.02245317	4.52925267	0.01894407	0.8919094	0.47448715
Nlrp4f	-0.1854572	-1.9884153	0.01892775	0.89195566	0.47448715
Poln	-0.1342842	-1.1123698	0.01892475	0.89196417	0.47448715
Fuca1	0.02973647	5.15110516	0.01891113	0.8920028	0.47448715
Ppic	0.04287888	4.60365247	0.01885946	0.89214946	0.47448715
Dao	0.22787772	-1.5808403	0.01885876	0.89215144	0.47448715
Ski	0.01524638	8.16273402	0.01885289	0.89216812	0.47448715
Otx2	0.09870959	1.57621485	0.01883568	0.89221703	0.47448715
Mgmt	0.08715504	-0.09654	0.01880902	0.89229283	0.47449675
Mst1r	0.09947598	-0.7294972	0.018557	0.89301213	0.47484853
Pvrl2	0.06447174	1.42364104	0.0184476	0.89332592	0.47498466
Dhcr24	0.02618056	5.09585216	0.0183851	0.89350563	0.47502823
Map4k4	-0.0188596	6.42734492	0.01837891	0.89352345	0.47502823
Fam189b	-0.0256144	4.60001513	0.01834577	0.89361886	0.47504822
Aff3	0.01908993	7.61446663	0.0183118	0.89371678	0.47506955
Dnajb9	-0.0256935	5.83039723	0.01828222	0.89380211	0.47508418
Snhg12	0.03068349	3.49717348	0.01820533	0.89402423	0.47516375
1700017B05I	0.03444932	2.86725022	0.01816437	0.89414278	0.47516375
Rbms2	0.02599609	5.91312169	0.01813915	0.89421582	0.47516375
Sumf1	0.02848145	5.03265388	0.01809445	0.89434542	0.47516375
Psmf1	0.02674317	4.76051962	0.01806922	0.89441862	0.47516375
Tmem62	-0.034772	3.23541605	0.01805076	0.89447225	0.47516375
C78339	-0.0375082	2.77947753	0.0180441	0.89449158	0.47516375
Atf7ip	-0.0141845	6.92989395	0.01802009	0.89456136	0.47516375
Cttnbp2nl	-0.0177641	6.16218878	0.01801946	0.89456318	0.47516375
Cd4	-0.0517319	3.24034563	0.01801443	0.89457782	0.47516375
Mad2l1	0.03343741	4.07790964	0.01801102	0.89458774	0.47516375
Sstr1	0.05514793	3.05387091	0.01784803	0.8950628	0.47537113
Syt6	-0.0349768	4.7974429	0.01781782	0.8951511	0.47537113
Garem	-0.0234275	4.79906231	0.01781762	0.89515169	0.47537113
Col7a1	-0.1336077	-1.240098	0.0177588	0.89532379	0.47543181
Zfp335	0.03136524	3.92791599	0.01769967	0.89549714	0.47549314

Vcpkmt	-0.0465831	1.7494766	0.01766	0.89561359	0.47552425
Dmrt2	0.14686624	-0.9402289	0.01760864	0.89576457	0.47557369
Col15a1	-0.0622751	1.46629283	0.0175815	0.89584442	0.47558536
Crym	-0.0247171	3.43461405	0.01756088	0.89590515	0.47558689
Arl11	0.15297529	-0.8594678	0.0174983	0.89608966	0.47563605
2210408F21I	0.04262861	2.4430017	0.01749022	0.89611351	0.47563605
1700012D01I	0.11477067	0.14562904	0.01743466	0.89627762	0.47569244
Fam222a	-0.048457	2.20401045	0.0174089	0.89635381	0.47570216
Atg10	-0.0302656	4.0415969	0.01727401	0.8967537	0.47588366
Rbm3os	-0.0942054	-0.0460533	0.01724285	0.8968463	0.47590207
H2-T10	0.04632854	1.18483638	0.01713338	0.89717233	0.47601674
Copg1	-0.0179443	7.25418824	0.01712387	0.89720068	0.47601674
Elp2	0.02268088	5.93591872	0.01711199	0.89723614	0.47601674
Cd53	-0.0344706	2.34394497	0.01705293	0.89741259	0.47607962
Fbxo11	0.01419952	8.26058227	0.01695459	0.89770706	0.47617909
Pnkp	-0.0427903	1.65228763	0.0169478	0.89772741	0.47617909
Adck4	-0.0678643	1.29627474	0.01693051	0.89777929	0.47617909
Tmem178	-0.0186361	5.34075358	0.016913	0.89783184	0.47617909
Wwc2	0.02331399	4.8312372	0.01686639	0.8979719	0.47622264
Trappc6a	0.07554919	1.50051565	0.01681882	0.89811504	0.4762462
5930438M14	0.10878747	-0.5977789	0.01681312	0.89813221	0.4762462
Nxph4	0.07191731	0.63504163	0.01674222	0.89834594	0.47630083
Atxn7l2	0.06254843	2.34763128	0.0167405	0.89835114	0.47630083
Pcdh8	0.03616377	2.58289504	0.01654544	0.89894165	0.47658317
Mfsd7b	-0.0339909	2.61069559	0.01645834	0.89920646	0.47669282
1700012D14I	-0.1130221	-0.1883919	0.01632395	0.89961646	0.47687941
Zfp334	-0.0179442	4.89297415	0.01627225	0.89977465	0.47690988
Spsb1	0.03822856	2.8969923	0.01626724	0.89978998	0.47690988
D930048N14	0.07469003	0.71953324	0.0162279	0.89991054	0.47691455
Nfib	0.01495682	8.11049038	0.01619798	0.90000234	0.47691455
H2-K2	0.11803963	-0.367524	0.01619444	0.90001319	0.47691455
Stxbp1	0.01795369	10.0420214	0.01618868	0.9000309	0.47691455
Ncr1	0.09896347	-0.1231929	0.01613842	0.9001853	0.47694841
Kifc1	-0.1208792	-1.4155674	0.01612367	0.90023067	0.47694841
Dhx38	-0.022586	4.56252447	0.01608749	0.90034206	0.47694841
Cdk5	0.02420292	4.20561656	0.01604936	0.90045955	0.47694841
Fam57a	0.05382219	1.66580717	0.01604771	0.90046467	0.47694841
Nasp	-0.0239111	4.75297334	0.0160398	0.90048905	0.47694841
Lrrc38	0.05111848	1.44435448	0.01603592	0.90050101	0.47694841
Ssrp1	0.02185819	6.70649479	0.01599917	0.90061445	0.47697776
Trim24	0.0176896	5.55712033	0.0159712	0.9007009	0.47699281
Foxn3	0.02047028	6.54156047	0.01592231	0.90085214	0.47703709
Tmem132a	-0.032093	2.66849841	0.01590666	0.90090061	0.47703709
A930006K02I	0.07051258	0.2417027	0.0158148	0.90118557	0.47713433
Gtl3	-0.0173838	4.59835817	0.01581005	0.90120034	0.47713433

Cdh9	0.04159681	3.91287303	0.01573759	0.90142577	0.47722294
Glp1r	0.07577974	0.0642864	0.01569442	0.90156031	0.47726342
Gpr22	-0.0219582	5.80525701	0.0156722	0.90162963	0.47726938
Slc39a14	0.02757399	3.35867201	0.01560284	0.90184639	0.47735338
Fam19a5	0.01736481	4.98488575	0.01553296	0.90206522	0.4774382
Ltbp1	0.03367981	4.37816882	0.0155146	0.90212282	0.4774382
Cyp4f17	0.14642534	-1.7790445	0.01547287	0.90225382	0.4774715
Fbxo18	-0.0160388	5.50909359	0.01545756	0.90230192	0.4774715
Oas1b	0.08564993	0.04731173	0.01541987	0.90242046	0.47750348
Ptpn1	0.01780075	4.77236984	0.01536955	0.90257896	0.4775566
Klhl8	-0.0239689	4.31491677	0.01528642	0.90284136	0.47763707
Kbtbd11	-0.0151269	7.70405204	0.01528455	0.90284726	0.47763707
3110043O21	0.0309874	3.97870213	0.01525879	0.90292873	0.47764543
A930001C03	0.18395739	-1.796291	0.01523757	0.90299589	0.47764543
Wtip	-0.0450042	2.38718565	0.01522446	0.90303742	0.47764543
Zswim6	0.01653336	6.10393689	0.01515792	0.90324843	0.47771291
Stk11ip	0.03378435	3.48361789	0.01514758	0.90328124	0.47771291
Tomm40	-0.027819	3.08970157	0.01502374	0.90367535	0.47787785
Myo5c	-0.0752611	1.43219009	0.01501306	0.9037094	0.47787785
Bgn	-0.0318469	8.30310633	0.01497706	0.90382432	0.47790787
Pigx	-0.035443	2.84092419	0.01492857	0.90397933	0.47795909
Trim23	-0.0175803	6.39756976	0.01490131	0.90406658	0.47796744
Snn	-0.0147686	6.63171243	0.01488314	0.90412478	0.47796744
Top3b	-0.0309318	3.52638291	0.01485753	0.90420686	0.47796744
Pik3r3	0.01377523	6.59840432	0.01485102	0.90422775	0.47796744
Cenpq	-0.0500109	2.30652887	0.01477007	0.90448777	0.47807414
Casq2	0.08455045	-0.2411714	0.01473089	0.90461389	0.47811006
Ptdss2	-0.0276862	3.24280538	0.01464522	0.90489023	0.47821586
Klf2	0.02699044	4.01005213	0.01463278	0.90493044	0.47821586
Irf2bp2	0.01332177	8.43053574	0.01434177	0.90587574	0.47858184
4833419F23	-0.0835694	-0.1610463	0.01432994	0.90591437	0.47858184
Gng10	-0.0286547	4.81533876	0.01430733	0.90598827	0.47858184
Lipt1	-0.0488983	1.47774105	0.0142733	0.90609957	0.47858184
Pak4	-0.0417743	4.81414536	0.01425867	0.90614749	0.47858184
Ccdc175	0.21615831	-1.7965656	0.01424109	0.90620507	0.47858184
Slc20a2	0.02564915	6.03436327	0.01423983	0.9062092	0.47858184
Jmjd7	-0.0957674	-0.6699958	0.01423938	0.90621066	0.47858184
Arl8b	-0.012702	7.95545664	0.01421356	0.90629531	0.47858184
Slc38a10	-0.025145	3.43456733	0.0142125	0.9062988	0.47858184
Gfod2	0.07557474	0.02465691	0.0142067	0.90631782	0.47858184
Arap3	0.05628154	1.59536031	0.01420551	0.90632173	0.47858184
Zfp37	0.02042103	6.80022442	0.01410098	0.90666531	0.47871545
Hs3st2	-0.0253995	4.34219434	0.01409311	0.90669123	0.47871545
Nphs2	0.03097639	3.92360048	0.01402584	0.90691309	0.47875671
Cep104	-0.0182505	4.65266436	0.0140169	0.90694264	0.47875671

Ccdc79	-0.0669339	0.91589966	0.01401644	0.90694414	0.47875671
Micu1	-0.0169317	4.45383813	0.01387755	0.9074041	0.47896876
Slc6a12	0.03981287	3.69420656	0.01383707	0.90753861	0.4789834
Prpf4	0.01713906	5.00342337	0.01383412	0.90754841	0.4789834
Ptgds	0.04474558	12.2504338	0.01375915	0.90779805	0.47906326
Gm16845	-0.0855955	0.69117642	0.01375368	0.90781628	0.47906326
Ap3m2	0.01607571	6.61854869	0.01371128	0.90795779	0.47910717
Lpar2	0.04968981	1.631953	0.01363353	0.90821788	0.47921365
Syk	0.05489869	1.46868149	0.01358333	0.90838622	0.47925488
D230025D16	-0.015127	5.65065119	0.01357546	0.90841264	0.47925488
Tshr	0.11794237	-0.4251946	0.01354411	0.90851796	0.47927968
4930579G18	-0.0832494	0.33076995	0.01344581	0.90884896	0.47940434
Rtel1	-0.03815	2.53186171	0.0134393	0.90887093	0.47940434
Tmem256	0.03398481	2.86839731	0.01342176	0.90893014	0.47940481
Uba2	0.01460246	5.79945367	0.01337975	0.90907212	0.47942612
Dnajc18	0.01666103	6.93329218	0.01337063	0.90910295	0.47942612
Khnyn	-0.0265698	2.936284	0.01335804	0.90914555	0.47942612
Rtn3	0.01685332	9.97579174	0.01333603	0.90922009	0.47943467
Lgalsl	-0.0132795	6.23959162	0.01331875	0.90927866	0.4794348
Agtr1a	-0.1755527	-1.2617721	0.01328816	0.9093824	0.47945874
Klf16	-0.0285683	2.45963519	0.01325121	0.9095079	0.47948371
Entpd1	-0.0287863	3.01205359	0.01323988	0.90954643	0.47948371
Tmem81	0.06487683	0.54016435	0.01301636	0.9103097	0.4798553
Stk25	0.01193302	6.73281195	0.01294896	0.91054116	0.47993242
Eda	0.03753785	2.48147763	0.01292456	0.91062509	0.47993242
2610524H06I	-0.0301848	2.15892431	0.0129228	0.91063118	0.47993242
Ifi203	-0.0422021	3.12728401	0.01284875	0.91088644	0.48002132
Dgkg	0.02637676	6.62842365	0.01282397	0.91097202	0.48002132
Gpn3	0.02800678	4.49154742	0.01282309	0.91097507	0.48002132
Zfp786	0.06550356	0.13871326	0.01265756	0.91154896	0.48029293
Phf12	-0.0130969	6.74279914	0.01261452	0.91169883	0.48033871
Deaf1	-0.0237287	4.04081843	0.01259905	0.91175273	0.48033871
Ccdc15	-0.0242663	4.26370537	0.01253568	0.91197398	0.48042448
Mir1188	-0.0817409	-0.5347815	0.01244368	0.91229616	0.48056245
Tppp	-0.0132975	8.81381514	0.01242754	0.91235282	0.48056245
Ankdd1b	0.02259572	3.20222513	0.01234809	0.91263221	0.4806788
Trdmt1	0.03565037	2.84535639	0.01230924	0.9127692	0.48072015
Mab21l1	-0.0504119	1.21648583	0.01226801	0.91291477	0.48074489
Cdc42ep5	-0.0414773	2.49276455	0.01226281	0.91293316	0.48074489
Strn3	-0.0129495	8.17349927	0.01222714	0.91305932	0.48078052
Trim8	-0.0167237	6.14101651	0.01218399	0.91321222	0.48083023
Dnmt3aos	0.15016722	-1.8159098	0.01216714	0.91327201	0.48083091
Cnst	0.01689313	6.16085831	0.01211251	0.91346609	0.48087185
AI854703	-0.0322923	3.07551616	0.01211231	0.91346679	0.48087185
Acad9	-0.0249761	4.13075264	0.01202634	0.91377315	0.4809326

Bcmo1	-0.1274095	-1.5031222	0.01201002	0.91383144	0.4809326
Pibf1	-0.0229537	4.56124547	0.01199709	0.91387764	0.4809326
Cables2	0.01905066	5.03028034	0.011994	0.91388867	0.4809326
Adal	0.01980151	3.96032848	0.01198171	0.91393264	0.4809326
Taf6l	-0.0529963	0.66481131	0.01198153	0.91393328	0.4809326
Car9	0.0881128	-0.7352023	0.01193503	0.91409974	0.4809894
2900097C17I	0.01949659	10.8249655	0.01184172	0.91443478	0.48110943
Pyroxd2	0.04350265	1.1896043	0.01181313	0.91453773	0.48110943
Mnd1	0.09067806	0.36796527	0.01180704	0.91455965	0.48110943
Atp6v0b	0.02065835	4.83149849	0.01180583	0.91456404	0.48110943
Exo1	0.08527677	-0.6416641	0.01178283	0.91464695	0.48110943
Mrps35	0.0271297	3.99390383	0.01177393	0.91467906	0.48110943
Zfp59	0.02744238	3.33000166	0.01172236	0.91486535	0.48117662
Nlgn1	-0.0196811	6.61629113	0.01166215	0.91508338	0.4812605
Smpdl3a	-0.024271	4.81997081	0.01164556	0.91514356	0.48126135
B4galt4	-0.0229445	4.72188672	0.01158495	0.91536375	0.48132693
G630025P09	-0.1582073	-1.6225258	0.01157901	0.91538537	0.48132693
Ap1b1	0.01667816	5.70390084	0.01155942	0.9154567	0.48133364
Cd63	0.03606549	4.98247981	0.01149065	0.91570753	0.48140973
Tmem109	0.0201983	3.48127732	0.01147927	0.91574912	0.48140973
C1qa	-0.1293807	-1.5955507	0.01147161	0.91577714	0.48140973
Itpripl2	-0.0206761	5.91294504	0.01145242	0.91584732	0.48141583
Rec8	-0.0713601	0.39427074	0.01140549	0.91601923	0.4814754
Olfml2b	0.06506877	0.63137112	0.01136954	0.91615115	0.48151395
Gpr108	-0.0303545	3.10882504	0.01134846	0.91622862	0.48152387
Adgb	0.10805367	-0.9356772	0.01129208	0.91643615	0.48160215
Gpr87	0.11931833	-1.7229467	0.01126323	0.91654254	0.48160953
Ubxn10	-0.0644702	0.82229865	0.01123679	0.91664017	0.48160953
Gabpb2	0.01439189	6.14422032	0.01122413	0.91668695	0.48160953
Igsf5	-0.202312	-1.9997255	0.01121858	0.91670747	0.48160953
Cpe	-0.0147435	10.0402984	0.01120893	0.91674318	0.48160953
2310009B15I	-0.0390339	2.1245251	0.01114664	0.91697392	0.48167532
Gins2	0.04059955	2.15849279	0.01114192	0.91699143	0.48167532
Stambp	0.01723308	4.3459198	0.0111277	0.91704423	0.48167532
Acer2	0.03369204	3.38377005	0.01107825	0.91722801	0.48174107
Adamts9	-0.0357468	2.69377646	0.01098623	0.91757119	0.48187414
Ptprn2	-0.0143092	7.30390433	0.01097889	0.91759864	0.48187414
Fasn	0.02061457	6.43402626	0.01091698	0.9178304	0.48194274
Rfx2	0.03011217	2.14871108	0.01091267	0.91784654	0.48194274
Zic5	0.04198262	1.62181319	0.01089243	0.91792249	0.48194876
Osbp	0.01287509	5.35473339	0.01087837	0.91797529	0.48194876
Sfxn4	-0.0312725	3.64508379	0.01084127	0.91811472	0.48199118
9230112J17F	-0.138092	-1.563652	0.01078448	0.91832866	0.48206123
Aamp	0.02079452	4.84802461	0.01076411	0.91840556	0.48206123
Sgk3	0.0159873	5.20054935	0.01074765	0.91846774	0.48206123

Gltscr1	0.01988815	4.38183036	0.01074367	0.91848276	0.48206123
Dnm1	-0.0164271	8.86763299	0.01064895	0.91884156	0.48221311
Nit1	-0.0239475	2.91999726	0.01063633	0.91888948	0.48221311
Ing2	-0.0159002	5.0945217	0.01061779	0.91895993	0.48221929
Tcaim	-0.0204656	4.43598128	0.01058466	0.91908602	0.48225466
Bin3	-0.0269208	2.93786398	0.01055583	0.91919586	0.48228151
Gm15987	-0.1466381	-1.7761642	0.01052701	0.91930585	0.48230843
Jade1	0.01400921	6.43364774	0.01050765	0.91937983	0.48231646
Psmc3ip	0.04322759	2.02747276	0.0104284	0.91968329	0.48244486
Gpc5	-0.0312602	3.53972772	0.01034396	0.92000794	0.48256051
Mfge8	0.03047655	3.1495473	0.01034053	0.92002118	0.48256051
Fam180a	0.03692309	5.14193717	0.01031328	0.92012624	0.48258482
Ubtd2	0.01592956	5.27685835	0.01025409	0.92035495	0.48260756
Mks1	0.06907061	0.20709525	0.0102522	0.92036226	0.48260756
Cep57	0.01660982	4.61047186	0.01024548	0.92038828	0.48260756
Plscr4	0.03059787	3.14324998	0.0102413	0.92040447	0.48260756
Carns1	-0.0450403	2.38018108	0.01017491	0.92066195	0.48271177
Ranbp10	0.01610993	4.71513143	0.01013501	0.92081709	0.48275514
Psrc1	0.03838885	1.47407062	0.01012344	0.92086214	0.48275514
Dennd5a	0.0104497	7.52315641	0.01009233	0.92098342	0.48278793
Scnn1a	-0.0293145	2.58433615	0.01001482	0.92128637	0.48291594
Col6a5	0.1360954	-1.8134956	0.00997537	0.92144101	0.4829662
Zfp953	0.02815129	2.98051226	0.00991866	0.92166383	0.48303038
Sp5	0.07859333	1.50954197	0.0099143	0.921681	0.48303038
Zfp558	0.03782201	2.74543963	0.00985669	0.92190813	0.48306036
Rdm1	0.0411288	2.51132514	0.00984392	0.92195853	0.48306036
Rspo1	-0.0254254	3.09497021	0.00984323	0.92196128	0.48306036
Capn3	-0.0393975	0.85097629	0.00983449	0.92199578	0.48306036
Tmem248	0.01447487	5.76332469	0.00982531	0.92203207	0.48306036
E2f3	-0.016868	5.37305131	0.00977491	0.92223164	0.48311307
Xrcc6bp1	0.04040717	1.74874407	0.00975588	0.92230711	0.48311307
Cacng4	0.05296621	1.11388818	0.00974205	0.92236201	0.48311307
Ldoc1l	0.01633248	4.91591851	0.00974059	0.92236781	0.48311307
Tspan9	0.02579191	3.49513706	0.00971079	0.92248626	0.48314432
Egfl7	-0.0277638	2.41048046	0.00963666	0.9227817	0.48326826
Mcmbp	-0.0118686	5.68264481	0.00954338	0.92315507	0.48338489
Dcst1	0.03688112	1.39971641	0.00954168	0.92316186	0.48338489
Cldn5	0.08955129	-0.2046789	0.00952941	0.92321112	0.48338489
Shmt2	-0.0257084	2.55247759	0.00951627	0.92326392	0.48338489
Cog8	0.02958865	2.01771711	0.00950206	0.92332104	0.48338489
Gm19557	-0.097966	-0.643446	0.00949305	0.92335727	0.48338489
Ccl6	0.06068234	0.33841368	0.00946752	0.92346006	0.48338605
Zfp493	-0.0371212	2.60744916	0.00945564	0.92350797	0.48338605
Prpf40b	-0.0265247	3.81498405	0.0094487	0.92353592	0.48338605
Msmo1	-0.0199412	4.99067555	0.00935591	0.92391108	0.48355162

Taf4b	0.02236811	2.90213099	0.00933914	0.9239791	0.48355642
Scn1b	0.01213854	5.60432202	0.00928613	0.92419445	0.48363833
Tchh	0.02542589	3.01158791	0.00924177	0.92437514	0.48368906
Heatr5a	0.01712856	4.46749891	0.00923345	0.92440908	0.48368906
Rassf7	0.07067274	0.14261191	0.00920093	0.92454186	0.4837008
Dap3	-0.0139729	4.8301408	0.00917011	0.92466794	0.4837008
Hdac5	-0.0190864	5.88492629	0.00915189	0.92474258	0.4837008
Osbpl7	-0.0275417	2.31354449	0.00914927	0.92475332	0.4837008
Mro	0.01615205	4.44287072	0.00914262	0.92478058	0.4837008
Tmem35	-0.0224389	3.85577349	0.00911029	0.92491329	0.4837008
Stil	0.05105547	0.88091538	0.00909936	0.92495821	0.4837008
Tox2	-0.0222851	2.75127914	0.00908505	0.92501704	0.4837008
Dhx30	0.02499589	4.70608379	0.00908139	0.92503211	0.4837008
Trf	-0.0205975	5.62323471	0.00907736	0.92504868	0.4837008
Ptges3l	0.05540712	1.05137096	0.00907002	0.92507888	0.4837008
Pdcd11	0.01481069	4.16139788	0.00905271	0.92515018	0.48370731
Ccno	0.09734043	-0.7397575	0.00902408	0.92526826	0.48373827
Cd300ld	-0.0872972	0.25936695	0.00899984	0.92536841	0.48375986
Il10ra	-0.0708577	0.14749932	0.00890375	0.92576664	0.48392144
Epb4.1l3	0.01253839	7.79596914	0.00888245	0.92585521	0.48392144
Cct6b	-0.1299648	-1.4905165	0.00886288	0.92593668	0.48392144
Klhl2	-0.0122794	6.90275888	0.00885931	0.92595155	0.48392144
Plcxd1	0.03792929	1.75679054	0.00884693	0.92600314	0.48392144
Aldh3a1	0.07955765	-0.7939376	0.00883568	0.92605004	0.48392144
Ptcd2	0.01756137	4.69005806	0.00882619	0.92608964	0.48392144
Creb3l4	-0.1417541	-0.9489596	0.00877418	0.92630705	0.48392791
Ccdc19	-0.0323034	2.03876038	0.00876406	0.92634943	0.48392791
Sema3e	-0.0382231	4.19611162	0.00875925	0.92636962	0.48392791
N6amt2	0.02274135	3.86164223	0.00875886	0.92637124	0.48392791
Foxn2	0.01750103	4.77039956	0.00874532	0.92642797	0.48392791
Hes7	-0.123872	-1.7035648	0.00873881	0.92645529	0.48392791
Nsun4	0.01978103	2.77881446	0.00871471	0.92655647	0.48395001
Cept1	-0.019803	3.88413645	0.00869815	0.92662608	0.48395561
Nmbr	-0.0622472	0.19861684	0.00864122	0.92686586	0.48405008
Nupl2	0.02513998	3.28139523	0.00855851	0.92721567	0.484202
Gns	-0.0174114	6.50749551	0.00853017	0.92733589	0.48423401
Atp6v1c2	-0.0758672	-0.1751808	0.00851293	0.92740913	0.48424149
Dync1i2	-0.0127196	7.48767004	0.00849452	0.92748745	0.48425162
Tdrd3	0.01255106	5.51620388	0.00847062	0.92758921	0.48425758
Lor	-0.0412509	0.3638543	0.00846417	0.9276167	0.48425758
5730403I07R	0.14599408	-1.970672	0.00843929	0.92772286	0.48428224
Col6a4	0.08039158	-0.2166068	0.00837081	0.92801585	0.48436328
Thap6	-0.0201778	3.86927998	0.00836832	0.92802652	0.48436328
Polr1c	-0.020505	4.05554449	0.0083617	0.9280549	0.48436328
BC020402	0.04373501	1.68090219	0.00831916	0.9282376	0.48442788

Daf2	0.03945448	0.64992593	0.00827951	0.92840836	0.484471
Dis3l2	0.01921914	3.42555374	0.00825743	0.92850358	0.484471
Trim28	0.01167994	5.90610985	0.00824911	0.92853949	0.484471
Cadm3	0.01513536	7.86896536	0.00824529	0.92855601	0.484471
Lingo1	0.01192683	7.59023862	0.00822663	0.92863667	0.48448233
Gcnt2	0.01421344	5.3346145	0.00819223	0.92878561	0.48448272
E530001F21I	-0.1161123	0.14723667	0.00818508	0.92881659	0.48448272
Smarca5	-0.011203	7.57245339	0.00817512	0.9288598	0.48448272
H2-DMa	0.04172503	2.02322094	0.00815791	0.92893451	0.48448272
Htr2c	-0.0189114	4.57506655	0.00815411	0.928951	0.48448272
Lrrc26	0.10234774	-1.5248642	0.00813162	0.92904877	0.48448272
6430571L13F	0.05897417	0.33278228	0.00813119	0.92905067	0.48448272
Gm3219	0.03545391	0.84330458	0.00811778	0.929109	0.48448272
Crtc2	-0.0160621	4.0438307	0.00807873	0.92927924	0.48453861
Rasgrp2	-0.0215734	2.56032541	0.00806617	0.92933407	0.48453861
Cars	-0.0211142	3.58290147	0.00803929	0.92945161	0.48456915
Iqca	0.07681486	-0.2712999	0.00793899	0.92989186	0.4847559
Sox7	0.04797954	0.68090057	0.00793083	0.92992779	0.4847559
Slc22a18	0.05561824	1.18189298	0.00787885	0.93015717	0.48484473
Exoc3l4	-0.0844526	-0.9109404	0.00780424	0.93048771	0.48498626
Schip1	0.01765559	3.86375504	0.00775304	0.93071551	0.48507424
Abcd1	0.02593761	3.7141478	0.00770541	0.93092807	0.48513195
Grpr	0.05539165	-0.1149348	0.00770178	0.93094429	0.48513195
Ets2	-0.0126249	5.6900743	0.0076395	0.93122332	0.48524659
Slc18b1	-0.0174737	5.1384207	0.00748199	0.93193414	0.48557476
Tigd3	0.05266766	-0.2656558	0.0074538	0.93206218	0.48557476
Lmx1b	-0.097787	0.19280357	0.00745019	0.93207859	0.48557476
Kcne1l	-0.0958809	-1.2949601	0.0074478	0.93208943	0.48557476
Upk1b	0.05036814	1.27671909	0.00740046	0.93230505	0.48560023
G6pc3	0.01907361	3.9418678	0.00739407	0.93233423	0.48560023
Rcc2	0.0110157	5.31857421	0.00737888	0.93240358	0.48560023
U2af1l4	0.02903112	2.09191891	0.00737273	0.93243169	0.48560023
Scx	0.05633672	-0.0094432	0.00737228	0.93243373	0.48560023
Gtpbp3	-0.018727	3.02973878	0.00735309	0.93252149	0.48561517
Fbxo34	-0.0146237	5.26287799	0.00730757	0.93273015	0.48569306
4931406H21I	-0.0783675	0.24026653	0.00729286	0.93279775	0.48569748
4930528A17I	0.11249498	-1.4834842	0.00726155	0.93294177	0.48570637
3110070M22	-0.0740497	-0.8977719	0.00726117	0.9329435	0.48570637
Ppp5c	0.01387242	4.63015193	0.00725063	0.9329921	0.48570637
Golim4	-0.0151308	5.93506605	0.00720654	0.93319561	0.48575817
Zfp68	-0.0145216	6.18202329	0.00719939	0.9332287	0.48575817
Myom1	-0.0603222	0.4210617	0.0071907	0.93326891	0.48575817
Cldn25	0.01501372	5.89007369	0.00717025	0.93336363	0.48577559
Tbc1d20	-0.0198888	4.21044894	0.00715796	0.93342059	0.48577559
Tnfrsf22	0.02933661	1.74743848	0.00712382	0.93357918	0.4858037

Ifi30	0.03580595	2.01187958	0.00712089	0.93359281	0.4858037
Ehmt2	-0.0131942	5.08226054	0.00709428	0.9337167	0.48583741
Sox1	-0.0166296	4.72724917	0.00704525	0.93394558	0.4859223
Ints4	0.01172914	5.51097562	0.0070284	0.93402439	0.4859223
B9d1	0.02379539	2.37419088	0.00702113	0.93405845	0.4859223
Tex261	-0.0221422	1.98762786	0.00699947	0.93416	0.4859223
Sec62	-0.0108087	7.55522414	0.00699617	0.93417546	0.4859223
Eps15	-0.0106297	8.62271066	0.00696528	0.93432059	0.48596704
Col4a4	-0.0856147	-0.8139241	0.0069203	0.93453248	0.48602849
Syt3	-0.0208034	3.66352254	0.00690535	0.93460308	0.48602849
Tusc1	-0.0168903	3.18417392	0.00690259	0.93461613	0.48602849
Podxl2	0.02924035	2.17688717	0.00687114	0.93476486	0.48606713
Gm11837	0.05994058	-0.748374	0.00685578	0.93483764	0.48606713
Mov10	-0.0233754	2.92325618	0.00684941	0.93486786	0.48606713
Sp9	0.02817317	1.30070133	0.00679245	0.93513862	0.48617715
Lama2	-0.0158462	5.21919044	0.00676484	0.93527026	0.48621483
Bmpr1a	-0.0157331	7.88324888	0.00672996	0.93543698	0.48624005
Zfp93	-0.0213008	3.83897095	0.00672993	0.93543708	0.48624005
Gpatch2l	0.01158504	5.15068383	0.00658657	0.93612683	0.48656781
Hras	0.01924534	5.0526213	0.00655915	0.93625964	0.48660607
Rorc	0.03909837	1.15378792	0.00650708	0.93651259	0.48668348
Sstr3	-0.015245	3.54620134	0.00649596	0.93656671	0.48668348
Nprl2	-0.0229241	2.467394	0.0064862	0.93661428	0.48668348
Orai3	0.01896276	5.00577325	0.00647981	0.93664545	0.48668348
Rps6kc1	-0.0123675	4.98005424	0.00644313	0.93682459	0.48674
Nr5a2	-0.1097483	-2.0178375	0.00642545	0.93691115	0.48674
Ubxn8	0.01764929	4.33700244	0.00642122	0.93693188	0.48674
Nos2	-0.1138596	-1.9593026	0.00639035	0.9370833	0.48678141
Srsf5	0.00850857	8.0887758	0.00638084	0.93713005	0.48678141
Slc26a4	-0.0479541	1.05431985	0.0063385	0.93733851	0.48683729
Thg1l	-0.0248682	2.94982883	0.00633494	0.93735608	0.48683729
Qsox1	0.02626701	2.23119922	0.00628204	0.93761761	0.48693628
Pm20d2	0.01290254	4.21869945	0.00626327	0.93771065	0.48693628
Sap30bp	-0.0166438	3.65865418	0.0062605	0.93772442	0.48693628
Arhgap25	0.01843906	3.64359913	0.00624119	0.9378203	0.48695531
Cldn2	-0.0509288	1.2896757	0.00619356	0.93805752	0.48704771
Igfbp2	-0.0230441	4.96392241	0.00616821	0.93818412	0.48708267
Iqcg	0.01926941	2.95988689	0.00610786	0.93848665	0.48720805
Arhgap15	0.01612229	3.82737943	0.00609641	0.93854417	0.48720805
Tmem219	-0.0318197	2.53294204	0.00607057	0.93867429	0.48724482
Rad51b	-0.0775628	-1.3350402	0.00603514	0.93885313	0.48730688
Nphp1	0.0164975	3.83450692	0.00601461	0.93895699	0.48731241
Gm9958	0.02847812	1.63163496	0.0060096	0.93898236	0.48731241
Tmem200c	0.02687942	1.70233929	0.00598628	0.93910064	0.48734302
Sh3gl3	0.01605134	5.61268157	0.00595986	0.93923492	0.48736653

2410006H16I	0.02415265	2.57226497	0.00595403	0.93926454	0.48736653
Gpn2	0.02593152	1.66041057	0.00589104	0.93958602	0.48747935
Exosc10	-0.0120546	4.86434649	0.00588622	0.93961069	0.48747935
Peo1	0.02399572	2.5381301	0.00585934	0.93974847	0.48747935
Sap130	0.00998311	6.64404836	0.00585054	0.93979365	0.48747935
Vstm2a	0.01109634	6.5331679	0.00584715	0.93981106	0.48747935
Dhrs11	-0.0454832	0.67137026	0.00584193	0.93983783	0.48747935
Wnt16	0.03799261	1.69469413	0.00580294	0.94003854	0.48751694
Col14a1	0.03822732	0.85800448	0.00579477	0.94008066	0.48751694
Cenpl	0.02610872	1.72929489	0.0057933	0.94008825	0.48751694
Ascl4	-0.0924264	-1.2625005	0.0057722	0.94019722	0.48754269
Fam50a	0.01764382	4.12554342	0.00574826	0.94032112	0.48757617
C530005A16I	0.01826191	3.24968215	0.00571925	0.94047162	0.48762033
Uso1	-0.0086159	6.42363592	0.00570617	0.9405396	0.48762033
Zfp87	0.01205298	5.56435072	0.00569759	0.94058425	0.48762033
Elmod3	-0.0233707	3.29131426	0.00567583	0.94069756	0.48764083
Rhog	-0.0191441	3.23796966	0.00566722	0.94074246	0.48764083
Gm13031	0.07993213	-0.4832155	0.00562343	0.94097141	0.48772875
Klk6	0.06559265	-1.3802806	0.00555604	0.94132546	0.48785299
Dok3	-0.0764514	-1.3432992	0.00555521	0.94132983	0.48785299
Hist1h4b	0.06753265	-1.8082194	0.00553793	0.941421	0.48786948
Lrrc3b	-0.0215115	3.63449655	0.00550666	0.94158629	0.48792437
Gja1	0.02222939	7.63703982	0.00548944	0.9416775	0.48793236
Cilp	-0.0598621	-0.1917374	0.00548135	0.94172043	0.48793236
Zfp708	0.01692663	3.49910848	0.00541108	0.94209448	0.48809379
Cmtm5	0.02584225	2.42396211	0.00539048	0.94220463	0.48809379
Cyp2j6	-0.0142507	4.00812163	0.00538164	0.9422519	0.48809379
Zbtbd6	0.06111661	0.54639363	0.00536638	0.94233368	0.48809379
Agpat5	-0.0099222	5.497355	0.00536151	0.94235985	0.48809379
Zfp444	-0.0165643	3.28469872	0.0053562	0.94238831	0.48809379
Slc25a34	0.04143345	0.06385238	0.00532859	0.94253674	0.4881399
Mios	0.01304403	4.46628771	0.00530847	0.94264513	0.48816528
Veph1	0.04761277	-0.1458469	0.00523017	0.94306891	0.48833628
Hk1	-0.0150838	5.81242358	0.00521278	0.94316343	0.48833628
Ankra2	-0.0145573	3.44615038	0.0052072	0.94319384	0.48833628
Ovca2	-0.0159447	3.35314287	0.00518931	0.9432913	0.48833628
Rhbdl1	-0.0303366	0.61950411	0.005189	0.94329303	0.48833628
Sox10	0.01559488	4.29225309	0.00518189	0.94333183	0.48833628
Zfp474	0.13722553	-1.4850592	0.00516596	0.94341882	0.48835056
Msto1	0.02932493	1.83431399	0.00514602	0.94352795	0.48837008
Tbc1d10c	0.06996396	-0.871725	0.00513563	0.94358485	0.48837008
Zfp608	0.01201988	5.16319009	0.00512653	0.94363479	0.48837008
Abca13	-0.0933657	-1.7270547	0.0051006	0.94377726	0.48838729
Slc35f5	0.01465128	4.54118939	0.00509885	0.94378688	0.48838729
Nmral1	0.02293373	2.8431498	0.00507033	0.94394403	0.48840149

1700123O21	-0.080769	-1.0875901	0.00506357	0.94398136	0.48840149
Cd180	0.02393454	2.7357235	0.00506153	0.94399259	0.48840149
Gas1	-0.0189625	5.78647272	0.00504716	0.94407205	0.48841185
Runx1t1	0.0091627	7.80809386	0.00499468	0.94436305	0.48845907
Nrf1	-0.0134191	3.87283049	0.00499198	0.94437806	0.48845907
Gpr124	0.01715398	4.91586024	0.00499098	0.94438359	0.48845907
Caps2	-0.0712981	-1.2710401	0.00498601	0.94441125	0.48845907
Rufy1	-0.0110846	4.69976452	0.00497053	0.94449746	0.48845907
Tmem43	0.01840172	3.88559186	0.00494764	0.94462523	0.48845907
Ndn	-0.0126024	6.22948305	0.00494726	0.94462731	0.48845907
Lhcgr	0.07755077	-1.0558066	0.00494521	0.94463876	0.48845907
Rpsa	0.01169787	6.48231852	0.00493239	0.94471046	0.48846542
Eif4e2	0.01058255	5.28784106	0.00488738	0.94496288	0.4885533
Grb10	-0.0080011	6.92938384	0.0048809	0.9449993	0.4885533
Map4	-0.007788	9.19688123	0.00485163	0.94516417	0.48860779
11-Mar	0.02733466	1.34585352	0.00478592	0.94553617	0.48870342
Tbc1d22b	-0.0143807	4.46307118	0.0047847	0.94554309	0.48870342
Ska2	-0.0196245	2.75900867	0.00477698	0.94558696	0.48870342
Gpr27	-0.0636077	-1.2313857	0.00477698	0.94558698	0.48870342
Rps6ka2	-0.0122806	5.22080832	0.00473763	0.94581117	0.48875823
Ncoa6	0.00970049	8.53285239	0.00473749	0.94581197	0.48875823
Gsdmd	-0.0260307	2.29812276	0.00468251	0.9461268	0.4888876
Ildr2	-0.0107636	9.87099209	0.00464947	0.94631693	0.4888876
Foxg1	0.01123832	6.13935789	0.00464792	0.94632585	0.4888876
Hist1h3f	-0.0606377	-1.7081125	0.00464659	0.94633351	0.4888876
Ints5	-0.0190566	3.12011438	0.00464204	0.94635973	0.4888876
Rbm15b	-0.0123779	3.8137189	0.00457488	0.94674858	0.48904749
4932416H05I	-0.0173948	2.63823692	0.00456805	0.94678825	0.48904749
Lzts2	0.01885597	2.82548724	0.00453578	0.94697625	0.48911387
Lynx1	-0.0095499	7.54082893	0.00447859	0.94731107	0.4892219
Lrfn4	-0.0238406	2.51329453	0.00447099	0.94735574	0.4892219
Car8	-0.0206313	2.79708353	0.00446959	0.94736398	0.4892219
E030025P04I	-0.0725525	-1.4199404	0.00445693	0.94743842	0.4892296
A530072M11	0.05008632	0.62588716	0.00443298	0.94757964	0.48924863
Tceal1	0.01089841	5.42049778	0.0044154	0.94768354	0.48924863
Dnase1l3	-0.0736642	-0.8624302	0.00441476	0.94768732	0.48924863
Gm5512	0.02090056	1.38961734	0.00441035	0.94771337	0.48924863
Fcgr1	-0.0516592	-0.627529	0.00439393	0.94781069	0.48925438
Gpc4	-0.012475	4.23406779	0.00438838	0.94784357	0.48925438
Gm6260	0.0303912	1.52096462	0.00437021	0.9479515	0.48925562
Axl	-0.0187302	5.27439036	0.00436794	0.94796502	0.48925562
Ppp1r21	-0.0083698	5.54478482	0.00434817	0.94808275	0.48928566
Surf1	0.01428098	4.01917473	0.00431339	0.94829048	0.48933814
Zscan2	-0.0301806	1.3090685	0.00431121	0.94830352	0.48933814
Zfp383	0.01858128	2.57221034	0.00429102	0.94842453	0.4893451

Cisd3	-0.0185315	2.82960951	0.00426864	0.948559	0.4893451
Cd83	0.01668148	2.18761984	0.00426097	0.94860513	0.4893451
Prox2	0.02567673	1.61354989	0.00425849	0.94862012	0.4893451
Mfng	-0.0444579	-0.3038503	0.00424986	0.94867212	0.4893451
Napepld	-0.0119513	6.43308313	0.00424467	0.94870341	0.4893451
Rnf44	0.00670754	6.73438254	0.0042335	0.94877086	0.4893451
Agpat9	0.02627845	1.27578134	0.00419367	0.94901207	0.4893451
Wdr18	-0.0106014	3.64050136	0.00419105	0.94902796	0.4893451
Slamf7	-0.0413009	1.12128087	0.00417705	0.94911305	0.4893451
Snx8	-0.0235103	1.59947853	0.00417181	0.9491449	0.4893451
Tfpt	0.019187	2.7330613	0.00415735	0.94923298	0.4893451
Itgb3bp	0.02086706	3.3353098	0.00415472	0.94924904	0.4893451
Irf9	0.01512171	3.88034125	0.00414987	0.94927863	0.4893451
Gm5083	0.07340744	-0.806053	0.0041474	0.9492937	0.4893451
Eomes	0.0402546	0.41617846	0.00414246	0.94932385	0.4893451
Ccdc135	0.05218266	-0.2724766	0.00414099	0.94933283	0.4893451
Cpne7	0.01979823	2.63343277	0.00413185	0.9493887	0.4893451
Slc25a43	-0.0643935	-1.5902955	0.00410667	0.94954293	0.48939391
2510003E04I	-0.0088857	7.09266894	0.00409507	0.94961416	0.48939993
Zfp780b	0.00959544	5.39516929	0.00405692	0.94984909	0.48947815
Rere	0.00693495	8.29609131	0.00405109	0.94988504	0.48947815
Uckl1	-0.0140948	3.45904809	0.00403031	0.9500136	0.48951371
Arl13b	-0.0112496	4.2966268	0.00398818	0.95027519	0.4896178
Msc	-0.076045	-0.979222	0.0039556	0.95047842	0.48969181
Tom1l1	-0.0122032	3.91602837	0.00394011	0.95057533	0.48969547
Zswim1	0.01132101	3.84826167	0.00392742	0.95065489	0.48969547
E430018J23F	-0.0215774	2.49952801	0.00392593	0.95066425	0.48969547
St7l	-0.0139336	4.22717222	0.00390485	0.95079665	0.48973298
I830012O16F	0.01710267	4.05990536	0.0038646	0.95105056	0.48983306
Zdhhc23	-0.0330704	1.06906349	0.00384134	0.95119789	0.48986696
1700007K13I	0.03788517	0.10950649	0.0038354	0.95123557	0.48986696
Siglece	-0.0532183	-0.5955054	0.00381218	0.9513832	0.48991229
Tnfrsf13c	0.0396776	0.45441152	0.00377725	0.95160615	0.4899964
Snrnp27	0.0132857	5.43105277	0.00372409	0.95194751	0.4900805
Zfp711	0.01432616	4.20917401	0.00372042	0.95197113	0.4900805
Mill2	-0.0354333	0.60879185	0.00371732	0.95199114	0.4900805
Smim13	0.00777772	8.64473928	0.00370723	0.95205624	0.4900805
AI182371	0.12954969	-2.1088921	0.00370547	0.95206762	0.4900805
Kti12	0.02072874	2.3447234	0.00367965	0.95223466	0.49013578
Zfp219	-0.0189174	1.95613144	0.00364696	0.95244706	0.49021441
BC055324	0.02909492	0.65282369	0.00357051	0.9529475	0.4904263
Ncl	0.00710261	8.89214441	0.00356463	0.95298617	0.4904263
Cenpo	-0.0233068	2.19298701	0.00355681	0.95303775	0.4904263
Gm5086	-0.0702605	-0.7664952	0.00354267	0.95313107	0.49044361
Gm17751	0.06563048	-0.8407761	0.00353299	0.95319507	0.49044584

Trp63	-0.0237731	2.24132109	0.00352355	0.95325752	0.49044727
Gm10649	0.0576922	-1.0484694	0.00350812	0.95335985	0.49044923
B3galnt1	0.00809309	4.8280408	0.00350499	0.95338068	0.49044923
Prx	0.04339611	-0.4765211	0.00348408	0.95351974	0.49049007
Gpr17	0.01704317	2.74550711	0.00346131	0.95367171	0.49053755
Adss	-0.0061738	7.09678092	0.00342007	0.9539482	0.49064906
Sco1	-0.012916	3.29472571	0.0034028	0.95406447	0.49067815
Atp6v0c-ps2	-0.0343428	-0.3216039	0.00339395	0.95412421	0.49067817
Ehbp1	0.0084009	6.77901696	0.00337078	0.95428084	0.49069111
Agpat6	-0.010247	4.13480936	0.00336494	0.95432043	0.49069111
Dbp	0.0153505	4.08973715	0.00335767	0.95436972	0.49069111
Cenph	0.04858092	-0.5446331	0.00335496	0.95438817	0.49069111
Zscan22	-0.0092332	5.38400301	0.00334595	0.95444935	0.49069187
Cfh	-0.0145389	7.13021819	0.003316	0.95465347	0.49076611
Ppib	-0.015425	2.66338172	0.00330079	0.95475745	0.49078887
Myct1	0.08572771	-1.6149048	0.0032639	0.95501067	0.49087401
Dgat1	0.02508659	1.99018369	0.00325928	0.95504252	0.49087401
Pdgfrl	-0.0214202	2.57959627	0.00324172	0.95516363	0.49090555
Bmx	-0.0262513	1.85250892	0.00322273	0.95529503	0.49091939
Pin1	0.01293012	3.03710293	0.00322056	0.95531001	0.49091939
Acbd7	-0.0523688	-1.2442013	0.00319666	0.95547598	0.49095253
Zfp618	-0.0193039	1.48784735	0.00319408	0.95549396	0.49095253
Sox2	0.01416823	3.57343566	0.00317861	0.95560172	0.49097721
Snrk	0.00675736	6.38919124	0.00315986	0.95573274	0.49098874
Wac	-0.0079215	8.63974807	0.00315241	0.95578489	0.49098874
Polr2i	0.01675197	3.0562357	0.00314977	0.95580338	0.49098874
Txnrd1	-0.0099121	4.89554205	0.00314022	0.95587034	0.49099245
Slc6a13	0.01617991	6.53886407	0.0031295	0.95594564	0.49099581
2810006K23I	-0.0102221	4.99404715	0.00312229	0.95599636	0.49099581
A630072M18	0.01279232	3.50751564	0.00309118	0.9562159	0.49100327
Apex1	-0.0517823	-1.045947	0.00308369	0.95626893	0.49100327
Ptger2	-0.0461674	-0.470083	0.00308316	0.95627267	0.49100327
Ikbip	-0.0151525	4.11084961	0.00308102	0.95628782	0.49100327
Slc25a54	-0.0551905	-0.925918	0.00307127	0.956357	0.49100327
Rnd2	-0.0232308	1.72906481	0.00306953	0.95636933	0.49100327
Cep112	0.01155631	3.64725574	0.00301485	0.95675931	0.49117281
Rab38	0.06234699	-1.447128	0.00298761	0.95695488	0.49122907
C030013G03	0.01374709	2.95740626	0.00298261	0.95699085	0.49122907
Gmip	-0.0181304	1.52538376	0.00297466	0.9570482	0.49122907
Vps41	0.00790417	7.02432777	0.00295383	0.95719869	0.49127563
Vil1	-0.0419461	-0.0528895	0.00294173	0.95728636	0.49128995
H2afy2	-0.011659	4.52595508	0.00292304	0.9574221	0.49132893
Maoa	-0.0085795	4.91687969	0.00289525	0.9576248	0.49140227
Ppt1	0.00721967	6.44486495	0.00285813	0.95789699	0.49151126
Fam73b	-0.0111555	3.66081619	0.00283594	0.95806063	0.49156454

Slc16a14	-0.0146787	4.04172305	0.00280292	0.95830522	0.49165934
Atg14	-0.0113373	4.2719661	0.00278791	0.95841696	0.4916716
Gdf5	-0.0509913	-1.6391325	0.00278364	0.95844877	0.4916716
Bex4	0.01156478	4.0910986	0.00276754	0.95856899	0.49169935
Wnt11	0.04725722	-1.0895182	0.00276039	0.95862251	0.49169935
Zfp81	-0.0099123	4.91312818	0.00274338	0.95875002	0.49173407
Rhob	-0.0069013	7.36944358	0.00271723	0.95894694	0.49180438
Zfp870	-0.0113229	3.74149517	0.00269642	0.95910425	0.49182021
Mccc1os	-0.0248563	0.88030954	0.00268378	0.95920011	0.49182021
3830408C21I	-0.0158805	2.47583336	0.00268108	0.95922068	0.49182021
Plau	-0.031436	-0.1551436	0.00267851	0.95924017	0.49182021
Rnf31	-0.0130332	3.23321975	0.00267367	0.959277	0.49182021
Mapkbp1	0.00925436	5.2659979	0.00265765	0.95939907	0.49185211
Ccdc122	0.01718681	2.24255344	0.00260936	0.9597693	0.49198287
Casp8ap2	-0.0057444	6.4656115	0.00260175	0.95982793	0.49198287
Muc2	0.07552012	-1.8954559	0.00259501	0.95987993	0.49198287
Depdc7	0.02268614	1.14205109	0.00259325	0.95989356	0.49198287
Olfr287	0.03768256	0.25108927	0.00257965	0.95999878	0.49198314
Cxcl12	-0.0080218	6.01762405	0.00257769	0.96001394	0.49198314
Scamp3	-0.0122077	3.63923408	0.00256999	0.96007366	0.49198314
Rpn1	-0.0085171	5.65877509	0.00253515	0.96034495	0.49208641
Gimap4	0.01890368	3.0918841	0.00252876	0.96039493	0.49208641
Lrrfip1	0.0069646	5.98272944	0.00251488	0.96050367	0.49211145
Chtf8	-0.0091676	5.11956091	0.00247612	0.96080897	0.49221755
Hoga1	0.0412224	-0.0458098	0.00247339	0.96083053	0.49221755
Map3k2	0.00652514	6.20693347	0.00245583	0.96096974	0.49223526
4632415L05I	0.00727191	5.48944619	0.00245392	0.96098488	0.49223526
Htr5a	0.01255266	5.37112254	0.00244305	0.96107131	0.49224885
Arid4a	-0.0077652	7.65718291	0.00241303	0.96131101	0.49234094
Nudt22	-0.0168309	1.45155025	0.0024037	0.96138582	0.49234857
Pttg1ip	0.01286162	6.25434244	0.00239365	0.9614666	0.49235557
2310009A05I	0.01615462	2.69505631	0.0023871	0.96151929	0.49235557
Tbc1d12	-0.0099865	3.91318309	0.00236988	0.96165826	0.49239606
Kcng3	-0.019929	3.24075379	0.00233091	0.96197453	0.49252732
Slc1a1	0.00762204	6.01790734	0.00229849	0.9622397	0.49263239
Rom1	-0.0187915	1.51486163	0.00229009	0.9623087	0.49263703
Tbxa2r	-0.0762783	-2.105866	0.00227148	0.96246199	0.49268482
Map3k8	0.01594901	1.90323526	0.00224855	0.96265185	0.49272929
Sult2b1	-0.0280771	0.36922458	0.00224651	0.96266876	0.49272929
Utp18	0.00781442	4.20392868	0.00223346	0.96277726	0.49275413
Per1	-0.0084511	5.52922724	0.00221507	0.96293066	0.49280196
Tmem169	-0.0102349	2.9231717	0.00219658	0.96308559	0.49284302
Gla	0.01125278	3.05440702	0.00218391	0.96319211	0.49284302
Lrrc1	-0.0110191	4.36593242	0.00218075	0.96321878	0.49284302
Rtkn	-0.0115218	3.44422276	0.00217043	0.96330582	0.49284302

Hemk1	-0.017082	1.69722115	0.00216985	0.96331072	0.49284302
Gm10638	-0.0350135	-0.6071244	0.00215085	0.96347162	0.49286775
Lancl3	0.01362944	3.95821715	0.0021452	0.96351961	0.49286775
Mast2	-0.0057631	6.29563596	0.00214292	0.96353895	0.49286775
Clstn1	-0.0068505	8.08403304	0.00213419	0.96361327	0.49287509
Epyc	0.03130953	-0.2199656	0.00210504	0.96386242	0.49289956
Ubxn2b	-0.0069749	6.02215154	0.00210436	0.96386826	0.49289956
Wdfy1	-0.0086113	4.67529189	0.00210325	0.96387774	0.49289956
AI197445	-0.0272378	0.52961767	0.00210054	0.96390099	0.49289956
Abcb1a	-0.0112142	5.71532328	0.00206237	0.9642303	0.49303729
Abhd13	-0.0060917	5.20449293	0.00205318	0.96431001	0.49304737
Afg3l1	0.01151988	3.28180885	0.00204168	0.96441	0.49305666
Usp45	0.00655717	6.85723407	0.00203085	0.96450447	0.49305666
Chd3	-0.0056485	7.99789304	0.00202774	0.96453167	0.49305666
P4ha3	-0.0188921	2.42550254	0.00202207	0.96458119	0.49305666
Nup88	0.00673825	5.69244822	0.00201672	0.96462813	0.49305666
Fer1l5	-0.0167541	1.38580258	0.00199924	0.96478163	0.49308142
Ccnb1	-0.0417984	-1.0194549	0.00199754	0.96479656	0.49308142
Mlf2	0.00505823	6.45549964	0.00195569	0.96516702	0.49319604
Abhd16a	0.00690546	4.50715658	0.00195359	0.96518573	0.49319604
2610008E11I	-0.0076986	5.29799621	0.00195189	0.96520086	0.49319604
Ngb	-0.0197043	0.84514126	0.00193965	0.9653101	0.4932212
Zbtb8a	-0.0157847	2.95987828	0.00190252	0.96564346	0.49336086
Spata7	-0.0076405	4.05031783	0.00187492	0.9658934	0.49345788
Fam198b	-0.0098559	3.62236405	0.00186538	0.96598029	0.4934716
Ssbp4	-0.0111917	2.9201445	0.0018577	0.96605034	0.49347672
Entpd2	0.02123165	0.55519252	0.00183513	0.96625702	0.49355162
Vat1l	0.00959707	5.41946063	0.00180786	0.96650857	0.49364943
Tceanc2	-0.0064135	4.63240654	0.00178085	0.96675947	0.49374689
Il21r	-0.039771	-0.8992308	0.00177092	0.96685225	0.49375072
Tcirg1	-0.0147305	1.52990941	0.0017672	0.96688711	0.49375072
Arf5	-0.0116965	3.03196978	0.00174916	0.96705645	0.49380651
Morc3	-0.009118	4.5466549	0.00173066	0.967231	0.49386067
Pltp	0.0124911	4.01039227	0.00172466	0.96728778	0.49386067
Xiap	0.00597509	7.12351358	0.00171628	0.96736729	0.49386067
Chsy1	-0.0061196	5.20745645	0.0017112	0.96741566	0.49386067
Rasl11a	0.02778566	0.00434263	0.00170623	0.96746295	0.49386067
Optn	-0.0074519	4.13149135	0.00169101	0.96760831	0.49388716
Ncs1	-0.0066346	6.14444869	0.00168729	0.96764396	0.49388716
Spata2l	-0.0088954	4.60710982	0.00168195	0.96769512	0.49388716
Wdr65	-0.0241577	1.0782569	0.00166682	0.9678407	0.4939308
Ap1s1	0.00978898	2.8383921	0.00165354	0.96796902	0.49396561
H2-DMb1	-0.0283822	0.53285769	0.00160979	0.96839533	0.49410921
2510039O18	0.01158173	3.40800812	0.00160785	0.96841435	0.49410921
Eif4ebp1	-0.0152757	1.68990599	0.00160618	0.96843077	0.49410921

Adprh	0.00600028	4.24486342	0.00159358	0.9685548	0.49414182
Actl6b	0.01146795	3.2826385	0.00156748	0.96881317	0.49420209
Gclm	0.00672083	5.32642741	0.00156606	0.96882736	0.49420209
Dyrk1a	-0.0044424	7.22479528	0.00155977	0.96888991	0.49420209
Bhlhe22	0.00641841	5.19943466	0.00155741	0.96891345	0.49420209
Ngrn	0.00713051	4.21976295	0.00153384	0.96914949	0.49428861
Vsig2	0.00829238	3.29637463	0.00152848	0.96920336	0.49428861
Rpap2	-0.0056853	4.70556522	0.00148236	0.96967136	0.49449661
Abcc3	0.02880473	-0.6679128	0.00147223	0.96977509	0.49451882
Lhx8	-0.0126813	2.6491906	0.00145248	0.96997841	0.49454633
2810454H06I	0.02891836	-0.9132212	0.00144903	0.97001402	0.49454633
Cstad	0.01185713	2.55510493	0.001448	0.97002476	0.49454633
Slc28a3	-0.0246938	1.09393552	0.00144365	0.97006972	0.49454633
Msr1	-0.0442088	-1.3220509	0.0014239	0.97027514	0.49459136
Map1s	-0.0085508	3.04419634	0.00142358	0.9702784	0.49459136
Map9	0.006233	7.90118263	0.00141492	0.97036892	0.49460683
Slc7a1	-0.0055021	5.28969118	0.00140306	0.97049332	0.49462276
Cckar	-0.0281605	-0.7734753	0.00140047	0.97052054	0.49462276
Hmha1	-0.0165166	1.25601341	0.00139271	0.9706023	0.49463376
Rpusd4	-0.010045	2.7187492	0.00138567	0.97067669	0.494641
Ptges2	0.00926792	2.3907811	0.00135373	0.9710164	0.49476293
Stx5a	-0.0072886	4.58266496	0.00135187	0.97103635	0.49476293
Pgam2	0.01887921	0.82968011	0.00132944	0.97127747	0.49482273
6330418K02I	0.01666011	1.11067126	0.00132573	0.97131754	0.49482273
Gnb5	0.00558632	6.22872661	0.00132418	0.97133434	0.49482273
Endod1	-0.0055653	4.92098218	0.0013117	0.97146965	0.49483542
Sypl2	-0.025187	0.14248168	0.00130758	0.97151449	0.49483542
Ifitm10	0.02390966	0.45607829	0.00130525	0.97153986	0.49483542
Lamp2	-0.0070803	7.76757349	0.00125462	0.97209703	0.49506577
Vars	0.0104478	2.45858868	0.00125165	0.97213012	0.49506577
Mtcp1	-0.0100674	3.03143394	0.00124781	0.97217282	0.49506577
Car7	0.01316683	1.42663876	0.00121461	0.97254539	0.49522481
Suv420h2	-0.0213297	-0.0499833	0.00120903	0.97260853	0.49522628
Sfrp5	0.05266876	-2.1405377	0.00118875	0.97283916	0.49525704
Rad54b	0.02876488	-0.1526747	0.00118803	0.97284734	0.49525704
Egln1	-0.0043492	6.86090147	0.00118782	0.97284972	0.49525704
Ushbp1	0.0207456	0.5480966	0.00114066	0.97339389	0.49548805
Hist1h4j	0.01480625	0.67386973	0.00113317	0.97348136	0.49548805
Pigb	0.01022778	2.54584533	0.00113292	0.97348434	0.49548805
Haus4	-0.013651	1.58054984	0.00112658	0.97355857	0.49549514
Bud13	-0.0087483	2.07170836	0.00110891	0.97376673	0.49554363
Slc7a7	0.01806754	1.12585442	0.00110826	0.97377443	0.49554363
Ccdc136	-0.0052607	5.29502051	0.00109833	0.97389208	0.49557282
Gm14015	-0.0436375	-0.754935	0.0010854	0.97404612	0.49562052
Slc7a8	-0.0061437	5.5378534	0.00104603	0.974521	0.49583146

Uros	0.00704641	3.53309665	0.00104024	0.9745916	0.49583668
Tgfa	-0.004132	5.15283698	0.00103018	0.97471471	0.49586862
Tert	-0.025399	-0.679246	0.00101499	0.97490181	0.49593311
Rgs22	-0.0206721	0.3420577	0.00098951	0.97521877	0.49598608
Cdca7	-0.0222244	0.33717584	0.00098893	0.97522594	0.49598608
Trim46	0.00675026	4.42013848	0.00098706	0.97524942	0.49598608
Fbxo2	-0.0072753	3.07285821	0.00098536	0.97527077	0.49598608
Pex10	0.00832808	2.67676415	0.00098242	0.97530767	0.49598608
Cd164l2	-0.0328957	-1.0215828	0.00096371	0.97554385	0.49607549
Tsn	0.00474942	6.2732336	0.00093512	0.9759092	0.49623057
Zfp467	0.00739656	4.00171468	0.0009147	0.97617359	0.49633431
Gm20324	0.02370139	-0.5352857	0.00090863	0.97625271	0.49634383
Dcdc2a	-0.0052307	7.4805659	0.00090385	0.97631531	0.49634495
Gm1604b	0.01044456	1.39017072	0.00088853	0.97651684	0.4964167
Zfp213	-0.0145887	1.09284585	0.00087565	0.97668757	0.49645668
Clec7a	0.02916382	0.80824858	0.00087349	0.97671629	0.49645668
Dhrs7b	-0.0105307	2.406399	0.00086364	0.97684791	0.49646919
Pik3cg	0.00791014	2.45335659	0.00086261	0.97686171	0.49646919
Trim3	0.00484043	4.52986376	0.00084966	0.97703607	0.4965271
Gpr55	-0.0183688	-0.1849099	0.00083444	0.9772426	0.49656219
Leprotl1	-0.0035082	6.11791754	0.0008314	0.97728401	0.49656219
Tmprss6	0.05162639	-2.147482	0.00082274	0.97740264	0.49656219
Fanca	-0.0101328	-2.154117	0.00082136	0.97742158	0.49656219
Mum1	-0.00955	2.98551717	0.00081614	0.97749336	0.49656219
Fut10	0.00624564	3.61978587	0.00081247	0.97754414	0.49656219
Hyou1	0.00578244	5.17947452	0.0008118	0.97755338	0.49656219
Srpx2	-0.0172041	0.24432946	0.00080926	0.97758844	0.49656219
Acacb	-0.0139685	1.28171749	0.00079155	0.97783493	0.4966185
Cd276	0.00886306	2.49201803	0.00079027	0.9778529	0.4966185
Ric3	0.00401034	6.1222974	0.0007883	0.97788056	0.4966185
Smim6	-0.0372743	-1.6185278	0.00077643	0.97804759	0.49663633
Prune	-0.0042974	4.58506646	0.00077543	0.97806184	0.49663633
Klri2	-0.0190496	0.40890264	0.00077295	0.97809694	0.49663633
Rab3c	0.0044343	9.29562799	0.00075855	0.97830187	0.4967097
Mir684-1	-0.0132604	0.23407638	0.00074152	0.9785467	0.49677274
Pcif1	-0.0045266	3.96278739	0.00074151	0.97854691	0.49677274
Pdp2	-0.0075396	3.55228606	0.00073653	0.97861905	0.49677867
Bphl	-0.0065876	4.07017953	0.00072398	0.97880192	0.49684082
A830019L24I	0.02592078	-0.6944655	0.00071563	0.97892441	0.49687231
Mas1	-0.0079103	2.57691821	0.00070718	0.97904919	0.49690496
Cecr5	-0.013723	0.6165469	0.00069259	0.97926641	0.49697471
Ccdc33	-0.0164041	0.10981215	0.0006885	0.97932764	0.49697471
Cd3e	0.01134523	2.30550467	0.00068582	0.97936803	0.49697471
Aftph	0.00323566	6.94774223	0.00065768	0.97979551	0.49716094
Esrra	-0.0095677	2.04728223	0.00065259	0.97987395	0.49717005

Rnf115	0.00359573	5.90660914	0.00064323	0.98001871	0.4971905
Slc39a10	-0.0033822	8.38104969	0.00063593	0.98013241	0.4971905
Bbs5	0.00559955	3.73149981	0.00063509	0.98014552	0.4971905
Ncf1	0.00649892	2.38759849	0.00063441	0.98015623	0.4971905
Nek8	0.015182	1.27127554	0.00061491	0.98046344	0.49731564
Efcab9	-0.0180434	-0.5491644	0.00060839	0.98056723	0.4973376
B230319C09	0.02273172	-1.0365313	0.00058835	0.98088996	0.49747058
E4f1	-0.0064826	2.50487204	0.00058095	0.98101051	0.49750102
Cdkl1	0.00842021	2.6430747	0.00056626	0.98125208	0.49758551
Tgoln1	-0.0037613	6.81995248	0.00056348	0.98129819	0.49758551
Rhov	0.01570013	1.2476218	0.00055775	0.9813935	0.49759881
Arhgef25	-0.0037553	5.31720204	0.00055463	0.9814455	0.49759881
Ntf3	-0.0150367	-1.2165419	0.00054936	0.98153385	0.49761291
Ubap2	-0.0027892	6.09196296	0.00053836	0.98171965	0.49767641
Gm13498	0.01359013	-0.4467189	0.00052956	0.98186971	0.49771216
Gskip	0.00463341	4.39527717	0.00052713	0.98191129	0.49771216
Gpc1	-0.0057966	3.38315005	0.00051584	0.98210602	0.49778017
Cct3	0.00321358	5.12571248	0.00050798	0.98224276	0.49781847
Tab1	0.00631593	2.84188839	0.00050456	0.98230274	0.49781847
Ubxn7	0.00230258	7.31127239	0.00049463	0.98247776	0.49786761
Pla2g5	0.01225744	1.43780082	0.0004922	0.98252083	0.49786761
Sp8	-0.0105467	0.59072781	0.00048215	0.98270009	0.49792774
Gm6787	0.02315273	-0.4742893	0.00047514	0.98282634	0.49796101
Myo1e	-0.0035277	4.55199139	0.00046679	0.98297778	0.49800704
Klhl7	-0.0029764	6.31339067	0.00045324	0.98322671	0.49809173
Col8a2	-0.0091292	2.09764553	0.00044893	0.98330653	0.49809173
F2rl2	0.00645011	1.92100183	0.00044785	0.98332674	0.49809173
Tmeff2	0.00287024	6.92265816	0.0004431	0.98341532	0.4981059
Stk38l	0.00262162	5.63816732	0.00043666	0.98353621	0.4981094
Exosc8	0.00371282	3.93105156	0.00043628	0.98354345	0.4981094
Triap1	-0.0078043	3.20084066	0.0004242	0.98377277	0.49816735
Krt18	0.0622112	-1.6566113	0.00042387	0.98377908	0.49816735
Mrpl19	-0.0030733	5.02303377	0.0004193	0.98386689	0.49818112
Fancc	0.00658056	1.8744647	0.00041085	0.98403013	0.49823308
Gm15708	0.01977994	-1.0342753	0.00039787	0.98428445	0.49833115
Ppp2r1b	0.0026521	5.52140425	0.00038525	0.98453563	0.49842762
Nhlh2	-0.0200404	-0.6575748	0.00037383	0.98476652	0.49848899
Aco2	0.00239345	7.41179208	0.00037326	0.98477817	0.49848899
Pnp2	-0.0129939	-0.4451936	0.0003634	0.98498065	0.4985104
Zmynd11	0.00213858	7.87641286	0.00035606	0.98513304	0.4985104
Prr5	0.00799592	1.40299752	0.00035304	0.98519616	0.4985104
Rtn1	0.0021309	10.4495925	0.00034869	0.98528763	0.4985104
Phtf1os	0.00681469	1.14798045	0.00034806	0.98530102	0.4985104
Traf3ip2	0.00506678	2.99233899	0.00034684	0.98532678	0.4985104
Polr3g	-0.0040502	3.14058005	0.00034602	0.98534399	0.4985104

Opn1mw	-0.0202474	-1.6765362	0.00034571	0.98535053	0.4985104
Mlh3	-0.0025854	5.77549953	0.00034464	0.98537337	0.4985104
Tle6	0.01138757	0.31341144	0.00034211	0.985427	0.4985104
Ggt5	0.0096833	1.43336164	0.00033242	0.98563491	0.4985849
Ptpmt1	-0.0031075	4.1804834	0.00032209	0.98585977	0.49865537
Gpr132	0.02202221	-1.4548746	0.00032047	0.98589556	0.49865537
Alox12	-0.0083788	1.23960461	0.0003162	0.98598967	0.49867228
Secisbp2l	0.00213476	7.76977179	0.00030918	0.98614614	0.49872072
Acvr1c	0.00382442	4.78138108	0.0003007	0.98633739	0.49877707
Abcb4	0.00970141	0.37409322	0.00029863	0.98638456	0.49877707
Tfr2	0.00864315	0.97778747	0.00029622	0.98643961	0.49877707
Tut1	-0.0054886	2.2972698	0.00028525	0.98669296	0.49885882
Spg7	-0.0041445	3.55351131	0.00028398	0.98672268	0.49885882
Inhbb	0.00821925	1.70768754	0.00027828	0.98685668	0.49887294
Prkce	-0.0023798	8.83402021	0.00027757	0.98687333	0.49887294
Col6a2	0.0052855	5.41390763	0.00027507	0.98693269	0.49887294
Eftud2	-0.0027985	5.01527971	0.00026849	0.9870898	0.4988966
Wscd2	0.00383287	3.52687442	0.00026781	0.98710606	0.4988966
Slc25a10	0.00716982	1.47873183	0.00026551	0.9871616	0.4988966
Fhod3	-0.003393	5.14905749	0.00025836	0.98733568	0.49895389
Pld4	0.00957972	-0.3204414	0.00023829	0.98783746	0.499134
Dph7	0.00298766	3.34348573	0.00023695	0.98787171	0.499134
Gm5795	0.02499467	-2.0561598	0.0002368	0.98787561	0.499134
Dtd2	-0.0039357	4.09246295	0.00023265	0.98798216	0.499134
Fkrp	-0.0022103	5.29122087	0.0002298	0.98805613	0.499134
Plk4	-0.0032308	3.65666331	0.00022834	0.98809413	0.499134
Itgb2	-0.006443	1.44270914	0.00022746	0.98811717	0.499134
D330050G23	0.00630669	1.95972551	0.00022291	0.98823647	0.49916358
Xpnpep3	-0.0025475	4.78115851	0.00021374	0.98848091	0.49925637
Eps8	-0.0021642	5.3883578	0.0002102	0.98857673	0.49927408
Dnase2a	-0.0102875	-0.3412987	0.00020267	0.98878334	0.49934774
Zfp639	0.00320736	4.46191698	0.00019275	0.98906113	0.49938825
Rubie	-0.0204675	-1.4883069	0.00019229	0.98907419	0.49938825
Srpk1	0.00169928	6.0743028	0.00019219	0.98907698	0.49938825
Prickle3	-0.0052526	2.54428717	0.00019115	0.98910658	0.49938825
Gm5796	-0.0098683	-0.9775086	0.00017894	0.98946033	0.49953617
Dysf	0.00956154	0.33250589	0.00017565	0.98955752	0.49953773
Rims4	0.01753164	-1.1305353	0.00017268	0.98964623	0.49953773
Fbxl22	-0.0171454	-0.6497519	0.00017251	0.98965124	0.49953773
Bcap29	-0.0020604	5.17023279	0.00017068	0.98970653	0.49953773
Il1r1	0.00281847	4.30797735	0.00016591	0.98985118	0.49955155
Rassf4	-0.0031707	3.13006963	0.00016577	0.98985548	0.49955155
Tm4sf20	0.01714376	-1.3584128	0.00016197	0.9899947	0.49959113
Plekhh2	0.00195409	5.65818336	0.00015881	0.99007079	0.49959886
Zkscan1	-0.0014713	7.6698238	0.00015443	0.99020862	0.49963773

Tdo2	0.0129768	-0.9958907	0.00014821	0.99040797	0.49970765
Tiam2	-0.0024094	4.92606776	0.00014262	0.99059048	0.49976378
Dnaic2	0.01362262	-1.540072	0.0001411	0.99064083	0.49976378
Angel2	-0.0016458	5.77778424	0.00013833	0.99073318	0.49977969
Fbxo25	-0.0019834	4.92372084	0.00013325	0.99090473	0.49981007
Nt5c2	0.00153114	5.54623857	0.00013134	0.99097009	0.49981007
Lrrc14	0.00300354	2.72830869	0.00013118	0.99097583	0.49981007
Akr1c18	0.00622021	1.31564521	0.00012525	0.9911819	0.49987339
Sephs2	-0.0022259	4.04139257	0.000123	0.99126156	0.49987339
1700010I14R	0.00609845	-0.0902284	0.00012237	0.99128385	0.49987339
Itsn2	0.0013833	7.26278222	0.00012018	0.99136252	0.4998824
Arhgap10	-0.0020119	4.4409268	0.00010975	0.9917457	0.50004493
Gm10509	0.00290297	2.66833887	0.00010702	0.99184887	0.50006628
Zfp408	-0.0015996	4.68741714	0.00010174	0.99205243	0.50013822
Abhd15	0.00924059	-1.3237041	8.67E-05	0.99266442	0.50041606
Card14	-0.0068893	0.21782031	8.51E-05	0.99273069	0.50041878
Ak7	0.00363736	1.73166403	8.33E-05	0.99280763	0.50042687
Gpr182	0.00314836	4.10662129	8.18E-05	0.99287344	0.50042935
Ndufaf6	0.00257204	1.59740847	5.78E-05	0.99401066	0.50095022
Zfhx4	-0.0011232	8.17332463	5.64E-05	0.99408076	0.50095022
Nubp2	0.0018045	3.47215544	5.61E-05	0.99409814	0.50095022
Dpp4	-0.0024065	5.50219731	5.44E-05	0.99419071	0.50095022
4833412C05I	0.00442154	0.25183559	5.40E-05	0.99421161	0.50095022
Mesdc2	0.00133607	6.39054405	4.75E-05	0.99457018	0.50109158
Fem1b	0.00082069	7.94531779	4.67E-05	0.99461409	0.50109158
Fem1c	0.00097974	5.33679925	4.18E-05	0.9949051	0.50116162
Ndufaf4	0.00095989	5.76558867	4.17E-05	0.99491188	0.50116162
5033404E19I	-0.0511655	-1.5114324	4.08E-05	0.99496963	0.50116162
Cd200r1	-0.004529	-0.6579988	4.03E-05	0.99499702	0.50116162
Serpinb1a	0.00243195	2.21914967	3.46E-05	0.99536324	0.50131535
4833418N02	-0.0020481	1.55173817	2.75E-05	0.99586669	0.50152206
Tctn1	0.00242036	2.90632425	2.61E-05	0.99597644	0.50152206
Polh	-0.0013635	2.48231141	2.58E-05	0.99599677	0.50152206
Katnal1	-0.0007117	6.85502897	2.55E-05	0.99601772	0.50152206
Msh3	0.00094118	4.12936455	2.21E-05	0.9962993	0.50160356
Kansl3	0.00066808	5.88337963	2.19E-05	0.99631227	0.50160356
Neo1	0.00064949	7.54444732	2.13E-05	0.99636267	0.50160356
Pecr	-0.0016588	1.25200424	1.97E-05	0.99649995	0.50162325
Nr3c1	-0.0004676	7.2766846	1.95E-05	0.99652386	0.50162325
C1qtnf4	0.00136021	2.03375182	1.80E-05	0.99665623	0.50163866
Gareml	0.00154143	2.21577455	1.78E-05	0.99667654	0.50163866
As3mt	-0.001028	3.54657067	1.57E-05	0.9968828	0.50171176
Cdkn2c	0.00149155	2.73098207	1.47E-05	0.99697701	0.50172844
St18	-0.0011166	3.77041997	1.25E-05	0.99721057	0.50181526
Micu3	0.00050413	7.58963231	1.01E-05	0.99749251	0.5018282

BC064078	-0.0019114	0.67834504	1.00E-05	0.99750279	0.5018282
Zfp346	0.0004811	4.78813054	9.86E-06	0.99752553	0.5018282
Zfp740	0.00057631	4.79995904	9.12E-06	0.99762009	0.5018282
Mir1b	-0.0117011	-1.1700818	8.89E-06	0.99765131	0.5018282
Cdk14	-0.0004092	6.91946638	8.86E-06	0.99765428	0.5018282
Dclk1	-0.0005143	10.5375499	8.79E-06	0.99766368	0.5018282
Cep162	-0.0003117	6.06887196	6.07E-06	0.99805844	0.50193751
Ift80	-0.0003513	4.88958403	6.06E-06	0.99806052	0.50193751
A430090L17I	0.00223277	0.76348656	6.04E-06	0.99806421	0.50193751
Zfp784	0.00054403	3.96359625	4.98E-06	0.99824194	0.50199617
Zfp945	0.0002874	5.18265876	3.54E-06	0.99851851	0.50208195
Fndc3a	0.00024321	8.08988479	3.46E-06	0.99853468	0.50208195
Gaa	0.00031902	5.861557	3.05E-06	0.99862367	0.50209598
Rgs14	-0.000475	2.19227833	2.04E-06	0.99887378	0.50217202
Prkcq	-0.0004795	2.94313215	1.78E-06	0.99894872	0.50217202
1600020E01I	-0.0008406	1.1376363	1.75E-06	0.99895821	0.50217202
Stau1	0.00015109	6.3092757	1.55E-06	0.99902034	0.50217254
Tysnd1	0.00020221	2.27809315	5.64E-07	0.99940807	0.50232743
Espn	-0.0002189	-0.5472656	4.73E-07	0.99945825	0.50232743
Olfr55	0.00666319	-1.2121881	3.49E-07	0.99953472	0.50232743
Ppp1r3fos	0.00054882	0.05384402	2.94E-07	0.99957295	0.50232743
Irgq	4.68E-05	7.40639249	1.10E-07	0.99973837	0.50237985
Carkd	-2.62E-05	3.50228562	2.09E-08	0.99988614	0.50242339
