# Supplementary material for: Mapping the physiological and molecular markers of stress and SSRI antidepressant treatment in S100a10 corticostriatal neurons
Source: Mol Psychiatry. 2019 Aug 20;25(5):1112–29. doi: 10.1038/s41380-019-0473-6 (PMC7031043; doi:10.1038/s41380-019-0473-6)
Supplement: Supplementary file 18 — Supplemental Table S2b [file 41380_2019_473_MOESM18_ESM.pdf]

Suppl Table 2b. Genelist representing the differentially expressed genes (4166) between the Sh and Sh+Flx anxious groups, in the context of all examined genes.

symbol	logFC	logCPM	F	PValue	FDR
Cstb	2.12514989	6.70738108	107.054117	1.86E-09	1.40E-05
Sccpdh	1.13880576	6.28810619	95.2511684	4.97E-09	1.86E-05
Nptx2	2.12896702	4.03919698	83.5984126	1.45E-08	2.76E-05
Ryr1	-4.0437925	2.63715206	83.4616409	1.47E-08	2.76E-05
Efhd2	1.23522722	6.73441745	81.0178166	1.87E-08	2.81E-05
Cdkn1a	1.39594543	5.39687725	68.5455145	7.07E-08	8.01E-05
Kcnip3	0.87889942	7.64498816	66.029914	9.46E-08	8.01E-05
Gm16523	-3.5391142	1.10422528	66.0159245	9.47E-08	8.01E-05
Unc13c	-1.17881	5.92405696	65.892915	9.61E-08	8.01E-05
Rgs13	6.72033626	0.46691348	64.9675375	1.07E-07	8.04E-05
Cck	0.99747324	7.75430441	63.9485641	1.21E-07	8.26E-05
Cycs	1.20390015	8.2438788	62.6173139	1.42E-07	8.90E-05
Ecm1	1.92798738	3.89611697	60.9380213	1.75E-07	9.69E-05
Hpcal4	1.20768267	9.96877456	60.5724084	1.83E-07	9.69E-05
Arl15	1.01325005	6.06996797	59.7485606	2.03E-07	9.69E-05
Tnnt2	1.93584142	4.613484	59.6289924	2.07E-07	9.69E-05
Omd	-1.2613191	5.17347677	58.6977596	2.33E-07	9.78E-05
Gng3	1.00520585	8.0666176	58.6333102	2.35E-07	9.78E-05
Tbrg3	-2.2325269	4.19389579	55.2458611	3.66E-07	0.00013914
Aldoa	0.87419969	9.15691313	55.154092	3.71E-07	0.00013914
Emd	1.07215763	5.92569873	54.3847389	4.12E-07	0.00014698
Medag	0.85298827	6.21870146	53.6590539	4.55E-07	0.00014698
Ptprz1	-1.3174092	6.78132457	53.5945355	4.59E-07	0.00014698
Atp6v1g2	0.91417377	9.45908367	53.2646873	4.80E-07	0.00014698
Zwint	0.77995103	6.89570744	53.119582	4.90E-07	0.00014698
Ttn	-2.3557704	3.64854809	51.7081163	5.97E-07	0.0001722
Mllt11	0.78099879	7.83720873	51.3247028	6.30E-07	0.00017508
6330415B21I	-2.1540021	3.14758581	50.8866351	6.71E-07	0.00017971
Cend1	0.86572908	7.05317087	50.5019856	7.09E-07	0.00017988
Clmn	-0.997865	5.98196232	50.3996416	7.19E-07	0.00017988
Sdpr	1.67090832	7.13197726	50.010712	7.61E-07	0.00018414
Vimp	1.09181277	6.1270351	49.7578903	7.89E-07	0.00018505
Lancl2	0.82326072	8.49514295	49.4534348	8.25E-07	0.00018759
Hrg	5.01029607	1.03122995	48.8884685	8.97E-07	0.0001978
Ywhaz	0.68169271	12.3202423	48.4295777	9.59E-07	0.00020129
Casr	2.81818126	1.89347907	48.3837235	9.66E-07	0.00020129
Cfl1	0.87302031	8.59102715	48.0875898	1.01E-06	0.00020331
Snap25	0.7735953	14.1167379	47.7957717	1.05E-06	0.00020331
Gm561	1.29166354	3.43496853	47.7797037	1.06E-06	0.00020331
Capn6	1.60324061	4.31627963	46.8487548	1.22E-06	0.00022812
Mbp	-1.4098768	7.0503577	46.5429983	1.27E-06	0.00023
Vsnl1	1.00439379	12.0201766	46.4743862	1.29E-06	0.00023
S100a6	2.07666351	5.94037353	45.5177584	1.49E-06	0.0002603
Thbs1	-2.156475	2.76445923	45.2331453	1.56E-06	0.00026316

Uchl1	1.01206838	6.11240315	45.1551	1.58E-06	0.00026316
Gm10471	-3.0096137	1.85774165	44.7131946	1.69E-06	0.00027588
Pik3c2b	-1.0683873	5.11271293	44.4917195	1.75E-06	0.00027596
Topaz1	5.97581074	0.39184056	44.4410222	1.77E-06	0.00027596
Plcg1	-1.1518586	4.07223723	44.2619153	1.82E-06	0.00027792
Rtl1	-2.1724263	2.9729589	44.1380829	1.85E-06	0.00027792
Meg3	-1.9116594	10.8517456	43.8718753	1.93E-06	0.00028424
Calm2	0.6937748	12.25381	43.5902529	2.02E-06	0.00029128
4930570G19	-1.9317516	3.53760825	43.4777522	2.06E-06	0.00029128
Chga	1.41802712	4.36510179	42.9756402	2.23E-06	0.00030898
Ftx	-1.7396513	4.04392197	42.7075421	2.33E-06	0.00030898
Anp32a	0.69108058	7.60656272	42.6773175	2.34E-06	0.00030898
Stip1	0.75458498	7.04168692	42.6610179	2.35E-06	0.00030898
Tars2	-1.5726995	2.52134656	42.4766884	2.42E-06	0.00031289
Stra6	2.397908	7.10475634	42.1408255	2.56E-06	0.00031966
Ppp2ca	0.66238191	8.45582872	42.1380566	2.56E-06	0.00031966
Nfrkb	-1.1641407	4.24815793	41.9385765	2.64E-06	0.00032488
6330403K07I	0.75836959	7.67692203	41.4107256	2.88E-06	0.00034379
Snca	0.74825345	7.68402649	41.4002997	2.89E-06	0.00034379
Actn2	-1.8352631	2.19011377	41.2365661	2.97E-06	0.00034587
Tagln3	1.01117922	6.38412678	41.1759192	3.00E-06	0.00034587
Ptchd4	-1.3142399	4.03406526	40.9478048	3.11E-06	0.00035384
Adamts3	-1.3894731	4.12886583	39.9173795	3.70E-06	0.00041131
Cox5a	0.86942124	6.35385492	39.7913486	3.78E-06	0.00041131
Bmyc	1.08901148	5.26490475	39.6908616	3.85E-06	0.00041131
Mall	4.51208273	0.83161331	39.6477219	3.88E-06	0.00041131
Rgs9	-1.5662587	5.09525429	39.6205579	3.90E-06	0.00041131
Gm21671	-3.4333313	0.9986675	39.5430964	3.95E-06	0.00041131
Nbas	-1.2603565	5.07413997	39.2261438	4.17E-06	0.00042836
Aox3	1.23550072	6.1406988	39.0804726	4.27E-06	0.00043331
Nrn1	0.81042915	7.30682903	38.7081001	4.56E-06	0.000456
Nrbf2	0.93430506	4.25797767	38.6131347	4.64E-06	0.00045749
Pdlim1	1.60930411	3.85893549	38.3946836	4.81E-06	0.00046907
Usp29	-1.0874564	5.56455857	38.2997649	4.90E-06	0.0004708
Coa3	1.00927044	4.39961295	38.1931324	4.99E-06	0.0004736
F5	-2.5289846	2.6508564	38.0066673	5.15E-06	0.00048295
Ndufa8	0.92122371	5.2594952	37.9243	5.23E-06	0.00048295
Adrbk2	-1.1353921	5.20441657	37.8699118	5.28E-06	0.00048295
Ndufab1	0.87863486	5.61527553	37.4350675	5.70E-06	0.00051529
Zfp804b	-2.1138066	2.52131327	37.292574	5.85E-06	0.00051679
Appl2	-0.7992776	6.30559206	37.2849142	5.86E-06	0.00051679
Eml6	-1.3691532	4.31941007	37.0602749	6.10E-06	0.00053167
Trnp1	0.86166419	6.17704548	36.9325138	6.24E-06	0.00053703
A230057D06	-2.0938923	3.28385481	36.8759685	6.30E-06	0.00053703
Ifitm1	1.61057019	5.15849788	36.8095801	6.38E-06	0.00053737

Prl	-6.4398849	1.48097504	39.5065134	6.46E-06	0.00053865
Aqp1	2.7963213	1.78608843	36.5527042	6.68E-06	0.00054607
Mdh2	0.69713782	7.24664761	36.5039779	6.74E-06	0.00054607
Pgk1	0.69488814	7.98381182	36.4764967	6.77E-06	0.00054607
Cemip	-1.1178197	3.50331555	36.2335996	7.07E-06	0.0005576
Gdap10	-2.1473612	2.40450832	36.1820971	7.14E-06	0.0005576
Myo9b	-1.0894963	4.49957266	36.176384	7.15E-06	0.0005576
Rad23b	0.62864338	7.99286749	36.1290363	7.21E-06	0.0005576
Lyrm7	0.91591645	4.50608836	36.0278224	7.34E-06	0.00056116
Plxnd1	-1.014357	4.68512081	35.9331804	7.47E-06	0.00056116
Cplx1	0.65535675	8.44920453	35.9021237	7.51E-06	0.00056116
BC048546	1.01165269	5.10503088	35.8725837	7.56E-06	0.00056116
Fat4	-0.8506439	5.96841046	35.8033257	7.65E-06	0.00056274
4933413L06F	-4.0132839	-0.1724786	35.6510249	7.87E-06	0.00056536
Srrm4os	-2.6244672	1.83106618	35.6460759	7.88E-06	0.00056536
Rap1gds1	0.59499849	8.5849631	35.6198741	7.91E-06	0.00056536
Irs4	-2.4459991	1.99826185	35.5136111	8.07E-06	0.00057108
Fam46a	0.85530486	5.75775238	35.4583153	8.15E-06	0.00057153
Dhrs1	0.95602434	5.70162098	35.3911078	8.25E-06	0.0005733
Atp6v1d	0.61555838	8.33107629	35.2763718	8.43E-06	0.00057799
Bcas1	-1.2852628	4.7132492	35.2006107	8.55E-06	0.00057799
Sorbs2os	-2.2076659	3.15431538	35.1987221	8.55E-06	0.00057799
Cnrip1	0.79385954	6.07897393	35.1025501	8.71E-06	0.00058079
Sqstm1	1.57688839	8.74120663	35.0754341	8.75E-06	0.00058079
Phlda1	0.95830815	7.72269091	35.0287746	8.83E-06	0.00058079
Pnpla1	-2.8962565	0.93716736	34.8564876	9.11E-06	0.00059449
Inhba	0.78830718	4.79140572	34.6920829	9.40E-06	0.00060773
Anapc1	-0.7731071	6.17805781	34.6057896	9.55E-06	0.00061234
Neat1	-1.0696581	5.07494143	34.4923418	9.76E-06	0.0006202
Mvk	1.33644662	2.941983	34.2649946	1.02E-05	0.00064184
Grik1	-1.8521113	2.80971496	34.1832176	1.03E-05	0.00064638
A330023F24I	-2.4000852	4.37251926	34.1062433	1.05E-05	0.00065043
Dynll1	0.86697633	8.0929737	34.0431424	1.06E-05	0.00065284
Atp8b2	-1.2691606	4.3200366	33.9307051	1.08E-05	0.00065841
Tnrc18	-0.7571765	5.12993566	33.8108092	1.11E-05	0.00065841
Dnah7b	-1.1865403	3.50336842	33.8092157	1.11E-05	0.00065841
Pkia	0.6335283	8.02784986	33.802421	1.11E-05	0.00065841
E030003E18I	1.58956279	2.39681955	33.7866628	1.11E-05	0.00065841
Gm10220	-2.5215778	1.69414283	33.5343677	1.17E-05	0.00068158
Map1a	-1.4462221	10.3099317	33.5232617	1.17E-05	0.00068158
Pcdhb16	-1.2541943	4.67358307	33.4813764	1.18E-05	0.00068178
Cd209c	-1.6613889	2.7033176	33.4329557	1.19E-05	0.00068229
Myo6	-1.0514741	6.39830014	33.3976821	1.20E-05	0.00068229
Nup155	-1.1214303	5.24826961	33.1966883	1.25E-05	0.00069887
Slc5a7	-1.0925172	4.15569526	33.1943287	1.25E-05	0.00069887

Hpca	0.78200026	8.12687707	33.0746234	1.28E-05	0.00070987
Dcc	-1.0253066	5.17733694	33.0346556	1.29E-05	0.0007101
Ptch1	-0.8907278	4.75473834	32.9022257	1.32E-05	0.00071897
Stmn2	0.6126056	7.85579106	32.8949008	1.32E-05	0.00071897
F8	-1.2445859	3.0977305	32.7745958	1.35E-05	0.00073063
Uqcrfs1	0.63134804	6.5995679	32.6917568	1.38E-05	0.00073717
Mapre3	0.73757135	7.32864478	32.6240878	1.39E-05	0.00074163
Atp5g1	1.09546791	5.41654017	32.5067236	1.43E-05	0.00075344
Gm15910	-1.5857655	4.50043733	32.3942423	1.46E-05	0.00076401
Eno3	1.5800274	3.21469551	32.2906015	1.49E-05	0.00076401
Mrps36	0.93696775	5.16856347	32.267576	1.49E-05	0.00076401
Cxx1c	0.76471147	5.91556429	32.2671037	1.49E-05	0.00076401
4930488L21F	-3.9537848	-0.5601524	32.2583979	1.50E-05	0.00076401
Las1l	-1.0904263	4.97411249	32.2231922	1.51E-05	0.0007641
Wisp1	1.65813361	1.94884932	32.1704402	1.52E-05	0.00076687
Csmd3	-1.5832567	5.37477156	32.0727748	1.55E-05	0.00077652
Hpcal1	1.06443528	4.60151312	32.0091423	1.57E-05	0.0007811
Ywhah	0.57079731	10.4947765	31.9253538	1.60E-05	0.00078888
Bdnf	1.08316013	4.26333758	31.8726255	1.62E-05	0.00079192
Rgs2	0.85829438	7.30623261	31.7358383	1.66E-05	0.00080763
Cap1	0.71230448	7.01686267	31.7077199	1.67E-05	0.00080763
Gm16702	-1.2698279	4.40077098	31.6032357	1.70E-05	0.00081925
Htra4	1.73618631	3.17792456	31.4628383	1.75E-05	0.00083708
Hprt	0.66556517	7.88764741	31.4086136	1.77E-05	0.00083862
Cox5b	0.88509517	6.31383891	31.3900777	1.78E-05	0.00083862
Ccpg1os	1.33473841	2.81273087	31.3472873	1.79E-05	0.00083916
Tfrc	-1.0049728	5.51718518	31.293169	1.81E-05	0.00083916
Zfp563	-0.9750924	4.894565	31.2624688	1.82E-05	0.00083916
Ywhab	0.57878466	10.3409979	31.2624274	1.82E-05	0.00083916
Basp1	0.77024898	9.41422249	31.2222417	1.84E-05	0.00084077
Dock10	-1.4697606	5.74765679	31.1819102	1.85E-05	0.00084245
Atp6v1e1	0.67130441	6.40075552	31.1031134	1.88E-05	0.00085071
Pisd-ps1	-1.7336609	5.75833039	31.0174971	1.92E-05	0.00086027
Jpx	-1.5459839	2.7866681	30.967147	1.93E-05	0.00086385
Vwa5b2	-1.650233	3.81022683	30.9078411	1.96E-05	0.00086905
1810058I24R	1.32857593	4.48426675	30.7672049	2.01E-05	0.00088393
Fetub	8.99090891	-0.5004889	39.6870577	2.02E-05	0.00088393
Gm3002	-1.995208	3.60229734	30.7133737	2.04E-05	0.00088644
Fmo1	0.97589624	6.87527009	30.6937994	2.04E-05	0.00088644
Gm14378	-3.996966	-0.2341426	30.6497896	2.06E-05	0.00088923
Ubr1	-1.0627184	6.15641405	30.6183886	2.08E-05	0.00088979
Eif2s2	0.62998791	7.44919119	30.587347	2.09E-05	0.00089031
1110059E24I	0.69078184	5.63787582	30.5557742	2.10E-05	0.00089097
Nicn1	0.65808995	6.46661073	30.4794483	2.14E-05	0.0008998
Idh3b	0.62391193	7.06719223	30.4121964	2.16E-05	0.00090233

Lmo7	-1.4484024	6.02077423	30.4106674	2.17E-05	0.00090233
Gm2115	1.48031825	3.08649973	30.231427	2.25E-05	0.00092918
Pou5f2	-5.891849	-0.1208998	30.2126816	2.25E-05	0.00092918
Phyh	0.69538954	7.28550816	30.1737622	2.27E-05	0.00093055
Herc2	-1.3017941	7.52920546	30.1520108	2.28E-05	0.00093055
Ndufs4	0.61703675	6.61749597	30.1047479	2.30E-05	0.00093451
Atpaf1	0.74117459	6.0288518	30.0599459	2.33E-05	0.00093806
AF357359	-1.6712453	2.62008413	30.011008	2.35E-05	0.00093897
Trank1	-1.6728906	6.22042844	30.0030511	2.35E-05	0.00093897
Rbfox3	0.60232838	7.81275432	29.9404246	2.38E-05	0.00094297
Wfs1	0.97425725	6.64987679	29.930853	2.39E-05	0.00094297
Sdhb	0.65907485	5.60717895	29.8646682	2.42E-05	0.00094839
C1qbp	0.70779469	5.21647744	29.8284256	2.44E-05	0.00094839
Vps28	0.82996138	5.80975147	29.8268401	2.44E-05	0.00094839
Ubl7	0.67745321	6.5759092	29.7833402	2.46E-05	0.00094948
Msl3	0.77619834	5.48611517	29.7689081	2.47E-05	0.00094948
Gm10677	-2.7544534	1.60301891	29.7464512	2.48E-05	0.00094948
Dnaja1	0.54382517	9.6899873	29.6817804	2.51E-05	0.00095735
Erbb4	-1.091049	4.58491755	29.6111594	2.55E-05	0.00096238
Otof	-2.2007052	2.91644019	29.5972183	2.56E-05	0.00096238
Cxx1a	0.77975341	6.02202972	29.5628649	2.58E-05	0.00096238
Arf1	0.67451238	8.31488361	29.5592751	2.58E-05	0.00096238
Shank1	-1.0795655	8.94138459	29.4979021	2.61E-05	0.00096988
Osgin2	0.93325692	5.95443226	29.3586156	2.69E-05	0.00099343
Sptbn2	-1.3781652	6.97958108	29.2410218	2.76E-05	0.00101307
Pdcd10	0.79210097	6.07404911	29.0549648	2.86E-05	0.00104351
Cul9	-1.3676002	3.96622826	29.0526279	2.87E-05	0.00104351
Plxna4os1	-4.6087387	-0.4873521	28.9877792	2.90E-05	0.00105119
Cab39l	0.80720056	5.7725496	28.9565937	2.92E-05	0.00105119
Pdzd2	-0.9543815	6.18780981	28.9486861	2.93E-05	0.00105119
Gm10538	-5.8020444	-0.6379455	28.9140343	2.95E-05	0.00105382
Mtx2	0.62850995	5.6916001	28.8493553	2.99E-05	0.00105591
Nckap5	-1.3837641	3.2134606	28.8342396	3.00E-05	0.00105591
Prkdc	-1.2222211	5.15821367	28.8292289	3.00E-05	0.00105591
Ano1	-1.8556381	1.54957846	28.8148367	3.01E-05	0.00105591
Ambra1	-0.9679156	5.77368778	28.7698338	3.04E-05	0.001061
Dstn	0.92174615	8.25470472	28.6957259	3.09E-05	0.00107272
Capg	1.55957029	2.58282	28.6640134	3.11E-05	0.00107495
Hsd11b1	2.15511094	1.67588969	28.5985075	3.15E-05	0.00108056
Comt	0.72648268	5.68667028	28.5959636	3.15E-05	0.00108056
Ddn	-0.7279613	6.89353307	28.5364931	3.19E-05	0.00108926
Hipk2	-0.6219076	7.03675887	28.3628223	3.31E-05	0.00112201
Mansc4	2.33615404	1.99202878	28.3542083	3.32E-05	0.00112201
Plekha5	-0.8616761	5.42071835	28.3221923	3.34E-05	0.0011246
Prkg1	-0.6850129	5.8376894	28.2116576	3.42E-05	0.00114625

Scn1a	-1.0054023	7.07931107	28.1512801	3.47E-05	0.00115352
Ddx26b	-1.5253323	4.50924521	28.1403693	3.48E-05	0.00115352
1810026B05I	-1.010066	3.78998523	28.1169103	3.49E-05	0.0011542
Fabp7	1.0909585	4.52045423	28.0730285	3.53E-05	0.00115678
Rbp4	1.29747343	4.08437903	28.0631906	3.53E-05	0.00115678
Srxn1	0.62930753	6.28059447	28.0450735	3.55E-05	0.00115678
Abca5	-1.0477978	5.65973686	27.9603645	3.61E-05	0.00117286
Dynlrb1	0.87768932	8.41788242	27.9219799	3.64E-05	0.00117746
Miat	-1.5407576	6.21309444	27.8506817	3.70E-05	0.00119049
Neurl1b	-1.3658263	4.89012946	27.7820103	3.75E-05	0.00120304
Cops3	0.70383272	5.48155789	27.7175476	3.81E-05	0.00121467
Nme1	0.70960156	6.28486745	27.6818224	3.83E-05	0.00121887
Pgbd5	0.6967869	7.53473833	27.6378881	3.87E-05	0.00122304
Zmym3	-0.9169882	6.02195042	27.6123809	3.89E-05	0.00122304
Ensa	0.69964682	8.89617653	27.6026799	3.90E-05	0.00122304
Vamp5	1.04822736	5.48863501	27.5881673	3.91E-05	0.00122304
Sh3gl2	0.85547115	9.33445628	27.5329509	3.96E-05	0.0012326
Metap1	0.69889874	6.1717032	27.419542	4.06E-05	0.00125804
Olfm2	0.91239476	5.18180106	27.3488204	4.12E-05	0.00127225
Necab1	0.70395042	7.85856048	27.3284916	4.14E-05	0.00127265
Homer2	-0.6795023	7.54094242	27.2031147	4.25E-05	0.00129813
Hspb6	0.94021373	4.31120956	27.1999236	4.26E-05	0.00129813
Zc3h15	0.53293733	7.05509728	27.0806284	4.37E-05	0.00132698
Firre	-1.9087441	3.39928148	27.0529463	4.40E-05	0.00132966
Uqcrq	0.87522352	4.16383216	26.986274	4.46E-05	0.00134379
Fkbp3	0.60504108	6.9856642	26.9149255	4.53E-05	0.00135951
Tomm70a	0.53779946	7.12662431	26.8636667	4.58E-05	0.00136827
Mrps33	0.62655205	6.8965535	26.8493891	4.60E-05	0.00136827
Orc1	-2.1922564	1.46819534	26.7655657	4.68E-05	0.00138822
4932438A13I	-1.3142715	7.50608328	26.655866	4.80E-05	0.00141659
Rab7	0.77085155	10.3187668	26.6175653	4.84E-05	0.00142302
Capzb	0.87462738	8.59633439	26.5222357	4.94E-05	0.00144303
Svep1	-1.4943046	2.75200356	26.5190891	4.94E-05	0.00144303
Smap2	0.54498266	7.69726981	26.4634212	5.01E-05	0.00144868
Zic4	1.17943072	6.26531762	26.4603365	5.01E-05	0.00144868
Lrp1b	-1.4298436	5.97905963	26.4490535	5.02E-05	0.00144868
Col3a1	1.31975551	5.0592517	26.4313413	5.04E-05	0.00144881
Ppl	1.29487272	2.44147578	26.4071689	5.07E-05	0.00145104
Med12l	-1.3163235	5.30820352	26.3842855	5.09E-05	0.00145289
Dhx37	-1.3086063	2.78793217	26.3662868	5.11E-05	0.00145318
Ankrd13d	-1.2778428	3.01863563	26.1550821	5.36E-05	0.00151144
Med23	-0.8062486	4.19630727	26.1355346	5.38E-05	0.00151144
Lrba	-0.895404	5.84648866	26.1178833	5.41E-05	0.00151144
Dzip1	-0.6601858	6.54829099	26.1086966	5.42E-05	0.00151144
4930579G24	-1.3357353	2.52831405	26.1018454	5.42E-05	0.00151144

Chchd10	0.81634424	5.60173779	26.0890513	5.44E-05	0.00151144
Ep400	-0.9635347	6.99752642	26.03967	5.50E-05	0.00152259
Nphp4	-1.7389101	2.08215996	25.9675035	5.59E-05	0.00154171
Uggt2	-1.2785987	4.89630415	25.8592953	5.73E-05	0.00157383
H2-T23	-0.8395989	4.24340416	25.7910953	5.82E-05	0.00159232
Smap1	0.57079104	6.28610141	25.7600608	5.86E-05	0.00159717
Zfp703	0.84516706	4.68493457	25.745295	5.88E-05	0.00159717
Trrap	-1.1725045	6.51894758	25.7236547	5.91E-05	0.00159919
Csnk1a1	0.50687777	7.62192174	25.6988259	5.94E-05	0.00160238
1700008O03	-1.3980881	2.94146089	25.669306	5.98E-05	0.00160731
Grik2	-1.1949971	5.18302285	25.6436328	6.01E-05	0.00161088
Gm20199	-2.6149105	2.13159792	25.5394561	6.16E-05	0.00164341
Nsun3	0.86334557	4.6415673	25.4957705	6.22E-05	0.00165387
Dnase1l2	-7.0500676	-0.4876255	25.4664747	6.26E-05	0.00165645
Intu	-1.082973	4.51360505	25.457715	6.27E-05	0.00165645
Cd84	-1.7172562	2.46459589	25.4041091	6.35E-05	0.00166573
Sfi1	-1.6141606	3.39486103	25.3734001	6.39E-05	0.00166573
Tanc2	-1.0988474	8.5132507	25.3706547	6.40E-05	0.00166573
Il17ra	-0.7510656	4.71235539	25.3648603	6.40E-05	0.00166573
Bag5	0.69961188	6.29016191	25.3480549	6.43E-05	0.00166573
Stmn1	0.66921378	8.45410907	25.341066	6.44E-05	0.00166573
Pkd1	-1.0012909	5.17481873	25.3220349	6.47E-05	0.00166721
Dtl	0.942228	4.00306842	25.2713305	6.54E-05	0.00168079
Cxxc5	0.73696384	6.44888195	25.2342727	6.60E-05	0.00168927
Penk	1.01193839	7.08273198	25.2131473	6.63E-05	0.00169165
Unc80	-1.264039	8.33898979	25.1496257	6.73E-05	0.00171054
Col9a3	-1.8523731	2.69052244	25.126794	6.76E-05	0.00171368
9330175M2(	-3.5360757	1.18172473	25.0346674	6.91E-05	0.00173638
Isyna1	1.09847461	6.19626698	25.0263525	6.92E-05	0.00173638
Cmya5	-1.355142	3.28475375	25.0252453	6.92E-05	0.00173638
Gm14827	-2.0783669	2.66352071	25.001206	6.96E-05	0.00174015
Ryr3	-1.8951045	5.30691319	24.9633485	7.02E-05	0.0017495
Zmat2	0.63933594	7.1416039	24.9368522	7.06E-05	0.00175434
Dynlt3	0.75957004	9.11236474	24.8333725	7.23E-05	0.00179065
Dst	-1.446295	9.14796673	24.7771858	7.33E-05	0.00180052
Golgb1	-0.6407418	7.98617775	24.7692992	7.34E-05	0.00180052
Gfod1	-0.6856056	6.81594251	24.7479108	7.38E-05	0.00180052
Vwa8	-0.9245052	5.28914095	24.7405998	7.39E-05	0.00180052
Lmtk2	-0.7984588	6.76082334	24.7365305	7.40E-05	0.00180052
Grin2c	-1.7731039	2.15088364	24.7244375	7.42E-05	0.00180052
Acot13	0.77505546	5.31541963	24.6721716	7.51E-05	0.0018124
Esd	0.74411638	6.08734854	24.6680263	7.51E-05	0.0018124
Rlf	-0.7898985	5.84215649	24.6383818	7.57E-05	0.00181901
Hsbp1	0.82068303	8.97240026	24.6086451	7.62E-05	0.00182199
Dlat	0.62042541	8.21845619	24.6036779	7.63E-05	0.00182199

Snhg11	-2.0135529	9.45745744	24.5529537	7.72E-05	0.00183767
Ephb1	-1.058822	3.65570156	24.5277321	7.76E-05	0.00184259
Mtpn	0.62570989	10.0811484	24.4948399	7.82E-05	0.00185085
Wdr52	-1.8288702	2.19941663	24.3975384	8.00E-05	0.00188608
Myo1b	-0.8113908	5.93592783	24.3866	8.02E-05	0.00188608
Tmem206	-1.0951401	4.40141597	24.3505552	8.09E-05	0.00189468
Psmd6	0.67815473	5.94318755	24.3401936	8.11E-05	0.00189468
Usp24	-0.8001687	7.4004889	24.2851803	8.21E-05	0.00191317
Taf1	-0.741171	6.83215299	24.2554777	8.27E-05	0.00192051
Mdm1	-1.2560868	3.14417576	24.2189927	8.34E-05	0.00192744
AA387883	-3.4587686	0.28824158	24.2095688	8.36E-05	0.00192744
Smim18	1.67623615	2.12277284	24.2004684	8.38E-05	0.00192744
Chmp4b	0.57424063	6.43768416	24.1123178	8.55E-05	0.00195759
Kmt2a	-0.9360549	8.41968347	24.1079644	8.56E-05	0.00195759
Gfap	1.06056772	4.21164272	24.0249764	8.73E-05	0.00197325
Morc2a	-0.5891442	6.17560802	24.0068565	8.77E-05	0.00197325
Arpp19	0.53472672	9.06957208	24.0039718	8.77E-05	0.00197325
Psmb1	0.65279999	6.01550234	23.9877286	8.80E-05	0.00197325
Malat1	-1.8749094	12.8518958	23.9860915	8.81E-05	0.00197325
Sptbn1	-1.1228955	10.099249	23.9849874	8.81E-05	0.00197325
3110035E14I	0.57411709	7.87383963	23.9839517	8.81E-05	0.00197325
Myo10	-0.6845577	5.70474364	23.8923505	9.00E-05	0.00201022
Grasp	0.87228651	4.23156831	23.8782191	9.03E-05	0.00201093
Klc1	0.6952975	7.49123601	23.8547199	9.08E-05	0.00201611
Atp5g3	0.70023722	8.20651786	23.8270164	9.14E-05	0.00202333
Creb3l1	1.16533586	5.25308453	23.8061763	9.19E-05	0.00202732
Akap8	-0.9629744	5.82462808	23.7640228	9.28E-05	0.00204159
Fam192a	0.7441733	5.25633001	23.7299747	9.36E-05	0.00205206
Syne1	-1.2589311	8.5747803	23.6560997	9.52E-05	0.00206284
Snrnp70	-0.8963036	5.73712397	23.6485536	9.54E-05	0.00206284
Pak1	0.55653263	9.92302229	23.6421201	9.55E-05	0.00206284
Ywhae	0.59314598	10.7332426	23.6240548	9.59E-05	0.00206284
Tmem44	-0.8131764	4.61437698	23.6238688	9.59E-05	0.00206284
Ndufa11	0.92343102	3.88211188	23.6230068	9.60E-05	0.00206284
Dnajb7	-6.4891451	-1.1210456	25.7077539	9.61E-05	0.00206284
Wipf3	0.74909994	7.87146541	23.6101385	9.62E-05	0.00206284
Ndufaf3	0.91107993	4.14631992	23.5933105	9.66E-05	0.00206519
Ano4	-0.9653072	3.81478993	23.499403	9.88E-05	0.00210575
Gm1966	-1.4125962	2.95962697	23.482702	9.92E-05	0.00210813
Timm8b	0.72286297	5.55387668	23.4327308	0.00010039	0.00212732
Hhip	-0.9208991	4.43848583	23.3975217	0.00010124	0.00212883
Rab3gap2	-1.0305529	6.46892476	23.3862314	0.00010151	0.00212883
Dgkz	-0.6353457	6.32633871	23.3841379	0.00010156	0.00212883
Pot1b	-1.1576934	3.44286884	23.3789609	0.00010169	0.00212883
Slc17a7	0.68422364	7.53302562	23.3647841	0.00010203	0.00212883

Hapln2	-2.4743816	1.13557707	23.346855	0.00010247	0.00212883
Lyst	-1.0746931	6.33483125	23.3279139	0.00010293	0.00212883
Gfra2	0.84460028	7.57978057	23.3167896	0.00010321	0.00212883
Pvrl3	-0.7635942	5.40676394	23.3154663	0.00010324	0.00212883
Cecr2	-0.9563427	3.27743897	23.3129053	0.0001033	0.00212883
Kcnj2	-0.7687272	5.54481899	23.2843526	0.00010401	0.00213489
Thap11	0.73900391	4.58550204	23.2780645	0.00010417	0.00213489
Col1a1	1.24493716	6.95686148	23.2591309	0.00010464	0.00213873
Snord19	-7.1815654	-1.6782624	25.2228219	0.00010625	0.00215825
Skida1	1.09767634	4.1254437	23.1899833	0.00010638	0.00215825
Gm14295	0.53102482	5.87168386	23.1682941	0.00010694	0.00215825
C130030K03l	-1.7051976	2.25501604	23.1647055	0.00010703	0.00215825
Gm5862	-2.6117892	0.79766452	23.1646518	0.00010703	0.00215825
Fam160a1	0.96646591	4.32997888	23.1384165	0.00010771	0.00216101
Hecw2	-1.0667405	6.36028967	23.1369408	0.00010774	0.00216101
Ehd3	0.61640892	7.70821092	23.1093686	0.00010846	0.00216955
Gm10389	-1.2215229	4.2490491	23.0847851	0.0001091	0.00217659
Gcnt1	1.41370121	2.91051144	23.0485143	0.00011006	0.00218533
Dcx	-0.8745334	5.69217413	23.0460164	0.00011012	0.00218533
Tenm1	-1.2980764	5.44457367	23.0084487	0.00011112	0.00219934
Inpp5d	-1.3524888	2.51991615	22.9805981	0.00011187	0.00220441
Lrrc45	-1.0302432	3.71480371	22.9770023	0.00011197	0.00220441
A730017L22l	-1.2959735	4.89894303	22.9466983	0.00011279	0.00220963
Tceal3	0.73686573	5.14347973	22.9352738	0.0001131	0.00220963
Atp11b	-0.7551144	6.31297214	22.9346251	0.00011311	0.00220963
Hars	0.56440057	5.89879143	22.9017356	0.00011401	0.00222144
Glt25d1	-0.9080706	3.99311375	22.8883824	0.00011438	0.00222283
Cyb5b	0.55728242	7.53246763	22.860342	0.00011516	0.00222641
Nqo2	0.80808625	5.67377076	22.860318	0.00011516	0.00222641
Itpr1	-0.8878959	8.99082868	22.84601	0.00011556	0.00222837
Rpgr	-1.0947484	4.34552002	22.8300058	0.00011601	0.00223127
Fam19a3	8.56811641	-0.4615225	24.7781913	0.00011658	0.00223651
Trerf1	-0.952607	5.40018908	22.7858013	0.00011725	0.00224373
Kcnh3	-0.9446924	3.65536925	22.7744166	0.00011758	0.0022442
Trappc9	-0.9934042	5.74787062	22.7578055	0.00011805	0.00224752
Arl6	0.72424702	5.70105526	22.7327318	0.00011877	0.00225548
Notch1	-1.5442125	2.82737551	22.6918124	0.00011995	0.00226797
Fam212b	0.67440885	6.76084735	22.6891127	0.00012003	0.00226797
Sdf2l1	1.24289601	2.66460887	22.5946962	0.00012281	0.00231472
Ppp1r16b	-0.8259343	6.6634523	22.553816	0.00012404	0.00231481
Nup214	-0.7471502	6.07547627	22.5424228	0.00012438	0.00231481
Bcan	-1.2253501	4.67204944	22.5340529	0.00012464	0.00231481
A630089N07	-1.7865508	8.10768397	22.5244528	0.00012493	0.00231481
Irs1	0.57976127	6.08048577	22.5237797	0.00012495	0.00231481
Lrrc55	-0.7628601	4.49946179	22.5184659	0.00012511	0.00231481

Napb	0.66012994	9.75654075	22.5139127	0.00012525	0.00231481
Tceal5	0.62493592	6.04172697	22.5097248	0.00012538	0.00231481
A330021E22I	-0.9613922	4.08752451	22.5026431	0.0001256	0.00231481
Slfn5	-1.1838554	4.46679279	22.455252	0.00012705	0.00233597
Pik3c2a	-0.6261598	6.15002745	22.415043	0.00012831	0.00235323
Agfg2	0.90660219	3.6596711	22.3823491	0.00012934	0.00236631
Capn1	0.79208854	4.19335936	22.3606435	0.00013002	0.00237311
Fam160b2	-0.6587837	6.58432787	22.2561432	0.00013339	0.00242785
Atp5o	0.89320665	6.67836527	22.2476764	0.00013367	0.00242785
Pnisr	-0.9555944	7.66635585	22.202842	0.00013515	0.00244877
Med12	-0.9098861	4.99139276	22.1527844	0.00013682	0.00247137
Slc34a2	2.57606755	0.83213554	22.1393712	0.00013727	0.00247137
Gabpa	0.62179486	6.71949932	22.1301058	0.00013759	0.00247137
Cobl	0.67802739	7.98664521	22.1154962	0.00013808	0.00247137
Ktn1	-0.5972421	7.13143191	22.1133978	0.00013815	0.00247137
Cadps2	-0.8368021	6.41826898	22.1061831	0.0001384	0.00247137
B3galnt2	-0.8965062	3.82718944	22.0972619	0.0001387	0.00247137
4930444F02I	-6.4955213	-0.6026053	22.063761	0.00013985	0.00248593
Akap11	-0.9582728	8.45941084	22.0530331	0.00014022	0.00248598
Myt1	-1.3247808	2.81373741	22.0444939	0.00014052	0.00248598
Osr2	3.81529077	-0.06452	22.0169472	0.00014147	0.00249293
Pak6	-0.9597466	3.74563752	22.0140751	0.00014157	0.00249293
1600029O15	2.06921112	1.62135196	21.9905438	0.0001424	0.0024963
Tmem218	1.15305451	3.03925079	21.9896222	0.00014243	0.0024963
Cyp26b1	-1.3029665	5.38657079	21.9506031	0.00014381	0.00251086
Psmd12	0.53878317	6.71043969	21.9471882	0.00014393	0.00251086
Atp6v1b2	0.607217	8.40068724	21.9325575	0.00014445	0.00251411
Hint1	0.73805641	5.21577441	21.8724834	0.00014662	0.00253253
Tmem40	4.23945404	-0.055011	21.8699069	0.00014671	0.00253253
Tm4sf20	-7.2502329	-1.3584128	23.6954605	0.00014676	0.00253253
Hectd1	-0.7805739	7.4322099	21.8656962	0.00014686	0.00253253
Cotl1	1.0393306	3.14023198	21.8511689	0.00014739	0.00253583
Arhgap6	0.83337969	4.73769687	21.8215502	0.00014848	0.00254831
Prex2	-0.7545537	7.37348837	21.8099827	0.0001489	0.00254831
Zswim5	-0.7758811	4.89871036	21.8036802	0.00014914	0.00254831
Amy1	-0.8230386	4.31067599	21.7867708	0.00014976	0.00255321
Ogt	-1.011776	7.68691781	21.7612719	0.00015071	0.0025631
4933424G05	-2.8076086	1.07474415	21.7521395	0.00015106	0.0025631
Lsm12	0.64193199	5.8603497	21.7352923	0.00015169	0.0025631
Zfp488	-1.473196	7.72018288	21.7347323	0.00015171	0.0025631
Gm20172	-3.1298255	0.71115803	21.690459	0.00015339	0.00258474
Ywhag	0.54004221	10.2179561	21.6723757	0.00015408	0.00258474
Adam4	-3.4189105	0.97597989	21.668311	0.00015424	0.00258474
Tet3	-0.7920306	6.03705093	21.654287	0.00015478	0.00258474
Akr1b10	0.7855961	3.77109963	21.6532168	0.00015482	0.00258474

Pdia3	0.75769731	7.51848694	21.6469613	0.00015506	0.00258474
Gm11549	0.6382484	6.88483758	21.6126146	0.00015639	0.00260116
2610301B20I	0.66474831	5.3130088	21.5542698	0.00015868	0.00263345
Cyfip1	-0.6403959	6.22946683	21.5315597	0.00015958	0.00264257
Gm16861	-1.7404035	2.44946528	21.5215495	0.00015998	0.00264335
1110037F02I	-0.7171243	5.5306821	21.46881	0.00016211	0.0026692
B4galnt2	-1.7077903	3.0710878	21.4532496	0.00016274	0.0026692
Tceal6	0.64319592	4.72825426	21.4528296	0.00016276	0.0026692
Panx2	-0.9875148	4.05650636	21.4475378	0.00016297	0.0026692
Dnajc7	0.51070453	6.56262307	21.4198191	0.00016411	0.00267655
Iqub	-2.0255817	1.53412581	21.4191468	0.00016413	0.00267655
Dock3	-1.2482358	7.93754843	21.3883251	0.00016541	0.00269145
Nron	-3.7633012	0.25872648	21.3766627	0.00016589	0.00269349
Trpv3	-5.2930628	-0.520925	21.3613702	0.00016653	0.002698
Shroom2	0.49512119	6.79998881	21.3366924	0.00016756	0.00270226
Smco1	-1.3375487	2.72570555	21.3302452	0.00016783	0.00270226
Daxx	0.76383779	3.87874798	21.329333	0.00016787	0.00270226
Plekhh1	-1.0205574	4.58905436	21.3062988	0.00016885	0.00271212
Baz2b	-0.5277606	6.99648662	21.2947213	0.00016934	0.00271421
Exph5	-1.2049334	6.36395724	21.2475335	0.00017136	0.00274074
Enoph1	0.64657711	4.85653078	21.2327913	0.000172	0.00274507
Atad2b	-0.757886	5.16211525	21.1794395	0.00017432	0.00277629
Ubr4	-1.0833246	7.17668192	21.1690678	0.00017478	0.00277766
Gapdh	0.54682022	9.52446363	21.1458859	0.0001758	0.0027874
Mdn1	-1.7653782	6.51506517	21.138419	0.00017613	0.0027874
Mob3c	0.94806989	4.65589937	21.1288712	0.00017656	0.00278824
Leng8	-1.1874288	6.48428617	21.0953723	0.00017806	0.00280602
Brwd3	-0.6998822	6.15172761	21.0561999	0.00017983	0.00282709
Xpo4	-0.8913605	4.12366709	21.0410868	0.00018052	0.00282709
Tmem194b	-2.3464947	2.10193843	21.0409127	0.00018053	0.00282709
Dear1	-2.6333216	1.65996176	21.0208075	0.00018145	0.00283546
Snrnp25	1.61681878	2.33329262	21.0127472	0.00018182	0.00283546
Gm12359	-3.4027334	0.15565247	20.9921848	0.00018277	0.00284435
Fam92a	0.61945458	6.562983	20.9786677	0.00018339	0.0028482
Olfr613	-1.5031213	5.43212935	20.9402801	0.00018519	0.0028701
Sulf1	1.07681567	5.8986324	20.917116	0.00018628	0.00287692
Mrpl30	0.76301509	5.69190516	20.9039628	0.0001869	0.00287692
Pcdhb20	-1.2308636	3.63432425	20.8984928	0.00018716	0.00287692
Hapln4	0.69296966	6.04615777	20.898481	0.00018716	0.00287692
Lrrc19	-3.4676698	0.34240193	20.8902146	0.00018755	0.00287707
Mmp17	0.84488001	5.60588234	20.865926	0.00018871	0.00288499
Utp20	-1.0822455	4.51937359	20.8622236	0.00018889	0.00288499
Fry	-1.2133776	8.22700154	20.8553144	0.00018922	0.00288499
Sntb1	1.5771004	1.79795292	20.8254681	0.00019066	0.00290049
Nnat	1.47829746	8.65677086	20.8167455	0.00019109	0.00290049

1500015O10	1.86811236	5.14687595	20.8103494	0.0001914	0.00290049
Fam184b	-1.5954254	2.75541386	20.7949079	0.00019215	0.00290137
Cxx1b	0.79585395	5.87807493	20.786893	0.00019255	0.00290137
Ssbp3	0.5538488	5.98619152	20.7813996	0.00019282	0.00290137
Serpinf1	1.37094165	5.60135265	20.7775404	0.000193	0.00290137
Zfp260	0.54929905	7.24177903	20.7569776	0.00019402	0.00291077
A130077B15	-1.4261707	7.65489008	20.7453791	0.00019459	0.00291356
Dbhos	-1.573715	3.35818867	20.724175	0.00019565	0.00292149
E330020D12I	-1.4839122	3.84757115	20.7072326	0.00019649	0.00292149
Tmem67	-1.1717656	3.81149428	20.7055688	0.00019658	0.00292149
Prrt2	0.56213594	7.21379978	20.7035435	0.00019668	0.00292149
Gm5531	-1.4194198	2.74342724	20.6813638	0.0001978	0.00293227
E330023G01	-1.8500574	1.42806035	20.6556447	0.0001991	0.00294473
Tpm1	0.71417156	9.356263	20.6468303	0.00019955	0.00294473
Nrgn	0.58914349	7.23116509	20.6416208	0.00019981	0.00294473
Pla2g4a	0.85225487	4.59834427	20.5947196	0.00020223	0.00297442
Trpm7	-0.6276198	6.58706459	20.5785821	0.00020306	0.00297664
5031426D15I	-2.0159314	3.58407533	20.5765113	0.00020317	0.00297664
Arf4	0.66122287	8.85386897	20.5474846	0.00020469	0.00299301
Ranbp2	-0.8653356	7.62672975	20.4920744	0.00020762	0.00302993
Ociad2	0.54954936	6.64314573	20.4827669	0.00020811	0.00303128
Ppia	0.76254088	9.86341578	20.4633781	0.00020915	0.0030405
Ift88	-0.8689282	4.21810903	20.4471273	0.00021003	0.00304564
Kifc2	-0.6788958	6.32616462	20.4385203	0.00021049	0.00304564
Ubr5	-0.7014295	7.85059962	20.4342322	0.00021072	0.00304564
Cox7b	0.78794103	7.33657115	20.4141389	0.00021181	0.0030508
Ferd3l	7.50494077	-1.5862539	22.0294033	0.00021189	0.0030508
Abcg1	-0.842193	4.94718893	20.4027737	0.00021243	0.00305273
Ttbk1	-1.0741562	4.05638659	20.3827799	0.00021353	0.00306261
Hist1h2bc	0.89630448	5.43187797	20.3660923	0.00021445	0.00306758
Slc6a17	0.6116132	8.25018407	20.3616508	0.00021469	0.00306758
4932413F04I	-3.5765157	0.1333129	20.3444018	0.00021565	0.00307538
Hebp1	0.90323367	3.58687395	20.3117704	0.00021747	0.00309065
Erh	0.88072588	5.18294234	20.3104597	0.00021754	0.00309065
Tyms	-1.0207386	3.66644618	20.2801216	0.00021925	0.00310904
Col19a1	-1.3458695	3.91312099	20.2512343	0.00022089	0.0031264
2310022B05I	0.65070189	6.31292069	20.2197958	0.0002227	0.00314045
Atm	-0.7487089	5.99784166	20.2141608	0.00022302	0.00314045
Unc13a	-1.3461452	6.96026908	20.2029852	0.00022367	0.00314045
Caprin2	-1.3179819	2.7154879	20.2017075	0.00022374	0.00314045
Mrpl17	0.61487393	5.65330269	20.1975559	0.00022398	0.00314045
Adcy5	-0.5031735	6.29168631	20.1313016	0.00022786	0.00318881
Ankrd46	0.59073087	6.77853635	20.1083507	0.00022921	0.00320186
9630001P10I	-2.6862512	0.21611996	20.0885357	0.0002304	0.00321237
Ap1s3	-1.218084	3.67176924	20.0635775	0.00023189	0.0032265

Taf10	0.91018224	4.15935946	20.0573254	0.00023227	0.0032265
Cdo1	1.07439598	6.85151754	20.0457795	0.00023297	0.00323021
Pcdhb6	-3.0429434	0.85978822	20.0136368	0.00023492	0.00325128
4933428G20	-2.2449319	1.69656661	19.9983563	0.00023585	0.00325821
Actg1	0.46290361	8.97424616	19.9741939	0.00023734	0.00326986
Gprasp1	-1.1647481	8.9720936	19.9705049	0.00023757	0.00326986
Tagln2	1.22301997	5.7784713	19.9586088	0.00023831	0.00327037
Pomp	0.73379708	7.36047553	19.9558337	0.00023848	0.00327037
Ugt8a	-0.8286615	5.3525216	19.9267739	0.00024029	0.0032821
Banf1	0.95411903	4.45274061	19.9117758	0.00024123	0.0032821
Sox2ot	-0.8078162	4.98179201	19.9087693	0.00024142	0.0032821
Minos1	0.70514075	6.48496504	19.9040423	0.00024172	0.0032821
Tbc1d30	-0.8068294	6.6190001	19.9029761	0.00024178	0.0032821
4930470H14I	-1.5539838	6.76646202	19.9002149	0.00024196	0.0032821
Bzrap1	-1.5659106	4.8099904	19.8840461	0.00024298	0.00329001
Daam1	-0.6926937	6.59201576	19.8738905	0.00024363	0.0032928
Pdap1	0.57650968	6.04391655	19.8643781	0.00024423	0.00329338
Fam199x	-0.7357313	4.77628233	19.8594261	0.00024455	0.00329338
Hmgb3	0.57565408	5.81681194	19.8473942	0.00024532	0.00329782
Atp13a1	-1.078777	2.84849454	19.8246982	0.00024677	0.00331149
Gde1	0.7051849	5.24696666	19.7974829	0.00024854	0.00332917
Mlxip	-0.6696673	5.16406634	19.7784303	0.00024978	0.00333983
Dot1l	-1.2327269	3.89011164	19.7690302	0.00025039	0.0033421
Polr3a	-0.8696047	3.97303105	19.7123576	0.00025414	0.00338604
Nmb	-3.5897407	0.12625906	19.6996503	0.00025498	0.00339131
Cntnap5b	-0.9050989	4.15925846	19.683233	0.00025608	0.00339991
Cyb5	0.83858674	5.88858328	19.6671091	0.00025717	0.00340829
Vwa1	0.93990775	4.20174091	19.6517025	0.00025821	0.00341606
Hmcn1	-1.0311886	3.75683083	19.6398607	0.00025901	0.00342016
Jph3	-0.6959192	5.02951187	19.6337183	0.00025943	0.00342016
Pcdhga10	-0.7671528	3.54483059	19.6146053	0.00026074	0.00343134
Nbea	-1.2290645	8.50315609	19.5979854	0.00026188	0.00343772
Insig2	0.52183257	6.13606135	19.5942094	0.00026214	0.00343772
Ppp2r2cos	-2.7403897	0.74501721	19.5783952	0.00026323	0.00344476
Slmo2	0.56750329	6.85782777	19.5675215	0.00026399	0.00344476
Ube2s	1.10920008	3.69044708	19.5665459	0.00026405	0.00344476
Met	0.96338063	3.05753534	19.5403378	0.00026588	0.00345875
Bri3bp	-0.6936743	5.72851111	19.5343766	0.0002663	0.00345875
1110008L16I	0.67535364	4.36276235	19.531368	0.00026651	0.00345875
Naa50	0.50850241	7.71159215	19.5218888	0.00026718	0.00346141
Prom1	-1.523001	2.67292899	19.506101	0.00026829	0.00346985
Slc4a4	-0.5683771	7.86424389	19.4762332	0.00027041	0.00349125
Cpm	1.01231728	4.42576325	19.4633681	0.00027133	0.00349136
Hp1bp3	0.43622193	8.1320659	19.4630894	0.00027135	0.00349136
Zyx	0.62448381	5.44226379	19.4333397	0.00027349	0.00351286

Arhgap12	-0.5503358	5.65828219	19.4192262	0.00027451	0.00351995
Fuk	-1.434052	1.56267441	19.4069281	0.0002754	0.00352538
Tial1	-0.6081901	5.9119415	19.3807765	0.00027731	0.00353204
1810037I17R	0.84206988	5.63404425	19.3726028	0.00027791	0.00353204
Nrep	0.69562781	7.95133817	19.3716867	0.00027798	0.00353204
Pcdhga3	-0.9354139	3.43958644	19.3686238	0.00027821	0.00353204
Cdan1	-0.9446796	3.37040138	19.3613884	0.00027874	0.00353204
Wsb1	-1.1753987	4.11688101	19.3557683	0.00027915	0.00353204
Blnk	1.08883612	3.30553498	19.3499001	0.00027959	0.00353204
Pitpna	0.43780719	8.72835352	19.3437222	0.00028004	0.00353204
Nsl1	-1.3479977	4.58000397	19.3307745	0.00028101	0.00353204
Mrps21	0.87309033	5.03713123	19.3156788	0.00028213	0.00353204
Pbld1	-1.1935696	3.46365228	19.3130092	0.00028233	0.00353204
4930511M06	-1.5533814	3.22956521	19.3074421	0.00028275	0.00353204
Znfx1	-0.7835837	5.36773482	19.3050465	0.00028293	0.00353204
Myh10	-0.9018617	8.54003851	19.3043344	0.00028298	0.00353204
Chd6	-0.7700386	7.19872025	19.3042697	0.00028299	0.00353204
Acot7	0.62338096	6.14942926	19.2944367	0.00028372	0.00353537
Adcy1	-0.8921506	8.75656607	19.2792102	0.00028487	0.00353993
St13	0.63193524	6.32199189	19.2770697	0.00028503	0.00353993
Rbp1	1.38883729	7.75562961	19.2635649	0.00028606	0.00354676
Vps13d	-1.0932268	6.62824952	19.2564156	0.0002866	0.00354763
Ppp3r1	0.48329178	10.0948401	19.2414224	0.00028774	0.00355072
Rad54l2	-0.5233197	5.63481962	19.2224888	0.00028919	0.00355072
Atf2	0.4561245	8.8748874	19.2193176	0.00028944	0.00355072
Hace1	-0.6359946	5.00898893	19.2186827	0.00028948	0.00355072
Macf1	-1.1220169	9.05836431	19.2136708	0.00028987	0.00355072
Gjc3	-0.9021421	5.44559443	19.2129605	0.00028993	0.00355072
Il1rap	-0.8819578	3.72863637	19.2076839	0.00029033	0.00355072
Zfp277	-1.1443054	6.31918205	19.2037453	0.00029064	0.00355072
Ybey	-1.0866751	4.3752205	19.1881368	0.00029184	0.003553
Kmt2c	-0.7331836	8.17683856	19.1861895	0.000292	0.003553
Pdk2	0.53949809	7.0969543	19.1755763	0.00029282	0.003553
Wdr13	-0.4541229	6.73099886	19.170245	0.00029324	0.003553
Nf1	-0.8500105	8.07663234	19.1571648	0.00029426	0.003553
Atp5j2	0.81778989	5.43706898	19.157032	0.00029427	0.003553
Tpm4	0.91947575	9.13505951	19.1558019	0.00029436	0.003553
Col11a1	-1.0598359	3.3666283	19.1526395	0.00029461	0.003553
Arhgef2	-1.0770058	5.56237743	19.1315221	0.00029627	0.00356729
Yipf3	0.68353081	4.45809091	19.1084056	0.0002981	0.00358357
Unc79	-1.1907989	5.96216454	19.0764268	0.00030065	0.00360846
Ccdc69	1.28962376	2.06926137	19.0673895	0.00030138	0.00361139
D930016D06	-1.1754995	4.25689725	19.0508141	0.00030272	0.00362079
Atp13a4	-1.7996128	2.28564418	19.0456973	0.00030313	0.00362079
Sulf2	0.55881025	6.53957386	19.0305672	0.00030436	0.0036259

Ubxn6	0.5804791	5.56864892	19.0284972	0.00030452	0.0036259
Chgb	0.72032427	9.20757245	19.0212494	0.00030511	0.00362717
Ak1	0.84924325	3.98628346	19.0140972	0.0003057	0.00362729
Cyfip2	-0.7922942	9.89947236	19.0092796	0.00030609	0.00362729
St8sia2	1.33168861	2.85033802	19.0020825	0.00030668	0.00362854
Adam10	-0.6129086	5.75542415	18.9942985	0.00030732	0.00363037
Inip	0.72614862	5.15802191	18.9636009	0.00030985	0.00365453
Nwd1	-0.8265233	5.14178275	18.9488419	0.00031108	0.00366323
Ryr2	-1.431794	8.03232556	18.9263972	0.00031295	0.00367953
Tpd52l1	0.88113608	5.00453111	18.8928273	0.00031578	0.00370196
4931430N09	-2.7441141	1.7189984	18.8920211	0.00031585	0.00370196
BC003965	0.55755733	6.03354468	18.8851586	0.00031643	0.00370299
Setd2	-0.6357347	7.47981448	18.8728961	0.00031747	0.00370809
Angel1	-1.9577471	1.42903695	18.8630255	0.00031831	0.00370809
Sh3bgrl3	0.73970117	6.73140754	18.853698	0.00031911	0.00370809
Haus2	0.60052384	5.4932092	18.8511317	0.00031933	0.00370809
Amer1	-0.8536039	4.17907539	18.8456145	0.0003198	0.00370809
Igf1r	-0.590418	6.90698274	18.8452968	0.00031983	0.00370809
Brox	0.5262534	5.69031614	18.8206832	0.00032195	0.00372649
Gm10845	-1.2997299	5.57078128	18.8153614	0.00032241	0.00372649
Ccdc134	-1.6812403	0.62097452	18.8064958	0.00032318	0.00372844
Pacsin2	0.56040997	6.2208514	18.8019686	0.00032357	0.00372844
Nid1	0.80969049	7.06377941	18.7957131	0.00032412	0.00372888
Gdap1l1	0.9430862	3.45144051	18.790114	0.00032461	0.00372888
Ahctf1	-0.715515	6.60380263	18.7666625	0.00032666	0.00373838
1700021F05I	0.67280594	4.52234889	18.7639751	0.0003269	0.00373838
F630111L10F	-1.6446084	2.56004193	18.7615508	0.00032711	0.00373838
Pkd2l1	-6.4990499	-1.3225536	18.7579364	0.00032743	0.00373838
Gm20752	-2.1466864	0.53927889	18.7503368	0.0003281	0.00374034
Ndufa6	0.65747673	5.13130613	18.7253741	0.00033031	0.00374687
C630031E19I	-2.2541931	1.84095033	18.7244865	0.00033039	0.00374687
Morf4l2	0.64382887	7.36995146	18.7240552	0.00033043	0.00374687
Prkd1	-1.2390376	2.50520455	18.7197468	0.00033081	0.00374687
2210016L21F	0.6412929	6.25796474	18.7157433	0.00033117	0.00374687
Thy1	0.56209963	7.45923875	18.685478	0.00033388	0.00375961
Baiap3	-2.1839614	1.53709293	18.6823485	0.00033416	0.00375961
Kmt2b	-0.8457883	4.65995809	18.6810272	0.00033428	0.00375961
Fut8	-0.8718285	6.99074698	18.6808064	0.0003343	0.00375961
Cir1	0.51983783	6.95479119	18.6752603	0.0003348	0.00375961
Wbp2	0.55182161	7.02579978	18.6568277	0.00033647	0.00377271
Dync2h1	-1.374469	6.15787971	18.6203202	0.0003398	0.00380438
Man2a2	-0.6109082	6.90128601	18.5870009	0.00034287	0.00383243
Plekha7	-0.9747078	2.98852326	18.5821022	0.00034333	0.00383243
Hdgf	0.68419215	6.54387813	18.561989	0.0003452	0.00384664
Gm17644	-1.6606613	7.68606543	18.55743	0.00034563	0.00384664

Med16	-0.6544393	5.29758617	18.5219718	0.00034896	0.00387799
Rbak	-1.3081318	3.25545728	18.4961175	0.00035141	0.00389751
Ank3	-1.1424682	9.13226804	18.4918528	0.00035182	0.00389751
AB041803	-1.9217774	2.42440817	18.482685	0.00035269	0.00389751
Ndufv2	0.61562636	6.21579475	18.4816373	0.00035279	0.00389751
Pappa	1.93990394	2.78235808	18.4408486	0.00035672	0.00393508
Kcnq1ot1	-1.7429294	8.05882548	18.4341832	0.00035737	0.00393641
Ctcfl	-1.5179419	3.10142924	18.4170923	0.00035903	0.00394893
Zfp445	-0.7036805	6.82886788	18.4027016	0.00036043	0.00395164
4933404O12	0.7747141	3.94674766	18.3992286	0.00036077	0.00395164
Vapa	0.56336284	7.18529711	18.3984132	0.00036085	0.00395164
Gnb4	-0.6977502	4.79186828	18.3867804	0.000362	0.00395381
Pdcd4	0.47508362	7.48491951	18.3808393	0.00036258	0.00395381
Commd6	0.91723823	5.30398125	18.3794455	0.00036272	0.00395381
Folh1	-1.8600733	1.91520935	18.3749771	0.00036316	0.00395381
Dock9	-0.7740729	6.49843439	18.3562984	0.00036501	0.00395817
Ankrd44	-0.8414898	5.2431102	18.355461	0.00036509	0.00395817
Gng2	0.43180837	7.99385618	18.3532987	0.00036531	0.00395817
Vps13c	-1.0900877	6.48139958	18.3496417	0.00036567	0.00395817
Adam12	0.80748165	5.7691662	18.3394618	0.00036669	0.00396231
Lrrc7	-1.1930734	8.01311351	18.3352148	0.00036711	0.00396231
Psma4	0.50126165	6.88952147	18.3214712	0.00036849	0.00396622
Cyp2f2	2.18525184	4.94083326	18.3210444	0.00036853	0.00396622
Pitpnm2os1	-3.7750517	-0.1977303	18.3050159	0.00037014	0.00397787
Cacna1b	-0.9040244	6.84283263	18.2566736	0.00037506	0.00401417
Rap2a	0.43526681	7.59364331	18.2466473	0.00037608	0.00401417
Hunk	-0.8621071	4.23948636	18.2441701	0.00037634	0.00401417
Heyl	0.83037969	6.00162945	18.2406491	0.0003767	0.00401417
Dcdc2c	-1.4339692	5.66716477	18.2404364	0.00037672	0.00401417
Dock7	-0.6879302	6.05798925	18.2403187	0.00037673	0.00401417
Supt6	-0.6847587	7.40632526	18.2348002	0.0003773	0.00401452
Eri1	0.61131476	5.71435781	18.2077965	0.00038009	0.00403851
Vopp1	0.55868914	5.84434405	18.1969534	0.00038122	0.00404477
Fryl	-0.7339521	7.5460087	18.1809317	0.0003829	0.00405679
4930547E14I	-5.4675949	-0.8269126	18.1644366	0.00038463	0.00406938
Tmem181b-ı	-1.2725669	5.11164669	18.1535622	0.00038577	0.00407576
Kcnh4	-1.8264143	1.83296226	18.1412038	0.00038708	0.00408382
Chit1	7.20733778	-1.5464265	19.4406122	0.0003881	0.00408495
Cyb5r4	0.52367845	5.78849428	18.1299345	0.00038828	0.00408495
Fancm	-0.8260204	4.04748168	18.094792	0.00039203	0.0041187
Sptb	-1.3243615	5.42552413	18.079049	0.00039373	0.00413073
Cntn2	-0.9266741	5.70335049	18.0701771	0.00039469	0.00413501
Slc25a35	0.83312545	4.99898898	18.0640513	0.00039535	0.00413619
Zc3h7b	-0.6139779	6.02032441	18.0563375	0.00039619	0.00413742
Stx12	0.5135119	7.93683339	18.0528143	0.00039657	0.00413742

A930011O12	-1.7673712	4.98274965	18.0406546	0.0003979	0.00414549
Dph5	-0.8674729	4.65440441	18.0267105	0.00039943	0.00415337
Dopey1	-1.0316413	5.58148673	18.0236356	0.00039976	0.00415337
Slc2a12	0.95699314	4.38770554	18.0150064	0.00040071	0.00415724
Mga	-0.7869073	7.82664413	18.0101769	0.00040124	0.00415724
Dcun1d1	0.47892508	6.8076592	17.9909818	0.00040337	0.00417347
Fam124a	-0.7661489	4.4110444	17.9780634	0.0004048	0.00418245
Tsg101	0.63385638	5.33394706	17.9729693	0.00040537	0.00418245
Txndc17	0.73666901	6.32172748	17.9681505	0.00040591	0.00418245
Thbd	1.26871661	8.3227459	17.9417824	0.00040886	0.00420338
Ube2a	0.70661887	6.34368022	17.9400408	0.00040906	0.00420338
Hey1	0.64306409	6.35228255	17.9339422	0.00040975	0.00420469
Hsd17b10	0.80214522	4.46645797	17.8863724	0.00041515	0.00425435
4930519F09I	-1.1790066	2.53184829	17.8445793	0.00041997	0.00429783
Top1mt	1.0319201	3.13439273	17.8236963	0.0004224	0.00430406
Kpna4	0.42874304	7.20475338	17.8230232	0.00042248	0.00430406
Zfp60	-0.5750618	6.37575966	17.8200832	0.00042282	0.00430406
Sst	0.77398649	6.90303756	17.8196312	0.00042287	0.00430406
Abi1	0.4621136	7.09800737	17.8100367	0.000424	0.00430592
Atp5f1	0.49363427	7.46224354	17.8082584	0.0004242	0.00430592
Ube2h	0.48788543	7.70872382	17.8023927	0.00042489	0.00430708
Tjp2	0.61329416	5.62012645	17.7833108	0.00042714	0.00432274
Col24a1	-3.0760443	0.43538325	17.7783731	0.00042772	0.00432274
2010107E04I	0.71708111	5.84260277	17.7746352	0.00042817	0.00432274
Eml5	-1.2941887	5.85855028	17.7623974	0.00042962	0.00433158
Gnas	0.51100498	10.8121039	17.7346766	0.00043293	0.00435454
Dicer1	-0.6406805	6.2937579	17.7300139	0.00043349	0.00435454
Elmo1	0.57927073	7.15388229	17.7266773	0.00043389	0.00435454
Naip5	-1.6724887	1.51567196	17.7239376	0.00043422	0.00435454
Fdft1	0.57006507	6.27779223	17.7187236	0.00043485	0.00435502
Higd2a	0.7353969	5.32361296	17.6991458	0.00043721	0.00436282
Gapvd1	-0.6306867	6.04140785	17.6987869	0.00043726	0.00436282
DQ267100	-3.0632001	0.15390844	17.6957772	0.00043762	0.00436282
Kdm5a	-0.6062065	7.06546638	17.6930488	0.00043795	0.00436282
Plec	-0.8131583	6.34716249	17.6872668	0.00043866	0.00436403
Prlr	-1.0481581	2.37048023	17.6723451	0.00044048	0.00437633
Mycbp2	-1.1230889	8.84042078	17.634278	0.00044516	0.00441698
Exosc1	0.83683452	4.68225896	17.6278337	0.00044595	0.00441698
Dkk3	0.67279876	8.69595645	17.6237807	0.00044646	0.00441698
Igf2r	-0.6888362	4.7242819	17.6200191	0.00044692	0.00441698
Crip2	0.63046522	4.55658148	17.589543	0.00045073	0.00444871
Wdr92	0.5112863	5.3582105	17.5795492	0.00045198	0.00445523
F420014N23	-1.8880499	0.69867719	17.5401652	0.00045696	0.00449793
Herc1	-1.2528341	8.10533263	17.5358572	0.00045751	0.00449793
Myo18a	-0.6568705	6.30847376	17.5255275	0.00045883	0.00450365

Bsn	-1.1200047	9.30856583	17.5185564	0.00045972	0.00450365
Myadm	0.49348823	7.63973607	17.5172134	0.0004599	0.00450365
Setd8	0.62572467	7.28392501	17.5020678	0.00046184	0.00451681
Cc2d1a	-1.2194929	2.91803253	17.4935307	0.00046294	0.00452169
Birc6	-1.0235821	7.93359802	17.4856989	0.00046395	0.00452365
Hivep1	-0.6112295	7.4051704	17.4826542	0.00046435	0.00452365
Gprin1	0.77012437	4.69339055	17.4660584	0.0004665	0.00453414
Arntl2	-1.5729144	2.04468903	17.4650549	0.00046664	0.00453414
Dlgap1	-0.7681784	8.83633306	17.453866	0.0004681	0.00454016
Pde1c	-1.0767899	4.17786531	17.4510399	0.00046846	0.00454016
Myeov2	0.72926423	4.74117315	17.442456	0.00046959	0.00454093
A730090N16	-2.946061	0.77945632	17.4411892	0.00046976	0.00454093
Dlg5	-0.6689646	5.22165872	17.4231119	0.00047213	0.00454777
Prpf8	-0.647293	8.26146319	17.4208367	0.00047243	0.00454777
Map3k6	1.32752449	1.76105039	17.4196099	0.0004726	0.00454777
Hfm1	-2.0043256	1.86536128	17.4174049	0.00047289	0.00454777
Gm17821	-0.7952959	9.70641566	17.401721	0.00047497	0.00455771
Ncald	0.51314435	7.7043195	17.3983439	0.00047542	0.00455771
Dcaf17	-0.7115602	4.46786314	17.3958714	0.00047574	0.00455771
Mrpl34	1.25846595	3.32687172	17.3882953	0.00047675	0.00456156
Tceb2	0.71710316	5.70765472	17.3751183	0.00047851	0.00456941
Ift22	0.87030354	4.09852226	17.3730402	0.00047879	0.00456941
Olfr856-ps1	-1.4832848	4.31338526	17.3576785	0.00048086	0.00458119
Tenm2	-1.0283541	7.00559318	17.354768	0.00048125	0.00458119
Hmbs	0.98991124	3.04214054	17.3493257	0.00048198	0.00458237
Stx1a	0.72084628	5.57076194	17.3398308	0.00048327	0.00458305
Taldo1	0.64133538	4.07313907	17.3352993	0.00048388	0.00458305
Eid1	0.49495852	8.03895077	17.3255677	0.0004852	0.00458305
Szt2	-1.0877511	3.93196404	17.3234653	0.00048549	0.00458305
Kidins220	-0.6431111	8.28762378	17.3183495	0.00048619	0.00458305
Usp49	-0.9172839	3.96459954	17.316811	0.0004864	0.00458305
Rit2	0.53104073	6.72674347	17.3155568	0.00048657	0.00458305
Zmat4	0.50310822	5.6454348	17.312798	0.00048694	0.00458305
Glrx	0.69962242	6.11625311	17.3082676	0.00048756	0.00458312
Atp2b2	-0.7663291	9.28140881	17.2961865	0.00048922	0.00459293
Ubc	0.66309483	6.71051976	17.2826399	0.00049108	0.00460465
Simc1	-0.6731759	4.18232201	17.2585693	0.00049441	0.00462213
Rsrc2	0.48528631	7.78253955	17.256014	0.00049476	0.00462213
Eif1b	0.58413705	5.81897077	17.2558154	0.00049479	0.00462213
Fam185a	0.81805522	3.69657058	17.2423486	0.00049667	0.00463165
Lsm2	1.21662057	4.23845865	17.239642	0.00049705	0.00463165
Rnf14	0.4315729	8.3873547	17.234008	0.00049783	0.00463323
Cox6a1	0.62963227	6.82536447	17.2183004	0.00050004	0.00464797
Vps13b	-0.9692344	6.99017199	17.2075317	0.00050155	0.0046563
Cacna1a	-0.8685239	6.4472544	17.1844374	0.00050482	0.00468086

4931403G20	-2.2526925	0.89301541	17.167936	0.00050717	0.00469685
Snx2	0.48948642	7.57439358	17.1607489	0.0005082	0.00470056
Ccdc108	-2.055834	1.32172893	17.1355166	0.00051183	0.0047148
Pfn1	1.02203012	6.68741467	17.1340365	0.00051204	0.0047148
Trappc3	0.66678407	5.00076725	17.1317029	0.00051238	0.0047148
Fanci	-1.2696842	2.90436969	17.1295115	0.00051269	0.0047148
Mprip	-0.5595544	7.49135185	17.1282075	0.00051288	0.0047148
Pcdhb15	-1.2658075	2.72245539	17.1189709	0.00051422	0.00471749
Ctla2a	-1.7165279	1.86945644	17.1137933	0.00051497	0.00471749
Tcea1	0.4953602	8.06123568	17.113168	0.00051506	0.00471749
Ppdpf	0.70354398	5.09338992	17.0962027	0.00051753	0.00473434
Ikbkap	-0.9346135	5.38567574	17.079004	0.00052005	0.00474694
Rab2a	0.44398307	9.67092269	17.07816	0.00052018	0.00474694
Sec22b	0.51429277	7.09426359	17.0666395	0.00052187	0.0047516
Ppp2r3d	-1.1743179	3.95125935	17.0660792	0.00052195	0.0047516
Clk1	-0.9068369	6.46913764	17.0508332	0.00052421	0.00476633
Chchd3	0.68105018	5.11662928	17.0415567	0.00052558	0.00477305
Gm15881	-3.2193402	0.39379139	17.0320168	0.000527	0.00478016
Fam163b	0.6126645	7.61194462	17.0170598	0.00052924	0.00479462
3010026O09	1.15325407	3.29919568	17.0026329	0.0005314	0.00480842
Sugp2	-1.1468194	3.99885619	16.9816305	0.00053457	0.00482963
Mrpl12	0.97025277	3.85456759	16.9785773	0.00053503	0.00482963
Zbtb48	1.84178652	1.61841727	16.9581448	0.00053814	0.00483876
Camk2a	-0.9213101	11.2091826	16.9554095	0.00053856	0.00483876
9530080O11	1.32795347	2.39329389	16.9528849	0.00053894	0.00483876
Akap9	-0.6385881	8.44979472	16.9509856	0.00053923	0.00483876
8430419L09I	0.67331339	5.70735474	16.9507371	0.00053927	0.00483876
Tmcc2	-0.7191113	4.78003844	16.9185894	0.00054421	0.00486357
Gm16675	6.18346135	-1.3070885	16.9172492	0.00054441	0.00486357
Dpm2	0.72035221	4.06808179	16.9154785	0.00054469	0.00486357
4930473A02I	-2.81734	0.53604873	16.9147685	0.0005448	0.00486357
Kcnj10	-0.7263011	5.29891954	16.9116709	0.00054528	0.00486357
Rab5c	0.57042101	6.19290957	16.9049166	0.00054632	0.00486712
Gprc5a	2.82241707	0.9928613	16.8935534	0.00054809	0.0048756
Smg1	-0.9564522	7.69714299	16.890422	0.00054857	0.0048756
Rnf217	0.6856007	4.29072797	16.8839776	0.00054958	0.00487875
Smim15	0.54756333	6.1523121	16.8719831	0.00055146	0.00488532
Gm14327	-1.5677534	2.97504976	16.8706943	0.00055166	0.00488532
Fgf1	0.64640754	7.40894278	16.8648927	0.00055257	0.00488532
Fam216a	0.55603986	5.4381747	16.86262	0.00055292	0.00488532
Cmah	-0.7078133	6.36639796	16.8464001	0.00055548	0.00490212
Ube2l3	0.5793658	7.51291036	16.8281417	0.00055837	0.00492185
4932411E22I	-1.2875999	2.56302696	16.8062127	0.00056187	0.00494263
Apobec4	-4.1426418	-0.0917944	16.8050833	0.00056205	0.00494263
Bptf	-0.7226057	8.32255731	16.7932341	0.00056395	0.00495079

4930509J09F	-4.2146011	-0.3195613	16.7910662	0.00056429	0.00495079
Agrn	-0.8354873	5.00625141	16.7848515	0.00056529	0.00495201
Cnot10	-0.8099699	4.35910618	16.7780914	0.00056638	0.00495201
Psmb2	0.72163577	5.36654514	16.7779006	0.00056641	0.00495201
Rab3gap1	-0.7400311	4.95786349	16.7404412	0.00057249	0.00499934
Calm3	0.45039525	8.7129131	16.6931244	0.00058028	0.00506142
Man2c1	-1.4582712	2.65960172	16.6789845	0.00058263	0.00506691
Plcb4	-0.7035281	7.05152563	16.6786945	0.00058268	0.00506691
Numa1	-0.4691067	5.77399352	16.674379	0.00058339	0.00506691
Ccdc82	-0.8383068	7.54247244	16.6730911	0.00058361	0.00506691
Spop	-0.4675952	7.72348367	16.6620644	0.00058545	0.00507703
Slco2a1	1.27132978	4.43168881	16.655795	0.0005865	0.00507741
Brca2	-0.8470636	3.83286062	16.6537202	0.00058685	0.00507741
Calm1	0.41465586	12.308041	16.6479296	0.00058782	0.00507924
Fkbp1a	0.60550663	9.78767574	16.6444019	0.00058842	0.00507924
Sod3	1.1032727	5.39044112	16.6390334	0.00058932	0.0050812
Ufd1l	0.46138989	6.36786093	16.6240759	0.00059185	0.00508406
Sptbn4	-1.6238127	3.60591124	16.6181352	0.00059285	0.00508406
Fat1	-0.6521245	6.13438627	16.6178158	0.00059291	0.00508406
Klf10	0.5605072	5.6640606	16.6174127	0.00059298	0.00508406
Mical3	-0.8754185	6.27076941	16.6149182	0.0005934	0.00508406
Selo	-1.3713333	1.26997923	16.6081777	0.00059455	0.00508406
Elavl4	0.57633422	6.90619641	16.6054753	0.00059501	0.00508406
Acyp2	0.6531707	4.62581168	16.6050921	0.00059507	0.00508406
Kif5a	-0.655163	9.49887627	16.5758982	0.00060007	0.00511788
Rab12	0.48363219	7.08669075	16.5740029	0.0006004	0.00511788
Polr3d	1.01496532	3.08164623	16.4943611	0.00061428	0.00523026
Fnip2	-0.7420842	4.74590785	16.4858251	0.00061579	0.00523165
2900011O08	0.53565567	7.725197	16.4855405	0.00061584	0.00523165
Rwdd1	0.59826467	5.23543726	16.4798915	0.00061684	0.00523423
Ndufa3	0.82742045	4.66141307	16.4724896	0.00061815	0.00523945
5031410I06R	-1.7281243	2.61793418	16.4626978	0.00061989	0.00524829
Il31ra	1.27316083	2.40872718	16.4506864	0.00062204	0.00526052
Aatk	-1.1232078	5.14712158	16.4364844	0.00062459	0.00527612
Spag5	-2.0913792	1.90163562	16.4290489	0.00062592	0.00528147
Trmt112	0.63411998	5.06178834	16.409037	0.00062954	0.00530603
Mfsd4	-0.9662782	7.06259956	16.402062	0.00063081	0.00530796
Psmb7	0.6298727	6.85311655	16.3999826	0.00063119	0.00530796
Ttc13	-1.0279564	3.19742116	16.392627	0.00063253	0.00531122
Catsper2	-1.3804212	1.9578967	16.3900805	0.00063299	0.00531122
Rian	-1.0542345	6.84491912	16.3713441	0.00063642	0.00533402
Rrp36	1.46050552	2.16322795	16.3478519	0.00064075	0.0053643
Nav1	-0.8923502	7.3625303	16.3347497	0.00064317	0.00537862
Prkar1b	0.51406013	7.74528638	16.31332	0.00064717	0.00540599
Son	-0.7481081	8.07903712	16.3036056	0.00064899	0.00541515

Ap1s1	0.97566485	2.8383921	16.2895956	0.00065162	0.00543109
Mtor	-0.8821073	6.58603647	16.2831599	0.00065283	0.00543516
Pomt2	-0.7672923	3.59827126	16.2634738	0.00065656	0.00546014
Ypel3	0.49603586	5.84479946	16.2542165	0.00065832	0.00546713
Ccna1	-5.4594467	-1.0159597	16.2513983	0.00065886	0.00546713
Ptgs2	0.94127695	4.12798815	16.2393391	0.00066116	0.00548019
Acot2	0.75108421	4.85835318	16.2324337	0.00066249	0.00548389
Pddc1	0.81995533	3.60516068	16.2293834	0.00066307	0.00548389
Serpinh1	1.01857481	4.25435092	16.2204752	0.00066479	0.00549201
Slc22a2	1.27131668	4.58244843	16.2142951	0.00066598	0.0054958
Mospd1	0.68958071	6.13396769	16.2058443	0.00066761	0.00549729
Celsr3	-1.6836172	4.73290508	16.205688	0.00066764	0.00549729
Fcho1	-1.1847096	2.7911638	16.1984556	0.00066904	0.00549729
Dusp14	0.63186592	5.93030232	16.1956206	0.00066959	0.00549729
Zc3h7a	-0.8723293	5.52498518	16.1910658	0.00067048	0.00549729
Gabrq	-1.341303	1.88100598	16.1906611	0.00067056	0.00549729
Chd8	-0.6424227	6.62880664	16.1843308	0.00067179	0.00550053
Gm5415	-2.1982047	2.10985562	16.1811017	0.00067242	0.00550053
Mmab	0.66614434	4.42183548	16.1641179	0.00067574	0.00552168
Dnaja4	0.65111925	4.57273453	16.1530265	0.00067792	0.00553346
Smad1	0.51763052	5.76445627	16.1418099	0.00068013	0.00554548
2010012O05	0.56081387	6.00612599	16.1339308	0.00068169	0.00554669
Gm12070	0.55176483	10.3711141	16.133488	0.00068178	0.00554669
Acss1	-0.7528009	3.75449704	16.129847	0.0006825	0.00554669
Synm	-0.6777978	5.50732263	16.1006672	0.00068831	0.00558788
B3gntl1	-0.9669252	2.62906946	16.092154	0.00069002	0.00559272
Snrnp200	-0.7673358	6.89140975	16.0902585	0.0006904	0.00559272
Sgcg	-4.1813581	-0.352772	16.0823958	0.00069198	0.00559948
Ppfia4	-0.7890873	3.62828696	16.0664792	0.00069519	0.00561941
Gabarapl1	0.5220585	8.09279506	16.0481663	0.00069891	0.00564119
Akap6	-1.0549983	8.14468972	16.0457986	0.00069939	0.00564119
Ccndbp1	0.63513007	6.68427437	16.0267215	0.00070329	0.00566119
Mif	0.76417538	5.21874154	16.0262802	0.00070338	0.00566119
Slc27a2	-1.4334864	1.809053	16.0168954	0.0007053	0.00566873
Lipt2	1.35155431	2.11350335	16.0122816	0.00070625	0.00566873
Ndufb5	0.60190156	6.73413816	16.0073623	0.00070727	0.00566873
D10Bwg1379	-1.0606471	7.06518929	16.0069556	0.00070735	0.00566873
Ccdc88c	-0.9496355	4.2524516	16.0009836	0.00070858	0.00566873
Ncf2	-1.5129553	2.47627398	15.9997051	0.00070885	0.00566873
4930431F12I	-1.5564398	2.63778753	15.9873088	0.00071142	0.00567871
Snai1	1.20805912	2.27376053	15.9863763	0.00071161	0.00567871
Mylk4	-1.9246849	1.16968029	15.967966	0.00071544	0.00570003
Arpc2	0.55595758	8.49607501	15.9636507	0.00071635	0.00570003
Cfl2	0.71330958	7.7484807	15.9626212	0.00071656	0.00570003
Rnf181	0.6869033	6.26670878	15.9524496	0.00071869	0.00571036

Tcf7	0.77783418	4.87830236	15.9491666	0.00071938	0.00571036
2410089E03I	-1.1514671	5.88888922	15.923335	0.00072484	0.00574755
Slco1a4	-0.9528037	4.88980651	15.9151945	0.00072656	0.00575517
Sat1	0.66230411	5.94072351	15.9086268	0.00072796	0.00576015
Zfp40	-0.7397026	4.31869583	15.8963517	0.00073058	0.00577478
Kmt2d	-0.7690872	7.22961619	15.8801977	0.00073404	0.00579604
Gm19434	-1.7376212	0.9469362	15.8686655	0.00073652	0.00580953
Hnrnpc	0.46131466	7.27085951	15.8623861	0.00073788	0.00581411
Tatdn3	-0.9645569	2.52536768	15.8557033	0.00073933	0.00581939
Tesc	1.01002453	3.01370342	15.8482027	0.00074095	0.00582044
Sptan1	-1.197135	9.21986903	15.8479322	0.00074101	0.00582044
Gnpda1	0.64418735	4.59177892	15.8362319	0.00074356	0.00583186
Timp2	0.66542696	8.97279797	15.834107	0.00074402	0.00583186
Polrmt	-1.1941491	2.23954961	15.8006691	0.00075135	0.00587846
Vwa3a	-1.8035487	2.05989752	15.7998583	0.00075153	0.00587846
Vegfa	-0.6339188	5.38698117	15.791158	0.00075345	0.00588239
Gldc	-1.0403936	2.46772824	15.7904833	0.0007536	0.00588239
Chaf1b	6.50886091	-1.7793604	16.802	0.00075573	0.00589285
Lamtor5	0.77093647	5.97963691	15.7596545	0.00076046	0.00592357
Ndufa12	0.60829977	5.67767073	15.7510471	0.00076238	0.00593241
Dgka	0.68272514	4.79442651	15.7441134	0.00076394	0.00593836
Acot1	0.67821161	3.95154939	15.732618	0.00076653	0.00595229
Gm7102	-1.3148641	5.46312547	15.7154043	0.00077042	0.0059748
Peg3	-0.8678429	8.84130121	15.7127572	0.00077102	0.0059748
Slc36a1os	-1.4635705	2.78054286	15.7090519	0.00077186	0.00597515
Pyroxd1	-0.7708938	3.35080755	15.7021286	0.00077343	0.00598116
P2ry1	0.92259375	4.02734054	15.695827	0.00077487	0.0059861
Pus10	-1.0693065	3.92504545	15.6884923	0.00077654	0.00599287
Snrpb	0.71397281	3.74360166	15.6798988	0.00077851	0.00600188
Ube2r2	0.63600279	8.34444785	15.6743712	0.00077978	0.00600405
Atp5c1	0.55595036	8.18712895	15.6717047	0.00078039	0.00600405
Dbi	0.82400514	7.09650929	15.6210899	0.00079213	0.00608065
Pdcd5	0.61893384	6.90457778	15.6130312	0.00079402	0.00608065
Nt5m	0.84999034	3.91316768	15.6119522	0.00079427	0.00608065
Grm5	-0.6568976	6.71237517	15.6114748	0.00079438	0.00608065
H2afx	0.83580522	3.05993906	15.6113779	0.0007944	0.00608065
Mccc2	-0.8456173	3.4833902	15.5888355	0.00079971	0.00611502
Kctd6	0.6259262	5.85232124	15.5831656	0.00080105	0.00611904
Mlip	-0.7753858	4.86704619	15.5774615	0.0008024	0.00611983
Capns1	0.80085982	9.23059355	15.5746949	0.00080306	0.00611983
Phax	0.56136561	5.38378733	15.5724012	0.0008036	0.00611983
Ndufb6	0.72645752	5.63196151	15.5474033	0.00080956	0.00615898
Fbxo22	0.48941764	6.86914648	15.5392847	0.00081151	0.00616754
Gabrb1	-0.8432395	3.55491588	15.5293735	0.00081389	0.00617504
Rgs19	0.78761415	4.53633584	15.5283345	0.00081414	0.00617504

Dzip3	-0.6882147	6.90177053	15.4733893	0.0008275	0.00625184
Gpd1l	0.44039369	7.02544809	15.4668272	0.00082912	0.00625184
Lrrc39	-1.1834431	2.13293728	15.4645964	0.00082966	0.00625184
1700028P14I	1.78974815	0.53112999	15.4643524	0.00082972	0.00625184
Tulp4	-0.4689644	8.14953768	15.4635846	0.00082991	0.00625184
Arntl	-0.6719095	3.98379063	15.458995	0.00083104	0.00625184
Csmd1	-1.140838	6.08621205	15.4579184	0.00083131	0.00625184
Chd3os	0.42375074	6.56164557	15.4501566	0.00083323	0.00625184
Dtd1	0.65600677	6.04522579	15.4485205	0.00083363	0.00625184
Eif5	0.41001051	8.75249106	15.4470978	0.00083398	0.00625184
Pcsk1	-0.9832809	3.26775986	15.4430071	0.00083499	0.00625184
Cep350	-0.7316218	6.68424156	15.4428843	0.00083503	0.00625184
Narfl	1.12142747	2.64718879	15.442565	0.0008351	0.00625184
AU040972	-2.2357327	1.08626308	15.4240274	0.00083971	0.00628007
Ptprk	-0.5061533	6.4903299	15.4128603	0.0008425	0.00629466
Lemd3	-0.6294641	4.50277454	15.3938186	0.00084728	0.00632182
Dync1h1	-1.6348826	9.33827441	15.3906211	0.00084809	0.00632182
Sfrp4	1.02892553	3.4712962	15.3883289	0.00084867	0.00632182
Gmnc	-3.4526668	-0.5135682	15.3840453	0.00084975	0.0063236
Pprc1	-0.8819623	3.91972088	15.3748349	0.00085208	0.00632913
Fez1	-0.5670669	5.52476133	15.3744387	0.00085218	0.00632913
Gm1979	-2.505746	0.01681008	15.3675221	0.00085393	0.00633186
Ppp2cb	0.43390202	7.09415398	15.3663377	0.00085423	0.00633186
Dock4	-0.8773154	6.18838306	15.3604227	0.00085574	0.00633675
Cdc42ep2	1.30918153	2.08049696	15.3526146	0.00085773	0.00634523
Zfp961	0.53568891	5.09700489	15.3453497	0.00085959	0.0063527
Gprasp2	-0.8032906	6.17426828	15.3345674	0.00086235	0.00636685
Mobp	-0.7936709	5.81453183	15.2904186	0.00087377	0.00644482
Dnajb6	0.42617676	7.95725082	15.2780124	0.00087701	0.00646005
Cdc42bpa	-0.5593344	9.05411707	15.2751968	0.00087775	0.00646005
Fhl2	0.56764047	5.79220638	15.2720956	0.00087856	0.00646005
Prdx5	0.77889194	5.90269109	15.2693459	0.00087928	0.00646005
Ctgf	0.81115613	5.68766544	15.2547328	0.00088312	0.00648194
Bud31	0.66095366	4.69115838	15.2496045	0.00088448	0.00648553
Tsc1	-0.7797065	5.94436256	15.2438516	0.000886	0.00649033
Snx13	-0.3968058	6.88848207	15.2308568	0.00088944	0.00650922
Sipa1l1	-0.8300373	8.88422936	15.2252445	0.00089093	0.00651378
Syn2	0.5700755	7.70541142	15.2172287	0.00089307	0.00652305
E330033B04I	-1.8513371	2.95242948	15.212726	0.00089427	0.00652548
Prr13	0.68260324	5.59039695	15.1962327	0.00089869	0.00654576
Pde10a	-0.8371998	7.06493354	15.1958462	0.0008988	0.00654576
Ppp1r9a	-1.0291177	8.91038892	15.150368	0.00091112	0.00662903
Cct6a	0.45410545	8.24498773	15.1308656	0.00091646	0.00665167
Hcfc1r1	0.77238317	4.47149002	15.1285491	0.00091709	0.00665167
Zfp182	-0.606891	4.90802302	15.1274855	0.00091738	0.00665167

Unc45a	1.17594163	2.75728444	15.1260651	0.00091778	0.00665167
Bora	-1.5355715	1.55862434	15.1167686	0.00092034	0.0066638
Car3	2.30566723	1.3697851	15.0932067	0.00092686	0.00669727
D130017N08	-0.8133081	3.83627959	15.0909723	0.00092749	0.00669727
Plxna2	-0.4854282	7.727418	15.0904266	0.00092764	0.00669727
Ap4e1	-0.903298	4.05886757	15.0711596	0.00093302	0.0067193
Lama3	-1.349558	2.5107146	15.0702474	0.00093327	0.0067193
Slco1c1	-1.0061715	4.33303442	15.0698813	0.00093338	0.0067193
Plxnb3	-1.510301	2.04041531	15.0650048	0.00093474	0.0067217
Cyp1b1	0.81538648	7.03186445	15.0613424	0.00093577	0.0067217
Cntnap5c	-2.1629402	1.45542887	15.0591197	0.0009364	0.0067217
Nop10	0.70194482	4.74626895	15.0498885	0.000939	0.00673007
Gm4432	-2.3450792	-0.0696758	15.0482523	0.00093946	0.00673007
Atp1a4	-2.9894253	-0.6699236	15.0454342	0.00094026	0.00673007
Plekhb1	-0.69528	5.81147658	15.0322844	0.00094398	0.00674666
Nbeal2	-1.1607298	1.80362844	15.030901	0.00094437	0.00674666
Lsm14b	0.5016431	5.55529305	15.0094633	0.00095048	0.00678384
Ube2j1	0.53865986	7.45386094	14.9969135	0.00095408	0.00679772
2610203C20I	-0.7806595	5.8366634	14.9958403	0.00095439	0.00679772
Xrcc4	0.81503123	3.43775911	14.9932003	0.00095514	0.00679772
2810408M09	0.82872495	3.2236678	14.988377	0.00095653	0.00680114
Pigr	-1.5155363	3.83540318	14.9851334	0.00095747	0.00680134
Cdk7	-0.5304093	6.25268145	14.9779678	0.00095953	0.00680958
Atp2c1	-0.5162586	6.56727985	14.9744801	0.00096054	0.0068103
Mpv17	0.66302782	5.95198813	14.9644405	0.00096345	0.00682325
Ankrd63	-0.9994329	4.06143589	14.9575654	0.00096545	0.00682325
Heatr5b	-0.7774668	5.48811715	14.9534724	0.00096664	0.00682325
Ndufc2	0.75439674	5.2850835	14.9525325	0.00096692	0.00682325
Nol10	-0.9749942	4.15900657	14.952524	0.00096692	0.00682325
Rasl10a	0.71824951	3.35772303	14.9403937	0.00097046	0.00684181
4930402H24I	-0.7111872	5.56917673	14.9358083	0.0009718	0.00684208
Snrpa1	0.61955286	4.24823877	14.9340352	0.00097232	0.00684208
Tpo	-5.8390068	-2.0661477	14.9288671	0.00097384	0.00684287
Nrk	1.77293772	1.47613572	14.9274391	0.00097426	0.00684287
Eif1a	0.65000419	6.62350168	14.9210805	0.00097613	0.0068496
Ndufa4	0.65129009	7.34644343	14.8994272	0.00098253	0.00688806
Pycard	1.00653748	2.9570236	14.8914862	0.00098489	0.00689162
Hbegf	1.4981816	1.72876483	14.8885791	0.00098575	0.00689162
Myl4	1.53396511	2.24549213	14.8884453	0.00098579	0.00689162
Pde1a	0.38384239	9.4446545	14.8702961	0.00099121	0.00691618
Setd1b	-0.5846779	5.46467722	14.868092	0.00099187	0.00691618
Tnfaip8l3	0.48078993	6.59912069	14.8674309	0.00099207	0.00691618
Ap1s2	0.58197824	6.31247399	14.855405	0.00099569	0.00692928
Hspa5	0.50130606	7.24254107	14.8550245	0.0009958	0.00692928
Ift20	0.6189937	6.78178911	14.8436765	0.00099922	0.00693052

Pim2	0.61145295	4.10662835	14.8398657	0.00100038	0.00693052
Emc4	0.47075798	6.33194098	14.8367388	0.00100132	0.00693052
Armcx4	-0.6233927	6.06988026	14.8314216	0.00100293	0.00693052
Btaf1	-0.736216	6.20105171	14.8313067	0.00100297	0.00693052
Psmd4	0.72936757	5.3258604	14.8304675	0.00100322	0.00693052
Hic2	-1.0810658	2.3305498	14.830163	0.00100332	0.00693052
Fam227a	-1.8336004	1.77176102	14.8283143	0.00100388	0.00693052
Sfr1	0.57637515	6.95960474	14.8227041	0.00100558	0.00693052
1500015L24R	-4.203103	-0.0251291	14.8209318	0.00100612	0.00693052
C77370	-1.0922879	6.42862139	14.8196703	0.00100651	0.00693052
Gosr2	0.44711962	6.62556428	14.817843	0.00100706	0.00693052
Mllt6	-0.5504074	6.01059421	14.8072306	0.00101031	0.00694645
Rgs11	-2.2697883	1.394385	14.7930981	0.00101464	0.00696986
Eid2b	0.61347667	4.42914406	14.7894139	0.00101577	0.00697126
Ptpn7	-1.0782465	3.28926987	14.7826674	0.00101785	0.00697516
Dmxl2	-1.0241781	8.52492844	14.7815324	0.0010182	0.00697516
Psma7	0.53876555	6.45837861	14.7785197	0.00101913	0.00697516
Gatad2b	0.41013557	7.58803378	14.7708042	0.00102152	0.00698512
Cd24a	-0.8126684	5.29033458	14.7672122	0.00102263	0.00698636
Chd2	-0.5195499	6.94922404	14.741669	0.00103058	0.00703006
Avpi1	0.82320776	3.24214639	14.7372995	0.00103195	0.00703006
Me2	0.61676721	6.29207004	14.7349283	0.00103269	0.00703006
2410076I21R	-2.0420161	0.47192172	14.7305265	0.00103408	0.00703006
Pclo	-1.1382004	10.1329204	14.7298084	0.0010343	0.00703006
Gpr88	-0.6851906	6.09706618	14.7287	0.00103465	0.00703006
Anln	-0.5358057	4.85821024	14.709058	0.00104084	0.00706313
Ubp1	-0.5875999	6.29825145	14.7072834	0.0010414	0.00706313
Zcchc12	-0.7707143	4.29286974	14.6968146	0.00104472	0.00707902
Cfp	-2.0710199	1.41500791	14.6939408	0.00104563	0.00707902
Zfhx2	-0.7131441	5.15266286	14.6877225	0.00104761	0.00707975
Grin2b	-0.7548619	7.05758381	14.6876688	0.00104763	0.00707975
Muc5b	-6.3638752	-1.4645111	15.5688241	0.00105238	0.00710327
Uty	-0.6574275	5.05926965	14.6679412	0.00105393	0.00710327
Gm4371	7.49364228	-0.7745846	14.6634001	0.00105539	0.00710327
4932418E24I	-2.6595896	-0.0372914	14.6628517	0.00105556	0.00710327
5730522E02I	-2.2324807	1.39875561	14.6619821	0.00105584	0.00710327
Atr	-0.7535613	4.55086409	14.6472945	0.00106057	0.0071287
Gm15401	-1.8521388	0.45559154	14.6413961	0.00106248	0.00713512
Tmem263	0.54402645	7.21113596	14.6355668	0.00106436	0.0071414
Spg11	-0.6758519	5.11874808	14.6318429	0.00106557	0.00714174
Sprr1a	7.41681031	-0.2489927	15.5208541	0.00106632	0.00714174
Paxbp1	-0.8385791	5.67637254	14.6218854	0.00106881	0.00714585
Sub1	0.53816743	8.79956942	14.621795	0.00106884	0.00714585
Mrpl36	0.80956947	5.37409144	14.6054529	0.00107417	0.00717512
Tnfaip8l1	1.2257109	2.81437686	14.5975456	0.00107677	0.00718604

Fam89b	1.12298672	2.77397986	14.5842195	0.00108115	0.00720889
Sh3bgr	5.67877972	-0.4642722	14.5777054	0.0010833	0.00721681
Ccnl2	-1.0570506	4.42696557	14.5624537	0.00108835	0.00724404
Exosc9	-0.6556029	4.0254313	14.5553166	0.00109073	0.00724747
Ppp2r3a	-0.546198	6.51542	14.5550953	0.0010908	0.00724747
Fbf1	-0.716016	4.05102096	14.5281343	0.00109982	0.00729749
Abat	-0.5809877	6.69238941	14.5267794	0.00110027	0.00729749
Itpr2	-0.5478272	5.33781271	14.5195267	0.00110272	0.00730722
Hivep3	-0.9887928	6.88983087	14.5130463	0.0011049	0.00731525
Mpc1	0.48230588	6.49887657	14.505674	0.0011074	0.00732529
Atp5k	0.68622581	5.92428404	14.5011691	0.00110892	0.00732892
Acyp1	0.79583682	5.19441005	14.4977327	0.00111009	0.00733017
Il1rapl1	-0.7702921	3.91836958	14.4917789	0.00111211	0.00733708
Tet2	-0.8340221	6.67525259	14.4844686	0.0011146	0.00734262
Schip1	0.75328537	3.86375504	14.4835634	0.00111491	0.00734262
Rab1	0.49035929	9.05096433	14.4722112	0.00111879	0.00736172
Timm17a	0.69395043	5.75091044	14.4672133	0.0011205	0.00736178
Armc1	0.44868644	7.59764091	14.4650519	0.00112125	0.00736178
Aprt	0.93644189	3.50790968	14.4635969	0.00112175	0.00736178
Ube2q1	0.42029761	7.10382136	14.4536436	0.00112517	0.00736986
Gigyf1	-1.3459005	2.40603753	14.4535375	0.00112521	0.00736986
Sugt1	0.56949684	5.59679068	14.4514586	0.00112592	0.00736986
Wbp4	0.44432113	6.16916851	14.4422099	0.00112912	0.00737976
Fbn2	-1.6122913	1.50799282	14.4384732	0.00113041	0.00737976
Eif4e3	0.55757792	5.53897092	14.4374255	0.00113077	0.00737976
Clip3	0.64586355	10.1860474	14.4357034	0.00113137	0.00737976
Csf2rb	-1.9122806	1.3256327	14.4260044	0.00113474	0.00739143
Vezt	-0.6295645	4.86885438	14.4248802	0.00113513	0.00739143
Cd86	-3.1372126	-0.1300237	14.4153218	0.00113846	0.00740669
Rhno1	0.94831487	2.51360383	14.4091466	0.00114062	0.0074083
Bloc1s3	0.87820644	3.16661035	14.4089662	0.00114068	0.0074083
Tctn3	-1.5468049	2.73399384	14.4036901	0.00114253	0.00741388
Fnip1	-0.6707608	5.8156585	14.3907303	0.00114708	0.0074328
Cuta	0.76209119	6.28943736	14.3875659	0.0011482	0.0074328
Fgf18	1.21712499	2.47317753	14.3863834	0.00114862	0.0074328
Nes	-0.6652638	4.26178602	14.3841262	0.00114941	0.0074328
Dctn2	0.47457077	5.87271617	14.3716481	0.00115382	0.00744294
Gm5523	0.58136038	5.24483222	14.3647658	0.00115627	0.00744294
Gm21119	-2.0406438	0.59824028	14.3643773	0.0011564	0.00744294
Pcdhgb4	-1.2086037	2.29278833	14.3641596	0.00115648	0.00744294
Wdr54	1.0042298	3.28234784	14.3599449	0.00115798	0.00744294
Adra1d	0.70060863	3.5059579	14.3560318	0.00115937	0.00744294
Nup85	-0.763783	3.70621767	14.3554544	0.00115958	0.00744294
Umps	0.65358147	4.08953276	14.3515405	0.00116097	0.00744294
Grin1os	-2.000693	0.24889645	14.3510722	0.00116114	0.00744294

Gm1943	0.8475864	3.5392695	14.346118	0.00116291	0.00744294
Iqsec2	-0.636385	5.69733138	14.3428479	0.00116408	0.00744294
Ndufc1	0.78622735	5.67207683	14.3411105	0.0011647	0.00744294
Arap2	-0.6533149	7.05856224	14.3380224	0.00116581	0.00744294
Loxl3	-1.4280958	1.77272486	14.3369863	0.00116618	0.00744294
Anxa5	1.0095374	8.20189293	14.3351918	0.00116682	0.00744294
Itpa	0.62515998	4.19649389	14.3351006	0.00116686	0.00744294
Znhit1	0.90838049	3.24479417	14.3254887	0.00117031	0.00745863
Tfcp2l1	0.91817426	7.02929852	14.3195128	0.00117246	0.0074608
H13	-0.7799707	4.82160613	14.3190275	0.00117264	0.0074608
Rheb	0.55071273	7.10790637	14.31181	0.00117524	0.00746821
Sumo2	0.72727394	8.73781098	14.3102913	0.00117579	0.00746821
Phyhipl	0.51743239	7.4588456	14.2861729	0.00118456	0.00751441
Clns1a	0.53403709	6.19127817	14.2847634	0.00118507	0.00751441
Inpp5f	-0.4104629	6.79477906	14.2711451	0.00119005	0.00753349
Med13l	-0.7770746	6.48937418	14.2710442	0.00119009	0.00753349
Vim	0.79356108	8.07016048	14.2613481	0.00119365	0.00754967
Rbbp5	-0.4881705	5.69643705	14.2499387	0.00119786	0.00756989
BC005764	-1.3798323	2.18317284	14.2450241	0.00119967	0.00757498
Adra1a	-0.7071043	4.48599076	14.2403869	0.00120139	0.00757945
Ankfy1	-0.5618954	6.00458421	14.218088	0.00120969	0.00762536
D930015E06l	-1.4001446	2.15529752	14.2032843	0.00121523	0.00765386
Mrpl48	0.65750659	4.45737419	14.1976878	0.00121733	0.00765913
Dennd1b	-0.6468396	4.59060003	14.1944538	0.00121855	0.00765913
5730409E04l	0.38084263	6.93327288	14.1825932	0.00122302	0.00765913
Focad	-0.6573364	5.28452876	14.1821844	0.00122317	0.00765913
Zdbf2	-1.0274725	5.97330262	14.1807989	0.0012237	0.00765913
Pdzd11	0.59786463	6.13967955	14.1807915	0.0012237	0.00765913
Asun	-0.5749972	4.53145219	14.1807524	0.00122371	0.00765913
Rbm6	-0.6019332	6.21619282	14.1793864	0.00122423	0.00765913
Pde8a	-0.5832123	4.07415265	14.1738044	0.00122635	0.00766058
Alms1	-0.8197325	5.05209571	14.1733778	0.00122651	0.00766058
Pafah1b3	1.59576659	2.03369673	14.1678741	0.0012286	0.00766058
Shprh	-0.8239935	5.96668018	14.1661677	0.00122924	0.00766058
Ewsr1	-0.7434834	7.69583582	14.160074	0.00123156	0.00766058
Gria2	-0.8447583	9.08586185	14.1581461	0.0012323	0.00766058
Cyb561a3	-0.7876004	3.48963815	14.1567168	0.00123284	0.00766058
4933402D24l	-3.0843815	0.32881142	14.1534963	0.00123407	0.00766058
Ppp2r3c	0.5117666	4.97086748	14.1523392	0.00123451	0.00766058
Itga8	-1.1605543	2.59275693	14.1519123	0.00123468	0.00766058
Tnfrsf23	1.55225141	1.09561265	14.1412226	0.00123877	0.0076796
Ermard	-0.7775837	3.92559189	14.1348438	0.00124121	0.00768407
Syne2	-0.5468275	5.49056442	14.134004	0.00124154	0.00768407
Ipw	-1.6537172	4.05705094	14.1293342	0.00124333	0.00768885
Huwe1	-0.8441848	9.48061089	14.1187934	0.0012474	0.00770763

Zfp580	0.70014422	3.72006209	14.0948546	0.00125668	0.00775413
Lphn2	-0.5042186	6.35521224	14.0940612	0.00125699	0.00775413
Asna1	0.6921061	6.49108005	14.0745699	0.00126461	0.00778918
Pcdhga12	-0.8983874	2.95173077	14.0724718	0.00126543	0.00778918
Gm15645	-1.3839671	1.94279585	14.0691587	0.00126673	0.00778918
Cox7a1	1.33127677	2.11222014	14.0683472	0.00126705	0.00778918
Atpif1	0.58455182	7.27261637	14.065532	0.00126816	0.00778918
Fbxl16	-0.6363465	7.27841856	14.063643	0.0012689	0.00778918
Gpd1	0.70823252	4.49827584	14.0411333	0.0012778	0.00783386
Tmem181c-ƥ	-1.3752012	4.26990538	14.0399428	0.00127827	0.00783386
Sec13	0.55466718	4.60747021	14.0308299	0.00128189	0.00784659
4930419G24	-1.7144228	1.826446	14.027576	0.00128319	0.00784659
Psmd8	0.55717293	5.10244073	14.0210145	0.00128581	0.00784659
Ylpm1	-0.9712979	8.12727214	14.0182637	0.00128691	0.00784659
Tmc7	-0.6719342	4.52991918	14.0165346	0.0012876	0.00784659
Ndfip1	0.63708583	7.96191732	14.0156561	0.00128795	0.00784659
Zar1l	2.19629215	0.48899529	14.0110144	0.00128981	0.00784659
Tcerg1l	-0.9863915	2.69450517	14.0103673	0.00129007	0.00784659
Ttll1	0.47487466	5.61818762	14.0098834	0.00129026	0.00784659
Ubqln1	0.40579192	7.10321703	14.0085232	0.00129081	0.00784659
Gm5464	-2.3974681	0.45420489	14.0047829	0.00129231	0.00784936
Rmi2	-1.1460191	2.19828046	13.9990137	0.00129463	0.00785709
Adcy2	-0.5774513	5.76768634	13.9835431	0.00130087	0.0078886
Sun1	-0.6768394	4.71651478	13.9658137	0.00130807	0.00792583
A230046K03	-0.7243467	6.37811693	13.9611306	0.00130998	0.00793098
Ran	0.41148181	7.71307405	13.9487042	0.00131505	0.00794332
Myl12b	0.68807009	7.8337359	13.9473	0.00131563	0.00794332
Clybl	0.72464494	4.35151724	13.9441615	0.00131692	0.00794332
Stmn3	0.67006157	5.74413792	13.9422268	0.00131771	0.00794332
Kank1	-0.8018321	3.58697731	13.9409943	0.00131822	0.00794332
Pycrl	0.91625362	2.65084745	13.9406226	0.00131837	0.00794332
Ssfa2	-0.5108783	5.4928508	13.9335284	0.00132129	0.00795451
Ssh3	-1.294315	1.67855805	13.9251927	0.00132472	0.0079688
Slc6a7	0.72898099	5.20504357	13.9163381	0.00132838	0.00798443
Ahi1	-0.7224335	6.88549225	13.9129413	0.00132979	0.00798577
Aldh1a2	0.92123813	9.18687312	13.9106602	0.00133074	0.00798577
Tprgl	0.51031437	6.93458413	13.8997033	0.00133529	0.00800188
Htt	-0.8691033	6.73840291	13.8990742	0.00133555	0.00800188
Ak3	0.59578511	8.15985105	13.8841342	0.00134179	0.00803285
Kif21b	-0.8478927	5.75344638	13.8723138	0.00134675	0.00805611
G2e3	-0.7719998	4.38339843	13.8683137	0.00134844	0.00805844
Ezh2	-1.100167	3.03078086	13.8651037	0.00134979	0.00805844
Slc9b2	0.96003854	4.25861711	13.8637384	0.00135036	0.00805844
Pan2	-0.9357602	3.5095207	13.8609409	0.00135155	0.00805897
Cops7a	0.63469837	6.91236937	13.8584368	0.0013526	0.00805897

Ankrd28	-0.4404877	6.58204557	13.8494402	0.00135641	0.00807004
Cxcr2	-0.8669252	4.02308333	13.8489624	0.00135661	0.00807004
Zzef1	-0.98081	5.30413241	13.8424299	0.00135938	0.00808012
1110008F13I	1.33493544	2.33546273	13.8336341	0.00136313	0.00809595
Ptges	1.03478595	4.28233446	13.827803	0.00136561	0.00810431
Ttc14	-1.1000668	6.58514574	13.8251418	0.00136675	0.00810464
Cpped1	0.4740676	6.46665598	13.8179215	0.00136984	0.00811492
Snora23	-2.7098326	0.98583809	13.8152821	0.00137097	0.00811492
Cyp2t4	-3.3548682	-0.8172949	13.8135159	0.00137173	0.00811492
Pafah1b2	0.39243829	8.35178325	13.807017	0.00137452	0.00812503
Cttnbp2	-0.8034321	6.72279741	13.8021763	0.0013766	0.00813094
Peak1	-0.7267113	6.77754492	13.7966137	0.001379	0.00813869
Cd300a	-1.0752966	3.03887199	13.7940819	0.0013801	0.00813874
Psap	0.59491727	8.49462902	13.7912443	0.00138132	0.00813958
Spag17	-4.5390024	0.24423401	13.7810935	0.00138572	0.00815908
Kif26b	-1.1905898	1.966585	13.7748627	0.00138843	0.0081686
Rpp30	0.63121895	3.992085	13.767637	0.00139157	0.0081807
Als2	-0.7034677	4.90617991	13.7647255	0.00139284	0.00818175
Prrt1	0.64328528	4.88909322	13.7595032	0.00139512	0.00818874
Ube2b	0.44963162	7.64249085	13.7559152	0.00139669	0.00818991
Cox6b1	0.91569858	5.47414806	13.7540565	0.00139751	0.00818991
Slc2a4rg-ps	-1.6818201	1.80381614	13.7474646	0.0014004	0.00819719
Myo5a	-1.0123083	9.77832343	13.7462428	0.00140094	0.00819719
E130311K13I	0.90966022	3.48396889	13.7417067	0.00140293	0.00820246
Asb14	-3.2263549	0.03318188	13.7387193	0.00140424	0.00820375
Nalcn	-0.5721916	6.39924881	13.7345538	0.00140608	0.00820379
Cela1	1.3867702	1.3190181	13.7337431	0.00140644	0.00820379
4933431G14	-2.2956611	0.30495939	13.7258331	0.00140993	0.00821259
Dhx35	-0.8599958	2.62896772	13.7253698	0.00141014	0.00821259
Mcts2	0.71309434	3.74713524	13.7148442	0.0014148	0.00823337
8430431K14I	-1.8308114	0.78650113	13.7048946	0.00141923	0.00824817
Mcee	0.80945985	4.05548428	13.7041855	0.00141954	0.00824817
Atad5	-0.8128108	3.88604756	13.6949014	0.00142369	0.00826585
Degs2	-1.5781573	1.57855019	13.6922376	0.00142488	0.00826637
Ppp2r1a	0.39649744	7.25493951	13.6849553	0.00142814	0.0082789
Dlx6os1	-0.7963933	3.48672029	13.6720266	0.00143396	0.00830618
2010015L04F	-0.8606139	3.55873562	13.6609268	0.00143897	0.00832878
Atp6v1c1	0.38259448	7.37129224	13.6503742	0.00144375	0.00835003
Cdk11b	0.39542003	7.39709748	13.639196	0.00144884	0.00837299
Phf20l1	-0.6465741	5.71701924	13.6357496	0.00145041	0.00837562
Lmcd1	1.49915357	2.10485348	13.6302739	0.00145291	0.00838361
Phka1	-0.8087135	4.255368	13.6189561	0.0014581	0.00840707
Fam111a	0.79031758	3.34294009	13.6134169	0.00146064	0.00841528
Capn5	0.51773062	5.56500213	13.604814	0.00146461	0.00843163
Fkbp1b	0.81499895	4.2158302	13.5975757	0.00146795	0.0084444

Zfyve28	-1.0423648	3.78121154	13.5930562	0.00147004	0.00844996
Glt8d1	-0.7068556	4.23810645	13.5878121	0.00147248	0.00845746
Abca1	-0.5690813	5.05854337	13.5751711	0.00147836	0.00848473
Ptprd	-0.6612421	8.31428597	13.5689047	0.00148128	0.00849502
Syt7	-0.7014321	6.93971431	13.5630469	0.00148402	0.00849817
Chp1	0.41562881	7.55719185	13.5628847	0.00148409	0.00849817
Yars2	0.64119532	4.04331491	13.5558808	0.00148738	0.00850692
Gdpd2	-1.4570228	2.27646471	13.554586	0.00148799	0.00850692
Gm20337	-1.5426082	2.04686625	13.5507788	0.00148977	0.00850692
Morc2b	-2.1739219	1.68893426	13.5484058	0.00149089	0.00850692
Slc44a1	-0.4552111	5.88927923	13.5475498	0.00149129	0.00850692
Myo1d	-0.6408669	4.42317551	13.5425993	0.00149363	0.00851375
Acrbp	-2.6310081	0.21321377	13.5382681	0.00149567	0.0085163
Lamtor3	0.71273198	5.61417099	13.5352539	0.00149709	0.0085163
Gtf3c1	-0.774685	6.48782816	13.533396	0.00149797	0.0085163
Pgam1	0.45670493	9.43813812	13.5320381	0.00149861	0.0085163
Tle1	-0.5625987	5.53266061	13.5287233	0.00150018	0.00851877
Msantd4	0.42813905	7.36767747	13.5218708	0.00150344	0.00852333
Llgl1	-0.6269179	3.95670421	13.5202816	0.00150419	0.00852333
4930449E18I	-3.5225575	-0.8202355	13.5198478	0.0015044	0.00852333
Clvs1	0.6449415	4.16527292	13.5108003	0.0015087	0.00854128
Ywhaq	0.44088691	9.00817021	13.5039772	0.00151196	0.00855077
Kbtbd8	-0.8615875	3.16545921	13.5025151	0.00151266	0.00855077
Psip1	0.48564094	8.79468226	13.4911652	0.0015181	0.00857301
Pcbp3	0.75869053	4.77316804	13.4890115	0.00151913	0.00857301
Pde6d	0.87404972	4.93270465	13.487156	0.00152002	0.00857301
Myo9a	-0.7093039	8.3603113	13.4754891	0.00152564	0.00859825
Uqcrh	0.71277011	7.05499761	13.4695864	0.0015285	0.00860664
Paip2	0.48222562	7.53393563	13.4676601	0.00152943	0.00860664
Gm15800	-1.151871	8.44724005	13.4548122	0.00153566	0.00863109
Cmpk1	0.50580428	7.47820594	13.4539641	0.00153607	0.00863109
Wfdc18	3.61336418	-0.5678841	13.4489951	0.00153849	0.00863821
Cox7c	0.71290562	7.31570746	13.4430721	0.00154138	0.00864795
Snx15	0.56850372	4.50132427	13.434073	0.00154578	0.00866616
Stx7	0.46591385	7.46022408	13.4303249	0.00154762	0.00866736
Ets1	0.63151497	4.0399327	13.4289243	0.0015483	0.00866736
Birc3	0.95843675	4.01387721	13.4236411	0.0015509	0.00867541
BC002163	0.63546433	3.58141497	13.4202279	0.00155258	0.00867833
Speg	-0.6101332	5.58177323	13.4069016	0.00155915	0.00870858
Arid1b	-0.7060282	6.24376872	13.3987054	0.00156321	0.00872475
Apc2	-0.9333385	4.60083919	13.3937907	0.00156565	0.00872588
Slc25a5	0.41144396	7.08642197	13.3936123	0.00156574	0.00872588
Acsl6	-0.5807901	6.0861071	13.3886559	0.0015682	0.00873191
Smarca2	-0.4395633	9.78810952	13.3867538	0.00156915	0.00873191
Ccdc43	0.60732522	4.71901413	13.3833468	0.00157085	0.00873488

Ppp1r11	0.64651692	4.56555625	13.3727444	0.00157614	0.00875784
Rab5a	0.46140145	5.49876439	13.3700826	0.00157748	0.00875875
Gm9159	-1.6603958	2.04989357	13.3616784	0.00158169	0.00877387
Crem	0.55347478	4.8377127	13.3588667	0.00158311	0.00877387
Mir8091	-2.9791864	-0.5753025	13.3526983	0.00158621	0.00877387
Tnik	-0.7817797	7.36927851	13.3524062	0.00158636	0.00877387
Anxa1	1.15031844	6.7899027	13.3513027	0.00158691	0.00877387
Cdc37	0.63859744	5.08594465	13.3507041	0.00158722	0.00877387
Foxred2	-0.8845317	2.99096199	13.3402235	0.00159251	0.00879666
2310039L15F	-1.3985241	2.85952207	13.3248886	0.00160029	0.00883315
Vamp2	0.44003635	10.2441072	13.3209615	0.00160229	0.00883768
Nxf7	3.25803552	0.83819956	13.3173962	0.00160411	0.00884121
Atp6v0d1	0.50922613	7.16095009	13.3139433	0.00160588	0.00884443
Srp54b	0.4060581	6.34362651	13.3080132	0.00160891	0.00884849
Nptx1	0.57903861	7.55312455	13.3078878	0.00160897	0.00884849
Psmb5	0.62261423	5.10193767	13.2842009	0.00162115	0.00890822
3110057O12	-0.8118378	3.00491508	13.2808065	0.0016229	0.00890822
Ahsa1	0.53181682	5.77367489	13.2796472	0.0016235	0.00890822
Hspb11	0.98188301	3.06169139	13.2775605	0.00162458	0.00890822
Srp68	0.51588463	5.94571397	13.2659362	0.00163061	0.00893018
Rasl10b	-0.9043579	3.49792742	13.2652445	0.00163097	0.00893018
Rhou	0.62537795	6.75176283	13.2619559	0.00163268	0.00893303
Trim56	0.77226382	3.52885382	13.2575835	0.00163496	0.00893896
Tmem86b	-1.9785892	0.33394382	13.2486144	0.00163964	0.00895803
Gm14092	-5.0394014	-1.0279516	13.2316334	0.00164854	0.00899558
Gje1	-1.4334425	1.15646148	13.2309373	0.00164891	0.00899558
5830418K08F	-0.8020469	5.57717409	13.2204374	0.00165444	0.00900828
Doc2a	1.05373034	2.81656322	13.2203502	0.00165449	0.00900828
Cep72	-1.9818103	1.07004551	13.2196843	0.00165484	0.00900828
Aldh3b2	2.1226122	-0.2498659	13.2001913	0.00166517	0.00905795
Dctn5	0.58718701	6.19143987	13.1863418	0.00167256	0.00909152
Ctc1	-0.6335979	4.13550488	13.1786675	0.00167666	0.00910724
Tiprl	0.47725845	6.36973233	13.1747641	0.00167876	0.00911201
Golm1	0.69600546	3.26257839	13.1614946	0.00168589	0.00914414
Snip1	0.93762116	3.26961063	13.1555694	0.00168909	0.00915486
Xcr1	-1.5264885	1.55130213	13.1467817	0.00169385	0.00917401
Clint1	0.39818263	6.88746452	13.1351531	0.00170016	0.00920157
Ppp1r1b	0.82262478	5.39614067	13.1317028	0.00170204	0.0092051
Utp14b	-0.7932332	4.91142659	13.1255873	0.00170538	0.00921649
Pigm	-0.7562184	4.17321797	13.1156235	0.00171083	0.00923929
Abcb6	-1.7937619	1.61675515	13.0974728	0.00172081	0.0092865
Pcdh20	-1.0185708	3.064212	13.0890108	0.00172548	0.00930503
Pou4f1	7.23560156	-1.0522652	13.8134939	0.00172809	0.00931239
Gpr165	-0.8985456	3.639751	13.0770411	0.00173211	0.00931504
9430041J12F	-1.6552777	1.8680772	13.07602	0.00173268	0.00931504

Daam2	-0.761522	5.00971729	13.0755576	0.00173294	0.00931504
Ddx18	0.50882961	5.05537199	13.0731053	0.0017343	0.00931504
Ift74	0.48237375	5.84316581	13.0722366	0.00173479	0.00931504
Lrrc57	0.54993623	5.83911175	13.0624168	0.00174026	0.00933676
Col5a2	0.83233916	2.99859565	13.0595424	0.00174187	0.00933676
Fmn1	-0.4810373	6.50161144	13.0582922	0.00174257	0.00933676
Ncdn	0.58635837	8.21970628	13.0537938	0.00174508	0.00934357
Megf10	-0.8893619	4.61145867	13.038185	0.00175385	0.00938049
Rsu1	0.72260541	6.85772679	13.0345638	0.00175589	0.00938049
Akap8l	-1.1519528	3.41002979	13.0341003	0.00175615	0.00938049
06-Sep	0.40171105	6.97292922	13.032634	0.00175698	0.00938049
Clcn7	-0.9445877	3.02906878	13.0213101	0.00176338	0.00940438
Fam214a	-0.5948982	5.23806352	13.0189856	0.0017647	0.00940438
Ppp3r2	-3.3885838	-0.420535	13.0172337	0.0017657	0.00940438
Psenen	1.04763485	5.2136911	13.012477	0.0017684	0.00940438
2810047C21I	1.42972557	1.87145865	13.0107746	0.00176937	0.00940438
Bard1	-1.4508803	1.56742693	13.0107496	0.00176938	0.00940438
Cspp1	-0.674673	4.72589044	13.0092535	0.00177023	0.00940438
Ldha	0.53093251	7.8797741	13.0010307	0.00177492	0.00941788
Dnah1	-0.9356847	3.06537276	13.0003912	0.00177528	0.00941788
Nupl1	0.48435682	6.08892636	12.9880819	0.00178233	0.00944856
Litaf	0.75778224	4.59247002	12.9821904	0.00178571	0.0094598
Tpi1	0.56608931	6.96436296	12.9698456	0.00179282	0.00949076
Cetn2	0.7577438	5.26854616	12.9640405	0.00179617	0.00950137
Neu3	-0.8604286	3.12335179	12.9619937	0.00179735	0.00950137
Aurkb	4.88875512	-1.0478296	12.9593141	0.00179891	0.00950288
Prr11	-0.8889897	3.12267496	12.9543846	0.00180176	0.00951128
Lrrc20	0.86580313	4.21284955	12.9490675	0.00180485	0.00952088
Gtf2f2	0.74608769	3.1438691	12.9392546	0.00181057	0.00954111
Nphs1	-1.194318	2.0604557	12.9377121	0.00181147	0.00954111
Kntc1	-3.5928058	0.00439838	12.9350281	0.00181304	0.00954111
Serf1	0.96072551	2.89627059	12.9333181	0.00181404	0.00954111
Ncbp2	0.47551845	6.54868575	12.9315908	0.00181505	0.00954111
Tekt5	-2.4716074	0.32940631	12.9289985	0.00181656	0.00954239
Smok4a	-1.6618365	1.7706538	12.9216354	0.00182088	0.00955838
Nat10	-0.7432792	3.42472653	12.9175679	0.00182327	0.00955939
Pcp4	0.47111146	8.83186666	12.9169699	0.00182362	0.00955939
Adam3	-6.4016385	-1.935804	13.623174	0.00182699	0.00956708
Bnip3l	0.51995473	8.24542799	12.9101493	0.00182764	0.00956708
Wdfy3	-0.9704144	7.86625625	12.9063175	0.0018299	0.00957223
Stk39	0.6458224	7.75126051	12.9028132	0.00183197	0.00957638
Cops4	0.45963873	6.02394846	12.9005336	0.00183332	0.00957676
Klf15	-0.8539772	4.25353879	12.8900413	0.00183954	0.00960255
Pop7	0.899143	2.62452614	12.8863499	0.00184173	0.00960732
Med15	0.43193936	6.51689822	12.874653	0.0018487	0.00963697

Myh3	-0.988583	2.04655776	12.8553315	0.00186028	0.00969059
Ubl4	0.39882321	6.74027419	12.8522706	0.00186212	0.00969265
H19	4.66175355	-1.5509996	12.8494242	0.00186383	0.00969265
A230073K19I	-1.6555369	4.54489843	12.8452885	0.00186633	0.00969265
4930539N22	-3.5626146	0.71910845	12.8445772	0.00186676	0.00969265
Abca2	-0.7515511	6.10425242	12.8428553	0.0018678	0.00969265
Lrp6	-0.4642458	6.82316275	12.8418117	0.00186843	0.00969265
Arhgap18	0.58313983	5.25575095	12.8383426	0.00187052	0.00969344
Khdrbs2	-1.1362004	1.68183347	12.8372846	0.00187116	0.00969344
Vps29	0.53778009	5.86087869	12.8333111	0.00187357	0.00969921
Fbxo42	-0.5014511	5.14758796	12.8288473	0.00187628	0.00970653
Fsip1	5.87375505	-1.5380426	12.8250992	0.00187855	0.00971161
Cox8a	0.63162924	7.4453694	12.8205368	0.00188133	0.00971925
Pno1	0.58037132	3.98519793	12.8154392	0.00188443	0.0097286
Dpm3	1.34598312	2.16360858	12.8089115	0.00188842	0.00974247
Kif20a	-1.1231468	1.644675	12.7942091	0.00189743	0.00978035
Stard13	0.50728784	5.39133096	12.7926844	0.00189837	0.00978035
Grsf1	0.41895416	6.82277294	12.7858241	0.00190259	0.00978698
9530091C08I	-1.6355756	4.62543707	12.7844449	0.00190344	0.00978698
Slco4c1	-1.2382769	2.13125651	12.7842391	0.00190357	0.00978698
Serac1	-0.745437	5.45659715	12.7749019	0.00190934	0.00980991
Ube2v2	0.44365785	6.88664878	12.7634784	0.00191642	0.00983524
Atg7	0.75848991	6.32718181	12.7627238	0.00191689	0.00983524
Vdac2	0.41407187	7.27146482	12.7592898	0.00191903	0.00983947
Isca2	0.53565908	5.90232956	12.7503235	0.00192462	0.00986139
Uprt	0.56895591	4.57891651	12.7467959	0.00192682	0.00986411
Khdrbs3	0.47191091	6.39263603	12.7452662	0.00192778	0.00986411
Rangap1	0.60608601	6.60553911	12.7398869	0.00193114	0.00987242
Ndufb10	0.58833409	5.91536809	12.7384666	0.00193203	0.00987242
Snx4	0.45779977	6.79050908	12.7349581	0.00193424	0.00987649
Atp5d	0.91972025	6.11576955	12.7330032	0.00193546	0.00987649
D7Ertd443e	-1.2049534	1.54308926	12.7243716	0.00194089	0.00989747
Rapgef1	-0.5325413	6.0110437	12.7211991	0.00194289	0.00990094
Dnajc30	0.58550475	4.96880301	12.7091041	0.00195054	0.00993316
Pcdhb21	-1.7203163	1.24682171	12.7061255	0.00195243	0.00993603
Med24	-0.5574876	4.25752126	12.7019401	0.00195509	0.00994281
Ldb2	0.46253121	6.02685681	12.6911781	0.00196194	0.00997089
Hivep2	-0.9620266	8.90992796	12.6804806	0.00196877	0.00999603
Ctsb	0.56370146	7.9144416	12.6792686	0.00196955	0.00999603
Dcdc2a	-0.6174533	7.4805659	12.674893	0.00197235	0.0100004
Vmn1r65	-1.4332057	1.14576224	12.6705921	0.00197512	0.0100004
Cdh19	-1.7239636	1.52153738	12.6686404	0.00197637	0.0100004
Fam178a	-0.3939083	7.06139048	12.6684835	0.00197647	0.0100004
Txnl1	0.43180916	7.14529889	12.6675411	0.00197708	0.0100004
Rai1	-0.5777171	5.70124103	12.6639154	0.00197941	0.01000327

Akap12	0.6128064	8.54412102	12.6625153	0.00198031	0.01000327
Cox6c	0.60587627	7.55381088	12.6591389	0.00198249	0.01000753
Cntnap4	-1.0573271	3.32376177	12.6467633	0.00199049	0.01003453
Serpinc1	-2.3729164	0.12159231	12.6467291	0.00199051	0.01003453
1700001K19I	-2.2895835	0.15610211	12.6419498	0.00199361	0.01004155
Zfp462	-0.6218974	6.71344293	12.6404574	0.00199458	0.01004155
C1ql3	0.55843188	6.79431107	12.6351966	0.001998	0.01005202
Zfp292	-0.5678255	7.57624967	12.6330486	0.0019994	0.01005231
Hspa8	0.40464014	10.7212684	12.6291712	0.00200193	0.01005827
Arhgap29	0.73274652	9.98480511	12.6256662	0.00200421	0.01006303
Rab26os	-1.7991487	0.98218832	12.6080133	0.00201578	0.01011432
Gm20063	0.62367141	3.75008213	12.5862997	0.0020301	0.01017101
Rsbn1l	-0.5000819	5.5308702	12.5842491	0.00203146	0.01017101
Klhl3	-1.0942156	2.05259147	12.5826309	0.00203254	0.01017101
Praf2	0.73036334	4.63199854	12.5816142	0.00203321	0.01017101
AU022252	0.9733439	2.42007903	12.5801252	0.0020342	0.01017101
Acp1	0.47776311	7.14125661	12.5782608	0.00203544	0.01017101
Yaf2	0.50958162	7.42070674	12.5748455	0.00203771	0.01017101
Rhox8	-1.5514679	1.56502683	12.5745185	0.00203792	0.01017101
Gm16853	-3.5638392	-0.9169055	12.5695869	0.00204121	0.0101753
Vmn1r58	-1.2303058	4.2997275	12.5691531	0.0020415	0.0101753
Cxxc1	0.48463907	5.98700297	12.5637998	0.00204507	0.01018633
Tyr	-1.2749787	2.55561045	12.5479562	0.00205568	0.01023238
Gja6	-3.117844	-1.1012232	12.5403808	0.00206077	0.01024465
Pdk3	0.48947453	5.03255319	12.5402294	0.00206087	0.01024465
Gpr171	-1.6692854	1.52802902	12.5361827	0.0020636	0.0102481
2900092D14I	-0.7371598	5.46346693	12.5346246	0.00206465	0.0102481
Mrps18b	1.11802744	2.96801777	12.5331238	0.00206567	0.0102481
Tacc3	1.16331772	2.22085714	12.5188246	0.00207535	0.01028931
Cacna1e	-0.858305	8.22203066	12.5090809	0.00208197	0.01031294
Caly	0.81884625	3.47990388	12.5060868	0.00208401	0.01031294
Rsf1	-0.5676697	7.58645342	12.5057584	0.00208423	0.01031294
Actb	0.55056942	9.97274988	12.4938515	0.00209237	0.01034638
Hcfc1	-0.5509583	6.90610262	12.4812792	0.002101	0.01037668
Atp5l	0.58003438	6.48418274	12.4808942	0.00210127	0.01037668
Zswim8	-0.6185165	5.28010502	12.4779061	0.00210332	0.01038001
Pcdhb17	-0.7042349	4.75954171	12.461149	0.0021149	0.01042493
Rhobtb2	-0.4465682	5.37579515	12.4607115	0.00211521	0.01042493
Cbr1	0.57827785	4.80828654	12.4538232	0.00211999	0.01044163
Cdkn2d	0.68323648	4.11351863	12.4453109	0.00212591	0.01045906
Sgol2	0.88999199	3.58539855	12.4447316	0.00212632	0.01045906
Lrrc61	0.53635812	5.11850472	12.4426166	0.00212779	0.01045946
Smpd4	-0.6442452	5.12357101	12.4381611	0.0021309	0.01046789
D8Ertd82e	-0.7410339	4.35499075	12.4278212	0.00213814	0.01049659
Csnk2b	0.55654571	5.72714885	12.418352	0.0021448	0.01052237

Alox8	-0.8265866	3.29180948	12.4105453	0.0021503	0.01054247
Cdk5rap2	-0.7404063	3.57249977	12.4024368	0.00215603	0.01056367
AW551984	-1.1014751	3.43531085	12.3980216	0.00215916	0.01057209
Cpsf4	-1.1589051	1.62507283	12.3891789	0.00216544	0.01059463
Slc12a5	-0.6506461	7.82459709	12.3875661	0.00216659	0.01059463
Ctdnep1	0.57011863	5.82205095	12.3768391	0.00217424	0.01062512
Park2	-0.8886201	3.05760267	12.3597834	0.00218647	0.01067791
Bnip3	0.48746862	6.08881487	12.3568129	0.0021886	0.01067964
Pdcd6	0.55550649	5.94969641	12.3553347	0.00218967	0.01067964
D630041G03	-0.8705558	4.20906915	12.3520079	0.00219207	0.01067968
1700048O20	1.0695426	1.87947772	12.3506325	0.00219306	0.01067968
Ddit4l	-0.5116306	4.93093377	12.3489878	0.00219424	0.01067968
Emp3	1.1865291	3.79766243	12.347429	0.00219537	0.01067968
Eif3a	-0.5481536	9.48235141	12.3410491	0.00219998	0.01068829
Stra13	0.88272954	4.00883163	12.3410414	0.00219999	0.01068829
Ccdc141	-0.6810506	4.28111604	12.337692	0.00220242	0.01069315
Rttn	-1.1785891	2.57433777	12.3243529	0.00221211	0.01073149
1810011O10	1.23164375	4.30148064	12.3228932	0.00221317	0.01073149
Slbp	0.49325325	5.38558167	12.308864	0.00222342	0.01077423
Ralgapa2	-0.8824497	4.62057579	12.3027061	0.00222794	0.01078462
Lst1	2.4844227	-0.2032257	12.30202	0.00222844	0.01078462
Rdh1	-1.3699902	2.07293096	12.2882182	0.00223861	0.01081854
Smn1	-0.7758665	4.31988517	12.2881845	0.00223863	0.01081854
Utp11l	0.76210501	4.11218496	12.2866303	0.00223978	0.01081854
Mcmdc2	-1.4053004	2.5289707	12.2770562	0.00224686	0.0108315
Rtn1	0.39956371	10.4495925	12.2720488	0.00225058	0.0108315
Cisd1	0.52726608	5.07555563	12.2712205	0.00225119	0.0108315
Tnrc6a	-0.4983875	6.73545539	12.2706486	0.00225162	0.0108315
Cep85l	-0.7919391	4.01500005	12.2697088	0.00225231	0.0108315
Cd300lg	-3.4644281	-1.1975361	12.2696831	0.00225233	0.0108315
Speer4b	-1.7391968	0.58808674	12.2693652	0.00225257	0.0108315
Cep19	0.4306064	5.80327152	12.2596612	0.00225979	0.01085321
Bzw1	0.62309188	8.32615048	12.257709	0.00226125	0.01085321
Hmmr	-0.8271031	3.27664422	12.2574747	0.00226143	0.01085321
Prkag3	-1.6658238	1.82360284	12.2514573	0.00226592	0.01086784
Gas2l3	-0.9244563	4.64263568	12.2487165	0.00226797	0.01087073
Mocs2	0.5193814	6.84766251	12.2429778	0.00227228	0.01088439
Mcf2	-1.2214108	3.13970048	12.2367854	0.00227693	0.01089972
Cluh	-0.591087	5.1625154	12.2341433	0.00227892	0.01090228
Maea	0.42743599	5.48426659	12.2309642	0.00228131	0.01090678
P2rx7	-1.2791336	2.03038646	12.227465	0.00228395	0.01091244
Tmem45b	-1.3652803	2.23267171	12.2206732	0.00228908	0.01092777
Lyg1	4.60696056	-1.5297551	12.2193669	0.00229007	0.01092777
B430319G15	1.28504972	1.96980397	12.2005968	0.00230433	0.01098881
Med27	1.05478088	3.23350047	12.1953321	0.00230834	0.01100097

BC005624	0.56953074	6.12337461	12.1860627	0.00231544	0.01102775
Ercc1	1.10946029	2.21367991	12.177463	0.00232204	0.01105217
Larp1b	0.7338499	4.07434066	12.1741742	0.00232456	0.01105719
Fabp5	0.6039388	4.98497235	12.1690553	0.00232851	0.01106893
E030024N20	0.48063139	5.67388708	12.1611468	0.00233461	0.01109093
Nlrc4	-5.5655343	-1.284615	12.1585193	0.00233665	0.01109356
Slc38a6	-1.1578339	3.17223248	12.1542357	0.00233997	0.01110229
Klhl1	-1.0251966	3.01700362	12.1509655	0.0023425	0.0111072
Ypel5	0.51721604	7.68390628	12.1490852	0.00234396	0.0111072
Nrp2	0.50809557	5.60289257	12.144323	0.00234766	0.01111772
Skil	0.40550667	7.34534605	12.135798	0.00235431	0.01114215
Crct1	6.16676738	-1.9746755	12.7683284	0.00235732	0.01114936
Srgap2	-0.4355627	6.07483362	12.1239731	0.00236356	0.01116485
Oxsr1	-0.4726011	5.96237437	12.1239569	0.00236357	0.01116485
Rassf10	1.02532494	2.070177	12.116535	0.0023694	0.01117349
Coro7	-0.7572855	4.16065774	12.1160873	0.00236975	0.01117349
Astn2	-0.8215643	3.40841357	12.1142182	0.00237122	0.01117349
H2afz	0.47875934	7.20723172	12.1140425	0.00237136	0.01117349
Slc18a2	-0.977021	2.87449392	12.1069753	0.00237692	0.01119269
A330076H08	-1.2499719	3.78922034	12.104537	0.00237885	0.01119472
Kcne3	3.07317227	-0.4643361	12.0974563	0.00238445	0.01121403
Pttg1	0.49332903	4.90101036	12.0950462	0.00238635	0.01121598
Lzts3	-0.4633504	5.7471234	12.0864652	0.00239316	0.01124094
Shcbp1l	-2.2805047	0.19433113	12.0768113	0.00240085	0.01126549
Slc22a12	-3.67079	0.35222828	12.0761305	0.00240139	0.01126549
Gm12709	-1.2083444	1.93978936	12.06024	0.00241411	0.01131806
Bbs2	-0.6627852	5.17881808	12.0515372	0.0024211	0.01133857
Mettl2	-0.8735622	4.13577789	12.0493005	0.00242291	0.01133857
1700047M11	-1.2301187	1.0395494	12.0491596	0.00242302	0.01133857
Sertad1	1.09798231	2.92452952	12.046953	0.0024248	0.01133982
Top2a	-1.1075123	2.36370115	12.0351077	0.00243437	0.0113775
A930013F10I	-1.8939335	1.71661462	12.0310294	0.00243768	0.01138586
Trappc1	0.62237653	5.99795277	12.0283835	0.00243983	0.01138593
C230004F18I	-1.1308451	3.88591467	12.0272719	0.00244073	0.01138593
Gm19757	-0.9851134	4.53340146	12.0240363	0.00244336	0.01139111
Zfp619	-0.8135261	2.83341509	12.0191166	0.00244736	0.01139387
Dopey2	-0.714187	5.69919646	12.0178518	0.00244839	0.01139387
Swi5	0.67084374	5.71478352	12.0168824	0.00244918	0.01139387
Stat2	-0.5366305	4.68576171	12.0158495	0.00245003	0.01139387
Pls1	-0.731626	4.03669189	12.0136071	0.00245186	0.011394
Zfr2	-0.787367	3.76353164	12.0120944	0.00245309	0.011394
Brip1	-0.9843944	3.21142349	12.0071794	0.00245711	0.01140561
Erlin2	-0.4737613	5.40469007	11.9998935	0.00246308	0.01142625
Atp5j	0.54117104	7.73128512	11.996482	0.00246588	0.01142707
Sptssb	0.86549459	4.63872135	11.9959674	0.00246631	0.01142707

Pkm	0.51776338	8.20468187	11.9904474	0.00247085	0.01143438
Irak4	0.92595886	3.52899172	11.9903434	0.00247093	0.01143438
H2afy	0.53791769	5.91929454	11.9868556	0.00247381	0.01143493
Fezf2	0.54406434	5.18090564	11.9865003	0.0024741	0.01143493
Them4	0.76240131	4.2544018	11.9751177	0.00248351	0.01145706
Psme4	-0.6283053	6.43544393	11.9739784	0.00248445	0.01145706
Lrpprc	-0.7944236	5.97469776	11.9735464	0.00248481	0.01145706
Tas2r137	-7.7924353	-1.9346366	13.5597392	0.00248638	0.01145706
Ghsr	-3.506343	-0.3539409	11.9701495	0.00248762	0.01145706
Ccdc92	0.45866495	6.70486455	11.9696344	0.00248805	0.01145706
Galk2	0.72418626	3.52257411	11.9539655	0.00250109	0.01150338
Gnai1	0.42792624	9.05039895	11.9533968	0.00250157	0.01150338
Eif4g2	0.50414694	10.874292	11.9506047	0.0025039	0.01150338
Gabarap	0.75675064	7.19834281	11.9484367	0.00250571	0.01150338
Creg2	0.5605457	7.62972907	11.9483552	0.00250578	0.01150338
BC030499	-1.5944817	2.4979529	11.9451011	0.0025085	0.01150884
Acaca	-0.7806679	5.67826567	11.9403474	0.00251249	0.01152008
Sumo1	0.59039035	7.58817477	11.9297546	0.00252139	0.01155384
Rad1	-0.8374766	4.79840405	11.9275268	0.00252327	0.01155538
Pnrc1	0.46653073	7.15483839	11.9230871	0.00252701	0.01156547
Adamts13	-0.8959635	4.12095028	11.9208005	0.00252894	0.01156725
Spp1	1.01757277	7.62210651	11.9181056	0.00253122	0.01157061
Rab10	0.42454678	8.51286959	11.9018948	0.00254497	0.01162047
2010204K13I	0.95468984	2.63947415	11.9015985	0.00254523	0.01162047
Apoo	0.53182362	4.83079787	11.8944533	0.00255131	0.01163892
Sgk2	-2.5501487	-0.36339	11.8907877	0.00255444	0.01163892
Scaf8	-0.4878636	5.88073503	11.889469	0.00255557	0.01163892
Gm6994	-2.4128454	0.04688228	11.8886465	0.00255627	0.01163892
Slc29a1	0.7896414	2.59628195	11.8877679	0.00255702	0.01163892
Gm4262	-0.8522149	3.20968272	11.8839262	0.00256031	0.01164682
Trpm2	-1.0816605	3.5551595	11.8722668	0.00257032	0.01168526
Iscu	0.64602698	6.3829146	11.8616908	0.00257944	0.01171268
Opa3	0.72385383	5.05372894	11.8616467	0.00257948	0.01171268
Kctd21	0.70652359	3.31513064	11.8595863	0.00258126	0.01171368
Uck1	0.68286076	3.16796925	11.8492245	0.00259023	0.01173321
Smc5	-0.7607031	5.74348268	11.8479206	0.00259136	0.01173321
C2cd4c	1.19302437	3.3817538	11.8475649	0.00259167	0.01173321
Kcnt1	-0.8477942	5.40537708	11.847396	0.00259182	0.01173321
Vma21	0.46677387	6.21773459	11.8421298	0.00259639	0.01174684
Ppih	0.86948578	3.16833844	11.8262662	0.00261024	0.01180234
Cramp1l	-0.4626583	6.39451023	11.8228422	0.00261323	0.01180396
Wdr6	-0.5742176	4.85577563	11.8222646	0.00261374	0.01180396
Car10	0.57635863	8.35833458	11.8178673	0.0026176	0.01181355
Krt26	-5.482278	-2.0467071	11.8158775	0.00261934	0.01181355
Selm	0.99009989	4.47033158	11.8135832	0.00262136	0.01181355

Cntnap3	-1.2725292	1.93386135	11.8126716	0.00262216	0.01181355
Oaz2	0.54541899	6.79732827	11.7993367	0.00263392	0.0118549
Tia1	-0.7376313	5.2628024	11.7986791	0.0026345	0.0118549
Baiap2l1	0.70886284	3.464308	11.793818	0.0026388	0.01185921
Pon3	1.15131667	3.32299925	11.7929192	0.0026396	0.01185921
Usmg5	0.58514973	5.86410141	11.7907254	0.00264154	0.01185921
Galnt16	-0.6735104	4.41989815	11.7874471	0.00264445	0.01185921
Arpc3	0.64638542	4.43972326	11.786665	0.00264515	0.01185921
Tfam	0.5787316	4.82690855	11.7836205	0.00264785	0.01185921
Depdc5	-0.6750195	5.25652569	11.7824762	0.00264887	0.01185921
Phlpp1	-0.5169736	6.22097017	11.7818064	0.00264947	0.01185921
Ppef2	-4.1808527	-0.8436793	11.7798402	0.00265122	0.01185921
Casp8	0.72476738	5.33351205	11.777656	0.00265316	0.01185921
Fam131c	4.582019	-0.4741631	11.7740246	0.0026564	0.01185921
9330102E08I	-0.7682414	4.04629306	11.7737386	0.00265666	0.01185921
Far2	-0.8518556	3.82220963	11.772687	0.00265759	0.01185921
Pin4	0.57316694	4.63656401	11.7722544	0.00265798	0.01185921
3110079O15	-4.6531996	-1.8125332	11.769059	0.00266083	0.01185921
Akr1b8	1.18443114	2.14255392	11.7667358	0.00266291	0.01185921
Hagh	0.67184506	4.40177537	11.7651188	0.00266436	0.01185921
Cgrrf1	0.59834831	3.86189549	11.7650706	0.0026644	0.01185921
Pgp	0.55140482	3.71346144	11.7638464	0.0026655	0.01185921
Zfyve27	-0.6170919	4.26992673	11.7617472	0.00266738	0.01186055
Rev3l	-0.6095753	7.29469965	11.7551911	0.00267326	0.01187607
Ttc1	0.5847744	6.00652084	11.7543293	0.00267404	0.01187607
Etl4	-0.4118393	7.93056652	11.7500621	0.00267788	0.01188212
Psmb4	0.62386689	5.59698976	11.7484708	0.00267931	0.01188212
Ino80	-0.5797774	5.08845141	11.747538	0.00268015	0.01188212
Cabin1	-0.5107851	5.24959159	11.744667	0.00268274	0.01188657
Klhl34	-1.1827796	5.45597322	11.7413917	0.0026857	0.01189264
Csrp2	0.64726142	4.57851133	11.7367355	0.0026899	0.01189347
Tcte2	0.86347177	2.88138795	11.7367171	0.00268992	0.01189347
Oip5	-2.8910312	0.22054863	11.7359241	0.00269064	0.01189347
Hipk4	-0.8354793	3.97058021	11.732274	0.00269394	0.01189785
Eef1a2	0.55111641	5.59721423	11.7286874	0.0026972	0.01189785
4930405J17F	-2.33644	1.00072485	11.7250625	0.00270049	0.01189785
Higd1a	0.47023972	6.64605834	11.7240827	0.00270138	0.01189785
Grk4	-1.0164752	4.63754829	11.7236463	0.00270178	0.01189785
Cdc123	0.47661294	6.25246	11.7227962	0.00270255	0.01189785
Zfp781	-0.600801	6.24306577	11.7225936	0.00270273	0.01189785
Cast	0.75141712	6.45665256	11.7206106	0.00270454	0.01189881
Numbl	0.58494751	4.00453307	11.7180333	0.00270689	0.01190216
Gas5	-0.394131	6.98860921	11.7122825	0.00271213	0.01191824
Gulp1	0.80770436	5.95941799	11.7004131	0.00272299	0.01195834
AI450353	-1.6219341	2.02107079	11.6988297	0.00272444	0.01195834

Rassf3	-0.5200641	5.51837471	11.6967196	0.00272638	0.01195984
Krr1	0.37323401	6.82468627	11.6933597	0.00272947	0.01196639
Rasgef1b	-0.5237787	5.48781559	11.690776	0.00273185	0.01196982
Palm2	-0.6853784	7.15217427	11.684822	0.00273733	0.01198685
Tspan13	0.72930539	8.54940171	11.6706922	0.0027504	0.01203705
Shc3	-0.9189852	3.93974062	11.6635149	0.00275707	0.01205919
Iqsec1	-0.6863525	7.84159001	11.6587322	0.00276152	0.01207162
Tspan2	-0.5564039	6.3946554	11.6564955	0.0027636	0.01207369
Asb11	-1.6678127	0.90161594	11.6471264	0.00277235	0.01209941
Xpo5	-0.6165735	4.68001948	11.6464376	0.002773	0.01209941
Ccrn4l	0.52373198	5.44062537	11.6450167	0.00277433	0.01209941
Gramd3	0.69338484	4.51568435	11.6412961	0.00277781	0.01210757
Cidea	6.80604533	-1.9543343	12.2282154	0.00278109	0.01210986
Trim43a	-2.2953996	-0.2022028	11.6372957	0.00278157	0.01210986
Car13	0.81296396	7.61595465	11.6285257	0.00278982	0.01213395
Snx10	0.4769612	6.49559681	11.6279733	0.00279034	0.01213395
Golph3	0.48385273	8.57650586	11.6230279	0.002795	0.0121472
Nhp2	0.95858426	3.21327063	11.6199781	0.00279788	0.01214735
Stk32a	-1.1886545	1.75903453	11.6195622	0.00279827	0.01214735
Dynll2	0.42908087	7.03477523	11.6070002	0.00281018	0.01219196
Mir350	-4.1772753	-2.0322071	11.5994538	0.00281735	0.01221604
Dnah5	-1.7836694	2.34714386	11.5920832	0.00282438	0.01223944
Pcnxl3	-0.5359501	4.69194894	11.586069	0.00283013	0.01225728
Chd5	-0.7349213	6.75626534	11.578928	0.00283698	0.01227983
Oxt	2.23223397	0.36731434	11.575976	0.00283981	0.01228502
Nhs	-0.7356677	3.61151607	11.5602783	0.00285494	0.01234334
Vbp1	0.53782907	5.18967613	11.5553465	0.00285971	0.01235685
Pcdhb19	-0.9091269	2.92157149	11.5449653	0.00286979	0.0123886
Zc3hav1	0.5688756	5.52843251	11.5443685	0.00287037	0.0123886
Strn	-0.3636011	6.48418196	11.5421893	0.00287249	0.01239062
Epha6	-0.6688884	4.797951	11.527355	0.00288696	0.01243709
Golga7b	0.75082154	4.36180067	11.5257994	0.00288848	0.01243709
Wdr17	-0.8351996	5.11350837	11.5240109	0.00289024	0.01243709
Rrm1	-0.574309	4.32761159	11.5236042	0.00289063	0.01243709
Slc35a2	-0.926561	3.20080837	11.522673	0.00289155	0.01243709
Pikfyve	-0.5831572	6.77056476	11.5207827	0.0028934	0.01243793
Nosip	0.5976138	4.60528241	11.5165499	0.00289756	0.01244866
Capza2	0.39590237	8.06231364	11.5118354	0.00290219	0.01245506
Rtca	0.51487064	5.33134198	11.5116572	0.00290237	0.01245506
Habp4	0.33716276	7.39970962	11.5080383	0.00290593	0.01246323
Tex9	-0.4667188	4.98234399	11.5032105	0.0029107	0.01247653
Ulk3	-0.7526323	3.94958032	11.5004231	0.00291345	0.0124812
Tmsb4x	0.65048674	8.89562074	11.4952662	0.00291855	0.01249592
Igf2bp3	-0.845674	4.2010688	11.4934225	0.00292038	0.01249661
Mir378b	-2.8855575	0.21148148	11.4830065	0.00293072	0.01253372

Gnaz	0.5160471	5.82216852	11.4804641	0.00293325	0.0125374
Yeats4	0.5318305	5.23072457	11.474809	0.00293889	0.01255435
0610010K14I	0.80706446	3.74429142	11.4696298	0.00294407	0.01256929
Scamp2	1.31604284	2.70673317	11.4597553	0.00295396	0.01260436
Ercc4	-0.6944705	3.83463509	11.4525118	0.00296124	0.01262824
Lca5l	-1.7396779	1.38066019	11.4481318	0.00296565	0.01263987
Atp5b	0.35904459	10.5802722	11.4375421	0.00297635	0.01267826
Greb1l	-0.925945	3.07983397	11.4285342	0.00298548	0.01270543
BC018507	-0.6912189	6.72558247	11.4279128	0.00298612	0.01270543
4933432I09R	-6.8259667	-0.7107174	11.4242023	0.00298989	0.01271124
Nek6	0.71467998	4.83374912	11.4232367	0.00299087	0.01271124
Trpc5	-0.7917345	4.12058133	11.4151671	0.0029991	0.01273898
Nin	-0.4741778	6.83042218	11.4083013	0.00300611	0.01276156
Plb1	1.32744532	2.38062734	11.4042134	0.0030103	0.01277211
Klhdc4	-0.800764	3.05653402	11.3996647	0.00301497	0.01278468
Klhdc1	-0.5908712	4.04135567	11.3964233	0.0030183	0.01278948
Ppp1r9b	-0.4513867	6.66886217	11.3952439	0.00301951	0.01278948
Vti1a	0.46748075	6.91696689	11.3880493	0.00302692	0.01281363
Lsmem1	5.83481876	-1.4809773	11.3817462	0.00303343	0.01283394
Myoc	-1.056978	2.14626982	11.3734781	0.00304199	0.01285612
Abracl	0.70056368	4.25687212	11.3733716	0.0030421	0.01285612
Ufl1	-0.4945899	5.79660196	11.3704375	0.00304514	0.01285706
Neurod6	0.58905604	6.14249421	11.3670334	0.00304868	0.01285706
Pcnx	-0.5367066	7.32151792	11.3663502	0.00304939	0.01285706
Atg2b	-0.7286604	6.04259617	11.3643892	0.00305143	0.01285706
Ankrd11	-0.379537	8.84875745	11.364293	0.00305153	0.01285706
Trp53bp1	-0.7459042	5.47033742	11.3632619	0.0030526	0.01285706
Fam208b	-0.474375	6.44869303	11.3606914	0.00305528	0.01286111
Gm10421	-3.7029532	0.08783032	11.3536117	0.00306267	0.01288158
Vdac1	0.37798966	8.05749857	11.35274	0.00306358	0.01288158
Nrip3	0.51984793	5.45211388	11.339933	0.003077	0.01292035
Lyplal1	0.91403176	2.81065316	11.3394359	0.00307752	0.01292035
Cpne2	0.53785801	4.41297001	11.3390102	0.00307797	0.01292035
Map4k2	-0.6165032	4.85664931	11.3308646	0.00308654	0.01294909
Btf3l4	0.37229542	7.05131846	11.3248983	0.00309283	0.01296777
Ncf4	3.79310054	-0.9556413	11.3232483	0.00309458	0.01296777
Micu2	0.50451918	5.20628011	11.3217338	0.00309618	0.01296777
Kdm5b	-0.6503851	5.90629015	11.3080274	0.00311071	0.01302137
Ccdc47	-0.3989912	7.07371652	11.3059738	0.00311289	0.01302166
Adam17	-0.6049122	4.19544606	11.3046967	0.00311425	0.01302166
Limch1	-0.9186253	5.83790073	11.3016522	0.0031175	0.01302796
Drp2	-0.8399699	5.99951499	11.2833416	0.00313708	0.01310248
Adam23	-0.5628401	7.15181729	11.2754785	0.00314553	0.01313047
Sik2	-0.4584393	5.84896474	11.2691665	0.00315233	0.01315154
Wdr3	-0.847882	3.22778779	11.2608769	0.00316128	0.01317866

Kdm3b	-0.3578344	7.03143235	11.2598993	0.00316234	0.01317866
Mfn1	-0.6076658	5.318936	11.2501754	0.00317289	0.0132117
Lgals3bp	0.85305309	4.89866269	11.2493456	0.00317379	0.0132117
Ctnnd1	-0.3945556	8.01841914	11.2461749	0.00317724	0.01321872
Plxnc1	-0.4362996	6.4050276	11.2377941	0.00318638	0.01324937
Zfp459	-1.1274019	3.00448716	11.222695	0.00320291	0.01331074
Utp15	-0.5966431	5.04699468	11.2160542	0.00321021	0.01333369
AF357425	-1.9130349	3.01202693	11.2142918	0.00321215	0.01333437
Gm16039	0.48034329	4.51962695	11.2112715	0.00321548	0.01334081
Snx1	0.44197942	5.72601252	11.2087383	0.00321827	0.01334502
Cul3	0.35439969	8.90574059	11.1995822	0.0032284	0.01336944
Rab11fip4os	-3.7281934	-0.7582432	11.1969409	0.00323133	0.01336944
4930512B01I	-1.8562984	0.91539927	11.1964669	0.00323185	0.01336944
Snapin	0.64974115	7.51446308	11.1962264	0.00323212	0.01336944
Deptor	0.40770075	7.41084037	11.1943381	0.00323421	0.01336944
Mir665	-3.2846866	-0.6775952	11.1931502	0.00323553	0.01336944
Gria3	-0.6069831	8.51083982	11.1921556	0.00323664	0.01336944
Csgalnact1	-0.8011268	3.77298761	11.1895757	0.0032395	0.01337392
Slc6a18	-1.515067	1.13879394	11.1859121	0.00324358	0.01338338
Itgam	-0.8907941	3.55949503	11.1831378	0.00324667	0.01338877
Slc15a2	-0.8906512	2.85421887	11.1785825	0.00325175	0.0134009
1700123L14F	-2.9342704	0.3657633	11.1773007	0.00325319	0.0134009
Ablim1	-0.3908021	6.74637897	11.170656	0.00326062	0.01342414
Zfp418	-0.7391907	3.63764889	11.1631994	0.00326898	0.01345119
AI462493	1.01007746	3.34834797	11.1584414	0.00327433	0.01346582
Ube2k	0.37241344	8.11082422	11.1464905	0.00328781	0.01351384
Clcn2	-0.6731908	3.90367884	11.1379423	0.00329749	0.0135462
Rwdd2b	1.2424502	2.25778466	11.1300533	0.00330645	0.0135693
Lmbr1l	-1.6139203	0.32699274	11.1298066	0.00330673	0.0135693
Cntn5	-1.4077115	2.35726734	11.1128118	0.00332613	0.01364143
Atp13a5	0.62271495	4.94737896	11.1085569	0.003331	0.01365396
Bambi	1.63728652	1.23573943	11.0972701	0.00334397	0.0136872
Vti1b	0.49582614	5.76983741	11.0968499	0.00334446	0.0136872
Mien1	0.70058452	5.3825051	11.0954421	0.00334608	0.0136872
Por	0.56531478	5.52808753	11.0943163	0.00334738	0.0136872
Lcmt1	0.58054592	4.85914656	11.092851	0.00334907	0.0136872
Cryl1	0.83888789	3.57111571	11.0918674	0.0033502	0.0136872
Twf1	0.53027457	7.41799396	11.0904139	0.00335188	0.0136872
2610001J05F	0.75474389	5.47852933	11.0879838	0.00335469	0.01369121
Rictor	-0.7317252	6.50234641	11.0850598	0.00335807	0.01369756
Per1	-0.5898928	5.52922724	11.0617578	0.00338516	0.01380054
Gbp8	1.48730911	1.80266192	11.0522103	0.00339633	0.01383855
Lrrtm4	-0.5714452	5.30938419	11.0378608	0.00341319	0.01389256
Idh3a	0.4191958	6.62865407	11.0377768	0.00341329	0.01389256
4930592I03R	-6.7594297	-1.8628536	11.5693114	0.00341883	0.01390269

Scube3	-2.3169773	1.4791475	11.0323491	0.00341969	0.01390269
Ogdh	-0.495813	7.67834973	11.0307101	0.00342163	0.01390269
9030624G23	-0.7230248	3.50587459	11.028426	0.00342433	0.01390269
Lin28b	-1.0289071	3.02215074	11.0278198	0.00342504	0.01390269
A330032B11	-0.9809695	2.15244353	11.0241875	0.00342934	0.01391262
Map3k15	-1.7306259	0.18468205	11.0167946	0.00343811	0.01394065
Itih2	0.75776886	5.04283293	11.0069675	0.00344981	0.01398052
Tgfb1i1	0.75664358	6.85124362	10.995669	0.00346331	0.01402603
Ube3a	-0.5148179	6.7638615	10.9944428	0.00346478	0.01402603
Arl16	0.52227849	4.2914429	10.9845248	0.00347668	0.01406453
Pard6b	0.85230027	3.34604039	10.9833985	0.00347804	0.01406453
Rprm	0.70827053	3.97565688	10.9773047	0.00348538	0.01408409
Clasrp	-0.9247232	2.54147042	10.9762679	0.00348663	0.01408409
Myd88	1.13913813	2.78060897	10.9716766	0.00349217	0.01409503
Krba1	-0.5778864	3.86026108	10.9695653	0.00349473	0.01409503
Adamts20	-1.5342475	2.22272247	10.9693597	0.00349497	0.01409503
Rbms1	0.48779264	6.26706979	10.9657871	0.0034993	0.01410489
Mapk8ip2	-1.1280536	4.63969142	10.9599782	0.00350634	0.01412569
Tomm34	0.4853856	5.83412048	10.9571436	0.00350979	0.01412771
Dck	0.68822681	4.99238985	10.954675	0.00351279	0.01412771
Ppil6	-1.5783988	2.35318332	10.9524814	0.00351546	0.01412771
Atg3	0.39341004	6.65030074	10.9522093	0.00351579	0.01412771
Tex35	-4.5692049	-1.7940402	10.9518215	0.00351626	0.01412771
Efhb	-1.482367	0.67924051	10.9472511	0.00352183	0.01414252
1700029J07F	-1.0570272	2.15905679	10.9406544	0.00352989	0.0141673
Etfa	0.488886	6.60591641	10.9361567	0.0035354	0.01417956
Ltbp4	-0.5596014	4.59000141	10.9350711	0.00353673	0.01417956
Ube4a	-0.5021295	5.74196263	10.9330601	0.00353919	0.01418187
Cc2d2a	-0.417512	5.78023341	10.9304715	0.00354237	0.01418702
Pou6f1	-0.5650963	5.0576674	10.9157292	0.00356053	0.01424477
Fig4	-0.5620715	4.3065797	10.9156772	0.00356059	0.01424477
Slc40a1	-1.0604586	2.11802595	10.9131452	0.00356372	0.01424969
Maob	-0.5992904	4.16422355	10.9085693	0.00356938	0.01426472
Mtmr3	-0.4559914	6.04740863	10.9038836	0.00357519	0.01428032
Coa4	1.15676813	2.07474742	10.8961381	0.00358481	0.01430927
Hspa1b	0.95833863	2.12639653	10.8947785	0.0035865	0.01430927
Sbf2	-0.4646426	7.08779668	10.89345	0.00358816	0.01430927
Smyd3	0.42732805	6.18558953	10.89085	0.0035914	0.01431458
Nudt10	0.66054871	3.41269048	10.8848054	0.00359894	0.01433267
Rfc2	0.83913402	3.34450161	10.8841542	0.00359976	0.01433267
BC051142	-1.7715577	1.26833807	10.8755375	0.00361054	0.01435574
Hrh3	0.58207778	4.7445627	10.8750155	0.0036112	0.01435574
5530401A14I	-2.2634817	0.01836977	10.8749421	0.00361129	0.01435574
Zfp873	0.63534049	3.36059903	10.8679815	0.00362004	0.01437977
Rif1	-0.6619975	5.78944348	10.8670788	0.00362117	0.01437977

D7Ertd715e	-1.234679	4.14583375	10.8637104	0.00362541	0.014389
5330413P13I	1.44622165	1.58644939	10.8610035	0.00362882	0.01439492
Gadd45g	0.98063895	2.06850127	10.8558332	0.00363535	0.01441319
Tmem115	1.05854	2.20816434	10.8512147	0.00364119	0.01442873
Nsmce2	0.50611346	5.28630791	10.8489154	0.00364411	0.01443158
Cts8	-1.7517728	0.00836896	10.8476091	0.00364576	0.01443158
Anapc13	0.76583517	5.13802789	10.8350168	0.00366176	0.01448729
Ttll4	-0.9828346	2.29826772	10.8315331	0.00366621	0.014493
Isy1	0.60630126	4.96121102	10.8308527	0.00366707	0.014493
Nktr	-0.5960022	7.07356696	10.8262276	0.00367298	0.01449844
Etohd2	-1.4473302	1.40307632	10.8241812	0.0036756	0.01449844
Soga1	-0.3862929	7.13274724	10.823764	0.00367613	0.01449844
Bbip1	0.44991937	5.93859455	10.8237258	0.00367618	0.01449844
Cartpt	-1.3138133	1.40408598	10.8212333	0.00367937	0.01450316
Trio	-0.6709426	7.7197757	10.8193655	0.00368176	0.01450316
Ralyl	0.38807433	7.08495102	10.817214	0.00368452	0.01450316
Gm20767	-1.6253466	1.1002341	10.8162217	0.0036858	0.01450316
Nsd1	-0.4597828	8.22829954	10.8152488	0.00368704	0.01450316
Kcnn3	-0.8198508	4.1629489	10.8009234	0.00370549	0.01456355
Mir8115	-2.921086	-1.2524233	10.8003073	0.00370628	0.01456355
Actn3	3.41949464	-0.3285975	10.7942142	0.00371416	0.01457924
Loxl2	-0.8138232	3.97633276	10.792922	0.00371583	0.01457924
Gucy1a3	-0.6088275	7.27725386	10.7918303	0.00371724	0.01457924
Akirin1	0.45012915	5.78929013	10.7912108	0.00371805	0.01457924
Ttc21b	-0.6806507	4.37167452	10.7864579	0.00372421	0.01459578
Gbp10	0.68229269	3.82201888	10.7838507	0.0037276	0.01460142
Ncapd3	-0.7418367	4.27461115	10.7801845	0.00373236	0.01460744
Cltb	0.5890831	5.79999966	10.7791402	0.00373372	0.01460744
Cops5	0.36232179	6.0242677	10.7778157	0.00373545	0.01460744
Srp19	0.43553506	6.44334797	10.7766825	0.00373692	0.01460744
Taf7l	-1.8520992	1.07692648	10.7744996	0.00373977	0.01461095
Colec12	-0.3918666	6.68445516	10.7671423	0.00374938	0.01463538
Alms1-ps2	-1.6750148	0.45228043	10.7667247	0.00374992	0.01463538
Pigp	0.68285718	4.8019085	10.7626417	0.00375527	0.01464494
Tspyl1	0.3396737	7.44783897	10.7618709	0.00375628	0.01464494
Myo16	-0.9091776	4.34876829	10.7490365	0.00377314	0.01469515
Dym	0.41941079	5.63051636	10.7476568	0.00377496	0.01469515
Abcc1	-0.8376702	3.88169339	10.7462164	0.00377686	0.01469515
Kcnj8	-2.4081242	0.66783399	10.746114	0.00377699	0.01469515
Acox3	-0.5861812	4.07254784	10.7442609	0.00377944	0.01469703
Nabp2	0.61940216	5.88498159	10.7346308	0.00379217	0.0147389
Samd4	-0.4716822	7.26723062	10.7300255	0.00379827	0.01475498
Zfp106	-0.3324192	9.16699433	10.7192043	0.00381266	0.01479897
Ak6	0.98116384	2.76185152	10.7185422	0.00381354	0.01479897
Eif4e	0.35455468	7.4070904	10.7089818	0.00382631	0.01484011

9330182L06F	-0.6340554	5.05696034	10.7076406	0.0038281	0.01484011
Parp9	0.81262485	3.39208783	10.7059046	0.00383042	0.01484145
Mapre2	0.35522174	9.8021625	10.6981931	0.00384077	0.01486259
Slc43a1	-1.9201081	0.58700747	10.6971898	0.00384212	0.01486259
Pbx3	0.75705668	7.274062	10.6971002	0.00384224	0.01486259
Myo1a	-4.78875	-1.8296905	10.695612	0.00384424	0.01486259
BC005561	-0.8332205	5.15199396	10.6944604	0.00384579	0.01486259
Gm12060	-4.430661	-1.0311945	10.688611	0.00385366	0.01488536
Rpl36al	0.73727915	7.29476133	10.6850507	0.00385847	0.01489625
Lrp4	-0.6580098	3.95895007	10.6818948	0.00386273	0.01490092
Syt5	0.56302544	5.40819515	10.6812135	0.00386365	0.01490092
Emc1	-0.477263	4.6600872	10.673393	0.00387424	0.0149233
Fbxo27	0.70694724	3.83541768	10.6716184	0.00387665	0.0149233
Rps6ka5	-0.9199003	3.29476778	10.6711265	0.00387732	0.0149233
Wdr59	-0.7388715	4.08083724	10.6708312	0.00387772	0.0149233
Hsd3b4	-1.9254548	0.76477743	10.6688909	0.00388035	0.0149233
Atp7a	-0.5276961	5.55625978	10.6681288	0.00388139	0.0149233
Txndc5	0.54470073	4.95852922	10.6619218	0.00388983	0.01494051
Timmdc1	0.60522493	4.29248554	10.6619114	0.00388985	0.01494051
Tmem82	-1.7442727	0.41533048	10.6588128	0.00389407	0.01494505
Tgm2	0.66379934	4.09520623	10.6581209	0.00389502	0.01494505
BC004004	0.78421869	5.27753471	10.6543579	0.00390015	0.01495712
Med21	0.80644241	5.66338597	10.6509791	0.00390477	0.01496718
Dpysl3	-0.3585745	6.30285504	10.6475317	0.00390949	0.01497762
N6amt2	0.75463301	3.86164223	10.6449097	0.00391309	0.01497892
Timm23	0.53871164	6.17913277	10.6443699	0.00391383	0.01497892
Csmd2	-1.0997118	4.84623264	10.6409703	0.00391849	0.01498609
Cbr4	0.64326327	4.17034349	10.637997	0.00392258	0.01498609
Cdc7	-1.2390665	2.8060365	10.63795	0.00392264	0.01498609
Naip2	-1.9165234	0.32866889	10.6371899	0.00392369	0.01498609
Gxylt1	-0.5255248	5.66834184	10.6328263	0.0039297	0.01499834
Brd8	-0.4348309	5.86129526	10.6319546	0.0039309	0.01499834
Prkcdbp	1.02439294	5.17983032	10.629975	0.00393363	0.01500113
Gng7	-0.6277853	5.43551438	10.6261434	0.00393892	0.01501367
9430008C03I	-1.3244036	1.71279956	10.6201633	0.00394719	0.01503068
Bach2	-0.711879	4.7164935	10.6187856	0.0039491	0.01503068
Pip4k2a	0.3499515	7.90645861	10.6185729	0.00394939	0.01503068
Anapc11	0.64965034	4.06063091	10.6102732	0.00396091	0.01506687
Gm9899	0.622218	3.86221393	10.6048371	0.00396847	0.01508799
1700007G11	-2.1584937	0.29803362	10.6022385	0.0039721	0.01509367
Cngb1	1.93796949	0.65399982	10.6008786	0.00397399	0.01509367
Gbf1	-0.5239177	5.9862477	10.5991616	0.00397639	0.01509513
Nr6a1	-0.8827147	3.0928936	10.5920463	0.00398634	0.01512523
Chrnb2	-0.6061319	3.9374046	10.5854369	0.0039956	0.01515272
Dkkl1	1.11847984	2.23243727	10.5785829	0.00400523	0.0151695

Thoc2	-0.5763339	7.06080005	10.5784983	0.00400535	0.0151695
Cdt1	2.72161481	-0.1894261	10.5777351	0.00400643	0.0151695
Gm6313	-1.4459575	1.20104026	10.5765369	0.00400811	0.0151695
Fyttd1	0.42658202	7.36104306	10.5739818	0.00401171	0.01517547
Gm19705	1.37839118	1.55104948	10.5707992	0.0040162	0.0151848
Fam103a1	0.45761661	7.87600911	10.5649018	0.00402454	0.01520864
Mnat1	0.62625149	4.19867211	10.5619234	0.00402875	0.01521004
Rap2b	0.45134803	5.93718849	10.560288	0.00403107	0.01521004
Kcnb1	-0.660589	8.01709243	10.5589602	0.00403295	0.01521004
Ube2d3	0.42820427	9.02053854	10.5589143	0.00403302	0.01521004
Ubn2	-0.5152118	7.06862462	10.5573102	0.00403529	0.01521097
Tmem181a	-0.7521997	3.45736147	10.5539789	0.00404002	0.01522116
Srp14	0.56410724	6.88944563	10.5412226	0.00405819	0.01528194
1700123M08	1.43942359	1.25179356	10.5394379	0.00406074	0.01528387
Akr7a5	0.96090452	2.3865428	10.5359147	0.00406578	0.01528585
Zfp866	-0.444324	5.11100105	10.5357934	0.00406596	0.01528585
Kpna2	0.41531426	5.1767939	10.5347944	0.00406739	0.01528585
1700109K24I	-2.4097165	0.84873822	10.5333527	0.00406945	0.01528595
Sdcbp	0.44167495	9.43623157	10.5306411	0.00407334	0.01529289
Ttc17	-0.5219569	4.84486367	10.5223392	0.00408526	0.01531653
Tubb4b	0.38007627	7.02528068	10.5220326	0.0040857	0.01531653
Dap	0.78668423	5.24106919	10.5219928	0.00408576	0.01531653
H2-T24	-0.763653	4.91991878	10.5187597	0.00409041	0.01531992
Pop4	0.73073538	4.0052	10.5185276	0.00409075	0.01531992
Ifitm2	1.11670437	7.3431538	10.5145903	0.00409642	0.01532734
Asb8	0.39517674	6.2702894	10.5143195	0.00409681	0.01532734
Efemp1	0.90398423	7.48332412	10.5119127	0.00410029	0.01533269
Polr2e	0.6341503	4.39097372	10.5005774	0.0041167	0.01537947
Bbs12	-1.2463737	1.27863567	10.5004399	0.0041169	0.01537947
D17Ertd648e	-1.5896118	0.72780669	10.4946183	0.00412535	0.01540339
Ovgp1	-2.0325594	0.34036295	10.4819635	0.0041438	0.01546457
Brk1	0.56255009	6.30129478	10.4782776	0.00414919	0.01547699
Rgsl1	-1.3936803	1.52849034	10.4669128	0.00416586	0.01553145
Lrrk2	-0.8073075	5.84619947	10.4602837	0.00417562	0.0155601
Brwd1	-0.5855119	6.9745461	10.45643	0.00418131	0.01557355
C030046E11I	-0.4992045	5.47917695	10.4495481	0.00419148	0.01560369
Lin37	0.56154919	4.4352741	10.4473942	0.00419467	0.01560781
Taf2	-0.4690801	5.91568039	10.4401792	0.00420537	0.01563798
Clec14a	-1.2571959	1.55869742	10.4391208	0.00420694	0.01563798
Prr18	-0.7649242	3.12852725	10.4371359	0.0042099	0.0156412
Dlst	0.38802769	6.53853897	10.4355225	0.0042123	0.01564237
Pola1	-0.9277809	2.66497059	10.4259418	0.00422658	0.01568765
B3gat2	-1.1363802	2.95666919	10.4063298	0.00425599	0.01578901
Qtrtd1	-1.0849135	2.08839529	10.3914697	0.00427843	0.01585786
Nop56	-0.5162647	6.54885014	10.3912334	0.00427879	0.01585786

Ift27	0.89202008	2.67768703	10.3898433	0.00428089	0.01585786
Fam73a	-0.4750677	6.03463599	10.383145	0.00429106	0.01588627
Cep97	-0.5009421	4.58543126	10.3808172	0.0042946	0.01588627
Prr15	2.03555928	0.78841463	10.3806058	0.00429492	0.01588627
Psme1	0.73929441	7.09575098	10.3772992	0.00429995	0.01589704
Txn2	0.72584479	4.68308684	10.3751106	0.00430328	0.0158985
Kif3c	0.4694688	6.41499445	10.3742584	0.00430458	0.0158985
Tcte1	2.21769769	0.71897727	10.3709935	0.00430956	0.01590906
Ace2	4.49208621	-0.716021	10.3639958	0.00432026	0.0159407
Plxna3	-1.2041316	2.16951874	10.3550337	0.004334	0.01598155
Rasgrf1	-0.7051135	8.33628821	10.3527035	0.00433758	0.01598155
Slc11a2	-0.5708597	4.99603711	10.3526139	0.00433772	0.01598155
Setx	-0.6685169	7.13268707	10.3452491	0.00434906	0.01600814
Gpaa1	-1.2894573	1.39963622	10.3448297	0.00434971	0.01600814
Zfp385c	-1.1042633	1.90571192	10.3437728	0.00435134	0.01600814
Lars	-0.4709521	5.12564252	10.3395688	0.00435783	0.01601904
Ephb2	-1.2834207	2.12447497	10.3390884	0.00435857	0.01601904
0610012G03	0.74540548	3.83728121	10.337467	0.00436108	0.01602015
Mff	0.37669586	7.56692729	10.3355656	0.00436402	0.01602015
Uba3	0.43378042	6.23925471	10.3344869	0.00436569	0.01602015
Btbd8	-1.585924	0.87581466	10.3332255	0.00436765	0.01602015
Hnrnpm	-0.4168182	6.77001369	10.3319949	0.00436956	0.01602015
Zfp12	-0.585525	4.74653812	10.3275871	0.00437639	0.01603739
Cox14	0.50336369	5.326743	10.3217205	0.00438552	0.01606296
Adarb2	-0.6340737	4.9735665	10.3197131	0.00438864	0.01606656
Gm16023	-1.3550762	0.95694866	10.3177291	0.00439173	0.01607004
Cds2	-0.4989913	7.73388419	10.3120836	0.00440054	0.01608776
Immp2l	2.00516699	-0.0527315	10.311878	0.00440086	0.01608776
Isca1	0.34589414	6.6628127	10.3003429	0.00441893	0.01614593
Scoc	0.46414577	8.74565605	10.2957894	0.00442608	0.01616419
Atp6v1g1	0.56601599	6.90417305	10.2875221	0.0044391	0.01620386
Ubtd1	1.86721206	-0.1061916	10.2853967	0.00444246	0.01620651
Nudt11	0.64558303	4.15227388	10.2843246	0.00444415	0.01620651
Cgn	-1.036575	2.17936625	10.2741934	0.00446019	0.01625147
Gabrg1	-0.8204751	3.40402134	10.2737991	0.00446081	0.01625147
Cyc1	0.59343475	6.32295405	10.271348	0.0044647	0.01625379
Nup93	-0.7889818	4.53506346	10.2706664	0.00446578	0.01625379
Slc7a15	-3.9351718	-1.0209344	10.2645423	0.00447552	0.01625679
Fbxw8	0.54317146	3.88757682	10.2644017	0.00447575	0.01625679
Lrch3	-0.5746997	4.78819514	10.2635913	0.00447704	0.01625679
Leo1	0.40984157	6.10134011	10.2635605	0.00447709	0.01625679
Grk1	-1.442196	1.35031602	10.2633358	0.00447744	0.01625679
Med31	0.71537886	3.37068714	10.259831	0.00448303	0.01626535
Galnt15	1.74055986	1.00556472	10.2591356	0.00448414	0.01626535
1700024B18I	-3.1916148	-0.3069596	10.2556359	0.00448973	0.01627775

Nsa2	0.44987389	6.72256089	10.2508548	0.00449737	0.01629461
Cry1	0.57697648	4.34636312	10.2484964	0.00450115	0.01629461
Smarce1	0.45257038	6.41595894	10.2477463	0.00450235	0.01629461
Snx12	0.44819094	7.72627146	10.2473002	0.00450306	0.01629461
Arsi	2.14550794	0.61766923	10.2302588	0.00453047	0.01638547
Fahd1	0.47847524	5.2997062	10.2289745	0.00453255	0.01638547
Gins4	0.74862157	4.78379394	10.2235587	0.0045413	0.01640524
E030030I06R	-1.106594	2.81872364	10.2228867	0.00454239	0.01640524
Iqck	-1.2423144	2.81758821	10.2202108	0.00454672	0.01641298
Cep152	-1.386879	1.78541853	10.2158599	0.00455377	0.0164227
Ola1	0.36050267	7.60669843	10.2149003	0.00455533	0.0164227
Kcnj12	0.83674186	3.19352967	10.2145026	0.00455598	0.0164227
Mrps2	0.45383696	4.70560518	10.2114609	0.00456092	0.01642649
Dnajb5	0.57266322	5.88746748	10.2111599	0.00456141	0.01642649
Pex12	0.57765314	4.06742378	10.2097216	0.00456375	0.01642703
Rgs17	0.52511108	8.03310869	10.2064565	0.00456906	0.01643102
Abcc5	-0.9786592	4.93688186	10.2063492	0.00456924	0.01643102
Tmem179	-0.6738562	3.41467723	10.2039475	0.00457315	0.01643722
Pabpc5	-1.0120114	2.29010092	10.1995826	0.00458028	0.01645494
Zfp131	-0.5349469	5.28940919	10.1969559	0.00458457	0.01646247
Ccer1	-5.5355892	-2.3162818	11.3771103	0.00458754	0.01646526
Fgf12	0.43721399	7.94471617	10.1871267	0.00460067	0.01649888
Mmp16	-0.5984205	5.36699332	10.1867404	0.00460131	0.01649888
Ndufv3	0.73150662	5.13708218	10.182773	0.00460783	0.01650806
Pdgfc	0.90809535	2.48770178	10.1825046	0.00460827	0.01650806
C030023E24I	-1.4214003	2.42704436	10.1811217	0.00461054	0.01650832
Mccc1	-0.7138052	3.58728736	10.1766456	0.00461791	0.01652629
Shc2	-0.6398303	4.57055209	10.1754	0.00461997	0.01652629
Kcnt2	-0.7661178	5.43269242	10.1738695	0.00462249	0.01652744
Commd10	0.57765124	4.67204562	10.1677863	0.00463254	0.01654067
Dnajc1	-0.5196369	5.08351019	10.1675815	0.00463288	0.01654067
Picalm	0.33055897	8.08333013	10.1665048	0.00463466	0.01654067
Slc35e1	-0.5714258	4.92752732	10.1662939	0.00463501	0.01654067
Cic	-0.4259783	6.64039487	10.1626334	0.00464108	0.01655443
Pcnt	-0.5726877	5.11387486	10.1590334	0.00464705	0.01656496
Sycp2	-1.4961413	2.48123862	10.1565212	0.00465122	0.01656496
Reep2	0.64574771	4.64945687	10.156161	0.00465182	0.01656496
Ikzf4	-0.4645565	4.19381993	10.1555339	0.00465286	0.01656496
B3gnt9	1.25393034	1.8098825	10.1466261	0.00466769	0.01660677
Pfn2	0.39564402	7.71573022	10.1448047	0.00467073	0.01660677
Rprd2	-0.4554368	7.26401555	10.1444965	0.00467125	0.01660677
Mrpl21	0.57739868	4.23155519	10.1429306	0.00467386	0.0166082
Park7	0.64803686	4.76409095	10.1399771	0.0046788	0.0166126
4930519G04	-1.0847977	2.31728697	10.1395406	0.00467953	0.0166126
Gm7361	-2.5899225	-0.8434527	10.1334512	0.00468973	0.01663343

Pcm1	-0.6030501	8.57560764	10.1332881	0.00469	0.01663343
Zfp704	-0.4525996	6.73180824	10.131524	0.00469296	0.01663343
Hnrnpab	0.59934911	7.5806322	10.1307468	0.00469427	0.01663343
Prelid1	0.62201116	5.79419782	10.1275927	0.00469956	0.01664106
Eif3k	0.6657991	4.33275932	10.1256284	0.00470287	0.01664106
Gtf2b	0.55449169	4.83713545	10.1255049	0.00470307	0.01664106
Myo7a	-1.1269487	2.71220591	10.1219785	0.00470901	0.0166542
Stx8	0.5993565	5.33836295	10.1151563	0.00472052	0.01668204
Mirg	-2.1315169	1.06503662	10.114677	0.00472133	0.01668204
Katnbl1	-0.6624092	4.70717542	10.1130266	0.00472412	0.01668403
Ucma	3.23804871	-0.7750345	10.1070326	0.00473426	0.01671199
Aida	0.42607395	6.45288865	10.1030997	0.00474093	0.01672767
Zfp169	-0.5993416	4.07761419	10.0968952	0.00475147	0.01674686
l7Rn6	0.52154049	6.09145303	10.0946608	0.00475528	0.01674686
Kctd12b	0.610753	4.98080591	10.0934413	0.00475736	0.01674686
Rnaseh2a	1.08008909	2.75816186	10.092973	0.00475815	0.01674686
Prdx6	0.45743726	6.75152676	10.0915434	0.00476059	0.01674686
1700030L20R	-1.447415	1.35267767	10.0912924	0.00476102	0.01674686
A4gnt	4.54045035	-1.4684835	10.0907179	0.004762	0.01674686
Ythdc2	-0.7039528	5.51634512	10.0888508	0.00476518	0.01675021
Zcchc2	-0.4634899	6.13430018	10.086044	0.00476998	0.0167592
Dennd6b	-1.3048887	2.8384596	10.0830998	0.00477501	0.01676904
Scn3a	-0.8813472	5.62374621	10.0805744	0.00477933	0.01677636
Vangl1	0.84502056	3.81104559	10.077971	0.00478379	0.01678417
Atp10b	-1.5016731	1.16803816	10.0736066	0.00479128	0.01680258
Trim2	0.48640264	9.04551566	10.0712407	0.00479535	0.01680898
Ints6	-0.5047602	4.96000102	10.0691391	0.00479896	0.01681379
Mir690	-5.0244472	-2.00979	10.0554623	0.00482255	0.01688856
Ubr2	-0.6310122	6.2756488	10.0494749	0.00483292	0.01690648
Mtx3	-0.503952	5.99207355	10.0477057	0.00483599	0.01690648
Pygl	-1.517265	1.09311284	10.047226	0.00483682	0.01690648
Stxbp2	-0.8303671	4.19154777	10.0471845	0.0048369	0.01690648
Sdhaf2	0.49221771	5.20430452	10.0460071	0.00483894	0.01690648
Kat7	0.40131891	5.9074198	10.0440358	0.00484236	0.01691057
Grem1	1.4136782	0.83619109	10.0407953	0.004848	0.01691797
Dnmt3a	-0.5010978	6.59707257	10.040222	0.004849	0.01691797
Terf2	0.38208935	6.26750373	10.0343383	0.00485925	0.01694586
Fbxl12	-0.8744259	2.3507409	10.0317984	0.00486368	0.01695331
Acot11	-0.6821891	3.82662615	10.0305245	0.00486591	0.01695331
Nomo1	0.53075772	5.3458177	10.0275107	0.00487117	0.01695593
Arhgdia	0.62617489	8.02589297	10.0275085	0.00487118	0.01695593
1110004E09I	0.66415754	5.77998357	10.0245447	0.00487636	0.01696611
Actr1a	0.39415908	6.16861461	10.0197853	0.00488471	0.01698726
Rbm12b2	-0.6747437	4.39119605	10.012919	0.00489677	0.01702132
Lsm10	0.88448767	2.36384022	10.0078159	0.00490576	0.01704466

Lpar3	-1.46305	2.27219078	10.0050777	0.00491059	0.01704906
Dnajc13	-0.6099296	6.26013549	10.0045008	0.00491161	0.01704906
Rnf187	0.49736552	6.80386205	10.0032348	0.00491384	0.01704906
Aplp1	0.46873144	8.34531989	10.0000174	0.00491953	0.0170609
Uqcr10	0.72406882	5.90447573	9.99852039	0.00492218	0.01706219
Ndufb9	0.59053947	6.52462575	9.99474482	0.00492886	0.01707748
Tmem132d	-0.7483228	3.96671457	9.98999134	0.00493729	0.0170978
Suco	-0.4506411	6.12057899	9.98886901	0.00493929	0.0170978
1700085C21I	-4.245973	-1.704339	9.98125536	0.00495283	0.01713342
Dr1	0.50118234	5.49912096	9.98051929	0.00495414	0.01713342
Ltv1	0.48882401	4.79175737	9.97664228	0.00496106	0.01714792
Aspm	-1.4833951	1.19774706	9.97560526	0.00496291	0.01714792
Mcm6	0.59045813	3.64528183	9.97199267	0.00496937	0.01716233
Psmc6	0.4381786	5.85723152	9.95774518	0.00499492	0.01723277
Stx6	0.50033199	4.9700372	9.95716008	0.00499597	0.01723277
Ankef1	-2.2857234	0.39636914	9.95593588	0.00499817	0.01723277
Mrpl51	0.46843312	5.05127658	9.95550348	0.00499895	0.01723277
Gm11127	-0.8117019	3.78729841	9.95136394	0.00500641	0.01723827
Dok7	-2.7123437	-0.7094101	9.95120321	0.0050067	0.01723827
Zfp184	-0.8706587	2.16563795	9.95079138	0.00500744	0.01723827
Cacybp	0.41479831	6.42728372	9.94876081	0.00501111	0.0172401
Mrpl32	0.71887623	3.95919809	9.94795028	0.00501257	0.0172401
Exoc2	-0.5090338	5.89155414	9.94452658	0.00501876	0.01725221
Dhx57	-0.8218866	5.85111194	9.94118843	0.0050248	0.01725221
Epg5	-0.8770042	5.48609808	9.94117595	0.00502482	0.01725221
Klc2	0.65206859	5.48248672	9.94091478	0.00502529	0.01725221
Arid3b	-1.1494468	2.17230131	9.93745347	0.00503156	0.01726584
Mettl16	0.52412826	5.66215822	9.93338957	0.00503894	0.01727756
Chdh	-1.3746185	1.47474422	9.93290719	0.00503981	0.01727756
Fam169b	-1.1213172	1.94048087	9.93176656	0.00504189	0.01727756
2610507B11I	-0.3506426	8.00325485	9.93033942	0.00504448	0.01727855
Tll1	0.76383853	3.55557792	9.9204495	0.0050625	0.01733236
Prpf19	0.42224437	7.10692766	9.90317229	0.00509415	0.01743277
Gon4l	-0.5312786	5.89696095	9.90190657	0.00509648	0.01743278
Klra2	-2.5700903	0.277556	9.89527874	0.00510869	0.01746555
Zfp398	-0.5798228	4.49869841	9.89333232	0.00511228	0.01746555
Rabif	0.5546476	5.84337403	9.89117837	0.00511626	0.01746555
Ankrd50	-0.4689116	5.18671778	9.89056181	0.00511739	0.01746555
B630005N14	0.36654706	6.47885509	9.89039488	0.0051177	0.01746555
Gadd45gip1	0.96610385	2.62818782	9.88529029	0.00512714	0.01748846
Cnot3	0.47709684	5.42677765	9.88424568	0.00512908	0.01748846
Abcb1b	-1.0137183	1.66778348	9.87727308	0.00514201	0.0175246
Pak7	-0.5987248	6.03121147	9.87590719	0.00514455	0.01752529
Tfap2a	0.75222634	3.94544385	9.86674024	0.00516162	0.01757545
Kctd1	-0.3832428	6.47743231	9.86487237	0.00516511	0.01757934

Klf14	-3.3981028	0.34924461	9.85893733	0.0051762	0.01759013
Ankmy2	0.35636328	6.26991117	9.85870952	0.00517662	0.01759013
Shank2	-0.8420512	6.98539914	9.85696675	0.00517989	0.01759013
Pfkfb2	-0.4250881	5.92260936	9.85689314	0.00518003	0.01759013
Tek	-0.9353038	2.9134999	9.85686074	0.00518009	0.01759013
Drd3	-2.8049845	-1.0424144	9.85565408	0.00518235	0.01759013
Fstl4	-0.6634382	3.03605396	9.84345288	0.00520526	0.01765993
Slc35d3	-0.9463502	1.68033819	9.84066755	0.00521051	0.01766974
Rasgrf2	-0.8881273	6.84330007	9.83794434	0.00521565	0.01767366
Mrpl24	0.93035526	2.82661338	9.83706126	0.00521732	0.01767366
Asgr1	1.06769139	6.16241364	9.83430348	0.00522252	0.01767366
Ankrd13c	0.3746282	6.39659034	9.83361694	0.00522382	0.01767366
Akirin2	0.51522806	6.60891221	9.83251874	0.0052259	0.01767366
Fam134a	0.45922587	5.4206259	9.83240533	0.00522611	0.01767366
Pomgnt1	-0.7742863	2.95261862	9.82971496	0.0052312	0.01767366
Plekhg5	-0.5494246	4.25616956	9.82939978	0.0052318	0.01767366
4930447C04I	-1.6036954	2.03600287	9.82883396	0.00523287	0.01767366
2310015B20I	1.45827122	1.96852753	9.82498649	0.00524017	0.01769009
Eprs	0.35994515	7.57236391	9.82378153	0.00524245	0.01769009
Spred3	-0.6518553	5.07279754	9.82135582	0.00524706	0.01769767
Kif23	-1.3953845	0.84121658	9.81585155	0.00525753	0.01772501
Enpp2	-0.4751552	8.24836656	9.80864011	0.00527128	0.01776338
Tcf12	0.48965404	7.35152925	9.79929836	0.00528915	0.01781561
Ppid	0.4126061	6.12373406	9.79488659	0.00529761	0.01783611
Kif1b	-0.5593219	9.93094244	9.79298436	0.00530127	0.01783855
Scn2a1	-0.7103552	8.05753643	9.79203294	0.0053031	0.01783855
Nudt8	1.88874328	0.48689699	9.78684737	0.00531308	0.01786411
Col11a2	-1.7748232	0.10976493	9.78167159	0.00532306	0.0178779
Aspa	0.50719114	6.03393762	9.78132396	0.00532373	0.0178779
Wipi1	0.66904333	4.24351571	9.78030108	0.0053257	0.0178779
Asap2	-0.5340426	6.25243567	9.77765337	0.00533082	0.0178779
Atxn10	0.33513134	7.66352388	9.77742658	0.00533126	0.0178779
Tuba4a	0.44470404	7.31249873	9.77670828	0.00533265	0.0178779
Ptpn13	0.45561129	6.26099968	9.77527437	0.00533542	0.0178779
Zfp804a	-0.6601378	5.92882127	9.77484935	0.00533624	0.0178779
Isl1	-1.5603539	1.28366596	9.77184562	0.00534206	0.01788856
Dera	1.28647764	2.55249595	9.77074486	0.0053442	0.01788856
Rnf208	0.45411725	4.92523072	9.76886278	0.00534785	0.0178928
Idh1	0.50239171	5.41998055	9.76694294	0.00535157	0.01789728
Naa38	0.80124492	3.65795244	9.76479027	0.00535576	0.01790329
2010111I01R	-0.5438319	4.66778239	9.75632305	0.00537224	0.0179495
Tnr	-0.7733712	5.08287813	9.75523346	0.00537437	0.0179495
Txndc9	0.41123925	5.80377906	9.75381867	0.00537713	0.01795073
Cdh13	0.78024693	4.87616683	9.74580322	0.0053928	0.01799505
Rps19bp1	0.73999505	2.88831036	9.74236565	0.00539954	0.01800768

Cyb561	0.41965985	4.44562997	9.74006183	0.00540406	0.01800768
Samd1	0.98091676	2.79565482	9.73967547	0.00540482	0.01800768
Ppp4r2	0.37826378	8.3018626	9.73897649	0.00540619	0.01800768
Scaf11	-0.3040994	7.60854134	9.72791622	0.00542796	0.01806223
Cript	0.51103877	7.53137225	9.72768588	0.00542841	0.01806223
Ampd2	-0.5612169	3.93877048	9.72698913	0.00542979	0.01806223
Nck2	0.53807166	4.64343953	9.72465041	0.00543441	0.01806621
Diap1	-0.4704094	4.50604362	9.72276659	0.00543813	0.01806621
Phpt1	0.74112693	3.28584852	9.72272579	0.00543821	0.01806621
Ppp1cb	0.35810588	9.23777984	9.71463502	0.00545423	0.01810256
Eya2	0.81970831	6.41533242	9.71428298	0.00545493	0.01810256
Skp1a	0.44418899	8.04762672	9.71305097	0.00545737	0.01810256
Asah2	-0.5176192	5.7264065	9.71232935	0.00545881	0.01810256
Hist1h1d	3.08194522	-1.3350052	9.70941195	0.0054646	0.01811377
Erich2	1.67036498	0.43049639	9.70535437	0.00547267	0.01813251
Ppip5k2	-0.5000455	5.30691516	9.70338449	0.0054766	0.0181375
A430078G23	-0.744516	3.71615754	9.69723726	0.00548886	0.01815155
Scarna3a	-3.2697613	-0.9411287	9.69678327	0.00548977	0.01815155
Ebf3	-1.0607958	1.46044412	9.69673571	0.00548986	0.01815155
Slc12a2	-0.4326992	6.5705165	9.69617173	0.00549099	0.01815155
Chrd	-0.9132478	2.10372602	9.69519693	0.00549294	0.01815155
Kdm7a	-0.4026895	7.30540922	9.68998773	0.00550336	0.01817756
Mir128-1	-1.8664695	1.50571919	9.68884315	0.00550565	0.01817756
Fibp	0.59044328	4.96051385	9.68371416	0.00551594	0.01820352
Txn1	0.61501523	6.41714117	9.68094541	0.00552151	0.01821387
Itga9	-0.6558282	3.28035532	9.67688436	0.00552968	0.018221
D130020L05I	-0.8039023	2.63523436	9.67630889	0.00553084	0.018221
Vasn	-0.5547311	3.78869828	9.67625005	0.00553096	0.018221
Acp5	3.05345819	-0.7192985	9.67293752	0.00553763	0.01823162
Gpbp1	0.38106419	7.99359467	9.67223947	0.00553904	0.01823162
Sspo	-4.3480907	-1.2452705	9.66873295	0.00554612	0.01824692
Serping1	0.90234295	7.04095034	9.66686577	0.0055499	0.01825133
Tmem251	0.64497498	3.7918645	9.66219757	0.00555935	0.01827439
Uhrf1bp1	-0.6949056	3.87314258	9.65618449	0.00557154	0.01830647
Ndufb4	0.7271333	5.90445898	9.64876048	0.00558664	0.01834804
Insr	-0.5294287	5.99010779	9.64638844	0.00559148	0.01835588
Gltpd1	0.562564	3.73848941	9.64013344	0.00560425	0.01838976
Unc5c	0.50621914	6.71418233	9.63847989	0.00560763	0.01839281
Eif4enif1	0.35012844	6.6647732	9.63541446	0.0056139	0.01839818
Mgp	1.08757387	8.28888369	9.63380232	0.00561721	0.01839818
Ddx55	-0.5176632	4.40733803	9.63253309	0.00561981	0.01839818
Ppapdc2	0.37523593	6.28486106	9.63234428	0.00562019	0.01839818
H2-Q4	-1.2052245	1.686645	9.63169293	0.00562153	0.01839818
Pts	0.54380947	5.2535485	9.62887354	0.00562732	0.01840909
Clic3	2.57611363	-0.5019365	9.62494632	0.00563539	0.01842745

Prkrip1	0.7847349	3.70442885	9.62067686	0.00564418	0.01844815
Il1bos	6.37059087	-2.0499981	10.0374407	0.00564804	0.01845229
Aldh5a1	-0.4726838	6.74500979	9.61767555	0.00565036	0.01845229
Atg10	0.69722309	4.0415969	9.6142388	0.00565746	0.01846742
Gm1821	0.64847965	4.9269643	9.60632727	0.00567383	0.0185128
Ces5a	4.81598688	-1.1757707	9.60354816	0.00567959	0.01852355
Rbm42	0.56905375	4.24635712	9.5969677	0.00569326	0.01856006
Kctd15	-1.1027019	2.12360408	9.59569681	0.00569591	0.01856062
Camkv	-0.5231262	6.00284055	9.59116437	0.00570535	0.01858331
Spata6	0.59471173	3.91402258	9.58465108	0.00571895	0.01860286
4833420G17	-0.5086686	4.88925606	9.58458989	0.00571908	0.01860286
Akap5	0.40041617	8.01809372	9.58421837	0.00571985	0.01860286
Mog	-0.9485756	2.85453928	9.58354133	0.00572127	0.01860286
Scnm1	0.68489209	4.02330932	9.58166392	0.0057252	0.01860757
Usp33	-0.457377	6.93210108	9.58000875	0.00572866	0.01861077
Rasl11b	0.48327889	5.61450664	9.57466066	0.00573988	0.01863913
Mrfap1	0.55209428	9.51155094	9.56950659	0.00575071	0.01866622
Adamts4	-1.1727986	1.78413118	9.56240722	0.00576566	0.01870667
Pglyrp3	6.20430353	-2.0173844	9.97318583	0.0057724	0.01872041
5430427O19	2.91058232	0.2311527	9.55579205	0.00577964	0.01872767
Gm15319	-1.7972264	0.17664311	9.5550888	0.00578113	0.01872767
Map1b	-0.8816984	12.0341584	9.55461831	0.00578212	0.01872767
Itpk1	0.58714746	3.82827909	9.55028488	0.0057913	0.01874931
Zfp871	-0.4251117	9.06013318	9.54399695	0.00580465	0.01878441
Hap1	-0.6681939	4.20512542	9.54106048	0.0058109	0.01879652
2010109A12I	-5.1685134	-2.023385	9.5266055	0.00584175	0.01888817
A530046M15	-1.7230483	0.47076079	9.52313865	0.00584918	0.0188994
Col9a2	1.01351752	2.67963195	9.52248052	0.00585059	0.0188994
Hecw1	-0.9132422	6.93926334	9.52145837	0.00585278	0.0188994
Rexo2	0.40864895	6.99962684	9.51837591	0.0058594	0.01891262
Acbd3	0.36233857	6.49257134	9.51473815	0.00586722	0.01892972
Bop1	0.82925693	2.95513326	9.51267771	0.00587165	0.01893588
Spag1	-0.7091734	2.86066119	9.50851153	0.00588063	0.01894765
E130309F12I	-0.8604995	2.72267869	9.50817991	0.00588134	0.01894765
Gimap1	-0.9372832	3.12082073	9.50590519	0.00588625	0.01894765
Grp	2.17178331	0.11469407	9.50529028	0.00588758	0.01894765
Gm4532	-4.7108459	-1.3773558	9.50512753	0.00588793	0.01894765
AI115009	1.70324393	0.65848687	9.50300403	0.00589252	0.01895428
Zfp507	-0.5000227	4.73466432	9.49823415	0.00590284	0.01897934
Klhl13	0.38245451	6.10968308	9.49696575	0.00590559	0.01898004
Exd2	-0.5176027	5.36403923	9.49387962	0.00591228	0.01899341
Tenm4	-0.6896329	5.97639143	9.48588245	0.00592966	0.01904108
Epdr1	0.51783278	5.86306948	9.4811712	0.00593992	0.01906251
Map3k19	-0.9780724	2.77347339	9.48048675	0.00594141	0.01906251
Strap	0.32291996	7.46723644	9.47701137	0.005949	0.01907619

Smim11	0.71957674	3.79796202	9.47620511	0.00595076	0.01907619
Nemf	-0.5014626	7.13975157	9.47333189	0.00595704	0.01908817
Polr3b	-0.5944425	4.43934886	9.47204288	0.00595986	0.01908905
Cldn1	1.48287946	3.08447347	9.46981219	0.00596475	0.01909655
Fnbp4	-0.4821054	5.76659596	9.46663487	0.00597171	0.01911069
Fat3	-0.8948191	8.06281523	9.46427902	0.00597689	0.01911908
Jmy	-0.3747725	7.18953639	9.45697185	0.00599296	0.01914413
Frem1	-1.7967209	1.21713709	9.4569483	0.00599301	0.01914413
Abhd5	0.55260407	6.67456623	9.45694647	0.00599301	0.01914413
Thap1	0.77532855	3.74336405	9.45607812	0.00599492	0.01914413
Lyl1	-2.2537434	-0.0769813	9.45217371	0.00600353	0.01916346
Fam178b	3.475265	-1.1721613	9.45088064	0.00600639	0.01916442
Stag3	-4.3397082	-1.6816123	9.44910138	0.00601032	0.0191688
Hspbp1	0.87494016	2.63907772	9.44580547	0.0060176	0.01917875
Ostf1	0.54071507	5.90732594	9.44537844	0.00601855	0.01917875
Commd3	0.44224421	5.0746158	9.44348804	0.00602273	0.01918394
1700063D05I	-0.9765779	1.83602218	9.44032824	0.00602974	0.01919809
4930480K15I	-1.6158207	2.7430116	9.43853895	0.00603371	0.01920258
Begain	-0.7194542	3.44830392	9.4340323	0.00604372	0.01922628
Tmem100	0.58812186	3.5969053	9.43097729	0.00605051	0.01923908
Ntmt1	0.86895194	2.14595237	9.42991826	0.00605287	0.01923908
Rtf1	0.33045793	8.31775041	9.42691669	0.00605956	0.01924274
H1f0	0.46009814	6.9773006	9.42673988	0.00605995	0.01924274
Rab3ip	0.46265636	5.14672072	9.42594894	0.00606172	0.01924274
Hcn2	-1.1488618	1.3127917	9.42368835	0.00606676	0.01925061
Ttc18	-1.8716253	0.60106621	9.42227982	0.00606991	0.01925244
Col1a2	0.755846	7.37951761	9.41733687	0.00608096	0.01927462
Agmat	2.8511149	-0.0296026	9.41685511	0.00608204	0.01927462
LOC1010557	-6.0226243	-1.8437998	9.81530237	0.00609131	0.01929586
Myoz3	-1.589054	1.1393035	9.41040483	0.0060965	0.01929684
Hscb	0.87677528	4.15092299	9.41028596	0.00609677	0.01929684
Slc43a2	-0.548472	5.45789386	9.40862859	0.00610049	0.01930048
Tubb6	0.89529519	3.92177905	9.39801708	0.00612438	0.01936789
Iqgap2	-0.5539596	4.97802915	9.39592666	0.0061291	0.01937465
Napg	0.37137376	6.73051535	9.39162943	0.00613881	0.01939717
Ncbp1	-0.4776287	4.95939243	9.37668035	0.00617273	0.01949615
Ntng1	-0.5335021	5.3774984	9.37535695	0.00617575	0.01949745
Arrdc2	1.30363064	1.72076775	9.366217	0.0061966	0.01954999
Zcchc14	-0.406298	6.32427807	9.36435013	0.00620087	0.01954999
Atp8b5	-3.3753491	-0.868689	9.36384016	0.00620203	0.01954999
BC061194	-1.8402002	0.34113822	9.36350036	0.00620281	0.01954999
S100a11	0.75922219	9.5078464	9.3611643	0.00620816	0.01955863
Gm16712	4.8572881	-1.9720096	9.3580377	0.00621533	0.01957299
Cntn3	-0.556399	5.52149284	9.35395202	0.00622471	0.0195943
Lipa	0.51372446	4.63551706	9.34720923	0.00624022	0.01963489

Mpc2	0.45878977	5.93669597	9.34583535	0.00624338	0.01963662
Ubqln4	0.43397438	6.15936586	9.34374734	0.0062482	0.01964353
Fkbp15	-0.4808536	4.74414784	9.33877382	0.00625969	0.01967139
Coq5	0.37224027	6.38058398	9.33522114	0.00626791	0.01968389
Senp7	-0.3410704	6.97076646	9.33478601	0.00626891	0.01968389
Kcnj16	-0.6525312	4.19006785	9.33289274	0.0062733	0.01968832
Gnb1	0.33688081	9.34935257	9.33191202	0.00627557	0.01968832
Spns1	-1.0717466	2.31003266	9.33005706	0.00627987	0.01969358
Rasal2	-0.5131364	7.19857233	9.32780494	0.0062851	0.01969445
Uchl5	0.36593441	6.67267395	9.32714354	0.00628664	0.01969445
Sp7	1.53802352	0.83037531	9.32654568	0.00628803	0.01969445
Lancl1	0.44280822	6.54720907	9.32317088	0.00629587	0.01971038
2410004B18I	0.45333522	5.13860267	9.32143325	0.00629992	0.01971038
Bcas1os2	-2.4288459	-0.1968734	9.32097032	0.006301	0.01971038
Mir181b-2	-3.9900847	-1.1861624	9.31813174	0.00630761	0.01972284
Mpdz	-0.406015	6.41183289	9.3105938	0.00632521	0.01976964
Adam33	-2.1090596	-0.0202813	9.30398999	0.00634068	0.01980972
Hmox1	0.86771349	2.83955747	9.30162665	0.00634622	0.01981879
D5Ertd579e	-0.4193184	7.30516997	9.29920748	0.0063519	0.01982634
Itm2a	0.84147042	6.66213787	9.29682856	0.0063575	0.01982634
Xkr6	-1.1115354	2.9998954	9.29640833	0.00635848	0.01982634
Pik3r4	-0.5102357	5.1104775	9.29585143	0.0063598	0.01982634
Cep95	-0.5545628	3.92792727	9.29497622	0.00636185	0.01982634
Fam193b	-0.9688797	3.14884989	9.2860906	0.00638281	0.01988338
Slc39a10	0.40845058	8.38104969	9.28391987	0.00638794	0.0198911
Stard8	-0.372996	5.63681122	9.27574555	0.0064073	0.0199418
Stx4a	0.53604534	6.39747626	9.27420024	0.00641097	0.0199418
Fam98b	0.48386173	6.91903186	9.27296825	0.00641389	0.0199418
Dennd5b	-0.6699028	6.59461121	9.27151038	0.00641736	0.0199418
Sdr39u1	0.52449878	4.98892235	9.27144502	0.00641751	0.0199418
Nup98	-0.3872351	6.39272742	9.2626682	0.00643841	0.01999846
Slx1b	-0.5491424	3.6325023	9.26100307	0.00644238	0.02000213
Setdb1	-0.6856531	4.51143675	9.25993845	0.00644493	0.02000213
Gapdhs	-1.6830175	1.29571558	9.25538084	0.00645582	0.02002765
Map7d2	0.44049108	7.71414111	9.24692035	0.0064761	0.02008226
Ifnar1	-0.4869431	5.75464592	9.24335404	0.00648467	0.02010052
Psmc1	0.39995004	6.37600681	9.23952499	0.00649388	0.02012076
Mink1	-0.5540564	5.58472022	9.23603208	0.0065023	0.02013853
Usp9x	-0.573543	9.85788988	9.22914909	0.00651892	0.02017039
Bloc1s5	0.89493896	3.3249666	9.22914024	0.00651894	0.02017039
Pcdhgb8	-1.3265241	1.14866749	9.22843409	0.00652065	0.02017039
Pwwp2a	-0.6041424	5.79995736	9.22628287	0.00652586	0.02017673
Hnrnpa1	-0.3903598	5.07049439	9.22458693	0.00652997	0.02017673
Nhlrc2	-0.5828449	3.76158255	9.22411602	0.00653111	0.02017673
Mmp19	1.07018709	2.61996574	9.22314465	0.00653346	0.02017673

Rnls	1.37548998	1.0564206	9.21985665	0.00654144	0.02019306
Decr1	0.63879936	5.39052888	9.21346929	0.00655697	0.02023267
Yap1	0.51707533	5.91019961	9.20291288	0.00658272	0.02030378
Klhl40	2.1935056	0.1157573	9.20176704	0.00658552	0.02030408
Gucy2f	-2.7011107	0.43556857	9.19958888	0.00659086	0.02031216
Otud6b	0.4701293	6.33504162	9.19590135	0.00659989	0.02033165
Rp9	0.59850509	4.0520469	9.19199487	0.00660948	0.02035283
Fip1l1	0.33661586	6.50639175	9.18819387	0.00661882	0.02037323
Tfg	0.34903937	6.88161521	9.18588733	0.0066245	0.02037616
Adra2b	-2.101239	0.18659244	9.18560063	0.0066252	0.02037616
Smox	0.83929509	2.88869692	9.18341964	0.00663058	0.02038325
Ccp110	-0.486342	5.8898063	9.18174104	0.00663471	0.02038325
Nefl	-0.4297122	8.72747445	9.18052528	0.00663771	0.02038325
Tmsb10	0.78980055	6.42642058	9.17921244	0.00664095	0.02038325
Lrp1	-0.4003027	7.3421971	9.17915554	0.00664109	0.02038325
Bai3	-0.6090934	6.39756936	9.17764883	0.00664482	0.02038633
Sphkap	-0.8117978	7.2290455	9.16923231	0.00666565	0.02043664
Kif13b	-0.5901439	3.67690673	9.16882208	0.00666666	0.02043664
Slc39a2	-2.3421425	0.47883261	9.16572196	0.00667436	0.02045187
Slc25a16	0.44653927	5.57022558	9.16242962	0.00668254	0.02046857
Armcx2	0.3657176	5.54431266	9.15408812	0.00670331	0.02052382
Tatdn1	0.48997052	4.60363615	9.15252039	0.00670722	0.02052742
Tdrp	0.58978275	5.87769403	9.14691526	0.00672123	0.02056191
Mamld1	0.4588799	6.24014348	9.14534589	0.00672516	0.02056554
Per3	-0.3839048	5.90032191	9.13930868	0.0067403	0.02059676
Sik1	-0.6362087	4.19170344	9.13908332	0.00674086	0.02059676
Sgsm2	-0.5788771	4.61831494	9.13197661	0.00675873	0.02064295
Tecpr2	-0.7799412	5.55760619	9.12888024	0.00676653	0.02065836
Erdr1	-0.7801483	3.40101231	9.12403166	0.00677877	0.0206873
Stk3	0.45207443	5.66440243	9.11766077	0.00679488	0.02072604
D630023F18	0.90103075	2.00640674	9.11683	0.00679699	0.02072604
Cdhr2	-3.5034204	-0.9457568	9.11535697	0.00680072	0.020729
Arhgap24	0.7278527	3.94176612	9.11224269	0.00680862	0.02074465
Kcnma1	-0.5897459	7.25180016	9.10910009	0.0068166	0.02075967
Clspn	-2.3903602	-0.1388371	9.10812363	0.00681909	0.02075967
D130043K22	-0.814899	3.37003417	9.10138558	0.00683625	0.02080346
Spef2	-1.6521774	0.09680215	9.09147578	0.00686157	0.02087206
Nelfe	0.74605513	3.95887873	9.08758073	0.00687155	0.02089395
4930429B21	0.50458747	5.16830204	9.08562001	0.00687658	0.02090077
D17H6S53E	-0.6781735	2.75255333	9.08375252	0.00688138	0.0209051
Thoc7	0.48834525	6.34780559	9.08226526	0.0068852	0.0209051
Med13	-0.3528628	9.08293275	9.08077359	0.00688903	0.0209051
Celf4	0.34768819	9.32146305	9.08072742	0.00688915	0.0209051
Jun	0.37183102	6.02552746	9.07640394	0.00690028	0.0209304
Olig2	0.63923519	3.43458375	9.072675	0.0069099	0.02094338

Ipo9	-0.426138	7.01402493	9.07257913	0.00691014	0.02094338
Mir3473	-3.0868359	-0.4214497	9.06887501	0.00691971	0.02095934
Gpr157	-1.1256671	2.14962224	9.06837678	0.006921	0.02095934
Fam122b	0.52443642	4.12560287	9.06445848	0.00693114	0.02097026
Cdca3	3.43548572	-0.5375831	9.06386328	0.00693268	0.02097026
Ccdc176	-1.3265642	2.36353421	9.06374319	0.00693299	0.02097026
Mir186	-3.4538458	-0.8361679	9.06020656	0.00694216	0.02098952
Spry1	0.82014723	3.19488316	9.05692734	0.00695067	0.02100192
Rsrp1	-0.6935987	6.29280834	9.05647008	0.00695186	0.02100192
Pebp1	0.50476338	8.27988098	9.05465121	0.00695658	0.02100774
Gan	-0.8270588	3.39614427	9.04985816	0.00696906	0.02103695
DQ267102	-2.7967563	-0.3130751	9.04524078	0.0069811	0.02106482
Tma7	0.52865005	6.81927028	9.04310485	0.00698668	0.02107317
Fundc2	0.52165595	7.72147033	9.03852542	0.00699865	0.02109234
Mrps12	0.79680162	3.59818794	9.03747819	0.00700139	0.02109234
Mcts1	0.42460732	5.13383329	9.03745014	0.00700147	0.02109234
Gm20748	1.25911237	1.58141484	9.03430845	0.0070097	0.02110068
Srsf6	-0.3513109	5.68150112	9.03424623	0.00700986	0.02110068
Ifitm3	0.94754679	7.69025273	9.02657445	0.00703001	0.02113992
AY512931	-1.4637715	1.78850411	9.0249193	0.00703437	0.02113992
Gtf2a2	0.49590998	4.82817	9.02438783	0.00703577	0.02113992
1700110I01R	-1.6743088	1.59061764	9.02431815	0.00703595	0.02113992
Llph	0.54912573	7.07072829	9.02392398	0.00703699	0.02113992
Kcng2	-1.8261088	0.29275489	9.02054705	0.00704589	0.02114989
Cln8	-0.5519623	3.78794767	9.02026096	0.00704664	0.02114989
Syndig1l	-0.8991148	2.34801023	9.01945558	0.00704877	0.02114989
Nudcd1	-0.5904964	3.65886042	9.01381778	0.00706366	0.0211861
Mfhas1	-0.4505557	5.25303798	9.00770798	0.00707984	0.02122485
Med10	0.56768835	3.50898414	9.0055632	0.00708553	0.02122485
Gm5441	-2.4773324	0.35213005	9.00532116	0.00708617	0.02122485
Lsm3	0.59141158	4.10836897	9.00467058	0.0070879	0.02122485
Arpp21	-0.4307714	8.75965728	9.00303746	0.00709224	0.02122937
Elovl7	-0.5892167	4.0967768	8.99916115	0.00710254	0.02125174
Cds1	-0.4961665	5.48304788	8.99382736	0.00711675	0.02128134
Mcm3ap	-0.7553167	4.45572644	8.99326325	0.00711825	0.02128134
Bag1	0.54032619	7.80584386	8.99225351	0.00712095	0.02128134
Serbp1	0.31455667	9.37863843	8.98709759	0.00713472	0.02131232
AF529169	-1.367383	1.49854143	8.98552327	0.00713893	0.02131232
Gm10069	-1.3603792	0.87244405	8.98518597	0.00713984	0.02131232
Rptoros	-4.1820213	-1.2315957	8.98298589	0.00714573	0.02131846
Mrps15	0.56464496	4.23879358	8.98229485	0.00714758	0.02131846
Tnk2	-0.5008162	5.09369377	8.9760532	0.00716433	0.02135992
Ranbp17	-0.6989272	3.12490601	8.97069342	0.00717874	0.0213944
Tmem246	-0.5162638	4.14919465	8.96443869	0.00719561	0.02143614
Dlc1	-0.4304672	6.60840981	8.96292182	0.0071997	0.02143983

Bag3	0.52648747	5.08106989	8.96026185	0.00720689	0.02145272
Gucy2c	3.04157279	-0.8766283	8.95374344	0.00722455	0.02149674
Cenpf	-0.8044209	2.51014231	8.95224305	0.00722862	0.02150032
Ntpcr	0.83754709	2.61603098	8.94998462	0.00723475	0.02151003
Glt8d2	0.62621889	3.02758039	8.94478709	0.00724888	0.02154351
Ddc	-1.1982164	1.34508419	8.93620314	0.00727229	0.02160452
Scg3	0.61782622	6.66561104	8.93342024	0.00727989	0.02161649
Zfand3	0.47499486	5.53411354	8.93247582	0.00728248	0.02161649
Shoc2	0.35131741	8.6086196	8.92912827	0.00729164	0.02161649
Notch3	-1.2662361	2.36530039	8.92802863	0.00729466	0.02161649
Usp34	-0.5414665	8.49626078	8.9274894	0.00729613	0.02161649
Fam69c	-1.7833686	0.11835775	8.92618679	0.00729971	0.02161649
Ssb	0.37615055	8.09090107	8.92588028	0.00730055	0.02161649
Lphn1	-0.4984625	8.35124724	8.92549449	0.00730161	0.02161649
Rnf113a2	0.53938072	4.72744651	8.92439821	0.00730461	0.02161649
Rac1	0.35079822	10.032103	8.92420941	0.00730513	0.02161649
Marf1	-0.4017951	7.87549306	8.91873571	0.00732018	0.02164017
Cd33	-0.6286808	3.1598662	8.9172675	0.00732422	0.02164017
Polr1a	-0.8955123	4.59391222	8.91721124	0.00732438	0.02164017
Ctse	-2.6376654	-0.3797391	8.91710156	0.00732468	0.02164017
Sorbs2	-0.7762954	7.36528757	8.91351987	0.00733455	0.0216608
Tango2	0.57436837	3.743236	8.90966268	0.00734519	0.02168371
Pkib	0.43682808	5.513234	8.90274505	0.00736433	0.02173165
Sgk1	0.40694569	6.58128598	8.9010243	0.0073691	0.02173717
Psma6	0.40054127	6.42066675	8.89664953	0.00738124	0.02174384
Trappc11	-0.4505791	5.46142792	8.89650746	0.00738163	0.02174384
Abcc3	-2.5805534	-0.6679128	8.89645626	0.00738178	0.02174384
Ulk4	-1.7067161	1.39967079	8.89603099	0.00738296	0.02174384
Eqtn	-2.6006409	0.10408834	8.89489498	0.00738611	0.0217446
Itga10	-0.786601	2.96327098	8.892635	0.0073924	0.02175457
Gm5124	0.46274544	5.01792414	8.88997057	0.00739981	0.02176785
Nlrp10	-2.2671055	-0.7884599	8.88741973	0.00740692	0.02177925
Tmprss5	-5.3213937	-1.6692367	8.88646736	0.00740958	0.02177925
Zdhhc17	-0.4716453	6.78753137	8.88545686	0.0074124	0.02177925
Nsmaf	-0.5345719	4.77577922	8.8831274	0.0074189	0.02178982
Paqr6	-0.9744502	2.09604064	8.88126442	0.00742411	0.02179658
Gm19466	-2.210791	-0.2042499	8.87637847	0.00743778	0.02182817
Gm8234	-0.7688898	3.69496776	8.87527927	0.00744086	0.02182867
Stc1	-0.9709982	2.90925401	8.87085608	0.00745327	0.02185652
Rnasek	0.6830087	7.09958463	8.86851928	0.00745983	0.02185838
Zbtb24	-0.5169345	5.10803084	8.86824572	0.0074606	0.02185838
Tfe3	0.49663879	5.30511724	8.86751938	0.00746264	0.02185838
Pura	0.30681279	6.97518795	8.86070174	0.00748184	0.02189904
Slc22a15	-0.7476754	2.70741686	8.86044687	0.00748256	0.02189904
Aqr	-0.5905478	5.22653794	8.85948057	0.00748528	0.02189904

Mast1	-0.5962149	4.88121181	8.85507888	0.00749771	0.02192685
Ints12	0.473277	4.73342716	8.85122502	0.00750861	0.02195017
Traf3	-0.5110856	5.05773297	8.84557408	0.00752463	0.02198842
Pdk4	-0.7815425	2.80876838	8.84409664	0.00752882	0.02199211
St8sia4	-0.8074887	3.27236278	8.84221691	0.00753416	0.02199914
Eya1	0.53963736	7.18135044	8.84086442	0.007538	0.0220018
D630045J12I	-0.4365387	6.85489924	8.83698281	0.00754905	0.02202547
Ube2n	0.30250176	7.43722179	8.83440257	0.0075564	0.02202777
Ddx42	-0.4266538	6.02745186	8.83374418	0.00755828	0.02202777
Dnah11	-2.1697766	-0.3051368	8.83361441	0.00755865	0.02202777
Rasa2	-0.4282507	5.06270366	8.82839743	0.00757354	0.02205636
Ralgapa1	-0.6805232	7.7368995	8.82811869	0.00757434	0.02205636
Mon2	-0.4759386	6.62046016	8.81898396	0.0076005	0.02211967
Foxo6	0.81239661	2.60702137	8.81847006	0.00760198	0.02211967
Pet112	-0.7382458	2.78670512	8.81366818	0.00761577	0.02214508
Sema6d	-0.4744561	6.16563267	8.81337493	0.00761662	0.02214508
Fadd	0.59547158	4.30094282	8.80876363	0.00762989	0.02217509
Apc	-0.5727801	9.68081644	8.79331475	0.00767456	0.02227766
Scn7a	0.52932254	4.22866684	8.79331353	0.00767456	0.02227766
Taok3	0.45801353	5.86871677	8.79242945	0.00767713	0.02227766
Zfp706	0.38846009	7.48004342	8.79191336	0.00767862	0.02227766
Smc6	-0.3308547	7.65803915	8.79142837	0.00768003	0.02227766
Twsg1	0.68488793	7.87788273	8.78840828	0.00768881	0.02229448
Cox7a2	0.59497174	6.00398457	8.78581798	0.00769634	0.0223077
Frzb	-0.7434417	3.21545134	8.78364109	0.00770268	0.02231745
Tbc1d9	-0.4719118	5.7477432	8.78135191	0.00770935	0.02231998
Edil3	0.54622405	6.55862889	8.78037499	0.00771219	0.02231998
Hsp90b1	0.44333902	9.12486298	8.78012705	0.00771292	0.02231998
Mrps23	0.54474754	4.23293926	8.77925807	0.00771545	0.02231998
Rara	0.74140905	5.15309412	8.77475608	0.0077286	0.0223494
Snx6	0.46642664	6.40291174	8.76740287	0.00775013	0.02240303
Aldh1a7	1.64020269	0.42021628	8.7634276	0.0077618	0.02242267
Wdr33	-0.5530472	5.10123031	8.76278515	0.00776369	0.02242267
Dnmt1	-0.5595249	5.72564765	8.76173732	0.00776677	0.02242267
Prrc2c	-0.5284325	10.0574127	8.76101614	0.00776889	0.02242267
Trpc6	-0.9521562	2.85249854	8.75930812	0.00777391	0.02242855
Tbck	-0.5862005	4.68675334	8.75776085	0.00777846	0.02243306
2700099C18I	-1.2171798	1.75363503	8.75667074	0.00778167	0.02243369
Tmem50b	0.37556728	5.85195413	8.74927442	0.0078035	0.02248796
Rgs6	-0.7429283	3.12285949	8.7405031	0.00782946	0.02254736
Npc1	-0.4895179	4.96851897	8.74028162	0.00783012	0.02254736
Gstm4	0.99069507	1.67802119	8.73145502	0.00785635	0.02261421
Eapp	0.45291237	5.25186188	8.73017042	0.00786018	0.02261655
Denr	0.42278258	5.7057541	8.72772404	0.00786747	0.02262162
Cep250	-0.6010364	4.7744097	8.72676054	0.00787034	0.02262162

Prkca	-0.5262429	8.12929589	8.72654411	0.00787099	0.02262162
Tnip1	0.64257431	4.09255579	8.72455033	0.00787694	0.02263005
Map3k10	-0.6236893	3.26749762	8.72068504	0.00788849	0.02265165
Lum	0.83057523	5.256301	8.72001417	0.0078905	0.02265165
Rilpl2	0.82725926	3.19208681	8.71743761	0.00789821	0.02266512
Gpr101	-1.0760993	2.41118593	8.71157183	0.0079158	0.02270691
Calb1	0.44428011	6.05115069	8.70984326	0.00792099	0.02271312
Dmd	-0.686904	7.88618675	8.70833023	0.00792554	0.02271697
Fam168a	0.29581394	8.13100082	8.70738154	0.00792839	0.02271697
Cnot1	-0.3884973	8.09961705	8.70547793	0.00793412	0.02272231
Atp6v1h	0.31119639	6.29465663	8.7039154	0.00793882	0.02272231
Agfg1	0.35747105	5.82001065	8.70357323	0.00793986	0.02272231
Nbeal1	-0.4843757	6.22589735	8.70273832	0.00794237	0.02272231
Cobll1	-0.5637311	4.89653014	8.70133325	0.00794661	0.02272576
Hydin	-2.0790494	0.40792578	8.69988463	0.00795098	0.02272959
Racgap1	0.71976666	3.21053084	8.6862005	0.00799239	0.02283926
Nefm	-0.657409	9.24354979	8.68224584	0.0080044	0.02286309
Jarid2	-0.4148603	5.84144929	8.68144894	0.00800682	0.02286309
Gm5607	-0.8393667	3.8160498	8.67909195	0.00801399	0.02287486
Ttc9c	0.47873183	6.09942812	8.67438947	0.00802832	0.02290704
Casp3	0.62122665	4.02964314	8.66613328	0.00805355	0.02297028
4930563F08I	-2.8687019	-0.1062883	8.65781485	0.00807905	0.02303427
Gm6682	0.39587678	5.04837045	8.65650514	0.00808307	0.02303698
B3galt5	-0.9057656	3.22309558	8.65448313	0.00808929	0.02304252
Krt18	-4.295178	-1.6566113	8.65336078	0.00809274	0.02304252
Hpgds	-1.0491122	2.40122513	8.65287765	0.00809423	0.02304252
Gabrd	-1.0804503	2.84095757	8.64797114	0.00810935	0.0230767
Ppa2	0.42264027	5.40210486	8.64698494	0.00811239	0.0230767
Ddah1	0.40180904	7.1291285	8.64404488	0.00812147	0.02308535
Tnfsf10	-0.7751307	3.01706064	8.64182085	0.00812834	0.02308535
Ints1	-0.6232613	4.56946716	8.64162855	0.00812894	0.02308535
Wnt7a	-1.2306994	1.63757621	8.64124457	0.00813013	0.02308535
4632427E13I	-1.4201183	1.56758032	8.64072891	0.00813172	0.02308535
Smarcd3	0.65272463	4.1281128	8.63975733	0.00813473	0.02308535
Aurkaip1	0.60485268	4.92854804	8.63903129	0.00813697	0.02308535
Murc	-2.9137015	-0.1710877	8.63782317	0.00814072	0.02308723
Pik3r6	1.43075487	1.61009317	8.62732377	0.00817331	0.02317092
Ptplad2	-0.7216942	3.7755559	8.62299506	0.00818679	0.02320037
Arhgap21	-0.5531591	8.18769952	8.62110719	0.00819268	0.02320828
Zmym6	-0.6957533	4.80463903	8.6140086	0.00821486	0.02326211
Fam21	-0.4659661	6.01400352	8.6130423	0.00821788	0.02326211
Dmxl1	-0.602648	7.30485153	8.60796485	0.0082338	0.02329836
Cntnap1	-0.5303846	5.86954492	8.60382573	0.00824679	0.02332079
Zbtb41	-0.3895587	6.45382066	8.6034603	0.00824794	0.02332079
Tuba1c	0.33794986	5.81028806	8.59982745	0.00825937	0.0233443

Klc4	0.9721739	2.29237103	8.596163	0.00827091	0.0233631
Mbnl2	0.36545154	9.1115468	8.59526316	0.00827375	0.0233631
Hdac9	-0.3454873	6.45270884	8.59475182	0.00827536	0.0233631
Col12a1	0.42681488	5.40904331	8.59135142	0.0082861	0.02338461
Mdp1	0.5193689	4.40685737	8.58900617	0.00829351	0.0233944
Fam198b	-0.6893069	3.62236405	8.58806118	0.0082965	0.0233944
Chd9	-0.4812813	7.98540391	8.58729536	0.00829892	0.0233944
Sod2	0.36174973	8.45926204	8.58571743	0.00830392	0.02339969
Uchl3	0.59604361	3.58882508	8.58387613	0.00830975	0.02340583
Srsf10	0.37074942	6.59195119	8.58224831	0.00831491	0.02340583
Atp9a	-0.5403338	4.80433389	8.58207649	0.00831546	0.02340583
Tlcd2	-0.9927593	1.37738879	8.57959905	0.00832332	0.02341752
Tomm7	0.45855559	4.6452699	8.57845107	0.00832697	0.02341752
Angptl1	-2.2576194	0.5520539	8.57781799	0.00832898	0.02341752
Mon1b	0.35685187	5.95988164	8.57269893	0.00834526	0.02344695
Trim39	-0.4969172	4.31409837	8.57256238	0.0083457	0.02344695
2810055G20	-0.8658471	2.25090386	8.57067929	0.0083517	0.02345004
Rasgrp3	-0.5842586	4.97241042	8.57025499	0.00835305	0.02345004
Sec23ip	-0.454985	5.45952368	8.56468003	0.00837084	0.0234912
Cdk19	-0.3802386	7.00391856	8.56020094	0.00838517	0.0235226
Sik3	-0.4390029	7.39081185	8.55799527	0.00839223	0.02353362
Nrd1	-0.3587146	7.74028781	8.55485981	0.00840229	0.02355301
Arhgap11a	0.79089837	3.16691669	8.55245388	0.00841001	0.02356397
Wash	-0.5726938	3.84877299	8.55168574	0.00841248	0.02356397
Tmem91	1.23461475	1.95762266	8.54922435	0.00842039	0.02356868
Pdgfrb	-0.8697908	4.4274663	8.5492079	0.00842045	0.02356868
Tac1	-0.693533	4.18556843	8.54708396	0.00842728	0.02357077
Mcam	-1.221227	1.72639642	8.54702305	0.00842748	0.02357077
Ccdc28a	0.86814784	2.50024667	8.54562475	0.00843198	0.023574
Iqsec3	-0.6175969	6.55451205	8.54471296	0.00843492	0.023574
Farp1	-0.3671369	5.39111264	8.53321531	0.00847206	0.023669
Zbtb34	-0.494383	6.30562841	8.52977716	0.00848321	0.02368239
Necap1	0.34185923	7.2058062	8.52913139	0.0084853	0.02368239
Cmtm3	0.72797598	3.14524451	8.52831112	0.00848796	0.02368239
Ddx5	-0.3699002	9.43719994	8.52784167	0.00848949	0.02368239
Tmem261	0.5680146	3.81384098	8.52152236	0.00851003	0.02373086
Cyp2e1	-1.1285246	1.04891713	8.51981049	0.0085156	0.02373332
Nup205	-0.6223608	3.99104559	8.51930861	0.00851723	0.02373332
Aldh1a1	0.69586182	7.64304832	8.51598187	0.00852808	0.02375472
Cetn3	0.48817975	7.87997825	8.50901593	0.00855084	0.02380927
Smndc1	0.52364185	5.33377647	8.50576421	0.00856149	0.02383007
Ubxn2a	0.39074911	6.98286005	8.50220376	0.00857316	0.0238486
Evi2a	-0.9329956	2.7935894	8.5017956	0.0085745	0.0238486
Tmem41a	0.63942501	3.74697865	8.49906015	0.00858348	0.02386474
Madcam1	4.63904914	-2.0561187	8.49679566	0.00859093	0.02387659

Srp9	0.49883127	6.79681905	8.49396543	0.00860024	0.02389362
Clu	0.76219309	7.27932893	8.49231801	0.00860567	0.0238988
Tenm3	-0.6040952	6.36800306	8.49146588	0.00860848	0.0238988
Chuk	-0.5745124	4.87758158	8.48750646	0.00862154	0.02392311
Pou5f1	-4.5514775	-2.4473957	8.48687906	0.00862362	0.02392311
Ndufa2	0.63467639	5.45859902	8.48556769	0.00862795	0.02392629
Qprt	1.20397284	1.21650241	8.48407495	0.00863288	0.02393112
Srp72	0.30029603	7.21966283	8.48293701	0.00863665	0.02393271
Gadd45a	0.64967108	4.16244323	8.48126393	0.00864219	0.02393586
Mterfd2	0.43783491	5.9146448	8.48066586	0.00864417	0.02393586
Plekhm2	-0.7397115	3.60509107	8.47724695	0.0086555	0.02395394
Dnajc12	0.73943241	3.61725097	8.47599359	0.00865966	0.02395394
Mrps25	0.4008578	4.61514581	8.47557243	0.00866106	0.02395394
Fastkd5	-0.5924282	3.34987176	8.47346224	0.00866806	0.02395394
Tex264	0.61072481	3.26062366	8.47343766	0.00866814	0.02395394
Acer1	-1.5301936	0.2981237	8.47292292	0.00866985	0.02395394
BC023829	0.69278491	3.99356688	8.47158515	0.0086743	0.0239574
Bmp3	0.65636026	5.23358809	8.46982884	0.00868014	0.02396471
Mst1	-3.7824452	-1.388693	8.46487102	0.00869666	0.02399811
Bex2	0.51073099	6.92183574	8.46427703	0.00869864	0.02399811
Ccnt2	-0.7202688	4.8957623	8.46267569	0.00870398	0.02400402
Igfn1	-0.9063707	4.3999423	8.4543889	0.00873169	0.0240663
Gpnmb	2.00343781	1.08924575	8.45400264	0.00873298	0.0240663
Hax1	0.54447749	5.40342421	8.45079752	0.00874372	0.02408706
Terf1	-0.6089149	3.52866775	8.44712351	0.00875606	0.02410385
Rnf7	0.51746136	7.98133013	8.4470677	0.00875625	0.02410385
Sbk1	-0.557733	4.16353769	8.44505085	0.00876303	0.02411366
Syt16	-0.4247949	6.11761101	8.44123081	0.00877588	0.02414018
Ppp1cc	0.33197747	7.62011982	8.44008783	0.00877973	0.02414192
Sdhc	0.5506345	6.84374404	8.43374278	0.00880114	0.0241899
Snrpg	0.59458511	5.054598	8.43241745	0.00880562	0.0241899
Gm21284	-1.66788	0.13904433	8.43205379	0.00880685	0.0241899
Htr2b	-1.4632951	0.19008035	8.42754445	0.00882212	0.02422295
Csf3r	-1.3835186	1.43917202	8.42235067	0.00883973	0.02426244
Chchd2	0.42947105	7.6487621	8.41993736	0.00884793	0.02427105
Ptges3	0.44164078	7.90167011	8.41952306	0.00884934	0.02427105
Ift172	-0.8638861	4.63791237	8.41734575	0.00885675	0.02428248
Nav3	-0.7201059	6.74141912	8.41511767	0.00886433	0.0242944
Gm10336	-0.6569167	4.45239221	8.41128126	0.00887741	0.02432136
Fam53b	0.57385323	4.28924641	8.40984319	0.00888232	0.02432592
Eny2	0.38261269	7.31050281	8.40516199	0.00889832	0.02436084
Khsrp	0.39031603	6.76865432	8.4026994	0.00890674	0.02437501
S100a10	0.89058273	5.84680724	8.39969441	0.00891704	0.02439429
Mtap7d3	1.18445215	1.90900847	8.3949832	0.00893321	0.02442962
Angpt2	0.63025591	3.99421813	8.39101607	0.00894685	0.02445486

Kat2a	0.40982828	5.89910999	8.3904039	0.00894896	0.02445486
Dusp9	1.56204215	0.92120224	8.38459196	0.00896899	0.02450067
Herc4	-0.5769994	4.61268462	8.38018168	0.00898423	0.0245194
Fzd3	-0.4034052	7.99007841	8.37941856	0.00898686	0.0245194
Gm9767	2.8830779	-1.0617499	8.37913432	0.00898785	0.0245194
9630033F20I	0.5662767	4.43530924	8.37882353	0.00898892	0.0245194
Pdzrn4	-0.8294796	2.404402	8.37441386	0.00900419	0.02455213
1700080N15	-2.6083783	0.14757437	8.37126392	0.00901512	0.02457299
Ly6g6e	3.42837069	-0.7640857	8.36169568	0.0090484	0.02465474
Thsd7b	-1.2525408	1.27825354	8.35211961	0.00908184	0.02473688
2700060E02I	0.3943913	5.83392768	8.34855613	0.00909432	0.02475651
Smad7	-0.701947	3.43137267	8.34813428	0.0090958	0.02475651
Alas2	-1.5092613	1.07308465	8.34699852	0.00909978	0.02475651
Speer4a	-1.6576391	0.36362606	8.34629499	0.00910225	0.02475651
Rnf34	0.49545047	5.2729918	8.34155702	0.00911889	0.02479001
Aimp2	0.65440338	3.29249552	8.34090631	0.00912118	0.02479001
Ercc5	-0.4356246	5.02331105	8.33849709	0.00912965	0.02479975
Chst11	0.43882543	5.6926055	8.3375295	0.00913306	0.02479975
Crocc	-1.2392866	2.06079015	8.3367961	0.00913564	0.02479975
Fxyd2	2.11478795	-0.1409644	8.33613107	0.00913798	0.02479975
Ccdc97	0.47287841	5.32492824	8.33231509	0.00915144	0.02481946
Tmem121	6.08213079	-1.3857168	8.3321949	0.00915187	0.02481946
Lhfp	0.6349852	6.11296999	8.32907938	0.00916287	0.02484033
Zfp236	-0.5342959	5.532951	8.32811984	0.00916626	0.02484055
9530059O14	-1.7756608	2.91368661	8.31804475	0.00920196	0.02491945
Znrf2	0.58799746	4.47525774	8.31698435	0.00920573	0.02491945
Psmd9	0.60037774	4.39542341	8.31618795	0.00920856	0.02491945
Nhp2l1	0.4201195	6.06380056	8.31615836	0.00920866	0.02491945
Dnajc5	0.3051127	7.92614819	8.3146721	0.00921395	0.02492475
Rpap3	0.41215458	4.49697845	8.31287268	0.00922035	0.02492794
Scara3	0.94328724	6.56283487	8.31247314	0.00922177	0.02492794
Mgst3	0.65924984	3.55588685	8.30710993	0.00924089	0.02497061
Col22a1	-2.6504897	-0.3577219	8.30512552	0.00924797	0.02497589
Sclt1	-0.5468105	4.66918407	8.30469636	0.0092495	0.02497589
Tstd1	-2.9863966	-1.3567026	8.29795162	0.00927363	0.02503203
Rsrc1	0.375958	5.32700455	8.29289102	0.00929177	0.02507199
Cyp19a1	-6.4742139	-1.5822901	8.60692442	0.00931295	0.02512008
Copg2	-0.5796325	5.85953571	8.27701044	0.00934898	0.02520686
Top2b	-0.3739484	8.14488493	8.27544839	0.00935463	0.02520686
Tmem60	0.46988962	5.36769151	8.27529019	0.0093552	0.02520686
Cspg5	-0.5034184	5.25798228	8.27295023	0.00936367	0.02522062
Ddx39b	0.39205066	5.43056837	8.27078281	0.00937152	0.02523271
Mia3	-0.4678903	6.29115697	8.26391916	0.00939643	0.0252856
Pip5kl1	-2.5356087	0.28413704	8.26351366	0.00939791	0.0252856
Tulp2	-1.6976686	0.43315833	8.26106825	0.0094068	0.02530046

Tmem159	0.74879794	4.62406698	8.25943196	0.00941276	0.02530741
1700071M16	-2.4601397	0.65191251	8.25458007	0.00943045	0.02534396
Vhl	0.42921343	4.8722494	8.25385039	0.00943311	0.02534396
Ankrd13b	-0.7258443	3.86267914	8.25137811	0.00944214	0.02534869
Gmnn	0.86889558	2.06183801	8.25023458	0.00944632	0.02534869
Il1rn	3.87249085	-1.1083452	8.25019561	0.00944646	0.02534869
Ccdc71	0.63683717	4.2507704	8.24966921	0.00944839	0.02534869
Igfbp6	0.90536789	4.92640641	8.24763994	0.00945581	0.02535954
Sdk2	-0.764512	3.60874328	8.24649632	0.00946	0.02536171
Ppa1	0.4302051	5.77835253	8.24544849	0.00946384	0.02536293
Ak5	0.47424616	6.20518148	8.24400722	0.00946912	0.02536803
Fam228a	-1.6894615	1.31402514	8.24248699	0.0094747	0.0253739
Zfp451	-0.5511135	5.17462745	8.23830588	0.00949005	0.02539587
Sned1	0.80040049	4.23200509	8.23758042	0.00949272	0.02539587
Phf14	-0.3359257	6.44424764	8.23748851	0.00949306	0.02539587
Spen	-0.4974849	6.87515701	8.23376062	0.00950677	0.0254235
Hells	-1.2211288	1.48357957	8.23167849	0.00951445	0.02543494
Dcaf5	-0.3659818	7.07539042	8.23043399	0.00951903	0.02543814
Cmc1	0.65297179	3.90903451	8.22353588	0.00954451	0.02548829
Cnot11	0.44333371	4.38164142	8.22297518	0.00954659	0.02548829
Naip6	-3.6720815	-2.0934829	8.2214986	0.00955205	0.02548829
Adamtsl1	-0.6829234	3.07913631	8.22089938	0.00955427	0.02548829
Scn4b	-0.6126602	4.78443793	8.22055291	0.00955555	0.02548829
Cyp4f15	-1.2754525	1.15049786	8.21984244	0.00955819	0.02548829
Trim12a	0.7435805	3.76202107	8.21852397	0.00956307	0.02549225
Atp2b1	-0.5247165	9.27705514	8.21552769	0.00957419	0.02551282
Tnks2	-0.3368046	8.33239635	8.21249142	0.00958546	0.02552552
Timm10b	0.82042699	2.6479208	8.21173697	0.00958827	0.02552552
Serp1	0.73143423	7.92018947	8.2114962	0.00958916	0.02552552
Dnajb4	0.34413662	8.41229707	8.20857253	0.00960004	0.02554305
Heatr1	-0.7309922	4.11923889	8.20789668	0.00960256	0.02554305
Terf2ip	0.39191659	6.07454767	8.20607027	0.00960936	0.02554882
Arl6ip1	0.31997615	7.00630343	8.20548607	0.00961154	0.02554882
Nrg3os	-1.9877881	0.39788992	8.20030966	0.00963086	0.02559111
Jazf1	0.5064802	5.2823902	8.19628872	0.0096459	0.02562199
Tox4	0.35728056	5.999696	8.19418867	0.00965376	0.02562637
Gm12338	0.62458736	4.54261356	8.19377971	0.00965529	0.02562637
Gda	-0.5789727	7.16852614	8.19311225	0.00965779	0.02562637
Zfp692	-1.2091826	2.60333688	8.18977115	0.00967032	0.02565054
Sh3bp1	0.69913907	3.23093993	8.18818847	0.00967627	0.0256543
AF357426	-2.2627301	-0.8974646	8.18757221	0.00967858	0.0256543
Dctn3	0.64930656	5.68725454	8.18029278	0.00970597	0.02571019
Anapc4	-0.4478393	5.48691874	8.18014708	0.00970652	0.02571019
Gm1976	0.54014353	4.49603841	8.17893271	0.0097111	0.02571324
1500012F01I	-0.6001265	3.6609381	8.17716849	0.00971776	0.02572179

Usp48	-0.6498556	5.49429636	8.17521963	0.00972511	0.02573218
Flnb	-0.3724061	6.1926105	8.17064361	0.00974242	0.02576887
B230118H07	0.47169755	5.48858594	8.1606453	0.00978034	0.02586006
Cdip1	0.32811652	6.7745802	8.15965631	0.0097841	0.02586088
Gucy2e	-1.1862892	1.54458021	8.15420271	0.00980486	0.02590664
Plxnb1	-0.6031818	4.42722131	8.15177811	0.00981411	0.02592194
Uqcr11	0.83163417	4.69448431	8.14906397	0.00982447	0.02594017
Tnfrsf12a	2.18165056	-0.8392961	8.13935061	0.00986166	0.0260292
Igsf3	-0.4640926	4.5003432	8.13225674	0.00988891	0.02609196
1700001J11F	-2.8776807	-1.5119809	8.1300161	0.00989754	0.02610353
Gm19897	-2.3668757	-0.0053827	8.12843462	0.00990363	0.02610353
Rragc	0.38191411	5.81121884	8.1284073	0.00990374	0.02610353
Tspan1	-4.3802131	-1.3200596	8.12396502	0.00992088	0.02613109
Fbxo28	0.33250667	6.1643748	8.12389135	0.00992116	0.02613109
Cog7	-0.6453353	4.52669569	8.12174302	0.00992947	0.02614378
Nipal4	-1.2322195	0.8634244	8.1173736	0.00994637	0.02617911
Ophn1	-0.5263418	4.9296589	8.11049209	0.00997307	0.02623245
Rab35	0.42623248	4.52165069	8.11034636	0.00997363	0.02623245
Akr1e1	0.41996954	5.1974539	8.10310744	0.0100018	0.02629731
Fam160a2	-0.4652166	6.65594544	8.10087934	0.01001049	0.02631093
Spag9	-0.3474821	8.37034525	8.09766286	0.01002304	0.02633471
Chp2	-1.2380308	0.84874003	8.09253465	0.0100431	0.02637816
Slc38a1	-0.35948	7.61637541	8.09068107	0.01005036	0.02638799
Rnf207	-1.9896841	0.9727215	8.08358949	0.01007819	0.02645181
Rptor	-0.4615131	5.34640575	8.0824435	0.0100827	0.02645438
Eif5a	0.69022745	7.19992216	8.08010995	0.01009188	0.02646921
Rtfdc1	0.54378463	5.58179619	8.07759291	0.01010179	0.02648595
Trappc8	-0.3782336	6.26284445	8.07308411	0.01011958	0.02652331
Med17	-0.4654848	3.9562245	8.06912609	0.01013522	0.02654843
Rufy2	-0.6623733	6.03410088	8.0684004	0.01013809	0.02654843
Naif1	1.64432705	0.03808639	8.06797345	0.01013978	0.02654843
Clip1	-0.4939786	7.59804925	8.0636877	0.01015676	0.0265836
Slc12a6	-0.3470242	6.78989424	8.06158823	0.01016508	0.02658515
Srp54a	0.30686306	7.11641345	8.06156018	0.0101652	0.02658515
Ndufa5	0.54203728	5.61427803	8.06085851	0.01016798	0.02658515
Gm19990	3.63772545	-0.756335	8.05824023	0.01017838	0.02659935
Mtmr4	-0.5380006	5.94243077	8.05761196	0.01018088	0.02659935
Phc3	-0.3781813	8.00497733	8.05594545	0.0101875	0.02659935
Gpi1	0.35987847	7.06928865	8.05592239	0.0101876	0.02659935
AI414108	-0.7762963	4.67017966	8.05235302	0.01020181	0.02662166
Pnldc1	-2.194921	-0.2701485	8.051248	0.01020621	0.02662166
Mme	-0.5481486	4.26111758	8.04949188	0.01021321	0.02662166
Sh3rf1	0.38041568	5.05297757	8.0493461	0.0102138	0.02662166
Ebf1	-0.6737647	4.28809844	8.04920684	0.01021435	0.02662166
Ifi27l2a	0.8783158	2.32156448	8.04843428	0.01021743	0.02662166

Il12rb2	-1.1511391	2.58358439	8.04632922	0.01022584	0.02663431
Mmadhc	0.5338506	5.8456984	8.04479822	0.01023196	0.02664099
Cdkn1b	0.59196519	4.09793414	8.04020765	0.01025033	0.02667507
Pa2g4	0.31171612	6.38809118	8.03975053	0.01025216	0.02667507
Ahrr	-3.5732874	-1.6587342	8.03213253	0.01028273	0.02673878
Gm7173	-2.1450057	-0.6906791	8.03187294	0.01028377	0.02673878
Gm6588	-3.3176468	-0.4709742	8.03038801	0.01028974	0.02674504
Dpcr1	3.12628159	-1.0147611	8.01811981	0.01033923	0.02686435
2900026A02I	-0.4490075	5.8920017	8.01523557	0.0103509	0.02688536
Wdr35	-0.5769226	4.69647187	8.00696009	0.01038447	0.02696323
Phf6	0.36166065	5.27930574	8.00577161	0.0103893	0.02696644
Plch2	-0.928418	3.25218423	8.00346668	0.01039868	0.02698144
Ttc23	0.7802444	2.80822105	7.99988449	0.01041327	0.02700996
Frem2	-1.0112494	1.81353757	7.99578226	0.01043001	0.02704402
Sae1	0.41617143	5.57085668	7.9932629	0.01044031	0.02705506
Myrf	-0.6714529	3.72404626	7.99297585	0.01044148	0.02705506
Mrpl55	0.70304342	3.61984209	7.98533058	0.01047279	0.02712041
Ubb	0.7653208	6.41461667	7.98409805	0.01047785	0.02712041
Clcn1	-1.9336157	0.05914403	7.98339141	0.01048075	0.02712041
Mta1	-0.4461261	4.54349994	7.98329146	0.01048116	0.02712041
Ahcyl1	0.35916443	9.39081939	7.98031142	0.01049341	0.02714273
Chchd4	0.56951925	4.78100268	7.97496049	0.01051544	0.02719033
Gpatch8	-0.3352746	8.04888298	7.97262493	0.01052507	0.02719825
Urod	0.40804487	5.0701814	7.97167472	0.01052899	0.02719825
Arhgdib	0.83611175	7.44678784	7.97158087	0.01052938	0.02719825
Smarca1	-0.4521976	5.63378935	7.96700793	0.01054827	0.02723767
Ttbk2	-0.423227	7.80790211	7.96577772	0.01055336	0.02724144
Abcc10	-1.2001826	0.96648343	7.96296376	0.01056501	0.02726203
Mdh1	0.31455164	8.81871868	7.96111387	0.01057268	0.02726203
Me3	-0.6746185	4.21230339	7.9609905	0.01057319	0.02726203
5031425F14I	4.24915193	-2.2204453	7.96034353	0.01057587	0.02726203
A330049N07	-1.7939457	-0.2488455	7.95310139	0.01060596	0.02732784
Uhrf2	-0.4218293	5.29468683	7.95244488	0.01060869	0.02732784
Tapt1	-0.3860829	5.13774678	7.9505178	0.01061671	0.02733913
2610005L07I	-0.4991905	6.35267353	7.94886828	0.01062359	0.02734744
Trip4	0.37403047	5.85807798	7.94553128	0.01063751	0.02737388
Ranbp1	0.37072278	5.21532742	7.94026386	0.01065953	0.02742113
Usp39	0.54581939	4.1659872	7.93870137	0.01066607	0.02742855
Ap3b1	-0.3686298	6.59633929	7.92675554	0.01071622	0.02754808
Amica1	1.23379655	1.3615346	7.92283934	0.01073272	0.0275768
Rnf185	0.41626582	5.79909455	7.92235855	0.01073475	0.0275768
Abca7	-1.1449701	1.81543376	7.91693281	0.01075766	0.02762619
Cs	0.29227236	7.85166602	7.9122375	0.01077753	0.02766775
St6galnac2	-1.1027658	1.27839149	7.90435923	0.01081096	0.02774408
Ankib1	-0.3201918	6.52754493	7.90222789	0.01082002	0.02775524

Rpp21	0.78848085	3.15334798	7.90159704	0.01082271	0.02775524
Rn4.5s	-1.3118629	0.52362576	7.90068298	0.0108266	0.02775573
Plekhg4	1.92416365	1.36026031	7.8968769	0.01084282	0.02777602
Slc25a12	0.34289199	7.49870517	7.89643801	0.01084469	0.02777602
Slc25a4	0.34171154	9.98946024	7.89549267	0.01084872	0.02777602
Prph	3.8924838	-0.8824467	7.89535166	0.01084932	0.02777602
Pef1	0.48999351	4.475448	7.89160185	0.01086534	0.02780754
Ubqln2	0.41529705	7.64015162	7.88395861	0.01089807	0.02787754
P4ha1	-0.4819251	3.95493008	7.88282345	0.01090294	0.02787754
Zfp157	-0.4649459	4.72711089	7.88206875	0.01090618	0.02787754
Parp2	0.40647168	5.19107671	7.88144773	0.01090885	0.02787754
Atp6v0e2	0.4479429	5.29601481	7.88037898	0.01091344	0.02787754
Tmem198b	1.39354376	0.79725695	7.88001645	0.01091499	0.02787754
Gpld1	-0.4477239	4.90241886	7.86919717	0.01096159	0.02796983
Slain1os	-1.8352172	1.00212883	7.86827338	0.01096558	0.02796983
Rsad1	0.67470317	3.20766424	7.86826511	0.01096562	0.02796983
Pla2g16	0.62461114	6.73766589	7.86816659	0.01096604	0.02796983
Vdac3	0.37949433	6.33344942	7.86549961	0.01097757	0.02798971
Cabyr	-0.7452856	4.12377264	7.86347294	0.01098633	0.02800254
Rmnd5b	0.38715386	4.74085418	7.86220265	0.01099183	0.02800704
Chmp2b	0.5375303	6.34152136	7.85891996	0.01100606	0.02803377
Cggbp1	0.40808406	7.33132985	7.85758219	0.01101186	0.02803903
Rcbtb1	-0.4656476	6.01330375	7.8566873	0.01101575	0.0280394
Rpl10a	0.73081276	8.07832466	7.85245528	0.01103413	0.02806433
Fkbp4	0.60229289	5.92966816	7.85212002	0.01103559	0.02806433
1700009P17I	1.12025095	1.70603759	7.85185097	0.01103676	0.02806433
Ndrg2	0.47507414	8.89316175	7.84766949	0.01105497	0.0280928
Cd320	-0.7716204	2.81476072	7.84651353	0.01106001	0.0280928
Usp40	-0.5555629	4.56375978	7.84602643	0.01106213	0.0280928
Eif2b2	0.66778181	4.10672235	7.84419891	0.01107011	0.0280928
Atp6ap1	0.46573293	7.63568664	7.84348617	0.01107322	0.0280928
Ndnf	-0.4585759	5.19802815	7.84298562	0.0110754	0.0280928
Gm5069	0.47188981	4.42178415	7.8427167	0.01107658	0.0280928
Med8	0.4303864	3.81984149	7.84240972	0.01107792	0.0280928
Cnga4	1.93524473	0.3023142	7.83754043	0.01109921	0.02812803
Trim66	-0.6430767	5.4375356	7.83751748	0.01109931	0.02812803
Sema4f	-0.6567776	4.77742819	7.83324007	0.01111806	0.02815872
C130060C02I	-1.8536982	0.02592742	7.83304102	0.01111893	0.02815872
Sez6l	-0.4216123	6.13618277	7.83094191	0.01112814	0.02817254
Crip1	1.1438597	2.42763612	7.82718141	0.01114467	0.02820485
Gm13889	0.93893061	1.54006208	7.82528429	0.01115301	0.02821525
Kdelr1	0.58305026	4.34912127	7.82453795	0.0111563	0.02821525
Ddx43	-3.6646938	-2.0649255	7.81467742	0.01119981	0.0283149
Magi3	-0.3691165	6.70809646	7.81389937	0.01120325	0.0283149
Psma3	0.37333287	6.99917801	7.81161719	0.01121335	0.02833088

Ndufb7	0.77289203	4.23432189	7.80826369	0.01122821	0.02835466
Osbpl6	-0.3843871	7.06424395	7.80778742	0.01123032	0.02835466
Scaper	-0.4988806	6.38242996	7.80635713	0.01123667	0.02836114
Sp100	0.63885752	5.07670591	7.80540222	0.01124091	0.0283623
Fam208a	-0.4876313	6.76686734	7.80300255	0.01125157	0.02837965
Dnajc11	0.5560356	3.98636206	7.79304888	0.01129591	0.0284679
Ap5s1	0.94289582	2.35698614	7.79242004	0.01129872	0.0284679
Rapgef3	-0.6671726	2.66865458	7.79192025	0.01130095	0.0284679
Snx19	-0.4683565	4.75984491	7.79071783	0.01130632	0.0284679
Rbm5	-0.426719	6.94168735	7.79042289	0.01130764	0.0284679
Zkscan6	-0.7683446	2.9109064	7.789974	0.01130965	0.0284679
Cnn1	3.39104517	-0.3466643	7.78919625	0.01131312	0.0284679
Camkmt	0.47356302	4.00481931	7.78732061	0.01132152	0.02847947
2010107G23	0.5308759	4.05042054	7.7861614	0.0113267	0.02848297
Tpgs2	0.33471252	6.19132369	7.78242407	0.01134345	0.02851529
Stoml2	0.56586955	4.28592508	7.78077246	0.01135087	0.02851529
Slc39a6	0.33001802	6.58109972	7.78075151	0.01135096	0.02851529
Gpr87	4.45042918	-1.7229467	7.77307517	0.01138548	0.02859242
Hspa2	-0.5395869	4.93156623	7.77211738	0.01138979	0.02859368
1700006F04I	-3.5143802	-0.7678995	7.76967018	0.01140082	0.0286118
Vcan	-0.8602567	4.12263198	7.76739317	0.0114111	0.02862153
Stk32b	0.81555955	2.80468229	7.76687879	0.01141342	0.02862153
Stab1	-1.475364	1.19212054	7.76627578	0.01141615	0.02862153
Cdk18	0.65220033	4.00573225	7.76535477	0.01142031	0.02862194
Bai1	-0.5172806	6.13424862	7.76386521	0.01142704	0.02862194
Hist1h2bn	0.97999959	3.01491365	7.76370751	0.01142776	0.02862194
Maged2	0.4612279	4.4644189	7.76190908	0.01143589	0.02863276
Prrx1	0.58672259	7.06862892	7.75949766	0.01144681	0.02863693
Zbtb6	-0.3837458	5.09024405	7.75838005	0.01145188	0.02863693
Cnnm2	-0.6381046	3.86824534	7.75833181	0.0114521	0.02863693
Nup88	-0.4232313	5.69244822	7.75817074	0.01145283	0.02863693
Pou3f3	-0.4449792	5.33868191	7.75406257	0.01147147	0.02867398
Trp53inp2	0.29429955	7.84324072	7.7528266	0.01147709	0.02867846
Zcrb1	0.3661935	6.95352091	7.74950536	0.01149219	0.02870155
Ppif	0.59278898	4.94539068	7.74871031	0.01149581	0.02870155
Nt5c	0.65754699	3.6139377	7.74827232	0.0114978	0.02870155
Nop16	0.92096951	2.38297078	7.74676489	0.01150467	0.02870914
Agap1	-0.4251526	6.71017495	7.74463238	0.01151439	0.02872384
Gpr162	-1.17032	2.14572844	7.74333032	0.01152033	0.02872911
Hist1h2bl	0.85301658	3.11037497	7.73906812	0.0115398	0.0287681
Slc1a2	-0.524301	10.9655463	7.7357216	0.01155512	0.0287967
Gria4	-0.4730828	6.76190526	7.729376	0.01158421	0.02885499
Tbc1d10b	0.39932867	4.60840359	7.7289435	0.0115862	0.02885499
Dgke	-0.5038757	5.5125218	7.72789928	0.011591	0.02885736
Zfp787	1.2028786	1.59554729	7.72686298	0.01159576	0.02885964

Snrpa	0.61418079	4.66726131	7.72584742	0.01160043	0.02886169
Gm17660	-3.8110255	-1.4513759	7.7245507	0.0116064	0.02886695
Coa6	0.50419875	3.73919613	7.71965278	0.01162896	0.02891349
Ccdc91	0.45313694	5.37046407	7.71622554	0.01164478	0.02894322
Wdr11	-0.5447239	5.39380015	7.71501497	0.01165037	0.02894753
Pcgf3	0.37517229	5.67129604	7.71372889	0.01165631	0.02895271
Ap3d1	-0.5203275	6.40065025	7.71130749	0.01166752	0.02897095
Map2	-0.5966436	8.3176101	7.7087295	0.01167946	0.0289893
Rbm43	0.50482506	4.53418318	7.7080434	0.01168264	0.0289893
Poll	1.20364864	0.87699302	7.70691579	0.01168787	0.02899268
Zkscan2	-0.4528584	4.76898938	7.70420425	0.01170045	0.0290143
Dcn	0.83966495	7.6761973	7.70190164	0.01171115	0.02903123
Cd34	-0.6551222	3.49869475	7.69592986	0.01173894	0.02909052
Txnl4b	0.71208572	3.25776878	7.69211284	0.01175675	0.02912502
Dpy30	0.63376449	4.81348672	7.69106699	0.01176163	0.0291275
Hibadh	0.5495126	5.59095261	7.68666747	0.0117822	0.02916881
Reep5	0.35412275	8.46075093	7.68400958	0.01179464	0.02918999
5830454E08I	1.93913308	0.68021135	7.68128639	0.01180741	0.02921194
Snord64	-1.6108749	0.11126049	7.67742605	0.01182554	0.02924439
Ccdc85c	-1.0731825	0.96337472	7.67683264	0.01182832	0.02924439
Carm1	0.42890763	5.24535522	7.67466073	0.01183854	0.02926
Hist1h1a	3.26431923	-1.2556486	7.67381533	0.01184252	0.02926019
B230206H07	-3.5696598	-1.6970281	7.67139555	0.01185391	0.0292787
Zic1	0.653656	8.07189603	7.66859452	0.01186712	0.02930167
Ppp4r4	-0.5996519	4.90833308	7.66673622	0.01187589	0.02930864
0610009B22I	0.65124194	5.56179021	7.66634118	0.01187775	0.02930864
Nop58	-0.4125432	6.14567815	7.66194458	0.01189853	0.02935026
Psg23	-1.5238377	1.27596816	7.66065336	0.01190465	0.02935568
Lhfpl3	-0.5808453	3.85531894	7.65624573	0.01192553	0.02939752
Tro	-0.6426502	6.41530895	7.65317587	0.0119401	0.02942378
4933431E20I	0.36123868	6.76028562	7.65171676	0.01194704	0.02943119
Nup133	-0.4180967	4.51006149	7.65073421	0.01195171	0.02943303
Dlgap5	-1.2833371	0.66188119	7.64482419	0.01197985	0.02949265
Gm5544	-4.6594124	-0.6670952	7.64303041	0.0119884	0.02950403
Wdr74	0.78486652	2.90109075	7.63937472	0.01200586	0.02952837
Il18	-0.4445257	4.1713392	7.63931086	0.01200617	0.02952837
Vps16	-0.7586272	4.44293353	7.63463682	0.01202853	0.02957367
Gtf2h3	-0.6001709	3.5832589	7.63279332	0.01203736	0.02958569
Plxnb2	-0.4431126	4.78627518	7.62671226	0.01206655	0.02964771
1110001J03F	0.85221756	2.66001857	7.62374897	0.0120808	0.02967301
Mir376a	-3.0751838	0.06383784	7.62288757	0.01208495	0.02967348
Hip1	-0.5431235	4.55084302	7.62032606	0.01209728	0.02969405
Gstt3	0.62980704	5.20221588	7.61745017	0.01211115	0.02971641
Pogz	-0.3673184	7.70921291	7.61628179	0.01211679	0.02971641
Zfp830	0.48905537	5.20672535	7.6159752	0.01211828	0.02971641

Taf8	0.64022321	2.78515592	7.61423825	0.01212667	0.02972726
Tmem150co:	-3.4404283	-0.5281174	7.61150864	0.01213987	0.0297499
Nyap1	-0.6984375	2.96489102	7.60721959	0.01216064	0.02979108
Stk10	-0.5921719	3.50792912	7.60544598	0.01216924	0.02980242
Tmem136	-0.5994446	3.41993937	7.59835991	0.01220368	0.029877
Aqp7	-1.7604687	0.05441588	7.59591361	0.01221559	0.02989252
Gcsh	0.50809418	5.68617672	7.59542203	0.01221799	0.02989252
08-Mar	-0.3529536	6.24265239	7.59003588	0.01224427	0.0299332
Sra1	0.47930567	5.14917602	7.58985627	0.01224515	0.0299332
Fam206a	0.46110495	5.91732222	7.58869303	0.01225083	0.0299332
Slc30a2	-0.785569	2.67361515	7.58829526	0.01225278	0.0299332
Psmb11	-1.2713772	1.33147065	7.58792905	0.01225457	0.0299332
Onecut2	-0.6583165	3.59482213	7.58146394	0.01228623	0.03000076
Slc25a11	0.41103729	5.65167589	7.57676851	0.01230928	0.03004089
Ddb2	-1.107339	1.05610744	7.57635399	0.01231131	0.03004089
Psmc2	0.30601365	6.27084943	7.57567012	0.01231468	0.03004089
Chrnb3	-1.3336288	1.28994325	7.57276389	0.01232898	0.030066
Prdm9	-0.9556147	1.62547008	7.56739569	0.01235544	0.03012073
Zfp414	0.57916245	4.50877681	7.56488613	0.01236783	0.03014115
Vps13a	-0.5290308	7.32010642	7.56365455	0.01237391	0.03014618
Otub2	0.58232825	3.60956246	7.55981225	0.01239292	0.03018269
Pcsk5	-0.6260547	4.26563116	7.55834873	0.01240017	0.03019055
Six4	-0.8040018	3.45258233	7.55247696	0.01242931	0.03025093
Pvalb	0.80392938	2.39315365	7.55172567	0.01243304	0.03025093
1700086O06	1.953941	-0.332981	7.55022108	0.01244052	0.03025802
Pla2g4e	-0.5135618	4.55148466	7.54951714	0.01244402	0.03025802
C030018K13I	-1.342936	1.52609964	7.54712871	0.01245591	0.03027711
Vmn2r84	-1.9851947	0.21344119	7.54607164	0.01246117	0.0302801
Lgals9	0.67685596	4.40799855	7.541318	0.01248488	0.03032788
Snapc4	-0.9323847	3.72206953	7.54048362	0.01248905	0.03032818
Prdx2	0.56797314	6.20444139	7.53843117	0.0124993	0.03034327
Zfp65	-0.554959	5.05369381	7.53708983	0.01250601	0.03034973
Helb	-0.6233803	3.42155083	7.53461217	0.01251841	0.03037
4930556M19	-1.2304237	1.12915762	7.53218014	0.0125306	0.03038973
Rab9	0.49067564	5.70015538	7.52557664	0.01256375	0.03046029
Rhoa	0.66210923	8.61857226	7.52332211	0.0125751	0.0304724
Pfdn1	0.72035171	3.51727609	7.52296909	0.01257687	0.0304724
Abhd12	0.38204306	6.33405157	7.51964403	0.01259362	0.03050313
Sqle	0.4078609	5.75304706	7.51465671	0.0126188	0.03055424
Cyp4a12b	-1.1943901	3.11657346	7.5131998	0.01262616	0.0305622
Cdc5l	0.27972029	6.18232915	7.51109107	0.01263683	0.03057815
Trappc12	0.3865662	5.73215981	7.50764103	0.0126543	0.03060281
Grk6	-0.6728844	2.89637392	7.50746845	0.01265517	0.03060281
Rnf111	0.2867824	7.07452193	7.50620849	0.01266156	0.03060839
Ccdc25	0.34575856	5.86118626	7.50299041	0.0126779	0.030638

Arx	-0.8254819	2.59868068	7.5004144	0.01269099	0.03065976
Zbtb16	-0.3699396	4.67160739	7.49326216	0.01272742	0.03073787
Bicc1	0.60856678	6.88745803	7.49183428	0.0127347	0.03074557
Gm6402	-0.7899891	1.77517024	7.48936019	0.01274734	0.03076617
Trnau1ap	0.49779266	3.70772269	7.48646082	0.01276217	0.03079052
Dnajc9	0.43292759	5.91835945	7.4857827	0.01276564	0.03079052
Zfp653	0.95464214	2.40126424	7.48463097	0.01277153	0.03079484
Lmln	-0.7945905	2.3864814	7.48171295	0.01278649	0.03082099
Slco5a1	-0.9429144	2.19075446	7.47638769	0.01281383	0.03087697
Rp2h	0.53208637	4.92032944	7.47332712	0.01282957	0.03090498
Prcc	0.57790606	4.29593695	7.46804813	0.01285677	0.03096057
Snap91	0.50514488	8.93722681	7.46466152	0.01287426	0.03099273
Marcksl1	0.5196069	4.29698089	7.45720835	0.01291284	0.03107562
Aasdh	-1.0060587	2.09204686	7.45522282	0.01292314	0.03109024
Slc7a4	0.73771635	2.98030875	7.4544399	0.0129272	0.03109024
Ascc3	-0.6498273	5.75611155	7.45026687	0.01294888	0.03112286
Akip1	0.84360179	2.97505457	7.45023275	0.01294906	0.03112286
Pea15a	0.37819317	7.66623059	7.44394788	0.01298179	0.03119154
D730045A05	-2.8504528	-0.3651192	7.44261199	0.01298876	0.03119829
4933406F09I	-5.4873602	-1.1412617	7.44144448	0.01299486	0.03120294
Pwp1	-0.5981768	4.19205255	7.44014126	0.01300166	0.03120929
Ppp1r10	0.34706441	5.87724125	7.43525528	0.01302722	0.03125291
Pdlim4	0.79360146	2.4719041	7.43507423	0.01302817	0.03125291
Sar1b	0.3884799	5.62421325	7.43266271	0.0130408	0.03127321
Eif4a3	0.43301429	5.00288545	7.43119	0.01304853	0.03128173
Tax1bp1	0.30383933	8.62016464	7.42943224	0.01305775	0.03129385
Rab3a	0.30804711	6.72958893	7.42404982	0.01308604	0.03135163
Gpank1	1.09014472	2.00662254	7.42043868	0.01310506	0.03138199
Ddhd2	-0.3502223	6.00364545	7.42005566	0.01310708	0.03138199
Tle4	-0.4526258	6.40043195	7.41839669	0.01311583	0.03139291
Dpy19l4	-0.4786489	4.18310181	7.41320153	0.01314327	0.03144856
Tax1bp3	0.82259986	4.64836874	7.40911981	0.01316487	0.03146529
Rbm17	0.3718866	6.6248111	7.40879528	0.01316659	0.03146529
6430503K07I	-2.7340469	-0.7179992	7.40878075	0.01316667	0.03146529
Xrn1	-0.4406591	6.04494522	7.40871085	0.01316704	0.03146529
Mirlet7bhg	-2.4152757	0.81513679	7.4073869	0.01317406	0.03147204
Csnk1g1	-0.336121	6.24794993	7.40391462	0.01319248	0.03149739
Rbm34	0.36581902	5.32933223	7.40380422	0.01319307	0.03149739
Gen1	-1.6205345	0.53316377	7.40177665	0.01320384	0.03150479
Scfd2	-0.7007208	2.97274159	7.40164028	0.01320457	0.03150479
Zbtb9	-0.8923695	2.9040012	7.39606985	0.01323421	0.03156549
Strbp	-0.4599687	9.34693261	7.39384197	0.01324609	0.03158378
Pml	0.4141402	4.59802592	7.38986457	0.01326733	0.03162436
Nrsn2	0.49408413	5.88585076	7.38832021	0.01327559	0.03162916
Ralb	0.52882194	4.37147083	7.38791082	0.01327778	0.03162916

Necab2	0.58909144	3.43692255	7.3868676	0.01328336	0.03163241
Grin2a	-0.5252747	6.61728008	7.38558056	0.01329025	0.03163877
Aen	0.55194415	4.24432563	7.38407811	0.01329829	0.03164788
Arhgef3	-0.494826	5.69113974	7.38156769	0.01331175	0.03165767
Insl6	1.34529739	1.1793507	7.38094578	0.01331509	0.03165767
Sssca1	0.77384827	2.92878654	7.38046841	0.01331765	0.03165767
Rpp14	0.43285045	5.40968028	7.38016323	0.01331929	0.03165767
Usp28	-0.7390758	3.5545743	7.37907078	0.01332515	0.03166158
Ttr	-1.1477992	5.00354118	7.37553008	0.01334418	0.03169676
Gfra4	0.48791395	4.35583022	7.37360092	0.01335456	0.03171137
Dhx9	-0.500305	7.76847638	7.37247681	0.01336062	0.03171571
Gjb2	0.63530663	9.09863828	7.37099834	0.01336858	0.03172458
Cops8	0.34130227	5.82412212	7.3691685	0.01337845	0.03173795
Scn8a	-0.6975272	8.83649402	7.3667971	0.01339125	0.03175827
Dach1	-0.9434998	2.04567433	7.35885669	0.0134342	0.03184005
B230217C12I	-0.3210545	5.85137644	7.35885338	0.01343422	0.03184005
Allc	-4.330771	-1.1534135	7.35317381	0.01346504	0.03189049
Msantd1	-2.8401288	-0.632476	7.353012	0.01346592	0.03189049
Map4k3	-0.3752045	6.59915635	7.3525819	0.01346826	0.03189049
Vmn2r29	-0.6553944	3.38591024	7.35157553	0.01347373	0.03189337
Ralgapb	-0.4354088	7.62537635	7.35061993	0.01347892	0.03189561
Oprk1	-0.8974842	3.81311892	7.34562078	0.01350615	0.03194995
Rapgef6	-0.3098864	6.7995315	7.34102216	0.01353125	0.03199351
1700017B05I	0.69808543	2.86725022	7.3406843	0.01353309	0.03199351
Fam101a	1.18593434	2.83924127	7.33935701	0.01354035	0.03200057
Slc30a7	-0.4823855	4.66352327	7.33551264	0.01356138	0.0320402
1700019G17	1.28947823	1.04324127	7.33409169	0.01356917	0.03204227
Vps11	-0.4642207	4.10357047	7.33379325	0.0135708	0.03204227
Pcolce	0.8166491	4.76115866	7.33126965	0.01358464	0.03206485
Mrps14	0.47836308	5.65995833	7.32746063	0.01360556	0.03210413
Zgrf1	-0.9210367	2.5509492	7.32657707	0.01361042	0.03210549
Emilin1	0.88713677	2.02283567	7.32428552	0.01362303	0.03212055
Scand1	2.61743268	-1.4286428	7.3238604	0.01362537	0.03212055
Mcf2l	-0.8290946	4.68359106	7.31358349	0.01368208	0.03224411
Ncoa3	-0.3885642	6.64765796	7.31127564	0.01369485	0.03225961
Sparc	0.79896174	8.82825556	7.30968376	0.01370367	0.03225961
2410007B07I	3.58051785	-1.9733839	7.30965714	0.01370382	0.03225961
Rab36	-0.435073	3.97702559	7.30789874	0.01371357	0.03225961
Ctsa	0.51954343	5.4161235	7.30776463	0.01371431	0.03225961
Tpd52	0.36563751	5.82796118	7.30773786	0.01371446	0.03225961
Fbxo8	0.40338607	4.84429171	7.30515252	0.0137288	0.03226443
Pkhd1l1	-3.2645154	-0.6430587	7.30487154	0.01373036	0.03226443
Scly	0.7593692	3.52715264	7.30383293	0.01373613	0.03226443
Dctpp1	0.8297915	1.63545479	7.30369027	0.01373693	0.03226443
Pigs	0.50199421	4.61576132	7.3034778	0.01373811	0.03226443

Lrp12	-0.4269305	4.10002591	7.30272081	0.01374231	0.03226443
2310007B03I	6.10200504	-1.8865621	7.9427806	0.01374821	0.03226817
Hlf	0.38443353	10.0220648	7.30049373	0.0137547	0.0322733
Cd59b	1.86096883	-0.4724651	7.29942685	0.01376064	0.0322743
Plekha2	0.5350969	5.21607089	7.2988715	0.01376373	0.0322743
Snrpe	0.51849182	4.52125606	7.29365179	0.01379283	0.03233243
Rnf219	0.35275512	5.20945198	7.28908536	0.01381834	0.03238212
Snap47	0.31079314	7.28616584	7.28752331	0.01382708	0.03239248
Atox1	0.75768163	4.36596324	7.28549521	0.01383844	0.03240811
Glmn	-0.7517004	3.94395078	7.28478883	0.0138424	0.03240811
Slc35b3	-0.7824287	2.18496086	7.28360815	0.01384902	0.03241349
Mgrn1	0.32502356	6.53038613	7.28223006	0.01385674	0.03242147
Kcnh5	-0.5666992	5.78019818	7.28130752	0.01386192	0.03242347
Kcnip2	-0.3456724	5.69882774	7.28014496	0.01386845	0.03242862
Pcmt1	0.30821262	6.42797914	7.27788003	0.01388117	0.03244132
Smarcb1	0.46188152	4.78304177	7.27763933	0.01388253	0.03244132
A230001M10	-1.5968435	0.24697136	7.27375335	0.01390439	0.0324823
1110008P14I	0.4128036	4.56807897	7.27130385	0.0139182	0.03250442
Hist1h2bg	1.49785533	0.15143316	7.25786498	0.0139942	0.03266489
Ranbp9	0.29371743	7.07801354	7.25748386	0.01399636	0.03266489
Morf4l1	0.37768502	8.47922785	7.25684733	0.01399998	0.03266489
Slc20a1	-0.5140267	5.34830117	7.25261269	0.01402403	0.03271085
Pvrl1	0.44742143	4.31616693	7.25041063	0.01403656	0.03272405
Galnt7	-0.587848	3.06937041	7.25008415	0.01403842	0.03272405
Smug1	0.41535644	4.24447993	7.24708729	0.01405549	0.03274388
Wdr90	-1.8959466	1.35133883	7.24680157	0.01405712	0.03274388
Ermap	-2.3121791	0.46988567	7.24603999	0.01406146	0.03274388
1700047A11I	-3.9495088	-1.7041524	7.2449363	0.01406776	0.03274388
Cryba2	3.34737163	-1.2182359	7.24476244	0.01406875	0.03274388
Kctd13	0.53907528	6.17116268	7.2439733	0.01407325	0.03274421
Mak	-1.229754	2.07930266	7.23626886	0.01411731	0.03283419
2700029M09	0.37037281	5.45364033	7.23568083	0.01412068	0.03283419
Guf1	-0.8497642	3.90420422	7.23109776	0.01414697	0.03287668
Nmrk1	-0.4876705	4.76005865	7.23096692	0.01414772	0.03287668
Sdad1	-0.373305	4.79160737	7.22772997	0.01416633	0.03290074
Fndc8	4.14668385	-2.2316128	7.22726324	0.01416901	0.03290074
Trim15	-2.716271	-1.2295396	7.22687662	0.01417123	0.03290074
Kcnh7	-0.5575395	6.28135969	7.2255413	0.01417892	0.0329084
Pcf11	-0.3616503	6.58616928	7.22206062	0.01419897	0.03294474
4930486F22I	-3.7757255	-1.2626018	7.22094067	0.01420543	0.03294954
Ntan1	0.47363951	5.48359563	7.21926321	0.01421511	0.0329618
Slc8b1	-0.9009478	1.35538975	7.21659306	0.01423054	0.03298433
Pfas	-0.6209099	3.81241102	7.21605911	0.01423362	0.03298433
Cyth2	0.5410814	4.26547245	7.21529338	0.01423805	0.0329844
Phlpp2	-0.5413619	5.35627201	7.21337666	0.01424914	0.0329999

Etv1	0.41429046	7.73736449	7.21080234	0.01426405	0.03302424
Trappc10	-0.3759915	5.8965905	7.20616649	0.01429095	0.03306291
Zfp36	0.79277265	4.95134142	7.20590769	0.01429245	0.03306291
Gtf2h2	0.4057469	4.9605787	7.20559283	0.01429428	0.03306291
Arhgap23	-0.4928061	5.68416057	7.20488597	0.01429838	0.03306291
Tshz2	-0.3296165	6.13773992	7.20177756	0.01431646	0.03309451
Tmem132b	-0.5500157	7.49325511	7.1988221	0.01433367	0.03311808
Piwil2	-3.0065282	-0.4778187	7.19851028	0.01433549	0.03311808
Sap18	0.50744992	6.70105069	7.19753369	0.01434118	0.03312103
Cpt2	0.59081887	3.28725638	7.19599662	0.01435014	0.03313131
Dpf2	0.34745125	5.81680931	7.19515414	0.01435506	0.03313131
Psmd13	0.57048498	3.96068932	7.19449979	0.01435888	0.03313131
Zfp64	0.62236512	2.7789355	7.1913329	0.01437738	0.0331638
Tmem161a	-0.7388503	3.19158888	7.18838862	0.01439461	0.03319333
Tubg1	0.51757727	4.38928208	7.18736993	0.01440058	0.03319688
Ifi27	0.62067186	4.907542	7.18634644	0.01440657	0.0332005
Ccdc159	-1.9612609	0.35808225	7.18058544	0.01444038	0.03326819
Rgcc	0.71403023	2.86685262	7.17858131	0.01445216	0.03328064
Maged1	0.3159007	8.26477941	7.17815669	0.01445466	0.03328064
Cadm3	0.44652333	7.86896536	7.1770647	0.01446108	0.03328521
Mapre1	0.39315813	7.8572816	7.17533468	0.01447127	0.03329844
Podxl	-0.3842608	4.29086336	7.17173723	0.01449247	0.03333701
Slitrk2	-0.5937156	5.37782692	7.1706188	0.01449907	0.03334196
Chrna4	-0.4751646	4.50706817	7.16557617	0.01452887	0.0333721
Dzank1	-0.4534904	8.55865494	7.16526864	0.01453069	0.0333721
Hnrnph2	0.29930925	7.21234144	7.16485559	0.01453313	0.0333721
A230072E10I	-1.7445047	0.01454516	7.16469779	0.01453407	0.0333721
Atf4	0.32501586	6.67721885	7.16463803	0.01453442	0.0333721
Cacna1h	-0.7539864	3.2704708	7.15940914	0.0145654	0.03342595
6820408C15I	2.92321528	-0.5140302	7.15772629	0.01457539	0.03342595
Cxcl5	-1.6360026	0.65863524	7.15769579	0.01457557	0.03342595
Puf60	0.37608624	6.5038711	7.15767498	0.0145757	0.03342595
Zfp715	-0.5060456	4.43571062	7.15691039	0.01458024	0.03342614
Wdhd1	-0.7794287	2.70306363	7.1552842	0.0145899	0.03343807
Pphln1	0.33193412	7.20904503	7.1540596	0.01459718	0.03344454
Fignl2	-1.2605643	1.1062992	7.15215541	0.01460851	0.03346028
Scml2	-1.6925818	-0.0223448	7.14984746	0.01462225	0.03347374
Nipbl	-0.366851	7.96640291	7.14967014	0.01462331	0.03347374
1110058L19I	0.47357313	4.25525354	7.1455371	0.01464796	0.03351391
Etf1	0.3307019	6.47593528	7.14522989	0.0146498	0.03351391
Mapk3	0.50578999	7.23936848	7.13671792	0.01470072	0.0336197
9530027J09I	-3.8780909	-0.9858513	7.13600398	0.014705	0.0336197
Cadm2	-0.4295266	8.70551438	7.13424299	0.01471557	0.0336336
Grid1	-0.7105549	2.92400738	7.12099318	0.01479532	0.03379301
Ltn1	-0.4272708	6.60022805	7.12097023	0.01479546	0.03379301

Polr2f	0.78798581	2.1390759	7.11988198	0.01480203	0.03379301
Snord118	-3.6269519	-1.5551468	7.11930501	0.01480552	0.03379301
Chd7	-0.6346107	4.64904099	7.1189211	0.01480784	0.03379301
Wnt9b	4.05143816	-1.9055539	7.11637854	0.01482321	0.03381781
Gdf9	-2.5464027	-0.9128164	7.11551318	0.01482845	0.03381947
Mfsd11	-0.6082677	3.18360502	7.10969622	0.0148637	0.03388957
Bhlhe22	0.43298213	5.19943466	7.10477821	0.01489358	0.03394738
Fam65b	-0.4988077	5.89319166	7.10389924	0.01489893	0.03394926
Rbbp7	0.34691127	7.41912342	7.10194319	0.01491084	0.03396607
Malsu1	0.44952664	4.73281261	7.10001688	0.01492258	0.03398249
Map7	-0.3903779	5.79155177	7.09925074	0.01492725	0.03398281
Muc6	-1.2365781	1.55308457	7.09579616	0.01494833	0.03402048
Zfp113	-0.5873635	4.27672379	7.09360361	0.01496173	0.03402519
Ranbp3	0.46344089	3.79785599	7.09288834	0.0149661	0.03402519
Dnah6	-1.6462067	0.7087383	7.09275132	0.01496694	0.03402519
Syt13	0.39715487	6.10051852	7.09248924	0.01496854	0.03402519
Wdr53	0.77140346	3.18469403	7.09020788	0.0149825	0.03404212
Pcdhga7	-0.7547125	2.71389991	7.08978899	0.01498507	0.03404212
Arl4a	0.4900169	7.29655897	7.08078216	0.01504034	0.03415735
Sh2d3c	0.42986734	4.98566397	7.07653264	0.0150665	0.0342064
Dact3	-0.3897611	4.11706436	7.07404264	0.01508185	0.03422483
2610507I01R	-0.3901459	4.73203653	7.07373582	0.01508375	0.03422483
Rab21	0.34884638	7.62913437	7.07197741	0.0150946	0.03423403
Ubr3	0.42579199	9.67637616	7.07160071	0.01509693	0.03423403
Tpd52l2	0.55201857	6.30856718	7.06959566	0.01510931	0.03425176
Prr14l	-0.4190554	7.45751808	7.06828158	0.01511744	0.03425983
Fam161a	1.02069346	1.83373933	7.0667418	0.01512697	0.03426897
Usp54	-0.288906	6.65042022	7.06615299	0.01513061	0.03426897
Atg5	0.41266272	4.61398251	7.06541177	0.0151352	0.03426902
Mybpc3	5.20493529	-2.0274697	7.06299875	0.01515015	0.03429253
Cnbp	0.40042976	9.17261251	7.06165515	0.01515849	0.03430104
Reln	-0.6970135	5.25469557	7.05219816	0.01521728	0.03442085
Caprin1	0.26209933	8.54069451	7.0516638	0.01522061	0.03442085
Pnpla3	-1.4079322	1.33916254	7.05031854	0.015229	0.03442944
0610030E20I	-0.398567	5.46102833	7.04842147	0.01524084	0.0344321
Has3	-1.2148428	1.97224757	7.04769947	0.01524534	0.0344321
Mctp1	-0.4118797	5.38954545	7.0474257	0.01524705	0.0344321
Spata21	4.06809282	-1.3681654	7.0468984	0.01525035	0.0344321
Klhl26	-0.5011009	3.63165044	7.04645286	0.01525313	0.0344321
Kif15	-1.2959183	1.06210072	7.04397053	0.01526864	0.03445676
Zfp652os	1.95812228	-0.6744618	7.04179058	0.01528229	0.03446663
Mrps6	0.57583824	4.25592822	7.04158302	0.01528359	0.03446663
Tmem145	-0.9626637	2.44946133	7.04106917	0.0152868	0.03446663
Cnot4	0.41876951	7.41731286	7.03843309	0.01530332	0.03449351
Lin7c	0.31884804	7.8688952	7.03553319	0.01532152	0.03452414

Gamt	1.24897722	1.99468364	7.03418512	0.01532998	0.03453285
4933421O10	-0.7038494	3.5009679	7.03052136	0.01535302	0.03457436
Fbxl14	-0.4375962	5.24253951	7.02947718	0.01535959	0.03457878
E030019B06I	-2.7673972	-0.5614128	7.02624076	0.01537999	0.0346143
Gpr65	-1.5930539	0.52277596	7.02192123	0.01540725	0.03466526
Selenbp1	-0.9155012	3.05446523	7.02044794	0.01541656	0.03467581
Cdc42bpb	-0.4241525	6.53365385	7.01549626	0.0154479	0.03473589
Pag1	-0.3963952	5.64446304	7.01426118	0.01545573	0.03474189
Smchd1	-0.4121408	6.18192511	7.01342541	0.01546103	0.03474189
Sbno1	-0.495787	8.41056419	7.01288401	0.01546446	0.03474189
Gm16907	-1.5122002	1.60754631	7.01160557	0.01547258	0.03474971
Srrt	-0.467934	4.88344828	7.00692214	0.01550234	0.03480613
Pnpo	0.59070721	3.78994723	7.00410814	0.01552025	0.03483592
Igfbp4	0.64728532	5.14860724	7.00166134	0.01553585	0.03486049
Usp11	-0.3974011	7.40496028	6.9953004	0.01557647	0.03493508
Emc2	0.41555147	6.48024617	6.99499845	0.0155784	0.03493508
Lin7b	0.51587392	3.68429884	6.99290437	0.0155918	0.03494434
Slc7a1	-0.3932014	5.28969118	6.9928973	0.01559185	0.03494434
Azin1	0.2968634	7.21388816	6.98929763	0.01561492	0.03497724
Cthrc1	0.63308604	2.88728204	6.98838239	0.01562079	0.03497724
Mafb	0.37504696	5.48543859	6.98825966	0.01562158	0.03497724
Ccdc90b	0.54494235	5.8537065	6.98740179	0.01562708	0.03497724
Lamc2	0.6331981	2.97337274	6.9863059	0.01563412	0.03497724
Kcnip1	0.64215002	4.52694561	6.98624539	0.01563451	0.03497724
Tpm3	0.34345594	7.43066738	6.98543693	0.0156397	0.03497843
Dynlt1b	0.67014369	4.20983588	6.98104758	0.01566793	0.03503112
Vamp8	1.22431871	5.10451775	6.97850398	0.01568431	0.0350573
Gng5	0.54541431	6.39957695	6.97362379	0.0157158	0.03511723
04-Mar	0.40346614	4.92876459	6.9710669	0.01573233	0.03512404
Crebrf	-0.3416242	7.2754805	6.9709878	0.01573284	0.03512404
Smagp	1.72051506	0.54925906	6.97097859	0.0157329	0.03512404
A230056P14I	-0.5353364	4.00572703	6.96962258	0.01574168	0.03512814
AI593442	-0.3664196	8.70047291	6.96924763	0.0157441	0.03512814
Samhd1	0.35082145	5.59678699	6.96800754	0.01575213	0.0351356
Ndufb11	0.52688349	5.59865569	6.96672702	0.01576043	0.03514366
Fus	-0.5457261	5.94252772	6.96583563	0.01576621	0.03514609
Tyk2	-0.7746846	2.82495666	6.96003891	0.01580384	0.03521951
Eno2	0.47116386	8.48693367	6.95895381	0.01581089	0.03522477
Polr2m	0.36565252	8.6440255	6.95431672	0.01584108	0.03526824
Rapgefl1	-0.3694992	6.31641623	6.95416873	0.01584205	0.03526824
Vps36	0.54352399	3.86772111	6.95379139	0.01584451	0.03526824
Fndc1	-0.9320211	1.61004126	6.95008673	0.01586868	0.03531156
Dtwd2	-0.7312528	2.91413883	6.94931266	0.01587373	0.03531234
Tceal8	0.60488414	7.27685964	6.94765905	0.01588454	0.0353259
Trub1	-0.4071058	4.67288102	6.94681114	0.01589009	0.03532776

Tmem5	0.44023856	4.60960499	6.94579095	0.01589676	0.03533213
Slc30a3	0.55384672	4.31403031	6.94285935	0.01591595	0.03536431
Chd1	-0.287452	6.5805177	6.93481322	0.01596877	0.03547116
Ttll9	-3.9615973	-1.3124561	6.93026281	0.01599873	0.03552719
Tmem88b	-0.4335963	5.67365952	6.92872706	0.01600885	0.03553915
Eif1	0.44776921	7.99043545	6.92662661	0.01602271	0.03555098
Brd7	0.32773796	5.86924557	6.92648269	0.01602366	0.03555098
Plxna1	-0.5588092	5.51998092	6.9249212	0.01603398	0.03556334
Lsm1	0.59826371	4.10099304	6.922637	0.01604908	0.03558631
Pgpep1	0.61028827	4.21898306	6.92145771	0.01605688	0.03559309
Scin	3.42407544	-0.5378997	6.91488798	0.01610042	0.03567906
Map1lc3b	0.45226612	7.87064155	6.9134812	0.01610976	0.03568921
C230029M16	6.07169813	-1.9497572	6.9123295	0.01611741	0.03569562
Arl14ep	0.33236875	6.37628164	6.91106335	0.01612582	0.03570372
Taf9	0.37091683	5.52362682	6.90624949	0.01615787	0.03575368
Lrig3	-1.1923619	1.08464464	6.90624142	0.01615792	0.03575368
Emc3	0.5082323	5.9107534	6.90487787	0.01616701	0.03576324
Cebpb	0.71894448	2.72160581	6.90256598	0.01618244	0.03578681
1700011I03R	1.87954613	-0.0163869	6.89824553	0.0162113	0.03584009
Nat8l	-0.5206199	6.37905119	6.89724675	0.01621799	0.03584429
Vamp4	0.28673167	6.82262268	6.89622217	0.01622484	0.03584889
Kndc1	-0.6788587	6.21769581	6.89487151	0.01623389	0.03585831
Gm16982	-2.0887383	0.00829823	6.89378235	0.01624119	0.03586387
C5ar2	-0.8640462	1.32805436	6.89179497	0.01625451	0.03588274
Ppfibp1	-0.3121803	6.07535407	6.88845763	0.01627692	0.0359111
2610207O16	-1.6906043	0.22999278	6.88845529	0.01627694	0.0359111
Rfx4	-0.696093	3.75945816	6.88711659	0.01628593	0.03591776
2310069B03	6.22991538	-1.3945449	7.11580463	0.01628953	0.03591776
Itpr3	-0.8670513	2.12556191	6.88050497	0.01633045	0.03599741
Bod1	0.44194959	6.10494432	6.87846356	0.01634423	0.03601718
Plcd4	-0.7569005	2.1931038	6.87536691	0.01636514	0.03603069
Zfp641	-0.8694089	3.02665455	6.87533915	0.01636533	0.03603069
Nefh	-0.7214891	5.51403652	6.874205	0.016373	0.03603069
Chchd6	0.59438833	3.43106469	6.87409293	0.01637376	0.03603069
Psmd3	0.43368679	4.38659442	6.87400197	0.01637437	0.03603069
Rpl27	0.4906171	6.01299865	6.87302924	0.01638095	0.0360346
Steap3	0.69038723	4.09819426	6.87163683	0.01639038	0.03604477
Crb2	2.66225905	-0.6515591	6.87033766	0.01639918	0.03605355
Fam126b	0.42001153	8.29445503	6.86809431	0.01641439	0.03607641
Capn7	-0.340157	6.54872315	6.86626335	0.01642681	0.03609314
Ppp2r5d	0.45487791	5.52700385	6.86457213	0.0164383	0.0361078
Nsg2	0.33674926	7.8674914	6.86319645	0.01644764	0.03611776
Tex13	-1.6418128	-0.5693191	6.8597062	0.01647139	0.03614789
Rab9b	0.32840748	6.46327609	6.85951136	0.01647272	0.03614789
Cped1	0.60414538	7.25689517	6.85831328	0.01648088	0.03614789

Tbc1d5	-0.3280114	6.45387232	6.85714711	0.01648883	0.03614789
Pcdhb18	-0.6524102	4.15419096	6.85704839	0.0164895	0.03614789
Yy2	-1.7237638	-0.0337332	6.85647671	0.0164934	0.03614789
Atl2	-0.4185644	6.17203477	6.85545345	0.01650038	0.03614789
Ptk2b	-0.4262979	7.47935581	6.85536453	0.01650098	0.03614789
Sec14l5	-3.1238389	-1.0991668	6.85481467	0.01650473	0.03614789
Usp19	-0.4301797	4.7584314	6.85350494	0.01651368	0.03615691
Galnt14	-0.9564676	2.27094978	6.85197829	0.0165241	0.03616547
Tlr2	3.08173911	-1.1053509	6.85152157	0.01652723	0.03616547
Mmp9	2.81833525	-0.7822995	6.84549413	0.01656848	0.03624517
Rab11a	0.32671894	6.56897151	6.84471878	0.0165738	0.03624623
Zfp385a	0.70181492	6.61742759	6.84307939	0.01658504	0.03626025
Kcnip4	0.362419	7.02681081	6.84175571	0.01659413	0.03626837
Vldlr	-0.4499626	6.06539724	6.84112956	0.01659843	0.03626837
Ppm1h	0.33259812	6.75681679	6.83973115	0.01660803	0.03627879
Mlx	0.62683936	3.4619609	6.83763497	0.01662245	0.03629567
Crebbp	-0.3260249	8.16522246	6.8372002	0.01662544	0.03629567
Grb7	1.263442	0.847069	6.83377946	0.01664899	0.03632017
Slx4ip	0.6479718	3.03855891	6.83353995	0.01665064	0.03632017
Macrod2	0.47229643	5.09283958	6.8334616	0.01665118	0.03632017
Acad10	-1.1810681	0.81067931	6.83054791	0.01667128	0.03633285
Ptgfrn	0.37251865	5.24899347	6.82992	0.01667561	0.03633285
Ccdc109b	1.01706553	1.96654742	6.82981889	0.01667631	0.03633285
Egfem1	-1.1108267	1.82446598	6.82981037	0.01667637	0.03633285
Zfp341	0.75462833	3.38565362	6.82679342	0.01669721	0.0363677
Polr3gl	-0.5840538	2.88161822	6.82573236	0.01670455	0.03637312
Bsg	0.74487175	7.36556229	6.82228516	0.01672841	0.03641451
E2f1	0.81288649	1.94559375	6.81544289	0.0167759	0.03650727
Ipo5	0.34800835	6.79794917	6.81436965	0.01678336	0.03651291
Gatc	0.44004426	7.36425707	6.81283134	0.01679406	0.0365256
Ccdc53	0.54445364	3.93266763	6.81041967	0.01681085	0.03655152
Zfand5	0.28327416	8.01763302	6.80907618	0.01682021	0.03656128
P2ry14	-1.0352052	1.47512137	6.80798478	0.01682782	0.03656723
Msx1os	-4.2207822	-1.7882486	6.80530274	0.01684654	0.0365973
Slc8a1	-0.5532977	9.18535327	6.80213528	0.01686867	0.03663478
Ttc37	-0.6135611	3.70231221	6.79974868	0.01688537	0.03665311
Dnajc2	0.36409728	6.43593183	6.79953256	0.01688689	0.03665311
Col10a1	-1.9679031	-0.5031777	6.79583004	0.01691283	0.03669881
Fam71e1	-1.4579787	0.25455267	6.79496028	0.01691893	0.03670143
4930539J05F	-1.3839331	0.32804557	6.79274157	0.01693451	0.0367246
Mtfr2	-3.6088356	-1.0937227	6.79018536	0.01695248	0.03675293
Napa	0.37784358	6.07574235	6.7893825	0.01695812	0.03675455
Cinp	0.52933025	4.26238492	6.78807959	0.01696729	0.0367638
Mpzl2	0.57135682	7.15656014	6.78478113	0.01699053	0.03680352
Acnat1	-1.7381553	0.1652493	6.7839108	0.01699667	0.03680619

Phip	-0.431218	7.35489708	6.78207854	0.01700959	0.03682184
Telo2	-1.1199383	1.49145732	6.78110319	0.01701648	0.03682184
Tm7sf3	-0.5099227	4.54772077	6.7804831	0.01702086	0.03682184
Fnbp1	-0.2977532	6.73205691	6.7793604	0.01702879	0.03682184
Atp8a1	-0.4547078	7.56623088	6.77873776	0.0170332	0.03682184
Rnf11	0.31513678	6.86253353	6.77871605	0.01703335	0.03682184
AI837181	0.48671467	4.19362542	6.77799838	0.01703842	0.0368222
Gm19461	-2.5311343	-0.8248495	6.77560944	0.01705533	0.03684812
Reps2	0.3068129	9.58647786	6.77061121	0.01709076	0.03691403
BC030336	-0.3651513	5.66464111	6.76869417	0.01710437	0.03692761
Zfp1	0.41719283	4.28516805	6.76815333	0.01710821	0.03692761
Psme3	0.28923751	6.7298075	6.76734981	0.01711392	0.03692761
Hid1	0.37535607	6.11254569	6.76695365	0.01711674	0.03692761
Jmjd1c	-0.4004242	8.21493046	6.76409155	0.0171371	0.03694402
Cnih3	0.3959204	5.92678748	6.76393792	0.01713819	0.03694402
Dgki	-0.604407	4.88385758	6.76380803	0.01713912	0.03694402
Mrps16	0.60858825	3.20787223	6.76232339	0.01714969	0.03695619
B830017H08	1.7357913	-0.2125688	6.75822372	0.01717892	0.03700856
Pbdc1	0.46314936	4.59042554	6.75661827	0.01719039	0.0370147
Chat	-1.1785237	1.53177093	6.75639313	0.017192	0.0370147
Gtf2ird1	-0.4951068	3.59592891	6.7557517	0.01719658	0.0370147
Setd6	-0.3894058	4.8372087	6.75089809	0.0172313	0.0370788
Rnf149	-0.4583754	5.59735815	6.74324578	0.01728621	0.03717618
Tgif1	0.73947757	2.7617842	6.74297411	0.01728816	0.03717618
Stxbp5l	-0.7712334	8.44935517	6.74224563	0.0172934	0.03717618
Tor1aip2	0.4387437	7.06303032	6.74183067	0.01729638	0.03717618
Cox6a2	0.92473053	1.09993149	6.74032209	0.01730724	0.03718729
Prkcq	-0.8795055	2.94313215	6.73973491	0.01731146	0.03718729
0610011F06I	0.52724063	3.61598656	6.73862947	0.01731942	0.03719374
Dnttip2	0.31480765	6.01952394	6.7370657	0.01733069	0.03720729
Slc6a8	0.35579954	6.34157848	6.73366169	0.01735525	0.03724935
Emx2os	-0.7366712	2.96091169	6.73129676	0.01737234	0.03727535
Pcdhga2	-0.9506796	2.1827769	6.72652687	0.01740685	0.03733768
Pogk	-0.2907616	6.21945239	6.72590714	0.01741134	0.03733768
Cyb5r1	0.67057722	2.67456624	6.72458809	0.0174209	0.03734751
Zbbx	-2.4172215	-0.6191186	6.72245142	0.0174364	0.03737006
Gm5136	-2.1079917	0.1255518	6.72129469	0.0174448	0.03737738
Sav1	0.35495218	6.59991401	6.72024066	0.01745246	0.0373831
Gata3	2.36377755	-0.4203698	6.71856593	0.01746463	0.03739093
Enox1	0.54459468	3.98225846	6.71818938	0.01746737	0.03739093
Uqcrb	0.42374169	6.81664099	6.71768126	0.01747106	0.03739093
Sfswap	-0.6226636	4.61788931	6.71442752	0.01749475	0.03743094
Eif2s3y	-0.6984103	5.08863465	6.71057432	0.01752284	0.03748036
Mrpl23	0.58301101	3.13313811	6.70649749	0.01755263	0.03753336
Dcaf4	0.62150125	3.15447989	6.70399244	0.01757096	0.03755481

Ccdc148	-0.4883439	4.10954772	6.70375843	0.01757267	0.03755481
Ndufa1	0.53874027	5.23904643	6.70251872	0.01758175	0.03756351
Pabpn1	-0.3592025	5.54509059	6.70181009	0.01758694	0.0375639
Ralbp1	0.31217061	6.77749448	6.69997238	0.01760041	0.03757854
Psmc3	0.3808814	5.5806721	6.69950932	0.01760381	0.03757854
Pou2f2	-0.786774	3.6192154	6.69858107	0.01761062	0.03758238
Arhgap28	0.61841	4.00882004	6.69742363	0.01761912	0.03758532
Cpd	-0.4185906	6.32169833	6.69692489	0.01762278	0.03758532
Dock6	-0.5045068	3.65249162	6.69634668	0.01762703	0.03758532
Pet2	-2.7652241	-0.6324574	6.69416681	0.01764305	0.03759836
Unc50	0.4059832	5.64596372	6.69349319	0.01764801	0.03759836
Tmem242	0.61419134	4.09529041	6.69305154	0.01765126	0.03759836
Znrf1	-0.3253494	6.14635925	6.69189969	0.01765973	0.03759836
Jakmip1	0.44148457	4.70511181	6.69186575	0.01765998	0.03759836
Zfp709	-0.5662238	4.13899333	6.69109123	0.01766569	0.03759836
Tmem170b	-0.3739982	7.50671763	6.69074568	0.01766823	0.03759836
Pde8b	-0.3968561	6.1734771	6.68903663	0.01768083	0.03761449
Zfp575	-0.7237968	2.81930145	6.68329164	0.01772324	0.03769403
Tap1	-1.0675344	1.05045136	6.68084511	0.01774133	0.03771353
Adck5	-1.1620396	1.01876887	6.68069242	0.01774246	0.03771353
AW046200	-2.1391671	0.14785655	6.67895812	0.0177553	0.03772314
Dazap2	0.39218161	8.82880673	6.67803832	0.01776212	0.03772314
Trappc2	0.46326895	3.89742879	6.67750138	0.0177661	0.03772314
Rad23a	0.51978959	4.96103067	6.67736614	0.0177671	0.03772314
2900052N01	-0.8481945	2.81930191	6.67619068	0.01777582	0.03773097
Nsf	0.37888416	9.99472431	6.67522625	0.01778297	0.03773547
Plp1	-0.5719764	8.66461957	6.67159484	0.01780994	0.03778201
Gpr183	-1.5057959	-0.160354	6.67054455	0.01781775	0.03778789
Glrx2	0.29770231	6.7348839	6.66680644	0.01784557	0.03782554
Nxf1	-0.3380413	5.93111729	6.66629668	0.01784937	0.03782554
Mrpl52	0.50294268	3.85555016	6.66612754	0.01785063	0.03782554
Fam174b	0.65302552	6.56798464	6.66370875	0.01786866	0.03785306
Il17rb	-2.0136214	-0.0002551	6.66054709	0.01789226	0.03789236
Cd6	2.6004802	-0.0356959	6.65618739	0.01792487	0.0379507
Gorasp2	0.35968911	7.01602837	6.65532032	0.01793136	0.03795373
Leprel1	-1.0317832	2.42918779	6.65340497	0.01794571	0.0379734
Dapk1	-0.4259678	5.9237312	6.65045796	0.01796782	0.03800945
Dnah9	-1.2483539	1.50568372	6.64955815	0.01797458	0.03801302
Edrf1	-0.5896948	5.06486854	6.64393048	0.01801689	0.03809178
Kcmf1	0.33772318	7.50021097	6.64156607	0.01803471	0.03810183
Ost4	0.63541304	7.07732292	6.64139764	0.01803598	0.03810183
Hspd1	0.24677739	8.27695858	6.64127695	0.01803689	0.03810183
Zfp457	-1.6736304	0.29169321	6.63959304	0.01804959	0.03811792
Bambi-ps1	2.35154282	-0.9567035	6.63700803	0.0180691	0.03814395
Enc1	-0.5851462	8.26105106	6.63565724	0.01807931	0.03814395

Arrdc3	-0.4773427	5.87080385	6.63522948	0.01808254	0.03814395
Lgr5	-0.7765931	2.48091856	6.63501357	0.01808418	0.03814395
Mtcl1	-0.6013486	5.72007567	6.63450695	0.01808801	0.03814395
Chmp1a	0.53232962	4.57431843	6.63392316	0.01809242	0.03814395
Tecpr1	-0.721281	3.46884039	6.63312836	0.01809844	0.03814591
Sult4a1	0.32093255	7.03888384	6.62484997	0.01816121	0.03826062
Bcl2	-0.4624834	6.6580827	6.62460579	0.01816306	0.03826062
Cnksr2	-0.6165743	9.57153435	6.62272332	0.01817737	0.03828002
Nme6	-0.9078287	1.77490388	6.62104551	0.01819014	0.03829615
Cox19	0.57418164	3.39470768	6.61825595	0.01821138	0.03833012
Shisa4	0.5578464	4.60312327	6.61168642	0.01826152	0.0384245
Kdm6a	-0.4561857	6.12837456	6.61103982	0.01826647	0.0384245
Exoc4	-0.3392953	5.87037846	6.60923191	0.0182803	0.03844281
Brsk1	-0.5311145	3.49277298	6.60377418	0.01832212	0.03851996
Mapkap1	0.30988076	6.5705397	6.60104695	0.01834306	0.03854497
Ctxn1	0.26417742	7.61037203	6.60024036	0.01834926	0.03854497
Tbce	-0.4606587	4.40846817	6.60015024	0.01834995	0.03854497
Gm12250	1.52305646	0.08217832	6.59954918	0.01835457	0.03854497
Ube2w	0.305432	6.24687381	6.59886409	0.01835984	0.03854525
9630013A20I	-0.9886967	1.48920394	6.59763702	0.01836928	0.03855089
1810062G17	-4.5676007	-1.2913555	6.59717851	0.01837281	0.03855089
Hdlbp	0.26157686	8.49839793	6.5965101	0.01837795	0.03855091
Lrrcc1	-0.432635	5.61702841	6.5952744	0.01838747	0.03855118
Frmd8	0.48384438	3.95943943	6.59502388	0.0183894	0.03855118
Ube2v1	0.41903491	6.89310435	6.59409188	0.01839658	0.03855118
Oat	0.44334482	8.127886	6.59360583	0.01840033	0.03855118
Ap2m1	0.30781243	7.80336144	6.59212524	0.01841175	0.03855118
Pnma1	1.05968266	1.58380208	6.59209057	0.01841202	0.03855118
Cndp2	0.42203615	4.36161415	6.59182542	0.01841406	0.03855118
Sap30	0.71354928	3.11921207	6.58931877	0.01843341	0.03857595
Tspan4	-0.8827137	2.48387872	6.58896139	0.01843618	0.03857595
1810013L24I	0.3133226	7.41829365	6.58660097	0.01845442	0.03859625
Lbr	-0.5374791	3.4404604	6.58608582	0.01845841	0.03859625
Engase	-1.0719315	0.75839057	6.58571034	0.01846131	0.03859625
Olfm4	4.40877368	-2.1604963	6.58183045	0.01849137	0.03864831
Wdr7	-0.5096289	7.54758209	6.5798899	0.01850642	0.03866118
Pnmal1	0.60761781	3.26626482	6.57911454	0.01851243	0.03866118
Drd1a	-0.481052	4.35712398	6.57904303	0.01851299	0.03866118
1500011K16I	0.67105628	3.942228	6.57412404	0.01855122	0.03873024
Shisa3	-0.8382114	3.96110094	6.57337109	0.01855708	0.03873169
Ubxn4	0.27733384	7.28314811	6.57211419	0.01856686	0.03874134
Scel	0.62013888	3.33749651	6.57056339	0.01857895	0.03875275
Kcnn2	-0.5000833	3.8382123	6.57008591	0.01858267	0.03875275
Clic4	0.53845411	8.64253682	6.56792664	0.01859951	0.03877709
Rrp7a	0.58852664	4.01122213	6.56223621	0.01864397	0.03885899

Pik3c3	-0.3477442	5.50158072	6.55919582	0.01866778	0.0388978
Thoc1	-0.4884313	4.76711734	6.55288644	0.01871729	0.03898871
Acsbg1	-0.4224055	4.57552639	6.55195826	0.01872459	0.03898871
Ppat	-0.4321126	4.38681869	6.55137095	0.0187292	0.03898871
Syt10	-0.75939	2.31250601	6.55099031	0.0187322	0.03898871
Med6	0.53663356	4.33643809	6.55016679	0.01873868	0.03899138
Myct1	-3.7258957	-1.6149048	6.5489696	0.0187481	0.03900017
Wbscr27	-0.3877622	4.58273635	6.54739478	0.0187605	0.03901515
Nucb2	0.39428962	5.26267907	6.54348437	0.01879134	0.03906682
Qrich1	0.2953549	6.48425595	6.54206976	0.01880251	0.03906682
5430417L22F	-0.3065199	6.02537351	6.54188533	0.01880397	0.03906682
Adcy8	-0.6820427	3.85815397	6.54160573	0.01880618	0.03906682
Klhl41	-1.1239372	1.5136362	6.53779482	0.01883632	0.03911687
BC030500	0.56500594	3.90057048	6.53680794	0.01884413	0.03911687
Ttyh1	-0.4652301	7.60305799	6.53658226	0.01884592	0.03911687
Nos3	-1.2820517	0.88492449	6.53493258	0.01885899	0.03913317
Nkx2-1	-1.0495364	1.90266197	6.53287986	0.01887526	0.03914378
Narg2	-0.5914165	3.98398958	6.53242795	0.01887885	0.03914378
Klhl17	-0.8764416	3.50189595	6.53215434	0.01888102	0.03914378
4930478L05F	-2.9685674	-1.0812398	6.53165645	0.01888497	0.03914378
Ngfrap1	0.41982933	6.52437935	6.52721561	0.01892026	0.03919893
Nob1	0.66420044	3.0220439	6.52669363	0.01892441	0.03919893
Arhgef18	-0.3947834	4.8838218	6.52633631	0.01892726	0.03919893
Atp5g2	0.6641668	4.07447845	6.52492825	0.01893847	0.03920175
Coch	0.46367455	7.79535415	6.52431858	0.01894332	0.03920175
Ppp1r8	0.90927987	3.99769124	6.52419596	0.0189443	0.03920175
Uchl4	-1.9083892	-0.6099704	6.52013118	0.01897671	0.03925799
4931406P16I	-0.375259	5.65674613	6.51846574	0.01899001	0.03927467
Grm7	-0.7508515	4.58101982	6.51733081	0.01899907	0.03928259
9030025P20I	-0.6352193	3.21124724	6.51188136	0.01904268	0.0393619
C230091D08I	-0.4821586	6.46143414	6.50964133	0.01906064	0.03937875
Arhgef28	-0.4472856	4.27869738	6.50901812	0.01906564	0.03937875
Serpina3n	0.99523846	2.68992215	6.50890046	0.01906658	0.03937875
Atp5sl	0.61261459	3.21692517	6.50763733	0.01907672	0.03938885
Med29	0.46135642	3.3883453	6.50667015	0.01908449	0.03939404
Cd248	0.72853022	4.35918922	6.50351469	0.01910985	0.03943042
Ctdsp1	0.50485087	6.68530634	6.50273271	0.01911614	0.03943042
Pik3cd	-0.6891619	2.8153947	6.50251659	0.01911788	0.03943042
Setmar	0.87921766	2.36982812	6.50098338	0.01913023	0.03944495
Cdr2l	0.52659715	3.23007911	6.500336	0.01913544	0.03944495
Chpf2	-0.7999747	2.28687468	6.49479055	0.01918018	0.0395263
Ptp4a3	0.6295955	3.47659249	6.49239819	0.01919951	0.03955528
Dthd1	-2.1887063	-0.4413479	6.48978637	0.01922064	0.03957792
Ncoa4	0.28755485	7.81367737	6.48973566	0.01922106	0.03957792
Ssna1	0.74081548	3.00827153	6.48856192	0.01923056	0.03958663

Eogt	-0.4039125	5.15256979	6.48748282	0.01923931	0.03959377
Camk4	-0.4165061	9.29810961	6.48209378	0.01928304	0.03967289
Naca	0.37266665	7.84367929	6.48117091	0.01929054	0.03967744
Cers3	4.26454556	-1.6244005	6.47677856	0.01932629	0.03974007
1300002E11I	-0.4113734	5.25378338	6.47443743	0.01934537	0.0397684
Ssu72	0.4349495	5.2620379	6.4721962	0.01936366	0.03979509
Irgm1	0.6142518	4.39098843	6.45946578	0.01946791	0.03999838
Gnao1	0.35163992	10.1861888	6.45362652	0.01951594	0.04008608
Pifo	-2.7970537	-0.2393641	6.45294013	0.01952159	0.04008672
Nup210	-0.8219176	3.04798879	6.45154013	0.01953313	0.04009943
Baz1b	-0.3032766	7.83348427	6.44552919	0.01958276	0.0401843
Ctnna1	0.43309919	7.91023598	6.4452357	0.01958518	0.0401843
Rab11b	0.32279511	9.08833055	6.44275134	0.01960574	0.04021086
Klhdc2	0.26306976	7.86071972	6.44214128	0.01961079	0.04021086
Kcnc1	-0.4660912	6.98292561	6.44172849	0.01961421	0.04021086
Fam115a	-0.4629839	6.74609982	6.43901104	0.01963673	0.04024565
Ccdc135	1.87379916	-0.2724766	6.43780134	0.01964677	0.04024565
Med26	0.63159782	2.57431246	6.43774041	0.01964728	0.04024565
Zeb2os	-0.918071	1.51606069	6.43243105	0.01969139	0.04032501
Ppp6c	0.48286057	6.26254495	6.43056221	0.01970695	0.04032766
Ep300	-0.3612547	8.10979494	6.43034731	0.01970874	0.04032766
5830416P10I	-1.3579603	0.35402887	6.43010943	0.01971072	0.04032766
Snf8	0.57632058	4.26851453	6.42969231	0.01971419	0.04032766
Vtcn1	1.70812596	-0.1211964	6.42806305	0.01972777	0.04034443
Grm1	-0.4815083	5.38932871	6.42497848	0.01975351	0.04038605
Eno1b	0.30970577	6.04849842	6.41277357	0.0198557	0.04057464
Aldh6a1	-0.3988551	5.93071237	6.41266432	0.01985662	0.04057464
Mir6369	-2.298951	0.57777275	6.4120266	0.01986198	0.04057464
Cish	1.22364354	1.17032559	6.41031109	0.01987639	0.0405843
Gcn1l1	-0.5692538	4.54736474	6.41017663	0.01987752	0.0405843
Zfp518a	-0.3561055	5.61763445	6.4078127	0.01989741	0.04061385
Smpd5	3.45045813	-2.011353	6.40269716	0.01994052	0.04069078
Fam101b	0.49859317	4.20018008	6.40071374	0.01995727	0.0407114
Gjd2	-0.836083	1.72208142	6.40021479	0.01996148	0.0407114
Abca8b	-0.8246551	3.33237728	6.39866872	0.01997455	0.04072697
Npas3	-0.565243	3.93110127	6.39594736	0.01999757	0.04075269
Sema4g	-0.7466111	2.77395805	6.39541376	0.02000209	0.04075269
Ints2	-0.5299284	4.08922005	6.39503991	0.02000525	0.04075269
Sec14l2	0.77538141	2.46746972	6.39366782	0.02001688	0.04075269
Naa16	0.54707794	4.04612996	6.39356218	0.02001777	0.04075269
Becn1	0.46984188	5.24781277	6.39273357	0.0200248	0.04075269
Cutc	0.57887663	3.81524854	6.39268681	0.02002519	0.04075269
N6amt1	0.33886555	5.66789646	6.39034493	0.02004506	0.0407637
Scrt1	-0.5389474	5.3381451	6.38997922	0.02004816	0.0407637
Sirpb1a	-3.2537249	-0.9216171	6.38893897	0.020057	0.0407637

Atp13a2	-0.6213947	4.17613048	6.38799732	0.020065	0.0407637
Prickle1	-0.4593413	5.67967014	6.38794685	0.02006542	0.0407637
Hdac7	-0.5169185	3.70229096	6.38788135	0.02006598	0.0407637
H2-K2	-2.5416827	-0.367524	6.3875686	0.02006864	0.0407637
Ppp1r12a	-0.3162307	7.45763336	6.38581443	0.02008355	0.04078295
Dancr	2.02972492	-0.0093493	6.38445978	0.02009508	0.04079531
Net1	0.35725985	5.59464004	6.38292918	0.02010811	0.04080587
Ergic1	0.33175378	6.53841747	6.38193558	0.02011658	0.04080587
Tango6	0.75304896	2.27746456	6.38193264	0.0201166	0.04080587
Stub1	1.31031035	0.86634592	6.3805846	0.02012809	0.04080989
Prrc1	0.38729011	6.03799627	6.38042389	0.02012946	0.04080989
Mis18bp1	-1.0957272	1.0692987	6.37644614	0.02016342	0.04086768
Ift122	-0.4986436	3.72749146	6.37485191	0.02017704	0.04088425
Fam216b	1.2751706	1.06230103	6.37232182	0.02019869	0.04091706
Rab33a	0.59081049	3.31455884	6.36671601	0.02024675	0.04098306
Adcy6	-0.4559327	4.09255721	6.36671087	0.02024679	0.04098306
Gm12504	-1.554585	0.86210628	6.36483919	0.02026286	0.04098306
Mdga2	-0.4435469	6.06321093	6.36340525	0.02027519	0.04098306
Gm14391	-1.3247014	1.93452359	6.362663	0.02028157	0.04098306
Il13ra1	0.54109202	4.87857504	6.36243305	0.02028355	0.04098306
Dstyk	-0.333637	5.49522802	6.36182234	0.0202888	0.04098306
Bmp1	-0.7380389	2.83587107	6.36178804	0.0202891	0.04098306
Psma1	0.4430867	5.5577464	6.36148723	0.02029168	0.04098306
Ccdc3	0.37777891	5.39949026	6.36063369	0.02029903	0.04098306
Itgae	3.65978619	-1.8146035	6.3590635	0.02031255	0.04098306
Bahcc1	-0.5608486	3.98732664	6.35898773	0.02031321	0.04098306
Stx3	-0.3877348	4.78234578	6.35866783	0.02031596	0.04098306
Ept1	-0.3348416	4.98612583	6.35845051	0.02031783	0.04098306
Sh3bp4	-0.543613	2.8722618	6.35837513	0.02031848	0.04098306
Spats2	0.53484209	3.28971686	6.35835204	0.02031868	0.04098306
Pdzd8	-0.2876498	7.10026311	6.35680788	0.020332	0.04099888
Actr1b	0.38328745	7.25929002	6.35536371	0.02034446	0.04101298
Arhgap39	-0.4192809	4.98563479	6.35384734	0.02035755	0.04101856
Mrpl18	0.47696492	5.44431311	6.35377657	0.02035816	0.04101856
Ell3	0.82895992	2.29649561	6.34677261	0.02041875	0.0411296
2810433D01	0.72150974	3.1447098	6.345538	0.02042945	0.04113841
Metap1d	-0.934756	2.28054549	6.34500265	0.0204341	0.04113841
Mrap	1.08926927	1.69198266	6.34383162	0.02044425	0.04114782
Arhgap33	-0.6255678	5.35412079	6.33657974	0.0205073	0.0412625
Ldhd	1.07579584	1.85823546	6.33578589	0.02051421	0.0412625
Fzd4	-0.7502889	2.98805303	6.33477675	0.020523	0.0412625
Fgfr3	-0.5140242	3.90040749	6.33469484	0.02052372	0.0412625
Ndufb8	0.45432202	4.70216124	6.33411881	0.02052874	0.0412625
Aamdc	0.50767509	4.22658645	6.33152984	0.02055132	0.0412929
Chsy3	-0.649201	2.91210775	6.33112335	0.02055487	0.0412929

Kif1a	-0.498993	9.884119	6.33048877	0.02056041	0.04129297
CK137956	1.49116684	0.78151713	6.3264872	0.02059539	0.04134795
Plk5	-1.3675833	1.09162456	6.32609685	0.02059881	0.04134795
1810043G02	0.58438205	2.74556179	6.31758499	0.02067345	0.04148005
Akt1s1	0.93852182	1.7893999	6.31733143	0.02067568	0.04148005
Bcr	-0.4759669	6.07003854	6.31585669	0.02068865	0.04149497
Arl2	0.61735625	3.71063299	6.31386709	0.02070615	0.04151898
Efr3b	-0.5150395	6.81278274	6.30602048	0.02077536	0.04164242
Gm20362	-1.3965688	0.71752703	6.30562878	0.02077882	0.04164242
Dgkh	-0.6932335	5.56521568	6.30487591	0.02078547	0.04164463
Phf19	1.59697351	0.71528443	6.30170871	0.02081349	0.04168062
Gabrg3	-0.7691244	4.67302753	6.30158985	0.02081455	0.04168062
Samd5	-0.5264438	3.98939051	6.29867053	0.02084041	0.04172128
Ccdc84	-1.4970471	1.12289914	6.29693022	0.02085585	0.04172739
Pak2	0.33318152	6.79178155	6.29680573	0.02085696	0.04172739
D830005E20	-1.3581593	-0.32603	6.29644553	0.02086015	0.04172739
Gm10125	-1.1783757	1.01722952	6.29454178	0.02087706	0.04174547
4933409K07	-0.4650389	7.36388262	6.29417436	0.02088033	0.04174547
Hn1l	0.71870476	4.36652087	6.28923804	0.02092424	0.04181422
Mpp1	0.314409	5.83676708	6.28880358	0.02092811	0.04181422
Edf1	0.6671245	6.41286507	6.288076	0.0209346	0.04181422
Myzap	0.79392396	4.17181973	6.28780535	0.02093701	0.04181422
Ap4b1	0.8768948	2.03441604	6.28693337	0.02094478	0.04181861
Il2ra	0.96889448	1.922807	6.28543808	0.02095812	0.04183411
Cadps	-0.5340045	8.58472785	6.28371376	0.02097351	0.0418537
Sod1	0.54757975	8.00215232	6.28276094	0.02098203	0.04185955
Mbtd1	-0.3843017	5.66318019	6.281972	0.02098908	0.04186248
Lacc1	0.51756972	4.5523204	6.27734112	0.02103051	0.04193195
Magoh	0.43031659	5.17826882	6.27683033	0.02103509	0.04193195
Tcam1	5.92571793	-1.9937716	6.46825256	0.02105758	0.04195978
Gm6583	-3.229393	-1.0788076	6.27349553	0.02106499	0.04195978
Ythdf3	0.29246631	7.18928412	6.27340246	0.02106583	0.04195978
Xlr4b	2.77578707	-0.3542584	6.27064671	0.02109058	0.04199792
Cfb	-0.93021	2.67106589	6.26992937	0.02109703	0.04199962
Pygo2	0.47311741	3.87133083	6.26267101	0.02116239	0.0421168
Eef1e1	0.47794002	3.33863197	6.26203433	0.02116814	0.0421168
Arpc5	0.40531542	7.34556386	6.26152514	0.02117273	0.0421168
Itga2	-2.5848626	0.13157257	6.25882181	0.02119715	0.04215081
Mkrn2	0.42087753	5.14989952	6.25838826	0.02120107	0.04215081
Olfm1	0.30718501	9.19770912	6.25514003	0.02123046	0.04219058
Serpina9	1.76339488	-0.6081491	6.254934	0.02123232	0.04219058
Zak	-0.3403709	6.48262938	6.24991332	0.02127784	0.04226982
Btbd19	-0.7979587	2.62610308	6.24877189	0.0212882	0.04227835
C4bp-ps1	-4.0079693	-1.5809795	6.2481994	0.0212934	0.04227835
Mea1	0.62891049	4.7171691	6.24724014	0.02130212	0.04228446

Prg4	0.69696682	8.48540854	6.24571832	0.02131595	0.04230073
Psmd10	0.71913385	4.10335909	6.2432598	0.02133832	0.04232826
Krt12	-0.4402566	4.21129125	6.24218523	0.02134811	0.04232826
C2cd5	-0.5183134	5.79741466	6.24158303	0.0213536	0.04232826
Snurf	-0.9519876	1.5189843	6.24121109	0.02135699	0.04232826
Pde6c	-3.4066507	-0.9940223	6.24109622	0.02135804	0.04232826
Neurod2	0.35300877	5.81515841	6.23985994	0.02136931	0.04233941
Iah1	0.71904083	3.18003593	6.23877287	0.02137923	0.04234787
Cbx3	0.37609443	8.36237764	6.23696122	0.02139576	0.04236945
Prrc2b	-0.3216126	8.7073686	6.23551427	0.02140898	0.04238444
Ift52	0.44225164	4.94907835	6.23104369	0.02144989	0.04244288
Kitl	-0.4565741	4.77964071	6.23054989	0.02145441	0.04244288
Dido1	-0.3697411	6.59201609	6.23043317	0.02145548	0.04244288
Fam227b	-1.2276746	0.68226812	6.21991454	0.02155209	0.04262276
Ino80c	0.48002141	5.69305021	6.2134488	0.02161173	0.04272943
Zfp160	-0.4512693	4.46987999	6.21215539	0.02162368	0.04274179
Apol7e	1.71778876	-0.2586059	6.20921346	0.02165089	0.0427843
Ubox5	0.59632859	3.32960985	6.20700949	0.0216713	0.04280801
Prpf39	-0.5841393	5.45336515	6.20612559	0.02167949	0.04280801
Lix1l	0.68721451	6.40813005	6.20606968	0.02168001	0.04280801
Tmem65	0.30253602	8.11504766	6.20077863	0.02172912	0.04289369
Mrpl13	0.42199754	4.72842033	6.19922348	0.02174358	0.0429052
1700016K19I	1.31049851	1.06684404	6.19892152	0.02174638	0.0429052
Crtc3	-0.4479647	4.93875182	6.19783236	0.02175652	0.0429081
Dscam	-0.430567	5.64786648	6.19753362	0.0217593	0.0429081
Tbp	0.30435866	5.33457464	6.19555161	0.02177776	0.04293321
Masp1	-0.8523097	1.67919418	6.19320852	0.0217996	0.04296498
Igtp	0.65264578	3.72896167	6.19079682	0.02182211	0.04299805
Vgf	0.75660536	3.63822286	6.18966983	0.02183263	0.04300749
Cacna1i	-0.5665059	4.05638113	6.18880655	0.0218407	0.04301209
Prkaca	0.37421683	7.24830415	6.18740931	0.02185376	0.04302652
Rps4l	0.64984213	1.95132353	6.18602575	0.02186671	0.04303336
Trpc1	-0.4663281	4.59398598	6.18581172	0.02186871	0.04303336
Pacrgl	0.70553749	3.30867818	6.18491676	0.02187709	0.04303856
Sgpl1	-0.5427247	4.29909087	6.18394371	0.02188621	0.0430452
Map7d1	0.38854439	6.98034278	6.18280589	0.02189687	0.04304539
Tpbg	0.75859264	3.35700631	6.18242007	0.02190049	0.04304539
Col23a1	0.66380081	5.23591138	6.18209703	0.02190352	0.04304539
Slc44a2	-0.4161848	5.08209519	6.18055405	0.02191799	0.04305957
4933427I22R	-3.6797042	-1.2003321	6.18010418	0.02192221	0.04305957
Tmsb15b2	1.77315129	-0.3630723	6.17887092	0.02193379	0.04306713
Vps4a	0.3655644	5.55781122	6.17847118	0.02193755	0.04306713
Ccdc104	0.33727998	7.65613033	6.17578352	0.02196281	0.04310545
Vmn2r-ps129	-1.6987865	0.85826362	6.16985253	0.02201868	0.04320379
Ssr4	0.51321478	3.42232035	6.16864833	0.02203004	0.04321478

Elmo3	0.99155567	1.38740745	6.16777325	0.0220383	0.04321968
Slc30a6	0.56199116	3.08987767	6.16639341	0.02205133	0.04323394
Mfn2	-0.3192623	6.63419644	6.16533536	0.02206133	0.04324224
Tns3	-0.3058045	6.05708022	6.16444577	0.02206974	0.04324743
Zfp783	-1.0659303	1.36514797	6.1623372	0.0220897	0.04326503
Trmt44	-1.3991334	0.66664518	6.16227735	0.02209026	0.04326503
Hn1	0.51944482	4.32365813	6.15917022	0.0221197	0.0433048
Skap2	0.46498397	4.03246123	6.15891591	0.02212211	0.0433048
Glt28d2	0.72948718	3.29985925	6.15137941	0.02219371	0.04343362
2310047M1(	0.71097407	2.67892349	6.14727867	0.02223278	0.04349872
Shisa7	-0.4907824	6.36471315	6.14606709	0.02224433	0.04350999
A730020M07	0.63499511	3.94037188	6.14517258	0.02225287	0.04351026
Psmg4	1.07628478	1.72007138	6.14483714	0.02225607	0.04351026
9830147E19I	-0.7684395	1.87966411	6.14398408	0.02226422	0.04351484
Mras	0.27186494	6.99979418	6.14173634	0.0222857	0.04354548
Tmem258	0.7081634	2.43606167	6.13799913	0.02232146	0.04359728
Dip2a	-0.5238899	5.45376591	6.13728031	0.02232835	0.04359728
Hrct1	4.46480514	-2.1066727	6.13714549	0.02232964	0.04359728
Tmem191c	-0.570108	4.21084663	6.13602229	0.02234041	0.04360695
Nlrp6	1.80807457	0.89625499	6.13401669	0.02235965	0.04363315
Cdc42	0.28863413	8.71371659	6.13065645	0.02239192	0.04368477
Chrna2	-2.102819	-0.6586616	6.12866478	0.02241108	0.04371077
Strip1	-0.4016837	4.37981101	6.12722323	0.02242495	0.04372646
Nat8	-1.8953871	0.23578524	6.12597773	0.02243695	0.04372721
Ankrd27	-0.3219759	5.38224598	6.12551326	0.02244143	0.04372721
Nhlrc1	0.4658862	4.10563353	6.12536808	0.02244283	0.04372721
Hexim2	0.69412708	2.30209558	6.12294409	0.0224662	0.04376139
Pde4dip	-0.4643087	7.48586352	6.12216627	0.02247371	0.04376464
Atp1b1	0.27184905	10.1417314	6.11840594	0.02251004	0.04381431
Gtf2h5	0.48998327	5.19964753	6.11831749	0.02251089	0.04381431
Sars2	0.8513558	1.56896706	6.11768148	0.02251704	0.04381491
Rtn4rl1	0.32103664	6.19990827	6.11612337	0.02253212	0.04383288
Gm4461	-1.8547238	0.55151926	6.11507305	0.02254229	0.04384129
Zfp422	0.38555664	6.39820939	6.10483221	0.02264173	0.04401286
Kif21a	-0.496259	8.49741799	6.10476888	0.02264234	0.04401286
Ctxn3	0.6315028	7.22561565	6.10417634	0.02264811	0.04401286
Kat2b	0.34755204	6.08005269	6.1019813	0.0226695	0.04402565
Lrp10	0.74151185	4.25497654	6.10195818	0.02266972	0.04402565
Nfu1	0.44694537	4.48020055	6.10119255	0.02267718	0.04402565
Zfp820	0.99826164	1.39759634	6.10091783	0.02267986	0.04402565
Zpr1	0.40809257	4.3602774	6.10008149	0.02268802	0.04402565
Nap1l5	0.28106314	7.1916082	6.09985428	0.02269024	0.04402565
Crybb1	3.30192154	-1.8920045	6.09928674	0.02269578	0.04402565
Zfp386	0.3520115	5.38364077	6.09623321	0.0227256	0.04407211
Xpo7	-0.2577471	7.18190454	6.09339494	0.02275336	0.04411454

Glo1	0.41609296	6.44821502	6.09069266	0.02277983	0.04415444
Gtf2e2	0.38632095	4.51126659	6.08870576	0.02279931	0.04418079
Romo1	0.770528	4.01738062	6.0866599	0.02281939	0.04420828
2700038G22	-1.4926058	-0.1344479	6.08310603	0.02285432	0.04425408
Mrpl11	0.49070956	4.64254819	6.083054	0.02285483	0.04425408
Epcam	-3.5294306	-0.4798403	6.07824965	0.02290214	0.04433425
1700018G05	-3.0682325	-0.9983156	6.07726972	0.02291181	0.04434152
Fmnl2	0.3414536	8.09144302	6.07666941	0.02291773	0.04434154
Ciita	-0.9178957	1.62985973	6.07508018	0.02293342	0.04436045
Klhdc7a	-0.8728478	2.71863597	6.07325121	0.02295149	0.04436407
Erich3	-0.8649376	3.68636656	6.07268033	0.02295713	0.04436407
Rgl2	-0.7337203	2.1835214	6.07263477	0.02295758	0.04436407
Fbxo45	0.30823121	6.25555646	6.07227071	0.02296118	0.04436407
Ankrd34a	0.37671	6.32298013	6.0718987	0.02296486	0.04436407
Prdm8	0.52715867	5.55077021	6.0691767	0.0229918	0.04440467
1110004F10I	0.44672491	6.35477478	6.06839548	0.02299954	0.04440589
Nap1l4	0.3734377	5.59025974	6.06791772	0.02300427	0.04440589
Col6a1	-0.4046484	5.05740719	6.06681222	0.02301523	0.04441562
Hspa1a	0.49791917	3.80987944	6.06513561	0.02303186	0.04443628
Gbas	0.36426713	6.53615095	6.06434288	0.02303973	0.04444003
Wdr20	-0.4769699	3.5679198	6.06320031	0.02305107	0.04445048
Arhgef10l	-0.576389	3.02220245	6.06041741	0.02307873	0.04449238
4930414L22I	0.60093643	3.17796153	6.05272541	0.02315537	0.04462866
Lgals3	1.07167705	1.77857783	6.05019893	0.02318061	0.04466582
Max	0.36515388	8.08866805	6.04529476	0.02322968	0.04474889
Vmn2r18	-3.9441312	-1.8672575	6.04340238	0.02324865	0.04477066
Ssr3	0.40401625	8.00002703	6.04278872	0.0232548	0.04477066
Gm3893	-0.5741136	5.8712045	6.04216478	0.02326106	0.04477066
4921504A21I	0.67929132	3.35380676	6.0417866	0.02326486	0.04477066
BC035044	1.35547829	0.39691978	6.04117597	0.02327099	0.04477097
Snx30	-0.3938161	5.50898575	6.03908855	0.02329195	0.04478881
Hiatl1	-0.4569582	4.0000402	6.03906373	0.0232922	0.04478881
Haus1	0.71914221	2.88420026	6.03810991	0.02330179	0.04479374
Larp6	0.48513875	3.96276884	6.03762068	0.02330671	0.04479374
Ctps2	-0.3077044	5.66905144	6.02997602	0.02338373	0.04491358
Cd7	3.31685674	-1.7326019	6.02986822	0.02338482	0.04491358
Chrnb1	-0.6244576	2.16982591	6.02935645	0.02338998	0.04491358
Ppp2r2b	0.31406505	6.76285514	6.02905618	0.02339301	0.04491358
Ccdc160	0.90096575	1.8488038	6.01767705	0.02350824	0.04512326
Tbpl1	0.26352198	6.41403785	6.01608607	0.0235244	0.04513723
Lgals12	-1.8453457	-0.2240564	6.01546401	0.02353072	0.04513723
Abca17	-2.46466	-0.2030973	6.01518394	0.02353357	0.04513723
Ckap2l	1.04360364	0.9087401	6.01317986	0.02355395	0.04516226
Il1f9	-0.9007526	2.29455078	6.01232612	0.02356264	0.04516226
Nudt14	1.19096033	1.06890462	6.01191752	0.0235668	0.04516226

Map3k9	-0.5497199	6.22637479	6.0113315	0.02357277	0.04516226
Gba2	-0.6620968	3.2358917	6.0109436	0.02357672	0.04516226
Ergic2	-0.425935	4.64021062	6.00915972	0.0235949	0.04518549
Mef2d	0.31900497	7.08971438	6.008313	0.02360354	0.04518549
Moxd1	-0.6677483	2.93073035	6.00712257	0.02361568	0.04518549
Mgat5b	-0.4510701	3.71645702	6.00654418	0.02362159	0.04518549
Cr2	-1.5517206	1.0796989	6.00626776	0.02362441	0.04518549
Pirt	5.7982164	-0.890339	6.00621077	0.02362499	0.04518549
Pde4c	-1.8135347	-0.1185333	6.00190596	0.02366899	0.04525811
Izumo4	-0.9523974	1.70919881	6.00099371	0.02367833	0.04526443
Ctsh	0.6181058	4.97746503	5.99740805	0.02371507	0.04532311
Eif1ax	0.46821295	6.23929247	5.99669696	0.02372236	0.0453255
BC052040	0.45836996	4.25305052	5.99228779	0.02376764	0.04540045
Itga3	-0.6120837	2.92621062	5.99006103	0.02379054	0.04543263
Gnpat	-0.3958455	5.40993141	5.98888796	0.02380262	0.04544412
Smc2	-0.4457008	4.51070417	5.98507897	0.02384188	0.04549552
Zbed5	0.57948339	3.8677752	5.98466019	0.0238462	0.04549552
1700123O20	0.5593426	3.87720246	5.98451118	0.02384774	0.04549552
A930017M01	-0.6672635	2.61294087	5.98372343	0.02385587	0.04549946
Srrm1	-0.2417821	7.56337242	5.98188492	0.02387486	0.04552028
Zfp433	-0.4551459	3.82422455	5.98149144	0.02387892	0.04552028
Gstp1	0.42035888	4.93816257	5.98079973	0.02388607	0.04552234
Cat	0.40694318	8.12823621	5.97974831	0.02389694	0.04553149
Cflar	-0.3023301	6.86384183	5.97757888	0.02391939	0.0455627
Slc4a11	2.75623889	-0.2079535	5.97442865	0.02395204	0.04561329
Fanca	-4.2308592	-2.154117	5.97338137	0.0239629	0.0456224
Pign	-0.4216032	4.09877498	5.97260977	0.02397091	0.04562606
Atp5e	0.62913373	5.23526752	5.97105987	0.023987	0.04563297
Psmb3	0.44738525	4.45844632	5.97095928	0.02398805	0.04563297
Zfp410	-0.4545283	4.00281026	5.97050281	0.02399279	0.04563297
Tpt1	0.48245539	9.33039457	5.96934834	0.02400479	0.04564422
Ttc3	-0.4400442	9.98771488	5.9649102	0.02405097	0.04570998
Tead4	-2.5525729	0.04047983	5.96485421	0.02405156	0.04570998
Epha3	-0.6163895	2.92841282	5.9636146	0.02406448	0.04571616
Slc25a17	0.45092401	5.69498941	5.96286617	0.02407228	0.04571616
Dpysl2	-0.3329318	8.72760806	5.96278811	0.02407309	0.04571616
Lonp1	-0.4158226	4.27239562	5.95996293	0.02410258	0.04576057
Baiap2	0.35496851	6.25207541	5.95930167	0.02410949	0.0457621
Josd1	0.32927321	5.03477143	5.95706523	0.02413286	0.04579489
Rfx8	-3.6843168	-1.9345285	5.95613475	0.0241426	0.04580177
Camk1d	0.29036614	8.32294529	5.95244209	0.02418127	0.04586354
Slc23a1	-1.0961842	0.69192188	5.95160266	0.02419007	0.04586863
Tgfa	-0.3148071	5.15283698	5.95044439	0.02420222	0.04588007
Med7	0.49830416	4.81836147	5.94598613	0.02424905	0.04595698
Psmc5	0.34289857	6.68950131	5.94541608	0.02425504	0.04595698

Cftr	-1.2326146	1.59461546	5.94423333	0.02426748	0.04596894
Gramd1b	-0.3763791	5.84688195	5.94169507	0.02429421	0.04600796
Klrd1	-1.6364672	-0.5107385	5.94063912	0.02430534	0.04601468
Zfp9	0.34382042	5.92135413	5.94019468	0.02431003	0.04601468
Hccs	0.45421505	4.98550885	5.93787404	0.02433451	0.0460494
Gpx1	0.70588247	6.58973415	5.93532162	0.02436148	0.0460888
Zfp369	-0.4319581	5.24517541	5.93418412	0.0243735	0.04609992
Slc38a4	1.1102335	1.56652704	5.93120046	0.02440508	0.04613713
Gdi2	0.25723955	8.14845822	5.93116316	0.02440548	0.04613713
5330426P16I	0.76087056	2.64642788	5.92936779	0.0244245	0.04614991
Pcdhb10	-1.0971828	1.33394292	5.92936395	0.02442454	0.04614991
Phxr4	-1.3671301	1.70294405	5.92627581	0.0244573	0.04620017
Zfp677	0.81330152	3.72824083	5.92463883	0.02447469	0.04622138
Vac14	0.45087036	4.71468339	5.92335721	0.02448831	0.04623546
Zfyve16	-0.4272772	4.90161948	5.92183873	0.02450446	0.04625431
Stox2	-0.437979	7.99509909	5.9207388	0.02451617	0.04626477
Arid1a	-0.4351484	8.05983056	5.91967628	0.02452748	0.0462724
Tpk1	0.5313553	4.03306475	5.91920064	0.02453255	0.0462724
Ccdc57	-0.7704366	1.83468668	5.91809174	0.02454437	0.04627675
Babam1	0.40493779	4.48843025	5.91733477	0.02455244	0.04627675
Pcbd1	0.72669273	1.94610971	5.91724764	0.02455337	0.04627675
Tagap1	-0.4001974	4.77504619	5.91460094	0.02458161	0.04631362
Nle1	-1.4115029	-0.0880185	5.91425757	0.02458527	0.04631362
Akr1c13	1.01274902	1.750315	5.91110047	0.02461902	0.04636554
Vstm2a	0.35308304	6.5331679	5.90816772	0.02465041	0.04641301
Scamp4	0.57016494	3.4873528	5.90718095	0.02466098	0.04642126
Pgap1	-0.4705653	5.42461788	5.90642889	0.02466904	0.04642479
Vezf1	0.36903582	7.58884059	5.90506997	0.02468361	0.04644056
Prr16	0.47977954	4.14023859	5.90410892	0.02469393	0.04644187
Mrpl42	0.38179454	5.90486063	5.90385143	0.02469669	0.04644187
Trap1	-0.4391427	4.50678277	5.90262027	0.02470991	0.0464536
Thsd7a	-0.3577769	6.78848442	5.90211689	0.02471532	0.0464536
Gm10791	-1.6967447	0.77777269	5.90024336	0.02473545	0.0464798
Prss22	2.5130089	-1.6286607	5.89891014	0.02474979	0.0464951
Eif3g	0.43627603	5.42210614	5.89650339	0.02477571	0.04653213
Gap43	0.34560597	7.23966044	5.8954065	0.02478753	0.04654267
Gmip	-0.7961281	1.52538376	5.8932482	0.0248108	0.04657472
Zfp189	0.52394396	3.26918649	5.89048953	0.02484059	0.04661546
Ppp1r7	-0.4103968	7.31260704	5.89008726	0.02484493	0.04661546
Pdzd9	-1.5000242	0.29976476	5.88656561	0.02488302	0.04667525
Unc5b	-0.708112	2.36938693	5.8809511	0.02494389	0.04677772
Nsun7	-1.0593963	2.25496462	5.87895684	0.02496554	0.0467898
Bnc2	0.55580556	7.67740496	5.8788091	0.02496715	0.0467898
Ucp2	0.72929541	7.46890025	5.87863535	0.02496904	0.0467898
E130215H24I	4.19852696	-1.8867216	5.87445388	0.02501453	0.04686333

Lhfpl4	-0.311446	6.02255288	5.87378653	0.02502179	0.04686524
2610203C22I	-1.7735302	0.00997097	5.86942582	0.02506935	0.04692748
Arr3	-3.0822439	-1.161148	5.86875976	0.02507662	0.04692748
Sh2b3	0.57939201	3.5835136	5.86866816	0.02507762	0.04692748
Gm7694	-0.7816584	1.83915516	5.86844618	0.02508005	0.04692748
Ptchd2	-0.7271934	2.78492461	5.86754006	0.02508994	0.04693429
Ostc	0.465491	4.63120836	5.86491347	0.02511866	0.0469763
Ncor2	-0.3798461	6.83000484	5.86399824	0.02512868	0.04698332
Sarnp	0.39759754	5.53756592	5.86212996	0.02514914	0.04700985
Gm5176	-2.0159604	-0.4659472	5.86055844	0.02516636	0.04702796
Slitrk3	-0.4845911	6.08726695	5.86003347	0.02517212	0.04702796
Fam126a	-0.4126885	4.10421944	5.85879313	0.02518573	0.04702796
Col4a3bp	-0.2653581	7.25980068	5.85817601	0.0251925	0.04702796
Lims1	0.45227958	7.53140052	5.85792467	0.02519526	0.04702796
Col16a1	-0.7692186	1.53962728	5.85781703	0.02519644	0.04702796
Mapk8ip3	-0.4912377	6.77528462	5.84943675	0.02528864	0.0471883
Dhodh	0.83161643	2.11767985	5.84795392	0.02530499	0.04720227
Npy1r	-0.4058476	5.20521963	5.84761704	0.02530871	0.04720227
Rarb	0.39574359	5.04062113	5.84458122	0.02534223	0.04725304
Nat9	0.73783786	1.99609858	5.84390364	0.02534972	0.04725526
Trmt13	-0.8938888	1.35786916	5.8428376	0.02536151	0.04726548
Arhgef7	0.34095909	6.71719714	5.84164764	0.02537467	0.04727827
Plekhm3	-0.4088216	6.33117024	5.83936805	0.02539991	0.04731355
Gfi1	4.89594257	-1.3103246	6.00888207	0.02541043	0.04732139
Ttc33	0.30111598	6.57140083	5.83776952	0.02541763	0.04732305
Yae1d1	0.3295666	6.50183287	5.83521514	0.02544597	0.04736406
Dcbld2	-0.348983	5.26479498	5.83013154	0.02550248	0.04745746
Uap1	0.32047488	5.8001539	5.82618612	0.02554643	0.04752747
Tomm5	0.4846539	4.59282901	5.82287269	0.02558341	0.04758071
Foxm1	1.13035117	1.41921814	5.8224852	0.02558774	0.04758071
Mmp23	-2.1221948	-0.4723101	5.82000011	0.02561552	0.04762056
Mex3d	-0.6194114	3.9163	5.81595472	0.02566081	0.04769295
Coq10b	0.41933111	5.61281997	5.81450087	0.02567711	0.04771142
Serp2	0.5516807	3.78297655	5.81328726	0.02569073	0.0477249
Fmn2	-0.5021356	6.65774574	5.81097505	0.02571669	0.0477613
4930469G21	-1.5784915	-0.1709537	5.8078662	0.02575164	0.04780394
Gkap1	0.46373465	5.17658871	5.80779968	0.02575239	0.04780394
Eid2	0.34443774	5.24405361	5.80376719	0.02579781	0.0478764
Ide	0.26249902	7.24622988	5.80317554	0.02580448	0.04787694
Abr	-0.3958136	7.69705062	5.79865403	0.02585553	0.0479598
Ap3s1	0.37977769	7.18832894	5.79462059	0.02590117	0.04802688
Selk	0.42657335	6.15916449	5.79432638	0.0259045	0.04802688
Pitpnm3	-0.3825344	6.08220485	5.79150432	0.02593649	0.04807193
Wnt3	-2.0805939	-0.8614481	5.7910523	0.02594162	0.04807193
Rpl6	0.39906713	7.75574068	5.78927756	0.02596177	0.04809738

4930565N06	-1.4734885	1.61358252	5.78767267	0.02598	0.04811927
Atp6v1f	0.60908842	4.75645672	5.78513208	0.02600889	0.04816089
Tmem178b	-0.3804109	7.54212313	5.78396229	0.0260222	0.04816236
Capn9	4.95350599	-2.3766796	5.9515622	0.02602252	0.04816236
Eno1	0.94596454	0.80270498	5.7815073	0.02605017	0.04819852
Tmem9b	0.32375098	5.44738389	5.78017355	0.02606538	0.04819852
Gsap	-0.6910862	2.23454519	5.78009752	0.02606625	0.04819852
Igdcc4	-0.324601	5.11809547	5.7799645	0.02606777	0.04819852
Abcc9	-0.9115356	3.11914776	5.77921015	0.02607637	0.04820256
Fam213a	0.53913317	7.66914769	5.77762819	0.02609443	0.04822406
Cad	-0.8810398	1.781203	5.77606772	0.02611226	0.04824512
Itsn1	-0.444534	7.01485215	5.77262953	0.02615159	0.04830589
Cct2	0.25028864	7.0039922	5.77184224	0.02616061	0.04831065
Ppara	-0.5828409	4.0984941	5.77101798	0.02617005	0.04831362
Cuedc2	0.51692448	4.43643921	5.7705775	0.0261751	0.04831362
Ccdc87	1.17668543	1.01600754	5.76908728	0.02619219	0.04833101
Psmb10	0.66402204	3.21934283	5.768632	0.02619741	0.04833101
Sdha	-0.2725547	8.6038196	5.76655279	0.02622127	0.04836315
Dnm3os	-1.2327359	1.87406229	5.76358065	0.02625543	0.04841424
Nae1	-0.3828067	5.057229	5.76178822	0.02627605	0.04842936
Elf3	-1.6822901	-0.0623376	5.7617459	0.02627654	0.04842936
Hnrnpu	-0.3104416	8.63388312	5.75997071	0.02629698	0.0484428
Spin2c	0.63702308	2.93583366	5.75898632	0.02630833	0.0484428
1810055G02	0.38466626	5.57403239	5.75869339	0.0263117	0.0484428
Ankrd32	-0.4866668	4.11258224	5.75854053	0.02631347	0.0484428
Tgm5	-2.3979917	0.06643321	5.75831035	0.02631612	0.0484428
Ago4	-0.8038026	3.22251351	5.75748244	0.02632567	0.04844848
BC017158	0.57698461	3.34202633	5.75563565	0.02634698	0.04847509
Soat2	-0.8317215	1.76274704	5.75511036	0.02635305	0.04847509
Bpgm	0.38199599	6.8275735	5.75325076	0.02637453	0.04848715
Atp9b	-0.6023537	3.99564467	5.75319905	0.02637513	0.04848715
Pole4	0.40655919	5.26961281	5.75286472	0.026379	0.04848715
Rph3al	1.11922028	1.23331176	5.75188789	0.02639029	0.04849603
Pcdh18	-0.5602957	3.19480884	5.74856557	0.02642876	0.04855481
Jakmip3	-0.7005183	4.95956322	5.74795904	0.02643578	0.04855583
Vmn2r85	-1.7227342	1.10954057	5.74652946	0.02645236	0.04857388
Snw1	0.32315429	6.48926014	5.74593761	0.02645922	0.04857388
Wwtr1	0.43098463	6.61329783	5.74543668	0.02646504	0.04857388
Plekho1	0.46101013	4.03851156	5.74084657	0.02651836	0.04863359
Isoc2a	0.74311634	1.96736771	5.74023877	0.02652543	0.04863359
Klhl18	-0.4437331	4.45705032	5.74017909	0.02652613	0.04863359
2700046A07I	0.81953186	2.66778136	5.73989123	0.02652948	0.04863359
Trp53	0.46211752	5.99533336	5.73984757	0.02652998	0.04863359
Jph4	-0.3130139	7.22585029	5.73921654	0.02653733	0.04863516
Ggct	0.51839606	4.48865281	5.73813547	0.02654991	0.04864635

Gna11	0.42782293	5.92150648	5.73703137	0.02656278	0.04865803
B3gnt3	2.37004425	-0.0407022	5.73596551	0.0265752	0.04866557
Trabd2b	0.59654861	6.3225332	5.73556506	0.02657987	0.04866557
Atp6ap2	0.33300397	7.25392313	5.73485383	0.02658816	0.04866888
Sde2	0.36880118	4.81445549	5.73151903	0.0266271	0.04872826
Trim43b	-1.9587079	-0.7916784	5.7306024	0.02663781	0.04873597
Gm608	-0.3252531	8.24058658	5.72918283	0.02665441	0.04875445
Cnn3	0.46705489	6.27032767	5.72844194	0.02666308	0.04875842
Anks1	-0.4603323	3.49404808	5.72590414	0.0266928	0.04880086
Ccdc101	0.4667398	4.2368717	5.72426525	0.02671201	0.04882408
Mrpl41	0.39575278	4.74946959	5.72330116	0.02672331	0.04883285
Hps5	-0.5656607	3.60626407	5.72200094	0.02673857	0.04883739
Grm3	-0.3588402	5.65836054	5.7210851	0.02674933	0.04883739
Nup160	-0.5010928	4.50080403	5.72096457	0.02675074	0.04883739
Rpe65	-1.898464	0.67821647	5.72087151	0.02675184	0.04883739
Nhsl2	-0.2870429	8.08117747	5.71620093	0.02680676	0.04892575
Wipf1	0.55359578	6.02779989	5.71457671	0.02682589	0.04894875
Pcdhb8	-1.0414866	1.09888164	5.71229836	0.02685275	0.04898585
Phldb1	-0.3892254	4.12078655	5.71114904	0.02686631	0.04899158
Cdsn	1.14304826	0.95293506	5.71092518	0.02686896	0.04899158
4933400F21I	-1.7091654	1.22189907	5.71032482	0.02687604	0.0489926
Sft2d3	-0.5229661	3.19211929	5.7093004	0.02688814	0.04900274
Nf2	0.34372701	6.09246586	5.70870534	0.02689517	0.04900365
2700062C07I	0.51552803	3.96942133	5.70624241	0.0269243	0.04903824
1700112E06I	1.02307915	1.17197641	5.70559371	0.02693197	0.04903824
Atp1a3	0.4397045	9.48253758	5.70544215	0.02693377	0.04903824
Dhtkd1	-1.3302579	1.49539204	5.70428617	0.02694745	0.04904098
Ash1l	-0.3267061	9.15914459	5.70372848	0.02695406	0.04904098
Chrac1	0.64487517	2.94502002	5.70363564	0.02695516	0.04904098
Adck2	-0.6879404	2.23540847	5.70264144	0.02696694	0.04904098
C230079O03	-3.6042342	-2.0034868	5.70255518	0.02696796	0.04904098
Mllt10	-0.3304409	6.2237758	5.6990165	0.02700995	0.04909584
Pyurf	0.53326921	5.11563781	5.69890886	0.02701122	0.04909584
Zdhhc21	-0.3694995	6.37299455	5.69532487	0.02705382	0.04916136
Asph	-0.3117785	6.75367526	5.69452916	0.02706329	0.04916665
Slc25a22	0.36522952	4.94675892	5.69375938	0.02707245	0.04917139
Tmem66	0.3653545	5.98328088	5.69215129	0.0270916	0.04919426
AI504432	-0.3970862	5.6203491	5.68858202	0.02713417	0.04925395
Timp4	-0.7124379	2.45770989	5.6882939	0.02713761	0.04925395
Fbxo44	0.44121656	4.46392844	5.6875381	0.02714663	0.04925525
Rnf112	-0.5796782	4.83507087	5.68713401	0.02715146	0.04925525
C1qb	0.92560418	2.39464549	5.68263794	0.02720522	0.04934085
Vmn2r87	-1.3424149	1.17231692	5.68035347	0.02723258	0.0493566
Kcnn1	-0.8443134	1.94683146	5.68006383	0.02723605	0.0493566
Mfsd8	-0.4955928	3.26371644	5.67978411	0.02723941	0.0493566

Ing4	0.43475856	3.88740656	5.67971606	0.02724022	0.0493566
Wwox	0.43938308	4.0488127	5.6790502	0.02724821	0.04935915
Rad9b	-1.4188912	0.48771744	5.67697503	0.02727311	0.04938614
Fam120b	-0.3504543	6.59916245	5.6767111	0.02727627	0.04938614
Tnfsf8	-2.7540204	-0.2182772	5.67363448	0.02731324	0.04944114
Klrg1	4.73722408	-2.0796857	5.67275607	0.02732381	0.04944643
Cmtr2	-0.7609383	2.13066756	5.67229529	0.02732935	0.04944643
Mdga1	-0.8317542	2.48382082	5.67082633	0.02734703	0.04945888
Tsc22d1	0.36076308	9.67766436	5.67062845	0.02734941	0.04945888
Abcc8	-0.753729	2.69792828	5.66908008	0.02736807	0.04947846
Hspa4l	-0.4943726	7.12134736	5.66863486	0.02737344	0.04947846
Rftn2	0.40593426	4.75878567	5.66574426	0.02740831	0.04952831
Ppp1r1c	-1.599195	1.07525846	5.66525405	0.02741422	0.04952831
Ctla2b	2.34755854	-0.6607534	5.6637195	0.02743276	0.04954987
AI467606	3.65664297	-1.3976377	5.66289717	0.0274427	0.04955589
Dcaf6	-0.4094152	6.91944124	5.65982726	0.02747984	0.04961102
Cep112	-0.5028957	3.64725574	5.65484803	0.0275402	0.04970802
Coil	0.60382187	3.23445408	5.65025588	0.027596	0.04979676
Gsta4	0.53172814	6.83520109	5.64926685	0.02760804	0.0497984
Zfp105	0.70843362	3.11122438	5.64909014	0.02761019	0.0497984
Lime1	-1.1570172	2.74346106	5.64261786	0.0276891	0.04992872
Cdk2ap2	0.5704833	3.39186092	5.64074225	0.02771201	0.04995803
C330024D21I	-1.5994851	0.122273	5.63899682	0.02773336	0.04997326
Per2	-0.4741569	5.05396561	5.63872518	0.02773668	0.04997326
Dip2c	-0.3854761	6.83602647	5.63841729	0.02774045	0.04997326
Nfkbiz	-0.8341935	2.15685519	5.63739959	0.0277529	0.0499837
Spc24	-0.8279698	2.43892939	5.63654112	0.02776342	0.04999063
P2ry12	0.55697423	3.67863473	5.63370366	0.0277982	0.05004124
Cdk2ap1	0.35183298	6.49375569	5.62999284	0.02784375	0.05011123
Adam22	-0.4057581	7.72163475	5.62904096	0.02785545	0.05012026
B020004J07F	-1.5860016	0.1922254	5.62816644	0.02786621	0.05012758
Epb4.1	-0.3240833	4.96364631	5.62755479	0.02787373	0.0501291
Bak1	0.61172679	3.90940784	5.6220129	0.02794201	0.05022802
Adi1	0.45416825	7.13727082	5.62200335	0.02794213	0.05022802
Ctr9	-0.359084	5.2111628	5.6204691	0.02796107	0.05025002
Twf2	0.50742166	3.43721528	5.61948725	0.02797319	0.05025977
Pir	0.45259822	3.46972287	5.61882083	0.02798143	0.05026252
Mapkapk5	-0.4138589	4.18382528	5.61688104	0.02800541	0.05028365
Zfp609	-0.2868104	7.47356055	5.61678538	0.02800659	0.05028365
Hnrnpk	0.28486988	7.92294978	5.61611948	0.02801483	0.0502864
Echdc3	1.04212357	1.11695065	5.61353116	0.02804688	0.05033188
Add2	-0.4234976	7.61685825	5.61289742	0.02805473	0.05033393
Snapc5	0.44740149	6.27476818	5.60996332	0.02809112	0.05038517
Slc25a24	0.58833659	5.32740564	5.60951234	0.02809672	0.05038517
Anapc15	0.66582061	2.43938327	5.6049358	0.0281536	0.05044614

Sacm1l	-0.3560423	6.00551482	5.6049347	0.02815362	0.05044614
Il11	-1.1671586	0.53524101	5.60464023	0.02815728	0.05044614
Tubgcp3	-0.3734519	4.42504064	5.60461273	0.02815762	0.05044614
Dpysl5	-0.611688	4.75965098	5.59950158	0.02822131	0.05053957
Vprbp	-0.3316366	6.75123308	5.59934638	0.02822325	0.05053957
Abca8a	-0.7686559	2.39900376	5.59705051	0.02825191	0.05057883
Tmem199	0.38746173	4.14860616	5.59643468	0.02825961	0.05058053
Pcdhb2	-1.2352761	1.80948339	5.59566892	0.02826918	0.05058559
Nphs2	0.61791752	3.92360048	5.5933273	0.02829847	0.05062593
4930578E11l	-1.6659075	-0.6153435	5.58798968	0.02836536	0.0507335
Islr	0.82409952	6.83435633	5.58653161	0.02838366	0.05075413
Tacc1	0.24963589	7.88929376	5.58508263	0.02840186	0.05076292
Eci3	1.27405295	0.66836361	5.58481903	0.02840518	0.05076292
Zfp397	-0.2546618	5.88909395	5.58452452	0.02840888	0.05076292
Anp32e	0.31979504	8.05480532	5.58249106	0.02843445	0.05079652
Mov10l1	-3.9139892	-2.089242	5.58146401	0.02844738	0.05080711
Ccdc132	-0.345515	6.47996057	5.58094421	0.02845393	0.05080711
Kdr	-0.576157	2.63592826	5.57970848	0.02846949	0.05081334
Grip1	-0.532448	4.08404271	5.57959181	0.02847096	0.05081334
Col26a1	-0.8791186	1.50329749	5.57798534	0.02849121	0.05083739
Pcdh9	-0.3776544	7.3123863	5.5715305	0.02857275	0.05096923
Kdm2a	-0.2826906	7.17003186	5.57101399	0.02857929	0.05096923
Lrp3	-0.4475124	4.92686059	5.57037848	0.02858733	0.05096923
Klk14	-1.4703709	-0.0143907	5.56946988	0.02859884	0.05096923
Sdk1	0.60114034	4.14071295	5.56870468	0.02860853	0.05096923
Igf2	-0.5857142	10.4379258	5.5685417	0.0286106	0.05096923
Ttpa	-0.9978703	1.48140709	5.56795928	0.02861798	0.05096923
2810403D21l	-1.5366888	0.25113302	5.56782048	0.02861974	0.05096923
Fbxo36	0.88253891	2.9116626	5.56730631	0.02862626	0.05096923
Atp5h	0.45447876	6.99955852	5.56654258	0.02863594	0.05097437
Lage3	0.66646447	3.39926527	5.56276201	0.02868394	0.0510477
Gsk3a	0.25823241	7.32149468	5.55883292	0.02873392	0.05112452
Ube2e3	0.38012543	7.65512624	5.55694926	0.02875792	0.05115508
Braf	-0.3134072	8.58365271	5.55445972	0.02878966	0.05119291
Gas2l1	0.56303003	3.8345885	5.55347396	0.02880225	0.05119291
Elp5	0.4460638	4.81554388	5.55335081	0.02880382	0.05119291
Mkrn1	0.30144653	6.7341484	5.55283207	0.02881044	0.05119291
Slc22a5	-0.6624507	2.58385577	5.55260782	0.02881331	0.05119291
Smarcad1	-0.4462666	5.2613754	5.55004984	0.028846	0.05123886
Zc2hc1a	0.33041857	7.30989653	5.54893801	0.02886022	0.05125199
Rogdi	0.40230261	4.61086762	5.54745307	0.02887923	0.05127036
Arhgap32	-0.483493	9.3418549	5.54706228	0.02888424	0.05127036
Rbx1	0.41304647	6.58727791	5.54650628	0.02889136	0.05127087
Bbs7	-0.4498246	4.04676063	5.54360072	0.02892861	0.05132484
Evc2	-0.8907302	2.24314415	5.54275527	0.02893946	0.05133195

Kifc1	-2.7311103	-1.4155674	5.54198593	0.02894934	0.05133733
Tnxb	-1.0075023	1.28082218	5.54031766	0.02897077	0.05135091
Smg8	-0.3600265	4.41716152	5.53999552	0.02897491	0.05135091
Rnf157	-0.4020644	7.48577637	5.53965849	0.02897924	0.05135091
Esyt2	-0.2852854	5.89773321	5.53925902	0.02898438	0.05135091
Zfp939	-0.8189977	2.6746555	5.53642299	0.02902087	0.05140342
Tmem230	0.31972461	6.66927818	5.53343396	0.02905939	0.05145949
Ttc39a	-1.2813523	1.32521746	5.53202038	0.02907763	0.05147964
Ksr2	-0.4565112	6.0655444	5.53089437	0.02909216	0.05149043
Smoc1	0.54731029	4.56734441	5.53048451	0.02909746	0.05149043
Spp2	-2.1901766	0.45542696	5.5294801	0.02911043	0.05150125
Adam32	-1.9248695	-0.4974968	5.52761498	0.02913454	0.05152444
Pik3ca	-0.2563705	7.01483984	5.52740363	0.02913728	0.05152444
5730405O15	-1.9699051	-0.293786	5.52413221	0.02917963	0.05157497
Scn11a	4.62594843	-1.9906575	5.52364408	0.02918595	0.05157497
Dusp23	0.82339476	2.79903088	5.52360343	0.02918648	0.05157497
Tspyl5	0.30157787	5.78190886	5.52208344	0.02920619	0.05159764
Aoah	-2.0861668	-0.6572058	5.51964114	0.02923788	0.05164147
Jup	0.37343471	5.22506196	5.51754569	0.02926511	0.05167739
Slc26a11	-0.8634949	2.08885462	5.51447207	0.02930509	0.05172548
Atp6v0a4	-1.4492251	0.14184209	5.51439198	0.02930614	0.05172548
Adcy10	-1.3138486	0.20138722	5.51374271	0.02931459	0.05172687
Taf11	0.37533586	5.26365295	5.51292286	0.02932527	0.05172687
Frmd3	-0.7892859	2.67448322	5.51200038	0.02933729	0.05172687
Tppp3	0.525911	3.54507337	5.51181431	0.02933972	0.05172687
Cdpf1	0.78314664	3.2397897	5.51154565	0.02934322	0.05172687
Zkscan16	-0.5272691	5.24616112	5.51115649	0.0293483	0.05172687
Mir1931	-2.7223808	-1.7815643	5.51061733	0.02935533	0.05172711
Foxj1	1.41703285	0.73725854	5.50801056	0.02938935	0.05176468
Tmem8b	-0.4157416	5.07765794	5.50777144	0.02939248	0.05176468
Cd209a	-1.0132471	3.352711	5.50739834	0.02939735	0.05176468
Islr2	0.63195854	2.79239509	5.50583823	0.02941774	0.05178843
Pcdh11x	-0.5313988	4.67107763	5.50507654	0.02942771	0.05179381
Gpsm1	-0.5674462	2.66469454	5.50349206	0.02944844	0.0518043
4933413G19	-3.4428166	-1.7289615	5.50340745	0.02944955	0.0518043
Kcnj9	-0.4238019	5.02242823	5.50295137	0.02945552	0.0518043
Gabra6	6.4340379	-2.0502817	5.65536024	0.02946733	0.0518043
Pisd-ps2	-1.1778178	1.82561188	5.50165058	0.02947256	0.0518043
A430107P09	-1.5572771	-0.089564	5.50145664	0.0294751	0.0518043
Slk	-0.3297929	8.16449595	5.50020189	0.02949155	0.05181077
Lamtor2	0.61582168	4.07058306	5.50012199	0.0294926	0.05181077
Zfp521	0.43373858	5.60118123	5.49945963	0.02950128	0.0518139
Peli3	1.77474402	-0.7958979	5.49788245	0.02952198	0.05183811
Fam96a	0.4712861	5.17674394	5.49655949	0.02953935	0.05185648
Prtg	-0.8653167	1.53552983	5.49542553	0.02955426	0.0518705

Zfp831	-0.6530925	5.24283615	5.49289832	0.0295875	0.05191669
Anxa4	0.54062875	6.49079403	5.48992088	0.02962671	0.05196534
Frem3	-1.7141341	0.105963	5.48836959	0.02964717	0.05196534
Ptprm	-0.5237206	5.20958612	5.48832959	0.02964769	0.05196534
Dffb	-1.2908034	1.45709339	5.487926	0.02965302	0.05196534
1700001D01I	-1.6748614	1.20263267	5.48787674	0.02965367	0.05196534
Plcb1	-0.4814226	8.78715465	5.48764055	0.02965679	0.05196534
Rps2	0.3770508	6.30221728	5.48550428	0.02968499	0.05200262
Acsl3	-0.2823391	7.2432512	5.48270531	0.02972199	0.05205528
Sel1l3	-0.6220888	4.96079136	5.48127106	0.02974097	0.05207636
Slc25a54	-2.5795436	-0.925918	5.47764044	0.02978908	0.05214843
Grhl1	-0.6047455	2.97171947	5.47321188	0.02984788	0.05223917
Rpgrip1l	-0.4849852	5.86432918	5.4723796	0.02985894	0.05224634
5530601H04I	-0.5875878	3.42913105	5.47012753	0.02988891	0.05228658
Mir325	-2.6244556	-1.4886438	5.46918491	0.02990146	0.05229634
Dexi	0.4204188	3.84167745	5.46375989	0.02997382	0.05241067
4930487H11I	-2.7830057	-1.2748223	5.45980377	0.0300267	0.05249091
Itga7	-1.8888702	-0.3260729	5.45647108	0.03007134	0.05255669
Ptgr1	0.86838636	2.92611121	5.45592904	0.03007861	0.05255715
Tbc1d25	0.57122081	3.07336258	5.45391184	0.03010567	0.05259218
Hyls1	0.65972108	2.25186842	5.45280712	0.0301205	0.05260584
Fam96b	0.59884359	2.67795125	5.45177349	0.03013438	0.05261784
Bbs10	-0.6127679	2.99343135	5.44763649	0.03019003	0.05270273
5730455P16I	0.27801623	7.14297718	5.44261535	0.03025772	0.05280345
Cox4i1	0.58494107	6.74873163	5.44231313	0.0302618	0.05280345
Vcl	0.36082421	6.50105909	5.43855826	0.03031254	0.05287968
Atg2a	-0.6201711	3.99208014	5.43762076	0.03032523	0.05288951
Gpc3	0.67899356	3.71078847	5.43415217	0.03037221	0.05295913
Snrpf	0.73828744	2.83226967	5.43313407	0.03038601	0.05297089
Pole	-0.9527472	1.08411934	5.43228438	0.03039754	0.05297868
Nap1l1	0.255464	8.68607673	5.429703	0.03043259	0.05302744
Tead3	0.66719212	2.73021835	5.42756522	0.03046165	0.0530627
Nedd8	0.55258182	5.60706888	5.427174	0.03046697	0.0530627
Rnpc3	-0.6122279	4.82241866	5.42423253	0.03050702	0.05312011
Zc3h12c	-0.2587136	6.25593924	5.42105032	0.03055041	0.05318332
Tm2d2	0.77598708	4.24607519	5.41793767	0.03059292	0.05323746
Ubl3	0.26659459	7.21135999	5.41773422	0.0305957	0.05323746
Srfbp1	0.466981	3.92073567	5.41600861	0.0306193	0.05326616
Ndufaf2	0.53833595	4.81039689	5.41362412	0.03065194	0.05331058
Sin3a	-0.2872086	6.53073228	5.41258129	0.03066623	0.05332307
Emp2	-0.4952497	4.33314643	5.41033564	0.03069702	0.05336424
Zfp809	0.36108178	4.84571026	5.409285	0.03071144	0.05337694
Nr1h2	0.59377667	4.09397517	5.40523441	0.0307671	0.05346129
Siah2	0.42277159	4.21540597	5.4043669	0.03077904	0.05346965
Tdrd7	-0.3587072	4.68204311	5.40078035	0.03082844	0.05354307

1700007J10F	-1.4457095	0.06999481	5.3997803	0.03084223	0.05355462
Fibin	-0.6553682	4.43120011	5.39901188	0.03085283	0.05356063
Megf6	-0.9907008	1.38357605	5.39720959	0.03087771	0.05359034
Zfp58	0.54903844	3.40158186	5.39673751	0.03088423	0.05359034
Recql	-0.450039	3.96390845	5.39602629	0.03089406	0.053595
Prkx	0.45610654	4.57559272	5.39506681	0.03090732	0.05359939
Stap1	-1.4324319	0.31756925	5.39480943	0.03091088	0.05359939
Rpl35	0.43779217	4.96906104	5.39370822	0.03092611	0.05360132
Mau2	-0.3711115	5.4300466	5.39363197	0.03092717	0.05360132
Kdm4c	-0.4807134	5.52623136	5.3929207	0.03093701	0.05360132
D330045A20	-1.6094207	-0.0948508	5.39219319	0.03094708	0.05360132
Cacna1c	-0.5165265	6.3128366	5.3921471	0.03094772	0.05360132
Helz	-0.444888	6.72444191	5.39150879	0.03095656	0.05360425
Scd1	-0.324874	6.0379921	5.38948876	0.03098456	0.05364035
LOC1026344	-1.140951	1.10325878	5.38649776	0.03102606	0.0536998
Timp1	1.30220518	1.52982041	5.38398949	0.03106092	0.05372641
Aatf	0.46253291	3.91732597	5.38324923	0.03107121	0.05372641
Spaca6	-1.2465531	2.96142993	5.38309709	0.03107333	0.05372641
Pcna	0.36438914	6.30910801	5.38284249	0.03107687	0.05372641
Prkab2	0.40232344	4.92458605	5.38281553	0.03107724	0.05372641
Zfp329	-0.3374634	6.15661559	5.38212854	0.0310868	0.05373055
Gem	1.64956898	2.8642039	5.38078715	0.03110548	0.05374954
Strn4	-0.3285731	5.44574804	5.38031009	0.03111212	0.05374954
Leprel2	-0.6076541	3.23305607	5.37959817	0.03112204	0.0537543
Adam1b	-1.6533833	0.2342261	5.37868774	0.03113473	0.05376384
Lrrc42	0.53367797	3.25022116	5.3777939	0.0311472	0.05377298
Ogg1	0.96741564	0.72943137	5.37443475	0.03119409	0.05384155
Tbcel	0.400294	5.2480881	5.37263128	0.0312193	0.05387267
Pnpt1	-0.5928889	3.92011407	5.37178582	0.03123113	0.05388068
Pitpnm2	-0.4001523	6.50109081	5.36863937	0.03127518	0.05394428
Cdc42se1	0.40437403	5.51171644	5.36701379	0.03129797	0.05396836
Glul	0.36816178	10.5718556	5.36627898	0.03130828	0.05396836
Ly96	0.60367001	3.76427015	5.36601143	0.03131203	0.05396836
Gm10653	-1.1843912	0.53454069	5.36559164	0.03131792	0.05396836
Ndufa13	0.65394314	5.844441	5.36431291	0.03133587	0.05398689
Arpc1b	0.75993175	6.03862312	5.36332904	0.03134969	0.0539983
Cdh11	-0.2651128	6.72503246	5.36171726	0.03137235	0.05400912
Acot6	0.64403184	2.70793181	5.36139621	0.03137687	0.05400912
Hmga2	0.43776735	3.850615	5.36134568	0.03137758	0.05400912
Prima1	0.77445655	1.40377764	5.36066056	0.03138721	0.05401332
Fam109a	-0.9728875	0.94421152	5.35967742	0.03140105	0.05402473
Eif4ebp3	-1.0342693	0.94907495	5.35835887	0.03141962	0.05404428
Pcdhgb1	-0.6534566	4.09657467	5.35694737	0.03143951	0.0540661
Slc12a8	-1.3206059	0.36052363	5.35489975	0.03146839	0.05410335
Ict1	0.58651788	4.30702121	5.3542902	0.03147699	0.05410574

Magel2	-1.9089855	0.39993086	5.35359977	0.03148673	0.0541101
Rnf4	0.27522976	6.47763243	5.34658725	0.03158593	0.05426813
Glra1	1.31825456	0.24685112	5.3441749	0.03162013	0.05431446
Scfd1	-0.3935449	4.64103708	5.3428136	0.03163945	0.05433001
Lmbr1	-0.3630751	5.0770825	5.34251675	0.03164367	0.05433001
Ephb6	-0.6019287	3.56772938	5.34078803	0.03166823	0.05435973
Ptp4a1	0.42929708	4.59349163	5.33991842	0.03168059	0.05436851
Tmcc3	0.34996299	6.34903671	5.33867896	0.03169822	0.05438632
Vwc2	0.61119717	3.14969717	5.3378884	0.03170947	0.05439318
Ube2e2	0.29642222	6.58122371	5.33720785	0.03171916	0.0543951
Ribc1	-1.550196	-0.2145785	5.33679102	0.03172509	0.0543951
Mfap1a	0.23805277	7.41490946	5.33442171	0.03175885	0.05444054
1110038B12I	-0.6646337	2.45169573	5.33293199	0.0317801	0.05446452
Dynlrb2	1.15552938	0.69709176	5.33104975	0.03180697	0.05449812
Znrd1	0.4801355	4.5581472	5.3301243	0.03182019	0.05450832
Apaf1	-0.4664078	3.97683856	5.32929989	0.03183198	0.05451606
Gpkow	0.31119571	6.8147221	5.32757297	0.03185668	0.0545459
Oser1	0.50880853	4.12300263	5.32623383	0.03187584	0.05454879
Ino80dos	-0.7425412	2.98122524	5.32602326	0.03187886	0.05454879
Clec3b	-1.2478199	0.90492489	5.32593127	0.03188017	0.05454879
Zcchc11	-0.3375209	6.83603884	5.32204881	0.03193583	0.05463155
Zcchc8	-0.4432111	3.8751708	5.32005392	0.03196446	0.05466698
Nudc	0.40597354	5.63005536	5.31959106	0.03197111	0.05466698
Bloc1s6	0.34761549	5.60075722	5.31466926	0.03204191	0.05477554
Slc35f6	0.76232755	1.93306923	5.31360573	0.03205723	0.05478925
Rnps1	0.25881742	6.25131145	5.31288666	0.03206759	0.05479448
Polg	-0.5097378	3.26366115	5.30959349	0.0321151	0.05482167
Mfap1b	0.26673609	6.89563488	5.30918539	0.032121	0.05482167
Eif4a1	0.26220624	7.37071226	5.30845176	0.03213159	0.05482167
Chmp2a	0.51441416	5.63235119	5.30810605	0.03213659	0.05482167
Sec16a	-0.3224163	6.43811088	5.30768444	0.03214268	0.05482167
Slc3a2	0.32167195	6.32439206	5.307625	0.03214354	0.05482167
Slc39a3	-0.5100521	3.21713689	5.30703306	0.0321521	0.05482167
Parp3	0.54237654	4.28637517	5.30674748	0.03215623	0.05482167
Lrrtm1	0.45562459	4.64472738	5.30673403	0.03215642	0.05482167
Xpot	-0.2706423	7.00853197	5.30672252	0.03215659	0.05482167
Dld	0.2761758	7.47831163	5.30377061	0.03219931	0.05488202
Rfng	-0.5455576	3.51082264	5.30275763	0.03221398	0.05488778
Rpl34	0.89945332	0.76180846	5.30252717	0.03221732	0.05488778
Gm10754	-0.8762865	1.74058134	5.3018672	0.03222689	0.05489161
Wdr34	-0.5274361	3.15942228	5.29981446	0.03225666	0.05492897
Luzp2	0.43966612	6.55836174	5.29934563	0.03226346	0.05492897
Ccbl1	-1.005385	1.5529635	5.29732092	0.03229287	0.05496655
Hoxd11	-1.7733854	-0.3836331	5.29642078	0.03230595	0.05497634
Dpep2	-3.695309	-1.7038124	5.29473652	0.03233044	0.05500555

Mturn	-0.2915473	6.37009665	5.29311955	0.03235398	0.05503311
B230216N24	-0.731545	2.24691743	5.29242747	0.03236406	0.05503777
Gsto1	0.55008769	4.19893539	5.29154174	0.03237696	0.05504724
Tmem158	0.42887599	4.10897851	5.28871424	0.03241819	0.05510485
B3gnt1	0.37811595	4.84392782	5.28753474	0.03243541	0.05512163
Tes	0.46874159	3.71867944	5.28489694	0.03247396	0.05517463
Slc12a9	-1.0333898	1.25442533	5.28283702	0.03250409	0.05521084
Zc3h11a	-0.2563204	6.83947605	5.28243454	0.03250998	0.05521084
Rpl7	0.42110415	6.91340087	5.27988719	0.0325473	0.0552617
Gm11744	-2.0309894	-0.4086737	5.27841899	0.03256883	0.05528574
Adrb3	-1.8509823	-0.894653	5.27764797	0.03258015	0.05529244
Wdfy4	-0.9796946	1.56055721	5.27624586	0.03260073	0.05531486
Zfp474	-3.6940389	-1.4850592	5.27530248	0.03261459	0.05531876
Gmfb	0.27961714	8.2222009	5.27508531	0.03261778	0.05531876
Slc4a3	-0.655762	3.42105113	5.27396096	0.03263431	0.05533428
Hspe1	0.34920036	6.34454213	5.26567557	0.03275639	0.05552873
Atxn7	-0.3365905	5.79439811	5.26401696	0.0327809	0.05555719
Akr1a1	0.47638904	7.81607609	5.26336888	0.03279048	0.05555719
Srcin1	-0.5981954	6.08474988	5.26303586	0.0327954	0.05555719
Fkbp8	0.46522392	5.10116843	5.2588937	0.03285671	0.05564848
Entpd2	-1.2291748	0.55519252	5.25657321	0.03289111	0.05567052
Prkar1a	0.2367982	9.6161682	5.25602895	0.03289919	0.05567052
Pxmp4	0.46450607	3.31185349	5.25600112	0.0328996	0.05567052
Pdcd2l	0.51706736	3.02662668	5.25581492	0.03290236	0.05567052
5830444B04	-1.1384589	1.32362467	5.25528858	0.03291018	0.05567052
Cyb5r2	-2.9330421	-1.4312352	5.25453728	0.03292133	0.05567052
Speer7-ps1	-1.4009108	0.64711457	5.25451461	0.03292167	0.05567052
Gpr155	-0.4087004	5.73513975	5.2533218	0.03293939	0.05567962
5430402O13	-1.8594121	-0.5565172	5.25315283	0.0329419	0.05567962
Fam129c	-2.3463766	-0.3552634	5.25137807	0.03296828	0.05571167
Hist3h2ba	2.10571245	0.02817008	5.24897995	0.03300398	0.05575942
Pdrg1	0.52145082	5.4404089	5.24846583	0.03301163	0.0557598
Nfkb1	-0.4136085	4.41632121	5.2466014	0.03303942	0.05579417
Nqo1	0.84540289	4.40171669	5.24586101	0.03305046	0.05580025
Lmo4	0.38967789	9.51337193	5.24392807	0.03307931	0.05583483
Gm13157	0.54032105	3.36192233	5.24322362	0.03308983	0.05583483
Poldip3	0.41195075	5.76822409	5.24293075	0.0330942	0.05583483
Arl5c	2.0156874	-0.6989785	5.24244196	0.03310151	0.05583483
Tmem202	2.22538847	0.48555398	5.24199687	0.03310816	0.05583483
Ccdc39	-0.564885	4.3535668	5.23851832	0.03316019	0.05591001
Klhl4	-0.361362	4.81035219	5.23617114	0.03319536	0.05595672
Serpine2	0.3519272	5.98452131	5.2351435	0.03321077	0.05596542
Piga	-0.7202052	3.12116202	5.23476729	0.03321641	0.05596542
Map4k4	-0.3185615	6.42734492	5.23433484	0.0332229	0.05596542
Tnfrsf1a	0.75719295	4.11588072	5.2318996	0.03325946	0.05601443

Oxld1	1.06956043	1.12722432	5.22962394	0.03329367	0.05605945
D330050I16F	-1.4735782	-0.4734021	5.2276014	0.0333241	0.05609811
Fbxo43	-1.4184887	-0.5212013	5.22641671	0.03334194	0.05611555
Gabpb2	-0.3105422	6.14422032	5.22520127	0.03336026	0.05613378
Cnot6l	-0.2765355	7.06788096	5.22060024	0.0334297	0.05623801
Dlx1as	-0.4923598	3.27813681	5.21766892	0.03347403	0.05629996
Cckar	-2.4411641	-0.7734753	5.21678034	0.03348748	0.05630995
Zfp770	0.28374221	6.22912452	5.2149846	0.03351468	0.05634306
Gm16062	1.48749132	-0.2612787	5.21340787	0.03353858	0.05637061
Wtap	0.27326688	6.35981409	5.21200911	0.0335598	0.05639364
Sfrp1	-0.5880854	6.64119233	5.20924679	0.03360175	0.05645149
Gstt1	0.62183953	4.87976353	5.20801958	0.03362041	0.05647019
Ern1	-0.8712762	2.21275338	5.2057699	0.03365464	0.05651503
Kpna1	0.28475054	6.91615196	5.20369548	0.03368624	0.05655544
Ube2i	0.318176	7.37592641	5.20172635	0.03371627	0.05659318
Samd4b	0.30032892	6.83146274	5.20060691	0.03373336	0.05660507
Nrbp2	-0.3552791	6.88218076	5.20027351	0.03373844	0.05660507
Pcdh10	-0.3833402	7.10637105	5.19870636	0.03376238	0.05663257
Hbs1l	-0.2983333	5.48903497	5.19736705	0.03378285	0.05664576
Hsd17b11	0.44110885	5.02271974	5.1972038	0.03378535	0.05664576
Ccdc166	0.55442966	3.31457511	5.19372253	0.03383864	0.05672195
Rbm33	-0.4313154	5.58997343	5.19324717	0.03384592	0.05672195
Ptger3	0.7706141	3.47970624	5.18952015	0.03390308	0.05678615
Clta	0.33893306	6.84018885	5.18928416	0.03390671	0.05678615
Parpbp	1.79946985	0.83810134	5.18926927	0.03390694	0.05678615
Gca	-0.3897552	6.18271984	5.18780054	0.0339295	0.05680468
Gm266	-1.1623138	0.6425385	5.18715322	0.03393944	0.05680468
Agl	-0.404663	5.63069025	5.18707025	0.03394072	0.05680468
Frmd7	-1.4844692	-0.1446833	5.18574802	0.03396105	0.05680642
Ttyh2	-0.6748246	2.6258952	5.18560085	0.03396332	0.05680642
Impdh2	0.40293919	3.73965062	5.18552534	0.03396448	0.05680642
Galnt6	-0.9247759	1.98311809	5.18493211	0.03397361	0.05680902
Cdk5rap1	-1.0606108	1.39925974	5.18324027	0.03399965	0.05683991
Tmem177	0.51609012	3.68336884	5.181116	0.03403239	0.05688196
Prkab1	0.59237545	3.39809455	5.18034376	0.0340443	0.05688759
Smc3	-0.323444	8.16643801	5.17991421	0.03405092	0.05688759
Rab4b	0.45686892	3.46362841	5.17840832	0.03407417	0.05691374
Rnf144b	-0.4125131	4.36927858	5.17424778	0.03413848	0.0570023
9230110C19I	0.56756985	3.02451979	5.17399522	0.03414238	0.0570023
Dph1	-2.6579327	-1.3555422	5.17150233	0.03418099	0.05703904
Midn	0.37809248	5.84324131	5.17117239	0.0341861	0.05703904
Timeless	1.20042254	2.14805561	5.17110133	0.0341872	0.05703904
Dlg1	-0.2999292	7.4792636	5.17009538	0.0342028	0.05705237
Pomgnt2	0.71502817	2.41069811	5.16849079	0.03422769	0.0570812
BC018242	0.39374952	4.75332154	5.16586523	0.03426846	0.05713649

Tcf20	-0.3465539	8.10599558	5.16439901	0.03429126	0.05716179
Ica1	-0.48975	4.14762539	5.16285458	0.03431529	0.05717721
Usp25	0.27160919	7.47059909	5.16282477	0.03431575	0.05717721
Cox4i2	-2.4419974	-1.5290112	5.16164596	0.0343341	0.05719508
Chd3	-0.2846249	7.99789304	5.16044294	0.03435284	0.05719911
Rgs10	0.41981047	4.32710585	5.16005088	0.03435895	0.05719911
Slc45a4	-0.4701374	3.61257026	5.16002248	0.0343594	0.05719911
Tmem87a	-0.4022367	4.44738721	5.15832104	0.03438593	0.05722845
Suclg2	0.45765813	5.58089658	5.15791402	0.03439228	0.05722845
B3glct	-0.3380377	5.47446189	5.15709948	0.03440499	0.05723691
Sri	0.33814981	6.57914909	5.15461874	0.03444374	0.05728867
Usp31	-0.4480075	7.46671707	5.15210066	0.03448313	0.05734146
Impa1	0.29951977	5.28422292	5.14731172	0.03455817	0.05745232
Arl8a	0.31751335	6.34525013	5.1468691	0.03456511	0.05745232
Rltpr	0.68386412	1.80579924	5.14566609	0.034584	0.05747098
Arhgef12	0.22877044	9.52684002	5.1450673	0.0345934	0.05747387
2410021H03I	-1.7042245	0.56287823	5.14159877	0.03464793	0.05755172
Commd7	0.39506461	4.6837121	5.1385857	0.03469538	0.05761778
Aldh1l2	-0.9599808	2.81231289	5.13692647	0.03472154	0.05762671
Foxo4	0.83287145	1.66540675	5.13673223	0.0347246	0.05762671
Cxcl1	6.29414508	-1.9014253	5.1367241	0.03472473	0.05762671
Map2k1	0.23405422	7.71419502	5.13616211	0.0347336	0.05762671
Snap29	0.33259461	6.39853214	5.13470979	0.03475652	0.05762671
Fcf1	0.41844525	4.74357637	5.13442859	0.03476097	0.05762671
Pxdn	-0.8326637	4.05497605	5.13407688	0.03476652	0.05762671
Plgrkt	0.51221409	4.07499512	5.13391483	0.03476908	0.05762671
A630075F10I	-1.7317689	-0.7287085	5.13386304	0.0347699	0.05762671
Abcd2	-0.5014775	4.77158187	5.13326368	0.03477937	0.05762967
Prpf38a	-0.4141954	4.10992073	5.13073535	0.03481935	0.05768318
Rbmx2	0.48992672	4.45926901	5.13001223	0.03483079	0.0576894
Vps45	-0.4540805	3.79900705	5.12865626	0.03485227	0.05769525
Syap1	0.55897794	5.92517928	5.12855936	0.0348538	0.05769525
4930451C15I	1.51196461	0.31137663	5.12810696	0.03486097	0.05769525
D030040B21	-1.3246514	-0.4700405	5.12784649	0.0348651	0.05769525
Tgs1	-0.4732445	6.52407515	5.12711412	0.0348767	0.05769984
Shank3	-0.4626331	4.59092802	5.12670136	0.03488325	0.05769984
Mpped2	0.35911591	6.62221731	5.12569822	0.03489916	0.05771343
Dennd1a	-0.3196627	5.51187402	5.12303701	0.03494141	0.05775811
Plk4	-0.4849694	3.65666331	5.12302639	0.03494158	0.05775811
Ankhd1	-0.3219227	7.51690378	5.12236196	0.03495213	0.05776283
Kxd1	0.48093289	4.23318675	5.12092785	0.03497493	0.05778778
Cntrl	-0.4872458	4.31281987	5.11948481	0.03499789	0.05779483
Mmachc	0.38152564	4.6196112	5.11900868	0.03500547	0.05779483
Vps54	-0.2840203	6.89711108	5.11885166	0.03500797	0.05779483
Nolc1	0.33219475	5.18570524	5.11872292	0.03501002	0.05779483

Wasl	0.26752485	8.11217707	5.11795901	0.03502218	0.05780219
Gpr37	-0.5681377	3.7003291	5.11470332	0.03507408	0.05787511
Otud4	-0.3037949	6.54656309	5.11236623	0.0351114	0.05792394
Erich1	0.61884543	2.96128345	5.1091707	0.03516248	0.05798693
Ufm1	0.31909905	6.00772638	5.10901083	0.03516504	0.05798693
Capn10	0.62096809	2.36488744	5.10793303	0.03518229	0.05800263
Gm13102	-2.2592964	-1.4191695	5.10711499	0.03519539	0.05801148
Pcdhb4	-0.8567345	2.06915542	5.10287123	0.03526344	0.05811087
Dbx2	0.67980636	1.98242717	5.10127616	0.03528906	0.05812929
Tmem260	-0.5135375	3.77287118	5.10026049	0.03530538	0.05812929
Bub1	-1.726623	0.38533996	5.09957673	0.03531637	0.05812929
Mief1	0.44085637	4.4905079	5.09898456	0.03532589	0.05812929
Gnl1	-0.2885992	5.89680834	5.09892658	0.03532683	0.05812929
Creb3l2	0.50655859	5.98241077	5.09883242	0.03532834	0.05812929
Apol7b	1.8032959	-0.4814013	5.09880009	0.03532886	0.05812929
Hes6	0.60348219	2.20106607	5.09751942	0.03534947	0.05815044
Rad51	1.20813066	0.79535411	5.09512769	0.03538799	0.05820105
Plp2	0.70907728	4.26104445	5.0943087	0.03540119	0.05821
Fastkd1	-0.6032181	2.74592134	5.09120843	0.03545122	0.05827948
Snx3	0.30454003	6.82295888	5.09064929	0.03546025	0.05828156
Pdcd11	-0.3522069	4.16139788	5.08905143	0.03548608	0.05831122
Ddah2	0.83116266	3.18176182	5.08847653	0.03549537	0.05831372
Rrp1	0.33409197	7.75798968	5.08588912	0.03553724	0.05836973
Clk4	-0.3900508	5.76616854	5.08380588	0.035571	0.05841238
Fhl1	0.28293358	7.70582581	5.08316474	0.03558139	0.05841666
Sncaip	0.54294697	5.65640111	5.08250103	0.03559216	0.05841715
Exd1	1.68187627	-0.3507734	5.0818443	0.03560281	0.05841715
Hist1h2bm	1.02914658	1.35635533	5.08170634	0.03560505	0.05841715
Kdm4a	-0.5289064	3.03882422	5.07899055	0.03564916	0.05847672
Omg	0.42472401	7.44931293	5.07781344	0.03566829	0.05849532
Efna3	0.82239125	1.5606198	5.07708336	0.03568017	0.05849633
Kcnd2	-0.3839251	6.04117674	5.07681683	0.0356845	0.05849633
Gpr137b-ps	-0.4672109	3.63692507	5.07365114	0.03573605	0.05856803
Med30	0.59570667	3.31133533	5.07237224	0.0357569	0.05857875
Sf3b5	0.58969137	3.86018362	5.07229177	0.03575821	0.05857875
AI314180	-0.3028126	7.16951713	5.07125426	0.03577514	0.0585808
Atrnl1	-0.2941505	6.22704218	5.07123555	0.03577544	0.0585808
C130021I20R	1.28464261	1.23443208	5.07047789	0.03578781	0.0585808
Kcnq2	-0.4111118	5.52989196	5.06984041	0.03579822	0.0585808
Rpain	0.53693871	2.84744177	5.0698225	0.03579851	0.0585808
Cdkn3	2.21480349	-0.6948635	5.06695966	0.03584529	0.05864456
Dcakd	0.54618184	4.5004566	5.06580377	0.0358642	0.0586627
Coq7	0.56714997	4.22424086	5.06490128	0.03587897	0.05866722
Mtbp	-1.0412787	1.38346682	5.06467906	0.03588261	0.05866722
Speer8-ps1	-1.5848734	0.0963112	5.06103323	0.03594235	0.05874657

Adam11	0.60857937	3.57860668	5.06044341	0.03595203	0.05874657
Shroom4	-0.6597024	3.13879811	5.06028482	0.03595463	0.05874657
Emc6	0.42696017	3.83980552	5.0564068	0.03601833	0.05883783
Magee1	-0.4483978	6.91827672	5.05425491	0.03605374	0.05888284
Gpr176	-0.7164798	2.61658925	5.05166882	0.03609633	0.05893958
Hist1h4d	0.71693049	2.81423744	5.04960139	0.03613043	0.05898242
Rsbn1	-0.2922063	6.12056606	5.04837201	0.03615072	0.05899474
Slc7a14	-0.3618413	7.01975861	5.04819103	0.03615371	0.05899474
Trim3	0.37278729	4.52986376	5.04712594	0.0361713	0.05901061
0610040F04I	-1.2908122	0.96442766	5.04625941	0.03618562	0.05902114
Dand5	0.46873056	3.55729578	5.04304325	0.03623883	0.05909161
Adh1	1.45627594	0.18930139	5.04269575	0.03624458	0.05909161
Cebpzos	0.50564612	2.99218171	5.04177705	0.0362598	0.05910357
Tet1	-0.3377249	6.20291372	5.04023039	0.03628543	0.05911632
Kif6	-1.3938241	0.20610313	5.04020508	0.03628585	0.05911632
Zfp511	0.77405326	2.57961167	5.03984899	0.03629176	0.05911632
Apbb1	0.3446344	5.52168404	5.03940365	0.03629914	0.05911632
Espn	-1.4788092	-0.5472656	5.03865963	0.03631149	0.05912083
Aoc2	-1.0438777	1.2423825	5.03828698	0.03631767	0.05912083
Ap2s1	0.58274323	3.45522275	5.03774516	0.03632667	0.05912264
Drd2	-0.7176324	3.84286227	5.03640164	0.03634898	0.05914591
Gpr116	-0.9108448	4.84174366	5.0359348	0.03635674	0.05914591
Trip11	-0.2546574	7.25368334	5.03439671	0.03638231	0.05917468
Urb1	-0.6633752	2.76951926	5.03366256	0.03639452	0.05918171
Abcc4	0.53224465	5.12241116	5.0328045	0.0364088	0.05919161
4930481A15I	1.13578459	1.32952791	5.03220753	0.03641874	0.05919161
Cope	0.41300558	4.71358402	5.03184904	0.03642471	0.05919161
Lrrc8b	-0.3630155	5.96179813	5.03140089	0.03643217	0.05919161
Npy	0.55904449	3.41690542	5.02777624	0.0364926	0.05927695
Dusp8	-0.4004372	5.57658294	5.02618842	0.03651911	0.05930717
Srebf1	-0.5164398	2.89281282	5.025526	0.03653018	0.0593123
Sf3a2	0.45699801	3.0888591	5.02469241	0.03654411	0.05932208
Tmem252	-1.2295281	-0.111035	5.02202118	0.03658879	0.05937456
Sesn3	-0.3004262	6.09215675	5.02181338	0.03659227	0.05937456
Bcat2	0.65877987	2.1273565	5.01718836	0.03666978	0.05948747
Casp4	1.04586484	0.78416586	5.01491467	0.03670796	0.05953652
Supt16	-0.4448626	6.65731618	5.01308752	0.03673867	0.05957345
Cdk10	-0.4352842	4.11816307	5.01088382	0.03677575	0.0596152
Rbbp9	0.40215032	6.4796997	5.01041546	0.03678364	0.0596152
Wiz	0.36229838	4.43720815	5.01014085	0.03678826	0.0596152
Zfp773	-1.2126447	1.1356325	5.00960566	0.03679728	0.05961693
Sptlc1	-0.4083947	3.62416493	5.00784506	0.03682695	0.05965212
Med4	0.48598036	3.6371704	5.00633306	0.03685245	0.05967394
Trib2	0.37312307	6.19818176	5.00610349	0.03685633	0.05967394
Pcsk2os1	-0.5992896	3.00792967	5.00356201	0.03689925	0.05973054

Mrpl20	0.53816369	4.0552747	5.00260413	0.03691544	0.05973633
Ppp1r13l	0.86614863	1.34262441	5.00240825	0.03691875	0.05973633
Lsm7	0.51967735	3.70808593	5.00103077	0.03694205	0.05976114
Lhpp	0.60306124	2.63005756	4.99749599	0.03700192	0.05984509
Mustn1	0.78179269	3.85996265	4.99453029	0.03705224	0.0599107
Adamts6	-0.8839684	1.791435	4.99390467	0.03706286	0.0599107
1700008I05R	2.73316428	-1.3148425	4.9936933	0.03706645	0.0599107
Fbxo38	-0.2804623	5.22070644	4.99110607	0.03711043	0.0599602
Frmd4a	-0.4567991	5.54003423	4.99067846	0.03711771	0.0599602
Smim14	0.45371252	7.54290449	4.99048162	0.03712105	0.0599602
Lrtm1	-1.3842918	3.1295136	4.98977685	0.03713305	0.05996666
Fxn	-0.6833401	2.26426653	4.98840284	0.03715644	0.05999152
Timm9	0.35513733	4.92435511	4.98770455	0.03716834	0.05999782
Thumpd1	0.3582421	5.95277989	4.98612379	0.03719529	0.0600284
Sgta	0.41474855	4.96266654	4.9852183	0.03721073	0.0600404
Tmtc2	-0.5792188	2.97770247	4.98305322	0.03724769	0.0600747
Prdm10	-0.6093187	2.96049665	4.98303509	0.037248	0.0600747
Pias4	0.72789264	1.41421414	4.9799424	0.03730087	0.06014703
Zfp287	-0.5516674	4.00793578	4.97871424	0.03732189	0.06016799
Pcdha8	-1.8914667	0.18925913	4.97816015	0.03733138	0.06017035
Dach2	1.60514805	0.10560805	4.97357226	0.03741003	0.06027594
Fam222b	0.26458519	6.75289279	4.97340196	0.03741296	0.06027594
Calr	0.31154782	6.40600111	4.97098184	0.03745453	0.06032996
Dhx36	-0.3771618	6.26856195	4.96861212	0.03749528	0.06037574
Kcnj5	2.49495512	-0.0883408	4.96839335	0.03749905	0.06037574
Lfng	-1.6134745	-0.3731663	4.96630997	0.03753493	0.06042054
Lysmd4	0.47148857	3.30306928	4.96497144	0.037558	0.06044471
Drap1	0.57366921	3.96958289	4.96422175	0.03757093	0.06045255
Zfp458	-0.6064814	4.61657535	4.96216441	0.03760643	0.0604967
Stx18	0.54659891	3.37978799	4.96131599	0.03762109	0.06049688
Sec23b	-0.4309815	4.50824447	4.96122404	0.03762268	0.06049688
Gm12657	0.54053757	3.51445235	4.95899447	0.03766122	0.06054587
U2surp	-0.2650771	7.73191875	4.95778303	0.03768218	0.06055878
Zfp654	-0.4174726	5.43811125	4.95759717	0.03768539	0.06055878
Dusp22	-0.3356006	4.8711312	4.95599306	0.03771317	0.06059044
Usp36	-0.4200268	4.61989909	4.95348179	0.0377567	0.06064739
Polr2h	0.58509472	4.08275577	4.95264009	0.0377713	0.06064884
Ttll7	-0.3428746	7.62182125	4.95207255	0.03778115	0.06064884
H2afj	0.62168282	3.5416245	4.95200025	0.03778241	0.06064884
Rxfp3	1.44029606	1.2141781	4.95113165	0.03779749	0.06064884
Cmpk2	0.32980416	4.55212567	4.95102242	0.03779939	0.06064884
Snapc2	0.50398053	4.22325126	4.95059101	0.03780688	0.06064884
Pyroxd2	0.87009018	1.1896043	4.95016955	0.0378142	0.06064884
Gm13375	0.762746	2.74227121	4.94761245	0.03785866	0.06070717
Arel1	-0.298404	6.26592541	4.94623933	0.03788256	0.06072625

Ankrd55	0.64091429	2.6483128	4.94501821	0.03790383	0.06072625
Fcer2a	-1.1735919	0.56786535	4.94483484	0.03790703	0.06072625
Ap3b2	-0.4992793	5.0760068	4.94461326	0.03791089	0.06072625
Ilf3	-0.2862054	5.74793852	4.94391576	0.03792305	0.06072625
Socs1	-3.1727161	-0.8725271	4.94385054	0.03792418	0.06072625
Rab1b	0.52327451	4.50357591	4.94367579	0.03792723	0.06072625
Tmppe	-0.631737	3.02552922	4.9431787	0.0379359	0.06072717
Zfp11	0.37068811	4.27418731	4.94074039	0.03797845	0.06078231
AI839979	-1.4873122	0.19634029	4.93951426	0.03799986	0.06079403
Smc1a	-0.3596025	8.10839609	4.93939311	0.03800198	0.06079403
Nfatc1	0.42700784	3.99991759	4.93807445	0.03802503	0.06081793
Gpcpd1	-0.313688	6.404355	4.93156072	0.03813912	0.06098527
A230050P20	-0.6565016	1.98851893	4.93117367	0.03814591	0.06098527
Tbk1	-0.386647	5.39326555	4.93030956	0.03816108	0.06099019
Tspan33	0.87953249	1.50651382	4.92962763	0.03817306	0.06099019
Entpd3	-0.7578632	1.63353035	4.92960888	0.03817338	0.06099019
Rala	0.24299216	6.48951825	4.9272957	0.03821404	0.06104214
Tubb5	0.29549226	7.90547831	4.92634356	0.03823078	0.06105589
Emid1	-1.1184776	0.7803174	4.92172772	0.03831209	0.06117272
Gtf3c3	-0.3561494	5.1667821	4.92057949	0.03833234	0.06119203
Itga6	-0.5677827	3.55203255	4.91920692	0.03835657	0.06121657
Lrch1	-0.4773095	4.25291968	4.91878456	0.03836403	0.06121657
Insrr	-1.6706307	0.39472325	4.91484378	0.03843371	0.0613147
Trmu	-0.8071074	2.08059847	4.90926881	0.03853253	0.06145928
Lrrk1	0.55322824	5.08688029	4.90833016	0.03854919	0.06146176
Hs3st5	1.01704279	1.6804255	4.90825843	0.03855047	0.06146176
Eif5a2	0.35122833	6.5726775	4.90741796	0.0385654	0.0614725
Nudcd2	0.51998566	3.02746944	4.90604717	0.03858976	0.06149827
Bhmt	3.11197869	-1.2367822	4.90517257	0.03860532	0.06150999
Rad50	-0.3927813	6.08377612	4.89796788	0.03873372	0.06170147
Evl	0.466915	4.22198237	4.89731153	0.03874545	0.06170704
Plin4	-1.1078695	0.99542139	4.89579536	0.03877254	0.06173708
Loh12cr1	0.57370889	2.95649361	4.89532926	0.03878087	0.06173725
1500011B03	0.39685645	5.03776438	4.89279208	0.03882627	0.0617964
Slc25a27	-0.4620857	4.58888176	4.892223	0.03883646	0.06179951
Srl	0.7001273	1.87903088	4.89113738	0.03885591	0.06181734
Cacna2d3	-0.4506441	5.15526663	4.89042784	0.03886863	0.06182446
Cyb5r3	0.62320575	7.41204742	4.88969643	0.03888174	0.06183221
Pcdhb22	-0.6032172	3.03876864	4.88918066	0.03889099	0.06183382
Ube2g1	0.31155337	6.83465287	4.88702307	0.03892972	0.06188227
Haus7	0.78021243	2.08117045	4.88491754	0.03896755	0.06191843
Mgat5	-0.4139489	4.25700485	4.88478588	0.03896992	0.06191843
Fam196a	-0.45734	4.27266502	4.8843795	0.03897723	0.06191843
Carf	-0.3613161	4.65339293	4.88360264	0.0389912	0.06192334
Ttc9b	0.61920186	3.14900253	4.88328971	0.03899683	0.06192334

Slc25a42	0.31217125	4.84202156	4.882538	0.03901036	0.06193171
Mir103-2	-1.8310887	0.14579881	4.88144531	0.03903004	0.06194984
1700052K11I	0.79322732	2.85164241	4.88005103	0.03905516	0.0619766
Zcchc17	0.32337963	5.21882703	4.87846964	0.03908367	0.06200188
Lingo1	0.29069039	7.59023862	4.87825087	0.03908762	0.06200188
C920025E04I	2.71873102	-1.1611135	4.87742845	0.03910246	0.06201231
Fut4	1.61871765	-0.2588624	4.87498599	0.03914658	0.06206915
Tmem143	0.68663899	2.62157586	4.87420348	0.03916072	0.06207845
Cfdp1	0.41371781	6.92855852	4.87241153	0.03919313	0.06211671
Enpep	-0.8079257	1.97642137	4.87083768	0.03922163	0.06214874
Vwa5a	0.38119465	5.49992937	4.8694642	0.03924651	0.06217504
Gm711	-3.6531028	-1.6191454	4.86806848	0.03927182	0.06220199
Pomc	-2.2554797	0.35685037	4.86719334	0.0392877	0.062214
Klf13	-0.2766676	7.23167154	4.86445661	0.0393374	0.06227955
Pcmtd2	-0.3832625	4.73890337	4.86388104	0.03934786	0.06228297
Tdrd6	-1.8433069	-0.5008325	4.8626241	0.03937071	0.062306
Sbds	0.30184597	6.7129318	4.86203637	0.03938141	0.06230978
Zdhhc3	-0.2997704	5.5882829	4.85930864	0.03943107	0.06237521
Adra1b	0.48005742	3.92520741	4.85445278	0.03951967	0.06250218
Lamp5	-0.3405176	5.75821944	4.85249982	0.03955537	0.06253969
Mrpl9	0.40422853	4.48985531	4.8522428	0.03956007	0.06253969
Zfp951	-0.5677462	2.46295741	4.85051968	0.0395916	0.06256285
Itgb8	-0.4819116	4.16989927	4.85021444	0.03959718	0.06256285
Asxl3	-0.7894442	4.1941141	4.84964599	0.03960759	0.06256285
Ankrd52	-0.3626614	5.89362456	4.84961964	0.03960808	0.06256285
Prorsd1	0.37985967	3.89631622	4.84743489	0.03964811	0.0626129
Hypk	0.46440724	6.55731281	4.84672192	0.03966119	0.06262037
Ptrf	0.62069188	6.57759735	4.8445944	0.03970023	0.06265621
Osr1	0.7454443	6.37133727	4.84450565	0.03970186	0.06265621
Sfxn3	0.31783359	7.52997578	4.84412018	0.03970894	0.06265621
Nip7	0.32503572	5.02357649	4.84235128	0.03974145	0.06269431
Il2rb	1.40550749	0.95630689	4.84137831	0.03975934	0.06270535
Kcnb2	-0.3995224	5.28418829	4.84106167	0.03976517	0.06270535
Map6d1	-0.3793503	5.02829765	4.83903995	0.03980238	0.06274126
4930545L23F	-1.9692215	0.66038367	4.83891579	0.03980467	0.06274126
Rhoj	0.5198808	5.38522687	4.83784389	0.03982442	0.0627592
Grina	0.3203049	7.00624105	4.8358178	0.03986177	0.06280488
Parva	0.38047351	7.42564118	4.83386487	0.03989782	0.0628427
1700008J07F	0.61682986	3.20091997	4.83359809	0.03990275	0.0628427
Alox12	-0.9962551	1.23960461	4.83315621	0.03991091	0.0628427
Mcm3	-0.8283736	0.97874568	4.83226999	0.03992729	0.06285529
Scp2	0.50122117	7.10915731	4.82839847	0.03999891	0.06295484
Tcf4	0.22470402	9.26007733	4.82655221	0.04003312	0.06299546
Mir124a-2	-1.7315139	-0.8553492	4.82545052	0.04005355	0.06301439
Rnf44	0.22625663	6.73438254	4.81676951	0.04021495	0.06325504

Mat2a	-0.2571485	7.87146733	4.81352534	0.04027545	0.06333692
Tec	0.52898635	5.07150878	4.81199568	0.04030401	0.0633618
Mzt2	0.56951859	2.97104028	4.8115395	0.04031254	0.0633618
Abcf1	0.27625843	6.76225071	4.81132173	0.04031661	0.0633618
Egln3	0.46544982	7.45130752	4.81076968	0.04032692	0.06336474
Pcdhb12	-0.7142998	2.49227365	4.80796791	0.04037934	0.0634338
4930594C11I	-2.0083466	1.31140351	4.8068141	0.04040094	0.06345446
1700052N19	-0.3981567	3.84405497	4.80318397	0.04046901	0.06354805
Ankrd24	-0.610808	2.49941167	4.8001945	0.04052516	0.06360166
AI413582	-0.4541952	3.26757408	4.80008736	0.04052717	0.06360166
Stbd1	0.43876357	4.08856523	4.79970238	0.04053441	0.06360166
Gpx8	0.64967898	5.63847575	4.79956158	0.04053706	0.06360166
5031439G07	-0.2860431	5.64957202	4.79839379	0.04055902	0.0636188
Bcap29	0.3467052	5.17023279	4.79807903	0.04056495	0.0636188
Nrxn2	0.33596241	5.40005457	4.79547185	0.04061404	0.0636686
Wbscr22	-0.480164	3.15028174	4.79531921	0.04061692	0.0636686
St8sia5	0.55522309	4.23302689	4.79504113	0.04062216	0.0636686
Gpr3	1.0258152	1.08837726	4.79366565	0.0406481	0.06369594
D8Ertd738e	0.72942484	3.18612086	4.79239586	0.04067205	0.06372017
Pik3r5	-0.5201421	2.80084635	4.79022785	0.040713	0.063771
Xpa	0.42213886	5.24649638	4.78836197	0.04074828	0.06381293
Timm8a1	0.41897243	5.39448299	4.78097968	0.04088819	0.06401866
Slc46a1	-0.7669074	1.24647663	4.78050738	0.04089715	0.06401935
Hmbox1	-0.3351847	5.1907916	4.77767619	0.04095097	0.06409021
Heca	0.36679918	4.53730829	4.77621014	0.04097887	0.0641205
Plag1	-0.6456404	2.70955177	4.77529241	0.04099634	0.06412787
Ccdc64	-1.1727217	0.49411901	4.77506489	0.04100068	0.06412787
Amer2	-0.4308231	4.18477025	4.77401229	0.04102073	0.06414586
Slc2a13	-0.3285679	6.84437649	4.77337794	0.04103283	0.0641514
Tmco5	1.20891971	0.61873959	4.7701759	0.04109393	0.06423353
Pik3cg	-0.592366	2.45335659	4.76962574	0.04110443	0.06423657
Nat6	0.60401332	3.18126994	4.76848376	0.04112626	0.06425729
Wnt7b	0.98628938	1.64236323	4.76656909	0.04116287	0.0642907
Plcb2	-0.8908734	1.39988177	4.76646956	0.04116478	0.0642907
Bre	0.3780309	4.14336878	4.76509241	0.04119114	0.06431848
Nup107	-0.4747467	3.78393651	4.76453929	0.04120173	0.06432163
Tmem150a	0.6084794	2.52339698	4.76280551	0.04123496	0.06433891
A330035P11I	0.8794697	1.93256336	4.76269171	0.04123714	0.06433891
Klf11	-0.4398073	4.21877842	4.76261901	0.04123853	0.06433891
Ppp3cb	0.24423916	8.65277141	4.7612612	0.04126458	0.06436616
Lrrc4	-0.3508769	5.96828391	4.76070976	0.04127516	0.06436928
Il33	-0.3782264	5.16812239	4.75889786	0.04130995	0.06441015
Usp27x	-0.3501089	4.93859851	4.75841798	0.04131917	0.06441114
Lima1	0.41145127	7.28145281	4.75794874	0.04132819	0.06441181
Kcns2	-0.4293211	3.87739497	4.75647161	0.0413566	0.06444269

Mmgt2	0.54919799	3.31545724	4.75578174	0.04136987	0.06444294
Ube2d2a	0.32268651	9.04656971	4.75557017	0.04137394	0.06444294
Vegfb	0.376441	4.6599426	4.75398635	0.04140444	0.06447705
Grik3	-0.4772523	5.57650509	4.7527604	0.04142806	0.06450045
Inha	-0.7443882	1.64997007	4.75222215	0.04143843	0.06450321
Gm15441	2.85448339	-1.1953439	4.75033506	0.04147483	0.06454648
Prdx3	0.38571948	5.17439889	4.74509761	0.04157605	0.06469058
Ppapdc1b	0.38246327	4.27415139	4.74272956	0.04162191	0.06473516
Ppt2	0.87837506	1.23233552	4.74272701	0.04162196	0.06473516
Kctd20	0.64831863	3.14629532	4.74207325	0.04163463	0.06473661
Ssr2	0.79336821	3.49506716	4.74178821	0.04164015	0.06473661
Rnf5	0.41886389	5.68227105	4.73887853	0.04169661	0.06481094
Relt	0.98445919	1.18477798	4.73731921	0.0417269	0.06484459
Mss51	-2.5608486	-0.9823114	4.73258391	0.04181903	0.06497431
Pabpc4	0.35632521	3.89769323	4.73212678	0.04182794	0.06497469
Ndufs7	0.41113018	5.10040801	4.72930425	0.04188298	0.06503663
Rnaseh2b	0.49962126	4.32882291	4.72919271	0.04188516	0.06503663
Zfp362	0.35074385	5.19322583	4.72802992	0.04190786	0.06505841
Sytl2	-0.3782563	5.78833679	4.72601435	0.04194724	0.06509887
Pmaip1	0.54910211	3.4226659	4.72561655	0.04195502	0.06509887
Pdcl	0.36639854	5.15656467	4.72536415	0.04195995	0.06509887
Tmem57	-0.2527921	6.12425202	4.72470177	0.04197291	0.06510551
Cipc	0.25499976	6.68247745	4.7233018	0.04200031	0.06513453
Mkrn3	-2.2235252	-0.2692378	4.72273585	0.04201139	0.06513825
Ly6a	-1.2313859	2.3561402	4.7217387	0.04203092	0.06514837
Adsl	-0.4657424	4.44688596	4.72151595	0.04203528	0.06514837
Ptprg	-0.3106483	6.91792331	4.72031464	0.04205883	0.06516733
Pld3	0.41201957	7.04304457	4.71976293	0.04206965	0.06516733
Tbrg1	0.45539668	4.80640048	4.71956271	0.04207358	0.06516733
Kdelr3	-1.0140158	1.07251434	4.71717093	0.04212053	0.06520864
Ccdc88b	1.15367177	0.13683675	4.71709422	0.04212204	0.06520864
Ndufs3	0.32546017	6.44442752	4.71687583	0.04212632	0.06520864
Gm10012	0.39381135	3.8279026	4.71589995	0.0421455	0.06521225
Trpm3	-0.3067285	6.82614017	4.71587203	0.04214605	0.06521225
Rprml	0.63911215	3.00857413	4.71515573	0.04216013	0.06522059
2610015P09I	-0.4346753	3.72388813	4.71416783	0.04217956	0.06523719
Hs3st2	0.46715523	4.34219434	4.70921202	0.04227718	0.06536567
Dhx15	-0.2335332	6.58366258	4.70906614	0.04228006	0.06536567
C4a	-1.762809	-0.5173054	4.70817599	0.04229762	0.06537935
Commd5	0.78587822	2.05512367	4.70767651	0.04230748	0.06538112
Mei1	-0.9808511	0.94362677	4.7042542	0.0423751	0.06547213
Rilpl1	0.38020644	5.94586796	4.70327952	0.04239438	0.06548843
Acmsd	-1.1881853	0.69188002	4.7016128	0.04242738	0.0655259
Slc4a7	-0.461673	3.91386654	4.70054915	0.04244845	0.06554495
Cep164	-0.4705305	3.31652975	4.69618696	0.04253498	0.06566506

C1d	0.3641602	4.9718572	4.69559901	0.04254666	0.06566957
Tbl3	0.73214872	2.07913153	4.69487156	0.04256112	0.06567837
F10	-1.8032277	-0.8615185	4.69391572	0.04258012	0.06569418
Ncor1	-0.3411891	9.19656886	4.69328196	0.04259273	0.06570011
Glis1	1.34105431	0.52950865	4.68902103	0.04267758	0.06581747
Ndufs8	0.58600062	4.82298406	4.68829323	0.04269209	0.06582632
Lta4h	-0.343067	4.27326009	4.6857466	0.04274292	0.06589114
Psg29	-1.3757722	0.02160437	4.68477802	0.04276227	0.06590743
Gpm6b	-0.3643855	8.47914724	4.68077966	0.04284225	0.06601713
Echdc1	0.70401473	1.77405329	4.67898559	0.04287819	0.06601796
Camsap2	-0.3496815	8.77325357	4.67885702	0.04288077	0.06601796
Srgap1	-0.5597844	5.14765201	4.67884418	0.04288103	0.06601796
Naa15	-0.2510076	6.62462264	4.67879299	0.04288205	0.06601796
Zfp280b	-0.3599337	4.89230233	4.67855661	0.04288679	0.06601796
Tbc1d8	-0.5547878	4.20495322	4.67799656	0.04289802	0.0660217
Klf9	0.29856951	8.08354523	4.67703879	0.04291724	0.06603772
Gm9839	-2.8175608	-0.419658	4.6741247	0.04297575	0.0661142
Gabrb2	-0.3731127	8.24512873	4.67358975	0.0429865	0.06611718
Kctd10	0.43239742	4.71801975	4.67151755	0.04302818	0.06616771
Fam210b	0.42801402	5.27692419	4.66944297	0.04306995	0.06621033
Rab2b	0.28446841	5.9805312	4.66870411	0.04308484	0.06621033
Cyb5d1	0.85146365	1.39283836	4.66852769	0.04308839	0.06621033
Slc26a7	0.79033449	4.94168083	4.66832719	0.04309244	0.06621033
Qpctl	0.58650902	2.65051681	4.66795055	0.04310003	0.06621033
Ndufa10	0.31224027	6.26299292	4.66600394	0.0431393	0.06624879
Bckdhb	-0.6959057	2.62445648	4.66541263	0.04315123	0.06624879
Gpam	-0.5078167	3.29092738	4.66539672	0.04315155	0.06624879
Nme2	0.46743467	6.80607066	4.66461335	0.04316737	0.06625951
Zc3h12d	1.78607776	-0.835249	4.66300987	0.04319977	0.06629565
Nmd3	-0.3696485	5.08200007	4.66237079	0.04321269	0.06629565
Prkag2os1	-1.7640558	-0.1707102	4.66213667	0.04321743	0.06629565
3110021N24	-0.5419249	2.41221087	4.65928139	0.04327522	0.06637073
Msrb2	0.38332689	4.28512107	4.65654198	0.04333075	0.06643445
Gstm6	0.70846389	2.2532875	4.65635772	0.04333449	0.06643445
Tcf25	0.22152482	8.4293522	4.65454214	0.04337134	0.06647736
Slc44a5	-0.8229015	1.67814149	4.65386295	0.04338513	0.06648492
Ebp	0.60613631	2.6593953	4.65269391	0.04340889	0.06650774
Cd48	-1.6055447	0.34257758	4.65084694	0.04344645	0.06654028
2610035F20I	0.51706535	3.01354408	4.65077705	0.04344787	0.06654028
Htr1b	-0.7618568	1.29237253	4.64994369	0.04346483	0.06655267
Gm9962	-1.0286152	0.50648119	4.64918287	0.04348032	0.0665628
6030443J06F	-0.8581433	1.76003475	4.64558686	0.04355362	0.06666141
Arid2	-0.2598854	6.70487632	4.644959	0.04356643	0.06666687
Bzw2	-0.4088296	3.56638894	4.6445413	0.04357496	0.06666687
Dnaja3	0.31876209	5.03685258	4.63882312	0.04369187	0.06683211

Paics	0.36632747	8.66683901	4.63793369	0.04371009	0.06684393
Fscn1	0.38456882	5.37348676	4.63757589	0.04371742	0.06684393
Nsun2	-0.2958517	5.15905274	4.63670135	0.04373535	0.06685771
Eri3	0.39575515	4.21132991	4.63560278	0.04375788	0.06686873
Rnf216	0.26124329	5.97522611	4.63544353	0.04376114	0.06686873
Scrn3	0.3555428	4.81804462	4.6350457	0.0437693	0.06686873
Grik4	-0.832997	1.91714354	4.63249121	0.04382176	0.06691123
Slc35b1	0.41110375	3.70442567	4.63247434	0.0438221	0.06691123
Inpp1	-0.6029782	3.03063513	4.63190011	0.0438339	0.06691123
Sec14l1	-0.2849404	6.38234486	4.63188876	0.04383414	0.06691123
Galnt5	-2.1277535	-1.0142029	4.63151982	0.04384172	0.06691123
Gps2	0.36333954	4.02309499	4.62964615	0.04388026	0.06692751
Tub	-0.469055	6.11922784	4.62910618	0.04389137	0.06692751
E130309D02I	0.41067554	4.011427	4.62873257	0.04389906	0.06692751
Gm14440	0.33926608	5.28812743	4.6286705	0.04390034	0.06692751
3110045C21I	2.33201791	-0.5811746	4.62848384	0.04390418	0.06692751
Thumpd2	-0.7264987	1.67259557	4.6283993	0.04390592	0.06692751
Pmepa1	0.29746543	7.36000559	4.62671338	0.04394065	0.06696685
Ccdc12	0.50250768	3.63339538	4.62505736	0.0439748	0.0669976
Usp17la	-1.3283101	0.44794742	4.62486846	0.0439787	0.0669976
Ints4	-0.3008445	5.51097562	4.62415286	0.04399346	0.06700649
Tubgcp6	-0.5281237	3.40036445	4.62264336	0.04402463	0.06704035
Ggact	0.5227908	5.86444957	4.62099588	0.04405867	0.06707857
Tmem205	0.79385103	1.69329535	4.61695813	0.04414224	0.06719216
Ets2	-0.3102557	5.6900743	4.6132296	0.04421956	0.06728421
Nckap5l	0.876873	1.45412957	4.61317706	0.04422065	0.06728421
Rfx1	-0.6170887	3.37654182	4.61214909	0.044242	0.06730304
Gm4890	2.80249034	-1.3919237	4.60996255	0.04428744	0.06735851
4921536K21I	-1.7559224	0.51703229	4.6085139	0.04431758	0.06739068
Hrh1	-0.5280346	2.61130733	4.60368384	0.04441823	0.06752372
Strada	-0.539075	3.65046623	4.60345211	0.04442307	0.06752372
Ccdc146	0.99314744	1.56847198	4.60300441	0.04443241	0.06752424
Xirp2	-0.9092289	2.38411212	4.60039719	0.04448687	0.0675933
Sec24a	-0.306194	5.49204325	4.59848526	0.04452685	0.06764035
Nanos1	0.27853337	5.23537647	4.59579024	0.04458328	0.06770981
Dnah10	-1.0255209	1.13140033	4.59532599	0.04459301	0.06770981
Zfp119a	0.84951194	1.13560126	4.59500906	0.04459966	0.06770981
Mrpl57	0.35594344	4.6920637	4.59343638	0.04463264	0.06773851
Trim59	-0.5804817	3.40018725	4.59324647	0.04463662	0.06773851
Eif3m	0.26989825	6.20702064	4.58998792	0.04470505	0.06782864
Hexim1	0.30146127	5.41505595	4.58781376	0.04475078	0.06788429
Fnta	0.36111795	6.63782903	4.58719414	0.04476382	0.06789034
Rasgrp4	-1.343921	-0.1143058	4.58664791	0.04477532	0.06789406
Mdm4	-0.2476626	6.15415626	4.58482439	0.04481374	0.06793857
Ung	0.73654227	2.10678737	4.57966149	0.04492271	0.06809002

Suv39h1	0.54810505	3.57991777	4.57910553	0.04493446	0.06809407
Bmp2	0.4949213	4.62517624	4.57815161	0.04495463	0.0681057
Tmem64	0.41639974	8.87291316	4.57788407	0.04496029	0.0681057
Zfp623	0.49985271	3.4205748	4.57722026	0.04497434	0.06811322
Rorc	-1.0613964	1.15378792	4.57616198	0.04499674	0.06813339
Dnajc8	0.3409899	6.22569928	4.57558762	0.04500891	0.06813806
2700046G09	-0.9276116	1.75503727	4.57450921	0.04503176	0.0681589
Relb	0.94618073	0.4167856	4.57366567	0.04504964	0.06816511
Aar2	0.55006319	3.78509195	4.57345826	0.04505404	0.06816511
Ints8	-0.34885	5.49096989	4.5712499	0.0451009	0.06822225
Plrg1	0.3421579	4.51438789	4.56613805	0.04520958	0.06837285
Tmc2	-1.3524222	-0.2558401	4.56394199	0.04525636	0.06842981
Jagn1	0.34439768	4.86171929	4.56320971	0.04527197	0.06843962
Nisch	-0.2983173	7.3684988	4.56230604	0.04529124	0.06845163
Csf2ra	-0.5469218	3.82945891	4.56184596	0.04530106	0.06845163
Thbs3	-0.985014	1.10642805	4.56155401	0.04530729	0.06845163
Gdf11	-0.9804431	1.80607405	4.56073378	0.0453248	0.06846429
Wdr19	-0.4486791	4.6043891	4.55966453	0.04534763	0.06846547
Nfasc	-0.3546404	7.70953215	4.55945158	0.04535218	0.06846547
Etaa1	0.44198818	4.87202741	4.55941511	0.04535296	0.06846547
Exosc2	-0.4621199	3.39289145	4.55761625	0.04539142	0.06850974
2310022A10I	0.46684749	3.59599026	4.55659065	0.04541336	0.06852907
Arhgap4	2.291412	-0.1392605	4.5556406	0.04543369	0.06854596
Gm684	0.48871727	3.50100373	4.55448174	0.04545851	0.06856962
Tdrd1	-1.2145602	0.84020245	4.55189217	0.04551403	0.06863955
Igbp1	0.39505994	5.79648607	4.55109793	0.04553107	0.06865145
Nutf2	0.52492726	3.14388551	4.5465697	0.04562838	0.06878434
1700049G17	-0.4872379	3.46919692	4.54354857	0.04569343	0.06886857
Baz1a	-0.4000689	4.11718974	4.54190106	0.04572895	0.06890825
Rpl4	0.31322326	8.80304344	4.54016933	0.04576631	0.06895071
Kansl1l	-0.3297781	5.50091386	4.5394076	0.04578276	0.06896164
Agk	-0.3660415	4.41723715	4.53856558	0.04580095	0.06897256
Maml2	0.37388361	5.76477574	4.53754249	0.04582306	0.06897256
Scpep1os	-1.7250124	-1.5246449	4.53742629	0.04582558	0.06897256
Gimap8	-0.8232902	1.21229968	4.53737012	0.04582679	0.06897256
Bloc1s1	0.71446848	5.14775056	4.53443239	0.04589036	0.06904116
Trappc4	0.51523206	3.52625341	4.5340093	0.04589952	0.06904116
Anapc16	0.53667515	5.99677279	4.53397533	0.04590026	0.06904116
Rnaseh2c	0.8495854	2.73847874	4.53349306	0.04591071	0.06904116
Megf8	-0.4686843	5.0083475	4.53313855	0.04591839	0.06904116
Gm14204	-0.7516362	2.45307692	4.5322998	0.04593657	0.06905465
Sfrp2	1.14019102	0.51677032	4.53099033	0.04596497	0.0690835
Cog1	-0.4189873	4.11567653	4.53039107	0.04597797	0.0690892
Naa60	0.25800768	6.11045487	4.52854954	0.04601796	0.06913544
Lcp2	0.48902566	3.02345756	4.52713174	0.04604877	0.06916787

Abra	-3.0691124	-0.3836444	4.52602741	0.04607278	0.06917181
Pcdhga5	-0.5070521	3.68672093	4.52593868	0.04607471	0.06917181
Edem3	-0.2854847	6.01959876	4.52573927	0.04607905	0.06917181
Slc25a51	-0.2468944	6.87552165	4.52376479	0.04612203	0.06922248
Arhgap26	-0.3513736	7.57515888	4.52316336	0.04613514	0.06922829
Fbxl3	0.24171582	7.46914678	4.51904355	0.04622499	0.06934926
Brsk2	-0.3609889	5.66464878	4.51699372	0.04626978	0.06940256
E130008D07I	-0.8670537	2.27454662	4.51530419	0.04630673	0.069444
6430548M08	0.32762831	6.91741558	4.51488412	0.04631592	0.069444
Pes1	0.3923535	4.47611042	4.51322464	0.04635225	0.069471
Ntn3	-1.0345313	1.52422669	4.51321569	0.04635245	0.069471
Spaca1	-1.6866857	0.59477305	4.5101529	0.04641959	0.06955773
Pqbp1	0.33093699	4.38254597	4.50813978	0.04646378	0.06961005
Tsfm	0.47061019	2.62494558	4.50701287	0.04648854	0.06963323
Tmem216	0.53689545	2.85697965	4.50630876	0.04650402	0.06964251
Ufc1	0.37363751	5.14966962	4.50371739	0.04656103	0.06971397
Osbpl8	-0.2353389	7.70354913	4.50010972	0.04664054	0.06981313
Gm2897	-0.517903	3.6036603	4.49986807	0.04664587	0.06981313
Slc38a11	-2.0015297	0.50440634	4.49607469	0.04672965	0.06992456
1700071K01I	-2.3256935	-0.3649096	4.49537073	0.04674521	0.0699339
Gps1	0.31825485	5.13884661	4.49473443	0.04675929	0.06994101
Pdss1	-0.7244994	2.13099445	4.4932899	0.04679126	0.06997488
Rrbp1	0.34074665	6.08677972	4.49168972	0.0468267	0.07000464
Tanc1	-0.2620209	5.909847	4.49154898	0.04682982	0.07000464
Kif9	-0.8890456	2.35054182	4.48999171	0.04686435	0.07002956
Chia1	-1.8750391	-0.1819364	4.48995476	0.04686517	0.07002956
Cdc14b	-0.4386561	4.35642075	4.48947925	0.04687572	0.07003138
Tspan15	-0.7076766	1.86957769	4.48667012	0.04693809	0.07010795
Ppm1k	-0.3350213	6.66579069	4.48632929	0.04694566	0.07010795
Serpinb9b	-2.1723459	-0.7571018	4.48331128	0.04701279	0.07019422
Cd226	-1.0771331	0.44264566	4.48281588	0.04702382	0.07019672
Zfp618	0.74440793	1.48784735	4.48019176	0.04708229	0.07027002
Pkhd1	2.82210735	-0.6882453	4.4791726	0.04710502	0.07028037
Prdx1	0.39229054	7.18344903	4.47904074	0.04710797	0.07028037
Fam117b	0.21481304	7.00148895	4.47754273	0.0471414	0.07030392
Lrrfip2	0.26599578	5.83690377	4.47682414	0.04715745	0.07030392
Zfp354c	-0.3037184	5.84331025	4.47679058	0.0471582	0.07030392
Rora	-0.2714416	8.37690505	4.47665448	0.04716124	0.07030392
Fut11	-0.4551362	3.90062084	4.47574113	0.04718165	0.070316
B230219D22	0.27730547	8.00694298	4.47515705	0.04719471	0.070316
Fam114a1	0.56962438	6.31411309	4.47496322	0.04719904	0.070316
Rcor1	0.33154195	5.10840281	4.47461462	0.04720684	0.070316
Sf3b6	0.36694773	5.00952642	4.4694611	0.04732226	0.07047393
9530077C05I	0.6696969	2.2458995	4.46681794	0.04738158	0.0705365
Gm9199	-1.3770519	-0.4350192	4.46613138	0.047397	0.0705365

Fam120c	-0.4269463	6.59807918	4.46610022	0.04739771	0.0705365
Scn1b	0.26614855	5.60432202	4.46591411	0.04740189	0.0705365
Triobp	0.52309132	6.03592652	4.46531379	0.04741538	0.07054258
Ngef	-0.345578	5.79286165	4.46402107	0.04744445	0.07056682
Msh6	-0.4236727	4.74445881	4.46375255	0.04745049	0.07056682
2700049A03	-0.4052949	3.7645438	4.4623776	0.04748143	0.07059884
Pex1	-0.4164277	4.55242646	4.46048794	0.047524	0.07064813
Ltk	-0.9670623	1.34450816	4.4591861	0.04755335	0.07067775
Nudt19	0.37151715	4.87249555	4.45776695	0.04758536	0.07070393
Ninj2	-1.915944	-0.9280136	4.45757009	0.04758981	0.07070393
Ube2m	0.47232555	3.10543801	4.4559625	0.04762611	0.07073478
Zfp710	-0.4349521	3.6340195	4.45577894	0.04763026	0.07073478
Ccnt1	-0.3350725	6.30201614	4.45539798	0.04763887	0.07073478
Myl6	0.43806484	7.87742737	4.45097481	0.04773895	0.07086935
Tada2a	-0.4091908	3.75529382	4.44863174	0.04779206	0.07093416
Sgcz	-1.0437358	1.8960049	4.44703225	0.04782835	0.07097399
Chml	-0.2672718	6.24860913	4.44596371	0.04785262	0.07099595
Fancf	0.69154346	3.01588235	4.44469436	0.04788146	0.07102469
Immp1l	0.32600904	5.4472159	4.4401348	0.04798523	0.07116455
Gm10825	-2.5764809	-1.3724712	4.43804842	0.0480328	0.07122101
Wnt9a	-0.5335246	3.82353206	4.43708054	0.04805488	0.07123968
Ptprt	-0.4637481	6.91884679	4.43597565	0.04808011	0.07126158
Rars	0.30740319	5.45583611	4.43560128	0.04808866	0.07126158
Fam214b	0.41029694	4.40234151	4.43474998	0.04810811	0.07127369
Hars2	-0.3852073	3.90856284	4.43441215	0.04811583	0.07127369
1700019D03	0.81470195	1.69475413	4.43386353	0.04812837	0.07127819
Gm20594	-0.8710091	0.45296374	4.4327398	0.04815407	0.07130218
Saysd1	-0.5806239	2.32347982	4.43223333	0.04816566	0.07130526
Ss18	0.31191938	5.34131109	4.43113403	0.04819083	0.07132844
Cacna1d	-0.5709434	5.10948348	4.42951709	0.04822787	0.07136918
Pdcd2	0.40341945	3.87021714	4.4272908	0.04827892	0.07143064
Il16	0.87304942	1.13578219	4.42652512	0.04829649	0.07144255
Mettl15	0.79537195	1.63250249	4.4253686	0.04832305	0.07146572
Bcl10	0.5679205	4.85800539	4.42501332	0.04833121	0.07146572
Whsc1l1	-0.2334427	7.71998622	4.42423826	0.04834902	0.07147796
Aes	0.36419804	6.35405243	4.42236125	0.04839219	0.07152338
Osmr	0.56587261	3.50329174	4.42207321	0.04839881	0.07152338
Ddx52	0.27559182	4.67861184	4.42105997	0.04842213	0.07154375
Luc7l	-0.34799	5.4931452	4.41947584	0.04845862	0.07155977
Itpka	-0.3896719	4.18836864	4.41942618	0.04845977	0.07155977
Chmp7	0.33861822	5.62002362	4.4193468	0.0484616	0.07155977
Sytl5	-0.4716032	4.73836367	4.41440021	0.04857574	0.07171421
Ulk1	0.31564548	6.46626402	4.41354017	0.04859562	0.07172944
Tnc	-0.8082808	1.7869043	4.41312401	0.04860524	0.07172953
Dnlz	0.4532014	3.76247593	4.41192977	0.04863287	0.07175617

Tnfaip6	-0.7104188	1.74255489	4.41010981	0.048675	0.07180077
C230052I12R	0.53124101	2.85948988	4.40979743	0.04868223	0.07180077
Ctsc	0.67626048	2.05099363	4.40893499	0.04870222	0.07181612
Car4	0.55968822	3.1795533	4.40834531	0.04871588	0.07182216
Zfp937	-0.3630452	5.13090645	4.40745049	0.04873663	0.07182465
Lcp1	-0.4441058	4.76173276	4.40744642	0.04873673	0.07182465
Sec31a	-0.2174966	7.07424304	4.40326901	0.04883373	0.07195347
Ndufs6	0.457398	5.1640407	4.40228032	0.04885672	0.07197321
Cpt1c	-0.5684099	3.36408473	4.40146814	0.04887562	0.07198275
Zufsp	-0.6794066	3.44116249	4.40083173	0.04889043	0.07198275
Tmem132a	0.53217076	2.66849841	4.40076473	0.04889199	0.07198275
Scg5	0.30931833	6.32416838	4.40017016	0.04890583	0.07198901
Mrpl38	0.47651912	3.37361832	4.39866203	0.04894096	0.07202659
Meig1	1.29012402	0.54987628	4.39564969	0.04901123	0.07210741
Dnajc28	0.36028258	3.87108935	4.39532928	0.04901871	0.07210741
G630071F17	-1.5381834	-0.2688964	4.39507172	0.04902472	0.07210741
Vcam1	-0.3755355	5.84331922	4.39388323	0.04905248	0.0721341
1700008F21I	-1.3050948	0.94287761	4.39324395	0.04906742	0.07214193
Ankub1	-1.472142	0.64183141	4.39193883	0.04909794	0.07216052
Rpl18a	0.42842362	7.4643089	4.39188021	0.04909931	0.07216052
Cd274	-0.5755044	3.76592191	4.39020423	0.04913853	0.07220402
Vars2	0.74173052	2.25968597	4.38967961	0.04915082	0.07220793
Vps8	-0.5875044	4.47332854	4.38918694	0.04916236	0.07221074
Arsj	0.97495681	0.86203898	4.38850971	0.04917822	0.0722199
Gabra1	-0.3132949	8.20508337	4.38720936	0.04920871	0.07224748
Kank4os	-2.6505853	-1.3756295	4.38681078	0.04921806	0.07224748
Cab39	0.23395225	7.71350092	4.38647665	0.04922589	0.07224748
Dnmbp	-0.4686655	3.65661278	4.38471411	0.04926726	0.07229405
A330050F15I	0.87296709	2.27249062	4.38283272	0.04931147	0.07234476
Vps9d1	-0.4176743	3.70412847	4.38005602	0.04937679	0.07242643
Arhgap19	0.52529871	3.4055371	4.37682203	0.04945299	0.07252402
Copz2	0.65080468	6.78348118	4.3743373	0.04951163	0.07259583
Sf3b1	-0.2608932	8.28905656	4.37370309	0.04952661	0.0726036
Cd46	-1.4676032	1.16625754	4.37177063	0.04957229	0.07265636
Rab6a	0.23230966	10.1161715	4.36908192	0.04963592	0.07272349
Znhit3	0.47565508	3.91894958	4.36901619	0.04963748	0.07272349
Ublcp1	0.27685011	6.44773241	4.36676884	0.04969074	0.0727873
Tor2a	0.65644703	1.89822862	4.36523224	0.04972719	0.07280621
Map1lc3a	0.44856384	4.80328015	4.3650368	0.04973183	0.07280621
Iqcj	-2.4873554	-1.5324239	4.36495792	0.0497337	0.07280621
Plch1	-0.5460944	3.10887711	4.36445616	0.04974561	0.07280621
Slc35a4	0.36734399	5.95206393	4.36401992	0.04975597	0.07280621
Pex19	0.29009007	6.27237414	4.36377127	0.04976188	0.07280621
Enpp6	-0.6479726	2.77992828	4.36245105	0.04979325	0.0728379
Dact2	0.61278877	2.14960065	4.36149024	0.04981609	0.07284669

Coprs	0.50951265	3.63211372	4.36122898	0.04982231	0.07284669
A730098P11I	0.28265033	6.70378136	4.36097315	0.04982839	0.07284669
Tex15	-0.6553713	2.37812707	4.3588385	0.0498792	0.07290676
Mfi2	2.17677151	-0.3768667	4.35825475	0.04989311	0.07291288
Gins1	1.54599527	0.15266599	4.35689234	0.04992558	0.07294612
Heatr2	0.35468601	4.21724217	4.35562946	0.0499557	0.07297591
Krt20	-0.6041275	3.87469395	4.35385282	0.04999811	0.07302275
Acbd7	1.95387131	-1.2442013	4.35313608	0.05001523	0.07302275
Fzd5	-0.8501639	2.67247355	4.35306339	0.05001696	0.07302275
Hsd3b2	-2.3937155	-1.284295	4.35230309	0.05003513	0.07302785
Crls1	0.38552979	4.55231975	4.35210233	0.05003993	0.07302785
Rbmx	-0.259332	6.76613628	4.34902696	0.05011351	0.07311531
Gcnt2	0.31976493	5.3346145	4.34878264	0.05011936	0.07311531
Ccl6	1.24713626	0.33841368	4.34794685	0.05013938	0.0731303
Zc4h2	0.32819619	4.71566266	4.34572644	0.05019261	0.0731937
Celrr	-1.4788634	0.30316582	4.34480702	0.05021467	0.07320563
Gdpd5	0.42103346	3.75002037	4.34457219	0.0502203	0.07320563
Slc26a8	-1.1250839	0.88939182	4.34353963	0.05024509	0.07322754
Zfp280c	-0.3612319	5.37018434	4.34245599	0.05027113	0.07325125
Gpd2	-0.2450644	6.9886585	4.34070857	0.05031314	0.07329822
Dcbld1	-0.7161152	1.98688471	4.33990331	0.05033251	0.07330647
LOC1010560	0.54042301	3.43306051	4.33961603	0.05033942	0.07330647
Gng11	0.60988488	5.55179355	4.33925494	0.05034812	0.07330647
Polr2g	0.38344662	5.38000688	4.33866668	0.05036228	0.07331286
Nudt16l1	0.33941382	4.1446376	4.33686251	0.05040574	0.07335843
Scarna3b	-2.7046865	-1.0973364	4.33655572	0.05041314	0.07335843
2700054A10I	-0.5537497	2.52735864	4.33610144	0.05042409	0.07336014
4833439L19I	0.35331446	7.79754832	4.33554055	0.05043762	0.07336559
Ptk2	-0.4126419	6.57805731	4.3343218	0.05046703	0.07339413
Wdr76	-0.6219802	2.1984456	4.33220918	0.05051805	0.07345409
Acot9	0.40980667	3.80085597	4.33134071	0.05053904	0.07347037
Asf1a	0.27184762	5.03613629	4.33036778	0.05056257	0.07349034
Kbtbd2	-0.256653	5.97260635	4.32927841	0.05058893	0.07349608
Zfc3h1	-0.430818	6.97548533	4.32875657	0.05060156	0.07349608
Zfp57	-0.833986	1.89585509	4.3286549	0.05060402	0.07349608
Postn	-0.5721221	2.19193298	4.328585	0.05060572	0.07349608
Eltd1	-0.844579	2.92488026	4.32798644	0.05062021	0.07350291
Robo3	-1.4851406	2.01714321	4.32641381	0.05065832	0.07354105
Slc10a3	0.67411804	2.47682987	4.32609327	0.05066609	0.07354105
Nol12	0.56424401	2.80881033	4.32448354	0.05070514	0.07358349
St6galnac3	-0.5009277	3.5054826	4.32377363	0.05072237	0.0735926
Rpe	0.35203327	5.42631599	4.32341672	0.05073103	0.0735926
Tmem200a	0.60762763	3.68035073	4.32128786	0.05078276	0.07365338
Fam210a	-0.2718676	6.44242624	4.32060873	0.05079927	0.07366309
Ncmap	-2.5404665	-0.974297	4.31966974	0.05082211	0.07368197

Snrpb2	0.34332521	5.68008037	4.31895319	0.05083955	0.07368747
Mrpl54	0.65820535	2.69776809	4.31841218	0.05085272	0.07368747
Robo2	-0.3395557	6.52649214	4.31830306	0.05085538	0.07368747
Pdhb	0.21805698	6.93228658	4.31727026	0.05088053	0.07370968
Ilvbl	-0.6562166	2.36640121	4.31589923	0.05091395	0.07374385
Mir344c	-2.6588424	-1.6073122	4.31430712	0.05095278	0.07375761
Ly75	-1.9833014	-0.5198203	4.31359682	0.05097012	0.07375761
Uck2	0.44612282	3.46702241	4.31356062	0.05097101	0.07375761
Acat1	0.28433821	7.12008456	4.31350513	0.05097236	0.07375761
Tbca	0.43113556	6.43366701	4.31349489	0.05097261	0.07375761
Kif13a	-0.2882425	5.84859562	4.30937336	0.05107335	0.07388912
Chn2	0.25811827	5.29458178	4.30805364	0.05110565	0.07392032
Gm5065	-2.310932	-1.8556745	4.30736042	0.05112263	0.07392032
Mknk1	0.37813228	4.31864868	4.30698577	0.05113181	0.07392032
Lrrc27	0.5804748	3.21464008	4.30688289	0.05113433	0.07392032
Fendrr	-2.2606219	-0.3723177	4.30590741	0.05115824	0.07394063
Agtr1b	-1.3449544	2.19031854	4.30483541	0.05118452	0.07396438
Vps39	-0.2714366	5.87363614	4.30350529	0.05121716	0.07399729
Slc5a6	-0.5074947	4.25431609	4.30101188	0.05127841	0.07407151
Mpeg1	-0.7232287	2.86253962	4.29978048	0.05130869	0.07408941
Slc22a3	-0.9884669	0.82570762	4.29956535	0.05131399	0.07408941
Rnf8	0.43149179	4.25082204	4.29889502	0.05133048	0.07408941
Ppp2r2a	-0.2396333	7.10196112	4.29877912	0.05133333	0.07408941
Ttll3	-0.7546854	1.83886483	4.29850052	0.05134019	0.07408941
Adamts18	-1.2743164	0.25730253	4.29693723	0.05137869	0.07413071
Anxa2	0.67473868	6.18191341	4.29636866	0.0513927	0.07413666
Slco1a5	2.37659198	-0.5127274	4.29515588	0.0514226	0.07416553
Dtna	-0.295975	7.13003161	4.29238347	0.05149102	0.0742389
Cog8	0.62897194	2.01771711	4.29229268	0.05149326	0.0742389
Rdh10	0.38988328	3.6843256	4.29091721	0.05152724	0.07427362
Cbx1	0.39153785	4.65570054	4.29042663	0.05153937	0.07427683
Sypl2	1.42979064	0.14248168	4.28843853	0.05158855	0.07433094
Zc3h10	0.63965523	3.0586151	4.28810793	0.05159674	0.07433094
Ggcx	-0.4602664	3.1388994	4.28604112	0.05164793	0.07436605
Doc2g	-2.1657961	-0.3671259	4.28596442	0.05164983	0.07436605
Spata2l	0.43743582	4.60710982	4.28592336	0.05165085	0.07436605
Zfp142	-0.3587277	5.2626512	4.28494773	0.05167504	0.07437759
Dnajb11	0.27838453	5.90712065	4.2848003	0.0516787	0.07437759
Tgds	-0.566921	2.74649204	4.28414442	0.05169497	0.07438673
Adar	-0.396327	6.00892083	4.28205329	0.05174688	0.07444715
Fndc3b	0.22632048	5.7564573	4.28082789	0.05177732	0.07447667
Pnrc2	0.4495666	7.27023271	4.2797091	0.05180514	0.07450239
Opn4	-1.8107362	-0.0898355	4.27921191	0.05181751	0.07450589
Hdac6	-0.5641699	3.12401288	4.27834363	0.05183911	0.07450837
Pip4k2c	0.31798276	6.1114067	4.27769241	0.05185532	0.07450837

Gm14393	0.55949527	2.67963904	4.27763928	0.05185665	0.07450837
Dph3	0.28650747	5.71694722	4.27754637	0.05185896	0.07450837
Zfp759	-0.413093	3.76817556	4.27579193	0.05190267	0.07455688
Ppp5c	0.33767281	4.63015193	4.27490644	0.05192474	0.07457431
Inpp5a	0.35536756	4.56294931	4.27410781	0.05194466	0.07458864
Irak2	-0.3814219	3.65460377	4.27172496	0.05200415	0.07465976
B3gat1	-0.3852427	6.02154827	4.27128217	0.05201521	0.07466136
Trim44	0.20309776	9.18709954	4.27049492	0.05203488	0.07467531
Mkks	0.38423712	5.11911302	4.26971293	0.05205443	0.07468297
Abhd11	0.46614106	3.15532683	4.26918301	0.05206769	0.07468297
Bank1	0.83883828	1.7660714	4.26887816	0.05207531	0.07468297
Atat1	0.2911205	5.68342495	4.26868905	0.05208005	0.07468297
Tsku	0.896439	2.14957255	4.26579847	0.05215243	0.07477247
Rbm4	-1.3444568	-0.5383219	4.26260562	0.05223252	0.07487299
Zbtb42	1.22718629	0.59707261	4.26066568	0.05228125	0.07492852
Jdp2	-0.3967372	3.35051052	4.2596729	0.0523062	0.07494997
BC049352	-2.3983811	-1.7776477	4.25863309	0.05233236	0.07497312
Tspan31	0.4422109	5.85510856	4.25706874	0.05237173	0.07501521
Phyhip	0.34054383	6.76786975	4.25384888	0.05245289	0.07511711
D930020B18	-2.8755734	-2.0613081	4.253367	0.05246504	0.07512018
Gpr63	-0.5248862	3.16893815	4.25241962	0.05248896	0.07512813
Raver1	0.41406495	6.00400555	4.25235334	0.05249063	0.07512813
BC039771	-0.8858639	0.57098261	4.2513654	0.05251558	0.0751495
Dmc1	-2.3964544	-1.4697402	4.25049956	0.05253746	0.07516647
Tbc1d7	0.40154382	4.04487966	4.24970236	0.05255761	0.07518096
Pkp1	1.08805436	1.20327792	4.24785475	0.05260435	0.07523347
A2m	0.82105797	1.67001198	4.24740549	0.05261572	0.07523539
Slc7a6os	0.47379236	3.9736553	4.2446329	0.05268596	0.07532147
Itfg3	0.66282085	1.93936477	4.24354279	0.05271361	0.07534519
Fras1	-0.6917566	4.82325395	4.24318699	0.05272264	0.07534519
Adam8	-2.229884	-0.6615818	4.24271266	0.05273468	0.07534804
Emx1	1.15609684	0.52695818	4.24095104	0.05277941	0.07539274
Igsf6	-0.9359846	1.69934988	4.24068925	0.05278606	0.07539274
Rtn3	0.2982558	9.97579174	4.2397064	0.05281104	0.07541406
Donson	-0.5408122	3.37025423	4.23803488	0.05285356	0.07545044
Tctex1d2	0.4377234	4.3958505	4.23661964	0.05288959	0.07545044
Ctcf	0.24676463	6.37279785	4.23646536	0.05289352	0.07545044
B4galnt4	-0.5945009	3.61833574	4.23640754	0.05289499	0.07545044
Arl9	3.07319877	-1.9153914	4.23627656	0.05289833	0.07545044
Bcas2	0.23577351	5.77682026	4.23569331	0.05291318	0.07545044
Ppap2a	0.47282051	3.7937364	4.23557514	0.0529162	0.07545044
Gdf3	-1.5283576	-0.750508	4.23554394	0.05291699	0.07545044
Frg1	0.28995684	4.81586465	4.23364795	0.05296533	0.07550501
Vmn2r86	-1.2457876	0.46302815	4.23286313	0.05298535	0.07551921
Pus1	0.68191826	1.66141706	4.23102443	0.0530323	0.07557176

Nmnat1	1.06242827	0.27411572	4.2270132	0.05313488	0.07569112
Zfp523	-0.3688037	4.07551606	4.22696015	0.05313624	0.07569112
Repin1	-0.357053	4.53713337	4.22636685	0.05315143	0.07569839
Sdf2	0.44555142	4.44281333	4.22539538	0.05317632	0.07571945
Fam89a	-3.3372482	-1.4200627	4.22444276	0.05320074	0.07573773
Thra	-0.3669303	5.82827563	4.22406937	0.05321031	0.07573773
Bclaf1	0.2138299	9.51920161	4.2225046	0.05325046	0.07573773
Gm7120	0.73524549	2.94204316	4.22244404	0.05325201	0.07573773
Gpr21	-1.0460479	0.86234498	4.22243224	0.05325231	0.07573773
Sstr4	0.72326266	4.50972522	4.22235153	0.05325439	0.07573773
Fam172a	0.28471145	5.60774182	4.22213935	0.05325983	0.07573773
1810026J23R	0.30430446	5.9224383	4.22057857	0.05329992	0.07578036
Mdfi	2.57654892	-1.4504208	4.21968221	0.05332295	0.07579875
Gpr89	0.45846944	3.42303375	4.21902108	0.05333995	0.07580167
1810041L15R	-0.4460063	5.52631739	4.21881621	0.05334522	0.07580167
Kctd4	0.46353395	5.06433096	4.21657907	0.05340279	0.07586911
Elac2	-0.6146848	2.88312398	4.21603481	0.05341681	0.07587465
Ank1	-0.5011445	5.05992668	4.21355056	0.05348084	0.07595122
Myo1e	-0.3362863	4.55199139	4.21082688	0.05355114	0.07603395
Dhx34	0.69281084	1.42848274	4.21050866	0.05355936	0.07603395
Ampd3	0.31469037	5.59985315	4.20933068	0.05358981	0.07603623
Rnf19b	0.33520797	4.34190555	4.20932936	0.05358984	0.07603623
Ythdf1	0.21242074	6.7367696	4.20926998	0.05359138	0.07603623
Kcnd1	-0.6737477	2.40261102	4.20634512	0.05366706	0.07612921
Mon1a	0.49653903	2.34691386	4.20539467	0.05369168	0.07613075
Trim16	-0.6337216	2.64917315	4.20538652	0.05369189	0.07613075
Klhl23	-0.3976354	5.1496477	4.20482678	0.05370639	0.07613075
Phf5a	0.47304207	4.82766972	4.20430727	0.05371986	0.07613075
H3f3a	0.49881484	8.1858611	4.20423352	0.05372177	0.07613075
Mrpl4	0.47513275	4.75516618	4.20395308	0.05372904	0.07613075
Fundc1	0.32362107	6.44940792	4.20319143	0.0537488	0.07614436
Tomm6	0.44497962	5.29163217	4.2024671	0.05376759	0.07614544
Ube2e1	0.46395985	4.09330134	4.20237968	0.05376986	0.07614544
Cd8a	-0.9896903	0.35350132	4.20131356	0.05379754	0.07615462
Spata18	-1.1040539	0.3596755	4.20097441	0.05380635	0.07615462
11-Sep	0.23755416	7.5978951	4.20095694	0.0538068	0.07615462
Arhgef17	-0.3261397	6.61858914	4.19929573	0.05384997	0.07620133
Slc25a21	-1.3481656	0.19478389	4.19862212	0.05386748	0.07621174
Glrx5	0.4391772	3.73372452	4.19770121	0.05389144	0.07623126
Tpm2	0.57481437	6.33843715	4.19643225	0.05392447	0.0762636
Gm2694	-1.3411195	0.04466497	4.1940791	0.05398578	0.07633592
Npr1	-1.0362451	0.10392755	4.19358205	0.05399874	0.07633985
Uba52	0.4246011	6.7196023	4.19310327	0.05401123	0.07634312
Aif1	0.94879368	2.15680055	4.19179824	0.05404528	0.07637686
Gm10785	-1.0125105	0.94385693	4.19124536	0.05405972	0.07638287

Pros1	0.49962449	5.1652713	4.1906982	0.05407401	0.07638867
Tlr9	-1.5500816	-0.3613039	4.18935719	0.05410905	0.07642378
Ust	0.56951546	5.5251111	4.18871141	0.05412593	0.07643324
Ddx21	-0.297454	4.80739924	4.18805332	0.05414314	0.07643665
Rpl23a	0.36770175	7.25485524	4.18756163	0.05415601	0.07643665
Elovl2	0.62382266	2.66654295	4.18745048	0.05415892	0.07643665
Samd8	-0.2752039	6.05743869	4.18534415	0.05421407	0.07649939
Abhd14a	-0.6430622	2.80306502	4.1849739	0.05422377	0.07649939
Shc1	0.45725834	6.42383923	4.18410864	0.05424645	0.07651699
Helq	-0.6900291	2.56920515	4.18330231	0.05426759	0.07653243
Lrrc16b	-1.179729	2.98873128	4.18166623	0.05431052	0.0765741
Wibg	0.88630531	2.09290631	4.18127137	0.05432089	0.0765741
Tmem131	-0.3126346	7.23294098	4.18100951	0.05432776	0.0765741
Gm15133	2.91647042	-1.759902	4.17885922	0.05438427	0.07663934
Dennd2c	1.31416579	-0.3429158	4.17657684	0.05444431	0.07670069
B930018H19	2.92784523	-0.9073337	4.17642738	0.05444825	0.07670069
Mtch1	0.27341757	6.41998609	4.17489529	0.0544886	0.07674215
Spata4	-2.4609768	-1.0776967	4.17442501	0.054501	0.07674215
Shb	0.62959348	1.70974903	4.17386498	0.05451576	0.07674215
Dvl2	-0.5939822	2.37289552	4.17375709	0.0545186	0.07674215
Gm5089	0.5458281	4.78982062	4.17286413	0.05454215	0.07676089
Ccdc158	1.97139234	-0.7985472	4.16801936	0.05467013	0.07692657
Iffo1	-0.564783	3.77255364	4.16665801	0.05470615	0.07696282
Hist1h1b	2.31712489	-1.8051552	4.16543447	0.05473855	0.07699396
Dnaja2	0.20310507	7.78861547	4.16358482	0.05478757	0.07704846
Hsd17b2	1.17524229	1.4829574	4.16296846	0.05480392	0.07705701
Sirt4	-0.7161558	1.73272007	4.161623	0.05483962	0.07709102
Ammecr1	-0.7373789	2.35776292	4.16128238	0.05484866	0.07709102
Usp13	-0.5909307	3.87018205	4.15877288	0.05491533	0.07716207
F2r	0.5303034	2.66735324	4.15860551	0.05491979	0.07716207
Nucb1	0.51814736	4.56765034	4.15709489	0.05495997	0.07720406
BC037704	1.03216123	1.27085945	4.15570217	0.05499704	0.07724168
Nudt6	0.47832235	3.7359988	4.15501551	0.05501534	0.07725291
Nabp1	0.39497307	3.64228668	4.15409158	0.05503996	0.07727302
Klk6	-2.219328	-1.3802806	4.15338082	0.05505891	0.07728516
Tmem55b	0.36984396	4.99186399	4.15276852	0.05507524	0.07729361
Zfp738	-0.3346288	5.23921593	4.15037099	0.05513924	0.07736896
Cxcr6	-1.4475974	0.03419362	4.14777235	0.0552087	0.07745193
Gltscr1l	-0.269953	6.39227417	4.14692159	0.05523146	0.07746938
Spcs1	0.37636489	5.36136485	4.14616832	0.05525162	0.07748317
Atp7b	-2.0347317	-0.6278342	4.14420669	0.05530417	0.07754237
Dgat1	-0.9059703	1.99018369	4.14285332	0.05534046	0.07757875
Tpgs1	0.66453402	3.14348779	4.1404143	0.05540593	0.07765601
Cav2	0.40532982	5.18438214	4.13918279	0.05543901	0.07767984
Gfpt1	-0.288155	6.60092891	4.13901073	0.05544364	0.07767984

Arhgef19	0.82825892	1.47445845	4.13829166	0.05546297	0.07769241
5930438M14	1.73661026	-0.5977789	4.13757674	0.0554822	0.0777005
Stam2	0.28846408	4.84893538	4.13730691	0.05548946	0.0777005
Cxxc4	0.33245518	6.13019003	4.13485144	0.05555557	0.07777855
Vps37d	0.92081553	0.98645161	4.13307196	0.05560354	0.07782004
Barx2	0.74918201	1.91715805	4.13291998	0.05560764	0.07782004
Myh1	1.25248376	0.52930343	4.13259776	0.05561633	0.07782004
R3hdm4	0.30059556	7.68370037	4.12976391	0.05569283	0.07791256
4930486L24I	3.37959692	-2.2845267	4.12931814	0.05570488	0.07791488
Gatad2a	0.34781819	5.71910595	4.12832976	0.0557316	0.07793771
Drc1	-0.4775258	3.18557095	4.12741563	0.05575632	0.07795776
Rin2	0.32622637	6.55233572	4.12622474	0.05578855	0.07797929
Syna	0.69291963	1.95238682	4.12607828	0.05579252	0.07797929
Gm5577	-1.8352741	1.02627357	4.12451722	0.0558348	0.07802385
Rufy1	-0.3198457	4.69976452	4.12347046	0.05586317	0.0780422
Ncstn	0.39308473	5.1058978	4.12297745	0.05587654	0.0780422
Fam3c	0.24601198	6.43511488	4.12260619	0.05588661	0.0780422
Got1	0.28137746	8.34719757	4.122498	0.05588954	0.0780422
Akr1c12	-2.3305492	-0.1706401	4.12013744	0.05595362	0.07811714
Pard6g	0.44352242	5.59374964	4.11885591	0.05598845	0.07815121
Ptpn2	-0.5020532	4.93947843	4.11758896	0.0560229	0.07818475
Tbc1d14	0.29674945	5.17225701	4.11702011	0.05603837	0.0781918
Lcorl	-0.3171682	5.13712707	4.11561797	0.05607654	0.07823051
Lrrc9	-0.9764192	1.59318661	4.11388916	0.05612364	0.0782706
Sord	0.39620119	4.3922817	4.11368076	0.05612933	0.0782706
Ddit3	0.407647	3.40717023	4.1134144	0.05613659	0.0782706
Foxj2	0.28445847	6.55270403	4.1115298	0.056188	0.07832773
Klhdc8a	0.43480084	3.8166038	4.11012649	0.05622632	0.07835375
Trpt1	0.47735444	3.12327704	4.11008116	0.05622755	0.07835375
Rbm12b1	-0.43311	4.6326872	4.10687679	0.05631516	0.07844926
Dab1	-0.368504	6.61765701	4.10680922	0.05631701	0.07844926
Ramp2	0.56092124	4.5868832	4.10429414	0.05638589	0.07853062
Got2	0.24273071	7.01775185	4.10346855	0.05640852	0.07854755
Phtf1os	0.74083901	1.14798045	4.10064414	0.05648602	0.07864087
Nrg4	-1.4662857	-0.4053342	4.09995008	0.05650508	0.07865281
1110059G10	0.45500454	4.20899495	4.09902319	0.05653055	0.07866022
Usp20	-0.4498071	3.79233137	4.09849805	0.05654498	0.07866022
Zmynd8	-0.2936984	6.82734109	4.09842204	0.05654707	0.07866022
Dock5	0.26646797	6.84778138	4.09778185	0.05656468	0.07866022
Iars	-0.3398152	6.46595723	4.09764499	0.05656844	0.07866022
Sema6a	-0.3653997	5.73162937	4.09746777	0.05657332	0.07866022
Jam2	0.27255261	5.78114302	4.09659862	0.05659723	0.07867888
Lynx1	0.2892352	7.54082893	4.09249337	0.05671035	0.07882152
Apol6	-1.275851	-0.189787	4.0917927	0.05672968	0.07882888
Lgals1	0.72192923	4.62330975	4.09153972	0.05673666	0.07882888

Xrcc2	-0.7239718	2.5538066	4.09019759	0.05677372	0.07886576
4930405A21I	-1.0653903	1.03742886	4.08980443	0.05678458	0.07886624
Cib1	0.75930093	1.9956303	4.08783048	0.05683914	0.07892741
Rgs16	0.40802748	3.66457542	4.08511049	0.05691442	0.07901732
Tdp2	0.45770974	3.61974665	4.08193326	0.05700251	0.07912497
Xbp1	0.28770041	6.19549155	4.08013543	0.05705242	0.0791796
Sall1	0.28507019	4.82359432	4.07902458	0.05708328	0.07920778
Dhx8	-0.2730126	5.10622165	4.07557374	0.05717929	0.07932632
Pram1	-0.9714204	0.92672815	4.07203483	0.05727793	0.07943694
Vash2	-0.7130329	1.75752496	4.07161603	0.05728962	0.07943694
Gpr45	0.5352387	2.23163796	4.07157407	0.05729079	0.07943694
Capza1	0.28978793	6.23692652	4.06976181	0.05734139	0.07949241
Fastkd2	-0.6488016	2.91665665	4.06567085	0.05745582	0.07963631
E030025P04I	-3.2051035	-1.4199404	4.06472011	0.05748244	0.0796585
Srsf3	0.28791062	7.78346233	4.06378608	0.05750862	0.07968006
1110002L01I	0.89189078	1.11753927	4.0621385	0.05755482	0.07972935
Api5	0.25564344	6.94753257	4.05976303	0.05762152	0.0798007
Extl1	-0.4259997	3.57333937	4.05954627	0.05762761	0.0798007
Crcp	0.39665278	4.59667811	4.05558703	0.05773897	0.07994016
Cxcl17	-2.4393437	-1.1080661	4.05435478	0.05777369	0.07997346
1810019D21I	2.32998261	-0.5570724	4.05386044	0.05778762	0.07997354
Vit	-0.9939734	2.21496262	4.05359618	0.05779507	0.07997354
Fam213b	0.3965451	4.12437467	4.05095091	0.0578697	0.08003788
Ehd2	0.56490216	4.92766503	4.05089147	0.05787137	0.08003788
Nkiras1	0.23941144	6.11160246	4.05081362	0.05787357	0.08003788
Elavl3	0.30446174	7.19794494	4.05000815	0.05789632	0.08005458
1700012B09I	1.65793218	-0.4327784	4.04962929	0.05790702	0.08005462
Lnx1	-0.4139889	4.01709519	4.04923817	0.05791807	0.08005514
Eef2	-0.2442131	8.35795737	4.04763863	0.0579633	0.08008574
Lamtor1	0.45315999	5.52992674	4.04753488	0.05796623	0.08008574
Gm15545	-1.8840593	-0.7285073	4.04732263	0.05797224	0.08008574
Gmps	-0.2771387	6.75866356	4.04488405	0.05804127	0.08016634
Fxyd7	0.64205713	2.00153272	4.04197663	0.0581237	0.08025324
2310067B10I	0.47485644	4.10431381	4.0419103	0.05812559	0.08025324
Rhobtb3	-0.3845514	4.45855426	4.03941011	0.05819658	0.08033648
A530058N18	-0.8611726	1.14834101	4.0377108	0.05824489	0.08036724
Fbxl7	0.53065809	6.20230065	4.03710021	0.05826226	0.08036724
Pcyt1b	0.35800325	5.32802086	4.03685284	0.0582693	0.08036724
Fam71d	-2.2263542	-1.4505967	4.03633079	0.05828416	0.08036724
Cdk14	0.27562358	6.91946638	4.03568124	0.05830265	0.08036724
Lnpep	-0.2231417	6.88376445	4.03563801	0.05830388	0.08036724
L2hgdh	0.23881819	6.11921602	4.03547701	0.05830847	0.08036724
Bcmo1	-2.9385988	-1.5031222	4.03530379	0.0583134	0.08036724
E330009J07I	-0.8960001	1.34869234	4.03523751	0.05831529	0.08036724
Ppp2r5a	0.29763897	5.75642456	4.03388836	0.05835373	0.08040544

Knop1	0.32872496	5.63143563	4.03302985	0.05837821	0.0804244
Eif3e	0.26665881	7.15623584	4.03226009	0.05840016	0.08042673
Npepps	-0.3276294	6.32023894	4.03191425	0.05841003	0.08042673
Fign	-0.4181131	4.7871539	4.03184294	0.05841207	0.08042673
Gfm1	-0.4253782	4.55150656	4.03035501	0.05845455	0.08047045
Reps1	0.27068462	6.00264591	4.02972239	0.05847262	0.08048056
Ptms	0.57152761	6.61621914	4.02898095	0.0584938	0.08049495
Ckmt1	0.3750397	5.28012258	4.02731995	0.0585413	0.08054554
2610020C07I	-1.0098231	0.53748742	4.02678897	0.0585565	0.08055167
Vkorc1	0.59891072	5.37488519	4.02355346	0.05864917	0.08066436
Neurl2	1.79071023	-0.3121361	4.02266533	0.05867464	0.08068325
Arl3	0.38792588	5.64605501	4.02188978	0.05869689	0.08068325
Snx9	0.37112242	4.81473648	4.02173452	0.05870135	0.08068325
Gm10409	-0.5007009	4.00369907	4.02157478	0.05870593	0.08068325
Pwp2	-0.6811263	2.67198387	4.02072988	0.05873019	0.08069803
Bcas3os1	-1.7217723	0.45882069	4.02016304	0.05874647	0.08069803
Slc25a37	-0.3099838	5.36740566	4.01986764	0.05875495	0.08069803
Nmrk2	-0.7940825	0.66187961	4.01970194	0.05875971	0.08069803
Gng8	-1.407211	-0.4881482	4.01913961	0.05877587	0.08070545
Ctps	-0.4156758	3.98879188	4.01680057	0.05884314	0.08078303
Adh7	1.05797811	1.41363985	4.01636141	0.05885578	0.0807856
Zfp326	-0.2711646	5.58681216	4.01213337	0.05897764	0.08093804
Adal	0.36131065	3.96032848	4.01145407	0.05899724	0.08095013
1700110K17I	-1.1265842	0.8249802	4.01095459	0.05901166	0.08095511
Col2a1	-2.3168151	-1.1175281	4.01036675	0.05902864	0.08096359
Hspa14	-0.3221184	4.2484401	4.00938505	0.059057	0.08098769
Ddx47	0.27758455	5.91726426	4.00877877	0.05907452	0.08099691
Tcp11l2	0.30589046	4.28628694	4.00733548	0.05911626	0.08103933
Nxpe4	-0.4626186	2.96070174	4.00660412	0.05913743	0.08104229
Gk5	0.55780804	1.99776092	4.00651422	0.05914003	0.08104229
Fancb	-1.1923558	0.97425989	4.00486885	0.05918768	0.08109277
Fem1c	-0.3035201	5.33679925	4.00403176	0.05921193	0.08111119
Gtpbp8	0.34732469	3.77131635	4.0022806	0.05926272	0.08116593
Pdha1	0.20735204	7.41076837	4.00146672	0.05928634	0.08118263
1700034G24	1.77801709	-0.0836493	4.00111475	0.05929656	0.08118263
Arhgap1	0.3281433	5.65617668	4.00041964	0.05931674	0.08119544
Hip1r	-0.5692173	3.19870087	3.99911108	0.05935476	0.08123267
Tsr2	0.30189806	4.95762152	3.99866877	0.05936762	0.08123544
Hps3	-0.469397	4.08700997	3.99608741	0.05944272	0.08132337
2310001H17I	2.18477264	-0.9587405	3.99524525	0.05946725	0.08134209
Phactr3	-0.2407089	6.15735084	3.99468137	0.05948368	0.08134973
Fuom	0.46269554	3.47949281	3.99398949	0.05950384	0.08136003
Acacb	-0.9874105	1.28171749	3.9934956	0.05951824	0.08136003
Pla2g4c	1.29590877	0.09718778	3.99330676	0.05952375	0.08136003
Il17rd	-0.685276	2.50258226	3.99111565	0.05958768	0.08143258

Rabggtb	0.30360819	6.33194334	3.98957703	0.05963262	0.08147916
Dctn6	0.38198548	6.37633636	3.98820697	0.05967268	0.08151903
Stxbp3a	-0.2770664	4.61286465	3.9876861	0.05968791	0.081525
Rbm11	0.44581235	3.26912102	3.98625348	0.05972984	0.0815531
Ctso	0.47969036	5.03282715	3.98598239	0.05973777	0.0815531
Sv2c	-0.4662767	4.80415195	3.98586871	0.0597411	0.0815531
Atg12	0.37078222	5.11943071	3.97855414	0.05995574	0.08183121
Cenpk	-0.9894738	1.17615186	3.97792882	0.05997413	0.08184142
Efhc2	-0.8990727	2.12178194	3.97524257	0.06005321	0.08193442
Btbd7	-0.3275208	5.67035502	3.97421269	0.06008355	0.08194684
Hipk3	-0.2037881	7.79073933	3.97414487	0.06008555	0.08194684
Ilk	0.38519733	6.00172694	3.97379763	0.06009579	0.08194684
Tie1	-1.7608286	0.39482434	3.97321513	0.06011297	0.08194684
Zcchc24	0.44718938	7.2701703	3.97308076	0.06011693	0.08194684
Akap2	-0.2463932	7.52593438	3.97206208	0.06014699	0.08197292
Pigg	-0.3981391	3.45784024	3.96980299	0.0602137	0.08203469
Fam20b	-0.3032961	4.91662611	3.96954127	0.06022143	0.08203469
Arg2	-0.6289582	2.92564472	3.96941664	0.06022512	0.08203469
Cars	-0.4692797	3.58290147	3.96901503	0.06023699	0.08203596
Fam221a	1.44249296	0.13123574	3.96854616	0.06025085	0.08203994
1700110C19I	-1.8868235	-0.1240952	3.96072751	0.06048256	0.08234051
Astn1	-0.3881759	8.03624661	3.95986122	0.0605083	0.08235204
Pkp2	-0.6439817	2.23426187	3.95934114	0.06052376	0.08235204
Asnsd1	0.43457075	5.29360696	3.95933399	0.06052397	0.08235204
Mavs	0.59936671	4.855431	3.95818842	0.06055803	0.08238344
Mybbp1a	-0.366576	4.39925614	3.95654323	0.06060699	0.08243509
Hadh	0.4144173	4.85452117	3.95498691	0.06065334	0.08248319
Snrpc	0.53852774	3.22601681	3.95317259	0.06070743	0.08253822
E2f6	0.29099437	5.50491294	3.95274059	0.06072032	0.08253822
Gpr84	-2.7263569	-1.0057645	3.95252271	0.06072682	0.08253822
Dyrk4	3.69203159	-2.242214	4.03725715	0.06074419	0.08254687
Aldh7a1	0.27734215	4.98129847	3.95081406	0.06077783	0.08256328
Gpr149	-1.1250221	0.73379147	3.95062405	0.06078351	0.08256328
Sall2	-0.298462	5.73749934	3.95043061	0.06078929	0.08256328
Erp44	0.38005	4.53609662	3.94884127	0.06083679	0.08261284
BC031181	0.39830446	6.05618873	3.9484122	0.06084962	0.08261531
Matr3	-0.3004039	8.16150937	3.94621728	0.06091531	0.08268953
Mrpl27	0.40330627	5.32071459	3.94100828	0.06107152	0.08288658
Ska3	-0.6986707	1.45807061	3.94051322	0.06108639	0.08289177
Trpm4	-0.7998548	2.0419583	3.93894584	0.0611335	0.08292464
Tmem119	0.6686079	3.49084428	3.93864935	0.06114242	0.08292464
5830417I10R	-0.3541212	6.11099523	3.93860404	0.06114378	0.08292464
Rbm3os	-1.509391	-0.0460533	3.93613309	0.06121815	0.08298104
Dnajb1	0.24649459	6.61552097	3.9361238	0.06121843	0.08298104
Pold1	-1.018871	0.57114693	3.93579948	0.0612282	0.08298104

1110032A03l	0.32188836	6.65671181	3.93551804	0.06123667	0.08298104
Ppp1r15b	-0.2469076	5.41122845	3.93538516	0.06124068	0.08298104
Glis2	0.39720646	5.21343482	3.93500416	0.06125216	0.0829816
Tmem223	0.38639818	3.72335312	3.93455153	0.0612658	0.0829851
Bmp2k	-0.3106749	5.06824754	3.9339452	0.06128408	0.08299487
Fam76a	0.3032616	5.87616487	3.93341267	0.06130014	0.08300164
Itfg1	0.25038803	7.75586571	3.93291686	0.0613151	0.08300691
Scara5	0.72875305	1.61318864	3.93162267	0.06135416	0.0830448
Fam43b	0.79100384	2.4659987	3.92827672	0.06145528	0.08316667
Mroh1	-0.5058535	4.29708474	3.92690914	0.06149667	0.08319657
Fam195b	0.58993817	2.5685986	3.92681364	0.06149956	0.08319657
Ankrd49	0.43991927	4.93883628	3.92560716	0.0615361	0.08322954
Ccdc171	-0.4602251	3.20507222	3.92527629	0.06154613	0.08322954
Svop	0.38746108	5.76057809	3.92333835	0.06160488	0.08328747
Mrpl35	0.25754769	5.08137465	3.92313115	0.06161117	0.08328747
Kcnk2	0.25057854	7.24702257	3.92193323	0.06164752	0.0833216
Pik3r2	0.3546371	4.18510452	3.92138176	0.06166427	0.08332922
Chchd5	0.52963912	2.63589755	3.92093781	0.06167775	0.08333243
Rnaset2b	0.52012513	4.08648288	3.92013659	0.0617021	0.08335031
Aph1b	-0.4460829	4.15526441	3.91796023	0.06176828	0.08342469
Meis2	-0.2855449	6.62560199	3.91722955	0.06179052	0.0834397
C1qtnf1	0.42717668	4.86034582	3.9160957	0.06182504	0.08345718
Zfand6	0.37310708	5.61016138	3.91607371	0.06182571	0.08345718
Mkx	0.41308997	5.3746159	3.91469647	0.06186768	0.08348198
Set	0.24221481	8.09528691	3.91466672	0.06186859	0.08348198
Calhm2	-0.7410603	2.54101821	3.91437528	0.06187747	0.08348198
Dhfr	-0.3795582	3.80304629	3.91396544	0.06188997	0.08348383
Pisd	-0.3348026	4.45308824	3.91140778	0.06196803	0.08357409
Sec61b	0.42352029	4.79413797	3.9089503	0.06204314	0.08365354
Mest	-0.4764884	6.37721065	3.90875073	0.06204924	0.08365354
Gtf2a1	0.229835	6.99718016	3.90357504	0.06220778	0.08385221
Grem2	0.32195445	5.2695346	3.90254441	0.06223941	0.08387977
Tomm22	0.3650773	5.96992969	3.90161766	0.06226787	0.08389673
Rcan1	0.31656524	5.88676338	3.90140603	0.06227437	0.08389673
Plk3	0.88020312	1.70869438	3.89984491	0.06232234	0.08394422
Ebf2	0.56777978	3.16521469	3.89953072	0.062332	0.08394422
Tor1b	-0.4208829	3.76440609	3.89907719	0.06234594	0.08394793
Tuba1b	0.22827629	9.23594797	3.89805568	0.06237737	0.08397517
Ormdl1	0.62193956	3.70147365	3.89712994	0.06240586	0.08398419
Mir3064	-1.3968995	-0.6415369	3.89711067	0.06240646	0.08398419
Tarbp2	-0.5347413	2.33320718	3.89544348	0.06245781	0.08402726
Coro1b	0.35080388	5.32204427	3.89534434	0.06246087	0.08402726
Ppp1r1a	0.44291711	7.06598969	3.88903609	0.06265564	0.08427417
P2ry6	-0.9064562	0.78746139	3.88759331	0.06270029	0.08431911
Mrc1	-0.8343462	2.19715843	3.8865427	0.06273282	0.08433471

Vcpkmt	0.68555008	1.7494766	3.88649249	0.06273438	0.08433471
2210408I21R	-0.4973431	4.27033096	3.88330167	0.06283331	0.08445181
Arfip2	0.28671349	5.19645534	3.88295695	0.06284401	0.08445181
Rgs22	-1.2427264	0.3420577	3.88212562	0.06286982	0.08447136
Mrpl16	0.28261131	5.6404914	3.87664598	0.06304024	0.08468372
Dedd2	0.83442507	1.48902123	3.87618276	0.06305467	0.08468372
Rnf2	0.29142613	5.42801567	3.87591649	0.06306297	0.08468372
Egfr	-0.4092549	5.96327822	3.87554429	0.06307457	0.08468372
Cnot7	0.26075326	6.74951294	3.87488426	0.06309515	0.08468372
Qtrt1	-0.5643295	2.71706356	3.87486956	0.0630956	0.08468372
B4galt1	0.45123397	4.19173818	3.87260612	0.06316623	0.08476334
Lrrtm3	-0.314781	4.97338521	3.87181778	0.06319085	0.08476889
Eif4h	0.22271972	7.04645059	3.8715924	0.06319789	0.08476889
Lxn	0.27139888	4.9324771	3.87138838	0.06320426	0.08476889
Slc30a5	-0.3379399	3.98366903	3.87037121	0.06323605	0.08479636
Sema3d	0.59320178	6.49635754	3.86899821	0.06327899	0.08483365
Dnajb9	0.38278705	5.83039723	3.8686119	0.06329107	0.08483365
Spred2	0.2612499	6.51431557	3.86839737	0.06329779	0.08483365
Ccna2	-0.6121053	1.98448009	3.86580654	0.06337893	0.08492723
Pthlh	0.62290325	2.27733895	3.86525716	0.06339615	0.08493514
4930412C18I	1.0292893	1.24851585	3.86235306	0.06348728	0.08504203
Zbed3	0.35114238	5.56461188	3.86153027	0.06351312	0.08504893
Necap2	0.56436648	3.41780728	3.8614672	0.06351511	0.08504893
Ralgps1	-0.375612	6.58154748	3.86092117	0.06353227	0.08505673
Phka2	-0.5681464	4.89890177	3.85988813	0.06356474	0.08507542
Cpsf1	-0.5121987	4.0570391	3.85975563	0.06356891	0.08507542
A230103J11I	0.71571291	1.99749863	3.85655778	0.06366958	0.08518591
P4ha2	-0.5866981	1.85672875	3.85633049	0.06367674	0.08518591
N4bp2l2	-0.227287	6.28193823	3.85605134	0.06368554	0.08518591
A230077H06	-0.8901629	2.28415411	3.85448768	0.06373484	0.08523666
Rps21	0.41436249	5.20132058	3.85376427	0.06375766	0.08525198
Inmt	0.71307224	3.49614324	3.8526716	0.06379216	0.08527542
Fhl3	0.80572099	2.04694674	3.85248888	0.06379793	0.08527542
Tsc22d4	0.42410601	3.17516185	3.85152382	0.06382841	0.08530097
Hist2h3b	-1.5751423	-1.2253731	3.84970822	0.06388581	0.08535682
Mgea5	-0.3041548	8.6334026	3.84948222	0.06389296	0.08535682
Rnf126	0.30465285	3.99171202	3.84888867	0.06391174	0.08536671
Rin1	-0.4609106	3.80022868	3.84814731	0.06393521	0.08538284
Diras2	-0.2426614	8.13275265	3.847192	0.06396546	0.08540804
Ppfia2	-0.30879	7.76153196	3.84588859	0.06400677	0.08544798
Gpn1	0.35882497	3.93186967	3.84351629	0.06408202	0.08553322
01-Sep	0.7113201	2.38936797	3.84218619	0.06412426	0.08556215
Hspb3	1.0911592	0.34003333	3.84211539	0.06412651	0.08556215
Plxdc1	0.42210772	2.91556988	3.84124433	0.06415419	0.08558386
Lnp	-0.3393932	6.97294153	3.84018267	0.06418794	0.08561366

Lhx2	0.31086481	5.46482425	3.8392993	0.06421604	0.08562106
Zfp945	-0.299786	5.18265876	3.83929074	0.06421631	0.08562106
Hspbap1	-0.6419114	2.22163898	3.83621147	0.06431438	0.08573657
Trmt6	0.31784749	4.64719553	3.83492123	0.06435552	0.08577618
Tmem117	-0.5037228	3.28497422	3.83342211	0.06440336	0.08582469
Apba3	-0.5709707	2.53870786	3.83036414	0.06450108	0.08593964
Has1	-1.2440031	0.34808261	3.82958655	0.06452595	0.08595752
Commd8	0.41899142	5.76987155	3.82902439	0.06454394	0.08596621
Nsdhl	0.3264649	4.56977349	3.82832987	0.06456618	0.08598056
Mgat4c	-0.5253727	2.88416955	3.82728288	0.06459971	0.08600995
Pcbd2	0.52602992	4.06968922	3.82495855	0.06467423	0.08609388
Pramel5	-2.2189967	-1.7145602	3.824393	0.06469238	0.08610276
Crnkl1	-0.340416	4.56709497	3.82377165	0.06471232	0.08611402
Tex12	-1.4396018	-0.1499423	3.82311854	0.06473329	0.08612021
Uggt1	-0.4470242	5.11580547	3.82291186	0.06473993	0.08612021
Cer1	2.75941505	-1.0477751	3.82201175	0.06476885	0.08612846
Slamf1	-2.4562161	-1.076657	3.82200401	0.0647691	0.08612846
Rac2	0.50796677	2.78924291	3.82109624	0.06479827	0.08614175
Dok5	-0.4383725	3.31033104	3.82097849	0.06480206	0.08614175
Chmp1b	0.36101468	5.41564145	3.81671747	0.06493924	0.08629897
Nlgn3	-0.3135652	5.98583432	3.81659028	0.06494334	0.08629897
Mrpl49	0.45707741	4.77436411	3.81580226	0.06496874	0.08631744
Taf9b	0.32302041	6.91473087	3.81393445	0.06502901	0.08638221
Rpl31	0.39835759	8.64150413	3.81321345	0.06505229	0.08639784
Ergic3	0.4398723	5.24047087	3.81265045	0.06507048	0.08640669
Galm	0.71945429	2.35655813	3.80948139	0.06517296	0.08652745
Clgn	-0.6331349	2.17288949	3.80851539	0.06520423	0.08655366
Zbtb11	-0.3121792	6.58427433	3.80694793	0.06525502	0.0865997
Zfp202	-0.7162929	1.45069473	3.80673223	0.06526201	0.0865997
Nespas	3.07543005	-1.9246916	3.80606799	0.06528354	0.08661296
Rnd1	-0.7612822	1.29585045	3.80516352	0.06531288	0.08663656
Il15ra	0.49708564	2.60517085	3.8047292	0.06532698	0.08663993
Tnks	-0.320194	5.86862362	3.8034197	0.06536949	0.08668099
Kcnc2	-0.3631084	5.91234506	3.80198397	0.06541614	0.08672752
Vsig10	-1.0139614	1.46708961	3.80118165	0.06544223	0.08674677
Acsl4	-0.2598226	6.37633032	3.79514087	0.06563902	0.08697918
Grap2	-0.8218826	1.1940081	3.79508774	0.06564075	0.08697918
Lrrtm2	-0.3337727	6.66013688	3.7942317	0.06566869	0.08698889
Yipf6	0.23660528	7.26621431	3.79415285	0.06567127	0.08698889
Gm101	-2.0985744	-1.1663423	3.79292083	0.06571151	0.08702682
4930428E07I	-1.7039882	-0.2484161	3.79229676	0.0657319	0.08703601
Gclm	0.33134375	5.32642741	3.7919987	0.06574165	0.08703601
Acbd4	-0.5842475	2.3604109	3.79072927	0.06578316	0.0870756
Tex26	1.48557904	-0.7564321	3.78986095	0.06581158	0.08708187
Cd93	-0.8451993	3.15480574	3.78980598	0.06581337	0.08708187

A930012L18I	-0.6660758	2.57284962	3.78952037	0.06582272	0.08708187
Tmf1	0.21084513	7.01464471	3.78886856	0.06584407	0.08709
Trim30a	-0.4343853	3.79956859	3.78841962	0.06585877	0.08709
Cgnl1	0.33999223	6.07090284	3.78826924	0.0658637	0.08709
Pdcd1lg2	-1.2874542	0.07482019	3.78499226	0.06597116	0.0871961
L3hypdh	0.61300522	2.99052929	3.78486014	0.0659755	0.0871961
Surf4	0.3798833	4.57231606	3.78475906	0.06597881	0.0871961
Ldoc1	2.29464689	-1.3494484	3.78358122	0.06601749	0.08723185
Uvssa	-0.3807121	4.85422542	3.7812746	0.06609332	0.0873051
Edc4	-0.5151727	3.93410082	3.78073599	0.06611103	0.0873051
Hepacam2	-1.4916806	0.47403687	3.78035994	0.06612341	0.0873051
Pcdhb14	-0.5277282	3.02501865	3.78013007	0.06613098	0.0873051
Usp21	0.2756	5.1328123	3.78012561	0.06613112	0.0873051
Dhps	0.64023862	3.07884382	3.77724275	0.06622609	0.08741509
Dpysl4	-0.5505522	2.8588484	3.77175544	0.06640729	0.08763884
Slc6a20a	-0.5959419	5.60574727	3.77134264	0.06642094	0.08764144
Zfp617	0.28752817	5.81358953	3.77072157	0.06644149	0.08765314
Leap2	-2.1208087	-0.4596987	3.76996619	0.0664665	0.08767071
Lyrm1	0.53552866	2.71571946	3.76819615	0.06652513	0.08773262
Mlkl	1.20422503	0.73842643	3.76676644	0.06657253	0.08777315
Dph6	0.23946839	6.23839124	3.7665634	0.06657926	0.08777315
Dcaf10	-0.3214294	5.20849913	3.76520684	0.06662428	0.08781706
4930430F08I	0.40124661	3.77965773	3.76385347	0.06666923	0.08786087
Fbxo40	1.81428932	-0.5305245	3.76242251	0.06671679	0.0879081
Mag	-0.5195137	2.55410134	3.76156851	0.06674519	0.08792603
Sf3b2	0.28653709	6.8265091	3.76130862	0.06675384	0.08792603
Foxc1	0.36789954	8.36033334	3.75909548	0.06682752	0.08800597
Ankle2	-0.2630206	5.23852826	3.75878119	0.066838	0.08800597
9430016H08I	0.43845177	3.49549773	3.75670915	0.06690708	0.08808147
Hspa12a	-0.2940304	8.26681948	3.75391429	0.06700039	0.08818884
Baalc	0.22990763	7.07479323	3.75332464	0.0670201	0.0881993
Itga4	-0.4180092	4.8409161	3.75228866	0.06705474	0.08822941
Phex	-0.7632877	1.95507507	3.75049713	0.06711469	0.0882928
Sidt1	-0.3684661	5.12946762	3.74931672	0.06715422	0.08832353
B4galnt1	0.32668802	5.39993322	3.7490967	0.06716159	0.08832353
Slc25a33	0.50506706	3.31598457	3.74855784	0.06717965	0.08833179
Prmt2	0.41097499	4.78853725	3.74737324	0.06721937	0.08836853
Sirt3	0.38030004	3.59719105	3.74566798	0.06727659	0.08842825
Itih3	-0.7951901	1.81272516	3.74460186	0.0673124	0.08844686
Tfb2m	0.31611542	5.65396134	3.7445443	0.06731433	0.08844686
Cops2	0.26687358	8.04261981	3.7436697	0.06734372	0.0884643
Mettl17	-0.7270702	1.69387304	3.74344741	0.06735119	0.0884643
Fgf5	0.61687825	2.67938052	3.74123572	0.06742559	0.08854652
Haus6	-0.392485	3.95219475	3.74064684	0.06744541	0.08855705
Fam107a	0.4308942	7.39578337	3.73798578	0.06753508	0.08865926

Sf1	0.22756219	6.7156002	3.73731785	0.06755761	0.08866587
Spsb4	-0.9473776	1.11843022	3.73713569	0.06756375	0.08866587
Zfp882	-0.3204258	4.53737201	3.73637861	0.0675893	0.08868229
Aip	0.42544805	5.1871287	3.73606427	0.06759991	0.08868229
Man1b1	-0.4260542	3.68547036	3.73531466	0.06762522	0.08869999
1500009C09I	0.31164672	5.50390501	3.73419212	0.06766315	0.08873422
Vta1	0.32965717	5.04032592	3.73372169	0.06767905	0.08873955
Sort1	-0.2773425	7.68974787	3.73289357	0.06770705	0.08875786
Cyp2j9	-0.6689575	2.44796972	3.73260909	0.06771667	0.08875786
Six3os1	-0.9117161	0.77632868	3.73182578	0.06774317	0.08876543
Rcn1	0.49027079	4.76303695	3.73173893	0.06774611	0.08876543
Lmbrd1	-0.2261279	6.75262406	3.73070859	0.06778099	0.08879562
4930479D17I	0.90573268	1.0864378	3.7302552	0.06779635	0.08880023
Gm6981	0.70175577	1.51002647	3.72982197	0.06781103	0.08880394
Errfi1	0.24485447	6.88726728	3.72685391	0.06791167	0.08892022
Aph1c	-0.4061827	3.93626938	3.72644567	0.06792553	0.08892027
Dyrk3	0.81939669	1.85283218	3.72604589	0.0679391	0.08892027
Mrpl1	-0.3514484	4.24105416	3.7258053	0.06794727	0.08892027
Gm5617	1.05145496	1.57345453	3.72518236	0.06796843	0.08893245
Serinc1	0.24681351	8.80303299	3.72203645	0.0680754	0.08905688
Gpr146	-0.4101445	4.1900935	3.72103199	0.06810959	0.08908607
Dnase1	-1.0595867	1.03143966	3.71962167	0.06815764	0.08912116
Myh8	2.25209593	-1.5295231	3.71954711	0.06816018	0.08912116
Smtnl2	-0.726754	1.14257697	3.71817869	0.06820684	0.08916662
Zfp54	0.93185556	1.59679142	3.71763877	0.06822526	0.08917516
Dad1	0.54651217	5.04388937	3.71706818	0.06824473	0.08918507
Metap2	0.21981473	7.30908151	3.71515242	0.06831015	0.08924394
Ccdc27	-2.0480157	-1.0113339	3.7150269	0.06831444	0.08924394
Btbd11	-0.3011579	4.43944584	3.71470427	0.06832547	0.08924394
Rbm18	0.2891542	6.24774405	3.71321703	0.06837632	0.08929481
Pecam1	-0.8935563	1.99038698	3.71069149	0.06846277	0.0893906
C2cd2	0.36812986	4.33129603	3.71014065	0.06848165	0.0893906
Klhl36	1.2642259	0.6160963	3.70962086	0.06849946	0.0893906
Tulp1	-3.0844376	-1.9917509	3.70912782	0.06851636	0.0893906
Exoc6b	-0.2249922	6.79077474	3.70905957	0.0685187	0.0893906
Abi3	-0.6404141	1.67972369	3.70898771	0.06852117	0.0893906
Pcdhb11	-1.1066576	0.96598835	3.70800917	0.06855473	0.08940615
Tbcb	0.41617634	4.55125127	3.70794533	0.06855692	0.08940615
Tmem69	-0.338217	4.43179247	3.70758105	0.06856942	0.08940691
Tspo	0.70156155	2.02514548	3.7066724	0.06860061	0.08943203
Chst2	-0.2413361	7.83723352	3.70601366	0.06862324	0.08944598
Tmem2	-0.2986763	4.21362306	3.70475662	0.06866643	0.08948673
Crk	0.21540809	7.7634951	3.70292972	0.06872926	0.08955306
Mir6920	-1.9839775	-1.5890956	3.70217732	0.06875516	0.08957124
Nr2c2ap	0.57414794	2.05884682	3.70124827	0.06878715	0.08959736

Alg13	-0.6053733	3.17797422	3.69824228	0.06889078	0.08971164
Rabac1	0.68322574	3.07237434	3.69800953	0.06889881	0.08971164
1500009L16I	0.35215948	4.68452919	3.69761324	0.06891249	0.08971388
Vnn1	-0.9641331	1.71551037	3.69700874	0.06893336	0.08972547
Spg21	0.45881363	5.13850679	3.69368835	0.06904812	0.08985303
Psmd7	0.21904276	6.76951316	3.69348034	0.06905531	0.08985303
Nufip2	0.23429933	7.74209213	3.69267241	0.06908327	0.08987382
Map3k4	-0.3411157	4.98220469	3.69154344	0.06912237	0.08990909
Smyd1	1.15598761	1.45294208	3.68968178	0.06918689	0.08996412
Hps1	-0.8044003	1.40435611	3.68936194	0.06919798	0.08996412
Prpf31	0.32288363	4.10967263	3.68928472	0.06920066	0.08996412
Tusc1	0.3896929	3.18417392	3.68587517	0.06931903	0.09010239
Gnb1l	1.3768186	0.36464636	3.68472895	0.06935887	0.09012683
Ppap2c	0.77238043	1.53986906	3.68464324	0.06936185	0.09012683
Tbl1x	0.20033372	6.44132166	3.68367559	0.06939551	0.09014953
Tor4a	-0.4586979	3.20994434	3.68294041	0.0694211	0.09014953
C330007P06I	0.31798521	7.68773185	3.68246645	0.0694376	0.09014953
Prnp	-0.286499	8.00642486	3.68243501	0.0694387	0.09014953
Tcf7l1	0.49477665	5.36479086	3.68241442	0.06943941	0.09014953
Fam188a	0.29509278	6.11642959	3.68174099	0.06946287	0.09015389
Nedd4	-0.1959432	9.5001887	3.68162794	0.06946681	0.09015389
2900041M22	1.26564199	-0.0298824	3.68088428	0.06949272	0.09017191
Tsc2	-0.380496	5.85466584	3.68000484	0.06952338	0.09019609
Npr3	-0.4710892	5.29409063	3.67574419	0.06967213	0.09037345
Smarcc2	0.21525484	8.25320197	3.67539063	0.06968449	0.09037385
Mettl6	0.49157808	3.84319082	3.674196	0.06972627	0.0904013
Dus2	-0.5599212	2.42156585	3.67409649	0.06972976	0.0904013
Nop14	-0.2764598	4.94818265	3.67343473	0.06975292	0.0904157
Herc6	-0.326273	5.29591099	3.67159642	0.0698173	0.090469
Alkbh7	0.6847534	1.95290312	3.67157186	0.06981816	0.090469
Unc93a	2.26133597	-0.8055965	3.67095005	0.06983995	0.09048161
Tnfrsf14	-3.2429171	-1.8318768	3.67043202	0.06985811	0.09048951
Gm13238	-1.0375396	0.09036922	3.66842006	0.0699287	0.0905653
Klf6	0.2380149	7.42428216	3.66703574	0.06997732	0.09061262
Ddx17	-0.3349849	8.05832796	3.66647854	0.0699969	0.09062233
Mad2l2	0.56219882	3.11655822	3.66546908	0.07003238	0.09065263
Ccne2	-0.7148246	1.99831562	3.6650838	0.07004593	0.09065453
Ascl1	-0.497518	3.37908203	3.66466258	0.07006075	0.09065806
Nfe2l1	0.26113662	7.82225763	3.66221566	0.0701469	0.09075388
Nhsl1	0.33192655	7.15377938	3.66143508	0.0701744	0.09077381
Dennd5a	-0.199127	7.52315641	3.66085194	0.07019496	0.09078475
4632428N05	-0.7525396	2.15941038	3.65923887	0.07025186	0.09084268
Tgtp1	0.49103321	3.77009538	3.65842658	0.07028054	0.09085164
Plekhg3	-0.3786769	3.33748503	3.65835642	0.07028302	0.09085164
Zfp266	-0.2122385	6.88720034	3.65787211	0.07030012	0.09085809

Gtf2ird2	-0.6204849	2.65349216	3.65665258	0.07034321	0.09089207
Akap14	-1.7967884	-0.9875609	3.65644223	0.07035065	0.09089207
Fsd1	0.66508825	2.29276353	3.65542413	0.07038665	0.0909194
Hmgn2	0.40925952	6.50993329	3.65485963	0.07040662	0.0909194
Shfm1	0.34698707	4.75682789	3.65481605	0.07040816	0.0909194
D930048N14	1.10350048	0.71953324	3.6544165	0.0704223	0.090922
Vapb	0.21052683	6.66791428	3.65309364	0.07046913	0.09094207
1700026D08	-0.9986027	0.68948357	3.65283669	0.07047823	0.09094207
Slc2a8	-0.7041849	1.62197619	3.65273132	0.07048197	0.09094207
Dsel	-0.3301784	5.03122799	3.65260806	0.07048633	0.09094207
Gm13483	-1.5498346	-0.7401471	3.65170528	0.07051833	0.0909677
Lyar	0.29774034	4.22170673	3.6510432	0.0705418	0.09098233
Rgn	-3.182084	-1.5682967	3.65061346	0.07055704	0.09098635
Gpr35	-1.3548562	0.11747477	3.6499082	0.07058206	0.09100297
Arvcf	-0.3971344	4.0585454	3.64926771	0.07060479	0.09100675
Slc17a8	0.58188294	2.68306358	3.64914187	0.07060926	0.09100675
Amph	0.31252261	6.92174511	3.64652781	0.07070213	0.09108985
Ooep	1.64676572	-1.0041709	3.64639301	0.07070692	0.09108985
Mcl1	0.28745474	8.67175862	3.64603845	0.07071953	0.09108985
Dlgap2	-0.3988523	6.60868622	3.64589471	0.07072464	0.09108985
Zfp316	-0.4724006	3.73283924	3.64561908	0.07073445	0.09108985
Pop5	0.37572658	4.39829903	3.64331096	0.07081661	0.09117641
Svip	0.23670331	5.08626736	3.64304801	0.07082597	0.09117641
Polr2a	-0.2465248	7.30718596	3.6412107	0.07089146	0.09124506
Taok1	-0.2036691	9.64326833	3.64074387	0.07090811	0.09125083
Mecr	0.9077746	1.30272389	3.63995881	0.07093613	0.09126281
Tmem63a	-0.4246399	4.55997756	3.63980112	0.07094176	0.09126281
Chrna5	-1.3478891	-0.3679875	3.63693603	0.0710441	0.09137881
Hebp2	0.39128021	3.40226414	3.63527879	0.07110338	0.09143937
Mrpl28	0.56275708	3.36146762	3.63300805	0.0711847	0.09152826
Rc3h1	-0.2144911	7.36999408	3.63194204	0.07122291	0.0915597
Ano2	-1.4201047	0.14009161	3.63164499	0.07123356	0.0915597
St6galnac5	-0.3271855	4.74676532	3.63092772	0.07125929	0.09157504
Ap1g2	-1.899913	0.00113336	3.63063155	0.07126992	0.09157504
Zfp438	0.35045592	3.94995783	3.62993246	0.07129501	0.09159159
C2cd2l	0.35524656	5.27762797	3.62935535	0.07131573	0.09160252
Pcdhgb7	-0.5512695	2.21993231	3.62843895	0.07134865	0.09161806
St3gal6	-0.3873901	4.08416693	3.62833852	0.07135226	0.09161806
Rpl24	0.32641798	6.6964983	3.62730413	0.07138944	0.09165011
H2-Q5	-1.3010103	-0.6068189	3.62656361	0.07141607	0.09166861
Nkx1-2	4.35471562	-1.9523449	3.80798657	0.07144443	0.09168934
Cand2	0.42124	3.67553313	3.62406819	0.07150589	0.09175154
Mphosph9	-0.4061045	4.87189749	3.62374966	0.07151736	0.09175154
Atf6	-0.2704909	6.94906502	3.62298508	0.07154492	0.09177119
Usp1	0.29605014	5.3274602	3.62242955	0.07156494	0.09178118

Fam118a	0.34099668	4.55151073	3.62202389	0.07157957	0.09178425
Sos2	-0.2517973	7.66871831	3.62143581	0.07160078	0.09179576
Col5a1	-0.4181945	3.40369357	3.62044708	0.07163646	0.09182581
Prickle2	-0.3415869	7.94042736	3.61983571	0.07165854	0.09183613
1110012L19I	0.62512984	3.3695072	3.61954604	0.071669	0.09183613
Syndig1	0.41938055	3.49911957	3.61718201	0.07175444	0.09191459
Chrm3	-0.4440452	3.59287046	3.61717374	0.07175473	0.09191459
Specc1	-0.2613565	6.56145607	3.61549599	0.07181544	0.09197664
Hs1bp3	-0.3512293	4.68705527	3.61484118	0.07183915	0.0919913
Sdhd	0.40768816	6.88424588	3.61445513	0.07185313	0.09199351
Mchr1	0.62098933	2.58860554	3.61270948	0.0719164	0.09205879
1700007L15I	-1.1802838	-0.0113701	3.6109504	0.07198022	0.09211262
Orai1	0.88429467	1.85472513	3.6108734	0.07198301	0.09211262
Alox15	-3.4330104	-1.1973716	3.60854055	0.07206775	0.09220533
Ssh2	-0.2655972	6.53010038	3.60812556	0.07208284	0.0922089
Unc93b1	-0.8331312	1.36127615	3.60615259	0.07215461	0.09228498
Rinl	0.82559094	1.37320554	3.60573147	0.07216994	0.09228885
Trappc5	0.40475273	4.15310119	3.60520244	0.07218921	0.09229775
Ndufaf4	0.27926271	5.76558867	3.60443541	0.07221715	0.09231774
Rdx	-0.2317192	7.03495233	3.60314686	0.07226411	0.09236204
Ndst1	-0.2586474	5.73500255	3.60211829	0.07230163	0.09236956
Hook2	0.88436003	1.55443116	3.60197176	0.07230698	0.09236956
Flot1	0.27960024	5.46448358	3.60188601	0.0723101	0.09236956
Pkd2l2	-0.4095315	3.72470118	3.60163536	0.07231925	0.09236956
Lrrc59	0.22955295	6.71192555	3.60077482	0.07235067	0.09239395
Dffa	0.2742555	5.08935317	3.60029667	0.07236813	0.09240052
Crhbp	0.70819837	3.05964508	3.59982445	0.07238538	0.09240681
Cd14	1.36355449	-0.0168069	3.59943179	0.07239972	0.0924094
Sftpc	3.14742959	-1.19407	3.59854116	0.07243228	0.09243523
A630020A06	-1.5287246	0.01746508	3.59575289	0.0725343	0.09254968
Acn9	0.46033839	2.90800262	3.59422664	0.07259022	0.09260528
Xab2	-0.3605772	3.85964232	3.59375415	0.07260754	0.09261162
Rims2	-0.4063232	6.98291186	3.59328689	0.07262468	0.09261773
9130019O22	-0.597831	2.12545968	3.59271932	0.07264549	0.09262135
Fam110b	0.43160534	3.61497942	3.59253634	0.07265221	0.09262135
Rspo1	-0.486819	3.09497021	3.59173448	0.07268163	0.09264312
Bach1	0.30733083	5.05667399	3.59069272	0.07271989	0.0926652
Unc13b	-0.3305755	6.53909414	3.5905898	0.07272367	0.0926652
Dmrt2	2.07258351	-0.9402289	3.5875354	0.07283596	0.09279252
Pcbp2	0.23594118	6.942454	3.5861971	0.07288522	0.09283952
Trpa1	-1.8839057	-0.9427308	3.58573015	0.07290242	0.09284566
Tnrc6b	-0.2647828	8.72502466	3.58349928	0.07298465	0.09293461
N4bp2	-0.4516481	3.4697598	3.58091718	0.07307996	0.09304017
Hint2	0.52838335	3.50760824	3.57928742	0.07314019	0.09308888
C730002L08I	0.64704518	2.30631274	3.57921053	0.07314304	0.09308888

Foxg1	0.31203018	6.13935789	3.57829053	0.07317706	0.09311639
Ccdc86	0.54048228	2.91362023	3.57650284	0.07324324	0.09318478
Tac2	0.8437479	1.01105244	3.57602395	0.07326097	0.09319154
Lypd2	-1.2064555	1.82578607	3.57438048	0.07332189	0.09325321
Agtr2	1.93318066	-1.1446577	3.57283601	0.07337918	0.09331027
Dagla	-0.298336	5.43864015	3.57177496	0.07341858	0.09334453
Ccdc142	-1.3322775	-0.4755545	3.56914864	0.07351619	0.0934528
St3gal1	0.28918183	5.20677659	3.56748387	0.07357814	0.0935157
Camk2n1	0.21801536	10.5133201	3.56683121	0.07360244	0.09353074
Acan	-1.9516662	-0.8003149	3.56638917	0.07361891	0.09353582
Flt4	-1.3339547	1.00648881	3.56566408	0.07364593	0.0935543
Blvra	0.54387526	2.55710238	3.56525319	0.07366124	0.09355792
Mbd3	0.31426675	4.4107566	3.56478471	0.07367871	0.0935599
Slc39a12	-0.8045726	2.63789016	3.56454242	0.07368775	0.0935599
Atp2a2	-0.2745651	9.64115715	3.5630116	0.07374486	0.09361657
2610008E11I	-0.3311108	5.29799621	3.5622469	0.07377342	0.09363697
Nxn	0.56295508	5.05717024	3.56166159	0.07379528	0.09364557
Cd28	1.17482762	0.22626198	3.56139714	0.07380516	0.09364557
Syt2	-0.503222	3.39364271	3.56027396	0.07384714	0.09368299
Pgr15l	1.4450974	0.38897474	3.55985423	0.07386283	0.09368706
Slc19a3	-1.4209093	0.32608447	3.55866186	0.07390744	0.09372779
Gm19710	-1.4745169	0.31012239	3.55417479	0.07407559	0.09391835
Chadl	0.73976225	1.07999139	3.55398387	0.07408275	0.09391835
Nagk	0.46641858	3.23250208	3.55237798	0.07414304	0.09397891
Nmt2	0.22892075	6.98586461	3.55148693	0.07417652	0.09400546
Nmnat2	-0.2567624	7.15343766	3.55067216	0.07420715	0.09400816
Ntsr1	0.78327528	1.74796493	3.55053175	0.07421243	0.09400816
Mrrf	0.33654258	3.972877	3.55009416	0.07422889	0.09400816
Zfp579	0.57702329	1.47293401	3.54989811	0.07423626	0.09400816
Rgs7bp	0.28139589	8.51584196	3.5494524	0.07425303	0.09400816
Blm	-0.7629593	2.1997472	3.54943059	0.07425385	0.09400816
Rab3il1	0.45335433	4.12135723	3.54890538	0.07427361	0.09401732
Sp110	0.52510327	3.39387382	3.54724246	0.07433623	0.09408071
2810408A11I	-1.5574335	-0.898105	3.54632548	0.07437079	0.0940916
Uqcc2	0.49057654	3.50855036	3.54631301	0.07437126	0.0940916
Txnl4a	0.29108689	6.03320287	3.54601563	0.07438247	0.0940916
P4hb	0.44343892	5.58337131	3.54497334	0.07442178	0.0941185
Tm2d1	-0.4405375	2.9591863	3.54478645	0.07442883	0.0941185
Fam129a	0.47683958	3.7238136	3.54392943	0.07446118	0.09413383
Ebi3	2.2030594	-1.0286193	3.54380019	0.07446605	0.09413383
Llgl2	1.16282632	-0.2605733	3.54332319	0.07448406	0.09414074
Cenpn	1.07371578	1.17111086	3.54239063	0.07451929	0.09415723
Inpp5j	-0.4834719	3.41493072	3.54216049	0.07452799	0.09415723
9930104L06F	0.52072426	3.10313452	3.54198091	0.07453477	0.09415723
Nfkbib	0.39193402	3.71176623	3.54129097	0.07456085	0.09416341

Hist3h2a	1.05354968	0.54765609	3.54118744	0.07456477	0.09416341
Gpx3	-0.5448508	3.06524729	3.53950064	0.07462857	0.09422812
Tefm	0.45047755	3.32562014	3.53760214	0.07470047	0.09430302
Lcor	-0.4598254	3.98024282	3.53595503	0.07476291	0.09436596
LOC1005036	0.57603816	3.67730937	3.53458048	0.07481506	0.0944159
Nufip1	0.41845734	4.1197829	3.53236888	0.07489906	0.09450601
Pdia4	0.30751488	4.46957625	3.53164022	0.07492676	0.09452506
Adap1	0.35286752	4.20432379	3.52860647	0.07504221	0.09465479
Dlk2	-0.6615396	1.71967125	3.5273115	0.07509155	0.09470111
Zfp959	-0.5697019	1.73695519	3.52572775	0.07515195	0.09473509
Rbm8a	0.3793411	5.27386236	3.52534704	0.07516648	0.09473509
Ebpl	0.677458	2.36077055	3.52521386	0.07517156	0.09473509
Fam47e	2.29978113	-1.255421	3.52509313	0.07517617	0.09473509
Rpl29	0.36638488	6.10243951	3.52464751	0.07519318	0.09473509
Nol3	0.45082029	2.90621969	3.52461895	0.07519427	0.09473509
Camta1	-0.407032	9.37254189	3.52400559	0.07521769	0.09474868
Trim35	0.2315724	6.9522191	3.52324945	0.07524658	0.09476916
5430435G22	0.51182113	4.44542886	3.5223069	0.0752826	0.09479774
Zmiz1	0.24100582	8.34258388	3.52199445	0.07529455	0.09479774
Hs3st3a1	-0.5254465	4.59110995	3.52028946	0.07535978	0.09485302
BC033916	-0.976494	0.31825089	3.5201455	0.07536529	0.09485302
Chpf	0.45876156	3.44592117	3.51985551	0.07537639	0.09485302
Nhlh1	1.57326334	-0.2689233	3.51912096	0.07540452	0.09486458
Zfp128	0.5218052	2.6900895	3.51895515	0.07541087	0.09486458
Ccni	0.2464307	8.52286496	3.51638913	0.07550924	0.0949724
Cmklr1	0.72931854	1.08346113	3.5158838	0.07552863	0.09498086
B230209E15	0.33567782	6.31479577	3.51525343	0.07555283	0.09499537
Chmp6	0.50937013	2.23547176	3.51481358	0.07556972	0.09500068
AI661453	0.75137941	1.73037844	3.51410746	0.07559684	0.09500557
D430036J16	-0.5788478	2.35604874	3.51402039	0.07560018	0.09500557
Ccdc42	1.71341532	-1.1815401	3.51372309	0.0756116	0.09500557
Birc5	-1.026358	0.28308296	3.51281795	0.07564639	0.09501297
Snx5	0.21686474	7.0692407	3.51253158	0.0756574	0.09501297
Ibtk	-0.2199366	5.74726595	3.51247096	0.07565974	0.09501297
A830018L16	0.30116489	6.95865729	3.51225195	0.07566816	0.09501297
Nup43	-0.5944545	2.47967835	3.51153508	0.07569573	0.09503169
Kit	-0.2695727	4.87311702	3.50982006	0.07576175	0.09509865
Gm15417	0.81749023	1.17185303	3.50829152	0.07582065	0.09513913
Polr2d	0.45666507	3.84926725	3.5080919	0.07582834	0.09513913
Zfp51	-0.4803529	4.1864609	3.50799567	0.07583205	0.09513913
Nmt1	0.22609673	5.26669908	3.50704564	0.07586869	0.09516918
Pi4kb	-0.3555125	4.34799035	3.50491055	0.07595111	0.09525664
Twist1	0.57071055	4.73255054	3.50181814	0.07607066	0.09539063
Rccd1	-0.7043102	1.48306808	3.49933597	0.07616678	0.09548722
Mettl21c	-2.1184639	-0.5973106	3.49903969	0.07617826	0.09548722

Rapgef5	0.23752484	6.3808839	3.49865495	0.07619317	0.09548722
E530011L22F	-0.4355395	2.52860136	3.498437	0.07620162	0.09548722
Fabp4	-2.6307098	-0.6828978	3.49818649	0.07621134	0.09548722
Garem	-0.3273093	4.79906231	3.49733762	0.07624426	0.09551252
Papss1	0.27077002	4.96505417	3.49663862	0.07627139	0.09553055
Neb	-0.9069538	2.55395156	3.49538321	0.07632013	0.09557565
Slc14a2	-1.8803092	-0.4003729	3.49427997	0.076363	0.09559136
Hdac3	-0.3521175	4.7598794	3.49413336	0.0763687	0.09559136
Myl6b	0.5840871	3.78639404	3.49384599	0.07637987	0.09559136
C230035I16R	1.72160271	0.05992777	3.49327234	0.07640217	0.09559136
Gm8787	-2.0302268	-1.3720546	3.49309413	0.07640911	0.09559136
Kbtbd7	-0.2959073	5.2378738	3.49309326	0.07640914	0.09559136
Lyrm2	0.34879619	4.85568116	3.49247715	0.07643311	0.0956054
Dis3l	-0.3282384	3.87216742	3.48838345	0.07659258	0.09578676
Eif2ak3	-0.4636039	3.26880954	3.48803101	0.07660633	0.09578676
Stard3	0.55683144	2.37085646	3.4877726	0.07661641	0.09578676
Limk1	0.50894344	2.85508073	3.48744505	0.07662919	0.09578678
Zdhhc5	0.28677849	5.9280113	3.48433677	0.0767506	0.09592256
Ppan	0.72662314	1.68015532	3.48284503	0.07680895	0.09597949
Ptger4	-1.1324857	0.6562272	3.48234173	0.07682865	0.09598811
BC029214	0.40725487	3.49030025	3.48081998	0.07688824	0.09604657
Kctd7	0.60850618	2.79768907	3.4794057	0.07694367	0.09609981
Nipsnap3b	0.38726714	3.68325496	3.47752492	0.07701746	0.09617595
Rnf141	0.3634689	4.23117867	3.47653798	0.07705621	0.09620002
Scrt2	0.41032294	3.35000913	3.47638097	0.07706238	0.09620002
Copa	-0.2763001	7.7765974	3.47444004	0.07713866	0.09627538
Srgn	0.4186135	5.14252766	3.47419191	0.07714842	0.09627538
Mrps17	0.38410014	4.15799816	3.47320274	0.07718734	0.09629318
Cep78	-0.5228973	2.62711095	3.47317698	0.07718835	0.09629318
Psma2	0.29599555	6.68666278	3.47183998	0.07724099	0.09634282
F8a	0.35998154	3.41659898	3.47026897	0.0773029	0.096404
Cox17	0.30880947	5.38108613	3.46944222	0.0773355	0.09642863
Slc4a1ap	0.26183113	5.65214781	3.46808036	0.07738923	0.09647959
Metrnl	0.5816736	2.14349993	3.4666065	0.07744744	0.09652332
Serf2	0.44342565	6.41739383	3.46654063	0.07745004	0.09652332
Grm2	-0.3405302	3.64352703	3.4660316	0.07747015	0.09653235
C130026I21R	0.84332258	1.06014838	3.46432034	0.07753782	0.09659079
Crmp1	-0.3088047	6.06433348	3.46419419	0.07754281	0.09659079
Coro1c	0.32182364	5.89259875	3.46359677	0.07756645	0.0966042
Ubl5	0.40619149	5.95117824	3.46325444	0.07758	0.09660504
Axin2	-0.3743869	4.98891397	3.45845832	0.07777013	0.09682572
Gm16973	-0.4998252	2.88073708	3.45707729	0.07782498	0.09687793
Ntrk2	-0.3556488	8.36611345	3.45668731	0.07784048	0.09688114
Tmem254a	-0.8011629	1.45185985	3.45497095	0.07790872	0.09694999
Ccdc51	0.87982154	0.54766976	3.4523644	0.07801249	0.09706302

Fbxo46	-0.5448218	1.66258558	3.44981633	0.07811409	0.09717332
Fbln1	0.56085108	4.89785489	3.4494231	0.07812978	0.09717672
1700012D14I	-1.6439793	-0.1883919	3.44828311	0.0781753	0.09718693
Pycr2	-0.5466194	2.68835934	3.44796323	0.07818808	0.09718693
Eif2a	0.21611655	6.74759483	3.44793284	0.07818929	0.09718693
Dnah7a	-0.485512	2.08891377	3.44791966	0.07818982	0.09718693
Tbx18	0.38409731	6.20995825	3.44719368	0.07821882	0.09720688
Ngdn	0.41959793	3.72143171	3.44681015	0.07823415	0.09720982
Zfhx2os	-1.1780371	0.39938693	3.44575293	0.07827643	0.09724624
Cnpy2	0.44098031	5.15196504	3.44513033	0.07830134	0.09726108
Pex11a	0.86465574	1.16454539	3.44423722	0.07833708	0.09728815
Lat	-2.0674619	-1.0664694	3.44388058	0.07835136	0.09728815
Rab43	0.35244918	5.4568672	3.44361399	0.07836204	0.09728815
Trim67	-0.7801877	1.22140215	3.44211063	0.07842227	0.09734682
Paf1	-0.2830906	5.95108344	3.44016595	0.07850027	0.09741955
Scai	-0.3062375	7.0689649	3.44000248	0.07850683	0.09741955
Mrpl3	0.25042137	5.42029151	3.43768837	0.07859978	0.09751875
Riiad1	1.05135107	0.2172966	3.43723579	0.07861797	0.09751996
Faf2	-0.3173759	4.40507578	3.43701725	0.07862675	0.09751996
Cpsf2	-0.2352992	6.35806903	3.43558805	0.07868424	0.09757513
2810474O19	-0.2534772	6.6693074	3.43460771	0.0787237	0.09760793
Fer1l5	-0.6806748	1.38580258	3.43345367	0.07877018	0.09763895
Zcchc9	0.25793925	5.82683185	3.43334009	0.07877476	0.09763895
Prmt5	0.2802786	4.74281688	3.43272165	0.07879969	0.09764917
Mapk10	0.25220239	9.60904329	3.43248975	0.07880903	0.09764917
Zfp346	0.28947139	4.78813054	3.43192768	0.0788317	0.09766111
Mbd2	0.29742434	7.38678796	3.43150565	0.07884872	0.09766607
2810004N23	0.53133418	4.55543889	3.4307597	0.07887882	0.09768722
Zfp444	0.42164996	3.28469872	3.43019728	0.07890152	0.09769921
Ppp2r4	0.27149858	5.70209698	3.42679023	0.0790392	0.09785354
Nxt2	0.32554492	6.76471218	3.42551118	0.07909096	0.0978889
Ell	-0.3333769	3.49996243	3.42543937	0.07909387	0.0978889
Oxr1	0.21969083	8.62710565	3.42409879	0.07914816	0.09793994
Lphn3	-0.3494199	6.76500608	3.4223547	0.07921886	0.09801126
Tln2	-0.2629009	6.80998264	3.42109171	0.0792701	0.09804428
Nt5c3b	0.38083304	3.23958664	3.42105261	0.07927169	0.09804428
Mcm10	-2.6474888	-1.3005088	3.42048907	0.07929457	0.09805641
Sncg	1.66110646	-0.3725225	3.41931786	0.07934214	0.09809906
Tnfaip8	0.45502071	5.03713846	3.41886006	0.07936074	0.09810589
Sestd1	-0.3475449	7.06403076	3.41795395	0.07939758	0.0981253
Myrip	0.34268011	6.38887838	3.41758971	0.07941239	0.0981253
Maf	0.33254488	7.20796103	3.41750883	0.07941568	0.0981253
Lmo3	0.28081584	7.13282891	3.41659441	0.07945289	0.0981551
Ubash3b	-0.2823546	5.78563294	3.41579513	0.07948542	0.09817913
Gm10474	-2.5988869	-1.5017368	3.41467966	0.07953086	0.09821126

Zfp345	-1.6257752	-0.7265107	3.41447945	0.07953902	0.09821126
Ddx28	0.4680161	2.5404257	3.4141923	0.07955072	0.09821126
Hnrnpul1	0.25147704	6.59340048	3.41348138	0.0795797	0.09822508
Spryd7	0.24513823	5.47150062	3.41327529	0.0795881	0.09822508
Fam135a	-0.3002397	5.76038231	3.4127295	0.07961036	0.09823639
5033404E19l	-3.1280006	-1.5114324	3.41180971	0.07964789	0.09826654
Zscan29	-0.3186653	5.40459204	3.41085177	0.079687	0.09829862
Cdkl2	0.25475956	6.06379876	3.40879159	0.07977118	0.09838628
Parp11	-0.4693525	3.76435497	3.40821723	0.07979467	0.09838669
Synj1	-0.4413478	9.53680777	3.40775764	0.07981347	0.09838669
Zfp27	-0.3916171	4.00866786	3.40767485	0.07981686	0.09838669
Tsr3	0.32770717	4.06406222	3.4073408	0.07983052	0.09838669
Wif1	1.66093577	-1.1356309	3.40718036	0.07983709	0.09838669
Ddx3y	-0.2277837	6.3508727	3.40485098	0.07993249	0.09846628
Csmd2os	-1.5381794	-0.3659152	3.40480914	0.0799342	0.09846628
Spin1	0.18238228	8.82687423	3.40445891	0.07994856	0.09846628
Gal3st3	0.31644748	6.01131671	3.40401457	0.07996678	0.09846628
Ccdc32	0.37603345	4.90145932	3.40349869	0.07998793	0.09846628
Ephb3	-0.680021	1.63705225	3.40345996	0.07998952	0.09846628
Ppfia3	-0.3807608	4.67632354	3.40323902	0.07999858	0.09846628
Mt2	0.43780561	5.17961798	3.40304143	0.08000669	0.09846628
Mnt	-0.2820286	5.01566522	3.40191664	0.08005285	0.09850693
F2rl2	0.55755991	1.92100183	3.40123295	0.08008092	0.09852531
Syngap1	-0.3096228	7.35111782	3.40018683	0.0801239	0.09855554
Med18	0.87159581	1.08521293	3.39999536	0.08013177	0.09855554
Ar	-0.3929719	4.30558559	3.39779481	0.08022228	0.09864777
Ptx3	2.01322521	-1.326802	3.39746545	0.08023583	0.09864777
Pi4ka	-0.355374	8.10487444	3.39721346	0.08024621	0.09864777
Commd9	0.64232695	2.15963691	3.39433541	0.0803648	0.09877736
Mark3	0.21001079	6.40504925	3.3873783	0.08065231	0.09911355
Calcoco1	0.21515431	7.47821036	3.38707798	0.08066474	0.09911355
Tmem208	-0.5717781	2.06204217	3.3861467	0.08070333	0.09914472
Slc39a8	-0.728541	2.78541867	3.38380517	0.08080043	0.09924776
Srrm2	-0.3298511	10.1848417	3.3833659	0.08081866	0.0992539
Tusc3	0.28934066	4.91653902	3.38114187	0.08091105	0.09935109
Foxr1	-1.4056248	-1.0863952	3.38024326	0.08094841	0.0993807
Ccr2	0.65922246	2.65618563	3.3797884	0.08096733	0.09938766
Dip2b	-0.3129459	7.80946321	3.37815061	0.08103549	0.09944027
Hist1h4k	0.64141518	1.94575363	3.37812172	0.0810367	0.09944027
Frmpd1os	-2.5253884	-1.7054424	3.37621336	0.08111621	0.09952155
Lrpap1	0.36428044	5.78732057	3.37474667	0.08117738	0.09958032
Ptcra	-1.3593449	-0.9409011	3.37330764	0.08123745	0.09963771
Alkbh1	0.42329533	4.29498988	3.37240182	0.08127529	0.09966782
Adh5	0.26989076	6.35142183	3.37052537	0.08135373	0.09974772
Tmem50a	0.33486554	5.6736444	3.36916302	0.08141074	0.09979899

Lin7a	0.25337351	7.6292927	3.36889035	0.08142216	0.09979899
Cpne8	-0.2577972	6.41143721	3.36824941	0.081449	0.0998074
Aars2	-0.7115812	1.65531529	3.36809117	0.08145563	0.0998074
Rhbdd1	-0.5466118	3.65204601	3.36737882	0.08148548	0.09982689
Gfm2	-0.5830804	3.58539059	3.36697908	0.08150223	0.09982689
Cdc6	1.06490107	0.74257693	3.36675888	0.08151146	0.09982689
Hnrnpd	0.19945316	8.29361653	3.36614763	0.0815371	0.09984198
Coro6	-1.1373693	1.88178598	3.36563173	0.08155874	0.09984761
Hspg2	0.34943798	3.48405132	3.36540348	0.08156831	0.09984761
Adora2b	-1.0014981	0.44978686	3.36507509	0.08158209	0.09984818
Urb2	-0.3867539	4.20458844	3.36200413	0.08171109	0.09998344
9530068E07I	0.42164137	6.71850553	3.36180979	0.08171926	0.09998344
Fuca2	0.28195625	4.54226025	3.36116679	0.0817463	0.099999
Gm16796	3.7445632	-2.0218992	3.36087353	0.08175864	0.099999
Mrps26	0.33436392	3.85237009	3.35982605	0.08180272	0.1000216
Mafg	-0.3545092	4.34596512	3.3595366	0.08181491	0.1000216
Chtf18	-2.0666585	-1.2665603	3.35948401	0.08181712	0.1000216
Phkb	-0.3014498	6.1319598	3.358007	0.08187935	0.10007648
Casp12	0.5864645	4.19056482	3.35760265	0.08189639	0.10007648
Thyn1	0.46159743	3.4539141	3.35746866	0.08190204	0.10007648
Ttc38	-0.3618279	3.77982838	3.35427723	0.08203671	0.10022471
Heatr6	-0.3151938	4.8189271	3.35344534	0.08207186	0.10023711
2310045N01	0.58165521	4.79237446	3.35340437	0.08207359	0.10023711
Jkamp	0.34324243	4.43764506	3.35229988	0.08212028	0.10027781
Slc9a9	-0.5610099	3.46094576	3.35166127	0.08214729	0.10029172
Mrps28	0.44499103	2.96171414	3.35084092	0.082182	0.10029172
Nploc4	0.26115811	5.5117451	3.35071314	0.08218741	0.10029172
Naa40	-0.4466866	2.94332388	3.35066221	0.08218957	0.10029172
Osbpl3	-0.4326646	5.09364473	3.35045075	0.08219852	0.10029172
Ascl2	1.83621442	-0.7872353	3.35005559	0.08221525	0.10029582
Cct4	0.24725706	5.9909585	3.34926067	0.08224892	0.10032058
Ascc2	-0.3936501	3.30241568	3.34884999	0.08226632	0.10032549
Best1	0.7084223	1.46628805	3.34798114	0.08230315	0.10034543
Bcl2l11	0.45038106	5.05409116	3.34783315	0.08230943	0.10034543
H2-Ke2	0.48562056	3.77446252	3.34680826	0.0823529	0.10036784
Nsun6	-0.4697492	2.75050169	3.34676896	0.08235456	0.10036784
Sgce	0.32860176	4.38630861	3.34435398	0.0824571	0.10047648
Mrpl2	0.51452538	3.1418661	3.34358158	0.08248993	0.10050015
Spint2	0.55381408	2.87373047	3.34238233	0.08254093	0.10054596
Tollip	0.23926417	6.59682381	3.34116109	0.0825929	0.10059293
Trpc3	-0.4057668	3.63334456	3.34035884	0.08262706	0.1006182
Fam171b	-0.2832918	7.11323488	3.33969335	0.08265541	0.10062519
Nol8	0.23455973	6.46452917	3.33959438	0.08265963	0.10062519
Hnrnpa0	0.20631844	7.6352573	3.33806168	0.08272497	0.10067304
Nme7	-0.4685813	3.52830339	3.33804263	0.08272578	0.10067304

5330434G04	-0.4411867	5.00213605	3.33765702	0.08274223	0.10067673
Gemin4	-0.4941001	2.31786914	3.33643486	0.08279439	0.10071471
Gemin8	0.41195906	3.45202186	3.33629639	0.0828003	0.10071471
Psmb6	0.3652105	4.90056129	3.33578244	0.08282225	0.10072507
Alox5ap	-0.7630339	1.4941033	3.33442345	0.08288031	0.10077935
Rnaseh1	0.58615834	2.79486791	3.33391197	0.08290218	0.1007896
Bet1	0.40645795	4.8666255	3.33209497	0.08297991	0.10086776
Sap130	0.23824061	6.64404836	3.33145182	0.08300745	0.10088488
Tbc1d23	-0.3409128	4.34883226	3.33014106	0.0830636	0.1009219
2310015A10	-0.5976037	2.92595868	3.33011259	0.08306482	0.1009219
Ctbp1	0.35126471	5.30065741	3.32813248	0.08314972	0.1010087
Gramd4	-0.3436208	4.03964658	3.32754543	0.08317492	0.10102295
Wdr18	-0.2991242	3.64050136	3.32638391	0.08322479	0.10105533
Wdr45b	0.31787527	4.47916037	3.32629683	0.08322853	0.10105533
Chd4	-0.2391637	7.44856066	3.32510719	0.08327964	0.10110104
Skp2	-0.5367713	2.8285553	3.32450729	0.08330543	0.10110296
Ccbl2	-0.5936474	2.49897631	3.32444338	0.08330818	0.10110296
Agap3	0.24036751	5.85847907	3.32338012	0.08335392	0.10113408
9330179D12	0.96245606	1.07492761	3.3232204	0.08336079	0.10113408
Edc3	0.47909547	3.0896471	3.32290445	0.08337439	0.10113422
Pls3	0.18923374	7.5080234	3.32231688	0.08339968	0.10114854
Pgm2	-0.3256474	4.06380993	3.32109	0.08345253	0.10119627
Cacng5	-0.5668509	2.68079669	3.31822878	0.08357591	0.10132952
Sbf1	-0.4278051	4.72245381	3.31636699	0.08365632	0.10139528
Taf1b	-0.2789595	4.16744644	3.31634684	0.08365719	0.10139528
Trh	-2.7892842	-1.6101563	3.31468375	0.08372909	0.10146603
4933427D14	-0.5697448	4.10754685	3.31228081	0.0838331	0.10157566
Cenpp	-0.5623965	2.28834247	3.31047438	0.08391139	0.10164533
Dbf4	0.63863505	1.82206078	3.31032883	0.0839177	0.10164533
Matn2	0.50987297	3.70694898	3.30893305	0.08397826	0.10170226
Gm14420	-0.2979698	5.40322491	3.3081347	0.08401292	0.10171518
Zfp207	0.19900917	7.90237065	3.3078345	0.08402595	0.10171518
Fbxo17	0.67522993	1.79926331	3.30775048	0.0840296	0.10171518
Tbc1d22a	0.35865444	3.48816352	3.3071088	0.08405748	0.1017325
Get4	0.36582292	3.89752722	3.30332125	0.08422224	0.10191547
Grhl2	-1.1585471	0.49224649	3.30258584	0.08425427	0.10193779
Rusc1	-0.3569819	5.8504456	3.3009112	0.08432727	0.10200372
Ptpn23	-0.2934148	4.86843946	3.30042469	0.08434849	0.10200372
Cst3	0.45546744	7.90737531	3.30040017	0.08434956	0.10200372
Pdxp	0.42202669	4.93016259	3.29950744	0.08438852	0.10203438
Nom1	-0.2941191	4.52773846	3.2957352	0.08455337	0.10220234
Entpd5	-0.3173927	4.33180174	3.29527945	0.08457331	0.10220234
Cdh1	0.59436935	6.50236452	3.29518693	0.08457736	0.10220234
Nexn	-0.3894249	5.68358116	3.29508243	0.08458193	0.10220234
Polr2l	0.36028828	3.98022412	3.29356009	0.08464859	0.10226641

Epha4	-0.2572009	7.2171443	3.29264136	0.08468885	0.10229857
Alg3	0.42461219	3.44367456	3.29176818	0.08472713	0.1023119
Apol7d	2.77264149	-1.7446552	3.29176707	0.08472718	0.1023119
Ogdhl	-0.689845	1.86515548	3.29145653	0.0847408	0.1023119
Gsx1	-2.51264	-1.7004673	3.29096862	0.0847622	0.10232127
Rnf24	-0.2674499	5.58235448	3.29036156	0.08478884	0.10233696
Abcg4	-0.4168894	4.08949237	3.28926393	0.08483704	0.10237866
Slc22a14	-3.8799004	-1.9966081	3.2880248	0.08489148	0.10242569
Wnk1	-0.195394	10.008663	3.28775546	0.08490332	0.10242569
Anxa7	0.23553748	6.45229665	3.28728641	0.08492394	0.10243009
Gtf2e1	0.39953236	3.80428153	3.28682005	0.08494445	0.10243009
Ptn	0.54112573	10.3823205	3.28653435	0.08495702	0.10243009
Tspan5	0.22325736	7.34070433	3.28643048	0.08496159	0.10243009
Krt7	2.89004741	-1.709268	3.28569843	0.0849938	0.1024416
Fkbpl	0.95241435	0.62039627	3.28559301	0.08499844	0.1024416
Uckl1	-0.3979061	3.45904809	3.28439987	0.08505098	0.10248845
Agpat2	0.87655389	1.41287467	3.28404662	0.08506654	0.10249074
Rad54b	-1.5512191	-0.1526747	3.28142027	0.08518235	0.10259891
Dcp2	-0.2282132	7.18498446	3.2813489	0.0851855	0.10259891
Gspt2	0.34452481	4.58635054	3.28108013	0.08519736	0.10259891
Fam109b	1.07865105	1.55754912	3.2806549	0.08521613	0.10260187
Snx25	-0.2594406	5.1798809	3.28040478	0.08522718	0.10260187
Dnal1	0.23543321	7.03713148	3.27911482	0.08528416	0.102654
Zmym1	-0.4545379	3.92520281	3.27809049	0.08532944	0.10269202
Slc29a3	-0.3338817	5.26561223	3.27620983	0.08541264	0.10277567
Ctsd	0.47004617	7.20349076	3.27395989	0.08551231	0.10287909
Slc19a1	-0.4920946	3.08317596	3.27361661	0.08552753	0.1028809
Srek1	-0.2765302	6.65390786	3.27277686	0.08556477	0.1029092
Hdx	-0.6378879	2.92416688	3.27149348	0.08562172	0.10293477
Plaur	1.95052818	-1.2438699	3.27148792	0.08562197	0.10293477
Dbndd2	-0.2288567	5.98223656	3.27137008	0.0856272	0.10293477
Tuba8	0.4531771	2.26077848	3.27040102	0.08567023	0.10297
Gyk	-0.5123845	4.36426834	3.26862553	0.08574915	0.10303494
Acot8	0.74973109	1.57010583	3.26856744	0.08575173	0.10303494
Ccnf	0.99999127	0.81005706	3.26787838	0.08578238	0.10303925
Jmjd6	0.37748962	2.98096008	3.26786921	0.08578279	0.10303925
Pdf	0.30725286	5.08672564	3.26752513	0.0857981	0.10304114
Ptgs1	0.42369665	3.44608602	3.26659646	0.08583944	0.10307428
Hsd3b3	-0.9837636	0.3184857	3.26603494	0.08586445	0.10308216
Rps25	0.43739533	7.66263212	3.265832	0.08587349	0.10308216
Apba1	-0.3041672	6.80136776	3.26473535	0.08592235	0.10312432
Nek9	-0.2134267	6.73170943	3.26375311	0.08596615	0.10316038
Chmp3	0.27677304	6.51614272	3.26245397	0.08602412	0.10319885
Palm3	-0.4914782	2.25433235	3.26241788	0.08602573	0.10319885
Ccdc167	-0.2405461	5.38308184	3.26145093	0.0860689	0.10323414

9430091E24I	0.83343689	1.9031491	3.26076356	0.08609961	0.10325446
Tmem42	-0.5169688	2.41919743	3.25964866	0.08614944	0.10326451
L3mbtl1	-0.9058866	1.58648883	3.25961434	0.08615098	0.10326451
Dnal4	0.44403499	2.43868295	3.25943436	0.08615903	0.10326451
Fezf1	-2.2616663	-0.6692118	3.25934424	0.08616306	0.10326451
Sult1a1	0.47335898	5.7934373	3.25848872	0.08620133	0.10329387
Setd5	-0.2326576	8.40831928	3.25777795	0.08623314	0.10331548
Zbtb7c	0.35841114	4.09881819	3.25746023	0.08624736	0.10331602
Skiv2l	-0.3473231	5.25710978	3.25678407	0.08627764	0.10333579
Fis1	0.35432661	3.99027107	3.25449311	0.08638032	0.10344226
Ankrd10	-0.3641269	3.86599986	3.25196129	0.08649397	0.10356182
Marveld2	1.42157248	0.62572504	3.24930529	0.08661337	0.10368824
Arhgef37	1.02020961	0.28806604	3.24861252	0.08664455	0.10370901
Dcdc2b	0.41463556	3.77536492	3.24807216	0.08666888	0.10371341
Fhl4	-1.068284	0.87634716	3.24791666	0.08667588	0.10371341
Pck2	0.45401029	3.2418545	3.24700631	0.08671688	0.10373021
Zeb1	-0.1755783	7.53527288	3.24699108	0.08671757	0.10373021
Aak1	-0.3087266	9.72626354	3.24583945	0.08676948	0.10377575
Tctn1	-0.9407593	2.90632425	3.24466499	0.08682245	0.10381342
Ube4b	-0.2162386	7.77348714	3.24427731	0.08683994	0.10381342
4930523C07I	-0.4557568	3.74449244	3.24422089	0.08684249	0.10381342
Gimap6	-0.8656617	1.78011228	3.24121054	0.08697848	0.10394269
Aars	0.29396815	5.61114746	3.24097541	0.08698911	0.10394269
Mtf2	-0.305338	5.55865667	3.24090707	0.0869922	0.10394269
Scg2	0.30848642	6.44632555	3.23919273	0.08706977	0.1040188
Efcab10	1.39605007	-0.4047209	3.23795115	0.08712599	0.1040694
Upf3a	0.37018648	5.8631165	3.23737728	0.087152	0.10408389
Igsf9	1.7067903	-0.311805	3.23527347	0.0872474	0.10418124
Zer1	-0.3161166	4.95266359	3.23483468	0.08726732	0.10418176
Prss23	0.40739932	3.46917784	3.23465193	0.08727561	0.10418176
Zfp385b	0.28057353	6.65665764	3.23428221	0.0872924	0.10418521
Sh3bp5	0.25656086	6.78191508	3.23279635	0.08735989	0.10421427
Ier5	0.28516621	6.19845767	3.23257172	0.0873701	0.10421427
Srsf7	-0.2138488	5.89883115	3.2325563	0.0873708	0.10421427
Tnfsf9	-3.1809798	-2.0132422	3.23211785	0.08739073	0.10421427
Atad1	0.21849367	7.21366824	3.23211715	0.08739077	0.10421427
4930500J02F	-1.9912799	-0.6155177	3.23191181	0.0874001	0.10421427
Prss54	2.32932633	-1.2922153	3.23141603	0.08742265	0.10422459
Dscaml1	-0.5962578	3.82701036	3.2309492	0.08744388	0.10423334
Armc2	-0.6287535	1.77471484	3.22964451	0.08750327	0.10428755
Plk1	-1.8121245	-0.9043404	3.22916048	0.08752531	0.10428881
Lrrc32	0.44331742	4.80677595	3.22901078	0.08753213	0.10428881
Gm15217	1.75704805	-1.6360095	3.22845377	0.0875575	0.10430248
Kctd2	0.27248049	5.52235383	3.22785681	0.08758471	0.10431832
Ppwd1	-0.3398982	3.72394038	3.22735859	0.08760742	0.10432881

Camkk1	0.36961203	4.88035327	3.22555737	0.0876896	0.1043922
Prmt1	0.57149302	2.8682413	3.22514561	0.08770839	0.1043922
Mtap	0.4295562	4.5968512	3.22497641	0.08771612	0.1043922
S100b	0.37943486	6.07697975	3.22497197	0.08771632	0.1043922
Mthfd1	-0.4222797	3.54331546	3.22170645	0.08786558	0.10455325
Pank4	-0.4589725	2.77003552	3.22003434	0.08794212	0.10462773
Gm20125	-2.8114937	-1.3196063	3.21885984	0.08799593	0.10467515
Hnrnpll	0.22440281	6.34844981	3.21812664	0.08802954	0.10469853
Tbc1d10a	0.75781699	1.66524401	3.21767777	0.08805013	0.10470043
Isg20l2	0.41288285	4.04315154	3.21731163	0.08806692	0.10470043
Scamp1	0.20789126	7.87224213	3.21717874	0.08807302	0.10470043
Nol4	0.28884884	6.37124719	3.21658525	0.08810025	0.10471621
4632415L05F	0.2626781	5.48944619	3.21490936	0.08817721	0.10475625
Cyp4a12a	-1.1883446	0.33010548	3.21483768	0.0881805	0.10475625
Tmem126b	0.37832804	4.18323739	3.21453451	0.08819443	0.10475625
Deb1	0.28503183	4.91334652	3.21417832	0.0882108	0.10475625
Vps18	-0.4066266	3.64210523	3.21405951	0.08821626	0.10475625
Aarsd1	0.41926801	3.70563603	3.2137856	0.08822885	0.10475625
Sv2b	0.31737647	9.37558707	3.21372369	0.0882317	0.10475625
Ndfip2	-0.2602022	6.27191141	3.21312221	0.08825936	0.10476265
Slc24a5	-0.4775313	2.28941633	3.21298516	0.08826566	0.10476265
Bivm	0.2472104	4.9555411	3.2126955	0.08827898	0.10476265
Nnt	-0.2522714	5.08269873	3.21196528	0.08831258	0.10476904
Slc7a10	-1.0509237	0.19958365	3.21176123	0.08832198	0.10476904
Ckap4	0.36390872	5.1576854	3.21166795	0.08832627	0.10476904
Ints10	-0.3736254	4.16093091	3.21033224	0.08838778	0.10482543
Foxp3	-1.5955504	-0.7855002	3.20890234	0.08845368	0.10488701
Arhgef33	1.31424368	-0.5838041	3.20828594	0.08848211	0.10490413
Sh3d19	0.28779387	6.73968738	3.20776993	0.08850592	0.10491577
Rae1	0.32914215	3.83274446	3.2064567	0.08856654	0.10497104
Rgs5	-0.444432	4.11792046	3.20504483	0.08863176	0.10502249
Slc38a10	0.3792952	3.43456733	3.20491115	0.08863794	0.10502249
Prr14	0.32590626	5.45232595	3.20441259	0.08866099	0.1050332
Abhd17b	0.29949463	5.76779969	3.20086157	0.08882535	0.10521013
Nfatc4	0.72723163	3.20919778	3.20058029	0.08883839	0.10521013
Tomm6os	-1.4915196	-0.1003295	3.19917722	0.08890344	0.10524763
Gm16386	-0.6220129	2.00126101	3.19916903	0.08890382	0.10524763
Sema5a	-0.2904618	6.42910481	3.19898959	0.08891214	0.10524763
Psg16	0.88727145	1.38269931	3.19824977	0.08894647	0.10527165
Scarb1	-0.7109124	2.48768877	3.19653532	0.08902608	0.10531434
Mrps7	0.30539698	5.25942909	3.19652519	0.08902655	0.10531434
Kif17	0.48084416	3.1648129	3.19640461	0.08903215	0.10531434
Taf13	0.2555037	6.57924145	3.19626367	0.0890387	0.10531434
Lrrc16a	-0.3420317	4.29144179	3.19527026	0.08908487	0.10535234
Plekha8	-0.3606337	4.32360688	3.19407177	0.08914061	0.10539477

Mtus2	-0.2189257	6.24056567	3.19389484	0.08914885	0.10539477
Elmo2	0.23365834	5.92396524	3.19180042	0.08924637	0.10549343
Pkig	-0.2602855	4.79792239	3.1890088	0.08937655	0.10561545
Olfr920	-2.21508	-0.1309598	3.18898297	0.08937775	0.10561545
Gabra5	-0.2578519	5.66006483	3.18859947	0.08939565	0.10561997
Asxl2	-0.2554192	6.49427482	3.1856236	0.0895347	0.10576759
Hectd2	-0.3636151	4.47598569	3.1845767	0.08958368	0.10580879
Gabrg2	-0.2736011	6.44501646	3.18380047	0.08962001	0.10583504
Rnf220	0.24540436	6.94879055	3.18304955	0.08965518	0.1058599
Rnf146	0.25712696	5.45688731	3.18236406	0.0896873	0.10588116
Gramd1a	-0.5089871	3.06794535	3.18202492	0.08970319	0.10588326
Gas2l2	-2.0465929	-0.998051	3.18014635	0.08979129	0.10597057
Coq10a	0.36424549	4.11376662	3.17701458	0.08993839	0.10612748
Foxj3	0.27296797	7.73374767	3.17563382	0.09000333	0.10618741
Top3b	-0.461042	3.52638291	3.17427779	0.09006716	0.10624601
Pitrm1	-0.269846	5.2643786	3.17350309	0.09010366	0.10627234
Med14	-0.3041859	7.67537787	3.1705536	0.09024275	0.10640373
Tamm41	-0.5833848	2.28639379	3.1705392	0.09024343	0.10640373
Dgcr14	0.36359485	3.75036642	3.16972656	0.09028179	0.10642732
Snord71	-2.9335247	-1.3463177	3.16946942	0.09029394	0.10642732
Etnppl	-0.5810618	2.95248482	3.16921411	0.090306	0.10642732
Asf1b	1.81548596	-1.5884799	3.16665782	0.09042685	0.10654063
Stoml1	0.4571972	3.03885234	3.1665795	0.09043055	0.10654063
Gga2	0.35775153	4.47761348	3.16446076	0.09053087	0.10663752
Tpp1	0.31890856	6.58033688	3.16424222	0.09054122	0.10663752
Hmgcl	0.45644305	2.57252918	3.16354568	0.09057423	0.10665155
Pi16	1.49289519	-0.6008267	3.16339083	0.09058157	0.10665155
Ppp1ca	0.40157823	5.51876175	3.16108057	0.09069117	0.10676384
Tank	0.3422701	6.08627914	3.1601638	0.09073471	0.10679327
Naip1	-2.5669179	-1.843122	3.15942814	0.09076966	0.10679327
Ficd	0.45997648	2.89199918	3.15919328	0.09078082	0.10679327
Zfp143	-0.4517878	3.39032296	3.15905355	0.09078746	0.10679327
Adipor1	0.30509276	6.28407623	3.15896249	0.09079179	0.10679327
Lrrc8e	-1.0328529	-0.1319681	3.15842353	0.09081741	0.10679327
Gm1987	-2.1554817	-1.4241787	3.15800622	0.09083726	0.10679327
Tph2	2.2472871	-1.2085196	3.15763871	0.09085474	0.10679327
Zadh2	-0.3082707	4.80412858	3.15743144	0.0908646	0.10679327
Fam72a	1.58823532	0.51817619	3.15729557	0.09087107	0.10679327
Itm2b	0.38047085	8.98079647	3.15629835	0.09091853	0.10679327
4933432I03R	1.60626025	0.10659377	3.15622604	0.09092198	0.10679327
Tmem14c	0.48707396	3.11534299	3.15597052	0.09093414	0.10679327
Arih1	-0.1928453	7.93081181	3.15582674	0.09094099	0.10679327
Ybx1	0.28244195	7.17213892	3.15565304	0.09094927	0.10679327
Plin3	0.39568768	3.68225391	3.15551058	0.09095605	0.10679327
Acadvl	0.35306562	4.90350757	3.15546563	0.09095819	0.10679327

3632454L22F	-0.8947263	0.7864583	3.15484287	0.09098786	0.10679392
Klhl22	-0.2870741	5.05935465	3.15443515	0.0910073	0.10679392
Scn10a	-2.4097493	-1.6089936	3.21164222	0.09102671	0.10679392
Dll1	-1.1816604	0.10744602	3.15401538	0.09102731	0.10679392
04-Sep	-0.2501589	5.93010736	3.15396035	0.09102993	0.10679392
Hdgfrp3	0.21646606	8.00669921	3.15337066	0.09105806	0.10681021
Enah	-0.2890001	8.35869406	3.15285816	0.0910825	0.10682218
Gak	-0.2360863	6.70953113	3.15101569	0.09117046	0.10686817
Gpx7	0.73644403	2.44494039	3.15079237	0.09118113	0.10686817
Map3k7cl	-1.1753404	0.50452359	3.15072227	0.09118448	0.10686817
Wdr83os	0.36378205	5.70252191	3.15061152	0.09118977	0.10686817
Gabrr2	-2.1663483	-0.870399	3.15034421	0.09120255	0.10686817
Tecr	0.35403839	7.85813035	3.15024697	0.09120719	0.10686817
Shisa5	0.43595724	5.06994187	3.14273044	0.09156723	0.10727327
Nwd2	-0.6289263	5.78589024	3.14206922	0.09159899	0.10729372
Poc1a	0.57672911	2.80503987	3.14101065	0.09164984	0.1073296
Gt(ROSA)26S	-0.4986485	2.76247461	3.14083598	0.09165824	0.1073296
Prr5l	-0.4875993	2.40754669	3.14014498	0.09169146	0.10734215
Ehd1	0.45863948	3.5760169	3.14001779	0.09169758	0.10734215
4930599N23	2.37100632	-2.0599011	3.13934395	0.09172999	0.10736334
Phb	0.36081094	3.81060631	3.13839983	0.09177542	0.10739976
Hmgb1	0.27238298	8.09298652	3.13649564	0.09186714	0.10749032
Fam167a	0.59583976	2.37068262	3.13610967	0.09188574	0.10749532
Usp50	-2.210228	-1.5371428	3.13580275	0.09190054	0.10749587
Prkra	0.35368561	3.57028419	3.13404803	0.09198519	0.10756914
Rras2	0.33792389	4.65178085	3.13390969	0.09199187	0.10756914
Fgfr4	-1.3658035	-0.6665554	3.13326484	0.092023	0.10758878
Ceacam2	-0.6140775	1.35594275	3.13202521	0.09208288	0.10762706
Cdk8	0.21896873	5.70955149	3.13199304	0.09208444	0.10762706
Atp2b3	-0.3571157	6.97569938	3.13072129	0.09214592	0.10766845
Clcn3	-0.237642	6.71782553	3.13066674	0.09214856	0.10766845
Fxyd4	-1.7034409	-0.3788599	3.12979857	0.09219056	0.10770075
Cdkn2aipnl	0.30445491	4.48162468	3.12948406	0.09220578	0.10770176
Rhof	0.4437859	3.497237	3.12771565	0.09229142	0.10778501
Fbxo31	0.47798605	3.94589921	3.12630988	0.09235957	0.1078478
Zfp28	-0.5066842	2.80136131	3.12387166	0.0924779	0.10796917
Galc	-0.4187402	3.76757953	3.12326165	0.09250753	0.10798696
Rbpms2	0.64976437	2.82537584	3.12114873	0.09261026	0.10809006
Exosc5	0.76530659	0.96836672	3.12076342	0.09262901	0.10809512
Pafah2	0.37919473	3.40663434	3.11977786	0.09267698	0.10813428
Znf512b	-0.2535022	4.93881231	3.11933061	0.09269876	0.10814287
Ahdc1	-0.2982448	4.66728877	3.11847134	0.09274062	0.10817489
Ap5m1	-0.4245828	3.37570823	3.11700191	0.09281226	0.10824161
Dhrs13	1.26376764	-0.3668726	3.11644737	0.09283931	0.10824564
Sp3os	0.56771932	3.10306267	3.11633959	0.09284457	0.10824564

Rph3a	-0.2895733	8.88862341	3.11488316	0.09291567	0.10830601
Atn1	0.22888649	7.89650302	3.11468731	0.09292523	0.10830601
Phc2	0.38603926	4.79911662	3.11364653	0.09297608	0.10832227
Eif5b	0.27085937	9.35457205	3.11341875	0.09298722	0.10832227
Rbbp8	0.40945688	4.13428988	3.11323196	0.09299635	0.10832227
Gigyf2	-0.2646234	6.95812281	3.1132199	0.09299694	0.10832227
Nudt15	0.63111596	1.94917808	3.11272617	0.09302108	0.10832355
Gm11944	-1.0465732	0.61037529	3.11260671	0.09302692	0.10832355
Ptprb	-0.6828021	5.31799751	3.11194567	0.09305926	0.10834438
Slc4a8	-0.3966854	6.84351407	3.11097143	0.09310694	0.10838307
Taf6	0.28761259	4.52925267	3.11018495	0.09314545	0.10838336
Ift81	-0.2111651	5.57400414	3.1100969	0.09314976	0.10838336
Mtrr	-0.3351959	4.01069066	3.11008113	0.09315053	0.10838336
Klf2	-0.3838248	4.01005213	3.10943907	0.09318199	0.10839873
Agr2	1.12620219	-0.1481052	3.10911992	0.09319763	0.10839873
Atp6v0a1	-0.2630053	7.21774793	3.10892682	0.09320709	0.10839873
Rrp8	0.37209449	4.19096313	3.10779292	0.09326269	0.10844658
Gria1	-0.3401947	6.49915439	3.10547043	0.0933767	0.10856231
Pold4	0.66607506	2.12316132	3.10457549	0.09342067	0.108568
07-Mar	-0.2811463	4.80727516	3.10441199	0.0934287	0.108568
Ypel4	0.63160157	2.45977121	3.10408842	0.09344461	0.108568
Ube2ql1	0.27098752	6.24484934	3.10403114	0.09344743	0.108568
Txk	1.86845845	-1.7280901	3.10389844	0.09345395	0.108568
AU023762	0.39401698	3.25929013	3.10172479	0.0935609	0.10867541
Kcnmb1	-1.8325715	-1.0258641	3.09979164	0.09365613	0.10876919
Nfatc2	-0.4229274	3.35874337	3.09905902	0.09369225	0.10879429
Sult6b1	0.88368246	0.88888743	3.09702031	0.09379286	0.10889208
Elf2	0.32421051	6.74349939	3.09676425	0.0938055	0.10889208
Large	0.28278509	6.42464331	3.09556756	0.09386462	0.10894385
Dclre1c	-0.4480938	5.24539351	3.09483848	0.09390066	0.10896882
Ptprn	-0.3327757	6.91147551	3.09427338	0.09392861	0.10897024
Ppp1r42	-1.4032125	-0.5472227	3.09422617	0.09393094	0.10897024
Gm16701	-1.0563416	0.69909148	3.09228732	0.0940269	0.10905382
Dolpp1	-0.56801	1.23214382	3.09218307	0.09403206	0.10905382
Tns1	0.21019096	6.61314246	3.09120968	0.09408028	0.10909288
Gpr19	-0.4436386	3.45953435	3.09077193	0.09410198	0.109101
Trim36	-0.4387838	3.36901537	3.09021528	0.09412957	0.109101
Stat1	0.2795933	5.50301959	3.08994231	0.09414311	0.109101
Pacrg	0.49394788	2.80278156	3.08968605	0.09415582	0.109101
Grm8	-0.6489791	2.66462651	3.08960159	0.09416001	0.109101
Ppp6r3	-0.2013966	6.4274008	3.08806814	0.09423611	0.10917232
Cdyl2	0.3197975	5.28059106	3.08630146	0.09432387	0.10925712
Rassf5	0.29049324	4.01244149	3.08513316	0.09438197	0.10930753
Pcsk2os2	-1.3522282	1.71436286	3.0836647	0.09445504	0.10937527
Ehd4	-0.3195122	4.01675613	3.08313661	0.09448134	0.10937548

C130026L21F	-0.798984	1.06076622	3.08307546	0.09448438	0.10937548
Rnase6	-2.1632208	-1.1019717	3.08264195	0.09450597	0.10938359
Mrps18c	0.38214208	4.53628145	3.08226286	0.09452486	0.10938507
Aox1	0.4344121	3.12249429	3.08203103	0.09453641	0.10938507
Rpl36	0.49024198	5.25284376	3.08044484	0.0946155	0.10945282
Ackr1	0.38274092	5.69606705	3.08027142	0.09462415	0.10945282
Rpl38	0.33104015	6.22195774	3.07911037	0.0946821	0.10950296
Hat1	0.28195429	5.05281116	3.07847811	0.09471367	0.10952259
Psat1	0.23767793	6.48892202	3.07596389	0.09483934	0.10964781
Scrib	-0.5433506	2.10448195	3.07572705	0.09485119	0.10964781
Tusc5	-2.1486371	0.35486227	3.07414535	0.09493036	0.10972242
Mlph	0.59614837	2.44175312	3.07330691	0.09497236	0.1097378
Mcoln1	-0.4616425	2.92515908	3.07329552	0.09497293	0.1097378
Tmed10	0.22989469	6.82479277	3.07240036	0.09501779	0.10977274
Setd4	-1.0183129	1.04697933	3.07185674	0.09504505	0.10978732
Pcnp	0.25418683	7.86009019	3.06978201	0.09514917	0.10989066
Cmtm4	-0.2280371	6.3562398	3.06841525	0.09521782	0.10995303
Tmco3	-0.3555667	3.76678227	3.06753248	0.0952622	0.10998734
Wnt5a	-0.3418383	5.5511028	3.06639537	0.0953194	0.11003644
Rd3	3.12902929	-1.8484215	3.06516245	0.09538146	0.11009114
Mast4	-0.2493242	6.6848335	3.06426442	0.0954267	0.11012641
Sfmbt2	-0.5979342	2.5418789	3.06327249	0.09547669	0.11016716
Mir6336	-1.9073546	-1.5248579	3.06278395	0.09550133	0.11017863
Npr2	-0.3026446	4.07112397	3.06116769	0.09558288	0.11025576
Hspb1	0.54816139	5.07250514	3.06062819	0.09561012	0.11027023
Musk	0.46807925	3.43332663	3.05937521	0.09567342	0.11032627
Otub1	0.39308965	5.7233998	3.05873081	0.09570599	0.11034032
S1pr4	-1.1688017	-0.8166348	3.05829727	0.09572791	0.11034032
Os9	-0.3650429	4.71647211	3.05826132	0.09572973	0.11034032
Prkcb	-0.2807056	11.0318329	3.05696615	0.09579526	0.11039888
Tbcd	-0.3529512	3.95613517	3.05624979	0.09583152	0.11041745
Flt3	-0.9797932	0.51524891	3.05606639	0.09584081	0.11041745
Pcdh15	-0.4380643	4.87020321	3.05530665	0.09587929	0.11043176
AI854517	-0.3672064	3.70653765	3.05524	0.09588267	0.11043176
Armcx5	0.23780199	5.36847734	3.05461199	0.09591449	0.11045145
Kif20b	-0.6540141	1.82200334	3.05362903	0.09596433	0.11049188
L1td1	0.4916405	2.81831236	3.0532934	0.09598136	0.11049339
Abcd4	-0.6114005	1.46587845	3.05279178	0.09600681	0.11049339
Camkk2	-0.3467115	6.00634976	3.0527322	0.09600983	0.11049339
D030047H15	-0.8918409	0.23058066	3.05238447	0.09602748	0.11049675
Fbxl18	-0.6755392	1.31069494	3.05173519	0.09606044	0.11051772
Uba1y	-1.6431179	-1.1650433	3.04912151	0.09619326	0.11065357
Rs1	-2.2042886	-0.2740352	3.04842932	0.09622848	0.1106771
Rps15	0.41276625	6.36645525	3.04653562	0.09632489	0.110771
A230009B12	-1.5930598	0.00823792	3.04617613	0.0963432	0.11077508

Cryz	0.47766249	2.82523574	3.04262824	0.09652418	0.11095058
Ofd1	-0.4088199	4.18802212	3.04260393	0.09652542	0.11095058
Gm17066	-0.3941658	4.80154573	3.04230223	0.09654083	0.11095129
Fmo5	0.39035541	3.72328381	3.04036575	0.09663979	0.11104801
Gm3435	-0.491381	2.79609154	3.03851853	0.09673431	0.11113959
Rbck1	0.34036926	4.11843858	3.03814814	0.09675327	0.11114436
Dvl3	0.22787046	6.09227167	3.03734131	0.0967946	0.1111748
Psmg1	0.491839	3.13073301	3.0357237	0.09687751	0.11124996
Suclg1	0.27622389	5.19103964	3.03548627	0.09688969	0.11124996
Ttc4	-0.2869995	3.91169362	3.03342345	0.09699556	0.11135448
Syvn1	0.34628903	4.83708926	3.03309622	0.09701237	0.11135673
Ddi2	0.55420355	2.7742584	3.03226303	0.09705518	0.11137438
Rgs12	-0.4471117	3.26343381	3.03215567	0.0970607	0.11137438
Gstp2	0.31818764	4.53982424	3.03193019	0.09707229	0.11137438
Trip12	-0.2195155	8.84253983	3.0300141	0.09717084	0.11145538
Polr3h	0.37470402	2.96034667	3.02977281	0.09718326	0.11145538
Ccnj	-0.4315942	2.89291347	3.02940376	0.09720226	0.11145538
Zglp1	-1.9423686	-1.4615656	3.02940258	0.09720232	0.11145538
Ppp2r1b	-0.2357314	5.52140425	3.02909147	0.09721834	0.11145671
Smarcal1	-0.3441854	6.14881675	3.02754772	0.09729787	0.11153085
Tmem255a	0.35342762	4.74803875	3.02697649	0.09732732	0.11154756
Hcn1	-0.3638538	7.36017206	3.02550851	0.09740305	0.11157534
Elk4	-0.2106632	6.40798049	3.02545803	0.09740566	0.11157534
Ccdc157	-0.376397	3.04729122	3.02539477	0.09740892	0.11157534
Zfp874b	0.32096616	4.64430705	3.0253533	0.09741106	0.11157534
Vps51	0.43707548	3.72065299	3.02340649	0.09751161	0.11166823
Cerk	-0.3742123	4.07824315	3.02320676	0.09752193	0.11166823
Map4k1	-0.8016986	0.43633808	3.02158612	0.09760573	0.11174713
Ercc6l2	-0.2553521	5.12584143	3.01919084	0.09772975	0.11187204
Pole3	0.50175071	2.9234239	3.0161809	0.09788585	0.1120194
Eif2ak1	0.22747072	6.22664605	3.01612205	0.0978889	0.1120194
Ppbp	-1.962193	-0.2778654	3.01579276	0.097906	0.1120194
Cmtm7	0.58465378	1.86623402	3.01528268	0.09793249	0.1120194
Cacnb1	0.25851199	5.12663718	3.01527	0.09793315	0.1120194
Tiparp	0.23742299	4.91605335	3.01403382	0.09799738	0.11207578
Mettl21a	0.49729424	2.89583709	3.01329718	0.09803568	0.11209355
Snrpd2	0.47606647	4.07892904	3.01316012	0.0980428	0.11209355
Rpl26	1.39397049	-1.291403	3.01259597	0.09807215	0.11210105
Gjc2	-1.0750816	0.31923454	3.0124595	0.09807925	0.11210105
Col20a1	-0.850484	1.24662046	3.01064638	0.09817364	0.11219184
Fam45a	0.26660913	4.9588035	3.00975593	0.09822004	0.11222776
Vash1	-0.4142729	3.42591804	3.00737474	0.09834423	0.11234871
Hif1a	-0.2612001	6.51471301	3.00715237	0.09835584	0.11234871
Kpnb1	0.20953501	8.29382347	3.00634078	0.09839822	0.11238
Fmnl1	0.35533555	5.94178596	3.0056526	0.09843417	0.11240394

Rnf165	-0.3231627	6.31500496	3.00525148	0.09845513	0.11241056
Ighmbp2	-0.5970865	1.95229387	3.00484282	0.09847649	0.11241056
Pon2	0.48229777	5.932534	3.00468166	0.09848491	0.11241056
Pced1b	-0.4018992	3.1020314	3.00333296	0.09855546	0.11247396
Dcun1d4	0.24577239	7.403709	3.0015915	0.09864663	0.11256089
Pdlim7	0.30652716	4.32830393	3.00109404	0.0986727	0.11257176
Dock8	-0.5228768	2.85605871	3.00083686	0.09868617	0.11257176
Pknox1	0.3188454	3.85227558	2.99888405	0.09878858	0.11267144
Edem2	0.63433462	1.59219095	2.99779692	0.09884564	0.11271938
Chkb	-0.5819447	1.33850918	2.99660359	0.09890832	0.11277283
Zfp354a	-0.3369688	3.36857815	2.99621659	0.09892866	0.11277283
Brpf3	-0.2295337	5.26307102	2.99595739	0.09894229	0.11277283
Mterfd1	0.30257471	5.07451548	2.99576028	0.09895265	0.11277283
N4bp2l1	-0.3708048	4.03004279	2.99530164	0.09897677	0.11278318
Lman1	-0.4165652	5.02057679	2.99387044	0.09905206	0.11285184
Adamts1	-0.375307	4.34942457	2.99222096	0.09913893	0.11293366
Adss	-0.1826504	7.09678092	2.99163993	0.09916955	0.1129426
Zdhhc20	-0.2868595	5.43041885	2.99150074	0.09917689	0.1129426
Fam189a1	-0.2702175	5.77277244	2.99080789	0.09921342	0.11295803
Neu4	-0.5326863	2.46054325	2.99043688	0.09923299	0.11295803
1600012H06I	0.3630574	4.59940473	2.99038706	0.09923562	0.11295803
1700028E10I	-1.4621562	-0.7948921	2.98813174	0.09935467	0.11307639
Pacsin3	0.40597925	3.80814932	2.98530734	0.099504	0.11319592
Trpc4	-0.406412	3.39938902	2.98512813	0.09951348	0.11319592
Ccl2	1.30510128	-0.4998618	2.98502607	0.09951889	0.11319592
Rnd3	-0.3424236	5.26196549	2.98479235	0.09953126	0.11319592
Gpatch11	0.31426882	5.43093597	2.98468221	0.09953709	0.11319592
Btf3	0.34830651	6.23544685	2.98443377	0.09955024	0.11319592
Prdx4	-0.3531167	3.41192748	2.98358722	0.09959508	0.11322974
AW822252	-1.3067706	0.07551761	2.98130252	0.09971619	0.11335026
2810442I21R	-2.5768644	-2.1376449	2.97804277	0.0998893	0.11352983
Fiz1	0.37404529	3.76023	2.97672127	0.09995958	0.11359249
Ppp1r2	0.22293293	8.03802023	2.9761601	0.09998944	0.11359527
Rrs1	0.24356423	4.79496528	2.97610619	0.09999231	0.11359527
Pcdhac1	-1.1938169	-0.0962579	2.97424569	0.10009139	0.11367774
Prrc2a	-0.2214719	7.7199155	2.9741739	0.10009521	0.11367774
Dnm3	-0.3669037	8.50630383	2.97320827	0.10014669	0.11371193
Pnn	-0.2932547	7.701744	2.97304045	0.10015564	0.11371193
Siva1	0.61411338	2.66080766	2.9726986	0.10017387	0.11371542
BC005537	0.23377712	7.01994452	2.97216404	0.10020238	0.11373058
Fam173a	0.44988683	3.09933044	2.97113113	0.10025751	0.11377593
Phf21b	-0.5952335	2.20298585	2.9705558	0.10028823	0.11379358
Cables1	-0.4316119	3.13166928	2.96961778	0.10033835	0.11383322
Oprl1	-0.3607557	3.3758433	2.96765907	0.10044308	0.11393482
Nkain1	-0.2753137	4.40543864	2.96625215	0.10051839	0.1139962

1810022K09I	0.27830924	4.99892239	2.96601451	0.10053112	0.1139962
Spata17	2.67543039	-1.2522356	2.96537549	0.10056535	0.1139962
Polr3k	0.2905571	6.06279642	2.96531785	0.10056844	0.1139962
Gm2061	0.75910519	1.29447359	2.96522937	0.10057318	0.1139962
Vcpip1	-0.244154	7.35241349	2.96349621	0.10066611	0.11408429
Parvb	0.44616938	3.54574773	2.9625123	0.1007189	0.11412061
Slc23a2	-0.2242546	7.23608384	2.96173247	0.10076077	0.11412061
Tmem185b	-0.5848651	3.21262042	2.96155025	0.10077056	0.11412061
Rragd	-0.1880231	7.20974552	2.96148913	0.10077384	0.11412061
Tiam2	0.33838137	4.92606776	2.96148195	0.10077423	0.11412061
6820431F20I	-0.2881507	8.79642571	2.96066323	0.10081821	0.11414723
Ctdspl	0.29964803	6.4620004	2.96047815	0.10082816	0.11414723
1700037C18I	1.05115816	0.04865188	2.95908855	0.10090288	0.11421458
Tmem87b	0.31259539	4.94297907	2.95831707	0.10094439	0.11424432
Fgfrl1	-0.4950001	2.25860153	2.95685927	0.10102288	0.11431591
Itpkc	0.53694792	2.14887091	2.95624039	0.10105623	0.11433639
Limk2	-0.2735958	5.20943889	2.954891	0.10112897	0.11440145
Fgfr1op2	0.21606137	7.74239464	2.95347129	0.10120558	0.11447084
Cd37	1.51180895	-0.4761001	2.95262346	0.10125136	0.11450079
Pip5k1a	-0.3147041	3.99435861	2.95240105	0.10126337	0.11450079
4930507D05I	1.68824563	-1.1882022	2.95213306	0.10127785	0.11450079
Catsperg1	-1.8603524	-1.0719641	2.95126665	0.10132467	0.11453647
Gm13547	-2.1854855	-2.4282955	2.95049444	0.10136643	0.1145664
Ankrd1	-1.7500045	-1.3890658	2.94873863	0.10146144	0.11465651
Mthfsd	-0.6564423	1.83132044	2.94791333	0.10150613	0.11468582
Abhd4	0.35688428	5.30739511	2.94754452	0.10152611	0.11468582
Lrrc4c	-0.2874404	8.09940003	2.94741289	0.10153325	0.11468582
Lsg1	0.29569168	4.02144153	2.94633158	0.10159186	0.11473475
Kdm2b	-0.2547514	4.37736772	2.94566115	0.10162822	0.11475854
Spock1	0.25263399	8.18431266	2.94287962	0.10177924	0.11491178
Nav2	-0.4078633	7.18840989	2.94234192	0.10180847	0.11492748
Zbtb49	-0.6331431	1.51435636	2.94186268	0.10183452	0.11493959
Pip5k1b	-0.525112	2.98888508	2.94144902	0.10185702	0.11494769
Prx	-1.2863531	-0.4765211	2.94072007	0.10189668	0.11497514
Zfp764	0.61002526	2.98678652	2.94029027	0.10192007	0.11498424
Zfp97	-0.2870823	4.12311113	2.93968955	0.10195277	0.11498803
Cnot2	0.21463702	6.21927661	2.93940086	0.10196849	0.11498803
Mir1188	-1.3324459	-0.5347815	2.93934781	0.10197138	0.11498803
Ncoa5	-0.3438195	4.6525009	2.93910249	0.10198474	0.11498803
4931429L15I	-1.7194397	-1.1998334	2.93847602	0.10201888	0.11500923
Sc5d	0.29936704	4.4301661	2.93659527	0.10212142	0.11507901
Derl3	1.76484526	-1.2662247	2.93647374	0.10212805	0.11507901
Tmem170	0.4447421	3.25063668	2.9360919	0.10214889	0.11507901
Aimp1	0.37789919	4.50395021	2.93596517	0.10215581	0.11507901
Acsl1	-0.2232398	6.51345693	2.93593441	0.10215748	0.11507901

Parp8	-0.3445721	4.80146209	2.93118453	0.10241712	0.11534819
Tbc1d31	-0.364277	3.31663818	2.93100054	0.10242719	0.11534819
Rrm2b	0.21696206	6.74187979	2.9296117	0.10250326	0.11541653
Slain1	-0.362087	4.91292818	2.92914387	0.1025289	0.11542807
Arl6ip4	0.36162356	4.90499571	2.92859081	0.10255922	0.11544488
Srrd	-0.7201417	1.36859612	2.92746787	0.10262081	0.11549688
Tada1	0.27907835	5.26378133	2.92621783	0.10268943	0.11554836
Scmh1	0.25241972	6.11744616	2.92607338	0.10269736	0.11554836
Ptpn5	0.26423907	5.9042918	2.92530256	0.10273971	0.11557867
Pfdn2	0.31511665	5.53679415	2.92489769	0.10276195	0.11558636
Ormdl3	0.42819658	4.20754267	2.92428799	0.10279547	0.11559157
Zbtb8os	0.47470912	4.06613915	2.92414583	0.10280329	0.11559157
Mir3473f	-2.1940752	-1.3801563	2.92397255	0.10281281	0.11559157
Psph	0.49899333	3.65078526	2.92359339	0.10283367	0.11559769
Lrfn1	-1.0306156	0.21389315	2.92307294	0.1028623	0.11560429
Pin1	0.38981825	3.03710293	2.92292639	0.10287036	0.11560429
Prdm11	-1.6814058	-0.5723408	2.9226116	0.10288769	0.11560644
Dbn1	-0.3333296	4.44211037	2.92168111	0.10293891	0.11564668
Filip1l	-0.2194812	5.30520595	2.92064215	0.10299615	0.11569365
Hsdl2	0.2909251	6.17222768	2.91921985	0.10307456	0.11576439
Tmem17	0.58184543	1.7627744	2.91880837	0.10309726	0.11577255
Hmg20a	0.34151788	5.9347233	2.91780576	0.10315259	0.11579075
Mrpl44	0.32711221	3.56917656	2.91779225	0.10315334	0.11579075
Chid1	-0.3300431	3.41051126	2.9174107	0.1031744	0.11579075
Rpl7a	0.31750436	7.31122517	2.91739617	0.10317521	0.11579075
Lrrc40	0.34606562	5.10632569	2.91691509	0.10320178	0.11580324
Auh	0.26364951	5.11797038	2.91557074	0.10327606	0.11586926
Rpl10	0.37116654	7.8633684	2.91503761	0.10330554	0.115885
Zbtb8a	-0.6205103	2.95987828	2.91446063	0.10333745	0.11590347
Zfp85os	0.48213072	2.59106711	2.91377219	0.10337555	0.11592886
Anapc7	0.37755101	3.5193806	2.91299914	0.10341834	0.11593869
Sh3bgrl2	0.32348619	4.30173649	2.91238992	0.10345208	0.11593869
Dek	0.27479434	8.40392104	2.91222917	0.10346099	0.11593869
Il12a	0.60404818	1.92491101	2.91206052	0.10347033	0.11593869
Hdac8	-0.592975	1.90663571	2.91203876	0.10347154	0.11593869
Ift46	0.3964232	4.57608285	2.91193935	0.10347705	0.11593869
Gpr12	0.40053259	4.05832662	2.91013416	0.10357713	0.11603349
Hmg20b	0.76734904	2.17156615	2.90948196	0.10361331	0.1160567
Rasa3	-0.2810541	5.2610341	2.90710058	0.10374557	0.11618749
Ppt1	0.23143244	6.44486495	2.90579326	0.10381826	0.11624948
Sorcs3	-0.4540293	4.18264428	2.90554764	0.10383192	0.11624948
Kif18b	1.99869009	-1.7836768	2.90050088	0.10411313	0.11654693
Acly	-0.2662462	7.0689687	2.89776707	0.10426584	0.11669451
Serinc5	-0.3309913	5.0789743	2.89743975	0.10428414	0.11669451
Bcl2a1b	0.65161218	1.45049734	2.89730569	0.10429164	0.11669451

Emc7	0.36329384	5.39308965	2.89607991	0.10436021	0.11673542
Pgls	0.66623185	1.35967136	2.89604648	0.10436208	0.11673542
Grb10	-0.1953202	6.92938384	2.89564302	0.10438467	0.11673542
9530052E02I	-1.8811816	-0.9840896	2.89553974	0.10439045	0.11673542
Fam19a1	0.22858333	5.87262786	2.89509486	0.10441536	0.11673621
Rpl21	0.34038886	7.82122317	2.89480933	0.10443135	0.11673621
Pcbp1	0.25299017	5.75618372	2.89466106	0.10443965	0.11673621
Grn	0.47234215	5.16360744	2.89407065	0.10447273	0.11673621
Nrg3	-0.2364472	6.24416023	2.89386031	0.10448452	0.11673621
Syf2	0.38562453	5.63618597	2.8937185	0.10449247	0.11673621
Nab1	0.19416688	6.61814642	2.89358252	0.10450009	0.11673621
Snap23	0.30435233	7.16619775	2.89322487	0.10452014	0.11674122
Aox2	2.80020144	-0.919169	2.89117582	0.1046351	0.1168471
Slc35g2	-0.3860615	3.02421412	2.89096317	0.10464704	0.1168471
Psapl1	-3.0654088	-1.7375642	2.89059557	0.10466768	0.1168471
Mis12	0.27592295	5.24682689	2.89042532	0.10467724	0.1168471
Inpp4b	-0.3656559	3.81977761	2.88999695	0.10470131	0.11685657
Prdm4	-0.3438651	4.99589618	2.88873984	0.10477196	0.11691803
Acat2	0.26697673	5.25145575	2.88502234	0.10498123	0.1171195
Dpcd	0.50640539	3.13768392	2.88497807	0.10498372	0.1171195
Map3k13	-0.4043891	5.16798129	2.88393135	0.10504273	0.11716791
Arl11	-2.2199995	-0.8594678	2.88101922	0.10520712	0.11733382
Ppp1r15a	0.66381958	2.79105364	2.88001281	0.105264	0.11737644
Gdap2	0.23695176	4.7954107	2.87978949	0.10527663	0.11737644
Myh14	-0.6452925	2.29748057	2.87851255	0.10534886	0.11743952
Hist2h2be	0.34587164	6.00580263	2.87721603	0.10542226	0.11748654
Igsf1	-1.4954781	0.70347853	2.87720224	0.10542304	0.11748654
Rap1b	0.2584977	7.25010556	2.87693767	0.10543803	0.11748654
Gm13807	2.14646187	-2.139352	2.87587369	0.10549832	0.11752993
Fam19a5	0.23630153	4.98488575	2.87569751	0.10550831	0.11752993
Arhgap30	-0.5848719	2.85648946	2.87449157	0.1055767	0.11757048
Cd59a	0.41400553	4.58348747	2.87434745	0.10558488	0.11757048
3110039I08R	-1.1504892	-0.0970578	2.87422668	0.10559173	0.11757048
Zfp942	-0.3497432	3.51107222	2.87363029	0.10562558	0.11757742
Accs	-0.4979862	2.8710364	2.87356455	0.10562931	0.11757742
Ddost	0.27971336	4.27040541	2.87166253	0.10573735	0.11767409
Cep89	0.49994991	3.02045372	2.87148348	0.10574753	0.11767409
Rprd1a	0.23440156	6.72617376	2.86986697	0.10583946	0.11775892
Rab22a	0.23064138	5.64374601	2.86947168	0.10586196	0.11776648
Slc19a2	-0.3952456	3.68181895	2.86904019	0.10588652	0.11777634
Igj	-0.8206817	1.01319306	2.86827431	0.10593013	0.11780738
Sox6	-0.3677345	5.00814424	2.8624332	0.10626345	0.11816055
9130401M01	0.27570784	4.78490337	2.86120708	0.10633357	0.118221
Pms2	-0.4060685	3.62544284	2.86048294	0.10637501	0.11822705
Gpr18	-1.4900795	-0.4416102	2.86039753	0.1063799	0.11822705

Mios	-0.3026	4.46628771	2.86028583	0.1063863	0.11822705
Kbtbd3	-0.3868219	3.29849578	2.85962729	0.106424	0.11823744
C130071C03I	-0.4958435	2.7042152	2.85880568	0.10647107	0.11823744
Sh3bp5l	0.34706	3.77831697	2.85875417	0.10647402	0.11823744
Nrsn1	-0.2169585	6.50508988	2.85857509	0.10648429	0.11823744
Pex2	0.37016929	4.2015927	2.85849315	0.10648898	0.11823744
Abca6	-0.6446672	1.65908555	2.85825082	0.10650287	0.11823744
Spns2	-0.3723155	3.5192407	2.85725056	0.10656023	0.11823744
Des	0.95623704	0.62764967	2.85723839	0.10656092	0.11823744
Atf6b	0.38980744	3.13973333	2.85704118	0.10657224	0.11823744
Snhg1	0.35787103	4.49501652	2.85699095	0.10657512	0.11823744
Pms1	-0.4401231	2.75300023	2.85674894	0.106589	0.11823744
Tgm3	-0.4374647	2.62821885	2.85663456	0.10659556	0.11823744
Nkx6-1	1.71968477	-0.5665665	2.85654765	0.10660055	0.11823744
Kcne4	-0.4652556	3.15263084	2.85586396	0.10663979	0.11826348
Ech1	0.4696238	3.948323	2.85555682	0.10665743	0.11826555
Fgf9	-0.3252511	4.84515337	2.85527959	0.10667335	0.11826572
Aven	0.61782345	1.31727111	2.84895043	0.10703756	0.11865197
Clic1	0.46959202	4.48526169	2.84816015	0.10708314	0.11867343
Gm6484	-2.3814221	-1.9123091	2.84806624	0.10708856	0.11867343
Nt5dc3	-0.2981868	5.92130142	2.84691423	0.10715504	0.11871716
Pter	0.34146888	3.4557309	2.84665846	0.10716981	0.11871716
Fbxo24	-1.1540232	-0.1138298	2.84656007	0.10717549	0.11871716
Ccdc93	-0.2704605	5.01806247	2.84506466	0.10726189	0.11878129
Dgkg	0.38366573	6.62842365	2.84500988	0.10726506	0.11878129
Vmn2r46	-1.5057046	-1.6613097	2.84308355	0.10737648	0.1188789
Car15	-0.7419397	2.46758281	2.84293802	0.1073849	0.1188789
Nagpa	0.51923211	2.8786944	2.84255267	0.10740721	0.11888605
Zfp36l1	0.30162639	7.72852485	2.84111053	0.10749074	0.11892847
Fam188b	-0.6722604	1.34550415	2.84088856	0.1075036	0.11892847
Mid1	0.38826461	4.12698169	2.84076104	0.10751099	0.11892847
Taf15	-0.290743	5.09488274	2.84048336	0.10752709	0.11892847
Erbb3	-1.4812807	-0.3009485	2.84033864	0.10753548	0.11892847
Stac2	0.24607372	5.87709308	2.84024927	0.10754066	0.11892847
Nek11	0.91567475	0.02576182	2.83936686	0.10759184	0.11896753
Cyth1	-0.4126116	3.89157414	2.8386876	0.10763125	0.11899357
Cog6	-0.2181666	5.57264426	2.83644023	0.10776178	0.11912032
Slc28a3	-1.0263838	1.09393552	2.83553977	0.10781413	0.11916063
Zrsr2	0.21397435	6.20356364	2.83492304	0.10785	0.11918271
Cysltr2	-1.8405845	-0.9832844	2.83161401	0.10804272	0.11936122
Ranbp6	-0.3212245	6.39165545	2.83115883	0.10806926	0.11936122
Slc4a2	0.43295896	3.65489648	2.83112846	0.10807103	0.11936122
Stk38	-0.2610397	4.80460911	2.83079339	0.10809057	0.11936122
Gm10046	-0.787387	0.33357784	2.83078437	0.1080911	0.11936122
Farp2	0.37143119	2.80240206	2.82902142	0.10819399	0.11945725

Rimklb	-0.3459879	3.68555585	2.82813955	0.1082455	0.11948393
Syt1	0.24237075	9.90610824	2.82806243	0.10825	0.11948393
Efcab12	-0.9047626	0.82845375	2.82762443	0.1082756	0.1194946
Gpt2	-0.2525586	4.97022842	2.82663841	0.10833325	0.11954064
Lyrm4	0.24438267	5.02712425	2.82498292	0.10843012	0.11962993
Cand1	-0.2574713	7.71060483	2.82409791	0.10848195	0.11966951
Lypla1	0.30241966	5.19566608	2.82350804	0.10851651	0.11969004
Cd164	0.40512546	7.83696566	2.82298915	0.10854692	0.11970598
Erap1	-0.5282665	3.54311035	2.82092014	0.10866829	0.11981593
Rbm47	0.34644789	4.57140377	2.82067988	0.10868239	0.11981593
Kpna3	0.22832727	7.16999286	2.82035367	0.10870155	0.11981593
Kcnf1	-0.3324509	5.28870068	2.82020091	0.10871052	0.11981593
Cct7	0.25496484	6.67429444	2.81920828	0.10876883	0.11986259
Mblac1	0.54329936	2.67170348	2.81854168	0.10880801	0.11987806
Trp53inp1	-0.2814177	5.78981049	2.81842565	0.10881483	0.11987806
C1ql2	1.95030231	-1.1618038	2.81706875	0.10889464	0.11992787
Gucy1a2	-0.3575362	7.95296243	2.81686396	0.10890669	0.11992787
Eif2s1	0.20438674	6.78122622	2.81684156	0.10890801	0.11992787
Psd2	0.34431364	4.92517248	2.81652656	0.10892655	0.11993068
09-Mar	0.61237562	1.83379373	2.81591296	0.10896267	0.11995285
Ankzf1	0.59705117	1.79473831	2.81549184	0.10898747	0.11996254
Taf12	0.44610683	4.10431954	2.81501779	0.1090154	0.11997568
Pdia6	0.29854966	4.75854562	2.81432672	0.10905613	0.12000289
Msln	0.71686244	2.22083589	2.81338013	0.10911194	0.12001963
Slc16a10	-0.4563477	2.28528914	2.81331425	0.10911582	0.12001963
Gm13582	-2.2075237	-1.3285864	2.81313588	0.10912635	0.12001963
Dpp7	-0.6462599	1.49041866	2.81298356	0.10913533	0.12001963
BC048403	0.31542824	4.04730673	2.81145274	0.10922569	0.12010139
Unc13d	1.69227828	-0.8819921	2.81100694	0.10925202	0.12011273
Marveld1	0.47727449	4.34164061	2.81030712	0.10929336	0.12013472
Slc25a32	0.33430989	3.88956618	2.8101264	0.10930405	0.12013472
Hs2st1	-0.2324485	6.7575588	2.80854044	0.10939783	0.12022018
Flywch1	0.37693114	3.82844992	2.80801736	0.10942878	0.12023658
Pcolce2	-0.8849085	1.14660412	2.80670729	0.10950635	0.12030418
Srf	-0.284476	4.85046671	2.80604989	0.10954529	0.12032935
Atp5s	0.24916746	4.66930354	2.80471587	0.10962438	0.12037678
Pacs2	-0.2019628	6.07020537	2.80469338	0.10962571	0.12037678
Eef1a1	0.28159498	10.3156722	2.80450943	0.10963662	0.12037678
LOC1005050:	-1.0747504	0.48424062	2.80343892	0.10970014	0.1204289
9530051G07	-0.880075	0.85505439	2.80288895	0.10973279	0.12044712
Tmco1	0.45816573	4.77673781	2.80165882	0.10980586	0.12050969
Zfp111	-0.3056219	5.16737656	2.80118572	0.10983398	0.12052291
Vps25	0.34348871	5.12728745	2.80072147	0.10986158	0.1205331
AA413626	-1.5866039	-0.6663741	2.80048897	0.10987541	0.1205331
Gm10406	-1.207133	-0.4766145	2.80020885	0.10989207	0.12053375

B2m	0.36931694	9.02542822	2.79913977	0.10995567	0.12057528
Magee2	-0.331593	4.78781903	2.79870457	0.10998158	0.12057528
Cyp27a1	-0.6506237	1.73152539	2.79869507	0.10998215	0.12057528
0610007P14I	0.33779602	4.25240262	2.79849229	0.10999422	0.12057528
Ndufaf1	0.43603726	3.33368147	2.79789158	0.11003	0.12059687
Bbs9	0.234005	5.07388828	2.79616302	0.11013302	0.12069215
Cyp46a1	0.29183768	4.74106673	2.79583175	0.11015277	0.12069617
09-Sep	0.35833355	5.57392956	2.79536674	0.11018051	0.12070893
Sema4c	0.86381724	0.62827725	2.79436425	0.11024034	0.12075684
Car5a	-1.17412	-0.2991194	2.7935199	0.11029077	0.12076232
Foxd1	0.46769617	5.52951661	2.79341234	0.11029719	0.12076232
Popdc3	-1.7198106	-0.7530781	2.79338562	0.11029879	0.12076232
Vav1	-0.9138928	0.25682111	2.79320231	0.11030974	0.12076232
Ky	-0.8594446	0.5283509	2.79241806	0.11035661	0.120796
Faf1	-0.2883786	4.73011911	2.79188499	0.11038848	0.12080091
Colgalt2	-0.5814389	1.4849493	2.79164146	0.11040304	0.12080091
Zhx1	0.20393131	8.00743807	2.79153513	0.1104094	0.12080091
Wnt4	0.26164838	5.58221254	2.79000326	0.11050107	0.12087535
Efcab1	0.39036741	3.56238764	2.7898596	0.11050967	0.12087535
Pcdha7	-1.2033087	-0.6336059	2.78938422	0.11053813	0.12088886
Nckap1l	-0.4087966	3.03439757	2.78696544	0.11068311	0.12102976
Tnrc6c	-0.2343999	6.9612226	2.78612657	0.11073344	0.12106715
Purb	-0.1967407	9.71124071	2.78335104	0.11090017	0.12123177
Ufsp1	0.60839996	1.89725205	2.78123258	0.11102763	0.12134425
Nars2	-0.4119525	3.83879504	2.78110312	0.11103542	0.12134425
Ccpg1	-0.2297334	6.52446713	2.78013714	0.1110936	0.12137975
Enkur	-0.547419	2.14024953	2.7800265	0.11110027	0.12137975
H2-Aa	-0.7441126	4.46773061	2.77922318	0.11114868	0.12141374
Abcg3	-2.4224667	-1.7768559	2.77897332	0.11116374	0.12141374
Usp4	-0.2473097	5.49972037	2.77823468	0.11120829	0.12144471
Acat3	-0.8943405	0.69937389	2.77777302	0.11123614	0.12145341
Amz1	0.56292913	1.78497787	2.77755824	0.1112491	0.12145341
9130221H12I	-0.3564343	3.48044409	2.77729765	0.11126483	0.12145341
Tmem210	-1.2151473	0.06352342	2.7768667	0.11129084	0.12146413
Trp63	-0.6826119	2.24132109	2.77647835	0.11131429	0.12147205
Cdh10	-0.327491	5.14094305	2.77579685	0.11135546	0.12149817
Ccnk	0.20675031	6.38171229	2.77554582	0.11137062	0.12149817
Ccnd2	-0.2259839	7.42819143	2.77435702	0.11144248	0.12154648
4933427E11I	2.33745245	-1.2907902	2.77419258	0.11145243	0.12154648
Zfp668	0.36336152	3.70242168	2.77400914	0.11146352	0.12154648
3110009E18I	0.87489448	1.10227185	2.77320318	0.11151228	0.12157634
Pgd	0.34139025	5.24899362	2.77297089	0.11152634	0.12157634
Lpar4	-0.6144775	2.27780024	2.77275317	0.11153952	0.12157634
Mylk3	-1.1277359	0.35676851	2.77212951	0.11157728	0.12159982
Dennd4b	-0.4324385	3.90586378	2.7716988	0.11160337	0.12161059

Mael	-1.2197638	0.65600567	2.77054161	0.11167349	0.12166932
Psmd5	0.23045459	5.36568857	2.76998281	0.11170737	0.12168856
Rbm22	-0.2622507	4.93733979	2.76881058	0.11177848	0.12174834
Cdh22	-0.7227713	1.76906801	2.76812549	0.11182007	0.12176449
Flt1	-0.3393403	4.99645471	2.76802368	0.11182625	0.12176449
Fasn	-0.3268273	6.43402626	2.76775566	0.11184252	0.12176449
Bsdc1	0.2556016	5.28190997	2.76747574	0.11185952	0.12176449
Larp7	0.2329217	5.74968368	2.76722979	0.11187446	0.12176449
Pp2d1	-0.8168925	0.70436595	2.76502103	0.11200874	0.12189294
Nanog	-1.1257064	-0.5025616	2.76265604	0.11215272	0.12203193
Zdhhc22	0.46187685	2.47904651	2.76101519	0.11225274	0.12211862
Nkain3	-0.5578162	1.994663	2.76081506	0.11226495	0.12211862
Pcdhb5	-0.4460144	1.85880226	2.76043833	0.11228793	0.12212591
Lgi1	0.24492987	7.46443041	2.76008847	0.11230928	0.12213142
Ppm1j	1.88530552	-1.8285793	2.75939012	0.11235191	0.12216006
Fez2	0.27375215	5.48917661	2.75880098	0.11238788	0.12216446
Cenpj	0.3168549	4.00138366	2.75879047	0.11238853	0.12216446
Spcs3	-0.2077145	6.30157083	2.75826004	0.11242093	0.12218198
Fbxl5	-0.2164207	5.6184531	2.75728207	0.1124807	0.12222923
Dpf3	-1.1228607	0.0628116	2.75557504	0.11258512	0.12230882
Dcun1d3	0.27072895	4.96451194	2.75555177	0.11258655	0.12230882
Acot10	-1.6440604	-0.5193226	2.75477316	0.11263421	0.12234288
Tmem11	0.38879549	3.33295823	2.75405976	0.11267791	0.12237262
Emc9	0.46102185	2.43498504	2.75319484	0.11273091	0.12241246
Kcna4	-0.3791396	5.66132831	2.75230224	0.11278564	0.12245417
2510003E04I	-0.2306262	7.09266894	2.75173459	0.11282047	0.12247425
Rrm2	-0.8096098	1.42874558	2.75121766	0.11285219	0.12249096
Gsdma	-2.8152754	-2.0985064	2.75064847	0.11288713	0.12251116
Ogfod3	0.65958087	2.08932277	2.74931874	0.11296881	0.12258206
Slc41a1	0.36323269	7.95331276	2.74764908	0.11307147	0.12267571
Stard10	0.37190497	3.3328825	2.74682411	0.11312223	0.12271304
Vat1	0.39038149	4.62141289	2.74553861	0.11320138	0.12278115
Coq9	0.31435414	4.37311304	2.74362562	0.1133193	0.12289127
Wbp1	0.40514557	3.50318168	2.74100238	0.11348123	0.12303868
Wscd1	-0.3378004	3.74854037	2.74071095	0.11349923	0.12303868
Zbtb38	-0.1960851	6.89674946	2.74062686	0.11350443	0.12303868
Cabp1	-0.4561594	2.88542464	2.73949442	0.11357444	0.12308979
Gm17296	-0.7558162	2.16490273	2.73929277	0.11358691	0.12308979
Coa5	0.24731315	7.00338621	2.73906807	0.11360081	0.12308979
Cbfa2t2	0.1807958	6.40881928	2.73739694	0.11370423	0.12318406
9430037G07	-0.5552791	2.05071542	2.73665072	0.11375046	0.12321634
Zdhhc7	0.43963304	3.13165854	2.73603404	0.11378867	0.12323496
Ppm1b	0.17274923	8.14640098	2.7358432	0.1138005	0.12323496
4831440E17I	-0.7213374	1.79053662	2.73405533	0.11391139	0.12332847
Ggps1	0.296857	6.34114367	2.73362277	0.11393823	0.12332847

Erc1	-0.1944138	7.67618624	2.73361693	0.1139386	0.12332847
Lactb	-0.320005	3.6918282	2.7333061	0.11395789	0.12332847
Oaz1	0.2790197	5.13371728	2.73312633	0.11396906	0.12332847
Ubald1	0.2994249	4.48217382	2.7328566	0.11398581	0.1233288
Pou2af1	0.54384631	4.8134834	2.73125688	0.11408521	0.12340737
Mbtps1	-0.2542228	5.43469457	2.73115856	0.11409132	0.12340737
3110040N11	0.43170023	3.1539725	2.72987233	0.11417133	0.1234744
Ppm1e	-0.2896259	8.03567188	2.72949366	0.11419489	0.1234744
Ramp3	-1.2553768	-0.3875601	2.72936868	0.11420267	0.1234744
5830415F09I	1.64913224	-0.7140421	2.72868546	0.11424521	0.12350259
Nifk	0.34551006	4.61760274	2.72747023	0.11432091	0.12354979
Map6	0.348655	5.70222243	2.72745575	0.11432182	0.12354979
Unc5d	-0.3281692	6.21275361	2.72692755	0.11435474	0.12356757
Art3	-1.5724627	-0.4051055	2.72639262	0.1143881	0.12357476
Fdx1	0.42258116	5.38919564	2.72629257	0.11439434	0.12357476
Rcsd1	0.41095477	4.59087504	2.72472793	0.11449197	0.12366242
Eci1	0.32406929	3.68755631	2.72232312	0.11464223	0.12380688
4933406I18R	-1.1285008	-0.502122	2.72171325	0.11468037	0.12383025
B4galt3	-0.5050408	1.94859098	2.72120355	0.11471226	0.12384685
Pex26	0.35220047	3.8248316	2.72080069	0.11473747	0.12385625
Taf1a	-0.4212825	3.23536216	2.72038009	0.1147638	0.12386684
Creb3	-0.5187386	3.08932762	2.71922101	0.1148364	0.12392736
Col6a3	0.53838268	3.31318191	2.71798307	0.11491399	0.12399326
Pde4d	-0.2810966	7.08529817	2.7175958	0.11493828	0.12400163
Pcdha6	-1.0404057	0.30558676	2.71713232	0.11496735	0.12401516
Clic6	-0.5278938	2.60919011	2.71662901	0.11499893	0.1240296
Kansl2	0.22769222	4.72657534	2.71614168	0.11502952	0.1240296
Fjx1	0.30017009	4.08630098	2.71612874	0.11503034	0.1240296
Cpne5	0.31525872	4.94584608	2.71574012	0.11505474	0.12403808
Creb1	0.1631676	7.03094245	2.71487694	0.11510896	0.1240787
Commd2	0.31842207	4.30203494	2.71439357	0.11513933	0.12409361
Calcr	-1.1116831	0.29845713	2.71311517	0.11521971	0.1241624
Ddx60	-0.8226812	1.88541566	2.71261606	0.11525111	0.1241784
Ppp1r16a	-0.4615242	2.41050644	2.71102027	0.11535158	0.1242688
Mboat1	-1.1029665	0.97998995	2.7106613	0.11537419	0.12427531
Cops7b	0.44232505	3.71950173	2.70889214	0.11548572	0.12436736
Cnih1	0.33792425	5.24449197	2.70877965	0.11549281	0.12436736
Dmrtc1a	0.76226426	0.89570495	2.70810017	0.11553568	0.12439567
Zfp788	-0.1932624	6.23732821	2.70765379	0.11556386	0.12440197
Ddx54	0.35013226	3.98407135	2.70748203	0.1155747	0.12440197
Foxc2	0.51125875	6.72668038	2.70675066	0.11562088	0.12442774
Alx3	0.56581893	3.2206397	2.7063778	0.11564444	0.12442774
Ccdc115	0.25565186	4.90636218	2.70631507	0.1156484	0.12442774
Ubxn1	0.42465016	5.407365	2.70546297	0.11570225	0.12446782
Tnnt3	2.67216251	-1.5294982	2.70510754	0.11572472	0.12447414

Inpp4a	-0.2984704	6.53634804	2.70476333	0.11574649	0.12447971
Wasf2	0.25271409	6.44383348	2.70444753	0.11576646	0.12448334
C1ql1	-0.5703382	2.81366461	2.70351763	0.1158253	0.12452876
Mettl10	0.34265276	5.06836517	2.70325375	0.11584201	0.12452887
2810442N19	-1.2173909	0.21262058	2.70186718	0.11592982	0.12460308
Selt	0.25218534	8.78450393	2.70157168	0.11594855	0.12460308
Ube2l6	0.39672674	5.58533511	2.70137724	0.11596087	0.12460308
Chn1	0.18447549	9.71703491	2.7002908	0.11602976	0.12465924
Ireb2	-0.2579149	6.4449494	2.69978657	0.11606174	0.12467575
AI847159	2.24453669	-1.8989295	2.69949061	0.11608052	0.12467807
Cyp11a1	-1.0472778	-0.1870064	2.69828153	0.11615728	0.12474265
Tssc1	-0.3291437	3.27212638	2.69702729	0.11623697	0.124802
Slc25a53	-0.535489	2.57101255	2.69688809	0.11624582	0.124802
Slc25a47	-1.2501158	-0.6245567	2.69658058	0.11626537	0.12480512
Olfr1372-ps1	1.1229614	-0.054429	2.69476536	0.11638085	0.12491121
Tubgcp2	-0.3004044	3.66953994	2.69443255	0.11640203	0.12491608
Nutm1	-2.3046011	-1.5031532	2.69270318	0.1165122	0.12501642
Wls	0.35164519	5.22275416	2.69066261	0.11664235	0.12513817
Rbm7	0.30440018	5.12838101	2.68946451	0.11671885	0.12520233
11-Mar	0.66030444	1.34585352	2.68912317	0.11674065	0.12520778
Adam28	-1.0094625	0.2058532	2.68865221	0.11677075	0.12520778
Nup62	0.27325897	5.49699393	2.68830585	0.11679288	0.12520778
Pum1	-0.1826047	6.90389033	2.68817972	0.11680095	0.12520778
Setd1a	-0.2689432	5.5857919	2.68807893	0.11680739	0.12520778
Sstr2	0.45078409	2.75647597	2.68615747	0.11693031	0.12531595
Rpl8	0.32374265	5.66174501	2.68597925	0.11694172	0.12531595
Gbp6	0.30768362	5.06947314	2.68456176	0.1170325	0.12539533
AI182371	-3.4215507	-2.1088921	2.68359852	0.11709424	0.12542946
Smim24	0.64440815	2.27280505	2.68354309	0.1170978	0.12542946
Cc2d1b	-0.3588183	3.62334227	2.68272324	0.11715038	0.12546786
Gdpd1	0.25527167	5.43781772	2.68199325	0.11719722	0.12550011
Trim33	-0.1741439	7.7052077	2.68143418	0.11723311	0.12552063
Nebl	0.23705088	6.61374962	2.68089157	0.11726796	0.12553242
Kcnq5	-0.3078993	6.25302514	2.68069886	0.11728034	0.12553242
Cnr2	-0.75512	0.95599685	2.68048109	0.11729433	0.12553242
Ercc3	-0.291818	4.2118526	2.67996464	0.11732751	0.12555002
Arpc4	0.42150767	5.34592983	2.67894423	0.11739311	0.1255883
Il10ra	-1.2498726	0.14749932	2.67888742	0.11739677	0.1255883
Atg4c	0.25724	6.30646857	2.67835865	0.11743078	0.12560677
Ankrd35	0.4015833	2.76425955	2.67738249	0.1174936	0.12565605
Foxo1	0.22102363	7.15529798	2.67666039	0.1175401	0.12568786
Fbrsl1	-0.3014652	4.62818827	2.67582222	0.1175941	0.12572767
Ttc30a2	0.97404855	-0.1179123	2.67263728	0.11779955	0.12592939
Tnfrsf10b	-0.39888	3.26675785	2.6715215	0.11787163	0.1259633
Gm19689	3.37454367	-1.7117989	2.71483345	0.11787399	0.1259633

Elfn2	-0.3006681	5.01552476	2.67136653	0.11788164	0.1259633
Trmt61b	0.38296097	3.3221804	2.67021331	0.1179562	0.12602502
Rabggta	-0.4055024	3.21503695	2.66969945	0.11798944	0.12604258
Gm16287	-0.6450323	1.40048103	2.66901748	0.11803358	0.12606707
Prrx2	0.78671414	3.43939736	2.66850885	0.1180665	0.12606707
A730043L09I	2.15466388	-2.1676103	2.66835088	0.11807673	0.12606707
A930007I19F	-2.005844	-1.0315165	2.6679703	0.11810138	0.12606707
Dpt	1.32835454	0.73137947	2.66785771	0.11810867	0.12606707
Gnpda2	0.27400696	5.96309501	2.66774845	0.11811575	0.12606707
Ist1	0.25213562	5.83351638	2.66752828	0.11813001	0.12606707
Junb	0.92916097	1.53956335	2.66619949	0.11821614	0.12613826
Myb	-0.7828155	0.61825725	2.66598032	0.11823036	0.12613826
C1qtnf7	0.64277502	4.36202515	2.66481611	0.11830589	0.12616977
Ccl3	2.75814488	-2.0153871	2.66478298	0.11830804	0.12616977
Cnst	0.25434439	6.16085831	2.66474748	0.11831035	0.12616977
Resp18	0.33191123	3.1582388	2.66437908	0.11833426	0.12617733
AF357355	-2.6205046	-1.001034	2.66401855	0.11835767	0.12618085
Acvr2a	-0.2558845	6.16874614	2.66381023	0.1183712	0.12618085
Zfp30	0.33501601	3.69679721	2.66272995	0.11844138	0.12623772
Fam105a	0.41960155	3.79205502	2.66217829	0.11847724	0.12625381
Gm2027	-1.0297732	0.23977782	2.66197992	0.11849014	0.12625381
Mycbpap	-0.7708438	0.72901751	2.66157394	0.11851654	0.12626401
Tm6sf2	-1.7877126	-1.4528194	2.66048578	0.11858735	0.12631691
Pacs1	0.20713373	6.74546738	2.66029322	0.11859988	0.12631691
Reep6	0.25956547	4.20138878	2.65991831	0.11862429	0.12632497
Cops6	0.26399053	5.82043294	2.65932035	0.11866323	0.12634851
Camsap1	-0.2597319	6.94819669	2.6590229	0.11868261	0.1263512
Pcdhgb5	-0.5177027	2.4519966	2.65797162	0.11875112	0.1264062
Nmur2	-2.7319924	-1.6835497	2.65603822	0.11887725	0.12652251
Fbxl17	-0.2239531	8.00944252	2.65456653	0.11897336	0.12660684
Hist1h4i	1.10405419	-0.2487635	2.65415281	0.11900039	0.12661764
Dmkn	1.20451923	-0.2987898	2.65324289	0.11905988	0.12666297
Papolg	-0.1922402	5.32489383	2.65195503	0.11914414	0.12673463
Scd2	-0.2029907	8.33435945	2.65127457	0.11918868	0.12676404
Rnf40	-0.3825946	3.98065516	2.6510015	0.11920657	0.12676509
Mpnd	0.43146858	3.83602864	2.65025952	0.11925517	0.1267988
Tnfaip2	-0.8574439	0.47359873	2.64777817	0.11941789	0.12695363
Sycp3	-0.6130421	2.03633713	2.64752298	0.11943464	0.12695363
Rfk	0.41785531	9.48783152	2.6470088	0.11946839	0.12697152
Hmgn3	0.27618991	6.929729	2.64639955	0.1195084	0.12698328
AU022754	-1.2637026	-0.2459675	2.64632477	0.11951332	0.12698328
Bc1	-2.8862134	-1.8486001	2.64595561	0.11953757	0.12699106
Ebf4	-0.960692	1.13461649	2.64432269	0.11964492	0.12707
Lgr6	1.53482621	-0.8661721	2.64431008	0.11964575	0.12707
Mrps18a	0.47263566	3.38640429	2.64401068	0.11966545	0.12707292

Olfml3	0.41224481	4.9645008	2.64340576	0.11970525	0.12708919
Ascl4	1.94394913	-1.2625005	2.64326295	0.11971465	0.12708919
Dnajb3	1.40415668	-0.1025041	2.64300142	0.11973187	0.12708948
Anxa8	0.60596577	3.46734733	2.64242001	0.11977015	0.12711213
Rdm1	-0.6741609	2.51132514	2.64063054	0.11988807	0.12721928
Parp14	-0.4260362	4.15536348	2.64033022	0.11990788	0.1272223
Prdm15	-0.4157967	3.40894954	2.63837963	0.1200366	0.12732403
Fbln2	-0.8045168	0.80247329	2.63794404	0.12006537	0.12732403
Lrig2	-0.2476277	5.05686369	2.63766013	0.12008412	0.12732403
Wdr78	-0.5333278	3.27238946	2.63753754	0.12009222	0.12732403
D930015M05	-1.4694096	0.26353852	2.63735154	0.12010451	0.12732403
Emr4	-2.2198964	-1.2104886	2.63714285	0.1201183	0.12732403
Katnb1	0.48666477	2.94699057	2.63707822	0.12012257	0.12732403
Dlec1	-2.0294461	-0.3980741	2.63656931	0.12015621	0.12734169
Hist2h4	0.98414607	0.30175153	2.63389668	0.12033305	0.12751109
Ccdc78	-1.0014651	-0.2894292	2.63289851	0.12039918	0.1275561
Phf21a	-0.2230382	6.60498082	2.63274223	0.12040954	0.1275561
Nudt16	0.30914888	4.68686686	2.63173392	0.12047639	0.1276089
C030034L19R	-1.9876728	-1.2749103	2.63106937	0.12052047	0.12761673
Sepp1	0.36771079	8.0461809	2.63084984	0.12053504	0.12761673
Dennd1c	-1.5269058	-0.2605778	2.63081723	0.1205372	0.12761673
C78339	-0.4360769	2.77947753	2.63059672	0.12055183	0.12761673
Ptpn22	0.52798335	2.35806896	2.63005557	0.12058776	0.12761834
Erlin1	-0.2750297	4.56877923	2.62990223	0.12059794	0.12761834
Meaf6	0.2516646	5.01797315	2.6296944	0.12061174	0.12761834
Hepacam	-0.3768669	4.23256353	2.62954892	0.1206214	0.12761834
Odf2l	-0.4187329	3.49166624	2.62923026	0.12064257	0.12762273
Ccdc102a	0.72078999	1.41072203	2.62813679	0.12071523	0.12766808
Pdk1	0.2116867	6.19472714	2.62804141	0.12072157	0.12766808
Rps13	0.30047081	5.90492291	2.62781688	0.1207365	0.12766808
Faim	0.28227328	5.76111039	2.62695218	0.12079401	0.12771089
Dclk2	-0.3522117	4.01234502	2.62589045	0.12086467	0.12776759
Pcx	-0.526168	2.01039584	2.62551408	0.12088973	0.12776856
Zbtb3	1.43277067	-0.3909106	2.62529517	0.12090431	0.12776856
Kif4	-0.6813387	1.16208681	2.62510935	0.12091668	0.12776856
Akap17b	-0.2590172	5.61061691	2.62476984	0.1209393	0.12776939
Fam173b	0.66195031	0.83055761	2.62458617	0.12095154	0.12776939
Stx2	-0.4678856	2.63517198	2.62376583	0.12100621	0.12780418
Lzic	0.34322469	4.1531194	2.62358085	0.12101854	0.12780418
Isoc2b	0.80918215	0.67709782	2.62292019	0.1210626	0.12781455
Marcks	0.27128806	9.44935108	2.62274723	0.12107414	0.12781455
Klhl6	-1.0816314	0.02189517	2.62266715	0.12107948	0.12781455
Samd9l	0.35641407	6.92484062	2.62103366	0.12118852	0.1278941
Uqcrc1	-0.2190791	5.02004495	2.62102739	0.12118894	0.1278941
Npas2	-0.4068422	5.29330213	2.62054192	0.12122137	0.12791033

Trpv6	0.59165088	1.64659565	2.61978412	0.12127201	0.12794576
H2-Ab1	-0.6641622	4.25397424	2.61866112	0.1213471	0.12799329
Gm10658	1.32473301	-0.5900214	2.61860012	0.12135118	0.12799329
4732471J01F	-0.6120461	1.65435426	2.61737428	0.12143321	0.1280618
Htr2a	0.36712999	4.42755385	2.61600065	0.12152521	0.12814081
Kcna2	-0.3250696	8.75410394	2.61529036	0.12157281	0.12816468
Cnnm3	-0.2590788	4.18017385	2.61515307	0.12158202	0.12816468
Fbxl21	-0.3344283	3.14489174	2.61449652	0.12162605	0.12819308
Sp6	1.62314487	-0.8815398	2.61367188	0.12168137	0.12823337
Fam114a2	0.32206663	5.08308205	2.61323037	0.12171101	0.12824658
Mfap5	-0.9675829	2.15899869	2.61222154	0.12177875	0.12829994
5730420D15I	-1.9812436	-0.8912027	2.6114562	0.12183017	0.12832895
Fam135b	-0.506764	4.6382602	2.61130253	0.1218405	0.12832895
Sostdc1	1.09107825	0.73496236	2.61088841	0.12186834	0.12834025
Spred1	0.20428591	8.20895939	2.61056486	0.12189009	0.12834515
Slc16a9	0.40698367	4.83671912	2.60758391	0.12209075	0.12853838
Sertad3	-1.1031634	0.88470497	2.60672641	0.12214854	0.12857639
Snx29	-0.5255147	2.1760274	2.60653968	0.12216113	0.12857639
4933433G19	-0.7651718	0.90088046	2.60612063	0.12218939	0.12858809
Sipa1l2	-0.4041734	4.53337432	2.6047985	0.12227859	0.12866391
Kcnrg	-1.0310092	1.30895024	2.60326055	0.12238246	0.12873993
Fli1	0.39128533	4.25282813	2.6032205	0.12238516	0.12873993
Entpd7	-0.3824075	4.12638199	2.6028647	0.12240921	0.1287454
Trappc2l	0.48459359	3.37912869	2.60263572	0.12242468	0.1287454
Mettl7a3	-1.3876048	-1.7645304	2.60048105	0.12257044	0.12888061
Cbln2	-0.4183513	3.41947887	2.59955333	0.12263325	0.12890071
Tmeff2	0.22123341	6.92265816	2.59949303	0.12263734	0.12890071
Adamts8	-1.0331074	-0.3330181	2.59943742	0.12264111	0.12890071
A530032D15	0.78990689	0.8682512	2.59873145	0.12268894	0.12892643
Hist1h2bk	1.54051579	-1.1580995	2.59856895	0.12269995	0.12892643
2010106C02I	-2.7328064	-1.616105	2.59702872	0.1228044	0.1290181
D3Ertd254e	-0.3230303	6.52483606	2.59618043	0.12286197	0.12906051
Gmfg	0.96804009	1.11371591	2.59571227	0.12289375	0.12907582
Paqr5	-0.5648767	3.21834626	2.59424999	0.1229931	0.12916208
Zdhhc8	-0.3350282	4.78650998	2.59356561	0.12303962	0.12919192
Mrpl22	0.39715144	3.03106697	2.59332538	0.12305596	0.12919192
Gatad1	0.20855131	7.04557195	2.59047532	0.12324998	0.12937751
Fa2h	-0.4297269	4.01003315	2.59004366	0.1232794	0.12939028
Dpy19l1	-0.1991703	6.7235325	2.58906498	0.12334612	0.1294422
Ctu2	0.61851338	1.72551479	2.58838746	0.12339234	0.12947259
Msl1	-0.1734768	8.01020206	2.58803854	0.12341615	0.12947458
Sncb	0.31526419	4.66624454	2.58781759	0.12343123	0.12947458
Abca9	0.30210055	6.25692304	2.58760099	0.12344602	0.12947458
Cttn	0.22002795	5.83338286	2.58547961	0.12359094	0.12960744
Arhgap5	-0.2209435	8.64702615	2.58524122	0.12360724	0.12960744

R74862	0.27681991	4.58028853	2.58466962	0.12364633	0.1296303
Gm14305	0.42714636	2.61102588	2.58289158	0.12376802	0.12973441
Twist2	0.92954795	0.8075385	2.58271342	0.12378022	0.12973441
Impg1	-1.0657102	0.27161682	2.58216027	0.12381811	0.12975599
Agpat6	0.28335285	4.13480936	2.58148047	0.12386469	0.12978668
Zkscan4	-0.5008109	2.2997498	2.58094033	0.12390172	0.12980735
Thrsp	0.56166788	3.17040787	2.5806745	0.12391995	0.12980832
Mal	-0.3035204	6.0909116	2.57962171	0.12399218	0.12986584
Clpx	0.21446621	5.3907089	2.57845969	0.12407196	0.12993125
Sema4d	-0.3904503	3.84624337	2.5777601	0.12412001	0.12996344
Psmg2	0.41203046	3.21681785	2.57716858	0.12416067	0.12998786
B230216G23	-1.4383343	-0.2643548	2.57512935	0.12430094	0.13010092
Bcar1	0.26042323	4.23651133	2.57496241	0.12431243	0.13010092
Otud5	0.22551386	5.85476213	2.57456264	0.12433995	0.13010092
Fam32a	0.25727158	6.0705098	2.57442719	0.12434928	0.13010092
Dennd4a	-0.2135683	7.418427	2.57424894	0.12436155	0.13010092
Stxbp5	-0.3207219	7.72459988	2.57408678	0.12437272	0.13010092
Zfp637	0.37778617	3.80381429	2.57159724	0.12454434	0.13026227
Dimt1	-0.290933	5.04268064	2.57083896	0.12459666	0.13029883
1110017D15I	-1.0313547	-0.1132766	2.56949405	0.12468954	0.13036678
Foxl1	0.97463026	0.5607768	2.56917645	0.12471148	0.13036678
Armc9	-0.3821047	3.36893747	2.56914334	0.12471377	0.13036678
Cwc15	0.27400161	6.787145	2.56859034	0.12475199	0.13038856
Wdr31	0.60271074	1.78852451	2.56816827	0.12478117	0.13040089
Dusp4	0.80635565	1.31826327	2.56567183	0.12495394	0.13056325
Myg1	0.43809078	3.22637071	2.56418389	0.12505705	0.13065278
Manbal	0.39908217	3.04796041	2.56279451	0.12515342	0.13073526
Lsr	0.45105512	3.45580772	2.5624784	0.12517535	0.13073997
Ift80	-0.2285399	4.88958403	2.56162487	0.12523461	0.13075832
Polr1d	0.28286916	6.27355112	2.56142618	0.12524841	0.13075832
Prpf18	0.24434896	4.94499553	2.56141129	0.12524945	0.13075832
Cpeb4	-0.2569696	8.15948239	2.56122111	0.12526266	0.13075832
Vamp3	0.30814866	7.50340041	2.55934135	0.12539332	0.13086766
Zfp239	0.22627195	5.66201513	2.55900486	0.12541672	0.13086766
Actr3b	-0.2406651	4.85592865	2.55896151	0.12541974	0.13086766
D10Jhu81e	0.32163373	3.58095012	2.55813021	0.12547759	0.13090982
0610031J06F	0.46673687	4.49173626	2.5577841	0.12550168	0.13091675
Rpn1	0.27163863	5.65877509	2.55737314	0.1255303	0.13092839
Nfil3	0.53971286	2.54597835	2.55655068	0.12558759	0.13096994
Spc25	0.7119462	1.57807401	2.55397631	0.12576713	0.13113893
Jag2	-0.6784646	0.88017764	2.55260721	0.12586273	0.13121323
Ppil3	0.41463257	3.79873045	2.55228286	0.12588539	0.13121323
Olfr691	2.0490022	-2.0704792	2.55220474	0.12589085	0.13121323
Cib2	0.67364822	2.27751293	2.55065309	0.12599934	0.13130805
Stat6	0.29501076	5.05259947	2.54962464	0.1260713	0.1313648

Clptm1	0.23684959	6.04131309	2.54916824	0.12610326	0.13137985
Lect1	-1.1640406	-0.5496984	2.54884878	0.12612563	0.13138491
Armc10	0.34785482	4.74546226	2.54772048	0.12620468	0.131449
Rin3	0.42340488	4.36278488	2.54734093	0.12623128	0.13145846
Zfp112	-0.6042348	2.14004169	2.54678012	0.12627061	0.13146892
Ehmt1	-0.3988222	4.40796228	2.54669784	0.12627638	0.13146892
Cybrd1	-0.5113907	2.11232125	2.54620387	0.12631103	0.13148674
Gpr125	-0.4465606	3.35494586	2.54510906	0.12638787	0.13154847
Rpp25l	0.60549146	2.05738718	2.54396237	0.12646841	0.13160982
Kcng4	0.62494427	1.67984873	2.54377039	0.1264819	0.13160982
Bmp8b	-2.2137556	-1.422884	2.54343872	0.12650521	0.13161582
Yme1l1	-0.2102926	6.23404001	2.54293328	0.12654074	0.13161844
Ippk	-0.2969157	3.8158183	2.54290362	0.12654282	0.13161844
Slc36a1	0.26361503	4.99323155	2.5415961	0.1266348	0.13169585
Insl5	-1.7921718	-1.3287275	2.58011969	0.12672468	0.13177105
Dlg3	-0.2815419	6.55474967	2.53961773	0.12677411	0.13180418
Glg1	-0.2256878	6.66224155	2.53723158	0.12694239	0.13196084
Adcy9	-0.2675341	6.22779904	2.53635508	0.12700427	0.13198222
Camk2n2	-0.2642956	4.57960333	2.53628824	0.12700899	0.13198222
Trp53bp2	-0.2131378	5.93993044	2.53606214	0.12702495	0.13198222
Socs2	0.27537527	5.3342442	2.53583708	0.12704085	0.13198222
Phf11b	1.10731746	0.71371584	2.53569453	0.12705092	0.13198222
Gm12942	-0.6453936	2.89548821	2.53335465	0.12721636	0.13213578
Kdm5c	-0.2897544	5.20889389	2.53244238	0.12728093	0.13218454
Smg6	-0.2300019	5.6233263	2.53114022	0.12737316	0.13226202
Ttc39b	-0.2791385	7.09488054	2.52872466	0.12754448	0.13241337
Pfdn4	0.39475747	3.92869864	2.52858722	0.12755423	0.13241337
Sgsm1	-0.468285	4.53170126	2.52738181	0.12763983	0.1324839
Armc8	0.20967038	7.05120599	2.52690651	0.1276736	0.13250062
Abcb7	-0.2745651	5.77587624	2.52657033	0.12769749	0.13250708
Mad2l1	0.38539297	4.07790964	2.52616979	0.12772596	0.13251829
Phyhd1	-0.505889	2.26409937	2.52418822	0.12786694	0.13264621
Fchsd1	-0.5492863	2.02506794	2.52349167	0.12791654	0.13267932
Hey2	-0.4691162	2.26917077	2.52253314	0.12798483	0.1327318
Epb4.1l2	0.21644511	7.5391637	2.52128595	0.12807375	0.13280566
Zfp658	-0.4634549	2.26400875	2.52093209	0.128099	0.13281219
Cdc42se2	0.18235463	6.29738969	2.52070127	0.12811546	0.13281219
Kcnk1	-0.253074	5.55611367	2.51997599	0.12816723	0.1328326
Ext2	0.5410742	5.05571587	2.51992927	0.12817056	0.1328326
Pkp4	-0.1898004	8.59015621	2.51948379	0.12820237	0.13284721
Sufu	0.2794214	4.10405418	2.51922227	0.12822105	0.13284821
Tigit	-1.9100701	-1.5637393	2.51638997	0.12842354	0.13303963
Dcaf12l2	1.2672712	-0.1083279	2.5156353	0.12847756	0.13307721
Eif3f	0.2553354	5.99474716	2.51273111	0.12868569	0.13327439
Spata33	1.4191003	-0.1889386	2.51105273	0.12880615	0.13336338

Mlec	0.20817456	6.89116658	2.51072062	0.12883	0.13336338
Zfp108	-0.4059924	3.07925753	2.51060398	0.12883838	0.13336338
Pcdha9	-1.3724909	0.22596979	2.51054352	0.12884273	0.13336338
Zfp446	0.37221839	3.31004203	2.51018399	0.12886856	0.13337171
Car12	0.40670928	3.31071986	2.50980096	0.12889608	0.1333818
Rps28	0.36586272	5.54608439	2.50919788	0.12893944	0.13339461
Tmx2	0.25229694	5.98579548	2.50913408	0.12894403	0.13339461
Dna2	-1.511145	-0.6860066	2.50793699	0.12903014	0.13345329
Endog	1.36665269	-0.8303844	2.50785092	0.12903633	0.13345329
Ap5z1	-0.5756836	2.61958372	2.50665203	0.12912265	0.13352415
Mlf2	0.18084529	6.45549964	2.5055129	0.12920473	0.13359061
Disp2	-0.2662504	7.01997622	2.5049098	0.12924821	0.13361715
Lct	-1.2689262	-0.2845821	2.50365403	0.1293388	0.13367846
Gpbp1l1	0.24654346	6.29620693	2.50359369	0.12934315	0.13367846
Rdh18-ps	-0.7430385	0.35944236	2.5026819	0.12940898	0.13372806
Zfp947	0.46853336	2.25516998	2.50209667	0.12945125	0.13375332
Efcab4b	-1.4711738	-1.4068119	2.50157887	0.12948866	0.13375979
Lrrc24	-0.6321145	0.76078617	2.50151641	0.12949318	0.13375979
Ugp2	0.18608902	6.43308734	2.49992511	0.12960824	0.13386022
Kif2a	0.19891426	7.65851713	2.4989575	0.12967827	0.13391411
Cars2	-0.5125304	2.19304104	2.49787898	0.12975638	0.13396317
Prkd3	0.25192753	5.66350149	2.49780825	0.12976151	0.13396317
Slc2a3	0.2533996	5.24964401	2.4972941	0.12979876	0.13398253
Kank4	0.43384426	3.92980639	2.49699335	0.12982056	0.13398253
Veph1	-1.1025094	-0.1458469	2.49681017	0.12983384	0.13398253
Cspg4	-0.4233465	2.74830297	2.4965635	0.12985173	0.13398256
Parn	-0.2464823	4.67553841	2.49629893	0.12987091	0.13398392
Ddx41	-0.3378997	3.8381311	2.49531467	0.12994232	0.13401455
Med22	0.32159087	3.78866602	2.49528834	0.12994423	0.13401455
Hsd3b7	0.39381212	2.68274283	2.49515099	0.1299542	0.13401455
Ahr	-0.346733	4.19486177	2.4946738	0.12998884	0.13403185
Creld2	-0.4600448	3.22286523	2.49438302	0.13000995	0.13403519
Antxr1	-0.2847159	6.12795934	2.49282993	0.13012279	0.13410757
Cd151	0.53806892	5.12328317	2.49270886	0.13013159	0.13410757
Gpr22	0.27753522	5.80525701	2.49247555	0.13014855	0.13410757
Med9	0.33514804	4.76136036	2.49241428	0.13015301	0.13410757
Uox	1.25050932	-0.8076337	2.49215561	0.13017182	0.13410757
Lrrc75b	-0.351532	4.44886064	2.49176146	0.13020049	0.13410757
Hsd17b7	-0.231992	5.00823565	2.49169524	0.1302053	0.13410757
Slc22a4	0.35408213	3.02269968	2.49062332	0.13028331	0.1341695
Mx2	0.9312918	0.06366285	2.48998244	0.13032997	0.13419913
Runx1	-0.511977	3.56880583	2.48813016	0.13046496	0.13431968
5930403L14F	-0.4392639	4.76609012	2.48786955	0.13048397	0.13432082
Zfp318	-0.3375001	6.73134452	2.48751181	0.13051006	0.13432924
Ppp2r5b	0.34083071	3.29609995	2.48704515	0.13054411	0.13433273

Itgav	-0.2178227	5.95813429	2.48664926	0.130573	0.13433273
Ybx3	0.33075998	7.39449024	2.48660829	0.13057599	0.13433273
Elovl4	0.36025968	4.81666935	2.4864838	0.13058508	0.13433273
Gm7854	-1.13859	0.00976066	2.48616201	0.13060857	0.13433848
Mta3	-0.2623546	5.56696149	2.48488484	0.13070187	0.13439503
Abca3	-0.3353628	4.11377564	2.48468914	0.13071617	0.13439503
Fosb	0.57438581	4.38460971	2.48433548	0.13074202	0.13439503
Dlg2	-0.274416	9.50865062	2.48411706	0.13075799	0.13439503
Kcnd3	-0.3390583	5.96922766	2.48410479	0.13075889	0.13439503
Mad2l1bp	0.5929564	1.72148209	2.48393833	0.13077106	0.13439503
Cd4	-0.6593801	3.24034563	2.48189464	0.1309206	0.13453029
Rps7	0.22681196	6.62288406	2.48087258	0.13099547	0.13457894
Nup50	-0.215732	5.45782005	2.48075332	0.13100421	0.13457894
Prdm12	-1.5176279	-0.1195421	2.48051367	0.13102177	0.13457894
Rwdd4a	0.24138532	5.61345418	2.48006302	0.13105481	0.13459444
Id3	0.46640543	6.0191475	2.47966596	0.13108392	0.13459791
Lpin3	2.51435887	-1.554597	2.47952761	0.13109407	0.13459791
Atxn7l1	0.2010316	6.14296575	2.47924013	0.13111515	0.13460114
Ago3	-0.2517502	6.0504347	2.47877079	0.13114959	0.13460717
Rpl39	0.37893906	7.34060567	2.47867084	0.13115692	0.13460717
Jade2	-0.2159198	5.26676133	2.47752938	0.13124072	0.13467474
Plscr1	0.4107036	4.17838695	2.47570736	0.1313746	0.1347937
Gemin5	-0.4126172	3.93796006	2.47542888	0.13139508	0.13479627
4933408N05	-1.4625985	-1.0389726	2.47457612	0.13145781	0.13484218
Fstl5	-0.3466662	4.1514189	2.4743098	0.13147741	0.13484385
Psmd11	0.29019047	5.94030805	2.47394462	0.13150429	0.13485298
Hpse	2.00617371	-1.4409293	2.47182784	0.13166023	0.13499002
Suds3	0.29913192	5.04632402	2.47161073	0.13167624	0.13499002
2210039B01I	-0.5548615	1.77921097	2.47139806	0.13169192	0.13499002
Efhc1	0.58074415	1.78039606	2.47070904	0.13174274	0.13502366
Rab30	0.24590148	4.8877884	2.47032184	0.13177131	0.13503449
Tex2	0.17725829	6.54955136	2.46932165	0.13184514	0.1350917
Spink8	1.12867085	-0.1941397	2.46880358	0.1318834	0.13511245
Nucks1	0.20040782	8.85000621	2.46851223	0.13190493	0.13511537
Rilp	1.2495408	-0.4836868	2.46826157	0.13192345	0.13511537
Fcrls	-0.7749391	1.54344306	2.46803366	0.13194029	0.13511537
Scube1	0.22683746	5.95682339	2.46766455	0.13196757	0.13511904
Dkc1	0.19705471	6.20082846	2.46739314	0.13198764	0.13511904
Dusp18	0.27101224	5.35682692	2.46725416	0.13199792	0.13511904
Jph1	-0.3339112	4.82841873	2.4659959	0.132091	0.13516922
Tmem129	0.38125103	3.06683303	2.46596808	0.13209306	0.13516922
Ttc39c	0.47452347	2.23883921	2.46566887	0.1321152	0.13516922
Sec62	0.20360827	7.55522414	2.46548746	0.13212863	0.13516922
Prss48	-1.7769729	-0.1717178	2.4650914	0.13215796	0.13516922
Rnf145	-0.2124885	5.73026321	2.46500736	0.13216418	0.13516922

Msx1	0.60872049	2.36231787	2.46488734	0.13217307	0.13516922
Erbb2ip	-0.1710106	7.94454887	2.46460283	0.13219414	0.13517234
Rgs8	0.28940612	6.68073307	2.46388451	0.13224737	0.13519071
Dab2ip	-0.2018598	6.24974934	2.46387394	0.13224815	0.13519071
Sim1	-1.3154148	-0.605711	2.46250012	0.13235002	0.13527641
Ly6e	0.209906	6.82283664	2.46100341	0.1324611	0.1353715
Noc3l	0.26759703	4.93042067	2.45995896	0.13253868	0.13543234
Mbip	-0.440749	3.81516301	2.45920327	0.13259485	0.13547128
Elof1	0.49458274	3.1789434	2.45875941	0.13262786	0.13548654
Arhgef5	0.38268142	5.78450739	2.45850708	0.13264662	0.13548726
Ptar1	-0.3098458	3.79022026	2.45725175	0.13274003	0.13554584
Vwc2l	0.42266147	3.42519573	2.45725073	0.13274011	0.13554584
Gm13710	0.88089302	0.50755034	2.45595575	0.13283655	0.13562586
Lpl	-0.4429438	4.20486379	2.45465476	0.13293353	0.1357064
BC029722	0.61361062	2.07928093	2.45428853	0.13296084	0.13571582
Anp32b	0.25583685	8.28371329	2.4536991	0.13300482	0.13572926
Ogfod1	-0.2205746	7.29648385	2.45362702	0.1330102	0.13572926
Neu1	0.3430489	3.96564936	2.45274508	0.13307603	0.13577796
Car8	0.4628517	2.79708353	2.45237293	0.13310382	0.13578785
2700070H01	-1.3174109	-1.1984218	2.45091886	0.13321248	0.1358802
Orc2	-0.2254152	5.78244805	2.45067671	0.13323058	0.1358802
AW549542	-0.9789063	-0.2372378	2.45017274	0.13326827	0.13590017
P2rx3	-0.7189811	1.27183356	2.44985822	0.1332918	0.13590568
Wnk4	0.47265322	4.36271897	2.44922018	0.13333954	0.13593589
Polq	-0.7093615	2.63055267	2.44714125	0.13349525	0.13607023
2210016F16	0.31588408	5.04382769	2.44697645	0.1335076	0.13607023
Gja3	3.15277825	-1.8770498	2.44644545	0.13354741	0.13609232
Piwil4	1.53480729	-0.897219	2.44589655	0.13358858	0.13611532
Slc35f2	-1.2163891	0.09581704	2.4456607	0.13360627	0.13611532
Cyp4f13	-0.6089734	1.16738394	2.44437168	0.13370302	0.13619539
Rgs9bp	1.59669426	-0.2906914	2.44380125	0.13374587	0.13622053
Krtcap2	0.34369247	3.33774937	2.44336059	0.13377897	0.13622656
Pars2	0.59742349	1.84573629	2.44323905	0.1337881	0.13622656
Melk	1.2298474	0.17902448	2.44214787	0.13387013	0.13629158
Nr1h3	0.6034195	2.61585111	2.44151748	0.13391755	0.13632135
Ube2t	1.69128779	-1.0713827	2.44120462	0.13394109	0.13632682
Sox11	-0.3264945	4.87642157	2.4398558	0.13404264	0.13640419
Pkdcc	-0.8489149	0.20160882	2.43971182	0.13405348	0.13640419
Tbx22	1.80656644	-1.4582454	2.43856839	0.13413964	0.13647335
Gm14057	-0.8910939	2.1774852	2.43774042	0.13420208	0.13651836
Lgalsl	0.17949812	6.23959162	2.43612762	0.13432379	0.13662364
Slx4	-0.2710653	5.16651281	2.43559868	0.13436373	0.13664574
Ptcd3	-0.3204259	5.21562764	2.43324509	0.13454165	0.13680813
Fam129b	0.38054888	5.3393645	2.4306964	0.13473464	0.13697647
Sirt1	-0.3201658	4.47245671	2.43057657	0.13474372	0.13697647

Prmt6	0.37286628	3.52929591	2.42930002	0.13484051	0.13705629
Eif4g1	-0.1743632	7.06173756	2.42863839	0.13489071	0.13708874
Ghitm	0.17313983	8.70164854	2.42826471	0.13491907	0.13709899
Rnf128	-0.5902365	1.67447049	2.42789755	0.13494694	0.13710874
Nkain4	-0.7287387	1.74106904	2.42666053	0.13504091	0.13718562
Heg1	-0.2494082	6.24919506	2.42486464	0.13517746	0.13730575
Zfp865	0.26747039	4.44992551	2.42444293	0.13520955	0.13731975
Pcp2	2.85477954	-1.5685669	2.42378588	0.13525956	0.1373341
Scarb2	0.40962479	4.27595107	2.42377634	0.13526029	0.1373341
Plekho2	0.38950766	4.99004047	2.4233244	0.13529471	0.13735045
1110007C09I	0.52137345	2.33907643	2.42258259	0.13535122	0.13738922
Gjb6	0.3604893	8.86000288	2.42220553	0.13537995	0.1373998
Gm867	-2.3509646	-1.991753	2.42187947	0.13540481	0.13740643
A430005L14I	0.4289702	2.80963571	2.42078991	0.1354879	0.13747216
Nim1k	0.34027694	4.19746749	2.42028926	0.1355261	0.13749232
Ccdc24	1.70319406	-0.9110974	2.41880367	0.13563954	0.13758879
Bcl11a	0.22446453	6.56535469	2.41713476	0.1357671	0.13769957
Epha10	-0.8667022	2.34216075	2.41597657	0.13585572	0.13777082
E130102H24I	-1.2792253	-1.0345136	2.41560438	0.13588421	0.13778109
Arfgap1	0.29472317	4.53177529	2.41360891	0.13603708	0.13790594
Fbxl22	-1.1220731	-0.6497519	2.4135173	0.13604411	0.13790594
Ganc	0.31252854	4.43255697	2.41205114	0.13615657	0.13800129
Map3k12	-0.2827872	5.62385011	2.41137967	0.13620811	0.13803488
Srsf12	0.34608887	4.02511157	2.41049721	0.13627589	0.13808491
Arnt2	-0.1876318	8.03920942	2.40908642	0.13638432	0.13817612
Wdr27	-2.0851169	-0.7713851	2.40781996	0.13648175	0.13825615
Rwdd3	-0.4946059	2.62408244	2.4065719	0.13657784	0.13833481
Zfp185	-0.5102123	3.37363519	2.40492311	0.13670492	0.13843168
Syt15	-0.8193483	1.38226934	2.40485221	0.13671039	0.13843168
Olfr692	-1.9822683	-1.3157961	2.40311518	0.13684442	0.1385487
Pcdhb13	-0.4615199	3.50224641	2.40227104	0.13690961	0.138596
Vdr	0.75478235	0.75906916	2.40200147	0.13693044	0.13859837
Ppfibp2	0.30037442	3.58271338	2.40153423	0.13696655	0.13861622
Hist1h2be	0.45488118	2.78253402	2.40106172	0.13700307	0.13863448
Cpsf6	-0.2901668	5.58266669	2.40058264	0.13704012	0.13863532
Ipmk	-0.22609	5.56169021	2.40057299	0.13704087	0.13863532
Dmtf1	0.20530948	6.44770029	2.3997709	0.13710292	0.13867456
Gdap1	-0.2971496	7.46345264	2.39959377	0.13711663	0.13867456
Cebpd	0.700187	1.35921377	2.39831886	0.13721534	0.13875568
Vstm5	0.52705942	2.30334241	2.39708586	0.13731089	0.13883359
Sowaha	-0.2525568	7.44815938	2.39586487	0.13740558	0.13891061
Rnf138rt1	-1.6122833	-1.5642962	2.39542666	0.13743959	0.13892626
Golga2	0.22342633	5.49769387	2.39423959	0.13753175	0.13895477
Gm13152	-0.7511172	1.28296953	2.39416713	0.13753738	0.13895477
Bet1l	0.49236629	3.14726464	2.39411176	0.13754168	0.13895477

Paxip1	-0.1845407	6.18251122	2.39410917	0.13754188	0.13895477
Col4a6	0.46471441	2.4222693	2.39369725	0.13757389	0.13896838
Fam46c	-0.4299904	2.4252796	2.39280396	0.13764332	0.1390198
Rnasel	-0.2722249	4.6797789	2.39179795	0.13772156	0.13903969
Lpin1	-0.2707043	4.6454142	2.39154453	0.13774128	0.13903969
Utrn	-0.188259	7.75997577	2.39133905	0.13775727	0.13903969
Bcap31	0.33131902	5.73327821	2.39122513	0.13776614	0.13903969
Tmc8	-1.8082856	-1.0856689	2.39106166	0.13777886	0.13903969
Cpsf7	-0.2605088	5.71114193	2.39096531	0.13778636	0.13903969
Cdv3	0.17996679	7.74731071	2.39088313	0.13779276	0.13903969
Alpk1	-0.4743384	2.5294278	2.39008722	0.13785474	0.13907558
Kcnmb2	-0.827308	0.84059901	2.38995022	0.13786541	0.13907558
Gtf2h4	0.65230902	1.44532771	2.38969053	0.13788564	0.13907729
Blcap	0.31862372	5.14326123	2.3894037	0.13790799	0.13908113
Lrp5	0.61559405	3.28031446	2.38826756	0.13799656	0.13915175
Tmem154	0.51381694	3.39839812	2.38710726	0.13808709	0.13919666
Bcs1l	-0.3692398	2.77376128	2.3870703	0.13808997	0.13919666
U2af1	-0.2975466	4.10283801	2.38698321	0.13809677	0.13919666
1110019D14I	0.40460016	2.68830163	2.38582406	0.13818729	0.13926918
Ift140	-0.5225358	3.26109083	2.38485273	0.13826319	0.13932696
4933430I17R	1.73218162	-1.6878358	2.38385593	0.13834114	0.13938678
Adamts2	-0.4176579	3.97568395	2.38320966	0.1383917	0.139419
Gins3	0.74572993	1.21726451	2.38286147	0.13841895	0.13942773
9330133O14	0.34666378	4.07579386	2.38214034	0.13847541	0.13944046
Chrnb4	-0.8447971	0.14843537	2.38194392	0.1384908	0.13944046
Rpp25	0.31122665	3.94710413	2.38178226	0.13850346	0.13944046
Zc3h8	-0.3866435	2.7597881	2.38168849	0.13851081	0.13944046
Ndufs2	0.24876223	6.6411601	2.38151326	0.13852453	0.13944046
Tnks1bp1	-0.3474633	3.62382755	2.38041892	0.1386103	0.13950807
Gbp2	0.48255009	4.51041072	2.38006905	0.13863774	0.13951697
Fth1	-0.1820388	9.06580264	2.37927753	0.13869983	0.13956073
6330408A02I	-0.2604357	3.61196677	2.37886503	0.1387322	0.13957458
Mtmr2	-0.2236748	6.65554123	2.37858086	0.13875451	0.1395783
Slc4a9	2.60372867	-1.3931945	2.37786065	0.13881106	0.13961647
Klk11	-3.0013868	-1.816411	2.41226976	0.13890255	0.13967945
Mvp	0.43069453	3.7879624	2.37658974	0.13891092	0.13967945
Sh2d2a	-0.6791487	1.19444342	2.37573916	0.13897781	0.13971411
Aldh3a2	0.18469246	6.15963536	2.37567771	0.13898264	0.13971411
Cytl1	1.83520211	-0.5109423	2.37432876	0.13908879	0.13980209
Pcp4l1	0.30209789	6.03176788	2.37333783	0.13916684	0.13986179
Shroom3	0.36959144	2.89460344	2.37269675	0.13921736	0.13989373
Chac2	0.36403673	3.90892416	2.37246127	0.13923592	0.13989373
A230056J06I	1.62462891	-0.1013602	2.37191405	0.13927906	0.13991834
Cenpq	-0.6206023	2.30652887	2.37068444	0.13937607	0.13999704
Ccl25	-0.7011869	1.70971134	2.37043657	0.13939564	0.13999795

08-Sep	-0.2226537	5.95391556	2.37017183	0.13941654	0.14000019
Snapc3	0.24429192	4.89748963	2.3692368	0.13949038	0.1400556
Dalrd3	0.42280707	3.33754619	2.36839348	0.13955703	0.14008659
Zdhhc9	0.22996568	6.30578159	2.36814935	0.13957633	0.14008659
Clcn4-2	-0.1897227	6.19279501	2.36813735	0.13957728	0.14008659
Zfp963	-0.4171335	2.52966685	2.36708129	0.1396608	0.14014435
05-Mar	0.1699877	6.50440038	2.36688873	0.13967604	0.14014435
Hnrnpr	0.16778958	7.8987067	2.36650187	0.13970665	0.14014435
Gosr1	0.26026071	5.27713659	2.36646517	0.13970956	0.14014435
Abcf2	0.24105201	5.35137765	2.36594506	0.13975073	0.14016691
Iqgap3	1.86024207	-0.6190251	2.36358961	0.13993739	0.14033536
Ptprv	-1.5216946	-0.9318184	2.36298542	0.13998532	0.14036347
Slc5a12	-0.9699555	0.11531533	2.36276452	0.14000284	0.14036347
Slc52a2	-0.9142141	0.70323119	2.36237452	0.1400338	0.14037574
F630042J09F	-1.5411786	-0.9915842	2.36185804	0.1400748	0.14039808
Cstf2	0.27262039	6.37257671	2.36081653	0.14015752	0.14045549
Phf23	0.30446668	4.64131352	2.36066559	0.14016952	0.14045549
Kdm6b	-0.2927204	5.61535267	2.3602481	0.1402027	0.14046997
4933439C10I	-0.6063197	1.82049563	2.35692477	0.14046717	0.14071615
Cyp2s1	0.55709862	3.8419522	2.35623721	0.14052196	0.14075224
Drg2	0.29324037	3.88772029	2.35499567	0.14062096	0.14083259
Tnni2	1.77662904	-1.3371784	2.35359428	0.1407328	0.14092086
Pcca	0.23352655	5.77101254	2.35342067	0.14074667	0.14092086
Fank1	-0.7175595	1.2666404	2.35239467	0.14082863	0.14098411
Acsl5	0.21269422	5.63286945	2.35201034	0.14085935	0.14099605
Slit2	-0.2164839	5.82072371	2.34983304	0.14103352	0.14115155
Adam18	-1.6671456	-0.7034302	2.34834818	0.14115246	0.14125174
Ankrd13a	0.24414933	4.90859285	2.34619839	0.14132486	0.1414054
Ap4s1	0.19982187	5.86757563	2.34592163	0.14134707	0.14140876
Tyw1	-0.6255425	2.0133922	2.34504564	0.14141741	0.14146026
Ica1l	-0.2214575	5.02285201	2.34074698	0.14176317	0.14178722
Zfp651	-0.3084535	4.59660151	2.33873354	0.14192547	0.14193063
Crebzf	-0.2743585	5.31094743	2.33845305	0.1419481	0.14193225
Gm20187	-1.212151	0.42513865	2.33824435	0.14196494	0.14193225
Rpl41	0.33140295	8.18340747	2.33765167	0.14201277	0.14196116
Tpcn2	-1.2440401	-0.307709	2.33622169	0.14212826	0.14204261
Ahnak	0.33832263	9.0753298	2.33596806	0.14214876	0.14204261
1700088E04I	2.29315127	-1.5563058	2.33593959	0.14215106	0.14204261
Vps26a	0.17878661	7.2527483	2.33488197	0.14223657	0.14209918
Tmem45a	-0.5780781	2.20741099	2.33454414	0.14226389	0.14209918
Ncs1	0.2460026	6.14444869	2.33453656	0.1422645	0.14209918
Oxct1	-0.1845841	8.33302897	2.33423536	0.14228887	0.1421046
Chtop	0.20659109	7.48466396	2.33322738	0.14237046	0.14215442
BC065397	-0.8783288	0.92368029	2.33291672	0.14239562	0.14215442
Atraid	0.2620365	4.54314961	2.33291672	0.14239562	0.14215442

Fam228b	-0.7595143	1.62913522	2.33126658	0.14252933	0.14226733
Nbn	0.23202321	4.74914227	2.33105296	0.14254665	0.14226733
Plbd1	-0.8897769	0.95484593	2.33051811	0.14259003	0.14228613
Bend3	-0.3619137	4.24324062	2.33026719	0.14261039	0.14228613
Ccdc129	1.09036706	-0.2345757	2.33008627	0.14262507	0.14228613
Snhg18	0.40262455	4.28839592	2.32988549	0.14264136	0.14228613
Rps29	0.24060071	6.7774653	2.32948621	0.14267377	0.14229954
Zfp532	-0.2072898	5.64739454	2.32729025	0.14285216	0.14245852
Gm19395	-2.1261691	-0.4158174	2.32571573	0.14298024	0.14256729
Chrm4	-0.5220467	2.00723448	2.32523791	0.14301913	0.14258712
Phrf1	-0.2828348	4.78880025	2.32379105	0.14313698	0.14268565
Ppp2r2d	0.24769707	4.71076883	2.32353866	0.14315755	0.14268719
Gspt1	0.16580684	7.58456715	2.32277491	0.14321982	0.14273029
Akap1	-0.3681169	3.33678222	2.32081565	0.14337971	0.14287065
Arfgef1	-0.2270297	7.73294247	2.32048625	0.14340661	0.14287848
Mtfmt	-0.4071484	3.0333309	2.31941577	0.14349408	0.14294664
Afap1	-0.2833909	7.61847143	2.3190922	0.14352053	0.142954
Fancg	-0.4277325	2.75922905	2.31682057	0.14370639	0.14311045
Tmem176a	0.49051731	4.32210892	2.3167063	0.14371575	0.14311045
Kcna1	-0.253047	7.66046982	2.31588162	0.1437833	0.14313999
Ak7	0.59647625	1.73166403	2.31587826	0.14378358	0.14313999
Slc30a9	-0.1994604	6.51316511	2.31521596	0.14383786	0.14317502
Vps37a	0.24460942	7.7321129	2.31467909	0.14388187	0.14319984
L3mbtl2	-0.2947381	3.73725995	2.31274585	0.14404051	0.14332966
Pfkl	0.28420585	4.18051187	2.31254585	0.14405694	0.14332966
Zdhhc1	-0.3458662	3.24992215	2.31222622	0.14408319	0.14332966
Hddc3	0.37774092	2.87659112	2.31215854	0.14408875	0.14332966
Ric3	0.2172812	6.1222974	2.31060034	0.14421682	0.14343804
Sox4	0.38140487	2.90899654	2.30981316	0.14428157	0.14348341
Ptprc	-0.6121602	1.9383955	2.30735305	0.14448416	0.14366583
Tbc1d32	-0.3449046	4.73628723	2.30670117	0.1445379	0.14370022
Gemin6	0.33632173	2.96628022	2.30617963	0.14458091	0.14372393
Kin	-0.2984551	4.30831348	2.30471235	0.144702	0.14381944
Maff	0.44927236	3.06441849	2.30455088	0.14471533	0.14381944
Pank2	0.23881729	5.50651976	2.30223893	0.1449064	0.14399025
Arhgap36	-0.9235442	0.91719473	2.30137906	0.14497754	0.14403464
Desi2	0.20277333	6.81310931	2.30118349	0.14499372	0.14403464
Stil	-0.7974455	0.88091538	2.30100279	0.14500868	0.14403464
Gm20139	-1.1768465	-0.1396949	2.30046563	0.14505315	0.14405054
Dapk2	0.74196541	0.52057744	2.30019844	0.14507528	0.14405054
Dlg4	0.19997235	7.45345862	2.29988239	0.14510146	0.14405054
Serhl	-1.1082711	-0.4036211	2.29988195	0.1451015	0.14405054
Dsc3	2.20523516	-1.3625269	2.29847976	0.14521771	0.14414684
Crispld2	-0.7117371	2.2841983	2.29776549	0.14527696	0.14418657
5430416N02	0.51527071	2.04785831	2.29747539	0.14530103	0.14419138

Taf6l	-0.7485911	0.66481131	2.29713971	0.14532889	0.14419995
Bola3	0.37182613	3.33328221	2.29604777	0.14541955	0.14426864
Ndufa9	0.25492151	6.21260257	2.29567779	0.14545029	0.14426864
Frmd6	-0.244961	5.61707454	2.29545054	0.14546917	0.14426864
Nbl1	0.56195569	6.51471903	2.29516467	0.14549293	0.14426864
Ttf2	-0.7024106	1.79361035	2.29514833	0.14549428	0.14426864
Arhgef9	-0.2151064	9.43024303	2.29456881	0.14554246	0.14429734
Pnp	0.27590465	4.58401959	2.29330368	0.1456477	0.14438226
Rgs4	0.20704398	9.56276024	2.29277589	0.14569163	0.14438226
Azi2	0.21889503	6.72420063	2.2926474	0.14570232	0.14438226
4930529M08	1.71922747	-0.9366219	2.29261397	0.14570511	0.14438226
Btrc	-0.220912	6.32207989	2.29155274	0.1457935	0.14445077
5330417C22I	-0.4132287	3.85966934	2.29009958	0.14591463	0.14455169
Kif5b	-0.1924983	8.37481273	2.28852366	0.14604614	0.14463225
Ppef1	-1.3110671	-0.7102362	2.28850928	0.14604734	0.14463225
Arl1	0.36803519	3.68099753	2.28843198	0.1460538	0.14463225
Sh3rf3	-0.3047261	5.61462486	2.28811197	0.14608052	0.14463556
Ndufa4l2	1.02028411	-0.0866325	2.28793029	0.14609569	0.14463556
E2f3	0.25522969	5.37305131	2.28750109	0.14613155	0.14465196
Mir568	-0.4055165	2.11864824	2.28623352	0.1462375	0.14471202
Tmem169	-0.3316407	2.9231717	2.28622969	0.14623782	0.14471202
Smyd2	0.26681393	5.5721474	2.2859574	0.1462606	0.14471202
Gtse1	-1.3298175	-1.4834709	2.28584183	0.14627026	0.14471202
2010300C02I	-0.2532142	5.50339108	2.28562168	0.14628868	0.14471202
Mina	0.40799874	2.58326857	2.28444965	0.14638677	0.14476465
Gnaq	0.15694483	8.68177688	2.28418843	0.14640865	0.14476465
Sfn	-1.7739999	-1.1509467	2.28399525	0.14642482	0.14476465
Slc35a3	-0.2333625	4.86209558	2.28388116	0.14643438	0.14476465
Utp6	0.20941535	6.67201553	2.2838335	0.14643837	0.14476465
Nadsyn1	-0.5363218	1.37774701	2.28215313	0.14657921	0.14486728
AI597479	0.23562184	5.87756521	2.28213401	0.14658081	0.14486728
4933407K13I	-0.7058642	1.84513764	2.2804988	0.14671802	0.14497632
Apln	-0.5356947	2.58939571	2.28035858	0.1467298	0.14497632
Ubac1	0.30696935	3.83876854	2.27938878	0.14681125	0.14502563
Tmem165	0.27822042	4.66271088	2.27912686	0.14683326	0.14502563
Fut9	-0.3025321	7.1307687	2.27907399	0.1468377	0.14502563
Tmem70	0.26079825	4.80019778	2.27829391	0.14690328	0.14507129
4932438H23I	-1.1237689	-0.1891643	2.2775632	0.14696474	0.14511027
Txndc16	-0.2360455	5.40969844	2.27718646	0.14699644	0.14511027
2310039H08I	0.54726487	2.04685104	2.27713479	0.14700078	0.14511027
Ctsz	0.38592164	3.99981656	2.27676045	0.14703229	0.14512227
Bcl11b	-0.3457026	6.10353451	2.27643038	0.14706008	0.1451306
Pnkd	0.20216641	6.53540609	2.27486767	0.14719172	0.14522869
Tmem231	0.33544982	2.88553283	2.27470861	0.14720512	0.14522869
Ltbp2	-2.1750578	-1.1453635	2.27432804	0.14723721	0.14522869

Ano10	-0.4087268	2.49026703	2.27428018	0.14724124	0.14522869
AA474331	-2.2038448	-1.6410653	2.27410202	0.14725627	0.14522869
Mlf1	1.15709157	-0.3012403	2.27265028	0.14737875	0.14533038
Tle6	0.99838984	0.31341144	2.26978508	0.14762085	0.14554998
Rrp1b	-0.2990551	3.45718733	2.26869156	0.14771338	0.14562207
Tmod4	-1.098477	-0.5114358	2.26617706	0.1479264	0.14581291
Fpgs	-0.782022	0.54372643	2.2647255	0.14804955	0.14591512
Nol6	-0.2487319	5.48247362	2.26432931	0.14808318	0.1459291
Hspb2	0.68570688	1.09885574	2.26314419	0.14818384	0.14600476
Snai2	0.53197292	3.53862021	2.26296714	0.14819888	0.14600476
6030458C11I	-0.3110296	4.92836648	2.26219054	0.1482649	0.14605061
Tyro3	-0.2559514	4.93736118	2.26184163	0.14829457	0.14606066
Ceacam20	-0.6130634	1.81222087	2.26124384	0.14834542	0.14609156
Cdkl1	0.52957543	2.6430747	2.2609107	0.14837377	0.1461003
Sbk3	-0.9209104	0.45675442	2.26010927	0.14844199	0.14614829
Acta1	0.69244582	0.90019356	2.25827505	0.14859827	0.14628296
Arhgap20os	-1.4702088	0.33173042	2.25770262	0.14864708	0.14630117
A330093E20I	-1.1708262	0.23437656	2.25760068	0.14865578	0.14630117
Gabra4	-0.2580909	5.85303945	2.25684194	0.14872051	0.14634568
Acpp	-0.6755146	4.28697878	2.25532339	0.14885018	0.14645406
Smco4	0.53707478	1.93044032	2.25375028	0.14898465	0.14656714
Tcp11l1	-0.2403256	5.22320502	2.25261522	0.14908176	0.14663323
4931428F04I	0.64354021	1.13749693	2.25250819	0.14909092	0.14663323
Enthd2	0.61841858	1.43722193	2.2518273	0.14914922	0.14667134
Aebp1	0.45575135	7.19862456	2.24971406	0.14933033	0.14683019
Eci2	0.29138018	5.4609683	2.249162	0.14937769	0.1468575
Pfdn5	0.39927327	5.7391927	2.24881674	0.14940731	0.14686738
Sp2	0.37415395	5.31463402	2.24727151	0.14954	0.14697855
Mllt1	-0.2764346	4.59534888	2.24654358	0.14960255	0.14702076
Aff1	0.22751099	7.01573603	2.24626086	0.14962685	0.14702175
Btbd16	-1.2811874	-0.8711988	2.24580199	0.14966631	0.14702175
Nlrp4f	-1.8447843	-1.9884153	2.24569616	0.14967541	0.14702175
Eif3d	-0.2482251	4.54766849	2.24562014	0.14968195	0.14702175
S1pr5	-0.5272309	2.05751533	2.24533889	0.14970614	0.14702626
Zic2	0.33083278	6.54778614	2.24481203	0.14975147	0.14705153
Glcci1	-0.1789176	6.88902066	2.24449982	0.14977834	0.14705866
Rtp3	-1.0571675	0.44595136	2.24288403	0.1499175	0.14715774
Tsn	0.23274411	6.2732336	2.2426704	0.14993591	0.14715774
Zfp91	0.16376727	7.46042978	2.2426449	0.14993811	0.14715774
Nlrp1a	-0.4583101	1.62889049	2.24103703	0.15007677	0.14727456
Smg9	0.43182655	2.90670721	2.24033394	0.15013745	0.14731483
Psd	-0.2797827	5.56008766	2.23881282	0.15026883	0.14742446
Snx18	0.20698275	6.1685314	2.23836074	0.1503079	0.14744351
Tmem183a	0.21998516	5.63463001	2.23573786	0.15053484	0.14764682
Rab15	0.17931108	6.31468885	2.23463141	0.1506307	0.14772153

Pde7a	-0.2771121	5.13023311	2.23274692	0.15079414	0.14786247
Dnajb12	0.25061558	3.90776884	2.23175559	0.1508802	0.14792752
Arl6ip5	0.25648503	3.89534564	2.23059186	0.1509813	0.1480073
Lrmp	-1.3208681	-0.0754907	2.23016249	0.15101862	0.14801465
9530036O11	-0.927343	-0.2627716	2.2300517	0.15102825	0.14801465
Gm11974	0.76559322	0.35866908	2.22964372	0.15106373	0.14803008
Dhdds	-0.2409934	4.89381283	2.22803993	0.15120329	0.14814192
2900008C10I	-1.0638137	0.21761927	2.22787834	0.15121736	0.14814192
Ccnl1	-0.4197468	4.7158139	2.22527643	0.15144414	0.1483447
Wnk3	-0.2808804	6.92545557	2.22426576	0.15153233	0.14841172
Syde1	0.40443886	5.18608369	2.22225602	0.1517079	0.14856427
Eya4	-0.5965242	2.43857973	2.22142824	0.15178029	0.14861575
Zfml	-0.2292116	7.89140434	2.22082631	0.15183295	0.14863498
Cdh8	0.24144088	5.4722849	2.22075081	0.15183955	0.14863498
Ddx56	0.42944236	3.08038876	2.22018759	0.15188885	0.14866384
Patl2	3.66302641	-1.4006495	2.25113819	0.15195626	0.14871041
Nid2	0.41148402	3.41703602	2.219117	0.15198261	0.1487168
Gm6525	-2.1182225	-1.3451882	2.21859638	0.15202823	0.14874204
Dio2	-0.2351849	5.92017166	2.21662097	0.15220148	0.14889213
Pid1	0.22553355	6.78804196	2.21626845	0.15223243	0.14890298
Ubap2l	0.18655146	8.54563331	2.21597995	0.15225776	0.14890833
Mfsd10	-0.6441047	0.81582909	2.21523193	0.15232345	0.1489176
Slc27a3	2.08303679	-1.6827004	2.21517437	0.15232851	0.1489176
AW209491	0.25249096	4.72485102	2.21513846	0.15233166	0.1489176
Rtn2	-0.4731674	2.61842012	2.21488942	0.15235354	0.1489176
Snrpn	0.5922148	0.80614836	2.21444279	0.1523928	0.1489176
Gad1	-0.3741125	7.57420596	2.21431136	0.15240435	0.1489176
Mrps34	0.37870976	3.02931867	2.21429037	0.15240619	0.1489176
Angptl3	-1.0909315	0.11043517	2.21402154	0.15242983	0.14892129
Copz1	0.28517679	6.13808659	2.21340834	0.15248375	0.14895458
Arl4c	-0.2706868	5.17834358	2.21303199	0.15251686	0.14896752
Tipin	0.46669161	4.23054188	2.21273404	0.15254308	0.14896955
Tle2	-0.5197805	1.5759767	2.212557	0.15255866	0.14896955
Dbt	-0.2524701	5.64794495	2.21195232	0.15261189	0.14900213
9330162012I	1.08380023	0.74118326	2.21139561	0.15266092	0.14901604
Dusp11	-0.201047	6.38920136	2.21099197	0.15269648	0.14901604
2310010J17F	0.73548128	0.76878002	2.21098966	0.15269668	0.14901604
Arhgap35	-0.1944436	8.93178848	2.21088051	0.1527063	0.14901604
Gpr85	0.28267087	3.9713865	2.21066309	0.15272546	0.14901604
Clvs2	-0.3453554	4.43268842	2.2104008	0.15274858	0.14901921
Lonrf3	0.23305889	5.72486877	2.20987273	0.15279513	0.14904525
Zscan18	-0.2947126	3.71227459	2.2081039	0.1529512	0.14917809
Zyg11b	-0.2455966	8.98696616	2.20698897	0.15304968	0.14925472
Ecscr	1.37430883	-0.6788505	2.20580663	0.15315419	0.14933723
Bcar3	0.39415566	2.67844132	2.20544026	0.15318659	0.14934939

Hoga1	1.22925569	-0.0458098	2.20496359	0.15322876	0.14934939
Rcn2	0.19956231	6.30450958	2.20468435	0.15325347	0.14934939
Wdr81	-0.3997286	3.02157766	2.20468187	0.15325369	0.14934939
Lipt1	0.58822861	1.47774105	2.20454043	0.15326621	0.14934939
Ndufa7	0.36673839	4.6453274	2.20355509	0.15335345	0.14940731
Galnt13	-0.2401554	5.74392475	2.20341918	0.15336548	0.14940731
Fbll1	0.49546655	2.23625039	2.20310454	0.15339336	0.14941506
Xylt1	-0.4623778	2.83464097	2.20270612	0.15342866	0.14943005
Slc6a11	-0.378313	4.54546055	2.20215411	0.15347759	0.14945829
Igfbp5	0.33952263	8.64917915	2.20156388	0.15352993	0.14946465
Pcsk7	-0.3708712	2.68672644	2.20141953	0.15354273	0.14946465
Msn	0.26119049	7.6261454	2.20140646	0.15354389	0.14946465
Zfp930	0.28976785	4.10431084	2.19954382	0.15370922	0.1496025
Csrnp1	0.61223934	1.76997254	2.1993617	0.1537254	0.1496025
Smg5	-0.2532033	4.39297691	2.19831411	0.15381849	0.14967368
4933416I08R	-1.5945701	-1.0641654	2.19736605	0.15390279	0.14971875
A330074K22I	-0.6614374	1.28954745	2.19711841	0.15392482	0.14971875
0610009O20	0.26870028	4.90817008	2.19697419	0.15393765	0.14971875
Fbxo3	-0.1720396	6.50548621	2.1968957	0.15394464	0.14971875
Galns	-0.7363407	0.66042022	2.19572689	0.15404869	0.14980052
Arf6	0.22880278	6.84791232	2.19371207	0.15422824	0.14995568
Fut10	-0.3242539	3.61978587	2.19342165	0.15425415	0.14996143
Tnfsf13b	-1.7884678	-1.2214902	2.19098519	0.15447166	0.15015343
Pqlc1	-0.2754238	3.33315234	2.19052895	0.15451244	0.15017361
Tlr13	-1.052887	0.9213886	2.18955543	0.15459948	0.15023173
Emg1	0.46538856	2.94861394	2.1894122	0.15461229	0.15023173
Auts2	-0.1807647	6.88281406	2.1889078	0.15465742	0.15025611
Psme2	0.35556432	5.28007112	2.18824087	0.15471711	0.15027988
Napepld	0.27076293	6.43308313	2.18814358	0.15472582	0.15027988
Trhr2	-0.98128	0.03532587	2.18796305	0.15474199	0.15027988
C530044C16I	2.01738545	-1.6134224	2.18723053	0.15480759	0.15030565
Sf3b4	0.26152886	4.65157292	2.1872194	0.15480859	0.15030565
Lincrna-cox2	-1.8864925	-1.3891797	2.18632353	0.15488888	0.15036414
Nlgn2	0.21401241	6.3200892	2.18595187	0.1549222	0.15037702
BC028528	0.56645074	1.6431558	2.18553849	0.15495927	0.15038006
Dapl1	0.33506706	5.38142757	2.18546995	0.15496542	0.15038006
Fam154b	0.6673442	0.89675354	2.18499627	0.15500791	0.15039342
Spi1	0.86332992	0.49073913	2.18486953	0.15501929	0.15039342
Tmem232	0.69766088	1.34682122	2.18413223	0.15508547	0.15043817
Ypel2	-0.1670868	6.38247528	2.18061306	0.15540182	0.15072555
4921515E04I	-1.4810408	-0.8939193	2.17989226	0.15546671	0.15075833
Socs5	-0.2004537	6.54267267	2.17979118	0.15547581	0.15075833
Igf2bp2	0.52562712	1.32033371	2.17830367	0.15560984	0.15086879
Arfip1	0.28976966	6.0988811	2.17767427	0.1556666	0.15089245
Spry4	-0.3544097	3.46186275	2.17758691	0.15567448	0.15089245

Ndufaf5	0.25266527	4.79767237	2.17603854	0.15581421	0.15100838
F930015N05	-0.3356782	3.60806078	2.17507834	0.15590094	0.15107292
Xkr8	-0.5811819	2.41378249	2.17485066	0.15592152	0.15107334
Muc2	2.78267534	-1.8954559	2.17446257	0.15595659	0.1510757
Cldn11	-0.3585148	6.53876508	2.17437809	0.15596423	0.1510757
Dynlt1f	-0.6029568	0.61524014	2.17305598	0.1560838	0.15113632
C1qtnf6	0.72226891	1.04805475	2.17296963	0.15609162	0.15113632
Gm11696	-0.5965688	1.18376986	2.17292101	0.15609602	0.15113632
Herpud2	0.22038981	6.21592798	2.17279519	0.1561074	0.15113632
Cyyr1	-1.3630503	0.36582894	2.17185925	0.15619214	0.15117977
Cblb	0.23692514	6.78147852	2.17185423	0.15619259	0.15117977
Fcgrt	0.50974711	3.55292364	2.17159583	0.15621599	0.15118291
Myo1f	-0.5881562	1.27857512	2.17081885	0.15628639	0.15121586
Cdhr3	-1.3376308	-0.2814062	2.17077509	0.15629036	0.15121586
C130050O18	-1.9488282	-2.0678571	2.16986781	0.15637261	0.15127593
Prkag1	0.22489743	4.21440019	2.16709059	0.15662472	0.15150028
Cep76	-0.3064114	3.92024491	2.16661856	0.15666761	0.15152224
Prrt3	0.61281625	1.74678824	2.16487759	0.15682596	0.15165583
Hnrnpul2	0.15427745	8.048024	2.16315068	0.15698321	0.15178833
Akap10	-0.227023	4.87047953	2.16261181	0.15703232	0.15181625
Hexb	-0.242826	4.74106074	2.16161841	0.15712291	0.15188425
Itgb6	1.74281135	-1.8874354	2.16138823	0.15714391	0.15188497
Chmp5	0.28445726	6.60861862	2.16048122	0.15722668	0.1519454
Csrnp3	-0.2924632	7.29463756	2.15999279	0.15727128	0.15195445
1700073E17I	-0.5848647	2.24183211	2.15984688	0.1572846	0.15195445
Ppp1r35	0.55568446	1.49108591	2.1597131	0.15729682	0.15195445
Tgfbrap1	-0.2050084	5.59622484	2.15851074	0.15740669	0.15204101
1700125H03I	-1.5128176	-0.4861726	2.15775857	0.15747547	0.15208786
Cep70	-0.3720715	4.03986928	2.1567503	0.15756772	0.15215737
Mrpl43	0.28148276	5.11496143	2.1560391	0.15763283	0.15219068
Nprl3	-0.3264757	3.06488781	2.1559303	0.1576428	0.15219068
Rpl15	0.24424801	7.96298966	2.15487525	0.15773946	0.1522644
P2rx6	-0.7690424	1.20179979	2.15225619	0.15797972	0.15247671
Nr5a2	-2.2210579	-2.0178375	2.15170184	0.15803063	0.15250622
Apod	0.48466718	10.3568368	2.15141418	0.15805706	0.1525121
Adgb	-1.5715577	-0.9356772	2.15053716	0.15813766	0.15256624
Peli1	0.20063424	5.95279181	2.1503611	0.15815385	0.15256624
Tmem25	-0.3849084	3.05454486	2.14991257	0.15819509	0.15258641
Map4	0.16395933	9.19688123	2.14961962	0.15822204	0.15259258
Neurl4	-0.2904261	4.54166249	2.14940078	0.15824217	0.15259258
Git2	0.20270714	5.44319972	2.1488613	0.15829182	0.15259514
Opa1	-0.2130235	7.78864065	2.14879658	0.15829777	0.15259514
Chm	-0.2176232	6.37748255	2.14864057	0.15831214	0.15259514
Adpgk	0.52793744	2.07154623	2.14831484	0.15834212	0.15259514
Ttll13	-1.4385124	-0.9046798	2.1482669	0.15834654	0.15259514

Gpr137b	0.32725512	2.9335845	2.14718336	0.15844635	0.15267171
Msantd3	0.43175072	2.9884941	2.14652907	0.15850666	0.15270555
Mapk1ip1l	0.22863827	8.02239861	2.14634045	0.15852405	0.15270555
Pcdhga9	-0.3953734	2.52573333	2.14613993	0.15854254	0.15270555
Clec18a	1.15135197	0.16717546	2.1454809	0.15860333	0.15274448
Zfp69	-0.7020677	0.82551149	2.14492488	0.15865464	0.15277428
Dfna5	0.31642795	4.32154807	2.14462217	0.15868258	0.1527815
Chl1	-0.3195569	7.47835662	2.14440245	0.15870286	0.1527815
Pdzd4	-0.2370027	5.59251781	2.14400987	0.15873912	0.15278724
Ccdc68	-0.7789691	0.39708641	2.14389681	0.15874956	0.15278724
Hmox2	0.31848171	5.03916106	2.14359215	0.1587777	0.15279472
Mrpl53	0.32849947	3.73890533	2.14293262	0.15883865	0.15283376
Tmem167b	0.33326504	6.20567732	2.14154443	0.15896702	0.15293766
1700001O22	-0.7573966	0.38566742	2.14085104	0.15903119	0.15296075
Celf6	0.37168853	2.68095841	2.14084439	0.1590318	0.15296075
Inpp5k	-0.2265715	4.16728098	2.14004603	0.15910572	0.15300224
Ddb1	-0.204738	6.36962205	2.13993633	0.15911588	0.15300224
Xlr	-0.7554015	0.44675069	2.13971782	0.15913612	0.15300224
Cacfd1	-0.2440563	4.71033613	2.13827804	0.15926957	0.15309283
5730559C18I	-1.8973606	-0.9736511	2.13826083	0.15927117	0.15309283
Swsap1	0.32912312	3.36427758	2.13697567	0.1593904	0.15318781
Slfn10-ps	-1.2844626	-0.7631627	2.13541071	0.15953574	0.153304
Ephb4	-0.5654496	3.24174514	2.1350645	0.15956791	0.153304
Rsl24d1	0.25468385	5.39693246	2.13501394	0.15957261	0.153304
Fam149b	0.21391282	5.38865691	2.13432314	0.15963684	0.15333025
Col17a1	-2.2524604	-1.226709	2.1342804	0.15964081	0.15333025
5730507C01I	0.37417141	3.12580485	2.13399202	0.15966763	0.15333535
Gm15446	-0.613501	1.42491026	2.13378372	0.15968701	0.15333535
Ahcy	0.31366291	3.87366617	2.13295735	0.15976391	0.15338956
Ankrd29	0.32431206	4.6037215	2.13106869	0.15993983	0.15353881
Etv4	-1.2796126	-1.3660121	2.13059287	0.15998418	0.15355058
Cstf2t	0.17204258	6.61119104	2.13043098	0.15999928	0.15355058
Tspan7	0.21111425	8.1016807	2.13027849	0.1600135	0.15355058
Leng1	0.43876649	4.27470386	2.12977379	0.16006058	0.15357611
Thop1	0.43409832	2.56012789	2.1290126	0.16013161	0.15362414
Micalcl	-0.5879662	1.66296183	2.12879846	0.1601516	0.15362414
Idh3g	0.18777939	6.41635125	2.12826355	0.16020155	0.15365165
Msh5	-2.3927147	-1.8124745	2.12805274	0.16022124	0.15365165
2310065F04I	2.7931572	-1.6420184	2.12748919	0.16027389	0.15367555
Slc9a5	-0.4641333	2.60183647	2.12734744	0.16028713	0.15367555
Tdgf1	-1.709463	-0.1595847	2.12526664	0.16048174	0.15382696
Gabarapl2	0.22379688	6.7008753	2.12522029	0.16048608	0.15382696
Efs	0.46745607	2.17687059	2.12402292	0.16059819	0.15390106
Sigmar1	0.28726786	3.01872859	2.12395643	0.16060442	0.15390106
Sgsh	0.55793591	1.3039999	2.12210669	0.16077782	0.1540352

Zfp687	-0.3242405	3.60778931	2.12202508	0.16078547	0.1540352
Hist1h2bb	1.20763632	-1.2061748	2.12154849	0.16083019	0.15405837
Zbtb2	0.21738594	4.67072501	2.12114749	0.16086782	0.15406758
Rpl31-ps12	0.28195353	4.06261921	2.12100841	0.16088088	0.15406758
Sharpin	0.30513211	3.8065174	2.12059808	0.1609194	0.1540848
Yipf5	0.18462701	5.41711625	2.12024623	0.16095245	0.15409677
0610043K17I	-0.4327732	1.52594547	2.11883371	0.16108519	0.15420417
Cirh1a	0.20893257	4.83870112	2.11815333	0.16114917	0.15422909
Kcnv1	-0.3343219	6.74877182	2.11811972	0.16115234	0.15422909
Atad2	-0.3573746	4.80944559	2.11682113	0.16127455	0.15432636
Smim7	0.30718124	6.30717233	2.11658264	0.16129701	0.15432816
Msl2	-0.1909195	7.27702736	2.11615115	0.16133765	0.15434736
Dtnbp1	0.259712	5.32169113	2.11583544	0.16136739	0.15435613
B3gnt5	-1.137391	0.09588602	2.11529743	0.16141809	0.15438494
Hgsnat	-0.2088134	5.17726335	2.11497934	0.16144808	0.15439393
Slc6a9	-0.3321576	4.07559452	2.11402998	0.16153761	0.15445987
Elf1	0.25849673	5.89231624	2.11307739	0.16162751	0.15452613
Zfp191	0.2686428	5.01105649	2.11268408	0.16166465	0.15454194
Vwf	0.56078723	2.07504973	2.11241494	0.16169007	0.15454654
Bex4	-0.330953	4.0910986	2.11154808	0.16177197	0.15460512
Il11ra1	-0.2401322	4.34286303	2.1112278	0.16180224	0.15461436
Pus7	-0.2976351	3.84631136	2.11060205	0.16186141	0.15463637
Morn2	0.41027177	2.89696583	2.11002435	0.16191605	0.15463637
Gm13298	-0.3033418	5.02359113	2.1099499	0.1619231	0.15463637
Gcm1	-1.5557106	-1.3960054	2.10978936	0.16193829	0.15463637
9930012K11I	-0.7156466	1.30483916	2.10968322	0.16194833	0.15463637
Mid1ip1	0.34647606	6.08785117	2.1096765	0.16194897	0.15463637
Gpalpp1	0.20943292	5.76288545	2.10917481	0.16199645	0.15466203
Mmgt1	0.23681929	4.66020871	2.10847231	0.16206297	0.15469215
Rapgef4	-0.2267161	7.31173449	2.10840602	0.16206925	0.15469215
Slc9a7	-0.3530167	4.25515932	2.10692323	0.16220978	0.15480659
Ctsw	-2.0595984	-2.2378359	2.10631756	0.16226722	0.15483615
4833417C18I	1.66171331	-1.3667908	2.10616135	0.16228204	0.15483615
9030204H09I	-1.4145021	-1.183812	2.10576011	0.16232011	0.15483968
Prdm6	0.42475228	5.62563107	2.10568733	0.16232702	0.15483968
Rps5	0.26708267	5.76664419	2.10516137	0.16237694	0.15486761
Serpinb1b	-0.9969873	-0.1128303	2.10489129	0.16240259	0.15487237
Glyr1	0.22526671	5.78046531	2.10396064	0.16249099	0.15493698
Fam53a	-0.327959	3.55264989	2.10355687	0.16252936	0.15495387
Klhl28	-0.3477626	3.90429028	2.10275216	0.16260587	0.15500128
Rps16	0.29949926	6.26269801	2.10218365	0.16265995	0.15500128
1700020L24I	-1.4841741	-1.6620476	2.10206044	0.16267167	0.15500128
Bbs1	-0.2746779	5.65451516	2.101825	0.16269408	0.15500128
Cchcr1	-0.7166689	0.99538075	2.1017907	0.16269734	0.15500128
Tinf2	0.40649645	2.67584235	2.10173049	0.16270307	0.15500128

Wdr5b	0.655913	1.16772343	2.10098413	0.16277412	0.15504928
Olfr1033	0.31559687	3.91660529	2.09997945	0.16286982	0.15510887
Entpd1	-0.3625932	3.01205359	2.0998905	0.1628783	0.15510887
Mgat4a	-0.2928411	5.48766177	2.09958702	0.16290723	0.15510887
Ebna1bp2	0.2373571	5.39896302	2.09945936	0.16291939	0.15510887
Parl	0.32930305	3.34903701	2.09895906	0.16296709	0.15513459
Nsg1	0.2389236	5.26403443	2.09843323	0.16301725	0.15516265
Zfp600	-1.135544	-1.1369565	2.09750988	0.16310536	0.1552088
Rreb1	-0.2977664	5.25007658	2.09749144	0.16310712	0.1552088
Lrriq1	-0.5212698	3.09680899	2.09671141	0.1631816	0.15524217
Gpr37l1	-0.4214626	3.0098003	2.09669078	0.16318357	0.15524217
Lor	0.64500551	0.3638543	2.0964685	0.16320481	0.15524268
Ssr1	0.1993354	7.3121515	2.09616241	0.16323405	0.15525081
2310068J16F	1.4369706	-0.8088933	2.0956361	0.16328435	0.15527105
Dusp26	0.19364107	6.07584242	2.09546938	0.16330029	0.15527105
Atp6v0c	1.47725047	-1.4626993	2.09522792	0.16332337	0.15527105
Slc5a3	-0.2191662	5.78479516	2.09507358	0.16333813	0.15527105
Htr1d	-0.7227916	1.15101983	2.09457775	0.16338555	0.15527715
Foxn3	0.23463932	6.54156047	2.09457365	0.16338595	0.15527715
Mettl13	-0.3981675	2.10148209	2.09364775	0.16347455	0.15534167
A330041J22F	-1.0667415	0.08516935	2.09264237	0.16357082	0.15537996
Sh3glb2	0.19944125	5.23675854	2.09260705	0.1635742	0.15537996
Nfe2l3	-0.4461346	2.48759013	2.09250376	0.1635841	0.15537996
2310069G16	-0.6774336	1.60202341	2.09236179	0.1635977	0.15537996
Acvrl1	-0.7064452	0.83253302	2.09206451	0.16362618	0.15538734
Cxcl11	2.49527576	-2.3691744	2.09144906	0.16368518	0.15541469
Xpnpep1	0.26319806	4.48513859	2.09107414	0.16372113	0.15541469
Elavl2	0.21268749	6.93896308	2.0909104	0.16373683	0.15541469
Ap2a2	0.17083837	6.88755799	2.09082245	0.16374526	0.15541469
Ncapd2	-0.4004192	2.67232371	2.0906594	0.1637609	0.15541469
Zfp384	0.20525432	5.17019792	2.09027016	0.16379825	0.15541469
Slc8a2	-0.2738273	5.55719983	2.09025181	0.16380001	0.15541469
Sema6c	-0.7873266	0.69316727	2.09000525	0.16382367	0.15541748
Sall3	-0.449517	1.87691425	2.08975562	0.16384763	0.15542055
Olfr113	-3.9584491	-2.0314591	2.11785503	0.16390394	0.15543965
Ccr7	2.00685191	-1.9770979	2.08911424	0.16390921	0.15543965
Rtbdn	-1.1421013	0.38635475	2.08737007	0.16407681	0.15557893
4921507L20F	-1.3171372	-0.5854421	2.08659524	0.16415133	0.15562992
Kcnj3	-0.2821113	5.04747371	2.08590066	0.16421817	0.15567361
Gm14322	0.2661571	4.29984094	2.08536987	0.16426928	0.15568938
Zfp799	-0.2163192	5.66244101	2.0851556	0.16428991	0.15568938
B9d2	0.52796227	1.8269852	2.08508116	0.16429708	0.15568938
Smim13	0.18479929	8.64473928	2.08288137	0.16450911	0.15585006
Traip	-0.9632549	0.84322021	2.08253728	0.1645423	0.15585006
Kat6b	-0.1647821	7.17037169	2.08247217	0.16454859	0.15585006

Uba5	0.18125966	5.87438216	2.08246019	0.16454974	0.15585006
Bcl7b	0.25794851	4.11000641	2.08221376	0.16457352	0.1558529
Nanos2	-2.412533	-2.2041108	2.08134028	0.16465785	0.15591308
Atp10a	-0.3389434	3.72381248	2.0809501	0.16469553	0.15592908
Clic5	-0.4940684	2.94378902	2.07966484	0.16481974	0.1560255
Fcgr3	0.72748669	1.06475756	2.07936	0.16484922	0.1560255
Tmub1	0.42575777	2.31800996	2.07925076	0.16485978	0.1560255
Zmpste24	0.29028767	5.50399362	2.07886594	0.164897	0.15604104
Tmem56	-0.2322898	6.25923531	2.07776965	0.1650031	0.15612175
Ssrp1	-0.2478672	6.70649479	2.07622018	0.16515319	0.15623595
Zfp808	0.24980857	3.85726606	2.07601845	0.16517275	0.15623595
Fkbp9	0.35961126	5.31413582	2.07584415	0.16518965	0.15623595
Nfic	0.22887164	7.69138183	2.07566405	0.16520711	0.15623595
Meis1	-0.6594578	1.67791817	2.07538219	0.16523444	0.1562421
Plaa	0.15523578	6.40093827	2.07514171	0.16525776	0.15624446
Glrb	0.18659874	6.74614551	2.07447748	0.16532221	0.15628569
Eno4	-0.565217	1.34689556	2.07362795	0.16540468	0.15632641
Lhfpl2	0.27421732	5.18302644	2.07360442	0.16540696	0.15632641
Ninj1	0.75188479	2.11751895	2.07311017	0.16545496	0.15633376
Mrpl46	-0.4020397	3.48417152	2.0729947	0.16546618	0.15633376
Fam115c	0.70201258	1.3926034	2.07260043	0.16550449	0.15633376
Fas	-1.131165	0.28238961	2.07250329	0.16551393	0.15633376
Kat5	0.21451432	4.68628449	2.07242515	0.16552152	0.15633376
Gm12185	-0.7162113	0.86068717	2.07223721	0.16553979	0.15633376
Zfp748	-0.2520211	4.81917853	2.07176093	0.16558609	0.15635744
Ube3c	-0.1986546	6.07983139	2.0715505	0.16560655	0.15635744
Phkg1	-0.5232539	1.630539	2.07111838	0.16564858	0.15637744
Aldh1l1	0.30705352	3.6319835	2.07050324	0.16570844	0.15639547
1810014B01l	0.43924106	2.62046531	2.07025139	0.16573295	0.15639547
Brms1	0.39226522	2.6401315	2.07022523	0.16573549	0.15639547
Pcdhga6	-0.4174055	2.36475453	2.0700517	0.16575239	0.15639547
Plekhs1	-1.1095133	-0.1763468	2.06965167	0.16579134	0.15639547
Mlh1	-0.3521079	3.81828075	2.06963695	0.16579277	0.15639547
Gimap3	0.52706605	2.71471156	2.06849946	0.16590359	0.15648032
Dact1	0.2895696	6.05623153	2.06789954	0.16596207	0.15650117
Ntn1	0.38892371	3.73349064	2.06784471	0.16596741	0.15650117
Nln	-0.2463683	4.43820929	2.06696586	0.16605314	0.15655161
C330006A16l	0.20770756	5.98893754	2.06686836	0.16606265	0.15655161
4930451G09	-0.5573213	1.59307176	2.06647209	0.16610132	0.15656839
Alg14	0.37846809	4.58603862	2.06553448	0.16619286	0.156635
Mybl1	0.2915083	4.22013849	2.06492146	0.16625275	0.15667176
Gm9079	-1.0057375	0.57744358	2.06367736	0.16637437	0.15674746
Ticam1	0.3185421	4.60652184	2.06355557	0.16638628	0.15674746
Cbln3	-0.6110322	1.1983502	2.0634585	0.16639577	0.15674746
Ubtf	0.19360047	5.83029552	2.06261919	0.1664779	0.15680245

Card6	0.27888401	4.85767612	2.06243469	0.16649596	0.15680245
Dgkk	-0.4700817	3.76416739	2.06159964	0.16657772	0.15685976
Zfpl1	0.40862821	2.51937251	2.06093102	0.16664322	0.15687897
Rsl1d1	0.18922264	6.66736682	2.0608832	0.16664791	0.15687897
Etv6	0.21151522	5.18970227	2.06075109	0.16666086	0.15687897
Rpl27a	0.21263603	6.97053072	2.0603716	0.16669805	0.156879
Tmem33	0.1690573	6.24306401	2.06032402	0.16670272	0.156879
Nelfb	0.24730057	5.02264515	2.05979272	0.16675481	0.15690834
Gabrb3	-0.2342623	8.62854624	2.05885032	0.16684727	0.15696552
2610020H08I	-0.7066049	0.73171678	2.05858939	0.16687288	0.15696552
Gm8979	-0.6025848	0.55509727	2.05853359	0.16687836	0.15696552
Dis3	-0.2459582	3.97753571	2.05814512	0.16691649	0.15698171
Dyrk1b	-0.3200108	3.09395663	2.05786814	0.16694369	0.15698761
Cdh5	0.37206735	5.44742373	2.05654143	0.16707405	0.15707193
Mnd1	1.15794498	0.36796527	2.05645063	0.16708297	0.15707193
Lrrc3b	0.41638956	3.63449655	2.05631629	0.16709618	0.15707193
Mrpl40	0.34761323	3.57713701	2.05576041	0.16715085	0.15710363
Airn	0.52625025	1.75210693	2.05532186	0.16719399	0.15712449
Fam184a	-0.2998457	4.85798108	2.05488183	0.16723729	0.15714549
AB124611	1.12046518	-0.6492218	2.0543347	0.16729115	0.15717641
Kcnk6	-0.6109628	1.35946233	2.05352811	0.16737058	0.15723136
Hist1h1e	0.52523853	2.92887352	2.05247641	0.16747423	0.15730902
Zscan2	-0.6665899	1.3090685	2.05142779	0.16757765	0.15734993
Ppp1r12b	-0.3862083	5.67617441	2.0514113	0.16757928	0.15734993
Gm3383	-0.8150636	-0.2915731	2.05139681	0.16758071	0.15734993
Naa30	0.19815258	5.57942222	2.05043932	0.16767522	0.15741896
Fgd4	-0.2325383	4.93470296	2.0498204	0.16773634	0.15745664
Pdpr	-0.3211785	3.89356158	2.04933287	0.1677845	0.15748214
Wdr47	-0.2853709	6.50006207	2.04904531	0.16781292	0.15748625
Cx3cr1	-0.3816489	3.35088241	2.04886376	0.16783087	0.15748625
Fzd1	0.32324936	6.13564224	2.04829506	0.16788709	0.1575193
Serpini1	-0.2216816	7.56108133	2.04749292	0.16796644	0.15757404
Trp53i13	-1.1519749	-0.2615405	2.04709364	0.16800595	0.1575914
1810034E14I	0.73470702	1.11510821	2.04604858	0.16810942	0.15764767
Cdc25a	0.36005796	2.73292837	2.04591843	0.16812231	0.15764767
Hmgcs1	-0.2395113	7.21088302	2.04584764	0.16812932	0.15764767
Cbll1	0.23611028	6.17443958	2.04563885	0.16815001	0.15764767
Gng12	0.26045439	6.58084897	2.04539324	0.16817434	0.15765079
Slc7a2	-0.3241557	6.92220245	2.04507418	0.16820597	0.15766073
Mpzl3	-1.3272579	-0.9425043	2.04475372	0.16823773	0.1576708
Chordc1	0.17466779	6.72134123	2.04417638	0.16829498	0.15770475
Rpl32	0.29011229	6.79791779	2.04383151	0.16832919	0.1577171
Slc16a4	-0.5949149	1.73228008	2.04305245	0.1684065	0.15776983
Rit1	0.28276169	4.74407317	2.04216371	0.16849474	0.15783279
Cygb	0.30738903	4.10908569	2.04137631	0.16857297	0.1578711

Gm2518	-1.0878129	-0.6249179	2.04132844	0.16857773	0.1578711
Dhrs4	0.32563177	3.26827748	2.04086289	0.16862401	0.15787686
Ddx25	-0.3360772	4.1774863	2.04074951	0.16863528	0.15787686
Pmp22	0.35503752	8.57001292	2.04063146	0.16864702	0.15787686
Trim28	0.18378128	5.90610985	2.04010597	0.16869928	0.15788384
Stk36	-0.6051062	1.23416764	2.04005227	0.16870462	0.15788384
Mob2	0.45948648	2.48552716	2.03980771	0.16872895	0.15788384
Fn3krp	0.24533258	4.38382354	2.03971008	0.16873867	0.15788384
Rdh14	0.25479346	5.61458882	2.03921896	0.16878754	0.15790987
Clpp	0.27129645	3.49623974	2.03900136	0.1688092	0.15791044
Rtkn2	-0.6969122	1.19225338	2.03861408	0.16884776	0.15792682
Rgl1	0.14929258	7.58694642	2.03805895	0.16890305	0.15795884
Scpep1	0.2998551	5.11264015	2.03778429	0.16893042	0.15796473
Afap1l2	0.37935775	2.61867956	2.03672578	0.16903593	0.15802825
S100a16	0.49901634	3.25723789	2.0366802	0.16904047	0.15802825
Pcdha11	-0.7345372	0.37164766	2.03586978	0.16912131	0.15808412
Pcdha2	-0.8849336	0.19844634	2.03530085	0.16917809	0.15811749
H2-DMa	0.67431519	2.02322094	2.03503308	0.16920482	0.15812277
Ccnd3	0.55844955	4.56294001	2.0346487	0.1692432	0.15813893
Shh	0.57686296	2.02679904	2.03421709	0.16928631	0.15815951
Rtkn	0.3511169	3.44422276	2.03382652	0.16932533	0.15817627
0610037L13F	0.29526202	4.97435407	2.03243709	0.16946424	0.158283
Prmt10	-0.4653309	3.33900613	2.03226165	0.16948179	0.158283
Gpc1	0.36466443	3.38315005	2.0318709	0.16952088	0.15829981
Esrrb	1.16991375	-0.3614553	2.03134143	0.16957387	0.15832052
B3gnt8	-1.1639315	-0.1537182	2.03122751	0.16958528	0.15832052
Dcun1d5	0.20016394	5.95831292	2.03020187	0.169688	0.1583967
Pcdha12	-0.6972138	1.20786734	2.02968239	0.16974005	0.15842558
Rpl19	0.26999748	7.38560878	2.02922544	0.16978586	0.15844862
F2	1.50761594	-1.044684	2.02863964	0.1698446	0.15848372
Hsf2	0.22262609	5.63223317	2.02761316	0.16994759	0.15854085
Smarcc1	0.14615759	7.39862034	2.0276082	0.16994809	0.15854085
Commd4	0.42684359	3.50891298	2.02629386	0.17008008	0.1586285
Dguok	0.40832383	2.97350383	2.02625136	0.17008435	0.1586285
1700013F07I	-0.7763581	-0.0736069	2.02515668	0.17019438	0.15871084
Lrrc49	-0.2987208	4.99706323	2.02495218	0.17021494	0.15871084
Dlgap3	-0.3160881	4.07743113	2.02446533	0.17026391	0.15873677
Gpsm2	-0.4094283	2.5646379	2.02381152	0.1703297	0.15877837
Ntn4	-0.533245	1.3451484	2.02332837	0.17037834	0.15879543
Lyve1	-0.5065743	3.43353616	2.02316906	0.17039438	0.15879543
Smad2	0.15978835	6.52510052	2.02299893	0.17041152	0.15879543
Cct8l1	-2.4790442	-1.1835794	2.02257083	0.17045464	0.15880532
Celsr2	-0.2292269	7.16649663	2.02247322	0.17046447	0.15880532
Cep162	-0.1799544	6.06887196	2.02216409	0.17049562	0.15881462
Mut	0.15614668	5.76690923	2.02166071	0.17054635	0.15884215

Slc25a1	0.41235511	3.82764877	2.020696	0.17064364	0.15889574
Eml2	-0.3489246	2.7595467	2.02067004	0.17064625	0.15889574
Acsf2	0.26195942	4.8861984	2.0195001	0.17076433	0.15898595
Rfxank	0.39506006	2.94693999	2.01869444	0.1708457	0.15903596
Ssc5d	0.44542622	2.55307744	2.01854835	0.17086045	0.15903596
Adamts12	0.45313926	2.48891366	2.0178944	0.17092654	0.15907774
Atrx	-0.2299348	9.86601145	2.01728603	0.17098805	0.15910197
Scn5a	-0.7651348	1.37007776	2.01721731	0.170995	0.15910197
Mrps24	0.35348307	3.95201043	2.01690051	0.17102704	0.15911205
B930059L03l	1.66661792	-1.1958104	2.01630436	0.17108736	0.15914843
Dus3l	-0.298608	3.75938333	2.01564797	0.1711538	0.15919049
Ppp3cc	0.22673629	5.04839543	2.0153109	0.17118793	0.1592025
Wipi2	-0.2699423	5.00363007	2.0148704	0.17123255	0.15922425
Uckl1os	-1.0438909	-0.4792258	2.01443857	0.1712763	0.1592452
Tram2	0.38248637	3.62401292	2.01368189	0.171353	0.15929676
Lpp	0.20850425	7.6917757	2.01238675	0.17148438	0.15937952
Gm20750	-1.5891983	-1.2381917	2.01222113	0.17150119	0.15937952
Tspan11	-0.460361	3.38958987	2.01201564	0.17152205	0.15937952
Prkrir	-0.1815155	5.34315048	2.01196676	0.17152701	0.15937952
Epn3	0.83019763	0.59555436	2.01056013	0.17166988	0.15949252
Anpep	0.39385362	6.47703909	2.01012614	0.17171399	0.15951374
Zhx2	0.27244965	6.38843228	2.0096909	0.17175825	0.15953509
1700028K03l	-0.6913167	2.08229983	2.0094653	0.17178119	0.15953665
Ntrk1	-1.2993316	-1.4088131	2.0089136	0.17183731	0.15956901
Exosc6	0.30869042	3.34859601	2.0075217	0.171979	0.15968082
Sirpb1b	-2.1077274	-1.704659	2.00680962	0.17205154	0.1597284
Kifc5b	-1.6002569	-1.1644293	2.00647425	0.17208572	0.15974036
Sumo3	0.28527855	6.45768741	2.00610078	0.17212379	0.15975593
Thap2	0.24125204	5.05056081	2.00573065	0.17216153	0.15977119
Rpl28	0.35957373	5.33779006	2.00551908	0.1721831	0.15977145
Zmat5	0.46566172	2.44656038	2.00409015	0.17232892	0.15988698
H1fx	1.17122084	-1.4640314	2.00315318	0.17242462	0.15995598
Zfp92	-0.5090119	2.40848391	2.0028499	0.17245561	0.1599561
Golga4	-0.1588077	7.9148528	2.00269424	0.17247152	0.1599561
Pde3a	-0.7357661	1.06521131	2.00252593	0.17248872	0.1599561
AI987944	0.30695367	3.80950383	2.00144214	0.17259954	0.16003909
Nr4a2	-0.5755425	6.88908107	2.00066911	0.17267865	0.16009264
Myadml2	-0.889958	0.67170072	1.99979993	0.17276764	0.16015536
Neto2	-0.2889682	5.74021452	1.99953885	0.17279438	0.16016035
Msc	-1.6757331	-0.979222	1.9991906	0.17283006	0.16017363
3110002H16l	-0.409463	3.3694138	1.99854168	0.17289657	0.16021547
Gm14326	-0.3371719	4.52824376	1.99787811	0.17296461	0.16024437
Lifr	-0.2534017	5.94162796	1.99782096	0.17297047	0.16024437
Cmtr1	-0.2927993	5.59587889	1.9969091	0.17306403	0.16031124
Lyg2	3.6451857	-1.660059	2.02287793	0.17312972	0.16035229

Cox7a2l	0.24881665	7.11294571	1.99561871	0.17319653	0.16039436
Hrsp12	0.28054979	4.29304503	1.99536955	0.17322213	0.16039827
Retn	1.7479611	-1.5550005	1.99470555	0.17329037	0.16044165
Mrpl14	0.45877348	3.03606529	1.99429369	0.17333271	0.16046105
Fam160b1	-0.2362704	5.00786603	1.99398363	0.1733646	0.16047077
Ksr1	0.2793616	3.86342717	1.99264661	0.17350218	0.16057831
Dusp3	-0.1650519	7.09315937	1.99232107	0.1735357	0.16058952
Rap1a	0.23073433	8.59777155	1.9917371	0.17359585	0.16062536
Gapt	1.27656758	-0.1591237	1.99011936	0.17376261	0.16075983
Ccdc163	-1.1756924	0.00792254	1.98965616	0.1738104	0.16078421
Ubxn11	0.89552931	1.41000936	1.98913269	0.17386442	0.16081435
Vgll4	0.43913323	2.97093093	1.98466501	0.17432631	0.1612217
Zscan26	-0.1504898	7.19874015	1.98429202	0.17436494	0.16123755
Stk32c	-0.3837946	2.62635104	1.98364243	0.17443225	0.1612799
Hs6st1	-0.249313	4.62727952	1.98324083	0.17447387	0.1612985
Xlr4c	1.63236387	-1.3636002	1.98251957	0.17454866	0.16132996
Rpl7l1	0.21007804	4.58990866	1.98239447	0.17456163	0.16132996
Cdr1	-0.3556264	9.91723274	1.98227441	0.17457409	0.16132996
Uaca	0.32550972	7.66103414	1.98208319	0.17459393	0.16132996
Fam43a	0.29676033	6.20468166	1.981415	0.17466327	0.16137416
Amotl2	-0.4142514	3.80934103	1.98052879	0.17475529	0.16143929
Bcl7a	0.25702518	4.93507583	1.97993272	0.17481721	0.16147661
Alg2	-0.1541901	7.44663579	1.97964776	0.17484682	0.16148408
Eps15l1	-0.2099535	5.75893508	1.9788689	0.1749278	0.16153898
Gpr150	-0.5973207	0.66432843	1.97758297	0.17506159	0.16164263
Rbm27	0.15417181	7.46391826	1.97665245	0.17515848	0.16171219
Fam73b	-0.2853252	3.66081619	1.97583006	0.17524417	0.16174612
Gltpd2	2.0681821	-1.9420875	1.97576678	0.17525076	0.16174612
Spink10	-1.3818377	-0.9899243	1.97567886	0.17525993	0.16174612
Gm11413	-1.4616169	-1.251623	1.97428751	0.17540503	0.16185201
Dhdh	0.23818073	6.4983225	1.97397405	0.17543774	0.16185201
Foxd2	0.53391947	2.72874182	1.97395819	0.1754394	0.16185201
Wdr70	-0.2946572	3.54999649	1.97255547	0.17558587	0.16196722
Rpf1	0.2417684	4.10670821	1.97223135	0.17561974	0.16197854
Cntln	-0.1857989	6.57104022	1.97013138	0.17583935	0.16214279
Siglece	-1.4367943	-0.5955054	1.97006622	0.17584617	0.16214279
Mroh7	-2.0902281	-0.4460366	1.96990867	0.17586266	0.16214279
Rb1	0.18662873	6.94098613	1.96871459	0.17598771	0.16220364
Stmnd1	-1.410869	-0.550832	1.96866138	0.17599328	0.16220364
Ckmt2	-1.7897683	-1.0422931	1.96865902	0.17599353	0.16220364
Fbxo30	-0.2888821	5.38115965	1.96788324	0.17607483	0.16225864
Tube1	-0.8746965	0.36225688	1.96749723	0.1761153	0.162276
Bmpr1b	-0.3207608	3.2590408	1.96694804	0.17617291	0.16230914
Smpd1	0.3039333	4.97641497	1.96646492	0.1762236	0.16231483
Dner	0.30592619	5.47834739	1.96634451	0.17623623	0.16231483

Metrn	0.97948424	-0.6406921	1.96625421	0.17624571	0.16231483
Zfp879	0.53995985	2.11439961	1.96606437	0.17626564	0.16231483
Radil	-0.5322933	2.13082272	1.96555274	0.17631935	0.16232178
Jak2	-0.1713291	6.25237217	1.96551448	0.17632337	0.16232178
Agbl3	0.32217514	3.1280662	1.96537421	0.1763381	0.16232178
Pggt1b	0.25690003	4.10302833	1.96497138	0.17638041	0.16233943
Zic5	0.55915669	1.62181319	1.96477959	0.17640056	0.16233943
Vps33b	0.2451762	5.18058997	1.96445116	0.17643508	0.16235128
Gstm1	0.32296804	7.64972735	1.96377161	0.17650651	0.16239709
Gm16596	-0.8526892	0.01562541	1.9629123	0.1765969	0.16244226
Fhl5	3.06857214	-2.2783668	1.96289302	0.17659892	0.16244226
Hcrtr2	-0.7274697	0.50982421	1.96238756	0.17665212	0.16247127
Fcer1g	0.41210827	3.20022187	1.96189028	0.17670447	0.16249949
Mageb16-ps	-1.3551402	-0.6955082	1.96166345	0.17672835	0.16250154
Wfdc2	1.32089771	-0.6818079	1.96134012	0.17676241	0.16251293
Xpo6	-0.1533894	6.87091952	1.96089937	0.17680884	0.1625357
Foxp4	0.3239181	4.89409226	1.96034197	0.17686759	0.16255563
Rpl22l1	0.27002258	5.0394012	1.96007976	0.17689523	0.16255563
Zfp869	-0.2408294	5.11244838	1.9600769	0.17689553	0.16255563
Haus8	0.62712224	1.04829652	1.95979565	0.17692519	0.16256297
Miip	-0.758826	0.92175841	1.95893813	0.17701564	0.16262616
02-Mar	-0.2348308	4.75045565	1.95864518	0.17704656	0.16263465
Rabgap1l	0.21237585	8.19784169	1.95800791	0.17711384	0.16267652
Mlst8	-0.3066328	2.91787372	1.95738204	0.17717994	0.16270237
Diras1	0.27583123	5.13546591	1.95733072	0.17718536	0.16270237
Agtpbp1	-0.2054694	8.9554073	1.95687882	0.17723311	0.16271407
Osgepl1	0.26517894	4.77018591	1.95679963	0.17724148	0.16271407
Trpc7	0.45070295	2.40421593	1.95433066	0.17750265	0.16293389
4930452G13	-1.9292921	-1.7082756	1.95362758	0.17757711	0.1629823
Vmn2r57	0.90303841	0.70186386	1.95206126	0.17774314	0.16311472
Scaf4	0.16764814	6.40873382	1.95152433	0.17780009	0.16314703
Bhlhb9	-0.1764254	5.47364275	1.95098471	0.17785735	0.16317961
Tmem245	-0.2374815	5.51547541	1.95047391	0.17791158	0.1632094
Fdps	0.32620106	3.83319565	1.9501691	0.17794395	0.16321142
Prep	-0.1912073	4.39806506	1.95004335	0.1779573	0.16321142
Snord4a	-0.6006073	0.58716041	1.94922791	0.17804394	0.16323996
Edn1	0.97921711	0.28649331	1.94922497	0.17804425	0.16323996
Cdkl3	-0.2944706	3.66734289	1.94913609	0.1780537	0.16323996
Gm4787	-1.1275873	2.161135	1.9488683	0.17808216	0.1632461
Birc2	0.19073918	5.4885812	1.94760524	0.17821649	0.16329722
Zfp263	-0.3072975	4.16060069	1.94753843	0.1782236	0.16329722
Wwc1	-0.2422937	4.29363113	1.9475383	0.17822362	0.16329722
Lims2	-0.4032834	2.67256742	1.94734731	0.17824394	0.16329722
Igfals	2.37125289	-2.2123969	1.94732072	0.17824677	0.16329722
AA414768	-0.3062365	3.09144475	1.94627967	0.17835761	0.1633788

Lrfn2	-0.4224334	1.80541065	1.94567137	0.17842241	0.16341821
Pcdhb3	-0.4200662	2.11823708	1.94533864	0.17845787	0.16342744
Ninl	-0.3577561	3.10125781	1.94516796	0.17847606	0.16342744
Taco1	0.50573158	1.58711343	1.94421286	0.1785779	0.16349132
2610002J02F	0.46820683	3.24413391	1.94410491	0.17858942	0.16349132
Gareml	-0.5137939	2.21577455	1.94347089	0.17865707	0.16349638
E030019B13I	-1.5330065	-1.3835577	1.94346382	0.17865782	0.16349638
Msh2	0.25141342	4.64569386	1.94344035	0.17866033	0.16349638
Spo11	1.31932959	0.07250482	1.94293347	0.17871444	0.16352595
Bms1	-0.1933455	6.0653261	1.9423785	0.1787737	0.16354828
Ticam2	-0.8702843	0.92254392	1.94229665	0.17878245	0.16354828
Cps1	-1.6689348	-1.1991951	1.94163788	0.17885283	0.16358036
Amdhd1	-1.7415345	-1.7095124	1.941342	0.17888446	0.16358036
Cep131	-0.3539732	2.86455836	1.94117593	0.17890221	0.16358036
Zfp647	0.50658479	1.3500515	1.94115225	0.17890474	0.16358036
Dmgdh	0.91239676	0.06703914	1.94049308	0.17897523	0.16362486
Tdrd5	-0.7455298	1.22862972	1.93990801	0.17903782	0.16365797
Gm17019	-1.3807101	-1.2206057	1.93949933	0.17908156	0.16365797
5830403L16F	1.22077206	-0.5332773	1.93948871	0.1790827	0.16365797
Eif2b4	0.34810056	3.19675848	1.93933912	0.17909871	0.16365797
Rgs18	1.02178666	-0.0944499	1.93833834	0.17920589	0.16373596
Adam21	-0.7536093	0.29743149	1.93781787	0.17926166	0.16376696
Smoc2	0.39192217	3.40767044	1.93669127	0.17938245	0.16384
Zfp282	-0.5165048	1.90740085	1.93666484	0.17938528	0.16384
Sgcb	-0.2478893	4.81756868	1.93624895	0.1794299	0.16386079
Pcdhgc4	0.78823059	0.9926195	1.93566937	0.1794921	0.16389764
Ddx1	0.1693749	6.99049267	1.93462458	0.17960429	0.16398013
4931414P19I	-0.8688726	1.11634917	1.93278717	0.17980181	0.16414048
Thbs4	-0.754891	0.57915578	1.93151932	0.17993825	0.16424506
Dzip1l	-0.2907241	3.47305147	1.9311173	0.17998155	0.16426459
Fbxo10	-0.3454775	4.18832971	1.9308971	0.18000526	0.16426625
Trip10	0.61977953	2.25482018	1.92992391	0.18011013	0.16430883
Mpg	0.47199836	1.96183919	1.9298932	0.18011344	0.16430883
Lypd6	-0.2551807	4.9247082	1.9298543	0.18011764	0.16430883
1200014J11F	-0.1863493	5.36314191	1.92929693	0.18017774	0.16434367
1700019A02I	-1.3928341	-1.7189132	1.92898382	0.18021151	0.16435448
Actl6b	-0.4017717	3.2826385	1.92833197	0.18028184	0.16439864
Phb2	0.22841294	5.0191574	1.92734415	0.18038849	0.16447555
Eif2b5	0.23124045	4.56639285	1.9271447	0.18041003	0.16447555
E130307A14I	-0.3669849	2.29238303	1.92671392	0.18045657	0.16449799
Ptpru	-0.3288284	3.24593205	1.926233	0.18050855	0.16452537
Zbtb45	-0.3918753	2.41541769	1.9257825	0.18055725	0.16453804
Hic1	0.4405045	3.26674791	1.92569868	0.18056631	0.16453804
2610524H06I	0.36767805	2.15892431	1.92499701	0.18064221	0.1645872
Mrps11	0.54210256	1.41683712	1.92456229	0.18068925	0.16461007

Xkr4	-0.3472132	4.24244498	1.92408136	0.1807413	0.1646375
Gm7609	-0.961273	-0.6325316	1.92244762	0.18091829	0.1647787
Tmem101	-0.5928479	2.47401716	1.92122823	0.18105052	0.16487912
Prrg4	-0.6494145	1.78377809	1.92078727	0.18109837	0.1648941
Rab11fip4	-0.2830204	5.52632492	1.92067153	0.18111093	0.1648941
Ero1l	-0.184742	6.37695427	1.92029206	0.18115212	0.16490943
Ripk1	0.3225672	3.73532796	1.91997994	0.18118601	0.16490943
Snx22	-0.8828567	-0.2486315	1.91990893	0.18119372	0.16490943
Oxnad1	0.37438647	3.78928905	1.91969625	0.18121682	0.16491044
Cd160	-0.7290683	1.22639695	1.91926856	0.18126328	0.16493271
Zfyve9	0.3076769	3.85483059	1.91844193	0.18135312	0.16497729
Mdk	0.42895881	3.66848016	1.91841309	0.18135626	0.16497729
Dus4l	0.39943319	2.58436004	1.91811853	0.18138829	0.16498642
Tti2	-0.2613351	4.5965	1.9179136	0.18141057	0.16498668
Mkl1	0.17776345	5.55924494	1.91727809	0.18147971	0.16502955
Nek3	-0.7493246	0.95365832	1.91606505	0.18161175	0.16512529
Sdhaf1	0.39622836	2.31989015	1.91584913	0.18163527	0.16512529
Stag2	-0.1699138	6.69822651	1.91543068	0.18168086	0.16512529
Txndc12	0.32594654	3.96438473	1.9153725	0.1816872	0.16512529
D3Ertd751e	0.22352568	5.72744736	1.91523409	0.18170228	0.16512529
Tpx2	0.53778098	1.79765562	1.91509835	0.18171707	0.16512529
Hipk1	-0.157319	8.53399763	1.91398002	0.181839	0.16521608
Dab2	0.251041	8.40747023	1.9132434	0.18191937	0.16526908
Siglech	-0.4770504	2.13107819	1.91234162	0.18201782	0.16533849
Pcyox1l	-0.5054735	1.9727679	1.91181002	0.18207589	0.16537121
S1pr3	-0.3083879	3.33109425	1.91138293	0.18212255	0.16539357
Asic3	-2.0565426	-2.0363089	1.93487764	0.18225955	0.16549795
Etv3	0.21716586	3.91685719	1.90986172	0.18228889	0.16550456
Smpd2	-0.4576003	2.31042198	1.90947899	0.18233077	0.16552255
Bcl2l13	-0.233665	5.02911328	1.90825024	0.18246531	0.16562464
Serpinb8	0.40358662	3.40522974	1.90800279	0.18249242	0.1656292
Slc13a1	-1.6646228	-1.3495689	1.9069651	0.18260615	0.16571238
Eif1ad	0.27998407	4.17200908	1.90554822	0.18276158	0.16583337
Zcchc7	-0.2343424	5.64380246	1.90440157	0.18288749	0.16592754
Rchy1	0.20658818	5.50211776	1.90415095	0.18291502	0.16593245
2810468N07	-0.3824225	2.59220216	1.90380141	0.18295343	0.16594723
Myh7	0.39387125	2.56956059	1.90297598	0.18304418	0.16600946
Polr2b	-0.1811065	6.23667482	1.90254202	0.18309191	0.16603268
4932411N23	-1.0473825	-0.9359805	1.9021965	0.18312992	0.16604707
9430018G01	-1.7111486	-1.5023933	1.90178746	0.18317493	0.16606782
Gm6251	0.63534799	0.42859106	1.90145454	0.18321158	0.16608097
E2f5	0.23017645	4.32218395	1.90048356	0.18331852	0.1661396
6330418K02I	0.60218105	1.11067126	1.90044452	0.18332282	0.1661396
Liph	-2.2761735	-1.6566254	1.90021805	0.18334777	0.1661396
Dtx1	0.26102679	4.62920153	1.90006305	0.18336485	0.1661396

Ezh1	-0.2471316	5.19034021	1.89977724	0.18339636	0.16614807
Bche	-0.3938165	7.2717699	1.89936492	0.18344181	0.16616919
Cdh18	0.38050195	3.95230194	1.89869669	0.18351551	0.16621588
Nek5	-1.9619842	-1.245548	1.89849539	0.18353772	0.16621592
Tsen2	-0.4224828	2.55053388	1.89803907	0.18358808	0.16624145
Cntnap2	-0.3017962	6.26469547	1.89759228	0.1836374	0.16626605
Zfp472	-0.4746205	1.75869423	1.89677046	0.18372816	0.16628254
Irf3	0.41043015	2.69589221	1.8964212	0.18376675	0.16628254
Socs7	-0.2116244	6.96844492	1.89629773	0.1837804	0.16628254
Zfp605	0.21694181	4.69555872	1.89627741	0.18378264	0.16628254
Slc6a1	-0.2411872	7.70563936	1.89623252	0.1837876	0.16628254
Fbln5	-0.5553151	4.21843074	1.89622333	0.18378862	0.16628254
Trappc6b	0.22253192	7.79666568	1.89601483	0.18381167	0.16628333
Gprc5c	0.43403946	3.07802592	1.89545756	0.18387328	0.16631197
Med11	0.45570583	2.92929786	1.89532745	0.18388767	0.16631197
S100a1	0.42306079	4.83217325	1.89505471	0.18391783	0.1663192
Aldh18a1	-0.320785	3.57618485	1.89312874	0.18413103	0.16643367
Tsc22d2	0.16462509	6.84533582	1.89295297	0.1841505	0.16643367
Tox	0.25787329	5.74876714	1.89290917	0.18415536	0.16643367
Slc35d1	-0.2273071	4.13099089	1.89282952	0.18416418	0.16643367
Gm14288	0.51843771	0.84512697	1.89273199	0.18417499	0.16643367
Cdc45	0.75033351	0.92619424	1.89270903	0.18417753	0.16643367
Lyrm9	-0.195597	5.68993301	1.89175667	0.1842831	0.16650901
Snd1	-0.1853786	5.02085632	1.88968643	0.18451284	0.16667898
Ccdc121	0.83841231	0.68327863	1.88966107	0.18451566	0.16667898
Rps6kc1	-0.2116937	4.98005424	1.88885303	0.18460543	0.16673999
B130034C11	0.86518499	0.87810322	1.88857451	0.18463638	0.16674787
8430408G22	-2.4928499	-1.1148148	1.88819558	0.18467851	0.16676584
6330403A02	0.22738754	7.47245341	1.88766057	0.18473801	0.16679949
2700089E24	0.19309791	8.43300585	1.88684273	0.184829	0.16682703
Tradd	0.69204004	0.67360552	1.88663411	0.18485222	0.16682703
Rps17	0.22919371	7.26328492	1.88630716	0.18488862	0.16682703
Gm5088	0.43944557	1.55621154	1.88623394	0.18489677	0.16682703
Crat	0.19516501	5.36198451	1.88617881	0.18490291	0.16682703
Nfe2	2.14288673	-1.1396766	1.88591957	0.18493178	0.16682703
Aplp2	-0.14511	8.46249735	1.88588172	0.18493599	0.16682703
Sec61a1	0.26605327	4.9649272	1.88578804	0.18494643	0.16682703
Wnt2b	-0.3281844	2.90172608	1.88453365	0.18508621	0.16693304
Copg1	-0.1884973	7.25418824	1.88400712	0.18514492	0.16696592
Cetn4	0.35668433	4.02178654	1.88372365	0.18517654	0.16697436
03-Mar	-1.0933877	0.04069443	1.88348003	0.18520372	0.1669788
Tal2	-2.4806935	-2.0176317	1.8832008	0.18523488	0.16698682
Klhl5	-0.2078273	5.72844719	1.88274365	0.1852859	0.16701275
Snrnp35	0.37889779	3.60208092	1.88193935	0.18537572	0.16706033
Mrps31	0.29019421	4.56028874	1.8818721	0.18538323	0.16706033

Eif2b1	0.2944836	4.79094889	1.88128234	0.18544913	0.16709963
Tcirg1	-0.4829339	1.52990941	1.88007764	0.18558382	0.16720092
Pde7b	-0.1498669	6.60018981	1.87986075	0.18560809	0.16720269
Sdc2	0.32924964	6.59430272	1.8791069	0.18569245	0.16725527
Cdh6	-0.2996291	2.79517801	1.87894078	0.18571104	0.16725527
Tmsb15b1	-0.5737449	1.17472549	1.87801213	0.18581504	0.16732885
Tmem184b	-0.21011	4.72598387	1.87712587	0.18591436	0.16738104
Larp4b	0.13593436	7.45689149	1.87709671	0.18591763	0.16738104
H3f3b	0.29910547	9.00360196	1.87681069	0.1859497	0.16738982
Lsm8	0.28071933	5.66105013	1.87494007	0.1861596	0.16755866
Thtpa	0.22406052	4.55589553	1.8746194	0.18619561	0.16756994
Gse1	-0.2594014	5.57081042	1.87437821	0.1862227	0.16756994
Tnfrsf25	-2.0255908	-1.3089208	1.87415671	0.18624759	0.16756994
Usp51	-0.6741771	1.25592002	1.87403303	0.18626148	0.16756994
Zscan20	-0.4029822	2.05490218	1.87381577	0.1862859	0.16757181
Naaa	-0.3386238	3.92316073	1.87257279	0.18642565	0.16767741
Zbp1	0.60308112	1.02666579	1.87217267	0.18647067	0.16769779
Mrpl10	0.23575102	4.71819044	1.8706751	0.18663927	0.1678293
BB557941	1.70397723	-0.6396822	1.87042328	0.18666764	0.16783469
Gls2	-0.4370329	2.43157506	1.86897116	0.18683133	0.16794688
Phf10	0.19687232	5.32366642	1.86891918	0.18683719	0.16794688
Gm3219	-0.5423283	0.84330458	1.86796427	0.18694494	0.16801219
Atxn7l3	-0.2392295	5.96367307	1.86784989	0.18695785	0.16801219
Adipor2	0.23239573	4.69808897	1.86748547	0.18699899	0.16801219
Hist1h4j	0.61496573	0.67386973	1.86748152	0.18699944	0.16801219
Pcdhb9	-0.4234763	2.25524528	1.86686384	0.1870692	0.168036
Aga	0.44451555	3.47617596	1.86672992	0.18708433	0.168036
Snord99	-1.9136396	-1.8805611	1.86665188	0.18709315	0.168036
Pltp	-0.4217138	4.01039227	1.86491401	0.18728963	0.16819233
Mapk13	-2.1368688	-1.634588	1.86429041	0.18736019	0.16822008
Rabl6	-0.2041016	5.81756979	1.86424464	0.18736537	0.16822008
Fndc7	1.87941286	-1.3887912	1.86368728	0.18742847	0.16825659
Etnk2	0.41691177	2.60113181	1.86296382	0.18751042	0.16831001
Rps26	0.31731761	5.54799771	1.86258143	0.18755375	0.16832876
Fgf2	0.49155498	1.59855045	1.86219174	0.18759792	0.16834826
3110052M02	-0.2134063	4.84073189	1.86135145	0.1876932	0.16841362
Bfsp1	0.85731442	0.95059007	1.86106374	0.18772584	0.16842276
Ppip5k1	-0.2932783	5.49143345	1.86076359	0.1877599	0.16843317
Gnptg	-0.2320176	4.63941865	1.86019669	0.18782425	0.16847075
Cpne1	0.18621395	5.90281002	1.85927515	0.18792891	0.16854447
Rps6ka6	0.41738055	2.62959887	1.8589121	0.18797016	0.16854594
Suv420h1	-0.152452	6.72862008	1.85886526	0.18797549	0.16854594
Lhx8	-0.4804976	2.6491906	1.85738993	0.18814324	0.1686762
Pilrb2	-1.7880775	-0.5024789	1.85617367	0.18828168	0.16878014
Atp2c2	0.83458641	0.00134164	1.85494396	0.18842178	0.1688529

Smc1b	-1.3777345	-0.7178383	1.85477438	0.18844111	0.1688529
Txlnb	-0.5019326	2.24854528	1.85476483	0.1884422	0.1688529
Plxna4	-0.2609007	7.02214531	1.85428665	0.18849672	0.1688529
Raver2	-0.305363	3.14227939	1.85425696	0.1885001	0.1688529
Dync1li1	0.23735267	5.04728039	1.85423949	0.1885021	0.1688529
Arhgdig	0.40822106	3.12759263	1.85357491	0.1885779	0.1688529
Epm2aip1	-0.2611647	7.97617275	1.85343699	0.18859364	0.1688529
Tceb3	0.21108156	6.00143541	1.85337802	0.18860037	0.1688529
Car7	0.52198166	1.42663876	1.85335696	0.18860277	0.1688529
Adm	-0.4116535	2.24747852	1.8532817	0.18861136	0.1688529
Nprl2	0.37663266	2.467394	1.85272277	0.18867516	0.1688529
Ahsa2	0.22576969	4.73143672	1.85255315	0.18869453	0.1688529
Gpr17	-0.4034413	2.74550711	1.85251095	0.18869935	0.1688529
Map4k5	-0.1635587	5.95929711	1.85250087	0.1887005	0.1688529
Klk10	-1.1277218	-0.2302173	1.851455	0.18881997	0.16893966
Camk2d	0.18460164	7.31796937	1.85071466	0.1889046	0.16898669
Msrb3	0.28143255	5.39694695	1.85060102	0.18891759	0.16898669
LOC381967	-1.0574718	-0.4098663	1.85034439	0.18894694	0.16899279
Phf13	0.31996727	3.5556069	1.84980381	0.18900878	0.16902794
Rnf169	-0.1512661	6.75839916	1.84897738	0.18910337	0.16908197
Cd3e	0.5945115	2.30550467	1.84888211	0.18911428	0.16908197
Cfhr2	0.72972706	0.69676622	1.84831607	0.18917911	0.16910525
Idua	-0.3096446	3.57886831	1.84800148	0.18921515	0.16910525
Amdhd2	0.48451226	1.22239547	1.84800147	0.18921515	0.16910525
Kdm5d	-0.3303191	4.88224008	1.84786762	0.18923048	0.16910525
Zfp39	-0.2026589	5.36008907	1.84745487	0.18927779	0.16912737
Blzf1	0.23846703	4.90929891	1.84718794	0.18930839	0.16913456
Aspn	-1.372892	-0.6780731	1.84682038	0.18935053	0.16913895
Zfp280d	-0.2583682	6.75746108	1.84675184	0.18935839	0.16913895
1700018A04I	1.9009345	-1.9461086	1.84632358	0.18940751	0.16916268
Dleu2	0.45876628	2.42882056	1.84581545	0.18946582	0.16919461
Thap3	-0.551093	2.29989503	1.84495626	0.18956445	0.16924574
Snx24	0.29380783	4.98465963	1.84492369	0.18956819	0.16924574
Esyt1	-0.3449178	3.45697832	1.8442056	0.18965068	0.16928462
Trim12c	0.29550388	5.55706008	1.84415168	0.18965688	0.16928462
Fastk	0.28056309	3.68472314	1.84390364	0.18968538	0.16928573
Ntrk3	-0.2538637	5.67850937	1.84374807	0.18970326	0.16928573
Haus3	0.2500488	4.11729922	1.84308754	0.1897792	0.16933336
Lurap1	-0.4596752	1.9634037	1.84279736	0.18981258	0.16934299
Chfr	-0.3984763	4.18743872	1.84212564	0.18988986	0.1693918
3010001F23I	-0.5761396	1.13950441	1.84189199	0.18991675	0.16939564
Dclk1	0.24076773	10.5375499	1.84151217	0.18996048	0.16939674
Rps6ka2	-0.2412153	5.22080832	1.84148899	0.18996315	0.16939674
Csgalnact2	-0.2823075	3.38256503	1.84015861	0.1901164	0.16949589
Arsb	0.18530676	7.01737196	1.84008512	0.19012487	0.16949589

Ptrh1	1.84221715	-1.2887047	1.83993542	0.19014212	0.16949589
E130112N10	-1.4827977	-0.6904843	1.83932808	0.19021215	0.16952181
Tmem110	-0.2969312	2.80599648	1.83916613	0.19023083	0.16952181
Mab21l3	-1.7808131	-1.79967	1.8390953	0.190239	0.16952181
Mmd	-0.1585677	7.33839218	1.83668042	0.1905178	0.16972998
Calcoco2	-0.7441125	-0.0155403	1.83667984	0.19051786	0.16972998
8030423F21I	-0.9150438	-0.8160297	1.83537859	0.1906683	0.16984383
Ftsj2	0.5831842	2.03862433	1.83415515	0.19080988	0.1699247
Ovca2	0.30042295	3.35314287	1.83412982	0.19081281	0.1699247
Spata1	-0.5926304	1.26760515	1.83400694	0.19082704	0.1699247
Idnk	0.36556592	3.48742722	1.83335783	0.19090222	0.16997146
Bloc1s4	0.37693152	4.26340494	1.83240921	0.19101215	0.17004916
Tldc1	0.55816581	1.15750519	1.83135912	0.19113393	0.17013738
Doc2b	0.36338849	4.21046912	1.8305599	0.19122669	0.17019975
Abcb10	-0.2950638	3.68773731	1.8296965	0.19132695	0.17025987
Eef2k	-0.2605233	4.34373641	1.82958738	0.19133963	0.17025987
Ccdc124	0.34731902	4.3329808	1.82918252	0.19138667	0.1702748
Faim2	0.18721311	6.72865198	1.82905223	0.19140181	0.1702748
Fgf10	-0.61275	2.46889666	1.82805742	0.19151747	0.17034128
Ackr3	0.31690582	3.93527382	1.82801887	0.19152195	0.17034128
Mex3c	0.1880788	5.21447884	1.82736435	0.1915981	0.17038881
Wdfy2	-0.543746	1.70188876	1.82641638	0.19170846	0.17045291
A330070K13I	-1.3880122	-0.6852585	1.82635474	0.19171563	0.17045291
Aloxe3	0.46778464	1.83074161	1.82613957	0.19174069	0.17045499
Morn3	-1.727572	-1.2840511	1.8256502	0.19179771	0.17048547
Pla2g12a	-0.3088281	3.72783429	1.82446375	0.19193601	0.17058819
Rhoc	0.38222849	3.48230513	1.82409496	0.19197903	0.17060621
Slfn2	-0.9678563	0.01257306	1.82305869	0.19209996	0.17069151
Amacr	0.28584881	3.65200596	1.82288261	0.19212052	0.17069151
Iqca	-1.211655	-0.2712999	1.82222727	0.19219706	0.17073929
Homer1	0.20174233	8.40981841	1.82178636	0.19224858	0.17074916
Pcyt1a	0.20844123	5.75957855	1.82174254	0.1922537	0.17074916
E030013I19R	1.3941864	-0.3495429	1.82017049	0.19243752	0.17088162
Cwh43	-1.4067318	-1.0775898	1.82007751	0.1924484	0.17088162
Cyp2b10	-1.8720086	-1.7136659	1.81922469	0.19254822	0.17095002
Zfp825	0.24011532	4.52217882	1.81896966	0.19257809	0.1709563
Fbxo41	-0.274435	5.17505856	1.81798441	0.19269351	0.17102702
C330021F23I	-0.3593683	2.50168453	1.81787951	0.19270581	0.17102702
Thnsl2	-0.4231772	1.59740363	1.81757634	0.19274135	0.17102702
Nptn	0.1827391	8.10613287	1.81751144	0.19274895	0.17102702
Mier1	0.1934647	6.44738241	1.81728505	0.1927755	0.17103034
Mir377	-1.5319288	-0.8233461	1.81602658	0.19292314	0.17114109
Rpn2	0.28251248	5.31139917	1.81524034	0.19301544	0.17120273
Hgs	0.28477573	4.12396268	1.81478813	0.19306856	0.17121983
Rap1gap	-0.3035987	4.12132355	1.81468751	0.19308038	0.17121983

4931440P22I	1.28035371	-1.4315636	1.81370969	0.19319531	0.17128141
Coa7	0.42454934	2.48376687	1.81370813	0.19319549	0.17128141
Il6st	0.22215789	7.38933824	1.81333594	0.19323926	0.17129997
Slc37a3	-0.2875052	4.04118976	1.81309349	0.19326778	0.17130501
Zcchc16	-0.3692647	2.91030763	1.81262193	0.19332326	0.17131824
Usp37	-0.2514737	5.63517387	1.81224672	0.19336741	0.17131824
Kirrel3	-0.356924	3.45483046	1.81221381	0.19337129	0.17131824
Sephs2	0.26003599	4.04139257	1.81219026	0.19337406	0.17131824
Pfkfb4	-0.5832686	1.19847148	1.81112313	0.19349972	0.17140933
Glipr1	1.4247035	-0.9652955	1.81090542	0.19352537	0.17141181
4833411C07I	-1.4852163	-1.3355056	1.81042825	0.19358161	0.17144137
Sntg2	-0.7033972	0.90102064	1.80947859	0.19369358	0.1715172
Fam179b	-0.1735051	6.53940217	1.80927169	0.19371799	0.1715172
Letm2	-0.3380524	3.25028569	1.80912048	0.19373583	0.1715172
Idi1	0.24825034	5.81647658	1.8078597	0.19388465	0.1716287
Zfp949	-0.2283288	4.59449231	1.8072773	0.19395345	0.1716587
9330159F19I	-0.2668279	6.85919471	1.80695962	0.19399099	0.1716587
Tmie	-0.4857879	1.66140643	1.80689092	0.19399911	0.1716587
Cnp	-0.2210877	6.61886777	1.80679808	0.19401008	0.1716587
Tex10	0.23119438	4.35355981	1.80600157	0.19410425	0.17171207
Crtap	0.50047277	4.08574639	1.80587017	0.19411979	0.17171207
Fam175a	-0.3525156	2.6092575	1.80554075	0.19415876	0.17171207
Casp6	0.39992187	2.37330272	1.80551373	0.19416196	0.17171207
0610009L18I	0.51923825	0.8437656	1.80444275	0.19428871	0.17180391
LOC106740	-0.280142	4.1747146	1.80402805	0.19433783	0.17182709
Fbn1	-0.3049814	3.99856872	1.80370515	0.19437608	0.17184065
Car2	-0.1586598	7.0909454	1.80309982	0.19444781	0.17186416
Cnot8	0.21612989	4.59883218	1.80309405	0.19444849	0.17186416
Snora31	-1.4228825	-1.2004144	1.80265939	0.19450002	0.17188945
AI197445	-0.8209278	0.52961767	1.80126384	0.19466557	0.1720025
Npbwr1	-1.0083203	0.40904425	1.80088176	0.19471092	0.1720025
Ppp1r14c	0.29321882	3.61494466	1.80077284	0.19472386	0.1720025
Caskin2	-0.3480547	2.60278845	1.80071444	0.19473079	0.1720025
Cdc73	0.18482017	6.15338114	1.80042979	0.19476459	0.1720025
Htatsf1	0.14234182	8.39789574	1.80042203	0.19476552	0.1720025
Wdyhv1	0.25384923	4.05496611	1.79838344	0.19500782	0.17219621
Epha7	-0.2448675	6.56712493	1.79818152	0.19503184	0.17219715
Bcl9	-0.1765426	6.42996014	1.79712016	0.19515816	0.1722884
Pik3ap1	0.43448346	3.13275307	1.79655004	0.19522606	0.17231351
Slc16a12	0.37005382	4.08482467	1.79633124	0.19525212	0.17231351
Nfkbie	0.54262478	2.13805308	1.7962879	0.19525728	0.17231351
Agpat3	-0.2375264	4.96856857	1.79610998	0.19527848	0.17231351
Erich6	-0.8231246	1.27272317	1.795597	0.19533962	0.17234718
Cilp2	0.94006708	0.61710162	1.79519912	0.19538706	0.17236876
Rai2	0.32387995	3.16974792	1.79494462	0.19541741	0.17237526

Amigo2	0.44642192	2.41715299	1.79246049	0.19571395	0.17260619
Tfpi	0.33180925	6.01795099	1.79221256	0.19574358	0.17260619
Ncoa2	-0.1963776	8.34814056	1.7921073	0.19575615	0.17260619
Lamp1	0.29867153	7.56325449	1.79198105	0.19577125	0.17260619
Kctd14	-0.7526603	-0.0124869	1.79163306	0.19581285	0.17262258
Ipo8	-0.2097498	5.43226085	1.79107876	0.19587913	0.17264867
Sugp1	0.23925692	4.58008137	1.79100066	0.19588848	0.17264867
Tspan18	0.49568751	2.75603736	1.7904782	0.19595099	0.17268348
Ccdc177	-0.4456307	2.74825824	1.78969727	0.19604447	0.17274556
Zc3hc1	0.40156514	2.28311213	1.78914443	0.19611068	0.17278361
Slc8a3	-0.2708359	3.92640813	1.78886553	0.19614409	0.17279242
Atxn7l3b	0.16564264	7.34105172	1.78850072	0.1961878	0.17279242
Shq1	-0.6103599	1.05411316	1.78848422	0.19618978	0.17279242
Phc1	-0.168656	6.3147553	1.78808027	0.1962382	0.17281477
Hdgfrp2	0.24753152	5.05959439	1.78744867	0.19631394	0.17286118
Ms4a6d	1.86703095	-1.2205621	1.78638149	0.196442	0.17293045
Shf	-0.6174634	1.94867076	1.78622897	0.19646031	0.17293045
Btn2a2	-1.7705977	-0.4733493	1.78621675	0.19646177	0.17293045
Myom1	0.97637398	0.4210617	1.78525349	0.19657746	0.17301198
Fech	0.18805236	7.02489319	1.78442019	0.19667761	0.17307982
Stard4	0.26055371	3.99959699	1.78341147	0.19679893	0.17316626
Slc25a15	0.29574002	3.41197835	1.78308507	0.1968382	0.17318051
Rdh13	-0.2560113	3.81621794	1.78284835	0.19686669	0.17318526
Mrpl50	0.26222497	5.7014081	1.7823494	0.19692676	0.17321779
Capn2	0.20443447	7.15307409	1.78210271	0.19695647	0.17321846
Calcrl	0.26969724	4.34254882	1.78140448	0.19704058	0.17321846
Mtmr12	-0.1516633	6.44446954	1.78135131	0.19704699	0.17321846
Ppargc1b	-0.4026114	3.29665832	1.78131408	0.19705147	0.17321846
Kdsr	-0.2258426	4.62322859	1.78129927	0.19705326	0.17321846
Sec24d	0.26139475	4.40749598	1.78119296	0.19706607	0.17321846
Rab3c	-0.213659	9.29562799	1.78092524	0.19709834	0.17322652
Gstz1	-0.2017277	4.65541876	1.78067167	0.19712891	0.17323309
Serpinb6a	0.34022005	5.68020965	1.78019213	0.19718673	0.17326361
Rbm38	-1.0865174	0.8031753	1.77966767	0.19725	0.1732989
Ybx2	-0.9403193	-0.0781036	1.77932724	0.19729108	0.17331274
Mettl5	0.29025673	3.45748939	1.77915423	0.19731196	0.17331274
Dennd3	-0.5322523	1.70226287	1.77890066	0.19734257	0.17331933
Dbnl	0.22428743	5.18181118	1.77847599	0.19739385	0.17333356
Glra3	-0.5914935	1.20109565	1.77838381	0.19740498	0.17333356
Arid5a	0.50628866	1.19531805	1.77766201	0.19749218	0.17338983
Zfp941	-0.2427801	4.53019868	1.77743951	0.19751908	0.17339314
Zswim1	-0.2430139	3.84826167	1.7767844	0.19759827	0.1734343
Thada	-0.2569361	5.05442858	1.77666924	0.1976122	0.1734343
Bhlha15	1.18323609	-0.1500511	1.77605189	0.19768688	0.17346407
Mpdu1	0.38820973	2.850041	1.7758398	0.19771254	0.17346407

Tas1r3	-0.8851677	-0.1782649	1.77581536	0.1977155	0.17346407
Adamts15	0.38866768	2.60173174	1.77515056	0.19779597	0.17351438
Tmod3	0.27282791	7.80433048	1.77422081	0.19790857	0.17357421
Zbtb43	-0.2835797	4.51846627	1.77408042	0.19792559	0.17357421
B330016D10	-0.9288118	0.44110558	1.77384829	0.19795372	0.17357421
1110038F14I	0.36177739	3.35510242	1.77382343	0.19795673	0.17357421
Crbn	-0.1763859	6.44617945	1.77125616	0.19826818	0.17381518
Gabra2	-0.2815968	4.85233987	1.77117622	0.19827789	0.17381518
3110070M22	1.19052116	-0.8977719	1.7708654	0.19831564	0.17381998
Zc3h13	-0.1883394	8.06484843	1.77074961	0.19832971	0.17381998
Clec7a	-1.4008181	0.80824858	1.77038034	0.19837457	0.17383899
Zfp59	-0.3371687	3.33000166	1.76819545	0.1986403	0.17404514
Paqr9	-0.2640533	5.44706084	1.76806459	0.19865622	0.17404514
Amer3	-0.3013586	3.42676157	1.76713274	0.1987697	0.17412422
Alg9	-0.3946379	2.99543005	1.7653028	0.19899278	0.17429928
1700034H15I	-0.5756282	2.06910052	1.7643485	0.19910924	0.17438093
D6Wsu163e	-0.2409957	4.54406392	1.76415119	0.19913333	0.17438167
Top1	0.18427191	8.05108416	1.76386054	0.19916882	0.17439239
Cenpe	-0.4119385	2.89436466	1.76354794	0.199207	0.17440546
BC024978	0.23675009	4.79122258	1.76311659	0.1992597	0.17443124
Cradd	0.31766964	4.87140784	1.7623953	0.19934786	0.17448806
Dock1	-0.1949369	5.76329246	1.76217937	0.19937426	0.1744908
Bcl6b	-0.7852945	0.05208606	1.76193612	0.19940401	0.17449648
Selenbp2	-0.9046158	-0.2173687	1.76161889	0.19944281	0.17451008
Hilpda	0.4002389	2.63433833	1.7611332	0.19950224	0.17454172
Sars	0.23279294	4.81691384	1.760452	0.19958563	0.17459431
Syn3	-0.2731806	6.89338645	1.760069	0.19963253	0.17461498
B4galt6	0.14334825	7.17140345	1.75875691	0.19979332	0.17473524
Arid5b	-0.1857493	6.23806089	1.75850231	0.19982453	0.17474217
Hk2	-0.3664843	2.80291057	1.75818286	0.19986371	0.17475605
1700034I23R	-1.1778617	-0.3781941	1.75792477	0.19989537	0.17476337
Csnk2a1	0.14812722	6.99510857	1.75759048	0.19993639	0.17477885
Eda	0.44067492	2.48147763	1.7573335	0.19996793	0.17478605
Tmx1	0.27060923	4.8957351	1.75688743	0.20002268	0.17481355
Tsga10	-0.222575	4.98707404	1.75668914	0.20004703	0.17481446
Adnp2	-0.2507783	4.66110951	1.75560287	0.20018048	0.1749107
A530013C23I	1.43109521	-1.4641545	1.75503608	0.20025015	0.17493602
Zzz3	0.1559605	7.29131947	1.75498768	0.2002561	0.17493602
Tppp	0.15774136	8.81381514	1.75437975	0.20033087	0.17498096
Fxyd5	0.39794933	6.75997088	1.75365456	0.20042011	0.17503852
Itgb7	-1.2392033	-1.4779286	1.75338876	0.20045283	0.17504671
Kdm4b	-0.3382123	2.7623584	1.75259754	0.20055026	0.17511142
C1ra	0.65044973	0.72250136	1.75231836	0.20058466	0.17512106
Dpp3	0.30144358	3.80938584	1.75176447	0.20065291	0.17512544
Zranb3	-0.3806146	2.7816883	1.75162592	0.20066999	0.17512544

Cul1	0.14075278	7.35300591	1.75156338	0.2006777	0.17512544
Stk25	0.13874066	6.73281195	1.75151999	0.20068305	0.17512544
Mynn	-0.1854478	5.5697216	1.75114682	0.20072907	0.17512949
Gna13	0.16900074	7.53995271	1.75110364	0.20073439	0.17512949
Amot	-0.2130165	4.95629387	1.7504418	0.20081603	0.17518035
Zmynd15	-0.8248194	0.33496792	1.74943744	0.20094001	0.17525255
Abtb1	-0.4565588	1.96667002	1.74934748	0.20095112	0.17525255
F3	0.30259263	5.60082471	1.74909446	0.20098237	0.17525255
Eif4a2	0.14344605	8.98994268	1.74901444	0.20099225	0.17525255
Tfap4	-0.53211	1.05252867	1.74857904	0.20104604	0.17527907
Atf5	0.34577262	3.26731139	1.74838718	0.20106975	0.17527937
Erg	-0.6451278	1.59530933	1.74649151	0.20130419	0.17546335
Slc24a1	-1.1170879	-0.0401307	1.74588522	0.20137925	0.17550838
Tmem132c	-0.3666052	2.32488406	1.74568337	0.20140424	0.17550977
Nudt17	-0.5648343	1.00296696	1.74531094	0.20145037	0.17552958
Pigl	-0.3413273	4.09399198	1.74430911	0.20157452	0.17561736
Mysm1	-0.1986029	6.25552941	1.74336339	0.20169181	0.17569913
Coq4	0.24265705	3.84372595	1.74256101	0.20179138	0.17576546
Adamtsl3	0.50208989	4.39157337	1.74218758	0.20183775	0.17576855
Cml1	0.60943625	1.30335737	1.74215502	0.20184179	0.17576855
Ggn	1.0069912	-0.0259875	1.74123491	0.20195609	0.17583417
Ube2z	0.22144504	6.14013848	1.74117101	0.20196403	0.17583417
Mocos	-0.5888646	1.42942326	1.74087178	0.20200122	0.17584614
Hjurp	-0.3870436	2.44062211	1.73954324	0.20216644	0.17596324
Rab32	0.40861857	3.17081687	1.73941293	0.20218265	0.17596324
Pvt1	-0.4379922	2.41868138	1.73891022	0.20224522	0.17599715
Mvd	0.39935042	1.82124357	1.73872296	0.20226854	0.17599715
Slc2a5	-0.9580926	-0.6597049	1.73827445	0.20232439	0.17602348
Acer2	0.43087172	3.38377005	1.73792985	0.20236731	0.17602348
Mllt4	-0.1354804	7.44020408	1.73791477	0.20236919	0.17602348
Rhbdd3	-0.7427338	-0.0468391	1.73764386	0.20240295	0.17602919
Gm15408	-1.172505	-0.4333354	1.73748539	0.20242269	0.17602919
15-Sep	0.28203951	6.70943786	1.73660599	0.20253233	0.17609648
9330188P03I	-0.9179568	0.9897814	1.73648812	0.20254703	0.17609648
Myom2	1.10977328	0.39220815	1.73477905	0.20276032	0.1762615
Gjc1	-0.5692596	1.80271383	1.73159467	0.20315849	0.17658716
Zcchc18	0.18261069	7.20061704	1.7303544	0.20331384	0.1766861
Mrpl15	0.35956705	4.9439857	1.73017904	0.20333581	0.1766861
Bmp4	0.42320618	7.4127385	1.72998735	0.20335984	0.1766861
Katnal1	0.18462434	6.85502897	1.72969274	0.20339677	0.1766861
Plcl2	-0.2651752	5.54410284	1.72959527	0.20340899	0.1766861
Dlx1	-0.3443057	3.88112312	1.72955826	0.20341363	0.1766861
Slc22a17	0.22001103	5.86933989	1.72929355	0.20344683	0.17669447
Adrb1	-0.36437	3.6706366	1.72786413	0.2036262	0.17682978
AU040320	-0.2859243	4.17222439	1.72706133	0.20372702	0.17685629

Sema4a	0.34130997	3.73889398	1.7270579	0.20372745	0.17685629
Eea1	0.17198106	7.47743477	1.72701703	0.20373259	0.17685629
Cyr61	0.73244794	2.43826021	1.72687017	0.20375104	0.17685629
Kcne1l	1.51896173	-1.2949601	1.72642327	0.2038072	0.17686739
Rad21	0.15088538	7.13610302	1.72639326	0.20381098	0.17686739
Psma5	0.20042234	5.72397389	1.72517915	0.20396366	0.17697941
Lox	0.40642192	3.27472047	1.72495883	0.20399138	0.17698299
Hs3st1	0.26217745	4.20816129	1.72347146	0.20417865	0.17712498
Pmvk	0.24804711	4.37566919	1.72293549	0.20424619	0.17716308
Patl1	0.18341747	6.17278137	1.72273502	0.20427145	0.17716451
Pds5b	-0.170625	7.92641985	1.72250872	0.20429998	0.17716877
Nek1	-0.2297042	6.39445689	1.72124534	0.20445934	0.17727033
Whsc1	-0.1490445	6.66122419	1.72111125	0.20447626	0.17727033
Prodh	-0.415264	2.14169972	1.7210183	0.20448799	0.17727033
Aldh3a1	1.10495708	-0.7939376	1.72031231	0.20457713	0.17732711
Gli3	-0.1869566	5.12944884	1.71958584	0.2046689	0.17738616
Glb1l2	-1.1704183	-0.4162008	1.71914984	0.204724	0.17741342
Efhd1	0.24926367	4.60422175	1.71701921	0.20499354	0.17762648
Setd3	0.13298439	6.79583478	1.71676536	0.20502568	0.17763381
Myl12a	0.37733818	6.85744348	1.71526614	0.20521565	0.17777786
Hemk1	-0.4806043	1.69722115	1.71480764	0.20527379	0.17780769
Prmt8	-0.1879154	7.02981199	1.71420061	0.20535079	0.17785386
Tmem234	0.24175094	4.73707194	1.7138214	0.20539892	0.17787301
Arl5b	-0.2822073	4.18578629	1.7136527	0.20542033	0.17787301
Rtp1	1.56702788	-1.5165248	1.7133736	0.20545576	0.17788316
Pvr	-0.3704879	2.53603465	1.71201418	0.20562846	0.17801213
Ptafr	1.32968087	-1.0445953	1.71046352	0.20582567	0.1781623
Tgtp2	0.35565483	3.77523968	1.70937276	0.20596454	0.17826193
Nek4	-0.2467711	4.4182294	1.70734481	0.20622303	0.17846506
Rft1	-0.4843061	2.59796167	1.70700357	0.20626656	0.17848215
03-Sep	0.16891272	8.9122498	1.70647203	0.2063344	0.17850679
Caml	0.24859087	3.67011036	1.70640752	0.20634264	0.17850679
Emp1	0.44836733	3.82778592	1.7056056	0.20644504	0.17857478
Gm14446	0.2940389	3.72874383	1.70445448	0.20659215	0.17868142
Iqch	-1.1122961	-0.5441629	1.70297511	0.20678139	0.17882449
Nrcam	-0.2273864	7.77680341	1.7027365	0.20681194	0.17883029
Ugdh	0.19330954	4.62494384	1.70234814	0.20686166	0.17885267
Gnl3	0.2330259	4.71939905	1.70204876	0.20690001	0.1788652
Gna14	-0.7940268	1.3106898	1.70158646	0.20695924	0.17889579
Nenf	0.25887185	4.26834623	1.7008635	0.2070519	0.17895526
Rpl35a	0.2795964	5.46905397	1.70048943	0.20709986	0.1789761
Scyl2	-0.2167003	5.8534477	1.70024137	0.20713168	0.17898297
Ckap5	-0.256231	7.87263961	1.69969267	0.20720208	0.17901273
Athl1	0.33649586	3.28005213	1.69953971	0.20722171	0.17901273
Topors	-0.1661797	6.03630215	1.69932881	0.20724878	0.17901273

4632434I11R	0.61014453	0.48499813	1.69922912	0.20726157	0.17901273
Prkag2	0.18999815	6.12234664	1.69896653	0.20729529	0.17902123
Tjp1	0.15581983	9.48507631	1.69766646	0.2074623	0.17913966
Rnf121	0.62847567	0.95704987	1.69723927	0.20751721	0.17913966
Mycn	-0.4152781	2.05725625	1.69713973	0.20753001	0.17913966
H2afv	0.32536851	5.31528394	1.69696534	0.20755243	0.17913966
Mrpl33	0.30884888	4.31556903	1.69662213	0.20759658	0.17913966
Hist1h4a	1.30252103	-1.4203992	1.69654638	0.20760632	0.17913966
Rnft2	0.27708012	4.54258712	1.69646057	0.20761736	0.17913966
Ppard	0.1942636	5.18174367	1.69638978	0.20762647	0.17913966
Fam195a	1.22816217	-1.2383846	1.69622749	0.20764735	0.17913966
Sbspon	-0.763103	0.73858241	1.6952589	0.20777203	0.17920771
Htatip2	0.461175	2.04140005	1.69524348	0.20777401	0.17920771
Bdh1	0.26863441	3.63196056	1.6928967	0.20807648	0.17944797
Gm8773	1.62218737	-1.5269507	1.69230965	0.20815224	0.17949266
Gas7	0.15685447	9.7921324	1.69112586	0.20830509	0.17958452
9030612E09I	-0.6989928	1.32594339	1.69094273	0.20832875	0.17958452
Lpcat4	-0.2098761	5.66208883	1.6909285	0.20833059	0.17958452
Mrpl39	0.20339152	5.38481267	1.69066358	0.20836482	0.17958661
Ism1	0.4351501	3.28477602	1.69053923	0.20838089	0.17958661
Lsp1	0.36967229	3.52750994	1.69034924	0.20840545	0.17958714
Stam	0.16686007	6.06935406	1.68978097	0.20847892	0.17962931
Zfp354b	-0.4583287	2.41234248	1.68960038	0.20850228	0.17962931
Ctnnb1	-0.141145	9.22493966	1.68852802	0.20864103	0.179701
Dmpk	0.31356568	3.50546501	1.6884788	0.2086474	0.179701
Rapgef2	-0.1871711	7.49215571	1.68830522	0.20866988	0.179701
Kctd12	0.14710087	7.42126274	1.68821688	0.20868131	0.179701
Ino80d	-0.1779572	7.35145253	1.68753761	0.20876929	0.17975612
Nrbp1	0.18549426	5.94088008	1.68688993	0.20885321	0.17980774
Cacna2d1	-0.2397943	7.62950505	1.68635879	0.20892207	0.17984638
Gabpb1	0.18883817	5.10050839	1.68584976	0.20898809	0.17988257
Pitpnm1	-0.3386212	3.35286185	1.68533836	0.20905444	0.17988831
Tmed9	0.15847285	6.26273586	1.68521259	0.20907076	0.17988831
Spopl	-0.2715194	3.63922646	1.68519041	0.20907364	0.17988831
Gm3414	-0.3327533	3.33853117	1.68490344	0.20911089	0.17988831
Hdhd3	0.83499011	0.2830346	1.68487437	0.20911466	0.17988831
Ccdc125	0.41249268	2.84858073	1.68407957	0.20921787	0.17994051
Fam120a	0.15151052	8.53748752	1.6840153	0.20922622	0.17994051
Acadm	0.27188508	5.06558011	1.683853	0.2092473	0.17994051
2610306M01	0.34363822	2.61735373	1.68188049	0.20950377	0.1801404
2410015M2C	0.37876487	2.73795877	1.68143034	0.20956235	0.18017012
Slc25a36	-0.1834332	4.98566063	1.68113946	0.20960022	0.18017608
Tmtc3	-0.2619186	5.40573068	1.68100814	0.20961732	0.18017608
Fnbp1l	0.20250554	8.5932555	1.68080536	0.20964372	0.18017813
Osbpl1a	-0.1567739	8.12679639	1.68047604	0.20968662	0.18019435

Yif1b	0.45289079	1.7760898	1.67962987	0.20979688	0.18026845
Dtymk	0.21990726	4.20043631	1.67931183	0.20983834	0.18027186
Ankrd16	-0.4789761	2.54010471	1.67913088	0.20986193	0.18027186
Lrrc8d	-0.2042455	5.77363906	1.67904647	0.20987294	0.18027186
Tcap	-0.6762713	0.86915898	1.67870731	0.20991718	0.18027849
Syt17	0.39111311	3.80397589	1.67856261	0.20993605	0.18027849
Lcn2	-2.7223742	-1.2419215	1.67836226	0.20996219	0.18027849
Fert2	-0.173513	4.96364727	1.67814558	0.20999047	0.18027849
Gm6297	-0.7601444	1.00891899	1.67787664	0.21002557	0.18027849
Bbx	-0.1661213	7.92283414	1.67784485	0.21002972	0.18027849
Wfdc17	0.70007582	0.93154725	1.67769798	0.21004889	0.18027849
Tst	0.31097693	4.00671627	1.67711293	0.21012528	0.18032342
Wdr45	0.24649331	4.46017267	1.67676469	0.21017077	0.18032344
Brix1	0.20361227	4.72605738	1.67669967	0.21017927	0.18032344
Plcl1	-0.2145895	5.43543837	1.67656072	0.21019742	0.18032344
Nup153	-0.1809154	6.16442377	1.67613486	0.21025307	0.18034064
Nckap1	-0.1889622	9.44861355	1.67603937	0.21026555	0.18034064
Cdk16	0.15915507	7.18779947	1.67467763	0.21044365	0.18047275
Kri1	-0.3421078	2.89361133	1.67377121	0.2105623	0.18054003
Tra2b	0.24492004	6.4036067	1.67371064	0.21057023	0.18054003
Gas2	-0.2726075	2.92876743	1.67325135	0.21063039	0.18056066
Hist1h2bp	0.93040073	-0.7282023	1.6731594	0.21064244	0.18056066
Baiap2l2	-1.1583443	-1.0733073	1.67227408	0.21075846	0.18063947
Acad8	0.20276077	4.24425617	1.67203721	0.21078952	0.18064545
Lmnb1	0.41253973	1.82116979	1.67173409	0.21082927	0.18065888
Camsap3	-0.2725211	3.48513944	1.67138534	0.21087502	0.18067744
Echs1	0.25817779	4.38260883	1.67016728	0.2110349	0.18079377
Prrt4	-1.0474835	-0.6122405	1.66966509	0.21110086	0.18082963
Klhl11	-0.242624	4.45954021	1.66910157	0.2111749	0.18085718
Pex11g	-1.2582593	-1.2928139	1.66905334	0.21118124	0.18085718
Sfmbt1	-0.1676287	5.97509888	1.66861077	0.21123942	0.18088636
Ctif	0.18402771	7.02124935	1.66823275	0.21128913	0.18090827
Parvg	0.5595436	1.48542068	1.66693247	0.21146023	0.1810341
Ndor1	-0.3372816	3.23268518	1.66593435	0.21159168	0.18112597
Ubfd1	0.15732976	6.94136484	1.66522396	0.21168531	0.18118544
Grb2	0.18038824	5.1641288	1.66501614	0.21171271	0.18118822
Pde5a	0.28157318	6.52878451	1.66449952	0.21178084	0.18122585
Acap2	-0.1865284	6.73780263	1.66418495	0.21182233	0.18124068
Fbxo32	-0.2410947	5.02528935	1.6639192	0.2118574	0.18125001
Lonp2	-0.1645307	5.557762	1.66368508	0.2118883	0.18125577
Atg101	0.39136572	2.8013093	1.66307924	0.21196828	0.18130352
Nsfl1c	0.22529477	5.06797912	1.66284008	0.21199987	0.18130986
Mtg2	-0.6388156	0.41438466	1.6626214	0.21202875	0.18131274
Gm9866	0.36978709	2.57794585	1.66244859	0.21205158	0.18131274
Rims1	-0.2202318	6.48495215	1.66202467	0.2121076	0.18133997

Kars	0.20757076	5.13841216	1.66140327	0.21218974	0.18137351
Samd12	0.33826173	3.24731974	1.66136212	0.21219518	0.18137351
Mfsd7b	-0.3413445	2.61069559	1.66081653	0.21226734	0.18141451
Gzma	-1.7620783	-0.5829634	1.66002732	0.21237177	0.18147098
Wdr77	-0.192601	5.07304581	1.65995161	0.21238179	0.18147098
Rad52	0.25222537	3.57807302	1.65886291	0.21252597	0.18157349
4930581F22I	-0.7513473	0.41831874	1.65853272	0.21256973	0.18158816
Ptbp2	0.20571438	6.37537676	1.658368	0.21259156	0.18158816
Klhl25	-0.363852	1.87790418	1.65789898	0.21265373	0.18162059
Map3k5	-0.310325	5.08153796	1.65575063	0.21293882	0.18182496
Sacs	-0.5827304	3.7593415	1.65573044	0.2129415	0.18182496
Cep192	-0.3356245	4.46641486	1.65427896	0.21313439	0.18194364
Dnpep	0.32123032	2.95419845	1.65417169	0.21314866	0.18194364
Zfp454	0.3535651	2.31027199	1.65413705	0.21315326	0.18194364
Aggf1	0.17588582	6.57041674	1.65360454	0.21322409	0.18197856
Eed	0.22518565	4.49116874	1.65330619	0.21326379	0.18197856
Il1rl2	-1.4884042	-1.0983278	1.65328247	0.21326695	0.18197856
Zbtb10	-0.1741836	5.46634052	1.65279917	0.21333127	0.18201274
Hcst	-1.7227681	-1.5368851	1.652516	0.21336897	0.1820242
Ppp4r1	-0.2395684	4.32499418	1.65054854	0.21363115	0.18222714
Akr1c18	0.70489134	1.31564521	1.65034525	0.21365826	0.18222954
Dnajc25	-0.4201018	2.95692566	1.64968351	0.21374655	0.18228412
Syp	0.2698161	8.81758959	1.64939341	0.21378526	0.18229641
Efnb3	-0.2820986	4.91469848	1.64912472	0.21382113	0.18230627
Nkx6-2	-0.7713672	0.35293073	1.64834309	0.21392552	0.18236615
Msi1	0.48988657	1.91976697	1.6482348	0.21393999	0.18236615
Spr	0.39987351	2.83179434	1.64722203	0.21407535	0.1824608
Tmem38b	-0.306384	3.18982367	1.64615424	0.21421818	0.1825618
Atad3a	-0.3276405	3.12139672	1.64586309	0.21425715	0.18257427
Apol9b	-1.0009853	-0.6994516	1.6453022	0.21433224	0.18261751
Pcnxl2	-0.3001469	4.8164733	1.64406475	0.21449803	0.18269288
5930412G12	0.75414125	0.82290441	1.64400855	0.21450556	0.18269288
Glb1	0.35993466	3.05622481	1.6439292	0.2145162	0.18269288
Gsto2	-1.2579419	-0.5104815	1.64382171	0.21453061	0.18269288
Whrn	0.32806544	3.21545349	1.64373321	0.21454248	0.18269288
Nfyc	0.24353023	4.60424449	1.64328481	0.21460261	0.18270805
Kalrn	0.18760432	11.7778886	1.64323715	0.214609	0.18270805
Nono	0.13188346	7.47503312	1.64302243	0.2146378	0.18271183
A330102I10F	-0.6769174	1.29368866	1.64176401	0.21480671	0.18283486
Htra3	-0.4680437	4.06121175	1.64149425	0.21484294	0.18284495
Tmem127	-0.1906144	6.90098997	1.64119011	0.21488379	0.18285898
Rab8a	0.37438827	4.84206691	1.64094497	0.21491673	0.18286626
Nadk2	0.20935747	4.75716011	1.64030257	0.21500307	0.18291898
Stk38l	0.16064985	5.63816732	1.64004782	0.21503732	0.18292737
Cecr6	-0.3138714	4.67755729	1.6384868	0.21524736	0.18307713

Ift57	0.15300476	5.92742361	1.63833237	0.21526815	0.18307713
Kif14	1.66778909	-1.3150309	1.63807595	0.21530269	0.18307713
Tmem18	-0.243302	4.31066498	1.6379814	0.21531542	0.18307713
B4galt5	-0.2342726	4.63843676	1.63783305	0.2153354	0.18307713
Enpp1	0.34835922	5.3449305	1.63669575	0.21548867	0.18318668
Dchs1	-0.3609928	2.92556451	1.63614467	0.21556298	0.18322909
Tubb2b	0.19702487	5.27879931	1.63478447	0.21574655	0.18336434
Sptssa	0.24648394	6.40935421	1.63448857	0.21578651	0.18337047
Upf2	-0.1499862	6.89054025	1.63436911	0.21580265	0.18337047
Klhdc8b	0.52738892	2.2070895	1.63372698	0.2158894	0.18339668
Kbtbd11	-0.1564686	7.70405204	1.63366946	0.21589718	0.18339668
1500004A13I	-0.2902248	4.96989979	1.63359796	0.21590684	0.18339668
Far1	-0.1827157	6.28201656	1.63264211	0.21603608	0.18347284
Col8a1	0.31613081	5.5011309	1.63257158	0.21604562	0.18347284
Gmpr	0.44173977	2.07266291	1.63239219	0.21606988	0.18347284
Pitpnb	0.15931593	6.1491041	1.6307684	0.2162897	0.18363871
Sepw1	0.3893773	5.57534825	1.63010345	0.2163798	0.18369441
Sh2d1b1	-1.0598435	0.8331285	1.62967462	0.21643793	0.18370355
D3Bwg0562e	-0.2099218	7.1409987	1.62966271	0.21643954	0.18370355
Hmgb2	0.27816732	4.01804394	1.62927206	0.21649251	0.18372772
Ccr4	-0.8827898	0.09339256	1.62846637	0.21660181	0.18379969
Ptov1	0.34852237	5.14555831	1.62804284	0.2166593	0.18380717
Mamdc2	0.86754869	0.03578776	1.62799829	0.21666535	0.18380717
Rps15a	0.16234864	7.20599227	1.62785985	0.21668414	0.18380717
Tcf7l2	0.26237938	6.41243746	1.62748974	0.2167344	0.18381432
Pou2f1	-0.1754673	5.80107812	1.62732838	0.21675632	0.18381432
Piezo2	-0.4022154	2.92854699	1.62725645	0.21676609	0.18381432
Gm8179	-1.3701273	-0.1915981	1.62703819	0.21679574	0.18381869
Gpr25	0.31458249	3.66236568	1.62615485	0.21691579	0.18388893
4933407L21F	0.75304792	0.14756745	1.62596439	0.21694169	0.18388893
Klri2	0.84193514	0.40890264	1.62588763	0.21695212	0.18388893
Ddt	0.35379667	3.74680858	1.62535735	0.21702425	0.18391361
Psen2	0.46140597	1.61387442	1.62531302	0.21703028	0.18391361
BC049635	-0.4022196	3.11311476	1.62492301	0.21708335	0.1839378
Ret	0.54163323	1.82544934	1.62446289	0.21714597	0.18395246
Hc	1.61486017	-1.9135058	1.62443561	0.21714969	0.18395246
Ccl19	-0.3255411	3.93998028	1.62359201	0.21726457	0.184029
Zfp955a	-0.1981199	5.05285549	1.62338891	0.21729225	0.18403165
Mpp6	0.26363385	8.59069649	1.6231318	0.21732728	0.18403237
Bhlha9	1.63122857	-1.2344932	1.62302261	0.21734216	0.18403237
Inpp5b	-0.1999616	4.90277006	1.62256314	0.2174048	0.18406463
Ecd	0.23723663	4.1883553	1.62212467	0.21746459	0.18408276
Cort	-1.5247399	-1.6899519	1.62204618	0.21747529	0.18408276
Zc3h6	-0.2378201	4.89306762	1.62179661	0.21750934	0.18408724
Ptp4a2	0.22602871	9.07797715	1.62147766	0.21755286	0.18408724

Tceal7	-1.018543	-0.1028315	1.62126486	0.2175819	0.18408724
Smim4	0.38583545	2.15559357	1.62111113	0.21760288	0.18408724
Fxr2	-0.2714028	3.71798348	1.6211082	0.21760328	0.18408724
Fkbp5	0.42725619	5.97414013	1.62038883	0.2177015	0.18413372
Pgap2	-0.2783164	4.20578138	1.62034619	0.21770733	0.18413372
Ank2	-0.229304	10.1838387	1.61948926	0.21782441	0.18420673
Polr3c	0.25889329	3.20865398	1.619355	0.21784276	0.18420673
Adat2	0.61734592	1.78734387	1.61843755	0.21796821	0.18429204
Fn3k	-0.3430507	3.23187119	1.61813264	0.21800993	0.18430653
Zscan21	0.26569136	4.60120424	1.61776772	0.21805986	0.18431288
Arid3c	-1.5324725	-1.2824569	1.61771871	0.21806657	0.18431288
Nat2	0.4032757	3.0417629	1.61709516	0.21815194	0.18436426
Cx3cl1	0.19740642	6.78151362	1.61690275	0.21817829	0.18436575
Spon2	-1.5939285	-0.6560705	1.6165201	0.2182307	0.18438927
Rnh1	0.26353273	4.45654197	1.61588484	0.21831775	0.18442182
Slc25a3	0.13902793	7.93988783	1.61588015	0.2183184	0.18442182
Hsd17b14	0.61937422	0.86079391	1.61519107	0.21841287	0.18448086
3830406C13I	0.19211617	6.13628386	1.61351993	0.21864221	0.1846423
Cyba	0.68842731	0.14828041	1.61343959	0.21865325	0.1846423
Kif27	-0.3920664	2.20180061	1.61305663	0.21870585	0.18466594
Samd3	-0.6247305	1.32817497	1.61256963	0.21877276	0.18467371
Nacad	-0.2967188	3.25361314	1.61255931	0.21877418	0.18467371
Cep170b	-0.1902745	7.77688155	1.61245211	0.21878891	0.18467371
Ndst2	-0.3004143	3.30347432	1.61221076	0.21882209	0.18468093
Psmc4	0.22421158	4.92455703	1.61106587	0.21897955	0.18478492
4921511C10I	-2.2926621	-1.6615581	1.61095674	0.21899456	0.18478492
Socs3	0.4893943	1.04320367	1.61040041	0.21907114	0.18482874
Nrros	-0.6877976	1.47144779	1.60971371	0.2191657	0.18488772
Fam102b	0.15074685	5.84785971	1.60949705	0.21919554	0.18489211
Bmp15	0.52809476	2.22693816	1.60865379	0.21931176	0.18496749
Noa1	0.19708322	4.42358664	1.60849084	0.21933422	0.18496749
Zc3h18	-0.2436408	3.91174905	1.6080474	0.21939537	0.18499826
Slc25a39	0.31193971	4.37736588	1.60687304	0.21955742	0.18509459
Zik1	-0.2761718	3.46310341	1.60686185	0.21955897	0.18509459
Usp32	-0.1861207	7.80025164	1.60548331	0.21974939	0.18523185
Casq1	-1.1036031	0.15794126	1.60532575	0.21977117	0.18523185
Gyg	0.16111557	5.57171995	1.60444025	0.21989361	0.18531422
Krit1	-0.2229121	5.84090534	1.60403707	0.21994939	0.18531555
Adc	-0.4741292	1.78714451	1.6039331	0.21996377	0.18531555
Igsf5	-2.0825048	-1.9997255	1.60389317	0.2199693	0.18531555
Prdm1	-0.5619985	2.00380264	1.60215969	0.22020935	0.18549695
Ssbp1	0.17512054	5.47566653	1.60153475	0.22029597	0.18554908
1500015A07I	-0.4630312	2.19845466	1.60065963	0.22041734	0.18561432
Dmrta1	0.50020728	3.04450842	1.60051622	0.22043724	0.18561432
Ik	0.13240159	8.56539055	1.60044117	0.22044766	0.18561432

Tspan9	0.32991939	3.49513706	1.60013094	0.22049071	0.18562973
Mfsd3	0.74242811	0.54359466	1.59958598	0.22056637	0.18563215
Brms1l	0.17457514	5.94753582	1.59946468	0.22058321	0.18563215
Trp53i11	0.35601897	8.13256173	1.59942207	0.22058913	0.18563215
Cml5	-0.7040703	0.85791997	1.59939732	0.22059257	0.18563215
4931406C07l	0.24507047	6.65583506	1.59807542	0.22077625	0.18575883
Arl4d	-0.4331438	2.09657324	1.59760994	0.22084098	0.18575883
Pgam5	0.19172926	6.09809971	1.59755448	0.22084869	0.18575883
Ostm1	0.18713751	5.44644216	1.59740825	0.22086903	0.18575883
Oc90	-1.7150993	-2.3165054	1.61579958	0.22088669	0.18575883
Sh3bp2	-0.5078561	1.47945493	1.59724539	0.22089169	0.18575883
Pth2r	1.63237675	-1.3312473	1.5957855	0.2210949	0.18590888
Phf8	-0.2133167	5.6399736	1.59539412	0.22114943	0.18592744
B230319C09l	1.16761058	-1.0365313	1.59527127	0.22116654	0.18592744
Chka	-0.2343992	4.53753609	1.59492595	0.22121467	0.18594705
Mt1	0.29869801	6.01750431	1.59447592	0.2212774	0.18597895
Herc3	-0.248842	7.65233924	1.59401857	0.22134118	0.18601171
Rgs3	0.29689074	4.10032418	1.59373962	0.22138009	0.18602357
Asb7	0.13437283	6.12224714	1.59335434	0.22143385	0.18603332
Nceh1	0.16016829	6.21461893	1.59330103	0.22144129	0.18603332
Als2cl	-0.5992501	0.5495359	1.59284668	0.22150471	0.18605384
Rmdn1	-0.2999971	3.73525051	1.59277065	0.22151533	0.18605384
Spag4	-1.4643121	-1.6186552	1.59243177	0.22156265	0.18607275
Epn2	0.13252663	6.4669456	1.59151808	0.2216903	0.18615911
Adamts5	-0.2867164	3.69374067	1.59103356	0.22175802	0.18619514
Trmt61a	-0.224575	4.52357401	1.59042291	0.22184342	0.18624261
Hif3a	-0.3752626	2.85379318	1.59027423	0.22186422	0.18624261
Ptpro	-0.2653034	3.42849331	1.58964025	0.22195294	0.18626091
Lpar5	1.33262071	-0.7769448	1.5895819	0.2219611	0.18626091
Spag6	0.42347283	1.99929427	1.58948209	0.22197508	0.18626091
Usp30	0.23586785	4.18557355	1.5894088	0.22198534	0.18626091
Limd2	0.22732084	4.46135496	1.58913896	0.22202312	0.18627177
Ptgr2	0.22283362	6.42291844	1.58868582	0.22208658	0.18630418
Ifnar2	0.27684431	5.47723388	1.58846131	0.22211803	0.18630973
Nr2f2	0.20163104	7.60255619	1.58700371	0.22232236	0.18646027
Dnajc15	0.23821044	4.25157973	1.58640898	0.2224058	0.18650939
Ctnnbl1	0.26761544	3.28848364	1.58568492	0.22250744	0.18657377
Dazl	-0.5129388	1.50788797	1.58544172	0.22254159	0.18658155
Upf3b	-0.2194218	5.6288383	1.58513479	0.2225847	0.18659571
Retnlg	2.13748151	-1.3288722	1.58496726	0.22260824	0.18659571
Tlr6	-1.8497682	-1.3341172	1.58419318	0.22271702	0.18662283
Mppe1	-0.5844371	0.69121084	1.58415932	0.22272178	0.18662283
Gm1564	-0.5436092	1.12274621	1.58359964	0.22280048	0.18662283
Cyp7b1	-0.308364	2.44135494	1.58356158	0.22280584	0.18662283
Nme4	0.9869882	0.20736977	1.58352022	0.22281165	0.18662283

Efcab5	-0.4827065	2.66556156	1.58341042	0.2228271	0.18662283
Wnt6	-0.5105816	3.15703096	1.58329256	0.22284368	0.18662283
Senp6	0.13222905	8.47980854	1.58327946	0.22284552	0.18662283
Recql5	0.41405273	2.43642197	1.58314464	0.22286449	0.18662283
Kptn	-0.465396	1.85330009	1.58288975	0.22290036	0.18663203
Bcl3	0.58787036	0.8728901	1.5818913	0.22304092	0.18672888
Ric8b	-0.2506753	5.65115964	1.58159148	0.22308316	0.18674339
Gm15987	-1.5578324	-1.7761642	1.58120044	0.22313825	0.18676867
Glce	-0.1942422	6.45973608	1.58038431	0.2232533	0.18683322
Plscr2	0.35481006	4.4211804	1.58005178	0.22330019	0.18683322
Mtmr9	-0.250686	3.64425542	1.58001453	0.22330545	0.18683322
Cmas	0.1311165	6.76787663	1.57985819	0.2233275	0.18683322
Gnai2	0.30438574	7.06060397	1.57961376	0.22336199	0.18683322
Ddx19b	-0.2224098	5.11197622	1.57959376	0.22336481	0.18683322
Canx	0.17585679	7.9635512	1.5787109	0.22348943	0.18691661
Mettl25	-0.4337834	2.64327942	1.57771676	0.22362986	0.18701321
Gle1	0.19034116	4.91011353	1.5773408	0.22368299	0.1870368
2310035C23I	-0.2217485	6.63927903	1.57694411	0.22373908	0.18706284
Rpl11	0.24882112	7.046423	1.57674118	0.22376777	0.18706598
Mn1	-0.2700258	4.58576522	1.57570714	0.22391407	0.18713765
Gm19619	-1.4797125	-1.2366144	1.57554582	0.22393691	0.18713765
Rab40b	-0.2442415	4.03718663	1.57548793	0.2239451	0.18713765
Lrrc18	-0.3518307	2.93618998	1.57526424	0.22397677	0.18713765
Kazn	0.19091619	6.12045193	1.57525387	0.22397824	0.18713765
2310036O22	0.25410029	4.55739908	1.57479612	0.22404307	0.18715031
Znrd1as	0.27328523	3.55287635	1.57479453	0.22404329	0.18715031
Syt12	-0.2288874	4.19268637	1.57424403	0.22412129	0.18719462
Senp5	-0.1528109	5.58786185	1.57202965	0.22443536	0.18743608
Nxph3	-0.8238718	-0.3782904	1.57138389	0.22452706	0.18747164
Mpped1	-0.1902525	6.04425152	1.57137777	0.22452793	0.18747164
Sco1	-0.2776968	3.29472571	1.57044573	0.22466036	0.1875449
Kcnk12	-1.0779743	-1.2942537	1.57040837	0.22466567	0.1875449
Cd82	0.84730883	1.51331837	1.5702261	0.22469158	0.18754566
Gimap9	1.03497197	0.14078881	1.56938104	0.22481177	0.18762391
Pspc1	-0.2270098	5.2308124	1.56921517	0.22483537	0.18762391
Mgat1	0.25562631	3.42055107	1.56902872	0.2248619	0.18762518
Hnf1b	1.17069857	-0.7988781	1.56881475	0.22489235	0.18762923
Atoh8	0.41133755	1.84341406	1.56864315	0.22491677	0.18762923
Nub1	0.1731214	5.30271563	1.56780922	0.22503552	0.18770742
Tob2	0.22613298	5.13169507	1.5676295	0.22506112	0.1877079
Kif5c	-0.2214489	9.59216499	1.56687831	0.22516818	0.1877763
Pigq	0.17577789	5.82510229	1.5658748	0.22531129	0.18787476
Camk1	0.24977279	3.80136889	1.5656151	0.22534834	0.18788478
Bbc3	0.72426126	-0.1095236	1.56520866	0.22540635	0.18791225
4930513N10	1.04810302	-0.4688363	1.56392237	0.22559005	0.1880417

Gm3985	-1.0418412	-0.8746755	1.56374831	0.22561493	0.1880417
Xpr1	-0.1728342	7.89515667	1.56359511	0.22563682	0.1880417
4732491K20I	-0.4530734	2.09539837	1.56293708	0.2257309	0.1880992
Arl10	-0.6060501	0.91584458	1.56261916	0.22577637	0.18811619
Cd302	0.25912393	4.11724662	1.56221854	0.22583368	0.18814305
Fam217b	-0.2193376	4.010407	1.56072421	0.22604763	0.18829117
Qsox1	0.41416175	2.23119922	1.5606194	0.22606265	0.18829117
Rnf10	0.18735816	5.83996032	1.56014645	0.22613043	0.18829117
4930427A07I	1.09788311	-0.0174891	1.56006038	0.22614276	0.18829117
Nxpe3	-0.340643	4.07112781	1.5599806	0.2261542	0.18829117
Ttl	-0.1524228	5.9524659	1.55992558	0.22616208	0.18829117
Hif1an	-0.2660781	4.74848081	1.55950387	0.22622255	0.18830431
4430402I18R	-0.3175217	2.61787943	1.55946538	0.22622807	0.18830431
Kank3	-0.5184904	1.87037182	1.55908554	0.22628255	0.18832876
Grip2	-0.4585265	2.18847827	1.5581998	0.22640965	0.18841364
Jund	0.22047828	5.02178906	1.55797593	0.22644179	0.18841948
Ctns	-0.3324095	2.65639179	1.55680718	0.22660968	0.18853826
Gm973	-0.3609498	2.46480324	1.55653542	0.22664874	0.18854985
Kdelr2	0.31707125	5.18839106	1.55632849	0.22667849	0.18855369
Nars	0.14039701	7.85975066	1.5559053	0.22673934	0.18855863
B230312C02I	-2.0269878	-1.0641934	1.55577265	0.22675842	0.18855863
Zfp867	-0.229861	4.15322404	1.55576273	0.22675985	0.18855863
Evc	-0.3600969	2.46743737	1.55550677	0.22679667	0.18856835
C030039L03F	-0.3239634	4.05436571	1.55483061	0.22689397	0.18861603
Apbb3	-0.4365834	2.08151078	1.55475879	0.22690431	0.18861603
Prss12	-0.3588582	1.85980198	1.55336464	0.22710512	0.18876204
Srgap3	-0.2299949	9.0810211	1.55293646	0.22716683	0.18877991
Rplp2	0.26495848	5.9945226	1.55281195	0.22718479	0.18877991
Zfp839	-0.1873187	5.26943064	1.55251321	0.22722786	0.18877991
Pam16	0.27809235	3.18915383	1.55247755	0.22723301	0.18877991
Kirrel2	-1.328683	-0.5200892	1.5523427	0.22725245	0.18877991
Entpd6	-0.3011418	3.09505747	1.55014766	0.22756934	0.18901082
8030462N17	0.16154003	5.47395335	1.55006822	0.22758082	0.18901082
Mrps30	0.31166443	3.23911452	1.5496794	0.22763701	0.18903656
Acad12	-0.9496893	-0.9552592	1.54798721	0.22788178	0.18921888
Mib2	-0.2819937	3.61288825	1.54649734	0.22809756	0.18937354
Coq3	0.26948456	3.37844803	1.5463526	0.22811854	0.18937354
Dmwd	-0.2183511	4.86534183	1.54551351	0.22824019	0.18945357
Drg1	0.24257951	5.31877549	1.54527841	0.22827429	0.18946091
Gm4922	-1.1416693	-1.3059537	1.5448164	0.22834133	0.18949256
Wdr1	-0.1608326	6.28277704	1.54458599	0.22837477	0.18949256
Dusp15	-0.3039238	2.85404897	1.54436391	0.228407	0.18949256
Sike1	0.14873056	6.60274024	1.54423916	0.22842511	0.18949256
Trp73	-1.0150302	-0.2048053	1.54408316	0.22844776	0.18949256
Sowahc	0.21294764	4.15343982	1.5439181	0.22847173	0.18949256

Txnrd2	-0.5759482	1.2978111	1.54379742	0.22848926	0.18949256
Fam198a	-0.505256	1.78053797	1.54320165	0.22857581	0.1895304
Upb1	-0.9830856	-0.4929598	1.5431355	0.22858542	0.1895304
Clec2l	-0.5069235	1.15729385	1.54283799	0.22862866	0.1895453
Rps18	0.35633722	6.22385773	1.5415755	0.22881226	0.18967655
Dag1	0.23037657	6.67986052	1.54116048	0.22887265	0.18970565
Plekha6	-0.1659778	6.85142814	1.54062227	0.228951	0.18973257
Fat2	0.44327871	2.77609445	1.5405899	0.22895572	0.18973257
Nfyb	0.18464254	5.3894868	1.53994335	0.22904989	0.18978965
Olr1	-0.8607834	-0.3912774	1.53927338	0.22914753	0.18984958
Zfp13	-0.5099013	1.79674284	1.53802818	0.22932913	0.18997905
Itgb1bp1	0.19001178	4.68775085	1.53767885	0.22938011	0.1900003
Gm2109	-2.1990015	-2.2724261	1.53674593	0.22951634	0.19009215
Rai14	0.22240682	6.49851665	1.5352912	0.22972895	0.19024724
Ccdc17	0.51975411	1.07721992	1.53425592	0.22988042	0.19035166
Nvl	-0.2196247	5.01649047	1.53289448	0.2300798	0.19049573
Pde11a	-0.8142773	-0.3706956	1.53232789	0.23016284	0.1905315
Acvr2b	-0.6449755	0.6247075	1.53225312	0.23017381	0.1905315
Otogl	1.24055119	-1.2053507	1.5308264	0.23038309	0.1906837
Fh1	0.18160825	5.26825445	1.5305711	0.23042057	0.19069368
Cask	-0.1500075	7.6195269	1.52994479	0.23051254	0.190747
Rhoq	0.21008542	6.22370793	1.52978606	0.23053585	0.190747
Leprel4	-0.3855091	2.95741642	1.529256	0.23061374	0.19079039
Tmem221	1.02180988	-0.4644933	1.52879685	0.23068123	0.19082518
Atp1b2	0.21301979	9.04784506	1.52821839	0.23076629	0.1908745
Chaf1a	-0.4641244	1.78037866	1.5274991	0.23087212	0.19094038
Slc1a4	0.26186633	4.33679528	1.5273311	0.23089685	0.19094038
Hnrnph1	-0.1190765	8.53090032	1.52664196	0.23099832	0.19100323
Atp2a3	-0.5521507	0.6266662	1.52617923	0.23106648	0.19103854
Prkce	0.1740076	8.83402021	1.52544288	0.231175	0.19109155
Mmp14	0.43062595	3.91261797	1.52539847	0.23118155	0.19109155
Ngf	0.60864348	0.84562194	1.52426568	0.23134863	0.19120412
Wdsub1	0.31798296	3.07045806	1.5241296	0.23136871	0.19120412
Wdr61	0.22940274	4.33102401	1.52350669	0.23146066	0.19125903
Tpr	-0.1623318	9.14637842	1.52273425	0.23157475	0.19133223
Sytl1	0.90455418	0.01323853	1.52225156	0.23164608	0.19135457
Pop1	-0.4054292	2.24893073	1.52220603	0.23165281	0.19135457
Zfp763	-0.2705405	4.04657361	1.52181545	0.23171055	0.1913812
Sgpp2	-0.2338612	3.73622858	1.52134023	0.23178083	0.19141606
Pou6f2	0.54073518	1.93296602	1.52118495	0.2318038	0.19141606
Man2b1	0.27779771	4.0911535	1.52086402	0.23185129	0.19143369
Timm13	0.4247937	2.54144414	1.52062738	0.23188631	0.19143369
Pithd1	0.22418274	4.77159265	1.52052326	0.23190172	0.19143369
Zfp964	-0.5381636	1.2599047	1.52025196	0.23194188	0.19143872
Lsm4	0.33748227	3.33172149	1.52013732	0.23195885	0.19143872

Pcnxl4	-0.2443705	4.64560216	1.51957151	0.23204264	0.19148681
Cep120	-0.1825711	6.33522438	1.5193774	0.2320714	0.19148948
Msrb1	0.25886079	4.22744188	1.51842211	0.23221298	0.19158523
Itm2c	0.28217034	7.70303371	1.51824313	0.23223952	0.19158605
Rftn1	-0.5264246	1.5953295	1.5163756	0.23251667	0.19179359
Cmbl	0.36732444	6.17617277	1.5154594	0.23265279	0.19187128
Gm5643	0.17402313	5.48793776	1.51539738	0.23266201	0.19187128
Tmem106c	0.43253947	3.83407144	1.51484047	0.23274481	0.19191846
Eif6	0.30258796	4.38628276	1.5137605	0.23290548	0.19202984
Rrp15	0.33582459	2.82371295	1.51334569	0.23296722	0.19205964
Ly6c1	-0.6265988	1.66203813	1.51284699	0.23304149	0.19209975
Lrrc8c	-0.2415015	4.05607155	1.5121527	0.23314493	0.19215862
Pdlim3	1.17151418	-0.6166203	1.51202382	0.23316414	0.19215862
Bdp1	-0.1914954	7.43458905	1.51036632	0.23341135	0.19234122
Flrt3	-0.1760479	5.81551562	1.50922314	0.23358205	0.19246075
Syt9	0.29121375	3.34875711	1.5087554	0.23365194	0.19248773
Nipa1	-0.2255217	4.82904993	1.50866053	0.23366612	0.19248773
Usb1	0.46685152	1.54034789	1.50754666	0.23383267	0.19258858
Col4a5	-0.3912539	3.29532106	1.5074984	0.23383989	0.19258858
Nubp2	0.30002758	3.47215544	1.50672028	0.23395633	0.19264258
Zcchc5	1.08882265	-0.4091356	1.50671704	0.23395682	0.19264258
Mki67	-0.2498089	3.76884703	1.50624897	0.2340269	0.19267913
Ints7	0.29958744	3.58778534	1.50576032	0.23410009	0.19268937
Cd83	-0.3143826	2.18761984	1.50569532	0.23410983	0.19268937
Mir872	1.95346611	-1.3539564	1.50565147	0.2341164	0.19268937
Slc29a2	0.70256949	0.35312579	1.50537509	0.23415782	0.19270231
Cdh2	0.22547294	6.47293761	1.50446768	0.23429385	0.19278175
Tbl1xr1	-0.1322058	7.63651991	1.50430717	0.23431792	0.19278175
Gstcd	-0.4594964	1.73762496	1.5042171	0.23433144	0.19278175
Igflr1	-1.2554425	-0.7968711	1.50383491	0.23438877	0.19280777
Fam3a	0.2548037	3.56031125	1.50326353	0.23447453	0.19285717
Mical1	0.3582498	2.58725045	1.50277514	0.23454786	0.19286581
Olfr464	-1.8142886	-1.9463999	1.50270352	0.23455862	0.19286581
Tmem59l	0.30018153	4.17803681	1.50267987	0.23456217	0.19286581
BC021785	-1.2413807	-1.6363028	1.50218918	0.23463589	0.19290528
Lats1	0.14915354	7.15099515	1.50161692	0.23472189	0.19295483
Wbp11	0.1500554	6.05145539	1.5013555	0.23476119	0.192966
Mrs2	-0.3123989	3.96128606	1.50107981	0.23480265	0.19297893
Dhx29	-0.2950824	4.80532999	1.50080026	0.23484469	0.19298472
Tmprss7	0.74747793	0.24829116	1.50069087	0.23486115	0.19298472
Tmem209	-0.2014262	4.22701157	1.50010688	0.23494903	0.19303578
Ache	0.38686022	2.62982925	1.49839889	0.23520628	0.19322598
Clcn6	-0.251832	3.55535968	1.49792715	0.23527739	0.19325228
Fndc4	-0.2723274	3.68251726	1.4978447	0.23528983	0.19325228
Pan3	-0.146375	6.70390034	1.49725128	0.23537933	0.19330463

Cacul1	0.15405621	6.85414444	1.49697717	0.23542069	0.19330801
Hs6st2	-0.2796071	4.82029221	1.49688241	0.23543499	0.19330801
Dsg2	-0.3357162	3.13330013	1.49615607	0.23554463	0.19337687
Crhr1	-0.4217946	1.89336019	1.49585147	0.23559063	0.19339347
Golga7	0.17437461	7.26421945	1.49562514	0.23562482	0.19340037
Egr4	-0.4047693	3.23099371	1.49517159	0.23569335	0.19343546
1700003M07	0.22785961	3.86364677	1.49450182	0.2357946	0.19349738
Supt3	-0.3402984	2.28573846	1.49428953	0.2358267	0.19350255
Epc1	-0.1490153	6.38698738	1.49405628	0.23586198	0.19351033
Myo18b	0.85257914	-0.6149514	1.49364115	0.23592478	0.19354069
Apba2	-0.2213963	4.81600432	1.49340255	0.23596089	0.19354914
Mettl4	-0.2213282	5.08264826	1.49289065	0.23603838	0.19359154
AW146154	0.33019746	3.00440561	1.49234209	0.23612145	0.1936385
Rrp12	0.25663299	3.92891724	1.49216875	0.23614771	0.19363886
Dda1	0.20216042	4.55962534	1.49198847	0.23617502	0.19364009
Adrm1	0.1854439	4.16478739	1.49171703	0.23621616	0.19365199
Papolb	-0.8568431	0.72027379	1.49155203	0.23624116	0.19365199
2610002M06	0.13691356	7.06876313	1.4905294	0.23639623	0.19375793
Spsb3	0.3307571	2.64510332	1.48990728	0.23649063	0.19381413
Prkaa1	0.15789431	5.69360873	1.48952607	0.2365485	0.19384038
Ccdc28b	0.26782555	3.40580795	1.48812051	0.23676203	0.19397821
Ccdc71l	0.20003813	5.24140435	1.48791081	0.23679391	0.19397821
Bmp5	0.36751646	5.86013317	1.48790836	0.23679428	0.19397821
Zfp365	-0.2258818	9.10482669	1.48729131	0.23688811	0.19403388
Prkar2b	0.16880403	5.63127322	1.48686935	0.23695231	0.19406527
Tsnax	0.14493031	7.46016546	1.48635947	0.23702991	0.19410763
Pnma2	0.18086034	6.40633816	1.48522814	0.2372022	0.19420111
Arid4a	-0.1939561	7.65718291	1.48514743	0.2372145	0.19420111
2810002D19	-0.3230589	3.26031947	1.48510009	0.23722172	0.19420111
Mtr	-0.4444158	2.80101481	1.48443519	0.23732306	0.19425992
Narf	-0.2097739	5.05031962	1.48428901	0.23734535	0.19425992
Diexf	-0.2228512	4.40759709	1.48403988	0.23738334	0.19426982
Pcmtd1	-0.1437942	8.28762032	1.48328756	0.23749812	0.19434255
Ifngr2	0.22336991	5.14388699	1.48187012	0.23771456	0.19449844
AI607873	0.46044019	1.61003838	1.48108408	0.2378347	0.19457552
Dcun1d2	0.19275513	5.52819333	1.48073357	0.2378883	0.19458764
Ly6h	0.52325909	0.87209353	1.48061887	0.23790584	0.19458764
Prelid2	1.25544762	-0.7033293	1.48032663	0.23795054	0.19458764
Srpk3	0.78101657	0.31649771	1.48021495	0.23796763	0.19458764
G730013B05	-0.7330037	0.35112344	1.4800794	0.23798837	0.19458764
Thap6	0.2654491	3.86927998	1.47989023	0.23801732	0.19458764
Crhr2	1.71590087	-1.7099164	1.47980015	0.2380311	0.19458764
S100pbp	-0.2027718	5.02257202	1.47655367	0.23852865	0.19497313
Adra2c	-0.397156	2.43547941	1.47570493	0.23865894	0.19505838
A4galt	0.33842886	2.77150386	1.47527403	0.23872513	0.19509121

Olfml2a	0.44192595	3.54799309	1.47476824	0.23880285	0.19513347
Mdm2	0.13039307	6.69822729	1.47390373	0.23893576	0.19520146
Gin1	-0.2859627	3.43491305	1.47388849	0.2389381	0.19520146
Sac3d1	0.42960402	2.31542067	1.47367077	0.23897159	0.19520756
Sqrdl	0.34744477	3.40384126	1.4734396	0.23900716	0.19521536
Tcf3	0.29052047	3.66483439	1.47298989	0.23907636	0.19523076
Ocln	-0.363569	2.73975676	1.4729788	0.23907807	0.19523076
3632451O06	0.28748101	4.23193063	1.47274913	0.23911342	0.19523837
6430550D23	-0.9925164	-0.2776649	1.4693015	0.23964493	0.19565106
Fosl2	-0.3825424	6.03954939	1.46887153	0.23971133	0.19568397
Kcnj15	1.42008655	-0.7769083	1.46777222	0.23988118	0.19580132
Ddx20	-0.3244909	3.71303709	1.46748218	0.23992602	0.19581661
Alkbh2	0.51214263	1.16747695	1.46691117	0.24001433	0.19586738
Lsm14a	0.16500539	8.08565336	1.46640116	0.24009325	0.19591046
Gm15412	-2.3290317	-1.650277	1.46605129	0.2401474	0.19593334
Nfat5	-0.1696147	8.73989418	1.46575743	0.2401929	0.19594914
Zfp449	0.26050571	5.20703437	1.46499855	0.24031044	0.19602372
Gpr126	0.27365022	3.94251813	1.46323606	0.24058372	0.19620951
Mypop	0.30828691	2.50815487	1.4630492	0.24061272	0.19620951
Bax	0.33284738	2.61546348	1.46302369	0.24061667	0.19620951
Timp3	0.31617294	9.35869266	1.46261378	0.2406803	0.19622881
Yars	0.19526072	5.01004981	1.46253412	0.24069267	0.19622881
Srsf2	0.15419298	8.34931977	1.46200479	0.24077487	0.1962745
Zfp524	0.75554865	-0.0109418	1.46176755	0.24081172	0.19628321
Adat1	-0.4065098	1.8778403	1.46129361	0.24088537	0.19630436
Tor1aip1	-0.1894635	7.34527973	1.4612637	0.24089001	0.19630436
Polr2c	0.25827679	4.23038674	1.46100546	0.24093015	0.19631575
Mks1	-0.8194499	0.20709525	1.46001395	0.24108435	0.19642005
Sh3glb1	0.14509406	8.20721644	1.45980105	0.24111748	0.19642571
Senp8	0.20819918	4.61752894	1.45943011	0.24117521	0.1964514
Zfp426	0.16597329	6.10522924	1.45906611	0.24123188	0.19647623
Nfatc2ip	-0.3172706	2.6767404	1.45868963	0.24129051	0.19650264
Frmpd4	0.19122221	7.77636696	1.45836828	0.24134057	0.19652208
Trim52	-1.3730921	-1.6565991	1.45797109	0.24140246	0.19655114
A630066F11	0.49362999	1.4922324	1.45674718	0.2415933	0.19667354
Nog	-0.7699888	-0.0920429	1.4566707	0.24160523	0.19667354
Serpina3g	1.32033677	-0.4356293	1.45644437	0.24164054	0.19668094
Mtch2	0.14581627	5.59819807	1.4560275	0.24170561	0.19671256
Fads1	0.18224427	6.22578949	1.45558325	0.24177496	0.19674766
Gm8615	0.28727834	2.82423481	1.45507842	0.24185381	0.19679047
AA543186	1.13426208	-1.0680148	1.45488563	0.24188394	0.19679364
Ntm	-0.1554119	7.47633413	1.45428475	0.24197785	0.19684869
Dapp1	0.28603212	4.05572574	1.4539466	0.24203072	0.19687035
Lrrc34	-1.415704	-1.0279857	1.45325154	0.24213943	0.19692516
Nek7	0.18783955	6.94421238	1.45318014	0.2421506	0.19692516

4930483K19I	0.89330619	-0.4326204	1.45262699	0.24223718	0.19697421
Bag2	-0.3829494	3.17330684	1.45216586	0.24230938	0.19701156
Emc10	0.23542067	5.07381744	1.45137934	0.24243259	0.19709038
Pcdha1	-1.1526773	-0.9128144	1.45121104	0.24245896	0.19709046
Ears2	-0.6969239	0.22658193	1.45061042	0.24255312	0.19712639
Dhx30	-0.3083513	4.70608379	1.45059385	0.24255572	0.19712639
Zbtb8b	-0.4755882	1.87284961	1.45035472	0.24259322	0.19713551
Kazald1	0.51226597	1.58188185	1.4498072	0.24267911	0.19718394
Sar1a	0.2182652	7.05937469	1.44923991	0.24276815	0.19721831
Wrap53	-0.5222245	1.41100641	1.44916556	0.24277982	0.19721831
Tmbim6	0.2324099	6.63596553	1.44903527	0.24280028	0.19721831
Pik3r1	0.15676662	8.09936598	1.44866633	0.24285821	0.19724401
Foxk1	-0.1577902	7.16862433	1.44698631	0.24312226	0.19741766
Gucy1b3	-0.194235	6.61329345	1.44697108	0.24312466	0.19741766
Gmds	0.28548991	3.36496846	1.446564	0.2431887	0.19744828
Tunar	0.35137941	3.21410415	1.44445098	0.24352144	0.19769705
Tmem30a	0.15690626	7.94358369	1.44409431	0.24357767	0.1977213
Ccdc127	0.1439042	6.5235289	1.44379905	0.24362423	0.19773769
Tonsl	0.77970074	0.46502871	1.44336148	0.24369324	0.19775472
Wnk2	-0.2486363	6.04766164	1.44333176	0.24369793	0.19775472
1700128F08I	-1.021491	-0.8445133	1.44276714	0.24378703	0.19780562
Cnnm4	-0.3726075	2.88400049	1.44242017	0.2438418	0.19782866
Card11	-1.005293	-1.0961286	1.44222138	0.24387319	0.19783273
Abca12	-1.2961983	-1.4656762	1.44196722	0.24391333	0.1978439
Ecel1	0.41125064	1.9455997	1.4414207	0.24399967	0.1978852
Zfp408	0.19036396	4.68741714	1.44131093	0.24401701	0.1978852
Zc3h12b	0.18492721	5.05571314	1.44107235	0.24405472	0.19789439
Igsf9b	-0.3465583	3.48985487	1.44084266	0.24409103	0.19790244
Stxbp4	0.17465362	7.33360469	1.44056315	0.24413522	0.19791687
Prpsap1	0.15517606	4.70373033	1.43893335	0.24439311	0.1980855
Zbtb18	-0.1632675	7.35838544	1.43884806	0.24440662	0.1980855
Milr1	-1.0546248	-0.3117248	1.43871987	0.24442692	0.1980855
Tmco6	0.41627012	1.74919843	1.43858141	0.24444885	0.1980855
Stambpl1	-0.2368771	3.56400113	1.43801002	0.24453937	0.19813745
Med1	-0.1602674	7.2607964	1.43782949	0.24456798	0.19813923
Adamts10	-0.3809438	2.08207284	1.43649148	0.24478016	0.19828971
Xrcc1	0.29579813	2.99943505	1.43544367	0.24494649	0.19840302
Fbxl8	-1.1612989	-0.7816331	1.43514457	0.244994	0.19842008
Jak1	-0.1594368	7.64637337	1.43378205	0.24521055	0.19857402
Myocd	1.99921434	-1.5673105	1.43350122	0.24525522	0.19858876
Epyc	-0.9003566	-0.2199656	1.43292471	0.24534694	0.19864159
D430019H16	-0.2110087	6.30631517	1.43274301	0.24537586	0.19864356
Npat	-0.1789331	5.99288499	1.43190108	0.24550992	0.19871656
Hcn3	0.43507916	1.66083011	1.43184397	0.24551901	0.19871656
Fbxl20	-0.140823	6.62663552	1.43146217	0.24557984	0.19874434

Kifap3	-0.2165233	8.45919916	1.43084958	0.24567747	0.19880191
Slc10a7	0.23763232	3.06877639	1.43032742	0.24576073	0.19884783
Fsd2	-1.4509192	-1.7199672	1.42990107	0.24582874	0.19885668
Wscd2	0.27735309	3.52687442	1.42988641	0.24583108	0.19885668
Tom1l2	0.15270716	7.57538087	1.42976034	0.2458512	0.19885668
Gsg1	1.29288364	-0.8084375	1.42903957	0.24596624	0.19892828
Gns	0.23245386	6.50749551	1.42864714	0.24602891	0.19895752
Anks3	-0.2913971	2.94614511	1.42820739	0.24609916	0.19899287
Nfxl1	-0.2793496	2.91886483	1.42804122	0.24612571	0.19899289
Hadhb	0.16359871	6.50866812	1.42741539	0.24622574	0.19905231
Ormdl2	0.64522873	1.43733539	1.42704574	0.24628485	0.19907864
Zfp846	-0.286521	3.9051803	1.42624968	0.24641221	0.19916013
Tspan17	0.23062902	3.27720702	1.42581678	0.2464815	0.19919467
Nup188	-0.2920124	3.73342481	1.42491128	0.24662652	0.1992904
Rel	0.19421839	4.52771932	1.42468384	0.24666297	0.19929682
Tmem160	0.59797197	1.07426555	1.42440078	0.24670833	0.19929682
Gm2011	-0.4733322	1.36350918	1.42417676	0.24674424	0.19929682
Pigv	-0.3554309	2.81035164	1.42398008	0.24677578	0.19929682
Trib1	0.18493511	5.66745826	1.42395534	0.24677974	0.19929682
Mir1191	-1.5829078	-1.5833267	1.42386719	0.24679388	0.19929682
Erf	-0.310863	3.33277045	1.42341889	0.24686578	0.19930357
AI464131	-0.3653444	2.93921916	1.42341589	0.24686626	0.19930357
Preb	0.22852119	5.17850217	1.42305813	0.24692366	0.19930357
Polr1b	-0.4584969	2.23088517	1.42288544	0.24695137	0.19930357
B430212C06I	-1.688146	-1.5430371	1.42282757	0.24696066	0.19930357
Dcaf12	0.2395073	5.27104991	1.42282138	0.24696166	0.19930357
Fdxacb1	-0.5510961	1.52243624	1.42205031	0.24708545	0.19936321
Lrrc71	-1.5058054	-1.4883196	1.42203002	0.24708871	0.19936321
Tmem80	-0.2968139	2.82179066	1.42103317	0.24724888	0.19946743
Taf4a	-0.1827795	4.74494453	1.4208952	0.24727106	0.19946743
4921511H03I	-1.6160697	-1.1054533	1.42036291	0.24735665	0.19949538
Rffl	-0.2986811	3.08964374	1.42034889	0.2473589	0.19949538
Flnc	0.43632496	2.89884862	1.41974066	0.24745675	0.19954336
Bysl	0.24099221	3.83428023	1.41964842	0.2474716	0.19954336
Kremen1	0.25828412	3.34457656	1.41927495	0.24753171	0.19957038
Wdpcp	0.20946644	3.98123373	1.41859834	0.24764066	0.19963058
2310057M21	0.20928264	4.09603702	1.41848073	0.2476596	0.19963058
Cd8b1	-0.732724	-0.329234	1.41809281	0.2477221	0.19963504
Ncl	-0.1415863	8.89214441	1.41796121	0.24774331	0.19963504
2410018L13I	-0.3392306	1.73556512	1.41795089	0.24774497	0.19963504
Cystm1	0.41405909	2.13882184	1.41711042	0.24788047	0.19972277
Elk1	-0.2015917	4.81294127	1.41672093	0.2479433	0.19974797
Slc26a5	-1.3750395	-1.149165	1.41658638	0.247965	0.19974797
Gstm5	0.24746823	5.97796216	1.41635658	0.24800208	0.19975639
Gm15328	-1.0676977	-0.036985	1.41588796	0.24807772	0.19977446

Acbd6	0.20837577	4.71462232	1.41588754	0.24807779	0.19977446
Klhl30	-0.5670853	0.76413979	1.41546497	0.24814602	0.19980796
Nol9	-0.2824366	3.57969393	1.41512443	0.24820103	0.1998308
Aagab	0.18842035	4.71518943	1.41476855	0.24825852	0.19985564
Il4ra	-0.7826538	0.15441314	1.41446912	0.24830692	0.19987315
Rassf4	-0.3066232	3.13006963	1.41388038	0.2484021	0.19990176
Qrfpr	-0.9049113	0.13814898	1.4136421	0.24844064	0.19990176
Myof	0.30687959	5.02471238	1.41363438	0.24844189	0.19990176
Trps1	-0.1951868	6.29006397	1.41337918	0.24848317	0.19990176
Phf7	0.7441581	0.91577903	1.41331673	0.24849327	0.19990176
Ccdc80	0.47950829	2.41847797	1.4132606	0.24850235	0.19990176
Rab3d	0.24585167	3.19566862	1.41258374	0.2486119	0.19996843
Proz	-0.5024662	1.46770786	1.41222975	0.24866922	0.19998719
Paqr3	-0.3777403	2.38706999	1.41211047	0.24868853	0.19998719
Ptgis	-0.3903376	2.96369474	1.41150178	0.24878714	0.20001836
3300002I08R	-0.8394431	-0.3547542	1.41149241	0.24878866	0.20001836
4930509E16I	-1.4271399	-1.2491604	1.4113774	0.24880729	0.20001836
Taok2	-0.1943462	5.310984	1.41058875	0.24893514	0.20009969
Atp6v0d2	1.32763867	0.3918407	1.41023731	0.24899214	0.20011247
Btbd2	-0.2401932	4.50350605	1.41016178	0.24900439	0.20011247
4930429F24I	0.63451263	0.47399518	1.40890506	0.24920837	0.20025494
Cdk5	0.22593792	4.20561656	1.40865	0.24924979	0.20026678
Dhrs3	0.31724329	4.64623871	1.40847744	0.24927782	0.20026785
Rer1	0.23345634	5.71875699	1.40758315	0.24942316	0.20034176
Snx33	-0.2887207	3.96125282	1.40758266	0.24942324	0.20034176
Cd79b	-1.4618064	-1.9913514	1.4067555	0.24955776	0.20041196
BC026585	0.78177451	0.725837	1.40671672	0.24956407	0.20041196
Pramef8	-0.2738981	4.01107825	1.40634591	0.24962441	0.20041769
Rgag4	-0.3464407	2.68256884	1.40634445	0.24962465	0.20041769
Gtdc1	-0.2018265	6.26817212	1.40600785	0.24967944	0.20044023
Ngly1	0.18474049	5.55152088	1.40563328	0.24974042	0.20046774
Mycbp	0.23400427	5.63413358	1.40455235	0.24991653	0.20058763
Tmem26	-1.3944925	-1.4309923	1.40392189	0.25001931	0.20064866
Sumf2	-0.3038697	2.65517025	1.4032613	0.25012708	0.20071037
Gm960	-0.947603	-0.5551087	1.40312255	0.25014972	0.20071037
Sema3c	0.22342609	3.8203494	1.40183913	0.25035927	0.20085702
Rxrb	0.28448759	3.23806349	1.40162285	0.25039461	0.20086389
Tnfrsf4	-2.1507199	-2.2440682	1.40136763	0.25043632	0.20087586
Npsr1	-0.5528289	1.3576542	1.40107593	0.250484	0.20089263
Lekr1	-0.3306362	2.74701292	1.400565	0.25056754	0.20093814
Dtx2	0.45770542	1.76024627	1.39978713	0.2506948	0.20101871
Rrnad1	-0.2427393	3.60625809	1.39961743	0.25072257	0.20101949
Mical2	0.17094414	8.91771607	1.39909537	0.25080804	0.20106652
Fam26f	0.72860793	1.22710449	1.39882801	0.25085182	0.20108013
Cd40	1.14159485	-1.0778425	1.39848225	0.25090846	0.20110405

Atp11c	-0.2348691	4.6841988	1.3979836	0.25099017	0.20114805
Rps20	0.21596101	6.34851348	1.39747913	0.25107288	0.20119283
Tmem19	0.23987294	3.56691785	1.39577461	0.25135258	0.20139545
Snx7	0.288595	4.97341229	1.3955822	0.25138417	0.20139925
Ago2	-0.1420235	7.24356027	1.39531184	0.25142858	0.20140417
Mmd2	0.24672299	4.00550522	1.39521795	0.25144401	0.20140417
Cul4a	0.13345239	7.55684241	1.39486222	0.25150246	0.20140835
Tap2	-0.6951555	0.2777487	1.39478133	0.25151575	0.20140835
H2-T10	0.41542125	1.18483638	1.39469595	0.25152978	0.20140835
Nrip2	0.46685027	2.07644589	1.3928853	0.25182761	0.20153763
Ppm1a	0.15527219	6.77648494	1.39287317	0.25182961	0.20153763
Gpr179	-1.2618071	-0.4016833	1.39286093	0.25183163	0.20153763
Gtpbp4	0.13297204	6.31664662	1.39285898	0.25183195	0.20153763
Ptprf	0.18068653	4.74980605	1.39274956	0.25184996	0.20153763
Parp16	0.66657966	1.90198188	1.39270583	0.25185716	0.20153763
Ufsp2	0.18862462	5.28542917	1.39257134	0.2518793	0.20153763
Lysmd1	0.28299035	3.29837465	1.39203929	0.25196693	0.20158624
Pdzd7	-0.5233346	1.33017718	1.3914495	0.25206411	0.20164249
Gm10560	-1.4362058	-1.0415094	1.39062748	0.25219964	0.20170045
Cntfr	0.36182036	4.14223416	1.3905606	0.25221067	0.20170045
Gm19522	0.35142424	2.40790063	1.39052079	0.25221724	0.20170045
Layn	0.47323463	1.44309849	1.39010832	0.25228528	0.20173336
Tcof1	-0.2153336	4.23575164	1.38964181	0.25236228	0.20177342
Ndrg1	0.26927085	7.57194374	1.38925198	0.25242663	0.20180337
Pigu	0.27701643	3.77721647	1.38858518	0.25253677	0.20185321
Poglut1	0.1623521	5.22591774	1.38854868	0.2525428	0.20185321
Ptpn1	-0.1692533	4.77236984	1.38782641	0.25266217	0.20192498
AK010878	-0.2356354	3.75420995	1.38767972	0.25268643	0.20192498
Tsen15	0.23011493	3.62941797	1.38678786	0.25283395	0.20194208
Wrb	0.15590017	5.3922587	1.38641471	0.2528957	0.20194208
Rbm19	0.36460557	2.26735387	1.38618836	0.25293317	0.20194208
Smarca5	-0.1458029	7.57245339	1.38610522	0.25294693	0.20194208
Gas8	0.31918033	3.60001775	1.38609557	0.25294853	0.20194208
Tlr3	-0.2221083	4.87896512	1.38603856	0.25295797	0.20194208
Steap2	0.16755701	4.92439914	1.3860188	0.25296124	0.20194208
Srsf1	-0.1316992	7.49633913	1.38598355	0.25296708	0.20194208
Alox12b	-0.5894789	2.01014161	1.38593655	0.25297486	0.20194208
Espl1	-1.1246327	-1.4992261	1.3859234	0.25297704	0.20194208
Htr5a	0.29763191	5.37112254	1.38521087	0.25309507	0.2020148
Dohh	0.31935687	3.27307213	1.3849132	0.25314439	0.20203267
4930579G18	-0.9375605	0.33076995	1.38466184	0.25318606	0.20204443
Zc2hc1c	-0.4856102	2.21582011	1.38432585	0.25324176	0.20205815
Noxo1	-0.9386981	-0.2666399	1.38414531	0.2532717	0.20205815
Mphosph10	-0.2007119	5.16500049	1.38399142	0.25329723	0.20205815
A330009N23	0.56553333	1.21981126	1.38351085	0.25337695	0.20205815

Satb2	-0.2060753	7.21935707	1.38348547	0.25338116	0.20205815
Zfp180	0.15529214	5.1210023	1.38337775	0.25339904	0.20205815
Rsph3a	0.19831526	4.43966848	1.38333013	0.25340694	0.20205815
2210404O09	-0.3737289	2.13870663	1.38325898	0.25341875	0.20205815
Rnf31	-0.2976175	3.23321975	1.38298833	0.25346367	0.20207073
Myef2	-0.203768	5.63887936	1.38276714	0.2535004	0.20207073
Rmnd5a	0.15493289	7.57797669	1.38267714	0.25351534	0.20207073
A630033H20	-0.947671	-0.6315604	1.3821548	0.2536021	0.20211841
Rps6	0.23961807	8.46228235	1.38139677	0.25372806	0.20219732
Krtcap3	-0.9170031	-0.9806215	1.38103979	0.25378741	0.20222313
D030028A08	0.43024656	1.33502954	1.38028429	0.25391308	0.20228873
Prrg1	-0.3522977	2.50080234	1.38022067	0.25392367	0.20228873
E2f4	0.21514282	4.54122018	1.37995544	0.25396781	0.2023024
Nudt2	0.29184231	3.19216054	1.37879904	0.25416037	0.2024343
Hsd17b12	0.31088675	3.64403364	1.37823776	0.2542539	0.20248729
Eftud2	-0.2005927	5.01527971	1.37745839	0.25438385	0.20254906
Cul7	-0.2651218	3.0716944	1.37737749	0.25439734	0.20254906
Tmem248	0.171762	5.76332469	1.37723153	0.25442169	0.20254906
Sh2b1	0.20225153	4.06126179	1.377125	0.25443947	0.20254906
Cbx4	-0.1990513	4.43718425	1.37688515	0.25447949	0.20255942
Krt73	1.18822119	-1.1365842	1.37658998	0.25452875	0.20257714
Itfg2	-0.2485876	3.37979046	1.37617325	0.25459832	0.20261101
Mst1r	-0.8984443	-0.7294972	1.37555702	0.25470124	0.20267142
Aasdhppt	-0.1696942	5.24523466	1.37495841	0.25480127	0.20272635
Prpf38b	-0.1557238	7.32605818	1.37470001	0.25484447	0.20272635
Rdh5	1.26679181	-1.2298068	1.37465881	0.25485136	0.20272635
Cndp1	-2.0575795	-1.3929104	1.37331263	0.25507656	0.2028563
Galnt9	0.2170893	4.68290137	1.37322306	0.25509155	0.2028563
Rab17	1.42079141	-1.5520423	1.37319741	0.25509584	0.2028563
Cnr1	0.1784674	6.5698156	1.37277409	0.25516672	0.20288973
Sdccag8	-0.1612366	6.24734401	1.3724097	0.25522775	0.20288973
Zmym2	-0.1919625	8.1176782	1.37219962	0.25526295	0.20288973
Cd9	0.38015836	4.85260339	1.37201437	0.25529398	0.20288973
Ak4	0.21461589	5.3752424	1.3719165	0.25531039	0.20288973
1190007I07R	-0.397569	2.18462635	1.37183473	0.25532409	0.20288973
Gm14169	0.37814704	1.90935333	1.37181609	0.25532721	0.20288973
Atp5a1	0.11731006	9.40853935	1.37023225	0.25559284	0.20307929
Gm11149	-1.1244308	0.11160232	1.36938765	0.25573464	0.20313655
Runx2	-0.2567037	4.70215214	1.36933593	0.25574333	0.20313655
Tfap2b	0.23487285	6.8869382	1.36931909	0.25574616	0.20313655
LOC1012436	-0.9330491	-0.5993772	1.36905626	0.2557903	0.20315011
Cdh3	-0.8309808	0.55645402	1.36868885	0.25585204	0.20315775
Alg1	-0.9532583	0.0254511	1.3686766	0.2558541	0.20315775
Nme5	0.28791318	3.39963312	1.36846742	0.25588925	0.20316416
Ccz1	0.18361734	4.8060253	1.36811073	0.25594921	0.20319027

Tram1	0.23161413	5.97538072	1.36752864	0.25604711	0.20324647
Coro2b	0.19356811	6.52167001	1.36641459	0.25623459	0.2033667
Smc4	-0.1962513	6.28417346	1.36630653	0.25625279	0.2033667
Ap2b1	0.15197616	7.86662395	1.36587604	0.25632529	0.20340272
Fam63b	-0.1415621	7.76590666	1.36516917	0.2564444	0.20347571
Pgm1	0.21313097	3.70359012	1.3649641	0.25647897	0.20348162
Bicd2	-0.1606946	6.4485102	1.36464429	0.2565329	0.20349105
Ccr5	0.58680608	1.19262425	1.3645718	0.25654512	0.20349105
Strip2	-0.342672	5.1919143	1.36433091	0.25658575	0.20350176
Trim65	0.27141153	4.06082884	1.36311782	0.25679048	0.2036426
Bace2	0.345233	3.59463179	1.36250348	0.25689424	0.20370335
Arhgap42	-0.1579087	5.70538165	1.36216925	0.25695071	0.20372659
Fggy	-0.3652024	2.85345833	1.36148566	0.25706626	0.20379667
Dgkq	-0.3901524	3.8686968	1.36128506	0.25710019	0.20380203
Ccdc116	0.69086219	0.07424863	1.36105457	0.25713917	0.20381139
Pik3c2g	-1.686844	-2.0787659	1.36032762	0.25726217	0.20388734
Trim14	0.59826341	2.15536847	1.35959503	0.25738621	0.20396409
Iars2	-0.1805218	5.53871229	1.35869914	0.257538	0.20406282
Anapc5	-0.1366723	6.32639075	1.3579626	0.25766288	0.20412248
Stac	0.86459872	0.1183344	1.35781471	0.25768796	0.20412248
Prkacb	0.13113897	9.17765633	1.35777363	0.25769493	0.20412248
Gpr123	0.21389653	6.43854164	1.3558716	0.25801783	0.20435668
Gp1ba	-1.1070424	0.3865583	1.35544275	0.25809071	0.20439282
Sardh	0.43773427	2.20150384	1.35460504	0.25823315	0.20446925
5930430L01F	0.27696717	4.70600952	1.35454405	0.25824352	0.20446925
Cd47	0.18658514	7.49962198	1.35439432	0.258269	0.20446925
Kl	0.19048902	5.31075688	1.3542149	0.25829952	0.20447184
Tril	-0.2304633	3.67077525	1.35401445	0.25833363	0.20447726
Mertk	-0.2858096	3.7656962	1.35273304	0.25855182	0.20461158
Kcnc4	0.25524851	4.33426305	1.35265793	0.25856462	0.20461158
Hsdl1	-0.2435241	4.27791316	1.35253733	0.25858517	0.20461158
Podxl2	0.41486865	2.17688717	1.3521416	0.25865261	0.20464336
Ccnh	0.16947742	4.97463151	1.35129259	0.25879738	0.20472239
Nlrp1b	-2.189939	-1.6327494	1.3512357	0.25880708	0.20472239
Usp14	0.15735782	6.97105052	1.35032502	0.25896249	0.20482373
Esco1	-0.1778348	5.65472216	1.35009795	0.25900126	0.20483279
Xlr3c	1.09649491	-1.4935788	1.34967419	0.25907363	0.20486843
Rcn3	0.52722738	3.12705558	1.34819115	0.25932712	0.20504727
Adam1a	-0.6262592	1.28166211	1.34801894	0.25935658	0.20504895
Ocrl	-0.1936437	6.61188684	1.34750048	0.25944529	0.20509746
Tacc2	-0.1638415	5.52330764	1.34716076	0.25950344	0.20510171
Eftud1	-0.2994419	3.10423985	1.34714962	0.25950534	0.20510171
Aox4	-0.727916	0.66151393	1.34584533	0.25972875	0.20525665
Zfp456	-0.3155334	3.08223601	1.34547516	0.2597922	0.20526457
Ccdc61	0.54053857	0.48748189	1.3454676	0.2597935	0.20526457

Npepl1	0.40187524	2.06684462	1.34530119	0.25982203	0.20526549
A830080D01	-0.2098726	4.31144439	1.34509671	0.25985709	0.20527157
Pof1b	0.6974305	0.09221184	1.34386333	0.26006872	0.20541712
Abt1	0.26837869	3.38623331	1.34339937	0.26014839	0.20545841
Wbp5	0.22361108	6.62007551	1.34296147	0.26022361	0.20549618
Fubp1	-0.1720668	7.1259254	1.34184466	0.26041558	0.20562613
Sec61g	0.26420707	4.7707202	1.34090628	0.26057702	0.20573195
Ubn1	-0.160236	5.81141917	1.34019568	0.26069936	0.20578323
St6gal1	0.2643203	6.19199686	1.33997376	0.26073759	0.20578323
Stat3	0.13763928	5.61785731	1.33995563	0.26074071	0.20578323
Atl1	-0.243938	6.2395318	1.33989183	0.2607517	0.20578323
Tmem161b	-0.3635936	3.57042843	1.33909247	0.26088945	0.20587028
Gm19557	-1.194666	-0.643446	1.33827737	0.26103001	0.20595953
Hist1h1c	0.52927424	2.87540411	1.33804105	0.26107078	0.20597003
Oas1g	-0.9336797	-0.3560232	1.3376797	0.26113314	0.20599757
Copb1	-0.1480633	6.10022922	1.33636389	0.26136037	0.20615514
Slc6a12	-0.4117632	3.69420656	1.33503221	0.26159061	0.20631505
Tmem125	0.60840245	0.65984188	1.33465492	0.26165589	0.20634484
Ttf1	-0.1676703	4.43710407	1.33443787	0.26169345	0.20635277
Crabp1	2.05935421	-1.4468871	1.33345325	0.26186394	0.20646551
Pm20d2	-0.1884424	4.21869945	1.33326624	0.26189634	0.20646935
Pkp3	1.31307224	-1.3205244	1.33298936	0.26194432	0.20647552
Lmod1	-0.2901415	5.0582383	1.33284243	0.26196979	0.20647552
Nsmce4a	0.22718746	4.58308296	1.33274454	0.26198675	0.20647552
Nudt18	0.2915965	3.81344971	1.33228311	0.26206675	0.20651687
Fbxl19	-0.2762979	3.63288	1.33172756	0.26216311	0.206564
Aoc3	-0.7160939	3.18181364	1.33159822	0.26218555	0.206564
Col6a5	-1.9512894	-1.8134956	1.33123628	0.26224836	0.206564
Rasa1	-0.1338223	6.61581885	1.33122212	0.26225082	0.206564
Luc7l3	-0.1532003	8.5707348	1.33114481	0.26226424	0.206564
Rngtt	-0.1711834	5.27968148	1.33066936	0.26234678	0.20659812
Xndc1	0.28032538	3.95280623	1.33057799	0.26236265	0.20659812
Tbccd1	0.19956907	4.58301558	1.33037013	0.26239875	0.20660486
Parp1	0.13332829	6.13886078	1.32987099	0.26248546	0.20665145
Lmo1	0.90896244	-0.3943841	1.32921264	0.2625999	0.20671984
Rc3h2	0.14591577	7.92859234	1.3285496	0.26271521	0.20676207
Shc4	-0.3099002	2.54008987	1.328478	0.26272767	0.20676207
Rab26	0.32689202	3.91471612	1.32842881	0.26273623	0.20676207
Alyref	0.29133787	2.80293546	1.32789912	0.26282841	0.20681291
Elovl6	-0.1873472	6.01508993	1.32737071	0.2629204	0.20684967
Glp1r	0.69006975	0.0642864	1.32731405	0.26293027	0.20684967
Wdr24	-0.3758735	2.33608061	1.32688049	0.26300579	0.20686025
Zfp827	0.16014353	6.30042239	1.32639638	0.26309015	0.20686025
Kcne2	-1.1800073	-0.9974753	1.32639624	0.26309018	0.20686025
Ing1	0.22590677	4.90266404	1.32631851	0.26310373	0.20686025

Fam150b	1.61247227	-1.1072825	1.32620689	0.26312318	0.20686025
Slmo1	-0.2423199	5.01944233	1.3262025	0.26312395	0.20686025
Kif16b	0.15246536	4.96535644	1.32612902	0.26313676	0.20686025
Hook3	-0.1355869	8.61231605	1.32579742	0.26319458	0.20688402
Ccdc110	0.99508774	0.36733429	1.32546845	0.26325195	0.20690744
Pbk	1.03571966	0.03747487	1.32517573	0.26330302	0.20692265
Sla	-0.2654164	3.91966931	1.32504132	0.26332647	0.20692265
Kcnd3os	-1.1507717	-1.0140629	1.32434881	0.26344735	0.20699595
1700007F19I	-0.6433205	0.05421021	1.32289107	0.26370204	0.20717437
Ash2l	-0.162015	5.00464747	1.321464	0.26395168	0.20734878
Prox1	-0.2003606	4.8871507	1.32091436	0.26404792	0.20739189
9430020K01I	-0.1271515	8.95856324	1.3207729	0.26407269	0.20739189
Thrb	0.16126113	7.02512197	1.32067691	0.26408951	0.20739189
Micall2	-0.7350346	0.41934992	1.32032839	0.26415056	0.20741786
Frk	0.28517249	4.02607557	1.32017252	0.26417788	0.20741786
Dok2	0.94729673	-0.7678923	1.31934986	0.26432209	0.20750263
Hrk	0.28902679	4.09783662	1.31924109	0.26434117	0.20750263
Efcab7	0.38701587	2.55634321	1.31820628	0.26452274	0.20761166
Sdccag3	-0.1892203	4.74850971	1.31813409	0.26453541	0.20761166
Gatm	-0.1432011	6.23725596	1.31776864	0.26459958	0.20763305
Fam26e	0.33237416	4.62726743	1.31766356	0.26461803	0.20763305
Ddr1	0.21355839	3.43016774	1.31746279	0.26465329	0.207639
Grtp1	-0.3358426	3.25590908	1.31717043	0.26470465	0.20765758
Ptpmt1	-0.1973634	4.1804834	1.3169233	0.26474808	0.20766993
Gm10865	-0.6414809	-0.4188497	1.31635769	0.2648475	0.20772619
Dynlt1a	-0.1819751	4.34694742	1.31590795	0.26492659	0.2077665
Pcdhgb2	-0.350487	2.11965788	1.31540548	0.26501499	0.20779006
Htr1f	-0.4998359	1.8051509	1.31536459	0.26502219	0.20779006
Ispd	0.28040016	3.40984449	1.3152649	0.26503973	0.20779006
Acy3	0.39987584	1.57757437	1.31466153	0.26514595	0.20785161
Hes5	0.42475882	1.57656207	1.31419237	0.26522858	0.20789466
Lss	-0.2293363	4.11244123	1.31402122	0.26525873	0.20789657
Manf	0.30258438	3.8192679	1.3136449	0.26532504	0.20792681
6030440G07	-0.8904076	-1.2399311	1.31339369	0.26536932	0.20793979
Eva1b	0.57897105	0.88173957	1.31185746	0.26564031	0.20813039
A530054K11I	-0.1709777	5.33622178	1.3112526	0.2657471	0.20819232
Aup1	-0.3522058	3.08808871	1.31108844	0.2657761	0.20819329
Styx	-0.2476389	4.25906461	1.31050078	0.26587993	0.20825288
Gxylt2	-0.2938888	3.90150015	1.31008333	0.26595371	0.20828892
Tmem151b	-0.2926217	4.64442946	1.30970751	0.26602017	0.20831922
Grrp1	-1.0859993	-1.7128194	1.30925989	0.26609934	0.20833964
Knstrn	0.64894321	0.64181907	1.30924601	0.2661018	0.20833964
Reep1	0.18360231	6.96590109	1.30889439	0.26616402	0.20836661
Nxnl1	1.83660905	-1.2919197	1.30807833	0.26630849	0.20845795
Pmm1	0.19355029	4.66168759	1.30786324	0.26634659	0.20846602

Naa20	0.20416363	4.99462686	1.30690201	0.26651692	0.20856361
BC022687	0.38652652	1.99778136	1.30683202	0.26652933	0.20856361
Mtfr1l	0.20367673	5.6179435	1.30668907	0.26655468	0.20856361
Rrp9	0.5100539	1.10828275	1.3064401	0.26659883	0.2085764
Ppp4c	0.43849859	2.05232108	1.30582166	0.26670854	0.20864047
A430033K04	-0.2087394	5.56796504	1.3054007	0.26678326	0.20867716
Fzd9	-1.0749057	-0.7379368	1.30460798	0.26692403	0.20874576
Vstm4	0.33348631	4.68068533	1.3045934	0.26692662	0.20874576
Ctsk	0.45096906	2.38531761	1.3043635	0.26696746	0.20875552
Rmst	-0.8069856	0.98297744	1.30420988	0.26699476	0.20875552
Tcta	0.25411179	4.4087972	1.30382926	0.2670624	0.20878665
2310030G06	0.42408741	2.80094313	1.30322672	0.26716954	0.20884864
Tox2	0.26585666	2.75127914	1.30227526	0.26733883	0.2089592
Aig1	0.33689347	3.24048573	1.30194668	0.26739733	0.20898315
Lamc3	0.33694153	2.59835371	1.30059643	0.26763789	0.20914125
Etohi1	0.22107104	4.21814731	1.30049828	0.26765539	0.20914125
Stmn4	0.25145826	6.7672225	1.30033756	0.26768404	0.20914186
Ppox	0.58040844	1.015352	1.29973881	0.26779083	0.20919595
Taf5l	-0.190271	4.1315046	1.29954217	0.26782592	0.20919595
Cdca4	0.30123252	2.88013428	1.29948039	0.26783694	0.20919595
Nell1	-0.2081328	4.05279299	1.29929919	0.26786928	0.20919942
Agbl5	-0.2943078	2.76543721	1.29901128	0.26792066	0.20921777
Snx17	0.2399251	4.55007244	1.2977161	0.268152	0.2093352
Hmgcr	-0.1870357	5.79284207	1.29770854	0.26815335	0.2093352
Crb1	-1.3291394	-0.9575645	1.29770067	0.26815476	0.2093352
Mc5r	-0.7391512	0.66592367	1.29748444	0.26819341	0.20934359
Galntl6	0.20064859	5.12805334	1.29574021	0.26850544	0.20953561
Aplnr	1.40329804	-1.2725046	1.29560021	0.2685305	0.20953561
Gpx4	0.34855525	4.24864188	1.29547253	0.26855336	0.20953561
2610028E06	-0.8539966	-0.2727317	1.2953576	0.26857394	0.20953561
Il15	0.40189853	1.90573341	1.29532894	0.26857908	0.20953561
Emcn	-0.8537902	0.59230906	1.29287178	0.26901961	0.20985747
Cstf3	-0.216066	5.29501296	1.29181236	0.26920984	0.20998403
Tmem39b	-0.4207269	1.82656269	1.29138768	0.26928615	0.21001027
Fam212a	0.99913777	-0.7309088	1.29131352	0.26929948	0.21001027
Zfp407	-0.1989203	4.98637286	1.2887059	0.26976867	0.2103543
Synpr	0.25758338	5.17764605	1.28851937	0.26980228	0.21035863
Piezo1	-0.4561782	1.30366693	1.28830529	0.26984085	0.21036684
Dnm1	0.18034747	8.86763299	1.28666502	0.27013665	0.21057556
Clec4g	1.19964893	-1.5949193	1.28620591	0.27021952	0.2106088
Mmp13	1.54352115	-1.9234618	1.28611769	0.27023544	0.2106088
Hcls1	-0.3648676	1.43159804	1.28595428	0.27026495	0.21060992
Stc2	-0.6821893	-0.1603115	1.28562941	0.27032362	0.21063376
4930404N11	0.67085593	0.04994917	1.2844091	0.27054416	0.2107837
Igdcc3	-0.8148239	0.90123757	1.28319461	0.27076389	0.21093299

Csnk2a2	0.20611303	5.19443205	1.28270736	0.27085211	0.21096974
Foxr2	-0.8270063	0.49524515	1.28262335	0.27086732	0.21096974
Nup37	-0.4933329	1.22698112	1.28243176	0.27090202	0.21097487
4933405O20	-1.801313	-0.526677	1.28201072	0.2709783	0.21101237
Cdc23	0.17153503	5.00918478	1.28178774	0.27101871	0.21102193
BC055324	-0.573746	0.65282369	1.28116504	0.2711316	0.21108792
Pias1	0.12352236	6.84402528	1.28002467	0.2713385	0.21122156
Aurka	0.65360646	-0.188556	1.27990858	0.27135958	0.21122156
Hddc2	0.22527789	3.66227842	1.27956329	0.27142227	0.21123212
Zxda	-0.193094	5.37995996	1.2795237	0.27142946	0.21123212
1110006O24	-0.972148	-0.6314302	1.27920389	0.27148755	0.21125541
Gm10416	-0.8136747	0.47683774	1.27866291	0.27158585	0.21130997
Ddx24	-0.1519567	5.66962142	1.2783398	0.27164458	0.21133375
Eif2s3x	-0.1458768	7.27309505	1.27815603	0.27167799	0.21133575
Gm9958	-0.4334916	1.63163496	1.27797578	0.27171077	0.21133575
Traf7	0.20961877	3.73534693	1.27786081	0.27173167	0.21133575
Slc36a2	-1.4934088	-1.7024372	1.27743146	0.27180978	0.21135926
Fam69a	0.17485818	4.66369222	1.27724792	0.27184317	0.21135926
Nol7	0.20241685	5.77233861	1.27696712	0.27189428	0.21135926
AA987161	-0.1656804	5.33446726	1.27693058	0.27190093	0.21135926
Utp18	0.18672256	4.20392868	1.27687601	0.27191086	0.21135926
Rpl17	0.19465175	6.86589986	1.27676565	0.27193095	0.21135926
Cep83	0.1628868	5.98075362	1.27604598	0.27206201	0.21141851
2610044O15	-0.206734	4.55994414	1.27603746	0.27206356	0.21141851
Arpc5l	0.18404891	5.82331496	1.27581485	0.27210412	0.21142813
Glod4	0.1489434	5.51592075	1.27559055	0.27214499	0.21143798
Add3	0.14628924	8.2735249	1.27415203	0.27240731	0.21161987
Hmga2-ps1	-0.6731633	0.57436353	1.27383846	0.27246453	0.21164241
Ccm2	0.22084677	3.74234732	1.27341812	0.27254127	0.21165138
Polr3e	-0.271501	3.83577343	1.27338757	0.27254685	0.21165138
Plagl1	-0.2392554	6.20316342	1.27331156	0.27256073	0.21165138
Dtwd1	0.44379388	2.03221828	1.27281464	0.27265149	0.21169241
Hdc	-0.8640784	-0.2146941	1.27271327	0.27267001	0.21169241
Serinc2	-0.5131302	0.7617066	1.27239233	0.27272865	0.21170836
Mgst1	0.46071626	3.65503979	1.27229197	0.27274699	0.21170836
Sgtb	0.15836659	8.05187458	1.27168108	0.27285868	0.21177313
Cisd3	0.31696477	2.82960951	1.27120837	0.27294514	0.21181143
Rbm26	-0.1473526	7.13250946	1.27110257	0.2729645	0.21181143
Cep85	-0.2350817	3.329014	1.2699514	0.27317523	0.21195302
Trdn	-0.6835185	0.16367472	1.26937303	0.27328118	0.21201331
Ipo11	-0.1733845	6.18942871	1.26862342	0.27341859	0.21209797
Zfp84	-0.1685667	5.15423552	1.26817735	0.2735004	0.2121395
Acss3	0.36724798	2.36379029	1.26799131	0.27353453	0.21214403
Gm16897	0.56727052	0.57425721	1.26736121	0.27365017	0.21221178
Nkd2	0.29080285	4.51143517	1.26683693	0.27374644	0.21226448

L3mbtl3	0.20990344	4.1829686	1.26592735	0.27391356	0.21237212
Rnf152	0.15155495	6.19103377	1.26542499	0.27400591	0.21242177
Bcl2l1	0.26684098	4.44907231	1.26429778	0.2742133	0.21254589
Abhd13	0.15097955	5.20449293	1.26424677	0.27422269	0.21254589
Naa25	-0.2018313	4.53536187	1.26397247	0.27427319	0.21256307
Fgl2	0.28019407	4.78953892	1.26371575	0.27432046	0.21257774
Ppp1r3e	-0.2497278	4.20352793	1.26297613	0.27445672	0.21266137
Npc1l1	-0.8580789	0.04696487	1.26238749	0.27456523	0.21271952
Ccdc181	0.18192166	5.18637453	1.26226137	0.27458849	0.21271952
Rps24	0.20161623	7.28769221	1.26202703	0.27463171	0.21273044
2610027K06I	-1.0294893	-0.8805224	1.26187742	0.2746593	0.21273044
B4galt2	-0.2737259	3.39230077	1.26159258	0.27471185	0.21274917
Sorcs2	-0.2386114	3.64157151	1.260401	0.27493184	0.21289756
Crtc1	-0.1729458	6.31936751	1.26018345	0.27497203	0.2129067
Tnfrsf18	-0.3909008	1.90014686	1.2597254	0.27505667	0.21295026
9530026P05I	0.53374728	0.5180559	1.25917656	0.27515813	0.21300683
Henmt1	-1.0073137	-0.5878007	1.25897069	0.2751962	0.21301431
Atp10d	-0.3419617	2.36718858	1.25863765	0.27525781	0.21304002
Dusp28	0.30894589	3.63802561	1.25823868	0.27533163	0.21307517
Degs1	0.21326951	6.56259852	1.25804333	0.27536779	0.21308117
Hkdc1	0.42244953	1.82969467	1.25758686	0.2754523	0.21312458
Pidd1	-1.1618113	-1.6530424	1.25732782	0.27550027	0.21313971
Morn4	0.19445109	5.72266138	1.25687464	0.27558423	0.21318267
Ykt6	0.19011267	5.12626291	1.25666506	0.27562306	0.21319073
Tlcd1	-0.4548223	2.1458999	1.2560625	0.27573476	0.2132412
Spata32	-0.7922422	-0.0808031	1.25590903	0.27576322	0.2132412
Nuak1	-0.1965378	5.19287171	1.25585314	0.27577359	0.2132412
Ppp1r14b	0.34240985	3.52403494	1.25561813	0.27581718	0.21325292
Sapcd2	1.37624964	-1.1384531	1.25469003	0.27598942	0.21336071
Cep128	-0.2769239	4.25132638	1.25456042	0.27601348	0.21336071
Fap	-0.4987018	1.52176877	1.25302184	0.27629935	0.21355969
Sipa1	0.3001382	2.6000308	1.25167317	0.27655027	0.21371153
Ttc29	-1.6695578	-2.3012411	1.25149701	0.27658306	0.21371153
Nfkbia	0.30717583	2.74707365	1.25122289	0.27663411	0.21371153
Rqcd1	0.19381523	4.95419579	1.25121927	0.27663478	0.21371153
Rnf39	0.38678486	1.8296328	1.25120061	0.27663826	0.21371153
Raf1	0.17074758	4.73108242	1.25078985	0.27671477	0.21374862
2900076A07I	-0.5159347	0.95672739	1.25008997	0.2768452	0.21380846
Batf2	1.25814736	-0.9426477	1.25006831	0.27684924	0.21380846
Mgl2	-0.654795	0.39839275	1.24961065	0.27693457	0.21385235
H2-DMb1	0.79131007	0.53285769	1.24933856	0.27698532	0.21386952
Plin2	0.42419485	3.42219361	1.24873912	0.27709718	0.21392112
Wdr4	-0.3625944	2.39994244	1.24846057	0.27714917	0.21392112
9330117O12	-0.4508267	1.48878023	1.24839381	0.27716164	0.21392112
Dgcr2	0.15703053	4.64335063	1.24836514	0.27716699	0.21392112

Trim43c	-1.1671651	-1.17469	1.24809269	0.27721787	0.21392112
Lrfn5	-0.2214248	5.23599728	1.24801042	0.27723323	0.21392112
Cenpo	-0.4379807	2.19298701	1.24791116	0.27725177	0.21392112
Tmem59	0.1936634	6.13848722	1.24718872	0.27738675	0.21400325
Stx1b	0.17008803	6.40618772	1.24684556	0.2774509	0.21403073
Scap	-0.2254062	4.49942401	1.24637628	0.27753866	0.21407641
Zfp74	0.20140683	5.97278665	1.244255	0.2779358	0.2143607
BC020402	-0.5402455	1.68090219	1.24319913	0.27813376	0.21449133
Tmbim4	0.23854785	4.59340577	1.24280459	0.27820778	0.21452156
Nedd4l	-0.2278013	8.72859637	1.2426853	0.27823016	0.21452156
Fam131a	0.18121077	5.2288787	1.2417341	0.27840874	0.21461888
4833424O15	-0.2044451	4.66209956	1.24170821	0.2784136	0.21461888
Brinp3	-0.1923804	4.53933013	1.24148292	0.27845592	0.21462945
Ppcs	0.31518182	2.41680731	1.24115495	0.27851755	0.21465489
Impact	0.12829985	7.83618296	1.24096727	0.27855282	0.21466002
Map10	-0.5175128	1.696296	1.24019982	0.27869711	0.21471779
Ido2	-0.3932622	2.00854124	1.24017561	0.27870166	0.21471779
Gm12429	-0.4472527	0.85949264	1.24003097	0.27872887	0.21471779
Mx1	-1.0380536	0.01692365	1.23995969	0.27874228	0.21471779
Tshb	-1.3285684	-1.2726407	1.23979175	0.27877387	0.21472007
Atxn2l	-0.1480204	6.83832865	1.23963841	0.27880272	0.21472025
Pih1d1	0.33487097	3.36208392	1.23890487	0.2789408	0.21480454
Cnnm1	-0.1681693	6.37053827	1.23843075	0.2790301	0.21485124
Ptma	0.227237	9.04456798	1.2379769	0.27911561	0.21488732
Cers2	0.24426844	6.07253664	1.237878	0.27913425	0.21488732
Dcst1	-0.4393743	1.39971641	1.2370615	0.27928819	0.21498247
Eif3i	0.15538325	5.80342661	1.23691851	0.27931516	0.21498247
Tbc1d24	0.19956425	6.35733208	1.23636573	0.27941946	0.21504068
4933411K20I	0.14957319	6.72775489	1.23529395	0.27962184	0.21517435
2010005H15I	1.50545088	-1.5641879	1.23444718	0.27978186	0.21519769
C230024C17I	1.77001278	-1.9188972	1.2344258	0.2797859	0.21519769
Nkx2-2os	-1.4344715	-0.6508866	1.23435789	0.27979874	0.21519769
Lat2	-0.7197914	-0.025745	1.23425208	0.27981875	0.21519769
Tbc1d13	0.23510284	4.34928635	1.23423991	0.27982105	0.21519769
Rab11fip3	-0.1451626	6.19412059	1.23422279	0.27982429	0.21519769
Prps2	0.15832992	6.17915191	1.23342756	0.27997471	0.2152913
Sdr9c7	-1.5914767	-1.6837212	1.23302832	0.28005027	0.21531351
Ubap2	0.13330199	6.09196296	1.23279828	0.28009382	0.21531351
Zfp358	0.24200256	3.17915035	1.23262252	0.2801271	0.21531351
Utp23	-0.2062995	4.38435016	1.23261092	0.2801293	0.21531351
Phlda3	0.34961342	2.61215372	1.23251687	0.28014711	0.21531351
Npm1	0.13049214	9.66262949	1.23199092	0.28024674	0.21532931
Cmip	0.16440635	9.08758755	1.23198176	0.28024847	0.21532931
Kcnq3	-0.1650075	5.53464675	1.23195374	0.28025378	0.21532931
Rbl1	-0.4850971	1.59039662	1.23150902	0.28033806	0.215372

Th	0.77565524	0.60102874	1.2312812	0.28038125	0.21538312
Pet100	0.35385938	3.54535669	1.23093429	0.28044703	0.2154116
Zfp551	-0.462518	2.48571282	1.23062193	0.28050628	0.21542261
Pkdrej	-0.7134746	0.50668192	1.23055593	0.2805188	0.21542261
Cdk9	-0.2011012	4.38479661	1.23002234	0.28062006	0.21547547
Gm10408	-0.7748432	-0.4186977	1.22989044	0.2806451	0.21547547
Fam78b	0.18969806	6.81848028	1.22928779	0.28075953	0.21550011
4921511I17R	-2.0316138	-2.4337037	1.22917264	0.2807814	0.21550011
Kifc3	-0.26186	3.01247194	1.22916016	0.28078377	0.21550011
Syngr1	0.15102188	6.81639169	1.22911635	0.28079209	0.21550011
Rbm3	0.21274165	7.90721489	1.22868954	0.28087319	0.21554029
Usp10	0.17826604	5.20254236	1.22718494	0.28115931	0.21573423
Mid2	-0.1685666	5.67538724	1.22705819	0.28118343	0.21573423
A930005H10	0.32112359	3.04340925	1.2264674	0.2812959	0.21579845
2610035D17	0.281056	3.31067357	1.22612559	0.281361	0.21582631
Ankk1	-0.7872742	-0.2694108	1.22508758	0.2815588	0.21595596
Irf7	0.69076857	0.15226717	1.22373032	0.28181773	0.21612779
Cse1l	0.13363224	6.72638057	1.22361129	0.28184045	0.21612779
Gnai3	0.18199076	6.40500411	1.22298096	0.28196083	0.216198
Mns1	-0.3263241	2.41699082	1.22281721	0.28199211	0.21619988
Calu	0.19966238	6.14089054	1.22258674	0.28203614	0.21621154
Dpagt1	0.31731164	3.3175558	1.22224219	0.28210199	0.21623993
Grhl3	-1.0254624	-0.2245876	1.22188707	0.28216988	0.21625746
Klhl9	-0.1251797	7.39308747	1.22178065	0.28219023	0.21625746
Lrfn3	0.2407105	3.13777682	1.22167019	0.28221135	0.21625746
Polg2	-0.6499299	0.97007582	1.22119082	0.28230305	0.21630195
Tcea3	0.27180874	3.93148528	1.22097232	0.28234486	0.21630195
Abcb8	0.19857519	4.05750279	1.22091455	0.28235591	0.21630195
Dedd	0.18869046	4.20891729	1.22057063	0.28242174	0.21633028
Smdt1	0.27244955	5.06212921	1.21938246	0.28264932	0.2164825
Gm1673	0.57088423	0.4194867	1.21878848	0.28276318	0.2165476
Bcam	0.38097781	3.57616704	1.21811912	0.28289157	0.2166238
Nyx	-0.9899956	-0.5550092	1.21721907	0.28306432	0.21673396
Ikzf2	0.21293426	4.23824318	1.21686987	0.28313138	0.21676318
Syncrip	0.12523863	7.70853875	1.21668105	0.28316766	0.21676883
Apobec1	-0.2834556	3.35042293	1.21598067	0.28330225	0.21684973
D17Wsu92e	-0.1166328	7.63463128	1.21456434	0.28357469	0.21703612
Cdr2	0.3246874	2.74416149	1.21412977	0.28365835	0.21704942
Efemp2	0.40100043	2.75571536	1.21412376	0.28365951	0.21704942
Itga5	-0.2697253	2.85314034	1.21398812	0.28368562	0.21704942
Cnih4	0.16163972	5.19353871	1.21387297	0.2837078	0.21704942
4930528D03	-0.7550222	-0.1350901	1.21287534	0.28390003	0.21717381
Eif3l	0.16033542	5.5720538	1.21272875	0.28392829	0.21717381
Pbxip1	0.31010771	5.41108187	1.21236605	0.28399823	0.2171976
St8sia3	-0.1870144	7.82621868	1.21226713	0.28401731	0.2171976

Themis	0.71442176	0.53572388	1.21123982	0.28421555	0.2173242
Mex3b	-0.2547115	3.37780557	1.21110903	0.2842408	0.2173242
Ddx58	0.23990694	5.01473816	1.21073834	0.28431239	0.21734439
Zfp777	0.33114966	2.08717096	1.21046348	0.28436548	0.21734439
Gja4	-0.7463546	-0.5079181	1.21045743	0.28436665	0.21734439
Noc4l	0.25735437	3.21503517	1.21037228	0.2843831	0.21734439
Supt4a	0.29965349	4.73820163	1.20973367	0.28450653	0.21740932
Pglyrp2	-2.3506046	-1.8629835	1.2096328	0.28452603	0.21740932
Nop2	-0.3175072	2.78938159	1.20928825	0.28459266	0.21741939
Sync	0.63187994	1.07307096	1.20926489	0.28459717	0.21741939
Ythdc1	-0.1416768	7.24314683	1.20888175	0.28467129	0.21745386
Btg2	0.35243919	3.76373562	1.20840903	0.28476277	0.21750159
Themis2	-0.5370407	1.61244666	1.20773351	0.28489356	0.2175281
Jak3	-0.7092402	-0.2724256	1.2077057	0.28489894	0.2175281
Syce1	-1.6821441	-2.0044352	1.20768565	0.28490283	0.2175281
Ppp3ca	0.15974318	9.63284311	1.2076307	0.28491347	0.2175281
Zfp760	0.17579534	5.25327047	1.20662118	0.2851091	0.21759811
Ddx23	0.16141443	5.43142983	1.20659295	0.28511457	0.21759811
6530402F18I	-0.4878661	1.42045997	1.20649876	0.28513284	0.21759811
Tsen34	0.21942572	4.22262638	1.20645017	0.28514226	0.21759811
Slc30a1	-0.1495462	5.11534121	1.20640918	0.2851502	0.21759811
Tmem150b	-1.4035361	-0.9969211	1.20618251	0.28519416	0.21760952
Fktn	-0.1621508	5.73022151	1.20585321	0.28525804	0.21762079
Rnf114	0.21540927	5.39363236	1.20580725	0.28526696	0.21762079
Timm50	0.41784521	1.68706968	1.20524899	0.2853753	0.2176813
Mef2a	0.12211418	8.26170554	1.20424433	0.2855704	0.21780676
Trappc3l	-1.7222365	-2.1751454	1.20410308	0.28559785	0.21780676
Actn4	-0.1259782	6.28219437	1.20379571	0.28565758	0.21782631
Stk17b	0.19771842	5.75517255	1.20367236	0.28568156	0.21782631
Adck3	0.26049869	2.71438383	1.2032981	0.28575433	0.21783824
Cdca7l	-0.6718441	0.60647303	1.20309468	0.28579389	0.21783824
Atp13a3	-0.1254965	7.15130974	1.20298503	0.28581521	0.21783824
Myt1l	0.18857671	8.60644459	1.20298011	0.28581617	0.21783824
02-Mar	0.26740733	5.33229017	1.20284522	0.28584241	0.21783824
Pld5	-0.2578998	4.2995414	1.20235322	0.28593815	0.21788907
Dpp8	-0.1061241	8.3975238	1.20198187	0.28601043	0.21792201
Zfp14	-0.2973713	3.10914981	1.20174957	0.28605566	0.21793253
H2-D1	0.22513949	5.17764564	1.20139534	0.28612466	0.21793253
Enox2	0.30325037	4.58699672	1.20137847	0.28612794	0.21793253
Fcrlb	-1.342305	-1.4451806	1.20122014	0.28615879	0.21793253
Gbp9	0.28419622	3.98239406	1.20098999	0.28620363	0.21793253
Gm20754	-0.5037956	0.72571659	1.20085011	0.28623089	0.21793253
Slc18b1	0.22197117	5.1384207	1.20076688	0.28624711	0.21793253
Pptc7	0.12600104	6.66629707	1.20071789	0.28625666	0.21793253
Igf2os	-0.3932549	3.56174372	1.19967052	0.28646091	0.2180659

Eif3h	0.17632592	6.19535446	1.19941037	0.28651167	0.21808241
C130074G19	0.1784784	6.86910222	1.19881338	0.28662821	0.21814898
Mir22hg	0.22416686	3.66681709	1.19844543	0.28670006	0.21818153
Trpm6	0.28281489	3.12694538	1.19748596	0.28688755	0.21828225
Sin3b	0.1961776	4.8061056	1.19747025	0.28689062	0.21828225
Aco1	-0.191626	4.27600672	1.19714844	0.28695354	0.21830799
Tmem135	0.12025564	6.07846201	1.19554227	0.28726786	0.21852495
Myh6	-0.6673677	0.26047312	1.19531991	0.28731142	0.21853592
Sox30	1.1373164	-1.0289941	1.1948718	0.28739921	0.21858053
Dok3	-1.0740705	-1.3432992	1.19447428	0.28747712	0.21859668
Cryzl1	0.17232139	5.36087716	1.19446611	0.28747872	0.21859668
Esam	-0.6236366	1.35258999	1.19425647	0.28751982	0.21860577
10-Sep	-0.2993984	2.8240973	1.19393683	0.2875825	0.21863027
Snrpd1	0.17266174	5.26247952	1.19379497	0.28761033	0.21863027
Tmem62	-0.2821166	3.23541605	1.19272697	0.28781992	0.21876742
Efna5	-0.1639332	6.08444567	1.19194574	0.28797337	0.21886188
Zfp296	-0.7868435	-0.1268593	1.19176322	0.28800923	0.21886696
E130317F20I	0.52337385	1.16468983	1.19084618	0.28818952	0.21898178
Isg20	0.40567654	1.67087983	1.19053823	0.2882501	0.21900563
AI846148	-0.2525879	2.95781087	1.18984573	0.28838638	0.21908698
Rab5b	0.14397358	7.01530066	1.18960823	0.28843314	0.21910032
Nlrp3	-0.9566421	-0.9564663	1.18890641	0.28857138	0.21914897
Zfp78	0.32186265	2.73225711	1.1888651	0.28857952	0.21914897
Vwa5b1	-0.6362689	0.4933153	1.18883814	0.28858483	0.21914897
Car14	-0.5973759	2.80967489	1.18840794	0.28866962	0.21918926
Spty2d1	0.16714012	4.96492595	1.1881446	0.28872153	0.21918926
Traf6	0.15077958	6.18016976	1.18812423	0.28872555	0.21918926
Galnt2	-0.213365	3.84837964	1.18786482	0.28877671	0.21920326
Ago1	-0.115546	6.22133921	1.18752582	0.28884357	0.21920326
Ipo13	-0.1790472	5.39730335	1.18744726	0.28885907	0.21920326
2210013O21	0.21443533	5.29079884	1.18725678	0.28889666	0.21920326
Gm13051	-1.0967817	-1.3911024	1.18713412	0.28892086	0.21920326
Zfyve21	0.23301237	4.02987159	1.18703819	0.2889398	0.21920326
Slc9a2	0.39807665	5.21957124	1.1869938	0.28894856	0.21920326
Il18bp	-0.3368347	3.28860402	1.1867009	0.28900638	0.21922496
Ccny	0.14098276	8.53740711	1.18616927	0.28911136	0.21926762
Qk	-0.1332664	9.3229642	1.1861031	0.28912443	0.21926762
Ifi47	0.3641377	3.77676149	1.18588607	0.28916731	0.21926762
Rab13	0.28653758	4.24254159	1.18582416	0.28917954	0.21926762
BC052688	-0.3391415	2.15406068	1.18461041	0.2894195	0.21940812
Myh7b	0.47890743	2.32790488	1.18448137	0.28944503	0.21940812
Dlk1	-0.3162692	3.41940784	1.18444317	0.28945258	0.21940812
Gprc5b	-0.2096771	4.83589244	1.18368231	0.28960316	0.21950008
Fhod1	-0.3627306	2.36053804	1.18253552	0.28983033	0.21965006
Tstd3	0.17677697	5.42823205	1.18202967	0.2899306	0.21970386

Cltc	-0.1855056	8.89667839	1.18185458	0.28996532	0.21970797
Flt3l	-0.4407165	1.70226686	1.1816052	0.29001478	0.21972325
2610018G03	-0.4297331	1.93661506	1.1811543	0.29010424	0.21976883
Sdc1	-0.4443272	2.17858721	1.18081371	0.29017184	0.21979784
Elf4	0.35787151	3.91616552	1.17903534	0.29052513	0.22004324
Fam83b	-0.9591199	-0.6156451	1.17860221	0.29061127	0.22008191
Rnf166	0.23617951	4.71944735	1.17848356	0.29063487	0.22008191
Ext1	0.13459642	5.68457115	1.17827796	0.29067577	0.22009066
Crebl2	0.21511166	5.09833326	1.17736039	0.29085841	0.22020672
Lamb1	0.18807286	4.17024606	1.17672578	0.29098482	0.22026047
Gad2	-0.2184949	8.8081103	1.1766113	0.29100763	0.22026047
Mtfp1	0.23493368	4.76527358	1.17645198	0.29103938	0.22026047
Srd5a1	-0.3965899	2.56381811	1.17641452	0.29104685	0.22026047
Rab37	-0.6217512	0.41174114	1.17615063	0.29109945	0.2202652
Irgm2	0.23852966	5.57099843	1.17608851	0.29111183	0.2202652
Cdc40	-0.1340175	6.87260442	1.17574688	0.29117995	0.22027373
Cntn1	-0.161879	8.31121589	1.17573748	0.29118183	0.22027373
Hcfc2	0.22940166	4.10084685	1.17417666	0.29149333	0.22046588
Usp18	0.39201849	1.69808751	1.1741702	0.29149462	0.22046588
Paqr7	-0.1638934	4.56034292	1.17379334	0.2915699	0.22050058
Lpo	-1.2170248	-0.8109951	1.1719412	0.29194025	0.2207584
Cd163	-0.5420555	1.35450107	1.17094907	0.29213889	0.22087435
Rbms2	-0.2079855	5.91312169	1.17088128	0.29215247	0.22087435
Tktl2	-1.4669902	-1.1117292	1.17030421	0.29226811	0.22093951
Crx	-0.9172245	-1.2279969	1.16998261	0.29233257	0.22096132
Atp2b4	-0.2053166	7.45255742	1.16986637	0.29235588	0.22096132
Fam98c	0.83969857	-0.7052618	1.16947481	0.29243441	0.22099841
1700037H04	0.2318217	4.31945239	1.16888784	0.29255218	0.22106514
Csnk1d	0.11997165	6.50616897	1.16814737	0.29270083	0.22112595
Brd9	-0.1297039	5.50512023	1.16807031	0.29271631	0.22112595
Ikbkb	0.21916821	4.30039297	1.16804654	0.29272109	0.22112595
D830030K20	-0.7260644	0.44318328	1.16767276	0.29279617	0.22115902
Fxr1	0.13285639	6.88681768	1.16753512	0.29282383	0.22115902
Nrp1	0.19345417	4.973746	1.16704187	0.29292297	0.22119315
Ehf	-0.6492981	0.60550469	1.16701683	0.292928	0.22119315
Pirb	-0.7302202	-0.2529225	1.16661441	0.29300892	0.22123199
Rbks	-0.6025046	1.44680274	1.16619369	0.29309355	0.22124055
Kdm3a	-0.15192	5.9982817	1.16593571	0.29314546	0.22124055
Sertm1	0.18494882	4.58370972	1.16589206	0.29315424	0.22124055
Mrto4	0.26738279	3.32261323	1.16582228	0.29316829	0.22124055
2810410L24	-0.6358216	-0.0493362	1.16577429	0.29317795	0.22124055
Htr2c	0.21833796	4.57506655	1.16567852	0.29319722	0.22124055
Shkbp1	0.55480361	0.80269498	1.16533929	0.29326552	0.22126982
Mfge8	0.33711537	3.1495473	1.16474566	0.29338508	0.22131185
Cldn12	-0.1587014	5.41596748	1.16471377	0.29339151	0.22131185

Apopt1	0.1564323	4.12066254	1.16462331	0.29340973	0.22131185
Faah	-0.2279005	4.14335949	1.16408561	0.2935181	0.22137133
C030029H02I	-0.5731696	1.08253575	1.16382535	0.29357057	0.22137899
Il7	1.37476158	-0.8826703	1.1637425	0.29358728	0.22137899
Ino80e	0.35660364	2.32870918	1.16339424	0.29365752	0.22138751
Rbm28	0.18205265	4.98405403	1.16339383	0.2936576	0.22138751
Cyp4f16	0.42625133	1.73480182	1.16301891	0.29373324	0.22142228
Spef1	0.42848736	1.53809466	1.16258158	0.2938215	0.22142621
Armc4	-0.8284784	-0.9967115	1.16252524	0.29383288	0.22142621
Npas4	-0.8778881	3.17988758	1.16243427	0.29385124	0.22142621
Aif1l	-0.3540569	2.10260785	1.16240808	0.29385653	0.22142621
Chodl	1.34921337	-1.4040797	1.1618769	0.2939638	0.22148479
Snx16	0.18809069	4.76565689	1.1612955	0.29408128	0.22154667
Naa10	0.33951187	2.32544461	1.16117812	0.294105	0.22154667
H2-Eb1	-0.503819	3.08413101	1.15934801	0.29447524	0.2218033
BC068157	0.24218103	5.92522908	1.1591889	0.29450746	0.22180529
6430573F11I	-0.2949337	2.57922385	1.15833755	0.29467993	0.2219129
Trit1	-0.2799666	3.53345895	1.15816914	0.29471406	0.22191633
Speer4e	1.09010827	-1.3641308	1.15784192	0.29478039	0.221944
Ect2	1.11364835	-0.849832	1.157145	0.29492175	0.22202814
Sipa1l3	-0.1610978	5.34766959	1.15595288	0.29516375	0.22218803
Ccl17	0.47462593	1.45136625	1.15568479	0.29521821	0.22219814
Smim20	0.27370915	3.66801231	1.15559517	0.29523642	0.22219814
Neurl1a	-0.2446586	4.46459299	1.15487436	0.29538292	0.2222861
Zfand2a	0.1489174	7.00570716	1.15437269	0.29548494	0.22234056
Rrad	1.16469994	-1.1122327	1.15406652	0.29554723	0.22236513
Zfp707	0.48421414	1.37092506	1.15340931	0.29568099	0.22244346
9430038I01R	-0.6749235	0.73306406	1.15324037	0.29571539	0.22244703
Tmem132e	-0.4452703	1.29276568	1.15249642	0.29586693	0.2225387
Abhd8	-0.1628014	4.56973272	1.15211983	0.29594368	0.22257411
Cst6	0.94771242	-0.7388505	1.15130727	0.29610937	0.22266054
Ubr7	0.14897881	4.90069223	1.15126515	0.29611797	0.22266054
Igsf10	-0.2598144	3.27417852	1.15091776	0.29618885	0.22269152
Slc39a5	-0.6526169	-0.5327547	1.15058564	0.29625663	0.22272016
Slc39a13	0.26372082	5.17208541	1.15029876	0.2963152	0.22272333
Gmcl1	-0.1782276	4.31263932	1.15027414	0.29632023	0.22272333
Mterfd3	0.2696709	2.97547655	1.14955433	0.29646725	0.22281151
Dpy19l3	-0.1908622	5.02698243	1.1486864	0.29664466	0.22292251
Tssk2	-1.5725375	-1.7611136	1.14825682	0.29673253	0.2229662
Grid2	-0.3612205	2.47251112	1.1472785	0.29693276	0.22308728
Sorbs3	0.26691439	7.07168681	1.14709932	0.29696945	0.22308728
Rab19	0.51380606	1.98296092	1.14703372	0.29698288	0.22308728
LOC171588	2.29914967	-1.1518178	1.1578987	0.29701552	0.22308945
Kctd17	0.25610453	4.42842515	1.146565	0.2970789	0.22311472
Slc7a7	-0.5877966	1.12585442	1.14527178	0.29734404	0.22329149

Gcat	-0.607191	0.83684208	1.1446289	0.29747596	0.22336819
Wdr55	0.18463217	4.11983613	1.14403246	0.29759842	0.22343778
Mecom	-0.5779805	1.34295964	1.14305146	0.29779999	0.22356674
Pdia5	-0.7592409	0.10203084	1.14263692	0.29788522	0.22359609
Itgb3	-0.3028362	2.3627299	1.14251659	0.29790997	0.22359609
Tnip3	0.5264102	1.79841068	1.14220002	0.29797509	0.22359609
Rasal3	1.03397037	-0.2015139	1.14214408	0.2979866	0.22359609
Slc1a1	0.16938615	6.01790734	1.14213668	0.29798812	0.22359609
1110015O18	-0.8169743	-0.9283855	1.14161602	0.29809527	0.22363353
Gabra3	0.14716638	5.47618171	1.14160451	0.29809764	0.22363353
Slfn1	1.03119911	-1.0410111	1.141187	0.2981836	0.22365739
Pnpla7	-0.3774006	2.12670054	1.14102269	0.29821744	0.22365739
Slc5a5	0.21139111	4.92902346	1.14101564	0.29821889	0.22365739
Tmem179b	0.33389185	3.23647778	1.14086342	0.29825025	0.22365854
Fam163a	-0.4810458	1.81468194	1.14043984	0.29833752	0.22370162
Dok6	0.37777842	2.04157033	1.1394238	0.29854699	0.22380159
Sstr3	0.20153801	3.54620134	1.13937095	0.29855789	0.22380159
Acss2os	-1.2956044	-1.5760205	1.13919216	0.29859478	0.22380159
Lrch2	-0.2400541	4.19729802	1.13905323	0.29862344	0.22380159
Gorab	0.21272458	3.49205678	1.13904664	0.2986248	0.22380159
Brd1	-0.1351582	6.0043742	1.13892527	0.29864985	0.22380159
Ccnb2	-0.8322501	-0.2245179	1.13866673	0.2987032	0.22381921
Rdh11	0.55801047	1.0106902	1.13823001	0.29879337	0.22386088
Fbxo7	-0.2257406	3.37786508	1.13810835	0.29881849	0.22386088
Sec23a	-0.1433665	6.49396383	1.13707344	0.29903233	0.2239987
Endod1	0.16392934	4.92098218	1.13681873	0.29908499	0.22401578
Plekha3	0.15776767	5.32988785	1.13635766	0.29918034	0.22404555
Spata45	-1.2928686	-0.5529667	1.13633772	0.29918447	0.22404555
Klhl24	-0.119287	6.75247327	1.13605666	0.29924262	0.22406672
Snhg7	-0.4008728	1.28793666	1.13438248	0.29958931	0.22430393
Zfyve26	-0.2206125	4.09534786	1.13402439	0.29966353	0.22432772
Prkcd	0.27324902	4.08542494	1.13394063	0.2996809	0.22432772
Mapk14	-0.1242657	6.53290444	1.13363425	0.29974443	0.22435289
Seh1l	0.19164118	5.64471097	1.13269102	0.29994013	0.22444478
Brd2	0.11265498	7.92473855	1.13265344	0.29994793	0.22444478
Slc13a5	-0.3228342	2.26735821	1.13260994	0.29995696	0.22444478
Rps27l	0.34783315	4.62792645	1.13230566	0.30002013	0.22446118
Gm527	-0.379075	2.04063156	1.13212406	0.30005785	0.22446118
Hspa1l	-0.3095237	2.37598543	1.13207208	0.30006864	0.22446118
Mfsd5	0.27206464	3.64305993	1.13147704	0.30019226	0.22453007
Susd5	0.37190839	2.31888443	1.13134066	0.3002206	0.22453007
1700023L04F	0.96240407	-0.0254087	1.12757731	0.30100415	0.22508604
Wdr25	0.29510726	1.99011313	1.12748208	0.30102401	0.22508604
Hnrnpa3	0.1206071	9.05026182	1.12582085	0.3013708	0.22532288
Gm3258	0.74386103	-0.8824314	1.12543187	0.30145208	0.22536119

Slc24a2	-0.1968966	9.69693903	1.12522051	0.30149625	0.22537176
Fbxo34	-0.1815323	5.26287799	1.12501922	0.30153833	0.22538075
Hsf5	1.24571133	0.24239207	1.12437718	0.30167261	0.22543178
2310002D06I	1.36962016	-1.6989495	1.12434814	0.30167868	0.22543178
Pih1d2	0.65347059	0.08336365	1.12426171	0.30169677	0.22543178
Trub2	0.28573049	3.0142733	1.12395439	0.30176108	0.22545738
Tubgcp4	-0.2059163	3.73131625	1.1235984	0.30183559	0.22549059
Gpr98	0.35745707	2.82382447	1.12251114	0.30206333	0.22563826
Smtn	0.3935757	2.3287142	1.1222464	0.30211882	0.22565723
Gpm6a	0.1732801	9.42964172	1.12194492	0.30218202	0.22568197
Ptpn3	0.17942408	6.04739918	1.12172322	0.30222851	0.22569422
Rprd1b	0.18593543	5.10640068	1.12142401	0.30229127	0.22571862
Rabep2	0.34894346	2.27559331	1.12113379	0.30235217	0.22574162
St8sia1	-0.177143	6.67540381	1.12073677	0.30243549	0.22578136
Tshz1	0.12433023	6.00307208	1.12052888	0.30247914	0.22579147
Nup62-il4i1	1.11967802	-1.2666709	1.12002637	0.30258467	0.22584777
Arl2bp	0.17671737	5.67878258	1.11942312	0.30271143	0.22589341
1700096K18I	0.49910348	1.18970557	1.11924377	0.30274913	0.22589341
Gm13749	-0.6902249	0.63489644	1.11920551	0.30275717	0.22589341
Clca1	-0.9678855	-0.4968022	1.11916219	0.30276627	0.22589341
Cux1	-0.1026939	8.04491016	1.11857633	0.30288948	0.22596286
Thap7	0.31859126	2.81002998	1.11820312	0.30296799	0.22599896
Wrnip1	0.17785655	5.40301787	1.11805907	0.30299831	0.22599909
Mrps5	-0.2991274	2.91157501	1.11767579	0.30307898	0.22603679
Stk24	0.17849877	7.31064576	1.11731767	0.30315439	0.22607055
Fam204a	0.2159285	5.54557337	1.11714255	0.30319127	0.22607558
Ranbp10	-0.1744108	4.71513143	1.11688639	0.30324523	0.22609335
Rapsn	-1.6112391	-2.5007466	1.1164751	0.3033319	0.22613549
Cd97	0.2884672	3.10544041	1.11532077	0.30357533	0.22625853
Vipr2	0.85138793	0.13129878	1.11528458	0.30358297	0.22625853
Rpl37a	0.23397403	5.95519467	1.11526338	0.30358744	0.22625853
Il17re	0.59834534	0.83772249	1.11484436	0.30367587	0.22630196
Osbpl5	-0.2050552	3.92473795	1.11466684	0.30371335	0.2263074
Vsig10l	-0.3789039	2.41814072	1.11438939	0.30377193	0.22632857
Eif4ebp2	-0.1800892	5.77871641	1.11301971	0.30406137	0.22652172
Tram1l1	-0.195349	4.25816909	1.11287517	0.30409194	0.226522
Ndufb3	0.19599138	5.49258129	1.11255992	0.30415862	0.22654917
Leprot	0.2859874	5.01098668	1.11208701	0.30425869	0.2265862
Acaa2	0.21576999	4.83542078	1.11203949	0.30426875	0.2265862
Clpb	-0.2383148	3.74008951	1.11125238	0.3044354	0.22666281
Pipox	-0.5408179	0.57711512	1.11117289	0.30445224	0.22666281
BC025920	0.42131916	1.46221459	1.11112552	0.30446227	0.22666281
Traf2	-0.4369919	1.32158657	1.11095389	0.30449863	0.22666739
Slc16a7	-0.2054959	4.68758201	1.11078791	0.3045338	0.22667107
Gpr139	0.77144038	-0.5549748	1.11043204	0.30460922	0.22670471

Mapt	0.13473595	6.89262027	1.11024458	0.30464896	0.2267118
Il5	1.60475438	-1.8544092	1.10999884	0.30470107	0.22672808
Rps6ka1	-0.3236283	2.51458449	1.10978054	0.30474737	0.22674004
Bmx	-0.4933554	1.85250892	1.10951507	0.30480368	0.22674586
Mir9-2	-1.2279134	-0.9702704	1.10945522	0.30481638	0.22674586
Cers4	-0.2546691	5.54302222	1.10931623	0.30484587	0.22674586
Flrt1	0.13451196	6.61301072	1.10875042	0.30496597	0.22680551
G6b	1.15806239	-1.0109044	1.10865354	0.30498654	0.22680551
Gm3230	-0.4720204	1.29743143	1.10808186	0.30510796	0.22687331
Fbln7	-0.4175024	4.07381577	1.10769522	0.30519012	0.22691191
Rab27b	0.17479745	5.41007318	1.10744143	0.30524406	0.22692952
Mapkapk2	0.22904978	4.27647673	1.10712274	0.30531182	0.2269574
Serpine3	-0.376587	1.95541913	1.10690806	0.30535747	0.22696147
Plcb3	0.24713539	4.33601191	1.1068125	0.3053778	0.22696147
Nos1	-0.2829152	3.24246223	1.10599556	0.30555163	0.22706512
Mapk8	0.16072122	7.5751734	1.10583974	0.3055848	0.22706512
Tmc6	0.58184261	0.25957733	1.10571867	0.30561058	0.22706512
Mrvi1	0.24270245	5.83222849	1.10558827	0.30563834	0.22706512
Gm10516	-0.3760676	2.08014304	1.10536137	0.30568667	0.22707853
D6Ertd474e	0.89721006	0.2199904	1.10519285	0.30572257	0.2270793
Riok1	0.23664607	3.87879186	1.10494414	0.30577555	0.2270793
Exosc3	0.20623594	4.95860279	1.10493024	0.30577852	0.2270793
Stab2	-1.223391	-1.1978547	1.10476976	0.30581272	0.22708222
Gm20597	1.2420776	-0.6734254	1.10461574	0.30584554	0.22708411
Camk2g	-0.170123	7.23186606	1.10432874	0.30590672	0.2270956
LOC1005034!	-0.8730352	-1.3524867	1.10425912	0.30592157	0.2270956
Tmed7	0.16373147	5.69417952	1.10403853	0.30596861	0.22710805
Alg5	0.33526167	3.24676587	1.10352667	0.3060778	0.22716661
Hexdc	-0.395773	1.95366144	1.10333846	0.30611796	0.22717394
Actl6a	-0.2116396	3.68241767	1.10308088	0.30617293	0.22719227
Syn1	0.24232294	10.2351077	1.1017459	0.30645808	0.22738136
Cul2	-0.1965295	5.27435675	1.10145717	0.3065198	0.22739082
Akr1c14	-0.2311307	3.92824185	1.10140263	0.30653146	0.22739082
Tlk2	0.13325802	7.11573256	1.10086292	0.30664688	0.22745395
Pask	-0.5726779	0.83317781	1.10060527	0.306702	0.22747234
A830019L24I	1.0664199	-0.6944655	1.10040047	0.30674582	0.22748235
Lin54	0.17220149	4.48532347	1.09961582	0.3069138	0.22758443
Tmem201	-0.2566118	3.96789872	1.09931291	0.30697869	0.22761004
Gm15787	-0.5247515	0.71033111	1.09863611	0.30712372	0.22767643
Sptlc2	-0.1469423	5.09740509	1.09861184	0.30712892	0.22767643
Tpra1	0.37008851	2.47467841	1.09839204	0.30717605	0.22768886
1700034F02I	-1.4294726	-1.1505467	1.09821583	0.30721383	0.22769437
Zfp68	-0.1780993	6.18202329	1.09796542	0.30726754	0.22771167
Mageb16	-0.7834844	1.09658276	1.09659569	0.30756154	0.22790704
Clmp	0.21037746	6.01158504	1.09604289	0.3076803	0.22793639

BC030307	0.50515948	0.74594489	1.09591209	0.30770841	0.22793639
Slc16a11	0.28713478	3.31827081	1.09581347	0.30772961	0.22793639
Mir143hg	-1.1100674	-1.3937044	1.09571192	0.30775144	0.22793639
Col4a2	0.19003663	4.28926856	1.09570429	0.30775308	0.22793639
Nanp	0.24045469	4.00038521	1.09484795	0.30793724	0.22802941
Ccdc149	-0.189565	4.44351434	1.09483754	0.30793947	0.22802941
Fam49a	0.13742211	8.23706648	1.09426758	0.30806213	0.22807886
Clasp2	-0.13729	8.0401354	1.09424465	0.30806707	0.22807886
Adam9	-0.1458632	4.9569587	1.09380787	0.30816111	0.22812597
Svil	0.20789311	4.74877453	1.09338541	0.30825211	0.22816059
Mlycd	0.21571628	3.16265208	1.09322528	0.30828661	0.22816059
Srrm4	-0.2120558	6.13290984	1.09316718	0.30829913	0.22816059
Pde1b	-0.1645754	5.52007197	1.09285504	0.3083664	0.22818787
Nfya	0.14038493	5.71546218	1.09269733	0.3084004	0.22819051
Gcdh	0.23599766	3.16647822	1.09228674	0.30848893	0.22823351
D330023K18	0.52198229	1.2343076	1.09194191	0.30856331	0.22825993
Pex11b	0.22031376	5.43222602	1.09177508	0.3085993	0.22825993
Slc6a20b	-0.3207916	1.71811984	1.09161426	0.30863401	0.22825993
Ripk2	0.22252276	4.3644406	1.0915476	0.30864839	0.22825993
Taf4b	0.24214178	2.90213099	1.091416	0.30867679	0.22825993
2510039O18	0.30042386	3.40800812	1.0909906	0.30876864	0.22828521
Mutyh	0.59767481	-0.0979963	1.09091942	0.30878401	0.22828521
Gtf2a1l	1.7418279	-1.665669	1.09083483	0.30880227	0.22828521
Ado	-0.1578007	5.37027576	1.09055589	0.30886252	0.22830725
Cdk2	-0.4252814	1.70180027	1.0899557	0.30899222	0.22838061
Triqk	0.27734211	3.52130228	1.08862239	0.30928059	0.22857123
Wdr16	0.779926	-0.2244289	1.0878809	0.30944113	0.22865523
Rhbdl1	-0.4431659	0.61950411	1.08781588	0.30945521	0.22865523
S1pr1	-0.2658123	5.64766175	1.08719481	0.30958977	0.22871071
Dapk3	0.39344954	1.76013072	1.08709914	0.3096105	0.22871071
Ncam1	0.17996394	7.88637421	1.08704716	0.30962177	0.22871071
Nek2	0.59565173	1.56168566	1.08650298	0.30973975	0.22875932
Cachd1	0.23742425	3.39907885	1.08639051	0.30976414	0.22875932
Polr2k	0.18171695	4.07922009	1.08632172	0.30977906	0.22875932
Cct5	0.12508597	6.33837543	1.08554921	0.30994668	0.22884278
Fam20a	0.24734523	2.57563194	1.08551967	0.30995309	0.22884278
Neo1	0.14741474	7.54444732	1.08466761	0.31013813	0.22893711
D1Ertd622e	0.13316952	5.78632366	1.08461753	0.31014901	0.22893711
Pom121	-0.1510092	6.32319514	1.08450976	0.31017242	0.22893711
Tfr2	-0.5252128	0.97778747	1.08428455	0.31022136	0.22895071
Zim1	-0.4444567	1.09994664	1.08390672	0.31030348	0.22898879
Panx1	0.38763713	2.45878713	1.0835608	0.3103787	0.22902176
Zfp661	-0.2473376	3.17375051	1.08293633	0.31051455	0.22908061
Ptprj	-0.1833363	6.83767348	1.08277596	0.31054945	0.22908061
Kcnj14	-1.3065249	-1.0669554	1.08277311	0.31055007	0.22908061

Lpar2	0.42565442	1.631953	1.0822352	0.31066717	0.22914446
Wbscr16	-0.3946778	1.98241993	1.08173551	0.31077601	0.22920068
Scnn1a	0.29251302	2.58433615	1.08160473	0.3108045	0.22920068
Cntn4	-0.2052509	5.2390942	1.0806021	0.31102306	0.22933931
Eml4	-0.1421924	6.15080929	1.08003778	0.31114617	0.22940753
Tlr5	1.0725695	-1.2240123	1.07889796	0.31139503	0.22954246
1110054M08	-0.4753735	1.12950431	1.07886036	0.31140325	0.22954246
Dctn1	-0.1985276	5.19929677	1.07877924	0.31142097	0.22954246
Mgmt	0.67468397	-0.09654	1.07844671	0.31149363	0.22957346
Gm4285	0.38235907	1.33931549	1.07763004	0.31167218	0.22968248
Eif4e2	0.15703577	5.28784106	1.07661482	0.31189434	0.22982362
Aaed1	0.27094247	4.32597343	1.07632723	0.31195731	0.22982815
Vill	-0.4119448	1.24115167	1.07630688	0.31196177	0.22982815
Trim63	-0.5069999	1.2032021	1.0760596	0.31201593	0.22984548
Nacc2	-0.1265341	6.50946676	1.07552362	0.31213337	0.22990941
Tmem128	-0.2451273	3.40362018	1.07529155	0.31218423	0.22991348
Dcaf12l1	-0.1518337	5.6089195	1.07521877	0.31220019	0.22991348
Ccdc15	-0.2348736	4.26370537	1.07505783	0.31223547	0.22991689
6330549D23	0.51378317	1.73944601	1.07401152	0.312465	0.23006333
Bid	-0.2611168	2.81864589	1.07345681	0.31258678	0.2300874
Trafd1	-0.2394925	5.08967615	1.07344737	0.31258886	0.2300874
Ccng1	0.13634728	8.15001711	1.07344344	0.31258972	0.2300874
Zfp688	0.48581205	1.2095014	1.07322167	0.31263843	0.23010067
Hspb8	0.25883818	6.19497759	1.07214253	0.31287558	0.2302413
Ilkap	0.18561347	4.44686146	1.07207294	0.31289088	0.2302413
Mir384	-1.4683209	-1.205553	1.07180673	0.31294943	0.23026179
Gm15663	0.28100796	3.1623083	1.07113346	0.31309756	0.23033117
Zhx3	0.14607972	6.21195891	1.07099627	0.31312776	0.23033117
Slc10a1	-1.3059592	-1.138606	1.07095956	0.31313584	0.23033117
Xpc	-0.2025507	3.9902574	1.07067341	0.31319884	0.23035492
E130309D14	-0.2064782	4.98591316	1.07020055	0.31330298	0.23039573
Armc5	0.25055316	2.58306319	1.06988661	0.31337215	0.23039573
Spag8	1.1534345	-1.5179381	1.06976567	0.3133988	0.23039573
Zdhhc6	0.21057101	4.61483211	1.06976196	0.31339962	0.23039573
Fbxw10	0.54830197	0.68857113	1.06972441	0.3134079	0.23039573
Ptchd1	-0.2505796	4.63115282	1.06931538	0.31349806	0.23043943
Apold1	-0.4543527	1.93923786	1.06854325	0.31366836	0.23054202
Gpr64	-0.6332149	1.17481498	1.06776875	0.31383932	0.23064507
Nkd1	0.22373706	4.85827505	1.06665698	0.31408494	0.23080297
Aco2	-0.1276526	7.41179208	1.06631891	0.31415968	0.23080965
Apol9a	0.98572991	-1.0042618	1.06631257	0.31416108	0.23080965
Ntf5	1.71690199	-1.8553363	1.06608687	0.31421099	0.23080965
9630028B13	-0.4568527	2.68441156	1.06596848	0.31423718	0.23080965
Optn	0.18640386	4.13149135	1.0659201	0.31424788	0.23080965
Gpr75	-0.2560183	3.63762434	1.0655102	0.31433857	0.23085366

Lhfpl1	0.95715286	-0.7826807	1.06490399	0.31447276	0.23092273
Cdc34	0.31091598	3.16329513	1.06480725	0.31449418	0.23092273
Epor	-0.5056603	0.39059471	1.06456542	0.31454774	0.23093945
Ifi44	0.30392696	3.33645629	1.06438076	0.31458864	0.23094687
Vtn	-0.2957164	6.16509271	1.0642354	0.31462085	0.23094791
Plekhh2	-0.1596576	5.65818336	1.06406952	0.3146576	0.23095229
Cul5	0.1161933	7.02475883	1.06385731	0.31470463	0.23096421
Trf	-0.2298213	5.62323471	1.06346809	0.31479092	0.23100494
Ccdc59	0.20189399	5.04935982	1.06325248	0.31483873	0.23101743
Ociad1	0.11949333	7.1434542	1.06241038	0.31502557	0.23113191
Plekhg2	0.24991211	4.20003844	1.06067896	0.31541019	0.23139147
Dnajc27	0.16014898	6.17751237	1.06051591	0.31544644	0.23139544
Senp2	0.10887586	6.40988616	1.06015565	0.31552657	0.23143158
Lrp2	-1.2258686	-1.6730941	1.05987961	0.31558798	0.23143384
Gpr4	-0.2823895	5.56002445	1.05986443	0.31559136	0.23143384
Arsk	0.27882616	3.59236562	1.05944644	0.31568438	0.23147943
Mansc1	0.23127562	4.31736911	1.05847808	0.31590003	0.23161492
Prc1	-0.495442	1.22386664	1.05802068	0.31600197	0.23166702
Sergef	-0.3730374	1.78404877	1.05780272	0.31605056	0.23167847
05-Sep	0.13422988	6.40078037	1.05767352	0.31607936	0.23167847
Atp6ap1l	-0.5419865	1.11229166	1.05735967	0.31614936	0.23170713
Zfpm2	-0.2911643	3.7385624	1.05709116	0.31620926	0.23172839
Zxdb	-0.1458622	5.19819493	1.0556479	0.31653148	0.23187964
1600002H07I	0.25997151	3.04316158	1.05554998	0.31655336	0.23187964
Hsph1	0.15888088	9.09662742	1.05551946	0.31656018	0.23187964
Ddx10	-0.1243853	5.55496738	1.05551515	0.31656114	0.23187964
Eid3	-1.4146397	-1.901409	1.05547462	0.3165702	0.23187964
Zfp746	0.16057311	4.31556848	1.05334481	0.31704661	0.23220592
Clca2	-1.0961654	-1.5911448	1.05304901	0.31711286	0.23223177
Alkbh6	0.26291887	3.12318257	1.05277163	0.317175	0.2322546
Trak1	-0.1221645	7.52589125	1.05077187	0.31762347	0.23252892
Zbtb5	-0.2803911	2.58687091	1.05072569	0.31763384	0.23252892
Tle3	0.15736129	5.22576563	1.05060181	0.31766165	0.23252892
Mir344b	-1.2998447	-2.0874716	1.05045724	0.31769411	0.23252892
Cactin	0.2658483	3.17297834	1.05041044	0.31770462	0.23252892
Dtnb	-0.1914865	4.6439669	1.05022814	0.31774556	0.2325362
Chrdl1	0.29489631	2.68327976	1.04964446	0.31787669	0.23260947
Yipf2	-0.3933117	1.33348163	1.04939496	0.31793277	0.23262781
Flad1	0.33947407	2.04431352	1.04840884	0.31815454	0.23276738
Gp1bb	-0.3442974	1.60154465	1.04815436	0.3182118	0.2327865
Rfc1	0.11780971	5.87408846	1.04793639	0.31826086	0.2327865
Shpk	0.65391578	0.61399241	1.04787901	0.31827378	0.2327865
Lin52	0.16027997	4.53970168	1.04741922	0.31837731	0.23283952
Rita1	-0.4088591	0.99608685	1.04629366	0.31863095	0.2330023
Qpct	0.42523153	1.21858287	1.04589837	0.31872009	0.23303273

Gm11128	-0.7498986	-0.8059278	1.04571522	0.3187614	0.23303273
BC053749	0.3851281	2.84818733	1.04569588	0.31876576	0.23303273
Zfa-ps	-0.4061618	1.11562698	1.04518671	0.31888066	0.23309401
Cdk15	-0.5263891	0.70685136	1.04480519	0.31896679	0.23313425
Zdhhc13	0.23961189	4.23415247	1.04450923	0.31903362	0.23316038
Nt5dc2	0.55395465	2.65514329	1.04384187	0.3191844	0.23324785
Tmem186	-0.2773642	2.68750825	1.04364944	0.3192279	0.23325691
Itgbl1	0.21856927	6.1219165	1.0433441	0.31929693	0.2332622
Cd36	-0.8171153	-0.390192	1.04334236	0.31929732	0.2332622
Gab3	-0.2679121	2.77288416	1.0431368	0.31934381	0.23327344
Ipcef1	0.16310681	7.00043625	1.04277405	0.31942586	0.23331066
Krt10	0.30663332	2.23718246	1.04255309	0.31947586	0.23331607
Mapk8ip1	-0.1510735	5.53140948	1.04241576	0.31950694	0.23331607
Slc25a19	0.226909	3.33440775	1.04232898	0.31952658	0.23331607
Cd44	0.23587757	3.13111967	1.04208499	0.31958181	0.23332233
Mib1	-0.153659	6.21587514	1.04201629	0.31959736	0.23332233
Ncoa6	0.14385955	8.53285239	1.04176983	0.31965317	0.23334036
C130083M11	-0.2987423	2.78221445	1.04162547	0.31968587	0.23334152
Dnph1	0.44429739	1.62737352	1.04127492	0.31976528	0.23337678
Tmem178	0.14588908	5.34075358	1.04079054	0.31987505	0.23343418
Oasl2	0.25212041	7.17688143	1.04046475	0.31994891	0.2334457
Marveld3	-1.4494293	-0.9875168	1.04044636	0.31995308	0.2334457
Rbms3	0.16158132	7.34695524	1.0398991	0.32007721	0.23351356
Snapc1	0.12714129	5.51212667	1.03975914	0.32010897	0.23351402
Chrm5	0.46845272	1.61380987	1.03921635	0.32023217	0.23358117
Pmpcb	0.13315132	5.02703198	1.03791858	0.32052699	0.23377349
Vars	0.30478326	2.45858868	1.0373489	0.32065653	0.23384523
Mtmr7	-0.2325504	5.04746558	1.03715107	0.32070153	0.23385531
Myo19	-0.3107532	2.91947592	1.03591757	0.32098231	0.2340373
Tpmt	0.25068055	3.69758478	1.03574683	0.3210212	0.23404291
Rps15a-ps6	-0.2630664	2.45714199	1.03556183	0.32106335	0.23405089
Eif2d	0.23449881	3.93549472	1.03534404	0.32111298	0.23406432
Cpox	0.1968756	5.72688531	1.03398048	0.32142393	0.23426822
Dusp7	0.12039821	6.05121561	1.03383715	0.32145664	0.2342693
Hs3st3b1	0.27448657	3.71015302	1.0336715	0.32149446	0.2342741
Antxr2	0.43358912	3.3378226	1.03307884	0.32162978	0.23431283
Rnf32	-0.2373745	3.43782625	1.03306005	0.32163407	0.23431283
Tfpt	0.2994868	2.7330613	1.0330283	0.32164133	0.23431283
Twistnb	-0.1370733	5.20112615	1.03269639	0.32171715	0.23434531
Tkt	-0.2068519	4.83904856	1.0313159	0.32203279	0.23450752
Zfp850	0.29093642	2.65478176	1.0312412	0.32204988	0.23450752
Cpxm2	-0.3227273	2.65931824	1.03120309	0.32205861	0.23450752
Gm14403	0.16686341	3.69756188	1.03117569	0.32206488	0.23450752
Cdk5r2	0.13011093	6.69943838	1.03099229	0.32210685	0.23451531
Cdc42ep3	0.19572068	3.36695661	1.03054595	0.32220902	0.23456694

Gm11992	0.77287537	-0.7542537	1.02995421	0.32234456	0.23464283
Ttc27	-0.2004574	3.37302631	1.02917052	0.32252417	0.23472211
Rpl23	0.16814631	7.17334771	1.02907189	0.32254679	0.23472211
Gm14436	0.16989772	5.06734564	1.02906952	0.32254733	0.23472211
Apex1	0.94280734	-1.045947	1.02826873	0.32273103	0.23483301
Arhgef15	0.32509841	2.8955382	1.02704279	0.32301254	0.23501505
Slc37a2	-0.7835177	-0.1409012	1.02591634	0.3232715	0.23518065
Nup54	0.1992237	3.41473095	1.02567607	0.32332678	0.23519805
Cckbr	0.18603158	4.6516168	1.02538857	0.32339293	0.23522336
Sgip1	-0.1668438	8.10724872	1.0247435	0.32354144	0.23528725
2810008D09	-0.5004612	0.70757999	1.02465104	0.32356273	0.23528725
Cd2bp2	0.1928473	4.98529416	1.02459837	0.32357486	0.23528725
Ralgps2	0.12585391	6.33811271	1.02411439	0.32368636	0.23534367
Dsg3	0.95402882	-0.7353267	1.02398921	0.32371521	0.23534367
Npnt	0.17759534	3.77911532	1.02370931	0.32377972	0.23536776
Fkbp10	-0.5309823	2.21110393	1.02350371	0.32382712	0.23537941
Naf1	0.18842015	4.34017599	1.02319367	0.32389862	0.23540857
Man1c1	0.15840195	6.0539353	1.02287971	0.32397104	0.23543839
Tspan32	-1.1360141	-1.3717372	1.02212193	0.32414594	0.23553482
Zfp652	0.13826048	6.34153792	1.02203272	0.32416653	0.23553482
2510002D24	0.26531982	3.33183668	1.02185554	0.32420745	0.23553971
Pgap3	0.86062819	-0.2730341	1.02173165	0.32423606	0.23553971
Tmtc1	0.15875705	8.45845134	1.02078597	0.32445459	0.23567564
Napsa	-1.0131065	-0.6509732	1.02046814	0.32452808	0.2357062
Opn3	0.25769266	3.12974357	1.01997405	0.32464238	0.23576638
Nuak2	1.26033588	-0.498086	1.01897614	0.32487338	0.23591131
Wac	0.14239645	8.63974807	1.01863512	0.32495238	0.23592875
Slc35e3	-0.1678451	4.481118	1.01855365	0.32497125	0.23592875
Sash1	-0.1264699	6.31869525	1.01830478	0.32502892	0.23592875
Fzd6	0.29657901	3.99486454	1.0182711	0.32503673	0.23592875
Bag6	0.17115365	5.6876848	1.0181937	0.32505467	0.23592875
Gm3696	-0.5155535	0.04726077	1.01788485	0.32512627	0.23595789
Pskh1	0.27460217	3.80027565	1.01770358	0.3251683	0.23595907
A430035B10	-0.265984	2.99546973	1.01760656	0.3251908	0.23595907
Dhx32	-0.2166184	4.10411335	1.01725789	0.32527168	0.2359846
Odf3b	0.79653356	0.00321497	1.0171836	0.32528891	0.2359846
Mxd1	0.1492694	6.02346775	1.01650455	0.32544651	0.23606076
Loxl4	-1.0163201	-1.1594294	1.01646012	0.32545683	0.23606076
Trmt5	0.23626382	3.47428555	1.01598084	0.32556814	0.23611866
Tnfaip1	0.1721503	6.03251067	1.01516813	0.325757	0.2362328
Tm9sf1	-0.2207798	3.98937282	1.01467903	0.32587073	0.23628554
Sh2d7	0.4810532	1.00357488	1.01454138	0.32590275	0.23628554
Tfdp2	0.11259671	6.23340646	1.01428084	0.32596337	0.23628554
Ppil2	-0.1644701	4.47776369	1.01418324	0.32598608	0.23628554
Prickle3	0.38510764	2.54428717	1.01417831	0.32598723	0.23628554

Exoc3	-0.1395354	6.57584827	1.01397615	0.32603428	0.23629681
Olfr99	1.33355927	-1.5113581	1.01378223	0.32607942	0.23630669
Ltbp1	-0.2736818	4.37816882	1.01304417	0.3262513	0.23640842
Slc41a2	-0.2034721	4.01969231	1.01258661	0.32635792	0.23646283
Akap13	-0.1285491	7.25756423	1.01207272	0.32647773	0.23651706
Fam122a	0.18294651	4.73155212	1.01199515	0.32649582	0.23651706
Pydc3	-0.5922309	0.73469597	1.01156981	0.32659503	0.23656608
Usp7	-0.1195612	7.09946502	1.01135493	0.32664517	0.23657956
Mr1	0.24448221	4.41173809	1.01108598	0.32670794	0.23660218
Sh3yl1	0.25912091	3.06946594	1.01073211	0.32679056	0.23663916
Map1s	-0.2279374	3.04419634	1.0104138	0.3268649	0.23667015
Coq2	0.16375887	4.82113311	1.00897763	0.3272006	0.23689035
Nr1d1	0.14216841	8.75075476	1.00807526	0.32741177	0.23699007
Zc3h4	0.15358984	5.87777576	1.00802139	0.32742439	0.23699007
Casq2	-0.7738747	-0.2411714	1.00798411	0.32743312	0.23699007
Ppp1r3g	-0.5279016	0.59363059	1.00601946	0.32789358	0.23730045
Msi2	0.1669352	9.31989194	1.00572734	0.32796213	0.23732716
Lmtk3	-0.5251792	1.61875562	1.00502535	0.32812692	0.23740775
Proca1	0.32515777	1.88584222	1.00498328	0.3281368	0.23740775
Dpf1	0.30555637	2.26390189	1.00323968	0.32854664	0.23768044
Fem1b	-0.120145	7.94531779	1.00311034	0.32857707	0.23768044
Snx32	0.18576404	4.27697155	1.00220746	0.3287896	0.23781124
Tmub2	-0.2017653	3.40390632	1.00171539	0.32890551	0.23787215
Avl9	-0.1567897	6.00225198	1.00094839	0.3290863	0.23797995
Apol8	-0.6248836	-0.2681025	1.00022555	0.3292568	0.2380803
1700024P16I	-0.6844749	0.72847317	0.99981539	0.3293536	0.23812734
Xylb	-0.2336498	3.45145933	0.99910847	0.32952054	0.23822508
Hectd3	-0.1852393	4.6912483	0.99872327	0.32961155	0.23826791
Dcp1b	-0.1951983	4.21256879	0.99850272	0.32966368	0.23828263
E4f1	-0.2700976	2.50487204	0.99836702	0.32969576	0.23828285
Zfx	0.15148129	6.22463205	0.99815094	0.32974685	0.23829682
Rars2	-0.188448	3.90870699	0.9971984	0.32997219	0.23838551
Clstn3	-0.2279306	4.49061883	0.99716273	0.32998063	0.23838551
Ddit4	0.29428507	2.67190027	0.99705892	0.3300052	0.23838551
Srsf4	0.2713554	2.37820875	0.99690718	0.33004112	0.23838551
Cog4	0.17081227	4.60369643	0.99685706	0.33005299	0.23838551
Pglyrp1	-0.4567121	0.74415633	0.9968264	0.33006025	0.23838551
Npy5r	0.51660003	0.98837721	0.9962567	0.33019518	0.23846
Lix1	0.14578456	4.84179322	0.99580286	0.33030272	0.23849254
Pnlip	-1.3276287	-1.7420603	0.9956841	0.33033087	0.23849254
Tmc3	-0.5308604	0.99473456	0.99566407	0.33033562	0.23849254
Abca13	-1.1955735	-1.7270547	0.99548038	0.33037916	0.23850103
Mttp	-0.3227538	2.01336206	0.99428804	0.33066202	0.23863332
B3gnt2	0.17953879	5.73961934	0.99423745	0.33067403	0.23863332
Fbxl12os	-0.4019681	2.29522123	0.99409596	0.33070762	0.23863332

Mpp3	-0.20772	3.41134994	0.99396152	0.33073954	0.23863332
AI506816	-0.9150148	-1.375879	0.99393007	0.33074701	0.23863332
Mfap3	-0.1601416	4.95890308	0.9939036	0.3307533	0.23863332
Rpl18	0.26517394	5.80811809	0.99375286	0.33078909	0.2386362
Zfp775	0.34328589	3.00237761	0.99353215	0.33084152	0.23865106
Mrps35	0.2386603	3.99390383	0.99314066	0.33093454	0.23869521
Nfe2l2	0.16717485	7.84925387	0.99295313	0.33097911	0.2387044
4930426L09F	0.49808387	0.84824692	0.99257817	0.33106825	0.23874574
Mllt3	0.13535748	7.72199041	0.99236424	0.33111912	0.23875947
Ttc26	-0.3252679	2.9379197	0.99197246	0.33121232	0.23877742
Lgi4	-0.4311128	1.45022589	0.99183763	0.3312444	0.23877742
Tlr4	0.26997842	4.43886686	0.99169966	0.33127724	0.23877742
Stat5a	-0.4863183	1.7590182	0.991552	0.33131238	0.23877742
Tm7sf2	-0.5318211	1.14682049	0.99153774	0.33131577	0.23877742
Srd5a3	-0.3613402	2.96750846	0.99145694	0.33133501	0.23877742
A930006K02I	-0.5432236	0.2417027	0.9911511	0.33140782	0.23880696
Elovl5	0.23029906	6.21453658	0.99096907	0.33145117	0.23881525
Efr3a	-0.139417	7.52803135	0.99065704	0.3315255	0.23884586
Gm1653	-1.0699168	0.50863292	0.99007866	0.33166333	0.23892222
Ano7	0.99177336	-1.3843268	0.98946876	0.33180876	0.23900403
1700030C10I	-0.4681767	1.00427737	0.98911353	0.33189351	0.23904212
Lrp11	0.15782498	5.96147788	0.98852031	0.3320351	0.23910523
Fam155a	0.15797073	6.69012916	0.98847934	0.33204488	0.23910523
Snhg12	0.22044955	3.49717348	0.9876895	0.33223354	0.23921812
Cenpc1	-0.1524994	5.59738244	0.98752162	0.33227366	0.23922404
4933434E20I	0.14843669	4.51298392	0.98726059	0.33233605	0.23923027
Mcfd2	0.21215456	4.96654256	0.98721857	0.3323461	0.23923027
Fam162a	0.21953564	5.00443547	0.98626466	0.33257426	0.23935424
Dcaf7	-0.1175604	7.9744213	0.98623173	0.33258213	0.23935424
Ghrl	-0.6803875	-0.638868	0.98536232	0.33279028	0.23948106
Kif22	-0.4341322	0.89356003	0.98504352	0.33286665	0.23951304
Cdca8	0.6605008	0.45926939	0.98489527	0.33290217	0.23951386
Was	-0.4503478	0.99596923	0.98477229	0.33293164	0.23951386
Grin3b	1.13280896	-1.7901405	0.98434	0.33303526	0.23956543
3110082J24F	-0.8541569	-0.6274529	0.98332458	0.33327885	0.23971766
Galk1	0.32340944	2.74754331	0.98306951	0.33334007	0.23973871
Mrpl45	0.24934913	3.64461514	0.98161264	0.33369008	0.23996743
Pbx1	0.1150895	9.2799897	0.98128212	0.33376955	0.24000157
Kctd8	-0.3842935	2.1487354	0.98109399	0.3338148	0.2400111
Dirc2	0.16921248	4.94217804	0.98004223	0.33406794	0.24017008
Fstl1	0.31812054	7.22418471	0.97969055	0.33415264	0.24020795
Map3k2	-0.1301312	6.20693347	0.9783673	0.33447161	0.24040001
Tmem55a	0.13880811	6.17654235	0.97829185	0.3344898	0.24040001
Abi3bp	-0.2153729	4.18280323	0.9781835	0.33451594	0.24040001
3000002C10I	0.24065541	2.14423524	0.9775837	0.33466069	0.24045605

Cyhr1	-0.1548841	5.5707049	0.97736416	0.3347137	0.24045605
Tmem71	0.85286276	0.03806179	0.97733809	0.33471999	0.24045605
Pcgf1	0.28237855	3.18836724	0.97732914	0.33472215	0.24045605
Nipa2	0.1661254	4.76445489	0.97684162	0.3348399	0.2405176
Bccip	0.18627195	5.57108292	0.97659383	0.33489977	0.24051821
Ctbp2	-0.1767395	4.21634869	0.97657273	0.33490487	0.24051821
Adarb1	-0.1634679	6.5564066	0.97545928	0.33517408	0.24066676
Ak8	0.85322962	-0.7773796	0.97545185	0.33517588	0.24066676
Soat1	0.13477056	5.47047829	0.9752953	0.33521376	0.24067092
Gpr83	-0.3172689	2.73427723	0.97516227	0.33524595	0.24067099
Nadk	0.17074309	5.45393474	0.97483759	0.33532453	0.24070437
Selplg	-0.510098	0.91735611	0.97464385	0.33537144	0.24070482
Slc30a10	-0.1887596	5.06742658	0.97456995	0.33538933	0.24070482
Cited1	0.43017672	1.57350636	0.97434453	0.33544392	0.24072097
Fut2	-0.6093154	0.57040956	0.97410301	0.33550242	0.24073992
Usf2	-0.1470927	4.98736857	0.9735721	0.33563107	0.2408092
Rps10	0.23629537	4.92802873	0.97336448	0.3356814	0.24082227
Gdf5	0.9659573	-1.6391325	0.97228462	0.33594334	0.24096955
Sfxn2	-0.2564561	2.52378903	0.97225334	0.33595093	0.24096955
Nuf2	-0.9500111	-0.0548779	0.97166118	0.3360947	0.24104962
Ppm1f	-0.1621076	4.58566477	0.97138473	0.33616184	0.24107472
Snord91a	-0.2389917	3.26393971	0.97019274	0.33645158	0.24122781
Irf9	-0.2290436	3.88034125	0.97014331	0.33646361	0.24122781
Mir28b	-1.1184022	-0.4985451	0.9701097	0.33647178	0.24122781
Nans	0.34189037	2.40515802	0.96992464	0.3365168	0.24123703
Fgf16	-1.1070215	-1.5914107	0.96964043	0.33658595	0.24126354
Orai2	-0.2114367	3.83792651	0.96868538	0.33681848	0.24140715
Abi2	-0.1213619	8.54721792	0.96851406	0.33686022	0.241414
Fam219aos	0.30464304	3.09071208	0.96805846	0.33697124	0.24147049
Ror2	0.55020459	0.84523662	0.9675795	0.33708801	0.2415311
Stmn1-rs1	-1.2651327	-1.1613984	0.96711344	0.3372017	0.24158948
Hspa4	-0.1468683	8.78386391	0.96683744	0.33726905	0.24161465
Tbx2	0.98685226	-1.4237405	0.96666611	0.33731086	0.24162154
Prkcsh	0.22682269	3.3401779	0.96554223	0.33758535	0.24179507
C1s1	-0.5871611	0.94045248	0.96526069	0.33765416	0.24182035
Znrf3	-0.1248302	5.9800087	0.9651288	0.3376864	0.24182035
Gm11627	0.42000401	1.40812479	0.96493495	0.3377338	0.24182035
Fcgr1	-0.7981925	-0.627529	0.96478388	0.33777074	0.24182035
Rlim	-0.1135824	7.70261019	0.96469831	0.33779167	0.24182035
Samd10	0.26687562	3.49672755	0.96460673	0.33781407	0.24182035
Abl2	-0.177855	5.655913	0.96424521	0.33790251	0.24186058
Cacnb4	-0.141072	9.03744874	0.96399408	0.33796397	0.24187394
Macc1	1.44686177	-2.0982567	0.96390545	0.33798567	0.24187394
Sec63	-0.1066101	7.07591059	0.96316868	0.33816608	0.24197996
Pnmal2	-0.1520806	6.34505682	0.96301743	0.33820313	0.24198339

2210015D19I	0.20762401	3.178976	0.96266871	0.33828859	0.24202145
Cwc25	-0.1638375	4.51326967	0.96138243	0.33860405	0.24222404
Phldb2	0.20490226	7.68725526	0.96063808	0.33878678	0.24233165
Rnase1	-1.335262	-0.8983568	0.96032426	0.33886387	0.24236368
Ssbp4	0.2538215	2.9201445	0.9600204	0.33893853	0.24239397
Fam63a	0.27076049	4.35498773	0.95940138	0.33909071	0.24247726
Farsa	0.3802027	1.36654708	0.95924057	0.33913026	0.24247726
Tmem14a	0.17299375	4.84503277	0.95915226	0.33915198	0.24247726
Mitf	-0.2063833	3.67275615	0.9570047	0.33968079	0.24283219
Dgcr6	0.24648895	3.95728815	0.95629024	0.33985698	0.2428644
Slc35e4	-0.4397629	1.114314	0.95626043	0.33986433	0.2428644
G6pdx	0.17740415	5.75914343	0.95622716	0.33987254	0.2428644
4930506M07	-0.1320088	5.40685335	0.95615384	0.33989063	0.2428644
Gbp7	0.18420976	4.86337133	0.95605176	0.33991582	0.2428644
5430421F17I	-0.5352121	0.8712816	0.95598029	0.33993345	0.2428644
Diablo	-0.2391047	4.26106158	0.9558516	0.33996521	0.2428644
AK129341	-0.2415485	5.21366025	0.95577201	0.33998485	0.2428644
Cox10	0.1608865	4.25664074	0.95492249	0.34019463	0.24299111
Kmo	0.52802325	0.99366397	0.9545431	0.34028837	0.24303492
Pdgfrl	-0.3722205	2.57959627	0.95405391	0.34040929	0.24309814
Rcan2	-0.100001	7.45083841	0.95351997	0.34054135	0.24314414
Acaa1a	-0.1921462	3.87713101	0.95349811	0.34054676	0.24314414
Wnt2	0.8459494	-1.0513622	0.95336759	0.34057905	0.24314414
Pabpc1l	1.73523782	-1.9515663	0.95326939	0.34060335	0.24314414
1700026L06I	-1.1329617	-1.4318368	0.95239432	0.34081999	0.24327563
Eme2	-0.3446254	1.98866727	0.95203257	0.3409096	0.24331445
Tmem168	-0.2349268	3.25320225	0.95191296	0.34093924	0.24331445
Skint3	-0.4664851	1.16596641	0.95150731	0.34103978	0.24336268
Them6	-0.2976492	2.62308151	0.95137851	0.34107172	0.24336268
Nipsnap1	0.15786649	4.73071935	0.95103645	0.34115654	0.24337951
A330069E16I	0.41281083	0.70240005	0.95100235	0.341165	0.24337951
Asphd1	0.24354686	2.41463671	0.95089093	0.34119264	0.24337951
Cdc26	0.26487249	4.58922415	0.95053111	0.34128191	0.24340095
Gm10768	-1.5089124	-2.1390661	0.97623511	0.34128758	0.24340095
Tmem144	-0.2713285	2.21110874	0.94982951	0.34145607	0.24349796
Trnt1	0.16373739	4.77161066	0.94950438	0.34153683	0.24352562
Ercc6	-0.212781	5.60019108	0.94941195	0.34155979	0.24352562
Slain2	0.12573594	6.73373401	0.94902232	0.34165661	0.2435715
2610528A11I	1.3219044	-1.5100274	0.94869644	0.34173762	0.2436061
Pbld2	0.56023436	0.65689591	0.94851864	0.34178182	0.24361446
Map3k11	0.21560159	3.78564312	0.94822496	0.34185486	0.24364337
Poldip2	0.16079679	5.13046729	0.94733852	0.34207546	0.24375502
Ubxn7	0.10073975	7.31127239	0.94728274	0.34208935	0.24375502
Irak1	-0.1066596	6.53281379	0.9472038	0.342109	0.24375502
Gpr56	-0.2581971	2.97073981	0.94705417	0.34214627	0.24375842

Camk2b	-0.1515164	8.06092179	0.94679364	0.34221116	0.2437815
Hsp90ab1	0.11624553	10.3381856	0.94652503	0.34227808	0.24378518
Pard3	0.13741933	5.12882172	0.94630523	0.34233285	0.24378518
Brat1	-0.2978926	2.28411883	0.94625938	0.34234428	0.24378518
Usp2	-0.1602932	5.72951967	0.9462512	0.34234632	0.24378518
Ptgdr	0.24201908	5.28157991	0.9455235	0.34252777	0.24388823
Msantd2	-0.3479145	3.04456159	0.94541007	0.34255606	0.24388823
Bcl2l15	-1.0936733	-0.0955266	0.94511603	0.34262942	0.24391368
Fau	0.2378926	5.68970486	0.94500099	0.34265813	0.24391368
Prkch	0.27876068	2.40953756	0.94483009	0.34270079	0.24391368
Cpne3	0.15260238	7.19702496	0.94474561	0.34272188	0.24391368
Slc25a20	0.22948845	4.78265158	0.94432635	0.34282656	0.24395246
Slc35g1	0.27628164	4.50834341	0.94403794	0.3428986	0.24395246
Prpf40a	0.13692443	6.39276281	0.94392363	0.34292716	0.24395246
Ntsr2	-0.2753159	3.21728665	0.94391826	0.3429285	0.24395246
Ccdc38	-0.6166236	-0.1987506	0.94387636	0.34293897	0.24395246
Ccdc6	0.12575831	6.7624702	0.94359861	0.34300838	0.2439787
Cp	0.17373028	7.00285072	0.9434255	0.34305164	0.24398634
Tra2a	0.14119675	6.08364828	0.94223022	0.34335062	0.24417582
Nov	0.31930065	7.77909032	0.94122416	0.34360255	0.24433182
Fgd2	0.50424565	0.37736609	0.94096799	0.34366674	0.2443543
Guk1	0.22210844	4.85729885	0.94075867	0.3437192	0.24436844
Kcnh8	-1.1951916	-1.0813013	0.94042055	0.34380397	0.24440554
Slc48a1	-0.166548	4.84507852	0.94020407	0.34385826	0.24441243
Kcnk3	-0.2792078	1.8758333	0.94012207	0.34387883	0.24441243
E130012A19l	-0.2580215	2.9411	0.93991143	0.34393167	0.24442683
Gpc4	0.18695305	4.23406779	0.93959301	0.34401157	0.24446045
Fgf13	-0.1583695	6.45146223	0.9393574	0.3440707	0.24446165
Zdhhc24	0.18667494	5.97825576	0.93932662	0.34407843	0.24446165
Traf3ip2	-0.2642869	2.99233899	0.93853855	0.34427635	0.24454198
U2af2	0.22576545	4.69481803	0.93845995	0.34429609	0.24454198
Casc3	-0.1614868	5.49386002	0.93842468	0.34430495	0.24454198
Usp38	-0.1659017	5.03510708	0.93835725	0.3443219	0.24454198
Pde9a	-0.3995579	2.03889486	0.93810077	0.34438636	0.24456461
Dmrta2	-0.7003163	-0.2709334	0.93762161	0.34450682	0.2446104
Pusl1	0.35156612	2.33169202	0.93758488	0.34451606	0.2446104
Dscr3	0.18392337	4.05739242	0.93742687	0.3445558	0.24461546
Fam118b	0.20149172	5.23855314	0.93709502	0.34463929	0.24464132
Rnf122	0.55026806	0.02224621	0.93702283	0.34465745	0.24464132
Mab21l2	-1.0003411	-0.8035876	0.93676035	0.34472351	0.24466506
Mtmr10	-0.1601455	4.27423478	0.93654072	0.34477879	0.24468115
Cstl1	0.86577504	0.25753104	0.93613635	0.34488062	0.24471443
Hsd17b4	-0.1667623	5.79095532	0.9360954	0.34489093	0.24471443
Ankfn1	-0.3898056	2.48226336	0.93562674	0.34500901	0.24477505
Dtx4	0.14799801	5.31482988	0.9354966	0.34504181	0.24477517

Amigo1	0.13699115	5.74003178	0.93513557	0.34513281	0.24481658
Ctnna3	-0.9278958	-0.1813405	0.93450735	0.34529126	0.24490581
Scarf1	-0.9163404	-0.7980304	0.9340528	0.34540596	0.244964
Ccdc73	-0.2564291	3.33422071	0.93386425	0.34545356	0.2449746
Tdg	-0.4468945	0.31915329	0.93350557	0.34554413	0.24501566
Cyp51	-0.1717131	5.33022634	0.93314036	0.34563638	0.24502359
Tmem41b	0.14519123	4.8261981	0.93313068	0.34563882	0.24502359
Epas1	-0.124354	8.09369916	0.93307339	0.3456533	0.24502359
Mlxipl	-0.4600449	0.95267483	0.93274249	0.34573692	0.24504038
Mospd3	0.27802319	5.64086596	0.9327211	0.34574233	0.24504038
Appl1	-0.118284	7.58721653	0.93190243	0.34594934	0.24514712
Klrg2	0.79744486	-0.5935712	0.93176768	0.34598343	0.24514712
Entpd4	-0.1432483	6.04676775	0.93172621	0.34599392	0.24514712
Golph3l	0.17326584	5.99608675	0.93160874	0.34602365	0.24514712
Ppp1r2-ps3	-0.596887	-0.4670166	0.93092545	0.34619661	0.24522642
Vav2	0.32322956	1.76348009	0.93090828	0.34620096	0.24522642
Kcnab1	0.14458266	5.30295476	0.93032908	0.34634768	0.24530718
Tmem98	-0.3242445	3.79390521	0.93004024	0.34642088	0.24533586
Map2k6	0.18278786	4.00294987	0.92943808	0.34657355	0.24541604
2700097O09	0.2646852	2.8756526	0.92933566	0.34659953	0.24541604
1700003F12I	-1.142501	-2.0074229	0.92893349	0.34670156	0.24545399
Zfp146	0.23603673	4.37017683	0.92886649	0.34671856	0.24545399
Tchp	-0.3310711	2.32131566	0.92832114	0.34685699	0.2455255
Rpl12	0.14400894	6.78598507	0.92821068	0.34688505	0.2455255
Hsf2bp	-0.4177826	2.07785124	0.92754654	0.34705376	0.24562174
Cdc14a	-0.2567257	4.04350205	0.92704468	0.34718132	0.24568884
Six1	0.35368099	5.37716334	0.92652573	0.3473133	0.24575906
Mgme1	-0.3187716	2.92595142	0.92602408	0.34744095	0.24582619
Rhod	0.41082171	1.72168286	0.92532873	0.347618	0.24590528
Sh2d4b	0.67580439	-0.1638562	0.92525752	0.34763614	0.24590528
Pnkp	-0.3167906	1.65228763	0.9251726	0.34765777	0.24590528
Ldhb	0.14067936	8.96055698	0.92507017	0.34768387	0.24590528
Polr3g	-0.2099094	3.14058005	0.92453217	0.34782098	0.2459441
Inf2	-0.1304496	6.07676718	0.92446635	0.34783775	0.2459441
Il17rc	0.37294386	1.90977284	0.92439438	0.3478561	0.2459441
Hist1h4c	0.70051323	-0.8188294	0.9243403	0.34786989	0.2459441
6330409D20	-0.4696777	0.73553757	0.92388999	0.34798474	0.2459802
Vip	-0.2509298	2.80295463	0.9238829	0.34798654	0.2459802
Eif4g3	-0.1422626	8.94854397	0.9236382	0.34804897	0.24600115
Gch1	-0.4280918	0.83721912	0.92330981	0.34813278	0.2460372
Ptpn4	-0.163799	7.20235562	0.92307725	0.34819215	0.24605598
Crtac1	0.27105911	3.20349585	0.92273535	0.34827945	0.24609449
Supt20	-0.1592091	4.95552167	0.92101435	0.34871938	0.24634697
Lag3	0.99113376	-1.3344653	0.9208371	0.34876474	0.24634697
Nr1i3	-0.9658556	-1.1087511	0.92079855	0.34877461	0.24634697

Ubxn10	-0.5938404	0.82229865	0.92069314	0.34880158	0.24634697
Ptpn11	-0.1092258	7.67249814	0.92047964	0.34885623	0.24634697
Ogn	-0.2393825	8.11652656	0.92037977	0.3488818	0.24634697
Fam171a1	-0.1589497	5.50197085	0.92026471	0.34891126	0.24634697
2700081O15	-0.1322559	6.20960645	0.92023724	0.3489183	0.24634697
Mob4	0.10599831	7.02416311	0.92018244	0.34893233	0.24634697
Slc39a1	0.24622822	5.19620193	0.91999268	0.34898093	0.24635041
Sgcd	-0.2803639	2.93559348	0.91990693	0.34900289	0.24635041
Chst15	-0.1637076	5.98406679	0.91973734	0.34904634	0.24635789
4931429I11R	-0.702149	-0.0218343	0.91947243	0.34911422	0.24637104
Rexo1	0.16623182	4.84694613	0.91940829	0.34913065	0.24637104
1810030O07	0.14286105	6.11730603	0.91863173	0.34932976	0.24647282
Rundc1	0.16144598	5.45873072	0.91855516	0.3493494	0.24647282
Prps1	0.11453576	5.87724468	0.9184614	0.34937345	0.24647282
Micall1	0.13998464	5.22167441	0.91809227	0.34946816	0.24651645
Mrm1	0.19663585	3.09996865	0.91774939	0.34955618	0.24654645
Itga11	0.27595644	2.90705307	0.91767053	0.34957642	0.24654645
Stpg1	0.4271207	0.68737858	0.91728408	0.34967567	0.24658557
Esm1	0.3499632	2.24517017	0.91706288	0.34973249	0.24658557
Arhgap15	0.19707547	3.82737943	0.91699807	0.34974914	0.24658557
Igf1	-0.1797971	5.25462119	0.91688458	0.3497783	0.24658557
1700101E01I	0.98348465	-0.8806146	0.91675571	0.34981142	0.24658557
Gsn	0.20264118	5.4563857	0.91662175	0.34984585	0.24658557
Jade3	0.28937848	4.31488406	0.91655894	0.349862	0.24658557
Cenpm	1.14198714	-1.5130958	0.91623218	0.34994601	0.24660048
Il27ra	1.02186765	-1.5039429	0.91616709	0.34996274	0.24660048
Sgol1	-0.4955509	0.8431864	0.91609309	0.34998177	0.24660048
Tcp1	0.09943148	7.08689799	0.91524189	0.35020078	0.2466693
Fbxo2	0.22147565	3.07285821	0.91524147	0.35020089	0.2466693
Ncan	-0.1639244	8.37898818	0.9151822	0.35021615	0.2466693
Rspo2	0.2890846	4.39083096	0.91512196	0.35023166	0.2466693
Kcnj11	-0.1980129	3.39176252	0.91507452	0.35024387	0.2466693
Zfp960	0.14807081	4.46068238	0.91487939	0.35029411	0.24668152
Mndal	-0.2663968	2.99456607	0.91399972	0.35052073	0.24681795
Xrn2	-0.1271688	5.65336846	0.91378956	0.35057491	0.24683234
Pcdhgb6	-0.3152501	2.14496782	0.91355847	0.35063449	0.24683234
Ear2	-0.9265794	-0.4067613	0.91344853	0.35066284	0.24683234
Lgmn	0.20993486	4.12100921	0.91337745	0.35068117	0.24683234
Tmed8	-0.1122614	6.24397623	0.91320731	0.35072506	0.24683234
Fancd2os	0.68048642	0.21805888	0.91315475	0.35073862	0.24683234
Ggnbp1	-1.0196054	-0.8377932	0.9125088	0.35090531	0.24692649
Limd1	0.22128781	5.16136736	0.91198583	0.35104035	0.24699834
E230029C05I	-0.5516541	0.54626243	0.91161042	0.35113733	0.24703684
Csrp2bp	0.1488533	4.95271622	0.9115191	0.35116093	0.24703684
Alox5	1.03307459	-1.3258855	0.91124097	0.35123281	0.24706424

Nudt21	0.18412629	4.7723066	0.91109807	0.35126976	0.24706706
Mip	0.91718816	0.37341419	0.91089117	0.35132325	0.24708152
Ror1	0.55305806	0.88849533	0.91067419	0.35137936	0.24709781
B230208H11	-0.9824351	-0.0817268	0.91043032	0.35144245	0.24711901
Hnrnpa2b1	0.09045525	8.6580106	0.90987706	0.35158563	0.24719651
Tfdp1	0.14093163	6.37155299	0.90935481	0.35172086	0.24726842
Nostrin	0.60349631	0.87042759	0.90866935	0.35189846	0.24737009
Notch2	0.22020623	7.07450311	0.90826158	0.35200417	0.24740645
Rbm15b	-0.1743819	3.8137189	0.90805768	0.35205705	0.24740645
Dolk	-0.2783549	2.47522989	0.90797536	0.3520784	0.24740645
Flywch2	-0.3422732	1.0455833	0.90796108	0.3520821	0.24740645
Tug1	0.10200573	8.05181296	0.90711999	0.35230036	0.24750813
Tox3	-0.209395	5.04677067	0.9069161	0.3523533	0.24750813
Creld1	0.24058297	3.59084989	0.90686475	0.35236664	0.24750813
Epm2a	0.40640006	1.61414237	0.90684196	0.35237256	0.24750813
Zfp955b	-0.134334	5.83765315	0.90670874	0.35240715	0.24750813
Nfkbid	-0.8769633	-1.1082854	0.90664085	0.35242479	0.24750813
Slc35a1	-0.207672	5.25560159	0.90640699	0.35248554	0.24752763
Htr5b	-0.9569098	-0.3098906	0.90584926	0.35263048	0.24760623
Ptprs	-0.1543261	6.88843335	0.90455229	0.35296788	0.24781994
Hapln1	0.24806021	3.8075648	0.90421386	0.35305599	0.24785861
Klrc1	0.736847	-0.657688	0.90367622	0.35319604	0.24793372
Actr6	0.23347191	3.63828272	0.90345616	0.35325339	0.24793505
Efcab9	-0.8148632	-0.5491644	0.90341532	0.35326403	0.24793505
Cyp3a13	-0.8820613	-1.1165901	0.90275636	0.35343584	0.24801758
Nfia	0.15148218	9.13850662	0.90263091	0.35346856	0.24801758
Tmem176b	0.27260188	5.40405704	0.90258393	0.35348081	0.24801758
Angptl2	-0.3026701	4.88653529	0.90146473	0.35377294	0.24819933
Rcc1	0.3605228	1.73423824	0.90121182	0.353839	0.24820341
Plod1	0.25870478	3.01885443	0.90118913	0.35384493	0.24820341
Tnnt1	-0.4202332	1.24684882	0.90102645	0.35388743	0.24821001
Caln1	0.16734304	4.57744271	0.90055363	0.354011	0.24827347
Klhl12	0.1534051	4.79415184	0.9000987	0.35412996	0.24833368
Ccdc94	0.34684307	1.59061397	0.89989044	0.35418443	0.24834866
Fkbp7	0.27190515	3.61304404	0.89898211	0.35442217	0.24845748
Eps8l1	0.22880304	2.75832042	0.8989732	0.3544245	0.24845748
Mybpc2	0.44912477	1.39858629	0.89891787	0.35443899	0.24845748
Ap1ar	0.10777932	6.76523956	0.89872042	0.35449071	0.24846391
Slitrk5	-0.1855071	5.86905806	0.89862992	0.35451441	0.24846391
Dtx3	0.16660602	5.05564026	0.89798666	0.35468297	0.24855882
Fam222a	0.35637163	2.20401045	0.8968939	0.35496958	0.24873644
Egr2	-0.499964	3.93185383	0.89649489	0.35507432	0.24878659
Pkd1l3	-0.4965514	0.8831859	0.89606173	0.35518807	0.24884305
Kcnj13	-0.2141697	6.22314713	0.89558246	0.35531399	0.24887114
Fbxo9	0.11219222	6.44977445	0.89556492	0.3553186	0.24887114

Tinagl1	-0.6104894	0.32428798	0.89553029	0.3553277	0.24887114
Dusp27	0.57874063	1.8053028	0.89451452	0.35559481	0.24903497
Fos	0.71388487	3.49210614	0.89433063	0.35564319	0.24903985
Pmel	1.03857425	-1.8486004	0.89420109	0.35567728	0.24903985
Ccdc23	0.32456874	3.16987757	0.89410955	0.35570137	0.24903985
Rnf125	-0.4123028	1.24006064	0.89290724	0.35601804	0.2492383
Trim30d	-0.2095293	3.62302109	0.89266045	0.35608309	0.24926057
Scd3	0.29817959	3.20431338	0.89165018	0.35634955	0.24942383
2210408F21I	0.29598646	2.4430017	0.89141658	0.35641121	0.2494437
Zfp53	0.22961778	2.75478349	0.88982694	0.35683118	0.24969758
Rps6kl1	0.32038839	2.37169747	0.88979156	0.35684053	0.24969758
Olig1	0.1685479	4.07031576	0.88916206	0.35700704	0.2497908
Arrdc4	0.29554569	2.72777521	0.88875699	0.35711425	0.24981978
Rimbp3	-0.5982972	0.85861884	0.88869301	0.35713118	0.24981978
Gm13446	0.28518633	2.27103668	0.88862804	0.35714838	0.24981978
Arxes1	0.27679894	2.41449458	0.88838092	0.35721381	0.24984226
Sft2d2	0.19922207	6.40335609	0.88810867	0.35728592	0.24986939
Zan	1.31446849	-2.1385419	0.88784671	0.35735533	0.24989463
Celf2	0.14183693	9.78204798	0.88690731	0.35760436	0.25004547
Pate2	-0.5430854	0.20605937	0.88665465	0.35767139	0.2500658
2310014L17F	-0.6291466	-0.1128098	0.88654641	0.35770011	0.2500658
Slitrk6	-0.456074	0.63049086	0.88625888	0.35777641	0.25009583
Mul1	0.21013727	3.14208276	0.88578292	0.35790277	0.25015293
Aldh1a3	0.71292787	-0.040302	0.88570001	0.35792479	0.25015293
LOC1005047(	0.75616558	-0.317366	0.88551994	0.35797262	0.25015727
Sez6	0.17922065	4.84745636	0.88542551	0.3579977	0.25015727
Fgd1	0.22533299	3.05365511	0.88520229	0.35805701	0.25015999
Btbd1	0.11010665	7.23030801	0.88499334	0.35811254	0.25015999
D830031N03	-0.1461307	5.26126611	0.88498623	0.35811443	0.25015999
Ccdc122	0.31602466	2.24255344	0.88490888	0.35813499	0.25015999
Trim37	-0.1484978	8.21341836	0.88443987	0.35825968	0.25020684
Rabepk	0.25915686	2.99144991	0.88440572	0.35826876	0.25020684
Rbm15	-0.1566687	3.93178982	0.88419569	0.35832462	0.25022255
Zfp958	-0.2157039	3.44409452	0.88391364	0.35839966	0.25024374
Aacs	-0.2411546	2.68904219	0.88375699	0.35844135	0.25024374
Kank2	0.18932482	7.56540758	0.88363336	0.35847425	0.25024374
Mgst2	-1.0303241	-1.4875532	0.88358021	0.3584884	0.25024374
Sfpq	-0.1308444	7.54784806	0.88333595	0.35855343	0.25026584
Ctsl	0.2381309	6.74069323	0.88309298	0.35861812	0.25027907
Stau1	-0.1140535	6.3092757	0.88258043	0.35875466	0.25027907
Gm4013	1.3582298	-1.6984571	0.88248769	0.35877938	0.25027907
Slc25a30	-0.4259333	1.14282659	0.88246086	0.35878653	0.25027907
Rpl14	0.22192401	6.55012026	0.88242209	0.35879686	0.25027907
Npl	0.437574	1.59114051	0.88239333	0.35880452	0.25027907
Slc26a10	-0.4463891	0.66412926	0.88238803	0.35880594	0.25027907

Senp1	-0.1578702	5.22528024	0.8821231	0.35887656	0.25030505
Agbl1	0.8697523	-0.8698047	0.88121887	0.35911774	0.25044998
Mta2	0.15764092	4.80843552	0.88024005	0.35937907	0.25060894
Bub1b	-0.5931937	0.47634977	0.87960477	0.35954884	0.25070402
Taf1c	-0.4549257	1.20429894	0.87934303	0.35961881	0.25071563
Trappc13	0.11003578	6.39477302	0.87929245	0.35963234	0.25071563
Foxk2	0.09366244	6.46145582	0.87911747	0.35967913	0.25072495
Cpxm1	-0.2859974	3.30145207	0.87837674	0.35987732	0.2508398
Snhg8	0.25481886	2.70202649	0.87794361	0.35999328	0.25089731
Cryab	0.22016463	7.16970998	0.877119	0.3602142	0.25102796
Egflam	0.24437335	3.52709281	0.87682084	0.36029413	0.25106033
Cox11	-0.1590124	3.91883494	0.87612072	0.36048192	0.25116785
Eda2r	-0.9672869	0.18129697	0.87535483	0.3606875	0.25128775
Mkln1	-0.1524971	6.15447147	0.87474021	0.3608526	0.25137943
Rb1cc1	-0.1456754	8.50531799	0.87415115	0.36101094	0.25146638
Efnb1	0.33223666	3.42047178	0.87341664	0.36120852	0.25158064
Zpbp	0.75396468	0.39865645	0.87297927	0.36132624	0.25163208
Nat1	0.412232	2.09121257	0.87289302	0.36134946	0.25163208
Aldh9a1	0.14014614	5.52377629	0.87206403	0.36157276	0.25174424
Gal	0.86470248	-0.4440306	0.87204592	0.36157764	0.25174424
Kif7	-0.4502199	1.49510603	0.87186707	0.36162585	0.25175444
Ap3m1	-0.1217092	5.66474037	0.87165108	0.36168407	0.25177161
2410002F23I	-0.2216765	3.20414754	0.87152467	0.36171816	0.25177197
Endou	0.31865916	1.87124236	0.87124723	0.36179298	0.25180068
Polr2j	0.35757643	3.17793511	0.87023772	0.36206542	0.25193593
Zfp938	0.18204256	4.44416906	0.87019255	0.36207761	0.25193593
Cd81	0.23988881	7.79192516	0.87015385	0.36208806	0.25193593
Dnajc19	0.15554755	4.74616253	0.87002484	0.36212291	0.2519368
AA986860	0.31343055	1.71718232	0.8698424	0.36217218	0.25194752
Nppc	-0.724769	-0.4988418	0.86969719	0.36221141	0.25194752
Arfgap3	0.19983063	4.63360754	0.86947553	0.36227131	0.25194752
Alpl	0.33390175	3.38132943	0.86945878	0.36227583	0.25194752
Amigo3	-0.9878848	-0.5059474	0.86934627	0.36230624	0.25194752
Tmem9	0.20586644	4.96445114	0.86909287	0.36237474	0.25196014
E530001F21I	-1.1136697	0.14723667	0.86903063	0.36239156	0.25196014
Tslp	1.17375105	-1.0755982	0.86887875	0.36243263	0.25196533
Nr2f6	0.26037547	2.59815813	0.86657796	0.36305557	0.25237502
Ddhd1	-0.1501926	6.49345786	0.86631383	0.36312719	0.25240141
Theg	0.7125354	-0.8968574	0.86574057	0.36328268	0.25248609
4833412C05I	-0.5710468	0.25183559	0.86462673	0.36358508	0.25264611
Lig3	0.16627785	4.99211735	0.86454807	0.36360645	0.25264611
Cdc25b	-0.2497978	2.97562223	0.86440998	0.36364397	0.25264611
Adprh	0.13972354	4.24486342	0.86439648	0.36364764	0.25264611
Ano5	-0.6551608	0.82467018	0.86415835	0.36371236	0.25266767
Oas3	-0.689911	0.6839544	0.86399716	0.36375617	0.25267471

Snord104	0.9571163	-1.7075835	0.86348442	0.3638956	0.25274301
Mmp2	0.44209181	1.32979393	0.86338777	0.36392189	0.25274301
Rhbdl3	-0.2595901	2.78035487	0.86285515	0.36406681	0.25282026
Dut	0.17903124	4.83592111	0.8626782	0.36411498	0.2528303
Gm16880	-1.1893345	-0.2276483	0.86245095	0.36417685	0.25283623
Nap1l3	0.15439934	5.81766145	0.86239922	0.36419094	0.25283623
Khnyn	-0.2138703	2.936284	0.86199756	0.36430034	0.25288674
Topbp1	-0.1636331	5.30103597	0.86182746	0.36434669	0.25288674
Wwp1	0.12601156	7.5347871	0.86172087	0.36437573	0.25288674
Mir5119	-1.0964574	-1.4890308	0.86153745	0.36442572	0.25288674
Mief2	0.40007338	1.96346521	0.86145212	0.36444898	0.25288674
Pak1ip1	0.14824994	4.15962948	0.8613898	0.36446597	0.25288674
Mpv17l2	0.32751559	2.69491878	0.86109997	0.36454499	0.25291818
Btbd9	-0.1686018	4.98296484	0.86062656	0.36467412	0.25298437
Tfap2d	0.8259658	-0.1952972	0.8604203	0.36473041	0.25300002
Fcgr2b	-0.694303	0.11290006	0.85977073	0.36490774	0.253077
Slc26a1	-0.7350262	-0.6319388	0.85973526	0.36491742	0.253077
Tmem43	0.24241524	3.88559186	0.8596431	0.36494259	0.253077
Zrsr1	0.0901723	6.95800016	0.85928652	0.36504001	0.25312115
Col4a4	0.94702631	-0.8139241	0.8587216	0.36519441	0.25320481
Gm16515	0.23995033	5.5106867	0.858223	0.36533077	0.25325469
Gpr132	-0.957388	-1.4548746	0.85821161	0.36533388	0.25325469
Tiam1	-0.1564817	6.45558787	0.85782819	0.36543879	0.25330401
Tnfrsf11b	-0.3865208	3.89176417	0.85753057	0.36552025	0.25333706
Pacsin1	0.13909765	6.57730162	0.85703134	0.36565695	0.25340293
Cdh4	0.20034701	3.48979286	0.85693681	0.36568285	0.25340293
Kcng1	0.67263673	0.10079227	0.85675557	0.3657325	0.25341393
Car11	-0.1410876	5.14702599	0.85621736	0.36588001	0.25349001
Trex1	0.35803159	1.23374519	0.85603323	0.36593049	0.25349001
Nrg1	-0.2486087	3.36162218	0.85598514	0.36594368	0.25349001
G0s2	0.4518156	1.60032905	0.85515689	0.3661709	0.25362398
Txnip	0.2244816	6.72781444	0.85490875	0.36623902	0.25364774
Ezr	0.1253705	6.23592406	0.85460292	0.36632299	0.25368248
Cks1b	0.36266007	3.42281938	0.85408044	0.36646652	0.25375845
Iqcb1	-0.1565999	4.50208082	0.8537993	0.36654379	0.25376296
1700025F24I	-1.4818518	-2.177585	0.85370796	0.3665689	0.25376296
Chil1	0.58573145	0.532544	0.85368748	0.36657453	0.25376296
Lrrn2	0.20646902	4.65525531	0.85333648	0.36667104	0.25379164
Qsox2	-0.2365111	2.93619289	0.85325042	0.3666947	0.25379164
Cct3	0.13177372	5.12571248	0.8531677	0.36671746	0.25379164
Tmem164	0.14251357	4.58664058	0.85301484	0.36675951	0.25379733
Rpl14-ps1	0.27525709	2.59739712	0.85282483	0.36681178	0.25381009
Zfp583	-0.3015475	3.39265592	0.85261308	0.36687006	0.25381943
Krt9	-0.1772496	3.95812675	0.8525299	0.36689295	0.25381943
Tmem29	-0.115508	4.81473657	0.8520802	0.36701676	0.25387531

Cep290	-0.140151	7.10751982	0.85199068	0.36704141	0.25387531
Rarres2	-0.7695913	0.59490737	0.85158826	0.36715227	0.25392857
Gal3st4	-0.4017431	1.56458374	0.85111538	0.3672826	0.25399529
Ccdc113	0.45877048	1.09575397	0.85067614	0.36740372	0.25405562
Prr5	-0.3897789	1.40299752	0.85033313	0.36749834	0.25408636
Ptk7	-0.3743126	1.28696047	0.85015503	0.36754748	0.25408636
Mef2c	-0.1486155	10.0758779	0.85014668	0.36754979	0.25408636
Atg16l2	-0.4462321	1.46873458	0.84994932	0.36760426	0.2541006
Sema3f	-0.4806818	0.80992883	0.84929507	0.36778491	0.25420205
Zfp810	-0.1483927	4.65836076	0.84869185	0.36795159	0.25429383
Zcchc10	0.23857026	3.6333882	0.84817378	0.36809483	0.25436938
Kctd3	-0.1673043	5.13243541	0.84799242	0.36814499	0.25438061
Asb1	-0.1686461	4.51615356	0.84732878	0.36832863	0.25448406
Rpp40	0.24190762	2.58937608	0.84652506	0.36855122	0.2546144
Fbxw15	-0.7799031	-0.9967257	0.84622462	0.36863447	0.25464846
Cdc42ep1	-0.4265784	2.57236804	0.84603772	0.36868628	0.2546608
St8sia6	-0.5417747	0.86685262	0.8456344	0.3687981	0.25471458
Mia	-0.5808406	-0.1530321	0.84509569	0.36894755	0.25479434
Acot5	0.77275807	0.37298916	0.8447808	0.36903495	0.25483124
Catsperd	-0.8402545	-0.7666412	0.84430134	0.36916808	0.2548997
Vps37c	0.25794255	3.47069224	0.84412007	0.36921843	0.254911
Tmem214	-0.1761622	4.44956222	0.84360974	0.36936023	0.25498544
Arg1	0.88115147	-0.9245313	0.84347208	0.3693985	0.25498839
Rad51ap1	-0.8054611	-0.3231284	0.84186751	0.36984495	0.25527307
Zfp35	0.13960241	4.4899564	0.84151677	0.36994264	0.25531701
Pja1	0.10966205	6.81192113	0.84128303	0.37000776	0.25533846
Ankrd66	1.51456878	-2.1740184	0.84104359	0.3700745	0.25536102
Aph1a	-0.2704346	2.94250574	0.84040726	0.37025192	0.25545478
Slc25a28	0.16832048	3.8932425	0.84031204	0.37027848	0.25545478
1700101I11R	0.75106543	0.31317228	0.84006203	0.37034824	0.25545627
6720489N17	-0.1968096	3.66694827	0.83998469	0.37036982	0.25545627
Cdh20	-0.2361802	3.92881565	0.83993816	0.3703828	0.25545627
Fam183b	0.81296015	0.14856088	0.83946935	0.37051367	0.25550477
BC037032	-0.5229662	0.53871092	0.83944218	0.37052125	0.25550477
Hmgn1	0.19183061	8.11437481	0.83891902	0.37066737	0.25558204
Casp7	0.33487421	2.71536491	0.8387534	0.37071365	0.25559045
Hmgcs2	-0.2246267	4.07339553	0.83857674	0.37076302	0.2555969
Tmem68	-0.128355	5.12180934	0.83847608	0.37079115	0.2555969
Zfp273	-0.2149092	3.24774517	0.83746588	0.37107368	0.25576815
Stt3a	0.14177091	5.16260031	0.83713354	0.3711667	0.25580876
Padi2	-0.2104554	4.03186539	0.83624162	0.3714165	0.25595741
Plekhf2	-0.1978361	4.63531593	0.83600127	0.37148386	0.25598031
AU041133	-0.2544622	2.83217733	0.8357244	0.37156147	0.25600801
Bpnt1	0.11400671	6.08443902	0.83561438	0.37159232	0.25600801
Orc4	-0.1234565	5.58400376	0.83514547	0.37172383	0.25606065

Fgd5	-0.5242731	1.84175982	0.8349866	0.37176841	0.25606065
Kcnh6	0.53578428	-0.1846566	0.83497691	0.37177113	0.25606065
Ap3s2	-0.1212299	6.62475984	0.83481667	0.37181609	0.25606811
Dcxr	0.61110939	0.43505574	0.8343442	0.37194873	0.25613594
Tbxas1	-0.9883046	-1.1977504	0.83392655	0.37206603	0.2561932
2410127L17F	0.19522763	4.94433774	0.83372638	0.37212227	0.2562084
Gpr133	0.29270801	2.64997106	0.83352381	0.3721792	0.25622408
Wee1	0.13561218	5.51696953	0.83326447	0.37225209	0.25625075
Rad51d	0.17188274	5.28271466	0.83309124	0.3723008	0.25626076
Pdp1	0.14908427	7.11894952	0.83281226	0.37237925	0.25629125
Flrt2	-0.1126781	7.45037819	0.83255487	0.37245166	0.25631756
Inpp5e	0.13865212	5.0308167	0.83237139	0.37250329	0.25632958
Chrna3	0.54458989	0.04976638	0.83220872	0.37254907	0.25633756
Gins2	-0.3570928	2.15849279	0.83201822	0.37260269	0.25635095
Pydc4	-1.05963	-1.4334036	0.83185553	0.3726485	0.25635895
Itpkb	0.22923512	3.35426576	0.83138609	0.37278071	0.25641591
Rbpms	0.26397552	5.27075179	0.83131882	0.37279966	0.25641591
Pyhin1	-0.5551952	1.688976	0.83114906	0.37284749	0.25641603
Csnk1g3	0.095587	7.45974778	0.83107557	0.3728682	0.25641603
Parm1	0.16221065	4.32365914	0.83082445	0.37293898	0.25644119
Dpys	-0.9109381	-0.9644051	0.83063493	0.37299241	0.25645442
Alx1	0.4769832	2.53238733	0.83015189	0.37312864	0.2565232
A630007B06	0.12805651	7.27132585	0.83003774	0.37316084	0.2565232
U2af1l4	0.29980512	2.09191891	0.82924439	0.37338477	0.25665362
Gm4980	-0.4303277	0.8216005	0.82890879	0.37347956	0.25669525
Klhl29	-0.235685	5.07510909	0.82857481	0.37357392	0.25673658
Mvb12b	-0.1194945	6.44063794	0.82803256	0.3737272	0.25681053
Lgi3	-0.2104347	3.63126066	0.82795195	0.37374999	0.25681053
Rnf123	-0.1545749	4.46546386	0.82765686	0.37383346	0.25684435
Ccdc107	0.28270415	2.78089312	0.8273546	0.37391897	0.25687958
Tsnaxip1	-1.3661831	-1.2806416	0.82719832	0.3739632	0.25688644
Tfip11	0.22383803	3.79497696	0.82696145	0.37403025	0.25689123
Col6a6	-1.2112547	-1.4085711	0.82693173	0.37403866	0.25689123
0610040J01F	-0.6366068	0.12504341	0.82557268	0.37442371	0.25711451
Cklf	0.41665102	0.98049407	0.82541656	0.37446798	0.25711451
Hltf	-0.1576238	4.91250401	0.82536063	0.37448384	0.25711451
Mcm5	0.40114692	0.90631205	0.82530059	0.37450087	0.25711451
Gss	0.22960681	3.45881164	0.82471318	0.37466752	0.25720227
Ldoc1l	0.14564591	4.91591851	0.82460833	0.37469728	0.25720227
Arhgap8	-0.8254107	-0.3610325	0.82434341	0.37477249	0.25723036
Lztfl1	0.13400464	6.37156356	0.82385543	0.37491107	0.25729559
Crot	-0.1300606	5.34776257	0.82376722	0.37493613	0.25729559
Pvrl2	-0.4523887	1.42364104	0.82289813	0.37518316	0.25744156
Flii	-0.1372773	5.38782624	0.82232475	0.37534627	0.25752993
Keap1	-0.1465753	4.39016593	0.82215139	0.37539561	0.25754022

Ccdc66	-0.1211162	5.59627486	0.82184199	0.37548368	0.25757541
Ankrd42	0.15878457	4.01704178	0.82173	0.37551557	0.25757541
Gm5801	0.56646476	-0.3960546	0.82129341	0.37563992	0.25763714
Rimkla	-0.2373519	3.67743877	0.82097902	0.3757295	0.25767109
Zfp41	-0.2607889	1.96882306	0.82087858	0.37575812	0.25767109
Setdb2	-0.2484839	2.9904605	0.82055928	0.37584915	0.25768818
Pdcd7	0.19206476	3.61848309	0.82051289	0.37586237	0.25768818
Ccdc55	0.11388122	6.97211916	0.82037172	0.37590263	0.25768818
Bace1	-0.1084602	5.79319228	0.82030926	0.37592044	0.25768818
Rhbdd2	0.17025044	4.33556615	0.82008012	0.3759858	0.25770943
Esrra	0.32783183	2.04728223	0.8194926	0.37615346	0.25780079
Gm4759	1.01726032	-0.9557459	0.81852133	0.37643086	0.25796734
Nrip1	-0.1401385	6.16891455	0.81823786	0.37651188	0.25799929
Rps4x	0.17963093	7.89493547	0.81714015	0.37682586	0.25819086
Wnt10a	0.29521223	1.65062776	0.81697732	0.37687247	0.25819921
Ccdc96	0.39966034	1.74268235	0.81663066	0.37697172	0.25824362
Gm15880	-1.4655321	-0.8696785	0.81566959	0.3772471	0.25838943
Gm12522	-0.4091416	1.18137555	0.81562418	0.37726011	0.25838943
Frmpd1	-0.1972086	2.76514959	0.81552726	0.3772879	0.25838943
Bmf	0.2893837	2.81806401	0.8151834	0.37738651	0.25841969
Spg7	-0.2231511	3.55351131	0.81513291	0.377401	0.25841969
Cyp4x1	-0.376918	0.99608208	0.81470456	0.3775239	0.25848025
Rfwd2	0.10467567	7.26114381	0.81437331	0.37761898	0.25852176
Glud1	-0.0919976	8.41518438	0.81413096	0.37768857	0.2585458
Tulp3	0.20174817	5.2594078	0.81396482	0.37773628	0.25855487
Nrap	-0.9723258	-1.1094914	0.81364379	0.37782851	0.2585944
Snhg4	-0.215312	3.32764924	0.81340704	0.37789654	0.25861737
Tbc1d4	-0.241787	3.07730751	0.81310932	0.37798212	0.25865234
D330050G23	-0.387374	1.95972551	0.81286234	0.37805314	0.25867734
Hist1h2bh	0.81091263	-0.7848318	0.81248283	0.3781623	0.25871254
Slc25a25	0.15284867	5.0207656	0.81244369	0.37817356	0.25871254
Snx11	-0.2058461	3.09313506	0.81151909	0.37843972	0.25885798
Cml3	0.33197617	1.66790898	0.81146542	0.37845518	0.25885798
Tmem203	0.22366782	2.72407557	0.81133482	0.3784928	0.25886011
Ccdc11	-0.5807174	-0.1982129	0.81121207	0.37852816	0.25886069
Timm21	-0.2104066	4.15049091	0.81095837	0.37860127	0.25888708
Cln5	0.22474704	6.00046903	0.81046697	0.37874292	0.25896034
Gpr107	-0.1885694	3.80767005	0.81024572	0.37880673	0.25898036
Yipf4	0.17068361	4.77633016	0.80987997	0.37891224	0.25900298
Oxa1l	0.15342388	5.17887572	0.80984003	0.37892377	0.25900298
Hhat	0.8919597	-0.7515552	0.80977203	0.37894339	0.25900298
Hnmt	0.15931844	5.1539492	0.80955215	0.37900685	0.25902275
Ruvbl1	0.17136067	4.59810199	0.80916941	0.37911735	0.25907466
E130310I04R	-1.4936176	-1.5761338	0.80901979	0.37916056	0.25908059
Cdk1	1.15780355	-1.4653054	0.80865542	0.37926582	0.25912891

1600020E01I	-0.4682224	1.1376363	0.80844808	0.37932574	0.25914624
Coq6	-0.3932262	1.27354156	0.80817731	0.379404	0.25915469
Abcf3	0.15595108	5.02089209	0.80816626	0.3794072	0.25915469
Aim1	0.26758273	3.96012799	0.8078815	0.37948953	0.25918733
Ccdc117	0.17826139	4.88265275	0.80752105	0.37959379	0.25923493
Tceb1	0.13770325	6.28248636	0.80718657	0.37969058	0.25927743
Gm10433	1.48536181	-1.6576177	0.80699057	0.37974731	0.25929256
Ttc32	-0.2923276	2.57335124	0.80585118	0.38007735	0.2594943
Fdxr	-0.3392328	1.38721774	0.80560487	0.38014876	0.25951943
Pnoc	0.67018119	-0.2246849	0.805198	0.38026675	0.25957635
Fzd8	0.39010788	1.75858584	0.8049291	0.38034476	0.25960598
Exo1	-0.7165823	-0.6416641	0.80468307	0.38041616	0.25963109
Iqcd	1.06486979	-1.7489565	0.80384459	0.38065962	0.25977362
Rfx7	-0.1202675	7.87409775	0.80307107	0.38088444	0.25988526
Plcxd1	-0.3713838	1.75679054	0.80298155	0.38091047	0.25988526
Gm6710	0.21326299	2.44380817	0.80292415	0.38092716	0.25988526
Pxk	-0.1348606	5.46625676	0.80260425	0.3810202	0.25990621
Hist1h2bj	0.84635474	-1.5692539	0.80258035	0.38102716	0.25990621
Ehhadh	0.38249759	1.10449656	0.80180444	0.38125298	0.26000214
Bok	0.18878101	4.24152508	0.80178219	0.38125947	0.26000214
6430584L05F	0.23847651	3.02541422	0.80173991	0.38127178	0.26000214
Tef	-0.0951831	6.79325453	0.80129553	0.38140121	0.26006676
Gm4349	-0.4906659	0.61440801	0.80068289	0.38157976	0.26016486
Ankrd34b	0.21111388	4.5032415	0.80054029	0.38162134	0.26016956
Rab7l1	0.37864528	4.22114935	0.80005126	0.38176398	0.26024315
2810454H06I	0.76638565	-0.9132212	0.79981457	0.38183304	0.26026658
Gm10941	0.87431054	-1.0465899	0.79943286	0.38194446	0.26029887
Banp	0.16709331	4.65768493	0.79941451	0.38194982	0.26029887
Klhl20	0.1538767	5.71641113	0.79894152	0.38208795	0.26036935
Rela	0.17907289	4.25629368	0.79880808	0.38212693	0.26037227
Chchd1	0.20834218	3.59794453	0.79858184	0.38219304	0.26037495
Gdf10	0.42153786	2.35877311	0.798557	0.3822003	0.26037495
Fntb	0.17822424	4.03066677	0.79820052	0.3823045	0.26039101
Clstn2	0.16758818	6.26457341	0.79803658	0.38235244	0.26039101
Hsd3b1	-2.2481981	-1.4652277	0.79802112	0.38235696	0.26039101
S100a4	0.40450689	3.08503307	0.79800142	0.38236272	0.26039101
Trip13	0.6770491	0.72257965	0.79763178	0.38247083	0.26044099
Zfp934	0.20818871	3.61369777	0.79688778	0.38268858	0.26056561
Slc1a6	-0.4747222	0.37276094	0.79546472	0.38310559	0.26082587
Trmt10b	0.18222037	3.70171068	0.79469808	0.38333052	0.26095533
Rab4a	0.13585139	4.98587144	0.7943861	0.38342211	0.26099399
Golga5	0.16754874	4.18905184	0.79426107	0.38345883	0.2609953
2610316D01I	0.33145801	2.20637587	0.7937869	0.38359812	0.26105463
Rasa4	0.75779037	-0.4240529	0.79362745	0.38364497	0.26105463
Pten	0.10398773	9.22052376	0.79355025	0.38366766	0.26105463

Ccdc184	0.18696278	3.20706414	0.7934148	0.38370747	0.26105463
Dbpht2	0.12789169	7.6516806	0.79337218	0.38372	0.26105463
Rad17	0.17787035	4.17621479	0.79319371	0.38377247	0.26106307
Prepl	-0.1788243	7.5494485	0.79309124	0.3838026	0.26106307
Nkrf	-0.1743255	5.84414015	0.79285977	0.38387068	0.26106307
Kctd9	0.14479933	4.95379289	0.79283813	0.38387704	0.26106307
Uevld	-0.1393377	5.20567585	0.79273823	0.38390643	0.26106307
Eva1a	0.4579257	1.13258846	0.79196935	0.38413272	0.2611828
Sec11a	0.23428105	4.0758586	0.79190344	0.38415213	0.2611828
Zmiz2	-0.1186538	6.4725955	0.79144151	0.38428818	0.26125162
Lins	-0.213452	3.94188219	0.79066105	0.38451822	0.26135809
Cnga2	1.21069825	-2.1500629	0.79694401	0.38453721	0.26135809
Rbm45	0.18907936	3.49260112	0.79055559	0.38454932	0.26135809
Krt80	-0.2853915	3.54297797	0.78971532	0.38479724	0.26146646
Egr3	0.14285686	7.83055763	0.78966854	0.38481105	0.26146646
1810032O08	0.67009881	-0.4284775	0.78955898	0.38484339	0.26146646
Micu1	0.12760879	4.45383813	0.78954272	0.3848482	0.26146646
Plcxd3	0.16958615	5.01837522	0.78917128	0.38495789	0.26150646
Dyrk1a	-0.0999951	7.22479528	0.78910315	0.38497801	0.26150646
Swt1	0.17185267	4.57133182	0.78898928	0.38501165	0.26150646
Slamf7	-0.5196176	1.12128087	0.78848515	0.38516063	0.26156487
Grin2d	0.27876568	1.80761077	0.78846231	0.38516739	0.26156487
Lhfpl5	0.93198183	-0.401819	0.78810465	0.38527313	0.261613
Ak2	0.19927964	4.85332912	0.78785605	0.38534666	0.26163924
Nfatc3	0.15637628	6.91576641	0.78773477	0.38538254	0.26163992
Nr1d2	0.11594275	7.42943897	0.78733265	0.38550154	0.26169703
Lzts1	0.27734442	2.77788599	0.78586407	0.3859366	0.26194508
Ripply2	0.62969009	-0.3361722	0.78579475	0.38595716	0.26194508
Boc	-0.1716919	3.53156235	0.7857457	0.3859717	0.26194508
Ppm1l	-0.1841156	4.72174536	0.78527095	0.38611252	0.26201694
Bckdk	0.14429513	4.30818593	0.78427604	0.38640787	0.26219364
1600002K03I	-0.5555841	0.25287878	0.78399599	0.38649106	0.26222638
Galr1	1.14449992	-1.8175192	0.7835891	0.38661199	0.26224842
Tnfrsf22	0.30551698	1.74743848	0.78358724	0.38661254	0.26224842
Plekhd1	0.86350957	-1.030758	0.78346182	0.38664983	0.26224842
Ccdc112	0.22770694	4.19145441	0.78341616	0.3866634	0.26224842
Tnni1	-0.9660656	-1.0624681	0.78309439	0.38675909	0.26228961
Cyp2u1	0.39668203	0.87259289	0.7825367	0.38692502	0.26237841
Ropn1l	0.90363704	-1.0281018	0.78241327	0.38696175	0.26237961
Chad	-1.2604411	-1.747927	0.78204065	0.38707269	0.26243111
Prpsap2	0.15718346	4.20561193	0.78151184	0.38723021	0.26249638
Uri1	0.11141027	6.632009	0.78148254	0.38723895	0.26249638
4930572O13	-0.666313	0.43251936	0.78118445	0.38732779	0.26253287
Clstn1	0.13076829	8.08403304	0.78065525	0.38748558	0.2626161
Zbed6	-0.0951691	6.82936848	0.78041634	0.38755685	0.26263461

Vps26b	0.09466941	7.41283833	0.78032897	0.38758292	0.26263461
Tmem120a	0.66094566	0.1265171	0.78004656	0.38766719	0.26266799
Aldoc	0.13879198	7.18074671	0.77966067	0.38778239	0.26272231
Asap3	-0.2967619	2.87495429	0.77928382	0.38789495	0.26277483
Col8a2	-0.4043867	2.09764553	0.77903839	0.38796828	0.26279137
Cers6	-0.1797246	5.97498481	0.77896763	0.38798942	0.26279137
Btbd3	-0.1312813	8.37597415	0.77846132	0.38814077	0.26287015
Xcl1	0.89995433	-0.5193357	0.77814515	0.38823533	0.26289668
4930583P06I	-1.1006793	-1.6649089	0.77809596	0.38825004	0.26289668
Cubn	0.22652971	5.30480592	0.77763129	0.38838908	0.26296709
Otulin	0.24365342	3.54519405	0.77708838	0.38855162	0.26301154
Tmem167	0.12358345	6.42597284	0.7770806	0.38855395	0.26301154
Fmo2	0.32150601	2.18109682	0.77700948	0.38857526	0.26301154
Alkbh5	0.12776165	6.397967	0.77694359	0.38859499	0.26301154
Clcn5	-0.3438016	2.3199123	0.7767969	0.38863893	0.26301755
Adck1	0.19834529	3.05850564	0.77616914	0.38882707	0.26309857
Ifi35	0.4382568	2.04781192	0.776038	0.38886639	0.26309857
Zfp560	-0.1931097	4.12025676	0.7758584	0.38892025	0.26309857
Spryd3	-0.2230322	4.21771774	0.77579215	0.38894012	0.26309857
Zfp536	-0.1161955	4.61730141	0.77578476	0.38894233	0.26309857
Sv2a	-0.1506554	5.86958726	0.77569557	0.38896909	0.26309857
Asb15	-0.6185079	0.58360885	0.77550923	0.38902499	0.26310728
Serpina1d	1.18998791	-1.5769784	0.77538667	0.38906176	0.26310728
Sox18	0.33958673	1.74663565	0.77530195	0.38908719	0.26310728
Ermp1	-0.1291634	5.74204652	0.77501756	0.38917255	0.26314128
A230072C01I	-0.2625306	3.64761711	0.77475012	0.38925284	0.26314299
Atp1a2	-0.2025272	11.2666227	0.77462672	0.3892899	0.26314299
Trmt10c	0.16635538	4.78226766	0.77453497	0.38931746	0.26314299
Dlx4	-1.4788124	-1.9372752	0.77444516	0.38934444	0.26314299
Mxra8	0.22988139	5.68300485	0.77439508	0.38935948	0.26314299
Pmm2	0.20238141	3.40285091	0.77430827	0.38938556	0.26314299
Trpc4ap	0.12111276	5.32545809	0.77302048	0.38977276	0.26338093
E130114P18I	-0.4178661	0.83679261	0.77269265	0.38987142	0.26342387
Gmppb	0.42936529	0.94314568	0.77176108	0.39015197	0.26358969
Tmem51	0.33233745	2.21709795	0.77163446	0.39019013	0.26359173
Znhit6	0.12868662	5.17501458	0.77135701	0.39027376	0.26362448
Pqlc3	-0.1948486	3.54648936	0.77114596	0.39033739	0.26363336
Dhrs11	0.50308255	0.67137026	0.77108028	0.3903572	0.26363336
C2cd3	-0.1800292	5.22226321	0.77034531	0.39057894	0.26375937
Psmg3	0.32753123	2.00134747	0.77009896	0.3906533	0.26378584
Hsp90aa1	0.12864824	10.9285529	0.76983766	0.3907322	0.26380783
Atcay	0.1536308	5.5480024	0.76969406	0.39077557	0.26380783
Arsa	0.25721461	2.4348493	0.76964174	0.39079138	0.26380783
Spint1	-0.6509136	-0.3384106	0.76951401	0.39082996	0.26381014
Sesn2	0.31954793	2.55196552	0.76923468	0.39091436	0.26384337

Lace1	-0.2017079	3.18675385	0.7685963	0.39110736	0.26394988
Greb1	-0.5182303	0.39296859	0.76840024	0.39116666	0.26396615
Wfdc15b	-0.9759188	-2.0026989	0.76806237	0.39126888	0.26399725
Alg10b	-0.1320638	5.55240241	0.76801528	0.39128313	0.26399725
Parp6	-0.1404472	5.88867392	0.76728806	0.39150331	0.26412205
Pole2	-0.568586	1.03558652	0.76702065	0.39158432	0.26413878
Sirt5	-0.2198002	2.29635344	0.76688251	0.39162618	0.26413878
Sirt6	-0.2396792	2.94757241	0.7668575	0.39163376	0.26413878
Gls	0.14980352	9.46596455	0.76666799	0.39169119	0.26415377
Ccdc22	-0.4276102	0.91836404	0.76547711	0.3920524	0.2643736
Fam84b	-0.1976044	2.78382697	0.76533147	0.39209661	0.26437965
Tshz3	-0.1256791	6.13421341	0.76462523	0.3923111	0.26450049
Olfml2b	-0.5084763	0.63137112	0.76444454	0.392366	0.26451374
Srm	0.18135067	4.05505289	0.76404662	0.39248695	0.2645715
Apex2	-0.5524799	1.18741274	0.76385664	0.39254472	0.26458666
Mpl	-0.5124848	1.52011049	0.76321461	0.39274003	0.26469452
Cbln1	0.32777115	1.84551883	0.7626309	0.39291773	0.26479049
Fam203a	0.29173224	2.59822023	0.76248126	0.3929633	0.26479742
Neu2	0.41937426	0.65232574	0.7619213	0.39313391	0.26488859
Ptpn12	-0.134637	6.02226526	0.76124097	0.39334134	0.26499487
Mc1r	-0.9107547	-1.8869924	0.76112223	0.39337757	0.26499487
2810408I11R	-0.5778273	0.38143534	0.76087342	0.39345348	0.26499487
Apbb1ip	-0.2082752	3.19513467	0.76082322	0.3934688	0.26499487
Six2	0.22227447	5.88949627	0.76071701	0.39350121	0.26499487
Pdlim5	0.18358099	7.39530716	0.76070916	0.39350361	0.26499487
Gpr115	0.30579945	1.61554284	0.7597695	0.39379056	0.2651643
Rcor3	0.1262047	5.44193821	0.75946809	0.39388267	0.26518804
Lpgat1	-0.1225941	7.83784343	0.75942279	0.39389652	0.26518804
Ap3m2	0.11965961	6.61854869	0.75909393	0.39399706	0.26523192
Stx11	0.3774968	2.10284213	0.75877527	0.39409452	0.26525461
Brinp1	-0.1336589	6.39757904	0.75875251	0.39410148	0.26525461
Tfb1m	0.24100398	2.80023338	0.7583315	0.3942303	0.26531751
Ptdss2	0.19819491	3.24280538	0.75817344	0.39427868	0.26532626
Alkbh3	0.23260842	3.75199751	0.75732234	0.39453934	0.26547786
Lancl3	0.25565932	3.95821715	0.75665651	0.39474344	0.26558194
Mir32	-1.4128838	-1.9347568	0.76254604	0.39476484	0.26558194
Mcemp1	0.6452796	0.11998169	0.75638602	0.3948264	0.26559575
Slc27a4	-0.1939107	3.48703901	0.75628897	0.39485617	0.26559575
Zfp667	0.204465	4.79433515	0.75589032	0.3949785	0.26565421
Nmbr	-0.5924638	0.19861684	0.75523753	0.39517894	0.26576519
Tmem51os1	0.66423962	-0.5113356	0.75493369	0.39527229	0.26579448
Slc14a1	0.25088511	2.67003188	0.7548651	0.39529336	0.26579448
Capn11	-1.015831	0.03771976	0.75401181	0.3955557	0.26592375
Casd1	-0.1321317	5.80115426	0.75400921	0.39555651	0.26592375
Stard6	-0.2623997	2.41474258	0.75364223	0.39566941	0.26595417

Srrm3	0.23802717	2.95902668	0.75363167	0.39567266	0.26595417
Cited2	-0.1299387	6.39891047	0.75346611	0.39572362	0.26596458
Smad9	0.13660202	5.40048518	0.75314283	0.39582314	0.26599473
Havcr2	0.41164017	1.70023594	0.75309005	0.39583939	0.26599473
Vps53	0.11004712	6.21974496	0.75259262	0.39599262	0.26605591
Fam104a	0.17936044	5.23095913	0.75256419	0.39600138	0.26605591
Ankrd9	0.46484325	0.40616197	0.75206514	0.39615519	0.26611537
Pla2g2d	0.64255498	-0.6830274	0.75204688	0.39616082	0.26611537
Nme3	0.20048972	3.44429198	0.75187173	0.39621482	0.26612781
Zbtb12	0.49944992	-0.2350096	0.75171648	0.39626271	0.26613614
Myo1c	0.19390713	5.19518825	0.75140177	0.39635979	0.26617751
Rnf182	-0.2824722	3.01037818	0.75110752	0.3964506	0.26621466
Smim22	-0.8980406	-0.4654635	0.7507545	0.39655958	0.26626401
Mcc	-0.1294398	6.54266634	0.75057827	0.396614	0.26627671
Dxo	0.22554745	2.09703573	0.74926811	0.39701894	0.26652473
Smarca4	-0.1590843	7.01594341	0.74908989	0.39707407	0.26653788
Farsb	0.11587043	5.68963609	0.74815107	0.39736469	0.26667479
Ccnd1	0.16783283	6.16639767	0.74810301	0.39737957	0.26667479
Cldn8	1.35117685	-2.2535441	0.74808652	0.39738468	0.26667479
Slc22a6	-0.245978	7.15639669	0.74795792	0.39742452	0.26667767
Snx14	-0.1440847	5.44398163	0.74733823	0.39761656	0.26678267
Rdh9	-0.5511987	0.40008975	0.74680131	0.39778307	0.26687052
Prokr1	-0.9411387	-0.1820214	0.74659591	0.3978468	0.2668894
Ttc22	-1.2623972	-1.6064773	0.74642413	0.3979001	0.26690129
Dyrk2	0.24895148	3.24863198	0.74587289	0.39807124	0.26699221
Kif3a	-0.1454261	8.10931872	0.74560673	0.39815391	0.26700878
Nfkb2	0.58533852	0.95481219	0.74556414	0.39816714	0.26700878
Prss16	1.008561	-1.0446287	0.74465093	0.39845099	0.26717525
Nsun4	0.18242018	2.77881446	0.74435689	0.39854246	0.26721269
Chst1	-0.1362897	5.89299389	0.74359431	0.39877981	0.26732748
Agmo	-0.4281902	1.67511286	0.74357784	0.39878494	0.26732748
Sprn	0.15048762	5.91404279	0.743344	0.39885776	0.26735241
Clrn1	-0.9543314	-1.0221017	0.74259562	0.39909097	0.2674625
Fam136a	0.16272248	3.84049672	0.74258807	0.39909333	0.2674625
Filip1	-0.2062564	3.97005883	0.74221251	0.39921043	0.26751709
Cxadr	0.1956328	6.56603292	0.74176996	0.3993485	0.2675857
Dgat2	0.1844678	3.75689684	0.74156707	0.39941182	0.26760423
Gm11201	-1.0405423	-0.2656289	0.74109321	0.39955977	0.26767944
Sspn	-0.2484601	4.45629155	0.74095829	0.39960191	0.26768377
Pou3f1	-0.246418	2.01277333	0.74009037	0.39987315	0.26784155
Cep57	-0.1412093	4.61047186	0.73959777	0.40002722	0.26792082
Mthfd2	-0.3881306	1.46340259	0.73922398	0.40014419	0.2679541
Mybpc1	0.39264677	0.97446557	0.73909323	0.40018512	0.2679541
Thsd1	-0.6187969	0.40548521	0.73902152	0.40020757	0.2679541
Ankrd33b	-0.1142476	6.30615254	0.73898246	0.4002198	0.2679541

Actr3	0.08780488	8.04242918	0.73883292	0.40026662	0.26796154
Dtx3l	-0.127397	4.87767254	0.73855265	0.4003544	0.26799638
Slc15a4	-0.2602752	2.35216293	0.73802234	0.40052057	0.26808369
Ttc30a1	0.4128906	1.65587813	0.73734734	0.40073223	0.26815003
Urgcp	-0.162035	4.39058297	0.7373263	0.40073883	0.26815003
Emb	0.24639436	6.18088226	0.73725377	0.40076158	0.26815003
St3gal2	0.11012216	5.92266657	0.73725033	0.40076266	0.26815003
Ethe1	0.28830298	2.6815575	0.73706526	0.40082073	0.26816496
Morc1	-1.403036	-1.4927498	0.73690131	0.40087218	0.26817547
Podn	0.20041267	5.0364247	0.7366963	0.40093653	0.26817816
Ptcd2	0.16201857	4.69005806	0.7366607	0.40094771	0.26817816
Enpp4	-0.1701544	4.28764148	0.73631692	0.40105566	0.26822645
Ece2	-0.2214589	2.54732572	0.73605033	0.4011394	0.26825711
Sfxn1	0.11088617	5.91828744	0.73586628	0.40119724	0.26825711
Stard3nl	0.14715956	4.46531517	0.73582952	0.40120879	0.26825711
F11r	-0.2585174	2.71136474	0.73533775	0.40136338	0.26833655
Ptplad1	0.09998492	6.68911998	0.73449074	0.40162985	0.26849064
Gpatch2	0.13639264	4.12318121	0.73437764	0.40166545	0.26849064
Tgif2	0.30396297	2.2958135	0.733281	0.4020109	0.26869761
C8g	0.61948455	0.37226714	0.7331057	0.40206616	0.26871061
2310009A05I	-0.281918	2.69505631	0.73265266	0.40220903	0.26878214
Toporsos	0.3116158	1.98353634	0.73240997	0.4022856	0.26880936
Lrrn3	-0.1142815	5.76530635	0.73128031	0.40264229	0.26902374
Polr2i	0.25173668	3.0562357	0.73101621	0.40272575	0.26905506
Tnfsf15	1.30610939	-1.6671084	0.73090501	0.4027609	0.26905506
2510009E07I	-0.1159876	6.8544813	0.73039981	0.40292064	0.2691378
Prkar2a	0.11057814	6.40143471	0.73023197	0.40297373	0.2691493
Fndc9	0.31258634	2.94928762	0.72955175	0.40318901	0.26925891
Ifngr1	0.21261357	3.18392937	0.72948664	0.40320963	0.26925891
Rbm14	0.25555409	3.06542224	0.72902828	0.4033548	0.26931602
Axin1	0.19709033	3.37018017	0.72896668	0.40337432	0.26931602
Vamp1	0.11168575	6.0370303	0.72882308	0.40341982	0.26931602
Arrb1	-0.1190434	7.40032448	0.72861059	0.40348716	0.26931602
Snta1	0.23157499	2.78513284	0.7284644	0.4035335	0.26931602
Tnfsf12	0.20761634	3.32585799	0.72843984	0.40354129	0.26931602
Ifih1	0.13729937	5.32452961	0.7284235	0.40354647	0.26931602
D2hgdh	-0.1579812	4.1262795	0.72818686	0.40362151	0.2693206
2010315B03I	0.15376946	4.22427496	0.72813368	0.40363837	0.2693206
Mtdh	-0.1110734	6.76294372	0.7280622	0.40366104	0.2693206
Ston2	-0.1943184	4.6411271	0.72783769	0.40373226	0.26934416
Syt3	0.21461681	3.66352254	0.72769961	0.40377607	0.26934943
Extl3	0.11360887	6.75161783	0.72758137	0.4038136	0.26935051
Tacr1	-0.1998588	3.12697226	0.72713984	0.40395375	0.26940214
Gjb1	-0.6750373	-0.6787146	0.72711125	0.40396283	0.26940214
R3hdm1	-0.1544735	9.58790115	0.72692525	0.4040219	0.26941468

Htr6	0.91592162	-1.4043342	0.72676243	0.40407362	0.26941468
Nrxn1	-0.1458265	9.77366721	0.72671283	0.40408938	0.26941468
Zfp712	-0.1742828	3.87900977	0.72623522	0.40424115	0.26949192
2900056M2C	0.15449539	7.95961071	0.72585908	0.40436074	0.26954769
Rrn3	-0.1023037	5.81564163	0.72549461	0.40447667	0.26960101
Tab1	0.23924678	2.84188839	0.72469592	0.4047309	0.26974649
Stag1	0.10068755	7.40633933	0.72430456	0.40485556	0.26975993
Gm5803	-0.6638485	-0.6594237	0.72426327	0.40486871	0.26975993
Bri3	0.17231376	4.3199298	0.72417536	0.40489673	0.26975993
Csrp1	0.20290824	7.56769806	0.72413045	0.40491104	0.26975993
Mphosph8	0.12908717	8.41146248	0.72406822	0.40493087	0.26975993
Blvrb	0.34977174	1.2397928	0.72328909	0.40517929	0.26989853
Arl6ip6	-0.1544983	4.59308749	0.72295146	0.40528701	0.26989853
N4bp3	0.4077015	1.3835988	0.72286909	0.4053133	0.26989853
Bicd1	-0.1297136	6.59984116	0.72280407	0.40533405	0.26989853
Mipol1	0.17314707	3.67320862	0.72274615	0.40535254	0.26989853
Ctsf	-0.27651	3.07466741	0.72273906	0.4053548	0.26989853
Aff2	0.16964062	5.87645456	0.72194863	0.40560723	0.27002776
Vps52	-0.1349219	4.55164951	0.7219059	0.40562088	0.27002776
Gm8801	0.46334882	-0.2353467	0.72173862	0.40567433	0.27003938
Lepre1	-0.2340487	2.22410944	0.72155449	0.40573319	0.27005459
Nhej1	0.61701935	1.11955873	0.72083185	0.40596428	0.27016187
Ccar1	0.11202108	8.06170419	0.72082525	0.40596639	0.27016187
Zw10	-0.3385337	2.49338319	0.72050383	0.40606925	0.27017353
Tbxa2r	1.09709523	-2.105866	0.72037124	0.40611169	0.27017353
Agtr1a	-1.2390395	-1.2617721	0.72029196	0.40613707	0.27017353
Mxi1	0.11120607	6.32122359	0.72014753	0.40618331	0.27017353
Xpnpep3	0.14819809	4.78115851	0.7200692	0.40620839	0.27017353
Lamp2	0.1731493	7.76757349	0.72001531	0.40622565	0.27017353
Bscl2	0.18404359	4.33923054	0.71998289	0.40623603	0.27017353
Rpusd2	0.14846076	4.33296508	0.71889383	0.40658504	0.27038167
Psmb8	0.31974943	2.65757263	0.7186035	0.40667816	0.27041962
Dnah8	-0.3997138	1.34852445	0.71827614	0.4067832	0.27046549
Pax6	0.26253439	2.24170717	0.71793714	0.40689201	0.27048869
Alkbh8	0.17783594	4.91475563	0.71788116	0.40690998	0.27048869
Gm10033	-0.2181478	4.59439636	0.71783042	0.40692627	0.27048869
Uqcrc2	0.08644745	6.7286354	0.71765691	0.40698199	0.27050176
Meox1	-0.7942837	-1.373733	0.71706139	0.40717332	0.27060495
Golga3	0.08712657	6.79705468	0.71665362	0.4073044	0.27064027
Dnase1l1	0.28472683	2.18178417	0.71664281	0.40730788	0.27064027
Zfp639	0.19968151	4.46191698	0.71649357	0.40735587	0.27064027
Lrif1	0.15729355	5.127569	0.7164472	0.40737078	0.27064027
Mex3a	0.2360207	2.55651255	0.71573022	0.40760148	0.27076955
Socs4	0.13466056	5.019161	0.71550986	0.40767242	0.2707927
Mesdc1	0.14193639	4.04770127	0.71494617	0.40785399	0.27088932

Cluap1	-0.1083733	5.50407573	0.71456346	0.40797733	0.27094725
D430042O09	-0.2072881	3.42769263	0.71422398	0.40808678	0.27099595
Nckipsd	0.17926721	4.12030892	0.7136978	0.40825652	0.27108467
Gm13251	0.27048198	2.15003669	0.71349921	0.40832062	0.271103
Fam161b	-0.1944277	3.87855137	0.71334023	0.40837194	0.271103
Xiap	-0.1217444	7.12351358	0.71325957	0.40839798	0.271103
Rad18	0.21011425	4.2203029	0.71316441	0.4084287	0.271103
Egln2	-0.2034988	4.09706298	0.71299284	0.40848411	0.27111579
Cenpv	0.18612915	2.83724603	0.71173322	0.40889123	0.27134021
Pcdh1	0.14050536	7.22796233	0.71172286	0.40889458	0.27134021
4930577N17	0.41904608	0.10867021	0.71147681	0.40897419	0.27136903
Kcnab2	-0.1536526	5.95193359	0.71129825	0.40903196	0.27138336
4930426D05	-0.8830125	-0.7480271	0.71045997	0.4093034	0.2714947
Wrn	-0.1087708	5.66555382	0.71045138	0.40930618	0.2714947
Crabp2	0.28100914	6.22136604	0.71044463	0.40930837	0.2714947
Hdac4	-0.1654146	5.07433914	0.71024961	0.40937155	0.27151261
Vpreb3	1.05066396	-1.2330033	0.70987646	0.4094925	0.27156881
Lrrn1	-0.1623184	6.20171724	0.70961596	0.40957696	0.27159686
Kti12	0.2857914	2.3447234	0.70952273	0.4096072	0.27159686
2700069I18R	0.39449841	0.80104733	0.70907187	0.40975346	0.27166983
Psmd1	-0.0886914	7.50243881	0.70887771	0.40981648	0.27168759
Npy2r	0.24310762	2.84536466	0.70863638	0.40989482	0.27171551
Orc5	-0.1936649	3.3294651	0.70771	0.41019576	0.27189098
Ccdc33	0.50916163	0.10981215	0.70755533	0.41024604	0.27190028
Odf2	-0.1622828	5.56183303	0.70665715	0.4105382	0.27203071
Slc7a11	0.17449519	9.83007909	0.70661767	0.41055105	0.27203071
Ccdc74a	0.2424338	2.01806722	0.70654746	0.4105739	0.27203071
Wasf1	-0.1487822	7.99226552	0.70650446	0.4105879	0.27203071
Ncr1	0.64132384	-0.1231929	0.70607829	0.41072666	0.27209861
Ackr4	-1.1011808	-1.4397916	0.70584523	0.41080257	0.27212487
Hadha	0.12370433	4.94884333	0.70542383	0.41093989	0.2721918
Tom1l1	-0.1685576	3.91602837	0.70479586	0.41114465	0.27230338
Sel1l	-0.121658	7.13736227	0.70391808	0.41143113	0.27244899
Crim1	-0.1550675	6.98334758	0.70389511	0.41143863	0.27244899
Il21r	-0.8279724	-0.8992308	0.70378845	0.41147347	0.27244899
Zfp689	-0.3193589	1.65049291	0.70220017	0.41199269	0.27276871
Kcns1	-0.2955338	1.60185337	0.70196621	0.41206926	0.27279533
Rnf167	0.2005387	4.18757965	0.70184416	0.41210921	0.2727977
Tubgcp5	-0.2108034	4.13761734	0.70161797	0.41218327	0.27282265
Serpinb10	-0.6218569	-0.6672712	0.70118713	0.41232439	0.27287086
Lrrc14	-0.2194475	2.72830869	0.70117348	0.41232886	0.27287086
1700124L16R	-1.0193436	-1.713115	0.70042053	0.41257567	0.27299455
Cd38	-0.2206564	2.81277897	0.70027154	0.41262453	0.27299455
Il5ra	-0.9197411	-0.831976	0.7002095	0.41264488	0.27299455
Mrps22	0.1213235	4.4030088	0.7001593	0.41266135	0.27299455

Ubxn2b	0.12269279	6.02215154	0.6995828	0.41285053	0.27303216
Slc24a3	0.13663317	6.88660705	0.69941576	0.41290537	0.27303216
Ncapg2	-0.2620348	2.56099685	0.69934212	0.41292955	0.27303216
Cdkn2aip	0.1531122	4.20203269	0.69931701	0.4129378	0.27303216
Ndufaf6	-0.2844436	1.59740847	0.69908881	0.41301274	0.27303216
B930003M22	-0.6000849	-0.1766797	0.69906012	0.41302217	0.27303216
Taf7	0.16732896	3.82423093	0.69894721	0.41305926	0.27303216
Kcnk13	0.39104991	0.83313891	0.69893557	0.41306308	0.27303216
Tpcn1	0.22464339	5.97215689	0.69883968	0.41309459	0.27303216
Anxa9	0.60864325	-0.5916334	0.69881703	0.41310203	0.27303216
Cks2	0.4425257	0.30209845	0.69876667	0.41311858	0.27303216
Gcc1	0.14224358	4.93927588	0.69834306	0.41325781	0.27310012
Nbr1	0.11280697	7.89776255	0.6982016	0.41330433	0.2731068
Imp3	0.18292516	4.80297023	0.69785838	0.41341721	0.27315733
S100a13	0.35080269	3.41422825	0.69758766	0.41350628	0.27317468
Mob1b	0.11714244	5.1918482	0.69747026	0.41354492	0.27317468
Setd7	0.10945761	8.66298225	0.69744654	0.41355273	0.27317468
Zfp664	0.12079046	7.69577042	0.69681609	0.41376031	0.27328774
Mapk1ip1	0.13191735	4.40151255	0.69647263	0.41387346	0.27333841
Ppp1r18	0.24221956	3.13725075	0.69620632	0.41396123	0.27336772
Swap70	0.12889274	4.83364218	0.69611684	0.41399073	0.27336772
C430049B03I	0.39113964	1.132541	0.69563272	0.41415038	0.27344907
Lingo2	-0.1544595	5.23399232	0.6946878	0.41446225	0.2736309
Slc38a2	0.22565459	9.37180083	0.69388381	0.4147279	0.27378219
Vmp1	-0.1241582	5.58892093	0.69365122	0.4148048	0.27380886
Slc6a14	-1.1637732	-1.0287029	0.69342903	0.41487828	0.27383326
Chn1os3	-0.6244613	-0.0760087	0.69318523	0.41495893	0.27384802
Otx1	0.25267843	2.08237612	0.69308222	0.41499301	0.27384802
Atg4d	-0.299655	2.16866913	0.69303042	0.41501015	0.27384802
Pam	-0.1211953	6.3274475	0.69275302	0.41510197	0.27386331
Glra4	-1.1181131	-1.6182774	0.692638	0.41514005	0.27386331
2810049E08I	-0.5946518	0.86852892	0.69262951	0.41514286	0.27386331
Tert	0.65056453	-0.679246	0.69222221	0.41527774	0.27389686
Pank1	-0.1100297	6.29469563	0.69218815	0.41528902	0.27389686
Zfpm1	0.2515924	1.76314135	0.69214521	0.41530325	0.27389686
Hvcn1	-0.584202	-0.3066528	0.69123364	0.4156054	0.27405722
Cyb5rl	-0.8326819	-0.4919336	0.69119124	0.41561947	0.27405722
Ddx19a	0.14613052	5.4407088	0.69066596	0.41579374	0.2741248
5830432E09I	0.79554635	-1.1119705	0.69066204	0.41579504	0.2741248
Pik3cb	-0.1303266	5.5200715	0.69033044	0.41590512	0.27417327
Kif24	-0.4518686	1.04121101	0.69018114	0.41595469	0.27418186
Naga	0.3368229	3.0896614	0.68998277	0.41602058	0.27420119
S100a9	0.67940046	0.04202695	0.68938564	0.416219	0.27430787
Zfp395	0.16747277	6.06510323	0.68909922	0.41631422	0.27434652
Map3k3	-0.1845919	3.27514667	0.68879773	0.41641449	0.27438586

Jrkl	0.16713726	3.30938128	0.68839846	0.41654734	0.27438586
Irgq	0.11322072	7.40639249	0.68835705	0.41656112	0.27438586
Exoc8	-0.1318219	4.65866988	0.6881005	0.41664652	0.27438586
Cacnb2	-0.1147141	6.33615313	0.68808125	0.41665293	0.27438586
Tnfrsf11a	-0.241613	2.63779918	0.68803168	0.41666944	0.27438586
P4ha3	0.34372833	2.42550254	0.68799096	0.416683	0.27438586
Wars2	-0.2307688	3.67473032	0.68794663	0.41669776	0.27438586
Tor1a	0.21462563	2.96947977	0.68793053	0.41670312	0.27438586
Dio3	0.74221423	-0.9980248	0.68696372	0.41702526	0.27457388
Gm10814	0.62308829	0.76249945	0.68677053	0.41708968	0.27458197
Myh15	-0.9764956	-1.1483054	0.68670733	0.41711076	0.27458197
2810417H13I	-0.5240022	0.64624972	0.68634254	0.41723245	0.27461995
Ndst4	0.2583181	3.24257886	0.68628651	0.41725114	0.27461995
Klrb1b	-0.8487701	0.72419676	0.68620514	0.41727829	0.27461995
Mbnl1	0.12618784	9.06154523	0.68602731	0.41733765	0.27463492
Alyref2	-0.2535748	1.75103512	0.68586658	0.4173913	0.27464613
Htra2	0.33768658	1.29477846	0.68536632	0.41755837	0.27471486
Acsm3	-0.7615472	-0.887876	0.6852604	0.41759375	0.27471486
Srpx	0.70777565	0.14940974	0.68522488	0.41760562	0.27471486
Cdyl	0.1334539	4.10914746	0.68496664	0.41769192	0.27474754
Prpf4b	0.11046827	7.35039184	0.68444548	0.41786616	0.27482677
Slc22a21	0.63368049	0.03731727	0.68438723	0.41788564	0.27482677
Mei4	0.51809558	0.42421138	0.68335321	0.41823171	0.27503025
Lif	-0.6900465	-0.9504071	0.68298263	0.41835585	0.27506634
Atp6v0c-ps2	0.48732016	-0.3216039	0.68297046	0.41835992	0.27506634
Peli2	0.13417326	5.70732848	0.68262867	0.41847447	0.27508163
Sap30bp	-0.1726072	3.65865418	0.68257335	0.41849301	0.27508163
Cald1	0.15784345	10.2790295	0.68257278	0.4184932	0.27508163
Cilp	0.66247753	-0.1917374	0.6821896	0.41862168	0.27510837
5031434O11	-0.8220637	0.19315469	0.68215651	0.41863278	0.27510837
Gm996	-0.1694736	4.57695993	0.68212333	0.41864391	0.27510837
Gm1141	1.42526592	-1.6419073	0.68175872	0.41876623	0.27516465
Morn1	-0.4744897	0.458594	0.68095242	0.41903693	0.27529917
G630025P09	-1.1282845	-1.6225258	0.68088612	0.4190592	0.27529917
Dnajb14	0.13662153	4.2317679	0.68082104	0.41908106	0.27529917
Rnft1	-0.151609	4.55113215	0.68013147	0.41931282	0.27540995
Tmem141	0.26697742	1.88506136	0.67999446	0.41935889	0.27540995
L3mbtl4	-0.4816041	0.00633733	0.67995543	0.41937201	0.27540995
Mir1954	-1.4761386	-1.8444918	0.68498316	0.41940356	0.27540995
Slc25a46	0.10161737	7.86801122	0.67977331	0.41943327	0.27540995
Whamm	-0.2678406	3.09783913	0.67917303	0.41963527	0.27547491
2810025M15	0.22760225	2.41788734	0.67917005	0.41963628	0.27547491
Srek1ip1	0.1313178	6.02457811	0.67911277	0.41965556	0.27547491
Plod2	-0.2409934	3.8469129	0.67904289	0.41967909	0.27547491
Rtcb	-0.1454534	4.1718067	0.67856472	0.41984013	0.27553667

Vmn2r1	0.98369833	-1.1488416	0.67839767	0.41989642	0.27553667
Sema3a	-0.1467295	6.40552977	0.67831456	0.41992442	0.27553667
Tada3	-0.1368212	5.0122739	0.67828378	0.4199348	0.27553667
Arap1	-0.2038125	3.70806444	0.67821834	0.41995685	0.27553667
Ciart	0.22161354	3.12972536	0.67798313	0.42003614	0.27556459
Lama1	-0.1956551	3.50150878	0.67768085	0.42013807	0.27560736
Zfp932	-0.1827982	4.46617735	0.67667566	0.42047729	0.27580577
Csf1r	-0.1815424	3.45706931	0.67644008	0.42055686	0.27583384
Nr2c2	-0.0834971	7.33174205	0.67625604	0.42061903	0.2758505
Gusb	-0.2138588	2.80361293	0.67534744	0.42092619	0.27602781
Ly86	-0.3177577	2.08681599	0.6750987	0.42101034	0.27605885
Epha8	-0.4503014	0.67208975	0.6742801	0.42128745	0.27620513
Anapc2	-0.1100218	4.74039092	0.67416435	0.42132666	0.27620513
Slc9a3r2	0.20669308	5.10276259	0.67402017	0.42137551	0.27620513
Atxn3	0.11896914	5.32775843	0.67393154	0.42140554	0.27620513
Zdhhc12	0.5030204	0.66138768	0.67389618	0.42141752	0.27620513
Icosl	0.31529119	1.45258805	0.67354883	0.42153525	0.27625041
Cnpy3	0.20433957	3.44903708	0.67335594	0.42160065	0.27625041
Atrn	-0.1299041	7.87994801	0.67329728	0.42162054	0.27625041
Pigo	-0.3021062	1.91965557	0.67325783	0.42163392	0.27625041
Leprotl1	0.09979753	6.11791754	0.67312325	0.42167956	0.27625619
Slit3	0.17877363	4.60546321	0.67283758	0.42177647	0.27629555
Unc5a	0.15451226	4.9158948	0.6724706	0.42190102	0.276353
Ivns1abp	0.10654536	7.54592213	0.67215912	0.42200678	0.27639814
Phf20	0.10965132	6.49914567	0.67201055	0.42205724	0.27640705
Pdss2	0.16669822	3.1149329	0.67113926	0.42235334	0.27657202
Hmces	-0.242854	2.01871036	0.6710524	0.42238288	0.27657202
Fopnl	0.15405932	4.94606246	0.67037441	0.42261354	0.27669891
Ston1	0.26641757	4.79793078	0.67023724	0.42266023	0.27670532
Rasl11a	-0.5616519	0.00434263	0.6699133	0.42277053	0.27675338
A930009A15	-1.0960243	-1.9983995	0.66963426	0.42286558	0.27679144
Rap1gap2	0.17910482	6.77560268	0.66940275	0.42294445	0.27681892
Hs3st4	0.12952115	5.45313509	0.66894566	0.42310026	0.27689674
Smim19	0.16997671	4.67526929	0.66871243	0.4231798	0.27692463
Exoc1	-0.1678732	5.66903012	0.6685671	0.42322937	0.27692678
Mis18a	-0.2320402	1.95977578	0.66848637	0.42325691	0.27692678
Arhgap27os3	-0.9947625	-1.6423363	0.66819057	0.42335785	0.27696866
Mcm9	-0.319455	2.0178278	0.66793406	0.42344541	0.27700178
Psmc3ip	-0.3399902	2.02747276	0.66736877	0.42363847	0.27710391
Ptger1	0.17656387	3.11132064	0.66723565	0.42368395	0.2771095
Tm4sf1	0.17439687	4.66416052	0.66688102	0.42380516	0.2771646
Dok4	-0.3131618	2.3734439	0.66665584	0.42388215	0.27719079
Psmd14	0.09712721	6.24716733	0.66628557	0.42400879	0.27724943
Nkain2	0.15248189	6.31843164	0.6657294	0.42419913	0.27734972
Tnpo2	0.10143199	5.98827346	0.66543459	0.42430008	0.27739154

Crlf3	0.23845737	3.11838493	0.6652248	0.42437193	0.27741434
Aass	-0.5264138	0.83297659	0.6645701	0.42459631	0.27751707
Cyp2j6	-0.1623102	4.00812163	0.66455034	0.42460308	0.27751707
Grhpr	0.29107078	2.24002637	0.66437911	0.42466179	0.27753127
Akna	0.17273544	3.34321702	0.66424476	0.42470787	0.2775372
Pigw	0.34229632	1.51908733	0.66397224	0.42480136	0.27755361
Mus81	0.23517026	2.15460591	0.66395583	0.42480699	0.27755361
Obfc1	0.2115501	2.73504634	0.66367024	0.424905	0.27756932
Bcl6	-0.0825923	6.10404496	0.66367012	0.42490504	0.27756932
Pla1a	0.5094958	0.99689346	0.6632252	0.4250578	0.2776339
Mylk	0.1466629	6.02107429	0.6631652	0.42507841	0.2776339
Arhgef16	0.8830964	-0.9134374	0.66305887	0.42511493	0.2776339
Dnah2	-0.326053	1.78466754	0.66291419	0.42516463	0.27764218
Krt25	-1.2161207	-1.5438074	0.66266054	0.42525179	0.27765246
Tspan14	-0.1464009	3.50768409	0.66265297	0.42525439	0.27765246
Mok	0.22868979	2.36552259	0.66216658	0.4254216	0.27773746
Xist	-0.625197	0.81075274	0.66196317	0.42549157	0.27774233
Prpf6	-0.1169688	5.5425876	0.6619296	0.42550311	0.27774233
Lmf2	0.21904212	2.46143487	0.66122758	0.42574471	0.27787585
Itih5	-0.1480462	6.2904773	0.66073521	0.42591429	0.27795705
Ankrd17	-0.1145139	8.9309109	0.66065122	0.42594323	0.27795705
Fancd2	0.30280844	1.26540415	0.6604494	0.42601277	0.27797825
Pou2f3	0.47904867	1.17603717	0.66017566	0.42610713	0.27801563
Lrrc56	-0.518372	0.36716053	0.65985794	0.42621669	0.27806293
Maml1	-0.1261268	5.19911972	0.65968919	0.4262749	0.27807672
Lcat	0.2872002	2.85756413	0.65920788	0.42644099	0.27816088
Tmem241	0.20634027	3.00809008	0.6590114	0.42650882	0.27818093
4833427F10I	-0.6217502	-0.7880059	0.65866869	0.42662717	0.27823393
Ino80b	0.51123675	-0.0544744	0.65839361	0.4267222	0.27827172
Trim41	-0.1180453	4.34501675	0.65735519	0.42708125	0.27846148
Nxpe2	-0.4478024	1.13167238	0.6573373	0.42708744	0.27846148
Col7a1	-0.994842	-1.240098	0.65661636	0.427337	0.27859998
Hyi	-0.3227435	1.56627172	0.65632739	0.4274371	0.27861921
Gskip	0.15959901	4.39527717	0.65631675	0.42744078	0.27861921
Ttk	0.73465942	-0.5704396	0.65491293	0.42792757	0.27891227
Pigf	-0.3009668	1.56171039	0.65451482	0.42806578	0.27897811
Clec2d	0.30796606	2.1306809	0.6540957	0.42821135	0.27904874
Cd63	0.27664021	4.98247981	0.65398608	0.42824944	0.27904932
Mapkapk3	0.37921161	2.24862019	0.65367363	0.42835803	0.27909584
Mfsd2b	-0.8375596	-0.5072159	0.65306372	0.42857013	0.27920978
Hsf1	0.11809422	5.47374936	0.65287502	0.42863578	0.27922293
Htr7	-0.2060648	3.40319101	0.65272149	0.42868921	0.27922293
Arhgef1	-0.1741679	4.30203653	0.65268477	0.42870199	0.27922293
Raph1	-0.1750793	7.73997908	0.65229692	0.42883702	0.27928663
Triap1	-0.2994909	3.20084066	0.65210811	0.42890278	0.2793052

Igfbp3	0.25568234	3.83518722	0.65160334	0.42907865	0.27939548
Zfp235	-0.2087918	3.30221274	0.65107064	0.42926438	0.27949215
Rassf7	0.57246306	0.14261191	0.65094652	0.42930767	0.27949608
Rxrg	-0.3157901	0.91500745	0.65024076	0.42955397	0.27963216
Paox	-0.4255585	0.96696285	0.65003097	0.42962723	0.27965558
Zfand4	-0.3689136	1.73462481	0.64962224	0.42977001	0.27970421
Zscan12	0.14201266	4.26561548	0.64960363	0.42977651	0.27970421
Flot2	0.11897815	4.8676707	0.64939786	0.42984842	0.27972674
Batf	-0.5903803	0.02424304	0.64925543	0.42989821	0.27972987
Phtf1	-0.1026374	5.56154343	0.64917077	0.42992781	0.27972987
Dgkd	-0.1582012	5.44293754	0.64880714	0.43005497	0.27975641
Afap1l1	-0.1617149	5.54664804	0.64878084	0.43006417	0.27975641
Tmem198	0.30466866	2.00437496	0.64873421	0.43008048	0.27975641
Nusap1	-0.3224137	2.16744972	0.64847764	0.43017024	0.27979054
Pex5	-0.1541522	3.90300021	0.64809314	0.43030482	0.27983502
Ccdc79	-0.448822	0.91589966	0.64806911	0.43031324	0.27983502
Ift43	0.26045258	3.32495798	0.64787407	0.43038153	0.27985517
Mmp12	0.9060134	-1.5258281	0.6473152	0.43057732	0.27994029
Gm10432	-0.3916614	1.42658681	0.64723374	0.43060587	0.27994029
Trim26	0.14042713	4.99294878	0.64718088	0.4306244	0.27994029
Gid4	-0.1003159	5.84590795	0.64648499	0.43086842	0.28007465
Ints5	0.22789923	3.12011438	0.64617249	0.43097808	0.28012166
Osgep	0.15943564	3.93937832	0.6459974	0.43103953	0.28013733
Tead1	-0.1267133	7.27776457	0.64578321	0.43111473	0.28016193
Fn1	0.21600753	8.00392047	0.6454123	0.43124501	0.28018607
Cd53	0.21238471	2.34394497	0.64536681	0.43126099	0.28018607
Serpinb1a	-0.3430512	2.21914967	0.6453584	0.43126394	0.28018607
Erlec1	-0.1096949	5.82466938	0.64477258	0.43146984	0.28026728
Hist1h4b	0.7191837	-1.8082194	0.64472143	0.43148782	0.28026728
Etfdh	0.13678586	5.16765696	0.6446839	0.43150102	0.28026728
Chic1	-0.1039852	6.36591201	0.64454675	0.43154925	0.28027433
Pla2g6	0.21546526	2.71901809	0.64432813	0.43162615	0.28028918
Med20	0.12915593	5.04033689	0.64426929	0.43164685	0.28028918
Fhdc1	-0.4166807	0.87332126	0.64371431	0.43184218	0.28039175
Tnfrsf19	0.12811535	5.27041797	0.64270167	0.43219893	0.2805991
Chst12	0.26411888	1.74990168	0.64227513	0.43234934	0.28067246
Gsdmc4	1.17842039	-1.6809568	0.64201626	0.43244067	0.28070745
Papd7	-0.1457138	4.69030791	0.6417685	0.4325281	0.2807279
Spata24	0.46695343	0.34077621	0.6417149	0.43254702	0.2807279
Phactr4	0.17577012	6.13209661	0.64158681	0.43259224	0.28073296
Mak16	0.1274988	4.98461037	0.6409185	0.43282828	0.28086184
Tbc1d12	0.16435948	3.91318309	0.6405001	0.43297616	0.2809335
Elmod2	-0.1579952	3.45349902	0.6399394	0.43317446	0.28103785
Kif19a	0.75059561	-1.1562717	0.6397536	0.4332402	0.2810562
P2rx5	0.87975533	-0.9221441	0.63950103	0.43332959	0.28108988

Rabl2	-0.1723718	3.87155561	0.63885447	0.43355856	0.28121409
2310003H01I	-0.3381759	0.9227629	0.63870592	0.4336112	0.28122391
Arfgef2	-0.1420835	6.1855433	0.63830331	0.4337539	0.28129214
Pomk	-0.1388653	4.76491231	0.63814265	0.43381087	0.28130232
Tmx4	0.11126166	8.3392014	0.63804756	0.43384459	0.28130232
Pbx4	0.44386101	0.13138919	0.63749214	0.43404164	0.28140576
Tcn2	0.24494807	4.22294844	0.63722738	0.43413562	0.28143279
Atrip	0.16861426	3.49551033	0.63716333	0.43415836	0.28143279
Gdi1	0.10613588	8.88966639	0.63683409	0.43427529	0.28146935
Ndp	0.51846028	1.01292183	0.63678906	0.43429128	0.28146935
Mcrs1	0.19380476	3.23936336	0.63668759	0.43432733	0.28146935
4933424G06	-0.5827227	0.47933401	0.63508823	0.43489614	0.28181362
D4Ertd617e	-1.1028446	-1.1719346	0.63472068	0.43502703	0.28187409
Pth1r	-0.4079983	1.07751311	0.63417645	0.43522094	0.28197538
Zfp747	0.14771931	3.70442029	0.63373389	0.43537873	0.28205325
Trmt1	-0.2398133	2.8026084	0.63326478	0.43554609	0.2821373
Fchsd2	-0.0957262	6.70699461	0.63143942	0.43619826	0.28251581
Lrrd1	-0.6871411	-0.3771125	0.6314185	0.43620574	0.28251581
Igip	-0.1244493	5.87735053	0.63093833	0.43637756	0.28260269
Ccdc173	0.22408029	3.05128335	0.63033771	0.43659263	0.28271757
Gm10649	0.74750214	-1.0484694	0.63017929	0.43664938	0.28272991
Tagap	-0.2255849	2.53749694	0.62984045	0.43677082	0.28278413
Ifi204	0.56086681	-0.3667039	0.62952156	0.43688515	0.28281012
Il1b	-0.9842365	-1.7587399	0.62951816	0.43688636	0.28281012
Syk	0.35536768	1.46868149	0.62936814	0.43694017	0.28282055
Abcb9	-0.3164297	1.61859793	0.62904666	0.4370555	0.2828589
Rassf1	0.23592777	3.92843638	0.62883344	0.43713202	0.2828589
Zfp758	-0.1675274	3.79792439	0.6288158	0.43713835	0.2828589
Cnn2	0.24059609	6.26796327	0.62878263	0.43715026	0.2828589
Plekhj1	0.27856136	2.19298864	0.62847091	0.43726217	0.28286642
Nr4a3	0.2455636	4.23225603	0.62843247	0.43727597	0.28286642
Wdr48	-0.1214048	5.5655483	0.62840457	0.43728599	0.28286642
Lias	0.12613661	4.59285949	0.62822878	0.43734913	0.28286642
Lhcgr	-0.9489843	-1.0558066	0.62822516	0.43735043	0.28286642
Klhl8	-0.1534452	4.31491677	0.62790559	0.43746525	0.2828981
Znf41-ps	-0.2168909	2.72156312	0.62787892	0.43747483	0.2828981
9030624J02F	0.09794227	5.22420034	0.62753684	0.43759779	0.28295322
Bai2	-0.1828031	4.57330281	0.62706403	0.43776783	0.28303877
Tekt2	0.90992406	-0.5607391	0.62672716	0.43788905	0.28309274
Yod1	-0.1424122	4.82945894	0.62612675	0.43810523	0.2832081
C1qc	0.39210284	2.26937786	0.62595508	0.43816707	0.28322366
Brap	0.1259886	5.40008628	0.62547655	0.43833953	0.28326157
Klf3	0.14404917	7.62085981	0.62544581	0.43835061	0.28326157
Saal1	-0.2506149	2.24950395	0.62543316	0.43835517	0.28326157
Plekhb2	0.09365196	6.30581925	0.62535747	0.43838246	0.28326157

Usp12	0.09591204	5.81733712	0.62521874	0.43843248	0.28326157
4932443I19R	-0.9390259	-1.4886467	0.62513	0.43846449	0.28326157
D17Wsu104e	0.29278216	2.07184343	0.62505915	0.43849004	0.28326157
Lactb2	0.18875899	3.85021558	0.62459545	0.43865735	0.28334524
Naglu	-0.3846435	1.20985482	0.6244141	0.43872281	0.28334722
Noc2l	-0.162034	4.00868273	0.62437769	0.43873595	0.28334722
Rgag1	0.36002959	0.93509014	0.62309676	0.43919878	0.28362045
Hspa13	0.13777884	4.88156484	0.62299762	0.43923464	0.28362045
Tigd5	-0.4517486	0.66060187	0.62261105	0.43937448	0.28368633
4930453N24	0.11722308	4.68066758	0.62100358	0.43995677	0.28403784
Vstm2b	0.18369537	3.40517909	0.62084504	0.44001426	0.28405051
Prokr2	0.36507686	1.92358666	0.62071946	0.44005982	0.28405547
Pxmp2	-0.2116261	2.49324457	0.62053673	0.44012611	0.28407382
Bhlhe41	0.13108131	7.69616051	0.61988797	0.44036161	0.28419932
Bcat1	-0.1310431	5.79615957	0.61979236	0.44039633	0.28419932
Aspg	-0.3576259	0.89639221	0.61961948	0.44045913	0.2842154
Naa35	0.10384415	6.08608385	0.61944474	0.44052262	0.28423191
Gm2a	0.22630337	5.00654987	0.61819297	0.44097784	0.28450116
1110034G24	0.32585592	1.72551301	0.61808357	0.44101767	0.28450238
Tmed5	0.14846744	5.54968377	0.61793564	0.44107152	0.28451266
Rell1	0.19834748	5.182429	0.61764631	0.44117688	0.28453367
Pced1a	-0.1547494	4.13256654	0.61763788	0.44117995	0.28453367
Fgd6	-0.1366089	6.33846098	0.61734511	0.44128661	0.2845664
Olfr55	-1.0616069	-1.2121881	0.6172903	0.44130658	0.2845664
Tnfaip3	0.24787083	2.85126605	0.61678061	0.44149237	0.28466174
Slc37a4	-0.231129	1.97965061	0.61625605	0.44168372	0.28476063
Tmem229a	-0.1309631	6.08972277	0.61614672	0.44172361	0.28476188
Ceacam1	0.23815527	2.96410889	0.61591875	0.44180682	0.28479105
Slc37a1	-0.3266067	0.7991249	0.61570438	0.44188509	0.28480825
Tmem219	0.30627005	2.53294204	0.61563767	0.44190945	0.28480825
Ncaph	0.62502853	-0.3833775	0.61495724	0.44215805	0.28494399
Fam181b	0.23674726	2.12299327	0.61425036	0.44241656	0.28508609
Fam132b	-0.6379455	-0.102842	0.61403	0.44249719	0.28511356
Fes	0.64200116	-0.3101794	0.61376409	0.44259452	0.28515111
Gsk3b	-0.0855455	9.51769032	0.61363795	0.44264071	0.28515111
Slirp	0.1359266	4.7971432	0.61355929	0.44266951	0.28515111
Pgam2	0.41044801	0.82968011	0.61324581	0.44278433	0.28520058
Rxfp1	-0.3667209	2.35363465	0.61303985	0.4428598	0.28521727
Gm2381	-0.8027632	-1.2024585	0.61296756	0.44288629	0.28521727
Ube2f	0.13625791	4.74670963	0.61220077	0.44316745	0.28535197
Mto1	0.16771552	3.51939617	0.61218966	0.44317153	0.28535197
Cebpa	-0.3459497	2.51636749	0.61085967	0.44365989	0.2856419
Epha5	-0.137934	6.13213742	0.60989007	0.44401646	0.28582519
Rest	0.17195316	4.90144498	0.60987831	0.44402078	0.28582519
Inadl	-0.136366	3.94804209	0.60915057	0.44428872	0.28597049

Id1	-0.2725479	3.82219196	0.60905817	0.44432275	0.28597049
Paip2b	0.10581071	5.25895448	0.60866499	0.44446764	0.28600955
Arhgap27	-0.2020371	3.24383541	0.60864958	0.44447332	0.28600955
Gm5	0.809489	-0.5194814	0.60858311	0.44449782	0.28600955
Rsph4a	0.24114808	2.93990736	0.60827495	0.44461144	0.28605812
Sertad4	0.21010683	5.75548275	0.60777749	0.44479495	0.28612371
Gpr135	0.71766695	-0.6005813	0.60768423	0.44482937	0.28612371
2810429I04R	-0.7771343	-1.2896192	0.60759713	0.44486152	0.28612371
Fen1	0.2061013	2.73471219	0.60751508	0.44489181	0.28612371
Dixdc1	-0.0802498	6.91295512	0.6073674	0.44494633	0.28612371
Exoc6	0.11441215	5.37342463	0.60734276	0.44495543	0.28612371
Gab1	-0.1015902	7.04320334	0.60727517	0.44498039	0.28612371
Plscr3	0.35039902	1.25531177	0.60715562	0.44502453	0.28612757
Sec24c	-0.1077412	6.32172526	0.60657765	0.44523808	0.28624026
Tex14	-0.7959802	-0.4598515	0.60634126	0.44532547	0.28624026
Nab2	0.15858634	5.10920248	0.60627364	0.44535047	0.28624026
Itprip	-0.6567354	-0.6277225	0.60626828	0.44535245	0.28624026
Dnaic2	-0.9892416	-1.540072	0.60570852	0.44555951	0.28633545
Gfpt2	0.14766147	4.19350323	0.60566156	0.44557689	0.28633545
Gm8300	0.6052893	-0.6615759	0.60554669	0.4456194	0.28633824
B3galnt1	0.10627851	4.8280408	0.60532502	0.44570146	0.28635337
Gpr55	0.51556808	-0.1849099	0.60527684	0.44571929	0.28635337
Apbb2	0.0938959	7.4120643	0.604438	0.44603006	0.28652848
Rpa1	0.11788405	4.58334867	0.60422819	0.44610784	0.2865539
C130060K24I	0.67919052	0.00503325	0.60358213	0.44634748	0.28668329
Rerg	-0.292572	2.91998122	0.60308656	0.44653145	0.28677689
Gpr1	1.05402516	-1.4864563	0.60217447	0.44687035	0.28696579
Mtus1	-0.0990279	6.68441201	0.60208913	0.44690209	0.28696579
Sec24b	-0.112065	7.02039347	0.60138506	0.44716401	0.28710089
1700021K19I	-0.1096518	5.53596705	0.60128629	0.44720077	0.28710089
Slc12a4	-0.4033765	1.46995527	0.601215	0.44722731	0.28710089
Zfp93	-0.1995054	3.83897095	0.60103518	0.44729426	0.2871193
Dync1i1	-0.1660673	5.12427395	0.60079537	0.44738357	0.28715206
Dcps	-0.2168684	2.22805824	0.60012813	0.44763222	0.28728707
Phactr2	0.11661109	8.26110327	0.5996716	0.44780247	0.28737175
Sox8	0.21072115	3.86742114	0.59903083	0.44804161	0.28750062
Fam110a	-0.371902	1.23693417	0.59886592	0.4481032	0.28751554
Csde1	0.07768341	9.14077136	0.59848818	0.4482443	0.28756743
LOC1026341(	-0.9692943	-1.4732897	0.59844419	0.44826073	0.28756743
Fam107b	0.20044298	5.28728601	0.59802767	0.44841642	0.2876427
Cwc22	-0.1157378	5.52773881	0.59772847	0.4485283	0.28768987
Snora81	-0.73702	-1.7249084	0.5975868	0.4485813	0.28769926
9030617O03	0.1954527	2.99011174	0.59679031	0.44887942	0.28786585
Cd276	-0.2418273	2.49201803	0.59581403	0.44924527	0.28805584
2700094K13I	0.21888499	3.19657688	0.59579483	0.44925247	0.28805584

Uxt	0.21891325	3.52008913	0.59560503	0.44932366	0.28807686
Lgi2	-0.257724	2.70263887	0.59533551	0.44942477	0.28811706
Med19	0.13904169	4.61208039	0.5950953	0.44951492	0.28812896
Il7r	-0.555149	-0.0123462	0.59508137	0.44952015	0.28812896
Tmem180	0.22627761	2.4419611	0.59466265	0.44967736	0.2881352
Dhx16	-0.2163137	2.95949162	0.59463987	0.44968592	0.2881352
Ccl28	0.32285374	1.68767328	0.59463669	0.44968711	0.2881352
Hacl1	-0.2481495	2.44761928	0.59459965	0.44970103	0.2881352
Mapk6	-0.0857005	7.5119382	0.59454393	0.44972195	0.2881352
Ap5b1	-0.6157125	-0.0106222	0.59372294	0.45003052	0.28830827
Cwf19l2	-0.1037309	5.62558779	0.59311248	0.45026018	0.28843077
Zfp791	0.2185553	2.83005514	0.59290525	0.45033818	0.2884561
Gm14164	-0.7495963	-0.9193449	0.59275473	0.45039485	0.28846777
Cd200r4	1.13169542	-2.3478976	0.5924972	0.45049184	0.28850526
Rpl13	0.13858404	7.37476852	0.59204269	0.4506631	0.28855547
Tmem196	-0.176247	3.16291533	0.59200389	0.45067772	0.28855547
Pfkfb1	-0.5822365	-0.0473381	0.59198286	0.45068565	0.28855547
9830166K06I	0.43826725	0.88567121	0.59134063	0.45092783	0.28866233
Atp1b3	0.16841636	8.8426417	0.59133616	0.45092952	0.28866233
Scgn	-1.0626565	-1.7738319	0.59091194	0.45108961	0.28874018
Shisa2	-0.2538337	2.48056302	0.59070129	0.45116914	0.28876644
Unc119b	0.19139528	4.03047117	0.58927372	0.4517087	0.28908712
Egfl7	-0.21758	2.41048046	0.58875361	0.45190554	0.28918842
Lipo1	0.17762809	4.94977586	0.58860481	0.45196188	0.2891998
Rnf135	0.20453052	2.75832843	0.58833503	0.45206405	0.28922428
Fam84a	0.11401881	4.98378711	0.58830018	0.45207726	0.28922428
Ppp2r5c	0.10044456	7.0535404	0.58816578	0.45212817	0.28922694
Hk1	-0.1595951	5.81242358	0.58799416	0.45219321	0.28922694
Evi5l	-0.1661154	3.32774126	0.58785442	0.45224617	0.28922694
Olfr316	-0.886126	-0.6026175	0.58783534	0.45225341	0.28922694
Mfap2	-0.4630718	-0.0144527	0.58778049	0.4522742	0.28922694
Lonrf2	-0.1226347	8.07291777	0.5876614	0.45231935	0.28923116
Hgf	-0.2909683	2.0473698	0.5864656	0.45277311	0.28949663
Akt2	0.13790103	5.97679673	0.58507825	0.45330049	0.28980913
Zfp790	0.14779982	4.40422388	0.58454353	0.45350402	0.28991454
Fam19a4	0.9515831	-1.4218659	0.58432317	0.45358794	0.28994348
Odc1	-0.0820028	6.42482973	0.58412951	0.45366171	0.28996593
AI662270	-0.3496361	0.58541783	0.58396256	0.45372532	0.28998188
Crkl	-0.092317	6.05458741	0.58353244	0.45388928	0.29002355
2500004C02I	-0.1647316	3.8040238	0.58352954	0.45389038	0.29002355
Stradb	-0.1192873	5.42089571	0.58328818	0.45398243	0.29002355
Acox1	-0.0932866	6.99373077	0.58322134	0.45400792	0.29002355
Trim17	0.3807841	0.58160471	0.58320948	0.45401245	0.29002355
Mum1	-0.2581526	2.98551717	0.58318311	0.45402251	0.29002355
Spryd4	0.26494842	2.31508092	0.58223486	0.45438448	0.29023006

Mtif3	0.16935733	3.36360623	0.58130422	0.45474019	0.29041054
Slc10a4	0.20518101	2.53480028	0.58129301	0.45474447	0.29041054
Ogfr	0.27115278	1.96457029	0.58080272	0.45493206	0.29045086
Cdkl4	-0.1457899	4.17292672	0.58079279	0.45493586	0.29045086
Rexo4	0.13724366	4.59770965	0.58072547	0.45496162	0.29045086
Pcdhac2	-0.1571162	4.03707919	0.58072321	0.45496249	0.29045086
Tha1	0.45599883	0.05886685	0.58014432	0.45518416	0.29056147
I730030J21R	0.95474339	-1.3284042	0.58006847	0.45521321	0.29056147
Rab10os	0.18163446	3.09165259	0.57914423	0.45556752	0.29076288
Gm13315	-1.0307333	-1.3209303	0.57847066	0.45582602	0.29090312
Hlx	0.93566011	-1.893623	0.57818471	0.45593583	0.29094844
Xlr3b	0.35642062	1.1616973	0.57775072	0.45610258	0.29103009
2210018M11	-0.12211	6.75589325	0.57746488	0.45621246	0.29107544
Ms4a6c	-0.4489127	0.37829737	0.57716868	0.45632637	0.29112336
Mkln1os	-0.2942635	1.23498685	0.57698373	0.45639751	0.29114399
Sigirr	-1.1288151	-1.8461738	0.57648831	0.45658819	0.29121909
Wasf3	0.11235496	5.95092442	0.57644067	0.45660653	0.29121909
Gorasp1	0.18539481	3.03344982	0.57637527	0.45663171	0.29121909
Cd2	0.82241297	-0.4858184	0.57621655	0.45669283	0.29123331
B3galt2	-0.1503515	5.41767912	0.5757996	0.45685346	0.29131098
Smim1	0.41985907	1.65134295	0.57557817	0.45693881	0.29132474
Chic2	0.1700493	3.94294952	0.57554209	0.45695272	0.29132474
Gmeb1	-0.101084	5.29855396	0.57530906	0.45704256	0.29135726
Zfp87	0.12027389	5.56435072	0.57520473	0.4570828	0.29135815
Samd15	-0.2804975	2.41371152	0.57423807	0.45745588	0.29155007
Fxyd1	0.19128523	3.46058344	0.57422317	0.45746163	0.29155007
Kcnq4	0.77290588	-1.0620554	0.57379628	0.45762654	0.2916304
Dpp10	0.13195052	6.87344246	0.57369262	0.4576666	0.29163115
Hdac11	-0.1125531	5.42574727	0.57324456	0.45783983	0.29169233
Morn5	1.10916753	-1.4756517	0.57318635	0.45786234	0.29169233
Oard1	0.1645185	4.57408272	0.5730399	0.45791899	0.29169233
Perp	0.2753435	6.65280061	0.5730097	0.45793067	0.29169233
Acaa1b	0.36556708	0.75719299	0.57294153	0.45795704	0.29169233
Acd	0.14990194	3.94948651	0.57279538	0.45801359	0.29170358
1810043H04I	0.31672931	1.96213509	0.57252216	0.45811934	0.29174616
Siah1a	0.10735955	5.65534911	0.57234198	0.4581891	0.29176582
Imp4	0.14733424	4.57065773	0.57209169	0.45828603	0.29180277
Mrpl19	0.11225105	5.02303377	0.57141847	0.45854692	0.2919441
Zfp868	0.14326439	4.89066314	0.57098096	0.4587166	0.29202029
Gga1	0.14999467	3.54956689	0.5709092	0.45874444	0.29202029
Wbscr17	-0.1621334	3.82824717	0.57046824	0.45891557	0.292074
E2f8	0.50121776	0.8932315	0.57035679	0.45895884	0.292074
Itgb3bp	-0.2447681	3.3353098	0.57032793	0.45897005	0.292074
Gatsl3	0.35159194	1.40357407	0.57029054	0.45898457	0.292074
Cenpt	0.23634978	2.40118375	0.57018294	0.45902635	0.29207582

Eif2ak4	-0.2251438	3.93746764	0.56962691	0.45924238	0.29215851
Adamts9	-0.2566062	2.69377646	0.5695858	0.45925836	0.29215851
Gm4951	-0.3018901	3.36598933	0.56945422	0.45930951	0.29215851
Hist1h3f	-0.6939761	-1.7081125	0.56944753	0.45931211	0.29215851
Nxf2	-0.8648367	-1.3173708	0.56912799	0.45943636	0.29217544
Cldn10	-0.2805343	1.85072512	0.56902923	0.45947478	0.29217544
Crnde	1.39767457	-1.7775101	0.56902779	0.45947534	0.29217544
Bend5	0.18394718	3.27714719	0.56880829	0.45956074	0.29217544
Ppp1r37	0.13740854	4.26330119	0.56877937	0.45957199	0.29217544
Trmt11	-0.2592666	1.67032125	0.56877826	0.45957242	0.29217544
Synb	-0.5841108	-0.6087097	0.56792784	0.45990354	0.29236117
Zfp26	-0.1188002	6.27134914	0.56770155	0.45999172	0.29238608
Rcor2	-0.2984195	1.62983719	0.56762724	0.46002068	0.29238608
Baz2a	-0.116086	6.12712443	0.56726585	0.46016156	0.29242443
Pnma3	-0.1987217	2.98502679	0.56719612	0.46018876	0.29242443
Epb4.1l1	-0.1102035	8.48495277	0.56716628	0.46020039	0.29242443
06-Mar	-0.1019447	8.79093608	0.56702855	0.46025411	0.29242443
Gphn	0.12197935	5.91839685	0.56688012	0.46031202	0.29242443
Zfp568	-0.1702796	3.27424501	0.5668727	0.46031492	0.29242443
Alg11	-0.115696	6.70173638	0.56663283	0.46040852	0.29245802
Slc35c1	-0.1700159	3.33152428	0.5665374	0.46044577	0.29245802
Rab42	0.75691405	-1.7234385	0.56637563	0.46050892	0.29247337
Ggnbp2	-0.0846734	7.36108644	0.5658512	0.46071375	0.29257868
Zbtb7a	-0.142214	6.03367033	0.56566203	0.46078767	0.29260086
Tmem200c	0.26099619	1.70233929	0.56556082	0.46082723	0.29260121
Lama2	-0.1449264	5.21919044	0.56461942	0.46119544	0.29279492
Sh3pxd2a	-0.1445961	7.07643607	0.56448251	0.46124903	0.29279492
2010109I03R	-0.6340968	-1.33369	0.56447695	0.46125121	0.29279492
Smim8	0.21182749	3.79567374	0.56438185	0.46128844	0.29279492
Vipr1	-0.2905559	2.42237839	0.56405105	0.46141798	0.29285236
Tut1	-0.2457444	2.2972698	0.56372476	0.46154582	0.29288211
Primpol	0.28951227	2.5564363	0.56370729	0.46155267	0.29288211
Nudt7	0.16933788	2.42761993	0.56363246	0.46158199	0.29288211
Scd4	-0.4545646	-0.175425	0.5634969	0.46163513	0.29289105
Spata19	-0.8427318	-2.0523574	0.56290052	0.46186901	0.29301466
Zkscan5	-0.2311848	3.89448709	0.56240914	0.46206186	0.29311222
Wbp1l	0.15160775	5.04040967	0.56189289	0.46226461	0.29321604
4833418N02	0.28510324	1.55173817	0.561289	0.46250197	0.29333678
Ccnyl1	0.11902254	5.32079068	0.56118046	0.46254466	0.29333678
Pkd2	-0.1740737	6.20222856	0.56111022	0.46257228	0.29333678
Zbtb25	-0.1613457	3.35792443	0.56049904	0.46281277	0.29345165
Ptch2	0.87817678	-1.929152	0.56045103	0.46283167	0.29345165
Ctbs	-0.1772162	3.00363502	0.56019816	0.46293124	0.29348534
Bcl7c	0.33803518	1.35263335	0.56002484	0.4629995	0.29348534
Abhd17a	0.16368354	3.66080097	0.56001804	0.46300218	0.29348534

Negr1	0.10260937	8.24486592	0.55979853	0.46308866	0.29351536
Rassf8	0.24192269	3.3170677	0.55940377	0.46324426	0.29358917
Mlc1	-0.2550296	3.17657548	0.55848963	0.4636049	0.29376783
Zfp322a	0.10170192	6.03840484	0.55848787	0.46360559	0.29376783
Dcaf8	-0.0898306	5.70611161	0.55839148	0.46364365	0.29376783
Ssx2ip	0.08616835	6.28551564	0.55809748	0.46375975	0.29378662
Idh2	0.17693324	4.04339643	0.55808694	0.46376391	0.29378662
Gm15708	0.69708179	-1.0342753	0.5579505	0.46381781	0.29378662
Sec11c	0.14096986	4.53769326	0.55791975	0.46382996	0.29378662
Anxa3	0.18179256	5.81377732	0.55762305	0.4639472	0.29379414
Tyw5	0.14191913	4.84978132	0.55758776	0.46396115	0.29379414
Slpi	-0.9937422	-1.3967674	0.557498	0.46399663	0.29379414
Nr2c1	0.13595863	4.14095446	0.55749327	0.4639985	0.29379414
Tmem194	-0.2662767	2.91223127	0.55696105	0.46420898	0.29390261
Srsf5	-0.0795513	8.0887758	0.5568287	0.46426134	0.29390716
Pcsk4	-0.2462572	1.48792254	0.55674482	0.46429453	0.29390716
BC030867	-0.8477692	-1.5114321	0.55622988	0.46449839	0.29401139
Upp2	0.17328235	3.38280111	0.55560407	0.46474634	0.29414352
AW112010	0.26588485	1.65671888	0.55547084	0.46479915	0.29415212
Pawr	0.21908057	5.34760824	0.55478343	0.4650718	0.29429985
Pfkp	-0.1425317	7.31963642	0.55460713	0.46514177	0.29431929
Scarf2	-0.433571	1.58556916	0.55447359	0.46519479	0.29432582
Gm16845	0.52334831	0.69117642	0.55429243	0.46526672	0.29432582
Ctage5	-0.0902375	5.84547173	0.55421363	0.46529801	0.29432582
Agtrap	0.24540787	4.03293785	0.55418588	0.46530903	0.29432582
Cdk5r1	-0.1234361	6.96452263	0.55364723	0.46552305	0.29441812
Sp140	0.11950104	4.89215056	0.55362106	0.46553345	0.29441812
St7	-0.1859452	3.42973863	0.55280498	0.46585801	0.29459855
Sdsl	0.79335088	-0.6387176	0.55223635	0.46608439	0.29471686
Mgat2	0.18315896	3.28423238	0.55139987	0.46641773	0.29489823
Nlrp5-ps	0.49678881	0.46725446	0.55131932	0.46644985	0.29489823
1110057K04I	0.10422731	5.54252814	0.55103069	0.46656497	0.29494615
Snx21	-0.289937	1.94297433	0.55054414	0.46675915	0.2950386
Pctp	0.23835378	2.59913689	0.55046718	0.46678987	0.2950386
Ruvbl2	0.12813859	4.49916799	0.54996747	0.46698946	0.29513988
Unk	-0.16887	3.96633081	0.54965008	0.4671163	0.29519517
Safb	0.0985145	6.30017107	0.54945388	0.46719474	0.29521987
Rpl22	0.16144462	6.20026759	0.5487155	0.46749013	0.29538164
Clk2	0.13110234	5.15025855	0.54856631	0.46754985	0.2953945
Slc35b2	-0.2816171	2.09245304	0.54832369	0.467647	0.29543099
Ube2o	-0.1602954	4.91134424	0.54815633	0.46771403	0.29544846
As3mt	-0.1865028	3.54657067	0.54785787	0.46783361	0.29545756
Glrx3	0.10619611	5.19640005	0.54784541	0.46783861	0.29545756
Sys1	0.18749053	3.2962455	0.54782546	0.4678466	0.29545756
Rbm4b	-0.1358253	4.34630072	0.54756047	0.46795282	0.29549976

Rfwd3	-0.0983025	5.39806734	0.54731655	0.46805063	0.29553664
Ephx4	0.11598506	5.18556437	0.54660684	0.46833541	0.29567907
Zswim7	0.21143346	2.12660425	0.54655796	0.46835503	0.29567907
Dusp10	-0.1923402	3.731895	0.54629645	0.46846005	0.29570185
Stt3b	-0.1015906	5.87106778	0.54627177	0.46846996	0.29570185
Ces2g	-0.2284661	3.49425668	0.5460714	0.46855045	0.29572778
Trim62	0.13290566	4.08115042	0.54591107	0.46861487	0.29574356
Fkbp11	0.71591128	-1.4184632	0.54571695	0.46869289	0.29576791
Fam186b	-0.9451724	-0.8546847	0.54528581	0.46886625	0.29585242
Tuba1a	0.08070786	9.67220925	0.54471001	0.46909795	0.29596605
Rnf19a	-0.0917539	6.03704394	0.5446422	0.46912525	0.29596605
Samd14	-0.2795734	2.17700552	0.54446858	0.46919515	0.29598526
Clec5a	0.48228457	-0.1262084	0.54434587	0.46924457	0.29599154
Mre11a	-0.2388777	2.75621419	0.54388698	0.46942945	0.29606177
Brf2	0.39612677	0.70218386	0.54387362	0.46943484	0.29606177
Spry2	-0.1029317	5.95328064	0.54346673	0.46959888	0.29610323
Ptges2	-0.1863304	2.3907811	0.54334159	0.46964935	0.29610323
Tbx3	-0.1646126	3.70014722	0.54319779	0.46970735	0.29610323
Adam5	-0.6196499	0.8451942	0.54305229	0.46976606	0.29610323
Gpc6	-0.1589888	7.3480851	0.54296395	0.4698017	0.29610323
Gm5083	-0.882162	-0.806053	0.54293212	0.46981455	0.29610323
Gypc	-0.2126322	5.39498822	0.54292889	0.46981585	0.29610323
R3hdm2	0.09427585	9.00287395	0.5429276	0.46981637	0.29610323
H2afy2	0.1593389	4.52595508	0.5421935	0.4701128	0.29626516
Snx20	0.32771083	0.86909136	0.541441	0.47041698	0.29643195
A530064D06	-0.7980406	-1.7685004	0.54120728	0.47051152	0.29646662
Clec2i	-0.8432102	-1.1682639	0.54097278	0.47060641	0.29650151
Gm14634	-0.5196166	-0.1042854	0.54072164	0.47070807	0.29653879
E2f7	0.88616389	-1.079622	0.54054976	0.47077766	0.29653879
Tmem240	0.2835374	0.89522004	0.54053366	0.47078418	0.29653879
AV039307	-0.4551756	0.33744871	0.54039072	0.47084207	0.29655036
Hspa9	-0.0854396	7.17277212	0.54024747	0.4709001	0.296562
Mamdc4	-0.6354887	-0.0726255	0.54005778	0.47097696	0.29658551
Mad1l1	0.22127055	2.26136408	0.53983974	0.47106533	0.29659872
Polb	-0.1556337	4.32665146	0.53981092	0.47107702	0.29659872
Eef1b2	0.13089197	7.031599	0.53950912	0.47119939	0.29665087
Mdfic	0.23951686	5.61031651	0.53896274	0.47142107	0.29676552
Tubb3	0.13367041	4.68233189	0.53833426	0.47167628	0.29690126
Ccdc120	-0.2307896	2.46031896	0.53808075	0.47177929	0.29694118
Hus1	-0.1300113	4.38056297	0.53780574	0.47189107	0.29695033
Uhrf1	0.47662466	0.39544064	0.53771943	0.47192616	0.29695033
Eps15	-0.093436	8.62271066	0.53770509	0.47193199	0.29695033
Cdkn2b	0.98450745	-1.6133933	0.53765548	0.47195217	0.29695033
Slc26a4	0.42914794	1.05431985	0.53722084	0.47212897	0.29703666
Srbd1	-0.1644761	3.49013912	0.53680935	0.47229646	0.29711711

Efcc1	-0.2095768	2.53421232	0.53622819	0.47253317	0.2972411
Kcnk10	-0.3009092	0.71105858	0.53574196	0.47273137	0.29734084
Nudt3	-0.1223345	6.12764947	0.53519559	0.47295425	0.29743181
Psen1	0.1188199	5.05000366	0.53519302	0.4729553	0.29743181
4732416N19	0.84832369	-1.9310366	0.53497394	0.47304472	0.29746311
Icam5	-0.1910085	2.88514723	0.53470236	0.47315561	0.2975079
Dusp2	0.66724826	-1.032968	0.53412684	0.47339074	0.29760168
Casc5	-0.5243239	0.48233608	0.53407972	0.47341	0.29760168
Gart	0.14139908	4.37755038	0.53394701	0.47346425	0.29760168
Enpp5	-0.0916481	7.09987463	0.53392032	0.47347516	0.29760168
Aldh1b1	-0.406438	0.04492689	0.5338071	0.47352146	0.29760168
Il1r1	0.15711835	4.30797735	0.53375492	0.4735428	0.29760168
Lrriq3	-0.4817458	0.57315114	0.5334842	0.47365353	0.29764207
Fgfbp1	0.27419411	4.61375719	0.53340379	0.47368643	0.29764207
Cyth4	-0.2577396	1.85872667	0.53316214	0.47378532	0.29767927
Otx2	-0.5104009	1.57621485	0.53248341	0.47406325	0.29780575
Ipo4	-0.130274	4.14921411	0.53247664	0.47406603	0.29780575
Sdf4	0.07802336	7.98580178	0.53236713	0.4741109	0.297809
Wdr82	0.08392201	6.39638875	0.53178905	0.47434788	0.29793291
Sec16b	-0.6929618	-0.2236277	0.53161315	0.47442003	0.29795328
Echdc2	-0.2348099	2.26378607	0.53136818	0.47452054	0.29799145
Scn9a	-0.2952591	2.68024145	0.52861775	0.47565147	0.29867666
Uspl1	0.09751131	5.10207478	0.52822272	0.47581428	0.2987013
Zfp383	-0.2063007	2.57221034	0.52816053	0.47583992	0.2987013
Stk19	-0.2714776	2.00526309	0.52815532	0.47584207	0.2987013
1700003M02	-0.4699797	0.2590926	0.52813607	0.47585	0.2987013
L1cam	-0.1733735	6.82476182	0.52799226	0.4759093	0.29871353
Adam15	-0.1308368	4.21539987	0.52745005	0.47613299	0.29882893
Madd	-0.1405199	6.89785884	0.52725232	0.4762146	0.29885514
Fbxo6	0.19939413	2.55592837	0.5262711	0.47661998	0.29908452
Abce1	-0.100551	6.26132422	0.52607087	0.47670277	0.29910691
Slc16a13	0.19962489	2.78603537	0.52595071	0.47675246	0.29910691
Fam166b	-0.6355352	-0.4522952	0.52589556	0.47677527	0.29910691
Fam221b	0.56186118	-0.509092	0.52525184	0.47704168	0.29924901
Arl8b	0.07719766	7.95545664	0.52502049	0.47713748	0.29926711
Fbxw5	0.13021652	5.41476069	0.52498948	0.47715032	0.29926711
Camk1g	-0.1863364	4.06859878	0.52460354	0.47731022	0.29932965
Nsun5	0.20774563	1.70862212	0.52444737	0.47737495	0.29932965
Ttll5	-0.1122916	5.3838044	0.52432495	0.4774257	0.29932965
Nat14	-0.2598421	1.17082962	0.52431635	0.47742927	0.29932965
Cmss1	-0.346703	2.41005536	0.52421006	0.47747334	0.29932965
Gfra1	-0.1605907	3.28474915	0.52417118	0.47748947	0.29932965
Ccdc65	0.23698398	2.03665474	0.52398454	0.47756687	0.29935316
Gm5468	0.37022496	1.88273939	0.52372465	0.4776747	0.29939359
Tcp10b	-0.8452721	-1.9858622	0.5236367	0.4777112	0.29939359

Prim2	0.1904012	2.69583079	0.52296725	0.47798917	0.29952742
4932416H05I	0.18441072	2.63823692	0.52293012	0.4780046	0.29952742
Disp1	-0.1838207	3.22651825	0.52180389	0.47847287	0.29979581
Rhobtb1	-0.2150473	2.61378833	0.52152462	0.47858912	0.2998436
Gm16677	-0.7967233	-1.2552854	0.52113738	0.47875038	0.29991958
Kif3b	-0.1089835	6.59547066	0.51982237	0.47929867	0.30023799
Nr2e1	0.19889619	3.69179722	0.5194864	0.47943892	0.30030077
Fam187b	-0.7177126	0.85249679	0.51930842	0.47951325	0.30032225
Gtpbp3	-0.157172	3.02973878	0.51893112	0.47967088	0.30039589
Dll4	-0.8743614	-1.5188964	0.51882774	0.47971409	0.30039787
Ankrd40	0.08009051	7.06114299	0.51866683	0.47978135	0.30041492
Hmgxb4	0.10176407	5.2954056	0.51848763	0.47985628	0.30043314
Mmp15	-0.3202431	1.11482654	0.51839312	0.4798958	0.30043314
Lingo3	0.20104916	3.01449084	0.51826249	0.47995044	0.30043314
Fkbp2	0.1391521	4.37876841	0.51821414	0.47997067	0.30043314
Rabep1	0.08523486	7.91370563	0.51767687	0.48019552	0.30053176
Rimbp2	-0.1730672	5.19509569	0.51757027	0.48024016	0.30053176
Pld6	-0.7677513	-1.921622	0.51755057	0.48024841	0.30053176
Cyld	-0.0905704	7.32138492	0.51722342	0.48038543	0.30059243
Kras	-0.1073176	8.1423861	0.51704965	0.48045824	0.30060637
Tmem184c	0.10253145	6.04331967	0.51697898	0.48048786	0.30060637
2010320M18	0.3070296	1.75692754	0.51676318	0.48057832	0.30063789
Mtx1	0.20181576	2.53885428	0.51655635	0.48066505	0.30065095
Atg9b	-0.4294839	0.83379581	0.51652223	0.48067936	0.30065095
Tymp	-0.2569488	2.53864225	0.51617621	0.48082451	0.30071667
Rgs14	0.23356501	2.19227833	0.51580557	0.48098008	0.30078888
Tab2	0.08474627	8.33770501	0.51556233	0.48108222	0.30082768
Cbfb	0.13727596	6.03301844	0.51545225	0.48112846	0.30083151
Arih2	-0.1123103	4.72467566	0.51515755	0.48125227	0.30088385
Pde12	0.16962025	3.23531426	0.51494523	0.48134151	0.30091457
Gm10536	-0.7042617	-1.3984401	0.51472776	0.48143295	0.30094665
Wdr75	-0.1475374	4.66345154	0.51441978	0.48156248	0.30100254
2900079G21	0.25446253	1.16559202	0.51401311	0.48173362	0.30108442
Slc13a4	-0.1806936	8.44182862	0.51362141	0.48189856	0.30116241
Opalin	-0.3188356	1.45829791	0.51285669	0.48222084	0.30133872
Rad51b	0.79097332	-1.3350402	0.51273126	0.48227374	0.30134667
Vps72	0.26801487	2.61179427	0.51243356	0.48239933	0.30136598
Arid4b	-0.1108164	7.72302548	0.5123807	0.48242163	0.30136598
Prickle4	-1.0263676	-1.8831369	0.5123723	0.48242518	0.30136598
Usp35	-0.2428403	1.87628475	0.51203787	0.48256634	0.30139258
Fsd1l	-0.1346997	6.17601251	0.51194779	0.48260437	0.30139258
Cpsf3l	-0.1861502	2.63719254	0.51192723	0.48261305	0.30139258
Mettl1	0.63294231	-0.7518971	0.51189072	0.48262847	0.30139258
Fancc	-0.2359144	1.8744647	0.51158109	0.48275925	0.30144916
2610307P16I	0.53001934	0.88520349	0.51124206	0.48290252	0.30149799

Snord47	-0.7364563	-1.5171434	0.51118709	0.48292576	0.30149799
Zfp366	0.50821505	-0.0905949	0.51110242	0.48296155	0.30149799
Zfp811	0.15983157	3.43051123	0.51101567	0.48299823	0.30149799
Tgfbr2	0.15628275	5.70117988	0.51065916	0.48314901	0.30156702
Insig1	0.08823044	6.28332118	0.51030006	0.48330098	0.30162354
Pdgfb	0.23941942	2.23334722	0.51025517	0.48331998	0.30162354
Chac1	-0.2277654	1.81672077	0.5099936	0.48343073	0.30163054
Wwp2	0.1409711	4.35375428	0.50997422	0.48343894	0.30163054
Tmprss6	1.06343133	-2.147482	0.5098766	0.48348028	0.30163054
Tvp23a	0.19163167	4.20461113	0.50984882	0.48349205	0.30163054
Ctss	-0.2085315	3.74244017	0.50966332	0.48357063	0.30165448
Insm1	-0.3018794	2.14257829	0.50952859	0.48362772	0.301665
2310034G01	0.51372358	0.91151971	0.50936994	0.48369496	0.30168186
Zfp367	0.15943164	3.52014188	0.50914565	0.48379005	0.30171608
Lrrn4cl	-0.1103661	4.95697795	0.50890989	0.48389003	0.30173063
Acadl	0.13894696	5.29580048	0.50890094	0.48389383	0.30173063
Crh	-0.4702413	0.14931193	0.50824635	0.48417162	0.30187876
Arf5	0.19843171	3.03196978	0.50798329	0.48428334	0.30192331
Ncln	-0.2309823	2.44640941	0.50729245	0.48457693	0.30208125
Zmym5	-0.0961632	6.59672674	0.50695632	0.48471989	0.30214525
Cpeb3	-0.1026163	7.55417114	0.50667789	0.48483836	0.30219399
Cttnbp2nl	-0.0941036	6.16218878	0.50589201	0.48517302	0.30237746
Cntnap5a	-0.1737302	4.3575796	0.50554985	0.48531884	0.30242979
Ing3	0.13151744	4.2107681	0.50550581	0.48533762	0.30242979
Oacyl	-0.5651253	0.29856542	0.5050091	0.48554946	0.30253666
Ccs	0.22761163	2.13602256	0.50442049	0.48580071	0.30266807
Cxcr5	-0.4068823	-0.2027588	0.5042263	0.48588365	0.30268981
Nif3l1	-0.1642009	3.38042311	0.50414984	0.48591631	0.30268981
Wdr60	-0.1411299	5.41442641	0.50403473	0.48596549	0.30269531
Pomt1	0.3068766	1.47726047	0.50387365	0.48603432	0.30271305
Nkx2-2	-0.368364	0.88378594	0.50370609	0.48610594	0.30273252
4930447N08	0.61550217	-0.703037	0.50351494	0.48618767	0.30274676
Etfb	0.17076045	4.37938077	0.50346384	0.48620952	0.30274676
Npc2	0.20768268	4.68547146	0.50328973	0.48628399	0.30276799
Sypl	-0.129164	6.38862586	0.50304688	0.48638789	0.30277915
Fabp3	0.15181398	3.7126582	0.5029915	0.48641159	0.30277915
Gzmb	-0.7165923	-0.3237874	0.50296481	0.48642301	0.30277915
Abhd2	-0.0981829	6.17205916	0.502627	0.48656762	0.30284404
Cox20	-0.1184867	5.02108414	0.50201537	0.48682964	0.30298198
Fbxw7	-0.0868365	8.24940269	0.50150335	0.48704916	0.30306825
Padi4	0.83666045	-2.0630303	0.5015018	0.48704982	0.30306825
Clec11a	-0.1843042	2.21982313	0.5014094	0.48708946	0.30306825
Ssh1	0.18644944	2.40579911	0.5013102	0.48713202	0.30306959
Myot	0.90115625	-2.0569196	0.50071143	0.48738903	0.30317421
Dse	0.23091767	3.90497036	0.50070054	0.4873937	0.30317421

Lrwd1	-0.2764992	1.80651946	0.50063601	0.48742142	0.30317421
Nop9	0.17816655	3.74602846	0.5003354	0.48755055	0.30321222
Gm7444	-0.4899732	0.29348424	0.50030555	0.48756338	0.30321222
Esrrg	0.15416353	6.04331139	0.49962914	0.48785417	0.30336791
Bnip1	0.25221047	2.16630286	0.49936754	0.48796672	0.30340129
Chek2	-0.2500676	2.34680415	0.49931634	0.48798875	0.30340129
Pigyl	0.33489781	3.02094125	0.49898599	0.48813094	0.30343962
Pip5k1c	-0.1001183	6.37559619	0.49898518	0.48813129	0.30343962
Pcdhb7	-0.2672971	2.24062595	0.49859956	0.48829737	0.30351771
Appbp2	-0.1106489	5.28876882	0.49838633	0.48838925	0.30354966
Parp12	0.14227226	4.35770763	0.49826417	0.48844189	0.30355723
Nmral1	-0.2268479	2.8431498	0.49814135	0.48849483	0.30356288
Ccar2	-0.1623469	4.23538832	0.49805533	0.48853192	0.30356288
Gm5086	-0.6871625	-0.7664952	0.497772	0.48865411	0.30361365
Slc27a1	-0.1914209	3.86559533	0.49750233	0.48877045	0.30366079
Mbd1	0.12182454	4.27586133	0.49718154	0.48890891	0.30370645
Sat2	0.19805983	2.12702867	0.49714448	0.48892491	0.30370645
Nrxn3	-0.1393836	8.1011022	0.49694191	0.48901238	0.30373178
Mtpap	0.11860321	4.90302838	0.4968625	0.48904668	0.30373178
Zfp473	-0.6706737	-0.3005322	0.49668522	0.48912327	0.30374909
Ehbp1l1	-0.1184234	4.25592144	0.49661053	0.48915554	0.30374909
Rnmtl1	-0.252197	2.06520423	0.49649055	0.48920739	0.30375614
Tysnd1	-0.1998851	2.27809315	0.49634118	0.48927195	0.30377108
Ccdc174	0.12092061	4.70050512	0.49545323	0.48965605	0.30398301
Mxra7	-0.2223939	2.78163918	0.49536479	0.48969434	0.30398301
Sult5a1	-0.581955	-0.535735	0.49527017	0.4897353	0.30398328
Fyco1	0.10204854	6.72397002	0.49503356	0.48983777	0.30402173
Snhg3	0.2540323	3.02494622	0.49490848	0.48989195	0.3040302
Irf6	0.20019151	3.74546148	0.49471358	0.4899764	0.30405745
Adcy3	-0.2235944	2.3116347	0.49435371	0.49013239	0.3041291
5830416I19R	1.03293894	-0.9376962	0.49398838	0.49029084	0.304184
Gm12992	0.26691261	1.37021767	0.49380622	0.49036988	0.304184
Slc7a6	-0.2475039	2.89527166	0.49380479	0.49037049	0.304184
Cd200	0.11758421	6.86633435	0.49377581	0.49038307	0.304184
Prps1l3	-0.107374	4.70122548	0.49338228	0.4905539	0.30423416
Ppp6r2	-0.1355755	4.59010303	0.49333479	0.49057452	0.30423416
Mpp2	0.08959213	6.71920698	0.49330928	0.49058561	0.30423416
Grwd1	0.28018056	1.41673485	0.49315252	0.49065369	0.30424331
Pde3b	-0.2572656	2.68835774	0.49308854	0.49068148	0.30424331
Efcab4a	-0.3398303	0.98823308	0.49273106	0.49083683	0.30431447
Gpr137c	-0.130026	4.87523066	0.49239801	0.49098162	0.30437909
Tal1	-0.4936593	0.26914581	0.49201786	0.491147	0.30445472
Apeh	0.25737804	2.13324778	0.49193098	0.4911848	0.30445472
Slc25a45	0.55418303	-0.4163997	0.49175917	0.49125958	0.30447592
Cpa6	-0.7948566	-1.1804844	0.49107882	0.4915559	0.3046344

Oscar	-0.4933199	0.10059127	0.4904341	0.49183699	0.30478342
Zdhhc18	0.19123317	3.24459797	0.49032048	0.49188655	0.30478895
Fam120aos	0.15763783	4.61883601	0.4899051	0.49206782	0.30487609
Sugct	0.27896628	1.49156978	0.48973565	0.49214181	0.30488564
Stard7	0.08513804	6.2672211	0.48968362	0.49216453	0.30488564
Mtm1	-0.2520865	2.68765722	0.48937579	0.49229899	0.30493565
Sh3tc2	-0.8268612	-1.284331	0.48931268	0.49232656	0.30493565
F830016B08I	-0.1209208	4.1638108	0.48902272	0.49245328	0.30497582
Nfs1	0.12252151	4.3159272	0.48897827	0.49247271	0.30497582
Tm9sf3	-0.0857825	7.28578233	0.48871391	0.49258831	0.30501003
Zfp85	0.17628476	2.44957893	0.48866594	0.49260929	0.30501003
2810029C07I	0.35094371	2.23293347	0.4883747	0.4927367	0.30506374
Sos1	-0.0889858	7.58363665	0.48742773	0.49315137	0.30525914
Fem1a	0.09556158	5.55688316	0.48738832	0.49316864	0.30525914
Gbx2	1.04796107	-1.7696169	0.48737521	0.49317438	0.30525914
Pmf1	0.28101195	3.35773183	0.48704951	0.49331716	0.30531208
Fbxw9	0.26864161	2.04056221	0.48699439	0.49334133	0.30531208
Pknox2	0.09324383	7.21215062	0.48660747	0.49351104	0.30539192
Mfsd7c	-0.3507828	1.40256348	0.48634949	0.49362426	0.30540861
Sntg1	0.12504539	5.58941701	0.48633515	0.49363055	0.30540861
Epha2	-0.7778361	-1.6460887	0.48626772	0.49366015	0.30540861
Iffo2	-0.1227435	4.67135398	0.48607005	0.49374694	0.30543711
Ndufaf7	-0.1045873	4.55722598	0.48571586	0.49390252	0.30550816
Cox15	0.11156968	5.47204878	0.4856044	0.49395149	0.30551326
Pianp	0.09756869	6.45296759	0.48517549	0.49414004	0.30559566
Proser2	-0.269296	2.69453176	0.48500715	0.49421407	0.30559566
Ndst3	-0.1663511	4.32585703	0.48500612	0.49421453	0.30559566
Nkx3-1	0.41160205	0.14710856	0.48489996	0.49426123	0.30559566
Polk	-0.1150669	5.18978043	0.48483816	0.49428842	0.30559566
Tmem38a	0.13982502	5.49186598	0.48472115	0.4943399	0.3056023
Katnal2	-0.5693746	-0.4732178	0.48442267	0.49447127	0.30565833
Ccdc14	-0.2376744	1.8649265	0.48427562	0.49453602	0.30566464
Tmem204	-0.1602101	3.94258334	0.48403753	0.49464088	0.30566464
Plek	-0.1132581	4.63025579	0.48399799	0.4946583	0.30566464
Pgs1	-0.128837	4.41509529	0.48383903	0.49472833	0.30566464
Polr1e	-0.2018283	1.80469132	0.48381152	0.49474046	0.30566464
Snupn	-0.1744247	3.39514449	0.48376193	0.49476231	0.30566464
Btla	-0.6556152	-0.3808945	0.48375192	0.49476672	0.30566464
Cpne9	-0.1942055	4.22777363	0.48359729	0.49483488	0.30566927
Ctu1	-0.2536777	1.55210896	0.48350824	0.49487413	0.30566927
Vcp	0.0779873	7.68924044	0.48345757	0.49489647	0.30566927
Tapbp	0.22945954	4.62292862	0.48321836	0.49500196	0.30570926
Bcl2l12	-0.4086335	0.23537933	0.48297208	0.4951106	0.30575118
Enpp3	-0.3589424	0.61980347	0.48275732	0.49520538	0.30578382
Xdh	0.1709707	3.2821717	0.48264703	0.49525406	0.30578382

Syngr2	0.30628878	1.68918734	0.48254846	0.49529758	0.30578382
Nts	-0.3655654	1.01467383	0.48241271	0.49535753	0.30578382
Enkd1	0.32544982	0.55606555	0.48239063	0.49536728	0.30578382
Chek1	-0.3383705	0.84374277	0.4819936	0.49554267	0.30586692
Frat2	-0.2563765	3.01946885	0.48187244	0.49559622	0.3058748
Acvr1c	0.1483352	4.78138108	0.48147383	0.49577245	0.30593949
Jtb	0.1215857	4.13897678	0.48145088	0.4957826	0.30593949
Pja2	-0.1014823	8.93374676	0.4811524	0.49591464	0.3059877
Bag4	-0.0996708	5.95107043	0.48108986	0.49594232	0.3059877
Apip	0.14411218	4.17678292	0.48089122	0.49603023	0.30601678
Atf3	0.51207145	1.54733601	0.48048226	0.49621132	0.30610332
Nudt5	0.21134528	2.90762845	0.48034131	0.49627376	0.30611666
Ccdc137	-0.1725194	4.23153916	0.48009124	0.49638457	0.30615984
Nova2	-0.1220453	7.26096089	0.47989516	0.49647149	0.30618177
Tmem53	0.35599502	1.62310313	0.47979633	0.49651531	0.30618177
Itgb5	0.18749815	4.05281799	0.47959607	0.49660412	0.30618177
Tssc4	0.17423402	2.47401417	0.47958398	0.49660948	0.30618177
Pgbd1	-0.1786307	2.33747517	0.47955076	0.49662422	0.30618177
Oaz3	-0.2487721	2.23339133	0.47907852	0.49683377	0.30628579
Magohb	0.19882457	2.81648979	0.47823844	0.49720693	0.30646687
Mfsd1	0.1658133	5.17209041	0.47819025	0.49722836	0.30646687
Ccdc138	0.20303252	2.60096319	0.47814141	0.49725007	0.30646687
Gtpbp2	-0.1417163	4.27138484	0.47804783	0.49729167	0.30646733
Pde4b	-0.1151604	7.9733752	0.47783827	0.49738486	0.30649281
2900097C17I	-0.1218094	10.8249655	0.47777113	0.49741473	0.30649281
Dhcr24	0.13534059	5.09585216	0.47755383	0.4975114	0.3065272
Mir6414	-1.1541725	-1.7663391	0.47740787	0.49757635	0.30654204
Tmem39a	-0.1177133	4.96808589	0.47713131	0.49769947	0.30655937
1700001L05F	-0.186408	4.34217344	0.47708349	0.49772076	0.30655937
Sass6	-0.151343	3.77705377	0.47698719	0.49776365	0.30655937
C030034I22R	-0.2498165	1.65412909	0.4769775	0.49776796	0.30655937
Gata6	0.98212775	-1.377922	0.4768676	0.49781691	0.30656435
Rab40c	-0.1757893	2.90796542	0.47655126	0.49795786	0.30662116
Sh2b2	-0.4371854	0.01806364	0.4764771	0.49799091	0.30662116
Pus3	0.17023659	3.36515777	0.47633955	0.49805223	0.30663374
Ptbp3	0.09973134	7.19156415	0.4761537	0.49813509	0.30664835
Apoe	-0.233053	6.97856543	0.47595899	0.49822193	0.30664835
Cercam	-0.2217185	1.93764476	0.47592861	0.49823549	0.30664835
Ppil1	0.13683592	3.77002774	0.47587439	0.49825967	0.30664835
Arhgef10	-0.1048978	5.27670448	0.47582804	0.49828035	0.30664835
2610100L16F	0.23170942	2.15137763	0.47554769	0.49840546	0.30670018
Gm15455	0.44082854	-0.8764265	0.47537014	0.49848472	0.30672379
Btg1	0.14102664	5.37506462	0.47508614	0.49861155	0.30675941
Tlr7	0.26869029	1.59115268	0.47505739	0.4986244	0.30675941
Slc12a7	-0.2996492	3.82914986	0.47495457	0.49867033	0.30676251

2900005J15F	0.28727246	2.24729623	0.4747465	0.4987633	0.30679455
Aim1l	-0.5052704	-0.2877581	0.474362	0.4989352	0.30687512
Nfib	-0.0808566	8.11049038	0.47418003	0.49901659	0.30689223
Prmt7	-0.1863828	3.30379527	0.47405886	0.49907079	0.30689223
Ebag9	0.07987355	5.98741643	0.47394653	0.49912106	0.30689223
Lmnb2	0.16501402	3.01885766	0.47392108	0.49913244	0.30689223
D430041D05	-0.1295874	7.66574254	0.47384255	0.49916759	0.30689223
Rnf20	-0.0946142	6.44450563	0.47361536	0.49926928	0.3069296
Gm16617	-0.8343528	-0.99522	0.47321425	0.49944893	0.3069569
Naalad2	0.27592181	1.33162238	0.47321169	0.49945007	0.3069569
Kdelc2	-0.130183	4.49541638	0.47320249	0.49945419	0.3069569
Rem2	-0.4054394	0.43792714	0.4729761	0.49955564	0.3069569
Trim25	0.1330243	6.41306615	0.47295605	0.49956462	0.3069569
Tep1	-0.2321726	2.96774896	0.47291213	0.4995843	0.3069569
Gm20110	-0.8155867	-1.7276857	0.47287682	0.49960013	0.3069569
Arl13b	-0.1222126	4.2966268	0.47275175	0.4996562	0.30696212
Avpr1a	0.67359172	-0.6701335	0.47267531	0.49969047	0.30696212
Mcidas	-0.6610331	-1.6433173	0.47238923	0.49981877	0.30699587
Slc17a6	-0.1157536	4.81099216	0.47237029	0.49982727	0.30699587
Itk	0.59383824	-0.1746427	0.47220895	0.49989965	0.30701519
Wdr96	-0.3270893	1.22536066	0.47203972	0.49997559	0.30703669
Ing2	-0.1058193	5.0945217	0.47192227	0.50002831	0.30704393
Slc45a3	0.53468446	0.25526345	0.47106294	0.50041433	0.30725582
Cebpz	0.07893172	6.19603558	0.46982552	0.50097111	0.30757251
A530072M11	0.52211528	0.62588716	0.46905354	0.50131902	0.3077254
Tigd3	0.4157576	-0.2656558	0.46903048	0.50132942	0.3077254
Il1a	-0.7009482	-1.0177028	0.46899988	0.50134322	0.3077254
Gm6568	0.29347645	0.79574085	0.46887713	0.50139858	0.30772767
Btbd10	-0.098681	5.98224862	0.46879402	0.50143607	0.30772767
Abhd11os	0.36136815	0.35444913	0.46871884	0.50146999	0.30772767
Ei24	-0.1151547	6.08275607	0.46823411	0.50168877	0.30783675
Il18r1	0.67101828	-0.6174719	0.46797641	0.50180515	0.30788297
Fermt3	-0.4347403	0.28907828	0.46779667	0.50188635	0.30790761
Syde2	-0.1871308	3.66367295	0.46760002	0.50197522	0.30793695
Galt	-0.1115252	4.43305	0.46725095	0.50213304	0.30800857
BB031773	0.63875429	-1.0355317	0.46706387	0.50221765	0.30803528
Siah1b	0.25948734	1.8626382	0.466696	0.50238411	0.30810282
Depdc7	-0.3022947	1.14205109	0.46663901	0.50240991	0.30810282
Peg10	0.12283872	6.36412456	0.46617173	0.50262151	0.30820738
Josd2	-0.231614	2.23009201	0.46596693	0.5027143	0.30822045
9130023H24	0.15777454	3.76212086	0.46594332	0.502725	0.30822045
Cd74	-0.257744	6.73494248	0.46584277	0.50277057	0.3082232
Zap70	0.7619127	-1.6527262	0.46556372	0.50289707	0.30825145
Adamts16	-0.397872	0.94222066	0.46555983	0.50289883	0.30825145
Polm	0.17190937	3.19492597	0.46530441	0.50301468	0.30827401

Prosc	0.09344001	5.53997079	0.46523459	0.50304635	0.30827401
Hspb7	-0.3577045	0.47307852	0.46520685	0.50305893	0.30827401
Lnx2	-0.1132184	4.43629105	0.46460374	0.5033327	0.30841658
Srsf11	-0.0912221	6.73223638	0.46419367	0.50351898	0.30849512
Rtn4r	0.12675304	4.37400648	0.46414052	0.50354313	0.30849512
Slc2a10	-0.3448272	0.93217711	0.46384593	0.50367704	0.30855196
Riok2	0.09362576	5.09861726	0.46340801	0.50387623	0.30864878
6430531B16I	0.65417053	-0.6821775	0.46325085	0.50394774	0.30866738
4930455C13I	-0.6208615	-0.7454366	0.46311619	0.50400903	0.30867971
Nupl2	0.18054116	3.28139523	0.46286355	0.50412406	0.30870934
Mterf1b	0.36492424	0.95309795	0.46282918	0.50413971	0.30870934
Mboat2	-0.1827119	4.15070648	0.46240395	0.50433343	0.30880276
Usp15	-0.0997431	6.41798032	0.46213799	0.50445466	0.30885177
Nasp	-0.1282111	4.75297334	0.46197603	0.50452852	0.30886796
Ldb1	-0.1247325	3.97055761	0.46189944	0.50456344	0.30886796
Efnb2	0.10029624	6.13005427	0.46176698	0.50462386	0.30887973
Aldh4a1	0.1469617	4.20524868	0.46128207	0.50484516	0.30898998
Bola1	0.24582791	1.69514703	0.46113986	0.50491009	0.30899527
Dhx38	-0.1180744	4.56252447	0.4610827	0.50493619	0.30899527
Olfr1417	-0.9358942	-2.0114826	0.4638565	0.50504774	0.30903832
Klhl15	-0.1914362	3.77533684	0.46061356	0.50515052	0.309076
Sparcl1	-0.1211984	8.69193104	0.46037352	0.50526025	0.30909485
Arglu1	-0.0734033	6.85567336	0.4603659	0.50526373	0.30909485
Egf	-0.8750247	-1.2277752	0.45936175	0.50572321	0.3093507
Def8	-0.1172026	4.42867502	0.45909631	0.50584479	0.30937518
Fignl1	0.583589	-0.7257423	0.45897008	0.50590263	0.30937518
Gm14015	-0.7782423	-0.754935	0.45895879	0.5059078	0.30937518
2410004P03I	0.2121824	2.3157078	0.45891428	0.5059282	0.30937518
Ubtd2	0.10753071	5.27685835	0.45842116	0.50615427	0.30948819
St6gal2	-0.1368889	4.83107162	0.45816953	0.5062697	0.30950871
Golim4	-0.1213483	5.93506605	0.45816814	0.50627034	0.30950871
Il1rapl2	-0.2020737	3.23313246	0.45804497	0.50632686	0.30951803
Armc6	-0.2172937	1.69641095	0.45711943	0.50675192	0.30973189
Ttyh3	-0.1193665	6.04932733	0.4571034	0.50675928	0.30973189
5730480H06I	-0.6161882	0.11818548	0.4567326	0.50692976	0.30979616
Arnt	0.09496023	6.02241584	0.45669505	0.50694703	0.30979616
1700102P08I	-0.6599351	-1.0640683	0.4564555	0.50705722	0.30983825
Surf1	0.14220534	4.01917473	0.45630723	0.50712544	0.3098547
Ajuba	-0.1538066	2.82632005	0.45581744	0.50735093	0.30994854
Pcsk1n	0.11579615	4.4277672	0.45574626	0.50738371	0.30994854
Cenph	-0.5871162	-0.5446331	0.45570443	0.50740298	0.30994854
Zfp133-ps	-0.3292293	0.55557483	0.45547785	0.50750737	0.30998706
Catip	-0.21178	2.46427014	0.45534213	0.50756992	0.31000003
Cxcl9	-0.7369932	-0.5124598	0.4547859	0.50782641	0.3101213
Orc3	0.07889706	7.85503594	0.45467568	0.50787726	0.3101213

Ppp4r1l-ps	0.2733181	1.82378909	0.45464265	0.5078925	0.3101213
Cda	0.74488174	-0.9011938	0.45440439	0.50800246	0.31016319
Irf4	-0.1795604	5.17414265	0.45421761	0.5080887	0.31018307
Asic1	-0.124263	4.84553209	0.45415476	0.50811772	0.31018307
Arsg	-0.1507498	3.08586952	0.45403943	0.50817098	0.31019034
Wdr8	-0.2177319	2.26818252	0.45381562	0.50827438	0.31021959
Fance	0.16611314	2.70443339	0.45375666	0.50830162	0.31021959
Tmem47	-0.1194197	7.50737928	0.45353311	0.50840494	0.3102574
Spag16	-0.3464649	0.42113633	0.4530735	0.50861747	0.31034874
Scml4	0.1588347	4.00497164	0.45303053	0.50863736	0.31034874
Sertad2	0.10114345	5.37338932	0.45269653	0.50879191	0.3104178
Olfr1393	-1.0306	-1.883936	0.45248108	0.50889166	0.3104534
Cited4	0.28512532	0.97690506	0.45236236	0.50894664	0.3104617
Acy1	-0.3288971	0.78264564	0.45213969	0.50904979	0.3104803
Pcyox1	0.12079547	5.62445195	0.45211782	0.50905991	0.3104803
Acp6	0.23248011	2.46566331	0.45159798	0.50930087	0.310602
Plcz1	-0.7147903	-0.6400122	0.4513338	0.5094234	0.31065147
Dgcr8	-0.111062	4.13811439	0.45119613	0.50948727	0.31066517
Urm1	0.20874589	2.19931317	0.45097368	0.50959051	0.3106925
Thnsl1	0.15428802	3.96352648	0.45092106	0.50961494	0.3106925
Eppk1	-0.6987879	-0.5770483	0.45075524	0.50969192	0.31070416
Unc119	0.28256248	1.76904327	0.45069737	0.5097188	0.31070416
Lhx9	0.64388718	-1.07217	0.45058225	0.50977226	0.31070416
Churc1	0.09870122	4.54674334	0.45052311	0.50979973	0.31070416
Tdrd3	-0.0915344	5.51620388	0.4500235	0.5100319	0.3108204
Katna1	0.18305657	3.60927627	0.44977599	0.51014699	0.31086528
Ush1g	-1.2642201	-1.4019684	0.44947076	0.51028898	0.31091689
Zranb1	-0.0971118	4.76917413	0.4494109	0.51031684	0.31091689
Wdr37	-0.0811149	7.17723915	0.44932669	0.51035603	0.31091689
Grcc10	0.24041806	3.80079278	0.44850394	0.51073921	0.31111378
Gm15413	-0.7088537	-0.5351781	0.44839594	0.51078955	0.31111378
Gramd1c	0.31699255	1.68330996	0.44836572	0.51080364	0.31111378
Aqp4	-0.1409146	5.58475126	0.44820231	0.51087982	0.31113492
Sirt7	-0.1652703	3.02721932	0.448106	0.51092473	0.31113701
Zfp286	-0.1801948	3.27813945	0.4467808	0.5115434	0.31148847
Rps8	0.13509005	7.08743797	0.44655543	0.51164875	0.31152733
Smim6	-1.1093803	-1.6185278	0.44616436	0.51183164	0.3116134
Mettl9	0.10724525	4.83069938	0.4459596	0.51192745	0.31164643
1700017G19	0.39218274	0.70194423	0.44584172	0.51198262	0.31165473
Txlna	-0.1010359	5.13475204	0.44573215	0.51203392	0.31166066
Atg16l1	-0.1570582	3.88649107	0.44553959	0.51212408	0.31169025
Dio3os	-0.9690819	-1.726407	0.44529968	0.51223645	0.31173335
B3gat3	-0.1614369	3.19066001	0.44515675	0.51230342	0.31174881
Msl3l2	-0.1551998	4.12858652	0.44506557	0.51234615	0.31174952
Ercc6l	-0.512101	-0.7930277	0.44432016	0.51269571	0.31192058

Cdon	-0.165454	5.1561861	0.44428878	0.51271044	0.31192058
Mrap2	0.37572724	1.52222856	0.44405163	0.51282174	0.31193779
Ovol1	0.51935282	-0.3744116	0.4440513	0.5128219	0.31193779
Pold2	0.27157962	2.57042042	0.44388038	0.51290215	0.31196131
Zkscan7	-0.284783	1.50064791	0.44376134	0.51295805	0.31197002
BC027231	-0.143462	3.97245081	0.44305179	0.5132915	0.3121475
Arfgap2	0.15706849	4.17412182	0.44282892	0.51339632	0.31217373
1810010H24	-0.2605942	1.16088715	0.44278312	0.51341786	0.31217373
Ddrgk1	0.13254143	4.10669404	0.44211443	0.51373261	0.31233979
Cdipt	0.10600053	4.67887572	0.44192702	0.51382088	0.31236814
Epn1	0.10790796	4.47923716	0.44169685	0.51392933	0.31240875
Slc39a9	-0.1037912	4.89213791	0.44114387	0.51419005	0.31254191
Kcnab3	0.10380193	5.49972719	0.44044774	0.5145186	0.31271628
Spin4	0.21814386	2.74782339	0.43954975	0.51494298	0.31294885
Dnase1l3	-0.7148255	-0.8624302	0.43900065	0.51520279	0.31305896
Zfp672	0.11228021	4.76502454	0.43899044	0.51520762	0.31305896
Lamtor4	0.29990763	1.98318415	0.43875379	0.51531967	0.31310168
Rpf2	0.10593409	4.36084234	0.43859645	0.51539419	0.31311958
Tktl1	-0.8107113	-1.6232973	0.43851532	0.51543262	0.31311958
Vipas39	0.09145124	4.82685102	0.4383351	0.51551801	0.3131461
Iglon5	0.14988726	3.56600338	0.43820756	0.51557846	0.31315745
Fbxo48	-0.5572783	-0.7738056	0.43800282	0.51567552	0.31319105
Dclre1b	0.1406842	3.22638578	0.43757396	0.51587894	0.31328923
Ripply3	-0.3117343	1.46341984	0.43734098	0.5159895	0.31330715
Pex3	0.10352415	5.12396252	0.43733576	0.51599198	0.31330715
Taf5	0.16342841	3.93774759	0.43723573	0.51603947	0.31331062
Gm7008	-0.438815	-0.366684	0.43699856	0.51615209	0.31335363
Tmem86a	0.25279576	4.25573713	0.43638764	0.51644238	0.3135045
Zfp114	0.40556496	0.38684237	0.43594496	0.51665292	0.31360693
Sf3b3	-0.0981228	5.27586135	0.43578173	0.51673059	0.31362869
Ms4a4c	0.88185545	-1.6384002	0.43568687	0.51677574	0.31363072
Znhit2	0.16477483	3.10195257	0.43558949	0.51682209	0.31363347
Kcna6	0.11523762	5.75091568	0.43510696	0.51705189	0.31371458
Fam24a	-1.1428052	-2.0965885	0.43509733	0.51705648	0.31371458
Shisa9	-0.1247451	6.09755279	0.43496683	0.51711866	0.31371458
Atp6v0b	-0.1258166	4.83149849	0.43495768	0.51712302	0.31371458
AI606473	0.52651345	-0.0018368	0.43401418	0.517573	0.31396217
Aldh2	0.13949844	5.30454069	0.43389836	0.51762829	0.31397031
Slc25a18	0.18728978	2.52180671	0.43348739	0.51782455	0.31403858
Rnf17	-0.376799	0.73325128	0.43348736	0.51782456	0.31403858
Gm4814	-0.7037883	-1.5837911	0.43335843	0.51788616	0.31405055
Ube2c	-0.3402765	0.65337984	0.43203805	0.51851776	0.3143978
Fam13b	-0.074536	6.9242022	0.43197189	0.51854945	0.3143978
Hist1h4h	0.64737758	-1.3963942	0.43189863	0.51858454	0.3143978
Pla2r1	0.39666563	-0.1361902	0.43167776	0.51869035	0.31443654

Pnpla6	-0.1618227	4.04739104	0.43084109	0.51909154	0.31465431
Cysltr1	-0.4385664	-0.4593609	0.43051398	0.51924854	0.31472405
Trmt2b	0.17247262	5.21430636	0.43029699	0.51935273	0.31476177
Pdpn	0.20680538	5.22471167	0.42996921	0.5195102	0.31478963
Lpcat1	-0.2003329	2.86572616	0.42996675	0.51951139	0.31478963
Aard	0.25646333	1.58846714	0.42993927	0.51952459	0.31478963
I830077J02R	0.30814464	0.98222716	0.42956847	0.51970284	0.31485555
Tob1	-0.0921048	6.21399761	0.42948638	0.51974231	0.31485555
2310033P09I	0.1696925	3.12529055	0.42945104	0.51975931	0.31485555
Lasp1	0.07227503	6.52538605	0.42916353	0.51989762	0.3149139
Lypla2	0.19075775	2.10910724	0.42898475	0.51998366	0.31494059
Gm15816	-0.445986	0.7008866	0.42834852	0.52029007	0.31510073
Ulk2	-0.0773403	7.46675213	0.42774893	0.52057912	0.31524621
Prss36	-0.3683245	0.86859551	0.42767592	0.52061434	0.31524621
Smad5	0.0866961	6.179103	0.42720831	0.52084	0.3153574
Lig1	0.11260661	4.07456712	0.42695641	0.52096164	0.31540559
Dnajb2	0.11513232	5.41496715	0.42670439	0.52108338	0.31545077
Aldob	-0.5108001	0.34713287	0.42661518	0.52112649	0.31545077
Misp	0.71100875	-1.123436	0.42647963	0.52119201	0.31545077
Il18rap	-0.6182411	-0.4302662	0.42645276	0.52120499	0.31545077
Zdhhc15	-0.18078	3.45046656	0.42635313	0.52125316	0.31545077
Hk1os	0.3641278	0.80907124	0.42619282	0.52133067	0.31545077
Osbp	0.08057765	5.35473339	0.42618425	0.52133482	0.31545077
Iqce	-0.1611709	3.35711486	0.42610596	0.52137268	0.31545077
Zfp110	-0.1282159	4.42279014	0.42545141	0.52168944	0.31561696
Hck	0.55887461	-0.4803346	0.42524303	0.52179036	0.31565256
4933413J09F	0.9897548	-1.5657156	0.42493381	0.52194018	0.31571773
Gm13293	-0.2982377	0.84650605	0.42458255	0.52211046	0.31577459
Ankdd1b	-0.1346342	3.20222513	0.42456623	0.52211838	0.31577459
Sun2	-0.1296326	6.58459989	0.42414654	0.52232197	0.31584814
A830009L08I	0.28005075	0.87970727	0.42414195	0.5223242	0.31584814
Cadm1	-0.1248875	6.13708656	0.42391667	0.52243354	0.31588159
Bbs5	0.14665679	3.73149981	0.42382132	0.52247983	0.31588159
Slfn9	0.24899273	1.62630599	0.42376775	0.52250584	0.31588159
Npm3	0.4390623	-0.8634113	0.4235343	0.52261923	0.31592467
Gid8	0.07636047	6.55454777	0.42343704	0.52266648	0.31592778
Fkbp14	0.16610492	4.22152797	0.4230699	0.52284491	0.31601017
Supv3l1	0.16312706	3.17870659	0.42276923	0.52299112	0.31607307
Golga1	0.08628001	5.51897548	0.42217762	0.52327903	0.3162216
Immt	-0.0905827	6.62963615	0.42174595	0.52348929	0.31632317
P2ry10	0.8719777	-1.3492971	0.4215877	0.52356641	0.31633916
Mecp2	-0.0829079	8.93917506	0.4215186	0.52360009	0.31633916
Poc1b	0.12244067	4.59320195	0.42130138	0.52370599	0.3163724
Carkd	0.12764918	3.50228562	0.42117676	0.52376677	0.3163724
Cyb561d1	-0.1311816	3.70922664	0.42114625	0.52378164	0.3163724

Adcyap1r1	0.1175293	6.44811207	0.42072432	0.52398752	0.31646915
Cybb	-0.4134857	0.85518632	0.42064507	0.5240262	0.31646915
Reck	0.1417899	5.35084657	0.4204397	0.52412648	0.31650423
Dpm1	-0.1132966	5.2982306	0.41980648	0.52443587	0.31665331
Mars	0.08997287	4.89170001	0.41976167	0.52445778	0.31665331
Slc9a6	0.09086401	6.2789716	0.41944067	0.52461476	0.3166973
Jrk	-0.2101589	2.19497887	0.41944001	0.52461508	0.3166973
Gstt2	0.30621683	2.13162719	0.41908203	0.52479025	0.31677756
Morc3	-0.1443231	4.5466549	0.41880967	0.52492359	0.31682166
Zfp101	0.1575191	2.54349396	0.41866424	0.52499482	0.31682166
Tmem130	0.12755456	5.16068055	0.41861514	0.52501887	0.31682166
Zfp3	0.15597592	3.98973026	0.41858781	0.52503226	0.31682166
Gnal	-0.1139929	8.43372102	0.41838678	0.52513076	0.31685561
Ncaph2	-0.1043387	4.2766549	0.41803247	0.52530444	0.31693492
Slc35f4	0.1749451	2.69234322	0.41789006	0.52537428	0.31695156
Rpap2	0.09532292	4.70556522	0.41761368	0.52550987	0.31700786
Osgin1	0.46819659	0.20312214	0.41687838	0.52587091	0.31720015
Pqlc2	0.32762923	0.36876817	0.41622528	0.52619197	0.31729015
Zscan22	0.10284947	5.38400301	0.41620896	0.5262	0.31729015
Fam132a	0.22222894	1.84484177	0.41616975	0.52621929	0.31729015
Sema3b	-0.3085367	2.712754	0.41614951	0.52622924	0.31729015
Ptprr	-0.1071534	4.20917954	0.41614471	0.5262316	0.31729015
Dlx5	-0.1842176	2.04843014	0.4158781	0.52636279	0.31734374
Tmem79	-0.7514937	-0.2191697	0.41548483	0.52655641	0.31743495
Rpia	0.15567252	2.92032853	0.41523065	0.52668162	0.31747885
Nrf1	0.12343359	3.87283049	0.4151087	0.52674171	0.31747885
Zp3r	-0.8093216	-1.4150992	0.41496404	0.52681301	0.31747885
Msra	0.16965772	3.48859937	0.41491453	0.52683742	0.31747885
Tmem63b	0.11123015	5.59252783	0.41490758	0.52684084	0.31747885
Arc	-0.261115	5.37353445	0.41372932	0.5274223	0.31780371
Nphp3	-0.1908259	2.49082718	0.41342781	0.52757128	0.31786795
Cenpi	-0.5700972	-0.5162075	0.41325043	0.52765896	0.31787158
Atg4b	-0.1292376	3.8982455	0.41324417	0.52766206	0.31787158
Tsacc	-0.4961431	0.05827724	0.4130442	0.52776094	0.31788372
Bcas3	-0.117644	4.43762814	0.41303202	0.52776696	0.31788372
Hist2h2bb	-0.616388	-0.8157757	0.41195709	0.52829909	0.31817868
Fam131b	-0.1250038	4.95506433	0.41181558	0.52836922	0.31819537
Zmynd10	-0.37931	0.25024876	0.41168627	0.52843332	0.31820842
Hyal1	0.22451261	3.56170097	0.41154247	0.52850461	0.31822581
Zfp956	0.25346594	2.29736313	0.4108779	0.52883433	0.31839878
G3bp1	0.09618317	5.14055908	0.41070118	0.52892207	0.31842605
Pus7l	-0.2730896	1.59734635	0.41054476	0.52899976	0.31843281
Rnf168	0.08544539	6.53820425	0.41050765	0.5290182	0.31843281
Purg	-0.1095818	6.01491215	0.4103244	0.52910924	0.31846206
Neto1	-0.1292075	7.50462645	0.41019381	0.52917413	0.31847556

Gm5148	0.14105589	3.95471613	0.40975413	0.52939275	0.31858157
Snx27	-0.072243	7.29329358	0.40939466	0.5295716	0.31866364
Vrk1	-0.1283141	3.86179314	0.40928844	0.52962447	0.31866989
Slc39a14	-0.1415441	3.35867201	0.40887244	0.52983162	0.31876393
Zc3h14	0.08071286	6.41769269	0.40880394	0.52986575	0.31876393
Bfsp2	0.54440898	-0.7331281	0.40861995	0.52995744	0.31879352
Eaf2	-0.2212204	1.50594615	0.40796193	0.53028556	0.31896532
Slc43a3	0.51075314	0.2159002	0.40754677	0.53049278	0.31906369
Susd1	-0.2532392	1.90334626	0.40746389	0.53053416	0.31906369
Top3a	0.25619357	0.99311995	0.40731386	0.53060909	0.31908317
Ccdc34os	-0.8343741	-1.6641624	0.40709936	0.53071626	0.31912203
Elac1	-0.0968364	5.18459079	0.40632078	0.53110557	0.31932515
Angel2	-0.0892554	5.77778424	0.4062536	0.53113919	0.31932515
Hnrnpf	0.12460589	6.78931311	0.40602325	0.53125449	0.31934568
Ace	-0.2155179	5.32388205	0.40601526	0.53125849	0.31934568
Ngfr	0.15422709	3.55633351	0.40592924	0.53130156	0.31934598
Efcab6	0.27318924	1.7914979	0.40568287	0.53142495	0.31939455
Aifm2	0.22058435	2.59545827	0.40532919	0.53160217	0.31947547
Chrm1	-0.0952612	5.75229557	0.40517986	0.53167703	0.31949486
Slc9a3r1	0.18884823	4.2153837	0.40488907	0.53182286	0.31955689
Gmppa	0.10589398	3.73672678	0.40442518	0.53205566	0.31967117
Slc6a13	-0.1850355	6.53886407	0.40432704	0.53210493	0.31967516
Larp1	-0.0767849	7.30000833	0.40357043	0.53248509	0.31986485
Lsm11	-0.1088819	4.47219831	0.40352895	0.53250595	0.31986485
Gm11346	-0.6968788	-0.647839	0.40327913	0.53263159	0.31990139
Spg20	-0.0943091	5.84138243	0.40323841	0.53265208	0.31990139
Crtc2	-0.1134501	4.0438307	0.4028564	0.53284432	0.31999123
Iba57	0.27793277	0.816488	0.40259839	0.53297424	0.32002701
Pln	-0.4256291	0.61041831	0.40256863	0.53298922	0.32002701
Pdhx	0.09056471	5.1461394	0.40240667	0.53307081	0.32003729
Dcaf15	-0.2588359	1.33317004	0.40236527	0.53309167	0.32003729
Incenp	0.14686623	3.47791435	0.40221712	0.53316633	0.32005649
Cacng3	0.08329894	5.64058481	0.4018968	0.53332781	0.32012781
Fam193a	-0.0919479	7.39228776	0.40165591	0.53344931	0.32016754
Gng4	0.09641619	5.47719063	0.40159636	0.53347935	0.32016754
1600014C10I	0.12426503	4.83188295	0.40127127	0.53364342	0.32021015
Mmrn2	-0.5635592	-0.3197743	0.40121614	0.53367125	0.32021015
Thpo	-0.2172707	2.09779978	0.40120193	0.53367842	0.32021015
Ccl27a	0.11318091	5.16769628	0.40107942	0.53374028	0.32022165
Fbrs	0.10512489	4.54391855	0.4009702	0.53379544	0.32022913
Cdca7	0.43086796	0.33717584	0.40085802	0.53385211	0.32023752
Gar1	0.10256823	4.14274439	0.40042684	0.53407002	0.32034262
LOC1008616	-0.4157775	-0.5326978	0.4002803	0.53414412	0.32034448
Gp49a	0.50133584	0.04712162	0.40025179	0.53415853	0.32034448
Ackr2	0.38891647	0.49083056	0.39998646	0.53429275	0.32039936

Gtf2i	-0.0716339	7.28734558	0.39982758	0.53437315	0.32040329
Sgsm3	-0.1375397	3.69861639	0.39980469	0.53438474	0.32040329
Mrps10	-0.1504506	2.80224304	0.39940969	0.53458473	0.32048174
Tmem189	0.17610355	2.5608425	0.39932609	0.53462707	0.32048174
Pbx2	0.11474928	4.84810198	0.39929318	0.53464374	0.32048174
Actn1	-0.1703118	4.61802019	0.39890113	0.53484242	0.32057521
Trim68	-0.2828309	1.24116995	0.39876275	0.53491258	0.32059165
Wdr41	-0.0887485	4.83009109	0.39841733	0.53508778	0.32067103
Gdnf	0.46031833	-0.5461555	0.39812914	0.53523404	0.32073306
Pde4a	-0.1181096	6.32580238	0.39803413	0.53528227	0.32073634
Shmt2	-0.1619414	2.55247759	0.39768571	0.53545923	0.32081674
Dpp9	0.10390807	4.59523009	0.39720867	0.53570168	0.32093637
Kcnq1	0.82657737	-1.3330321	0.39684114	0.53588861	0.32102272
Utp14a	0.12565475	3.98157565	0.39669351	0.53596374	0.32103108
Kif11	-0.2164762	2.2910576	0.39664552	0.53598816	0.32103108
4930563E18l	-0.9870948	-1.5944678	0.39648874	0.53606797	0.32105325
Ankrd37	-0.2506948	1.64700432	0.39631325	0.53615733	0.32108113
Zfp943	-0.1488746	3.03883785	0.39584807	0.53639433	0.32119742
Zfp61	-0.1729888	3.04587609	0.39543055	0.53660721	0.32129898
Rnf38	0.06985154	7.97757377	0.39534746	0.53664959	0.32129898
Bckdha	0.21895668	1.13284598	0.39484549	0.53690579	0.32142671
Tspan3	0.11563092	7.0022232	0.39457829	0.53704225	0.32148275
Neil1	-0.4138477	-0.0590332	0.39413588	0.53726834	0.32159242
Zswim6	0.08429244	6.10393689	0.3938587	0.53741008	0.3216516
Tada2b	-0.104105	4.39056583	0.39356413	0.5375608	0.32171614
Asns	-0.122636	5.25667555	0.39319926	0.53774759	0.32180225
Pigk	-0.1209039	5.13219269	0.39297772	0.53786106	0.32183763
Pzp	-0.5941673	-0.8954508	0.39291632	0.53789252	0.32183763
Slc35c2	0.24559759	1.83053402	0.3927513	0.53797708	0.32185523
Tk1	-0.6362336	-0.8897527	0.39269147	0.53800774	0.32185523
Lpar1	0.16055162	6.85129751	0.39260079	0.53805423	0.32185737
Macrod1	0.4177432	0.16466757	0.39249826	0.53810679	0.32186315
Ciz1	0.09219983	5.70076852	0.39222957	0.5382446	0.32190221
Zfp72	-0.1859073	3.12617452	0.39220357	0.53825793	0.32190221
Tmsb15l	0.30510733	1.11771483	0.39178952	0.53847042	0.32200362
Cntd1	0.16328081	2.33775579	0.39169	0.53852153	0.32200851
Hspa12b	0.16681139	3.62175018	0.39139047	0.53867537	0.32205563
Tmed3	0.19341776	2.97606323	0.39136938	0.5386862	0.32205563
Golt1b	0.10768744	5.46758268	0.39126168	0.53874155	0.32206305
Gopc	-0.0764011	6.07453978	0.39094346	0.53890512	0.32213517
Zfp334	-0.0879275	4.89297415	0.39075724	0.53900088	0.32216674
AF251705	0.53088914	-0.5359717	0.39066083	0.53905047	0.32217071
Asah1	-0.0964511	5.68220169	0.39055994	0.53910238	0.32217606
Mark1	0.08182115	5.97795323	0.39027553	0.53924876	0.32221241
Nhlrc3	0.2737738	0.51559575	0.39027483	0.53924911	0.32221241

Kat8	-0.1209398	3.25460002	0.38999586	0.53939276	0.32226643
Fmr1	-0.0740859	6.28502288	0.38993241	0.53942545	0.32226643
Cept1	-0.1326222	3.88413645	0.38977776	0.53950512	0.32227533
Qdpr	-0.0809425	5.53356808	0.38973672	0.53952627	0.32227533
She	-0.3342776	1.34917998	0.38964643	0.53957279	0.32227746
Ccl7	0.60605208	-0.4119397	0.38953486	0.5396303	0.32228614
Igsf11	0.0920182	5.86683619	0.38921464	0.53979541	0.32235909
Abcb4	-0.3741807	0.37409322	0.38908513	0.53986222	0.32237332
Satb1	0.10815908	7.71689747	0.3886026	0.54011126	0.32248858
Dbr1	-0.1223538	3.96110004	0.38848041	0.54017436	0.32248858
Peg13	0.08499912	8.15164275	0.38845902	0.5401854	0.32248858
Taf1d	-0.1161445	3.99055053	0.38835527	0.540239	0.32248858
Fgf23	-0.6941144	-1.0142725	0.38829488	0.54027019	0.32248858
C1qa	-0.6217122	-1.5955507	0.38804226	0.54040073	0.32254083
Cdk12	-0.0776282	7.26615932	0.38792613	0.54046076	0.322551
Matk	0.14807412	3.61966561	0.38776017	0.54054657	0.32257655
4833422C13I	-0.2457277	2.31396796	0.38732424	0.54077209	0.32267615
Ranbp3l	0.12617327	7.77261109	0.38727125	0.54079952	0.32267615
Cbr2	0.2789642	1.3138646	0.38713091	0.54087217	0.32269383
Pi4k2a	0.10922565	4.91082753	0.38696637	0.54095737	0.322719
Zfp52	0.11089017	4.08566603	0.38677856	0.54105464	0.32273764
3110001I22R	-0.2715995	1.10874887	0.38673992	0.54107466	0.32273764
Casc4	0.08665416	8.2105552	0.38644684	0.54122654	0.32280256
Cebpg	0.07967132	6.39864754	0.38601635	0.54144977	0.32291003
Usf1	0.18556485	2.76170238	0.38586677	0.54152738	0.32293064
Mthfr	-0.1908769	2.89904496	0.38569287	0.54161763	0.32295878
Ctxn2	0.27285186	1.98381935	0.38521753	0.54186447	0.32308028
Ddx3x	-0.0595027	8.72107366	0.38512565	0.5419122	0.32308307
Foxo3	0.08699713	6.59802806	0.38491219	0.54202314	0.32312353
Wbscr25	0.6165854	-0.9108051	0.38471712	0.54212456	0.32313298
Angpt1	0.14459551	4.59662105	0.38471597	0.54212515	0.32313298
Brinp2	-0.1041614	4.4590988	0.38459111	0.54219009	0.32314601
Elmsan1	0.08414426	5.92131118	0.38448568	0.54224492	0.32315301
Cyb5d2	0.13079044	3.33322216	0.38440035	0.54228932	0.32315379
Wdr65	-0.3702972	1.0782569	0.38410981	0.54244052	0.32320239
Mal2	0.0746997	6.39667895	0.38407808	0.54245704	0.32320239
Gm6654	0.31075833	0.80954439	0.38380093	0.54260135	0.32324065
Slc33a1	-0.1361277	3.78701826	0.383755	0.54262528	0.32324065
Gata2	-0.4261907	0.77249264	0.38365627	0.54267671	0.32324065
Hnrnpdl	0.06591661	7.47776317	0.38359765	0.54270725	0.32324065
Emr1	0.39268738	0.09863425	0.38354111	0.54273672	0.32324065
Zfp7	0.19871625	2.73567721	0.38331183	0.54285622	0.32326383
Vat1l	-0.1359517	5.41946063	0.38330107	0.54286183	0.32326383
Prss57	1.03923685	-1.3924371	0.38316726	0.54293159	0.32327971
Prss35	-0.3210781	0.77546363	0.38292644	0.5430572	0.32331295

Ehbp1	0.08959705	6.77901696	0.38289496	0.54307362	0.32331295
Lats2	0.09929119	6.82253489	0.38208139	0.54349838	0.32354015
0610010F05l	0.10982828	5.99731277	0.38195401	0.54356494	0.3235541
Dos	0.10026612	6.40590854	0.38173413	0.54367987	0.32358804
Cyp4f17	-0.7062015	-1.7790445	0.38167988	0.54370823	0.32358804
Wipf2	0.07736957	7.56041348	0.38151418	0.54379489	0.32361394
Wtip	0.2208737	2.38718565	0.38106979	0.54402741	0.32372663
Cpb2	-0.9690936	-1.7620521	0.38070675	0.5442175	0.32381406
Snn	-0.0762366	6.63171243	0.38058712	0.54428017	0.32382566
Pld4	0.37810899	-0.3204414	0.38046684	0.5443432	0.3238342
Ints9	0.09632388	4.3612064	0.38038195	0.54438768	0.3238342
Deaf1	-0.129541	4.04081843	0.38026902	0.54444688	0.3238342
Thoc5	0.13939054	3.55371757	0.38023025	0.5444672	0.3238342
Hnrnph3	0.09081767	4.49209724	0.38013501	0.54451714	0.32383822
Fam207a	0.16012606	3.37033488	0.37993301	0.54462307	0.32387554
Alg8	0.11934484	3.33045314	0.37968077	0.54475541	0.32392544
Gclc	0.08158368	6.26031202	0.3795847	0.54480583	0.32392544
Sstr1	0.24386748	3.05387091	0.37942318	0.54489062	0.32392544
Fam20c	-0.2137175	2.99503013	0.37937259	0.54491718	0.32392544
Hfe	-0.2595513	3.40174727	0.37936172	0.54492289	0.32392544
Drd5	-0.384722	0.25089244	0.37899012	0.54511808	0.3240134
Ldlrad4	-0.100373	4.36462641	0.37891557	0.54515725	0.3240134
Gm3500	-0.3702713	-0.3763363	0.37876335	0.54523726	0.32403184
Smcr8	-0.0965019	5.78459802	0.37862504	0.54530997	0.32403184
Neurl3	0.32132836	1.02709041	0.37861003	0.54531787	0.32403184
Slc7a8	0.11151058	5.5378534	0.37834087	0.54545943	0.32407427
Arhgap44	-0.0795094	6.68127894	0.37826236	0.54550074	0.32407427
Yipf1	-0.1539147	3.00966955	0.37822787	0.54551888	0.32407427
Map2k7	-0.1213093	5.36639003	0.37799571	0.54564107	0.32412119
Gm5795	0.84799011	-2.0561598	0.37691798	0.54620895	0.32443283
Nxph2	0.38648465	0.00279407	0.37633877	0.54651462	0.32458869
1810020O05	0.42293619	-0.8442492	0.37598739	0.54670022	0.32467321
Ncapg	-0.5630494	-0.3341187	0.37572366	0.54683959	0.32473027
Sephs1	-0.096316	5.67359731	0.37549197	0.54696209	0.32473775
Slc39a11	-0.2036977	1.72164444	0.37545765	0.54698024	0.32473775
Cabp7	0.54760069	-1.1968149	0.37538568	0.54701831	0.32473775
Pkmyt1	0.3946904	-0.4137615	0.37534332	0.54704071	0.32473775
Zfp335	-0.1446328	3.92791599	0.37529053	0.54706864	0.32473775
Fto	-0.0734804	7.00755629	0.37473912	0.54736051	0.32488529
Ripk3	-0.6296651	-0.6316331	0.37428565	0.54760075	0.32500218
Itpripl2	-0.1182163	5.91294504	0.37410612	0.54769592	0.32502099
Arhgef25	0.1002294	5.31720204	0.37406238	0.54771911	0.32502099
Slc16a1	-0.1190319	5.03789291	0.37394209	0.54778291	0.32503313
Eri2	-0.1240762	4.18640994	0.37328494	0.54813164	0.32520417
Thumpd3	-0.0997894	4.77480916	0.37323557	0.54815786	0.32520417

Rnmt	0.07751183	5.92020427	0.37272981	0.54842657	0.32533785
Brf1	-0.1004074	4.23692636	0.37145198	0.54910661	0.32570684
Pabpc4l	0.2694695	2.23419676	0.37139794	0.5491354	0.32570684
Il6ra	-0.202474	2.57813041	0.37060499	0.54955826	0.3259147
Ivd	0.09593499	4.16781787	0.37050377	0.54961228	0.3259147
LOC1008622(	0.19144847	2.56939459	0.37049642	0.5496162	0.3259147
Clec1a	0.26715539	1.93760054	0.36982537	0.54997461	0.32610145
Nfkbil1	-0.3859069	-0.2143319	0.36967931	0.55005268	0.32611124
Cdc42ep5	0.23211092	2.49276455	0.36958222	0.55010459	0.32611124
Caps2	-0.6519505	-1.2710401	0.36955052	0.55012154	0.32611124
Papd4	-0.116236	5.05321007	0.36937165	0.55021719	0.32614217
Folr2	0.64927534	-0.8304092	0.36902193	0.55040432	0.32621329
Derl1	-0.1058521	5.09007702	0.36898486	0.55042416	0.32621329
D030056L22I	0.11850596	4.86622	0.36812352	0.55088558	0.32646097
Gm14379	-0.5214068	-0.7781729	0.36794793	0.55097974	0.32649097
Apmap	-0.1341801	4.89839302	0.36769104	0.55111755	0.32654684
Atg13	0.07145513	5.92728263	0.36752372	0.55120734	0.32657424
Eif4ebp1	0.2293914	1.68990599	0.36731433	0.55131975	0.32661505
Cmtm8	0.43082153	0.26693661	0.36693769	0.55152206	0.3267091
Stk11ip	0.16645504	3.48361789	0.36644464	0.55178711	0.32680321
Zfhx4	-0.0902804	8.17332463	0.36639265	0.55181508	0.32680321
Plxdc2	-0.0737411	6.74706993	0.36631922	0.55185458	0.32680321
A830082N09	-0.1554105	4.43826558	0.36630048	0.55186466	0.32680321
1700102H20I	0.87212994	-1.2506463	0.36623618	0.55189925	0.32680321
Ndufs1	-0.1004524	6.10677345	0.36615614	0.55194232	0.32680321
Tlr8	0.86945315	-1.0531026	0.36606502	0.55199136	0.32680644
Zic3	0.27295184	1.36545791	0.36597166	0.55204161	0.3268104
Ubiad1	0.20041678	1.89560882	0.36563416	0.55222335	0.32689219
Angptl7	0.33858532	1.56096418	0.36553572	0.55227638	0.32689779
Cdhr1	-0.1608344	2.46622717	0.36516364	0.5524769	0.32695364
Krt222	0.08519149	6.63809537	0.36514392	0.55248754	0.32695364
Cd109	-0.2244149	3.07290153	0.36511802	0.55250151	0.32695364
Ccdc63	0.83681511	-2.0957611	0.36491959	0.55260851	0.32699117
Ppargc1a	-0.095067	7.95652804	0.36433742	0.55292269	0.32713032
Igfbp7	-0.1529092	5.10106368	0.36432223	0.55293089	0.32713032
Tctn2	0.14705816	2.69486415	0.3642292	0.55298113	0.32713424
Amz2	-0.1049193	4.75288145	0.36413746	0.55303069	0.32713776
Rag1	-1.2786363	-1.9203626	0.36347339	0.55338963	0.32732427
Ppp1r3b	-0.1399685	4.93530695	0.36319354	0.55354102	0.32734356
Car5b	-0.1803486	1.96853498	0.36317659	0.5535502	0.32734356
Tmem173	0.23103435	1.61094865	0.36312563	0.55357777	0.32734356
6230400D17I	-0.4131873	0.25397357	0.36309051	0.55359678	0.32734356
Zfp870	-0.1290495	3.74149517	0.36291879	0.55368974	0.32734561
St18	-0.1731085	3.77041997	0.36291103	0.55369394	0.32734561
Kdelc1	0.15076468	2.8426093	0.36270854	0.55380359	0.32734561

Rsad2	0.29329591	1.25882069	0.36269404	0.55381145	0.32734561
Pafah1b1	0.07067165	9.15130596	0.36258556	0.55387021	0.32734561
Gm13826	0.14363918	2.96121639	0.36256567	0.55388099	0.32734561
Plce1	0.07951248	4.90128955	0.36252	0.55390573	0.32734561
Zfp37	0.10365339	6.80022442	0.36215845	0.5541017	0.3274285
Akap7	-0.0878835	6.50819349	0.36200855	0.55418299	0.3274285
Tab3	-0.0762477	6.0446983	0.36196763	0.55420518	0.3274285
Trim45	-0.1814348	2.49172665	0.36187712	0.55425428	0.3274285
Itgb1	0.12344521	6.34118709	0.36183846	0.55427525	0.3274285
Vgll3	-0.1810085	2.95816261	0.36177831	0.55430789	0.3274285
Col14a1	-0.3072057	0.85800448	0.3598299	0.55536704	0.32802729
Ercc8	-0.2075589	2.35817298	0.35975277	0.55540905	0.32802729
Cenpu	-0.3249147	-0.0263099	0.35946971	0.55556326	0.32809254
Sumf1	-0.128281	5.03265388	0.35888639	0.55588134	0.32825454
Shcbp1	-0.5304868	-0.5415224	0.35861758	0.55602804	0.32831103
Gpr6	-0.7445541	-1.4290837	0.35855072	0.55606454	0.32831103
Tmtc4	-0.1015819	4.07511298	0.35822748	0.55624106	0.32836974
Nxph4	0.35027777	0.63504163	0.35820832	0.55625153	0.32836974
1700040L02F	0.19853229	1.83594238	0.35804038	0.55634329	0.32839807
Elavl1	0.06908163	6.63422728	0.35786845	0.55643726	0.32841108
Pex7	0.10272146	4.47396907	0.35783986	0.55645289	0.32841108
Stat4	0.18432674	1.82879885	0.35744081	0.55667112	0.32851403
Zfp516	-0.0940517	5.28253629	0.35728859	0.55675441	0.32852091
Snora30	-0.6663098	-1.4576189	0.35722134	0.55679122	0.32852091
Oprd1	0.13890228	4.16299304	0.3571794	0.55681418	0.32852091
4930579K19I	0.81481986	-1.3326872	0.35676504	0.55704107	0.32859169
Tubg2	0.11727317	4.31544614	0.35673756	0.55705613	0.32859169
Tmcc1	0.05993322	6.94096167	0.35672038	0.55706554	0.32859169
Cenpl	-0.1992621	1.72929489	0.35644058	0.55721887	0.32865628
Opn1mw	-0.5963067	-1.6765362	0.35598235	0.55747016	0.32877865
B930025P03I	0.6345411	-1.1895272	0.35590023	0.55751522	0.32877937
2200002D01I	0.31286125	0.63943445	0.35537784	0.557802	0.32892264
A730046J19F	-0.3997402	0.59754944	0.3552589	0.55786734	0.32892266
Ankle1	-0.871792	-1.8448087	0.35521814	0.55788973	0.32892266
Nmnat3	-0.1901707	2.54700516	0.3550175	0.55799999	0.32894058
Gngt2	-0.3058048	0.72351475	0.35500324	0.55800783	0.32894058
Sms	0.07095235	7.09061847	0.35465652	0.55819847	0.3290271
Pcsk6	0.19011057	1.62600231	0.35413949	0.558483	0.32916895
Hyal2	-0.2446925	1.18618416	0.35352718	0.55882032	0.32929509
Tuft1	-0.3546833	0.44373696	0.35351384	0.55882768	0.32929509
A730056A06	0.24234289	1.59917993	0.35351195	0.55882871	0.32929509
Lrfn4	-0.2150356	2.51329453	0.35331745	0.55893595	0.32931154
Cplx3	0.22967394	1.32700046	0.35329214	0.55894991	0.32931154
Ids	0.08310602	9.64471167	0.35322246	0.55898834	0.32931154
Fam58b	0.15867034	4.09447951	0.35301565	0.55910243	0.32935289

Sfxn5	-0.1131804	4.82337831	0.35288986	0.55917184	0.32936792
Asb2	0.47769316	0.02670531	0.35270081	0.5592762	0.32940352
Rbm20	-0.1821823	2.41221173	0.35246281	0.55940763	0.32945506
Gm20753	-0.6570322	-1.593677	0.35231393	0.55948988	0.32945932
Ifit3	0.15157063	5.94234365	0.35229073	0.5595027	0.32945932
Cd3g	-0.8656593	-1.3715452	0.35198744	0.55967034	0.32953216
Bves	-0.4151379	-0.6052803	0.35179045	0.55977926	0.32957043
Rraga	0.09870032	6.03591811	0.35155503	0.5599095	0.32962124
Rad9a	-0.1274407	3.49725422	0.35141519	0.55998689	0.32964092
Mrpl47	-0.1015987	3.55239049	0.35131752	0.56004095	0.32964688
Map3k14	0.26631696	1.77007305	0.35112438	0.5601479	0.32968396
Snph	-0.115636	5.88306559	0.35094069	0.56024964	0.32971797
Trim9	-0.1201903	8.22979145	0.35046689	0.56051224	0.32984189
Desi1	0.07771584	5.52702697	0.35038378	0.56055833	0.32984189
Icam1	0.28750091	1.87701251	0.35032288	0.56059211	0.32984189
Rps6ka4	0.18236587	2.71149669	0.35015595	0.56068471	0.32984596
Rnf26	-0.1580036	3.12694688	0.35007959	0.56072708	0.32984596
Lama5	0.17920443	1.76974396	0.35007263	0.56073095	0.32984596
Tcerg1	-0.07869	6.61335519	0.349977	0.56078402	0.32985131
Mettl18	-0.2532664	2.00591038	0.34976215	0.5609033	0.3298956
Otud7b	-0.0698807	7.25464734	0.34962103	0.56098166	0.32991582
Dhx40	-0.0835355	5.37711587	0.34919117	0.56122051	0.33000469
Rassf6	-0.3561816	0.63128141	0.34919075	0.56122075	0.33000469
Nhlrc4	-0.5554295	-0.4151963	0.34867989	0.56150487	0.3301423
Cisd2	0.08119963	6.17756167	0.34861174	0.56154279	0.3301423
Cxcl16	0.21265266	3.95304305	0.34798856	0.56188981	0.33032042
Pou3f2	0.10435736	4.41732911	0.34787366	0.56195384	0.33033218
Atic	0.11326895	4.23843957	0.34743483	0.56219851	0.33045011
Ikzf1	-0.1296149	4.34495436	0.34735132	0.56224509	0.33045159
Zbtb1	0.09146411	4.59540125	0.34727082	0.56229001	0.3304519
Necab3	0.12061161	5.96483057	0.34719249	0.56233372	0.3304519
Atxn1	-0.093989	8.6929335	0.34707134	0.56240133	0.33046574
Ttc19	-0.0871521	6.26122451	0.34656894	0.5626819	0.33060471
Trpv2	0.13428833	3.07012746	0.34591564	0.56304716	0.33072898
Rab31	0.10074621	5.56481474	0.34576789	0.56312983	0.33072898
Pdik1l	-0.1093084	3.96548955	0.34575754	0.56313562	0.33072898
Exoc3l4	-0.4997332	-0.9109404	0.34568724	0.56317497	0.33072898
Rfc5	-0.1776782	2.67648638	0.34566128	0.5631895	0.33072898
Mmaa	-0.1554085	3.75448196	0.34556701	0.56324227	0.33072898
Nol11	-0.1075247	4.28240854	0.34549863	0.56328055	0.33072898
Rps11	0.1273911	6.85286099	0.34545046	0.56330753	0.33072898
Ddx49	0.28537839	0.76089997	0.34541938	0.56332493	0.33072898
C330027C09l	-0.2242209	1.86514799	0.34533022	0.56337486	0.33072898
Col6a4	-0.6084079	-0.2166068	0.34532392	0.56337839	0.33072898
Klf4	-0.1411758	6.47978248	0.34521769	0.5634379	0.33073803

Oscp1	-0.1363	3.51831819	0.34508828	0.56351041	0.33075471
Magi2	-0.1009952	7.42040035	0.34482767	0.56365648	0.33081456
Isoc1	0.10293848	5.90672511	0.34430609	0.56394905	0.33096038
Rasgrp1	0.10911091	9.64199137	0.34416192	0.56402998	0.33098197
Exosc8	0.10401768	3.93105156	0.34402428	0.56410726	0.33100143
Cep55	-0.6709534	-1.7708971	0.34386797	0.56419504	0.33102705
Oas1a	-0.4011577	0.68271435	0.34366682	0.56430805	0.33106746
Nras	0.07507269	7.27390837	0.3430684	0.56464453	0.33123895
Phf11c	-0.5587508	-1.5857981	0.34290866	0.56473441	0.33124788
Spata9	-0.136606	3.51825626	0.34288441	0.56474806	0.33124788
Ano6	0.12073043	6.43153776	0.34259295	0.56491214	0.33129695
Mctp2	-0.1881926	1.62389427	0.3425789	0.56492005	0.33129695
2410004N09	-0.2602671	0.54396546	0.34249051	0.56496983	0.33130024
Sf3a3	0.09459892	4.70077619	0.34227782	0.56508965	0.3313446
Siae	-0.1068206	4.5606751	0.34218448	0.56514225	0.33134954
Atp6v1c2	-0.4938691	-0.1751808	0.34206402	0.56521015	0.33136345
5031425E22I	-0.1497163	3.42439837	0.34112568	0.56573959	0.33164793
Dmrt3	-1.4010472	-1.5248204	0.34099223	0.56581497	0.3316662
Ier5l	0.54498139	-1.2465365	0.34086966	0.56588422	0.33167408
Ssbp2	-0.081292	6.26453336	0.34081192	0.56591685	0.33167408
5730403I07R	0.96812709	-1.970672	0.34054623	0.56606703	0.33173618
Ccl5	-0.2798677	1.43448762	0.33953321	0.56664039	0.33199361
Cacng8	0.35035674	0.70465337	0.33948827	0.56666585	0.33199361
Mro	-0.099016	4.44287072	0.3394814	0.56666974	0.33199361
Klhdc3	0.09087112	4.44002084	0.33941055	0.56670988	0.33199361
Tmem126a	0.10041416	4.47075064	0.33937931	0.56672759	0.33199361
Plbd2	0.09073444	5.28181595	0.33918507	0.56683769	0.33203217
Grm4	-0.1284887	4.02745174	0.33858185	0.56717989	0.3321951
Spats2l	0.08280268	5.09041211	0.33853865	0.56720441	0.3321951
Gm2762	-0.8830886	-1.6727129	0.33990075	0.56758106	0.33238974
Ggt7	-0.1534101	2.63432091	0.33764601	0.56771159	0.33239688
Slc44a3	0.54812776	-1.1805436	0.33761417	0.5677297	0.33239688
Tmem255b	-0.7339695	-0.9751836	0.33758051	0.56774884	0.33239688
C5ar1	0.5309952	-0.0568141	0.33754243	0.5677705	0.33239688
Gm9776	-0.1625113	2.31677585	0.33740976	0.56784597	0.33241513
Nek8	-0.36156	1.27127554	0.33732469	0.56789438	0.33241752
Ccdc170	0.29697853	0.66336915	0.33708051	0.56803336	0.3324334
Zfp768	0.18959466	2.42165946	0.33706369	0.56804294	0.3324334
Cd1d1	-0.6822344	-1.2586938	0.33699166	0.56808395	0.3324334
Etv5	-0.1012411	6.16652977	0.33696563	0.56809878	0.3324334
Pcdh17	-0.1108314	6.97977252	0.33675169	0.56822063	0.33246129
Tspyl2	-0.0944805	6.06718009	0.33667598	0.56826377	0.33246129
Cldn2	-0.3835995	1.2896757	0.33662562	0.56829246	0.33246129
Rsph3b	0.10450636	4.40296319	0.33653372	0.56834483	0.33246129
Ubxn8	0.12842116	4.33700244	0.33649303	0.56836803	0.33246129

Tifab	-0.298235	1.68083859	0.33627102	0.5684946	0.33250939
2810001G20	0.17208946	4.81931109	0.33575345	0.5687899	0.33265618
Fzd10	0.26079273	1.03771457	0.33559644	0.56887954	0.33268266
Zc3h3	0.17685543	2.62834499	0.335378	0.56900431	0.33272969
B3gnt6	0.94399909	-1.3576428	0.33509941	0.5691635	0.33275624
Nudt12	0.21797234	2.84423276	0.33508543	0.56917149	0.33275624
Clec2f	0.49105308	-0.7657779	0.33503015	0.56920309	0.33275624
Hibch	-0.1143123	4.11210801	0.33498804	0.56922717	0.33275624
Ehmt2	-0.0906454	5.08226054	0.33440074	0.56956314	0.33292448
Lrrc26	-0.6408594	-1.5248642	0.33432986	0.56960371	0.33292448
Xlr4a	-0.3951884	0.03908689	0.33423833	0.56965612	0.33292916
Garnl3	-0.1308469	5.19900696	0.33412319	0.56972205	0.33293028
Extl2	0.08593775	6.08279579	0.33407998	0.5697468	0.33293028
Stxbp1	0.08144117	10.0420214	0.33394097	0.56982643	0.33293908
B930041F14	0.09964562	5.11013948	0.33386796	0.56986826	0.33293908
Nfix	0.07299948	8.37197493	0.3338213	0.569895	0.33293908
Cmc2	0.11475503	3.25622659	0.33291863	0.57041276	0.3332156
Ten1	0.14214445	3.27363178	0.33277512	0.57049516	0.33323779
Ccm2l	0.58993258	-0.5703278	0.33250262	0.5706517	0.33330293
Dhx33	-0.1273049	4.90226004	0.33230126	0.57076742	0.33330293
Cog5	0.12374034	4.63452983	0.33227298	0.57078368	0.33330293
Pcdha10	-0.4041536	-0.3966167	0.3322717	0.57078441	0.33330293
Lcmt2	-0.1632566	2.52533972	0.33211022	0.57087726	0.3333312
Esr1	0.28648654	1.10288743	0.33193977	0.57097529	0.33333963
Cep104	-0.0889217	4.65266436	0.33193059	0.57098057	0.33333963
Gyltl1b	-0.8031211	-1.9403739	0.33173142	0.57109517	0.33336921
Bean1	0.10048759	3.92752223	0.33168806	0.57112012	0.33336921
Lrrc51	0.24252229	2.09673844	0.33147527	0.57124262	0.33341426
Cpeb2	-0.0657264	7.36638977	0.33139959	0.57128619	0.33341426
Elk3	0.09613395	4.99464242	0.33109229	0.57146322	0.33349163
Abhd16a	0.09016802	4.50715658	0.33099452	0.57151956	0.33349856
Cep44	0.14178172	2.75179763	0.33082882	0.57161507	0.3335067
Gm5105	-0.5559063	-0.6224718	0.33081606	0.57162244	0.3335067
Cirbp	0.18136238	4.87862209	0.33058731	0.57175435	0.33355772
Urah	-0.4060597	0.09704359	0.33040245	0.57186101	0.333594
Ndufb2	0.09981074	3.99487828	0.33019967	0.57197804	0.33360979
Klrk1	0.36710526	0.53259997	0.3301712	0.57199448	0.33360979
Tmem106a	-0.3362601	1.6543206	0.33008113	0.57204648	0.33360979
Klhdc9	0.11176607	3.65695043	0.33000375	0.57209116	0.33360979
Ginm1	0.1451561	5.23454548	0.32996676	0.57211253	0.33360979
Dock2	-0.1613044	2.3581505	0.32987315	0.5721666	0.33360979
Map3k1	-0.0917435	5.10802838	0.32981638	0.57219939	0.33360979
Mov10	0.16040541	2.92325618	0.32944287	0.57241526	0.33366668
4930539E08I	0.14099848	3.15092154	0.3293557	0.57246566	0.33366668
Sppl3	-0.0750344	5.25228808	0.32928259	0.57250794	0.33366668

Sla2	0.36380361	-0.1584206	0.32927179	0.57251419	0.33366668
4930578C19I	-0.9231457	-1.5017189	0.32926283	0.57251937	0.33366668
Kctd11	0.20020465	3.32432119	0.3291293	0.57259661	0.33368577
Ccdc36	-0.6185362	-1.4751331	0.32903264	0.57265254	0.33369244
D19Bwg1357	0.08099114	5.51629324	0.32846566	0.57298081	0.3338578
2010107G12	-0.8385285	-1.5028494	0.32812892	0.57317596	0.33394556
Cep63	0.11663654	5.47835818	0.32781252	0.57335944	0.33402652
Tbc1d2b	-0.092992	4.3888856	0.32749933	0.57354118	0.33410645
Thsd4	0.17252494	6.29216156	0.32713682	0.57375168	0.33420312
Bcor	-0.0792476	5.37892961	0.32668772	0.57401268	0.33432918
Hsbp1l1	0.20773257	1.59126542	0.32647007	0.57413925	0.33437694
Porcn	0.1106024	4.36489916	0.32621627	0.57428692	0.33443698
Rps23	0.11103214	7.24528783	0.3258609	0.57449382	0.33450402
Col5a3	-0.7526054	-1.0560599	0.32580899	0.57452405	0.33450402
Ano3	0.10685089	7.88990707	0.32578879	0.57453582	0.33450402
Rplp0	0.11271047	6.63245504	0.32570673	0.57458363	0.33450589
Bend4	-0.1108638	4.40579276	0.32535839	0.57478664	0.33459811
Ltf	0.34335267	0.23885672	0.32419075	0.57546821	0.33496887
Rfxap	0.13857225	4.03711884	0.32376489	0.5757172	0.3350878
Ccdc34	0.09468105	6.08041037	0.32367775	0.57576817	0.33509147
Slc1a3	-0.0997842	7.23936215	0.32342105	0.57591839	0.33514345
Rassf2	-0.118429	7.49587503	0.32337246	0.57594683	0.33514345
Hira	-0.092849	4.13802974	0.32295964	0.5761886	0.3352406
Ryk	-0.1237794	4.46530085	0.32287393	0.57623882	0.3352406
Maoa	-0.0905184	4.91687969	0.32284577	0.57625532	0.3352406
Gm10548	-0.2242881	1.74627685	0.3227545	0.57630882	0.3352406
1110046J04F	-0.2461789	1.55816368	0.32270601	0.57633724	0.3352406
Rnf103	-0.070319	6.23756175	0.32246752	0.57647708	0.33526879
Csdc2	0.08925938	6.84565661	0.32242106	0.57650433	0.33526879
4930593A02I	-0.6539599	-1.3030035	0.3223947	0.57651979	0.33526879
Hnf1a	0.55989999	-0.9538867	0.32222217	0.57662101	0.33530166
Ipo7	0.0892716	5.83228562	0.32153772	0.57702293	0.33550936
Loxl1	0.22550351	1.84589249	0.32101989	0.57732738	0.33564272
Pex10	-0.1502649	2.67676415	0.32099543	0.57734177	0.33564272
Anks6	-0.1575536	2.0881777	0.32043603	0.57767105	0.33580812
Bub3	0.07467335	5.8548539	0.32026509	0.57777175	0.33584063
4930506C21I	-0.3632758	-0.2857268	0.31967645	0.57811877	0.33601631
Smurf2	0.06960524	7.06923446	0.31950985	0.57821707	0.3360474
Trim32	0.08331348	7.59726018	0.31895001	0.57854763	0.33621347
Zbtb4	0.06008341	7.52108843	0.31861382	0.57874632	0.33630288
Mcur1	-0.1264114	5.21029881	0.31842093	0.57886038	0.3363431
C1qtnf4	0.18652476	2.03375182	0.31797615	0.57912356	0.33645861
Bmper	-0.1241905	3.17057497	0.31782005	0.57921599	0.33645861
Nt5c3	0.07635119	5.14275865	0.31781045	0.57922167	0.33645861
Gm13629	-0.207838	1.52851678	0.3177819	0.57923858	0.33645861

Arhgap17	-0.1108378	4.57259103	0.31763138	0.57932774	0.33648434
Susd4	0.08609318	4.42295152	0.31705196	0.57967121	0.33665777
Ptbp1	0.12573626	6.00333761	0.31668412	0.57988947	0.33671583
Rnf214	-0.0662485	6.33644603	0.31665412	0.57990728	0.33671583
Atpaf2	-0.1221734	3.16296147	0.31660212	0.57993815	0.33671583
Camta2	-0.0839852	7.54902242	0.31658092	0.57995074	0.33671583
Gm16432	-0.3187461	1.11354933	0.31632622	0.580102	0.33676433
Gpr108	-0.1562607	3.10882504	0.31628911	0.58012405	0.33676433
Itga2b	-0.3584892	-0.091508	0.31612361	0.58022238	0.33678152
Sp4	-0.069276	5.9452757	0.31600313	0.58029399	0.33678152
Il1rl1	-0.5018929	-0.1980598	0.31597877	0.58030848	0.33678152
Trim11	-0.1351507	2.75592502	0.31592784	0.58033876	0.33678152
Pias3	-0.1352257	2.71943935	0.3158558	0.58038159	0.33678152
Obscn	0.30284131	0.39962601	0.31571862	0.58046317	0.33678152
Ttpal	0.09409861	5.12525269	0.31571061	0.58046794	0.33678152
C1rl	0.47367826	0.26255936	0.31538585	0.58066118	0.33686319
Zfp467	-0.1390971	4.00171468	0.31529299	0.58071645	0.33686319
Dleu7	-0.2259911	1.26264563	0.31519543	0.58077454	0.33686319
Apof	0.43895388	-0.0834847	0.31517224	0.58078835	0.33686319
4930505A04I	0.44094526	-0.1434764	0.31430235	0.58130683	0.33713004
B130024G19	0.26383341	1.1616731	0.3142243	0.5813534	0.33713004
Naprt1	-0.3284728	0.45155741	0.31417428	0.58138325	0.33713004
Lamc1	0.08182414	6.02517539	0.31366945	0.58168466	0.33725751
Spata7	-0.0998453	4.05031783	0.31365485	0.58169338	0.33725751
Tgoln1	0.08909851	6.81995248	0.31358024	0.58173795	0.33725751
Zfp442	-0.1186364	3.50310262	0.31306647	0.58204509	0.3374095
Slc9a4	0.82988863	-1.2674155	0.31285284	0.5821729	0.33745751
Wars	0.08933683	5.11435786	0.31243944	0.58242039	0.33757488
Sytl3	0.24392657	0.95486318	0.31219518	0.58256672	0.33763361
Commd1	0.14743581	4.15830275	0.31106465	0.583245	0.33800059
Lrrc36	0.43230395	-0.0502323	0.31092344	0.58332983	0.33802364
Edn3	-0.1110499	6.20018642	0.31064995	0.58349421	0.33808697
Gm11517	-0.5753823	-1.0909567	0.31059167	0.58352925	0.33808697
Notum	0.17823216	1.64644829	0.31038802	0.58365173	0.33810989
Gpsm3	-0.2758371	2.31143864	0.310376	0.58365896	0.33810989
Cdk13	-0.0620846	7.17706312	0.31025156	0.58373382	0.33812714
Zfp281	0.08564308	6.16581119	0.30985638	0.58397171	0.33823882
Wdtc1	-0.0882594	5.20720695	0.30860659	0.58472537	0.3386492
Ddx6	-0.0572265	8.14171641	0.30803309	0.58507188	0.33874224
Bmi1	0.08330966	6.05149084	0.30800209	0.58509062	0.33874224
Prss53	-0.8180629	-1.4968797	0.30798098	0.58510338	0.33874224
Jsrp1	0.47924439	-0.75672	0.30795579	0.58511862	0.33874224
Pank3	0.07050187	7.493322	0.3078733	0.5851685	0.33874224
Msh3	-0.1149792	4.12936455	0.30784481	0.58518573	0.33874224
Mastl	-0.2464805	1.51160158	0.30781771	0.58520213	0.33874224

Rex2	-0.544728	-1.6257951	0.30743631	0.58543291	0.33884968
Ina	0.09062159	6.3206916	0.30720245	0.58557451	0.33889723
Elp4	0.08723975	4.2881464	0.30715142	0.58560542	0.33889723
Igsf21	-0.1456652	3.61207176	0.30703963	0.58567314	0.33890826
Usp8	0.05935691	7.66784425	0.3069708	0.58571485	0.33890826
Lrrc38	0.22395283	1.44435448	0.30666745	0.58589872	0.33898851
Nkg7	0.66253116	0.02728111	0.30657861	0.58595259	0.33899353
Pebp4	-0.4599757	-0.8767812	0.30642218	0.58604748	0.33902228
Med9os	-0.594981	-1.163347	0.30557182	0.58656384	0.33929482
Nap1l2	-0.1150102	5.39456633	0.30545455	0.58663512	0.33930989
Qars	0.103652	3.78645225	0.30488423	0.58698205	0.33947096
Csk	0.15595667	2.98253537	0.30484798	0.58700411	0.33947096
0610010B08I	0.12484721	4.54974306	0.30459725	0.58715678	0.33953308
4930404I05R	-0.7316406	-1.5477678	0.30435226	0.58730603	0.3395932
Zbtb7b	0.17285619	2.97171509	0.30416826	0.58741818	0.33963187
Kif2c	0.61485512	-1.5242468	0.30390543	0.58757846	0.33969684
Gm9833	-0.1803964	1.15406897	0.3038355	0.58762111	0.33969684
Sema6b	0.16348959	2.90012105	0.30346123	0.58784953	0.3398027
Tbc1d1	-0.0852876	4.55994656	0.30319973	0.58800924	0.33986882
BC049715	0.64976339	-0.4258807	0.30312496	0.58805492	0.33986904
Agps	0.06556581	7.19752054	0.30284981	0.58822308	0.33993804
Mfap3l	0.11127817	5.63323855	0.30278136	0.58826494	0.33993804
Actrt3	0.56999671	-0.9276285	0.30266702	0.58833486	0.33995225
Ptpn6	0.273468	0.89948557	0.30252252	0.58842325	0.33995235
Stk35	-0.0804999	5.14102998	0.30251857	0.58842566	0.33995235
Sox13	-0.1778354	3.36118298	0.30240681	0.58849405	0.33996567
Harbi1	0.21213049	2.1471248	0.30211543	0.58867241	0.34004252
Efna4	0.28837289	1.84046717	0.30198979	0.58874936	0.34005005
Gm715	0.22442908	1.23912547	0.30194612	0.58877611	0.34005005
Olfr78	-0.5055523	-0.7827399	0.30176757	0.5888855	0.34008256
Gpr151	-0.7824641	-1.6206207	0.30170626	0.58892308	0.34008256
Sgms1	-0.0708769	5.94055763	0.30147076	0.58906745	0.34013975
Smarca5-ps	-0.1756552	1.23453082	0.30132079	0.58915943	0.34016667
Ip6k2	-0.091118	4.0375015	0.3012376	0.58921046	0.34016996
Gm6548	0.1541206	3.51256874	0.3008218	0.58946567	0.34028816
Tbc1d15	-0.0783923	5.20688553	0.30075625	0.58950592	0.34028816
Smco3	-0.1346551	2.69433529	0.30056645	0.58962252	0.34029899
4930404H11I	-0.8816032	-1.2410107	0.30051744	0.58965263	0.34029899
Nfx1	-0.0659314	6.44274509	0.30050416	0.58966079	0.34029899
9530082P21I	-0.0849566	5.19462914	0.30034701	0.58975737	0.34032855
Dppa2	0.90185397	-1.8299176	0.30014952	0.5898788	0.34037026
Hykk	0.1115658	5.34662653	0.30008187	0.5899204	0.34037026
Ero1lb	-0.1221451	3.76443278	0.29992926	0.59001428	0.34038513
Tns4	-0.2731743	1.63779668	0.29989246	0.59003693	0.34038513
Impg2	-0.6101178	-0.3888372	0.29979292	0.59009818	0.34039428

Erbb2	-0.3261241	1.57463106	0.29921349	0.59045501	0.34052478
Xaf1	-0.1285277	3.44495522	0.29917466	0.59047894	0.34052478
Gsr	0.07173618	5.88993082	0.29913626	0.59050261	0.34052478
Rnf13	0.09205381	7.06736367	0.29910033	0.59052475	0.34052478
Gm7904	-0.9601081	-1.4868777	0.29900105	0.59058596	0.34052478
Siah3	0.25221504	0.8095347	0.29898349	0.59059678	0.34052478
Slc35d2	-0.2774412	0.16421298	0.2988467	0.59068113	0.34054724
Nlrx1	-0.241926	1.86726911	0.29820496	0.59107718	0.34074939
Pagr1a	0.11469065	3.04353143	0.29811761	0.59113113	0.3407543
Gm11437	-0.5871891	-1.1134879	0.2979837	0.59121386	0.3407758
Slc35e2	-0.0975886	5.59113456	0.29769859	0.59139008	0.34084316
Htr1a	-0.1172406	3.55770126	0.29764762	0.5914216	0.34084316
Sp9	-0.1869447	1.30070133	0.29743465	0.59155332	0.34088754
Timd4	-1.0390506	-2.1091428	0.29734548	0.59160849	0.34088754
Gm14325	0.08555147	4.34187906	0.29730274	0.59163493	0.34088754
Sytl4	-0.2348239	2.35993316	0.29717681	0.59171287	0.34090139
Mypn	-0.2646363	1.79093751	0.29705059	0.59179101	0.34090139
E430025E21I	-0.0725706	6.34156917	0.29704364	0.59179531	0.34090139
Mcm4	-0.1029999	4.37157769	0.29604835	0.59241224	0.34123057
Iqgap1	0.0665439	6.34663726	0.29579239	0.59257111	0.3412788
Gipc1	0.17737242	3.03328594	0.29576685	0.59258697	0.3412788
Ankrd54	-0.1578635	2.01306305	0.29543447	0.59279343	0.34134656
BC003331	0.06660016	6.91312747	0.29543092	0.59279563	0.34134656
Snrpd3	0.1653846	2.63607777	0.29514631	0.59297254	0.34142222
Afmid	0.24975866	1.12223607	0.29412686	0.59360711	0.34174731
Memo1	0.09170154	4.53854241	0.29409291	0.59362827	0.34174731
Tmem81	0.29670441	0.54016435	0.29382068	0.59379797	0.34176975
Tmod2	0.06336038	9.94559003	0.2937927	0.59381542	0.34176975
Gp5	-0.587083	-1.3607598	0.29375678	0.59383783	0.34176975
Arfrp1	-0.1176327	3.99464723	0.2936929	0.59387767	0.34176975
Sp3	0.07383053	6.67392446	0.29359664	0.59393772	0.34176975
Mob3a	0.13547245	2.63458704	0.293592	0.59394061	0.34176975
Cacng7	-0.0850539	5.83701289	0.29335616	0.5940878	0.34180419
Vmac	0.15513932	3.71146309	0.29331399	0.59411412	0.34180419
Tph1	0.68232102	-0.7652572	0.29321554	0.59417559	0.34180419
Slc35g3	-0.837036	-1.5128325	0.29320411	0.59418273	0.34180419
Clp1	-0.1348269	2.44874484	0.29298204	0.59432143	0.34185741
Magi1	-0.094492	6.35945009	0.2928554	0.59440056	0.34185741
Klhdc10	-0.0693318	6.89725649	0.29283714	0.59441198	0.34185741
Tprn	-0.1956023	2.34731961	0.29246476	0.59464479	0.34196056
Slc6a6	-0.0653168	7.94764577	0.29234462	0.59471995	0.34196056
Mmp28	0.41682756	0.01821936	0.29233161	0.59472809	0.34196056
Lypd6b	-0.1199149	3.94970159	0.29218999	0.59481671	0.3419853
Maz	-0.1021315	3.87511503	0.29179501	0.59506402	0.34210127
6330416G13	-0.1127996	3.76233321	0.29150501	0.59524573	0.34216159

Tjap1	-0.242422	1.35414695	0.29148198	0.59526017	0.34216159
Brd4	0.06856641	8.73212684	0.29138994	0.59531787	0.34216854
1700123O21	-0.6052543	-1.0875901	0.29081289	0.5956799	0.34235039
Kcnc3	-0.1323519	5.24132334	0.29022106	0.59605168	0.34253782
Rem1	-0.6455179	-0.5359513	0.29008629	0.59613642	0.34256027
Tmem163	-0.1342508	2.75960425	0.28962789	0.59642481	0.34269974
Slc16a14	0.14946815	4.04172305	0.28952913	0.59648698	0.34270921
Wdfy1	-0.0979418	4.67529189	0.28935967	0.5965937	0.34274427
Cox6b2	-0.233462	2.88656334	0.2891121	0.59674967	0.34280762
Ankrd12	-0.086948	9.70283078	0.28901099	0.59681339	0.34281797
Prr12	-0.0841485	5.7528392	0.28881531	0.59693676	0.3428193
Nubp1	-0.1708692	2.29977372	0.28867851	0.59702304	0.3428193
Surf2	0.13433128	5.00252869	0.28860715	0.59706806	0.3428193
Arhgap25	-0.1206537	3.64359913	0.28857918	0.59708571	0.3428193
Fuz	-0.3160573	0.47719722	0.28856934	0.59709191	0.3428193
Kif1c	-0.1130348	5.6443033	0.28846375	0.59715854	0.3428193
Casz1	0.22507753	2.11849461	0.28845791	0.59716223	0.3428193
Brd3	0.07059593	6.20578828	0.28842409	0.59718357	0.3428193
Chst13	0.59331379	-0.7207541	0.28835528	0.59722701	0.3428193
Exo5	0.13644293	3.74028073	0.28814668	0.59735872	0.34286866
Capn3	-0.2139556	0.85097629	0.28804841	0.59742079	0.34287806
Hmha1	-0.2364388	1.25601341	0.28784701	0.59754804	0.34292485
Irs2	0.13214913	4.35157986	0.28695162	0.59811448	0.34322366
Myc	-0.129141	3.6606208	0.28685869	0.59817334	0.34322833
Lemd1	-0.6508936	-1.7102109	0.28679431	0.59821412	0.34322833
Gnb2	0.11840826	3.61938385	0.28662469	0.59832159	0.34326373
Gab2	0.08417668	6.02991746	0.28640489	0.59846092	0.34331741
Gnl2	-0.0706339	5.97776156	0.28596691	0.59873875	0.34345053
Pla2g5	-0.3057703	1.43780082	0.28511339	0.59928098	0.34373528
Hmgb1-rs17	-0.2331161	0.38820929	0.28478813	0.59948789	0.34380539
Trib3	0.73571179	-1.1817498	0.28477715	0.59949488	0.34380539
Gm5126	-0.1572483	2.50523899	0.28453144	0.59965129	0.3438688
Ms4a1	0.74115248	-1.7458976	0.28414913	0.59989483	0.34398216
Mpv17l	-0.0812	5.64672728	0.28403515	0.59996748	0.34398878
Plscr4	-0.1611842	3.14324998	0.28398713	0.59999809	0.34398878
Synpo	-0.0676169	6.50701661	0.28373535	0.60015865	0.34400732
Pias2	-0.0687528	6.01353074	0.28372662	0.60016423	0.34400732
Spata3	-0.5015691	-0.8067021	0.28365709	0.60020858	0.34400732
Stx5a	-0.1049501	4.58266496	0.2835873	0.60025311	0.34400732
Lrrc8a	0.06682901	6.15744372	0.28357317	0.60026213	0.34400732
Aplf	-0.0947616	4.27157919	0.28350054	0.60030848	0.34400732
2210416O15	0.39755418	-0.6375409	0.28336538	0.60039476	0.34400732
Sh3bgrl	-0.0900061	8.852275	0.28336139	0.60039731	0.34400732
Ppie	0.19106268	1.3935998	0.28315057	0.60053194	0.34403824
Zswim3	-0.2086511	2.11516617	0.28310914	0.6005584	0.34403824

Gm20743	0.55014537	-1.8229197	0.28306147	0.60058886	0.34403824
Gm3716	0.4159541	-0.725553	0.28297002	0.60064728	0.34404543
Creg1	0.12417865	4.80450992	0.28270334	0.60081775	0.34407369
Skap1	0.35109703	-0.0607999	0.28265017	0.60085175	0.34407369
Tsta3	-0.1998277	2.29746459	0.28260545	0.60088035	0.34407369
Helz2	-0.2590138	1.98281562	0.28255665	0.60091156	0.34407369
Fah	-0.1167861	3.18799279	0.28253414	0.60092596	0.34407369
Dpp6	-0.1006798	6.35339394	0.28240324	0.6010097	0.34409537
Runx1t1	0.06891586	7.80809386	0.28191266	0.60132377	0.34424891
Lrguk	0.12174427	3.24587054	0.2817517	0.6014269	0.34428168
Mapk1	0.06539991	9.09375929	0.28156886	0.60154408	0.34432248
Grin3a	-0.1151932	4.72129811	0.28142375	0.60163713	0.34433451
Fblim1	0.21763546	3.58566952	0.28139292	0.6016569	0.34433451
Ccnb1	0.47746965	-1.0194549	0.28130295	0.6017146	0.34434126
Gm6086	-0.5808123	-1.5354807	0.28123076	0.60176091	0.34434149
Csrnp2	-0.0854674	5.41423429	0.2808955	0.60197609	0.34443036
Krcc1	0.13865921	7.09047023	0.28084574	0.60200804	0.34443036
Palb2	-0.283339	0.75081152	0.2807535	0.60206727	0.34443797
Ncoa7	0.0759294	6.32087926	0.28046729	0.60225116	0.3445169
Rcl1	-0.1913926	2.11762534	0.2803948	0.60229775	0.34451728
Tmem237	0.14747221	3.40213193	0.28016067	0.60244829	0.34455181
Mphosph6	0.12989188	4.50521503	0.28015805	0.60244998	0.34455181
Calr3	-0.3233578	-0.0376434	0.28003479	0.60252926	0.34457088
Robo4	-0.3277118	0.64692429	0.27971986	0.60273194	0.34463534
Prkd2	-0.1647538	2.88515912	0.27971686	0.60273387	0.34463534
Scx	0.34400847	-0.0094432	0.27920622	0.60306282	0.34479715
Gtf3c5	0.11949522	3.13958845	0.27875074	0.60335655	0.3449388
Zkscan17	-0.1202812	3.86490782	0.27830163	0.60364649	0.34507825
Ecsit	0.08209752	3.81187568	0.27783056	0.60395092	0.34522598
Dsc2	0.38389562	-0.1495148	0.27773278	0.60401416	0.34523582
Glb1l	0.1355764	3.33240544	0.27754884	0.60413315	0.34527752
Cuedc1	-0.1491492	2.60927741	0.27740736	0.6042247	0.3452889
Zfp428	-0.2556419	0.8567422	0.27737581	0.60424513	0.3452889
Mzb1	-0.6925084	-1.791709	0.27711984	0.60441087	0.3453433
Gm8580	0.31311709	-0.6333046	0.27704642	0.60445843	0.3453433
Prdm5	-0.1376455	3.03414188	0.27701553	0.60447843	0.3453433
Erich5	-0.4626196	-0.7867221	0.27676365	0.60464166	0.34538597
Usp42	-0.0945302	4.91321096	0.27675817	0.60464522	0.34538597
Rnf138	-0.1065799	3.49491587	0.27653962	0.60478692	0.34542854
Agbl2	-0.1968607	1.56639521	0.27648825	0.60482024	0.34542854
Scube2	-0.266846	1.10588406	0.27637256	0.60489529	0.34542854
Mroh8	0.3785779	-1.0031828	0.2763269	0.60492492	0.34542854
Zfp248	-0.1058694	4.558211	0.27628828	0.60494998	0.34542854
Kcnk9	-0.1864583	2.87097366	0.27614822	0.60504088	0.34545015
Gpr160	0.35947756	0.01767484	0.27606633	0.60509404	0.34545015

Erc2	0.09422424	8.10052414	0.27600899	0.60513128	0.34545015
Glra2	-0.1124619	3.31837041	0.27585724	0.60522983	0.34545015
Pik3ip1	-0.1442435	4.0136335	0.27580819	0.6052617	0.34545015
Gna15	0.69245703	-1.6031651	0.27580443	0.60526414	0.34545015
Renbp	-0.2407294	2.99842113	0.27543399	0.60550491	0.34556127
M6pr	0.09516511	6.22491652	0.27513634	0.60569852	0.34564547
Slamf9	-0.6001141	-0.3339572	0.27505462	0.6057517	0.34564952
C2cd4d	0.7469799	-2.1321454	0.2749389	0.60582702	0.3456556
Exoc7	0.08904433	4.63607834	0.27484595	0.60588753	0.3456556
Ushbp1	0.33969548	0.5480966	0.27476103	0.60594283	0.3456556
E230016K23I	0.50707605	-0.708949	0.27475513	0.60594668	0.3456556
Stk4	0.05938284	5.92312332	0.27436186	0.60620291	0.34577548
Inhbb	-0.2447963	1.70768754	0.27420466	0.60630541	0.34580764
Mbnl3	-0.2193126	1.24336771	0.27395678	0.60646709	0.34584491
C030037D09I	-0.271125	1.34915979	0.27380497	0.60656617	0.34584491
Dnm2	-0.072105	5.25761461	0.27376617	0.60659149	0.34584491
Endov	-0.0916883	4.5500775	0.27373426	0.60661232	0.34584491
Rbl2	0.05988392	7.24597562	0.27371388	0.60662563	0.34584491
Pim3	-0.1380338	2.92316168	0.27364111	0.60667314	0.34584491
Spata2	0.09522895	5.49015829	0.27355594	0.60672875	0.34584491
Epha1	-0.9701757	-1.3427968	0.27353935	0.60673959	0.34584491
Clec4a2	-0.3689906	-0.2856982	0.27326741	0.60691726	0.3459199
Nrm	0.24728656	0.49915898	0.27302043	0.60707871	0.34598563
Arrdc1	-0.2379638	0.91740947	0.27288607	0.60716659	0.34600943
Gm1123	-0.7260914	-2.3133754	0.27279848	0.60722389	0.34601579
Ttll12	-0.114289	4.13670622	0.27203349	0.60772484	0.34627494
N4bp1	0.06170338	7.19119929	0.27183496	0.60785499	0.3462973
Gsg1l	-0.0802499	5.847959	0.27169943	0.60794388	0.3462973
Pex13	-0.0701632	5.35870662	0.27169286	0.60794819	0.3462973
Sirpa	0.07149707	7.17435197	0.27169202	0.60794874	0.3462973
Setbp1	-0.063596	6.75198855	0.27113538	0.60831411	0.34647912
Foxf2	0.15427028	2.76111386	0.27096978	0.6084229	0.34651477
T2	-0.3416126	-0.1876592	0.27065659	0.60862877	0.34658612
Bmp7	0.14217048	7.77650848	0.27063862	0.60864059	0.34658612
Ltbp3	0.11223973	4.64029279	0.27052648	0.60871434	0.34660181
1190002N15	-0.0856058	5.34921163	0.2704471	0.60876656	0.34660523
Aldh16a1	0.40488195	0.16075163	0.27028953	0.60887025	0.34663795
Zmym4	-0.0720089	7.67947104	0.26988056	0.60913954	0.34674054
Prrg2	0.35990084	-0.1605994	0.26987548	0.60914288	0.34674054
Ganab	0.09798034	5.05845587	0.26960545	0.60932083	0.34681551
B230217O12	-0.1843364	1.77765777	0.26912985	0.60963453	0.34689536
Msr1	0.65389084	-1.3220509	0.2691011	0.6096535	0.34689536
Rhob	0.06873011	7.36944358	0.26904564	0.60969011	0.34689536
Adcy4	0.34012344	0.47949154	0.2690167	0.60970921	0.34689536
Irf8	0.21227721	1.6282917	0.2689545	0.60975027	0.34689536

Pepd	-0.1056874	3.62132166	0.26889448	0.60978991	0.34689536
Dph2	-0.1543671	1.76580914	0.26885349	0.60981698	0.34689536
Cd3eap	0.11788039	3.59037109	0.26883214	0.60983107	0.34689536
Ube2d1	0.084027	6.92626022	0.26836728	0.61013825	0.34701192
Zfp944	0.1049257	3.91730998	0.26833386	0.61016034	0.34701192
Cecr5	0.27753292	0.6165469	0.26831205	0.61017477	0.34701192
Aldh3b1	-0.2772071	0.86317787	0.26800457	0.61037816	0.34710127
4930444P10I	-0.6504406	-1.494696	0.26781219	0.61050549	0.34713658
Col4a1	0.11595591	3.65295433	0.26764949	0.61061322	0.34713658
Armc3	-0.479726	-0.9834486	0.26761335	0.61063716	0.34713658
Ppfia1	-0.0568338	6.96011551	0.26758213	0.61065783	0.34713658
Plac9b	-0.1382858	3.93696673	0.26751512	0.61070222	0.34713658
Plekhh3	-0.2165443	1.54505035	0.26749145	0.61071791	0.34713658
C920006O11	-0.1794085	1.74888489	0.26715806	0.61093888	0.34722392
Pgr	0.09466173	5.93249371	0.26711998	0.61096413	0.34722392
Actr5	0.38023741	0.08199314	0.26693584	0.61108627	0.34726702
Sh3tc1	0.54374038	-1.176216	0.26658901	0.61131645	0.34736663
Rpl9	0.09533636	7.1941389	0.26653219	0.61135418	0.34736663
Mcph1	0.11359914	5.21063922	0.26644256	0.6114137	0.34737414
Bst2	0.31341037	1.61087395	0.2663028	0.61150655	0.34740058
Ccdc89	-0.3282639	0.33416161	0.26604263	0.61167946	0.34745218
Acot12	-0.6079896	-1.5535456	0.26600084	0.61170725	0.34745218
Gtf3a	0.14890474	2.55083537	0.26595331	0.61173885	0.34745218
Rps3a1	0.09276813	7.93110802	0.26588745	0.61178265	0.34745218
Slc25a26	-0.1069707	3.09585486	0.26560998	0.61196725	0.34753071
Rpap1	-0.1279843	2.8673749	0.26544439	0.61207748	0.34755095
Mgll	-0.0765052	5.42694072	0.26541725	0.61209555	0.34755095
Galnt11	-0.1158602	3.29136292	0.26521823	0.61222809	0.34759989
6430571L13F	0.34551747	0.33278228	0.26511978	0.61229369	0.34761083
Agap2	-0.0784034	6.9295941	0.26496653	0.61239581	0.34764152
Eepd1	-0.1356695	2.48443429	0.2648996	0.61244043	0.34764152
Ccnc	0.07736136	5.84716191	0.2648112	0.61249937	0.34764866
Abca4	-0.2010497	3.09333688	0.26413259	0.61295224	0.34787939
Uap1l1	-0.2210825	4.09503862	0.26405705	0.6130027	0.3478817
2810428I15R	0.17579681	2.82113477	0.2639861	0.6130501	0.34788228
1810062O18	-0.3506101	0.63893738	0.26386677	0.61312984	0.34790121
4930432K21I	0.29880668	-0.3257549	0.26299448	0.61371343	0.34817372
Rlbp1	-0.2200514	1.98670262	0.26297177	0.61372864	0.34817372
Trip6	0.1506144	3.17019165	0.26290961	0.61377027	0.34817372
Tomt	-0.2415093	0.54964426	0.26287157	0.61379575	0.34817372
Rpl30	0.30569256	0.13246401	0.26268145	0.61392315	0.34821965
P2rx4	0.1320748	3.21501847	0.26254368	0.6140155	0.3482457
Xpo1	0.06801036	6.55374024	0.26246675	0.61406709	0.34824863
Slc52a3	0.21476631	1.79376373	0.26228314	0.61419024	0.34829214
Klkb1	-0.7764049	-1.2638815	0.26187123	0.61446673	0.34842259

Ube2cbp	-0.4173484	0.2999942	0.26166029	0.61460842	0.34847659
Acads	0.44510376	-0.6553317	0.26146867	0.6147372	0.34851688
Rfx2	0.14792596	2.14871108	0.26141631	0.6147724	0.34851688
C630043F03I	0.11232362	3.2595682	0.26124347	0.61488862	0.34855642
Rfx5	0.07979054	4.85278337	0.26095811	0.61508062	0.34863891
Adprhl2	-0.4859781	-0.4313696	0.26081132	0.61517943	0.34864487
Itgax	-0.4528697	-0.4385055	0.26080442	0.61518408	0.34864487
Nrg2	0.45727047	-0.8635565	0.26071257	0.61524593	0.34865358
Ell2	-0.0669317	6.02742888	0.26029529	0.61552709	0.34878656
Agpat9	0.20139382	1.27578134	0.25997634	0.61574219	0.34886071
Rundc3a	0.08700292	5.33651374	0.25996334	0.61575096	0.34886071
Kcna5	-0.2431212	0.85548491	0.25977652	0.61587704	0.34890579
Tekt1	0.41261021	-0.1937425	0.25958569	0.61600587	0.34895242
Zfp948	0.11788971	3.61939955	0.2594214	0.61611685	0.34896776
Btd	0.15328261	3.78710427	0.25940787	0.61612599	0.34896776
Pcbp4	-0.1319077	2.70007356	0.25905298	0.61636585	0.34907726
Gadl1	0.39316182	-0.5549758	0.25872148	0.6165901	0.3491779
Jade1	-0.0693863	6.43364774	0.25856357	0.61669698	0.34921206
Med28	0.10442158	5.31342009	0.25837225	0.61682654	0.34925906
Tk2	-0.1444624	3.29112277	0.25822679	0.61692508	0.34928849
Cyp20a1	0.16824521	2.77855337	0.25770601	0.61727816	0.349444
Cit	0.08097206	7.31494479	0.25768427	0.61729292	0.349444
Rab11fip2	-0.0596854	6.93761623	0.25746516	0.61744161	0.34947636
Inhbe	-0.8667732	-1.9838091	0.25745493	0.61744856	0.34947636
Psme2b	-0.1438769	3.17254541	0.2573941	0.61748985	0.34947636
Trim8	0.07658859	6.14101651	0.25715002	0.61765562	0.34954381
Stoml3	0.76379616	-1.6978164	0.25705256	0.61772184	0.34955491
Tnfrsf9	0.55825126	-1.4474965	0.25688831	0.61783347	0.34958812
Asic4	-0.3068634	-0.0935885	0.25682908	0.61787374	0.34958812
Ldlr	0.08887859	3.69142439	0.25642368	0.61814951	0.34968839
Atp8b4	-0.4863261	-0.8469891	0.25639639	0.61816808	0.34968839
Ccdc13	-0.4268284	-0.3812154	0.256363	0.6181908	0.34968839
Myh4	-0.5181247	-0.373539	0.25628454	0.61824422	0.34969223
Osbpl9	0.07546879	6.74841289	0.25616891	0.61832295	0.3497104
Rabgef1	0.10462492	4.26776228	0.25599158	0.61844374	0.34975234
Tceanc2	0.07582428	4.63240654	0.25567916	0.61865667	0.34984638
Gys1	0.19219395	1.99857252	0.25522343	0.61896758	0.34999582
Xrcc6bp1	0.21036091	1.74874407	0.25506241	0.61907751	0.35000434
Fermt1	0.48527037	0.35393899	0.25500196	0.61911879	0.35000434
Sowahb	-0.1115261	3.51482256	0.25499635	0.61912262	0.35000434
Acr	-0.384318	-0.0750512	0.25457908	0.61940776	0.35012149
Spats1	0.27071689	0.64257121	0.25453029	0.61944112	0.35012149
Epc2	-0.0557171	7.43690002	0.25448446	0.61947245	0.35012149
H2-Ke6	0.20804902	2.38317371	0.25441999	0.61951655	0.35012149
Calr4	-0.5799695	-1.2051204	0.25427706	0.61961432	0.35015036

Yes1	0.08399159	4.78123407	0.25395852	0.61983235	0.35024719
Opn1sw	0.42853179	0.40682071	0.25380668	0.61993635	0.35027957
Spa17	0.19951854	2.41346414	0.2535244	0.62012978	0.35036247
Vps4b	0.07014701	5.43696052	0.2531409	0.62039278	0.35046223
Gm765	0.14238902	2.83983776	0.25313068	0.6203998	0.35046223
Klf16	-0.1248089	2.45963519	0.25272389	0.62067906	0.35059358
Rnd2	-0.2119439	1.72906481	0.25260732	0.62075914	0.35061241
Dnmt3b	-0.3214999	0.32546079	0.25248368	0.6208441	0.350634
Zfp646	0.09888733	3.74038985	0.25203995	0.62114923	0.35075882
4930524B15	-0.8179804	-1.5930791	0.2520263	0.62115862	0.35075882
Elp2	-0.0874294	5.93591872	0.25194717	0.62121307	0.35076316
Vps35	0.05959085	6.730399	0.2513608	0.6216169	0.35096476
Cd244	0.61633074	-0.6517929	0.25114621	0.62176483	0.35099844
Efna2	0.28602276	0.54814271	0.25113851	0.62177014	0.35099844
Alg6	-0.1237047	3.97261279	0.25101244	0.6218571	0.35102111
Lrrc2	-0.2077625	1.76997162	0.25078752	0.62201229	0.3510823
Dars	0.07003691	5.23673454	0.25007879	0.62250189	0.35130768
Rps9	0.11928849	6.45473868	0.25007391	0.62250526	0.35130768
Uso1	-0.0570884	6.42363592	0.24999223	0.62256175	0.35131312
Lrrc3	0.11711624	3.66983848	0.24983116	0.62267316	0.35134956
Zfp599	-0.1463498	2.83120872	0.24975686	0.62272456	0.35135214
Zkscan8	-0.0765421	6.10581544	0.24816438	0.62382876	0.35194865
G630090E17	-0.7146719	-1.1328509	0.24809691	0.62387564	0.35194865
Rell2	-0.1848442	1.68782175	0.24794356	0.62398222	0.3519823
Cd72	-0.4230093	-0.3622226	0.24782934	0.62406163	0.35200063
Fbxl15	0.33298841	-0.4412051	0.24775871	0.62411075	0.35200187
Tmem150c	0.11354642	4.08315618	0.24760083	0.62422058	0.35202949
Tmem151a	-0.1006058	4.63055063	0.24737247	0.62437951	0.35202949
Fbxw2	-0.075755	5.59019633	0.24733691	0.62440427	0.35202949
Gnptab	-0.0813228	6.15644286	0.2472856	0.62443999	0.35202949
Tgfbi	-0.2791159	2.29086337	0.24726447	0.62445471	0.35202949
Ccsap	-0.1190219	3.95722994	0.24724043	0.62447145	0.35202949
Mc4r	-0.4373596	-0.7315664	0.24721634	0.62448822	0.35202949
Dnaaf1	0.65782525	-1.8853075	0.24702622	0.62462067	0.35207769
Hdac1	0.09677284	5.29501835	0.24694392	0.62467801	0.35208356
Tmed2	-0.0888266	5.71179949	0.24664558	0.62488602	0.35217405
Tesk2	0.14304582	2.09730237	0.24657898	0.62493248	0.35217405
Eral1	0.10990447	3.54601251	0.24646266	0.62501363	0.35218418
Svopl	-0.407827	-0.2633265	0.24639506	0.6250608	0.35218418
Apoa1bp	0.10236328	3.85453329	0.24635138	0.62509129	0.35218418
Riok3	0.06965962	6.33207179	0.2460042	0.62533372	0.35226353
Irs3	-0.1899599	2.20963821	0.24600077	0.62533612	0.35226353
Zfp512	-0.0711195	5.07894091	0.24590469	0.62540325	0.35226353
B630019K06	0.10646024	3.26317026	0.24587417	0.62542458	0.35226353
BC100451	0.67981298	-1.5581313	0.24581356	0.62546693	0.35226353

Apool	0.19505232	2.40628015	0.2456721	0.62556582	0.35229277
D030045P18I	0.46874516	-0.5537525	0.24554367	0.62565563	0.3523169
Kansl1	0.05154969	6.95826193	0.24525615	0.62585681	0.35238659
Ccl22	0.55458398	0.31341969	0.24523254	0.62587334	0.35238659
Cep135	-0.1187704	4.32391521	0.24514209	0.62593666	0.3523867
Hnrnpl	-0.0787118	5.22655387	0.24509804	0.6259675	0.3523867
Celsr1	-0.1482721	2.35191803	0.24492601	0.62608798	0.35242808
Stk16	0.10622957	4.51350464	0.24468059	0.62625996	0.35247259
Ttll11	-0.1122685	4.20607825	0.24467907	0.62626103	0.35247259
10-Mar	-0.3846278	-0.6737566	0.24458441	0.62632739	0.35248349
Cyp4f14	-0.5818923	-0.5174918	0.24434851	0.62649284	0.35255015
Ntng2	0.17998525	1.99714029	0.24426362	0.6265524	0.35255722
Tpst1	-0.0835567	5.01559497	0.2437941	0.62688207	0.35269135
Rpusd4	0.13092134	2.7187492	0.24379022	0.62688479	0.35269135
Gga3	0.07476583	5.38664136	0.24343077	0.62713745	0.35280703
Caap1	0.11310299	2.76923956	0.2432956	0.62723252	0.35283406
Gpr34	0.14414748	2.07134329	0.2432065	0.62729521	0.35284286
Utp3	0.06401906	6.12821502	0.24313193	0.62734768	0.35284591
Fancl	-0.1150324	2.71916364	0.24303135	0.62741848	0.35285928
Adssl1	0.14357777	2.68975469	0.24292545	0.62749304	0.35285982
Nlgn1	0.08968692	6.61629113	0.24289635	0.62751353	0.35285982
Nkiras2	-0.4126202	0.14816501	0.24262147	0.62770716	0.35292072
Ap1g1	-0.080367	7.3243594	0.24260904	0.62771592	0.35292072
Mepce	-0.0868598	4.31707559	0.24236479	0.62788811	0.35299035
Lgals8	0.07252101	6.12087827	0.24218262	0.62801659	0.35299035
Fam159b	0.59219229	-2.0502647	0.24214182	0.62804538	0.35299035
Fam229b	-0.1362723	2.47483404	0.24198411	0.62815668	0.35299035
Dclre1a	0.12186007	3.56079589	0.24194673	0.62818306	0.35299035
Pdzk1ip1	-0.2619212	1.63263449	0.24194646	0.62818325	0.35299035
Klra9	0.60505655	-0.9669091	0.24192552	0.62819804	0.35299035
Tmem200b	0.34575684	-0.2351335	0.24189974	0.62821624	0.35299035
B3galt6	0.17308864	2.33602443	0.24148388	0.62850999	0.35311306
Ablim2	-0.0814806	6.07377197	0.24145733	0.62852876	0.35311306
Gm4925	-0.4037775	-0.0737922	0.24101495	0.62884162	0.35326237
Rmnd1	0.16282934	1.31481141	0.24090596	0.62891875	0.35327924
Ric8	-0.0825479	5.22884109	0.24079459	0.62899759	0.35329707
Tspyl3	0.07074892	4.6678116	0.24067475	0.62908246	0.35330689
Ptpn9	-0.0703194	5.88928776	0.24060983	0.62912844	0.35330689
Mfsd7a	-0.7695994	-0.7476039	0.2405704	0.62915637	0.35330689
Fars2	0.14133677	2.72872315	0.24043961	0.62924904	0.35333248
Amfr	0.07384138	5.82024607	0.24037256	0.62929657	0.35333272
Plekhf1	-0.1658473	3.09606806	0.24003361	0.62953692	0.35341514
Slmap	0.05974088	8.96692926	0.23996793	0.62958352	0.35341514
Ifi205	0.41248948	-1.2619821	0.23995866	0.6295901	0.35341514
Mcm7	0.12988418	2.98666999	0.23987752	0.62964768	0.35341514

Retnla	0.28198535	1.0485688	0.23983347	0.62967895	0.35341514
Cyp2c44	0.57332431	-0.9963417	0.23960741	0.62983945	0.35347878
Lpar6	0.15068132	2.23537527	0.23921876	0.63011562	0.35357785
Sarm1	-0.1463429	3.71037135	0.23914908	0.63016516	0.35357785
Ppic	0.1546216	4.60365247	0.23914254	0.63016981	0.35357785
Artn	0.69722385	-2.0795552	0.23909373	0.63020452	0.35357785
Slc46a3	-0.1953709	2.39258348	0.23875319	0.63044681	0.35368733
Ubald2	0.14465476	2.32642476	0.23860318	0.6305536	0.35372079
Tmed1	0.25936685	1.04176317	0.23846112	0.63065478	0.35373712
Rspo4	0.53972796	-1.1341276	0.2384201	0.63068401	0.35373712
Pm20d1	0.16160072	2.06002401	0.2383637	0.63072419	0.35373712
Gbp11	-0.4178538	-0.1374851	0.23827239	0.63078926	0.35374192
Sez6l2	0.08650954	5.43861925	0.23817179	0.63086096	0.35374192
Slc25a13	-0.2535786	0.32074734	0.2381532	0.63087422	0.35374192
Lpcat2	-0.1674855	2.55061214	0.23778051	0.63114004	0.35386453
Scamp3	0.11763865	3.63923408	0.23768073	0.63121126	0.353878
Zfp251	0.10258763	3.78981328	0.23739251	0.63141707	0.35396694
Timm10	0.13545261	3.84783903	0.23715467	0.63158702	0.35403575
Fmnl3	0.13921103	3.58003564	0.23708067	0.63163993	0.35403895
Haus5	-0.1752842	1.62049443	0.23694438	0.63173738	0.35406712
Bad	0.21058147	2.66095758	0.23686161	0.63179658	0.35407384
Hsf4	-0.1886315	1.34637394	0.23657727	0.63200006	0.35416141
Slc2a4	-0.3297628	0.01525547	0.23645177	0.63208992	0.35418531
Olfr539	-0.4522947	-0.4667982	0.23612522	0.63232386	0.35428993
Atf7	0.11226243	4.26147139	0.2360243	0.6323962	0.354304
Ccrl2	-0.2290396	0.53143071	0.23579476	0.63256082	0.35436976
Glp2r	-0.2537025	1.68436136	0.2356741	0.63264739	0.35438161
Tmem8	-0.1264041	2.32998663	0.2356336	0.63267645	0.35438161
Steap4	-0.5288191	0.17579235	0.23554494	0.63274008	0.35439079
Fgfr2	-0.0954333	6.83047451	0.23540201	0.6328427	0.35439702
Igsf8	0.08293613	5.06803153	0.23539782	0.63284571	0.35439702
Tbcc	0.10086093	3.28109105	0.23530779	0.63291037	0.35440678
Foxp1	0.05468608	8.77787499	0.23515259	0.63302187	0.35443233
Rabl3	0.08722828	4.40385949	0.23511274	0.63305051	0.35443233
Celf5	0.07305425	6.86974412	0.23487415	0.63322202	0.3545019
Fam13a	-0.0795061	3.99578644	0.23479457	0.63327926	0.35450749
Pelo	0.11940384	3.1943276	0.23467118	0.63336801	0.35453072
Armcx1	-0.0649856	5.69029124	0.23453625	0.63346512	0.35455861
Ggt5	0.25887259	1.43336164	0.23444155	0.63353328	0.35457031
A730017C20	0.06451245	5.8893004	0.23425113	0.63367041	0.3546206
Dennd2d	-0.3908808	-0.2058797	0.23409007	0.63378644	0.35465006
Rims3	0.25283411	0.8032777	0.23404681	0.63381762	0.35465006
Xpnpep2	-0.4677567	0.25433481	0.23384278	0.6339647	0.3547059
Vps41	-0.0703432	7.02432777	0.23370498	0.63406408	0.35473505
Cdc42ep4	0.12877739	6.00259451	0.23348772	0.63422084	0.35479629

Mpp7	0.08320644	4.60590607	0.23337512	0.63430213	0.3548153
Ccdc153	0.18098891	1.80355475	0.23320474	0.63442516	0.35485766
6030419C18I	0.16631227	2.38184951	0.23304399	0.6345413	0.35489615
Mast3	-0.0736495	7.30129697	0.23297225	0.63459314	0.35489869
Tspyl4	0.06100831	8.38403276	0.2328474	0.63468339	0.3549227
Frat1	0.18978794	1.57041128	0.23260422	0.63485927	0.35499275
Id2	0.10677939	6.09138023	0.23254336	0.6349033	0.35499275
Col27a1	-0.198009	2.12821318	0.23240362	0.63500443	0.35502283
Aqp11	-0.1500492	1.77804299	0.23224125	0.63512198	0.35506209
6720483E21I	-0.5682088	-1.7520889	0.23204742	0.63526238	0.35510422
Sh3rf2	-0.1141093	3.63581108	0.23199929	0.63529726	0.35510422
Olfm3	0.08473134	4.62868668	0.23183311	0.6354177	0.35510422
Slit1	0.13698194	2.95992299	0.23182407	0.63542425	0.35510422
Fam151b	0.18412531	1.36776781	0.2317475	0.63547977	0.35510422
Rps19-ps3	0.22043729	0.61789326	0.23174527	0.63548139	0.35510422
Gsta3	-0.2825816	0.88015063	0.23157555	0.63560449	0.35512663
Tardbp	0.05589446	7.45746874	0.23155943	0.63561618	0.35512663
Rab6b	0.0587006	10.4152573	0.23128021	0.63581882	0.3552134
Fxyd6	0.09090405	5.08938502	0.23089244	0.6361005	0.3553443
1700024G13	-0.8618368	-1.5267944	0.23052774	0.63636568	0.35546596
Nup35	-0.1079127	3.28593471	0.23037846	0.6364743	0.35548939
9330151L19I	0.10571917	4.78112007	0.23033986	0.6365024	0.35548939
Slc35f5	-0.0998456	4.54118939	0.23025521	0.63656402	0.35549733
Tmem147	0.10759688	3.44846783	0.22966017	0.63699757	0.35570916
Gm5512	-0.1506054	1.38961734	0.22960451	0.63703816	0.35570916
Tacr3	-0.1979693	1.90532315	0.22948163	0.63712779	0.35573273
Slc2a1	-0.107431	4.64273172	0.22927684	0.63727723	0.35576702
Khk	-0.2368207	2.21443771	0.2292675	0.63728405	0.35576702
Uxs1	0.08754181	3.5382047	0.2291543	0.6373667	0.35578667
Capn12	-0.4080306	-0.7080199	0.22870665	0.63769376	0.35594276
Tnpo3	-0.0660064	6.1065511	0.22861145	0.63776337	0.35595512
Smarcd2	-0.1546028	3.09274541	0.22846892	0.63786761	0.35598682
Tbr1	-0.0888088	6.77187326	0.22828198	0.63800441	0.35603667
Tsen54	-0.3309783	-0.256233	0.22815363	0.63809836	0.35605771
Trim46	-0.1024104	4.42013848	0.22807387	0.63815676	0.35605771
Grpel2	-0.0900696	4.21739814	0.22799216	0.63821661	0.35605771
Pttg1ip	0.12872954	6.25434244	0.22791689	0.63827175	0.35605771
Clec16a	-0.073084	6.3092255	0.22790641	0.63827943	0.35605771
Ccdc175	-0.8043662	-1.7965656	0.22777905	0.63837276	0.35608329
Pak3	-0.086381	7.31845417	0.22748688	0.63858698	0.35617629
Dnmt3aos	-0.7274878	-1.8159098	0.22734992	0.63868745	0.35620585
Aifm3	-0.0807514	5.53542131	0.22719436	0.63880162	0.35624207
Rpa3	0.13473026	3.1345464	0.22711869	0.63885717	0.35624207
Gm20300	0.06331787	7.24327955	0.22706735	0.63889487	0.35624207
Pi15	-0.3647452	0.75635518	0.22670002	0.63916475	0.35636606

Epsti1	-0.2452882	1.15801116	0.22605325	0.63964059	0.35660486
Cbx6	-0.0654583	6.62613416	0.22597862	0.63969555	0.356609
Ect2l	-0.6174635	-0.6733218	0.22572167	0.63988486	0.35668802
Ldlrap1	0.22635892	1.14273587	0.22557077	0.6399961	0.35672352
4930590J08F	-0.4361398	-0.7086782	0.2253601	0.64015148	0.35677184
Dmp1	-0.3099713	0.28825488	0.22532429	0.6401779	0.35677184
Ucp3	0.43423648	-0.2060946	0.2251027	0.64034145	0.35683647
G6pc3	0.10559943	3.9418678	0.22502709	0.64039727	0.35684107
Mpp5	0.06000469	6.90625661	0.2246969	0.64064121	0.35691227
Rint1	-0.086089	4.06276788	0.22468239	0.64065193	0.35691227
Gstk1	0.12722193	3.26041485	0.22464889	0.64067669	0.35691227
Sepn1	-0.1378531	3.45527712	0.22459658	0.64071536	0.35691227
BC064078	-0.2928022	0.67834504	0.22398126	0.64117066	0.35713937
Ch25h	-0.5393807	-1.5622507	0.2238081	0.64129892	0.35718429
Tbc1d22bos	-0.6065842	-1.0139922	0.22360233	0.64145142	0.3572427
Hes1	0.10271218	3.68862672	0.22352138	0.64151144	0.3572496
Dhx58	0.26560317	0.55327898	0.22339328	0.64160645	0.35727599
Rpl36a	0.10592834	6.48678995	0.2228393	0.64201768	0.35746513
Eml3	0.13961006	2.79223939	0.22280734	0.64204142	0.35746513
Mblac2	-0.0874191	6.02592494	0.22267257	0.64214157	0.35749435
Arhgap9	-0.3475463	0.95300811	0.2225883	0.64220421	0.3575027
Sh2d1a	-0.7190529	-1.5924025	0.22202158	0.64262584	0.35771086
D630032N06	-0.5797311	-0.9869433	0.22170215	0.64286378	0.35779664
Pgm3	-0.0917477	4.00884134	0.22168665	0.64287534	0.35779664
Atp6v1a	0.06949876	9.34063743	0.22151686	0.64300191	0.35783906
Gabbr2	-0.0838652	7.12221704	0.22145644	0.64304696	0.35783906
Itch	-0.0519013	7.05379418	0.2213895	0.64309689	0.3578403
Zfp62	-0.0737361	6.19282385	0.22115229	0.64327388	0.35790853
Slc31a1	0.06851921	5.88304645	0.22109728	0.64331494	0.35790853
Cerkl	-0.3026007	-0.1444446	0.22096635	0.64341269	0.35791792
Sphk2	-0.0911869	4.07618968	0.22094686	0.64342725	0.35791792
Wdr83	0.12294972	2.6962975	0.22078422	0.64354874	0.35795896
Ccdc81	0.38793398	0.12320534	0.22065871	0.64364253	0.35798458
Sox9	0.0826102	5.66087096	0.22053113	0.6437379	0.35801108
Slc32a1	-0.1051181	4.04629258	0.22040877	0.6438294	0.35803542
Btbd6	0.08055878	4.22448594	0.2201551	0.64401918	0.35811441
Cgref1	-0.1371027	2.1814467	0.22002618	0.64411569	0.35814153
Fhod3	0.09858367	5.14905749	0.21988384	0.64422229	0.35815825
Snrnp48	0.07403631	4.90671398	0.2198567	0.64424262	0.35815825
Ndc80	-0.3926946	-0.3951648	0.21978442	0.64429677	0.35815825
S1pr2	-0.2111332	1.73585486	0.21973107	0.64433674	0.35815825
Pnck	0.08171705	3.71850949	0.2195853	0.64444599	0.35816733
Hyou1	-0.100421	5.17947452	0.21955382	0.64446959	0.35816733
Morc4	0.11391615	2.89235091	0.21951817	0.64449632	0.35816733
Actr2	0.05050204	8.88066286	0.21945336	0.64454492	0.3581678

9430021M05	-0.1072385	4.43883393	0.21906155	0.64483891	0.35830462
Asgr2	-0.7117224	-2.1466861	0.21899633	0.64488787	0.35830529
Trove2	0.0634332	7.07330955	0.21877424	0.64505469	0.35837143
Cd52	-0.1590817	2.35416589	0.21827915	0.64542694	0.3585474
Cd209b	-0.6156423	-1.5381916	0.21822587	0.64546702	0.3585474
Heph	0.24922067	2.0407408	0.21770186	0.64586163	0.35874003
Secisbp2l	-0.0565745	7.76977179	0.21759968	0.64593865	0.35875625
Hsd11b2	0.67139101	-1.1414281	0.21752841	0.64599238	0.35875953
Uba6	0.08318412	5.70518127	0.21732168	0.6461483	0.35881955
Galnt4	0.16500272	2.3252378	0.21697422	0.64641055	0.35893862
Nxt1	0.15399391	2.54312673	0.21636387	0.64687187	0.35914864
N28178	-0.0859667	7.04077347	0.21630125	0.64691924	0.35914864
Zfand1	0.1080401	4.29971391	0.2162548	0.64695439	0.35914864
Prune2	0.06014272	6.64228343	0.21622055	0.64698031	0.35914864
Mthfs	-0.1363083	2.33692126	0.21595064	0.64718465	0.35923549
Barhl2	-0.3915343	-0.0727471	0.21578056	0.64731349	0.35928042
Gm5796	-0.3313291	-0.9775086	0.21567487	0.64739358	0.35928545
Eomes	0.29708905	0.41617846	0.21564222	0.64741834	0.35928545
Mtmr6	0.05307414	7.40515423	0.21547204	0.64754736	0.35933047
Cwc27	-0.0697434	5.33572549	0.21532692	0.64765744	0.35936497
Ddo	0.13020853	3.49965367	0.21505456	0.64786416	0.35945035
Fam179a	-0.411923	-0.4688586	0.21499795	0.64790715	0.35945035
Malt1	-0.1059007	3.60673066	0.21481294	0.64804768	0.35948123
Uimc1	-0.08159	4.62464259	0.21479849	0.64805866	0.35948123
St6galnac6	0.09933007	4.07207896	0.21472364	0.64811554	0.3594862
Gm5547	0.65332481	-1.9888501	0.21453629	0.64825797	0.35953861
Ccdc50	0.06777043	7.37499426	0.21421724	0.64850069	0.35964664
Six3	0.11851022	3.04223827	0.2139638	0.64869364	0.35972705
Fzd2	0.13393162	4.20920458	0.2135194	0.64903233	0.35988826
Sgk3	0.07184874	5.20054935	0.21312492	0.64933335	0.36002856
Adra2a	0.12033878	4.09654235	0.21289609	0.64950811	0.36005184
Ctnnal1	-0.078722	4.46199785	0.21285925	0.64953625	0.36005184
Slc13a3	0.16690302	6.93531399	0.21284556	0.64954671	0.36005184
Cnot6	-0.0729083	6.42197688	0.21277855	0.64959792	0.36005184
D2Wsu81e	0.12467362	2.04981063	0.21275578	0.64961532	0.36005184
Dnajc16	-0.1144688	3.07893277	0.21239905	0.64988811	0.36017642
Szrd1	0.10281649	4.64014995	0.21222319	0.65002268	0.36019752
Hs6st3	-0.1162322	2.87786613	0.2121502	0.65007856	0.36019752
Rpl3	0.0838347	8.2228053	0.21213858	0.65008746	0.36019752
Tex261	0.12177734	1.98762786	0.21206474	0.650144	0.36019752
Lonrf1	0.07157501	6.32971245	0.21203566	0.65016627	0.36019752
Apobec2	0.95961061	-1.4578593	0.21188542	0.65028136	0.36023467
Yif1a	0.19437346	1.70687004	0.21177859	0.65036322	0.36025342
Procr	0.29206729	1.61350465	0.21159308	0.65050544	0.36030025
Gemin2	-0.1403629	2.12116911	0.21154302	0.65054383	0.36030025

Eef1g	0.08630602	7.72429687	0.21137014	0.65067646	0.36032296
Tmem222	0.15727352	4.53161946	0.21136435	0.6506809	0.36032296
B3galt1	0.05443658	5.80671302	0.21128865	0.650739	0.36032853
Chst10	0.08890769	3.87552992	0.21108701	0.6508938	0.36038765
A930003A15	0.63970883	-1.9242753	0.21038155	0.65143612	0.3606613
Zdhhc14	0.08881348	4.30619587	0.21018982	0.65158371	0.36070057
Ubac2	-0.1120326	2.53218994	0.21016446	0.65160324	0.36070057
Smu1	0.06837199	5.43062054	0.20995892	0.65176155	0.36076159
Lrrc48	0.14826185	2.39259325	0.20980638	0.65187911	0.36080003
Ndrg4	-0.0614054	10.805371	0.20970284	0.65195894	0.36081759
Fbxl6	-0.21448	0.94416263	0.20945611	0.65214924	0.36086247
R3hcc1l	-0.0733532	4.57641189	0.20941762	0.65217894	0.36086247
Exosc4	0.1100367	3.40495254	0.20941064	0.65218433	0.36086247
Brpf1	-0.0699909	4.65622633	0.20929301	0.65227512	0.36088608
Pamr1	-0.0879059	4.03100599	0.20879882	0.65265689	0.36107067
Agbl4	0.18512773	2.42252129	0.2086684	0.65275774	0.36108337
Myh13	0.54756443	-1.4079411	0.20858691	0.65282077	0.36108337
Scn2b	-0.0707285	6.88841078	0.2085824	0.65282426	0.36108337
Rmdn2	-0.0975631	3.71864916	0.20822386	0.65310176	0.36121023
Sppl2a	-0.0711483	6.38309585	0.20792491	0.65333336	0.36129424
Mcm8	-0.0954324	3.21512786	0.20786662	0.65337855	0.36129424
Gm13031	-0.493929	-0.4832155	0.20780095	0.65342945	0.36129424
4930503L19F	-0.1344667	3.03546724	0.20775652	0.6534639	0.36129424
Grid2ip	0.2522949	1.10690443	0.20771708	0.65349449	0.36129424
Ajap1	0.07192395	5.20149619	0.20749069	0.65367012	0.36133557
Upk1b	-0.291506	1.27671909	0.2074638	0.65369099	0.36133557
Draxin	-0.6456595	-1.4922165	0.20739506	0.65374434	0.36133557
Slc30a4	-0.0575482	6.40576099	0.20731712	0.65380485	0.36133557
Gba	0.08421829	3.90078188	0.20731037	0.65381009	0.36133557
Sox10	-0.0988314	4.29225309	0.20711343	0.65396305	0.36136163
Zmat3	0.06594737	9.37056188	0.20698125	0.65406576	0.36136163
Insc	0.25466359	0.14504822	0.20695966	0.65408254	0.36136163
Rtp4	0.15764631	3.93435672	0.20695017	0.65408992	0.36136163
Orai3	0.10677336	5.00577325	0.20693963	0.65409811	0.36136163
Ubl4b	-0.3211477	0.06162456	0.20687732	0.65414655	0.36136178
Bbox1	-0.255999	0.58517396	0.20679233	0.65421263	0.36137167
Syt11	-0.0717842	8.29352381	0.20644653	0.65448167	0.36147194
Smad6	0.16071128	2.58406296	0.20643515	0.65449053	0.36147194
Mreg	-0.1479024	2.40495326	0.20614504	0.65471647	0.3615701
Ntf3	0.7577691	-1.2165419	0.20590407	0.65490428	0.36164719
Fzr1	-0.1361511	2.83729254	0.20581954	0.65497019	0.36165697
Tbata	0.32441226	-0.0328198	0.20539262	0.65530334	0.36181429
Zfp429	0.13177074	2.01858964	0.20532465	0.65535642	0.36181697
Anapc10	-0.0869845	4.5033661	0.20520937	0.65544647	0.36182535
Tyrobp	-0.1799845	1.37625052	0.20504645	0.65557379	0.36182535

4933416C03l	-0.7945912	-1.228785	0.20501865	0.65559552	0.36182535
9230112J17F	0.64510723	-1.563652	0.20496998	0.65563356	0.36182535
Rarg	0.12160375	2.85799831	0.20484405	0.65573204	0.36182535
Ddr2	-0.1171718	6.27923198	0.20471044	0.65583656	0.36182535
Gm15713	-0.4461989	-0.6500398	0.2046933	0.65584997	0.36182535
Maneal	0.07789043	4.31987879	0.20461563	0.65591075	0.36182535
Cbl	0.0559508	6.56782542	0.20460584	0.65591841	0.36182535
Ikbke	-0.2529793	0.5804378	0.20459329	0.65592823	0.36182535
Ftsj1	-0.0831081	3.73079047	0.20451483	0.65598965	0.36182535
Spire1	-0.0768868	7.44840204	0.20449896	0.65600207	0.36182535
Ube2q2	0.06081463	6.36693787	0.2044737	0.65602186	0.36182535
Rpp38	-0.129818	2.56177142	0.20444171	0.65604691	0.36182535
C77080	0.09374283	5.81398474	0.20430648	0.65615282	0.36185716
Dlgap4	-0.0562615	7.19902997	0.20408177	0.6563289	0.36192766
Ankrd6	-0.0887424	5.51365967	0.20394981	0.65643237	0.36195811
Parp4	-0.0657194	5.70580261	0.20382747	0.65652832	0.36198441
Rsl1	0.13464096	2.89096568	0.20365887	0.65666062	0.36201585
4930447A16I	0.68302869	-1.7228355	0.20363179	0.65668187	0.36201585
Hdac2	-0.0527102	7.14680496	0.20343781	0.65683417	0.3620732
Amn	0.30853178	-0.2705762	0.20333901	0.65691178	0.36207639
Pccb	0.08026798	4.70569849	0.20330757	0.65693649	0.36207639
Gnat1	0.38068848	-0.4817534	0.20321077	0.65701255	0.36209171
Sec22c	-0.0778284	4.63441973	0.20309731	0.65710174	0.36209599
Pnpla8	0.0574649	6.88366748	0.20307213	0.65712154	0.36209599
Ick	0.06253126	6.31242919	0.20301667	0.65716514	0.36209599
Rasd1	-0.2671592	0.3412436	0.20282126	0.65731885	0.36214576
Pygb	0.06302135	6.4839507	0.20270938	0.6574069	0.36214576
Tirap	0.11287774	4.4284544	0.20254091	0.65753953	0.36214576
E2f2	0.17995362	3.00064518	0.20250559	0.65756735	0.36214576
Mplkip	-0.0698058	4.05352396	0.2025002	0.65757159	0.36214576
Mapkbp1	-0.0807418	5.2659979	0.20243906	0.65761975	0.36214576
Nxph1	0.08084634	4.83149152	0.20243305	0.65762449	0.36214576
Chst9	0.38033928	-0.8220098	0.20241121	0.65764169	0.36214576
Serpinb6b	0.14503506	5.19949551	0.20228812	0.65773868	0.36217258
Sdr42e1	-0.1036757	2.65229571	0.20222442	0.65778888	0.36217364
Mir425	-0.5251215	-1.6929276	0.20199273	0.65797157	0.36224764
Cdc25c	0.47426109	-1.6134283	0.20172342	0.65818409	0.36233805
Gimap7	0.34906466	0.15483927	0.20152951	0.6583372	0.36239574
Mogs	-0.1806731	1.72178949	0.20124622	0.65856107	0.36249237
E130006D01I	0.54115707	-2.0148907	0.20105965	0.6587086	0.36254698
Pola2	-0.1479917	1.80786801	0.20094769	0.65879718	0.36256913
Gprin3	0.09691082	3.67896331	0.20088009	0.65885067	0.36257197
Frmd4b	0.06993818	5.2760679	0.20074941	0.65895411	0.36260229
Cnih2	0.08532169	3.54137883	0.20064901	0.65903362	0.36261944
Mtmr1	-0.0660809	5.14613504	0.20044478	0.65919541	0.36264275

Eml1	-0.0536845	5.94191498	0.20043998	0.65919922	0.36264275
B3galt4	-0.4013205	-0.7880389	0.20041246	0.65922103	0.36264275
Hrasls	0.07662937	5.07368425	0.20027559	0.65932952	0.36265976
Cpq	-0.1331855	4.27189071	0.20025148	0.65934864	0.36265976
Msto1	0.17723593	1.83431399	0.19992219	0.65960987	0.36277685
Abhd15	0.42753997	-1.3237041	0.19967192	0.65980859	0.36285954
Plekha1	0.05574731	6.73437233	0.19953986	0.65991351	0.36289063
Tbc1d9b	-0.0557445	6.59204366	0.19931445	0.66009268	0.36294251
H2-M3	0.17345741	2.40479271	0.19929944	0.66010462	0.36294251
Ccne1	0.15138818	1.77384286	0.19920818	0.6601772	0.36295113
Rhov	0.28757923	1.2476218	0.19915807	0.66021706	0.36295113
Fmod	0.12928443	7.83887884	0.19874501	0.66054588	0.36310528
Dusp19	0.08373295	4.78410897	0.1984273	0.66079908	0.36319555
Gimap5	0.27106827	1.56612691	0.19841746	0.66080692	0.36319555
Ephx1	-0.1295686	4.19076574	0.19830267	0.66089846	0.36321925
Cldn25	-0.0793979	5.89007369	0.19822653	0.6609592	0.36322601
Fam57b	0.15584907	2.79398044	0.19787623	0.66123882	0.36333281
Hist1h2ak	-0.4863819	-1.7770005	0.19786172	0.66125041	0.36333281
Serinc4	-0.5149886	-1.0261286	0.19773033	0.66135537	0.36335763
Pot1a	0.07753575	5.1867932	0.19768392	0.66139245	0.36335763
Mier2	-0.1816765	1.225176	0.19743452	0.66159183	0.36344054
Gli2	-0.1060091	3.37357665	0.19730932	0.66169198	0.36346894
Mdc1	-0.0666784	5.55897763	0.19677406	0.66212055	0.36364481
Usp43	-0.3536632	1.0019832	0.19674775	0.66214164	0.36364481
Impa2	-0.3275369	-0.1342386	0.19669446	0.66218435	0.36364481
Stap2	0.20225947	1.64210429	0.19666738	0.66220606	0.36364481
Tgm1	-0.6276402	-1.4017659	0.19634344	0.66246586	0.36376085
Trhde	-0.0926678	5.81450051	0.19586748	0.66284805	0.36394407
Ddx46	0.05009536	7.32957545	0.1956419	0.66302938	0.36401699
Nlrc5	0.33033841	0.00570201	0.19539993	0.66322403	0.36409721
Gm13498	0.26613864	-0.4467189	0.19522962	0.66336112	0.36414581
St7l	0.09838947	4.22717222	0.19513533	0.66343705	0.36416085
Usp53	-0.0593338	6.92540021	0.19468491	0.66380007	0.36433345
Tusc2	-0.0605978	4.92345459	0.19462264	0.66385029	0.36433436
Gpr182	0.15789985	4.10662129	0.19441091	0.66402114	0.3643892
Ttc9	0.07625422	4.23542392	0.19437842	0.66404737	0.3643892
Psma8	0.31849168	0.17721155	0.19412613	0.66425112	0.36447434
Gm14005	0.27659444	0.45134043	0.19385083	0.66447363	0.36456976
Itgb2	-0.1934965	1.44270914	0.19367141	0.66461875	0.3646015
Vstm2l	0.24210287	-0.1881095	0.19365912	0.66462869	0.3646015
Pdcl3	0.09948283	4.225778	0.19354318	0.66472251	0.36462179
Glipr2	0.1683139	4.05857951	0.19349329	0.66476289	0.36462179
Gm1045	-0.5387921	-1.1786568	0.19329993	0.66491947	0.36468101
Cpne4	-0.0728972	6.36856692	0.19313795	0.6650507	0.36472632
Tomm40l	0.07279124	3.94998471	0.19293271	0.66521707	0.36479089

Amn1	0.09191553	4.58958325	0.19263015	0.66546253	0.36489882
Nos2	0.66511883	-1.9593026	0.19254859	0.66552874	0.3649038
Il22ra1	-0.1481134	1.58253079	0.19249912	0.66556891	0.3649038
Ngrn	0.08169858	4.21976295	0.19236718	0.66567607	0.36493588
Caskin1	0.08347507	5.76529907	0.19223398	0.66578429	0.36496853
Ndc1	-0.1145591	2.89070847	0.19211192	0.66588351	0.36499625
Traf3ip3	0.40527202	-0.8302029	0.19203272	0.6659479	0.36500487
BC006965	-0.1867199	1.79044994	0.19169766	0.66622051	0.36511995
Pdgfa	-0.0630628	5.81482077	0.19165504	0.66625521	0.36511995
Id4	-0.0738112	5.33668979	0.19118699	0.66663654	0.36528383
Arhgef4	-0.0770119	5.08822958	0.19110257	0.66670539	0.36528383
Nradd	0.31027625	0.29732932	0.19105065	0.66674773	0.36528383
Ahcyl2	0.06626743	8.1213262	0.19104905	0.66674903	0.36528383
Tdrkh	-0.0945175	4.42683345	0.19067969	0.6670505	0.3654223
Dsp	-0.1971902	1.66527481	0.1904487	0.66723919	0.36549898
Lair1	-0.1423065	3.29310948	0.19033354	0.66733333	0.36552385
Tex40	0.20010083	2.10217503	0.19012574	0.66750326	0.36554845
Ccr9	-0.1176424	3.73349126	0.19011216	0.66751438	0.36554845
Txnrd3	0.10788033	3.37677223	0.19009988	0.66752442	0.36554845
Zfp771	0.23571421	0.69994245	0.18988623	0.66769927	0.36559381
Ccdc62	-0.19956	1.89830832	0.18985809	0.66772231	0.36559381
Mknk2	0.07978444	3.96354222	0.18980512	0.66776569	0.36559381
Rps27a	0.07086791	6.62252119	0.18976051	0.66780221	0.36559381
Mfsd6	0.07800225	7.207852	0.18968695	0.66786247	0.36560012
Frmpd3	-0.2218087	1.33049436	0.18937233	0.66812033	0.36570437
Gas6	-0.0791219	4.3991346	0.18933564	0.66815042	0.36570437
Apcdd1	0.13714013	3.98764952	0.18922863	0.6682382	0.36572573
Arap3	0.20956576	1.59536031	0.18912041	0.66832699	0.36574764
Jmjd8	0.10094563	3.88038761	0.18886144	0.66853961	0.36583731
Ndel1	0.07346344	4.91685716	0.18850116	0.66883568	0.36597263
Tmem229b	-0.1106304	3.13060807	0.18838876	0.66892812	0.36599651
App	0.05472559	8.66509883	0.18787602	0.66935023	0.36620075
Zfp174	-0.0863746	3.76830348	0.18775546	0.66944958	0.36622839
Slc38a3	0.10362422	4.10921612	0.18757571	0.66959777	0.36628275
Cenpb	0.1009603	4.12701979	0.18745268	0.66969926	0.36631156
D730005E14	0.55595431	-1.8001265	0.18720485	0.6699038	0.36639672
Cxcl14	0.08537153	3.20959099	0.18703785	0.67004173	0.36644544
Slc17a5	-0.0800934	3.89423497	0.18692732	0.67013305	0.36646867
Mapk11	-0.1059626	3.69421599	0.18659041	0.67041162	0.36659428
Spice1	-0.0958499	3.42151264	0.18642166	0.67055127	0.36664391
Rnpepl1	-0.0885249	4.18973917	0.1862892	0.67066094	0.36666674
Cdnf	-0.1601342	2.31471702	0.18625317	0.67069078	0.36666674
Lama4	-0.0811566	4.07808699	0.18584896	0.67102575	0.36679889
Maml3	-0.0726853	4.82223032	0.18584349	0.67103029	0.36679889
Gtf3c4	-0.0656403	5.27152828	0.18543552	0.67136883	0.36693655

Fzd7	-0.1313621	5.93664349	0.18541516	0.67138574	0.36693655
Pou3f3os	0.08672816	3.71496871	0.18533866	0.67144927	0.36693655
Zfp109	0.10427521	3.49465105	0.18530432	0.6714778	0.36693655
Stk40	-0.0647418	4.15938639	0.1850156	0.67171775	0.36704093
Cyp2r1	-0.2157873	0.36074522	0.18493915	0.67178133	0.36704894
Slc35f1	0.07863701	7.28824329	0.18478855	0.67190661	0.36709065
LOC1026324	-0.9374204	-1.9097647	0.18441687	0.67221607	0.36723297
Perm1	-0.3806584	-0.4116374	0.18432049	0.67229638	0.3672501
Palmd	-0.0760275	5.6060739	0.18416173	0.67242871	0.36728775
Traf1	0.29108079	0.98828572	0.18412034	0.67246323	0.36728775
Clip2	-0.1190937	3.95451113	0.18399997	0.67256362	0.36730046
Gm3086	-0.335098	0.26584689	0.18397503	0.67258443	0.36730046
1700094D03	-0.1967101	1.92607072	0.1838383	0.67269853	0.36733603
Ppme1	-0.0697811	5.17759303	0.18373767	0.67278253	0.36735516
Prpf3	0.07548517	4.35903595	0.18363511	0.67286818	0.36737518
Rps14	-0.1451565	4.3577063	0.18333022	0.67312295	0.36748753
Bphl	0.1027478	4.07017953	0.18300518	0.67339483	0.36760921
Zfp593	0.20237489	0.84499048	0.18285323	0.67352204	0.3676519
Cers5	0.0595768	4.92314804	0.18270226	0.67364848	0.36768727
Ccdc19	-0.1471349	2.03876038	0.18265884	0.67368486	0.36768727
Bmp6	0.12129252	6.6331763	0.18259319	0.67373987	0.36769054
Fam76b	0.09404511	4.65486334	0.18246469	0.67384758	0.36772257
Mospd2	0.06929032	5.1513818	0.18193661	0.6742907	0.36792648
Cdadc1	-0.0524723	6.00419115	0.18185448	0.67435968	0.36792648
Ppp1r3f	-0.0773254	4.33312591	0.18184411	0.6743684	0.36792648
Gkn3	0.60917532	-1.1762182	0.18174061	0.67445536	0.36794716
Prmt3	0.07656447	4.30951432	0.18113313	0.67496638	0.3681792
Grin1	-0.0825446	6.12469084	0.18111831	0.67497886	0.3681792
1700012D01	-0.3476576	0.14562904	0.18086679	0.67519075	0.36826801
Zmynd11	0.04822909	7.87641286	0.18062281	0.67539646	0.36835342
BC051226	0.44256231	-1.6089049	0.18050117	0.67549908	0.3683826
Card10	-0.2393677	0.38311934	0.18029646	0.67567187	0.36845005
Cbfa2t3	-0.0736301	4.7943287	0.17994771	0.67596651	0.36858393
Ccdc77	-0.0991708	3.53799165	0.17953341	0.67631698	0.36872513
Nde1	-0.1166907	3.81609371	0.17946117	0.67637814	0.36872513
Grk5	0.06686214	5.33355787	0.17944437	0.67639237	0.36872513
Gm12216	-0.3568288	-0.754129	0.17940806	0.67642311	0.36872513
C030013G03	0.10999059	2.95740626	0.17935123	0.67647124	0.36872513
Ccdc30	0.11338131	3.32439912	0.17927673	0.67653435	0.36872646
Tmem192	0.12813745	2.30239886	0.1792323	0.676572	0.36872646
Esf1	-0.0493998	7.18536144	0.17905388	0.67672322	0.36876473
Klrb1c	-0.4554729	-0.3976589	0.17900162	0.67676754	0.36876473
Polr3f	0.06656348	4.75486577	0.17897548	0.6767897	0.36876473
3200001D21	0.37232524	0.18461242	0.17825673	0.67739997	0.36907044
H60b	0.29013415	0.46473753	0.1779302	0.6776777	0.36917375

Mas1	0.12347177	2.57691821	0.17787379	0.67772572	0.36917375
Ltbr	-0.1577703	2.37578847	0.17785238	0.67774394	0.36917375
Ccno	-0.4314042	-0.7397575	0.17780245	0.67778644	0.36917375
Nudt1	-0.3200907	0.61450683	0.17772848	0.67784943	0.36918125
2610034B18I	0.1118421	4.17684086	0.17764041	0.67792445	0.3691953
Sox21	0.10544593	3.44466163	0.17751094	0.67803476	0.36922857
Plagl2	-0.0802447	5.17042877	0.17703197	0.67844326	0.3694242
Mapk9	-0.0509499	8.50240878	0.17680088	0.67864059	0.36950483
Elovl1	0.15425573	3.3782113	0.17663613	0.67878136	0.36950596
Prap1	-0.6904869	-1.6095757	0.17660478	0.67880816	0.36950596
B4galt7	-0.1965221	1.05384379	0.1765805	0.67882891	0.36950596
Pitpnc1	-0.0490399	6.72428963	0.17656788	0.6788397	0.36950596
Ifit1	0.10185038	6.03124522	0.17642469	0.67896214	0.36954579
Ppp1r3c	0.08147004	6.08555093	0.17635558	0.67902126	0.36955115
Ap4m1	-0.1826661	1.14471779	0.17580903	0.67948927	0.36974195
Safb2	0.06675595	5.15612092	0.17574945	0.67954033	0.36974195
Psd4	-0.2924208	0.02147733	0.17563384	0.67963946	0.36974195
Kcnk5	-0.1810059	2.33406875	0.17563181	0.6796412	0.36974195
Zfp874a	0.07098523	4.88317352	0.17562632	0.67964592	0.36974195
Lrrc41	0.07626737	4.24108537	0.17560105	0.67966758	0.36974195
Btbd17	0.20892338	0.43753418	0.17530019	0.67992576	0.36981417
Cpz	-0.6069135	-1.0697219	0.17528476	0.679939	0.36981417
Diap2	0.06405408	7.4642199	0.175274	0.67994824	0.36981417
Rbpj	0.04994842	6.51270234	0.17513041	0.68007156	0.36985443
Ghr	0.08550885	5.29795125	0.17492215	0.68025053	0.36992494
Hist1h2bf	0.34035446	-0.8437802	0.17483925	0.68032181	0.36993688
Ldb3	0.15492166	2.06945824	0.17477255	0.68037917	0.36994126
Zdhhc16	0.1140824	2.26251779	0.1744124	0.68068911	0.37008296
Tmem134	-0.1073209	2.61010811	0.17422049	0.68085443	0.37014601
Dusp5	-0.2559673	-0.2021188	0.17395975	0.6810792	0.37023028
Pelp1	-0.0977992	3.28073169	0.17392618	0.68110816	0.37023028
Champ1	0.0684012	4.80465221	0.17380231	0.68121502	0.37024357
Gm5535	-0.8127267	-1.7777729	0.17378344	0.6812313	0.37024357
Orc6	0.07112408	4.67622612	0.1736459	0.68135003	0.37028126
Bin3	-0.1093471	2.93786398	0.173588	0.68140002	0.37028161
Xylt2	0.17415777	2.16298136	0.17350695	0.68147002	0.37029282
Ptrh2	-0.0615921	4.96435401	0.17329509	0.68165309	0.3703301
Kcp	-0.3345584	0.03723685	0.17315658	0.68177285	0.3703301
D5Ertd605e	-0.4584887	-1.4829157	0.17315143	0.6817773	0.3703301
Tbc1d16	-0.0710906	4.73877911	0.17307783	0.68184096	0.3703301
Adcy7	-0.1961766	1.81924374	0.17305085	0.6818643	0.3703301
Kdm1b	0.08393255	4.2157133	0.1730369	0.68187637	0.3703301
Smpdl3b	0.36255911	-0.2309417	0.17302784	0.6818842	0.3703301
Ammecr1l	0.05067172	5.73285666	0.172882	0.68201042	0.37037183
Rims4	-0.4723147	-1.1305353	0.17273206	0.68214023	0.37041551

Tbx21	-0.5231687	-0.7649604	0.17245842	0.68237733	0.37051744
Gpr158	-0.0689119	8.18522937	0.17239483	0.68243247	0.37052055
5033406O09	-0.6722929	-0.5531035	0.17220958	0.68259315	0.37056258
Mbd6	-0.1220464	3.97290676	0.17219167	0.68260868	0.37056258
Clock	-0.053085	7.60340753	0.17212201	0.68266913	0.37056858
Mrgprf	-0.2522794	2.03239922	0.17186543	0.68289191	0.37066269
Slc4a10	-0.0566781	8.98850848	0.17175203	0.68299043	0.37068934
Rock1	-0.0678585	8.00893157	0.17146608	0.68323903	0.37079744
Ndufv1	0.07924531	4.74009569	0.17134669	0.68334291	0.37082698
Mtmr11	0.08738	3.419479	0.17118142	0.68348676	0.37086005
Ccdc155	-0.304479	-0.6128863	0.17106621	0.6835871	0.37086005
Gm16894	0.1714724	1.2872279	0.17106275	0.68359011	0.37086005
Irf2	0.07141402	5.24195731	0.17099525	0.68364891	0.37086005
Anxa6	0.07474266	5.87471101	0.17099281	0.68365103	0.37086005
Nipal3	0.0866809	4.4565347	0.17084066	0.68378363	0.37090516
Gm6787	0.44909137	-0.4742893	0.17075571	0.68385769	0.37091851
Trim7	-0.236515	0.29043078	0.17040584	0.68416295	0.37103402
Pcdhga11	-0.1309111	3.13527731	0.17038088	0.68418474	0.37103402
Gnpnat1	0.07187714	4.78318396	0.1703144	0.68424279	0.37103402
Manea	0.06675858	5.51538336	0.17028494	0.68426852	0.37103402
Use1	0.11802823	4.73330947	0.17018331	0.6843573	0.37105534
Uhrf1bp1l	-0.0747926	7.76480923	0.1700493	0.68447441	0.37109201
Phgdh	-0.1345988	2.53464317	0.16985755	0.68464207	0.37115609
Furin	0.12305023	2.94407601	0.16966448	0.684811	0.37122084
Lrrc58	0.06934692	10.1415831	0.16957077	0.68489303	0.37123458
Dao	-0.8076167	-1.5808403	0.16952247	0.68493532	0.37123458
Ttc34	-0.2982279	-0.1334546	0.16935193	0.6850847	0.3712685
Cdk6	-0.1951313	2.18186163	0.16933801	0.6850969	0.3712685
Sox2	-0.1062412	3.57343566	0.16911496	0.68529243	0.37134763
Arhgap20	0.05382575	8.42146037	0.16901981	0.68537588	0.37136603
Armcx3	-0.046508	7.26398778	0.16864199	0.68570753	0.37149466
Adamtsl5	-0.0978164	2.76696844	0.16860013	0.6857443	0.37149466
Mzt1	0.05463103	5.93690151	0.16853203	0.68580413	0.37149466
Slc20a2	0.08874965	6.03436327	0.16852379	0.68581137	0.37149466
Wdr43	0.06556982	4.90443713	0.16844822	0.68587779	0.37150381
Syne3	-0.2840305	0.83033734	0.16838218	0.68593584	0.37150843
Chd1l	0.09664517	3.10993716	0.16828562	0.68602075	0.37152759
Sart1	0.07171864	4.71131357	0.16811802	0.68616819	0.37156421
Ttc7b	0.07522803	8.19378473	0.16807917	0.68620238	0.37156421
Dhrs7b	-0.1463434	2.406399	0.1679809	0.68628888	0.37156421
Cmtm5	0.14121521	2.42396211	0.16793165	0.68633225	0.37156421
Gemin7	0.10697555	3.2202262	0.16792734	0.68633604	0.37156421
Crygs	-0.636744	-2.0379571	0.16742522	0.68677858	0.37177696
F730043M19	-0.1872381	0.97539026	0.16730998	0.68688025	0.37180455
Heatr9	-0.5892363	-1.4993693	0.16725511	0.68692868	0.37180455

Bud13	-0.1072566	2.07170836	0.16703242	0.68712531	0.37188415
Tasp1	0.05909011	5.96691952	0.16685219	0.68728456	0.37192647
Crybg3	-0.0865675	4.24807861	0.16683169	0.68730269	0.37192647
Ang	0.20609789	2.28939294	0.16675638	0.68736926	0.37193567
Phkg2	0.13136642	2.60356202	0.16666608	0.68744912	0.37195205
Fam78a	0.23060263	0.64030231	0.16645942	0.68763197	0.37202415
Itsn2	-0.0514294	7.26278222	0.16633513	0.68774201	0.3720334
Gnat2	0.72838079	-1.6727266	0.16632808	0.68774826	0.3720334
Irf2bp1	0.07411077	3.91958908	0.16623569	0.68783008	0.37205083
Tmem95	-0.5876014	-2.4731922	0.16596209	0.68807256	0.37215515
Rab18	0.05849181	7.34877302	0.16585827	0.68816462	0.37217811
Glyctk	-0.1975294	1.38324795	0.16576734	0.68824529	0.37219491
Fkrp	0.05924234	5.29122087	0.1653838	0.68858584	0.37235222
Lsm6	0.05929895	5.28972953	0.16527543	0.68868214	0.37236089
Mpi	0.07334416	4.47507128	0.16524883	0.68870578	0.37236089
Pdzk1	-0.1086251	2.85859714	0.16517153	0.68877451	0.37236089
Abcb1a	0.10052198	5.71532328	0.16514239	0.68880042	0.37236089
Tyw3	-0.11677	3.04954393	0.16482005	0.68908722	0.37248909
Ankrd34c	0.08146713	4.32065125	0.16459539	0.68928731	0.37252615
Acss2	0.12361129	4.31701525	0.16458036	0.68930071	0.37252615
Scrn1	-0.0739373	5.45736724	0.16457579	0.68930478	0.37252615
Acad11	0.06889493	4.50550654	0.1643642	0.68949338	0.37260124
Adck4	0.20417366	1.29627474	0.16425979	0.6895865	0.37262471
Cry2	-0.0587362	6.33826493	0.16404904	0.68977457	0.3726826
Cnbd2	-0.1187565	2.38608532	0.16402842	0.68979298	0.3726826
Csad	-0.0713507	4.22252035	0.16361238	0.69016469	0.37285657
Ano8	-0.1929914	0.67743509	0.16354556	0.69022444	0.37286199
6330419J24F	0.10650591	3.50590208	0.16346225	0.69029896	0.3728754
Cys1	-0.0940901	5.15008941	0.16339781	0.69035661	0.37287969
Mir684-1	-0.1977399	0.23407638	0.16309023	0.69063198	0.37300156
Ubash3a	0.49025803	-1.2589354	0.1629745	0.69073568	0.37303071
Fbl	0.0861524	4.29211646	0.16273114	0.69095386	0.37312168
A730020E08I	0.12898912	2.69495924	0.1621649	0.69146226	0.37333046
Tmem72	0.52909461	-1.1869953	0.16214845	0.69147704	0.37333046
Ankrd45	0.0620776	6.27744958	0.16213426	0.6914898	0.37333046
Impad1	-0.0481359	7.02690267	0.16198531	0.69162372	0.37337589
Slc25a23	-0.0561564	8.69692611	0.16169712	0.69188304	0.37346357
Clec4a1	-0.3708701	-0.821454	0.16169416	0.6918857	0.37346357
Sf3a1	0.07198882	5.62274131	0.16137908	0.69216954	0.37356808
Rubie	-0.4910271	-1.4883069	0.1613664	0.69218096	0.37356808
9130024F11I	-0.0907789	4.23553446	0.16131341	0.69222873	0.37356808
Usp6nl	0.05801686	5.91250472	0.1611445	0.69238105	0.37362341
Ppp1r26	-0.074212	4.18231138	0.16106053	0.69245682	0.37363741
Ptpn18	0.48331839	-1.6842723	0.16079837	0.69269349	0.37370263
Rps6ka3	0.04869529	7.88393158	0.16076711	0.69272172	0.37370263

4933426M11	-0.0741689	5.71371529	0.16076111	0.69272715	0.37370263
Ier3	0.10764037	4.1511689	0.16013456	0.69329379	0.37398142
Zfp622	-0.0734449	4.68996095	0.15998982	0.69342487	0.37402523
Pcdhga8	-0.1108632	2.67989961	0.15988834	0.69351681	0.37404793
Galnt10	0.15608193	1.9081187	0.15980604	0.69359141	0.37406127
Glis3	0.09975756	3.09215531	0.15970908	0.69367931	0.37408178
Tubb4a	0.06724934	10.3898448	0.15953605	0.69383627	0.37413953
Abhd6	-0.0745325	4.18091732	0.15936358	0.69399282	0.3741472
Trim13	0.11980583	2.67528309	0.15932062	0.69403182	0.3741472
Pabpc1	0.04871533	8.19210904	0.15925278	0.69409344	0.3741472
Zfp655	0.05611277	5.7401676	0.15921473	0.694128	0.3741472
Cd180	0.13521269	2.7357235	0.15917568	0.69416348	0.3741472
Rbfox1	0.05627499	9.25183504	0.15913594	0.69419959	0.3741472
Gpn2	-0.133658	1.66041057	0.15913588	0.69419964	0.3741472
Tmem116	-0.233585	-0.0018565	0.15888745	0.6944255	0.37424204
Mfap4	0.14167313	3.76607458	0.15867407	0.69461965	0.37429578
Pard3b	-0.0876315	3.32297747	0.15866819	0.69462501	0.37429578
Grpel1	0.07739042	4.23880688	0.1585317	0.69474928	0.37433585
Pcdha3	0.40585351	-0.4062687	0.15834356	0.69492068	0.37440131
Rfx3	0.08248941	7.27365649	0.15801521	0.69522011	0.37453573
Pcdh12	0.79074015	-1.9877816	0.15789826	0.69532684	0.37456633
Psmb9	-0.1571607	3.32181791	0.15770701	0.6955015	0.37463351
Herpud1	-0.0718401	4.41628755	0.15752642	0.69566651	0.37469549
Slc26a2	0.11969741	6.09247466	0.15742225	0.69576176	0.37471989
2310011J03F	0.09557516	2.93835107	0.15733938	0.69583755	0.3747338
Otud3	0.14404837	1.74259567	0.15720881	0.69595702	0.37477123
Rps3	0.09502793	6.97730527	0.15699892	0.69614919	0.37483988
Fcrl6	-0.2509454	0.28566598	0.15696043	0.69618444	0.37483988
Acsf3	0.15857491	1.50699388	0.15682908	0.69630479	0.37487777
Unkl	-0.0728034	5.40387752	0.15666157	0.69645835	0.37493354
Vps33a	-0.0566563	6.28335802	0.15657491	0.69653784	0.37494942
Chpt1	0.07558172	5.44121006	0.15631432	0.696777	0.375044
Pbrm1	0.04325093	7.48916556	0.15622756	0.69685668	0.375044
Spidr	-0.1204137	2.25997588	0.15622011	0.69686352	0.375044
Obsl1	-0.0908782	3.21369127	0.15611261	0.69696229	0.37506059
Gtf3c6	0.08899815	5.50182977	0.15607771	0.69699436	0.37506059
Rhoh	0.23945886	-0.0962766	0.15589251	0.69716463	0.37511993
Sobp	-0.0455875	7.44545726	0.15582609	0.69722572	0.37511993
1700027H10I	-0.2558905	0.39054886	0.15579464	0.69725465	0.37511993
Eng	-0.0799587	3.29847141	0.15570594	0.69733627	0.37513693
Cd80	0.18642385	1.40087992	0.15563857	0.69739828	0.37514339
Slc38a7	-0.1062367	2.39339797	0.15556145	0.69746928	0.37515468
Irf5	-0.2067946	0.96066234	0.15544637	0.69757526	0.37518479
Pnpla2	0.10036905	4.47056317	0.15525725	0.69774955	0.37525162
Zfp330	0.06645218	6.1249446	0.15517532	0.69782509	0.37525197

A930001C03I	-0.5097222	-1.796291	0.15514803	0.69785026	0.37525197
Pgf	0.271959	1.21134176	0.15480433	0.69816743	0.37539561
Cherp	0.05054979	5.54780331	0.15474809	0.69821937	0.37539663
Oraov1	-0.0924717	3.70779662	0.15456846	0.69838534	0.37545895
Tmem30b	0.20872124	2.72185465	0.15449998	0.69844864	0.37546607
Nr3c1	-0.0416676	7.2766846	0.15443328	0.69851031	0.37547232
Atl3	0.05380028	7.4225482	0.15432315	0.69861216	0.37550016
Klf8	-0.0809819	2.88497504	0.15408155	0.69883577	0.37559343
Heatr3	-0.0693472	4.64907755	0.15393556	0.69897098	0.37563919
Map3k7	0.04238287	6.60263861	0.15371045	0.69917962	0.3757244
A830010M2(	-0.0741692	8.6443168	0.15346352	0.69940869	0.37580209
Chst8	0.20297355	0.65193605	0.1534466	0.69942439	0.37580209
Prob1	-0.1378248	1.69995626	0.15338926	0.69947762	0.37580378
Gzf1	-0.0575234	5.15141052	0.15328424	0.69957514	0.37582079
Klhl35	0.37705967	-0.7426236	0.15315702	0.69969331	0.37582079
Esyt3	0.12018872	2.46429092	0.1530795	0.69976536	0.37582079
Zfp518b	0.04886183	5.59134857	0.15305253	0.69979043	0.37582079
Fhad1	0.13189813	2.56366346	0.15301574	0.69982462	0.37582079
Ndn	0.06887119	6.22948305	0.15299489	0.69984401	0.37582079
Eef1d	0.06139731	5.27726507	0.1529777	0.69985999	0.37582079
Gaa	0.07166616	5.861557	0.15278866	0.70003583	0.3758883
Tbl2	0.07606445	3.49257687	0.15267036	0.70014592	0.37589801
Il20rb	0.46644629	-0.6847299	0.15266154	0.70015414	0.37589801
Daglb	-0.0925602	3.13088302	0.15256942	0.70023991	0.37591716
2310040G24	0.29643973	0.08428143	0.15236798	0.70042757	0.3759856
Casc1	-0.2956696	-0.6692017	0.15232497	0.70046765	0.3759856
Gstm2	0.13107649	5.29499627	0.15219434	0.70058945	0.37602407
Mgat3	0.05340989	6.48403666	0.15201287	0.70075874	0.37608803
Prrg3	-0.0450229	6.63317947	0.151885	0.7008781	0.37612517
Trmt2a	-0.0880607	3.786557	0.15180095	0.70095659	0.3761332
0610038B21I	0.44377859	-1.8223318	0.15176159	0.70099335	0.3761332
Prpf40b	-0.1058025	3.81498405	0.15129021	0.70143407	0.37634276
Gpr68	0.07837641	3.49668745	0.15111071	0.70160211	0.376406
Gm6938	-0.3561319	-0.7268445	0.15085409	0.70184253	0.37650805
Trem2	0.28752397	-0.4979594	0.1506848	0.70200127	0.37656628
Gpr153	0.10646796	3.00274264	0.15045487	0.70221704	0.37665509
Rap2c	0.0427276	6.85442395	0.14997026	0.70267241	0.37687239
Zfp952	-0.0580437	4.62373598	0.14990467	0.70273411	0.37687854
Tubb2a	0.07217239	7.72927064	0.14980492	0.70282797	0.37690193
Zfp862-ps	0.11010373	2.31865458	0.14961408	0.70300764	0.37692691
Pfkfb3	-0.0633988	4.54080087	0.14960139	0.7030196	0.37692691
Heatr5a	0.06866094	4.46749891	0.14959533	0.70302531	0.37692691
Abhd3	-0.1149711	3.71128761	0.14934102	0.70326496	0.37701756
Rab20	0.54971508	-2.0306466	0.14930927	0.7032949	0.37701756
Parp10	0.13666475	1.9135946	0.14923788	0.70336222	0.37702288

Map2k4	0.05309713	7.68792526	0.14919218	0.70340533	0.37702288
Ddx11	-0.3001091	0.10747502	0.14888855	0.70369194	0.37711126
Lmo2	0.07838407	4.39602433	0.14888732	0.70369311	0.37711126
Gtf3c2	-0.0638475	6.34947437	0.14885773	0.70372106	0.37711126
Mafk	0.12419698	3.20017283	0.14861052	0.70395468	0.37720501
Dnajc4	-0.1216139	2.40710364	0.14854874	0.7040131	0.37720501
Dnajc14	-0.0848123	4.30884159	0.14843375	0.70412188	0.37720501
Rcan3	0.05785148	5.12241842	0.14842682	0.70412843	0.37720501
2410006H16I	-0.1211802	2.57226497	0.14836449	0.70418742	0.37720501
Zbtb37	0.12116084	2.91738233	0.14835361	0.70419771	0.37720501
Acot3	0.28931442	0.33841072	0.14828451	0.70426312	0.37721311
Irf2bpl	-0.057313	5.74511218	0.14820096	0.70434224	0.37722855
Wdr86	0.30160356	1.31056023	0.14801617	0.7045173	0.37729537
Rhebl1	-0.2404685	0.05855139	0.14787006	0.70465581	0.37734261
Tbkbp1	-0.120395	2.13818558	0.14778832	0.70473333	0.37735718
Ppifos	-0.4537529	-1.5463159	0.14727888	0.70521704	0.37758416
Rplp1	0.07941305	5.20971012	0.14721084	0.70528172	0.37758416
Adcyap1	0.09759706	2.9020933	0.14716403	0.70532622	0.37758416
Tigd2	-0.0663268	4.43055657	0.14713001	0.70535857	0.37758416
Flcn	-0.0555601	4.56344802	0.1470451	0.70543934	0.37760045
Otud7a	-0.130605	3.09168606	0.14677414	0.70569724	0.37771155
Pla2g4d	-1.5361833	-2.0625272	0.14730016	0.70591183	0.37779944
Rhpn1	0.15556409	0.76521571	0.14631287	0.70613692	0.37788032
Cd300lb	-0.5974952	-1.3241389	0.14625339	0.70619368	0.37788032
4930525G20	-0.125787	2.28997565	0.14623201	0.70621407	0.37788032
Shisa6	-0.085255	5.30408921	0.14617034	0.70627293	0.37788486
Dnajc10	0.04756249	6.34267739	0.14597598	0.70645854	0.37794573
A930004D18	0.13691612	2.82111606	0.14594571	0.70648747	0.37794573
Lsm5	-0.1445946	1.66590589	0.14588525	0.70654524	0.37794968
Ppm1g	0.05927179	4.91335133	0.14566515	0.70675567	0.37799445
Ppp1r13b	-0.0582274	5.6077684	0.14563861	0.70678105	0.37799445
Sh3pxd2b	-0.0635815	4.59955708	0.14562052	0.70679836	0.37799445
Lsamp	-0.0894837	5.43219533	0.14558695	0.70683048	0.37799445
Rsph1	0.1385334	2.13736563	0.14547337	0.70693917	0.37802143
Lrig1	0.0850228	3.267394	0.14542891	0.70698174	0.37802143
Ifitm10	0.24907493	0.45607829	0.14521362	0.70718794	0.37810474
Zbtb22	0.09048458	3.3208382	0.14477221	0.70761127	0.37830411
Zfp81	0.07166715	4.91312818	0.14458098	0.70779491	0.37837532
Faxc	-0.0590158	7.8893427	0.14445417	0.70791674	0.37841349
Snrnp40	0.07803504	4.37384869	0.14440131	0.70796756	0.37841368
Slc4a5	-0.3184007	0.04024916	0.14423155	0.7081308	0.37847397
I830012O16I	0.10047073	4.05990536	0.14411323	0.70824465	0.37850785
Rpl13a	-0.0587596	7.00463303	0.14399207	0.70836128	0.37854321
Rundc3b	0.06769243	4.81308097	0.1436146	0.70872502	0.37870295
Supt7l	0.06054283	5.59171236	0.14357711	0.70876117	0.37870295

Pxdc1	0.11081819	3.64415578	0.14343682	0.70889651	0.37873517
Dennd6a	0.05181419	6.86793009	0.1433888	0.70894286	0.37873517
Ccdc88a	-0.0627758	9.01307016	0.14331794	0.70901126	0.37873517
Tmem109	0.0714754	3.48127732	0.14330534	0.70902342	0.37873517
Acad9	0.08662478	4.13075264	0.14325241	0.70907454	0.3787355
Ildr2	-0.0600528	9.87099209	0.14313083	0.70919198	0.37877126
Erp29	0.08920797	4.632178	0.14306989	0.70925087	0.37877574
Stau2	0.0486675	7.12925765	0.14283143	0.70948144	0.3788719
Rps19	0.08769878	4.67855439	0.14267462	0.70963317	0.37892596
Trim27	0.05742499	4.48871746	0.14243825	0.70986208	0.3790096
Ppig	0.06133334	9.2691446	0.14240701	0.70989236	0.3790096
Pla2g15	0.15329752	2.06800813	0.14235642	0.70994138	0.3790096
Ido1	0.17350597	1.31686761	0.14211274	0.71017767	0.37910876
Cenpw	0.15283074	1.57348839	0.14201331	0.71027416	0.37913329
Rbfox2	0.04557937	7.5197972	0.14195391	0.71033182	0.37913708
Det1	-0.13018	1.32187188	0.14170661	0.71057202	0.3791976
Vsx2	-0.6289605	-1.7368724	0.14169584	0.71058248	0.3791976
Tmx3	-0.0625157	5.80840114	0.14168105	0.71059685	0.3791976
Ramp1	0.09110778	4.14651163	0.14161509	0.71066097	0.37920484
Smim5	-0.2632678	-0.0767975	0.14150744	0.71076564	0.37921754
Ptpn21	-0.0703569	4.8586225	0.14148663	0.71078588	0.37921754
Rhot2	0.06825882	5.09631847	0.14126309	0.7110034	0.37930126
Drosha	0.04732735	6.89887873	0.1412012	0.71106366	0.37930126
Rnf139	0.05476516	5.09145467	0.14116953	0.7110945	0.37930126
Capn15	-0.1101439	1.8302036	0.14097656	0.71128251	0.37936453
Erv3	-0.6811922	-1.2482462	0.14094399	0.71131425	0.37936453
Wfdc1	0.18155408	2.89032144	0.14077617	0.7114779	0.37942483
Fam134c	-0.1195123	2.95746964	0.1405488	0.71169979	0.37951618
9330159M07	-0.1099959	2.13112621	0.1403394	0.71190432	0.37959826
Letmd1	0.06325211	4.8416147	0.14018448	0.71205575	0.37964083
Prelp	0.08935093	8.14582054	0.14004685	0.71219037	0.37964083
Coasy	-0.081947	3.65694836	0.13999427	0.71224181	0.37964083
Mylip	0.08226376	4.11546844	0.13999044	0.71224556	0.37964083
Ccser1	-0.1160524	3.31125484	0.13993664	0.71229821	0.37964083
R3hcc1	-0.0803162	4.26773556	0.13993029	0.71230443	0.37964083
Osbp2	-0.0597473	5.65116238	0.13989557	0.71233842	0.37964083
C2	0.14867416	2.81686402	0.13966861	0.71256071	0.37971171
Gm16740	-0.3244453	-0.806682	0.13965641	0.71257266	0.37971171
Atad3aos	0.26588634	0.04760216	0.13958384	0.71264378	0.37972263
Mfsd2a	-0.1609212	1.27973298	0.13949729	0.71272864	0.37972389
Rps6kb2	-0.1067701	2.55862419	0.13947818	0.71274738	0.37972389
Stxbp3b	-0.2718099	-0.9580444	0.13930182	0.71292038	0.37977217
C1galt1c1	0.08471874	4.60541063	0.13925915	0.71296226	0.37977217
Afg3l2	0.05617352	5.7591495	0.13906261	0.71315524	0.37977217
Adora3	0.42313486	-1.5513716	0.13903362	0.71318373	0.37977217

Cdk4	0.08980248	4.37868643	0.13902824	0.71318901	0.37977217
Gm5595	-0.1079139	2.83552425	0.13901844	0.71319864	0.37977217
Myo5b	-0.0982489	4.19877001	0.13901809	0.71319898	0.37977217
Notch4	-0.1572148	1.02824809	0.13897327	0.71324302	0.37977217
Hras	0.08806194	5.0526213	0.13879901	0.71341432	0.37983642
Pdp2	-0.104314	3.55228606	0.13863635	0.71357434	0.37988345
Klhl14	0.15391102	1.43658351	0.13860627	0.71360394	0.37988345
Chchd7	-0.0830011	3.63145787	0.13840553	0.71380158	0.37994546
Frs2	-0.0414003	7.4298957	0.13838507	0.71382174	0.37994546
Prr3	0.05965885	4.200943	0.13826545	0.7139396	0.37998123
9130008F23I	0.31072411	-0.5012529	0.13804919	0.71415283	0.38004432
Adamts17	-0.1734895	2.62311081	0.13802895	0.71417279	0.38004432
Oas1b	0.2703089	0.04731173	0.13799111	0.71421012	0.38004432
Epb4.1l4b	-0.0878762	3.33579708	0.13784339	0.71435592	0.38009347
Cyb561d2	-0.1604004	1.76477958	0.13779487	0.71440383	0.38009347
Ptplb	0.06112628	5.00768767	0.13760078	0.71459557	0.38016852
Mlh3	0.05146107	5.77549953	0.13736684	0.71482687	0.38023488
Rgs7	0.05560146	7.1722786	0.13732464	0.71486863	0.38023488
Dnajc21	-0.058706	7.02084378	0.13732086	0.71487236	0.38023488
Gpc5	0.11272471	3.53972772	0.13718468	0.71500714	0.3802796
Epb4.1l5	-0.0625257	4.6767128	0.13706175	0.71512888	0.38031738
0610040B10I	0.24294769	0.65165979	0.13695373	0.71523589	0.38031987
Upp1	-0.402051	-1.5056241	0.13688669	0.71530233	0.38031987
Mri1	0.14958525	1.14525126	0.13686532	0.71532351	0.38031987
Tmem243	0.08222385	4.06310175	0.1368254	0.71536309	0.38031987
Klhl33	0.46849496	-1.944118	0.13680122	0.71538706	0.38031987
Tex30	0.16114398	2.38532697	0.13654216	0.71564408	0.38042954
Prpf4	0.0536937	5.00342337	0.13606232	0.71612087	0.38065603
Prkg2	-0.0649903	4.33622435	0.13590954	0.71627288	0.38070985
Fbxo11	0.04014129	8.26058227	0.13547435	0.71670641	0.38088919
Xkrx	-0.1299368	2.04510831	0.13542695	0.71675368	0.38088919
Smim12	0.11038723	2.10273062	0.13541799	0.71676262	0.38088919
Ptpra	0.04852622	6.44475606	0.1351351	0.71704491	0.38096813
Sart3	0.082527	4.68295842	0.13505022	0.71712969	0.38096813
Gm3604	-0.0884623	2.73309904	0.13498582	0.71719403	0.38096813
Spcs2	0.07154079	5.45084433	0.13497737	0.71720247	0.38096813
Phtf2	0.05752052	5.23459006	0.13496789	0.71721195	0.38096813
Tbc1d19	-0.0587416	5.83751372	0.13496393	0.7172159	0.38096813
1810044D09I	-0.3559723	-1.5291417	0.13490878	0.71727101	0.38097043
Men1	0.0822694	4.03317558	0.13478925	0.71739051	0.38100692
Diap3	-0.2315343	0.48127226	0.13471436	0.71746541	0.38101972
Frrs1l	-0.0574525	7.44286109	0.13464599	0.71753382	0.38102908
Scyl3	-0.0572902	5.298139	0.13459347	0.71758638	0.38103001
Pcsk2	-0.0603016	7.2390638	0.13442731	0.71775273	0.38108586
Palm	-0.0539066	4.89436797	0.13433881	0.71784138	0.38108586

Thap4	0.0740176	4.20500576	0.13433623	0.71784397	0.38108586
Zmat1	-0.0606813	5.17673557	0.13404252	0.71813842	0.3812152
BC055402	-0.6040344	-2.4646339	0.13394442	0.71823685	0.38124047
Mms19	-0.0969187	3.6826746	0.13363829	0.71854428	0.38137443
Plk2	0.06118219	7.04814951	0.1335418	0.71864126	0.38137443
Pmpca	-0.0419197	6.05823535	0.13354132	0.71864174	0.38137443
Ifnlr1	0.4220154	-0.4183534	0.13335451	0.71882962	0.38144715
2410016O06	-0.0927333	3.82287711	0.13325995	0.71892477	0.38147066
Klf5	0.05908333	6.62739111	0.13312772	0.7190579	0.38151431
Krt19	0.1386633	1.77903333	0.13242264	0.71976905	0.38186462
Optc	0.39126913	-1.6157922	0.13236241	0.7198299	0.38186989
A230070E04	-0.0811054	4.56051307	0.13203316	0.72016281	0.38201604
Clk3	-0.060322	4.70824977	0.13197459	0.72022208	0.38201604
Gfy	0.67004572	-1.9307506	0.13193894	0.72025816	0.38201604
Kdm1a	-0.0580553	5.91142414	0.1318423	0.72035601	0.38204011
Pgrmc1	0.07463386	7.58892138	0.13179351	0.72040542	0.38204011
Trim23	-0.0520712	6.39756976	0.13135598	0.720849	0.38224833
Slc25a40	-0.0870392	3.38294368	0.13120216	0.72100515	0.3823041
Rhog	0.09534008	3.23796966	0.13093844	0.7212731	0.38241915
2610305D13	-0.2107089	0.60259501	0.1308295	0.72138387	0.38242645
Serpina10	-0.5946326	-1.9734045	0.13082462	0.72138883	0.38242645
2610037D02	-0.234826	0.16203569	0.13072232	0.72149292	0.3824546
D630013N20	-0.6285965	-0.8906889	0.13050071	0.72171852	0.38253541
Cd27	0.41970913	-1.5497778	0.1304599	0.7217601	0.38253541
Sgms2	-0.1420928	3.45145195	0.13042236	0.72179835	0.38253541
Ctdsp2	0.06712443	8.00564391	0.13029417	0.721929	0.38257762
Shmt1	-0.356438	-0.4443665	0.1301965	0.7220286	0.38258689
Mbd4	0.08608771	4.40401113	0.13017698	0.7220485	0.38258689
Col28a1	0.34412158	-0.372539	0.13000089	0.72222818	0.38265507
Palld	0.12721035	1.61944209	0.12991444	0.72231644	0.3826748
Ikzf5	0.05190481	5.07149542	0.12975684	0.72247744	0.38273306
Ocm	0.29065181	-0.9293338	0.12960117	0.72263656	0.38279033
Tnnc1	0.14593232	1.94600666	0.12920379	0.72304327	0.38295881
A330048O09	-0.2836109	0.44010957	0.12916899	0.72307892	0.38295881
Tcf24	-0.1835079	1.26987938	0.12914082	0.72310779	0.38295881
Tcfl5	0.48439654	-1.4827412	0.12892236	0.72333173	0.38298336
Psmf1	0.07146937	4.76051962	0.12887549	0.72337982	0.38298336
Larp4	-0.0441946	7.14921979	0.12884399	0.72341212	0.38298336
Slc35a5	-0.058171	5.44790196	0.1288326	0.72342382	0.38298336
C4b	-0.0985866	2.77120039	0.12880991	0.7234471	0.38298336
Gbp3	0.08703192	4.32332538	0.12879688	0.72346047	0.38298336
Smek1	-0.0520664	5.62499511	0.12867356	0.72358705	0.38302014
Vwa9	-0.0596007	4.48041734	0.12862972	0.72363207	0.38302014
Ctnna2	-0.0504885	6.28819644	0.12852838	0.72373616	0.3830482
Alk	0.2020868	0.73476148	0.12826004	0.72401202	0.3831614

Lrrc4b	-0.069137	4.57026457	0.12822098	0.7240522	0.3831614
C1qtnf2	-0.1961079	1.96562314	0.1280565	0.72422148	0.38322395
Ppm1d	0.05751388	5.13616871	0.12791066	0.72437168	0.38323942
Cacna1g	0.07873581	4.47879731	0.12783393	0.72445074	0.38323942
Cct6b	0.54735824	-1.4905165	0.12777204	0.72451453	0.38323942
Dnaaf2	0.10489449	3.59941112	0.127741	0.72454653	0.38323942
Hist1h2ai	0.29968052	-1.5684036	0.12773363	0.72455413	0.38323942
Fermt2	-0.0365745	7.13789339	0.12772091	0.72456725	0.38323942
Foxl2	-0.401582	-0.4672663	0.12768105	0.72460835	0.38323942
Lpcat3	0.08568193	3.53870214	0.12754688	0.72474676	0.38327353
Zfp740	-0.0680223	4.79995904	0.12748567	0.72480992	0.38327353
Proser1	0.05941725	5.5896054	0.12746998	0.72482611	0.38327353
Dnajc18	0.05148926	6.93329218	0.12737256	0.7249267	0.3832997
Usp46	-0.0417975	7.22034114	0.12715176	0.72515483	0.38339329
Arhgap22	0.25337465	0.14062593	0.12693362	0.72538043	0.38348554
Aff3	0.05016026	7.61446663	0.1263213	0.72601486	0.3837939
Fam53c	0.05000557	5.54866896	0.12615003	0.72619263	0.38382608
Zfp954	-0.1303409	1.98684143	0.12605812	0.72628809	0.38382608
Hmgcll1	-0.0779499	3.35418801	0.12604431	0.72630243	0.38382608
Gm1604b	-0.1253646	1.39017072	0.12602011	0.72632757	0.38382608
Htr4	-0.1636361	0.69245636	0.12601624	0.72633159	0.38382608
1700018L02F	-0.2388421	0.78479279	0.12575612	0.72660201	0.38394193
Pdxk	0.05169644	7.60065812	0.12542855	0.72694298	0.38409505
Shd	-0.1148167	2.34245362	0.12526798	0.72711031	0.3841564
Syt14	-0.1663203	1.37972022	0.12521462	0.72716595	0.38415874
Bgn	0.09263226	8.30310633	0.12485157	0.72754481	0.38428656
Ngb	-0.159626	0.84514126	0.12484727	0.72754931	0.38428656
Rtn4ip1	0.0985168	3.29227625	0.12483552	0.72756157	0.38428656
Atxn1l	-0.0471335	6.29541844	0.124706	0.7276969	0.38431047
Tmem138	-0.128034	1.17105868	0.12469412	0.72770932	0.38431047
Ecm2	0.08405859	5.03263301	0.12452222	0.72788905	0.38437833
Stat5b	-0.0671267	4.00803531	0.12446204	0.727952	0.38438451
Gm17762	0.29090766	-0.4665116	0.12398048	0.7284564	0.38462378
Gm13242	-0.3103855	-0.9866561	0.12391852	0.72852138	0.38462937
Sell	0.47778934	-1.0459632	0.12387261	0.72856953	0.38462937
Adk	-0.0597952	4.8192347	0.12369566	0.72875524	0.38470033
Stx17	0.05195592	5.51081247	0.12359302	0.72886303	0.38471307
B430010I23F	0.33909272	-0.1740072	0.12355671	0.72890119	0.38471307
Zfp382	-0.0774669	4.01271086	0.12348861	0.72897274	0.38471307
Map2k5	-0.0792376	3.60652918	0.12346006	0.72900275	0.38471307
Ppp1r21	-0.044546	5.54478482	0.12342861	0.72903581	0.38471307
Fbxo5	-0.1981147	0.50880187	0.12331066	0.72915983	0.38473708
Mettl3	-0.0737643	3.85454975	0.12326694	0.72920582	0.38473708
Rxra	0.08895202	5.7142349	0.12323904	0.72923517	0.38473708
Sphk1	-0.1206963	4.58508224	0.12312908	0.72935089	0.38475784

Eps8l2	-0.1709859	1.20284362	0.12310417	0.72937711	0.38475784
Slc25a10	-0.1618593	1.47873183	0.12298708	0.72950041	0.38479583
Ogfrl1	-0.0490592	7.93342895	0.12290152	0.72959055	0.38481631
4921524J17F	-0.1133408	4.04418156	0.12263709	0.72986936	0.3849363
Taz	0.08157583	3.1171464	0.12251964	0.72999331	0.38496626
Hps6	-0.1410539	1.04365059	0.12248603	0.7300288	0.38496626
Col6a2	0.11426754	5.41390763	0.12200684	0.73053526	0.38520625
Sprtn	-0.1149535	1.57573087	0.12183087	0.73072154	0.38527739
4930452B06I	0.08533104	3.51300428	0.12150198	0.73107008	0.38532345
Cbr3	-0.098206	2.12842456	0.1214317	0.73114463	0.38532345
Cep83os	0.05257157	5.37662296	0.12142761	0.73114898	0.38532345
Nelfa	-0.0554313	4.89453732	0.12141303	0.73116445	0.38532345
Fam189a2	0.19152408	0.55392825	0.121393	0.7311857	0.38532345
Ccdc103	-0.275063	-0.0614749	0.12136708	0.7312132	0.38532345
Cyth3	0.0627391	6.99938131	0.12132316	0.73125981	0.38532345
Trim71	-0.3805849	-0.8253599	0.12130864	0.73127523	0.38532345
Clec9a	-0.3054931	-0.0266248	0.12130276	0.73128147	0.38532345
Rn45s	-0.0814273	14.5621267	0.12126405	0.73132257	0.38532345
Tgfb1	0.10906479	2.17365205	0.1210696	0.73152912	0.38539851
Hmgxb3	0.07906071	3.94378601	0.12103322	0.73156778	0.38539851
Sox7	0.19227137	0.68090057	0.12094132	0.73166549	0.3854057
Maf1	-0.0653908	4.57616593	0.12092373	0.73168419	0.3854057
Phykpl	-0.0858391	3.26191407	0.12080914	0.73180608	0.3854271
Atf7ip	-0.0367021	6.92989395	0.12070082	0.73192136	0.3854271
Ppp1r3fos	0.40231048	0.05384402	0.12069916	0.73192313	0.3854271
Efna1	-0.2235515	0.04723327	0.12069239	0.73193034	0.3854271
Cbwd1	0.06482222	4.13995644	0.12047559	0.73216126	0.3855006
Map3k8	-0.1138393	1.90323526	0.12043827	0.73220103	0.3855006
Gm5820	0.13990155	1.83755461	0.12038645	0.73225626	0.3855006
Tfeb	-0.1584723	1.47424651	0.12036841	0.7322755	0.3855006
Rnf6	0.03685963	6.81073045	0.12027117	0.7323792	0.38552814
Med25	-0.080807	2.48573837	0.12012799	0.73253197	0.3855815
Raly	0.10730721	2.78369417	0.11987115	0.73280629	0.38569883
Usp16	0.05046963	6.12332536	0.11949647	0.73320706	0.38586711
Myl9	-0.1128387	5.49638529	0.11947607	0.7332289	0.38586711
Slc7a3	-0.1409673	1.30527275	0.11927475	0.73344454	0.38595352
Creb5	-0.057406	4.20964926	0.11921655	0.73350692	0.38595927
Mtmr14	-0.086587	3.11934636	0.11908654	0.73364634	0.38600555
Nudt9	0.06825244	5.37527833	0.11903243	0.73370438	0.38600902
3110007F17I	-0.1736927	1.17181934	0.11894105	0.73380245	0.3860201
Tacstd2	-0.2519757	0.91133709	0.11891689	0.73382838	0.3860201
Zc3h12a	0.27454779	-0.5978045	0.11869644	0.73406515	0.38610089
Alpk2	-0.4532824	-1.4775081	0.11867806	0.7340849	0.38610089
Mcat	0.09957862	2.54081818	0.11860093	0.73416781	0.38611743
Sag	0.25301067	-0.7599543	0.1185151	0.73426011	0.38613344

Gfer	-0.0915662	3.00280689	0.11847689	0.73430121	0.38613344
Wdr95	-0.5285107	-1.3915746	0.11831699	0.73447328	0.38619685
Tshr	-0.3512307	-0.4251946	0.11796212	0.73485564	0.38637082
Slc38a5	-0.3537977	-1.1945764	0.11782093	0.73500795	0.38642381
BC016579	0.50195029	-1.2504356	0.11777046	0.73506241	0.38642536
Tfcp2	0.05711827	3.79045747	0.11767669	0.73516365	0.3864515
Rasgef1a	-0.053013	6.81493714	0.11756939	0.73527954	0.38648534
Mrgbp	0.17818947	0.49676204	0.11748783	0.73536768	0.3864974
Eya3	-0.0543906	5.04768282	0.1174372	0.73542241	0.3864974
Rnf150	-0.0587514	7.33779721	0.11739592	0.73546703	0.3864974
Zfat	0.10483956	2.02412204	0.1173575	0.73550858	0.3864974
Chrm2	-0.1302249	1.96358718	0.11730327	0.73556724	0.38650115
Pygm	0.10442967	3.78732599	0.11702736	0.7358659	0.38660891
Fhit	-0.1982975	1.2402851	0.11699969	0.73589588	0.38660891
Trabd	-0.1175495	2.25259154	0.11697101	0.73592695	0.38660891
Rhbdf1	0.12039525	1.5426094	0.11688215	0.73602325	0.3866129
Adamtsl2	0.2703862	-0.4198794	0.11686888	0.73603763	0.3866129
Gm11837	-0.2411299	-0.748374	0.11649657	0.73644158	0.38678241
Tead2	0.11675436	2.48379968	0.1164353	0.73650813	0.38678241
Efcab11	-0.3049516	-0.5475158	0.11642895	0.73651503	0.38678241
Fgf11	0.0504594	6.14600193	0.11634129	0.73661028	0.38679139
Nr3c2	0.05199534	5.95236095	0.11627962	0.7366773	0.38679139
Gzmm	0.42112723	-1.6068137	0.11627087	0.73668682	0.38679139
Mark2	0.05184373	6.32552604	0.11613978	0.73682937	0.38681828
Cdh9	0.10886628	3.91287303	0.11608883	0.7368848	0.38681828
Bex1	-0.0726468	5.22081363	0.11608155	0.73689273	0.38681828
Gm4477	-0.2386519	-0.8045183	0.11600115	0.73698023	0.38683714
Cdkal1	0.07009924	4.0708894	0.1158995	0.73709091	0.38686817
Tenc1	0.07181894	5.97022186	0.11582237	0.73717492	0.38687102
Robo1	0.0568875	7.25073871	0.11579614	0.7372035	0.38687102
Strn3	0.03983635	8.17349927	0.11572296	0.73728326	0.38687102
Cct8	-0.0419062	6.86970819	0.11570518	0.73730264	0.38687102
Capsl	-0.1769626	1.18901442	0.11543437	0.73759805	0.38695453
Zfp608	-0.0562905	5.16319009	0.11543246	0.73760013	0.38695453
Tatdn2	-0.0747026	4.03065392	0.11541741	0.73761656	0.38695453
Isg15	0.11390017	1.64213574	0.11532979	0.73771223	0.38696919
Stx16	-0.0384923	6.02270895	0.11529734	0.73774768	0.38696919
Tgfbr3	0.08214076	6.77981759	0.11489801	0.73818428	0.38716379
Ptger2	0.28027957	-0.470083	0.11486363	0.7382219	0.38716379
Bap1	-0.058873	4.87571872	0.11465788	0.73844723	0.38725489
Sec61a2	-0.0773046	4.92170337	0.11454622	0.7385696	0.38729199
Gcnt4	-0.0791744	4.22489231	0.11446106	0.73866298	0.38731013
Lysmd3	-0.0622549	4.76163054	0.1144205	0.73870747	0.38731013
Syce2	-0.1336052	1.79897998	0.11435509	0.73877923	0.38731408
Rsph9	-0.1547938	2.03446966	0.11431953	0.73881826	0.38731408

Gm10190	-0.2710009	-0.2077484	0.11385236	0.73933156	0.38755608
Arhgef11	0.04377643	6.85563668	0.11378804	0.73940232	0.38756609
Chrna7	0.1498049	1.221174	0.11360146	0.73960772	0.38762757
Slc16a8	0.40057606	-1.1791877	0.11358763	0.73962296	0.38762757
Casp1	-0.141968	1.62809504	0.1134422	0.7397832	0.38767281
Mettl11b	0.54417846	-2.3411546	0.11341549	0.73981263	0.38767281
Sirt2	0.06483924	6.66112863	0.11331337	0.73992524	0.38770473
Zfp300	-0.0922908	2.71413456	0.11299572	0.74027586	0.38786136
Psd3	-0.0548024	9.82749616	0.11292953	0.740349	0.38787258
Ppm1m	0.07906009	3.80344664	0.11273996	0.74055857	0.38795528
Rab11fip5	-0.0513086	5.95716067	0.11260193	0.7407113	0.38800819
Hrh2	-0.1183767	2.16821105	0.11229812	0.74104782	0.38815737
Mtcp1	-0.0952433	3.03143394	0.11219415	0.74116309	0.38818387
Slc9a8	-0.0695505	4.08268611	0.11215915	0.74120192	0.38818387
Lrrc23	0.21057452	0.84062034	0.11191603	0.74147176	0.38827462
St6galnac4	0.10501809	2.46563388	0.11184834	0.74154695	0.38827462
Sympk	0.07496337	4.57832281	0.11181205	0.74158727	0.38827462
Apitd1	0.3025067	-0.4734113	0.11178668	0.74161547	0.38827462
Myo5c	-0.2030461	1.43219009	0.11177001	0.741634	0.38827462
Ubap1l	-0.4320654	-1.8406109	0.11148806	0.74194758	0.38840713
9430083A17I	-0.1861208	1.31944819	0.11144935	0.74199066	0.38840713
BC037034	0.06738602	3.84047215	0.11131279	0.74214274	0.38845963
Gm609	-0.4416613	-1.8422175	0.11103003	0.74245794	0.3885975
Amd1	-0.1796084	-0.1636389	0.11093344	0.74256572	0.3886268
Atp8a2	-0.1081285	3.82763017	0.11071254	0.7428124	0.38872878
Ccdc130	0.12114706	2.27195659	0.11060483	0.74293279	0.38875025
Mapk7	-0.0846555	2.64550853	0.11055783	0.74298533	0.38875025
Fuca1	-0.0724522	5.15110516	0.11050311	0.74304654	0.38875025
Zkscan3	0.06494471	3.32695568	0.11048504	0.74306675	0.38875025
Mfng	0.22641038	-0.3038503	0.11044408	0.74311257	0.38875025
Nck1	-0.0617362	4.98985231	0.11030706	0.74326592	0.38880337
Gpr20	-0.3522533	-1.386732	0.11016781	0.74342188	0.38883737
Mat2b	0.03913474	7.44759251	0.11015646	0.74343461	0.38883737
Mgat4b	-0.0798108	2.87867883	0.11008069	0.74351951	0.38884019
Rere	0.03617146	8.29609131	0.11005915	0.74354366	0.38884019
Kctd18	-0.0538488	4.36837927	0.10969474	0.74395255	0.38897383
D16Ertd472e	0.06064319	6.32211038	0.10963804	0.74401624	0.38897383
Lyz2	-0.1084803	3.56935062	0.10961344	0.74404388	0.38897383
Tbc1d8b	-0.0662245	4.33544602	0.10958221	0.74407897	0.38897383
1700001L19I	-0.094906	3.38356868	0.10957591	0.74408605	0.38897383
H2-K1	0.08038761	5.06587241	0.1095543	0.74411034	0.38897383
Trim47	0.1609289	2.25336164	0.10914444	0.74457146	0.38918775
Trmt1l	0.04641057	4.88441546	0.1090639	0.74466219	0.38920806
Mir6236	-0.136301	4.65422758	0.10879288	0.74496776	0.38934064
Mtrf1l	0.06687793	4.04188522	0.10856265	0.74522768	0.38944935

Mbd5	-0.0515281	7.79862583	0.10839245	0.74542002	0.38952273
Dnajc17	0.13227958	2.30473884	0.10817329	0.74566794	0.38962514
Elmod3	-0.1035108	3.29131426	0.10794681	0.74592444	0.38973202
Pgrmc2	0.04709881	5.49575319	0.10775939	0.74613692	0.38981588
Wnt11	0.34640165	-1.0895182	0.10755307	0.74637108	0.38989927
Agt	0.21189634	1.29022148	0.10752411	0.74640396	0.38989927
Gpr161	-0.2204378	-0.3219541	0.107474	0.74646088	0.38989927
Ifltd1	-0.0769416	5.40439857	0.10743565	0.74650445	0.38989927
Etnk1	-0.0361202	8.62575492	0.10735674	0.74659412	0.38991896
Mb21d2	-0.0560169	5.16656613	0.10727172	0.74669078	0.38994229
Asap1	-0.0483015	7.9708348	0.10711896	0.74686456	0.38997333
Cpne6	0.0784477	5.07405814	0.10710169	0.74688422	0.38997333
2510049J12F	-0.2020231	1.13682001	0.10708239	0.74690618	0.38997333
Card14	0.24222785	0.21782031	0.10654351	0.74752043	0.39026688
Ctdspl2	0.04196734	6.57527525	0.1064213	0.74765998	0.39030305
Pigt	-0.0552246	5.31439876	0.10639171	0.74769378	0.39030305
9230114K14I	-0.1101365	1.94223052	0.10610984	0.74801602	0.39044409
Invs	-0.0581069	4.82609087	0.10597452	0.74817089	0.39049776
Foxq1	-0.2402823	1.12475046	0.10585562	0.74830704	0.39054165
Rasip1	-0.0942981	2.4086378	0.10566582	0.74852459	0.39059348
Slc25a29	-0.1626171	0.83597894	0.10562069	0.74857634	0.39059348
Rpl37	0.06332545	6.35124797	0.10559425	0.74860668	0.39059348
Ftl1	0.06394907	6.19724188	0.10556052	0.74864537	0.39059348
Ccdc85b	0.05898776	4.38375005	0.10552672	0.74868416	0.39059348
Qrsl1	-0.0813707	2.65418748	0.10547003	0.74874922	0.39059348
Col15a1	0.15575612	1.46629283	0.10544878	0.74877361	0.39059348
Thrap3	0.03931095	7.46786872	0.10537612	0.74885705	0.39059348
Bloc1s2	0.07923654	3.29573558	0.1053605	0.74887499	0.39059348
Ctf1	0.1226479	2.64594289	0.10519873	0.74906087	0.39063917
Xxylt1	-0.0710787	3.52555456	0.10518005	0.74908234	0.39063917
Stim1	-0.060938	4.00960311	0.10514832	0.74911882	0.39063917
Usp5	0.06258511	4.66250852	0.10504041	0.74924295	0.39066629
Man2b2	-0.0931975	3.67084702	0.10497763	0.74931518	0.39066629
Ttc25	-0.2695301	-0.1515874	0.10496729	0.74932708	0.39066629
Amt	0.1283114	1.03484184	0.10474692	0.74958087	0.39077145
Tom1	0.07114757	4.10098346	0.10464814	0.74969472	0.39080365
Gcc2	-0.0556967	7.54554474	0.10457465	0.74977946	0.39082066
Sorcs1	-0.0756626	4.97864272	0.10451041	0.74985357	0.39083213
9330158H04I	0.40054383	-1.188079	0.10437809	0.75000628	0.39087267
Fam69b	0.09266615	3.04810245	0.10427162	0.75012924	0.39087267
Rnf41	0.04479014	5.35339329	0.10426466	0.75013729	0.39087267
Phospho2	0.05422679	5.00546569	0.10423604	0.75017035	0.39087267
Cstad	0.09923518	2.55510493	0.10421739	0.7501919	0.39087267
Alas1	0.04704195	4.46677621	0.10403828	0.75039897	0.39094482
Klf12	0.03678124	7.35340552	0.10400747	0.75043461	0.39094482

C330013E15I	0.18146295	0.41328429	0.10391221	0.75054483	0.39097509
Arrb2	-0.0682146	4.08125527	0.10386273	0.75060211	0.39097778
Sybu	-0.0580321	5.28269704	0.10373994	0.7507443	0.39102469
Glt1d1	0.13138585	1.04808471	0.10358886	0.7509194	0.39108874
Efcab14	-0.0490383	7.24510589	0.10347232	0.75105456	0.39113104
Pmfbp1	-0.3221067	-1.3142859	0.10331417	0.75123812	0.39113104
Adora2a	-0.1755922	1.89798399	0.1032547	0.75130718	0.39113104
Ttc8	0.04568919	4.70920459	0.10324123	0.75132282	0.39113104
Phf11a	0.22398569	-0.4520695	0.10322534	0.75134128	0.39113104
Arl5a	0.04278845	6.9303259	0.10320869	0.75136063	0.39113104
Gm6225	-0.3134858	-1.1707404	0.1032044	0.75136561	0.39113104
Pigx	-0.0941592	2.84092419	0.10292422	0.75169138	0.39124159
Dock11	0.05279893	5.48991872	0.10290343	0.75171557	0.39124159
Klhl2	0.04184594	6.90275888	0.10288721	0.75173445	0.39124159
Gck	0.26350934	-0.9810828	0.10257431	0.7520989	0.39140411
Socs6	-0.0504689	4.27814036	0.10248148	0.75220714	0.39143329
Abcd3	0.04063844	6.90680663	0.10222657	0.75250466	0.39156094
Klre1	0.3464503	-1.0160264	0.10217647	0.75256318	0.39156423
Adprm	0.0821147	3.70174473	0.10212167	0.75262721	0.39157039
Kcnh1	-0.0587895	6.07146337	0.10169903	0.75312168	0.39180047
Pvrl4	0.12158941	1.55218341	0.10164953	0.75317967	0.39180347
Zfp566	-0.115459	1.84360935	0.10158942	0.7532501	0.39181293
Cxcr4	-0.342818	-1.3127137	0.10149495	0.75336086	0.39184337
Cfh	0.08088145	7.13021819	0.10142266	0.75344564	0.3918603
Arpc1a	-0.0480131	6.16045961	0.10089705	0.75406309	0.39214517
1700015F17I	-0.3950283	-0.8664059	0.10084995	0.75411851	0.39214517
Rdh12	-0.1756499	0.1413352	0.10082302	0.7541502	0.39214517
Fgf14	-0.0421971	6.28372255	0.10064123	0.75436426	0.39222929
Arv1	-0.1540795	0.87145989	0.10053031	0.75449498	0.39224893
Kctd5	0.08457984	2.84732475	0.10052043	0.75450663	0.39224893
Muc15	-0.1569289	2.10105816	0.10044325	0.75459763	0.39226787
Fam98a	-0.0438084	5.72445129	0.10039548	0.75465397	0.39226787
Tmem35	0.07454092	3.85577349	0.10035652	0.75469994	0.39226787
Dus1l	0.0926553	3.06335656	0.1002694	0.75480275	0.39229413
Slc22a8	0.07829218	8.06097155	0.10005398	0.7550572	0.39239919
Nell2	-0.0502963	6.79545258	0.09968857	0.75548953	0.39258822
Mapk12	0.10687436	1.78775596	0.0996581	0.75552562	0.39258822
Plekhg1	-0.0509237	5.36137989	0.09959579	0.75559943	0.39259938
Bnip2	0.05412587	7.06219684	0.09947567	0.75574181	0.39261886
Syngr3	0.05010858	5.08592985	0.09945436	0.75576707	0.39261886
Gpr173	-0.0852514	2.88554124	0.09943171	0.75579393	0.39261886
Micu3	0.05011327	7.58963231	0.09925549	0.75600303	0.39268845
Tspan12	0.09331984	3.01681319	0.09923059	0.75603259	0.39268845
Bbs4	-0.0392587	5.56476616	0.09912019	0.75616371	0.39272936
Pdyn	0.07993423	3.32308681	0.09880374	0.75654	0.39287612

Tnip2	-0.0685973	3.05890154	0.09879446	0.75655104	0.39287612
Fam86	0.0905745	3.38812708	0.09872033	0.7566393	0.39289475
Rad51ap2	-0.1736467	1.2965391	0.09867129	0.75669769	0.39289788
Asprv1	-0.1960288	0.50430577	0.09860913	0.75677174	0.39290913
Lpxn	0.35486115	-1.4889475	0.09856403	0.75682548	0.39290984
Tnpo1	-0.0385996	6.64202774	0.09844614	0.75696601	0.3929556
Nsmf	0.04859608	5.93049297	0.09823494	0.75721803	0.39303546
Fam13c	-0.0562023	4.49680051	0.09816591	0.75730046	0.39303546
A630023P12I	-0.3595409	-1.2681202	0.09814924	0.75732037	0.39303546
Sntn	0.23159781	-0.1784704	0.09814165	0.75732943	0.39303546
Pdlim2	-0.0930442	3.29156799	0.09808123	0.75740163	0.39304573
Tbx20	0.28951248	-1.4327561	0.09777862	0.75776354	0.39318559
Zfp772	-0.0616337	4.258458	0.09775194	0.75779548	0.39318559
Slco3a1	-0.0596422	4.96501028	0.09772445	0.75782839	0.39318559
Adamtsl4	-0.0792722	2.8491448	0.09754388	0.7580447	0.39327062
Txnrd1	-0.0536331	4.89554205	0.09748905	0.75811043	0.39327752
Spsb2	0.15753563	0.96639361	0.09735561	0.75827049	0.39333335
Mfsd9	-0.1764451	1.13707931	0.09713034	0.75854095	0.39342286
Pgm2l1	0.06403209	10.1785393	0.09712453	0.75854793	0.39342286
Timm22	0.06332479	3.87699275	0.09707186	0.75861122	0.39342848
1700034J05F	0.5415341	-1.6206311	0.09696696	0.75873732	0.39346667
9930111J21F	-0.1065674	3.80923068	0.09689916	0.75881887	0.39346842
Sox5	0.05327203	5.99055607	0.09685813	0.75886823	0.39346842
Abhd14b	-0.0674319	5.31205164	0.09683335	0.75889805	0.39346842
C1qtnf3	0.46859718	-2.2217414	0.09677247	0.75897133	0.39347922
1700025G04	0.04199313	6.95265813	0.09671201	0.75904413	0.39348977
Ube2j2	-0.0739872	2.89842445	0.09645304	0.75935622	0.39362435
Rfesd	-0.0589987	4.17403698	0.096264	0.75958433	0.39371539
Nccrp1	-0.3371972	-1.1306751	0.09617627	0.75969028	0.3937431
Zfp598	-0.0487183	4.07853737	0.09603822	0.7598571	0.39380235
Rasgef1c	0.07529793	3.5194525	0.09598122	0.75992602	0.39381086
Plekhg6	0.31999771	0.0553454	0.0958459	0.76008972	0.39386848
Zfp120	-0.0718882	4.37521183	0.09578619	0.760162	0.39387873
Ms4a6b	0.27075927	0.48023595	0.09566863	0.76030436	0.3939208
Mccc1os	0.14776641	0.88030954	0.09556251	0.76043296	0.3939208
Edaradd	0.38969904	-1.1186257	0.09555401	0.76044327	0.3939208
1600016N20	0.25270908	-0.9326504	0.09554577	0.76045326	0.3939208
3110039M20	-0.1415733	2.13060271	0.09544318	0.76057767	0.39394281
Gm17751	-0.348808	-0.8407761	0.09542413	0.76060078	0.39394281
Prim1	-0.0939997	2.94450573	0.09528458	0.76077014	0.3939899
Zfp708	-0.0711354	3.49910848	0.09526267	0.76079675	0.3939899
Chtf8	0.0570815	5.11956091	0.09515649	0.76092572	0.39402949
Ptpn14	-0.0727621	6.35989005	0.09500904	0.76110495	0.39404761
Mrc2	0.10491301	4.96143287	0.09499064	0.76112733	0.39404761
Slc6a15	0.05836222	4.70351203	0.09496183	0.76116237	0.39404761

BC021891	0.12722604	1.57565952	0.09495487	0.76117083	0.39404761
2410131K14I	-0.08837	1.90497599	0.09481852	0.76133677	0.39410632
Stox1	-0.1096644	1.31133111	0.09474815	0.76142246	0.39411282
Msx2	0.13642828	2.29540939	0.09470479	0.76147527	0.39411282
Lrrc14b	-0.0886177	2.80334172	0.09467879	0.76150695	0.39411282
Add1	-0.0408123	7.6835705	0.09456254	0.76164864	0.39413737
9130019P16I	0.19095801	0.2995342	0.09452159	0.76169857	0.39413737
Cntf	-0.2962326	-1.5165918	0.09451056	0.76171202	0.39413737
Crym	0.05712787	3.43461405	0.09419086	0.76210229	0.39431211
Mtrf1	-0.0685542	3.13216346	0.09411791	0.76219145	0.39433104
Nedd9	0.04646518	4.36676939	0.09404052	0.76228606	0.3943528
Ank	0.04271753	6.15385939	0.09397716	0.76236357	0.394356
Nyap2	0.05372292	5.85461771	0.09394342	0.76240485	0.394356
D10Wsu102e	-0.0438811	5.43301154	0.09390655	0.76244997	0.394356
Tspan6	0.08470914	3.9849307	0.09373332	0.76266209	0.39443851
Acta2	0.08353568	3.71239408	0.09358296	0.76284638	0.39448515
Ccdc106	-0.1213749	1.77355217	0.0935375	0.76290214	0.39448515
Nrde2	-0.1193256	2.0474504	0.09349383	0.76295571	0.39448515
Uba7	-0.1657302	0.63843414	0.09348819	0.76296262	0.39448515
Hexa	0.09515642	3.74109112	0.09315599	0.7633706	0.39463075
Ydjc	-0.1098336	1.79528566	0.09314127	0.7633887	0.39463075
Dll3	-0.3254131	-1.1968643	0.0931304	0.76340205	0.39463075
Slc25a38	0.0703142	2.90799022	0.09299721	0.76356588	0.39468825
Mms22l	0.15636657	0.73336835	0.09286646	0.76372682	0.39474423
Zdhhc4	-0.0676087	2.59386182	0.09258796	0.76407006	0.39489443
Spata22	0.36458685	-0.4635355	0.09243191	0.76426262	0.39496674
Lefty1	-0.1248001	1.93851202	0.09238316	0.76432281	0.39497064
Rab3b	0.04192971	5.12020472	0.09232867	0.76439012	0.39497821
Klrb1f	-0.22776	-0.1107929	0.09211123	0.76465889	0.39508987
Atg9a	-0.0521663	5.13802662	0.09199636	0.76480103	0.39512598
Arcn1	0.02987825	7.30276195	0.09196963	0.76483412	0.39512598
Wdr89	0.11033705	1.09939631	0.09192058	0.76489486	0.39513014
Emx2	0.07487137	2.96208256	0.09151931	0.76539236	0.3953397
Cyp39a1	-0.0876178	3.46184472	0.09150837	0.76540593	0.3953397
Tcf19	-0.1067696	2.42159714	0.09139305	0.76554914	0.39535119
Ss18l1	-0.0437535	6.11108159	0.09136846	0.7655797	0.39535119
Zswim4	0.08260373	2.37334394	0.09136315	0.76558628	0.39535119
Smo	0.09056134	6.95353589	0.09130838	0.76565435	0.39535912
Eif3c	0.0325406	8.03434266	0.09101479	0.76601957	0.39552048
2810021J22F	-0.0575799	4.0634388	0.0909116	0.76614809	0.39555961
Cryga	-0.4141961	-1.8631762	0.09064419	0.76648151	0.39568227
Haus4	0.11798418	1.58054984	0.09061981	0.76651193	0.39568227
Ctnnd2	0.0518459	9.36130243	0.09059419	0.76654391	0.39568227
Yrdc	0.06372705	3.48619489	0.09043882	0.76673793	0.39575519
Slc2a9	-0.331103	-0.8794348	0.09032887	0.76687536	0.39579889

Fam175b	-0.0469136	4.53151692	0.09025212	0.76697133	0.39582119
Gatsl2	-0.0395608	6.17021355	0.08976893	0.76757661	0.39610631
Mrps27	-0.0701021	3.35582252	0.08966305	0.76770947	0.39614762
9930111J21F	0.09295512	2.9542156	0.08944973	0.76797743	0.39625863
Pdgfd	0.11109318	3.02315085	0.08928278	0.76818738	0.39629108
Zfp821	0.06465829	3.53873423	0.08927411	0.76819829	0.39629108
Pum2	-0.0320567	8.19361958	0.0892507	0.76822775	0.39629108
Angptl6	0.19011369	-0.1215775	0.08920001	0.76829155	0.39629108
Zfp212	-0.0813319	2.92232215	0.08918291	0.76831308	0.39629108
Ptgs2os	-0.1429797	0.78329448	0.08914779	0.7683573	0.39629108
Gm20324	0.24151533	-0.5352857	0.08885467	0.76872675	0.39645437
Rce1	-0.1980031	0.41775303	0.08864539	0.76899094	0.39653953
Zfp503	0.06458733	3.92476051	0.08864011	0.7689976	0.39653953
Bco2	-0.210884	0.95810225	0.08845326	0.76923377	0.39663405
Pcdhgc3	0.09382703	4.17040003	0.0884033	0.76929697	0.39663937
Col4a3	0.19598925	0.00043083	0.0883067	0.76941921	0.39665057
Sorl1	-0.0568358	6.15766545	0.08830257	0.76942443	0.39665057
Galnt12	-0.136933	0.82133625	0.08800845	0.76979709	0.39681541
Aaas	0.07501431	3.0916257	0.08792244	0.76990619	0.39682105
Cpeb1	-0.0564094	5.01021217	0.0879164	0.76991384	0.39682105
Gtf2f1	0.05066794	5.9128178	0.08783396	0.77001848	0.39684772
Mzf1	0.154878	0.2685717	0.08778587	0.77007954	0.39685192
Scn3b	-0.0465413	5.74038972	0.08760204	0.77031312	0.39694502
Acvr1	0.05589404	4.31727452	0.0874003	0.77056976	0.39704999
Zfp800	0.05394146	5.95938566	0.08720543	0.77081797	0.39714737
Slc25a44	-0.0365543	5.90625202	0.08715569	0.77088138	0.39714737
Tmem74b	0.2387599	-1.2658424	0.08712731	0.77091757	0.39714737
Grb14	0.07668924	3.48487065	0.08685806	0.77126119	0.397229
Gltp	0.06292457	5.52220233	0.0868354	0.77129013	0.397229
Ip6k1	-0.0481334	5.79046022	0.08679095	0.77134693	0.397229
Dbil5	-0.3510965	-1.4337472	0.0867724	0.77137063	0.397229
Rassf9	-0.1520453	0.90130565	0.08673994	0.77141211	0.397229
Fam219a	0.06014539	3.871757	0.08672742	0.77142812	0.397229
Tcaim	0.05867034	4.43598128	0.08669506	0.77146949	0.397229
Cby1	0.10654397	3.21791179	0.08667146	0.77149967	0.397229
Dlx2	-0.1146777	0.88539876	0.08655374	0.77165026	0.39727927
Aste1	-0.0892508	2.36355824	0.08633841	0.77192602	0.39739396
Abl1	0.05677364	5.32165928	0.0862269	0.77206897	0.39744028
Olfr558	0.56269573	-1.0715801	0.08611399	0.77221381	0.39748756
Pear1	-0.0970675	3.42299803	0.08581145	0.77260243	0.39766031
Fads2	0.08001044	3.20624724	0.08557882	0.77290176	0.39776625
Acap3	-0.0481336	4.76959602	0.0855476	0.77294195	0.39776625
Dctd	-0.1264134	1.64541764	0.0855279	0.77296733	0.39776625
Ikbkg	0.03507561	5.88354635	0.08535847	0.77318569	0.39782893
Rasd2	0.05080611	4.98101848	0.08531899	0.7732366	0.39782893

Tmco4	0.12106101	2.26886504	0.08530997	0.77324824	0.39782893
Nova1	-0.0402233	7.15070647	0.08521245	0.77337407	0.39786638
Cd79a	-0.1215259	2.13856601	0.0849756	0.77368	0.39799647
Ifitm6	-0.1951057	0.49823614	0.08467931	0.77406335	0.39816207
Tmem238	0.2023424	-0.4145217	0.08464479	0.77410806	0.39816207
Zfp317	-0.0581816	4.05380451	0.08451459	0.77427679	0.39822155
Tceanc	-0.0810242	3.2960577	0.08435398	0.77448512	0.39830138
Ccdc18	0.14333277	1.71139588	0.0842343	0.77464051	0.39835398
Fam60a	0.05921523	3.5921575	0.08418411	0.7747057	0.3983602
Cables2	0.04980862	5.03028034	0.08401492	0.77492564	0.39841897
Tmc4	-0.0647453	3.05233319	0.08400115	0.77494355	0.39841897
Meiob	-0.300814	-0.9393233	0.08397364	0.77497933	0.39841897
Kynu	-0.3384412	-0.5723934	0.083883	0.77509729	0.39844849
Usp3	-0.0469232	4.04294891	0.08384791	0.77514299	0.39844849
Psmd2	-0.0416286	5.80504036	0.08375147	0.77526859	0.39847155
Fra10ac1	0.0458196	4.40983291	0.08373191	0.77529408	0.39847155
Oas1c	0.12279658	1.37818016	0.08356346	0.77551369	0.39855711
Ccdc60	-0.2746854	-0.8743788	0.08344104	0.77567345	0.39861191
Trdmt1	-0.0937728	2.84535639	0.08337729	0.77575669	0.39862737
Phf2	-0.0300602	6.53371212	0.08327502	0.77589031	0.39866873
Bin1	0.04925929	5.36717275	0.08270419	0.77663773	0.39902543
Ccdc151	0.13032087	0.63614155	0.08261937	0.77674903	0.39903745
Tor3a	0.09362742	3.71345958	0.08260529	0.77676751	0.39903745
Npdc1	-0.0491812	4.46292027	0.0824295	0.7769984	0.39910349
Git1	-0.0412629	6.87270412	0.08242639	0.77700249	0.39910349
Papd5	-0.0451324	6.11240003	0.0823245	0.77713644	0.39914497
Cass4	0.18003593	0.49765302	0.08217441	0.77733392	0.39921349
1700010I14R	0.15801273	-0.0902284	0.08210639	0.77742348	0.39921349
Ube3b	-0.0434416	6.09056482	0.08208808	0.77744759	0.39921349
Slc25a34	-0.1541007	0.06385238	0.08205341	0.77749327	0.39921349
Mrgpre	0.07864353	3.56459171	0.08196632	0.77760805	0.39921349
Nt5c1a	0.14389765	0.79955439	0.08194122	0.77764113	0.39921349
Gpr174	0.35194736	-1.3478259	0.08192064	0.77766827	0.39921349
Nek10	0.19726941	0.42378789	0.08189991	0.7776956	0.39921349
Lrp8	-0.0757466	5.85580177	0.08179526	0.77783367	0.3992496
Emc8	0.03805704	5.95088228	0.08176591	0.7778724	0.3992496
Clec4a3	-0.3207354	-0.2808366	0.0813613	0.77840719	0.39949675
Ifit2	0.05592689	5.61040824	0.0811208	0.77872575	0.39962114
H6pd	-0.0692194	3.62020881	0.08102336	0.77885497	0.39962114
Spock2	0.04801101	8.11714131	0.0810093	0.77887362	0.39962114
Bhlhe40	0.03375552	7.52034272	0.08098913	0.77890038	0.39962114
9930021J03R	-0.0458045	8.19119709	0.08095873	0.77894072	0.39962114
Cpe	-0.0402131	10.0402984	0.08093728	0.77896919	0.39962114
Cbx8	-0.1803414	0.15420196	0.08073838	0.77923337	0.39972932
Ptdss1	-0.0437129	4.8909108	0.08068475	0.77930465	0.39973855

Tpp2	0.04833953	6.74275554	0.08043592	0.77963576	0.39986183
Uvrag	0.03786703	5.56574296	0.08042403	0.7796516	0.39986183
Gpatch2l	0.04046691	5.15068383	0.08034344	0.77975896	0.39986798
Blmh	0.04438813	5.01121896	0.080335	0.7797702	0.39986798
Rab11fip1	0.06028381	3.66126826	0.08014346	0.78002563	0.3998856
Tbrg4	-0.0526553	3.84983377	0.08013916	0.78003137	0.3998856
4930520O04	-0.3556713	-1.0839657	0.08009802	0.78008627	0.3998856
Mthfd2l	0.10829747	2.19025458	0.08006678	0.78012798	0.3998856
Phf11d	-0.1221477	1.52265086	0.08006622	0.78012872	0.3998856
Cacng4	-0.1557372	1.11388818	0.08004993	0.78015048	0.3998856
Crispld1	-0.0640477	3.89178035	0.08002953	0.78017772	0.3998856
Ttc30b	0.05607757	3.5675271	0.0798931	0.78035999	0.39995169
Chmp4c	0.38336884	-1.6063499	0.07983565	0.7804368	0.3999532
Slc36a4	0.04776529	4.97063688	0.07981115	0.78046956	0.3999532
Mvb12a	0.08375601	1.88568296	0.0794593	0.7809407	0.40016729
Pigz	0.09097703	2.17195228	0.07940708	0.78101071	0.40017584
Oxtr	0.08539049	3.55185533	0.07927818	0.78118365	0.40019664
Zbtb17	-0.0824042	2.67988355	0.07926303	0.78120399	0.40019664
Sppl2b	-0.1124963	1.50331952	0.07925753	0.78121137	0.40019664
Kansl3	-0.040163	5.88337963	0.07901541	0.78153668	0.4003238
Slitrk4	-0.0489293	6.29654105	0.07899336	0.78156633	0.4003238
Hhex	-0.1138448	1.7525512	0.07874633	0.78189884	0.40039487
Bmpr1a	0.05382171	7.88324888	0.0786881	0.7819773	0.40039487
Cdkn2c	-0.1062602	2.73098207	0.07868501	0.78198147	0.40039487
Tmem220	-0.084443	2.86846777	0.0786736	0.78199684	0.40039487
Kcng3	-0.1144916	3.24075379	0.07865012	0.78202849	0.40039487
Gimap4	-0.1048321	3.0918841	0.07861071	0.78208162	0.40039487
2010002M12	0.07749676	2.29318535	0.07858072	0.78212206	0.40039487
Fgfr1	0.06265028	6.04436897	0.07857328	0.7821321	0.40039487
Esrp2	0.2283657	-0.9592268	0.07840964	0.78235294	0.40048059
Agpat1	0.05182105	4.8021746	0.07832934	0.7824614	0.40050878
Cst7	-0.4002952	-1.9428477	0.07813543	0.78272355	0.40059578
9930014A18	-0.1474609	0.8415086	0.07808431	0.78279273	0.40059578
Oasl1	0.24909276	-1.023746	0.07808247	0.78279521	0.40059578
Plekha4	0.15390523	0.34010636	0.07804571	0.78284498	0.40059578
Npas1	0.19786281	-0.7981625	0.07798257	0.78293046	0.40061219
Edem1	-0.0365661	4.94744188	0.07769417	0.78332142	0.40075976
Poc5	0.04421679	3.97921674	0.07769102	0.7833257	0.40075976
Snora28	-0.3586084	-1.4223794	0.07744628	0.7836581	0.40087384
Pigh	-0.1030346	2.03473925	0.07742826	0.78368261	0.40087384
Sox12	-0.0695868	2.99499347	0.07740843	0.78370957	0.40087384
Uqcc1	0.06018203	3.87512325	0.07736954	0.78376245	0.40087384
Tln1	-0.0535756	6.04945015	0.07700935	0.78425296	0.40108797
Sgpp1	-0.0347081	6.5640417	0.07698362	0.78428804	0.40108797
Gpr61	0.09366306	1.32576376	0.07688768	0.78441892	0.40112756

Gm6260	-0.1284174	1.52096462	0.07668095	0.78470125	0.40117577
Elmod1	0.04418441	7.65046911	0.07667336	0.78471162	0.40117577
Poln	-0.2587663	-1.1123698	0.07666671	0.78472072	0.40117577
Pla2g7	-0.0978593	3.67048988	0.07666202	0.78472713	0.40117577
Serpinb6c	-0.1405913	2.72569528	0.07648904	0.7849637	0.40126937
Dnajc24	0.05261981	3.81975444	0.07632692	0.78518569	0.40130155
Tmem259	-0.0767375	3.37626986	0.07632389	0.78518984	0.40130155
Gtsf1	-0.3111239	-1.7485368	0.07628118	0.78524838	0.40130155
Wnt16	-0.1408675	1.69469413	0.07626776	0.78526677	0.40130155
Map9	0.0457368	7.90118263	0.07624779	0.78529414	0.40130155
4921531C22I	0.08672303	2.74290352	0.07597189	0.78567272	0.40146766
E230016M11	-0.2028635	0.20227686	0.0758938	0.78578001	0.40149513
Idi2	-0.1884311	-0.0722269	0.07549336	0.78633113	0.40174936
Ppcdc	-0.0785709	2.87627421	0.07544509	0.78639767	0.40175599
Abhd17c	0.04211548	5.2820418	0.07532558	0.7865625	0.40181284
Pcgf6	-0.0744316	3.32218114	0.07517619	0.78676876	0.4018735
Lig4	-0.0512176	4.33541158	0.07516198	0.78678839	0.4018735
Slc16a3	0.23648505	-1.1228918	0.07500062	0.78701143	0.40193622
Celf1	0.03897399	8.13798273	0.07499562	0.78701835	0.40193622
Atxn7l2	0.12731103	2.34763128	0.07480249	0.78728566	0.40204536
Spag7	-0.0542705	5.28595099	0.07475368	0.78735328	0.40205252
Dpyd	0.06655232	3.2659172	0.07449281	0.78771507	0.40214428
Gsdmd	0.09959989	2.29812276	0.07443925	0.78778944	0.40214428
Sh3gl3	-0.0554834	5.61268157	0.07442729	0.78780605	0.40214428
Suz12	0.03401533	6.88359314	0.07434527	0.78792	0.40214428
Fbxo21	0.0442152	5.66249624	0.07433507	0.78793417	0.40214428
Lrtm2	-0.0521696	5.89053661	0.07430575	0.78797492	0.40214428
Nelfcd	0.05528747	3.75889393	0.07429426	0.78799089	0.40214428
4933412O06	-0.1692057	1.25724567	0.07429378	0.78799157	0.40214428
Letm1	0.04652072	5.94169306	0.07427658	0.78801547	0.40214428
Gm10584	0.25621067	-1.1520838	0.07423341	0.78807551	0.40214756
Gm6277	0.08878057	2.28629519	0.07416442	0.78817148	0.40215096
Ptpla	-0.0750607	2.93398082	0.07412849	0.78822148	0.40215096
Def6	-0.1428793	0.86126945	0.07410127	0.78825937	0.40215096
AW495222	0.09457824	1.74746184	0.0740447	0.78833814	0.40215096
Mettl22	0.09173723	2.59116622	0.07403602	0.78835022	0.40215096
Fam219b	-0.0661196	3.70312131	0.07395932	0.78845708	0.40217812
Nus1	-0.0311803	6.88160066	0.07391458	0.78851945	0.40218259
Nr1h4	-0.2937248	-0.4247243	0.07373237	0.78877362	0.40228488
Sox1	-0.0537739	4.72724917	0.07361533	0.78893705	0.40233952
01-Mar	0.05204024	5.07859103	0.07355216	0.78902534	0.40233952
Mkl2	-0.0535422	9.41262042	0.07350299	0.78909407	0.40233952
D630003M21	-0.1962109	0.44311259	0.07350211	0.7890953	0.40233952
Anks1b	-0.0467679	8.50588551	0.07341888	0.78921171	0.4023428
Tesk1	0.05388437	4.95553184	0.07340752	0.7892276	0.4023428

Oaf	0.11429682	1.012557	0.07338248	0.78926265	0.4023428
Lax1	-0.2769732	-0.2643545	0.07313758	0.78960568	0.40246592
Zfp719	0.04620077	5.71662256	0.07313345	0.78961147	0.40246592
Gm10789	-0.3331169	-1.9141048	0.07303377	0.78975128	0.40250983
4932441J04F	0.22933382	-0.8893644	0.07286554	0.78998745	0.40260284
Samm50	0.0502005	3.50730105	0.07270932	0.79020703	0.40268739
Kbtbd4	0.04609943	4.48817103	0.07253142	0.7904574	0.40270025
Mir17hg	-0.4520251	-1.6099372	0.07251193	0.79048486	0.40270025
Rbm48	-0.0506802	3.43229954	0.07250951	0.79048826	0.40270025
Ring1	-0.048851	4.47181518	0.07250332	0.79049698	0.40270025
Atmin	-0.0322268	6.26839051	0.07250069	0.79050069	0.40270025
Eefsec	-0.0796823	2.69284834	0.07243794	0.79058909	0.40271793
Vasp	0.09356165	3.48114641	0.07234346	0.79072229	0.40275843
Clnk	-0.2371615	-0.6436547	0.07225627	0.79084528	0.40279373
Dnaaf3	0.24126792	-1.1739159	0.07203073	0.79116383	0.40291865
Ska1	0.49901238	-1.8990028	0.07198569	0.7912275	0.40291865
Sh3gl1	0.06506717	3.29642853	0.07196858	0.7912517	0.40291865
Pcdhga1	0.10104632	1.75969594	0.07153111	0.79187137	0.40317983
Fyb	0.07078325	4.4935111	0.07151153	0.79189915	0.40317983
Aamp	0.05367253	4.84802461	0.07146257	0.79196864	0.40317983
Plekhn1	-0.1902791	-0.0644064	0.07145485	0.79197959	0.40317983
Ndnl2	0.06175143	3.46944245	0.07139492	0.79206469	0.40319579
2900055J20F	0.07851816	3.12873871	0.07118345	0.79236528	0.40332143
Ggh	-0.0748202	3.23462189	0.07106734	0.79253052	0.40337817
Fam35a	0.09092106	2.54139424	0.07085486	0.7928333	0.40348607
Sepsecs	0.09170876	2.25079891	0.07084308	0.79285009	0.40348607
Trim24	0.03722699	5.55712033	0.0707766	0.79294493	0.40349067
Slc24a4	-0.0976609	2.83603818	0.07076134	0.79296671	0.40349067
Rnf144a	-0.0469641	4.77117646	0.07067471	0.79309037	0.40352622
Cox16	-0.056068	4.60842813	0.07043345	0.79343523	0.4036743
Asb4	-0.1695828	0.34542722	0.07034554	0.79356103	0.40371092
Rnf43	-0.0794939	3.23811628	0.07026411	0.79367765	0.40374287
Cpb1	0.1171358	2.22765188	0.07010744	0.79390222	0.40382972
Txndc15	0.04818913	4.33369455	0.07003578	0.79400501	0.40382993
AU019823	0.04803902	5.02484557	0.07003211	0.79401029	0.40382993
Gpr27	-0.2555349	-1.2313857	0.06962559	0.79459458	0.40408433
Zfp553	-0.0700229	3.70563064	0.06960915	0.79461823	0.40408433
Hpgd	-0.0724915	3.95251446	0.06957172	0.79467214	0.40408435
Rcc2	-0.0338044	5.31857421	0.06949329	0.79478512	0.40409871
Zbtb21	0.04158535	5.05564308	0.06946358	0.79482793	0.40409871
Zwilch	0.12312867	1.4763998	0.06942966	0.79487684	0.40409871
Fam57a	-0.1072252	1.66580717	0.06937466	0.79495614	0.40409871
Slc25a43	0.32453794	-1.5902955	0.06936524	0.79496974	0.40409871
Asb13	0.04331731	4.60256161	0.0692457	0.79514224	0.40412793
Pygo1	0.04276919	4.86566756	0.06922217	0.79517622	0.40412793

Nudt4	0.0489753	9.94797792	0.06921343	0.79518884	0.40412793
Rbm10	0.04059315	4.92040676	0.0690513	0.79542312	0.40416764
Cnpy4	-0.0459265	4.55218705	0.0690355	0.79544597	0.40416764
Calb2	0.09870769	1.07864787	0.06901398	0.7954771	0.40416764
Srr	0.04044795	6.94514693	0.06895421	0.79556356	0.40416764
Rnf170	0.03959348	5.90689747	0.06894112	0.7955825	0.40416764
Tagln	0.1001629	3.30791348	0.06893576	0.79559026	0.40416764
1700007K13I	-0.1600558	0.10950649	0.06884653	0.79571945	0.4042059
Ankrd39	-0.0977883	1.7336555	0.06875046	0.79585863	0.40424922
Cpt1a	-0.0449356	5.93109532	0.06852004	0.79619288	0.40431894
Zfp644	0.03878225	7.40811311	0.0685183	0.79619542	0.40431894
Fbxl2	-0.0572011	4.66631756	0.06849213	0.79623341	0.40431894
Star	0.07514459	2.52896689	0.06848673	0.79624126	0.40431894
Lurap1l	-0.0666149	2.60578454	0.06847012	0.79626539	0.40431894
Dync1li2	0.02754142	8.01244482	0.06841542	0.79634485	0.40433192
Ppp1r12c	-0.0346274	5.32770825	0.06834254	0.79645079	0.40435834
Mettl7a1	0.05513386	6.05892047	0.0681358	0.79675162	0.40448369
Aim2	0.11444113	2.19248865	0.06809679	0.79680845	0.40448517
Cdc42bpg	-0.1015074	1.33986723	0.06789135	0.79710797	0.40460983
Tmem256	0.07699887	2.86839731	0.06773626	0.7973344	0.40466615
Galnt3	-0.3188398	-0.752167	0.06770838	0.79737513	0.40466615
Cytip	-0.0847217	2.74174815	0.06770454	0.79738075	0.40466615
Susd2	-0.0525474	5.00079841	0.06765585	0.79745191	0.40467488
Ccdc67	0.28125832	-1.3627201	0.06760463	0.7975268	0.40468551
Gpr141	-0.3508266	-0.5865768	0.06731126	0.79795634	0.40487519
Dnali1	0.15833371	0.12482844	0.06727068	0.79801584	0.40487519
Pxn	-0.042728	5.22610762	0.06723415	0.79806941	0.40487519
Tsr1	-0.0441232	6.03519977	0.06718915	0.79813542	0.40487519
Scyl1	0.04112292	4.19231876	0.06716527	0.79817047	0.40487519
Epb4.1l4a	-0.0843234	1.53030088	0.06711434	0.79824523	0.40488573
Pf4	-0.2551572	-1.4000512	0.06707088	0.79830904	0.40489068
Lamb2	0.08231442	4.32199266	0.06703418	0.79836294	0.40489068
Tbc1d10c	0.24384706	-0.871725	0.06695954	0.79847263	0.40491893
Zfp513	0.08682291	1.85284122	0.06690812	0.79854824	0.4049299
Jakmip2	0.04168997	6.83067057	0.06682111	0.79867623	0.40493234
St3gal5	-0.0406211	6.4731257	0.06680857	0.79869468	0.40493234
Zbtb39	0.04400345	4.42513232	0.06679477	0.798715	0.40493234
Traf3ip1	0.03616468	4.88522269	0.06672102	0.79882358	0.40496002
Bdh2	0.09222448	3.09510841	0.06656464	0.79905404	0.40501134
Pcgf2	-0.0523551	5.09123752	0.06655157	0.79907332	0.40501134
Synpo2	0.0697598	3.37274146	0.06654243	0.7990868	0.40501134
Slc26a6	0.19638357	-0.1905844	0.0664676	0.7991972	0.40503993
Srprb	-0.0555454	3.49806931	0.06639011	0.79931159	0.40504586
A930024E05I	0.14860077	0.66919933	0.06636942	0.79934214	0.40504586
Gli1	0.12114554	1.65310861	0.06634996	0.79937089	0.40504586

Rasl12	0.18438421	-0.0654377	0.06620079	0.79959137	0.40508707
Cabp4	0.31041721	-0.8660315	0.06616647	0.79964214	0.40508707
Sema4b	-0.0633119	2.77315186	0.06615615	0.79965741	0.40508707
Ifitm7	-0.2434325	-1.6271057	0.06611769	0.79971431	0.40508707
Akt1	-0.0474242	5.08357645	0.06611234	0.79972224	0.40508707
Hfe2	-0.3380124	-1.659473	0.06605692	0.79980427	0.40508868
D130040H23	0.06866406	2.95254379	0.06603724	0.79983341	0.40508868
Dok1	0.20700015	-0.2343909	0.0659961	0.79989434	0.40509219
Best3	0.22706993	-0.0664537	0.06565259	0.80040394	0.4053229
Klhl42	-0.0306031	6.43862941	0.06542294	0.80074541	0.40546844
Paqr4	-0.0593591	3.91087184	0.06531048	0.80091286	0.40547446
Myh2	-0.2021545	0.20706863	0.06529932	0.80092949	0.40547446
Syne4	-0.2854593	-1.3494945	0.06529138	0.80094133	0.40547446
Btc	-0.5741641	-1.977228	0.06524365	0.80101245	0.40547446
Ankra2	0.05142027	3.44615038	0.06523352	0.80102756	0.40547446
Wdr12	0.05308564	4.64403414	0.06518425	0.80110101	0.40548428
Hs3st6	-0.2191055	-0.5819177	0.06514663	0.80115711	0.40548531
Oprm1	0.12084209	1.91344994	0.06506929	0.80127252	0.40550905
Zfp420	-0.0419511	4.40160952	0.06504275	0.80131213	0.40550905
Pxylp1	0.05597588	3.30128163	0.06461391	0.80195351	0.40578905
Xrcc5	0.05161329	4.92129584	0.06457777	0.80200768	0.40578905
Nox4	-0.1105631	1.22891877	0.0645644	0.8020277	0.40578905
Nhlh2	0.25279271	-0.6575748	0.06445346	0.80219407	0.40584585
5031414D18I	0.22262024	-1.0537703	0.06440042	0.80227365	0.40585874
Aspdh	-0.1917729	0.11307525	0.06433042	0.80237876	0.40587638
Kiz	0.04933379	4.925296	0.06430512	0.80241674	0.40587638
Stom	0.11983906	5.20970168	0.06415003	0.80264986	0.40596692
Shroom1	-0.0933196	1.58595977	0.06381467	0.80315495	0.4061569
Traf5	0.10365286	1.45352908	0.06379558	0.80318375	0.4061569
Impdh1	-0.0748734	2.57882557	0.06379282	0.80318791	0.4061569
Apobr	0.18125629	0.34536625	0.06368823	0.80334575	0.40620933
Irak3	0.08852142	2.79377996	0.06348696	0.80364989	0.40633573
Csnk1e	-0.0379289	6.14590417	0.06299963	0.80438844	0.40668174
4921534H16I	-0.4124649	0.0222078	0.06274535	0.80477501	0.4068238
Tars	-0.0410843	4.55001334	0.06274345	0.80477789	0.4068238
Sntb2	-0.0372267	5.67243718	0.06266539	0.80489673	0.40685645
1700020I14R	-0.0386192	6.32617778	0.06234927	0.80537881	0.4070727
Gm5113	-0.0622923	4.18435604	0.06217243	0.80564906	0.40718186
Actr8	0.04126543	4.68296503	0.06209596	0.80576605	0.40718234
Spata5	-0.0525101	4.23811925	0.06208243	0.80578675	0.40718234
Ikzf3	0.07618153	2.779429	0.06206537	0.80581286	0.40718234
Sbsn	-0.0898569	1.79275705	0.0619681	0.80596182	0.40721758
Acbd5	0.02509637	7.15738997	0.06194895	0.80599117	0.40721758
Dis3l2	0.05381545	3.42555374	0.06181827	0.8061915	0.40729137
Egr1	-0.0623766	9.14900846	0.06173106	0.80632534	0.40733155

Neil2	0.20914172	0.05266386	0.06168528	0.80639563	0.40733962
Gpr52	0.08986076	1.71756204	0.06155416	0.80659711	0.40741396
Frs3	-0.0828197	2.42524315	0.06143923	0.80677391	0.40745036
Vmn2r118	0.34779463	-1.2699865	0.06143273	0.80678392	0.40745036
Ccl9	-0.0829002	1.82352796	0.06140142	0.80683211	0.40745036
Lbh	0.06291473	6.3473381	0.06136393	0.80688983	0.40745207
Thoc6	0.11062435	1.21006605	0.06131956	0.80695818	0.40745374
Ccdc9	0.05678411	2.73401385	0.06129127	0.80700176	0.40745374
Htra1	0.05503831	3.91348664	0.06125219	0.807062	0.40745673
Gpatch3	0.1771388	-0.187732	0.06120353	0.80713703	0.40746718
Gpc2	-0.2072256	0.06735224	0.06108703	0.80731678	0.4075305
Lrrc1	-0.05817	4.36593242	0.06105095	0.80737249	0.4075312
Clec10a	-0.261439	-1.1430793	0.06086749	0.80765602	0.40761087
Dbndd1	-0.088805	1.76124318	0.06086111	0.80766588	0.40761087
BC017643	-0.0784721	2.48039427	0.06084335	0.80769336	0.40761087
Ccdc162	-0.1538839	0.22297356	0.06073276	0.80786453	0.40766983
Ppap2b	-0.0542226	6.43322826	0.06069185	0.80792789	0.40767438
Meis3	0.06113288	3.60716697	0.06047693	0.80826113	0.4078151
Pla2g3	0.19763179	-0.0423008	0.06001326	0.80898222	0.40814525
Bola2	0.08197367	2.45401443	0.05998627	0.80902428	0.40814525
Olfr287	0.19040036	0.25108927	0.05973264	0.80942008	0.40831746
Brca1	0.1176322	1.08808677	0.05959206	0.80963983	0.40838154
Tmbim1	-0.0514217	5.07240249	0.05955621	0.80969592	0.40838154
Slc16a5	0.17566294	-0.6751443	0.05954694	0.80971043	0.40838154
Hbp1	0.03121654	6.72167701	0.05948869	0.8098016	0.40840006
Zfp574	0.05036913	4.88659637	0.05943138	0.80989136	0.40841787
Hcrtr1	-0.294235	-1.4360629	0.05938359	0.80996624	0.40842817
Jmjd7	-0.1919154	-0.6699958	0.05932201	0.81006277	0.40844939
Slc9a1	0.04456624	5.26187894	0.05890005	0.81072565	0.40875615
Papss2	0.0453367	5.79153678	0.05878444	0.8109077	0.40882047
Fam65a	-0.0471008	7.33207848	0.05862893	0.8111529	0.40887985
Pigc	-0.0844084	3.10018614	0.05857218	0.81124246	0.40887985
Skiv2l2	0.03227929	6.77451449	0.05857139	0.81124371	0.40887985
Timm17b	-0.1080201	2.74288703	0.05849911	0.81135784	0.40887985
Gm17801	-0.2610728	-0.6337183	0.05849686	0.8113614	0.40887985
Zfhx3	0.02903207	7.2958918	0.0584547	0.811428	0.40887985
1700029I15R	-0.1961084	-0.841606	0.05845117	0.81143359	0.40887985
9330020H09I	0.18721634	-1.0428225	0.05843348	0.81146155	0.40887985
1190005I06R	0.23232881	-1.2426018	0.05839495	0.81152246	0.40888307
Nacc1	0.02827303	6.99277199	0.05826893	0.81172182	0.40895605
Bcl2l2	0.02753953	6.25153777	0.05788867	0.81232482	0.40923139
Gcfc2	-0.0833562	2.09808493	0.05785555	0.81237744	0.40923139
Samsn1	-0.1309824	0.62142411	0.05762913	0.81273757	0.40938531
Sft2d1	-0.0488212	3.36527521	0.05752041	0.81291078	0.40940125
Pkn1	-0.0904957	2.85347814	0.05751059	0.81292642	0.40940125

Bend6	0.03584741	6.64558195	0.05743234	0.81305121	0.40940125
Gm17769	-0.1157281	0.14104306	0.05741983	0.81307116	0.40940125
Prune	0.03650071	4.58506646	0.05735851	0.81316902	0.40940125
Cntrob	-0.0856152	2.14754063	0.05734193	0.81319548	0.40940125
Gm5141	-0.0699576	3.06213134	0.0573375	0.81320255	0.40940125
Zcchc4	-0.2015253	-0.1139491	0.05733544	0.81320584	0.40940125
B9d1	0.06676776	2.37419088	0.05720238	0.81341841	0.40948079
Mmp24	-0.0782604	2.48675089	0.05713114	0.81353233	0.40951065
Crlf2	-0.1164541	0.43132701	0.05691695	0.81387529	0.4096558
Ncoa1	0.02610432	8.10415337	0.05685832	0.81396928	0.40967562
Cbx5	-0.0280468	9.15622761	0.05680476	0.81405521	0.40969138
Suv420h2	-0.147161	-0.0499833	0.05671233	0.81420356	0.40973855
Ube2g2	0.04675731	4.67683069	0.05665205	0.8143004	0.40975979
Zxdc	0.03558133	5.4891973	0.05655217	0.81446095	0.40980537
Lrrc75a	0.05337547	3.25761938	0.05652775	0.81450023	0.40980537
Ttc16	-0.3331658	-1.5557674	0.05645747	0.81461332	0.40983478
Mtf1	0.02904125	5.99906572	0.05636932	0.81475528	0.40987871
Ap1m1	0.05685828	3.73255191	0.05627312	0.81491033	0.40992636
Gm15760	0.08213708	2.13793902	0.05624275	0.8149593	0.40992636
Dync1i2	0.0327161	7.48767004	0.05614738	0.8151132	0.40997489
Tfec	-0.3572376	-1.7338427	0.056085	0.81521393	0.40997489
Ttc28	-0.036902	5.76228808	0.05601039	0.8153345	0.40997489
Pcdha5	-0.1213961	0.8341099	0.05600745	0.81533925	0.40997489
Cbln4	0.04280734	4.04057016	0.05597352	0.81539411	0.40997489
Lclat1	-0.0335573	6.05359353	0.05594936	0.81543318	0.40997489
Cox8b	-0.3076976	-1.4825481	0.05591622	0.81548679	0.40997489
Ptprn2	0.03212542	7.30390433	0.05588066	0.81554433	0.40997489
Hhatl	0.13451426	0.58083269	0.0558786	0.81554766	0.40997489
Rab14	-0.02624	7.96281547	0.05567551	0.81587668	0.41007978
Mettl14	-0.0379428	5.82265542	0.05565773	0.81590551	0.41007978
Rbm39	-0.0259738	7.64977397	0.0556486	0.81592033	0.41007978
Itpripl1	-0.1378523	1.21223054	0.05543416	0.81626851	0.41022729
6430562O15	0.31907303	-1.7198949	0.05533861	0.81642388	0.41026828
C1galt1	-0.0456174	4.66589561	0.05531674	0.81645946	0.41026828
Cant1	0.05727021	3.30876225	0.05527515	0.81652715	0.41027481
Gm13212	0.07571777	1.56577892	0.05520525	0.81664098	0.41030452
Serpinb9	0.04717616	7.10563105	0.05503491	0.81691866	0.4103914
Xrcc3	-0.0588104	3.43280101	0.05499906	0.81697716	0.4103914
Oxsm	-0.06711	3.16295024	0.05499303	0.81698702	0.4103914
Hdac10	0.08156626	1.601888	0.05496502	0.81703273	0.4103914
Snrnp27	0.05056754	5.43105277	0.05458058	0.81766157	0.41066353
Gpr82	0.34413304	-1.234143	0.0545669	0.81768399	0.41066353
Tarsl2	0.04998437	4.5568024	0.05452782	0.81774804	0.4106682
A930011G23	-0.1771581	-0.3035177	0.05444545	0.81788315	0.41070856
Lrsam1	0.0598088	3.61904986	0.05433252	0.81806857	0.41077417

Gal3st1	-0.1340076	0.20452388	0.05420312	0.81828125	0.41081641
Grap	0.11480837	0.96999009	0.05419242	0.81829885	0.41081641
Suv39h2	-0.0618918	3.0955712	0.05418139	0.81831699	0.41081641
St3gal3	0.05142144	3.19153663	0.0541117	0.81843168	0.41082037
Igf2bp1	-0.3885684	-1.5499557	0.05411005	0.81843441	0.41082037
Sox17	0.11113606	1.86351752	0.05404503	0.81854147	0.41084662
1700086L19F	-0.0598116	3.09670287	0.05399598	0.8186223	0.41085532
Cplx2	0.03780239	9.63292106	0.05395124	0.81869605	0.41085532
Inca1	0.09170508	0.79195085	0.05393482	0.81872313	0.41085532
4930550C14I	0.12259612	1.73402426	0.05385112	0.81886121	0.41089712
Gm4944	-0.0392098	4.17769705	0.05375217	0.81902459	0.41091504
Carns1	0.1028585	2.38018108	0.05374628	0.81903433	0.41091504
Maats1	-0.1695784	0.57079124	0.05372998	0.81906126	0.41091504
Rorb	-0.0388676	8.50372588	0.05366775	0.81916411	0.41093916
Serpind1	-0.0722083	4.92559936	0.05349767	0.81944556	0.41105286
Man2a1	0.0287683	6.07197675	0.0534063	0.81959696	0.41110132
4833419F23I	-0.162951	-0.1610463	0.05336374	0.81966752	0.41110471
Zfp526	0.05768982	3.07793303	0.05332978	0.81972385	0.41110471
D15Ertd621e	0.02602539	7.09753655	0.05330309	0.81976813	0.41110471
Rac3	0.20666013	-0.9383691	0.05324665	0.81986182	0.41112421
Gbgt1	0.14197069	0.52653388	0.05316234	0.82000186	0.41115184
Senp3	-0.0387428	4.80653401	0.05314748	0.82002654	0.41115184
Cdc37l1	0.02718416	6.61550498	0.05308737	0.82012649	0.41117446
Zfand2b	-0.1011559	1.87465094	0.0530221	0.82023505	0.41120141
Cbx2	-0.1157347	1.04972225	0.05297204	0.82031838	0.4112157
Elfn1	0.05404992	4.83375736	0.05293566	0.82037895	0.41121858
Mxd4	0.08610679	2.4850677	0.05284705	0.82052659	0.41122402
Stxbp6	-0.0360315	6.56505081	0.0528302	0.82055468	0.41122402
Nphp1	-0.0486262	3.83450692	0.05280039	0.8206044	0.41122402
Trmt10a	0.05434397	2.92068667	0.05279279	0.82061707	0.41122402
Ccdc136	0.03673514	5.29502051	0.05273325	0.8207164	0.41122402
Clcc1	-0.042804	4.36557929	0.05273186	0.82071872	0.41122402
Pllp	-0.0556109	2.36712096	0.05268564	0.82079586	0.41123204
Zkscan14	0.09959434	2.42938013	0.05265659	0.82084437	0.41123204
Patz1	0.03796364	4.85691423	0.05255581	0.82101278	0.41126164
Comp	-0.2224779	-1.3445254	0.05255561	0.82101311	0.41126164
Gm13139	0.07641947	2.32441717	0.05251189	0.82108622	0.4112708
Zmynd12	-0.2427165	-1.5953784	0.05246846	0.82115886	0.41127538
Wsb2	-0.0243559	7.7269333	0.05244088	0.82120502	0.41127538
Hdhd2	0.03224386	5.52610197	0.05217525	0.82165021	0.41147087
Kirrel	-0.0604906	4.57305455	0.05212475	0.82173498	0.41148585
Acvr1b	-0.0398007	6.06409219	0.05202115	0.82190901	0.41149113
4930528A17I	-0.314535	-1.4834842	0.05201682	0.82191629	0.41149113
Prex1	-0.0425157	5.9249427	0.05201343	0.82192199	0.41149113
Aff4	-0.0254515	8.98795918	0.05198789	0.82196494	0.41149113

Casp9	-0.0392593	4.67728021	0.05193338	0.82205661	0.41150064
Cxcl10	-0.0996421	2.81711053	0.05191136	0.82209365	0.41150064
4931440F15I	-0.185421	-0.2478327	0.0518167	0.82225301	0.41155294
Plac8	0.28702981	-1.2201908	0.05177108	0.82232987	0.41156395
Hr	0.07677266	3.0286007	0.05170743	0.82243717	0.41159019
Zeb2	-0.033391	8.89773701	0.05166985	0.82250054	0.41159445
Itgb4	-0.0973371	3.19530324	0.05154588	0.82270978	0.41166441
Angptl4	0.08691253	2.19976334	0.05149403	0.82279738	0.41166441
Gm3558	-0.168528	-1.2723541	0.05148953	0.82280499	0.41166441
Plcd3	-0.0550786	3.08169908	0.05138479	0.8229821	0.41172556
Thbs2	0.06167721	5.06590599	0.05132442	0.82308425	0.41174921
Ccdc183	-0.1289576	0.60674389	0.05127973	0.82315993	0.4117596
Snx8	-0.0829515	1.59947853	0.0512177	0.82326501	0.41178471
S100a8	-0.2775053	-1.1417502	0.05110714	0.82345247	0.41183428
4930515G01	0.23530446	-0.7787779	0.0510945	0.82347393	0.41183428
Smpdl3a	0.05179274	4.81997081	0.05104028	0.82356596	0.41185285
Mterf1a	-0.115651	1.18052134	0.05099079	0.82364999	0.41186741
Vav3	-0.0511929	3.29633522	0.0508803	0.82383779	0.41193386
Lmna	0.05153744	3.66476007	0.05071129	0.82412545	0.41205023
Zfp2	-0.0389027	4.45091169	0.05033564	0.82476664	0.41233321
Lyrm5	-0.0403189	5.17912218	0.05031534	0.82480137	0.41233321
Prr32	-0.182541	-0.3942978	0.05027618	0.82486838	0.41233923
Pde6a	-0.072092	2.04928689	0.05016071	0.82506609	0.41241059
Enho	0.09881683	0.55148826	0.05008204	0.82520094	0.4124505
Nfam1	-0.1412874	1.15050389	0.05002634	0.82529647	0.41247077
A730036I17F	-0.263659	-1.0930303	0.04998068	0.82537484	0.41248246
1500017E21I	-0.2816987	-1.8060662	0.04992889	0.82546376	0.41249941
Rock2	-0.0331464	10.0961384	0.04981943	0.82565189	0.41256594
Srebf2	0.0439308	5.20482012	0.04959459	0.82603896	0.41273186
Cep57l1	-0.0568936	2.90026587	0.04942125	0.82633801	0.41285378
Ccdc169	0.22275582	-2.2268421	0.04932432	0.82650548	0.41289147
4930538K18I	0.14261908	0.49385641	0.04931388	0.82652353	0.41289147
Slc29a4	0.08838039	1.10904848	0.04925794	0.82662027	0.4129123
Spon1	0.03829902	5.35663672	0.0489886	0.82708685	0.4130912
AI118078	-0.1585611	-0.438439	0.0489771	0.8271068	0.4130912
Rab34	0.05755136	4.25800237	0.04895587	0.82714363	0.4130912
Fpgt	-0.0356373	4.93554178	0.04884922	0.82732883	0.41313005
Il17d	0.08269602	0.95456877	0.04884764	0.82733157	0.41313005
Cstf1	-0.0547403	2.83750905	0.0486588	0.82766	0.41326052
Sema3e	0.08828494	4.19611162	0.04863409	0.82770303	0.41326052
Tbx15	-0.0514535	6.7955854	0.04856635	0.82782104	0.41328316
Car9	0.18674006	-0.7352023	0.04854482	0.82785856	0.41328316
Cd22	0.34949528	-2.1449166	0.04848219	0.82796778	0.41331017
M1ap	-0.3607334	-1.5333077	0.04843757	0.82804562	0.41332153
Rbmxl1	-0.0337515	5.34589188	0.04825706	0.82836095	0.41339941

Asphd2	0.05413214	2.68261618	0.04819666	0.8284666	0.41339941
Otop2	0.21114385	-0.9323722	0.04819628	0.82846726	0.41339941
Ctnnbip1	0.03808576	3.99076024	0.04813776	0.82856969	0.41339941
Gm20751	-0.2061502	-1.2658381	0.04813586	0.82857302	0.41339941
Scaf1	0.03778853	4.68206565	0.04811391	0.82861146	0.41339941
Ipp	0.04940961	3.30453463	0.04810459	0.82862778	0.41339941
Mtss1l	-0.0299149	7.05287022	0.04809616	0.82864254	0.41339941
Clasp1	-0.0320894	7.49695129	0.04805127	0.82872119	0.41341115
Wwc2	-0.0389656	4.8312372	0.04800889	0.82879548	0.41342072
Papola	-0.0266236	8.23868163	0.04795259	0.82889423	0.41344073
Traf4	-0.0527801	2.52163121	0.04792319	0.82894582	0.41344073
Aebp2	-0.0251741	6.84929857	0.04785554	0.82906459	0.41347119
5430405H02I	-0.0851398	1.41100039	0.04782563	0.82911713	0.41347119
2610034M16	0.14130489	0.1506247	0.04773715	0.82927265	0.41352125
Lars2	0.03618112	13.4159353	0.04759986	0.82951425	0.41361423
Lefty2	0.25938235	-1.3405723	0.04754603	0.8296091	0.41363403
Ankrd26	0.03308684	5.96875239	0.04712056	0.83036065	0.41398123
Ccdc114	0.16780243	0.152478	0.04702021	0.83053844	0.41401847
2300009A05I	-0.0662747	2.43271049	0.04699408	0.83058475	0.41401847
Dap3	0.03161167	4.8301408	0.04698496	0.83060093	0.41401847
Mylpf	-0.1774191	-0.1585606	0.04674979	0.83101851	0.41416914
Dph7	-0.0421279	3.34348573	0.04673857	0.83103844	0.41416914
Ckb	-0.0337412	7.92797394	0.04671851	0.83107412	0.41416914
Tm2d3	-0.0483862	3.24777264	0.04669044	0.83112406	0.41416914
Lenep	0.10172825	1.41408675	0.04661071	0.83126597	0.41421234
Rabgap1	-0.0251985	7.271955	0.04648513	0.83148975	0.41428664
Vsig2	0.04581228	3.29637463	0.04644721	0.8315574	0.41428664
H2-Ob	0.22266778	-0.2005902	0.04643411	0.83158076	0.41428664
Ovol2	0.07365057	1.7981292	0.04612686	0.83212994	0.41447128
Taf3	0.02900269	6.44110544	0.04612101	0.83214042	0.41447128
Dctn4	0.02540679	7.41932633	0.04611094	0.83215845	0.41447128
Rbm46	0.13037995	1.57546975	0.04610315	0.83217239	0.41447128
1110020A21I	0.09867105	1.0658075	0.04599766	0.83236144	0.41452295
Slc11a1	-0.1893326	-0.4192487	0.0459836	0.83238665	0.41452295
Pcdh8	0.05959402	2.58289504	0.04559915	0.83307765	0.41483952
Arf2	0.03697104	6.06373611	0.0455171	0.83322551	0.41488561
Kcnj6	-0.0469163	5.88929856	0.04546013	0.83332827	0.41490577
Cdkl5	-0.0368194	8.25420541	0.04542709	0.83338789	0.41490577
Sec14l3	-0.2083434	-0.5228344	0.0454027	0.83343193	0.41490577
Toe1	0.0576682	2.66854513	0.04527973	0.83365409	0.41494448
Ripk4	-0.1710036	-0.0521389	0.04526961	0.83367239	0.41494448
Synrg	-0.0260962	6.70088302	0.04522222	0.83375811	0.41494448
Osbpl10	-0.0846723	2.18542351	0.04521032	0.83377964	0.41494448
BC068281	-0.0532347	2.29279265	0.04520665	0.83378628	0.41494448
07-Sep	-0.0298916	9.11917513	0.04495805	0.83423677	0.41514113

Fbxl4	-0.0415067	3.57468846	0.04460041	0.83488715	0.41538818
Stim2	0.03756452	5.92475523	0.04459682	0.83489369	0.41538818
Gm10778	-0.0467535	3.91403951	0.04459372	0.83489934	0.41538818
Gpr26	-0.0681986	3.43552285	0.0445315	0.83501277	0.41541139
Lck	-0.1465179	0.1777984	0.04450739	0.83505675	0.41541139
Mark4	0.04393778	3.63180136	0.04447474	0.83511633	0.41541348
Oas2	-0.1311828	0.13255888	0.04431058	0.83541622	0.4155351
Grpr	0.13284182	-0.1149348	0.04411837	0.83576809	0.41568255
Tbc1d17	-0.070108	2.57286383	0.04405143	0.83589081	0.41571603
Rgp1	-0.0332728	4.49069946	0.04398714	0.83600878	0.41574713
Pink1	0.02193605	7.52866259	0.04384897	0.83626262	0.4158458
Zfp119b	0.10134614	0.7155587	0.04372331	0.83649383	0.4159332
Kpna6	-0.0315234	7.46646261	0.0435524	0.83680886	0.41606226
Klhl10	-0.2305259	-0.8904987	0.04341538	0.83706189	0.41616049
Rybp	-0.0282719	6.64734823	0.04326568	0.83733882	0.41627058
Dars2	0.05902607	3.49596305	0.04321407	0.8374344	0.41627364
Tufm	-0.0405996	3.65762386	0.04317138	0.83751351	0.41627364
Aptx	0.02998542	5.00670793	0.043144	0.83756428	0.41627364
Nkap	0.03837597	5.26887566	0.04311405	0.83761981	0.41627364
Trp53rk	-0.1017842	2.02455285	0.04309894	0.83764785	0.41627364
Me1	0.02646218	5.34578068	0.04308272	0.83767795	0.41627364
Timm44	-0.0463447	3.40871495	0.04299706	0.83783699	0.4163251
Rtn4rl2	0.09134535	0.92144409	0.04288135	0.83805208	0.4164007
Cpsf3	0.03172944	5.09229648	0.04285551	0.83810016	0.4164007
Wfikkn2	0.09762148	2.97892266	0.04278605	0.83822945	0.41641473
Yy1	0.0281965	5.38509177	0.04278069	0.83823943	0.41641473
Nit2	0.05666224	2.27151986	0.04268159	0.83842411	0.41643555
Mthfd1l	-0.0529291	3.49817884	0.04267337	0.83843945	0.41643555
Fam180a	0.0761299	5.14193717	0.04265438	0.83847487	0.41643555
Gmpr2	-0.0518006	3.44926098	0.04262564	0.83852848	0.41643555
Slc35f3	-0.029541	5.58481233	0.04260438	0.83856816	0.41643555
Tmod1	0.03534353	4.73124694	0.04257959	0.83861443	0.41643555
Uros	-0.0450914	3.53309665	0.04249659	0.83876949	0.41648498
Fbxo16	0.06360669	2.08289641	0.04233413	0.83907341	0.41660831
Stk11	0.04954512	5.51305633	0.04212497	0.83946562	0.41675153
Rbm25	-0.0302046	8.74047397	0.04211396	0.83948629	0.41675153
Zfp276	-0.0573675	2.6787564	0.04209147	0.83952854	0.41675153
Eif3b	-0.0370423	3.89030819	0.04181887	0.84004144	0.41697855
Atp2a1	-0.2273414	-0.7557197	0.04172919	0.84021056	0.4170349
D630024D03	0.26905599	-1.7869437	0.04157734	0.84049733	0.41713272
Eif2b3	-0.0485405	2.84573605	0.04154179	0.84056456	0.41713272
Ppp6r1	-0.0346402	4.81027619	0.04153655	0.84057447	0.41713272
Mb21d1	-0.0971497	0.81745462	0.04145796	0.84072319	0.41717893
Lbp	0.0952804	3.35016585	0.04139688	0.84083887	0.4171813
Gm14405	-0.0996751	0.15617204	0.04138333	0.84086455	0.4171813

Fan1	0.06815196	1.65864258	0.04136542	0.84089848	0.4171813
Dusp6	-0.0449377	5.38548791	0.04133802	0.84095043	0.4171813
Prr24	0.03504369	4.53434013	0.0412188	0.84117666	0.41726593
Klhl32	-0.0794136	0.85298259	0.04117547	0.84125898	0.41727917
Mageh1	-0.0399336	4.38329685	0.04112183	0.84136091	0.41728901
Gpr50	-0.2544018	-1.5457712	0.0410995	0.84140337	0.41728901
Sema5b	-0.0733214	3.53938769	0.04106423	0.84147047	0.41728901
Cd2ap	0.0303475	7.43091632	0.04104802	0.84150133	0.41728901
C130046K22I	0.06201881	2.95526203	0.0409708	0.84164834	0.41733432
Kcnk4	-0.1050652	0.31919566	0.04078922	0.84199465	0.41747844
Acer3	-0.0542424	3.65386616	0.04070515	0.84215526	0.41750773
Rfc3	0.06937551	1.77971401	0.04070004	0.84216503	0.41750773
Tubd1	0.08937807	1.56401701	0.04064689	0.84226667	0.41753052
Fam50a	-0.0466439	4.12554342	0.04056552	0.84242241	0.41758013
Man1a2	-0.0249687	7.88075799	0.04043434	0.8426738	0.41759373
Gtpbp10	-0.0389285	4.49151741	0.04041821	0.84270474	0.41759373
Cldn5	-0.1832303	-0.2046789	0.04041456	0.84271175	0.41759373
Gramd2	0.16250767	-0.4528712	0.04039332	0.8427525	0.41759373
Evi5	0.02314686	7.79948001	0.04037022	0.84279685	0.41759373
Zfp423	-0.0296458	6.14028828	0.04036266	0.84281135	0.41759373
Tmem88	-0.0720413	2.02488157	0.04034798	0.84283954	0.41759373
Vil1	0.14814156	-0.0528895	0.04021271	0.84309951	0.41769495
Mipep	0.0451112	4.032367	0.03984481	0.84380891	0.41797274
Gm4841	0.07082512	2.36667917	0.03983006	0.84383743	0.41797274
Atxn2	-0.03112	8.07817741	0.03981736	0.84386197	0.41797274
Sdcbp2	-0.1443042	-0.7021209	0.03980643	0.84388311	0.41797274
Fbxw17	0.07182354	2.00307824	0.03965828	0.84416988	0.41808002
Rep15	-0.2789018	-1.3864379	0.03963698	0.84421116	0.41808002
Cog2	0.03849982	3.48085586	0.03959638	0.84428986	0.41809081
Ednra	0.03803156	5.01863621	0.03956641	0.844348	0.41809081
Nt5dc1	0.0571977	3.02824271	0.03953953	0.84440016	0.41809081
Sh2d5	0.03811531	4.63060511	0.03950275	0.84447154	0.41809856
Nr4a1	-0.1069239	4.92205442	0.03945255	0.84456903	0.41811922
Pde2a	-0.0361971	6.77089871	0.03941191	0.84464802	0.41812167
Nup210l	-0.2481518	-0.3714807	0.03939266	0.84468545	0.41812167
Zfp46	0.02554767	5.51714231	0.03934977	0.84476885	0.41812856
1110051M20	-0.0360667	4.1589489	0.03932819	0.84481085	0.41812856
Wnt5b	0.08688459	1.1476272	0.03927809	0.84490837	0.41814924
Xrra1	0.22362166	-1.5196995	0.03919357	0.84507306	0.41820315
Rnf180	-0.0612784	2.8396008	0.03912316	0.84521039	0.41824352
A830052D11	-0.1092168	0.26860189	0.03902425	0.84540352	0.41831149
Myo3b	-0.1000255	0.40957379	0.03878925	0.8458634	0.41848681
Adam19	0.0337938	4.64126525	0.03875224	0.84593596	0.41848681
Lypd1	0.04344063	4.83227986	0.03873657	0.8459667	0.41848681
Tmpo	0.03546959	5.86284667	0.03870776	0.84602322	0.41848681

Gper1	0.07234542	1.96462985	0.03870085	0.84603678	0.41848681
Gne	0.02694407	5.51174018	0.03856795	0.8462978	0.41858832
Runx3	0.0961777	0.86058091	0.03852807	0.84637621	0.4185995
Spdl1	0.14766351	-0.1312964	0.03838959	0.84664886	0.41870674
Tifa	-0.0545961	3.62939513	0.03835234	0.84672229	0.41871545
Ciapin1	0.03058519	4.72236937	0.03820083	0.84702131	0.41882491
Ggta1	-0.0788282	2.39992791	0.03818363	0.84705529	0.41882491
Gpn3	-0.0480223	4.49154742	0.03813504	0.84715135	0.41884479
Mapk4	0.02827359	7.79315389	0.03803406	0.84735117	0.41891597
Reep4	0.13526638	0.28493167	0.03793726	0.84754299	0.41898319
Mir1b	0.36671068	-1.1700818	0.03780878	0.84779796	0.4190774
Il34	0.03760283	3.78935797	0.03778496	0.84784529	0.4190774
Kcns3	0.06471912	1.97479653	0.03771907	0.84797627	0.41911452
Mettl20	-0.0568971	2.58159646	0.0376023	0.84820867	0.41920177
Kcna3	0.08210277	1.38186323	0.03744158	0.84852918	0.41933254
Ascc1	0.05660475	3.49080484	0.03730052	0.84881107	0.41944422
Ablim3	-0.0226938	5.35289374	0.03696186	0.84949013	0.41975212
Slc31a2	-0.0554799	5.11246442	0.03684682	0.84972151	0.4198381
Lepr	0.04079861	5.63711491	0.03681973	0.84977606	0.4198381
Asb6	0.04811113	2.52934525	0.03677599	0.84986418	0.41985398
Otud1	-0.0332699	6.12233648	0.03665279	0.85011266	0.41994908
Dkk2	-0.259396	-0.8060264	0.03657972	0.85026023	0.41999432
Stk33	-0.078399	1.62331277	0.03650257	0.85041621	0.42004371
Abcg2	-0.0387489	5.21468383	0.03639512	0.85063374	0.42012349
Casp8ap2	-0.0214527	6.4656115	0.03626805	0.85089142	0.42020622
Cd68	0.06643154	2.3580621	0.03625728	0.85091329	0.42020622
Mesdc2	0.03781119	6.39054405	0.03620587	0.85101769	0.42022043
Rtel1	-0.0612503	2.53186171	0.03618107	0.85106808	0.42022043
1700105P06I	0.25763123	-1.8469964	0.03615734	0.85111632	0.42022043
Zfp493	-0.0706902	2.60744916	0.03610699	0.8512187	0.42022043
Tmem123	0.04521821	5.06646632	0.03610252	0.8512278	0.42022043
Eif4b	0.02000045	7.59682671	0.03607777	0.85127817	0.42022043
Mroh5	0.14242239	-0.7387819	0.03603413	0.85136701	0.42023663
Msmo1	-0.0390674	4.99067555	0.03592047	0.85159867	0.42032332
Cnppd1	0.04673952	4.12454066	0.03587282	0.8516959	0.42034365
AI427809	-0.132764	1.2099746	0.03584375	0.85175527	0.42034529
E030011O05	-0.1577374	-0.8651926	0.03579258	0.85185979	0.42036922
Smek2	-0.0226613	6.69850331	0.03575262	0.85194147	0.42038089
Surf6	0.03606119	3.73763035	0.03572619	0.85199553	0.42038089
Ermn	0.02862317	6.68337228	0.03566746	0.85211573	0.42041255
Hint3	0.0359751	4.30640939	0.03545506	0.85255127	0.42059909
Ccng2	-0.0383146	4.33906316	0.03541102	0.85264174	0.42059909
Evi2a-evi2b	0.16302562	-1.3671055	0.03540114	0.85266203	0.42059909
Pinx1	-0.0437862	3.43679909	0.03535178	0.85276353	0.42061045
Mt3	-0.1464675	-1.0707301	0.0353354	0.85279722	0.42061045

Prom2	0.23781975	-1.9620922	0.03525716	0.85295828	0.42062928
Actg2	-0.1790056	-0.8348818	0.0352436	0.85298622	0.42062928
Fv1	0.10456123	0.54461146	0.03523516	0.85300361	0.42062928
Spn	0.12942958	-0.2228099	0.03519998	0.85307611	0.42063737
Rhbdf2	-0.177645	-1.7055015	0.03517279	0.85313218	0.42063737
Pld1	-0.0403446	5.52894528	0.03514512	0.85318925	0.42063786
Itga1	-0.0333588	4.19163823	0.03498911	0.85351147	0.42076907
Rnase4	-0.050212	5.15514776	0.03493709	0.8536191	0.42079447
Rab33b	-0.0247497	5.90779528	0.0348641	0.85377022	0.42084132
Slc22a23	0.02545749	6.19523421	0.03482789	0.85384525	0.42084453
Prkci	-0.0216541	7.35728916	0.03478723	0.85392955	0.42084453
4933406C10I	0.18640356	-1.0047284	0.03474967	0.85400747	0.42084453
Pard6a	0.07395041	2.15301672	0.03472755	0.85405339	0.42084453
Zbtb20	0.02411368	6.86078302	0.03472569	0.85405726	0.42084453
Fam71b	-0.2594841	-1.7554449	0.03467078	0.8541713	0.42087308
Tbc1d22b	0.03867786	4.46307118	0.03463277	0.85425028	0.42088435
Dydc2	0.17128307	-0.8900281	0.0345151	0.85449512	0.42097733
Cxcl12	0.02916815	6.01762405	0.034384	0.85476842	0.42108432
Pald1	-0.0682354	1.79750332	0.03430208	0.85493946	0.42111366
Slfn8	0.06157977	3.41249401	0.03430169	0.85494027	0.42111366
Ifrd2	-0.0875326	1.23966364	0.03425932	0.85502881	0.42112962
Gm13034	-0.1631072	-1.6862613	0.03419429	0.85516483	0.42116896
Zfp697	0.03131403	4.89969601	0.03411982	0.85532075	0.4212181
Gm4788	0.09887339	0.00703406	0.03409233	0.85537836	0.42121882
Cdh7	0.03749633	3.87185716	0.03401642	0.85553754	0.42126955
Rps12	0.04270283	6.21202793	0.03394981	0.85567738	0.42131075
Zfp597	0.02610269	4.89162682	0.03377138	0.85605268	0.42146787
Nod1	-0.0601156	2.07213642	0.03359681	0.85642084	0.42154857
Gmeb2	-0.0497218	2.96301519	0.03358266	0.85645072	0.42154857
Snhg5	-0.0360381	3.73901185	0.03357045	0.85647653	0.42154857
Neurod1	0.04830822	3.51486705	0.03356164	0.85649513	0.42154857
Tpst2	-0.0512139	2.1402579	0.03356049	0.85649757	0.42154857
Gm4961	-0.1826247	-1.7273926	0.0334499	0.85673144	0.42163601
Aspscr1	-0.0657928	2.7002338	0.03339075	0.8568567	0.42165627
Irf2bp2	-0.0203231	8.43053574	0.0333469	0.85694963	0.42165627
Gale	-0.1373436	-0.2678959	0.03331458	0.85701815	0.42165627
Ttc5	0.02780647	4.61761479	0.03330958	0.85702876	0.42165627
AW011738	0.06431021	2.60334174	0.03329785	0.85705366	0.42165627
Xlr3a	-0.100172	0.44850432	0.03322509	0.85720811	0.42170459
Nkapl	0.10345027	0.25498264	0.03313086	0.8574084	0.42177547
Dusp16	0.03807862	4.92493437	0.03297997	0.85772973	0.42190587
Snhg10	-0.0848217	0.7763282	0.03276529	0.85818825	0.42210051
Tbc1d20	-0.0416715	4.21044894	0.03274187	0.85823836	0.42210051
Arf3	-0.0288579	10.0859814	0.03271576	0.85829424	0.42210051
Iqcc	-0.0580534	2.65551747	0.03265169	0.8584315	0.42214033

Eva1c	0.07002104	2.79785056	0.03256256	0.85862266	0.42220451
Zfp595	-0.0329195	3.35783293	0.03251326	0.8587285	0.42220451
Rps15a-ps4	-0.0675828	1.32191386	0.03251216	0.85873086	0.42220451
Lrrc10b	0.06130386	3.93809863	0.03227711	0.85923669	0.42242552
Tmem108	0.05383565	3.46789091	0.03217976	0.85944675	0.42249498
Gm16532	-0.1204066	1.04913494	0.03215945	0.85949062	0.42249498
Rad51c	0.07629718	1.97713349	0.03203356	0.85976282	0.42260109
Ddx31	0.07073364	1.36652736	0.03193457	0.85997726	0.4226788
Fam220a	-0.027102	4.2702704	0.03174788	0.86038261	0.42280441
Dnah17	-0.1612194	-1.109053	0.03171627	0.86045136	0.42280441
Ppapdc1a	0.12291784	-0.1787357	0.0316871	0.86051484	0.42280441
AW549877	-0.0232719	7.65307398	0.03168194	0.86052608	0.42280441
Tmem106b	0.02323814	7.56207036	0.03167125	0.86054935	0.42280441
Fam49b	-0.0226886	6.79964942	0.0316613	0.86057101	0.42280441
Kif18a	-0.0949119	0.99561614	0.03146533	0.86099844	0.4229867
Dcp1a	-0.0246623	5.44539344	0.03141836	0.86110109	0.42300943
Upf1	-0.0330089	4.27540549	0.03138972	0.86116372	0.42301249
Hmgn5	0.02857781	7.16198763	0.03132551	0.86130425	0.42305382
Sec1	-0.1191014	-0.0709414	0.03118129	0.86162042	0.42318141
Lin9	0.04923796	3.01299053	0.03107109	0.8618625	0.42327259
1700003E16I	-0.0870975	0.61505747	0.03100544	0.86200693	0.42331423
Exoc5	0.021259	6.2944493	0.03095757	0.86211236	0.42331423
Fitm2	-0.1078903	0.590713	0.03094798	0.86213348	0.42331423
Pcsk9	0.10249634	-0.0783968	0.03093004	0.86217302	0.42331423
8430427H17I	0.02151246	6.53254101	0.03078945	0.86248326	0.42340173
Ilf2	0.03134032	5.70695281	0.03077206	0.86252167	0.42340173
Rev1	-0.0281506	4.38275609	0.03074341	0.86258501	0.42340173
Pigb	-0.0526737	2.54584533	0.03073602	0.86260136	0.42340173
Ppp2r2c	0.02593193	9.02177401	0.0307215	0.86263346	0.42340173
Pofut1	0.03712472	3.36432094	0.03061656	0.86286576	0.42348804
Lamb3	0.21695136	-1.016154	0.0305049	0.8631134	0.42358187
Ttc12	0.09766265	1.39047069	0.03044166	0.86325386	0.42362309
Lrrfip1	-0.0241861	5.98272944	0.03037397	0.86340436	0.42366923
Tceal1	0.02854668	5.42049778	0.03034749	0.86346329	0.42367043
Nt5c2	-0.0232369	5.54623857	0.03027456	0.86362571	0.42369766
Carhsp1	-0.0518346	5.37283522	0.03026371	0.8636499	0.42369766
Gpr156	0.09172283	1.13406632	0.0302366	0.86371034	0.42369766
Cyp4v3	0.04593015	4.68104747	0.03022118	0.86374473	0.42369766
Gfod2	-0.1090561	0.02465691	0.03006182	0.86410069	0.42384456
Asb3	-0.035637	4.02289855	0.02992915	0.86439776	0.42395299
Sorbs1	-0.0176565	7.52415737	0.02991263	0.86443479	0.42395299
Fgf7	0.06679304	1.59761899	0.02976484	0.86476663	0.42406518
Plcg2	-0.0513664	1.88534038	0.0297604	0.86477661	0.42406518
Trim6	0.17349145	-0.5497775	0.02964179	0.86504355	0.42416835
Zfp36l2	0.03182447	6.13442197	0.02956787	0.86521019	0.42422233

Sbno2	-0.0554206	1.71964168	0.02953323	0.86528837	0.42423294
Gpr137	-0.0418445	3.18893614	0.0293084	0.86579685	0.42443073
Zfr	0.02385113	8.62368606	0.02930482	0.86580497	0.42443073
Rgma	0.04229314	4.29821178	0.02923814	0.86595617	0.42447711
Hirip3	0.02427543	5.06218827	0.02914658	0.86616409	0.42453466
Aktip	-0.0227991	6.09517602	0.0291366	0.86618676	0.42453466
5830418P13I	0.11311805	0.17613779	0.02900406	0.86648839	0.42465475
Srpk2	0.01999173	7.79659098	0.02885781	0.86682203	0.4247433
Laptm4a	-0.0352137	8.0527121	0.02883294	0.86687885	0.4247433
Rpgrip1	0.1476311	-0.9646415	0.028817	0.86691529	0.4247433
Ap2a1	-0.0354787	4.06991428	0.02880423	0.86694448	0.4247433
H2-DMb2	0.21052322	-1.6585688	0.02880086	0.86695218	0.4247433
Myh11	-0.1174504	2.52897226	0.02876359	0.86703743	0.42475732
Pim1	-0.0918797	0.33204306	0.02868723	0.86721227	0.42481523
Ska2	0.04726096	2.75900867	0.02865108	0.86729512	0.42482807
2810013P06I	-0.034054	4.65624148	0.02861211	0.86738451	0.42484412
Rgl3	-0.0956577	0.42338172	0.02855407	0.86751774	0.42487077
BC031361	0.04731956	2.8200458	0.02853907	0.86755221	0.42487077
Wdr44	0.02652397	4.89162934	0.02846145	0.86773066	0.42493042
1700001K23I	0.21882161	-1.6193535	0.02839788	0.86787699	0.4249381
Smyd5	0.04225943	3.00264503	0.02837497	0.86792976	0.4249381
Bst1	0.20983339	-1.3908768	0.02835681	0.86797162	0.4249381
Ldlrad3	0.04702863	5.77201379	0.02835624	0.86797293	0.4249381
Gm10635	-0.1037087	0.85486743	0.02824934	0.86821958	0.42503111
Foxn2	-0.0307372	4.77039956	0.02819046	0.86835562	0.42506997
Gm12191	0.05792965	1.2385392	0.02810154	0.86856138	0.42513018
Tas1r1	0.16162165	-0.3951764	0.02808833	0.86859198	0.42513018
AI854703	-0.0475781	3.07551616	0.02797468	0.86885549	0.42520406
Vrk2	-0.1053171	0.45857261	0.02797434	0.8688563	0.42520406
1700030K09I	0.07280769	1.60509031	0.02789273	0.86904585	0.42524134
Rnf115	0.02366736	5.90660914	0.02789072	0.86905054	0.42524134
Slc39a7	-0.0356515	4.19422238	0.02786835	0.86910254	0.42524134
Fgr	0.10241895	0.32425747	0.02778638	0.86929333	0.42530696
Cep68	-0.030209	5.0034212	0.02763161	0.86965433	0.42544112
Adrbk1	0.02804747	5.09070677	0.02758294	0.86976808	0.42544112
4930563E22I	0.10330308	0.91985291	0.0275789	0.86977751	0.42544112
A630072M18	-0.0383174	3.50751564	0.02757168	0.86979441	0.42544112
Gm11186	-0.2291671	-1.441342	0.02751354	0.86993045	0.42547991
Scrg1	-0.0873443	1.44732958	0.02733408	0.87035128	0.42565282
Ankrd23	0.12569654	-0.7013477	0.02726801	0.87050658	0.42565282
Dram1	-0.0889359	0.62220117	0.02724542	0.87055971	0.42565282
D11Wsu47e	0.04377754	2.51229817	0.02724272	0.87056606	0.42565282
Phactr1	0.02253295	7.94788212	0.02724203	0.8705677	0.42565282
Slc2a6	-0.0836572	1.04842691	0.0271774	0.87071985	0.42569947
Galnt18	0.04963219	2.29504213	0.02711629	0.87086393	0.42574216

Mettl7a2	-0.0512081	1.73110516	0.02684462	0.87150636	0.42602846
Smpd3	-0.0273091	4.76558049	0.02674308	0.87174733	0.42609603
Prox2	0.06723321	1.61354989	0.02673851	0.87175818	0.42609603
Zfp217	-0.0493833	3.61460677	0.02670658	0.87183405	0.42610535
Sil1	0.05374626	2.5779536	0.02664594	0.8719783	0.42611493
Mtss1	0.02762864	6.68507177	0.02662741	0.8720224	0.42611493
Lpin2	-0.0250574	6.58671134	0.02662648	0.87202463	0.42611493
Aifm1	0.03542373	4.10845366	0.02657934	0.87213692	0.42611493
Aftph	0.02058857	6.94774223	0.02657901	0.8721377	0.42611493
Wdr46	-0.0433479	4.10368222	0.02650455	0.87231527	0.42617393
Trhr	0.06666573	1.50417131	0.02647423	0.87238765	0.42618154
C3ar1	0.05484616	2.03882037	0.02643531	0.8724806	0.42619919
Gpt	0.10376789	0.46254604	0.02638115	0.87261011	0.42622571
Gm5431	-0.0806395	0.93549401	0.02634295	0.87270153	0.42622571
Homer3	0.04316903	1.81476363	0.02631745	0.87276259	0.42622571
Bmpr2	0.02381445	9.84150137	0.02629096	0.87282604	0.42622571
AI429214	-0.0507392	3.37119213	0.0262704	0.87287533	0.42622571
Ndufs5	0.14324479	-1.4944173	0.0262702	0.87287581	0.42622571
Ifrd1	0.02628761	5.97236501	0.02623828	0.87295235	0.42623534
Myo1h	0.12057886	-1.0196732	0.02613589	0.87319823	0.42632764
Srsf9	0.03290107	4.93587337	0.02606653	0.87336505	0.42638134
2810459M11	-0.0631952	1.84804127	0.02597887	0.87357624	0.42638805
Uba2	0.02061239	5.79945367	0.02597861	0.87357688	0.42638805
Spdya	0.07630702	0.69594156	0.02596045	0.87362066	0.42638805
Mamstr	-0.0961582	0.20664591	0.02593765	0.87367566	0.42638805
St5	0.0377453	4.99487358	0.02591243	0.87373654	0.42638805
Pcgf5	0.03237464	4.11613292	0.0258985	0.87377018	0.42638805
Kdm8	0.06968694	1.41305886	0.02589581	0.87377669	0.42638805
Il13ra2	-0.080255	1.98597913	0.02584019	0.8739111	0.4264259
Arhgap31	0.02654049	6.53570096	0.02576325	0.87409726	0.42647749
1700030J22F	-0.0420697	3.65914995	0.02573142	0.87417436	0.42647749
F13a1	-0.0782469	1.59449827	0.02570765	0.87423197	0.42647749
Fbxl13	0.16334919	-0.9624995	0.02570259	0.87424423	0.42647749
Foxred1	-0.0412222	3.05111748	0.02561527	0.87445612	0.42655311
Ghdc	0.04484431	2.22972725	0.02554455	0.874628	0.42660921
Dbp	-0.0426486	4.08973715	0.02536836	0.87505727	0.42679084
Fbxw4	0.04129931	2.72014757	0.0252924	0.87524283	0.42683095
Fndc5	0.02340824	5.00331875	0.02528358	0.87526439	0.42683095
Gm16576	0.08045806	0.70651927	0.02526484	0.87531021	0.42683095
Rps6kb1	0.01894509	7.12851419	0.02522416	0.87540976	0.42685174
Grik5	0.03501039	5.30620687	0.02515154	0.87558767	0.42690824
C530005A16I	0.03836692	3.24968215	0.02513042	0.87563944	0.42690824
Pemt	-0.1483326	-0.981126	0.02502645	0.87589471	0.42700494
Acp2	0.01999024	5.57459214	0.02495564	0.87606887	0.42705279
Tmem253	-0.1850077	-1.077582	0.02490557	0.87619219	0.42705279

Trak2	0.02094423	6.83064815	0.0248903	0.87622982	0.42705279
Hes7	0.20901266	-1.7035648	0.02486787	0.87628513	0.42705279
Pecr	-0.0620993	1.25200424	0.02485411	0.87631905	0.42705279
Ptges3l	-0.0880883	1.05137096	0.02484786	0.87633446	0.42705279
Cep41	0.05361926	2.89860501	0.02477777	0.8765075	0.42708875
Wnt1	-0.1402682	-1.3555042	0.02477185	0.87652212	0.42708875
Ciao1	0.02276474	5.39363612	0.02473016	0.87662519	0.42711123
Megf9	-0.0241964	5.961198	0.02465169	0.8768194	0.42717811
1810024B03I	-0.1566929	-0.6292529	0.02455643	0.87705557	0.42725242
Kif26a	0.04548397	2.41192325	0.02454423	0.87708585	0.42725242
Smad4	-0.0195614	5.86879899	0.02446663	0.87727866	0.42731859
9130011E15I	-0.0323881	3.63260374	0.02440017	0.87744402	0.42732597
Cln6	0.07625334	1.96632768	0.02438521	0.87748128	0.42732597
Poli	-0.029855	3.37439859	0.02434808	0.87757382	0.42732597
Eif2ak2	0.03097594	5.80721038	0.02434402	0.87758393	0.42732597
Parg	0.02062108	5.62381836	0.02434096	0.87759156	0.42732597
Cux2	-0.0315302	4.80386625	0.0243233	0.8776356	0.42732597
Fam196b	-0.0559716	1.52133932	0.02420646	0.87792737	0.42742011
Ifi30	0.06483079	2.01187958	0.02420024	0.87794291	0.42742011
Zfp94	-0.0446907	2.68639115	0.0240551	0.87830639	0.42754222
Gna12	-0.0237544	5.26986265	0.02405457	0.87830772	0.42754222
Lzts2	0.0431721	2.82548724	0.02402743	0.87837581	0.42754762
Spata5l1	0.12939163	-0.7576528	0.02368289	0.87924372	0.4279423
Secisbp2	0.0318018	3.83289147	0.02355597	0.87956507	0.42804468
Bfar	0.02569519	5.04974187	0.02355474	0.87956819	0.42804468
Lingo4	0.16807683	-1.408254	0.02349785	0.87971253	0.42808715
Plcxd2	0.0260091	7.79209282	0.02343597	0.87986971	0.42813587
Snrk	0.01837177	6.38919124	0.02336995	0.88003765	0.4281774
Gbp4	0.04140308	2.90450644	0.02335755	0.88006922	0.4281774
Tnfaip8l2	0.13411307	-0.6473809	0.02328772	0.88024719	0.42822281
Ddx39	0.04960313	2.37267461	0.02327613	0.88027674	0.42822281
E430018J23F	-0.0522365	2.49952801	0.02317528	0.8805343	0.42831419
Unc45b	0.19755061	-0.6686464	0.02315788	0.88057878	0.42831419
Zfp592	-0.0226399	5.65613648	0.02310354	0.88071785	0.42835406
Bcl2a1d	0.06213561	1.18069673	0.02305551	0.88084089	0.42838613
Ndrg3	0.01523484	8.73840103	0.02300724	0.8809647	0.42841856
Pex5l	0.02502363	6.82776309	0.02295259	0.88110503	0.42842676
Pcid2	0.03824768	4.26634617	0.02295198	0.88110658	0.42842676
Luzp1	0.01902211	8.22082905	0.02291696	0.8811966	0.42842676
Sltm	0.02036497	7.70883421	0.02289508	0.88125288	0.42842676
Sp8	-0.0724879	0.59072781	0.02288954	0.88126713	0.42842676
Gnb2l1	0.02378369	5.840013	0.02280117	0.88149474	0.42850964
Gm20257	-0.0565505	2.45904281	0.022699	0.88175843	0.42861005
Kcnmb4	-0.0271154	3.94159509	0.02263133	0.8819334	0.4286476
Numb	0.02486903	5.65734998	0.02261056	0.88198717	0.4286476

Agpat4	0.02871794	5.19971773	0.02260286	0.88200712	0.4286476
Sema7a	-0.0237851	5.34240719	0.02254122	0.88216684	0.42869746
Treml2	-0.1647105	-0.6834042	0.02248882	0.88230281	0.42873575
Sdc4	0.04963646	5.26521449	0.02242517	0.88246817	0.42878622
Rad54l	-0.0719798	0.81036565	0.02240487	0.88252098	0.42878622
Oma1	0.02966962	3.22056231	0.02234112	0.88268692	0.42883907
Ppp2r5e	0.02065998	6.40327889	0.02229974	0.88279478	0.42886369
Fastkd3	-0.025944	3.79209498	0.02212225	0.88325853	0.4290612
Megf11	0.02483174	5.52647012	0.02207071	0.88339354	0.42907263
Atg14	0.03079295	4.2719661	0.02206599	0.88340592	0.42907263
Tcea2	-0.0426696	2.74191191	0.02204021	0.88347353	0.42907263
Cage1	0.0730358	1.19686726	0.02202598	0.88351087	0.42907263
Paqr8	0.02073705	5.11849638	0.021884	0.88388404	0.42922607
Slc25a14	-0.0294159	4.89905277	0.02182718	0.88403373	0.42926318
Zdhhc2	-0.0208811	5.21262082	0.02181157	0.8840749	0.42926318
1700109H08	-0.1930876	-1.4188016	0.02176453	0.884199	0.42927132
Fgfr1op	0.0228982	4.38467889	0.02176184	0.88420612	0.42927132
Neurod4	0.09662703	0.44780469	0.021583	0.88467929	0.4294522
Ing5	0.02866148	4.683819	0.02156414	0.88472933	0.4294522
Stard5	-0.0454297	3.55374453	0.02155617	0.88475045	0.4294522
Tmem63c	-0.0408855	4.14277226	0.02147818	0.88495758	0.42949402
Asl	0.04799436	2.93065126	0.02145935	0.88500764	0.42949402
Kcnj4	-0.03502	3.36991447	0.02145858	0.88500969	0.42949402
Arhgap10	0.02810519	4.4409268	0.02143755	0.88506563	0.42949402
Ythdf2	-0.02207	5.07872959	0.021295	0.88544554	0.42965017
Egfl6	0.09555609	0.88646447	0.02125649	0.88554839	0.42965017
P2ry13	-0.0513568	2.42328425	0.02125243	0.88555926	0.42965017
Ier2	0.06204568	1.23049555	0.02118464	0.88574055	0.42971034
Fam154a	-0.1233739	-1.9889816	0.02108291	0.88601321	0.42978791
Plcd1	0.04600143	2.01051143	0.02108223	0.88601503	0.42978791
Sfxn4	-0.0439631	3.64508379	0.020915	0.8864647	0.42997823
4921507P07	0.08510486	0.29850225	0.02086646	0.88659556	0.4300139
AA465934	-0.0741105	0.237487	0.02082009	0.88672071	0.43004679
6030407O03	-0.1378302	-0.8430597	0.02077039	0.88685502	0.43008412
Cr1l	0.02715156	5.40021269	0.0205538	0.88744224	0.43034108
Tlr1	-0.1781588	-0.9829122	0.02050144	0.88758468	0.43037442
Cdc16	0.02462893	4.94816157	0.02047026	0.88766961	0.43037442
Polr1c	-0.0303264	4.05554449	0.0204653	0.88768311	0.43037442
Slc47a1	0.05194776	5.38233735	0.02034017	0.88802455	0.43051213
Oplah	-0.0423165	1.8057182	0.02026994	0.88821667	0.43057219
Jmjd4	-0.0250105	4.63418793	0.020238	0.88830414	0.43057219
Hcn4	0.05253714	1.30114906	0.02023198	0.88832064	0.43057219
Dcaf11	0.02019839	4.86651126	0.0201295	0.88860185	0.43068067
Arhgef26	-0.028569	4.33599906	0.02009442	0.88869828	0.43069958
Rom1	0.0545945	1.51486163	0.02005523	0.8888061	0.430724

Src	0.03481023	3.27587709	0.02002231	0.88889677	0.43074011
Rab23	-0.0198656	5.37717903	0.01996806	0.88904634	0.43075779
Map2k3os	0.12120021	-0.8604769	0.01995634	0.88907869	0.43075779
Ccdc152	-0.0437779	1.90916393	0.01994661	0.88910553	0.43075779
Copb2	-0.0145734	7.23618966	0.01986728	0.88932475	0.43081308
Myh9	-0.0195296	6.86419916	0.01983138	0.8894241	0.43081308
Zcchc6	0.01472226	7.22987234	0.01982461	0.88944285	0.43081308
Kdm4d	0.11593254	-0.1753256	0.01980977	0.88948394	0.43081308
Rcbtb2	-0.0267867	4.36717018	0.01980151	0.88950681	0.43081308
Dsg1c	0.11751949	-0.8061699	0.0197522	0.88964355	0.43085149
Hcar1	0.05335346	4.37220881	0.01953641	0.89024387	0.43108669
Pycr1	0.08767727	0.14120247	0.01953632	0.89024413	0.43108669
Pik3r3	-0.0157971	6.59840432	0.01951183	0.89031248	0.43109196
Lmf1	-0.0400547	2.93702703	0.01941519	0.89058259	0.43114901
G3bp2	-0.0194322	9.47815298	0.01938478	0.89066775	0.43114901
Hhipl1	-0.1481503	-1.2036781	0.01938055	0.8906796	0.43114901
C920009B18I	0.06703714	0.99448876	0.01937682	0.89069004	0.43114901
Nthl1	0.08702336	0.3650133	0.0193502	0.89076464	0.43114901
Atp6v0a2	-0.0233355	5.13639764	0.01933027	0.89082053	0.43114901
Lap3	0.02110073	5.62660117	0.01932595	0.89083266	0.43114901
Pgm5	0.03543443	6.55807452	0.01922931	0.8911041	0.43125256
9430076C15I	-0.1563858	-1.4963144	0.01911364	0.89142994	0.43137331
Mtg1	-0.0409474	2.77387314	0.01909382	0.89148586	0.43137331
St3gal4	0.05039044	3.96093969	0.01907955	0.89152613	0.43137331
Zfp750	0.06622173	2.36164251	0.01902467	0.89168122	0.43141014
Map2k2	0.02823644	4.35852667	0.01901193	0.89171726	0.43141014
Mesp2	0.08799188	1.96370812	0.01898008	0.89180741	0.43142593
Arid3a	0.04595683	2.96326975	0.01881336	0.89228049	0.43162695
Akr1c21	-0.2026235	-1.1791494	0.01876034	0.89243138	0.43167211
Zfp786	0.08149778	0.13871326	0.01871863	0.89255024	0.43170176
Egln1	0.01723333	6.86090147	0.01867277	0.8926811	0.43171085
Cdh15	0.15957907	-1.396928	0.0186717	0.89268414	0.43171085
Sp1	-0.0164789	7.09172639	0.0185571	0.89301185	0.43184149
AA415398	-0.0423901	2.98746953	0.01847153	0.89325722	0.43193231
Bend7	-0.0582708	0.64875533	0.01844294	0.89333933	0.43194417
Asrgl1	0.0247923	5.83523342	0.01841843	0.89340977	0.43195038
Dnajc6	0.01806838	8.53886935	0.01836935	0.89355098	0.4319576
Tstd2	-0.0351008	2.78070103	0.0183499	0.89360697	0.4319576
Cth	0.0716619	0.94286867	0.01834713	0.89361496	0.4319576
Reep3	-0.027598	7.27510265	0.01833322	0.89365503	0.4319576
Tgm4	-0.0595593	1.23746859	0.01829242	0.89377268	0.43198663
Smurf1	0.02124847	5.13253771	0.01819733	0.89404738	0.43209156
Gdpgp1	0.02633387	4.8161016	0.01815885	0.89415874	0.4321138
Lrrc29	0.13837667	-0.596089	0.01814164	0.89420861	0.4321138
Sec22a	0.03397889	2.98563507	0.01809309	0.89434937	0.43215398

Ccr10	0.1604544	-1.0131679	0.01805123	0.89447086	0.43217055
Jag1	0.03106059	3.72956607	0.01804159	0.89449889	0.43217055
Ntn5	0.07477483	0.36897687	0.01800969	0.89459159	0.4321875
Cdk20	-0.0679896	1.18876467	0.01796996	0.89470719	0.43219872
Adap2	-0.0383421	3.73538247	0.01795587	0.89474822	0.43219872
D430020J02F	-0.0920828	0.56245737	0.01794234	0.89478766	0.43219872
Als2cr12	0.10718054	-1.0692108	0.01789022	0.89493962	0.43224429
Rnf130	0.01559168	7.06320133	0.01785057	0.89505537	0.43227236
3110082I17R	0.04217495	2.68629787	0.01779578	0.89521556	0.43232189
Tomm20	-0.0139268	7.75160618	0.01771149	0.89546246	0.43241328
Ednrb	-0.0326267	3.98408025	0.01759932	0.89579198	0.43254457
Snhg6	-0.0480371	1.68394643	0.01752307	0.89601659	0.43262517
Cadm4	-0.0365896	3.23482238	0.01750056	0.89608299	0.43262939
Kcnn4	0.23431744	-1.6284853	0.01745154	0.89622775	0.43265681
Lef1	-0.0259125	4.50686433	0.01743054	0.89628982	0.43265681
Dnaic1	-0.058345	0.56456003	0.01740324	0.89637056	0.43265681
Ptpre	-0.0255033	5.24251314	0.01739651	0.89639049	0.43265681
Cdh12	-0.0267576	6.34900079	0.01737659	0.89644946	0.43265681
1700113A16I	-0.0293244	3.91329545	0.0173643	0.89648587	0.43265681
Qser1	-0.0209529	6.91275708	0.01728205	0.89672982	0.43272745
Tlk1	-0.0133872	7.60864649	0.01727606	0.89674761	0.43272745
Dgkb	-0.0252246	7.92645672	0.01696425	0.89767808	0.43309104
Mmp11	0.07638993	1.13783293	0.01696031	0.89768991	0.43309104
Suox	0.02966464	4.24986719	0.01694344	0.8977405	0.43309104
Uhmk1	0.0165391	6.47143497	0.01694031	0.89774988	0.43309104
Kcnu1	0.06951494	0.58323831	0.01690093	0.89786809	0.43309104
Tapbpl	-0.0249233	3.28314003	0.01689488	0.89788626	0.43309104
BC018473	0.16846804	-1.5335454	0.01687488	0.89794637	0.43309104
Rgs20	-0.0274141	5.70952215	0.01686936	0.89796297	0.43309104
5031434C07I	0.17424449	-1.6976484	0.01690688	0.89807972	0.4331195
Exosc10	-0.0203515	4.86434649	0.01679114	0.89819841	0.43312218
Zfp607	-0.0438003	2.48139595	0.01679036	0.89820075	0.43312218
Pex16	-0.060203	0.74110707	0.01667996	0.89853402	0.43324858
Tdo2	-0.1409213	-0.9958907	0.0166653	0.89857838	0.43324858
Cln3	-0.0378413	2.55672215	0.01659437	0.89879318	0.4333003
Rspo3	-0.0381614	7.1031934	0.01659173	0.89880118	0.4333003
Foxp2	0.02241922	7.30765241	0.01654777	0.89893456	0.43331668
Celf3	-0.0236137	5.51005772	0.01653224	0.89898173	0.43331668
A430105I19F	0.03606567	3.33664494	0.01652344	0.89900846	0.43331668
Dak	0.04503654	2.76656113	0.01647312	0.89916147	0.43336259
Cul4b	0.01524781	7.03109806	0.01641198	0.89934769	0.43342449
Zfp324	0.03809852	3.0189689	0.01634801	0.89954294	0.43346585
Gm128	0.10417437	-0.8928464	0.016346	0.89954909	0.43346585
Nrarp	-0.0418998	2.7117854	0.01629521	0.89970438	0.43351283
Thg1l	0.039856	2.94982883	0.01622147	0.89993026	0.43356862

Peo1	-0.0418698	2.5381301	0.01621968	0.89993575	0.43356862
Kat6a	-0.0141256	8.11258679	0.01618963	0.90002798	0.43357894
Exog	-0.0324538	2.92209593	0.01617504	0.90007277	0.43357894
Xrcc6	0.0304248	3.3703194	0.01603474	0.90050465	0.43372811
Dmtn	-0.0220743	6.22968677	0.01602942	0.90052108	0.43372811
Gm2382	0.03521466	2.975679	0.01601813	0.90055591	0.43372811
Smim3	0.03652687	3.12589515	0.0159832	0.9006638	0.43375222
Map2k3	0.0327984	3.88687435	0.01590935	0.90089229	0.43379504
Plod3	-0.0411497	2.62282767	0.01588812	0.90095805	0.43379504
Acadsb	-0.0182422	6.82862526	0.01585279	0.90106763	0.43379504
4933428C19I	0.11883514	-0.7452506	0.01582913	0.90114107	0.43379504
3830408C21I	-0.0392293	2.47583336	0.01582767	0.90114559	0.43379504
Ccdc85a	0.02033855	5.81768813	0.01580643	0.9012116	0.43379504
Cacng2	0.01735529	5.27518469	0.01579211	0.90125609	0.43379504
Cox18	-0.030904	2.90177609	0.01579052	0.90126105	0.43379504
Dnajb13	-0.1406149	-1.4063093	0.01578617	0.90127456	0.43379504
Lman2	-0.0194052	5.61149008	0.01575358	0.90137597	0.43379504
Tsc22d3	-0.0301147	6.56716066	0.0157148	0.90149676	0.43379504
Opcml	-0.0213699	8.17137437	0.015714	0.90149927	0.43379504
Noxred1	0.13040959	-0.8251038	0.01571232	0.90150449	0.43379504
Cdkn1c	-0.0363203	5.69488742	0.01567081	0.90163399	0.43380243
Spire2	0.03076843	2.52442626	0.01567032	0.9016355	0.43380243
Fam81a	0.01897648	5.71032253	0.01562627	0.9017731	0.43382237
2310002F09I	0.18681861	-1.5177749	0.01562003	0.90179262	0.43382237
Rarres1	-0.1304553	-0.6069795	0.01558401	0.9019053	0.43384876
Ddx51	-0.0202464	3.89237777	0.015483	0.90222199	0.43397327
Smarcd1	-0.01677	5.84974477	0.01541483	0.90243631	0.4340237
Wisp2	-0.1432938	-1.02055	0.01540135	0.90247875	0.4340237
Ctdp1	0.02166914	3.88503483	0.01534492	0.90265661	0.4340237
Hyal3	0.06849891	0.5348794	0.01534252	0.9026642	0.4340237
Zfp711	-0.0290646	4.20917401	0.01534141	0.90266771	0.4340237
Synj2	-0.022461	6.45483524	0.01532214	0.90272852	0.4340237
Spsb1	-0.0376023	2.8969923	0.01531736	0.9027436	0.4340237
Hopx	0.03824797	3.04404463	0.01528623	0.90284196	0.4340237
Olfr239	-0.141371	-1.2311747	0.01527417	0.90288008	0.4340237
Ccdc40	-0.0813276	0.06775867	0.01526615	0.90290544	0.4340237
Tc2n	0.06286205	0.86585266	0.01524169	0.90298285	0.43403028
Rab27a	0.02600366	3.40232344	0.01521501	0.90306734	0.43403028
Mtif2	-0.0181148	5.36245845	0.01520701	0.90309272	0.43403028
Dennd4c	-0.0181828	6.04642157	0.01515	0.90327357	0.43408939
Tprkb	0.01520447	5.39818019	0.01512234	0.90336144	0.4341038
Gm15706	-0.0381154	1.90207161	0.01504389	0.90361111	0.43419597
Pcdh7	-0.0185384	8.15354378	0.01500443	0.90373695	0.43422862
Rxfp2	0.08251912	-0.5855475	0.01494055	0.90394099	0.43428909
Zfp455	0.02924529	3.18800045	0.0149288	0.90397859	0.43428909

Trp53cor1	-0.0746214	-0.0263745	0.01490729	0.90404744	0.43429435
Rhpn2	-0.0378313	4.0440724	0.01475453	0.90453779	0.43450208
Ptgfr	-0.0374536	4.72271228	0.0147052	0.90469669	0.43455058
Aqp9	0.1477101	-1.2215807	0.01468633	0.90475751	0.43455197
8430429K09I	0.0241368	4.21523201	0.01463863	0.90491152	0.434588
Gnb5	0.01824568	6.22872661	0.01460362	0.90502474	0.434588
Mybl2	0.1002254	-1.316841	0.01459333	0.90505801	0.434588
Zfp935	0.02411157	4.49689751	0.01455715	0.90517519	0.434588
Mif4gd	0.03159918	3.18581163	0.0145513	0.90519415	0.434588
G530011O06	-0.033332	4.65973078	0.0145358	0.90524441	0.434588
Lyn	-0.0341519	3.40651406	0.01452601	0.90527616	0.434588
Klhl21	-0.0260435	4.54310308	0.01451989	0.905296	0.434588
Dnm1l	0.01605933	8.30233516	0.01449833	0.90536598	0.43459378
Ogfod2	-0.0292441	3.487368	0.01438487	0.90573514	0.43474316
Spast	-0.0145276	6.53538806	0.01430991	0.90597985	0.43480741
1700084C01I	-0.0432964	1.65505672	0.01430835	0.90598492	0.43480741
Dysf	0.09316369	0.33250589	0.01423041	0.90624006	0.43490204
Exoc3l	-0.0511344	1.39302069	0.01415897	0.90647453	0.43497101
Rec8	-0.0781388	0.39427074	0.01413862	0.90654143	0.43497101
4930467D21I	-0.1386632	-1.3164276	0.01412462	0.90658748	0.43497101
Col18a1	-0.0415198	1.82900347	0.01411604	0.90661573	0.43497101
Ncam2	-0.0202056	7.16289986	0.0139442	0.90718308	0.43521537
02-Sep	-0.0165769	7.89735262	0.01376671	0.90777283	0.43544545
Slco2b1	-0.0397279	2.70595602	0.0137592	0.90779787	0.43544545
Thoc3	-0.0296251	4.17805033	0.0137353	0.90787761	0.43544545
Ccnjl	0.06388316	1.08242256	0.01371611	0.90794166	0.43544545
Derl2	-0.0228651	3.56033296	0.01371274	0.90795292	0.43544545
Zfp213	-0.0574784	1.09284585	0.01366891	0.90809943	0.4354535
Pak4	-0.0402366	4.81414536	0.01365844	0.90813449	0.4354535
Mbtps2	0.02041125	4.92830405	0.01365564	0.90814386	0.4354535
Fgfbp3	0.03161651	2.68347118	0.01361605	0.90827647	0.43546279
Mdh1b	0.12658864	-1.1226519	0.0136152	0.90827932	0.43546279
Slc16a6	-0.0269868	3.11581184	0.01355359	0.90848608	0.43553408
Tnfrsf1b	-0.0669394	1.11957083	0.01351786	0.90860623	0.43556384
Zbtb26	0.0170974	4.37235312	0.01346498	0.90878432	0.43562137
Fam83h	-0.0413832	1.56831649	0.0134406	0.90886655	0.43563295
Cdc27	-0.0161732	7.83018118	0.01341904	0.90893934	0.43563697
Pilra	0.08574769	-0.5315319	0.01340239	0.90899559	0.43563697
Minpp1	0.01839508	4.91685676	0.01338613	0.90905054	0.43563697
Frmd5	0.02375035	5.14701112	0.01336729	0.90911426	0.43563697
Dyx1c1	-0.0416639	2.02333161	0.01335221	0.9091653	0.43563697
Zfyve20	0.01793434	5.76894164	0.01330926	0.90931082	0.43567886
Nr2f1	0.01853514	6.76098752	0.01329204	0.90936924	0.43567903
Fahd2a	-0.0318384	2.7954796	0.01325386	0.90949891	0.43568913
Trim21	-0.0301058	3.83998389	0.01325163	0.90950649	0.43568913

Pstpip1	0.14023577	-0.5833353	0.01318489	0.90973359	0.43577009
Vps37b	0.0263283	3.12214722	0.01314111	0.9098829	0.43581115
Rras	0.03648233	4.72190055	0.0131071	0.90999906	0.43581115
Rnf113a1	0.03178199	1.95448335	0.01308503	0.91007452	0.43581115
Fam174a	0.02054817	5.07082992	0.01306979	0.91012666	0.43581115
Fam169a	-0.0257149	5.76125406	0.01305965	0.91016136	0.43581115
Gbp5	0.05237811	2.13971357	0.01305774	0.91016789	0.43581115
Sult2b1	0.06327491	0.36922458	0.0130022	0.9103583	0.43586961
Srpr	-0.0250735	6.37658832	0.01298824	0.9104062	0.43586961
Zscan25	-0.1192019	-0.360117	0.01295894	0.91050687	0.43588999
Ap1b1	-0.0173342	5.70390084	0.012928	0.91061329	0.43591311
Nsmce1	0.04333636	3.55079185	0.01288134	0.91077401	0.43593344
Zfp606	0.01974916	4.59360883	0.01286958	0.91081456	0.43593344
Btk	0.1011817	0.14467041	0.01284831	0.91088795	0.43593344
Nt5e	0.03122137	4.27306299	0.01284824	0.91088821	0.43593344
Ccser2	0.01236551	8.27618302	0.01278797	0.91109653	0.43600532
Cdh24	0.10547038	-1.4470112	0.01268056	0.911469	0.43611825
Dazap1	-0.0242866	3.48253625	0.01267621	0.91148412	0.43611825
4931403E22I	-0.2112672	-1.295699	0.01266965	0.91150693	0.43611825
Prkaa2	-0.0155595	7.2938567	0.01263989	0.91161046	0.43613997
Gm1715	0.15761284	-1.7361002	0.01257992	0.91181948	0.43616698
Mettl23	-0.0323215	2.83220101	0.01255853	0.91189415	0.43616698
Efcab2	0.01910898	3.97879449	0.01255426	0.91190906	0.43616698
Tgfb2	-0.0209377	4.28430239	0.0125385	0.9119641	0.43616698
Nlk	-0.015968	8.55890243	0.01251307	0.91205305	0.43616698
Hsd17b13	-0.136919	-1.215818	0.01250801	0.91207074	0.43616698
Gas1	0.0298825	5.78647272	0.0125071	0.91207394	0.43616698
Wdr5	0.03676375	3.23638315	0.01236852	0.91256031	0.43637175
Gpatch1	-0.0152286	5.0819513	0.01228443	0.91285677	0.43648569
Mcm2	-0.0315545	2.2388127	0.01224727	0.91298808	0.43650999
Osbpl2	-0.0148459	5.46258645	0.01223713	0.91302399	0.43650999
Papln	-0.1249468	-0.9964692	0.01218043	0.91322484	0.43653105
Osbpl11	-0.0199866	4.16970836	0.01217544	0.91324254	0.43653105
Armc7	0.03523745	2.3542711	0.01217542	0.9132426	0.43653105
Coro1a	-0.0244591	4.02559701	0.01213252	0.91339494	0.43654892
2810006K23I	0.02012074	4.99404715	0.01211663	0.91345145	0.43654892
1700003D09I	0.10867078	-0.5231101	0.01211574	0.91345459	0.43654892
C030006K11I	0.0311112	2.96509353	0.01197188	0.9139678	0.43676636
Dusp1	0.03137502	6.28480359	0.01195527	0.91402723	0.43676694
Pfn4	-0.0377282	1.95490093	0.01192743	0.914127	0.43678679
Lilrb4	-0.067116	0.43424165	0.01187261	0.91432372	0.43685296
Anxa11	-0.0178574	3.947233	0.01182648	0.91448964	0.4369044
Zcwpw1	-0.0551017	0.6780819	0.01177102	0.91468957	0.43695398
Pold3	0.01757746	4.88174881	0.01176538	0.91470992	0.43695398
Itgal	-0.0607018	0.64269499	0.01174091	0.9147983	0.43696838

Cep170	-0.0172885	7.48685712	0.01172014	0.91487336	0.4369764
Ralgds	-0.0128656	5.87226687	0.01152847	0.91556948	0.43725558
Dsn1	-0.0384334	1.38617472	0.01152712	0.91557444	0.43725558
Fst	0.06532504	0.61537621	0.01148992	0.91571022	0.43729258
C130036L24I	0.0800604	-0.1689288	0.01146058	0.91581748	0.43731597
Slc45a1	0.02987675	2.61914513	0.01142048	0.91596429	0.43731731
Zfp780b	-0.0160801	5.39516929	0.01140929	0.91600529	0.43731731
Wdr62	0.05726783	1.01093731	0.01139984	0.91603995	0.43731731
Sfrp5	0.19097203	-2.1405377	0.01139615	0.91605349	0.43731731
Zfp933	-0.0191794	4.53456843	0.01137183	0.91614275	0.43733209
Pip4k2b	0.01452466	7.02044146	0.01128484	0.91646283	0.43741775
Col25a1	0.01969542	5.59930305	0.01126577	0.91653317	0.43741775
Laptm5	0.0414892	3.30029559	0.01123992	0.91662859	0.43741775
Tomm40	0.02401409	3.08970157	0.01123689	0.9166398	0.43741775
Foxf1	0.13756036	-1.9447467	0.01123641	0.91664156	0.43741775
Tmed4	-0.0240331	5.53251403	0.01122816	0.91667206	0.43741775
Lipg	-0.0456716	1.29673501	0.01120224	0.91676792	0.43742775
Hook1	0.0275761	5.07421021	0.01119097	0.91680964	0.43742775
H2-Q1	0.03065507	6.0697146	0.01111534	0.91709011	0.43753373
Kctd16	0.02649001	3.81506031	0.01109233	0.91717564	0.43753475
Prkcz	0.01411292	6.3385358	0.01108339	0.9172089	0.43753475
Dnttip1	-0.0290121	2.98877728	0.01103019	0.91740709	0.43760146
Gm7457	-0.1281718	-0.9471554	0.01097671	0.91760676	0.43766888
Fam117a	-0.0445831	3.77824039	0.01091531	0.91783667	0.4377507
Fcho2	0.01555026	6.99913835	0.010866	0.91802174	0.43781113
Zcchc3	-0.0219732	5.00157565	0.0108103	0.91823134	0.43788325
Gla	0.02525849	3.05440702	0.01077208	0.91837546	0.43792414
Dnajc3	0.02031943	6.76679914	0.01075	0.91845884	0.43793366
Fbxo4	-0.0327489	3.73373503	0.01073589	0.91851218	0.43793366
Rufy3	0.0151876	8.36759982	0.01067575	0.91873988	0.43801438
Pparg	-0.0420686	1.72385308	0.01065165	0.9188313	0.43803012
Prdm16	0.02919161	3.06366429	0.01061097	0.91898587	0.43807597
Leng9	0.04253984	1.11170176	0.01059313	0.91905375	0.4380771
Acin1	-0.0119477	5.86499961	0.01057967	0.91910503	0.4380771
Rbm12	0.01434722	5.25852923	0.01053401	0.91927912	0.43813224
Gm10440	-0.1878808	-1.531503	0.01048911	0.91945073	0.43818618
Pkn2	-0.0139297	6.06826145	0.01045253	0.91959077	0.4382164
Slc38a9	0.02601338	4.58404155	0.01044204	0.91963096	0.4382164
Gltscr2	0.02430542	5.54829414	0.01040516	0.91977253	0.43822452
Lrrc6	-0.0221678	3.88873187	0.01039543	0.91980989	0.43822452
Hdac5	0.0204353	5.88492629	0.01039195	0.91982326	0.43822452
Fam134b	-0.0171882	5.61864022	0.01037333	0.91989487	0.4382308
Irak1bp1	-0.0177596	5.9075469	0.01034892	0.91998885	0.43824774
Ddx27	-0.0163406	4.1812579	0.01031788	0.92010849	0.4382769
Tnfrsf21	0.02010814	6.54315861	0.01027105	0.92028933	0.4383184

Krt77	0.09091885	-0.5571166	0.01026506	0.92031249	0.4383184
Mir6390	-0.1085318	0.08973674	0.01024116	0.92040499	0.43833463
Ccdc58	0.02376321	3.0272777	0.01009857	0.92095906	0.4385517
Tti1	-0.019085	4.735674	0.01008498	0.92101209	0.4385517
Abcc6	0.10021084	-0.8892767	0.01007881	0.92103619	0.4385517
Zbtb33	-0.0158722	5.58479729	0.01005186	0.92114145	0.43857398
Khdrbs1	0.011843	6.94592177	0.00991351	0.92168411	0.4388045
Gm5860	0.02860385	2.07446429	0.0098853	0.92179523	0.43880962
Olfml1	0.02248752	4.58663499	0.00985239	0.92192508	0.43880962
Hlcs	-0.0181468	4.42716132	0.009849	0.92193849	0.43880962
Sh3d21	-0.0519769	1.01929013	0.00982056	0.92205085	0.43880962
Ccdc8	0.08690207	-0.2951193	0.00980658	0.92210619	0.43880962
Flna	0.01623803	5.69101565	0.00977463	0.92223275	0.43880962
Six5	0.03663196	3.32592734	0.00977233	0.92224187	0.43880962
Trim34b	0.06928656	-0.8356453	0.00976982	0.92225181	0.43880962
Zbed4	0.02178774	4.47113745	0.00976593	0.92226723	0.43880962
Adhfe1	-0.0194763	5.11547406	0.00975248	0.92232058	0.43880962
Zfp219	0.03138717	1.95613144	0.00974801	0.92233833	0.43880962
Tacr2	0.07585785	0.30130535	0.00971482	0.92247022	0.43881925
Bcl9l	0.01767857	5.44706175	0.00971348	0.92247557	0.43881925
Pcdhgc5	-0.0221793	4.78780458	0.00967388	0.92263319	0.43883273
Dclk3	0.01811781	5.48919709	0.00967316	0.92263607	0.43883273
Yeats2	0.01390057	5.57972497	0.0096526	0.92271804	0.43883273
Nit1	-0.0234896	2.91999726	0.00964762	0.92273792	0.43883273
Ccdc126	-0.0319184	2.28289532	0.00955117	0.92312381	0.43898842
AA388235	-0.0223368	3.22421595	0.00952648	0.92322292	0.43900772
Tmem236	0.16382729	-1.7102304	0.00950331	0.92331601	0.43902416
Slc1a5	-0.0327732	2.10216484	0.00945845	0.9234966	0.43908219
Ints3	-0.0147906	5.30231358	0.00940799	0.92370031	0.43908336
Slc22a18	-0.0634246	1.18189298	0.00940715	0.92370368	0.43908336
Bcdin3d	-0.050669	0.61609728	0.00940142	0.92372684	0.43908336
Jam3	0.02743581	2.89043088	0.00938256	0.92380315	0.43908336
C7	-0.0515503	0.35357518	0.00936852	0.92385999	0.43908336
Dennd2a	-0.0181179	4.23267622	0.00935483	0.92391544	0.43908336
Decr2	-0.013646	5.53858173	0.00934406	0.92395913	0.43908336
Ddx4	0.04418922	0.98895315	0.00934204	0.92396734	0.43908336
P4htm	0.02574582	2.39224684	0.00930744	0.92410777	0.43912228
Ptcd1	-0.0202873	3.44479309	0.00929066	0.92417602	0.43912689
Uba1	0.01131334	7.91621844	0.00924494	0.92436221	0.43918552
Nampt	-0.0124765	7.31073047	0.00923163	0.9244165	0.43918552
Ppapdc3	0.0306484	2.36618951	0.00919973	0.92454677	0.43919501
Txlng	0.01352778	5.65176157	0.00919422	0.9245693	0.43919501
C920021L13I	0.03502995	1.96554996	0.00916932	0.92467117	0.43919501
Ifi203	0.03447034	3.12728401	0.00916485	0.92468949	0.43919501
Cd300ld	-0.0844096	0.25936695	0.00915515	0.92472922	0.43919501

Tmem120b	-0.0786266	0.44603953	0.00911904	0.92487733	0.43923754
Ttc7	-0.0287854	3.14285201	0.00908893	0.92500107	0.43926849
6720468P15I	0.15057695	-0.4914859	0.00906248	0.92510992	0.43929238
Slco4a1	0.0442965	1.80494302	0.00881184	0.92614958	0.43973291
Magt1	-0.0190837	5.22966798	0.00881057	0.92615488	0.43973291
Krt1	-0.0414188	1.74797374	0.00869701	0.92663087	0.43993106
Tnfrsf13c	-0.0616652	0.45441152	0.00867893	0.92670694	0.43993933
1700066M21	0.01320011	4.79759952	0.00862581	0.9269309	0.4400178
Gpatch4	0.01386412	4.88868949	0.00851362	0.9274062	0.44021557
E330011O21	-0.0605273	-0.1778107	0.00845415	0.92765943	0.44030791
Iws1	0.01140398	6.44071015	0.00838395	0.92795955	0.44039833
Nipal2	0.02813908	2.85416739	0.00838213	0.92796734	0.44039833
Eaf1	0.0140509	5.06304878	0.00834802	0.9281136	0.44040014
Mcmbp	0.01109474	5.68264481	0.00834319	0.92813437	0.44040014
Vegfc	-0.0286854	2.74930106	0.00833399	0.92817388	0.44040014
Zfp319	-0.0209418	3.1469779	0.00832652	0.92820599	0.44040014
Vrk3	0.03086016	2.8399183	0.00826946	0.92845168	0.44048885
Rab39	-0.0495253	0.57454384	0.00818408	0.92882092	0.44061555
Mocs1	-0.0255949	4.31887356	0.00818055	0.92883623	0.44061555
Gabbr1	-0.0147464	7.35813942	0.00814178	0.92900459	0.44066424
P2rx1	0.1168932	-2.3316039	0.00812988	0.92905636	0.44066424
Zbtb40	-0.036102	2.50864861	0.00809141	0.92922393	0.44069474
Lrrc47	-0.0297819	2.66498574	0.00808815	0.92923815	0.44069474
Exosc7	-0.0208002	3.35910013	0.00804621	0.92942131	0.44072966
Pld2	-0.0319057	2.40034598	0.00804439	0.92942929	0.44072966
Mrpl37	0.01609804	4.02283892	0.00799157	0.92966068	0.44081152
Zfp148	0.01024819	7.82606425	0.00796385	0.92978246	0.44084139
Slc50a1	0.02311133	3.05410095	0.00790901	0.93002396	0.44092802
Gm6815	-0.0631136	-0.9340087	0.00784883	0.93028997	0.44102627
Bcorl1	0.01353318	4.54898799	0.00781609	0.93043511	0.44104152
Srpx2	0.053454	0.24432946	0.00781059	0.93045951	0.44104152
Amotl1	-0.0139155	6.88193783	0.00778301	0.93058209	0.44104152
Gripap1	-0.014595	4.91768759	0.00778012	0.93059493	0.44104152
1700019L03F	0.06615024	-0.6529311	0.00776722	0.93065236	0.44104152
Mill2	0.04854994	0.60879185	0.00776215	0.93067492	0.44104152
Wdr73	-0.0140312	4.41027299	0.00774754	0.93074001	0.4410445
Chsy1	0.01293355	5.20745645	0.00768205	0.93103255	0.44112704
2310009B15I	0.03227116	2.1245251	0.00767756	0.93105266	0.44112704
Ubap1	0.01309319	5.09841286	0.00766909	0.93109061	0.44112704
Ephx2	-0.0161838	3.85688729	0.00763059	0.93126335	0.44118102
C87436	0.02367513	3.60586379	0.00760773	0.93136609	0.44120182
4931408D14I	0.04917272	0.71792381	0.00752664	0.9317319	0.44134724
Kmt2e	0.01126024	9.52673944	0.00745787	0.93204369	0.44143043
Plvap	-0.0461652	0.6205832	0.00745284	0.93206652	0.44143043
Sesn1	-0.0115288	6.4168834	0.00744899	0.93208406	0.44143043

Lhx6	0.01557699	4.21620547	0.00737399	0.93242591	0.44156445
Nubpl	0.02425658	2.80469616	0.00725963	0.93295062	0.44171492
Fam19a2	-0.0132791	5.53212453	0.00725486	0.93297257	0.44171492
Frrs1	-0.0474319	1.18737088	0.0072479	0.93300468	0.44171492
Cd200r1	-0.0604244	-0.6579988	0.0072363	0.93305815	0.44171492
2810403A07I	-0.014641	6.56468175	0.0072295	0.93308954	0.44171492
Mpst	-0.0487559	0.44164435	0.0072279	0.93309696	0.44171492
Il2rg	0.0630926	0.43366703	0.00718089	0.93331432	0.44178994
Tm6sf1	0.02789669	1.87347446	0.00716301	0.93339717	0.44180127
1700007P06I	-0.0594985	-0.667829	0.00714288	0.93349059	0.44181761
Ppil4	-0.0116987	5.95392326	0.00704991	0.93392375	0.44199474
1700028J19F	0.07175165	-1.2807606	0.00702048	0.9340615	0.44203204
Gchfr	-0.0610283	0.21337602	0.00699484	0.9341817	0.44206104
Gipc2	0.03450082	1.46851857	0.0069659	0.93431766	0.44209749
Zfp558	-0.0313481	2.74543963	0.00692571	0.93450696	0.44215355
Rfc4	-0.0271763	1.9149827	0.00691574	0.93455404	0.44215355
Clptm1l	-0.0169077	4.51045933	0.00688937	0.93467862	0.44218461
1600023N17	0.07375026	-0.9773858	0.00684352	0.9348958	0.44222464
Tspan8	0.02944105	4.35901052	0.00683627	0.93493024	0.44222464
Igfbp2	0.02442837	4.96392241	0.00683419	0.93494011	0.44222464
Trpv4	0.05931351	-0.4879297	0.00680466	0.93508048	0.44225112
Dhcr7	0.01680331	3.72930431	0.00678805	0.93515957	0.44225112
Mtag2	0.08444027	-0.504034	0.00678524	0.93517295	0.44225112
Fgd3	-0.073153	-0.2428206	0.00674921	0.93534488	0.44230454
Pcif1	-0.0136387	3.96278739	0.006731	0.93543199	0.44231785
Pdzrn3	0.01042765	7.30350977	0.00671439	0.93551152	0.44232143
Txndc11	0.01604841	3.87622766	0.00670459	0.93555846	0.44232143
Syt6	-0.0211516	4.7974429	0.0066925	0.93561646	0.44232143
AW554918	-0.0118342	4.9547317	0.00665914	0.93577675	0.44236932
Tgfb3	-0.0250497	4.69475048	0.00661998	0.93596544	0.44241408
4932435O22	-0.1649295	-1.8514079	0.00660887	0.93601907	0.44241408
Cacna1f	0.08552997	-0.5969001	0.00660281	0.93604835	0.44241408
Mocs3	0.05432357	-0.9356207	0.00657457	0.93618492	0.44242712
Apobec3	0.02732641	2.31423405	0.00657271	0.93619392	0.44242712
Prkcg	-0.0122655	8.20544702	0.00654127	0.93634636	0.44247129
Dram2	-0.0146969	5.47488685	0.00652082	0.93644574	0.44249015
Klf7	0.01178111	6.5193186	0.00650879	0.93650425	0.44249015
Tmem28	-0.0321141	1.70191866	0.00649067	0.93659251	0.44250398
Zdhhc23	0.04229995	1.06906349	0.00645377	0.93677258	0.44256118
Osbpl7	-0.0240403	2.31354449	0.00642561	0.93691036	0.4425984
4930526I15R	-0.0352792	1.65572054	0.00641238	0.9369752	0.44260115
Phf1	-0.0264272	2.86487896	0.00637352	0.93716604	0.44266343
Sdc3	0.01223959	5.2388551	0.00635174	0.93727324	0.44266566
Adora1	0.01170036	7.10715741	0.00634859	0.93728878	0.44266566
A130010J15F	0.0238087	3.56718103	0.00626971	0.93767873	0.44279351

Smg7	-0.0083694	8.02274237	0.00626887	0.93768287	0.44279351
Mier3	0.01212991	5.08618564	0.00625805	0.93773658	0.44279351
Tma16	0.01814186	3.76072172	0.00623229	0.93786456	0.44282607
Icam4	-0.0601612	-0.0270766	0.00620513	0.9379998	0.44286204
Rbbp4	-0.0108684	5.94783195	0.00608095	0.93862197	0.44311801
Rnf25	0.02666428	2.08902688	0.00607329	0.9386606	0.44311801
Rwdd2a	-0.0190958	2.99713776	0.00606167	0.93871917	0.44311801
Tmem175	-0.0119286	4.99426847	0.00599023	0.93908057	0.44317246
Naaladl1	-0.0468755	0.48937503	0.00596468	0.9392104	0.44317246
Pex6	0.01823103	3.79924889	0.00596255	0.93922121	0.44317246
Slc9b1	0.10044032	-0.7893292	0.00595887	0.93923994	0.44317246
Cbs	-0.0161697	3.17066703	0.00594942	0.93928804	0.44317246
Sucla2	0.00868653	6.74457014	0.00594491	0.93931098	0.44317246
Chst3	0.06076874	0.66964206	0.00594471	0.93931201	0.44317246
Fam171a2	0.01912742	2.68908278	0.0059445	0.93931311	0.44317246
5730508B09I	-0.02116	2.79684429	0.00592073	0.93943429	0.44317246
Iqcg	-0.0189604	2.95988689	0.00592028	0.93943657	0.44317246
C530008M17	-0.0151796	5.06620149	0.00591092	0.93948439	0.44317246
2310061I04R	-0.0142225	4.20453282	0.00589504	0.93956553	0.44318287
Slitrk1	0.0103663	7.08121807	0.0058604	0.93974303	0.44323191
Gm15421	-0.0233004	1.85128343	0.0058517	0.93978768	0.44323191
Dcaf13	0.01116096	4.45411103	0.00581328	0.93998528	0.44329723
Rbm41	0.01397371	4.52288136	0.0057725	0.94019568	0.44336858
Fam189b	0.01424722	4.60001513	0.00572294	0.94045245	0.44346179
Tmem37	-0.0602925	0.73722853	0.00570423	0.94054968	0.44347758
Gm6623	-0.0217167	1.5751644	0.00569376	0.94060418	0.44347758
Tvp23b	0.01433557	4.76558333	0.00564833	0.94084112	0.44356141
Rragb	0.01084714	4.62507117	0.00558978	0.94114795	0.44367818
Csnk1g2	-0.0150874	5.59108536	0.00556713	0.94126707	0.44370646
Cbx7	-0.0150419	4.92502466	0.00555052	0.94135458	0.44371983
Arhgef40	0.01428027	3.63309355	0.00549838	0.94163012	0.44382181
Mob1a	-0.0161431	3.39147199	0.00546301	0.9418178	0.44386004
Elp3	-0.0103092	5.3579593	0.0054608	0.94182957	0.44386004
Cnep1r1	-0.0101916	5.90933978	0.0054288	0.94199991	0.44386773
Wdr26	0.00821076	8.56156612	0.00542502	0.9420201	0.44386773
Dync2li1	0.0167132	3.59751117	0.00541968	0.94204858	0.44386773
Ppp1r3d	0.03405359	1.13591682	0.00540581	0.94212264	0.44386773
Homez	0.01132948	4.36385714	0.00540224	0.94214174	0.44386773
Csf1	0.0165806	4.87426401	0.00537853	0.94226859	0.44388329
Zranb2	-0.009041	8.01362559	0.00537395	0.94229311	0.44388329
4933412E12I	-0.0254463	1.84800618	0.00535423	0.94239888	0.44390523
1700023F06I	-0.0949912	-1.5503012	0.00533397	0.94250778	0.44390664
Elp6	-0.0262721	2.98309569	0.00533166	0.94252022	0.44390664
Dnajc5b	-0.0722973	-0.5513479	0.00531339	0.9426186	0.4439251
Galnt1	-0.0119732	6.23301899	0.00529499	0.94271786	0.44394398

Nudt22	0.02443927	1.45155025	0.00522225	0.94311195	0.44410168
Gstm3	0.02543676	1.53275135	0.00519136	0.94328015	0.44415301
Zfp275	-0.011662	5.65090821	0.00517821	0.9433519	0.44415744
Lipe	-0.0234528	2.00487894	0.00516794	0.94340798	0.44415744
A930015D03	-0.0310652	1.37320484	0.00513463	0.94359035	0.44421541
Fbxo33	0.01239648	4.8653221	0.00511373	0.94370503	0.44424085
2310061J03F	-0.028749	2.3589709	0.00510322	0.94376283	0.44424085
Cldn22	-0.0999934	-1.6312496	0.00507914	0.94389546	0.4442754
Tgfbr1	0.01249849	5.32494421	0.00506609	0.94396742	0.4442814
Plekhm1	0.01027566	5.38139561	0.00501783	0.94423448	0.44434733
Figf	-0.0347353	1.88956967	0.00501011	0.94427733	0.44434733
Cacnb3	-0.0089523	6.89215654	0.00500869	0.94428521	0.44434733
2900009J06F	0.07146951	-0.6726362	0.00497579	0.94446814	0.44440553
Ckap2	0.04806245	0.36365987	0.00495515	0.94458328	0.44443183
Irf1	0.02256876	2.9745514	0.00491803	0.94479087	0.44450162
Pou3f4	0.03045358	0.57012501	0.0048799	0.94500492	0.44457444
Rasal1	0.02376218	3.28396186	0.00483968	0.94523165	0.444638
Stamos	0.06640761	-1.1689577	0.00483492	0.94525857	0.444638
Supt5	0.01206064	5.64203307	0.00480285	0.94544012	0.44469551
Bin2	0.02023249	3.02808699	0.00478186	0.94555922	0.44472365
Gm16938	0.02359443	2.17302732	0.00474632	0.9457616	0.44477604
Cdk5rap3	0.0170592	3.15904172	0.00473251	0.94584042	0.44477604
Strc	-0.0820618	-1.2857172	0.0047311	0.94584851	0.44477604
Spdef	-0.1143952	-1.9795163	0.00471223	0.94595642	0.44479891
Luc7l2	-0.0099279	7.39714737	0.00468723	0.94609972	0.4448384
Tjp3	0.05824541	-0.8532734	0.00467107	0.94619255	0.44484794
Armcx6	-0.0386579	0.96305597	0.00466306	0.94623862	0.44484794
4931406H21I	0.06394933	0.24026653	0.00462473	0.94645967	0.44492398
Trim34a	-0.0207587	3.15628048	0.00457038	0.94677474	0.4450442
Paip1	0.01015968	6.24463162	0.00454147	0.94694308	0.44509544
Xk	-0.0139811	5.23281007	0.00450976	0.94712831	0.44513275
Clip4	0.01035734	5.24150222	0.00450757	0.94714113	0.44513275
Mast2	-0.0083511	6.29563596	0.00449332	0.94722462	0.44514409
Tchh	0.01768744	3.01158791	0.00447847	0.94731177	0.44515717
Slfn3	0.06668007	-0.2510446	0.00445184	0.94746843	0.44520289
Alg12	-0.023839	2.79775609	0.00438738	0.9478495	0.44534473
Atg4a	0.02219666	3.5910716	0.00438073	0.94788903	0.44534473
Chst14	-0.0280606	1.60237469	0.00437055	0.94794946	0.44534523
Ttc39d	0.10050076	-1.637163	0.00433135	0.94818308	0.44542151
Lrrc73	0.0211046	2.43181403	0.0043234	0.94823059	0.44542151
Mettl8	-0.0164165	3.77776576	0.00430348	0.94834984	0.44544963
Cacna2d4	-0.030657	1.19254656	0.00427903	0.94849651	0.44545601
Zbtb14	-0.0108002	5.14679904	0.00427369	0.94852865	0.44545601
Rnf215	-0.0178364	2.67262694	0.00427154	0.94854156	0.44545601
3110043O21	0.0165938	3.97870213	0.00421201	0.94890082	0.44559683

Hrc	0.06125498	-0.3257219	0.00417749	0.94911037	0.44566733
Creb3l4	-0.096685	-0.9489596	0.00412661	0.9494208	0.44569921
Ms4a4b	-0.0622713	-0.5138973	0.00411274	0.94950571	0.44569921
Calml4	-0.0268583	1.39227829	0.0041072	0.94953967	0.44569921
Rpsa	-0.0106685	6.48231852	0.00410476	0.94955467	0.44569921
Cnksr3	0.01947709	1.78761728	0.00410116	0.94957674	0.44569921
LOC1016697	-0.0829128	-0.978259	0.00409844	0.94959343	0.44569921
Pstpip2	0.01209638	3.88083727	0.00409832	0.94959417	0.44569921
5430416O09	0.09468798	-1.8621924	0.00404483	0.94992377	0.44576139
Pds5a	0.00859445	6.86280437	0.00403628	0.94997661	0.44576139
Foxd2os	0.01898539	2.51119551	0.00403626	0.94997672	0.44576139
Rbm24	-0.0142081	3.97278208	0.00403293	0.94999735	0.44576139
Rpusd1	-0.0133378	2.98295012	0.00402154	0.9500679	0.44576139
Cdca5	-0.0763507	-1.4969449	0.00401907	0.95008321	0.44576139
Ccdc150	0.02305456	-1.6023388	0.00394753	0.95052886	0.44594259
Fam168b	-0.0083769	8.42329081	0.00392578	0.95066513	0.44597863
Popdc2	0.04241215	0.81035692	0.00389152	0.95088059	0.4460298
Pstk	-0.0147551	4.18438685	0.00388954	0.95089312	0.4460298
Gm10638	-0.0472605	-0.6071244	0.00387696	0.95097245	0.44603911
Ptpdc1	0.00845667	5.41754893	0.00384837	0.95115332	0.44609606
Laptm4b	-0.008655	5.34753559	0.00383486	0.95123901	0.44610835
Smad3	-0.0088708	6.75745112	0.00377192	0.95164029	0.44626864
C230037L18	-0.0304309	1.60175458	0.00371007	0.95203787	0.44642718
3110062M04	0.02781417	0.70944072	0.0036704	0.95229468	0.44651969
Zbtb46	0.02662149	1.31475956	0.0036566	0.95238435	0.44652764
Cd164l2	0.06387797	-1.0215828	0.0036327	0.95254001	0.44652764
A830082K12	0.0087468	6.61205122	0.00363199	0.9525446	0.44652764
Kremen2	-0.0404337	-0.7455648	0.00363121	0.95254974	0.44652764
Cdk17	0.00899415	7.87426799	0.00358775	0.95283419	0.44663307
Spaca5	-0.0695187	-1.6395404	0.00348801	0.95349359	0.44691423
Stambp	0.00960523	4.3459198	0.00345892	0.95368772	0.44694985
Zbtbd6	-0.0480685	0.54639363	0.00345876	0.95368876	0.44694985
Cntn6	-0.022881	3.21774274	0.00342278	0.95392998	0.44701881
Cdca2	0.07543101	-0.3892356	0.00341904	0.95395509	0.44701881
Sash3	-0.0298468	1.90364415	0.00337469	0.95425437	0.4471107
Atp6v0e	-0.0161067	4.75238444	0.00336996	0.95428639	0.4471107
Zfp784	-0.0144716	3.96359625	0.00335892	0.95436127	0.4471107
Gtpbp1	0.01395528	4.45862357	0.0033506	0.95441778	0.4471107
Ece1	-0.0096714	5.98034349	0.00333888	0.95449744	0.4471107
Abcd1	0.01722772	3.7141478	0.00333569	0.95451914	0.4471107
AI317395	0.07026444	-0.8851693	0.00332133	0.95461705	0.4471107
A330040F15	-0.0461535	-0.8518091	0.00331972	0.95462802	0.4471107
Sidt2	0.00795761	5.19264066	0.00328873	0.95484004	0.44715963
Dnase2a	0.03623742	-0.3412987	0.00328703	0.95485172	0.44715963
Cacna2d2	-0.0125331	4.99637062	0.00326949	0.95497219	0.44718813

Ikbip	-0.0155803	4.11084961	0.00324675	0.95512889	0.44722448
Iigp1	-0.0148576	4.08486279	0.00324059	0.95517145	0.44722448
Myom3	-0.0527738	-0.2197565	0.00323231	0.95522868	0.44722448
Nos1ap	0.01499201	3.69490422	0.00321917	0.9553197	0.44723886
Slu7	0.00795038	5.97942903	0.00321047	0.95538	0.44723886
Scamp5	0.00960607	6.44709612	0.00320209	0.95543826	0.44723886
Arxes2	-0.0133008	3.63078278	0.00315343	0.95577774	0.44736986
Phf12	-0.0065178	6.74279914	0.00312514	0.9559763	0.44741345
Araf	-0.006829	7.24708396	0.00312317	0.95599016	0.44741345
Coro2a	-0.0128443	5.32555707	0.00309102	0.95621707	0.44749173
Tdrd9	-0.1311239	-1.9587224	0.00308254	0.95627709	0.4474919
Ptgds	0.02152048	12.2504338	0.00305374	0.95648158	0.44753009
B4galt4	0.011832	4.72188672	0.00305212	0.95649311	0.44753009
Aqp6	0.06566019	-1.3654759	0.00304586	0.95653769	0.44753009
Srpk1	-0.0067512	6.0743028	0.00303408	0.95662177	0.44754151
Cd163l1	-0.0636311	-1.1743733	0.00301463	0.95676088	0.44757868
Lmx1b	0.06078653	0.19280357	0.00299631	0.95689234	0.44760973
Zmynd19	0.02318683	1.55240992	0.00298513	0.9569727	0.44760973
Fam149a	0.00733954	5.22175722	0.00297424	0.95705118	0.44760973
3425401B19I	-0.0204738	3.75183628	0.0029722	0.95706594	0.44760973
Trmt12	0.01281605	3.27438155	0.0029408	0.95729305	0.4476845
Usp22	-0.0066588	7.05458543	0.00293362	0.95734516	0.4476845
Dvl1	-0.0101962	4.07621373	0.00292411	0.95741429	0.44768891
Fbxo25	0.00913903	4.92372084	0.00291091	0.95751041	0.44770595
Dpp4	0.0174027	5.50219731	0.00289751	0.95760824	0.44770765
Mitd1	0.01441755	3.3516068	0.00289407	0.95763341	0.44770765
Gldn	0.03044985	0.93540306	0.00287997	0.95773665	0.44772801
Esr2	-0.0252209	0.69385445	0.00286061	0.95787878	0.44776655
Alad	-0.0140695	2.53834977	0.00285233	0.95793972	0.44776713
Lman2l	0.0121332	3.23296751	0.00284327	0.95800652	0.44777045
1700120C14I	0.04389821	-1.1689078	0.00282591	0.95813474	0.4477805
Ddx59	-0.0156838	2.5780165	0.00281633	0.95820574	0.4477805
Serpine1	-0.0395129	-0.2823831	0.00281614	0.9582071	0.4477805
Pappa2	0.02071873	2.28047774	0.00278008	0.95847535	0.44786532
Gm10767	0.0161306	1.92261135	0.00276475	0.95858984	0.44786532
Mum1l1	-0.029242	1.70224745	0.00276108	0.95861733	0.44786532
Lysmd2	-0.0083577	4.50476051	0.00274622	0.95872874	0.44786532
Ccbe1	0.01109891	3.84561048	0.00273847	0.95878693	0.44786532
Fibcd1	0.03539284	0.43586962	0.00273746	0.95879451	0.44786532
Arhgef6	0.00765488	6.45736664	0.00273439	0.95881764	0.44786532
Synj2bp	0.00598048	7.03416657	0.00272689	0.95887408	0.44786532
Rab38	0.06882452	-1.447128	0.00272001	0.95892594	0.44786532
Wdr91	-0.0129987	3.08481468	0.0026949	0.95911582	0.44786819
Zbtb44	0.00655629	7.23538293	0.00269354	0.95912607	0.44786819
Emilin2	-0.0564032	-1.0549577	0.00269234	0.95913523	0.44786819

Cyp2j12	-0.0397172	-0.5687111	0.00268729	0.95917354	0.44786819
Gpr180	0.01184746	3.9525995	0.00267977	0.95923062	0.44786819
Lrrc28	0.00851895	4.49518742	0.00265857	0.95939202	0.44791567
Cd55	-0.0126147	5.98307271	0.00263587	0.95956559	0.44794939
Ddx50	0.00640465	6.27247617	0.00263351	0.95958369	0.44794939
Lztr1	0.00612001	5.7292613	0.00260929	0.95976981	0.4480084
Cenpa	0.02948094	1.06374212	0.00257807	0.96001097	0.44809308
Zfp612	0.00891928	7.4305425	0.00255736	0.96017183	0.44814028
Snord42a	-0.0395007	-1.1470432	0.0025329	0.96036255	0.44818699
Fads3	-0.0102774	3.57480128	0.00252921	0.9603914	0.44818699
Rbbp6	-0.0057052	9.12289896	0.00249547	0.96065624	0.44824743
Vkorc1l1	0.00545076	6.22933451	0.00249085	0.96069266	0.44824743
Ccdc105	0.05320832	-0.7318402	0.00248265	0.96075735	0.44824743
LOC1000389	0.05457004	-1.5510624	0.00248232	0.96075995	0.44824743
Pcdh19	0.00889881	6.24158074	0.00245897	0.96094483	0.44830581
Rusc2	-0.008918	6.58476708	0.00244303	0.96107148	0.44832339
Zfp90	-0.0095446	3.80356774	0.00243919	0.96110205	0.44832339
Rgmb	0.00876002	4.71335	0.00242406	0.9612228	0.44835184
Rpa2	0.01309582	3.4815477	0.00235482	0.96178011	0.44858389
Eps8	0.00713208	5.3883578	0.00232749	0.9620024	0.44865107
Akt3	0.00644216	8.0782817	0.00232242	0.96204375	0.44865107
E130308A19	0.00824782	5.17680017	0.00230824	0.96215976	0.44866701
Cog3	-0.0072392	5.52673204	0.00230362	0.96219757	0.44866701
Pick1	-0.0093928	3.57902935	0.00228601	0.96234223	0.44870657
Cap2	-0.0084488	8.29989561	0.00224987	0.96264085	0.44881791
Htr3a	0.02556992	0.66237424	0.00223693	0.96274835	0.44884014
Hiat1	0.00620866	6.3790195	0.00222036	0.96288648	0.4488547
Mpzl1	-0.0107401	3.20946085	0.00221883	0.96289926	0.4488547
Spock3	0.00706446	5.22420083	0.00219687	0.96308316	0.44891253
Pibf1	-0.0102274	4.56124547	0.00216901	0.96331779	0.44899399
Pdcd6ip	0.00517566	7.45387281	0.00214106	0.96355474	0.44907653
Zfyve1	-0.0073842	4.19700661	0.00210948	0.96382436	0.44913802
Prok2	0.04761742	-1.3584056	0.00210585	0.96385542	0.44913802
Brcc3	-0.0071516	5.43400162	0.00210459	0.96386631	0.44913802
Tmem215	0.01590153	3.3389055	0.00208125	0.96406706	0.44920366
Cd247	0.0643095	-1.5631675	0.00206845	0.96417767	0.4492273
Tbc1d2	0.03512252	-0.1847319	0.00201291	0.96466145	0.44942479
Asxl1	-0.0077861	5.35960439	0.00199649	0.96480577	0.44946411
Apoa2	-0.0427555	-1.2367279	0.00198194	0.96493417	0.44949601
Pdxdc1	-0.0051745	6.24525789	0.00197503	0.96499536	0.4494966
Mob3b	0.00804643	6.71471211	0.00196575	0.96507765	0.44950702
Agpat5	-0.006075	5.497355	0.00195354	0.9651862	0.44952966
Rab28	0.00617377	5.36756956	0.00193644	0.96533881	0.44957283
Usp47	0.00440126	7.1442615	0.00192592	0.96543302	0.44958879
Soga3	0.00780213	6.51710607	0.00188243	0.96582521	0.44972686

Tdp1	0.01266722	2.43650373	0.00187977	0.9658494	0.44972686
Rbfa	-0.0126866	2.63680944	0.00184409	0.96617479	0.44978283
Mrps9	0.0094289	3.40868777	0.00184056	0.96620722	0.44978283
Gm6498	0.03289272	-1.0240939	0.00183943	0.9662176	0.44978283
Slc41a3	0.0166502	2.46873405	0.0018382	0.96622882	0.44978283
Smr3a	-0.0537298	-0.5832651	0.00183378	0.96626942	0.44978283
Ercc2	0.01680032	1.24485289	0.00181625	0.96643095	0.44980269
Gltscr1	0.0081109	4.38183036	0.00180529	0.96653237	0.44980269
Actr10	0.00493157	7.66079374	0.00180078	0.96657411	0.44980269
Pradc1	-0.0120014	2.72955504	0.00179874	0.96659311	0.44980269
Bod1l	-0.0058431	8.59520974	0.00179671	0.9666119	0.44980269
Rnpep	-0.0101873	3.44437432	0.00178373	0.9667327	0.44981178
Comtd1	-0.016599	1.05456235	0.00177566	0.96680794	0.44981178
Pnp2	-0.0288224	-0.4451936	0.0017753	0.96681132	0.44981178
Syt4	0.00720206	7.23979806	0.00176829	0.96687685	0.44981436
Rasgrp2	-0.0099951	2.56032541	0.00174178	0.96712595	0.44990235
9430015G10	0.01454983	2.45774709	0.00171524	0.96737721	0.44997588
Nlrc3	0.03057209	-0.9379213	0.00171242	0.96740399	0.44997588
2810032G03	0.01435502	2.61698762	0.00170072	0.96751552	0.44999985
Tnfsf13	-0.0434704	-1.1613654	0.00167198	0.96779102	0.45009899
Ppp1r36	-0.0342627	-0.2099402	0.00166599	0.96784866	0.45009899
Gm166	-0.0182153	0.64345671	0.00165447	0.96795995	0.45012283
Man1a	-0.0082773	5.19943443	0.00162795	0.96821761	0.45020245
Zfp628	0.01214735	2.2903361	0.00162451	0.9682512	0.45020245
Crlf1	-0.0303629	-0.4769437	0.00160663	0.96842635	0.45025598
Zfp389	-0.0481525	-1.5357067	0.00158926	0.96859741	0.45026545
3110056K07I	0.01023129	2.46016357	0.00158809	0.96860896	0.45026545
Pwwp2b	0.0143249	2.38647075	0.00158058	0.96868318	0.45026545
Nnmt	0.03498504	-0.2419865	0.00158022	0.96868682	0.45026545
Zfp82	0.01420845	1.45211517	0.0015145	0.9693445	0.45054324
Rmi1	0.00556451	5.67301753	0.00148277	0.96966714	0.45066528
A430090L17I	-0.0443195	0.76348656	0.00147687	0.96972754	0.45066542
Fbxo47	-0.0170612	1.25856779	0.00143706	0.97013816	0.45080567
Ada	0.01529294	1.31544401	0.00143423	0.97016753	0.45080567
Pi4k2b	-0.0129928	2.02012472	0.00143018	0.97020961	0.45080567
Lyz1	-0.0247988	1.04164161	0.00141927	0.97032348	0.45082389
C2cd4b	0.02203005	-0.0762654	0.00141491	0.97036901	0.45082389
Mboat7	0.00560247	5.19985747	0.0014064	0.97045826	0.45083743
Ocel1	0.01432647	1.7625037	0.00135664	0.97098528	0.45105432
Klhl7	-0.0049991	6.31339067	0.00132982	0.97127336	0.45116021
Sh3kbp1	0.00505452	6.38425475	0.00130972	0.97149124	0.45123347
Abtb2	0.01245074	2.54631764	0.00128319	0.97178133	0.45131671
Fsbp	0.04213082	-1.8040217	0.00128233	0.97179078	0.45131671
Zfyve19	-0.0067751	3.28719945	0.00127573	0.97186341	0.4513225
Dusp12	0.01136961	2.30696083	0.00124497	0.97220453	0.45142844

Gtf2h1	-0.0066148	5.09621696	0.00124431	0.97221189	0.45142844
Gbe1	0.00643175	3.96139429	0.00121831	0.97250363	0.45151221
Gm15055	-0.0417042	-1.4464587	0.00121542	0.97253629	0.45151221
Il10rb	0.00940313	3.04627877	0.00121218	0.97257288	0.45151221
Ugcg	-0.0059164	6.93736904	0.00120013	0.97270952	0.4515321
Tmem107	-0.0115969	1.71680392	0.00119779	0.97273611	0.4515321
Ski	0.00383751	8.16273402	0.00118982	0.9728269	0.4515463
B130006D01	-0.0481178	0.52011361	0.00115005	0.9732847	0.45173084
Zc3hav1l	0.00621587	6.00486285	0.00113961	0.97340619	0.45175927
Mab21l1	0.01468367	1.21648583	0.00113043	0.97351348	0.45178112
Zfp229	-0.0075629	3.67899273	0.00111226	0.97372716	0.45185232
Lad1	-0.0430688	-1.4804742	0.00108711	0.97402574	0.45194593
Haghl	-0.013611	0.89350603	0.00108513	0.97404937	0.45194593
Prcp	-0.0067292	3.71054934	0.00107683	0.97414881	0.45194785
Rab24	-0.0054437	5.10408853	0.00107473	0.97417401	0.45194785
Lmbrd2	-0.0050947	6.12720119	0.00105442	0.97441906	0.45203358
Gucy2g	-0.0127213	1.35849486	0.00104891	0.97448602	0.45203669
Dmap1	-0.0076848	3.50122349	0.00104063	0.97458688	0.45205552
Mpp4	0.0236117	-1.0414371	0.00101896	0.97485277	0.45213229
Fubp3	-0.0047198	5.88427179	0.00101732	0.97487295	0.45213229
Cdc20	-0.025558	-0.3598917	0.00099768	0.97511661	0.45221734
Gm12505	-0.0090115	1.77653996	0.00096144	0.97557257	0.45240083
Bhmt2	0.0420317	-0.5181588	0.0009539	0.97566855	0.45241737
Rab8b	-0.0041077	6.34753689	0.00094253	0.97581396	0.45243327
Dtd2	-0.0077934	4.09246295	0.00094179	0.97582348	0.45243327
Rtn4	-0.0039974	9.9353984	0.00092738	0.97600899	0.45249131
Ptprh	-0.0257029	-0.9851058	0.00090729	0.97627031	0.45258449
Casp2	0.00534738	3.86831317	0.00085861	0.97691542	0.45285557
Gpr124	-0.0071395	4.91586024	0.00084403	0.97711227	0.45289373
Pofut2	0.00776766	3.73010993	0.00084357	0.97711849	0.45289373
Ncf1	0.00742692	2.38759849	0.0008363	0.97721723	0.45289844
Icmt	-0.0041857	4.61061729	0.00083394	0.9772494	0.45289844
Atp11a	-0.0042436	6.90085178	0.000825	0.97737163	0.45290072
Tm9sf4	-0.0048997	4.70297856	0.0008245	0.97737846	0.45290072
Nupr1	0.00849184	6.67529031	0.00081711	0.97748004	0.45290072
Gm10509	0.00804274	2.66833887	0.00080857	0.97759802	0.45290072
Slc2a2	-0.0246248	-0.36637	0.00080503	0.97764704	0.45290072
Pfkm	0.00495804	5.87960746	0.00079956	0.9777231	0.45290072
Cmtm6	-0.0089497	4.86642247	0.000798	0.97774486	0.45290072
Rpl5	-0.004918	8.2506935	0.0007934	0.97780914	0.45290072
Gstm7	-0.0076863	4.40010318	0.00079257	0.97782069	0.45290072
A630001G21	-0.0178442	0.62575873	0.00078702	0.97789851	0.45290072
Polh	-0.0074597	2.48231141	0.0007836	0.97794649	0.45290072
Evpl	0.01412037	1.2386368	0.00078131	0.97797884	0.45290072
Fndc3a	-0.0035757	8.08988479	0.00076499	0.97820993	0.45297977

Fyn	0.00326108	6.00425844	0.00075059	0.97841592	0.45304719
Zgpat	0.01026674	1.82987523	0.00074503	0.97849605	0.45305633
Slc18a3	0.0207078	0.02036782	0.00073674	0.97861588	0.45307232
Bcl2a1a	-0.013324	0.16812951	0.0007343	0.97865138	0.45307232
1810021B22I	0.01678912	0.54550243	0.00072704	0.97875721	0.45309335
Zfp691	-0.0097048	2.53282717	0.00071857	0.97888125	0.45312281
Axl	0.00776904	5.27439036	0.00071335	0.97895803	0.45312952
Afg3l1	0.00686436	3.28180885	0.00070618	0.97906405	0.45312952
Gucd1	0.00755323	3.58927075	0.00070113	0.97913907	0.45312952
A330076C08I	0.02283115	0.12744797	0.00070004	0.97915517	0.45312952
Fam133b	-0.0036015	5.13952641	0.00068696	0.97935076	0.45312952
Sap30l	0.00556556	3.93973451	0.00068269	0.97941502	0.45312952
Tyrp1	0.06666081	-1.86409	0.0006816	0.97943155	0.45312952
2610318N02	-0.0012542	-2.0397248	0.00067307	0.97956064	0.45312952
1110032F04I	-0.0082563	2.18019869	0.00067118	0.97958931	0.45312952
Mettl24	-0.0172421	-0.0698367	0.00067031	0.97960252	0.45312952
Cpa2	-0.0165646	-0.4612285	0.00066958	0.97961373	0.45312952
Gng10	0.00615074	4.81533876	0.00066912	0.97962065	0.45312952
Cav1	-0.0063786	6.14573405	0.00065617	0.97981875	0.45319321
Rab39b	-0.0039304	6.74306171	0.00064636	0.97997012	0.45320439
Csf2rb2	-0.0193682	-0.1943771	0.0006437	0.98001137	0.45320439
Gm10790	-0.0200858	-0.5039736	0.00064234	0.98003257	0.45320439
Zfp946	0.00692515	2.87394696	0.000639	0.98008459	0.45320439
Plau	0.01503137	-0.1551436	0.00062237	0.98034525	0.45329698
Atp1a1	0.00397061	7.63614666	0.00061434	0.98047248	0.45332787
1110065P20I	-0.0147823	0.87558483	0.00060336	0.98064773	0.45335896
Mars2	-0.0101611	2.29555454	0.00060247	0.98066206	0.45335896
Fam102a	-0.0038811	5.70098183	0.00059788	0.9807358	0.45335896
Ate1	0.00334319	6.22399955	0.00059505	0.98078147	0.45335896
Gtl3	0.00331569	4.59835817	0.00057536	0.98110196	0.45347916
Smyd4	-0.0063248	2.62051006	0.00057022	0.98118661	0.45349034
Ptrhd1	-0.0047247	3.71099187	0.00056505	0.98127206	0.45350189
D230025D16	-0.0030357	5.65065119	0.00054698	0.98157385	0.45361342
C330018D20I	0.00447172	3.75577288	0.00052238	0.98199293	0.45377913
Klhl38	-0.0155269	0.04729006	0.00051512	0.98211856	0.45380923
Gm15698	-0.0041567	-1.4406106	0.00050383	0.98231551	0.45382207
Ypel1	0.0057929	3.12855469	0.00049625	0.98244904	0.45382207
Pdpk1	-0.0023395	7.65899046	0.00049588	0.98245548	0.45382207
Nedd1	0.00656374	2.72720241	0.00049421	0.98248508	0.45382207
Lrp2bp	-0.0150217	0.78292561	0.00049339	0.98249972	0.45382207
Nudt13	-0.0055138	2.66711577	0.00049284	0.98250935	0.45382207
Slc7a5	0.00427848	4.14321219	0.00048742	0.98260589	0.45383872
Spata13	0.00339549	4.58809525	0.00046627	0.98298742	0.45398698
Lrrn4	0.03059668	-0.7844833	0.00044711	0.98334043	0.45412206
Muc1	0.0149903	-0.6133025	0.00043851	0.98350144	0.45416845

Col13a1	0.00815817	2.28792226	0.00041122	0.98402299	0.45438133
Tmem97	-0.0068638	2.13363819	0.00040674	0.98411022	0.45439363
Rspry1	0.00315905	4.75303855	0.00040055	0.9842317	0.45442175
Manba	0.00706881	2.7862483	0.00039198	0.9844012	0.45447204
Gpr111	-0.0113244	-2.0538352	0.00038286	0.98458373	0.45450288
Specc1l	0.00269424	6.43890441	0.00038259	0.98458917	0.45450288
Gja1	0.00594249	7.63703982	0.00037378	0.98476764	0.45452326
Gm20939	-0.0041263	3.27695277	0.0003734	0.98477532	0.45452326
Zfp629	-0.0037809	4.52124725	0.00037145	0.9848151	0.45452326
Usp45	0.00278031	6.85723407	0.00036559	0.98493529	0.45455076
Ticrr	0.01586032	-0.3232316	0.00034977	0.98526479	0.45467485
Tmeff1	-0.0033519	5.11783559	0.00034582	0.98534822	0.45468538
Nmi	0.00626098	3.19911829	0.00034069	0.9854574	0.45470779
Nynrin	-0.0029605	5.67264852	0.00033124	0.98566038	0.45473375
Cyp2d22	-0.0044851	3.38887593	0.00032967	0.98569458	0.45473375
Daf2	-0.0080055	0.64992593	0.00032741	0.98574353	0.45473375
Atp8b1	0.00509436	2.4639012	0.00032518	0.98579225	0.45473375
Dhrs7	-0.0049098	3.8290547	0.00032406	0.98581676	0.45473375
Psrc1	-0.0069102	1.47407062	0.00031673	0.98597792	0.45476424
Lgr4	0.00260263	5.69464346	0.00031555	0.98600412	0.45476424
Mcu	0.00390617	4.64332139	0.00031262	0.98606936	0.45476637
Dlx6	0.00752842	0.88559198	0.00030656	0.98620488	0.45480091
Alcam	-0.003659	9.49713943	0.00028657	0.98666224	0.45498386
Cd99l2	-0.0030822	5.75979085	0.00027995	0.9868171	0.45500371
Rpusd3	0.00889885	0.91871578	0.00027955	0.9868266	0.45500371
Rslcan18	0.00497468	2.38677572	0.00027567	0.98691826	0.455018
Dnd1	-0.0159297	-1.5052537	0.00027234	0.98699752	0.45502658
Gm6537	-0.0178097	-1.9734939	0.00025683	0.98737325	0.45517182
Gars	0.00182842	6.01948736	0.00025412	0.98744014	0.45517468
4933408B17I	0.04697398	-1.1572245	0.00024395	0.98769377	0.45523687
Slc16a2	0.00232975	5.39027333	0.00024385	0.98769641	0.45523687
Gm10354	-0.0356687	-2.0395813	0.00024077	0.98777432	0.4552448
Acot4	0.01131888	0.0646272	0.00023118	0.98802026	0.45533017
B3gnt7	-0.0189358	-0.8629206	0.00022523	0.98817547	0.45537373
Fbxw11	-0.0018291	7.36807432	0.00021178	0.98853398	0.45549608
Slc35b4	-0.0019404	5.64591633	0.00021073	0.98856242	0.45549608
Ly6c2	0.01247839	-0.5892945	0.00019666	0.98895072	0.45564701
Gnl3l	0.00169514	8.04128811	0.00019319	0.98904874	0.45566418
Plat	0.0038297	4.7598382	0.0001875	0.98921112	0.455711
Sp5	0.00968975	1.50954197	0.0001791	0.9894555	0.45579559
Wapal	-0.001356	7.56975924	0.00017001	0.98972662	0.45588538
Inppl1	-0.0028428	4.24130284	0.00016851	0.98977197	0.45588538
Clec12a	0.01485553	-0.500665	0.00016112	0.98999874	0.45596183
Pcyt2	0.00470993	1.66845225	0.0001561	0.99015571	0.45598206
Kcnh2	0.00394524	2.32121856	0.00015583	0.99016422	0.45598206

Zfp953	0.00334309	2.98051226	0.00014677	0.99045467	0.45608781
Atf1	-0.0018026	7.58170167	0.00014243	0.99059664	0.45612519
Gadd45b	0.00294449	2.14979483	0.00013752	0.99076009	0.45617245
Lemd2	-0.0038139	2.11383672	0.00013214	0.99094265	0.4562285
Trappc6a	0.00624657	1.50051565	0.00012623	0.99114778	0.45629493
Tmem104	0.0023779	3.95676381	0.00011943	0.99138929	0.45635511
Adnp	0.00137434	7.86024029	0.00011877	0.99141307	0.45635511
Vsx1	-0.0033474	3.86348811	0.00011603	0.99151261	0.45635511
Alkbh4	0.00426656	1.04714267	0.00011578	0.99152184	0.45635511
Mnda	0.00758976	-0.6338785	0.00011318	0.99161775	0.45636039
Avp	-0.0123658	-1.658286	0.00011217	0.99165498	0.45636039
Zfp940	-0.0025314	3.31935635	0.00010767	0.99182432	0.45641031
Asic2	0.00176878	5.16739371	0.00010537	0.9919119	0.45641343
Mycl	0.00300151	2.03148345	0.00010431	0.99195277	0.45641343
Tmem74	0.00394452	1.24584302	9.93E-05	0.99214945	0.45647592
Ftsj3	0.00161353	5.00058319	9.58E-05	0.9922878	0.45648888
Rps27	0.00519275	-0.165867	9.55E-05	0.99229933	0.45648888
Fam217a	-0.0086815	-0.6141347	9.33E-05	0.99239009	0.4564902
Myo1g	0.00780221	0.13617966	9.12E-05	0.99247711	0.4564902
Wdr36	-0.0018049	3.83820002	9.10E-05	0.99248475	0.4564902
Cdh23	-0.0085755	-1.1264785	8.61E-05	0.99268803	0.45653049
Rmdn3	0.0016609	3.79942027	8.60E-05	0.99269408	0.45653049
Gng13	0.00355376	1.38099842	7.77E-05	0.99305293	0.45663698
Hinfp	-0.0027708	1.97206335	7.66E-05	0.99310499	0.45663698
Epb4.1l3	0.00116017	7.79596914	7.62E-05	0.99312317	0.45663698
Bahd1	0.00215515	4.09485007	7.52E-05	0.99316913	0.45663698
Lca5	0.0017643	4.18042977	7.06E-05	0.99337782	0.4566846
Crip3	-0.0566119	-1.9973046	6.98E-05	0.99341832	0.4566846
Ier3ip1	-0.0014064	5.53606747	6.77E-05	0.99351726	0.4566846
Chrna1	-0.0030476	2.07646178	6.76E-05	0.9935218	0.4566846
Avil	-0.0043266	-1.4523535	6.54E-05	0.99362626	0.4566846
Pex14	0.00231798	2.54592797	6.49E-05	0.99365359	0.4566846
Kiss1r	-0.00457	0.15055536	6.40E-05	0.99369887	0.4566846
Vangl2	-0.002404	1.96473114	5.83E-05	0.99398641	0.45677056
Zfp931	0.00181721	2.57628972	5.74E-05	0.99402827	0.45677056
Rangrf	-0.0021077	2.89797601	5.59E-05	0.99410923	0.45677056
Mfsd12	-0.0058094	-1.2930054	5.55E-05	0.99412948	0.45677056
Lrrc17	0.00435527	0.06156484	5.20E-05	0.99431789	0.45678356
Zkscan1	-0.0008506	7.6698238	5.17E-05	0.99433733	0.45678356
Phf3	0.00085528	8.18853207	5.16E-05	0.99434045	0.45678356
Adrb2	-0.0030852	2.24218647	4.77E-05	0.99455797	0.45685551
Mtfr1	-0.001764	3.94927546	4.01E-05	0.99501081	0.45700679
Gc	-0.208445	-2.7059228	3.95E-05	0.99505763	0.45700679
Gm13308	0.00785033	-1.3918595	3.91E-05	0.99507007	0.45700679
Prdm2	0.00083079	7.02812682	3.46E-05	0.99536695	0.45711515

Rhot1	0.00074504	6.11619502	2.93E-05	0.99573468	0.45725603
Tm9sf2	-0.0008088	5.92029723	2.81E-05	0.99582376	0.45726894
Nudcd3	-0.000586	6.41496489	2.68E-05	0.99591991	0.4572851
Prss8	-0.0400536	-2.1549414	2.27E-05	0.99624958	0.45738304
Pcdhga4	-0.0016477	1.68857709	2.23E-05	0.99628014	0.45738304
Ppib	0.00125589	2.66338172	2.19E-05	0.99631614	0.45738304
Hps4	0.00133381	2.44925576	1.82E-05	0.9966416	0.45749342
Brdt	-0.0008739	4.23195234	1.78E-05	0.99667854	0.45749342
Cpne7	0.0010448	2.63343277	1.23E-05	0.99724184	0.45772397
Fbxo18	0.00043182	5.50909359	1.11E-05	0.99737527	0.4577326
E030018B13I	-0.0165065	-1.4538829	1.10E-05	0.99738267	0.4577326
Serinc3	0.00049156	8.71528027	9.01E-06	0.99763447	0.45781209
Abhd10	0.00054874	4.33952825	8.68E-06	0.99767796	0.45781209
Rgs1	-0.0030932	-0.295751	7.13E-06	0.99789642	0.45788433
Eln	-0.0006808	2.43103705	4.64E-06	0.99830285	0.4580428
Alx4	0.00051912	5.70558227	4.12E-06	0.99840037	0.45805953
Asb5	-0.000997	0.5376072	3.29E-06	0.99857034	0.45809747
Ccdc37	-0.0012482	0.91688166	3.13E-06	0.99860521	0.45809747
Gpr62	-0.0008429	0.89676421	1.76E-06	0.99895401	0.45822946
Cyp4b1	6.78E-05	-1.0150473	1.51E-06	0.99903171	0.45823708
Retsat	-0.0002841	2.56790834	7.99E-07	0.99929583	0.4583302
Pdgfra	7.23E-05	5.49716989	1.35E-07	0.99971049	0.45847635
Cwf19l1	8.98E-05	3.90492963	1.12E-07	0.99973672	0.45847635
