# Supplementary material for: Mapping the physiological and molecular markers of stress and SSRI antidepressant treatment in S100a10 corticostriatal neurons
Source: Mol Psychiatry. 2019 Aug 20;25(5):1112–29. doi: 10.1038/s41380-019-0473-6 (PMC7031043; doi:10.1038/s41380-019-0473-6)
Supplement: Supplementary file 19 — Supplemental Table S3 [file 41380_2019_473_MOESM19_ESM.pdf]

Suppl Table S3. List of genes (389) affected by single housing and normalized by Flx.

symbol	Sh_logFC	Sh_FDR	Flx_vs_Sh_logFC	Flx_vs_Sh_FDR	max_FDR
Prl	11.49910345	0.007517013	-6.912934967	0.001298739	0.007517013
Nrbf2	-1.019413645	0.007517013	0.689274374	0.004692414	0.007517013
Gm561	-1.139087412	0.015919177	1.093780376	0.001299161	0.015919177
Grin1os	3.00906483	0.018063529	-2.024987962	0.011228166	0.018063529
Cox5b	-0.915688063	0.018063529	0.87574177	0.001916545	0.018063529
Pik3c2b	0.898244241	0.018063529	-0.864075918	0.00191865	0.018063529
Lyrm7	-0.874026787	0.018063529	0.639439994	0.007062683	0.018063529
Gng3	-0.727457428	0.018063529	0.734158607	0.001650257	0.018063529
6330403K07I	-0.666845126	0.018063529	0.667483705	0.0016475	0.018063529
Snx2	-0.650327193	0.018063529	0.663114197	0.001651308	0.018063529
1110059E24I	-0.68689767	0.018063529	0.609825452	0.003338413	0.018063529
Stmn1	-0.72193538	0.018063529	0.537528996	0.008568601	0.018063529
Gm14295	-0.60422602	0.018063529	0.474511315	0.006296393	0.018063529
Thap11	-0.807208224	0.019908727	0.709034554	0.004486232	0.019908727
Fam160b2	0.724333634	0.019908727	-0.581391619	0.007456026	0.019908727
Mllt11	-0.558084651	0.022123525	0.48227842	0.005528225	0.022123525
Mtap7d3	-2.251970079	0.019908727	1.292750335	0.02268552	0.02268552
Coa3	-0.831824964	0.023025115	0.864740942	0.002168318	0.023025115
Pitpnm2os1	4.552491921	0.024425128	-2.657182359	0.016671909	0.024425128
1700028P14I	-2.298177072	0.024425128	2.040272983	0.005354402	0.024425128
Nrn1	-0.65070423	0.024425128	0.495212594	0.010562436	0.024425128
Med23	0.783080701	0.024732786	-1.036940491	0.000599232	0.024732786
Plxnd1	0.829734252	0.024732786	-0.785896486	0.004434559	0.024732786
Mrps33	-0.596326309	0.024732786	0.689005734	0.001650257	0.024732786
Tnnt2	-1.215603133	0.024887125	1.520098546	0.001025121	0.024887125
Nrgn	-0.692025293	0.018139125	0.39553488	0.025483204	0.025483204
Ppp2r2cos	3.09993696	0.025985692	-2.546212244	0.007977206	0.025985692
Orc1	1.993722944	0.025985692	-1.610011037	0.008612672	0.025985692
AA413626	6.642325864	0.027150847	-8.113426445	0.00271372	0.027150847
Sh3bgr	-7.096315235	0.027150847	5.772729952	0.009832201	0.027150847
Gm5464	2.941803397	0.027150847	-2.494985798	0.00774449	0.027150847
Sema4g	1.427395909	0.027150847	-1.196407868	0.011098618	0.027150847
Erbb4	0.940336147	0.027150847	-1.321136078	0.000599232	0.027150847
Ptchd4	0.972181831	0.027150847	-1.07112972	0.00271372	0.027150847
Bmyc	-0.802822885	0.027150847	1.013174898	0.001298739	0.027150847
Bzw2	0.908026421	0.027150847	-0.865940713	0.005006121	0.027150847
Medag	-0.543892055	0.027150847	1.03924865	6.25E-05	0.027150847
Efhd2	-0.641404108	0.027150847	0.731073599	0.002045772	0.027150847
Ndufa6	-0.719746194	0.027150847	0.606512565	0.008846654	0.027150847
Adam10	0.671152141	0.027150847	-0.593877998	0.006898519	0.027150847
Med24	0.737457119	0.027150847	-0.47743458	0.025435928	0.027150847
Slc35b1	-0.882963786	0.029344832	0.68858569	0.01324405	0.029344832
Nfrkb	0.792856856	0.030000852	-0.66774047	0.009832201	0.030000852
Snca	-0.607474247	0.019908727	0.340290767	0.030723143	0.030723143
Adamts3	0.996095905	0.031599729	-0.943145584	0.00740694	0.031599729

Cycs	-0.683619131	0.031599729	1.081834733	0.00034486	0.031599729
Katnbl1	0.950781668	0.031817311	-0.630074784	0.026743328	0.031817311
Sfi1	1.411313503	0.031997119	-1.450727886	0.004391538	0.031997119
Gosr2	-0.523967654	0.032144325	0.547307194	0.004192998	0.032144325
BC048546	-0.858224522	0.023025115	0.479193532	0.032404834	0.032404834
Gpr155	0.819755922	0.030000852	-0.514322507	0.032869738	0.032869738
Gabrq	1.520300305	0.033576392	-2.024702223	0.001648507	0.033576392
Ryr1	1.533491336	0.033819954	-2.511964215	0.000389346	0.033819954
Bora	1.862743432	0.033819954	-1.906277313	0.004006895	0.033819954
AB041803	1.925657475	0.033819954	-1.551342453	0.013954322	0.033819954
Gm20748	-1.885394215	0.033819954	1.576922358	0.011098618	0.033819954
Gjd2	1.509785218	0.033819954	-1.880190261	0.00204219	0.033819954
Vwa5b2	1.301115276	0.033819954	-1.69873853	0.001650257	0.033819954
Emd	-0.641886881	0.033819954	1.898527286	3.68E-07	0.033819954
Rilpl2	-1.245192414	0.033819954	1.130147703	0.008262925	0.033819954
Col11a1	1.088097468	0.033819954	-1.145061695	0.004150298	0.033819954
Gldc	1.161581553	0.033819954	-0.989837083	0.012567203	0.033819954
Me3	1.061819569	0.033819954	-0.779597152	0.022840794	0.033819954
Mapre3	-0.56356468	0.033819954	0.799461008	0.000986503	0.033819954
Tnrc18	0.578488845	0.033819954	-0.545970864	0.007023658	0.033819954
Gm16039	-0.63052853	0.033819954	0.415056718	0.031238833	0.033819954
Strn	0.470547377	0.033819954	-0.499576441	0.004391178	0.033819954
Zmym3	0.758036412	0.034179293	-0.955666096	0.002056357	0.034179293
Tagln3	-0.681976436	0.034179293	0.709927449	0.004949048	0.034179293
Nxf7	-3.83124101	0.034275592	3.248373614	0.012403407	0.034275592
4933413L06F	2.71390537	0.034275592	-2.877048177	0.004718282	0.034275592
E330023G01	1.776715465	0.034275592	-2.184563885	0.002985108	0.034275592
Rinl	-1.872813735	0.034275592	1.976532485	0.004593423	0.034275592
Syndig1l	1.318764596	0.034275592	-0.932101732	0.023709683	0.034275592
Mrpl12	-1.037517334	0.034275592	0.889431298	0.011098618	0.034275592
Pkd1	0.857717516	0.034275592	-0.941331149	0.004434559	0.034275592
Abca5	0.856664229	0.034275592	-0.922718972	0.004274122	0.034275592
Cntn3	0.783628042	0.034275592	-0.860769607	0.004099687	0.034275592
Zfp746	-0.67458836	0.034275592	0.451241574	0.031446693	0.034275592
Atf4	-0.522774202	0.034275592	0.594198604	0.003338413	0.034275592
Vti1b	-0.630728748	0.034275592	0.453771754	0.024165212	0.034275592
C430049B03I	-2.044899592	0.035248898	1.373392288	0.02953153	0.035248898
Leng8	1.07045333	0.035248898	-1.346646547	0.002186558	0.035248898
Mdm1	1.091419266	0.035248898	-1.266007904	0.004006895	0.035248898
Ets1	-0.736966306	0.035248898	0.904074778	0.002264101	0.035248898
Slc4a4	0.54285964	0.035248898	-0.615032395	0.003596646	0.035248898
Cotl1	-0.953524121	0.035376764	1.035586775	0.004391538	0.035376764
Ccdc43	-0.707168361	0.035376764	0.61566714	0.013170407	0.035376764
Fam19a3	-7.5213543	0.035603784	7.903549525	0.006041857	0.035603784
Atp1a4	3.381762249	0.035603784	-2.416672323	0.020598576	0.035603784
Shcbp1l	2.888566243	0.035603784	-2.397798083	0.01288825	0.035603784

Il1rap	0.848026405	0.035603784	-1.09216321	0.002045772	0.035603784
Ptprz1	0.74679071	0.035603784	-1.110311301	0.001188689	0.035603784
Ndufa11	-0.803746517	0.035603784	0.951876155	0.003277847	0.035603784
Ano4	0.83249707	0.035603784	-0.591721852	0.027768531	0.035603784
Trnau1ap	-0.76406936	0.035603784	0.658726133	0.012900226	0.035603784
Msl3	-0.597087168	0.035603784	0.528686309	0.011583639	0.035603784
Rap2a	-0.423294075	0.035995184	0.399162265	0.009408339	0.035995184
Psmd6	-0.574548391	0.03605736	0.658443801	0.003826237	0.03605736
F420014N23	1.867307302	0.036179983	-2.190829413	0.003338413	0.036179983
Fam184b	1.371263529	0.036179983	-1.140223026	0.017234903	0.036179983
Usp29	0.73619797	0.036179983	-1.285045839	0.00034486	0.036179983
Adcy5	0.464246207	0.036179983	-0.400576794	0.013544602	0.036179983
Skap2	-0.938903652	0.024425128	0.518005636	0.036341025	0.036341025
4930579G24	1.077039484	0.036937252	-1.142356799	0.005827316	0.036937252
Agrn	0.838595417	0.036937252	-1.200503414	0.001650257	0.036937252
Nelfe	-1.00515148	0.036937252	0.946245465	0.009848229	0.036937252
Ndufs4	-0.461324475	0.036937252	0.514361526	0.004718282	0.036937252
Ccpg1os	-0.974436273	0.037092741	0.954649704	0.008746989	0.037092741
Tmem136	0.900026747	0.037092741	-0.766001953	0.016403071	0.037092741
Higd2a	-0.71872029	0.037092741	0.594578161	0.017159788	0.037092741
Chmp4b	-0.477543631	0.037092741	0.462648288	0.009085263	0.037092741
Mpc1	-0.519238687	0.037092741	0.404822979	0.021378417	0.037092741
Stx1a	-0.841250126	0.025985692	0.472242618	0.037325094	0.037325094
Pcnx	0.646378453	0.038068542	-0.583315029	0.012403407	0.038068542
Bag5	-0.563253321	0.038225577	0.742714618	0.002091788	0.038225577
Ncapd3	0.930550649	0.038311294	-0.895349865	0.009408339	0.038311294
Muc6	2.013940768	0.038367421	-1.600939832	0.015852724	0.038367421
Kif26b	1.312615056	0.038367421	-1.539383197	0.005352798	0.038367421
Rab5a	-0.511636872	0.038367421	0.379704549	0.026750462	0.038367421
Rtl1	1.289104009	0.03848806	-2.213970707	0.000556276	0.03848806
Bambi	-1.959233129	0.03848806	1.433576443	0.028445829	0.03848806
Actn2	1.129101307	0.03848806	-1.406001348	0.002924215	0.03848806
Hscb	-1.172030846	0.03848806	1.132775528	0.008993761	0.03848806
Mief1	-0.791847069	0.03848806	0.550730988	0.033712643	0.03848806
Dnajc30	-0.664389634	0.03848806	0.620542427	0.011003972	0.03848806
Cabin1	0.602800318	0.03848806	-0.404717835	0.038099477	0.03848806
Stmn2	-0.430122031	0.03848806	0.417944222	0.009376393	0.03848806
0610009L18I	-1.954680212	0.024425128	1.043491449	0.03892322	0.03892322
Bcas1	0.86828343	0.039060622	-1.329461904	0.001298739	0.039060622
Chaf1b	-6.534914212	0.039095331	5.831989895	0.013605575	0.039095331
Batf2	-4.604963192	0.039095331	3.065652796	0.035325188	0.039095331
Gm16907	2.4714276	0.039095331	-2.433333243	0.009245241	0.039095331
Zar1l	-2.341862329	0.039095331	2.070302407	0.015020882	0.039095331
Sntb1	-1.38708532	0.039095331	1.894929735	0.001937745	0.039095331
Poll	-1.711868228	0.039095331	1.405574108	0.019130969	0.039095331
Ftx	1.035323586	0.039095331	-2.056682696	0.000192716	0.039095331

Snrnp25	-1.395824059	0.039095331	1.633639663	0.004679798	0.039095331
Gpaa1	1.628851641	0.039095331	-1.313368385	0.019683142	0.039095331
Meg3	1.094498103	0.039095331	-1.702765674	0.001259044	0.039095331
Lrp1b	1.073700731	0.039095331	-1.218119794	0.005576138	0.039095331
Tmem67	1.009893198	0.039095331	-0.972391052	0.010688436	0.039095331
Dnah7b	0.831352591	0.039095331	-1.140566647	0.001651308	0.039095331
Utp11l	-0.848635915	0.039095331	0.816005602	0.011098618	0.039095331
Gbp10	-0.812935306	0.039095331	0.838318038	0.008911482	0.039095331
Nat10	0.814956202	0.039095331	-0.767360769	0.011554203	0.039095331
Pnisr	0.789667086	0.039095331	-0.747670268	0.012035505	0.039095331
Exosc1	-0.784982642	0.039095331	0.717723115	0.013858897	0.039095331
Nwd1	0.752858479	0.039095331	-0.737834275	0.010336839	0.039095331
Lrrtm4	0.683603172	0.039095331	-0.700659276	0.008122805	0.039095331
Pcnxl3	0.619376275	0.039095331	-0.763236006	0.003578872	0.039095331
Dgke	0.724409479	0.039095331	-0.622309593	0.018991006	0.039095331
Fam199x	0.652109448	0.039095331	-0.690007659	0.007220111	0.039095331
Mospd1	-0.686275159	0.039095331	0.650877994	0.010995127	0.039095331
Tial1	0.545371513	0.039095331	-0.78871646	0.001608793	0.039095331
Myo9b	0.706560198	0.039095331	-0.613444169	0.016519325	0.039095331
Cnrip1	-0.535698461	0.039095331	0.726665418	0.001990163	0.039095331
Smpd4	0.721487708	0.039095331	-0.525882178	0.031286318	0.039095331
Rheb	-0.579172317	0.039095331	0.667445315	0.004845377	0.039095331
Dzip3	0.682550409	0.039095331	-0.554535887	0.022065543	0.039095331
Pomp	-0.642471836	0.039095331	0.560966353	0.016532923	0.039095331
Arhgap39	0.659101126	0.039095331	-0.537788281	0.020521416	0.039095331
4833420G17	0.66559179	0.039095331	-0.455777621	0.03563708	0.039095331
Rab36	0.648813739	0.039095331	-0.45536722	0.033611995	0.039095331
2610301B20	-0.558559512	0.039095331	0.509969625	0.013518739	0.039095331
Ndufa12	-0.610628259	0.039095331	0.45248132	0.028937947	0.039095331
Ppp2ca	-0.403523142	0.039095331	0.601181647	0.001298739	0.039095331
Tceal6	-0.546087075	0.039095331	0.437220985	0.022501016	0.039095331
Zmat2	-0.5001579	0.039095331	0.420004785	0.019213966	0.039095331
Ccdc47	0.470891369	0.039095331	-0.331212903	0.034310845	0.039095331
Tspan33	-1.564724107	0.039363477	1.146853237	0.031286318	0.039363477
Grwd1	-1.545682823	0.039363477	1.116715802	0.033306375	0.039363477
Pisd-ps1	1.181273193	0.03938931	-1.61653333	0.002176856	0.03938931
Ube4a	0.5889815	0.03938931	-0.592785224	0.009559987	0.03938931
C030046E11	0.590280644	0.03938931	-0.464635515	0.024824809	0.03938931
Ufd1l	-0.439715542	0.03938931	0.55075987	0.003359824	0.03938931
Atp6v1g2	-0.48199295	0.03938931	0.448848028	0.013012475	0.03938931
Pcdhga3	0.82716633	0.03940335	-1.197762723	0.002168318	0.03940335
Sumo1	-0.663184705	0.03940335	0.67049808	0.010170589	0.03940335
Usp24	0.623337288	0.039459588	-0.702699199	0.006296393	0.039459588
Cfl1	-0.484442534	0.039459588	0.511067813	0.008061635	0.039459588
Banf1	-0.840977682	0.039095331	0.57782045	0.039471095	0.039471095
H19	-4.98016568	0.039550782	4.848037614	0.011304213	0.039550782

Svep1	1.107221336	0.039550782	-0.987585024	0.015991288	0.039550782
Lhpp	-1.045953487	0.039550782	1.020880626	0.010995127	0.039550782
Nob1	-1.011022612	0.039550782	0.95169365	0.0125932	0.039550782
Chrac1	-1.068636653	0.039550782	0.839669034	0.024651958	0.039550782
Med31	-0.862476584	0.039550782	0.977377934	0.005987738	0.039550782
Lmo7	0.980841464	0.039550782	-0.850601413	0.018530868	0.039550782
Rfc2	-0.982799877	0.039550782	0.822996752	0.020861355	0.039550782
Med12	0.743694649	0.039550782	-0.942279671	0.003338413	0.039550782
Taf10	-0.780126537	0.039550782	0.900693067	0.005781534	0.039550782
Ift22	-0.801277697	0.039550782	0.732118201	0.015845459	0.039550782
Srfbp1	-0.764118782	0.039550782	0.736613333	0.011820351	0.039550782
Cyb5	-0.724309152	0.039550782	0.70512883	0.011866797	0.039550782
Fancm	0.752381184	0.039550782	-0.674618416	0.01757078	0.039550782
Cln8	0.709996959	0.039550782	-0.677700818	0.012166448	0.039550782
Ndufa8	-0.570861052	0.039550782	0.701050422	0.004274122	0.039550782
Ift20	-0.617053039	0.039550782	0.62407317	0.009832201	0.039550782
Dynlt3	-0.578580036	0.039550782	0.636747758	0.007273831	0.039550782
Arl6	-0.580842839	0.039550782	0.612230562	0.008993761	0.039550782
Anapc1	0.499086866	0.039550782	-0.687777837	0.002264101	0.039550782
Prrc1	-0.588056815	0.039550782	0.58144325	0.01052111	0.039550782
Pign	0.671126625	0.039550782	-0.486906852	0.033805771	0.039550782
D630045J12F	0.565520951	0.039550782	-0.569114827	0.01052111	0.039550782
Psmb1	-0.514686709	0.039550782	0.597196691	0.005528225	0.039550782
Haus2	-0.533301143	0.039550782	0.563288708	0.008056926	0.039550782
Ensa	-0.50622745	0.039550782	0.525063199	0.009158252	0.039550782
Ptprk	0.489895948	0.039550782	-0.52669554	0.007840571	0.039550782
Cplx1	-0.416517572	0.039550782	0.32164781	0.028933971	0.039550782
Fam178a	0.424355891	0.039570981	-0.329508257	0.027976367	0.039570981
Plcg1	0.654052773	0.039978462	-0.731220058	0.00667274	0.039978462
Glt8d1	0.727960974	0.039990206	-0.981596581	0.00271372	0.039990206
Mbp	0.774035978	0.040189766	-1.074118539	0.002456409	0.040189766
Atg5	-0.58480138	0.040189766	0.50255417	0.019683142	0.040189766
2010015L04F	1.033982166	0.033819954	-0.626138669	0.040333118	0.040333118
Polrmt	1.128371318	0.040336509	-1.026175541	0.015461689	0.040336509
Tpd52l1	-0.764309429	0.040383836	0.835786492	0.008993761	0.040383836
Chchd6	-0.855246191	0.040383836	0.640884758	0.033157689	0.040383836
Ep400	0.704631313	0.040383836	-0.75296554	0.008721891	0.040383836
Plin4	2.274618221	0.035248898	-1.357676512	0.040570093	0.040570093
Abcg1	0.69948973	0.040977592	-0.750366512	0.009158252	0.040977592
D130043K22I	1.033041878	0.040189766	-0.723596237	0.041039778	0.041039778
Gm16675	-5.617806098	0.041153393	6.576809534	0.005987738	0.041153393
Tmem26	6.164635837	0.041153393	-4.718137894	0.018530868	0.041153393
Slc2a4rg-ps	1.704809736	0.041153393	-1.86001789	0.00740694	0.041153393
Cstb	-0.743596237	0.041153393	2.464245859	7.51E-07	0.041153393
Trrap	0.847017927	0.041153393	-0.877569934	0.01028441	0.041153393
Rpgr	0.858925268	0.041153393	-0.8501392	0.012542302	0.041153393

Eif3k	-0.785746269	0.041153393	0.850511255	0.008503474	0.041153393
Ccer1	7.191934904	0.04155866	-7.223928912	0.008348062	0.04155866
Gm16853	3.867368432	0.041701618	-2.890997173	0.038016984	0.041701618
Rasl11b	-0.619736983	0.039095331	0.414054612	0.041939768	0.041939768
Ube2b	-0.449869839	0.042122471	0.381382071	0.022862867	0.042122471
Gm16523	1.482176745	0.042227443	-2.123020873	0.002498886	0.042227443
Rgs9	0.909287339	0.042227443	-1.216542718	0.003994905	0.042227443
Tia1	0.775429015	0.042227443	-0.654082964	0.02324221	0.042227443
Rp9	-0.734281501	0.042227443	0.561325401	0.033157689	0.042227443
Gabarapl1	-0.481069255	0.042227443	0.634514578	0.003410248	0.042227443
Cox5a	-0.508910891	0.042227443	0.603119209	0.005827316	0.042227443
Cisd1	-0.558117821	0.042227443	0.464840287	0.024512648	0.042227443
Timm8b	-0.552725089	0.042227443	0.43099244	0.031245658	0.042227443
Pak1	-0.422022904	0.042227443	0.324467669	0.032905195	0.042227443
Csrp2	-0.700719602	0.042346541	1.031135124	0.002045772	0.042346541
Slc46a1	1.33948036	0.042375447	-1.099802753	0.024021694	0.042375447
Pou4f1	-7.427482906	0.042405465	7.297143136	0.012796843	0.042405465
Dnah1	0.963265516	0.042405465	-0.88303233	0.016519325	0.042405465
1700021F05I	-0.574434434	0.042405465	0.579474445	0.011419732	0.042405465
Ywhah	-0.37108848	0.042405465	0.375733937	0.011481361	0.042405465
Ypel3	-0.453396927	0.042432377	0.363084454	0.028696373	0.042432377
Tbrg3	1.021424942	0.042529096	-1.772305534	0.00123253	0.042529096
Tmem251	-0.766604351	0.042529096	0.833142418	0.008835803	0.042529096
Trmt112	-0.577037696	0.042529096	0.581879288	0.011913278	0.042529096
Cend1	-0.445564658	0.042529096	0.5554224	0.004718282	0.042529096
Lrba	0.642321402	0.042822241	-0.877404753	0.002924215	0.042822241
Zyx	-0.519825969	0.043179994	0.868203471	0.001025121	0.043179994
Acbd3	-0.430943115	0.04332218	0.453178872	0.010747499	0.04332218
Adra1a	0.689357619	0.043411571	-0.647466103	0.015845459	0.043411571
Oxt	-3.359410479	0.024425128	1.730170731	0.043428667	0.043428667
Ddn	0.497845012	0.043614562	-0.715591027	0.002258197	0.043614562
Gm10677	1.790034147	0.043658584	-1.511192936	0.024082937	0.043658584
Sptbn4	1.423130093	0.043658584	-1.391810542	0.014422526	0.043658584
Chrd	1.087440147	0.043658584	-1.210922328	0.0078438	0.043658584
Eif1b	-0.51382531	0.043658584	0.578959314	0.008525424	0.043658584
Rnf185	-0.541230361	0.043658584	0.548599749	0.011968664	0.043658584
Gabpa	-0.481070794	0.043658584	0.572883851	0.006106932	0.043658584
Bcan	0.939105039	0.043763464	-1.266298004	0.005521076	0.043763464
Cdan1	0.784107834	0.043763464	-1.053264663	0.003277847	0.043763464
Fgf16	6.540207923	0.044050805	-4.393314662	0.018241344	0.044050805
Gm19990	-4.699983956	0.044050805	4.342281956	0.017473686	0.044050805
4930449E18I	3.279703566	0.044050805	-2.785679787	0.019239476	0.044050805
Serpinc1	2.413158612	0.044050805	-2.908917049	0.006108443	0.044050805
Rgsl1	1.514166881	0.044050805	-1.583073046	0.010505556	0.044050805
Thsd7b	1.622548005	0.044050805	-1.298739345	0.028100258	0.044050805
Trank1	1.066961503	0.044050805	-1.474032828	0.003338413	0.044050805

2310047M10	-1.049506874	0.044050805	1.227745112	0.00740694	0.044050805
Kbtbd8	0.868127325	0.044050805	-1.173470252	0.003082402	0.044050805
Pcdhb3	1.131986029	0.044050805	-0.887821431	0.030072291	0.044050805
Dtwd2	1.043889909	0.044050805	-0.732465549	0.041672086	0.044050805
Ubr4	0.825933891	0.044050805	-0.902028338	0.009158252	0.044050805
Ahi1	0.698783432	0.044050805	-0.826128584	0.006623643	0.044050805
Atr	0.70823714	0.044050805	-0.642601715	0.018779445	0.044050805
Vps28	-0.550711336	0.044050805	0.769107393	0.002910191	0.044050805
Serac1	0.757184629	0.044050805	-0.54517784	0.043802031	0.044050805
Simc1	0.59016457	0.044050805	-0.706421192	0.005987738	0.044050805
Bud31	-0.613489189	0.044050805	0.669165514	0.009185552	0.044050805
Ppia	-0.607511382	0.044050805	0.674525496	0.008870903	0.044050805
2310022B05	-0.522001805	0.044050805	0.749209286	0.002468976	0.044050805
Atad2b	0.594249609	0.044050805	-0.563199394	0.016322751	0.044050805
Leo1	-0.462295094	0.044050805	0.59378266	0.004486232	0.044050805
Nicn1	-0.428771052	0.044050805	0.540299057	0.004949048	0.044050805
Med12l	0.898108952	0.044183624	-1.14283408	0.005114735	0.044183624
Ndufab1	-0.513797231	0.044183624	0.80660545	0.001650273	0.044183624
Atp7a	0.580471175	0.044183624	-0.692181669	0.006460544	0.044183624
Nup214	0.56567745	0.044183624	-0.598644195	0.010597092	0.044183624
Atpaf1	-0.484978204	0.044183624	0.393892986	0.030072291	0.044183624
Ttn	1.075404597	0.044351217	-1.129532804	0.010505556	0.044351217
Notch1	1.154133765	0.044475524	-1.508323859	0.004486232	0.044475524
Bmpr1b	0.831806428	0.044475524	-1.02693935	0.005528225	0.044475524
4931430N09	3.144131341	0.027150847	-1.513173078	0.044825864	0.044825864
1700009P17	-1.45776484	0.045067646	1.270579563	0.022318844	0.045067646
Mir872	-5.565773764	0.045377012	4.556666772	0.028937947	0.045377012
Pcdhb22	0.987826162	0.045377012	-1.309113261	0.004679798	0.045377012
Plekhg5	0.629737453	0.045377012	-0.879303121	0.003103288	0.045377012
Fstl4	0.761622647	0.045377012	-0.72597621	0.016322751	0.045377012
Hprt	-0.421611474	0.045377012	0.531645472	0.005352798	0.045377012
Clybl	-0.693373104	0.045433003	0.535813347	0.036341025	0.045433003
F630111L10	1.369530568	0.043763464	-0.973310086	0.045606874	0.045606874
C1qbp	-0.461820607	0.045635971	0.49503415	0.010497183	0.045635971
Celsr3	1.437022596	0.045853434	-1.851162276	0.005528225	0.045853434
Bri3bp	0.557566703	0.045853434	-0.617112067	0.010562436	0.045853434
Ralyl	-0.514579753	0.034179293	0.302905812	0.046084234	0.046084234
Gabrb1	0.90320498	0.035603784	-0.545761735	0.046256774	0.046256774
AI462493	-1.280318789	0.035603784	0.777305272	0.046262541	0.046262541
Cdca3	-4.573873592	0.039095331	2.896574686	0.046331423	0.046331423
5830418K08	0.780990246	0.046348053	-0.748858913	0.01663447	0.046348053
Snapc5	-0.67096989	0.046453529	0.499394637	0.043085256	0.046453529
Fam103a1	-0.49885767	0.046453529	0.500025419	0.014308387	0.046453529
4930419G24	1.642186676	0.046465693	-1.503415808	0.020967517	0.046465693
Omd	0.574760148	0.04658219	-0.988928411	0.001137218	0.04658219
1110008L16	-0.542689681	0.046671254	0.545872596	0.013598567	0.046671254

Casp4	-1.673334321	0.046744401	1.635088817	0.016048644	0.046744401
Tnc	1.360484431	0.046744401	-1.136074648	0.026206166	0.046744401
Isoc2a	-1.101074058	0.046744401	1.02151444	0.01924992	0.046744401
Nckap5	0.909137929	0.046744401	-1.090447672	0.008503474	0.046744401
Gabrg1	0.913789234	0.046744401	-0.998337476	0.011196289	0.046744401
Gm16702	0.784705232	0.046744401	-1.085111865	0.003338413	0.046744401
Nup155	0.645443164	0.046744401	-0.976443462	0.002131753	0.046744401
Myd88	-1.227251823	0.046918597	1.253875747	0.01324405	0.046918597
Timeless	-2.047427386	0.039550782	1.339051773	0.046984064	0.046984064
Cox14	-0.574850875	0.043658584	0.402576836	0.047214524	0.047214524
Calcr	2.560135037	0.047375008	-1.846126994	0.040547144	0.047375008
Trim56	-0.763586292	0.047375008	0.959446578	0.00518714	0.047375008
Sdf2l1	-0.915814578	0.047375008	0.792172349	0.025682072	0.047375008
Mlxip	0.53014965	0.047375008	-0.549246314	0.012565184	0.047375008
Eri1	-0.503534793	0.047451551	0.519103732	0.01288825	0.047451551
Gfi1	-7.032475575	0.047524603	7.717269149	0.010301972	0.047524603
Cers3	-5.973677306	0.047524603	4.86536801	0.032001665	0.047524603
Sqstm1	-0.906551613	0.047524603	1.660574896	0.000791463	0.047524603
Ift27	-0.979022824	0.047524603	1.105546359	0.008904098	0.047524603
Ints2	0.732001203	0.047524603	-0.648746291	0.023409383	0.047524603
Pomt2	0.684784537	0.047524603	-0.545059268	0.031593172	0.047524603
Ndufb10	-0.576464242	0.047524603	0.639869798	0.009940194	0.047524603
Sde2	-0.535348112	0.047524603	0.650069041	0.006572872	0.047524603
Atrnl1	0.459164663	0.047524603	-0.45195012	0.015972747	0.047524603
Paqr8	0.494212867	0.047524603	-0.371644947	0.042658256	0.047524603
BC023829	-0.843056148	0.046453529	0.60565799	0.047562722	0.047562722
Sorcs3	1.123836599	0.036179983	-0.68235966	0.047575246	0.047575246
Metap1	-0.465710934	0.047660713	0.463148105	0.017012846	0.047660713
Jph3	0.548068553	0.047856773	-0.786388649	0.002924215	0.047856773
Ncf4	-3.94530377	0.047908194	3.21760772	0.031681961	0.047908194
4632427E13I	1.867084565	0.047908194	-1.502449828	0.025639895	0.047908194
Coa4	-1.219492018	0.047908194	1.517804191	0.006329729	0.047908194
1810011O10	-1.222088536	0.047908194	1.395134144	0.008993761	0.047908194
AF357359	1.0439386	0.047908194	-1.502546394	0.003256901	0.047908194
Selo	1.180463429	0.047908194	-1.265287991	0.011098618	0.047908194
Zzef1	0.898272918	0.047908194	-0.876277272	0.017671194	0.047908194
Sdk2	0.933764874	0.047908194	-0.738519858	0.037461109	0.047908194
Cntnap1	0.646339756	0.047908194	-0.739977548	0.007929404	0.047908194
Mrps15	-0.65748385	0.047908194	0.627924984	0.018529826	0.047908194
Ppp1r11	-0.618598404	0.047908194	0.626110584	0.014419418	0.047908194
Asah2	0.562049949	0.047908194	-0.565277525	0.014823595	0.047908194
Dusp14	-0.545366894	0.047908194	0.57582399	0.012244812	0.047908194
Vegfb	-0.601663656	0.047908194	0.466849802	0.03931004	0.047908194
Ubp1	0.532431466	0.047908194	-0.528322954	0.015935284	0.047908194
Ddx26b	0.975374583	0.048111284	-1.187555479	0.006460544	0.048111284
2010204K13I	-0.964824345	0.048111284	0.801012939	0.030424595	0.048111284

Fnbp4	0.545027163	0.048111284	-0.534631131	0.016519325	0.048111284
Lphn3	0.653437313	0.04813012	-0.597334084	0.024259924	0.04813012
Pate2	2.109124752	0.048253413	-1.705875922	0.030861699	0.048253413
Ptprn	0.657596824	0.048302918	-0.580956442	0.028418662	0.048302918
Pkp2	1.14394474	0.048576523	-1.112363843	0.022096142	0.048576523
Prkch	-0.964462861	0.048576523	0.8207237	0.029049252	0.048576523
Pcdh11x	0.787724854	0.048576523	-0.979381732	0.006594218	0.048576523
Itga10	0.918105114	0.048576523	-0.838623344	0.021698027	0.048576523
Sp100	-0.785931171	0.048576523	0.867528185	0.010938384	0.048576523
Fnip2	0.617260136	0.048576523	-0.679371728	0.010648176	0.048576523
Myl12b	-0.635649717	0.048576523	0.592064837	0.02134132	0.048576523
Cbr1	-0.551972126	0.048576523	0.463263471	0.030652552	0.048576523
Ankrd13c	-0.41312516	0.048576523	0.557510668	0.004391178	0.048576523
Ogt	0.733396611	0.049217091	-0.933191114	0.006055737	0.049217091
Mdn1	1.259959	0.049309986	-1.516728582	0.008122805	0.049309986
Ttll1	-0.436594536	0.049309986	0.427645428	0.017410432	0.049309986
Galnt16	0.674798794	0.049570401	-0.914928277	0.005827316	0.049570401
Cyb5r4	-0.421459631	0.049608601	0.433869678	0.014386013	0.049608601
Gdap10	1.101622009	0.04970599	-1.458499922	0.005983664	0.04970599
Ssh3	1.235633944	0.04970599	-1.122985708	0.019793083	0.04970599
Tars2	0.813315743	0.04970599	-0.705838066	0.027248386	0.04970599
Rab12	-0.405727175	0.04970599	0.439428526	0.011913278	0.04970599
